# A comprehensive mechanistic model of adipocyte signaling with layers of confidence

**DOI:** 10.1038/s41540-023-00282-9

**Published:** 2023-06-07

**Authors:** William Lövfors, Rasmus Magnusson, Cecilia Jönsson, Mika Gustafsson, Charlotta S. Olofsson, Gunnar Cedersund, Elin Nyman

**Affiliations:** 1 Department of Biomedical Engineering, Linköping University, Linköping, Sweden; 2 Department of Mathematics, Linköping University, Linköping, Sweden; 3 School of Medical Sciences and Inflammatory Response and Infection Susceptibility Centre (iRiSC), Faculty of Medicine and Health, Örebro University, Örebro, Sweden; 4 School of Bioscience, Systems Biology Research Center, University of Skövde, Skövde, Sweden; 5 Department of Health, Medicine and Caring Sciences, Linköping University, Linköping, Sweden; 6 Department of Physics, Chemistry and Biology, Linköping University, Linköping, Sweden; 7 Department of Physiology/Metabolic Physiology, Institute of Neuroscience and Physiology, The Sahlgrenska Academy at University of Gothenburg, Gothenburg, Sweden; 8 Center for Medical Image Science and Visualization (CMIV), Linköping University, Linköping, Sweden

**Keywords:** Dynamic networks, Time series, Computer modelling

## Abstract

Adipocyte signaling, normally and in type 2 diabetes, is far from fully understood. We have earlier developed detailed dynamic mathematical models for several well-studied, partially overlapping, signaling pathways in adipocytes. Still, these models only cover a fraction of the total cellular response. For a broader coverage of the response, large-scale phosphoproteomic data and systems level knowledge on protein interactions are key. However, methods to combine detailed dynamic models with large-scale data, using information about the confidence of included interactions, are lacking. We have developed a method to first establish a core model by connecting existing models of adipocyte cellular signaling for: (1) lipolysis and fatty acid release, (2) glucose uptake, and (3) the release of adiponectin. Next, we use publicly available phosphoproteome data for the insulin response in adipocytes together with prior knowledge on protein interactions, to identify phosphosites downstream of the core model. In a parallel pairwise approach with low computation time, we test whether identified phosphosites can be added to the model. We iteratively collect accepted additions into layers and continue the search for phosphosites downstream of these added layers. For the first 30 layers with the highest confidence (311 added phosphosites), the model predicts independent data well (70–90% correct), and the predictive capability gradually decreases when we add layers of decreasing confidence. In total, 57 layers (3059 phosphosites) can be added to the model with predictive ability kept. Finally, our large-scale, layered model enables dynamic simulations of systems-wide alterations in adipocytes in type 2 diabetes.

## Introduction

A major challenge within biomedical research, from clinical studies to drug development, is how to handle and make optimal use of the increasing availability of omics data, e.g., proteomics, phosphoproteomics and transcriptomics. The analysis of large-scale biological data is today often done within the field of bioinformatics using methods to construct biological networks. These networks are often constructed using prior knowledge that can be found in databases, where the interactions in the databases often are classified with a level of confidence^1–4^. These constructed biological networks can typically not be used to simulate dynamic time-resolved scenarios, e.g., to predict the clinical effect of a treatment or the change in signaling inside cells in response to new drugs. Such predictions are instead the strength of systems biology models based on ordinary differential equations (ODEs).

Systems biology ODE-based models are built on mechanistic details known about the system at study, such as intracellular signaling pathways. Furthermore, ODE-based modeling is the most common framework to model biological systems when time-resolved data is available^5^. This is evident in e.g., the BioModels database^6^ which contains over 1000 manually curated models, where over 80% of the models are ODE-based. Also, there exists ODE-based models for most biological systems, which are often partially overlapping and not interconnected.

The ODE-based models are often constructed using a set of kinetic rate equations, and these rate equations need to be fed with numerical values for the corresponding kinetic rate determining constants. These rate constants are model parameters that are usually unknown since they are often impossible to measure explicitly, and must instead be estimated based on indirect time-resolved measurements (e.g., measurements of the level of a protein). A central challenge with large ODE-based models is thus how to handle the large number of unknown parameters and how to estimate the parameter values.

This parameter identification challenge has been approached using adjoint sensitivities^7^ which improves the scaling of the parameter estimation problem with the number of parameters, together with the use of a sparse linear solver. The authors speed up the optimization process ~40,000 times for a model with ~4000 free parameters^7^. This development is important to go from smaller to larger ODE-based models. However, methods developed to estimate parameters for large ODE-based models still cannot handle the whole omics scale.

Other methods to handle semi-large protein data using ODE-based models have been developed^8–11^. These approaches use information-rich multi-perturbation data to create data-driven ODE-based models. These methods have been used to predict the response of new drug candidates and rank their ability to overcome drug resistance. Even though these methods can handle a large number of parameters, for all possible interactions between states, they are limited to ~200 proteins since all possible interactions are tested. Also, the information-rich perturbation data used in these methods is rare and costly to obtain experimentally.

One approach to avoid the estimation problem is to use parameter-free Boolean logical models instead of ODE-based models. Such models have been used to study large protein-activity datasets^12,13^. Parameter-free Boolean logical models are useful in the search for biologically relevant interactions between proteins and can thus be used for hypothesis generation when it comes to new interactions. However, parameter-free Boolean logical models are typically not used to explain behaviors seen in times series data, to predict time resolved effects of new drugs/perturbations or to simulate scenarios, which are important aspects in biomedical research. However, there are other more advanced approaches to Boolean modeling, which uses continuous time simulations and transition rates described by real numbers^14,15^.

Another solution to the large parameter estimation problem is to divide the problem into smaller sub-problems, adding a single protein/gene (and corresponding data) at a time, preferably starting from an established core model. The additions of new proteins/genes can then be done in parallel, substantially reducing the computation time. We have earlier developed a method to do such a model expansion^16^. However, this method does not take the confidence of included interactions into account and can only add a single layer of new interactions, ignoring the chain-like nature of signaling pathways.

One final approach is to develop smaller, partially overlapping, models and to interconnect them to yield a large model. In this way, the development of each submodel corresponds to a manageable parameter estimation problem, each resulting in a submodel of high confidence. Unfortunately, this process is time-consuming and there is no guarantee that the submodels can be combined.

In summary, it is an increasingly common situation to have large-scale omics data that need to be analyzed, and to have partially overlapping small ODE-based models, which are of high confidence, but which cannot be used to analyze the omics data. One challenge is to use the existing data to establish a core model of high confidence, and another challenge is to have a differentiated view of the variables that are not in core model with different degrees of confidence. One example of such a situation is adipocyte signaling.

Adipocyte signaling controls multiple processes of the adipocyte, and transmits the response to biologically important regulators such as insulin and adrenaline. Dysregulations in the adipocyte signaling network can lead to complications such as type 2 diabetes (T2D). Thus, understanding adipocyte signaling is central to understanding metabolic diseases such as type 2 diabetes, a disease for which the prevalence is increasing globally.

Within adipocyte signaling, we have developed several partially overlapping models for different aspects of the adipocyte, such as glucose uptake, lipolysis, and adiponectin secretion^17–22^. These models have not been combined into a single high confidence core model. Furthermore, large-scale phosphoproteomics data is available from 3T3-L1 adipocytes, which has not been analyzed using scalable mechanistic approaches. Adipocyte signaling is therefore a good use-case to solve an increasingly common problem.

**Fig. 1:**
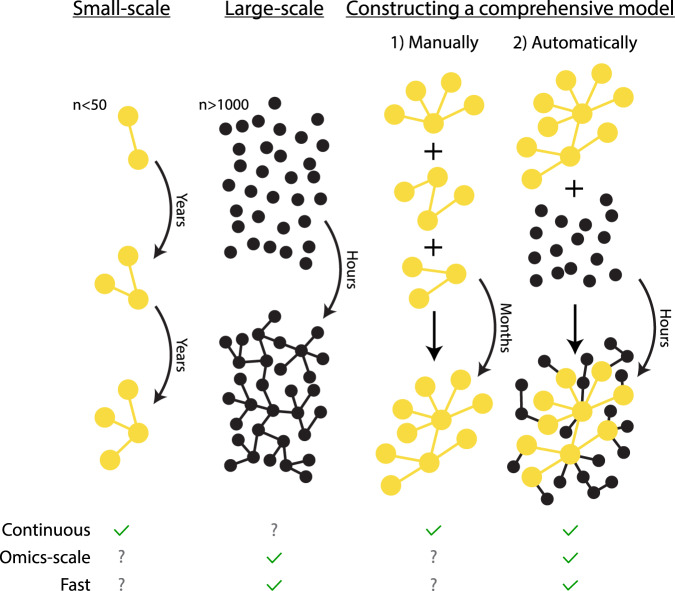
Overview of traditional approaches and the developed approach. In the field of systems biology, typically small mechanistic models of high confidence are developed. These dynamical models can be used to make time continuous predictions such as to simulate new experiments, e.g., a new drug intervention. However, they are slow to develop and are thus not suitable for analyzing omics data. Conversely, large omics data are commonly analyzed using statistical models within the field of bioinformatics using tools that are much faster than the slow development of the mechanistic models. However, the networks generated from such methods are typically not (time) continuous, and the individual interactions are often put together to a network in a less rigorous manner than in the small scale mechanistic models. In our approach, we first manually combine three separate mechanistic models into a connected core model. This core model is then expanded automatically using omics data and lists of interactions. During this expansion, we gradually introduce lower confidence additions. Thus, we end up with an expanded model with a highly confident core with layers of gradually decreasing confidence, with the possibility to simulate experiments on a large portion of the phosphoproteome. Relative to the ordinary way of developing mechanistic models, this expansion is fast, and is able to construct a model consistent with data and prior knowledge within a time-frame of days rather than years.

Here, we present a method to create comprehensive mechanistic models with layers of confidence, starting with the interconnection of three partially overlapping models into a core model containing detailed interactions of high confidence. From there, we add large-scale data in layers starting from high confidence interactions to purely data-driven interactions (Fig. 1). When adding these layers, we use a parallel, pairwise approach with a manageable computation time.

## Results

**Fig. 2:**
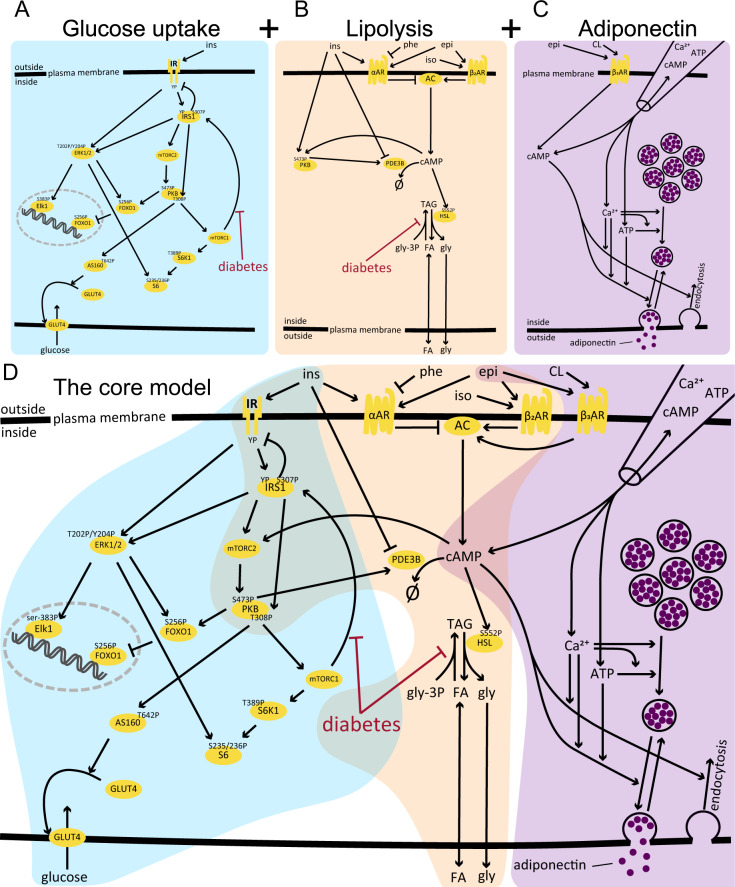
The submodels and the connected core model of adipocyte signaling. By connecting models of **A** glucose uptake in response to insulin (ins), **B** fatty acid (FA) and glycerol release in response to *α*- and *β*_2_-adrenergic receptor signaling, and **C** adiponectin secretion in response to cAMP, ATP and Ca^2+^ (added inside the cell with a pipette) and extracellularly stimulated *β*_3_-adrenergic receptor signaling a core model (**D**) was created. The model includes key differences between signaling normally and in type 2 diabetes. Additional inputs to the models include phentolamine (phe), isoprenaline (iso), epinephrine (epi), and CL 316243 (CL).

We first established a core model of adipocyte signaling (Fig. 2D), based on our earlier work^18,21,22^. The included earlier works consists of three independent models. Firstly, a glucose uptake model^18^ that includes major insulin signaling pathways of adipocytes, based on data from both non-diabetic and type 2 diabetic patients (Fig. 2A). Secondly, a lipolysis model^22^ that describes the release of fatty acids and glycerol in response to *α*- and *β*_2_-adrenergic receptor agonists and inhibitors, as well as the anti-lipolytic effect of insulin (Fig. 2B). The lipolysis model includes intracellular signaling intermediaries and is also based on data from both non-diabetic and type 2 diabetic patients. Finally, an adiponectin release model^21^ that unravel the mechanisms of adiponectin release in response to epinephrine, and a *β*_3_-adrenergic receptor agonist, as well as intracellular signaling intermediaries. This adiponectin release model includes patch-clamp experiments of adiponectin vesicle exocytosis in clonal 3T3-L1 adipocyte cell line, as well as confirming studies in adipocytes from non-diabetic patients (Fig. 2C).

### The crosstalk between different pathways in adipocyte signaling

To connect the models, we searched for crosstalk between the included signaling pathways (Fig. 2). There are clear crosstalk between glucose uptake and lipolysis, through the signaling pathways of both insulin and *α*- and *β*-adrenergic receptors. A central node for this crosstalk is cAMP, which is stimulated by *β*-adrenergic receptors and inhibited by *α*-adrenergic receptors, and in turn affects the phosphoinositide 3-kinase inhibitor (PI3K*α*)^23^. PI3K*α* is also involved in the insulin – glucose uptake signaling network. Furthermore, insulin will lead to the activation of PKB which in turn activates phosphodiesterase 3B (PDE3B). At unphysiologically high insulin concentrations there is also an additional inhibitory effect from insulin to PDE3B (ref. ^23^). PDE3B is involved in the breakdown of cAMP, which in turn is a central mediator in both adiponectin secretion and lipolysis^20,24^.

### The creation of a core model from previous models and data

The three separate models^18,21,22^ were connected in two steps. Firstly, we connected the lipolysis model^22^ with the glucose uptake model^18^. This was done by replacing the insulin action (*Ins*_1_) and the protein kinase B (PKB) equations from the lipolysis model with the insulin receptor and PKB from the glucose uptake model, and by having cAMP from the lipolysis model activate the mammalian target of rapamycin complex 2 (mTORC2) in the glucose uptake model. This interaction of cAMP to mTORC2 is a simplified mechanism of the activation of PI3K with subsequent activation of mTORC2. Secondly, we connected the newly combined lipolysis–glucose uptake model with the adiponectin model^21^. In essence, we combined the model by letting the *β*_3_-adrenergic receptors from the adiponectin model activate adenylyl cyclase (AC) from the lipolysis model instead of directly leading to a production of cAMP. All details regarding the connection of the models and the connected core-model are found in the Supplementary Information, in the “Model equations and parameter values” section.

### The core model can reproduce all previously used data

We tested the core model by separating the experimental data used in the previous works^18,21,22^ into an estimation set and a testing set. To be able to compare the combined core model to the ingoing individual models, we divided the data in the same way as done in the previous works. In practice, this meant that we used the data for adiponectin release stimulated by the *β*_3_-adrenergic receptor agonist CL 316243 in the presence of intracellular ATP^21^, and the data for the phosphorylation of hormone-sensitive lipase (HSL) in response to stimulation with insulin and isoprenaline^22^, for model testing and thus removed this testing data from the set of estimation data. After estimating the model parameters using the estimation data, we found the best agreement to be acceptable, supported by a *χ*^2^-test (${v}_{est}^{* }=616.50\, < \,{\chi }^{2}(p=0.05,df=582)=639.23$). We then estimated the model uncertainty by finding the maximal and minimal simulation values in each time point for each experiment, while simultaneously requiring the agreement with the estimation data to be sufficiently good (${v}_{est}^{* }\, < \,{\chi }^{2}(p=0.05,df=582)$), described in detail in the “uncertainty estimation” section. While estimating the model uncertainty, we also collected the parameter set giving the best agreement with the testing dataset. We found that the best model agreement to the testing data was statistically acceptable (${v}_{test}^{* }=20.65\, < \,{\chi }^{2}(p=0.05,df=16)=26.30$). The full description and graphical representations of the model agreement to data is given in the Supplementary Information, in the “Recreation of the model agreement to data and tests from previous works” section.

**Fig. 3:**
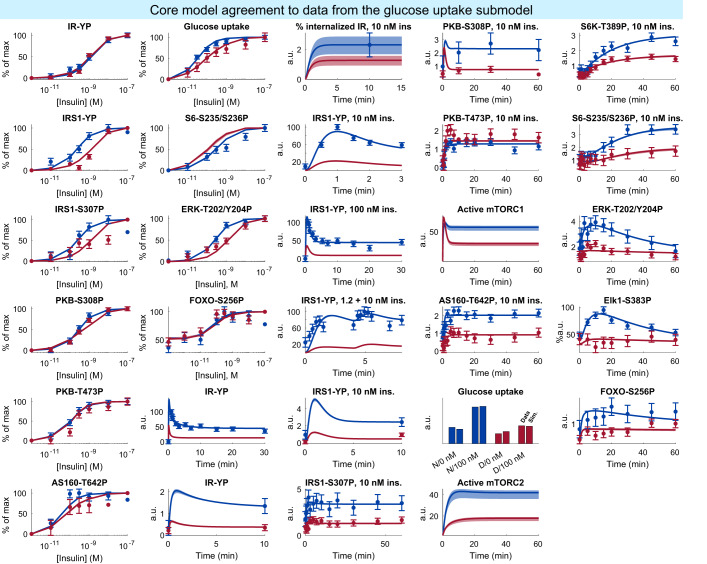
Model agreement with the insulin signaling data. Data comes from isolated human adipocytes stimulated with insulin in different doses and for different times^18^. In all panels, lines represent the model simulation with the best agreement to data, the shaded areas represent the model uncertainty, and experimental data points are represented as mean values with error bars (SEM). Simulations and experimental data in red correspond to experiments under type 2 diabetic conditions, and in blue under non-diabetic conditions. The model agreement was assessed with a *χ*^2^-test, as described in the “Statistical analysis” section.

**Fig. 4:**
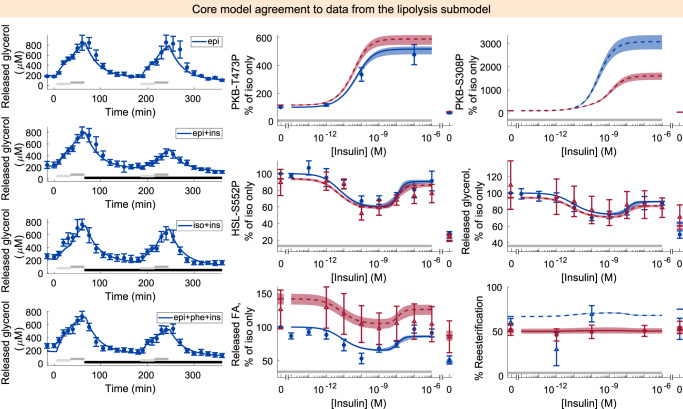
Model agreement with the lipolysis data. Released glycerol (left) was measured using microdialysis in the adipose tissue in situ^43^. All other data (middle, right) of released glycerol, released fatty acid (FA), phosphorylation of hormone-sensitive lipase (HSL) on Serine 552, and phosphorylation of protein kinase B (PKB) on serine 308 or threonine 473 were measured in isolated human adipocytes^23^. In all panels, lines represent the model simulation with the best agreement to data, the shaded areas represent the model uncertainty, and experimental data points are represented as mean values with error bars (SEM). Simulations and experimental data in red correspond to experiments under type 2 diabetic conditions, and in blue under non-diabetic conditions. Dashed lines and experimental data with open triangles were not used to estimate the model parameters. Light/dark gray horizontal bars indicate adrenergic stimulation with epinephrine (epi) or isoprenaline (iso), and black horizontal bars in the left figures indicate insulin (ins) stimulation. The model agreement was assessed with a *χ*^2^-test, as described in the “Statistical analysis” section.

**Fig. 5:**
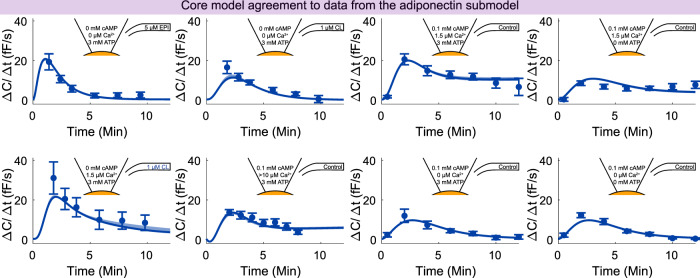
Model agreement with the adiponectin exocytosis data. Data represent patch-clamp capacitance recordings in 3T3-L1 adipocytes^22^. In all panels, lines represent the model simulation with the best agreement to data, the shaded areas represent the model uncertainty, and experimental data points are represented as mean values with error bars (SEM). Stimulation included epinephrine (EPI), CL *β*_3_-adrenergic receptor agonist CL 316243 (CL), cAMP, calcium (Ca^2+^), and ATP. The model agreement was assessed with a *χ*^2^-test, as described in the “Statistical analysis” section.

After testing the model, we included both the estimation and testing data into an extended set of data. We tested if the model could explain the extended set of data sufficiently well. We found the model agreement to the extended data to be acceptable (${v}_{ext}^{* }=654.81\, < \,{\chi }^{2}(p=0.05,df=598)=656.00$), and thus decided to use the connected model as a core for further extensions. The model agreement to the extended data is shown in Figs. 3–5. We again estimated the model uncertainty by finding the maximal and minimal simulation values in each time point for each experiment, while simultaneously requiring the agreement with the extended set of data to be sufficiently good (${v}_{ext}^{* }\, < \,{\chi }^{2}(p=0.05,df=598)$), described in detail in the “uncertainty estimation” section.

Finally, we tested if the connection rendered any large changes in the individual submodels, by testing the connected model’s ability to explain the individual datasets. The combined model, when trained on the full set of data, was slightly worse at describing the individual datasets, relative to the individual models trained on only the individual datasets. Neither the agreement to the glucose uptake dataset (${v}_{glu}^{* }=378.14\, < \,{v}_{ext}=410.95\, < \,{\chi }_{glu}^{2}=441.28$), the lipolysis dataset (${v}_{lipo}^{* }=163.20\, < \,{v}_{ext}=178.60\, < \,{\chi }_{lipo}^{2}=181.77$) nor the adiponectin release dataset (${v}_{adi}^{* }=40.0\, < \,{v}_{ext}=65.3\, < \,{\chi }_{adi}^{2}=69.8$) lead to the rejection of the connected model when using the parameter set trained to the full dataset.

### The core model can reproduce both the normal and T2D condition using the same set of parameters

The values kinetic parameters of the model were kept constant between the normal and type 2 diabetic condition simulations. The type 2 diabetic condition was simulated in the same way as in the original works by changing the initial values for 6 states in the model: (1) IR was reduced by 45%^17^, (2) GLUT4 was reduced by 50%^17^, (3) FOXO1 was reduced by 45%^18^, (4) AS160 was reduced by 55%^18^, and 6) S6 was reduced by 52%^18^. Furthermore, two parameters were used to reduce the effect of mTORC1 on IRS1-S307 phosphorylation^17^ and the reesterification of fatty acids to triglyceride (TAG)^22^, symbolized by the red arrows with flat arrowheads in Fig. 2. These two scaling parameters were allowed to vary between 0 (full blockage) and 1 (no effect) during the parameter estimation, but where kept constant in the simulation of the type 2 diabetic condition. We refer to the original works^17–19,22,23^ for the rationale on these changes in the type 2 diabetic condition.

### The connected core model can predict the effect of the crosstalk: the effect of isoprenaline on glucose uptake

**Fig. 6:**
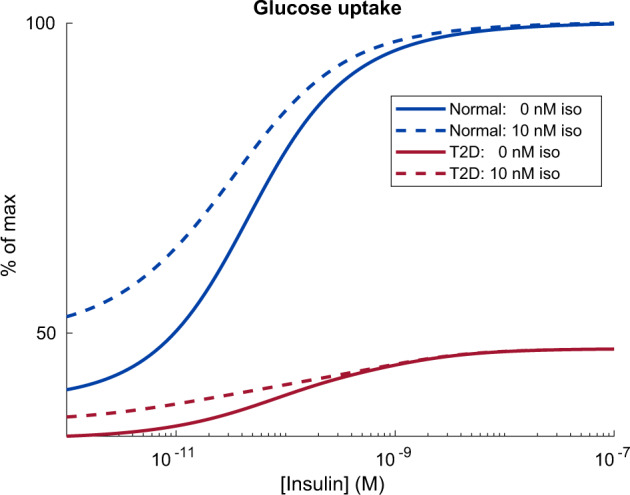
Predicting the effect of isoprenaline on glucose uptake. The model predicts that 10 nM isoprenaline (iso) increases the glucose uptake both without insulin and with low doses of insulin. However, above 10 nM insulin, glucose uptake is maximal and cannot further be augmented by isoprenaline. Lines correspond to the simulated glucose uptake as the response to insulin stimulation, where the uptake is expressed as the percentage of the maximal uptake. Solid lines correspond to the glucose uptake in the absence of isoprenaline, and dashed lines correspond to glucose uptake in the presence of 10 nM isoprenaline. Both insulin and isoprenaline were added at time point 0, and the response was measured after 30 min. Blue and red lines correspond to normal or type 2 diabetic (T2D) conditions respectively.

With the connected core model, we can now make predictions how inputs from one submodel affect the function of another submodel. For example, we can now predict how isoprenaline stimulation affects the glucose uptake. We did this by predicting the dose response of insulin on the glucose uptake, with or without isoprenaline added (Fig. 6). Our predictions show that 10 nM isoprenaline increases the glucose uptake both without insulin and with low doses of insulin. However, above 10 nM insulin, glucose uptake is maximal and cannot further be augmented by isoprenaline. Similar observations have been observed in e.g., rat adipocytes^25^. Furthermore, our predictions also show the same effect of isoprenaline on glucose uptake in the type 2 diabetic condition, but the augmenting effect is halted at a lower concentration of insulin. This prediction highlights the benefits of connecting the submodels, by enabling simulations that was not previously possible by the individual submodels.

### From the core model to the comprehensive mechanistic model of adipocyte signaling

To go from the core model to the comprehensive adipocyte, we use phosphoproteomic data for the time-resolved insulin response in 3T3-L1 adipocytes^26^. The phosphoproteomic dataset contains 37,248 phosphosites on 5705 proteins, of which 15% are insulin responding. After removing sites with only a single repeat in any time point, the dataset contained 5909 sites on 1937 proteins. Prior knowledge on possible protein–protein interactions was collected using the python package OmniPathDB^3^, which compiles a list of interactions from several sources, such as Reactome and PhosphoSitePlus. The list includes a confidence level in the sense of number of primary sources that have studied each interaction.

We first decided whether to employ a top-down or a bottom-up approach. By filtering the list of interactions to only include interactions where phosphoproteomic data were available, we could estimate the size of the maximal model if constructed using all available interactions with data. Such a model would contain 6642 states and 59169 parameters, assuming each phosphorylation site can switch between a phosphorylated and an unphosphorylated state, returning to the unphosphorylated state using a single parameter, and that each input in the list of interactions would contribute with one parameter. Clearly, the amount of unknown parameters would be too many to optimize using even the most state-of-art methods. We thus conceived a bottom-up approach instead.

We divided the phosphoproteomic data into two sets as done by the authors in ref. ^26^: one set with sites responding to insulin stimulation and another set with sites not responding to insulin stimulation, based on if the sites are effected by inhibitors of PI3K and PKB. We refer to these datasets as *responders* or *nonresponders* respectively. For our model expansion, we started with the responder data.

**Fig. 7:**
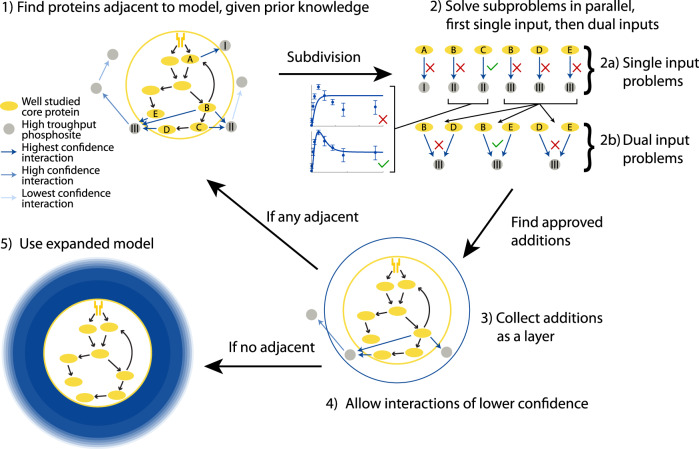
Overview of the developed approach. At the core of the method is our connected model. Outside the core connected model, there exists phosphoproteomic data (gray circles) not covered by the model. Using lists of interactions and our automatic model expansion algorithm we are able to add parts of this phosphoproteomic data, for proteins adjacent to the core model to create an expanded model that can be used to simulate scenarios. In step 1, the method find all phosphosites that are adjacent to the model using a set of allowed interactions. The possible additions are subdivided into independent parallel subproblems. In step 2, the methods tests the possible additions, first using only a single input in step 2a, then using dual inputs in step 2b. The additions are evaluated based on the agreement to data, exemplified using the two time-series plots, where dots with error bars correspond to experimental data and solid lines correspond to simulations of the new addition using inputs from the model. If the agreement is good enough, the potential addition is added to the model. After step 2, all approved additions are collected and added as a new layer in the model in step 3. Including the new layer, the method checks for additional possible additions. If any adjacent site exists, the method returns to step 1 and finds all possible additions. If no possible additions exist, the method allows additional interactions of a lower confidence in step 4 and again checks for possible additions. When interactions of all confidence levels have been included and there exists no more adjacent sites that can be added to the model, the expansion is stopped. Finally, the expanded model can used to simulate new scenarios in step 5.

Using a pairwise approach, we expand the core model towards the comprehensive mechanistic model in multiple layers (Fig. 7). We refer to the additions of all adjacent sites as the addition of a *layer* of sites to the model. For the first layer, we start with the core model and add phosphorylation sites by identifying the sites that are adjacent to the core model given the prior knowledge about the interactions. We start with interactions of the highest confidence, i.e., with the most (20) primary sources. From that level of confidence, we test in a random order if the phosphorylation sites can be added using interaction from the core model as input. Once no more adjacent proteins could be added to the layer, we save the layer and use the layer to find new adjacent sites. This layered addition of sites is continued until no more sites could be added to the model.

Once no more sites could be added to the model, we also included the interactions with the second-highest number of primary sources and attempted to add adjacent sites. We continued to include interactions with a lower confidence in a stepwise manner until no more sites could be added even with the interactions with the lowest number of primary sources. Using the responder data and all interactions, we added 254 sites to the model.

**Fig. 8:**
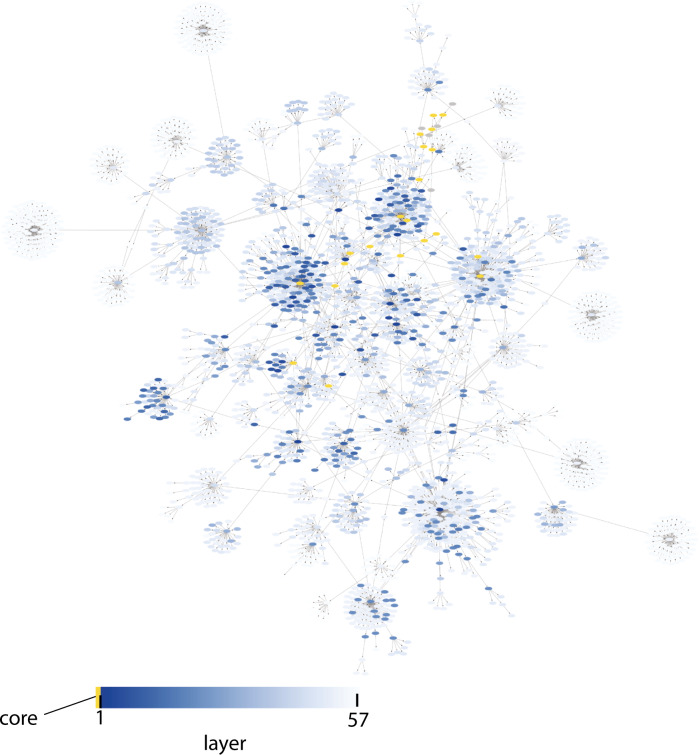
The comprehensive model of adipocyte signaling. The network structure of the expanded model, color coded by the order addition for the 60 added layers in blue. The core model is shown in yellow. Additions with high confidence is shown in dark blue, and additions with lower confidence in light blue. The full network is also available in Supplementary Software 1. The simulations and experimental data for all phosphosites in the model are available in the Supplementary Information, in the “Time series of all sites added to the model” section.

We then used all of the data (including the nonresponders), and again started with the interactions with the highest number of primary sources. Again, we step-wise included interactions with a lower number of primary sources. Using all data and all interactions, we could add 1957 additional sites, to a total of 2211 sites. When no more interactions could be added using all the data and all the interactions, we continued to add sites not yet included in the model using a data-driven approach. Here, we created a new list of potential interactions, based on the agreement between the simulations of the sites in the expanded model with the experimental data of the sites that had not been added. Using the data-driven approach, we could add 848 additional sites, to a total of 3059 sites structured into a total of 57 layers (Fig. 8). The final expanded model contains 6288 states and 6537 parameters. The parameter values were trained using 24472 time-resolved data points with a single input (insulin stimulation) and using 5201 data points with two other independent input signals (insulin stimulation combined with either the MK or LY inhibitor) as validation.

We collected the added proteins into a list, and sorted the list by the first expansion layer in which any of the protein’s phosphorylation sites were added. We then ran the list through gProfiler^27^ as an ordered query with mus musculus as the target organism. As a result, “response to insulin" (GO:0032868, *p*-value: 2.2 ⋅ 10^−30^) and “insulin signaling” (KEGG:04910, *p*-value: 3.9 ⋅ 10^−28^) were identified as important terms. For comparison, we also ran the same test with the same proteins but in a randomized order, without finding the terms as important (“response to insulin”, GO:0032868, *p*-value: 1.8 ⋅ 10^−21^ and “insulin signaling”, KEGG:04910, *p*-value: 1.1 ⋅ 10^−9^). In summary, these results indicate that our method is indeed able to capture the relevant pathways for insulin signaling.

#### Predicting inhibitions

**Fig. 9:**
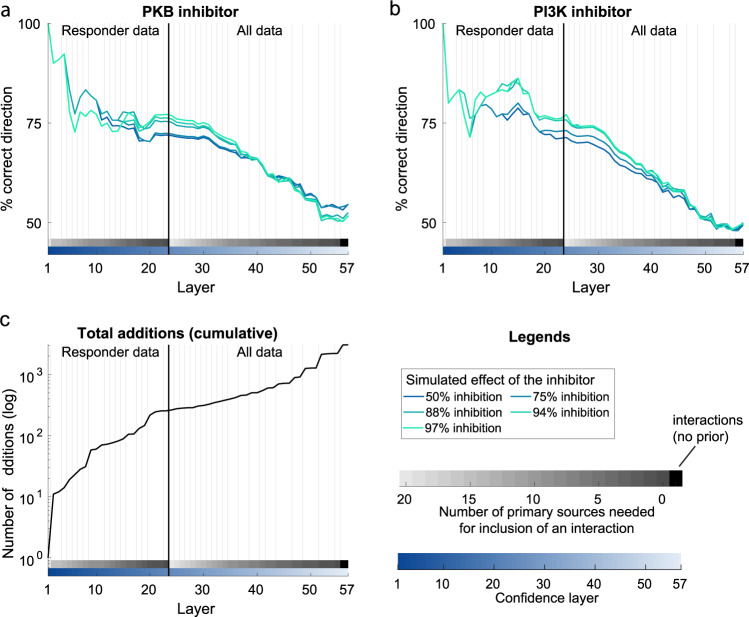
The predictive ability of the comprehensive model. For each added layer, we compute the predictive ability of the model using independent inhibitor data. **a** shows the ability of the model to correctly predict the direction (inhibitory or stimulatory) for the effect of PKB inhibition at 20 min after insulin stimulation, for the automatically added phosphosites. **b** shows the ability of the model to correctly predict the direction (inhibitory or stimulatory) for the effect of PI3K inhibition at 20 min after insulin stimulation, for the automatically added phosphosites. **c** shows the total number of added phosphosites. Vertical black lines correspond to when additional (nonresponder) data and purely data-driven interactions were allowed.

To test the validity of the expanded model, we made predictions by simulating the change in the insulin response with a PKB inhibitor and with a PI3K inhibitor, and compared the predictions to experimental data for the insulin response in the presence of the PKB inhibitor MK2206 and the PI3K inhibitor LY294002, gathered in the same study as the large-scale time series data^26^. Since the model was expanded using both interactions and data with different levels of confidence, we evaluate the model’s predictive capabilities on a layer-to-layer basis. The simulated time series of all phosphorylation sites, and the effect of the inhibitors, can be found in the Supplementary Information, in the “Time series of all sites added to the model” section. The model is able to correctly predict the direction of the two separate inhibition experiments in a majority of all sites, in all confidence layers (Fig. 9). Note that the model predictions are the most accurate when the most confident interactions and data are used, and that the accuracy of the predictions fall as the confidence is decreased (when going from left to right in Fig. 9a, b). Since the model expansion is done using the most confident interactions and data first - and then gradually decrease in confidence - it is clear that additions with higher confidence give the most accurate model predictions (left to right in Fig. 9a, b).

#### Simulating type 2 diabetes

**Fig. 10:**
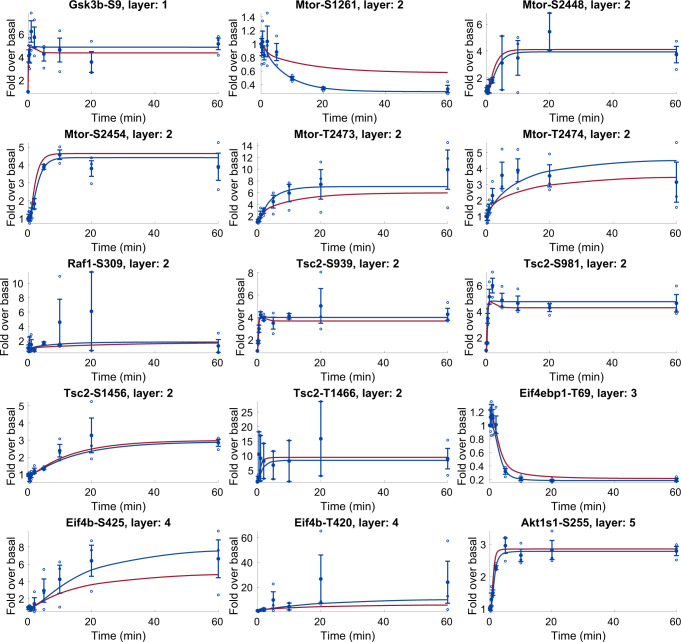
Simulations and data for the first 15 added sites of the comprehensive model. In all panels, blue lines represent model simulation with the best agreement to data and experimental data points are represented as mean values with error bars (SEM). Individual data points are shown as empty circles. Red lines are simulations of the type 2 diabetic conditions, and these simulations are model predictions without corresponding data. All model simulations of the effect of type 2 diabetes are available in the Supplementary Information, in the “Time series of all sites added to the model” section. The model agreement was assessed with a *χ*^2^-test, as described in the “Statistical analysis” section.

**Fig. 11:**
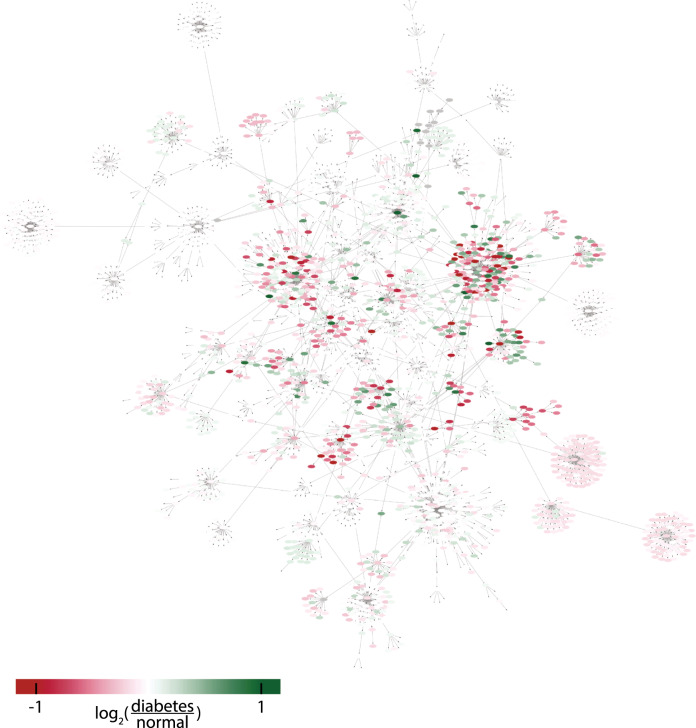
The effect of type 2 diabetes propagated to all sites in the expanded model. The color code goes from red corresponding to decreased signaling, to green corresponding to increased signaling in type 2 diabetes. White corresponds to no, or low, effect. The full network is also available in Supplementary Software 1. The simulations and experimental data for all phosphosites in the model is available in the Supplementary Information, in the “Time series of all sites added to the model” section.

Since the connected model used as a core for the automatic model expansion could simulate type 2 diabetes, we can now simulate how the effect of type 2 diabetes would spread to a large portion of the phosphoproteome. We evaluated the effect of type 2 diabetes by simulating the response to insulin, but now in a type 2 diabetic condition. In practice, we simulated the type 2 diabetic condition as described in the section “The core model can reproduce both the normal and T2D condition using the same set of parameters”. The time-resolved comparison between normal and type 2 diabetic conditions are shown for the first 15 added phosphosites in Fig. 10, and for all phosphosites in the Supplementary Information, in the “Time series of all sites added to the model” section. Furthermore, we quantified the change between normal and type 2 diabetic conditions by finding the fold change relative to the normal condition at *t* = 20 min (Fig. 11). The quantified effect is also available in Supplementary Data 1.

## Discussion

In this work, we present a methodology for automatic model expansion from a dynamic core model to include large-scale data and keep track of the level of confidence of both data and interactions (Fig. 7), showcased in the context of adipocyte signaling. In the methodology, the new data is added in parallel in layers, to reduce computation time and allow for thousands of added time-resolved measurements. To create a connected high-confidence core model of adipocyte signaling, we have connected major events related to lipolysis, glucose uptake, and adiponectin release into a connected model (Fig. 2). This connected model, developed herein, agrees with numerous observations from adipocytes (Figs. 3–5). From the core model, we use our methodology to add layers of experimentally measured phosphosites^26^ based on a list of possible interactions, including level of confidence, compiled using OmniPathDB^3^. The developed methodology allows for combining detailed mechanistic models, common in the field of systems biology, with large-scale data, traditionally analyzed in the field of bioinformatics, thus acting as a bridge between such methods.

The biological relevance of a comprehensive model of adipocyte signaling is related to type 2 diabetes. Insulin resistance is a hallmark of type 2 diabetes, and the core of the model describes in detail the intracellular insulin resistance of adipocytes (Fig. 3). A major function of adipocytes is to act as an energy reserve for other organs, i.e., to store and release fatty acids. The release of fatty acids is increased in type 2 diabetes, due to a decrease in reesterification (Fig. 4). Also, high levels of circulating adiponectin has been associated with a reduced risk of type 2 diabetes^28^, and an increased insulin sensitivity^29^. All of these processes have already been established in detail in models, and here we provide a connection including crosstalk that allow for their simultaneous simulations. Even more importantly, we provide a link between these highly established processes to the whole phosphoproteome, where the model allows anyone to simulate, e.g., the effect of a drug. We provide a type 2 diabetes version of the model (Figs. 10 and 11), and therefore the model can be used to predict the effect of anti-diabetic drugs throughout the adipocyte. In several efforts (e.g., refs. ^30,31^), plasma omics measurements of diabetic patients have been related to clinical parameters using statistical models. Such models provide important insight to relations between clinical parameters and specific proteins and/or genes, and can be used for classifications of patient groups. Such purely statistical models cannot, however, go beyond the data used for training to e.g., simulate the effect of new treatments.

The created comprehensive model has a layered level of confidence based on both interactions and data (Fig. 8). Most of the data from the insulin signaling models^18,22^ comes from isolated primary adipocytes from non-diabetic and type 2 diabetic patients, where protein levels and activities have been measured using antibodies and the western blot technique. The adiponectin release and phosphoproteome data instead comes from the 3T3-L1 adipocyte cell line, where the release of adiponectin was measured using a patch-clamp technique, and the protein phosphorylations were measured with mass-spectrometry. Furthermore, the small-scale data (western blot and patch-clamp) are typically more reliable since there are more repeats (typically *n* = 5−15) compared to the large-scale phosphoproteomic data (*n* = 0−3) per measurement. The additions in the expanded model is based on interactions with varying degrees of confidence. During the expansion, we first start with the interactions with the highest confidence, and then incrementally allow interactions with lower confidence (Fig. 9).

The development of new mechanistic ODE-based models for biological systems is a long and iterative process. The development of an ODE-based model typically starts with finding or collecting data, and formalizing a hypothesis based on the observation in the data for some biological system. The hypothesis is translated into a mathematical model, which is tested and refined, typically in many steps. The development of the models included in the core model here, as well as the gathering of corresponding data, have taken us and our collaborators decades of work. Thus, it is not feasible to cover the entire phosphoproteome with consistent high quality data and mechanistic ODE-based models. The developed automated methodology, therefore, provides a reasonable trade-off between confidence in the model and the coverage of the phosphoproteome (Fig. 7).

The most confident part of the model is the connected core model, which agrees with the full set of original experimental data sufficiently well (Figs. 3–5), supported by a *χ*^2^-test: ${v}_{ext}^{* }=654.81\, < \,{\chi }^{2}(p=0.05,df=598)=656.00$. However, at the same time the optimal cost (${v}_{ext}^{* }$) is close to the threshold of rejection (from 654.81 to 656.00). This means that only changes in the parameter values resulting in a maximum cost increase by 1.19 is accepted when estimating the model uncertainty. In other words, a cost increase by around 0.2%. This narrow range of acceptable increases of the cost before rejection directly translates to a model uncertainty that is also narrow. We do not believe this narrow uncertainty to be the result of failed optimizations, but rather due to that only a small increase in the cost is allowed. Consequently, the model is given a larger freedom with the estimation dataset (Supplementary Figs. 1–3), due to fewer data being used, resulting in a larger uncertainty. Simultaneously, the best agreement to either the estimation or full datasets are highly similar, since extended set of data consists mostly of data from the estimation dataset. Perhaps alternative thresholds of rejection should be considered, such as allowing a cost increase from the optimal cost corresponding to one degree of freedom (*χ*^2^(*p* = 0.05, *df* = 1) = 3.84). Anyway, the model is accepted and large-scale which provides a key and necessary criterion for a valuable explanatory model. Furthermore, we showed the extended model’s predictive capability using independent inhibition data which indeed yielded largely correct predictions, which further strengthens the validity of the large-scale predictions.

We showcased the usefulness of the model by predicting the change in phosphorylation levels in type 2 diabetic condition. These predictions could not be tested against experimental data and are as such predictions of lower reliability. When time-resolved experimental data of phosphoproteome changes in response to insulin in the type 2 diabetic condition becomes available, such data can be used to test the model and to increase its reliability. While experimental data are the gold standard for observing the behavior of biological systems, the model could still be useful even when such data are eventually available. Since the data is typically time-discretized while the model is time-continuous, it can be used for predicting what is happening in between experimentally measured data points, or what happens given different conditions, for example, a different insulin dose.

Another way to increase the reliability of the full expanded model could be to further refine the core model by including additional processes and origination points for further expansion. While the core model explains processes like glucose uptake, lipolysis, and adiponectin release, it does not currently contain other important processes like leptin and resistin secretion, or insulin stimulated lipogenesis. Including such additional processes would improve both the core model and the expanded model, and are relevant future extensions of the model.

The presented expanded model is a model that is consistent with both experimental data and prior knowledge, but it also is not the final model for the phosphoproteome. Rather, the model is one acceptable explanation the dynamics of a large part of the insulin stimulated phosphoproteome. Training the model on additional large-scale time-resolved data, including additional perturbation data, when such data becomes available will result in a different (and more reliable) expanded model. We here present a way of integrating experimental data from different conditions into a cohesive framework that is consistent with prior knowledge.

While the developed method is able to generate an extended model that is consistent with both experimental data and prior knowledge, there are still some limitations to the models. Firstly, some core model is necessary to start the automatic expansion. In practice, this might not be an issue, since most signaling pathways have at least some small-scale detailed model available. Furthermore, it would also be possible to start with a minimal core as long as the model can respond to the inputs (such as responding to insulin in this case). Still, the use of a reliable core model is also a strength of the method, because it establishes a reliable foundation for the construction of the expanded model, and it increases that chance that the model can capture the intricate dynamics of biological systems. Another limitation is related to the availability of large-scale data and prior knowledge of the interactions in the studied system. Unfortunately, large-scale datasets measured using a high number of repeats and at many time points are not available, and detailed information on the interactions in the system are also typically not available in the prior knowledge. Instead, the prior knowledge typically only contains information on the level of which proteins interact with each other, and not how phosphorylations on specific sites on proteins influence the phosphorylation of sites on other proteins.

The developed method herein resembles our previously developed method (LASSIM^2^). The previous method also consisted of a core model used to describe additional constituents of a biological system. The most significant change in the current developed method, is that we now expand the model in an iterative fashion, and that we now use prior knowledge to guide the model expansion. We now also use prior knowledge of the interactions with different levels of reliability, and keep track of the corresponding reliability of the expanded model.

A remaining issue in the method developed herein is that the expanded model will always be constructed in a feed-forward manner. In other words, that new additions will not feed back to previous additions. Such feedbacks are potentially important for the dynamic behavior of many measured phosphosites, and such phosphosites will not be correctly represented with the method. To further develop the method to include key feedback signals would therefore be an important future step.

Alternative attempts at modeling the phosphoproteome have been made, such as the machine learning clustering analysis in the original work that collected the phosphoproteome data used herein^26^, or the PhosR method for identifying “signalomes” from the same research group^32^. Other attempts include the multi-omics analysis method MEPHISTO^33^, or the employment of protein-protein interaction databases to infer time-dependent kinase-substrate relationships^34^. However, to the best of our knowledge, none of these methods are able to explain the time dynamics of the phosphoproteome using a mechanistic approach. Furthermore, they are not able to simulate new situations and make predictions, something our expanded model is able to do.

Another approach to model human adipocytes on a large scale is the use of genome-scale metabolic models^35^. Such models provide another view on adipocyte changes in obesity and type 2 diabetes. These models integrate genomics, transcriptomics, and proteomics data and apply to a fixed metabolic map based on known human metabolic reactions. Such models are complementary to the work herein since they do not consider the signaling pathways, and our approach does so far not include metabolic reactions. We are currently gathering labeled metabolite data for metabolic flux analysis from human adipocytes. These data will be integrated in the next version of the comprehensive adipocyte.

The developed method is not limited to adipocyte signaling, and could be applicable to other biological systems which have a signaling cascade originating from a well-define core, perhaps most simply other systems of signal transduction via phosphorylations, where large-scale experimental time-series data is available. Some such examples could be FGF21 signaling in mice^36^, or human stem cell differentiation^37^.

In summary, we here present a method that can scale small-scale dynamic models to the omics level, while still preserving the dynamic qualities of the model. Our method can integrate data from different sources into a cohesive framework, and can be further extended when additional data becomes available in the future. The expanded model can be used to predict events, such as perturbations by an inhibitor or a drug, and the changes to the signaling network as a response to a disease. Furthermore, the method should be applicable to other biological systems where detailed small-scale models, and omics-level data, are available.

## Methods

### Data collection and filtration

We used publicly available data, both data previously used to develop our models of glucose uptake, lipolysis, and adiponectin release^17,18,21,22^ and from other sources^26^.

For the newly used phosphoproteomic data^26^, we excluded sites where any time point had less than two repeats and then calculated the mean and standard error of the mean. For the inhibition data, sites with less than two repeats, or with an unclear effect of the inhibition (such that the uncertainty covered both an increase and a decrease) were ignored when the prediction accuracy was calculated. Furthermore, we divided the data into two groups (*responders* and *nonresponders* to insulin) based on the original classification done by the authors of the phosphoproteome work^26^.

### Quantifying the model agreement to experimental data

In order to evaluate the model’s performance, we quantified the model agreement to data using a function typically referred to as a cost function. In this case, we used the normalized sum of squared residual as cost function (Eq. (1)).

1 $$v(\theta )=\mathop{\sum}\limits_{t}{\left(\frac{{y}_{t}-{\hat{y}}_{t}(\theta )}{SE{M}_{t}}\right)}^{2}$$

Here, *v*(*θ*) is the cost for the set of parameter values *θ*, equal to the sum of the normalized residual over all measured time points, *t*; *p* is the parameters; *y*_*t*_ is the measured data and ${\hat{y}}_{t}(\theta )$ is the model simulations; *SEM*_*t*_ is the standard error of the mean for the measured data.

### Statistical analysis

To reject models, we used the *χ*^2^-test with a significance level of 0.05. We used 582 degrees of freedom for the original training data leading to a threshold for rejection of *χ*^2^(*p* = 0.05, *df* = 582) ≈ 639.23. For the extended set of experimental data including all the experimental data from the connected model we used 598 degrees of freedom, resulting in a threshold for rejection of *χ*^2^(*p* = 0.05, *df* = 598) ≈ 656.00. Any combination of model and parameter set that results in a cost (Eq. (1)) above the threshold must be rejected. If no parameter set exists for a model that results in a sufficiently low cost, the model structure must be rejected. We assumed the measurement noise to be additive and normally distributed.

For the automatic model expansion, we allowed additions to the model where the cost for the tested phosphorylation site was below *χ*^2^(0.05, 8) ≈ 15.5.

### Optimization and software

We used MATLAB R2021a (MathWorks, Natick, MA) and IQM tools (IntiQuan GmbH, Basel, Switzerland), a continuation of SBTOOLBOX2^38^, for modeling. IQM tools uses CVODES^39^ to numerically integrate the ODEs. The parameter values were estimated using the enhanced scatter search (eSS) algorithm from the MEIGO toolbox^40^. We allowed the parameter estimation to freely find the best possible combinations of parameter values, within boundaries. Both the optimal parameter values and the bounds for the parameter values are given in Supplementary Tables 1–3.

### Uncertainty estimation

To estimate the model uncertainty, we employed the formulation of the uncertainty estimation problem as described in ref. ^41^ and implemented in refs. ^21,22^. In short, we estimated the model uncertainty by maximizing or minimizing a specific model prediction $\hat{p}$ (such as the simulation of an experiment at a specific time point), while requiring that the model agreement with data is sufficiently good (the cost being less than the *χ*^2^-limit) (Eq. (2)).

2 $$\begin{array}{ll}{{{\rm{minimize}}}}&\hat{p}\\ {{{\rm{subject}}}}\,{{{\rm{to}}}}&v(\theta )\le {\chi }^{2}\end{array}$$

Here, some prediction $\hat{p}$ is minimized to find the lower bound on the value of the prediction, while requiring the cost *v*(*θ*) to be below the *χ*^2^-threshold. To get the upper bound of the prediction, the problem in Eq. (2) can be solved as a maximization problem. In practice, we solved the maximization problem as a minimization problem by changing the sign of the objective function (Eq. (2)) to $-\hat{p}$. Furthermore, we relaxed the constraint (Eq. (2)) into the objective function as a penalty term offset by $| \hat{p}|$, scaled with the absolute value of the prediction given the optimal parameter set $\hat{p}({\theta }^{* })$ if *v*(*θ*) > *χ*^2^ (Eq. (3)).

3 $$\begin{array}{ll}{{{\rm{minimize}}}}&\hat{p}+penalty\\ {{{\rm{subject}}}}\,{{{\rm{to}}}}&penalty=\left\{\begin{array}{ll}| \hat{p}| +| \hat{p}({\theta }^{* })| \cdot (1+| v(\theta )-{\chi }^{2}| ),&{{{\rm{if}}}}\,v(\theta ) > {\chi }^{2}\\ 0,&{{{\rm{otherwise}}}}\end{array}\right.\end{array}$$

### Automatic model expansion

To do the automatic model expansion, we created an algorithm which is outlined as pseudocode in Algorithm 1. In essence, we create an expanded model by iteratively adding proteins to the core model by identifying the proteins that are closest to, but not yet in, the model, and test if they can be added to the model in a pairwise fashion. This was done using phosphoproteomic data collected using mass-spectrometry^26^, and a list of protein-protein interactions compiled using the python package OmniPathDB^3^. In detail, we find all proteins that are adjacent to the model, i.e., having at least one direct interaction in the list of interactions going from the model to the proteins. Using the list of adjacent proteins and corresponding prior knowledge interactions, we test if the proteins can be added to the model one by one (in parallel). In practice, each addition of an adjacent protein is tested using three different types of interactions. Firstly, we test if the interaction can be a single pairwise interaction corresponding to a phosphorylation, a dephosphorylation, or a saturated phosphorylation of the adjacent protein. Secondly, we test if the interaction must be a double interaction with two different inputs (double phosphorylation, double dephosphorylation or one phosphorylation with one dephosphorylation). Lastly, we test if the interaction results in a phosphorylation leading to a subsequent secondary state (such as an internalization) before returning to the unphosphorylated state. The adjacent proteins and interactions are tested by comparing the model simulation of the adjacent protein with the available experimental data. All adjacent proteins where the model simulations agree with the data sufficiently well are added to the model. We refer to the addition of the adjacent proteins that agree with data sufficiently well as the addition of a *layer* to the model. Once one such layer have been added to the model, we repeat the process of finding and adding another layer of adjacent proteins. For subsequent addition of layers, proteins in the previous layers are also used to find the new adjacent proteins.

#### Algorithm 1

Automatic model expansion

*I* ← prior knowledge of protein-protein interactions

*P*_*M*_ ← all proteins in model

*P*_*A*_ ← all proteins adjacent to model, given *I* and *P*_*M*_

$$valid\_interactions={{\emptyset}}$$

*layer* ← 0

**while** $${P}_{A}\ne {{\emptyset}}$$ **do**

** for** *p*_*A*_ in *P*_*A*_ **do**

*  I*_*A*_ ← interactions from *P*_*M*_ to *p*_*A*_ in *I*

**  for** *i* in *I*_*A*_ **do** ⊳ Interactions already tested are not tested again

*   valid* ← test_pairwise(*i*)

**   if** *valid* **then**

*    valid*_*interactions* = *valid*_*interactions* + *i*, **return**

**   end** **if**

**  end** **for**

**  if** not *valid* **then**

*   i*_1_ ← best *i* from pairwise loop

**   for** *i*_2_ in *I*_*A*_ **do**

*    valid* ← test_double(*i*_1_, *i*_2_)

**    if** *valid* **then**

*     valid*_*interactions* = *valid*_*interactions* + *i*_1_ + *i*_2_, **return**

**    end** **if**

**   end** **for**

**  end** **if**

**  if** not *valid* **then**

**   for** *i* in *I*_*A*_ **do** ⊳ Interactions already tested are not tested again

*    valid* ← test_extra_state(*i*)

**    if** *valid* **then**

*     valid*_*interactions* = *valid*_*interactions* + *i*, **return**

**    end** **if**

**   end** **for**

**  end** **if**

**  if** *valid* **then**

*   P*_*M*_ = *P*_*M*_ + *p*_*A*_,

**  end** **if**

** end** **for**

* P*_*A*_ ← all proteins adjacent to model, given *I* and *P*_*M*_

* layer* = *layer* + 1

**end** **while**

## Supplementary information

Supplementary Information

Supplementary Software 1

Supplementary Data 1

nr-reporting-summary

## Data Availability

All experimental data used in this work is publicly available data which have previously been published^17–19,23,24,26,42,43^.

## Code availability

The experimental data as well as the complete code for data analysis and modeling are available at https://github.com/willov/comprehensive-adipocyte(DOI: 10.5281/zenodo.7696571) and is mirrored at https://gitlab.liu.se/ISBgroup/projects/comprehensive-adipocyte. The model equations and best parameter values are also available in the Supplementary Information, in the “Model equations and parameter values” section.
