# Supplementary material for: A comprehensive mechanistic model of adipocyte signaling with layers of confidence
Source: NPJ Syst Biol Appl. 2023 Jun 7;9:24. doi: 10.1038/s41540-023-00282-9 (PMC10244124; doi:10.1038/s41540-023-00282-9)
Supplement: Supplementary file 4 — nr-reporting-summary [file 41540_2023_282_MOESM4_ESM.pdf]

nature portfolio

Corresponding author(s): William Lövfors

Last updated by author(s): Mar 3, 2023

Reporting Summary

Nature Portfolio wishes to improve the reproducibility of the work that we publish. This form provides structure for consistency and transparency in reporting. For further information on Nature Portfolio policies, see our Editorial Policies and the Editorial Policy Checklist.

Statistics

For all statistical analyses, confirm that the following items are present in the figure legend, table legend, main text, or Methods section.

n/a	Confirmed	
☐	☒	The exact sample size (*n*) for each experimental group/condition, given as a discrete number and unit of measurement
☒	☐	A statement on whether measurements were taken from distinct samples or whether the same sample was measured repeatedly
☐	☒	The statistical test(s) used AND whether they are one- or two-sided<br>*Only common tests should be described solely by name; describe more complex techniques in the Methods section.*
☒	☐	A description of all covariates tested
☐	☒	A description of any assumptions or corrections, such as tests of normality and adjustment for multiple comparisons
☐	☒	A full description of the statistical parameters including central tendency (e.g. means) or other basic estimates (e.g. regression coefficient) AND variation (e.g. standard deviation) or associated estimates of uncertainty (e.g. confidence intervals)
☐	☒	For null hypothesis testing, the test statistic (e.g. *F*, *t*, *r*) with confidence intervals, effect sizes, degrees of freedom and *P* value noted<br>*Give P values as exact values whenever suitable.*
☒	☐	For Bayesian analysis, information on the choice of priors and Markov chain Monte Carlo settings
☒	☐	For hierarchical and complex designs, identification of the appropriate level for tests and full reporting of outcomes
☒	☐	Estimates of effect sizes (e.g. Cohen's *d*, Pearson's *r*), indicating how they were calculated

Our web collection on statistics for biologists contains articles on many of the points above.

Software and code

Policy information about availability of computer code

Data collection	No software for data collection was used
Data analysis	We used MATLAB R2021a (MathWorks, Natick, MA) and IQM tools (IntiQuan GmbH, Basel, Switzerland), a continuation of SBTOOLBOX2, for modeling. IQM tools uses CVODES to numerically integrate the ODEs.<br>The parameter values were estimated using the enhanced scatter search (eSS) algorithm from the MEIGO toolbox.<br>The complete code for data analysis and modeling are available at https://github.com/willov/comprehensive-adipocyte and is mirrored at https://gitlab.liu.se/ISBgroup/projects/comprehensive-adipocyte.

For manuscripts utilizing custom algorithms or software that are central to the research but not yet described in published literature, software must be made available to editors and reviewers. We strongly encourage code deposition in a community repository (e.g. GitHub). See the Nature Portfolio guidelines for submitting code & software for further information.

Data

Policy information about availability of data

All manuscripts must include a data availability statement. This statement should provide the following information, where applicable:

- Accession codes, unique identifiers, or web links for publicly available datasets
- A description of any restrictions on data availability
- For clinical datasets or third party data, please ensure that the statement adheres to our policy

All experimental data used in this work is publicly available data. The data files used in the project is available in the code repository.
The original data is available in:
Brännmark, C. et al. Insulin Signaling in Type 2 Diabetes: Experimental and Modeling Analyses Reveal Mechanisms of Insulin Resistance in Human Adipocytes. Journal of Biological Chemistry 288, 9867–9880 (Apr. 5, 2013).

Rajan, M. R., Nyman, E., Kjølhede, P., Cedersund, G. & Strålfors, P. Systems-Wide Experimental and Modeling Analysis of Insulin Signaling through Forkhead Box Protein O1 (FOXO1) in Human Adipocytes, Normally and in Type 2 Diabetes. Journal of Biological Chemistry 291, 15806–15819 (July 22, 2016).

Komai, A. M., Brännmark, C., Musovic, S. & Olofsson, C. S. PKA-independent cAMP Stimulation of White Adipocyte Exocytosis and Adipokine Secretion: Modulations by Ca2+ and ATP. The Journal of physiology 592, 5169–5186 (Dec. 1, 2014).

Komai, A. M. et al. White Adipocyte Adiponectin Exocytosis Is Stimulated via B3-Adrenergic Signaling and Activation of Epac1: Catecholamine Resistance in Obesity and Type 2 Diabetes. Diabetes 65, 3301–3313 (Nov. 2016).

Stich, V. et al. Activation of A2-Adrenergic Receptors Blunts Epinephrine-Induced Lipolysis in Subcutaneous Adipose Tissue during a Hyperinsulinemic Euglycemic Clamp in Men. American Journal of Physiology-Endocrinology and Metabolism 285, E599–E607 (Sept. 1, 2003).

Jönsson, C., Castor Batista, A. P., Kjølhede, P. & Strålfors, P. Insulin and β-Adrenergic Receptors Mediate Lipolytic and Anti-Lipolytic Signalling That Is Not Altered by Type 2 Diabetes in Human Adipocytes. Biochemical Journal 476, 2883–2908 (Oct. 15, 2019).

Human research participants

Policy information about studies involving human research participants and Sex and Gender in Research.

Reporting on sex and gender	This work only uses previously published data.
Population characteristics	This work only uses previously published data.
Recruitment	This work only uses previously published data.
Ethics oversight	This work only uses previously published data.

Note that full information on the approval of the study protocol must also be provided in the manuscript.

Field-specific reporting

Please select the one below that is the best fit for your research. If you are not sure, read the appropriate sections before making your selection.

☒ Life sciences ☐ Behavioural & social sciences ☐ Ecological, evolutionary & environmental sciences

For a reference copy of the document with all sections, see nature.com/documents/nr-reporting-summary-flat.pdf

Life sciences study design

All studies must disclose on these points even when the disclosure is negative.

Sample size	This work only uses previously published data. The sample sizes and rationale are detailed in the original works.
Data exclusions	For the phosphoproteomic data, we excluded sites where any time point had less than two repeats.

Replication	This work only uses previously published data. We did not perform any reproducibility tests.
Randomization	This work only uses previously published data. We did not perform any additional randomization.
Blinding	This work only uses previously published data. For this work, additional blinding is not relevant.

Reporting for specific materials, systems and methods

We require information from authors about some types of materials, experimental systems and methods used in many studies. Here, indicate whether each material, system or method listed is relevant to your study. If you are not sure if a list item applies to your research, read the appropriate section before selecting a response.

Materials & experimental systems

n/a	Involved in the study	
☒	☐	Antibodies
☒	☐	Eukaryotic cell lines
☒	☐	Palaeontology and archaeology
☒	☐	Animals and other organisms
☒	☐	Clinical data
☒	☐	Dual use research of concern

Methods

n/a	Involved in the study	
☒	☐	ChIP-seq
☒	☐	Flow cytometry
☒	☐	MRI-based neuroimaging
