# Supplementary material for: A comprehensive mechanistic model of adipocyte signaling with layers of confidence
Source: NPJ Syst Biol Appl. 2023 Jun 7;9:24. doi: 10.1038/s41540-023-00282-9 (PMC10244124; doi:10.1038/s41540-023-00282-9)
Supplement: Supplementary file 1 — Supplementary Information [file 41540_2023_282_MOESM1_ESM.pdf]

Supplementary information to
"A comprehensive mechanistic model of adipocyte signaling with varying layers of confidence"

William Lövfors[1,2,3,✉1], Rasmus Magnusson[4], Cecilia Jönsson[1,5], Mika Gustafsson[6], Charlotta S. Olofsson[7], Gunnar Cedersund[1,3,8,*,✉2], and Elin Nyman[1,*,✉3]

[1]Department of Biomedical Engineering, Linköping University, Linköping, Sweden
[2]Department of Mathematics, Linköping University, Linköping, Sweden
[3]School of Medical Sciences and Inflammatory Response and Infection Susceptibility Centre (iRiSC), Faculty of Medicine and Health, Örebro University, Örebro, Sweden
[4]School of Bioscience, Systems Biology Research Center, University of Skövde, Skövde, Sweden
[5]Department of Health, Medicine and Caring Sciences, Linköping University, Linköping, Sweden
[6]Department of Physics, Chemistry and Biology, Linköping University, Linköping, Sweden
[7]Department of Physiology/Metabolic Physiology, Institute of Neuroscience and Physiology, The Sahlgrenska Academy at University of Gothenburg, Gothenburg, Sweden
[8]Center for Medical Image Science and Visualization (CMIV), Linköping University, Linköping, Sweden
[*]These authors contributed equally

[✉1]william.lovfors@liu.se
[✉2]gunnar.cedersund@liu.se
[✉3]elin.nyman@liu.se

April 5, 2023

Contents

1 Recreating the model agreement to data and tests from previous works. 3
1.1 The combined model can explain all previous data 3
1.2 The combined model can accurately predict all previous validation data 7
1.3 Lipolysis 7
1.4 Adiponectin 7
1.5 Glucose uptake 8

2 Model equations and parameter values. 11
2.1 Connecting the submodels 11
2.2 Description of the equations for the connected model 14
2.3 The glucose uptake submodel 15
2.4 The lipolysis submodel 20
2.5 The adiponectin submodel 23
2.6 The kinetics of the additions in the expanded model 25
2.7 Parameter values 27
2.8 Initial values 31

3 Time series of all sites added to the model 34
3.1 How to interpret the figures. 34
3.2 Insulin stimulation, normal and in type 2 diabetes 36
3.3 Insulin stimulation, normal and with the PKB inhibitor 3095
3.4 Insulin stimulation, normal and with the PI3K inhibitor 6154

1 Recreating the model agreement to data and tests from previous works.

This document shows that the combined model can recreate model agreement with data and the validations from the previous modelling works. We first trained the model to the dataset without the subsets previously used for validations. In practice, this meant that we divided the full data set into a training data set and a testing data set. The testing data set consisted of the data for adiponectin release stimulate by CL and ATP from citeadiponectin2, and the phosphorylation of HSL in response to stimulation with insulin and isoproterenol from [1]. The training data set consisted of the rest of the data, i.e. all data not in the testing data set. We first trained the model on the training data set and tested if the model agreement to data was sufficiently good. We then tested the models predictive power by simulating the experiments corresponding to the data in the testing data set, and again tested if the agreement to data was sufficiently good. For the validation from the insulin signaling – glucose uptake work [2, 3], we recreated the inhibitions with rapamycin, torin and PD184352.

1.1 The combined model can explain all previous data

We first tested if the model could explain the set of training data sufficiently well. This was done by optimizing the model parameters such that the model agreement to data was as good possible. The model agreement to data was estimated using the *objective function* defined in Eq. (1).

$$v(\theta) = \sum_{t} \left(\frac{y_t - \hat{y}_t(\theta)}{SEM_t} \right)^2 \tag{1}$$

Here, v is typically referred to as the cost, and it is equal to the sum of the normalized residual over all measured time points, t; θ is the parameters; y_t is the measured data at time t and $\hat{y}_t(\theta)$ is the model simulations at time t; SEM_t is the standard error of the mean for the measured data at time t.

The better the agreement between the model simulations and the data becomes, the lower the cost ($v(\theta)$) becomes. To test if the agreement is sufficiently good, a χ^2-test is used. If the cost is greater than the χ^2-statistic ($v(\theta) > \chi^2$) the model given the parameter values θ must be rejected. If the cost given the *optimal* parameters θ^* is greater than the χ^2-statistic ($v(\theta^*) > \chi^2$) then the model structure must be rejected. Conversely, if we can find a parameter set resulting in a cost lower than the χ^2-statistic ($v(\theta) < \chi^2$) we deem the model sufficiently good.

Here, we found the best cost (v^*) for the combined model when trained to the set of training data to be below the threshold of rejection ($v^*_{train} = 616.50 < \chi^2(p = 0.05, df = 582) = 639.23$). The model agreement to the training data is shown in Supplementary Figures 1 to 3.

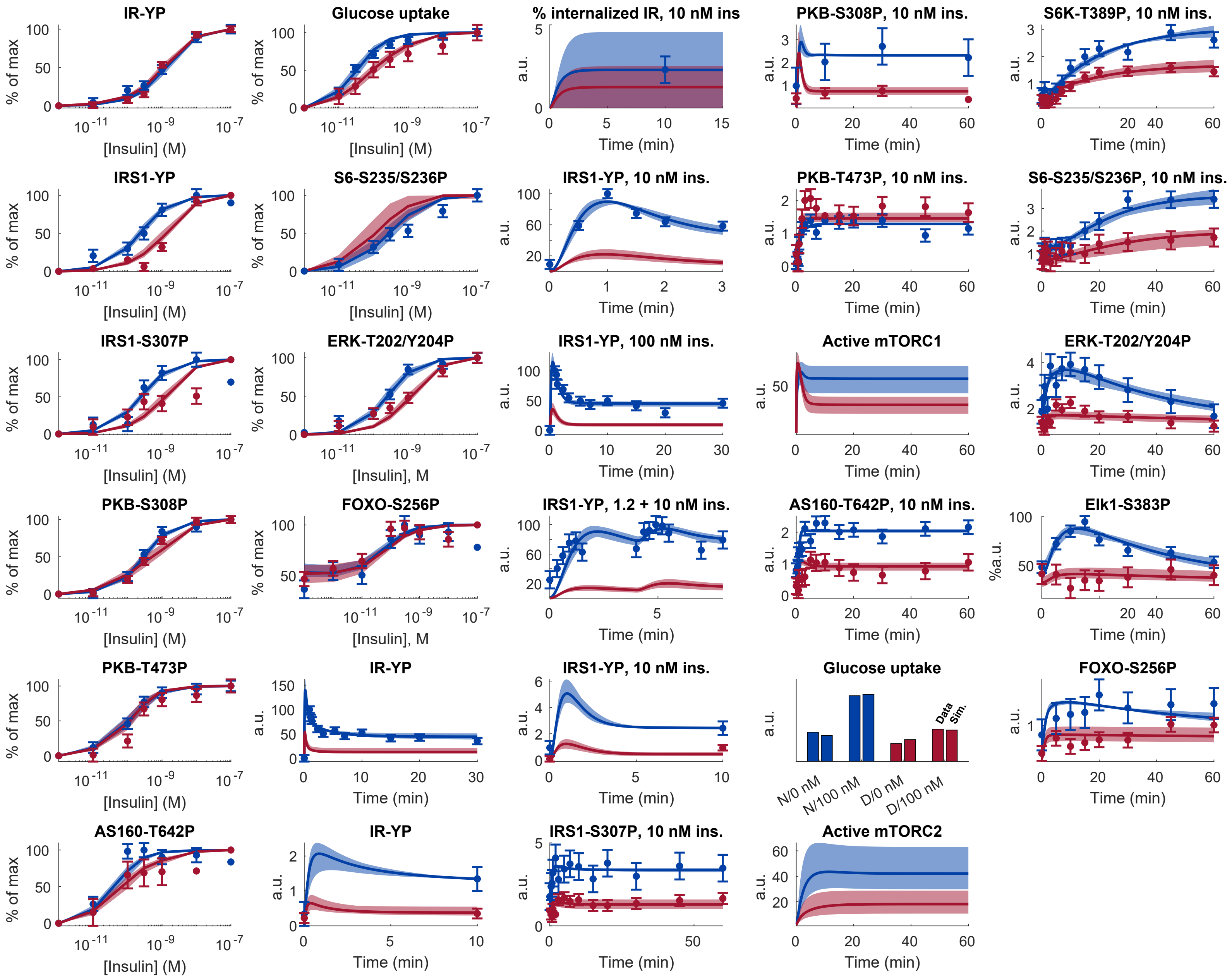

Supplementary Figure 1: Model agreement with data from [2]. Data comes from isolated human adipocytes stimulated with insulin in different doses and for different times [2]. In all panels, lines represent the model simulation with the best agreement to data, the shaded areas represent the model uncertainty, and experimental data points are represented as mean values with error bars (SEM). Simulations and experimental data in red corresponds to experiments under diabetic conditions, and in blue under non-diabetic conditions.

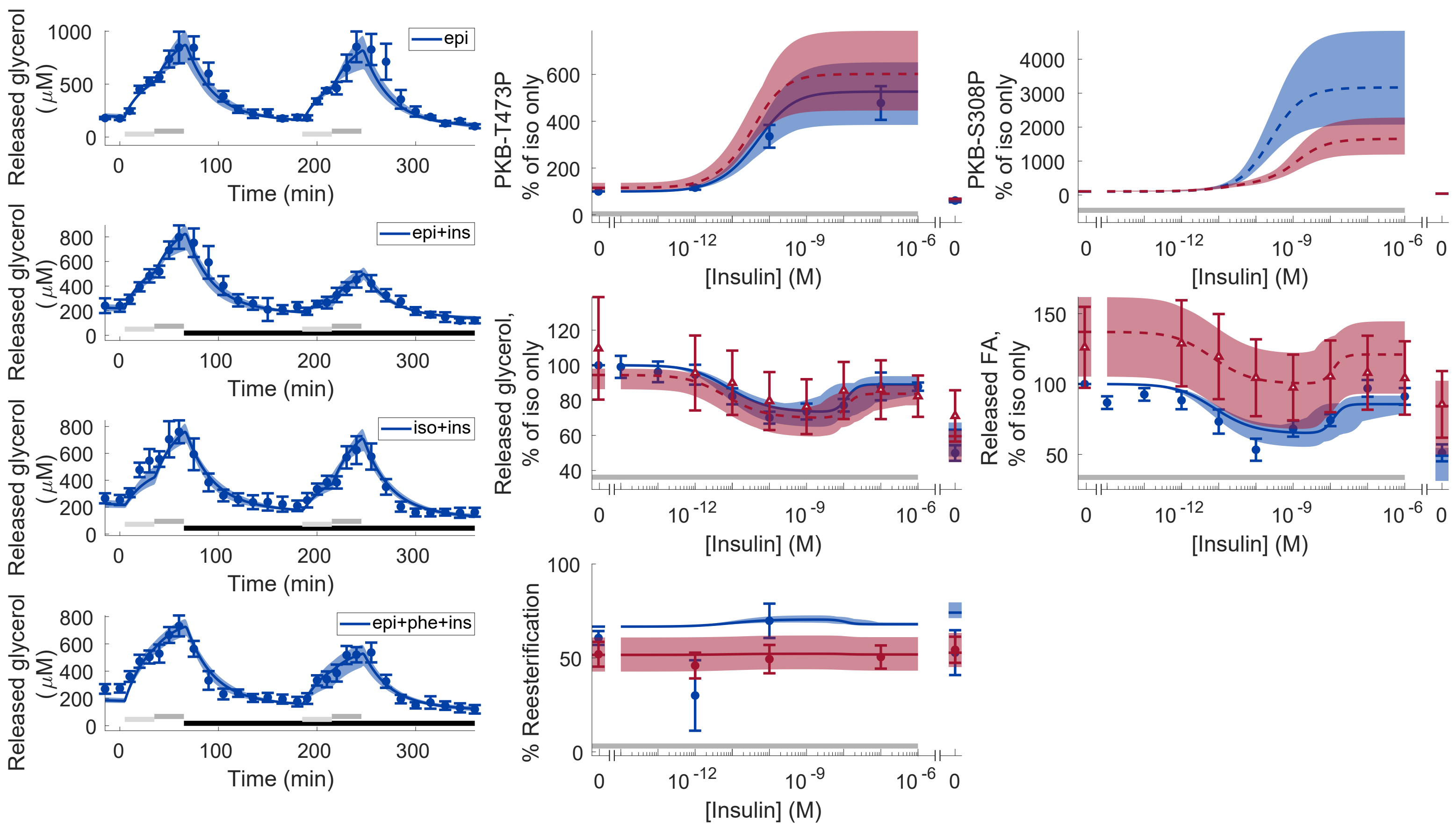

Supplementary Figure 2: Model agreement with lipolysis data from [4]. Released glycerol (left) was measured using microdialysis in the adipose tissue in situ [5]. All other data (middle, right) was measured in isolated human adipocytes [4]. In all panels, lines represent the model simulation with the best agreement to data, the shaded areas represent the model uncertainty, and experimental data points are represented as mean values with error bars (SEM). Simulations and experimental data in red corresponds to experiments under type 2 diabetic conditions, and in blue under non-diabetic conditions. Dashed lines and experimental data with open triangles were not used to estimate the model parameters. Light/dark gray horizontal bars indicate adrenergic stimulation, and black horizontal bars in the left figures indicate insulin stimulation. epi - epinephrine, ins - insulin, iso - isoprenaline, FA - released fatty acids

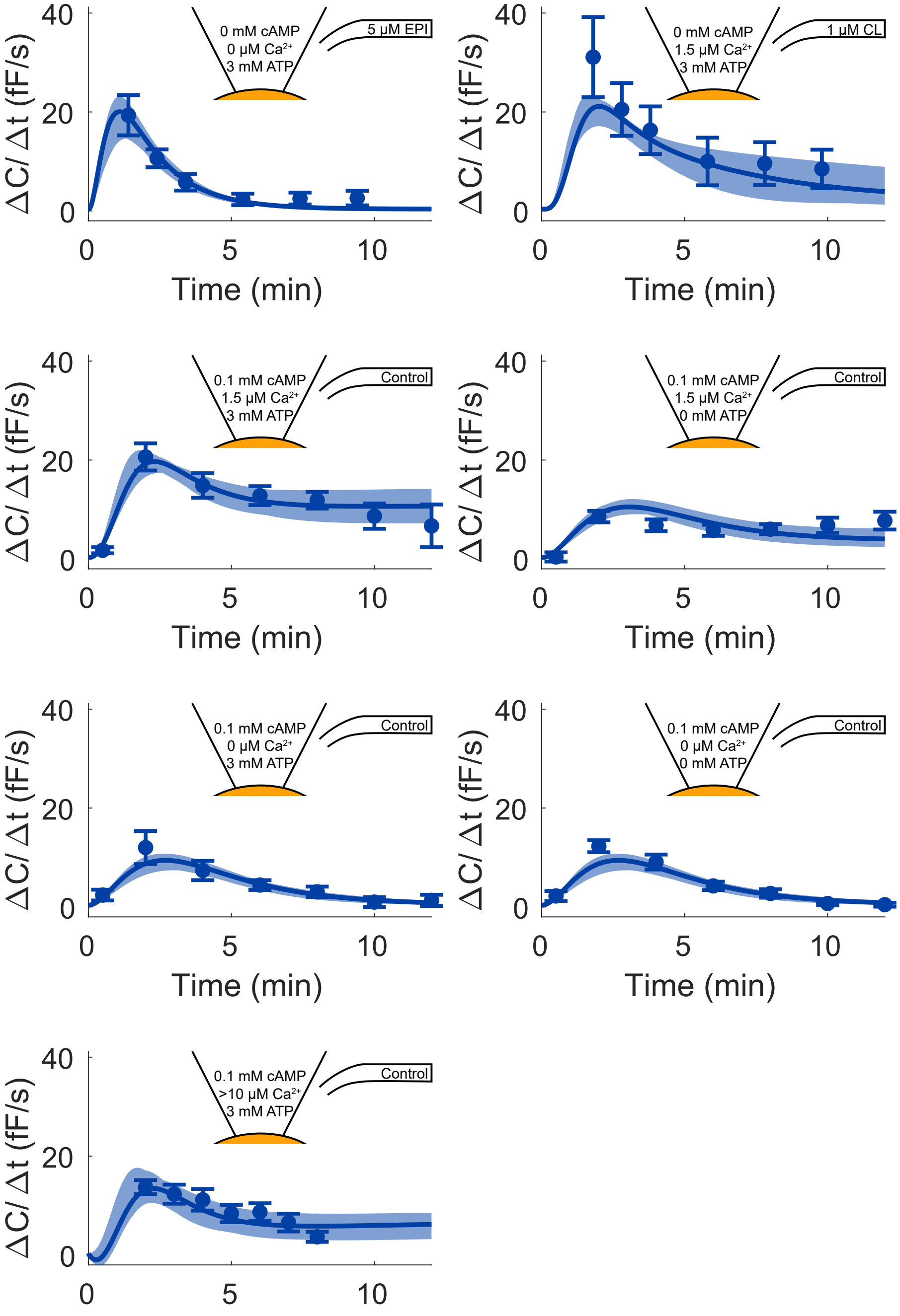

Supplementary Figure 3: Model agreement with the adiponectin release data used in [1]. Data represent patch-clamp capacitance recordings in 3T3-L1 adipocytes [1]. In all panels, lines represent the model simulation with the best agreement to data and experimental data points are represented as mean values with error bars (SEM). EPI - epinephrine, CL - β3-adrenergic receptor agonist CL 316243, Ca^{2+}- Calcium.

1.2 The combined model can accurately predict all previous validation data

The validation data set consisted of the data for adiponectin release stimulate by CL and ATP from citeadiponectin2, and the phosphorylation of HSL in response to stimulation with insulin and isoproterenol from [1].

1.3 Lipolysis

We used the dose response for HSL phosphorylation (HSLp) as validation data in the original lipolysis work [1]. Here, we have also excluded the HSLp data from the pool of training data and used the HSLp data as testing data. We retrained the model to the limited training data and predicted the dose response of HSLp. The model prediction and experimental data is shown in Supplementary Figure 4A.

1.4 Adiponectin

We used the exocytosis time series data for stimulation with 1 µM CL and 3 mM ATP (CL+ATP dataset) as validation data in the work of the adiponectin submodel [6]. Here, we also excluded the CL+ATP dataset from the pool of training data and used the CL+ATP dataset for model testing. We retrained the model to the limited pool of training data and predicted the exocytosis when stimulated with CL+ATP. The model prediction and experimental data is shown in Supplementary Figure 4B.

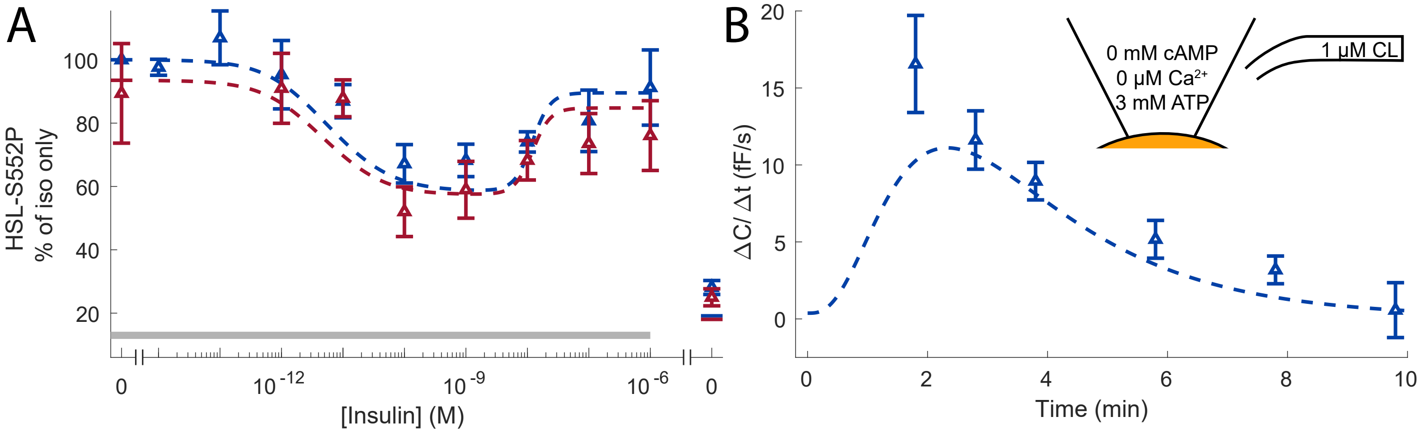

Supplementary Figure 4: Recreation of the model validation from the previous lipolysis work ([1, Fig. 4 and S2 Fig.]) and adiponectin work ([6, Fig. 4]). In both panels, lines represent the model simulation with the best agreement to data, and experimental data points are represented as mean values with error bars (SEM). Simulations and experimental data in red corresponds to experiments under diabetic conditions, and in blue under non-diabetic conditions. Dashed lines and experimental data with open triangles were not used to estimate the model parameters.

1.5 Glucose uptake

As in the original work, we simulated the effect of the inhibitions ranging from 50% to 93% inhibition. First, we recreated the validations from [3]. Here, we predicted the effect of the mTORC1 inhibitor rapamycin on the phosphorylation of of S6-S235/236P, IR-YP, IRS1-307P, AS160-T642P, PKB-S473P and FOXO1-S256P. Both model predictions and experimental data are shown in Supplementary Figure 5. In short, rapamycin should have an inhibiting effect on mTORC1, and thus on IRS1-307P, IR-YP and S6-S235/236P. At the same time, rapamycin should not have an inhibiting effect on mTORC2 and downstream phosphorylations. Thus, PKB-473P, AS160-T642P and FOXO1-S256P should not be inhibited.

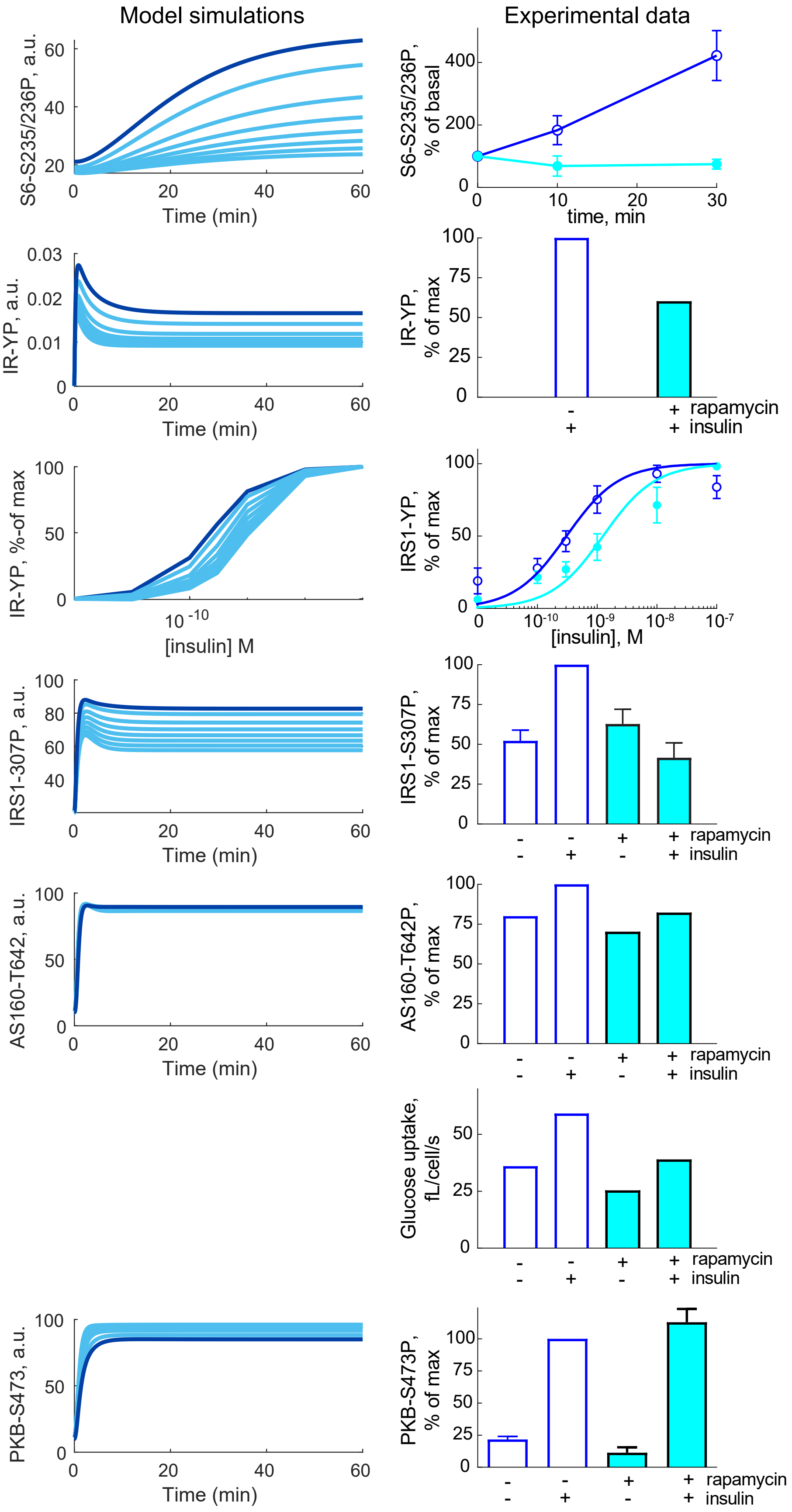

Supplementary Figure 5: Recreation of the model validation in [3, Fig. 7].

We then recreated the validations from [7]. Here, the mTORC1 inhibitor rapamycin was again used, as well as the mTORC1 and mTORC2 inhibitor torin, and the MEK inhibitor akti.

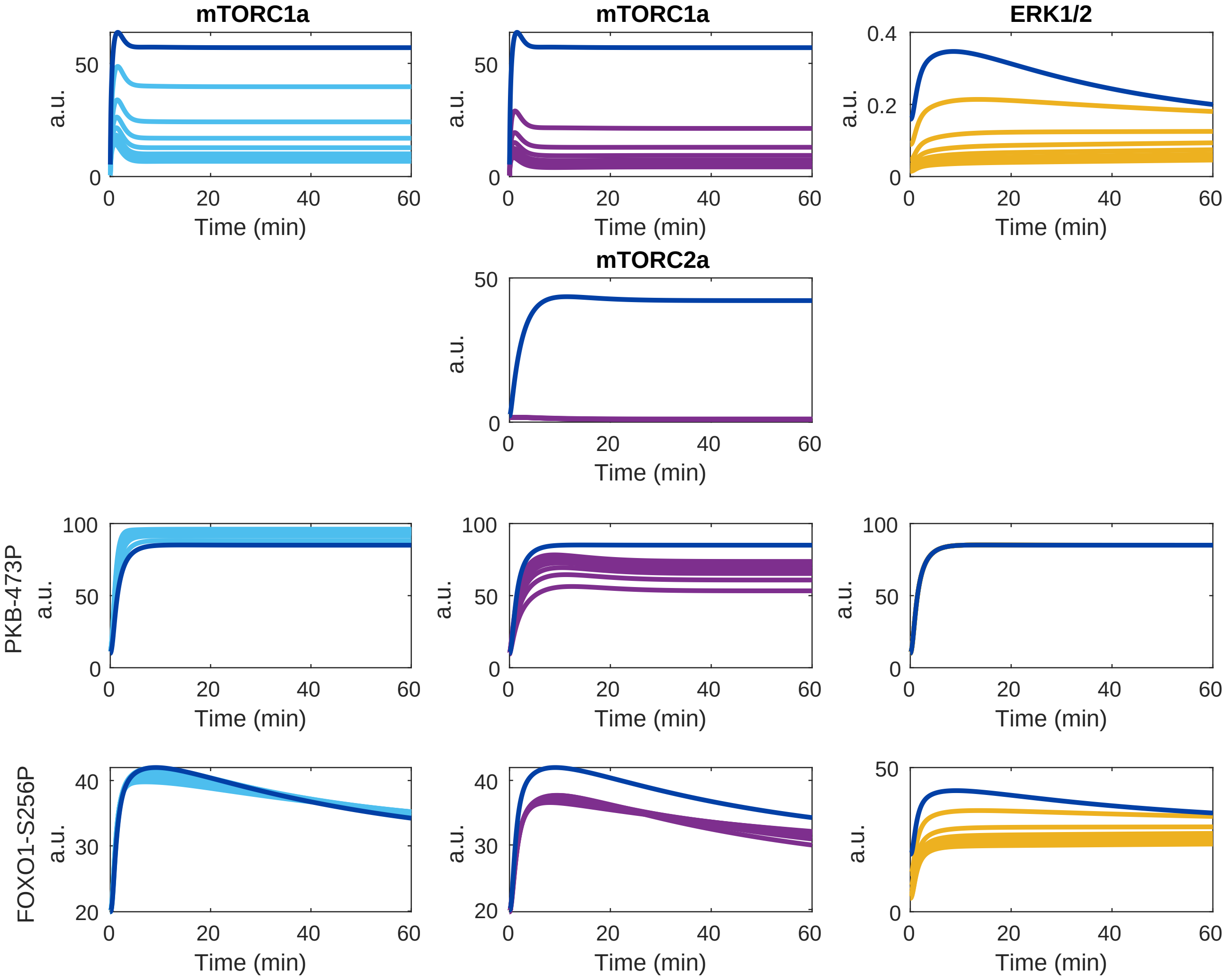

Supplementary Figure 6: Recreation of the model validation in [2, Fig. 11].

2 Model equations and parameter values.

The connected model is based on ordinary differential equations (ODEs), and is a combination of three different previously published models for glucose uptake [2], adiponectin secretion [6] and lipolysis [1]. A typical ODE used in this work looks similar to Eq. (2).

$$\begin{aligned} d/dt(x) &= -va + vb \\ va &= ka \cdot x \\ vb &= kb \cdot input \end{aligned} \tag{2}$$

Here, x is a state in model, va and vb are reaction rates, ka and kb are rate determining parameters, and $input$ is some input to the state. In other words, the amount of the state x is decreased by the reaction va with the speed ka, and increased by the reaction vb with the speed kb depending on some input $input$.

In all ODEs in this document, vGx and kGx corresponds to reaction rates and rate parameters for the glucose uptake submodel, vLx and kLx to the lipolysis submodel and vAx and kAx to the adiponectin submodel. Many of the states in the model correspond to different versions of the same thing (e.g., a phosphorylated version of a protein and an unphosphorylated version). For most of these versions, the phosphorylated/activated version spontaneously return the unphosphorylated/inactive version. These spontaneous returns to the base version will typically not be detailed.

The final connected model is shown in Supplementary Figure 9, and the equations are given in Equations (10) to (37). However, we will first highlight the changes made to the original submodel equations due to the connection of the submodels before we go through the equations for the connected model.

2.1 Connecting the submodels

For these three submodels, there are important points of crosstalk that had not previously been addressed. The glucose uptake submodel [2] and lipolysis submodel [1] have an overlap in the insulin receptor and subsequent signaling intermediaries leading up to the phosphorylation of PKB-S473 (Supplementary Figure 7). In the lipolysis model, we had previously simplified the signalling pathway from the insulin receptor to PKB as a direct interaction (action-1 in Supplementary Figure 7).

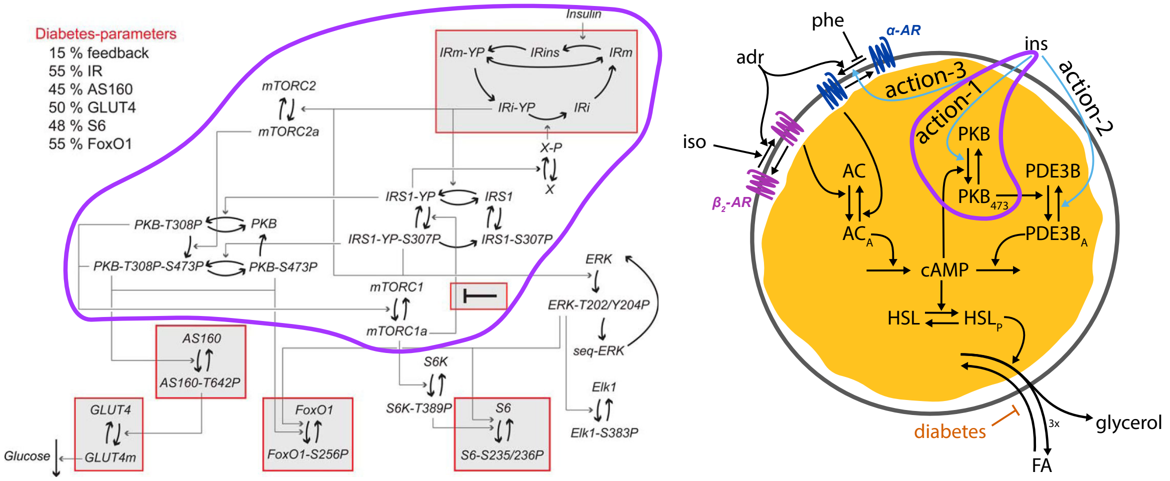

Supplementary Figure 7: The glucose uptake model [2] and the lipolysis model [1]. The overlap between the models is highlighted in purple. Both model figures have previously been published under a CC-BY license.

To connect these two submodels, we updated the lipolysis submodel to use the insulin signalling pathway from the glucose uptake submodel instead of insulin action-1. In practice, we removed the equations for PKB in the lipolysis model, and changed the input to Phosphodiesterase 3B (PDE3B) from PKB_{473} as in the lipolysis model to $(PKB\_T473P + PKB\_S308P\_T473P)$ from the glucose uptake model. The equations for PDE3B used in the original lipolysis work is given in Eq. (3).

$$
\begin{aligned}
d/dt(PDE3B) &= -vL2a + vL2b \\
d/dt(PDE3Ba) &= vL2a - vL2b \\
vL2a &= kL2a \cdot \boldsymbol{PKB473} \cdot PDE3B \\
vL2b &= kL2b \cdot PDE3Ba \cdot Ins\_2
\end{aligned} \tag{3}
$$

The updated reaction rate equations ($vL2a$, $VL2b$) for PDE3B after the models had been connected is given in Eq. (4).

$$
\begin{aligned}
vL2a &= kL2a \cdot \boldsymbol{(PKB\_T473P + PKB\_S308P\_T473P)} \cdot PDE3B \\
vL2b &= kL2b \cdot PDE3Ba \cdot Ins\_2
\end{aligned} \tag{4}
$$

Furthermore, we added the indirect effect of cAMP on PKB (a simplified mechanism of the activation of PI3K) from the lipolysis model as an activation of mTORC2 in the pathway from the glucose uptake model, shown in bold in Eq. (5).

$$
\begin{aligned}
d/dt(mTORC2) &= -vG5c + vG5d \\
d/dt(mTORC2a) &= vG5c - vG5d \\
vG5c &= mTORC2 \cdot (kG5c \cdot IRi\_YP + \boldsymbol{kL1a \cdot cAMP}) \\
vG5d &= kG5d \cdot mTORC2a
\end{aligned} \tag{5}
$$

In addition to the overlap between the glucose uptake and lipolysis submodels, there is also an overlap between the lipolysis submodel and the adiponectin secretion submodel(Supplementary Figure 8). This overlap is cAMP.

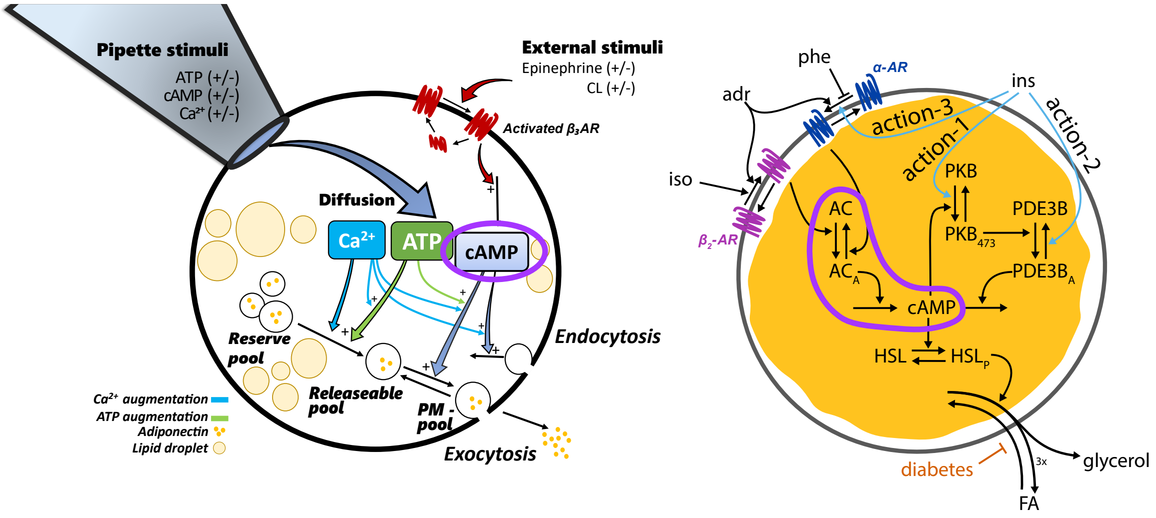

Supplementary Figure 8: The adiponectin model [6] and the lipolysis model [1]. The overlap between the model is highlighted in purple. Both model figures have previously been published under a CC-BY license.

To connect the lipolysis and adiponectin submodels, we combined the reactions relating to cAMP. Furthermore, the simplified effect from the β_3 receptors to directly lead to cAMP production was instead directed to the activation of the adenylyl complex (AC) in the lipolysis submodel. The equations for cAMP from the adiponectin submodel is given in Eq. (6).

$$\begin{aligned} d/dt(cAMP) &= vApip\_cAMP + vA6a - vAdegcAMP \\ vApip\_cAMP &= kADiffcAMP \cdot (pipcAMP - cAMP) \cdot pip \\ vA6a &= kA6a \cdot BETA3a \\ vA6b &= kAdegcAMP \cdot cAMP \end{aligned} \tag{6}$$

The equations for cAMP from the lipolysis submodel is given in Eq. (7).

$$\begin{aligned} d/dt(cAMP) &= vL6a - vL6b \\ vL6a &= kL6a \cdot ACa \\ vL6b &= kL6b \cdot PDE3Ba \cdot cAMP \end{aligned} \tag{7}$$

The equations for cAMP for the connected model is given in Eq. (8).

$$\begin{aligned} d/dt(cAMP) &= vApip\_cAMP - vA6b + vL6a - vL6b \\ vApip\_cAMP &= kADiffcAMP \cdot (pipcAMP - cAMP) \cdot pip \\ vA6b &= kAdegcAMP \cdot cAMP \\ vL6a &= kL6a \cdot ACa \\ vL6b &= kL6b \cdot PDE3Ba \cdot cAMP \end{aligned} \tag{8}$$

Here it should be noted that ACa is now activated by both the β_2 receptor from the lipolysis submodel and β_3 from the adiponectin submodel. The equations for the combined AC activation

is given in Eq. (9).

$$
\begin{aligned}
d/dt(AC) &= -vL5a + vL5b \\
d/dt(ACa) &= vL5a - vL5b + vA5a \\
vL5a &= kL5a \cdot BETA2a \cdot AC \\
vL5b &= kL5b \cdot ALPHAa \cdot ACa \\
vA5a &= kA5a \cdot BETA3a
\end{aligned} \tag{9}
$$

2.2 Description of the equations for the connected model

Here we will go through all the equations for the fully connected model Supplementary Figure 9. For readability, we will, as much as possible, go through the signalling pathways in the glucose uptake submodel first, then the lipolysis submodel and lastly the adiponectin submodel. Please note that, the model equations are explained in greater detail in the original works [1, 2, 6]. Also note that all model parameters with names starting with k are rate determining parameters, for which the parameter values are estimated based on the experimental data. These rate parameters will not be mentioned in detail below.

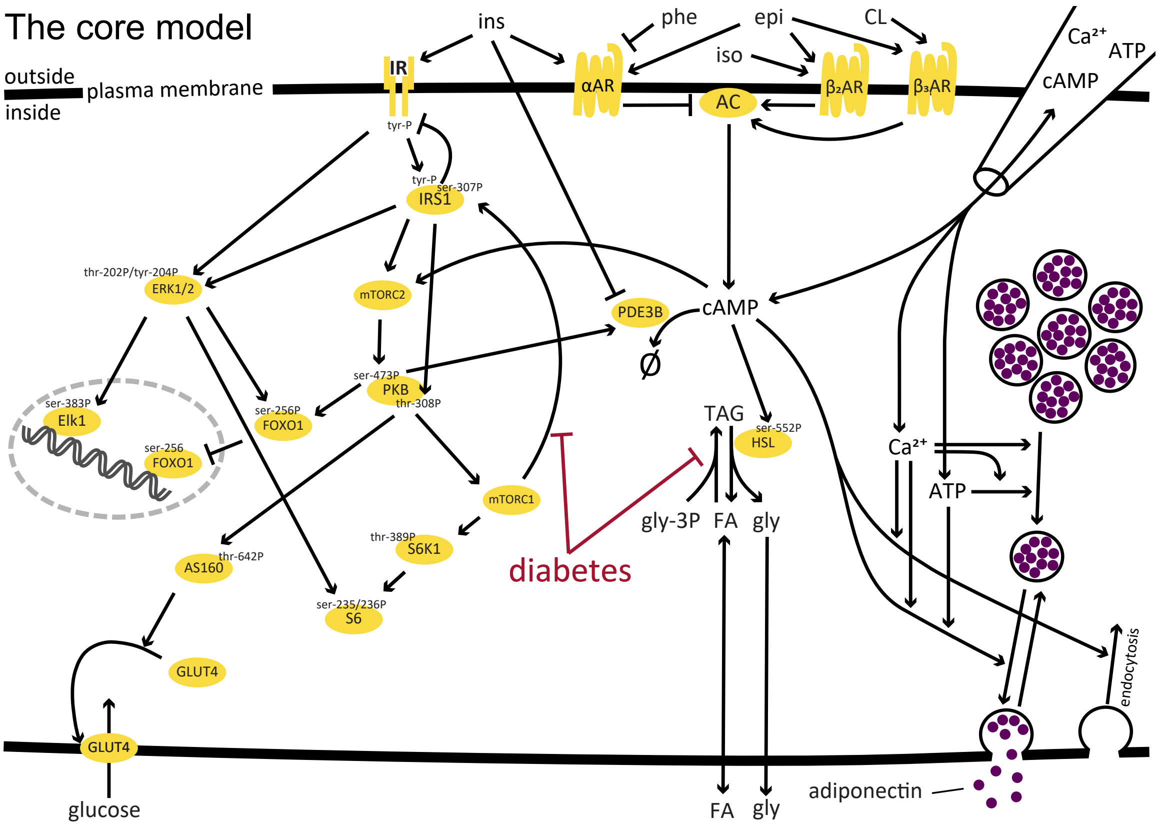

Supplementary Figure 9: **The core model of adipocyte signaling**. The core model includes crosstalk between glucose uptake in response to insulin, fatty acid and glycerol release in response to alpha- and beta-adrenergic receptor signaling and adiponectin secretion in response to cAMP, ATP and Ca2+ (added into the cell with a pipette).

2.3 The glucose uptake submodel

This section describes the equations of the insulin signalling submodel, based on the model developed in [2]. Note that all reactions and parameters below are renamed from vx and kx to $v\boldsymbol{G}x$ and $k\boldsymbol{G}x$ relative to the original model in [2].

In the glucose uptake submodel, insulin binds to the insulin receptor, leading to phosphorylation of the insulin receptor. To a lesser extent, the insulin receptor can also be phosphorylated without the presence of insulin. The phosphorylated insulin receptor can either return to the unphosphorylated state directly, or be internalized. The internalized receptor will be dephosphorylated, and then return to the plasma membrane. This dephosphorylation is controlled by some unknown protein X. In the model, the states correspond to the following: IR (and IRm in Supplementary Figure 7) – the unphosphorylated insulin receptor in the plasma membrane, IR_YP – the phosphorylated insulin receptor, $IRins$ – the insulin receptor bound to insulin, IRi_YP – the internalized phosphorylated insulin receptor, and IRi – the internalized insulin receptor. The equations for the insulin receptor are described in Eq. (10).

$$\begin{aligned}
d/dt(IR) &= -vG1a - vG1basal + vG1r + vG1g \\
d/dt(IR_YP) &= vG1basal + vG1c - vG1d - vG1g \\
d/dt(IRins) &= vG1a - vG1c \\
d/dt(IRi_YP) &= vG1d - vG1e \\
d/dt(IRi) &= vG1e - vG1r \\
\\
vG1a &= kG1a \cdot IR \cdot ins \\
vG1basal &= kG1basal \cdot IR \\
vG1c &= IRins \cdot kG1c \\
vG1d &= IR_YP \cdot kG1d \\
vG1e &= IRi_YP \cdot kG1f \cdot X_P \\
vG1g &= IR_YP \cdot kG1g \\
vG1r &= IRi \cdot kG1r
\end{aligned} \tag{10}$$

Downstream of the insulin receptor is the insulin receptor protein 1 (IRS1). IRS1 can be phosphorylated on tyrosine sites, and on serine 307. Note that in the model, IRS1 can be phosphorylated on both the tyrosine sites and the serine 307 site. The tyrosine phosphorylations are driven by the internalized and tyrosine phosphorylated IR, and the serine 307 phosphorylation on the already tyrosine phosphorylated IRS1 is induced by the activated mammalian target of rapamycin complex (mTORC) 1. Finally, IRS1 can spontaneously be phosphorylated on serine 307, and can spontaneously become dephosphorylated. In the model, the states correspond to: $IRS1$ – unphosphorylated IRS1, $IRS1\_YP$ – tyrosine phosphorylated IRS1, $IRS1\_S307P$, and $IRS1\_YP\_S307P$ – the IRS1 with both tyrosine phosphorylation and serine 307 phosphorylation. The equations for IRS1 are given in Eq. (11)

$$
\begin{aligned}
d/dt(IRS1) &= vG2b + vG2g - vG2a - vG2basal \\
d/dt(IRS1\_YP) &= vG2a + vG2d - vG2b - vG2c \\
d/dt(IRS1\_YP\_S307P) &= vG2c - vG2d - vG2f \\
d/dt(IRS1\_S307P) &= vG2basal + vG2f - vG2g \\
\\
vG2a &= IRS1 \cdot kG2a \cdot IRi\_YP \\
vG2b &= IRS1\_YP \cdot kG2b \\
vG2c &= IRS1\_YP \cdot kG2c \cdot mTORC1a \cdot diabetes \\
vG2d &= IRS1\_YP\_S307P \cdot kG2d \\
vG2f &= IRS1\_YP\_S307P \cdot kG2f \\
vG2basal &= IRS1 \cdot kG2basal \\
vG2g &= IRS1\_S307P \cdot kG2g
\end{aligned} \tag{11}
$$

In the model, there is a protein that is still unidentified, which is denoted "protein X". This protein is a step in between IRS1 and IR, which drives the dephosphorylation of the internalized and tyrosine phosphorylated IR. protein X is activated by the tyrosine phosphorylated IRS1. The model states correspond to: X – inactive protein x, and X_P – activated protein X. The equations for the unknown protein X are given in Eq. (12).

$$
\begin{aligned}
d/dt(X) &= vG3b - vG3a \\
d/dt(X\_P) &= vG3a - vG3b \\
\\
vG3a &= X \cdot kG3a \cdot IRS1\_YP \\
vG3b &= X\_P \cdot kG3b
\end{aligned} \tag{12}
$$

In the model, the signaling pathway leading up to the activation of mTORC2 have been simplified to a direct interaction from IR to mTORC2. mTORC2 is activated by IR that is both internalized and tyrosine phosphorylated. The model states correspond to: $mTORC2$ – inactive mTORC2, and $mTORC2a$ – activated mTORC2. The equations for mTORC2 is given in Eq. (13).

$$
\begin{aligned}
d/dt(mTORC2) &= -vG5c + vG5d \\
d/dt(mTORC2a) &= vG5c - vG5d \\
\\
vG5c &= mTORC2 \cdot (kG5c \cdot IRi\_YP + kL1a \cdot cAMP) \\
vG5d &= kG5d \cdot mTORC2a
\end{aligned} \tag{13}
$$

Downstream of IRS1 lies protein kinase B (PKB/Akt). Tyrosine phosphorylated IRS1 induces phosphorylation of PKB on threonine 308, and the dually phosphorylated IRS1 induces phosphorylation of PKB on threonine 308 on PKB already phosphorylated on serine 473. Finally, activated mTORC2 induces the serine 473 phosphorylation of PKB already phosphorylated

on threonine 308. In the model, the dually phosphorylated PKB must first lose its threonine 308 phosphorylation, before the phosphorylation on serine 473 can be removed. Both single phosphorylated PKB can lose its phosphorylation. All dephosphorylations happen spontaneously. The model states corresponds to: PKB – unphosphorylated PKB, PKB_S308P – PKB phosphorylated on serine 308, PKB_T473P – PKB phosphorylated on threonine 473, and PKB_S308P_T473P – PKB phosphorylated on both serine 308 and threonine 473. The equations for PKB is given in Eq. (14).

$$
\begin{aligned}
d/dt(PKB) &= -vG4a + vG4b + vG4h \\
d/dt(PKB\_S308P) &= vG4a - vG4b - vG4c \\
d/dt(PKB\_T473P) &= -vG4e + vG4f - vG4h \\
d/dt(PKB\_S308P\_T473P) &= vG4c + vG4e - vG4f \\
\\
vG4a &= kG4a \cdot PKB \cdot IRS1\_YP \\
vG4b &= kG4b \cdot PKB\_S308P \\
vG4c &= kG4c \cdot PKB\_S308P \cdot mTORC2a \\
vG4e &= kG4e \cdot PKB\_T473P \cdot IRS1\_YP\_S307P \\
vG4f &= kG4f \cdot PKB\_S308P\_T473P \\
vG4h &= kG4h \cdot PKB\_T473P
\end{aligned} \tag{14}
$$

Downstream of PKB is mTORC1. In the model, mTORC1 is activated by both PKB only phosphorylated on threonine 308, and by dually phosphorylated PKB phosphorylated on both threonine 308 and serine 473. The models states correspond to: $mTORC1$ – inactive mTORC1, and $mTORC1a$ – activated mTORC1. The equations for mTORC1 is given in Eq. (15).

$$
\begin{aligned}
d/dt(mTORC1) &= vG5b - vG5a \\
d/dt(mTORC1a) &= vG5a - vG5b \\
\\
vG5a &= mTORC1 \cdot (kG5a1 \cdot PKB\_S308P\_T473P + kG5a2 \cdot PKB\_S308P) \\
vG5b &= mTORC1a \cdot kG5b
\end{aligned} \tag{15}
$$

Also, downstream of PKB is Akt substrate of 160 kDa (AS160). AS160 is phosphorylated on threonine 642 induced by PKB either phosphorylated on only serine 473 or both serine 473 and threonine 308. The model states correspond to: $AS160$ – unphosphorylated AS160, and $AS160\_T642P$ – AS160 phosphorylated on threonine 642. The equations for AS160 is given in Eq. (16).

$$\begin{aligned}
d/dt(AS160) &= vG6b - vG6a \\
d/dt(AS160_T642P) &= vG6a - vG6b \\
\\
vG6a &= AS160 \cdot (kG6a1 \cdot PKB_S308P_T473P + kG6a2 \cdot PKB_T473P) \\
vG6b &= AS160_T642P \cdot kG6b
\end{aligned} \tag{16}$$

AS160 when phosphorylated on threonine 642 leads to the translocation of glucose transporter 4 (GLUT4) to the plasma membrane. The model states correspond to: $GLUT4m$ – GLUT4 when translocated to the membrane, and $GLUT4$ – GLUT4 internalized in the cell. The equations for GLUT4 is given in Eq. (17).

$$\begin{aligned}
d/dt(GLUT4m) &= vG7a - vG7b \\
d/dt(GLUT4) &= -vG7a + vG7b \\
\\
vG7a &= GLUT4 \cdot kG7a \cdot AS160_T642P \\
vG7b &= GLUT4m \cdot kG7b
\end{aligned} \tag{17}$$

When GLUT4 have been translocated to the plasma membrane, it enables the uptake of glucose from the extracellular environment. In addition to the GLUT4 dependent uptake, there is also a GLUT4 independent uptake. The model state for glucose that have been taken up is $GLUCOSE$. The equation for the glucose uptake is given in Eq. (18).

$$\begin{aligned}
d/dt(GLUCOSE) &= vG8 \\
\\
vG8 &= kG8 \cdot GLUT4m \cdot gluc/(kGmG4 + gluc) + kGglut1 \cdot gluc/(kGmG1 + gluc)
\end{aligned} \tag{18}$$

In addition to the glucose uptake, the *glucose uptake* model also details additional functions, such as the phosphorylation of forkhead box protein O1 (FoxO1) and ETS Like-1 protein (Elk1), via the phosphorylation of extracellular signal-regulated kinases 1/2 (ERK1/2). In the model, ERK1/2 phosphorylation on either of threonine 202 and tyrosine 204 is induced by both the internalized tyrosine phosphorylated IR and the IRS1 phosphorylated on both tyrosine sites and serine 302. After the phosphorylation of ERK1/2, it is sequestered and then returned to the unphosphorylated state. The model states correspond to: ERK – unphosphorylated ERK1/2, ERK_T202_Y204P – ERK1/2 phosphorylated on either of threonine 202 and tyrosine 204, and $seqERK$ – sequestered ERK1/2. The equations for ERK1/2 is given in Eq. (19).

$$
\begin{aligned}
d/dt(ERK) &= -vG10a - vG10basal + vG10c \\
d/dt(ERK\_T202\_Y204P) &= vG10a + vG10basal - vG10b \\
d/dt(seqERK) &= vG10b - vG10c \\
\\
vG10basal &= kG10basal \cdot ERK \\
vG10a &= kG10a1 \cdot ERK \cdot IRi\_YP + kG10a2 \cdot ERK \cdot IRS1\_YP\_S307P \\
vG10b &= kG10b \cdot ERK\_T202\_Y204P \\
vG10c &= kG10c \cdot seqERK
\end{aligned}
\tag{19}
$$

ERK1/2 when phosphorylated on either of threonine 202 and tyrosine 204, will induce the phosphorylation of Elk1 on serine 383. The model states correspond to: $Elk1$ – unphosphorylated Elk1, and $Elk1\_S383P$ – Elk1 phosphorylated on serine 383. The equations for Elk1 is given in Eq. (20).

$$
\begin{aligned}
d/dt(Elk1) &= -vG11a + vG11b \\
d/dt(Elk1\_S383P) &= vG11a - vG11b \\
\\
vG11a &= kG11a \cdot Elk1 \cdot ERK\_T202\_Y204P \\
vG11b &= kG11b \cdot Elk1\_S383P
\end{aligned}
\tag{20}
$$

In addition to Elk1, ERK1/2 also induces the phosphorylation of FoxO1 in the model. FoxO1 is phosphorylated on serine 256, induced by both Elk1/2 when phosphorylated on either of threonine 202 and tyrosine 204, and PKB phosphorylated on either tyrosine 473 or both tyrosine 473 and threonine 308. The model states correspond to: $FOXO$ – unphosphorylated FoxO1, and $FoxO1\_S256P$ – FoxO1 phosphorylated on serine 256. The equations for FoxO1 is given in Eq. (21).

$$
\begin{aligned}
d/dt(FOXO) &= -vG12a + vG12b \\
d/dt(FOXO\_S256P) &= vG12a - vG12b \\
\\
vG12a &= (kG12a1 \cdot (PKB\_T473P + PKB\_S308P\_T473P) \\
&\quad + kG12a2 \cdot ERK\_T202\_Y204P)/(kGm12 + FOXO) \cdot FOXO \\
vG12b &= kG12b \cdot FOXO\_S256P
\end{aligned}
\tag{21}
$$

ERK when phosphorylated on either threonine 202 or tyrosine 204, also leads to activation of the *ribosomal protein S6* (S6), in combination with the S6 kinase, *ribosomal protein S6 kinase* (S6K). In the model, S6K is phosphorylated on threonine 389, induced by the activated mTORC1a. The model states correspond to: $S6K$ – unphosphorylated S6K, and $S6K\_T389P$ – S6K phosphorylated on threonine 389. The equations for S6K is given in Eq. (22).

$$
\begin{aligned}
d/dt(S6K) &= vG9b - vG9a\\
d/dt(S6K\_T389P) &= vG9a - vG9b\\
\\
vG9a &= S6K \cdot kG9a \cdot mTORC1a\\
vG9b &= S6K\_T389P \cdot kG9b
\end{aligned} \tag{22}
$$

In the model, S6K which is phosphorylated on threonine 389 phosphorylates S6 on serine 235 and 236. The phosphorylation of S6 is also induced by ERK phosphorylated on threonine 202 and tyrosine 204. The model states correspond to: $S6$ – unphosphorylated S6, and $S6\_S235\_S236P$ – S6 phosphorylated on any of the serine sites at 235 and 236. The equations for S6 is given in Eq. (23).

$$
\begin{aligned}
d/dt(S6) &= vG9d - vG9c\\
d/dt(S6\_S235\_S236P) &= vG9c - vG9d\\
\\
vG9c &= S6 \cdot kG9c1 \cdot S6K\_T389P + kG9c2 \cdot S6 \cdot ERK\_T202\_Y204P\\
vG9d &= S6\_S235\_S236P \cdot kG9d
\end{aligned} \tag{23}
$$

2.4 The lipolysis submodel

This section describes the equations of the lipolysis submodel, based on the model developed in [1]. Note that all reactions and parameters below are renamed from vx and kx to $v\boldsymbol{L}x$ and $k\boldsymbol{L}x$ relative to the original model.

The lipolysis – with subsequent fatty acid and glycerol release – is in the model controlled using two different inputs: insulin which inhibits the lipolysis, and catecholamines which induce the lipolysis.

In the model, the lipolysis increasing effect of catecholamine stimulation is transmitted via the activation of the β_2 adrenergic receptor (β_2AR). In the model, the β_2AR is activated by both epinephrine (epi) and isoprenaline (iso). Due to the difference in receptor activation for the same concentration of epinephrine and isoprenaline, we introduced a scaling term for isoprenaline ($isoscale$, set to be around 10 times as strong). The model states correspond to: $BETA2$ – the inactive β_2AR, and $BETA2a$ – the activated β_2AR. The equations for the β_2 adrenergic receptor is given in Eq. (24).

$$
\begin{aligned}
d/dt(BETA2) &= -vL4a + vL4b\\
d/dt(BETA2a) &= vL4a - vL4b\\
\\
vL4a &= (kL4a \cdot (iso \cdot isoscale + epi) + kL4a2) \cdot BETA2\\
vL4b &= kL4b \cdot BETA2a
\end{aligned} \tag{24}
$$

In addition to the activation of the β_2AR, epinephrine also activates the α_2 adrenergic receptor (α_2AR). The α_2AR is also activated by insulin, and the activation is inhibited by the inhibitor phentolamine (phe). The model states correspond to $ALPHA2$ – the inactive α_2AR, and $ALPHA2a$ – the activated α_2AR. The equations for the αAR is given in Eq. (25).

$$\begin{aligned}
d/dt(ALPHA) &= -vL3a + vL3b \\
d/dt(ALPHAa) &= vL3a - vL3b \\
\\
vL3a &= (kL3a \cdot Ins_3 \cdot epi + kL3a2) \cdot (1 - phe_effect \cdot phe) \cdot ALPHA \\
vL3b &= kL3b \cdot ALPHAa
\end{aligned} \tag{25}$$

Activation of the β_2AR leads to activation of the adenylyl cyclase (AC), and activation of the α_2AR deactivates AC. Additionally, AC can be activated by the β_3 adrenergic receptor (β_3AR), which is also activated by epinephrine and was originally part of the adiponectin submodel (not detailed yet). The model states correspond to: AC - inactive AC, and ACa - active AC. The equations for the adenylyl cyclase is given in Eq. (26).

$$\begin{aligned}
d/dt(AC) &= -vL5a + vL5b \\
d/dt(ACa) &= vL5a - vL5b + vA5a \\
\\
vL5a &= kL5a \cdot BETA2a \cdot AC \\
vL5b &= kL5b \cdot ALPHAa \cdot ACa \\
vA5a &= kA5a \cdot BETA3a
\end{aligned} \tag{26}$$

When AC is activated, it will lead to the production of cAMP. In addition to the production, cAMP will also be degraded. In the model, this is done either passively of by activated PDE3B. Finally, in the model, cAMP can also diffuse between the cytosol and an attached pipette if a patch-clamp experiment is simulated (originally part of the adiponectin submodel, and not detailed yet). The equation for cAMP is given in Eq. (27).

$$\begin{aligned}
d/dt(cAMP) &= vL6a - vL6b - vA6b + vApip_cAMP \\
\\
vL6a &= kL6a \cdot ACa \\
vL6b &= kL6b \cdot PDE3Ba \cdot cAMP \\
vA6b &= kAdegcAMP \cdot cAMP \\
vApip_cAMP &= kADiffcAMP \cdot (pipcAMP - cAMP) \cdot pip
\end{aligned} \tag{27}$$

As mentioned, the degradation of cAMP is controlled by PDE3B. The activation of PDE3B is in turn induced via PKB phosphorylated on either only threonin 473, or phosphorylated on both threonine 473 and serine 308, originally induced by insulin stimulation. Furthermore,

there is another insulin action at high concentrations of insulin that leads to the deactivation of PDE3B (Eq. (31), Ins_2). The model states correspond to: $PDE3B$ - inactive PDE3B, and $PDE3Ba$ - activated PDE3B. The equations for PDE3B is given in Eq. (28).

$$\begin{aligned} d/dt(PDE3B) &= -vL2a + vL2b \\ d/dt(PDE3Ba) &= vL2a - vL2b \\ \\ vL2a &= kL2a \cdot (PKB\_T473P + PKB\_S308P\_T473P) \cdot PDE3B \\ vL2b &= kL2b \cdot PDE3Ba \cdot Ins\_2 \end{aligned} \tag{28}$$

When the cAMP levels rise, it triggers a signaling cascade leading to the activation of lipases that hydrolyze the triacylicglycerides (TAGs) into fatty acids (FAs) and glycerol. In the model, this signaling cascade is simplified as only the activation of HSL. In the model, HSL phosphorylation on serine 552 is induced by the increased levels of cAMP. The model states correspond to: HSL - unphosphorylated HSL, and $HSLp$ - HSL phosphorylated on serine 552. The equations for HSL is given in Eq. (29).

$$\begin{aligned} d/dt(HSL) &= -vL7a + vL7b \\ d/dt(HSLp) &= vL7a - vL7b \\ \\ vL7a &= kL7a \cdot cAMP \cdot HSL \\ vL7b &= kL7b \cdot HSLp \end{aligned} \tag{29}$$

Once HSL is activated, it will lead to the breakdown of TAGs into fatty acids and glycerol which are subsequently released. Both fatty acids and glycerol will be cleared away from the extracellular environment in the *in vivo* setting, but not in the *in vitro* setting. Some fatty acids will be reesterified into TAGs again. In the model, the pool of TAGs is assumed to be unchanged during the timescale of the model simulations. Furthermore, the glycerol-3P needed to reform TAGs from FAs are also assumed to be unlimited. The model states correspond to: Gly - released glycerol, and FA - released fatty acids. The equations for the release of glycerol and fatty acids is given in Eq. (30). Note that $kLclear$ was set to 0 in the *in vitro* setting.

$$\begin{aligned} d/dt(Gly) &= vL8a - vL8b \\ d/dt(FA) &= 3 \cdot vL8a - vL8c \\ \\ vL8a &= kL8a \cdot HSLp \\ vL8b &= kLclear \cdot Gly \\ vL8c &= (kLclear + kL8c \cdot diab\_reest) \cdot FA \end{aligned} \tag{30}$$

We modelled the other two insulin actions described in the works by Jönsson et al. [4] and Stich et al. [5] as two separate sigmoidal functions, both dependent on the concentration of insulin. The dose dependent insulin actions are given in Eq. (31).

$$
\begin{aligned}
Ins_2 &= 100 + \frac{min_2 - 100}{1 + (ins/EC50_2)^{n_2}} \\
Ins_3 &= 100 + \frac{min_3 - 100}{1 + (ins/EC50_3)^{n_3}}
\end{aligned} \tag{31}
$$

2.5 The adiponectin submodel

This section describes the equations of the lipolysis submodel, based on the model developed in [6]. Note that all reactions and parameters below are renamed from vx and kx to $v\boldsymbol{A}x$ and $k\boldsymbol{A}x$ relative to the original model.

In the model, vesicles containing adiponectin is moved from a large reserve pool of vesicles, to a releasable pool, and is then attached to the plasma membrane. Finally, the vesicles can undergo exocytosis. The experimental data, and thus the model measurement equation, is the membrane capacitance which corresponds to the size of the cell. The size is increased when vesicles undergo exocytosis. The size can also shrink during endocytosis. The exocytosis and endocytosis is controlled by the intracellular mediators cAMP, Ca^{2+} and ATP. cAMP have previously been discussed as a part of the lipolysis submodel (Eq. (27)). In the context of the adiponectin submodel, cAMP can also be produced as a result of the activation of the β_3 adrenergic receptor (β_3AR) and controlled directly via a patch-clamp experiment. In the patch-clamp experiment, cAMP is infused in the cell using the attached pipette, and the cAMP levels can thus be controlled.

The β_3 adrenergic receptor (β_3AR) is activated by epinephrine and the synthetic analogue CL 316,243 (CL). The activated β_3AR will then be desensitized before returning to the inactive state. The model states correspond to: $BETA3$ – inactive (β_3AR), $BETA3a$ – activated (β_3AR), and $BETA3de$ – desensitized (β_3AR). The equations for the β_3AR is given in Eq. (32).

$$
\begin{aligned}
d/dt(BETA3) &= vA4c - vA4a \\
d/dt(BETA3a) &= vA4a - vA4b \\
d/dt(BETA3de) &= vA4b - vA4c \\
\\
vA4a &= kA4a \cdot (epi + kACL \cdot CL) \cdot BETA3 \\
vA4b &= kA4b \cdot BETA3a \\
vA4c &= kA4c \cdot BETA3de
\end{aligned} \tag{32}
$$

In addition to cAMP, two other intracellular mediators were controlled using the patch-clamp setup: Ca^{2+} and ATP. All mediators can diffuse between the pipette and the intracellular environment, and then can be removed from the intracellular environment (by being sequestered, degraded, etc.). Due to the large differences in the volume of the pipette and the cell, the concentration change in the pipette due to diffusion was scaled as a fraction of the cell volume and the pipette volume ($kAVcell/kAVpip$). The model states correspond to: Ca – intracellular Ca^{2+}, $cAMP$ – intracellular cAMP, $pipCa$ – Ca^{2+} in the pipette, $pipATP$ – ATP in the pipette, and $pipcAMP$ – cAMP in the pipette. The equations for the intracellular mediators Ca^{2+} and ATP, and the pipette, is given in Eq. (33).

$$
\begin{aligned}
d/dt(Ca) &= vApip\_Ca - vAremCa \\
d/dt(ATP) &= vApip\_ATP - vAdegATP \\
\\
d/dt(pipCa) &= -vApip\_Ca \cdot kAVcell/kAVpip \\
d/dt(pipATP) &= -vApip\_ATP \cdot kAVcell/kAVpip \\
d/dt(pipcAMP) &= -vApip\_cAMP \cdot kAVcell/kAVpip \\
\\
vApip\_Ca &= kADiffCa \cdot (pipCa - Ca) \cdot pip \\
vApip\_ATP &= kADiffATP \cdot (pipATP - ATP) \cdot pip \\
vAremCa &= kAremCa \cdot Ca \\
vAdegATP &= kAdegATP \cdot ATP \\
vApip\_cAMP &= kADiffcAMP \cdot (pipcAMP - cAMP) \cdot pip
\end{aligned}
\tag{33}
$$

The reserve pool of vesicles (Res) was assumed to not change during the short timescale of the adiponectin release experiments. The equation for the reserve pool is given in Eq. (34).

$$
d/dt(Res) = 0 \tag{34}
$$

The vesicles will migrate from the reserve pool to a releasable pool, augmented by Ca^{2+} and ATP. Then, the releasable vesicles will attach to the plasma membrane, controlled by cAMP and augmented by Ca^{2+} and ATP. The vesicles attached to the plasma membrane can then undergo exocytosis or return to the releasable pool. The model states correspond to: Rel – the releasable pool, and PM - the vesicles attached to the plasma membrane. The equations for releasable pool is given in Eq. (35).

$$
\begin{aligned}
d/dt(Rel) &= vARes\_Rel - vARel\_PM + vAPM\_Rel \\
d/dt(PM) &= vARel\_PM - vAPM\_Rel - vAPM \\
\\
vARes\_Rel &= ((Ca/(kAm + Ca)) \cdot (kACa2 + kAATP2 \cdot ATP) + kARelBasal) \cdot Res \\
vARel\_PM &= cAMP \cdot (kAcAMP + (Ca/(kAm + Ca)) \cdot ATP \cdot kACaATP) \cdot Rel \\
vAPM\_Rel &= kArel \cdot PM \\
vAPM &= kAexo \cdot PM
\end{aligned}
\tag{35}
$$

In addition to the exocytosis, cAMP also induces endocytosis. The endocytosis is also augmented by Ca^{2+}. The equation for the endocytosis is given in Eq. (36)

$$
d/dt(Endo) = vACa\_Endo - vAEndo \tag{36}
$$

The vesicles undergoing exocytosis leads to increased levels of adiponectin in the extracellular environment. Since the experiments are *in vitro*, we assume that no adiponectin is cleared. Also, we assume that the released adiponectin will diffuse too far away form the cell to be affected by the endocytosis. The equation for the released adiponectin is given in Eq. (37).

$$\begin{aligned} d/dt(Adiponectin) &= vAPM \\ \\ vACa\_Endo &= kACacAMP \cdot cAMP \cdot Ca \\ vAEndo &= kAEndo \cdot Endo \end{aligned} \tag{37}$$

2.6 The kinetics of the additions in the expanded model

During the automatic expansion, we test additions with fixed set of kinetics and directions. These include mass-action and Michaelis-Menten kinetics, as well as phosphorylations and dephosphorylations. We first try with a single input. If this does not work, we try with two inputs, the best single input plus one extra. And finally, if no dual input combination works, we include a third alternative, corresponding to a three pool state where the phosphorylation site can become unavailable for a short while. These different alternatives make up all the possible variations of dynamics in the additions. We will now go through the equations that constitute these combinations. For simplicity, we will refer to the input phosphosite as Ap and Bp, the unphosphorylated target protein as P, and the phosphorylated target protein as Pp. We will also refer to inputs leading to phosphorylations as being "positive", and inputs leading to dephosphorylations as being "negative". The rate determining parameters will be referred to as kp for reactions that lead to a phosphorylation, and kd for reactions that lead to a dephosphorylation.

It should be noted that over the course of the insulin stimulation, an addition that is classified as being phosphorylated by Ap can still lead to a decreased level in phosphorylation in Pp if the input phosphosite is decreasing over time (leading to a "lowered" input). The inverse is also true for additions classified as dephosphorylations. If the levels of Ap decrease in such a case, the levels of Pp will increase.

Phosphorylation with a single input

Increasing levels of Ap leads to increasing levels in Pp, until the amount of P runs out.

$$\begin{aligned} d/dt(P) &= -v1 \\ d/dt(Pp) &= v1 \\ v1 &= Ap \cdot kp \cdot P - kd \cdot Pp \end{aligned} \tag{38}$$

Dephosphorylation with a single input

Increasing levels of Ap leads to decreasing levels in Pp.

$$
\begin{aligned}
d/dt(P) &= -v1 \\
d/dt(Pp) &= v1 \\
v1 &= kp \cdot P - Ap \cdot kd \cdot Pp
\end{aligned} \tag{39}
$$

Phosphorylation with a Michaelis-Menten kinetic, with a single input

Increasing levels of Ap leads to increasing levels in Pp, up until a certain level of Ap (determined by km).

$$
\begin{aligned}
d/dt(P) &= -v1 \\
d/dt(Pp) &= v1 \\
v1 &= \frac{Ap}{(km + Ap)} \cdot kp \cdot P - kd \cdot Pp
\end{aligned} \tag{40}
$$

Phosphorylation with double positive inputs

Increasing levels of both Ap and Bp leads to increasing levels in Pp, until the amount of P runs out.

$$
\begin{aligned}
d/dt(P) &= -v1 \\
d/dt(Pp) &= v1 \\
v1 &= P \cdot (Ap \cdot kp + Bp \cdot kp2) - Pp \cdot kd
\end{aligned} \tag{41}
$$

Dephosphorylation with dual negative inputs

Increasing levels of both Ap and Bp leads to decreasing levels in Pp.

$$
\begin{aligned}
d/dt(P) &= -v1 \\
d/dt(Pp) &= v1 \\
v1 &= P \cdot kp - Pp \cdot (Ap \cdot kd + \cdot Bp \cdot kd2)
\end{aligned} \tag{42}
$$

Dual input with one positive and one negative input

Increasing levels of Ap and Bp can lead to both increasing or decreasing levels in Pp, based on the levels of both Ap and Bp, and the ratio of the rate determining constants kp and kd.

$$
\begin{aligned}
d/dt(P) &= -v1 \\
d/dt(Pp) &= v1 \\
v1 &= P \cdot Ap \cdot kp - Pp \cdot Bp \cdot kd
\end{aligned} \tag{43}
$$

Phosphorylation with an inactivation, with a single input

Here, the state for the inactive (unavailable) state is referred to as Pu. The inactivation is increased by a rate kuf and returned to the basal state with a rate of kub. Increasing levels of both Ap and Bp leads to increasing levels in Pp, until the amount of P runs out.

$$
\begin{aligned}
d/dt(P) &= -v1 + v3 \\
d/dt(Pp) &= v1 - v2 \\
d/dt(Pu) &= v2 - v3 \\
v1 &= Ap \cdot kp \cdot P - kd \cdot Pp \\
v2 &= kuf \cdot Pp \\
v3 &= kub \cdot Pu
\end{aligned}
\tag{44}
$$

2.7 Parameter values

This section gives the optimal parameter values for the connected model both when estimated to the estimation data set (columns θ^*_{est}), and to the total data set (columns θ^*_{tot}). Furthermore, the bounds used in the optimization for all parameters are also given (columns *lowerbound* and *upperbound*). The parameter values and optimization bounds for the glucose uptake submodel are given in Supplementary Table 1 and Supplementary Table 2. The parameter values and optimization bounds for the lipolysis submodel are given in Supplementary Table 3. The parameter values and optimization bounds for the adiponectin submodel are given in Supplementary Table 4.

Parameter	θ^*_{est}	θ^*_{tot}	lower bound	upper bound
$kG1a$	$1.2696 \cdot 10^{-1}$	$1.2501 \cdot 10^{-1}$	$1 \cdot 10^{-8}$	$1 \cdot 10^{6}$
$kG1basal$	$2.2526 \cdot 10^{-4}$	$2.2526 \cdot 10^{-4}$	$1 \cdot 10^{-8}$	$1 \cdot 10^{6}$
$kG1c$	$1.7234 \cdot 10^{-1}$	$1.7224 \cdot 10^{-1}$	$1 \cdot 10^{-8}$	$1 \cdot 10^{6}$
$kG1d$	$9.9995 \cdot 10^{5}$	$9.9997 \cdot 10^{5}$	$1 \cdot 10^{-8}$	$1 \cdot 10^{6}$
$kG1f$	$2.0184 \cdot 10^{1}$	$2.0267 \cdot 10^{1}$	$1 \cdot 10^{-8}$	$1 \cdot 10^{6}$
$kG1g$	$5.3850 \cdot 10^{-8}$	$3.1332 \cdot 10^{-8}$	$1 \cdot 10^{-8}$	$1 \cdot 10^{6}$
$kG1r$	6.5382	6.4662	$1 \cdot 10^{-8}$	$1 \cdot 10^{6}$
$kG2a$	$7.2702 \cdot 10^{1}$	$7.2938 \cdot 10^{1}$	$1 \cdot 10^{-8}$	$1 \cdot 10^{6}$
$kG2c$	$2.0619 \cdot 10^{3}$	$2.0024 \cdot 10^{3}$	$1 \cdot 10^{-8}$	$1 \cdot 10^{6}$
$kG2basal$	$7.2956 \cdot 10^{-2}$	$7.1708 \cdot 10^{-2}$	$1 \cdot 10^{-8}$	$1 \cdot 10^{6}$
$kG2b$	$3.7448 \cdot 10^{3}$	$3.7448 \cdot 10^{3}$	$1 \cdot 10^{-8}$	$1 \cdot 10^{6}$
$kG2d$	4.8589	4.8512	$1 \cdot 10^{-8}$	$1 \cdot 10^{6}$
$kG2f$	$9.1342 \cdot 10^{-1}$	$9.1327 \cdot 10^{-1}$	$1 \cdot 10^{-8}$	$1 \cdot 10^{6}$
$kG2g$	$2.9109 \cdot 10^{-1}$	$2.8790 \cdot 10^{-1}$	$1 \cdot 10^{-8}$	$1 \cdot 10^{6}$
$kG3a$	$8.6395 \cdot 10^{1}$	$8.5992 \cdot 10^{1}$	$1 \cdot 10^{-8}$	$1 \cdot 10^{6}$
$kG3b$	$1.0026 \cdot 10^{-1}$	$1.0452 \cdot 10^{-1}$	$1 \cdot 10^{-8}$	$1 \cdot 10^{6}$
$kG4a$	$6.7328 \cdot 10^{2}$	$6.7328 \cdot 10^{2}$	$1 \cdot 10^{-8}$	$1 \cdot 10^{6}$
$kG4b$	$9.6161 \cdot 10^{4}$	$9.6157 \cdot 10^{4}$	$1 \cdot 10^{-8}$	$1 \cdot 10^{6}$
$kG4c$	$6.2629 \cdot 10^{3}$	$6.1511 \cdot 10^{3}$	$1 \cdot 10^{-8}$	$1 \cdot 10^{6}$
$kG4e$	$4.8452 \cdot 10^{-2}$	$4.8419 \cdot 10^{-2}$	$1 \cdot 10^{-8}$	$1 \cdot 10^{6}$
$kG4f$	7.7143	7.3032	$1 \cdot 10^{-8}$	$1 \cdot 10^{6}$
$kG4h$	$9.1781 \cdot 10^{-2}$	$9.1778 \cdot 10^{-2}$	$1 \cdot 10^{-8}$	$1 \cdot 10^{6}$
$kG5a1$	3.0828	2.9201	$1 \cdot 10^{-8}$	$1 \cdot 10^{6}$
$kG5a2$	$1.3173 \cdot 10^{4}$	$1.3540 \cdot 10^{4}$	$1 \cdot 10^{-8}$	$1 \cdot 10^{6}$
$kG5b$	$2.3183 \cdot 10^{1}$	$2.3184 \cdot 10^{1}$	$1 \cdot 10^{-8}$	$1 \cdot 10^{6}$
$kG5d$	$1.4851 \cdot 10^{-1}$	$1.4851 \cdot 10^{-1}$	$1 \cdot 10^{-8}$	$1 \cdot 10^{6}$
$kG5c$	6.4703	6.3330	$1 \cdot 10^{-8}$	$1 \cdot 10^{6}$
$kG6a1$	$1.9667 \cdot 10^{-1}$	$1.9198 \cdot 10^{-1}$	$1 \cdot 10^{-8}$	$1 \cdot 10^{6}$
$kG6a2$	$1.1012 \cdot 10^{-7}$	$1.0513 \cdot 10^{-7}$	$1 \cdot 10^{-8}$	$1 \cdot 10^{6}$
$kG6b$	$2.2643 \cdot 10^{-1}$	$2.3368 \cdot 10^{-1}$	$1 \cdot 10^{-8}$	$1 \cdot 10^{6}$
$kG7a$	$2.5980 \cdot 10^{-6}$	$2.6309 \cdot 10^{-6}$	$1 \cdot 10^{-8}$	$1 \cdot 10^{6}$
$kG7b$	$1.6018 \cdot 10^{3}$	$1.6330 \cdot 10^{3}$	$1 \cdot 10^{-8}$	$1 \cdot 10^{6}$
$kG8$	$3.7913 \cdot 10^{4}$	$3.8195 \cdot 10^{4}$	$1 \cdot 10^{-8}$	$1 \cdot 10^{6}$
$kGglut1$	$2.0747 \cdot 10^{-2}$	$2.0805 \cdot 10^{-2}$	$1 \cdot 10^{-8}$	$1 \cdot 10^{6}$
$kGmG4$	$1.1159 \cdot 10^{1}$	$1.1071 \cdot 10^{1}$	$1 \cdot 10^{-8}$	$1 \cdot 10^{6}$
$kGmG1$	$8.8508 \cdot 10^{-1}$	$8.8402 \cdot 10^{-1}$	$1 \cdot 10^{-8}$	$1 \cdot 10^{6}$
$kG9a$	$1.1660 \cdot 10^{-6}$	$1.1786 \cdot 10^{-6}$	$1 \cdot 10^{-8}$	$1 \cdot 10^{6}$
$kG9b$	$4.9870 \cdot 10^{-2}$	$4.9403 \cdot 10^{-2}$	$1 \cdot 10^{-8}$	$1 \cdot 10^{6}$
$kG9c1$	$4.2332 \cdot 10^{-1}$	$4.1739 \cdot 10^{-1}$	$1 \cdot 10^{-8}$	$1 \cdot 10^{6}$
$kG9c2$	$1.9737 \cdot 10^{-2}$	$1.9038 \cdot 10^{-2}$	$1 \cdot 10^{-8}$	$1 \cdot 10^{6}$
$kG9d$	$3.2024 \cdot 10^{-2}$	$3.2662 \cdot 10^{-2}$	$1 \cdot 10^{-8}$	$1 \cdot 10^{6}$

Supplementary Table 1: **Parameter values related to the glucose uptake submodel.**

Parameter	θ^*_{est}	θ^*_{tot}	lower bound	upper bound
$kG10a1$	$9.4534 \cdot 10^{-7}$	$2.4530 \cdot 10^{-7}$	$1 \cdot 10^{-8}$	$1 \cdot 10^{6}$
$kG10a2$	$8.2693 \cdot 10^{-4}$	$8.3407 \cdot 10^{-4}$	$1 \cdot 10^{-8}$	$1 \cdot 10^{6}$
$kG10basal$	$9.5726 \cdot 10^{-3}$	$9.5430 \cdot 10^{-3}$	$1 \cdot 10^{-8}$	$1 \cdot 10^{6}$
$kG10b$	$1.8650 \cdot 10^{-1}$	$1.8514 \cdot 10^{-1}$	$1 \cdot 10^{-8}$	$1 \cdot 10^{6}$
$kG10c$	$2.5402 \cdot 10^{-4}$	$2.5572 \cdot 10^{-4}$	$1 \cdot 10^{-8}$	$1 \cdot 10^{6}$
$kG11a$	$9.2964 \cdot 10^{-8}$	$9.2595 \cdot 10^{-8}$	$1 \cdot 10^{-8}$	$1 \cdot 10^{6}$
$kG11b$	$3.9138 \cdot 10^{-1}$	$3.9123 \cdot 10^{-1}$	$1 \cdot 10^{-8}$	$1 \cdot 10^{6}$
$kG12a1$	$1.0188 \cdot 10^{3}$	$1.0328 \cdot 10^{3}$	$1 \cdot 10^{-8}$	$1 \cdot 10^{6}$
$kG12a2$	$4.9313 \cdot 10^{5}$	$4.9310 \cdot 10^{5}$	$1 \cdot 10^{-8}$	$1 \cdot 10^{6}$
$kG12b$	$1.9751 \cdot 10^{1}$	$1.9997 \cdot 10^{1}$	$1 \cdot 10^{-8}$	$1 \cdot 10^{6}$
$kGm12$	$1.7943 \cdot 10^{4}$	$1.7943 \cdot 10^{4}$	$1 \cdot 10^{-8}$	$1 \cdot 10^{6}$
$diabetes$	$5.5870 \cdot 10^{-2}$	$5.6831 \cdot 10^{-2}$	0	1

Supplementary Table 2: **Parameter values related to the glucose uptake submodel.**

Parameter	θ^*_{est}	θ^*_{tot}	lower bound	upper bound
$kLdrift$	$2.3544 \cdot 10^2$	$2.3859 \cdot 10^2$	$1 \cdot 10^{-8}$	$1 \cdot 10^6$
$kL1a$	6.3543	6.5066	$1 \cdot 10^{-8}$	$1 \cdot 10^6$
$kL2a$	$2.0167 \cdot 10^{-2}$	$2.0173 \cdot 10^{-2}$	$1 \cdot 10^{-8}$	$1 \cdot 10^6$
$kL2b$	$1.5134 \cdot 10^{-2}$	$1.5133 \cdot 10^{-2}$	$1 \cdot 10^{-8}$	$1 \cdot 10^6$
$kL3b$	$2.6030 \cdot 10^3$	$2.6139 \cdot 10^3$	$1 \cdot 10^{-8}$	$1 \cdot 10^6$
$kL3a$	$1.3271 \cdot 10^2$	$1.4695 \cdot 10^2$	$1 \cdot 10^{-8}$	$1 \cdot 10^6$
$kL3a2$	$2.5765 \cdot 10^2$	$2.5765 \cdot 10^2$	$1 \cdot 10^{-8}$	$1 \cdot 10^6$
$kL4a$	$1.1421 \cdot 10^{-2}$	$1.1415 \cdot 10^{-2}$	$1 \cdot 10^{-8}$	$1 \cdot 10^6$
$kL4a2$	$2.3029 \cdot 10^{-4}$	$2.3122 \cdot 10^{-4}$	$1 \cdot 10^{-8}$	$1 \cdot 10^6$
$kL4b$	2.1055	2.1060	$1 \cdot 10^{-8}$	$1 \cdot 10^6$
$kL5a$	$2.5570 \cdot 10^{-1}$	$2.5654 \cdot 10^{-1}$	$1 \cdot 10^{-8}$	$1 \cdot 10^6$
$kL5b$	7.5766	7.5763	$1 \cdot 10^{-8}$	$1 \cdot 10^6$
$kL6a$	$8.4296 \cdot 10^{-2}$	$8.4395 \cdot 10^{-2}$	$1 \cdot 10^{-8}$	$1 \cdot 10^6$
$kL6b$	$2.3022 \cdot 10^{-2}$	$2.3084 \cdot 10^{-2}$	$1 \cdot 10^{-8}$	$1 \cdot 10^6$
$kL7a$	$7.2582 \cdot 10^2$	$9.0592 \cdot 10^2$	$1 \cdot 10^{-8}$	$1 \cdot 10^6$
$kL7b$	$1.3458 \cdot 10^1$	$1.2935 \cdot 10^1$	$1 \cdot 10^{-8}$	$1 \cdot 10^6$
$kL8a$	$2.9542 \cdot 10^2$	$2.6349 \cdot 10^2$	$1 \cdot 10^{-8}$	$1 \cdot 10^6$
$kL8c$	$2.2077 \cdot 10^{-2}$	$2.5507 \cdot 10^{-2}$	$1 \cdot 10^{-8}$	$1 \cdot 10^6$
$kLclear$	$3.7677 \cdot 10^{-2}$	$3.6746 \cdot 10^{-2}$	$1 \cdot 10^{-8}$	$1 \cdot 10^6$
$isoscale$	$1.2000 \cdot 10^1$	$1.2000 \cdot 10^1$	8	$1.2000 \cdot 10^1$
phe_effect	$8.9227 \cdot 10^{-1}$	$8.8912 \cdot 10^{-1}$	$6 \cdot 10^{-1}$	1
$min2$	$2.0854 \cdot 10^1$	$2.0854 \cdot 10^1$	0	$1 \cdot 10^2$
$min3$	$9.8712 \cdot 10^{-3}$	$9.8712 \cdot 10^{-3}$	0	$1 \cdot 10^2$
$EC502$	1.0882	1.0720	-5	3
$EC503$	$8.2934 \cdot 10^{-2}$	$6.6394 \cdot 10^{-2}$	-5	3
$n2$	3	2.1674	$5 \cdot 10^{-1}$	3
$n3$	3	2.9723	$5 \cdot 10^{-1}$	3
$diab\_reest$	$9.6659 \cdot 10^{-2}$	$1.2111 \cdot 10^{-1}$	0	1

Supplementary Table 3: **Parameter values related to the lipolysis submodel.**

Parameter	θ^*_{est}	θ^*_{tot}	lower bound	upper bound
$kArel$	$2.0500 \cdot 10^{-7}$	$3.2440 \cdot 10^{-7}$	$1 \cdot 10^{-8}$	$1 \cdot 10^{6}$
$kAexo$	$6.6957 \cdot 10^{-1}$	$6.6957 \cdot 10^{-1}$	$1 \cdot 10^{-8}$	$1 \cdot 10^{6}$
$kACaATP$	$2.9179 \cdot 10^{1}$	$2.9226 \cdot 10^{1}$	$1 \cdot 10^{-8}$	$1 \cdot 10^{6}$
$kAcAMP$	$1.8864 \cdot 10^{1}$	$1.8864 \cdot 10^{1}$	$1 \cdot 10^{-8}$	$1 \cdot 10^{6}$
$kACa2$	$1.1695 \cdot 10^{-3}$	$1.1638 \cdot 10^{-3}$	$1 \cdot 10^{-8}$	$1 \cdot 10^{6}$
$kAATP2$	$9.2660 \cdot 10^{-4}$	$9.2651 \cdot 10^{-4}$	$1 \cdot 10^{-8}$	$1 \cdot 10^{6}$
$kAEndo$	$8.0992 \cdot 10^{5}$	$9.9826 \cdot 10^{5}$	$1 \cdot 10^{-8}$	$1 \cdot 10^{6}$
$kACacAMP$	$6.0089 \cdot 10^{2}$	$6.0768 \cdot 10^{2}$	$1 \cdot 10^{-8}$	$1 \cdot 10^{6}$
kAm	$1.2535 \cdot 10^{-8}$	$1.2535 \cdot 10^{-8}$	$1 \cdot 10^{-8}$	$1 \cdot 10^{6}$
$kARelBasal$	$1.0534 \cdot 10^{-4}$	$1.0749 \cdot 10^{-4}$	$1 \cdot 10^{-8}$	$1 \cdot 10^{6}$
$kAdegcAMP$	$2.0201 \cdot 10^{-6}$	$1.8269 \cdot 10^{-6}$	$1 \cdot 10^{-8}$	$1 \cdot 10^{6}$
$kAremCa$	$5.5039 \cdot 10^{-5}$	$4.8329 \cdot 10^{-5}$	$1 \cdot 10^{-8}$	$1 \cdot 10^{6}$
$kAdegATP$	$2.2781 \cdot 10^{-1}$	$2.4347 \cdot 10^{-1}$	$1 \cdot 10^{-8}$	$1 \cdot 10^{6}$
$kADiffcAMP$	$2.7110 \cdot 10^{-1}$	$2.7104 \cdot 10^{-1}$	$1 \cdot 10^{-8}$	$1 \cdot 10^{6}$
$kADiffCa$	$1.6417 \cdot 10^{4}$	$1.6417 \cdot 10^{4}$	$1 \cdot 10^{-8}$	$1 \cdot 10^{6}$
$kADiffATP$	$5.3773 \cdot 10^{-1}$	$4.9675 \cdot 10^{-1}$	$1 \cdot 10^{-8}$	$1 \cdot 10^{6}$
$kA4a$	$1.7383 \cdot 10^{4}$	$1.7384 \cdot 10^{4}$	$1 \cdot 10^{-8}$	$1 \cdot 10^{6}$
$kACL$	$2.4186 \cdot 10^{-5}$	$2.4874 \cdot 10^{-5}$	$1 \cdot 10^{-8}$	$1 \cdot 10^{6}$
$kA4c$	$1.0638 \cdot 10^{-8}$	$1.0001 \cdot 10^{-8}$	$1 \cdot 10^{-8}$	$1 \cdot 10^{6}$
$kA4b$	2.8284	2.8344	$1 \cdot 10^{-8}$	$1 \cdot 10^{6}$
$kA5a$	3.2559	3.2559	$1 \cdot 10^{-8}$	$1 \cdot 10^{6}$
$kAVpip$	$2.2330 \cdot 10^{-5}$	$2.3223 \cdot 10^{-5}$	$2 \cdot 10^{-5}$	$6 \cdot 10^{-5}$
$kAVcell$	$1.6689 \cdot 10^{-13}$	$1.6613 \cdot 10^{-13}$	$8.6400 \cdot 10^{-15}$	$1.7168 \cdot 10^{-13}$

Supplementary Table 4: **Parameter values related to the adiponectin submodel.**

2.8 Initial values

The model was given arbitrary initial values and then simulated without any inputs until steady state was reached to get a stable set of inti before any experiment was started. In most cases, the values are expressed as percentages. In other words, all state of a component in the model sum to 100. Note that the initial values for the mediators in the pipett ($pipCa$, $pipcAMP$, and $pipATP$) was set to the corresponding experimental concentrations during the start of the simulation of the adiponectin experiments (after steady state), and was set to 0 during all other experiments. The initial values used - before simulation to steady state - are given in Supplementary Table 5, Supplementary Table 6, and Supplementary Table 7 for the glucose uptake, lipolysis, and adiponectin submodels respectively.

State	Initial values
IR	100
IR_YP	0
$IRins$	0
IRi_YP	0
IRi	0
$IRS1$	100
$IRS1\_YP$	0
$IRS1\_YP\_S307P$	0
$IRS1\_S307P$	0
X	100
X_P	0
PKB	100
PKB_S308P	0
PKB_T473P	0
PKB_S308P_T473P	0
$mTORC1$	100
$mTORC1a$	0
$mTORC2$	100
$mTORC2a$	0
$AS160$	100
$AS160\_T642P$	0
$GLUT4m$	0
$GLUT4$	100
$GLUCOSE$	0
$S6K$	100
$S6K\_T389P$	0
$S6$	100
$S6\_S235\_S236P$	0
ERK	100
ERK_T202_Y204P	0
$seqERK$	0
$Elk1$	100
$Elk1\_S383P$	0
$FOXO$	100
$FOXO\_S256P$	0

Supplementary Table 5: **Initial values for the glucose uptake submodel.**

State	Initial values
$BETA2$	100
$BETA2a$	0
$ALPHA$	80
$ALPHAa$	20
AC	80
ACa	20
$PDE3B$	80
$PDE3Ba$	20
$cAMP$	0
HSL	80
$HSLp$	20
Gly	0
FA	0

Supplementary Table 6: **Initial values for the lipolysis submodel.**

State	Initial values
$BETA3$	100
$BETA3a$	0
$BETA3de$	0
Ca	0
ATP	0
Res	99
Rel	1
PM	0
$Endo$	0
$Adiponectin$	0
$pipCa$	0
$pipATP$	0
$pipcAMP$	0

Supplementary Table 7: **Initial values for the adiponectin submodel.**

3 Time series of all sites added to the model

3.1 How to interpret the figures.

All figures show the time series of a phosphosite added to the model during the model expansion. In all figures, dots and error bars corresponds to the experimental data (mean and SEM), and lines correspond to model simulations. The different simulation and data are colored coded based on what conditions are being applied. For normal conditions, the simulations and data are blue. Simulations under type 2 diabetic conditions are red. An example is shown in Supplementary Figure 10. For simulations of experiments with the PKB inhibitor MK2206 (MK) and the PI3K inhibitor LY294002 (LY), the simulations are shifting from the "normal blue" to turquoise corresponding to the amount of inhibition exerted by the inhibitor. En example is shown in Supplementary Figure 11. The color blue for normal simulations shift from a dark blue to a light blue the further away from the core model a site is added, corresponding to the confidence of the addition. Compare the color for the normal simulations in Supplementary Figure 11 and Supplementary Figure 12.

The title of a figure tells which phosphosite that was used an input to the added phosphosite (input -> added). Furthermore, the title also gives the cost, corresponding to the model agreement to data (cost: value). The cutoff for acceptable agreement was set to $cost = \chi^2(p = 0.05, df = 8) \approx 15.5$. Finally, the title also gives which layer the phosphosite was added to.

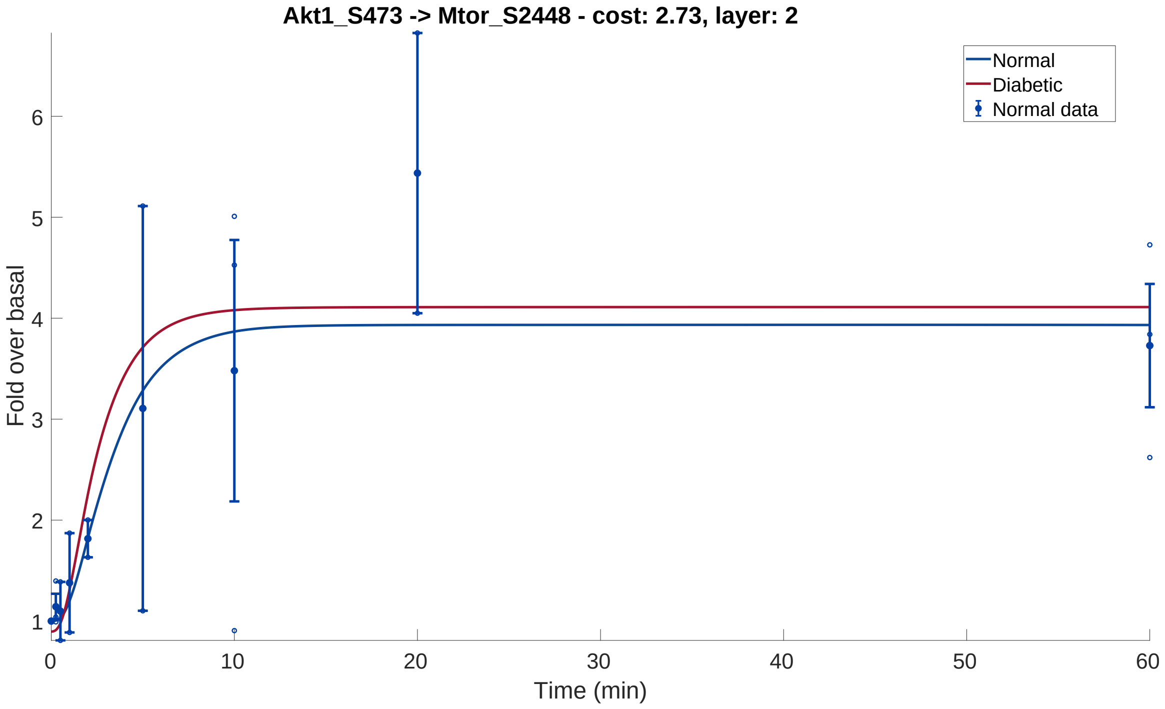

Supplementary Figure 10: **Simulation of Mtor-S448, in response to insulin stimulation normally and in type 2 diabetes.**

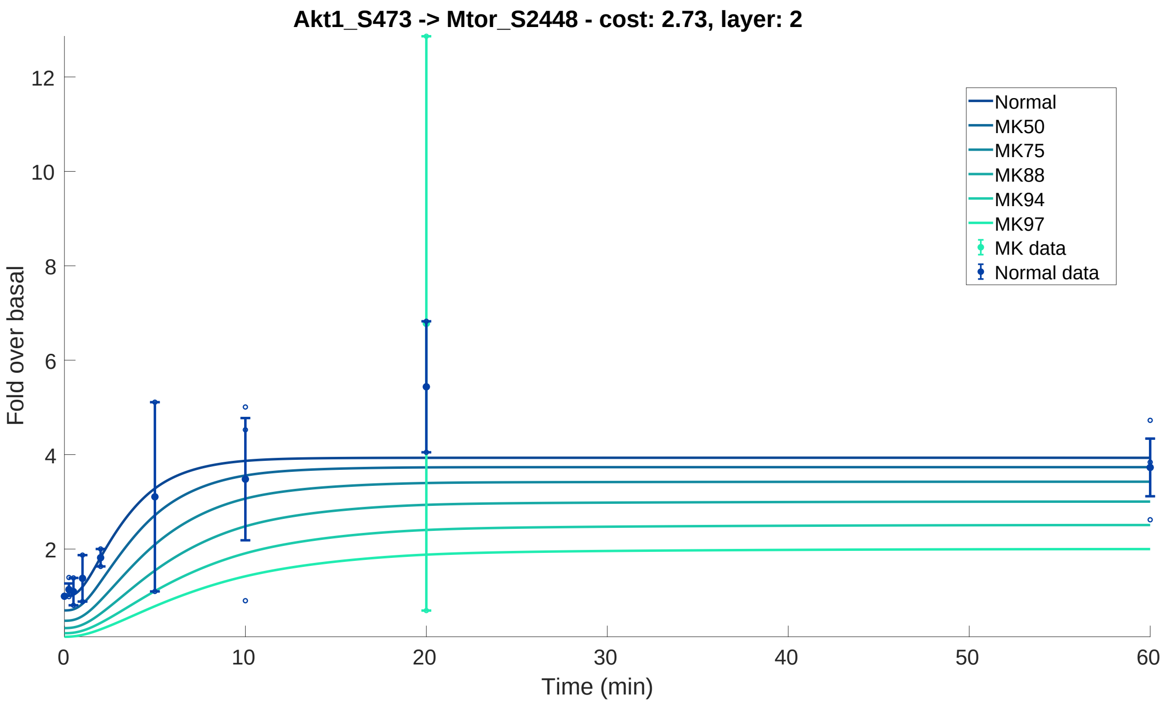

Supplementary Figure 11: **Simulation of Mtor-S448, in response to insulin stimulation normally and with the inhibitor MK2206.**

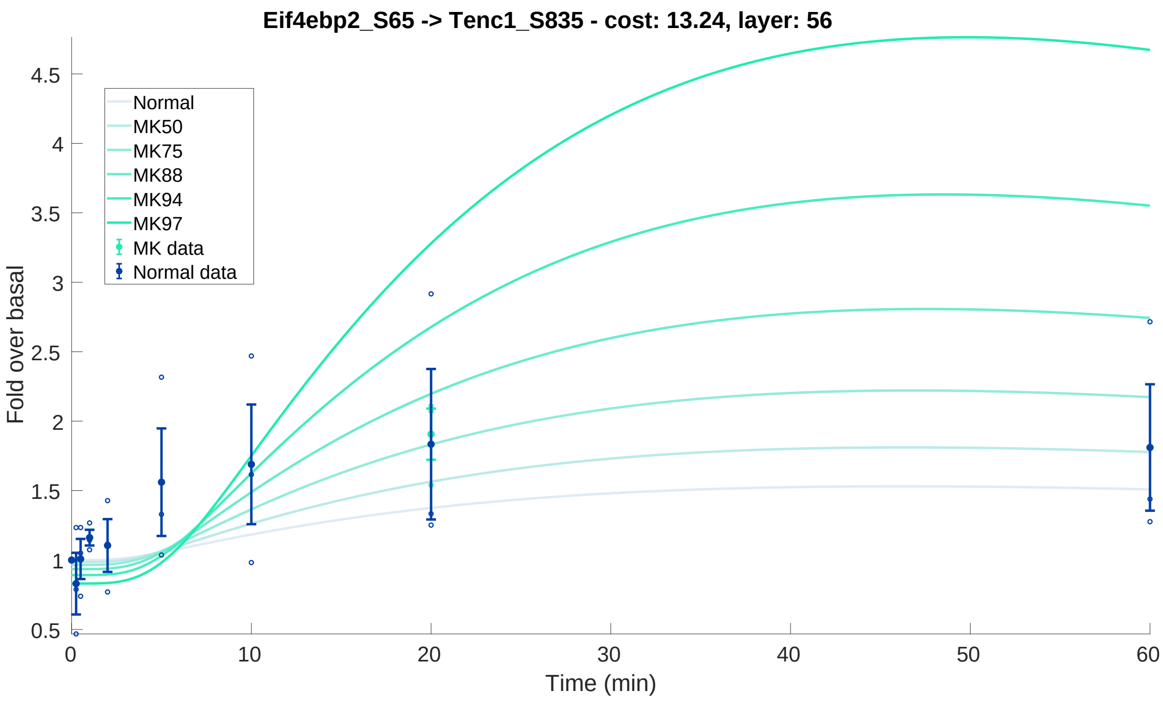

Supplementary Figure 12: **Simulation of Znf318-S214 - a phosphosite added in a distant layer - in response to insulin stimulation normally and with the inhibitor MK2206.**

3.2 Insulin stimulation, normal and in type 2 diabetes

The following pages give all simulations of insulin stimulation normally and in type 2 diabetic conditions.

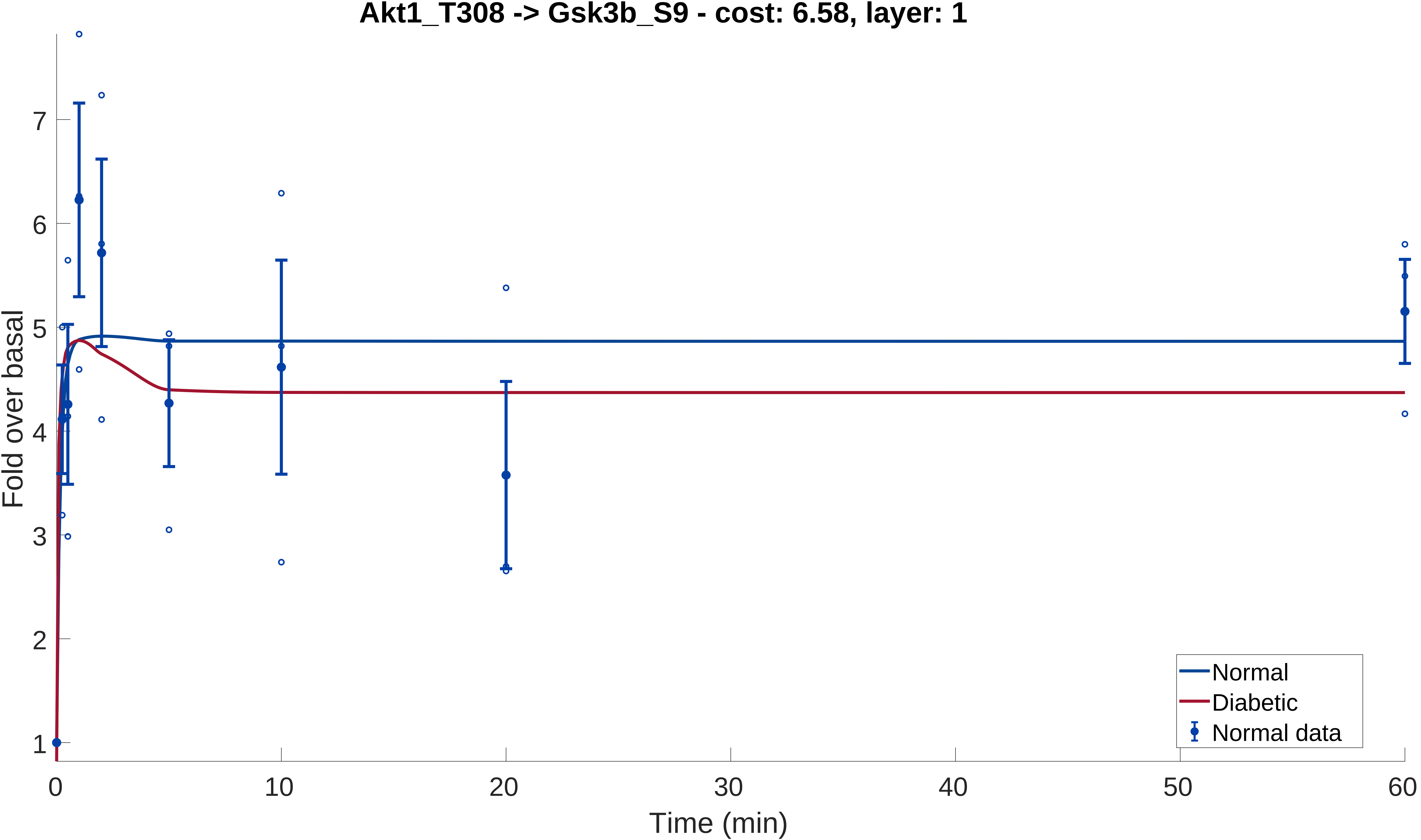

Akt1_T308 -> Gsk3b_S9 - cost: 6.58, layer: 1
Fold over basal
1
2
3
4
5
6
7
0
10
20
30
40
50
60
Time (min)
Normal
Diabetic
Normal data

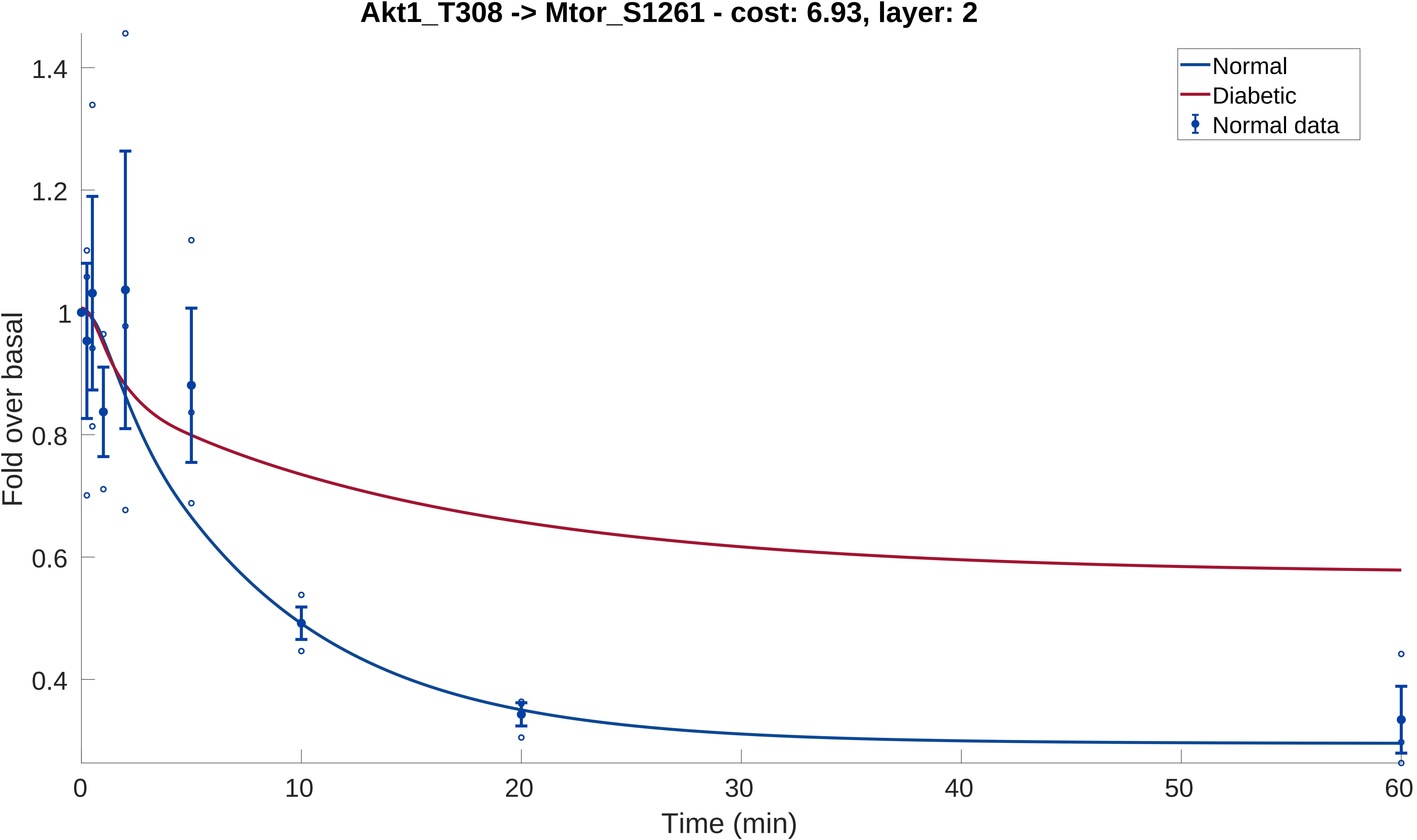
Akt1_T308 -> Mtor_S1261 - cost: 6.93, layer: 2
Normal
Diabetic
Normal data
Fold over basal
Time (min)
1.4
1.2
1
0.8
0.6
0.4
0
10
20
30
40
50
60

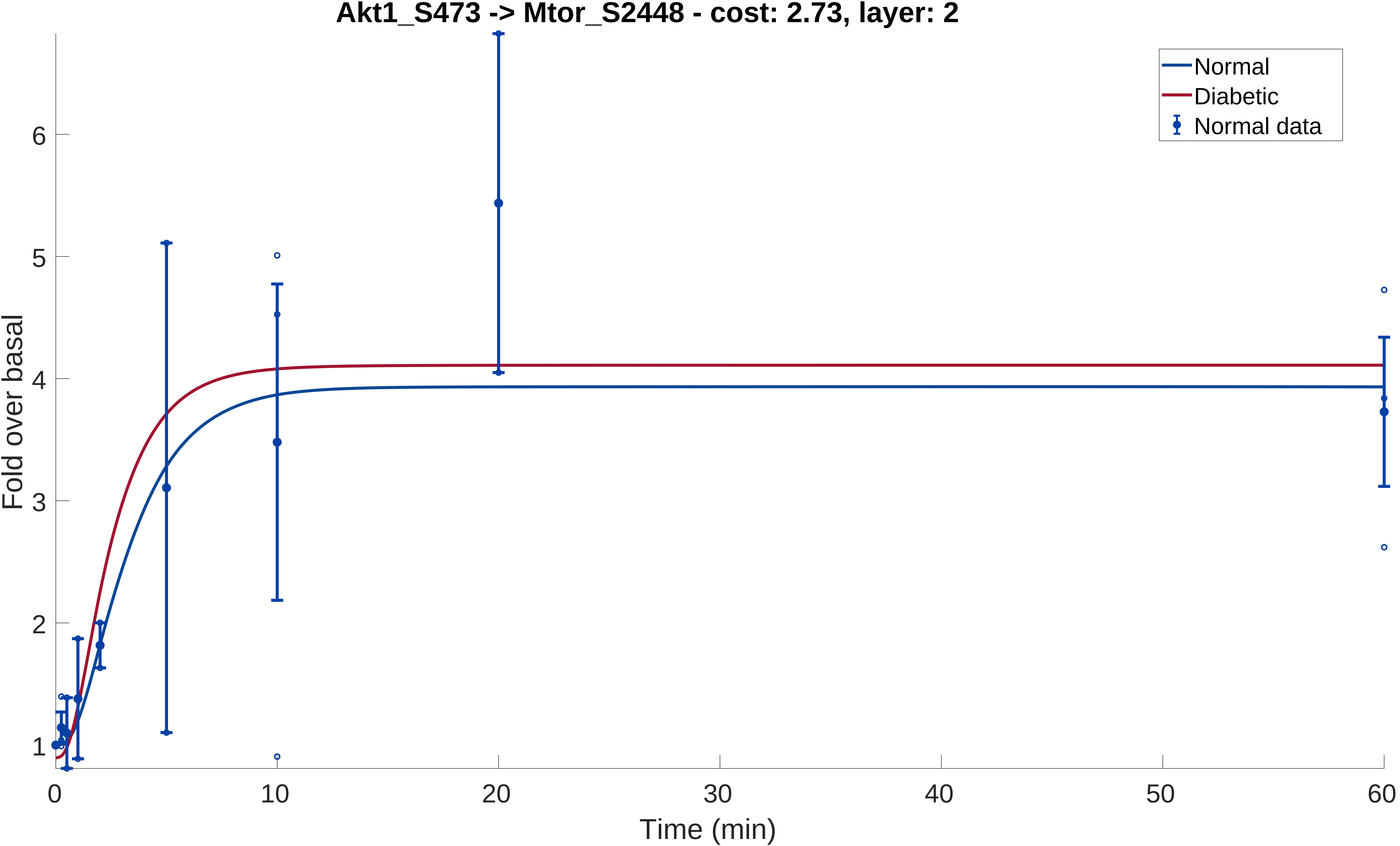
Akt1_S473 -> Mtor_S2448 - cost: 2.73, layer: 2
Normal
Diabetic
Normal data
Fold over basal
Time (min)
1
2
3
4
5
6
0
10
20
30
40
50
60

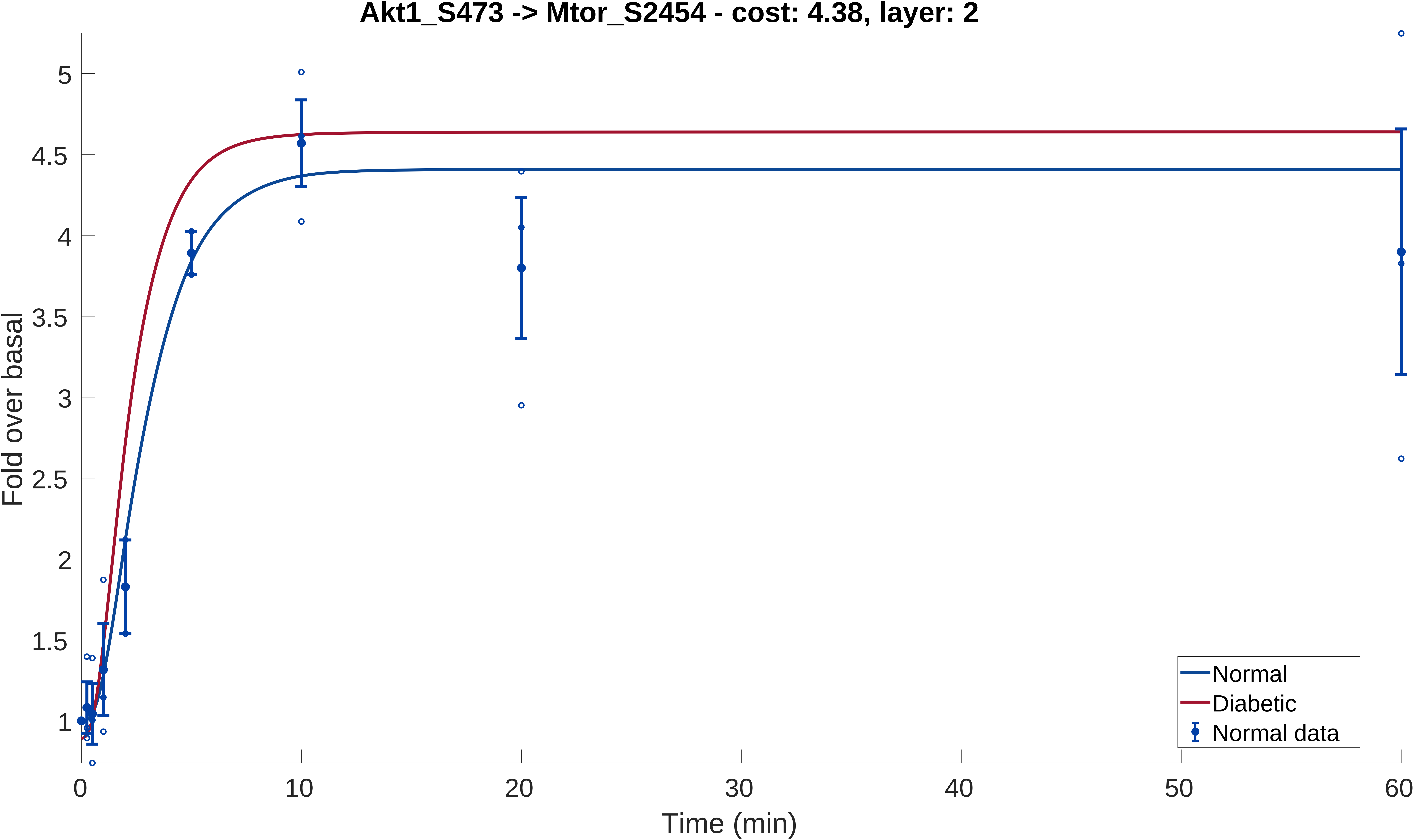
Akt1_S473 -> Mtor_S2454 - cost: 4.38, layer: 2
Fold over basal
Time (min)
0
10
20
30
40
50
60
1
1.5
2
2.5
3
3.5
4
4.5
5
Normal
Diabetic
Normal data

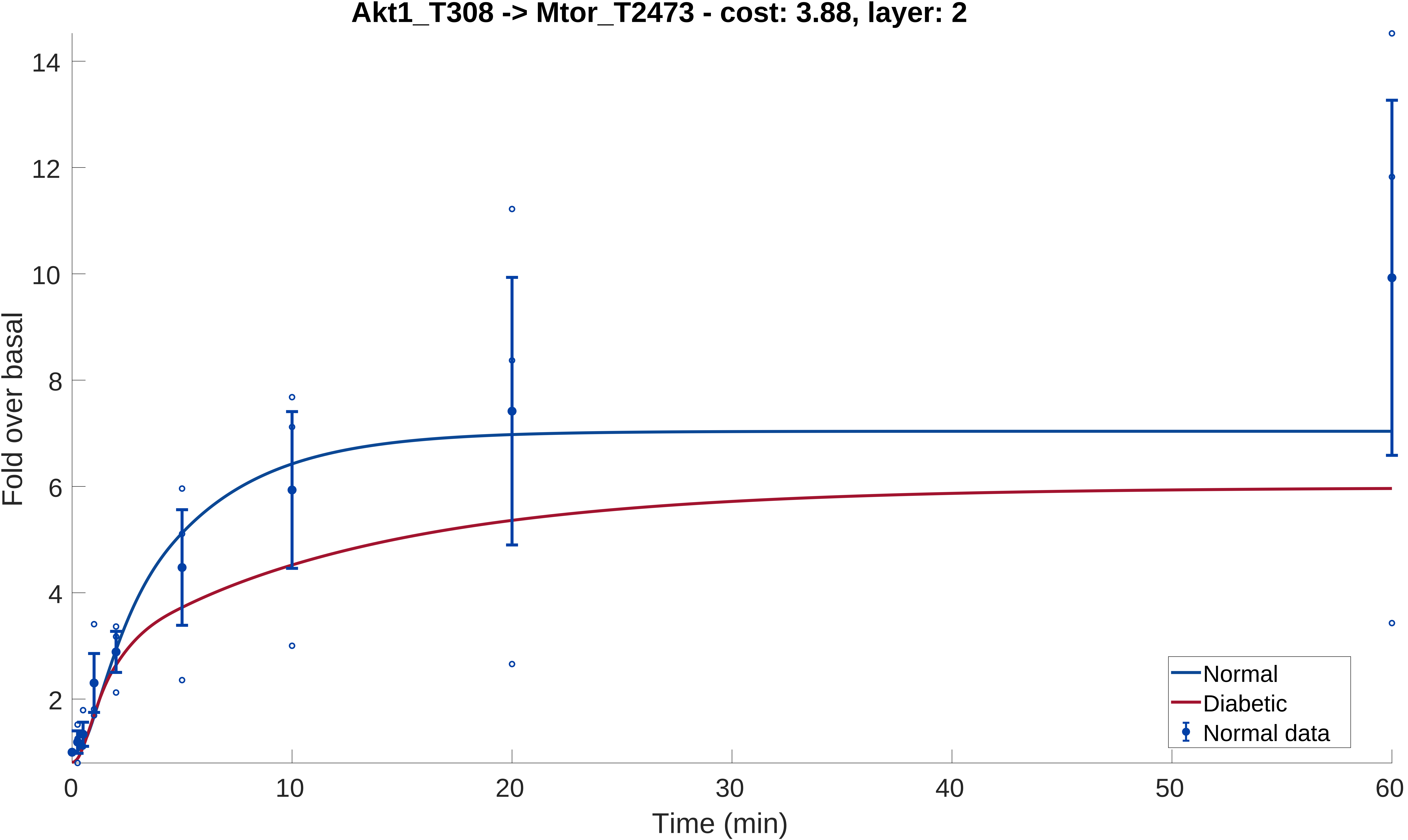

Akt1_T308 -> Mtor_T2473 - cost: 3.88, layer: 2
Fold over basal
Time (min)
0
2
4
6
8
10
12
14
0
10
20
30
40
50
60
Normal
Diabetic
Normal data

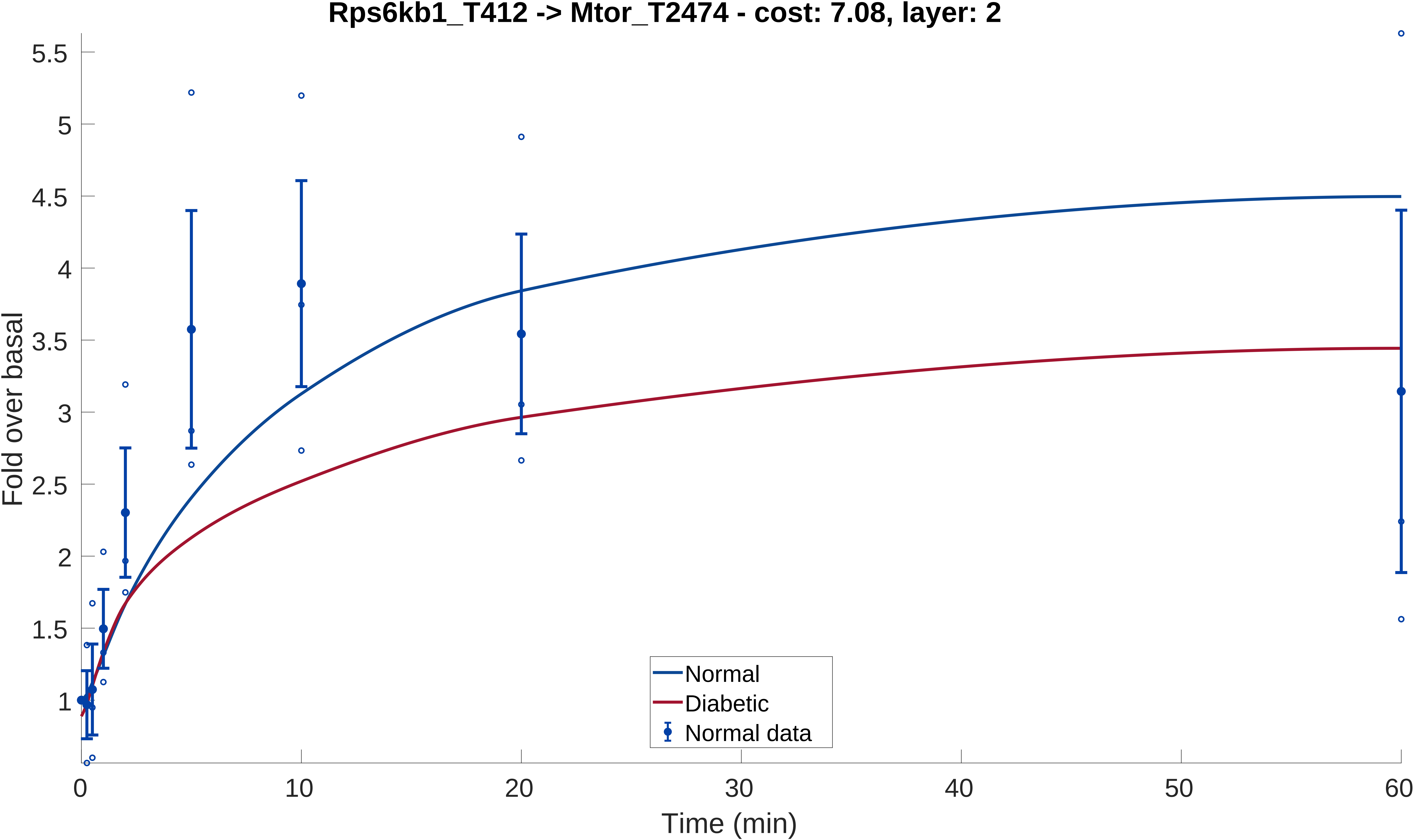
Rps6kb1_T412 -> Mtor_T2474 - cost: 7.08, layer: 2
Fold over basal
Time (min)
5.5
5
4.5
4
3.5
3
2.5
2
1.5
1
0
10
20
30
40
50
60
Normal
Diabetic
Normal data

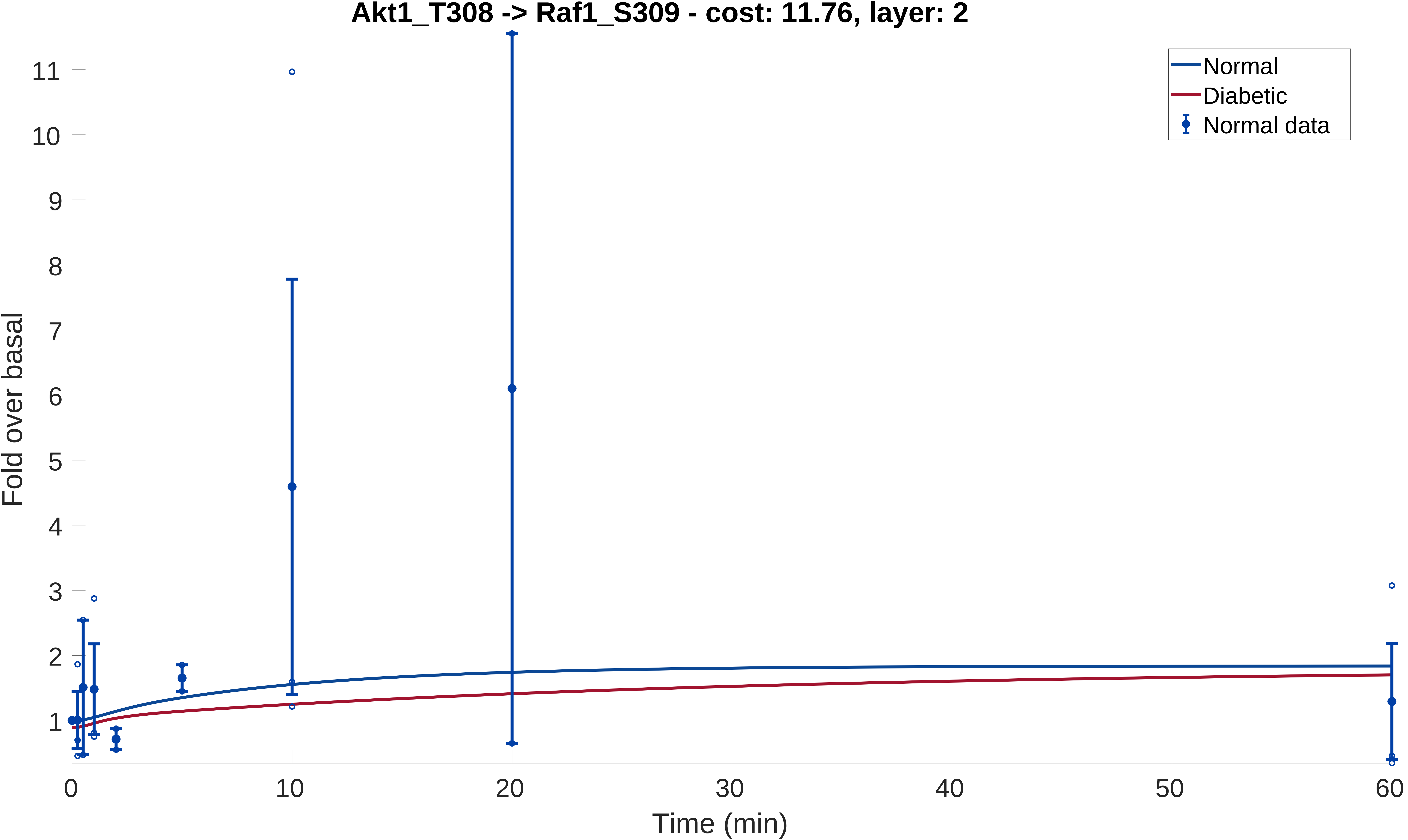

Akt1_T308 -> Raf1_S309 - cost: 11.76, layer: 2
Normal
Diabetic
Normal data
Fold over basal
Time (min)
0
10
20
30
40
50
60
1
2
3
4
5
6
7
8
9
10
11

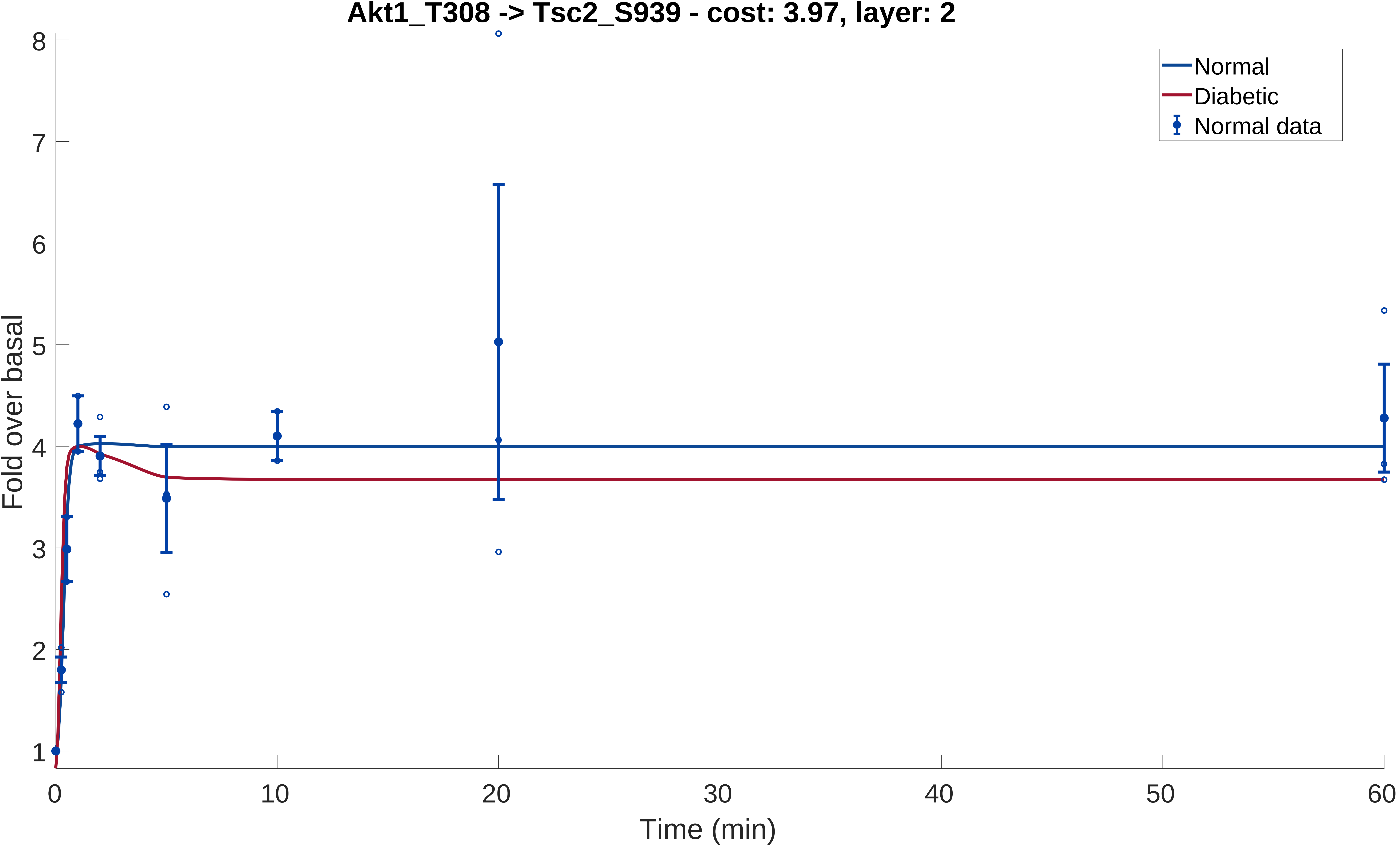
Akt1_T308 -> Tsc2_S939 - cost: 3.97, layer: 2
Normal
Diabetic
Normal data
Fold over basal
Time (min)
1
2
3
4
5
6
7
8
0
10
20
30
40
50
60

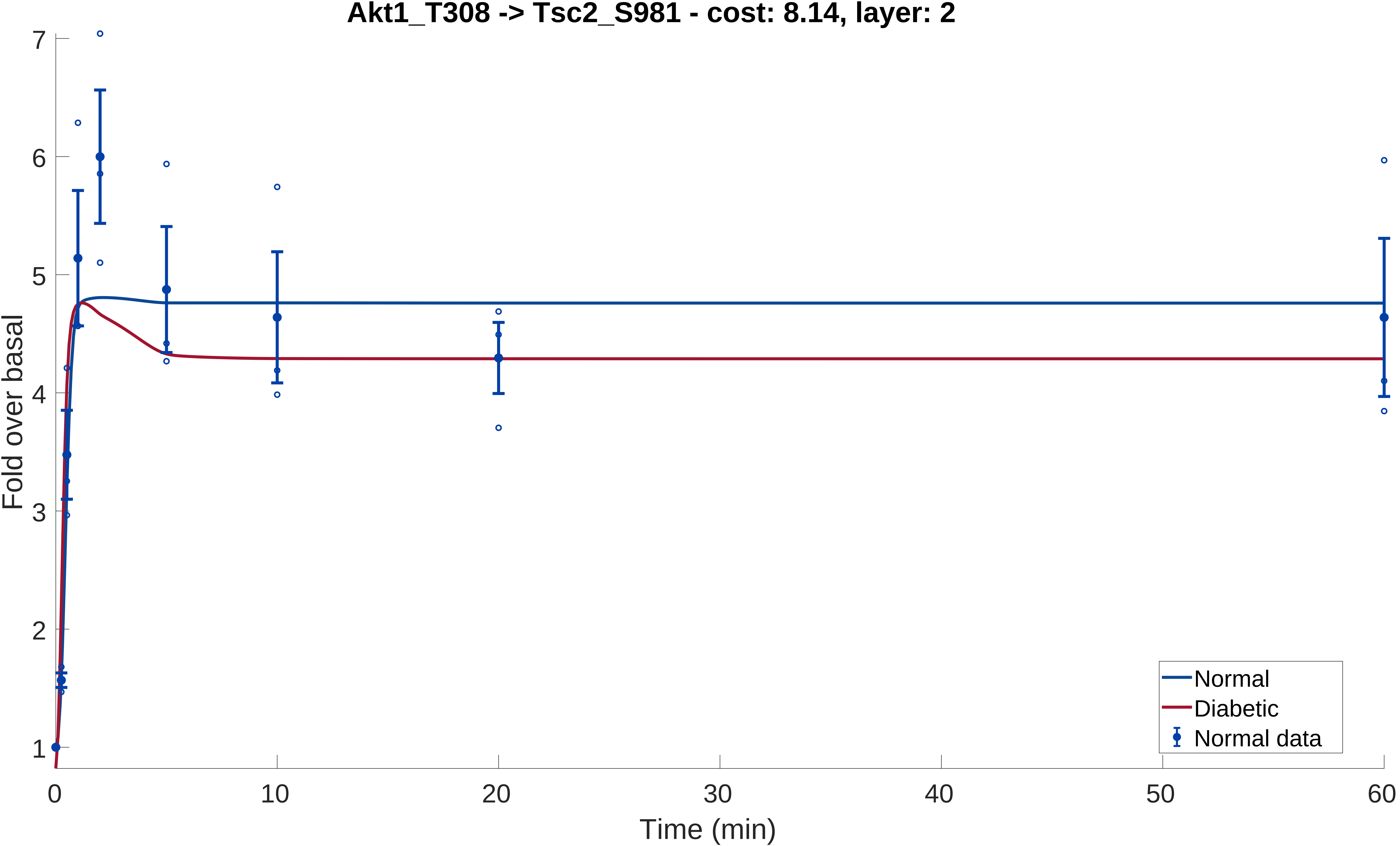
Akt1_T308 -> Tsc2_S981 - cost: 8.14, layer: 2
Fold over basal
Time (min)
1
2
3
4
5
6
7
0
10
20
30
40
50
60
Normal
Diabetic
Normal data

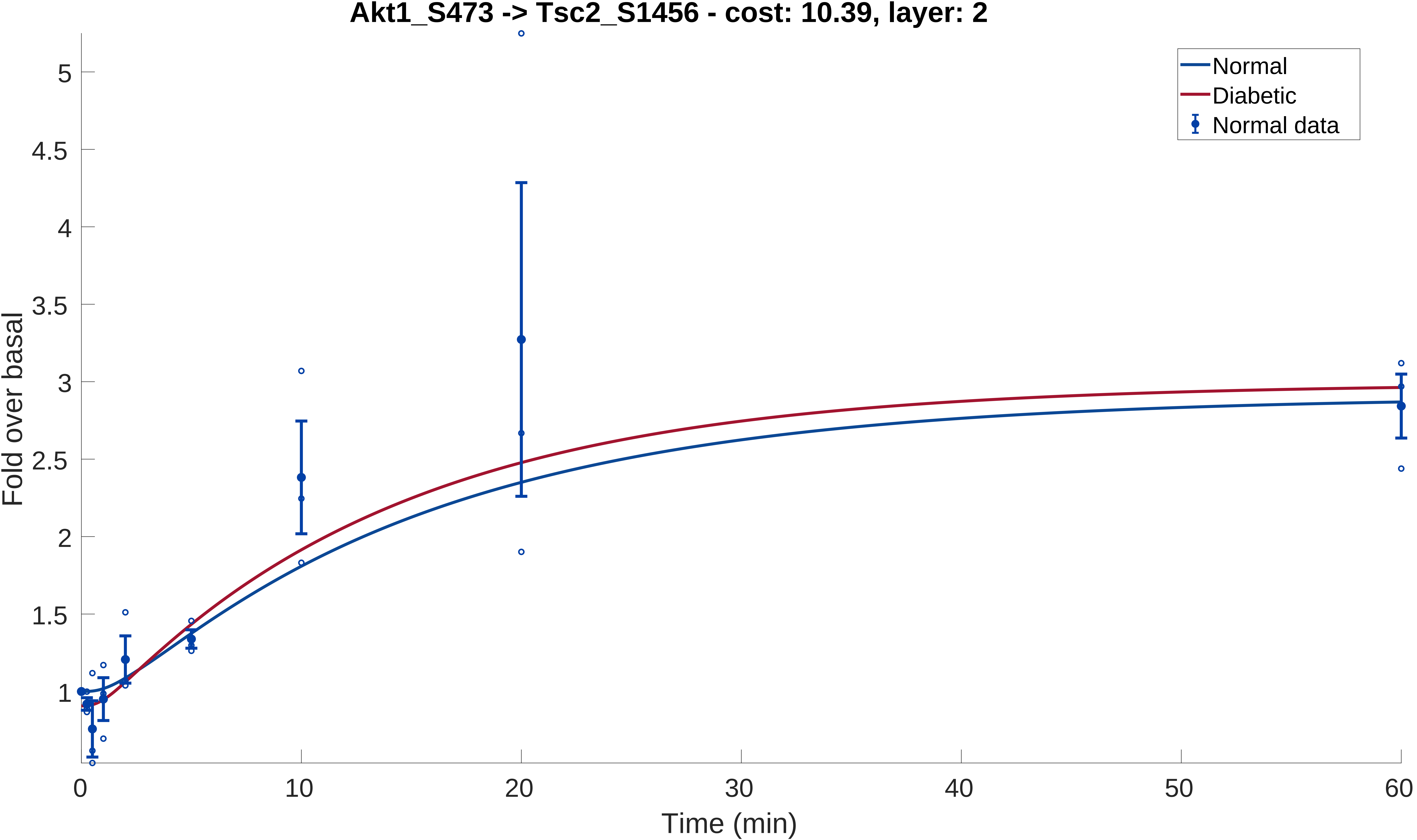

Akt1_S473 -> Tsc2_S1456 - cost: 10.39, layer: 2
Normal
Diabetic
Normal data
Fold over basal
Time (min)
5
4.5
4
3.5
3
2.5
2
1.5
1
0
10
20
30
40
50
60

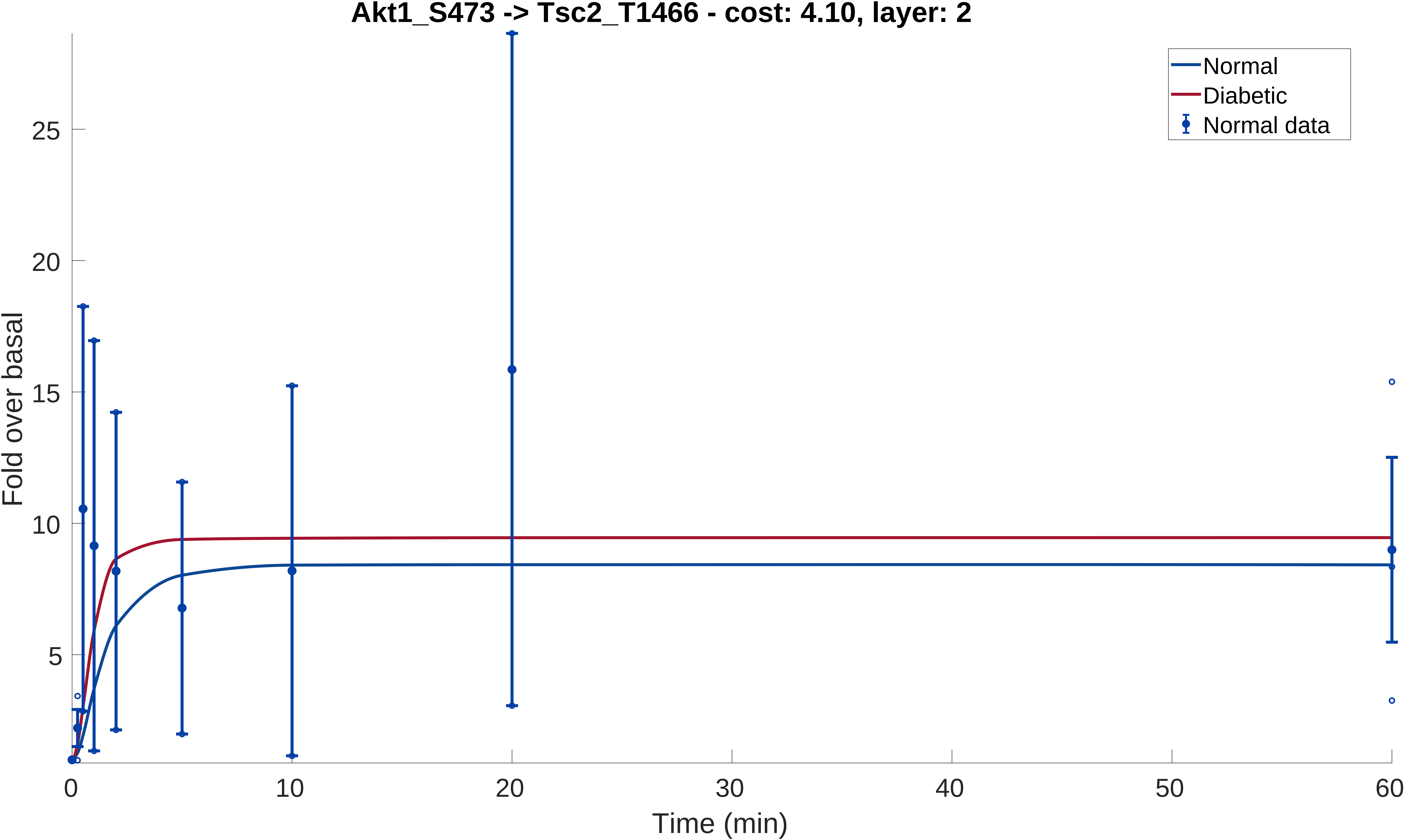

Akt1_S473 -> Tsc2_T1466 - cost: 4.10, layer: 2
Normal
Diabetic
Normal data
Fold over basal
Time (min)
0
10
20
30
40
50
60
5
10
15
20
25

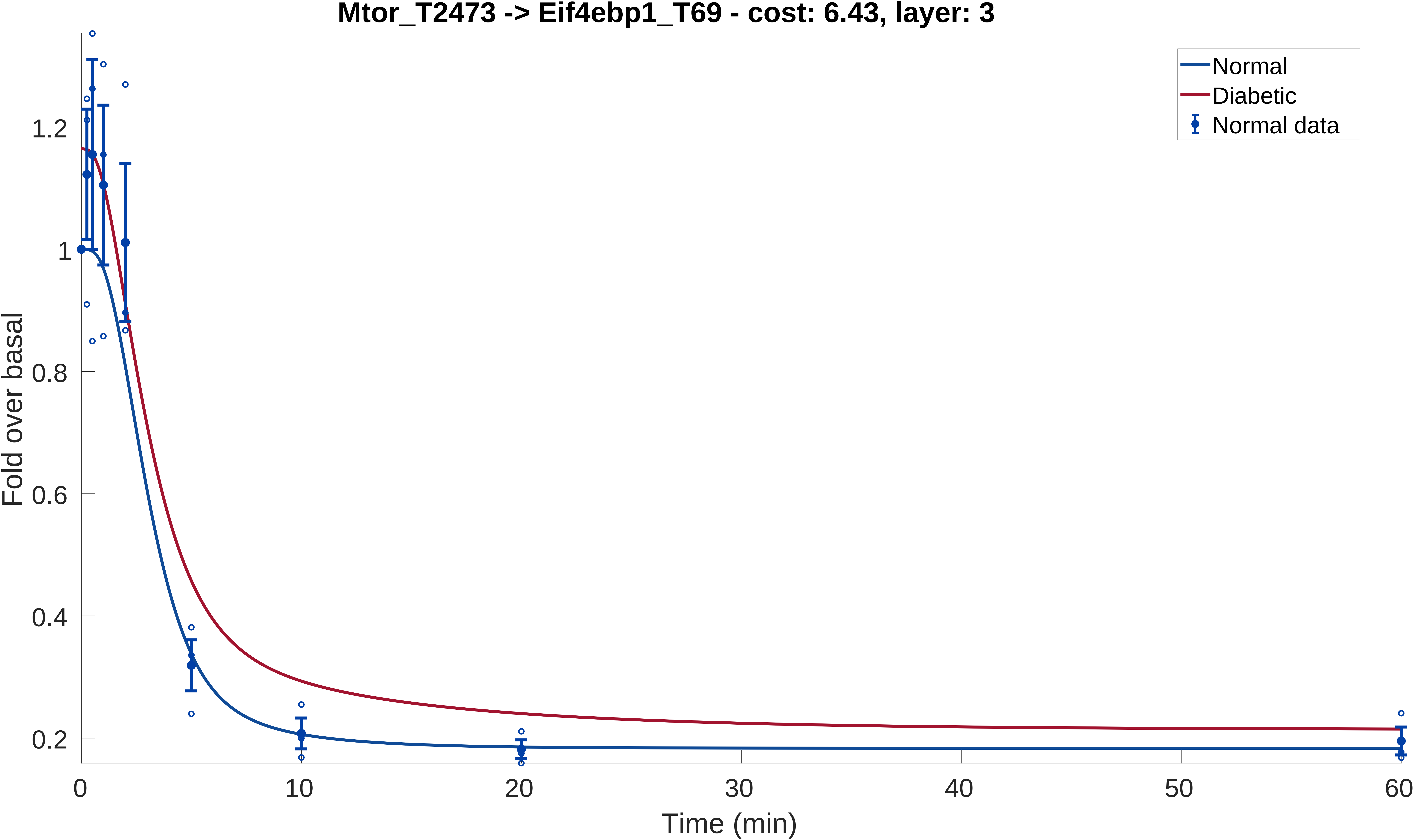

Mtor_T2473 -> Eif4ebp1_T69 - cost: 6.43, layer: 3
Normal
Diabetic
Normal data
Fold over basal
1.2
1
0.8
0.6
0.4
0.2
0
10
20
30
40
50
60
Time (min)

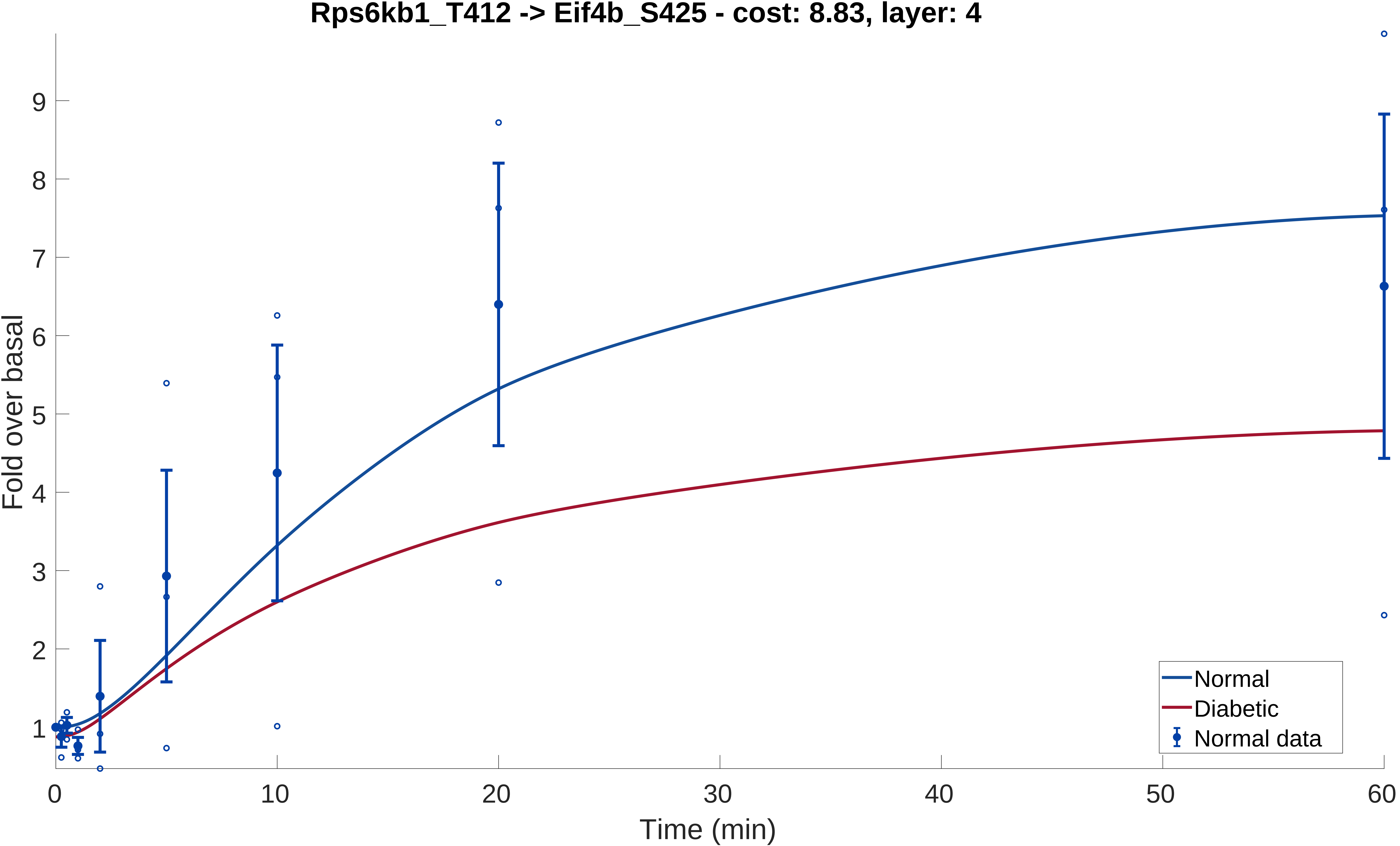

Rps6kb1_T412 -> Eif4b_S425 - cost: 8.83, layer: 4
Fold over basal
9
8
7
6
5
4
3
2
1
0
10
20
30
40
50
60
Time (min)
Normal
Diabetic
Normal data

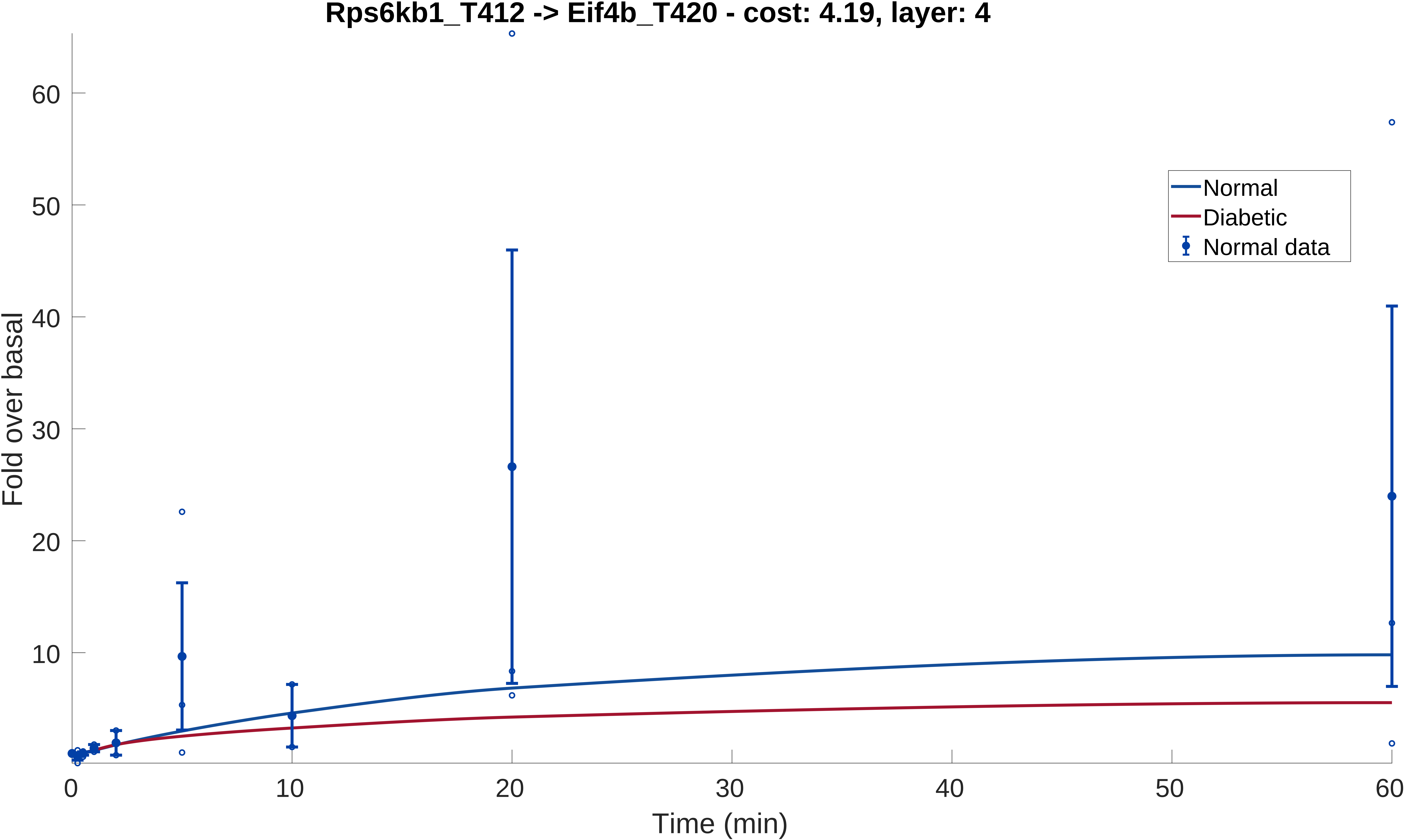
Rps6kb1_T412 -> Eif4b_T420 - cost: 4.19, layer: 4
Fold over basal
Time (min)
Normal
Diabetic
Normal data
0
10
20
30
40
50
60

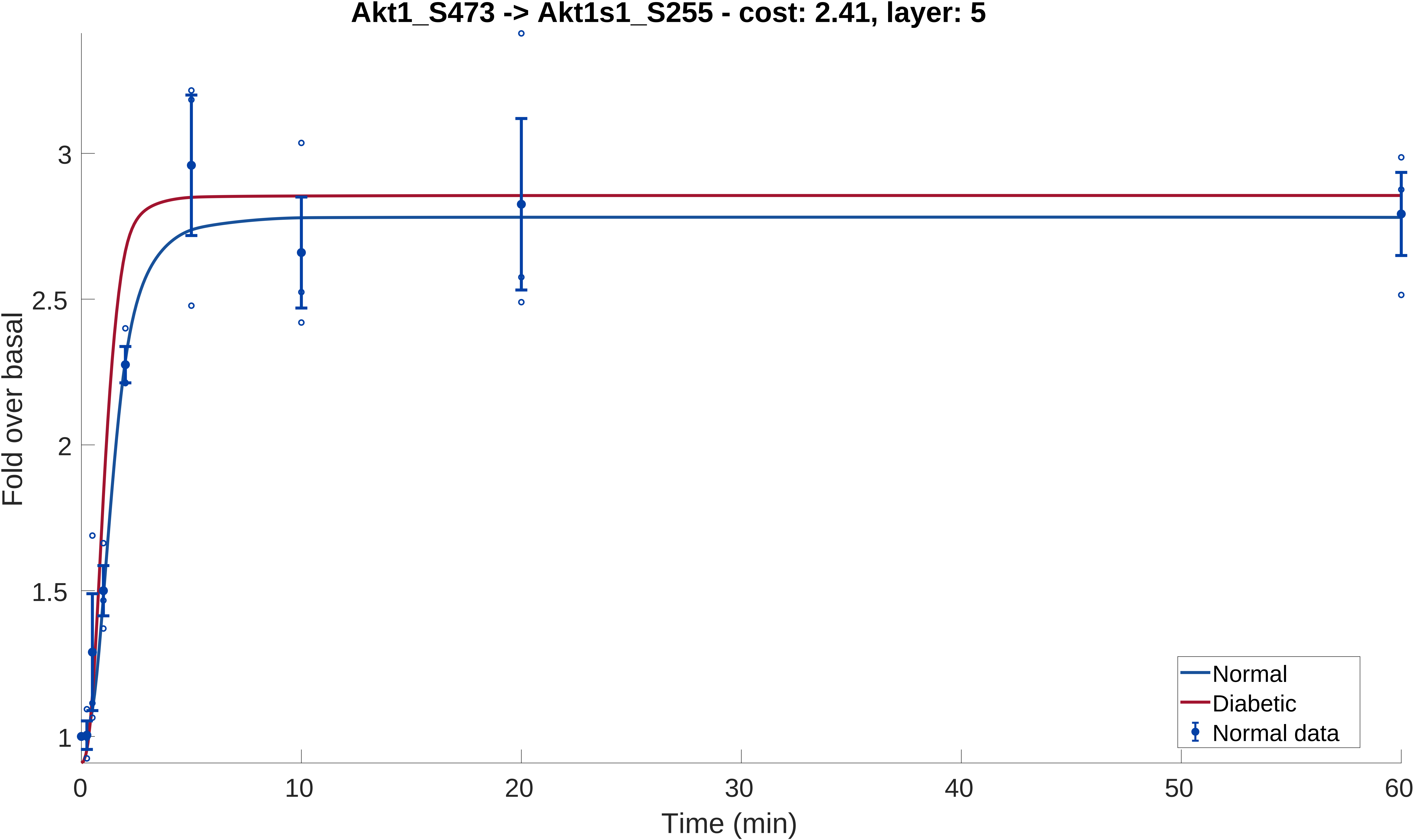

Akt1_S473 -> Akt1s1_S255 - cost: 2.41, layer: 5
Fold over basal
1
1.5
2
2.5
3
0
10
20
30
40
50
60
Time (min)
Normal
Diabetic
Normal data

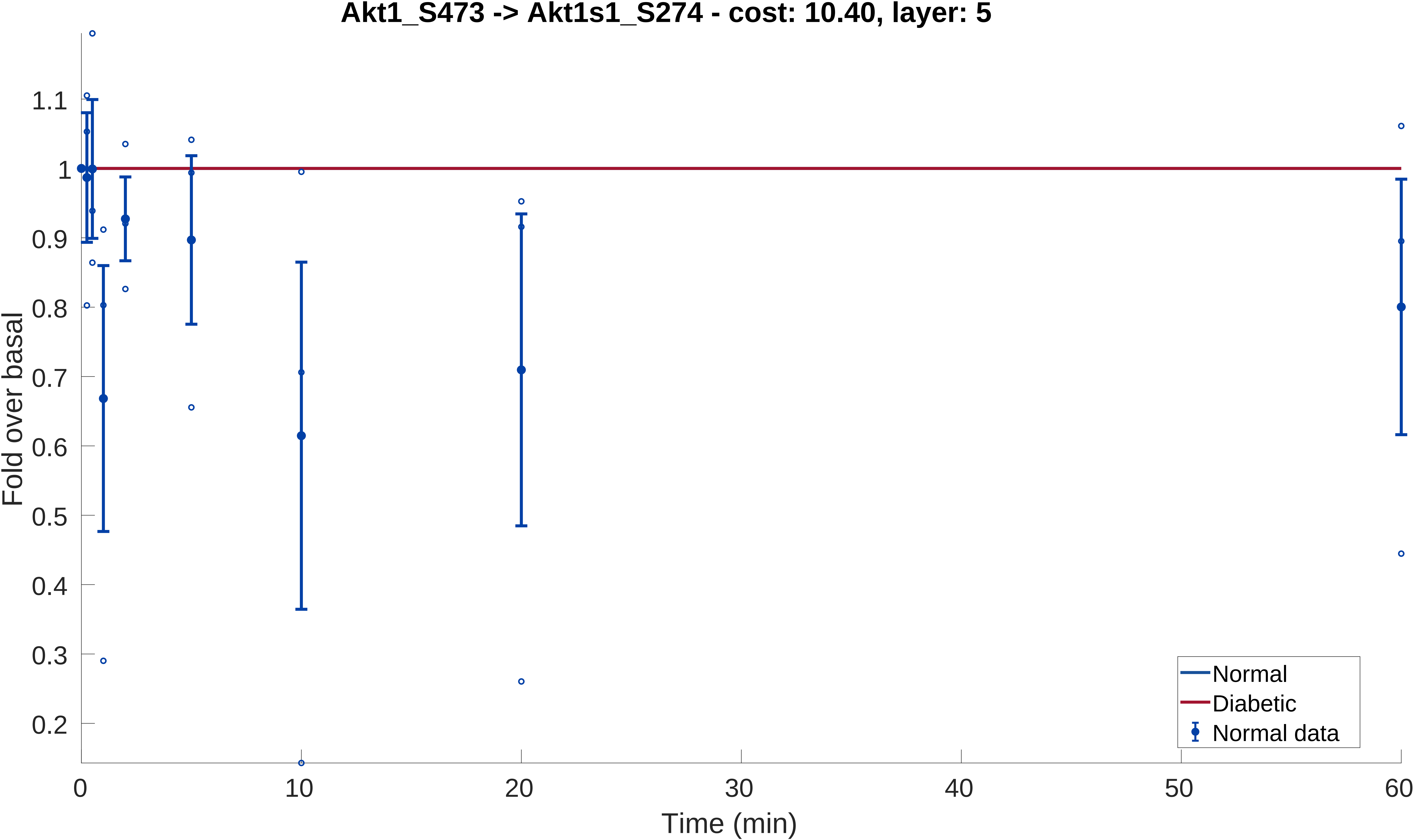
Akt1_S473 -> Akt1s1_S274 - cost: 10.40, layer: 5
Fold over basal
Time (min)
1.1
1
0.9
0.8
0.7
0.6
0.5
0.4
0.3
0.2
0
10
20
30
40
50
60
Normal
Diabetic
Normal data

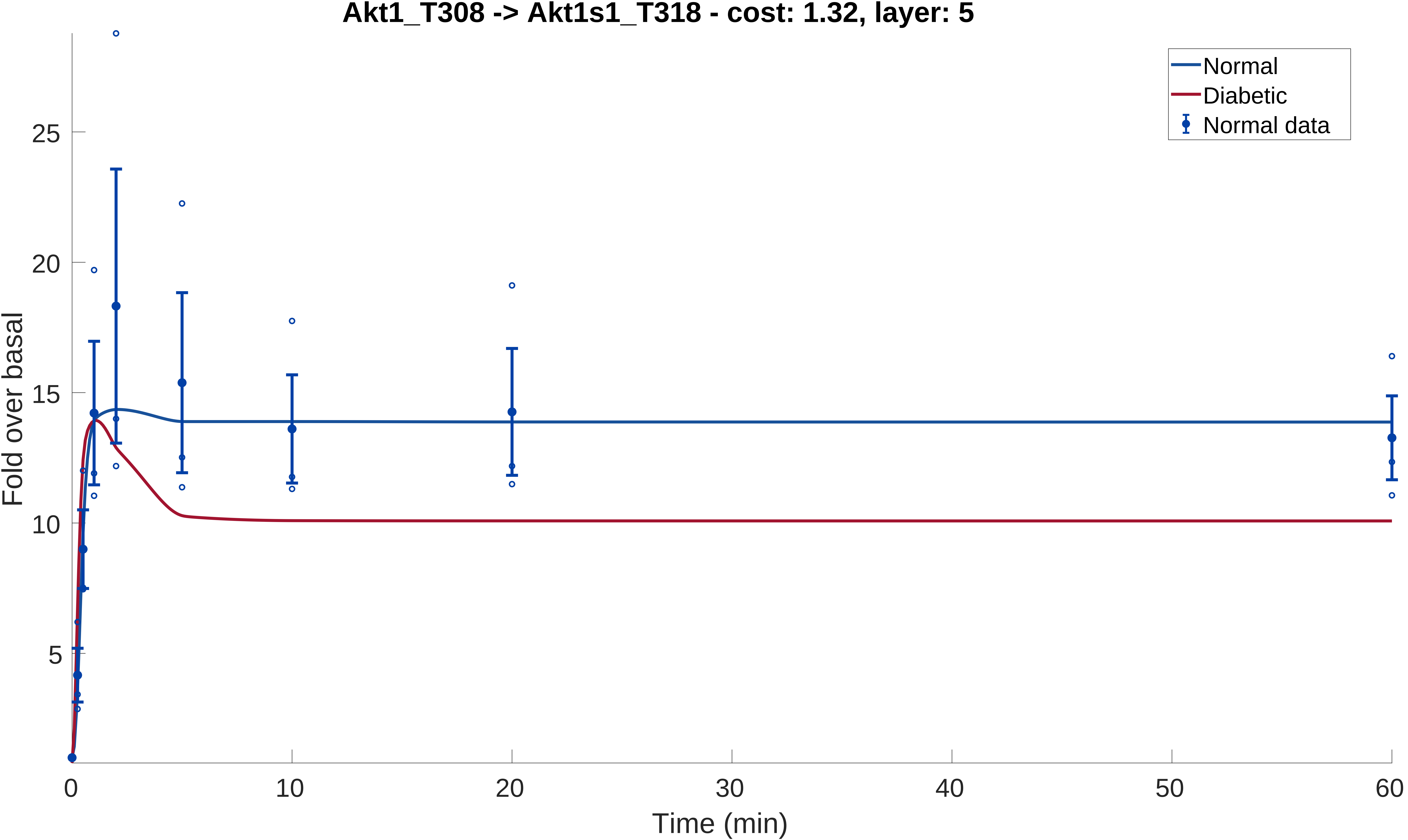
Akt1_T308 -> Akt1s1_T318 - cost: 1.32, layer: 5
Normal
Diabetic
Normal data
Fold over basal
Time (min)
0
10
20
30
40
50
60
5
10
15
20
25

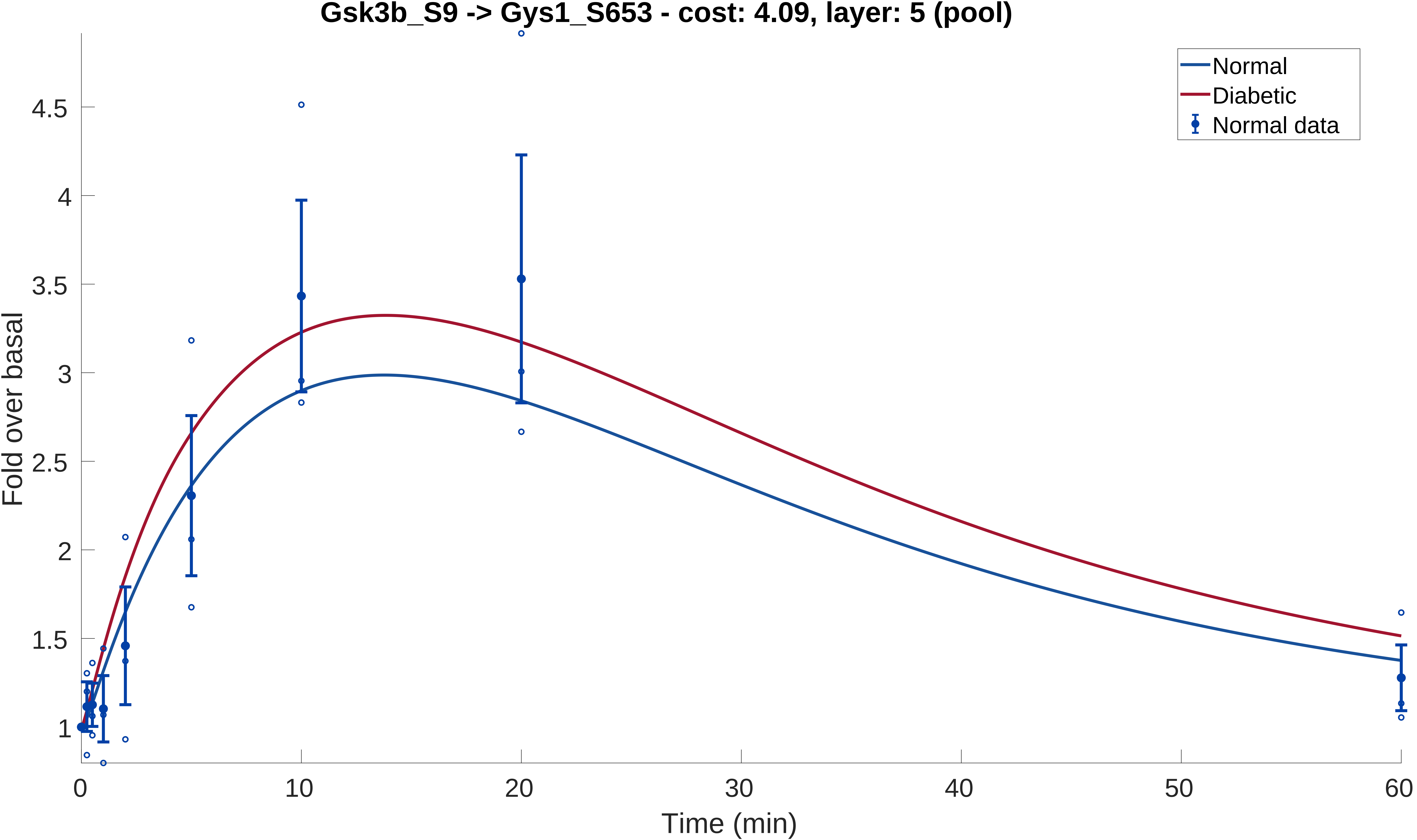

Gsk3b_S9 -> Gys1_S653 - cost: 4.09, layer: 5 (pool)
Normal
Diabetic
Normal data
Fold over basal
Time (min)
1
1.5
2
2.5
3
3.5
4
4.5
0
10
20
30
40
50
60

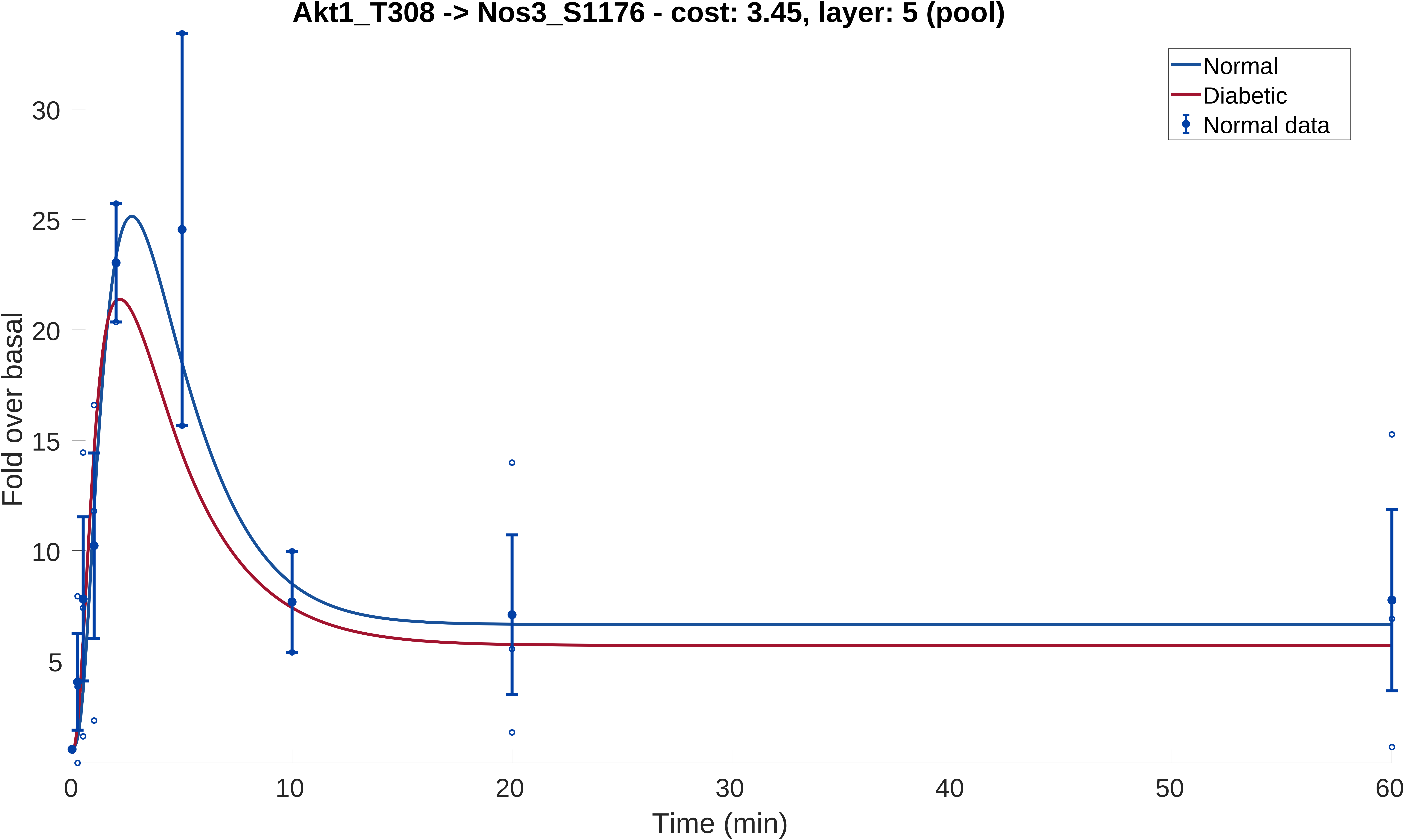

Akt1_T308 -> Nos3_S1176 - cost: 3.45, layer: 5 (pool)
Normal
Diabetic
Normal data
Fold over basal
Time (min)
0
10
20
30
40
50
60
5
10
15
20
25
30

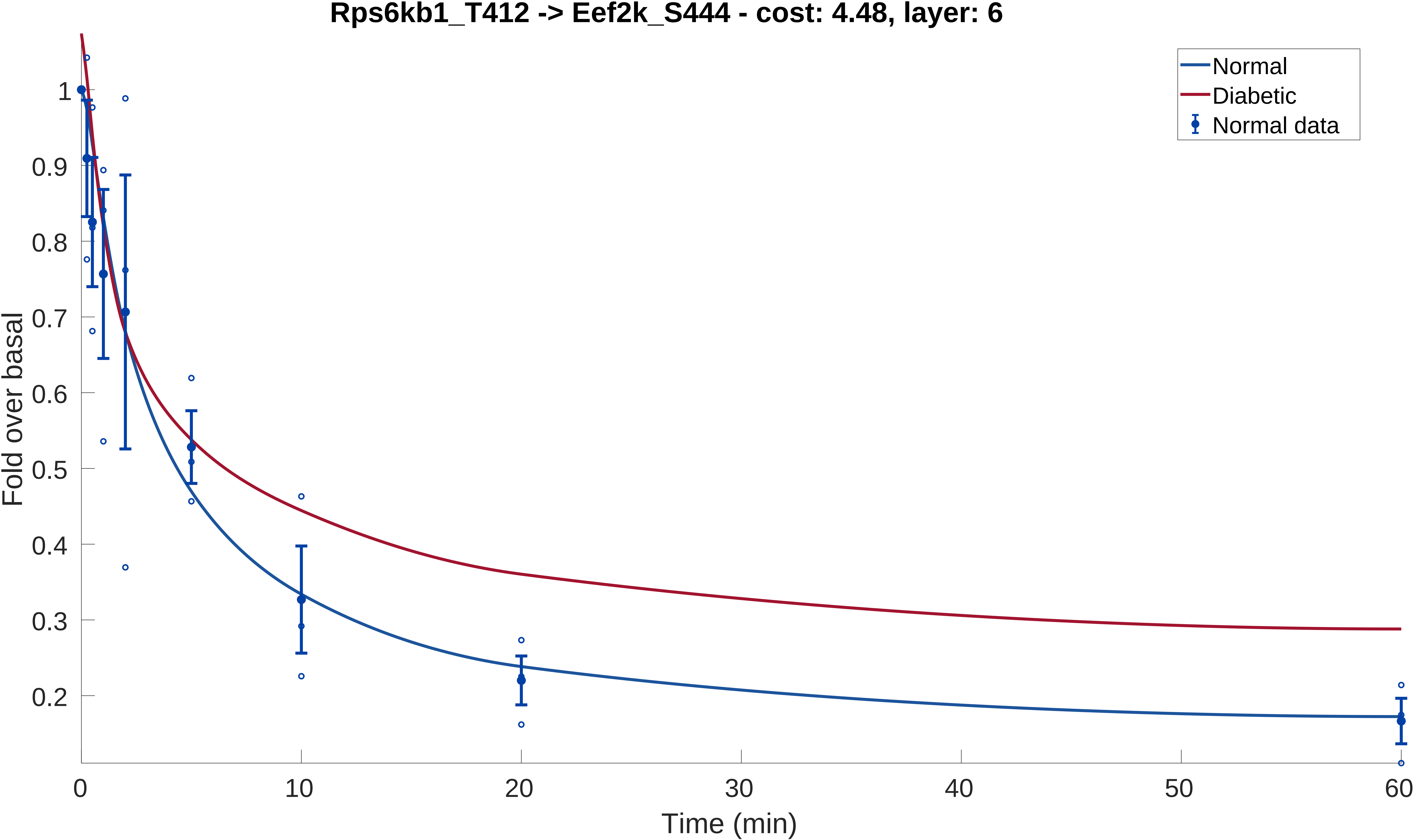
Rps6kb1_T412 -> Eef2k_S444 - cost: 4.48, layer: 6
Fold over basal
Time (min)
Normal
Diabetic
Normal data
0
10
20
30
40
50
60
0.2
0.3
0.4
0.5
0.6
0.7
0.8
0.9
1

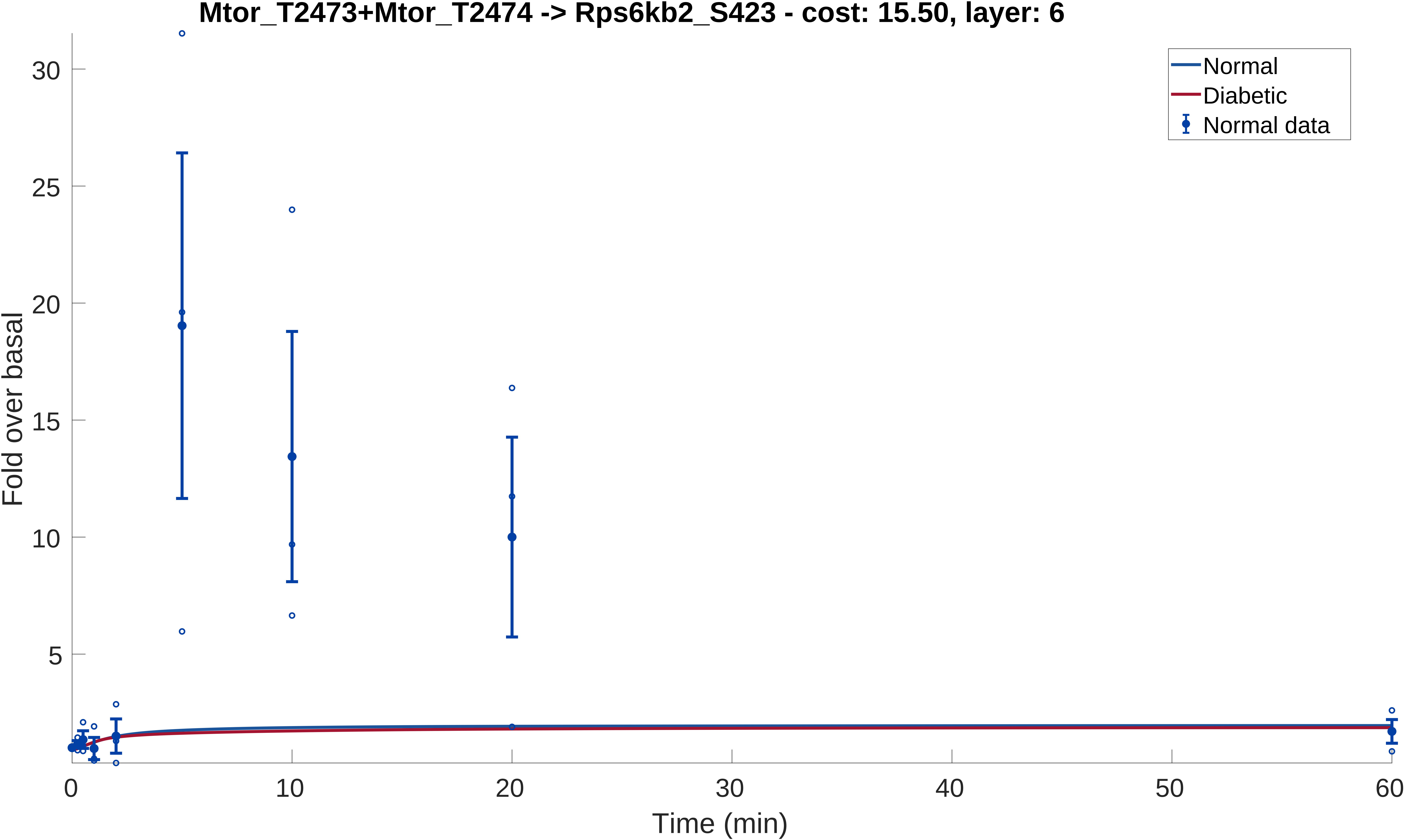

Mtor_T2473+Mtor_T2474 -> Rps6kb2_S423 - cost: 15.50, layer: 6
Normal
Diabetic
Normal data
Fold over basal
30
25
20
15
10
5
0
10
20
30
40
50
60
Time (min)

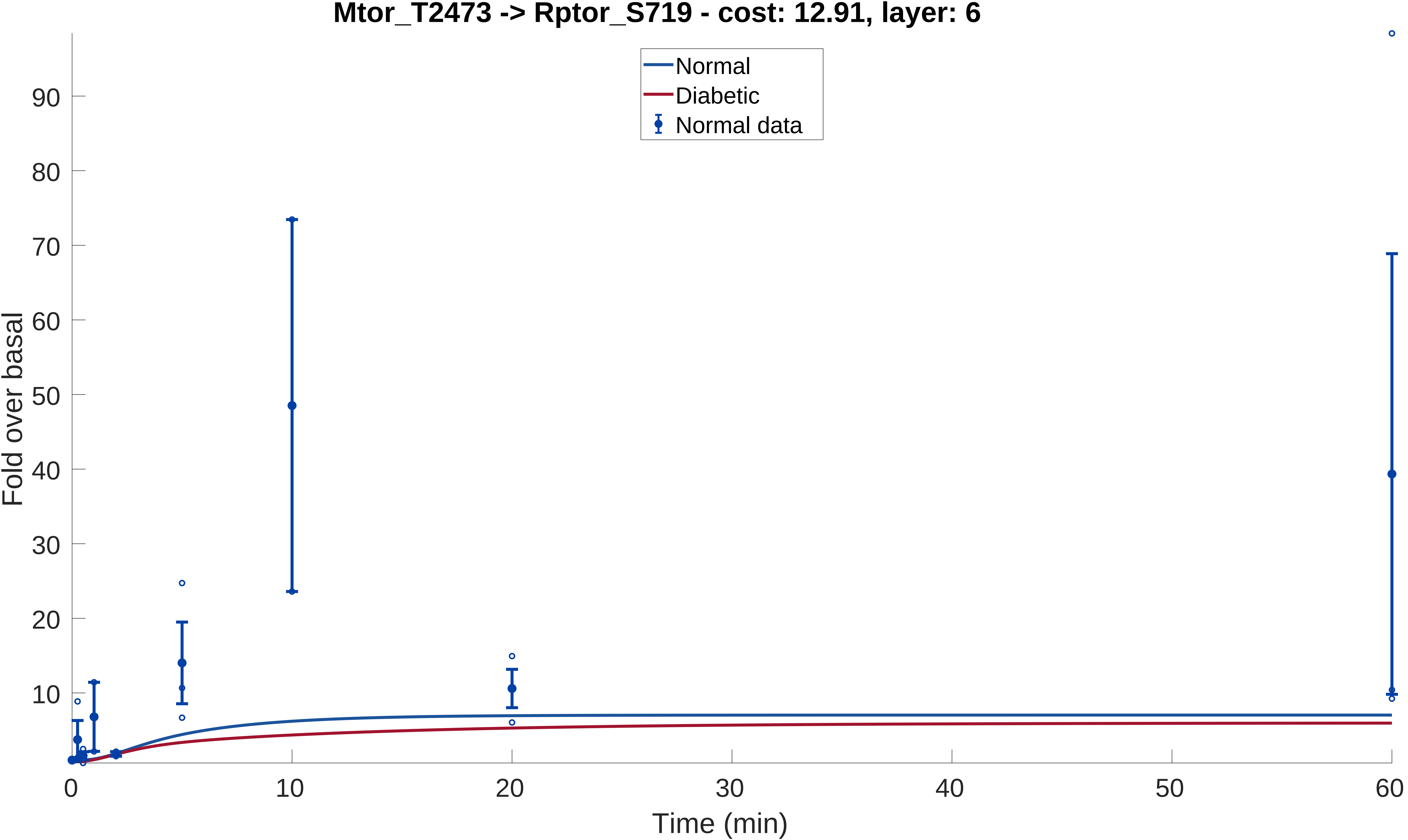
Mtor_T2473 -> Rptor_S719 - cost: 12.91, layer: 6
Normal
Diabetic
Normal data
Fold over basal
Time (min)
0
10
20
30
40
50
60
70
80
90

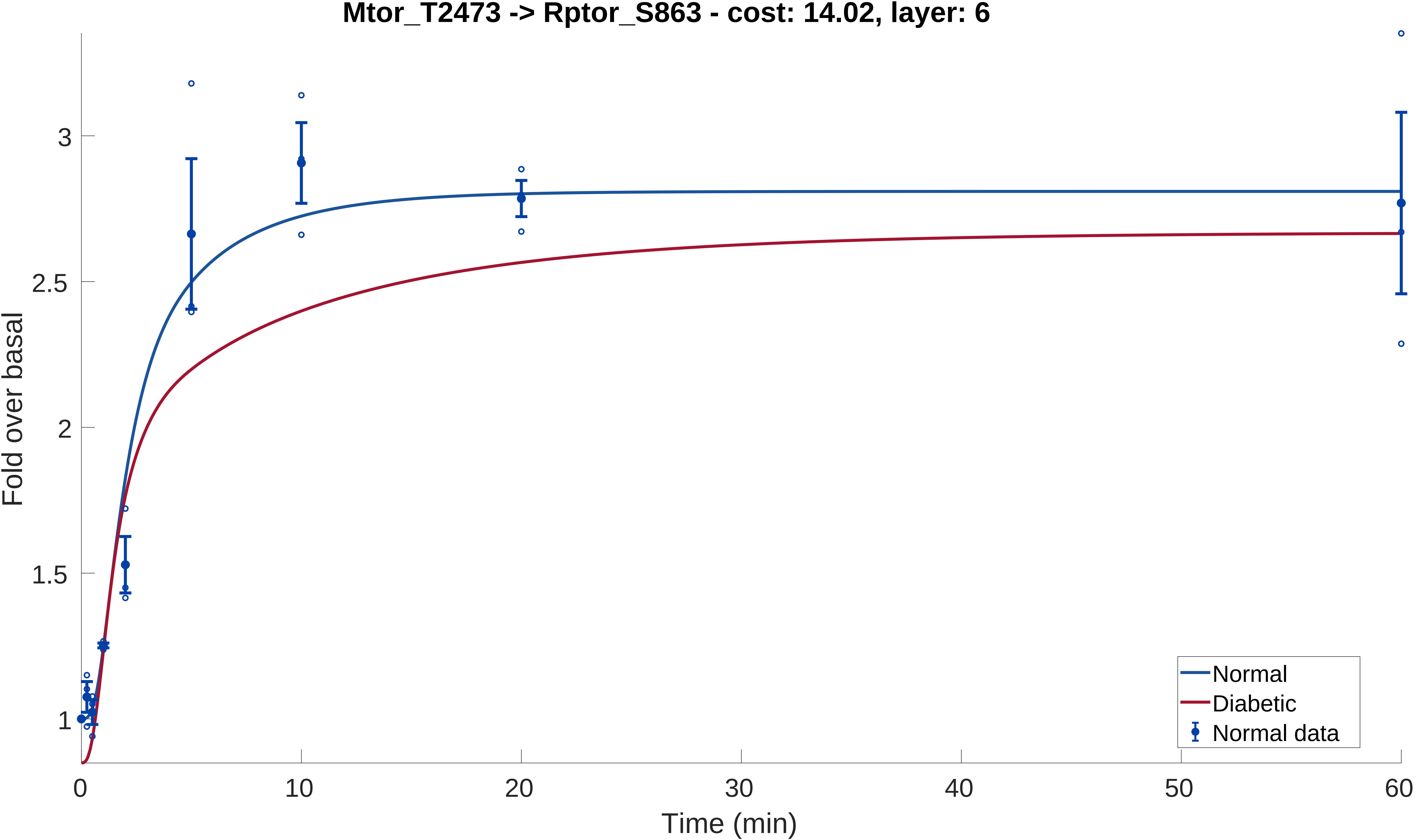

Mtor_T2473 -> Rptor_S863 - cost: 14.02, layer: 6
Fold over basal
Time (min)
0
10
20
30
40
50
60
1
1.5
2
2.5
3
Normal
Diabetic
Normal data

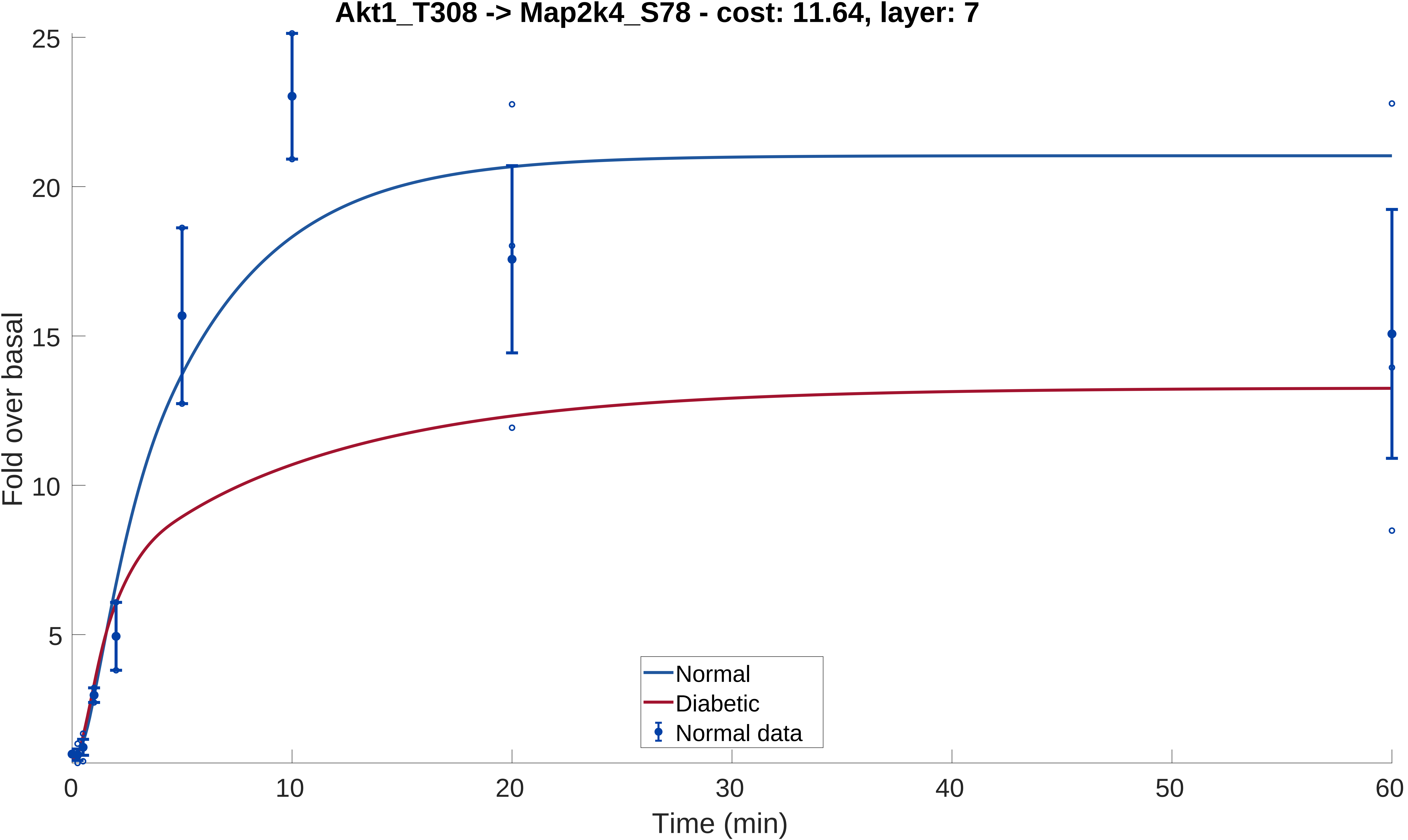

Akt1_T308 -> Map2k4_S78 - cost: 11.64, layer: 7
Fold over basal
25
20
15
10
5
0
10
20
30
40
50
60
Time (min)
Normal
Diabetic
Normal data

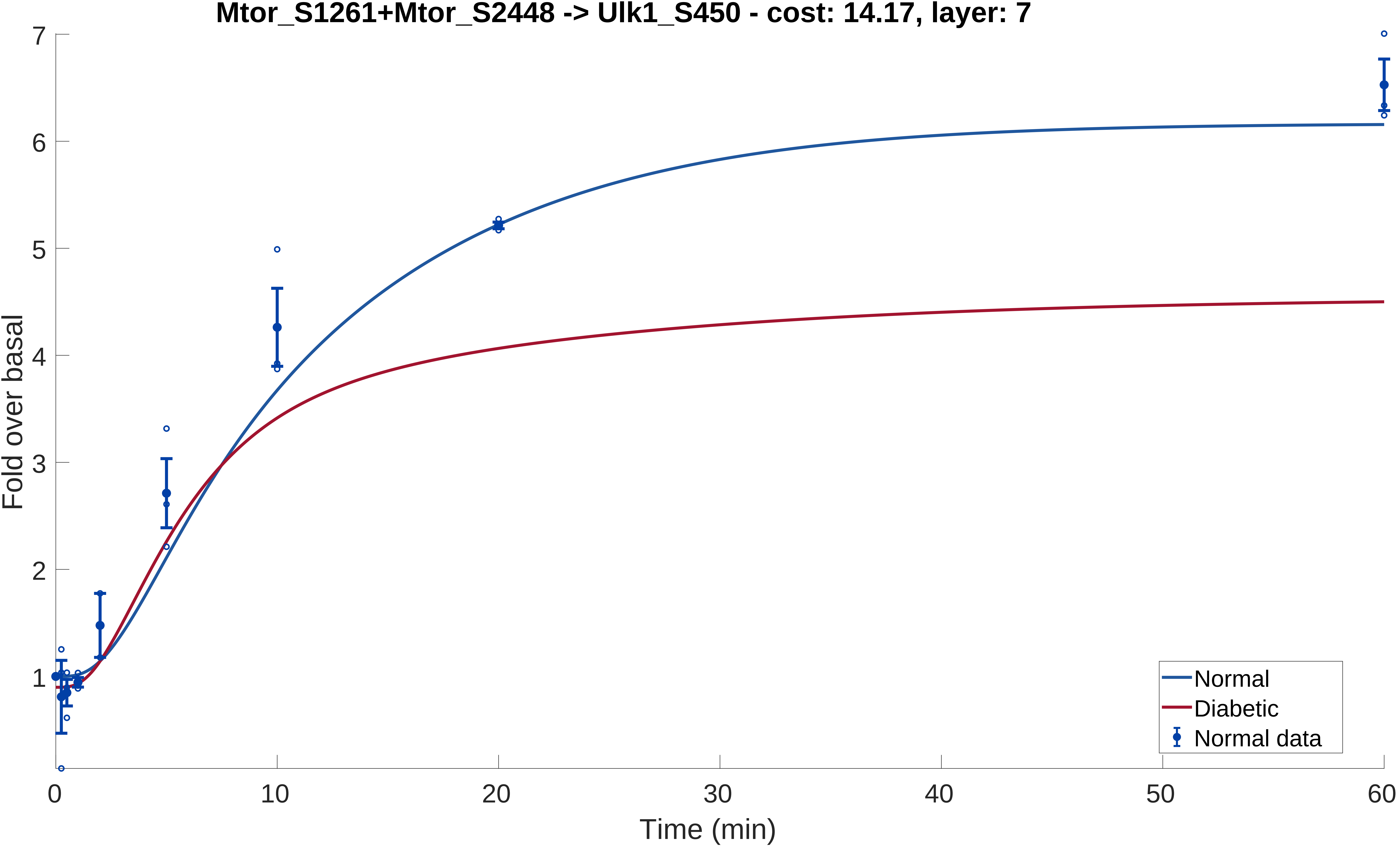

Mtor_S1261+Mtor_S2448 -> Ulk1_S450 - cost: 14.17, layer: 7
Fold over basal
Time (min)
0
10
20
30
40
50
60
1
2
3
4
5
6
7
Normal
Diabetic
Normal data

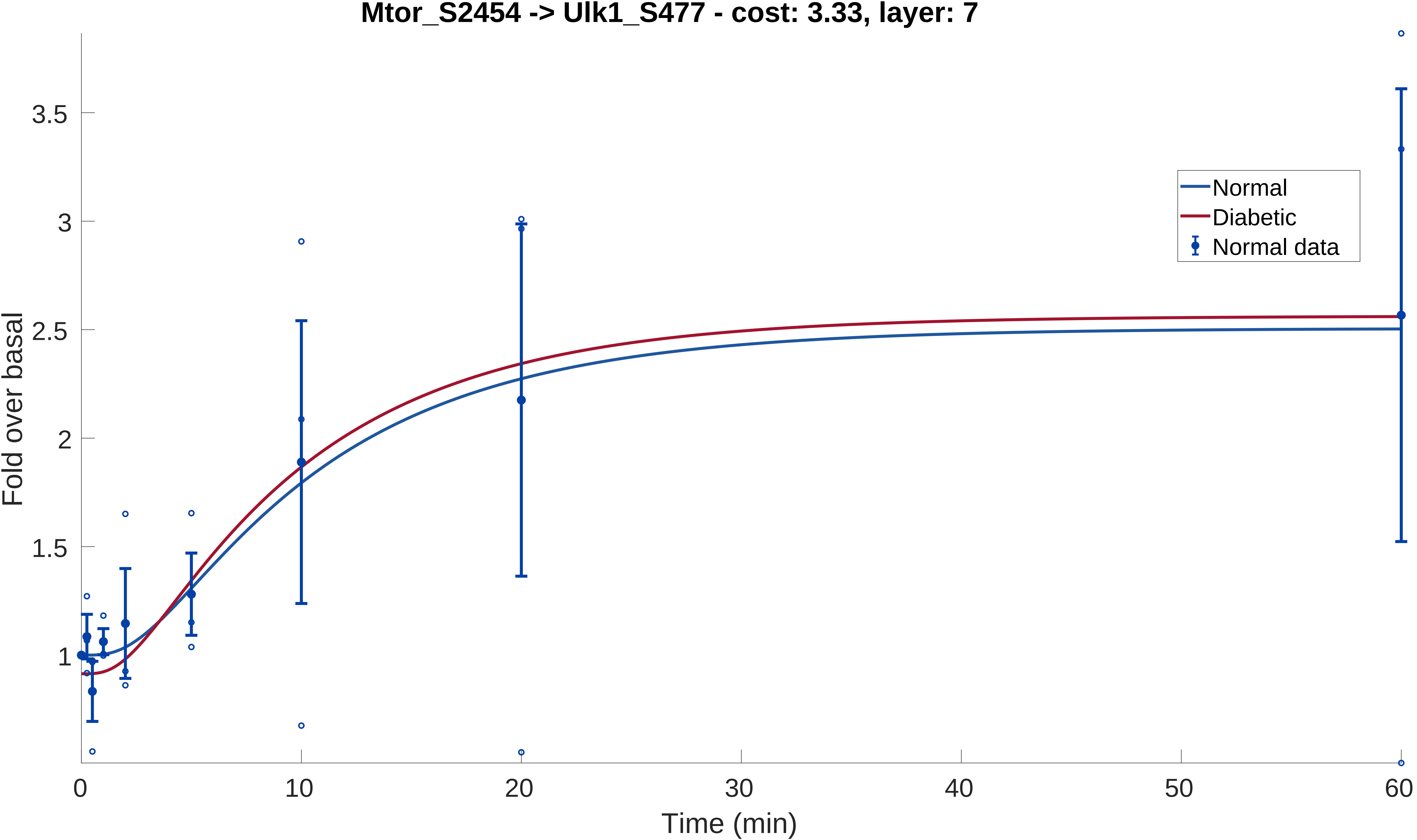

Mtor_S2454 -> Ulk1_S477 - cost: 3.33, layer: 7
Fold over basal
3.5
3
2.5
2
1.5
1
0
10
20
30
40
50
60
Time (min)
Normal
Diabetic
Normal data

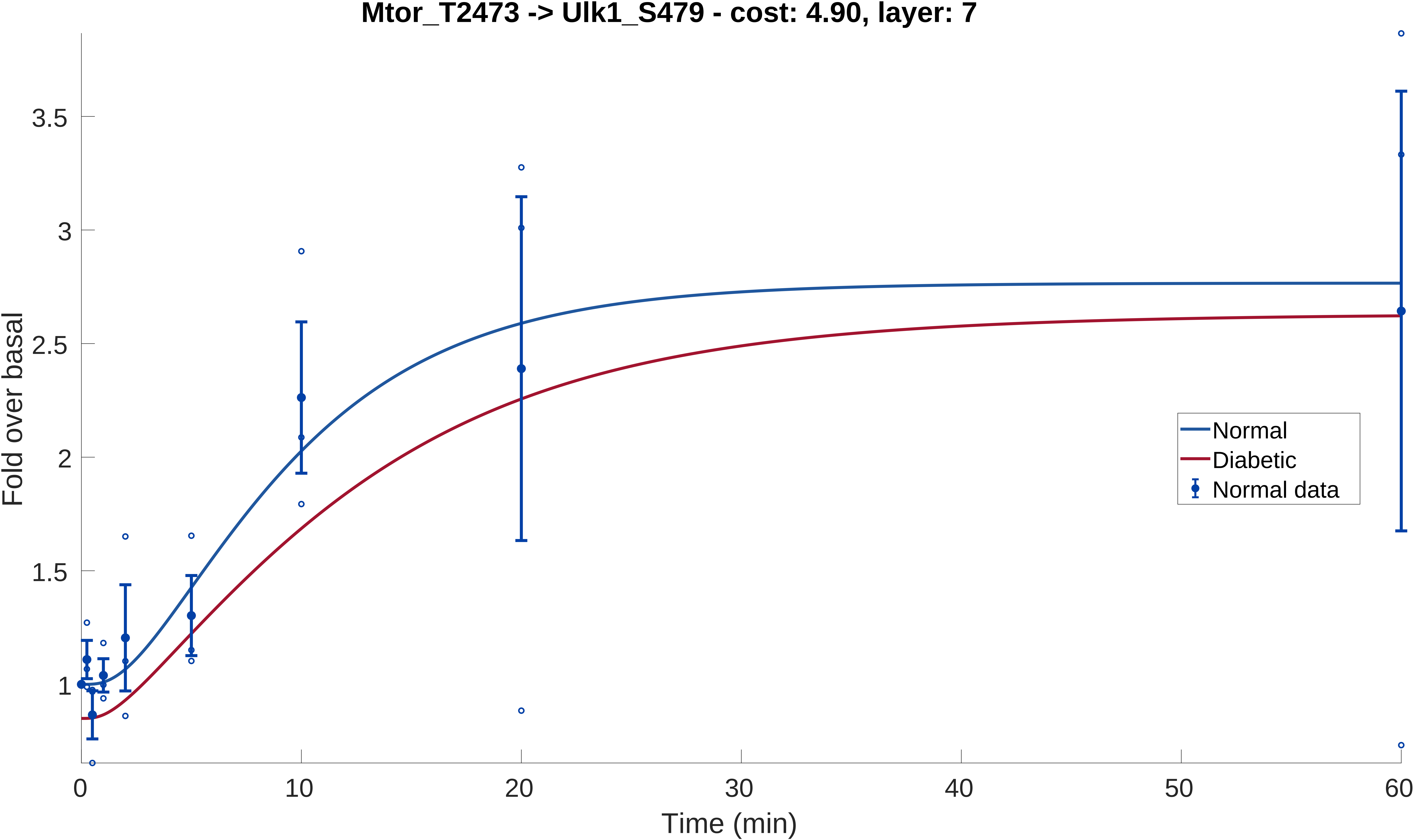

Mtor_T2473 -> Ulk1_S479 - cost: 4.90, layer: 7
Fold over basal
3.5
3
2.5
2
1.5
1
0
10
20
30
40
50
60
Time (min)
Normal
Diabetic
Normal data

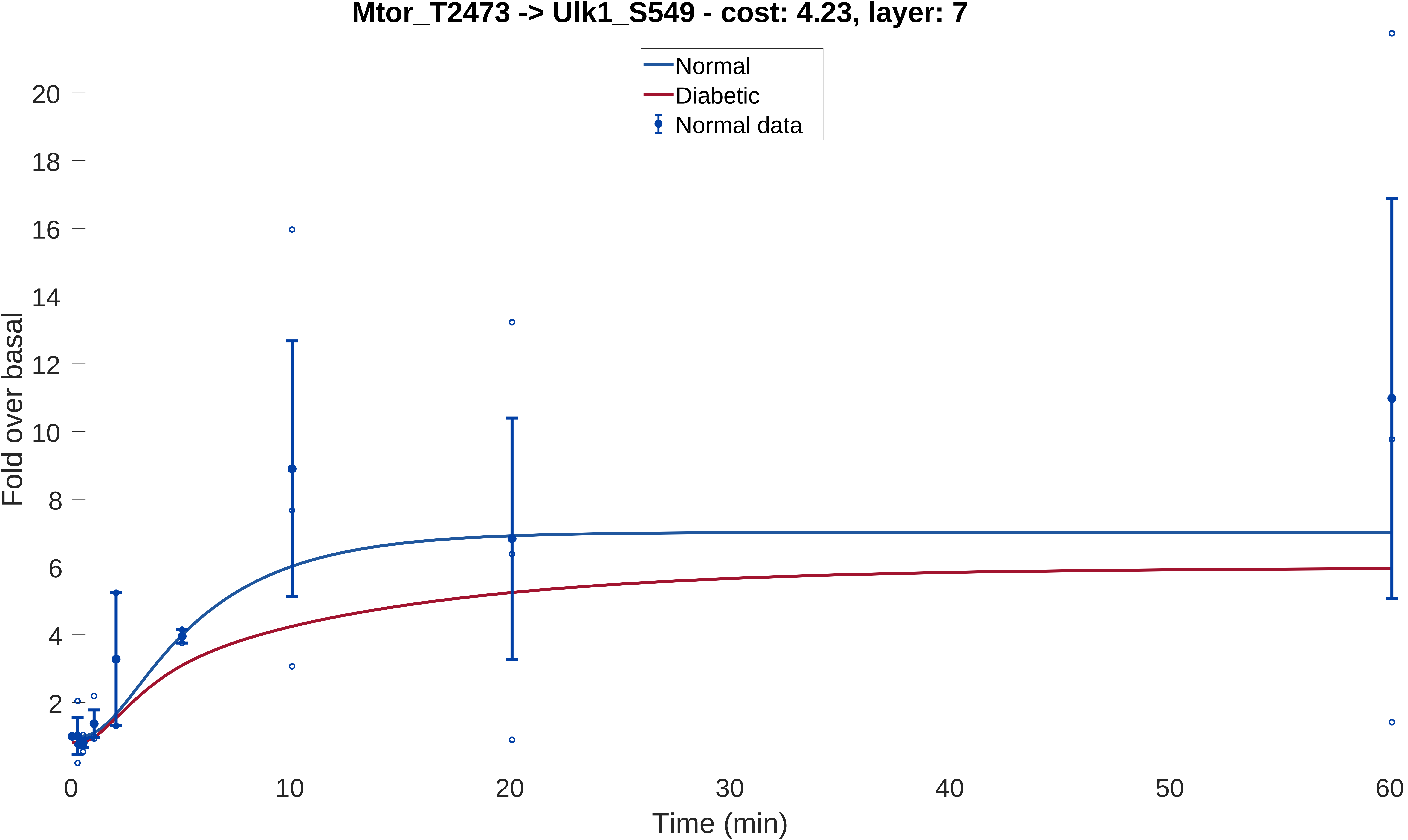

Mtor_T2473 -> Ulk1_S549 - cost: 4.23, layer: 7
Normal
Diabetic
Normal data
Fold over basal
2
4
6
8
10
12
14
16
18
20
0
10
20
30
40
50
60
Time (min)

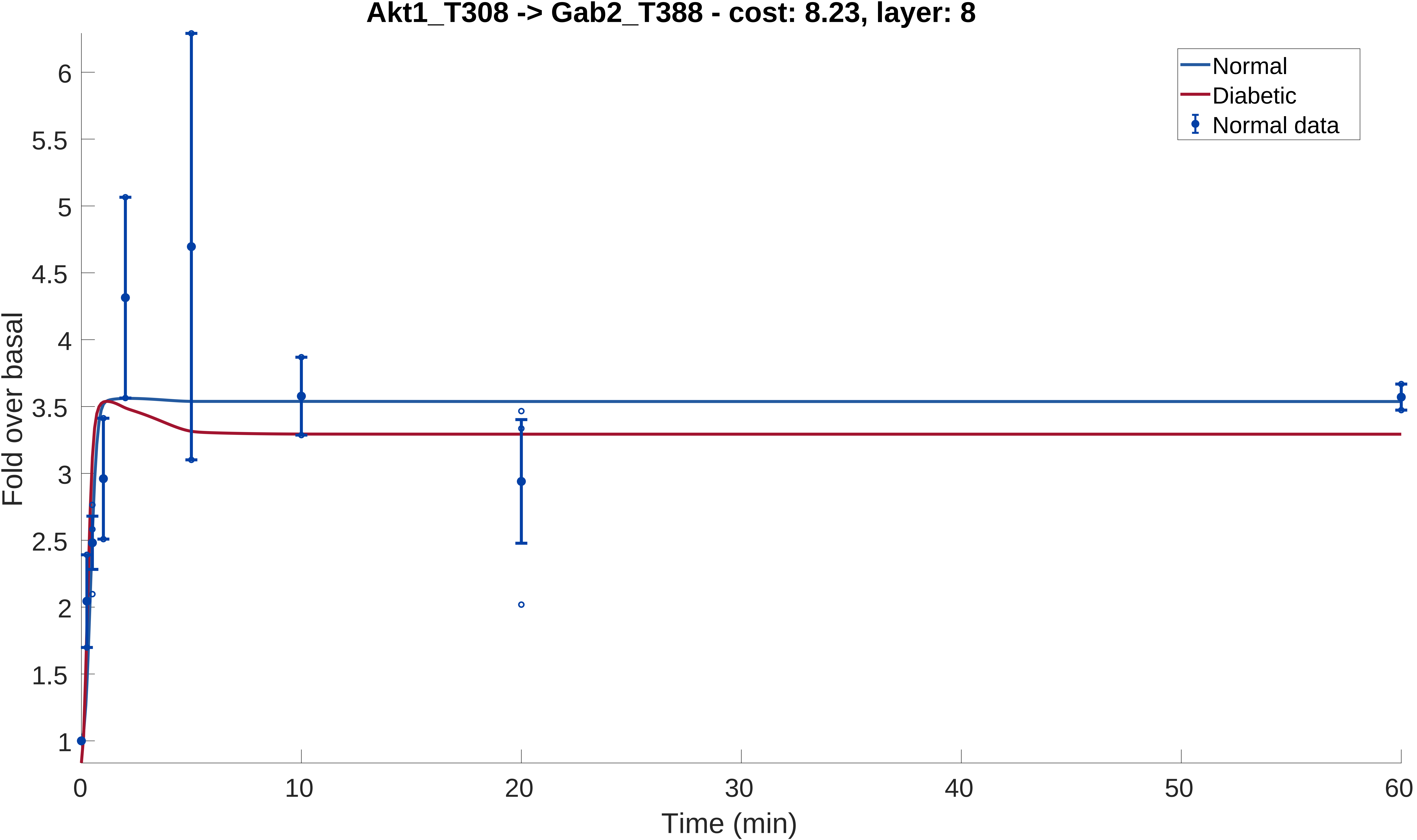
Akt1_T308 -> Gab2_T388 - cost: 8.23, layer: 8
Normal
Diabetic
Normal data
Fold over basal
Time (min)
1
1.5
2
2.5
3
3.5
4
4.5
5
5.5
6
0
10
20
30
40
50
60

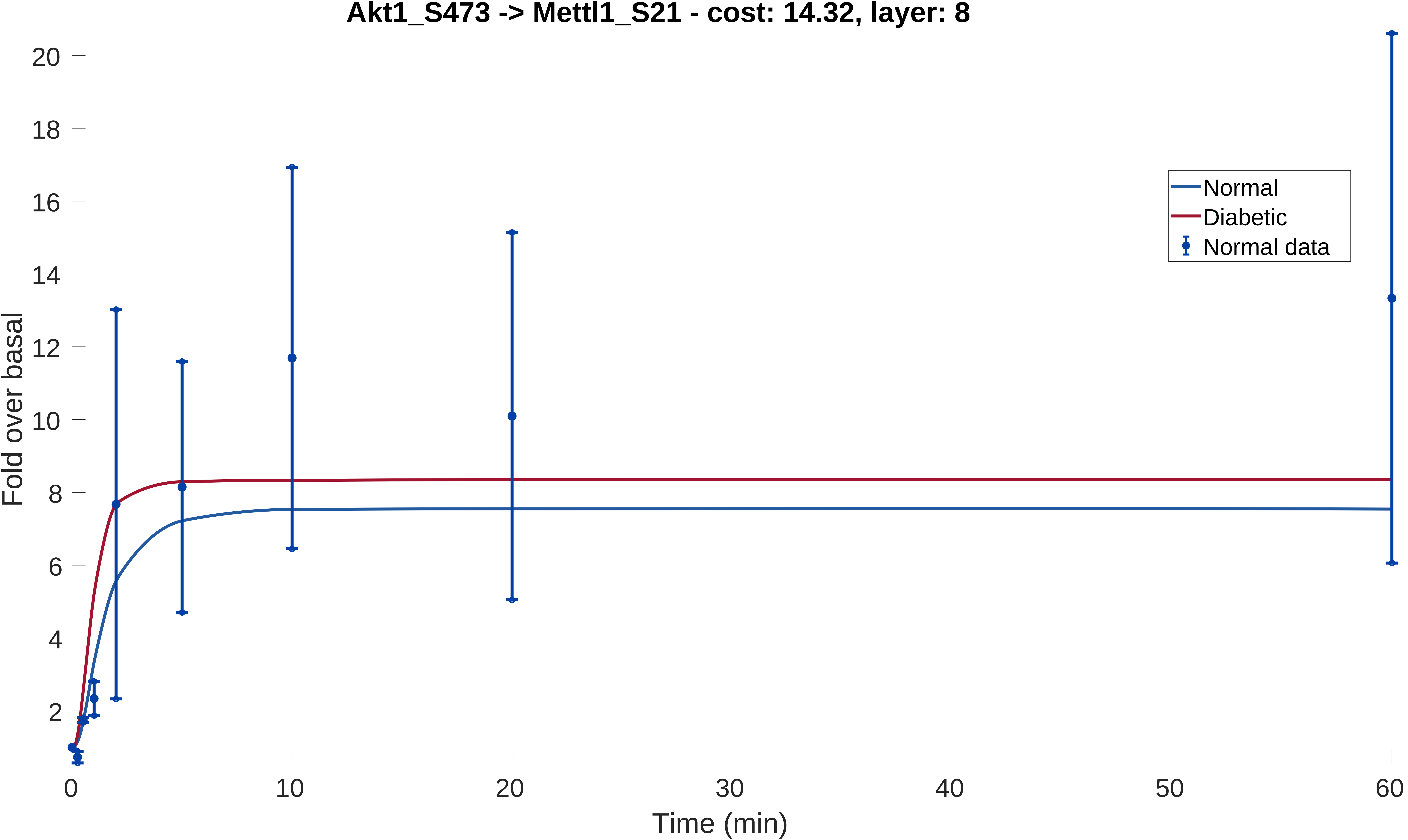
Akt1_S473 -> Mettl1_S21 - cost: 14.32, layer: 8
Fold over basal
Time (min)
Normal
Diabetic
Normal data
0
10
20
30
40
50
60
2
4
6
8
10
12
14
16
18
20

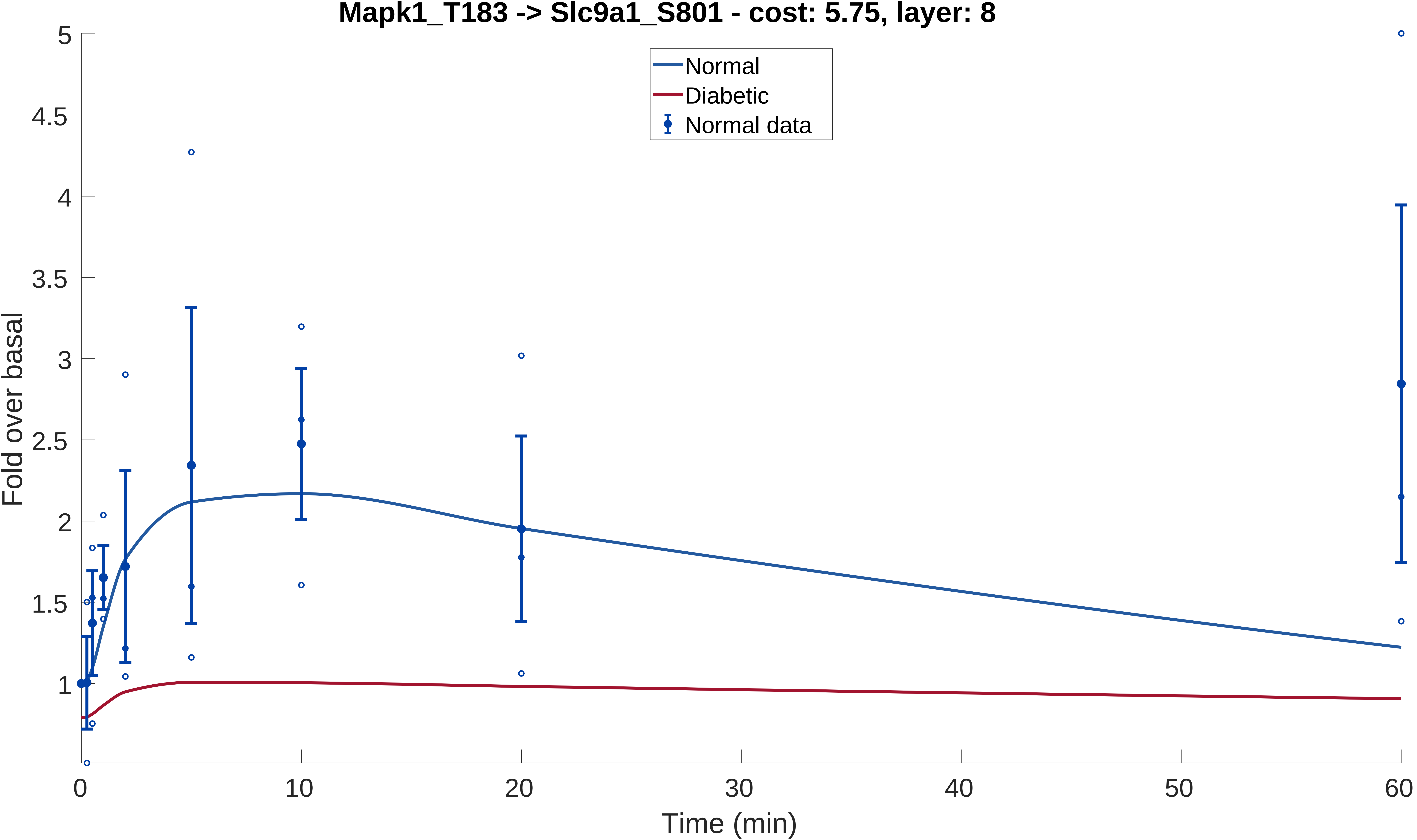
Mapk1_T183 -> Slc9a1_S801 - cost: 5.75, layer: 8
Normal
Diabetic
Normal data
Fold over basal
Time (min)
5
4.5
4
3.5
3
2.5
2
1.5
1
0
10
20
30
40
50
60

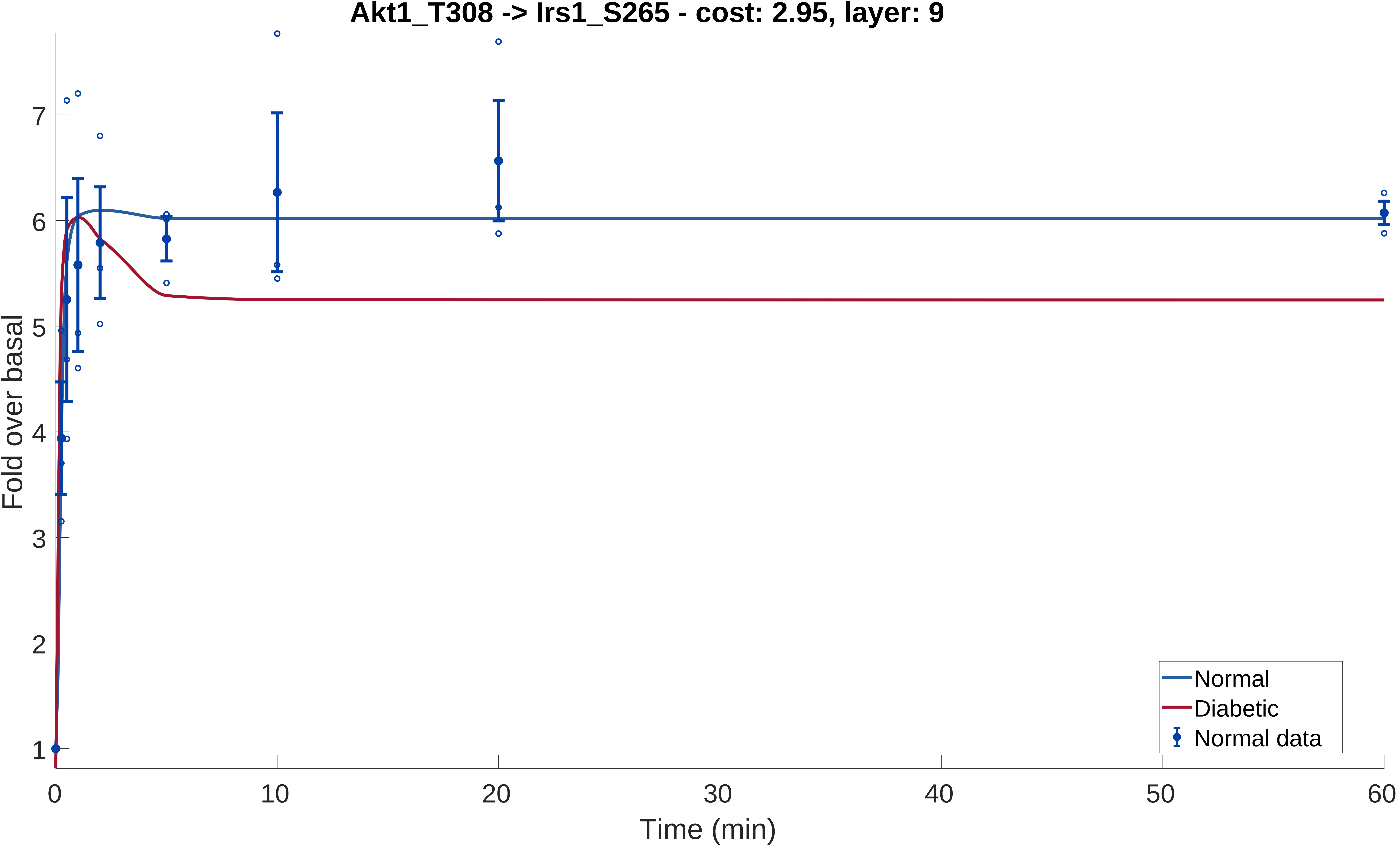

Akt1_T308 -> Irs1_S265 - cost: 2.95, layer: 9
Fold over basal
Time (min)
1
2
3
4
5
6
7
0
10
20
30
40
50
60
Normal
Diabetic
Normal data

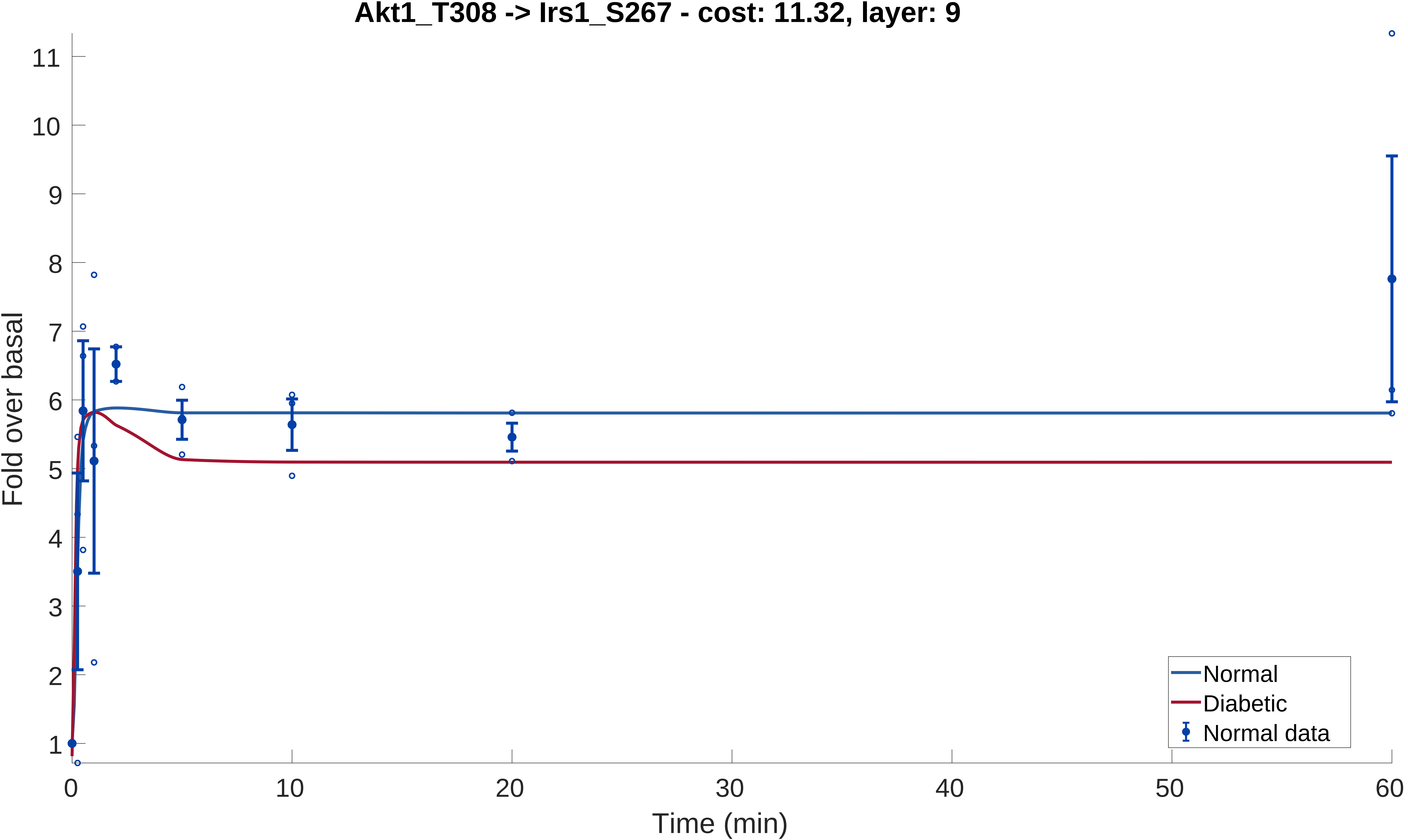
Akt1_T308 -> Irs1_S267 - cost: 11.32, layer: 9
Fold over basal
Time (min)
Normal
Diabetic
Normal data
0
10
20
30
40
50
60
1
2
3
4
5
6
7
8
9
10
11

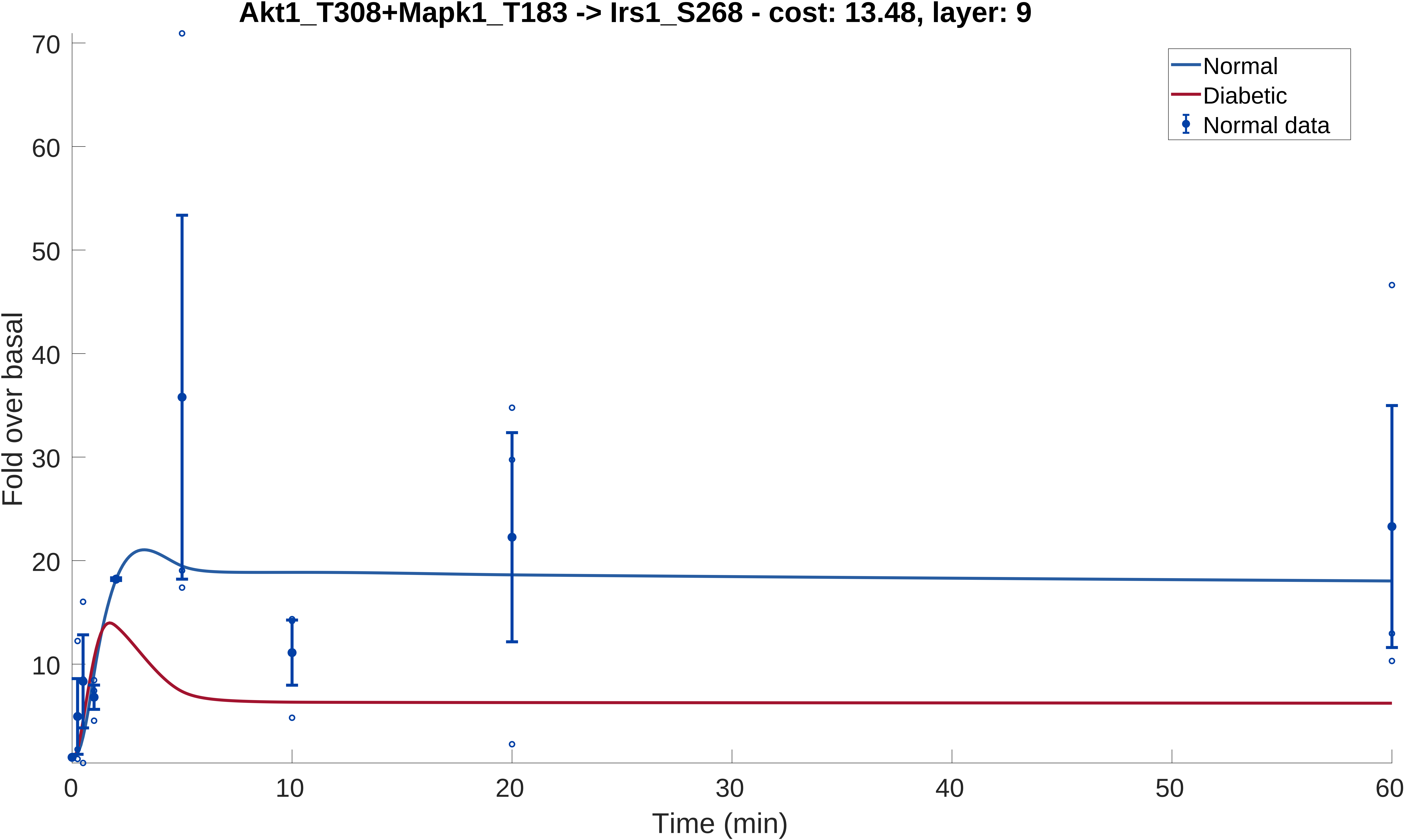
Akt1_T308+Mapk1_T183 -> Irs1_S268 - cost: 13.48, layer: 9
Fold over basal
Time (min)
Normal
Diabetic
Normal data
0
10
20
30
40
50
60
70

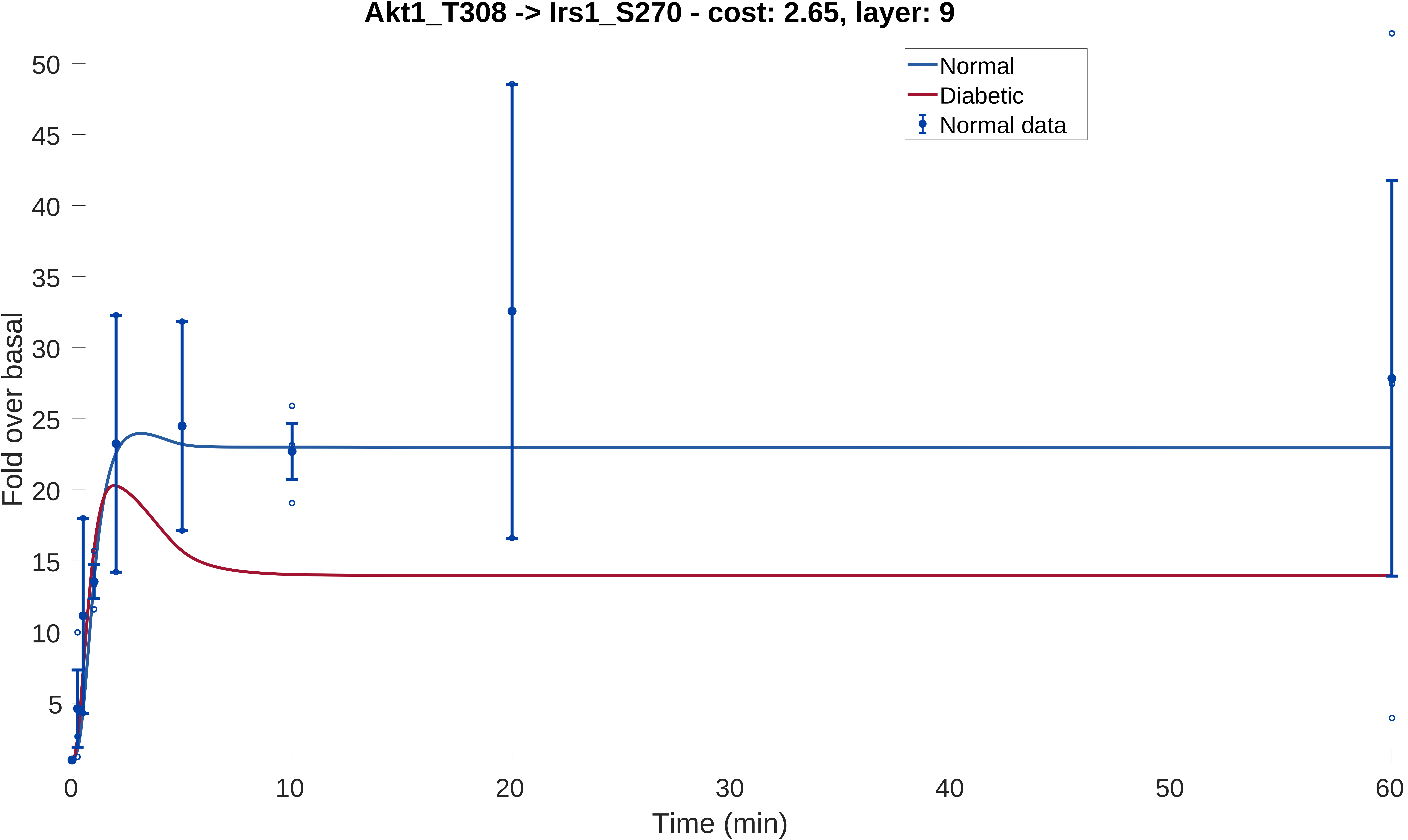

Akt1_T308 -> Irs1_S270 - cost: 2.65, layer: 9
Normal
Diabetic
Normal data
Fold over basal
Time (min)
0
10
20
30
40
50
60
5
10
15
20
25
30
35
40
45
50

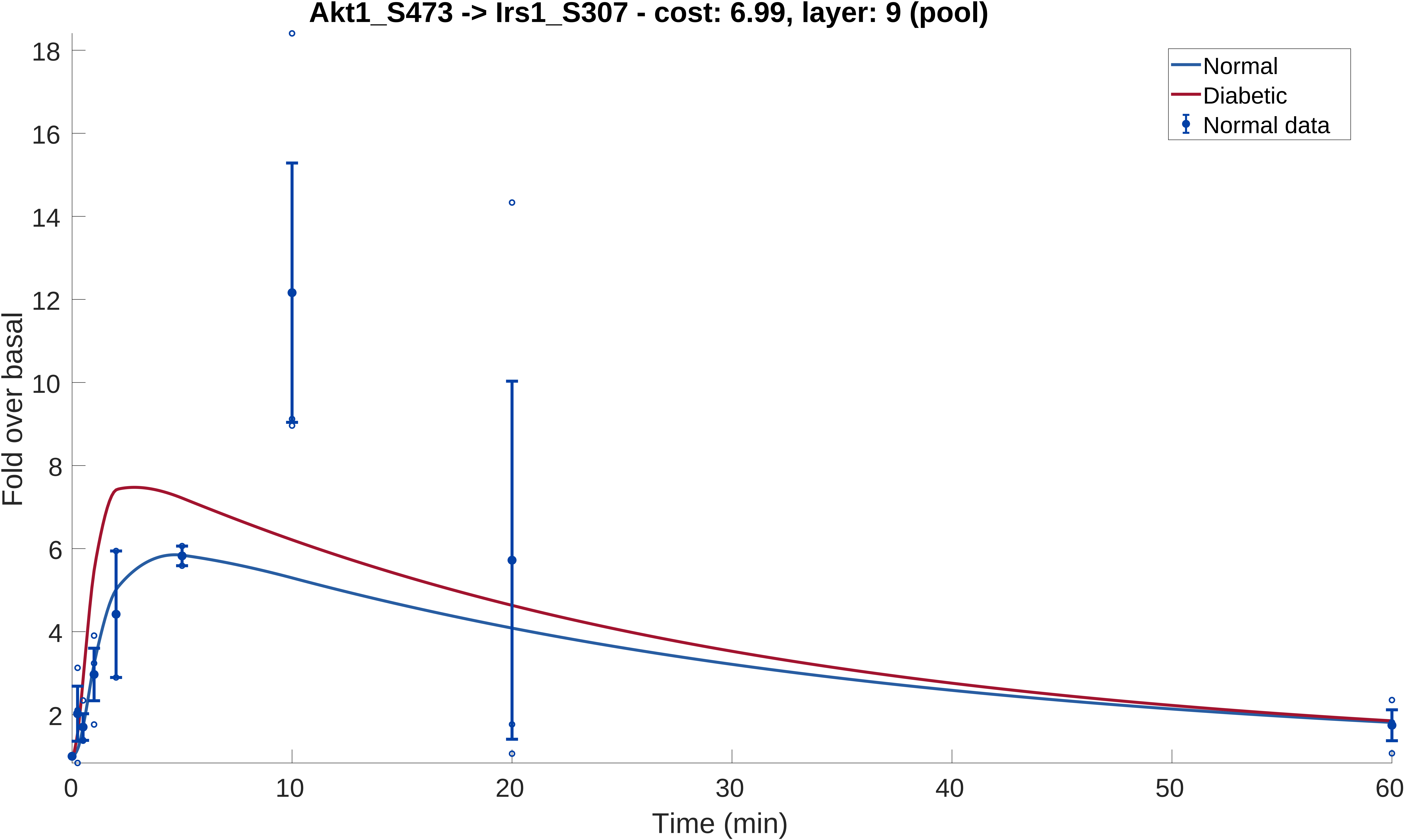

Akt1_S473 -> Irs1_S307 - cost: 6.99, layer: 9 (pool)
Normal
Diabetic
Normal data
Fold over basal
Time (min)
0
10
20
30
40
50
60
2
4
6
8
10
12
14
16
18

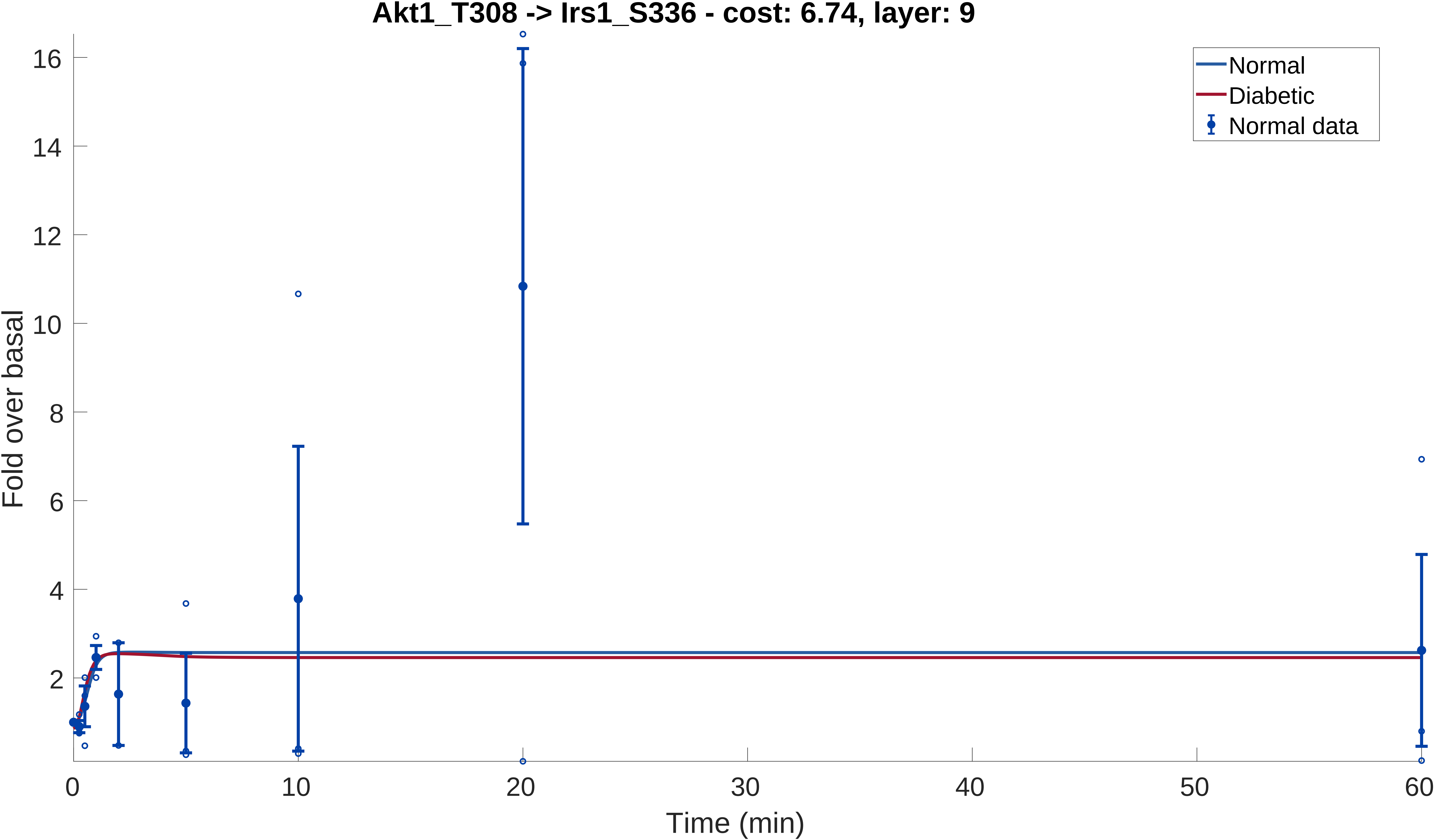

Akt1_T308 -> Irs1_S336 - cost: 6.74, layer: 9
Normal
Diabetic
Normal data
Fold over basal
Time (min)
0
10
20
30
40
50
60
2
4
6
8
10
12
14
16

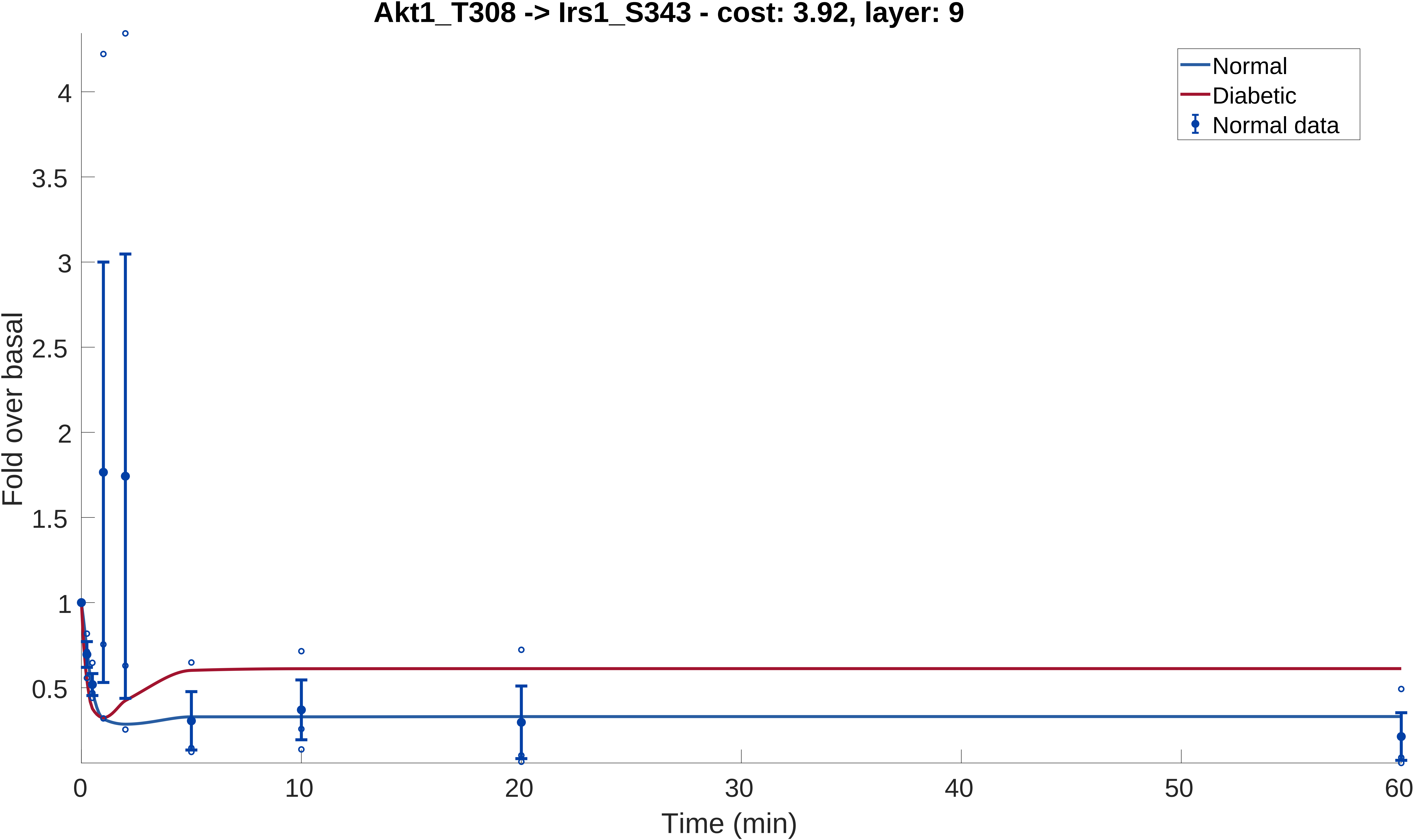

Akt1_T308 -> Irs1_S343 - cost: 3.92, layer: 9
Normal
Diabetic
Normal data
Fold over basal
4
3.5
3
2.5
2
1.5
1
0.5
0
10
20
30
40
50
60
Time (min)

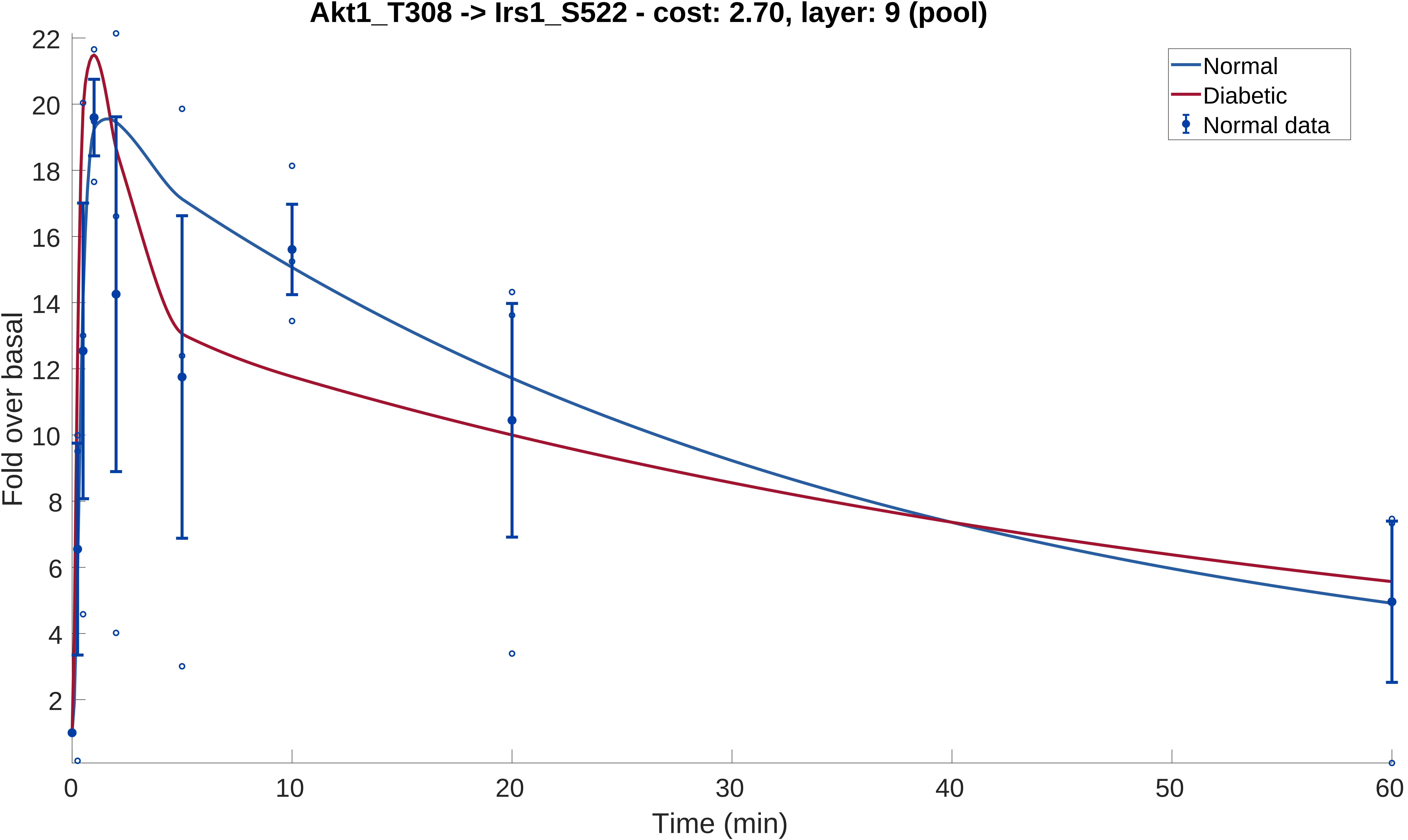
Akt1_T308 -> Irs1_S522 - cost: 2.70, layer: 9 (pool)
Normal
Diabetic
Normal data
Fold over basal
Time (min)
22
20
18
16
14
12
10
8
6
4
2
0
10
20
30
40
50
60

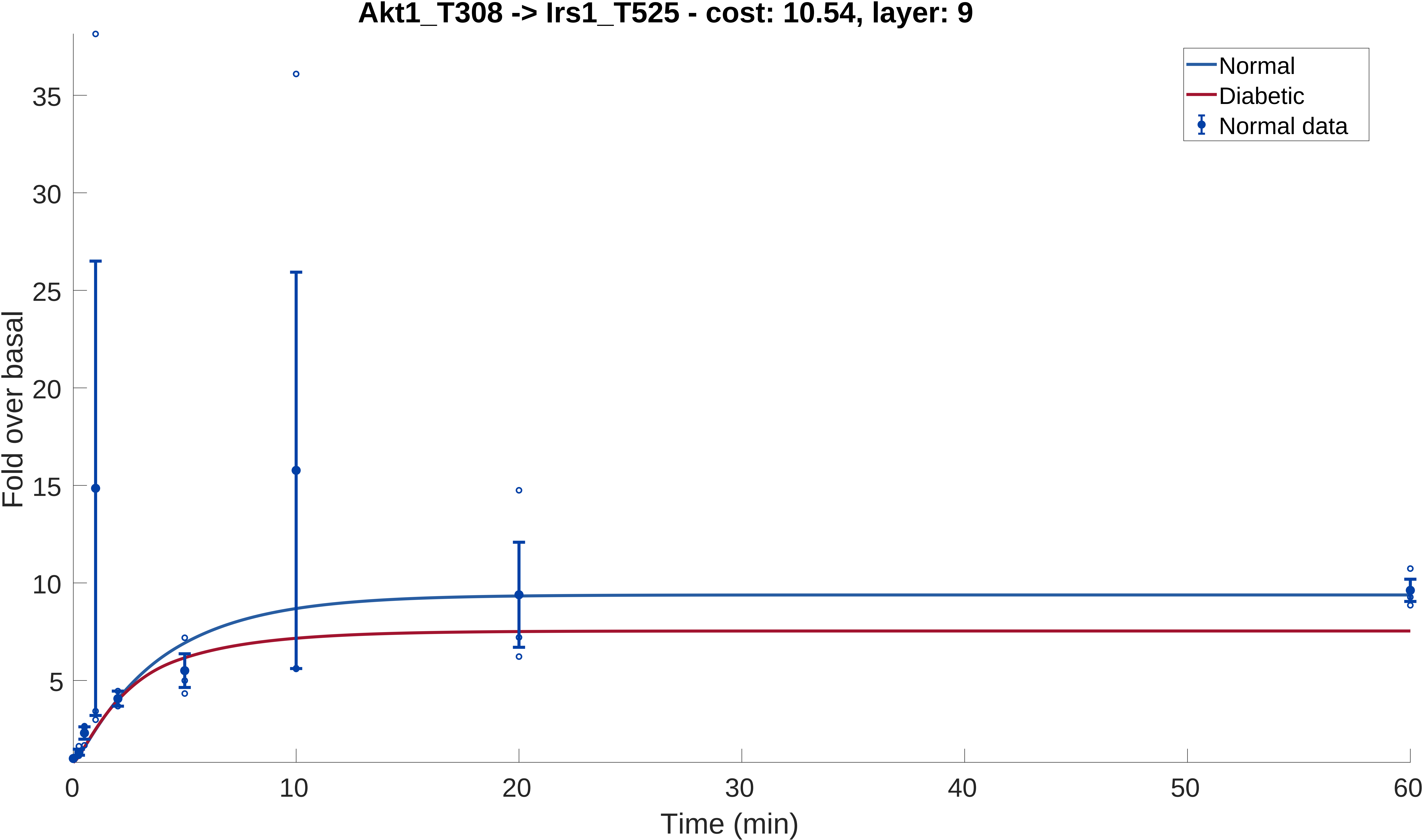

Akt1_T308 -> Irs1_T525 - cost: 10.54, layer: 9
Normal
Diabetic
Normal data
Fold over basal
Time (min)
35
30
25
20
15
10
5
0
10
20
30
40
50
60

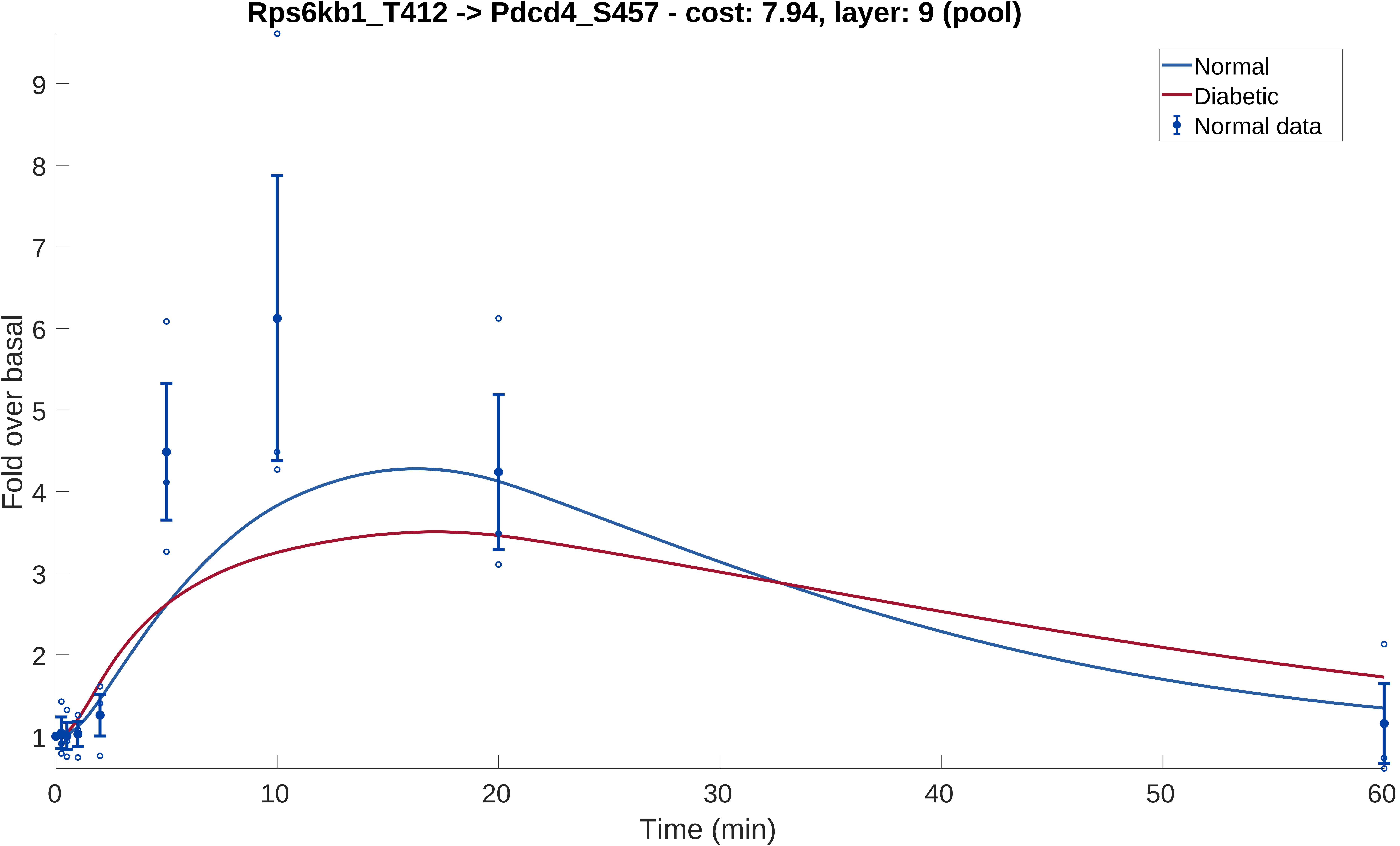

Rps6kb1_T412 -> Pdcd4_S457 - cost: 7.94, layer: 9 (pool)
Normal
Diabetic
Normal data
Fold over basal
Time (min)
1
2
3
4
5
6
7
8
9
0
10
20
30
40
50
60

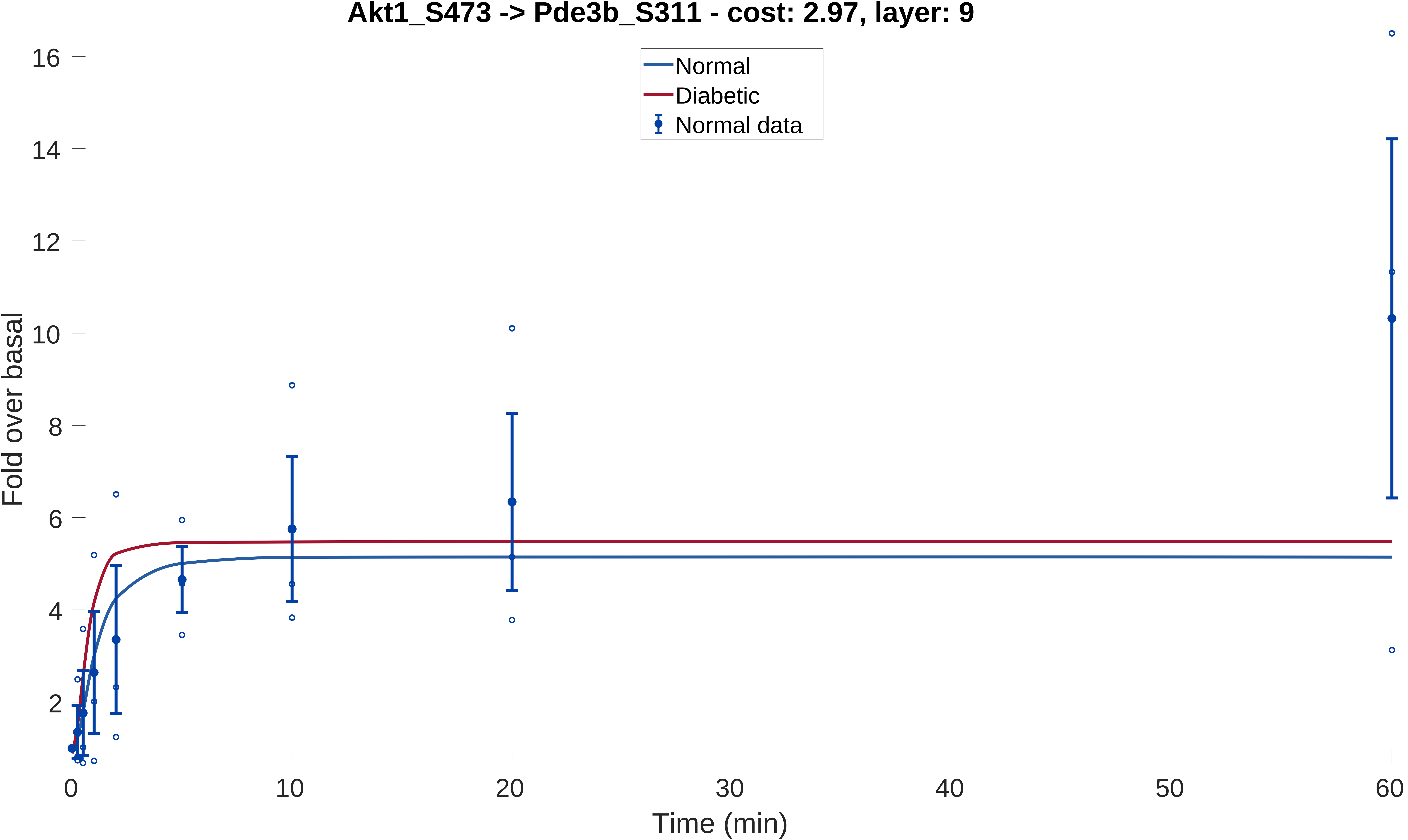
Akt1_S473 -> Pde3b_S311 - cost: 2.97, layer: 9
Normal
Diabetic
Normal data
Fold over basal
Time (min)
0
10
20
30
40
50
60
2
4
6
8
10
12
14
16

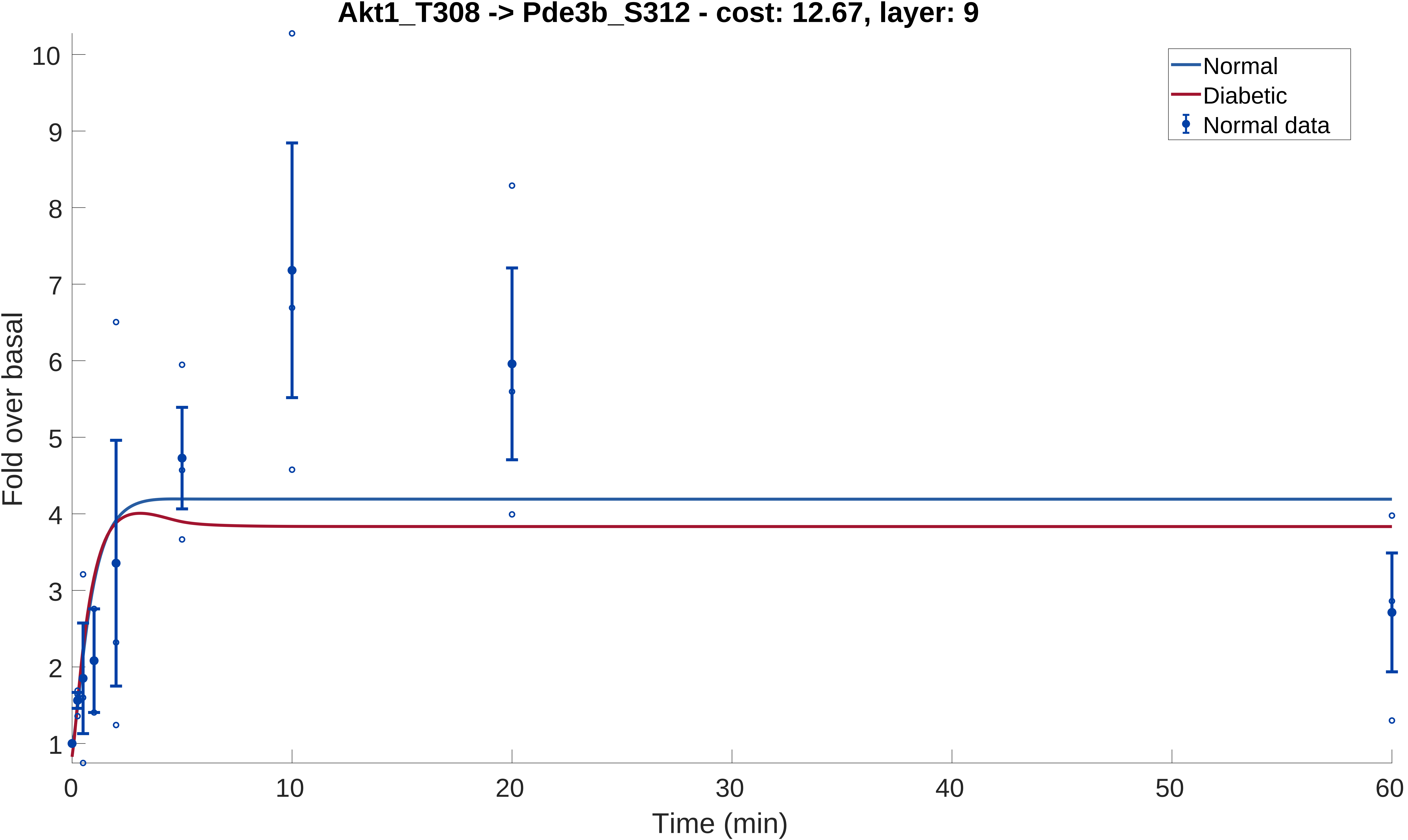

Akt1_T308 -> Pde3b_S312 - cost: 12.67, layer: 9
Normal
Diabetic
Normal data
Fold over basal
Time (min)
1
2
3
4
5
6
7
8
9
10
0
10
20
30
40
50
60

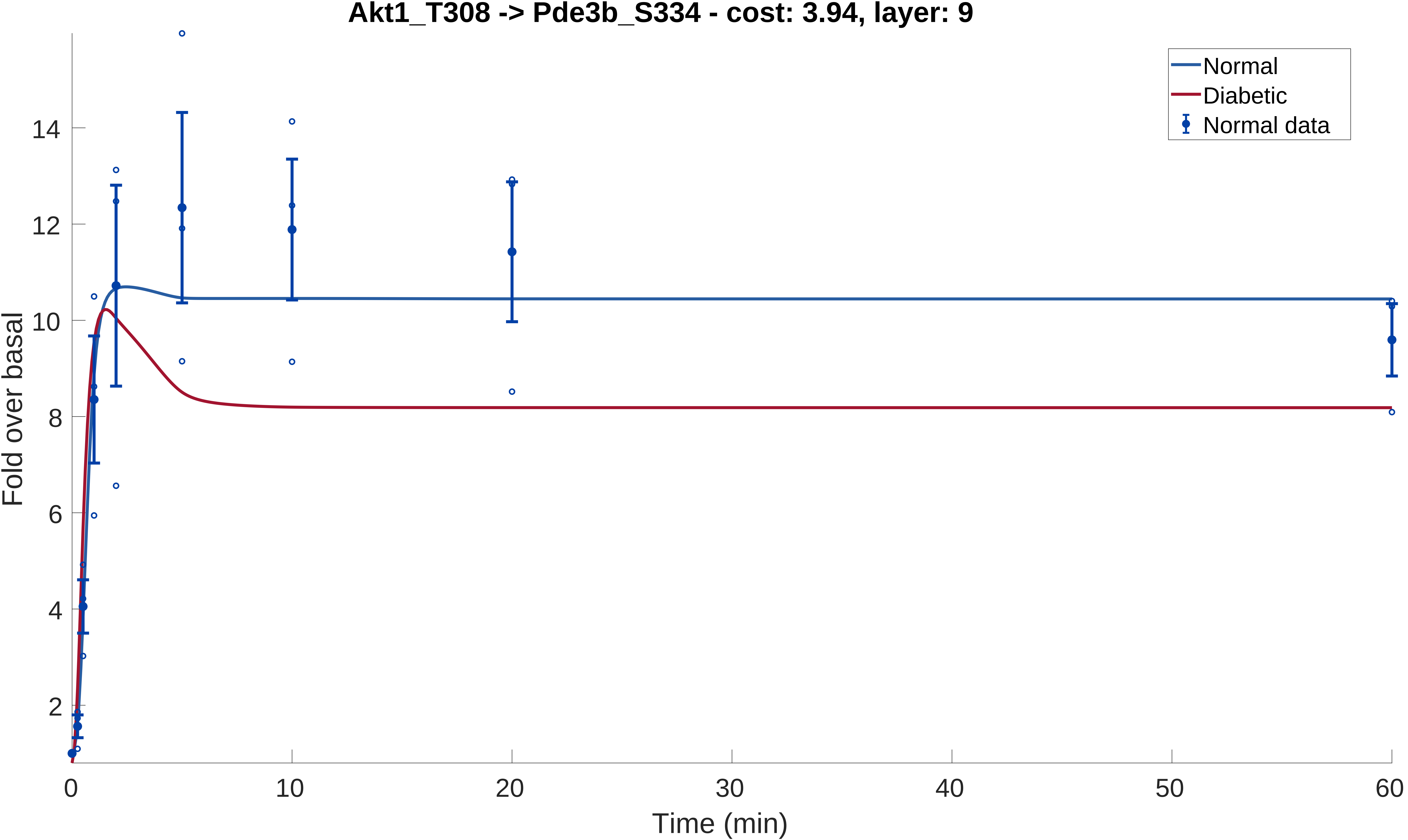

Akt1_T308 -> Pde3b_S334 - cost: 3.94, layer: 9
Normal
Diabetic
Normal data
Fold over basal
Time (min)
2
4
6
8
10
12
14
0
10
20
30
40
50
60

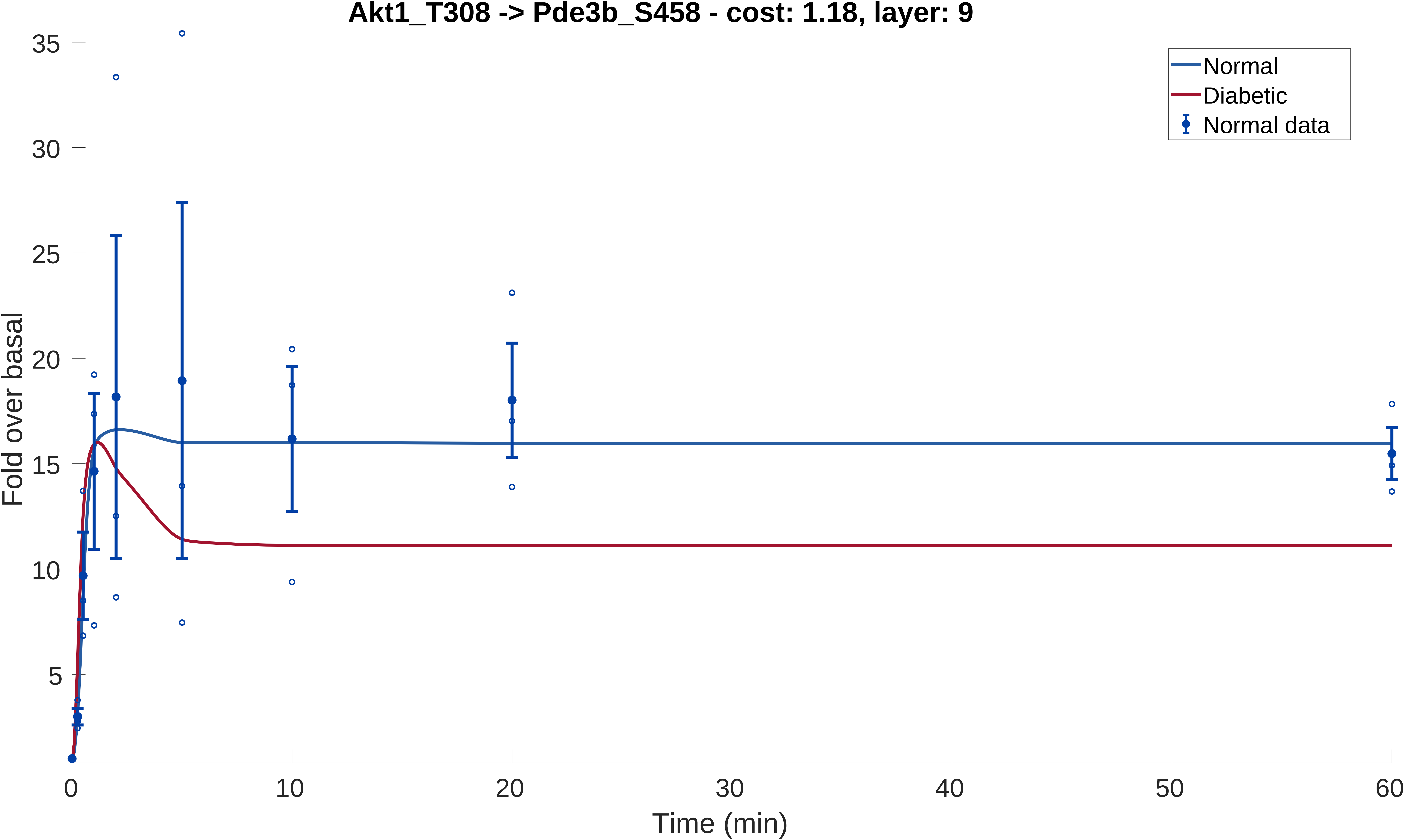

Akt1_T308 -> Pde3b_S458 - cost: 1.18, layer: 9
Normal
Diabetic
Normal data
Fold over basal
Time (min)
0
10
20
30
40
50
60
5
10
15
20
25
30
35

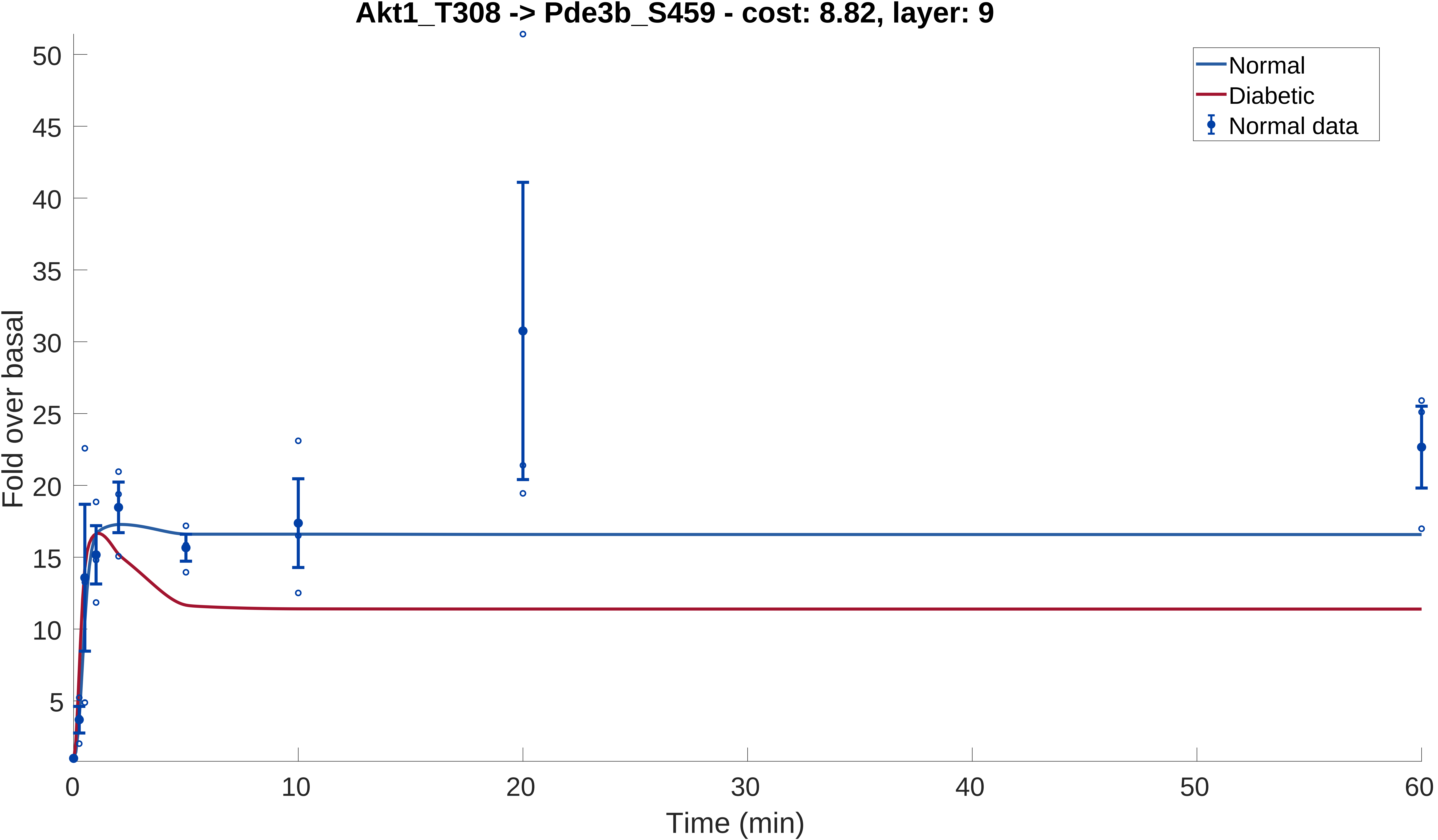

Akt1_T308 -> Pde3b_S459 - cost: 8.82, layer: 9
Normal
Diabetic
Normal data
Fold over basal
Time (min)
0
10
20
30
40
50
60
5
10
15
20
25
30
35
40
45
50

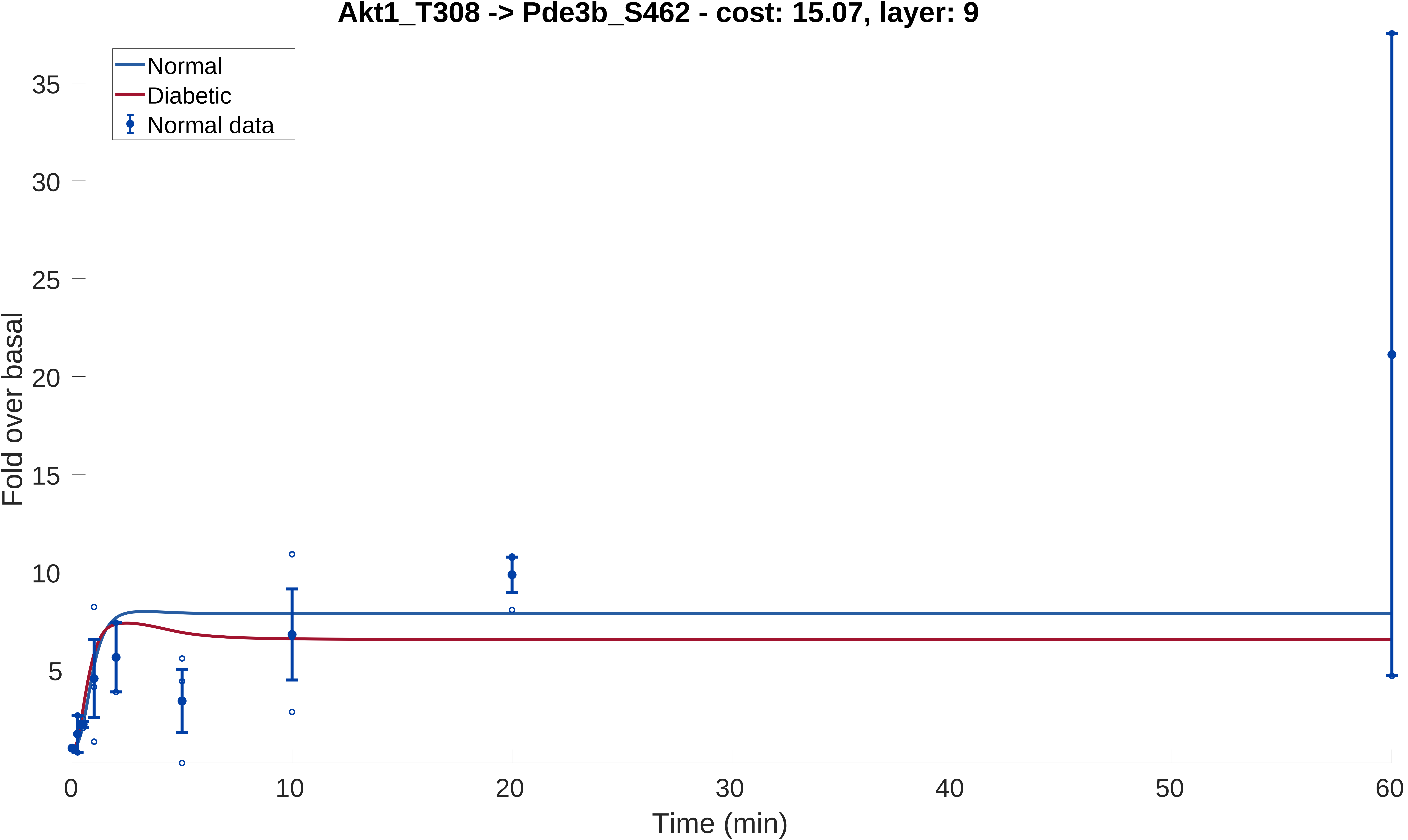
Akt1_T308 -> Pde3b_S462 - cost: 15.07, layer: 9
Normal
Diabetic
Normal data
Fold over basal
Time (min)
0
10
20
30
40
50
60
5
10
15
20
25
30
35

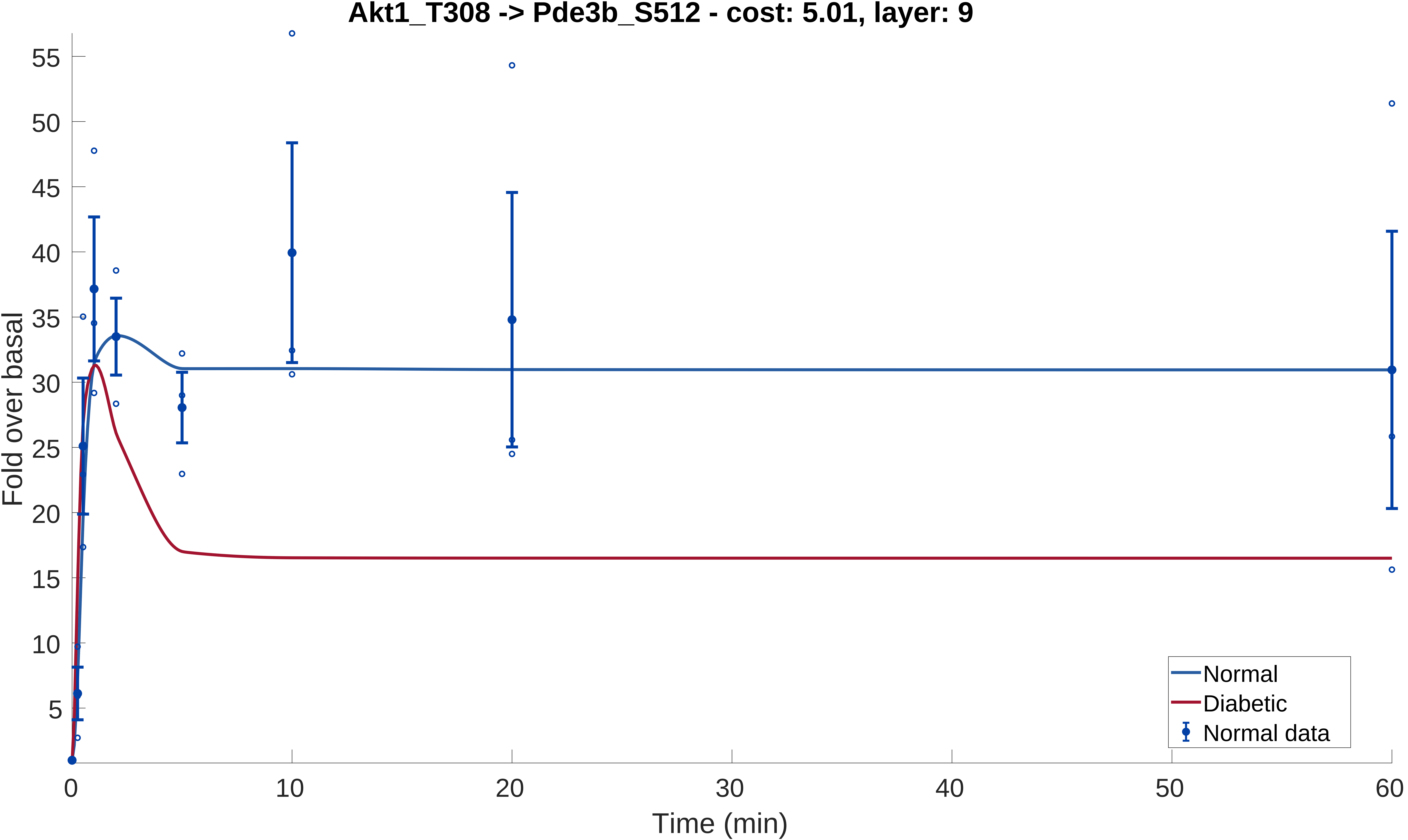

Akt1_T308 -> Pde3b_S512 - cost: 5.01, layer: 9
Fold over basal
55
50
45
40
35
30
25
20
15
10
5
0
10
20
30
40
50
60
Time (min)
Normal
Diabetic
Normal data

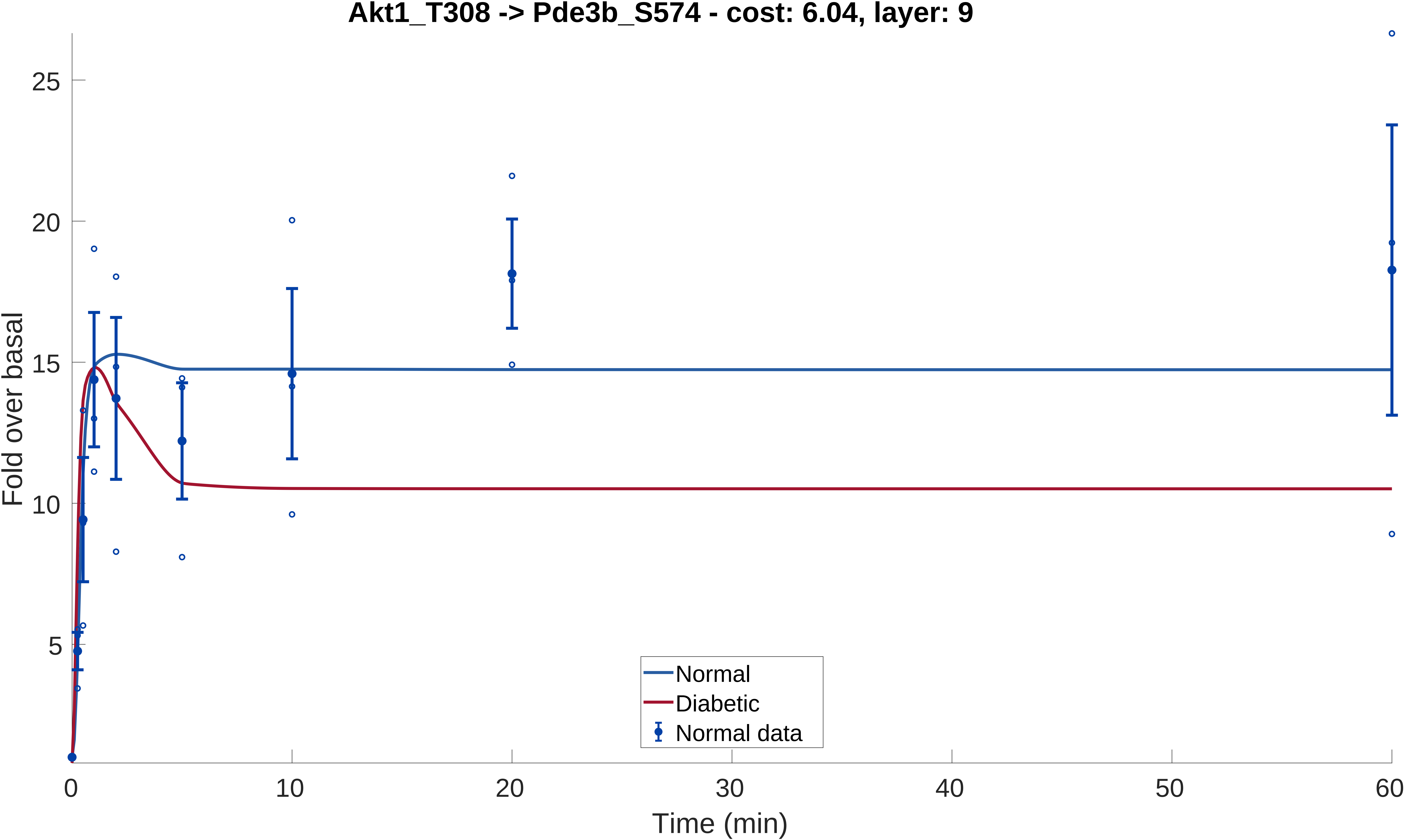
Akt1_T308 -> Pde3b_S574 - cost: 6.04, layer: 9
Fold over basal
25
20
15
10
5
0
10
20
30
40
50
60
Time (min)
Normal
Diabetic
Normal data

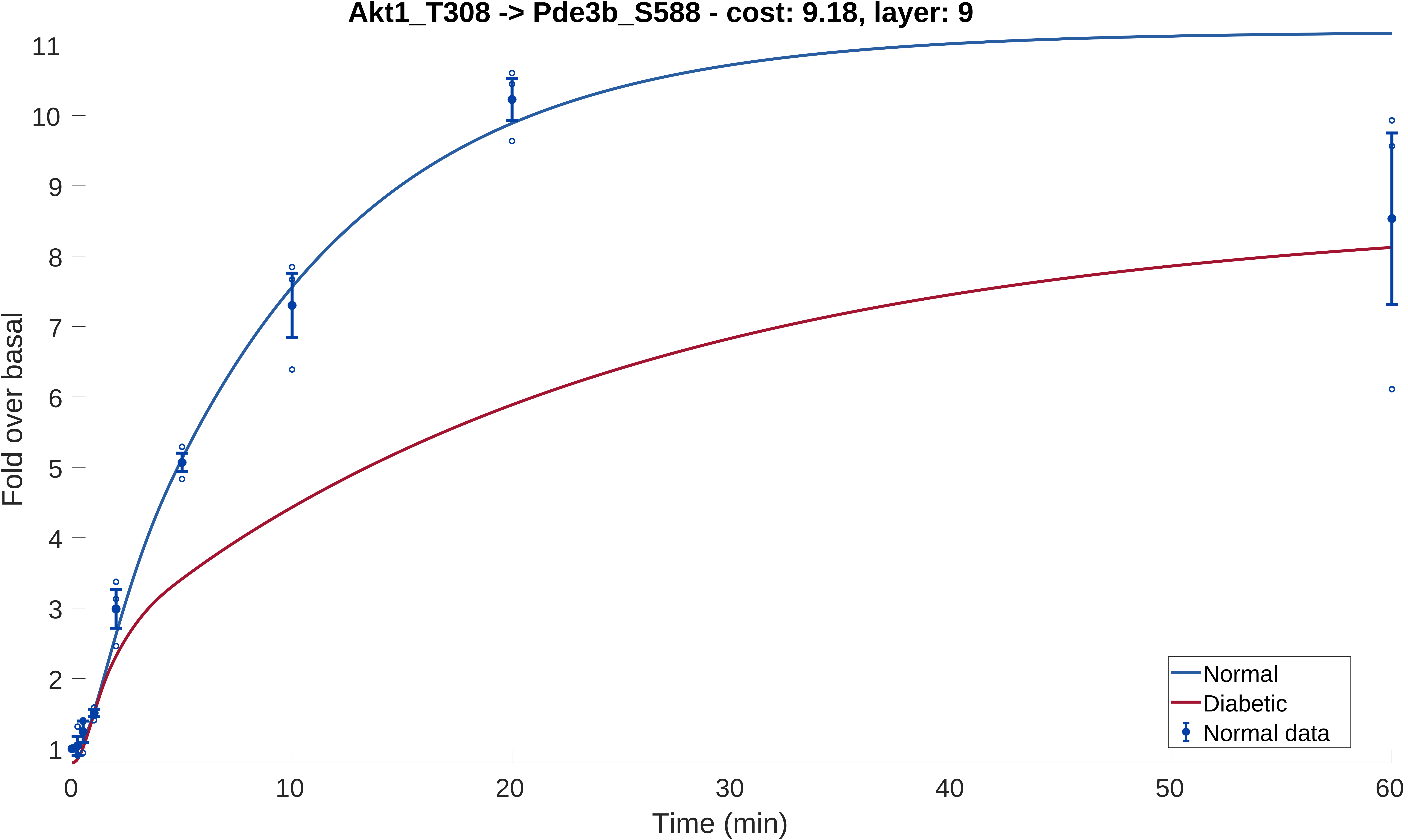

Akt1_T308 -> Pde3b_S588 - cost: 9.18, layer: 9
Fold over basal
Time (min)
0
10
20
30
40
50
60
1
2
3
4
5
6
7
8
9
10
11
Normal
Diabetic
Normal data

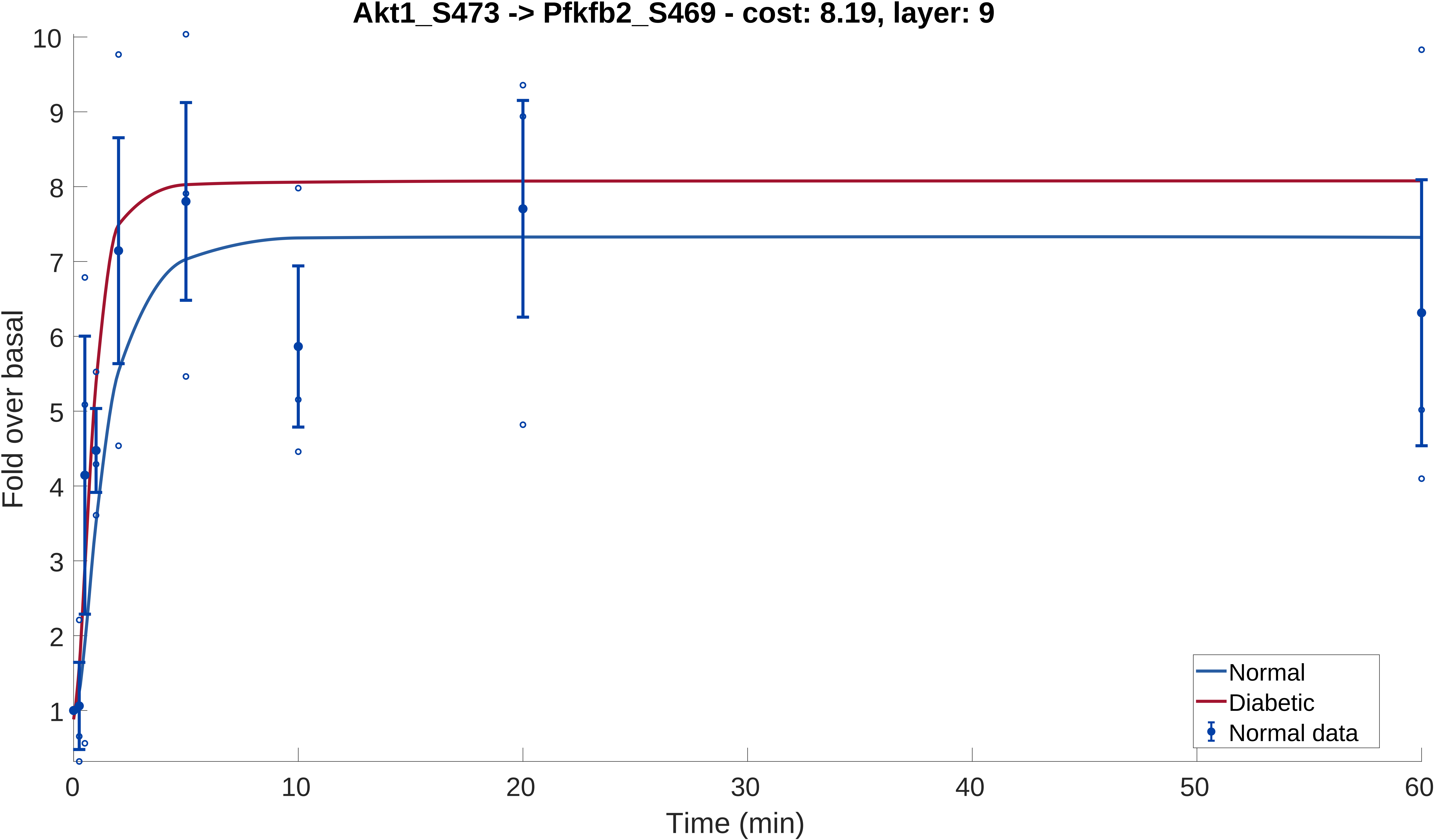

Akt1_S473 -> Pfkfb2_S469 - cost: 8.19, layer: 9
Fold over basal
Time (min)
0
10
20
30
40
50
60
1
2
3
4
5
6
7
8
9
10
Normal
Diabetic
Normal data

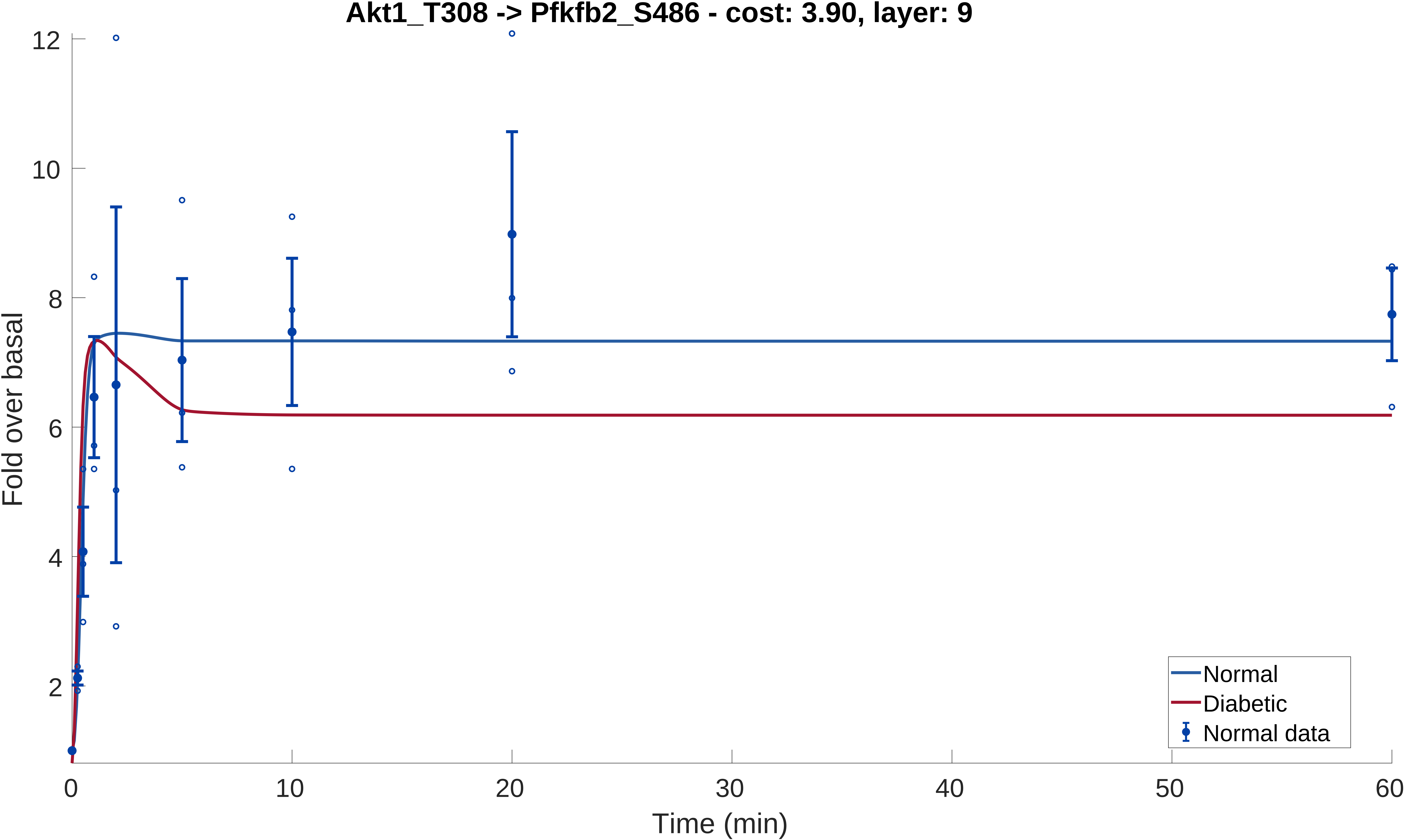
Akt1_T308 -> Pfkfb2_S486 - cost: 3.90, layer: 9
Fold over basal
Time (min)
Normal
Diabetic
Normal data
0
10
20
30
40
50
60
2
4
6
8
10
12

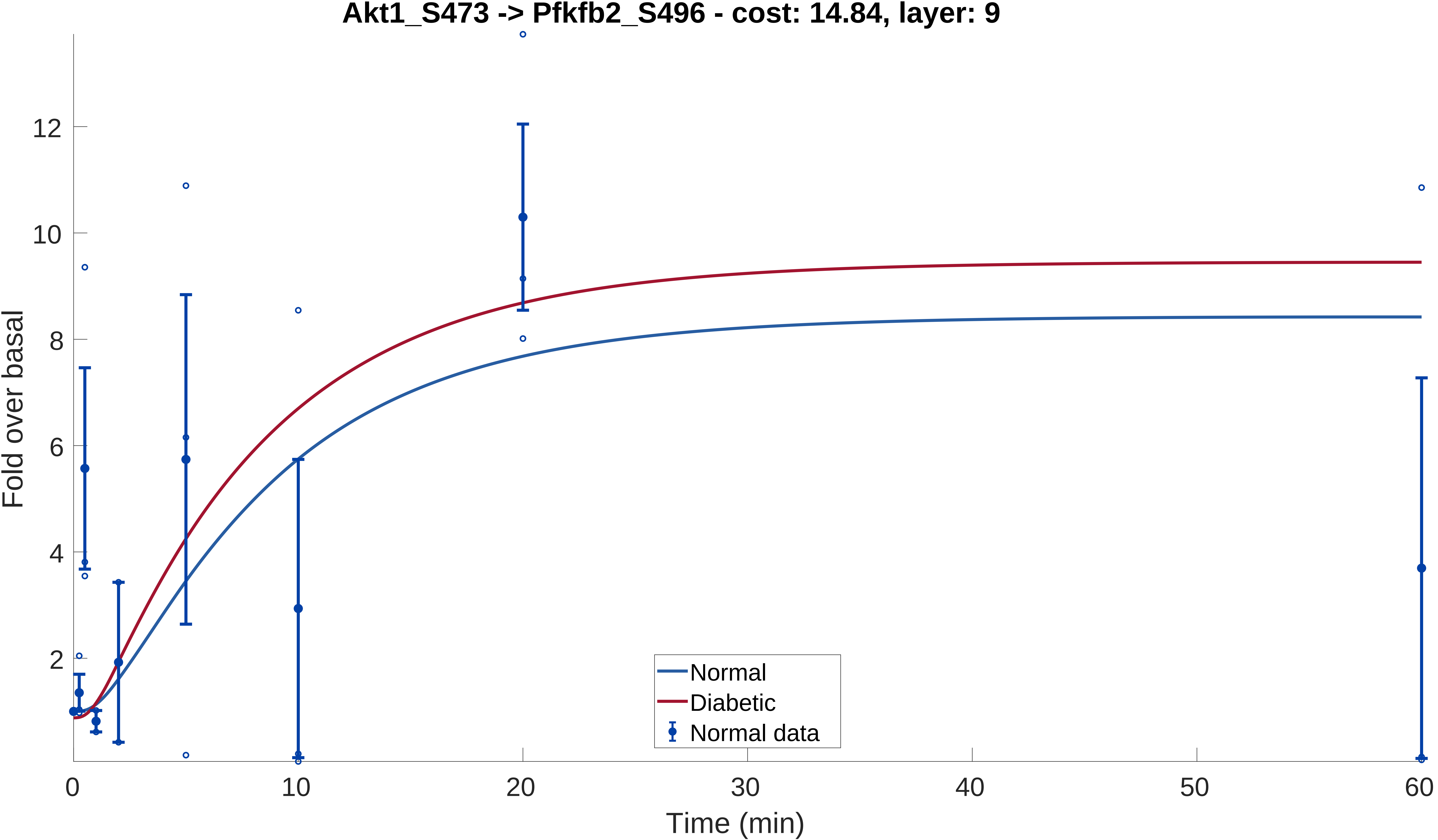

Akt1_S473 -> Pfkfb2_S496 - cost: 14.84, layer: 9
Fold over basal
12
10
8
6
4
2
0
10
20
30
40
50
60
Time (min)
Normal
Diabetic
Normal data

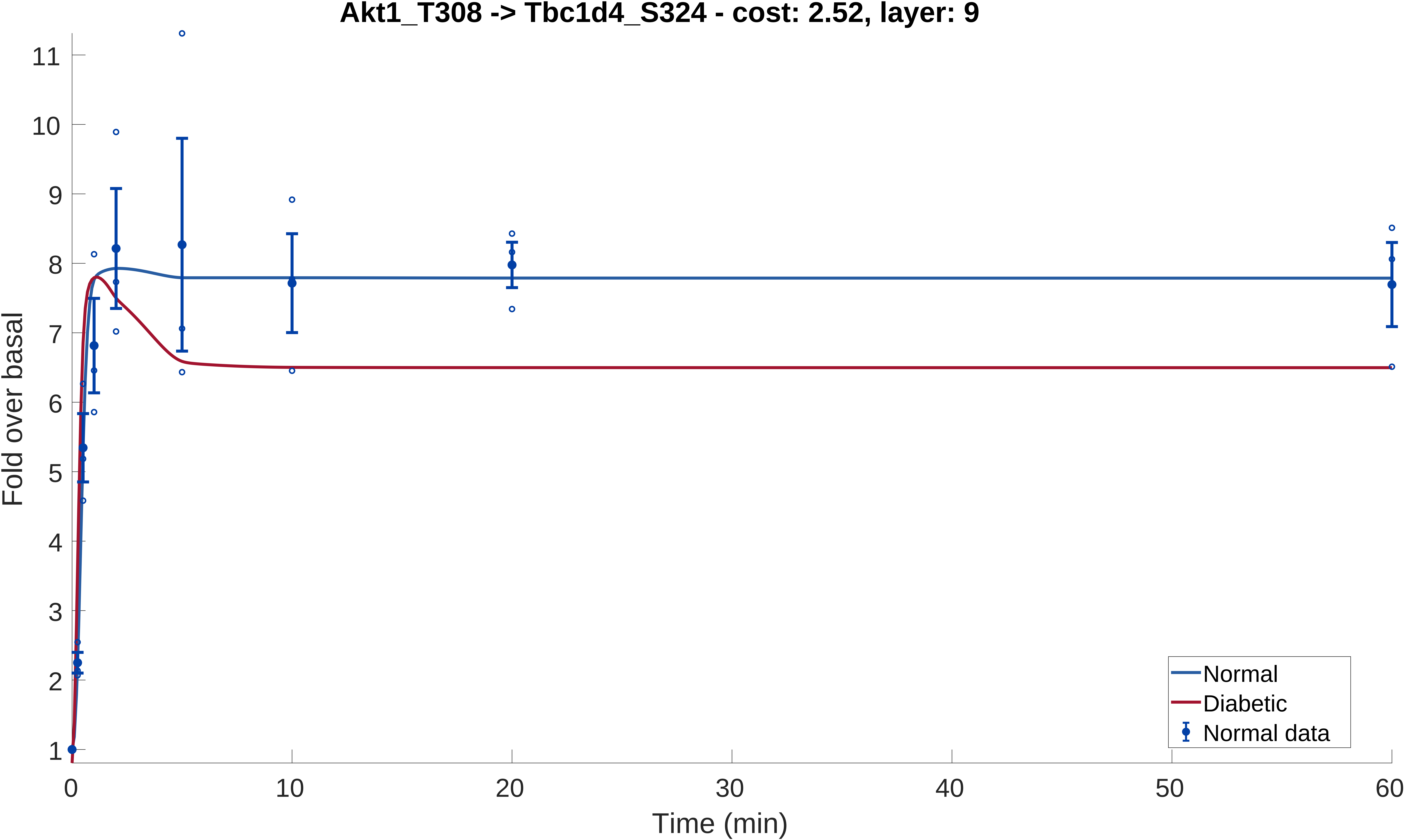

Akt1_T308 -> Tbc1d4_S324 - cost: 2.52, layer: 9
Fold over basal
Time (min)
Normal
Diabetic
Normal data
1
2
3
4
5
6
7
8
9
10
11
0
10
20
30
40
50
60

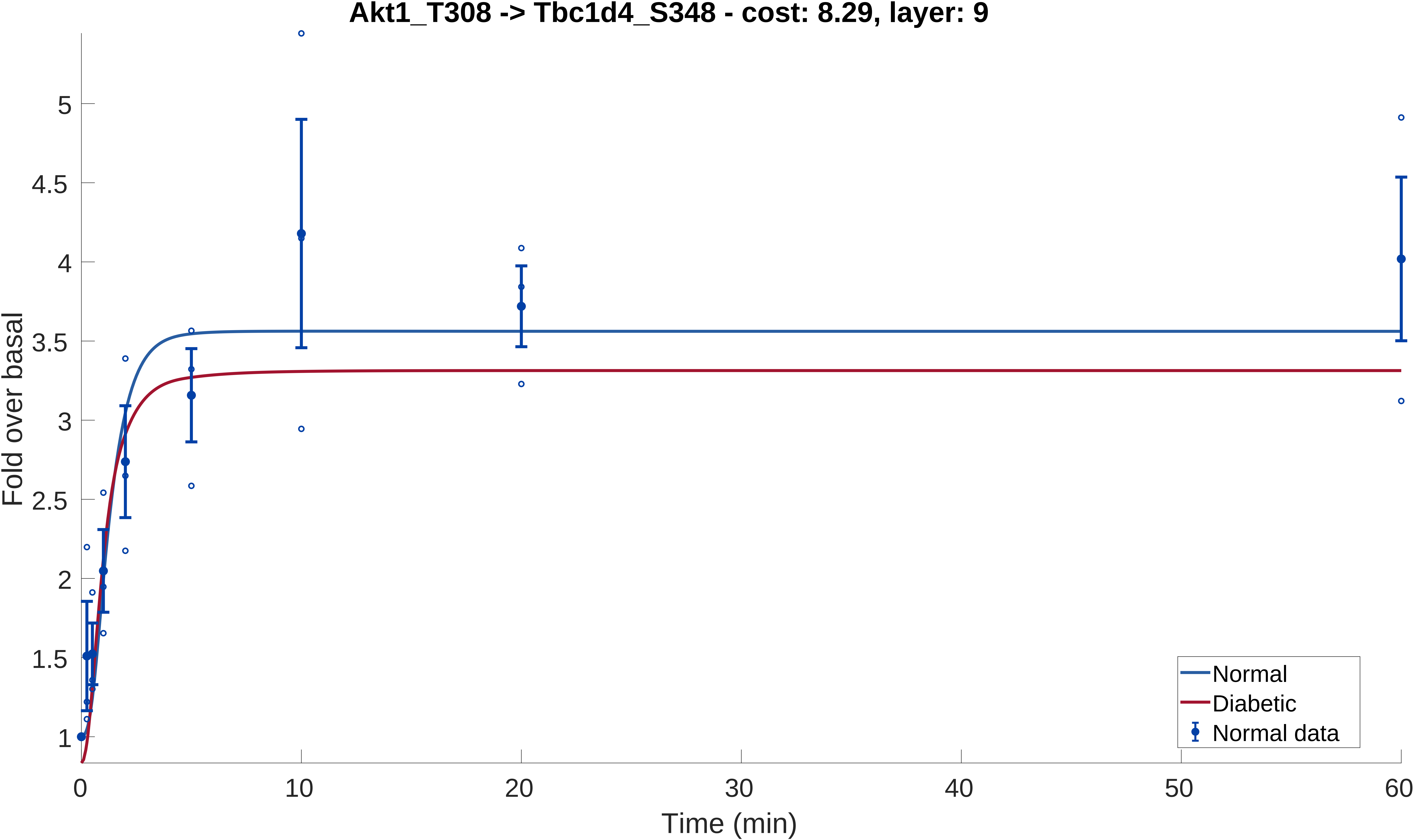

Akt1_T308 -> Tbc1d4_S348 - cost: 8.29, layer: 9
Fold over basal
1
1.5
2
2.5
3
3.5
4
4.5
5
Time (min)
0
10
20
30
40
50
60
Normal
Diabetic
Normal data

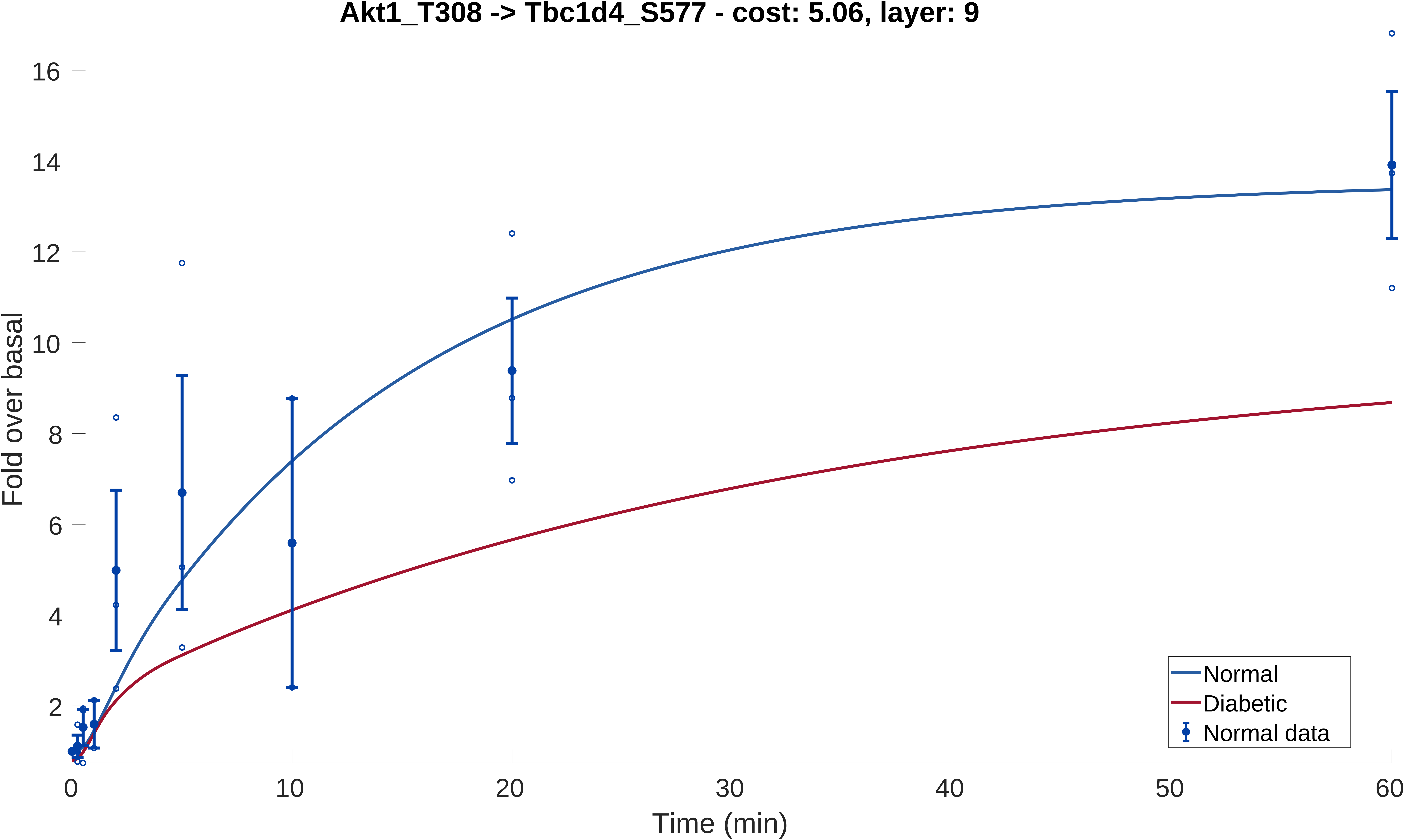

Akt1_T308 -> Tbc1d4_S577 - cost: 5.06, layer: 9
Fold over basal
Time (min)
Normal
Diabetic
Normal data
0
10
20
30
40
50
60
2
4
6
8
10
12
14
16

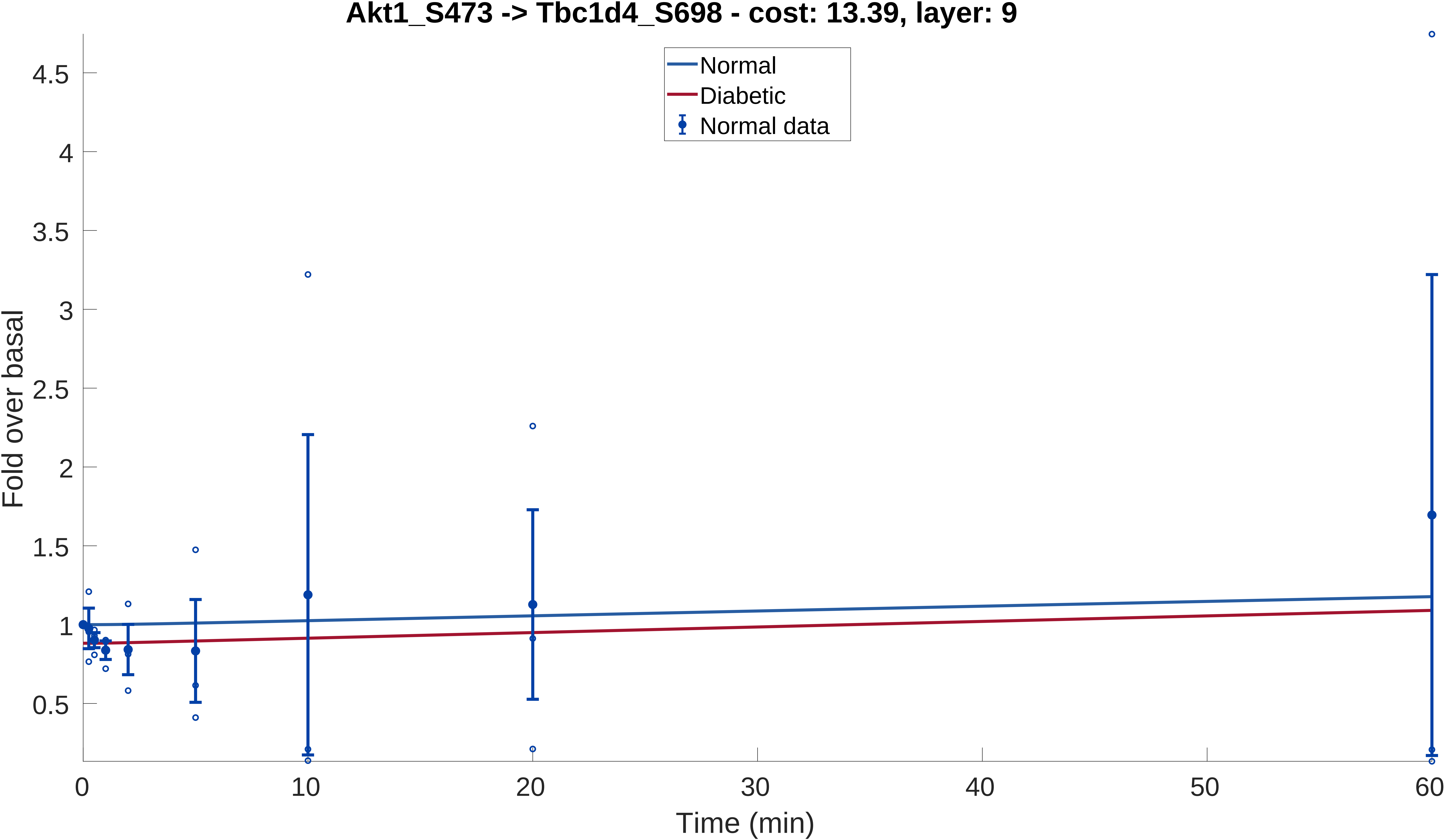

Akt1_S473 -> Tbc1d4_S698 - cost: 13.39, layer: 9
Normal
Diabetic
Normal data
Fold over basal
Time (min)
4.5
4
3.5
3
2.5
2
1.5
1
0.5
0
10
20
30
40
50
60

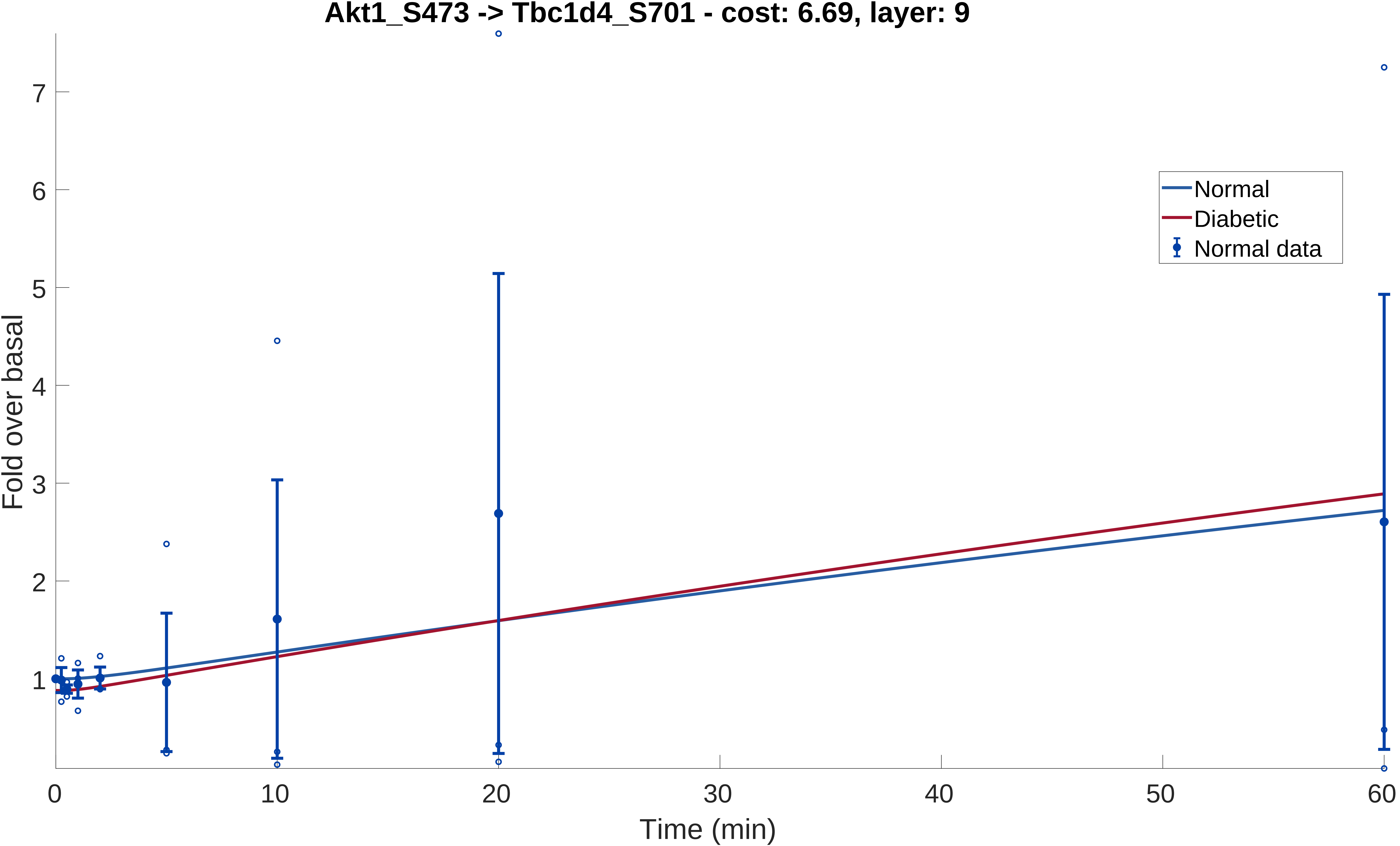
Akt1_S473 -> Tbc1d4_S701 - cost: 6.69, layer: 9
Normal
Diabetic
Normal data
Fold over basal
Time (min)
0
10
20
30
40
50
60
1
2
3
4
5
6
7

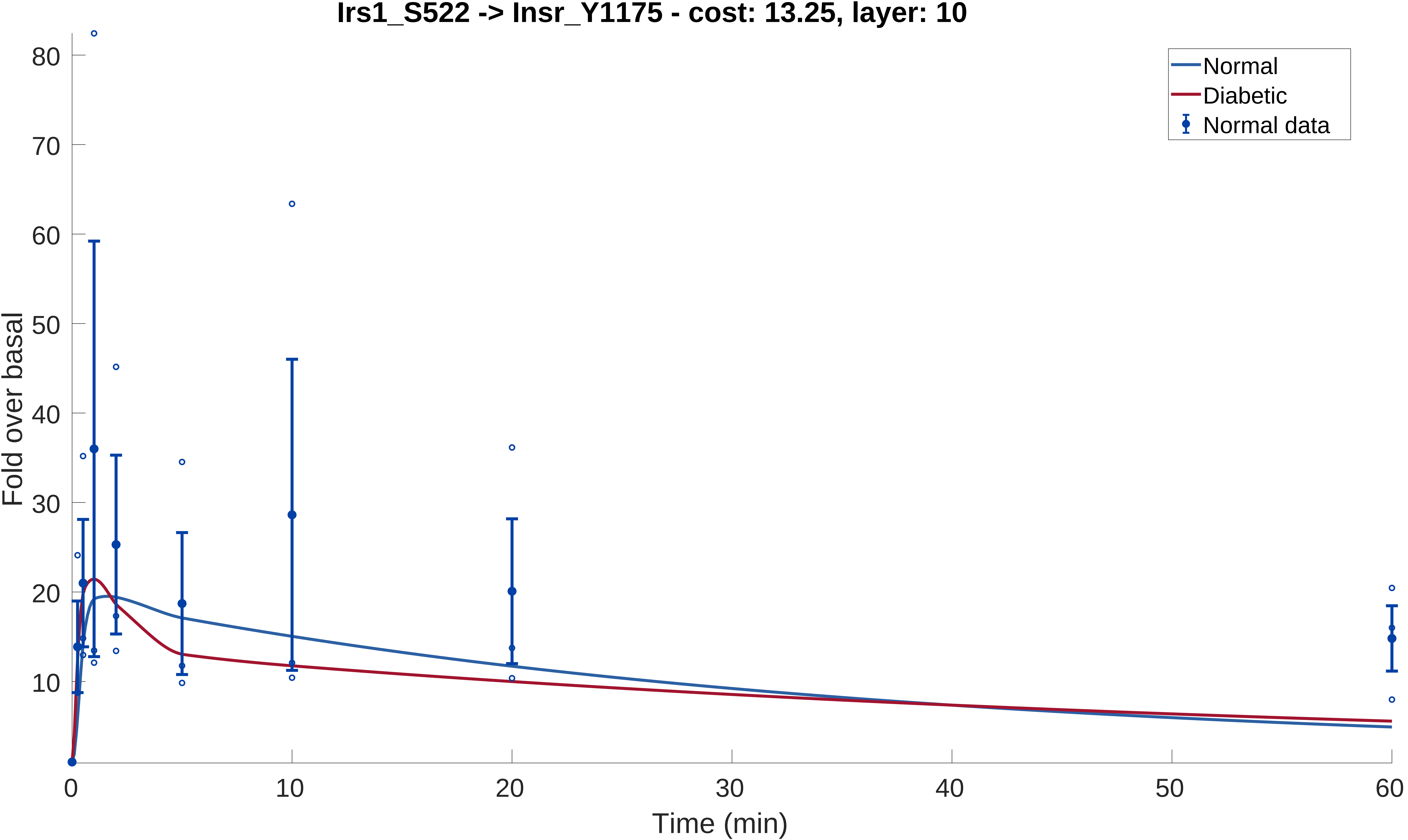
Irs1_S522 -> Insr_Y1175 - cost: 13.25, layer: 10
Normal
Diabetic
Normal data
Fold over basal
Time (min)
0
10
20
30
40
50
60
10
20
30
40
50
60
70
80

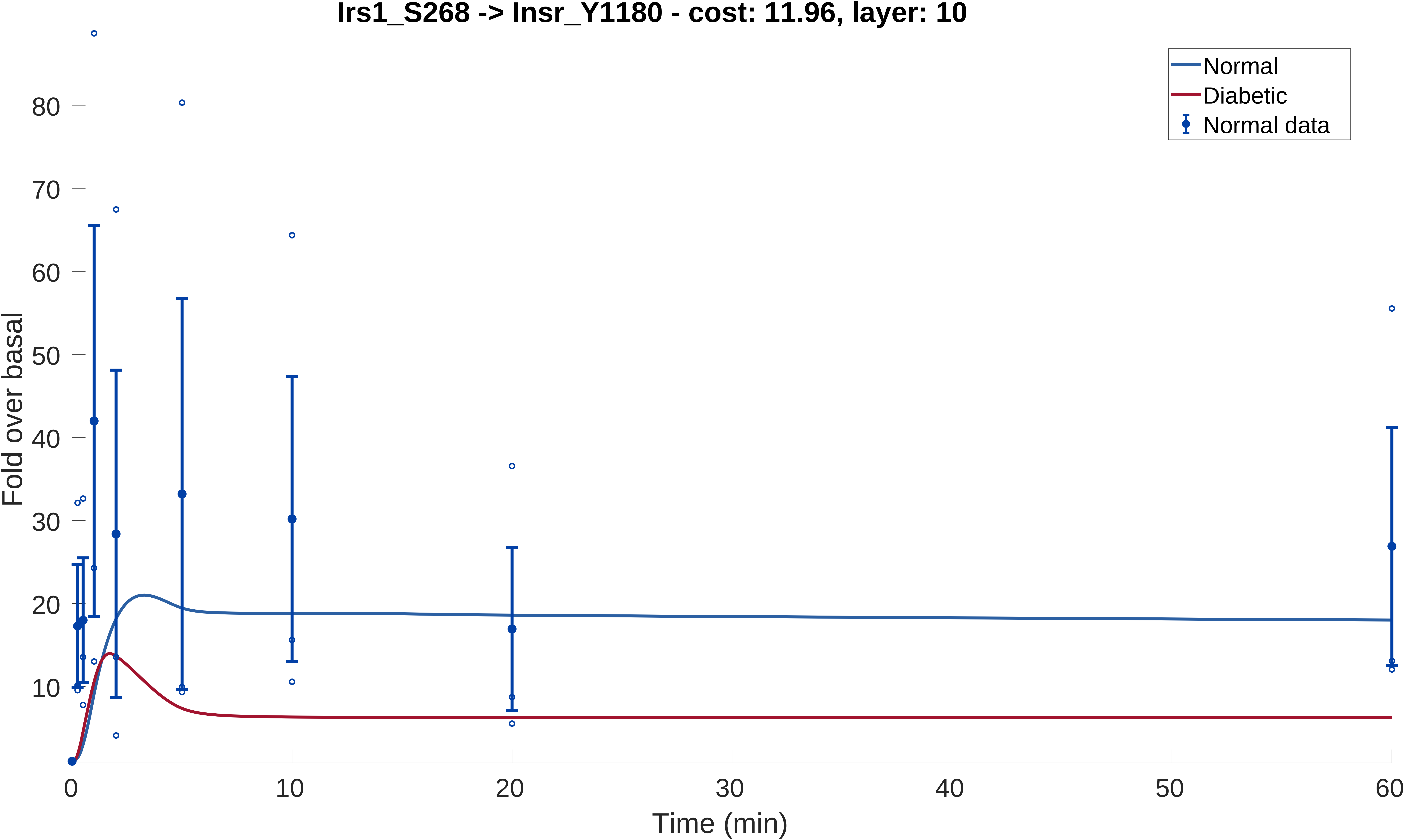

Irs1_S268 -> Insr_Y1180 - cost: 11.96, layer: 10
Normal
Diabetic
Normal data
Fold over basal
Time (min)
0
10
20
30
40
50
60
70
80

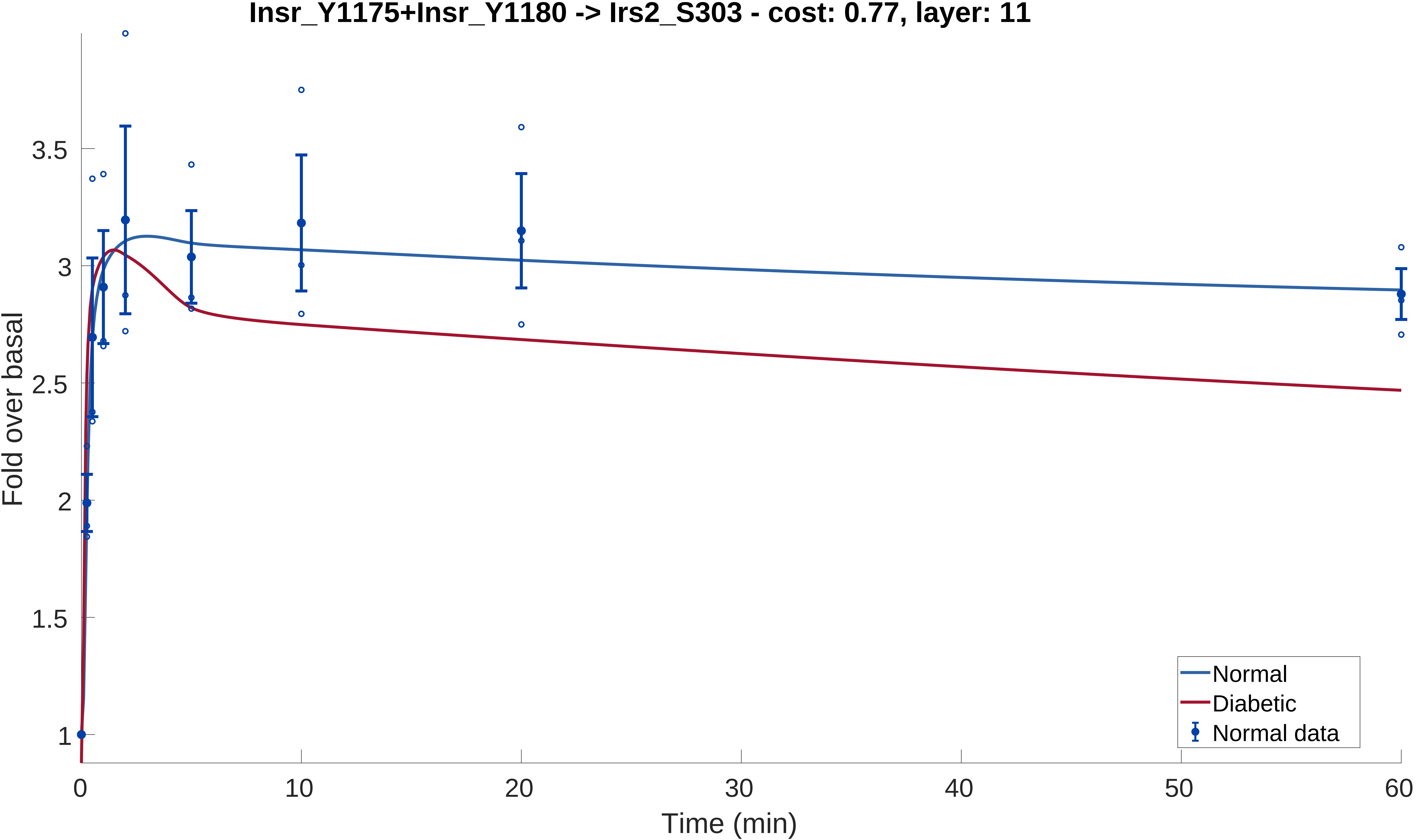

Insr_Y1175+Insr_Y1180 -> Irs2_S303 - cost: 0.77, layer: 11
Fold over basal
3.5
3
2.5
2
1.5
1
0
10
20
30
40
50
60
Time (min)
Normal
Diabetic
Normal data

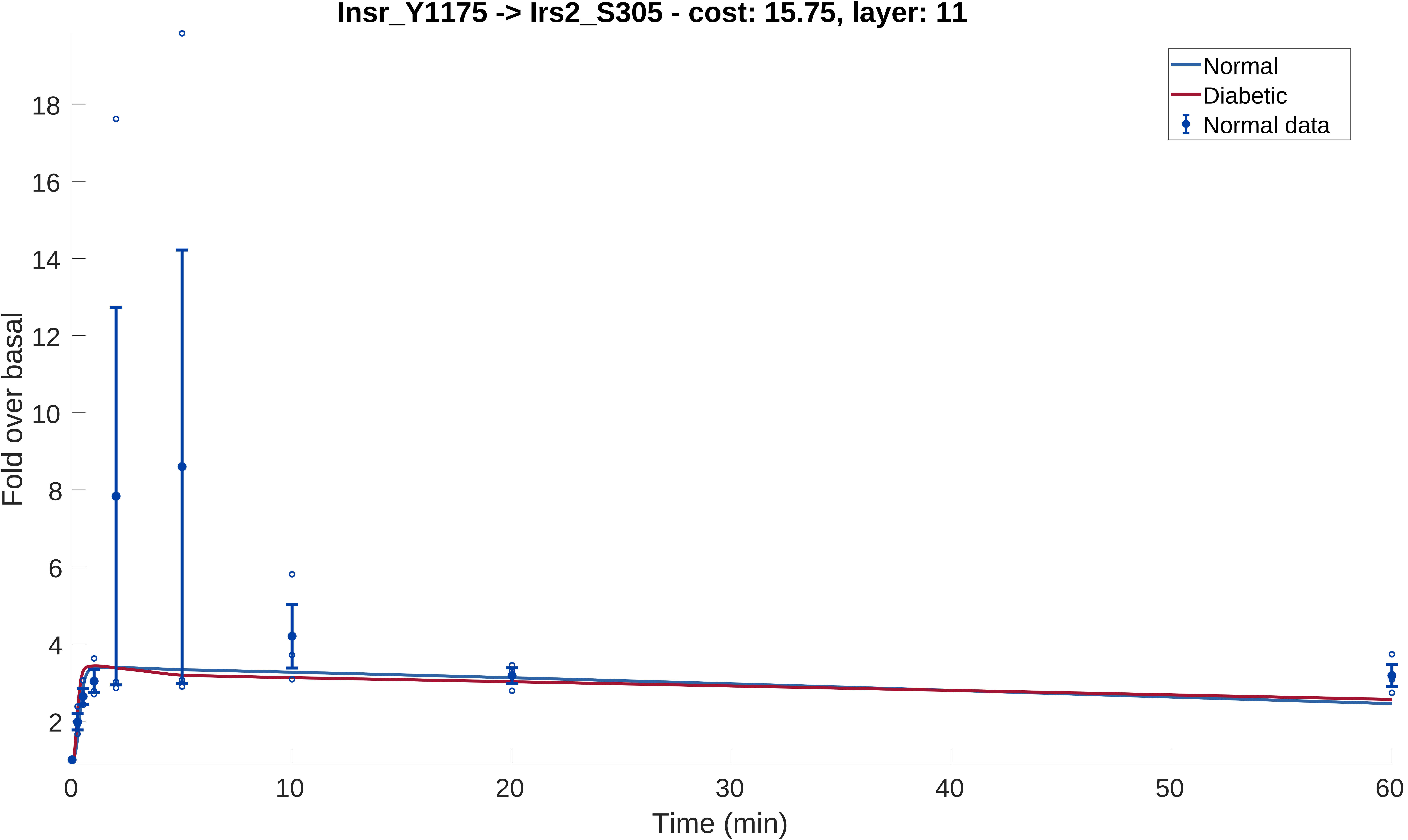

Insr_Y1175 -> Irs2_S305 - cost: 15.75, layer: 11
Normal
Diabetic
Normal data
Fold over basal
18
16
14
12
10
8
6
4
2
0
10
20
30
40
50
60
Time (min)

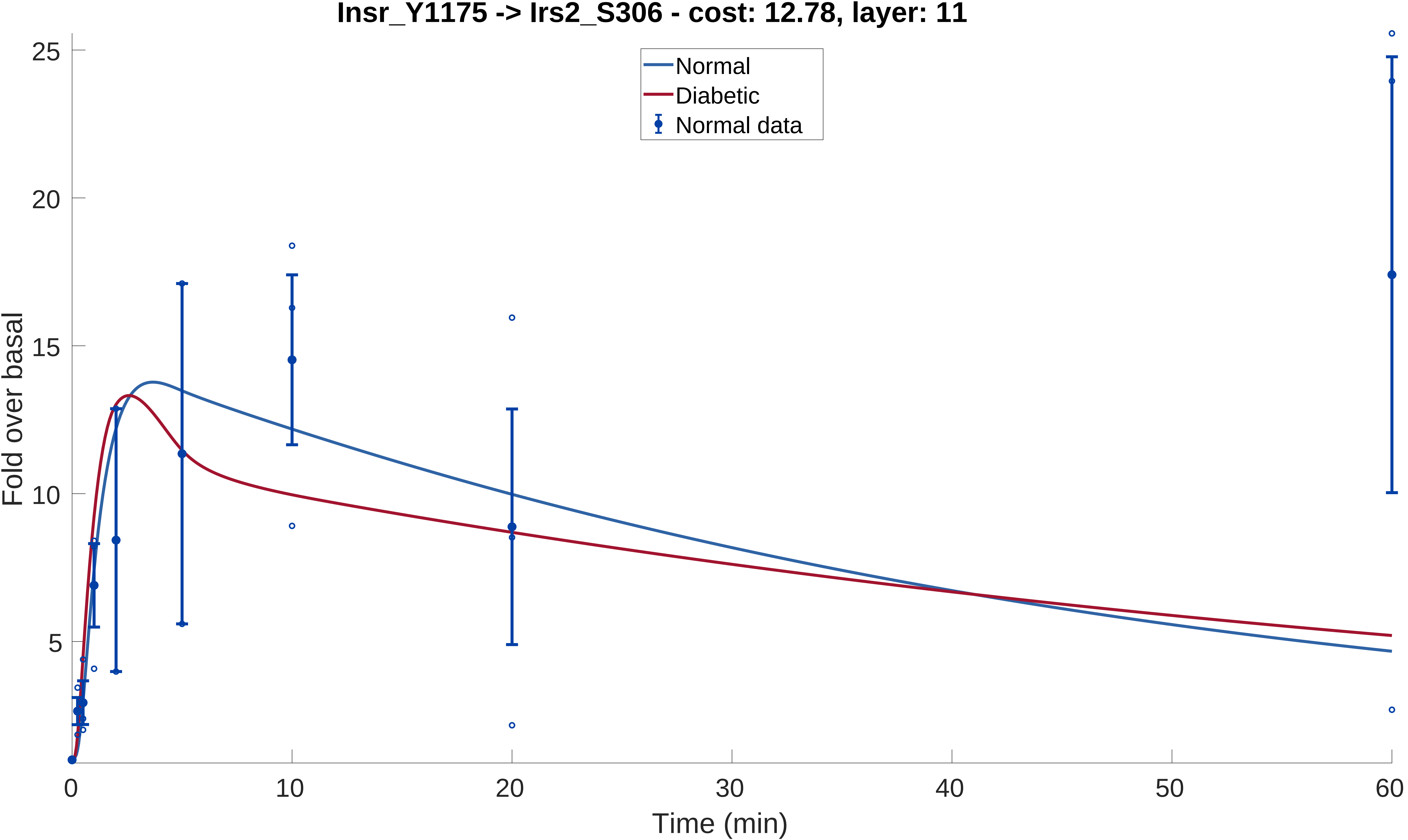

Insr_Y1175 -> Irs2_S306 - cost: 12.78, layer: 11
Normal
Diabetic
Normal data
Fold over basal
25
20
15
10
5
0
10
20
30
40
50
60
Time (min)

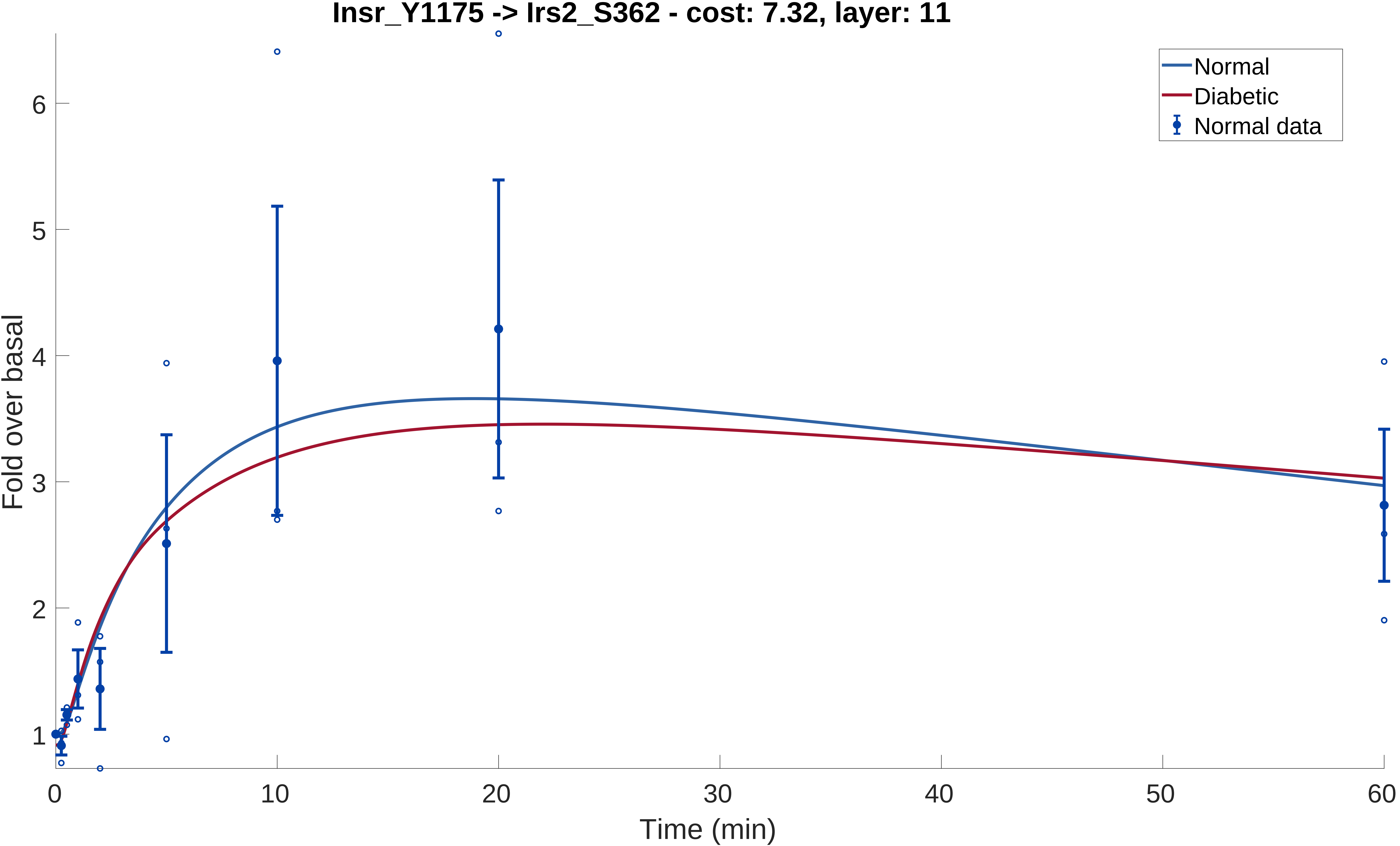
Insr_Y1175 -> Irs2_S362 - cost: 7.32, layer: 11
Normal
Diabetic
Normal data
Fold over basal
Time (min)
1
2
3
4
5
6
0
10
20
30
40
50
60

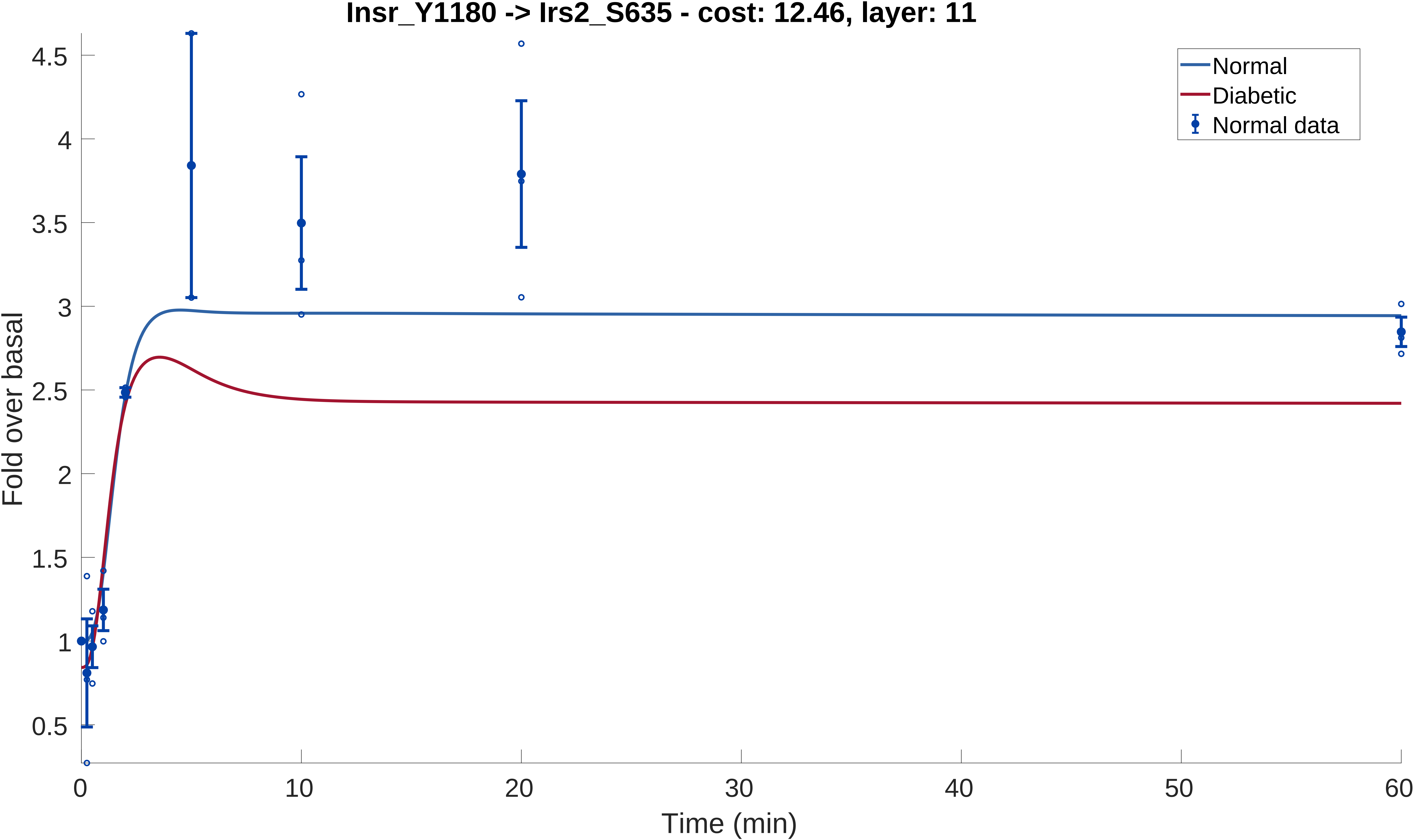

Insr_Y1180 -> Irs2_S635 - cost: 12.46, layer: 11
Fold over basal
Time (min)
Normal
Diabetic
Normal data
0.5
1
1.5
2
2.5
3
3.5
4
4.5
0
10
20
30
40
50
60

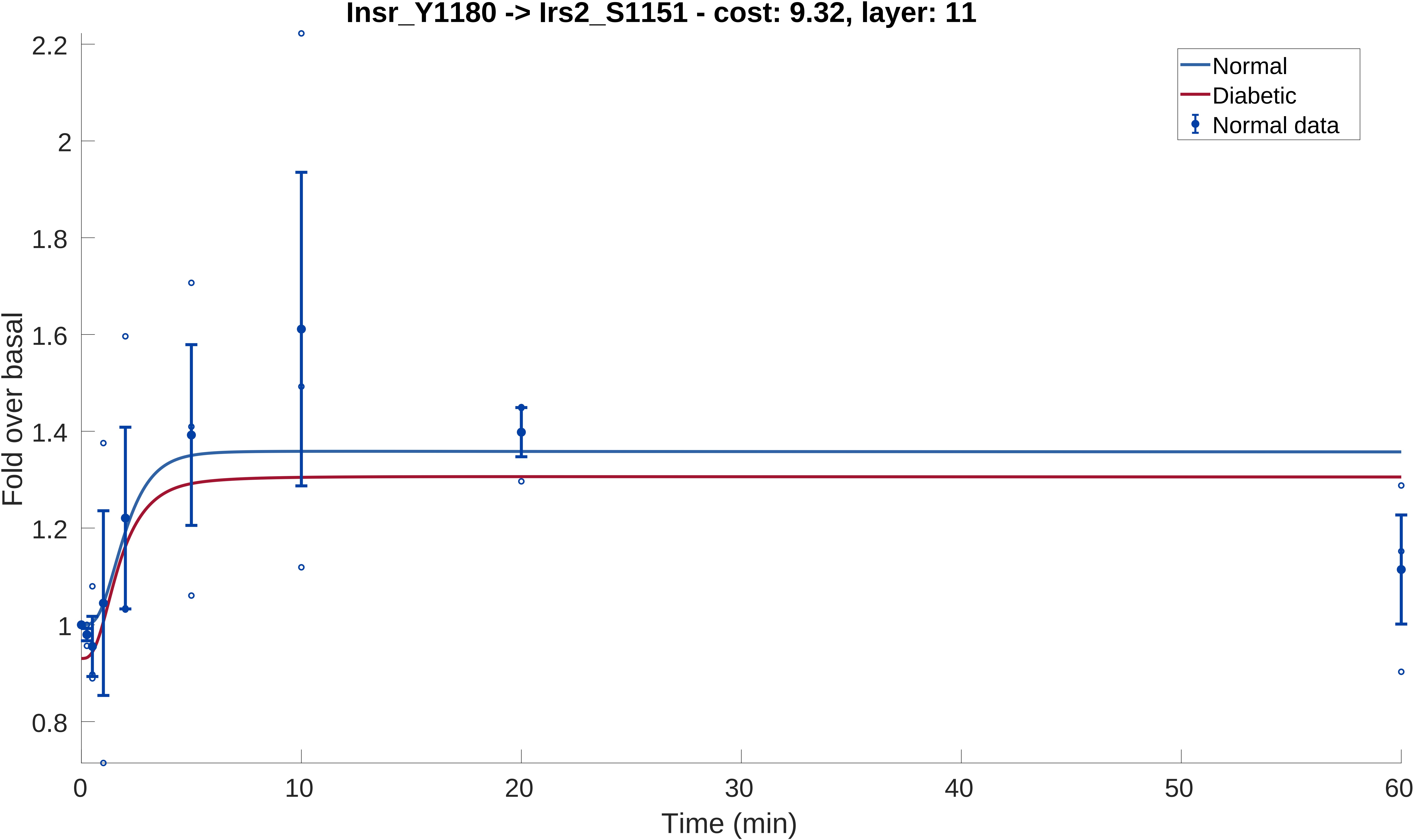
Insr_Y1180 -> Irs2_S1151 - cost: 9.32, layer: 11
Normal
Diabetic
Normal data
Fold over basal
2.2
2
1.8
1.6
1.4
1.2
1
0.8
0
10
20
30
40
50
60
Time (min)

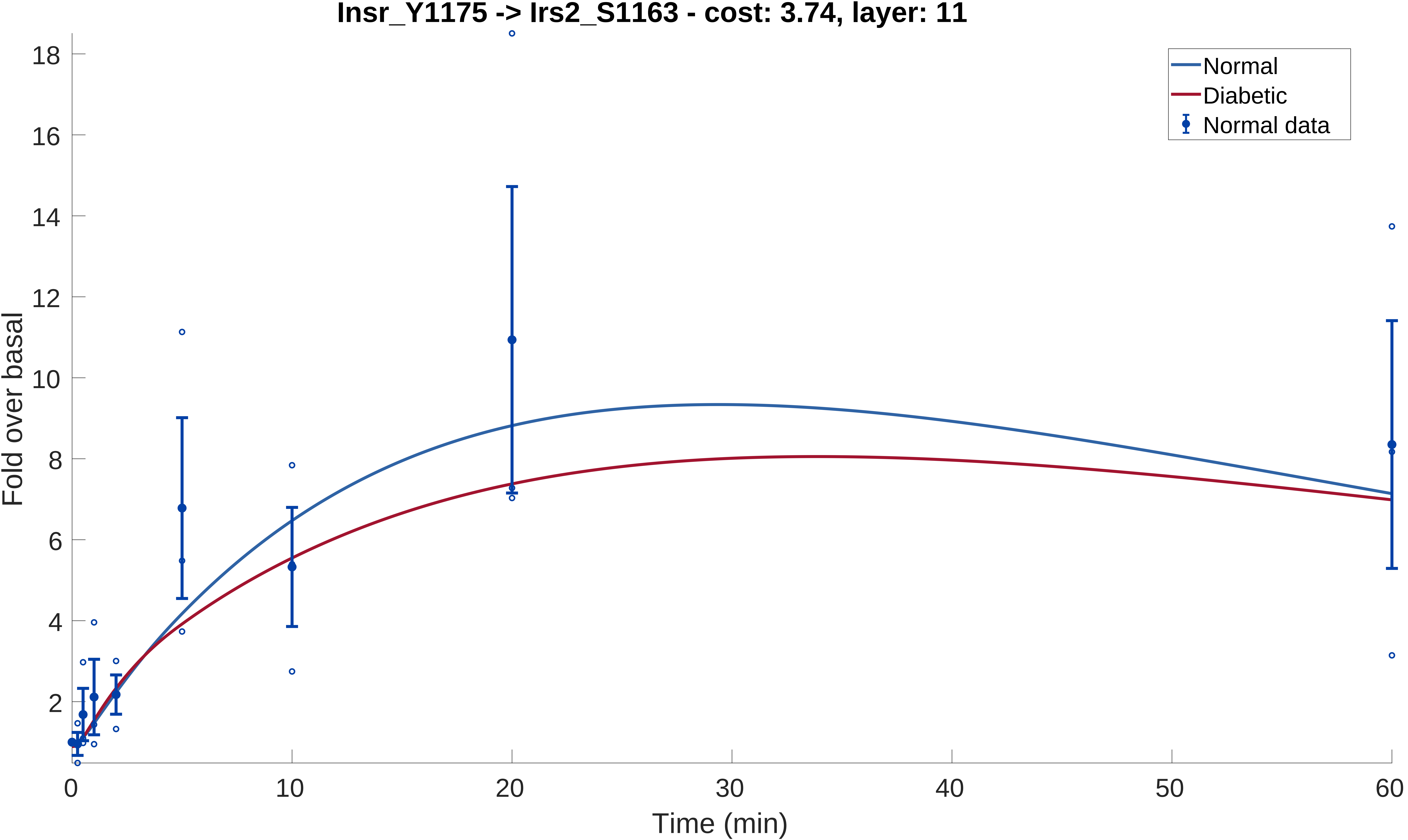

Insr_Y1175 -> Irs2_S1163 - cost: 3.74, layer: 11
Normal
Diabetic
Normal data
Fold over basal
Time (min)
0
10
20
30
40
50
60
2
4
6
8
10
12
14
16
18

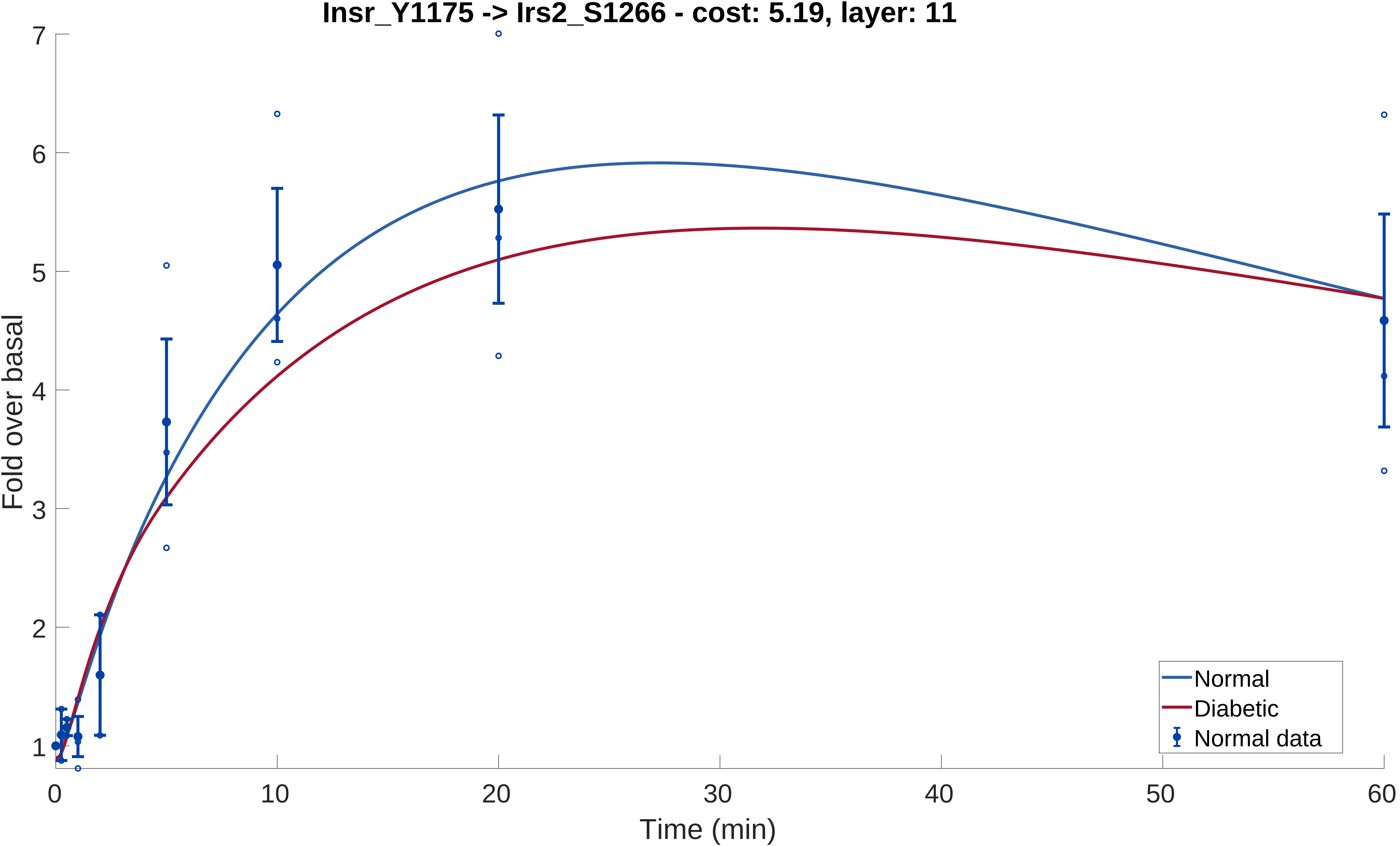
Insr_Y1175 -> Irs2_S1266 - cost: 5.19, layer: 11
Fold over basal
Time (min)
Normal
Diabetic
Normal data
0
10
20
30
40
50
60
1
2
3
4
5
6
7

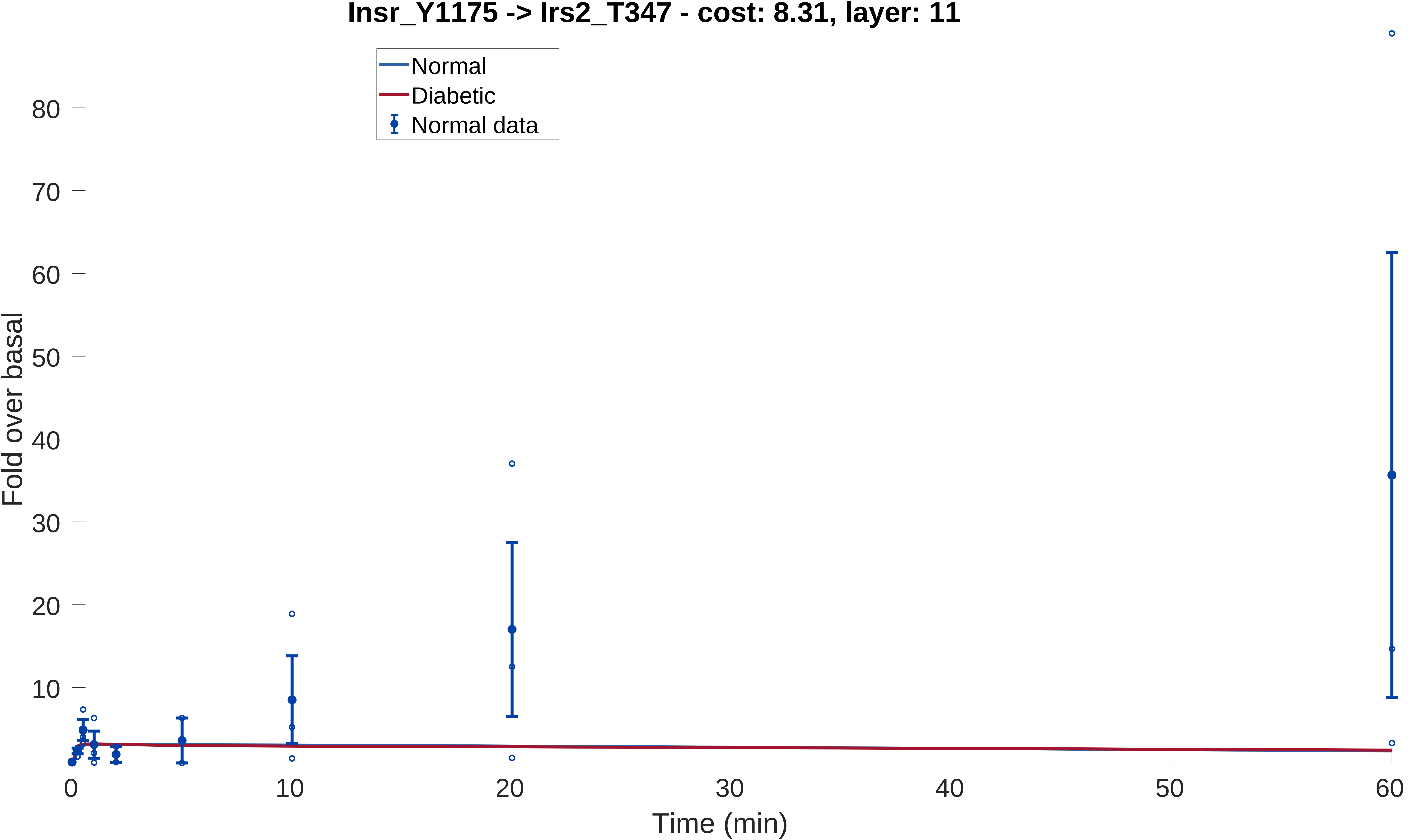

Insr_Y1175 -> Irs2_T347 - cost: 8.31, layer: 11
Normal
Diabetic
Normal data
Fold over basal
Time (min)
0
10
20
30
40
50
60
70
80

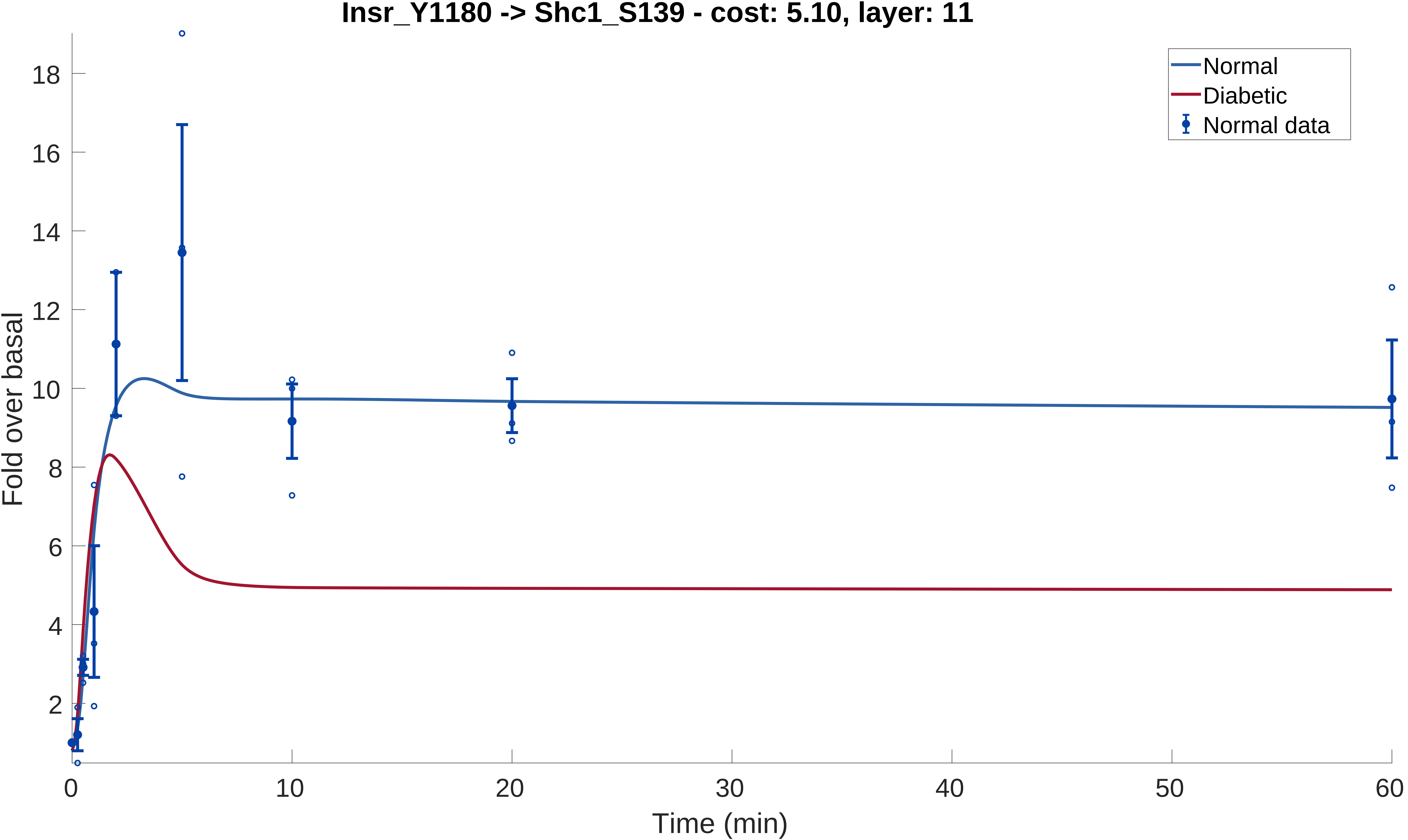
Insr_Y1180 -> Shc1_S139 - cost: 5.10, layer: 11
Normal
Diabetic
Normal data
Fold over basal
Time (min)
2
4
6
8
10
12
14
16
18
0
10
20
30
40
50
60

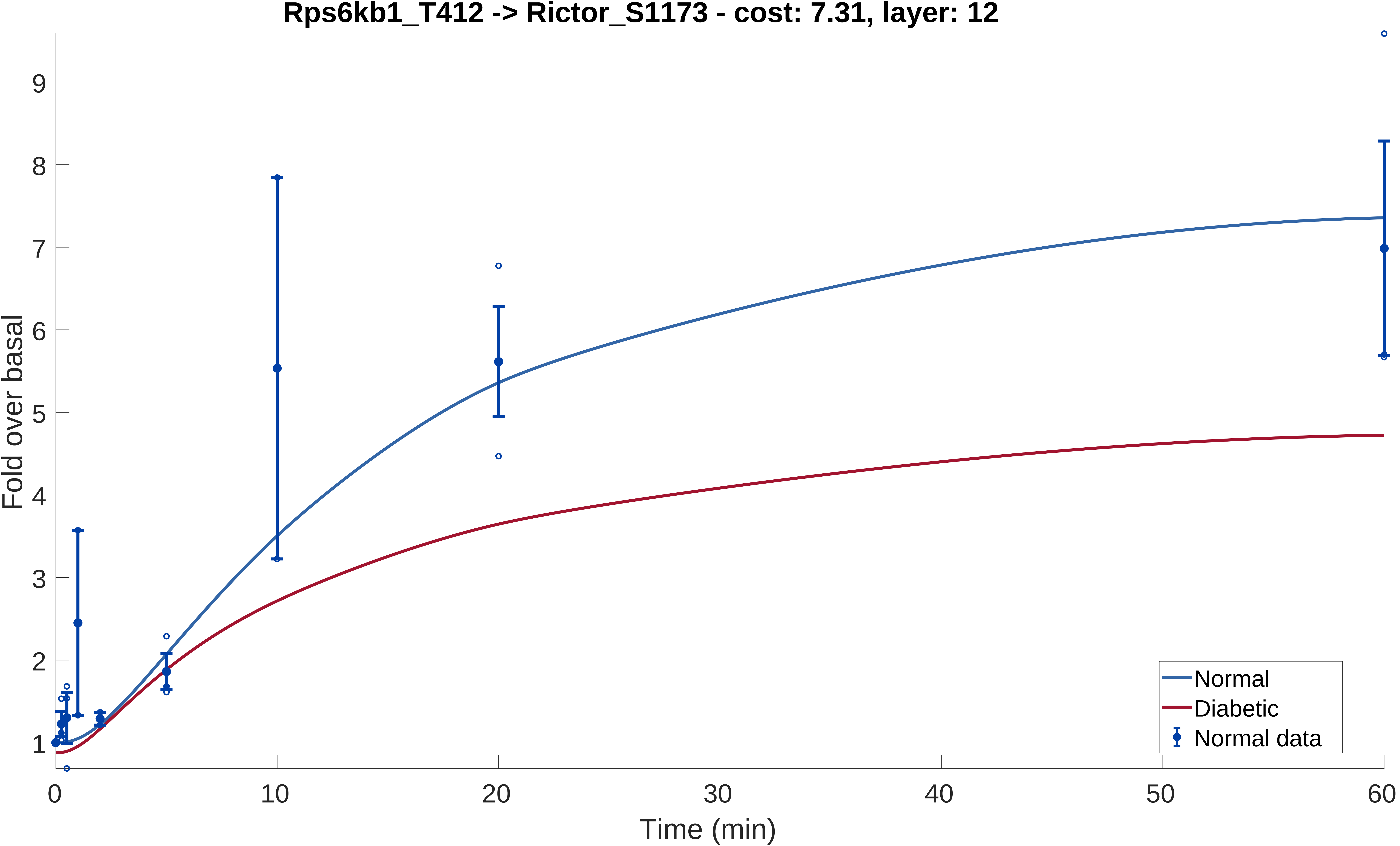

Rps6kb1_T412 -> Rictor_S1173 - cost: 7.31, layer: 12
Fold over basal
9
8
7
6
5
4
3
2
1
0
10
20
30
40
50
60
Time (min)
Normal
Diabetic
Normal data

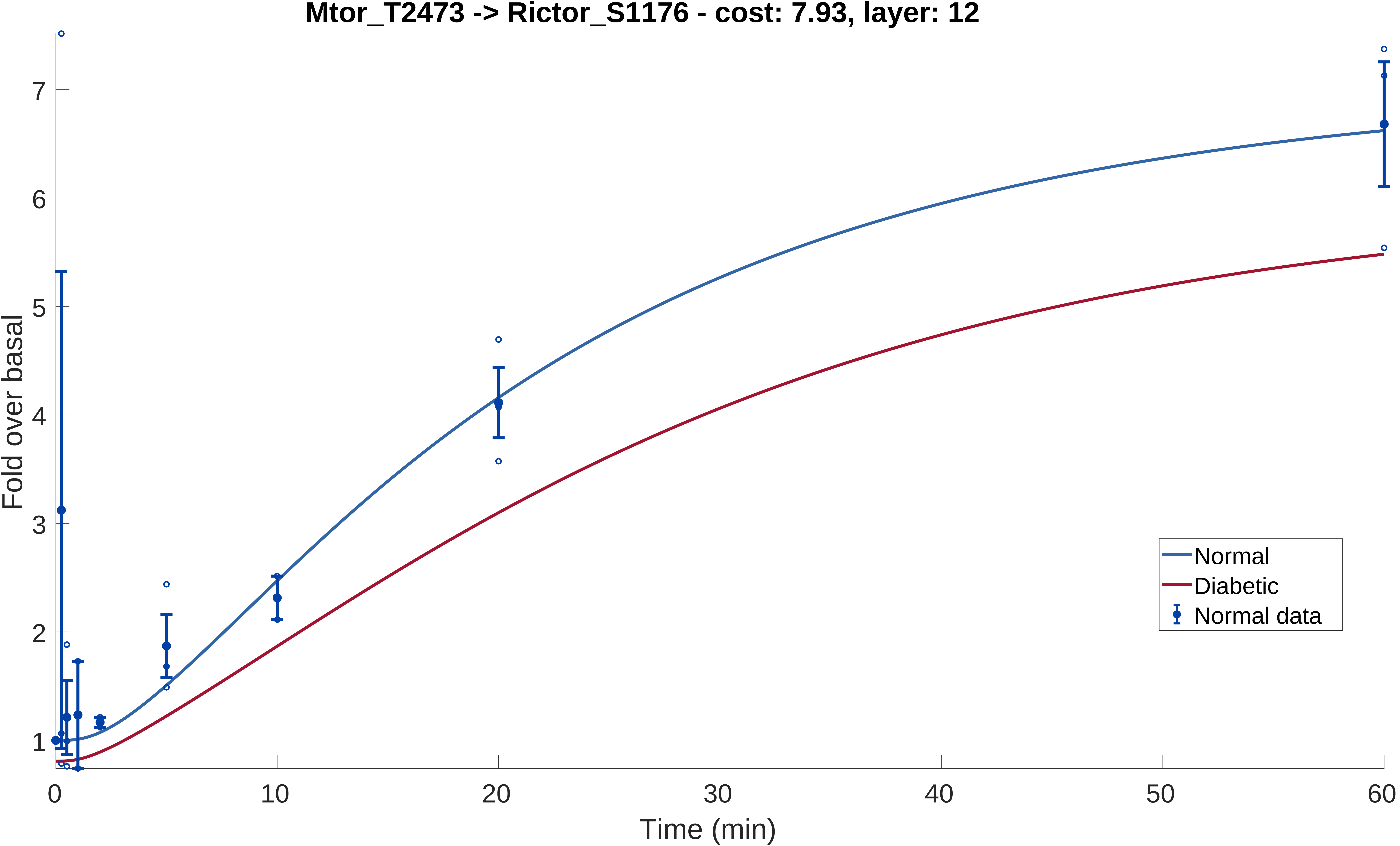
Mtor_T2473 -> Rictor_S1176 - cost: 7.93, layer: 12
Fold over basal
Time (min)
Normal
Diabetic
Normal data
0
10
20
30
40
50
60
1
2
3
4
5
6
7

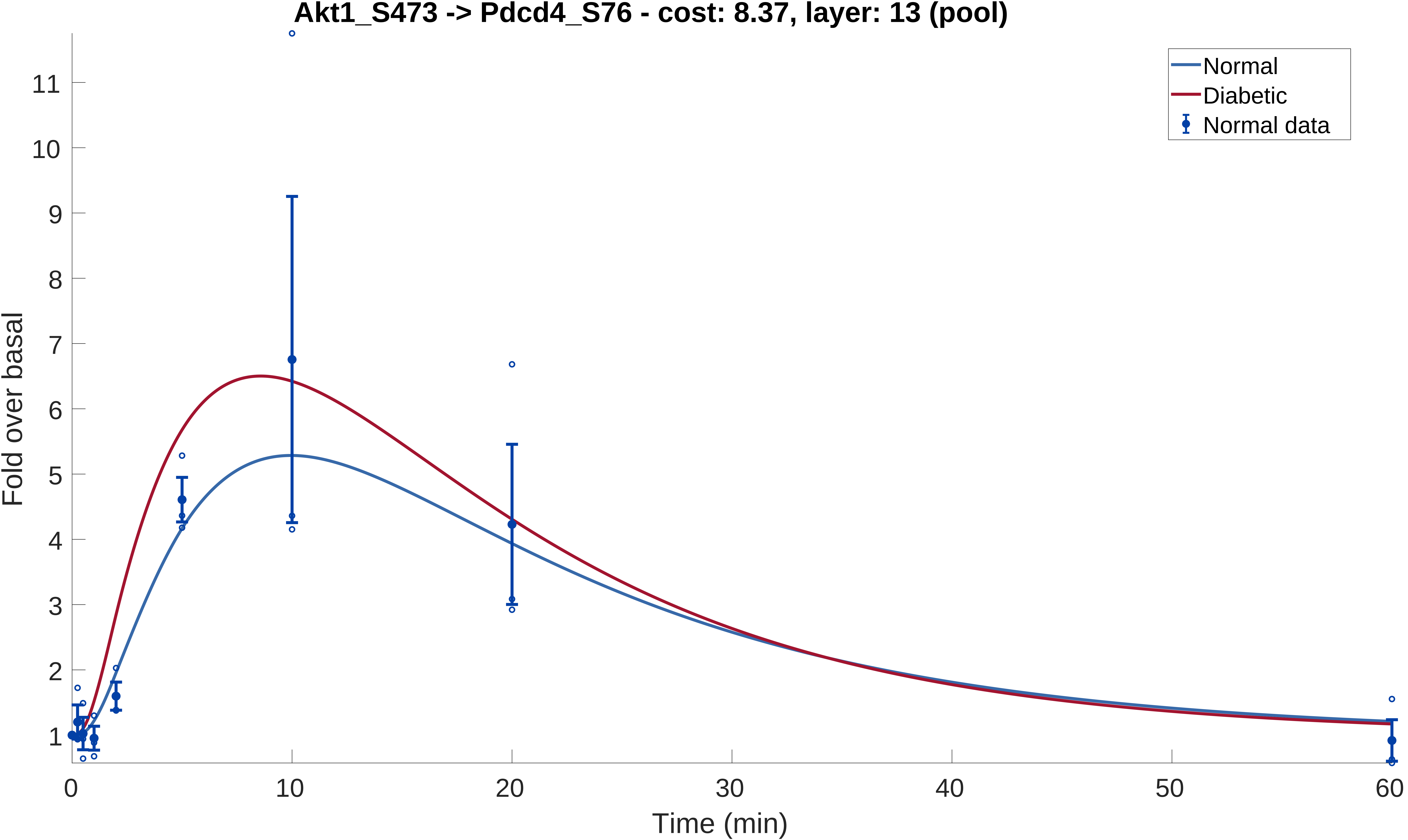
Akt1_S473 -> Pdcd4_S76 - cost: 8.37, layer: 13 (pool)
Normal
Diabetic
Normal data
Fold over basal
Time (min)
0
10
20
30
40
50
60
1
2
3
4
5
6
7
8
9
10
11

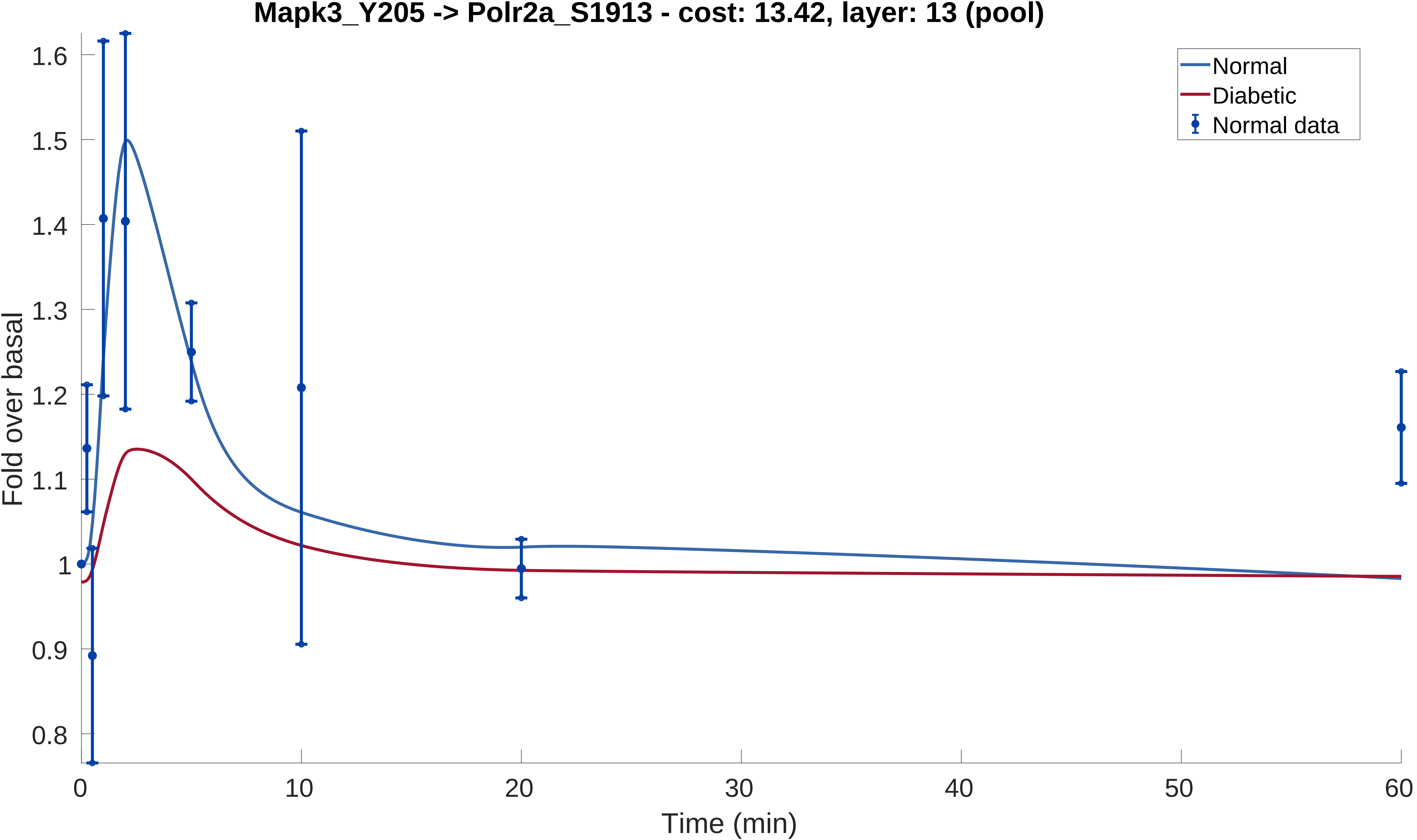
Mapk3_Y205 -> Polr2a_S1913 - cost: 13.42, layer: 13 (pool)
Normal
Diabetic
Normal data
Fold over basal
1.6
1.5
1.4
1.3
1.2
1.1
1
0.9
0.8
0
10
20
30
40
50
60
Time (min)

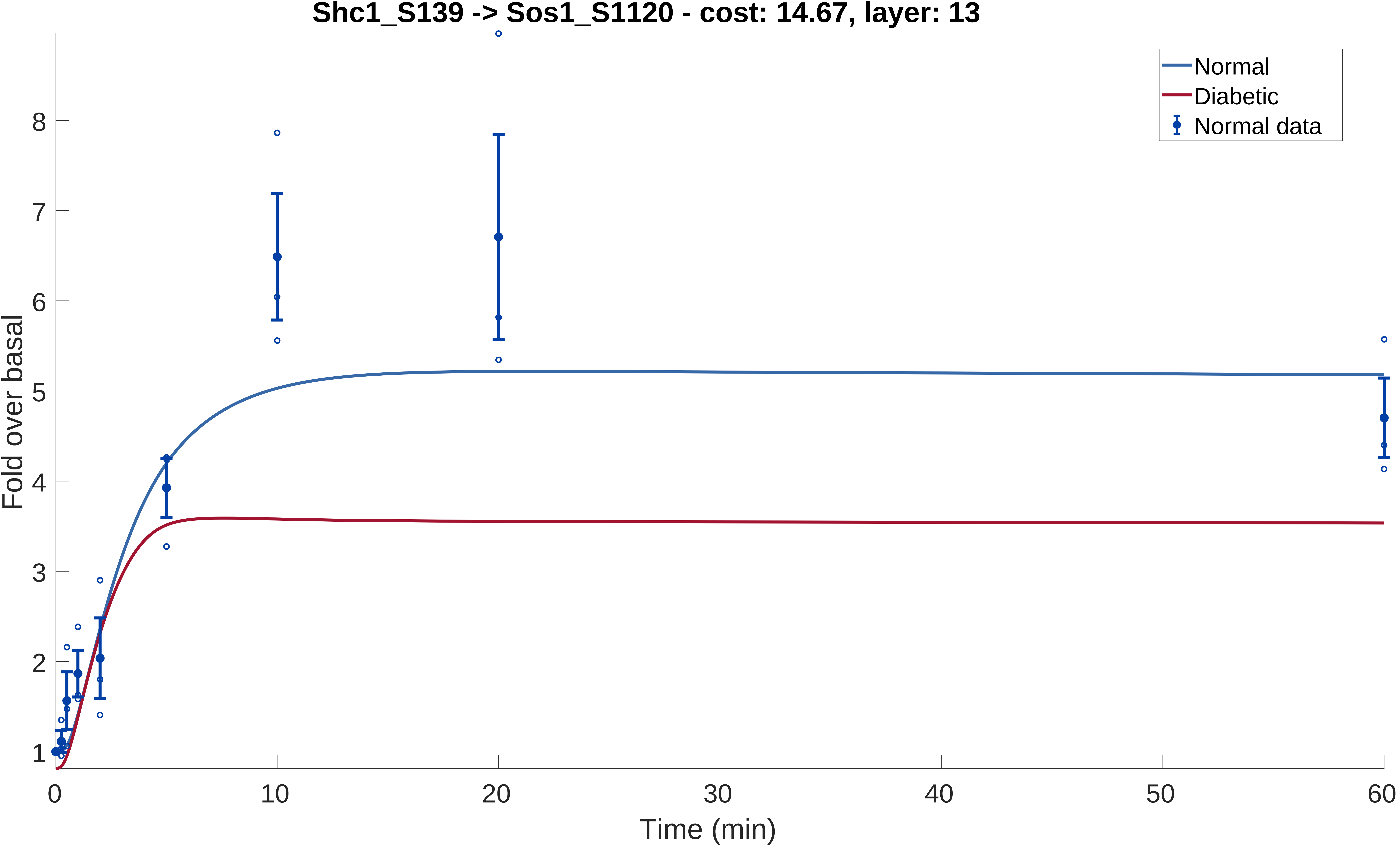
Shc1_S139 -> Sos1_S1120 - cost: 14.67, layer: 13
Normal
Diabetic
Normal data
Fold over basal
1
2
3
4
5
6
7
8
0
10
20
30
40
50
60
Time (min)

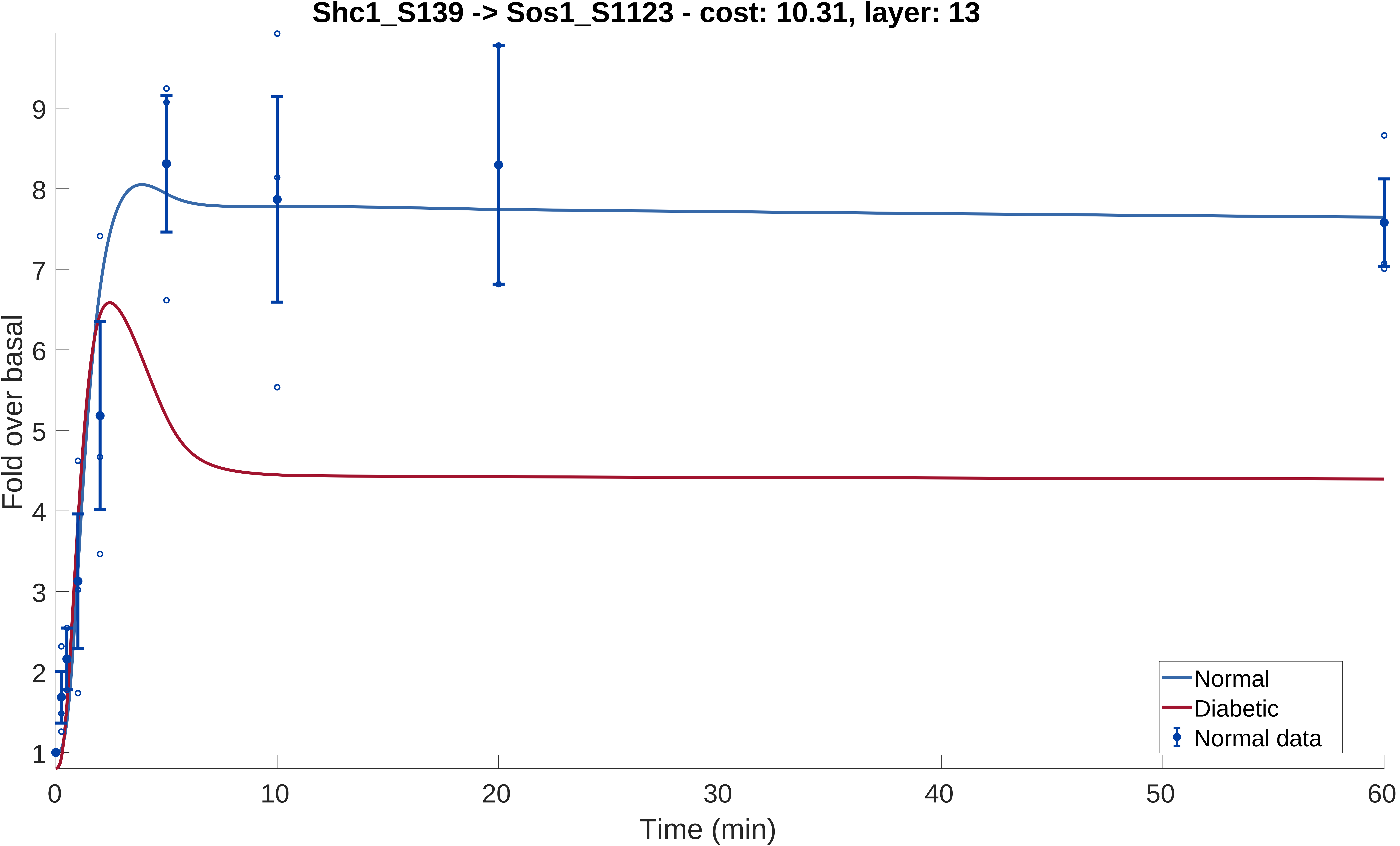
Shc1_S139 -> Sos1_S1123 - cost: 10.31, layer: 13
Fold over basal
Time (min)
1
2
3
4
5
6
7
8
9
0
10
20
30
40
50
60
Normal
Diabetic
Normal data

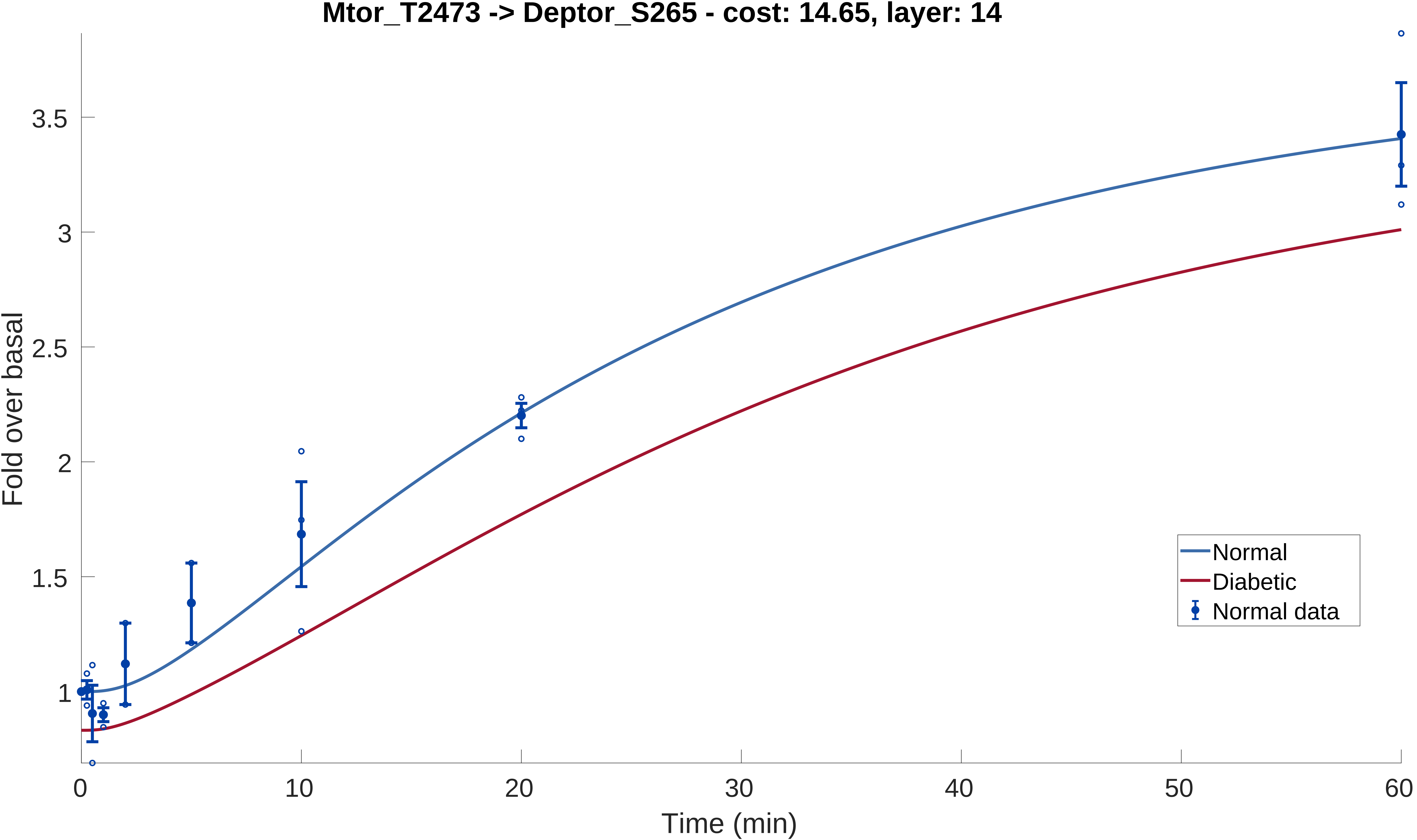

Mtor_T2473 -> Deptor_S265 - cost: 14.65, layer: 14
Fold over basal
Time (min)
3.5
3
2.5
2
1.5
1
0
10
20
30
40
50
60
Normal
Diabetic
Normal data

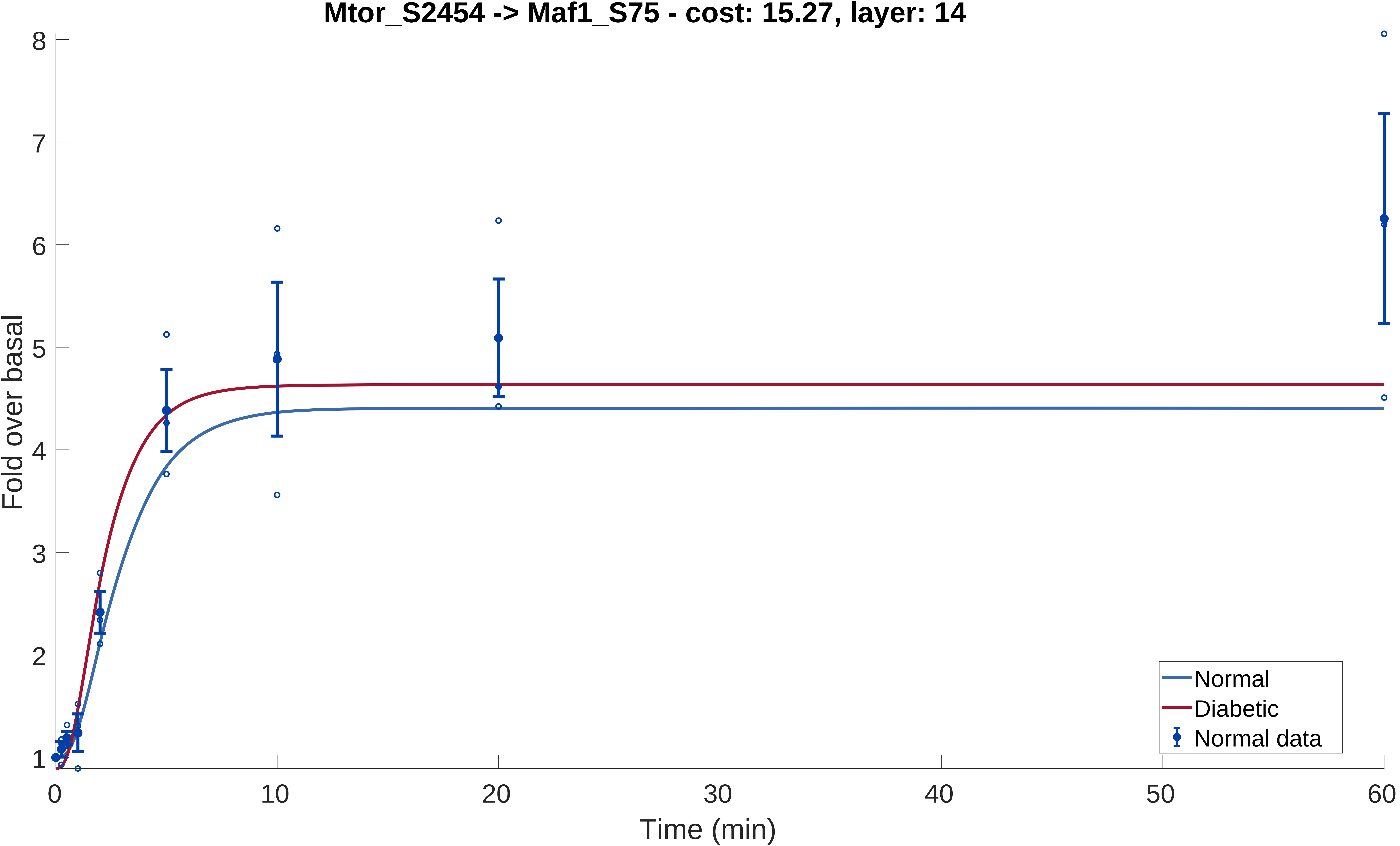

Mtor_S2454 -> Maf1_S75 - cost: 15.27, layer: 14
Fold over basal
Time (min)
0
10
20
30
40
50
60
1
2
3
4
5
6
7
8
Normal
Diabetic
Normal data

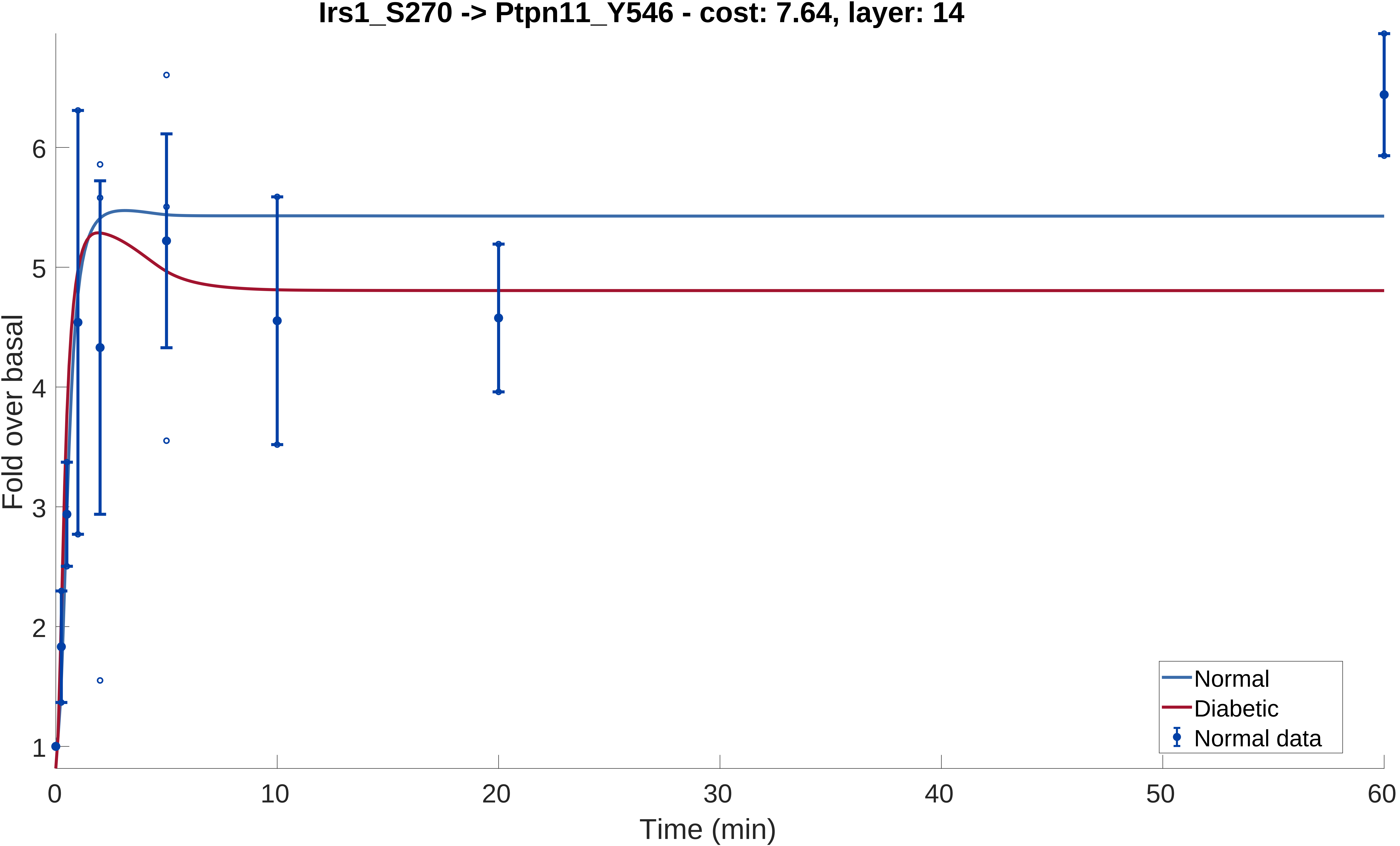

Irs1_S270 -> Ptpn11_Y546 - cost: 7.64, layer: 14
Fold over basal
Time (min)
0
10
20
30
40
50
60
1
2
3
4
5
6
Normal
Diabetic
Normal data

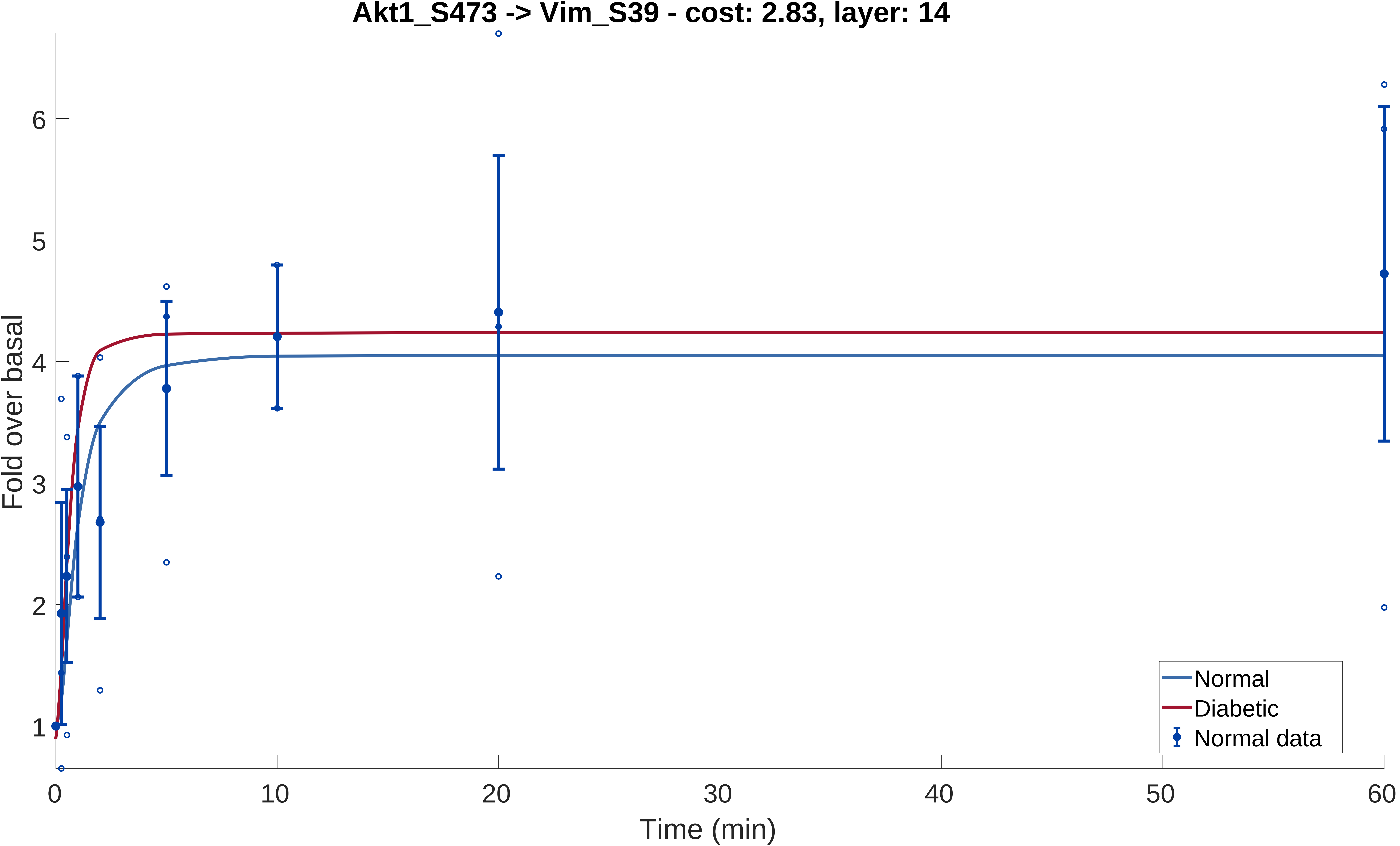

Akt1_S473 -> Vim_S39 - cost: 2.83, layer: 14
Fold over basal
1
2
3
4
5
6
0
10
20
30
40
50
60
Time (min)
Normal
Diabetic
Normal data

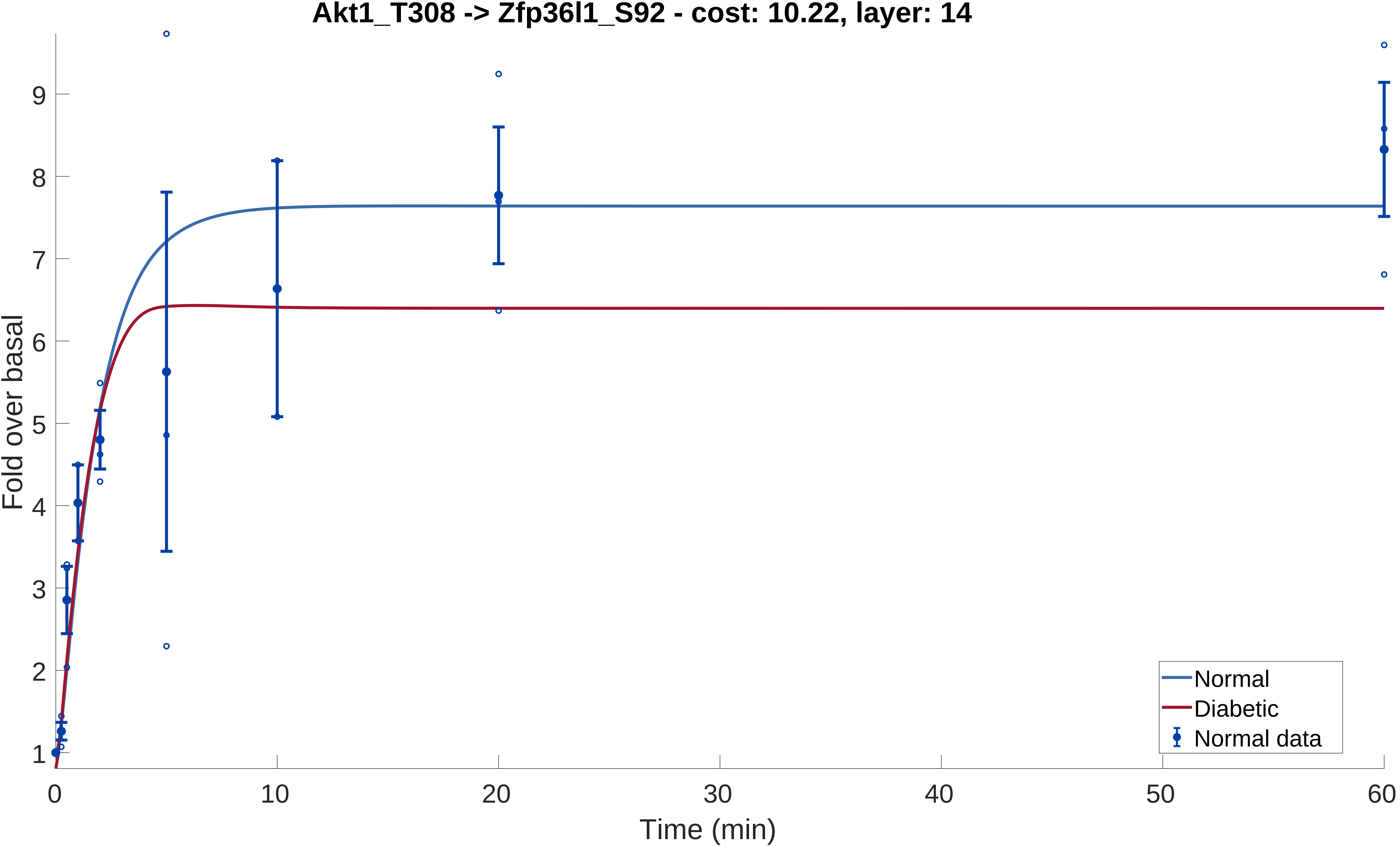

Akt1_T308 -> Zfp36l1_S92 - cost: 10.22, layer: 14
Fold over basal
Time (min)
1
2
3
4
5
6
7
8
9
0
10
20
30
40
50
60
Normal
Diabetic
Normal data

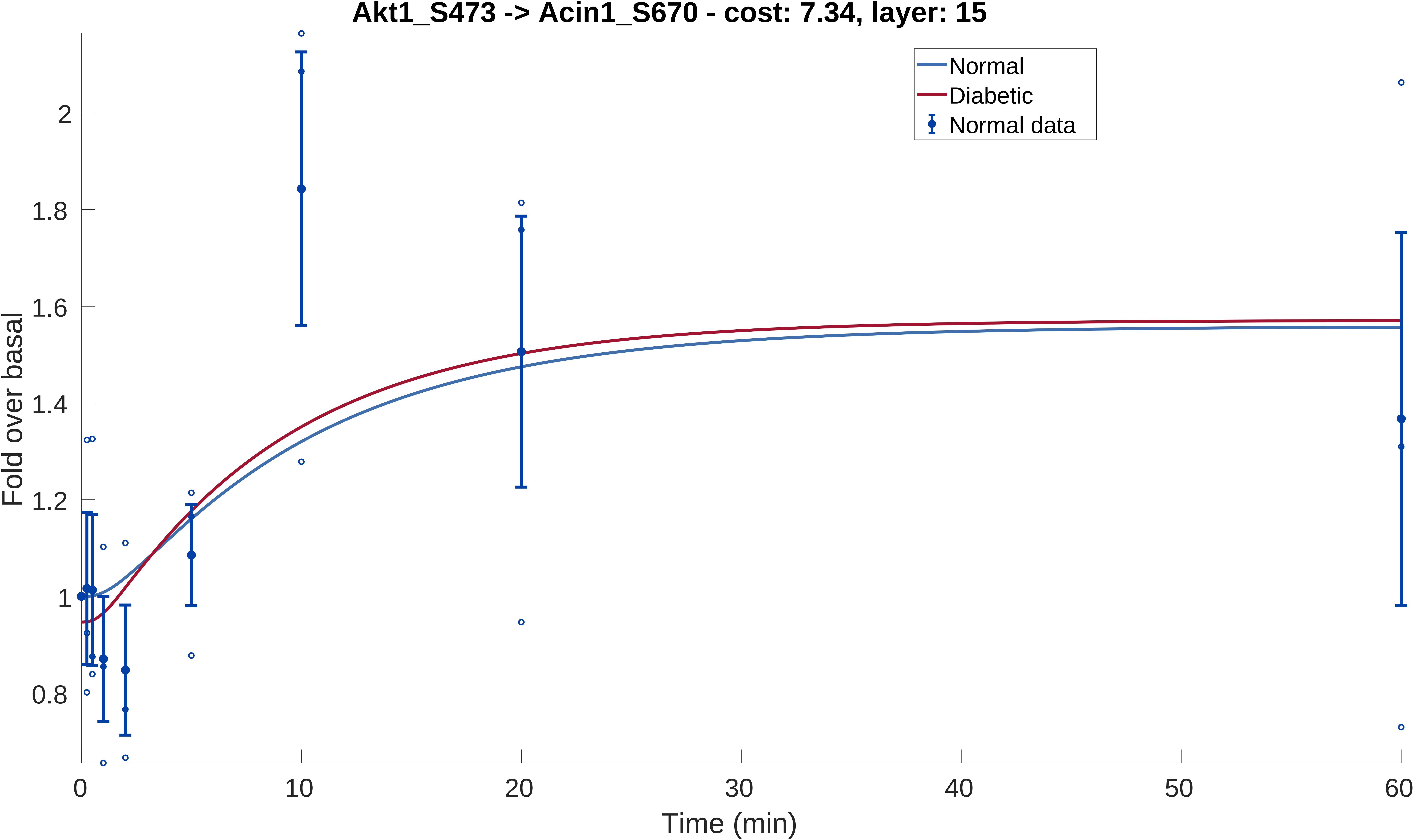
Akt1_S473 -> Acin1_S670 - cost: 7.34, layer: 15
Normal
Diabetic
Normal data
Fold over basal
Time (min)
0.8
1
1.2
1.4
1.6
1.8
2
0
10
20
30
40
50
60

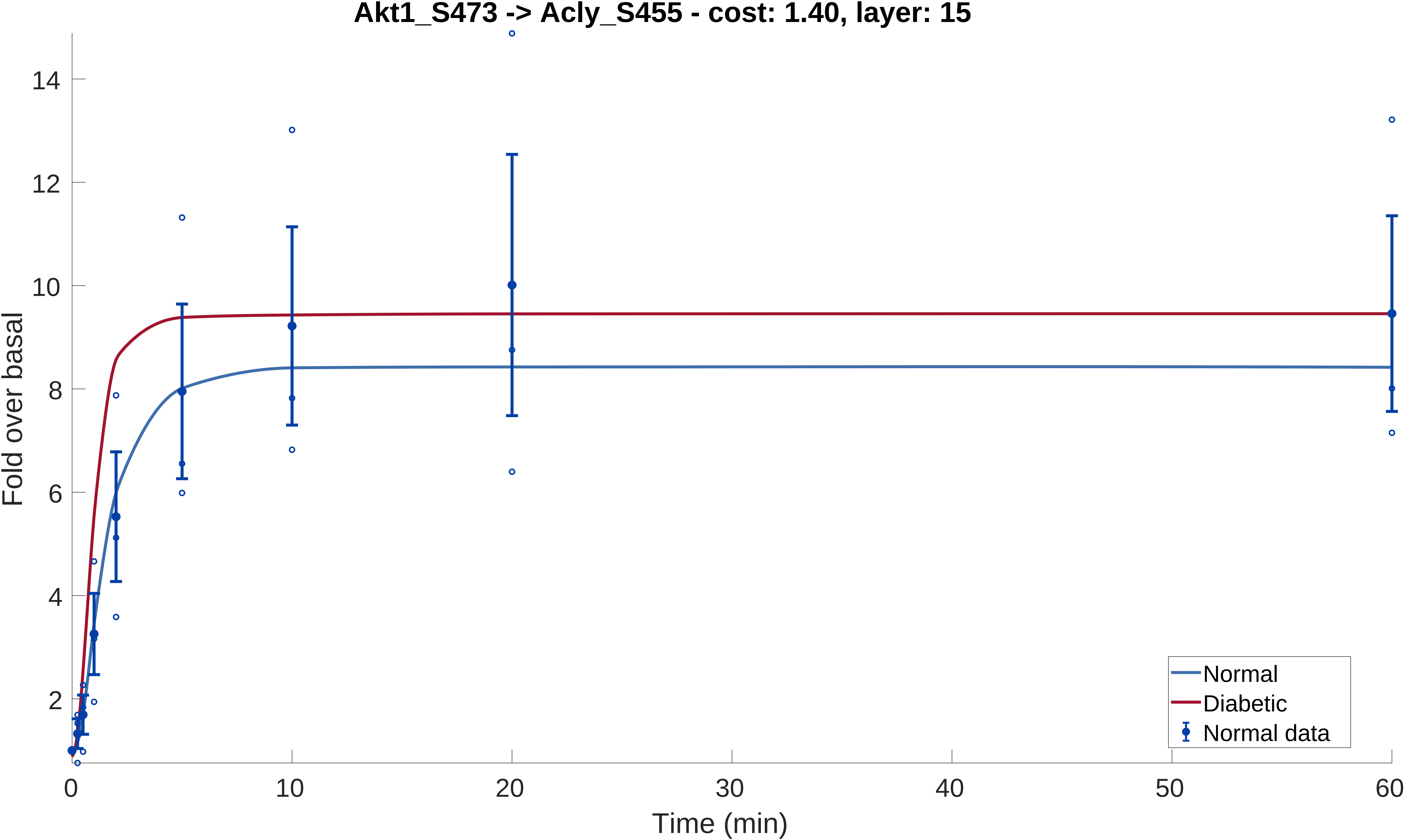
Akt1_S473 -> Acly_S455 - cost: 1.40, layer: 15
Fold over basal
Time (min)
Normal
Diabetic
Normal data
0
10
20
30
40
50
60
2
4
6
8
10
12
14

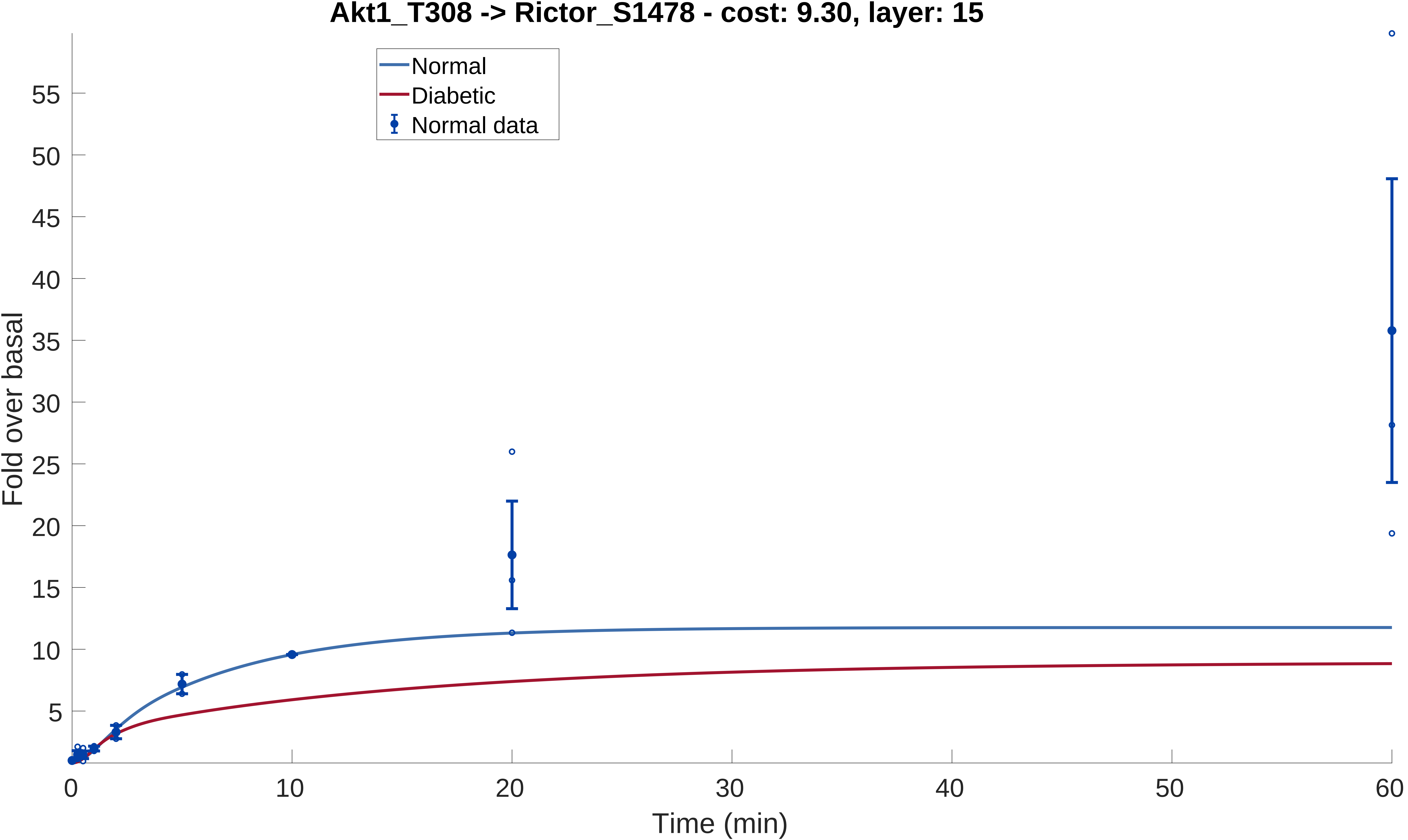
Akt1_T308 -> Rictor_S1478 - cost: 9.30, layer: 15
Normal
Diabetic
Normal data
Fold over basal
Time (min)
5
10
15
20
25
30
35
40
45
50
55
0
10
20
30
40
50
60

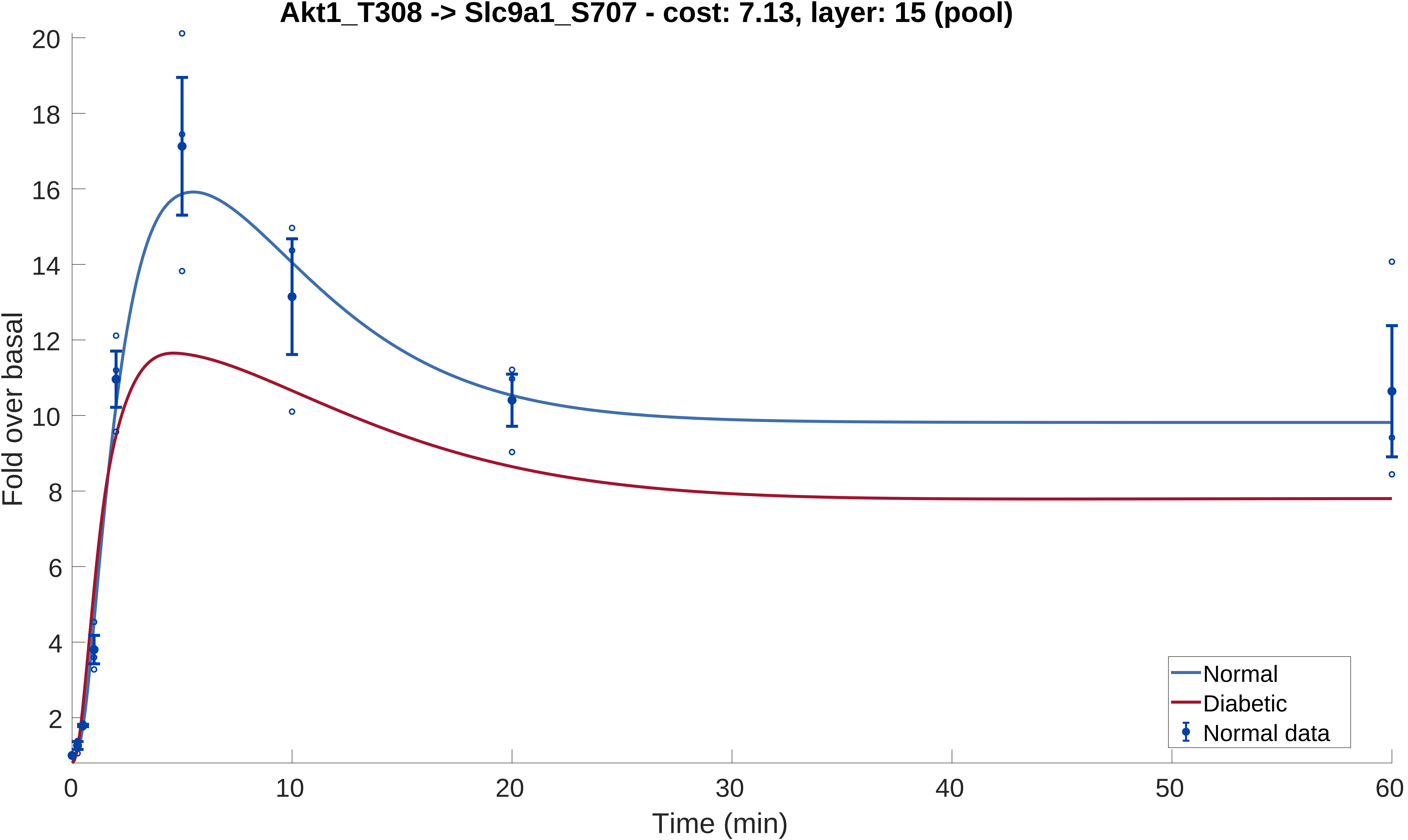
Akt1_T308 -> Slc9a1_S707 - cost: 7.13, layer: 15 (pool)
Fold over basal
Time (min)
20
18
16
14
12
10
8
6
4
2
0
10
20
30
40
50
60
Normal
Diabetic
Normal data

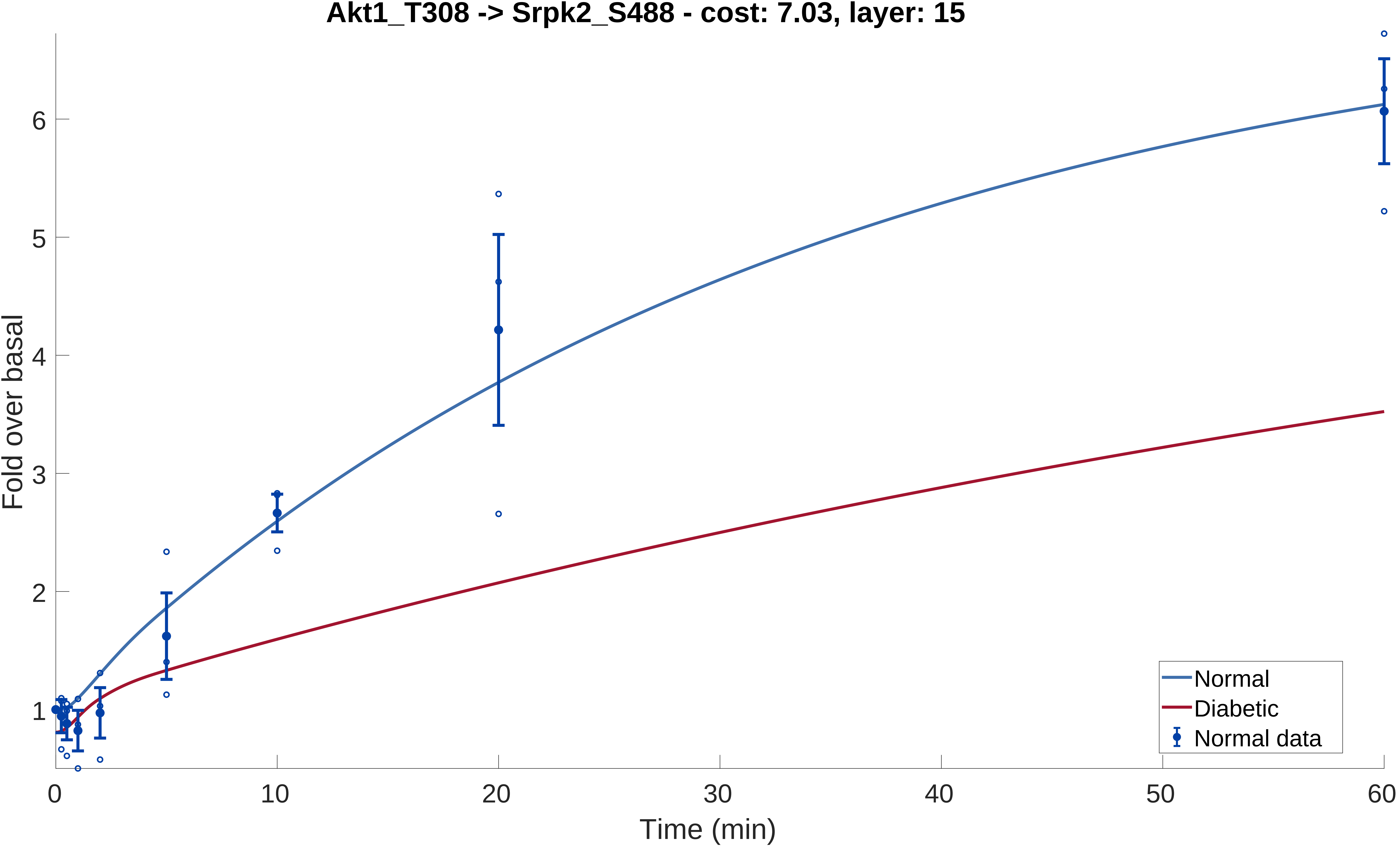

Akt1_T308 -> Srpk2_S488 - cost: 7.03, layer: 15
Fold over basal
Time (min)
0
10
20
30
40
50
60
1
2
3
4
5
6
Normal
Diabetic
Normal data

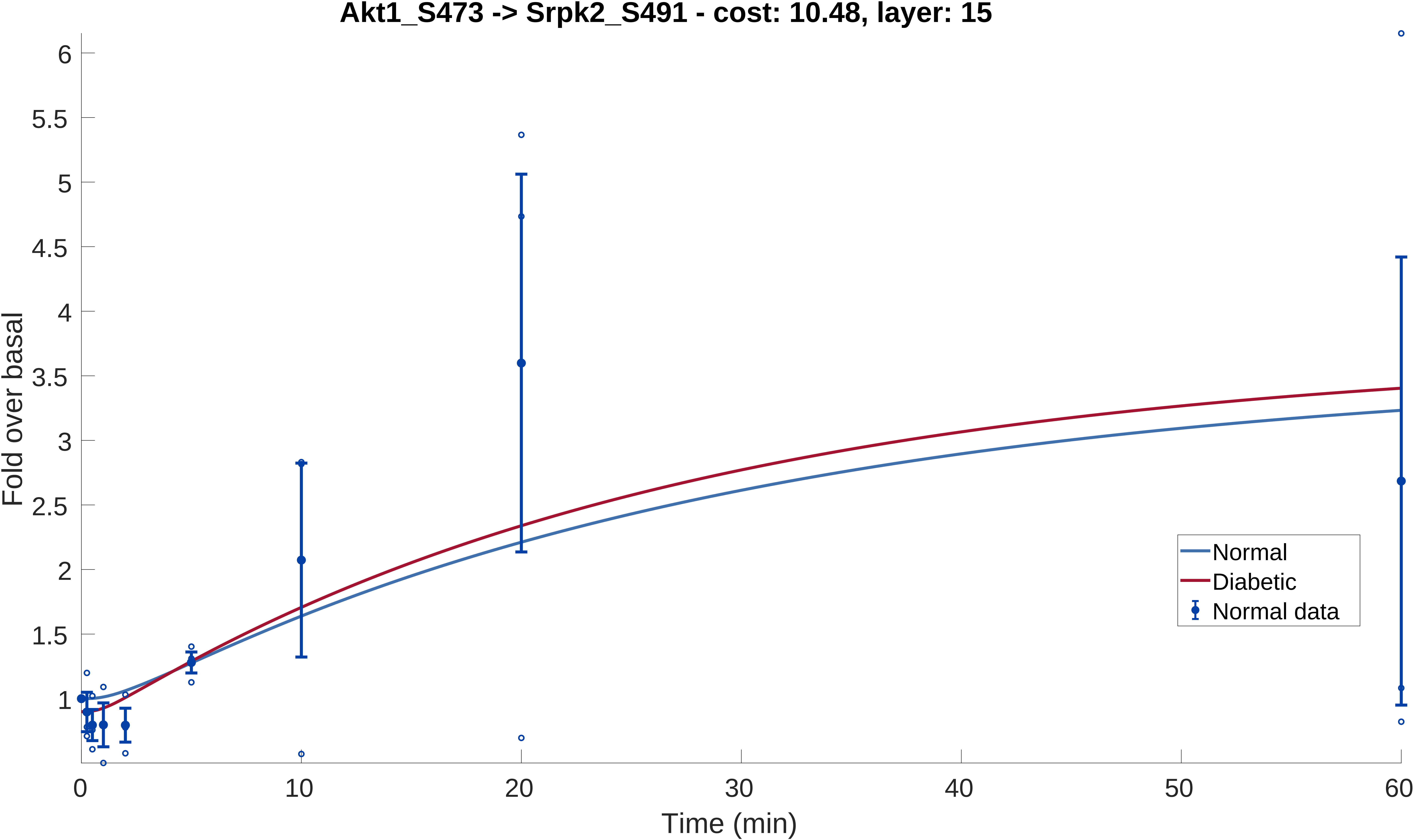

Akt1_S473 -> Srpk2_S491 - cost: 10.48, layer: 15
Fold over basal
Time (min)
0
10
20
30
40
50
60
1
1.5
2
2.5
3
3.5
4
4.5
5
5.5
6
Normal
Diabetic
Normal data

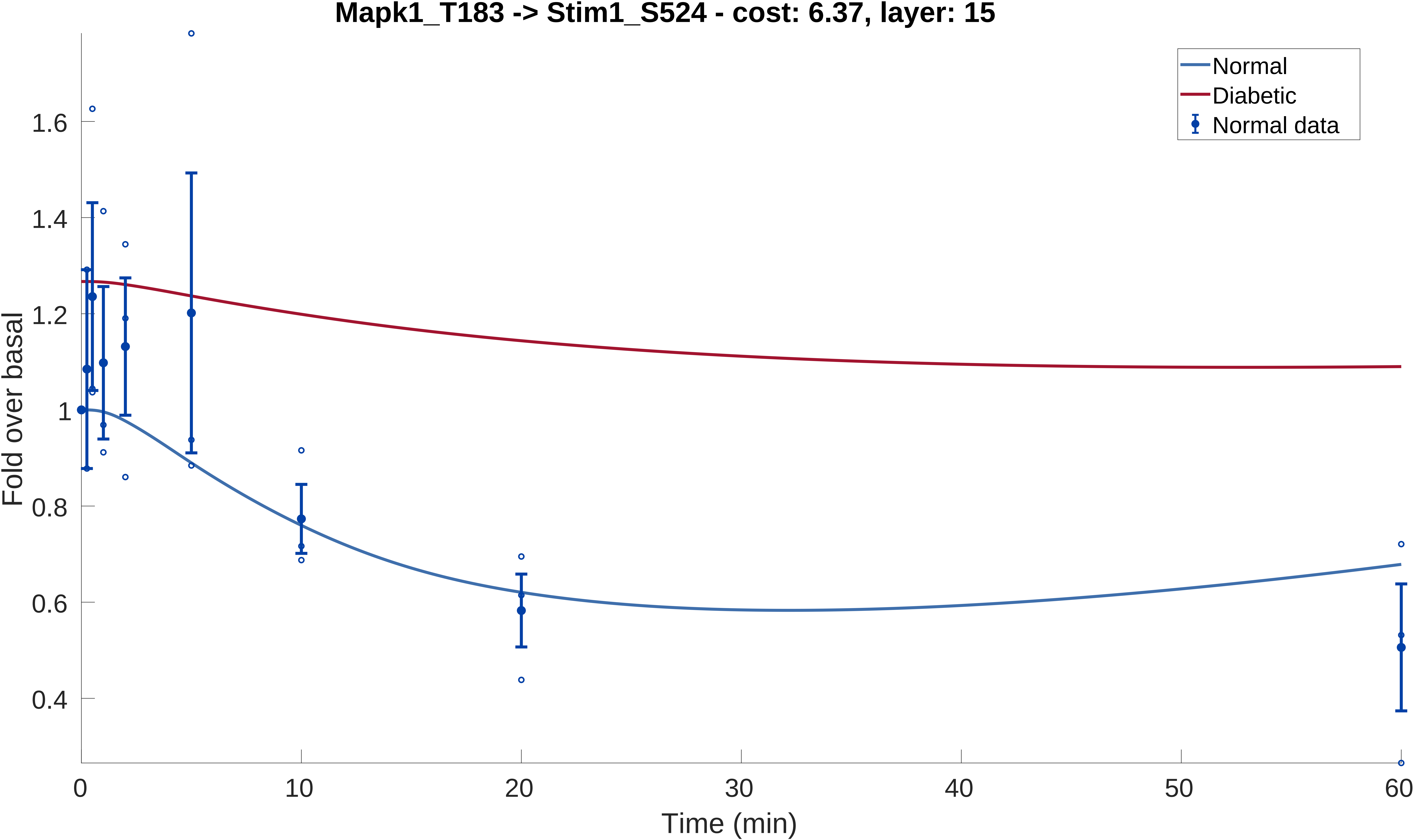

Mapk1_T183 -> Stim1_S524 - cost: 6.37, layer: 15
Normal
Diabetic
Normal data
Fold over basal
1.6
1.4
1.2
1
0.8
0.6
0.4
0
10
20
30
40
50
60
Time (min)

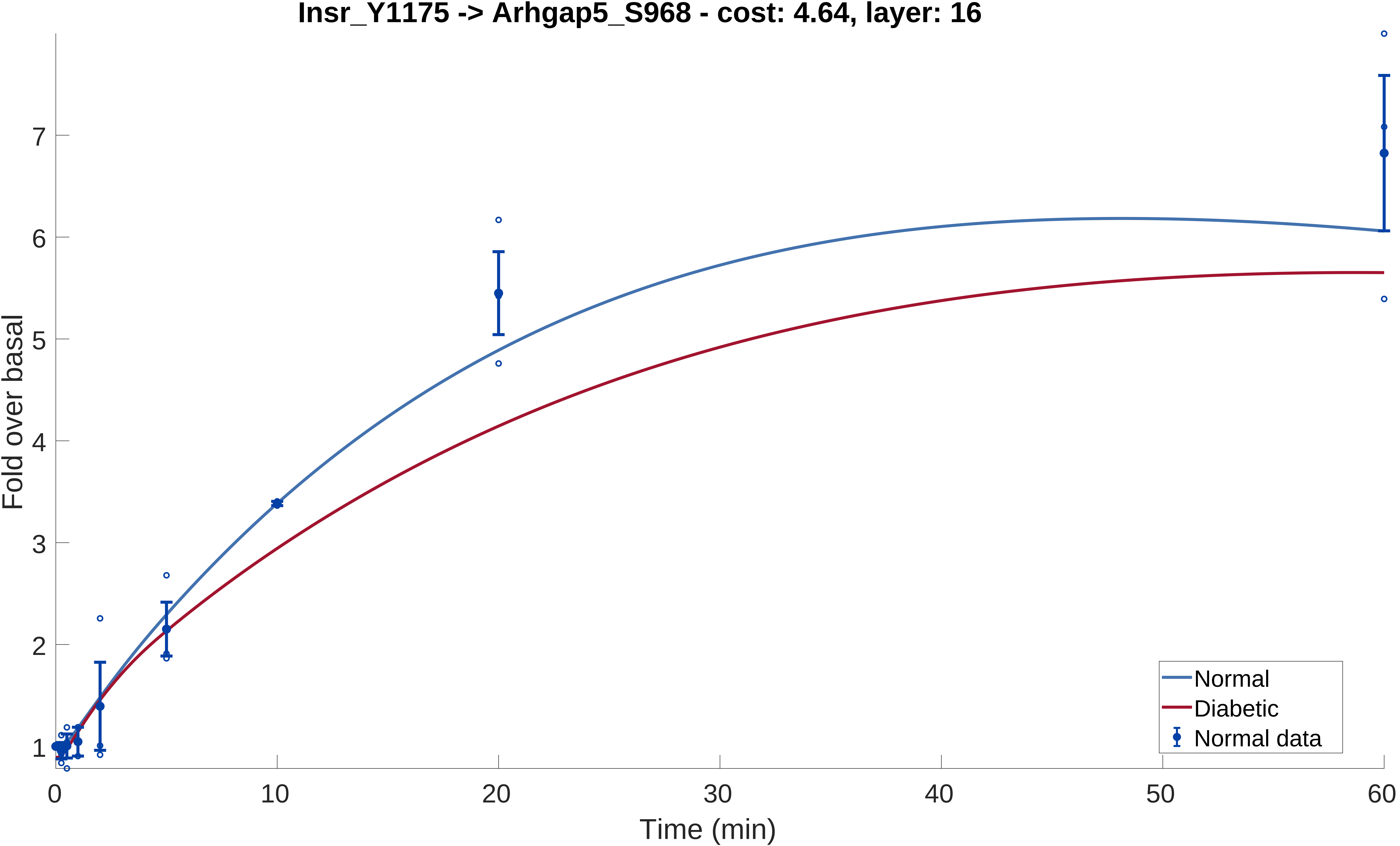

Insr_Y1175 -> Arhgap5_S968 - cost: 4.64, layer: 16
Fold over basal
Time (min)
1
2
3
4
5
6
7
0
10
20
30
40
50
60
Normal
Diabetic
Normal data

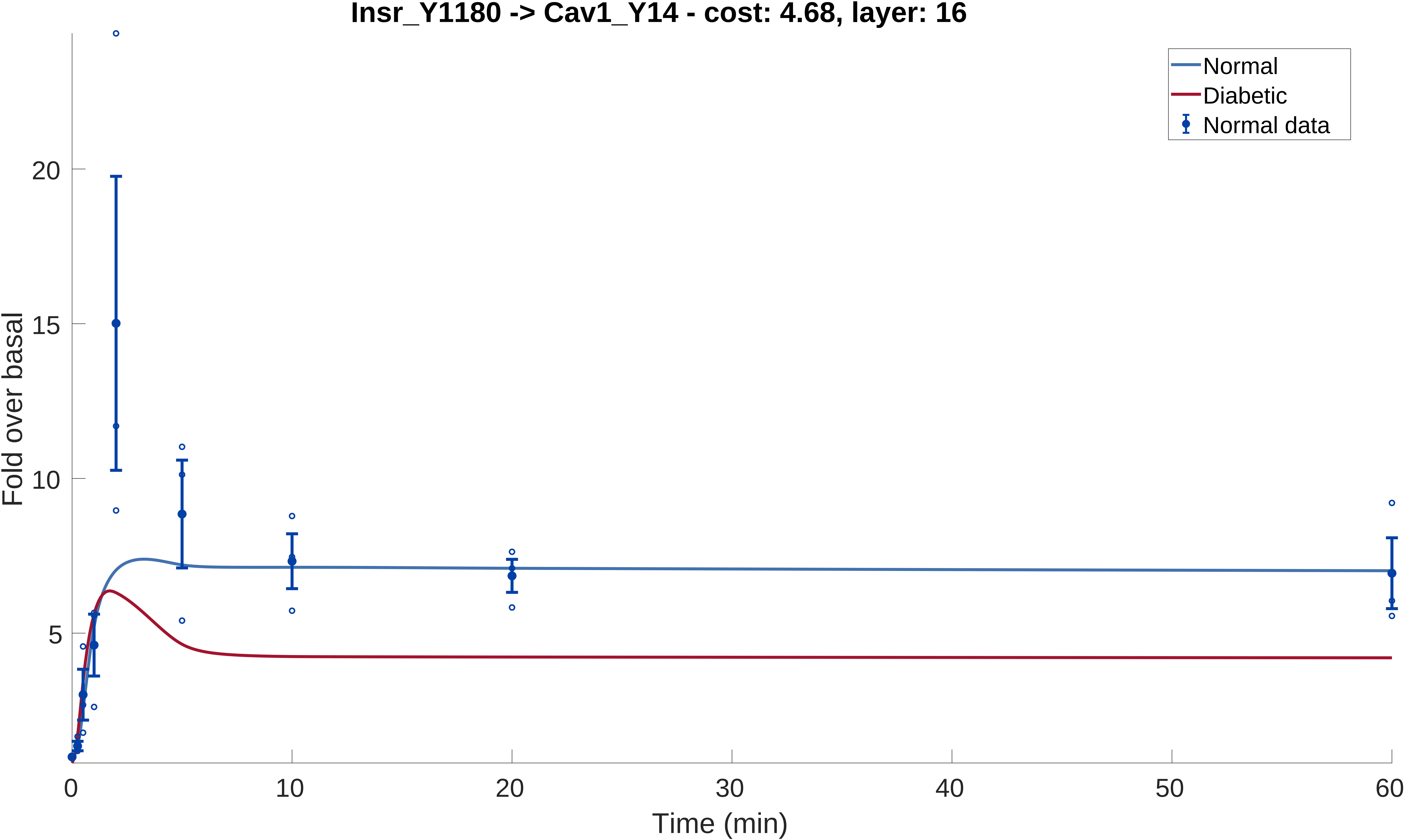

Insr_Y1180 -> Cav1_Y14 - cost: 4.68, layer: 16
Normal
Diabetic
Normal data
Fold over basal
Time (min)
20
15
10
5
0
10
20
30
40
50
60

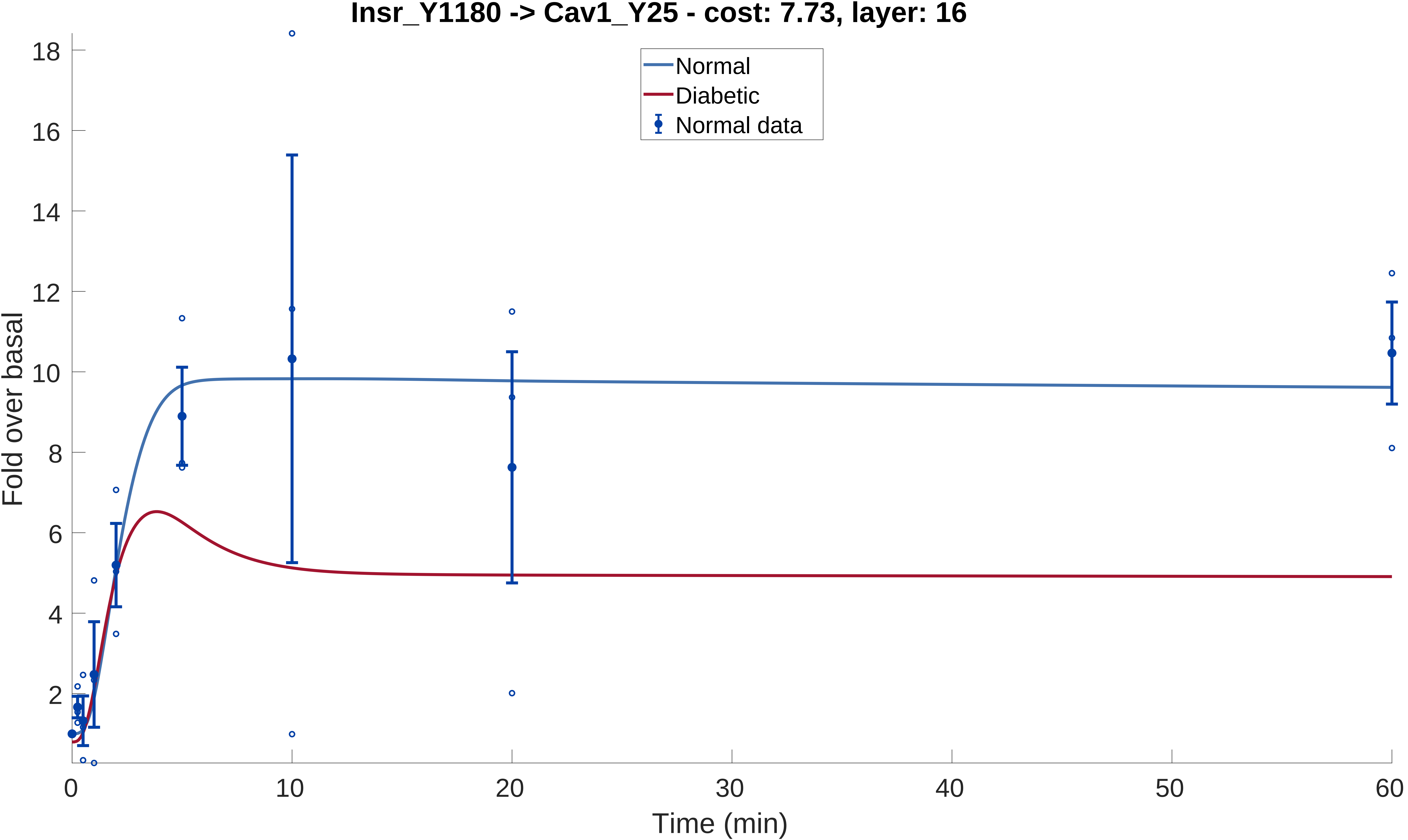
Insr_Y1180 -> Cav1_Y25 - cost: 7.73, layer: 16
Normal
Diabetic
Normal data
Fold over basal
Time (min)
0
10
20
30
40
50
60
2
4
6
8
10
12
14
16
18

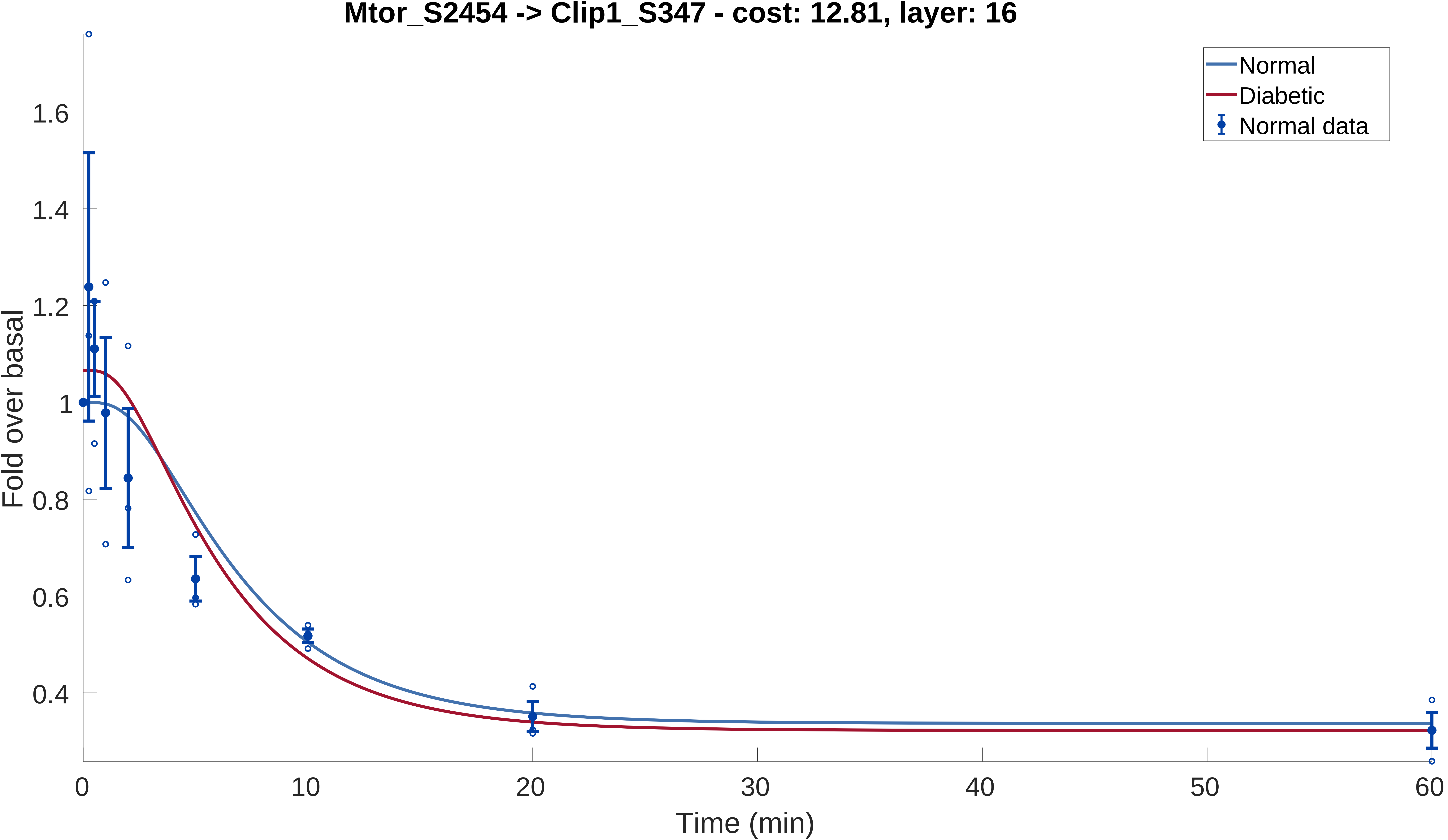
Mtor_S2454 -> Clip1_S347 - cost: 12.81, layer: 16
Fold over basal
1.6
1.4
1.2
1
0.8
0.6
0.4
0
10
20
30
40
50
60
Time (min)
Normal
Diabetic
Normal data

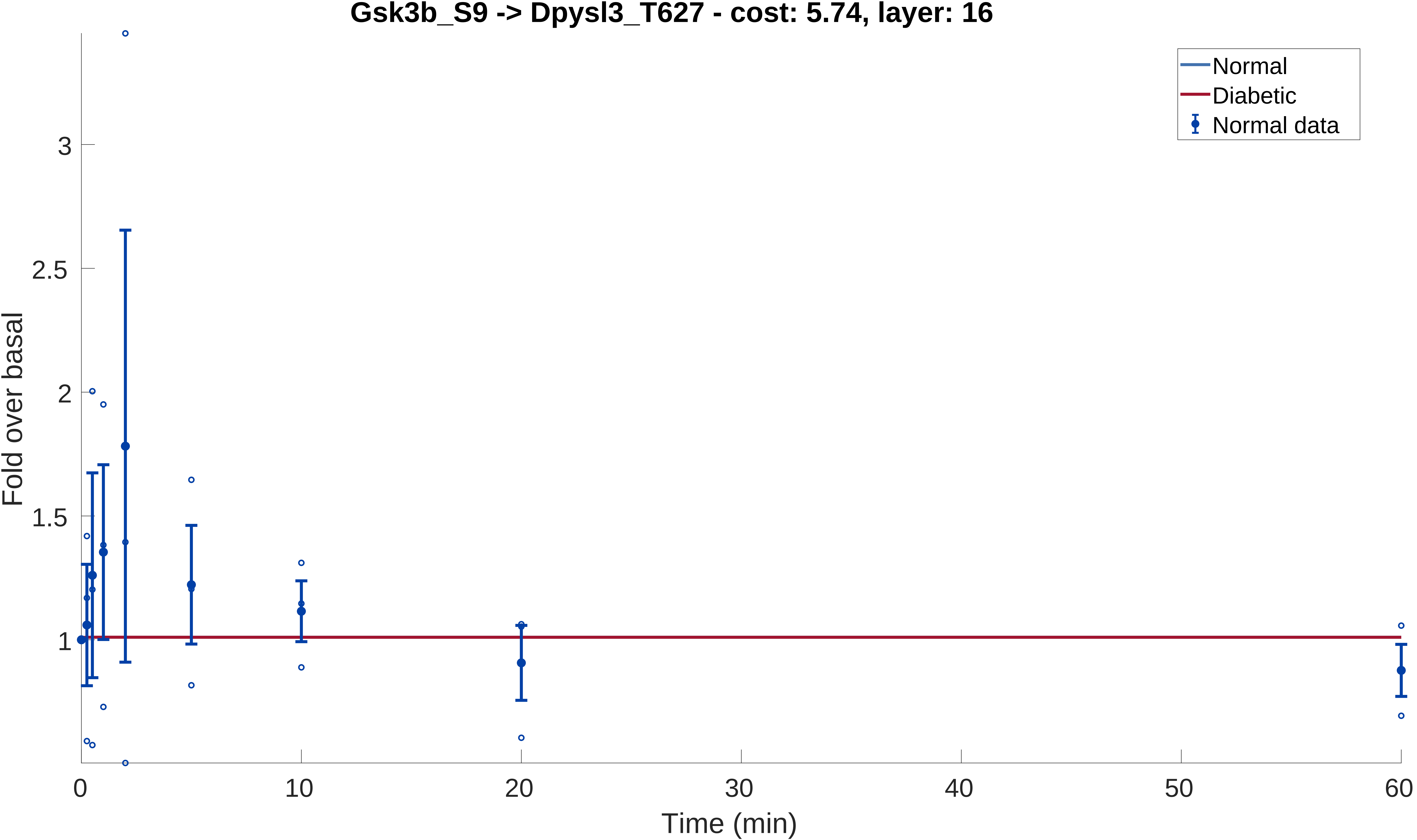

Gsk3b_S9 -> Dpysl3_T627 - cost: 5.74, layer: 16
Normal
Diabetic
Normal data
Fold over basal
3
2.5
2
1.5
1
0
10
20
30
40
50
60
Time (min)

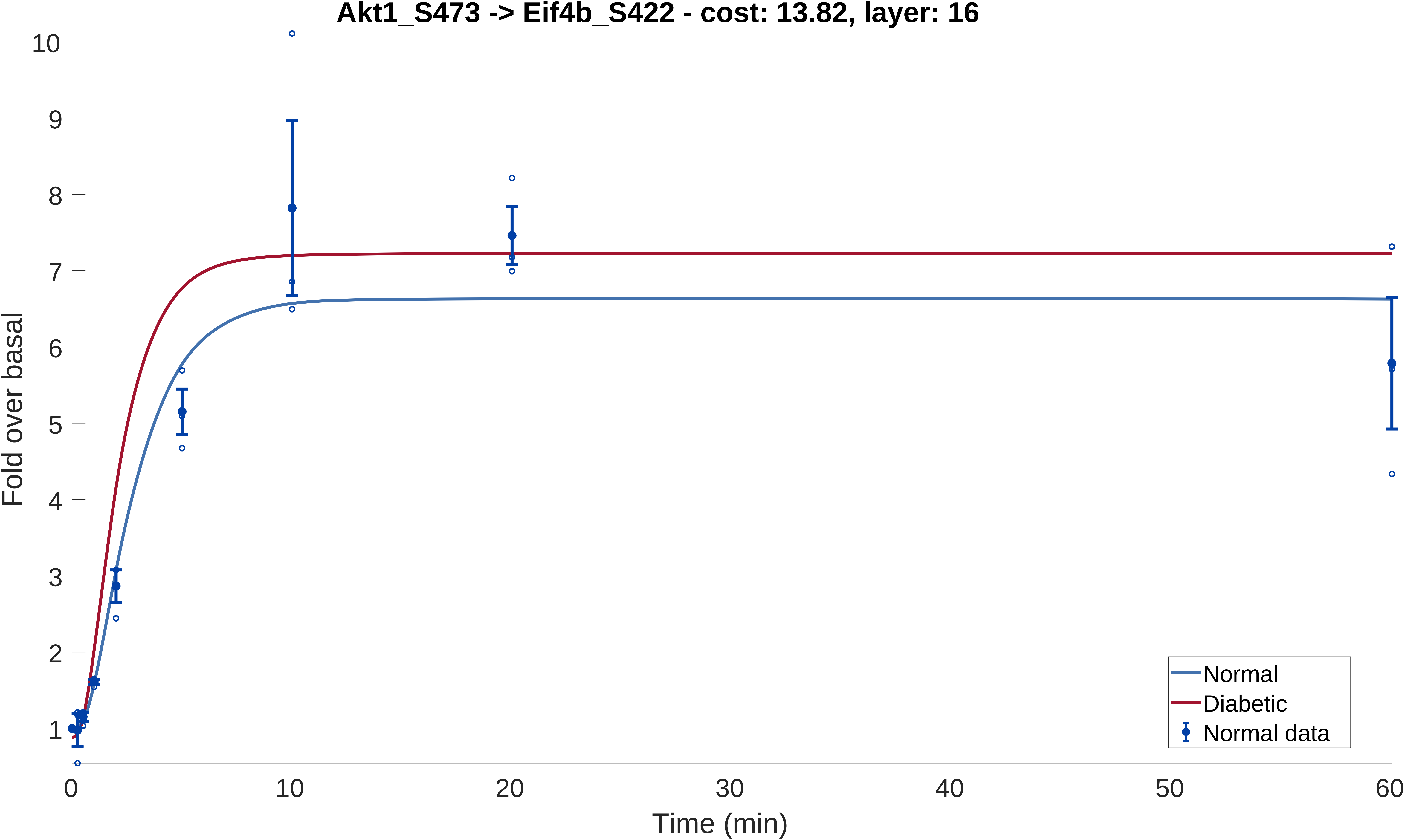

Akt1_S473 -> Eif4b_S422 - cost: 13.82, layer: 16
Fold over basal
Time (min)
1
2
3
4
5
6
7
8
9
10
0
10
20
30
40
50
60
Normal
Diabetic
Normal data

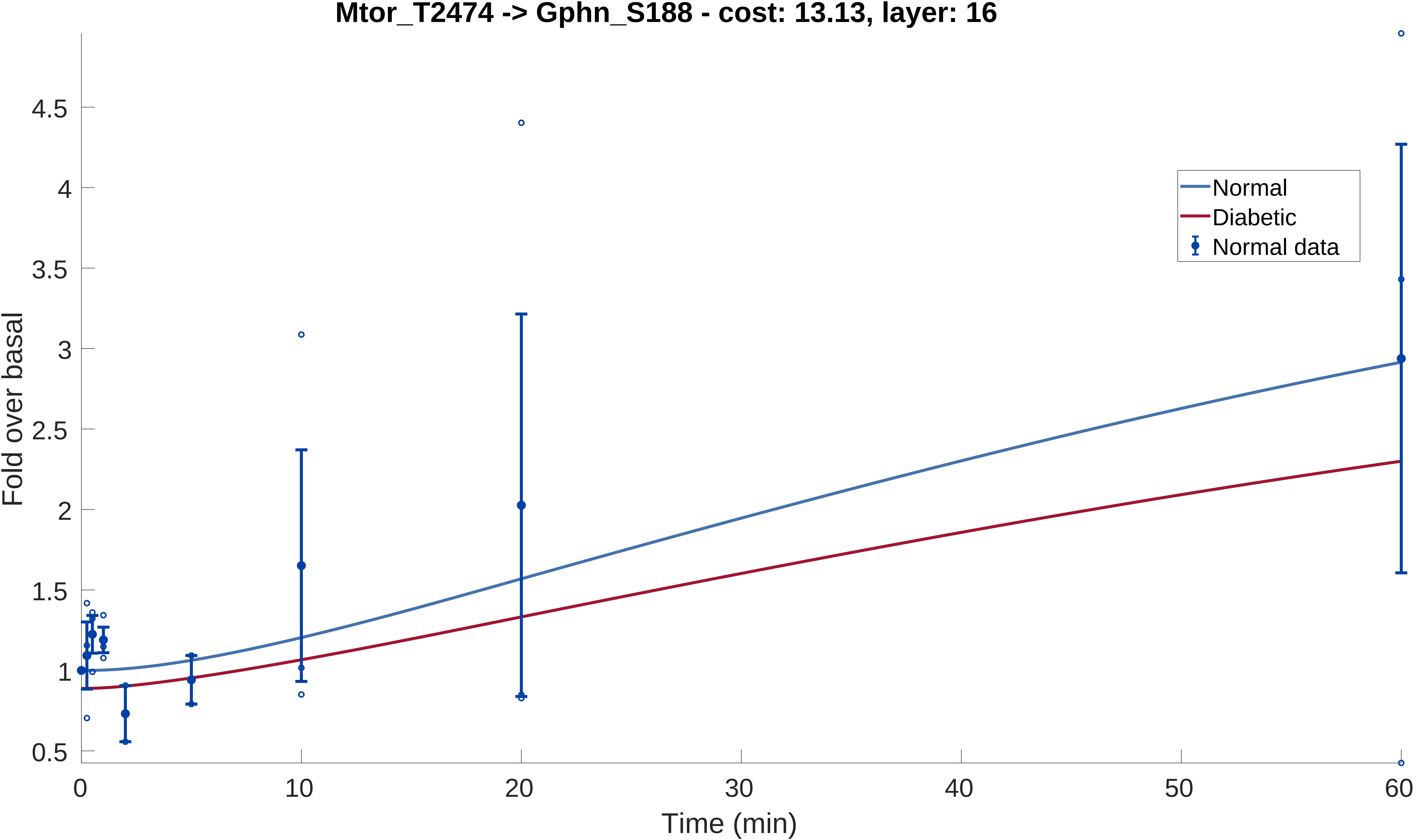

Mtor_T2474 -> Gphn_S188 - cost: 13.13, layer: 16
Normal
Diabetic
Normal data
Fold over basal
Time (min)
0.5
1
1.5
2
2.5
3
3.5
4
4.5
0
10
20
30
40
50
60

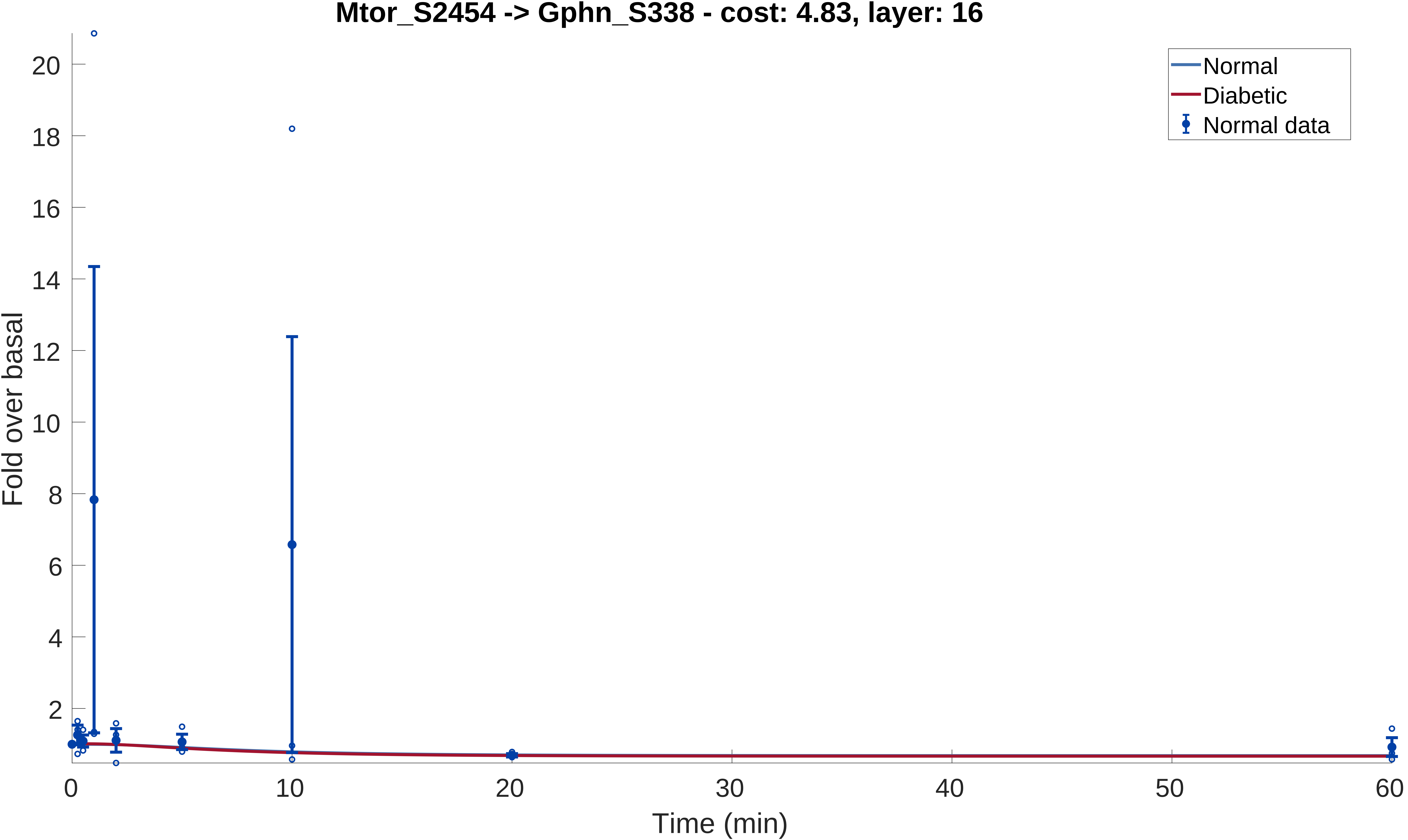

Mtor_S2454 -> Gphn_S338 - cost: 4.83, layer: 16
Normal
Diabetic
Normal data
Fold over basal
Time (min)
0
10
20
30
40
50
60
2
4
6
8
10
12
14
16
18
20

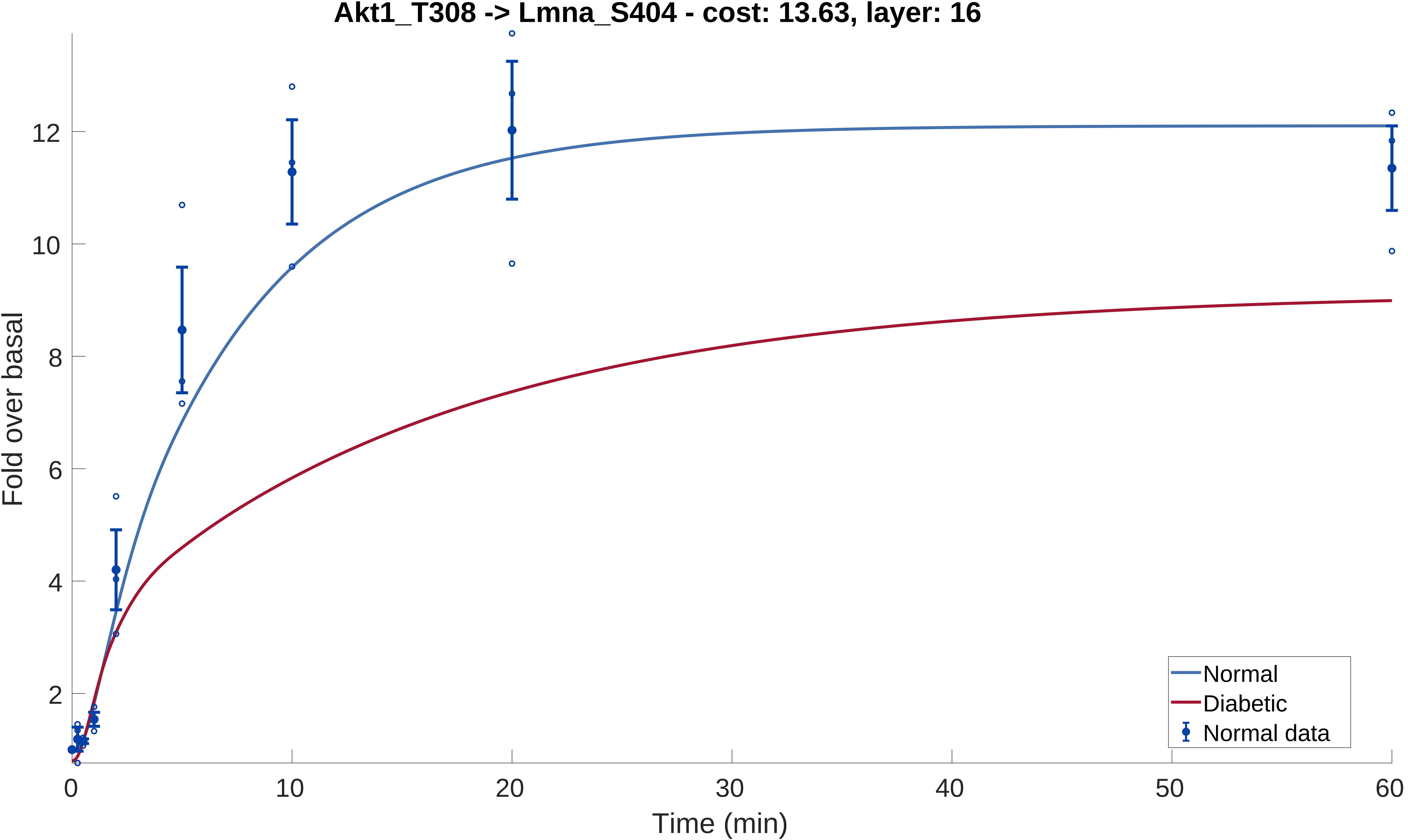

Akt1_T308 -> Lmna_S404 - cost: 13.63, layer: 16
Fold over basal
12
10
8
6
4
2
0
10
20
30
40
50
60
Time (min)
Normal
Diabetic
Normal data

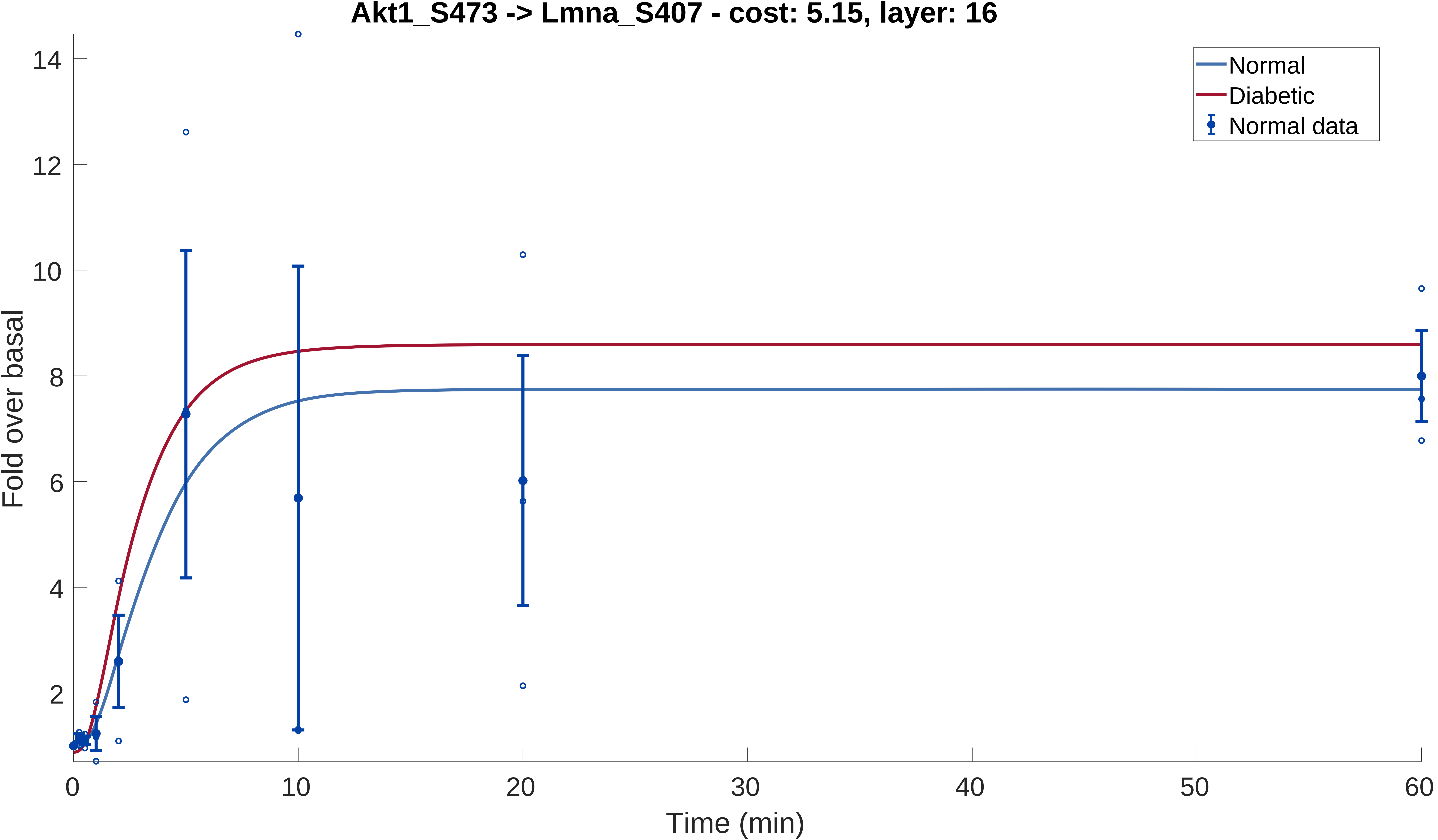
Akt1_S473 -> Lmna_S407 - cost: 5.15, layer: 16
Normal
Diabetic
Normal data
Fold over basal
Time (min)
14
12
10
8
6
4
2
0
10
20
30
40
50
60

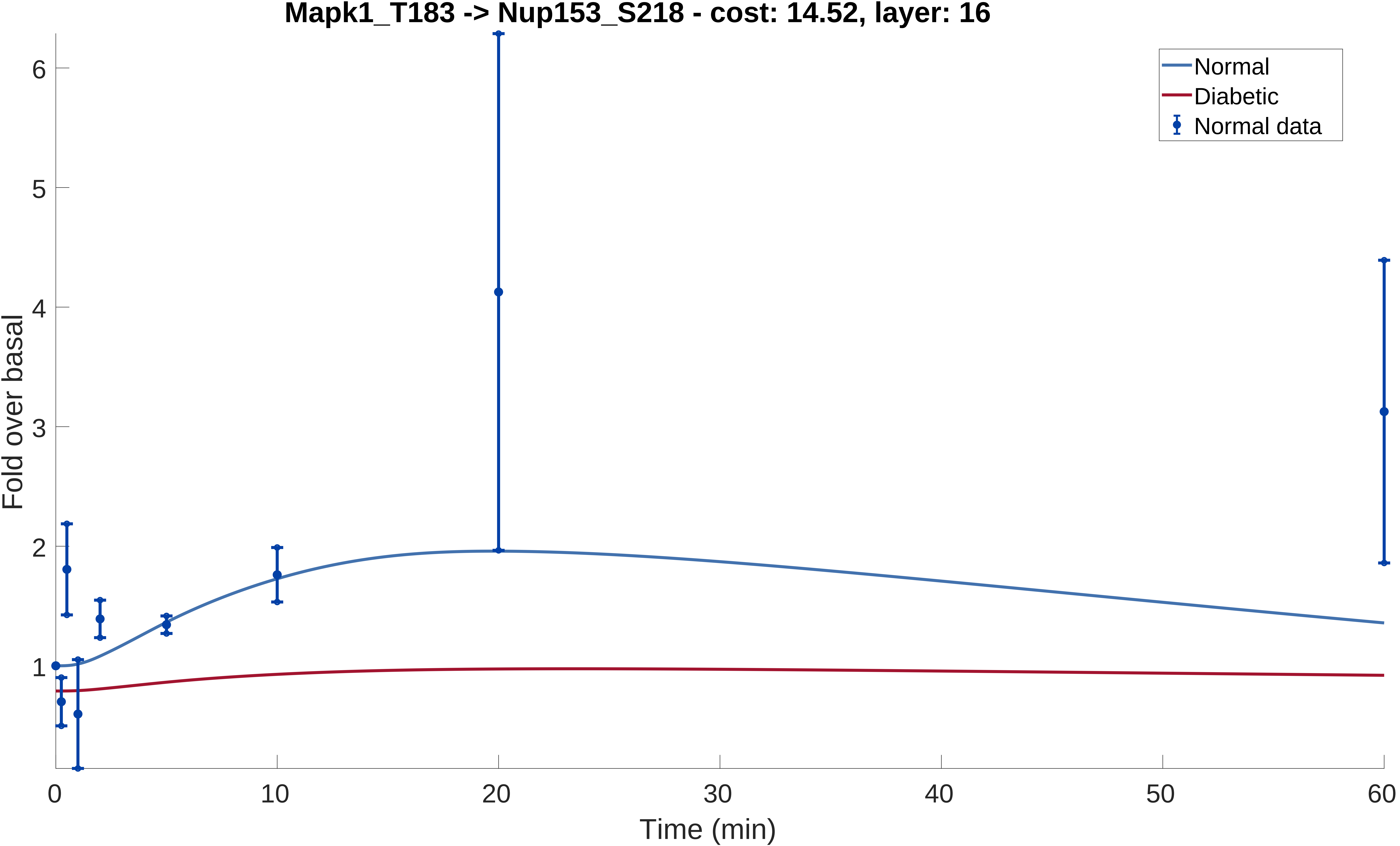

Mapk1_T183 -> Nup153_S218 - cost: 14.52, layer: 16
Normal
Diabetic
Normal data
Fold over basal
Time (min)
0
10
20
30
40
50
60
1
2
3
4
5
6

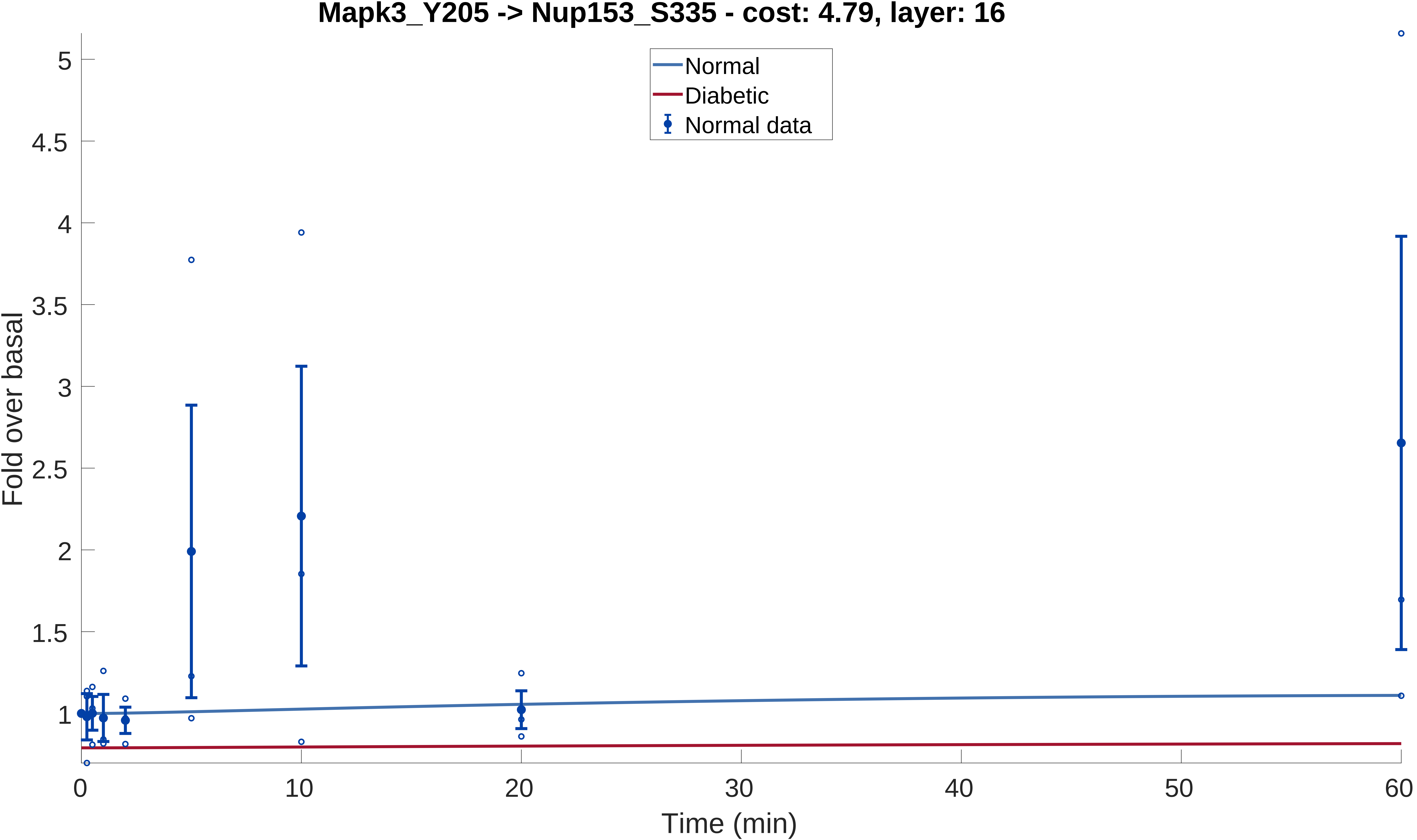

Mapk3_Y205 -> Nup153_S335 - cost: 4.79, layer: 16
Normal
Diabetic
Normal data
Fold over basal
Time (min)
0
10
20
30
40
50
60
1
1.5
2
2.5
3
3.5
4
4.5
5

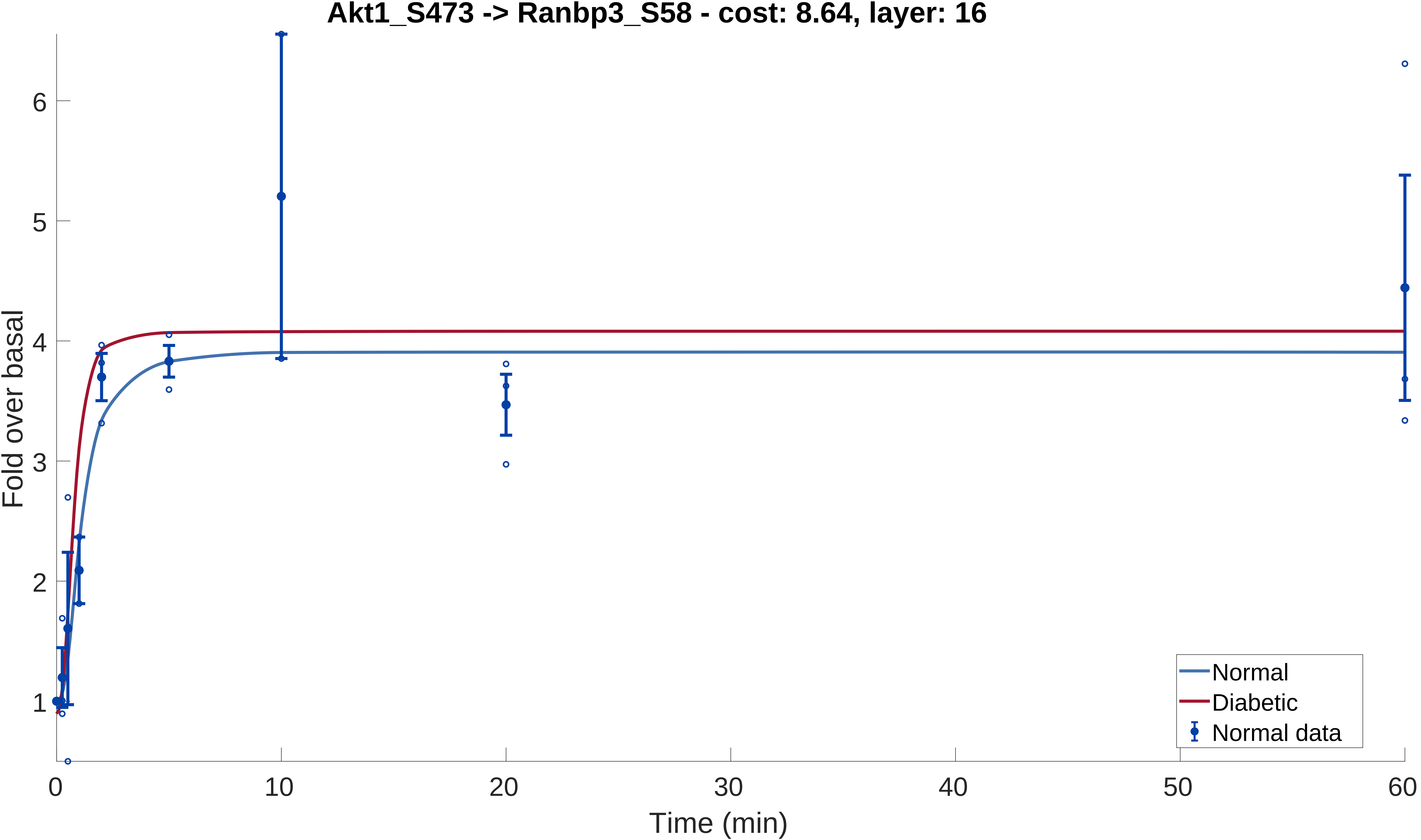

Akt1_S473 -> Ranbp3_S58 - cost: 8.64, layer: 16
Fold over basal
Time (min)
Normal
Diabetic
Normal data
0
10
20
30
40
50
60
1
2
3
4
5
6

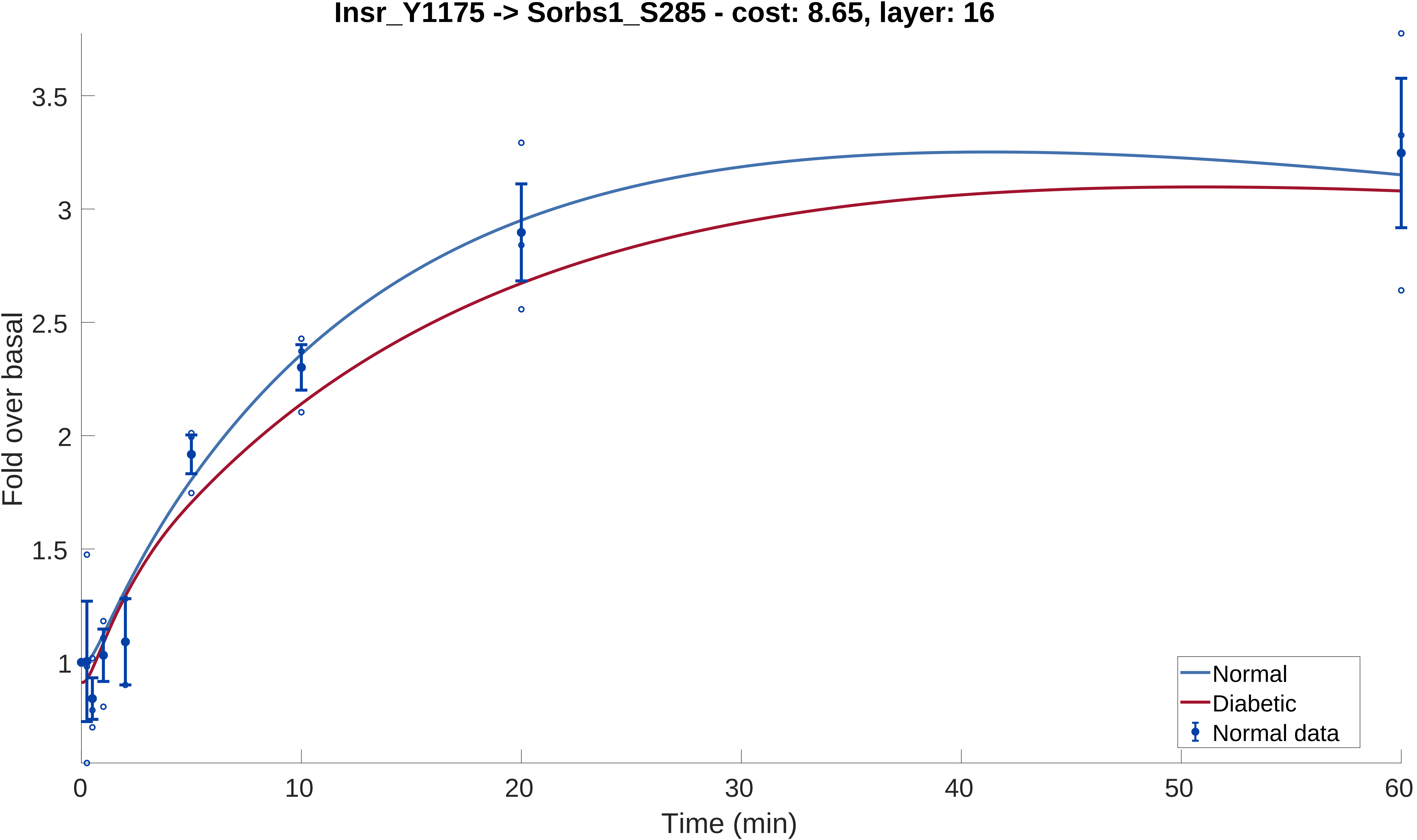

Insr_Y1175 -> Sorbs1_S285 - cost: 8.65, layer: 16
Fold over basal
3.5
3
2.5
2
1.5
1
0
10
20
30
40
50
60
Time (min)
Normal
Diabetic
Normal data

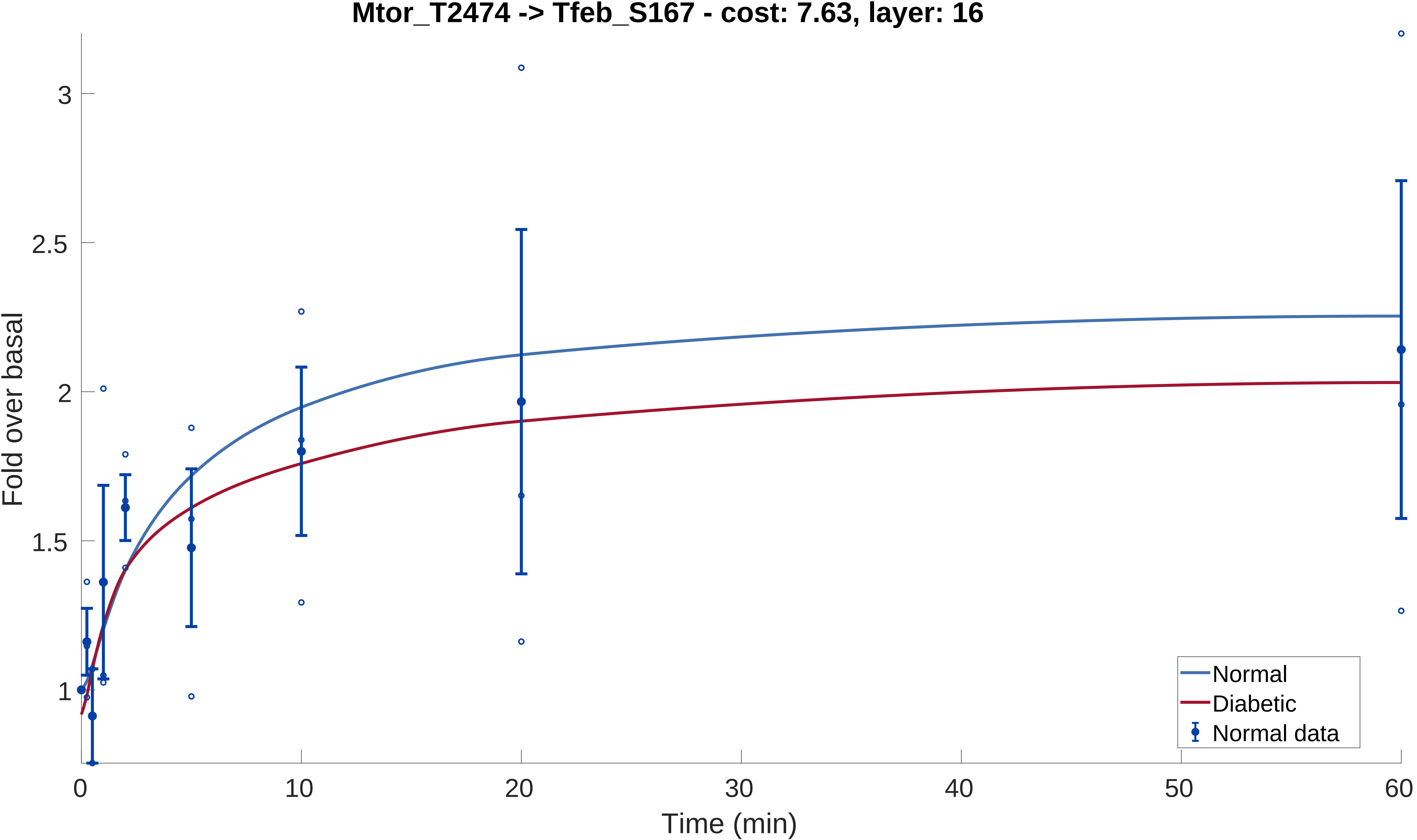

Mtor_T2474 -> Tfeb_S167 - cost: 7.63, layer: 16
Fold over basal
3
2.5
2
1.5
1
0
10
20
30
40
50
60
Time (min)
Normal
Diabetic
Normal data

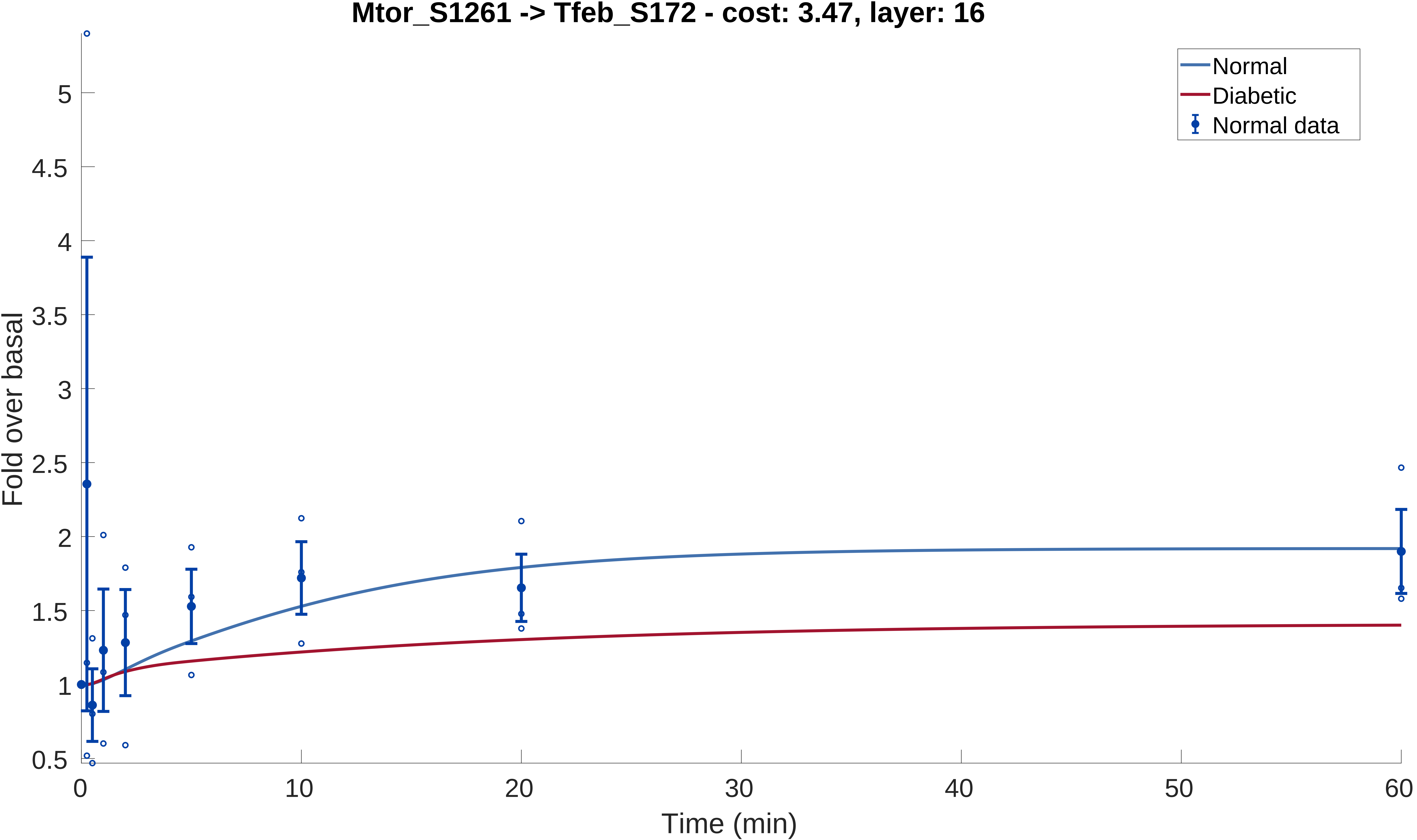
Mtor_S1261 -> Tfeb_S172 - cost: 3.47, layer: 16
Normal
Diabetic
Normal data
Fold over basal
5
4.5
4
3.5
3
2.5
2
1.5
1
0.5
0
10
20
30
40
50
60
Time (min)

Mtor_T2474 -> Tfeb_S180 - cost: 8.51, layer: 16

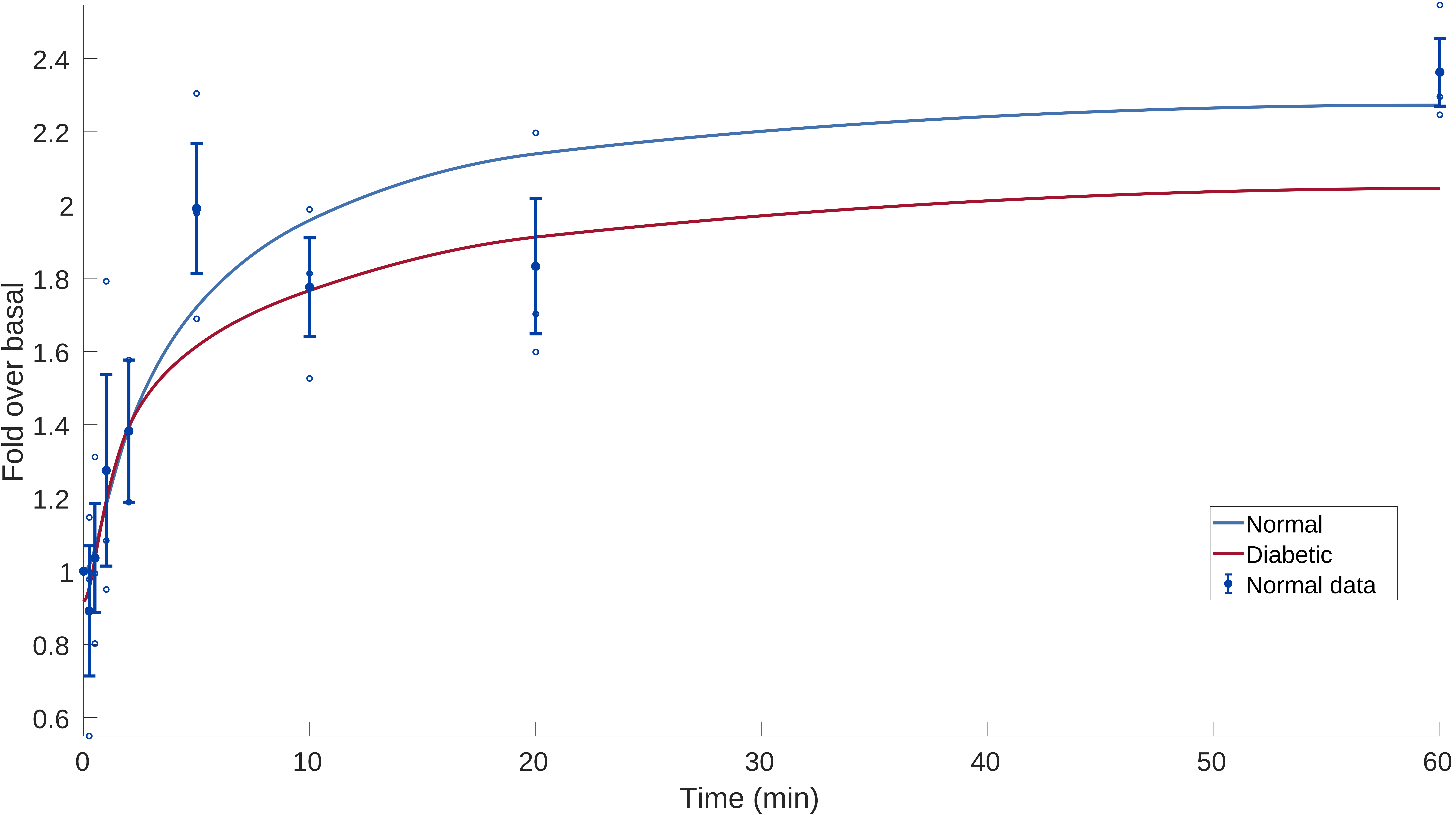

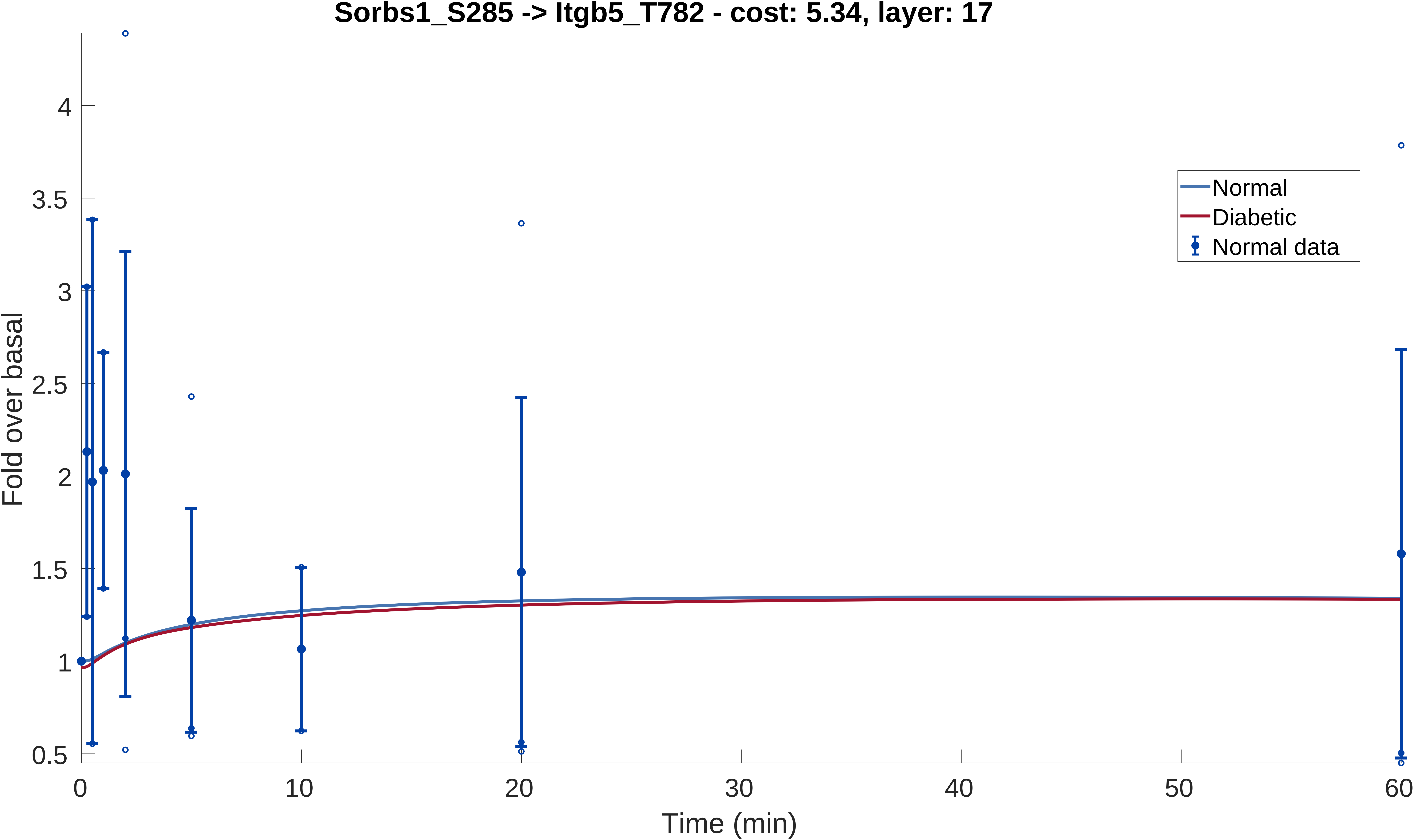

Sorbs1_S285 -> Itgb5_T782 - cost: 5.34, layer: 17
Fold over basal
0.5
1
1.5
2
2.5
3
3.5
4
0
10
20
30
40
50
60
Time (min)
Normal
Diabetic
Normal data

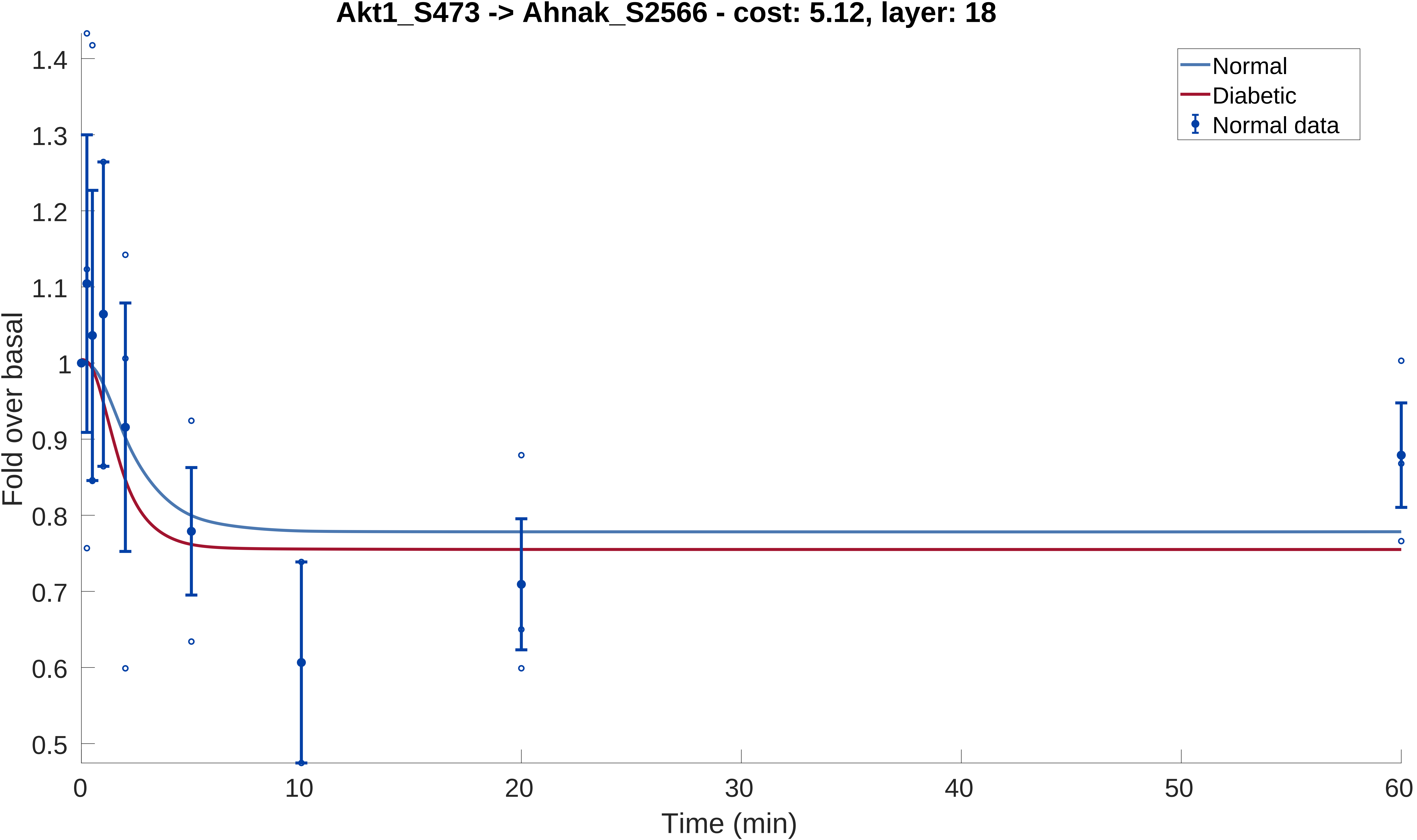
Akt1_S473 -> Ahnak_S2566 - cost: 5.12, layer: 18
Normal
Diabetic
Normal data
Fold over basal
Time (min)
1.4
1.3
1.2
1.1
1
0.9
0.8
0.7
0.6
0.5
0
10
20
30
40
50
60

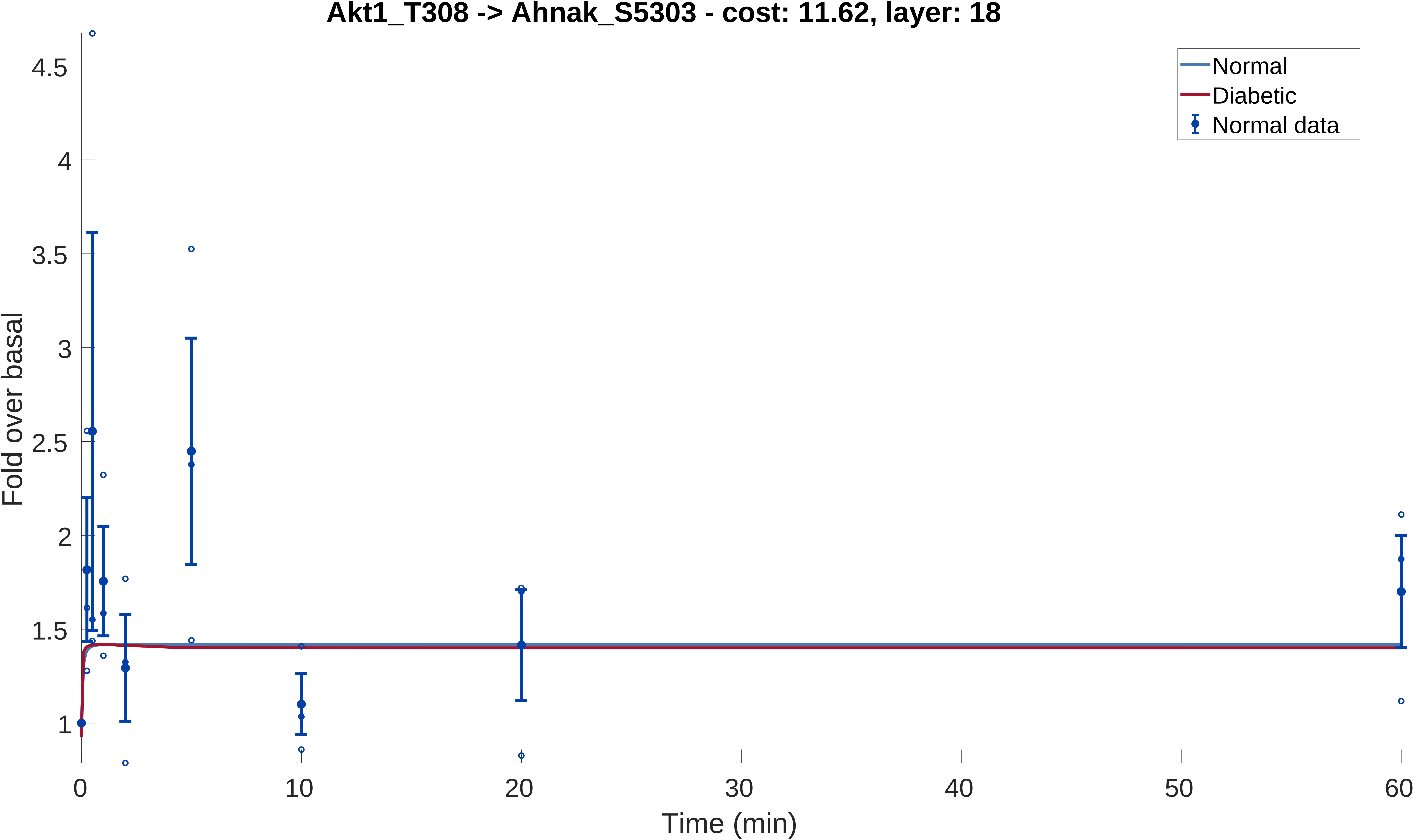

Akt1_T308 -> Ahnak_S5303 - cost: 11.62, layer: 18
Fold over basal
Time (min)
Normal
Diabetic
Normal data
4.5
4
3.5
3
2.5
2
1.5
1
0
10
20
30
40
50
60

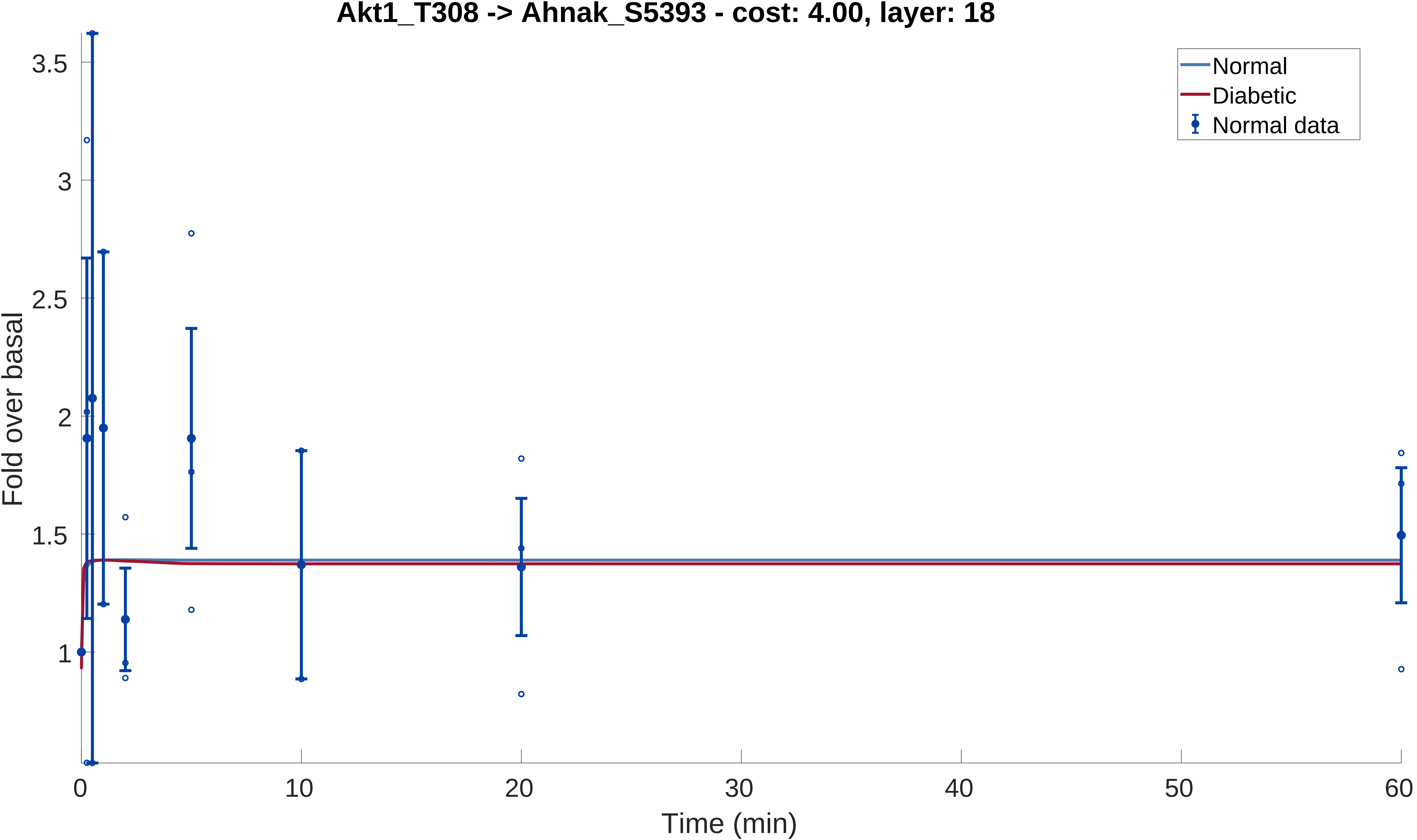

Akt1_T308 -> Ahnak_S5393 - cost: 4.00, layer: 18
Normal
Diabetic
Normal data
Fold over basal
Time (min)
3.5
3
2.5
2
1.5
1
0
10
20
30
40
50
60

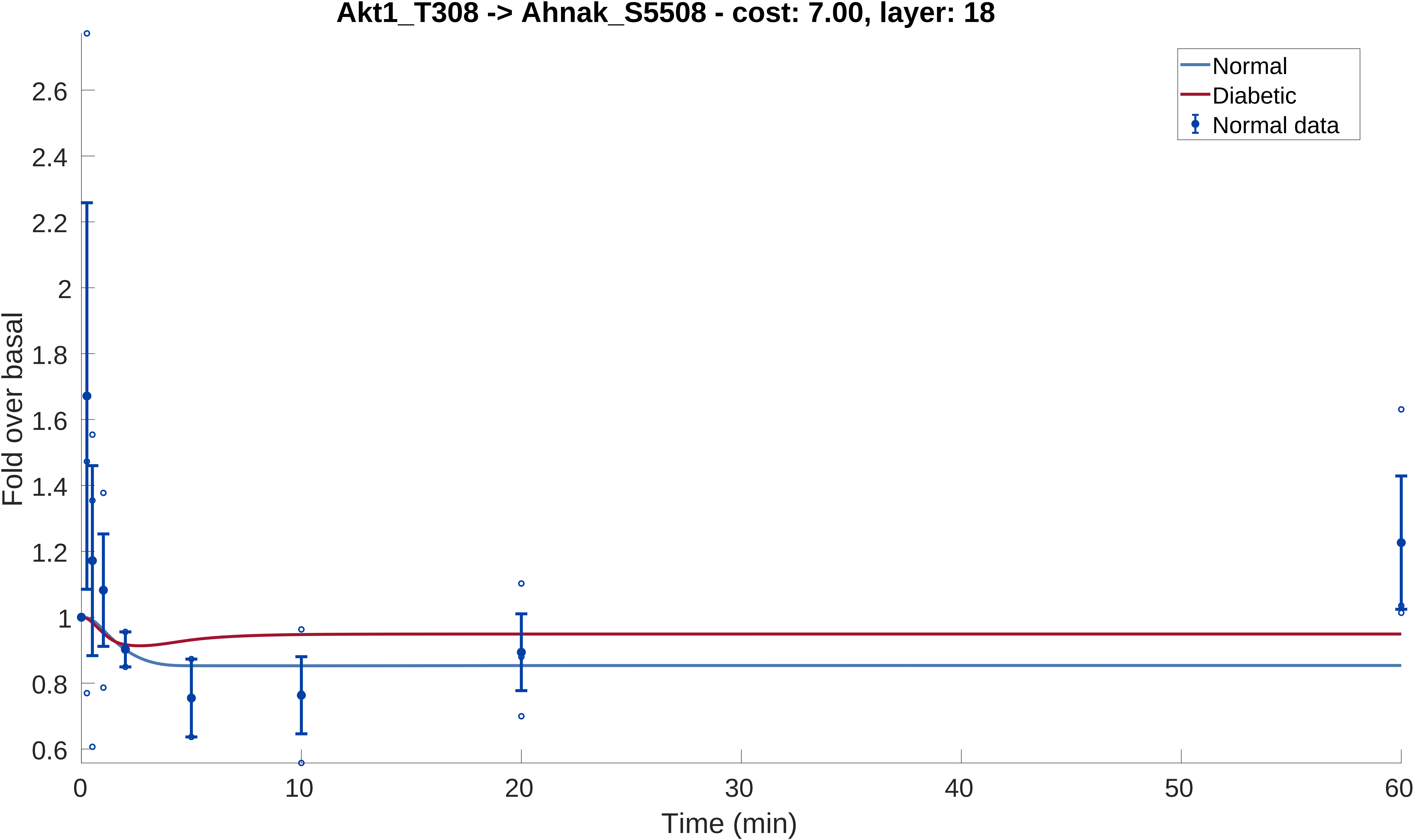

Akt1_T308 -> Ahnak_S5508 - cost: 7.00, layer: 18
Normal
Diabetic
Normal data
Fold over basal
Time (min)
0.6
0.8
1
1.2
1.4
1.6
1.8
2
2.2
2.4
2.6
0
10
20
30
40
50
60

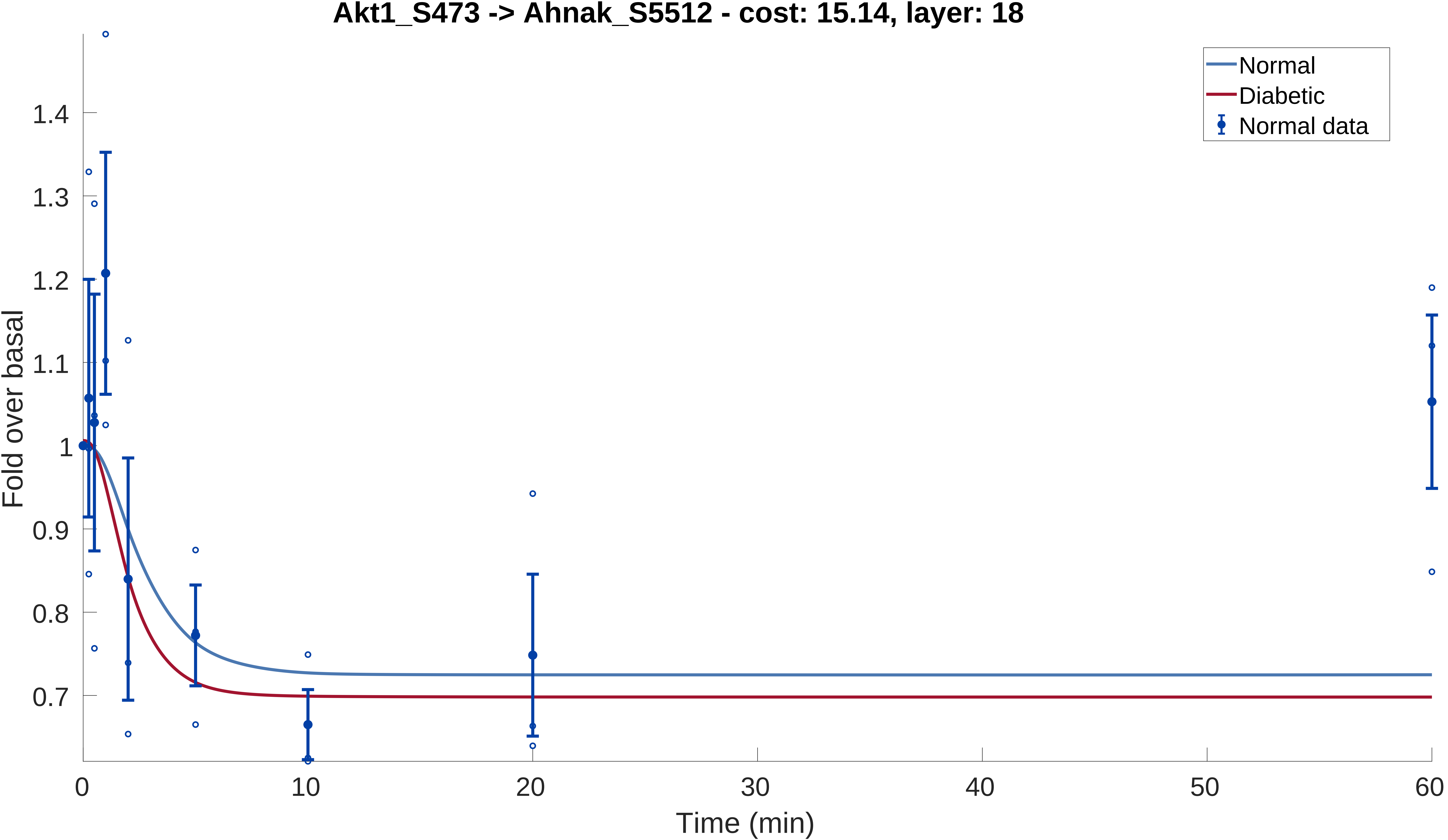

Akt1_S473 -> Ahnak_S5512 - cost: 15.14, layer: 18
Normal
Diabetic
Normal data
Fold over basal
Time (min)
1.4
1.3
1.2
1.1
1
0.9
0.8
0.7
0
10
20
30
40
50
60

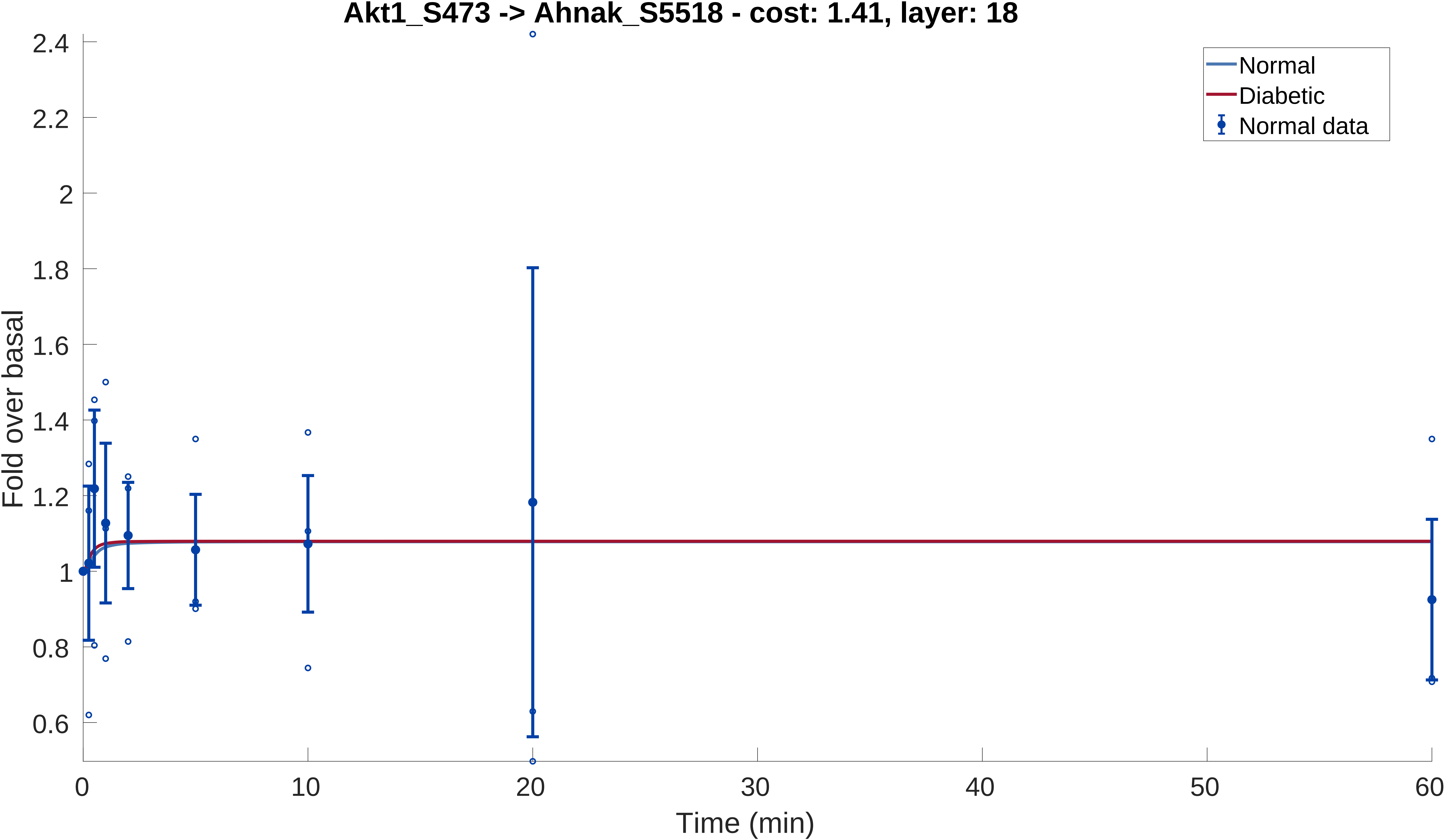

Akt1_S473 -> Ahnak_S5518 - cost: 1.41, layer: 18
Normal
Diabetic
Normal data
Fold over basal
Time (min)
2.4
2.2
2
1.8
1.6
1.4
1.2
1
0.8
0.6
0
10
20
30
40
50
60

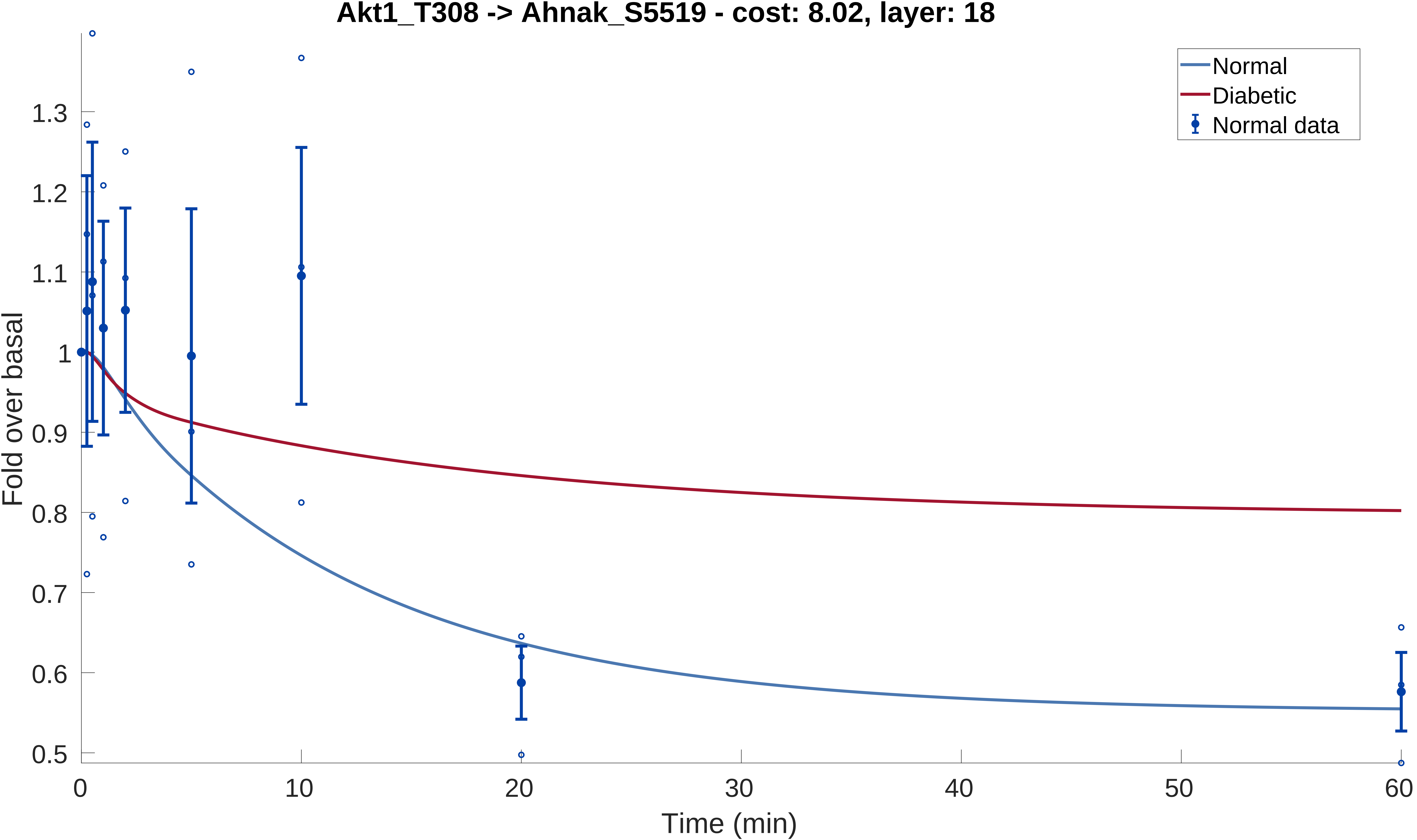

Akt1_T308 -> Ahnak_S5519 - cost: 8.02, layer: 18
Normal
Diabetic
Normal data
Fold over basal
Time (min)
1.3
1.2
1.1
1
0.9
0.8
0.7
0.6
0.5
0
10
20
30
40
50
60

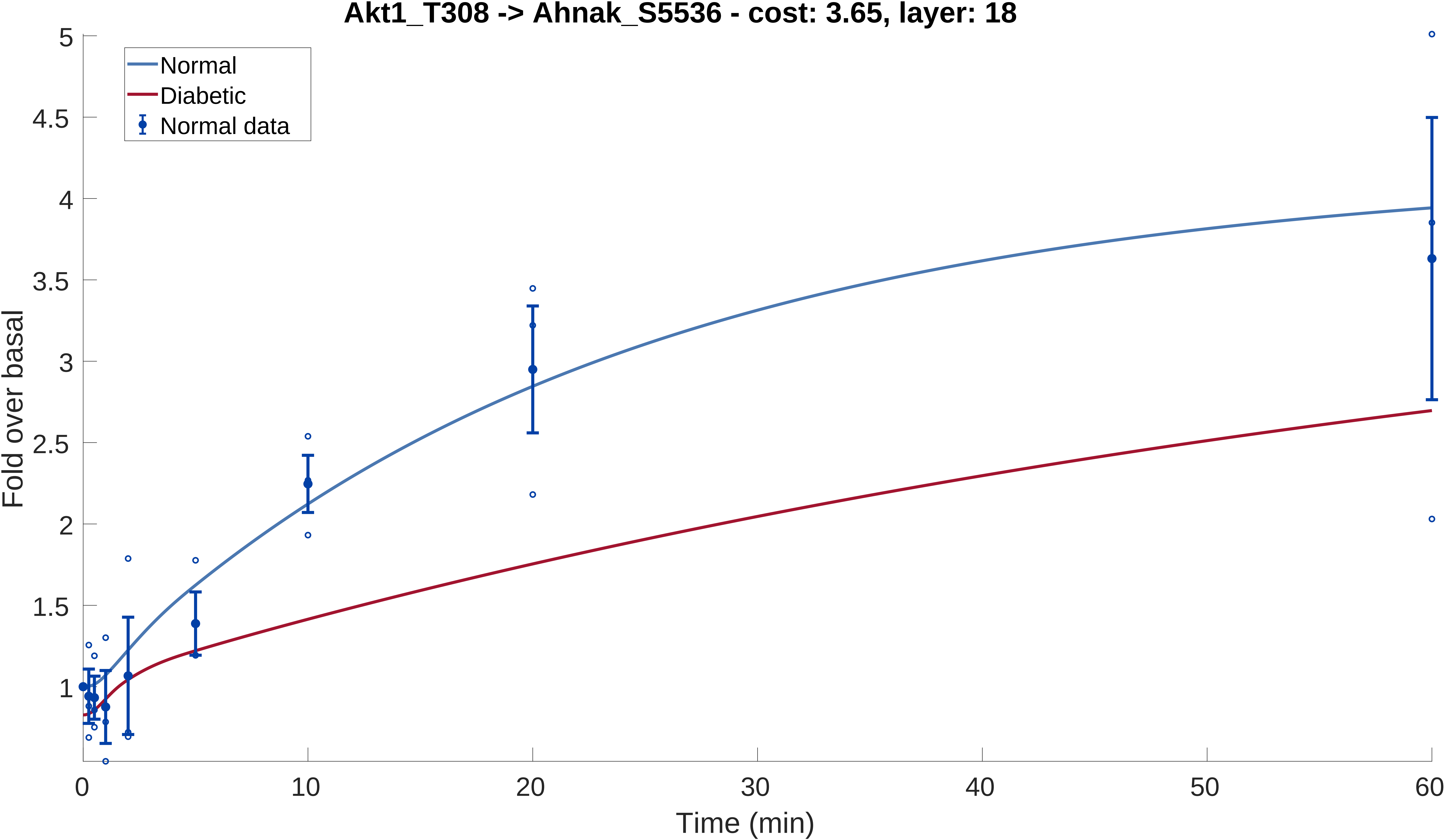

Akt1_T308 -> Ahnak_S5536 - cost: 3.65, layer: 18
Normal
Diabetic
Normal data
Fold over basal
Time (min)
5
4.5
4
3.5
3
2.5
2
1.5
1
0
10
20
30
40
50
60

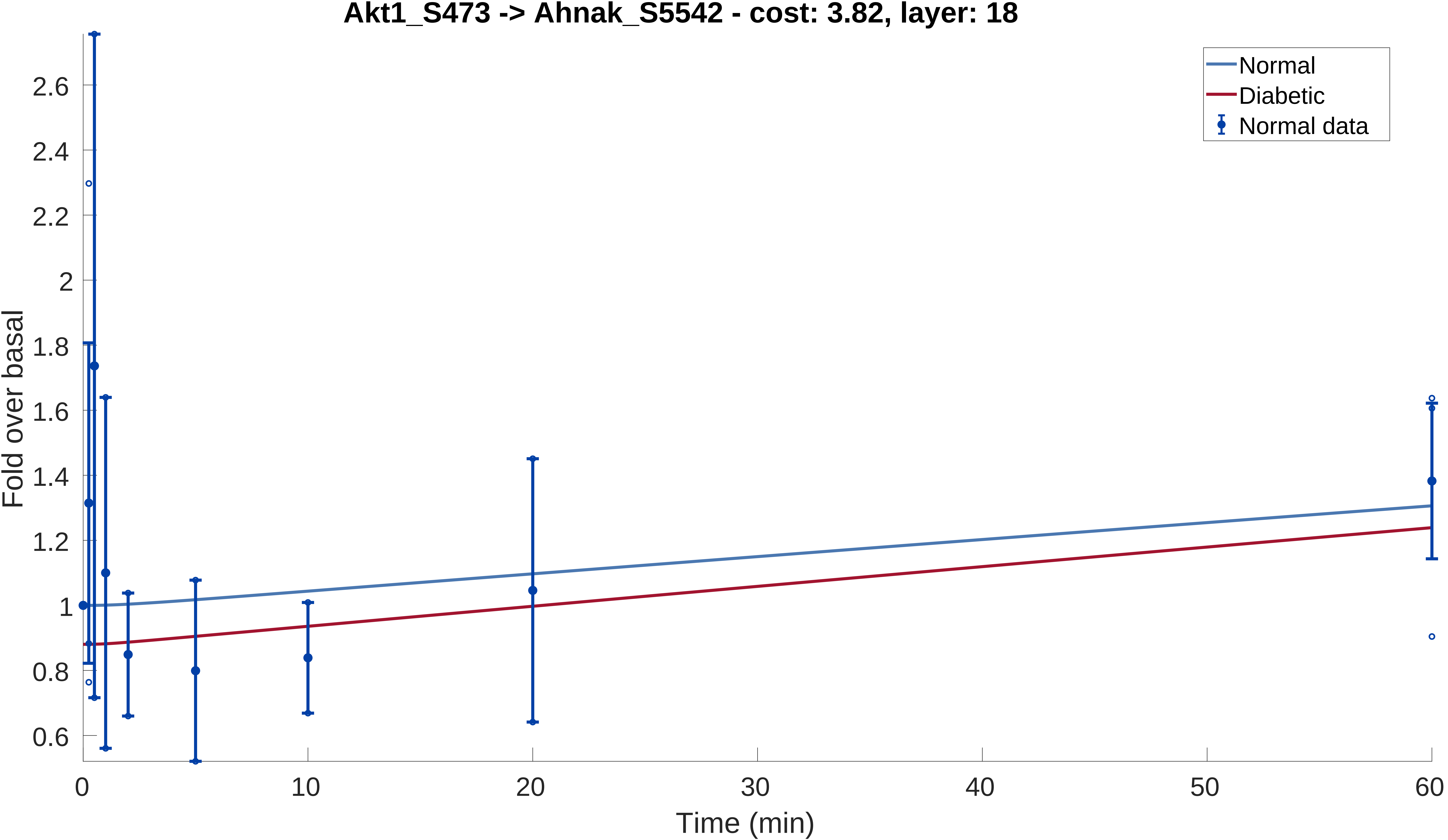

Akt1_S473 -> Ahnak_S5542 - cost: 3.82, layer: 18
Normal
Diabetic
Normal data
Fold over basal
2.6
2.4
2.2
2
1.8
1.6
1.4
1.2
1
0.8
0.6
0
10
20
30
40
50
60
Time (min)

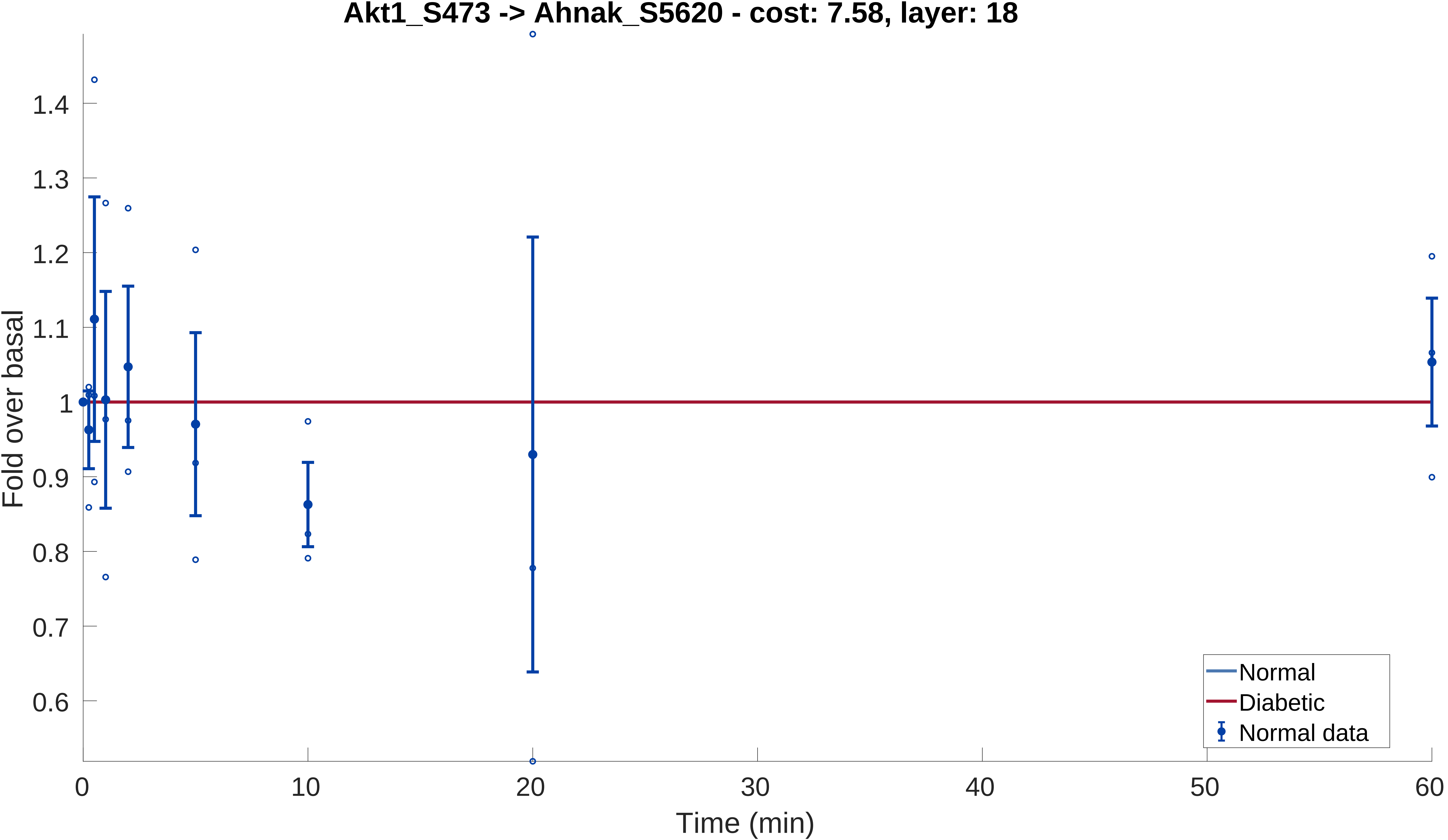

Akt1_S473 -> Ahnak_S5620 - cost: 7.58, layer: 18
Fold over basal
1.4
1.3
1.2
1.1
1
0.9
0.8
0.7
0.6
0
10
20
30
40
50
60
Time (min)
Normal
Diabetic
Normal data

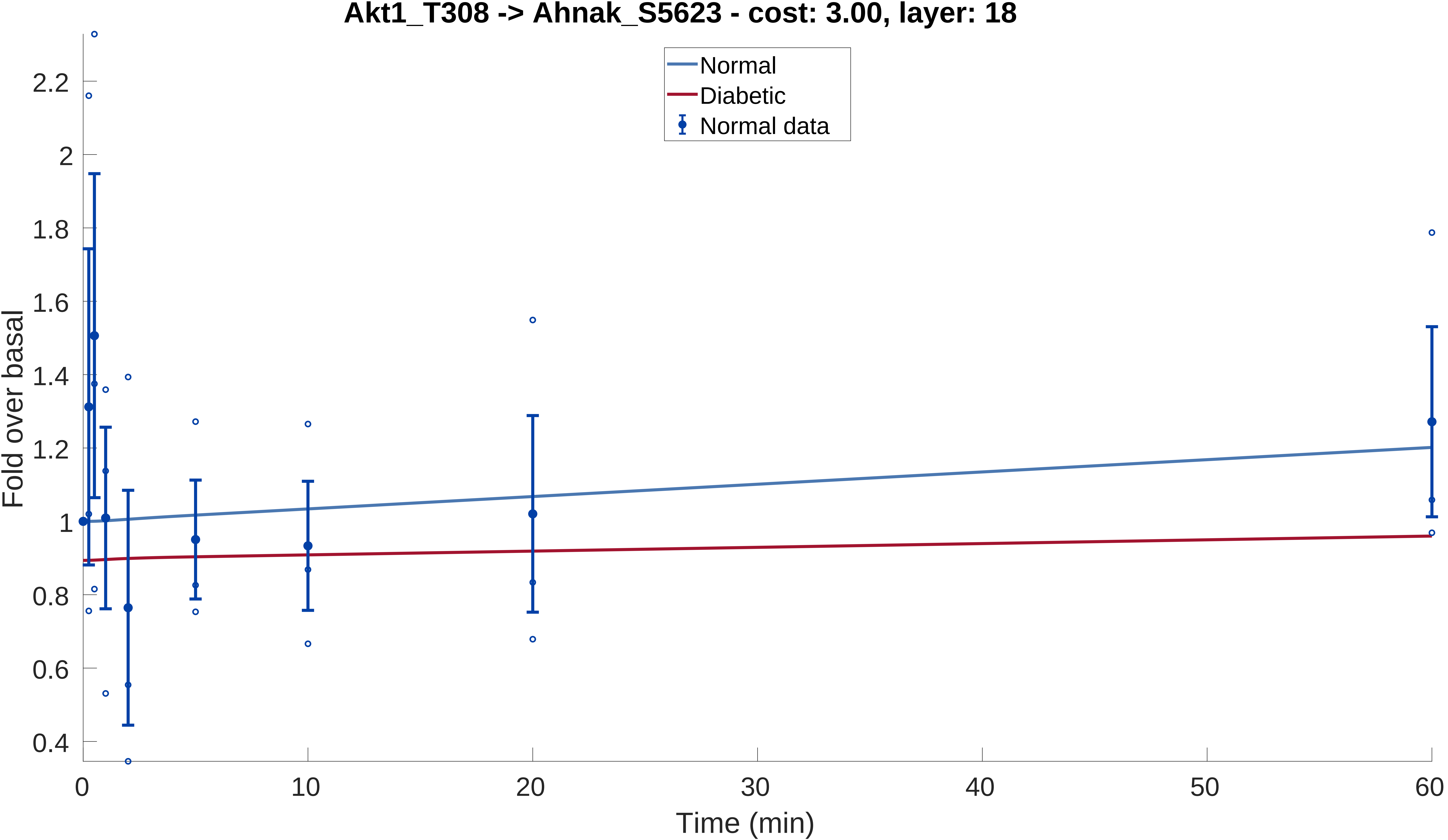
Akt1_T308 -> Ahnak_S5623 - cost: 3.00, layer: 18
Normal
Diabetic
Normal data
Fold over basal
0.4
0.6
0.8
1
1.2
1.4
1.6
1.8
2
2.2
0
10
20
30
40
50
60
Time (min)

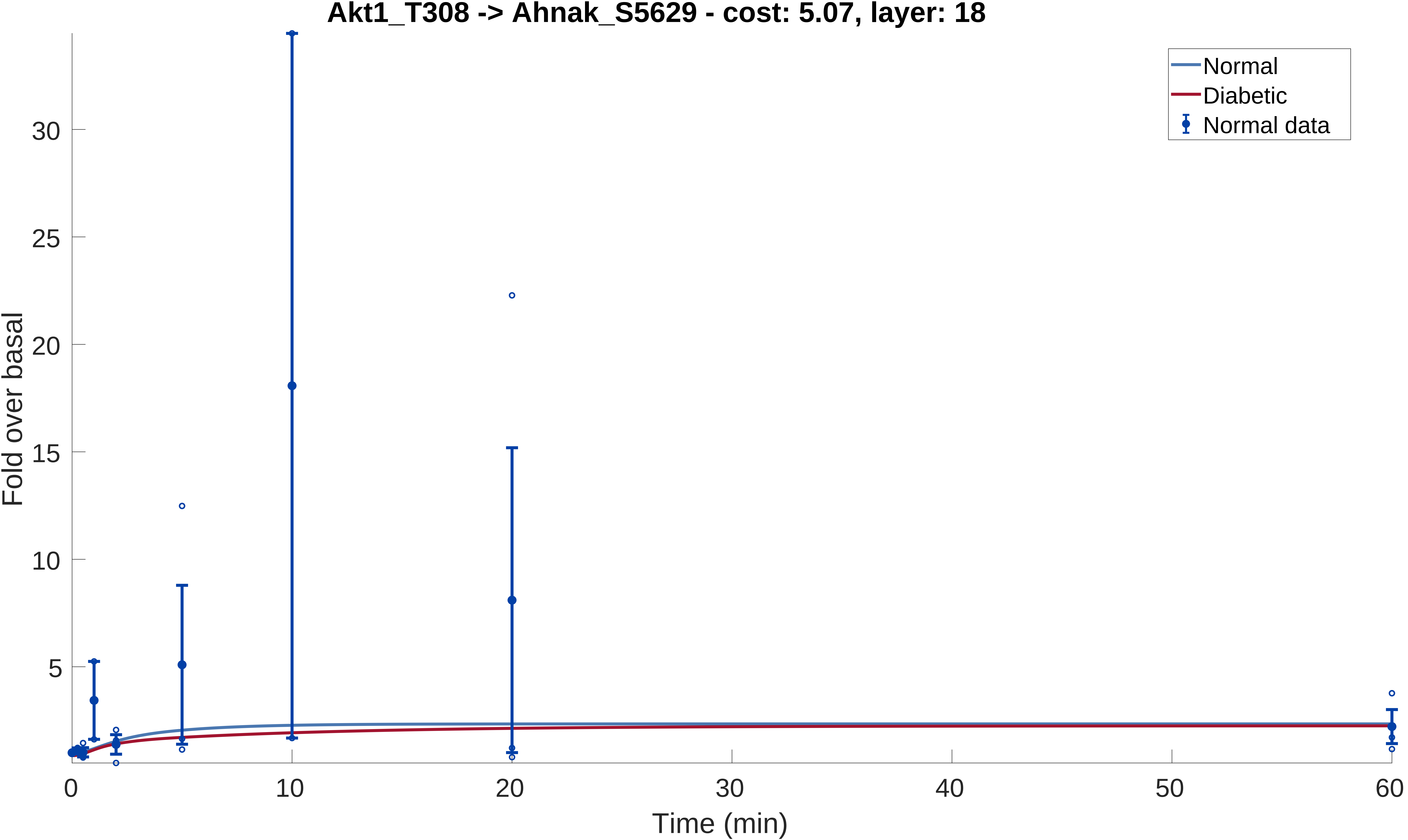
Akt1_T308 -> Ahnak_S5629 - cost: 5.07, layer: 18
Normal
Diabetic
Normal data
Fold over basal
Time (min)
0
10
20
30
40
50
60
5
10
15
20
25
30

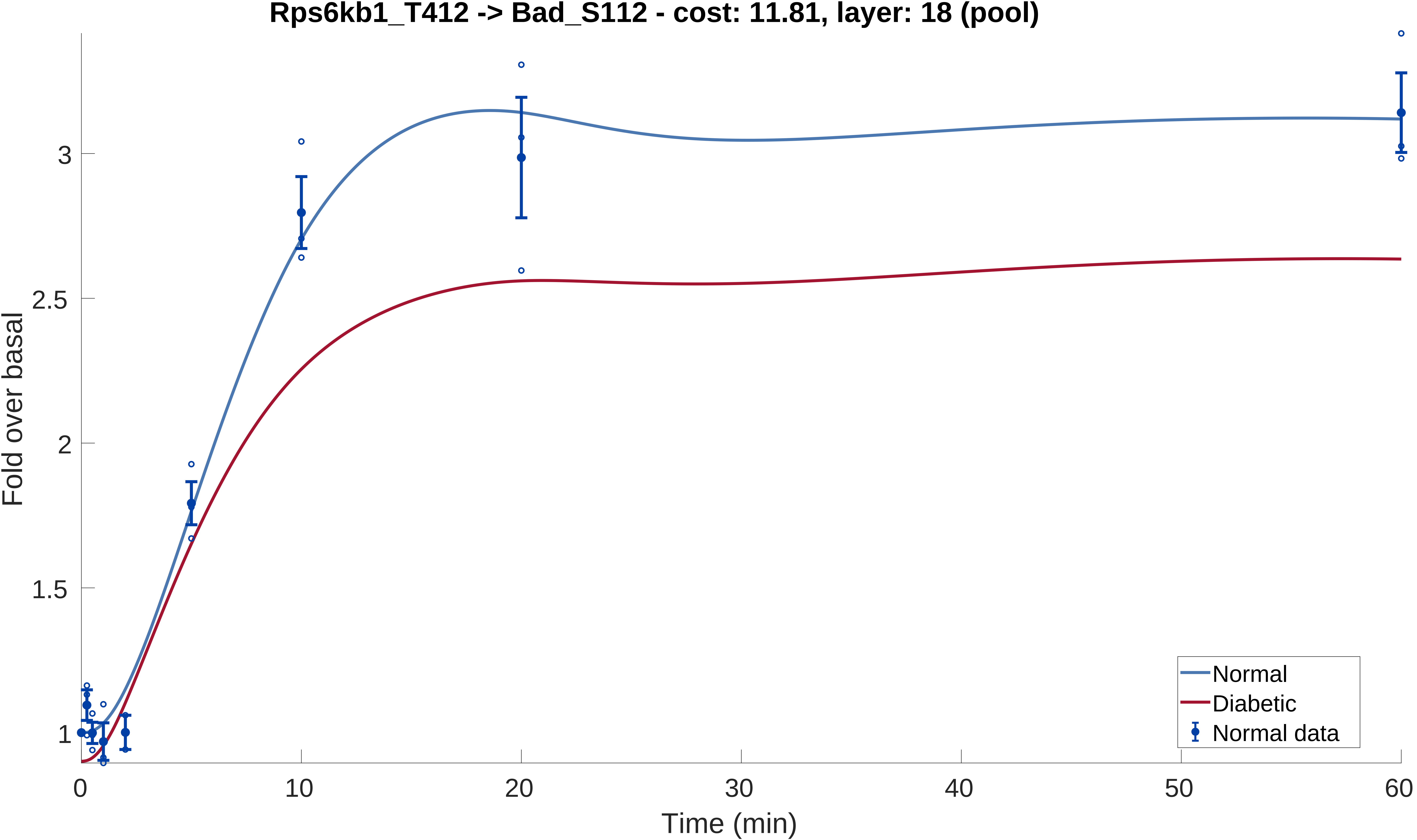

Rps6kb1_T412 -> Bad_S112 - cost: 11.81, layer: 18 (pool)
Fold over basal
3
2.5
2
1.5
1
0
10
20
30
40
50
60
Time (min)
Normal
Diabetic
Normal data

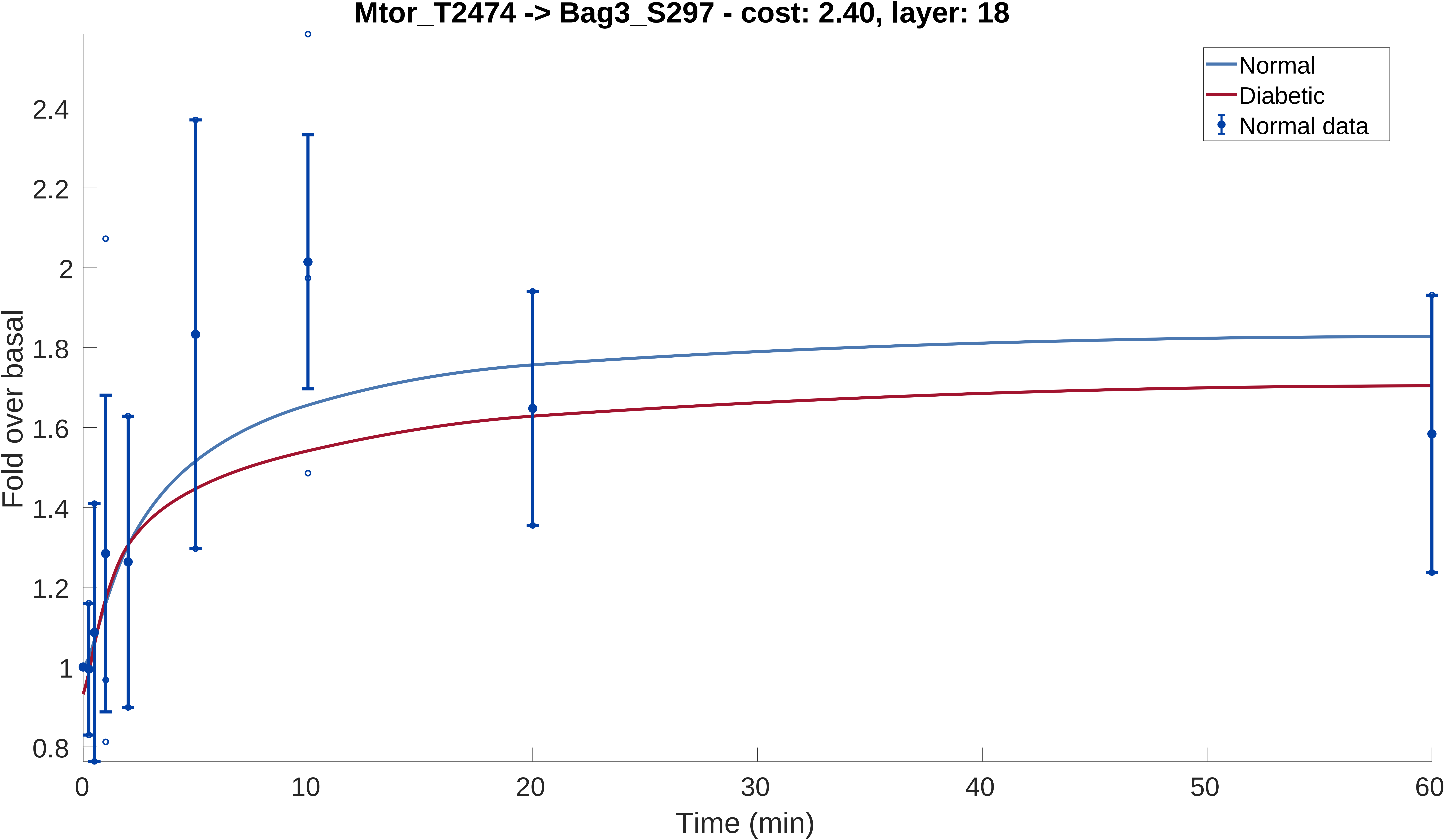

Mtor_T2474 -> Bag3_S297 - cost: 2.40, layer: 18
Normal
Diabetic
Normal data
Fold over basal
2.4
2.2
2
1.8
1.6
1.4
1.2
1
0.8
0
10
20
30
40
50
60
Time (min)

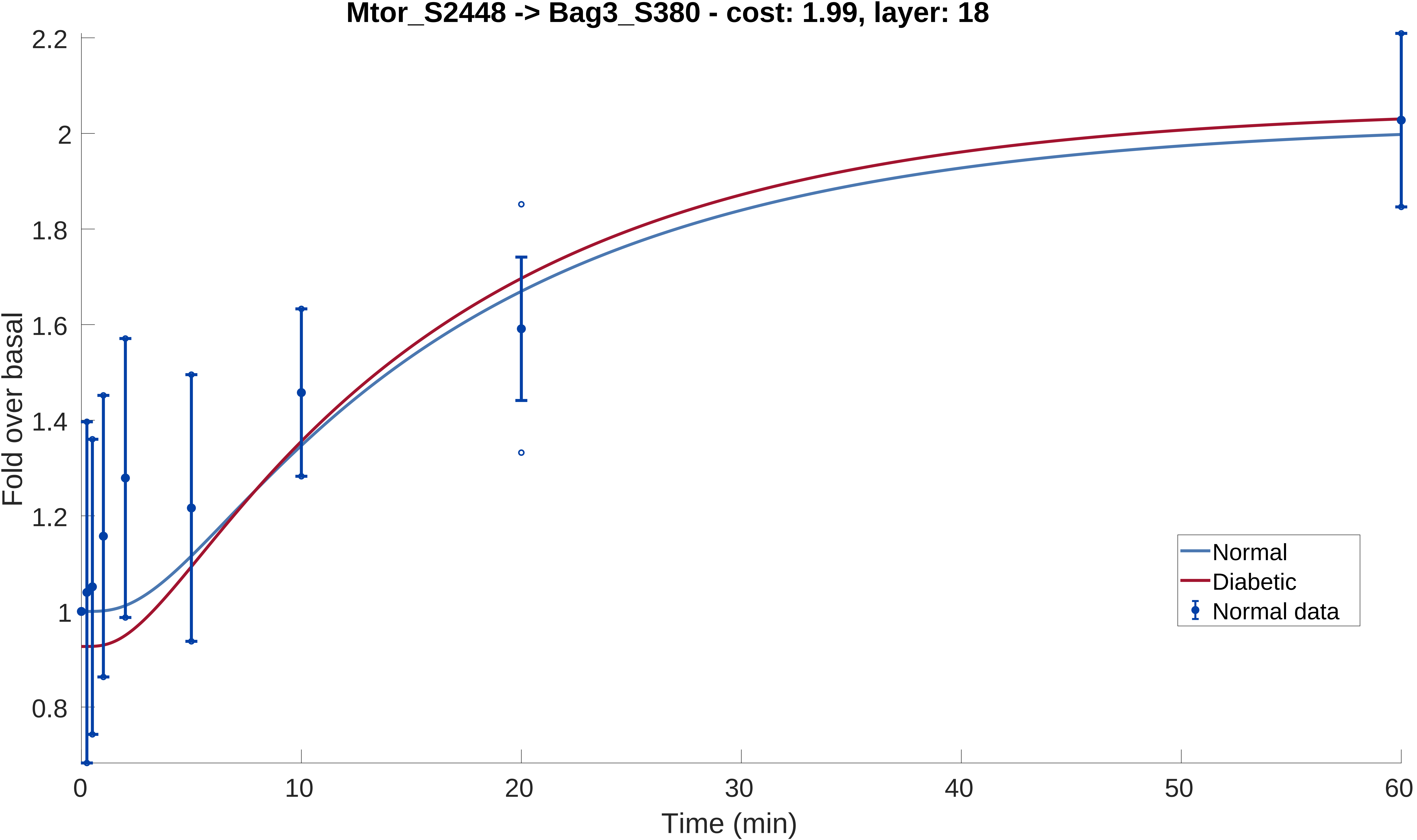

Mtor_S2448 -> Bag3_S380 - cost: 1.99, layer: 18
Fold over basal
Time (min)
2.2
2
1.8
1.6
1.4
1.2
1
0.8
0
10
20
30
40
50
60
Normal
Diabetic
Normal data

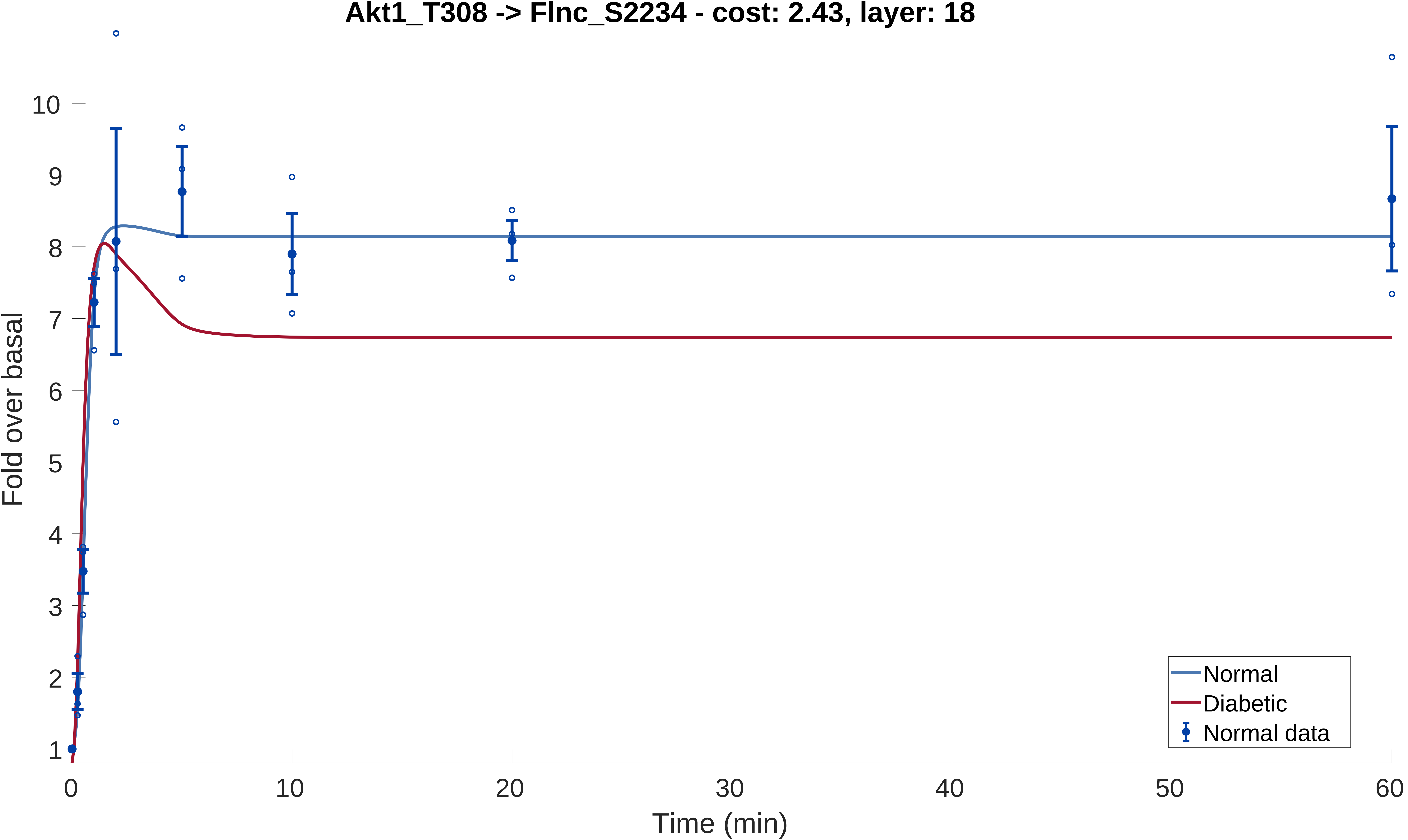

Akt1_T308 -> Flnc_S2234 - cost: 2.43, layer: 18
Fold over basal
Time (min)
1
2
3
4
5
6
7
8
9
10
0
10
20
30
40
50
60
Normal
Diabetic
Normal data

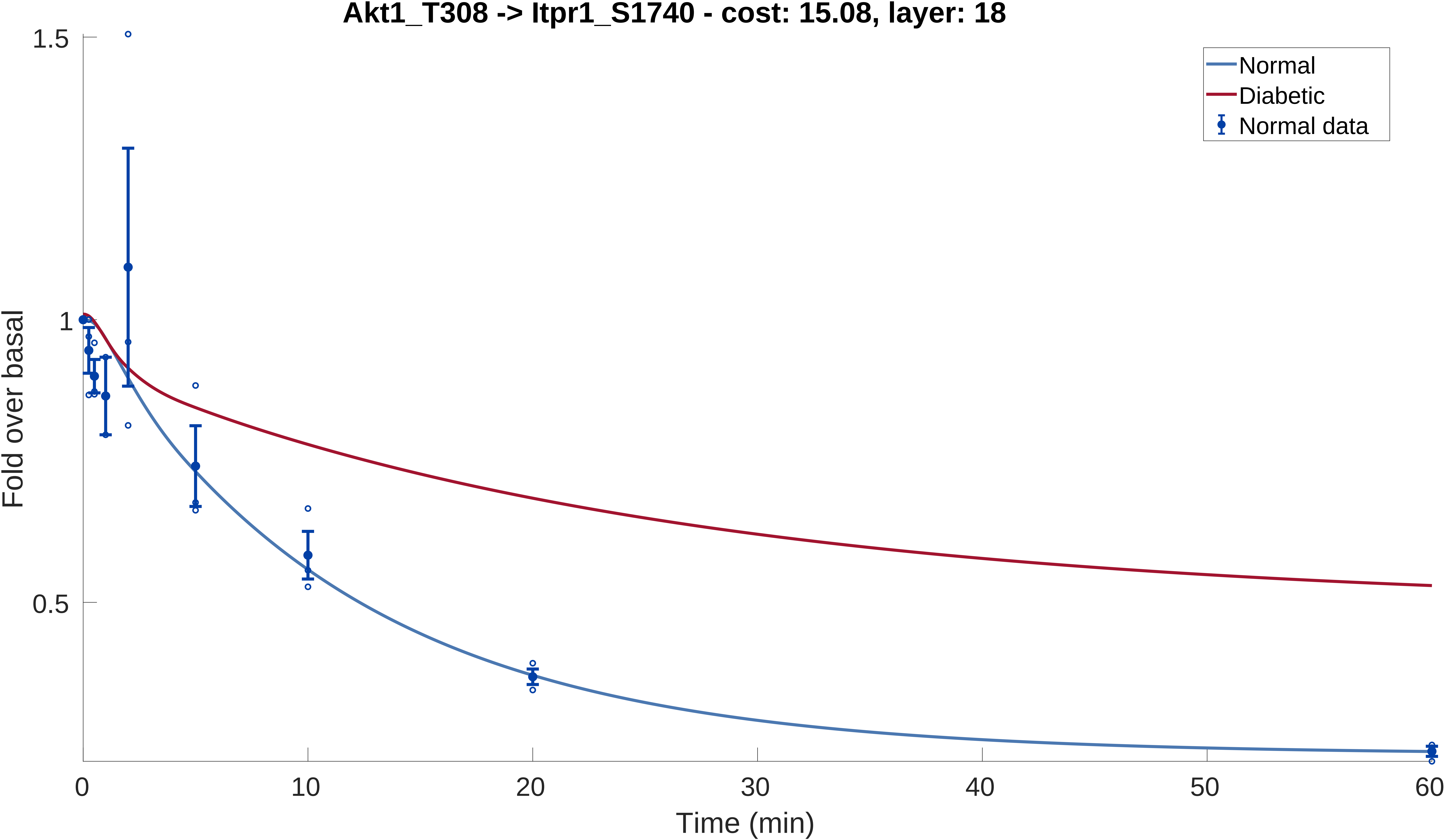

Akt1_T308 -> Itpr1_S1740 - cost: 15.08, layer: 18
Normal
Diabetic
Normal data
Fold over basal
1.5
1
0.5
0
10
20
30
40
50
60
Time (min)

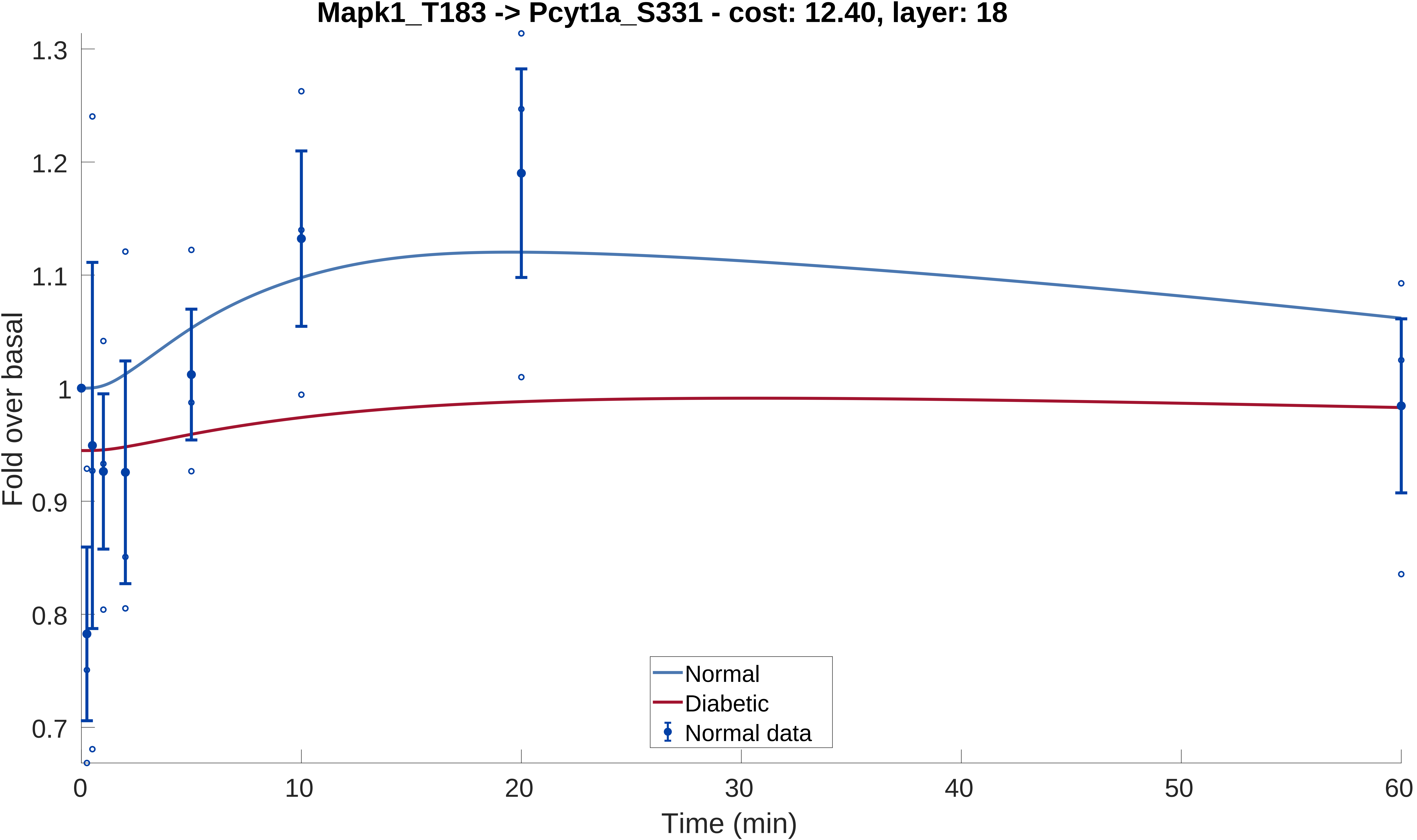
Mapk1_T183 -> Pcyt1a_S331 - cost: 12.40, layer: 18
Fold over basal
Time (min)
0.7
0.8
0.9
1
1.1
1.2
1.3
0
10
20
30
40
50
60
Normal
Diabetic
Normal data

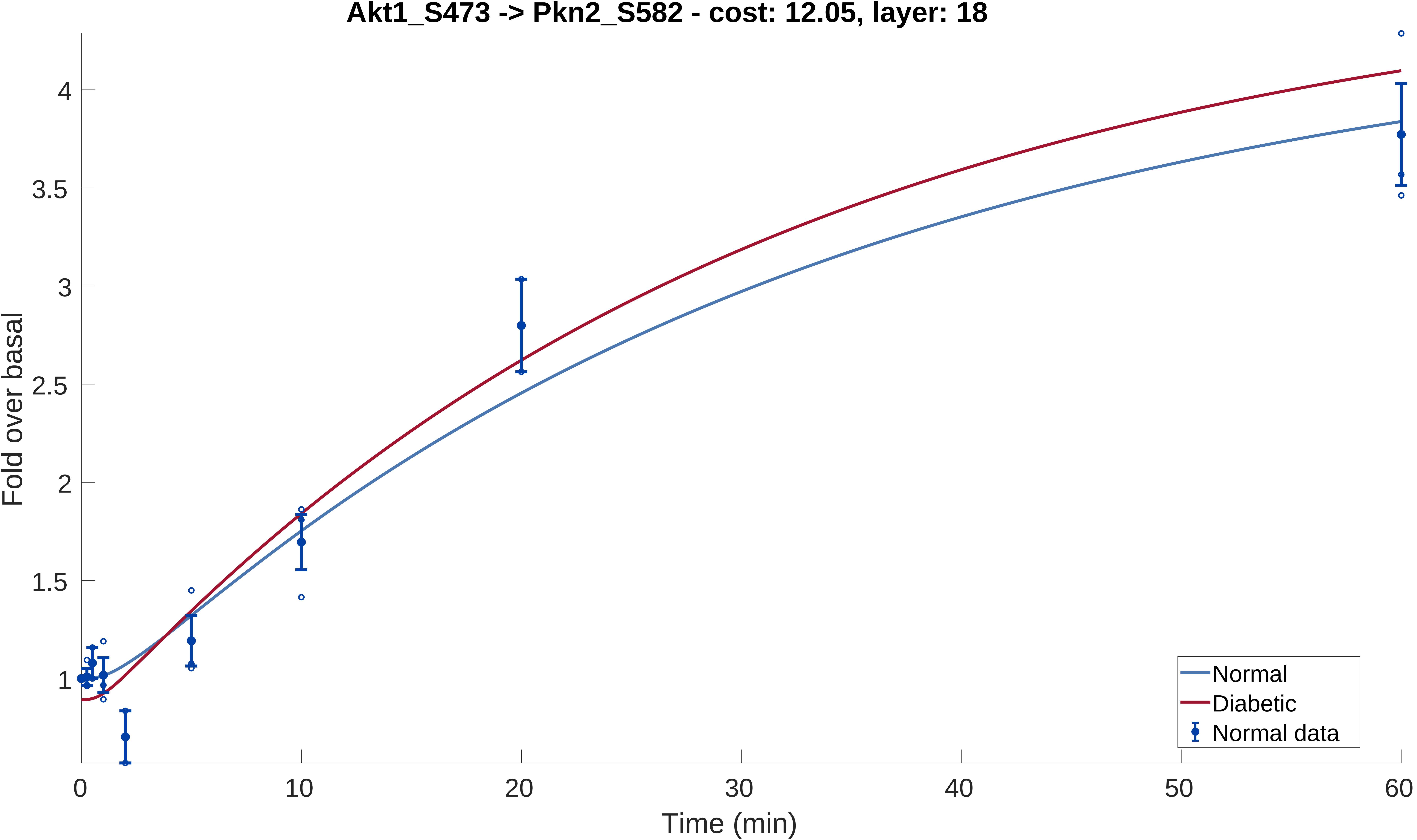
Akt1_S473 -> Pkn2_S582 - cost: 12.05, layer: 18
Fold over basal
Time (min)
4
3.5
3
2.5
2
1.5
1
0
10
20
30
40
50
60
Normal
Diabetic
Normal data

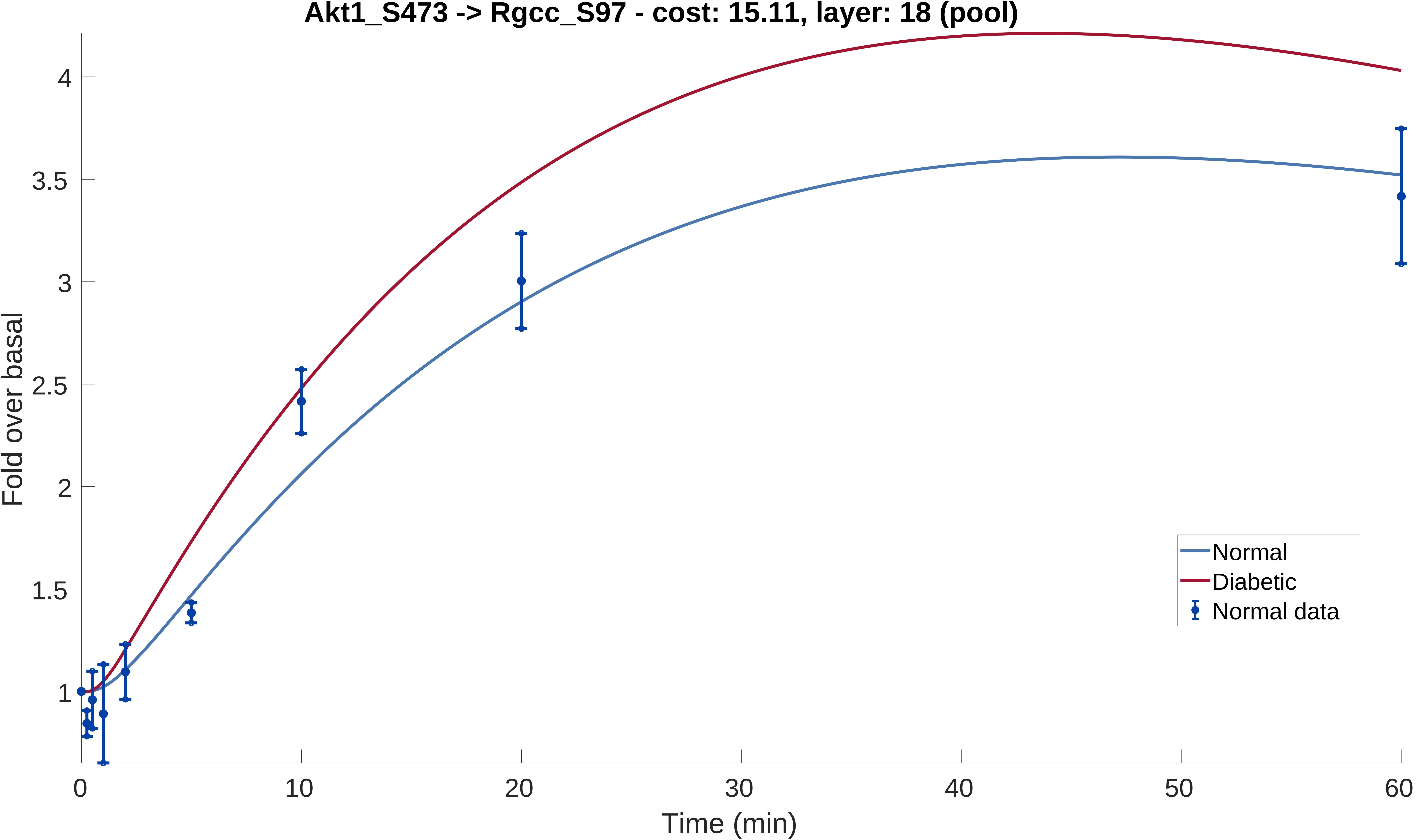
Akt1_S473 -> Rgcc_S97 - cost: 15.11, layer: 18 (pool)
Fold over basal
Time (min)
4
3.5
3
2.5
2
1.5
1
0
10
20
30
40
50
60
Normal
Diabetic
Normal data

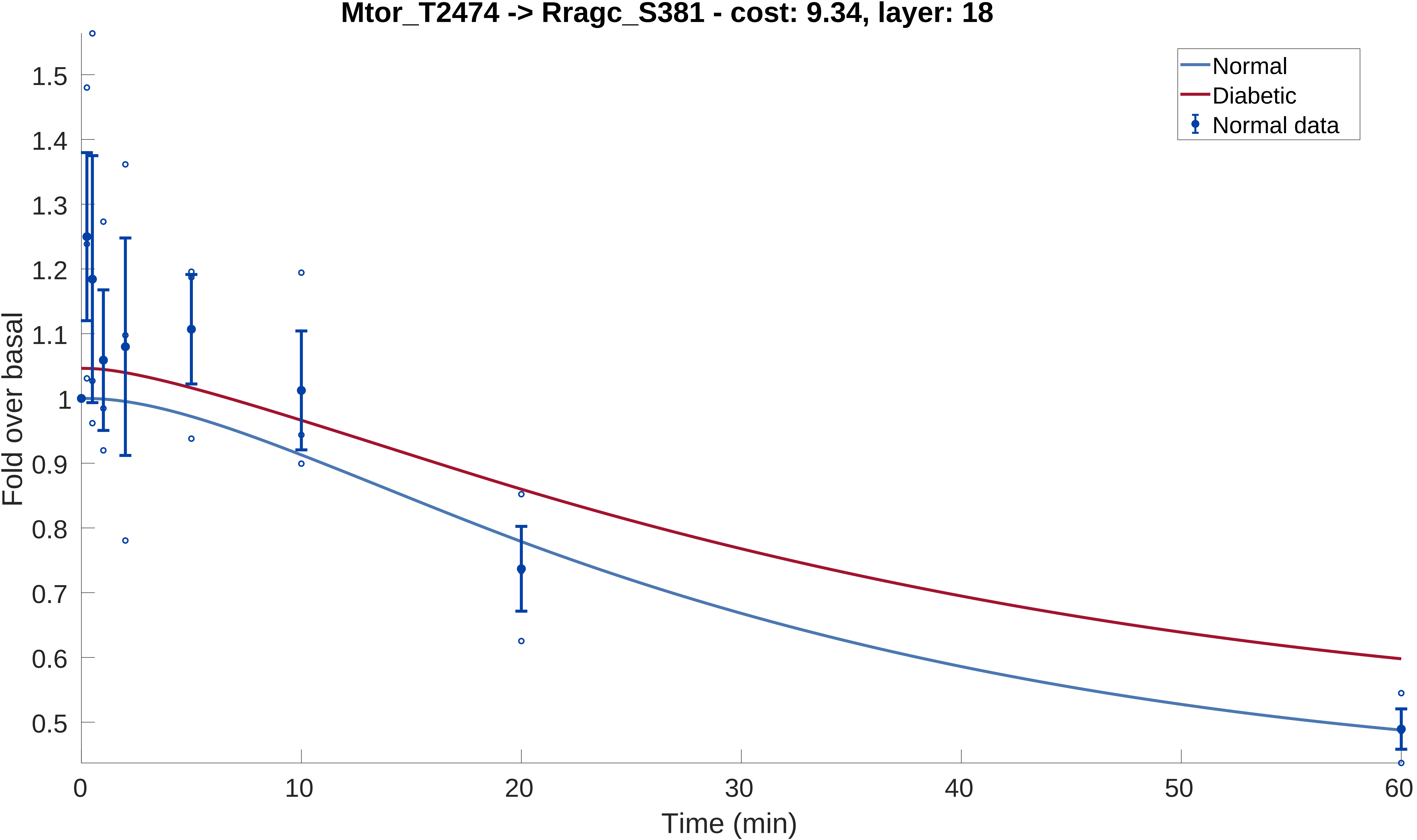

Mtor_T2474 -> Rragc_S381 - cost: 9.34, layer: 18
Normal
Diabetic
Normal data
Fold over basal
Time (min)
0.5
0.6
0.7
0.8
0.9
1
1.1
1.2
1.3
1.4
1.5
0
10
20
30
40
50
60

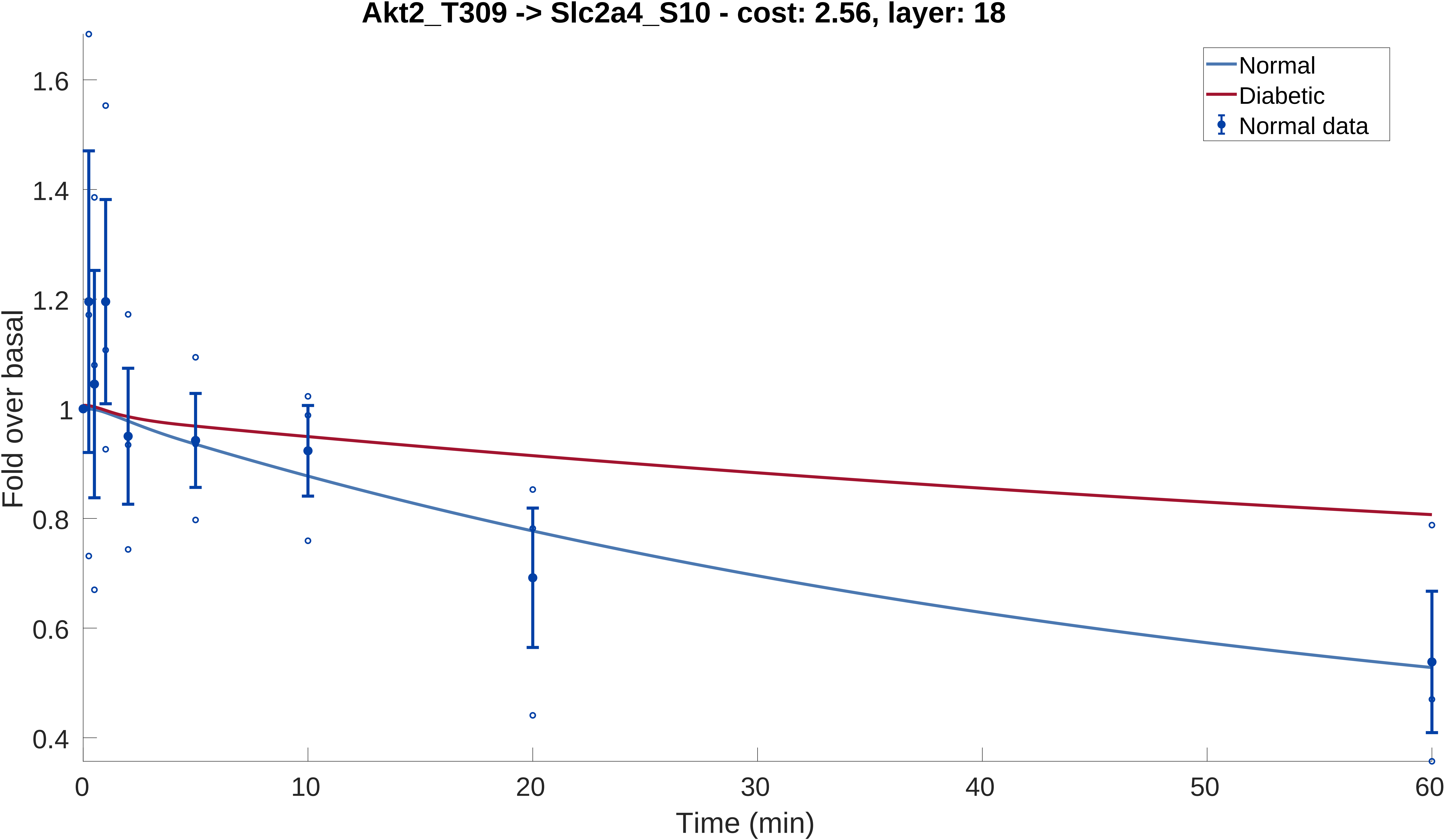

Akt2_T309 -> Slc2a4_S10 - cost: 2.56, layer: 18
Normal
Diabetic
Normal data
1.6
1.4
1.2
1
0.8
0.6
0.4
Fold over basal
0
10
20
30
40
50
60
Time (min)

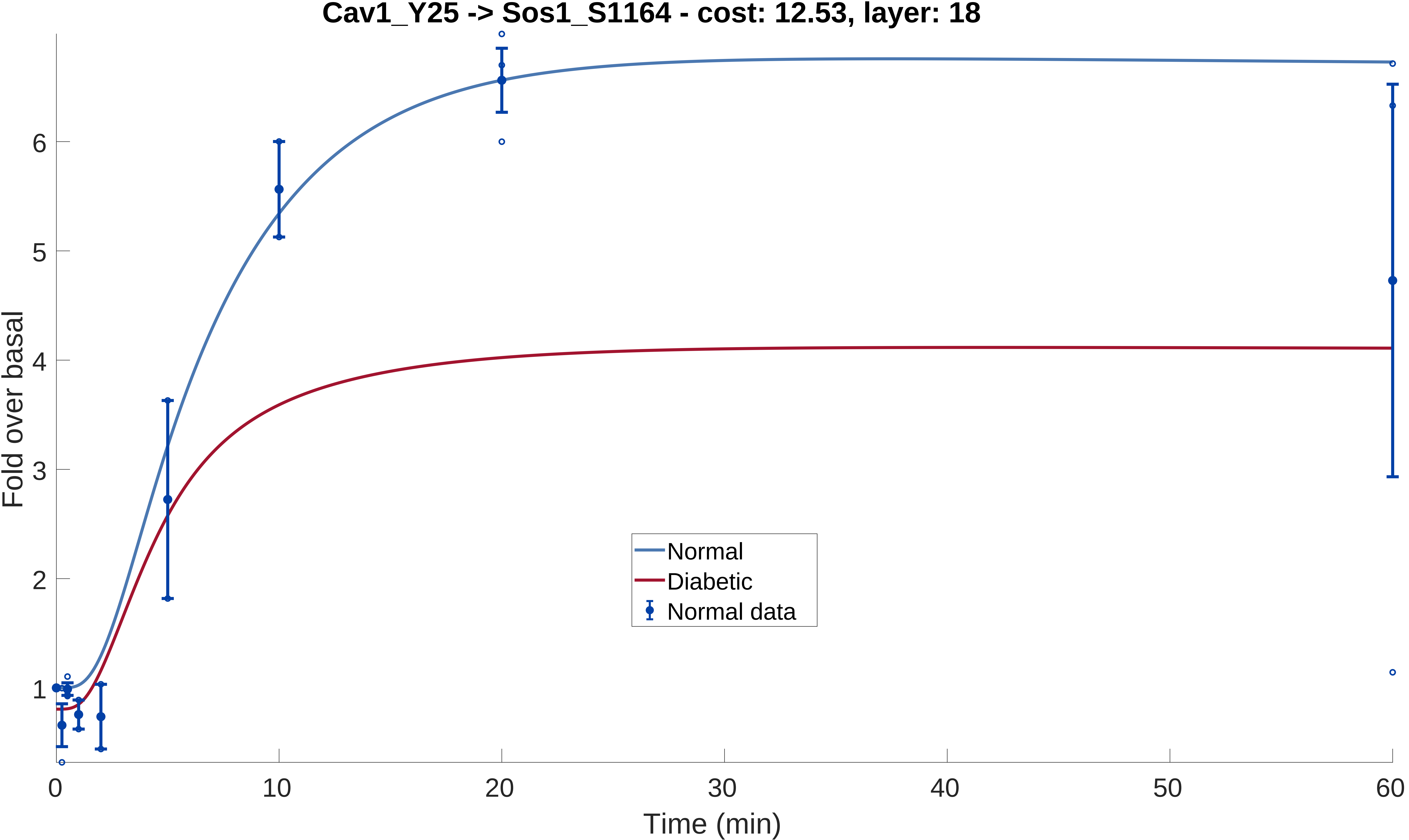

Cav1_Y25 -> Sos1_S1164 - cost: 12.53, layer: 18
Fold over basal
Time (min)
Normal
Diabetic
Normal data
0
10
20
30
40
50
60
1
2
3
4
5
6

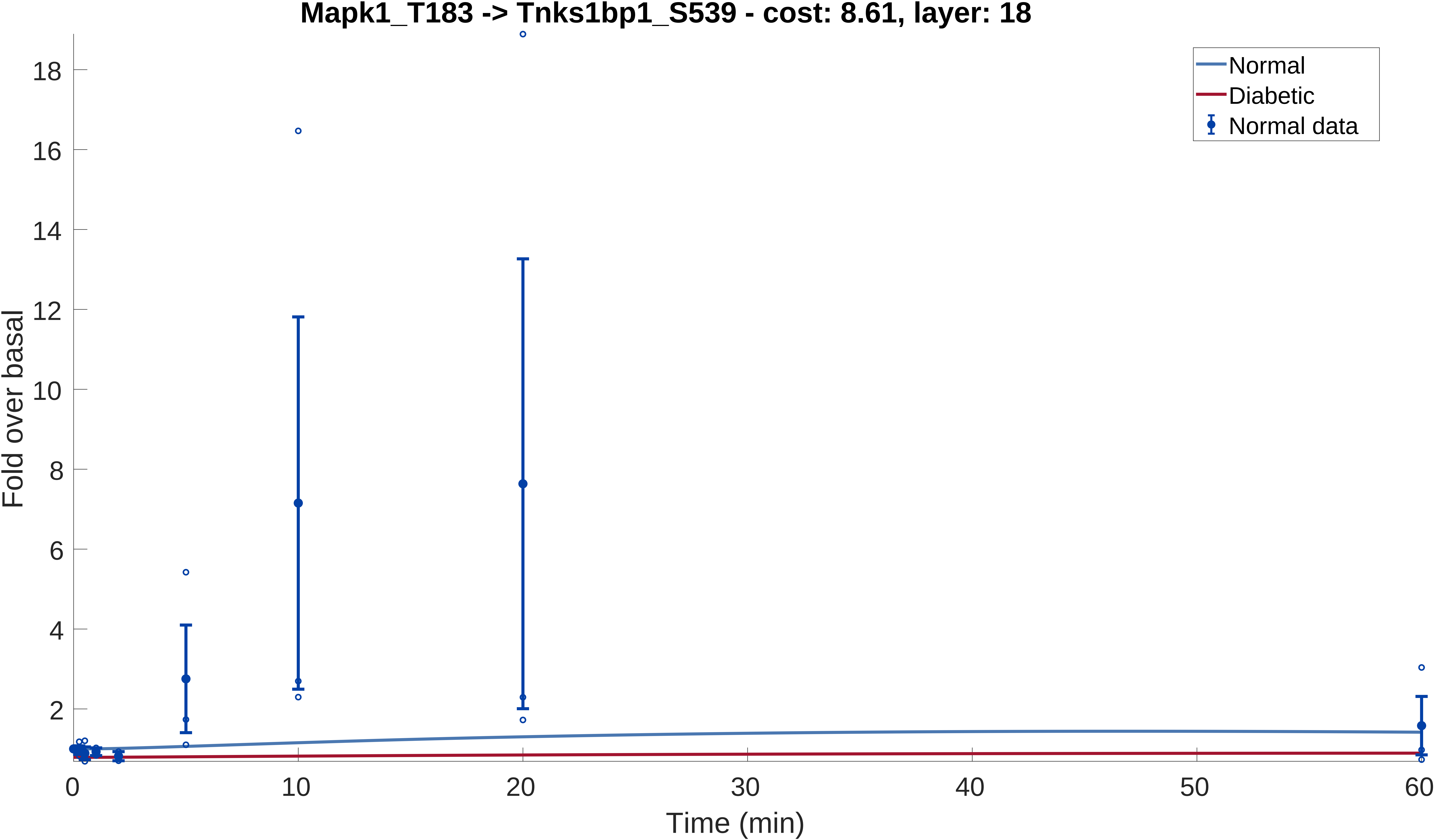

Mapk1_T183 -> Tnks1bp1_S539 - cost: 8.61, layer: 18
Normal
Diabetic
Normal data
Fold over basal
18
16
14
12
10
8
6
4
2
0
10
20
30
40
50
60
Time (min)

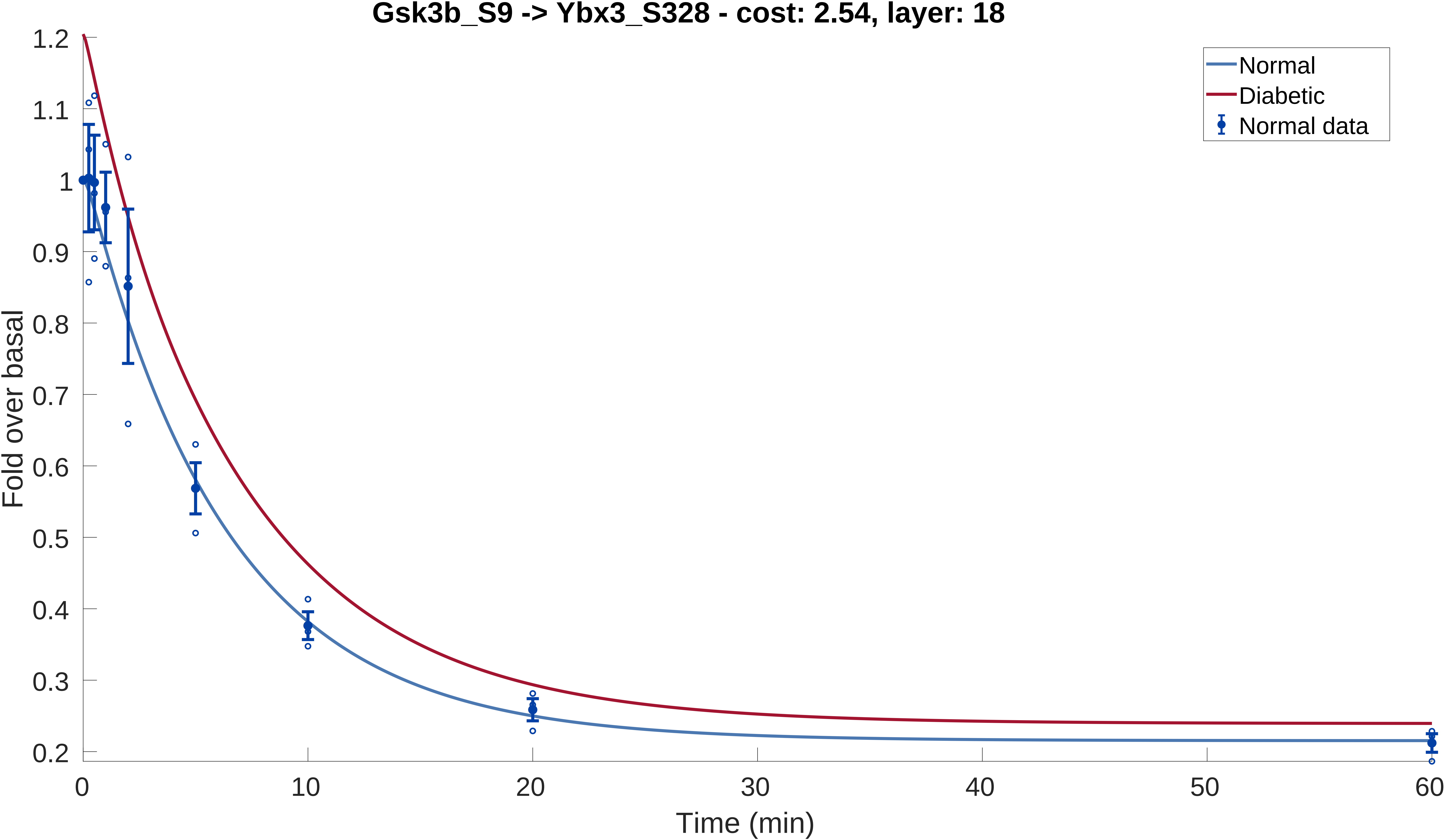

Gsk3b_S9 -> Ybx3_S328 - cost: 2.54, layer: 18
Normal
Diabetic
Normal data
Fold over basal
1.2
1.1
1
0.9
0.8
0.7
0.6
0.5
0.4
0.3
0.2
0
10
20
30
40
50
60
Time (min)

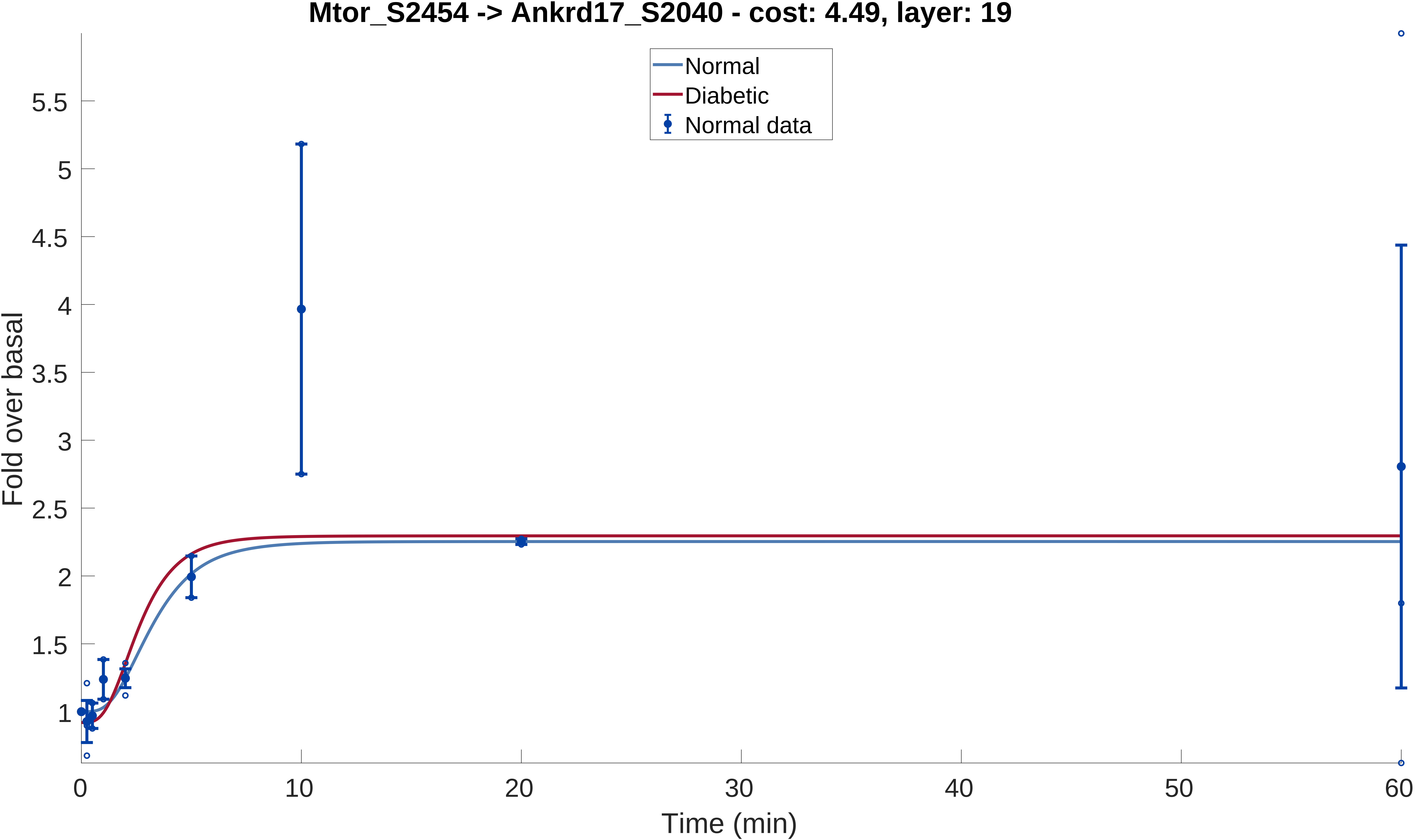

Mtor_S2454 -> Ankrd17_S2040 - cost: 4.49, layer: 19
Normal
Diabetic
Normal data
Fold over basal
1
1.5
2
2.5
3
3.5
4
4.5
5
5.5
0
10
20
30
40
50
60
Time (min)

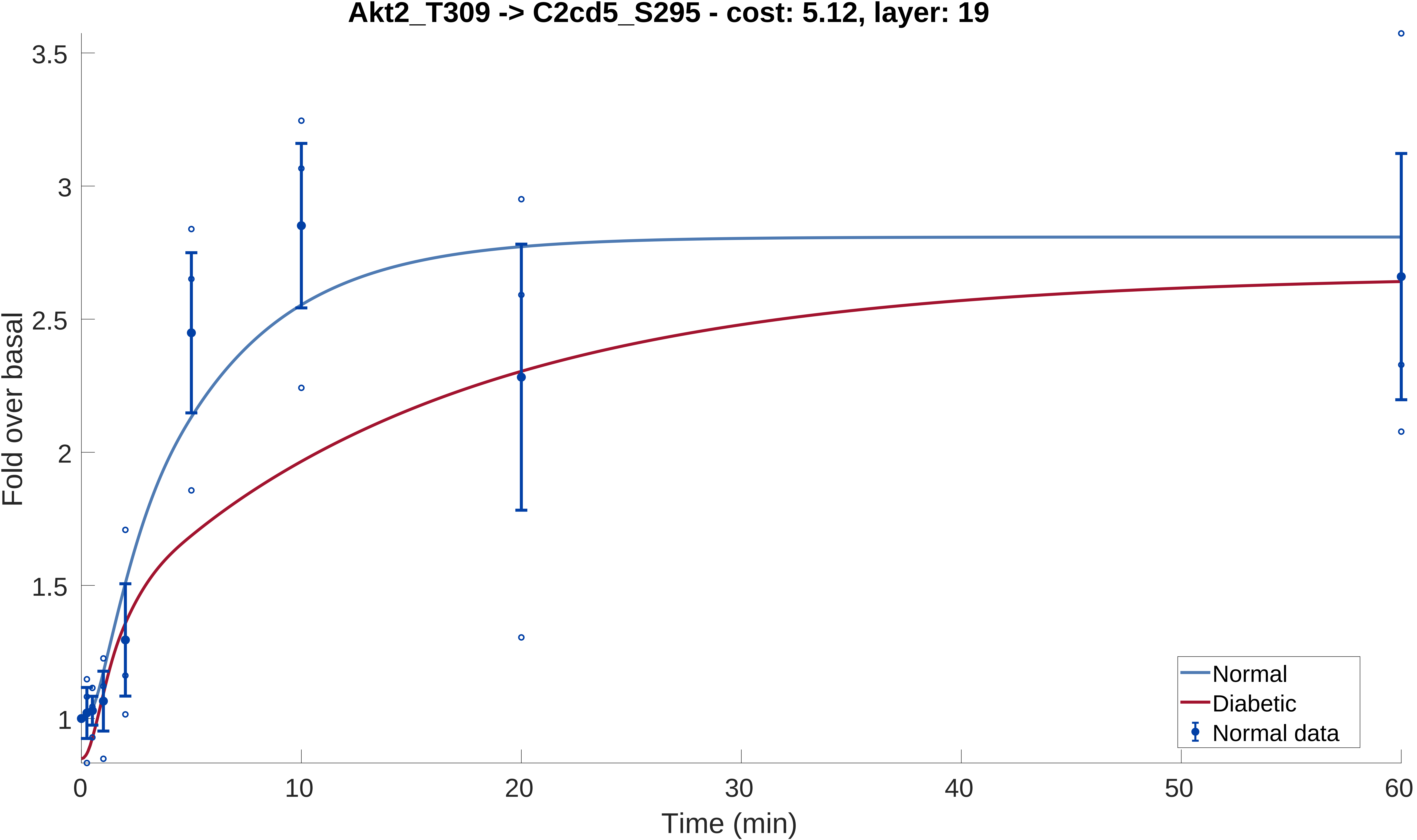
Akt2_T309 -> C2cd5_S295 - cost: 5.12, layer: 19
Fold over basal
3.5
3
2.5
2
1.5
1
0
10
20
30
40
50
60
Time (min)
Normal
Diabetic
Normal data

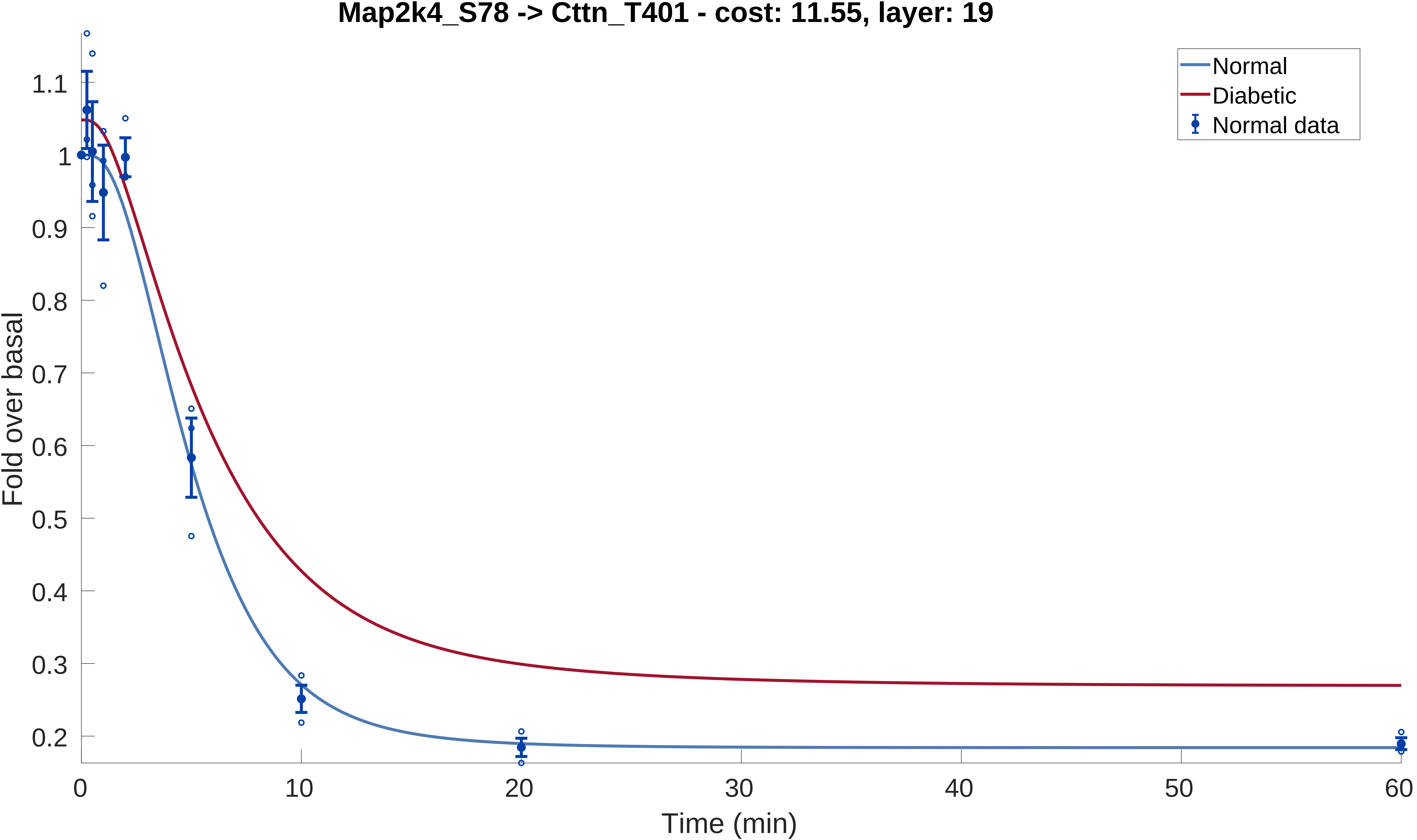
Map2k4_S78 -> Cttn_T401 - cost: 11.55, layer: 19
Normal
Diabetic
Normal data
Fold over basal
Time (min)
0
10
20
30
40
50
60
0.2
0.3
0.4
0.5
0.6
0.7
0.8
0.9
1
1.1

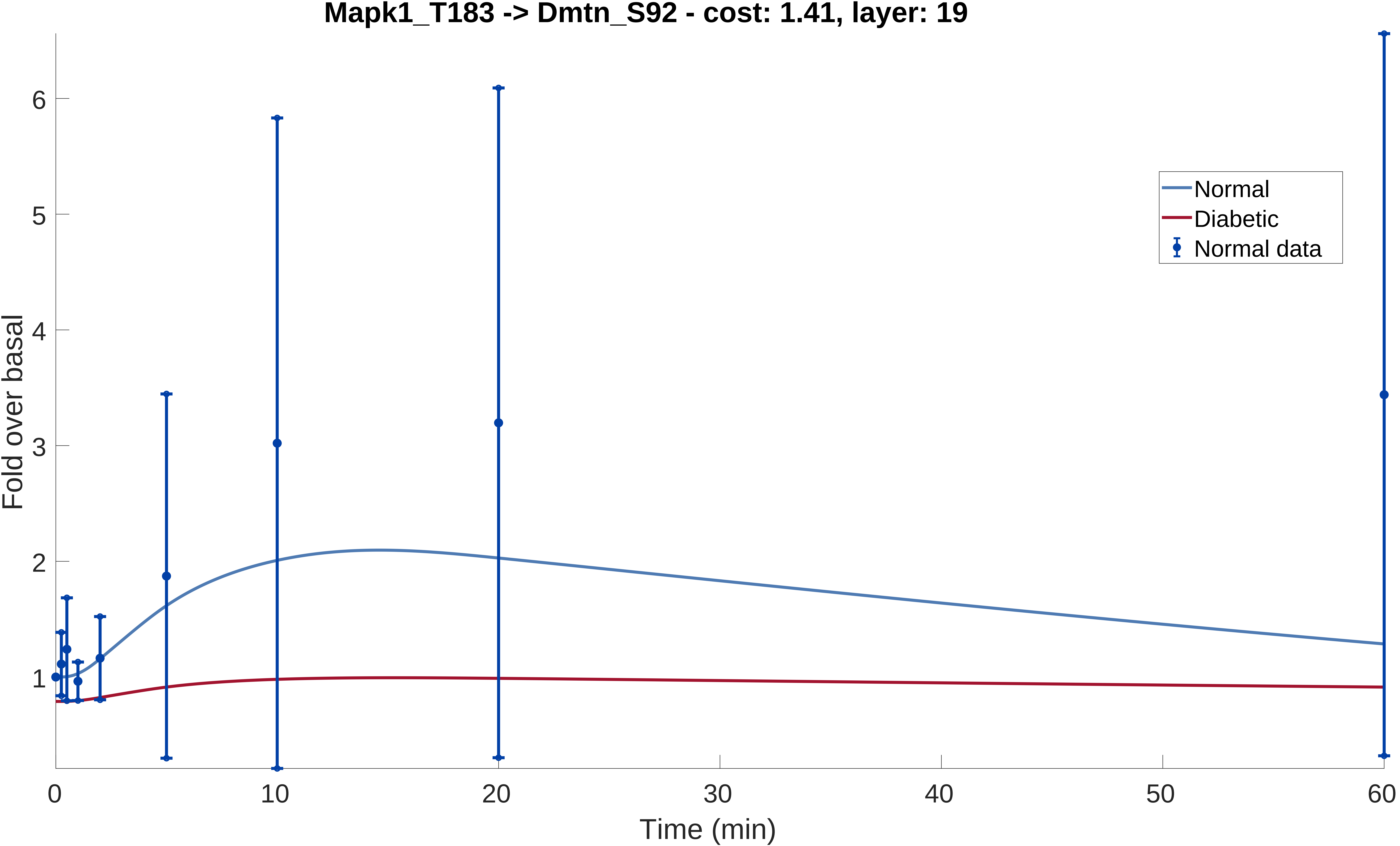

Mapk1_T183 -> Dmtn_S92 - cost: 1.41, layer: 19
Normal
Diabetic
Normal data
Fold over basal
Time (min)
0
10
20
30
40
50
60
1
2
3
4
5
6

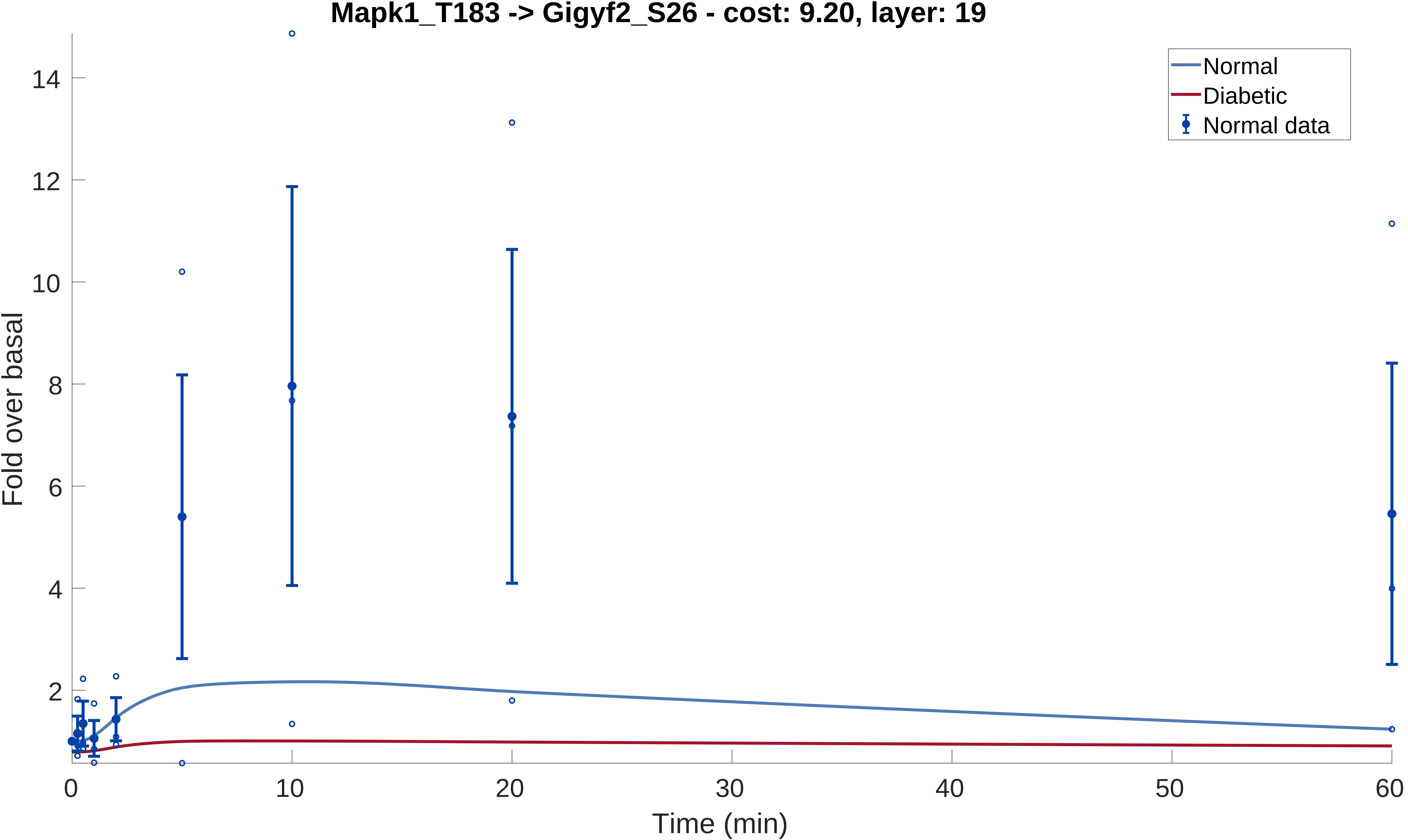
Mapk1_T183 -> Gigyf2_S26 - cost: 9.20, layer: 19
Normal
Diabetic
Normal data
Fold over basal
Time (min)
0
10
20
30
40
50
60
2
4
6
8
10
12
14

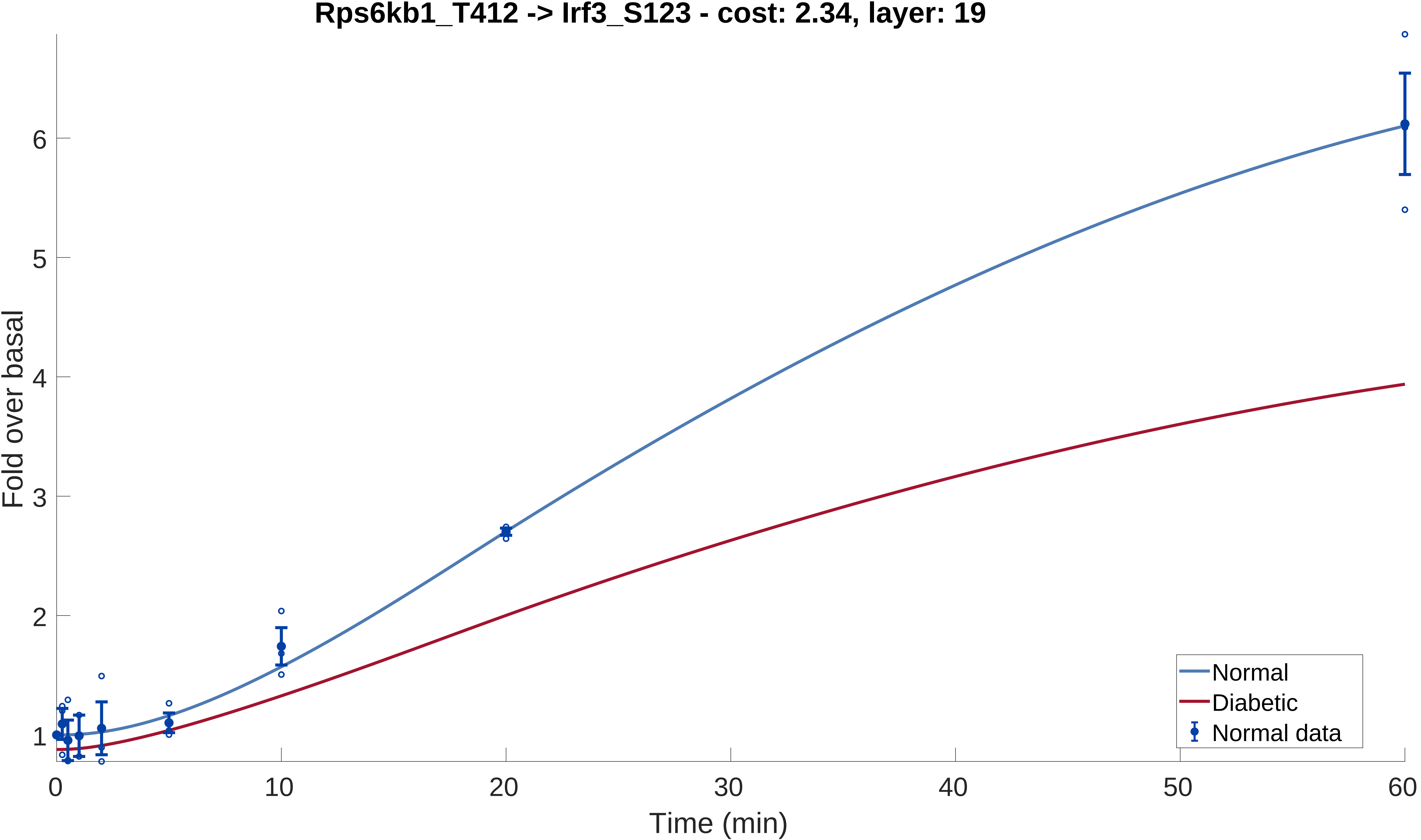
Rps6kb1_T412 -> Irf3_S123 - cost: 2.34, layer: 19
Fold over basal
Time (min)
Normal
Diabetic
Normal data
1
2
3
4
5
6
0
10
20
30
40
50
60

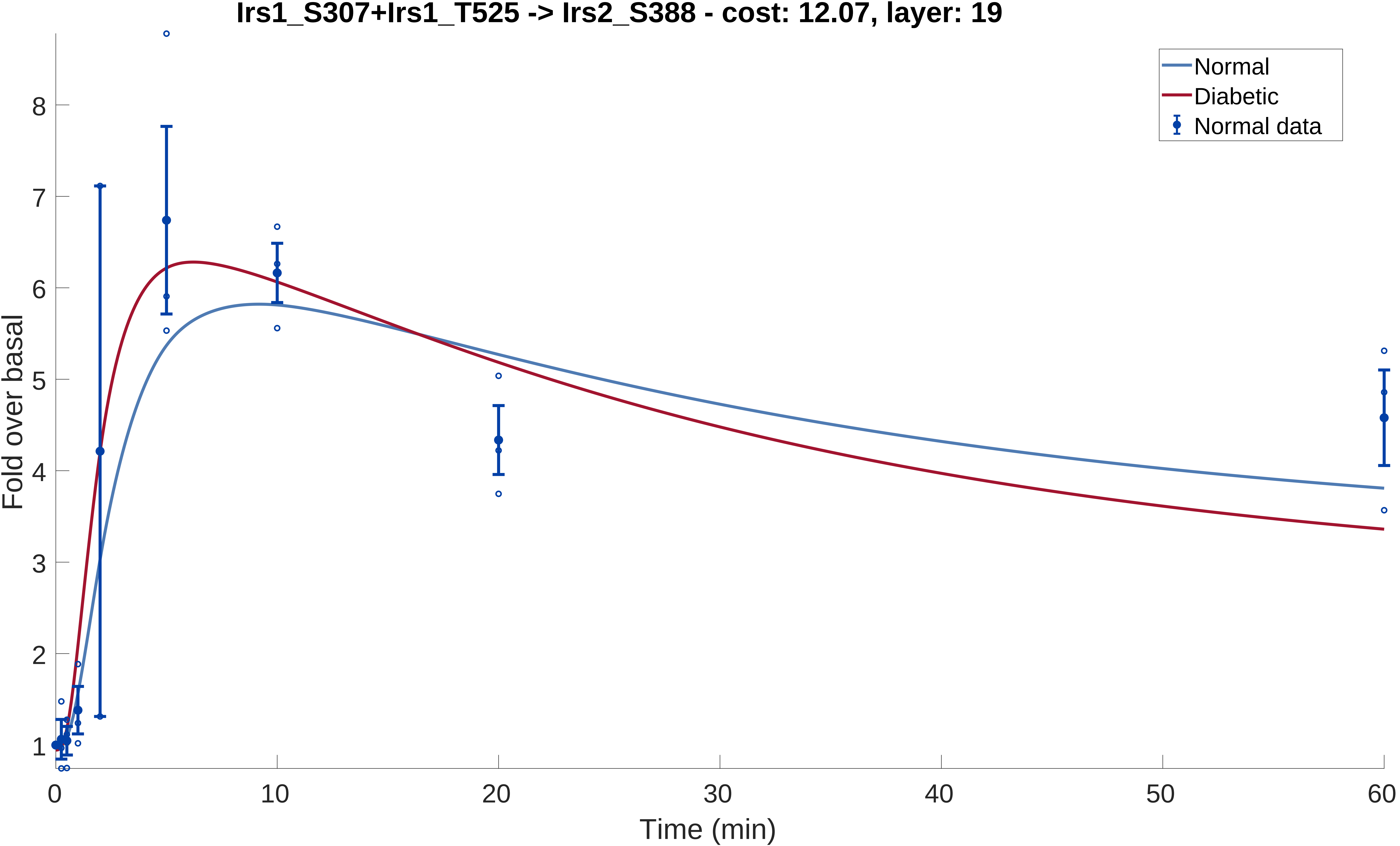

Irs1_S307+Irs1_T525 -> Irs2_S388 - cost: 12.07, layer: 19
Normal
Diabetic
Normal data
Fold over basal
Time (min)
1
2
3
4
5
6
7
8
0
10
20
30
40
50
60

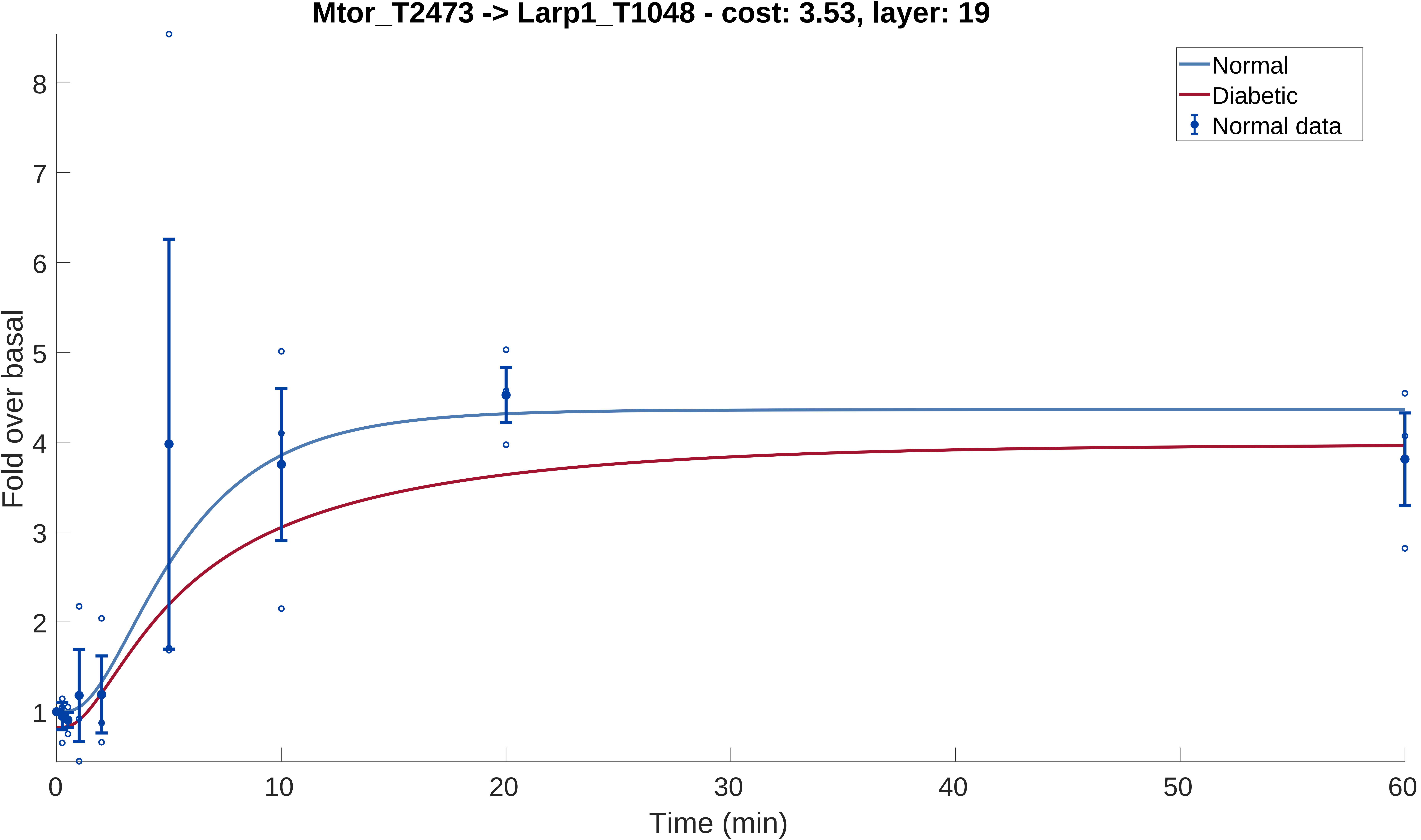

Mtor_T2473 -> Larp1_T1048 - cost: 3.53, layer: 19
Normal
Diabetic
Normal data
Fold over basal
Time (min)
1
2
3
4
5
6
7
8
0
10
20
30
40
50
60

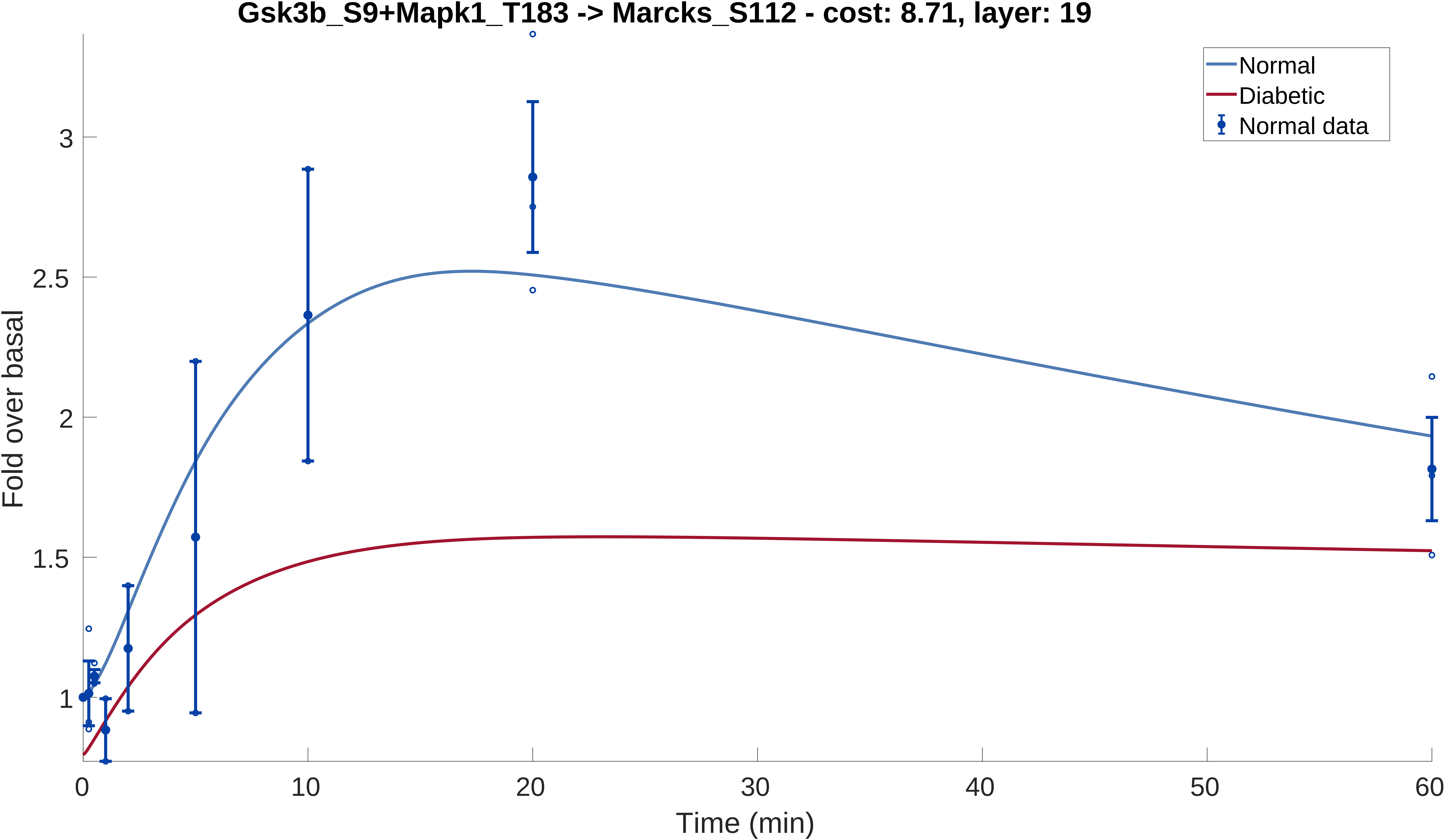

Gsk3b_S9+Mapk1_T183 -> Marcks_S112 - cost: 8.71, layer: 19
Normal
Diabetic
Normal data
Fold over basal
1
1.5
2
2.5
3
0
10
20
30
40
50
60
Time (min)

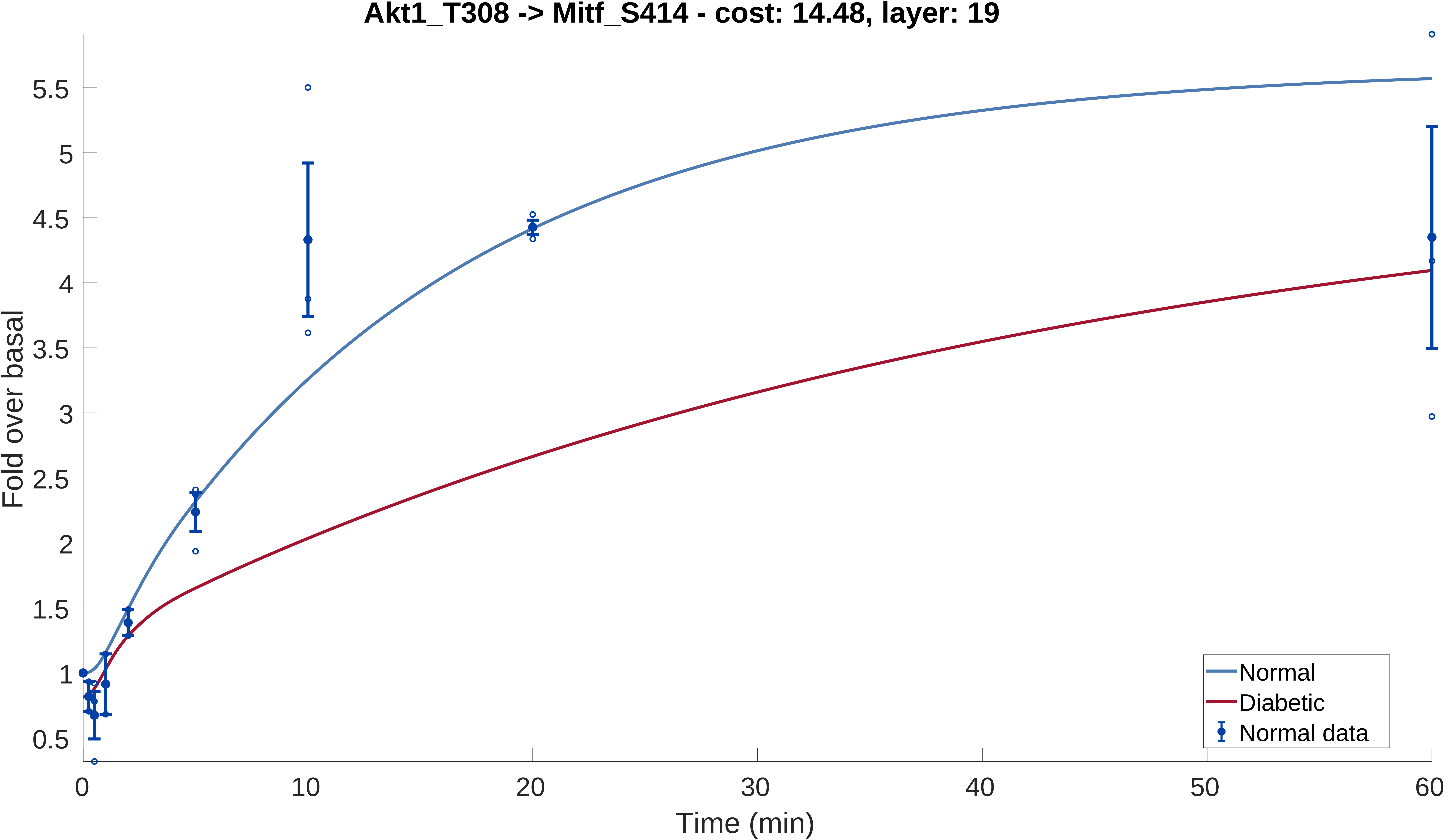
Akt1_T308 -> Mitf_S414 - cost: 14.48, layer: 19
Fold over basal
5.5
5
4.5
4
3.5
3
2.5
2
1.5
1
0.5
0
10
20
30
40
50
60
Time (min)
Normal
Diabetic
Normal data

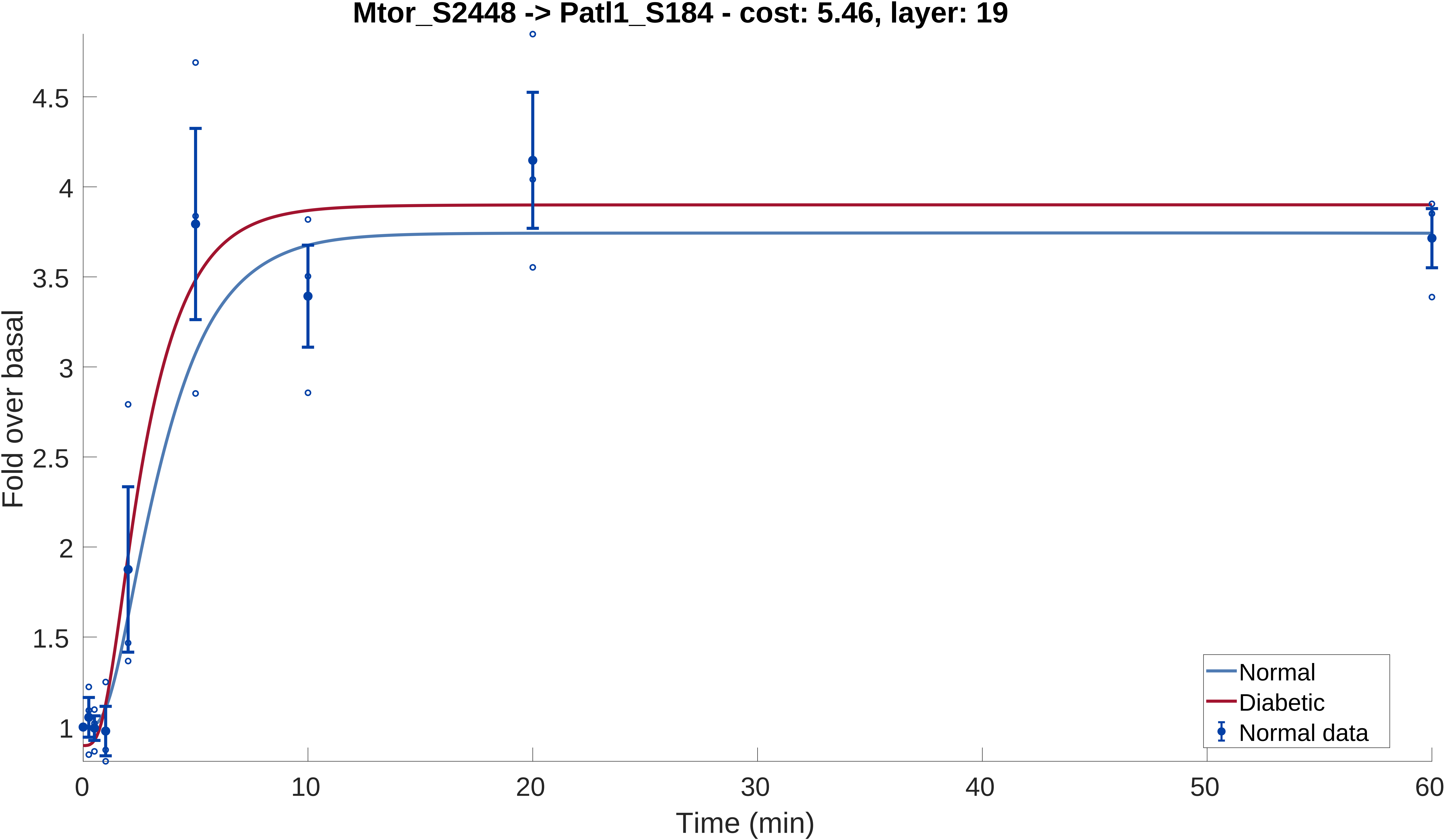

Mtor_S2448 -> Patl1_S184 - cost: 5.46, layer: 19
Fold over basal
4.5
4
3.5
3
2.5
2
1.5
1
0
10
20
30
40
50
60
Time (min)
Normal
Diabetic
Normal data

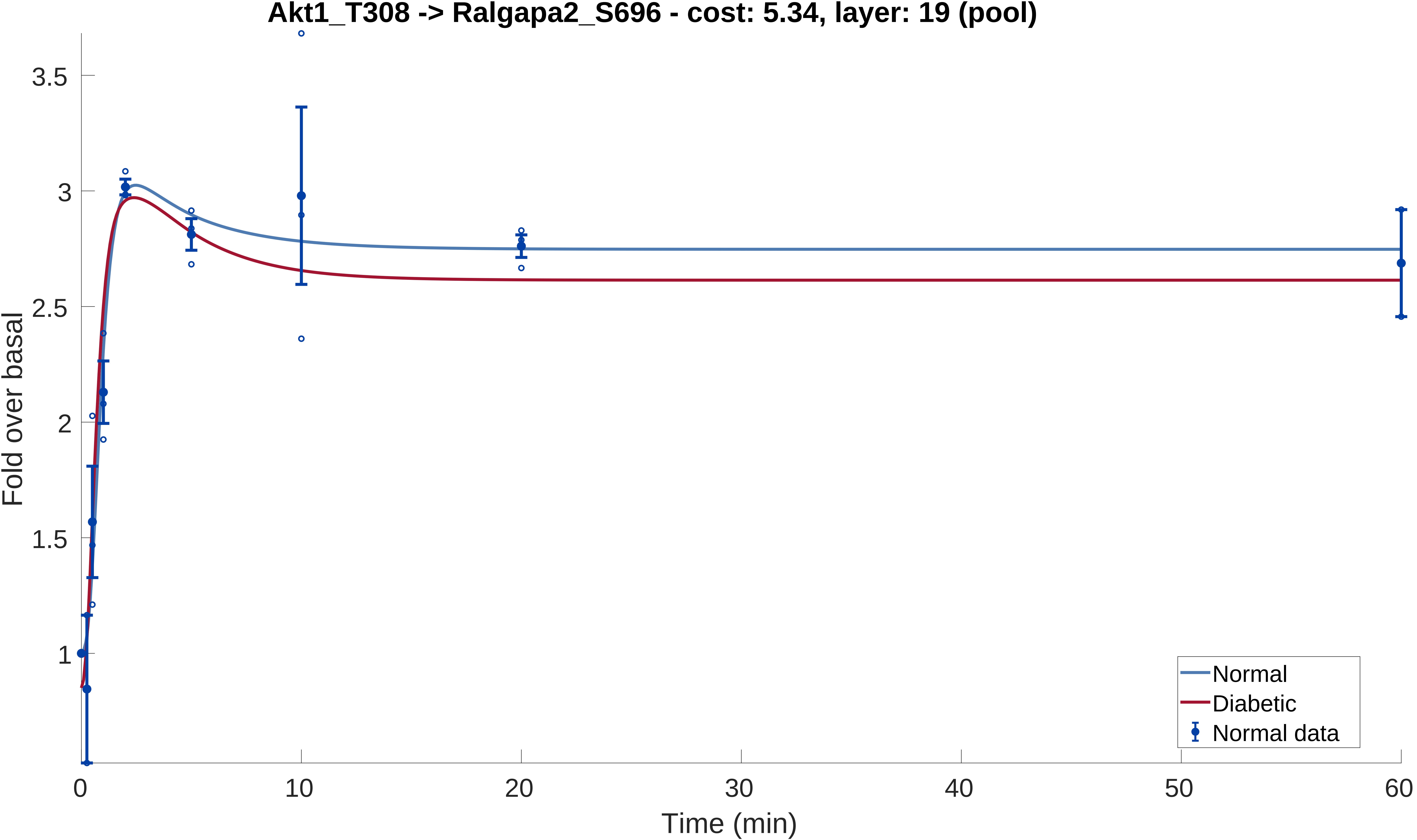

Akt1_T308 -> Ralgapa2_S696 - cost: 5.34, layer: 19 (pool)
Fold over basal
3.5
3
2.5
2
1.5
1
0
10
20
30
40
50
60
Time (min)
Normal
Diabetic
Normal data

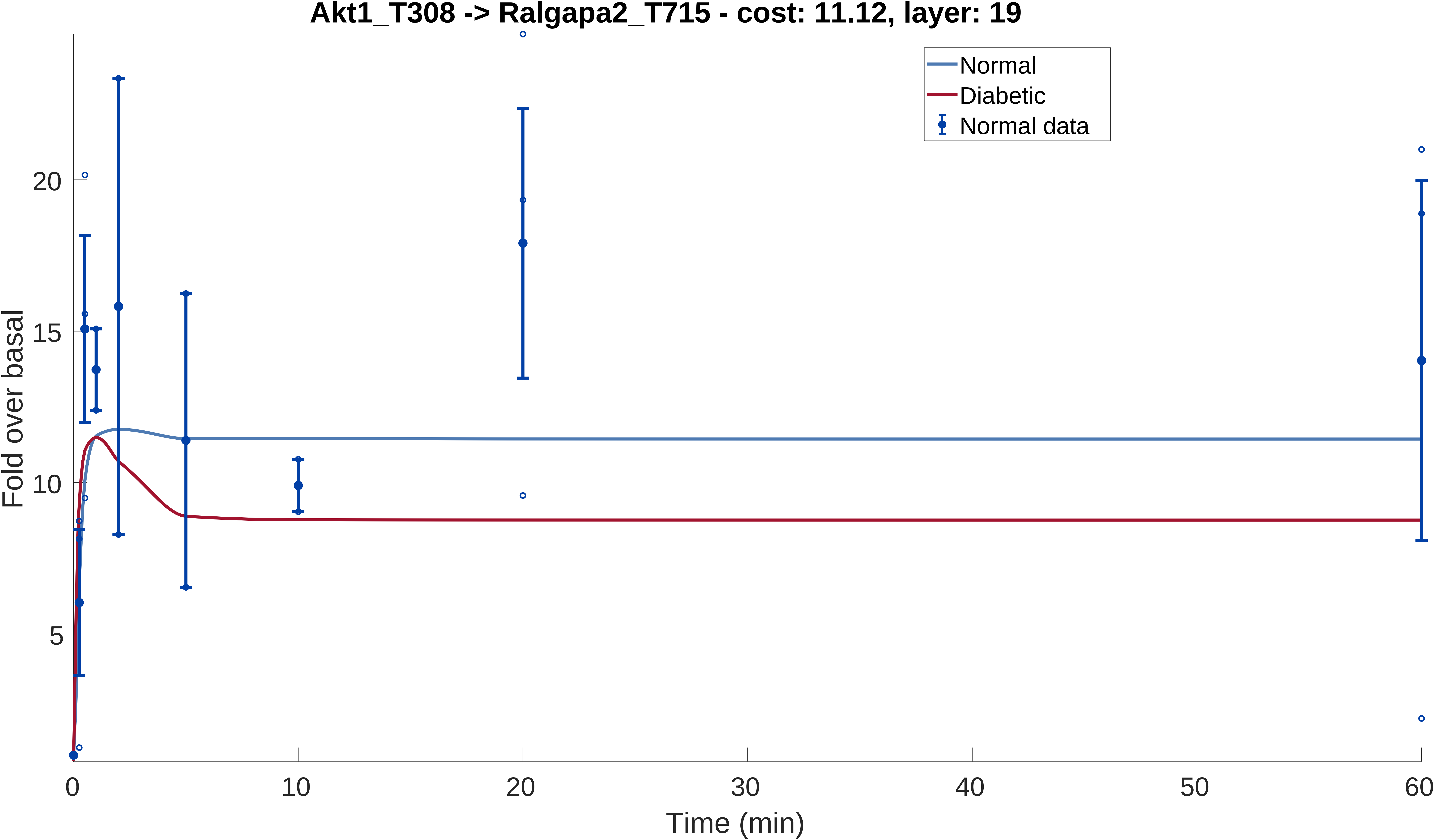

Akt1_T308 -> Ralgapa2_T715 - cost: 11.12, layer: 19
Normal
Diabetic
Normal data
Fold over basal
20
15
10
5
0
10
20
30
40
50
60
Time (min)

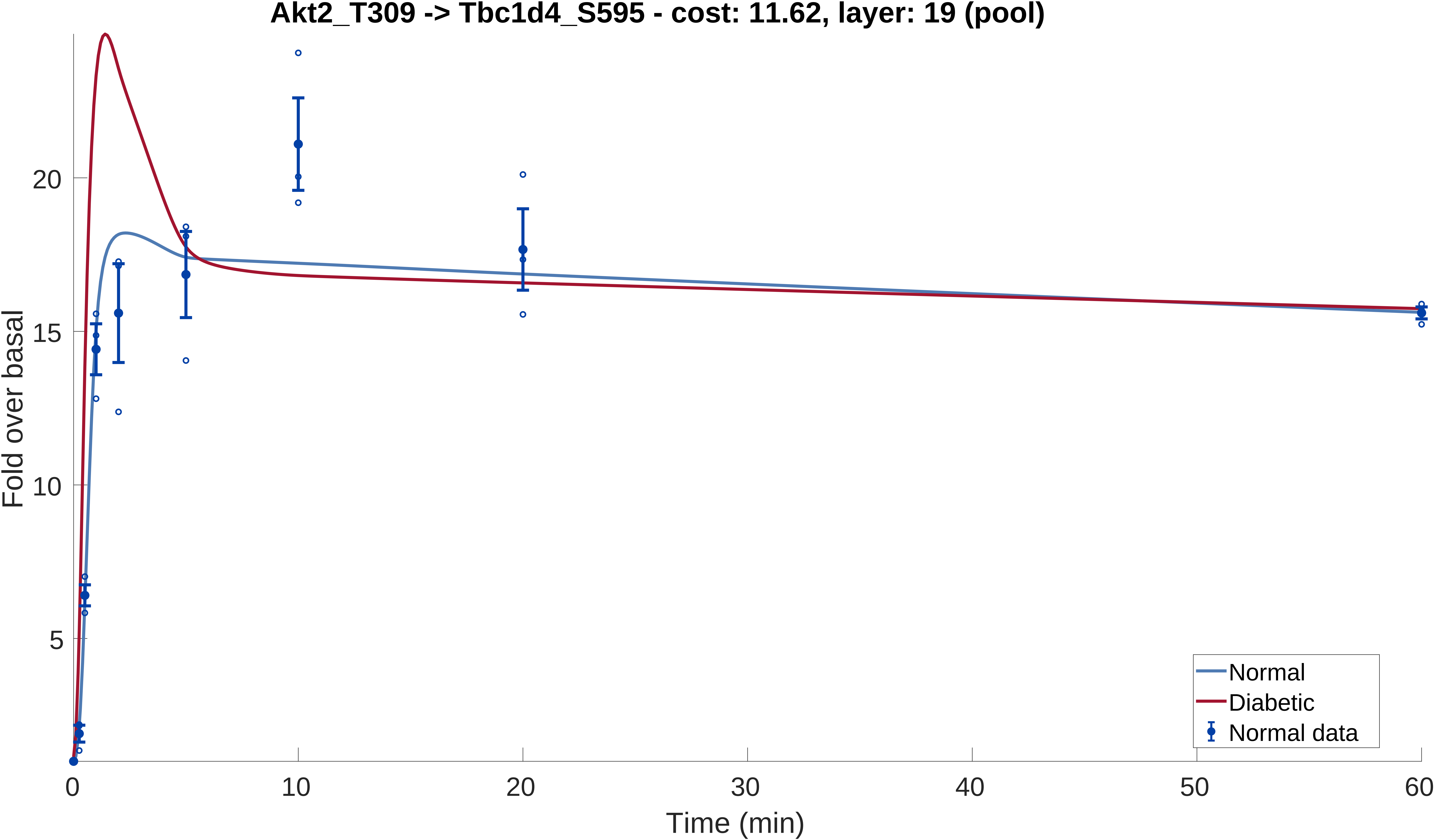

Akt2_T309 -> Tbc1d4_S595 - cost: 11.62, layer: 19 (pool)
Fold over basal
20
15
10
5
0
10
20
30
40
50
60
Time (min)
Normal
Diabetic
Normal data

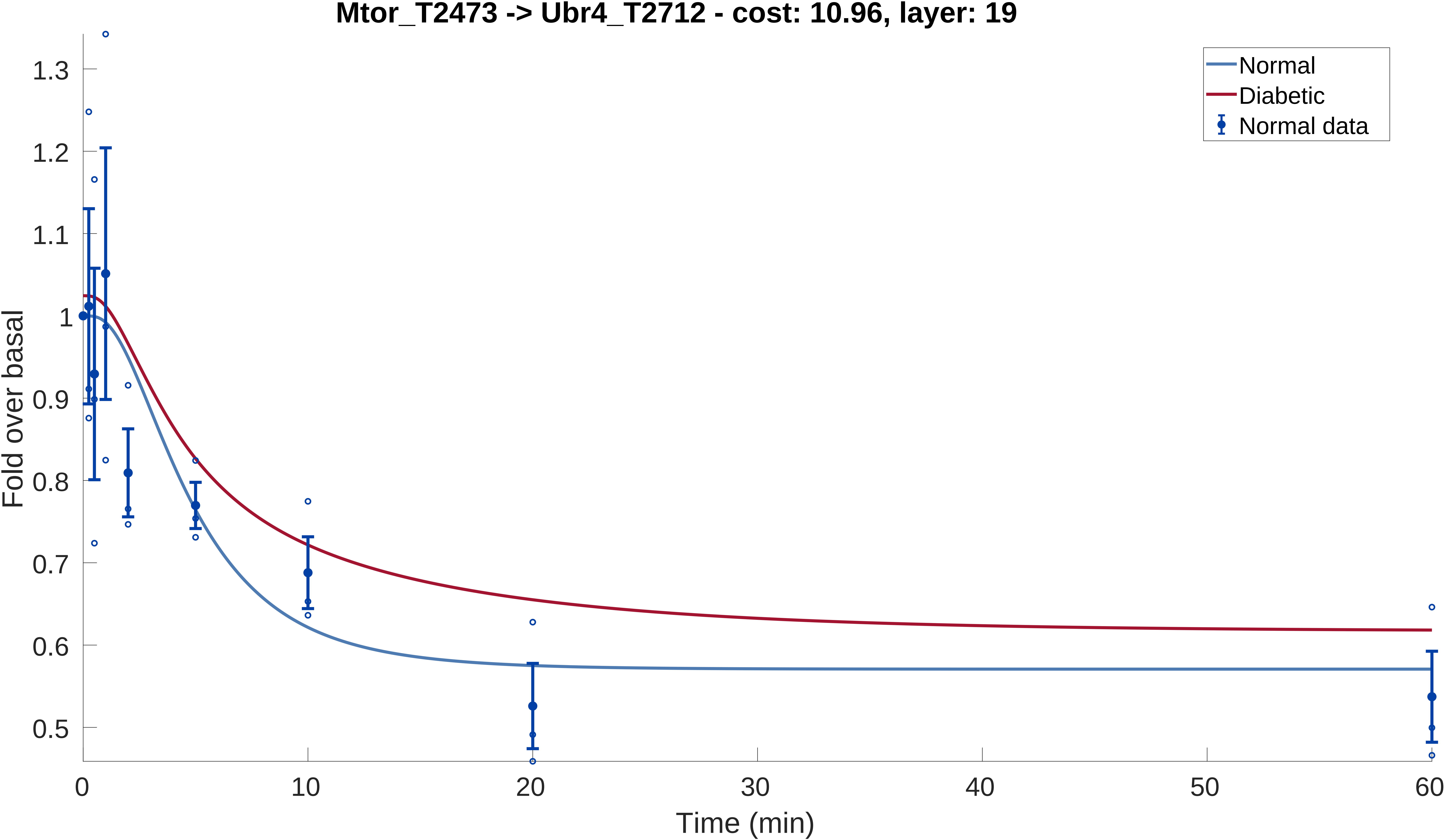

Mtor_T2473 -> Ubr4_T2712 - cost: 10.96, layer: 19
Normal
Diabetic
Normal data
Fold over basal
1.3
1.2
1.1
1
0.9
0.8
0.7
0.6
0.5
0
10
20
30
40
50
60
Time (min)

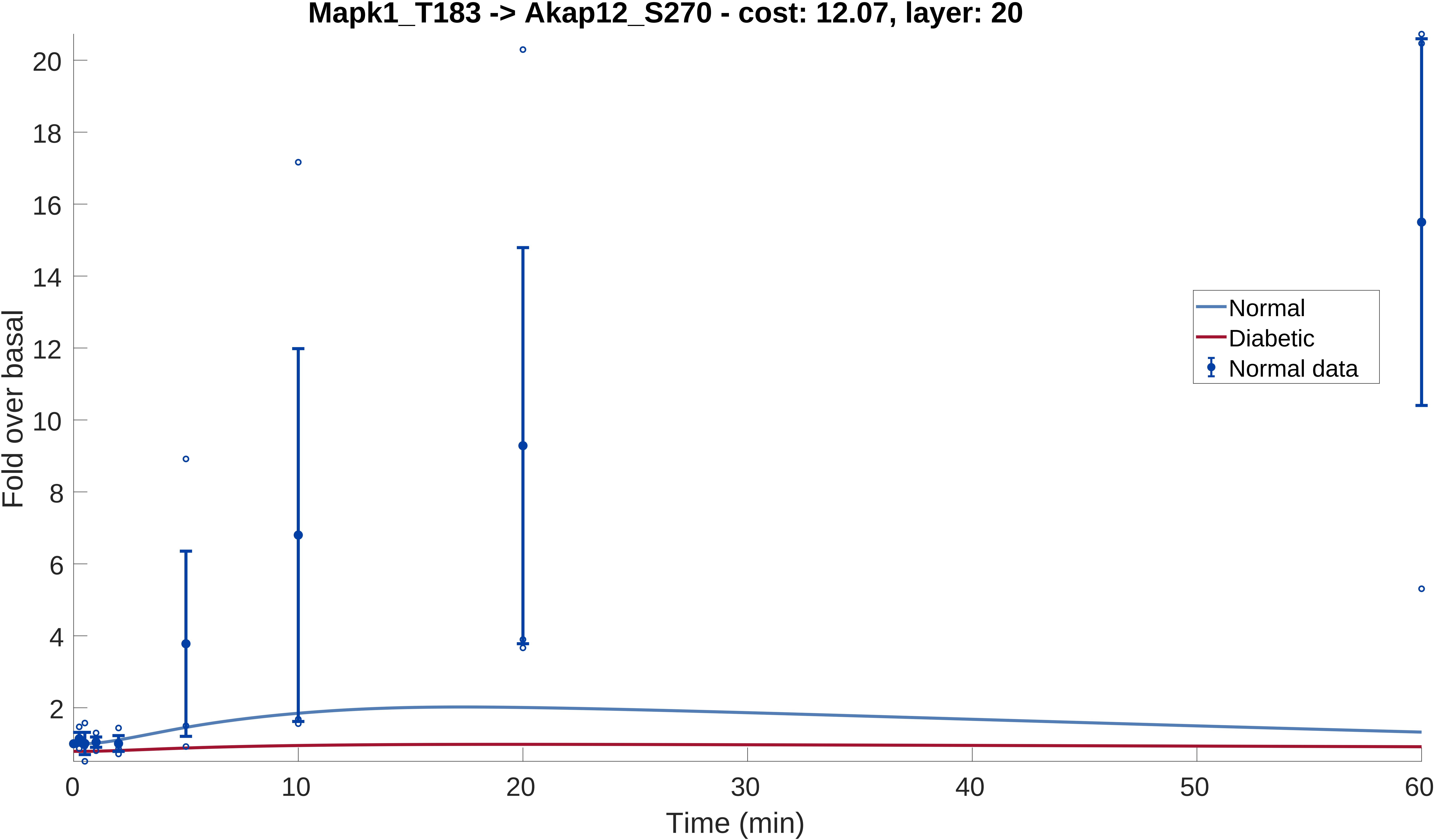

Mapk1_T183 -> Akap12_S270 - cost: 12.07, layer: 20
Fold over basal
Time (min)
Normal
Diabetic
Normal data
0
10
20
30
40
50
60
2
4
6
8
10
12
14
16
18
20

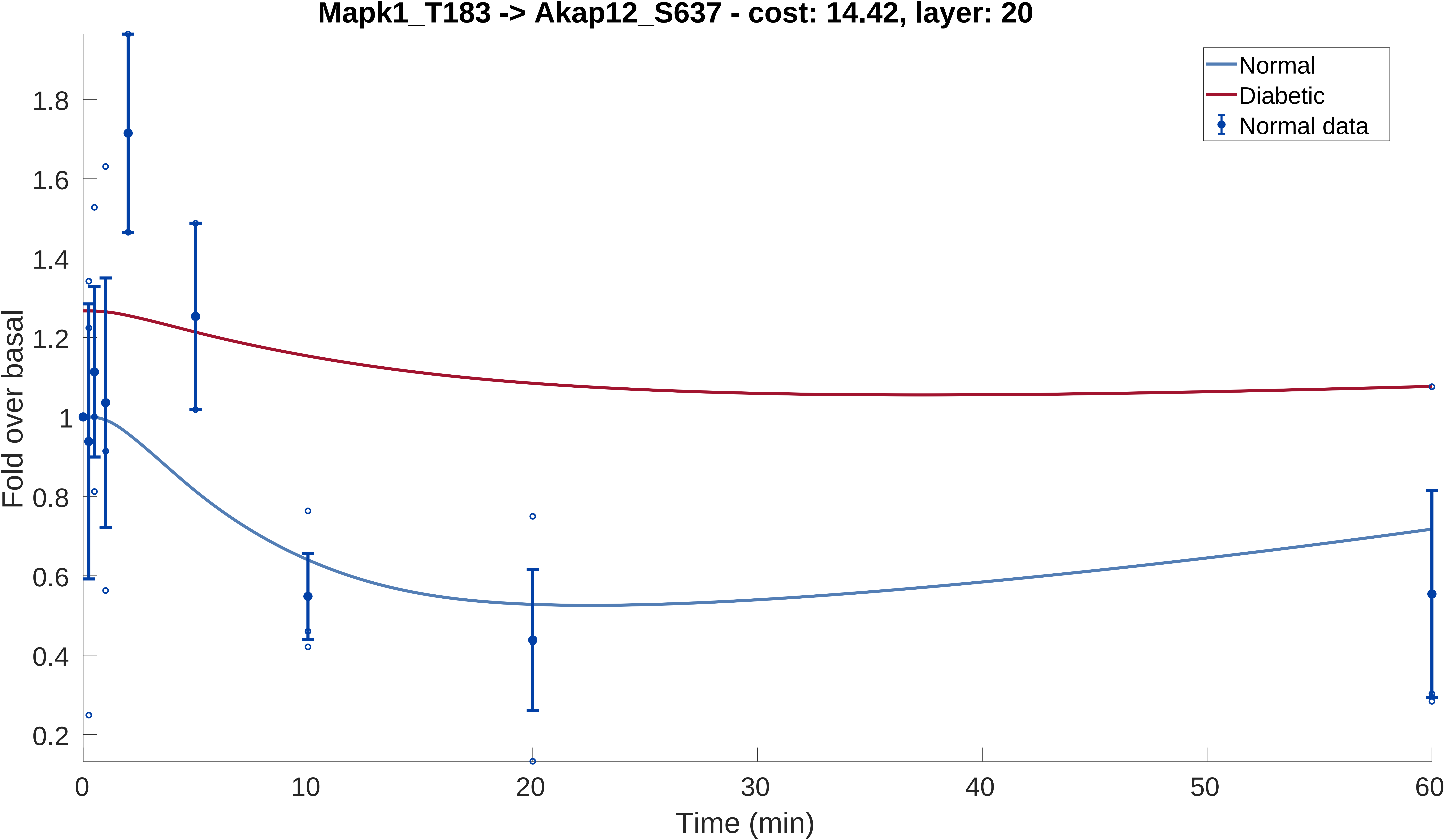
Mapk1_T183 -> Akap12_S637 - cost: 14.42, layer: 20
Normal
Diabetic
Normal data
Fold over basal
Time (min)
0.2
0.4
0.6
0.8
1
1.2
1.4
1.6
1.8
0
10
20
30
40
50
60

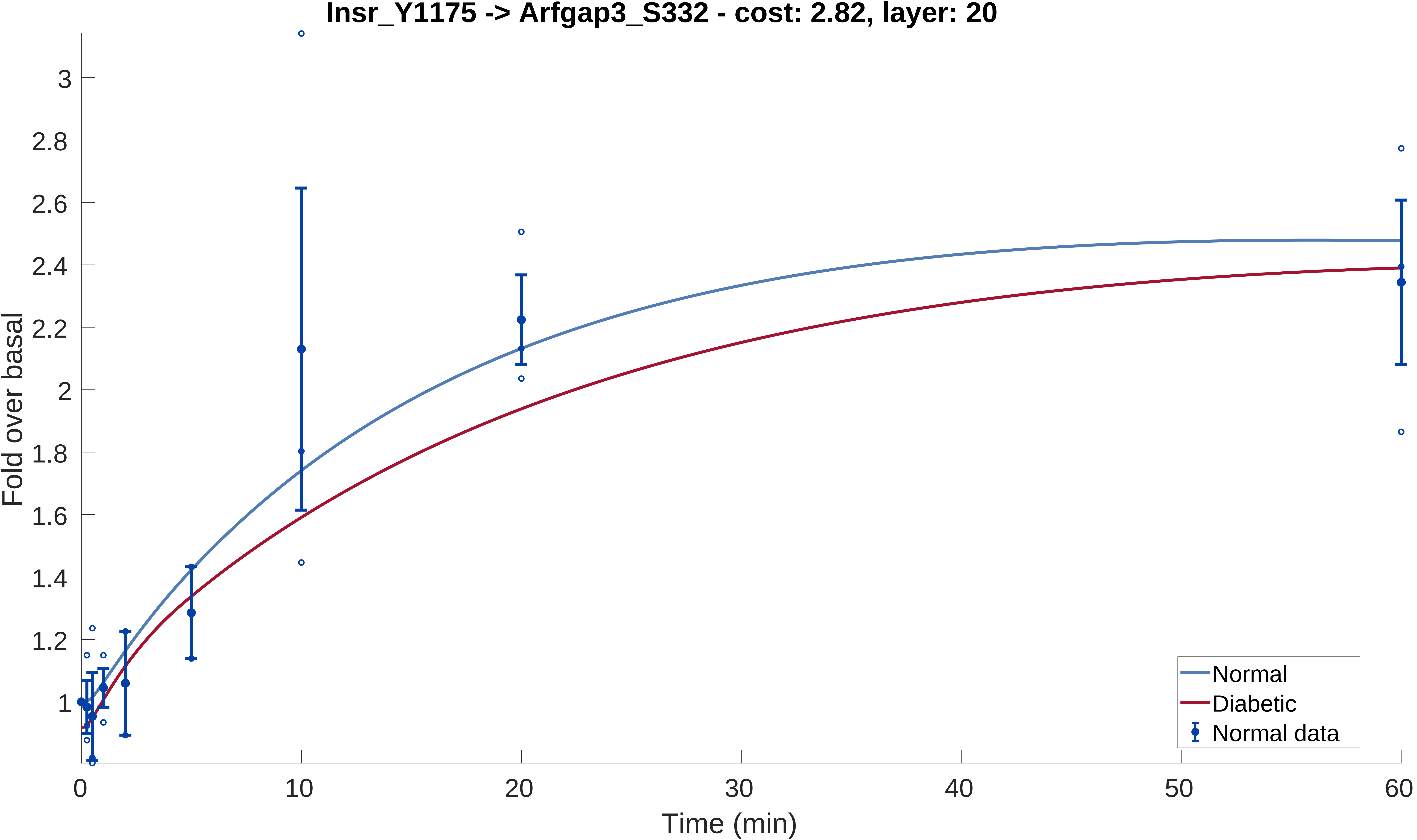

Insr_Y1175 -> Arfgap3_S332 - cost: 2.82, layer: 20
Fold over basal
Time (min)
Normal
Diabetic
Normal data
3
2.8
2.6
2.4
2.2
2
1.8
1.6
1.4
1.2
1
0
10
20
30
40
50
60

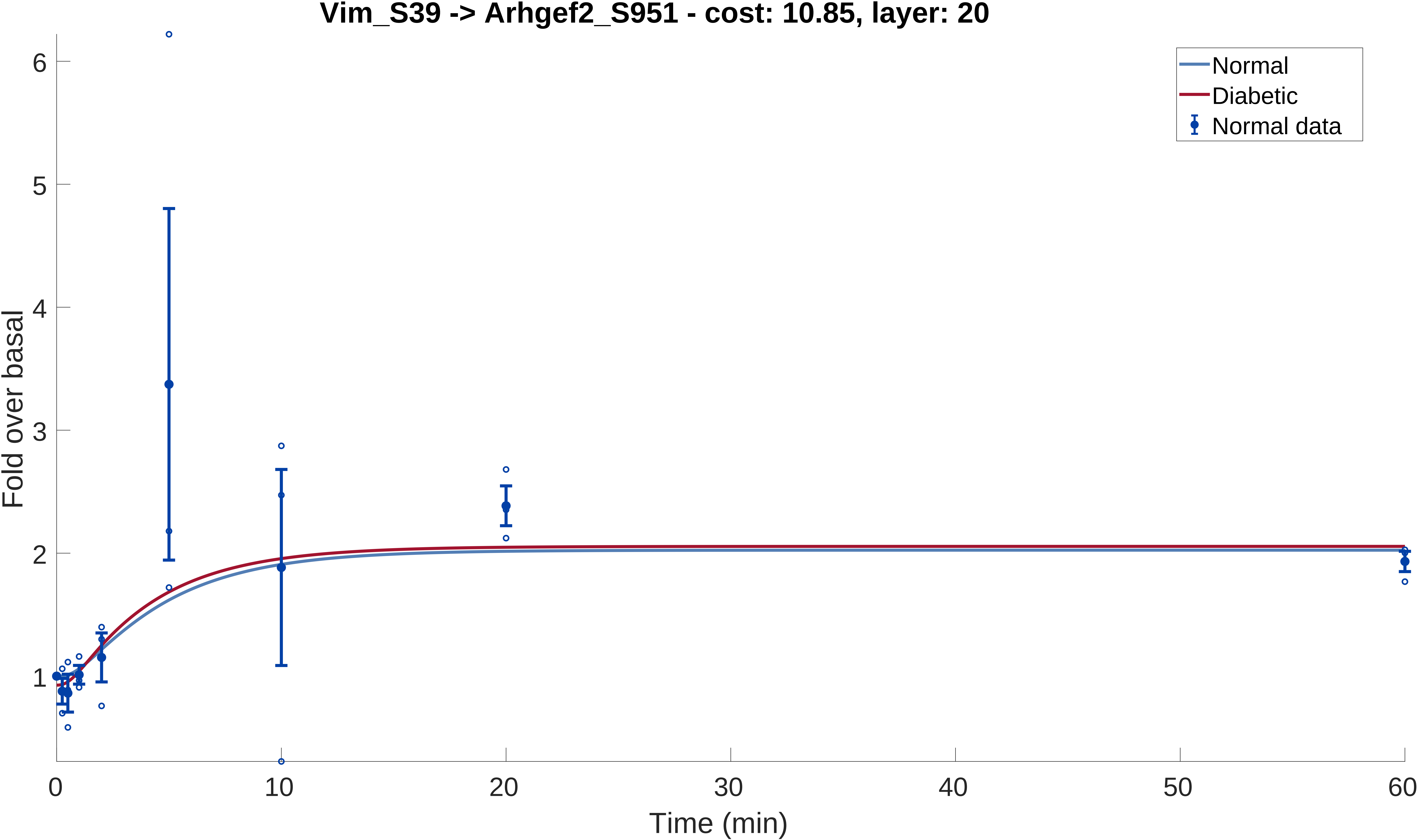
Vim_S39 -> Arhgef2_S951 - cost: 10.85, layer: 20
Normal
Diabetic
Normal data
Fold over basal
Time (min)
0
10
20
30
40
50
60
1
2
3
4
5
6

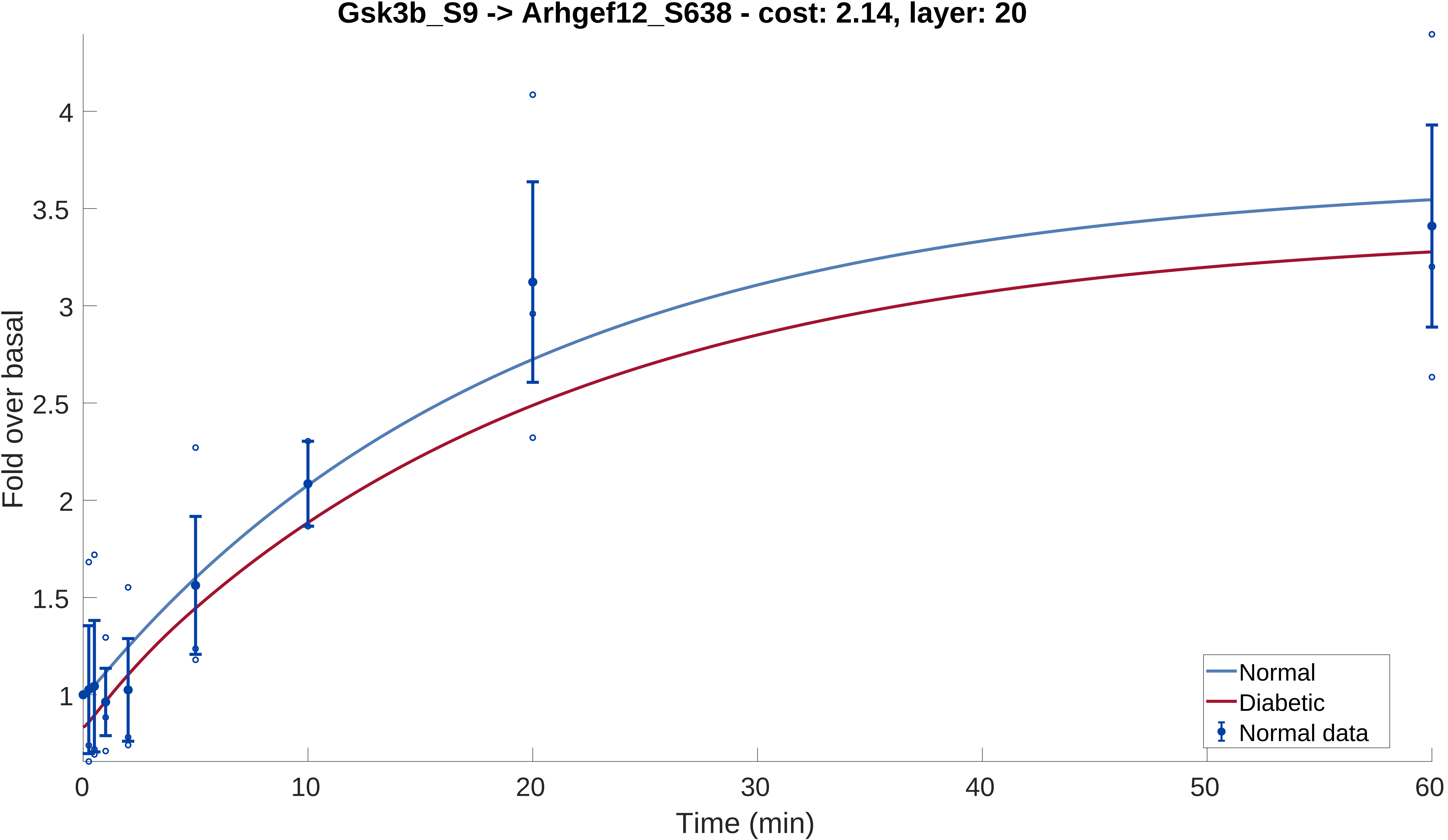

Gsk3b_S9 -> Arhgef12_S638 - cost: 2.14, layer: 20
Fold over basal
4
3.5
3
2.5
2
1.5
1
0
10
20
30
40
50
60
Time (min)
Normal
Diabetic
Normal data

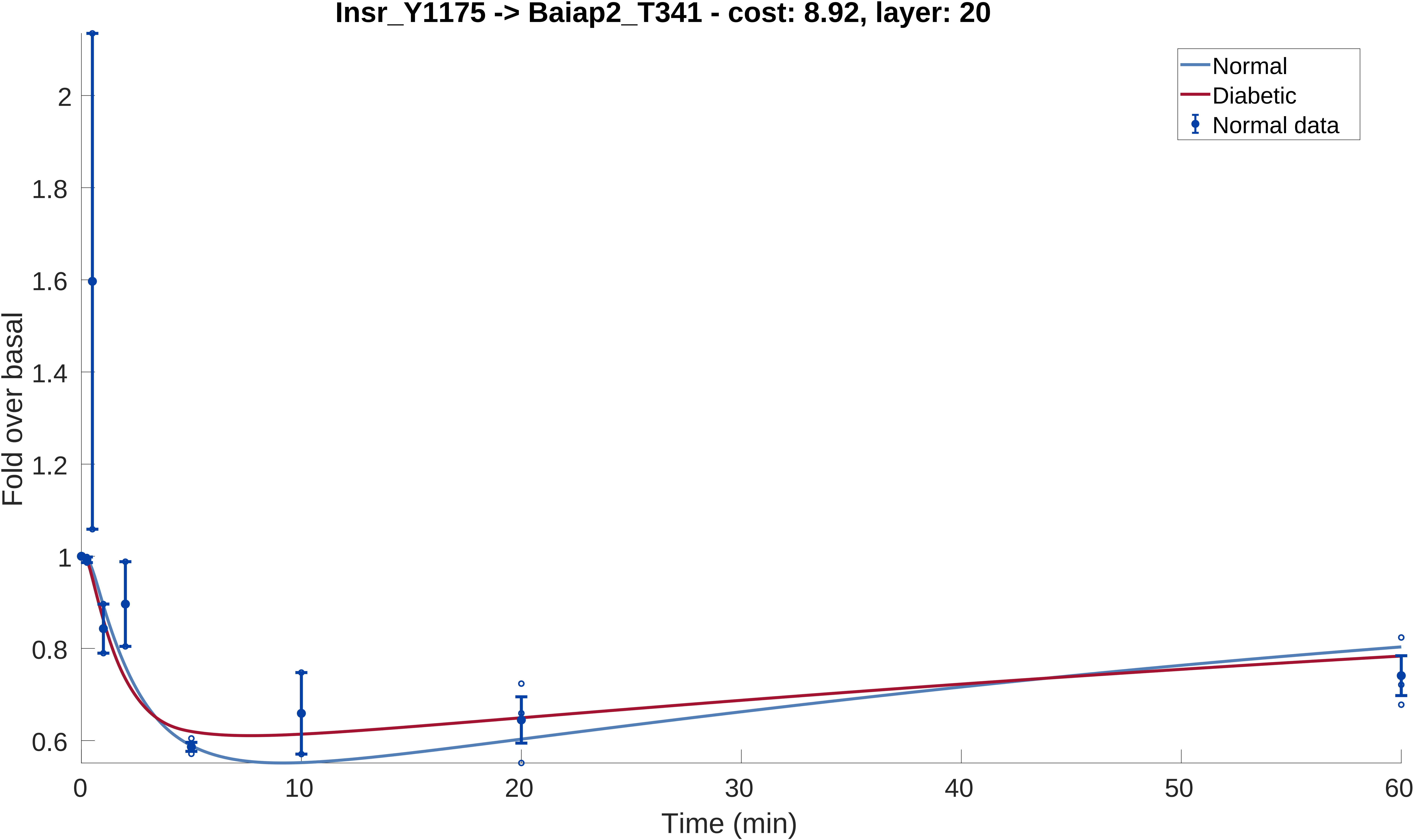

Insr_Y1175 -> Baiap2_T341 - cost: 8.92, layer: 20
Normal
Diabetic
Normal data
Fold over basal
2
1.8
1.6
1.4
1.2
1
0.8
0.6
0
10
20
30
40
50
60
Time (min)

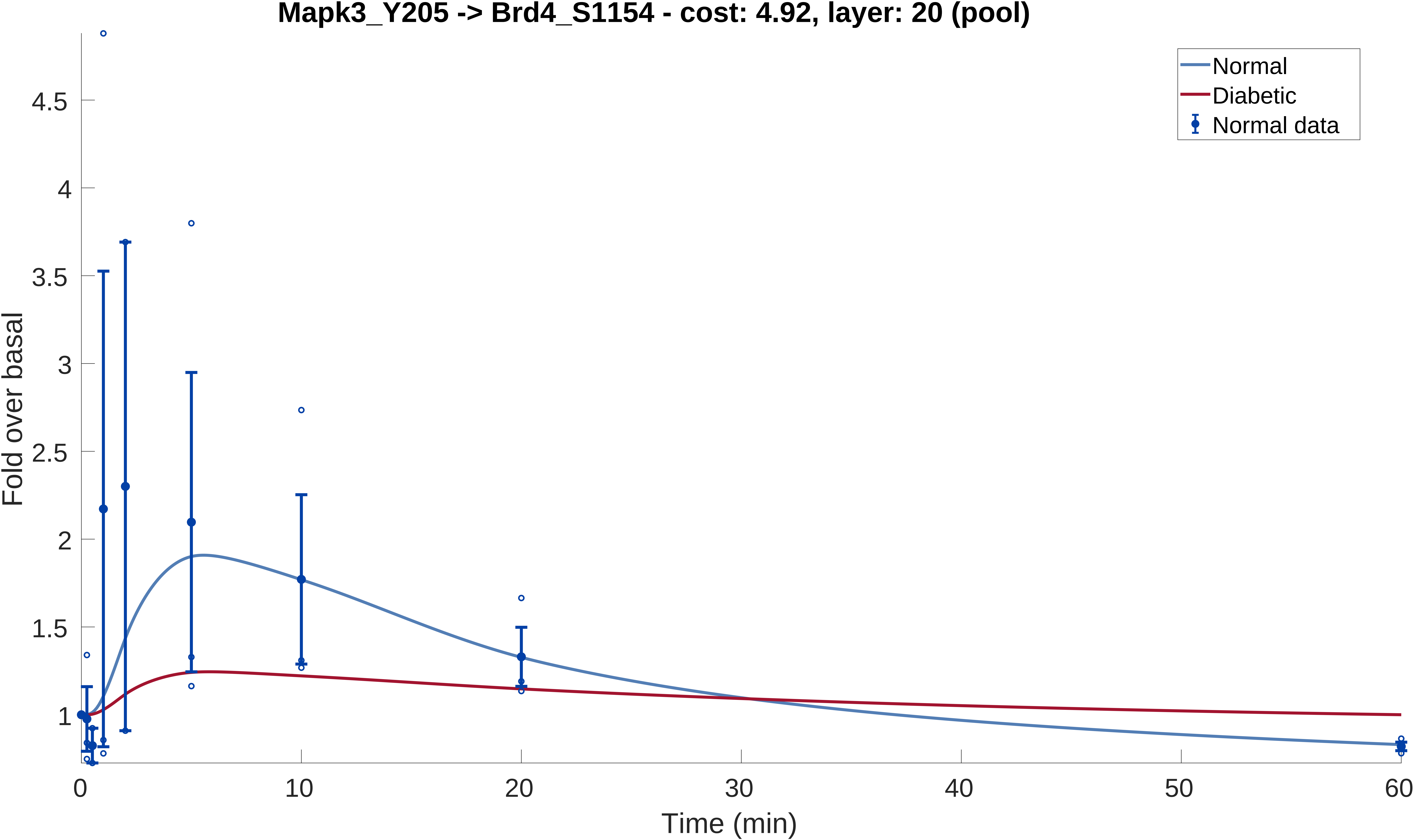

Mapk3_Y205 -> Brd4_S1154 - cost: 4.92, layer: 20 (pool)
Normal
Diabetic
Normal data
Fold over basal
Time (min)
4.5
4
3.5
3
2.5
2
1.5
1
0
10
20
30
40
50
60

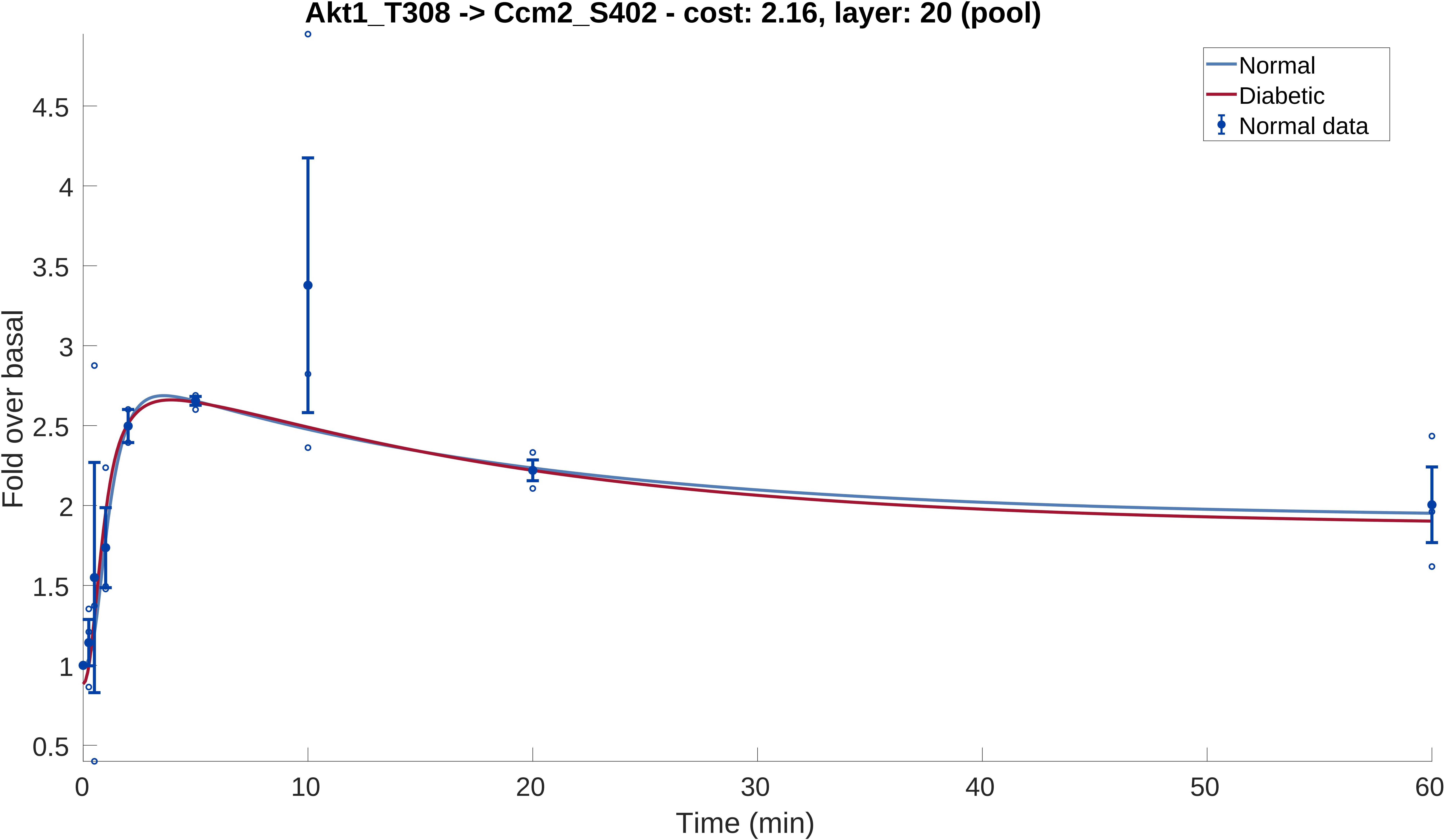

Akt1_T308 -> Ccm2_S402 - cost: 2.16, layer: 20 (pool)
Normal
Diabetic
Normal data
Fold over basal
Time (min)
0.5
1
1.5
2
2.5
3
3.5
4
4.5
0
10
20
30
40
50
60

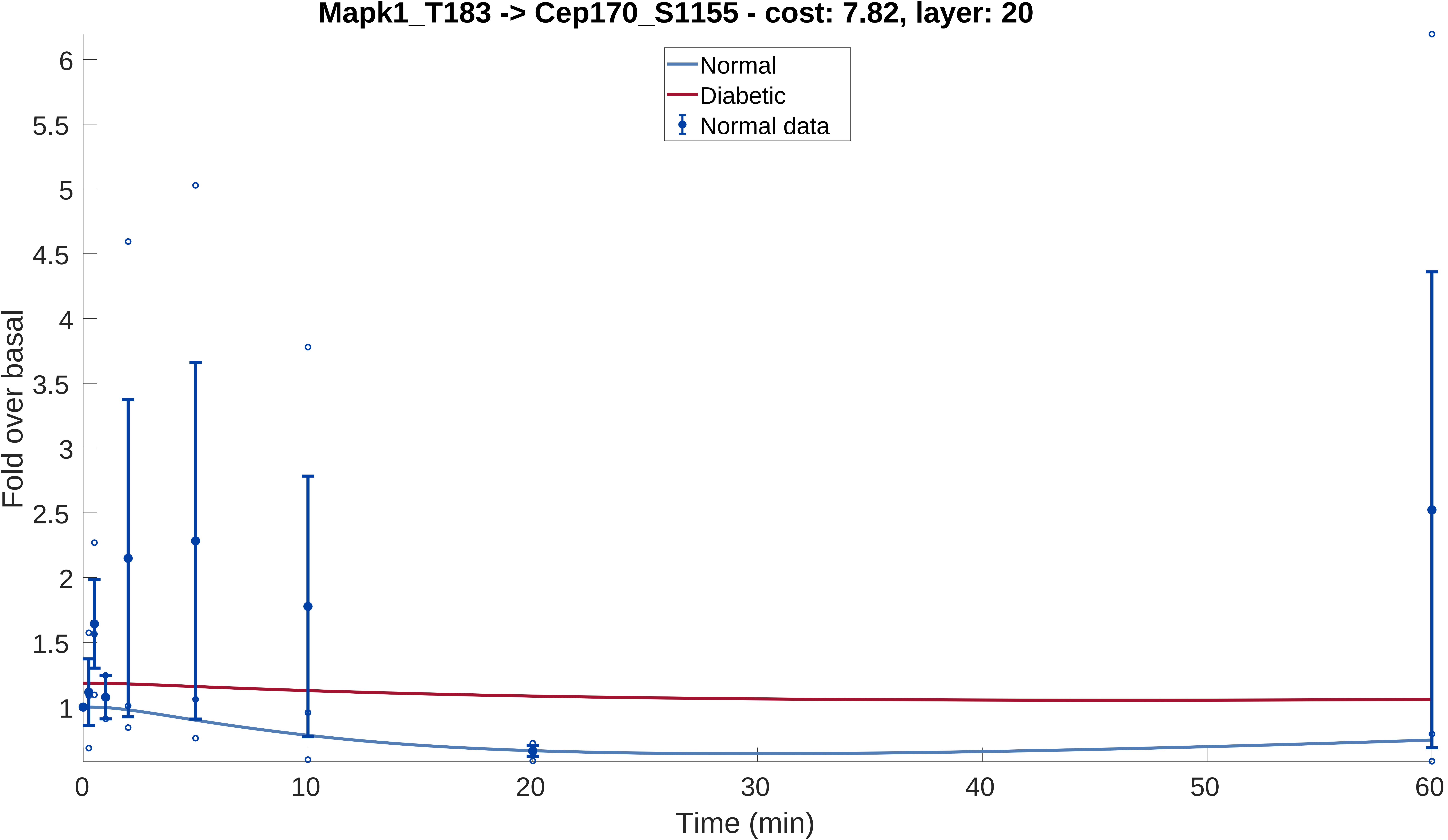

Mapk1_T183 -> Cep170_S1155 - cost: 7.82, layer: 20
Normal
Diabetic
Normal data
Fold over basal
Time (min)
1
1.5
2
2.5
3
3.5
4
4.5
5
5.5
6
0
10
20
30
40
50
60

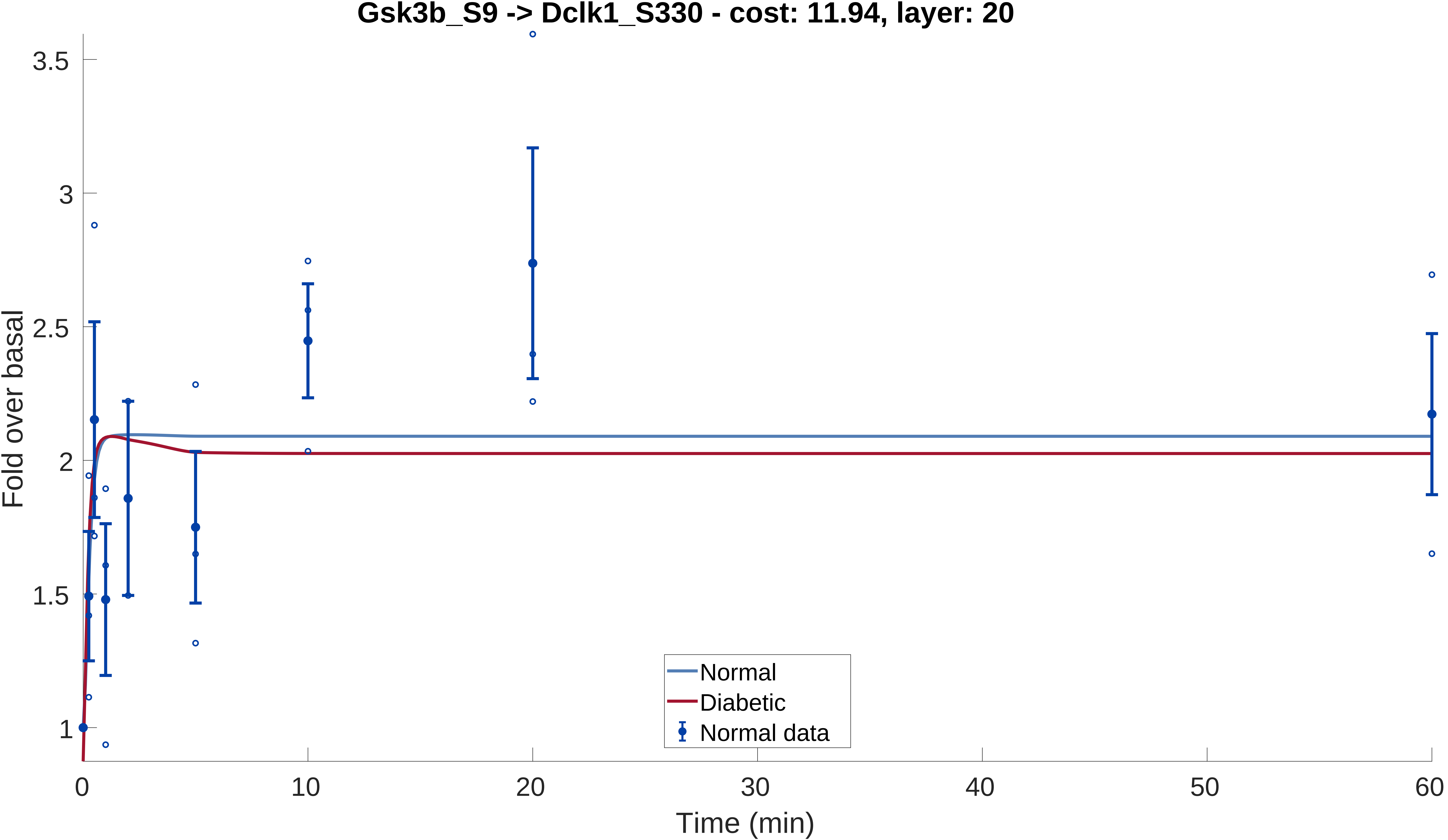

Gsk3b_S9 -> Dclk1_S330 - cost: 11.94, layer: 20
Fold over basal
3.5
3
2.5
2
1.5
1
0
10
20
30
40
50
60
Time (min)
Normal
Diabetic
Normal data

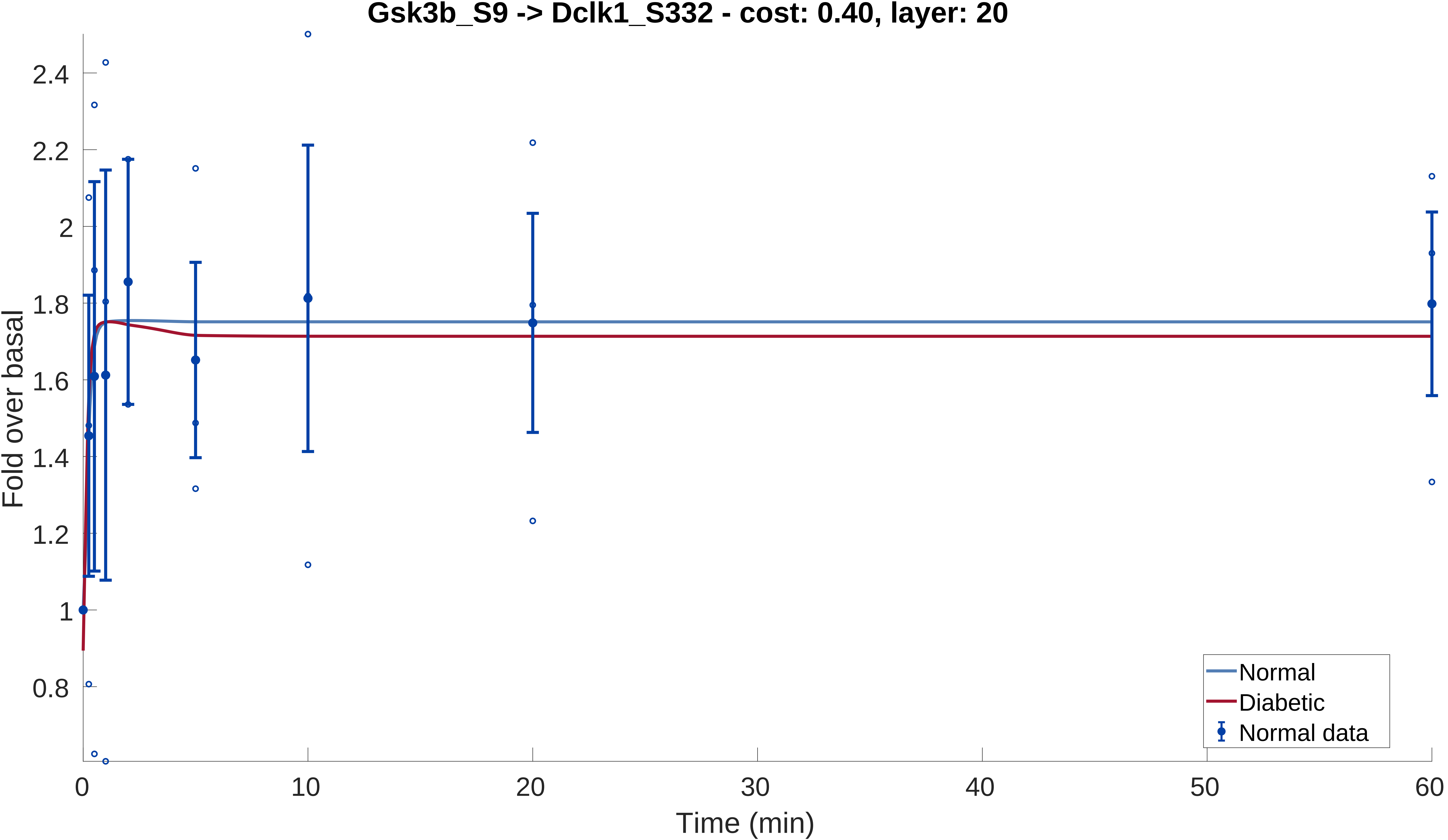
Gsk3b_S9 -> Dclk1_S332 - cost: 0.40, layer: 20
Fold over basal
2.4
2.2
2
1.8
1.6
1.4
1.2
1
0.8
0
10
20
30
40
50
60
Time (min)
Normal
Diabetic
Normal data

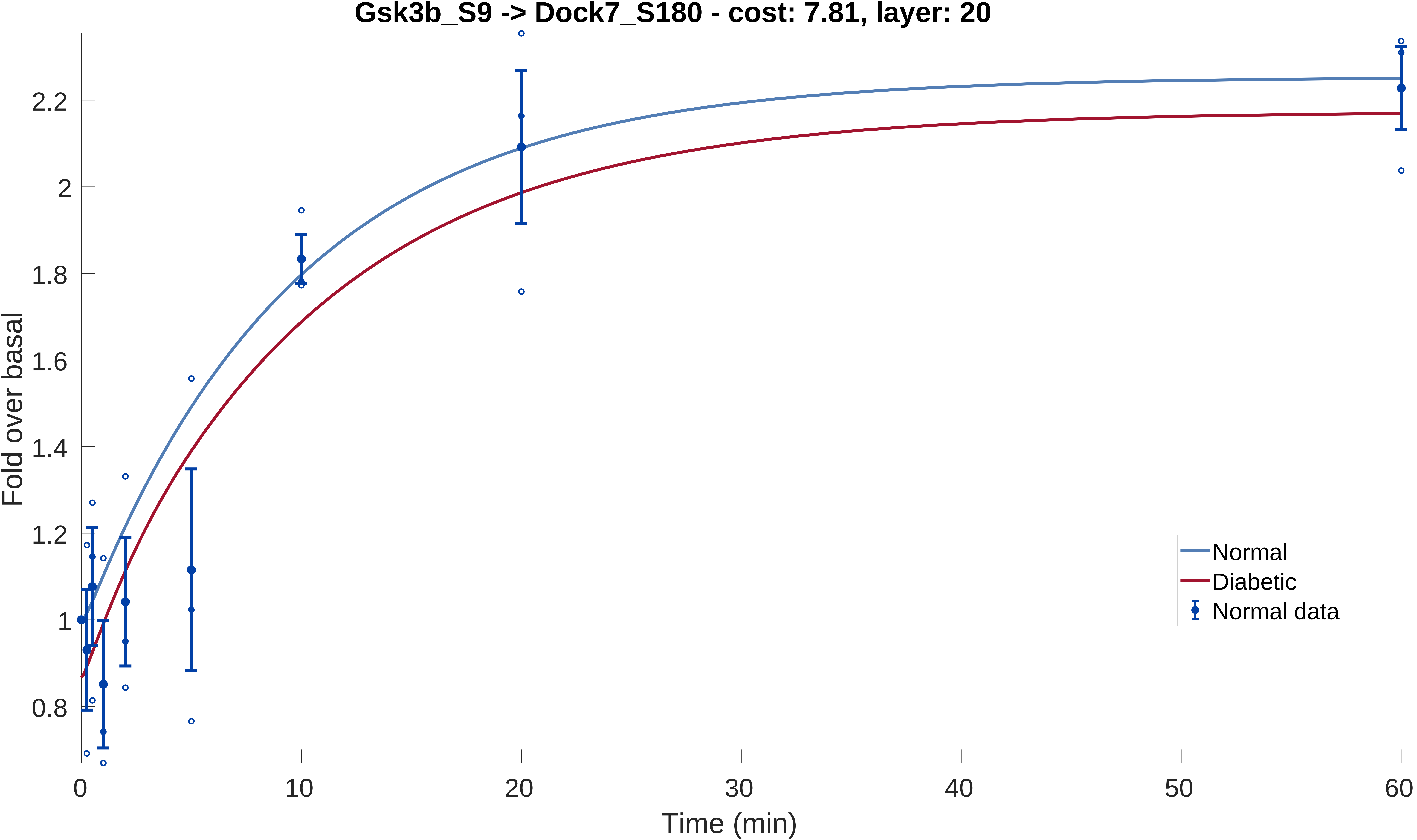

Gsk3b_S9 -> Dock7_S180 - cost: 7.81, layer: 20
Fold over basal
2.2
2
1.8
1.6
1.4
1.2
1
0.8
0
10
20
30
40
50
60
Time (min)
Normal
Diabetic
Normal data

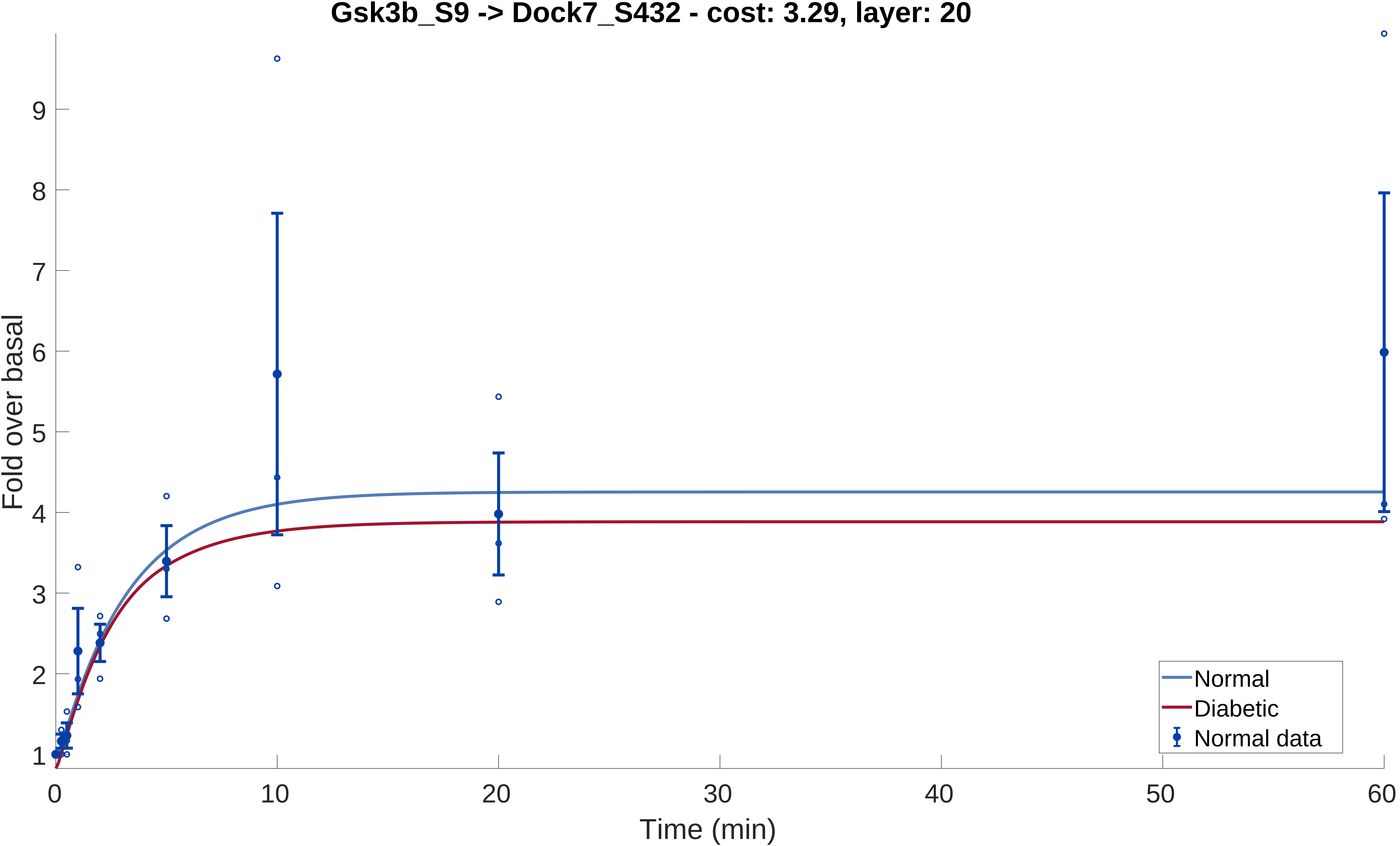

Gsk3b_S9 -> Dock7_S432 - cost: 3.29, layer: 20
Fold over basal
Time (min)
Normal
Diabetic
Normal data
1
2
3
4
5
6
7
8
9
0
10
20
30
40
50
60

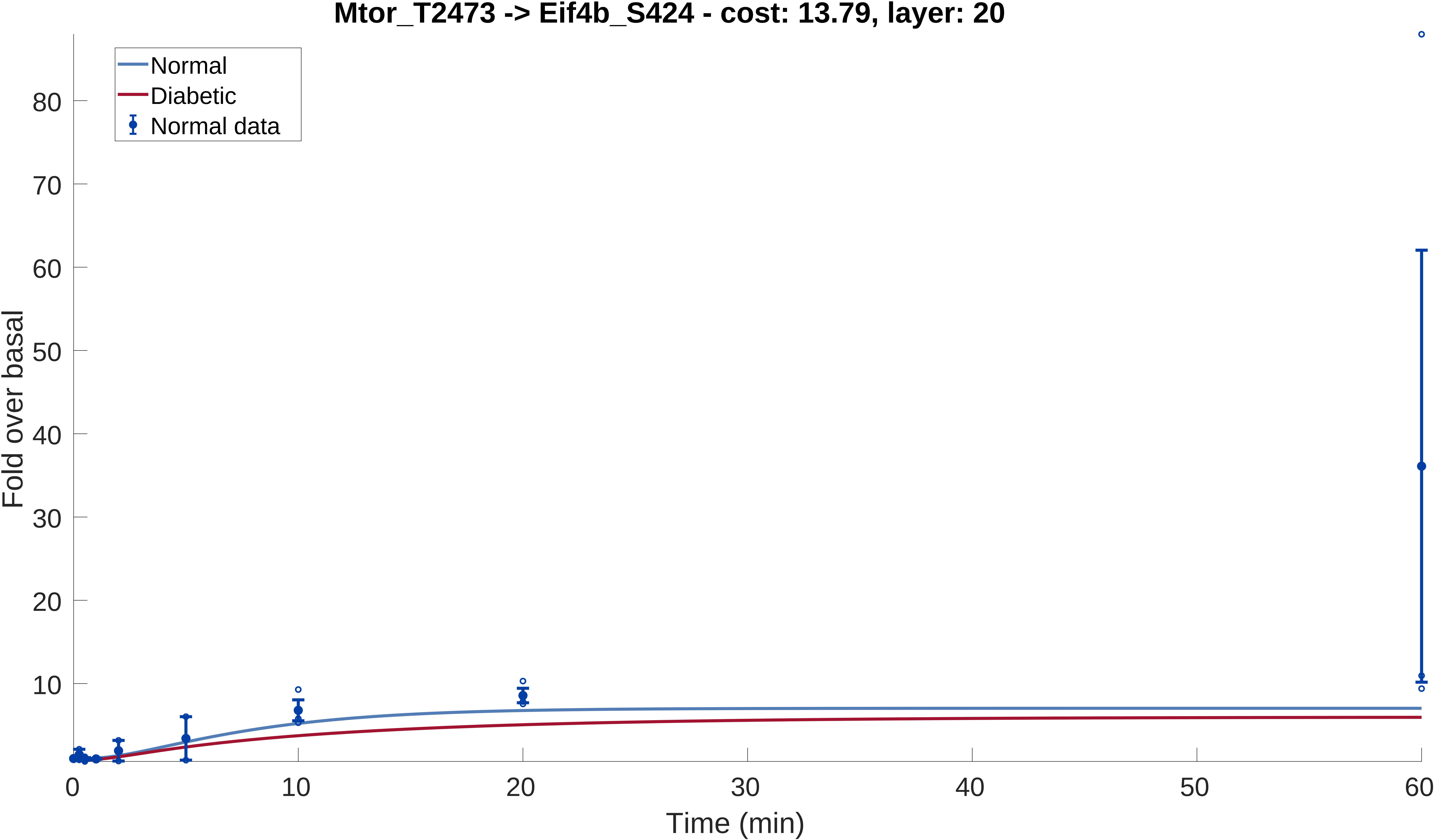

Mtor_T2473 -> Eif4b_S424 - cost: 13.79, layer: 20
Normal
Diabetic
Normal data
Fold over basal
Time (min)
0
10
20
30
40
50
60
70
80

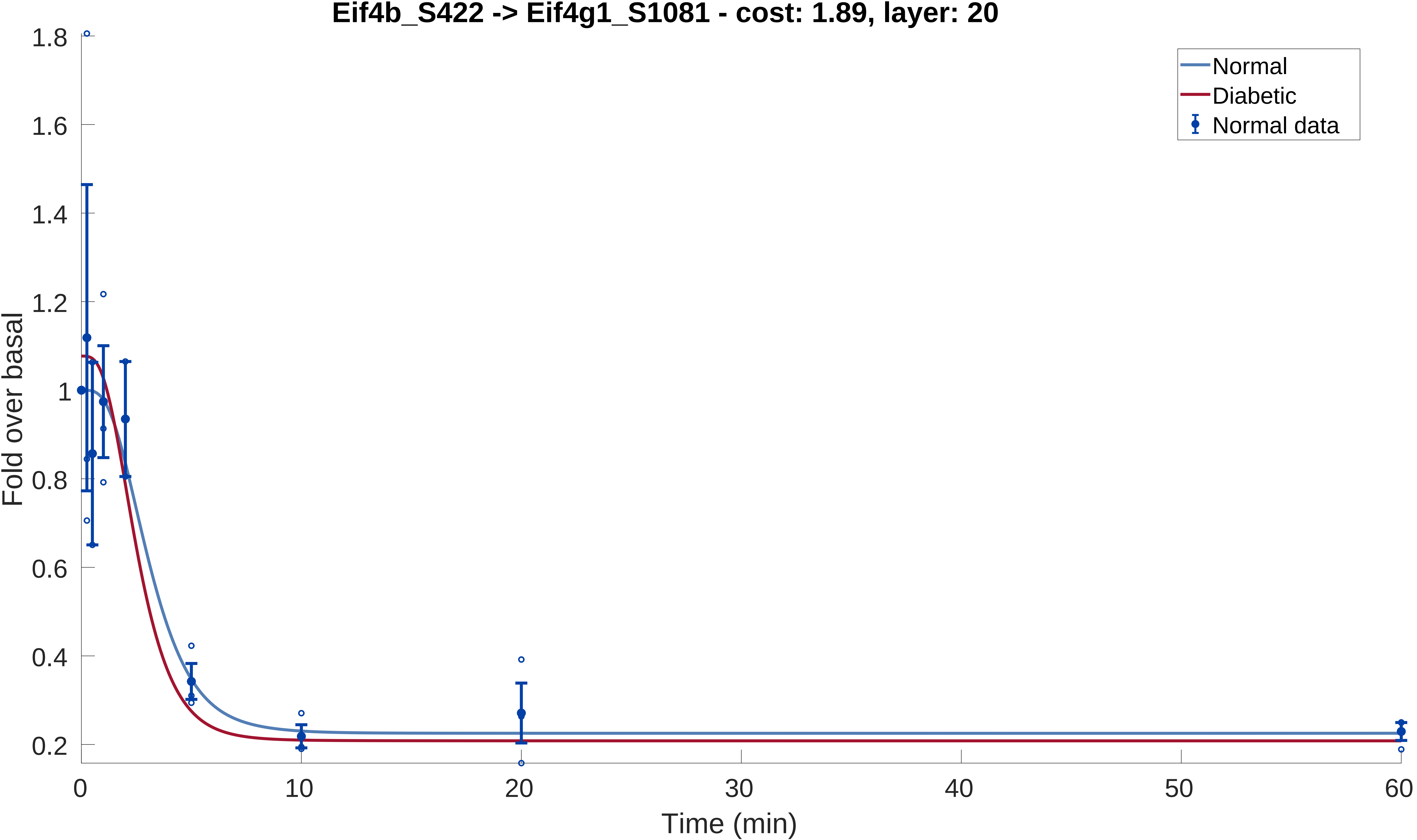

Eif4b_S422 -> Eif4g1_S1081 - cost: 1.89, layer: 20
1.8
1.6
1.4
1.2
1
0.8
0.6
0.4
0.2
Fold over basal
0
10
20
30
40
50
60
Time (min)
Normal
Diabetic
Normal data

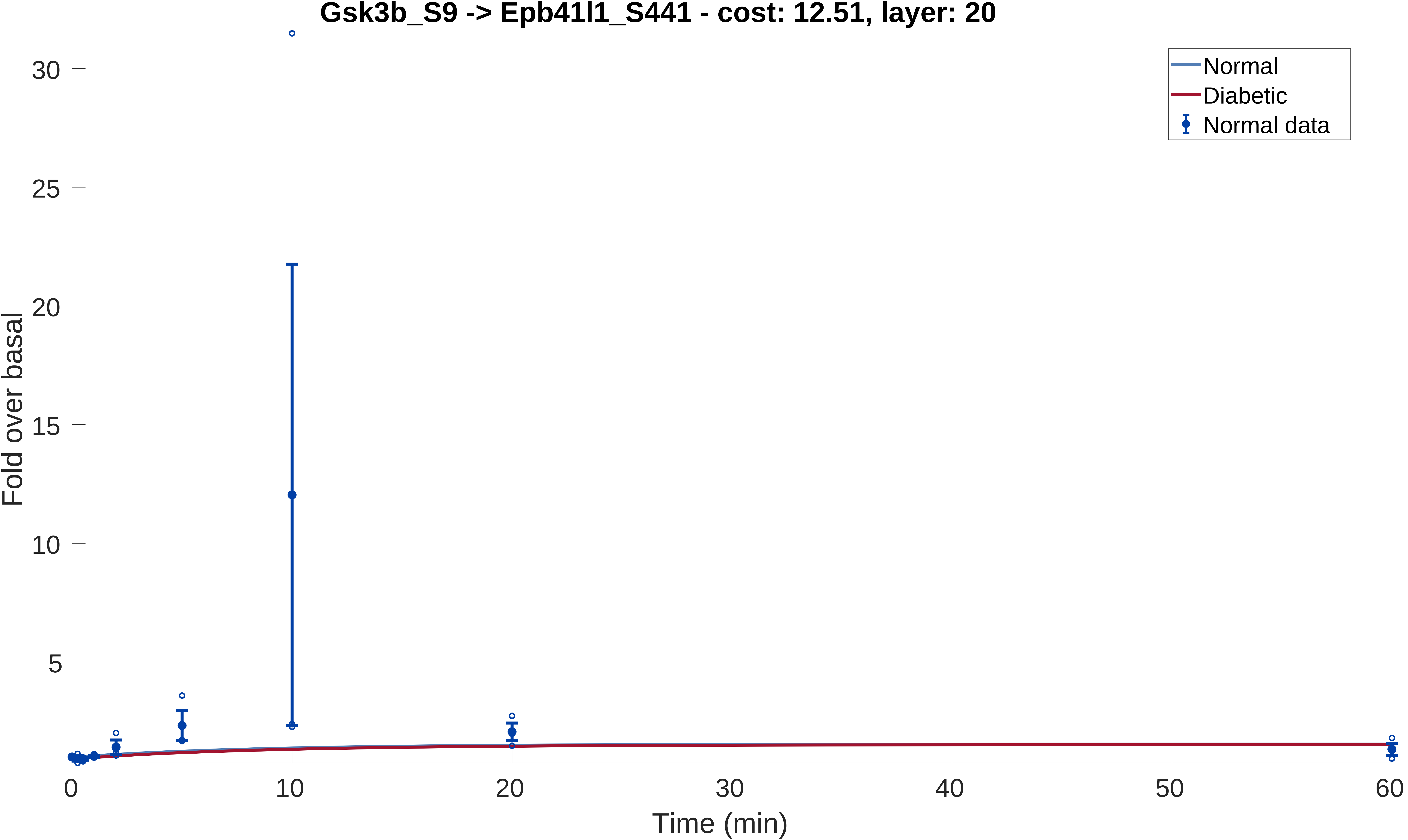

Gsk3b_S9 -> Epb41l1_S441 - cost: 12.51, layer: 20
Normal
Diabetic
Normal data
Fold over basal
Time (min)
0
5
10
15
20
25
30
0
10
20
30
40
50
60

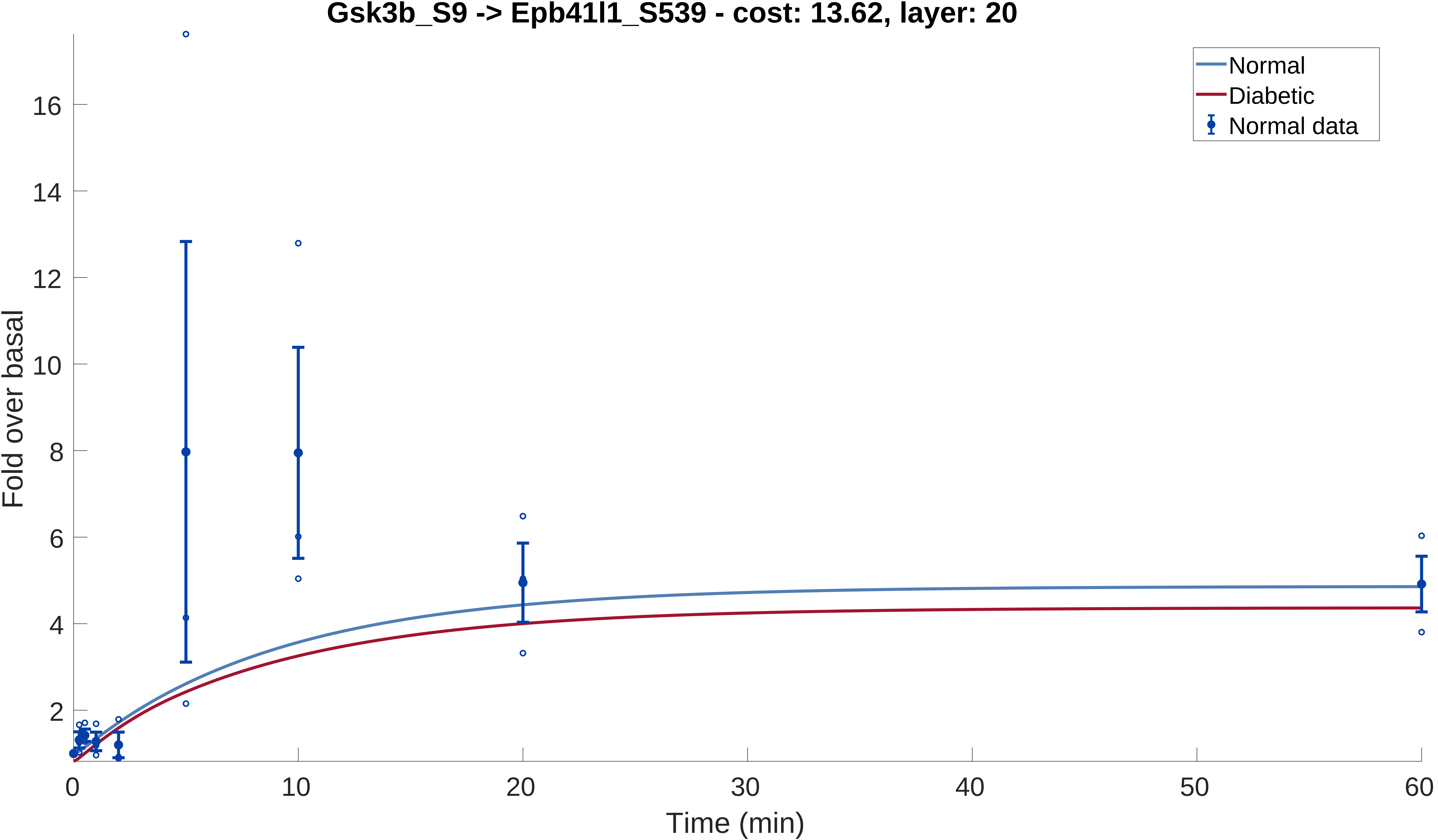
Gsk3b_S9 -> Epb41l1_S539 - cost: 13.62, layer: 20
Normal
Diabetic
Normal data
16
14
12
10
8
6
4
2
0
10
20
30
40
50
60
Fold over basal
Time (min)

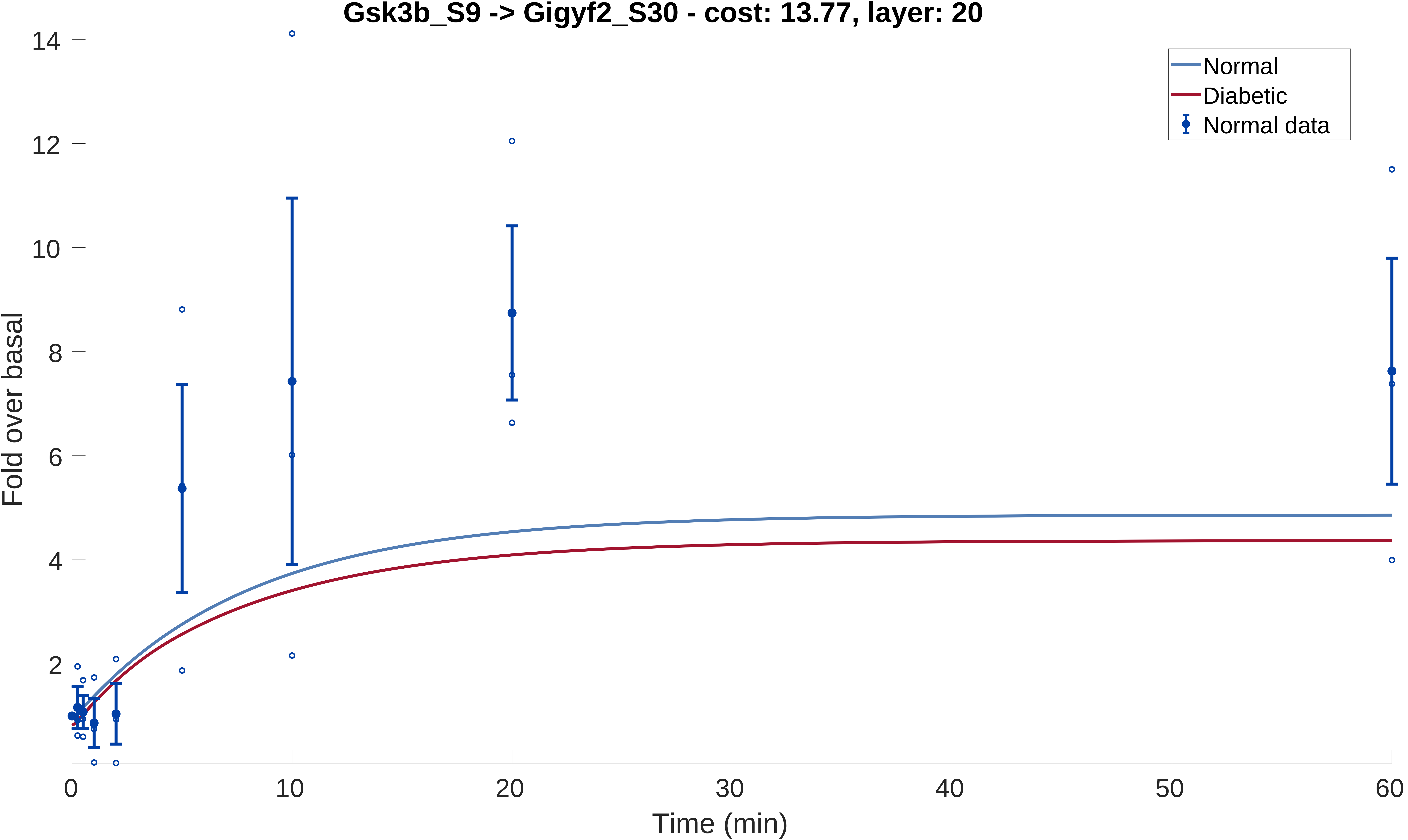

Gsk3b_S9 -> Gigyf2_S30 - cost: 13.77, layer: 20
Normal
Diabetic
Normal data
Fold over basal
Time (min)
0
10
20
30
40
50
60
2
4
6
8
10
12
14

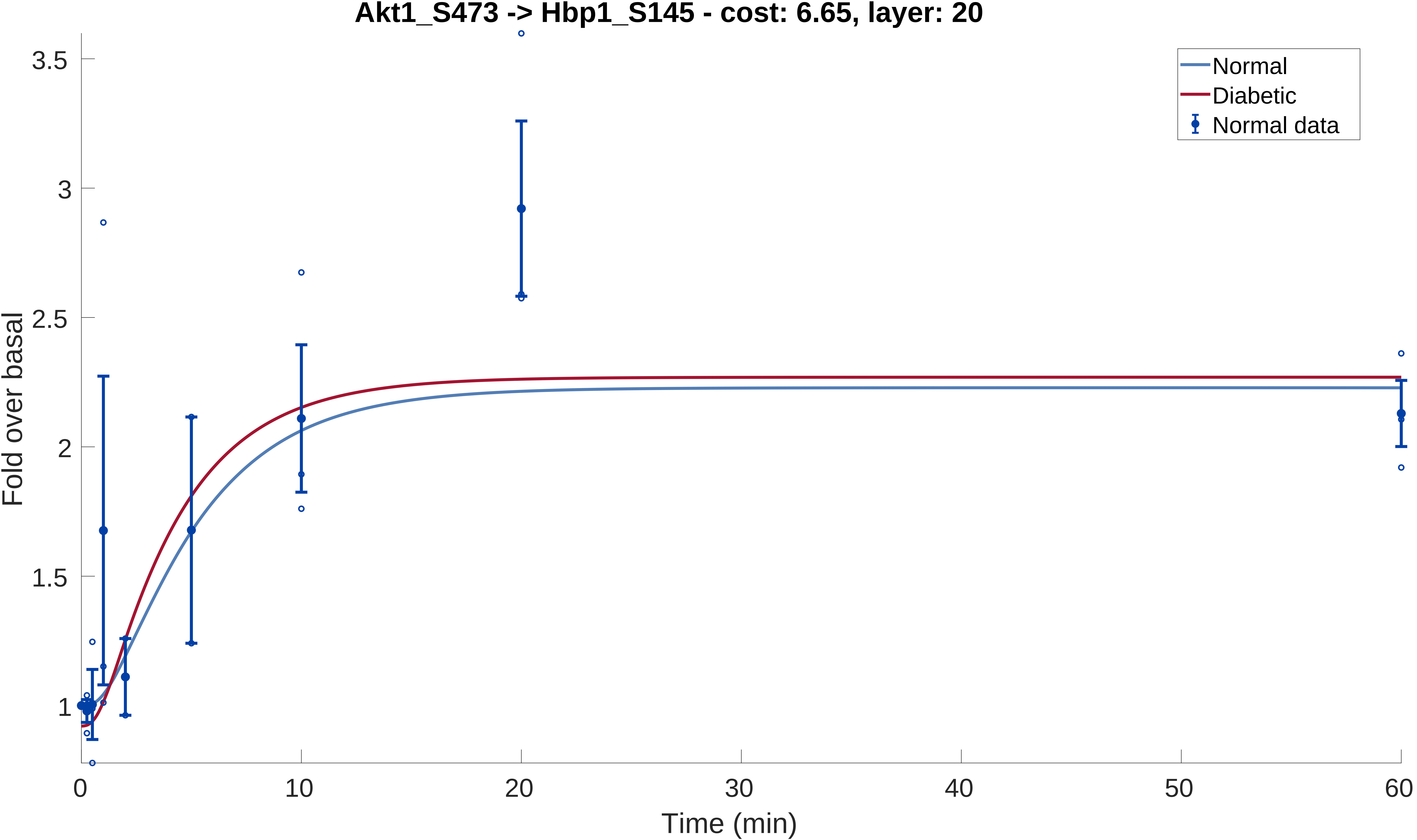

Akt1_S473 -> Hbp1_S145 - cost: 6.65, layer: 20
Normal
Diabetic
Normal data
Fold over basal
Time (min)
3.5
3
2.5
2
1.5
1
0
10
20
30
40
50
60

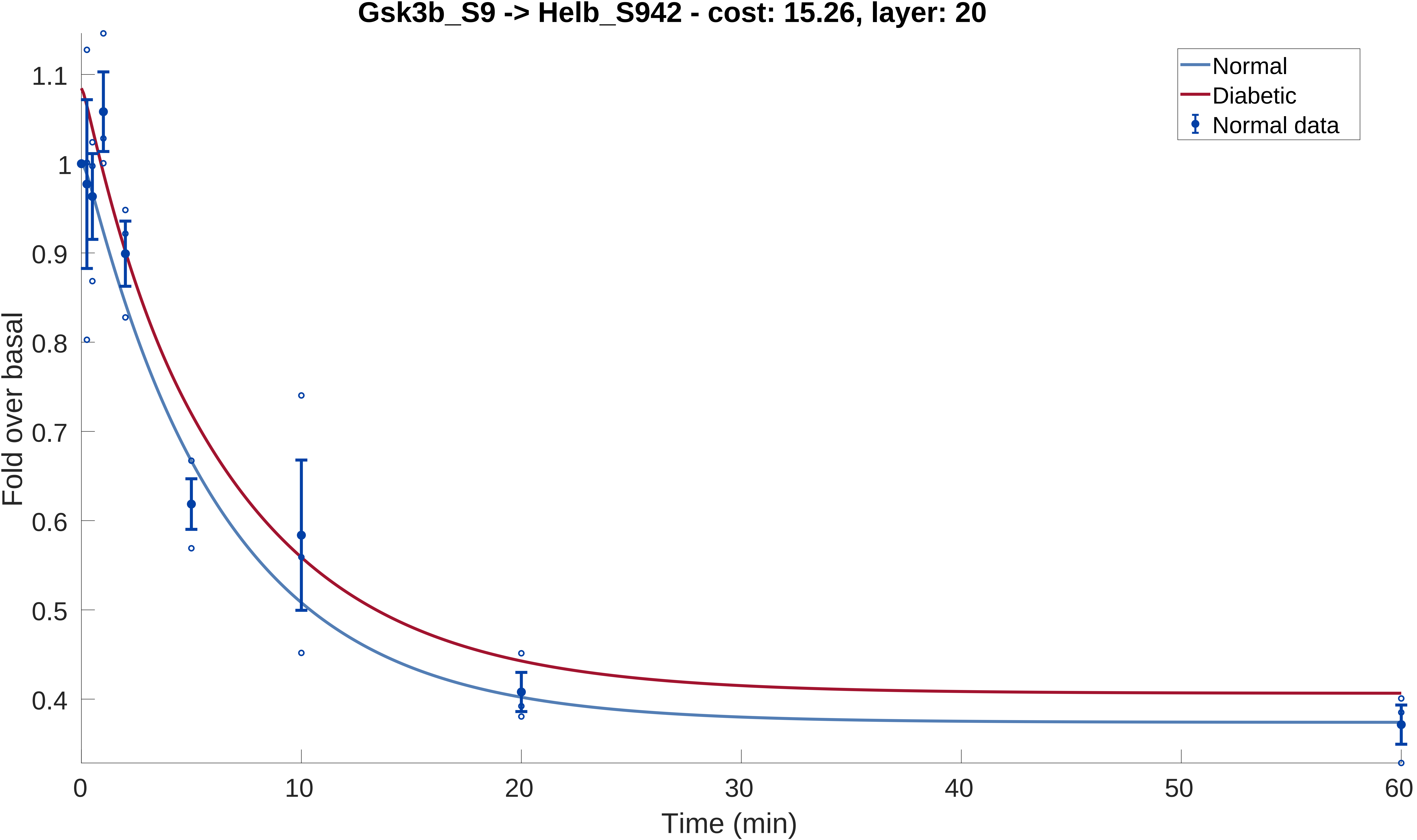

Gsk3b_S9 -> Helb_S942 - cost: 15.26, layer: 20
Normal
Diabetic
Normal data
Fold over basal
Time (min)
1.1
1
0.9
0.8
0.7
0.6
0.5
0.4
0
10
20
30
40
50
60

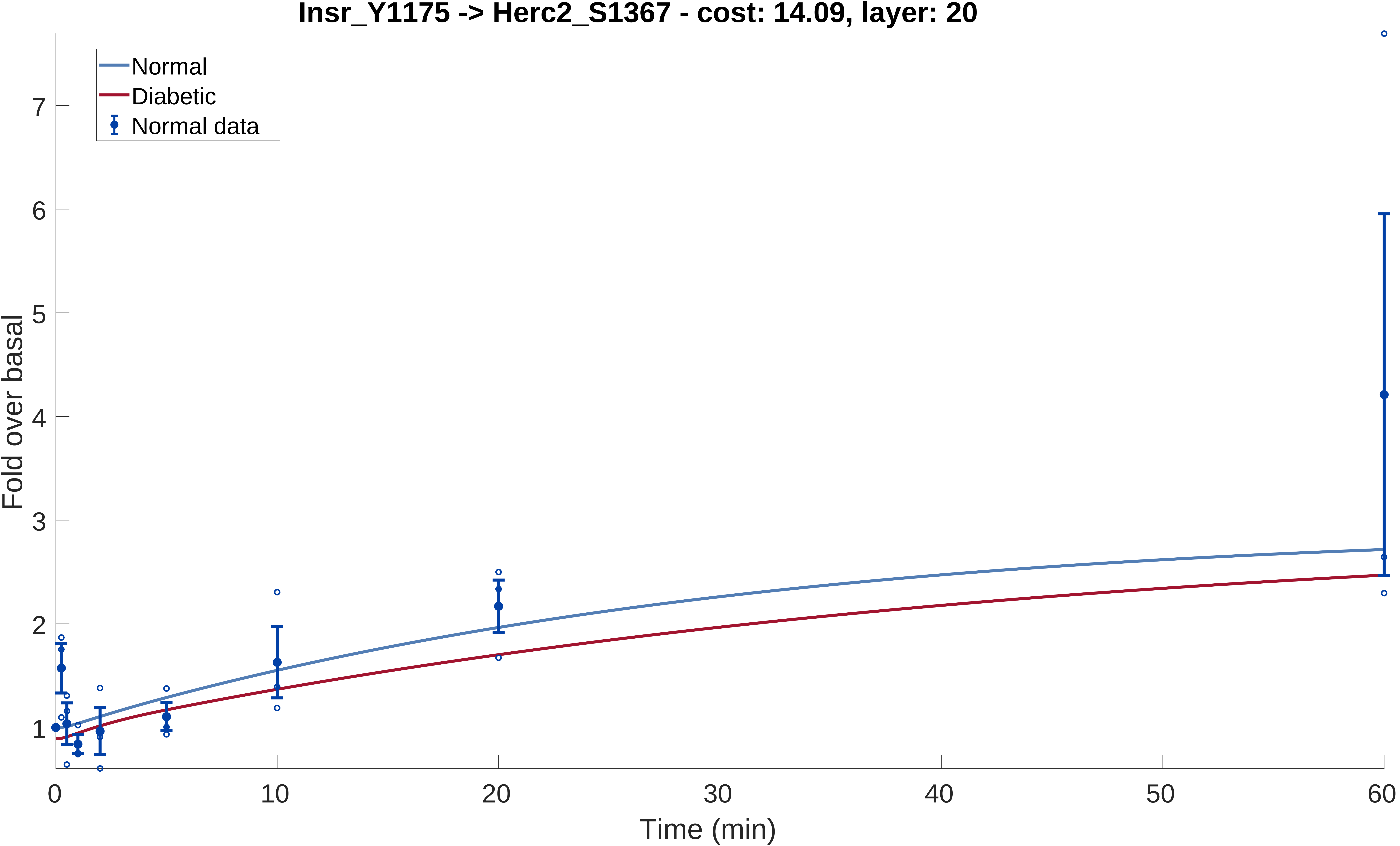

Insr_Y1175 -> Herc2_S1367 - cost: 14.09, layer: 20
Normal
Diabetic
Normal data
Fold over basal
Time (min)
1
2
3
4
5
6
7
0
10
20
30
40
50
60

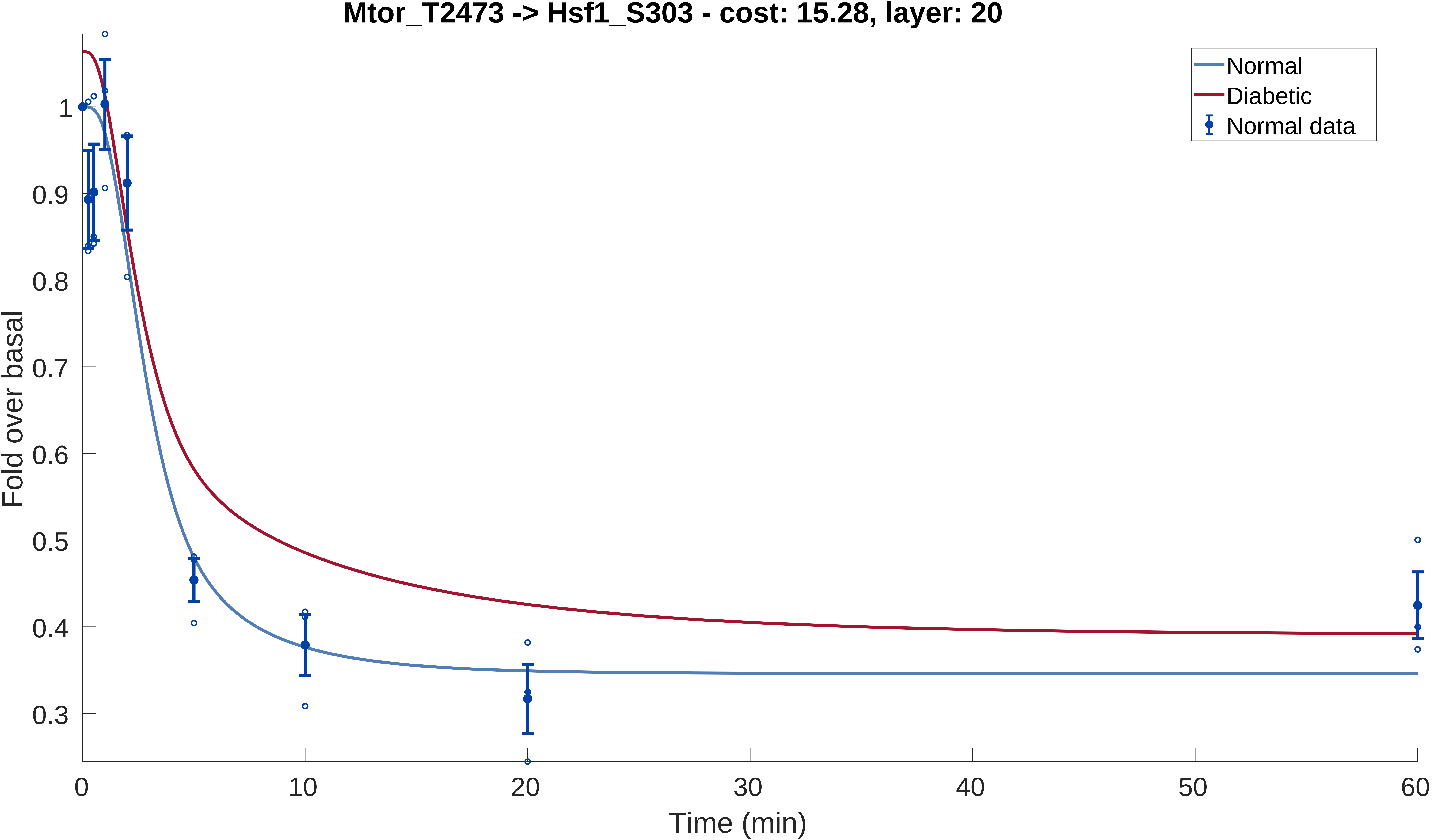

Mtor_T2473 -> Hsf1_S303 - cost: 15.28, layer: 20
Normal
Diabetic
Normal data
Fold over basal
1
0.9
0.8
0.7
0.6
0.5
0.4
0.3
0
10
20
30
40
50
60
Time (min)

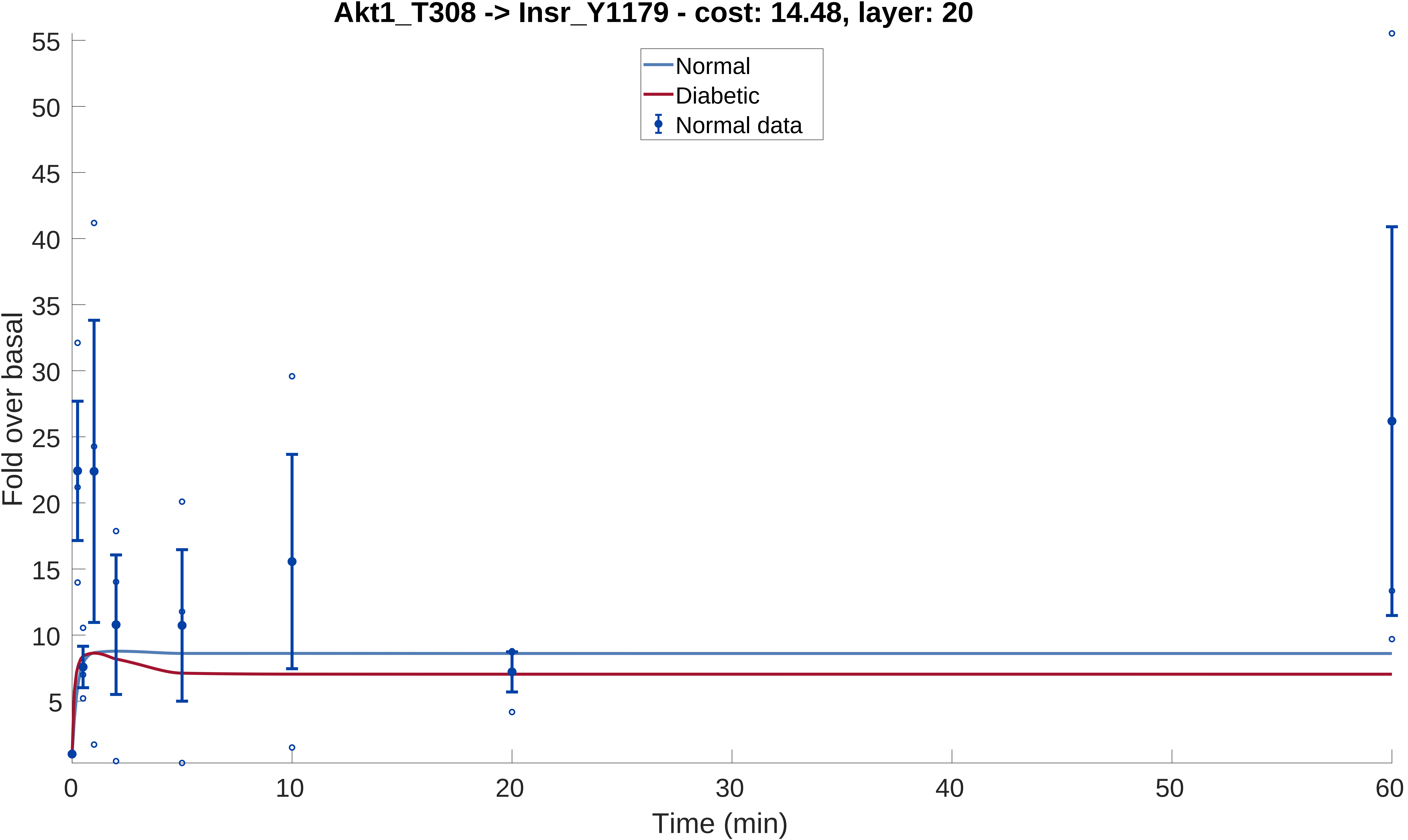

Akt1_T308 -> Insr_Y1179 - cost: 14.48, layer: 20
Normal
Diabetic
Normal data
Fold over basal
Time (min)
0
10
20
30
40
50
60
5
10
15
20
25
30
35
40
45
50
55

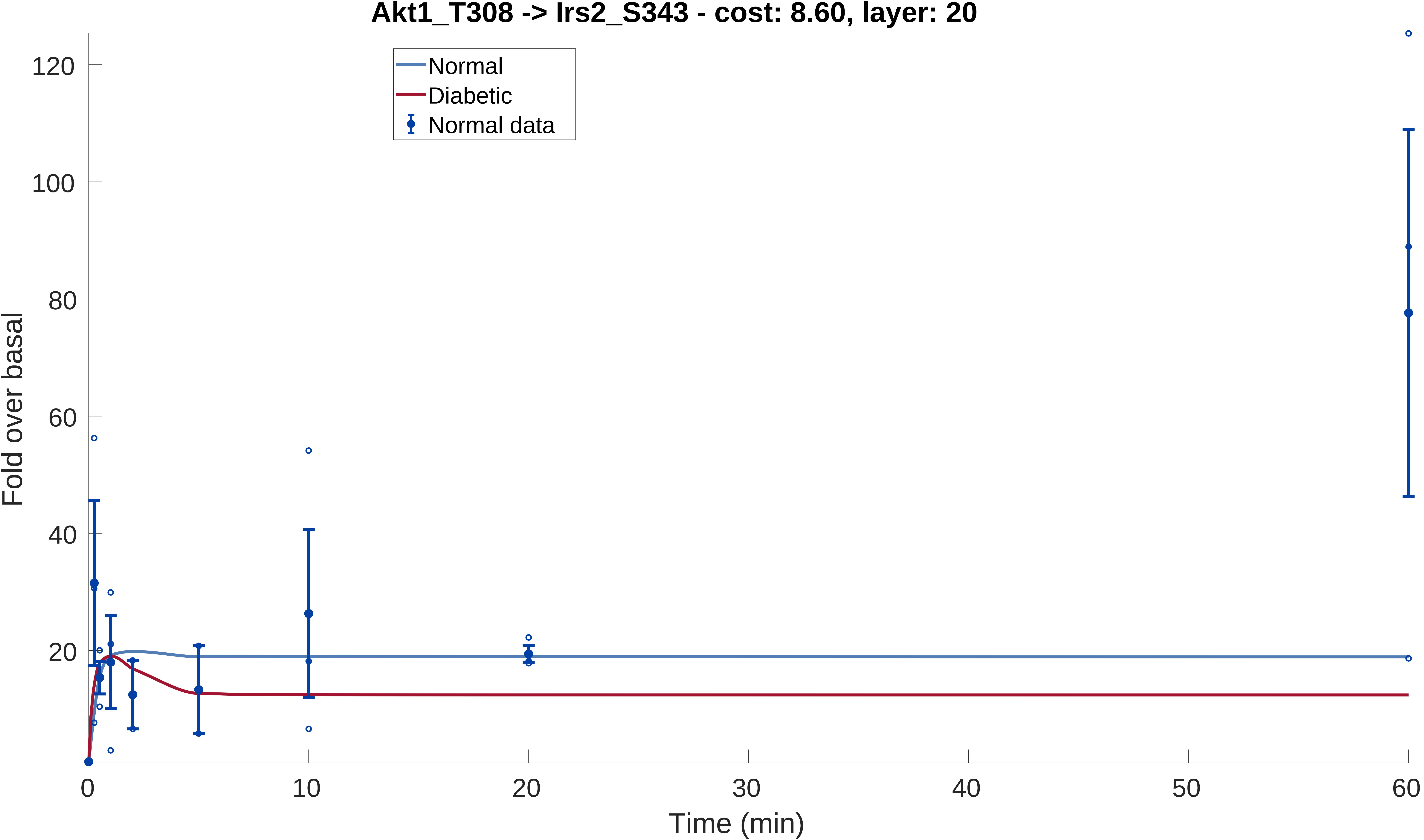
Akt1_T308 -> Irs2_S343 - cost: 8.60, layer: 20
Normal
Diabetic
Normal data
Fold over basal
Time (min)
0
20
40
60
80
100
120
0
10
20
30
40
50
60

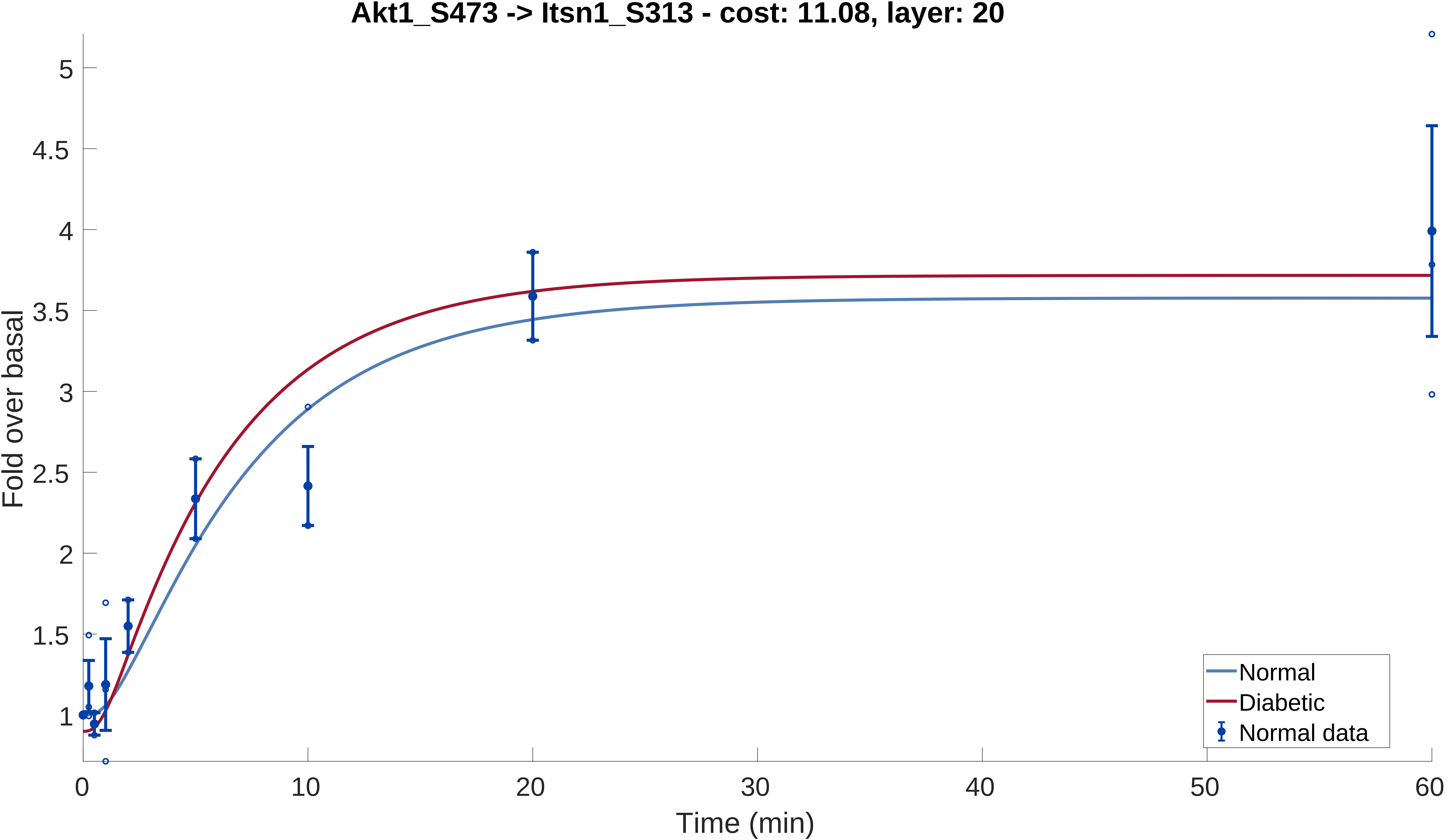

Akt1_S473 -> Itsn1_S313 - cost: 11.08, layer: 20
Fold over basal
Time (min)
0
10
20
30
40
50
60
1
1.5
2
2.5
3
3.5
4
4.5
5
Normal
Diabetic
Normal data

Akt3_T305 -> Iws1_S667 - cost: 4.80, layer: 20

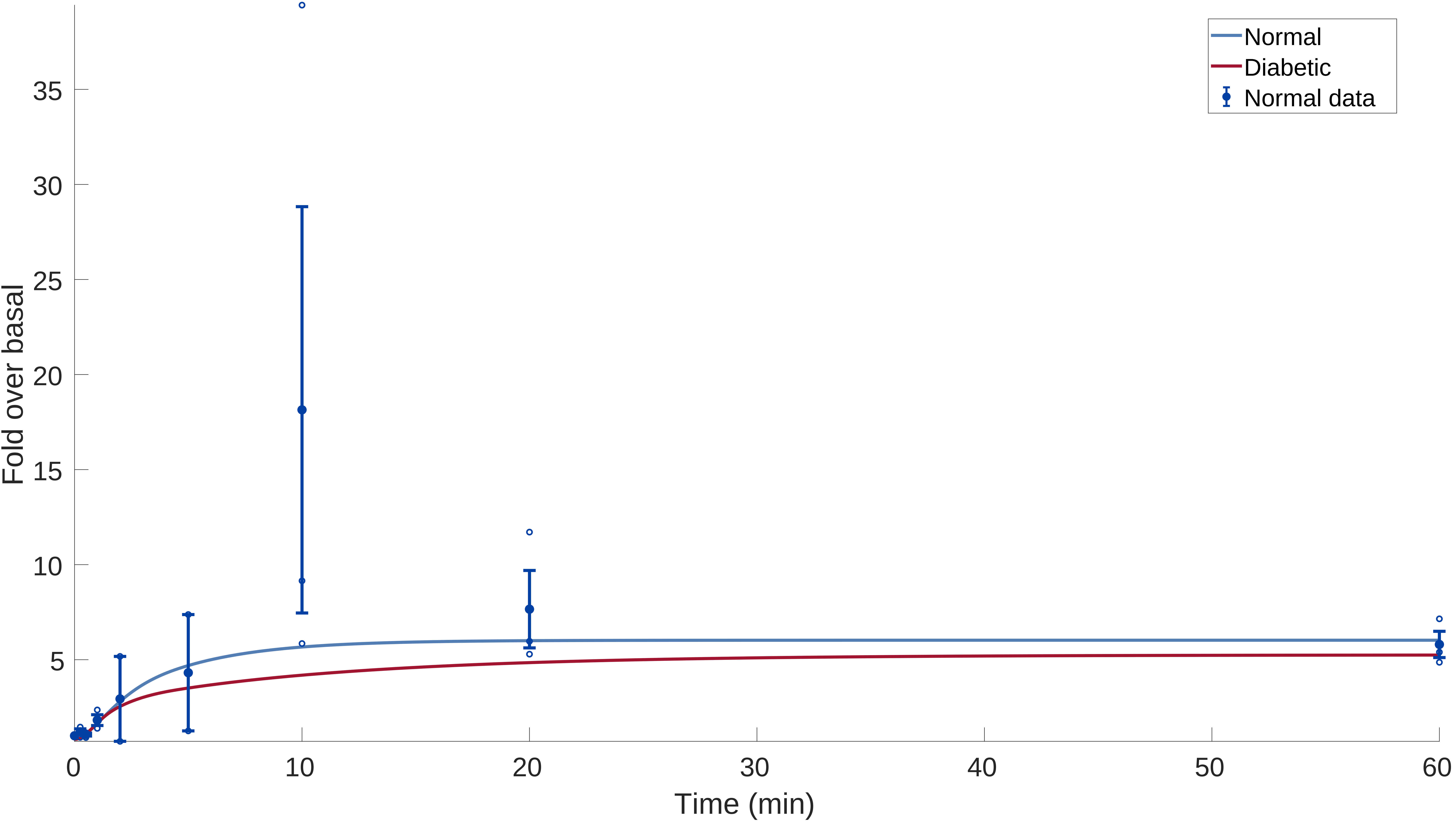

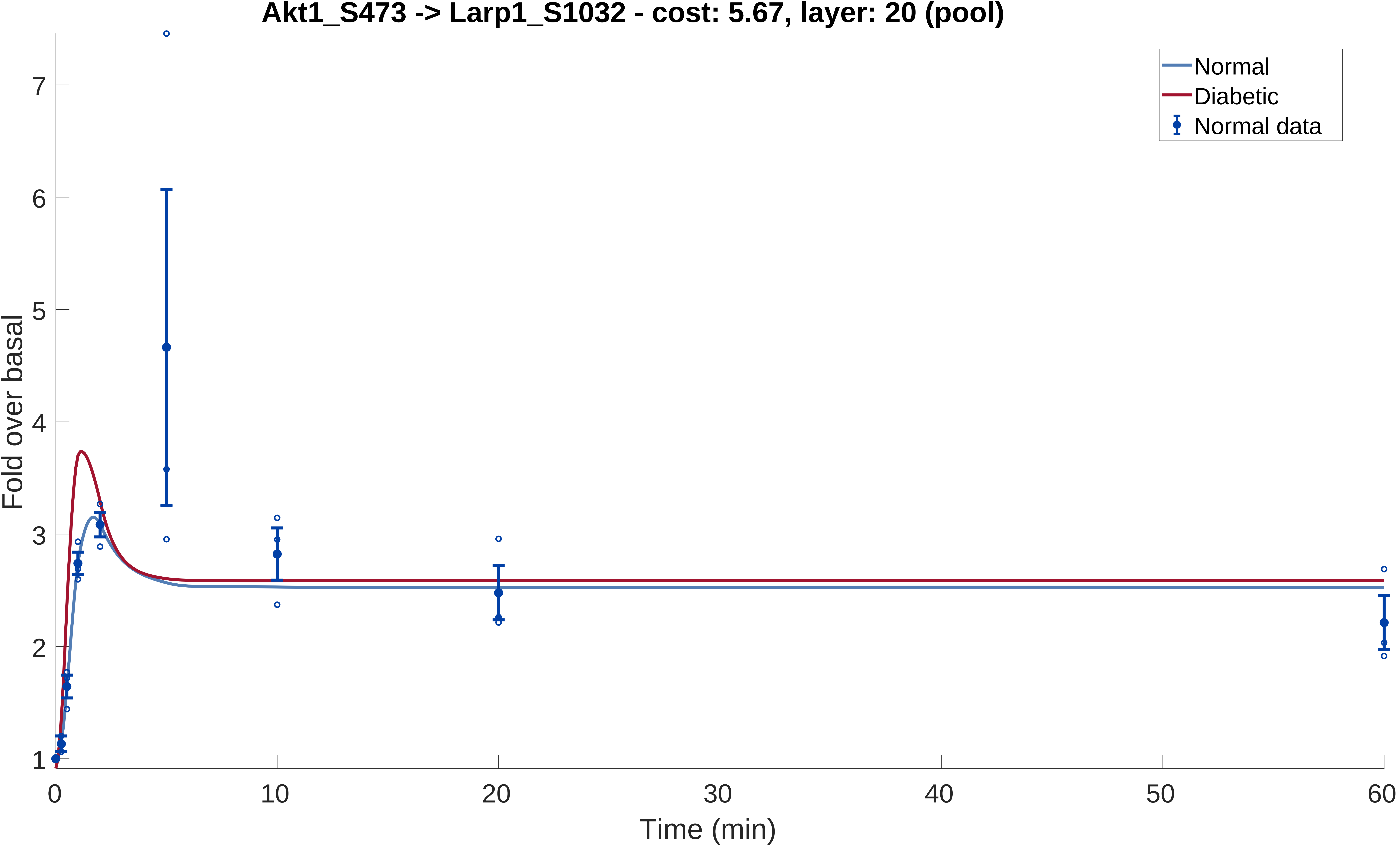

Akt1_S473 -> Larp1_S1032 - cost: 5.67, layer: 20 (pool)
Normal
Diabetic
Normal data
Fold over basal
Time (min)
1
2
3
4
5
6
7
0
10
20
30
40
50
60

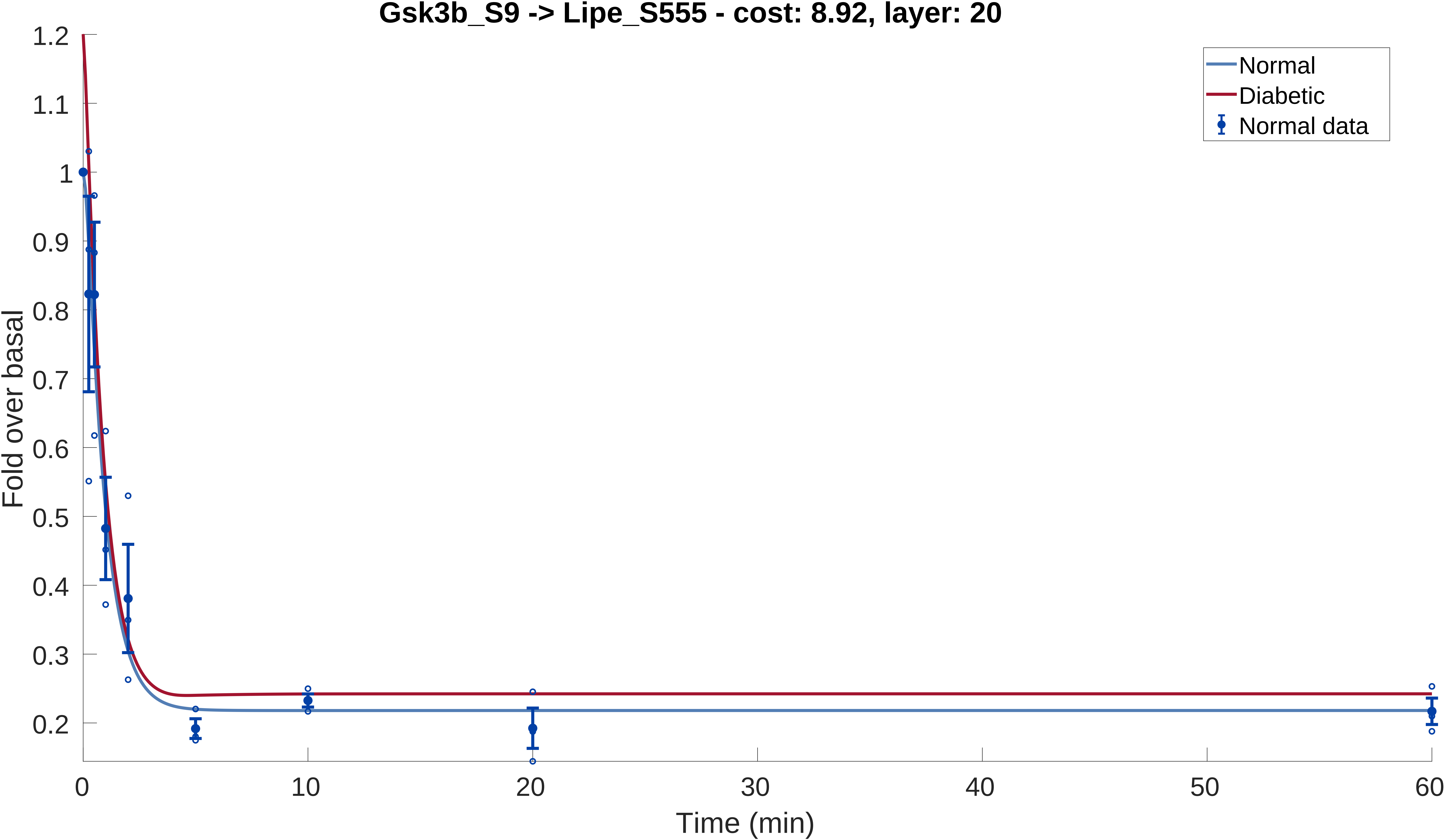

Gsk3b_S9 -> Lipe_S555 - cost: 8.92, layer: 20
Normal
Diabetic
Normal data
Fold over basal
Time (min)
1.2
1.1
1
0.9
0.8
0.7
0.6
0.5
0.4
0.3
0.2
0
10
20
30
40
50
60

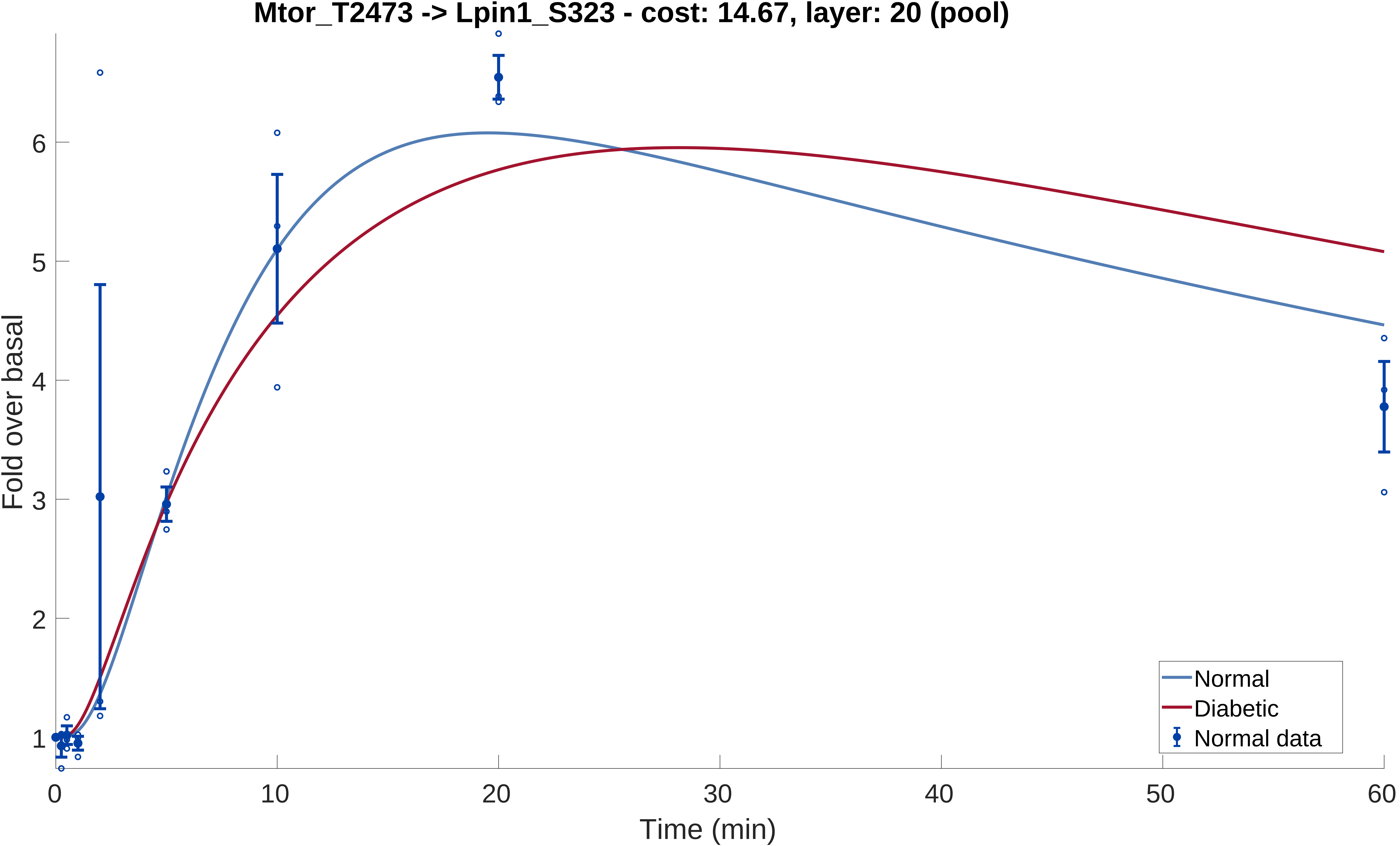
Mtor_T2473 -> Lpin1_S323 - cost: 14.67, layer: 20 (pool)
Fold over basal
1
2
3
4
5
6
0
10
20
30
40
50
60
Time (min)
Normal
Diabetic
Normal data

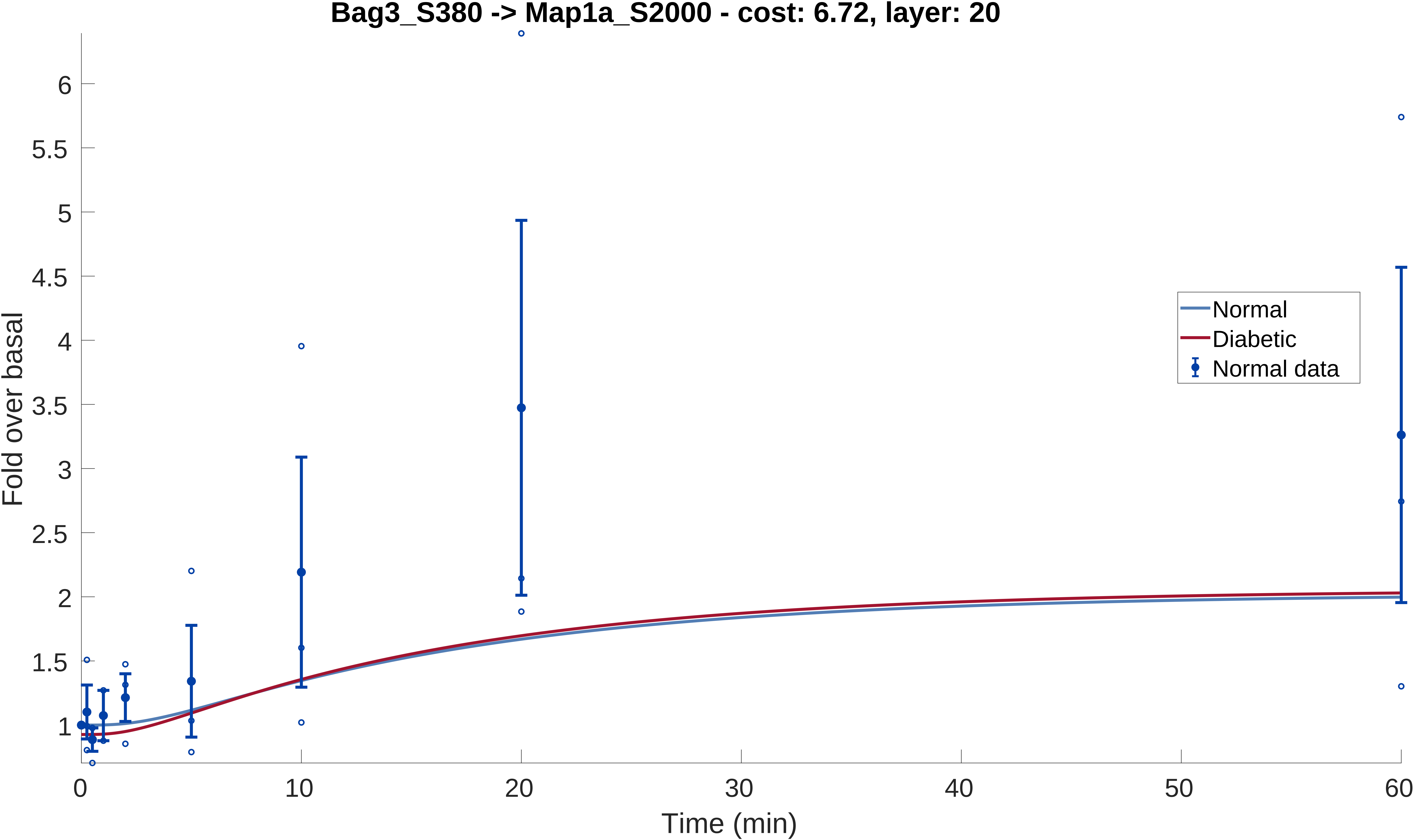

Bag3_S380 -> Map1a_S2000 - cost: 6.72, layer: 20
Fold over basal
Time (min)
Normal
Diabetic
Normal data
0
10
20
30
40
50
60
1
1.5
2
2.5
3
3.5
4
4.5
5
5.5
6

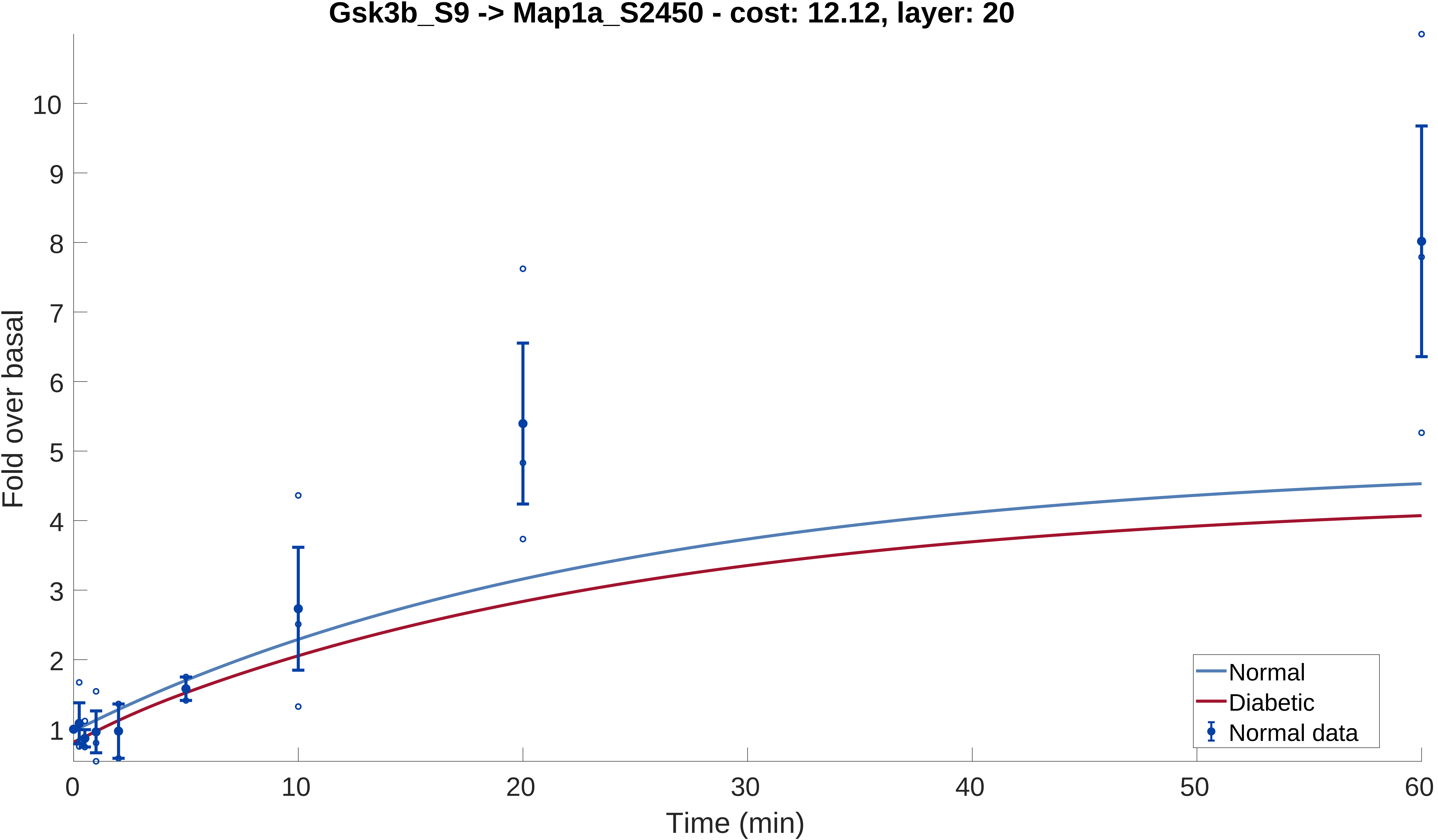

Gsk3b_S9 -> Map1a_S2450 - cost: 12.12, layer: 20
Fold over basal
Time (min)
Normal
Diabetic
Normal data
0
10
20
30
40
50
60
1
2
3
4
5
6
7
8
9
10

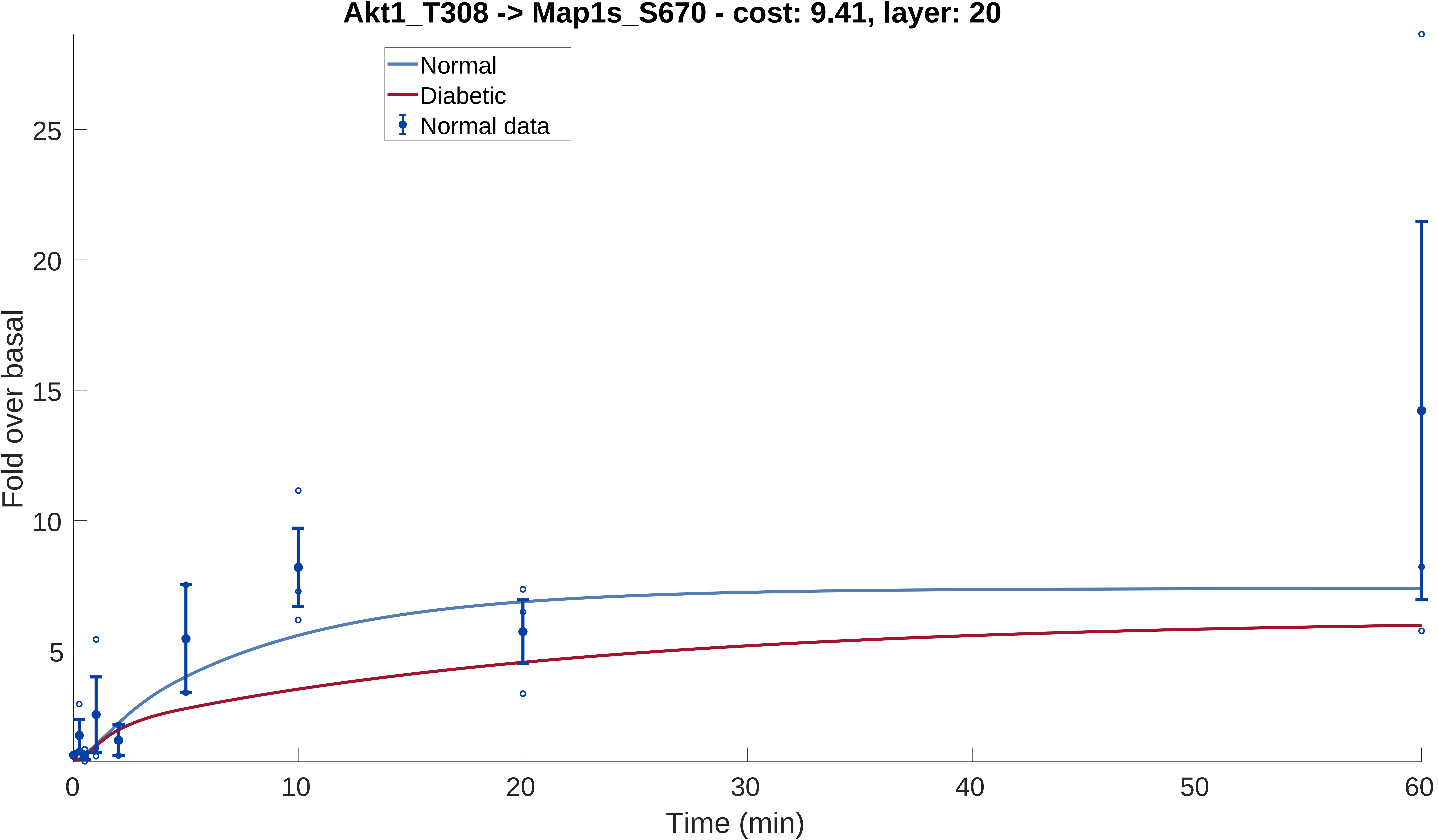

Akt1_T308 -> Map1s_S670 - cost: 9.41, layer: 20
Normal
Diabetic
Normal data
Fold over basal
Time (min)
25
20
15
10
5
0
10
20
30
40
50
60

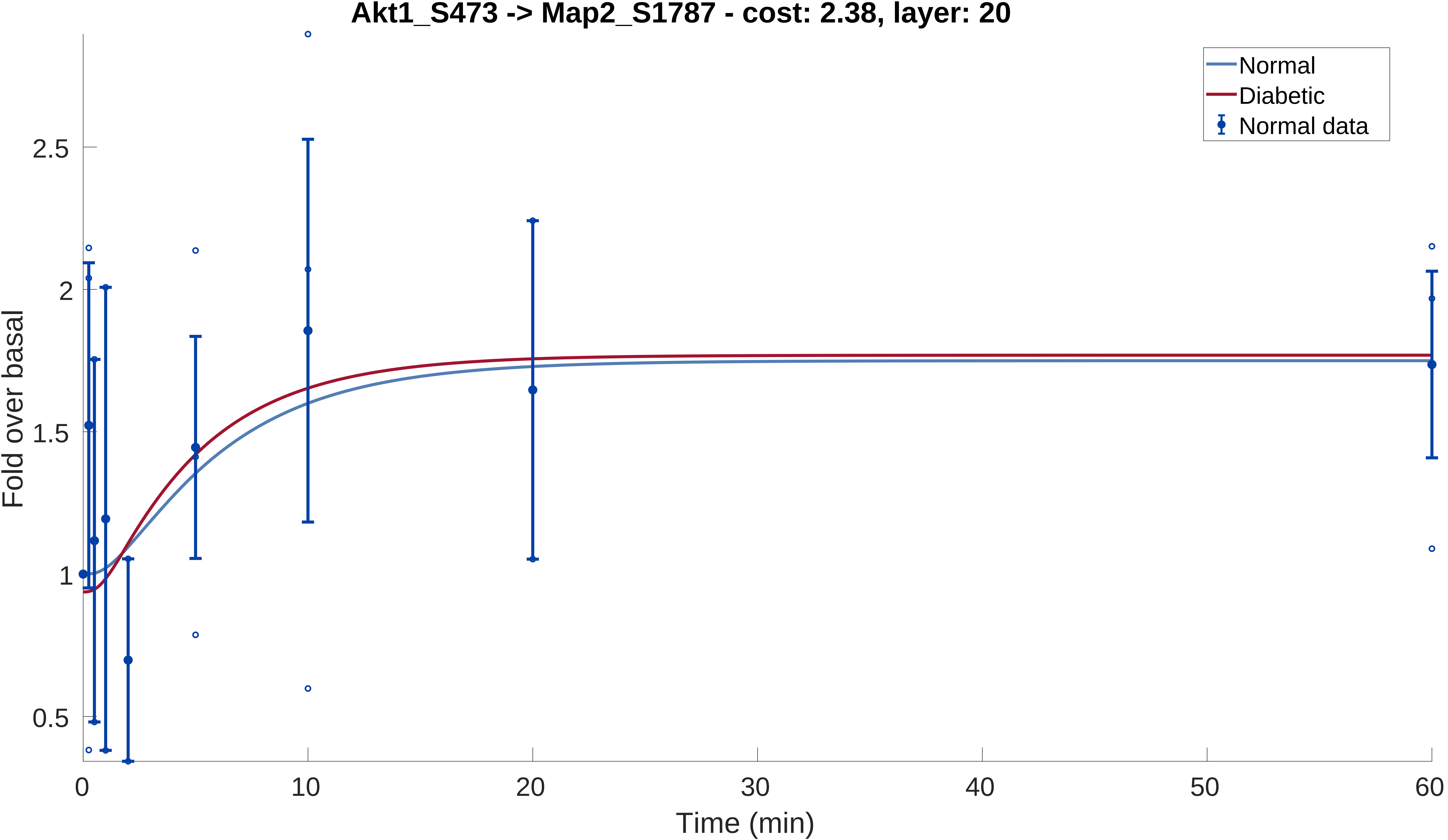

Akt1_S473 -> Map2_S1787 - cost: 2.38, layer: 20
Normal
Diabetic
Normal data
Fold over basal
2.5
2
1.5
1
0.5
0
10
20
30
40
50
60
Time (min)

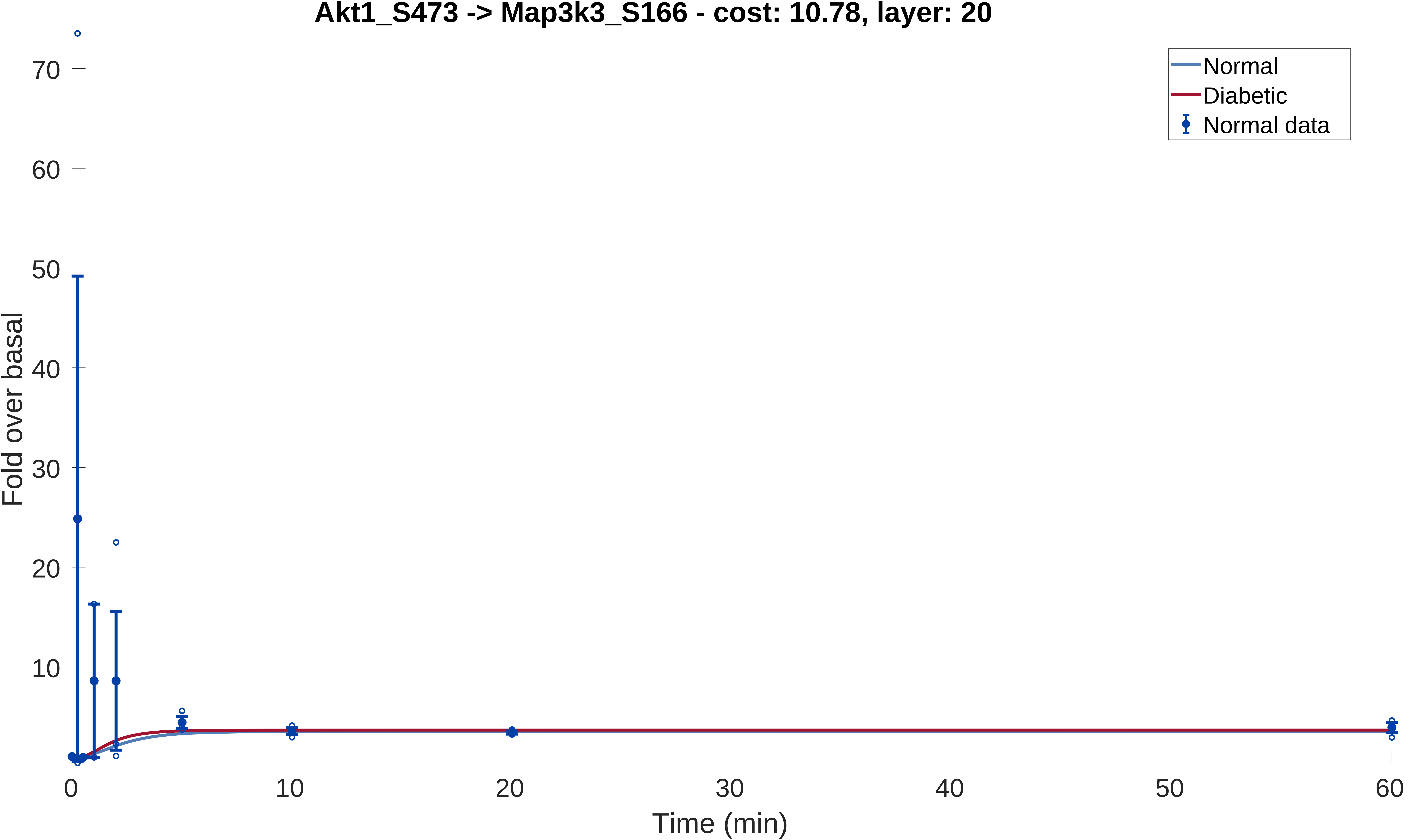

Akt1_S473 -> Map3k3_S166 - cost: 10.78, layer: 20
Normal
Diabetic
Normal data
Fold over basal
Time (min)
0
10
20
30
40
50
60
70

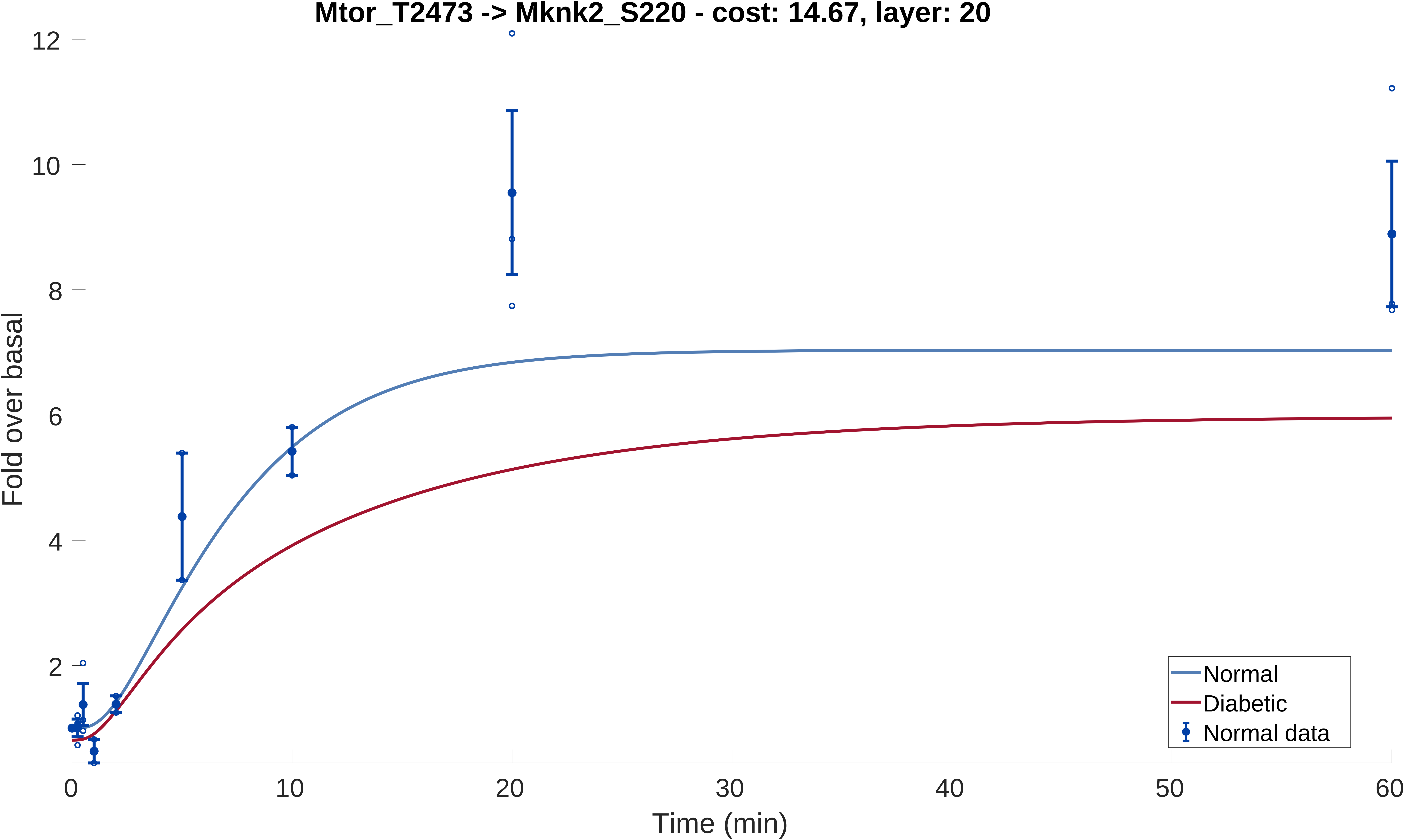
Mtor_T2473 -> Mknk2_S220 - cost: 14.67, layer: 20
Fold over basal
Time (min)
0
10
20
30
40
50
60
2
4
6
8
10
12
Normal
Diabetic
Normal data

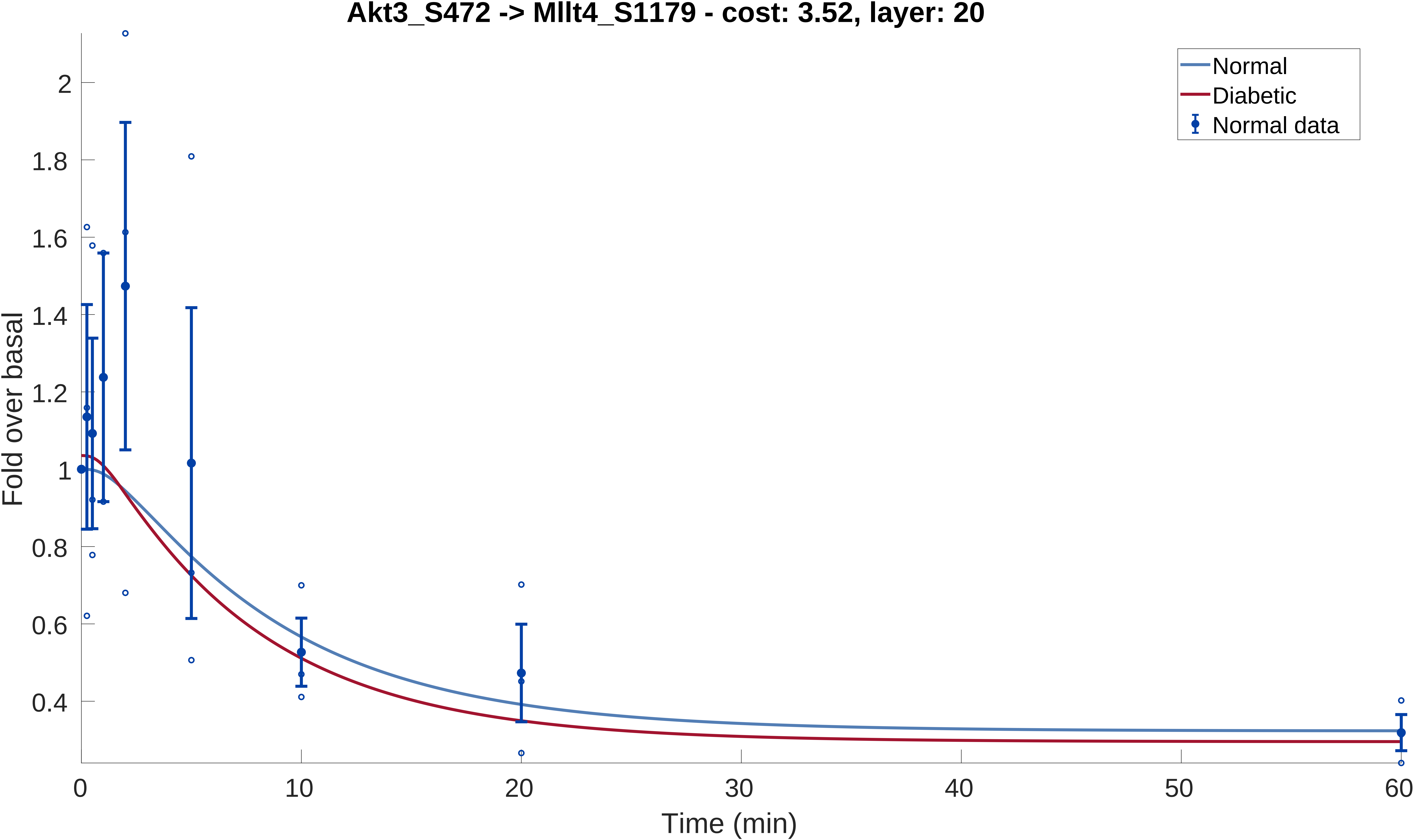

Akt3_S472 -> Mllt4_S1179 - cost: 3.52, layer: 20
Normal
Diabetic
Normal data
Fold over basal
Time (min)
0.4
0.6
0.8
1
1.2
1.4
1.6
1.8
2
0
10
20
30
40
50
60

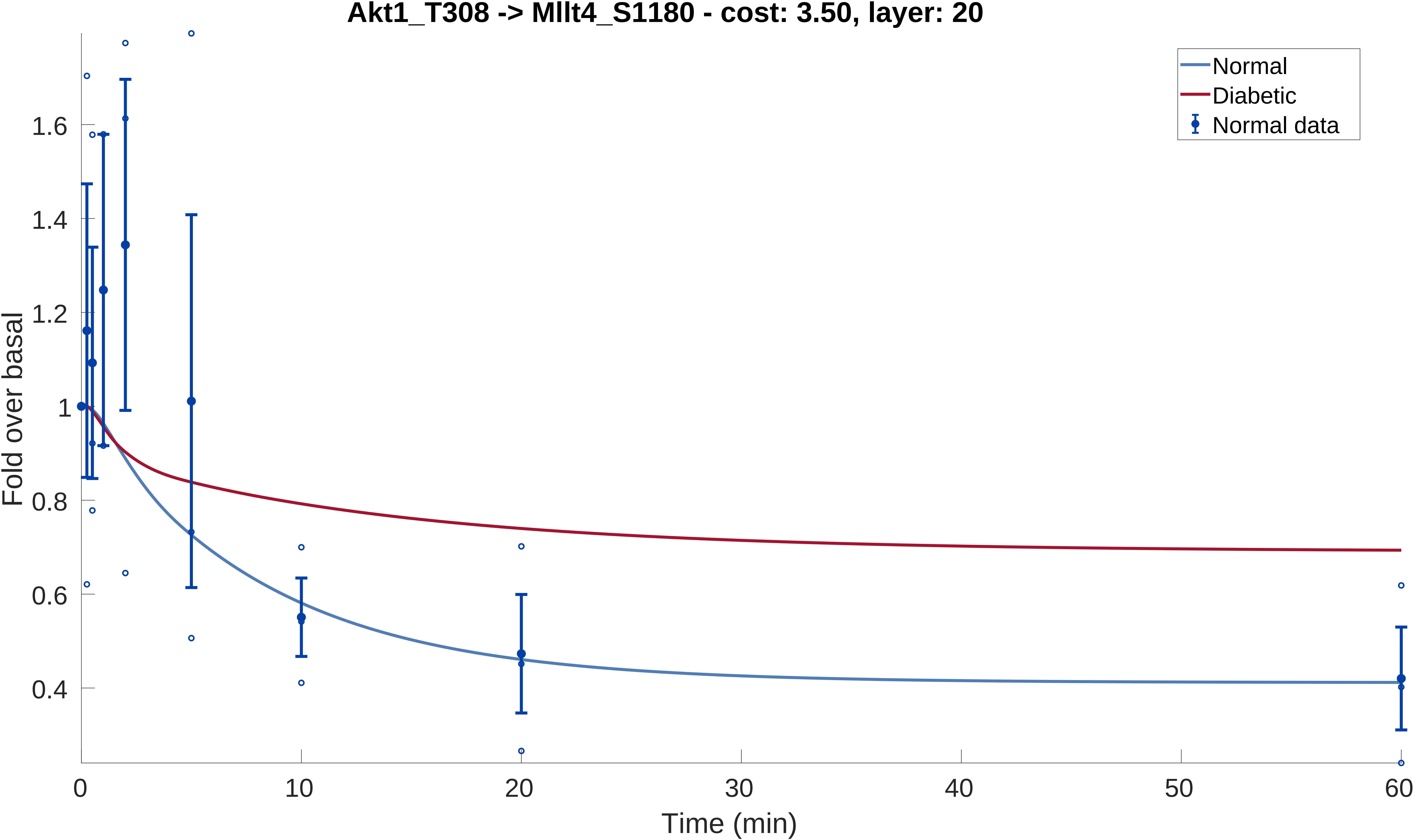

Akt1_T308 -> Mllt4_S1180 - cost: 3.50, layer: 20
Normal
Diabetic
Normal data
1.6
1.4
1.2
1
0.8
0.6
0.4
Fold over basal
0
10
20
30
40
50
60
Time (min)

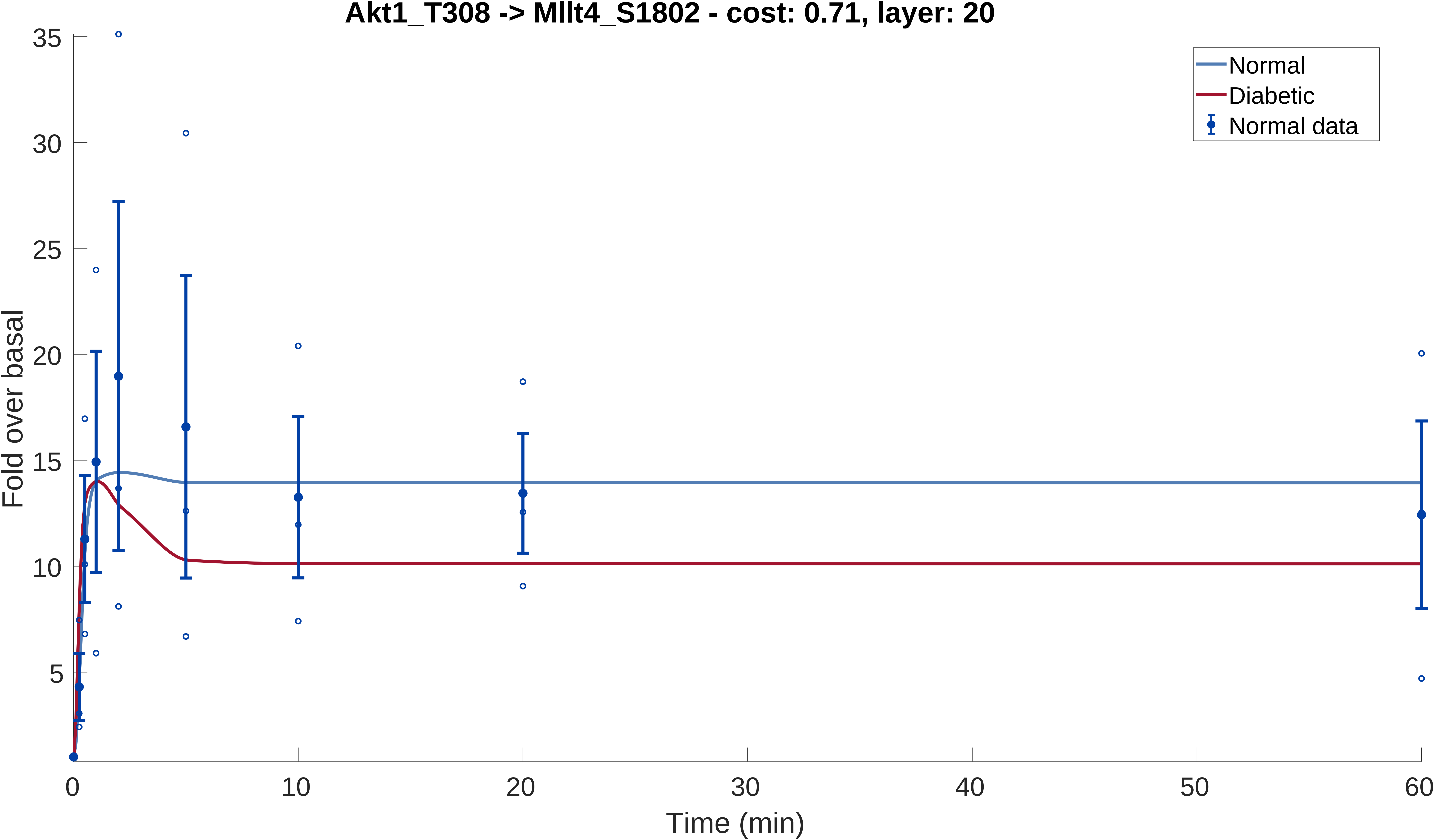

Akt1_T308 -> Mllt4_S1802 - cost: 0.71, layer: 20
Fold over basal
Time (min)
Normal
Diabetic
Normal data
35
30
25
20
15
10
5
0
10
20
30
40
50
60

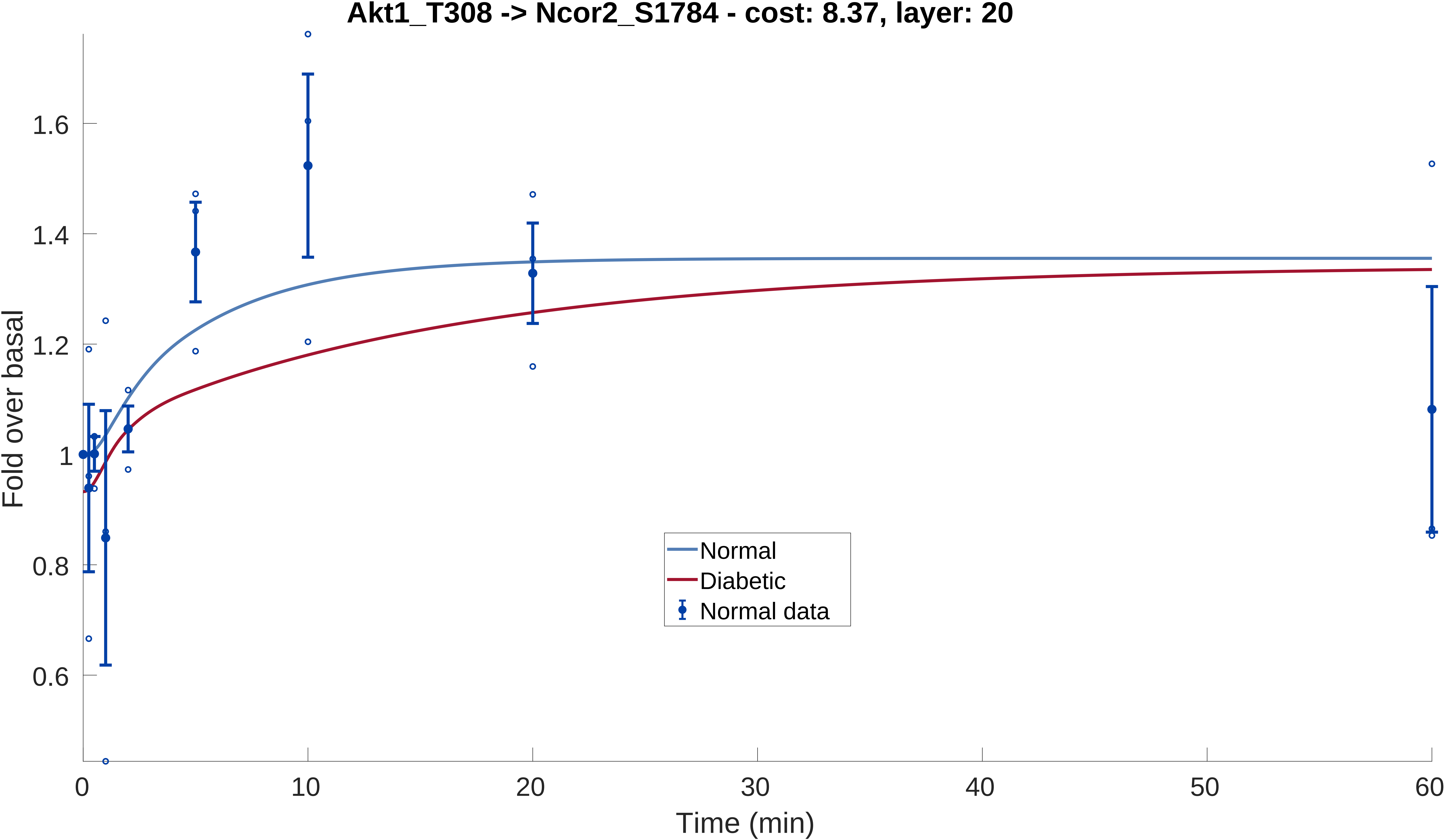
Akt1_T308 -> Ncor2_S1784 - cost: 8.37, layer: 20
Fold over basal
1.6
1.4
1.2
1
0.8
0.6
0
10
20
30
40
50
60
Time (min)
Normal
Diabetic
Normal data

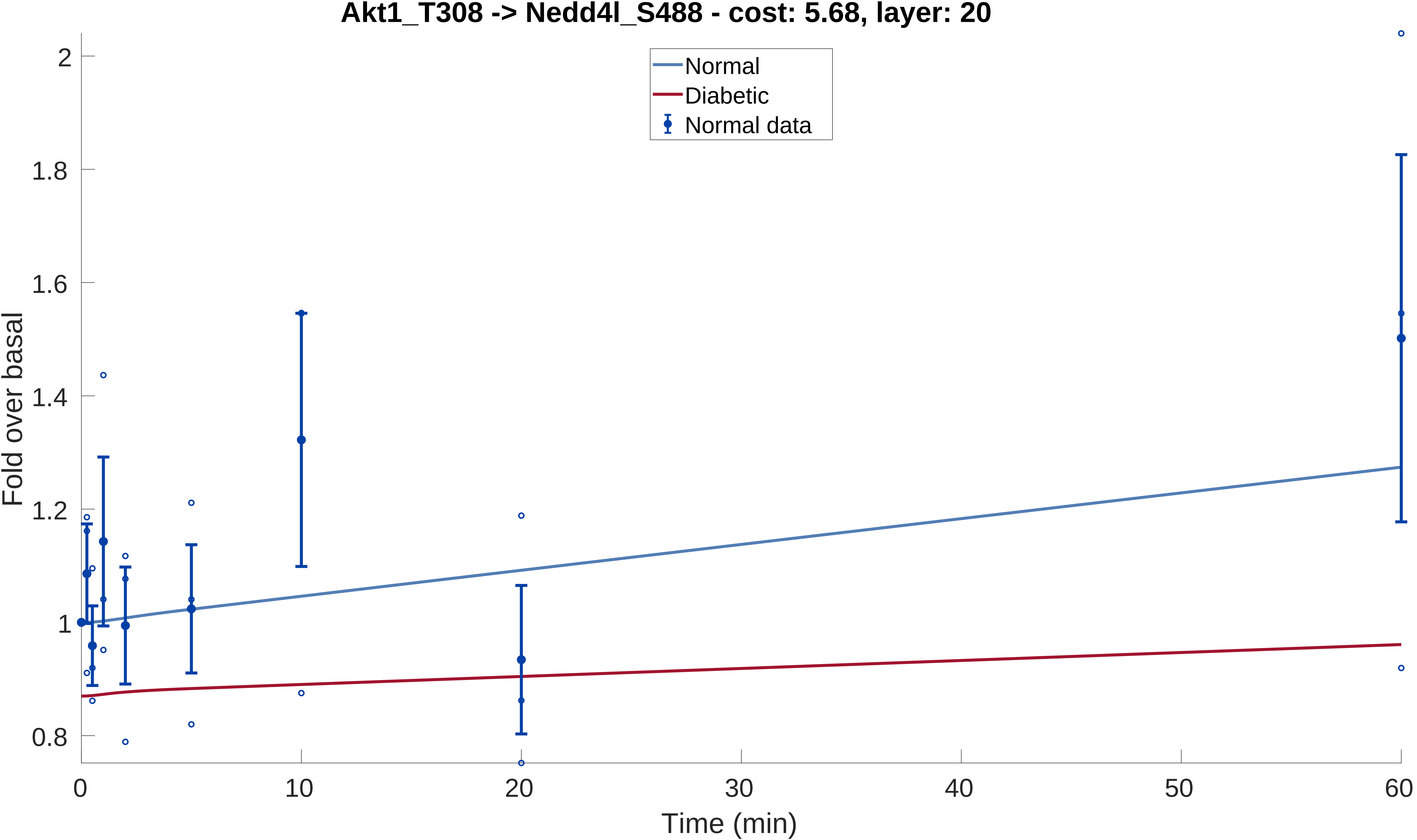

Akt1_T308 -> Nedd4l_S488 - cost: 5.68, layer: 20
Normal
Diabetic
Normal data
Fold over basal
Time (min)
0.8
1
1.2
1.4
1.6
1.8
2
0
10
20
30
40
50
60

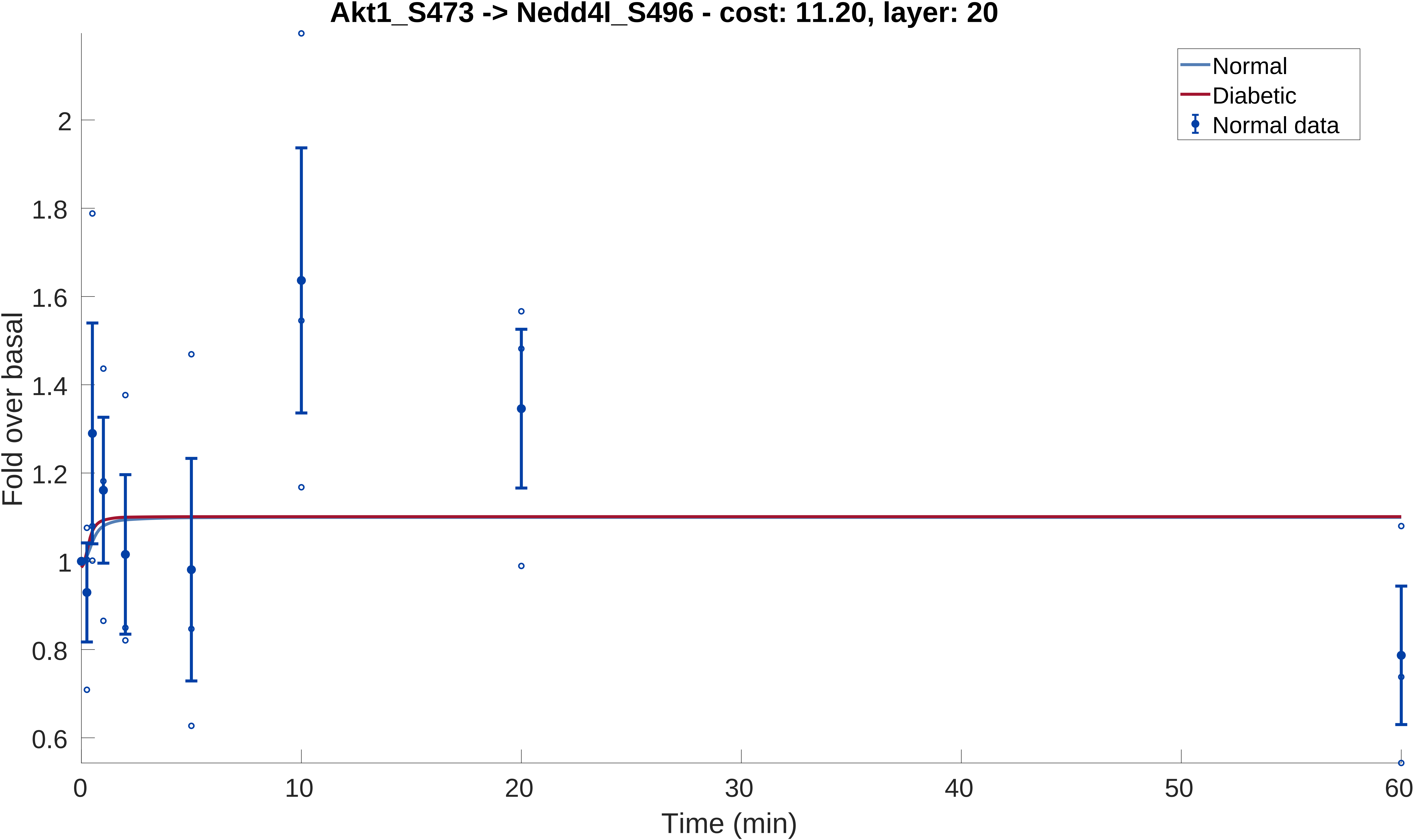

Akt1_S473 -> Nedd4l_S496 - cost: 11.20, layer: 20
Normal
Diabetic
Normal data
Fold over basal
Time (min)
0.6
0.8
1
1.2
1.4
1.6
1.8
2
0
10
20
30
40
50
60

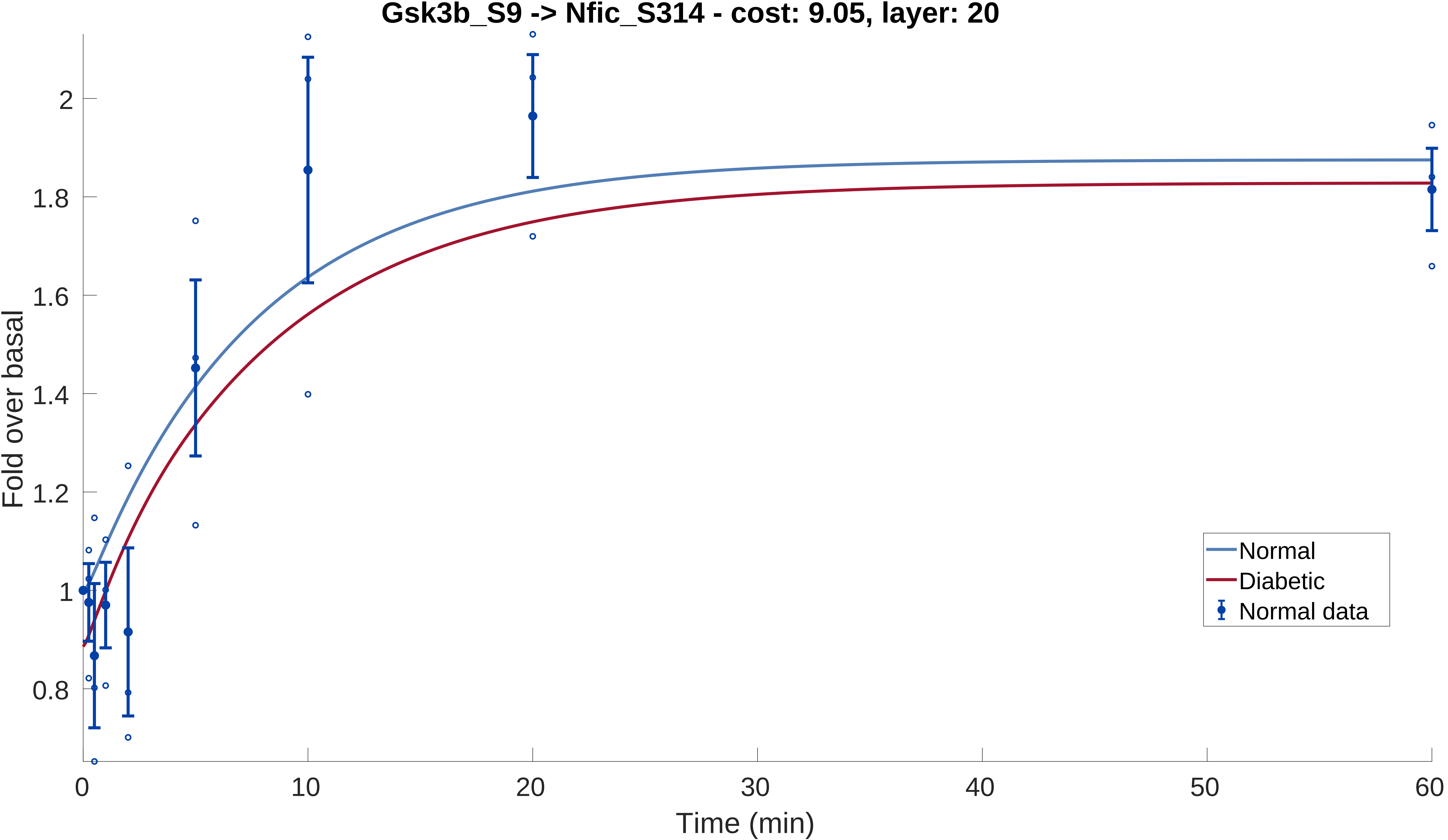

Gsk3b_S9 -> Nfic_S314 - cost: 9.05, layer: 20
Fold over basal
0.8
1
1.2
1.4
1.6
1.8
2
0
10
20
30
40
50
60
Time (min)
Normal
Diabetic
Normal data

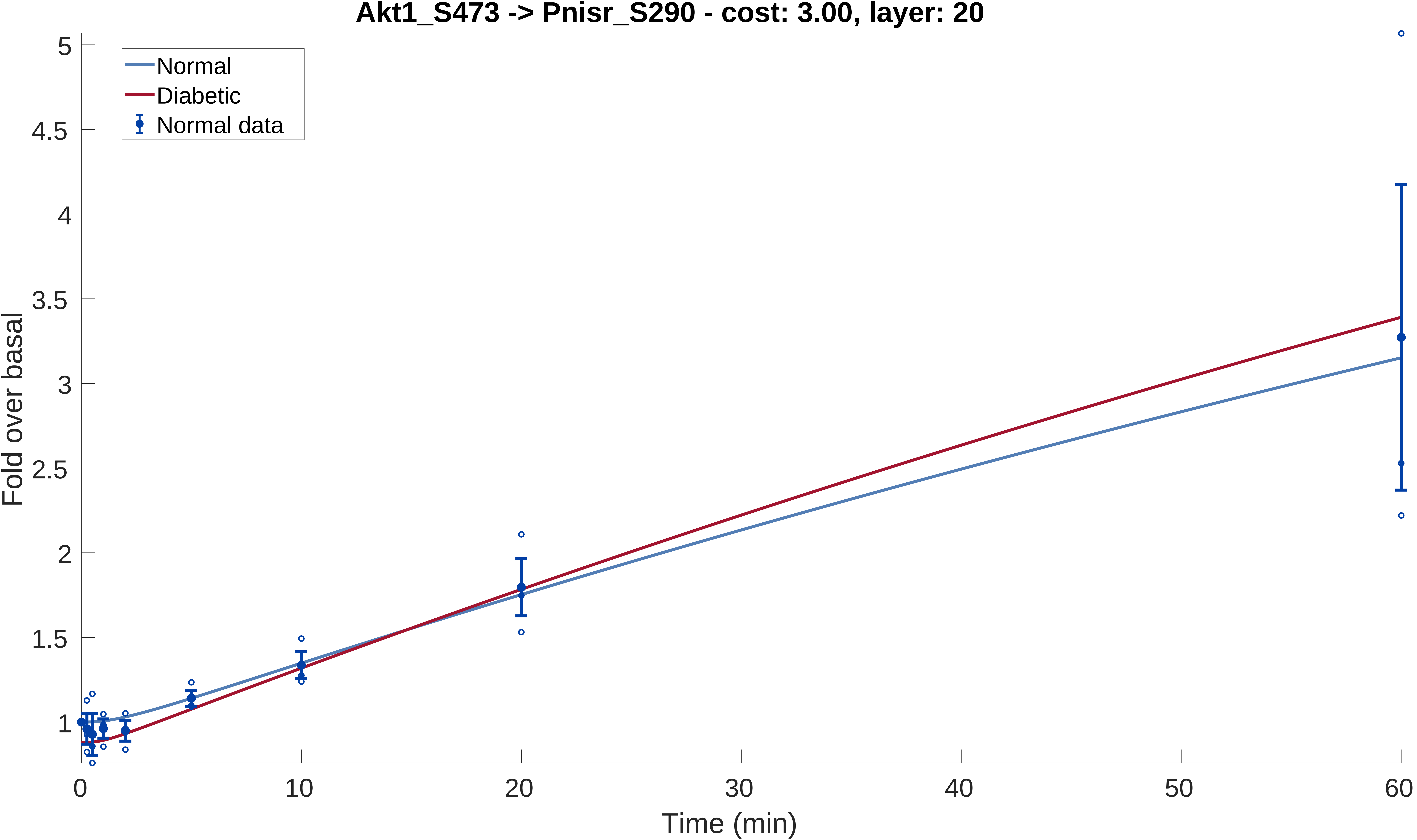

Akt1_S473 -> Pnisr_S290 - cost: 3.00, layer: 20
Normal
Diabetic
Normal data
Fold over basal
Time (min)
5
4.5
4
3.5
3
2.5
2
1.5
1
0
10
20
30
40
50
60

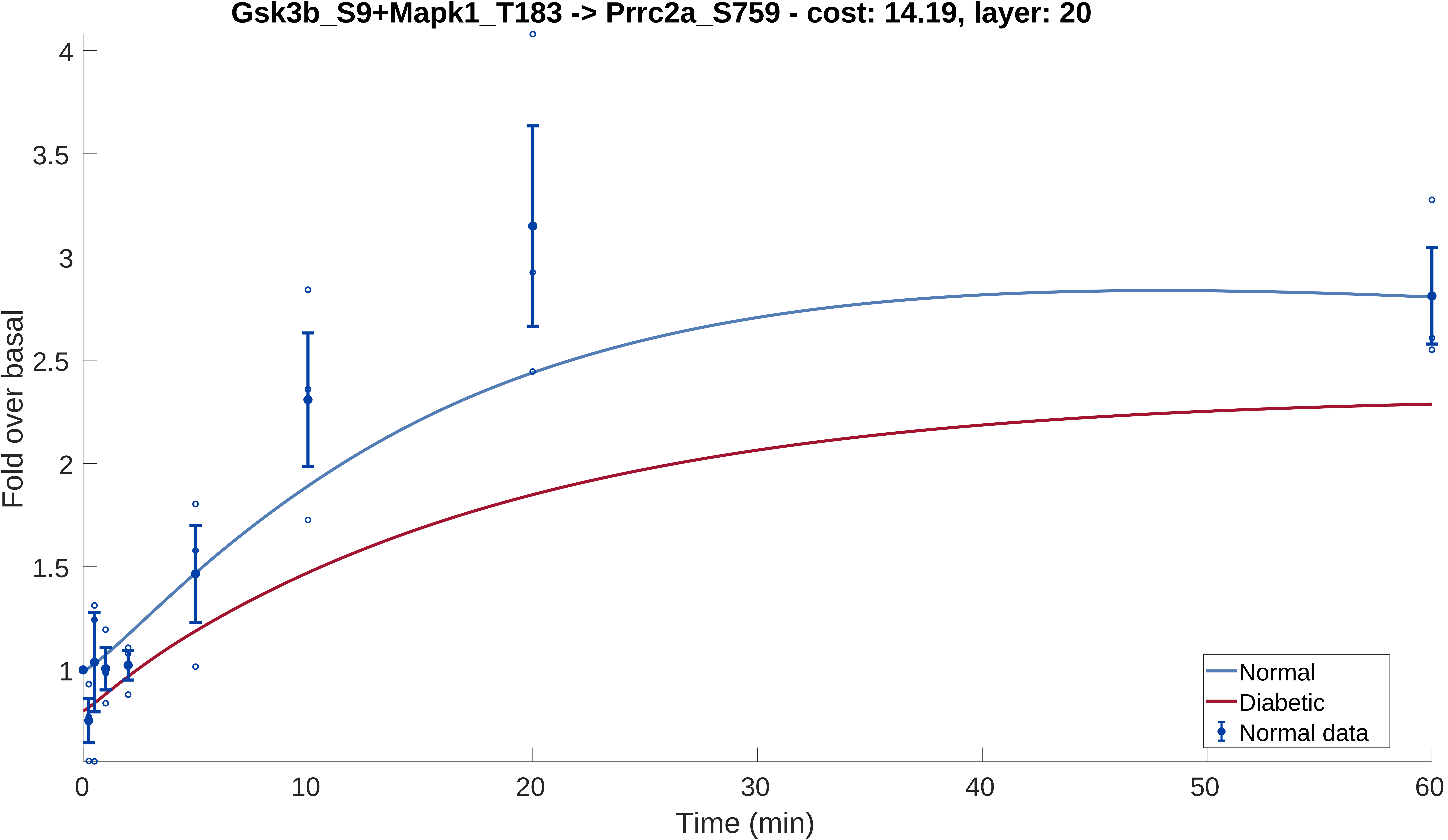
Gsk3b_S9+Mapk1_T183 -> Prrc2a_S759 - cost: 14.19, layer: 20
Fold over basal
Time (min)
0
10
20
30
40
50
60
1
1.5
2
2.5
3
3.5
4
Normal
Diabetic
Normal data

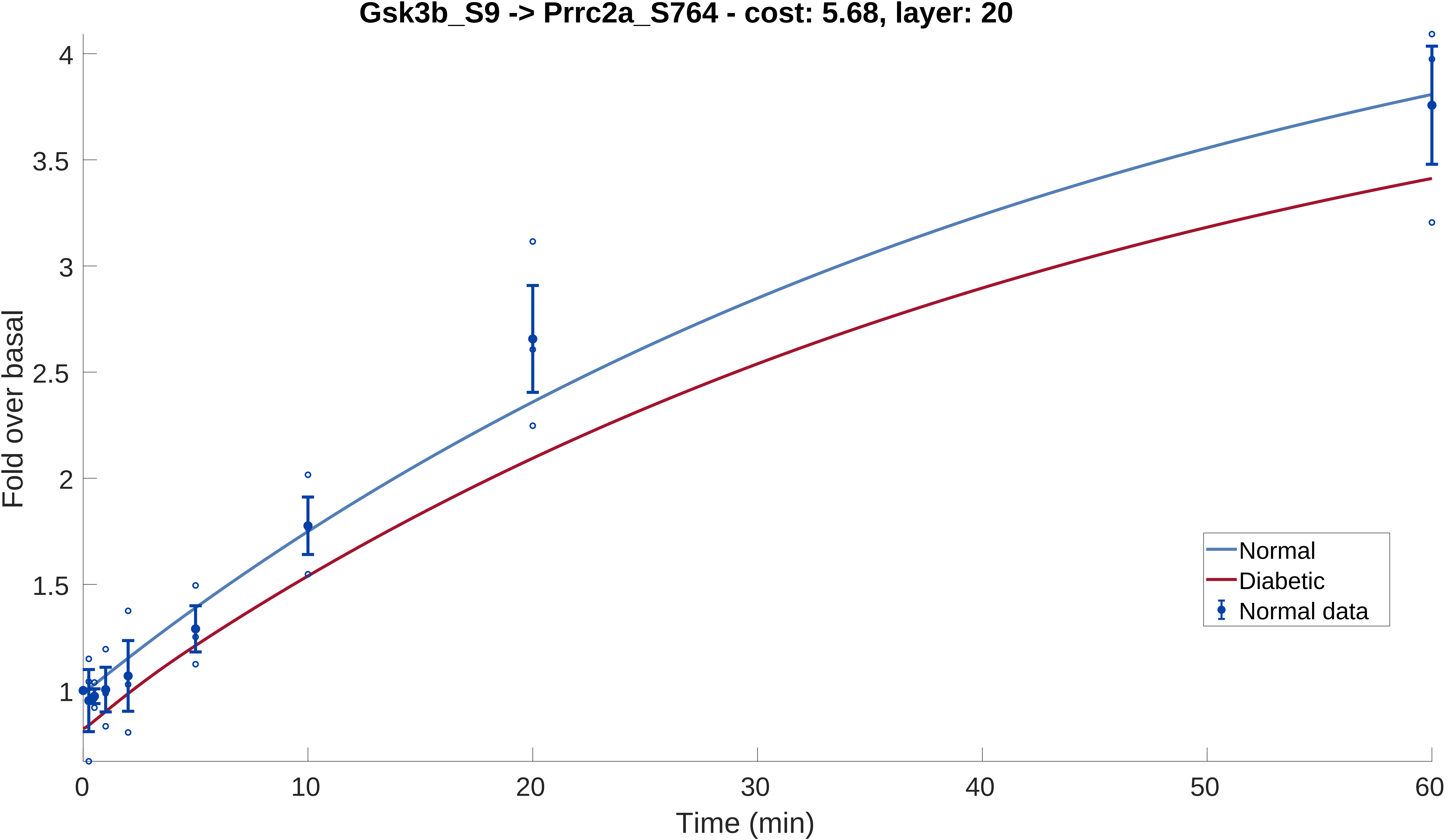

Gsk3b_S9 -> Prrc2a_S764 - cost: 5.68, layer: 20
Fold over basal
4
3.5
3
2.5
2
1.5
1
0
10
20
30
40
50
60
Time (min)
Normal
Diabetic
Normal data

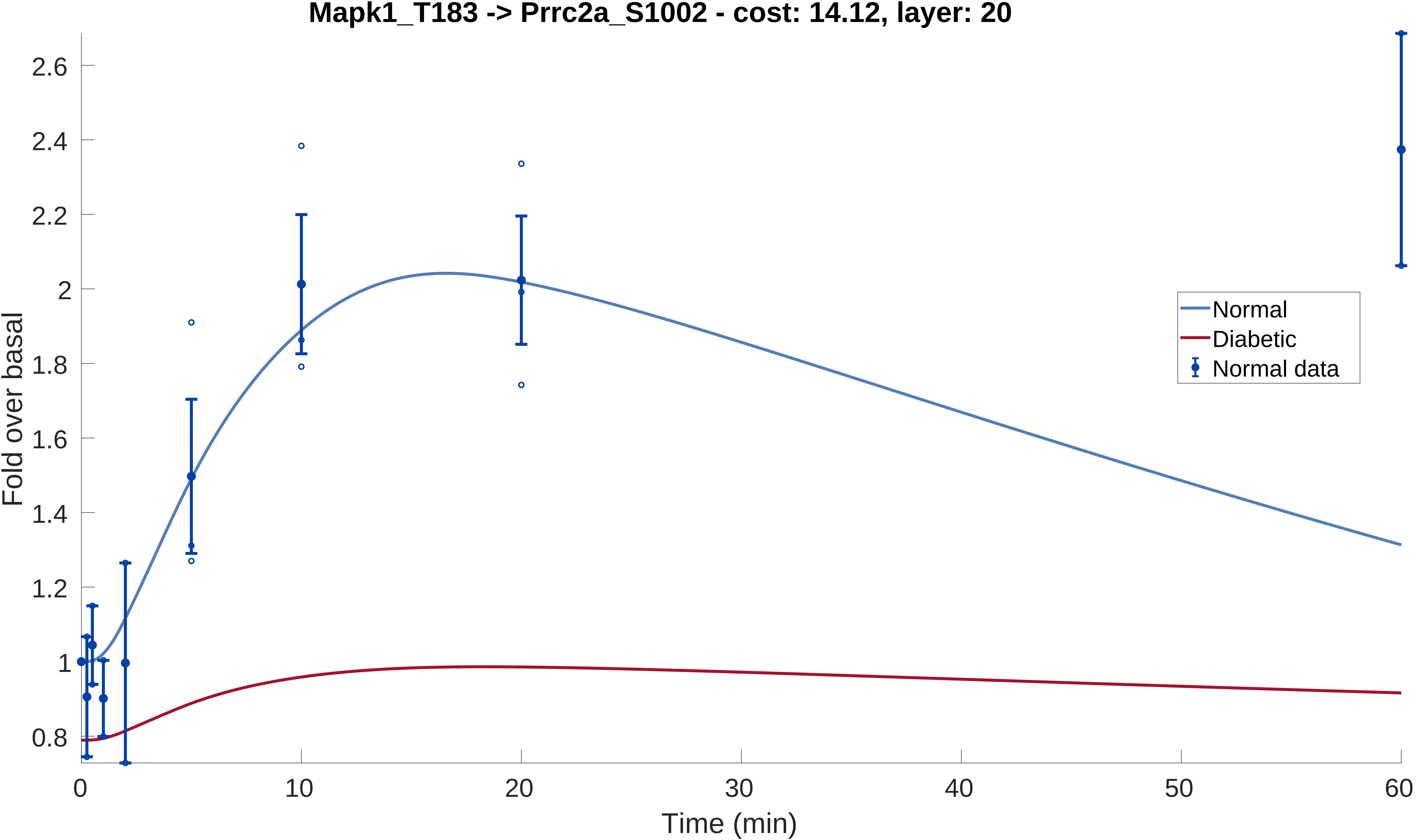
Mapk1_T183 -> Prrc2a_S1002 - cost: 14.12, layer: 20
Fold over basal
2.6
2.4
2.2
2
1.8
1.6
1.4
1.2
1
0.8
0
10
20
30
40
50
60
Time (min)
Normal
Diabetic
Normal data

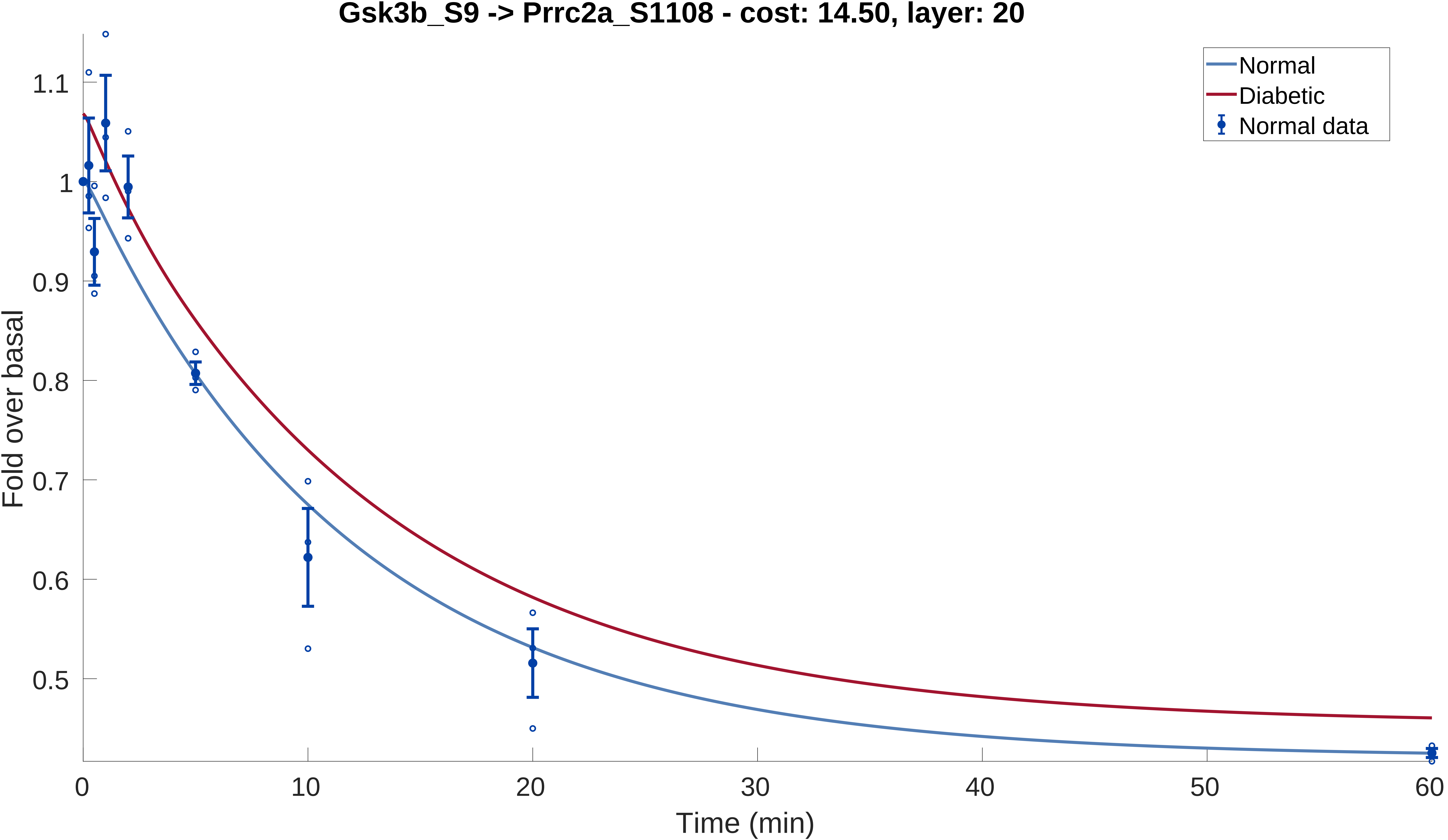
Gsk3b_S9 -> Prrc2a_S1108 - cost: 14.50, layer: 20
Normal
Diabetic
Normal data
Fold over basal
1.1
1
0.9
0.8
0.7
0.6
0.5
0
10
20
30
40
50
60
Time (min)

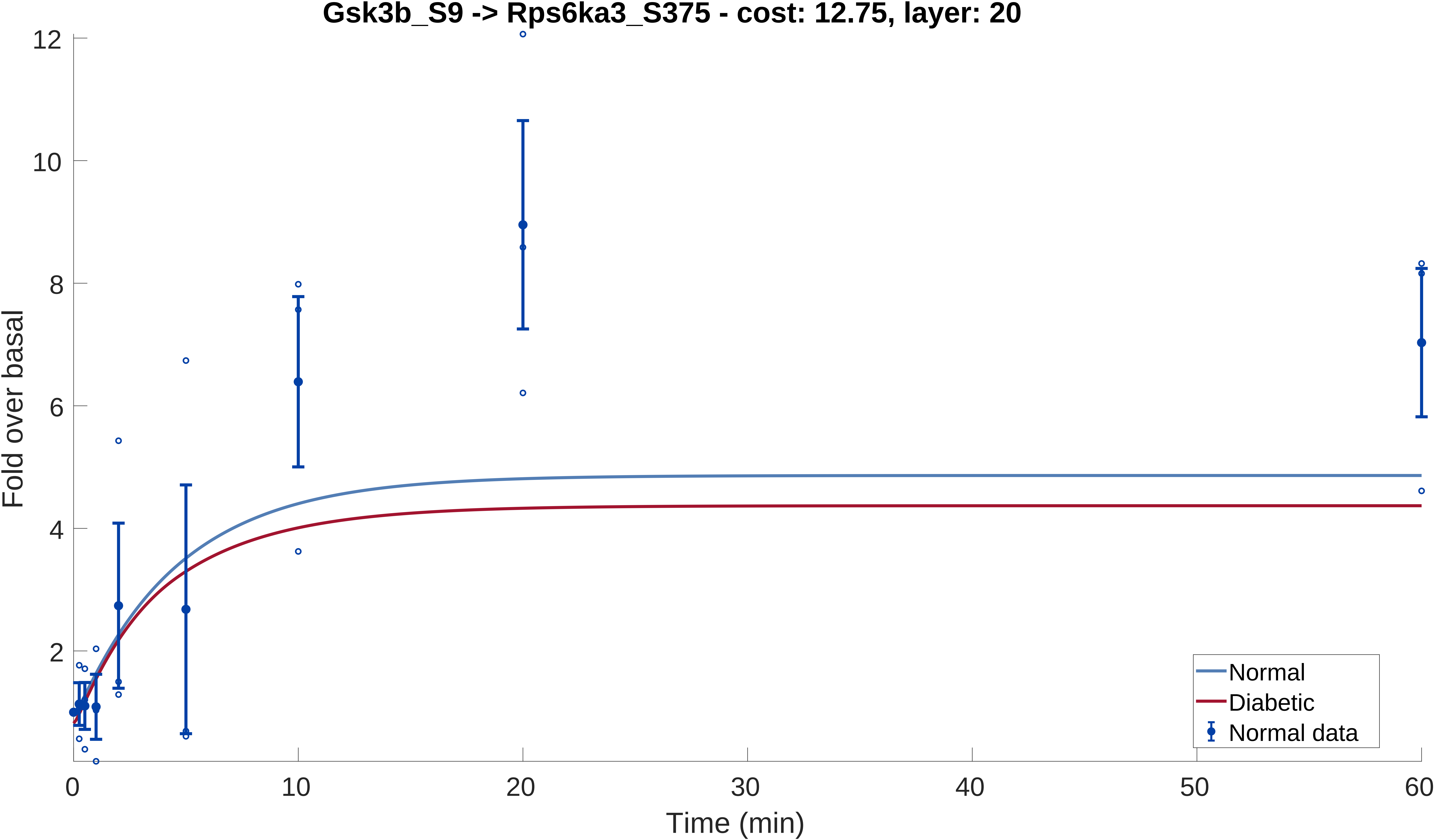

Gsk3b_S9 -> Rps6ka3_S375 - cost: 12.75, layer: 20
Fold over basal
Time (min)
0
2
4
6
8
10
12
0
10
20
30
40
50
60
Normal
Diabetic
Normal data

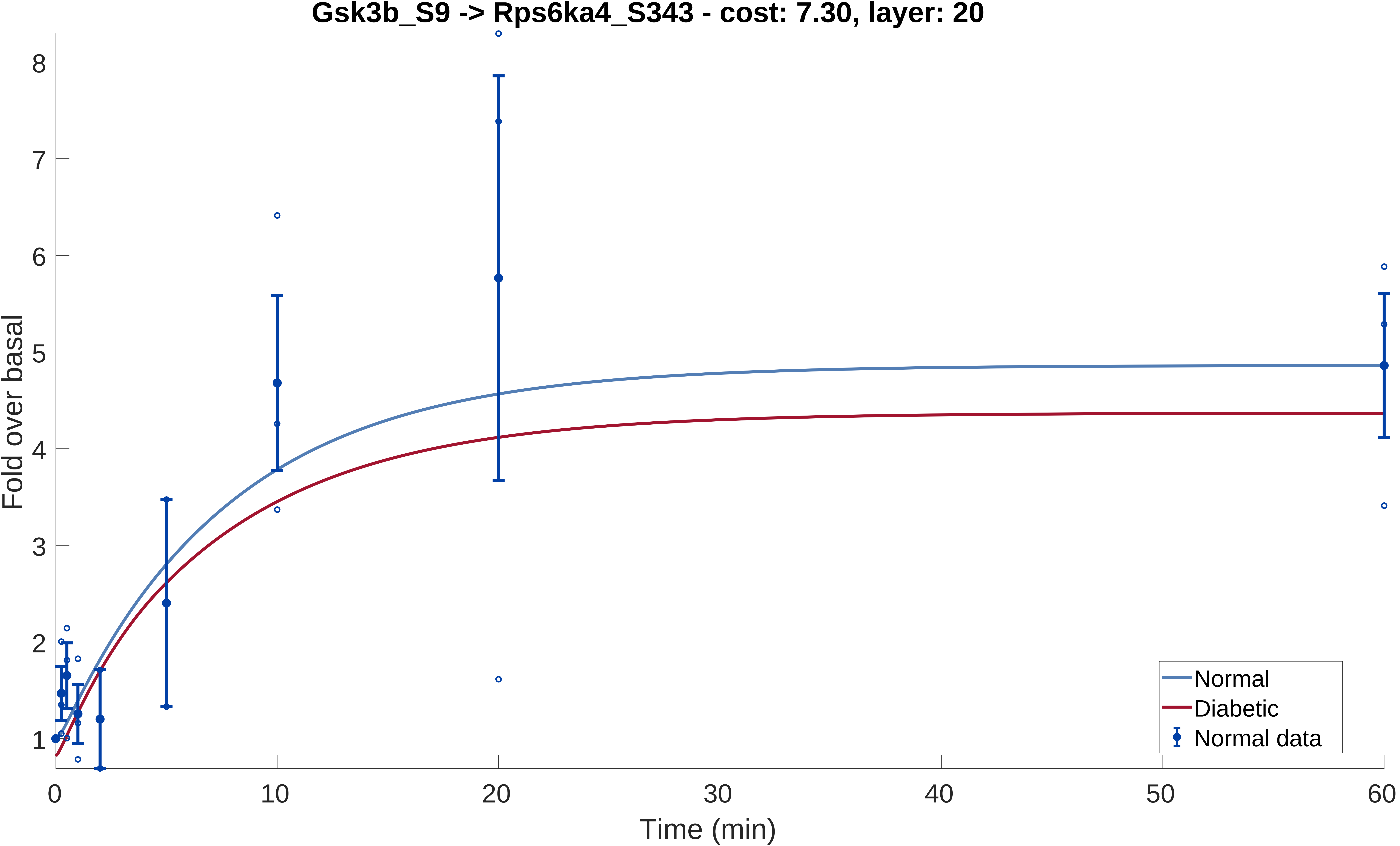
Gsk3b_S9 -> Rps6ka4_S343 - cost: 7.30, layer: 20
Fold over basal
Time (min)
1
2
3
4
5
6
7
8
0
10
20
30
40
50
60
Normal
Diabetic
Normal data

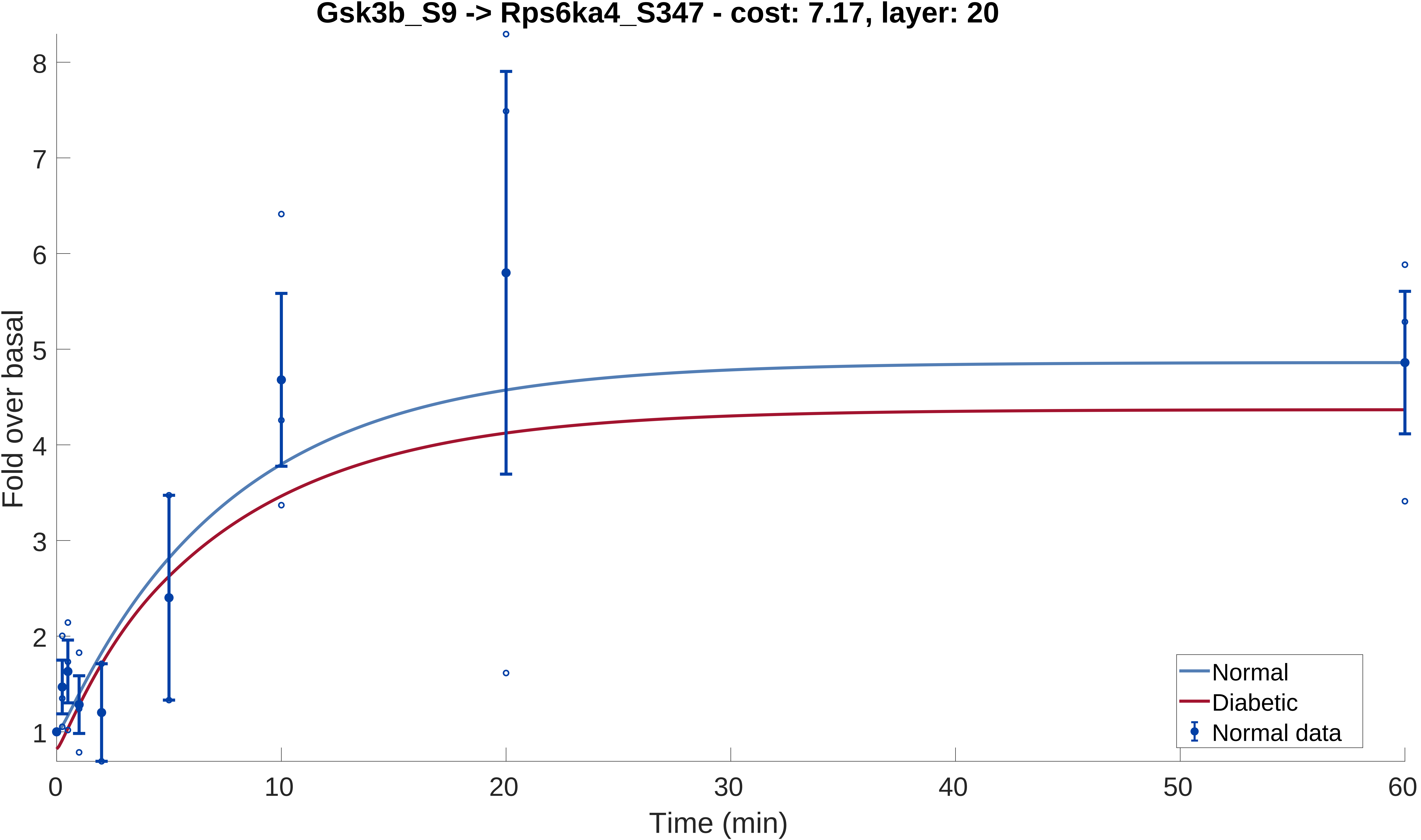

Gsk3b_S9 -> Rps6ka4_S347 - cost: 7.17, layer: 20
Fold over basal
Time (min)
1
2
3
4
5
6
7
8
0
10
20
30
40
50
60
Normal
Diabetic
Normal data

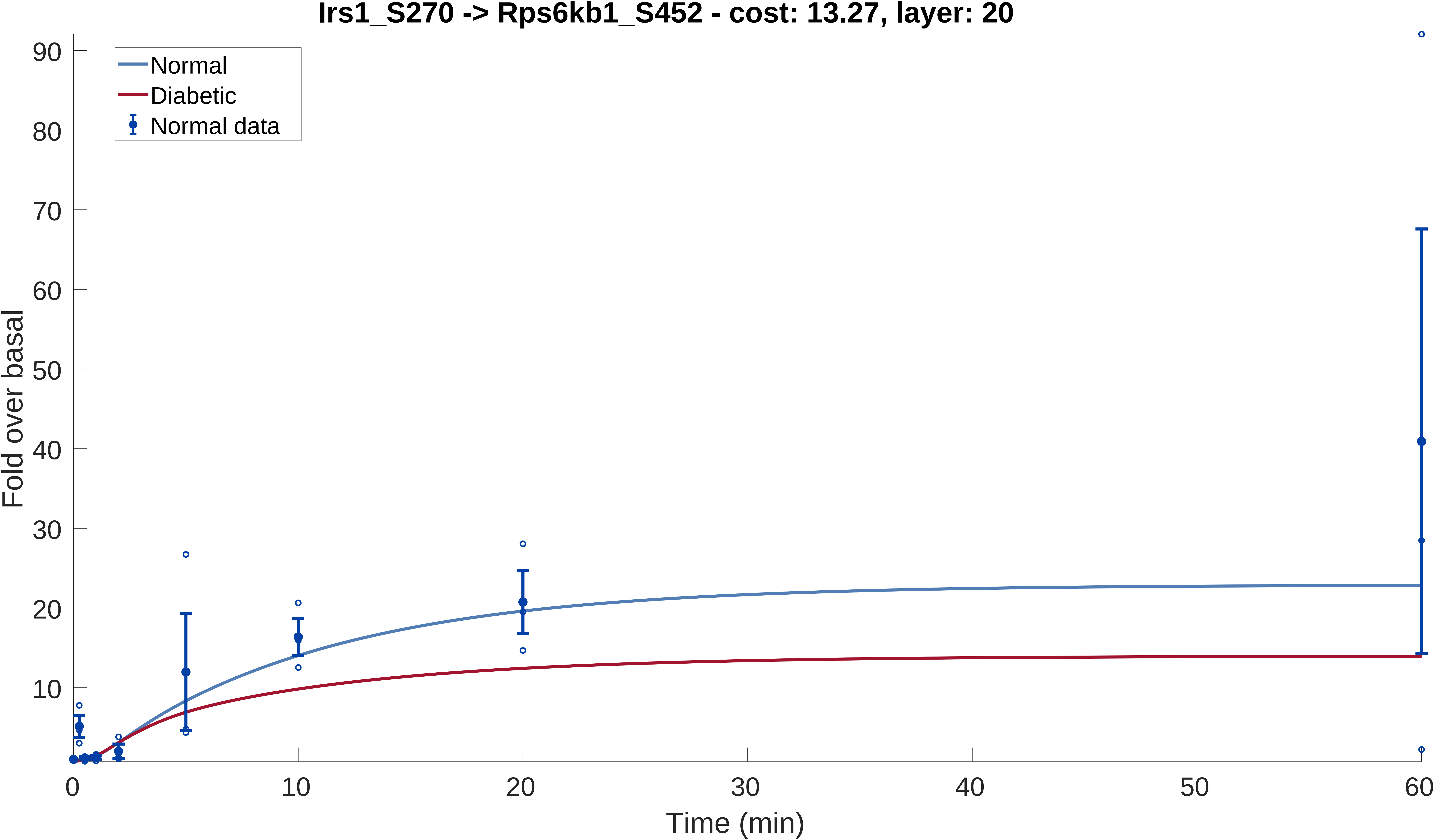

Irs1_S270 -> Rps6kb1_S452 - cost: 13.27, layer: 20
Normal
Diabetic
Normal data
Fold over basal
Time (min)
0
10
20
30
40
50
60
70
80
90

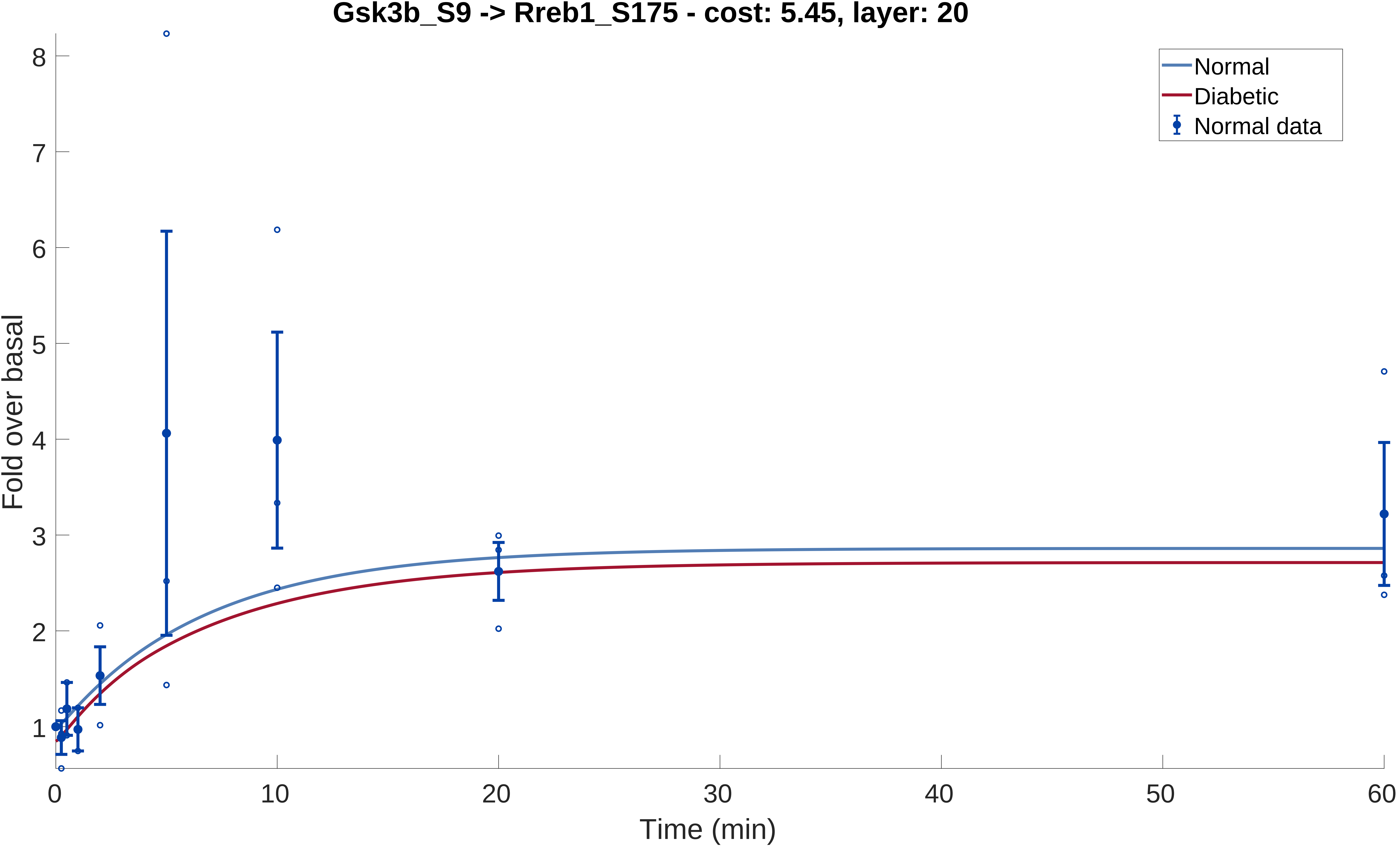

Gsk3b_S9 -> Rreb1_S175 - cost: 5.45, layer: 20
Normal
Diabetic
Normal data
Fold over basal
Time (min)
1
2
3
4
5
6
7
8
0
10
20
30
40
50
60

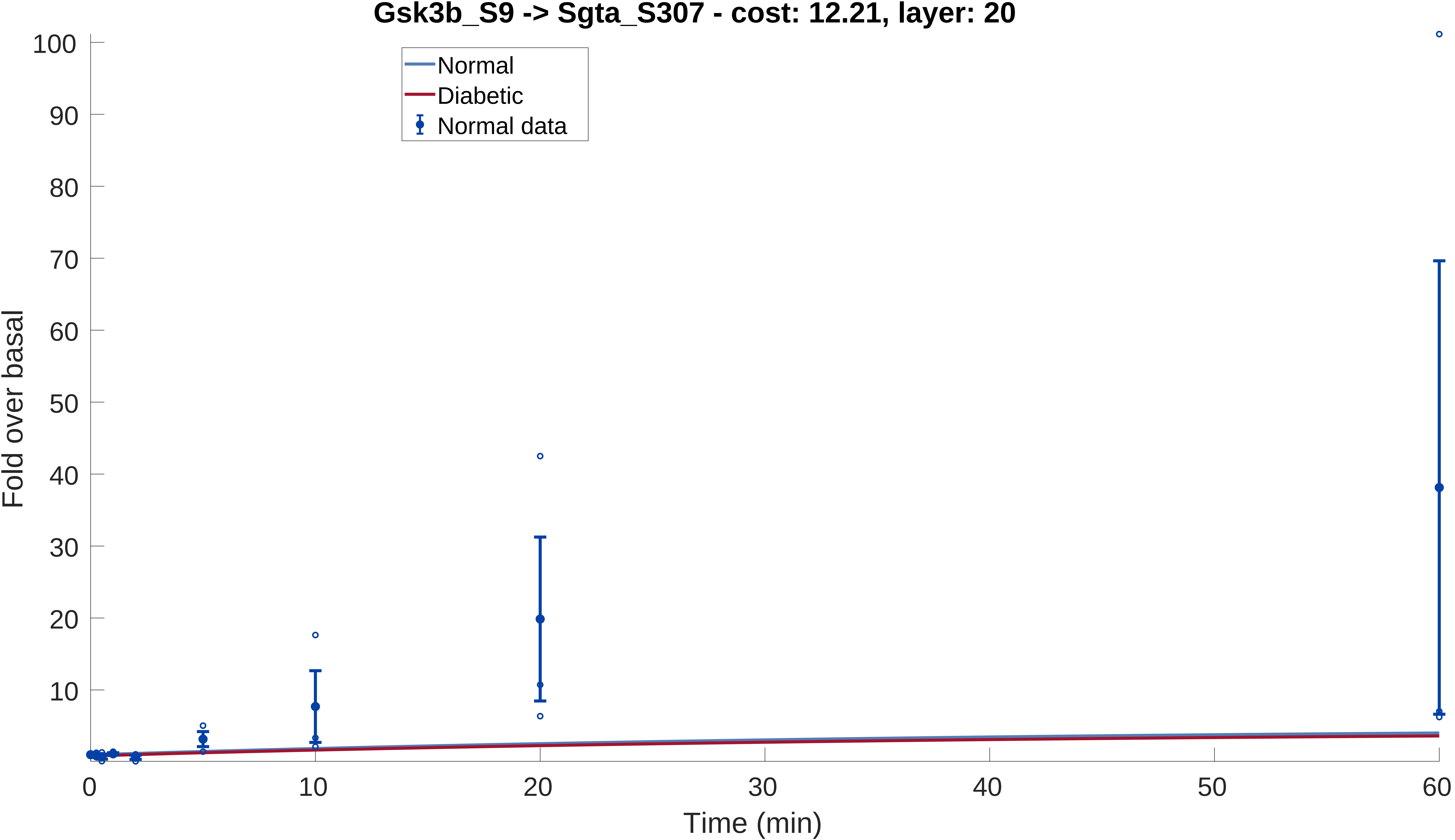

Gsk3b_S9 -> Sgta_S307 - cost: 12.21, layer: 20
Normal
Diabetic
Normal data
Fold over basal
Time (min)
0
10
20
30
40
50
60
70
80
90
100

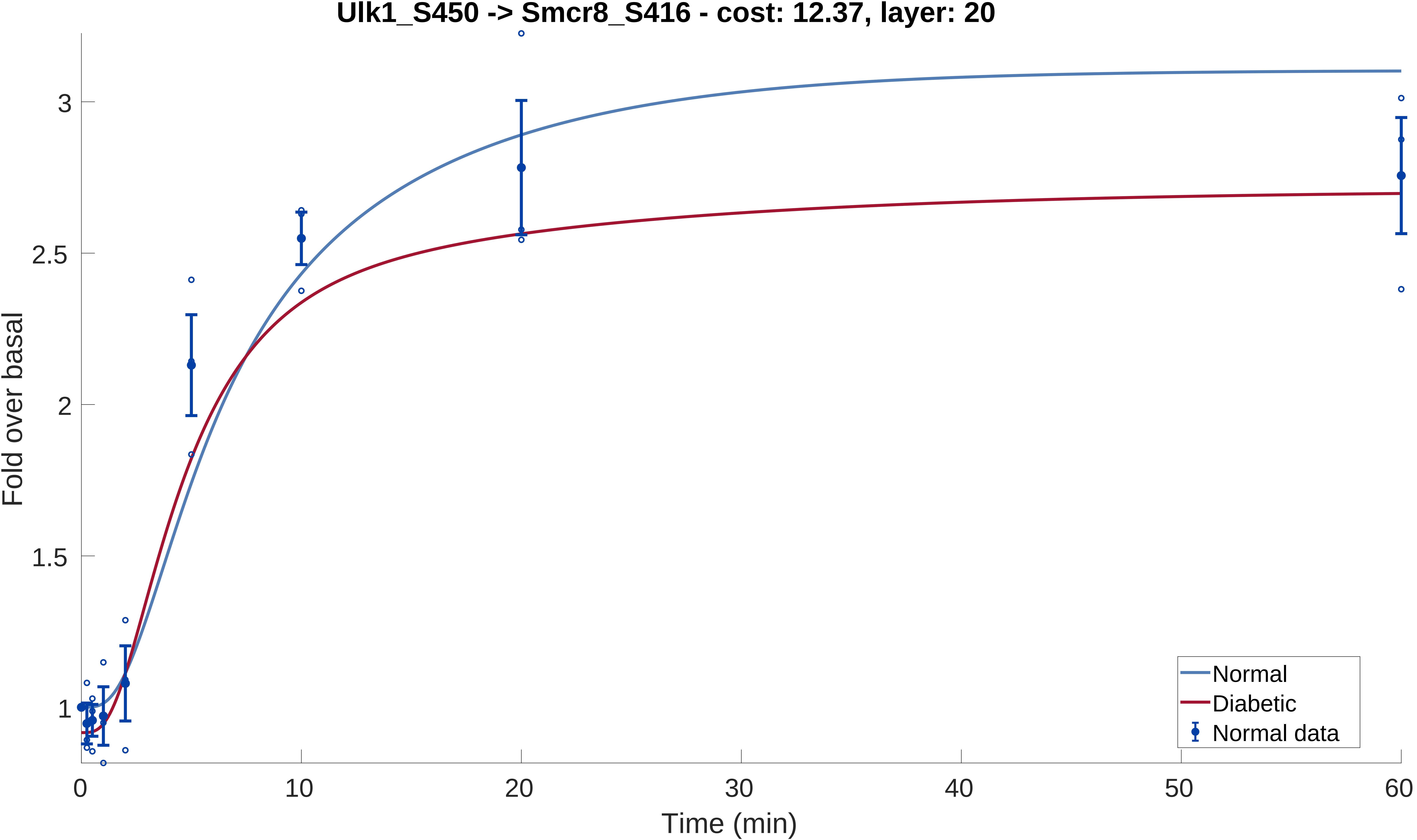

Ulk1_S450 -> Smcr8_S416 - cost: 12.37, layer: 20
Fold over basal
Time (min)
0
10
20
30
40
50
60
1
1.5
2
2.5
3
Normal
Diabetic
Normal data

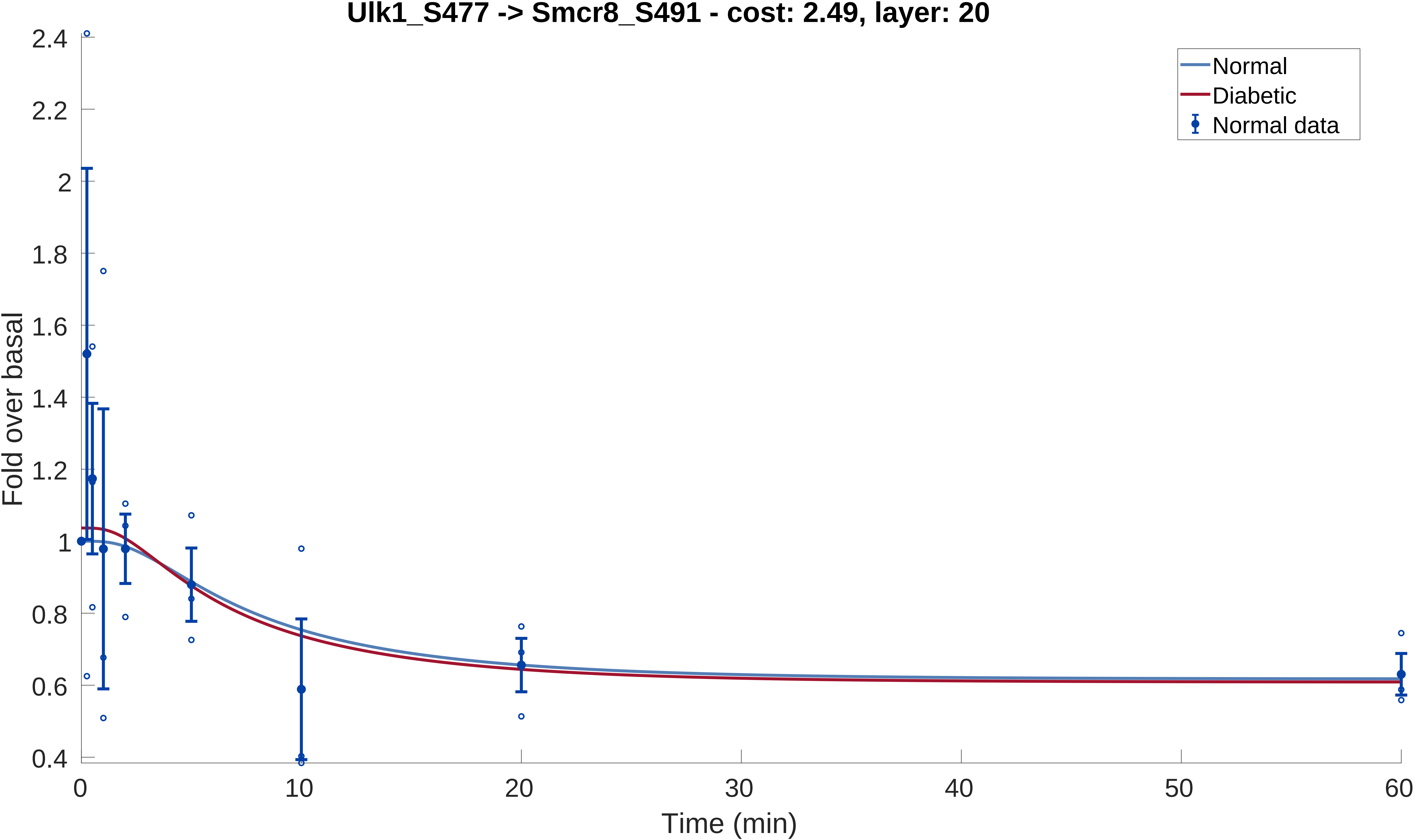

Ulk1_S477 -> Smcr8_S491 - cost: 2.49, layer: 20
Normal
Diabetic
Normal data
Fold over basal
Time (min)
0.4
0.6
0.8
1
1.2
1.4
1.6
1.8
2
2.2
2.4
0
10
20
30
40
50
60

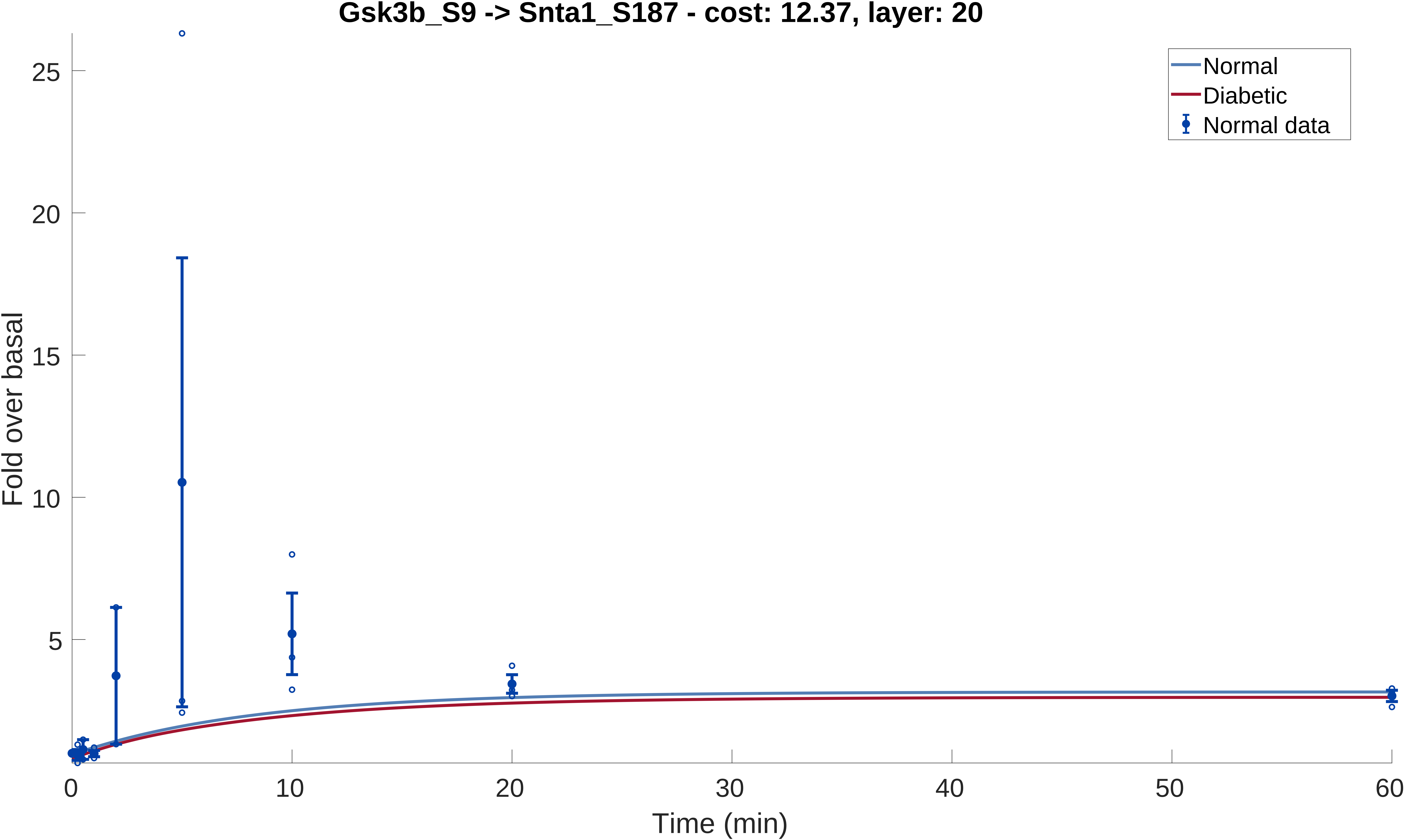

Gsk3b_S9 -> Snta1_S187 - cost: 12.37, layer: 20
Normal
Diabetic
Normal data
Fold over basal
25
20
15
10
5
0
10
20
30
40
50
60
Time (min)

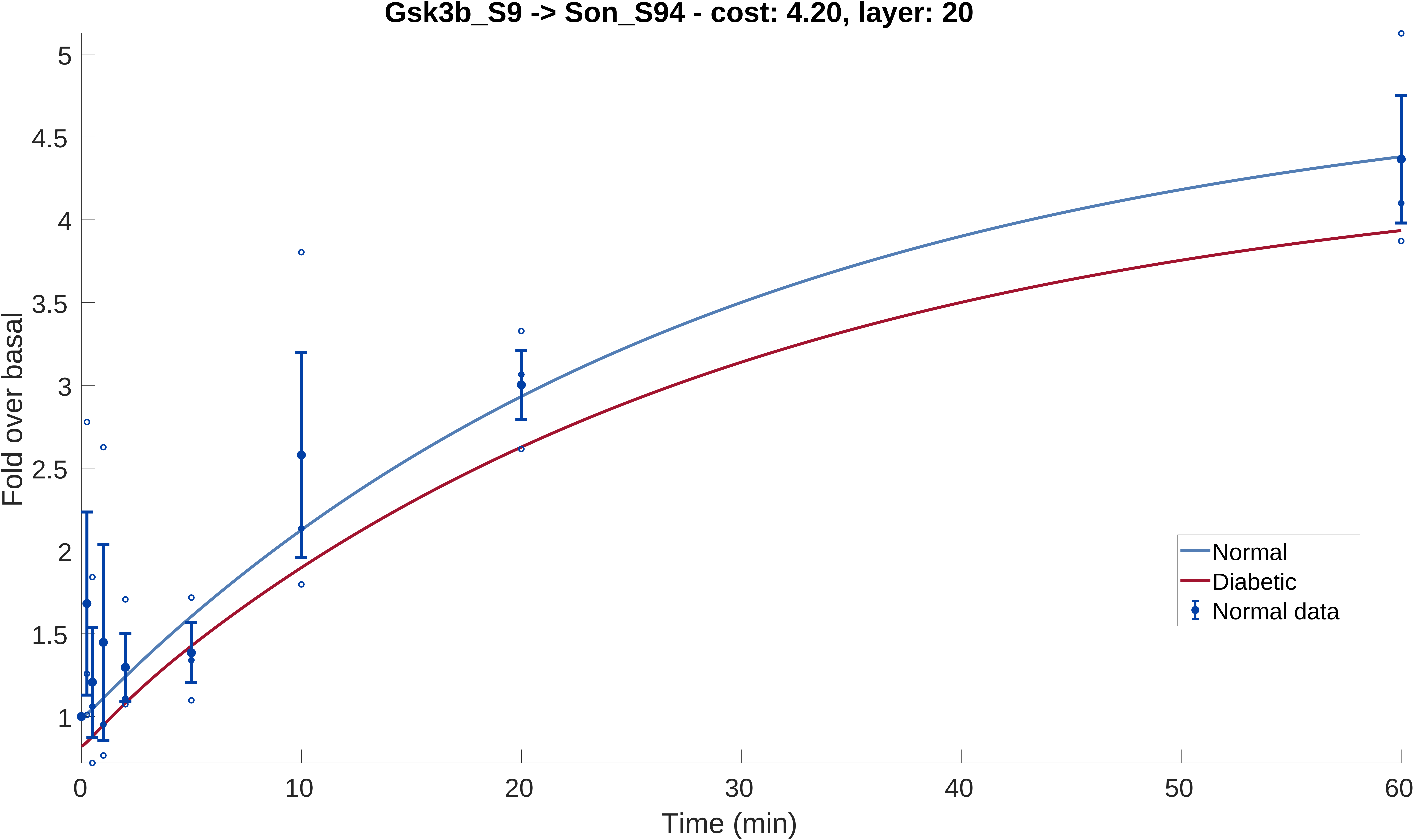

Gsk3b_S9 -> Son_S94 - cost: 4.20, layer: 20
Fold over basal
Time (min)
0
10
20
30
40
50
60
1
1.5
2
2.5
3
3.5
4
4.5
5
Normal
Diabetic
Normal data

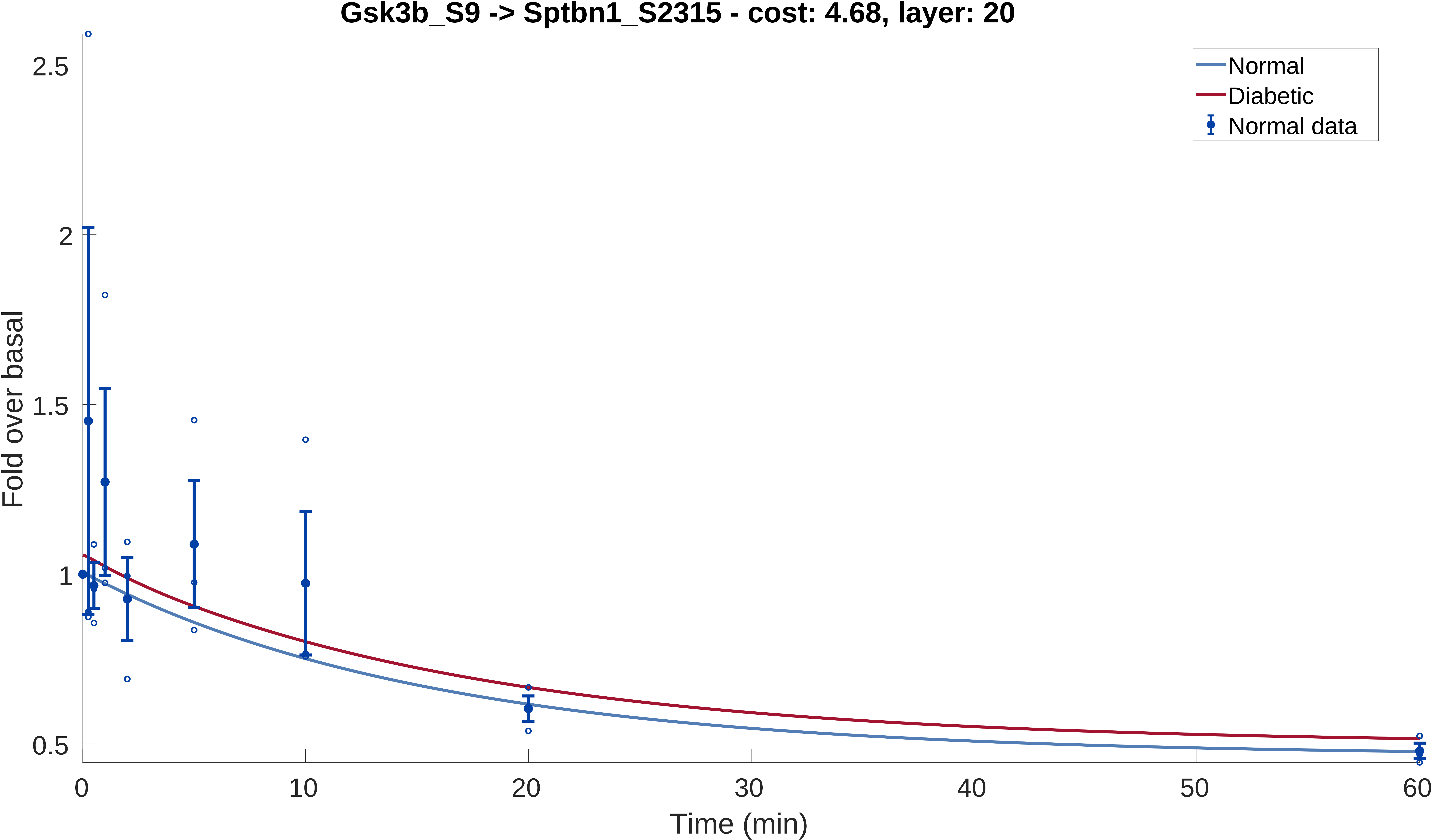
Gsk3b_S9 -> Sptbn1_S2315 - cost: 4.68, layer: 20
Fold over basal
2.5
2
1.5
1
0.5
0
10
20
30
40
50
60
Time (min)
Normal
Diabetic
Normal data

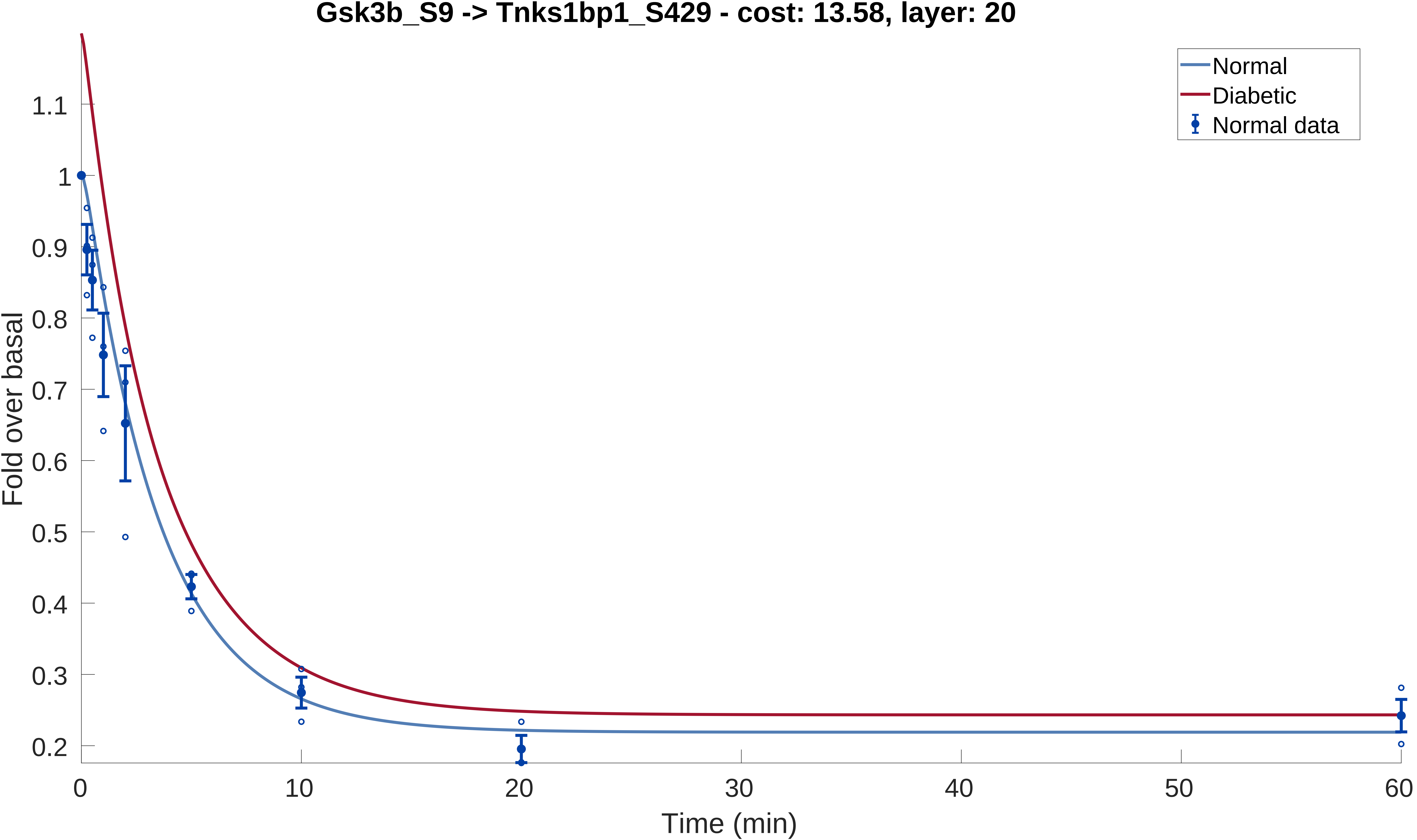

Gsk3b_S9 -> Tnks1bp1_S429 - cost: 13.58, layer: 20
Normal
Diabetic
Normal data
Fold over basal
Time (min)
1.1
1
0.9
0.8
0.7
0.6
0.5
0.4
0.3
0.2
0
10
20
30
40
50
60

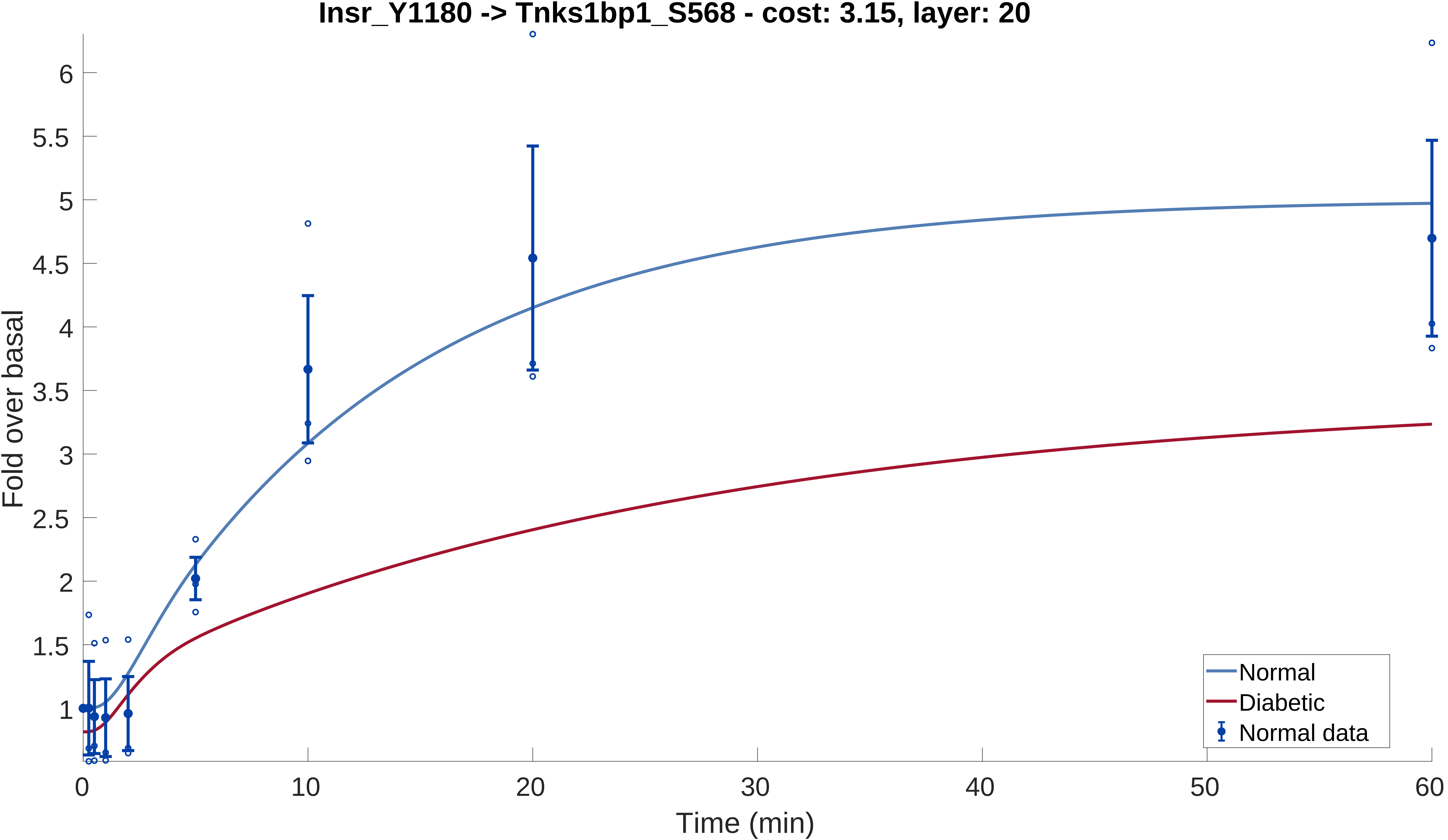

Insr_Y1180 -> Tnks1bp1_S568 - cost: 3.15, layer: 20
Fold over basal
Time (min)
Normal
Diabetic
Normal data
0
10
20
30
40
50
60
1
1.5
2
2.5
3
3.5
4
4.5
5
5.5
6

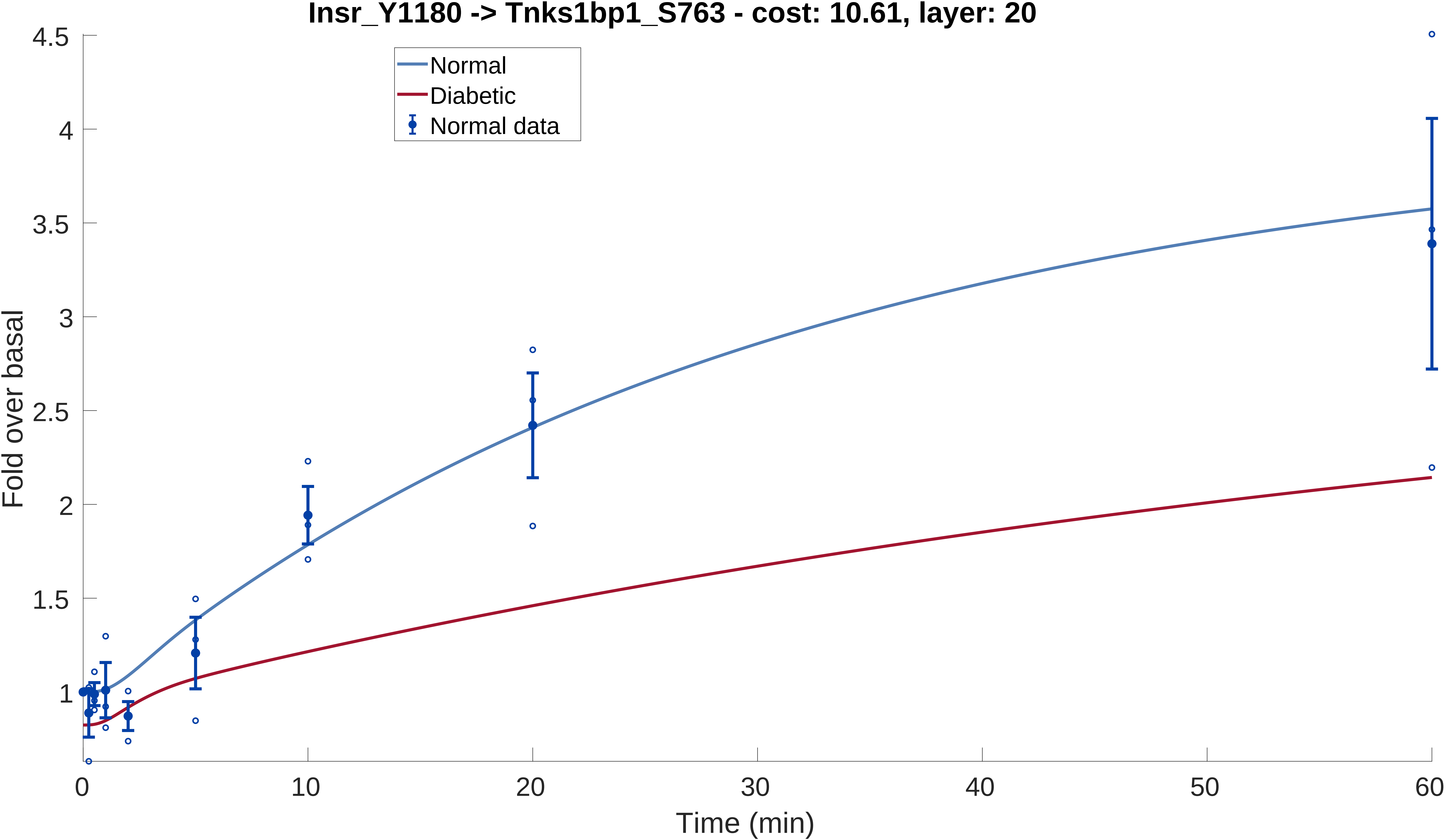

Insr_Y1180 -> Tnks1bp1_S763 - cost: 10.61, layer: 20
Normal
Diabetic
Normal data
Fold over basal
Time (min)
4.5
4
3.5
3
2.5
2
1.5
1
0
10
20
30
40
50
60

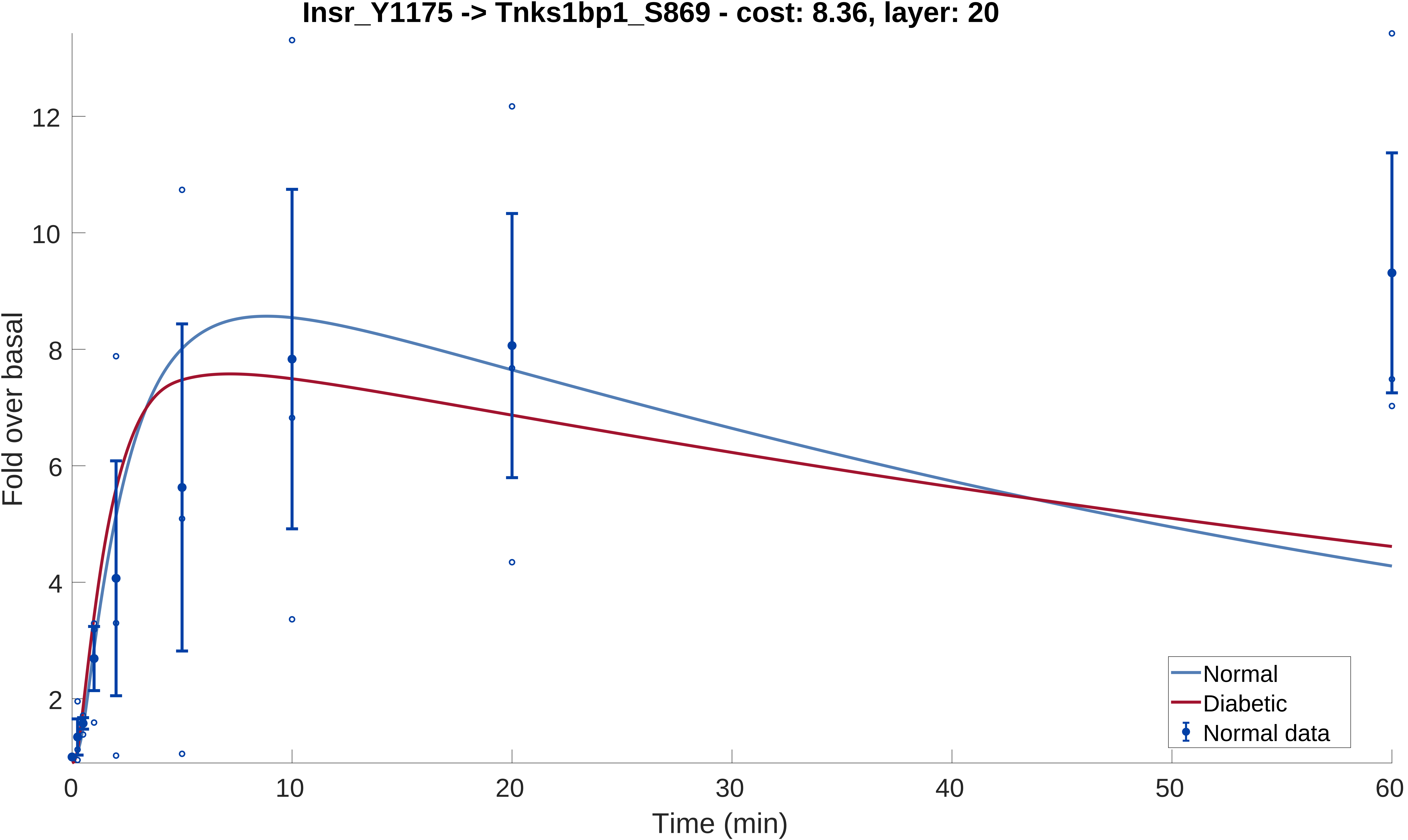

Insr_Y1175 -> Tnks1bp1_S869 - cost: 8.36, layer: 20
Fold over basal
12
10
8
6
4
2
0
10
20
30
40
50
60
Time (min)
Normal
Diabetic
Normal data

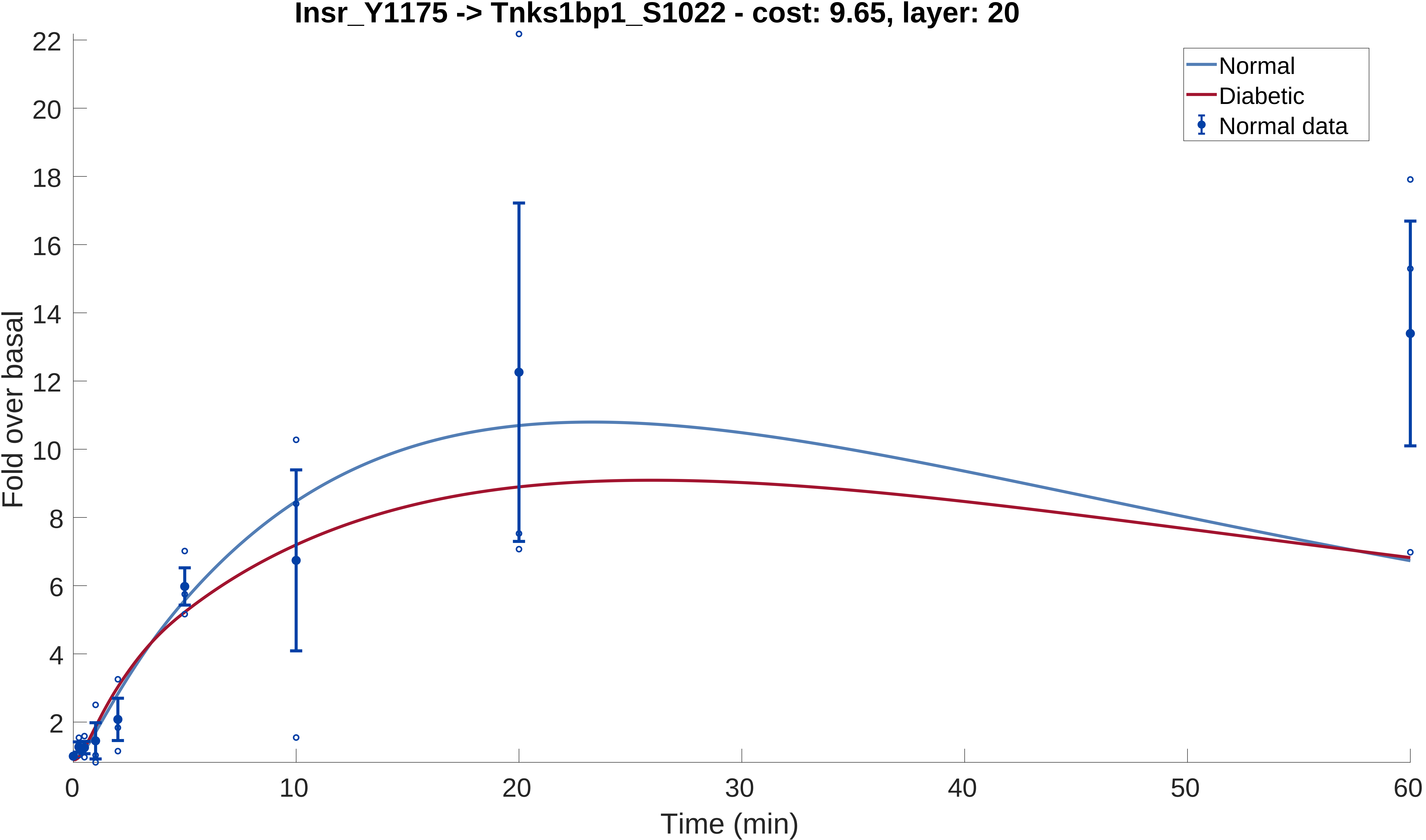
Insr_Y1175 -> Tnks1bp1_S1022 - cost: 9.65, layer: 20
Normal
Diabetic
Normal data
Fold over basal
Time (min)
2
4
6
8
10
12
14
16
18
20
22
0
10
20
30
40
50
60

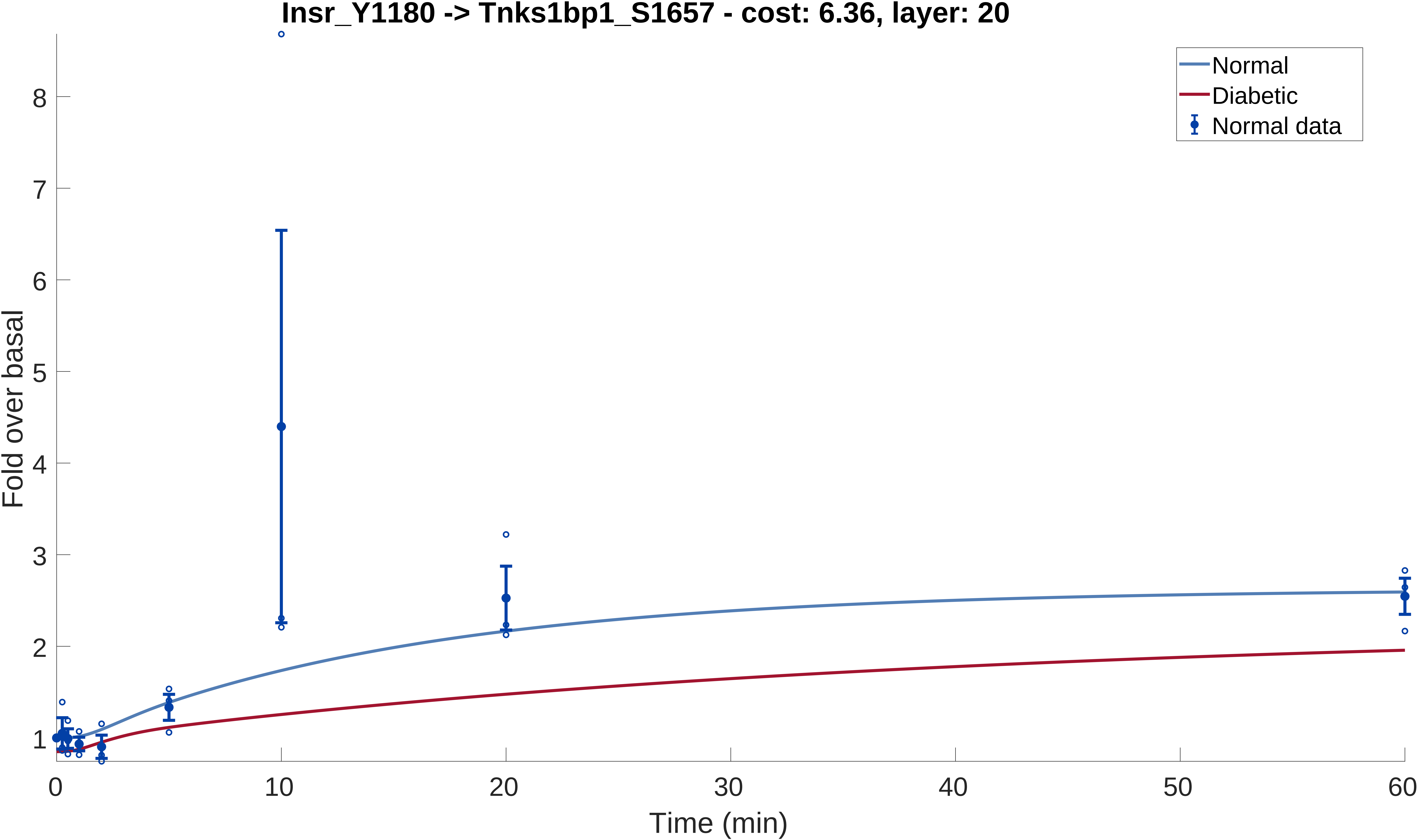

Insr_Y1180 -> Tnks1bp1_S1657 - cost: 6.36, layer: 20
Normal
Diabetic
Normal data
Fold over basal
1
2
3
4
5
6
7
8
0
10
20
30
40
50
60
Time (min)

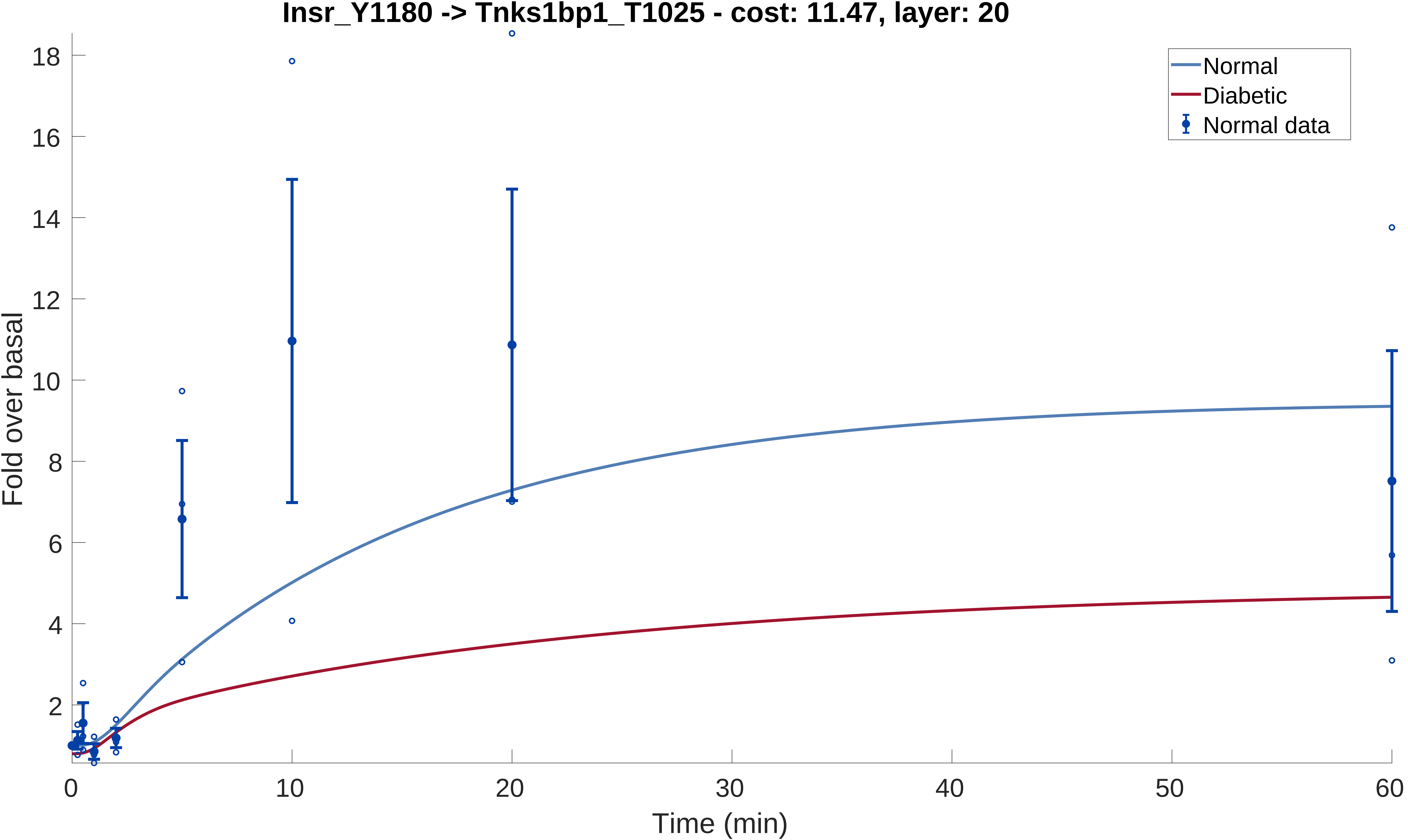
Insr_Y1180 -> Tnks1bp1_T1025 - cost: 11.47, layer: 20
Normal
Diabetic
Normal data
Fold over basal
Time (min)
2
4
6
8
10
12
14
16
18
0
10
20
30
40
50
60

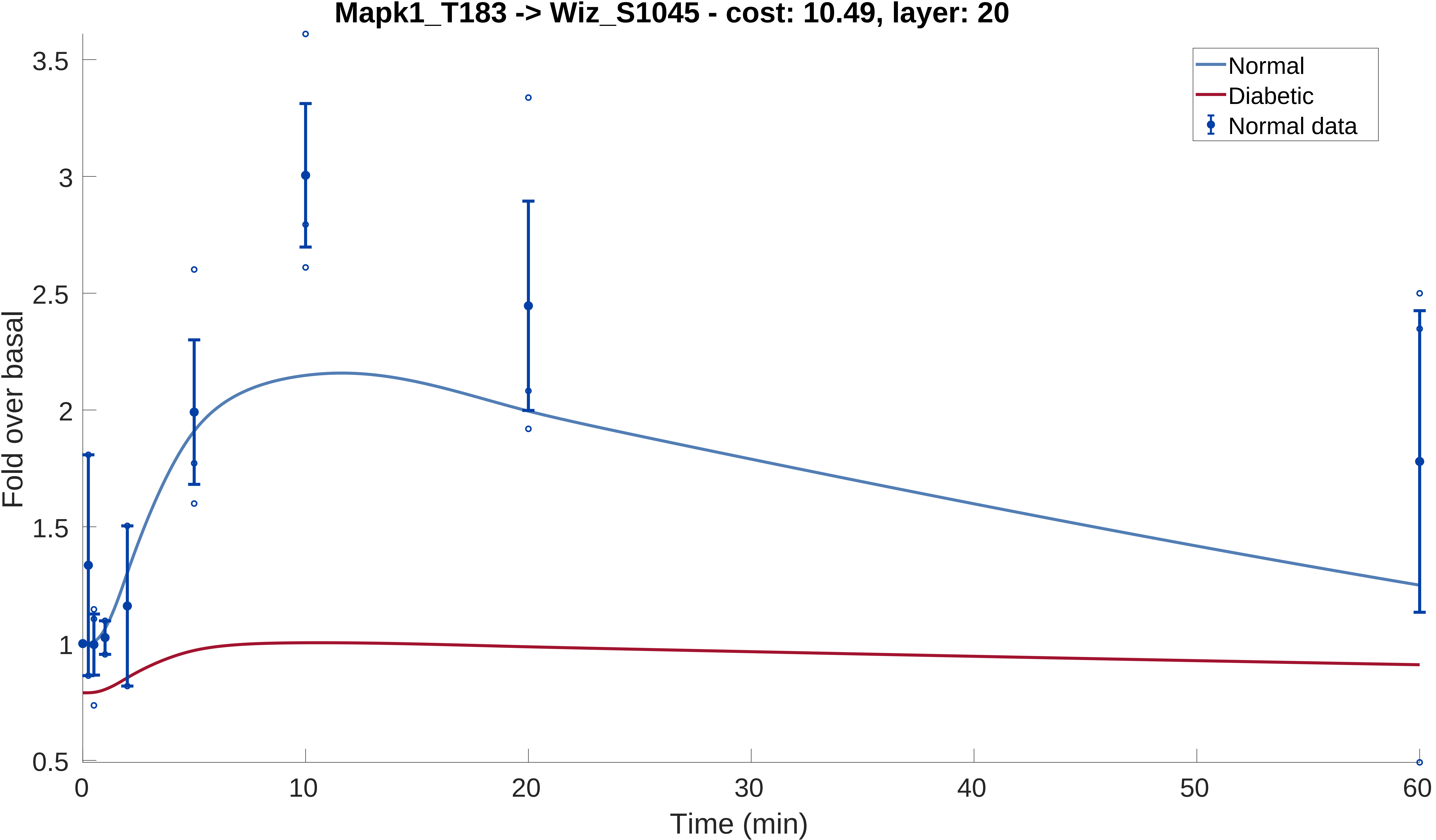

Mapk1_T183 -> Wiz_S1045 - cost: 10.49, layer: 20
Normal
Diabetic
Normal data
Fold over basal
Time (min)
0.5
1
1.5
2
2.5
3
3.5
0
10
20
30
40
50
60

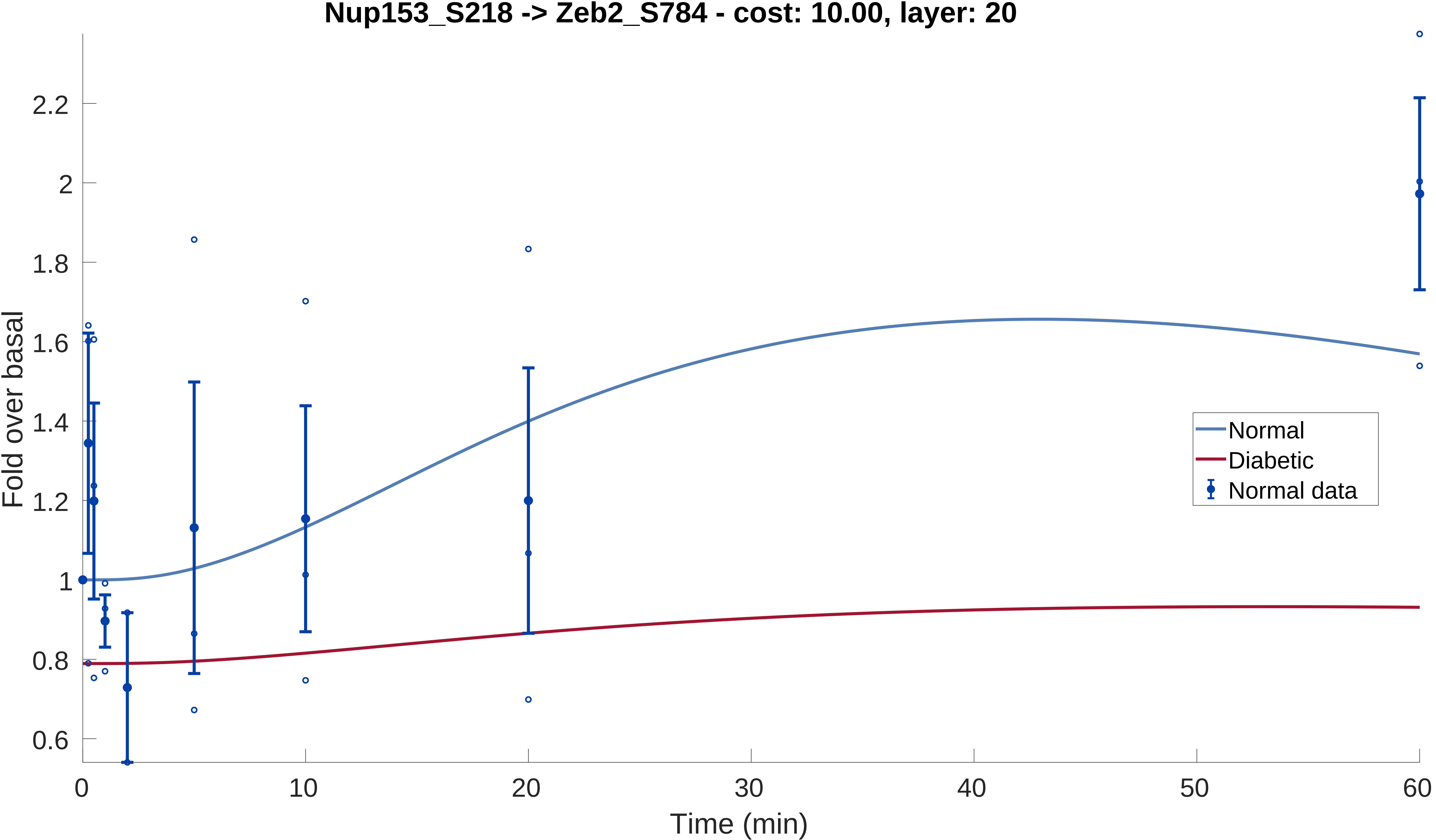
Nup153_S218 -> Zeb2_S784 - cost: 10.00, layer: 20
Fold over basal
Time (min)
Normal
Diabetic
Normal data
0.6
0.8
1
1.2
1.4
1.6
1.8
2
2.2
0
10
20
30
40
50
60

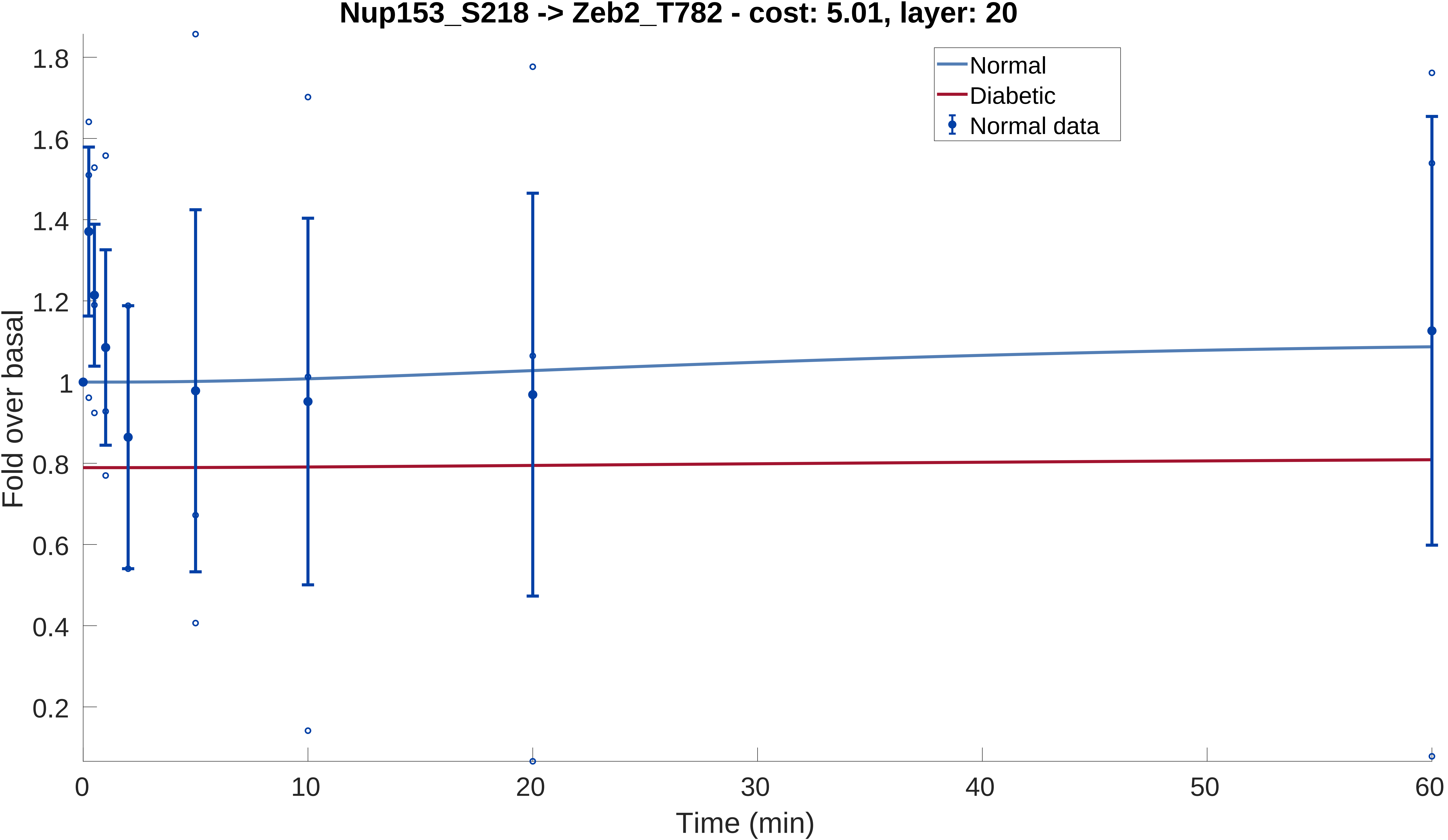
Nup153_S218 -> Zeb2_T782 - cost: 5.01, layer: 20
Normal
Diabetic
Normal data
Fold over basal
Time (min)
0.2
0.4
0.6
0.8
1
1.2
1.4
1.6
1.8
0
10
20
30
40
50
60

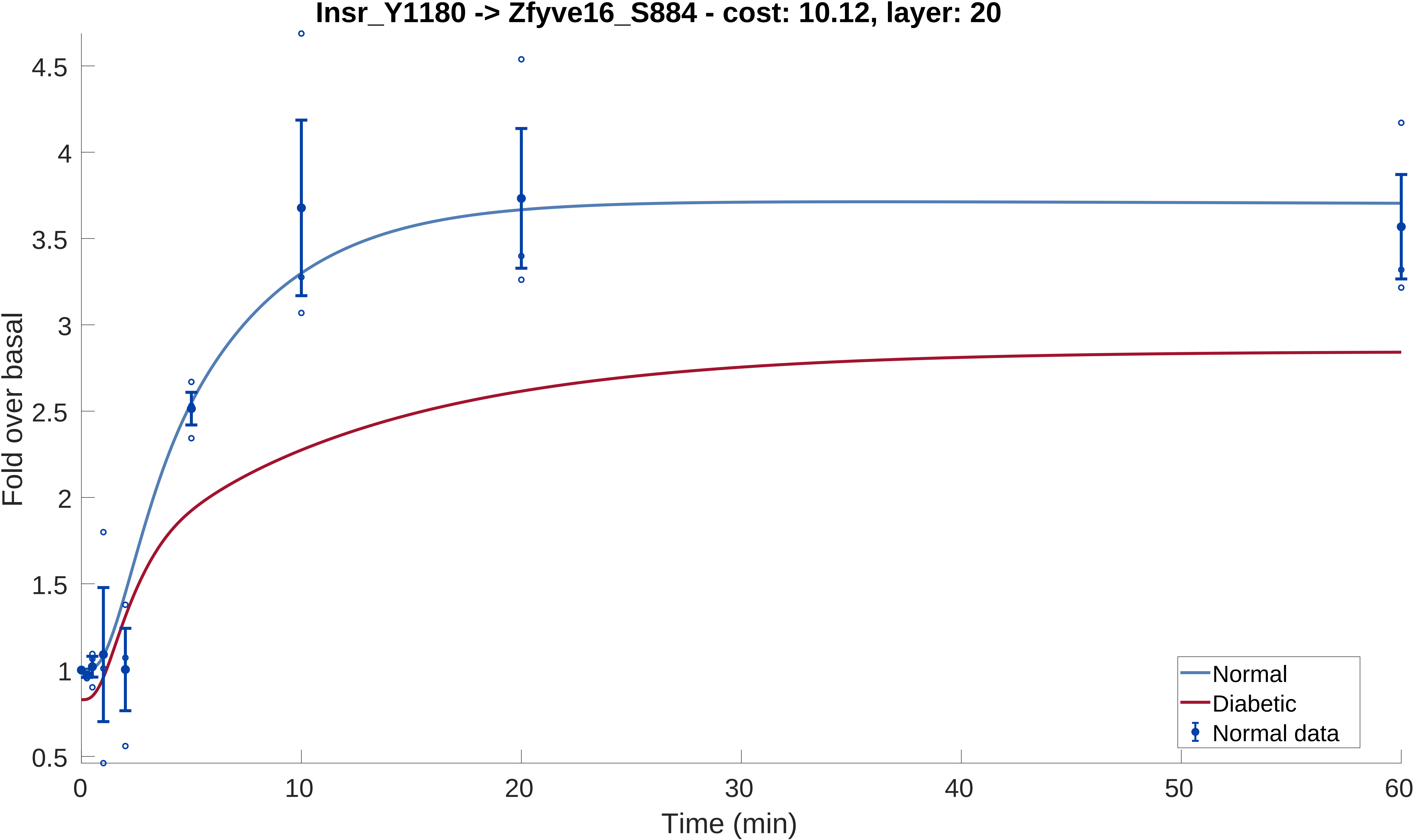

Insr_Y1180 -> Zfyve16_S884 - cost: 10.12, layer: 20
Fold over basal
4.5
4
3.5
3
2.5
2
1.5
1
0.5
0
10
20
30
40
50
60
Time (min)
Normal
Diabetic
Normal data

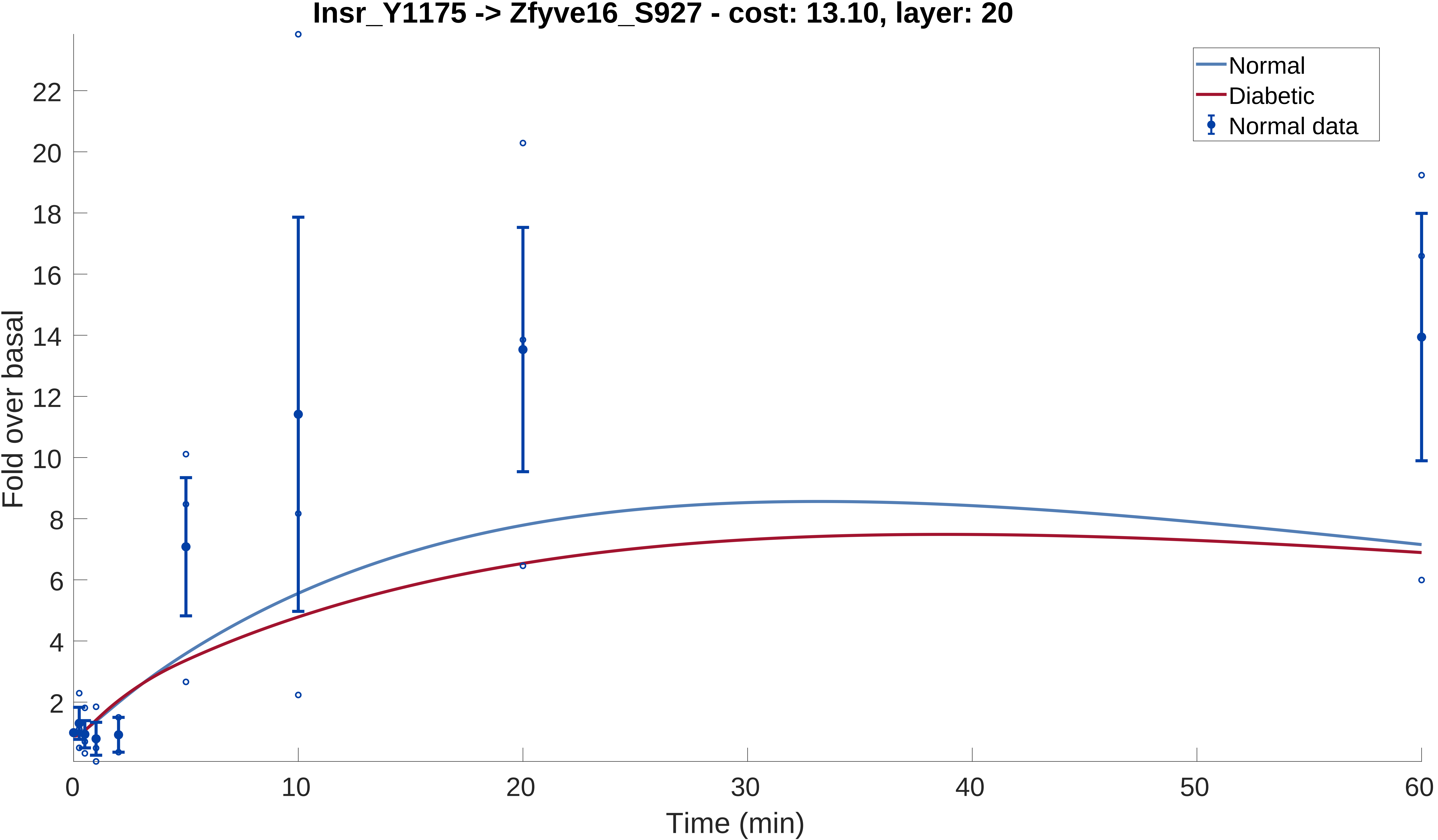

Insr_Y1175 -> Zfyve16_S927 - cost: 13.10, layer: 20
Normal
Diabetic
Normal data
Fold over basal
2
4
6
8
10
12
14
16
18
20
22
0
10
20
30
40
50
60
Time (min)

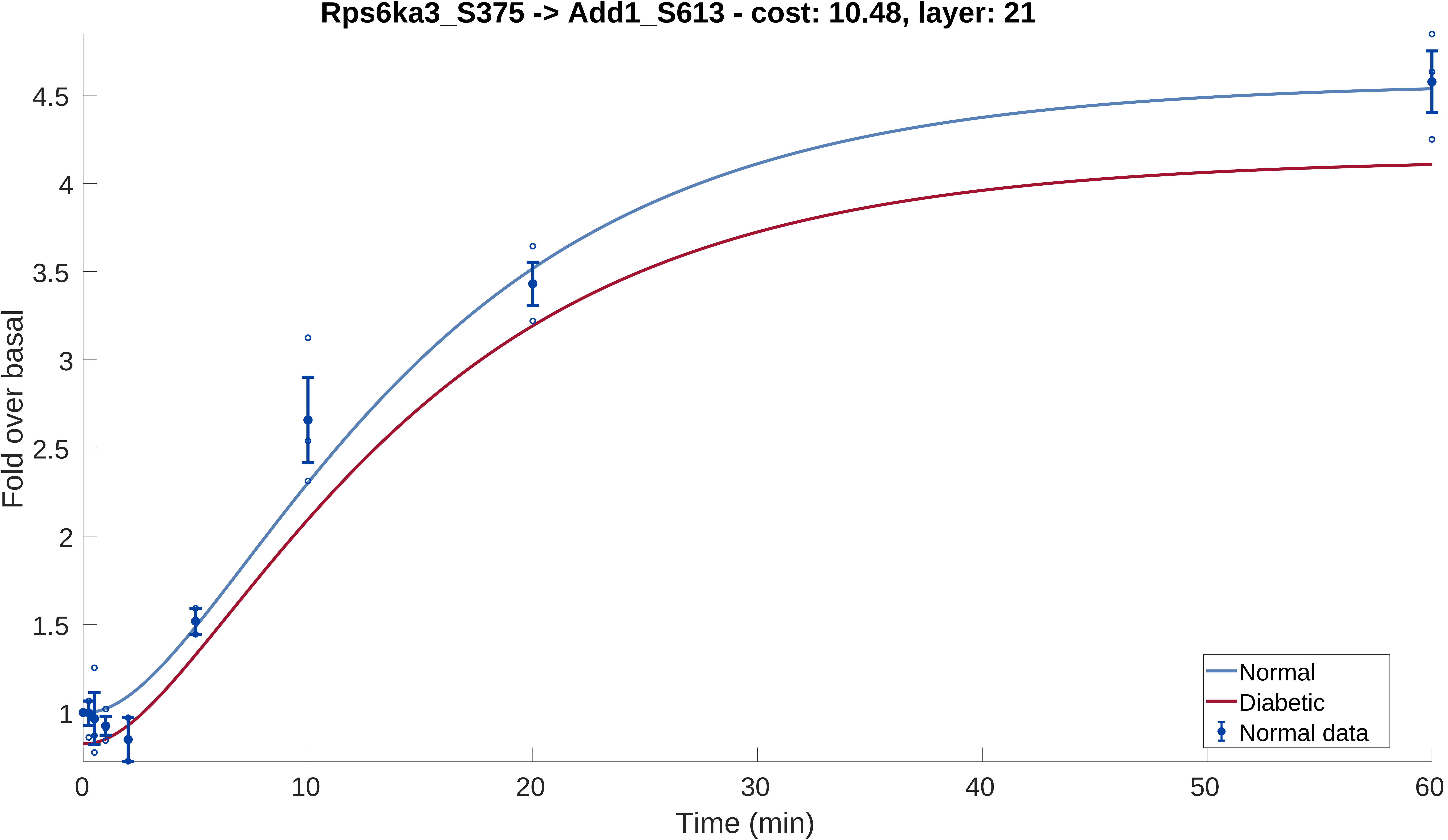

Rps6ka3_S375 -> Add1_S613 - cost: 10.48, layer: 21
Fold over basal
4.5
4
3.5
3
2.5
2
1.5
1
0
10
20
30
40
50
60
Time (min)
Normal
Diabetic
Normal data

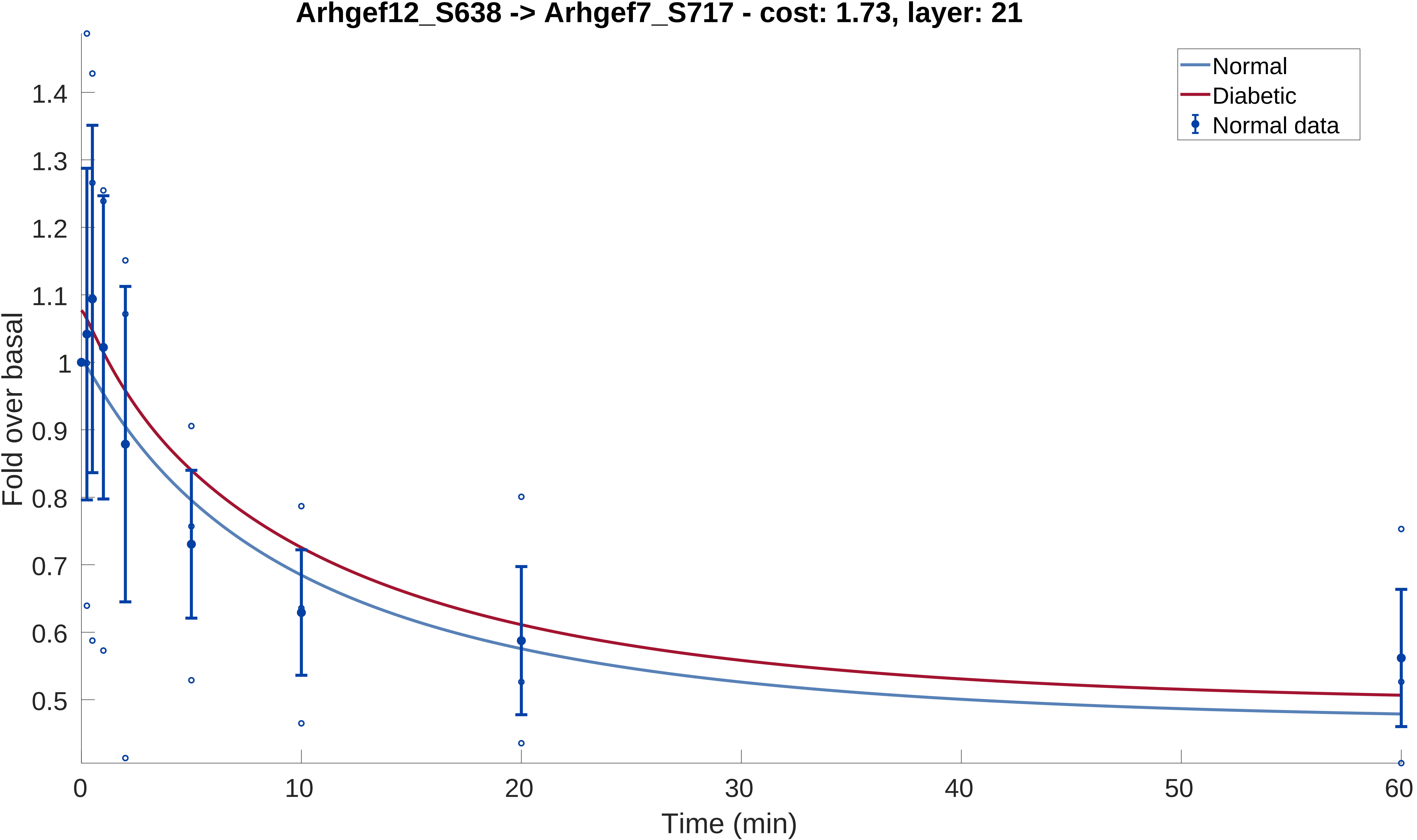

Arhgef12_S638 -> Arhgef7_S717 - cost: 1.73, layer: 21
Normal
Diabetic
Normal data
Fold over basal
Time (min)
0
10
20
30
40
50
60
0.5
0.6
0.7
0.8
0.9
1
1.1
1.2
1.3
1.4

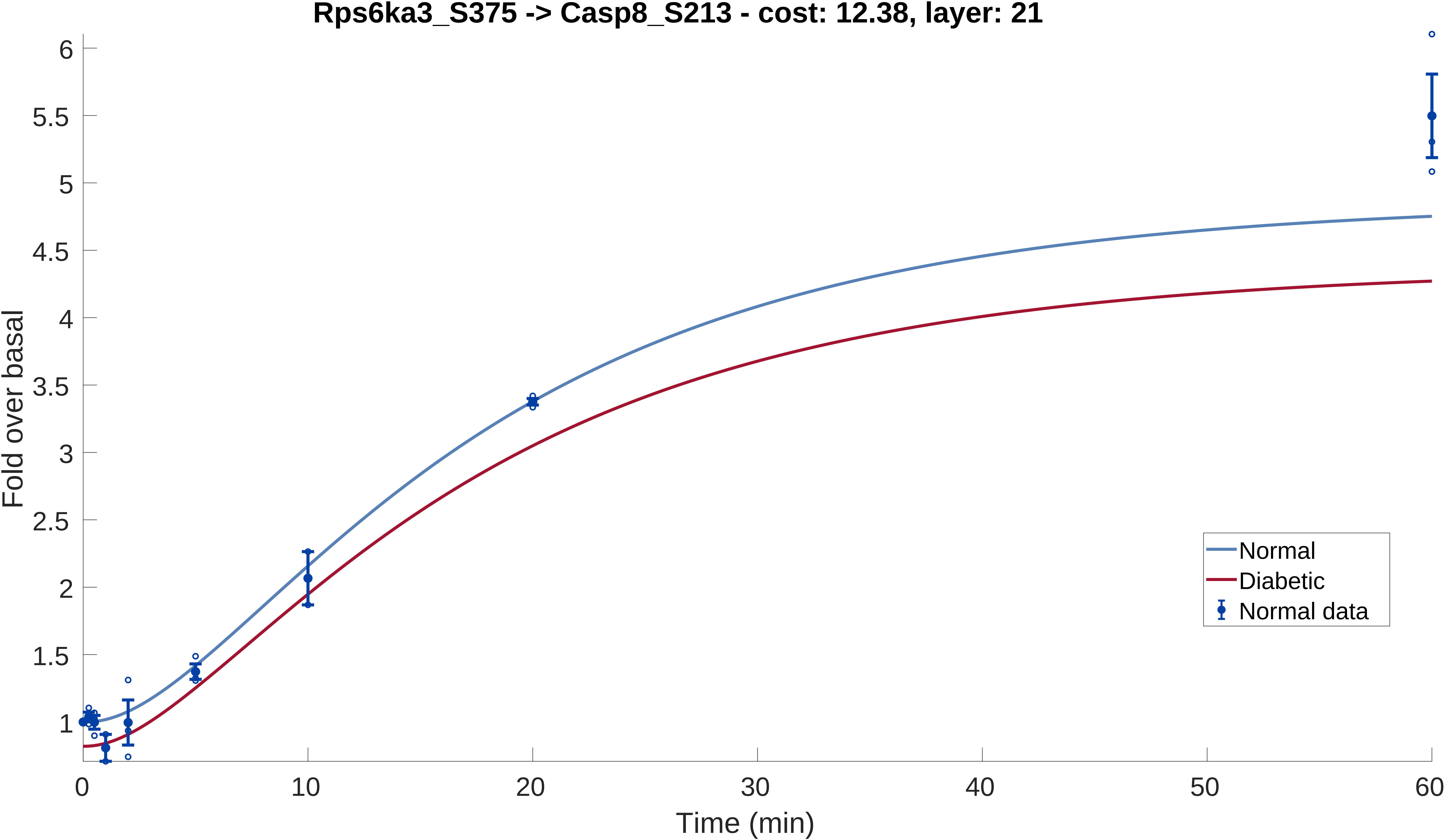

Rps6ka3_S375 -> Casp8_S213 - cost: 12.38, layer: 21
Fold over basal
Time (min)
Normal
Diabetic
Normal data
0
10
20
30
40
50
60
1
1.5
2
2.5
3
3.5
4
4.5
5
5.5
6

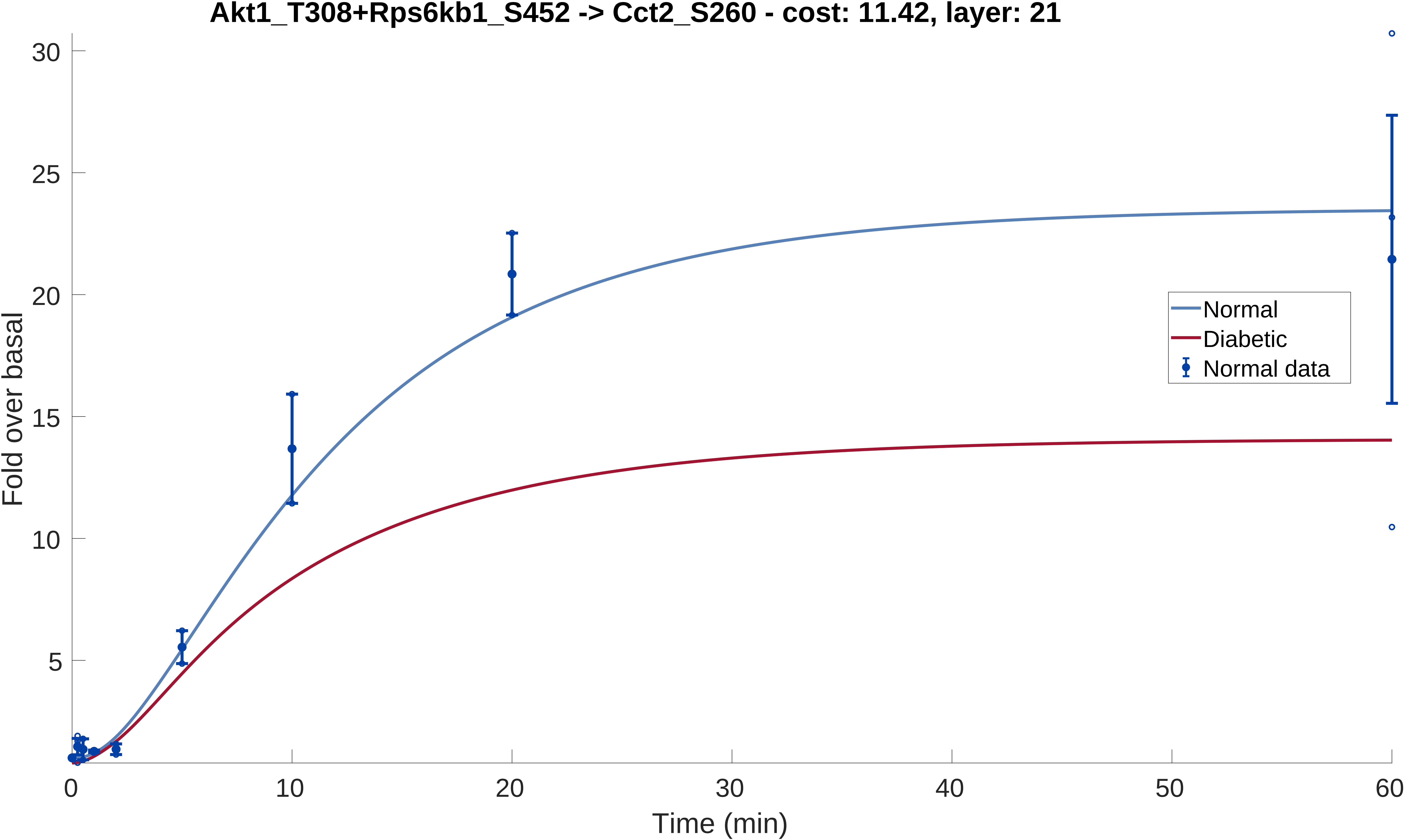

Akt1_T308+Rps6kb1_S452 -> Cct2_S260 - cost: 11.42, layer: 21
Fold over basal
Time (min)
Normal
Diabetic
Normal data
0
5
10
15
20
25
30
0
10
20
30
40
50
60

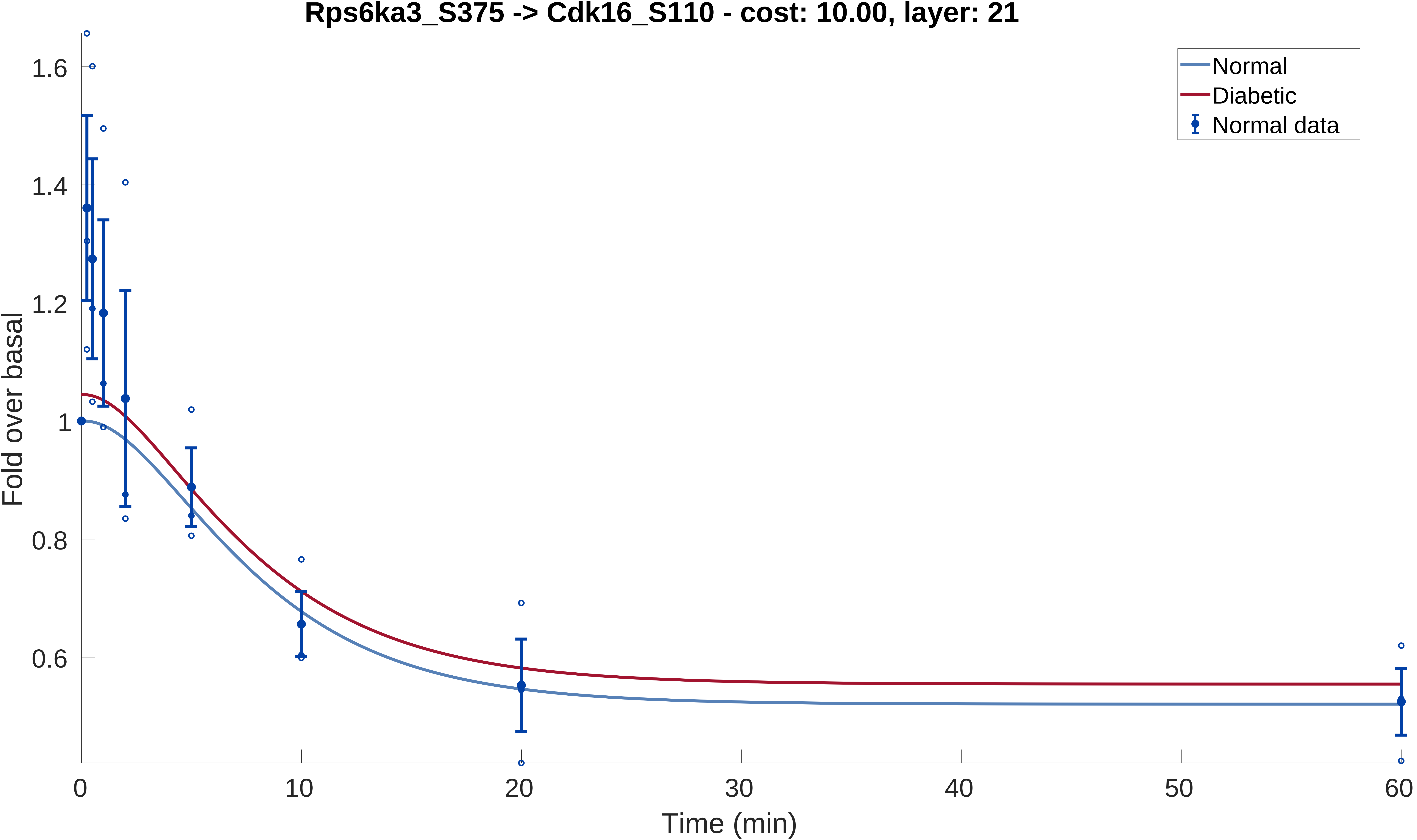
Rps6ka3_S375 -> Cdk16_S110 - cost: 10.00, layer: 21
Fold over basal
1.6
1.4
1.2
1
0.8
0.6
0
10
20
30
40
50
60
Time (min)
Normal
Diabetic
Normal data

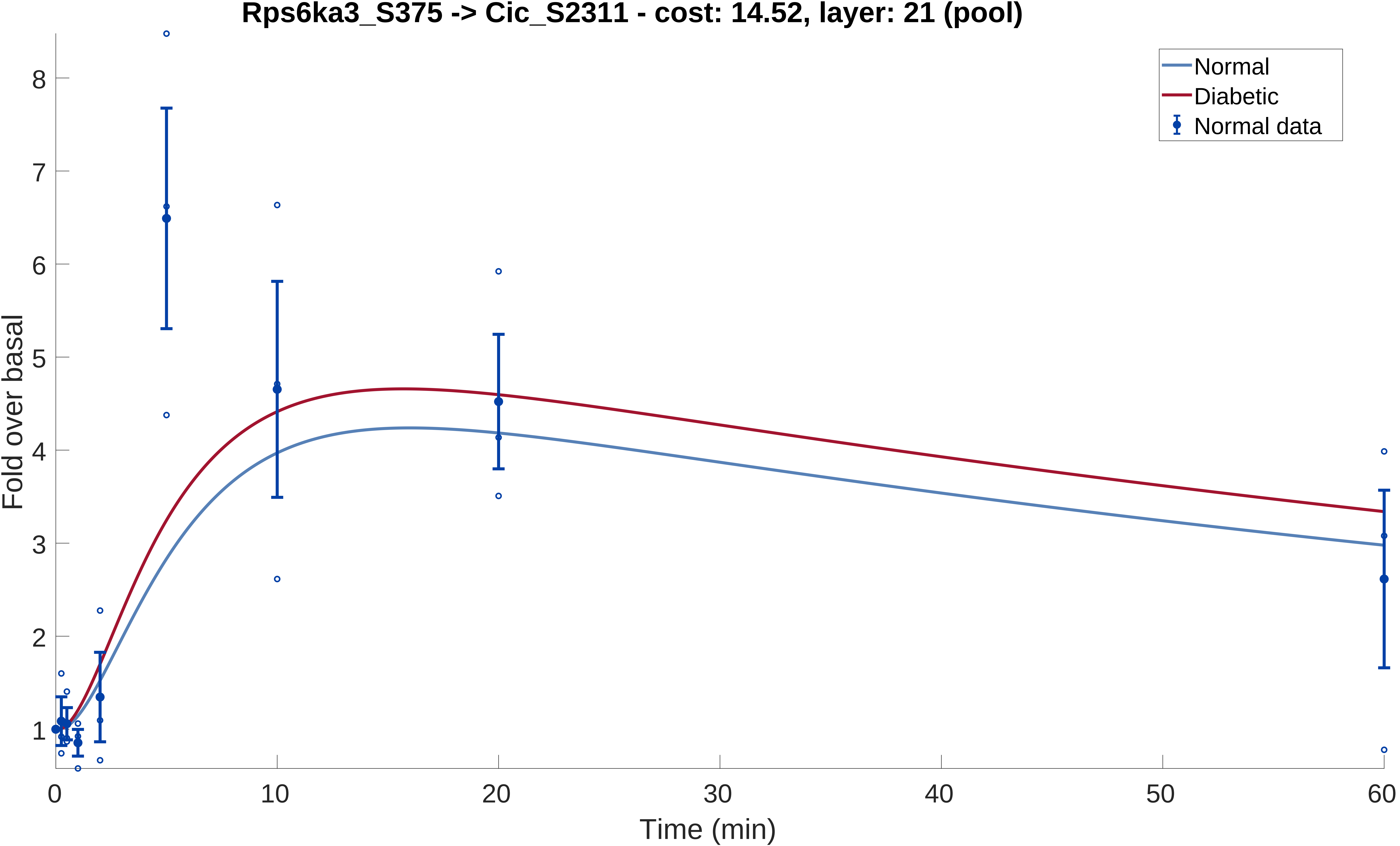

Rps6ka3_S375 -> Cic_S2311 - cost: 14.52, layer: 21 (pool)
Normal
Diabetic
Normal data
Fold over basal
Time (min)
1
2
3
4
5
6
7
8
0
10
20
30
40
50
60

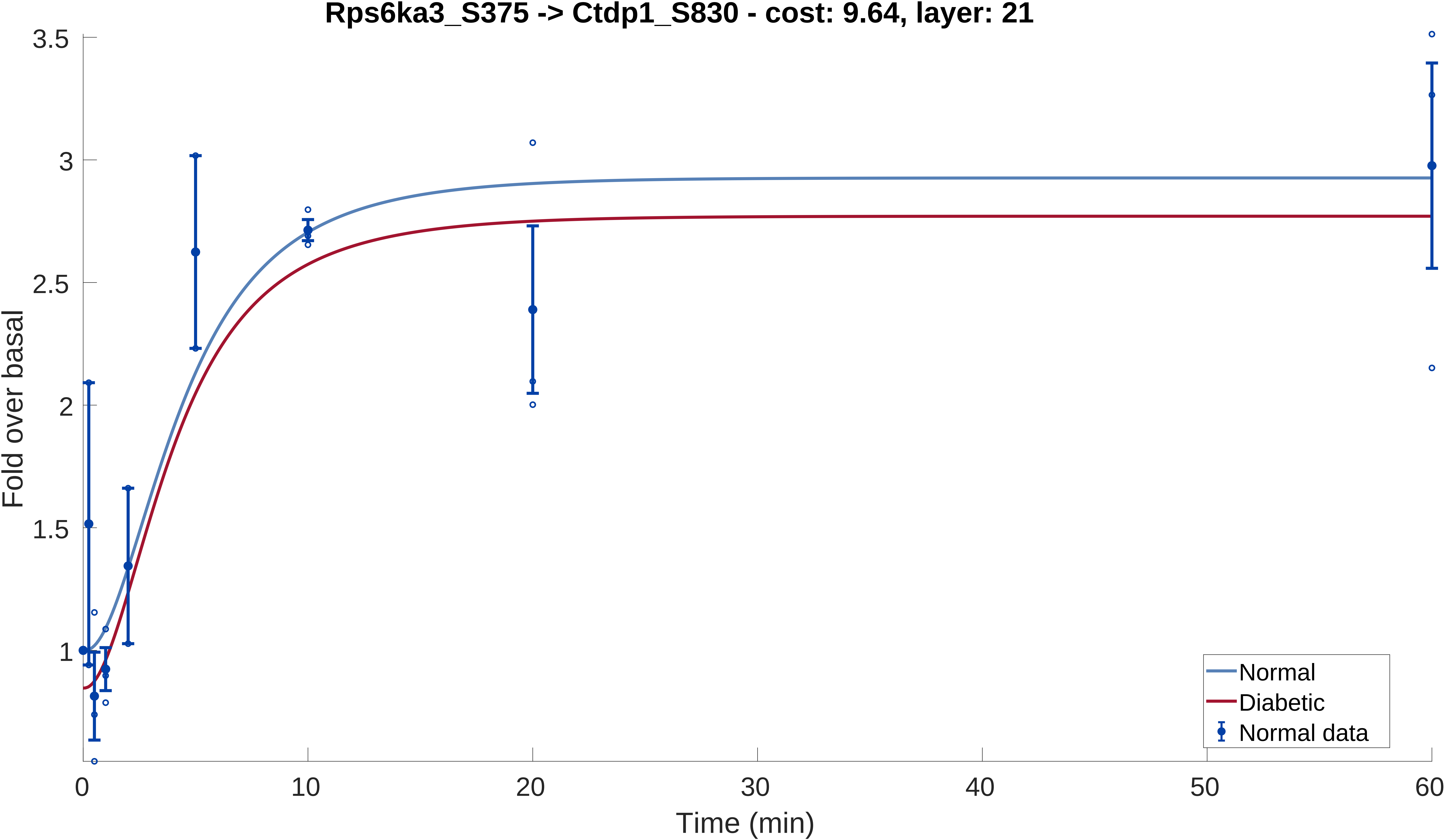
Rps6ka3_S375 -> Ctdp1_S830 - cost: 9.64, layer: 21
Fold over basal
3.5
3
2.5
2
1.5
1
0
10
20
30
40
50
60
Time (min)
Normal
Diabetic
Normal data

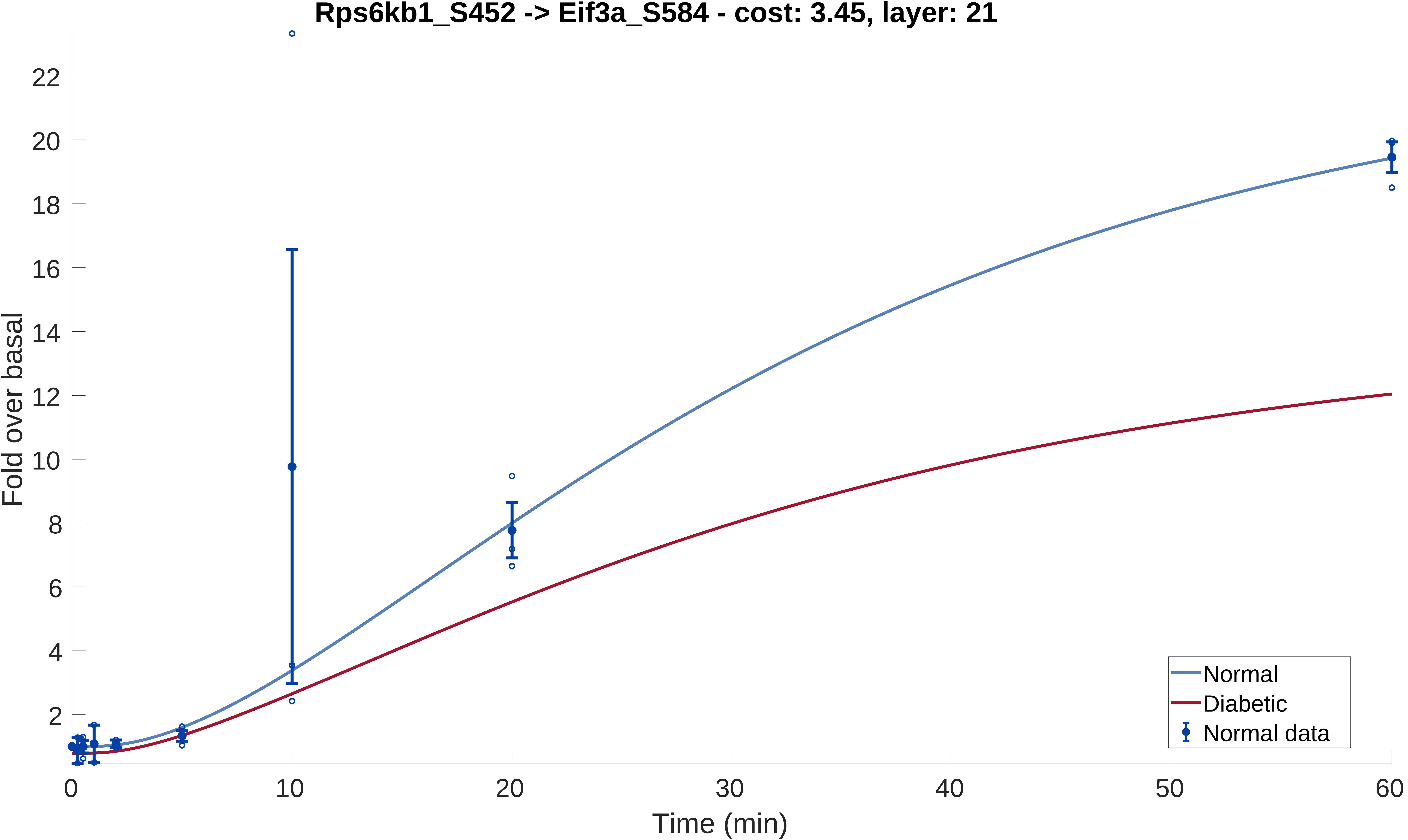

Rps6kb1_S452 -> Eif3a_S584 - cost: 3.45, layer: 21
Fold over basal
2
4
6
8
10
12
14
16
18
20
22
Time (min)
0
10
20
30
40
50
60
Normal
Diabetic
Normal data

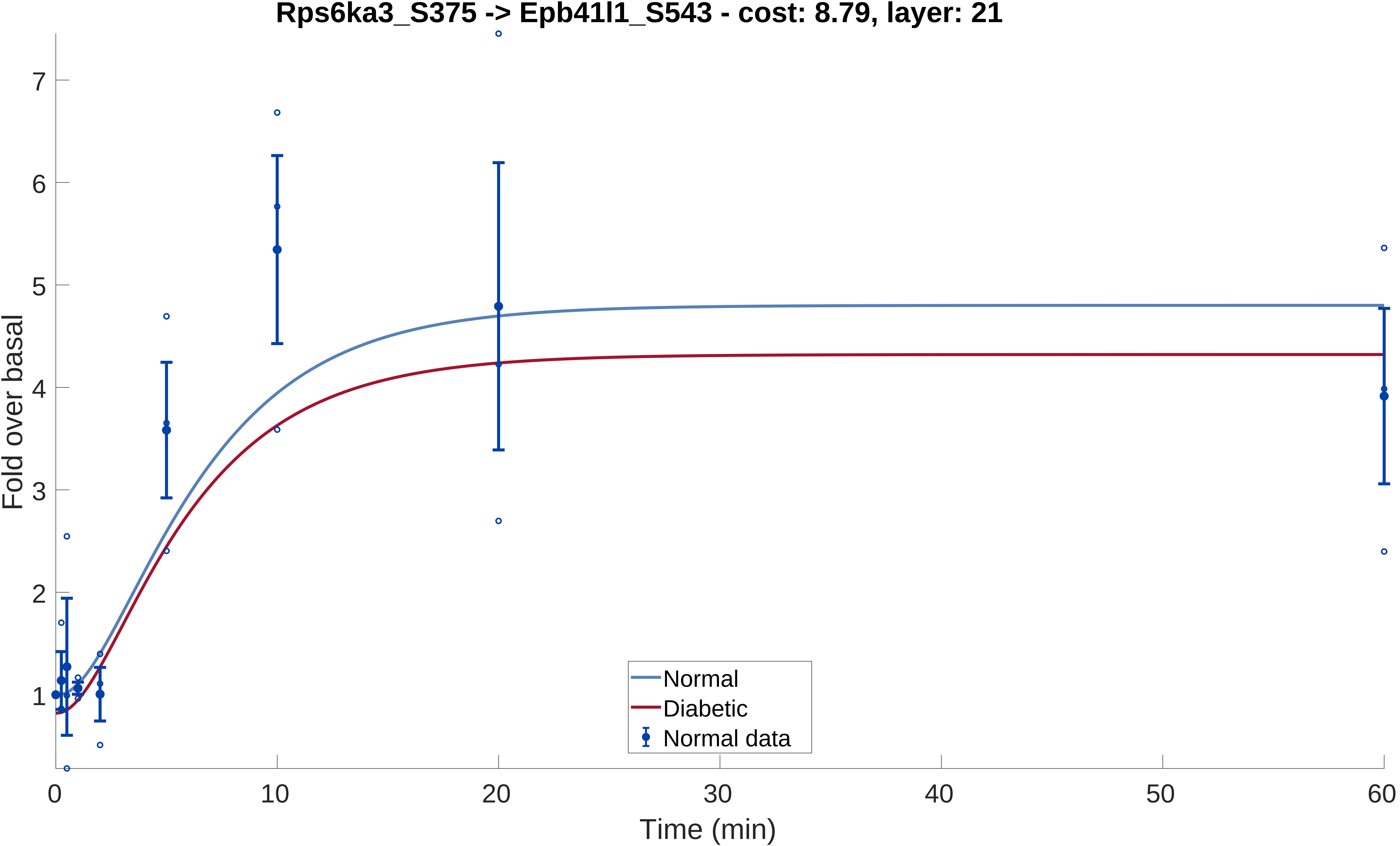
Rps6ka3_S375 -> Epb41l1_S543 - cost: 8.79, layer: 21
Fold over basal
Time (min)
Normal
Diabetic
Normal data
0
10
20
30
40
50
60
1
2
3
4
5
6
7

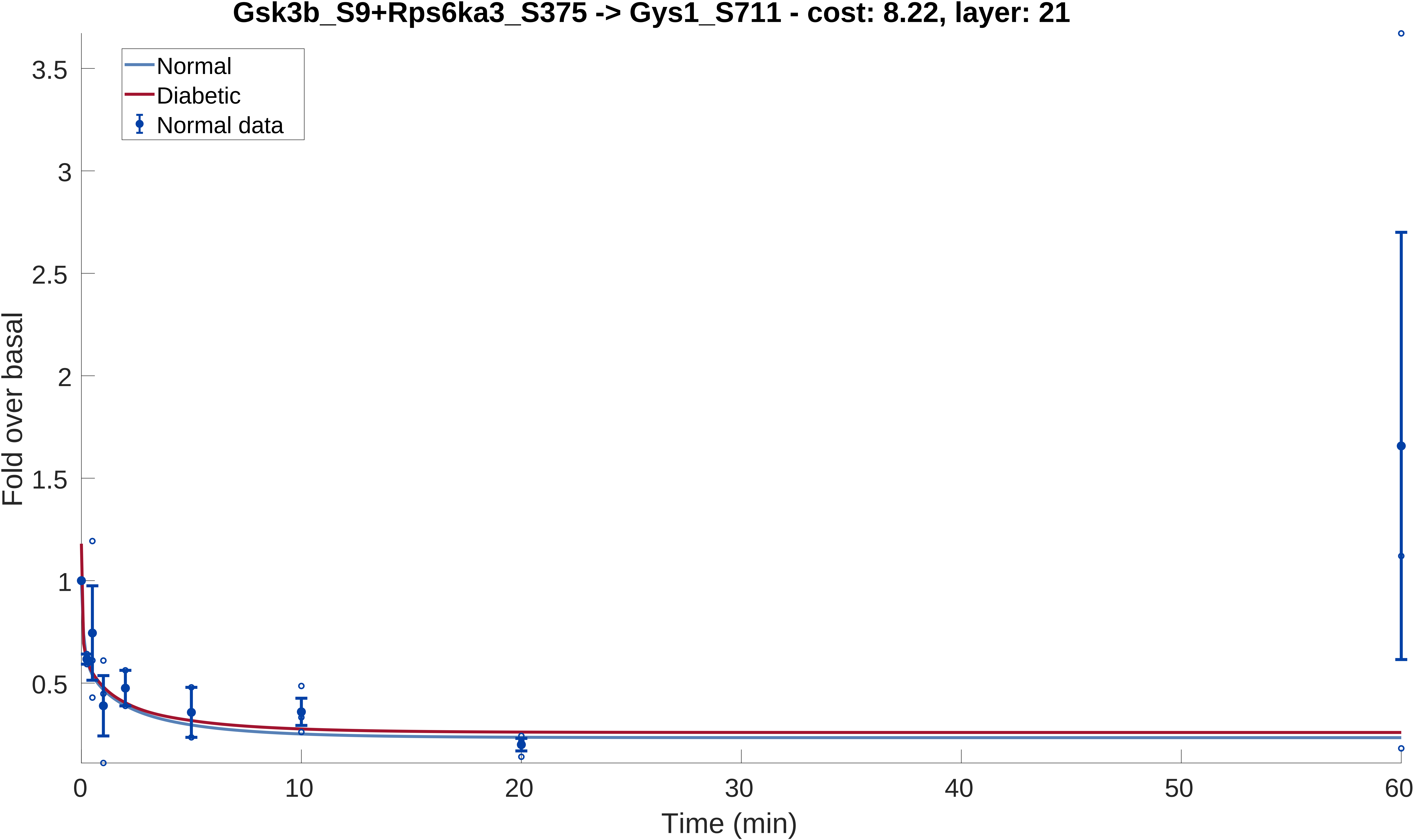

Gsk3b_S9+Rps6ka3_S375 -> Gys1_S711 - cost: 8.22, layer: 21
Normal
Diabetic
Normal data
Fold over basal
3.5
3
2.5
2
1.5
1
0.5
0
10
20
30
40
50
60
Time (min)

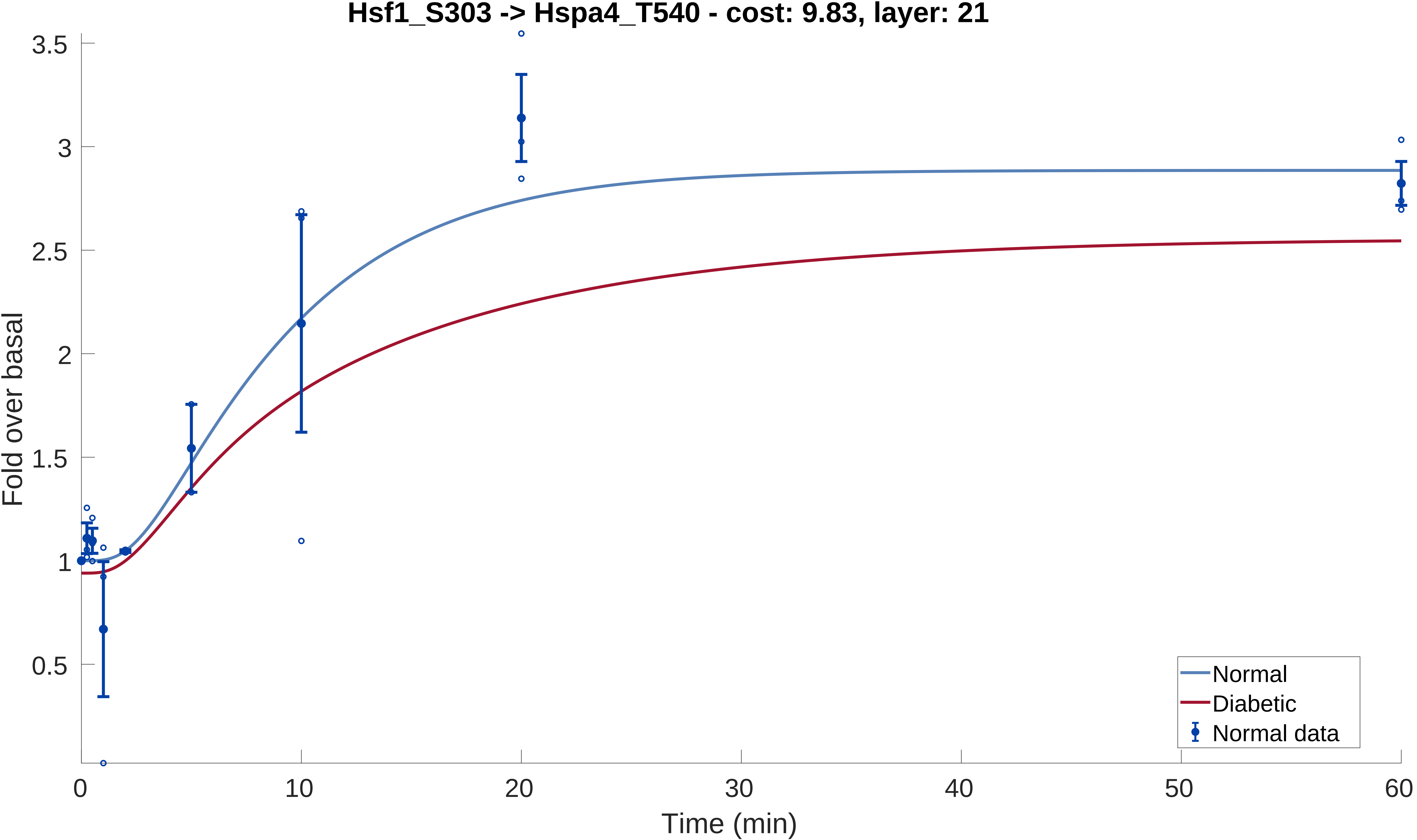

Hsf1_S303 -> Hspa4_T540 - cost: 9.83, layer: 21
Fold over basal
3.5
3
2.5
2
1.5
1
0.5
0
10
20
30
40
50
60
Time (min)
Normal
Diabetic
Normal data

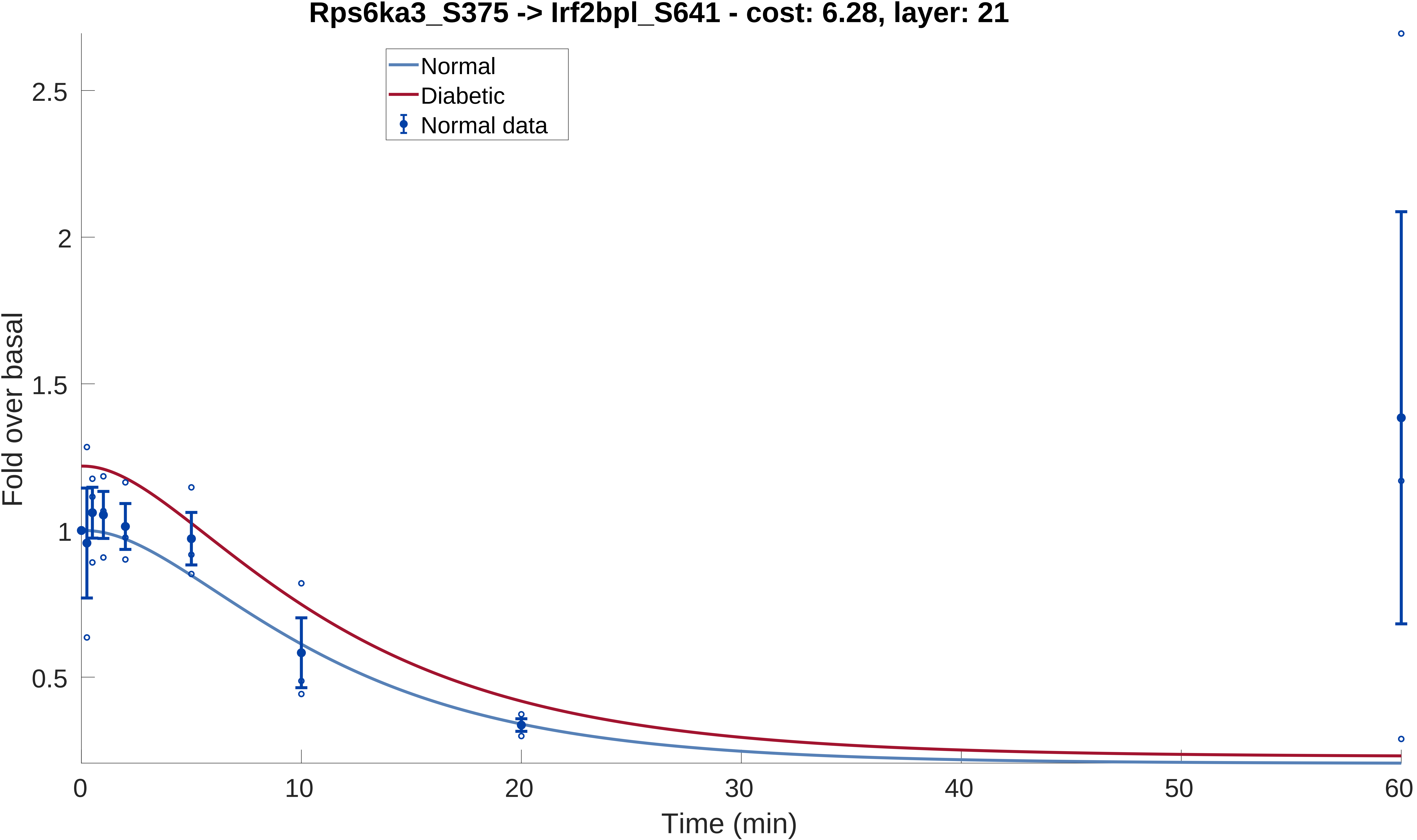

Rps6ka3_S375 -> Irf2bpl_S641 - cost: 6.28, layer: 21
Normal
Diabetic
Normal data
Fold over basal
2.5
2
1.5
1
0.5
0
10
20
30
40
50
60
Time (min)

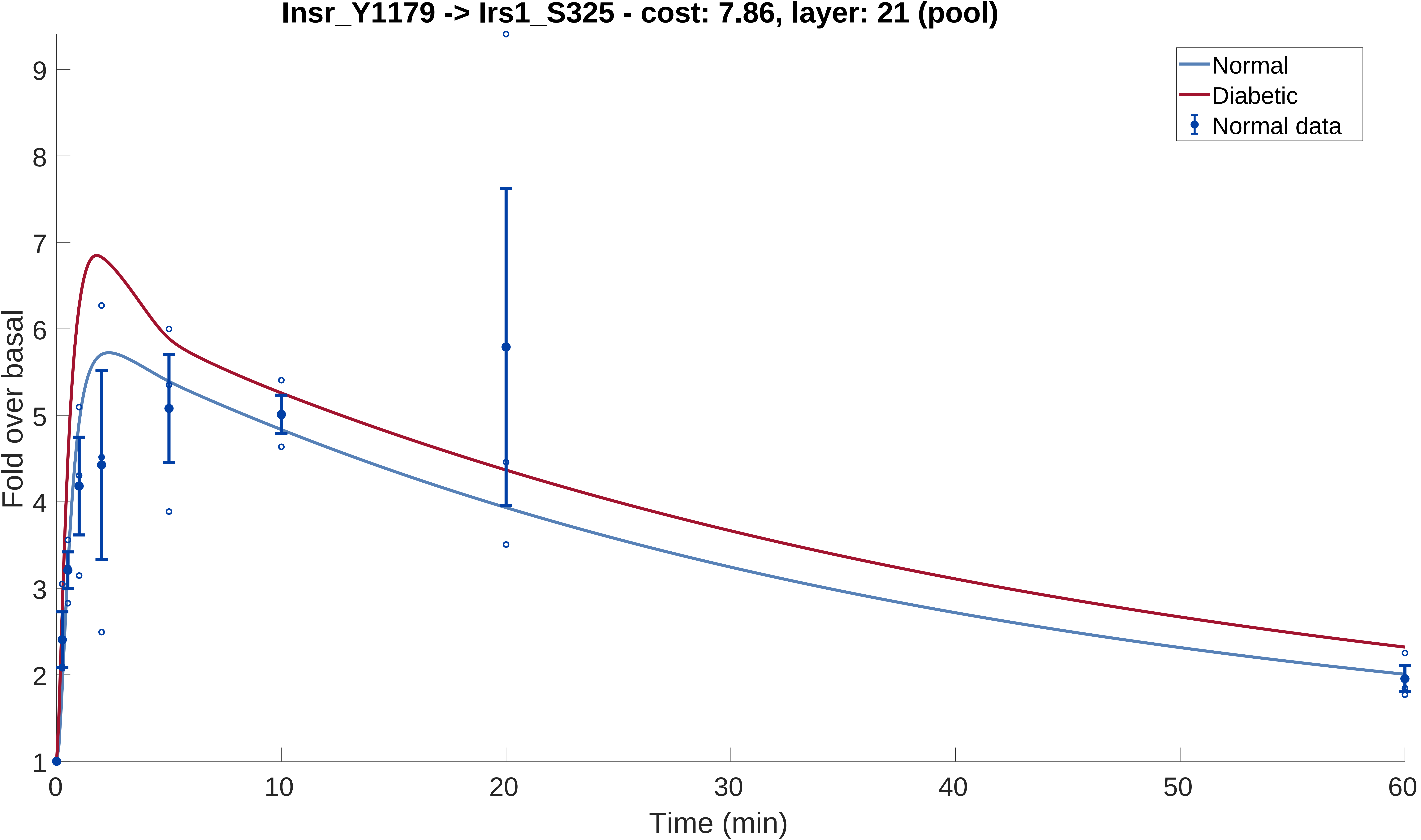
Insr_Y1179 -> Irs1_S325 - cost: 7.86, layer: 21 (pool)
Normal
Diabetic
Normal data
Fold over basal
Time (min)
1
2
3
4
5
6
7
8
9
0
10
20
30
40
50
60

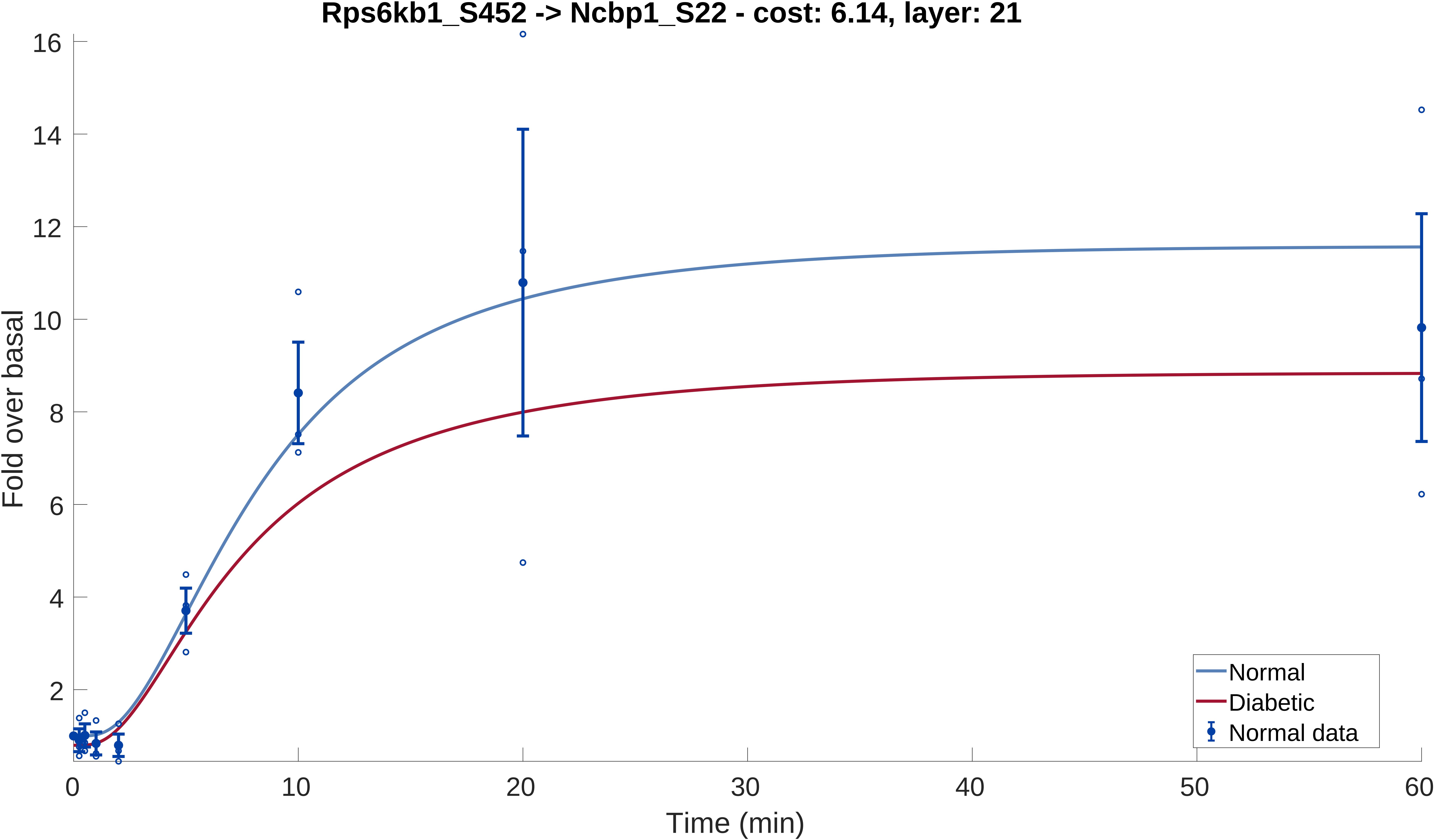

Rps6kb1_S452 -> Ncbp1_S22 - cost: 6.14, layer: 21
Fold over basal
16
14
12
10
8
6
4
2
0
10
20
30
40
50
60
Time (min)
Normal
Diabetic
Normal data

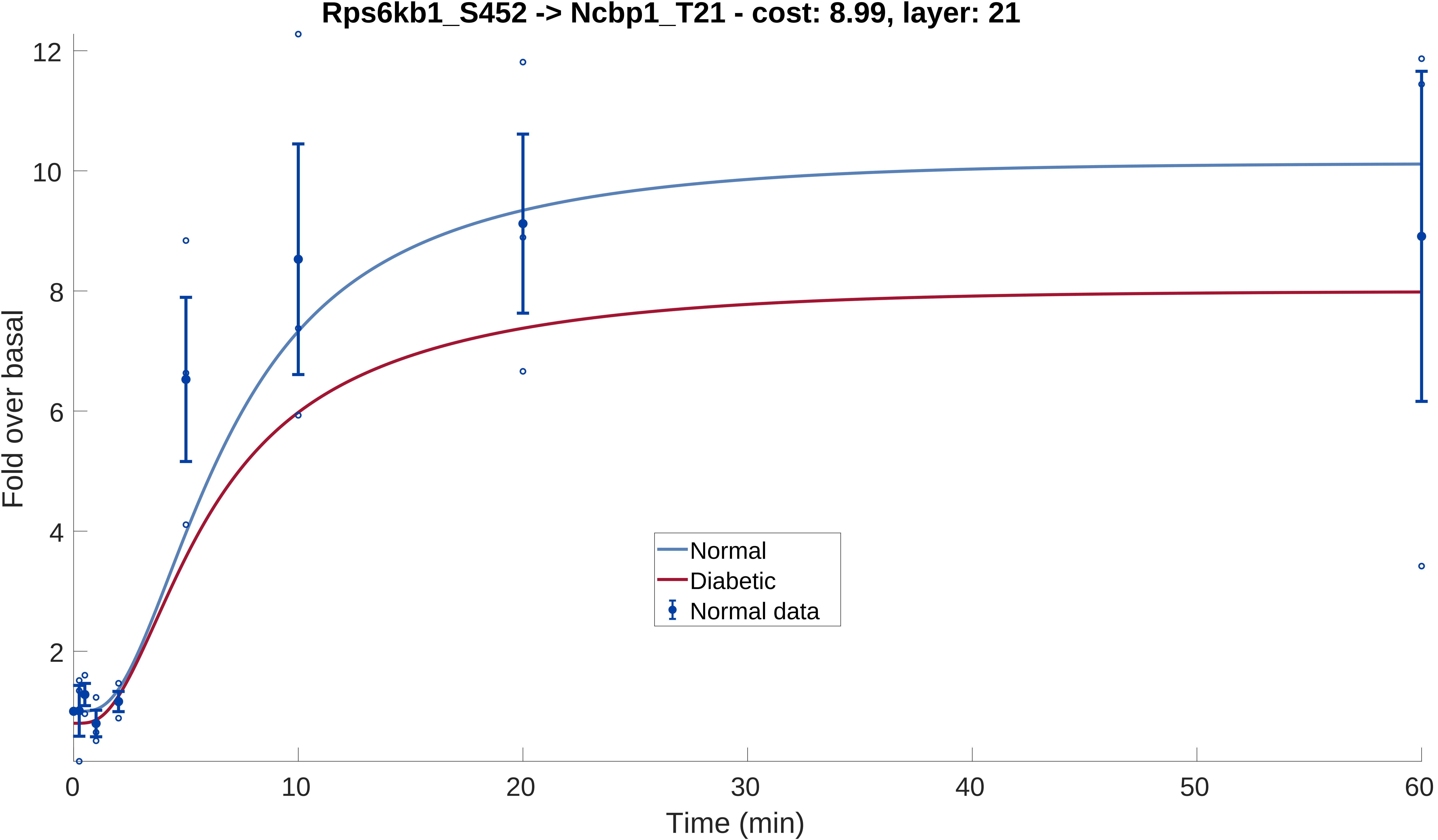

Rps6kb1_S452 -> Ncbp1_T21 - cost: 8.99, layer: 21
Fold over basal
12
10
8
6
4
2
0
10
20
30
40
50
60
Time (min)
Normal
Diabetic
Normal data

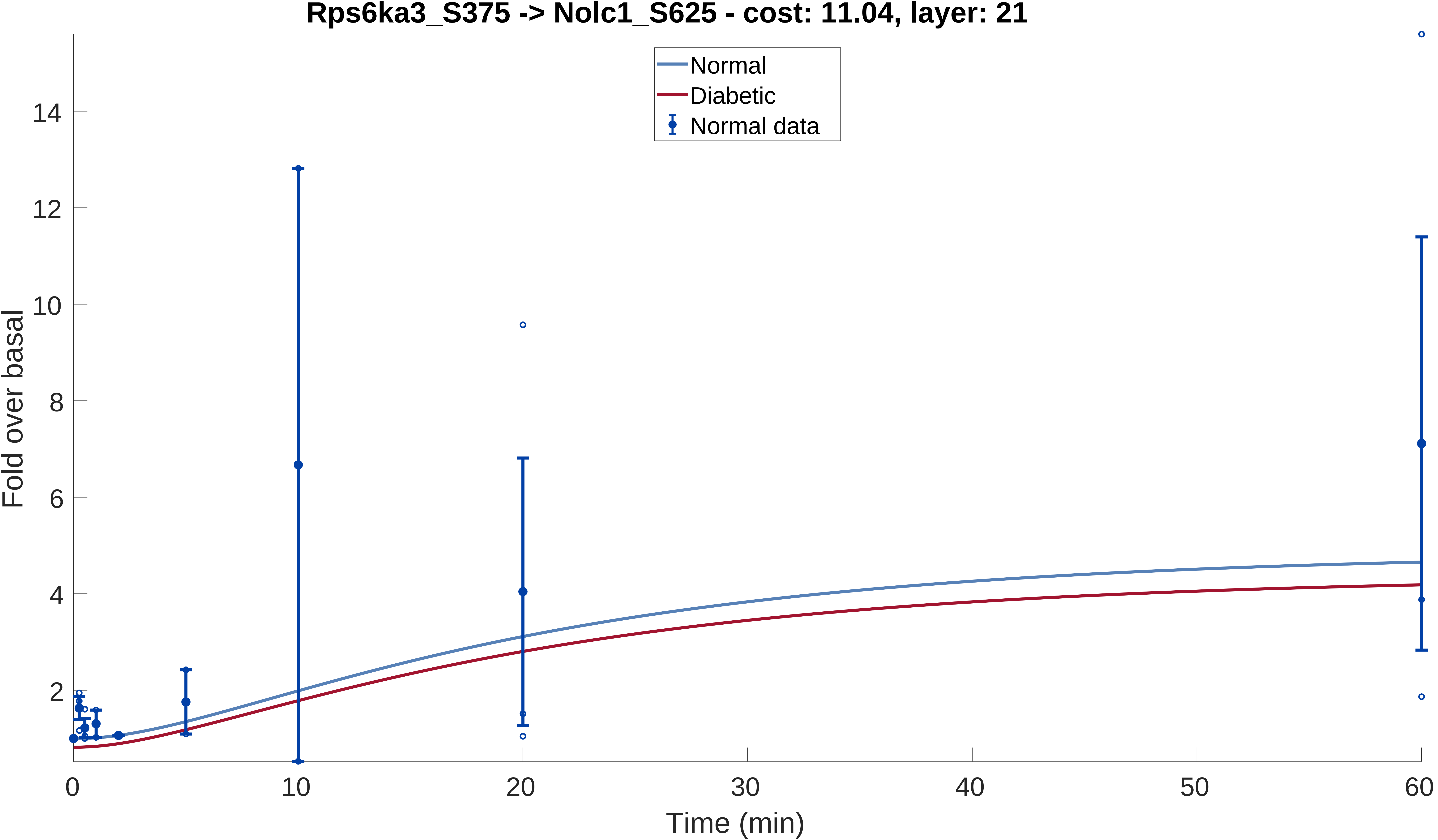

Rps6ka3_S375 -> Nolc1_S625 - cost: 11.04, layer: 21
Normal
Diabetic
Normal data
Fold over basal
14
12
10
8
6
4
2
0
10
20
30
40
50
60
Time (min)

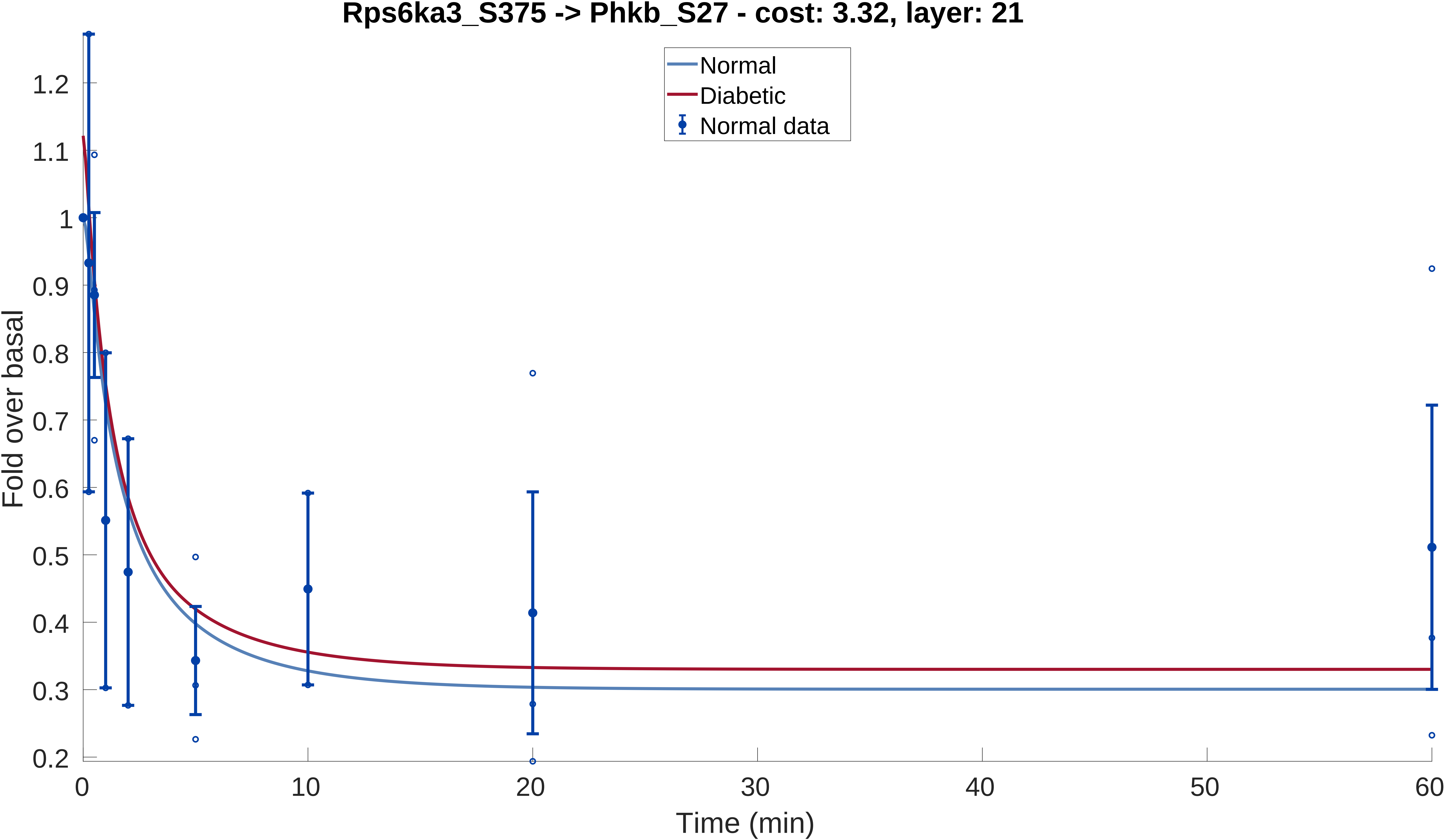

Rps6ka3_S375 -> Phkb_S27 - cost: 3.32, layer: 21
Normal
Diabetic
Normal data
Fold over basal
1.2
1.1
1
0.9
0.8
0.7
0.6
0.5
0.4
0.3
0.2
0
10
20
30
40
50
60
Time (min)

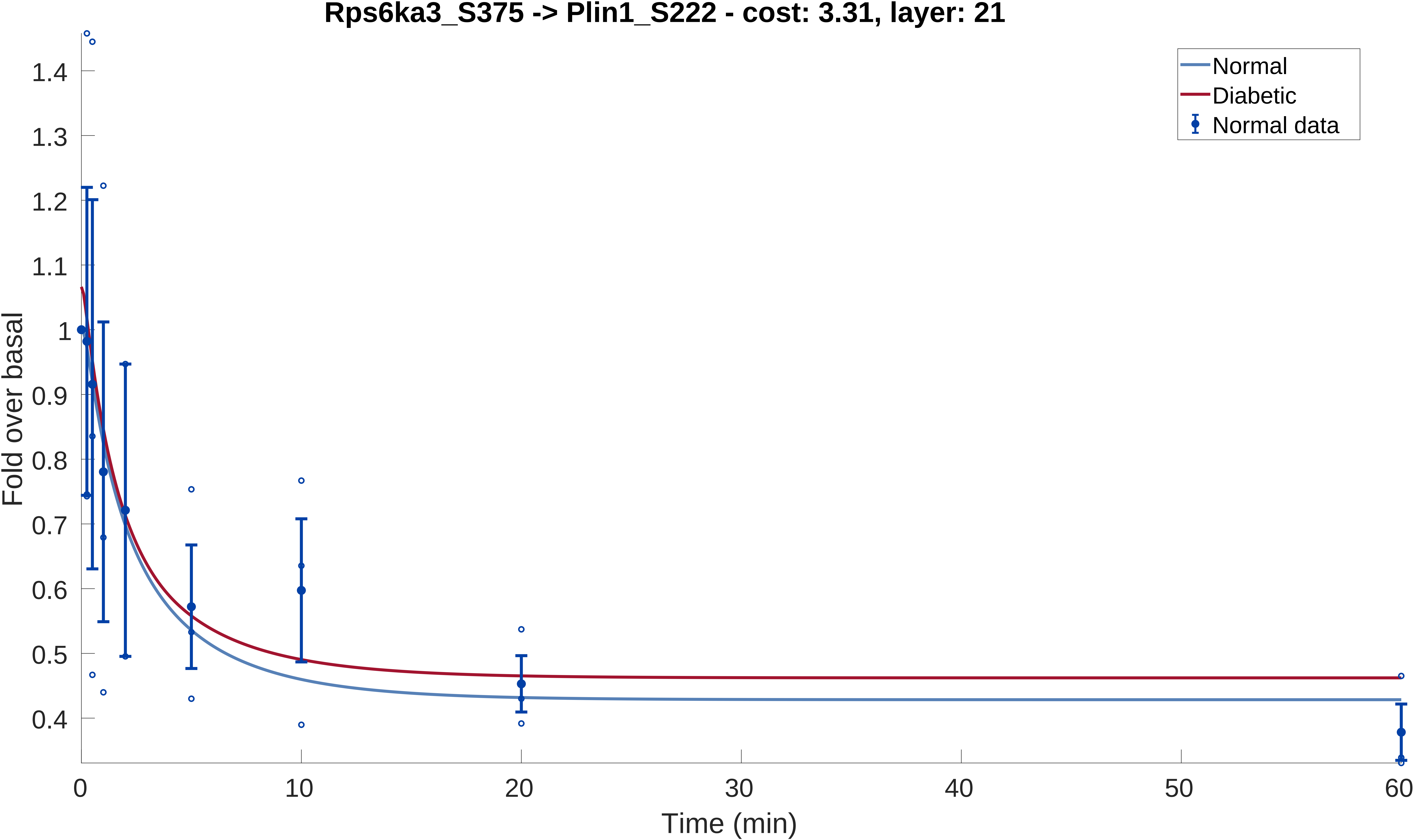

Rps6ka3_S375 -> Plin1_S222 - cost: 3.31, layer: 21
Normal
Diabetic
Normal data
Fold over basal
1.4
1.3
1.2
1.1
1
0.9
0.8
0.7
0.6
0.5
0.4
0
10
20
30
40
50
60
Time (min)

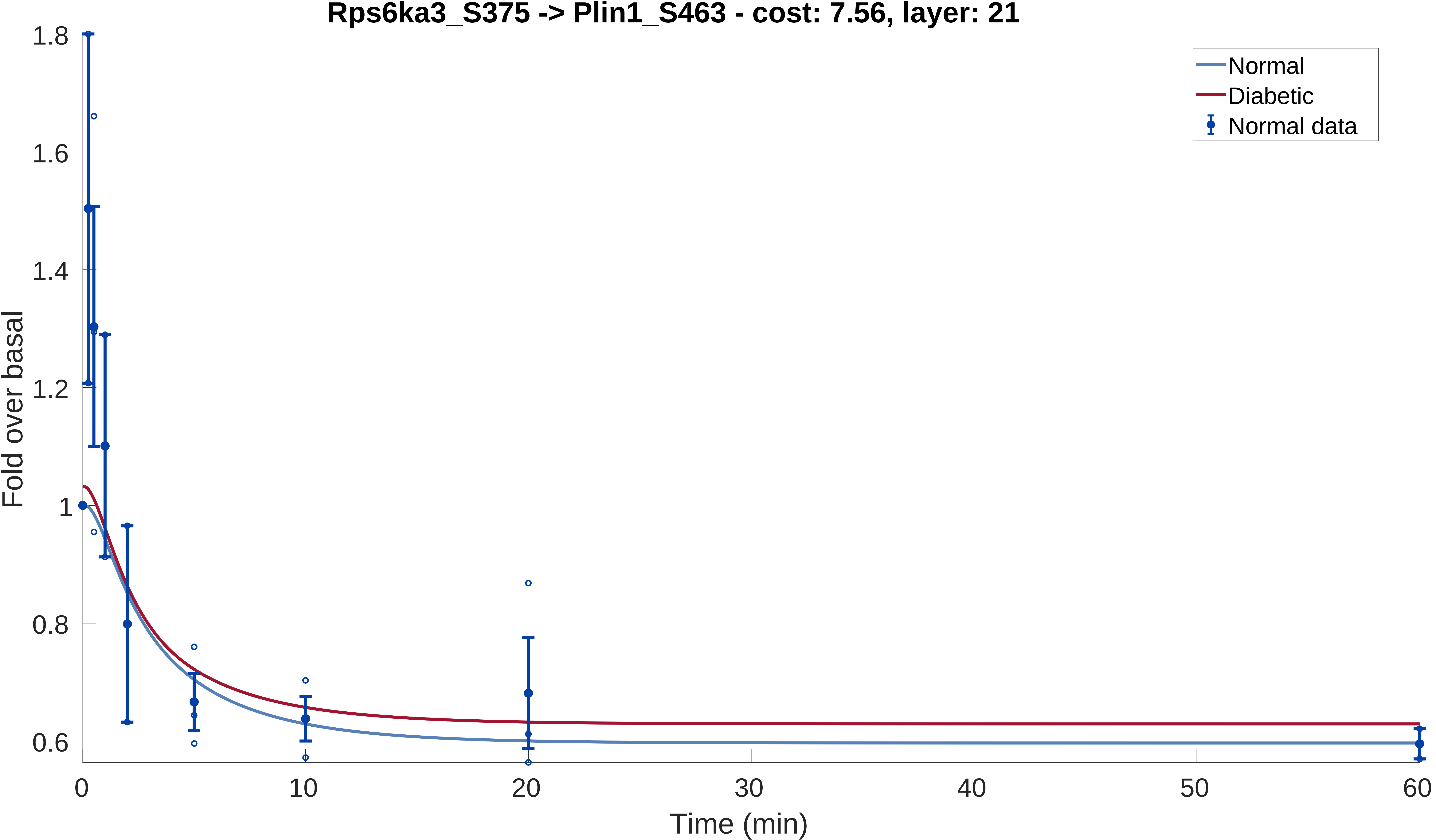

Rps6ka3_S375 -> Plin1_S463 - cost: 7.56, layer: 21
Normal
Diabetic
Normal data
Fold over basal
1.8
1.6
1.4
1.2
1
0.8
0.6
0
10
20
30
40
50
60
Time (min)

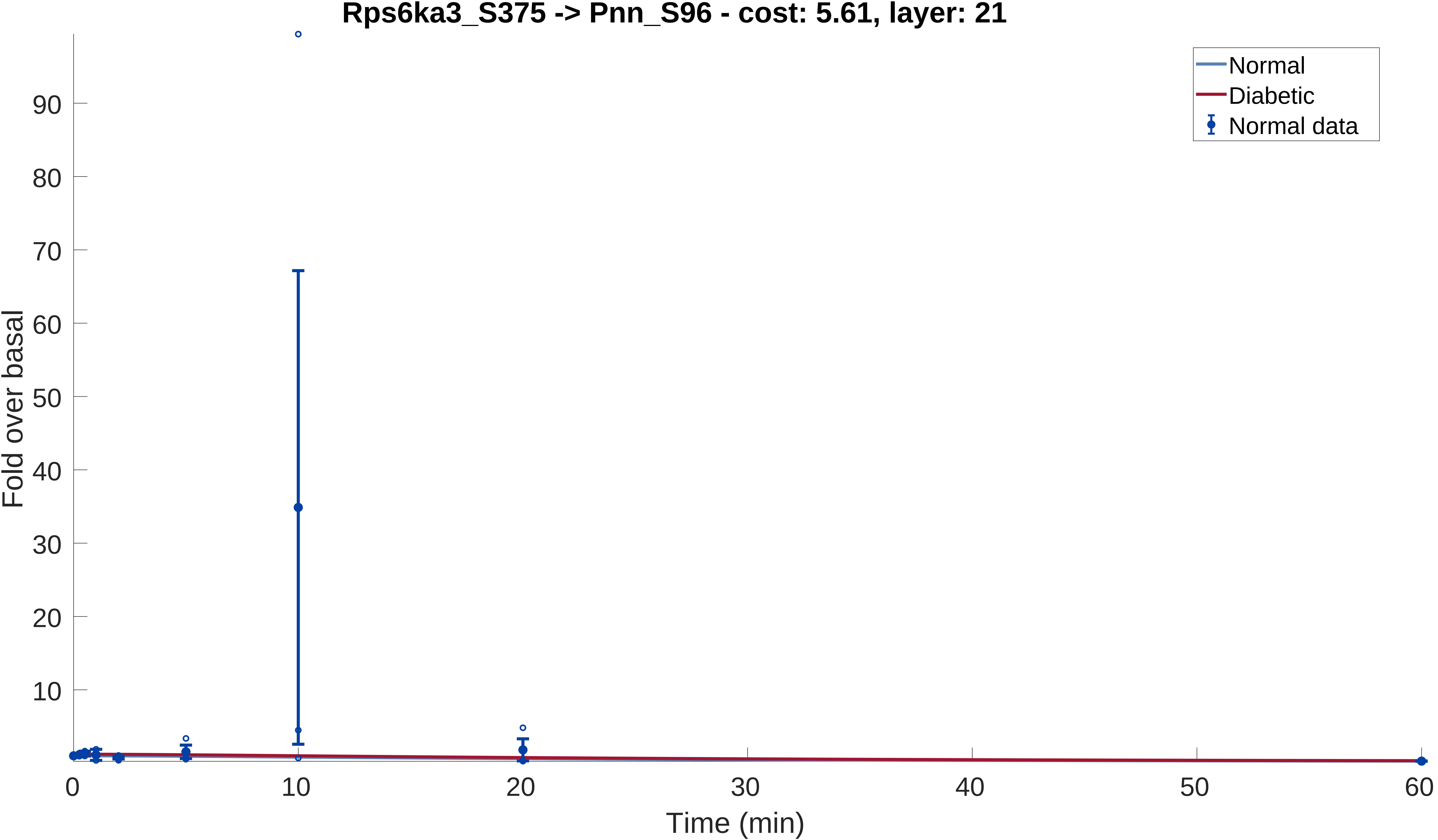
Rps6ka3_S375 -> Pnn_S96 - cost: 5.61, layer: 21
Normal
Diabetic
Normal data
Fold over basal
90
80
70
60
50
40
30
20
10
0
10
20
30
40
50
60
Time (min)

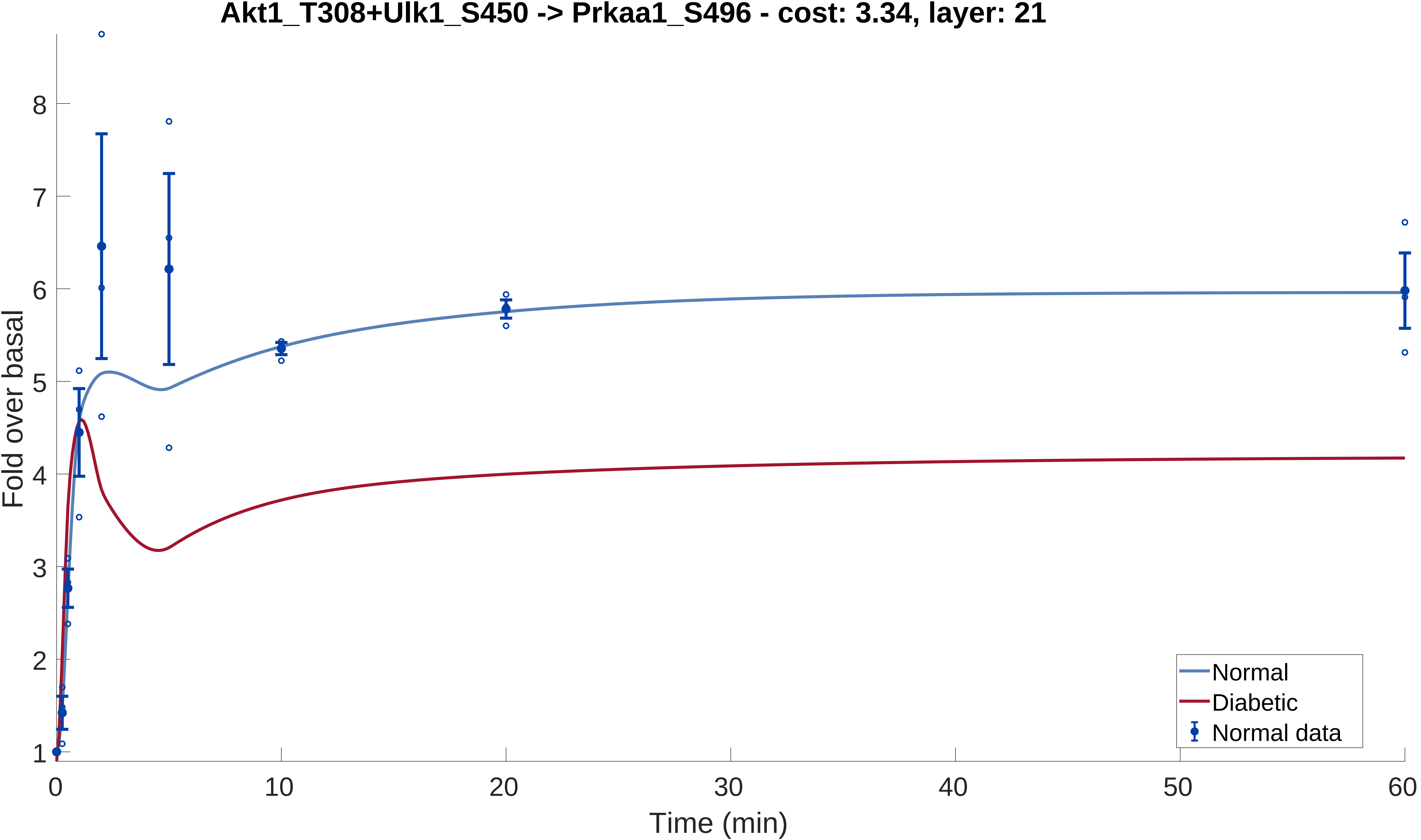

Akt1_T308+Ulk1_S450 -> Prkaa1_S496 - cost: 3.34, layer: 21
Fold over basal
Time (min)
0
10
20
30
40
50
60
1
2
3
4
5
6
7
8
Normal
Diabetic
Normal data

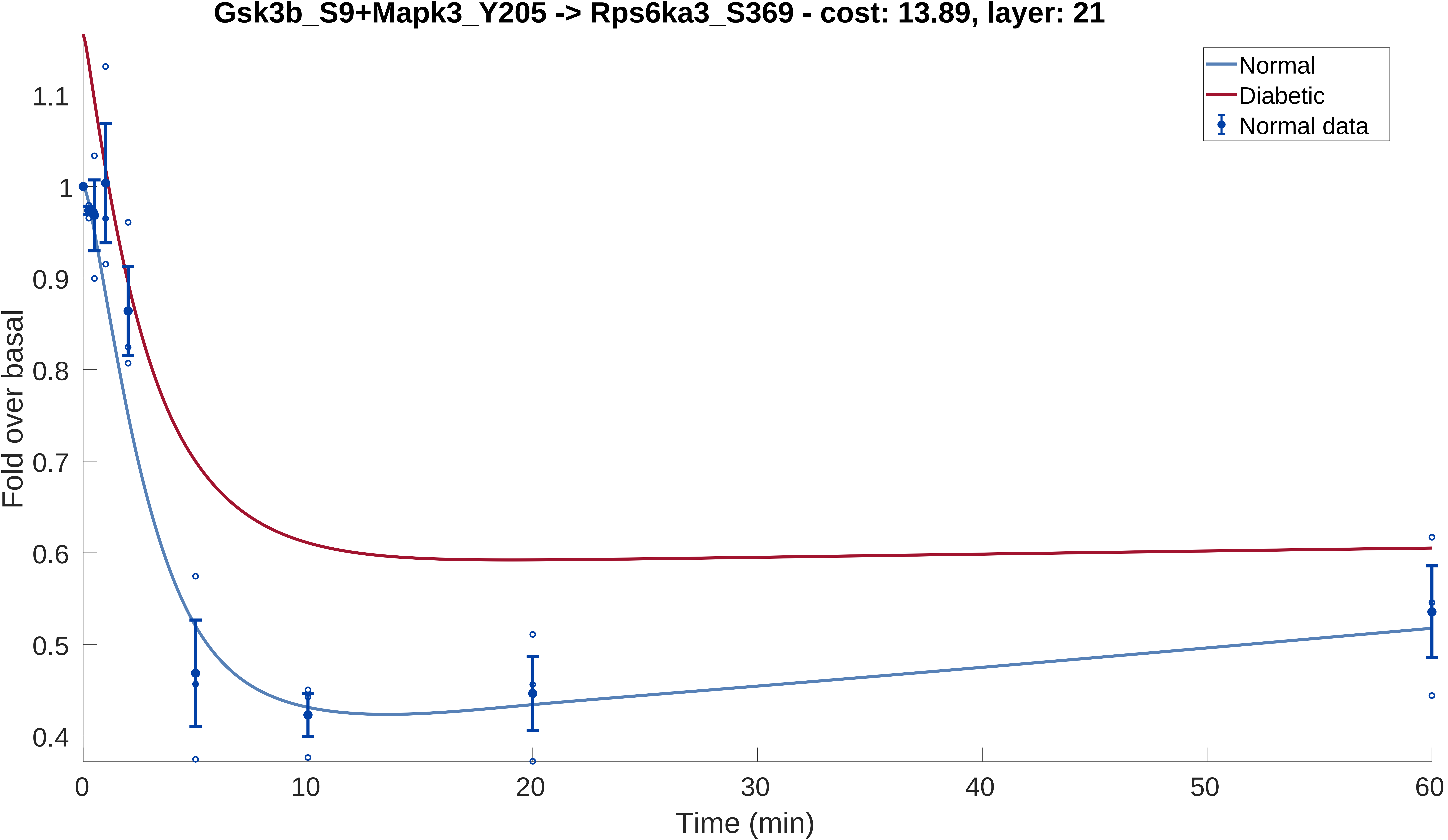
Gsk3b_S9+Mapk3_Y205 -> Rps6ka3_S369 - cost: 13.89, layer: 21
Normal
Diabetic
Normal data
Fold over basal
1.1
1
0.9
0.8
0.7
0.6
0.5
0.4
0
10
20
30
40
50
60
Time (min)

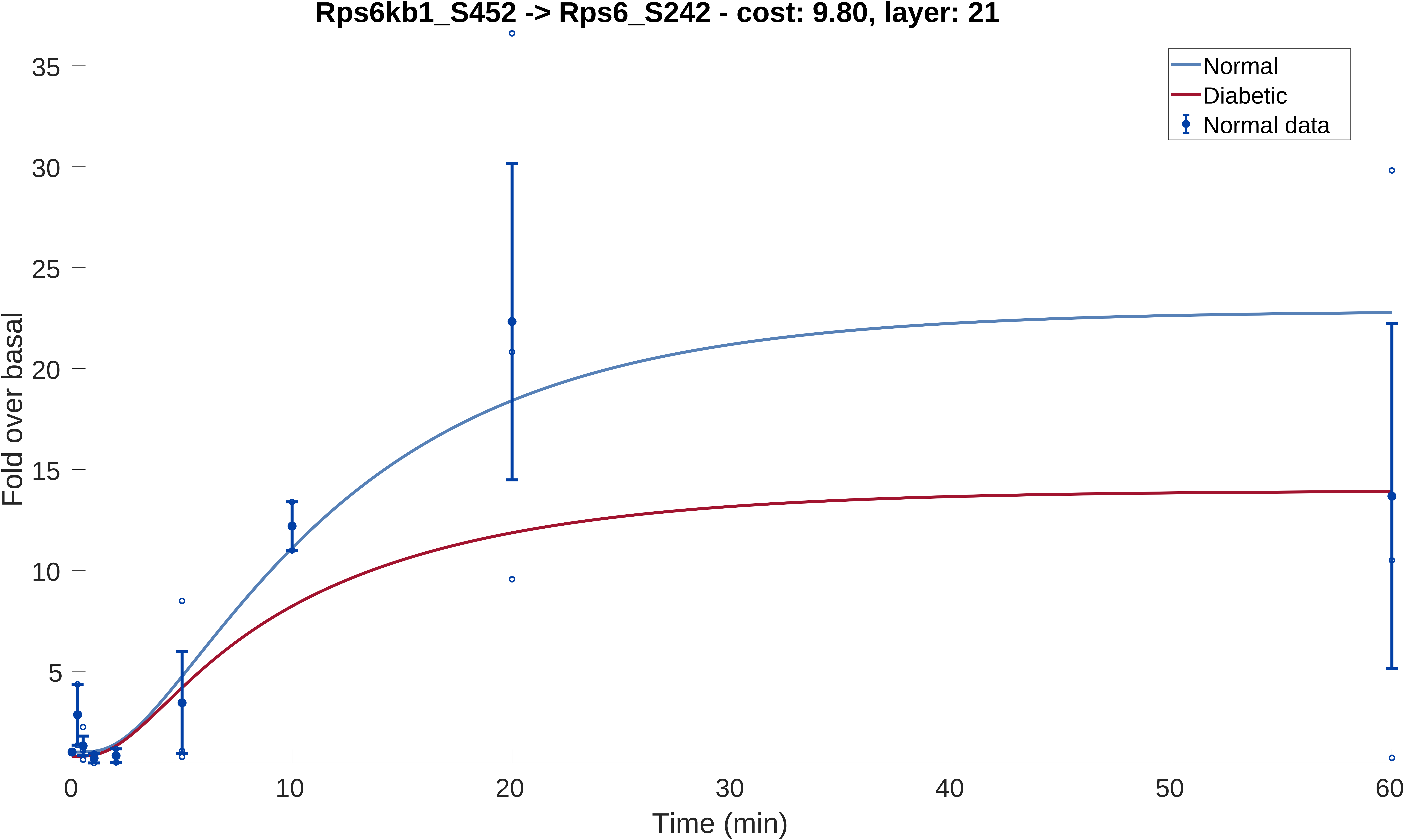

Rps6kb1_S452 -> Rps6_S242 - cost: 9.80, layer: 21
Normal
Diabetic
Normal data
Fold over basal
Time (min)
0
10
20
30
40
50
60
5
10
15
20
25
30
35

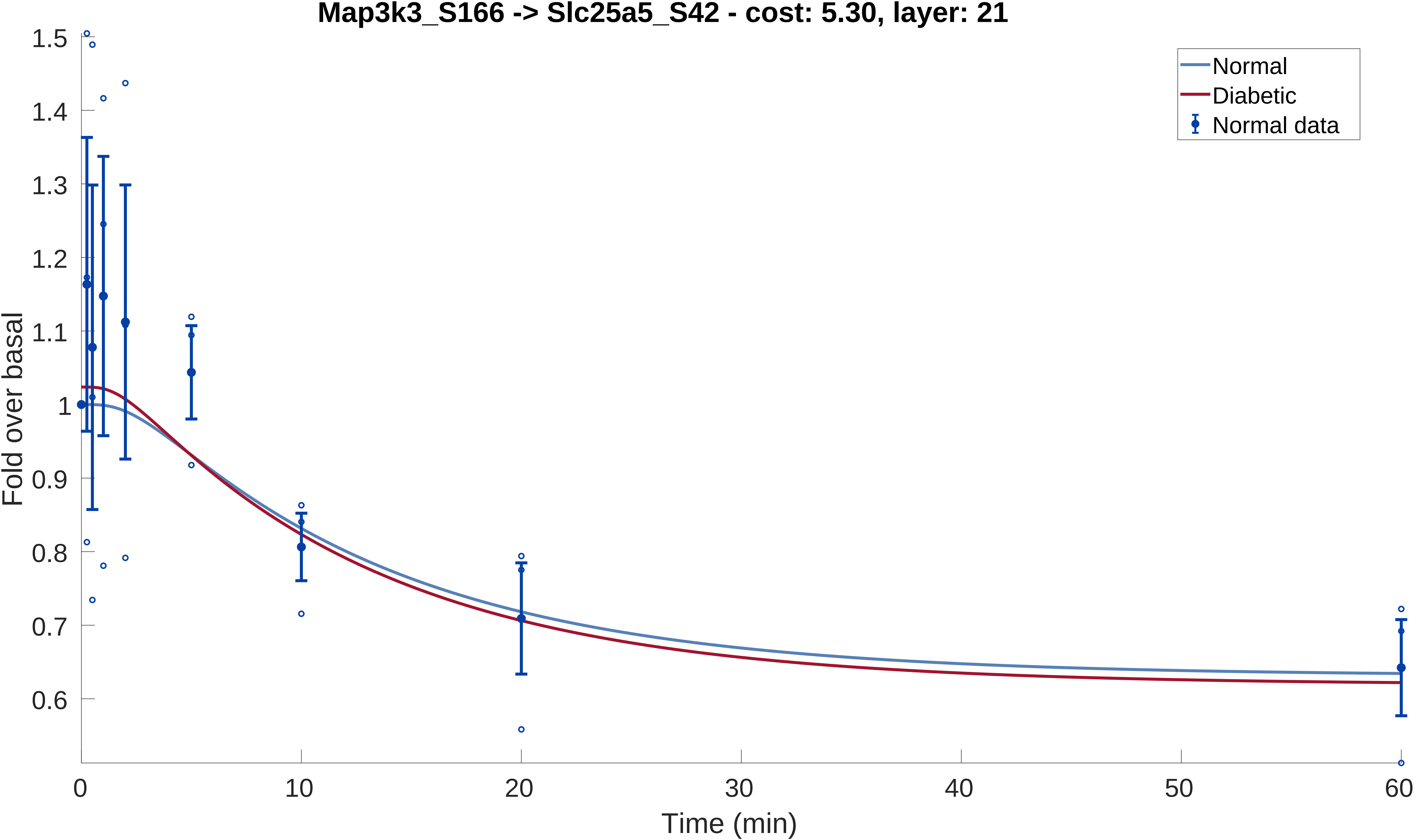
Map3k3_S166 -> Slc25a5_S42 - cost: 5.30, layer: 21
Normal
Diabetic
Normal data
Fold over basal
Time (min)
1.5
1.4
1.3
1.2
1.1
1
0.9
0.8
0.7
0.6
0
10
20
30
40
50
60

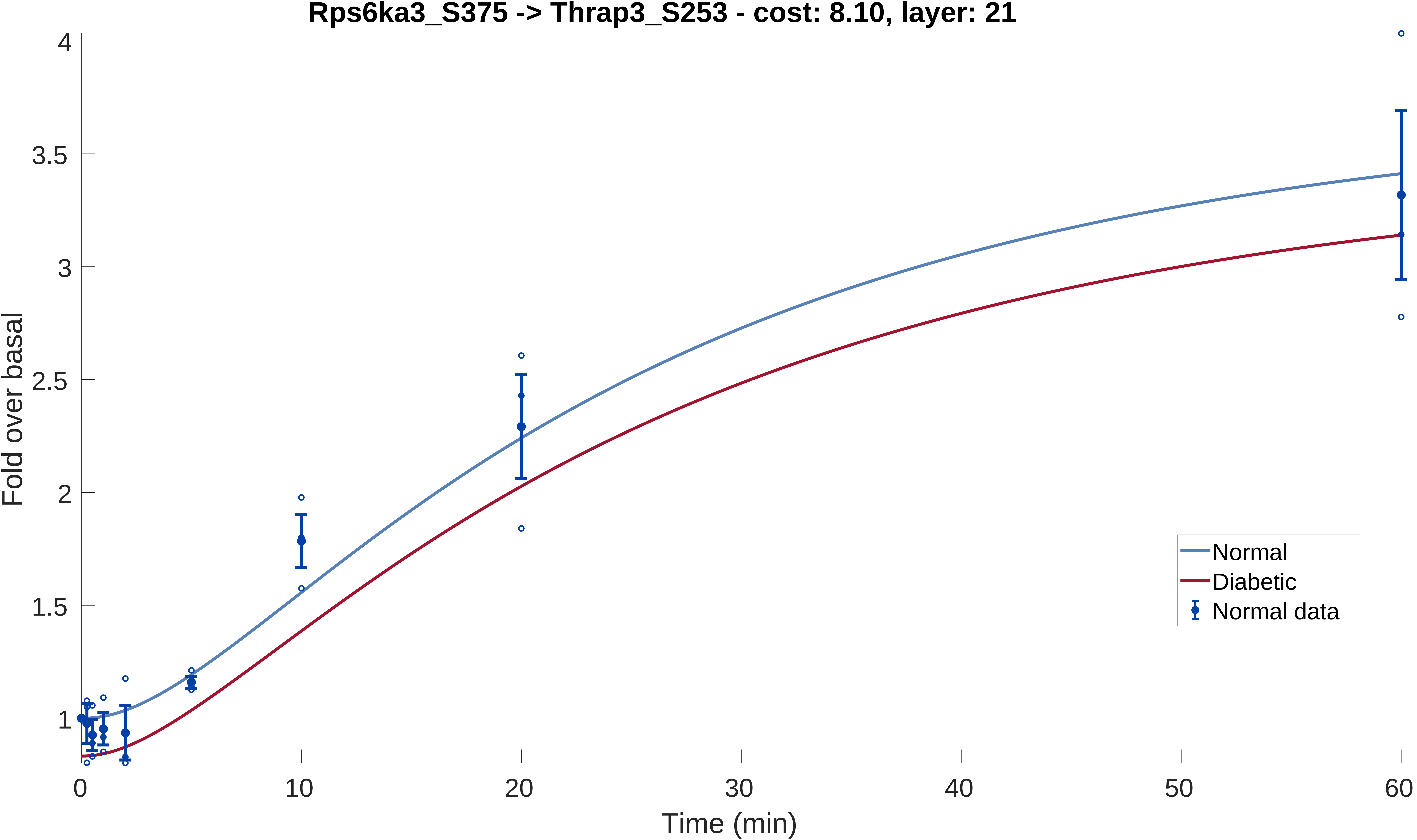

Rps6ka3_S375 -> Thrap3_S253 - cost: 8.10, layer: 21
Fold over basal
Time (min)
0
10
20
30
40
50
60
1
1.5
2
2.5
3
3.5
4
Normal
Diabetic
Normal data

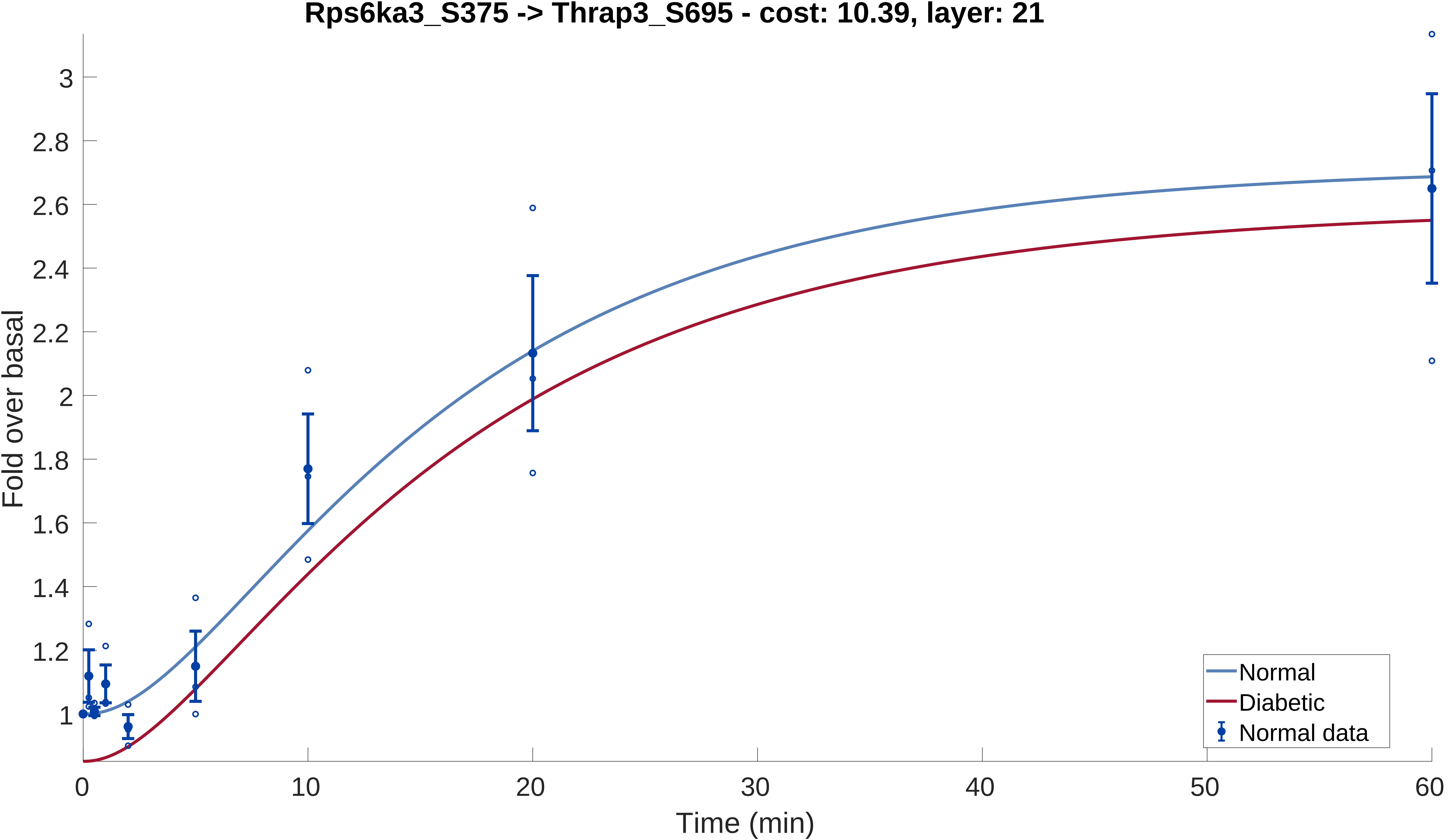

Rps6ka3_S375 -> Thrap3_S695 - cost: 10.39, layer: 21
Fold over basal
3
2.8
2.6
2.4
2.2
2
1.8
1.6
1.4
1.2
1
0
10
20
30
40
50
60
Time (min)
Normal
Diabetic
Normal data

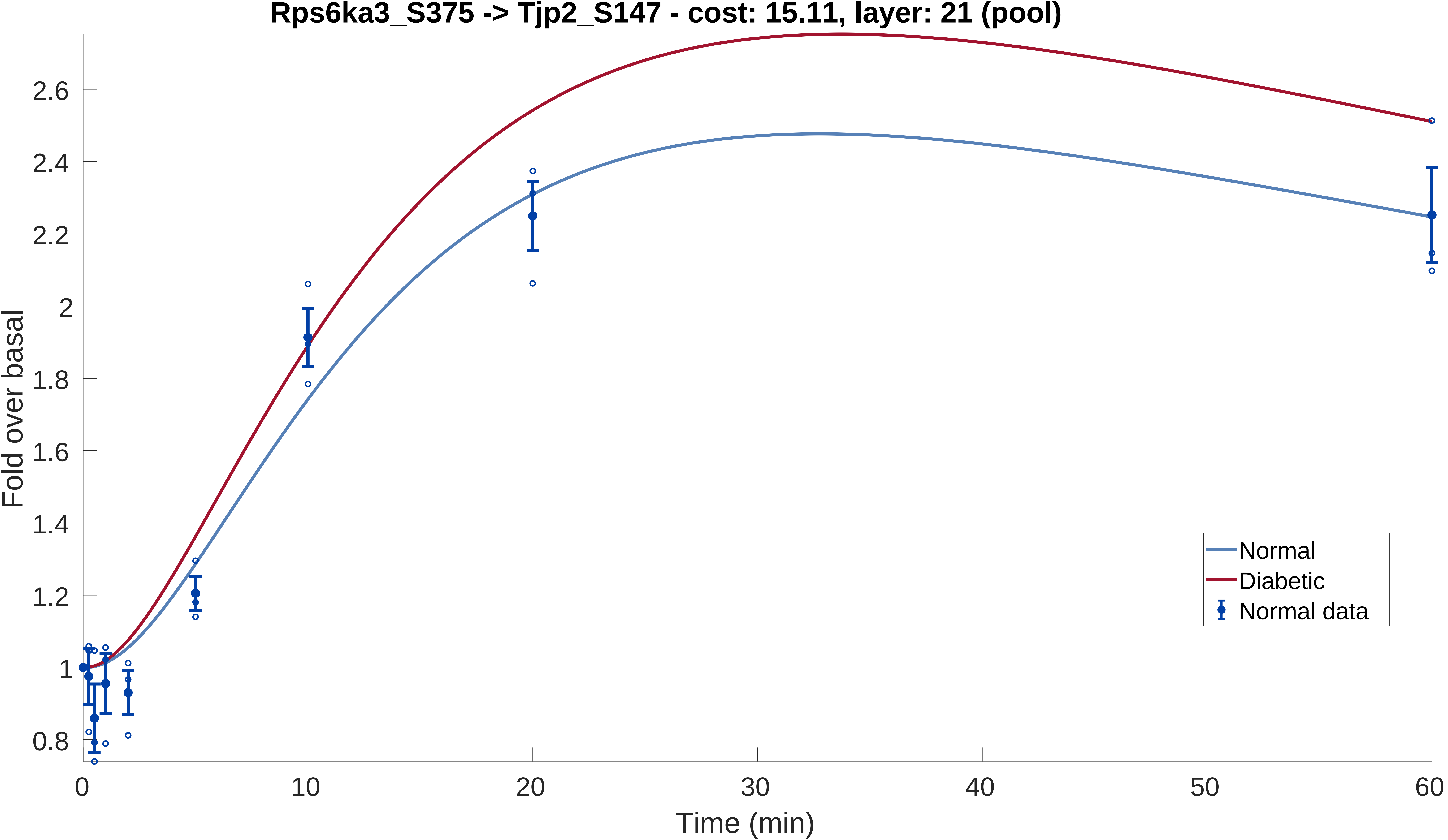

Rps6ka3_S375 -> Tjp2_S147 - cost: 15.11, layer: 21 (pool)
Fold over basal
2.6
2.4
2.2
2
1.8
1.6
1.4
1.2
1
0.8
0
10
20
30
40
50
60
Time (min)
Normal
Diabetic
Normal data

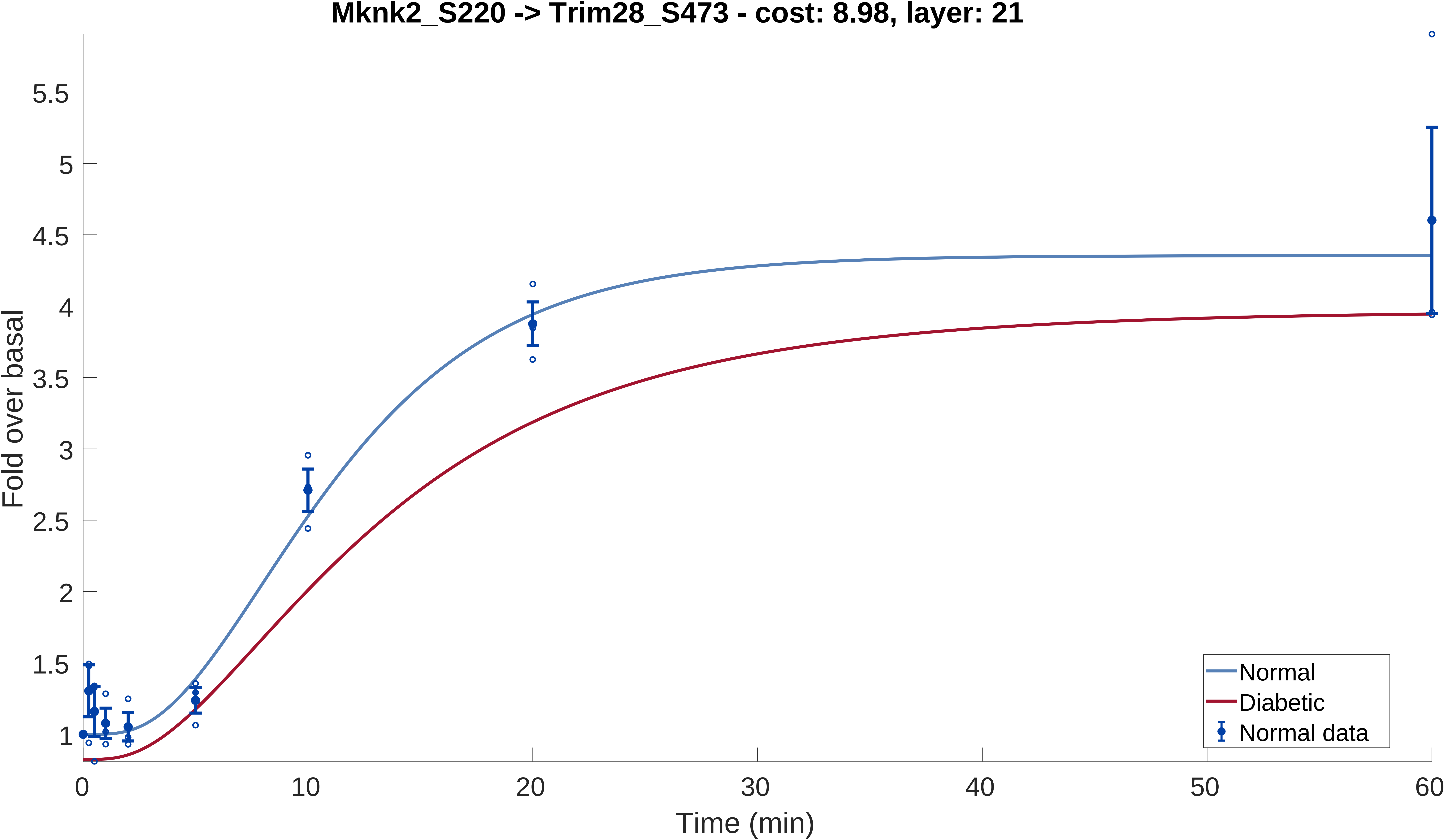

Mknk2_S220 -> Trim28_S473 - cost: 8.98, layer: 21
Fold over basal
5.5
5
4.5
4
3.5
3
2.5
2
1.5
1
0
10
20
30
40
50
60
Time (min)
Normal
Diabetic
Normal data

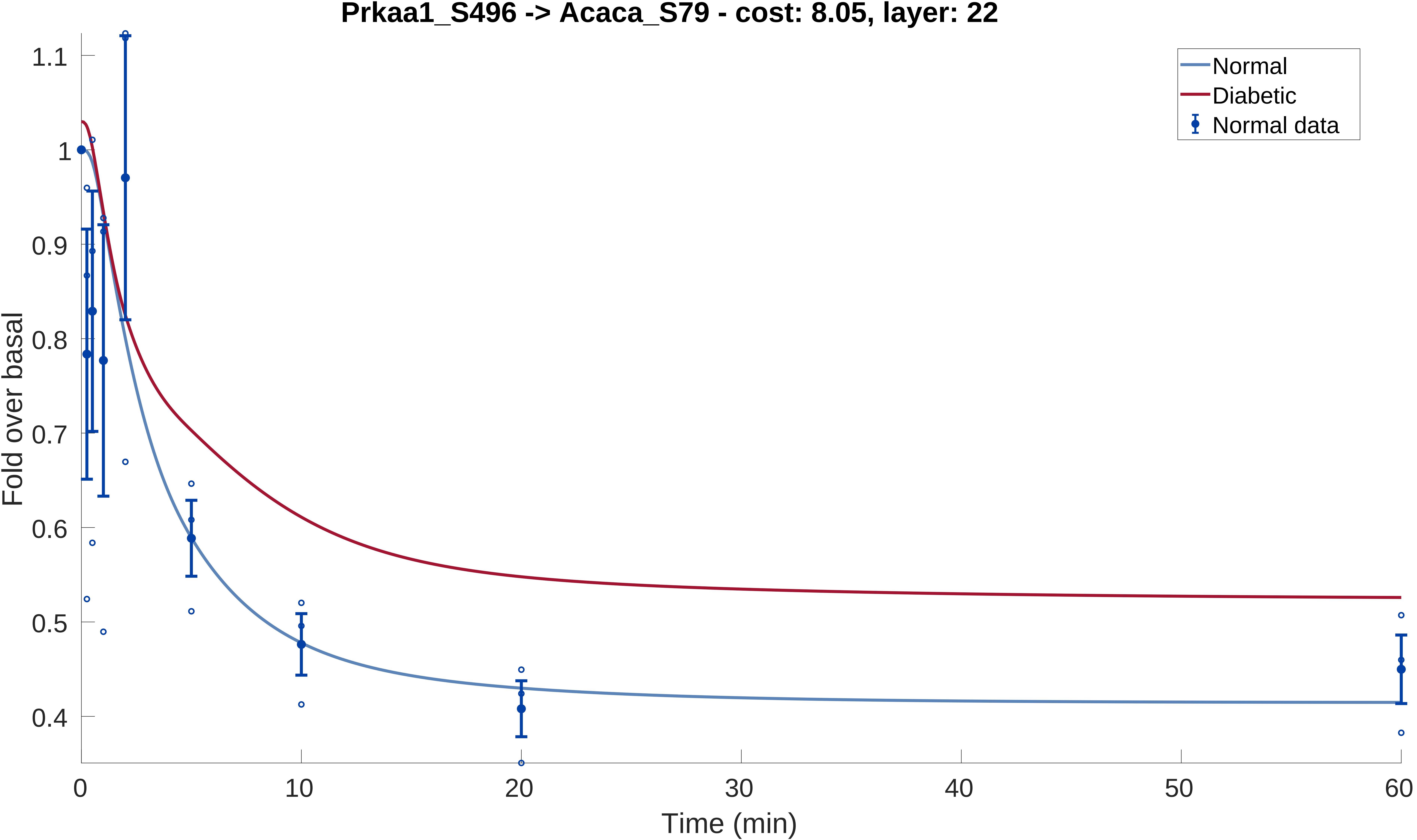

Prkaa1_S496 -> Acaca_S79 - cost: 8.05, layer: 22
Fold over basal
Time (min)
Normal
Diabetic
Normal data
1.1
1
0.9
0.8
0.7
0.6
0.5
0.4
0
10
20
30
40
50
60

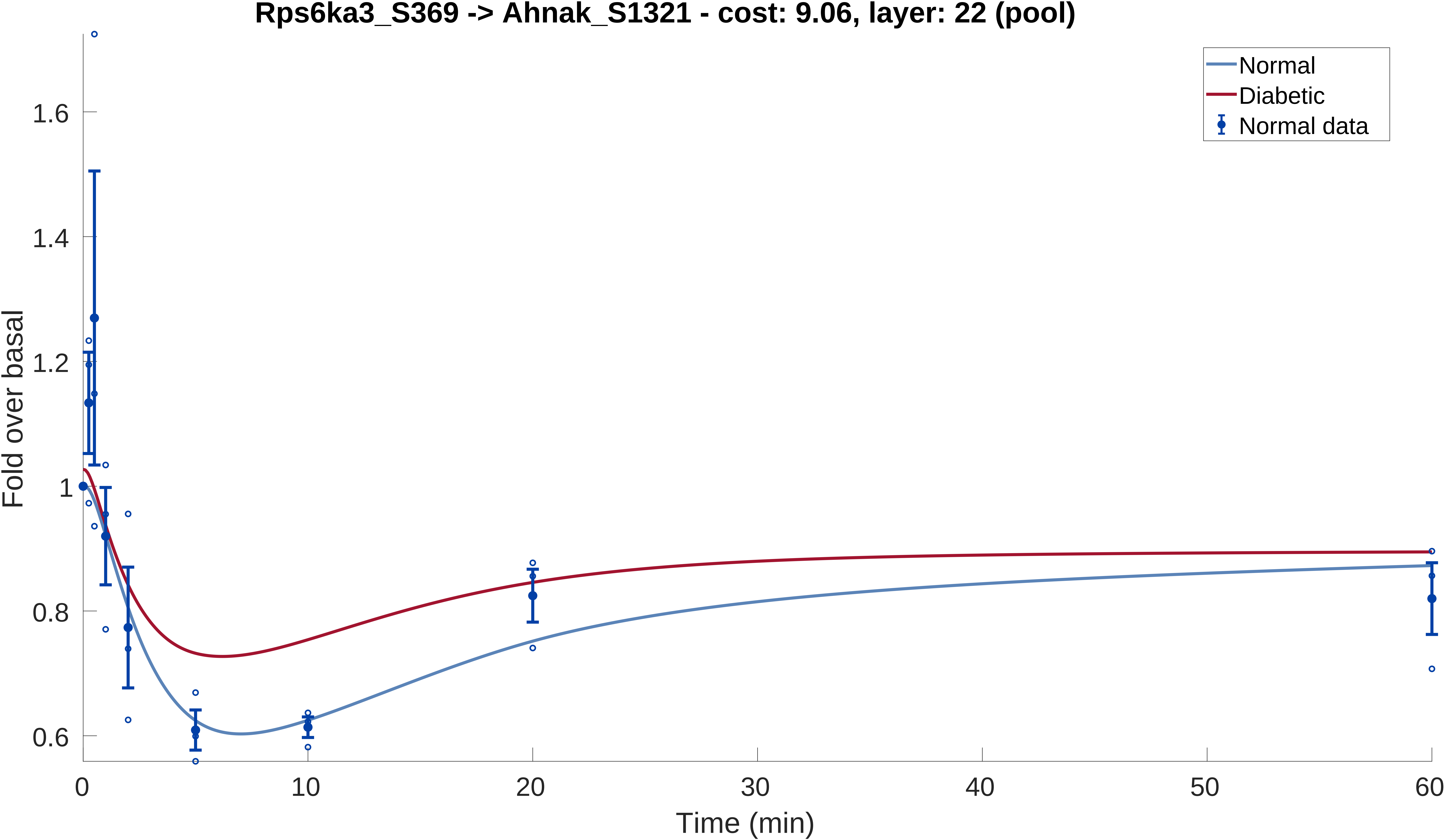

Rps6ka3_S369 -> Ahnak_S1321 - cost: 9.06, layer: 22 (pool)
Normal
Diabetic
Normal data
Fold over basal
1.6
1.4
1.2
1
0.8
0.6
0
10
20
30
40
50
60
Time (min)

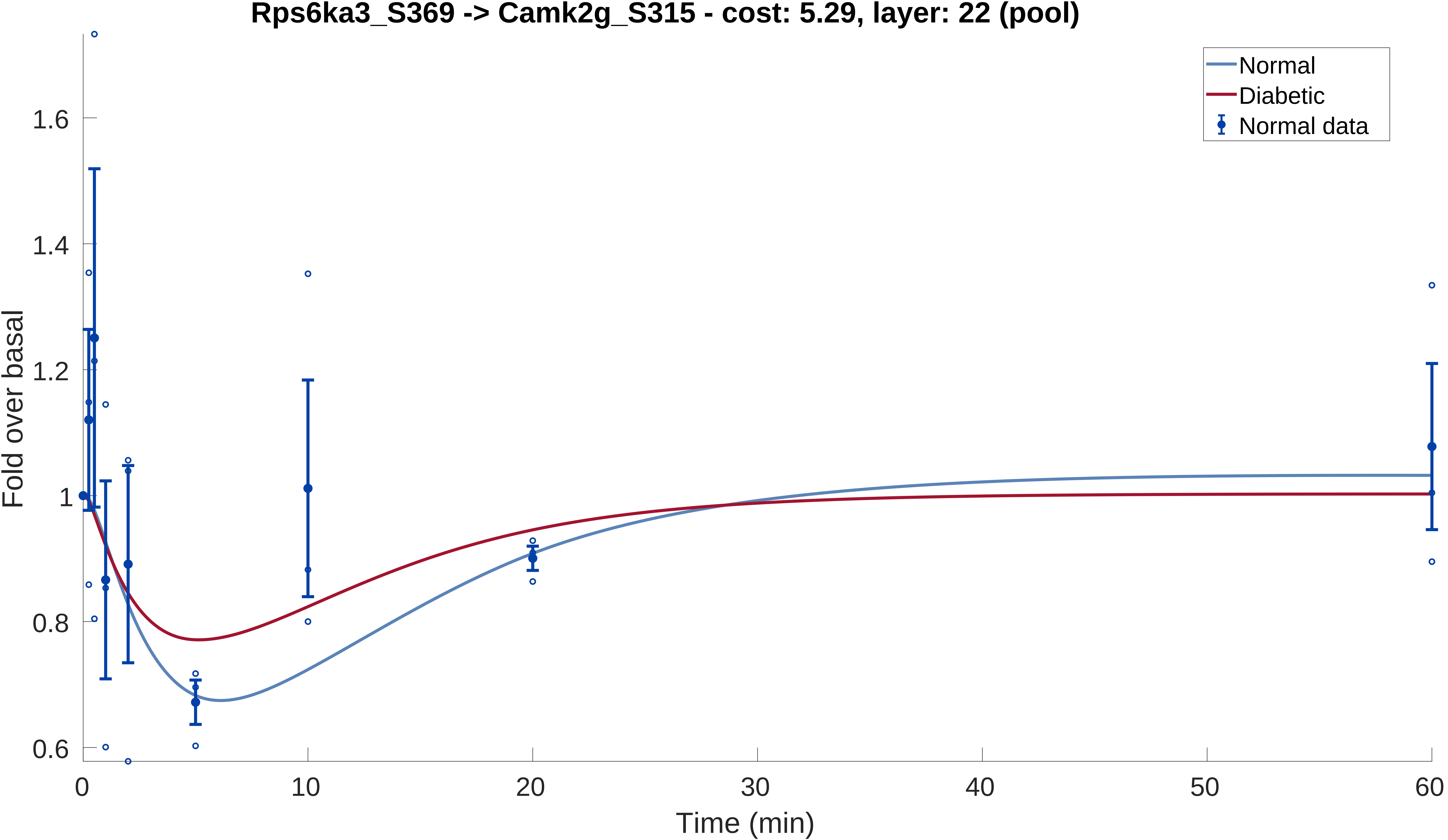

Rps6ka3_S369 -> Camk2g_S315 - cost: 5.29, layer: 22 (pool)
Normal
Diabetic
Normal data
Fold over basal
1.6
1.4
1.2
1
0.8
0.6
0
10
20
30
40
50
60
Time (min)

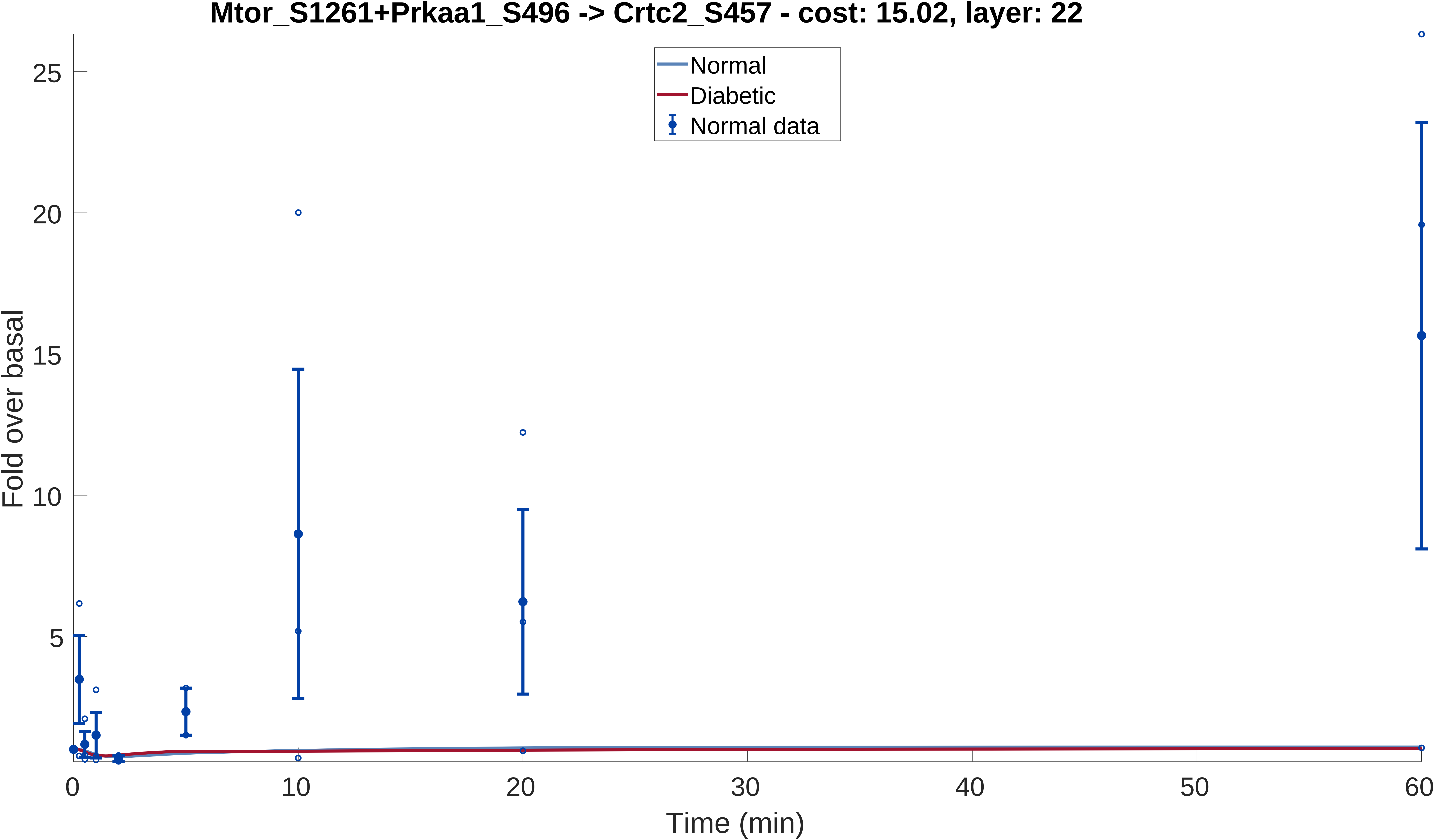

Mtor_S1261+Prkaa1_S496 -> Crtc2_S457 - cost: 15.02, layer: 22
Normal
Diabetic
Normal data
Fold over basal
Time (min)
0
10
20
30
40
50
60
5
10
15
20
25

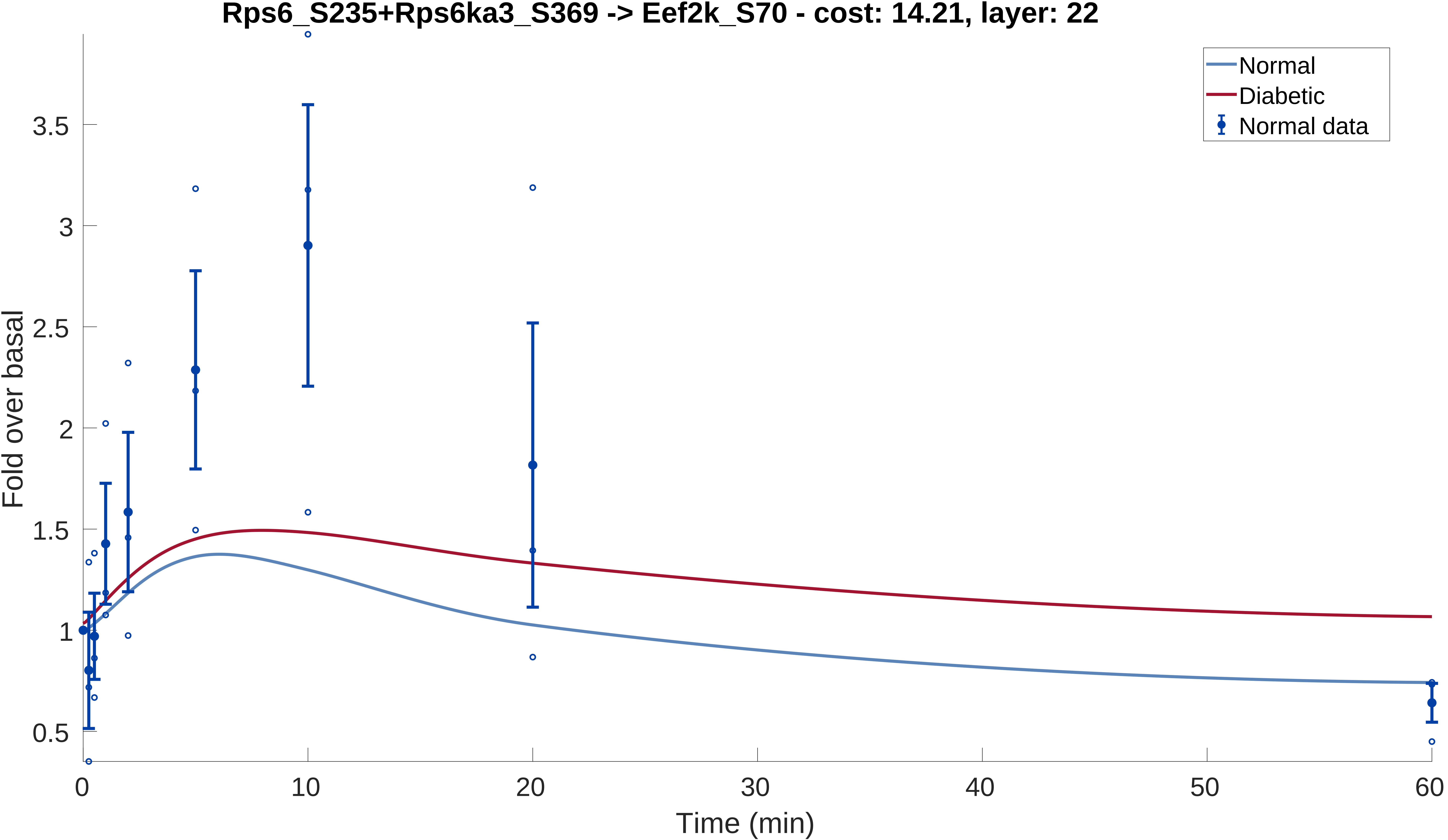
Rps6_S235+Rps6ka3_S369 -> Eef2k_S70 - cost: 14.21, layer: 22
Normal
Diabetic
Normal data
Fold over basal
0.5
1
1.5
2
2.5
3
3.5
0
10
20
30
40
50
60
Time (min)

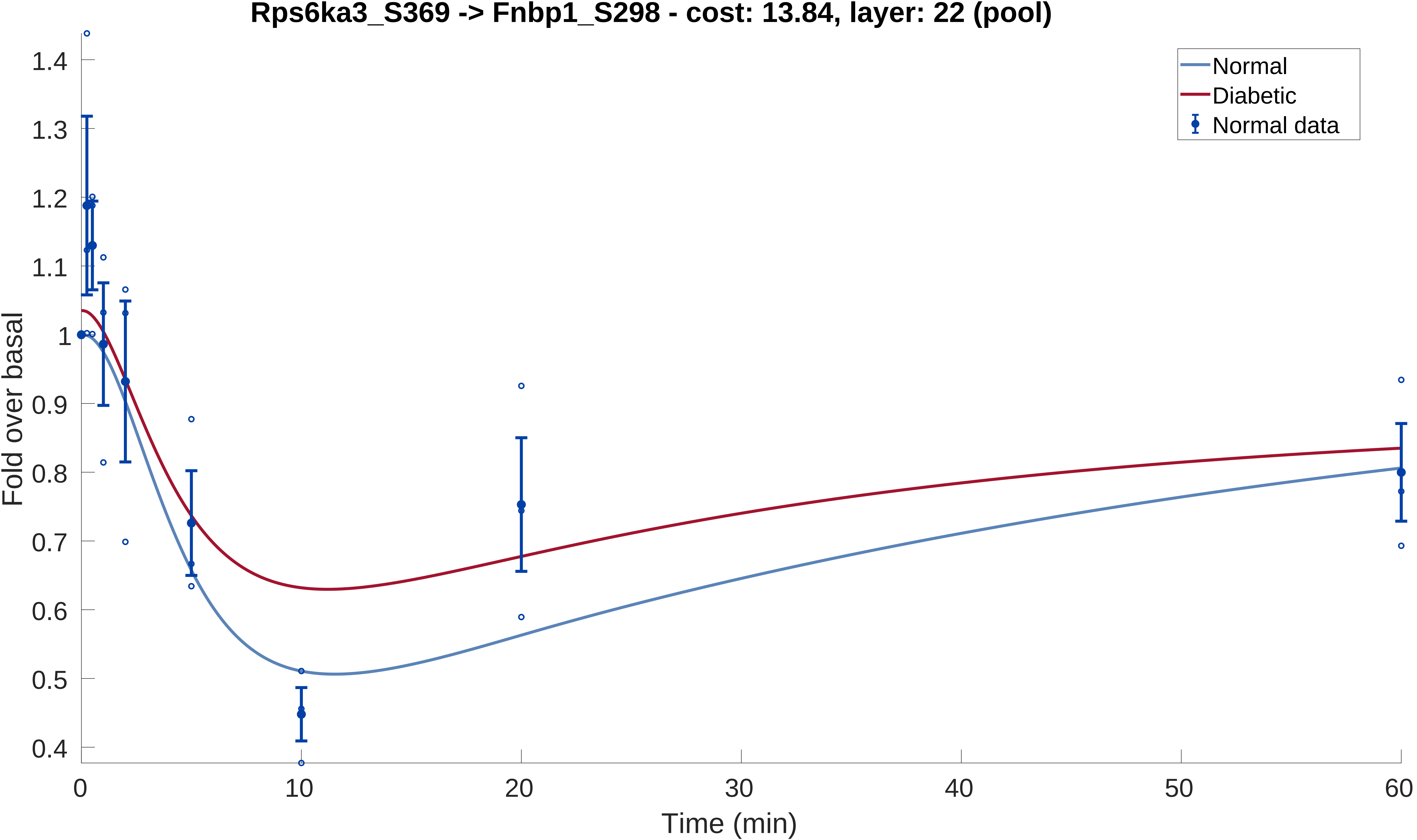

Rps6ka3_S369 -> Fnbp1_S298 - cost: 13.84, layer: 22 (pool)
Normal
Diabetic
Normal data
Fold over basal
1.4
1.3
1.2
1.1
1
0.9
0.8
0.7
0.6
0.5
0.4
0
10
20
30
40
50
60
Time (min)

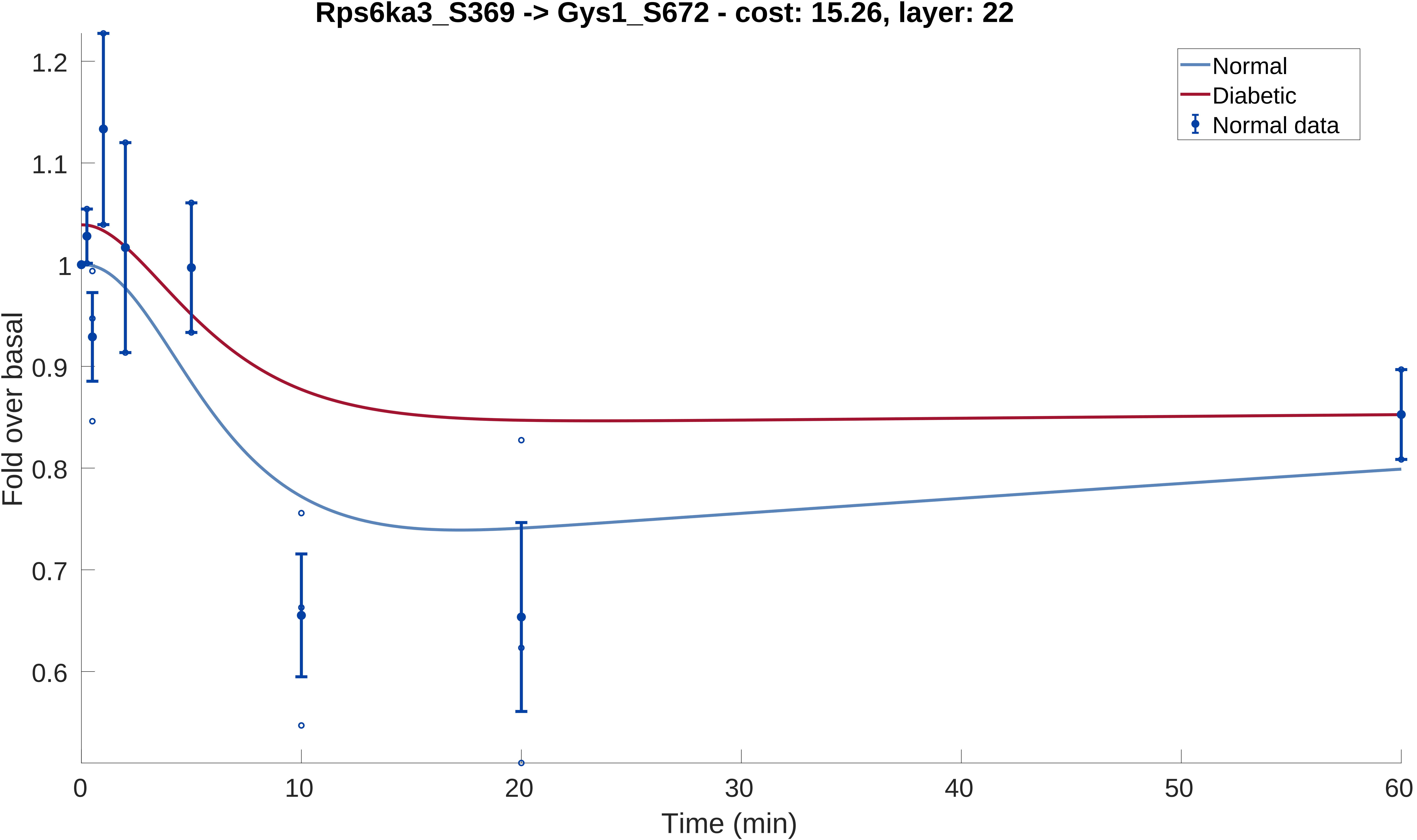

Rps6ka3_S369 -> Gys1_S672 - cost: 15.26, layer: 22
Normal
Diabetic
Normal data
1.2
1.1
1
0.9
0.8
0.7
0.6
Fold over basal
0
10
20
30
40
50
60
Time (min)

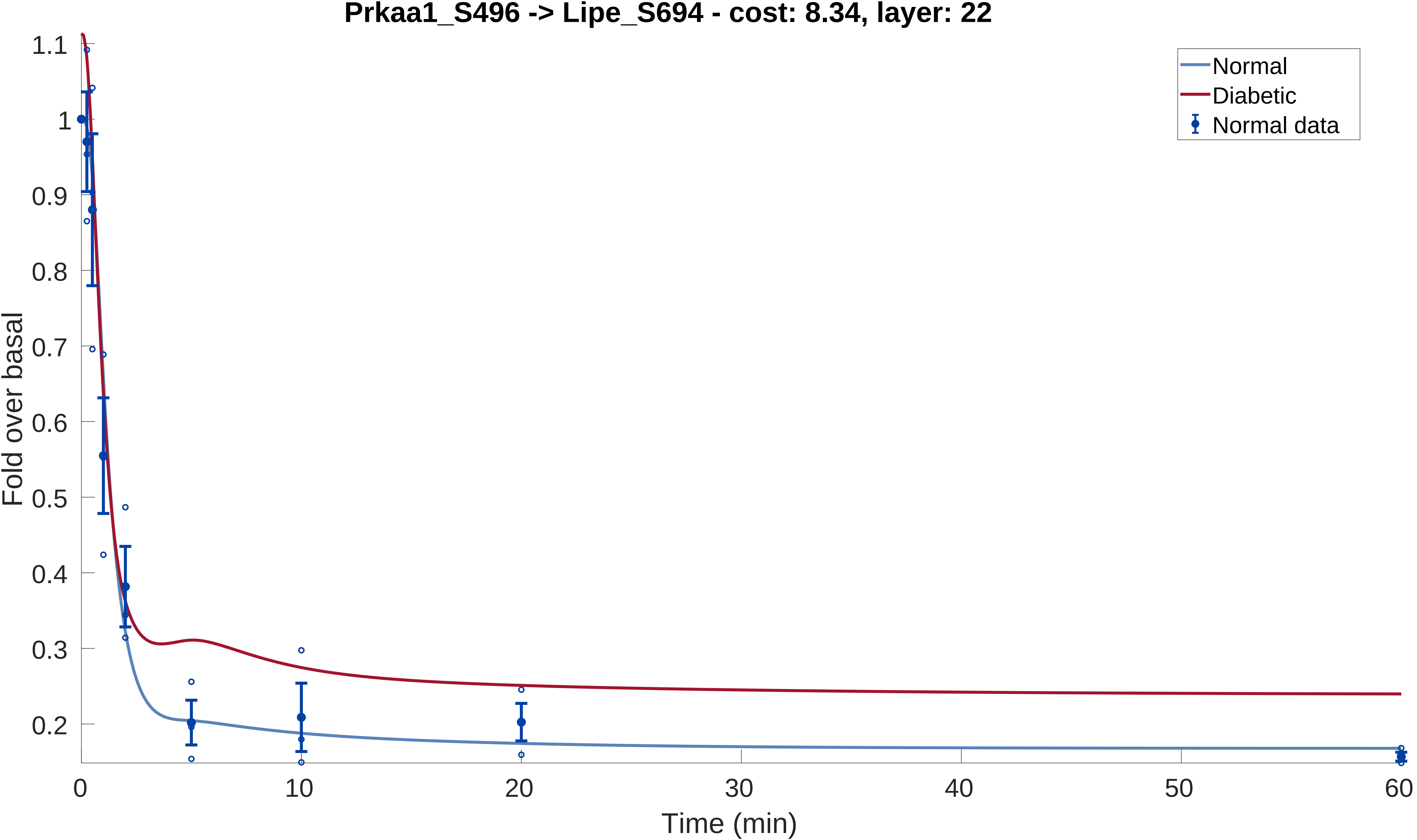

Prrkaa1_S496 -> Lipe_S694 - cost: 8.34, layer: 22
Normal
Diabetic
Normal data
Fold over basal
Time (min)
1.1
1
0.9
0.8
0.7
0.6
0.5
0.4
0.3
0.2
0
10
20
30
40
50
60

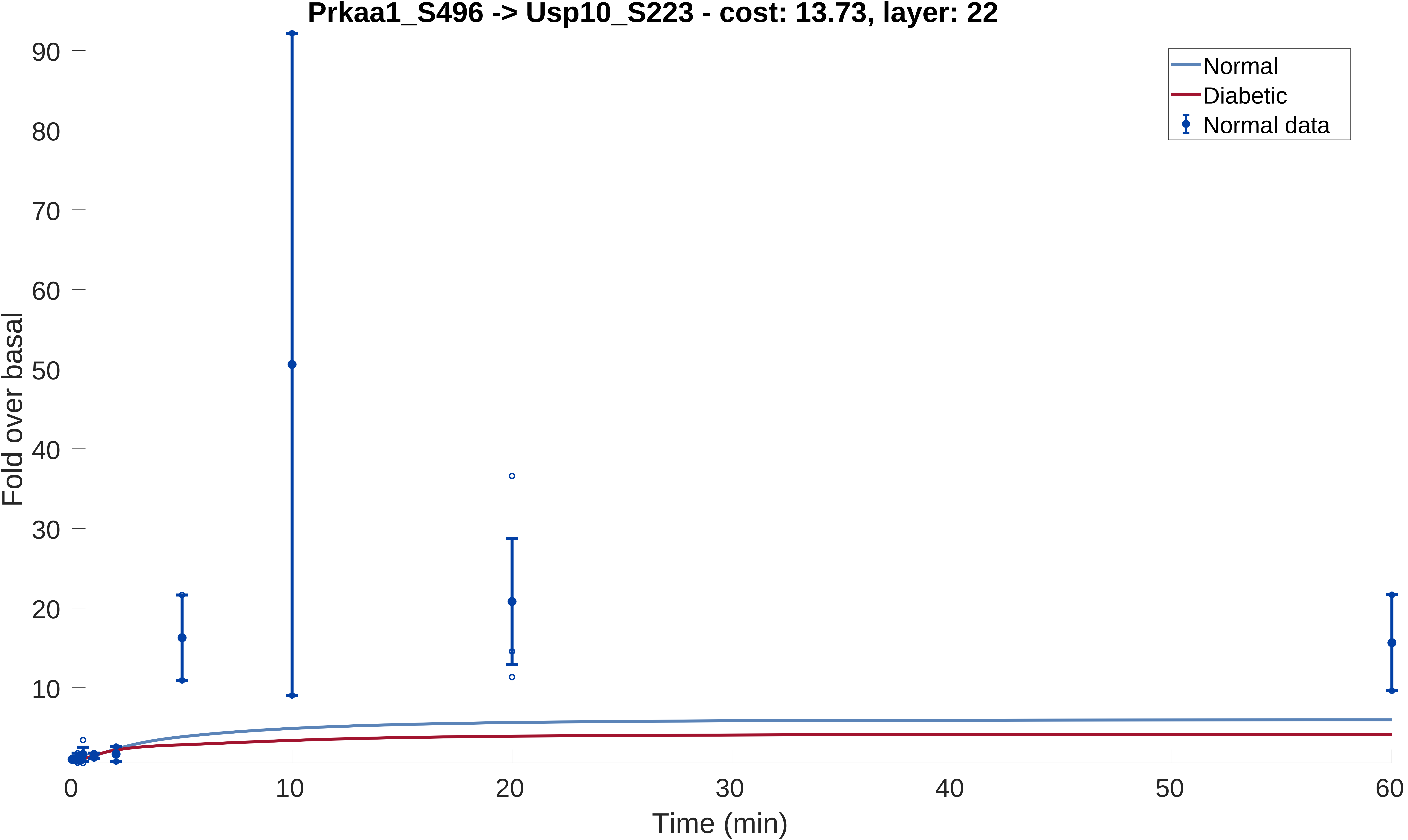

Prkaa1_S496 -> Usp10_S223 - cost: 13.73, layer: 22
Normal
Diabetic
Normal data
Fold over basal
Time (min)
0
10
20
30
40
50
60
70
80
90

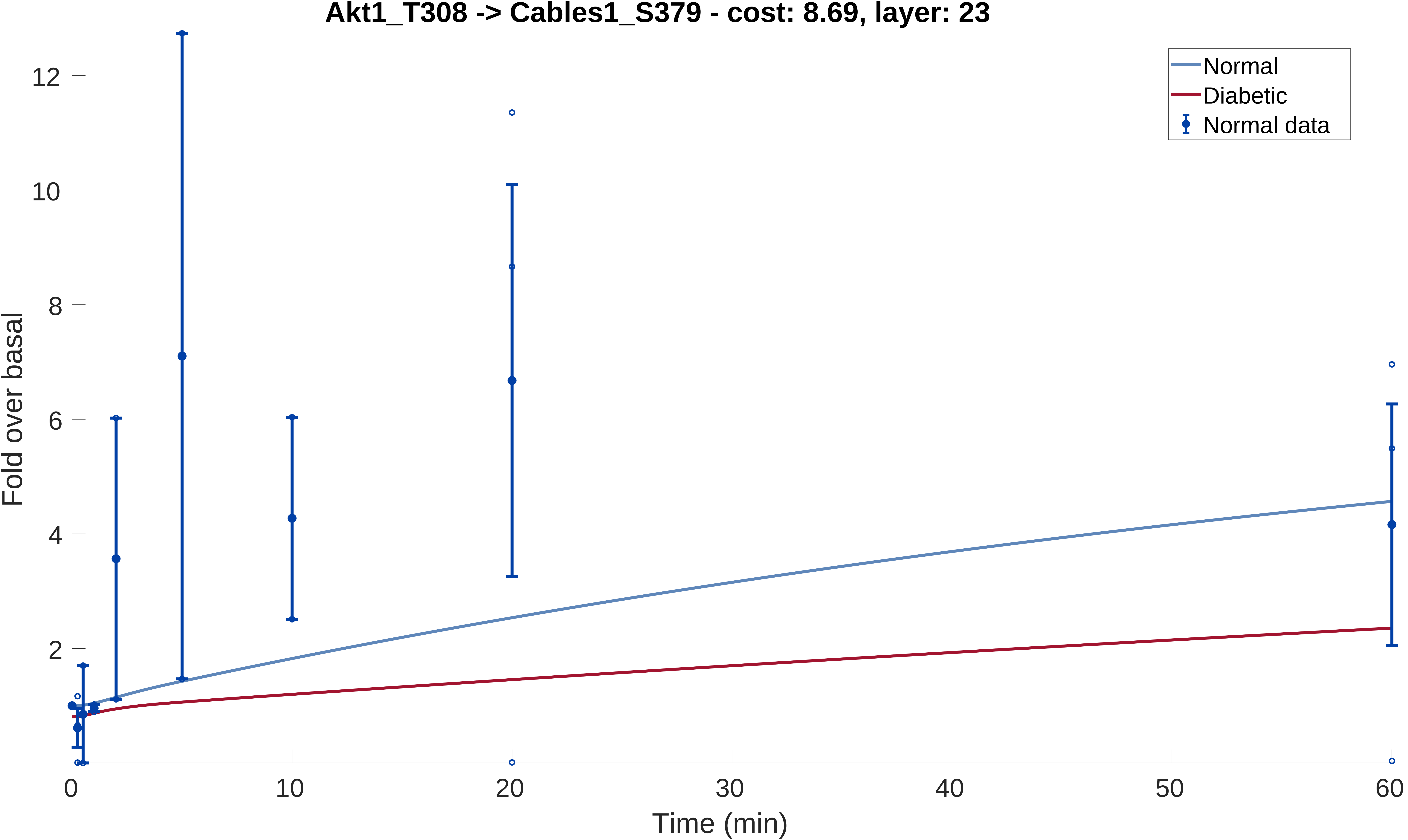

Akt1_T308 -> Cables1_S379 - cost: 8.69, layer: 23
Fold over basal
Time (min)
0
2
4
6
8
10
12
0
10
20
30
40
50
60
Normal
Diabetic
Normal data

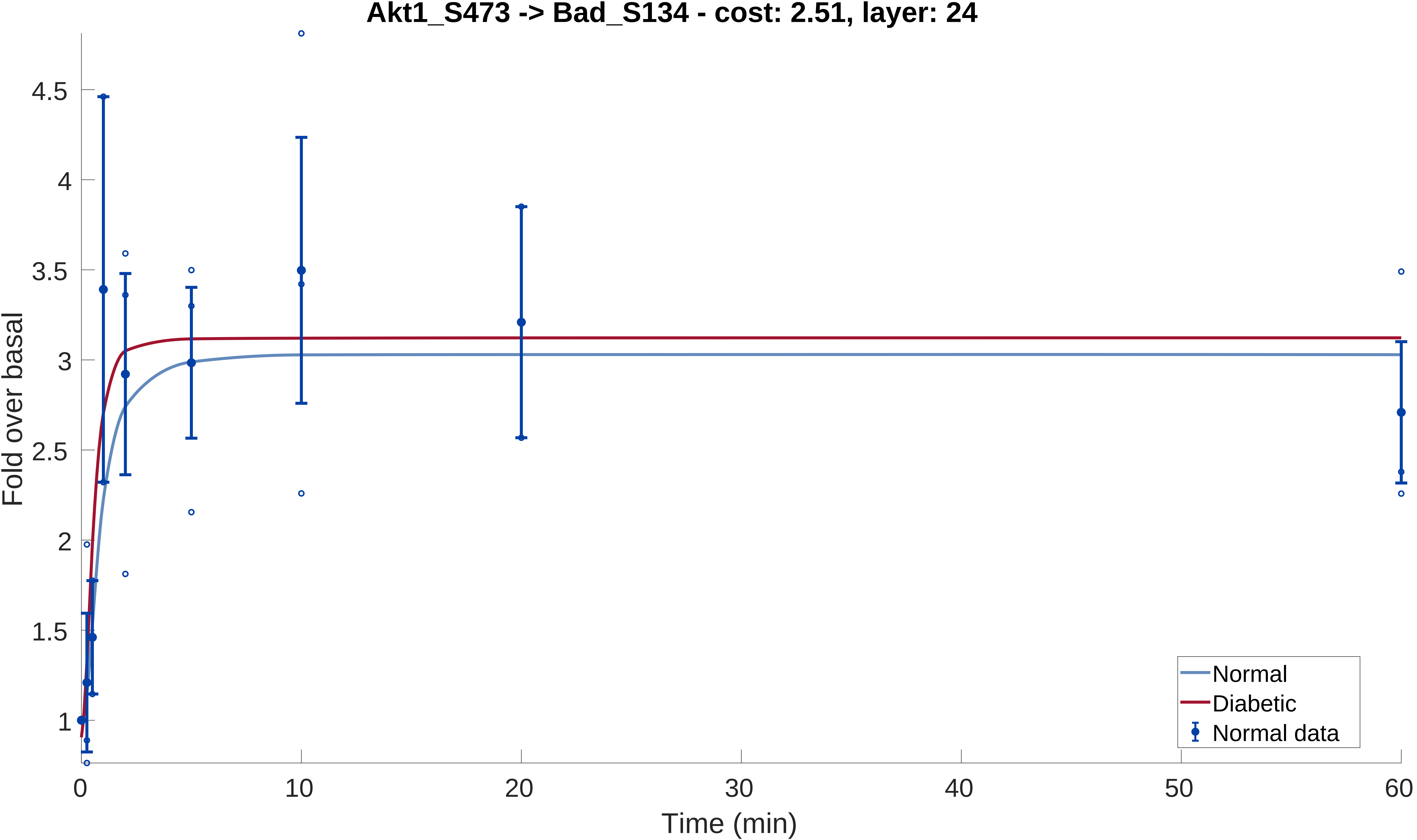

Akt1_S473 -> Bad_S134 - cost: 2.51, layer: 24
Fold over basal
4.5
4
3.5
3
2.5
2
1.5
1
0
10
20
30
40
50
60
Time (min)
Normal
Diabetic
Normal data

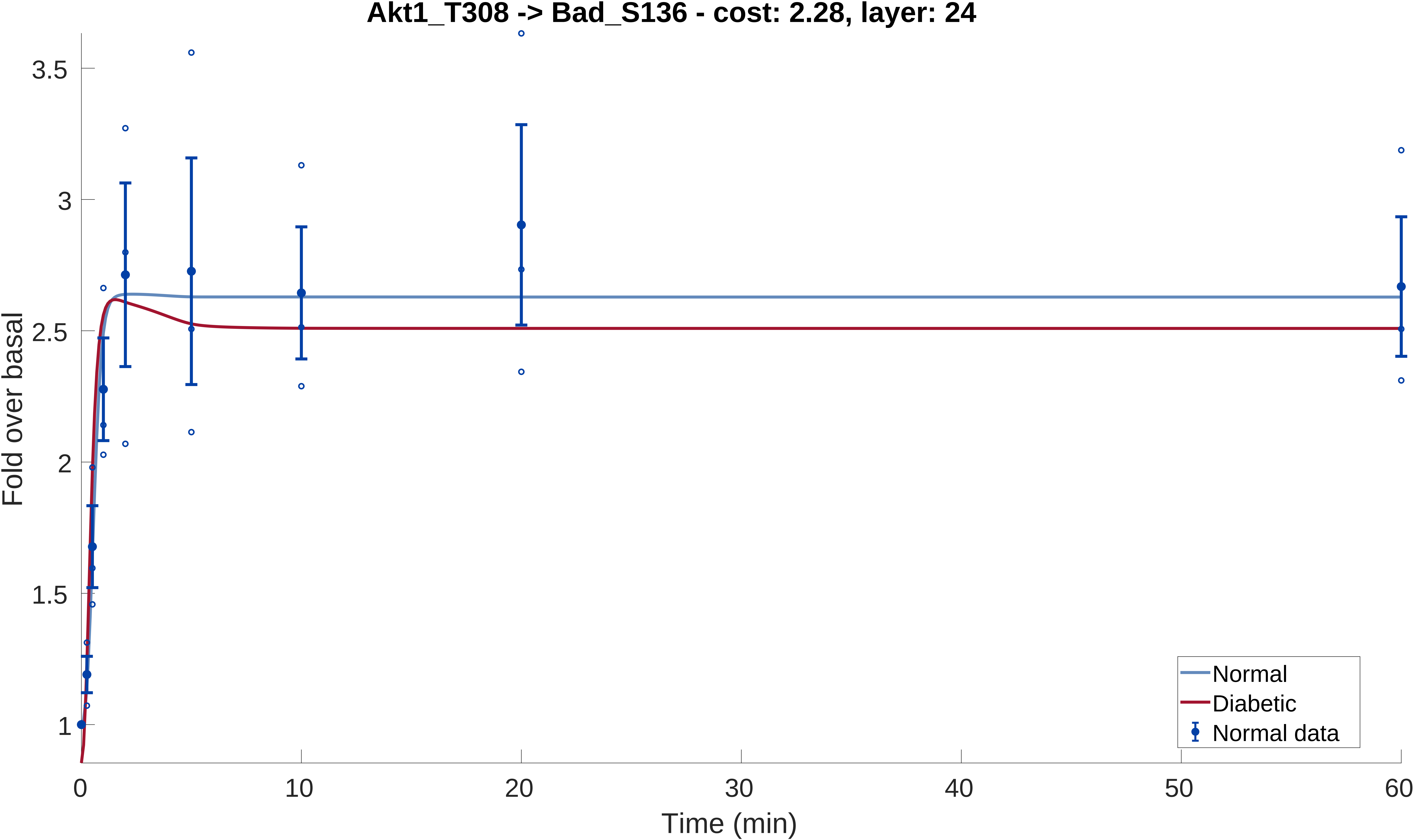
Akt1_T308 -> Bad_S136 - cost: 2.28, layer: 24
Fold over basal
Time (min)
3.5
3
2.5
2
1.5
1
0
10
20
30
40
50
60
Normal
Diabetic
Normal data

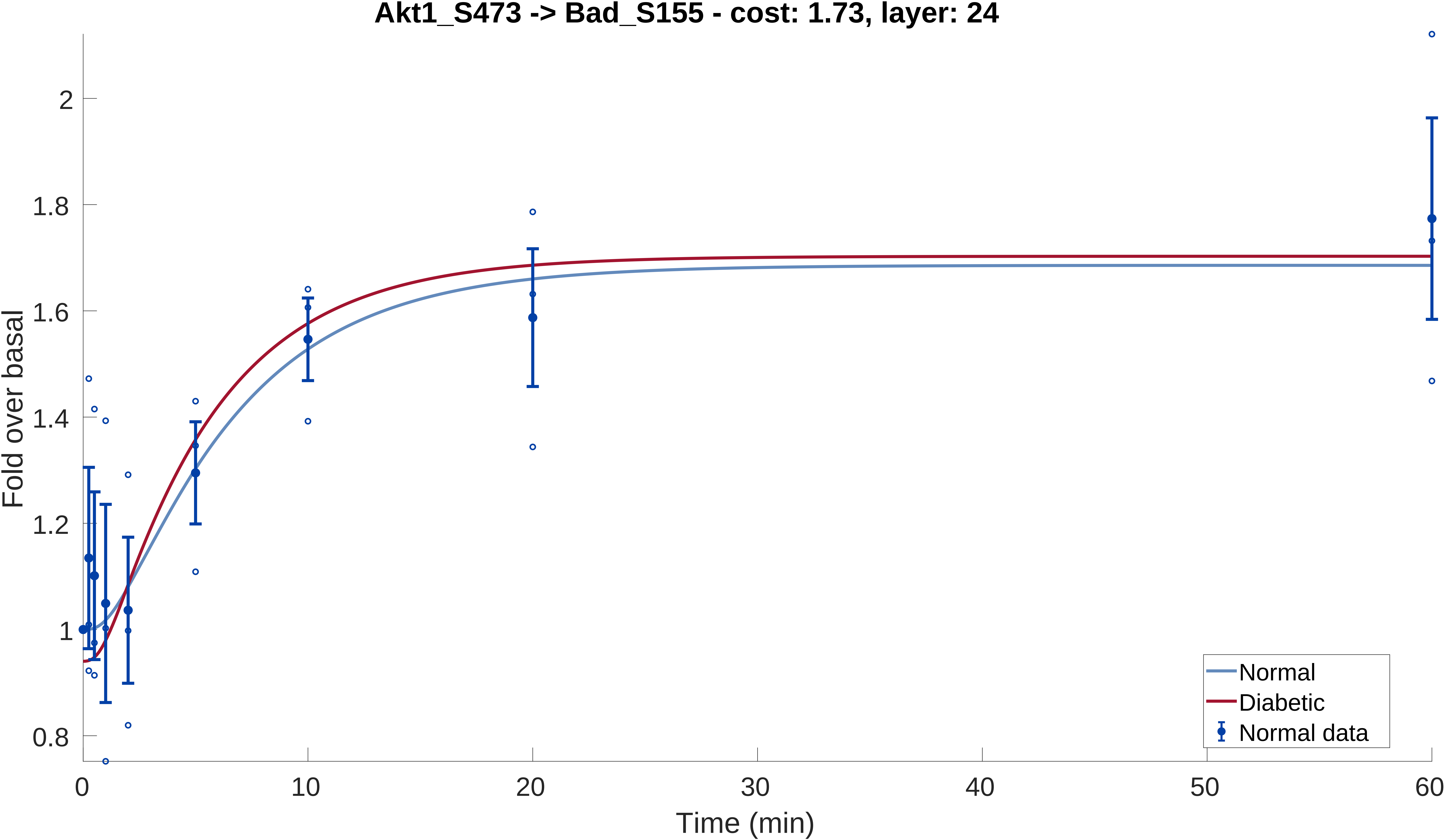

Akt1_S473 -> Bad_S155 - cost: 1.73, layer: 24
Fold over basal
2
1.8
1.6
1.4
1.2
1
0.8
0
10
20
30
40
50
60
Time (min)
Normal
Diabetic
Normal data

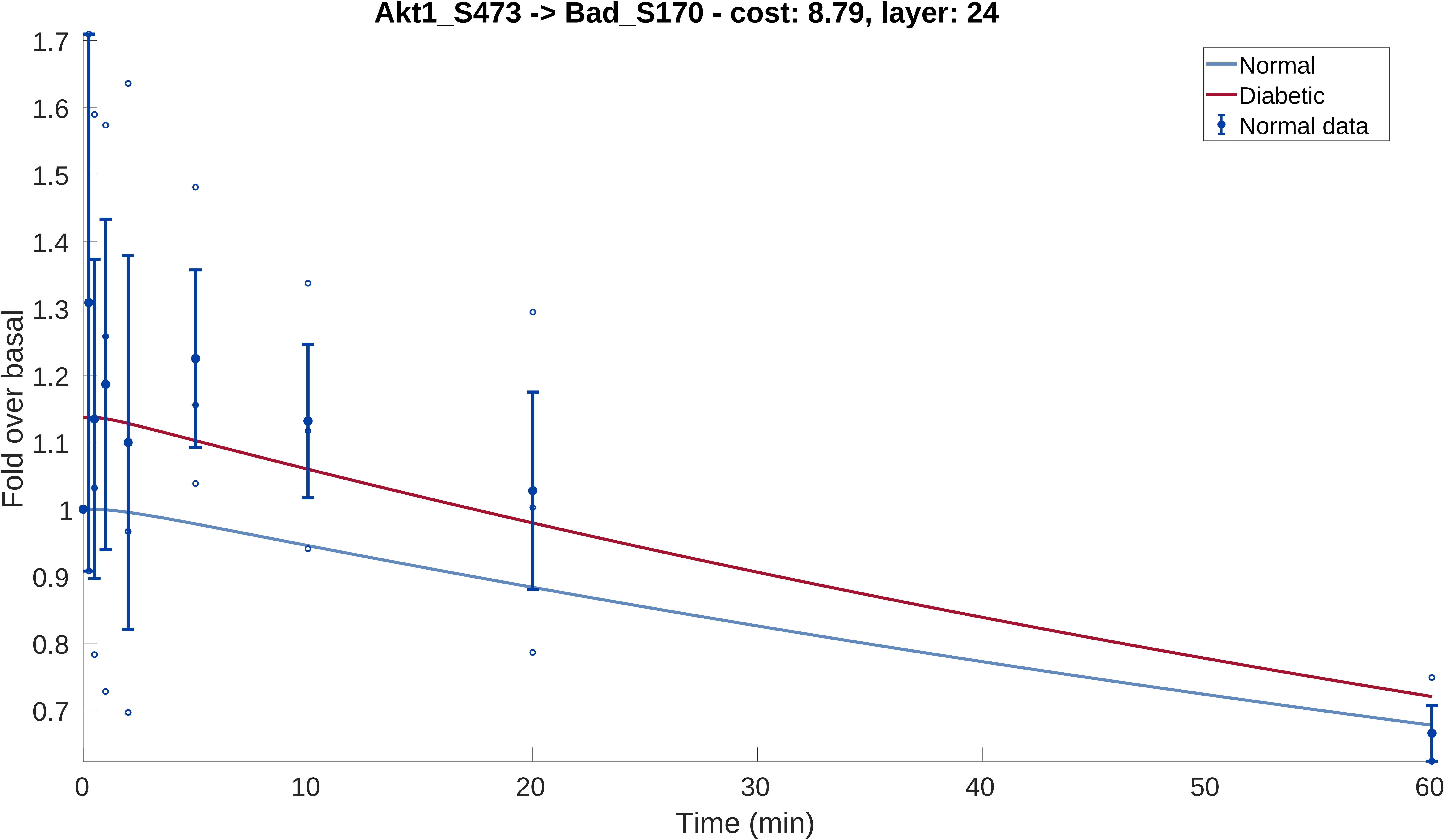

Akt1_S473 -> Bad_S170 - cost: 8.79, layer: 24
Fold over basal
1.7
1.6
1.5
1.4
1.3
1.2
1.1
1
0.9
0.8
0.7
0
10
20
30
40
50
60
Time (min)
Normal
Diabetic
Normal data

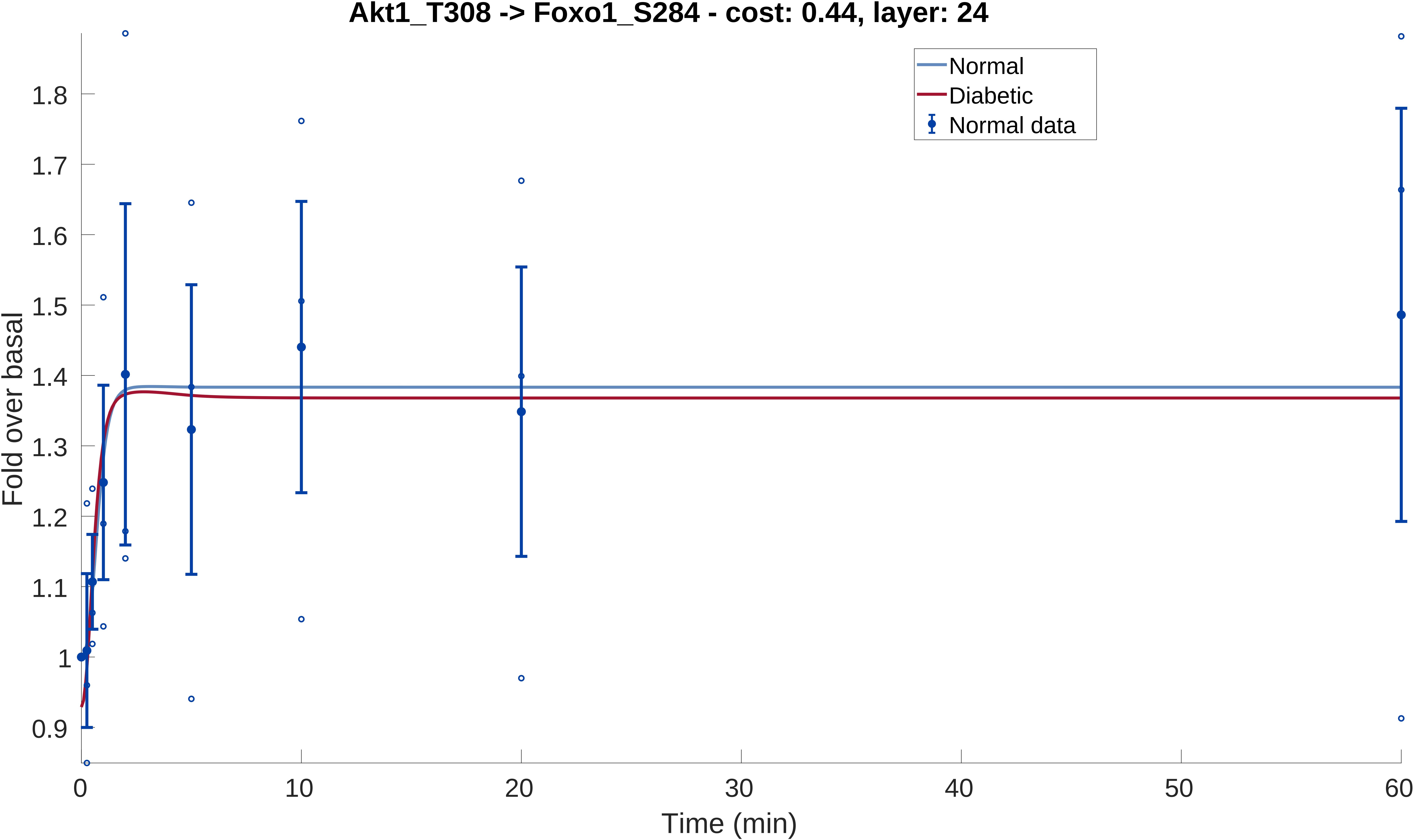
Akt1_T308 -> Foxo1_S284 - cost: 0.44, layer: 24
Normal
Diabetic
Normal data
Fold over basal
Time (min)
0.9
1
1.1
1.2
1.3
1.4
1.5
1.6
1.7
1.8
0
10
20
30
40
50
60

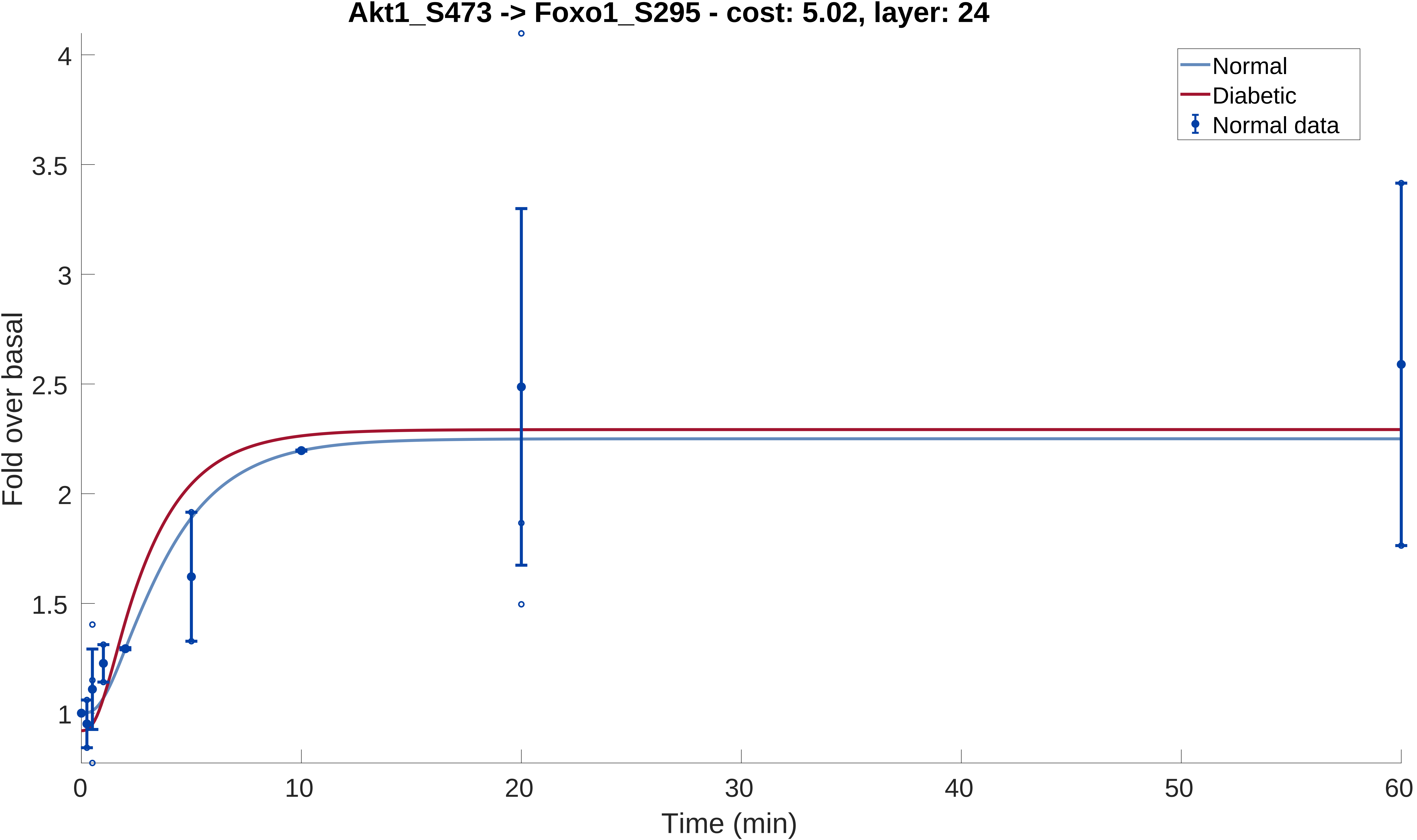
Akt1_S473 -> Foxo1_S295 - cost: 5.02, layer: 24
Normal
Diabetic
Normal data
Fold over basal
Time (min)
4
3.5
3
2.5
2
1.5
1
0
10
20
30
40
50
60

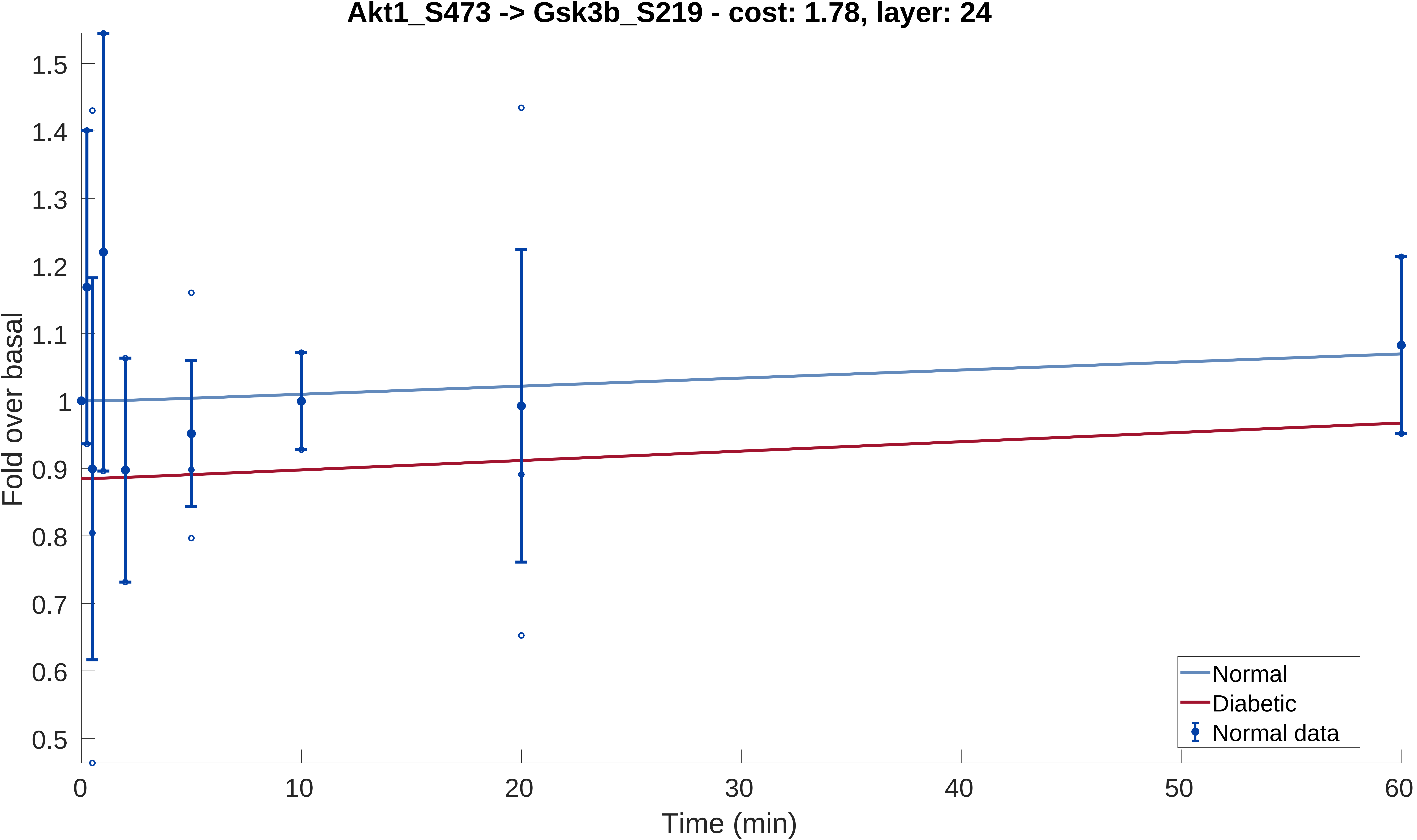

Akt1_S473 -> Gsk3b_S219 - cost: 1.78, layer: 24
Fold over basal
Time (min)
0.5
0.6
0.7
0.8
0.9
1
1.1
1.2
1.3
1.4
1.5
0
10
20
30
40
50
60
Normal
Diabetic
Normal data

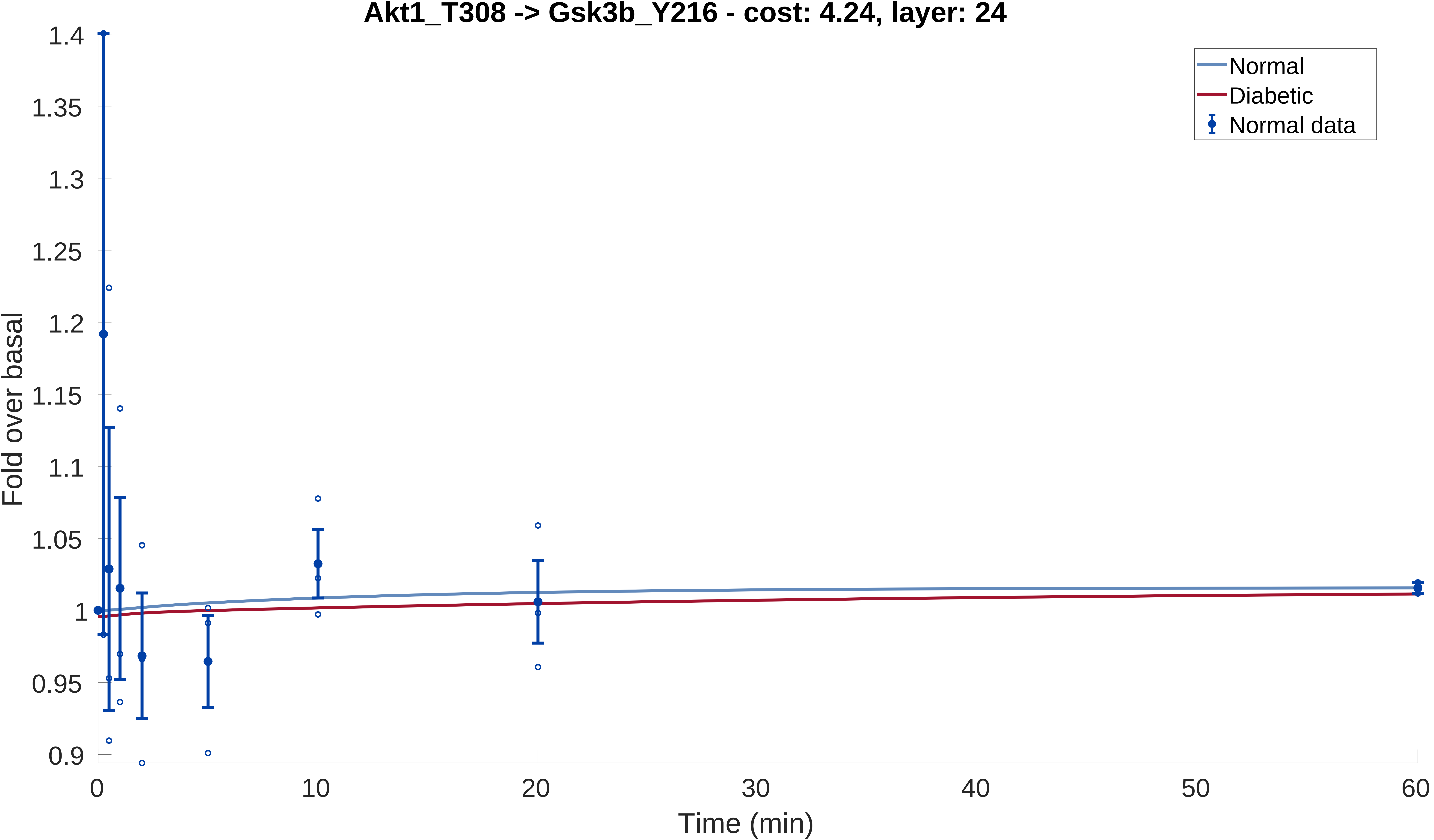

Akt1_T308 -> Gsk3b_Y216 - cost: 4.24, layer: 24
Normal
Diabetic
Normal data
Fold over basal
Time (min)
1.4
1.35
1.3
1.25
1.2
1.15
1.1
1.05
1
0.95
0.9
0
10
20
30
40
50
60

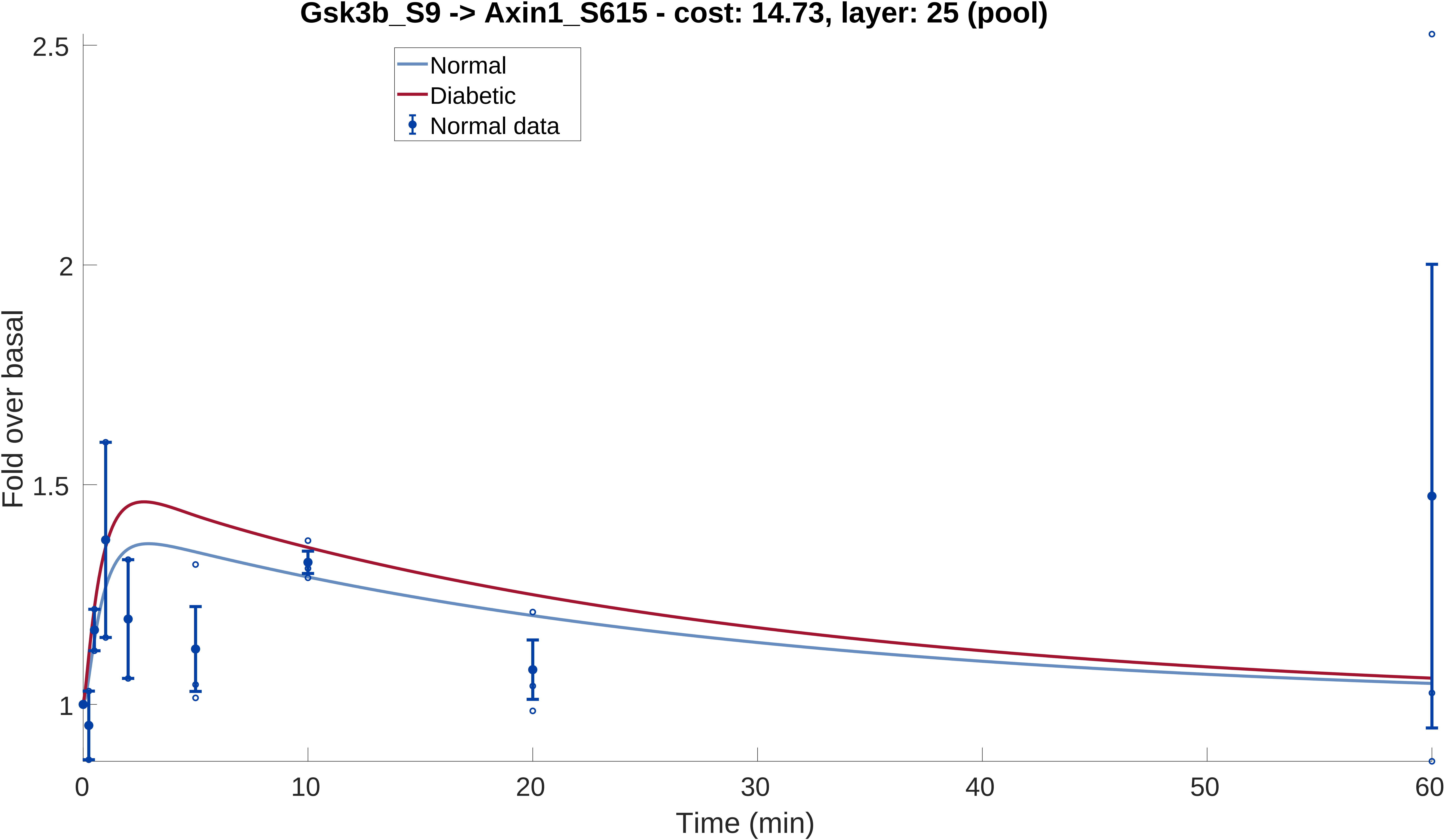

Gsk3b_S9 -> Axin1_S615 - cost: 14.73, layer: 25 (pool)
Normal
Diabetic
Normal data
Fold over basal
2.5
2
1.5
1
0
10
20
30
40
50
60
Time (min)

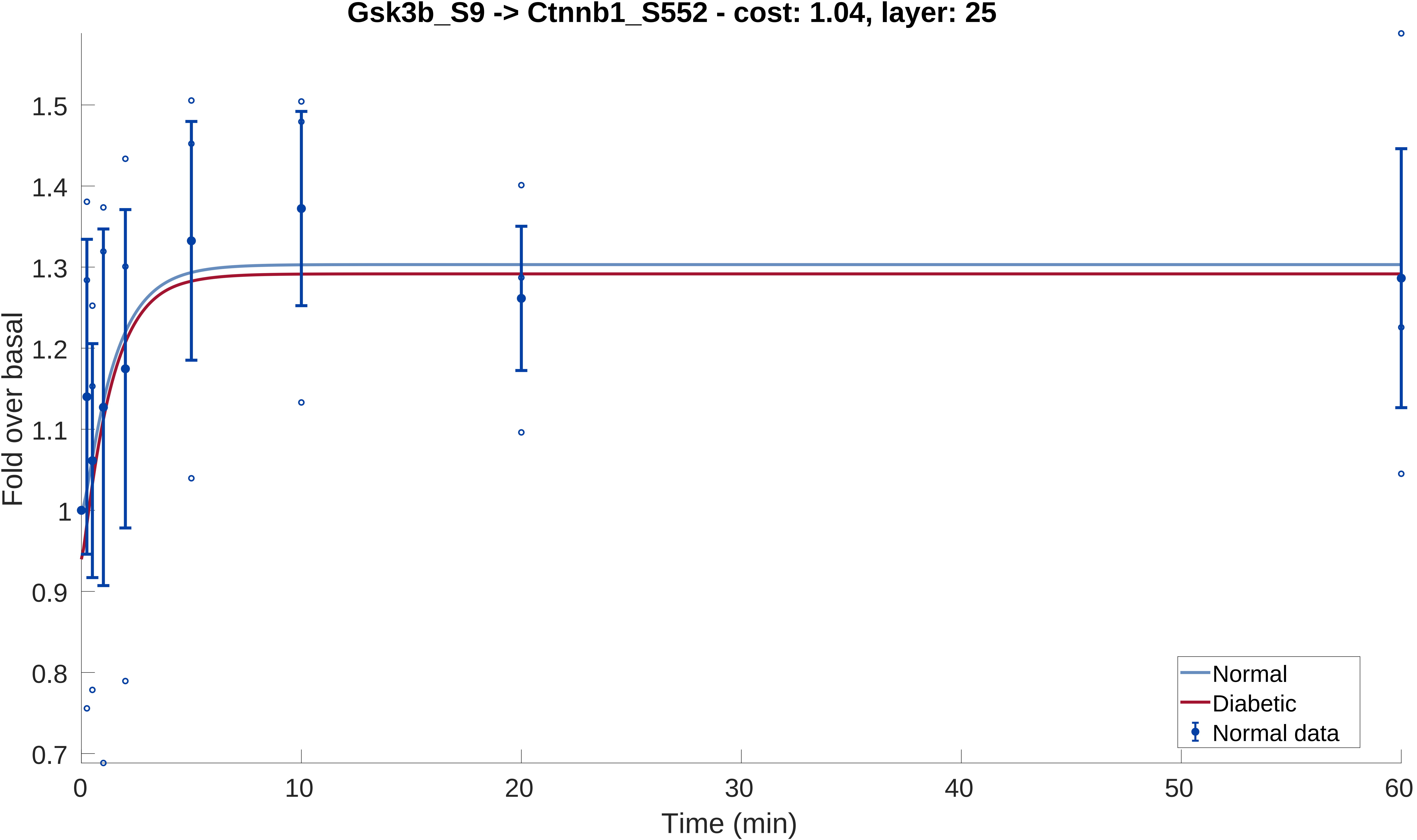

Gsk3b_S9 -> Ctnnb1_S552 - cost: 1.04, layer: 25
Fold over basal
1.5
1.4
1.3
1.2
1.1
1
0.9
0.8
0.7
0
10
20
30
40
50
60
Time (min)
Normal
Diabetic
Normal data

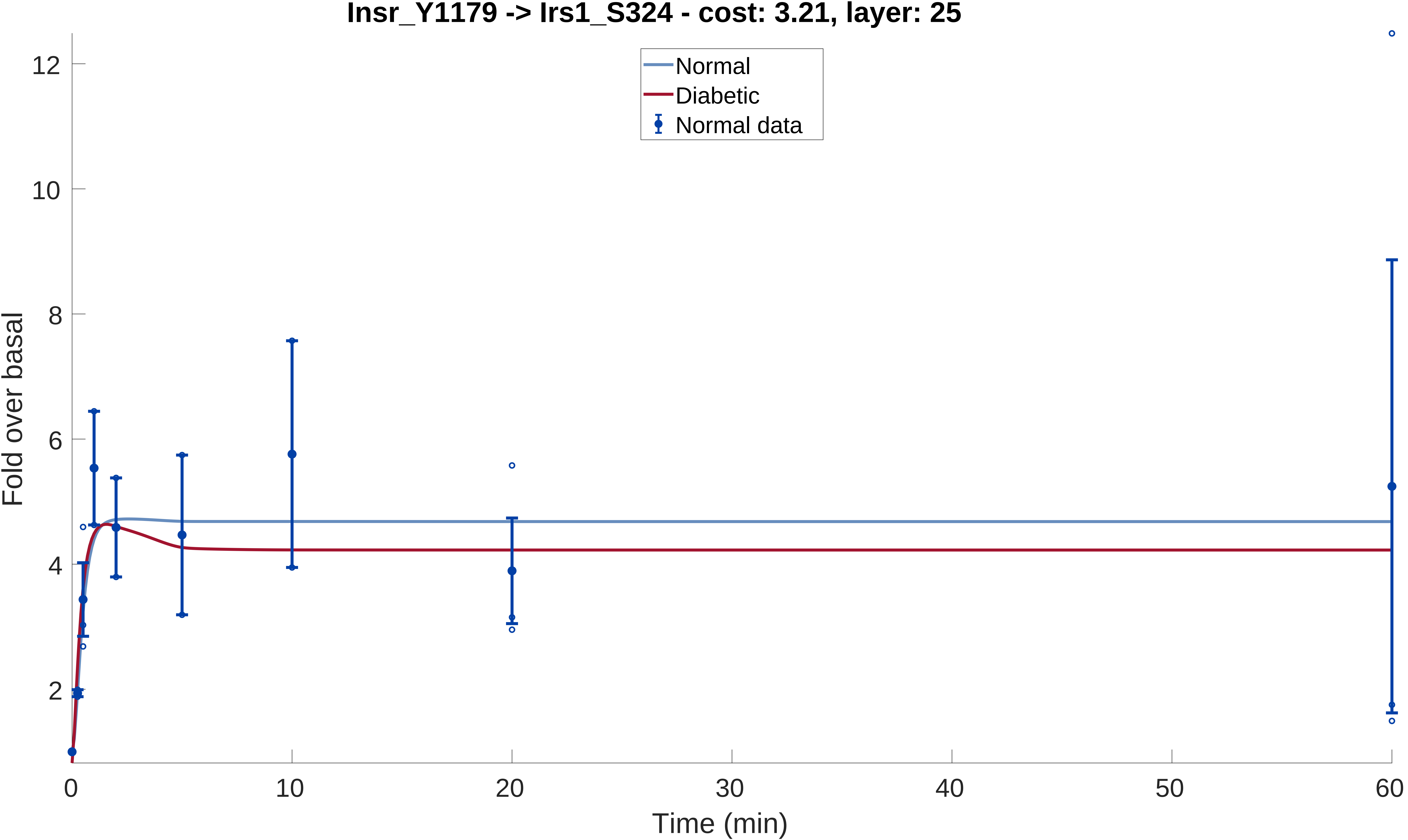

Insr_Y1179 -> Irs1_S324 - cost: 3.21, layer: 25
Normal
Diabetic
Normal data
Fold over basal
12
10
8
6
4
2
0
10
20
30
40
50
60
Time (min)

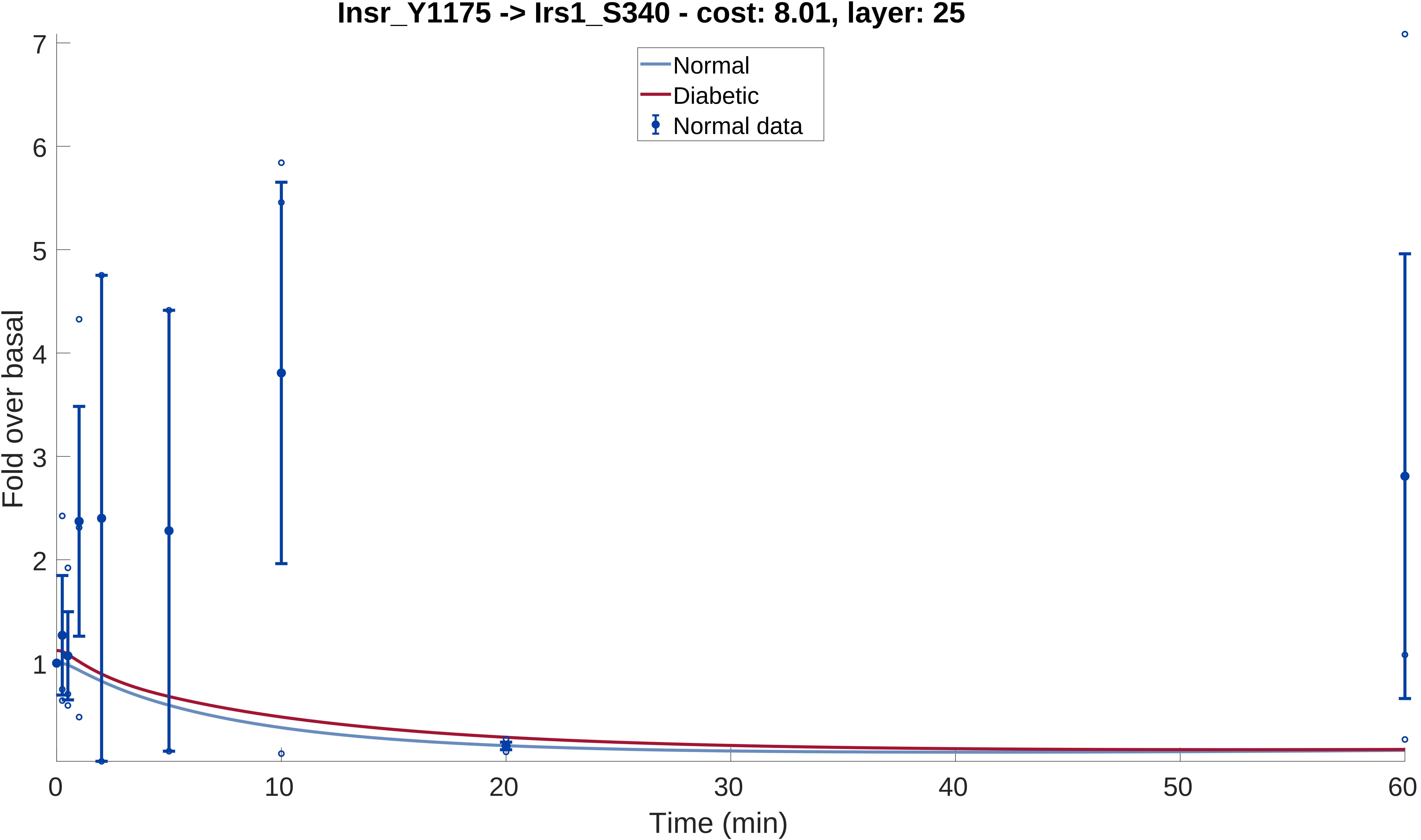

Insr_Y1175 -> Irs1_S340 - cost: 8.01, layer: 25
Normal
Diabetic
Normal data
Fold over basal
Time (min)
0
10
20
30
40
50
60
1
2
3
4
5
6
7

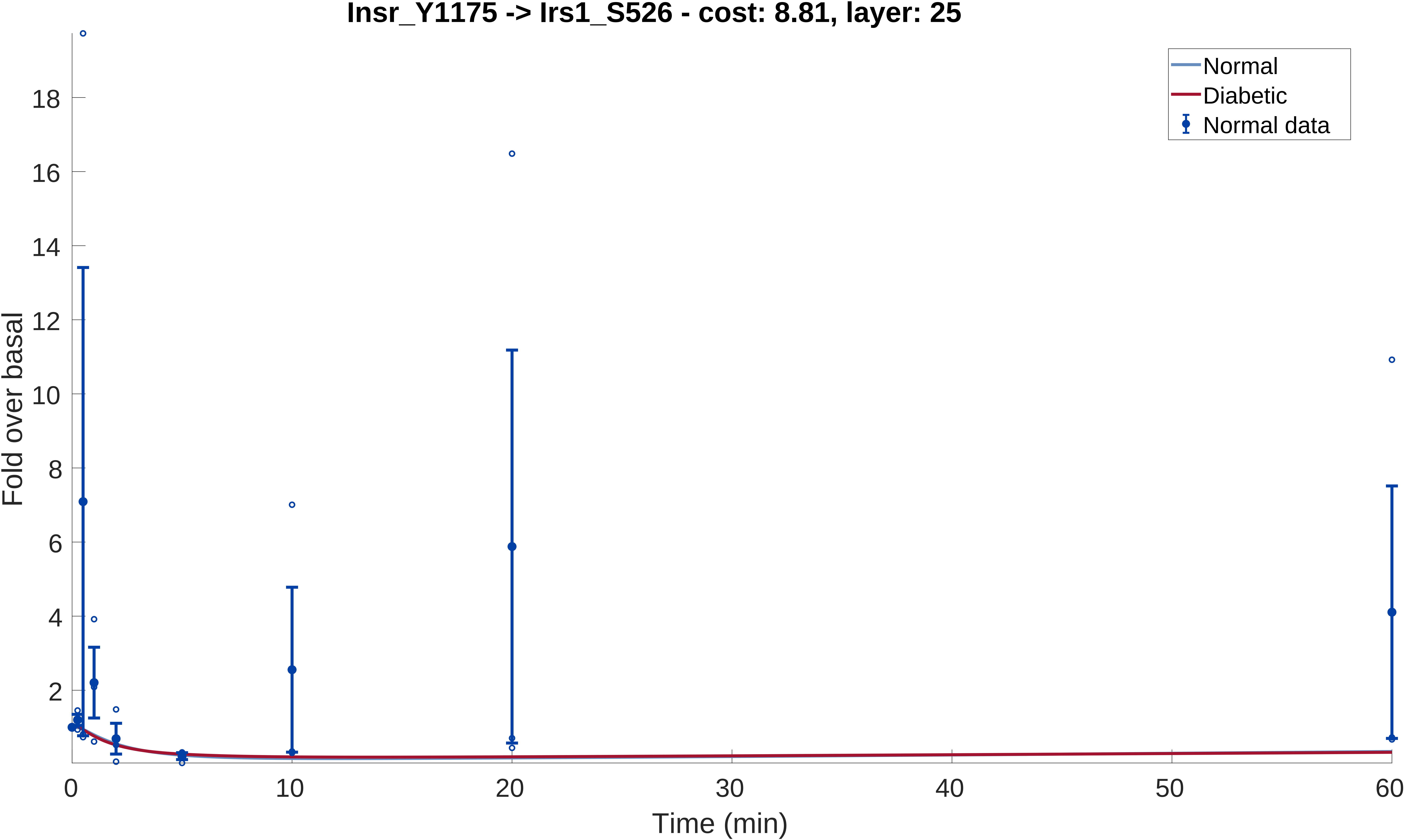

Insr_Y1175 -> Irs1_S526 - cost: 8.81, layer: 25
Normal
Diabetic
Normal data
Fold over basal
Time (min)
18
16
14
12
10
8
6
4
2
0
10
20
30
40
50
60

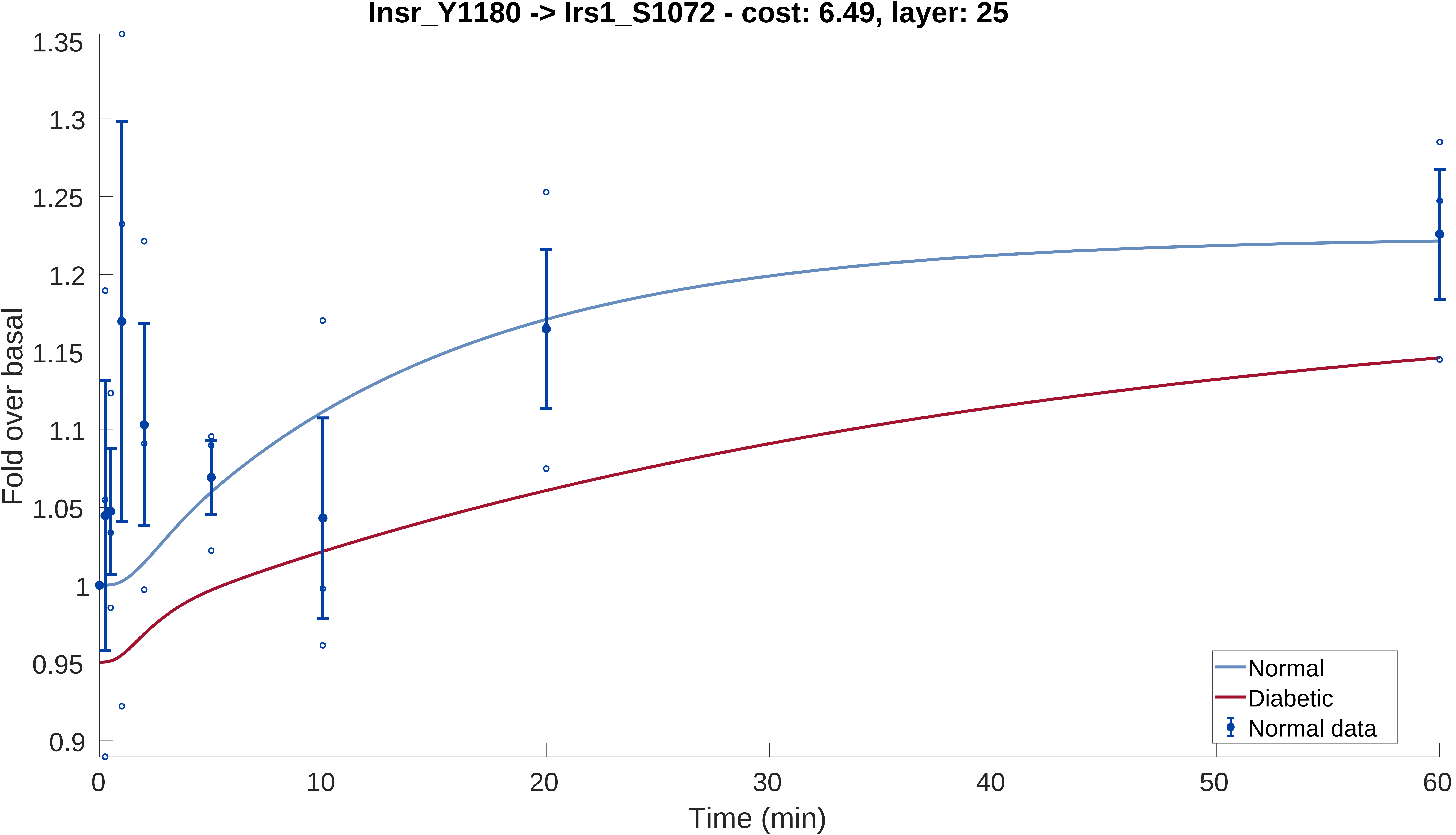
Insr_Y1180 -> Irs1_S1072 - cost: 6.49, layer: 25
Fold over basal
Time (min)
1.35
1.3
1.25
1.2
1.15
1.1
1.05
1
0.95
0.9
0
10
20
30
40
50
60
Normal
Diabetic
Normal data

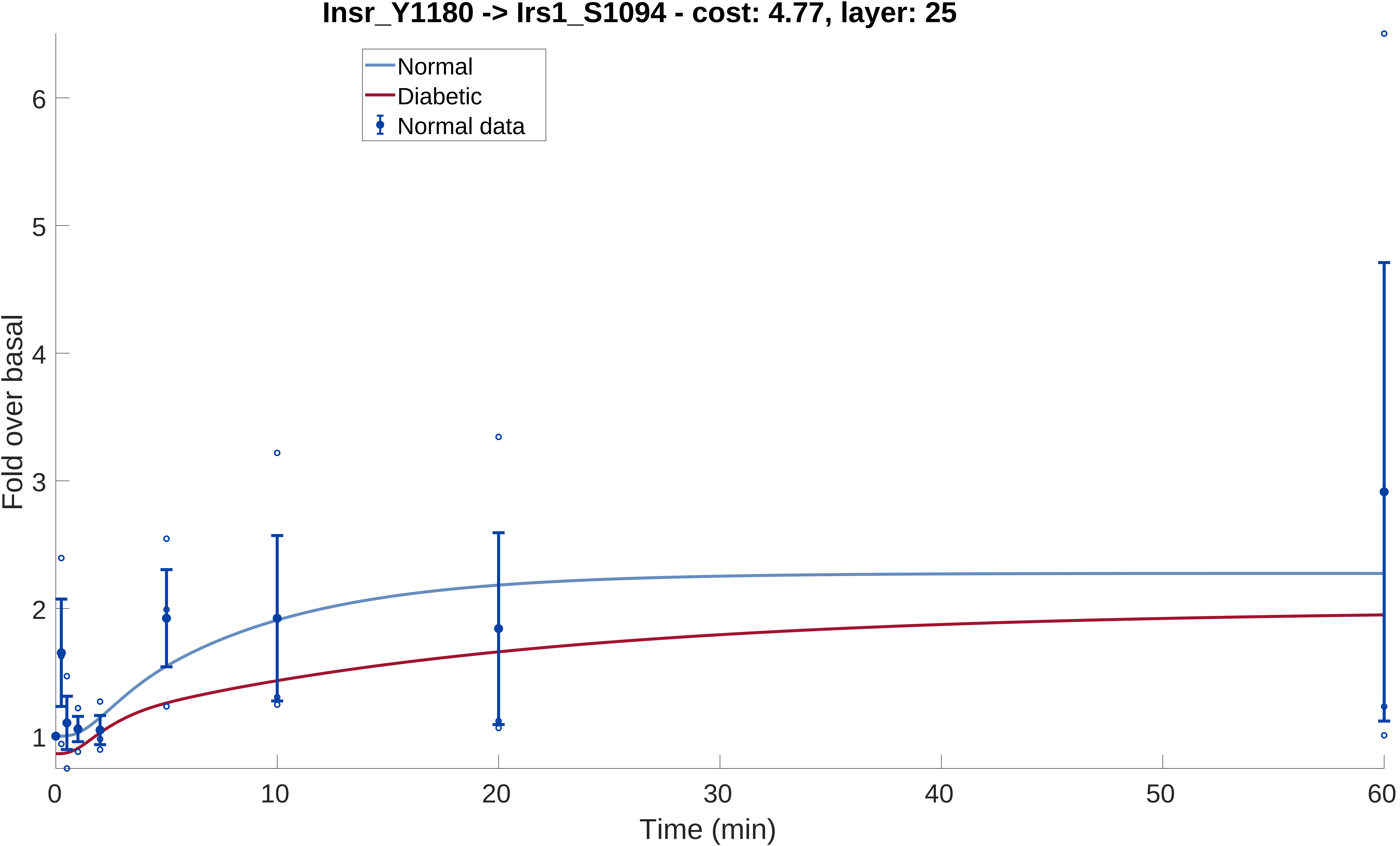
Insr_Y1180 -> Irs1_S1094 - cost: 4.77, layer: 25
Normal
Diabetic
Normal data
Fold over basal
Time (min)
1
2
3
4
5
6
0
10
20
30
40
50
60

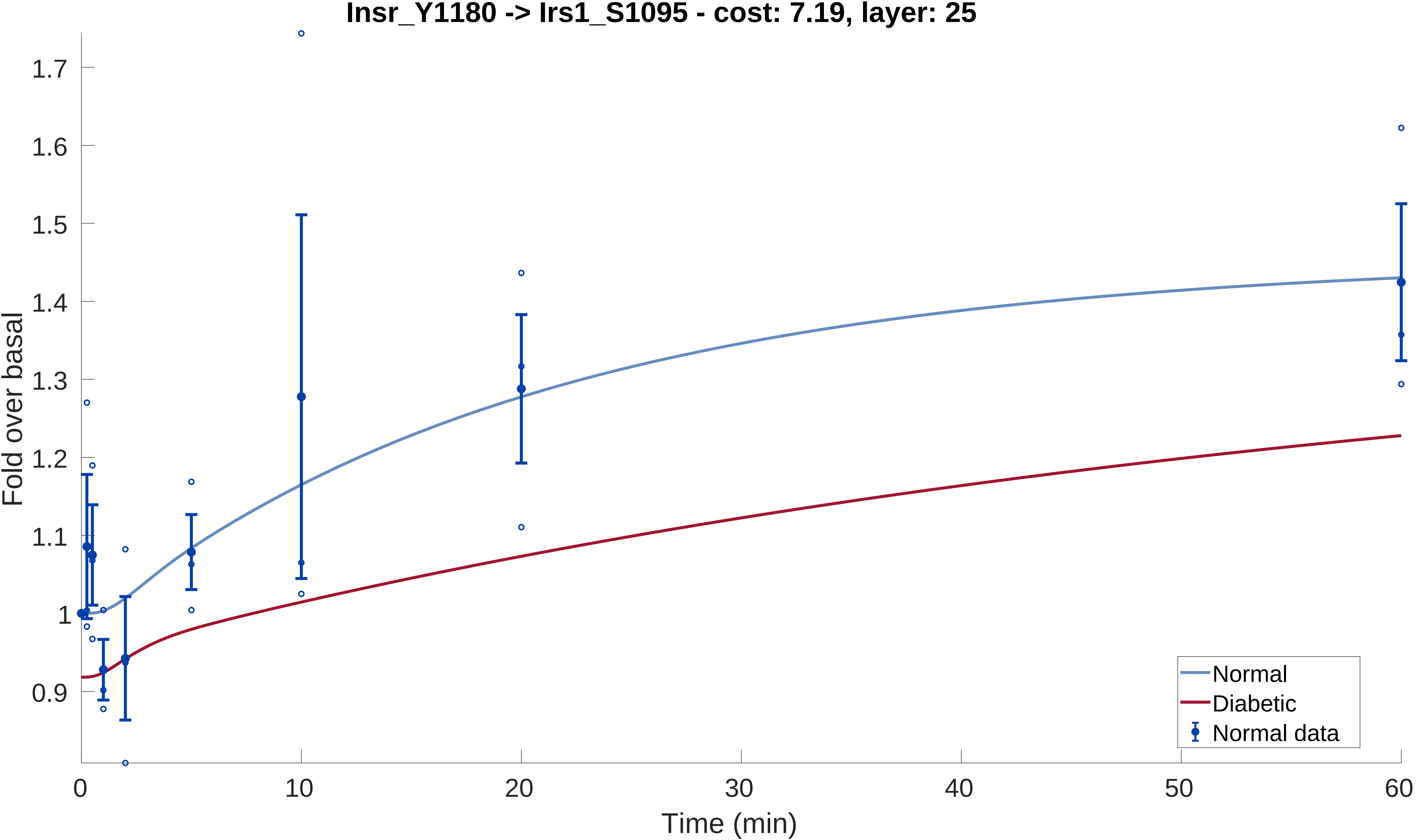

Insr_Y1180 -> Irs1_S1095 - cost: 7.19, layer: 25
Fold over basal
Time (min)
0.9
1
1.1
1.2
1.3
1.4
1.5
1.6
1.7
0
10
20
30
40
50
60
Normal
Diabetic
Normal data

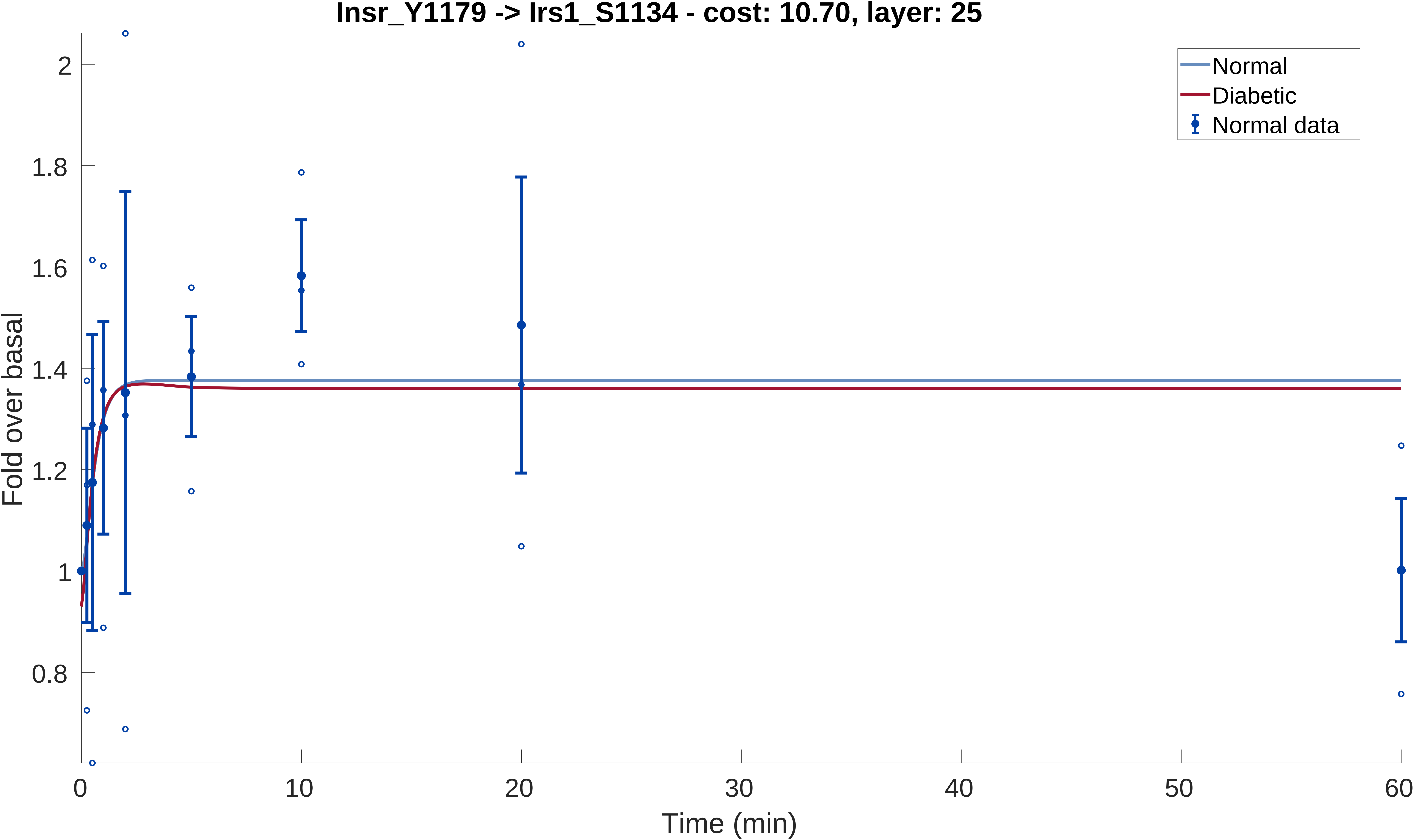
Insr_Y1179 -> Irs1_S1134 - cost: 10.70, layer: 25
Normal
Diabetic
Normal data
Fold over basal
2
1.8
1.6
1.4
1.2
1
0.8
0
10
20
30
40
50
60
Time (min)

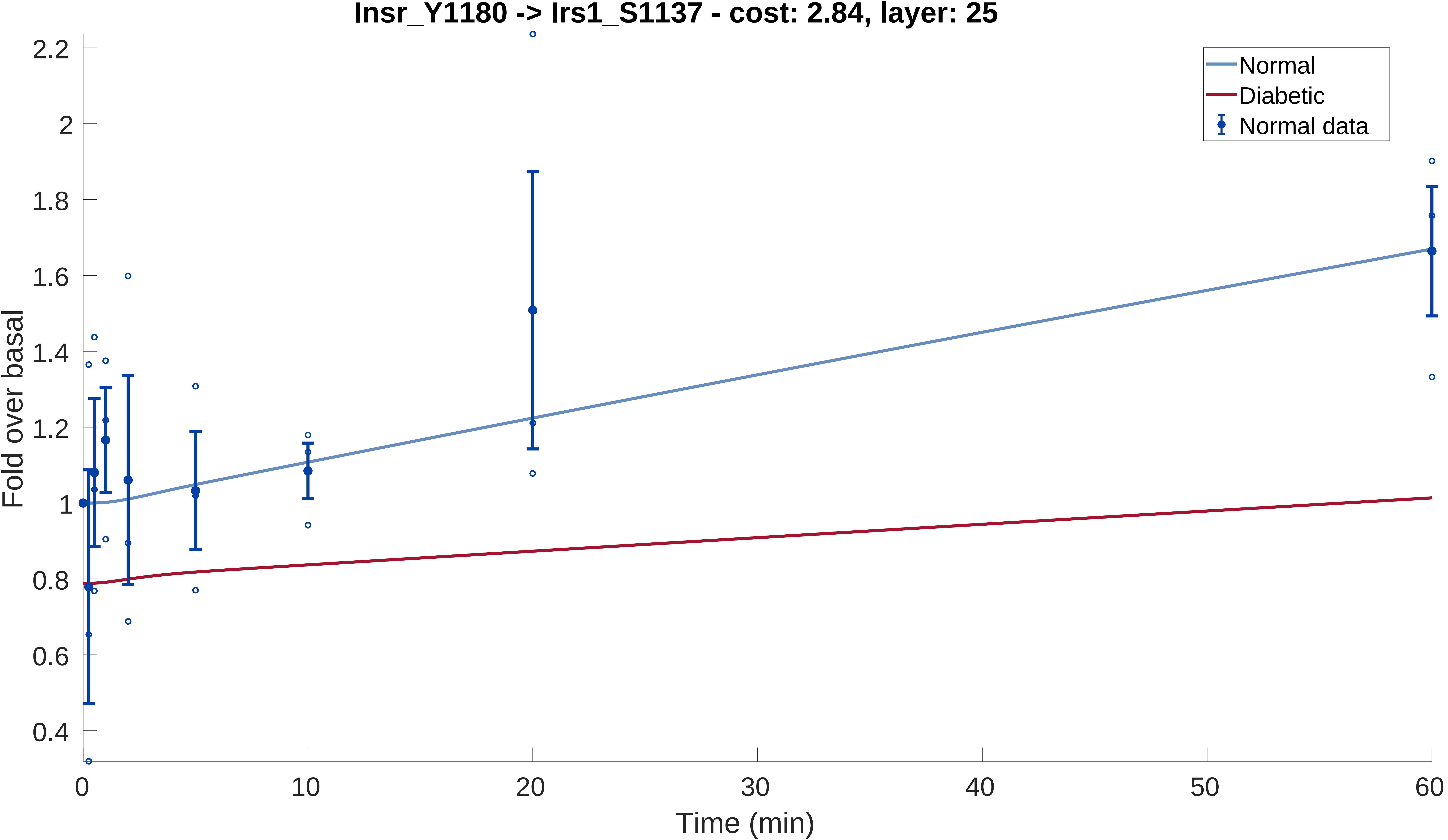
Insr_Y1180 -> Irs1_S1137 - cost: 2.84, layer: 25
Normal
Diabetic
Normal data
Fold over basal
Time (min)
2.2
2
1.8
1.6
1.4
1.2
1
0.8
0.6
0.4
0
10
20
30
40
50
60

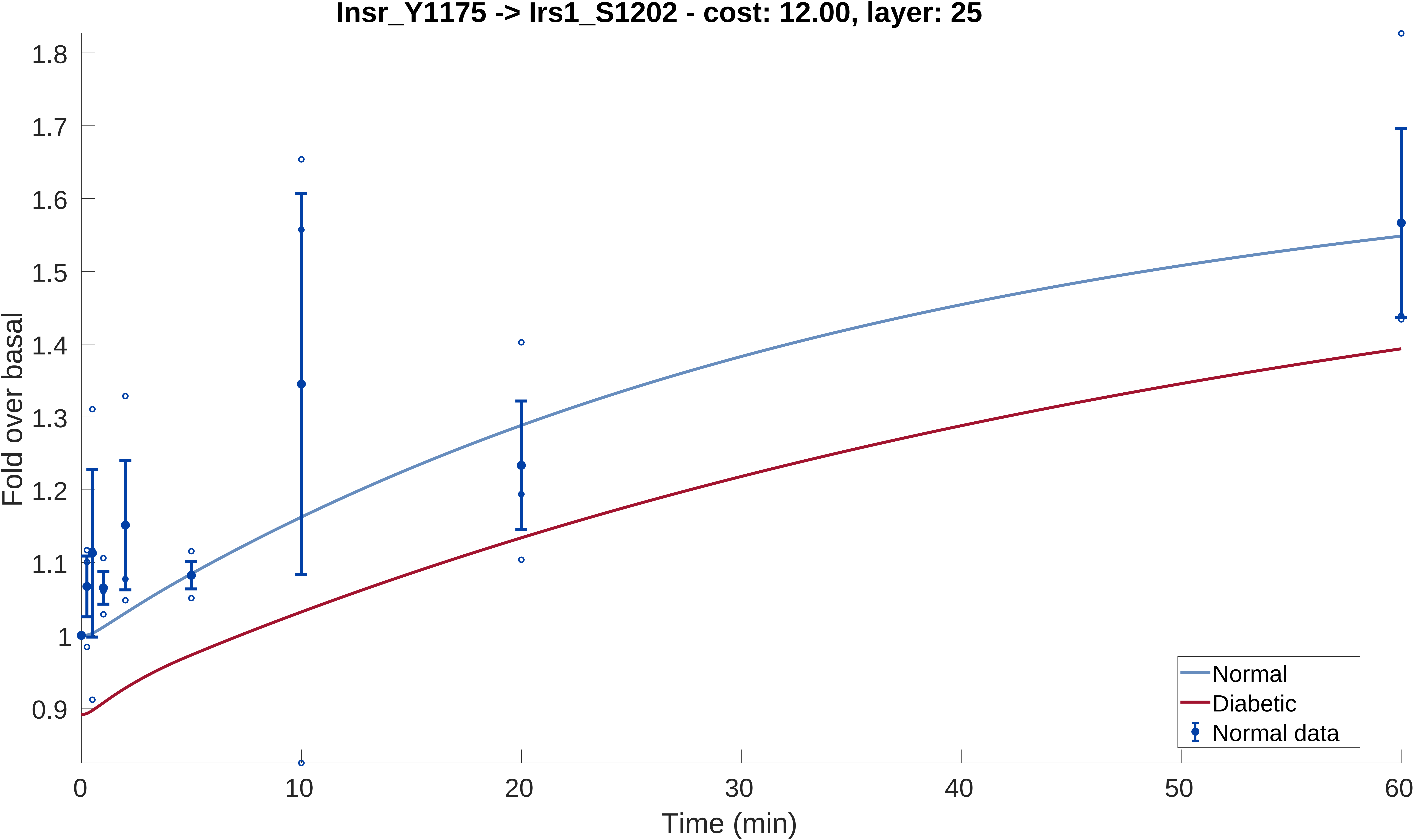

Insr_Y1175 -> Irs1_S1202 - cost: 12.00, layer: 25
Fold over basal
1.8
1.7
1.6
1.5
1.4
1.3
1.2
1.1
1
0.9
0
10
20
30
40
50
60
Time (min)
Normal
Diabetic
Normal data

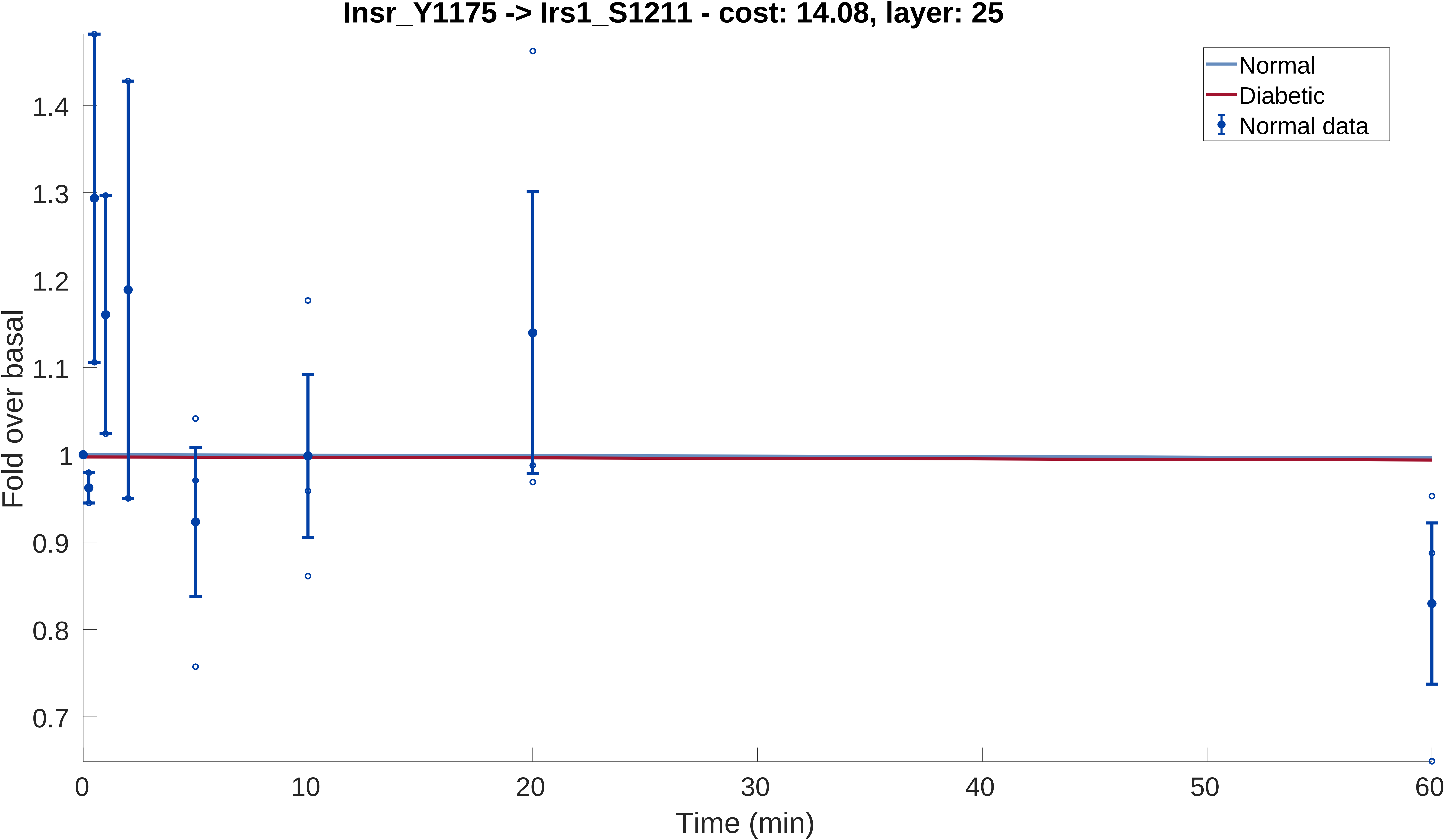
Insr_Y1175 -> Irs1_S1211 - cost: 14.08, layer: 25
Normal
Diabetic
Normal data
Fold over basal
Time (min)
1.4
1.3
1.2
1.1
1
0.9
0.8
0.7
0
10
20
30
40
50
60

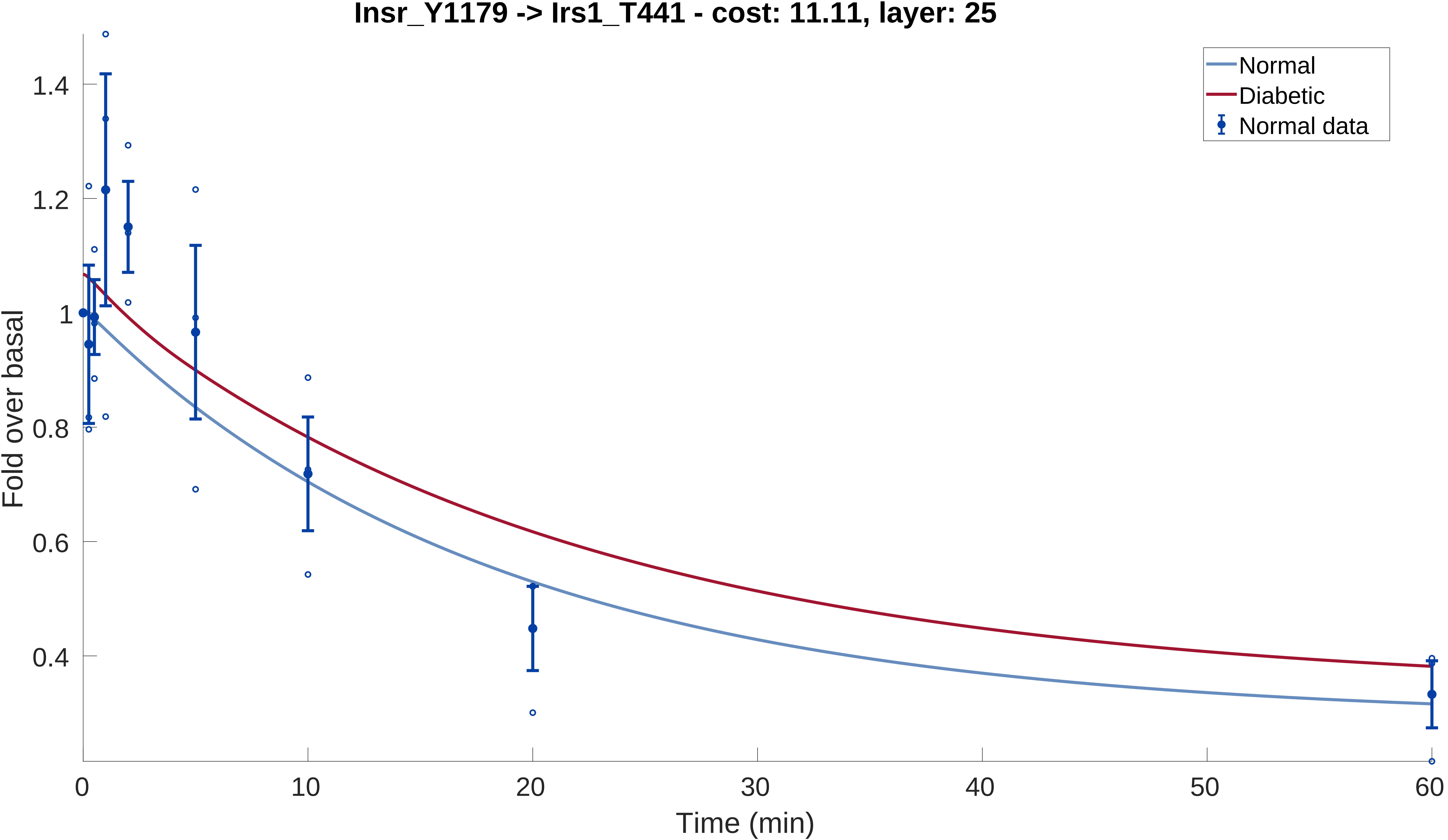
Insr_Y1179 -> Irs1_T441 - cost: 11.11, layer: 25
Normal
Diabetic
Normal data
Fold over basal
1.4
1.2
1
0.8
0.6
0.4
0
10
20
30
40
50
60
Time (min)

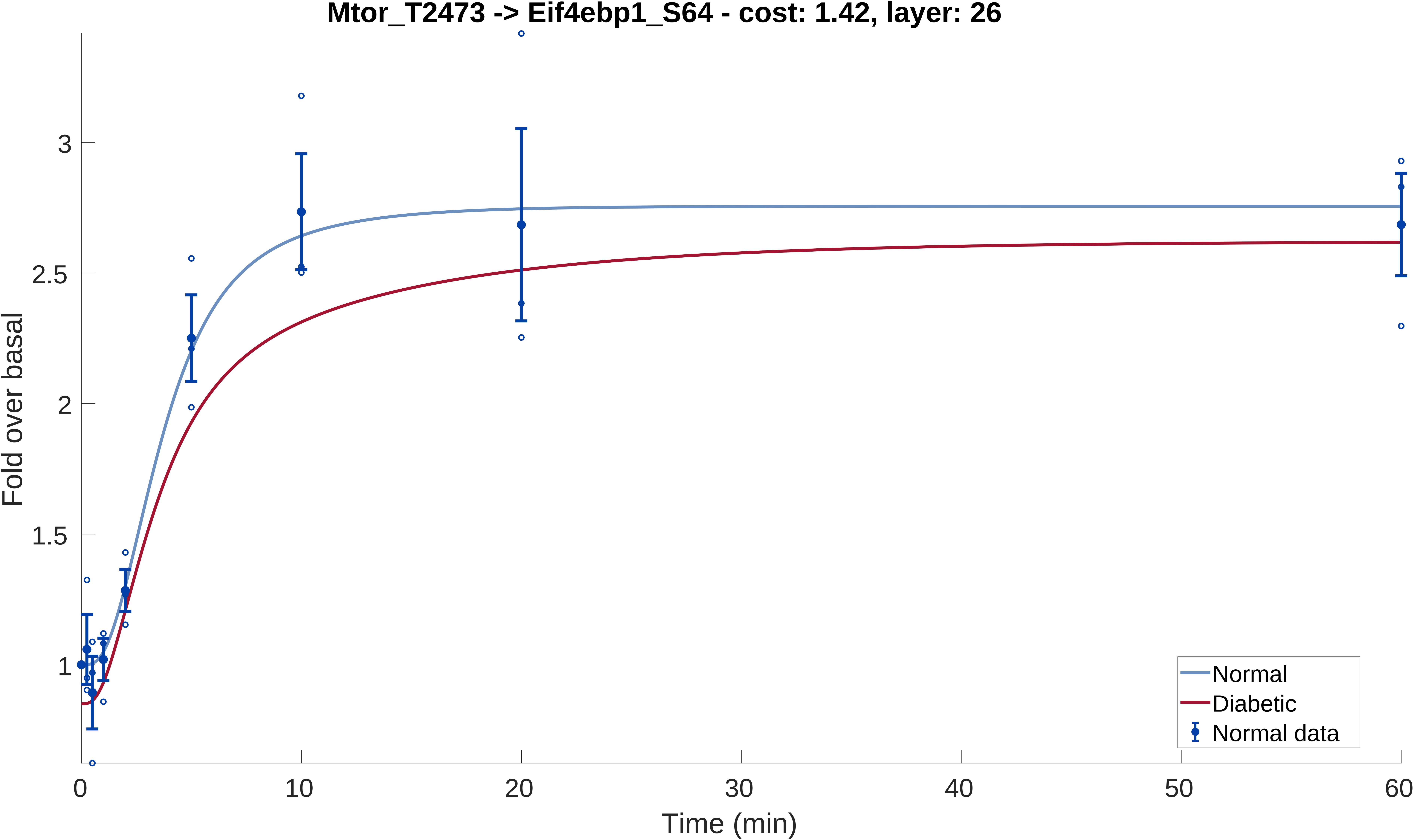
Mtor_T2473 -> Eif4ebp1_S64 - cost: 1.42, layer: 26
Fold over basal
3
2.5
2
1.5
1
0
10
20
30
40
50
60
Time (min)
Normal
Diabetic
Normal data

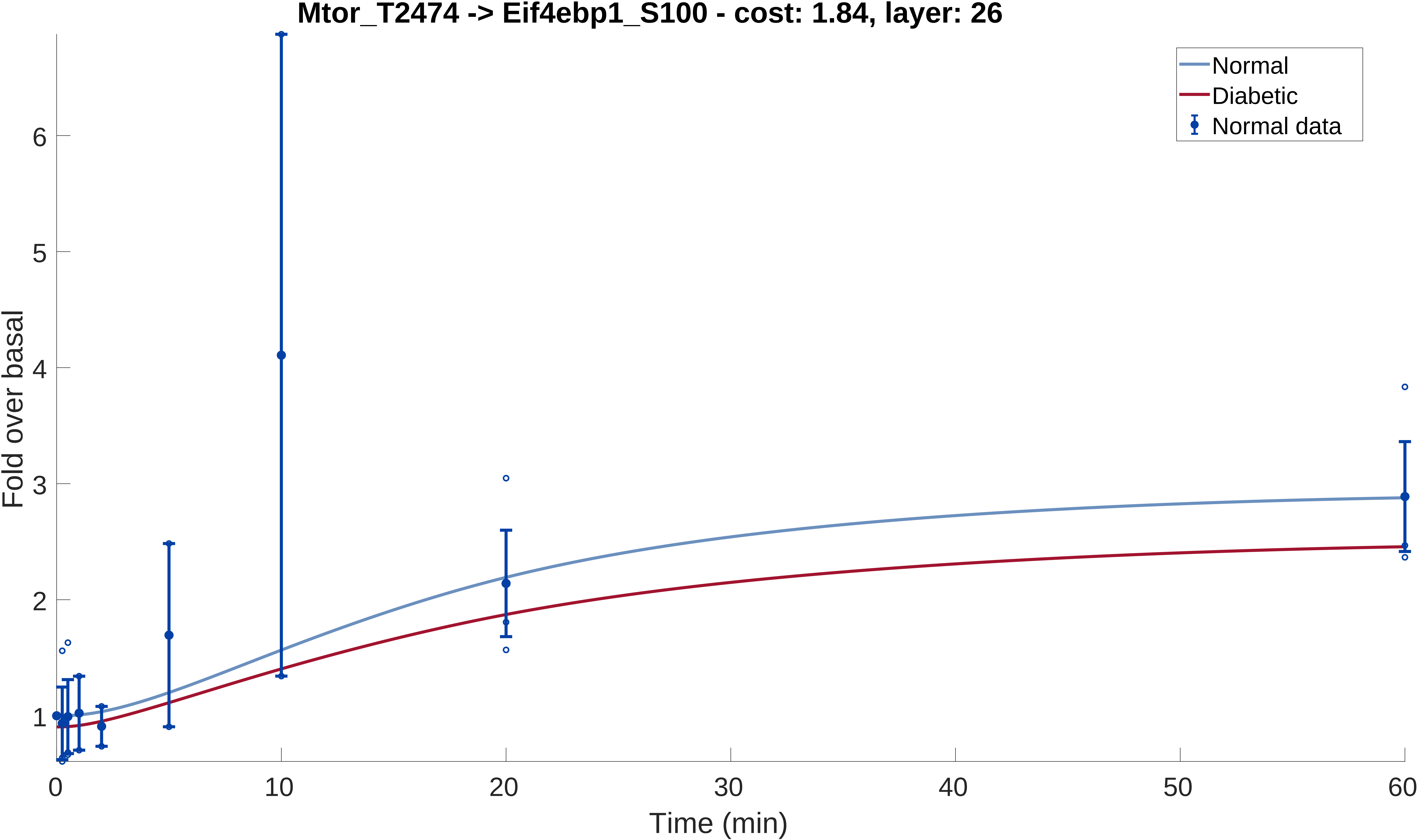

Mtor_T2474 -> Eif4ebp1_S100 - cost: 1.84, layer: 26
Normal
Diabetic
Normal data
Fold over basal
Time (min)
1
2
3
4
5
6
0
10
20
30
40
50
60

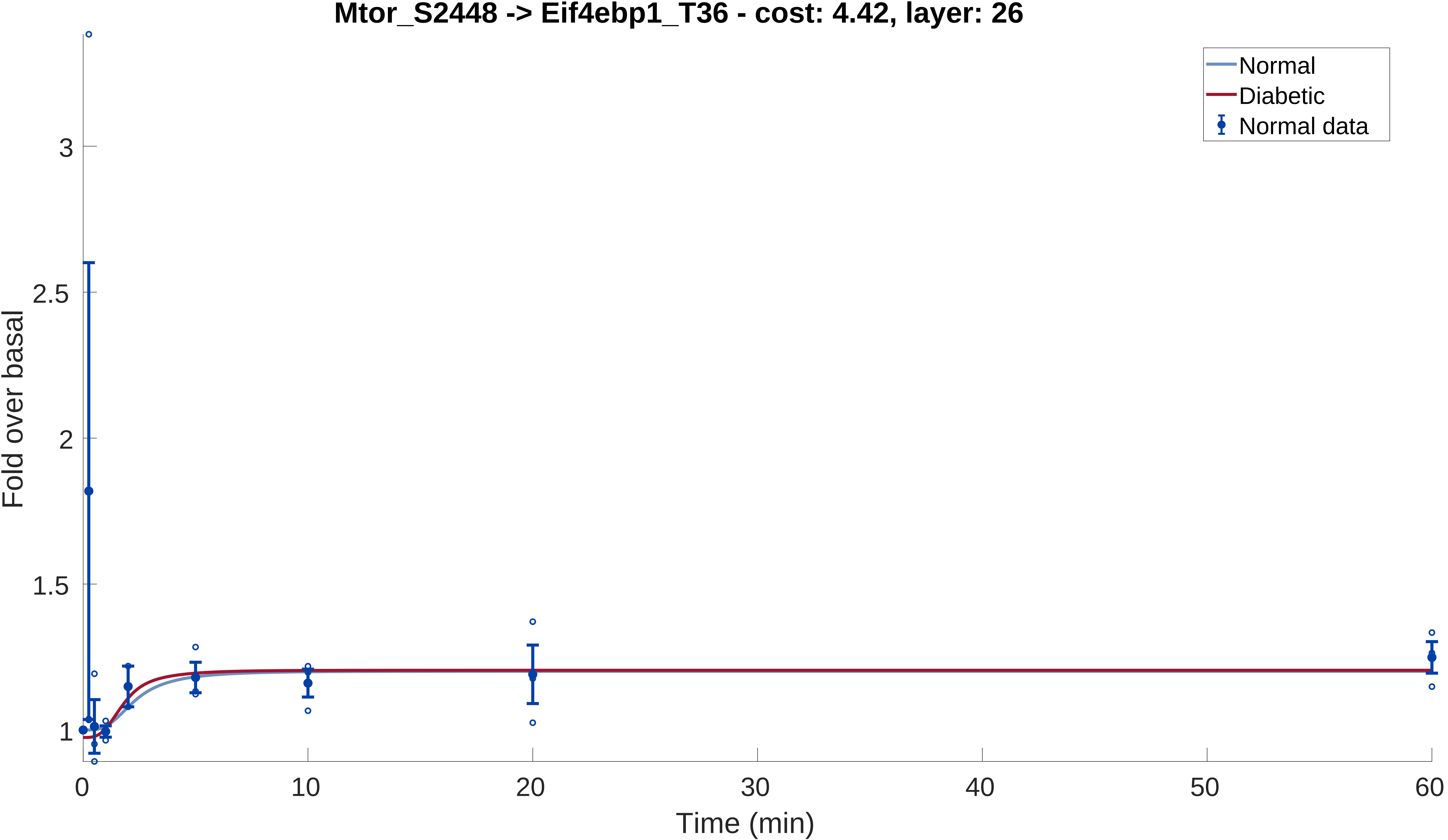

Mtor_S2448 -> Eif4ebp1_T36 - cost: 4.42, layer: 26
Normal
Diabetic
Normal data
Fold over basal
3
2.5
2
1.5
1
0
10
20
30
40
50
60
Time (min)

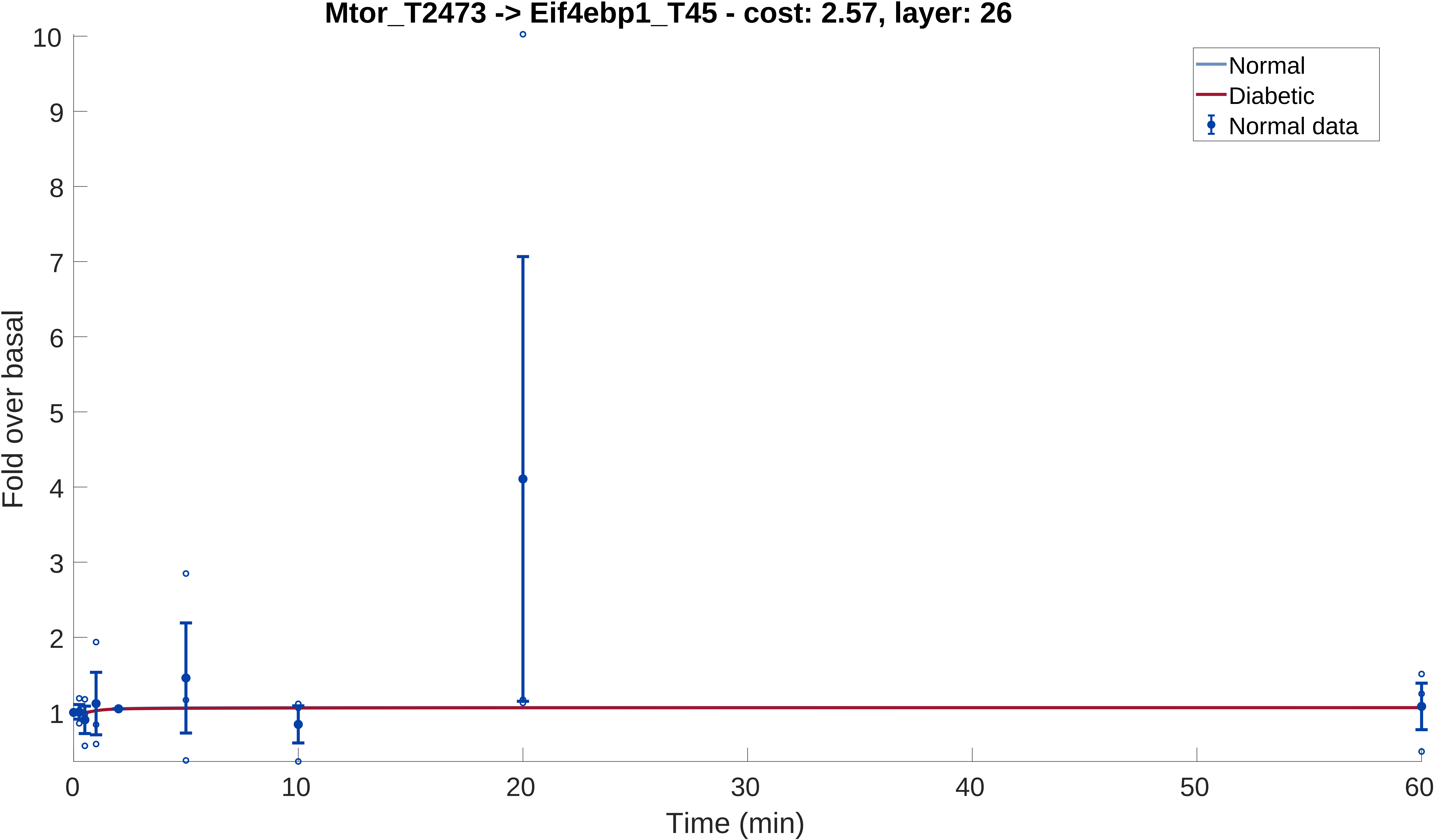
Mtor_T2473 -> Eif4ebp1_T45 - cost: 2.57, layer: 26
Normal
Diabetic
Normal data
Fold over basal
Time (min)
10
9
8
7
6
5
4
3
2
1
0
10
20
30
40
50
60

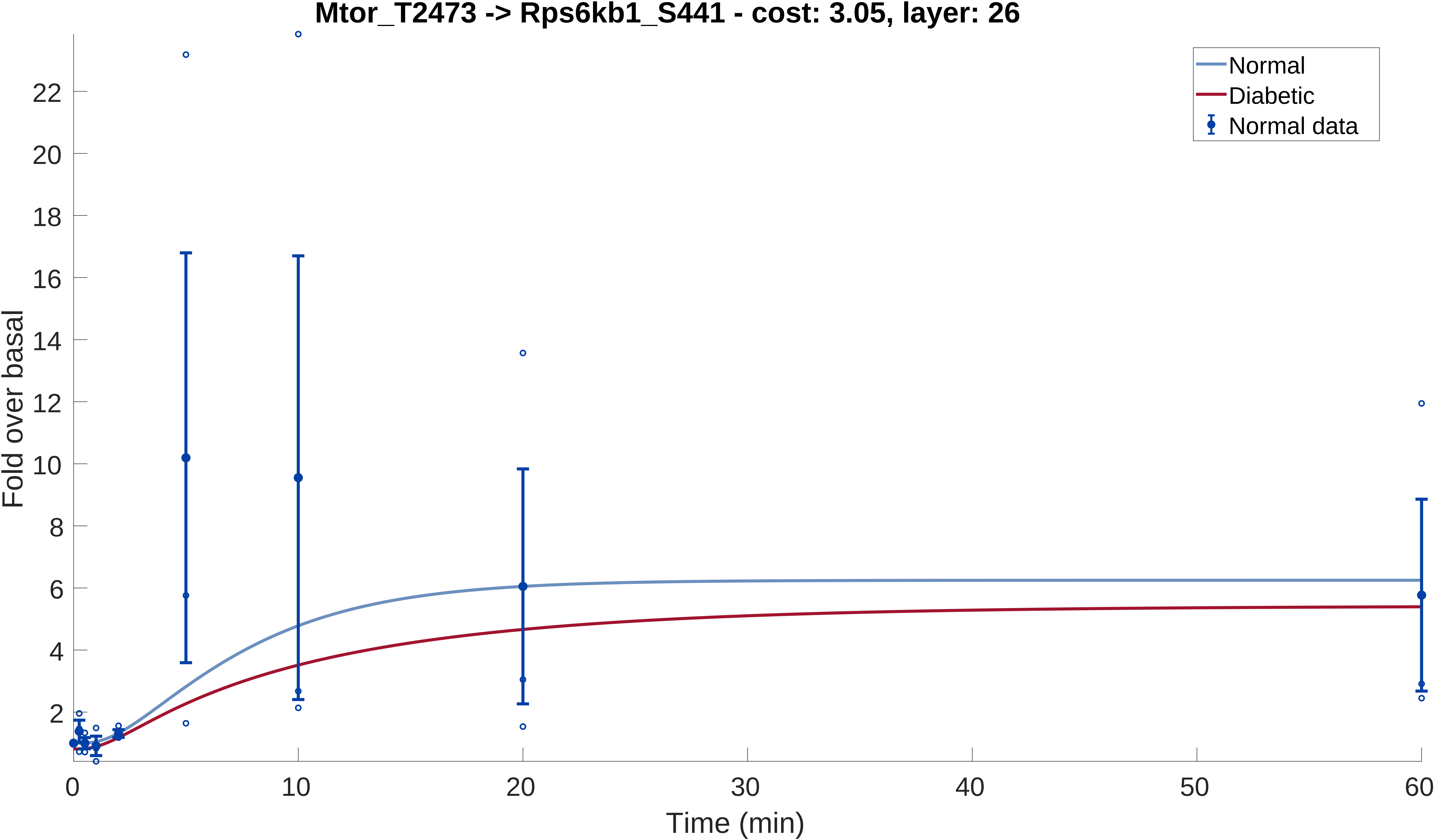
Mtor_T2473 -> Rps6kb1_S441 - cost: 3.05, layer: 26
Normal
Diabetic
Normal data
Fold over basal
22
20
18
16
14
12
10
8
6
4
2
0
10
20
30
40
50
60
Time (min)

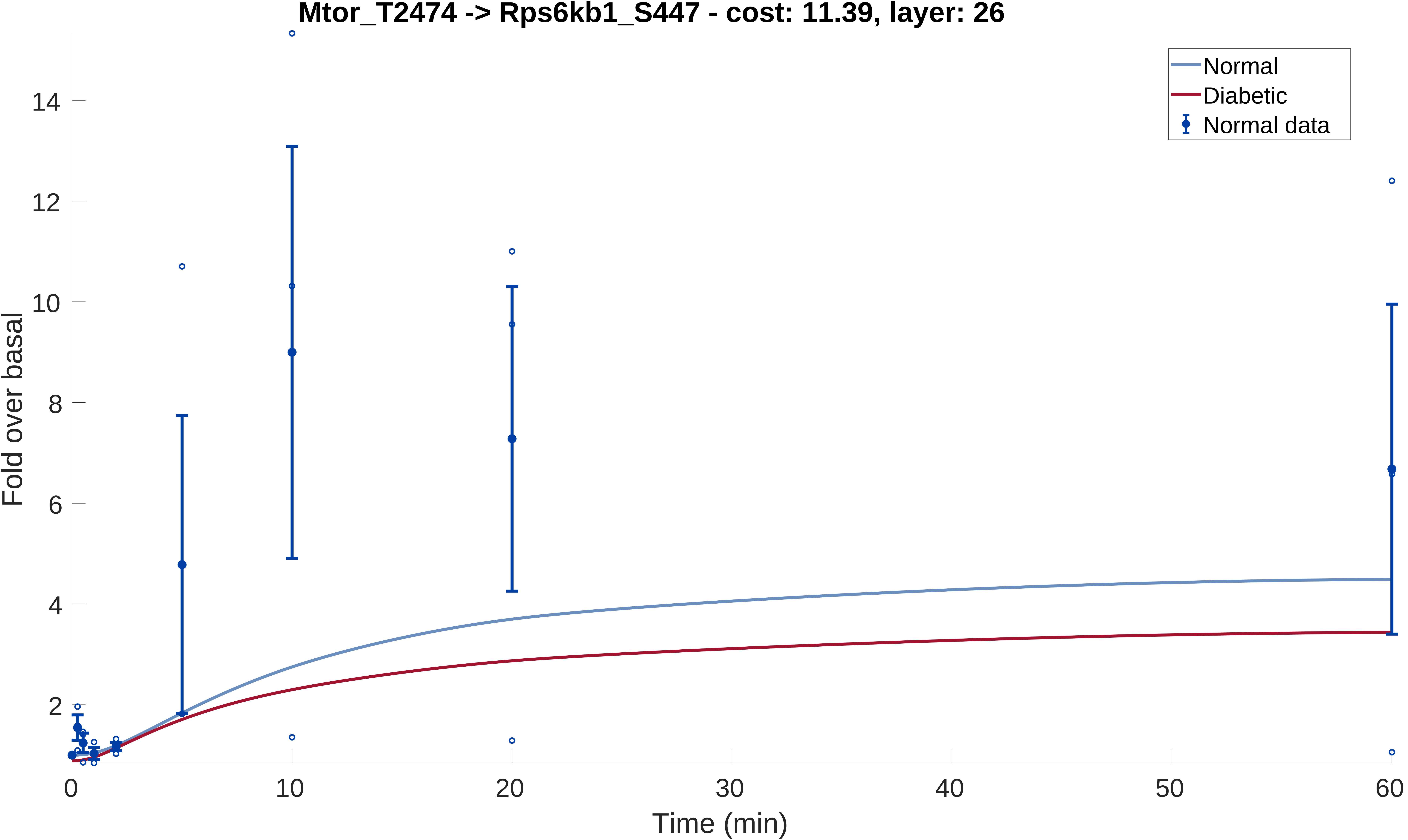

Mtor_T2474 -> Rps6kb1_S447 - cost: 11.39, layer: 26
Normal
Diabetic
Normal data
Fold over basal
Time (min)
0
10
20
30
40
50
60
2
4
6
8
10
12
14

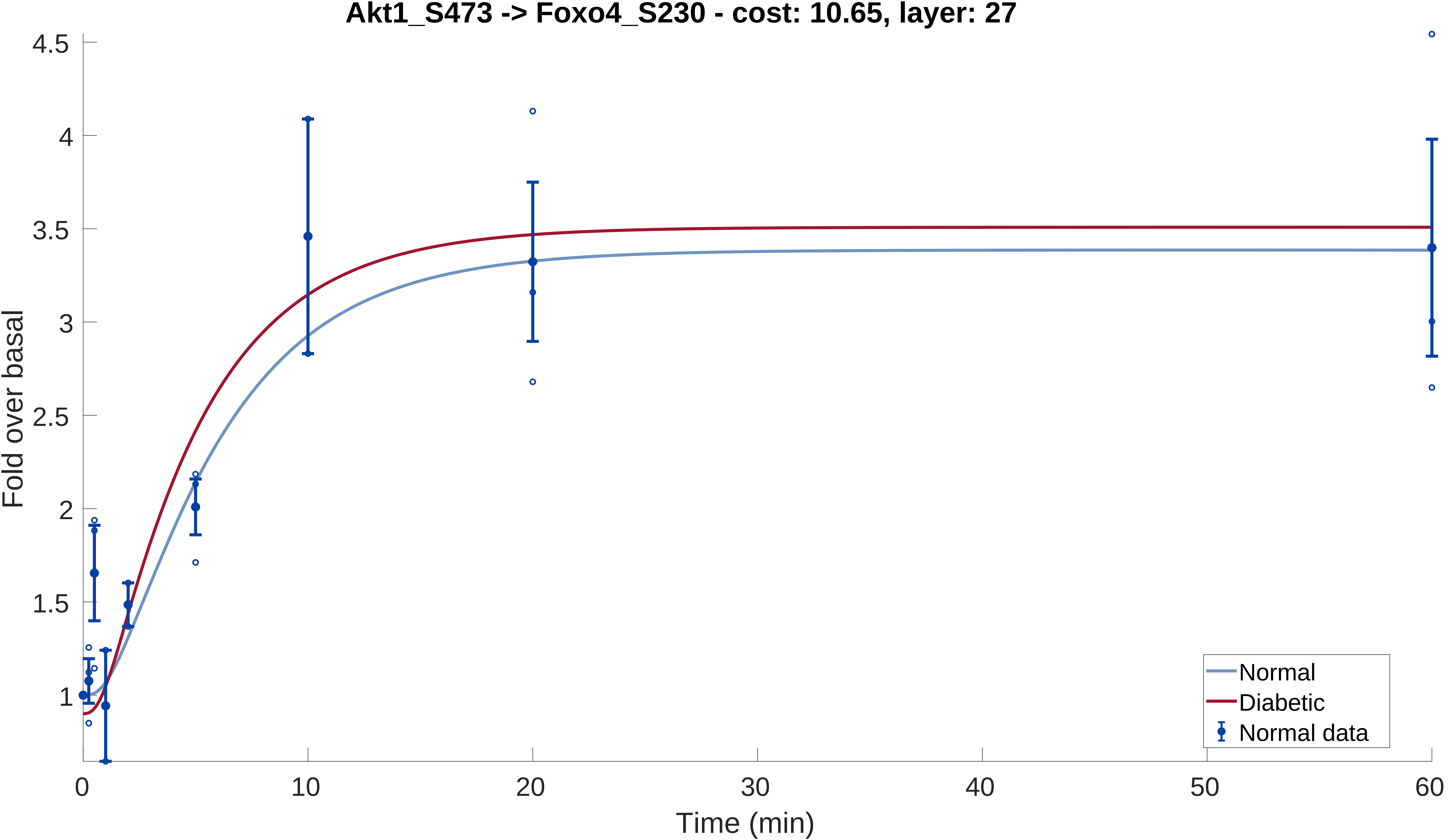
Akt1_S473 -> Foxo4_S230 - cost: 10.65, layer: 27
Fold over basal
Time (min)
0
10
20
30
40
50
60
1
1.5
2
2.5
3
3.5
4
4.5
Normal
Diabetic
Normal data

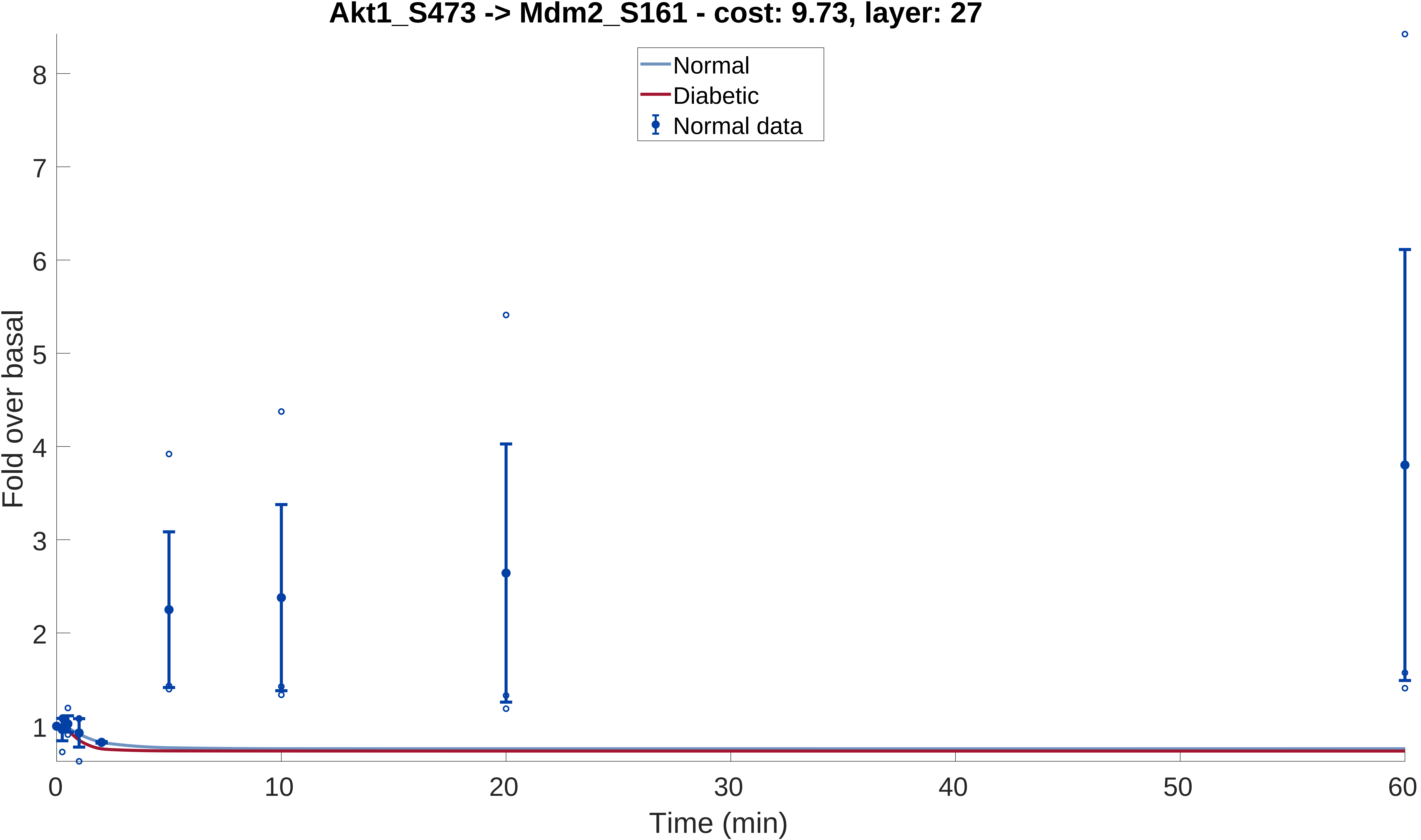

Akt1_S473 -> Mdm2_S161 - cost: 9.73, layer: 27
Normal
Diabetic
Normal data
Fold over basal
1
2
3
4
5
6
7
8
0
10
20
30
40
50
60
Time (min)

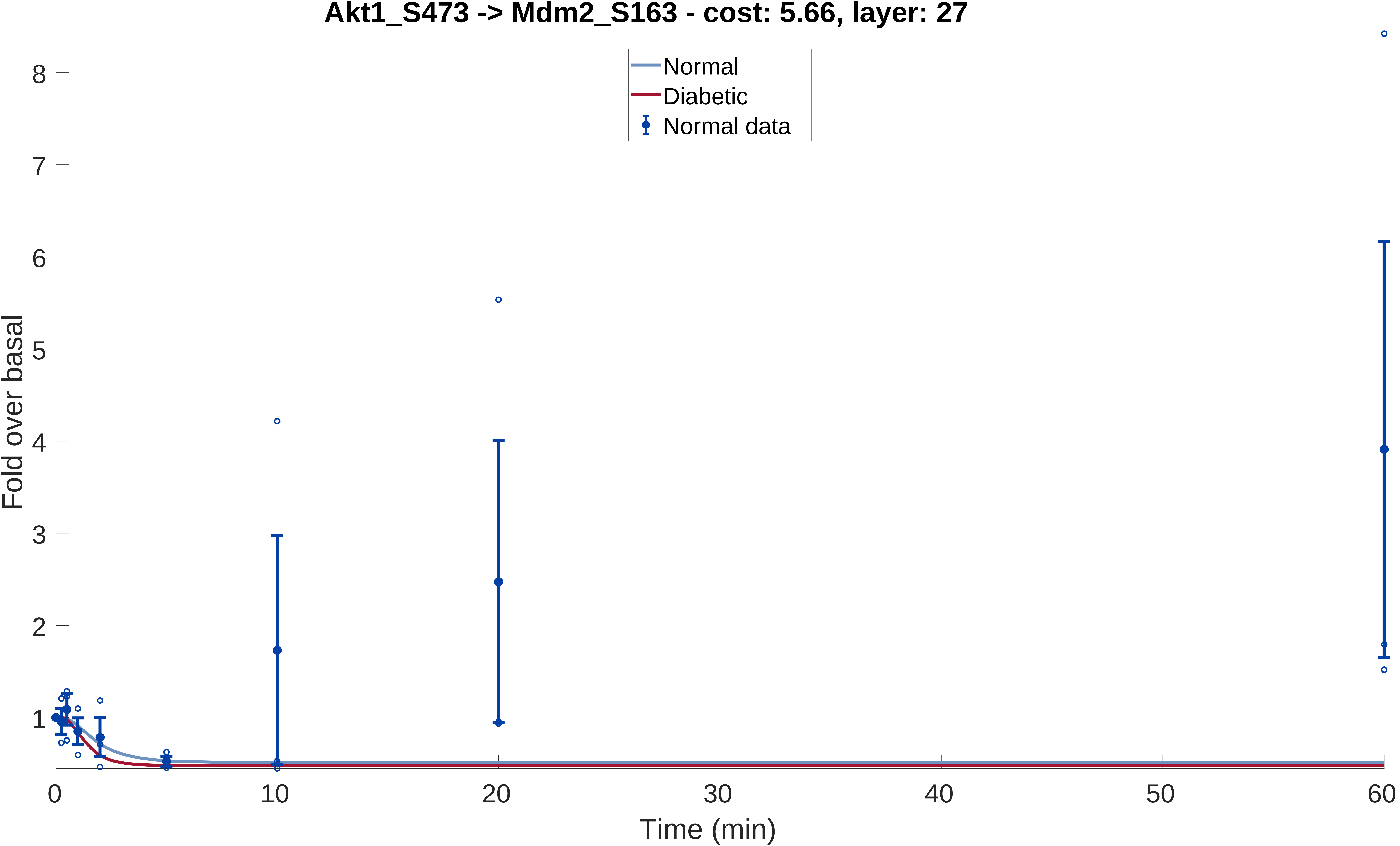

Akt1_S473 -> Mdm2_S163 - cost: 5.66, layer: 27
Normal
Diabetic
Normal data
Fold over basal
Time (min)
1
2
3
4
5
6
7
8
0
10
20
30
40
50
60

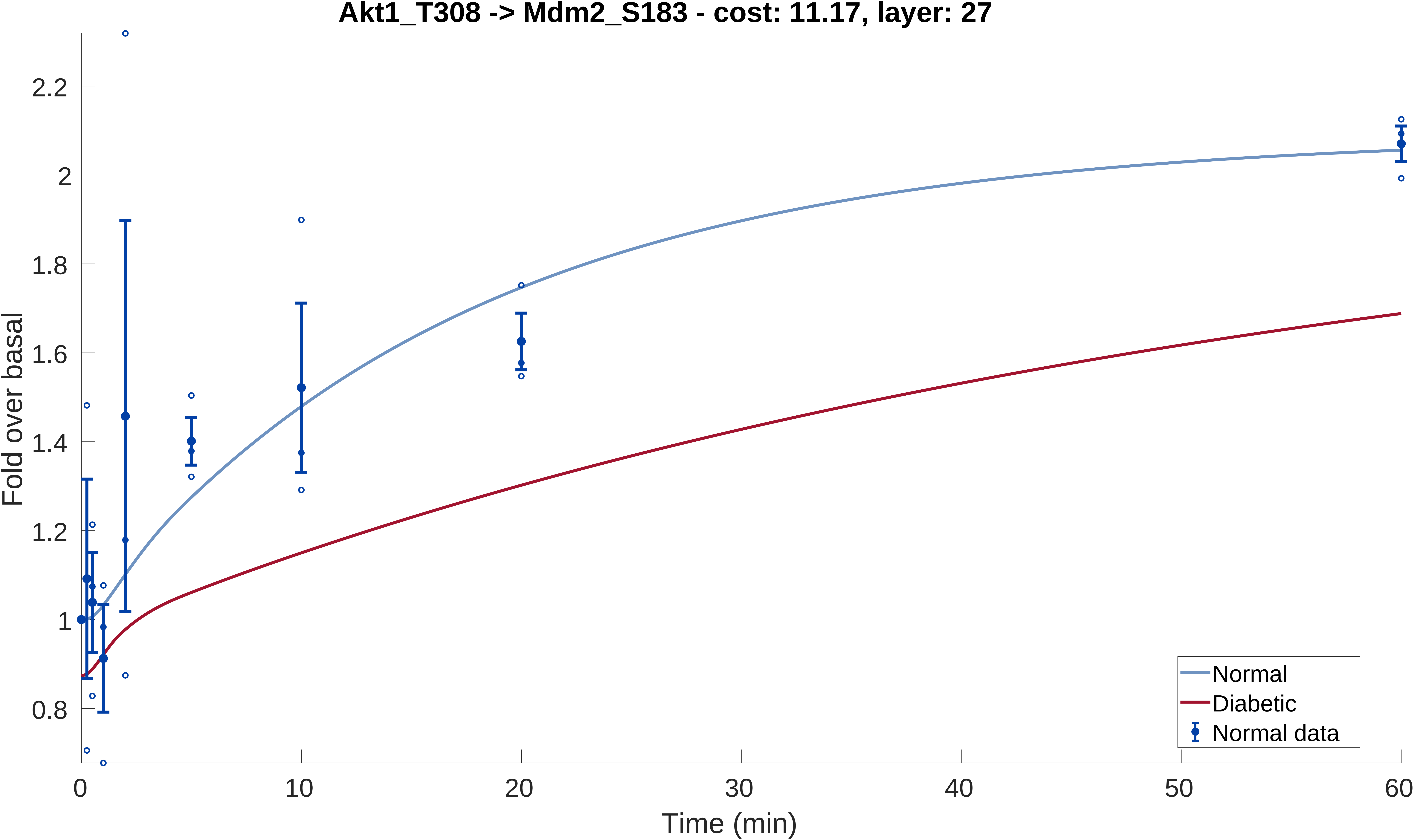

Akt1_T308 -> Mdm2_S183 - cost: 11.17, layer: 27
Fold over basal
2.2
2
1.8
1.6
1.4
1.2
1
0.8
0
10
20
30
40
50
60
Time (min)
Normal
Diabetic
Normal data

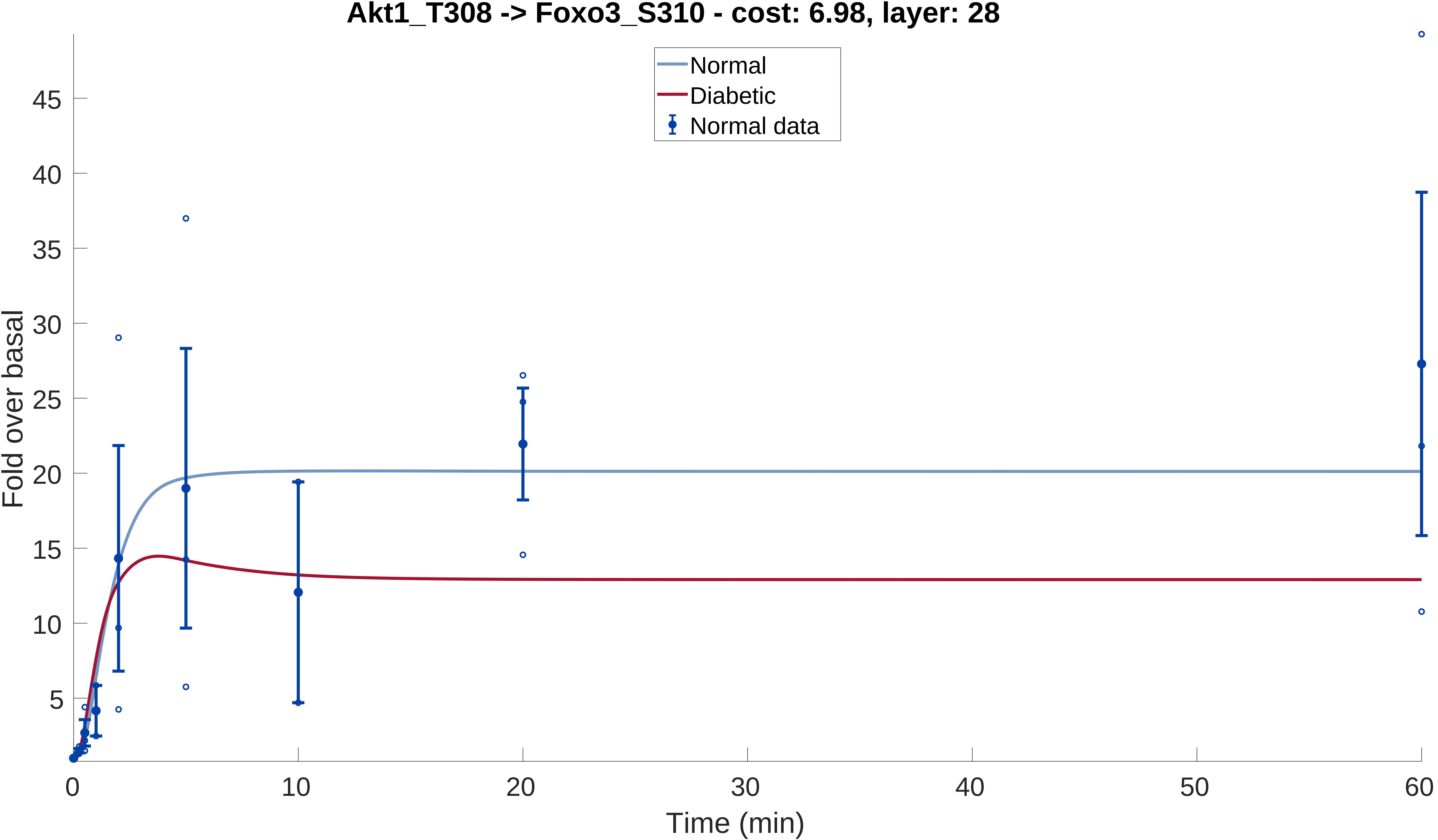
Akt1_T308 -> Foxo3_S310 - cost: 6.98, layer: 28
Normal
Diabetic
Normal data
Fold over basal
Time (min)
45
40
35
30
25
20
15
10
5
0
10
20
30
40
50
60

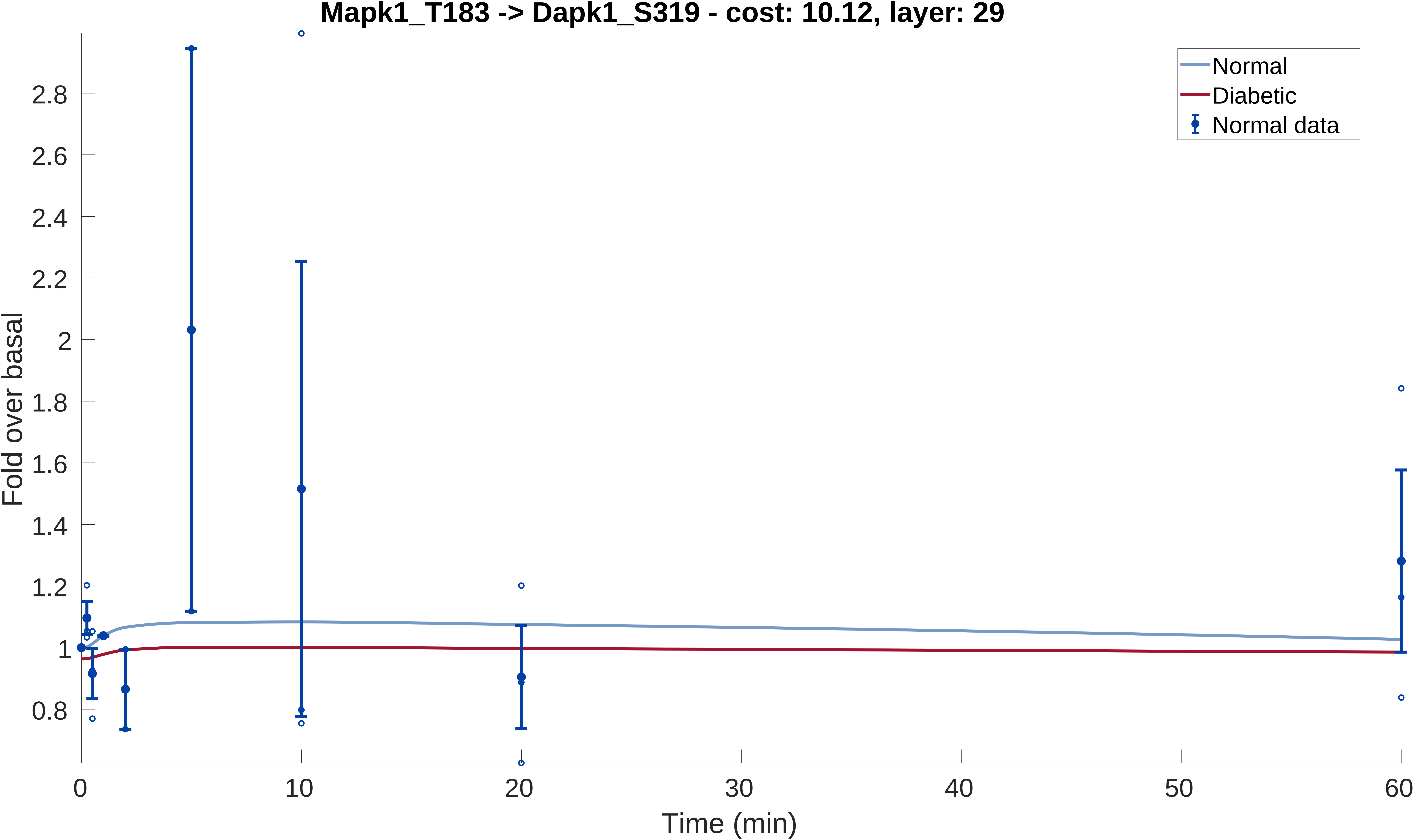

Mapk1_T183 -> Dapk1_S319 - cost: 10.12, layer: 29
Normal
Diabetic
Normal data
Fold over basal
2.8
2.6
2.4
2.2
2
1.8
1.6
1.4
1.2
1
0.8
0
10
20
30
40
50
60
Time (min)

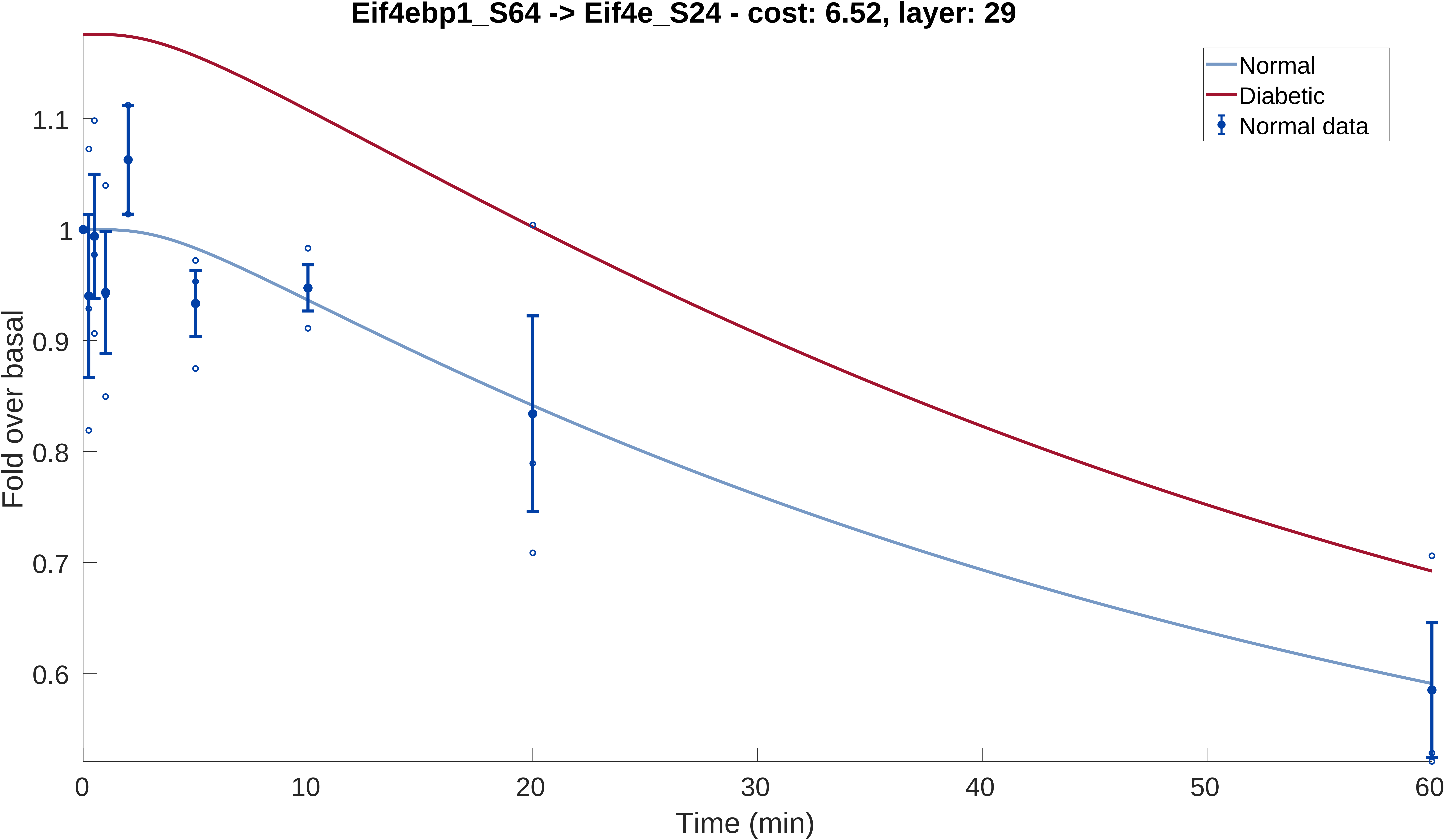

Eif4ebp1_S64 -> Eif4e_S24 - cost: 6.52, layer: 29
Normal
Diabetic
Normal data
Fold over basal
1.1
1
0.9
0.8
0.7
0.6
0
10
20
30
40
50
60
Time (min)

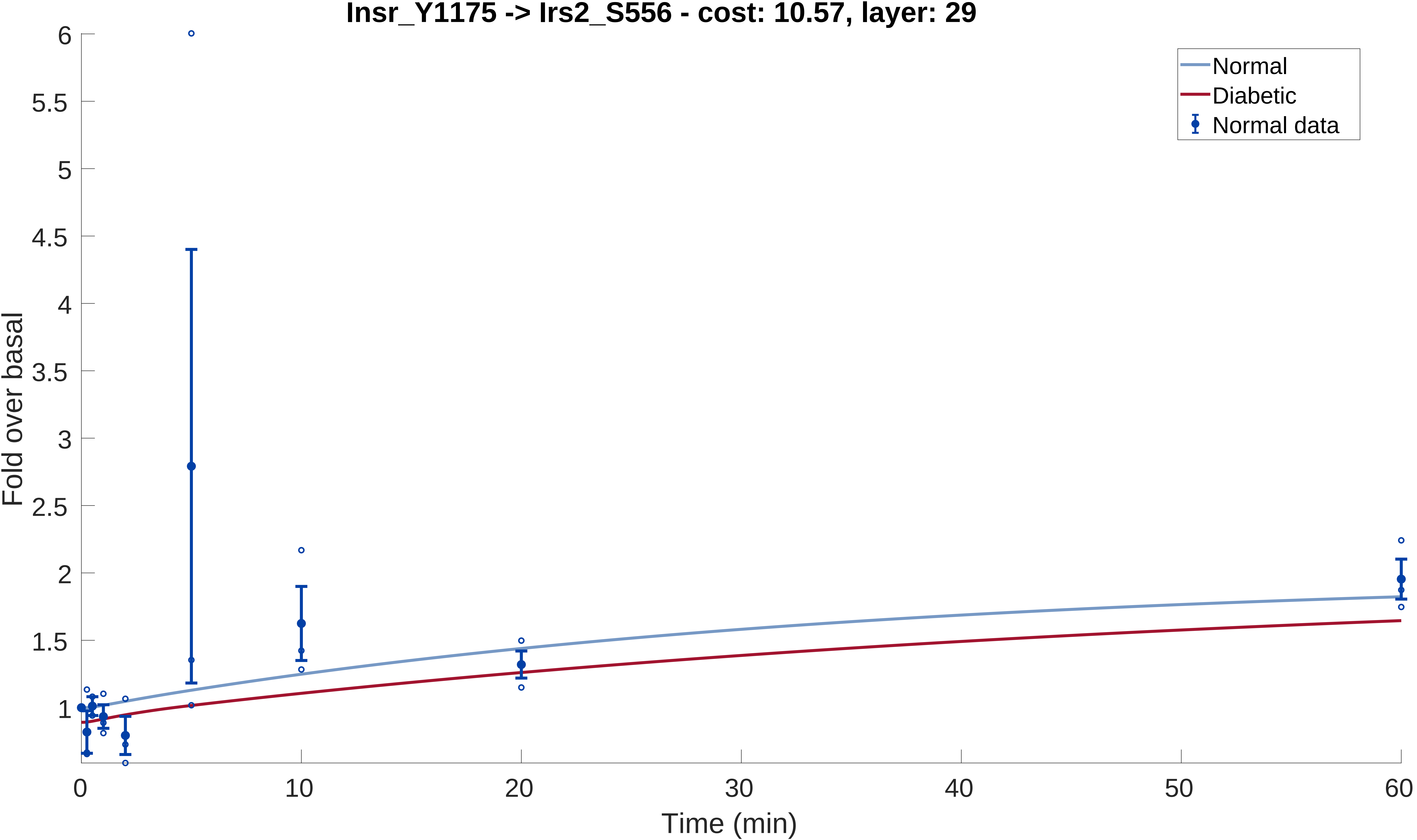

Insr_Y1175 -> Irs2_S556 - cost: 10.57, layer: 29
Normal
Diabetic
Normal data
Fold over basal
Time (min)
6
5.5
5
4.5
4
3.5
3
2.5
2
1.5
1
0
10
20
30
40
50
60

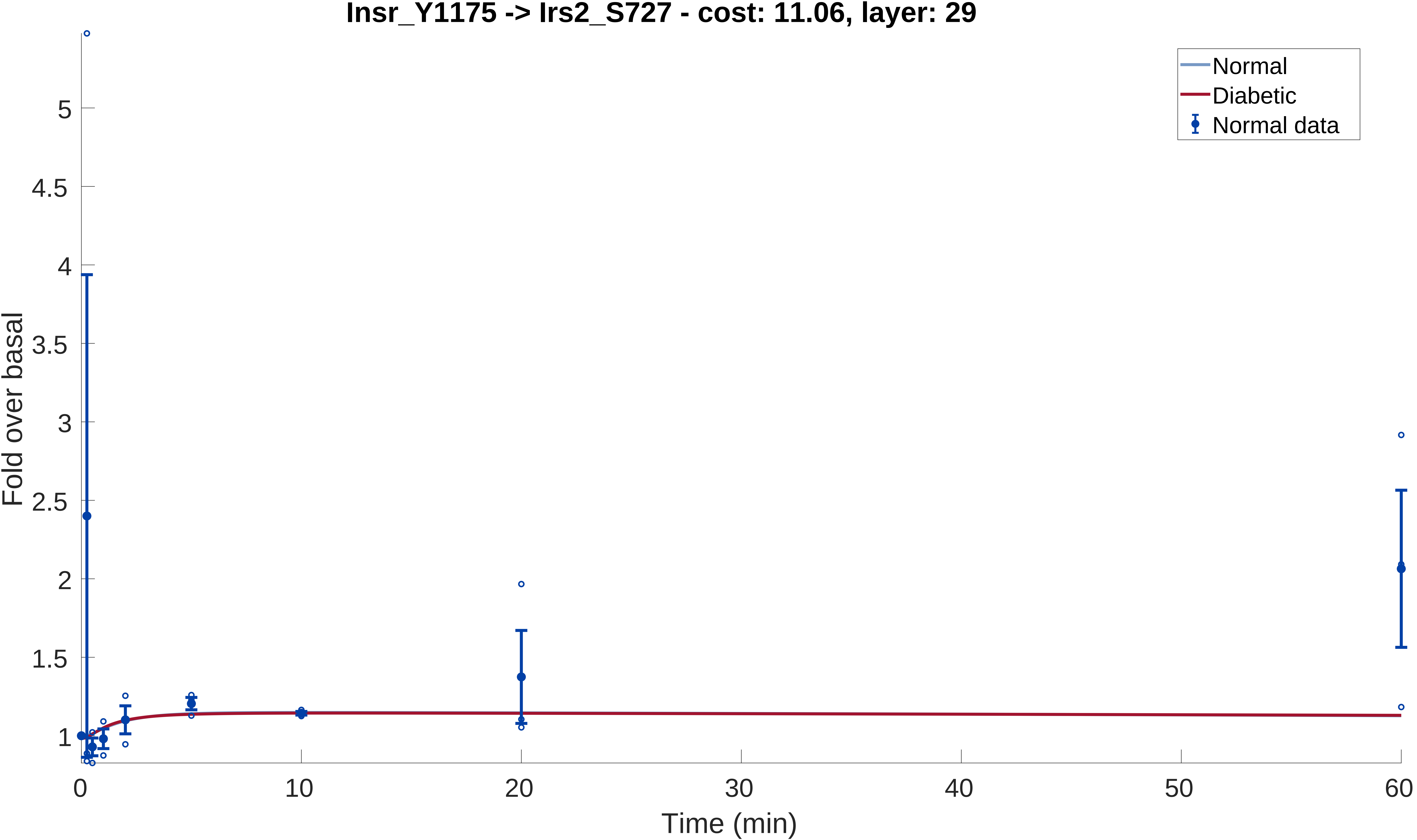

Insr_Y1175 -> Irs2_S727 - cost: 11.06, layer: 29
Normal
Diabetic
Normal data
Fold over basal
5
4.5
4
3.5
3
2.5
2
1.5
1
0
10
20
30
40
50
60
Time (min)

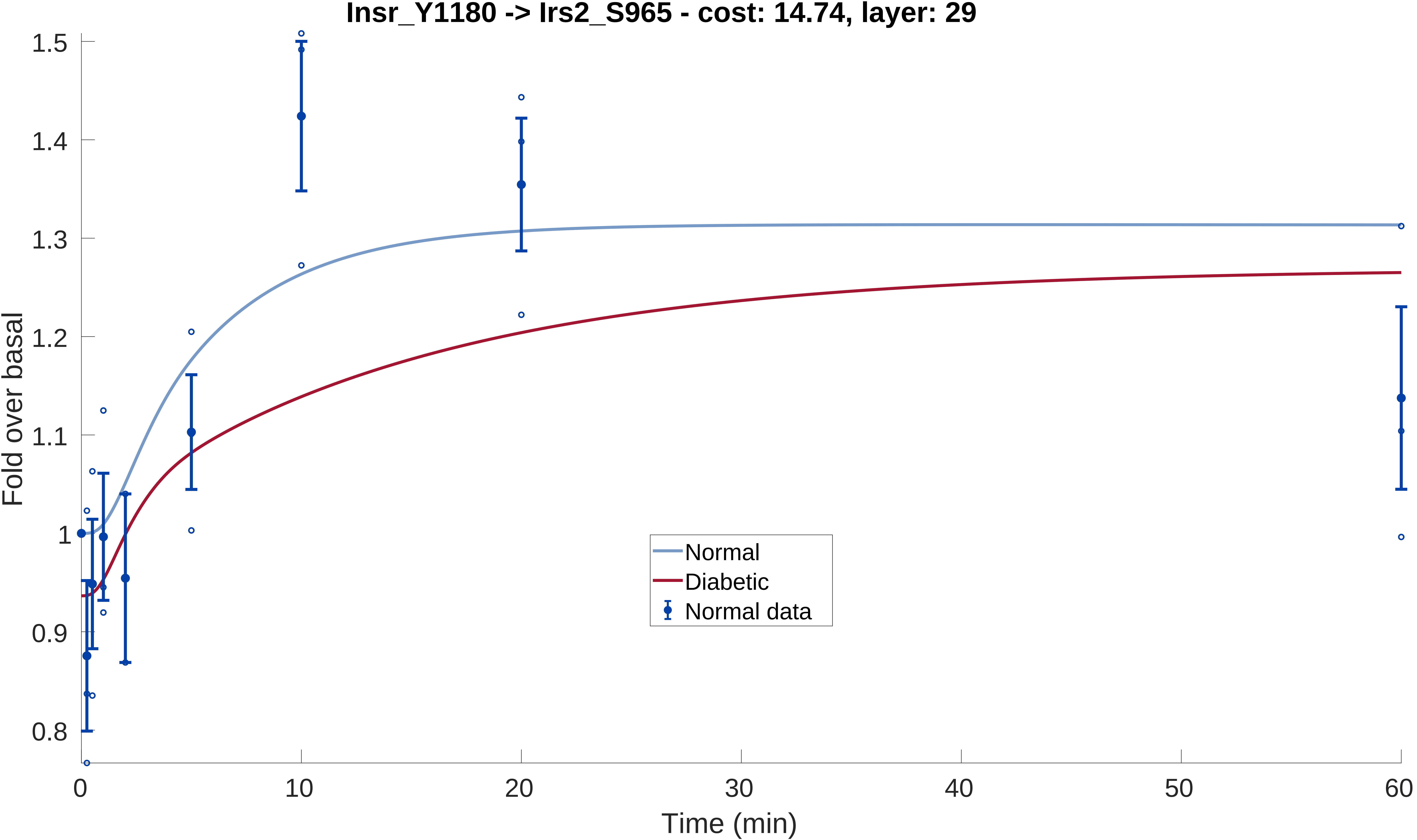
Insr_Y1180 -> Irs2_S965 - cost: 14.74, layer: 29
Fold over basal
Time (min)
Normal
Diabetic
Normal data
0.8
0.9
1
1.1
1.2
1.3
1.4
1.5
0
10
20
30
40
50
60

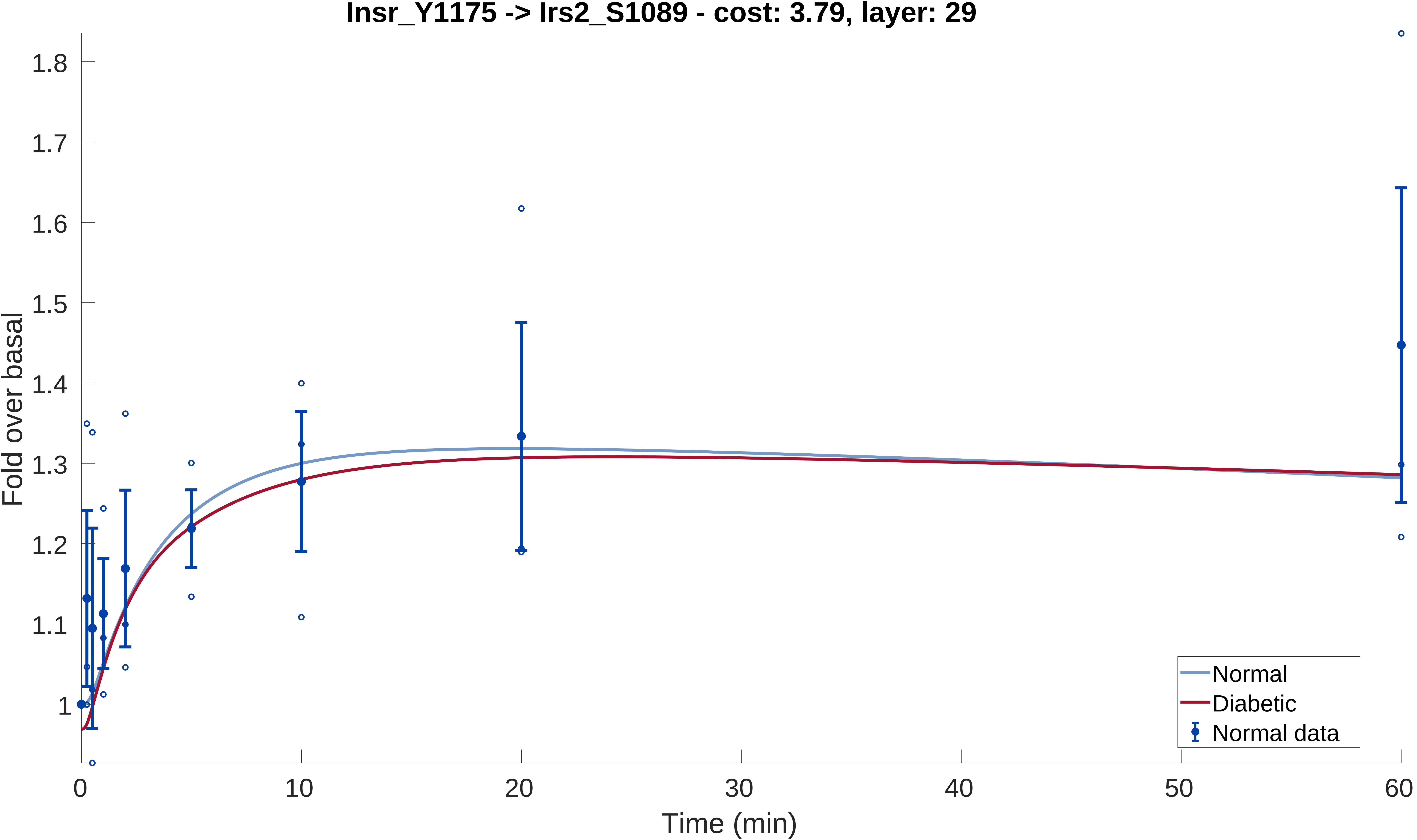

Insr_Y1175 -> Irs2_S1089 - cost: 3.79, layer: 29
Fold over basal
1.8
1.7
1.6
1.5
1.4
1.3
1.2
1.1
1
0
10
20
30
40
50
60
Time (min)
Normal
Diabetic
Normal data

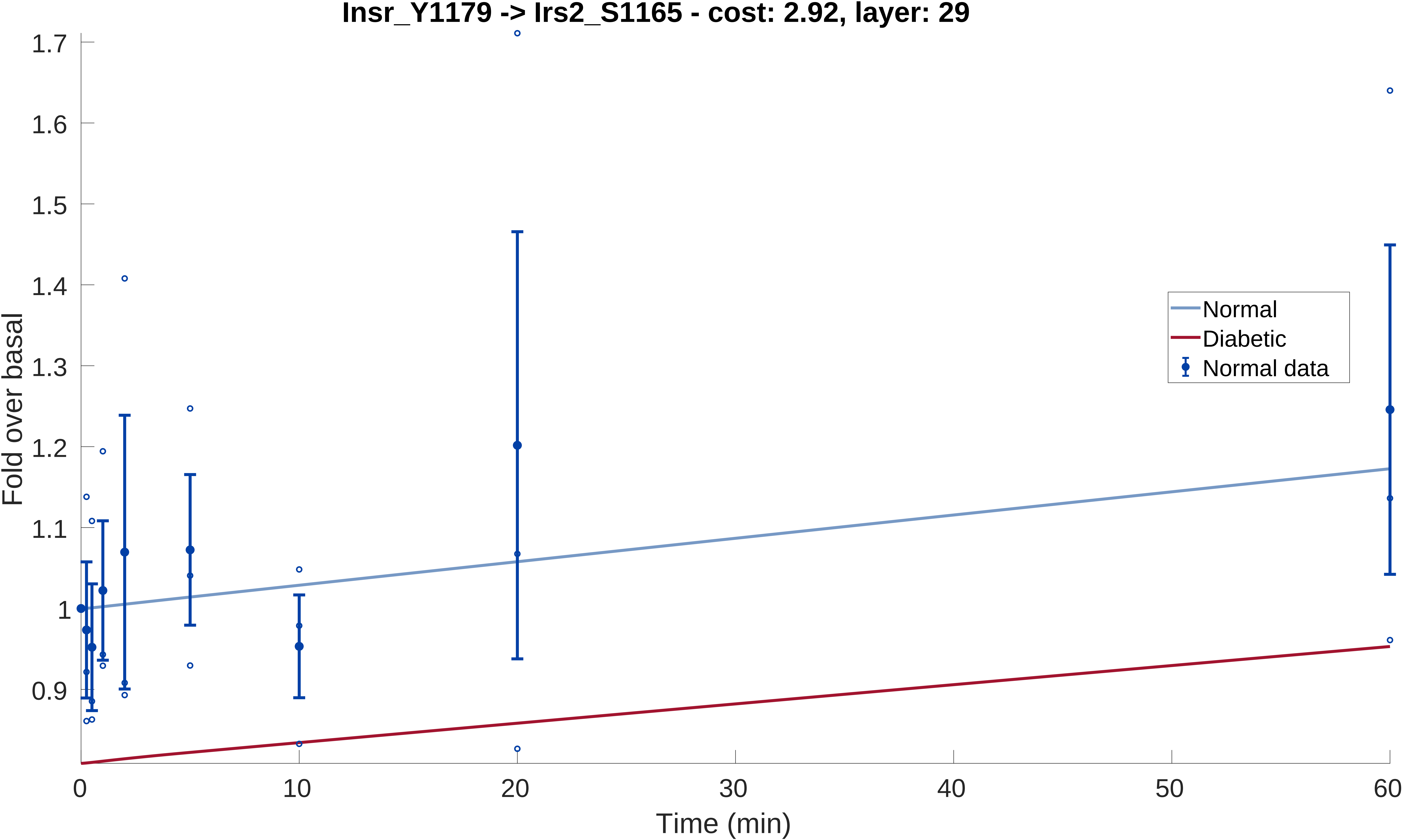

Insr_Y1179 -> Irs2_S1165 - cost: 2.92, layer: 29
Fold over basal
1.7
1.6
1.5
1.4
1.3
1.2
1.1
1
0.9
Time (min)
0
10
20
30
40
50
60
Normal
Diabetic
Normal data

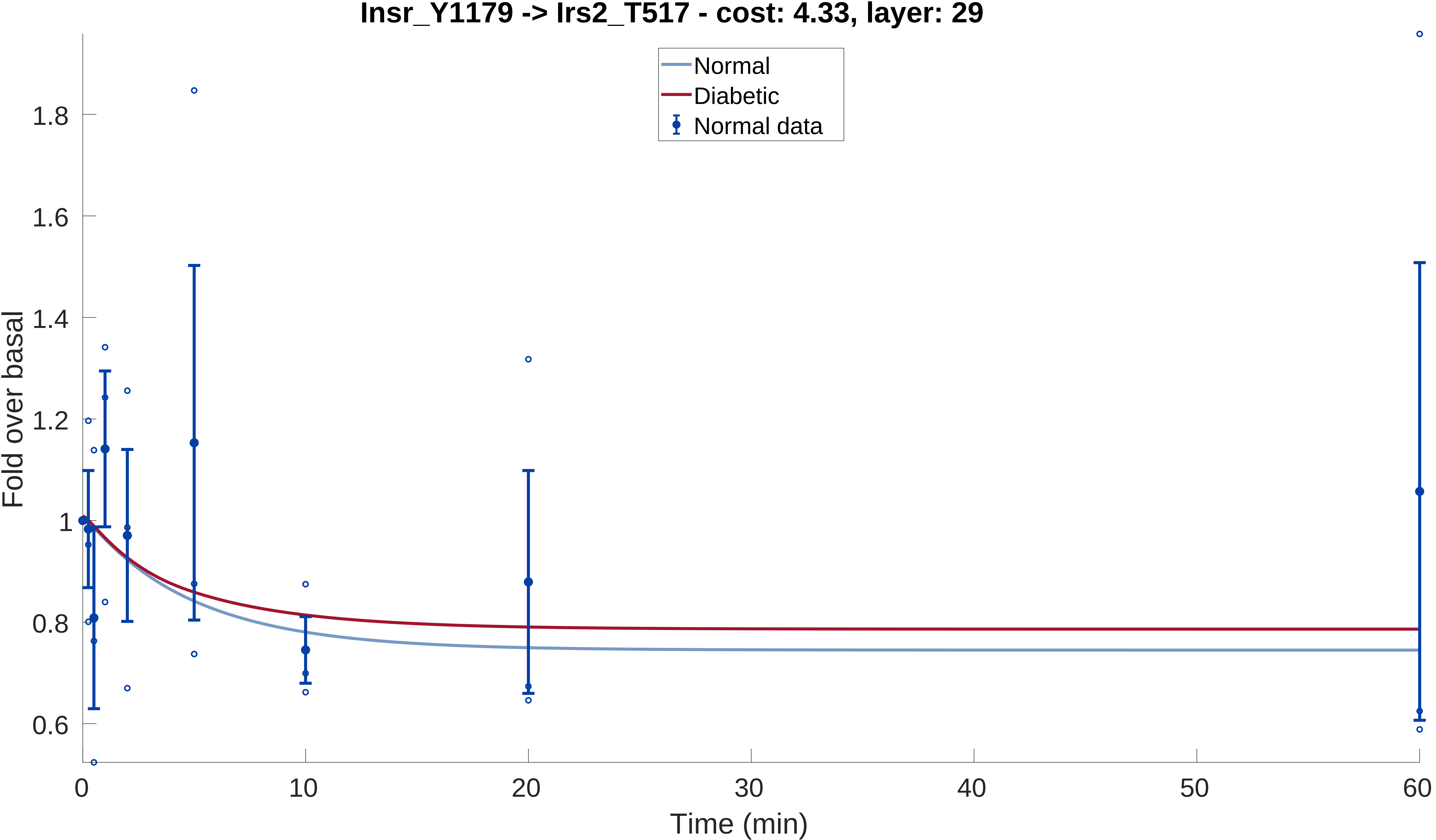
Insr_Y1179 -> Irs2_T517 - cost: 4.33, layer: 29
Normal
Diabetic
Normal data
Fold over basal
1.8
1.6
1.4
1.2
1
0.8
0.6
0
10
20
30
40
50
60
Time (min)

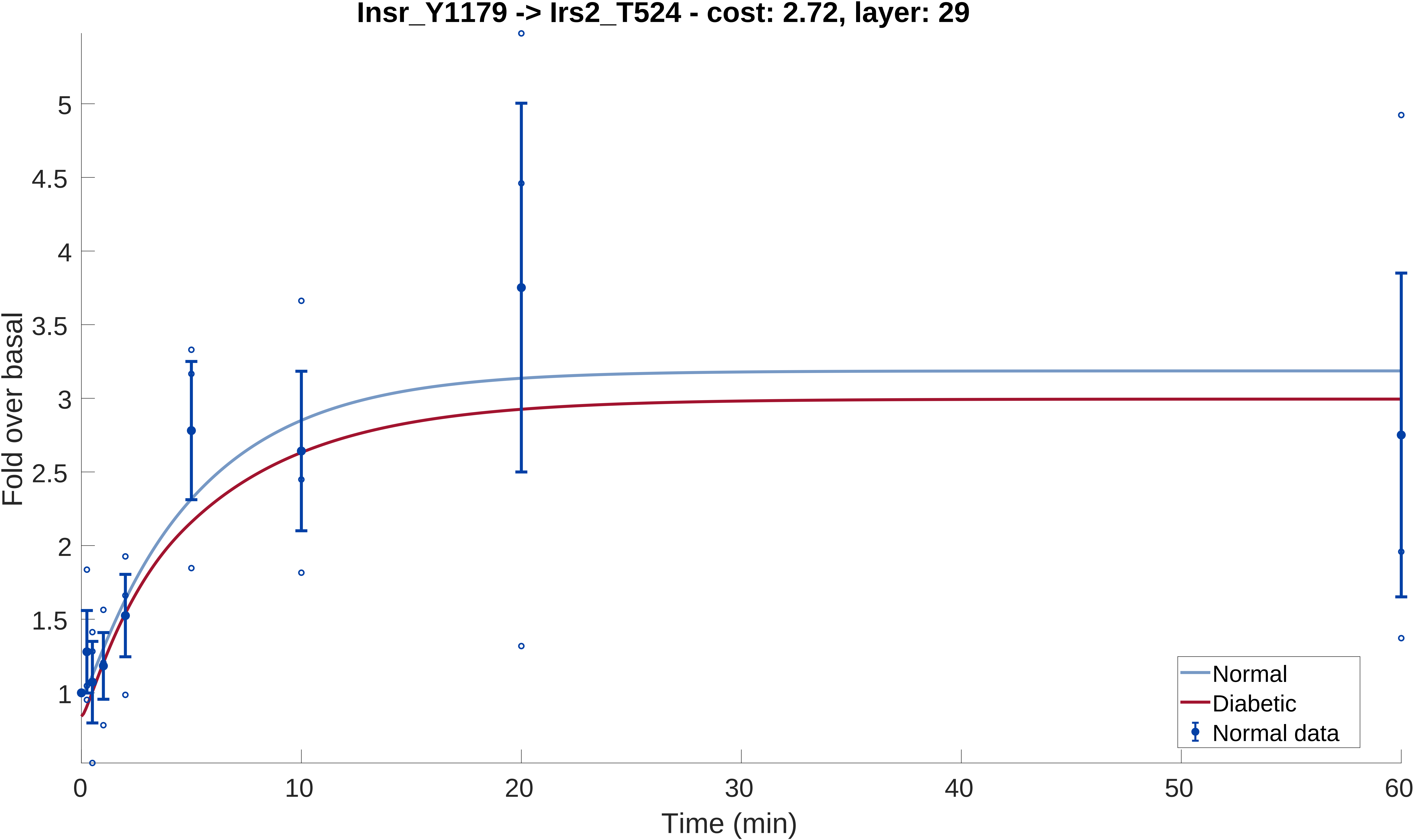
Insr_Y1179 -> Irs2_T524 - cost: 2.72, layer: 29
Fold over basal
5
4.5
4
3.5
3
2.5
2
1.5
1
0
10
20
30
40
50
60
Time (min)
Normal
Diabetic
Normal data

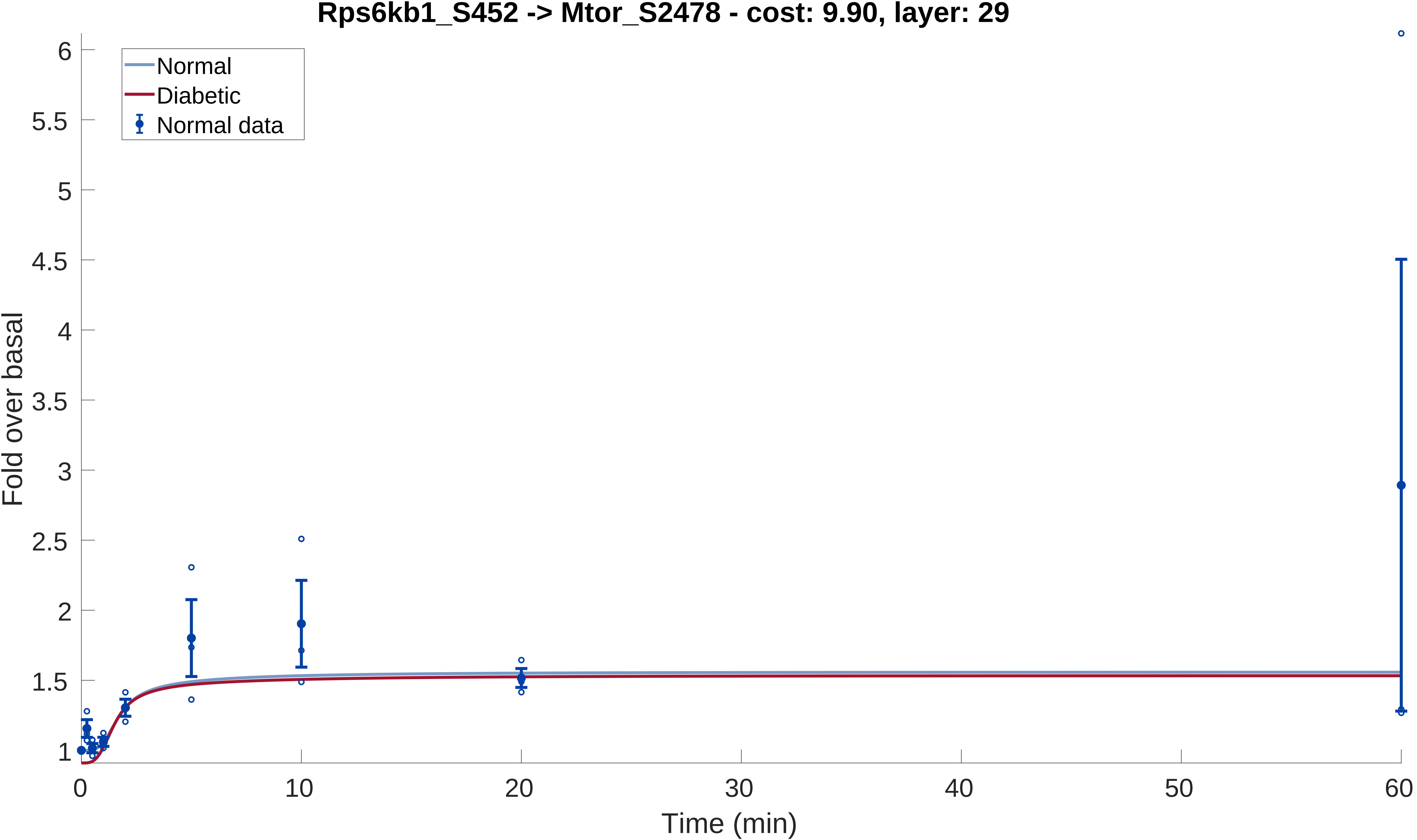
Rps6kb1_S452 -> Mtor_S2478 - cost: 9.90, layer: 29
Normal
Diabetic
Normal data
Fold over basal
Time (min)
1
1.5
2
2.5
3
3.5
4
4.5
5
5.5
6
0
10
20
30
40
50
60

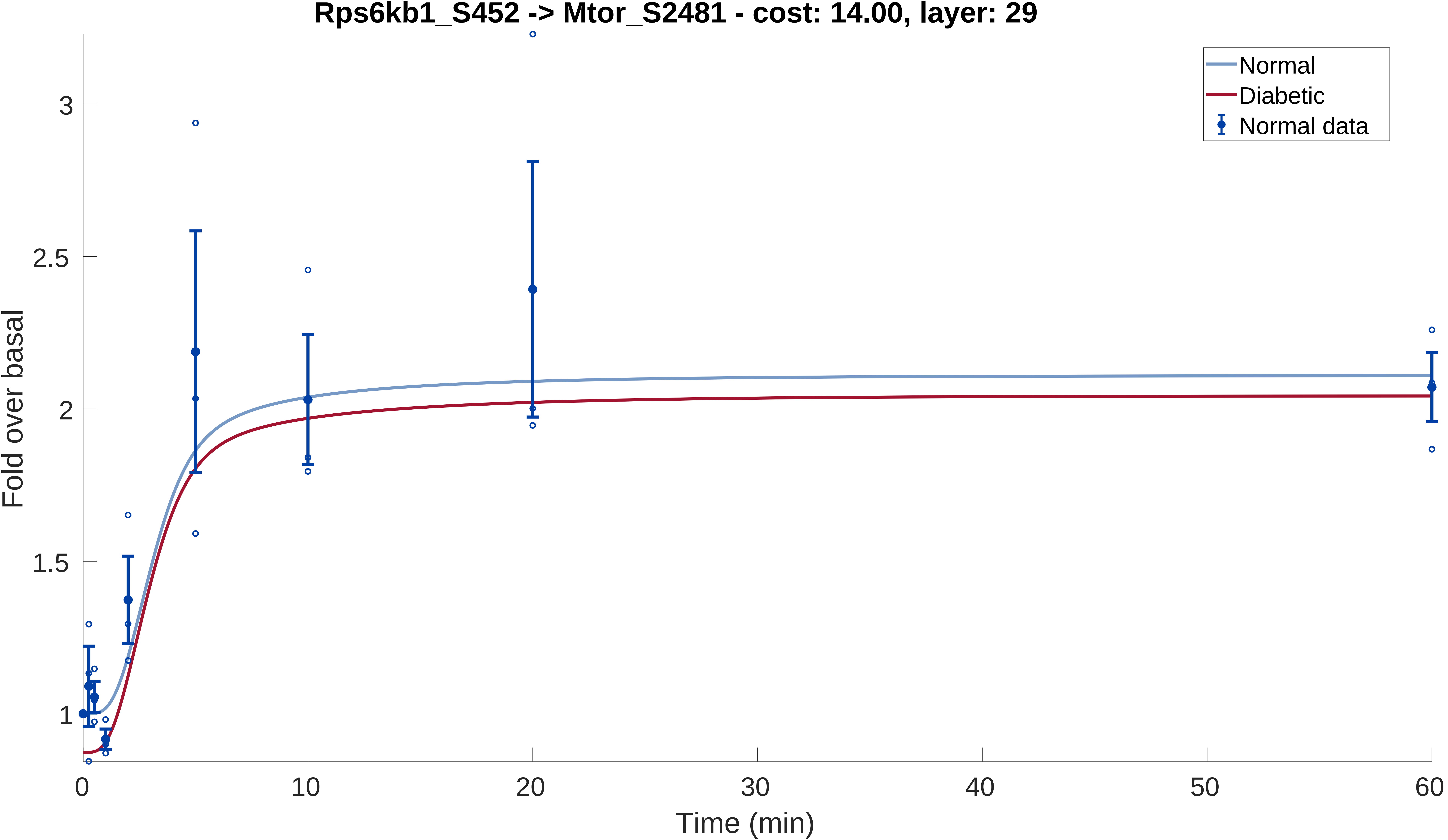
Rps6kb1_S452 -> Mtor_S2481 - cost: 14.00, layer: 29
Normal
Diabetic
Normal data
Fold over basal
3
2.5
2
1.5
1
0
10
20
30
40
50
60
Time (min)

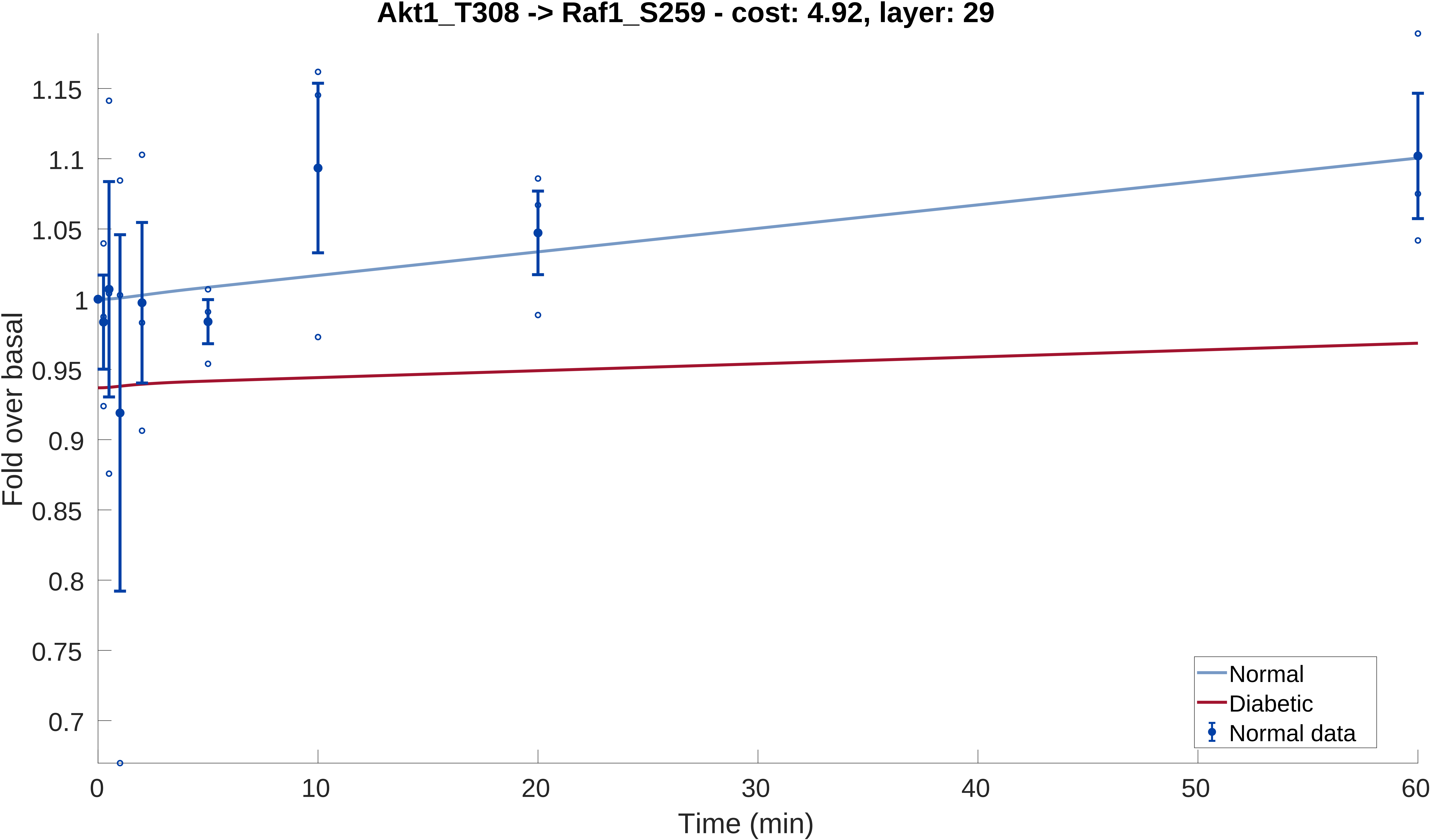

Akt1_T308 -> Raf1_S259 - cost: 4.92, layer: 29
Fold over basal
1.15
1.1
1.05
1
0.95
0.9
0.85
0.8
0.75
0.7
0
10
20
30
40
50
60
Time (min)
Normal
Diabetic
Normal data

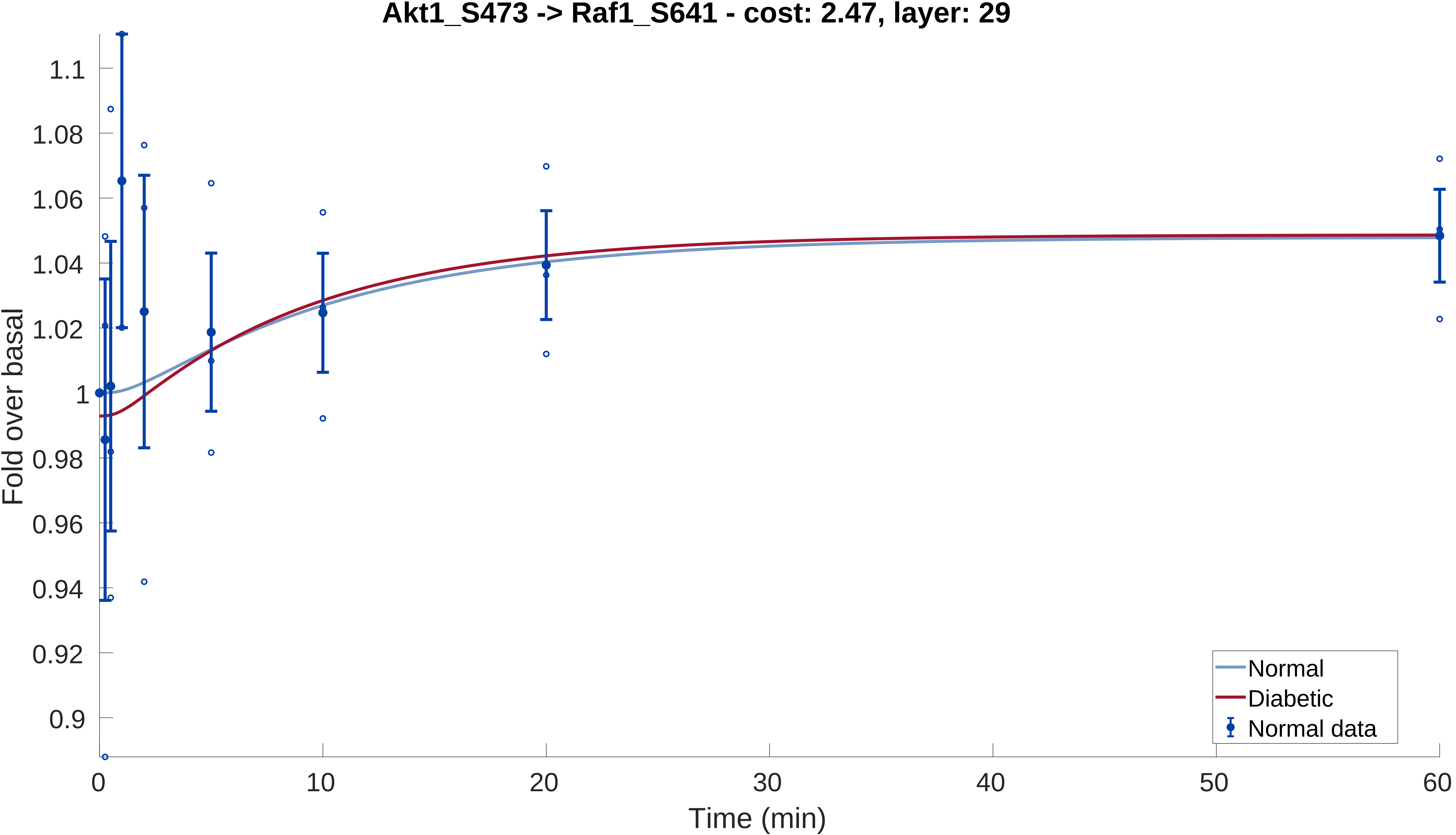

Akt1_S473 -> Raf1_S641 - cost: 2.47, layer: 29
Fold over basal
1.1
1.08
1.06
1.04
1.02
1
0.98
0.96
0.94
0.92
0.9
0
10
20
30
40
50
60
Time (min)
Normal
Diabetic
Normal data

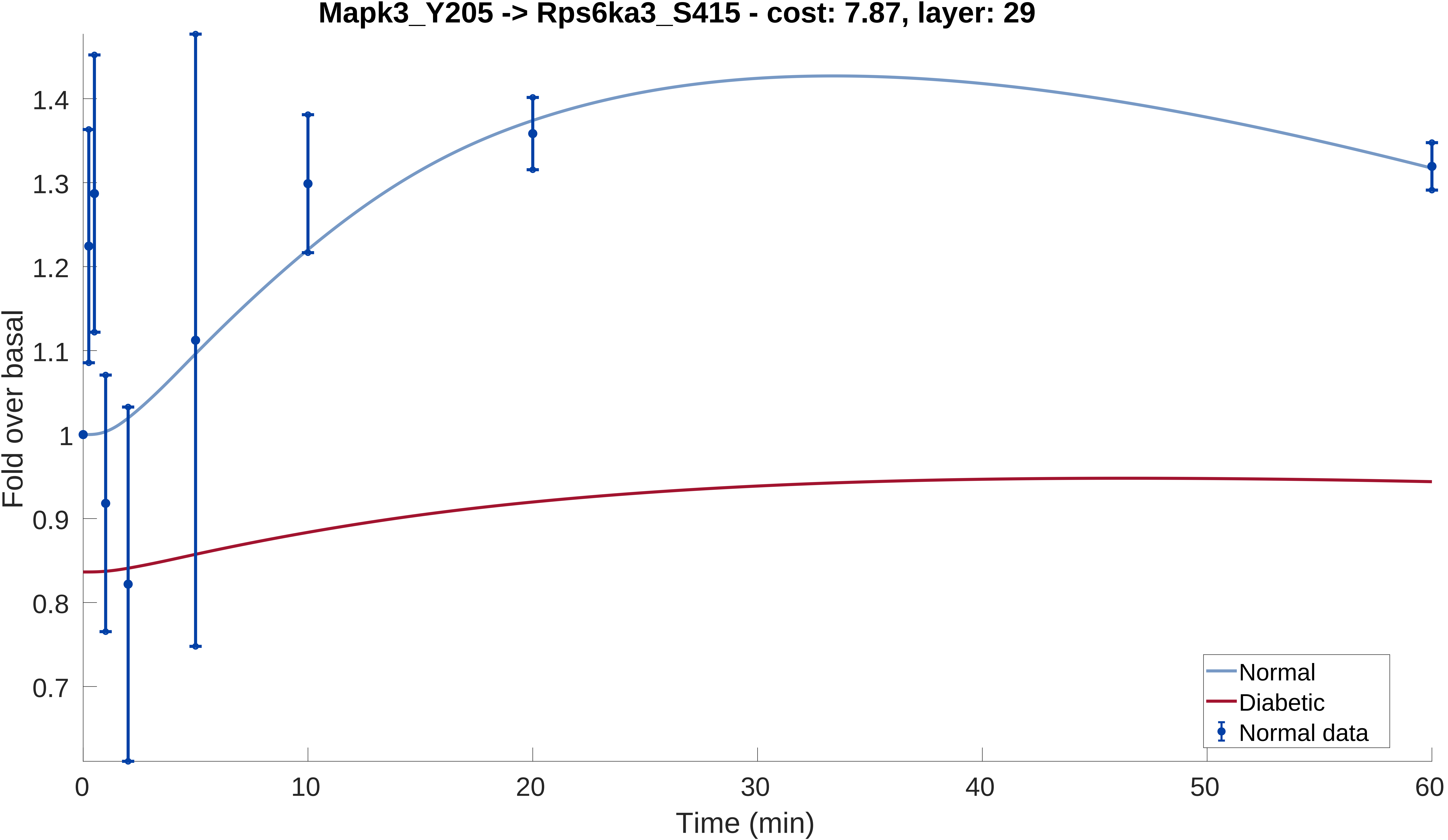

Mapk3_Y205 -> Rps6ka3_S415 - cost: 7.87, layer: 29
Fold over basal
1.4
1.3
1.2
1.1
1
0.9
0.8
0.7
0
10
20
30
40
50
60
Time (min)
Normal
Diabetic
Normal data

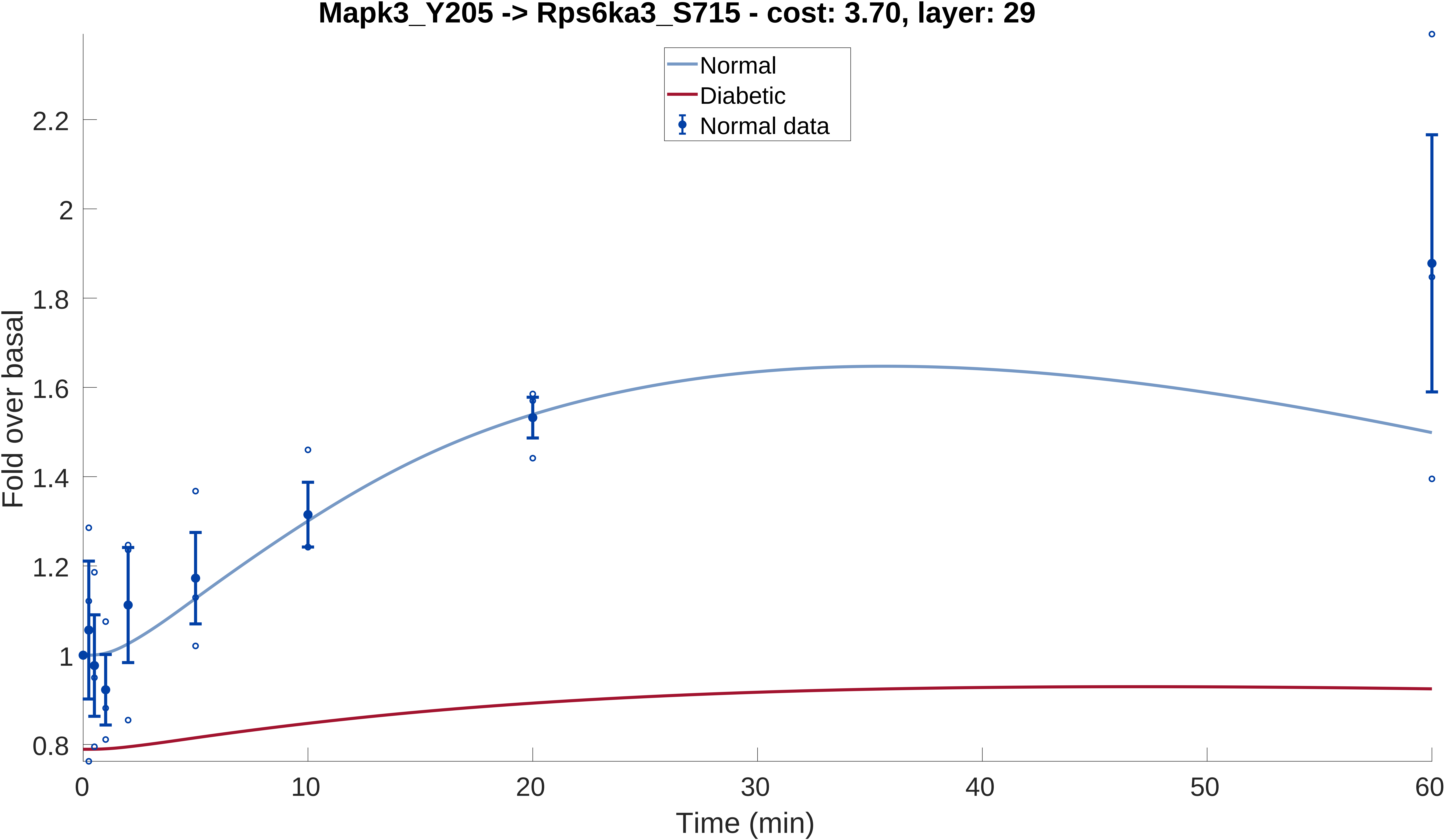
Mapk3_Y205 -> Rps6ka3_S715 - cost: 3.70, layer: 29
Normal
Diabetic
Normal data
Fold over basal
0.8
1
1.2
1.4
1.6
1.8
2
2.2
0
10
20
30
40
50
60
Time (min)

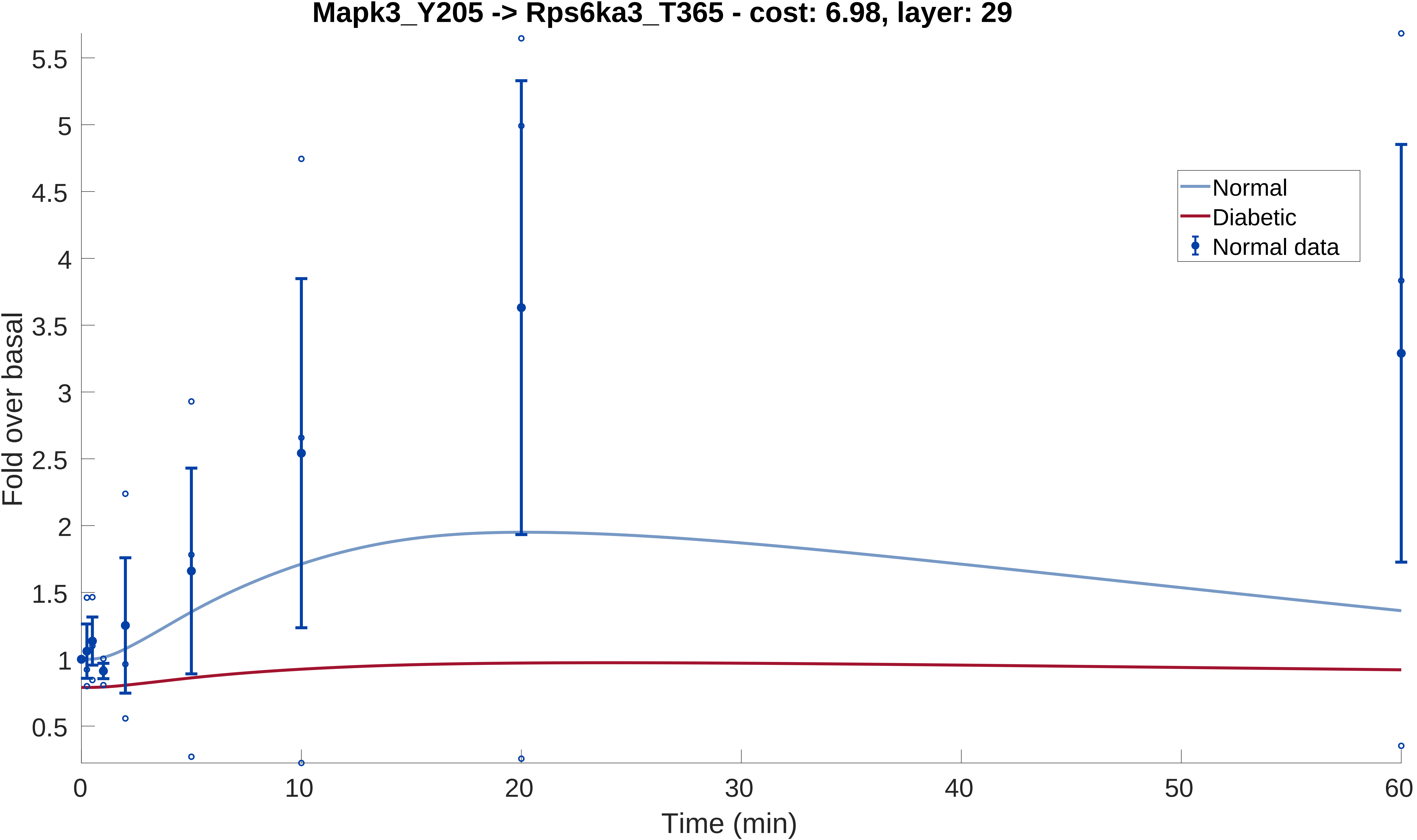
Mapk3_Y205 -> Rps6ka3_T365 - cost: 6.98, layer: 29
Fold over basal
5.5
5
4.5
4
3.5
3
2.5
2
1.5
1
0.5
0
10
20
30
40
50
60
Time (min)
Normal
Diabetic
Normal data

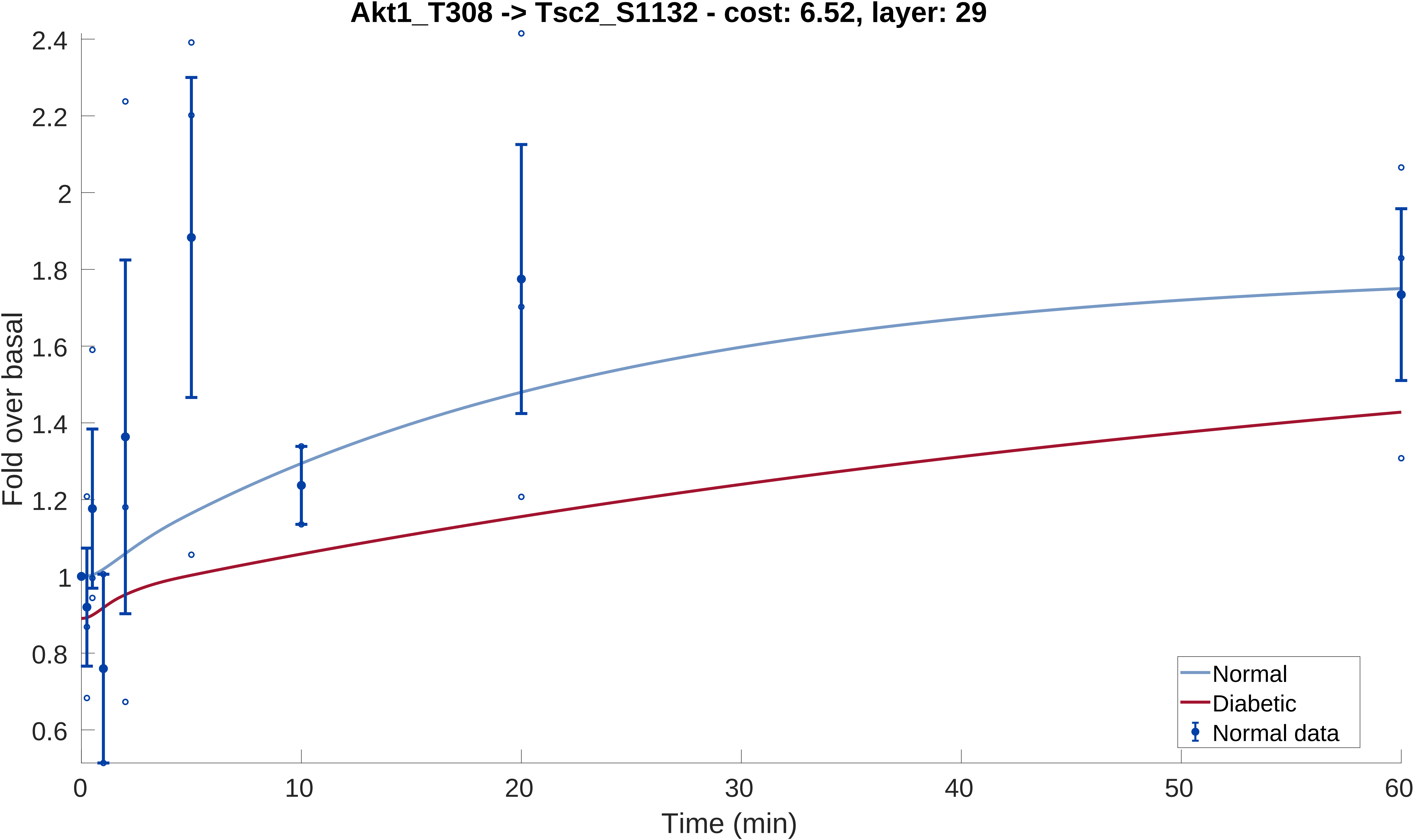

Akt1_T308 -> Tsc2_S1132 - cost: 6.52, layer: 29
Fold over basal
2.4
2.2
2
1.8
1.6
1.4
1.2
1
0.8
0.6
0
10
20
30
40
50
60
Time (min)
Normal
Diabetic
Normal data

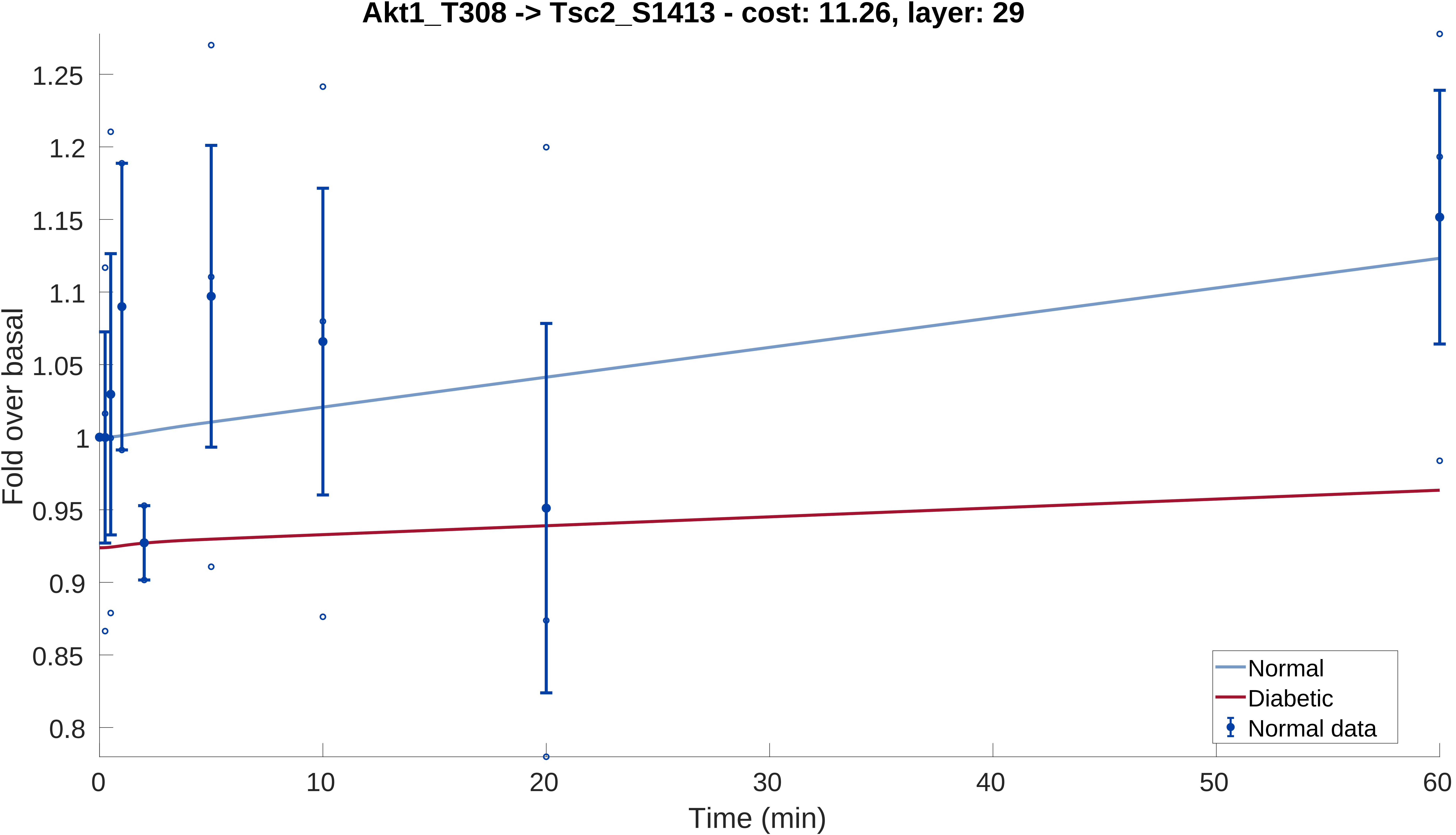
Akt1_T308 -> Tsc2_S1413 - cost: 11.26, layer: 29
Fold over basal
1.25
1.2
1.15
1.1
1.05
1
0.95
0.9
0.85
0.8
0
10
20
30
40
50
60
Time (min)
Normal
Diabetic
Normal data

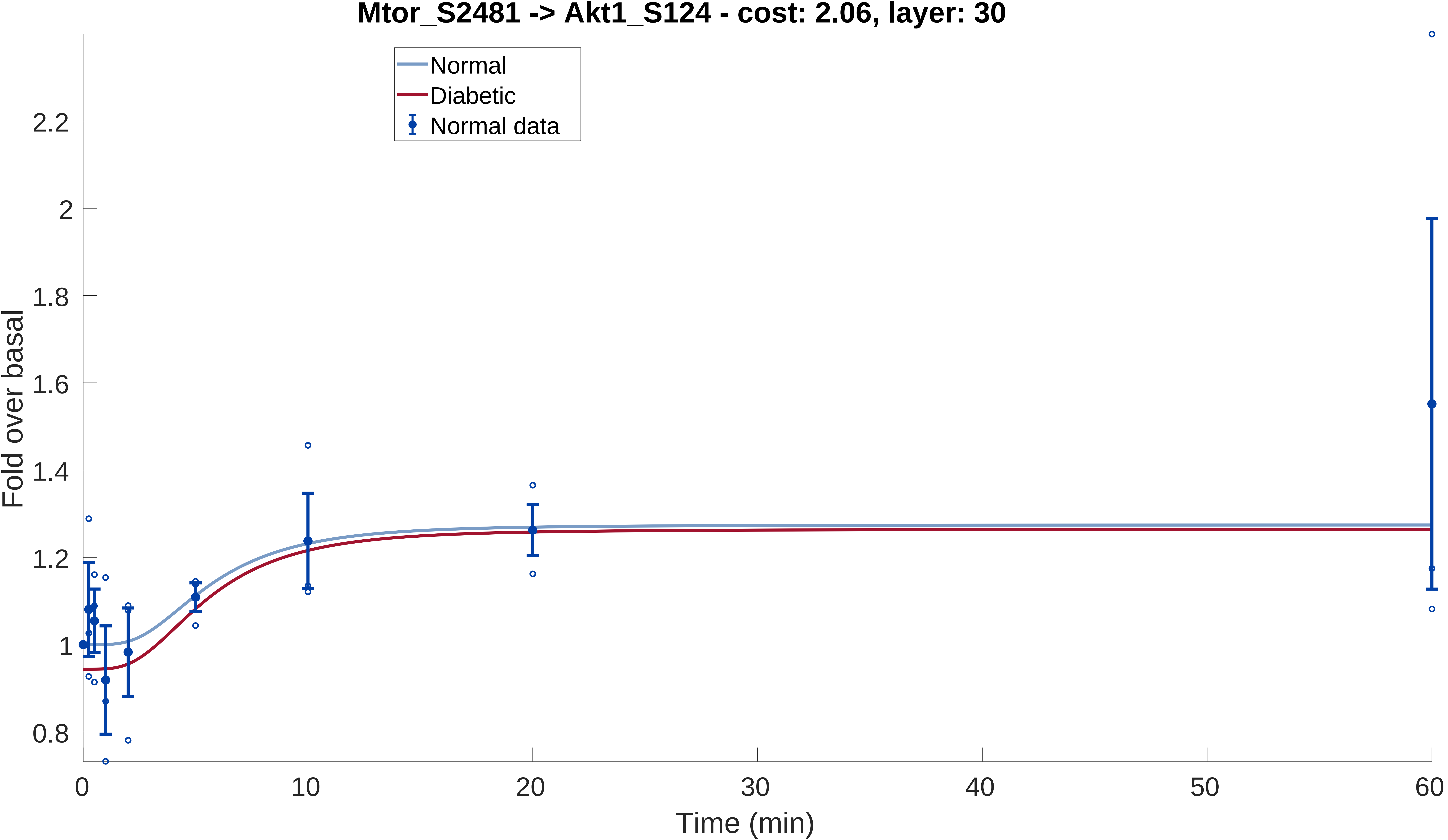
Mtor_S2481 -> Akt1_S124 - cost: 2.06, layer: 30
Normal
Diabetic
Normal data
Fold over basal
2.2
2
1.8
1.6
1.4
1.2
1
0.8
0
10
20
30
40
50
60
Time (min)

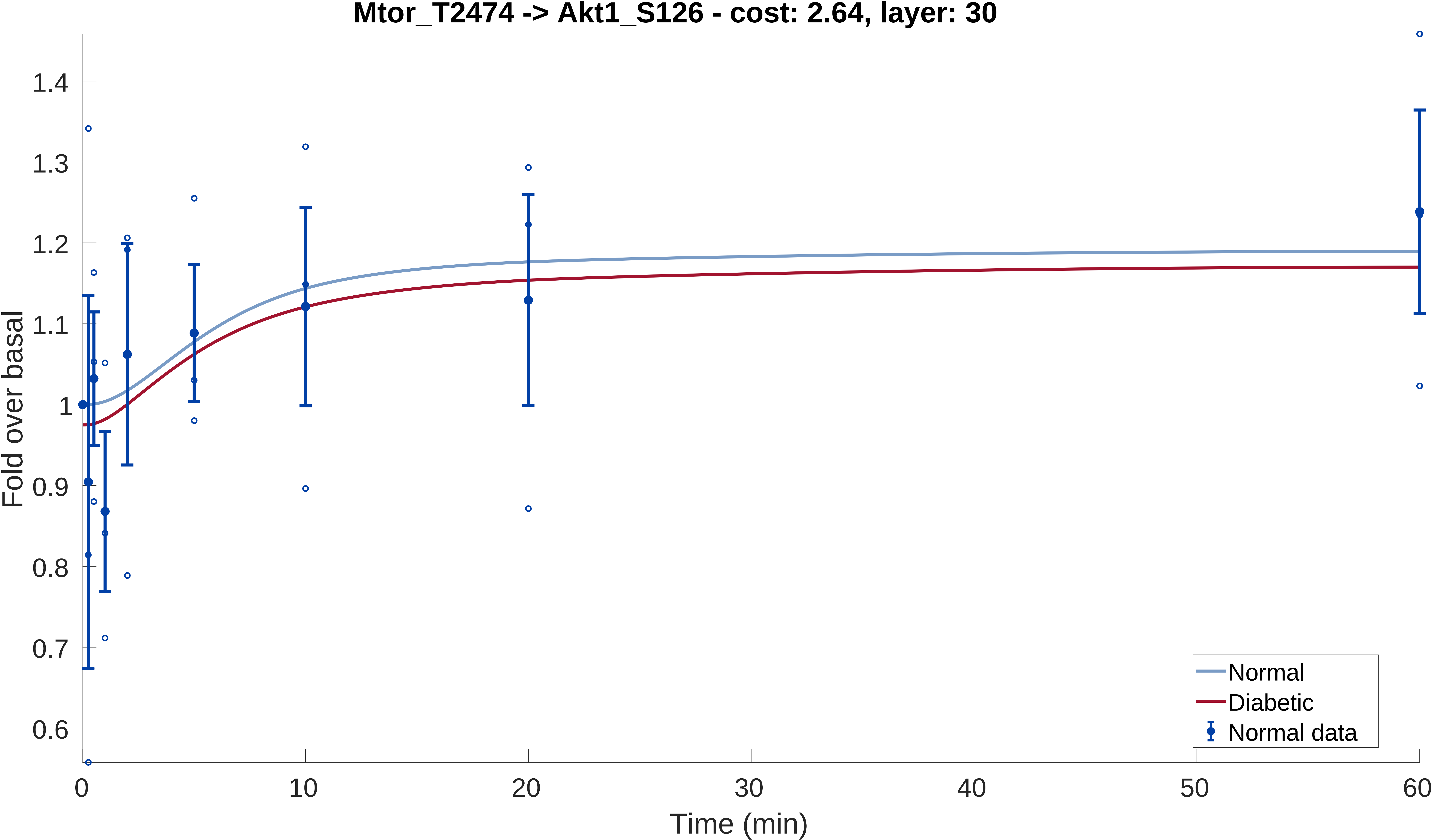

Mtor_T2474 -> Akt1_S126 - cost: 2.64, layer: 30
1.4
1.3
1.2
1.1
1
0.9
0.8
0.7
0.6
Fold over basal
0
10
20
30
40
50
60
Time (min)
Normal
Diabetic
Normal data

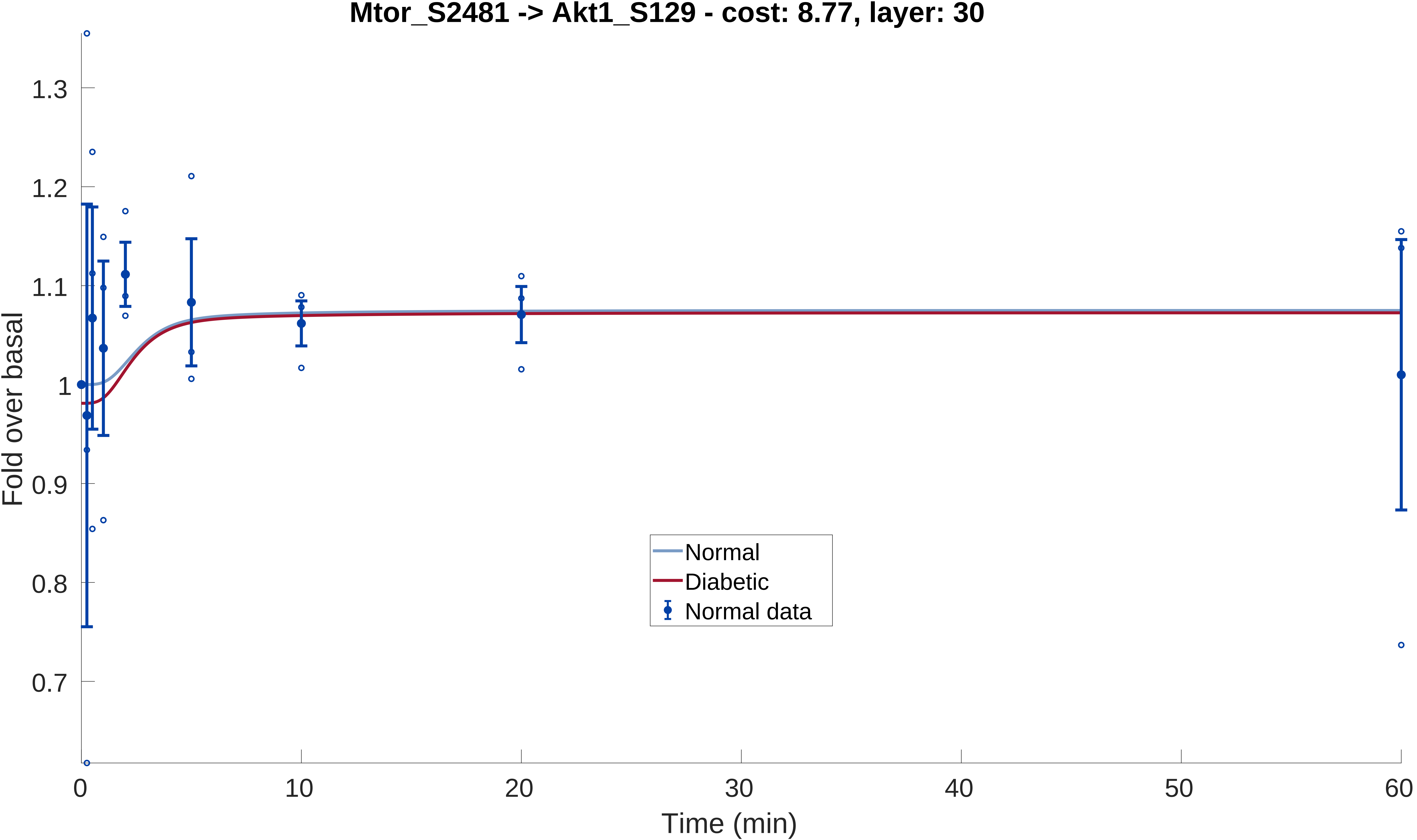

Mtor_S2481 -> Akt1_S129 - cost: 8.77, layer: 30
Fold over basal
1.3
1.2
1.1
1
0.9
0.8
0.7
0
10
20
30
40
50
60
Time (min)
Normal
Diabetic
Normal data

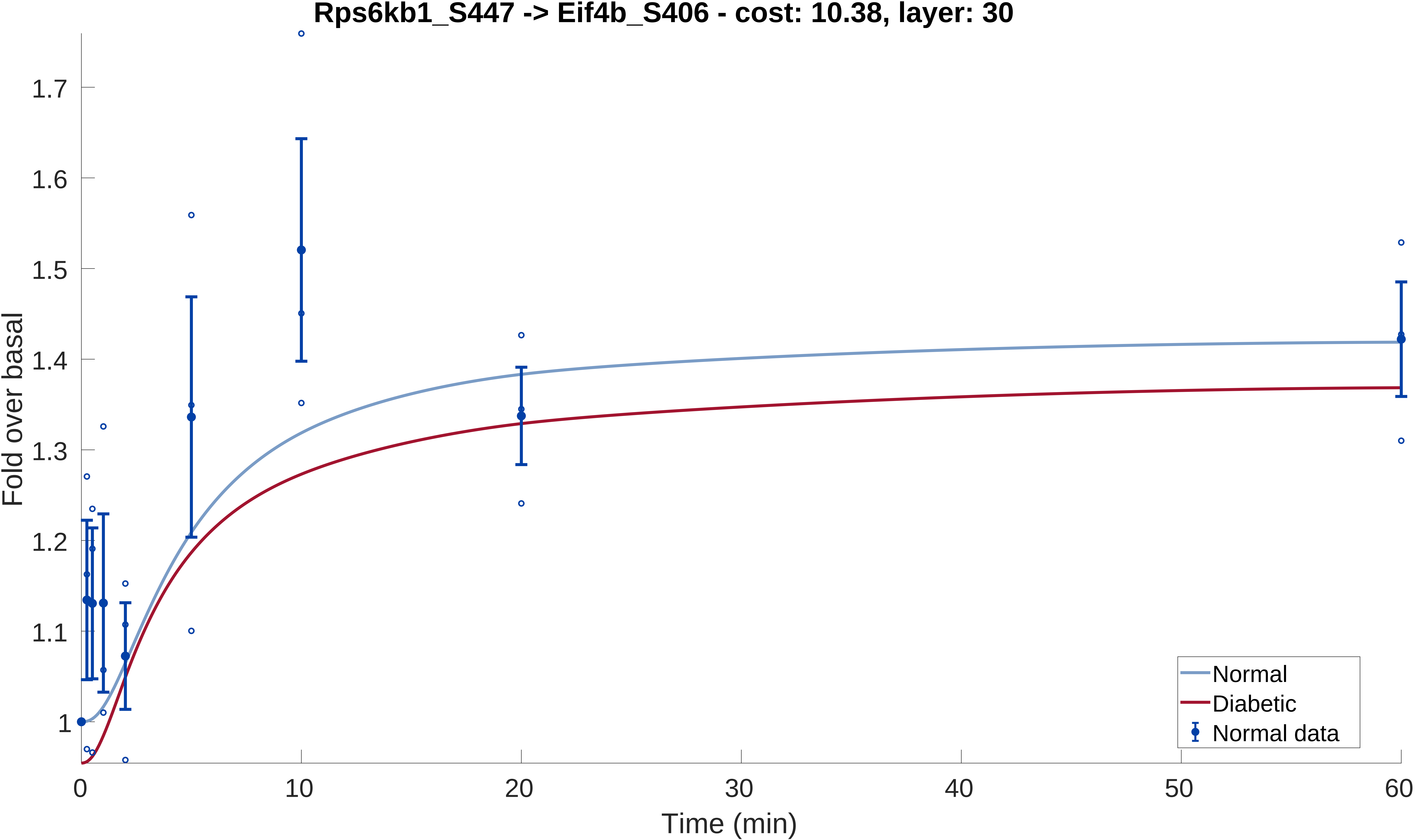

Rps6kb1_S447 -> Eif4b_S406 - cost: 10.38, layer: 30
Fold over basal
1.7
1.6
1.5
1.4
1.3
1.2
1.1
1
0
10
20
30
40
50
60
Time (min)
Normal
Diabetic
Normal data

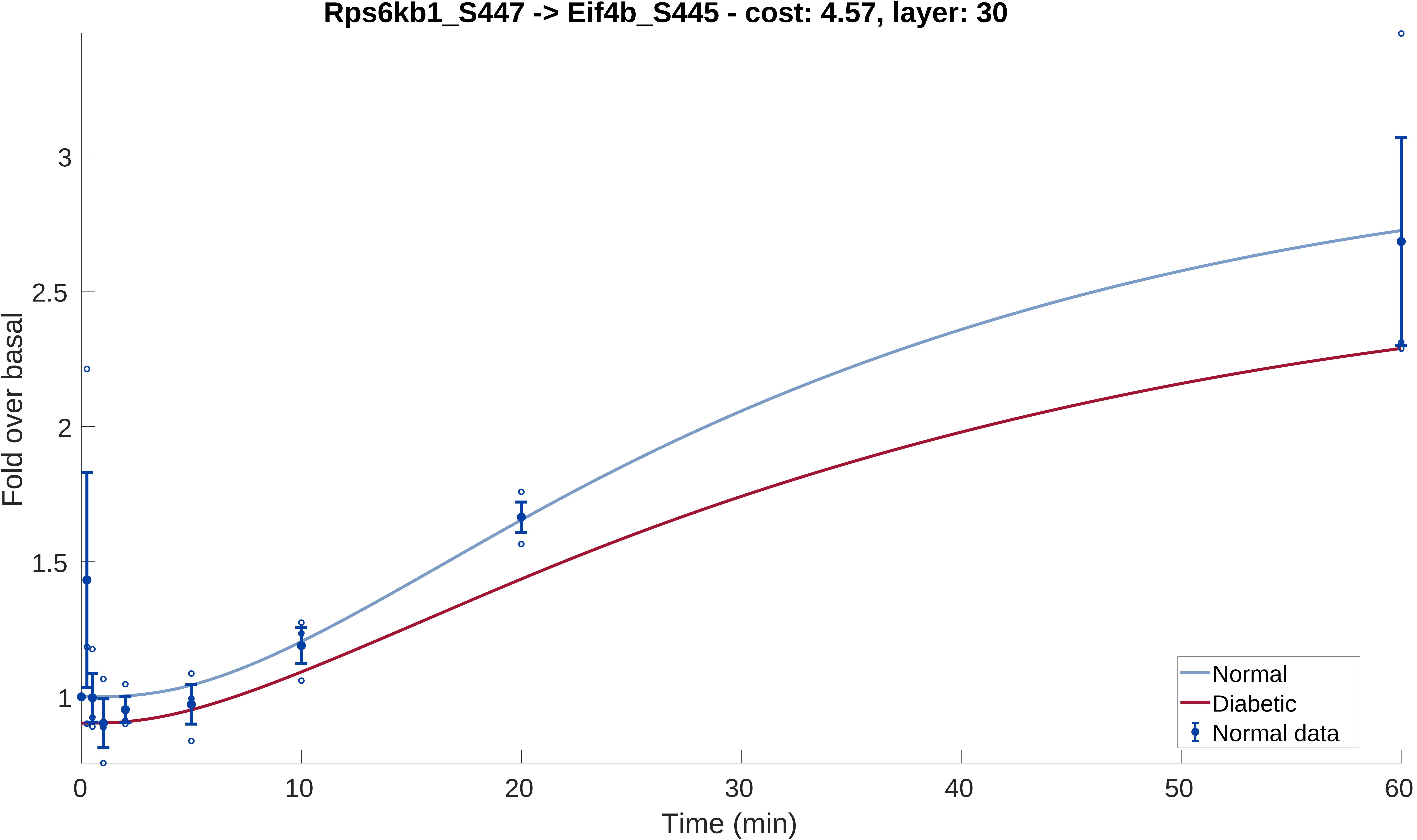

Rps6kb1_S447 -> Eif4b_S445 - cost: 4.57, layer: 30
Fold over basal
Time (min)
0
10
20
30
40
50
60
1
1.5
2
2.5
3
Normal
Diabetic
Normal data

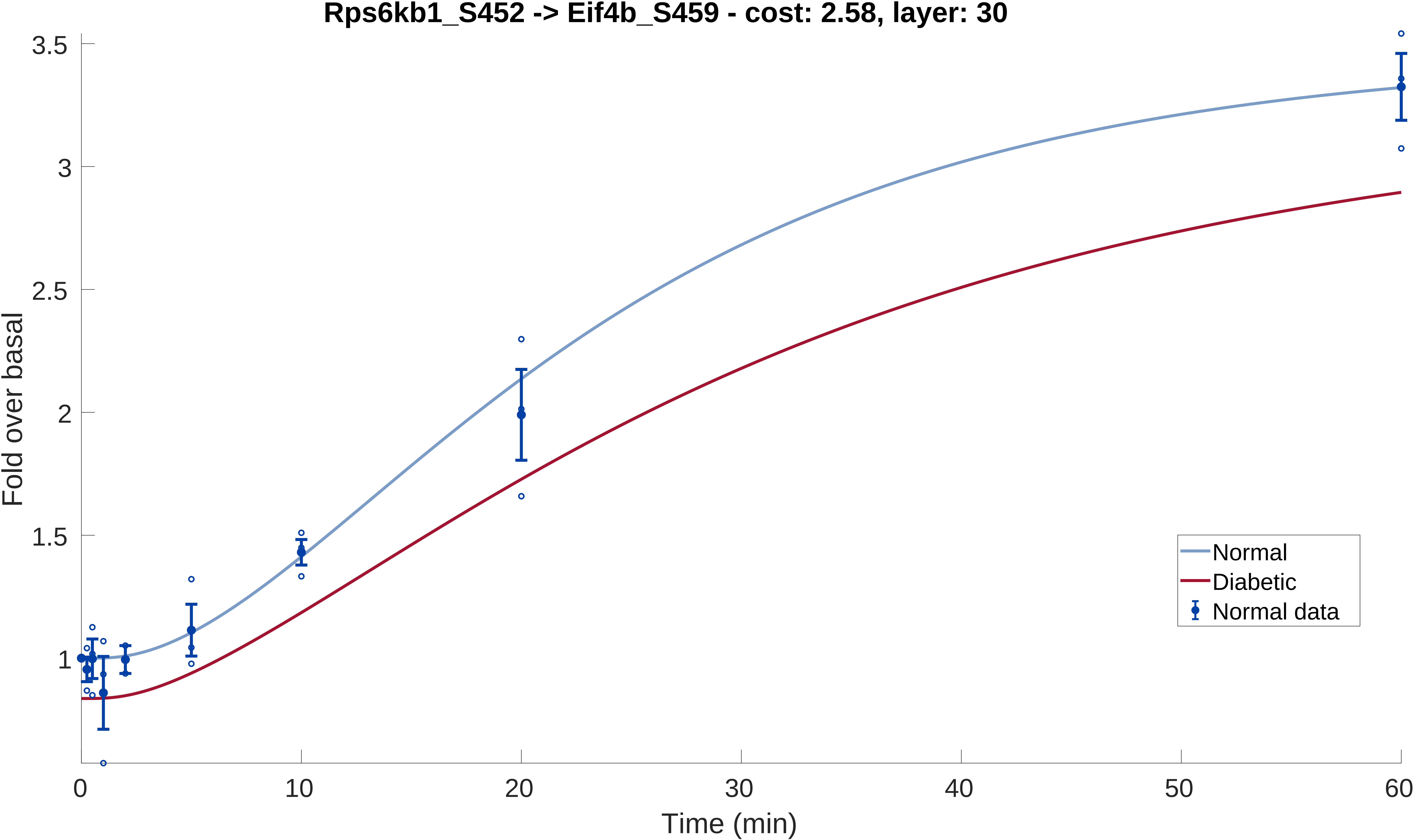
Rps6kb1_S452 -> Eif4b_S459 - cost: 2.58, layer: 30
Fold over basal
3.5
3
2.5
2
1.5
1
0
10
20
30
40
50
60
Time (min)
Normal
Diabetic
Normal data

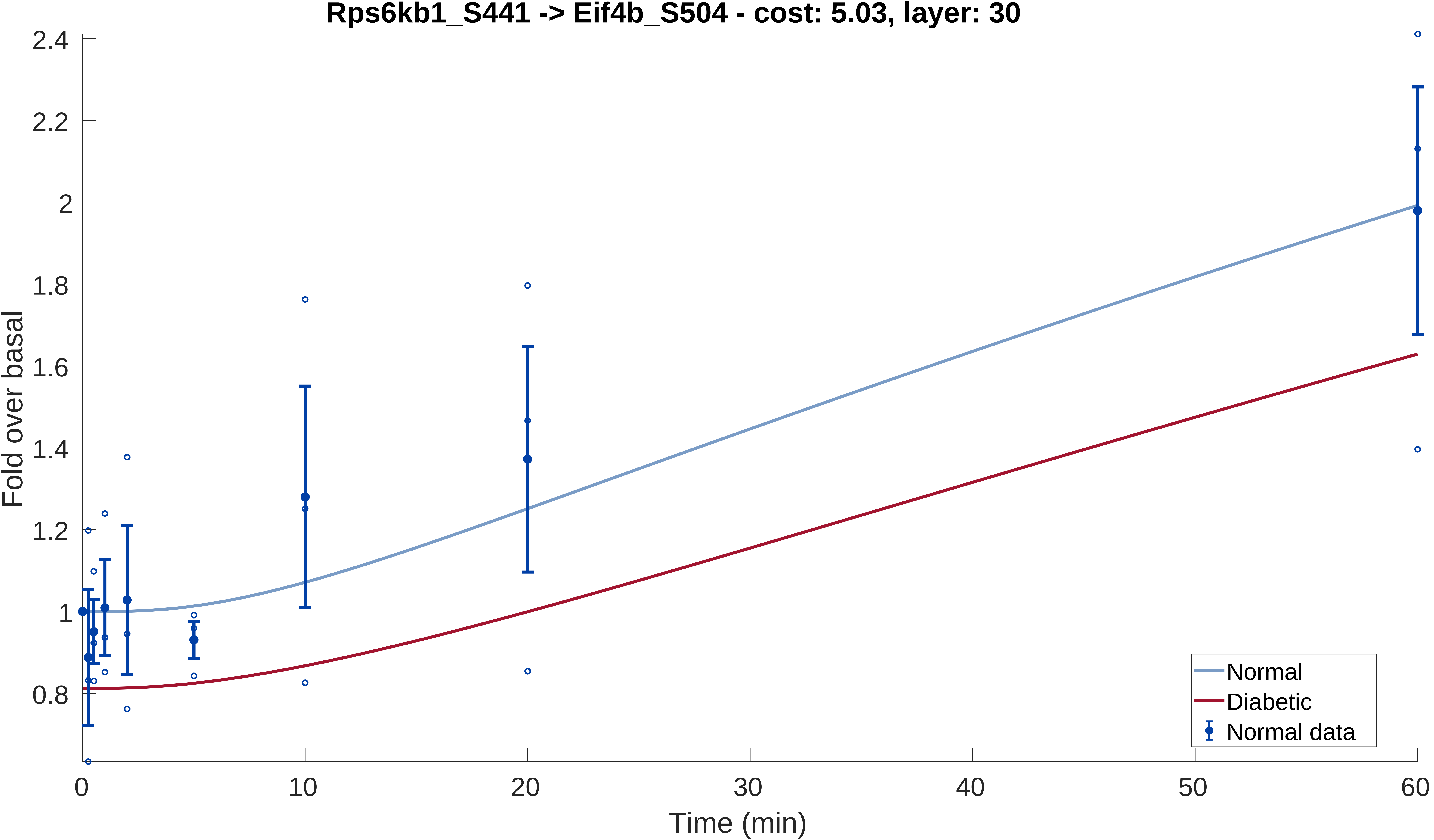
Rps6kb1_S441 -> Eif4b_S504 - cost: 5.03, layer: 30
Fold over basal
2.4
2.2
2
1.8
1.6
1.4
1.2
1
0.8
0
10
20
30
40
50
60
Time (min)
Normal
Diabetic
Normal data

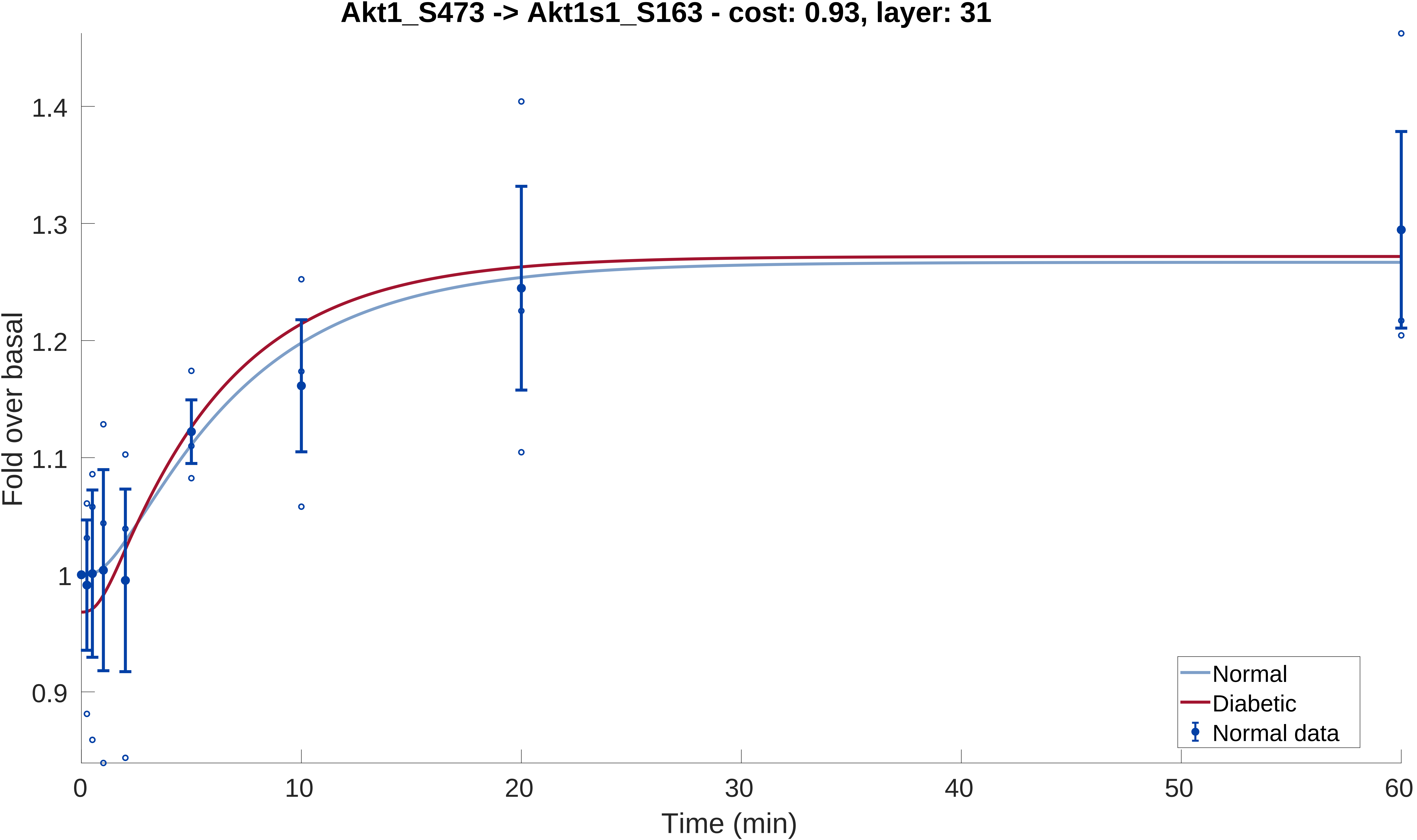
Akt1_S473 -> Akt1s1_S163 - cost: 0.93, layer: 31
Fold over basal
1.4
1.3
1.2
1.1
1
0.9
0
10
20
30
40
50
60
Time (min)
Normal
Diabetic
Normal data

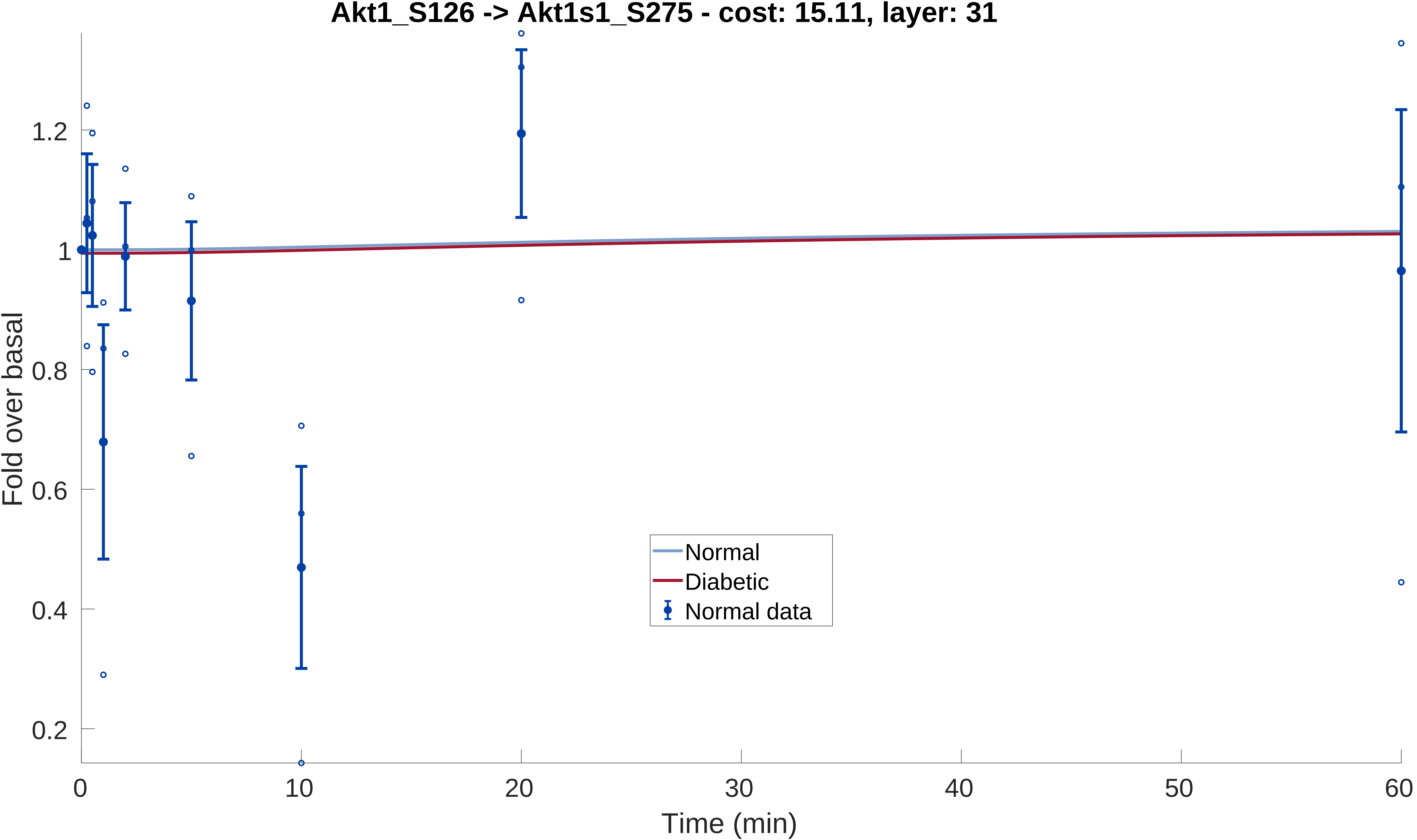

Akt1_S126 -> Akt1s1_S275 - cost: 15.11, layer: 31
Fold over basal
1.2
1
0.8
0.6
0.4
0.2
0
10
20
30
40
50
60
Time (min)
Normal
Diabetic
Normal data

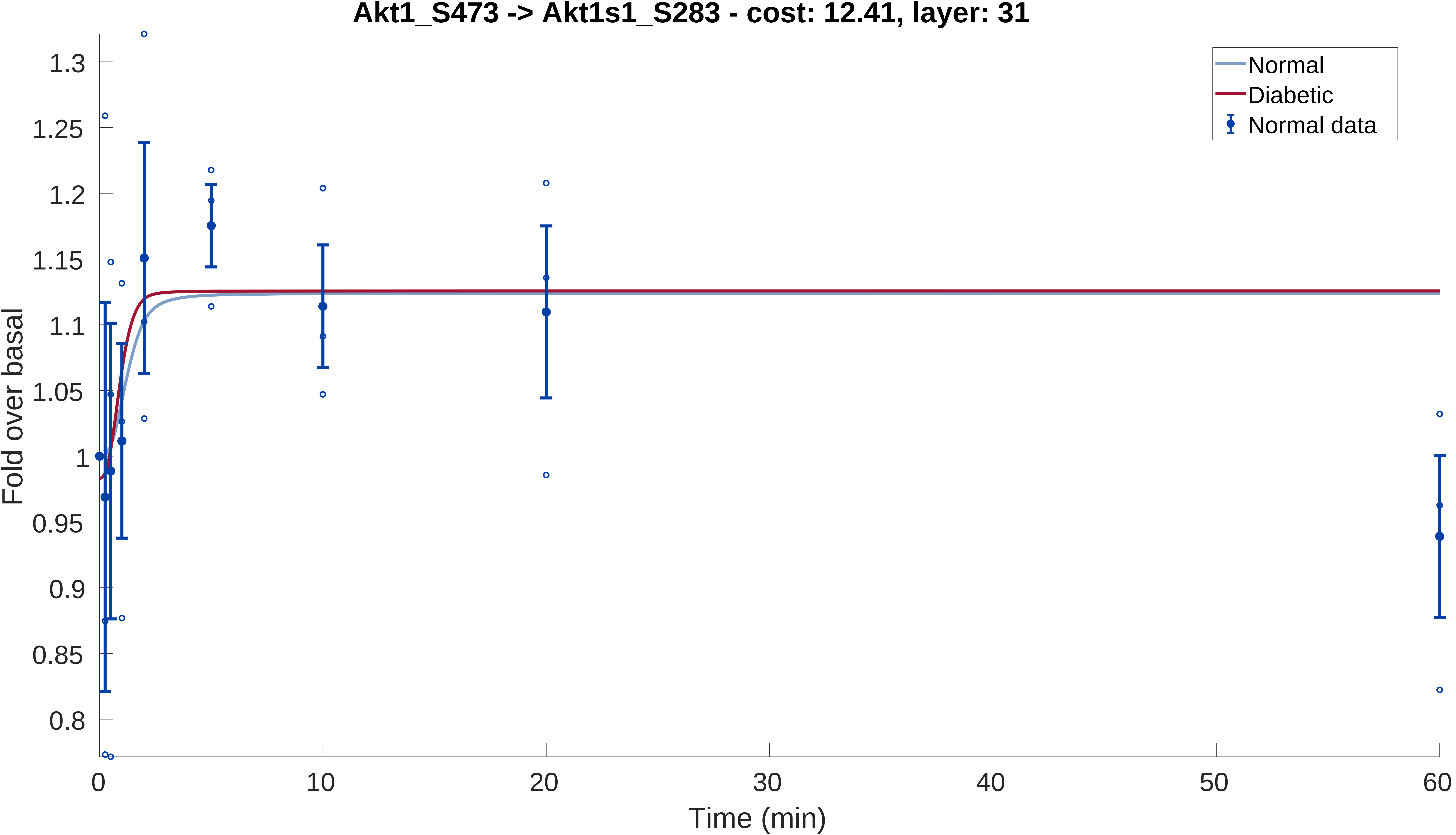

Akt1_S473 -> Akt1s1_S283 - cost: 12.41, layer: 31
Normal
Diabetic
Normal data
Fold over basal
Time (min)
1.3
1.25
1.2
1.15
1.1
1.05
1
0.95
0.9
0.85
0.8
0
10
20
30
40
50
60

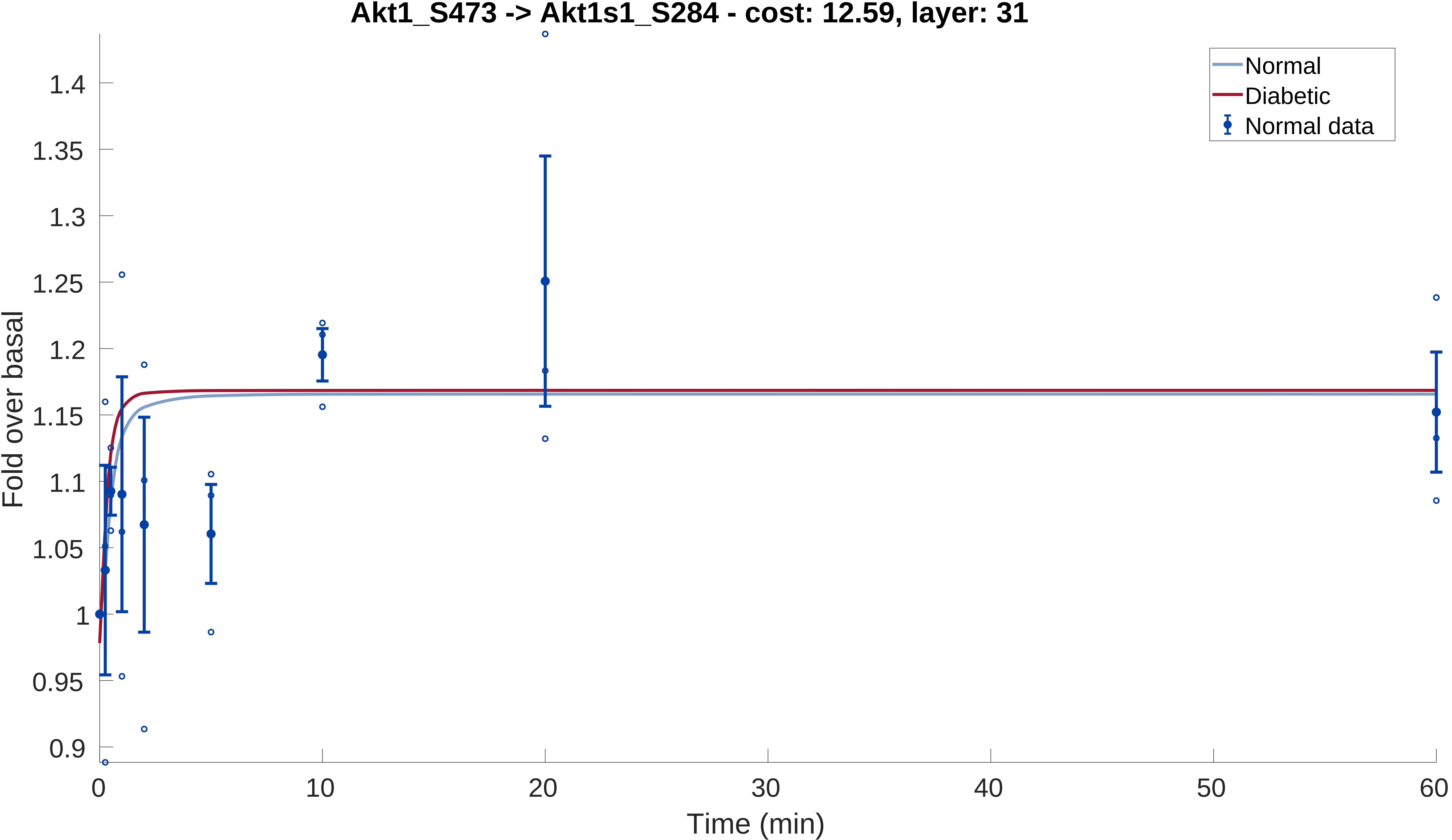

Akt1_S473 -> Akt1s1_S284 - cost: 12.59, layer: 31
Normal
Diabetic
Normal data
Fold over basal
1.4
1.35
1.3
1.25
1.2
1.15
1.1
1.05
1
0.95
0.9
0
10
20
30
40
50
60
Time (min)

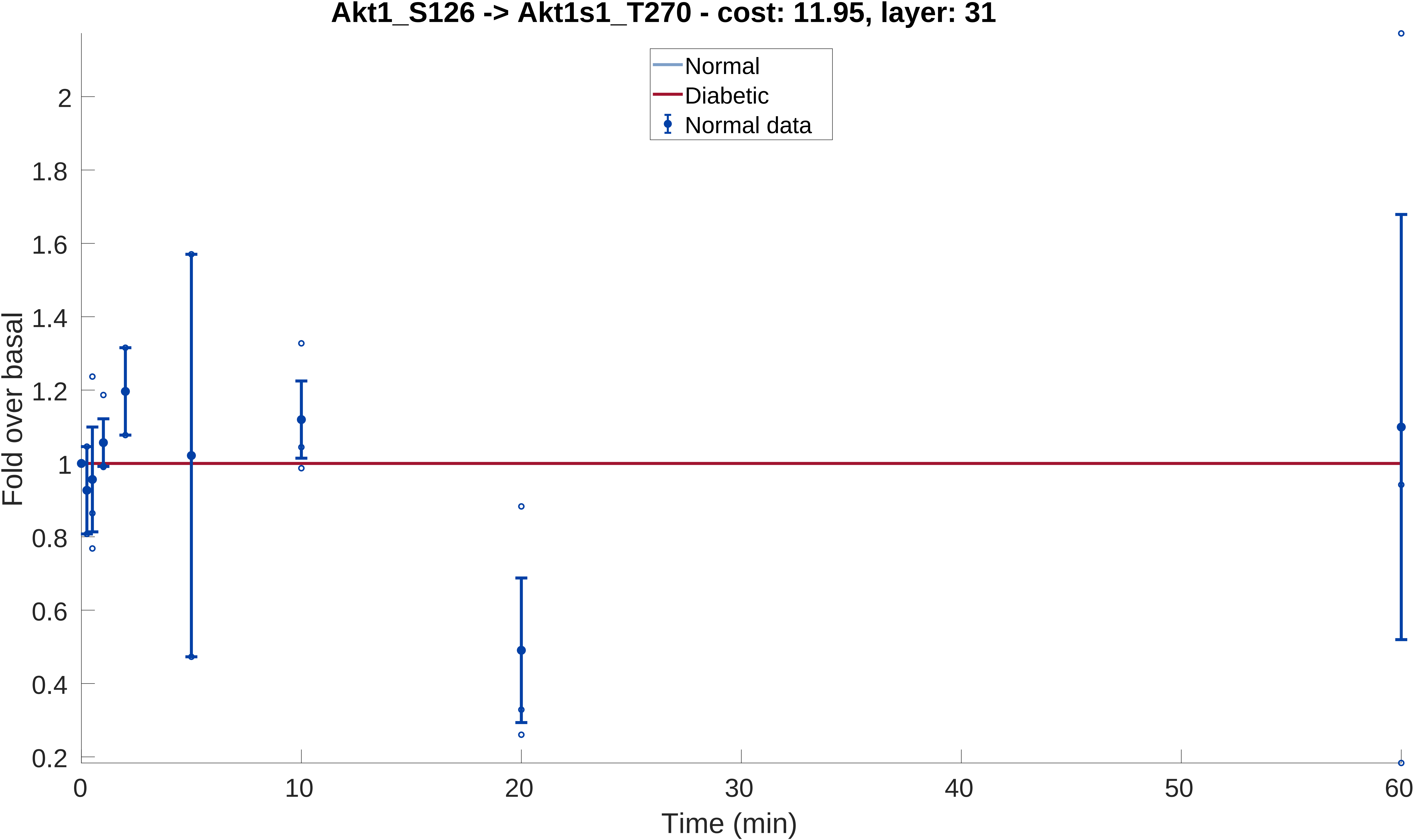

Akt1_S126 -> Akt1s1_T270 - cost: 11.95, layer: 31
Normal
Diabetic
Normal data
Fold over basal
Time (min)
0.2
0.4
0.6
0.8
1
1.2
1.4
1.6
1.8
2
0
10
20
30
40
50
60

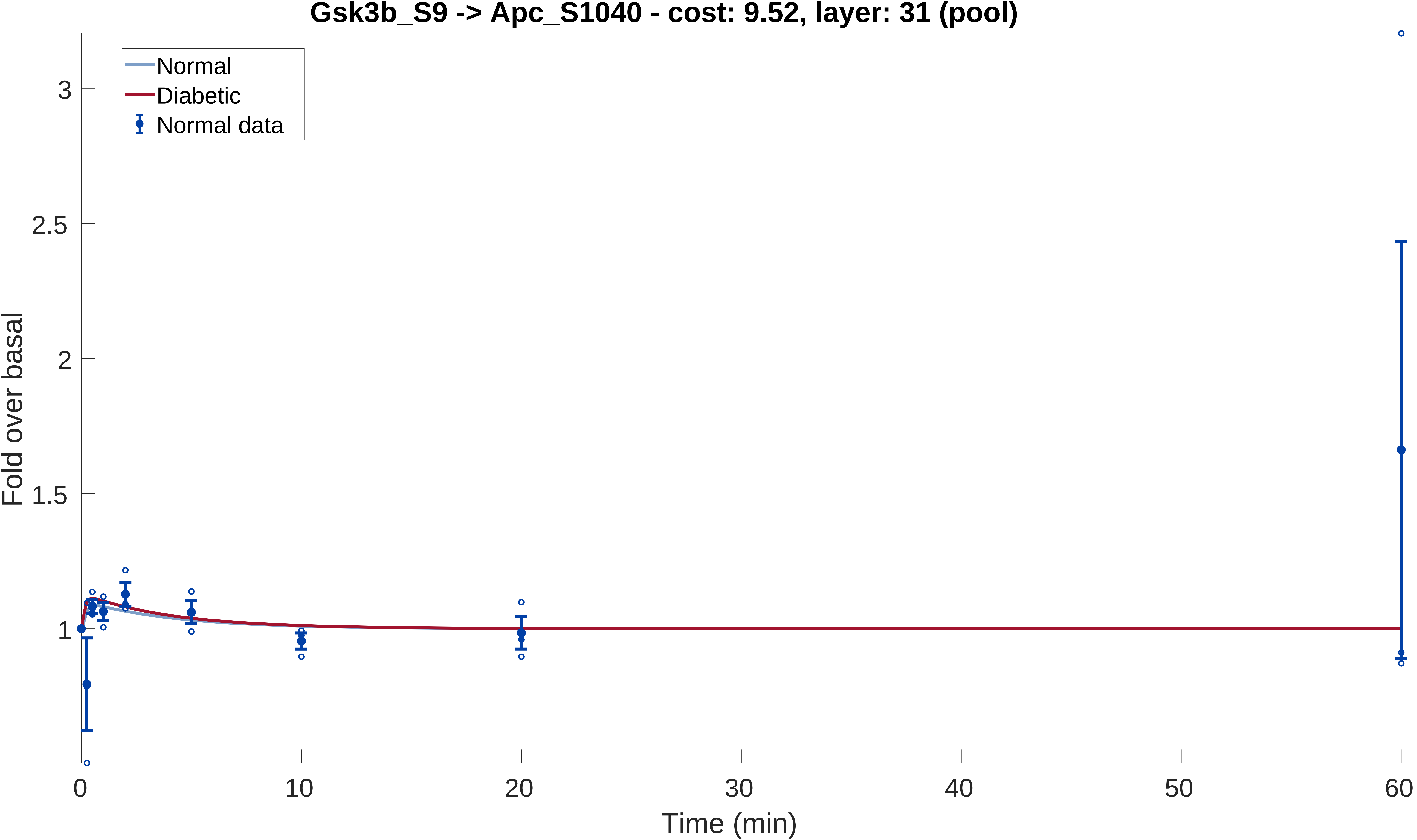

Gsk3b_S9 -> Apc_S1040 - cost: 9.52, layer: 31 (pool)
Normal
Diabetic
Normal data
Fold over basal
Time (min)
3
2.5
2
1.5
1
0
10
20
30
40
50
60

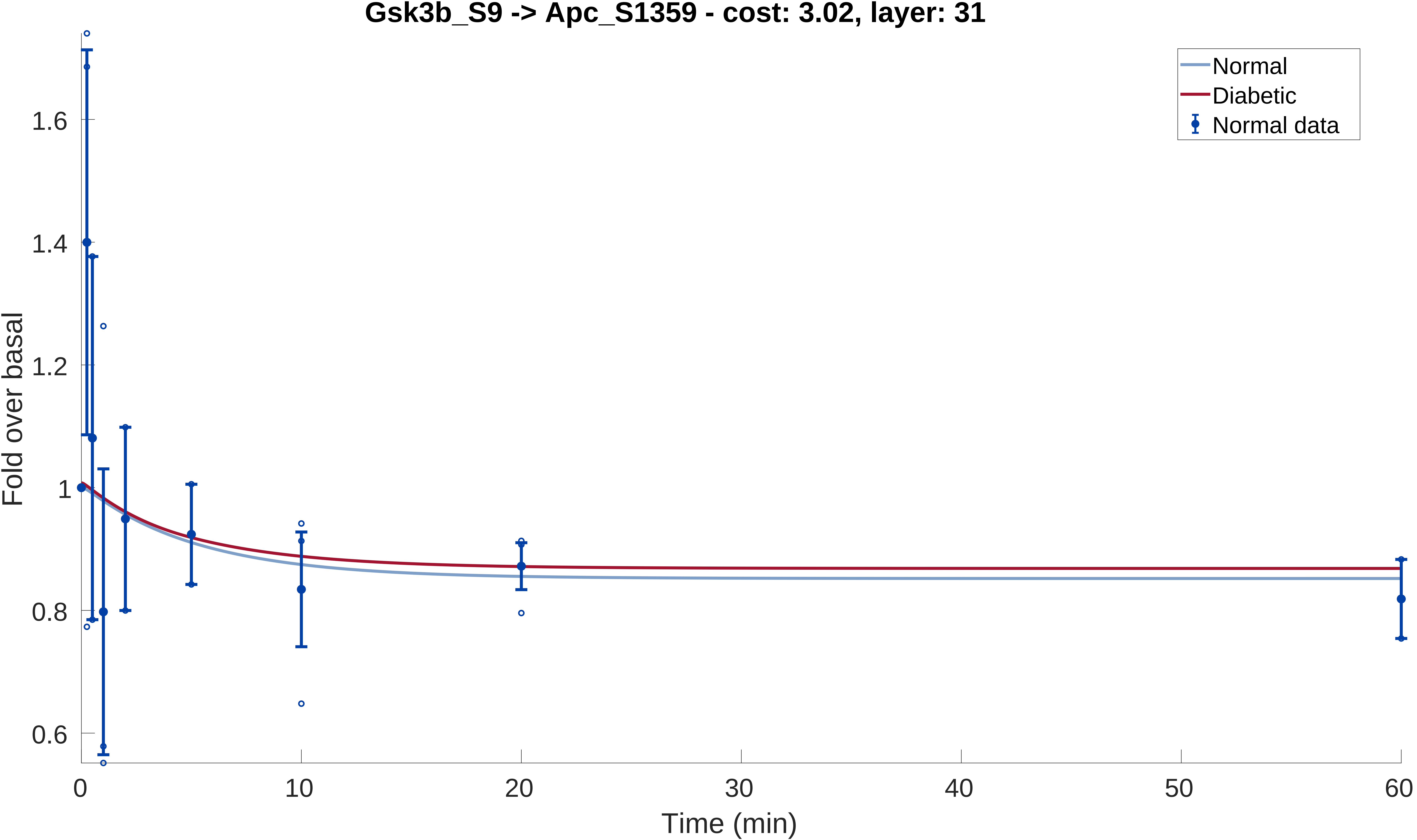
Gsk3b_S9 -> Apc_S1359 - cost: 3.02, layer: 31
Normal
Diabetic
Normal data
Fold over basal
1.6
1.4
1.2
1
0.8
0.6
0
10
20
30
40
50
60
Time (min)

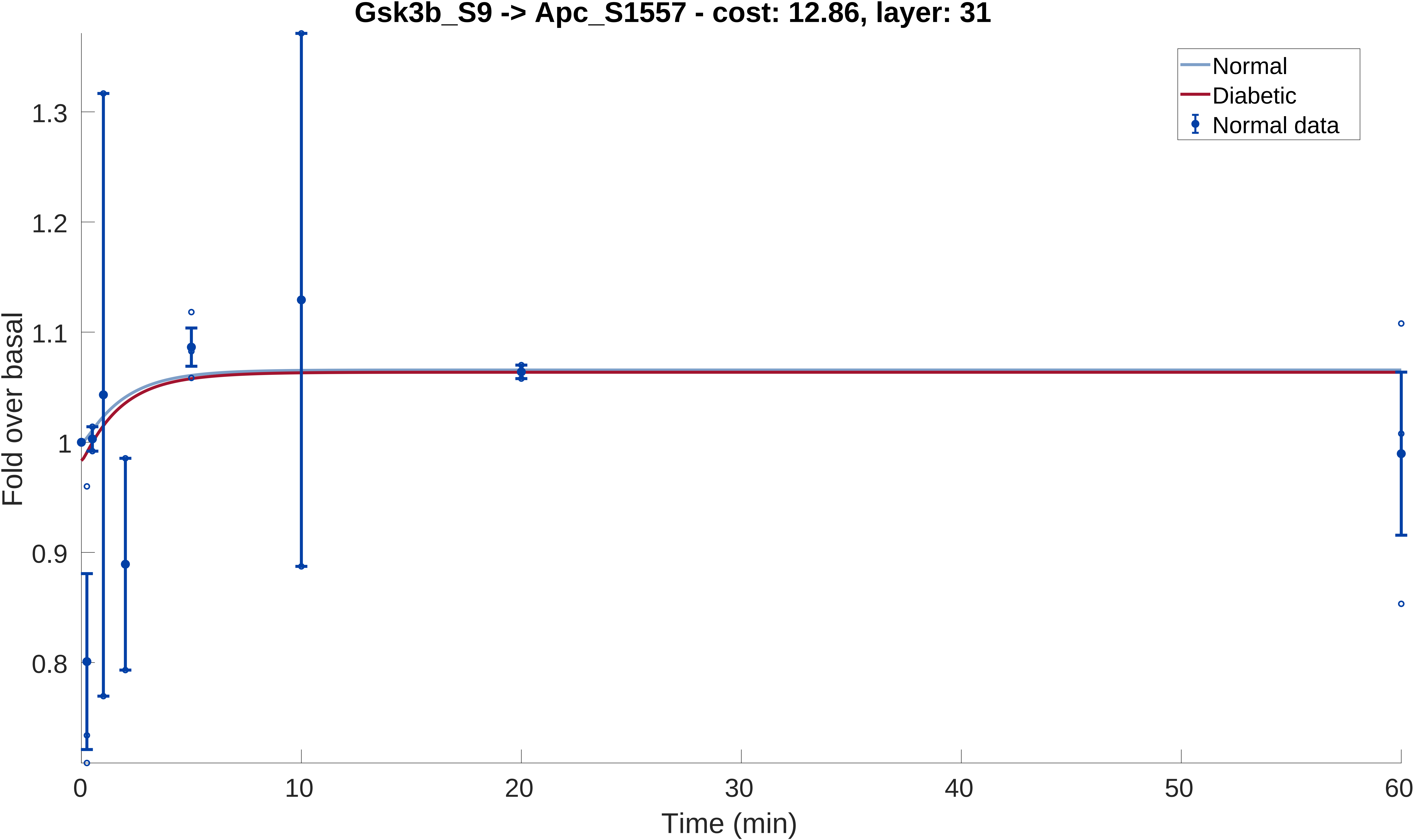

Gsk3b_S9 -> Apc_S1557 - cost: 12.86, layer: 31
Normal
Diabetic
Normal data
Fold over basal
1.3
1.2
1.1
1
0.9
0.8
0
10
20
30
40
50
60
Time (min)

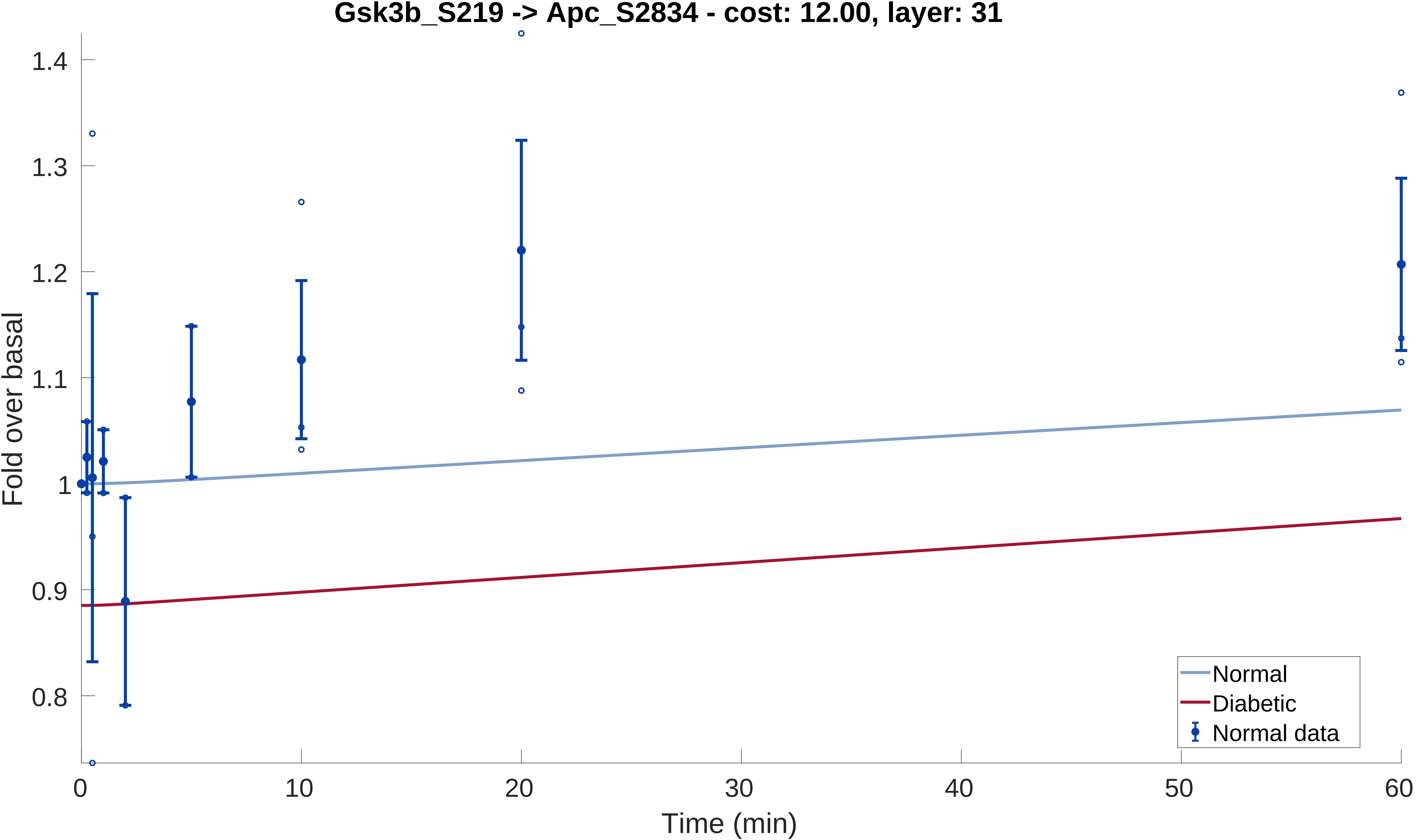

Gsk3b_S219 -> Apc_S2834 - cost: 12.00, layer: 31
Fold over basal
1.4
1.3
1.2
1.1
1
0.9
0.8
0
10
20
30
40
50
60
Time (min)
Normal
Diabetic
Normal data

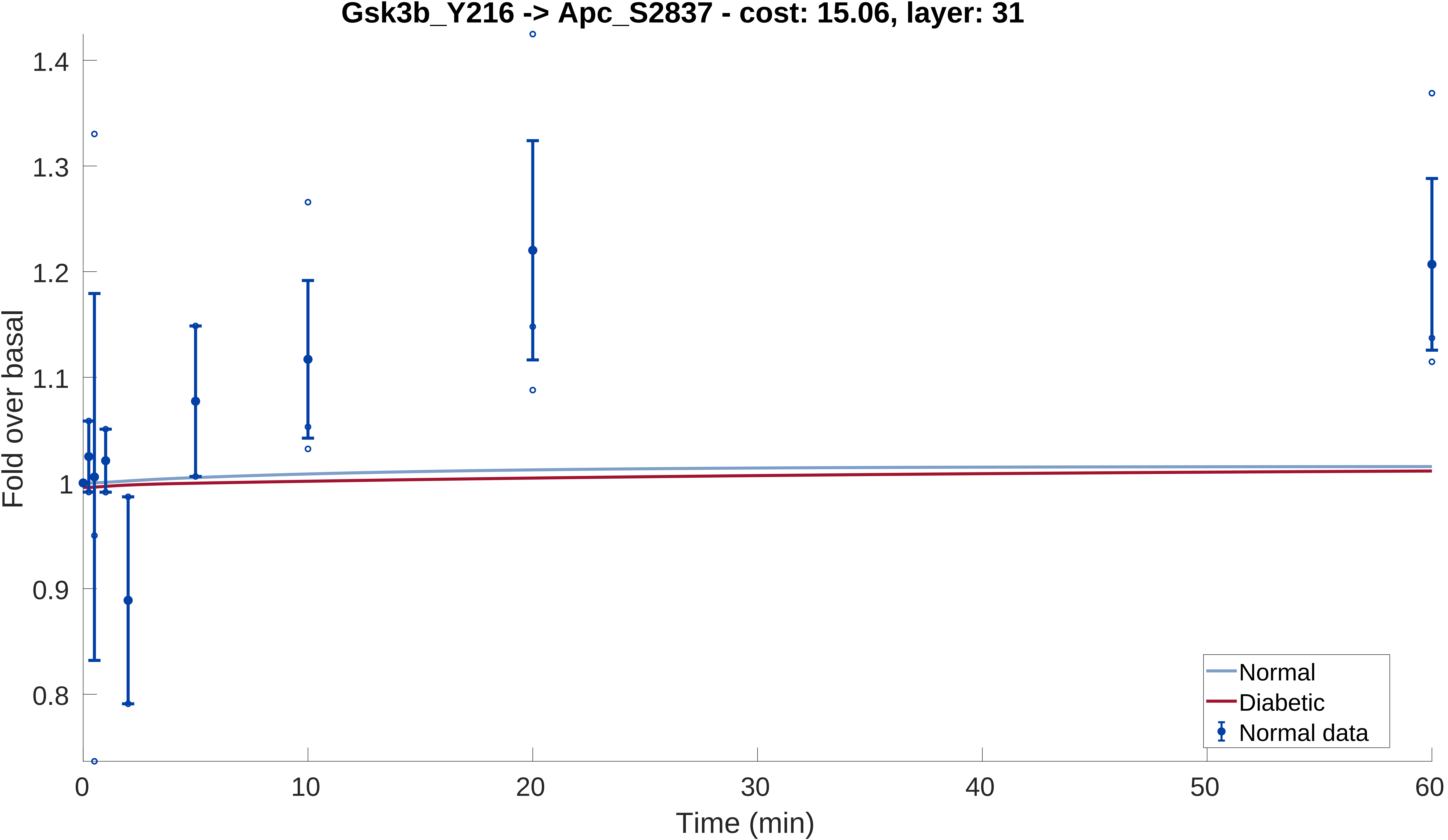
Gsk3b_Y216 -> Apc_S2837 - cost: 15.06, layer: 31
Fold over basal
1.4
1.3
1.2
1.1
1
0.9
0.8
0
10
20
30
40
50
60
Time (min)
Normal
Diabetic
Normal data

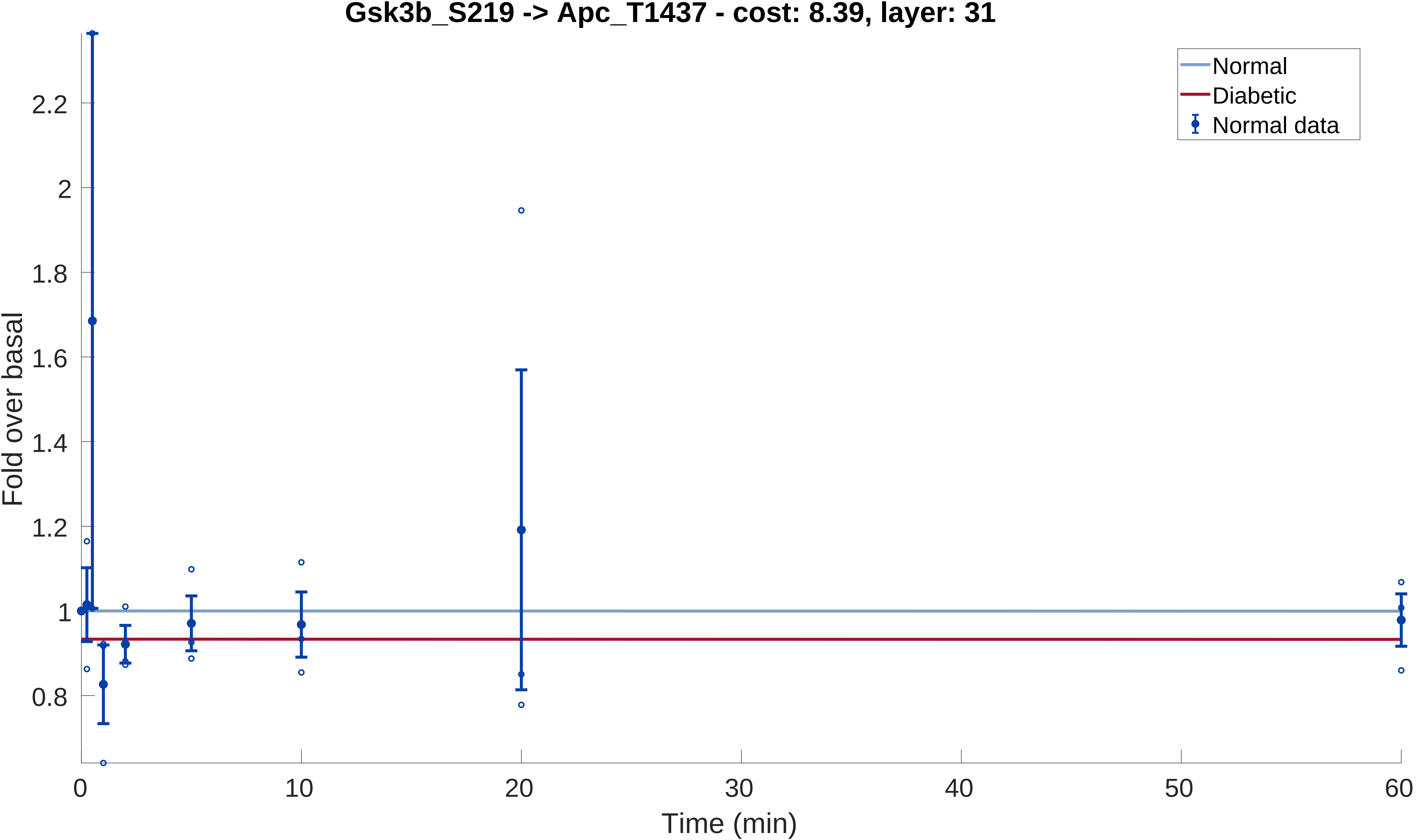

Gsk3b_S219 -> Apc_T1437 - cost: 8.39, layer: 31
Normal
Diabetic
Normal data
Fold over basal
2.2
2
1.8
1.6
1.4
1.2
1
0.8
0
10
20
30
40
50
60
Time (min)

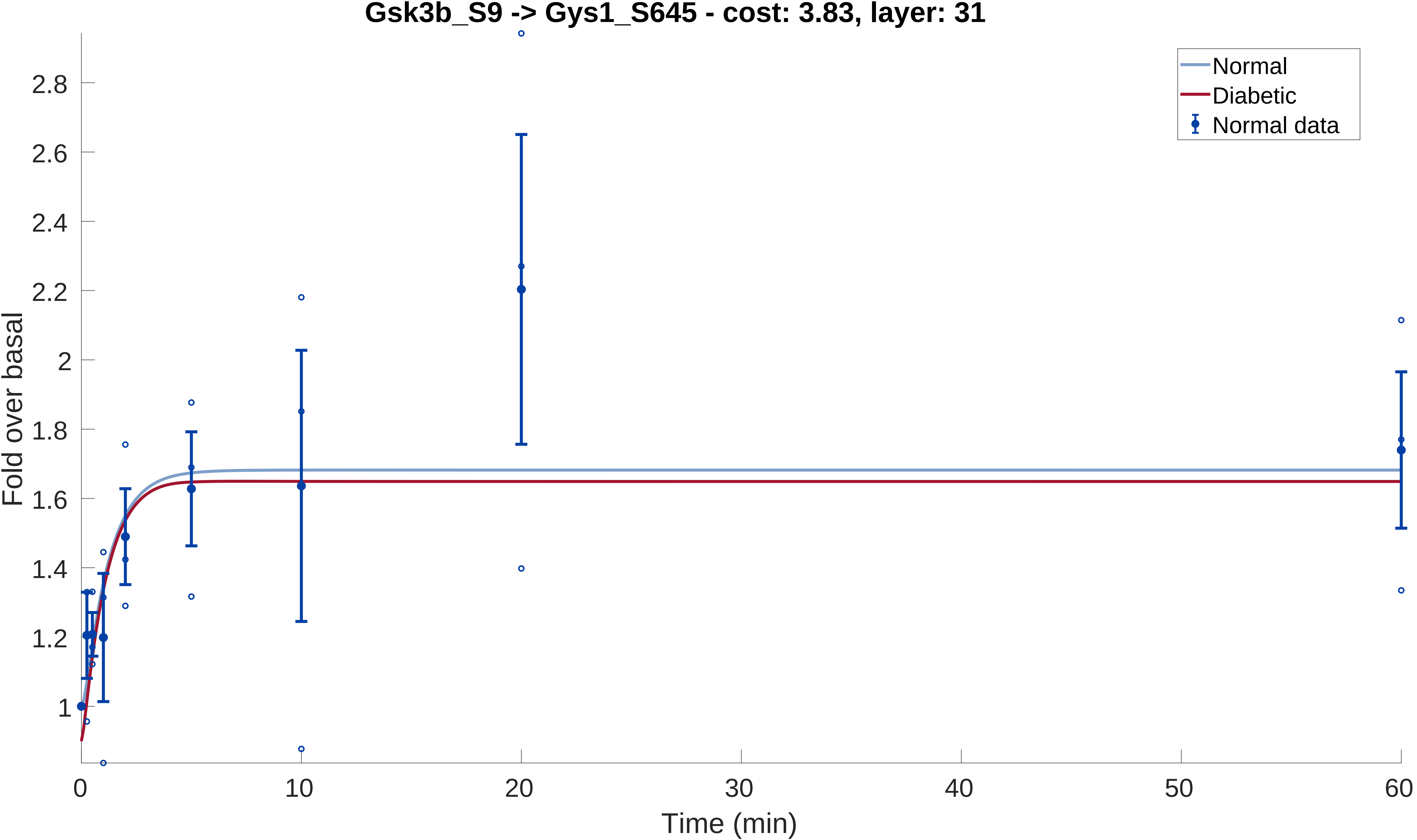

Gsk3b_S9 -> Gys1_S645 - cost: 3.83, layer: 31
Normal
Diabetic
Normal data
Fold over basal
Time (min)
2.8
2.6
2.4
2.2
2
1.8
1.6
1.4
1.2
1
0
10
20
30
40
50
60

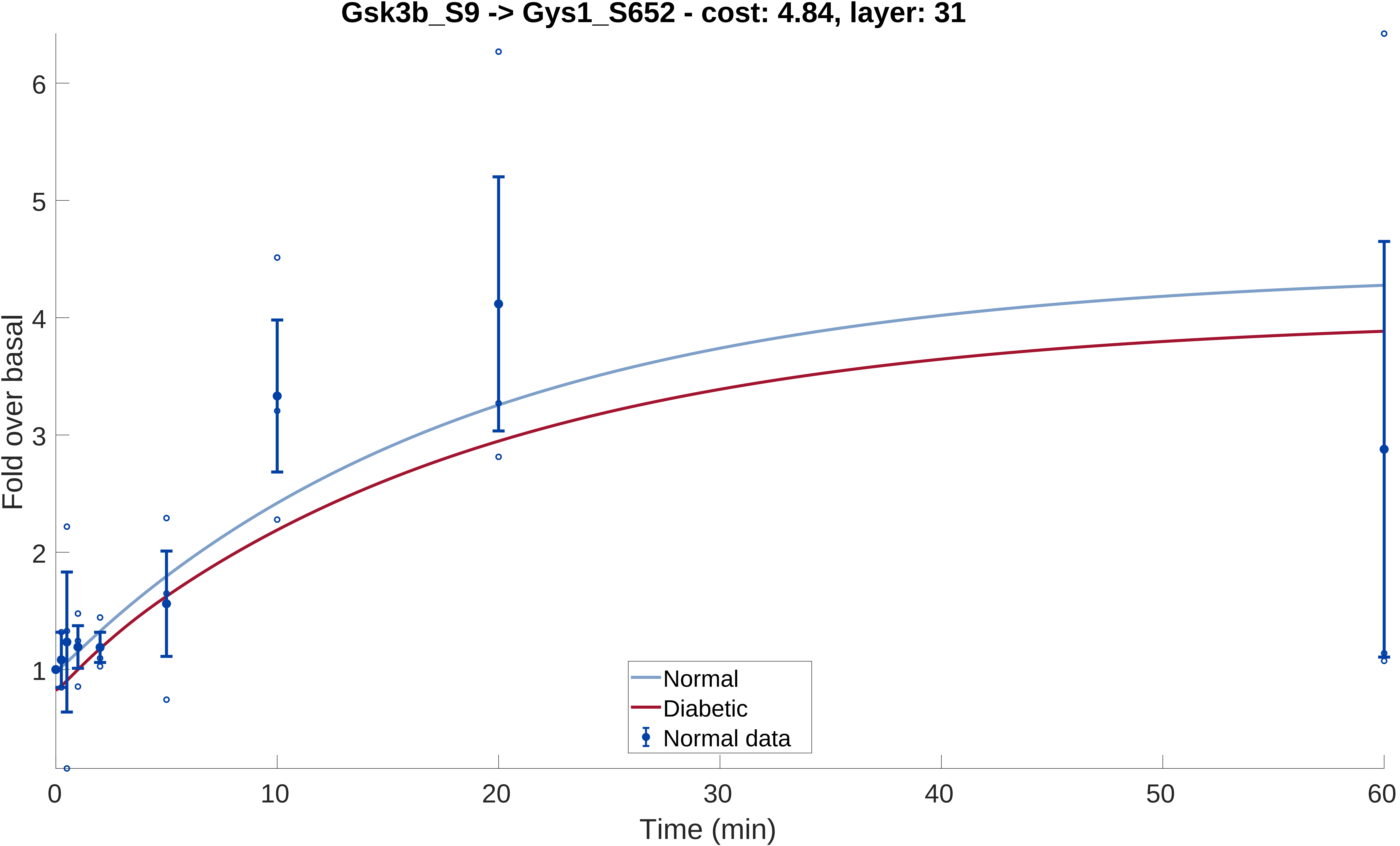

Gsk3b_S9 -> Gys1_S652 - cost: 4.84, layer: 31
Fold over basal
Time (min)
Normal
Diabetic
Normal data
0
10
20
30
40
50
60
1
2
3
4
5
6

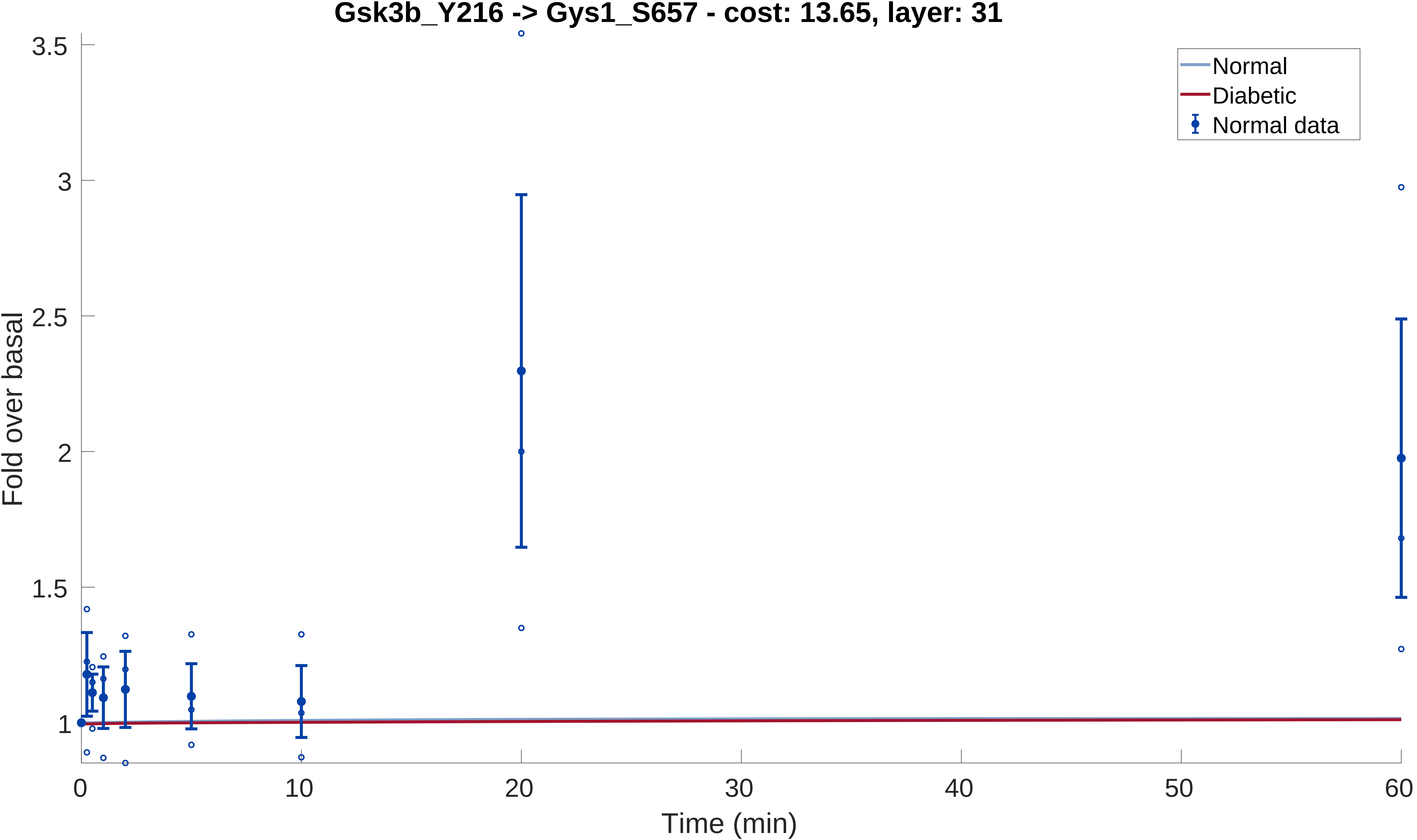
Gsk3b_Y216 -> Gys1_S657 - cost: 13.65, layer: 31
Fold over basal
Time (min)
Normal
Diabetic
Normal data
3.5
3
2.5
2
1.5
1
0
10
20
30
40
50
60

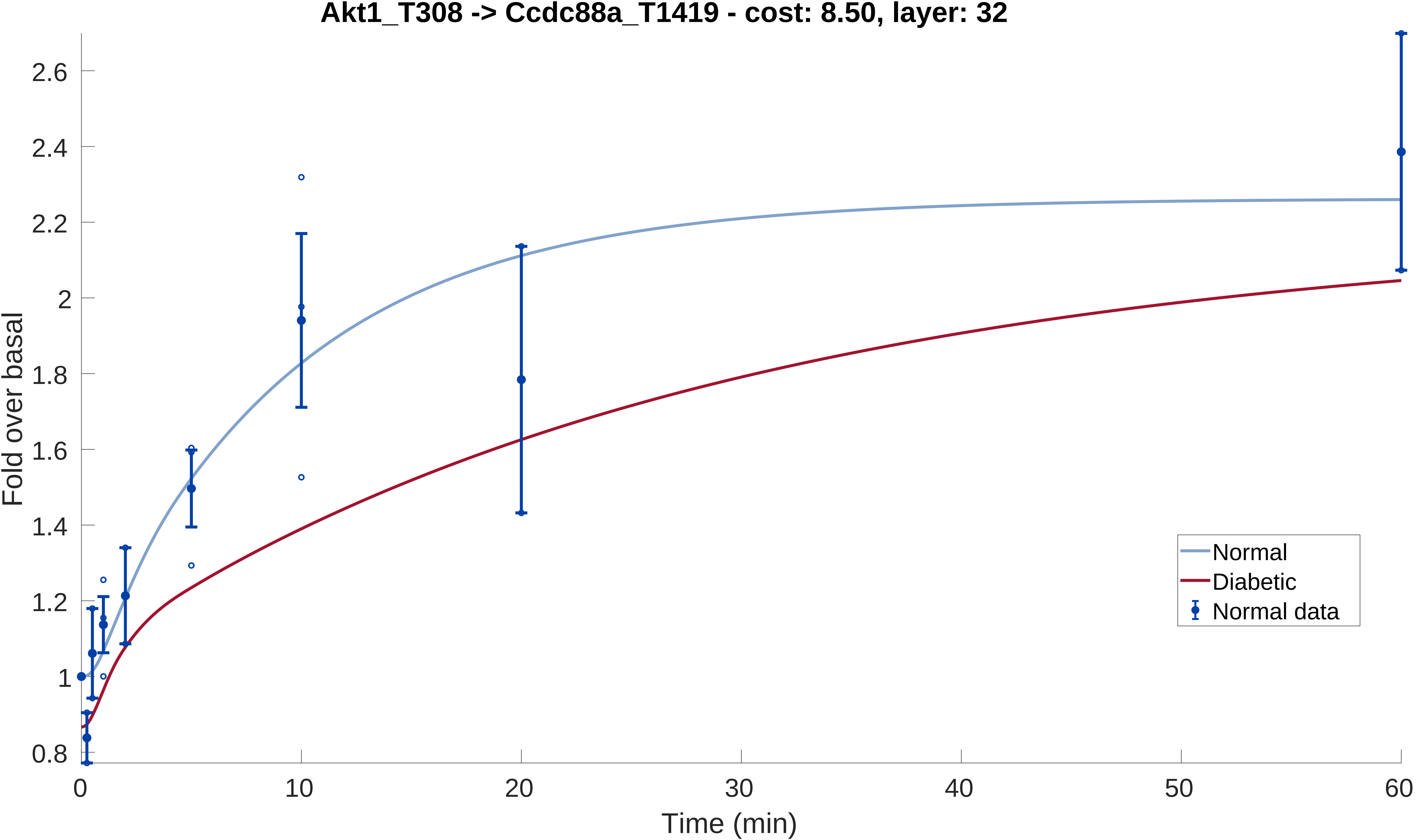

Akt1_T308 -> Ccdc88a_T1419 - cost: 8.50, layer: 32
Fold over basal
2.6
2.4
2.2
2
1.8
1.6
1.4
1.2
1
0.8
0
10
20
30
40
50
60
Time (min)
Normal
Diabetic
Normal data

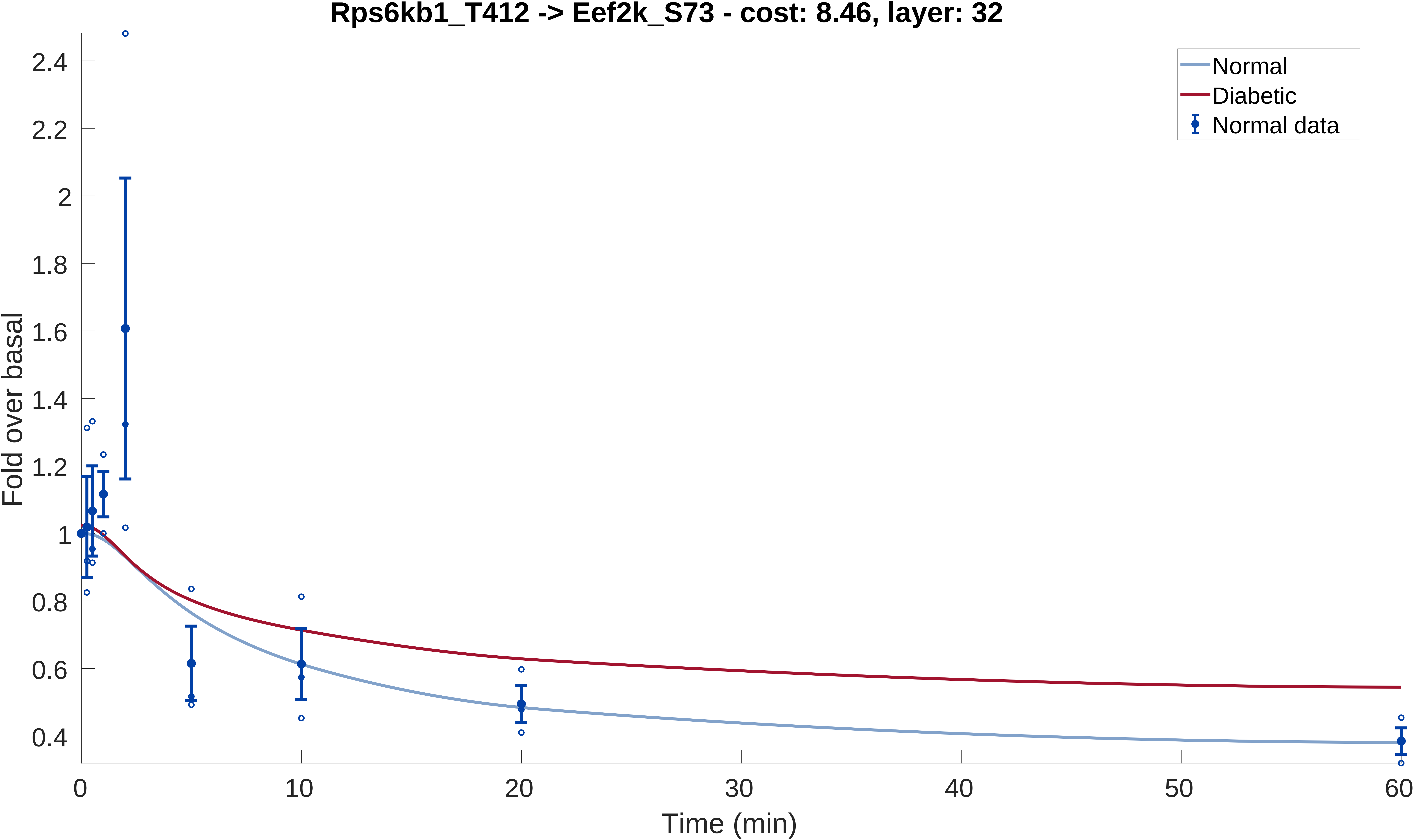

Rps6kb1_T412 -> Eef2k_S73 - cost: 8.46, layer: 32
Normal
Diabetic
Normal data
Fold over basal
Time (min)
0.4
0.6
0.8
1
1.2
1.4
1.6
1.8
2
2.2
2.4
0
10
20
30
40
50
60

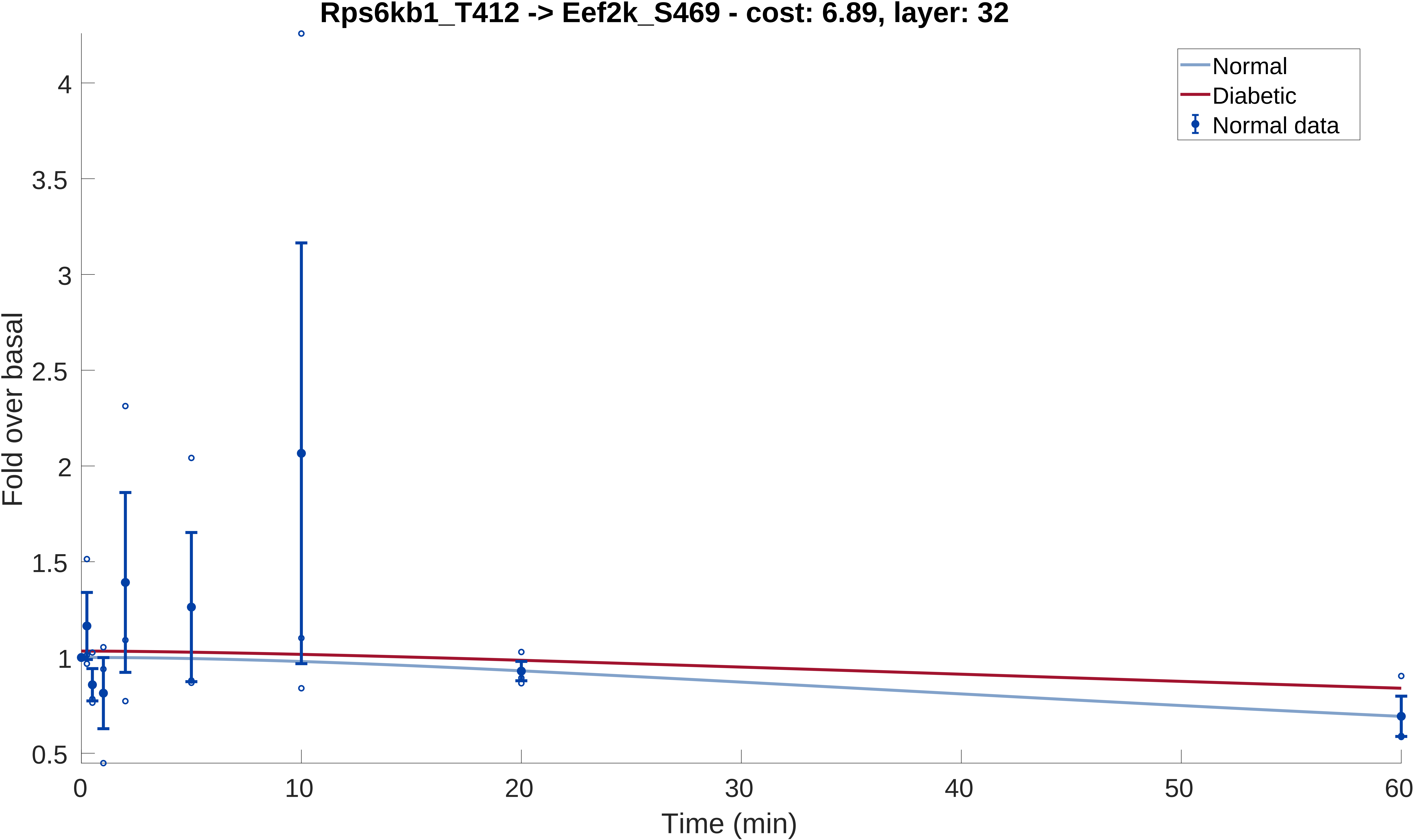

Rps6kb1_T412 -> Eef2k_S469 - cost: 6.89, layer: 32
Normal
Diabetic
Normal data
Fold over basal
0.5
1
1.5
2
2.5
3
3.5
4
0
10
20
30
40
50
60
Time (min)

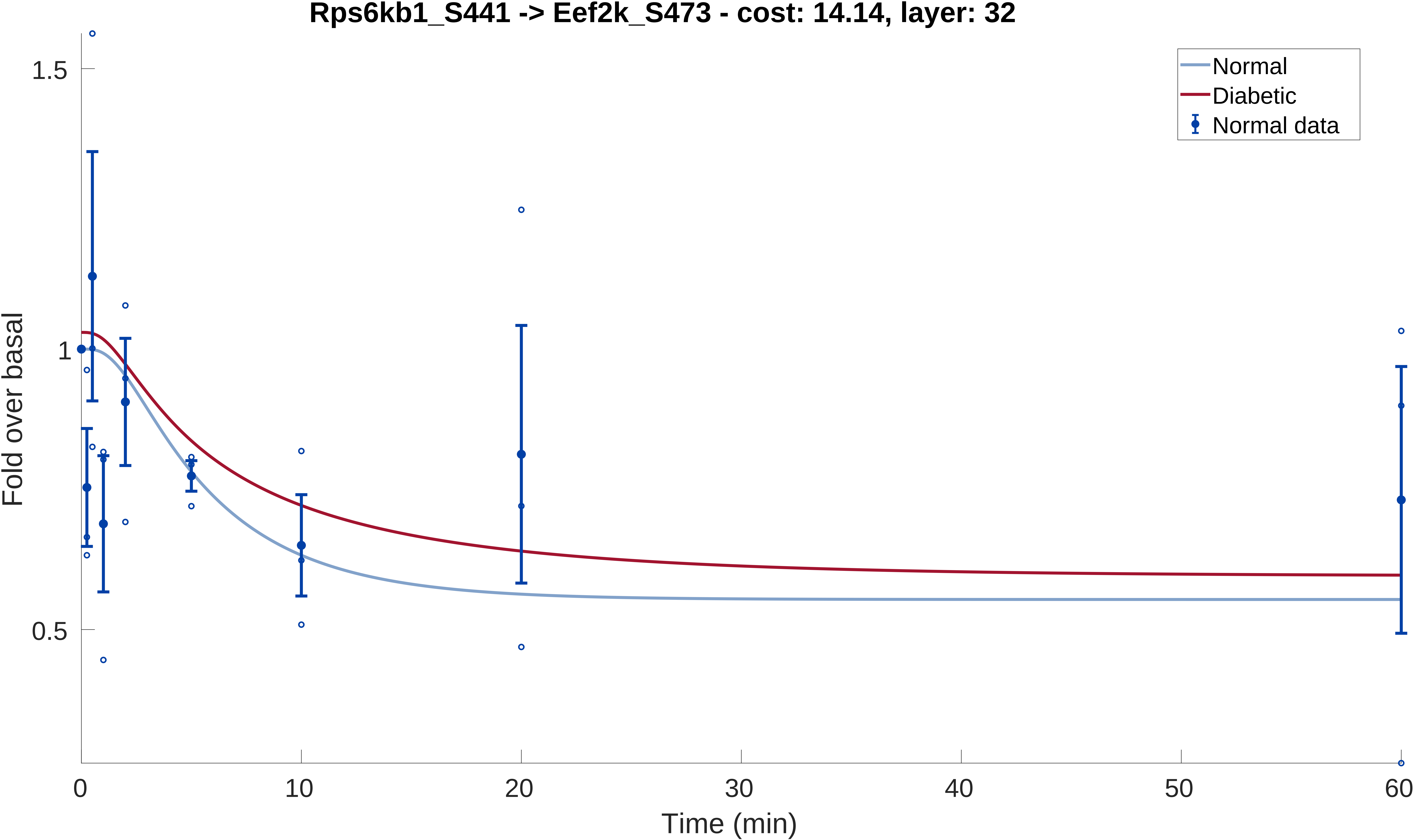
Rps6kb1_S441 -> Eef2k_S473 - cost: 14.14, layer: 32
Normal
Diabetic
Normal data
Fold over basal
1.5
1
0.5
0
10
20
30
40
50
60
Time (min)

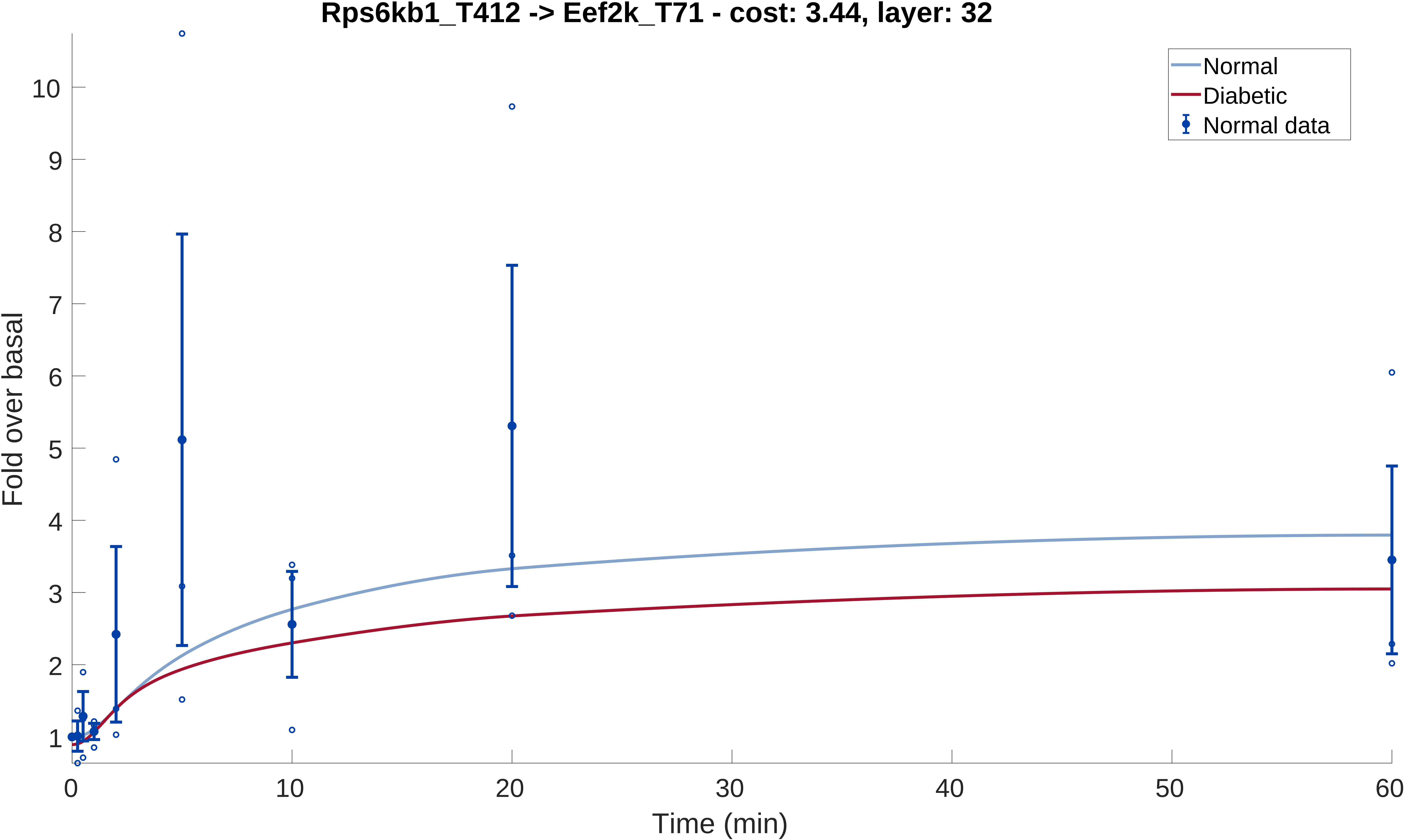

Rps6kb1_T412 -> Eef2k_T71 - cost: 3.44, layer: 32
Normal
Diabetic
Normal data
Fold over basal
Time (min)
1
2
3
4
5
6
7
8
9
10
0
10
20
30
40
50
60

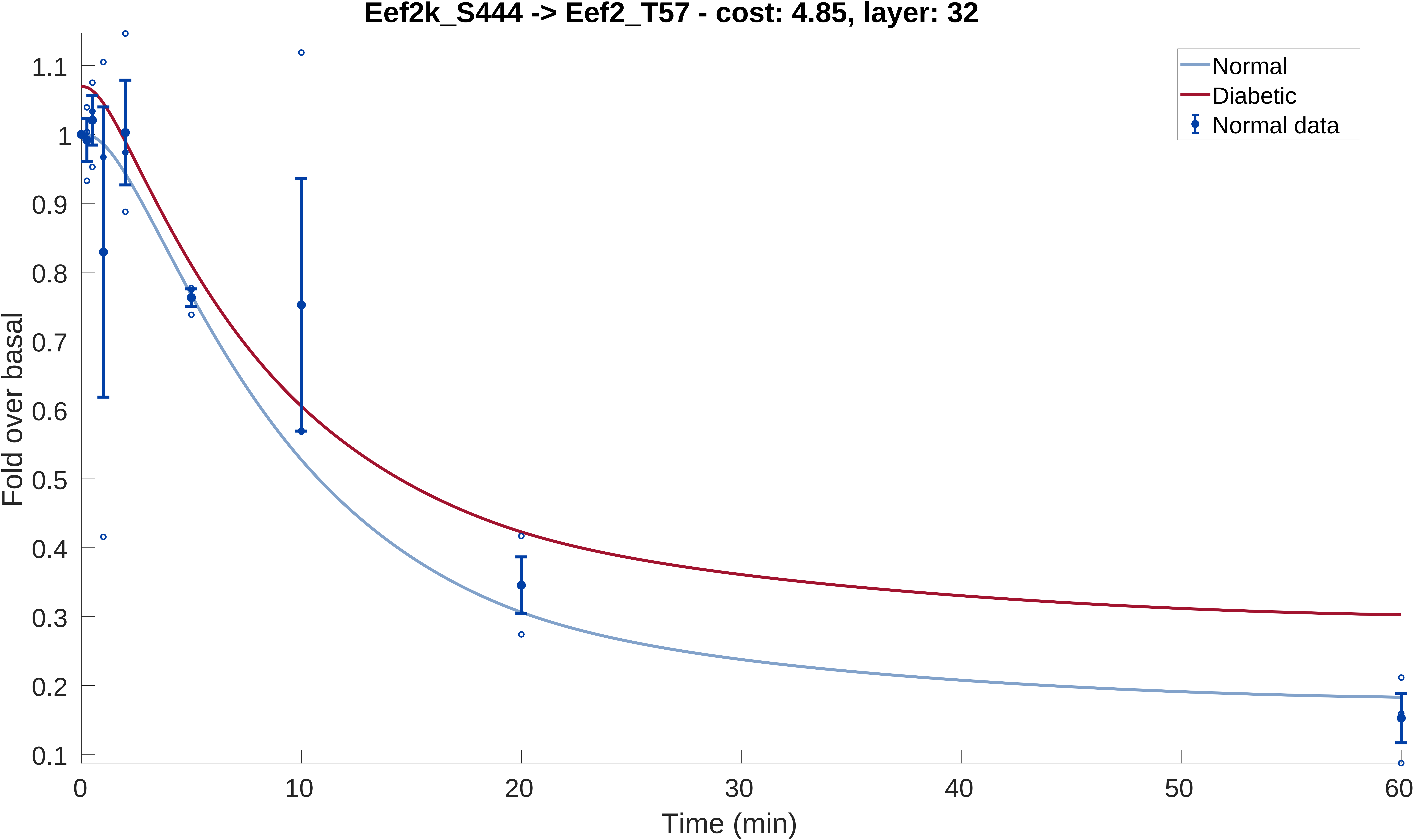
Eef2k_S444 -> Eef2_T57 - cost: 4.85, layer: 32
Normal
Diabetic
Normal data
Fold over basal
Time (min)
0.1
0.2
0.3
0.4
0.5
0.6
0.7
0.8
0.9
1
1.1
0
10
20
30
40
50
60

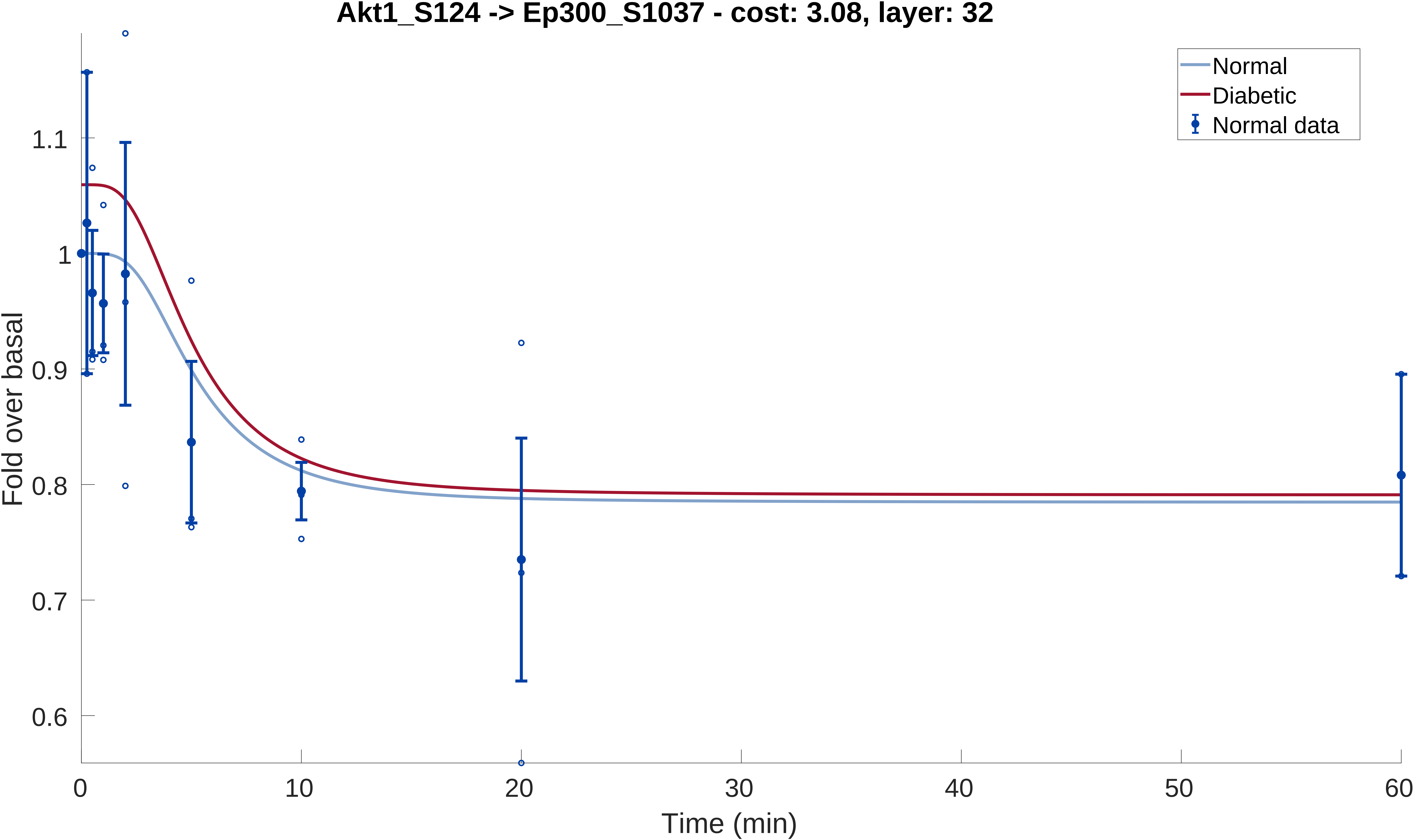

Akt1_S124 -> Ep300_S1037 - cost: 3.08, layer: 32
Normal
Diabetic
Normal data
Fold over basal
1.1
1
0.9
0.8
0.7
0.6
0
10
20
30
40
50
60
Time (min)

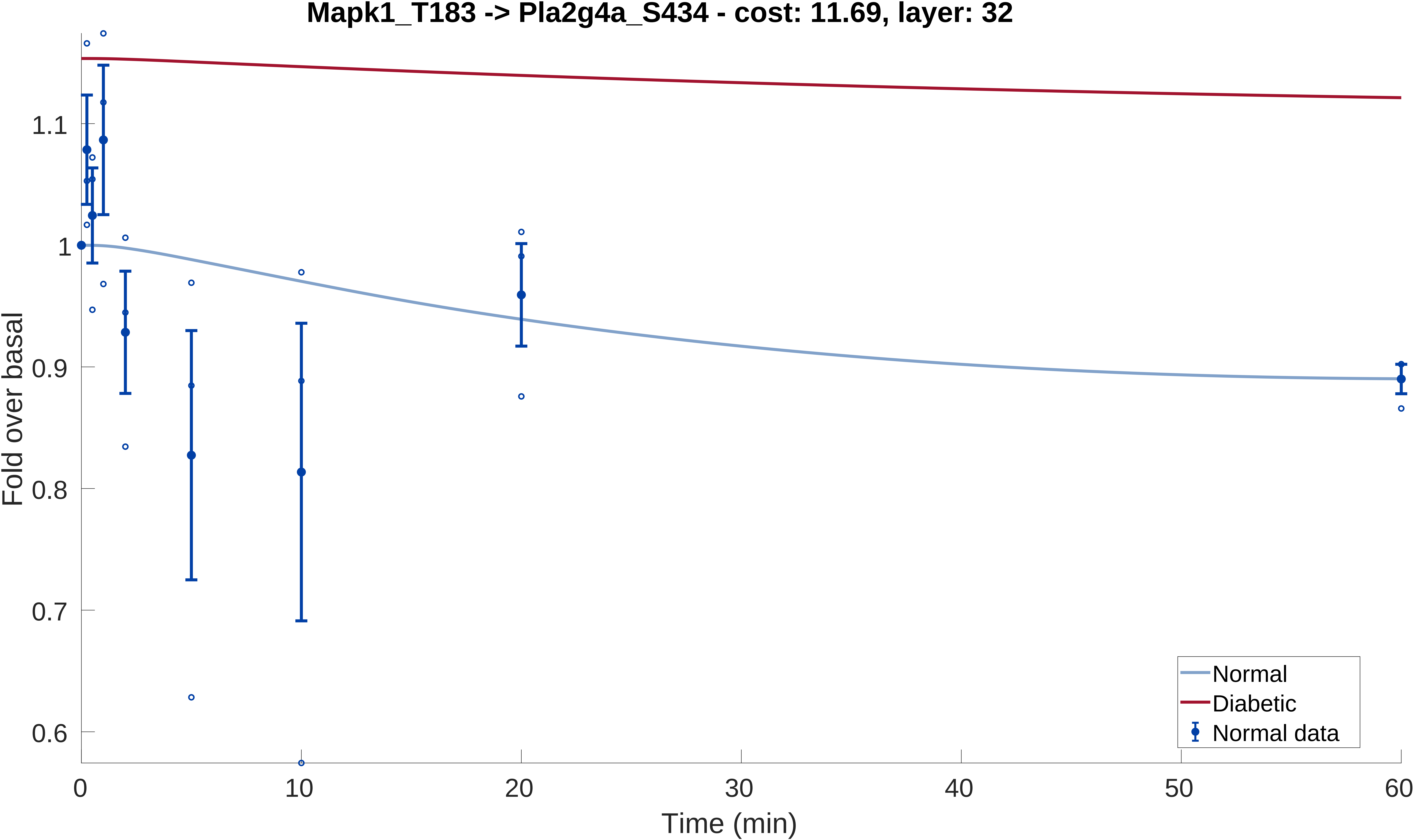

Mapk1_T183 -> Pla2g4a_S434 - cost: 11.69, layer: 32
1.1
1
0.9
0.8
0.7
0.6
Fold over basal
0
10
20
30
40
50
60
Time (min)
Normal
Diabetic
Normal data

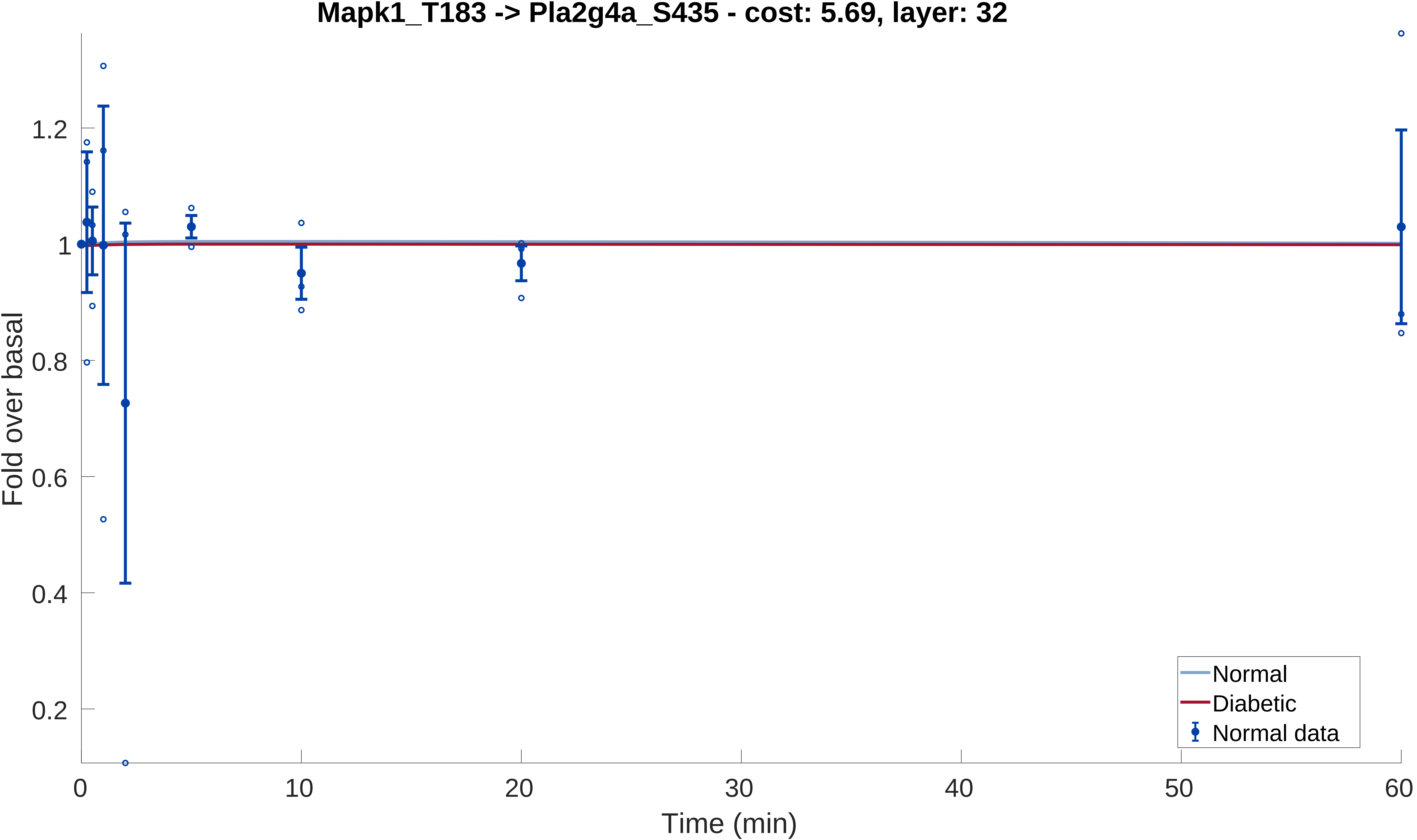

Mapk1_T183 -> Pla2g4a_S435 - cost: 5.69, layer: 32
Fold over basal
1.2
1
0.8
0.6
0.4
0.2
0
10
20
30
40
50
60
Time (min)
Normal
Diabetic
Normal data

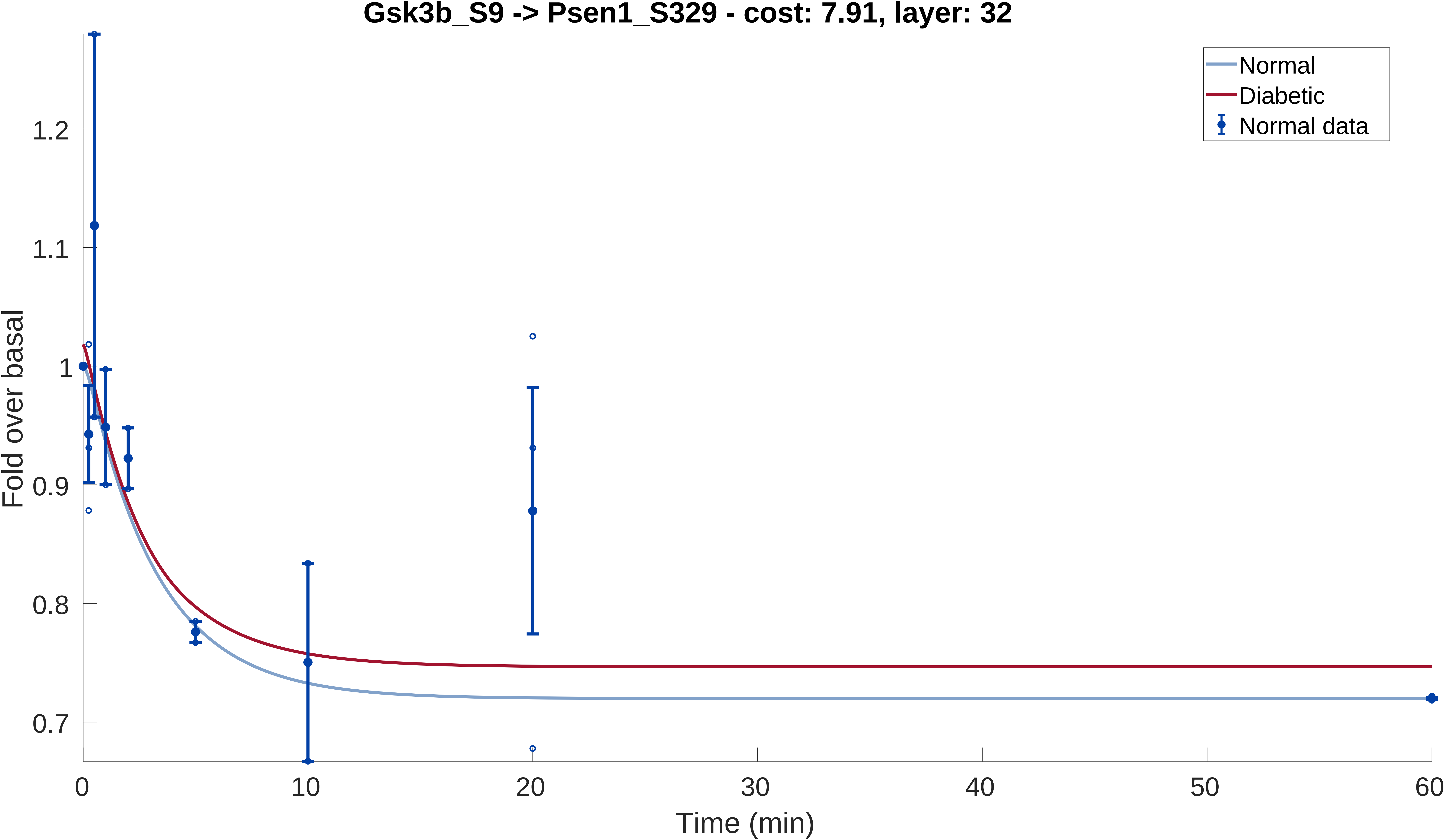
Gsk3b_S9 -> Psen1_S329 - cost: 7.91, layer: 32
Normal
Diabetic
Normal data
Fold over basal
1.2
1.1
1
0.9
0.8
0.7
0
10
20
30
40
50
60
Time (min)

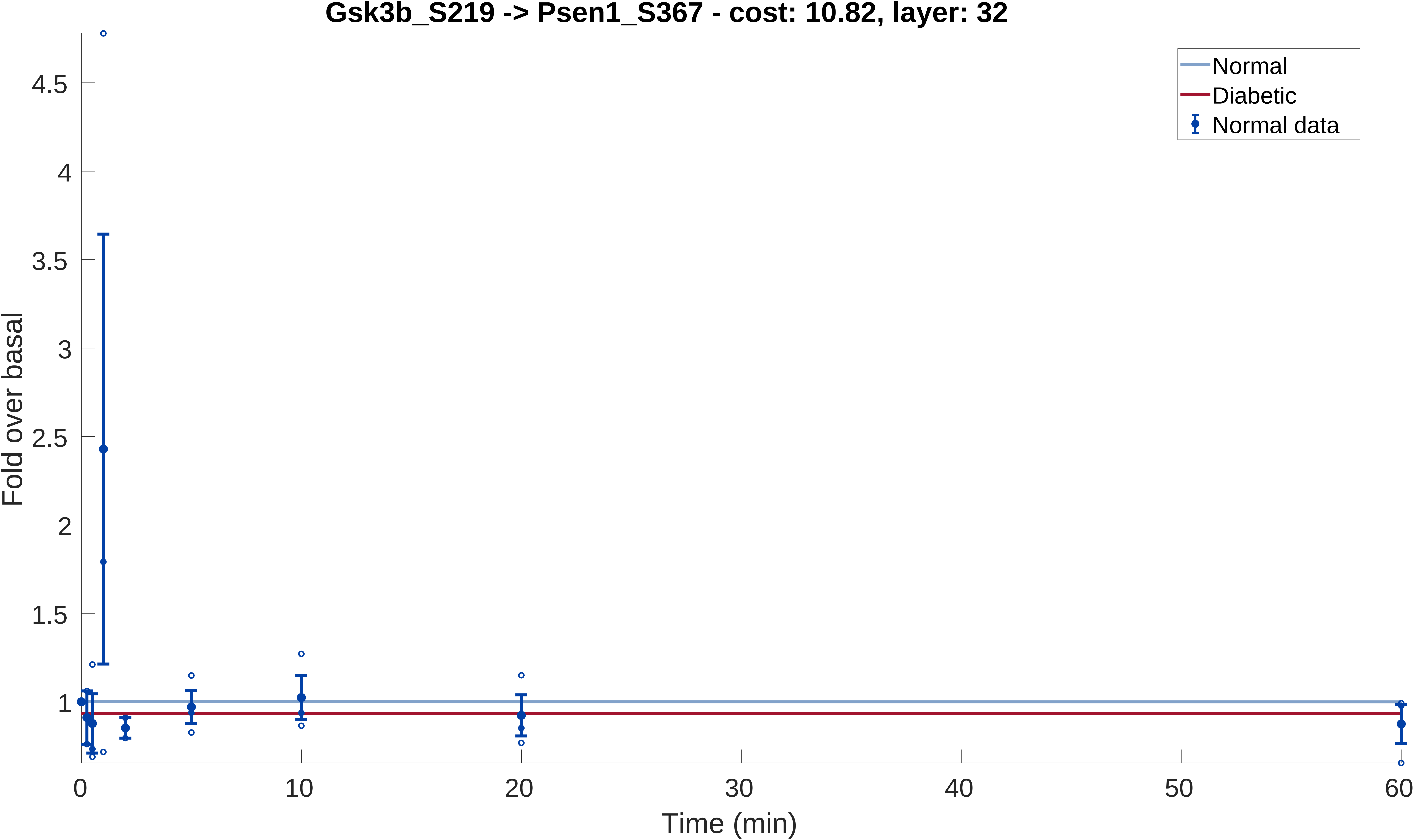
Gsk3b_S219 -> Psen1_S367 - cost: 10.82, layer: 32
Normal
Diabetic
Normal data
Fold over basal
4.5
4
3.5
3
2.5
2
1.5
1
0
10
20
30
40
50
60
Time (min)

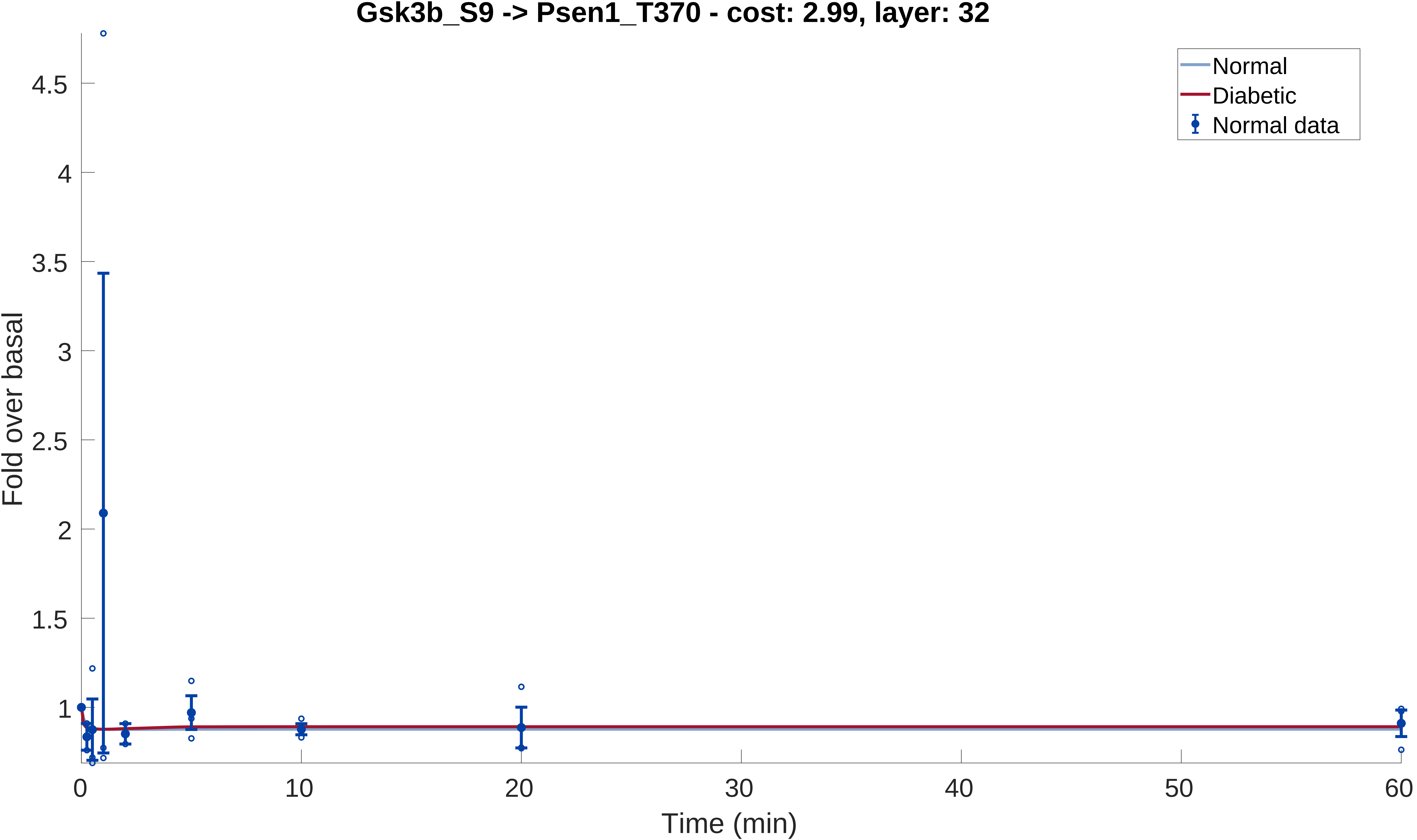

Gsk3b_S9 -> Psen1_T370 - cost: 2.99, layer: 32
Normal
Diabetic
Normal data
Fold over basal
Time (min)
4.5
4
3.5
3
2.5
2
1.5
1
0
10
20
30
40
50
60

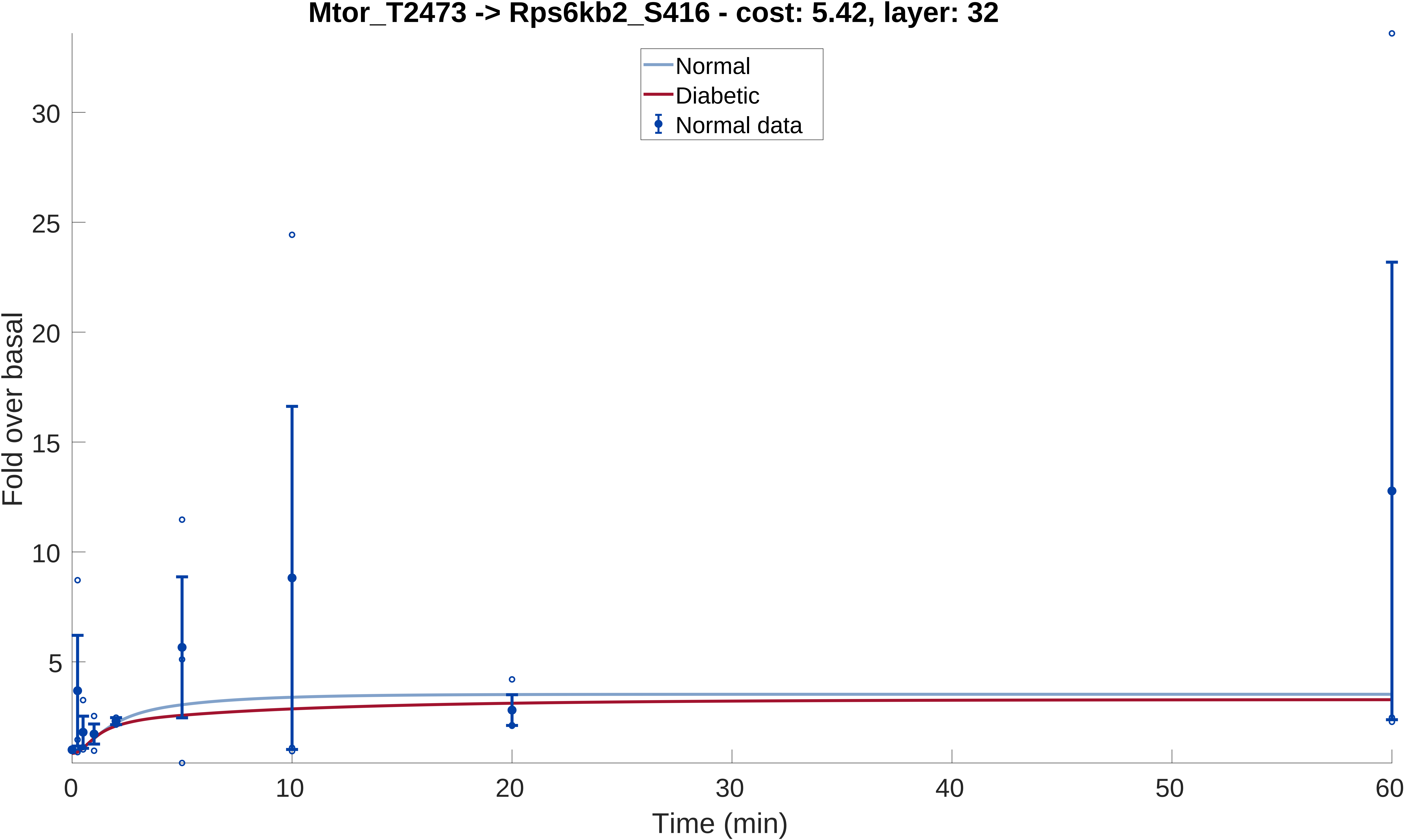

Mtor_T2473 -> Rps6kb2_S416 - cost: 5.42, layer: 32
Normal
Diabetic
Normal data
Fold over basal
Time (min)
0
10
20
30
40
50
60
5
10
15
20
25
30

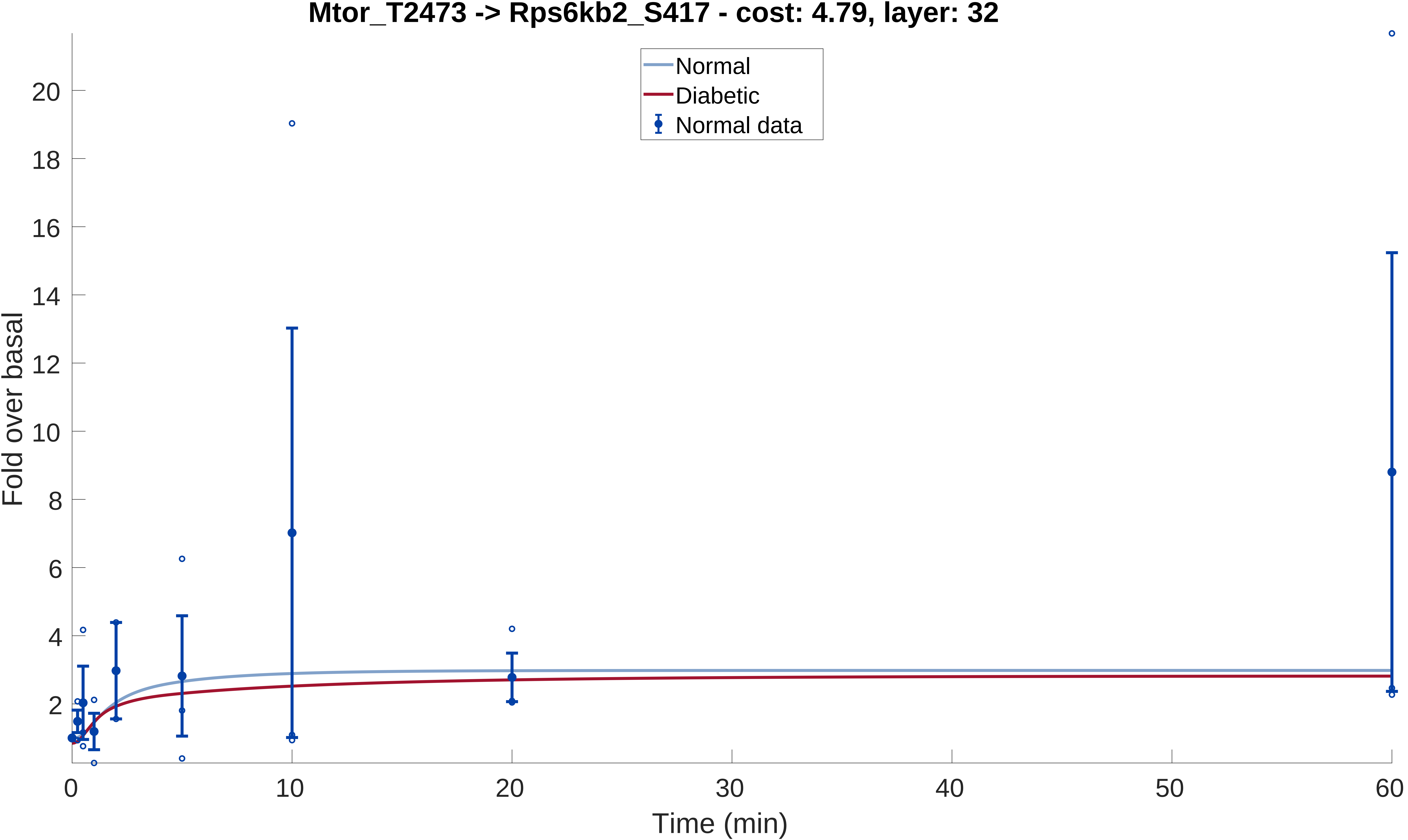

Mtor_T2473 -> Rps6kb2_S417 - cost: 4.79, layer: 32
Normal
Diabetic
Normal data
Fold over basal
Time (min)
0
10
20
30
40
50
60
2
4
6
8
10
12
14
16
18
20

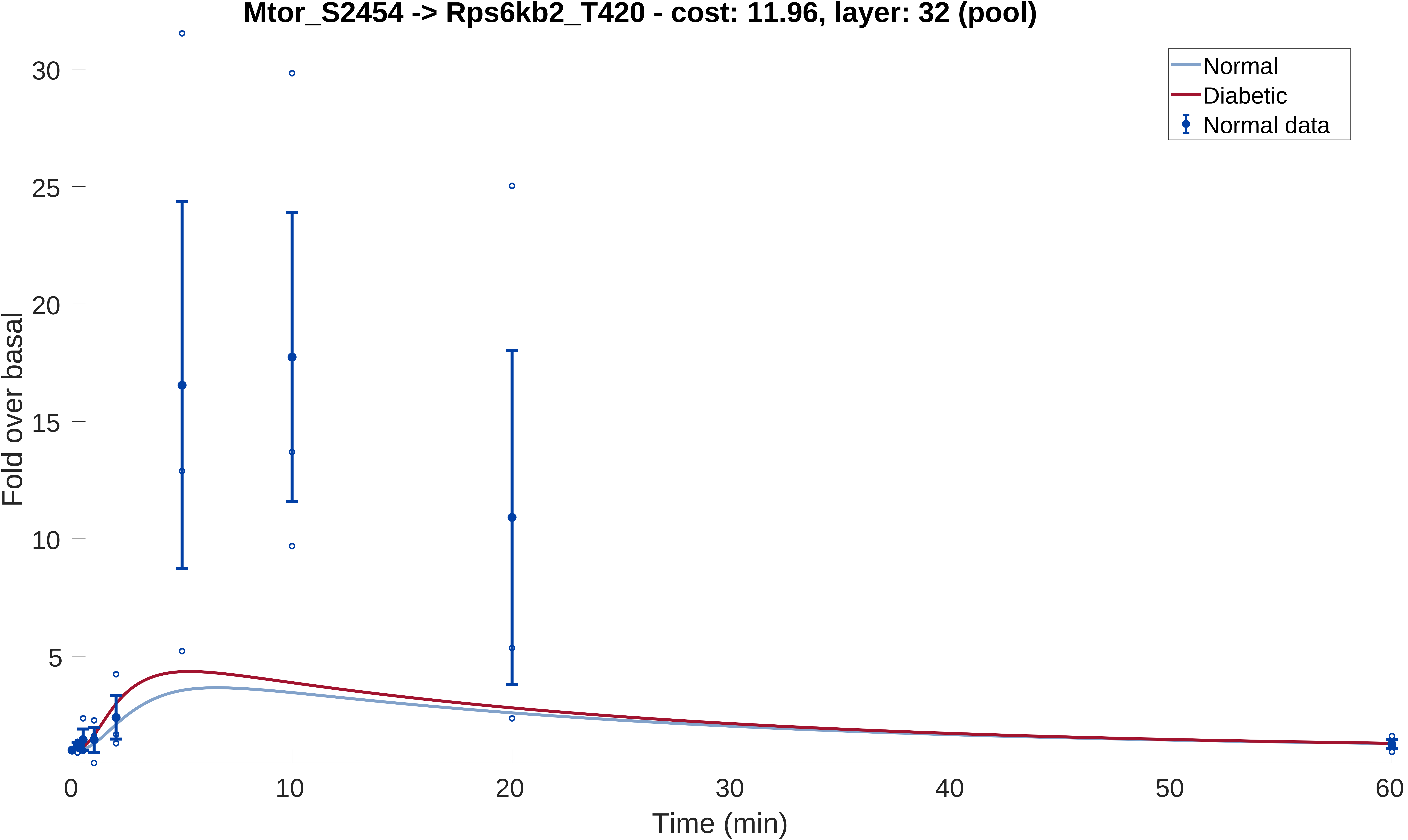

Mtor_S2454 -> Rps6kb2_T420 - cost: 11.96, layer: 32 (pool)
Normal
Diabetic
Normal data
Fold over basal
Time (min)
0
10
20
30
40
50
60
5
10
15
20
25
30

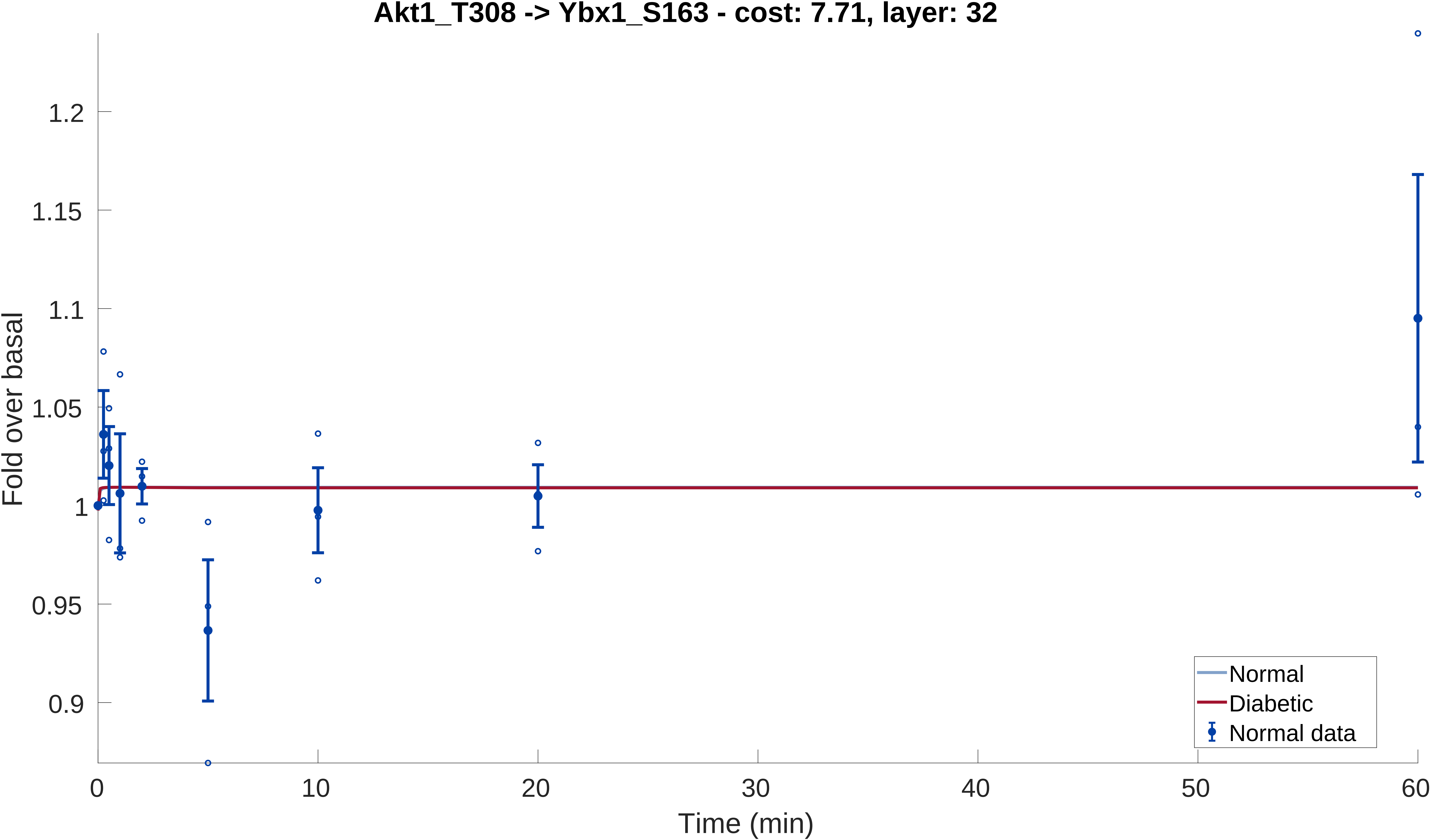

Akt1_T308 -> Ybx1_S163 - cost: 7.71, layer: 32
Fold over basal
1.2
1.15
1.1
1.05
1
0.95
0.9
0
10
20
30
40
50
60
Time (min)
Normal
Diabetic
Normal data

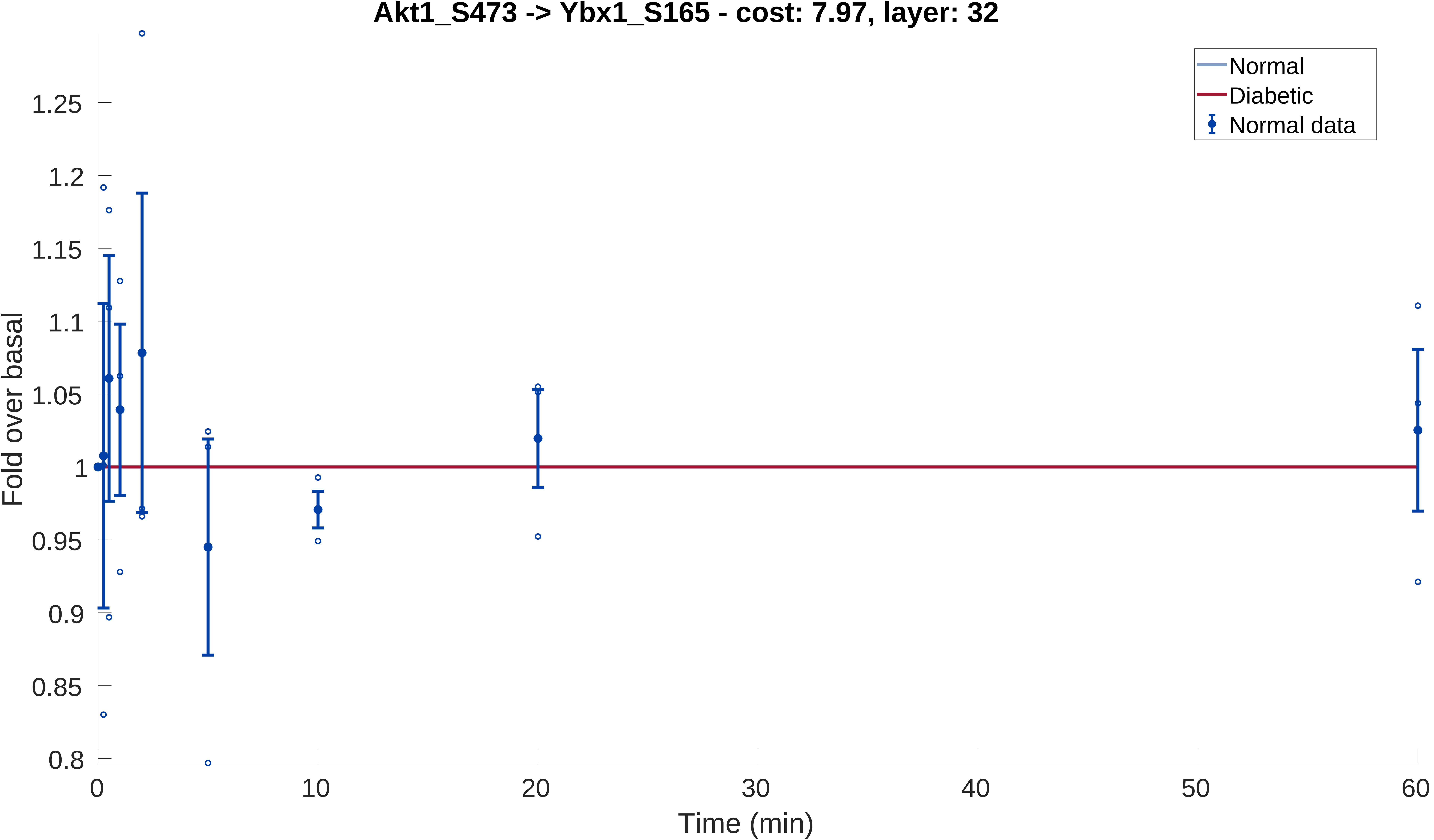
Akt1_S473 -> Ybx1_S165 - cost: 7.97, layer: 32
Normal
Diabetic
Normal data
Fold over basal
1.25
1.2
1.15
1.1
1.05
1
0.95
0.9
0.85
0.8
0
10
20
30
40
50
60
Time (min)

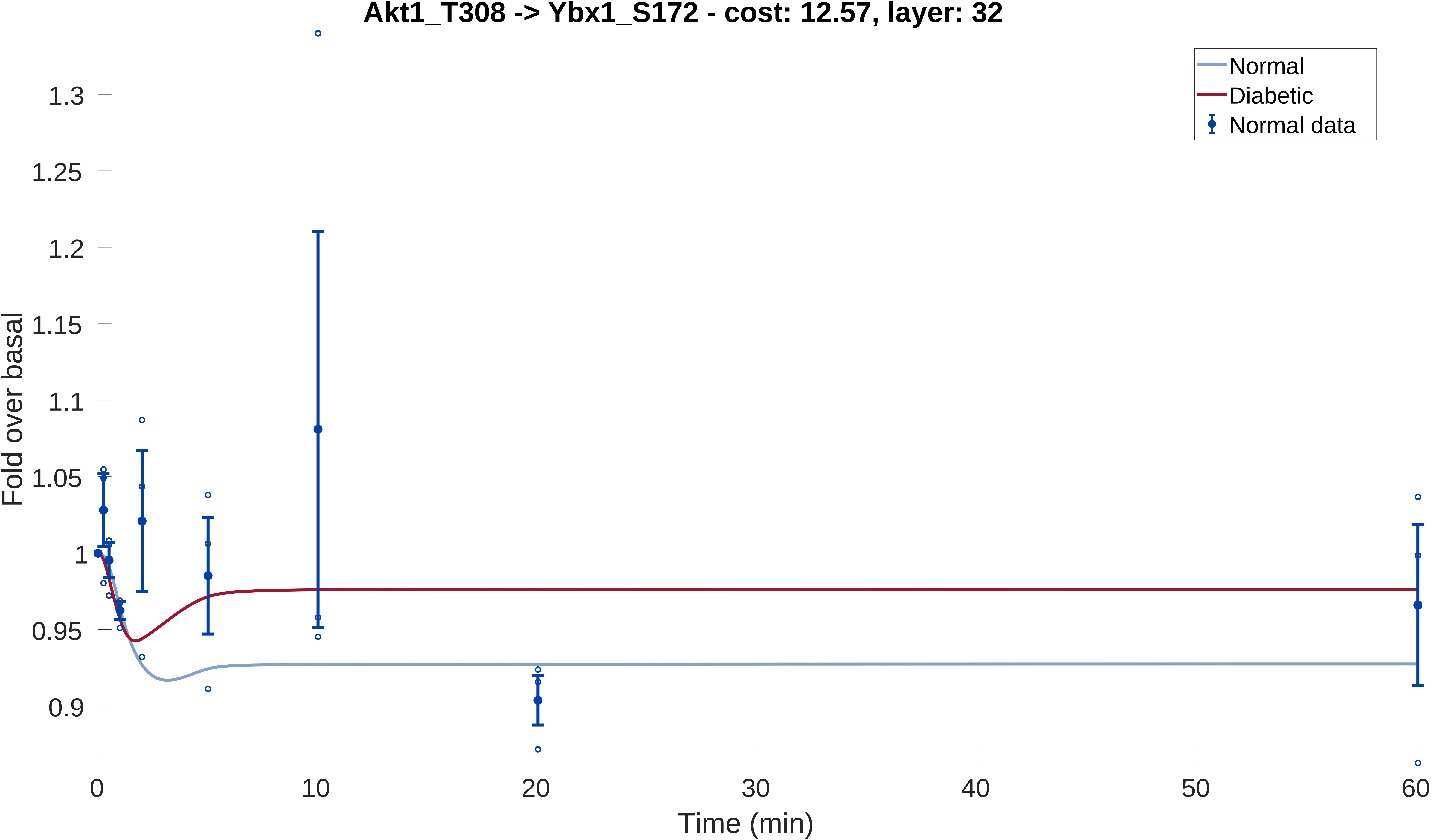
Akt1_T308 -> Ybx1_S172 - cost: 12.57, layer: 32
Normal
Diabetic
Normal data
Fold over basal
1.3
1.25
1.2
1.15
1.1
1.05
1
0.95
0.9
0
10
20
30
40
50
60
Time (min)

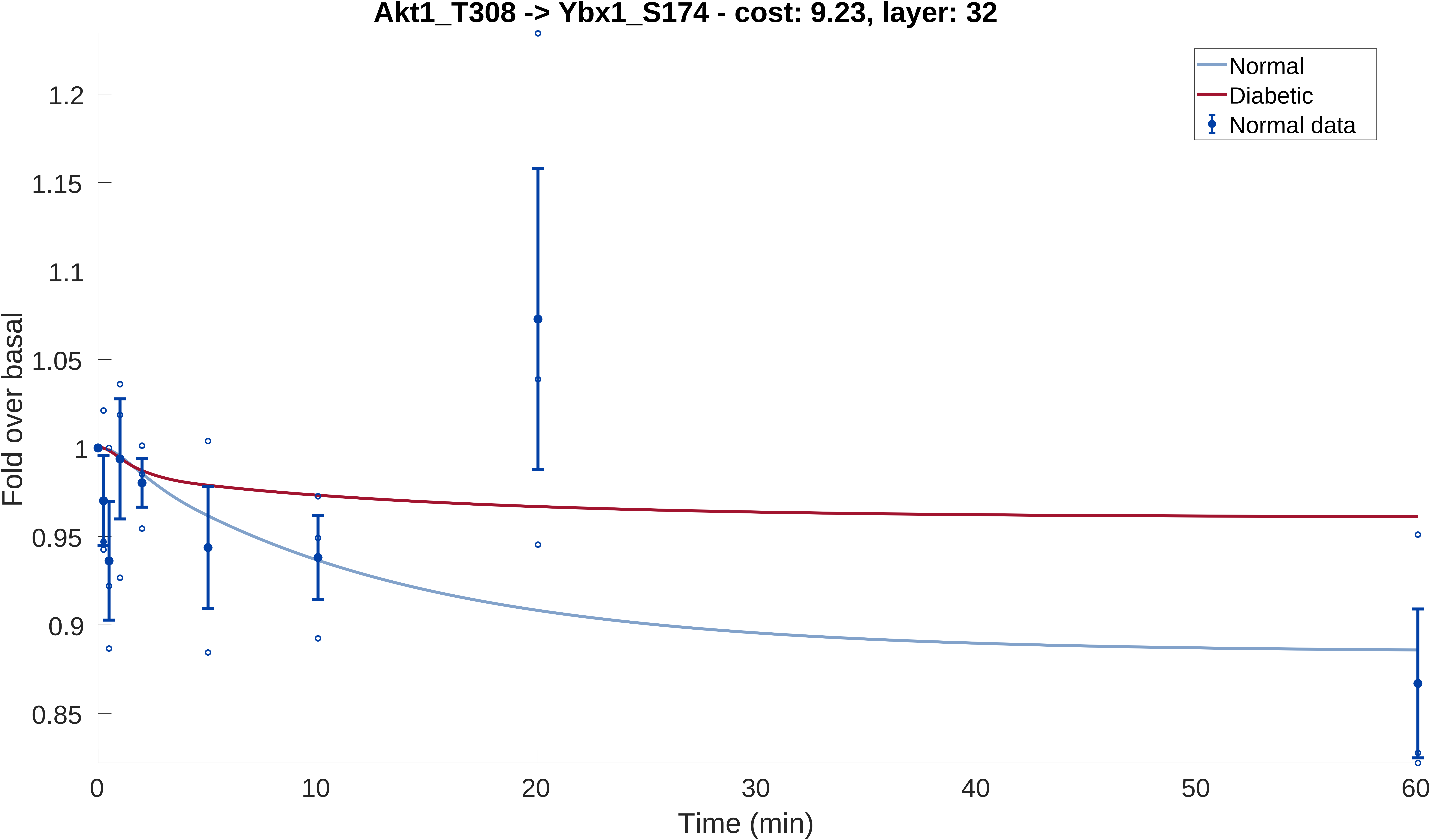

Akt1_T308 -> Ybx1_S174 - cost: 9.23, layer: 32
Normal
Diabetic
Normal data
1.2
1.15
1.1
1.05
1
0.95
0.9
0.85
Fold over basal
0
10
20
30
40
50
60
Time (min)

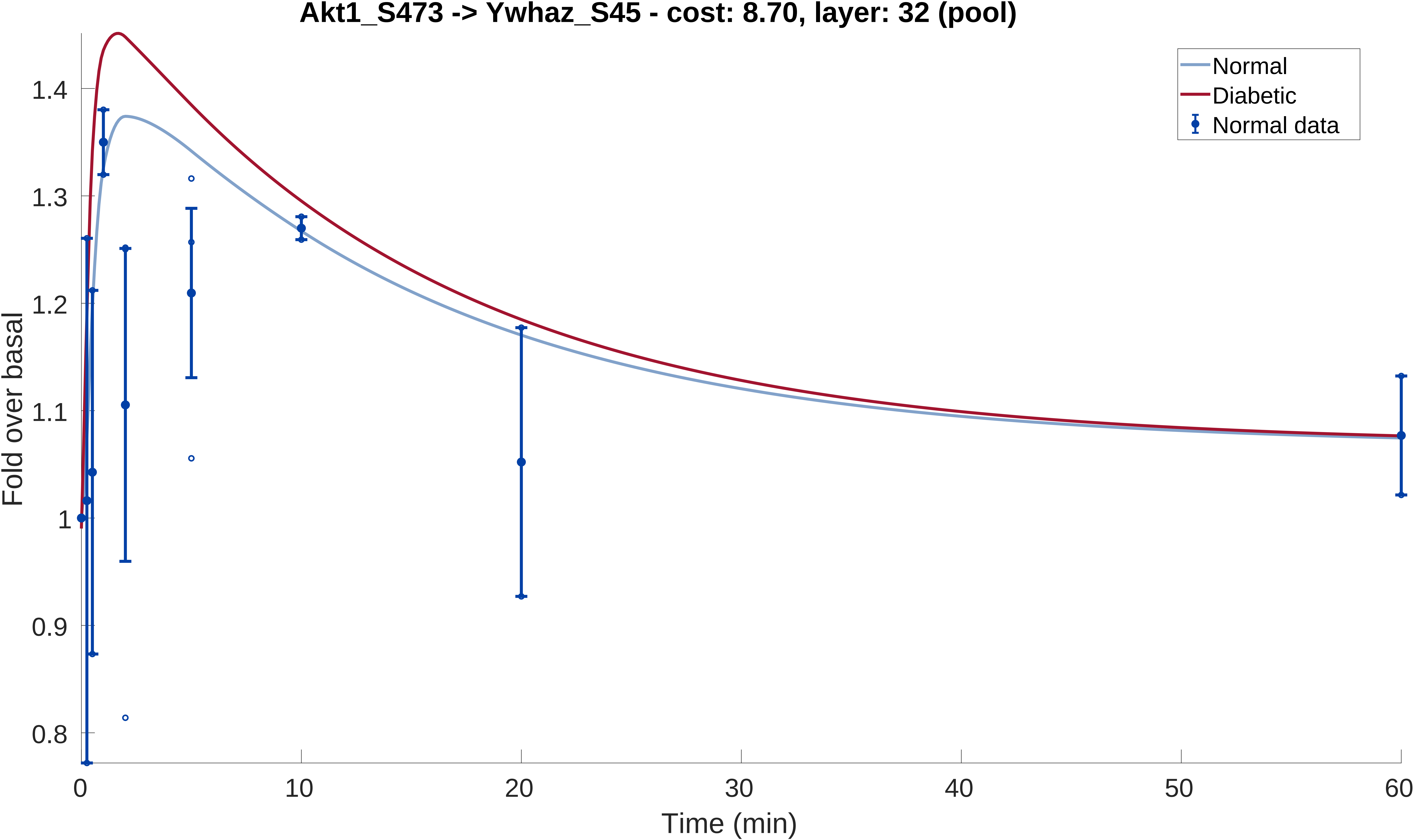

Akt1_S473 -> Ywhaz_S45 - cost: 8.70, layer: 32 (pool)
Normal
Diabetic
Normal data
Fold over basal
1.4
1.3
1.2
1.1
1
0.9
0.8
0
10
20
30
40
50
60
Time (min)

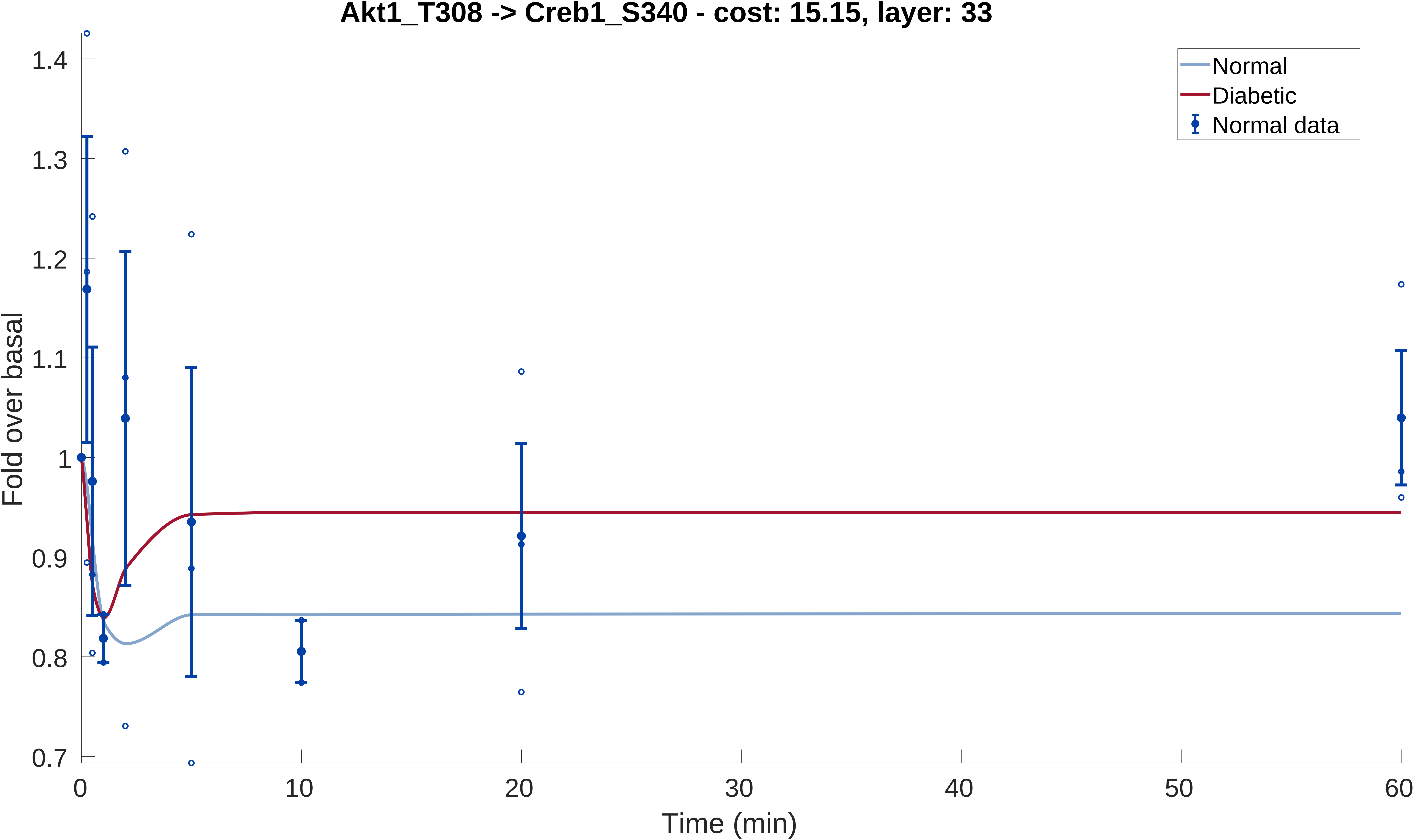
Akt1_T308 -> Creb1_S340 - cost: 15.15, layer: 33
Normal
Diabetic
Normal data
Fold over basal
1.4
1.3
1.2
1.1
1
0.9
0.8
0.7
0
10
20
30
40
50
60
Time (min)

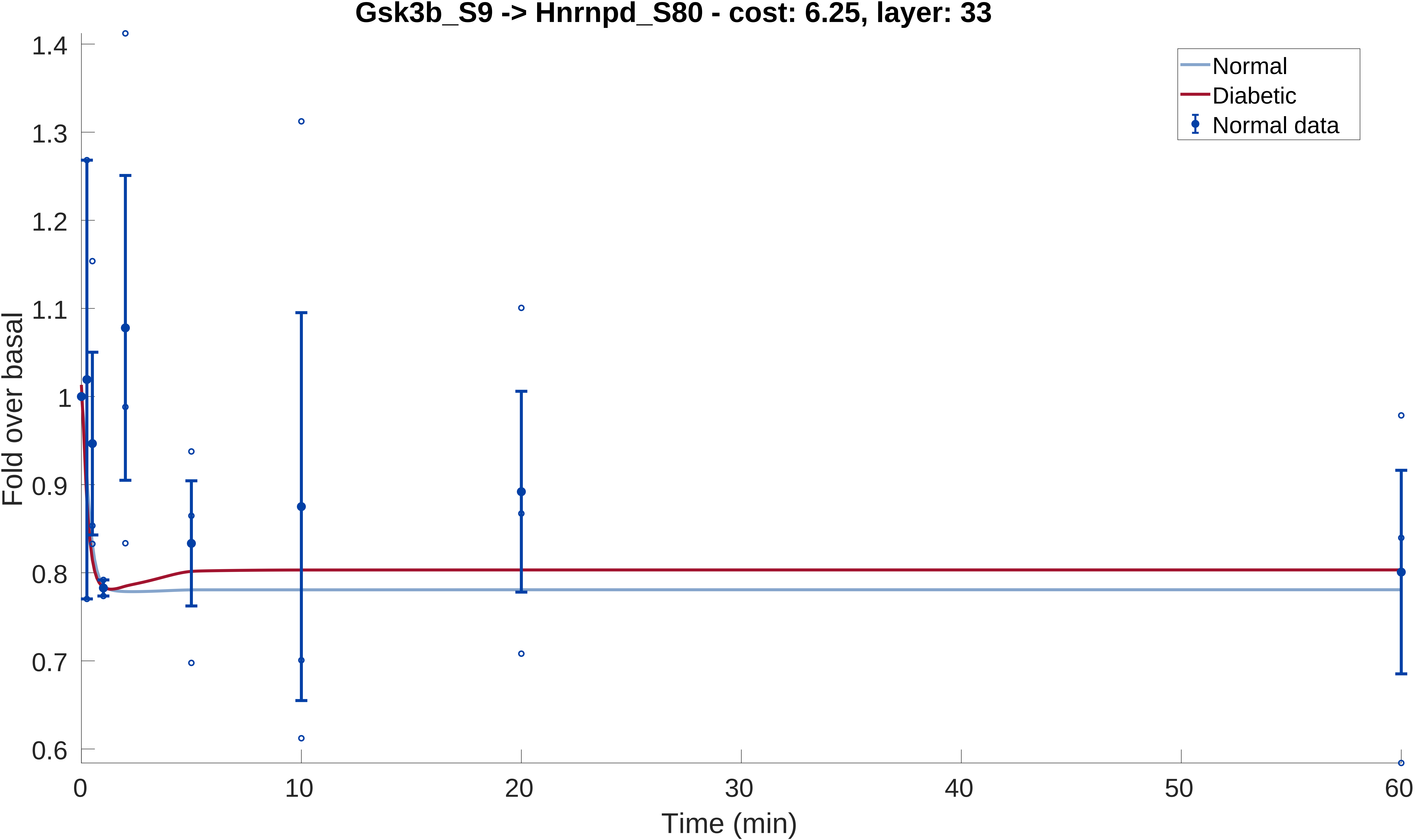

Gsk3b_S9 -> Hnrnpd_S80 - cost: 6.25, layer: 33
Fold over basal
1.4
1.3
1.2
1.1
1
0.9
0.8
0.7
0.6
0
10
20
30
40
50
60
Time (min)
Normal
Diabetic
Normal data

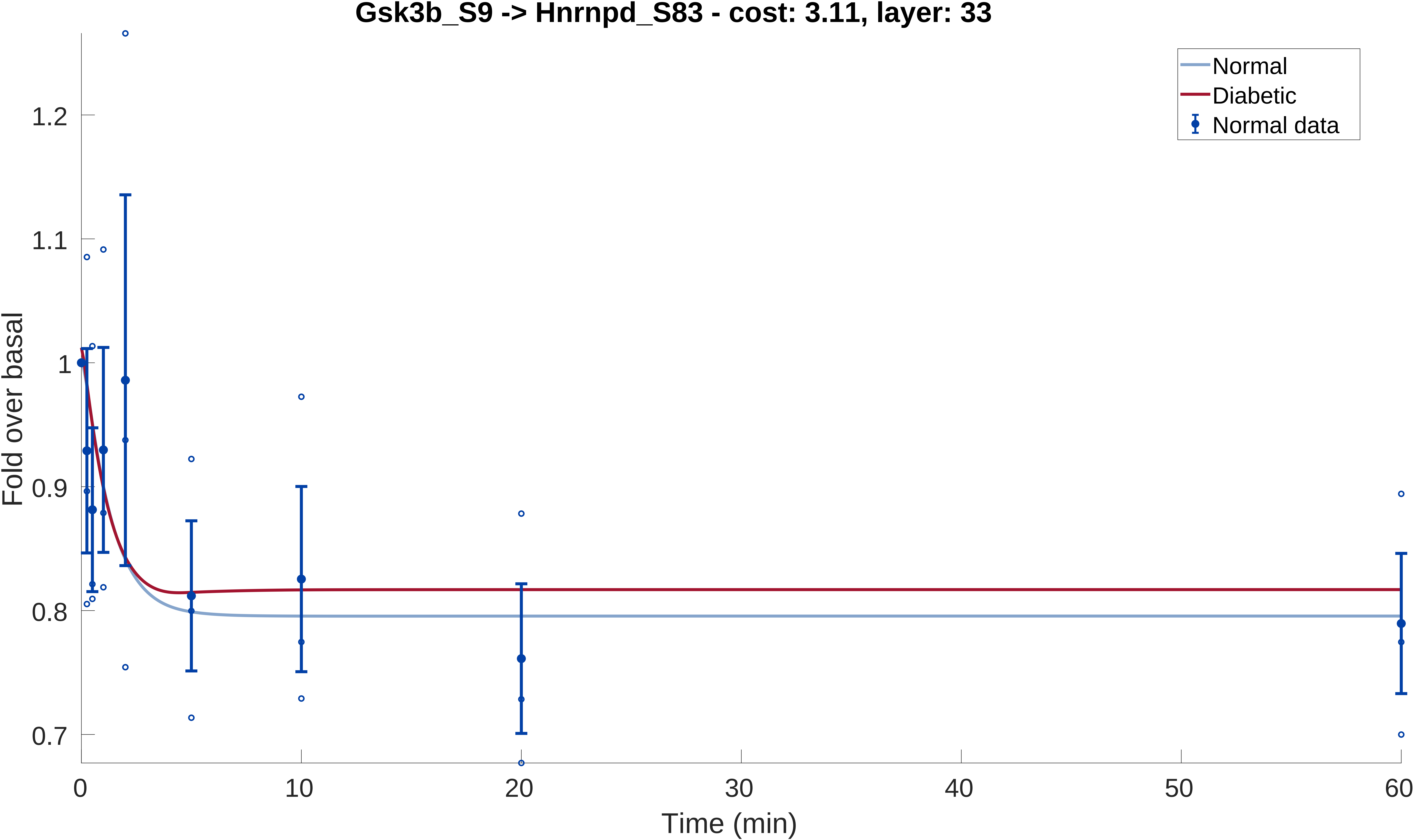
Gsk3b_S9 -> Hnrnpd_S83 - cost: 3.11, layer: 33
Normal
Diabetic
Normal data
Fold over basal
1.2
1.1
1
0.9
0.8
0.7
0
10
20
30
40
50
60
Time (min)

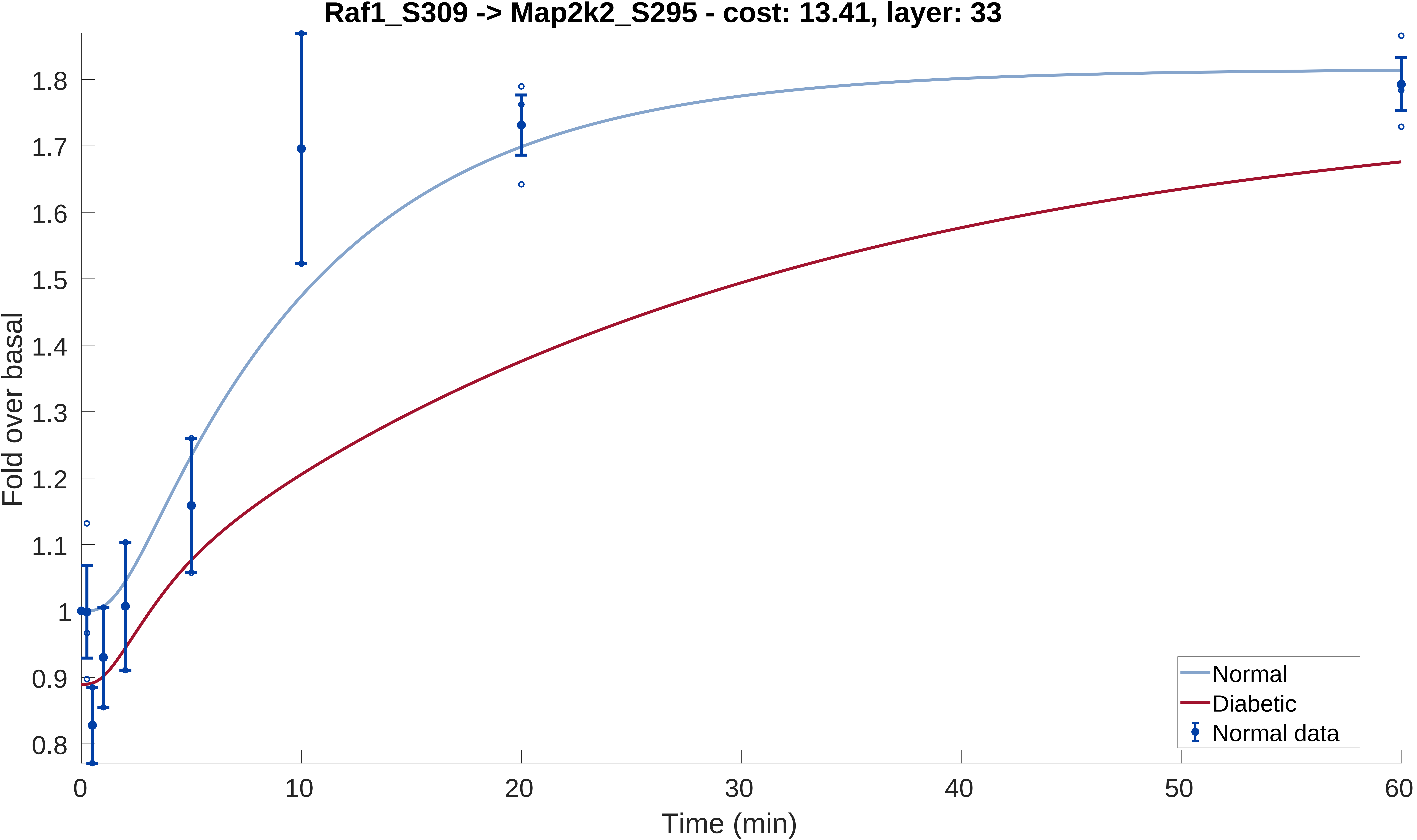
Raf1_S309 -> Map2k2_S295 - cost: 13.41, layer: 33
Fold over basal
1.8
1.7
1.6
1.5
1.4
1.3
1.2
1.1
1
0.9
0.8
0
10
20
30
40
50
60
Time (min)
Normal
Diabetic
Normal data

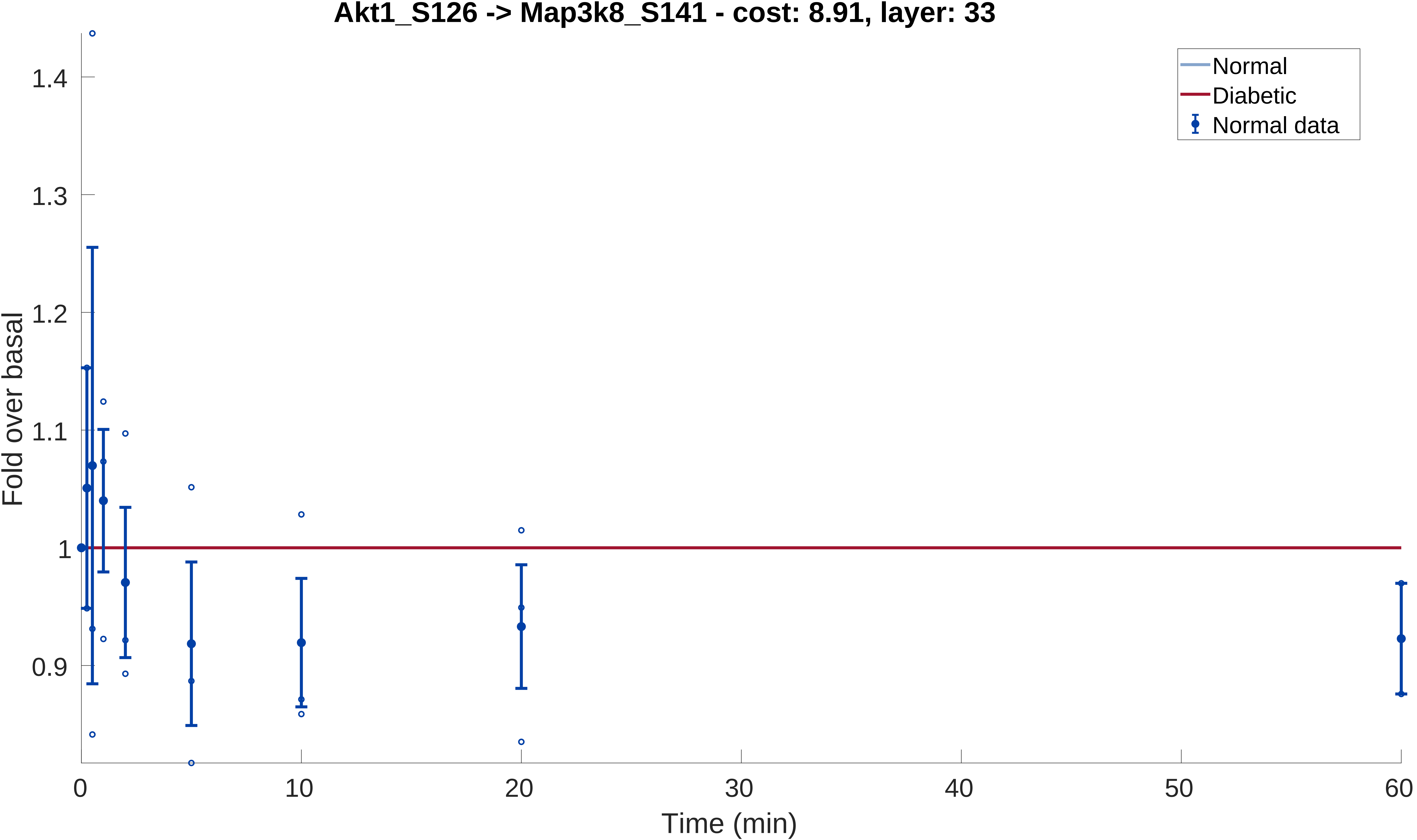
Akt1_S126 -> Map3k8_S141 - cost: 8.91, layer: 33
Normal
Diabetic
Normal data
1.4
1.3
1.2
1.1
1
0.9
Fold over basal
0
10
20
30
40
50
60
Time (min)

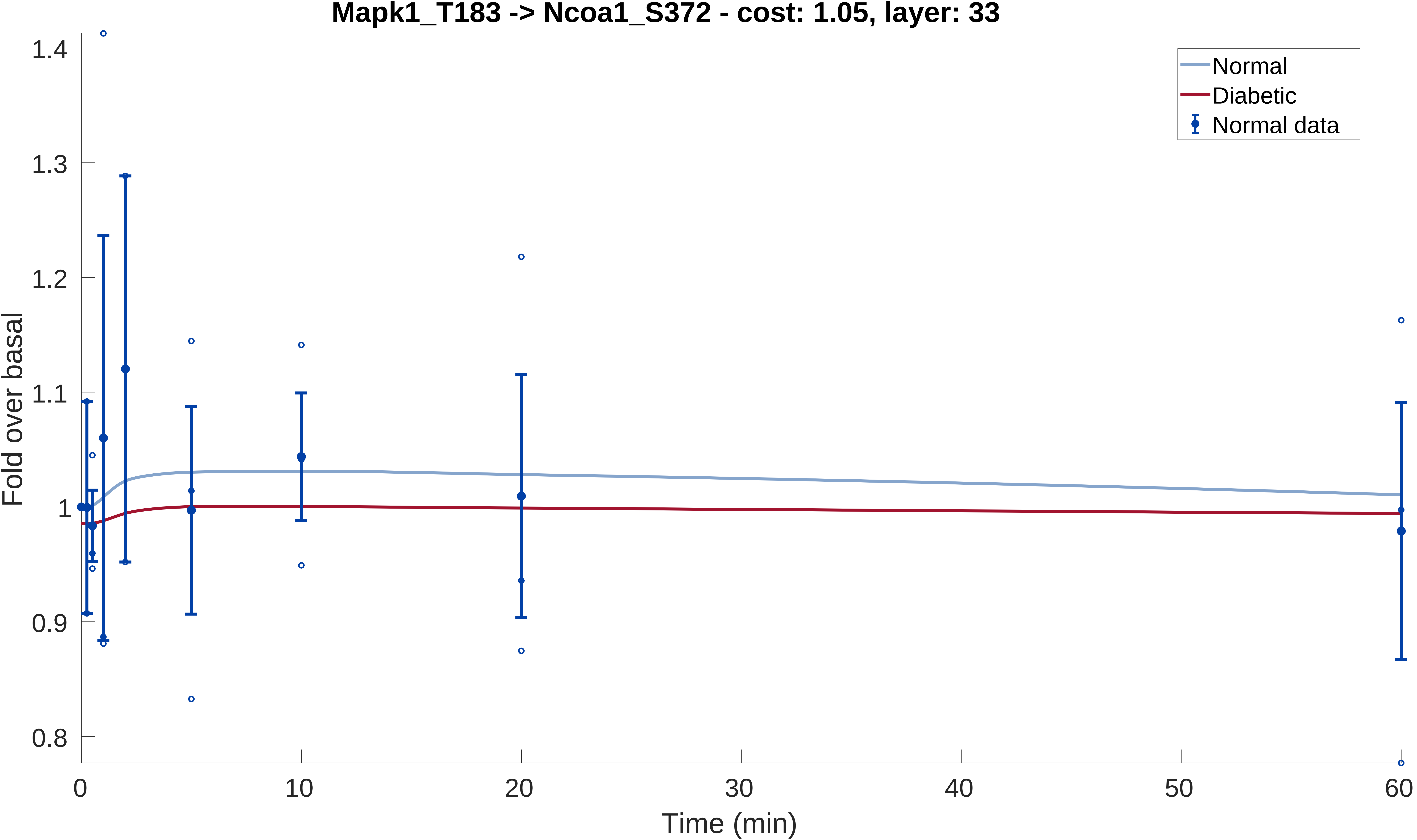
Mapk1_T183 -> Ncoa1_S372 - cost: 1.05, layer: 33
Fold over basal
Time (min)
Normal
Diabetic
Normal data
1.4
1.3
1.2
1.1
1
0.9
0.8
0
10
20
30
40
50
60

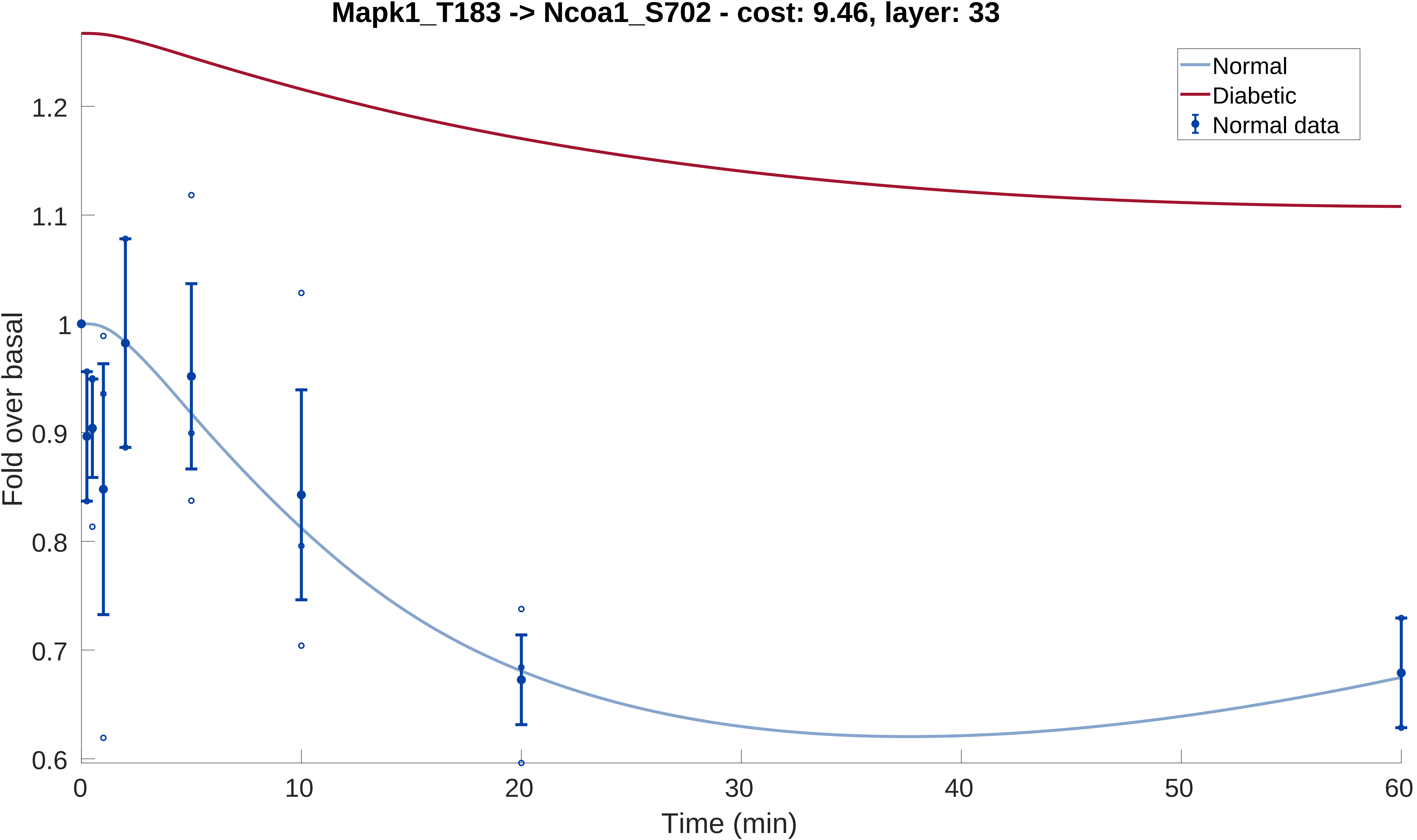
Mapk1_T183 -> Ncoa1_S702 - cost: 9.46, layer: 33
Normal
Diabetic
Normal data
Fold over basal
Time (min)
1.2
1.1
1
0.9
0.8
0.7
0.6
0
10
20
30
40
50
60

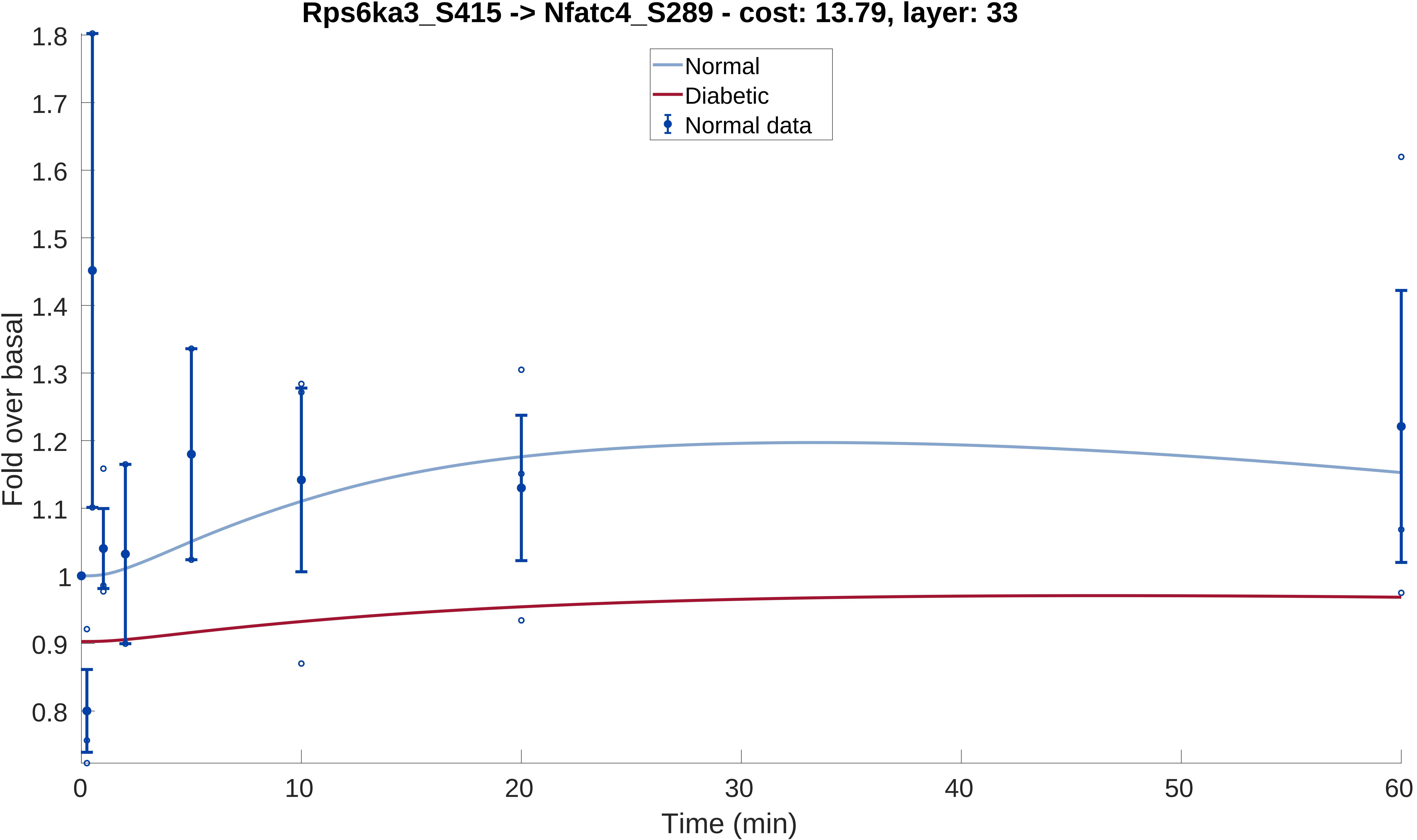
Rps6ka3_S415 -> Nfatc4_S289 - cost: 13.79, layer: 33
Normal
Diabetic
Normal data
Fold over basal
Time (min)
0.8
0.9
1
1.1
1.2
1.3
1.4
1.5
1.6
1.7
1.8
0
10
20
30
40
50
60

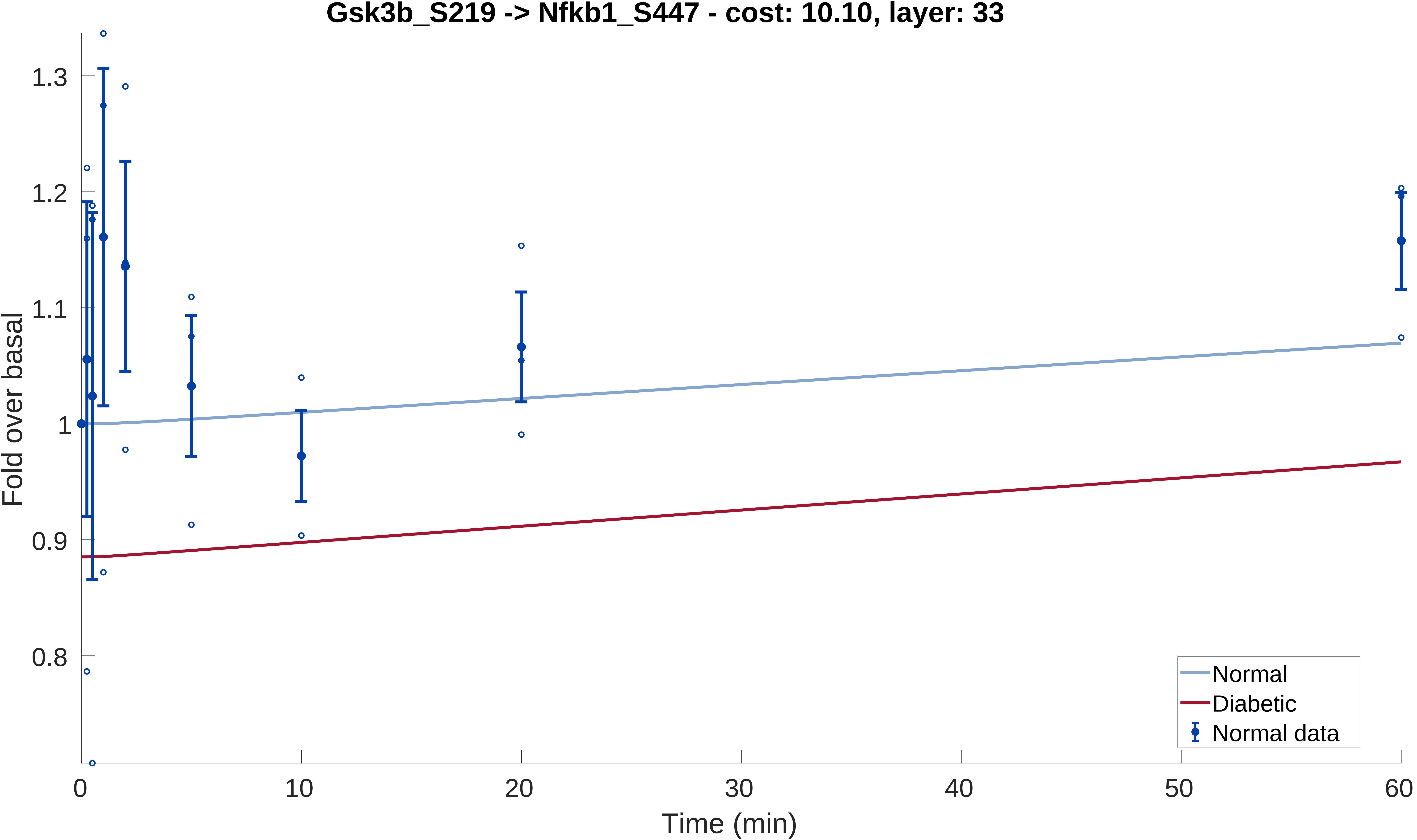

Gsk3b_S219 -> Nfkb1_S447 - cost: 10.10, layer: 33
Fold over basal
1.3
1.2
1.1
1
0.9
0.8
0
10
20
30
40
50
60
Time (min)
Normal
Diabetic
Normal data

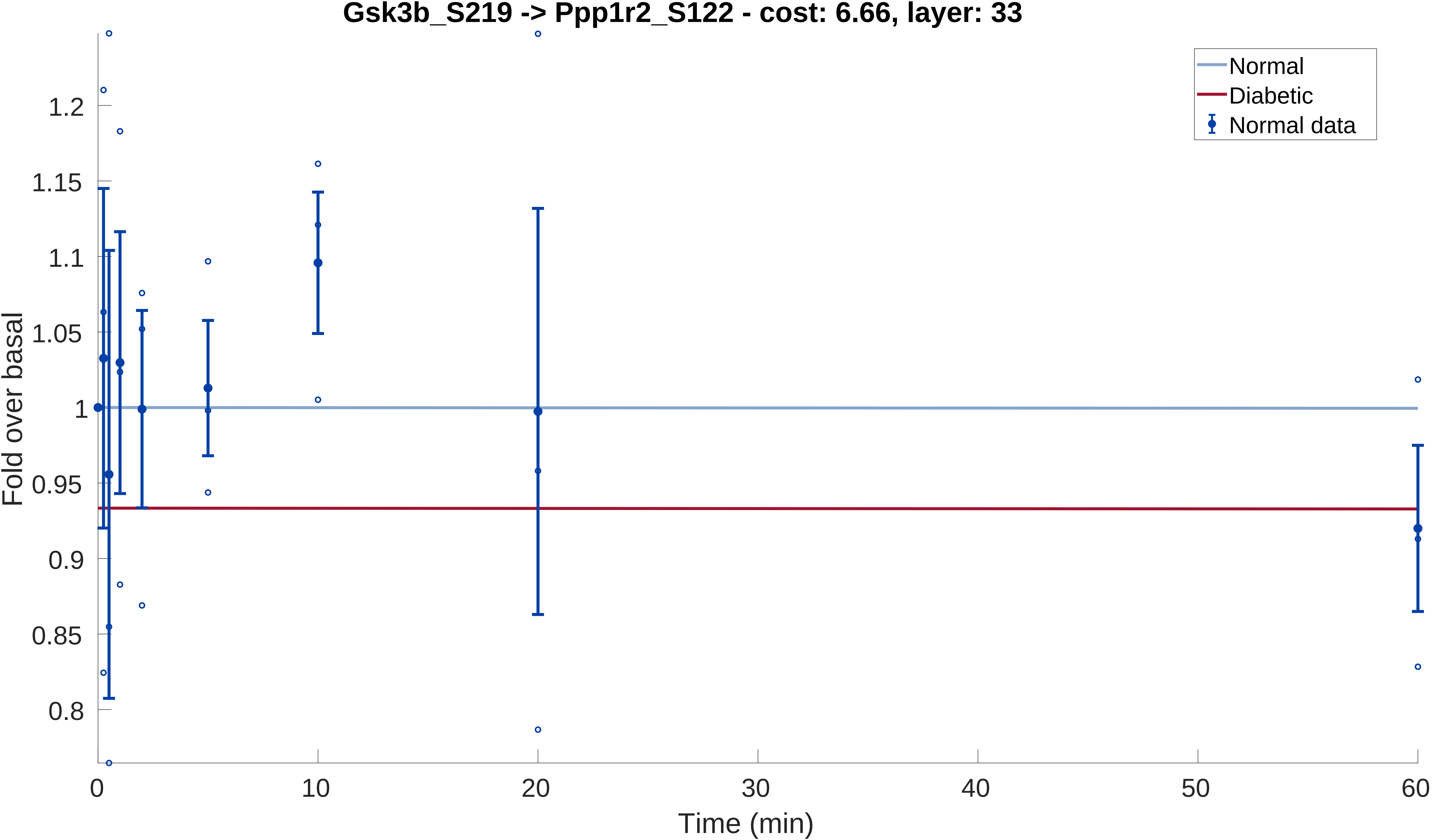

Gsk3b_S219 -> Ppp1r2_S122 - cost: 6.66, layer: 33
Normal
Diabetic
Normal data
Fold over basal
1.2
1.15
1.1
1.05
1
0.95
0.9
0.85
0.8
0
10
20
30
40
50
60
Time (min)

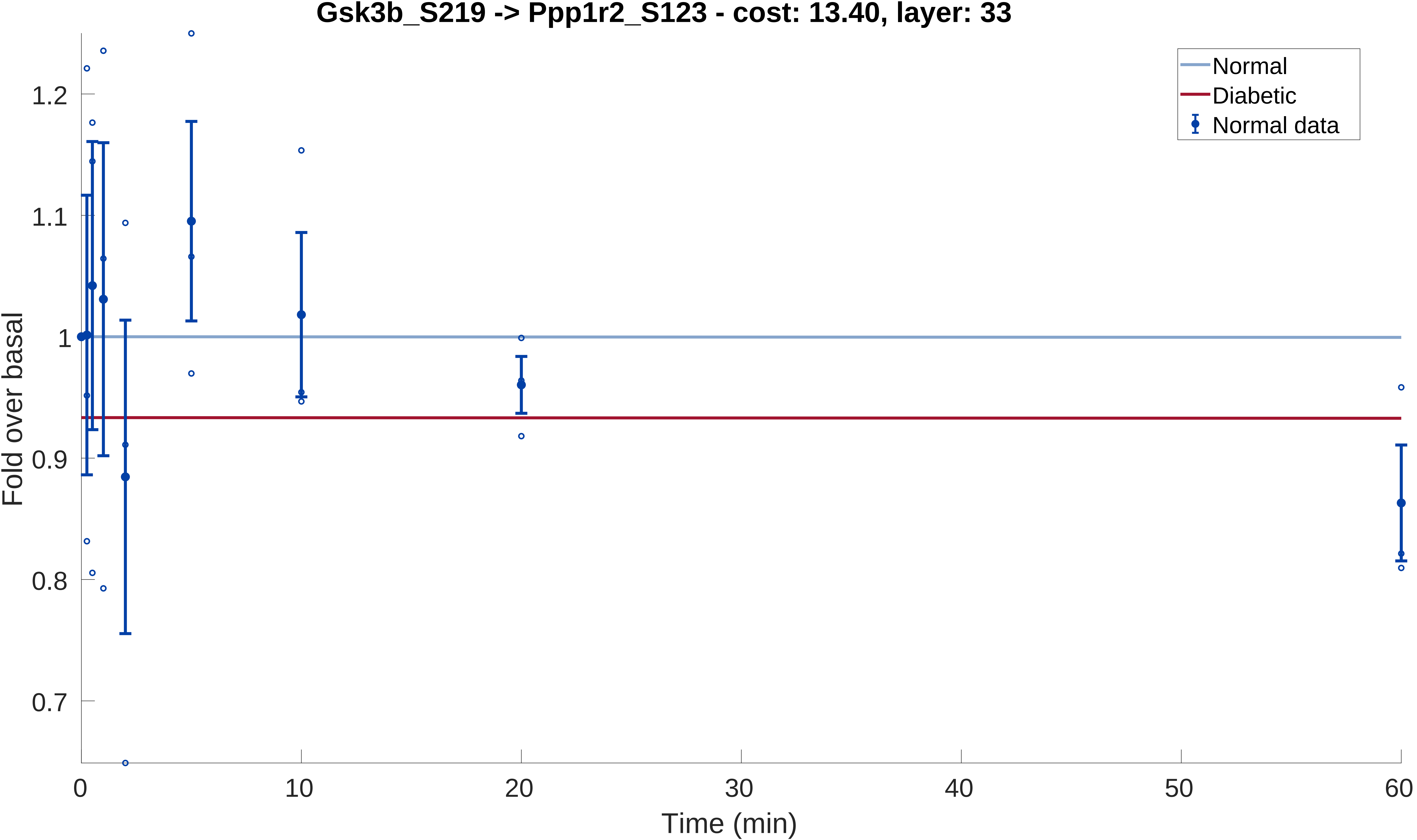

Gsk3b_S219 -> Ppp1r2_S123 - cost: 13.40, layer: 33
Normal
Diabetic
Normal data
Fold over basal
Time (min)
1.2
1.1
1
0.9
0.8
0.7
0
10
20
30
40
50
60

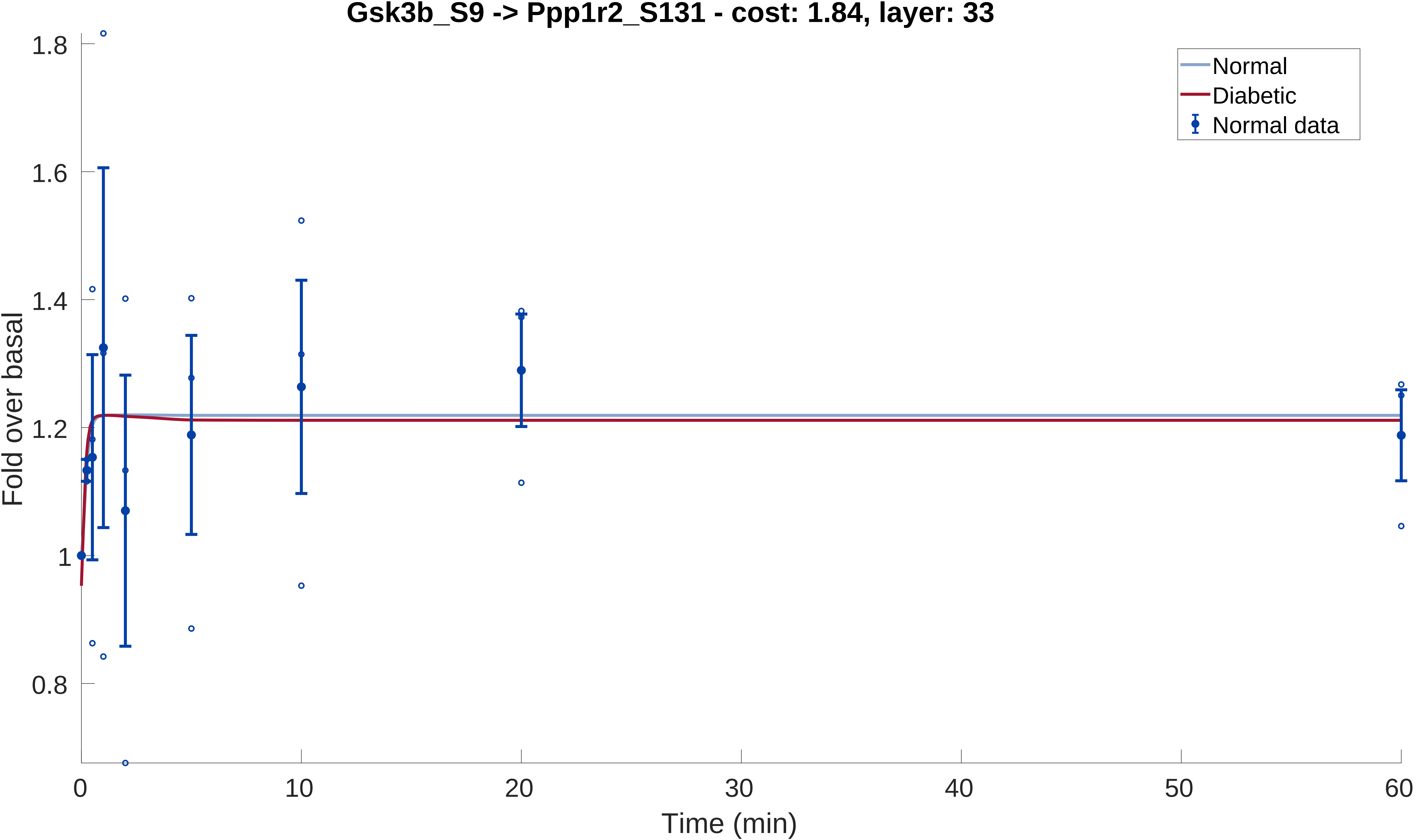

Gsk3b_S9 -> Ppp1r2_S131 - cost: 1.84, layer: 33
Normal
Diabetic
Normal data
Fold over basal
Time (min)
1.8
1.6
1.4
1.2
1
0.8
0
10
20
30
40
50
60

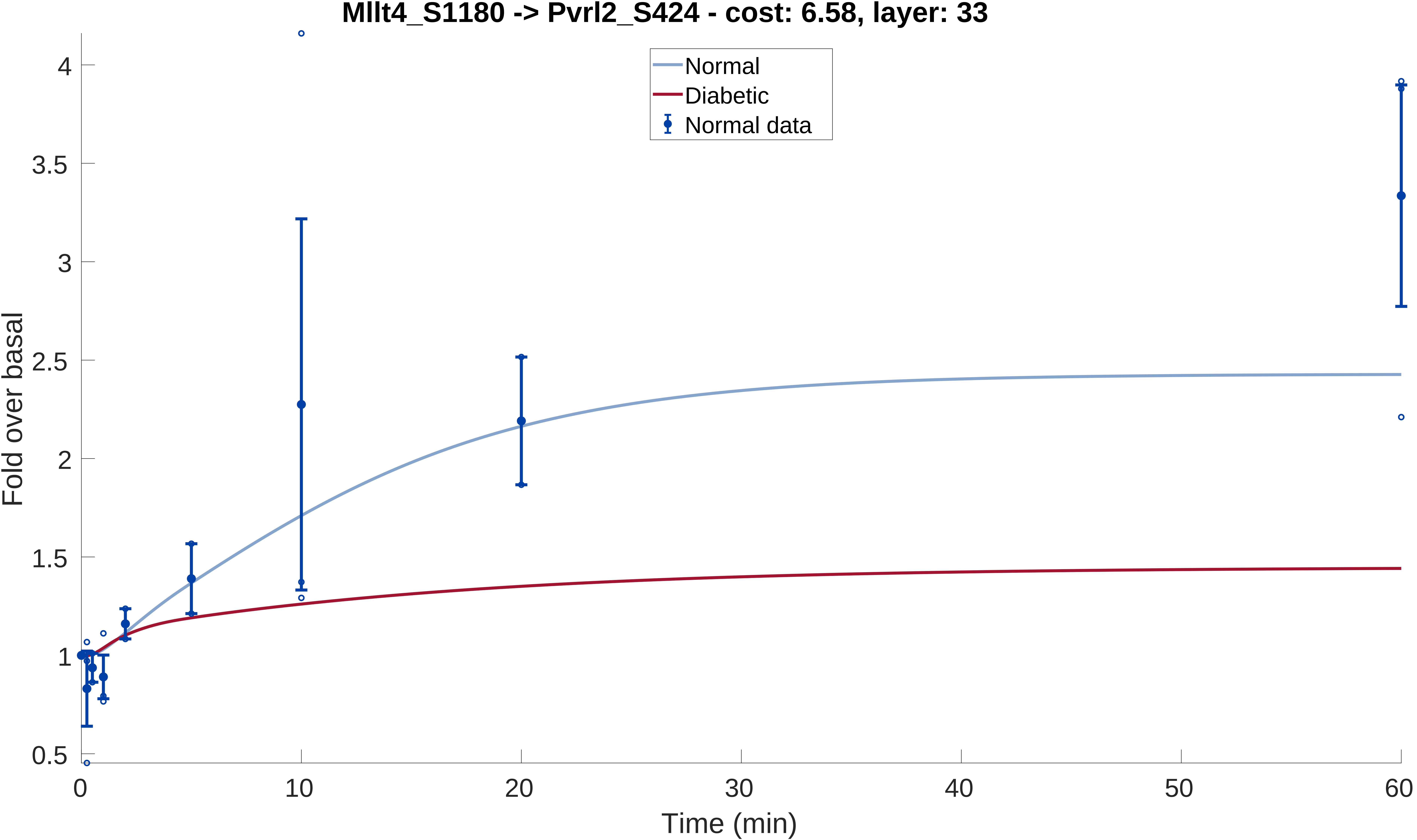

Mllt4_S1180 -> Pvrl2_S424 - cost: 6.58, layer: 33
Normal
Diabetic
Normal data
Fold over basal
Time (min)
0.5
1
1.5
2
2.5
3
3.5
4
0
10
20
30
40
50
60

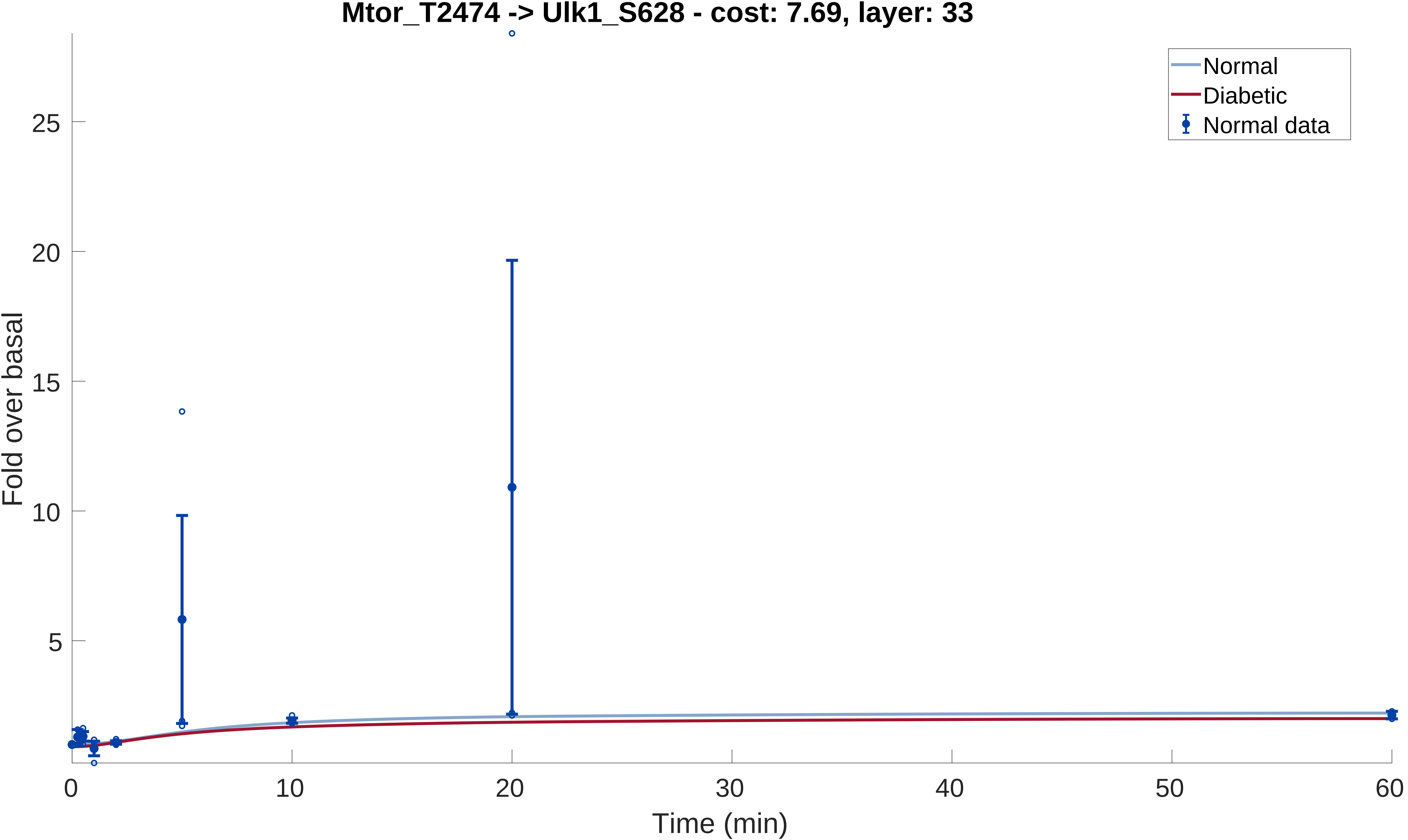

Mtor_T2474 -> Ulk1_S628 - cost: 7.69, layer: 33
Normal
Diabetic
Normal data
Fold over basal
25
20
15
10
5
0
10
20
30
40
50
60
Time (min)

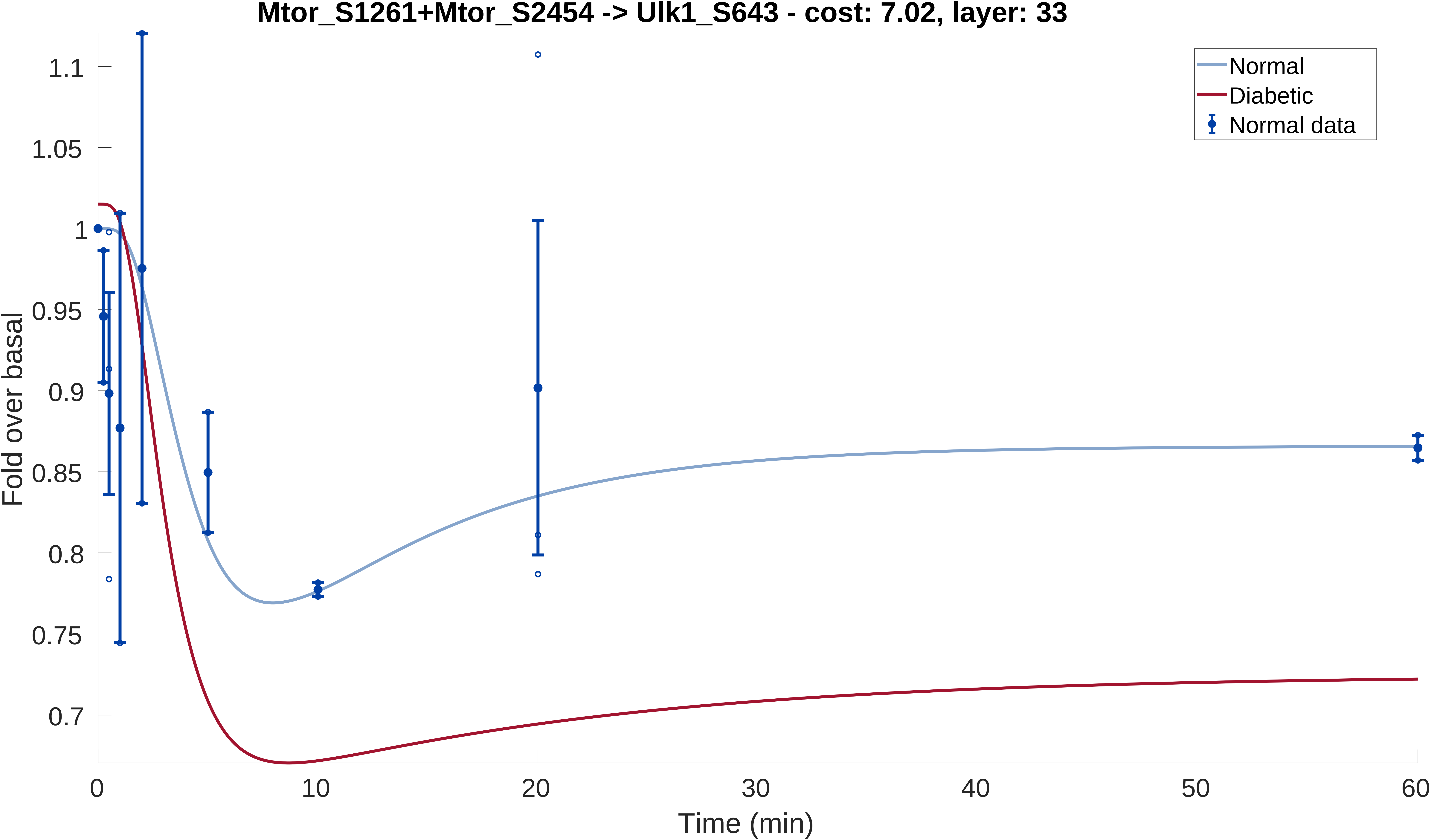

Mtor_S1261+Mtor_S2454 -> Ulk1_S643 - cost: 7.02, layer: 33
Normal
Diabetic
Normal data
Fold over basal
1.1
1.05
1
0.95
0.9
0.85
0.8
0.75
0.7
Time (min)
0
10
20
30
40
50
60

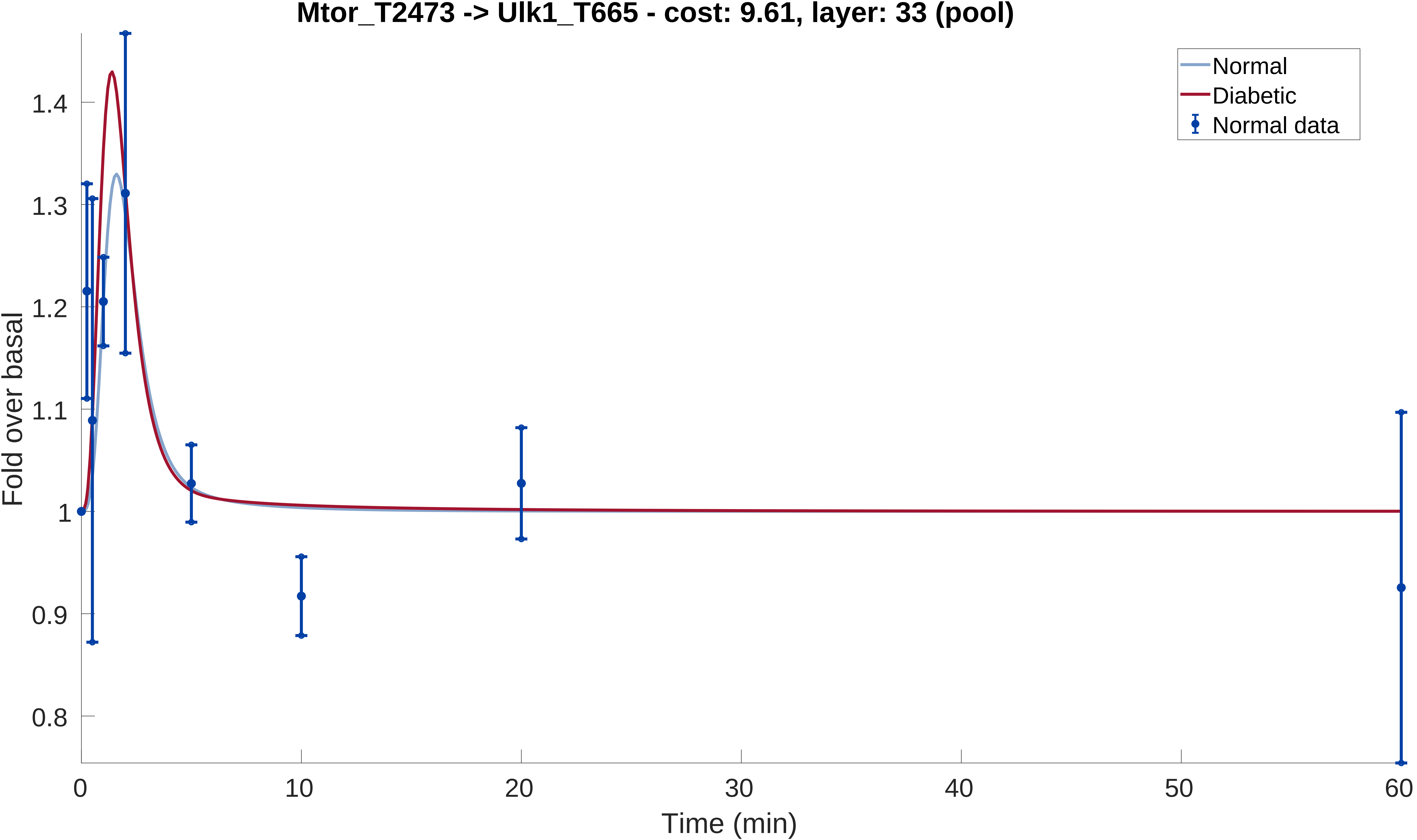

Mtor_T2473 -> Ulk1_T665 - cost: 9.61, layer: 33 (pool)
Normal
Diabetic
Normal data
Fold over basal
1.4
1.3
1.2
1.1
1
0.9
0.8
0
10
20
30
40
50
60
Time (min)

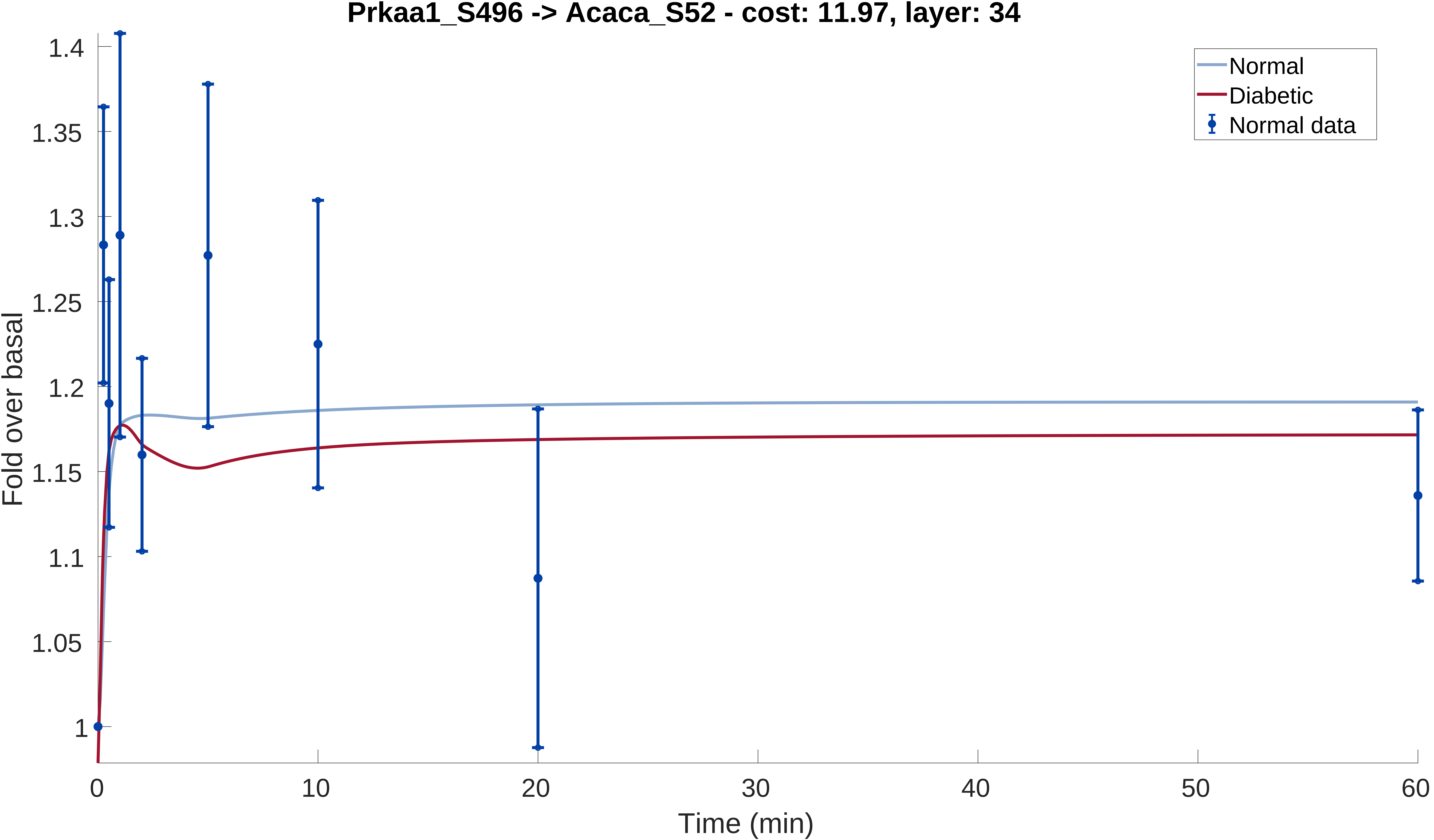
Prkaa1_S496 -> Acaca_S52 - cost: 11.97, layer: 34
Normal
Diabetic
Normal data
Fold over basal
1.4
1.35
1.3
1.25
1.2
1.15
1.1
1.05
1
0
10
20
30
40
50
60
Time (min)

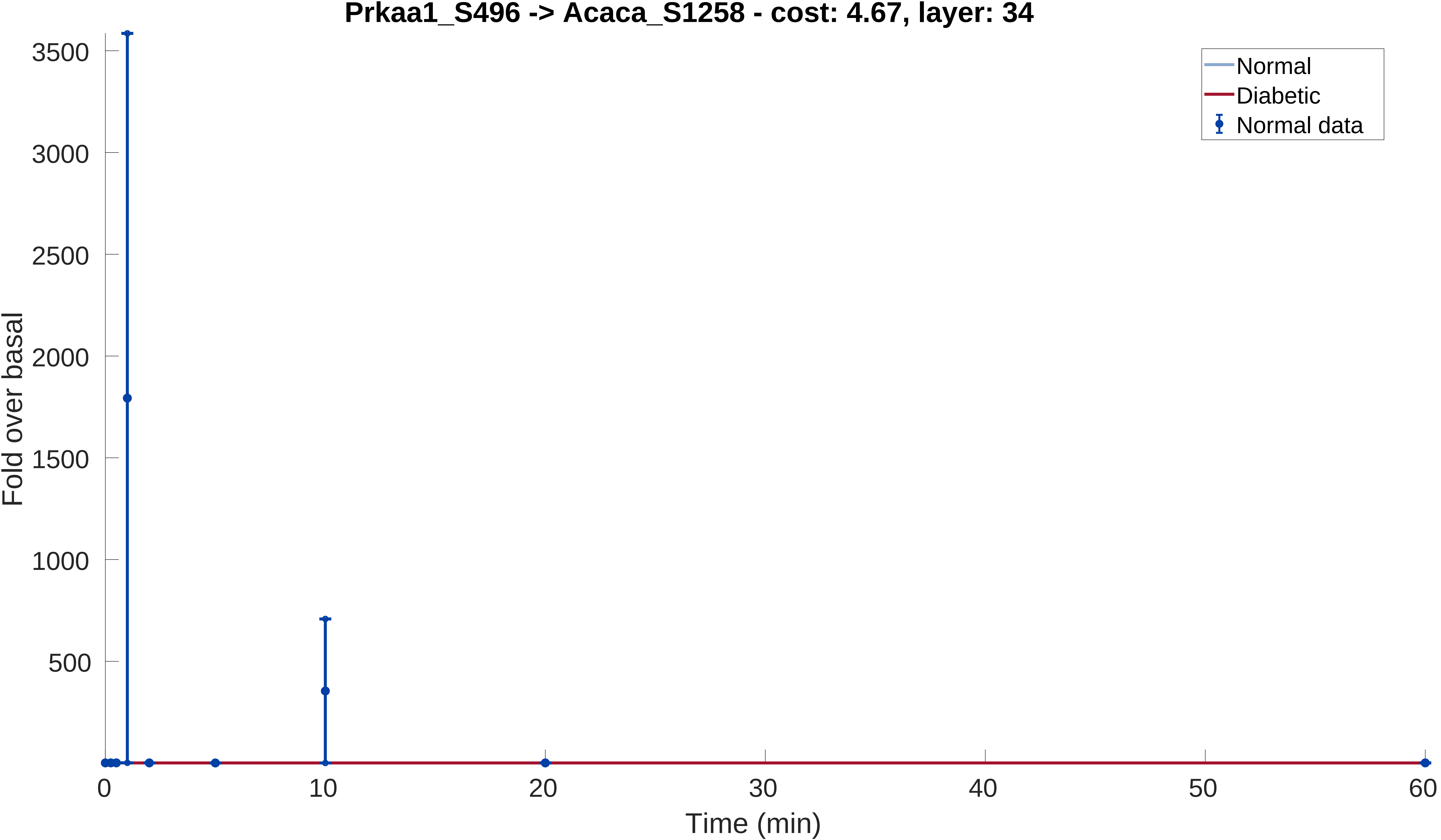

Prkaa1_S496 -> Acaca_S1258 - cost: 4.67, layer: 34
Fold over basal
3500
3000
2500
2000
1500
1000
500
0
10
20
30
40
50
60
Time (min)
Normal
Diabetic
Normal data

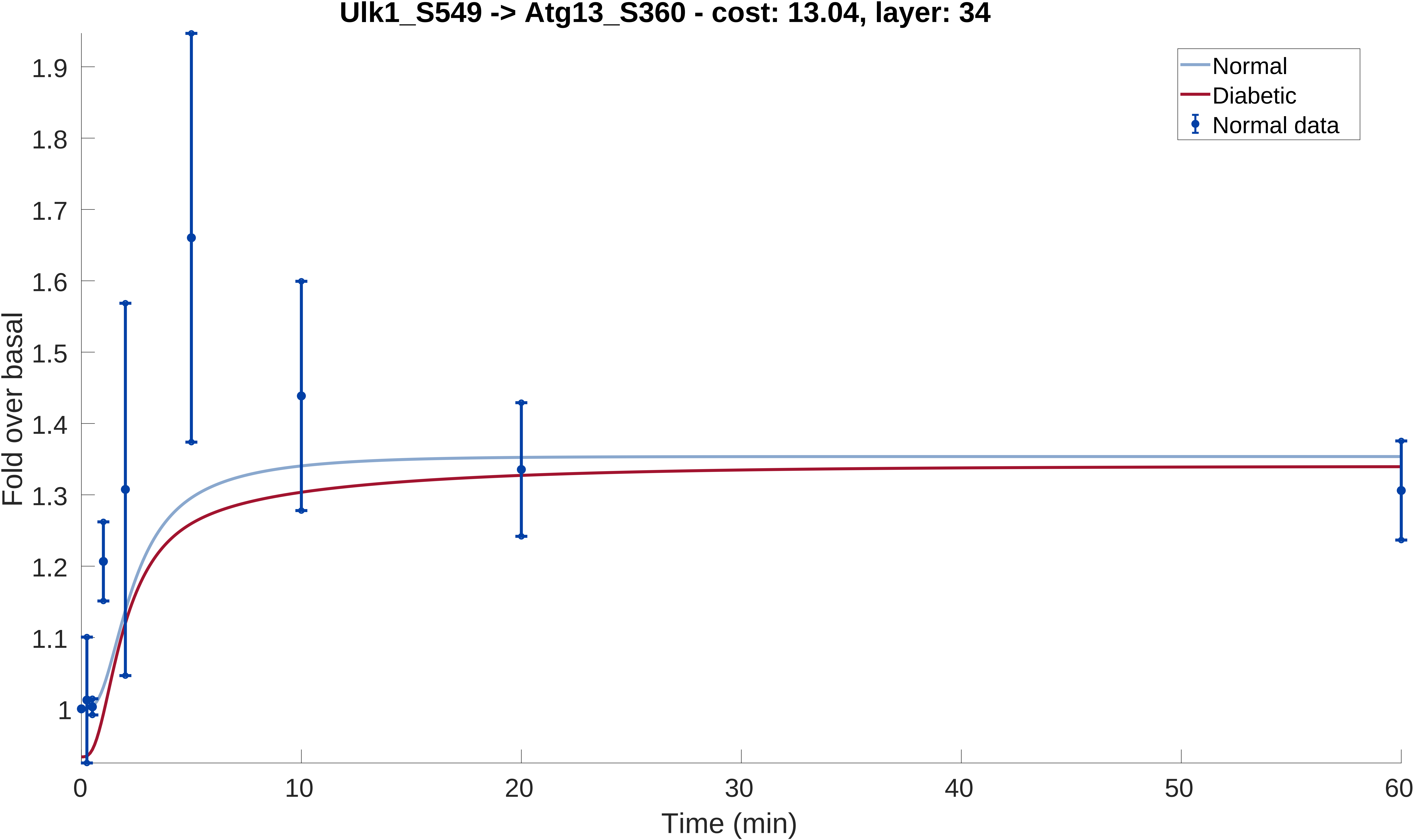

Ulk1_S549 -> Atg13_S360 - cost: 13.04, layer: 34
Normal
Diabetic
Normal data
Fold over basal
1.9
1.8
1.7
1.6
1.5
1.4
1.3
1.2
1.1
1
0
10
20
30
40
50
60
Time (min)

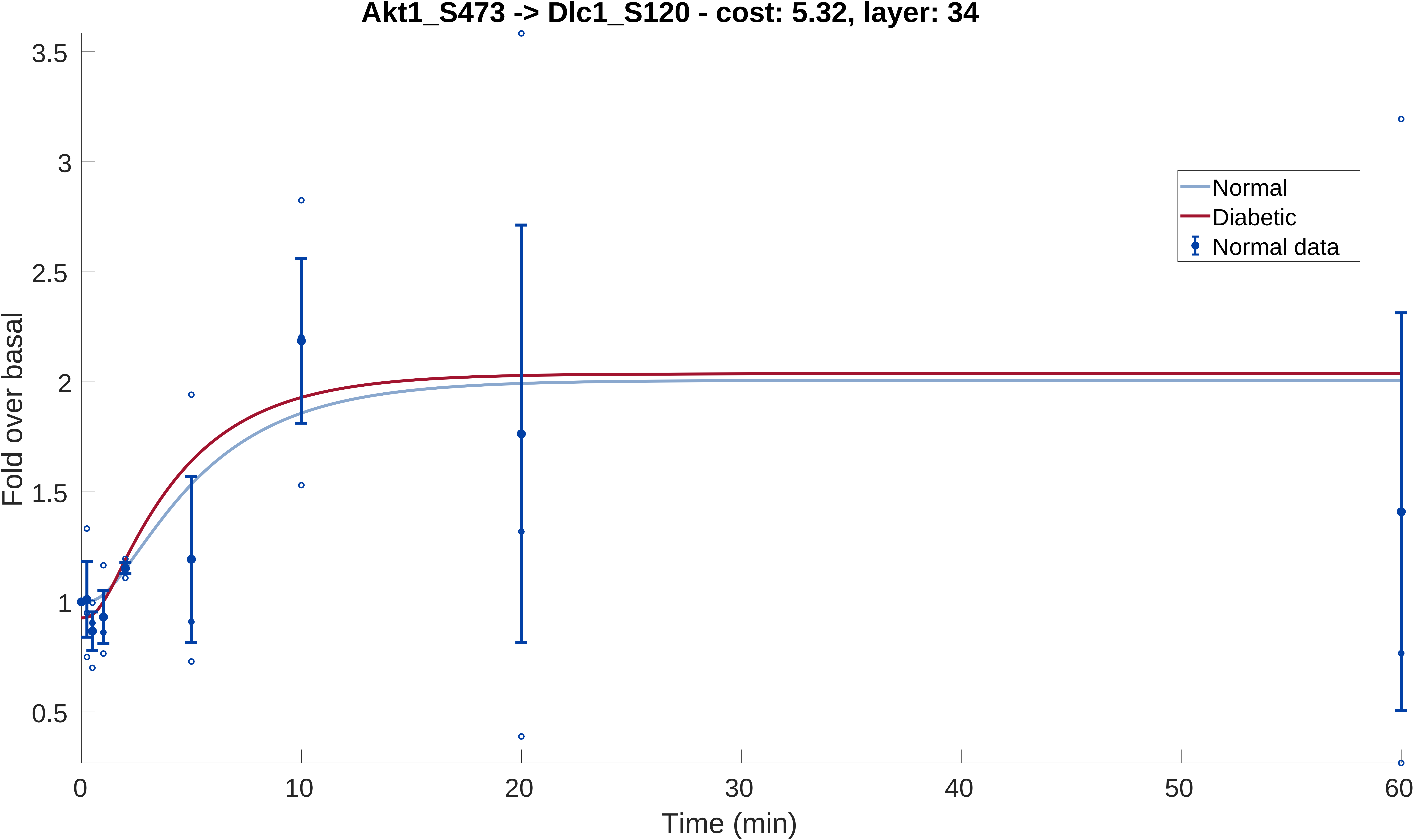

Akt1_S473 -> Dlc1_S120 - cost: 5.32, layer: 34
Fold over basal
3.5
3
2.5
2
1.5
1
0.5
0
10
20
30
40
50
60
Time (min)
Normal
Diabetic
Normal data

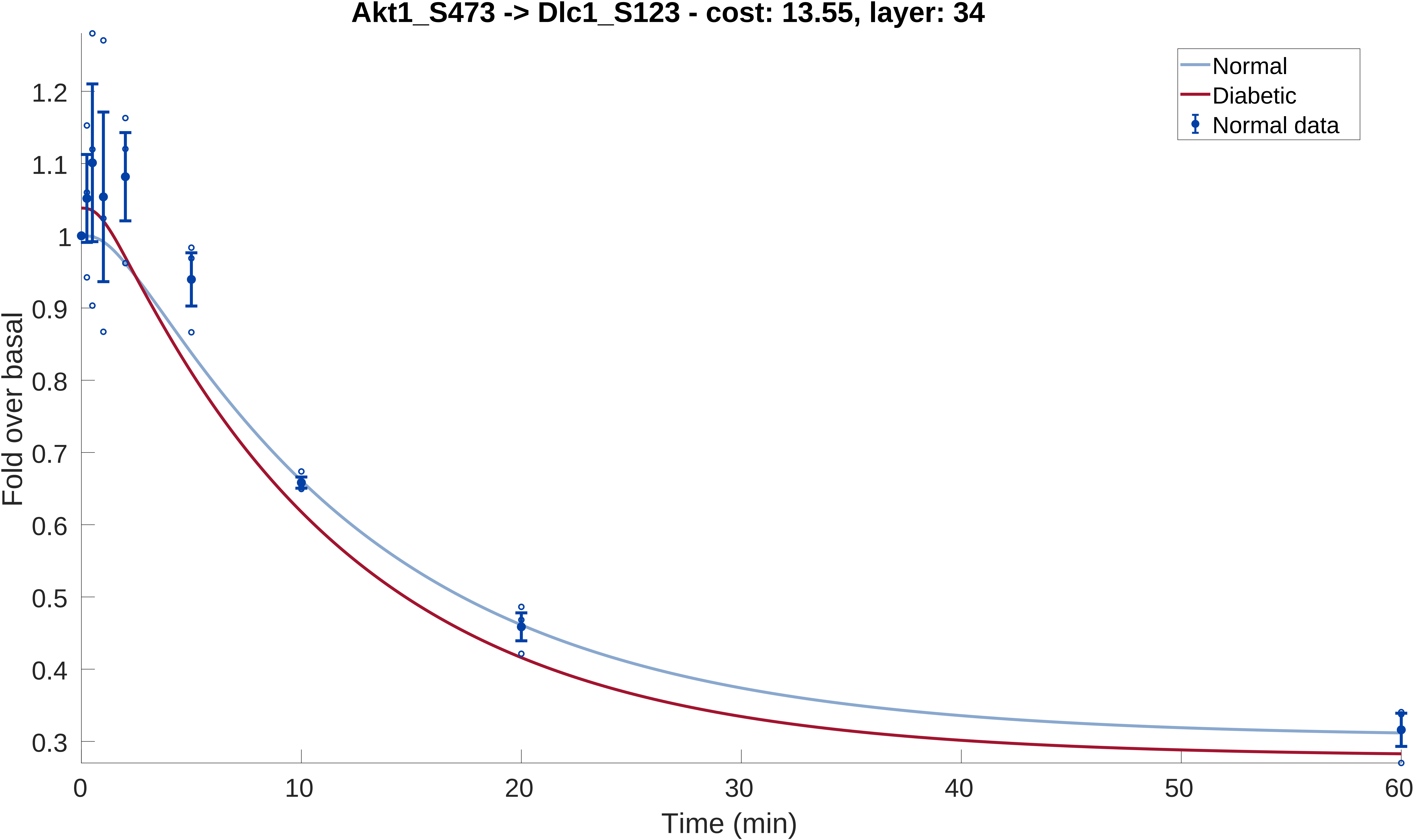
Akt1_S473 -> Dlc1_S123 - cost: 13.55, layer: 34
Normal
Diabetic
Normal data
Fold over basal
Time (min)
1.2
1.1
1
0.9
0.8
0.7
0.6
0.5
0.4
0.3
0
10
20
30
40
50
60

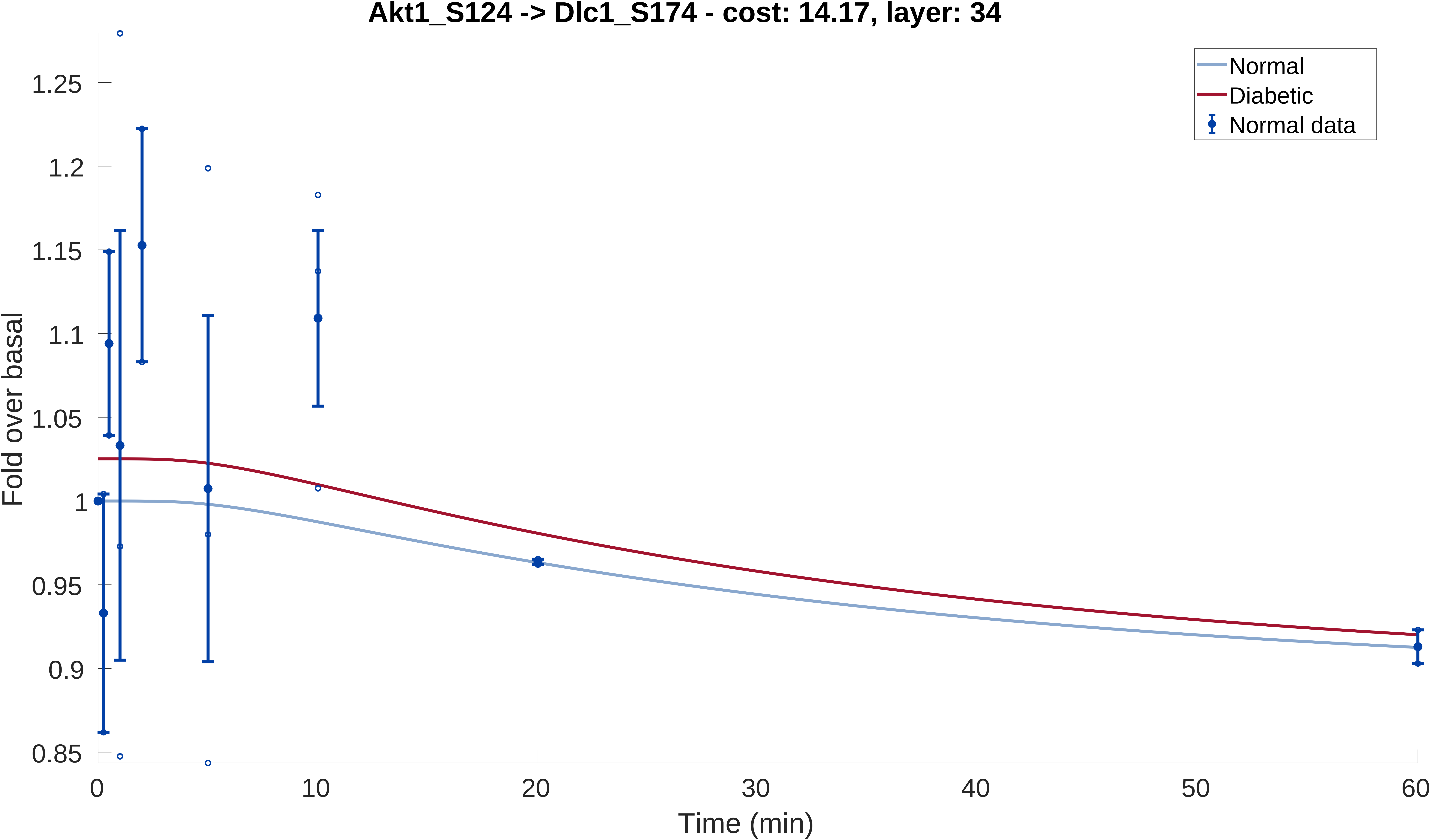

Akt1_S124 -> Dlc1_S174 - cost: 14.17, layer: 34
Normal
Diabetic
Normal data
Fold over basal
1.25
1.2
1.15
1.1
1.05
1
0.95
0.9
0.85
0
10
20
30
40
50
60
Time (min)

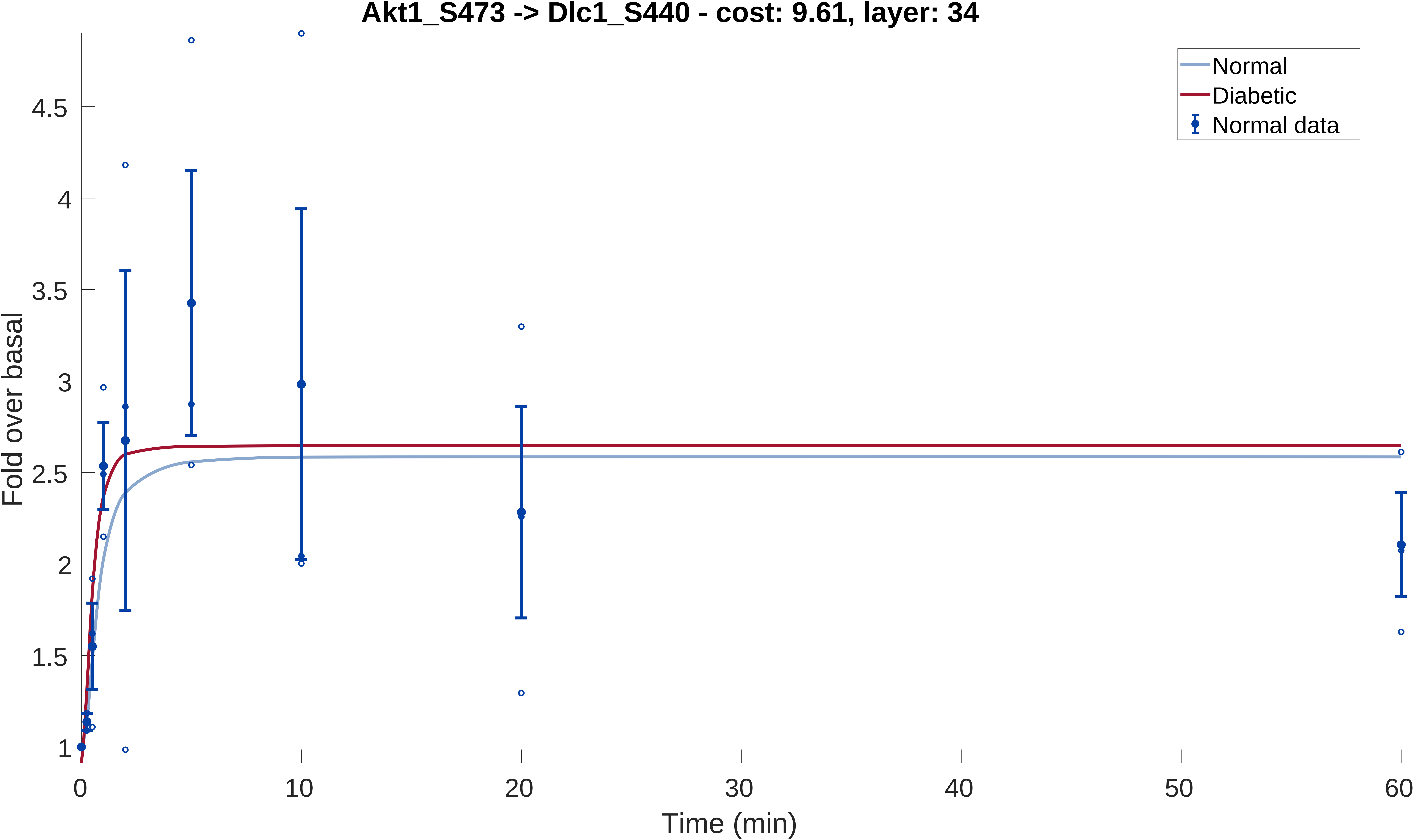

Akt1_S473 -> Dlc1_S440 - cost: 9.61, layer: 34
Normal
Diabetic
Normal data
4.5
4
3.5
3
2.5
2
1.5
1
0
10
20
30
40
50
60
Fold over basal
Time (min)

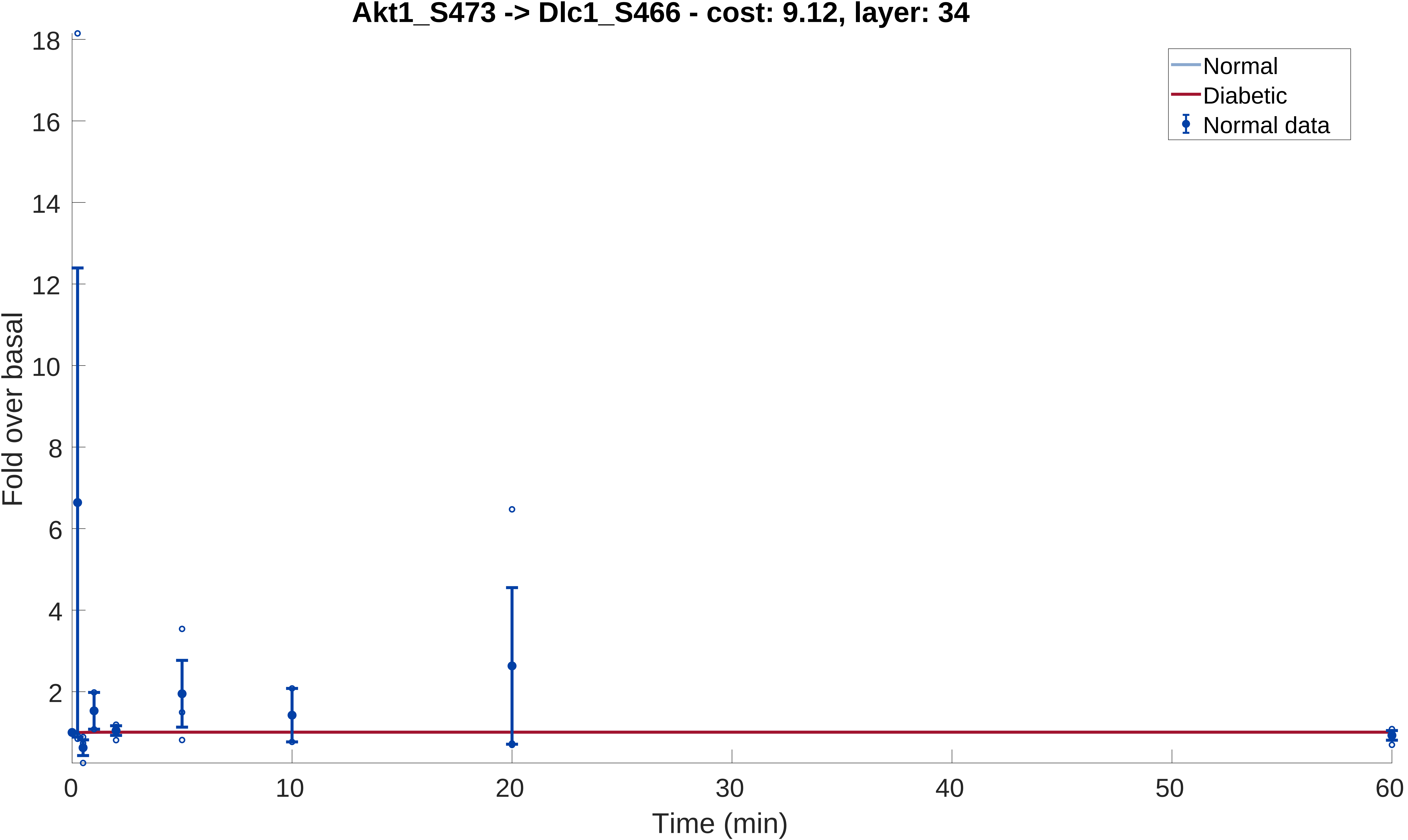

Akt1_S473 -> Dlc1_S466 - cost: 9.12, layer: 34
Normal
Diabetic
Normal data
Fold over basal
Time (min)
18
16
14
12
10
8
6
4
2
0
10
20
30
40
50
60

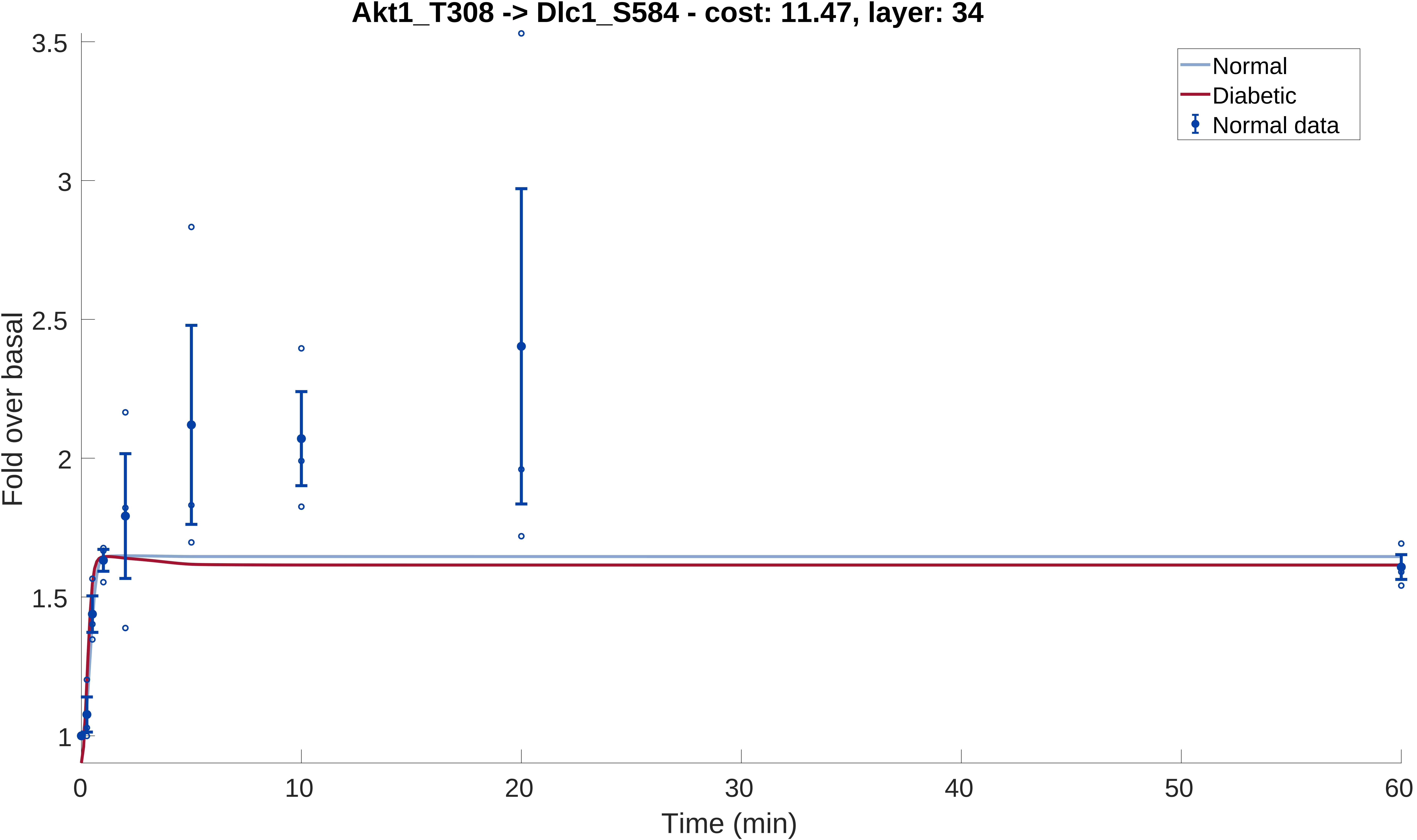

Akt1_T308 -> Dlc1_S584 - cost: 11.47, layer: 34
Normal
Diabetic
Normal data
Fold over basal
Time (min)
3.5
3
2.5
2
1.5
1
0
10
20
30
40
50
60

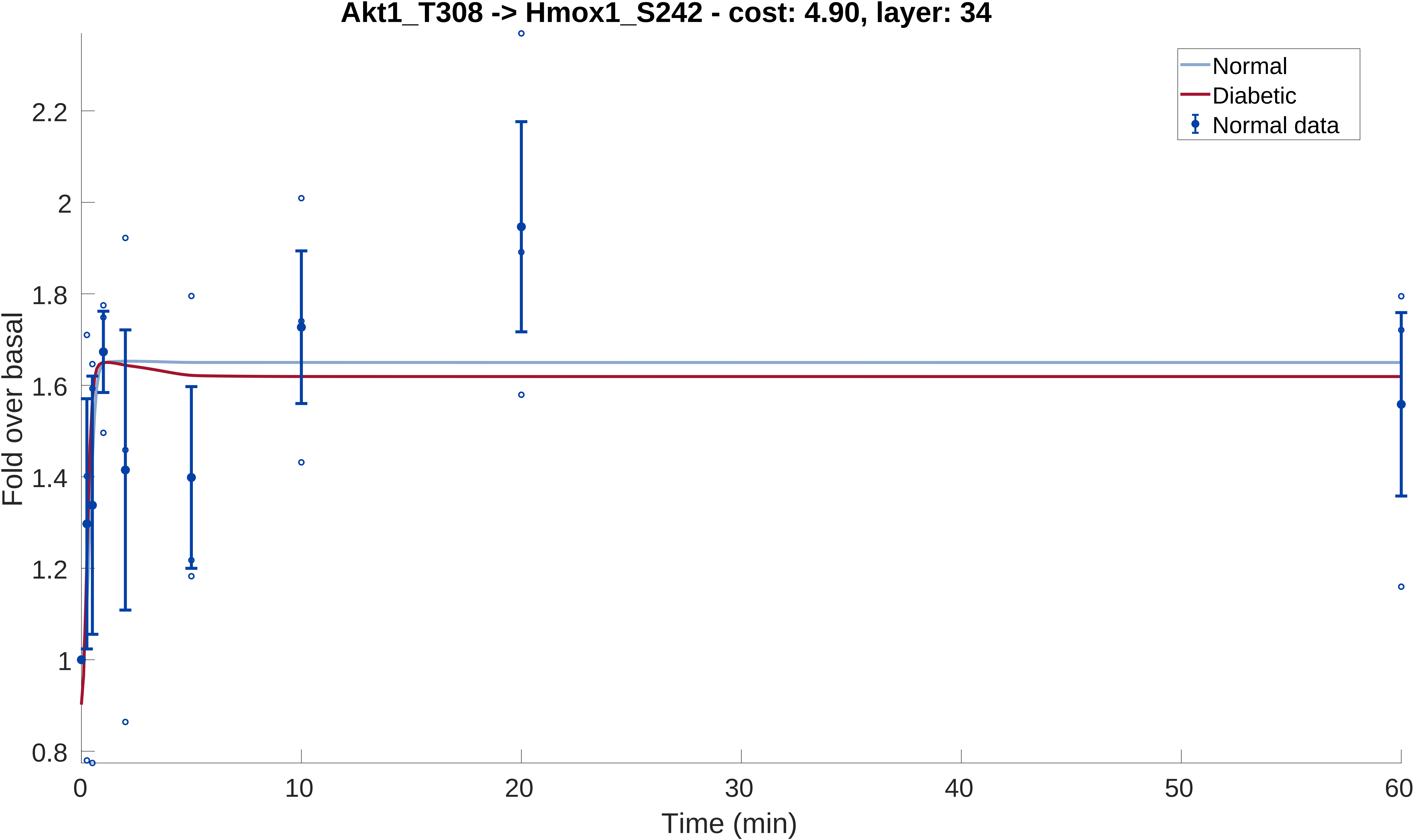

Akt1_T308 -> Hmox1_S242 - cost: 4.90, layer: 34
Normal
Diabetic
Normal data
Fold over basal
Time (min)
0.8
1
1.2
1.4
1.6
1.8
2
2.2
0
10
20
30
40
50
60

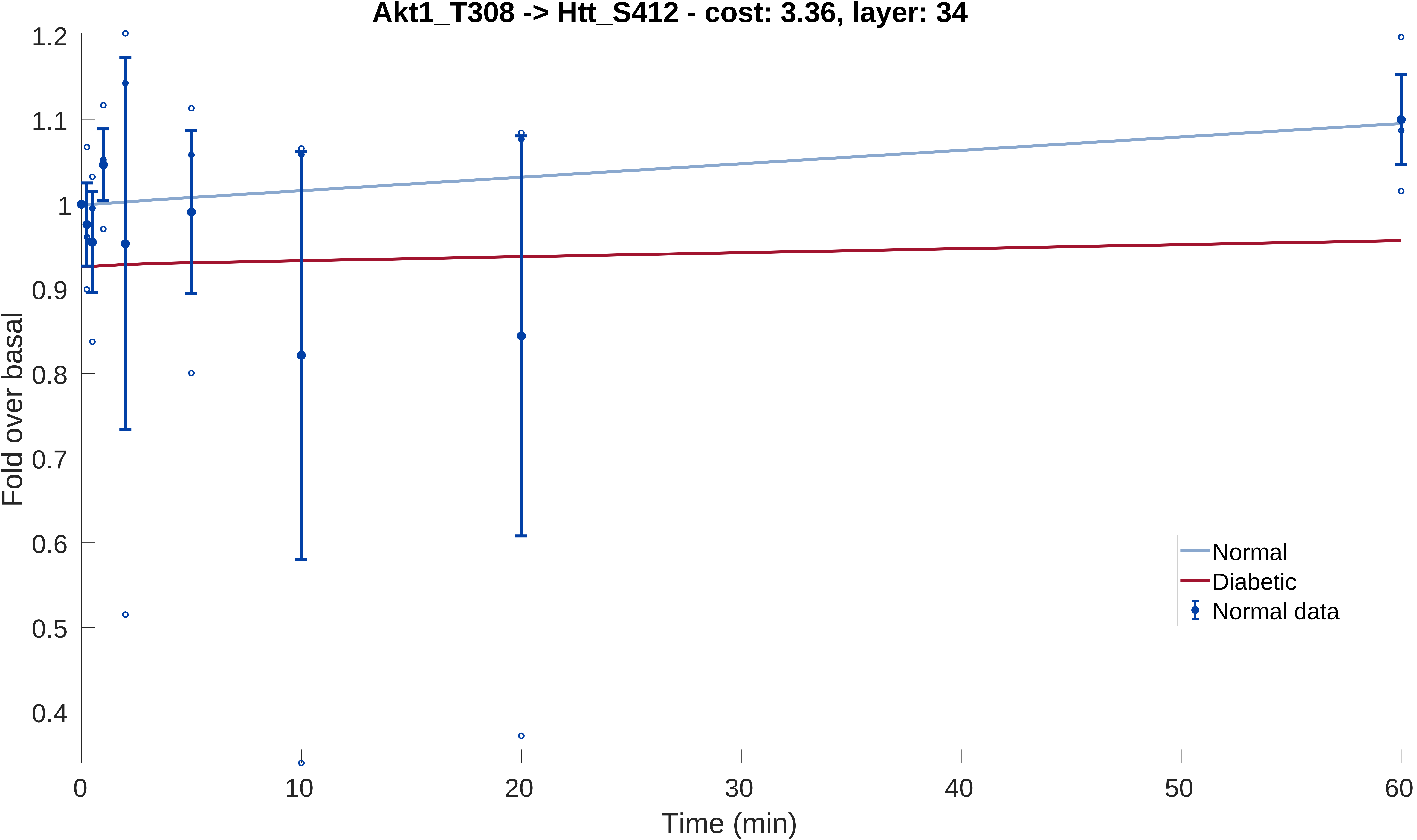
Akt1_T308 -> Htt_S412 - cost: 3.36, layer: 34
Fold over basal
Time (min)
1.2
1.1
1
0.9
0.8
0.7
0.6
0.5
0.4
0
10
20
30
40
50
60
Normal
Diabetic
Normal data

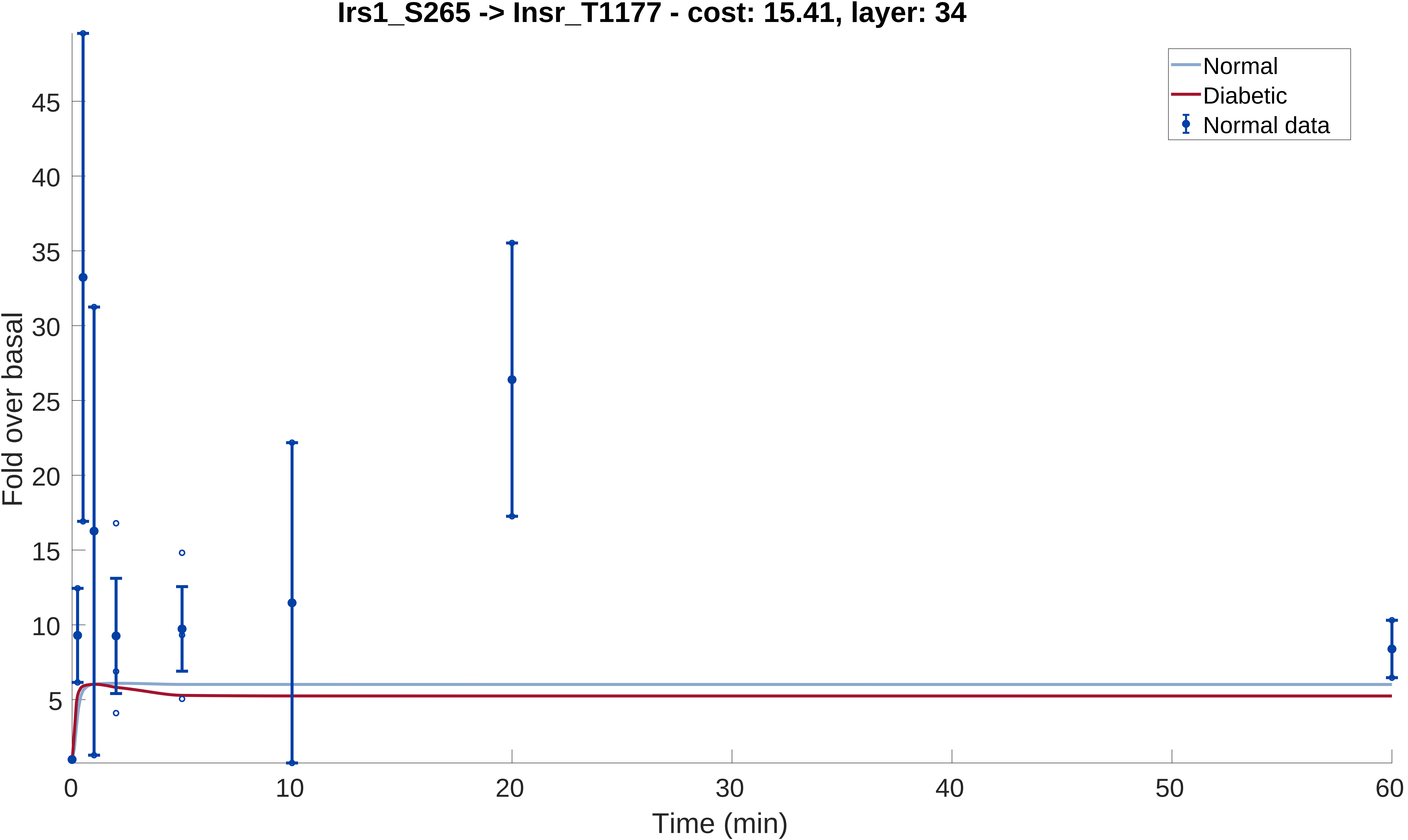

Irs1_S265 -> Insr_T1177 - cost: 15.41, layer: 34
Normal
Diabetic
Normal data
Fold over basal
Time (min)
0
10
20
30
40
50
60
5
10
15
20
25
30
35
40
45

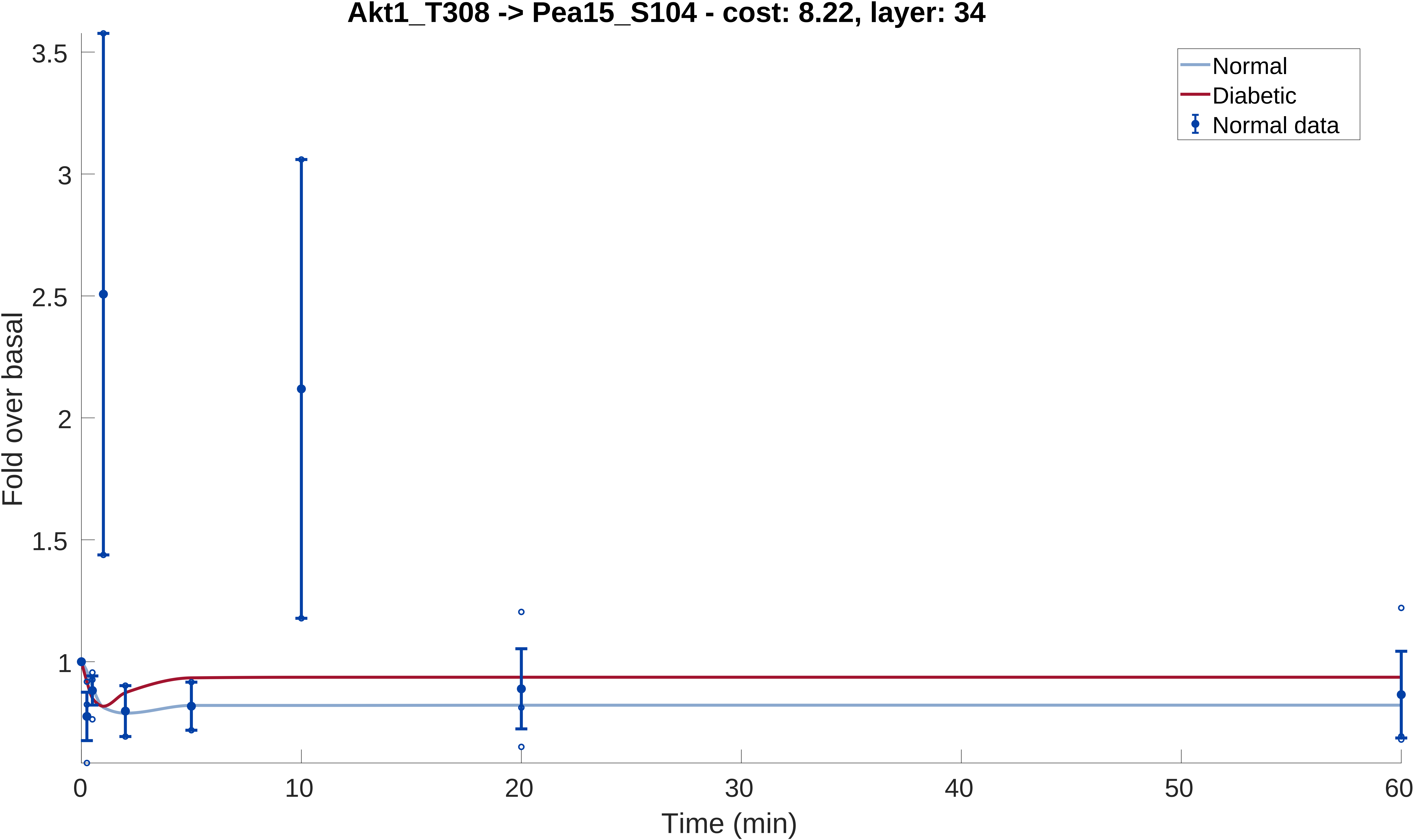
Akt1_T308 -> Pea15_S104 - cost: 8.22, layer: 34
Fold over basal
3.5
3
2.5
2
1.5
1
0
10
20
30
40
50
60
Time (min)
Normal
Diabetic
Normal data

Ptpn11_Y546 -> Ptk2b_S375 - cost: 12.42, layer: 34

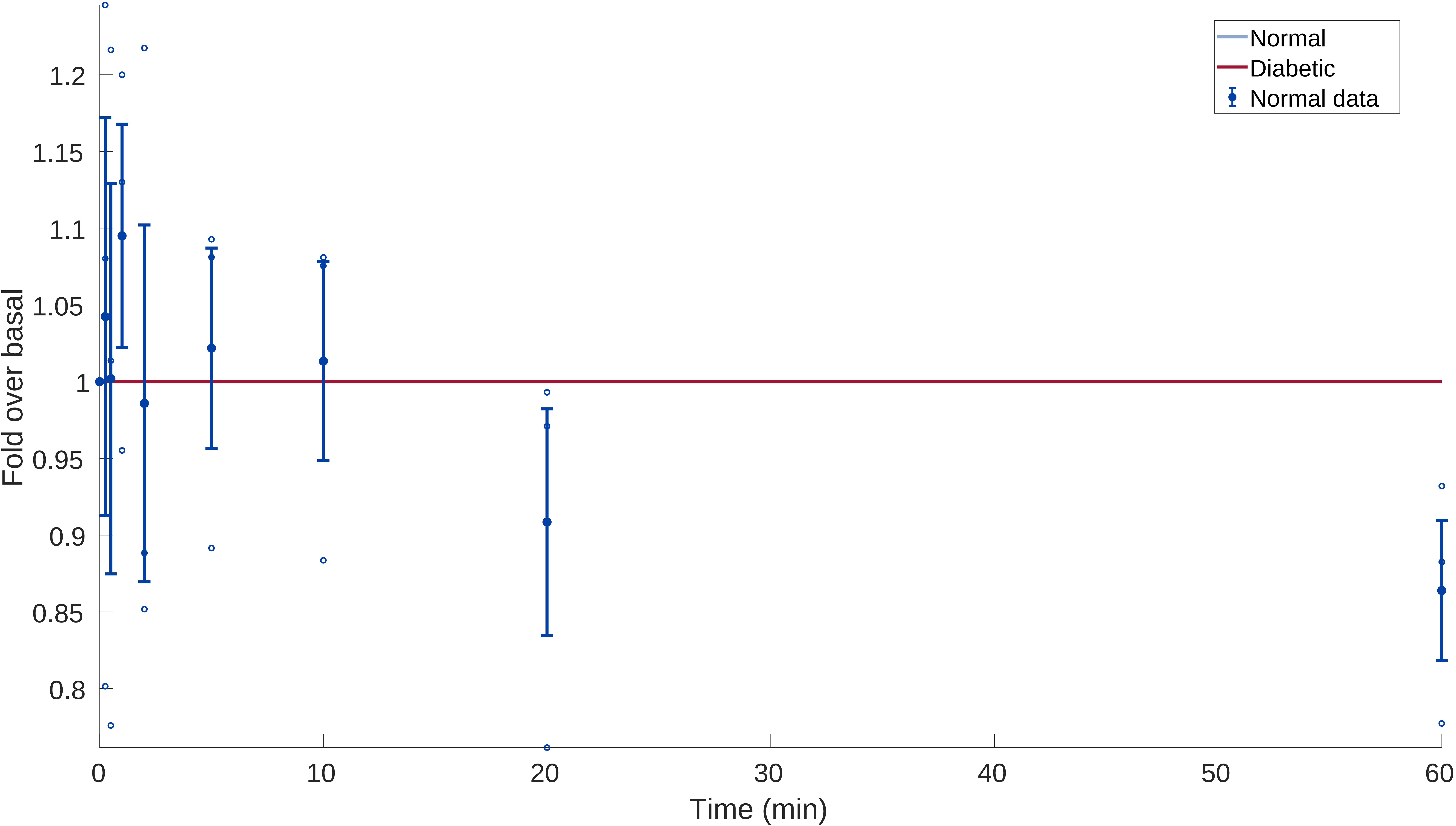

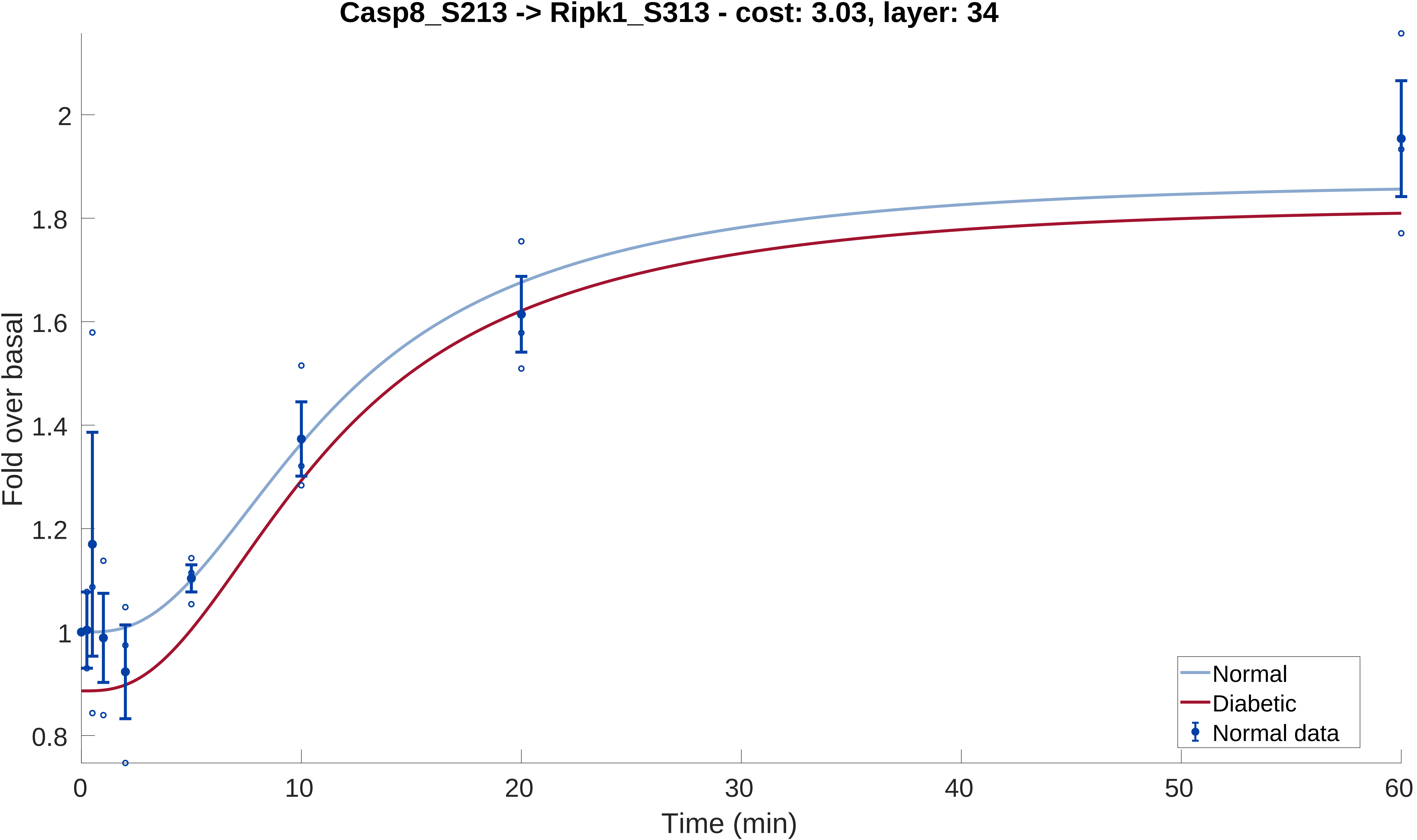

Casp8_S213 -> Ripk1_S313 - cost: 3.03, layer: 34
Fold over basal
0.8
1
1.2
1.4
1.6
1.8
2
0
10
20
30
40
50
60
Time (min)
Normal
Diabetic
Normal data

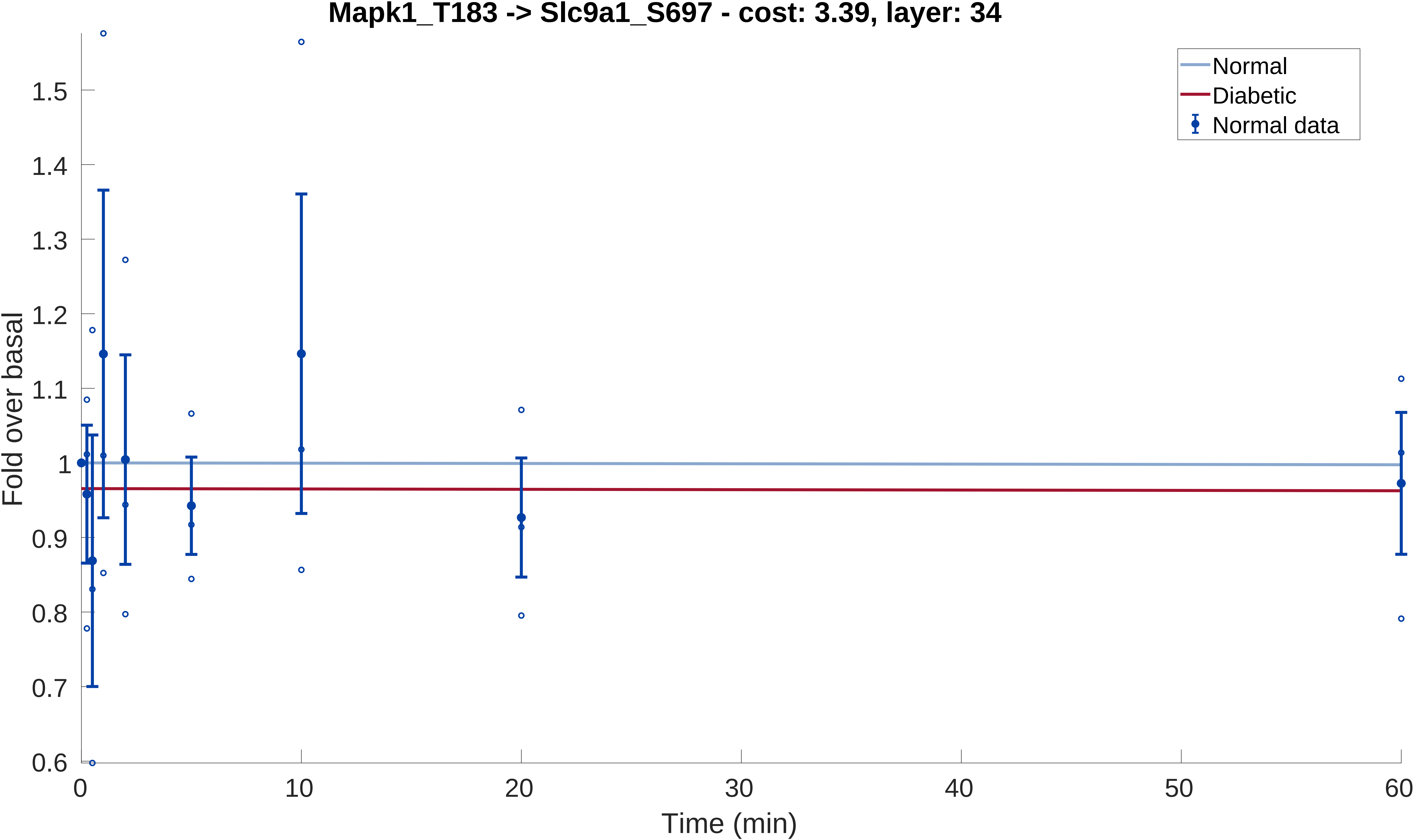
Mapk1_T183 -> Slc9a1_S697 - cost: 3.39, layer: 34
Normal
Diabetic
Normal data
Fold over basal
Time (min)
0.6
0.7
0.8
0.9
1
1.1
1.2
1.3
1.4
1.5
0
10
20
30
40
50
60

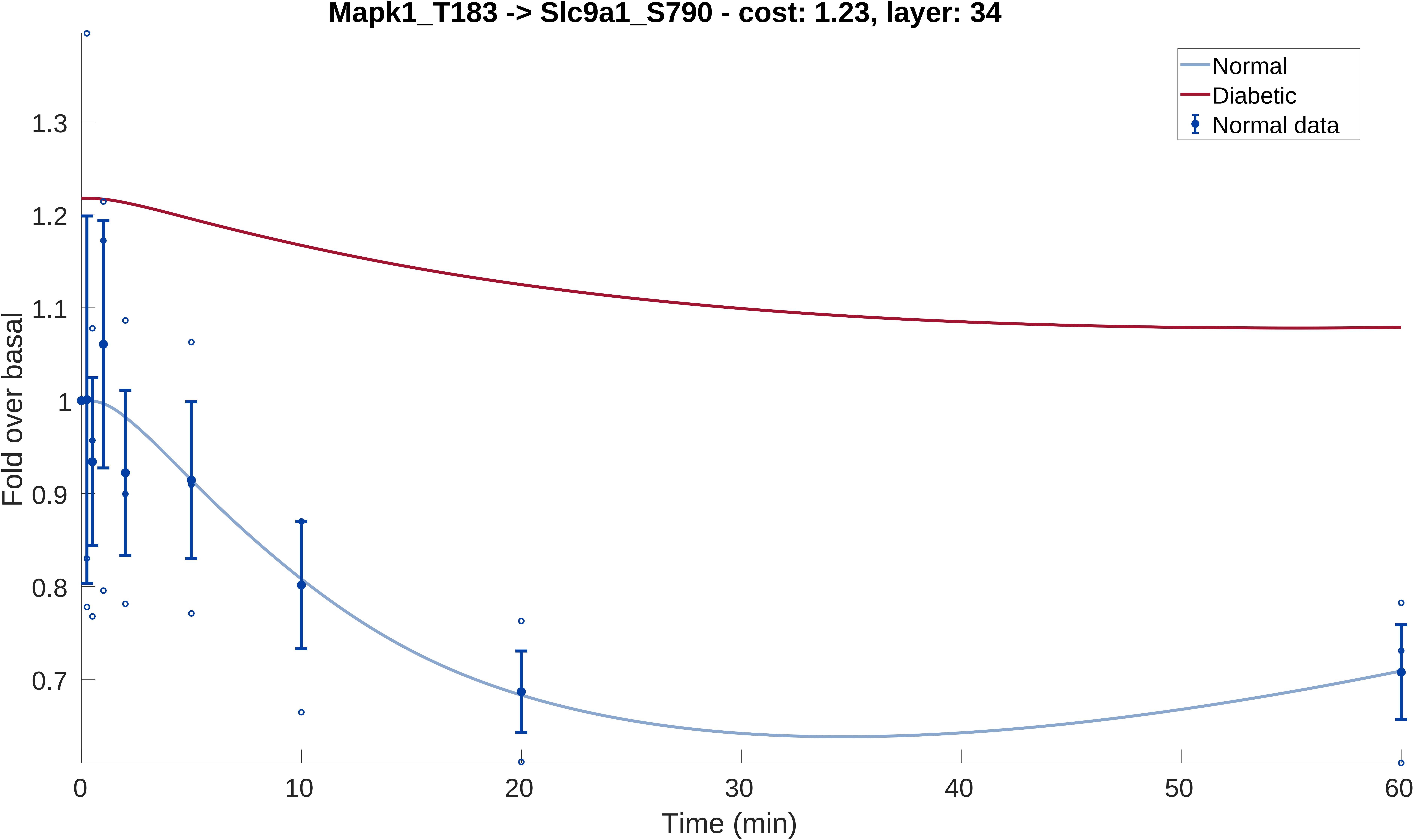

Mapk1_T183 -> Slc9a1_S790 - cost: 1.23, layer: 34
Normal
Diabetic
Normal data
Fold over basal
1.3
1.2
1.1
1
0.9
0.8
0.7
0
10
20
30
40
50
60
Time (min)

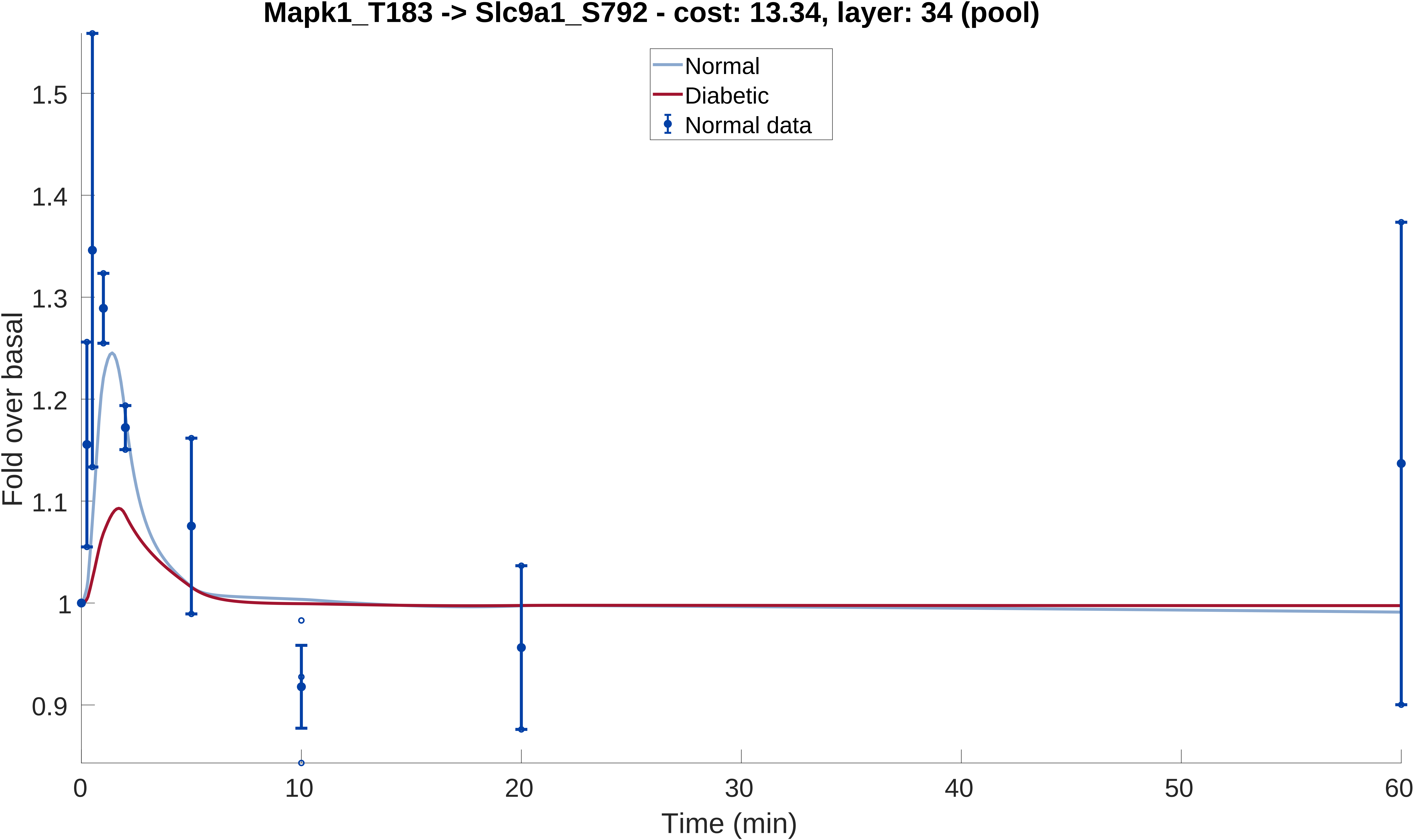
Mapk1_T183 -> Slc9a1_S792 - cost: 13.34, layer: 34 (pool)
Normal
Diabetic
Normal data
Fold over basal
1.5
1.4
1.3
1.2
1.1
1
0.9
0
10
20
30
40
50
60
Time (min)

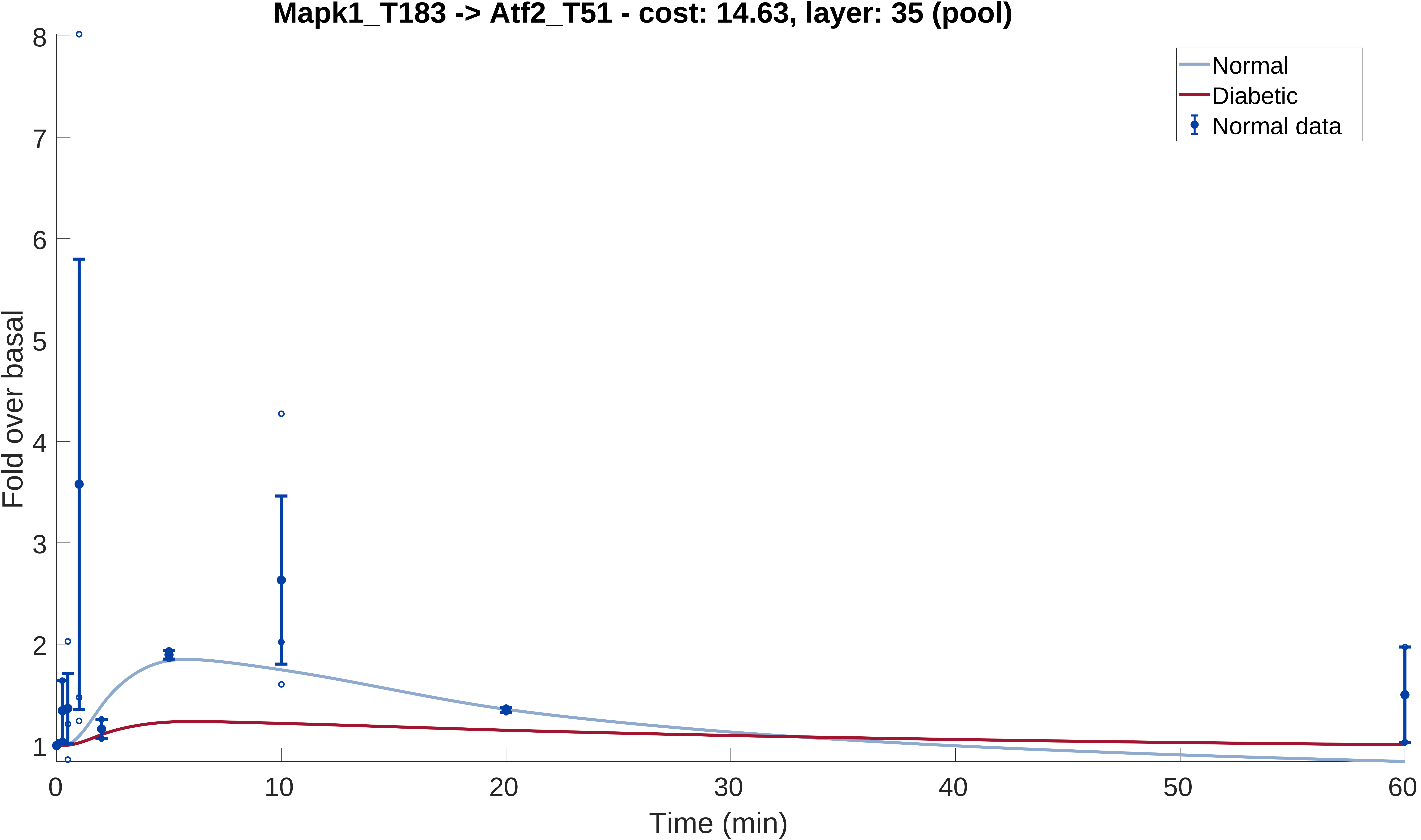
Mapk1_T183 -> Atf2_T51 - cost: 14.63, layer: 35 (pool)
Fold over basal
Time (min)
Normal
Diabetic
Normal data
0
10
20
30
40
50
60
1
2
3
4
5
6
7
8

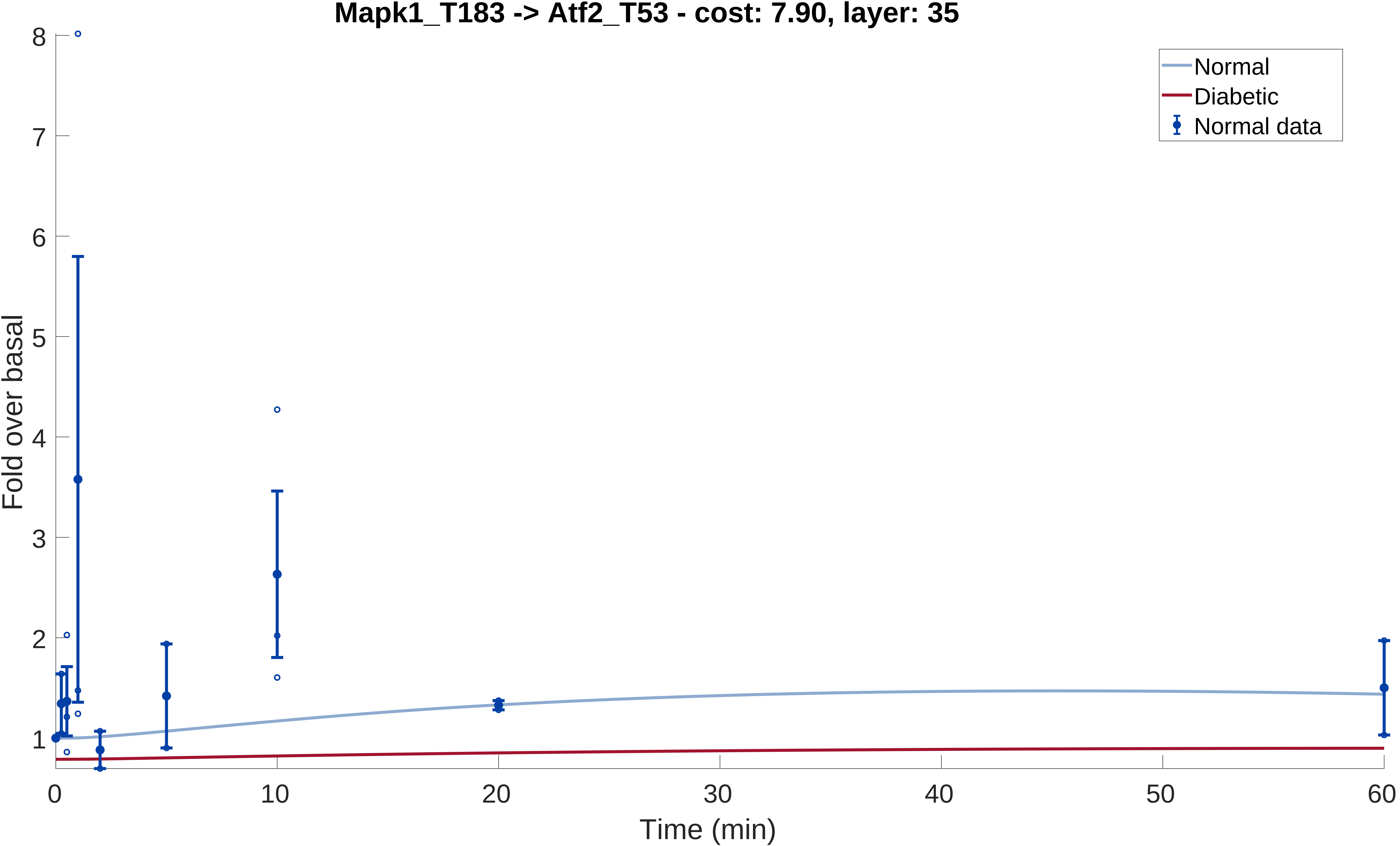

Mapk1_T183 -> Atf2_T53 - cost: 7.90, layer: 35
Normal
Diabetic
Normal data
Fold over basal
Time (min)
0
10
20
30
40
50
60
1
2
3
4
5
6
7
8

Akt1_S129 -> Braf_S348 - cost: 14.11, layer: 35

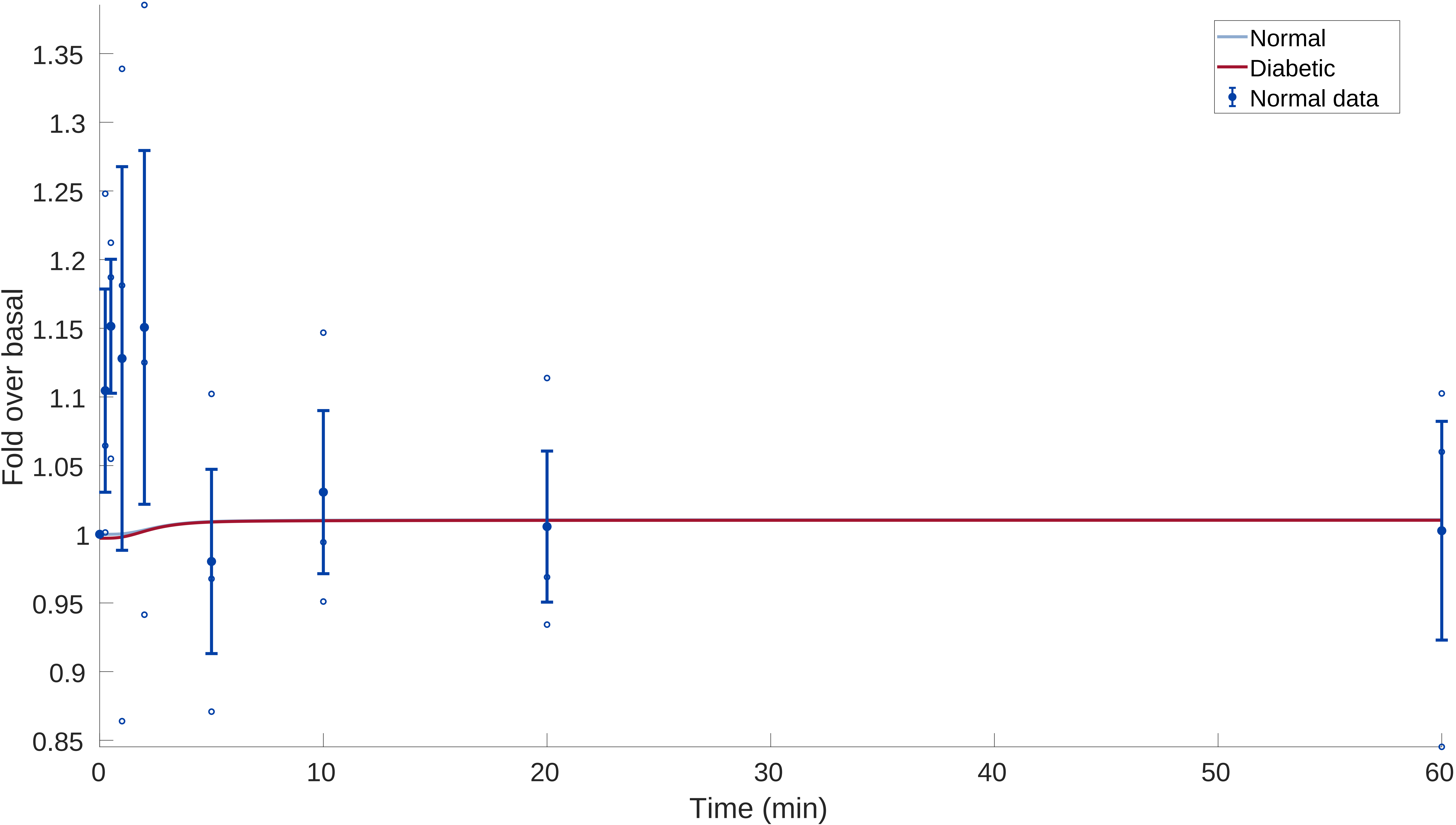

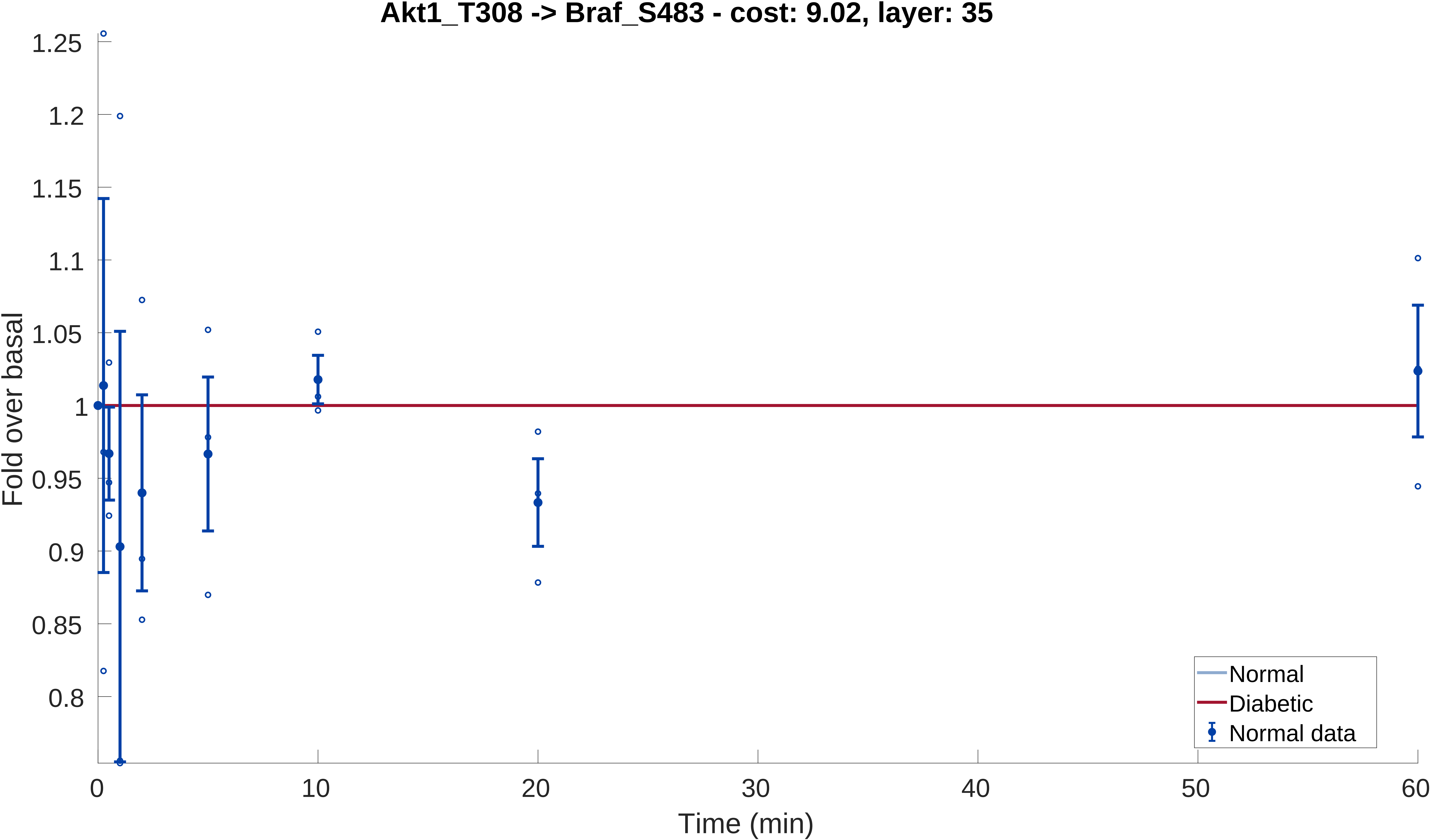
Akt1_T308 -> Braf_S483 - cost: 9.02, layer: 35
Fold over basal
Time (min)
1.25
1.2
1.15
1.1
1.05
1
0.95
0.9
0.85
0.8
0
10
20
30
40
50
60
Normal
Diabetic
Normal data

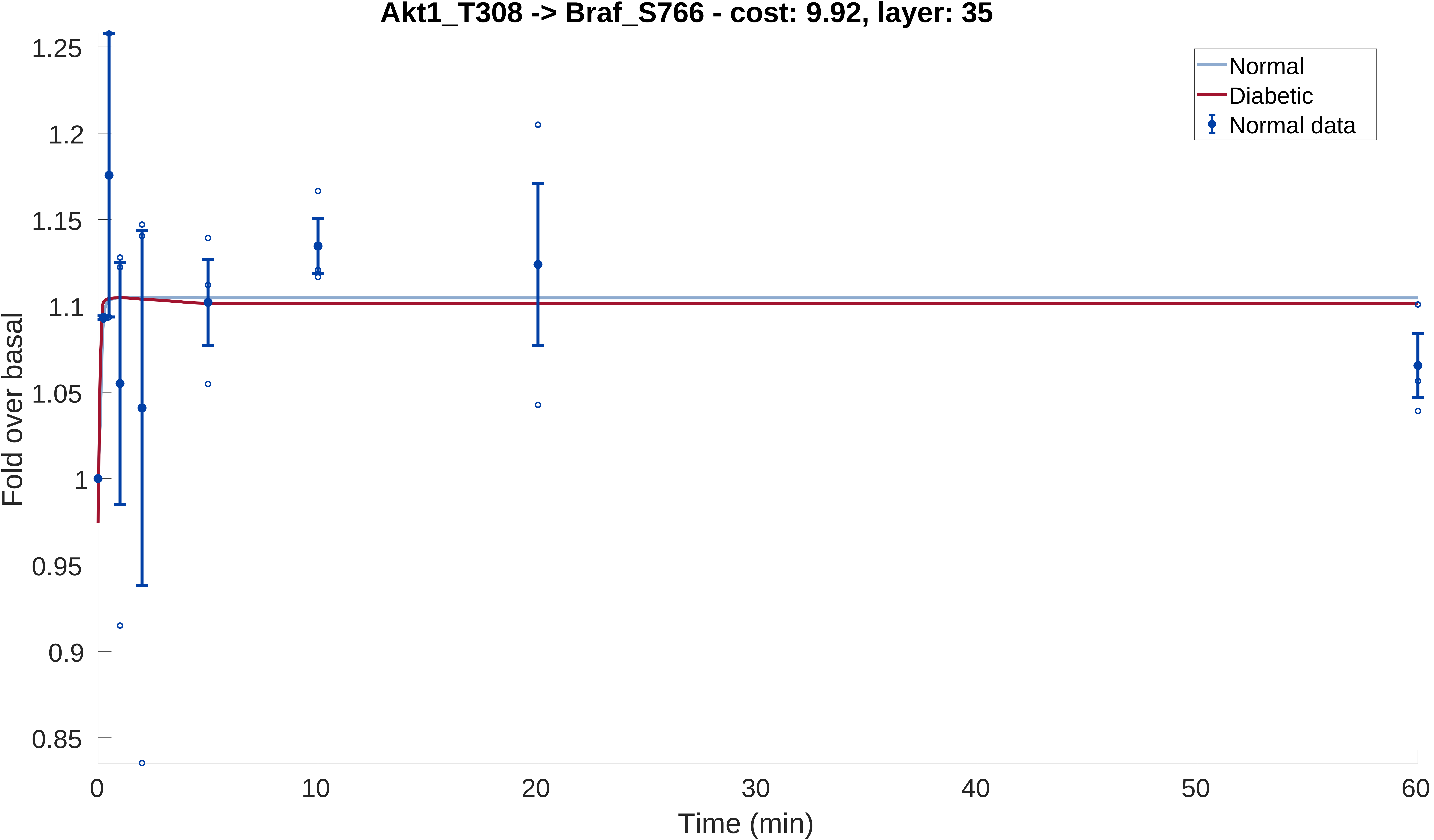

Akt1_T308 -> Braf_S766 - cost: 9.92, layer: 35
Normal
Diabetic
Normal data
Fold over basal
Time (min)
1.25
1.2
1.15
1.1
1.05
1
0.95
0.9
0.85
0
10
20
30
40
50
60

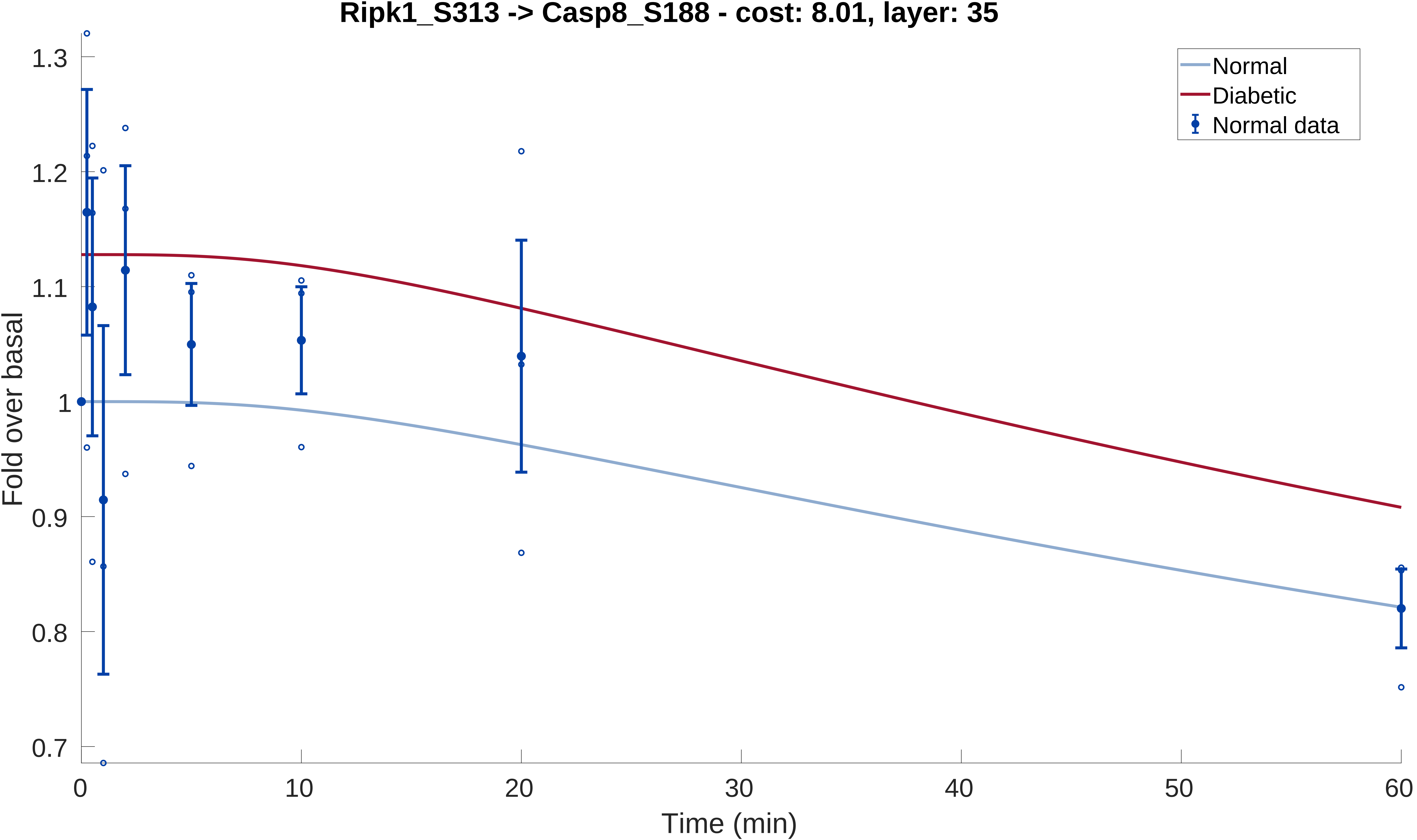
Ripk1_S313 -> Casp8_S188 - cost: 8.01, layer: 35
Normal
Diabetic
Normal data
Fold over basal
Time (min)
1.3
1.2
1.1
1
0.9
0.8
0.7
0
10
20
30
40
50
60

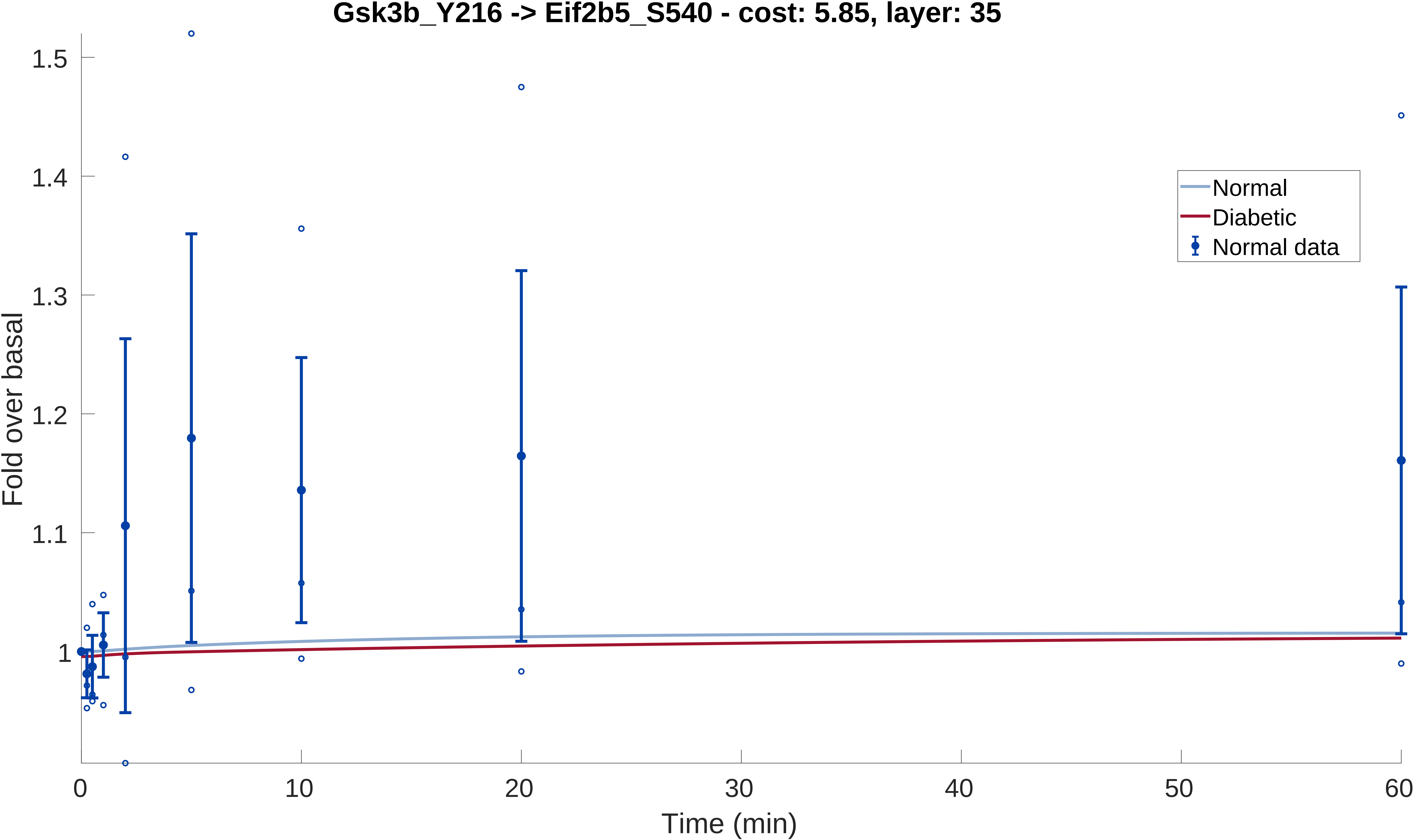

Gsk3b_Y216 -> Eif2b5_S540 - cost: 5.85, layer: 35
Fold over basal
1.5
1.4
1.3
1.2
1.1
1
Normal
Diabetic
Normal data
0
10
20
30
40
50
60
Time (min)

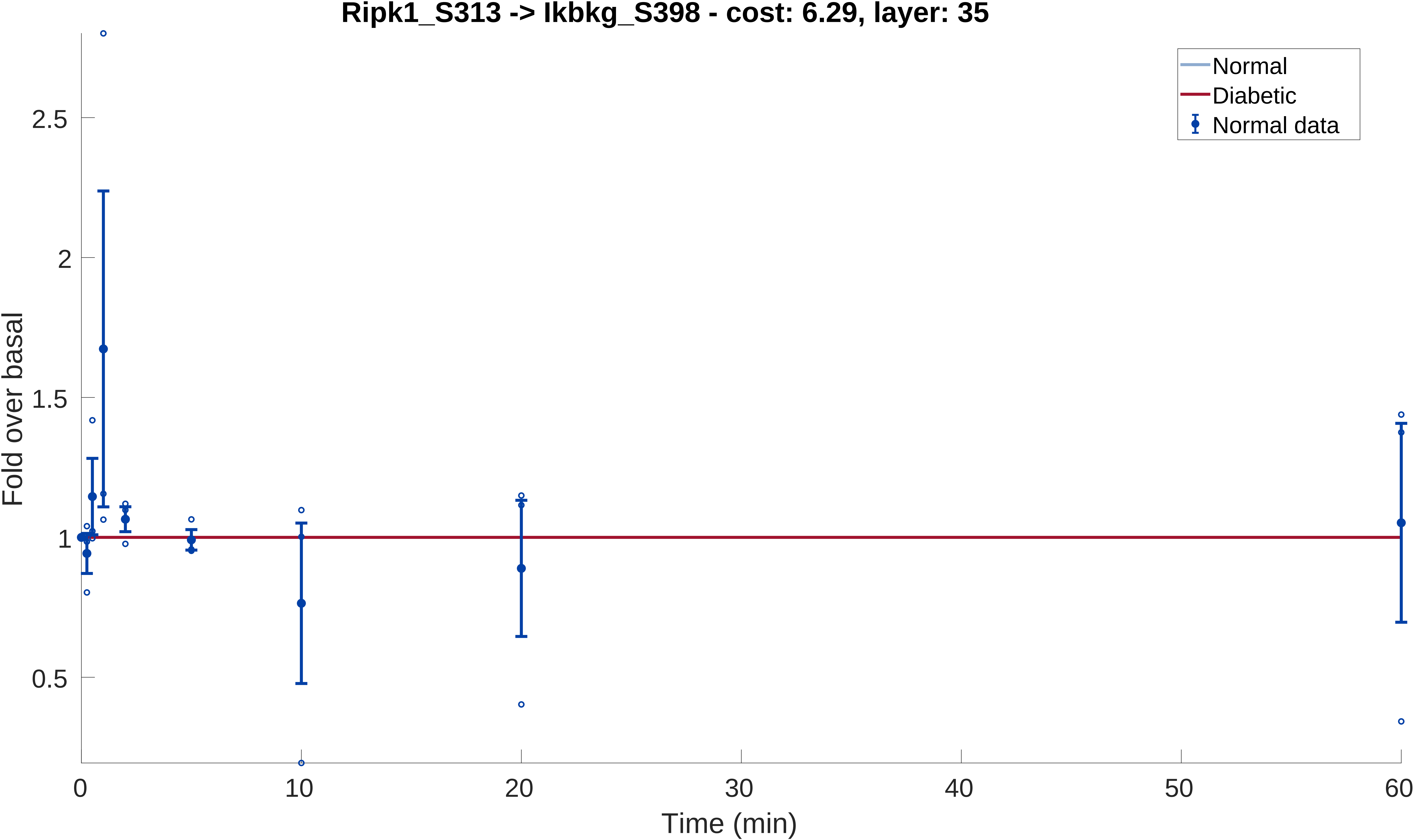

Ripk1_S313 -> Ikbkg_S398 - cost: 6.29, layer: 35
Normal
Diabetic
Normal data
Fold over basal
Time (min)
0.5
1
1.5
2
2.5
0
10
20
30
40
50
60

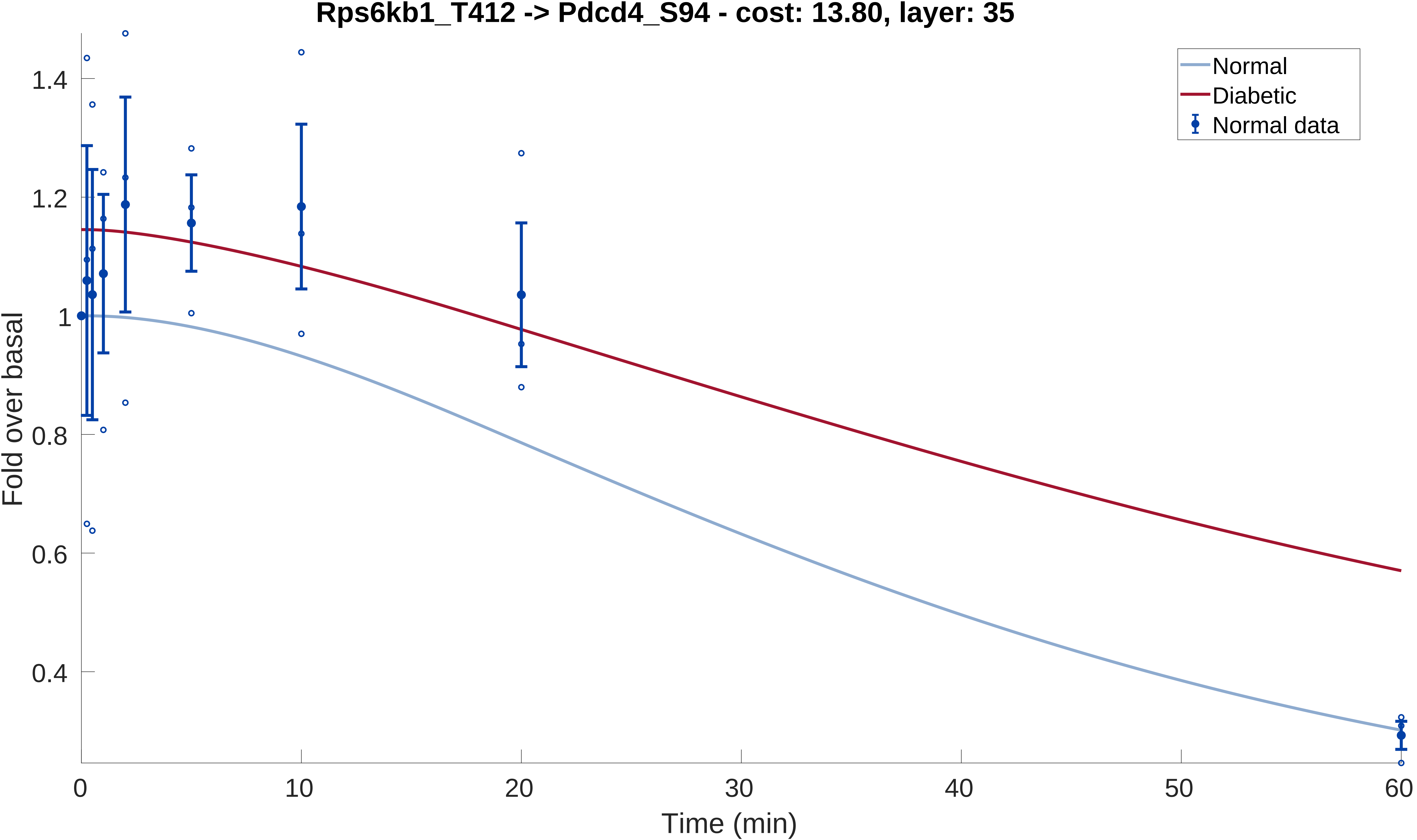

Rps6kb1_T412 -> Pdcd4_S94 - cost: 13.80, layer: 35
Normal
Diabetic
Normal data
Fold over basal
1.4
1.2
1
0.8
0.6
0.4
0
10
20
30
40
50
60
Time (min)

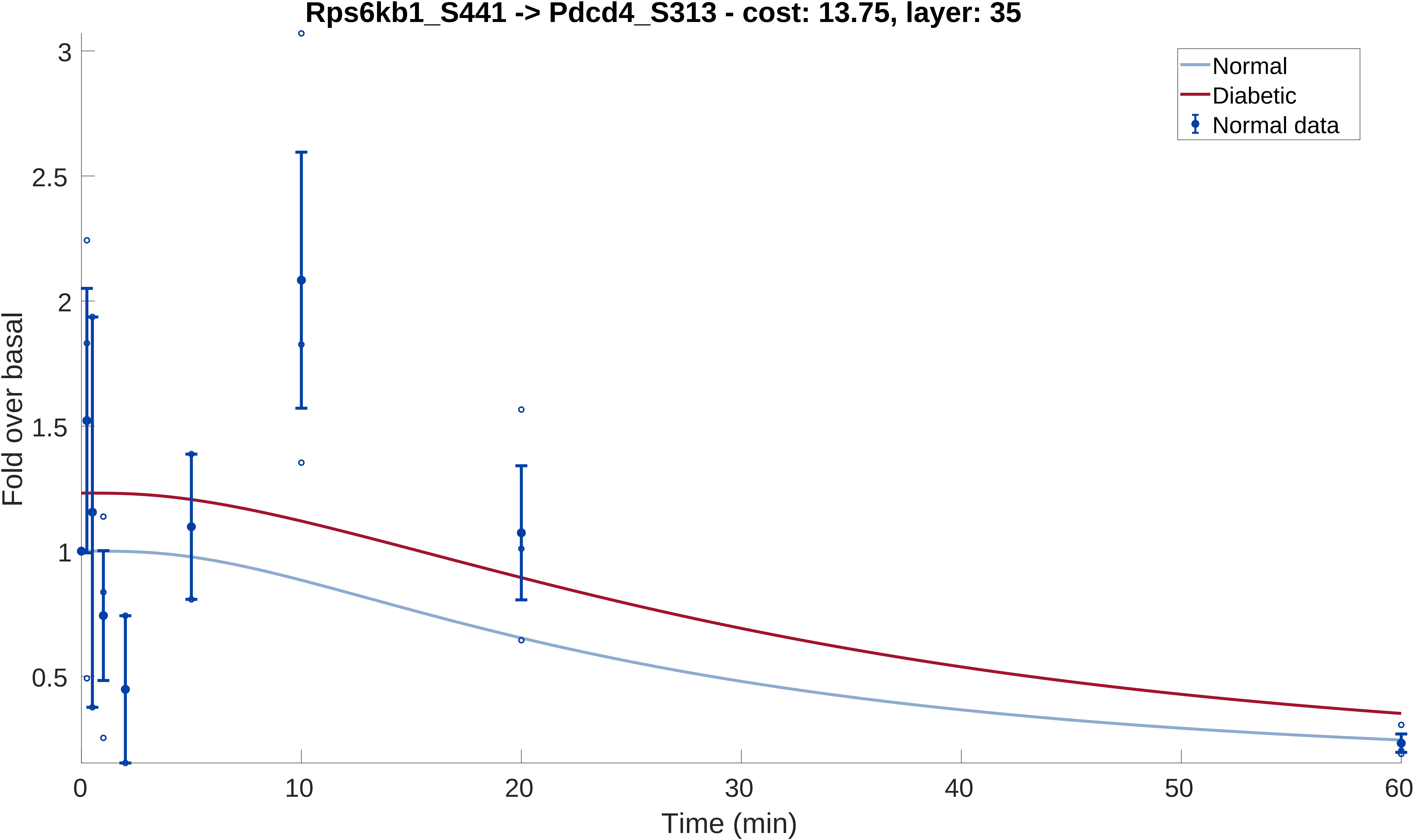
Rps6kb1_S441 -> Pdcd4_S313 - cost: 13.75, layer: 35
Normal
Diabetic
Normal data
Fold over basal
Time (min)
0
10
20
30
40
50
60
0.5
1
1.5
2
2.5
3

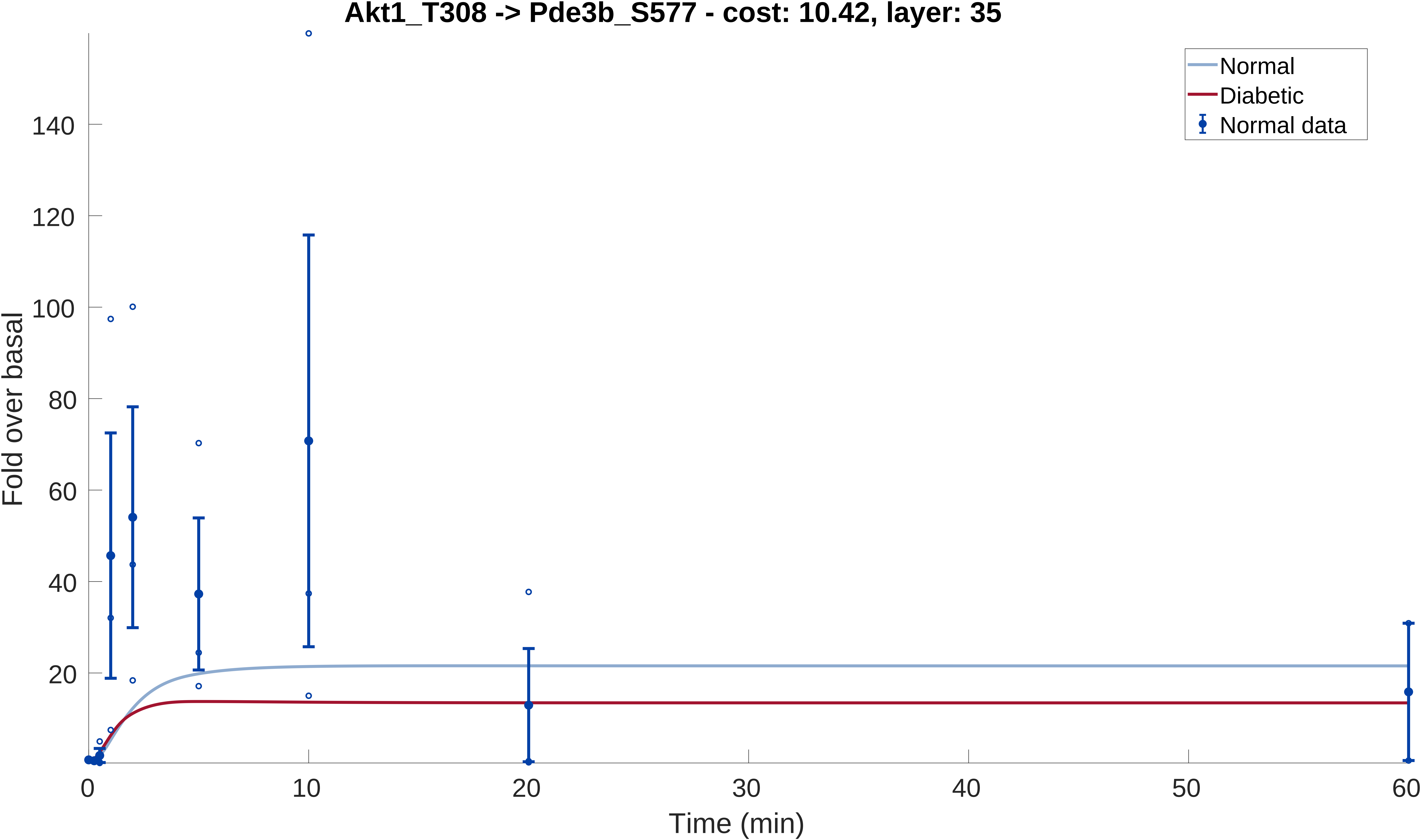
Akt1_T308 -> Pde3b_S577 - cost: 10.42, layer: 35
Normal
Diabetic
Normal data
Fold over basal
Time (min)
0
10
20
30
40
50
60
20
40
60
80
100
120
140

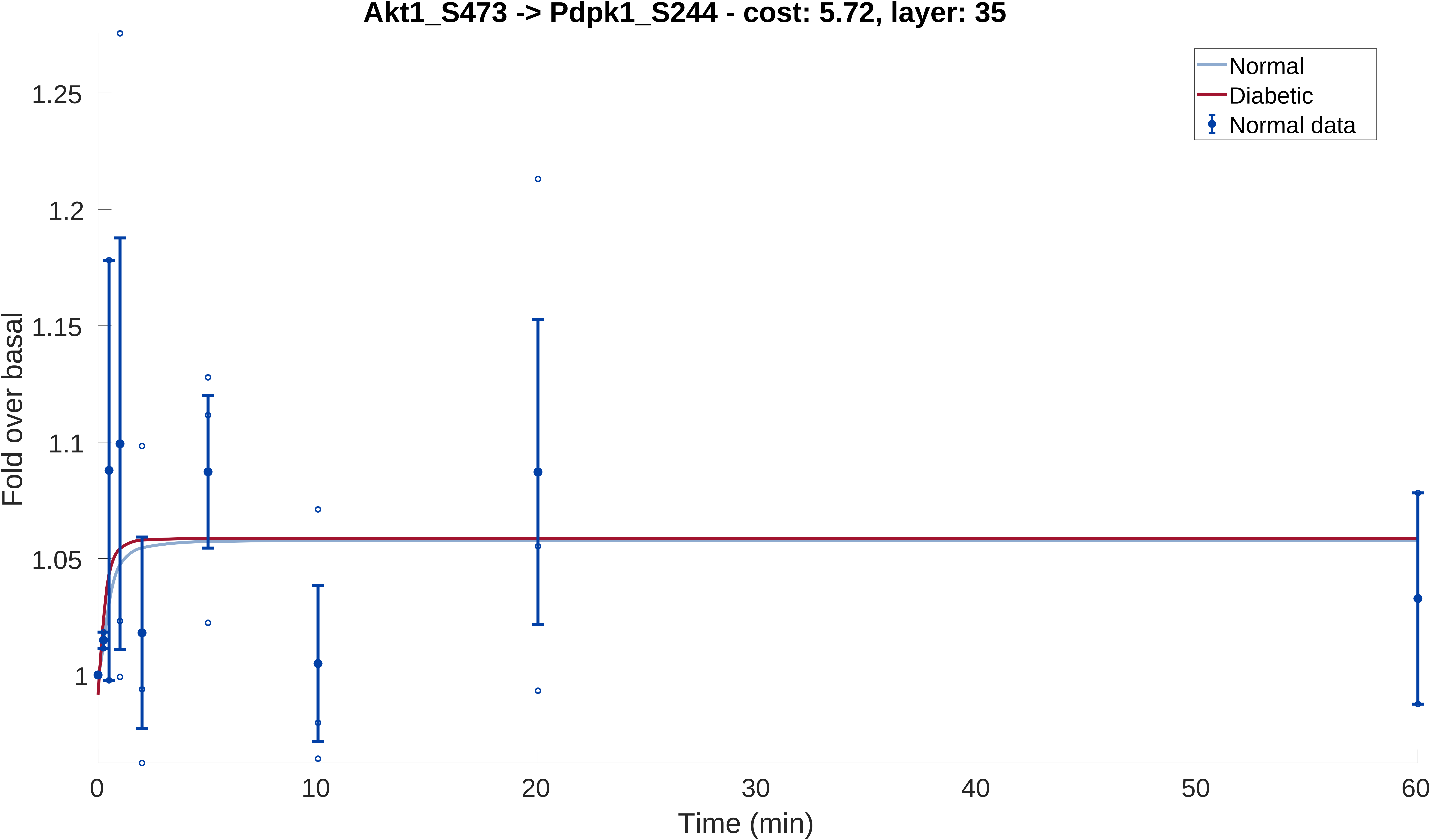

Akt1_S473 -> Pdpk1_S244 - cost: 5.72, layer: 35
Normal
Diabetic
Normal data
Fold over basal
1.25
1.2
1.15
1.1
1.05
1
Time (min)
0
10
20
30
40
50
60

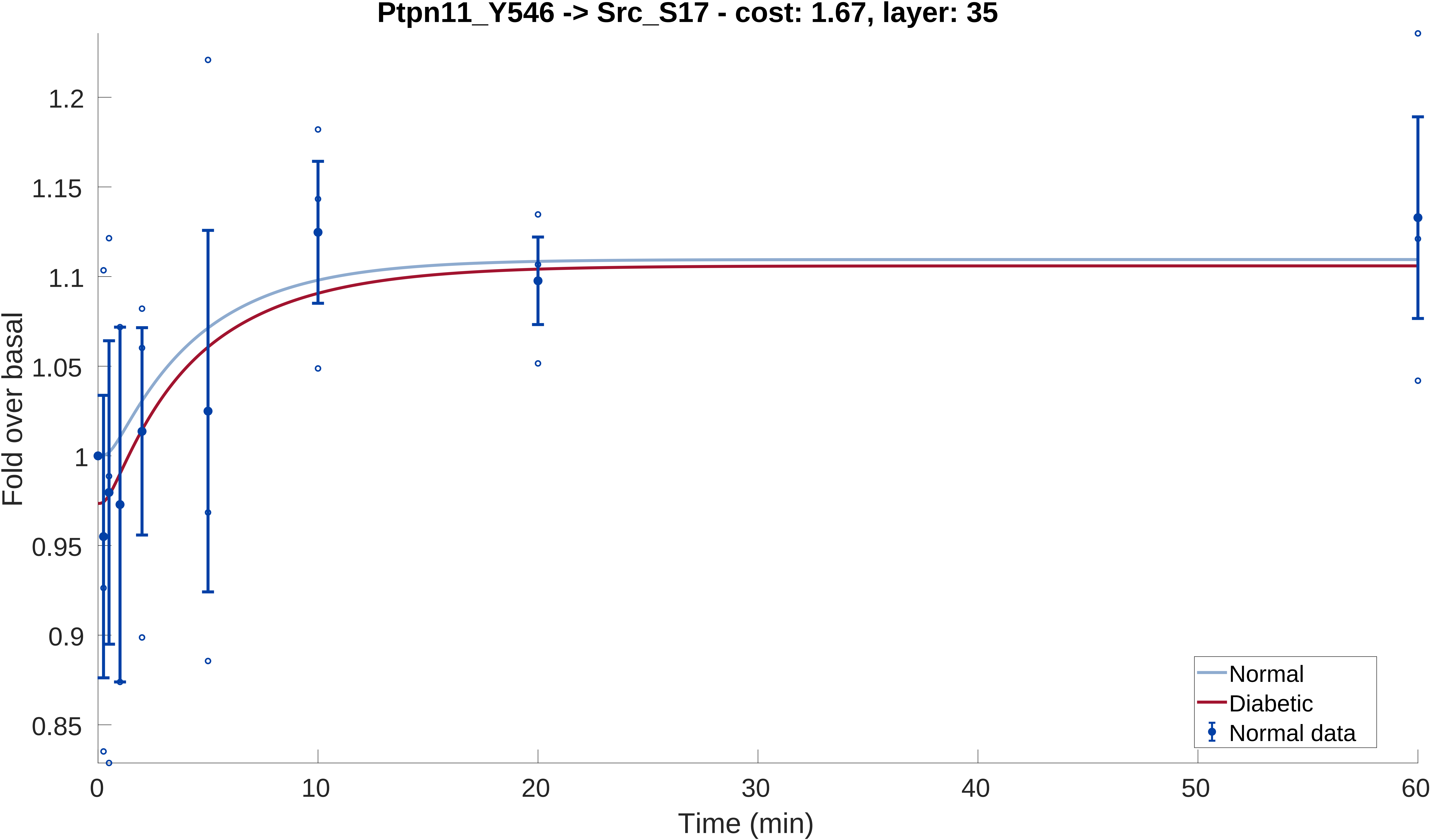

Ptpn11_Y546 -> Src_S17 - cost: 1.67, layer: 35
Fold over basal
1.2
1.15
1.1
1.05
1
0.95
0.9
0.85
0
10
20
30
40
50
60
Time (min)
Normal
Diabetic
Normal data

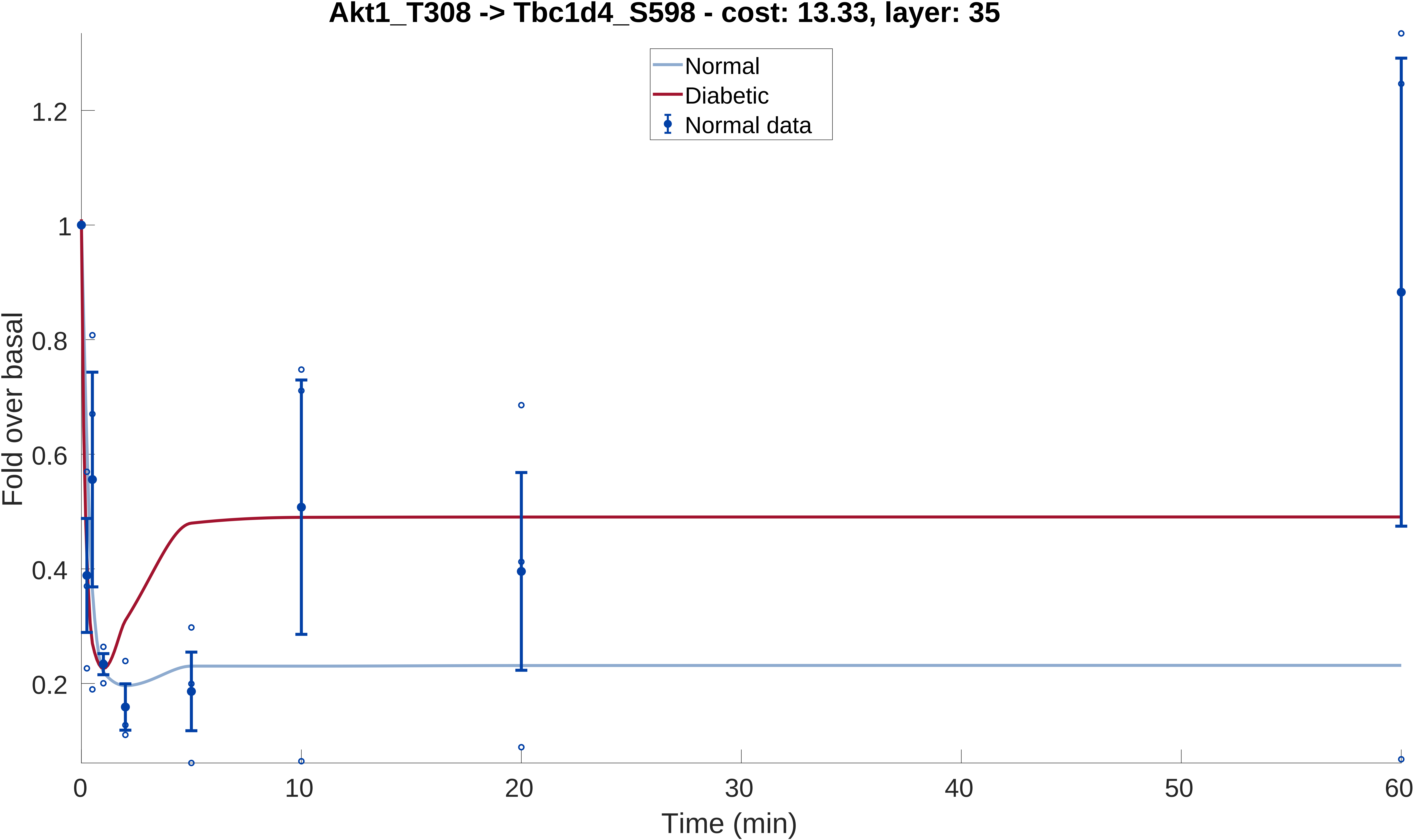

Akt1_T308 -> Tbc1d4_S598 - cost: 13.33, layer: 35
Normal
Diabetic
Normal data
Fold over basal
1.2
1
0.8
0.6
0.4
0.2
0
10
20
30
40
50
60
Time (min)

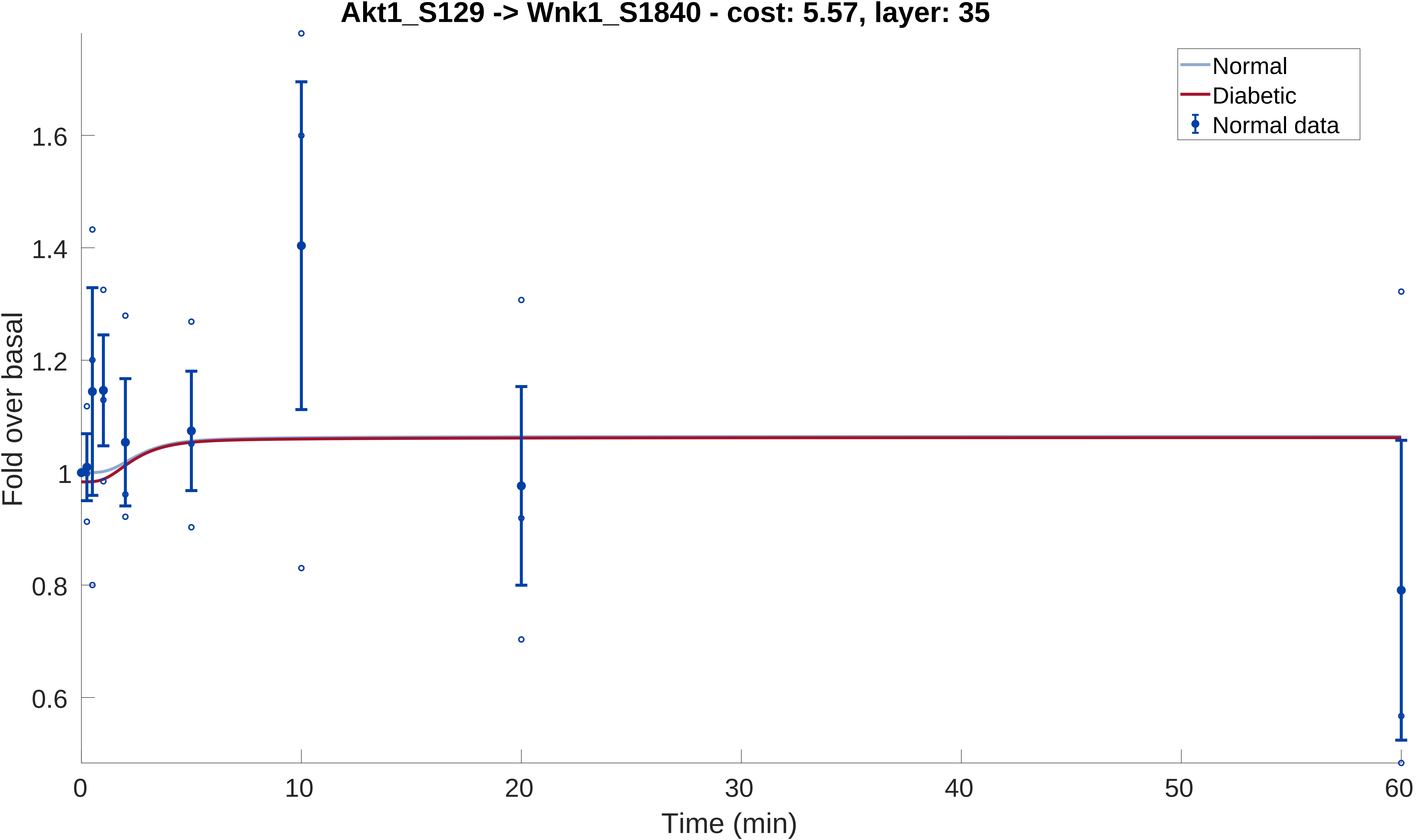
Akt1_S129 -> Wnk1_S1840 - cost: 5.57, layer: 35
Normal
Diabetic
Normal data
1.6
1.4
1.2
1
0.8
0.6
Fold over basal
0
10
20
30
40
50
60
Time (min)

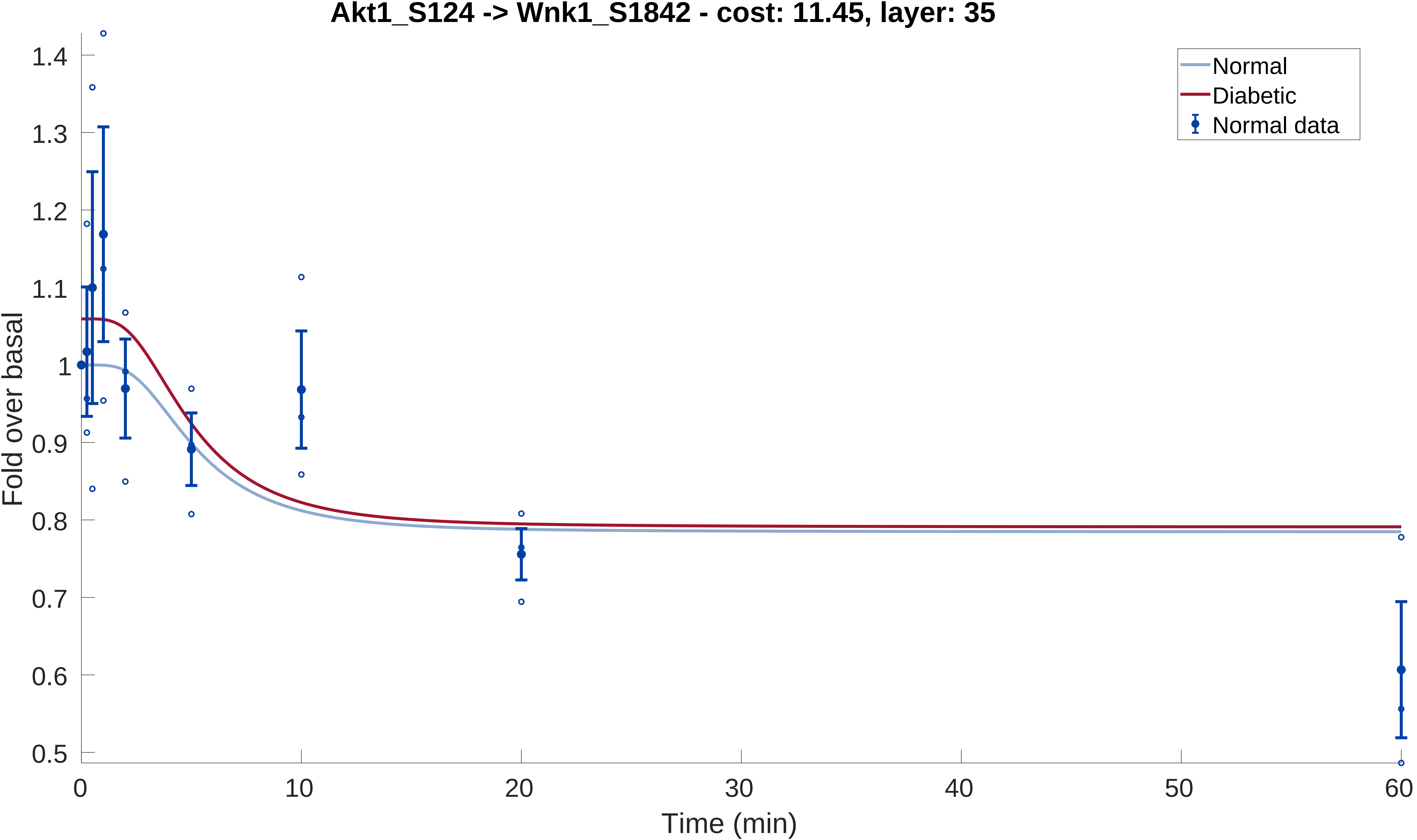

Akt1_S124 -> Wnk1_S1842 - cost: 11.45, layer: 35
Normal
Diabetic
Normal data
1.4
1.3
1.2
1.1
1
0.9
0.8
0.7
0.6
0.5
Fold over basal
0
10
20
30
40
50
60
Time (min)

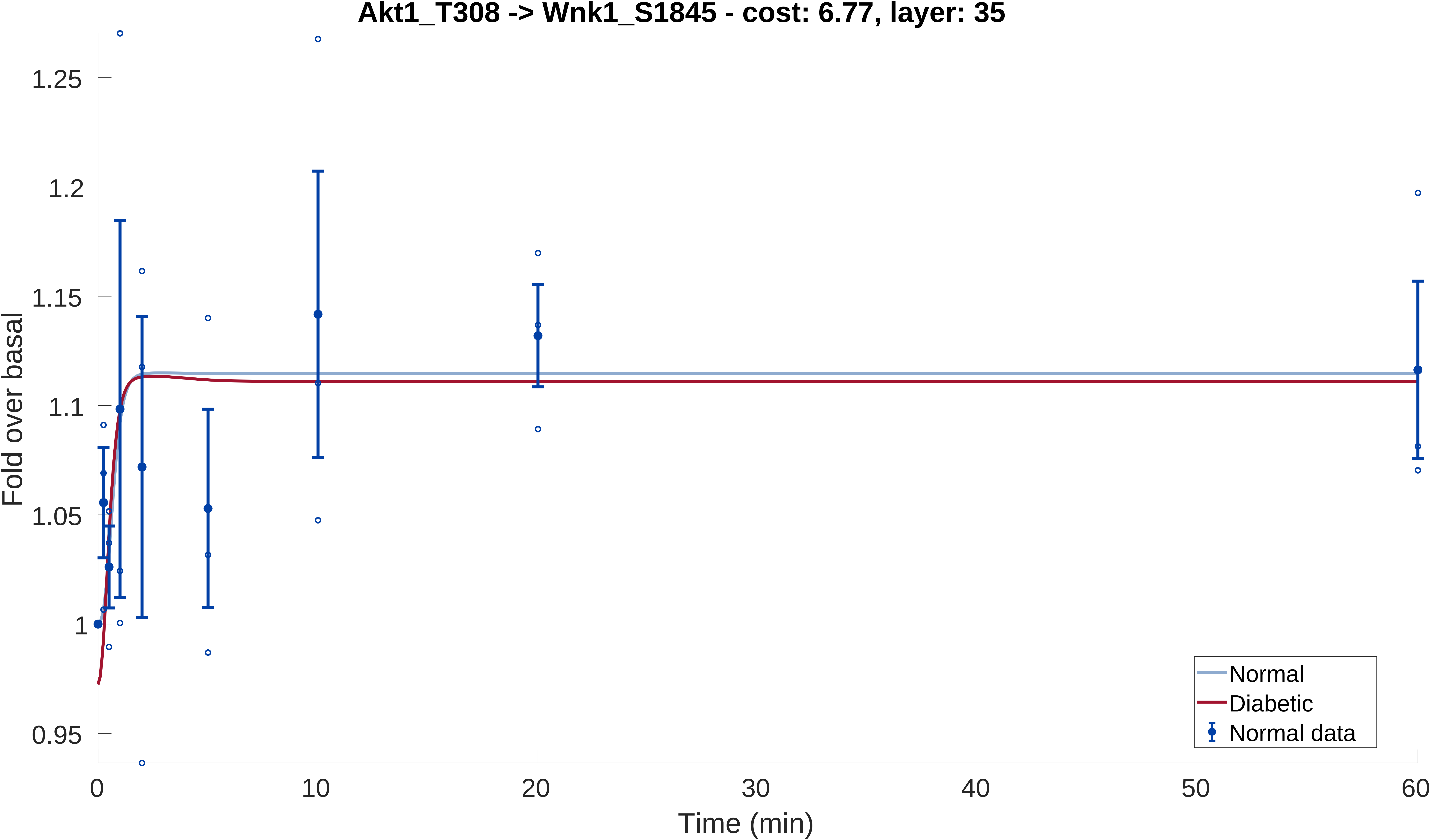

Akt1_T308 -> Wnk1_S1845 - cost: 6.77, layer: 35
Fold over basal
1.25
1.2
1.15
1.1
1.05
1
0.95
0
10
20
30
40
50
60
Time (min)
Normal
Diabetic
Normal data

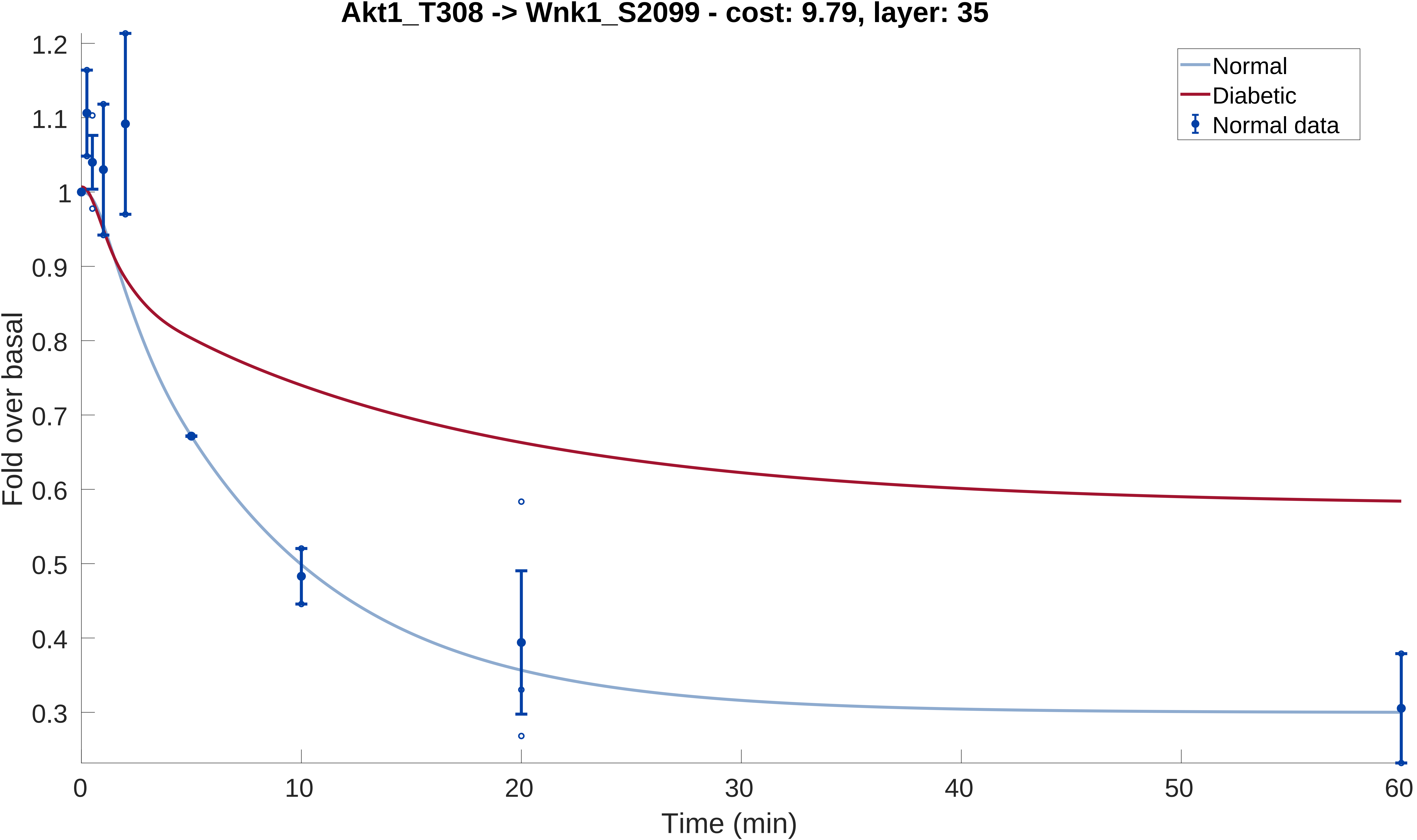

Akt1_T308 -> Wnk1_S2099 - cost: 9.79, layer: 35
Normal
Diabetic
Normal data
Fold over basal
1.2
1.1
1
0.9
0.8
0.7
0.6
0.5
0.4
0.3
0
10
20
30
40
50
60
Time (min)

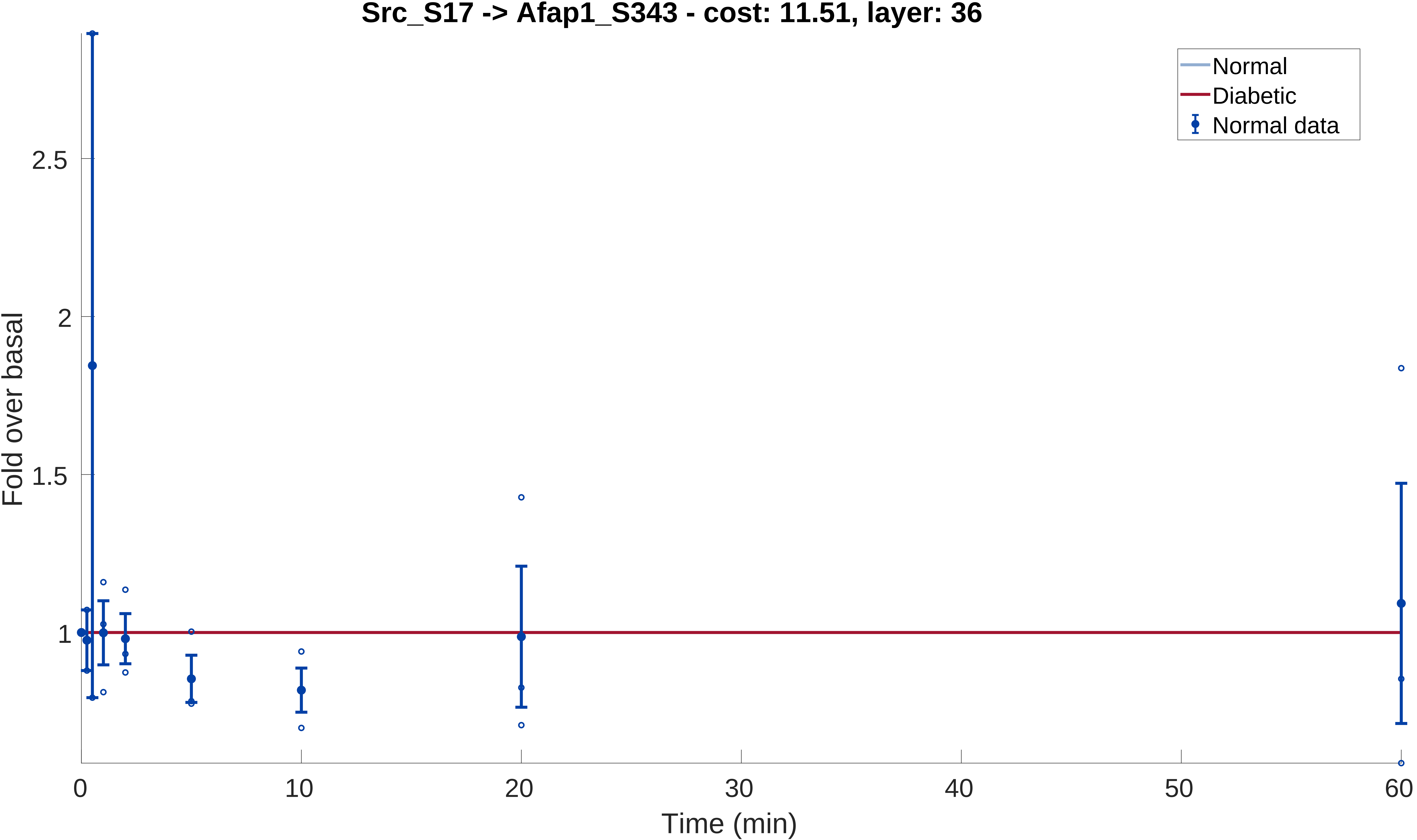
Src_S17 -> Afap1_S343 - cost: 11.51, layer: 36
Normal
Diabetic
Normal data
Fold over basal
2.5
2
1.5
1
0
10
20
30
40
50
60
Time (min)

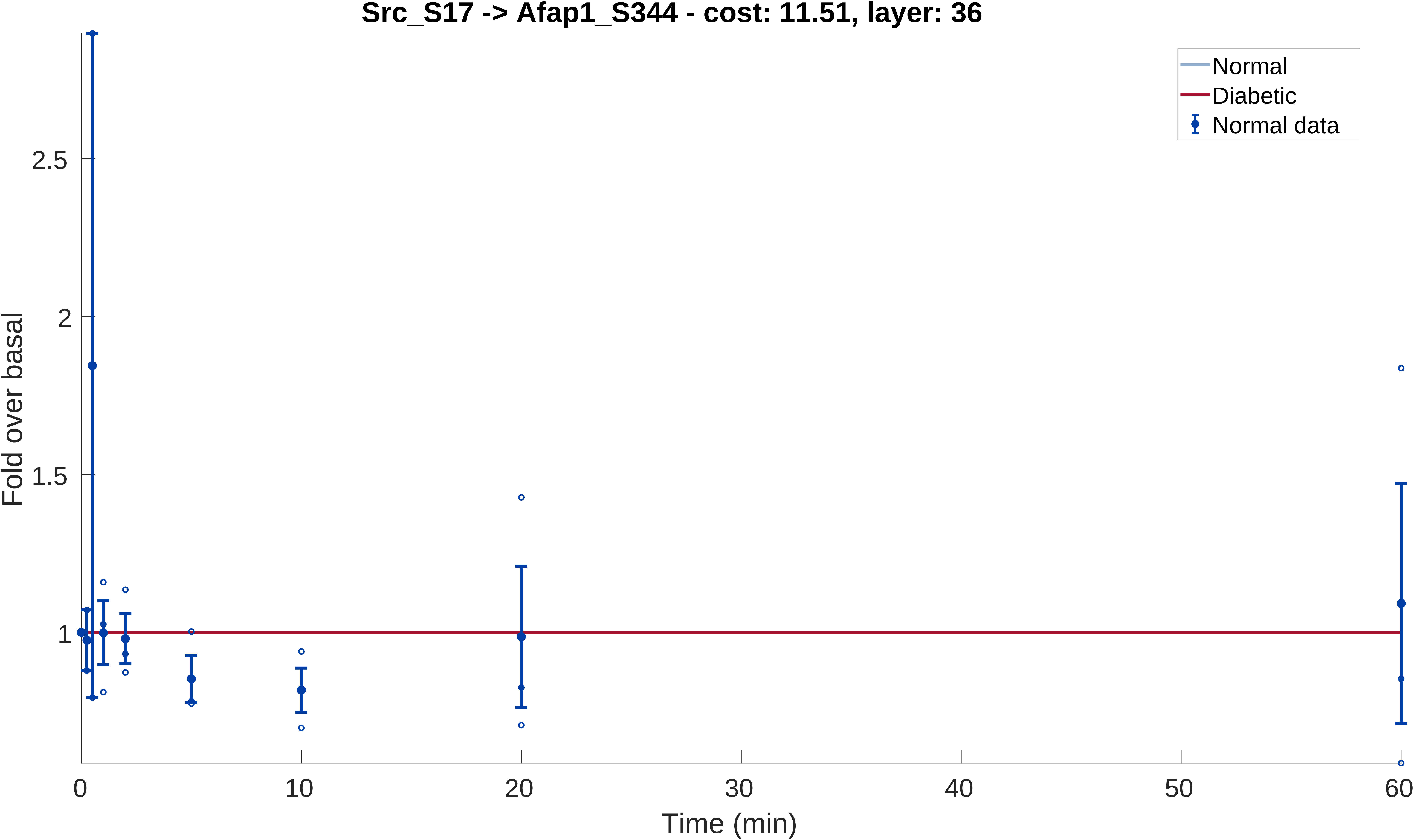
Src_S17 -> Afap1_S344 - cost: 11.51, layer: 36
Normal
Diabetic
Normal data
Fold over basal
2.5
2
1.5
1
0
10
20
30
40
50
60
Time (min)

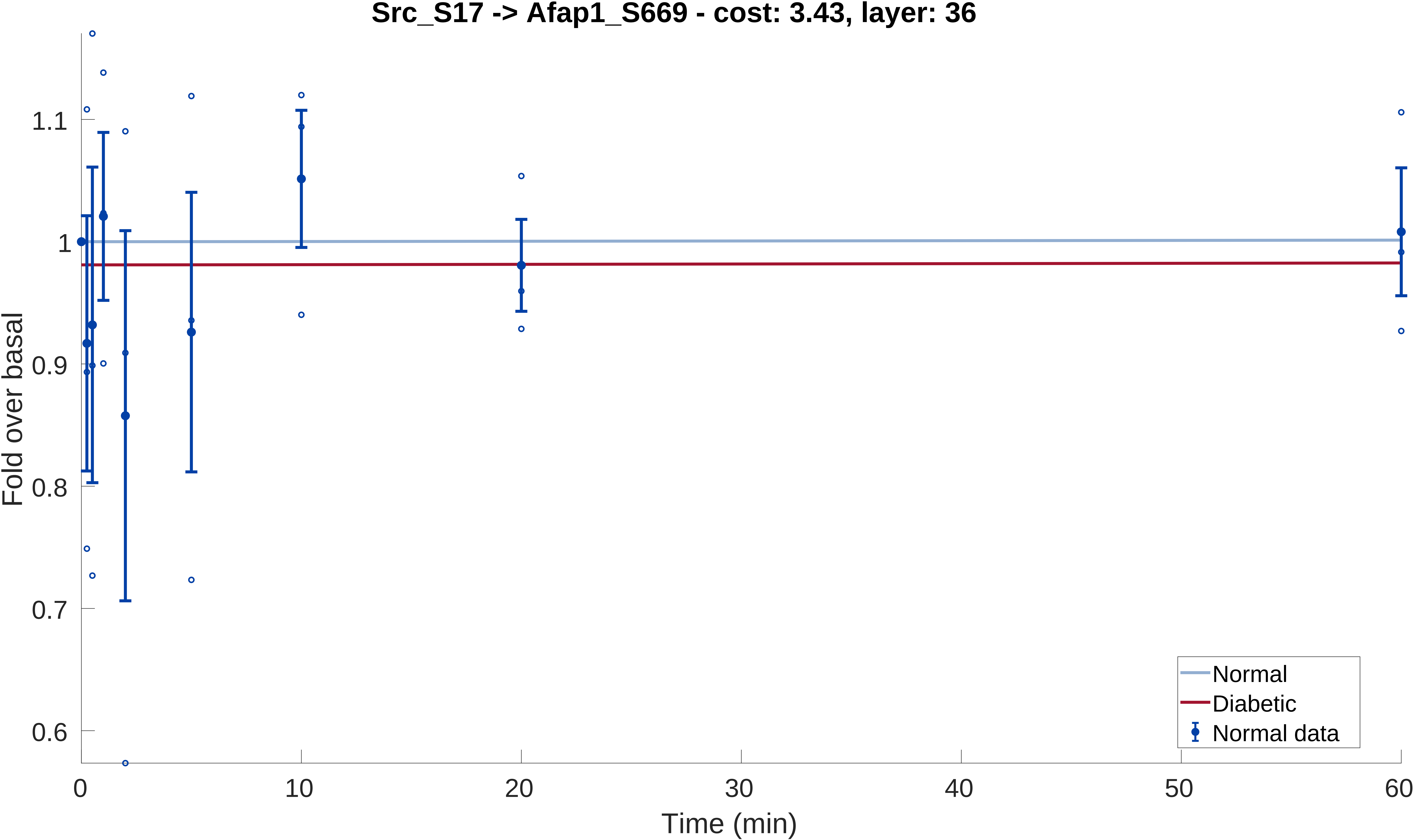

Src_S17 -> Afap1_S669 - cost: 3.43, layer: 36
Fold over basal
1.1
1
0.9
0.8
0.7
0.6
0
10
20
30
40
50
60
Time (min)
Normal
Diabetic
Normal data

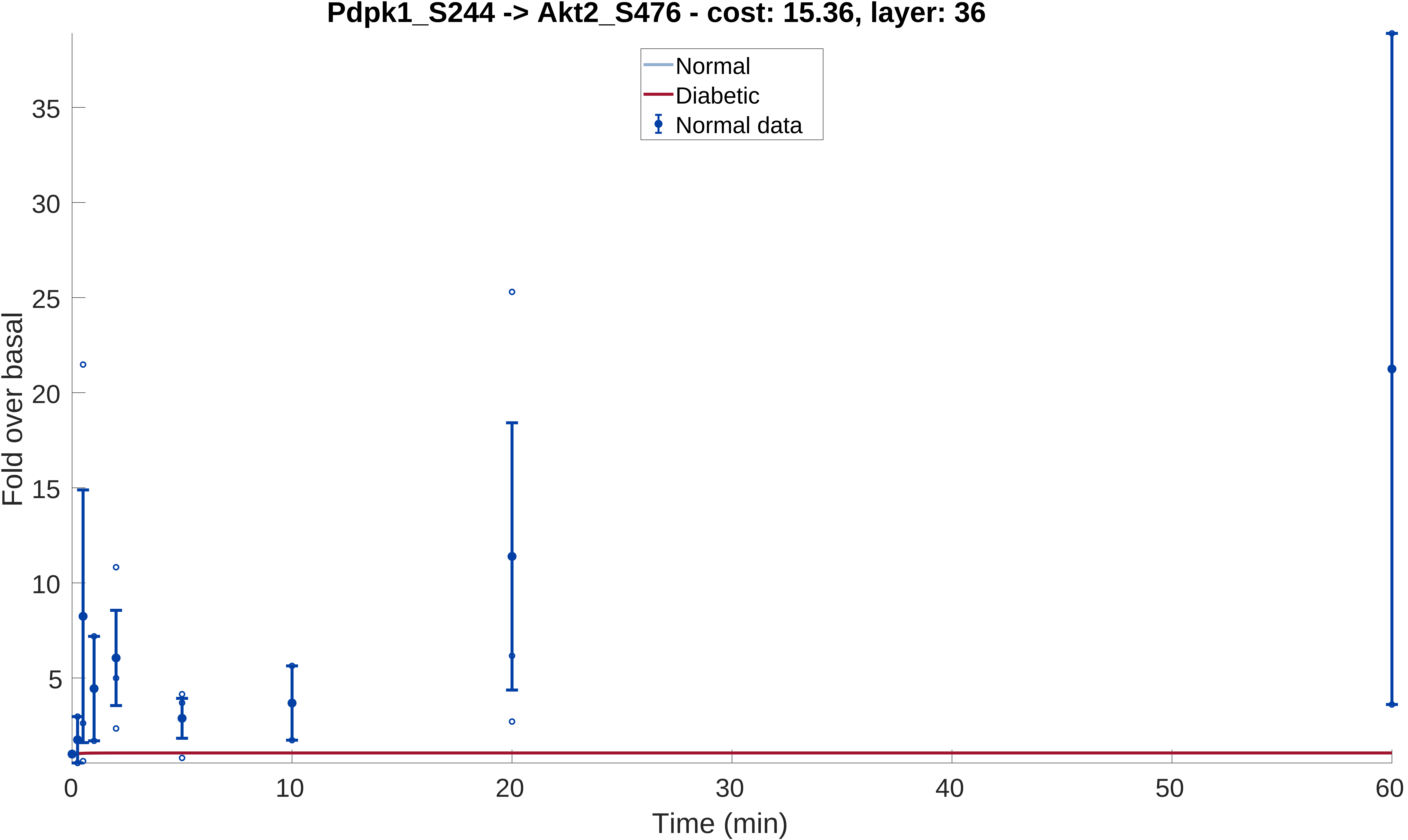

Pdpk1_S244 -> Akt2_S476 - cost: 15.36, layer: 36
Normal
Diabetic
Normal data
Fold over basal
5
10
15
20
25
30
35
0
10
20
30
40
50
60
Time (min)

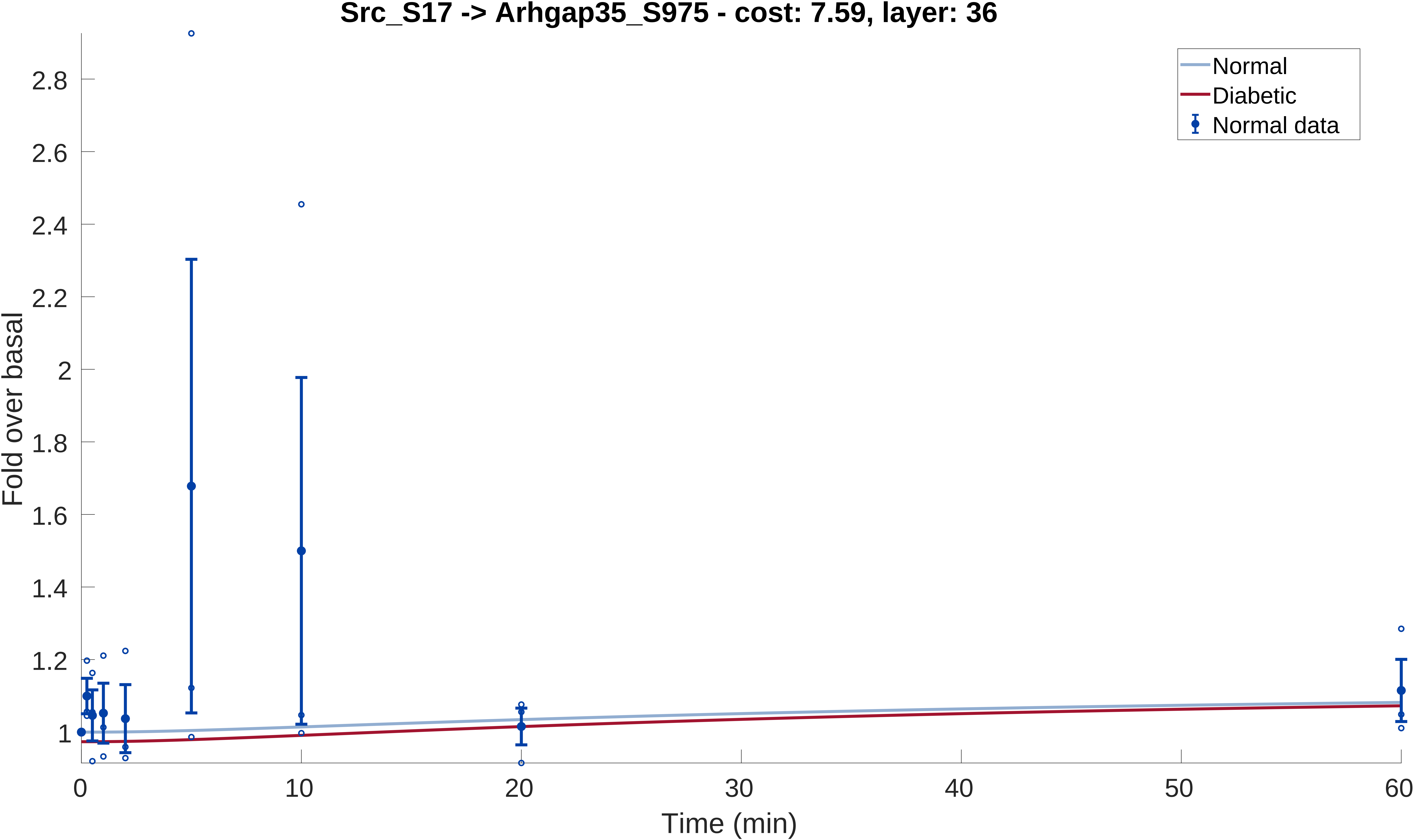

Src_S17 -> Arhgap35_S975 - cost: 7.59, layer: 36
Normal
Diabetic
Normal data
Fold over basal
Time (min)
2.8
2.6
2.4
2.2
2
1.8
1.6
1.4
1.2
1
0
10
20
30
40
50
60

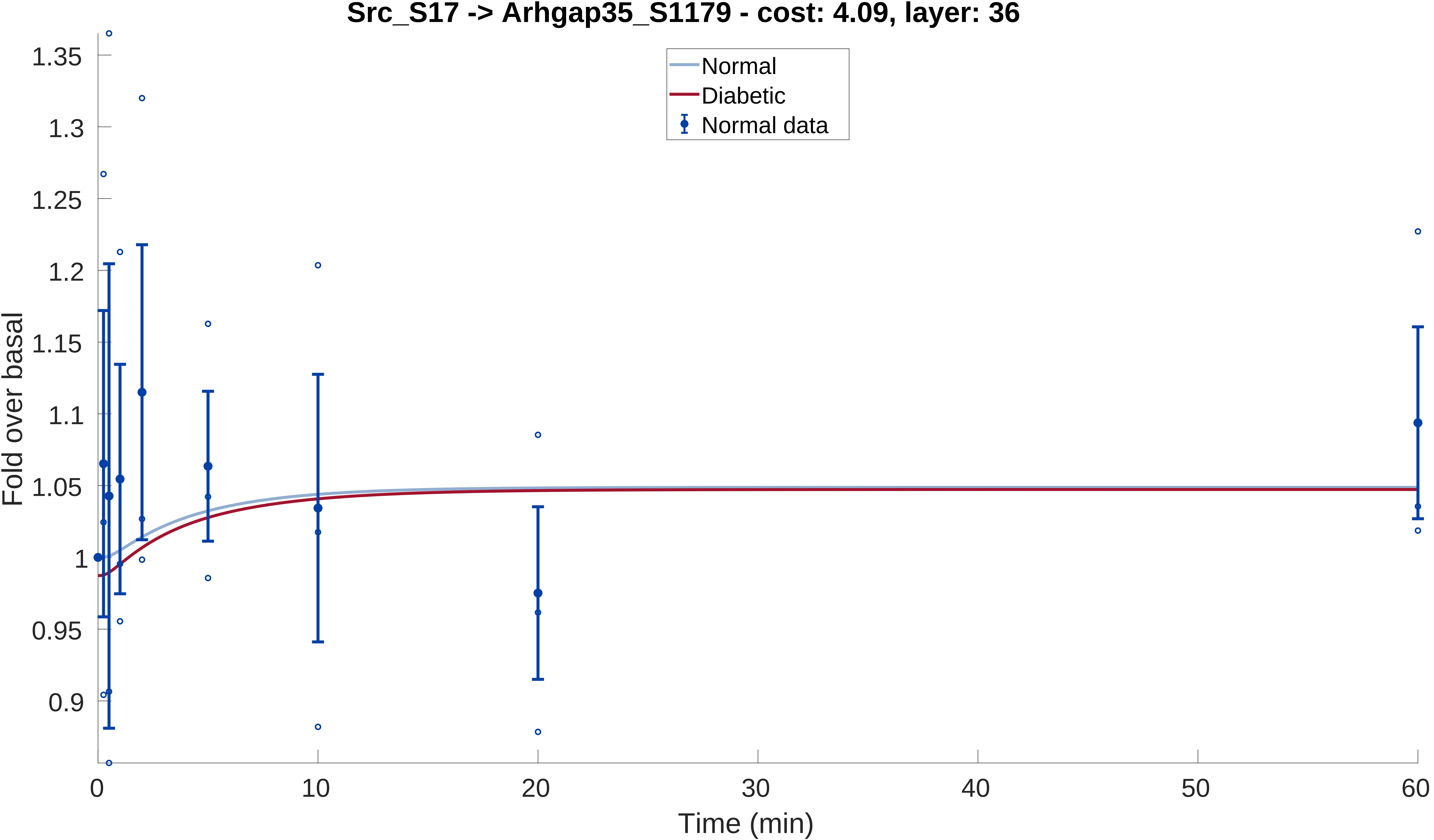

Src_S17 -> Arhgap35_S1179 - cost: 4.09, layer: 36
Normal
Diabetic
Normal data
Fold over basal
1.35
1.3
1.25
1.2
1.15
1.1
1.05
1
0.95
0.9
0
10
20
30
40
50
60
Time (min)

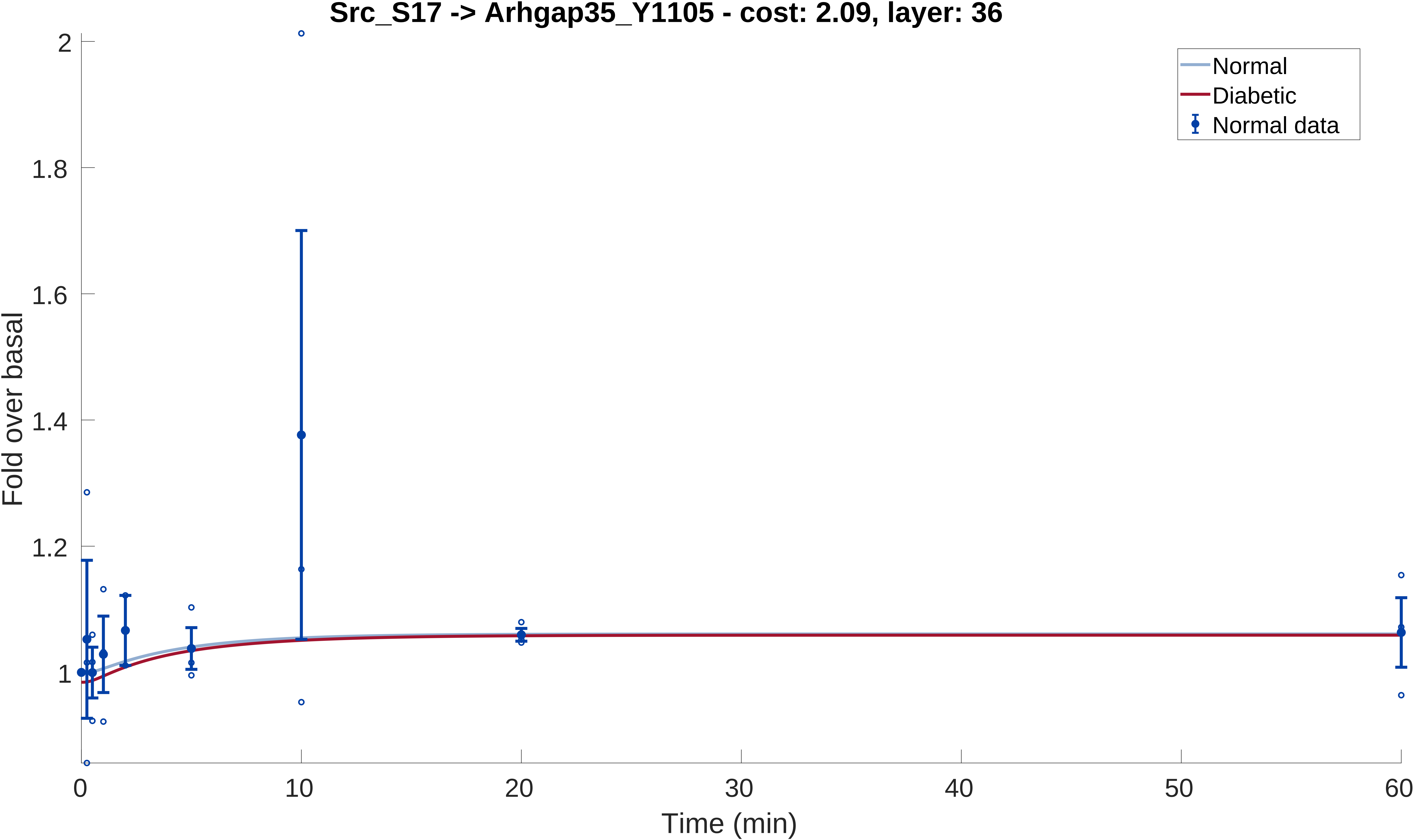
Src_S17 -> Arhgap35_Y1105 - cost: 2.09, layer: 36
Normal
Diabetic
Normal data
Fold over basal
2
1.8
1.6
1.4
1.2
1
0
10
20
30
40
50
60
Time (min)

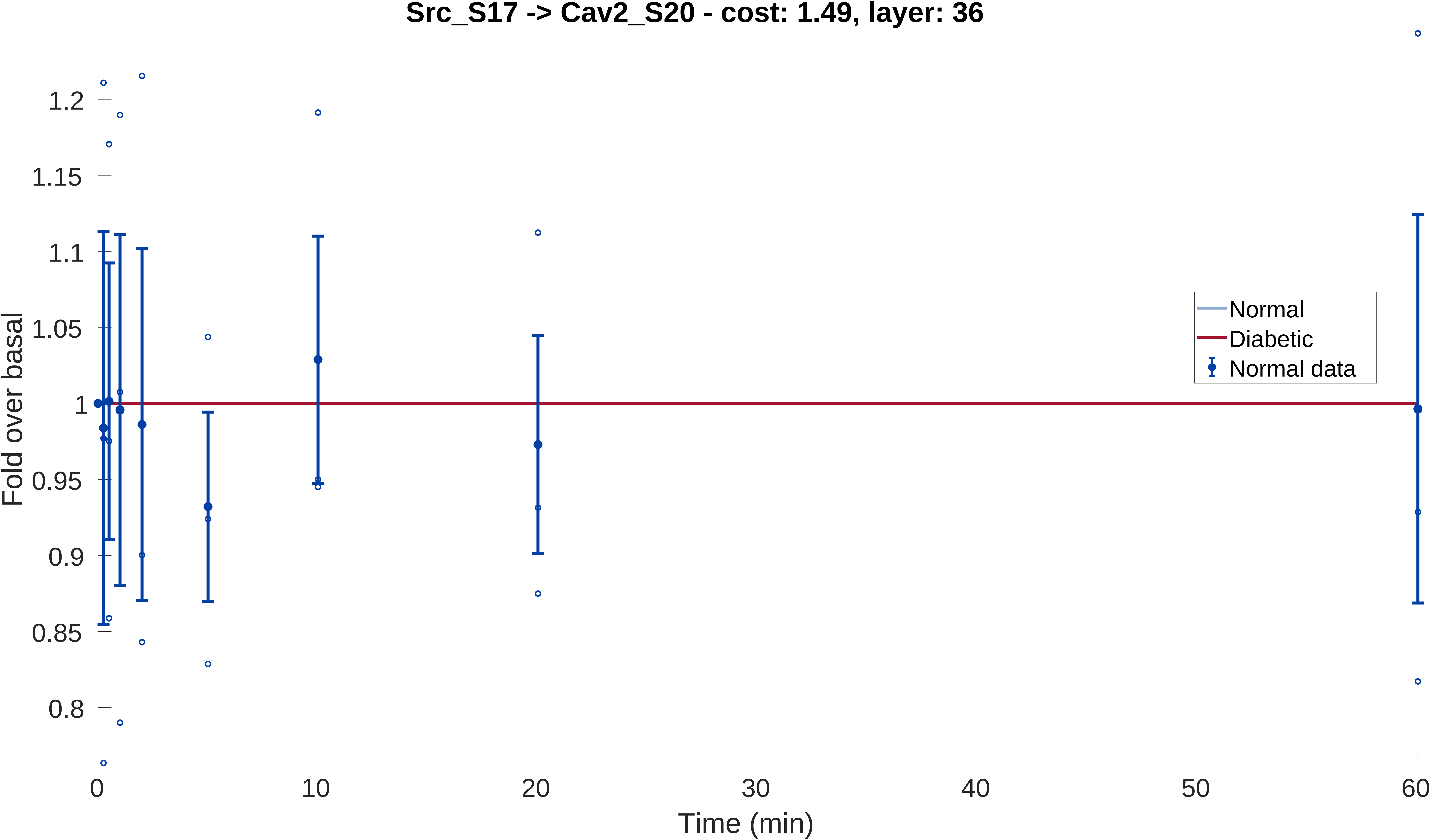
Src_S17 -> Cav2_S20 - cost: 1.49, layer: 36
Fold over basal
1.2
1.15
1.1
1.05
1
0.95
0.9
0.85
0.8
0
10
20
30
40
50
60
Time (min)
Normal
Diabetic
Normal data

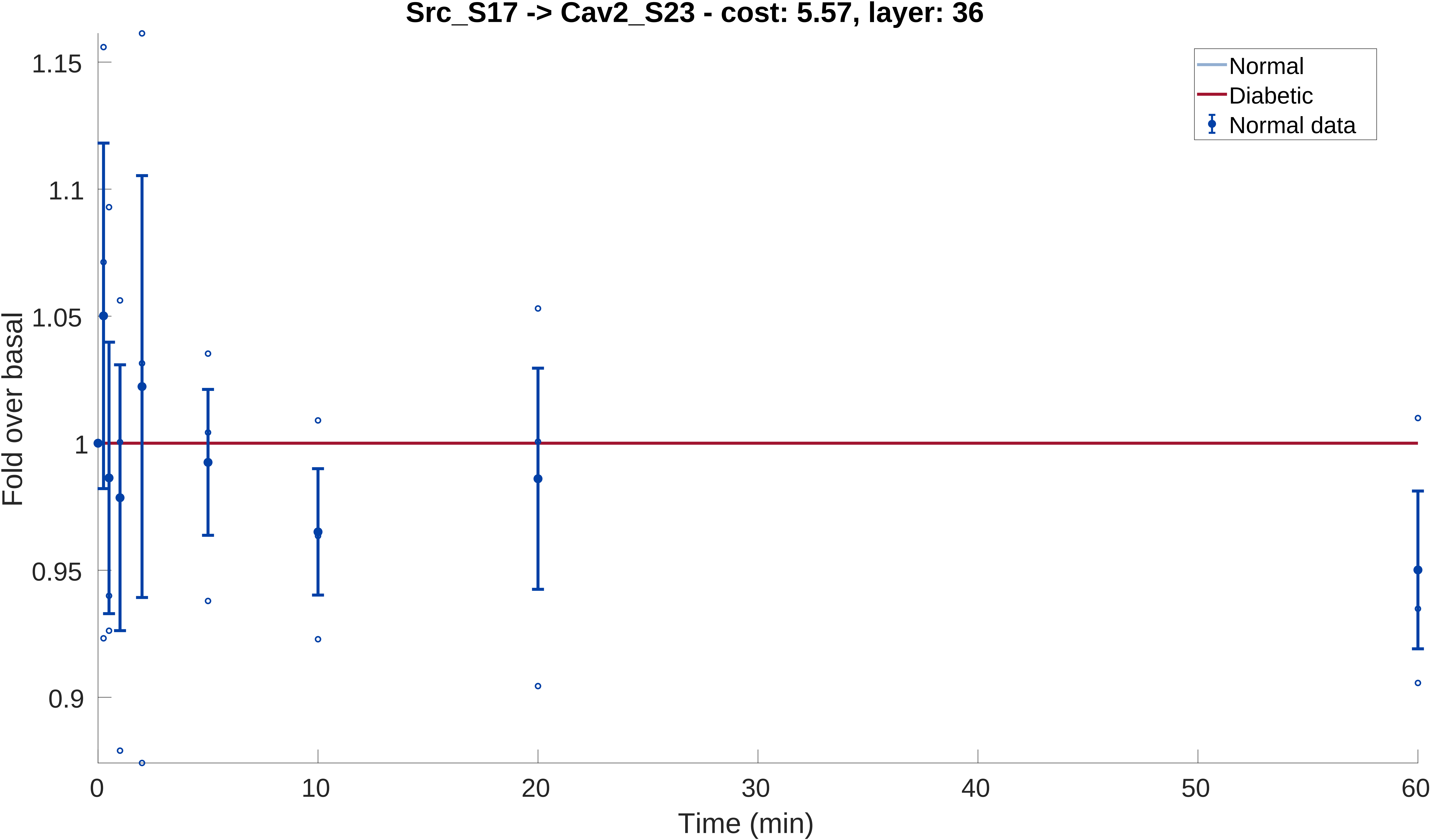

Src_S17 -> Cav2_S23 - cost: 5.57, layer: 36
Normal
Diabetic
Normal data
Fold over basal
1.15
1.1
1.05
1
0.95
0.9
0
10
20
30
40
50
60
Time (min)

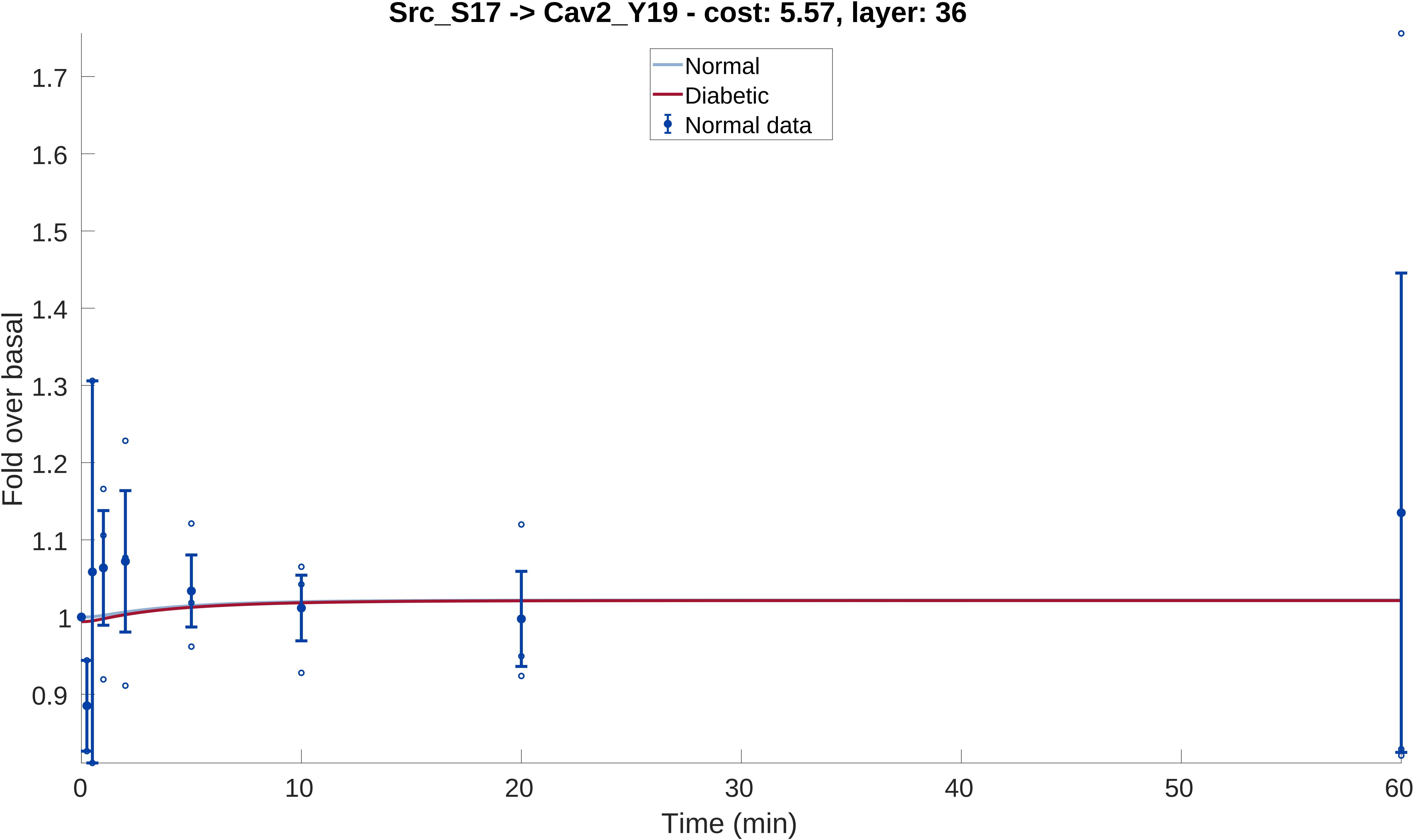
Src_S17 -> Cav2_Y19 - cost: 5.57, layer: 36
Normal
Diabetic
Normal data
Fold over basal
1.7
1.6
1.5
1.4
1.3
1.2
1.1
1
0.9
0
10
20
30
40
50
60
Time (min)

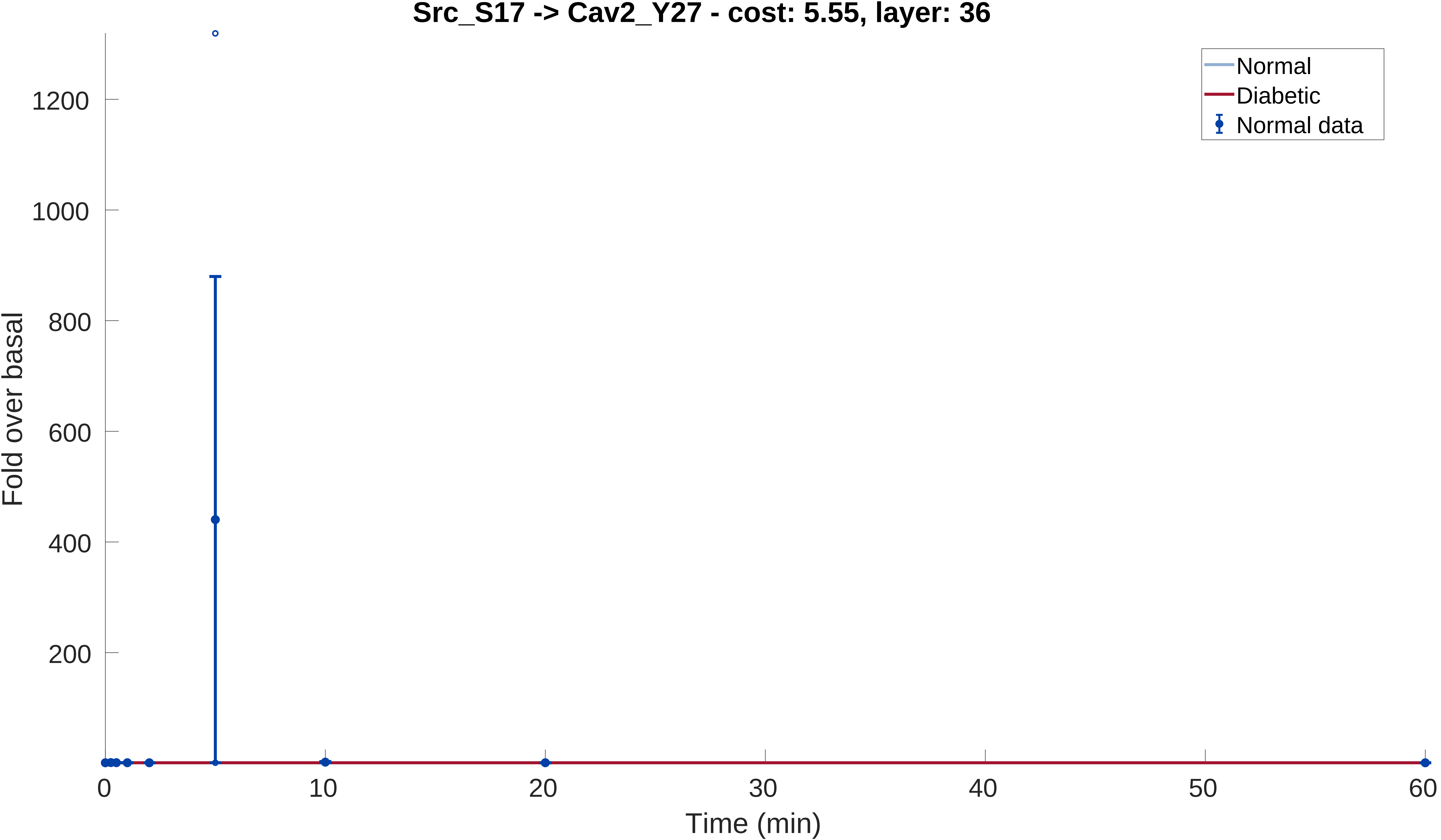
Src_S17 -> Cav2_Y27 - cost: 5.55, layer: 36
Normal
Diabetic
Normal data
Fold over basal
Time (min)
1200
1000
800
600
400
200
0
10
20
30
40
50
60

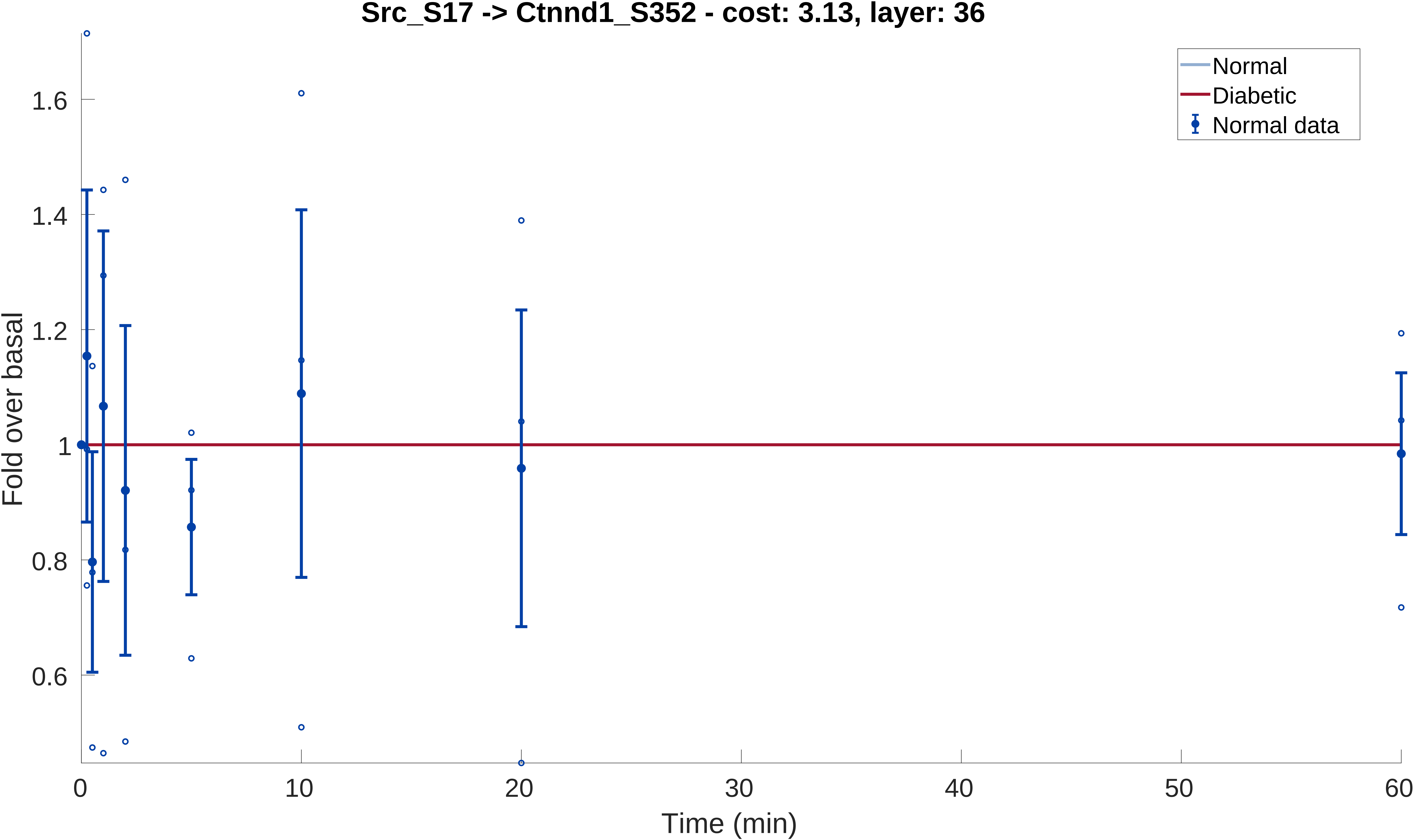

Src_S17 -> Ctnnd1_S352 - cost: 3.13, layer: 36
Normal
Diabetic
Normal data
Fold over basal
1.6
1.4
1.2
1
0.8
0.6
0
10
20
30
40
50
60
Time (min)

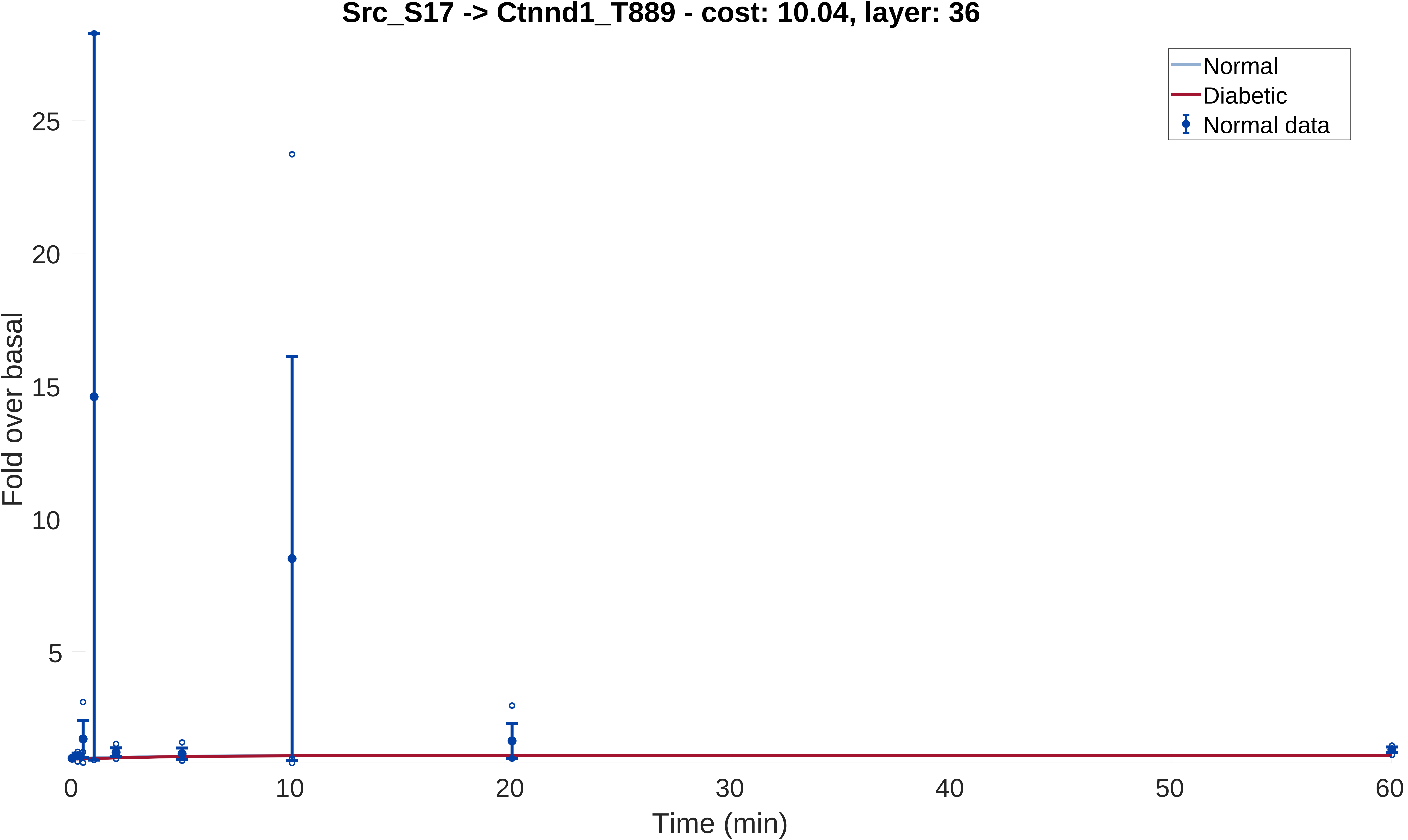

Src_S17 -> Ctnnd1_T889 - cost: 10.04, layer: 36
Normal
Diabetic
Normal data
Fold over basal
Time (min)
0
5
10
15
20
25
0
10
20
30
40
50
60

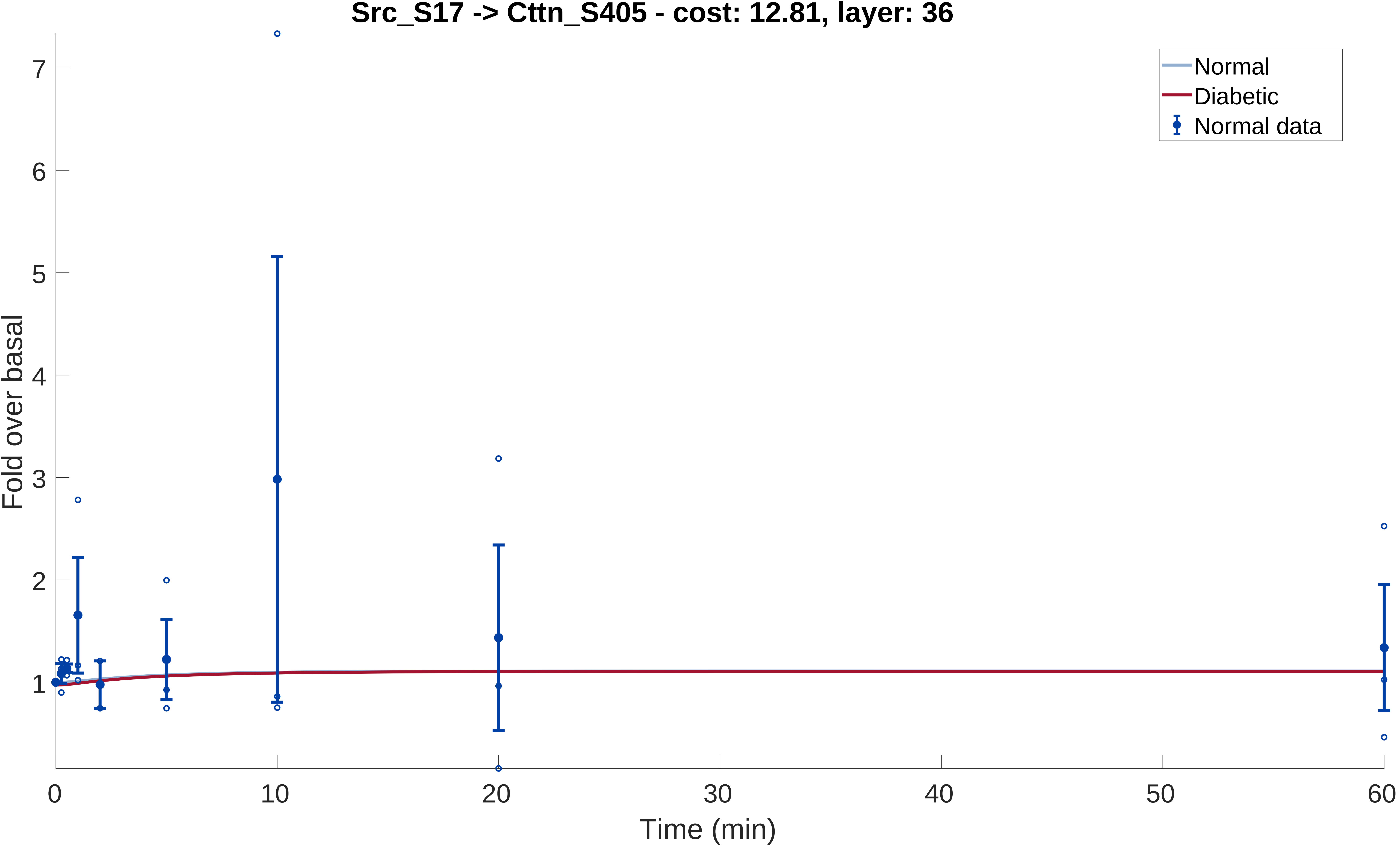

Src_S17 -> Cttn_S405 - cost: 12.81, layer: 36
Normal
Diabetic
Normal data
Fold over basal
Time (min)
1
2
3
4
5
6
7
0
10
20
30
40
50
60

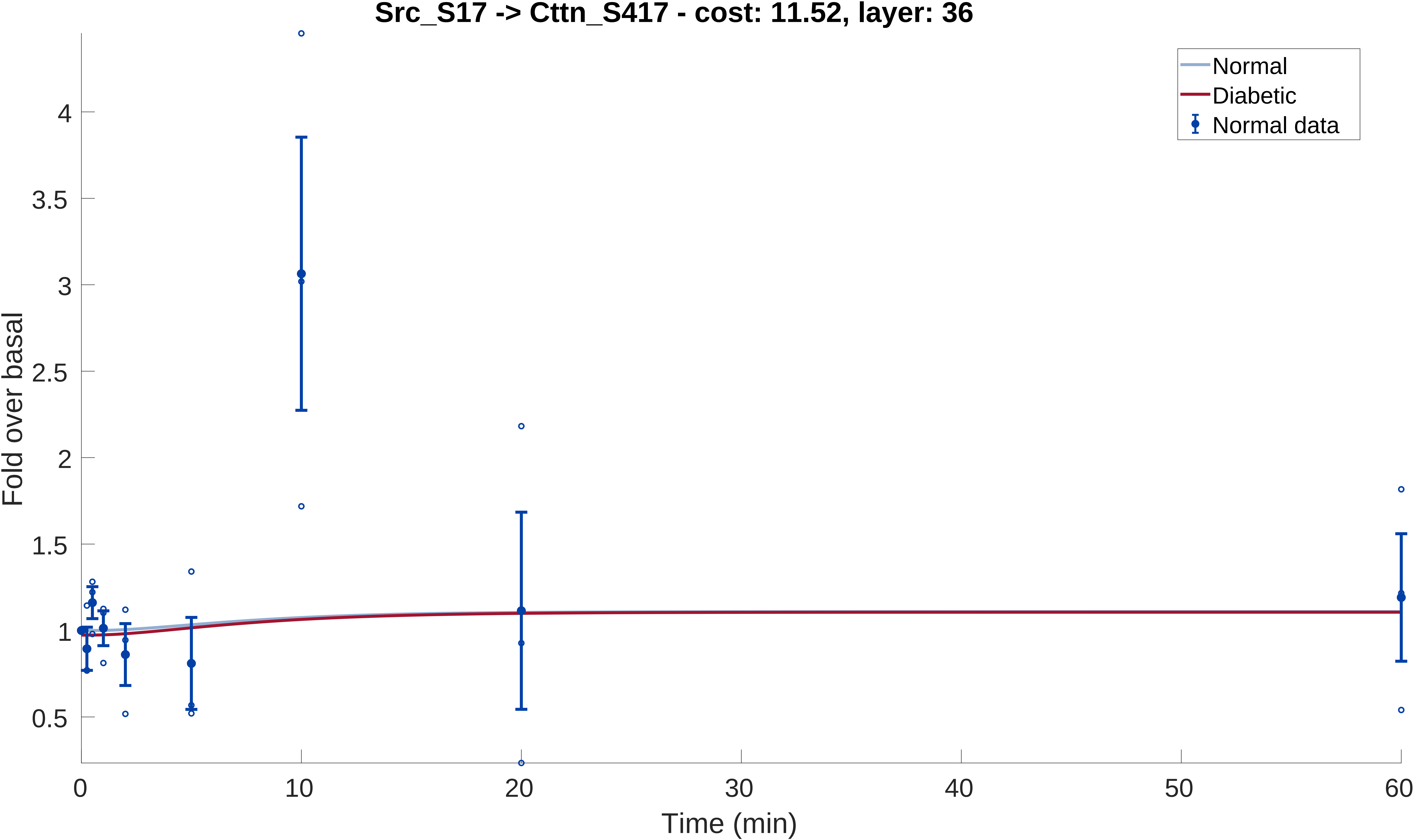

Src_S17 -> Cttn_S417 - cost: 11.52, layer: 36
Normal
Diabetic
Normal data
Fold over basal
4
3.5
3
2.5
2
1.5
1
0.5
0
10
20
30
40
50
60
Time (min)

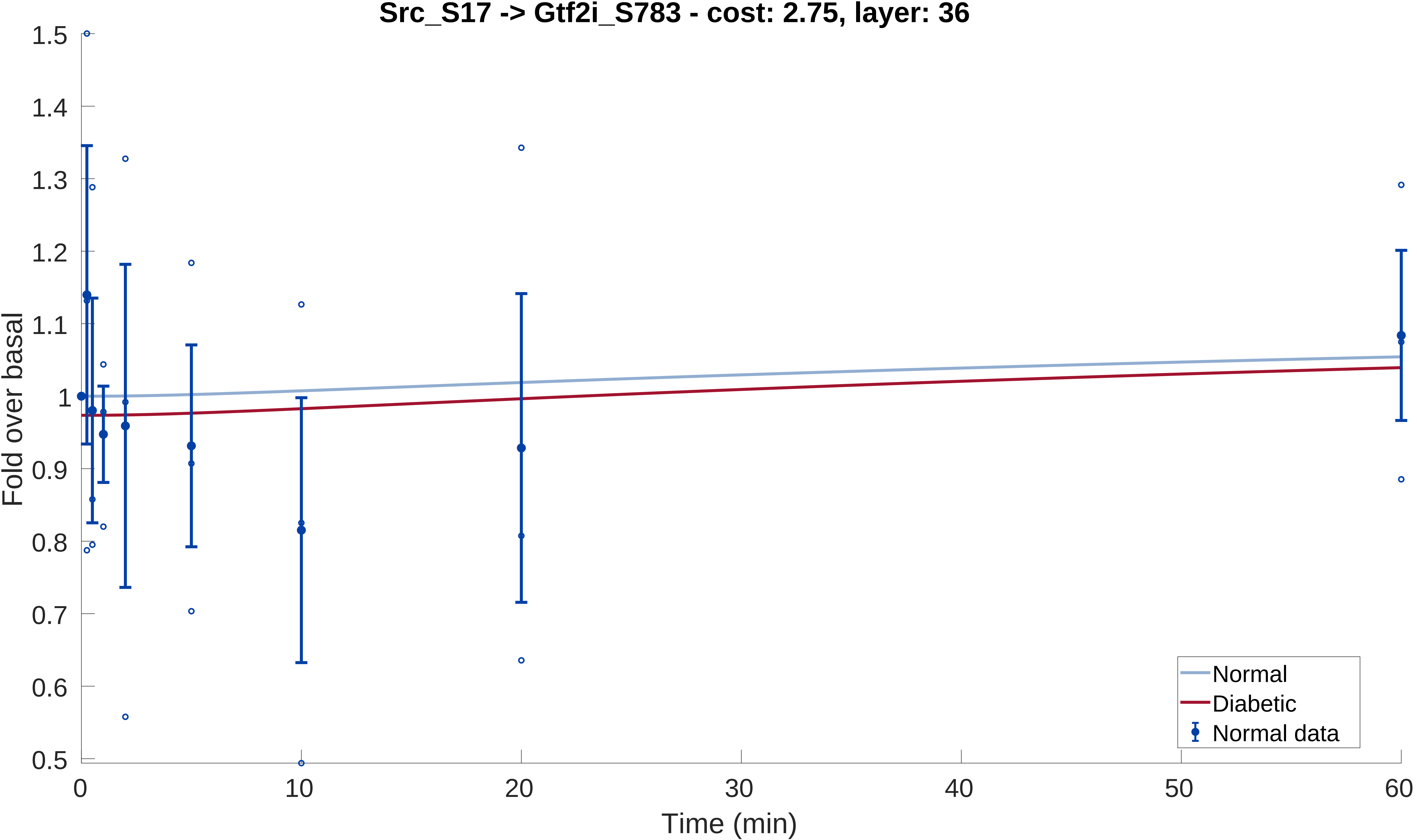
Src_S17 -> Gtf2i_S783 - cost: 2.75, layer: 36
Fold over basal
Time (min)
1.5
1.4
1.3
1.2
1.1
1
0.9
0.8
0.7
0.6
0.5
0
10
20
30
40
50
60
Normal
Diabetic
Normal data

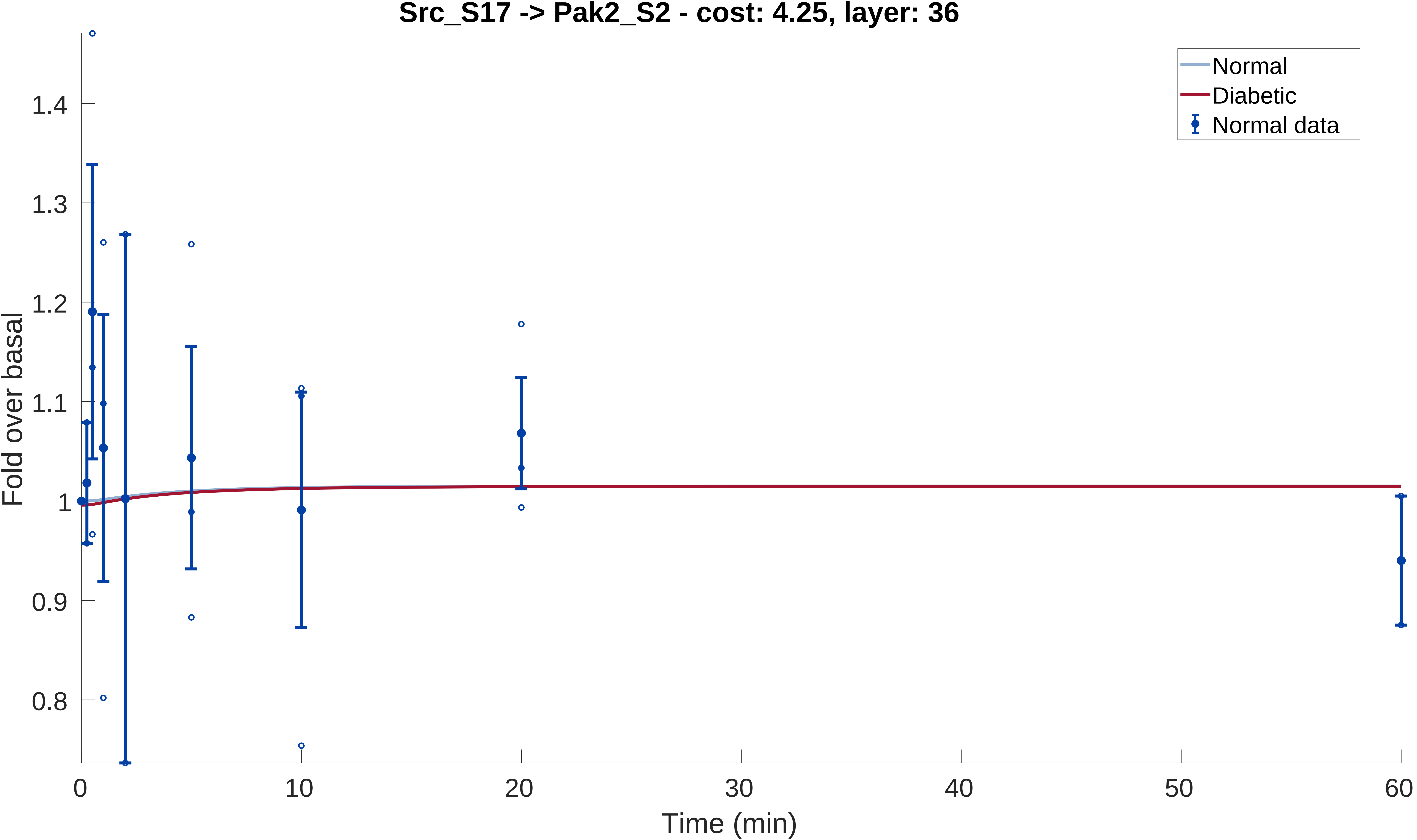
Src_S17 -> Pak2_S2 - cost: 4.25, layer: 36
Normal
Diabetic
Normal data
Fold over basal
1.4
1.3
1.2
1.1
1
0.9
0.8
0
10
20
30
40
50
60
Time (min)

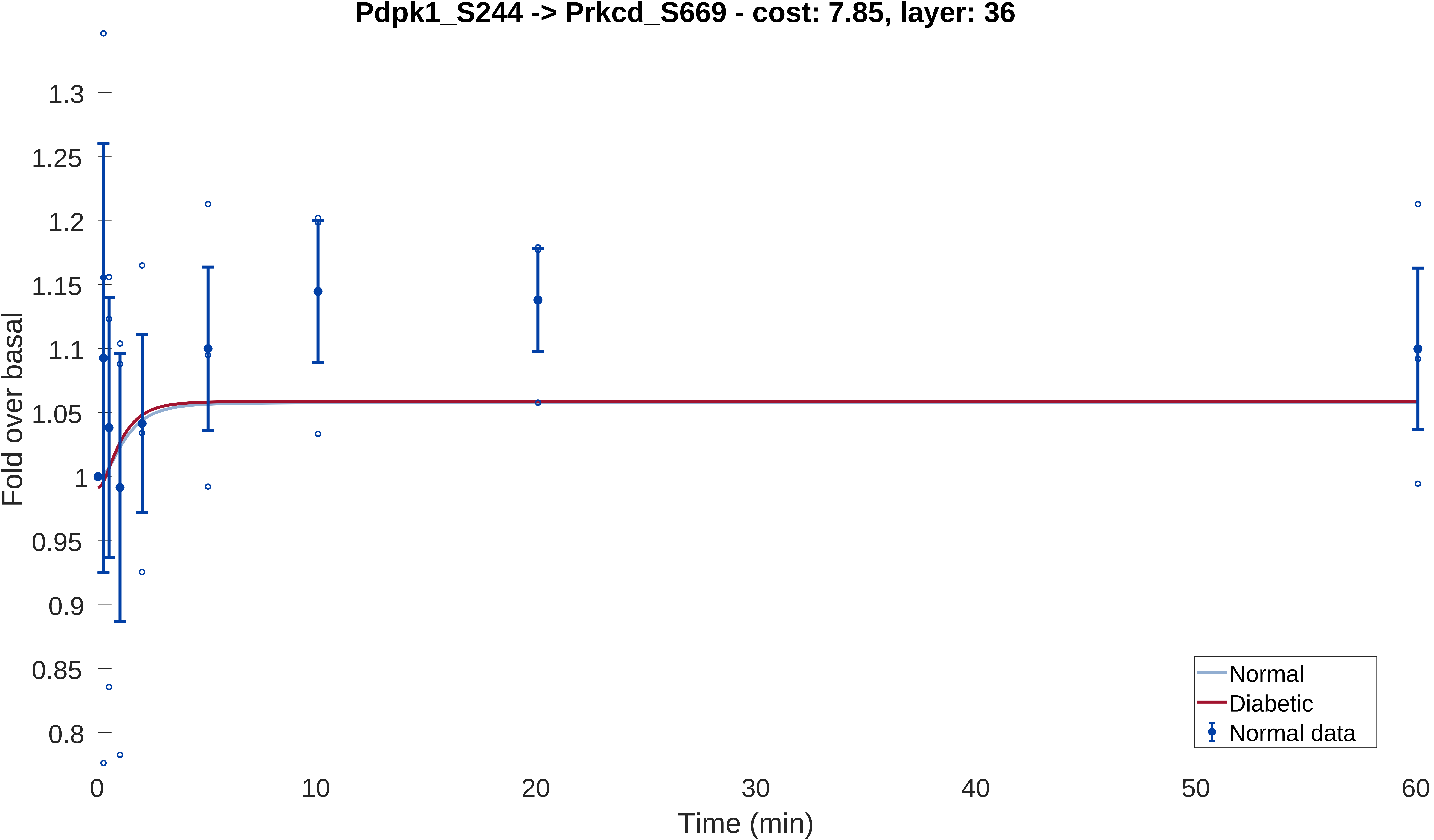
Pdpk1_S244 -> Prkcd_S669 - cost: 7.85, layer: 36
Fold over basal
Time (min)
1.3
1.25
1.2
1.15
1.1
1.05
1
0.95
0.9
0.85
0.8
0
10
20
30
40
50
60
Normal
Diabetic
Normal data

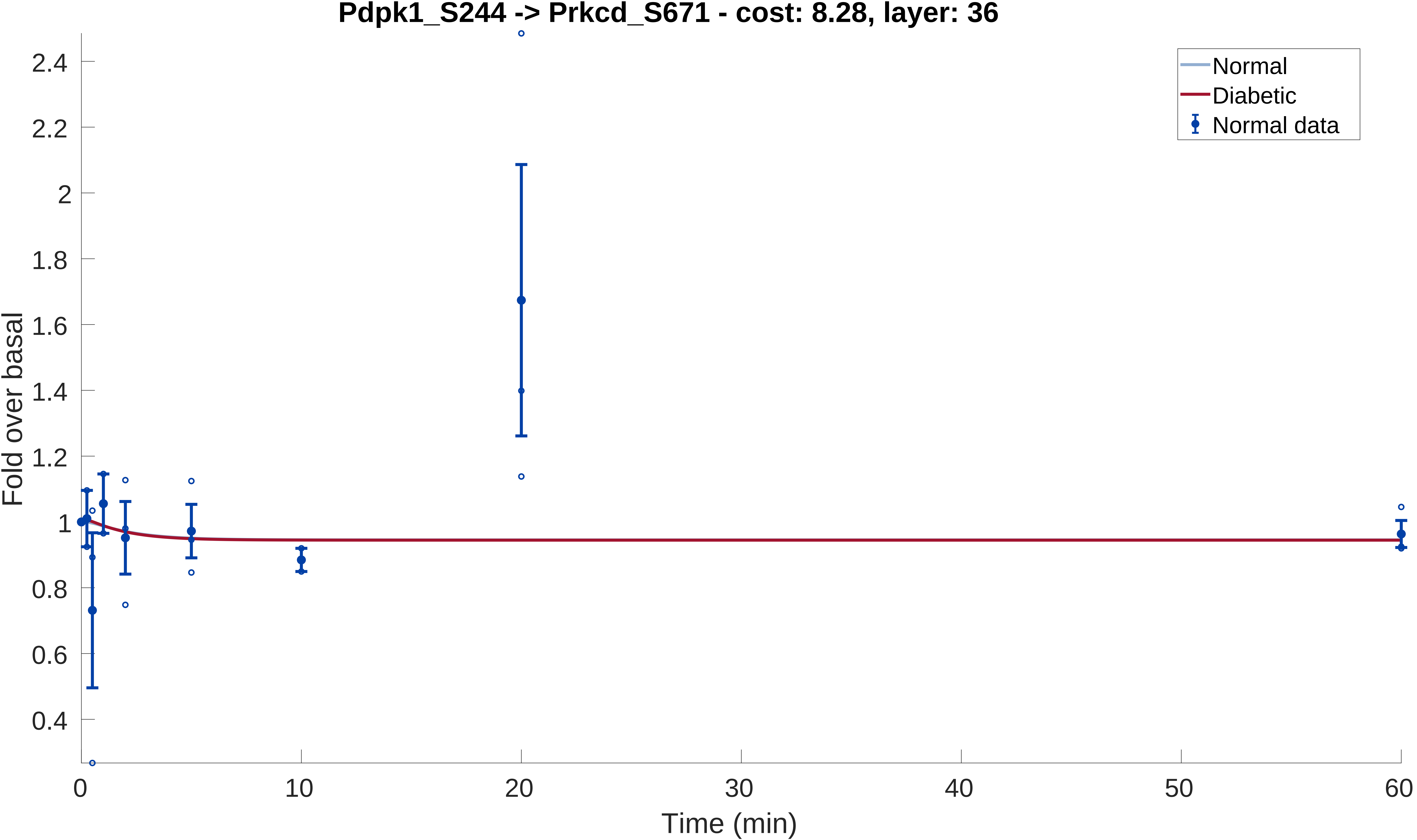

Pdpk1_S244 -> Prkcd_S671 - cost: 8.28, layer: 36
Normal
Diabetic
Normal data
Fold over basal
Time (min)
2.4
2.2
2
1.8
1.6
1.4
1.2
1
0.8
0.6
0.4
0
10
20
30
40
50
60

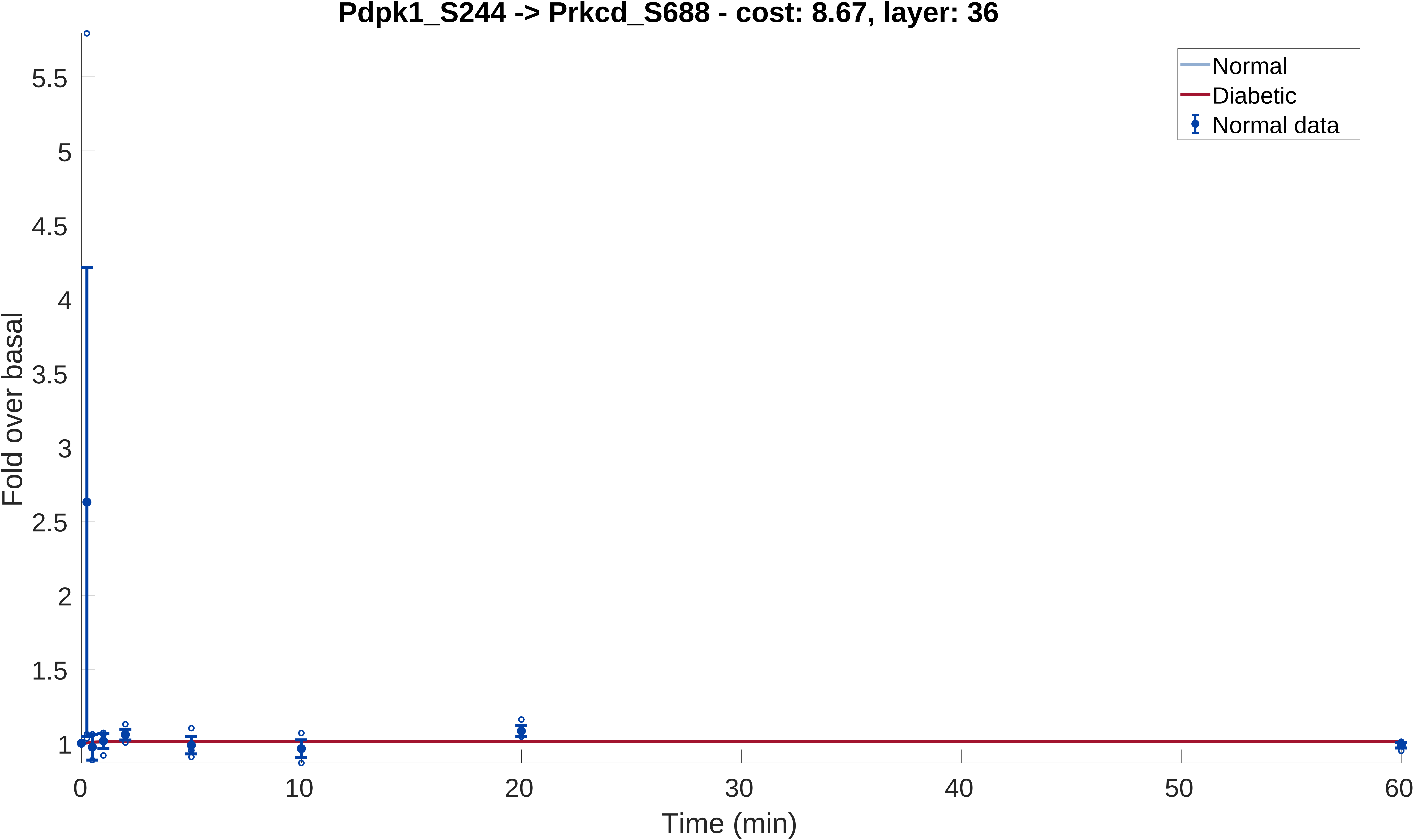
Pdpk1_S244 -> Prkcd_S688 - cost: 8.67, layer: 36
Normal
Diabetic
Normal data
Fold over basal
5.5
5
4.5
4
3.5
3
2.5
2
1.5
1
0
10
20
30
40
50
60
Time (min)

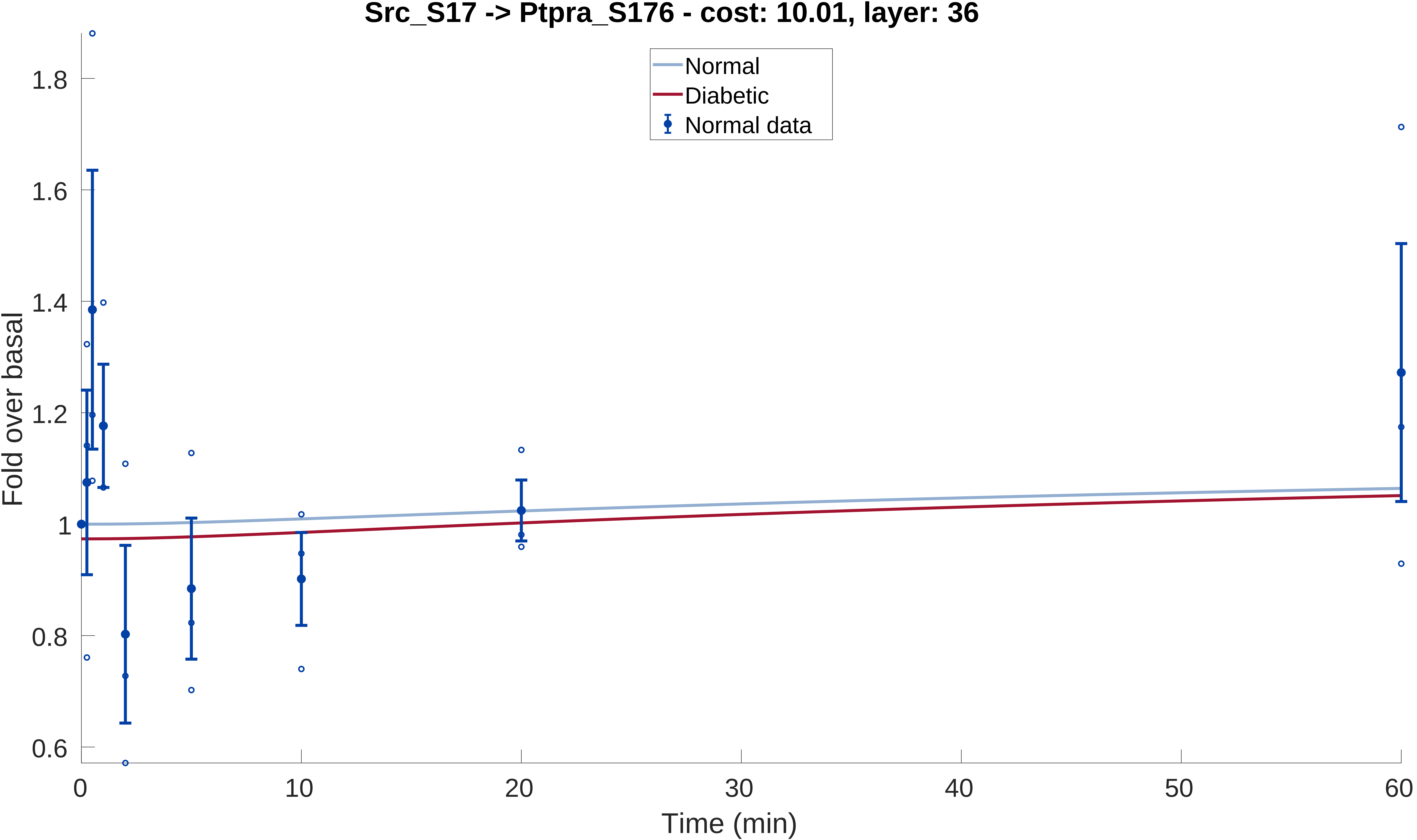

Src_S17 -> Ptpra_S176 - cost: 10.01, layer: 36
Normal
Diabetic
Normal data
Fold over basal
1.8
1.6
1.4
1.2
1
0.8
0.6
0
10
20
30
40
50
60
Time (min)

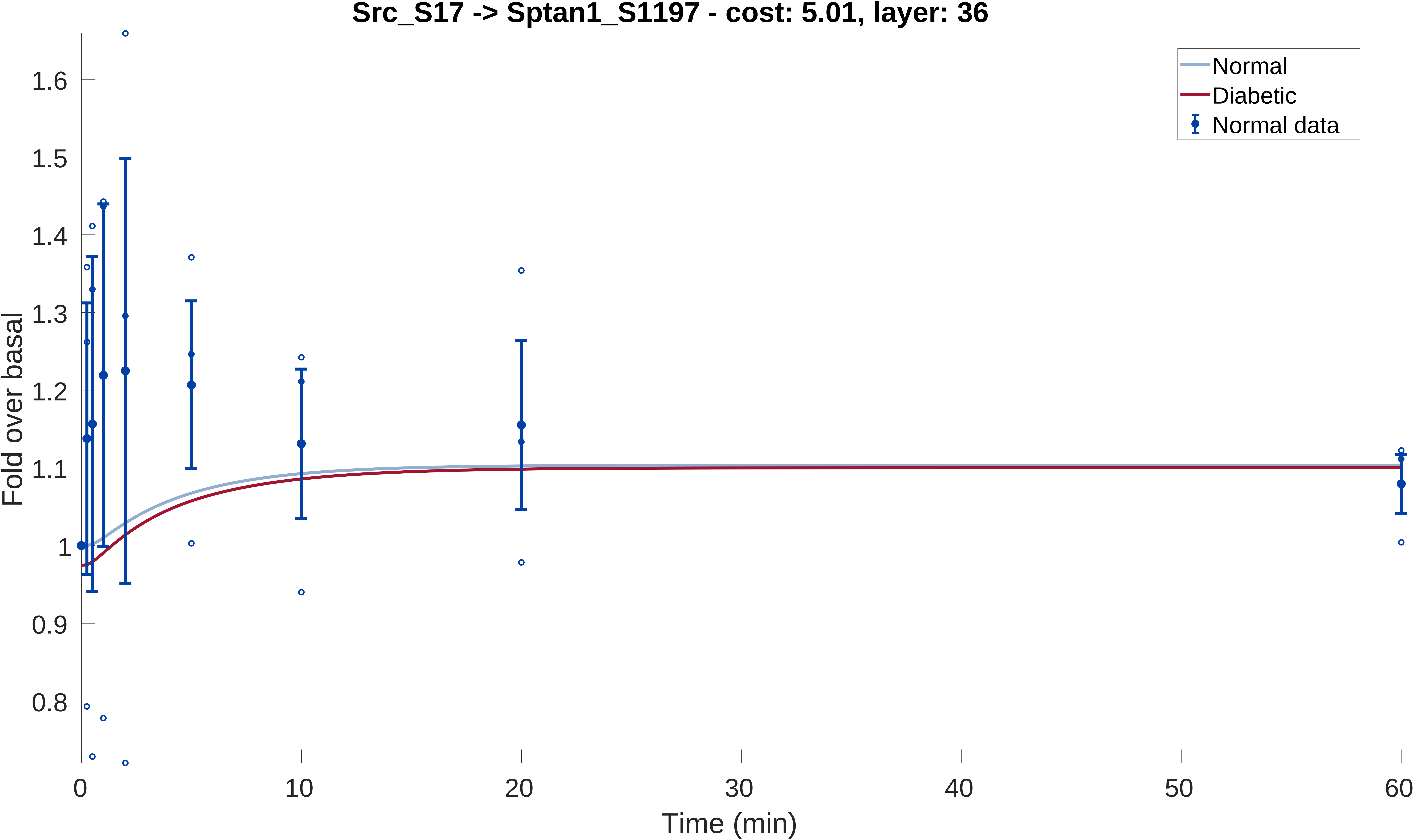

Src_S17 -> Sptan1_S1197 - cost: 5.01, layer: 36
Normal
Diabetic
Normal data
Fold over basal
Time (min)
0.8
0.9
1
1.1
1.2
1.3
1.4
1.5
1.6
0
10
20
30
40
50
60

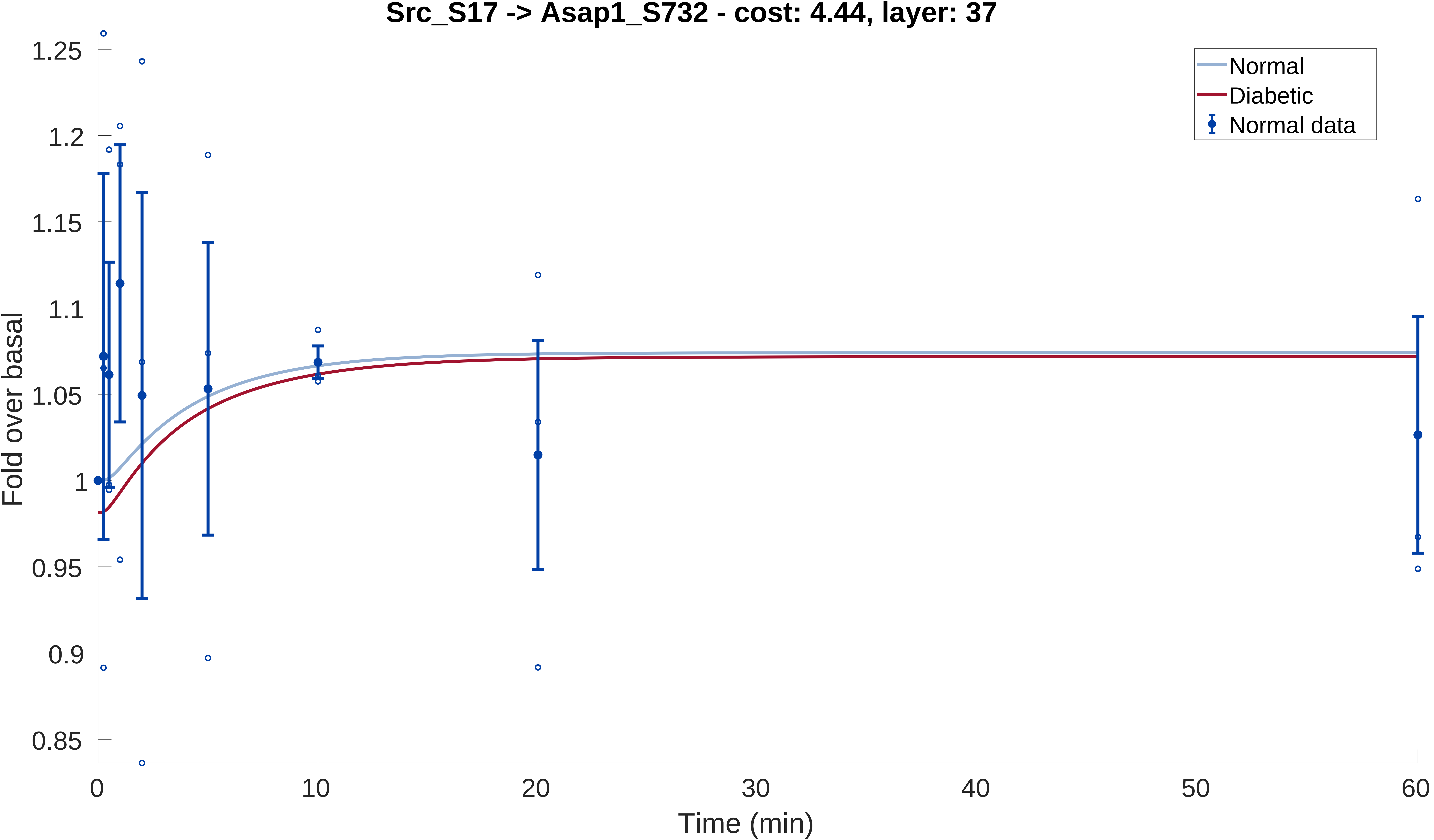

Src_S17 -> Asap1_S732 - cost: 4.44, layer: 37
Normal
Diabetic
Normal data
Fold over basal
1.25
1.2
1.15
1.1
1.05
1
0.95
0.9
0.85
0
10
20
30
40
50
60
Time (min)

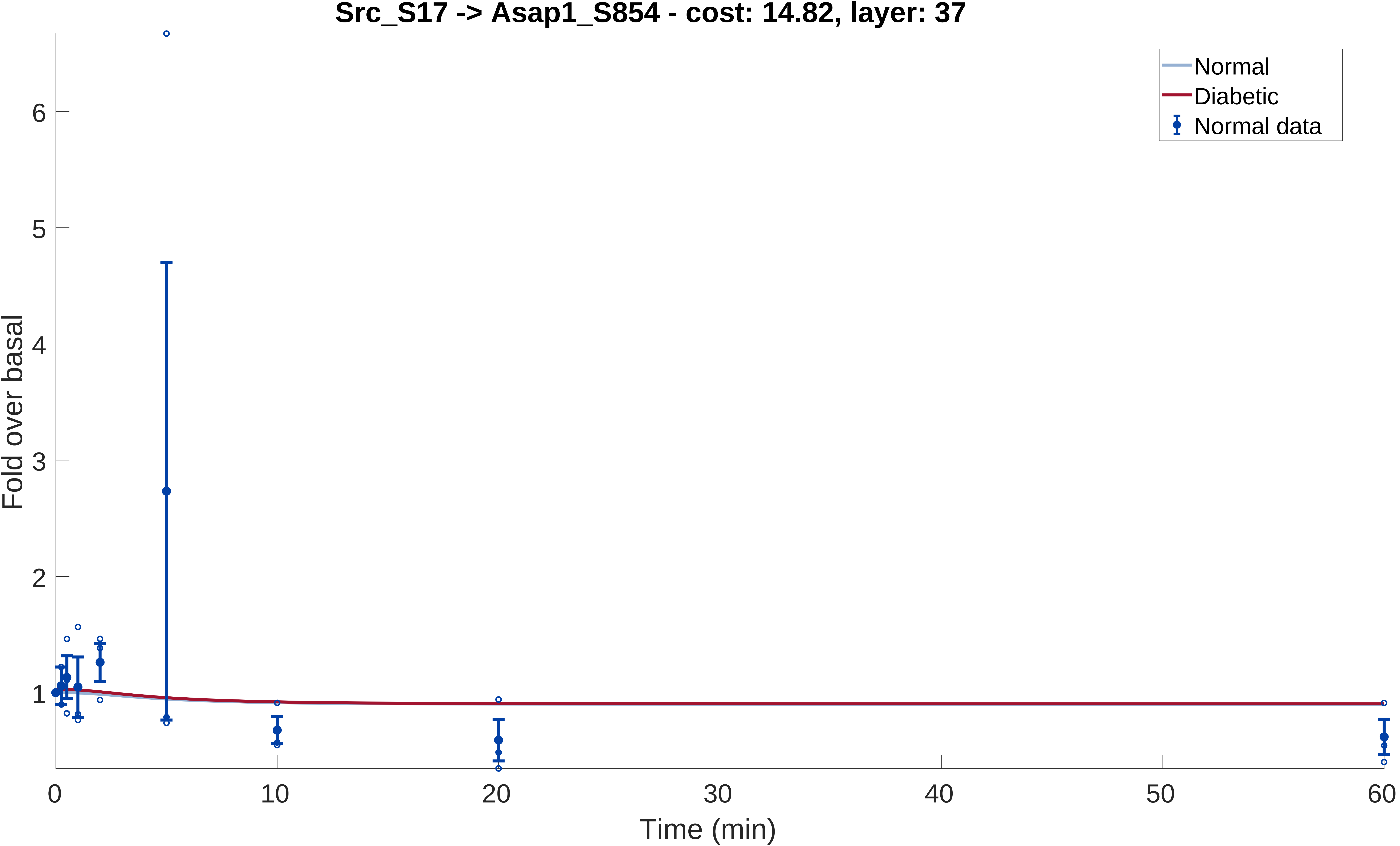

Src_S17 -> Asap1_S854 - cost: 14.82, layer: 37
Normal
Diabetic
Normal data
Fold over basal
Time (min)
0
10
20
30
40
50
60
1
2
3
4
5
6

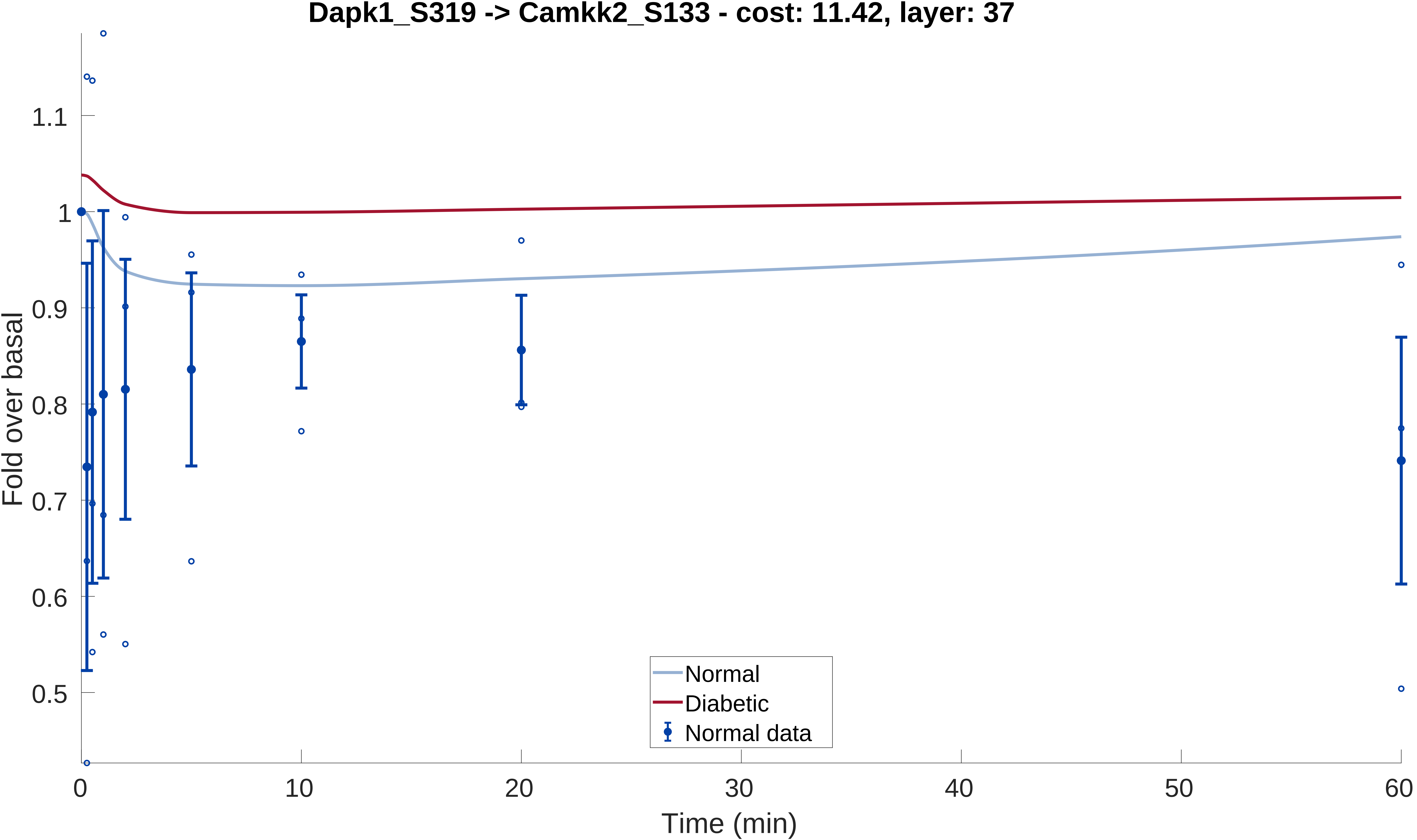

Dapk1_S319 -> Camkk2_S133 - cost: 11.42, layer: 37
Fold over basal
1.1
1
0.9
0.8
0.7
0.6
0.5
0
10
20
30
40
50
60
Time (min)
Normal
Diabetic
Normal data

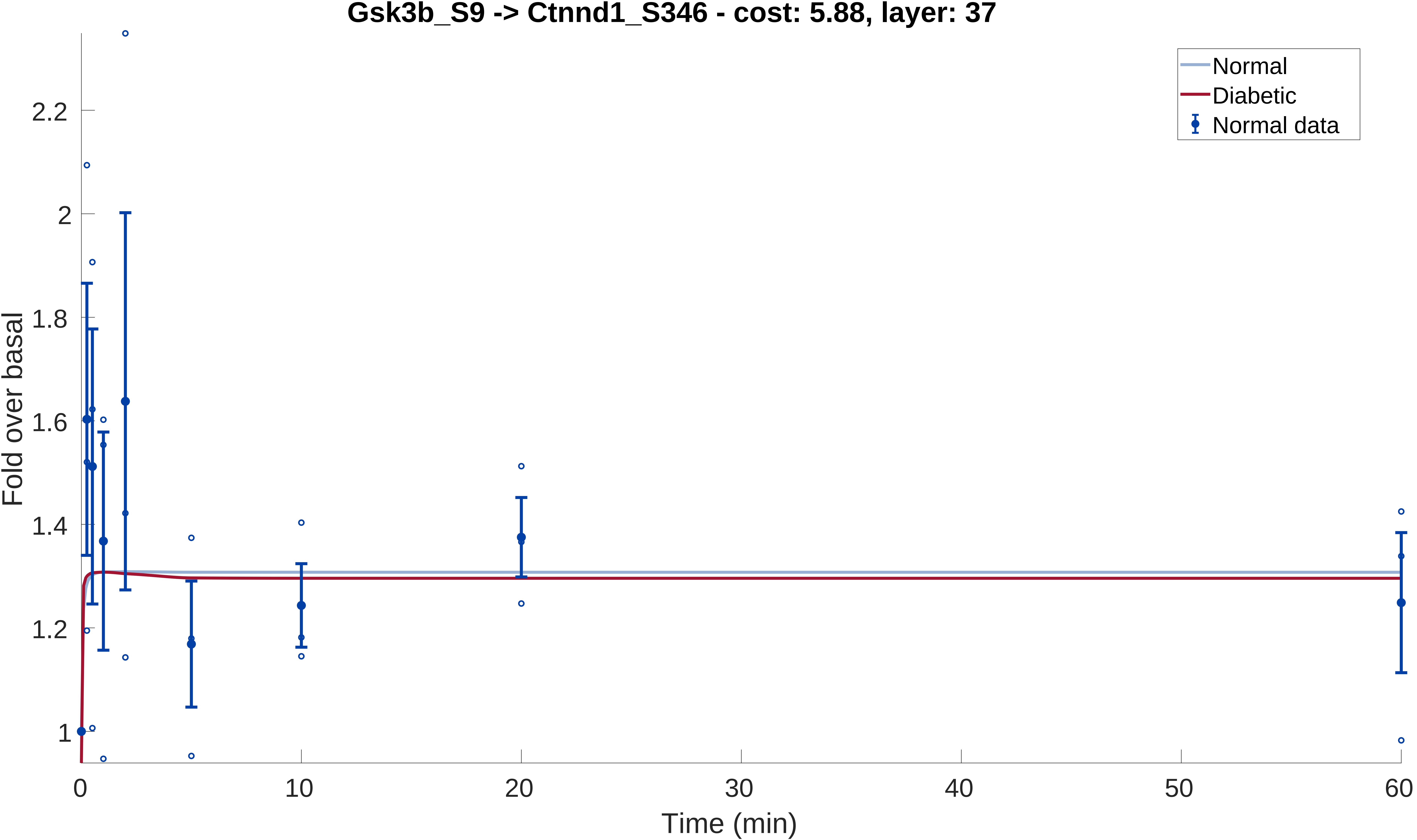
Gsk3b_S9 -> Ctnnd1_S346 - cost: 5.88, layer: 37
Normal
Diabetic
Normal data
Fold over basal
Time (min)
2.2
2
1.8
1.6
1.4
1.2
1
0
10
20
30
40
50
60

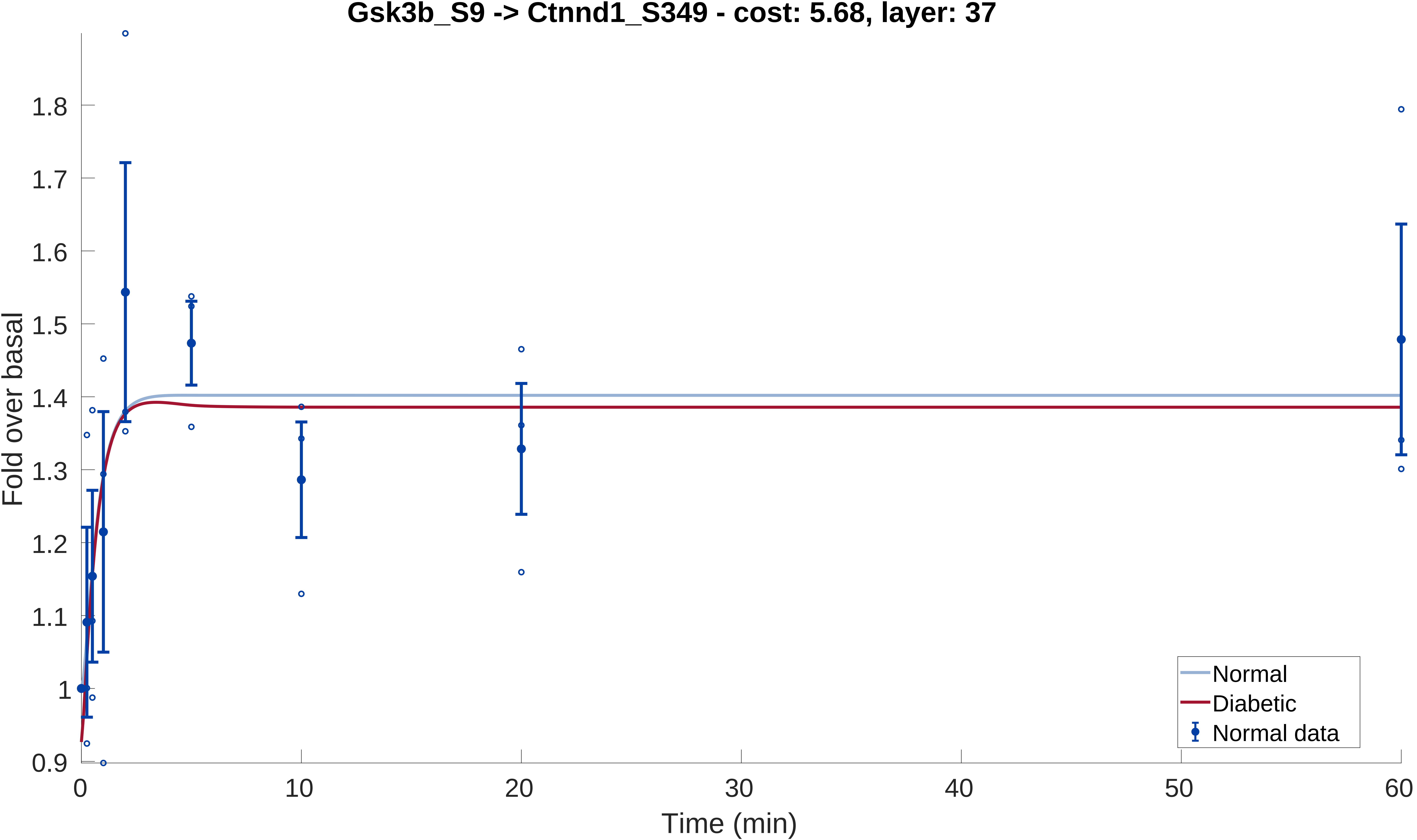

Gsk3b_S9 -> Ctnnd1_S349 - cost: 5.68, layer: 37
Fold over basal
1.8
1.7
1.6
1.5
1.4
1.3
1.2
1.1
1
0.9
0
10
20
30
40
50
60
Time (min)
Normal
Diabetic
Normal data

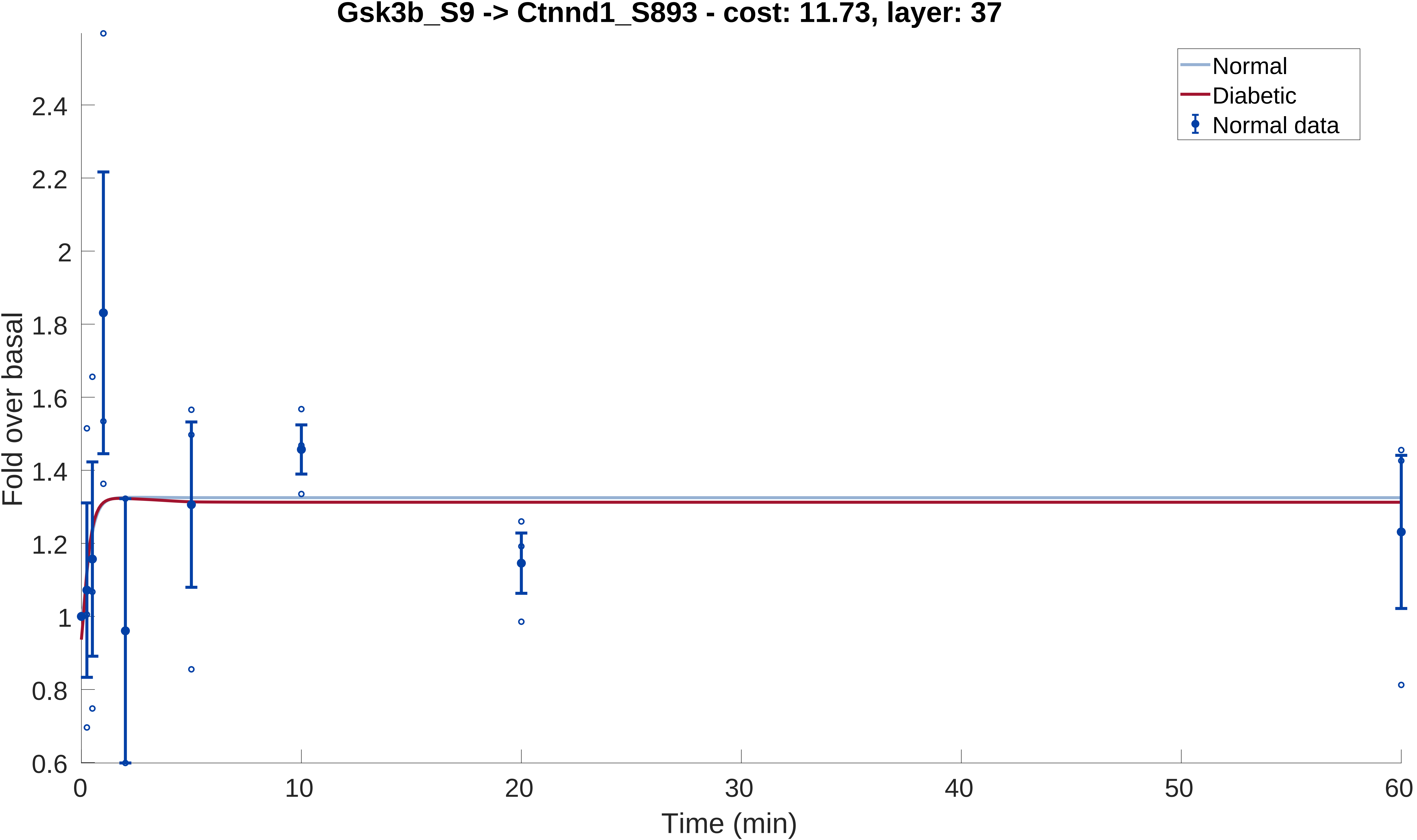

Gsk3b_S9 -> Ctnnd1_S893 - cost: 11.73, layer: 37
Normal
Diabetic
Normal data
Fold over basal
Time (min)
0.6
0.8
1
1.2
1.4
1.6
1.8
2
2.2
2.4
0
10
20
30
40
50
60

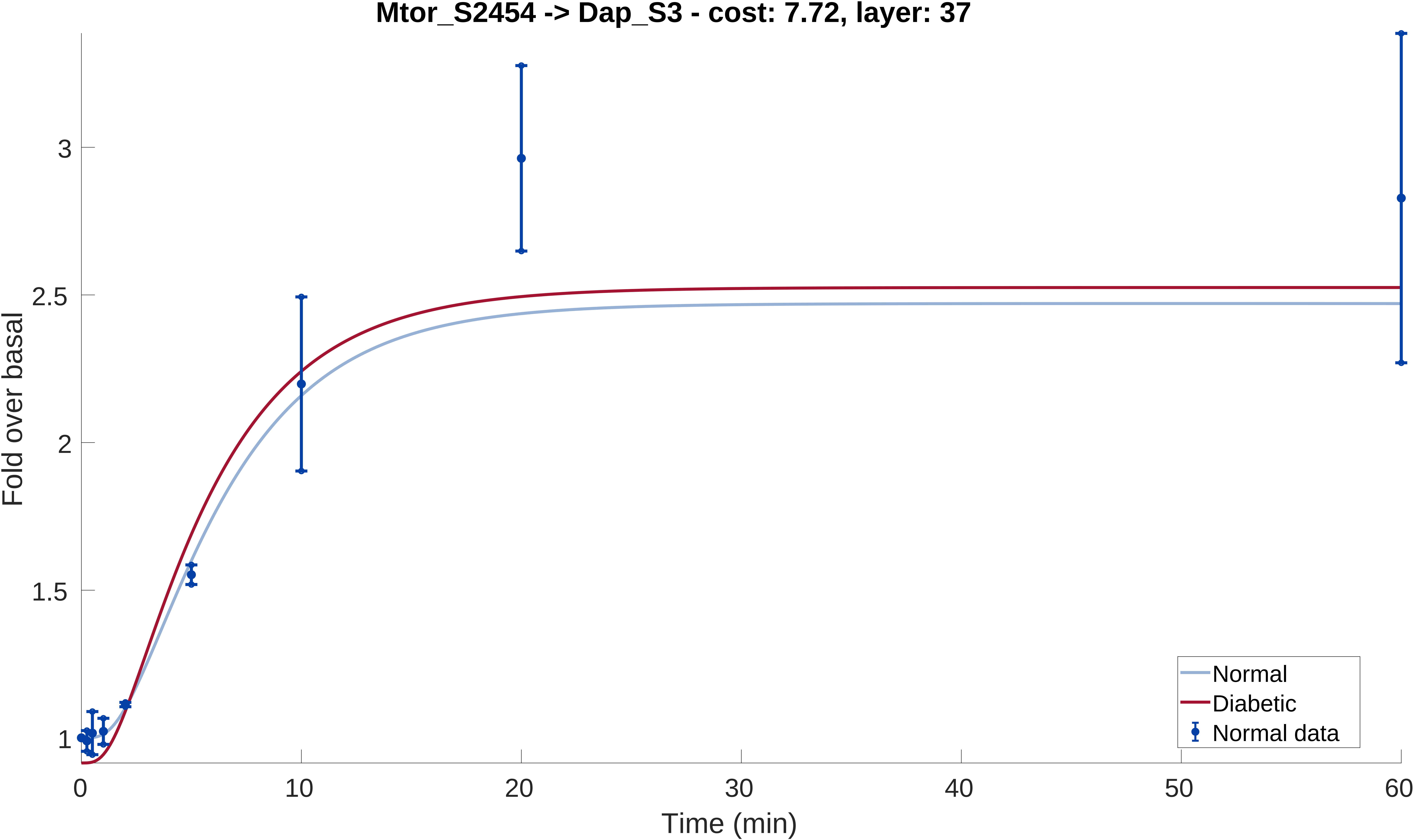

Mtor_S2454 -> Dap_S3 - cost: 7.72, layer: 37
Fold over basal
Time (min)
Normal
Diabetic
Normal data
0
10
20
30
40
50
60
1
1.5
2
2.5
3

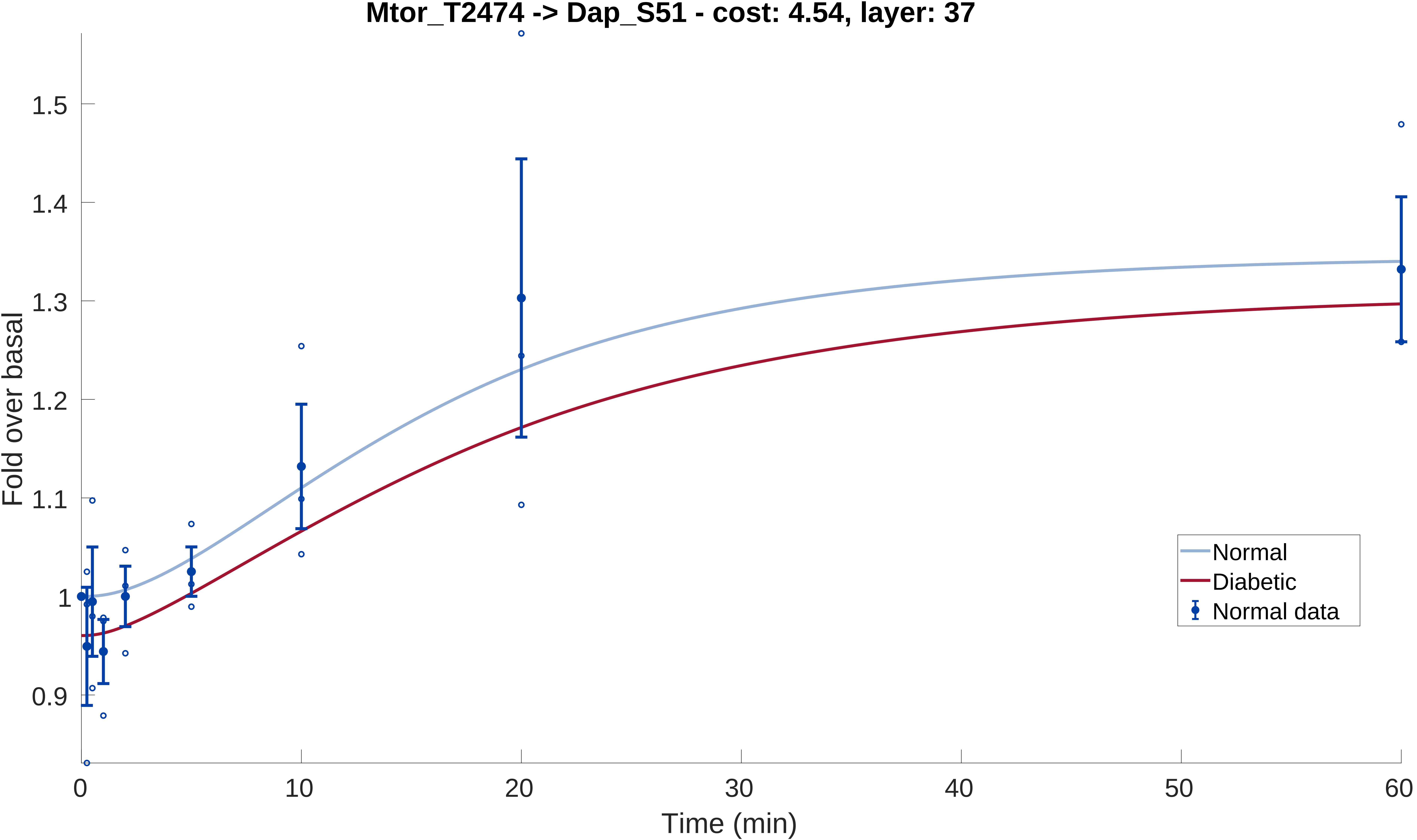
Mtor_T2474 -> Dap_S51 - cost: 4.54, layer: 37
Fold over basal
1.5
1.4
1.3
1.2
1.1
1
0.9
0
10
20
30
40
50
60
Time (min)
Normal
Diabetic
Normal data

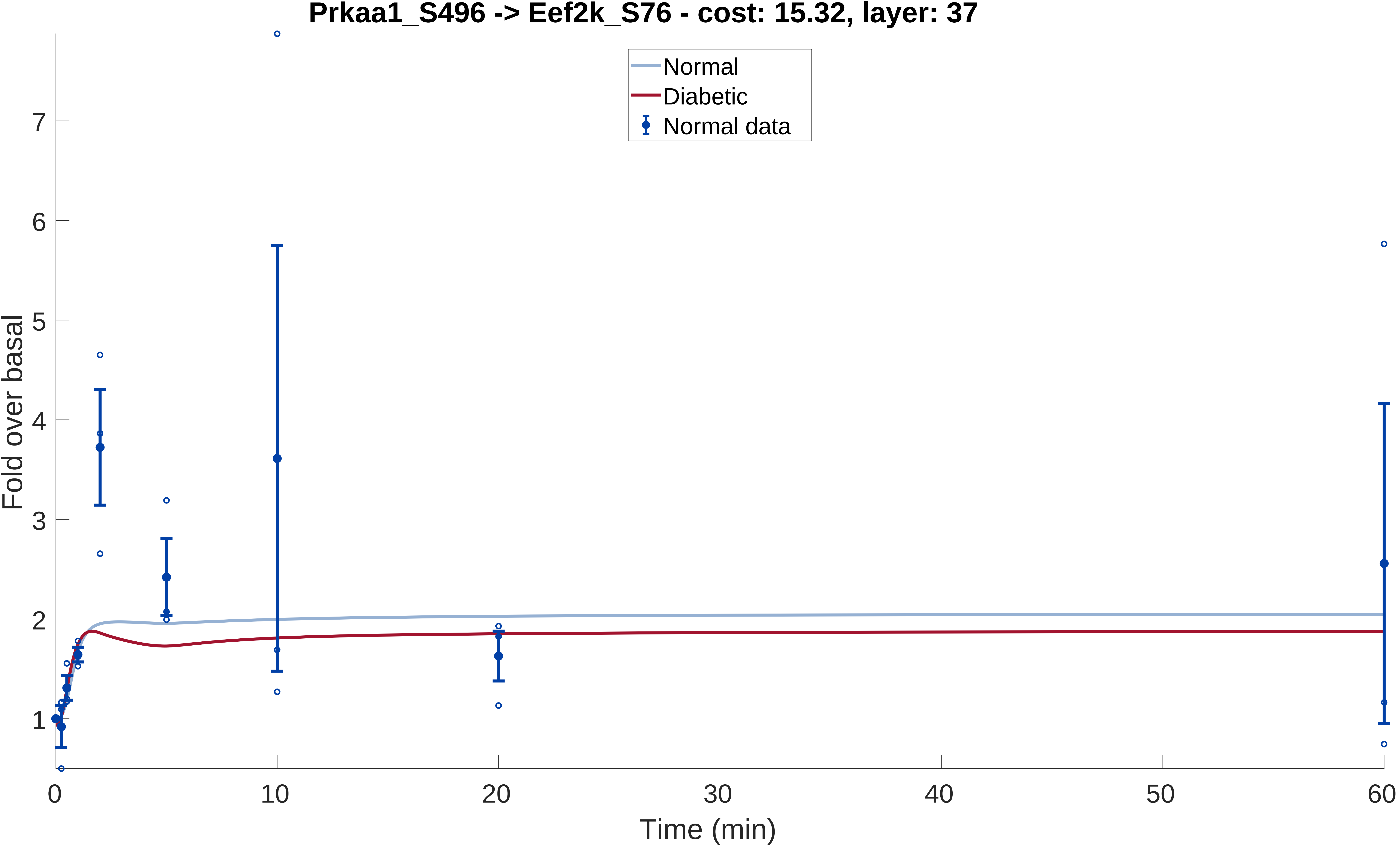

Prkaa1_S496 -> Eef2k_S76 - cost: 15.32, layer: 37
Normal
Diabetic
Normal data
Fold over basal
1
2
3
4
5
6
7
0
10
20
30
40
50
60
Time (min)

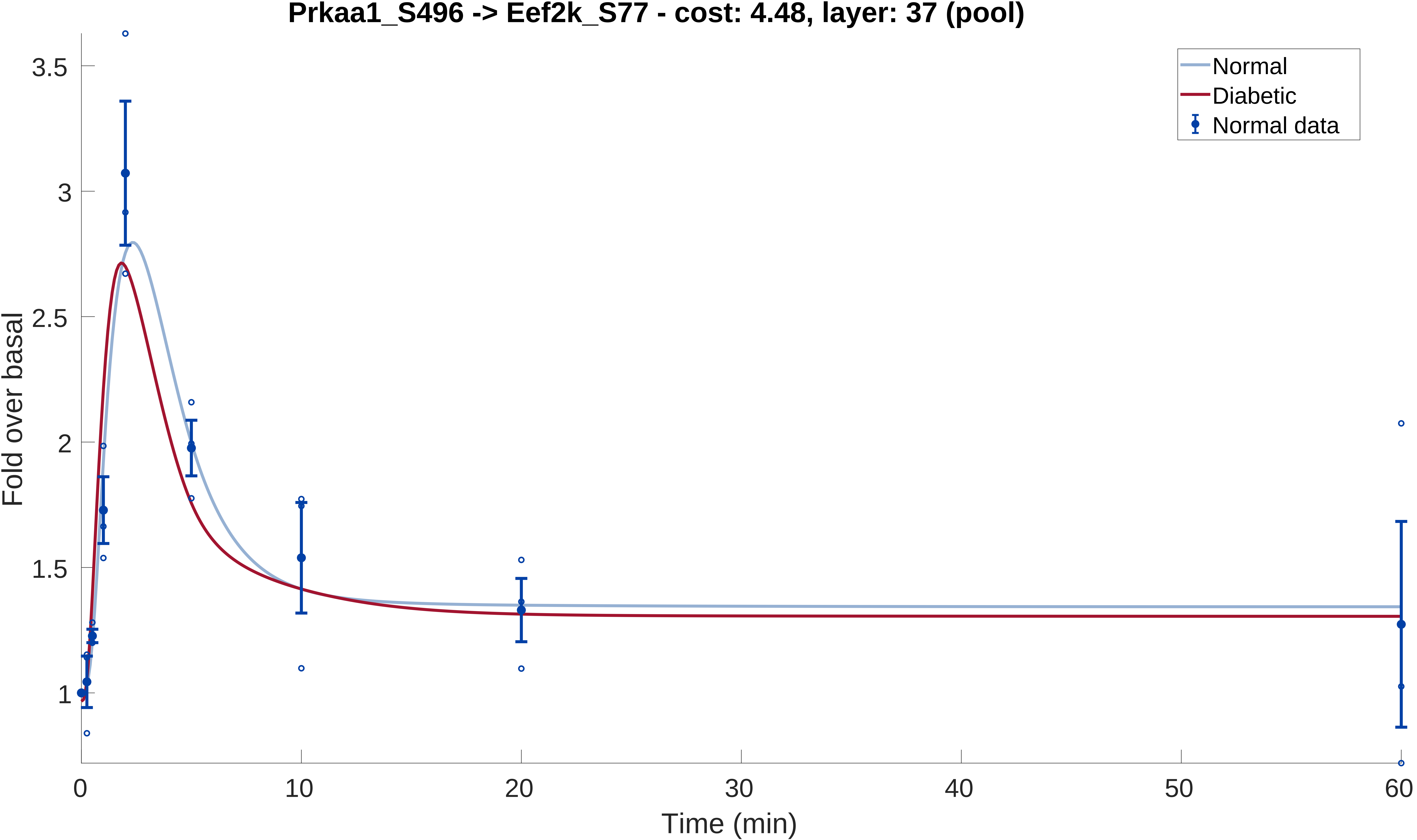

Prkaa1_S496 -> Eef2k_S77 - cost: 4.48, layer: 37 (pool)
Normal
Diabetic
Normal data
Fold over basal
3.5
3
2.5
2
1.5
1
0
10
20
30
40
50
60
Time (min)

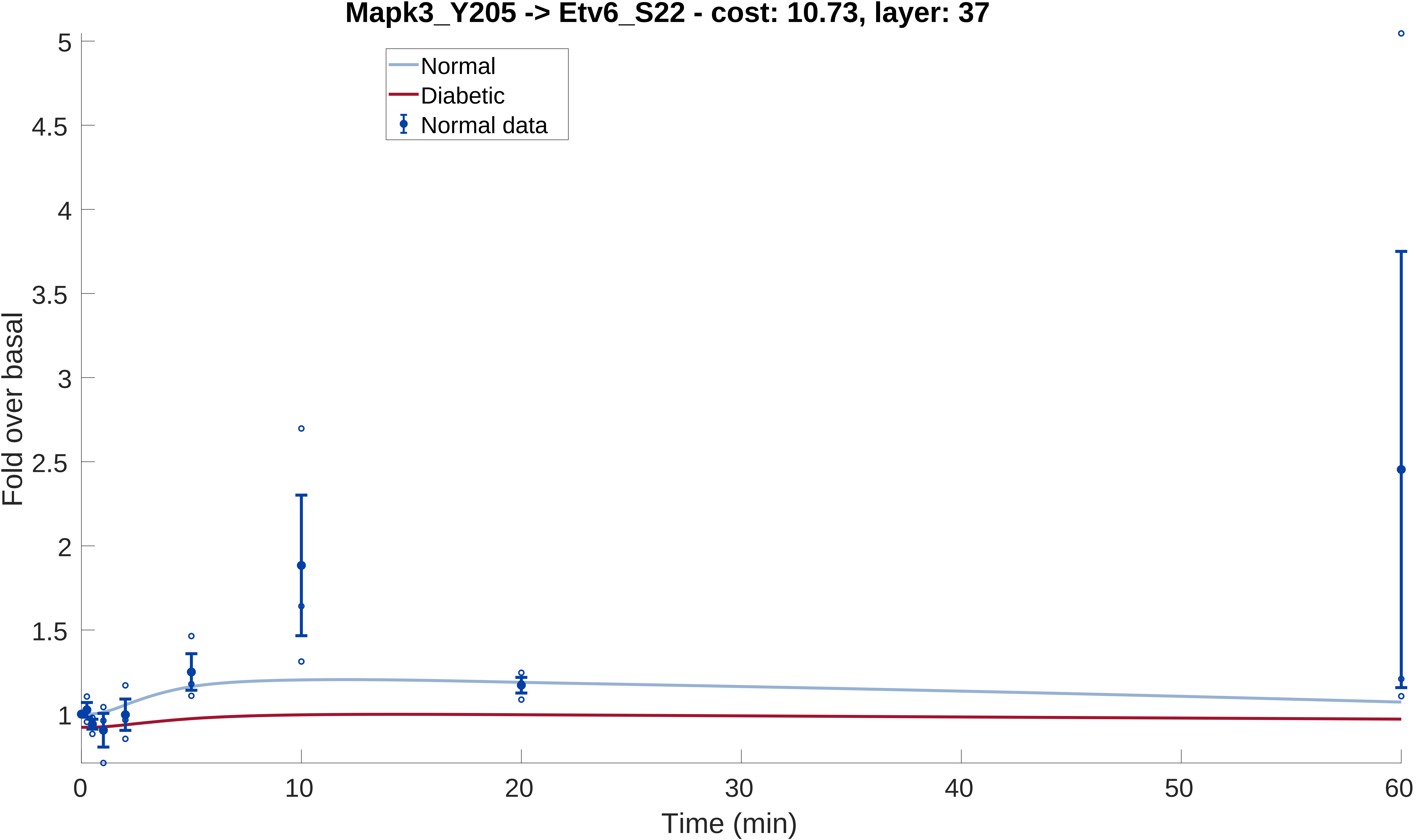

Mapk3_Y205 -> Etv6_S22 - cost: 10.73, layer: 37
Normal
Diabetic
Normal data
Fold over basal
Time (min)
5
4.5
4
3.5
3
2.5
2
1.5
1
0
10
20
30
40
50
60

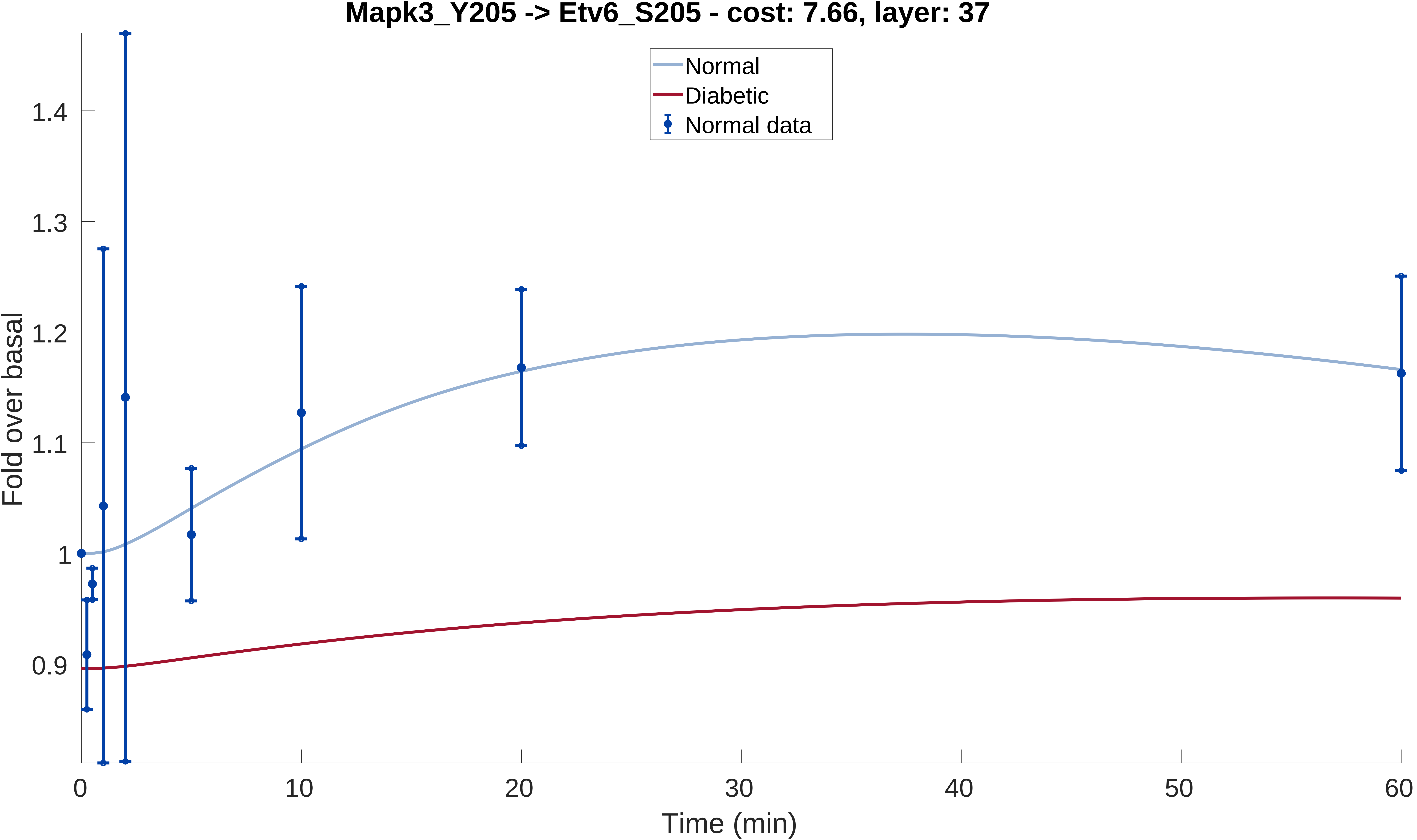

Mapk3_Y205 -> Etv6_S205 - cost: 7.66, layer: 37
Normal
Diabetic
Normal data
Fold over basal
1.4
1.3
1.2
1.1
1
0.9
0
10
20
30
40
50
60
Time (min)

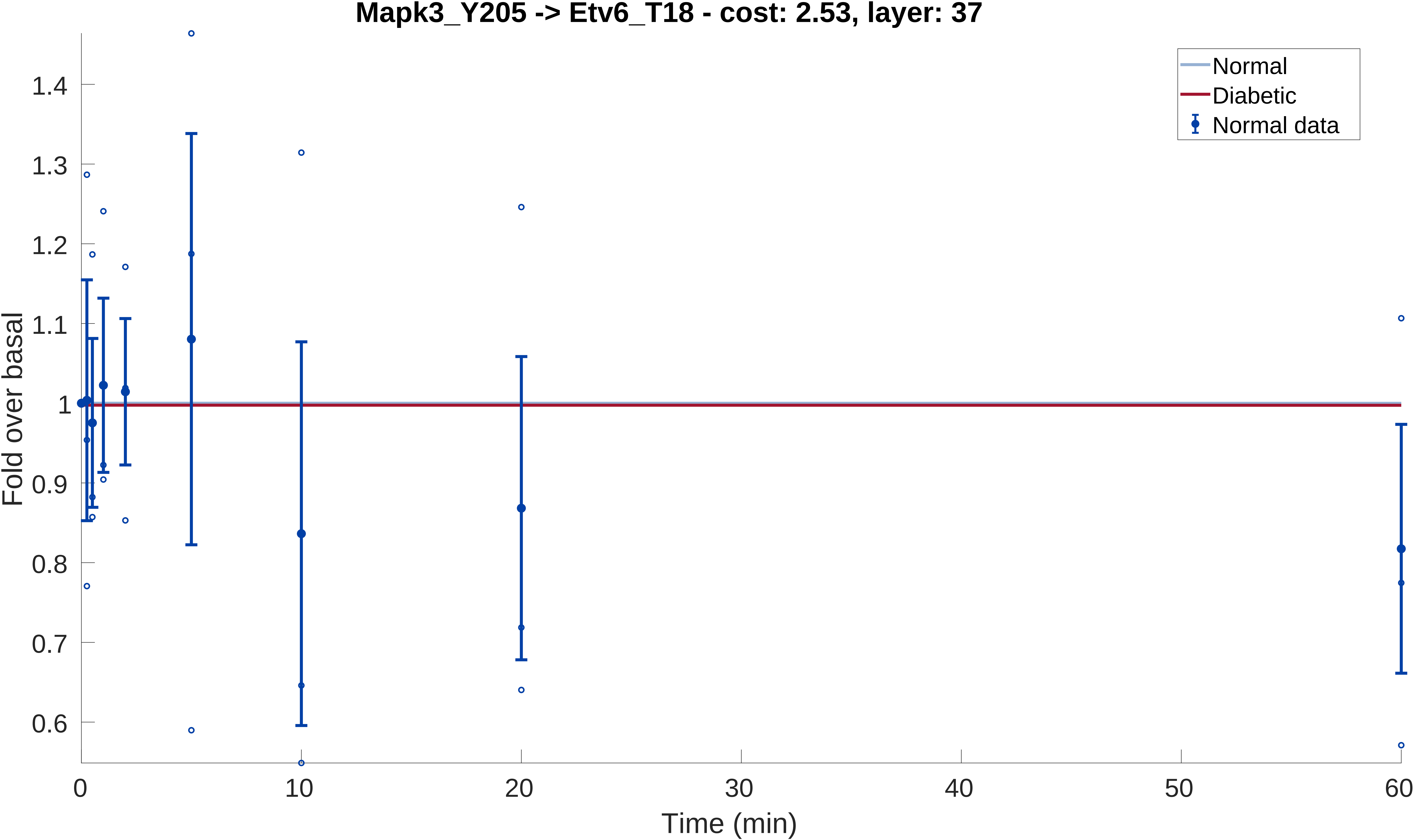

Mapk3_Y205 -> Etv6_T18 - cost: 2.53, layer: 37
Normal
Diabetic
Normal data
Fold over basal
Time (min)
1.4
1.3
1.2
1.1
1
0.9
0.8
0.7
0.6
0
10
20
30
40
50
60

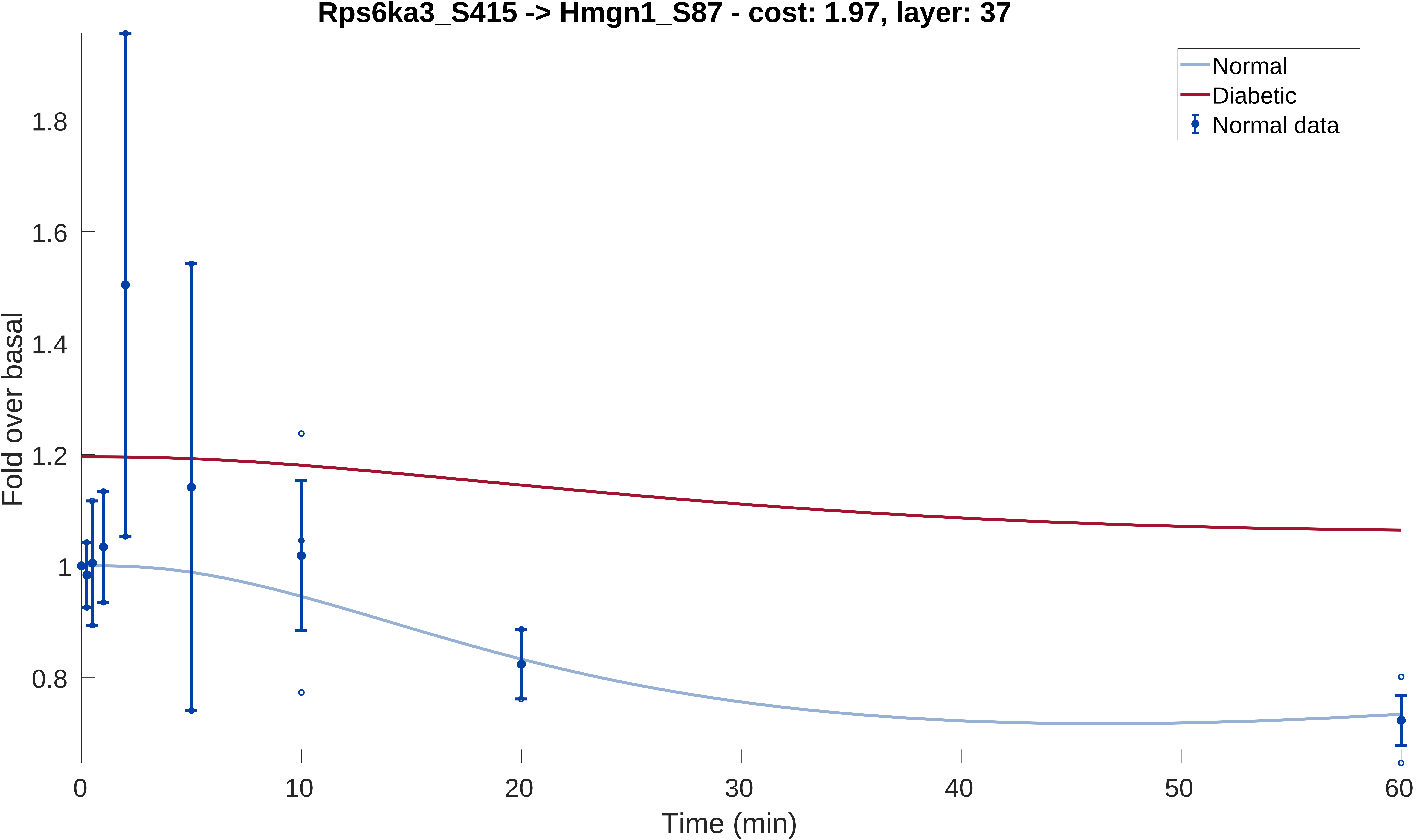

Rps6ka3_S415 -> Hmgn1_S87 - cost: 1.97, layer: 37
Normal
Diabetic
Normal data
Fold over basal
1.8
1.6
1.4
1.2
1
0.8
0
10
20
30
40
50
60
Time (min)

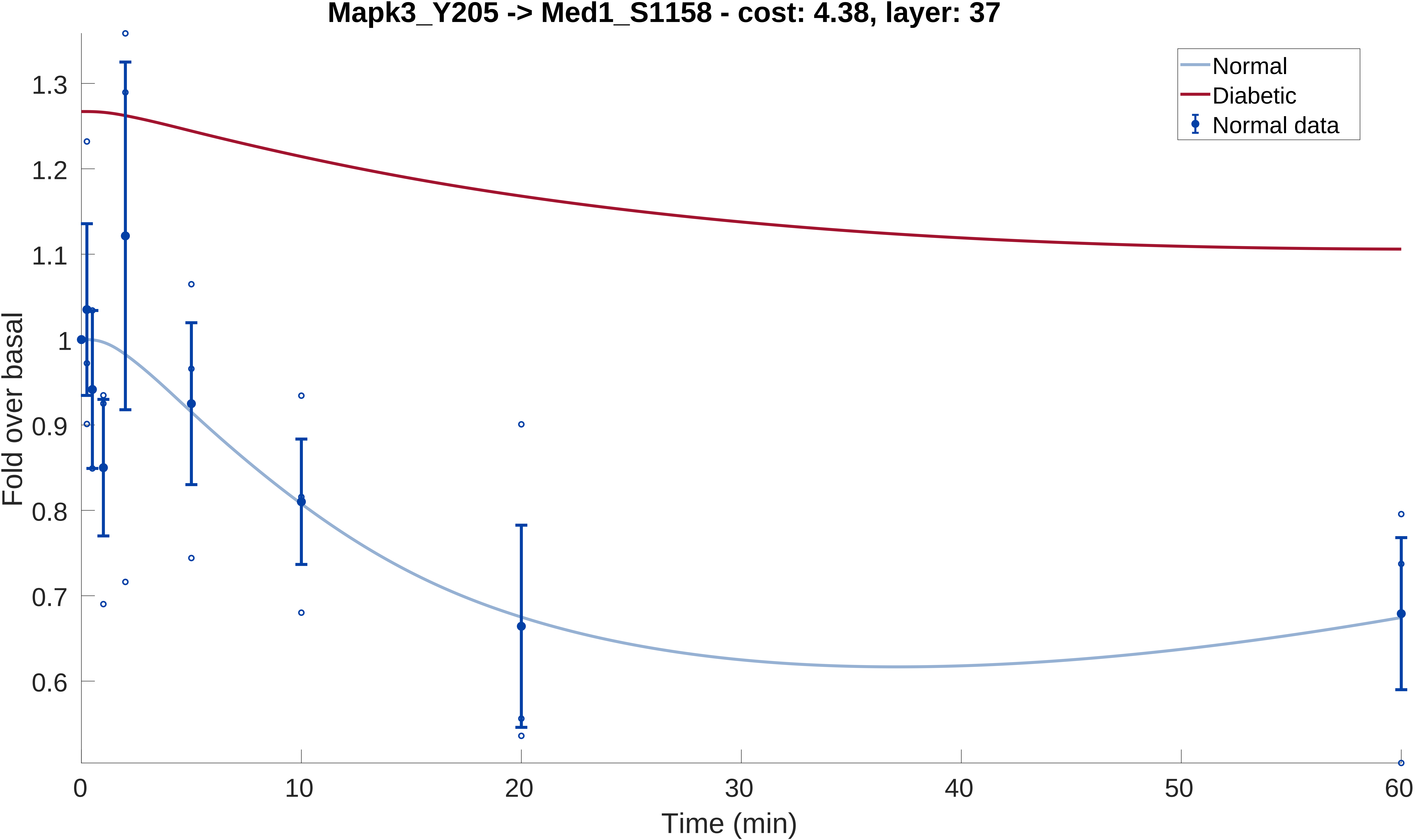
Mapk3_Y205 -> Med1_S1158 - cost: 4.38, layer: 37
Normal
Diabetic
Normal data
Fold over basal
1.3
1.2
1.1
1
0.9
0.8
0.7
0.6
0
10
20
30
40
50
60
Time (min)

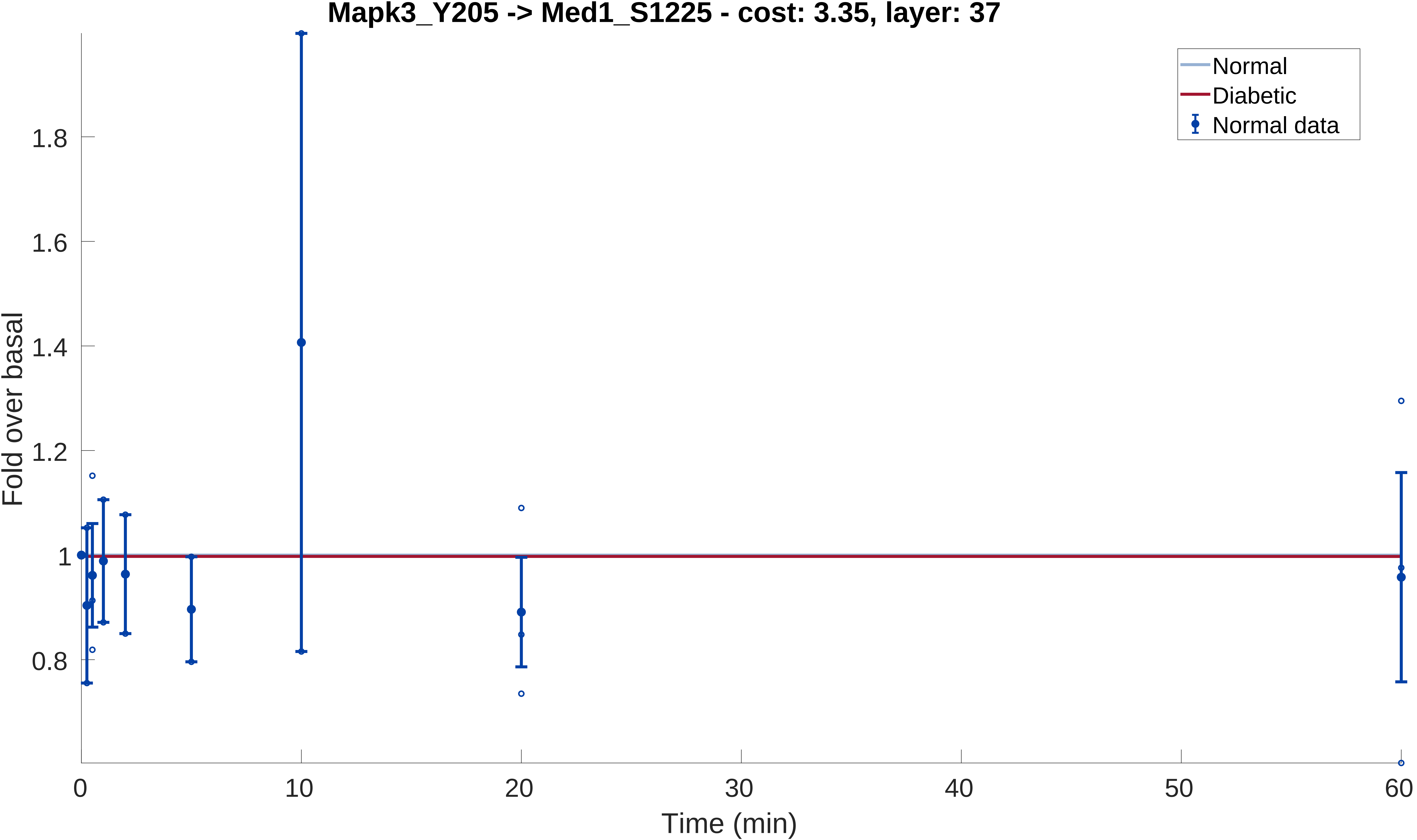

Mapk3_Y205 -> Med1_S1225 - cost: 3.35, layer: 37
Normal
Diabetic
Normal data
Fold over basal
1.8
1.6
1.4
1.2
1
0.8
0
10
20
30
40
50
60
Time (min)

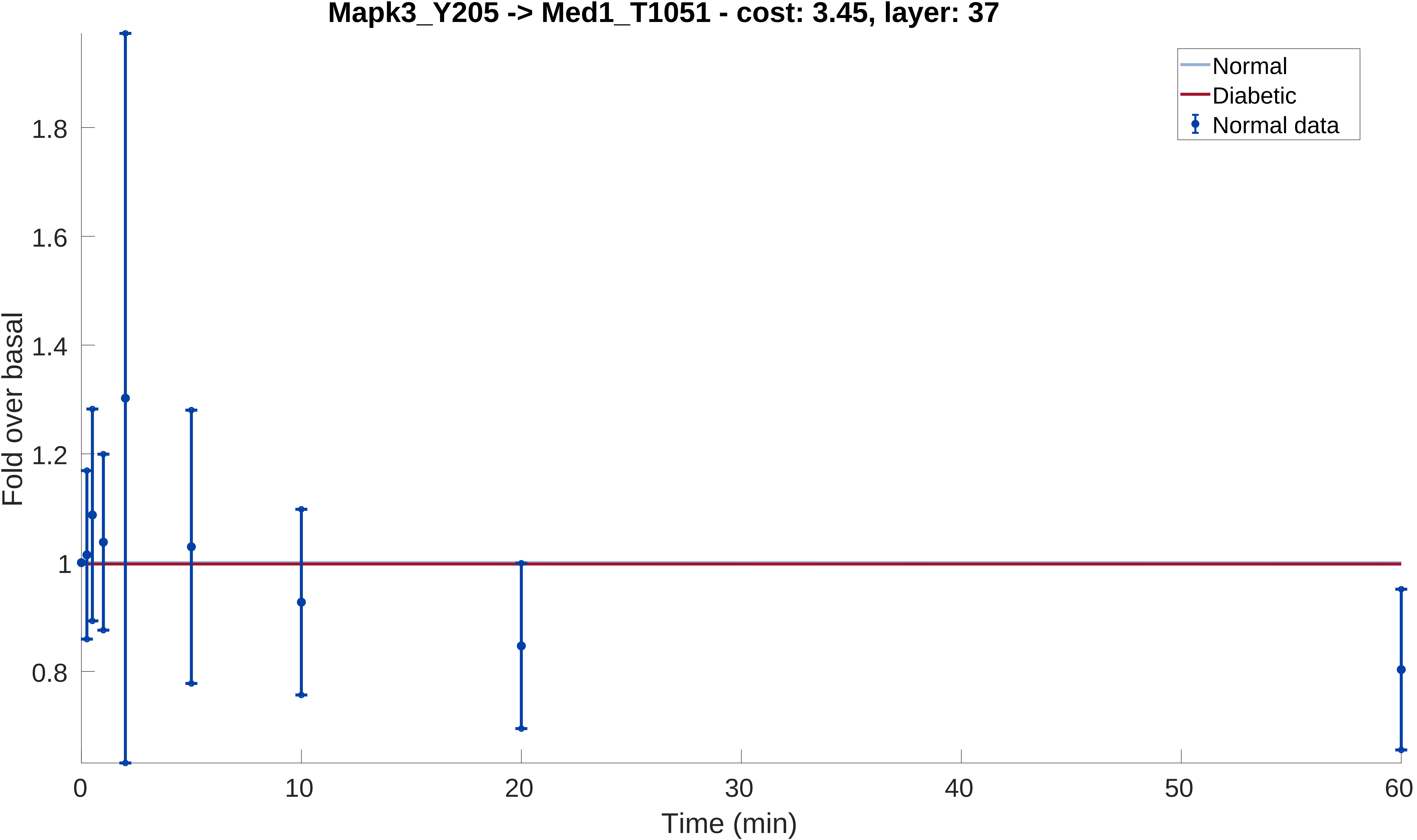
Mapk3_Y205 -> Med1_T1051 - cost: 3.45, layer: 37
Normal
Diabetic
Normal data
Fold over basal
Time (min)
1.8
1.6
1.4
1.2
1
0.8
0
10
20
30
40
50
60

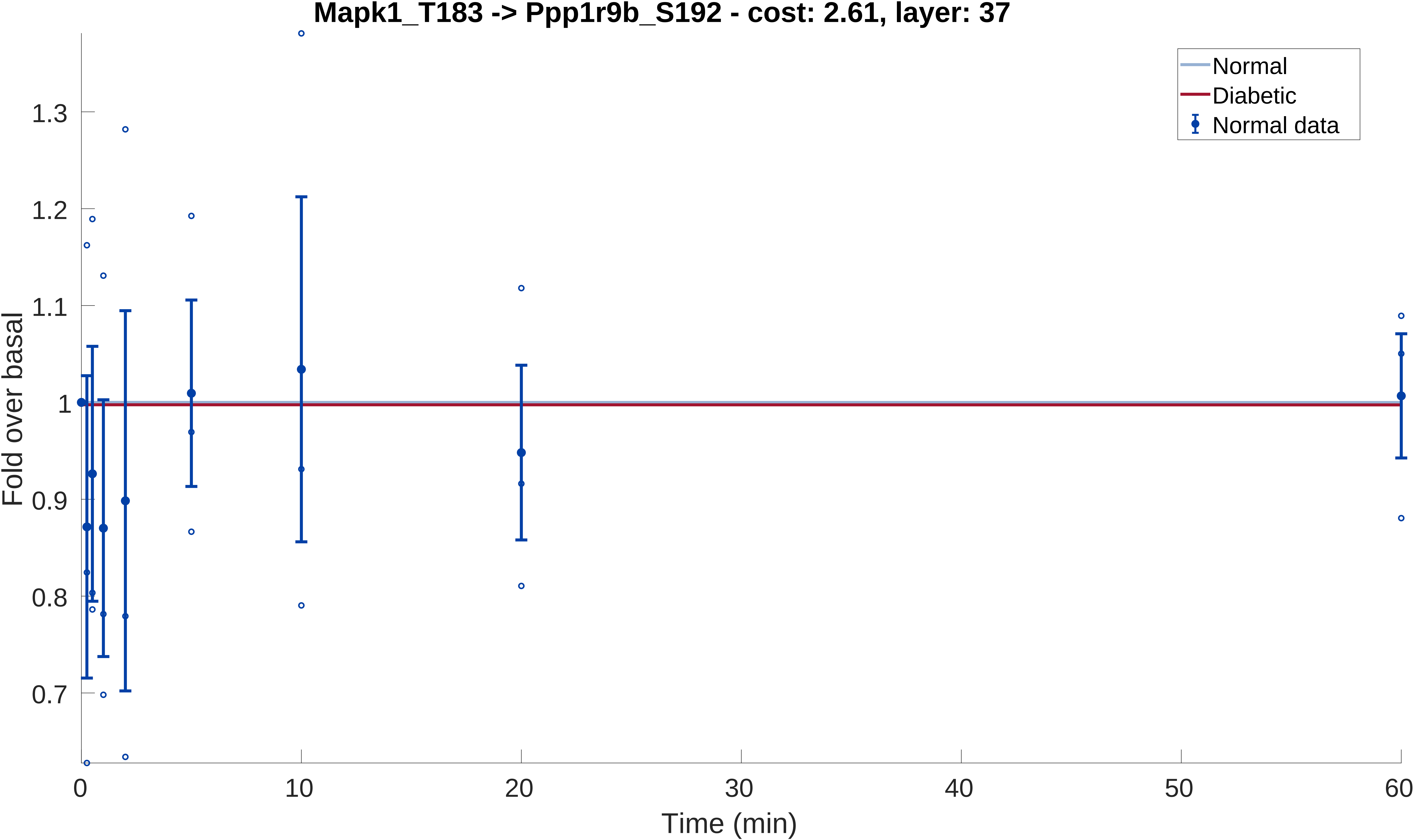

Mapk1_T183 -> Ppp1r9b_S192 - cost: 2.61, layer: 37
Normal
Diabetic
Normal data
Fold over basal
Time (min)
1.3
1.2
1.1
1
0.9
0.8
0.7
0
10
20
30
40
50
60

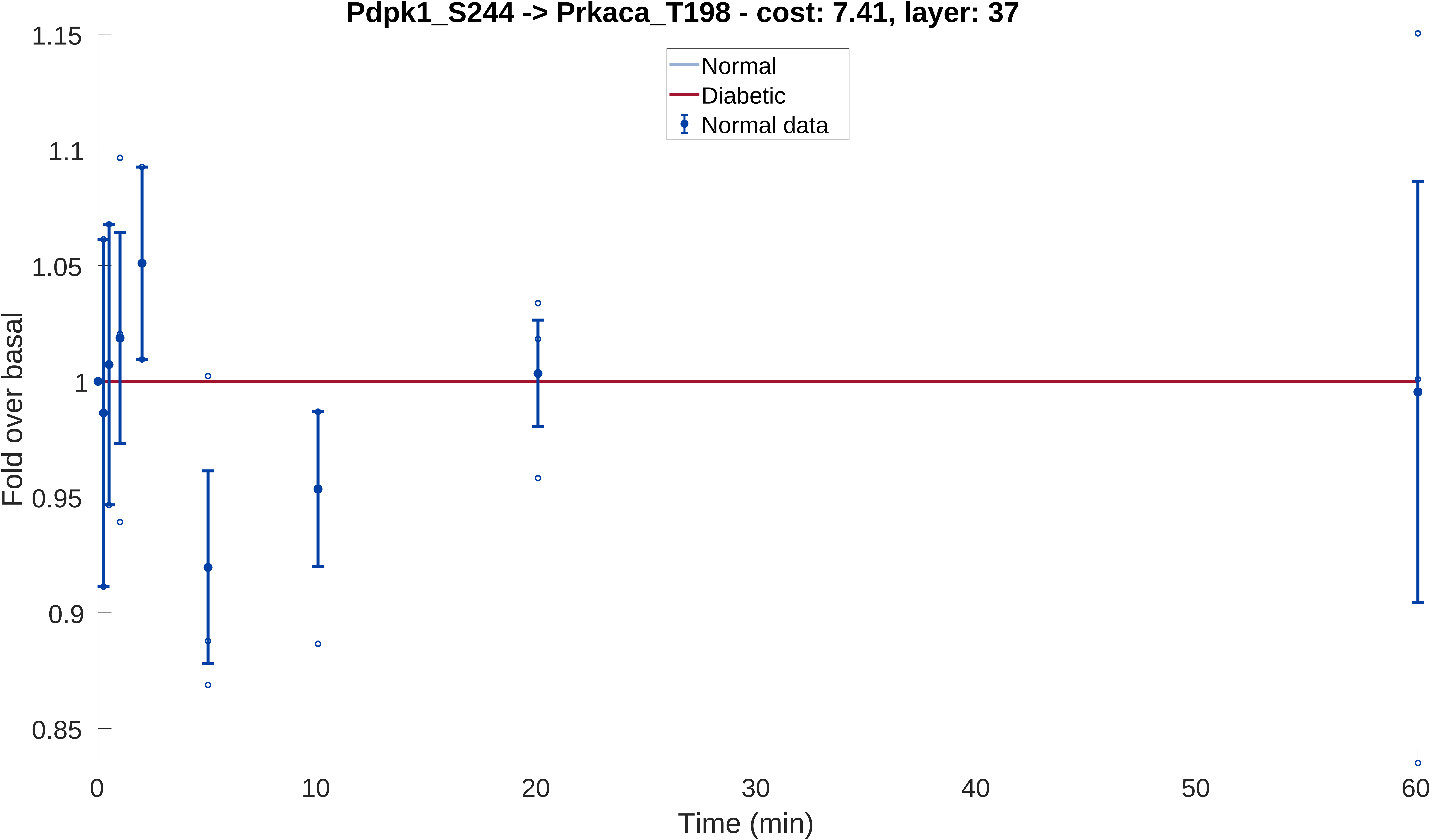

Pdpk1_S244 -> Prkaca_T198 - cost: 7.41, layer: 37
Normal
Diabetic
Normal data
Fold over basal
1.15
1.1
1.05
1
0.95
0.9
0.85
0
10
20
30
40
50
60
Time (min)

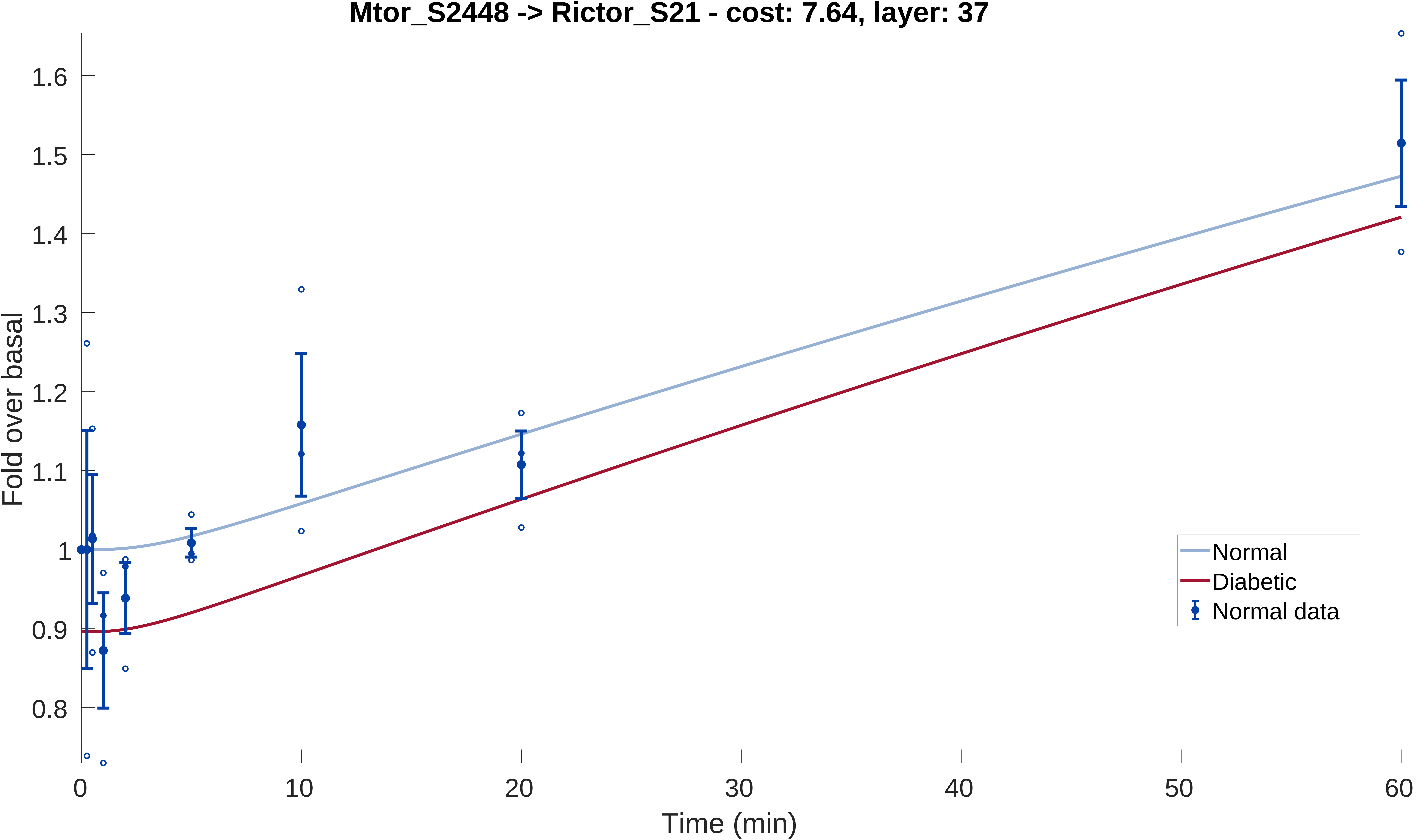

Mtor_S2448 -> Rictor_S21 - cost: 7.64, layer: 37
Fold over basal
1.6
1.5
1.4
1.3
1.2
1.1
1
0.9
0.8
0
10
20
30
40
50
60
Time (min)
Normal
Diabetic
Normal data

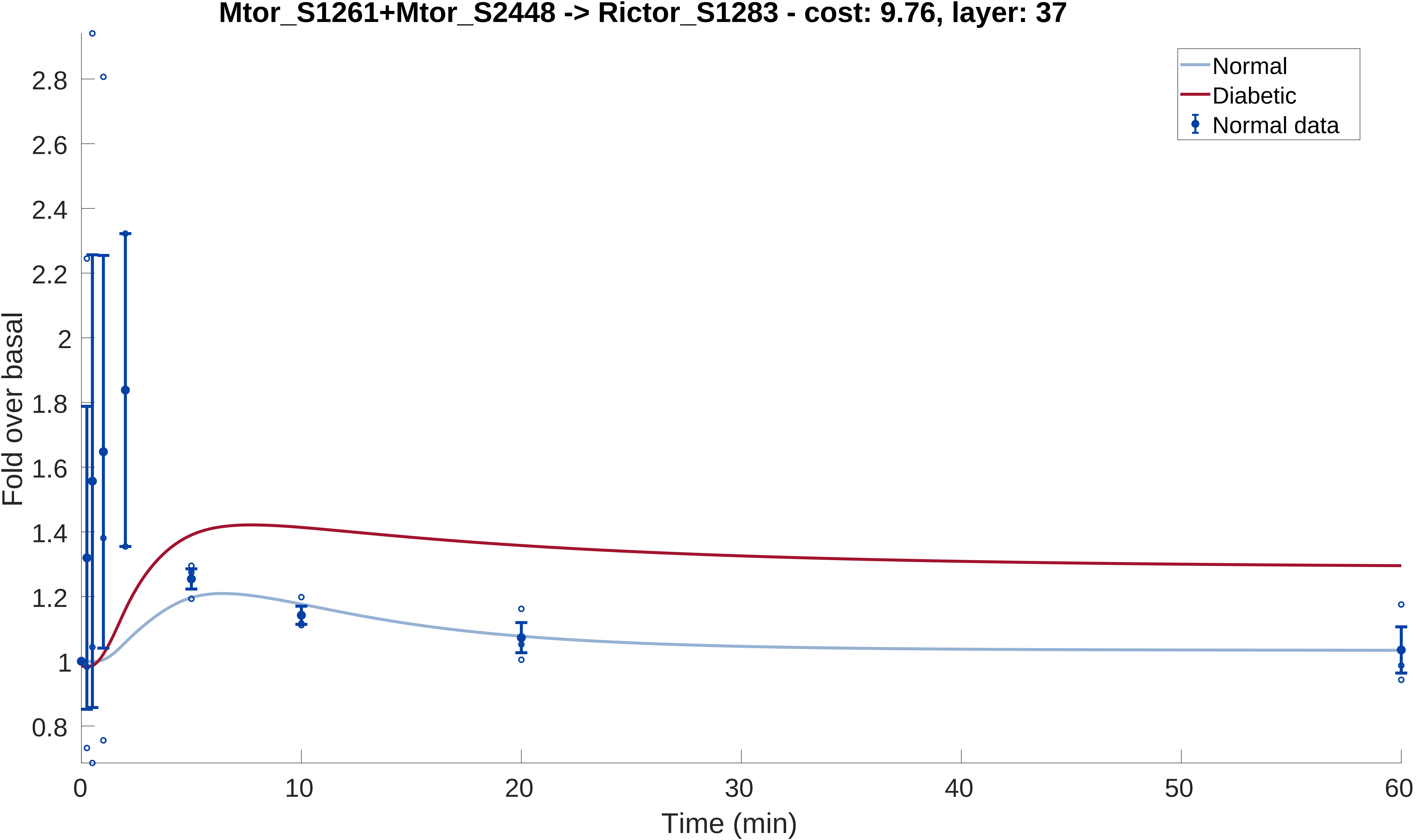

Mtor_S1261+Mtor_S2448 -> Rictor_S1283 - cost: 9.76, layer: 37
Normal
Diabetic
Normal data
Fold over basal
Time (min)
0.8
1
1.2
1.4
1.6
1.8
2
2.2
2.4
2.6
2.8
0
10
20
30
40
50
60

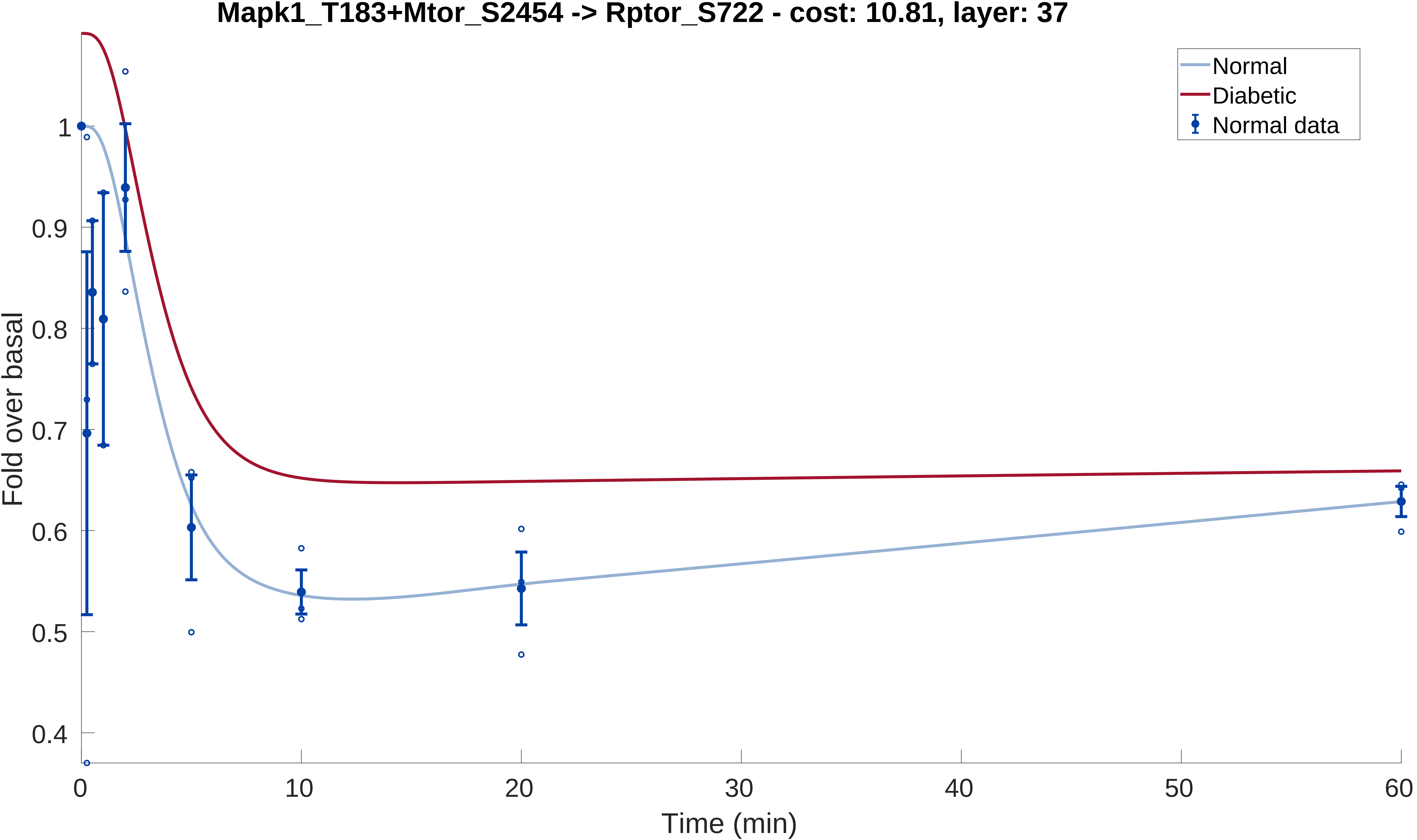

Mapk1_T183+Mtor_S2454 -> Rptor_S722 - cost: 10.81, layer: 37
Normal
Diabetic
Normal data
Fold over basal
1
0.9
0.8
0.7
0.6
0.5
0.4
0
10
20
30
40
50
60
Time (min)

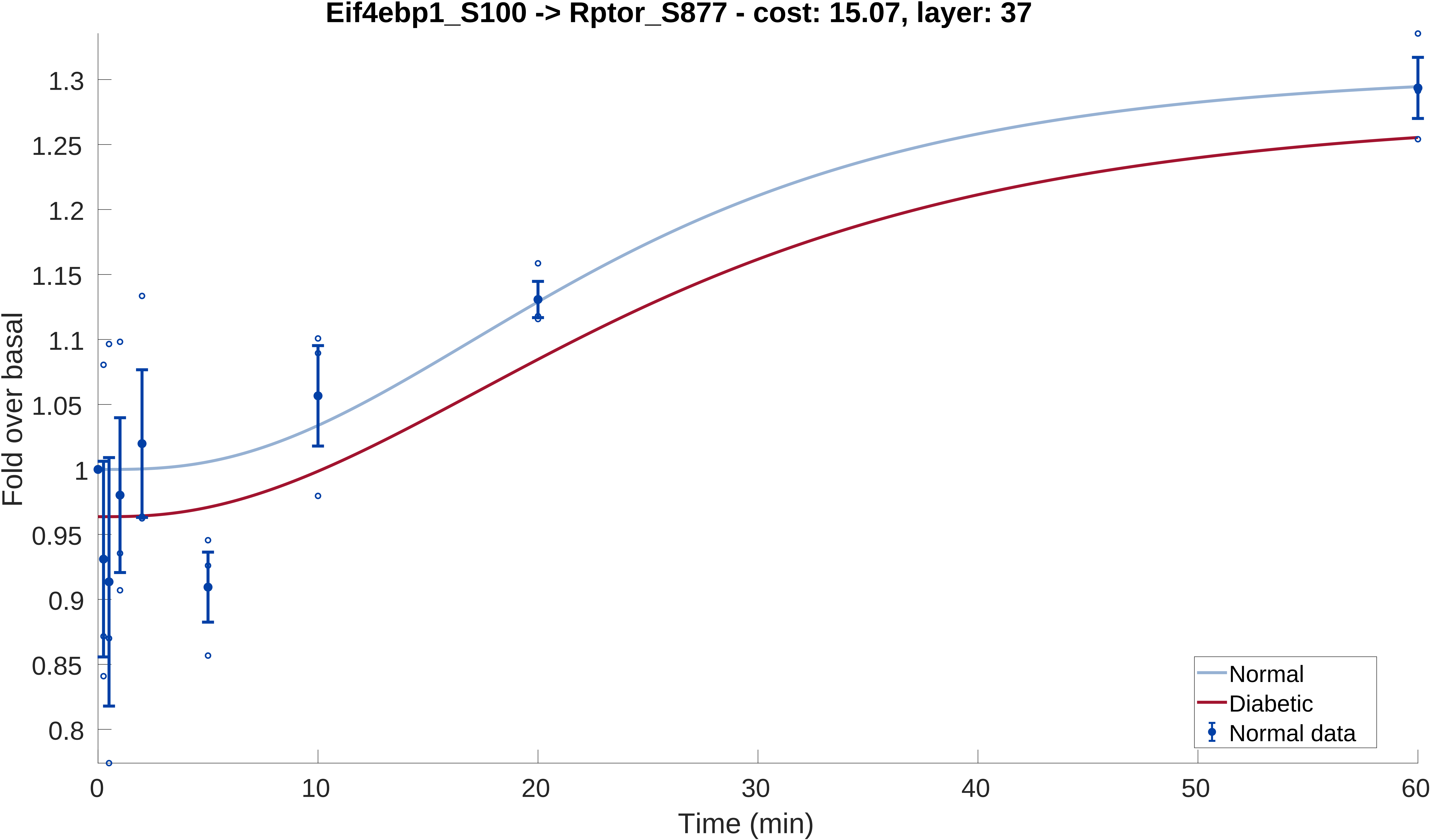

Eif4ebp1_S100 -> Rptor_S877 - cost: 15.07, layer: 37
Fold over basal
1.3
1.25
1.2
1.15
1.1
1.05
1
0.95
0.9
0.85
0.8
0
10
20
30
40
50
60
Time (min)
Normal
Diabetic
Normal data

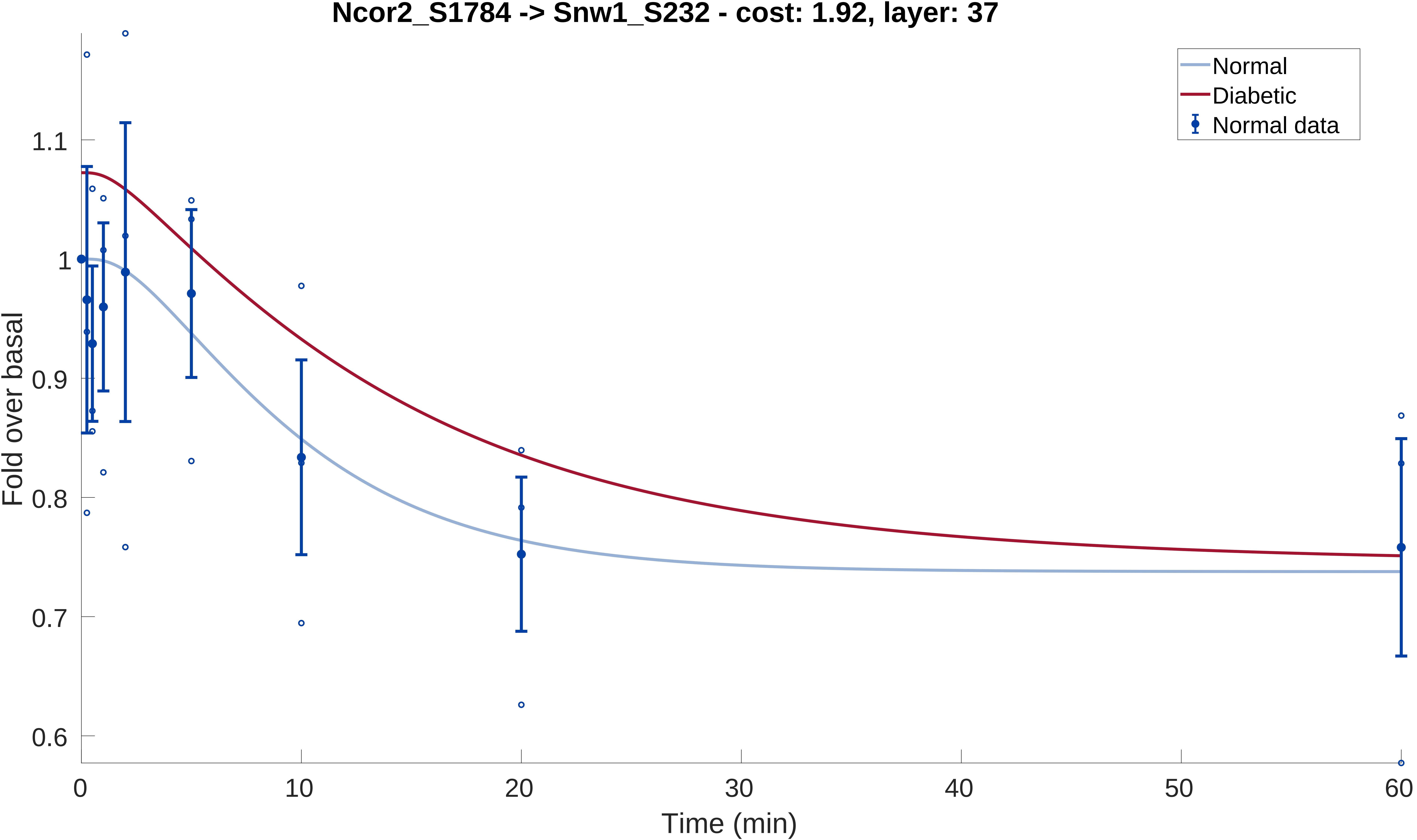
Ncor2_S1784 -> Snw1_S232 - cost: 1.92, layer: 37
Normal
Diabetic
Normal data
Fold over basal
Time (min)
1.1
1
0.9
0.8
0.7
0.6
0
10
20
30
40
50
60

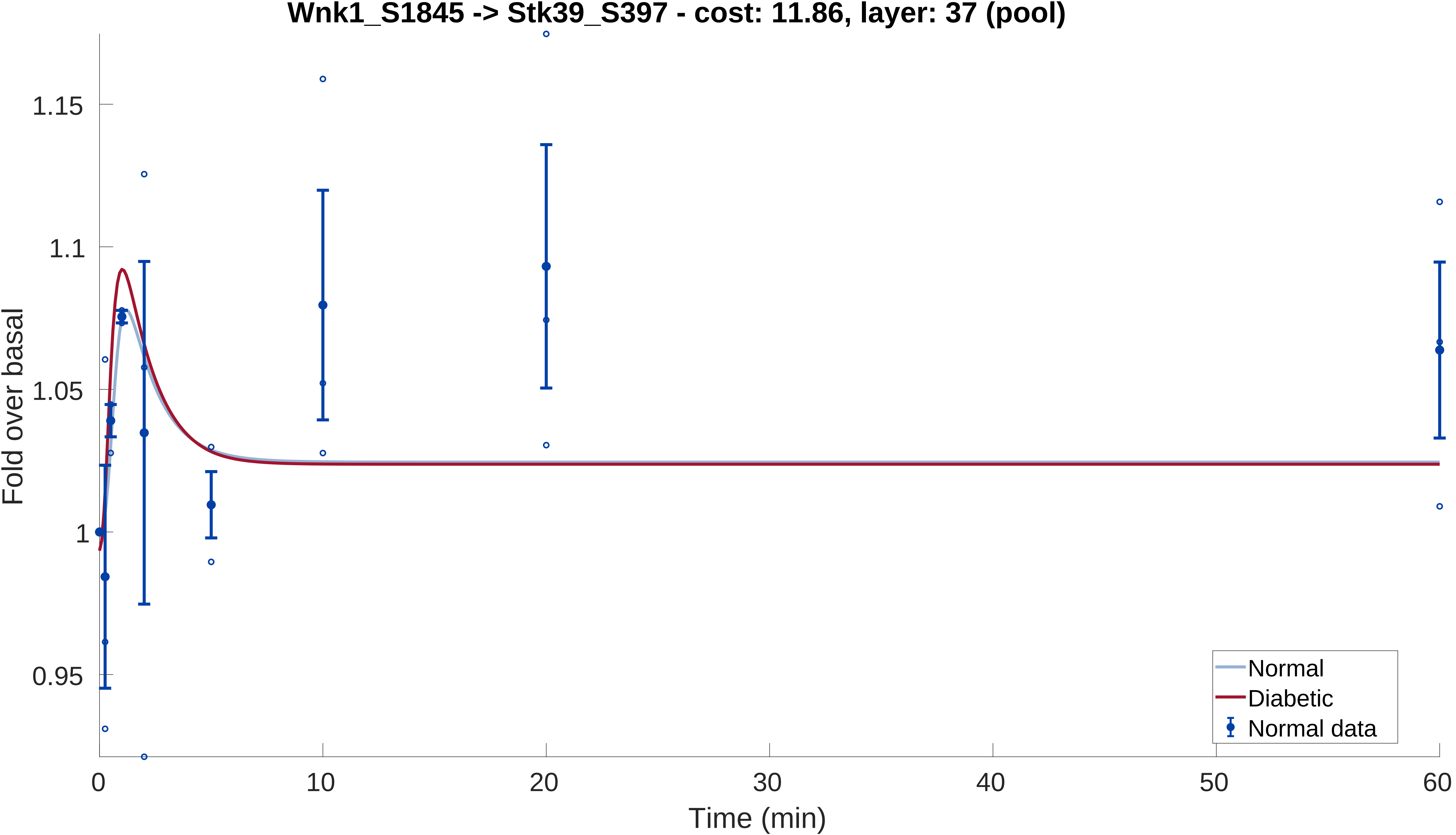
Wnk1_S1845 -> Stk39_S397 - cost: 11.86, layer: 37 (pool)
Fold over basal
1.15
1.1
1.05
1
0.95
0
10
20
30
40
50
60
Time (min)
Normal
Diabetic
Normal data

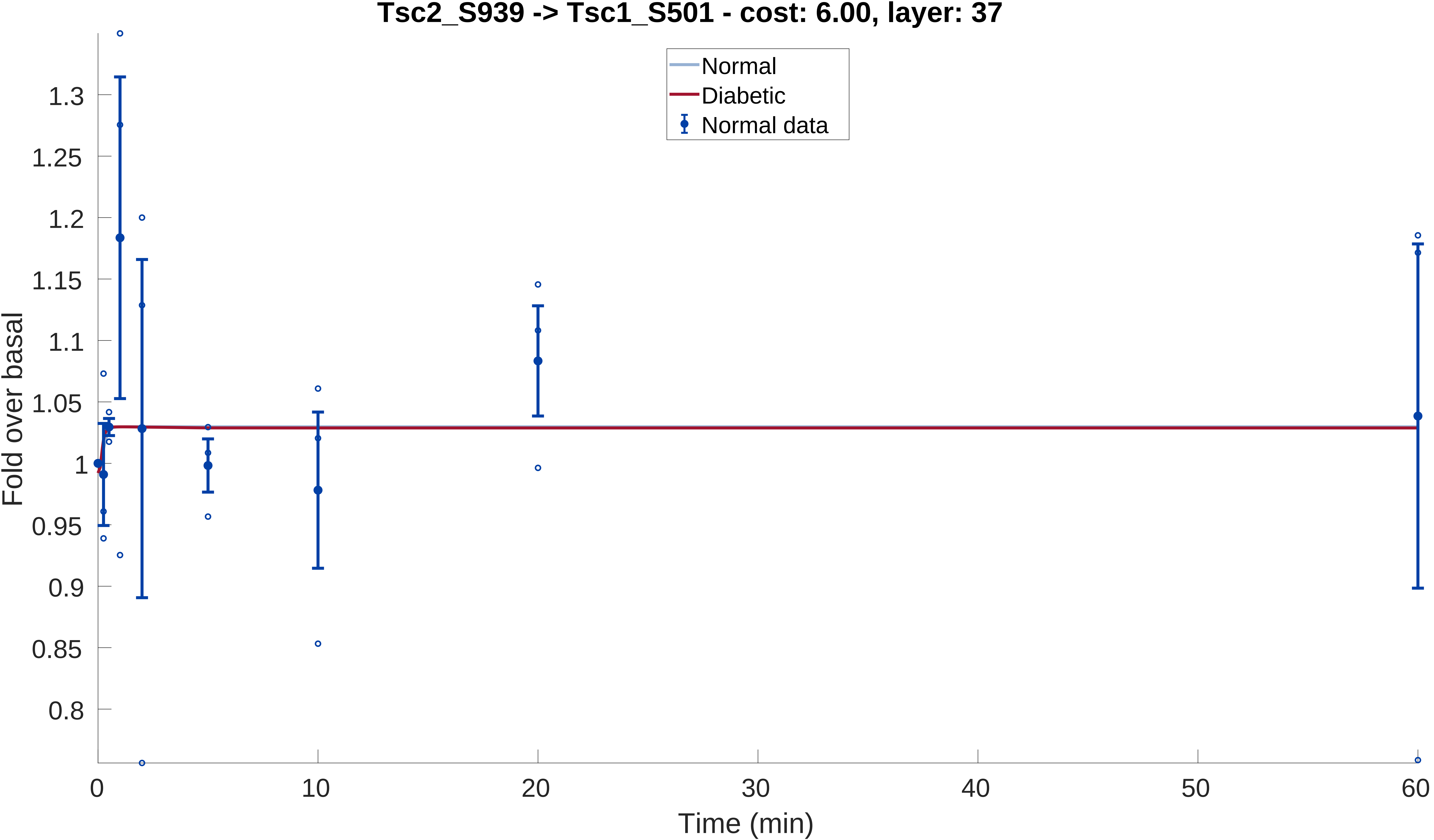

Tsc2_S939 -> Tsc1_S501 - cost: 6.00, layer: 37
Normal
Diabetic
Normal data
Fold over basal
1.3
1.25
1.2
1.15
1.1
1.05
1
0.95
0.9
0.85
0.8
0
10
20
30
40
50
60
Time (min)

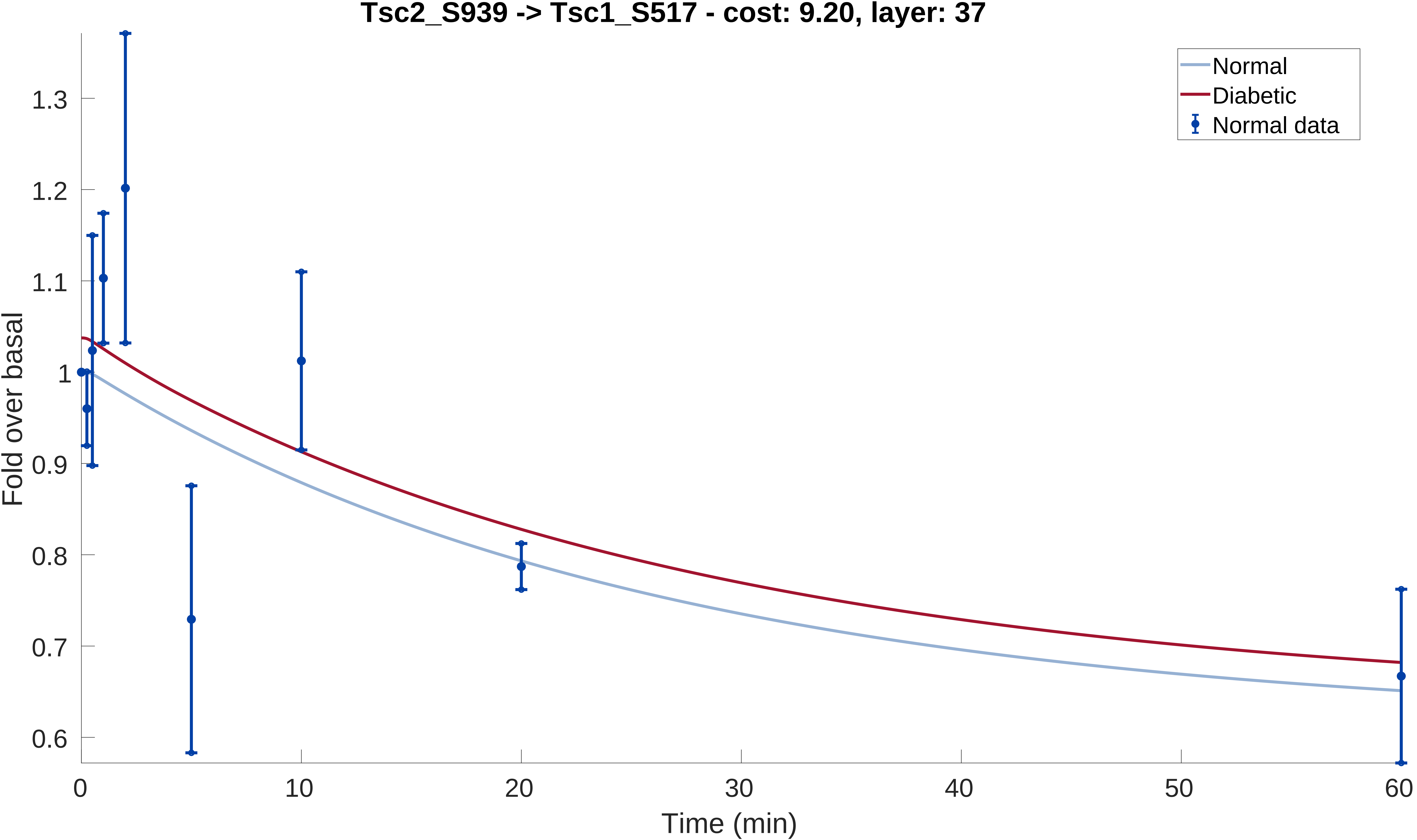

Tsc2_S939 -> Tsc1_S517 - cost: 9.20, layer: 37
Normal
Diabetic
Normal data
1.3
1.2
1.1
1
0.9
0.8
0.7
0.6
Fold over basal
0
10
20
30
40
50
60
Time (min)

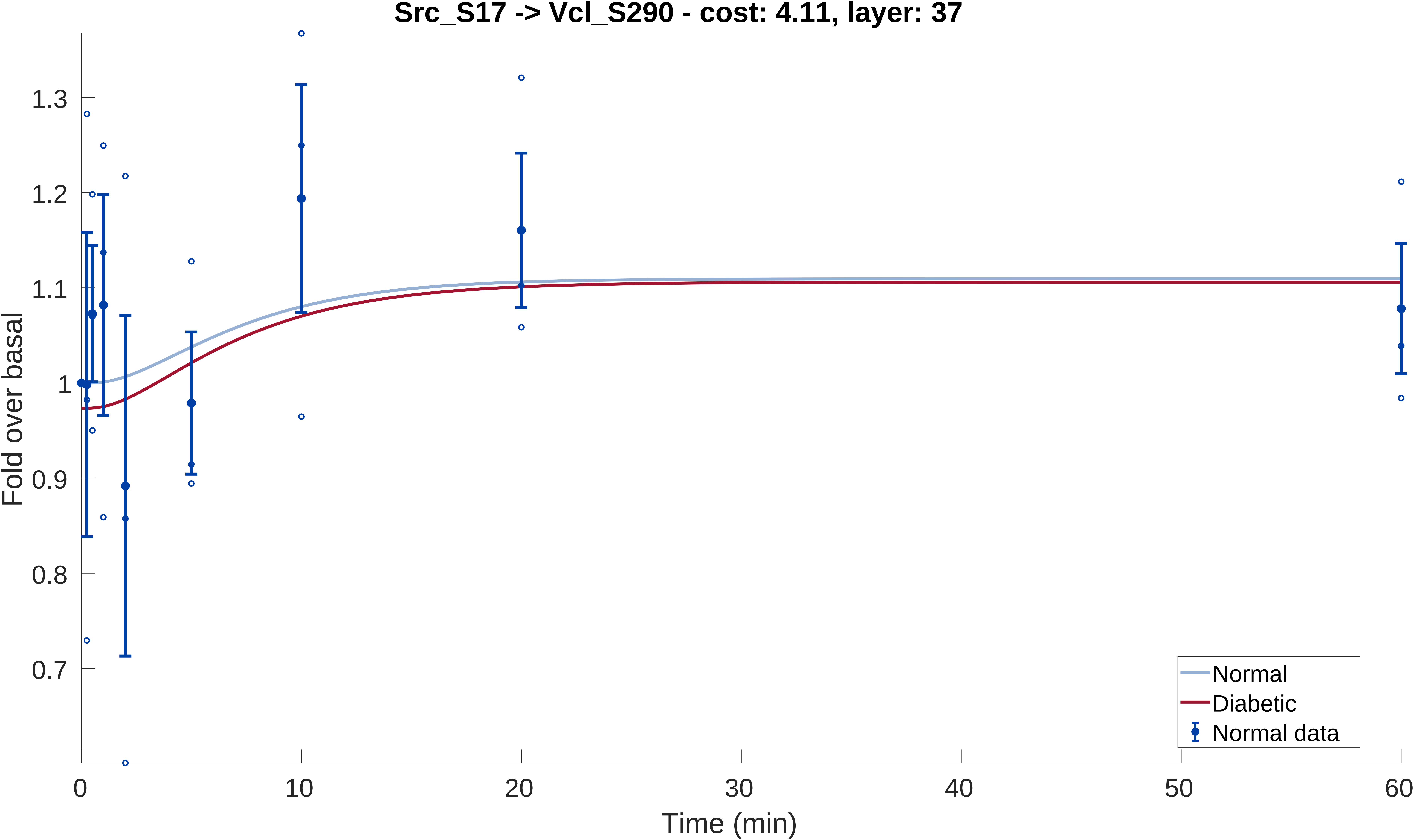

Src_S17 -> Vcl_S290 - cost: 4.11, layer: 37
Fold over basal
1.3
1.2
1.1
1
0.9
0.8
0.7
0
10
20
30
40
50
60
Time (min)
Normal
Diabetic
Normal data

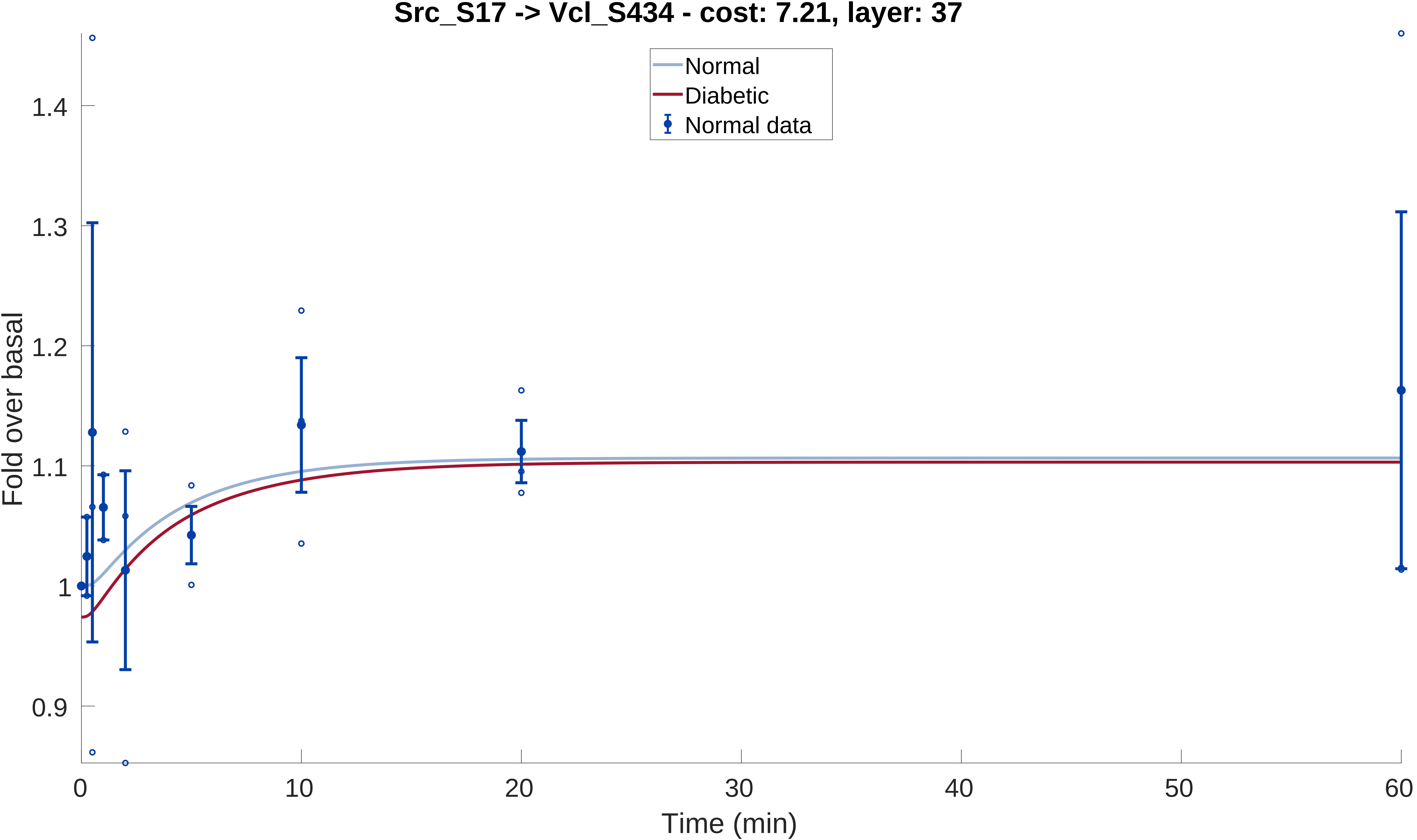

Src_S17 -> Vcl_S434 - cost: 7.21, layer: 37
Normal
Diabetic
Normal data
Fold over basal
Time (min)
0.9
1
1.1
1.2
1.3
1.4
0
10
20
30
40
50
60

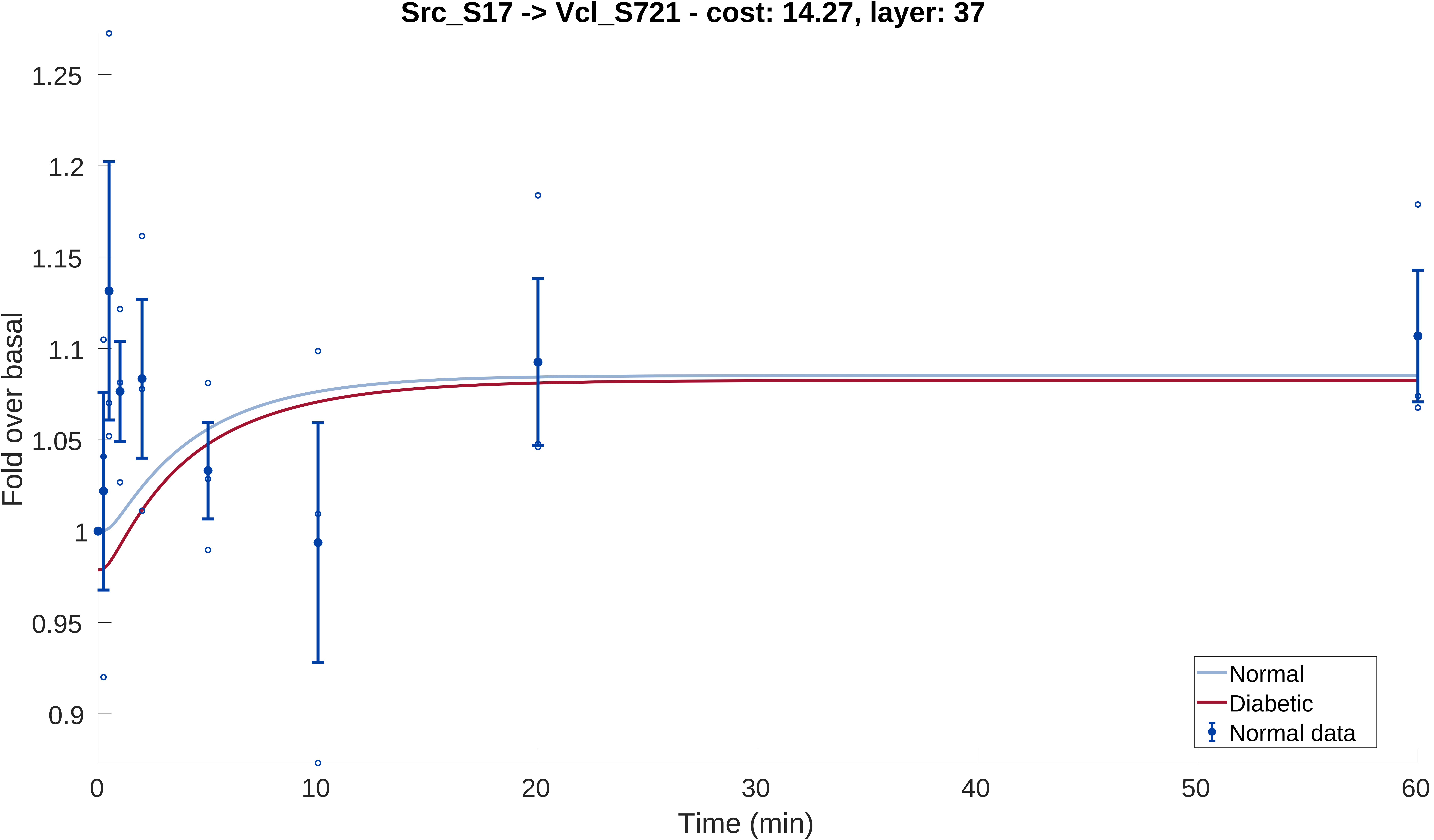

Src_S17 -> Vcl_S721 - cost: 14.27, layer: 37
Fold over basal
1.25
1.2
1.15
1.1
1.05
1
0.95
0.9
0
10
20
30
40
50
60
Time (min)
Normal
Diabetic
Normal data

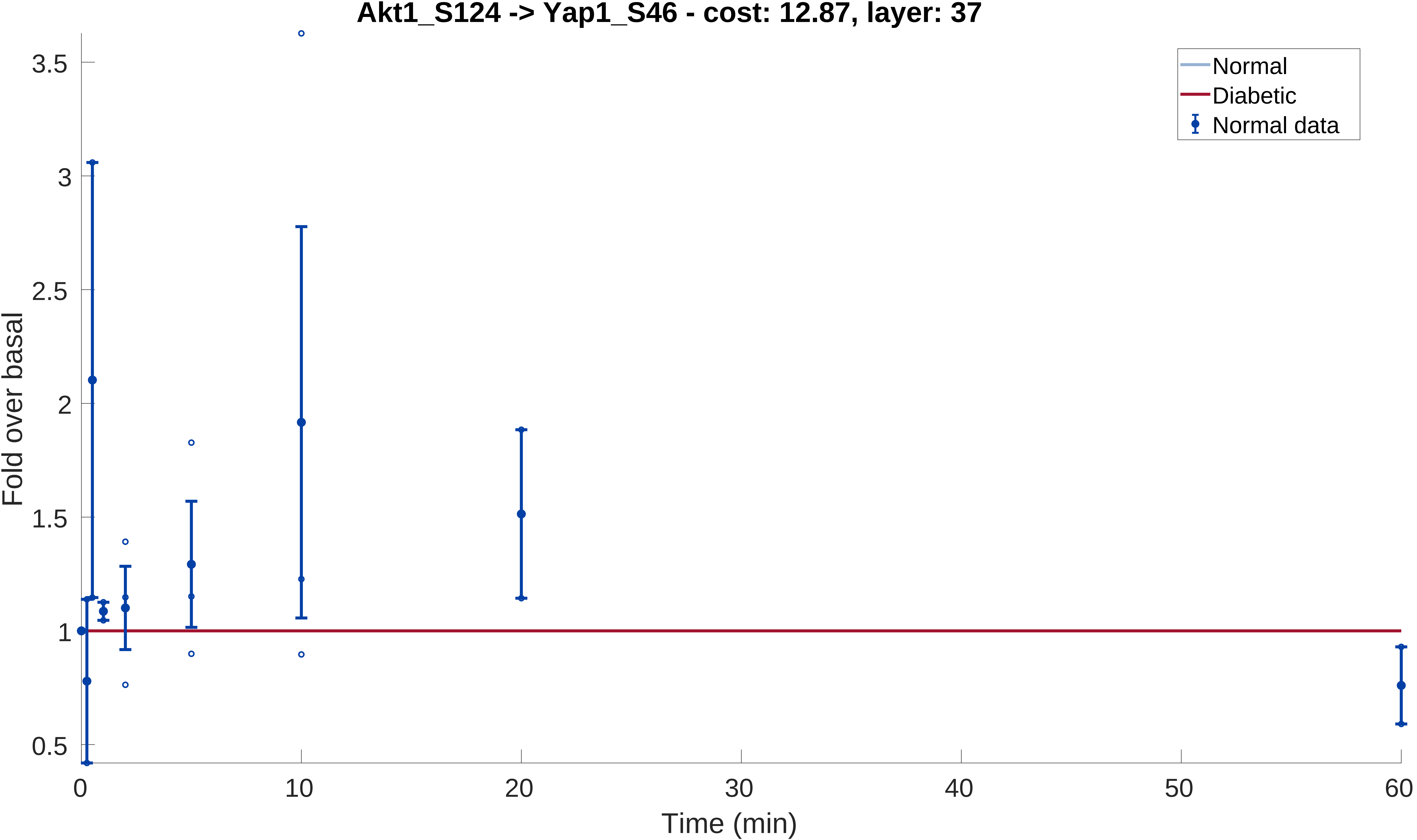
Akt1_S124 -> Yap1_S46 - cost: 12.87, layer: 37
Normal
Diabetic
Normal data
Fold over basal
Time (min)
3.5
3
2.5
2
1.5
1
0.5
0
10
20
30
40
50
60

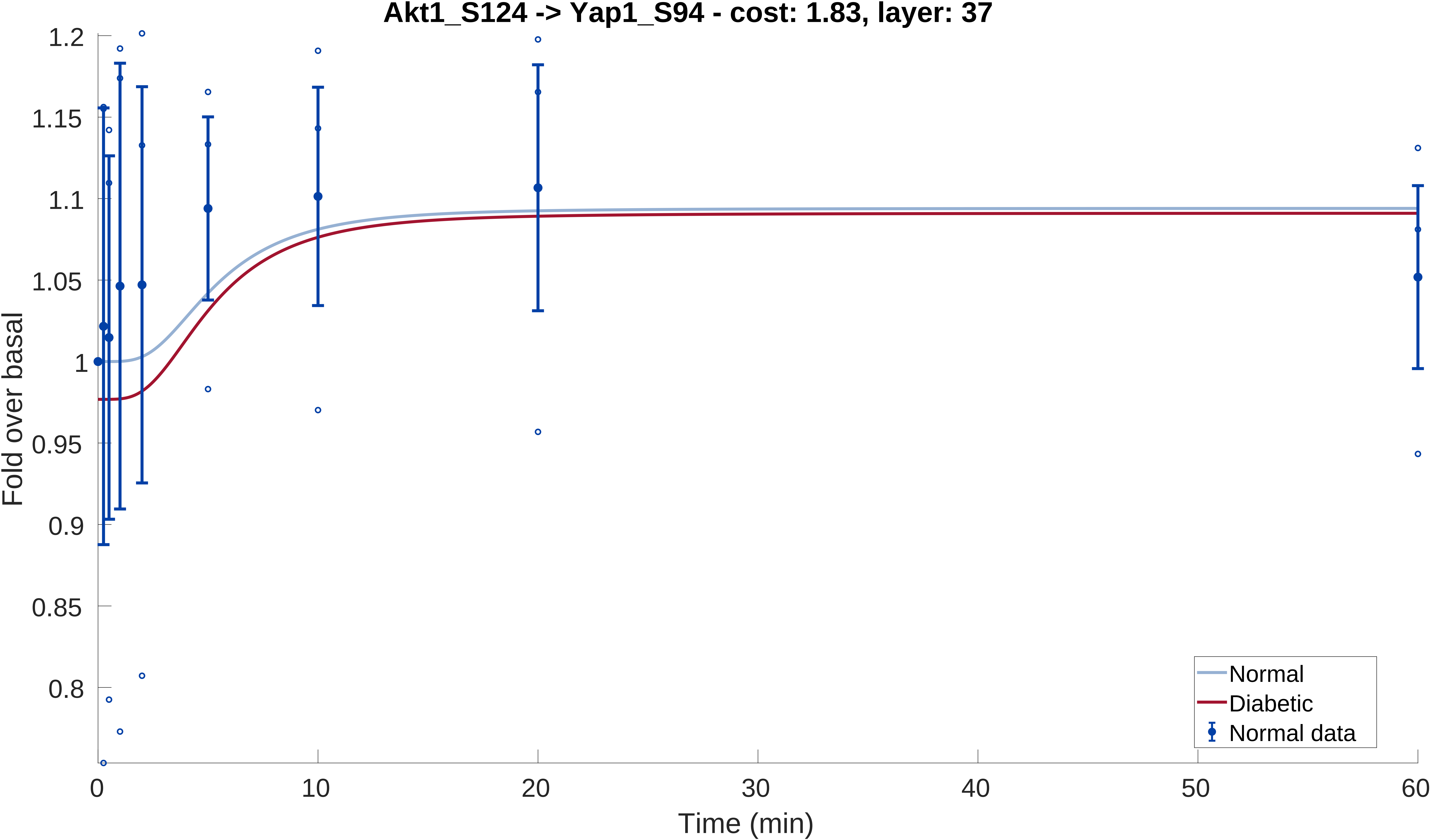
Akt1_S124 -> Yap1_S94 - cost: 1.83, layer: 37
Fold over basal
1.2
1.15
1.1
1.05
1
0.95
0.9
0.85
0.8
Time (min)
0
10
20
30
40
50
60
Normal
Diabetic
Normal data

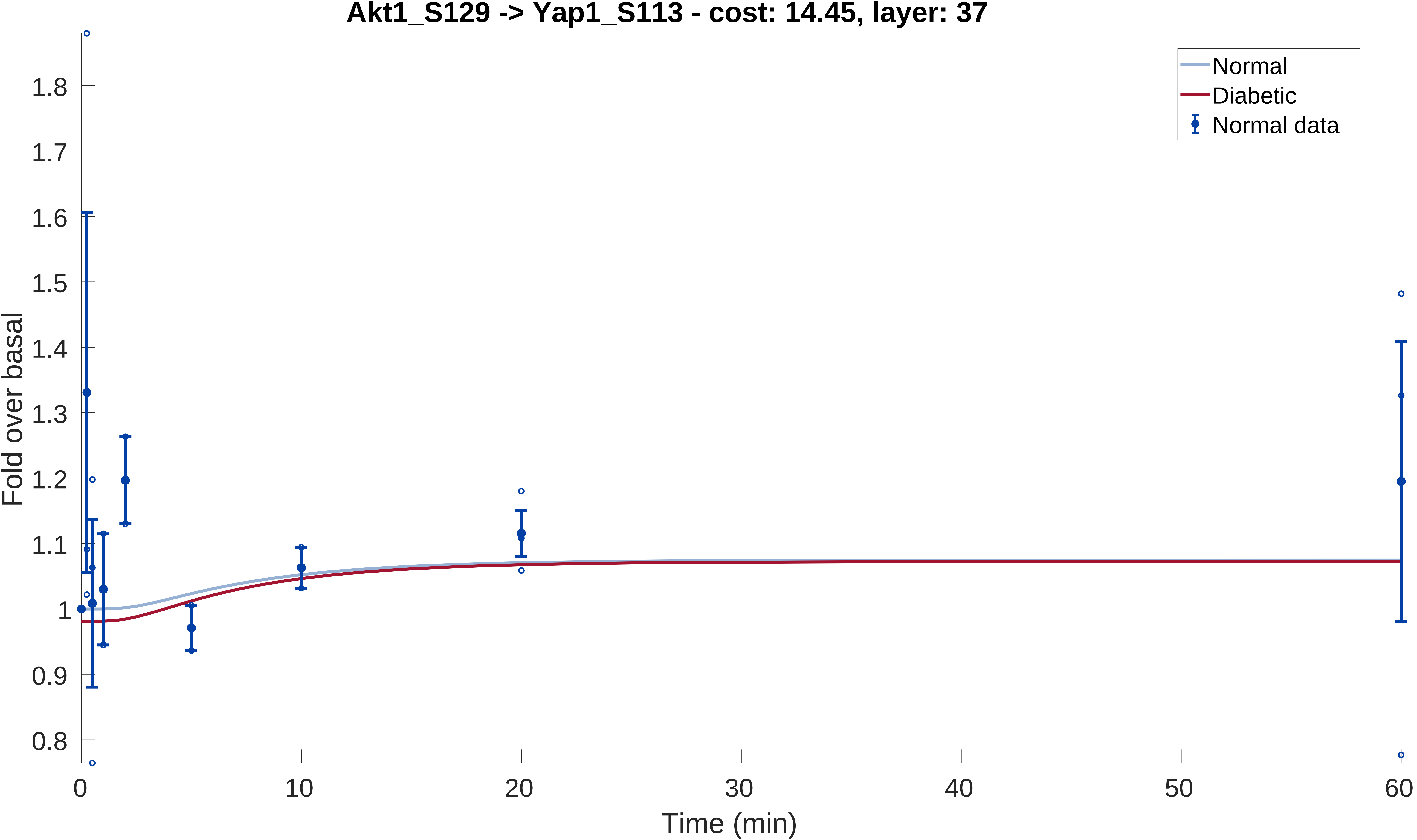
Akt1_S129 -> Yap1_S113 - cost: 14.45, layer: 37
Normal
Diabetic
Normal data
Fold over basal
Time (min)
1.8
1.7
1.6
1.5
1.4
1.3
1.2
1.1
1
0.9
0.8
0
10
20
30
40
50
60

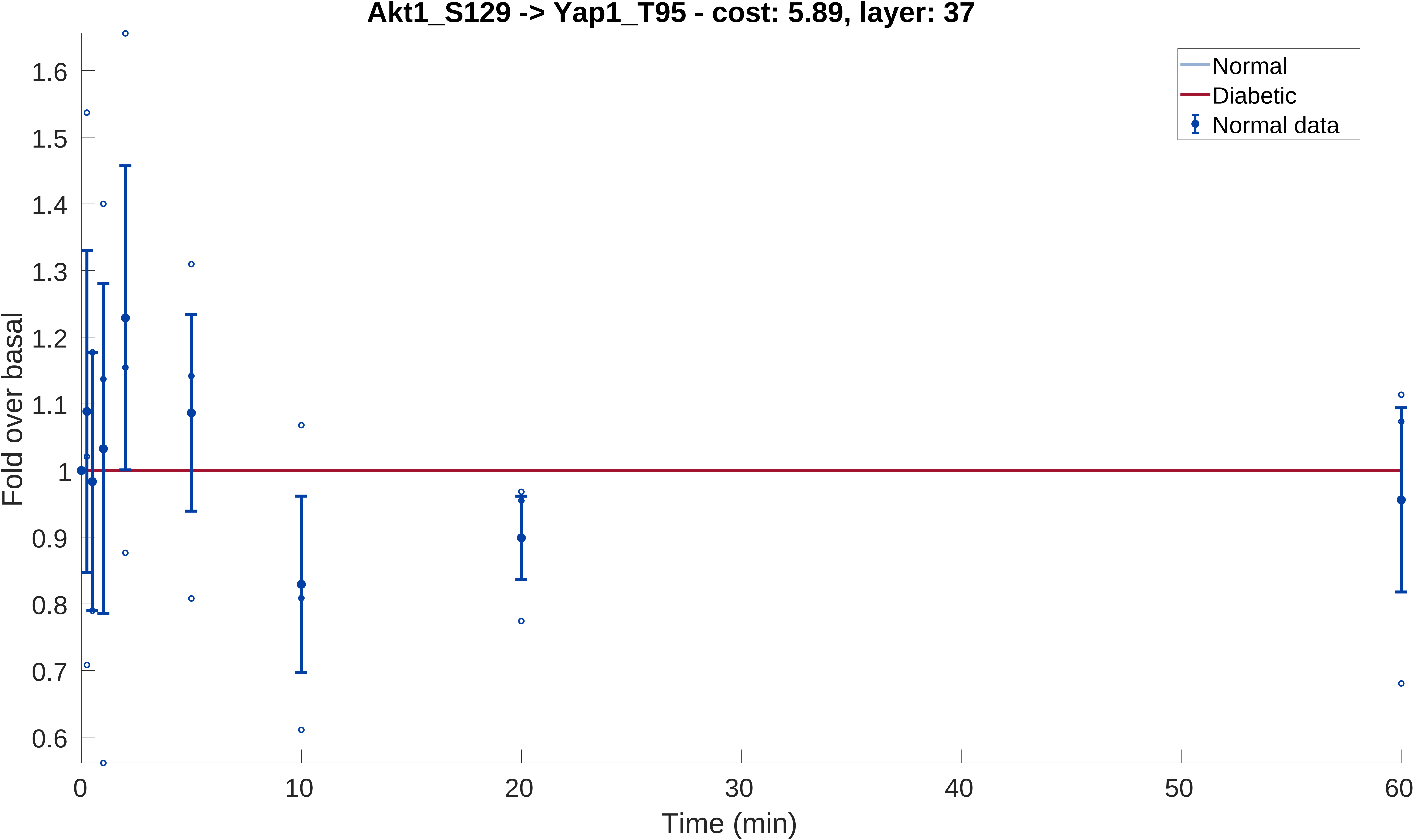

Akt1_S129 -> Yap1_T95 - cost: 5.89, layer: 37
Normal
Diabetic
Normal data
Fold over basal
1.6
1.5
1.4
1.3
1.2
1.1
1
0.9
0.8
0.7
0.6
0
10
20
30
40
50
60
Time (min)

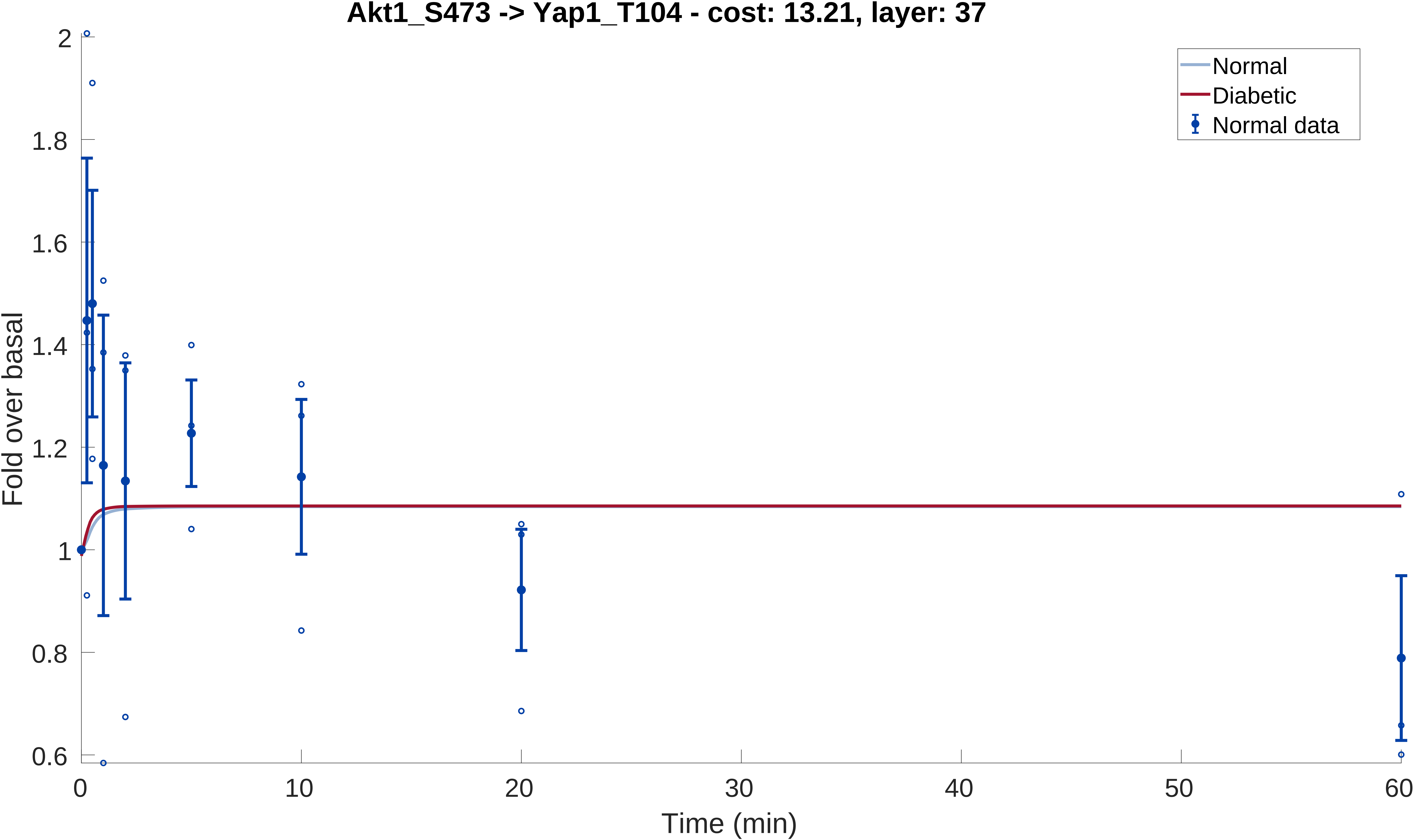

Akt1_S473 -> Yap1_T104 - cost: 13.21, layer: 37
Normal
Diabetic
Normal data
Fold over basal
Time (min)
2
1.8
1.6
1.4
1.2
1
0.8
0.6
0
10
20
30
40
50
60

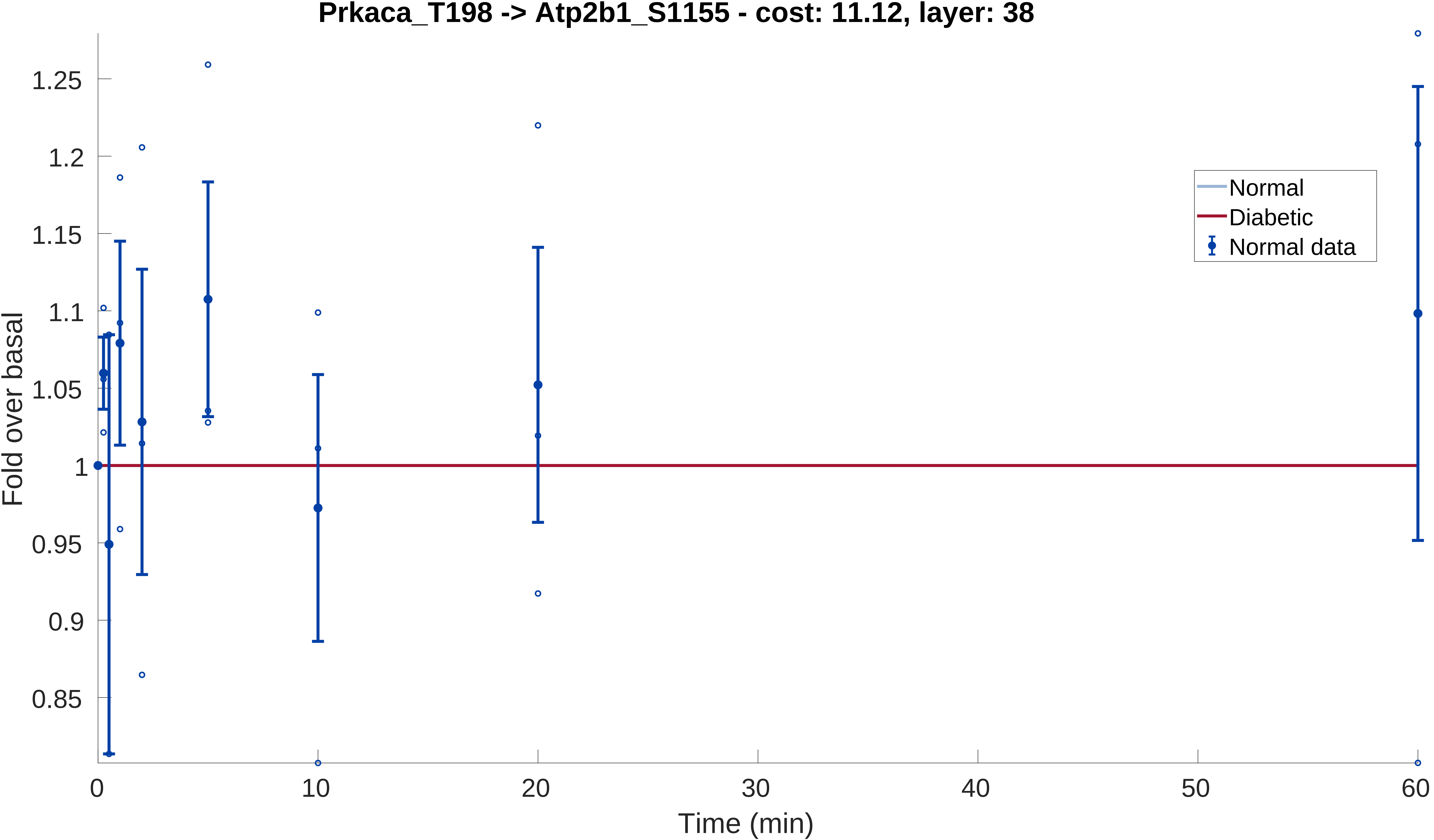

Prkaca_T198 -> Atp2b1_S1155 - cost: 11.12, layer: 38
Fold over basal
1.25
1.2
1.15
1.1
1.05
1
0.95
0.9
0.85
0
10
20
30
40
50
60
Time (min)
Normal
Diabetic
Normal data

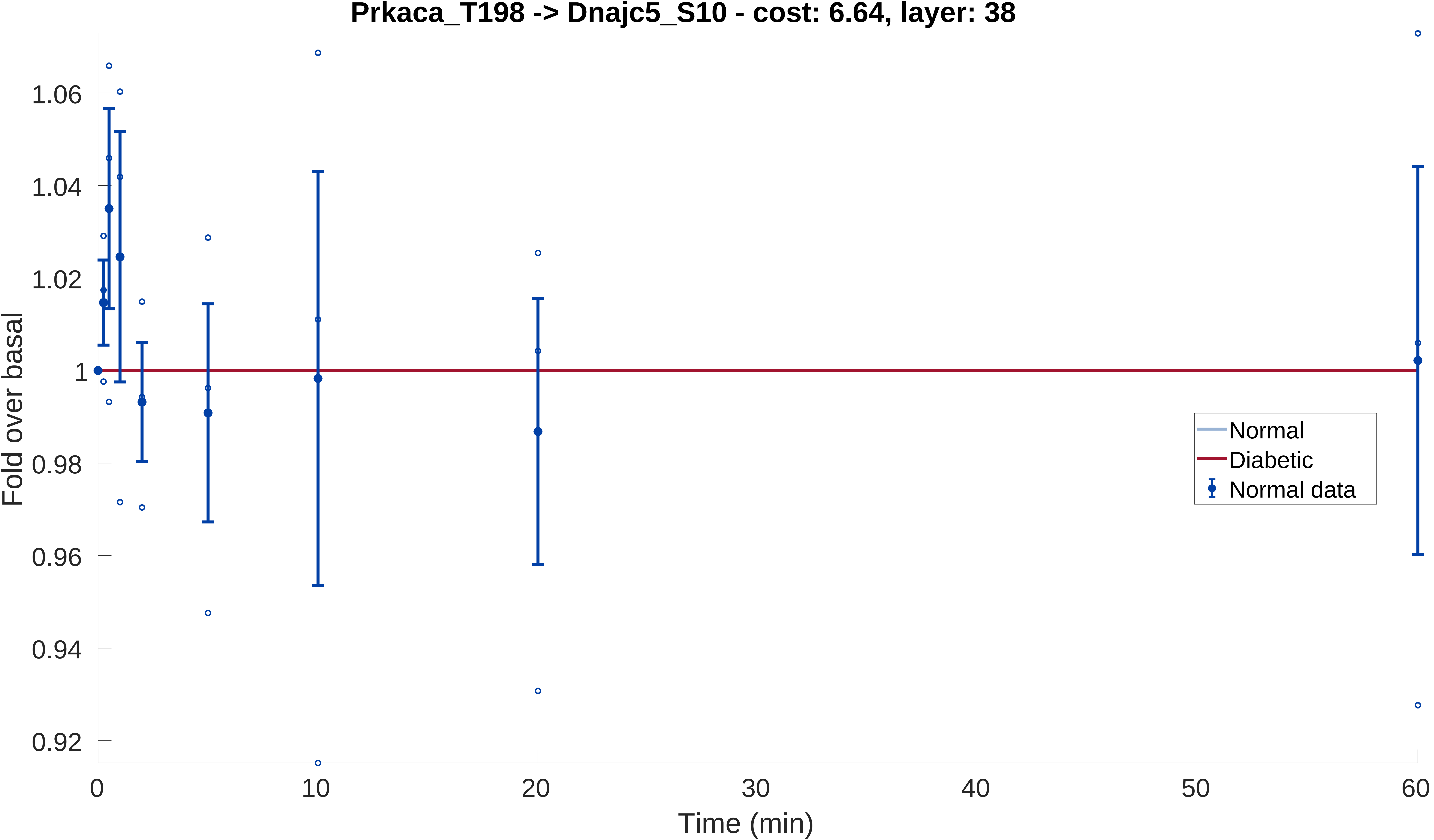

Prkaca_T198 -> Dnajc5_S10 - cost: 6.64, layer: 38
1.06
1.04
1.02
1
0.98
0.96
0.94
0.92
Fold over basal
0
10
20
30
40
50
60
Time (min)
Normal
Diabetic
Normal data

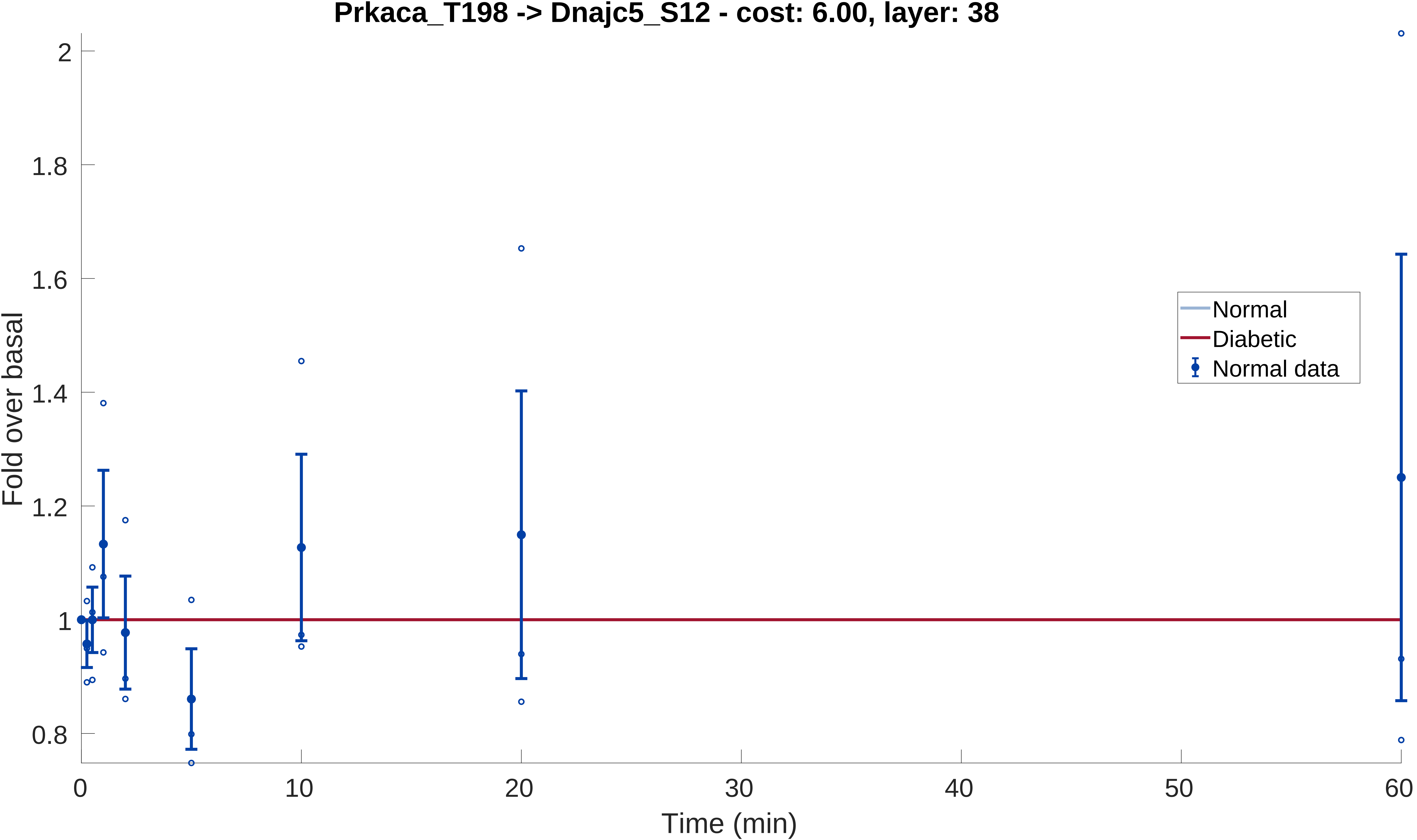
Prkaca_T198 -> Dnajc5_S12 - cost: 6.00, layer: 38
Fold over basal
2
1.8
1.6
1.4
1.2
1
0.8
0
10
20
30
40
50
60
Time (min)
Normal
Diabetic
Normal data

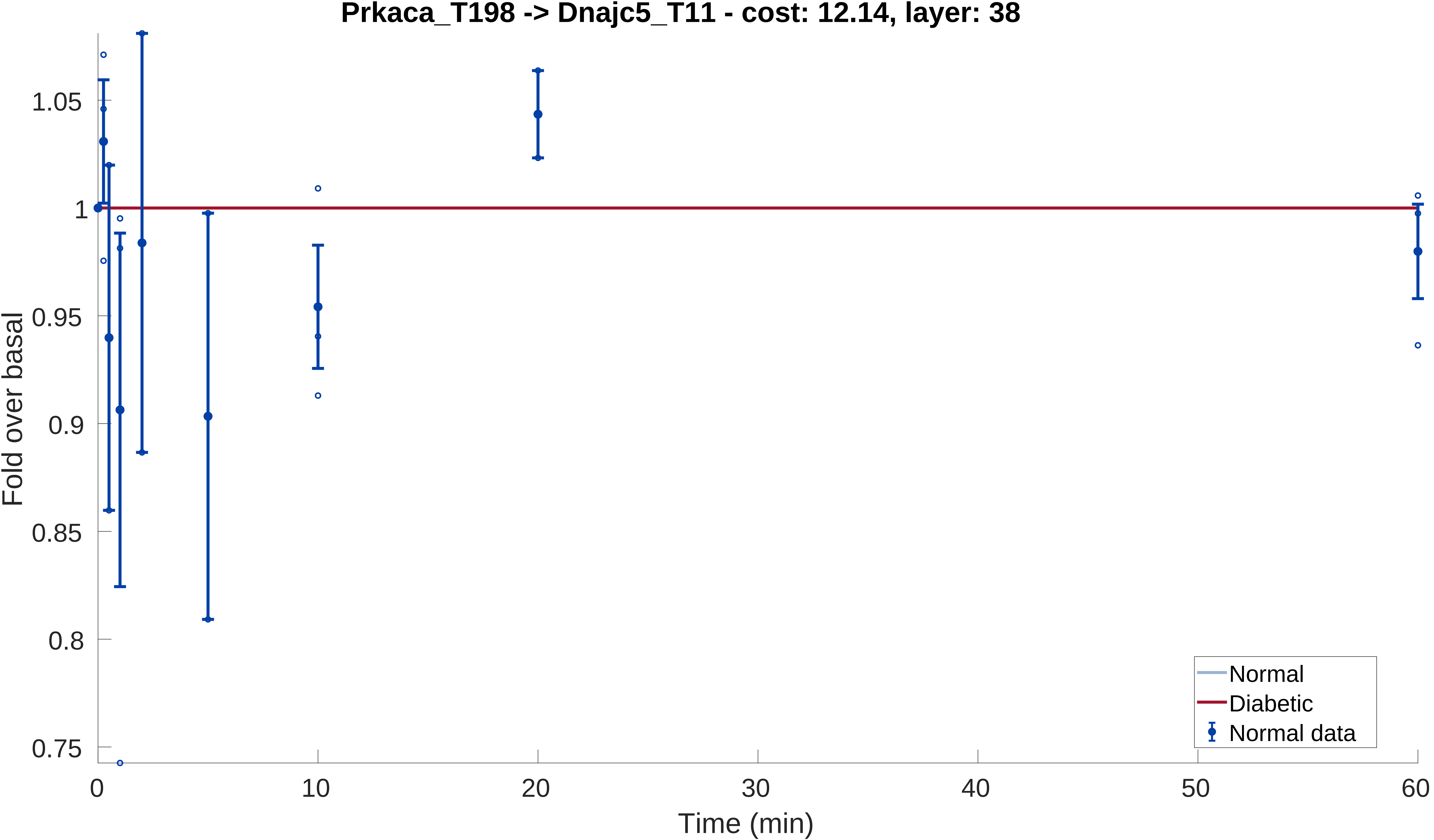

Prkaca_T198 -> Dnajc5_T11 - cost: 12.14, layer: 38
Fold over basal
1.05
1
0.95
0.9
0.85
0.8
0.75
0
10
20
30
40
50
60
Time (min)
Normal
Diabetic
Normal data

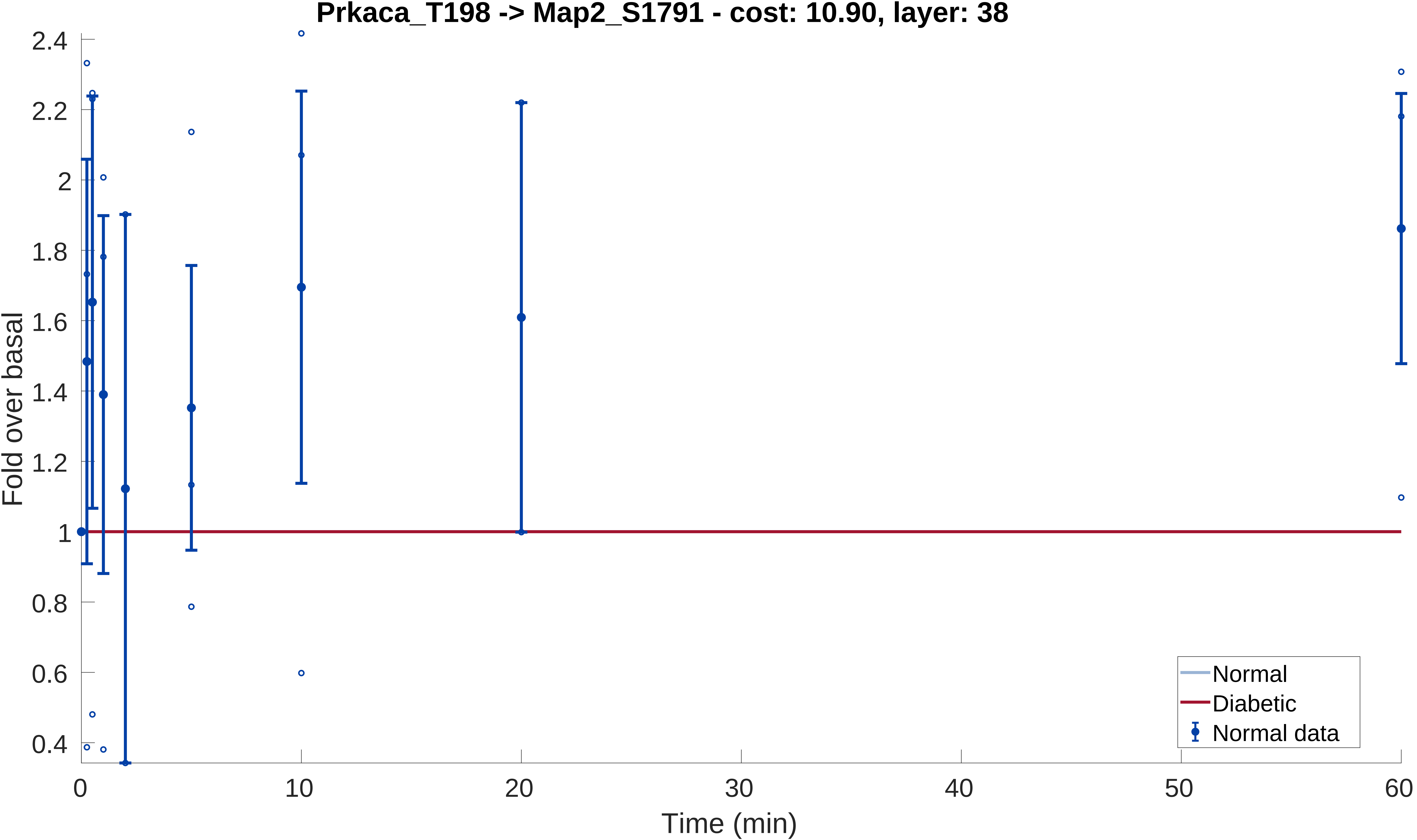
Prkaca_T198 -> Map2_S1791 - cost: 10.90, layer: 38
Fold over basal
2.4
2.2
2
1.8
1.6
1.4
1.2
1
0.8
0.6
0.4
0
10
20
30
40
50
60
Time (min)
Normal
Diabetic
Normal data

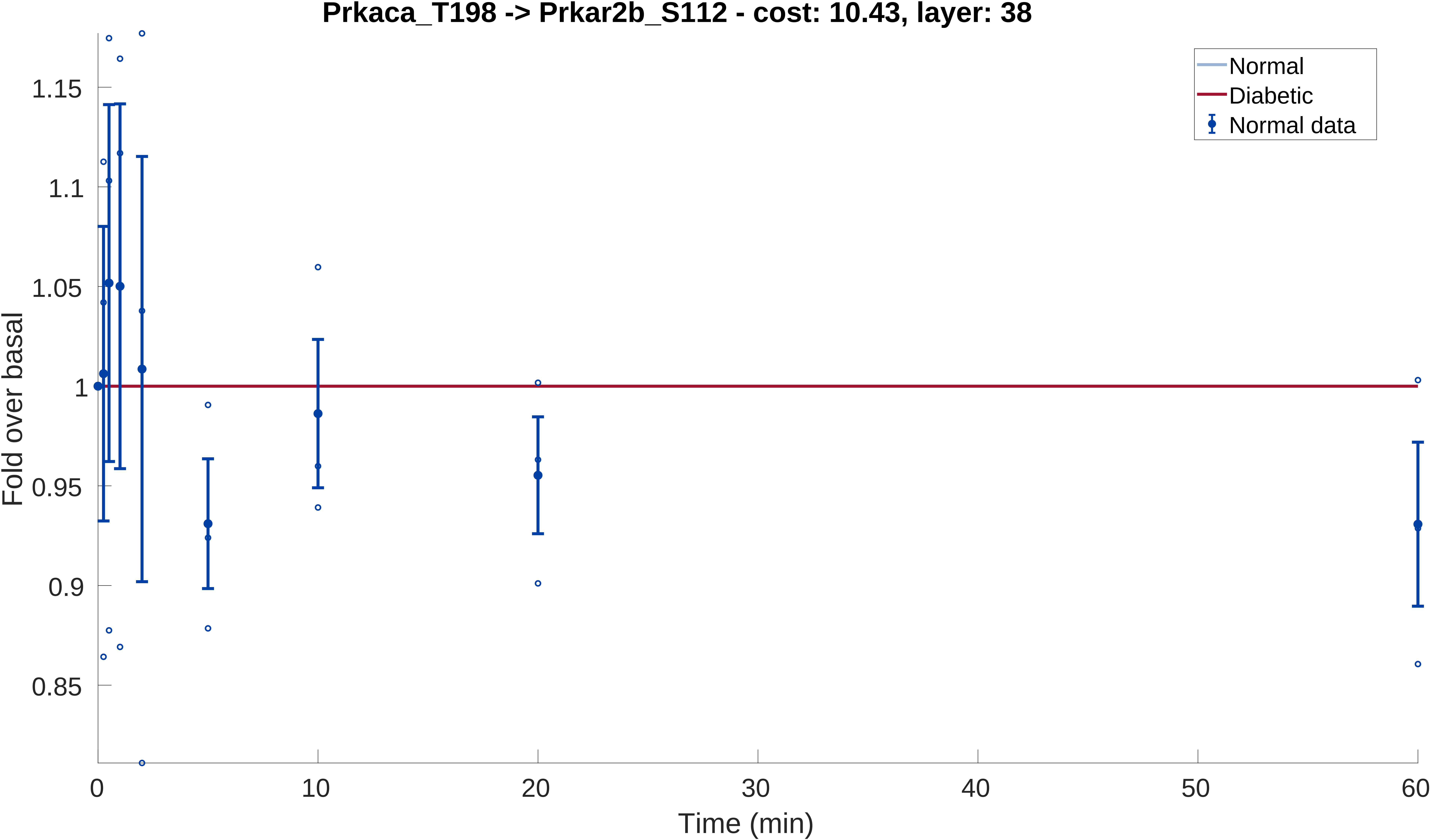
Prkaca_T198 -> Prkar2b_S112 - cost: 10.43, layer: 38
Normal
Diabetic
Normal data
Fold over basal
1.15
1.1
1.05
1
0.95
0.9
0.85
0
10
20
30
40
50
60
Time (min)

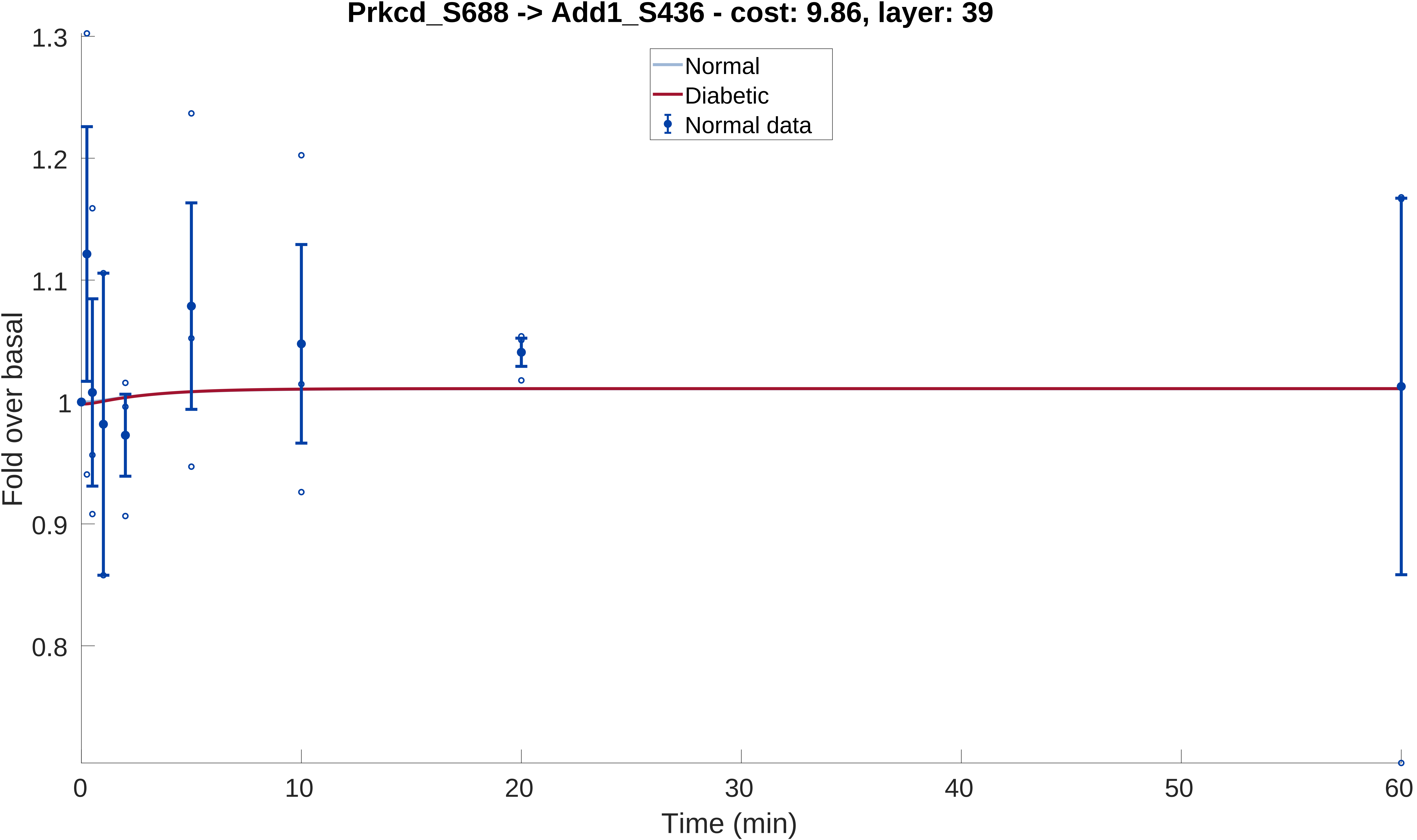

Prkcd_S688 -> Add1_S436 - cost: 9.86, layer: 39
Normal
Diabetic
Normal data
Fold over basal
1.3
1.2
1.1
1
0.9
0.8
Time (min)
0
10
20
30
40
50
60

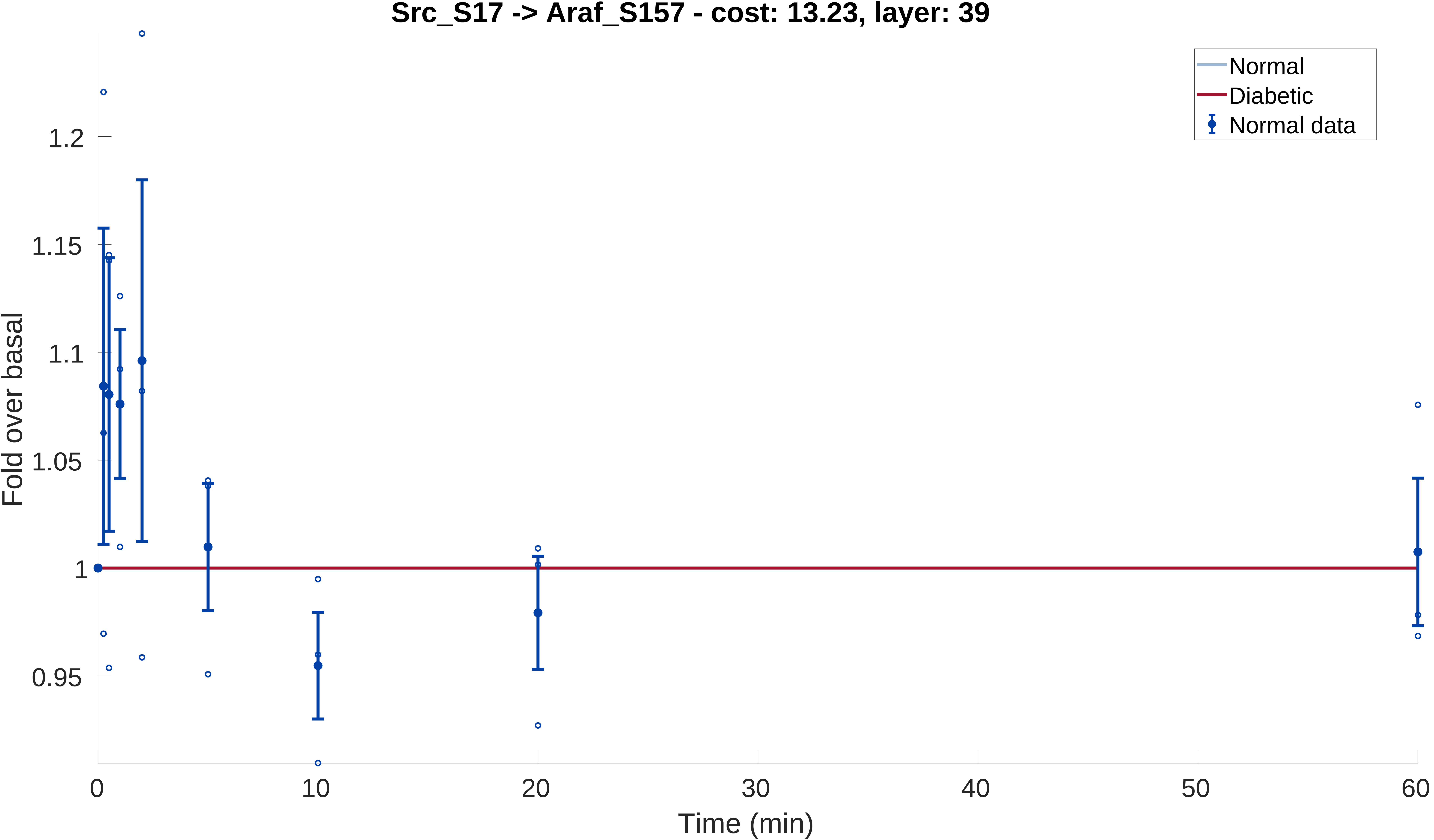

Src_S17 -> Araf_S157 - cost: 13.23, layer: 39
Normal
Diabetic
Normal data
Fold over basal
1.2
1.15
1.1
1.05
1
0.95
0
10
20
30
40
50
60
Time (min)

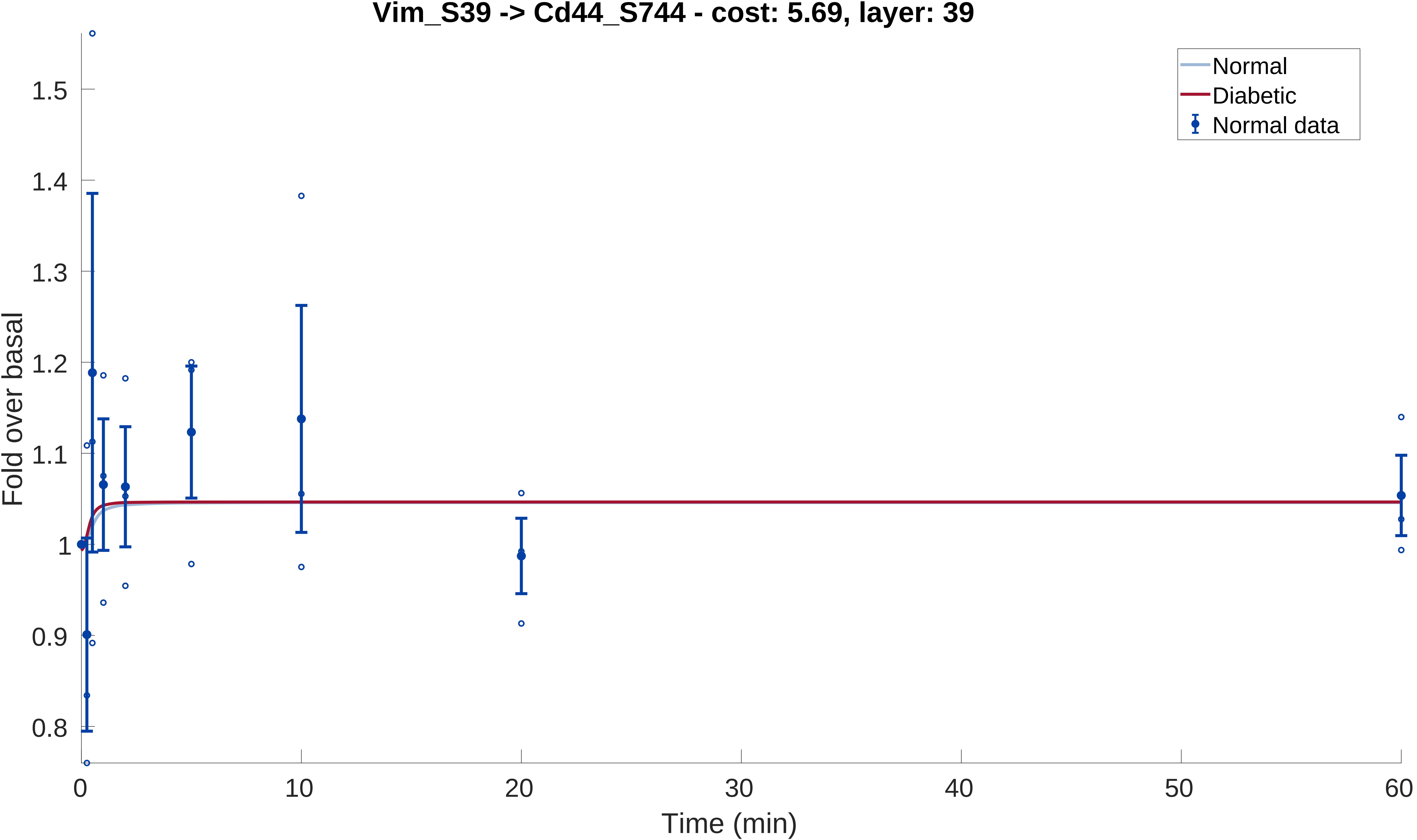

Vim_S39 -> Cd44_S744 - cost: 5.69, layer: 39
Normal
Diabetic
Normal data
Fold over basal
Time (min)
0.8
0.9
1
1.1
1.2
1.3
1.4
1.5
0
10
20
30
40
50
60

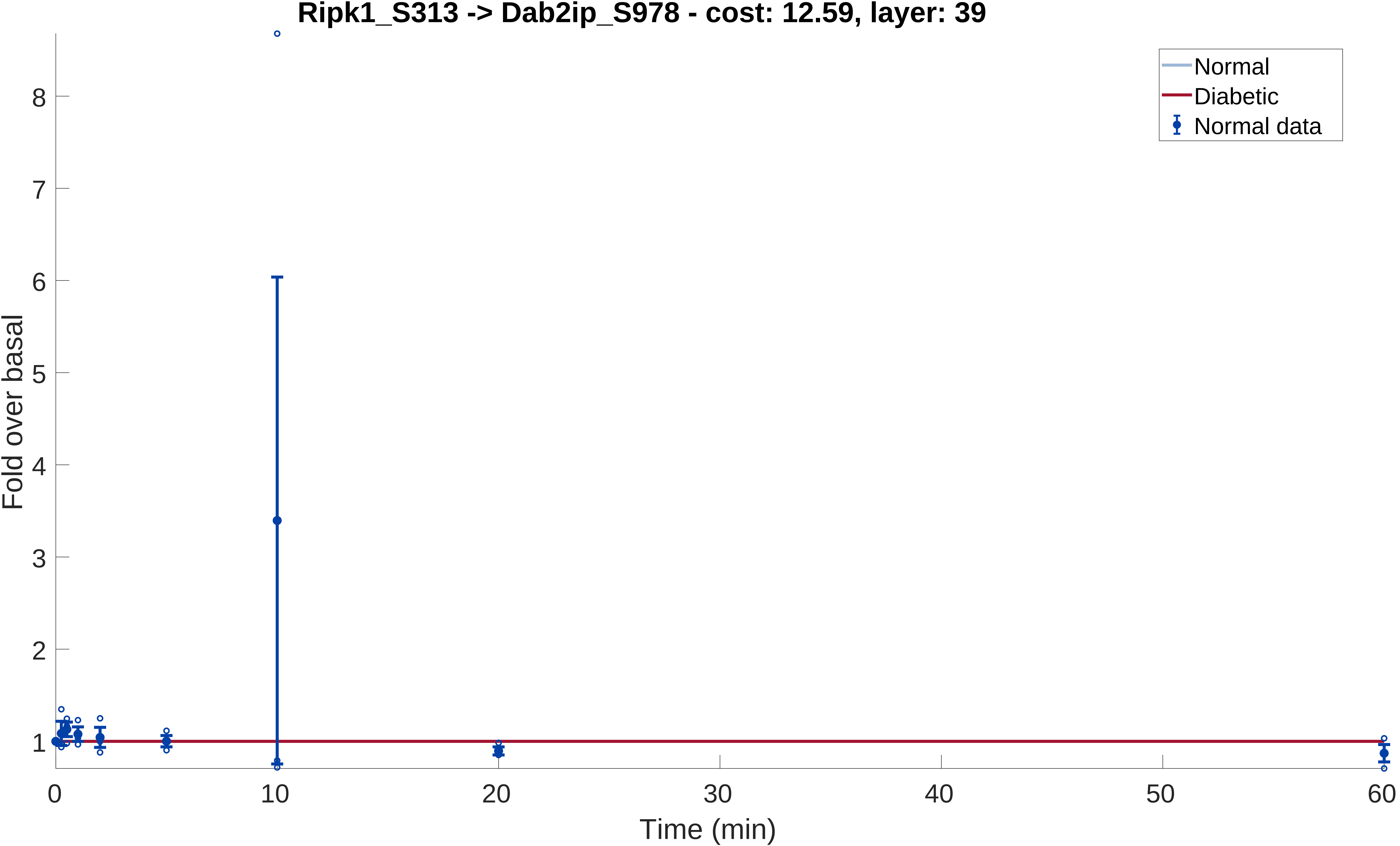

Ripk1_S313 -> Dab2ip_S978 - cost: 12.59, layer: 39
Normal
Diabetic
Normal data
Fold over basal
Time (min)
1
2
3
4
5
6
7
8
0
10
20
30
40
50
60

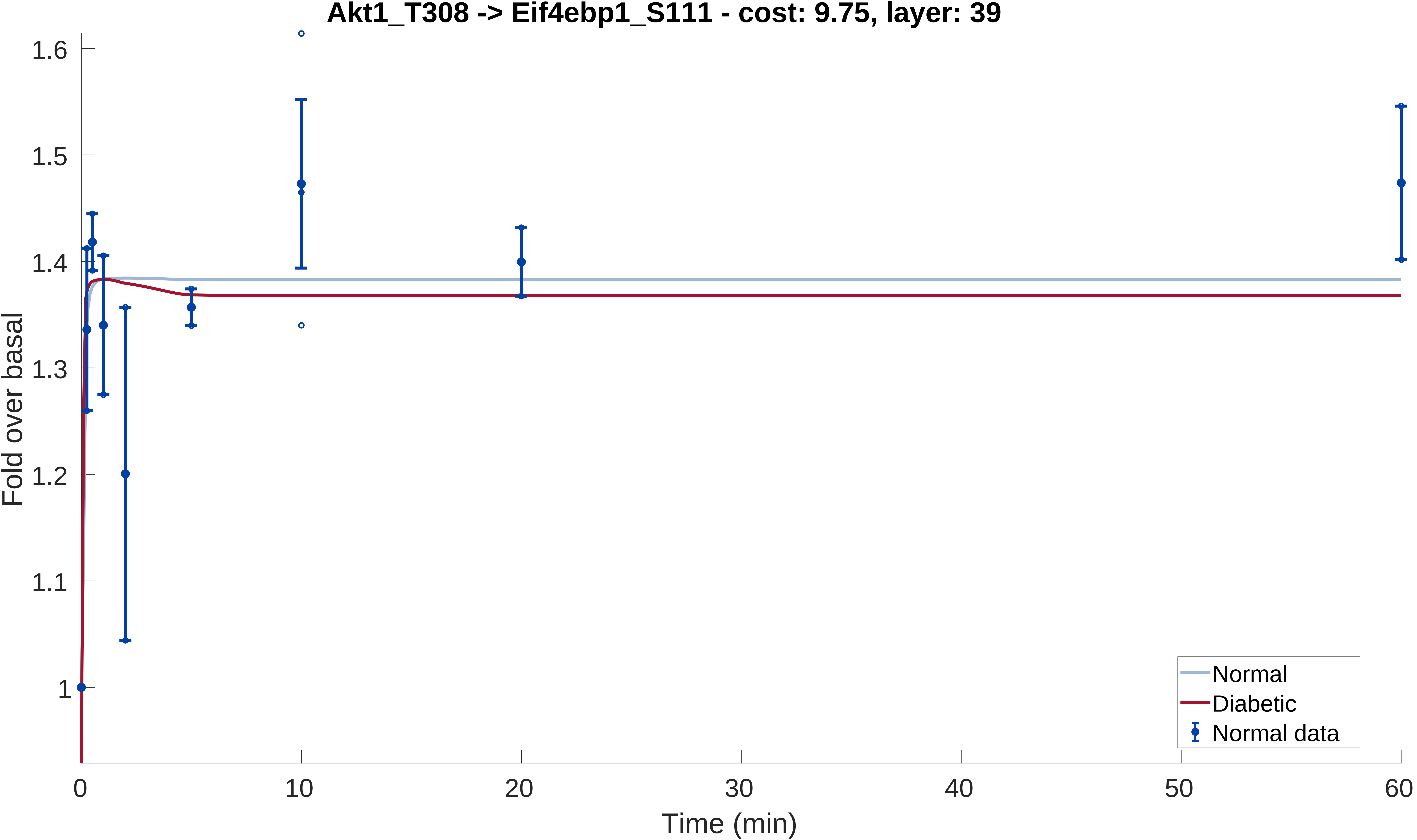

Akt1_T308 -> Eif4ebp1_S111 - cost: 9.75, layer: 39
Fold over basal
1.6
1.5
1.4
1.3
1.2
1.1
1
0
10
20
30
40
50
60
Time (min)
Normal
Diabetic
Normal data

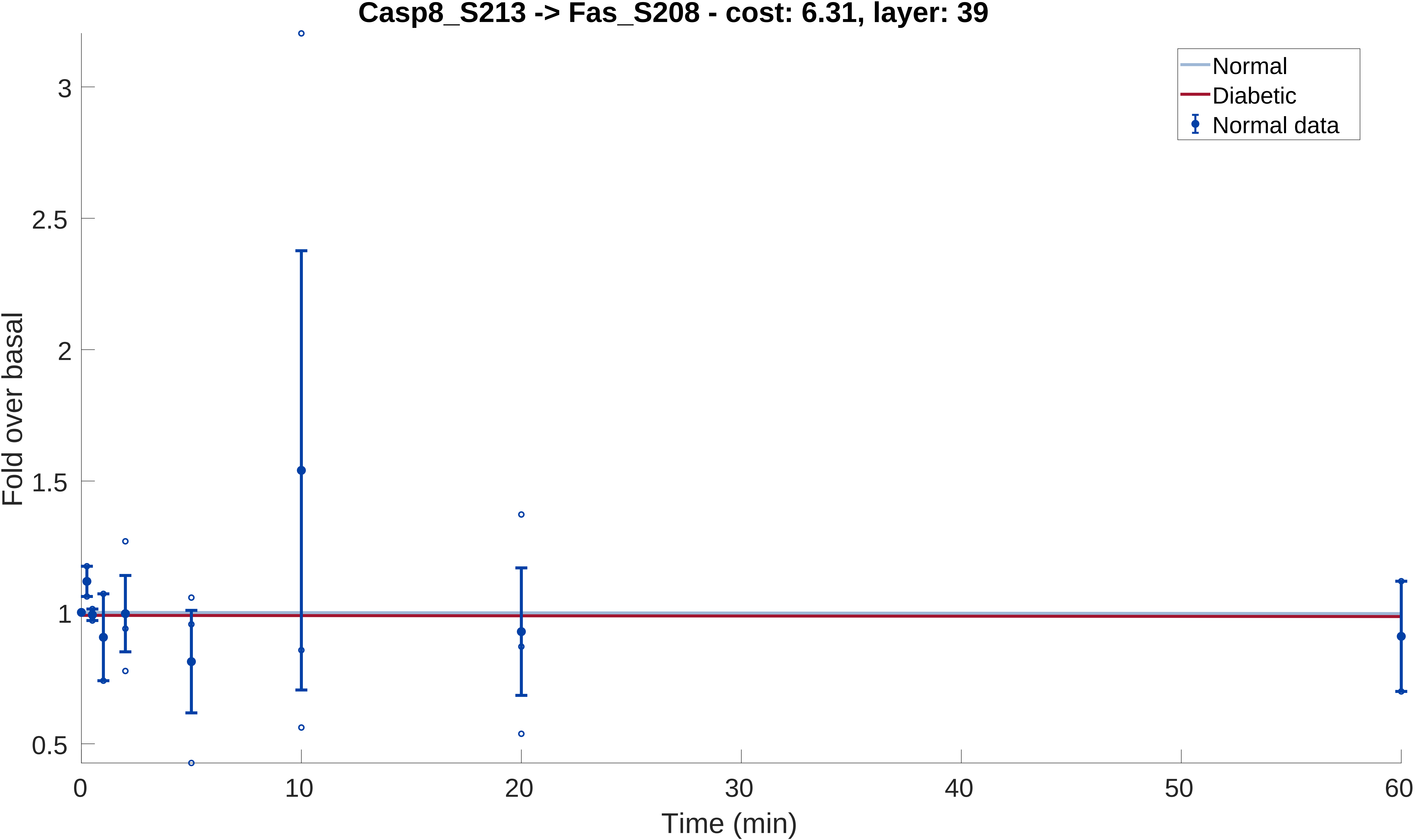
Casp8_S213 -> Fas_S208 - cost: 6.31, layer: 39
Normal
Diabetic
Normal data
Fold over basal
Time (min)
0.5
1
1.5
2
2.5
3
0
10
20
30
40
50
60

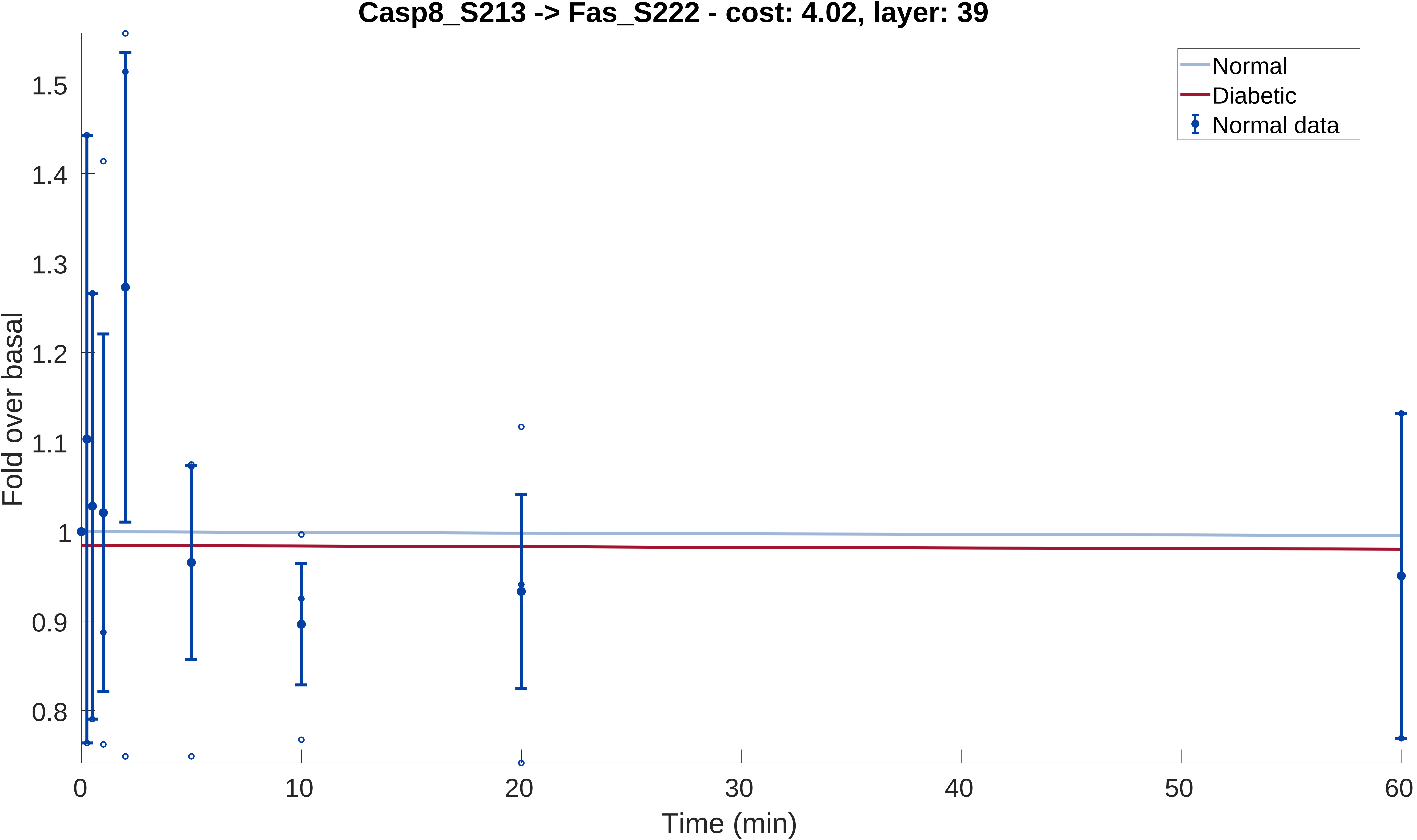

Casp8_S213 -> Fas_S222 - cost: 4.02, layer: 39
Normal
Diabetic
Normal data
Fold over basal
Time (min)
1.5
1.4
1.3
1.2
1.1
1
0.9
0.8
0
10
20
30
40
50
60

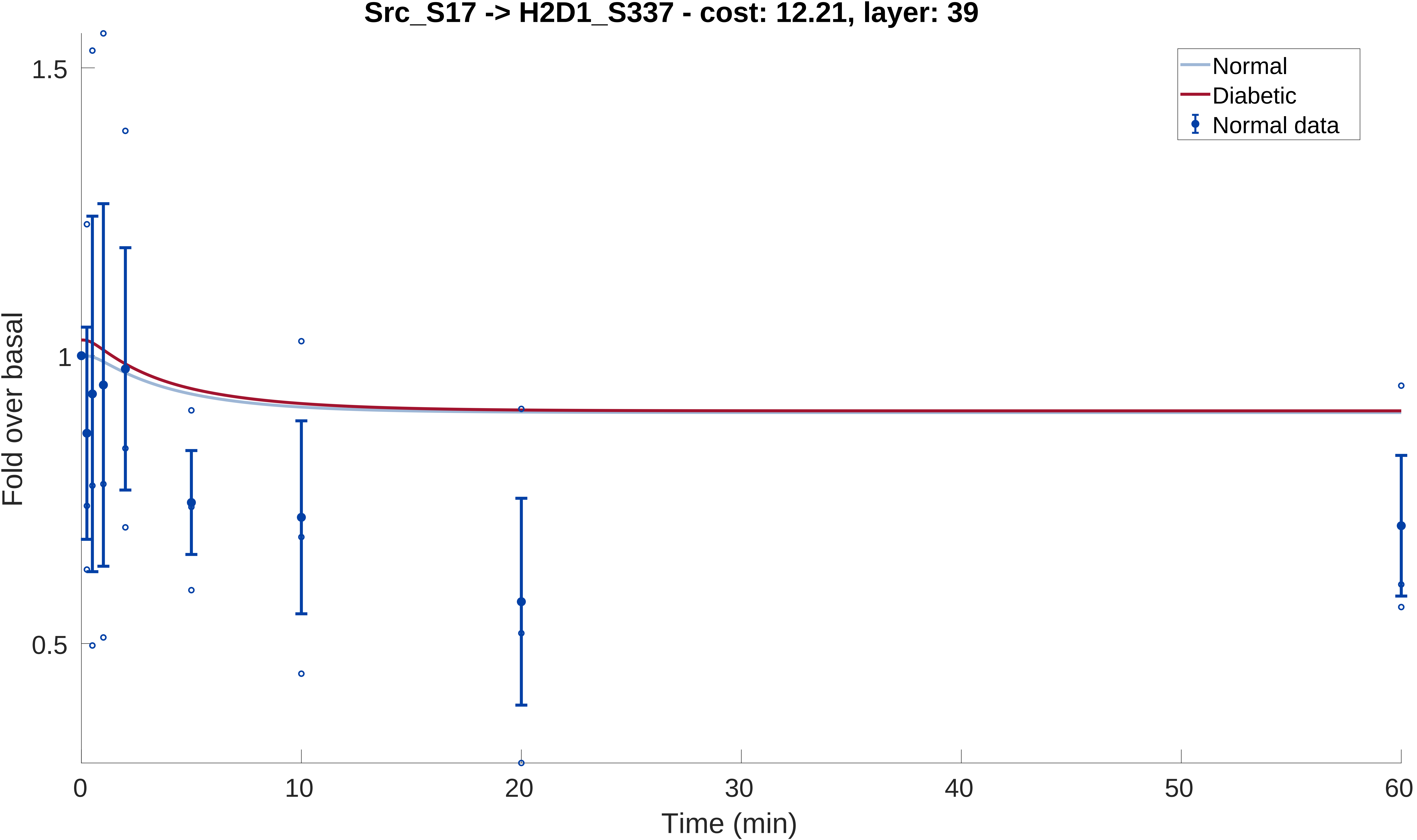

Src_S17 -> H2D1_S337 - cost: 12.21, layer: 39
Normal
Diabetic
Normal data
1.5
1
0.5
Fold over basal
0
10
20
30
40
50
60
Time (min)

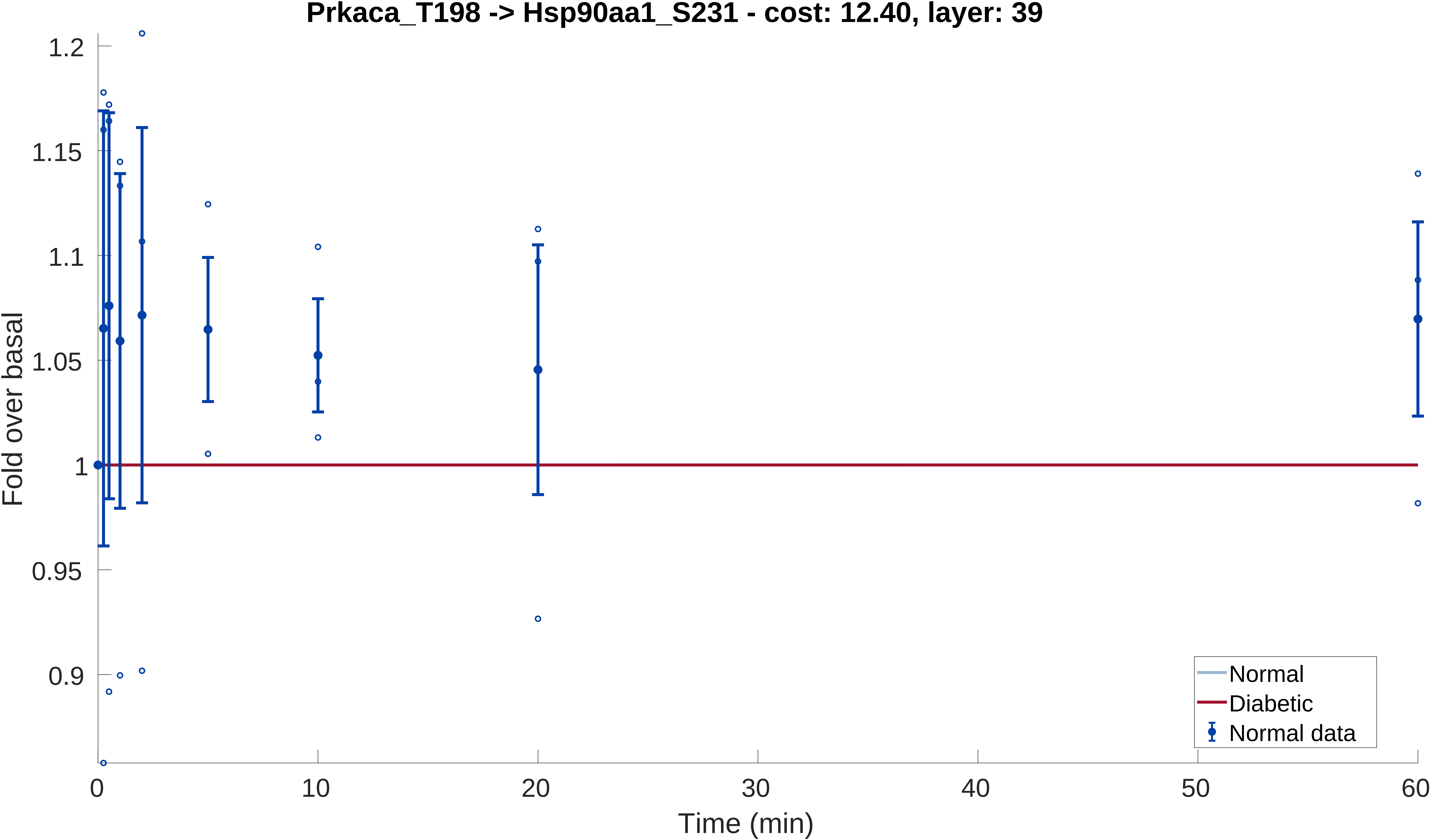

Prkaca_T198 -> Hsp90aa1_S231 - cost: 12.40, layer: 39
Fold over basal
Time (min)
1.2
1.15
1.1
1.05
1
0.95
0.9
0
10
20
30
40
50
60
Normal
Diabetic
Normal data

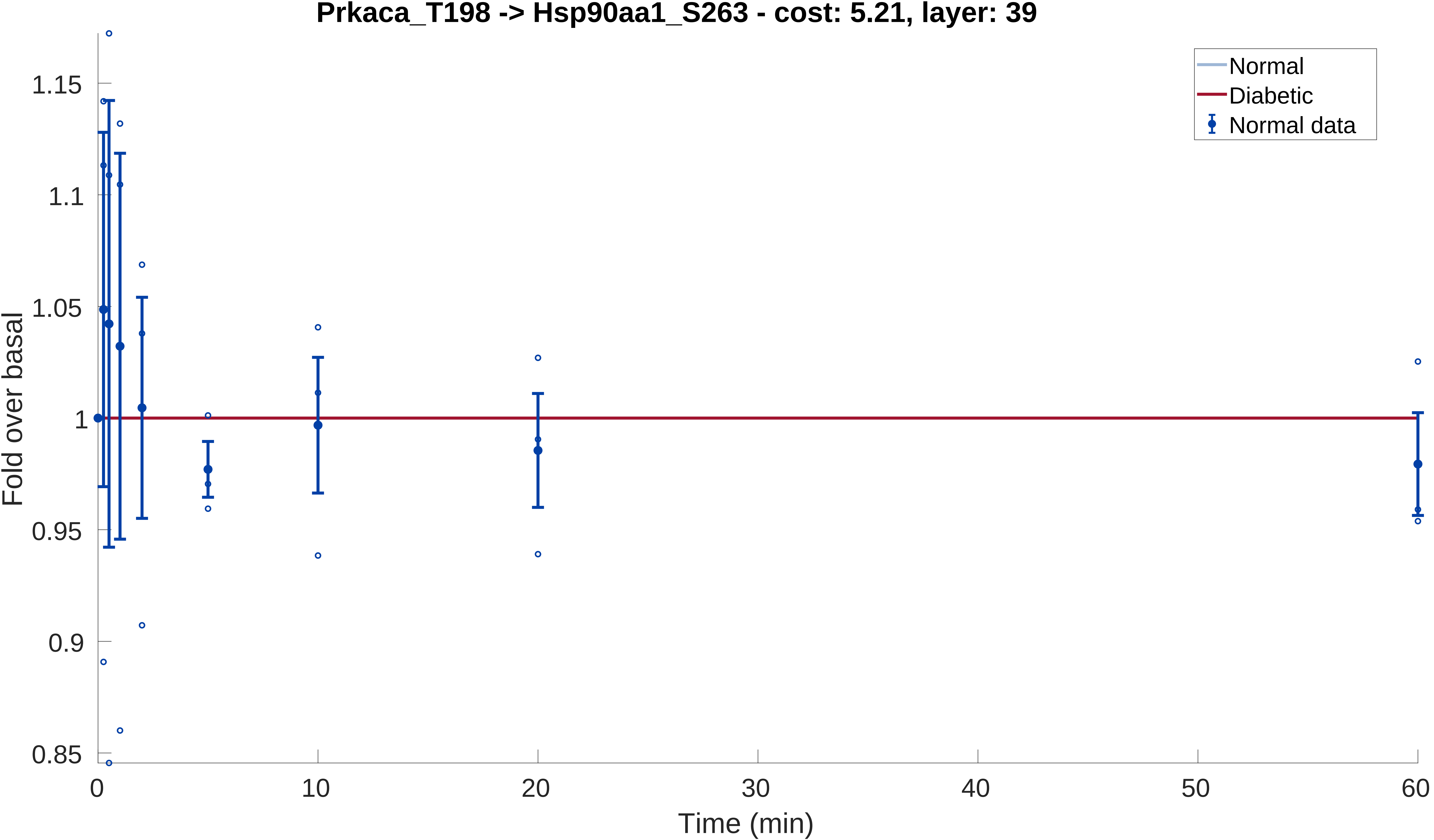
Prrkaca_T198 -> Hsp90aa1_S263 - cost: 5.21, layer: 39
Normal
Diabetic
Normal data
Fold over basal
Time (min)
1.15
1.1
1.05
1
0.95
0.9
0.85
0
10
20
30
40
50
60

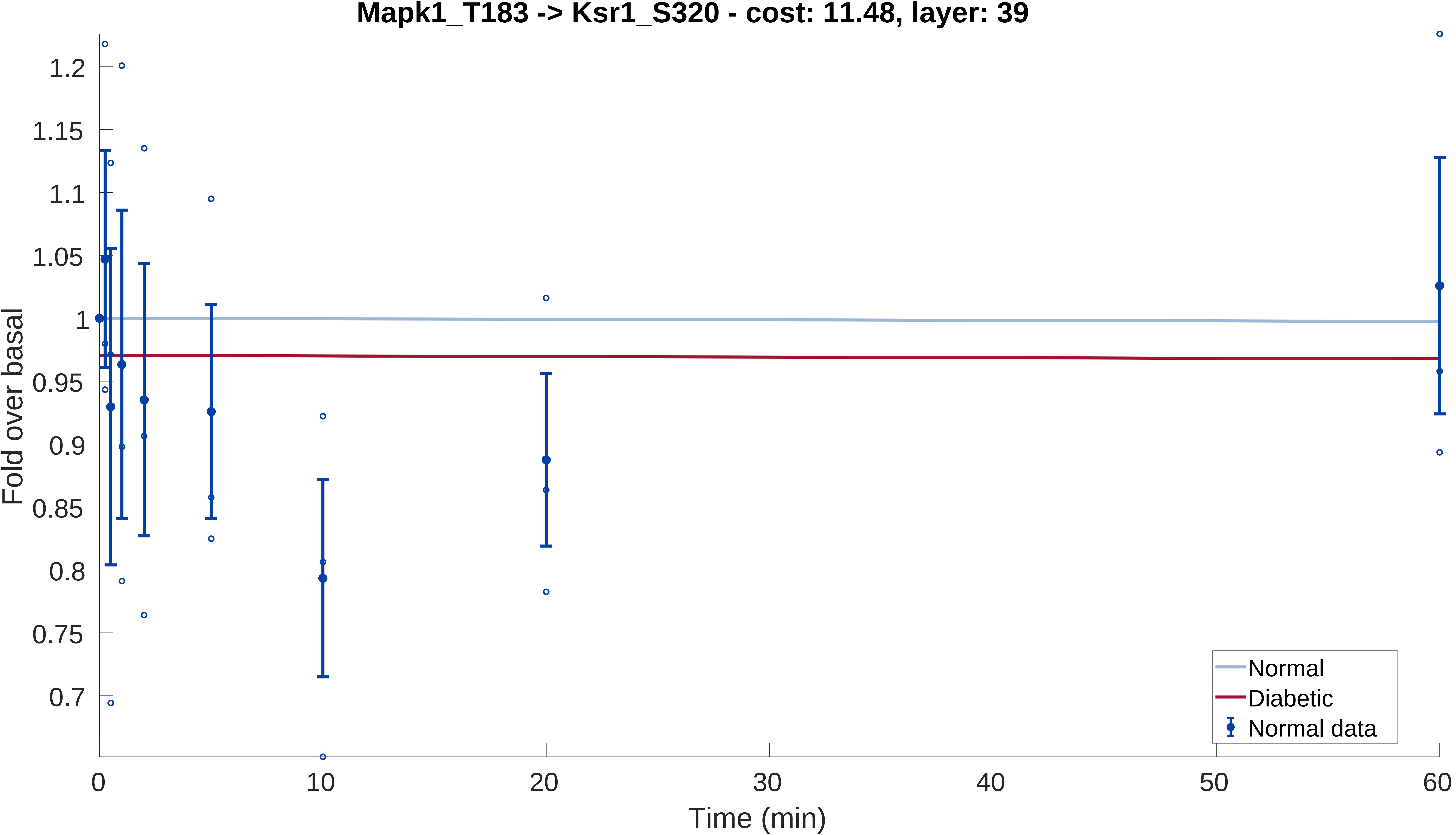

Mapk1_T183 -> Ksr1_S320 - cost: 11.48, layer: 39
Fold over basal
Time (min)
1.2
1.15
1.1
1.05
1
0.95
0.9
0.85
0.8
0.75
0.7
0
10
20
30
40
50
60
Normal
Diabetic
Normal data

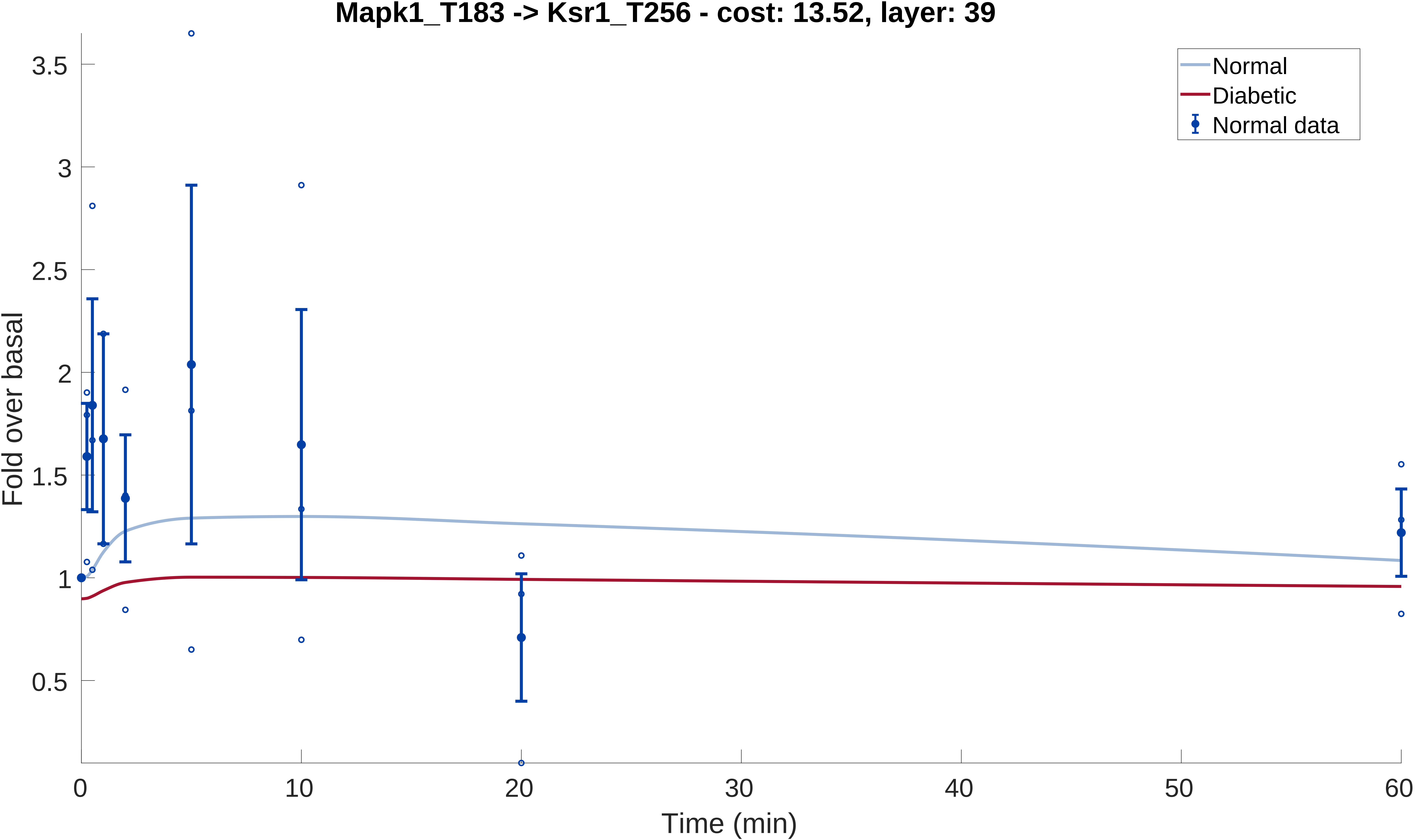
Mapk1_T183 -> Ksr1_T256 - cost: 13.52, layer: 39
Fold over basal
Time (min)
Normal
Diabetic
Normal data
3.5
3
2.5
2
1.5
1
0.5
0
10
20
30
40
50
60

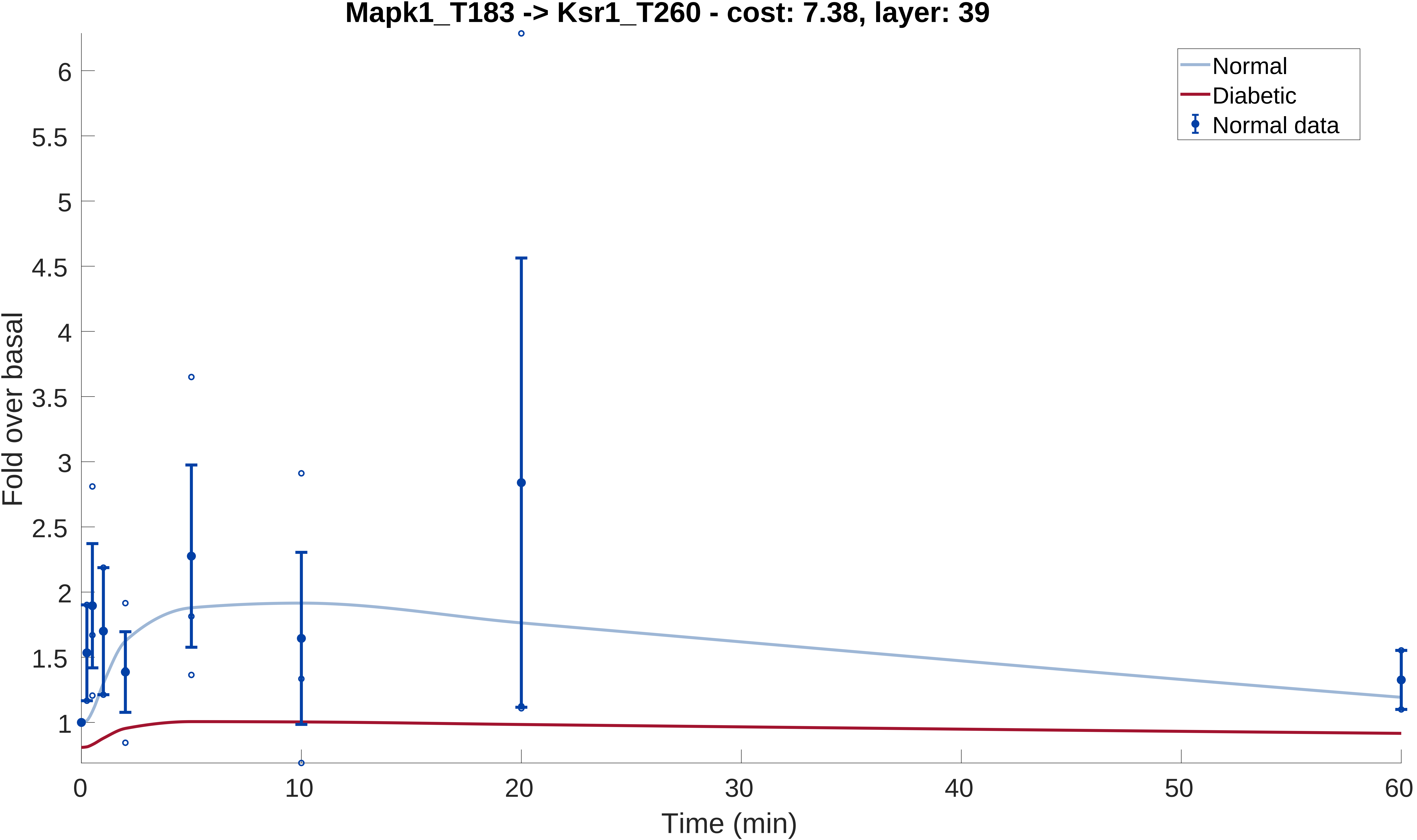
Mapk1_T183 -> Ksr1_T260 - cost: 7.38, layer: 39
Normal
Diabetic
Normal data
Fold over basal
Time (min)
6
5.5
5
4.5
4
3.5
3
2.5
2
1.5
1
0
10
20
30
40
50
60

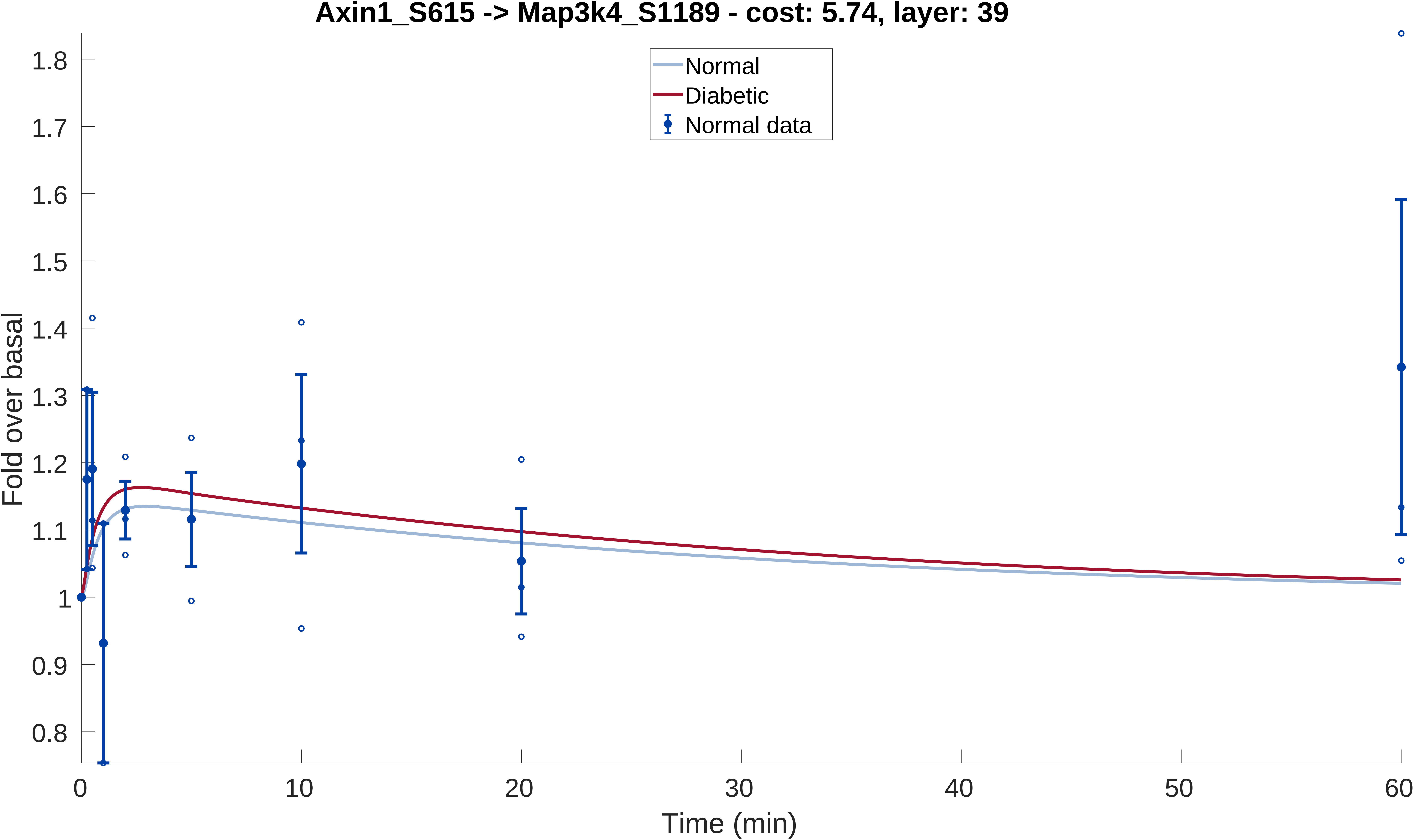

Axin1_S615 -> Map3k4_S1189 - cost: 5.74, layer: 39
Normal
Diabetic
Normal data
Fold over basal
1.8
1.7
1.6
1.5
1.4
1.3
1.2
1.1
1
0.9
0.8
0
10
20
30
40
50
60
Time (min)

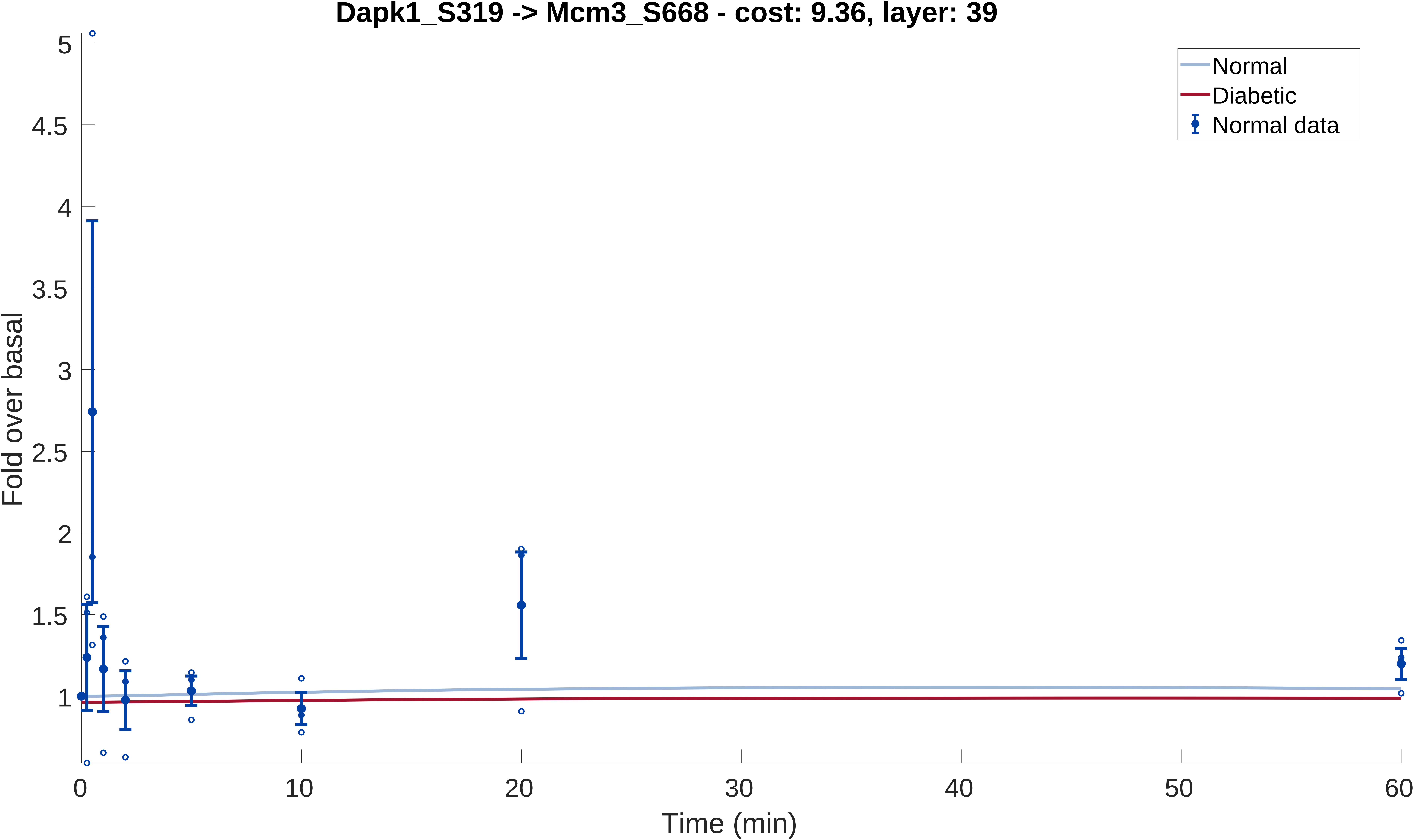
Dapk1_S319 -> Mcm3_S668 - cost: 9.36, layer: 39
Normal
Diabetic
Normal data
Fold over basal
Time (min)
5
4.5
4
3.5
3
2.5
2
1.5
1
0
10
20
30
40
50
60

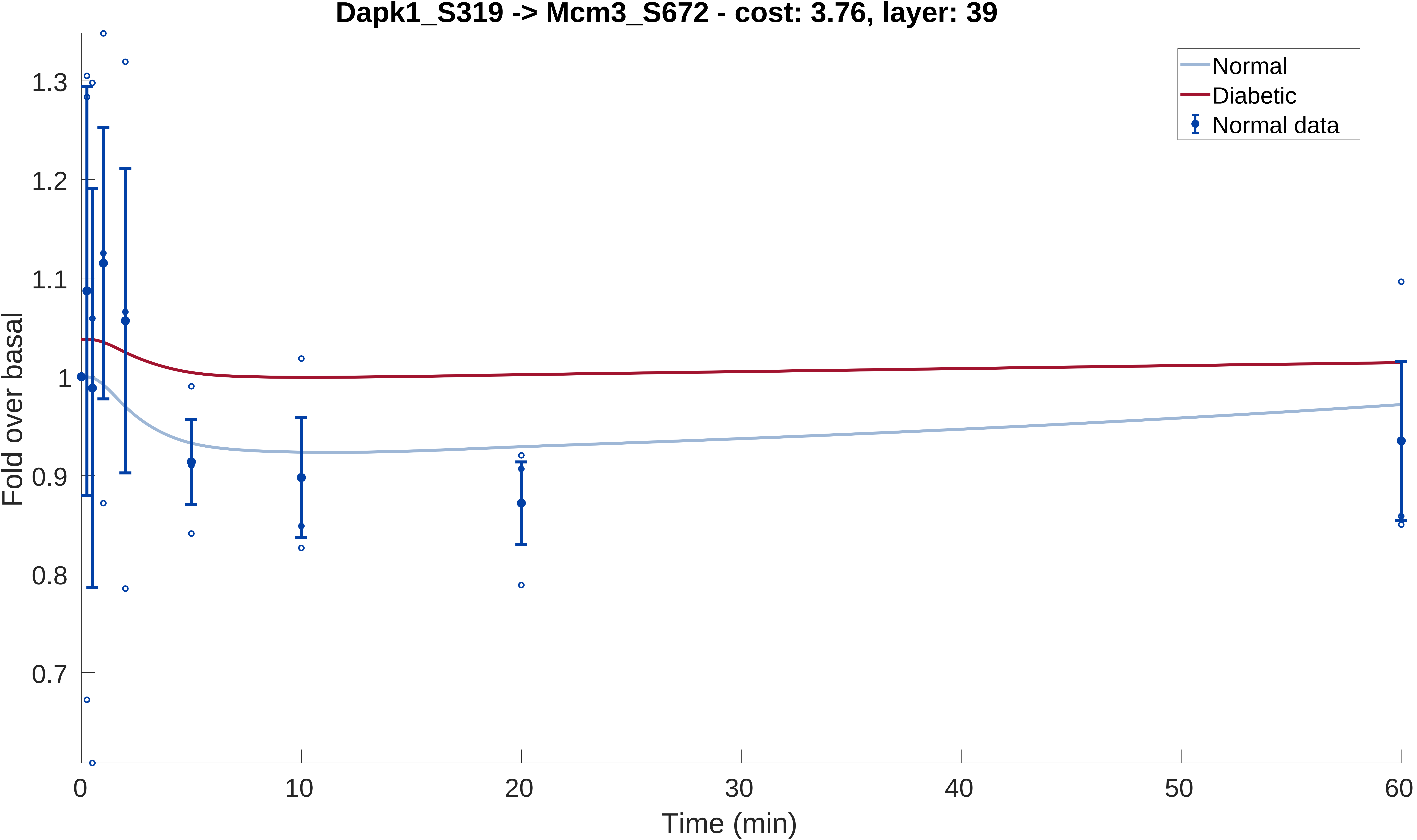

Dapk1_S319 -> Mcm3_S672 - cost: 3.76, layer: 39
Normal
Diabetic
Normal data
Fold over basal
1.3
1.2
1.1
1
0.9
0.8
0.7
0
10
20
30
40
50
60
Time (min)

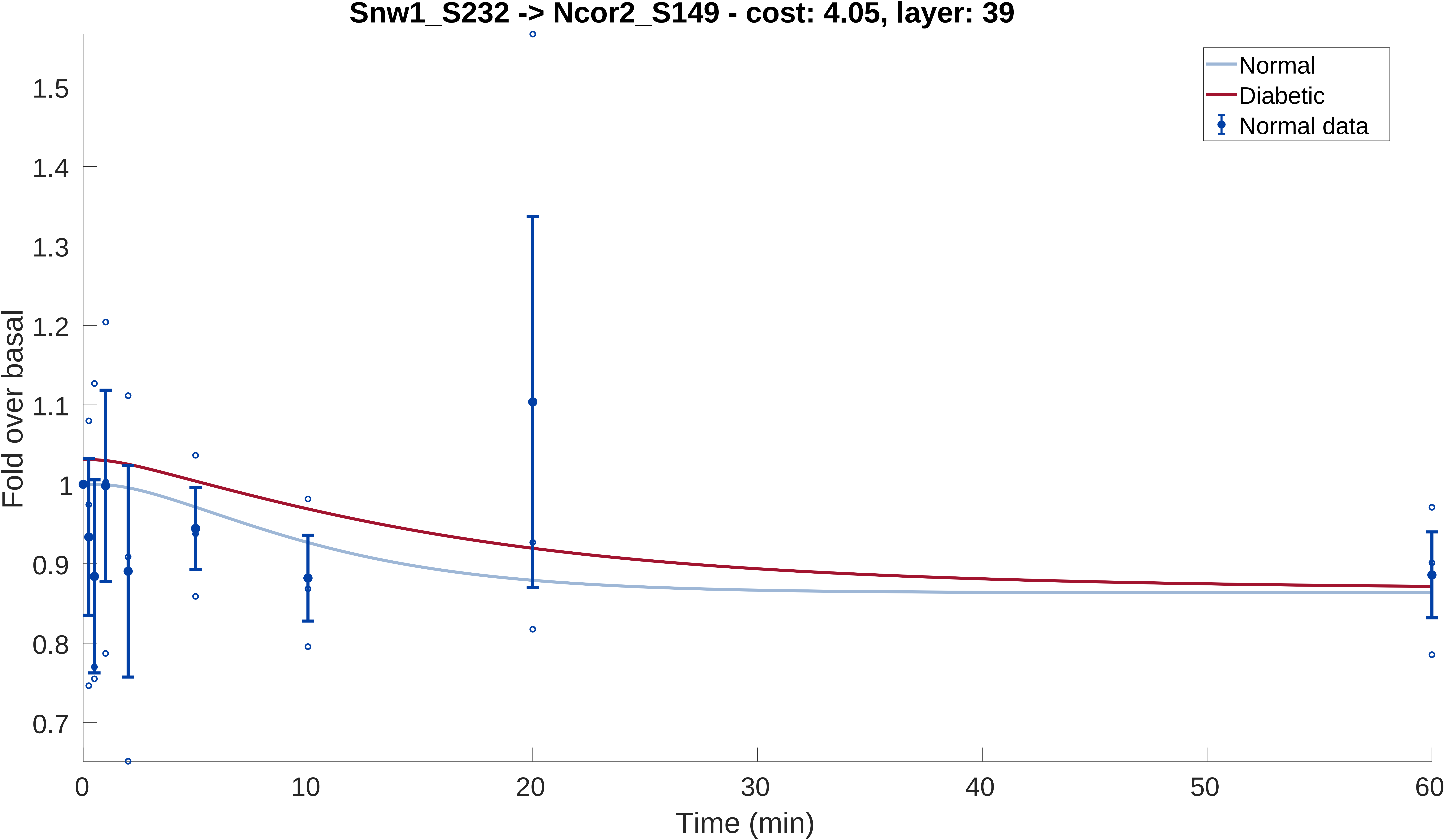
Snw1_S232 -> Ncor2_S149 - cost: 4.05, layer: 39
Normal
Diabetic
Normal data
Fold over basal
Time (min)
0.7
0.8
0.9
1
1.1
1.2
1.3
1.4
1.5
0
10
20
30
40
50
60

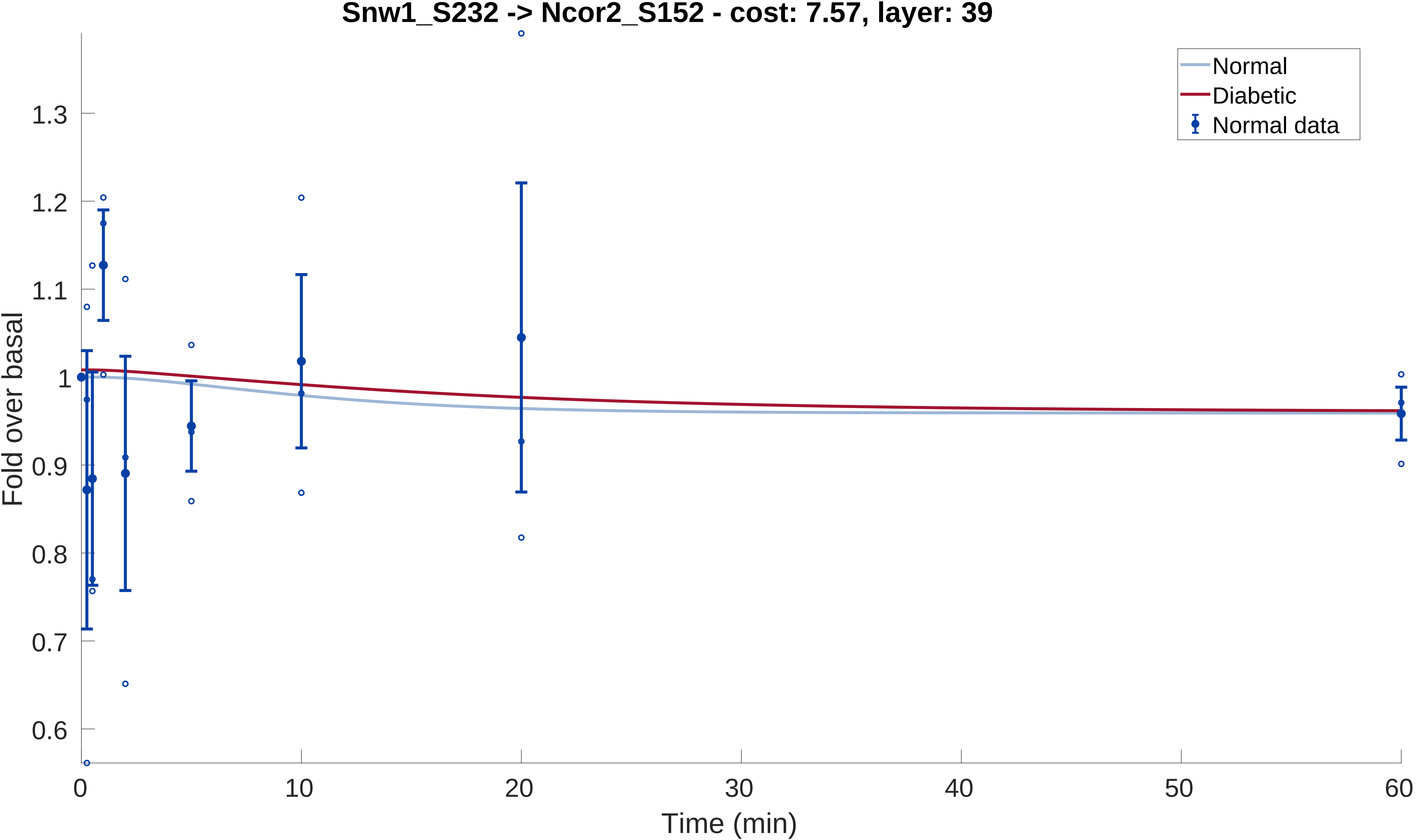

Snw1_S232 -> Ncor2_S152 - cost: 7.57, layer: 39
Normal
Diabetic
Normal data
Fold over basal
Time (min)
0.6
0.7
0.8
0.9
1
1.1
1.2
1.3
0
10
20
30
40
50
60

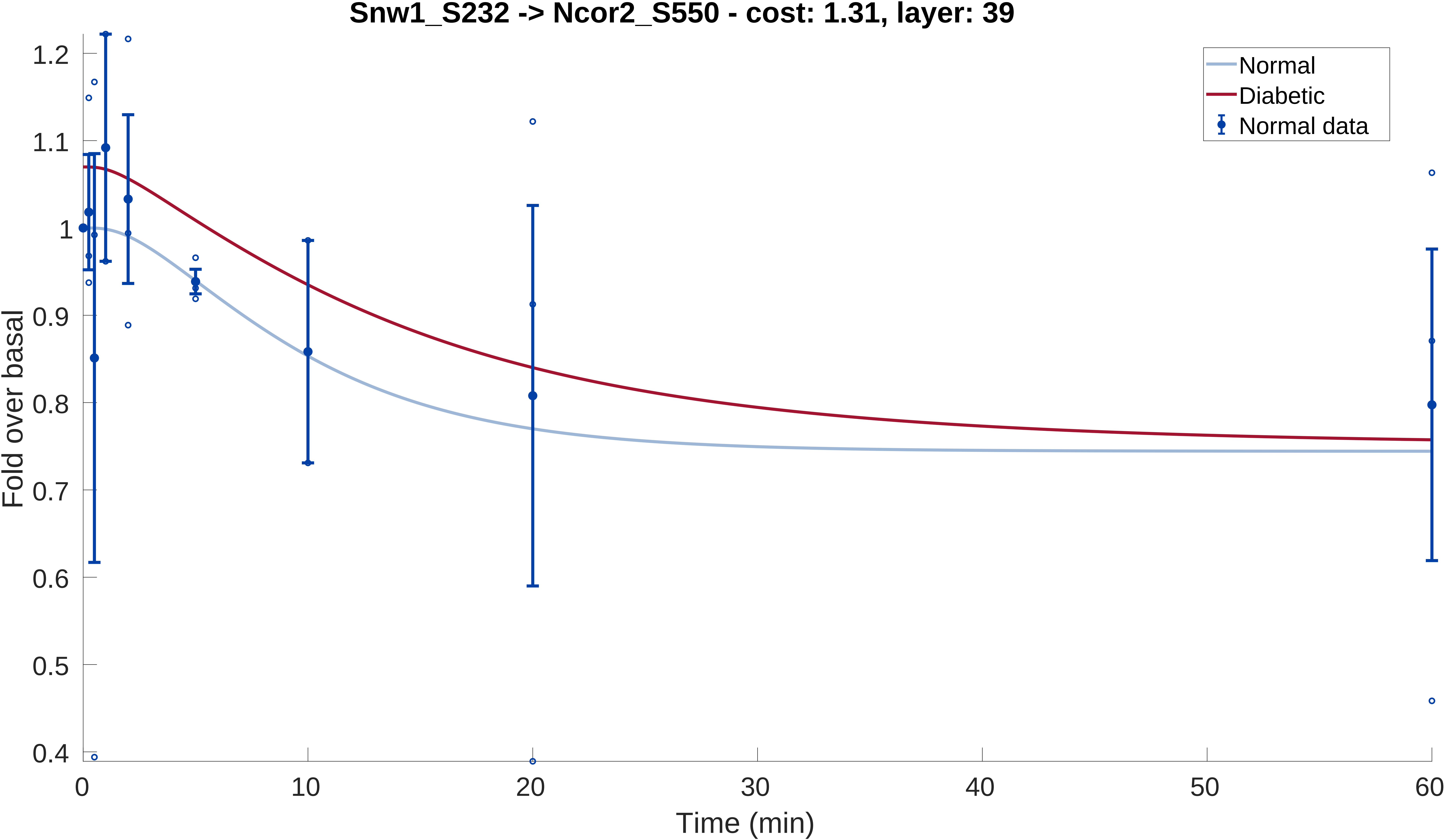

Snw1_S232 -> Ncor2_S550 - cost: 1.31, layer: 39
Normal
Diabetic
Normal data
Fold over basal
1.2
1.1
1
0.9
0.8
0.7
0.6
0.5
0.4
0
10
20
30
40
50
60
Time (min)

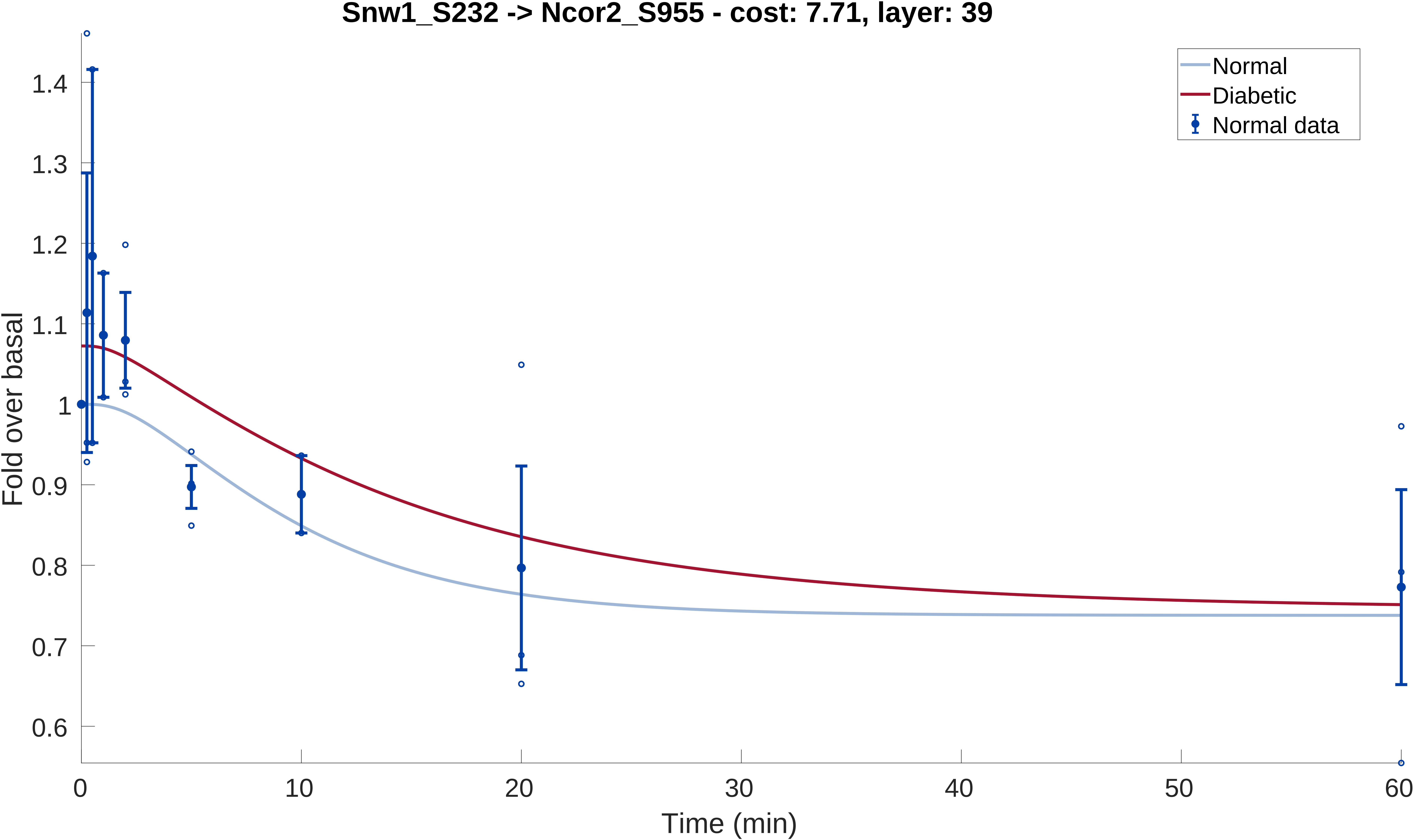
Snw1_S232 -> Ncor2_S955 - cost: 7.71, layer: 39
Normal
Diabetic
Normal data
Fold over basal
1.4
1.3
1.2
1.1
1
0.9
0.8
0.7
0.6
0
10
20
30
40
50
60
Time (min)

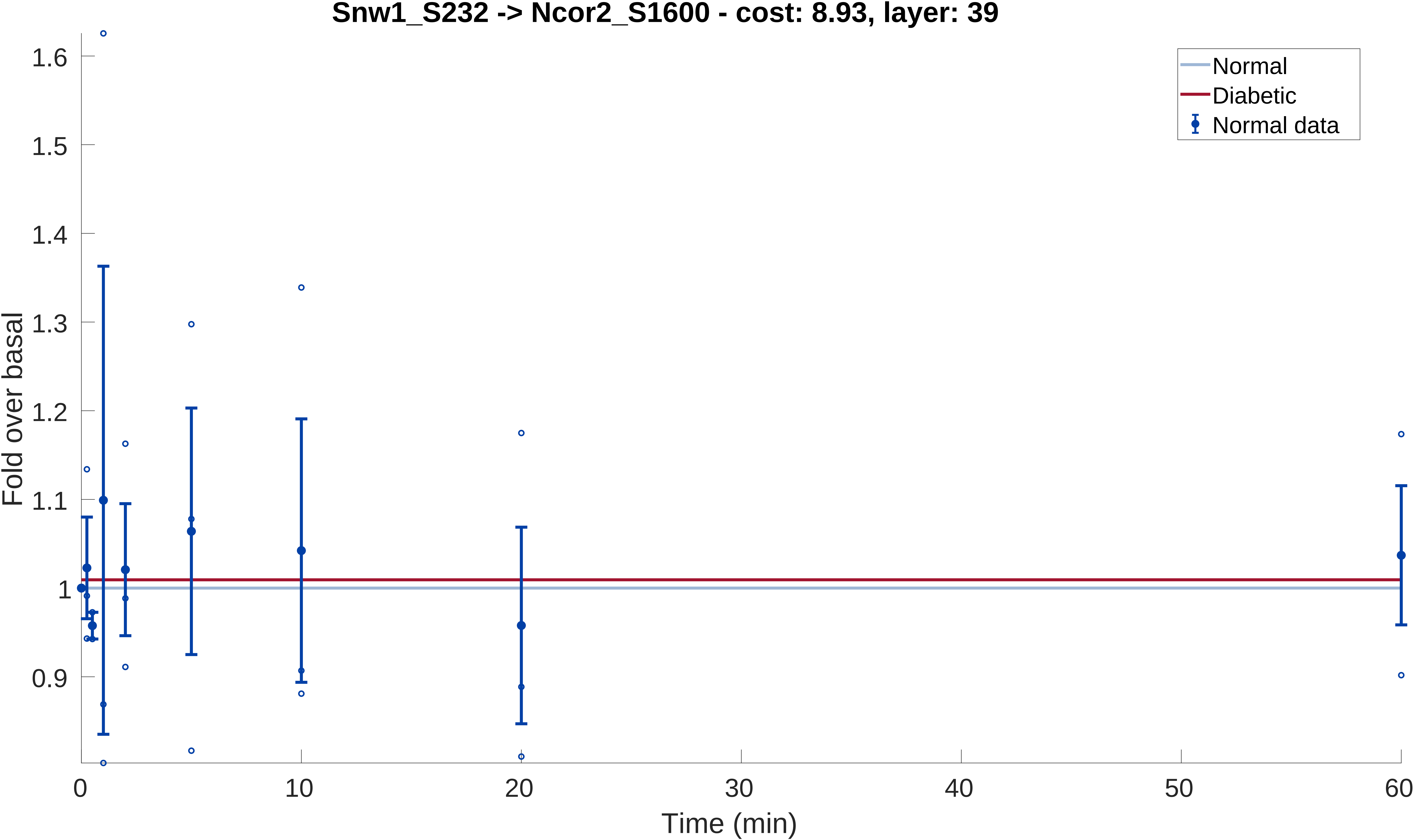

Snw1_S232 -> Ncor2_S1600 - cost: 8.93, layer: 39
Normal
Diabetic
Normal data
Fold over basal
Time (min)
1.6
1.5
1.4
1.3
1.2
1.1
1
0.9
0
10
20
30
40
50
60

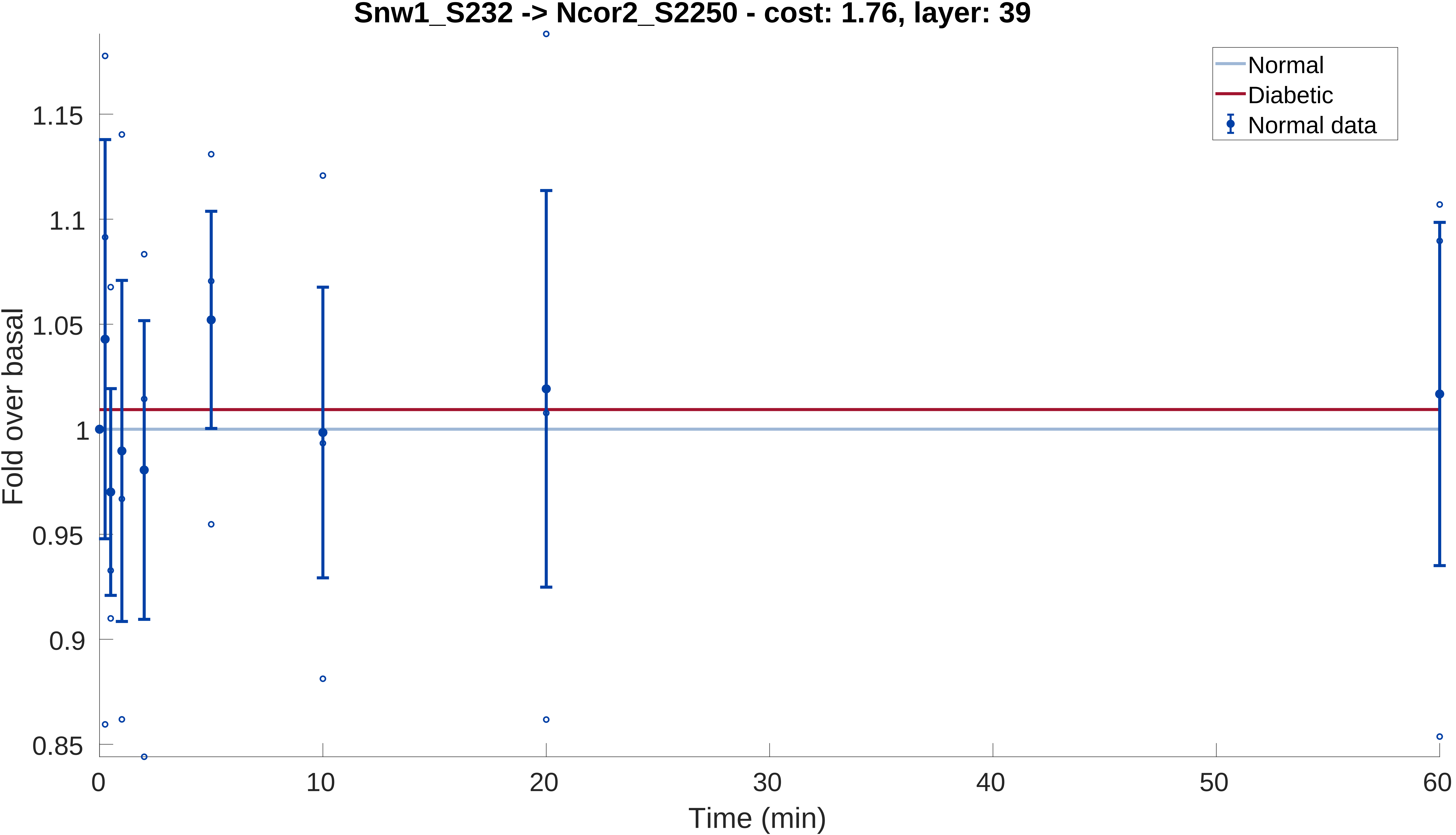

Snw1_S232 -> Ncor2_S2250 - cost: 1.76, layer: 39
Normal
Diabetic
Normal data
Fold over basal
1.15
1.1
1.05
1
0.95
0.9
0.85
0
10
20
30
40
50
60
Time (min)

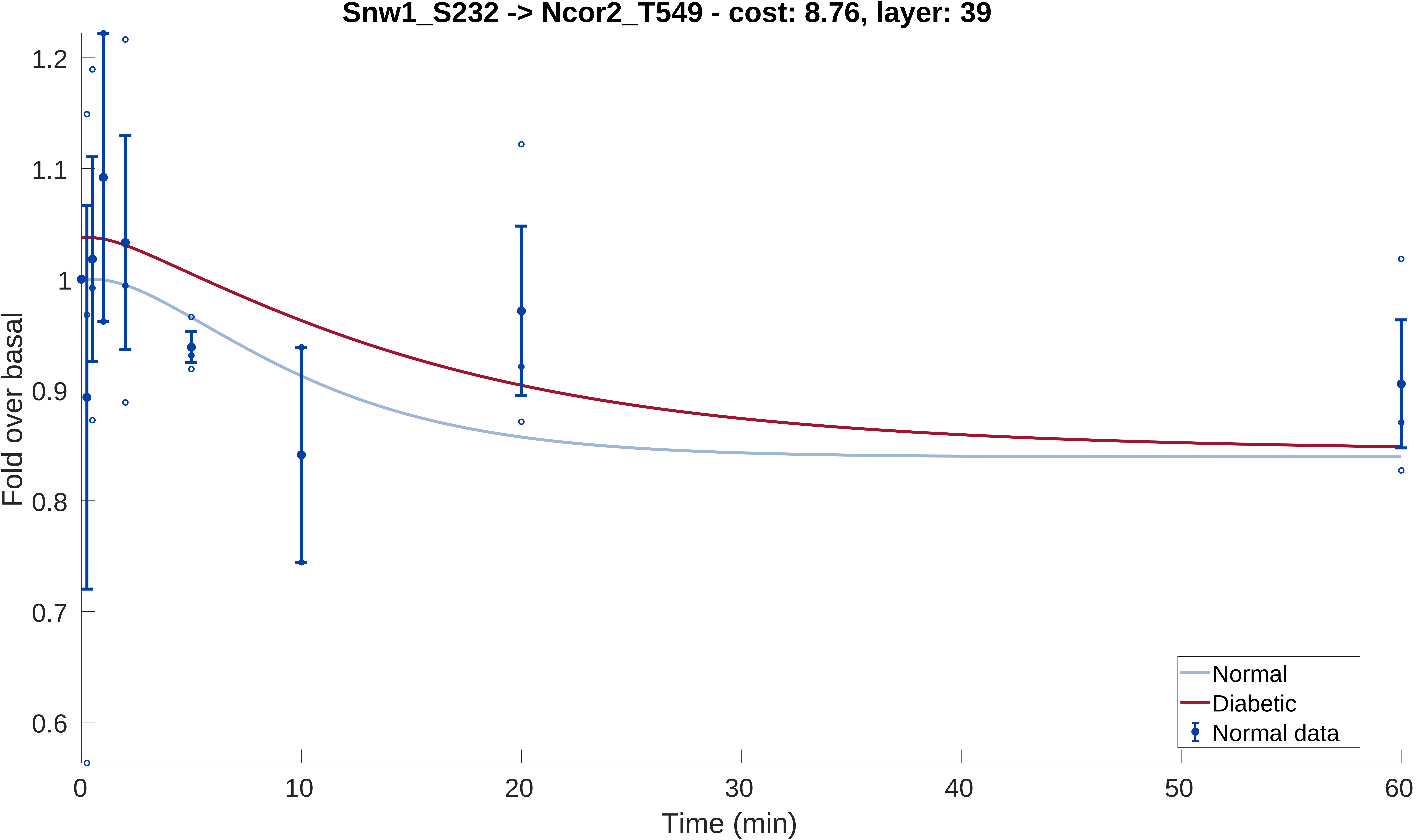
Snw1_S232 -> Ncor2_T549 - cost: 8.76, layer: 39
Fold over basal
Time (min)
1.2
1.1
1
0.9
0.8
0.7
0.6
0
10
20
30
40
50
60
Normal
Diabetic
Normal data

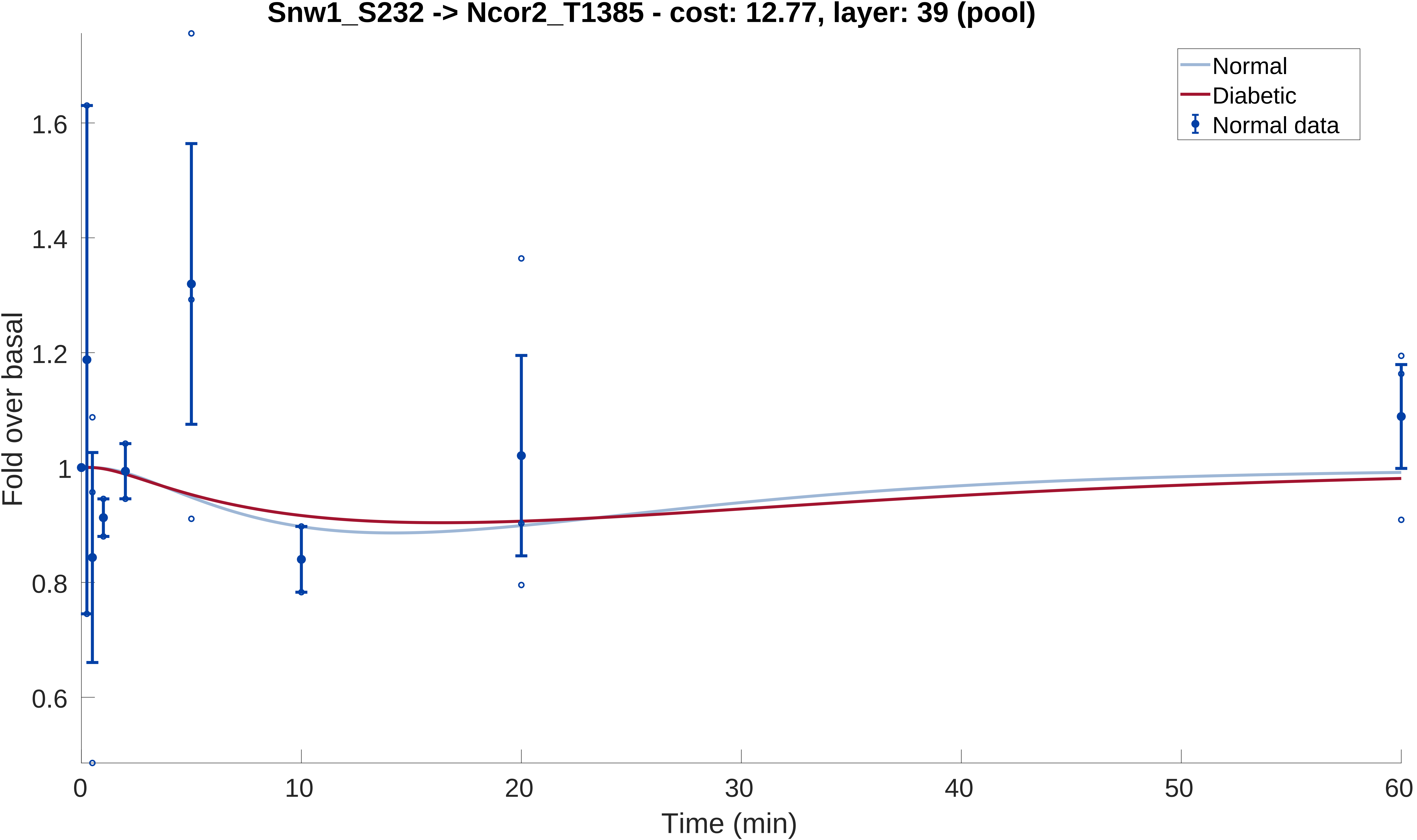

Snw1_S232 -> Ncor2_T1385 - cost: 12.77, layer: 39 (pool)
Normal
Diabetic
Normal data
Fold over basal
Time (min)
0.6
0.8
1
1.2
1.4
1.6
0
10
20
30
40
50
60

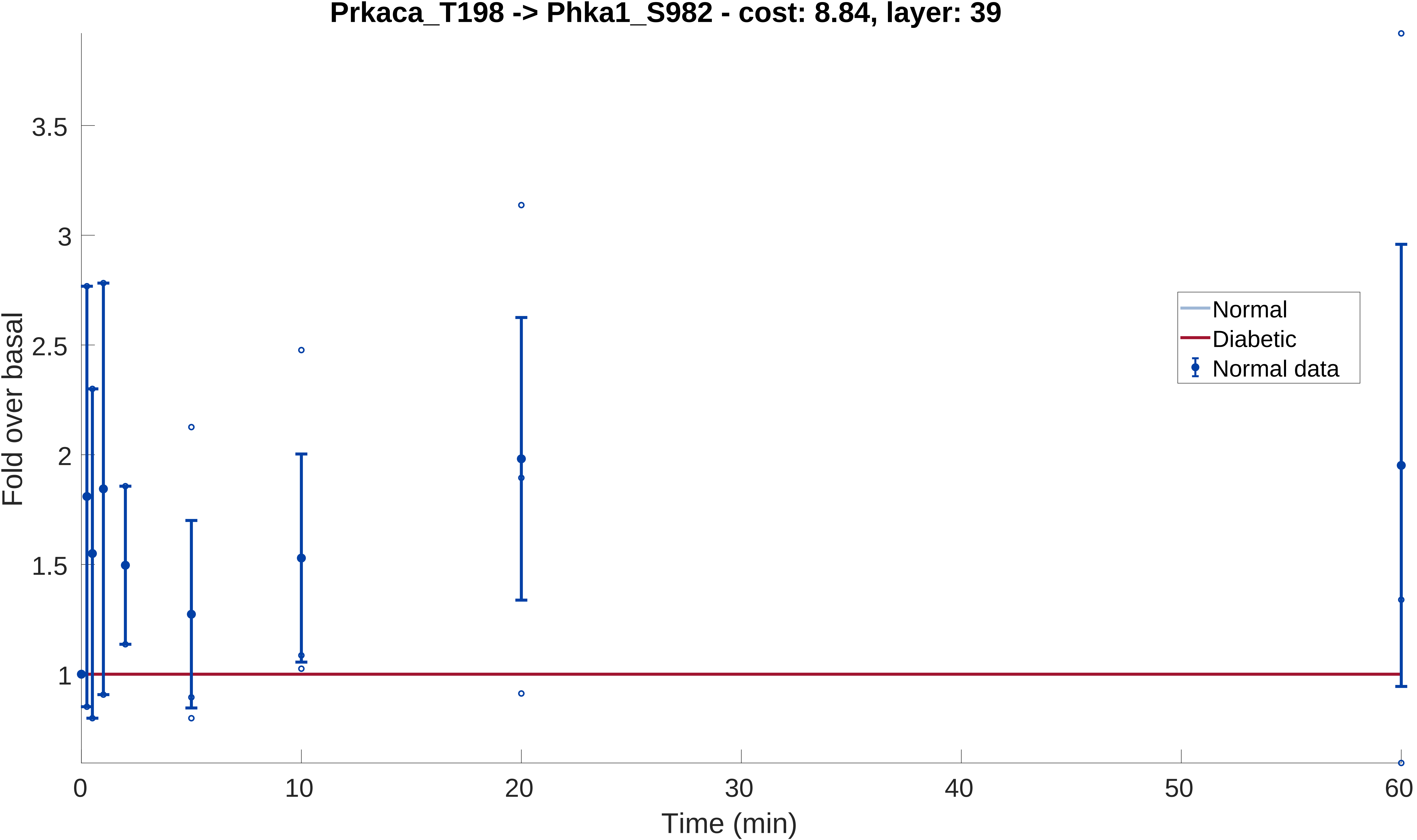

Prkaca_T198 -> Phka1_S982 - cost: 8.84, layer: 39
Fold over basal
3.5
3
2.5
2
1.5
1
0
10
20
30
40
50
60
Time (min)
Normal
Diabetic
Normal data

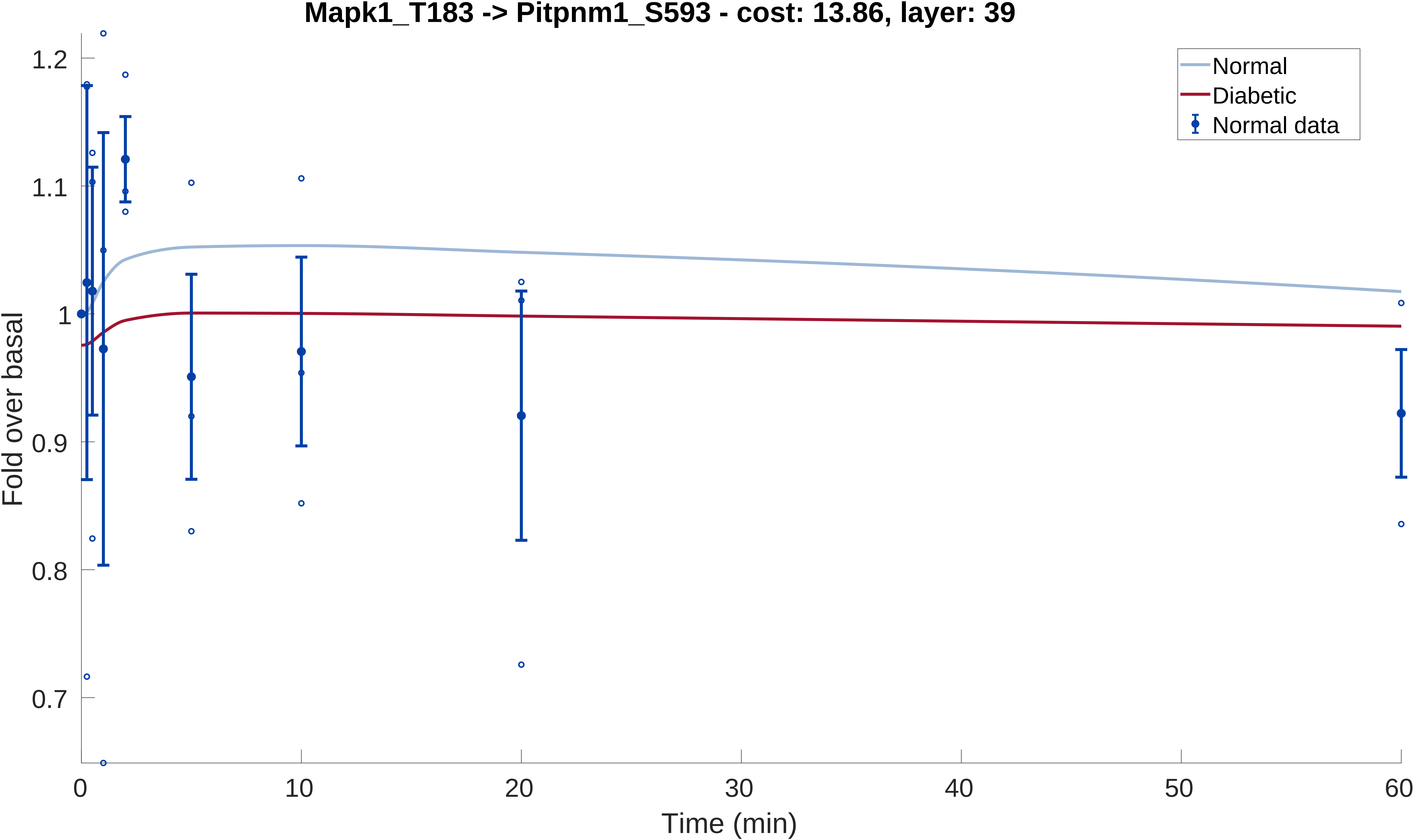

Mapk1_T183 -> Pitpnm1_S593 - cost: 13.86, layer: 39
Normal
Diabetic
Normal data
Fold over basal
1.2
1.1
1
0.9
0.8
0.7
0
10
20
30
40
50
60
Time (min)

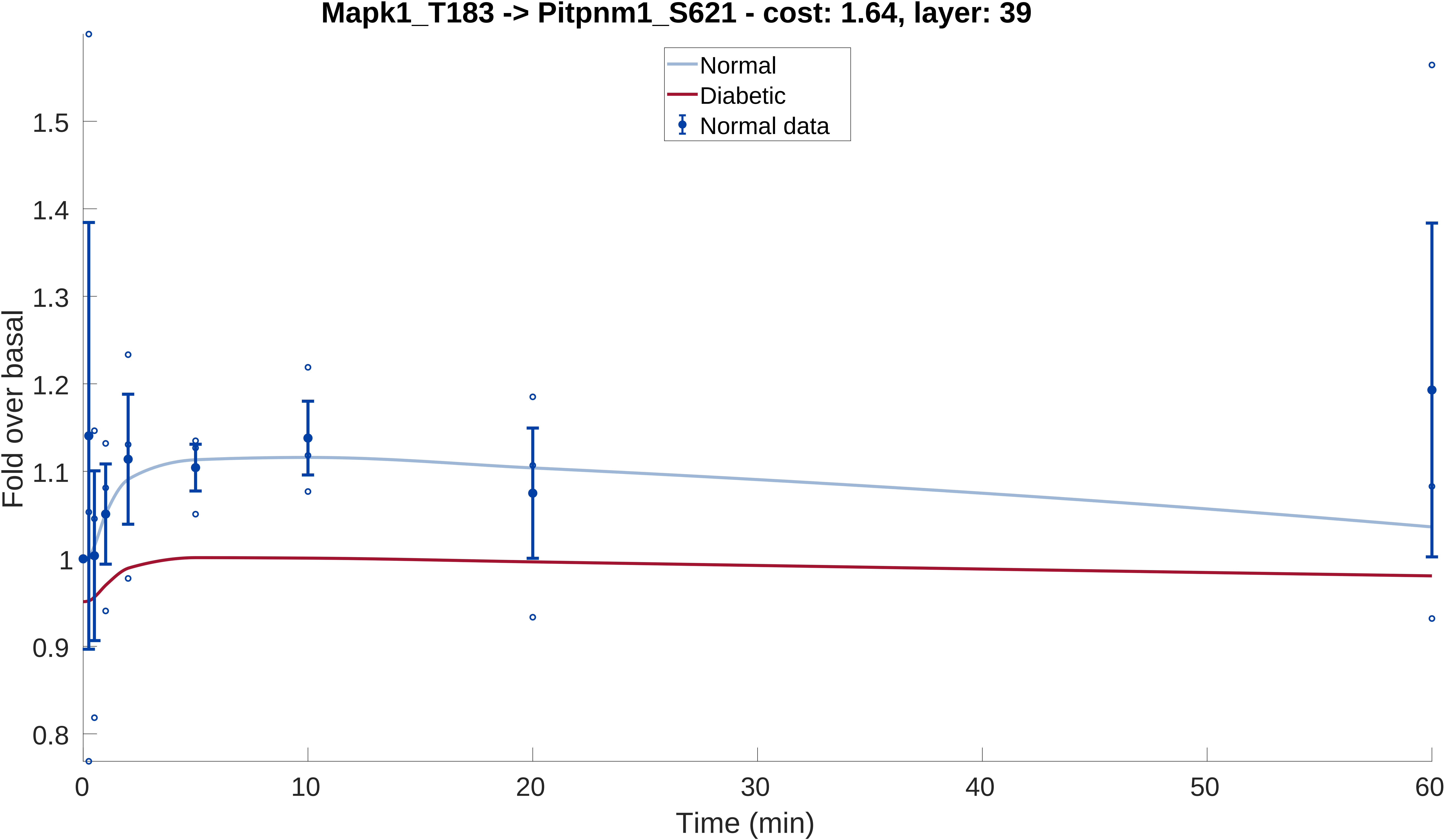

Mapk1_T183 -> Pitpnm1_S621 - cost: 1.64, layer: 39
Normal
Diabetic
Normal data
Fold over basal
Time (min)
1.5
1.4
1.3
1.2
1.1
1
0.9
0.8
0
10
20
30
40
50
60

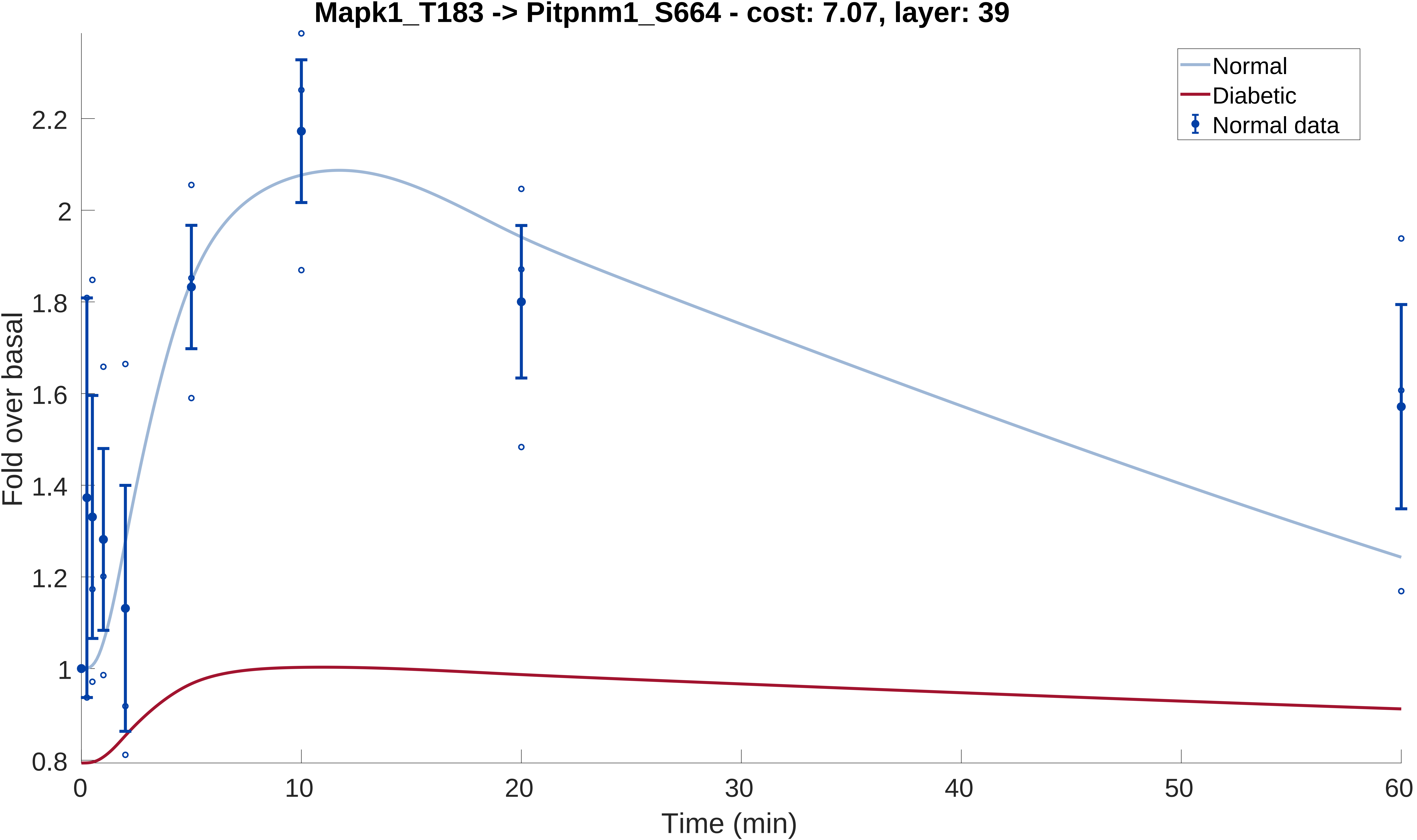

Mapk1_T183 -> Pitpnm1_S664 - cost: 7.07, layer: 39
Normal
Diabetic
Normal data
Fold over basal
0.8
1
1.2
1.4
1.6
1.8
2
2.2
0
10
20
30
40
50
60
Time (min)

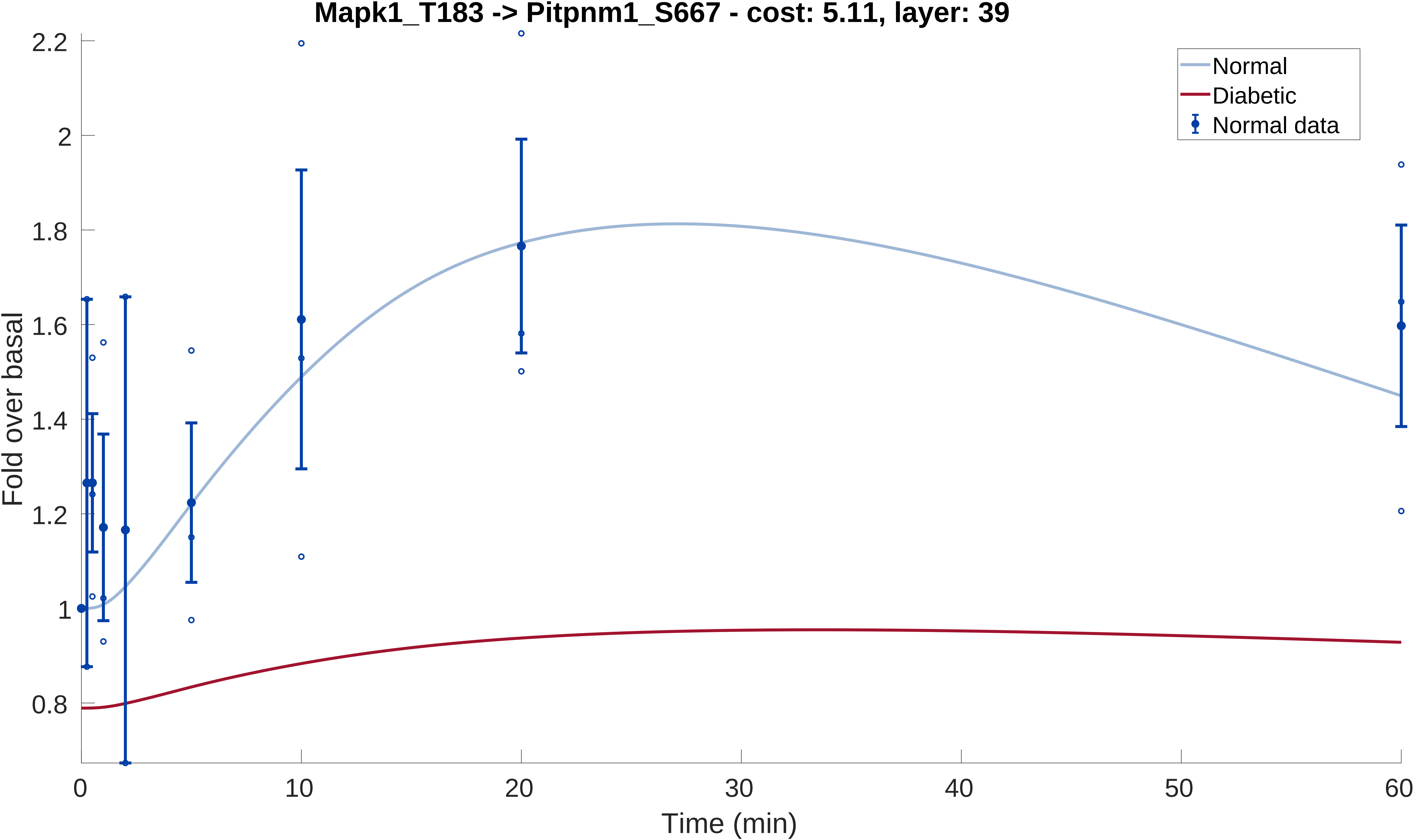

Mapk1_T183 -> Pitpnm1_S667 - cost: 5.11, layer: 39
Normal
Diabetic
Normal data
Fold over basal
0.8
1
1.2
1.4
1.6
1.8
2
2.2
0
10
20
30
40
50
60
Time (min)

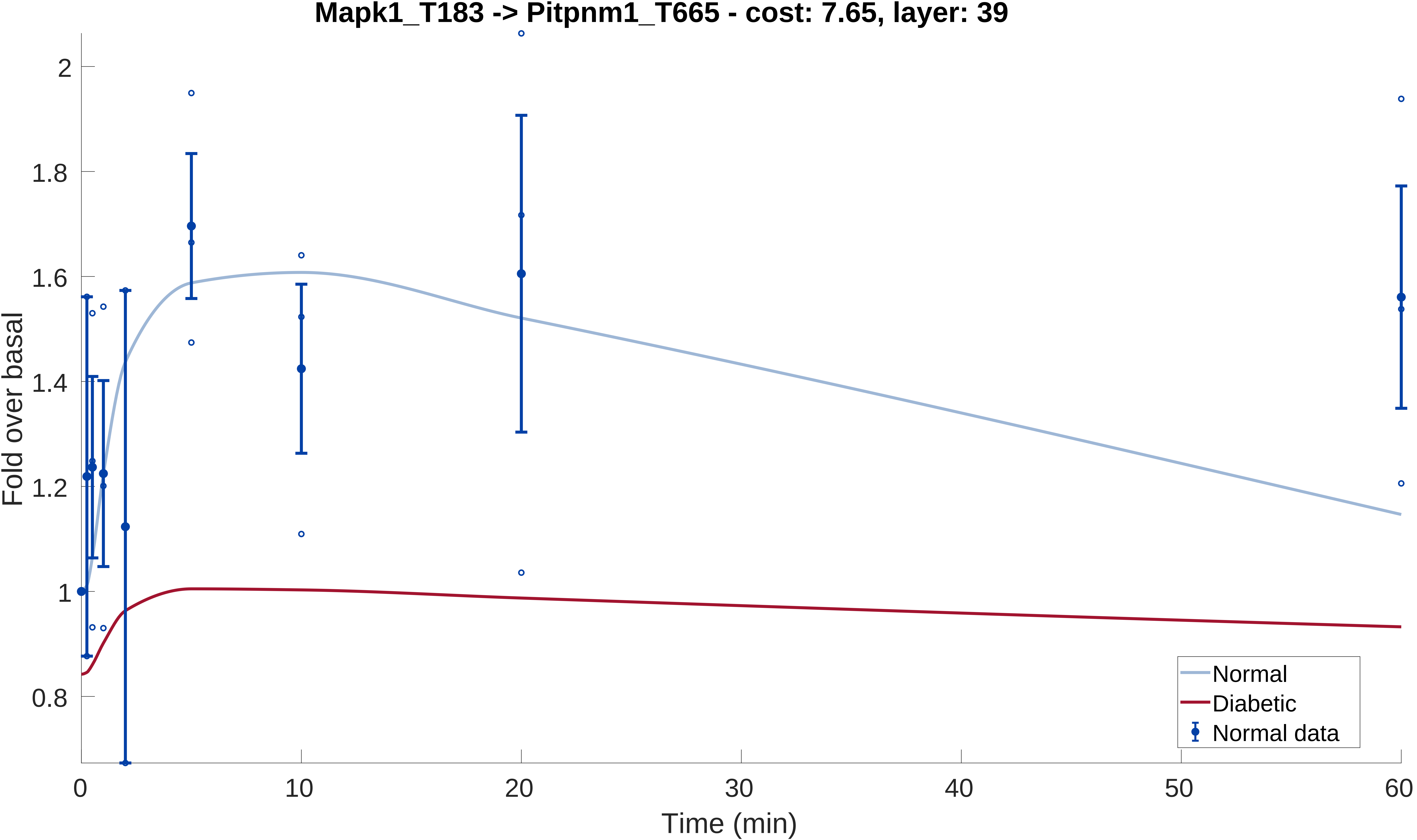

Mapk1_T183 -> Pitpnm1_T665 - cost: 7.65, layer: 39
Fold over basal
2
1.8
1.6
1.4
1.2
1
0.8
0
10
20
30
40
50
60
Time (min)
Normal
Diabetic
Normal data

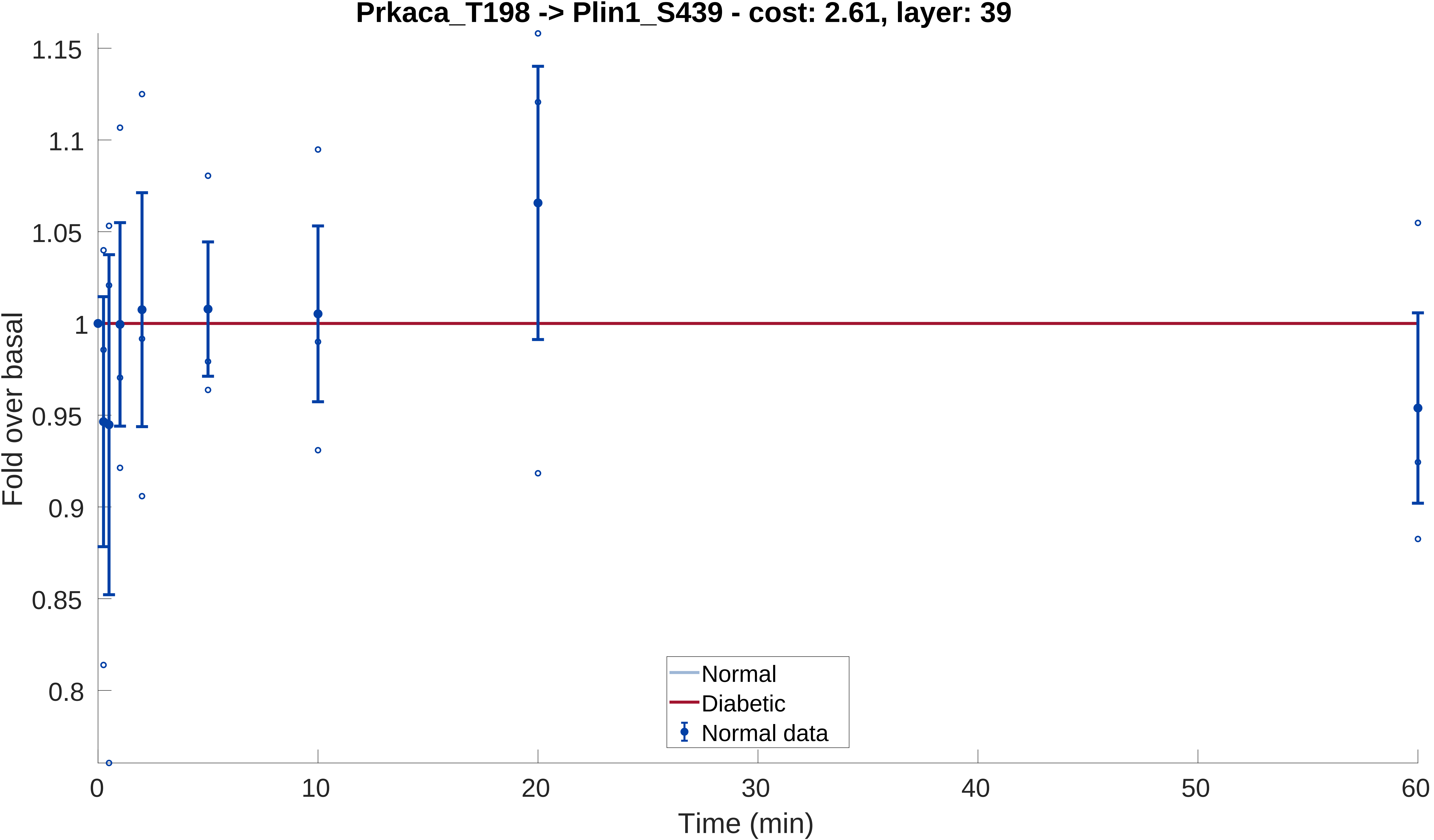

Prkaca_T198 -> Plin1_S439 - cost: 2.61, layer: 39
1.15
1.1
1.05
1
0.95
0.9
0.85
0.8
Fold over basal
0
10
20
30
40
50
60
Time (min)
Normal
Diabetic
Normal data

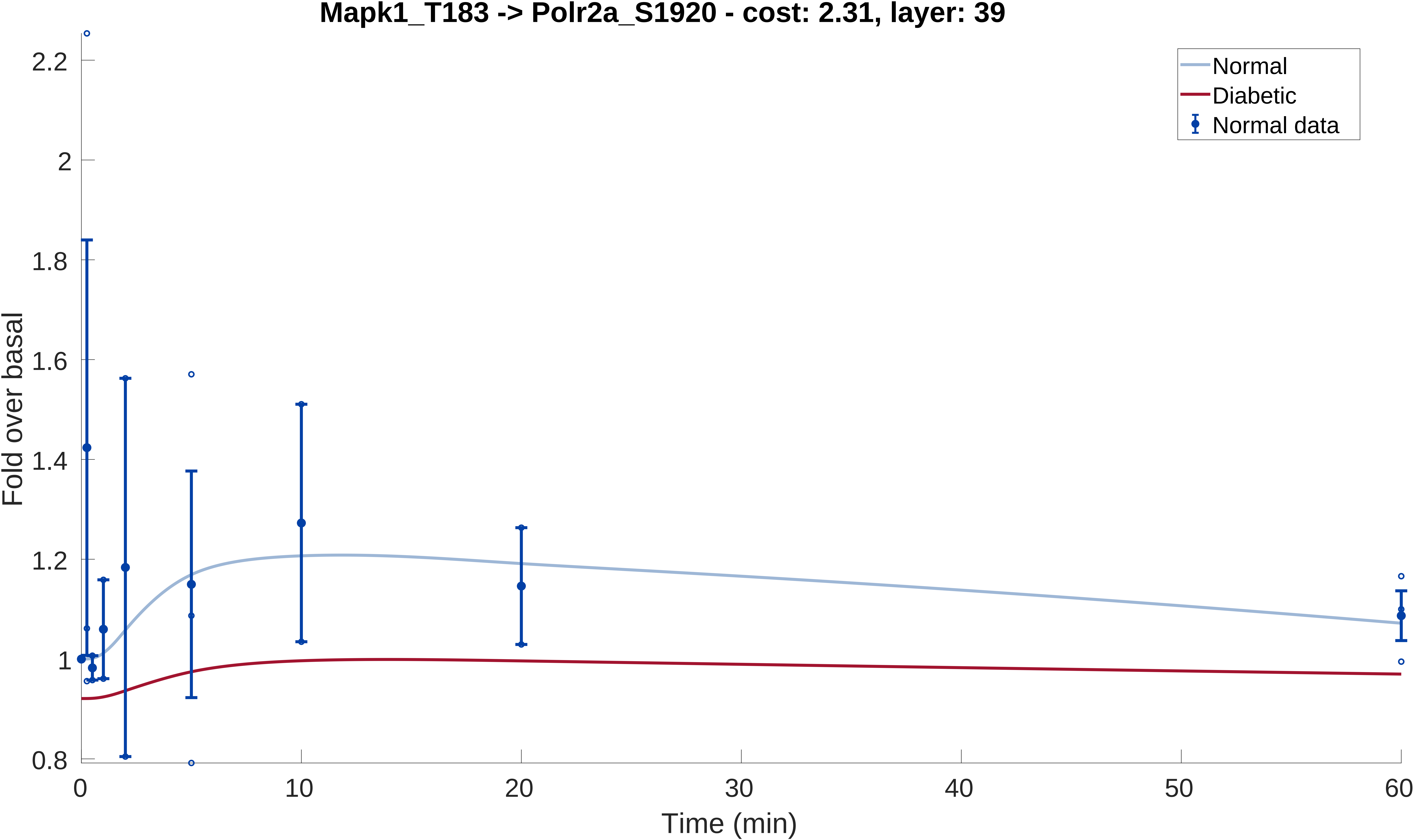
Mapk1_T183 -> Polr2a_S1920 - cost: 2.31, layer: 39
Normal
Diabetic
Normal data
Fold over basal
Time (min)
0.8
1
1.2
1.4
1.6
1.8
2
2.2
0
10
20
30
40
50
60

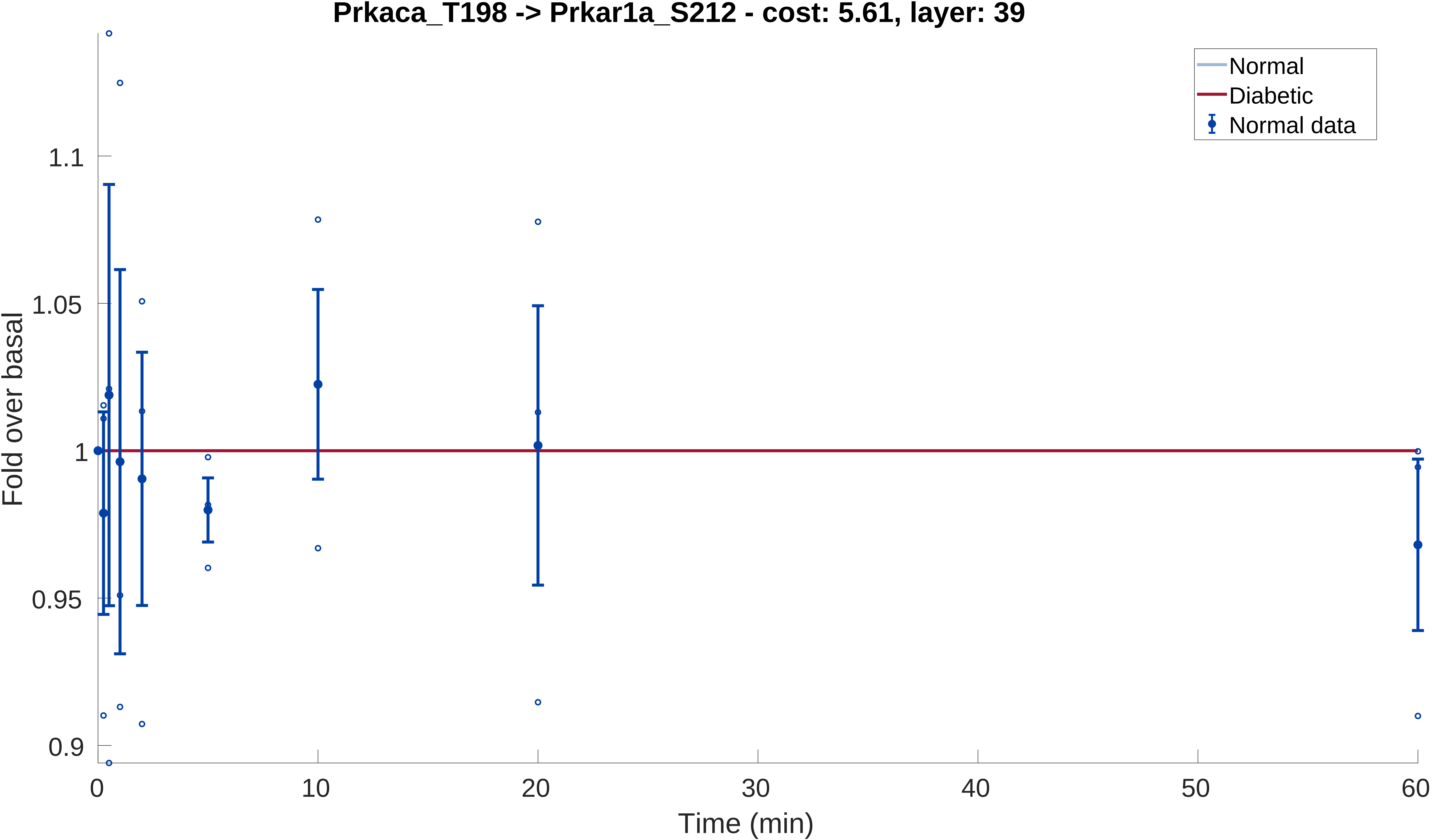

Prkaca_T198 -> Prkar1a_S212 - cost: 5.61, layer: 39
Normal
Diabetic
Normal data
Fold over basal
Time (min)
1.1
1.05
1
0.95
0.9
0
10
20
30
40
50
60

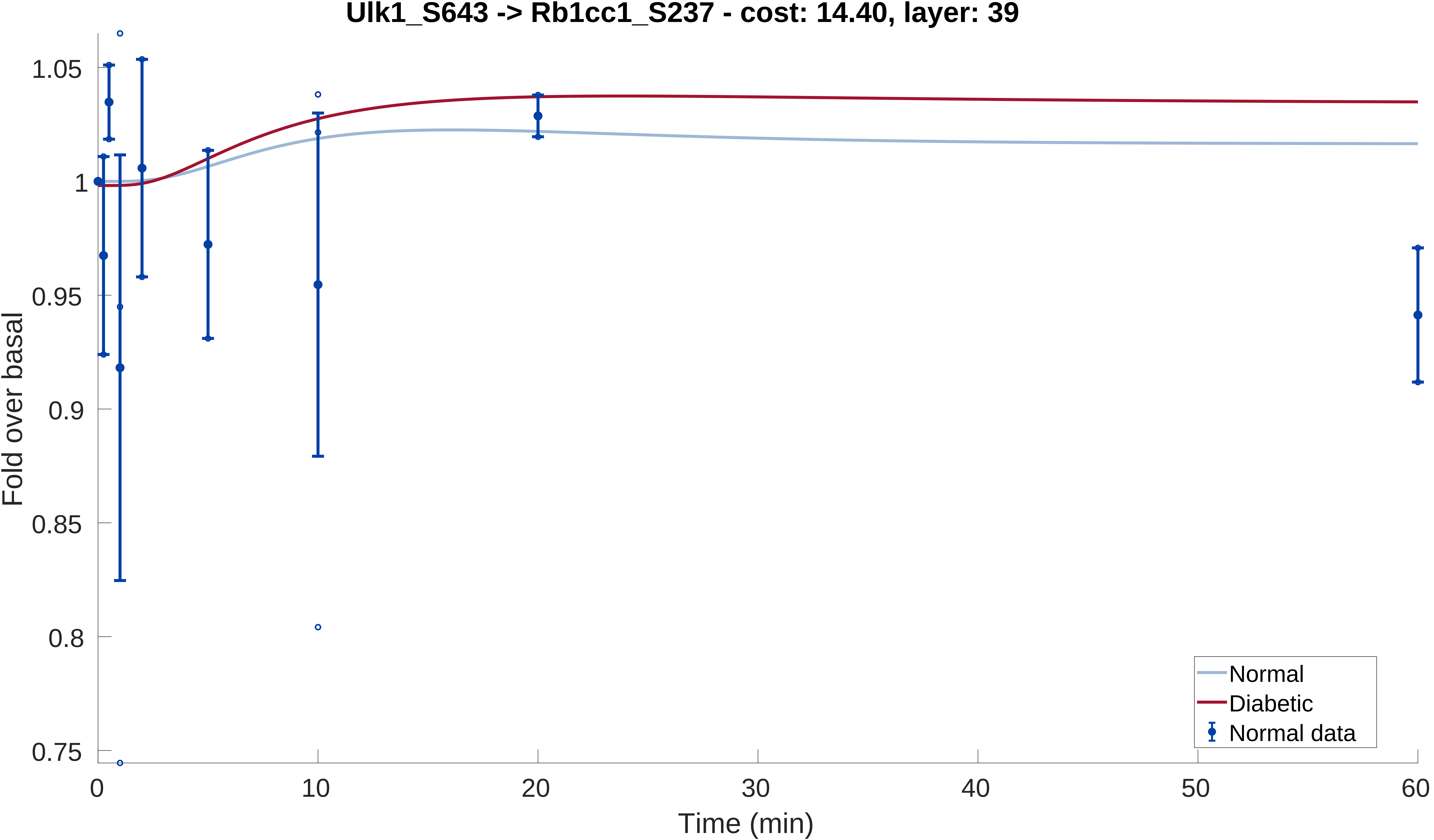

Ulk1_S643 -> Rb1cc1_S237 - cost: 14.40, layer: 39
Fold over basal
1.05
1
0.95
0.9
0.85
0.8
0.75
0
10
20
30
40
50
60
Time (min)
Normal
Diabetic
Normal data

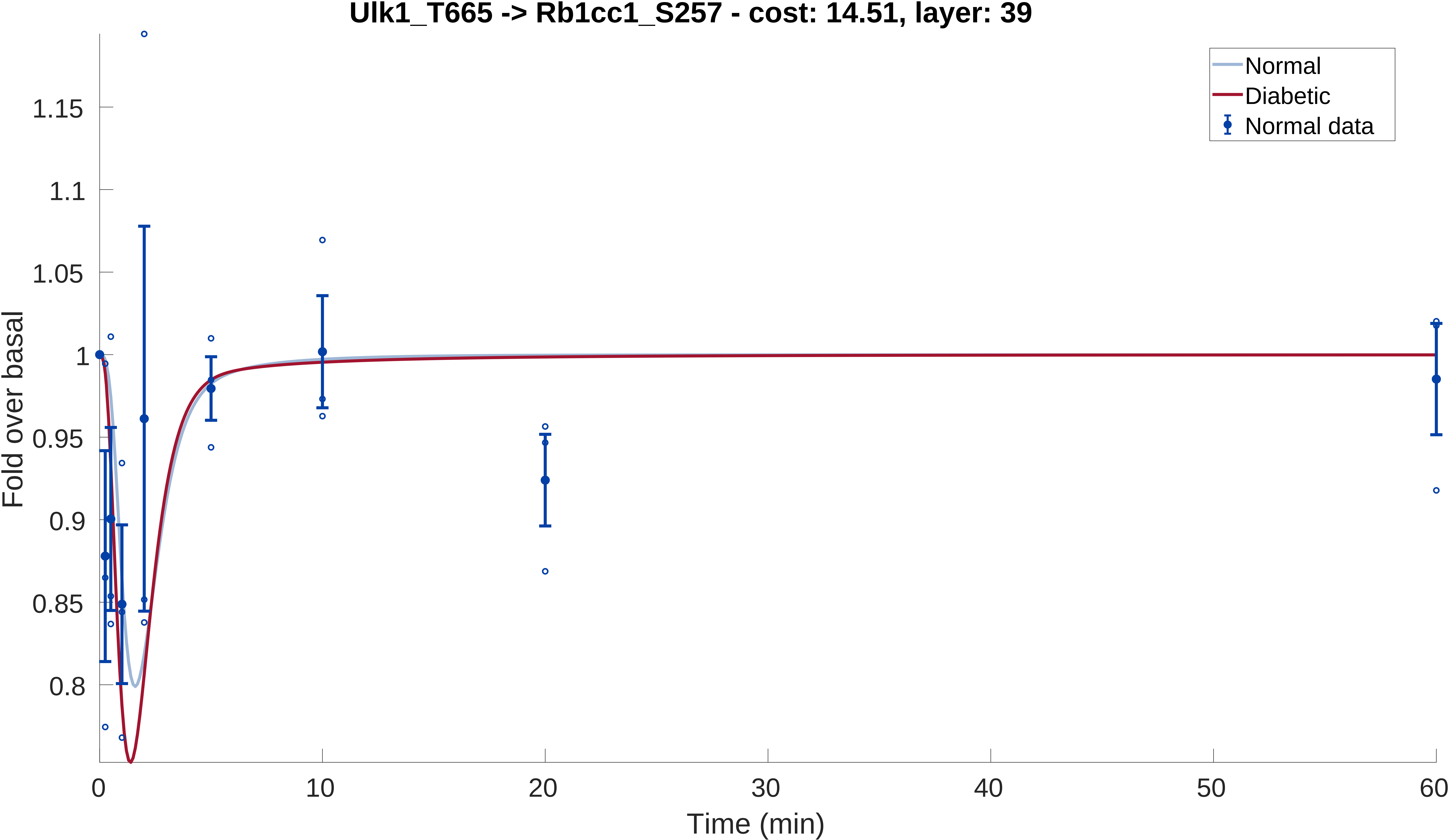

Ulk1_T665 -> Rb1cc1_S257 - cost: 14.51, layer: 39
Normal
Diabetic
Normal data
Fold over basal
1.15
1.1
1.05
1
0.95
0.9
0.85
0.8
0
10
20
30
40
50
60
Time (min)

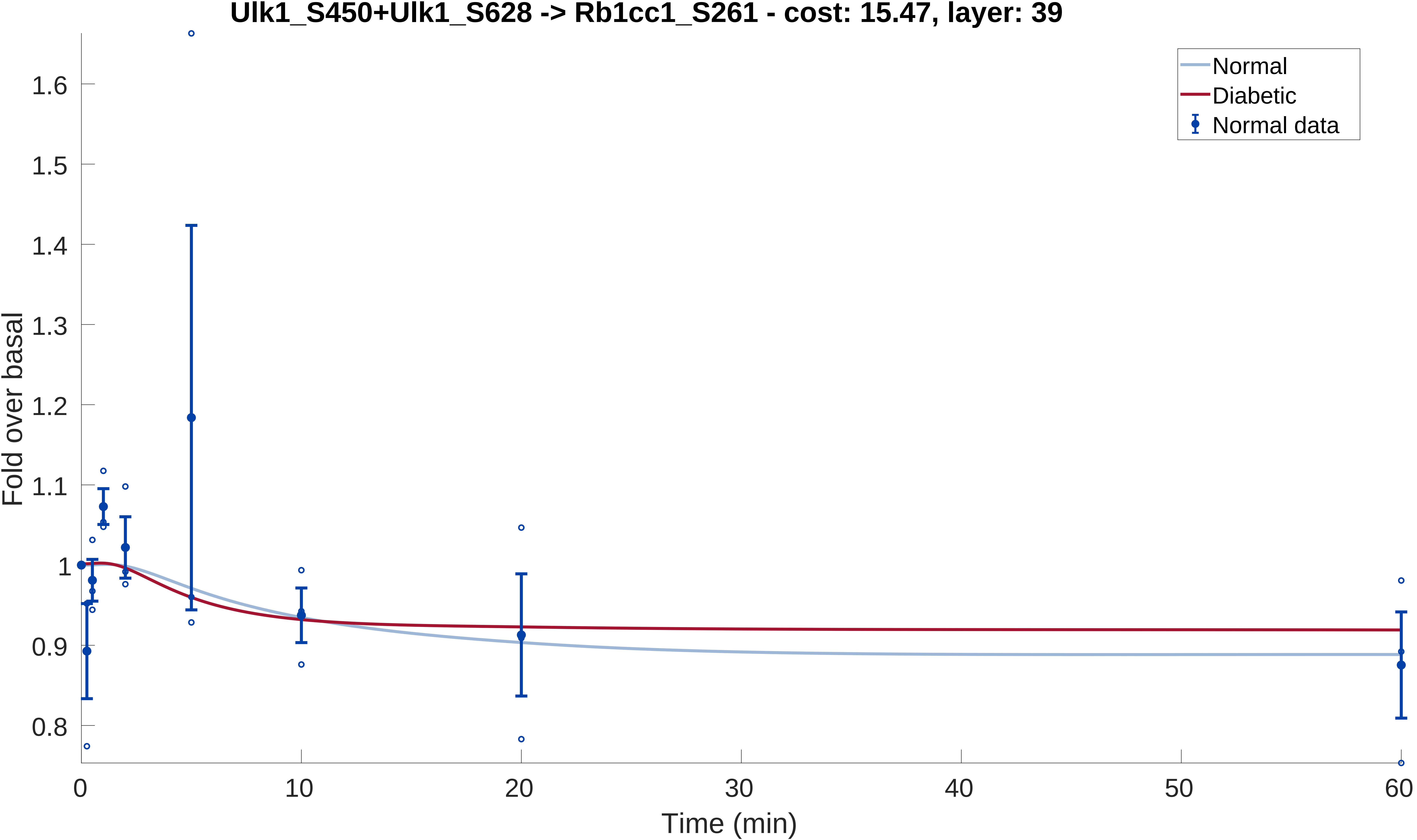
Ulk1_S450+Ulk1_S628 -> Rb1cc1_S261 - cost: 15.47, layer: 39
Normal
Diabetic
Normal data
Fold over basal
1.6
1.5
1.4
1.3
1.2
1.1
1
0.9
0.8
0
10
20
30
40
50
60
Time (min)

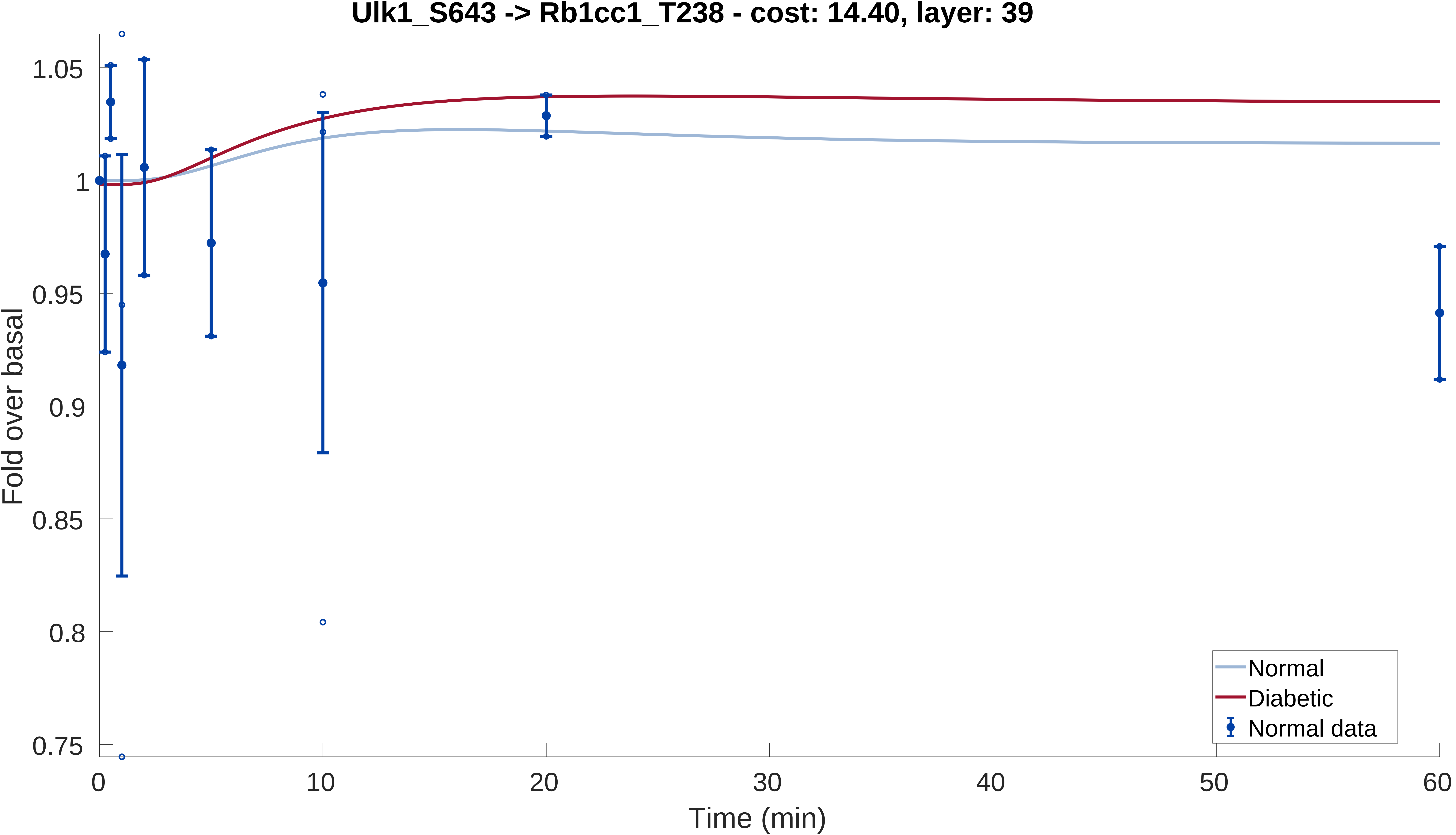

Ulk1_S643 -> Rb1cc1_T238 - cost: 14.40, layer: 39
Fold over basal
1.05
1
0.95
0.9
0.85
0.8
0.75
0
10
20
30
40
50
60
Time (min)
Normal
Diabetic
Normal data

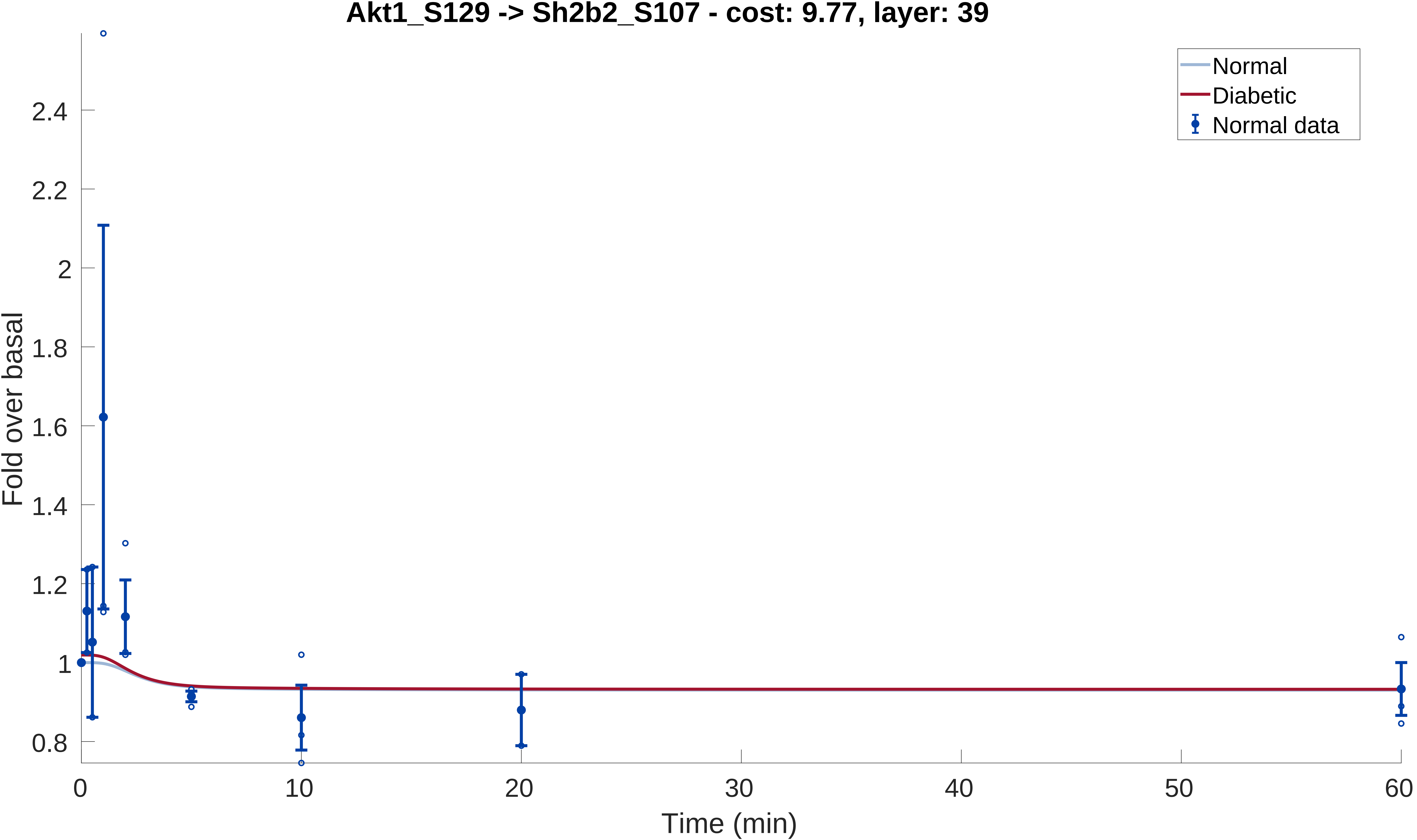

Akt1_S129 -> Sh2b2_S107 - cost: 9.77, layer: 39
Normal
Diabetic
Normal data
Fold over basal
2.4
2.2
2
1.8
1.6
1.4
1.2
1
0.8
0
10
20
30
40
50
60
Time (min)

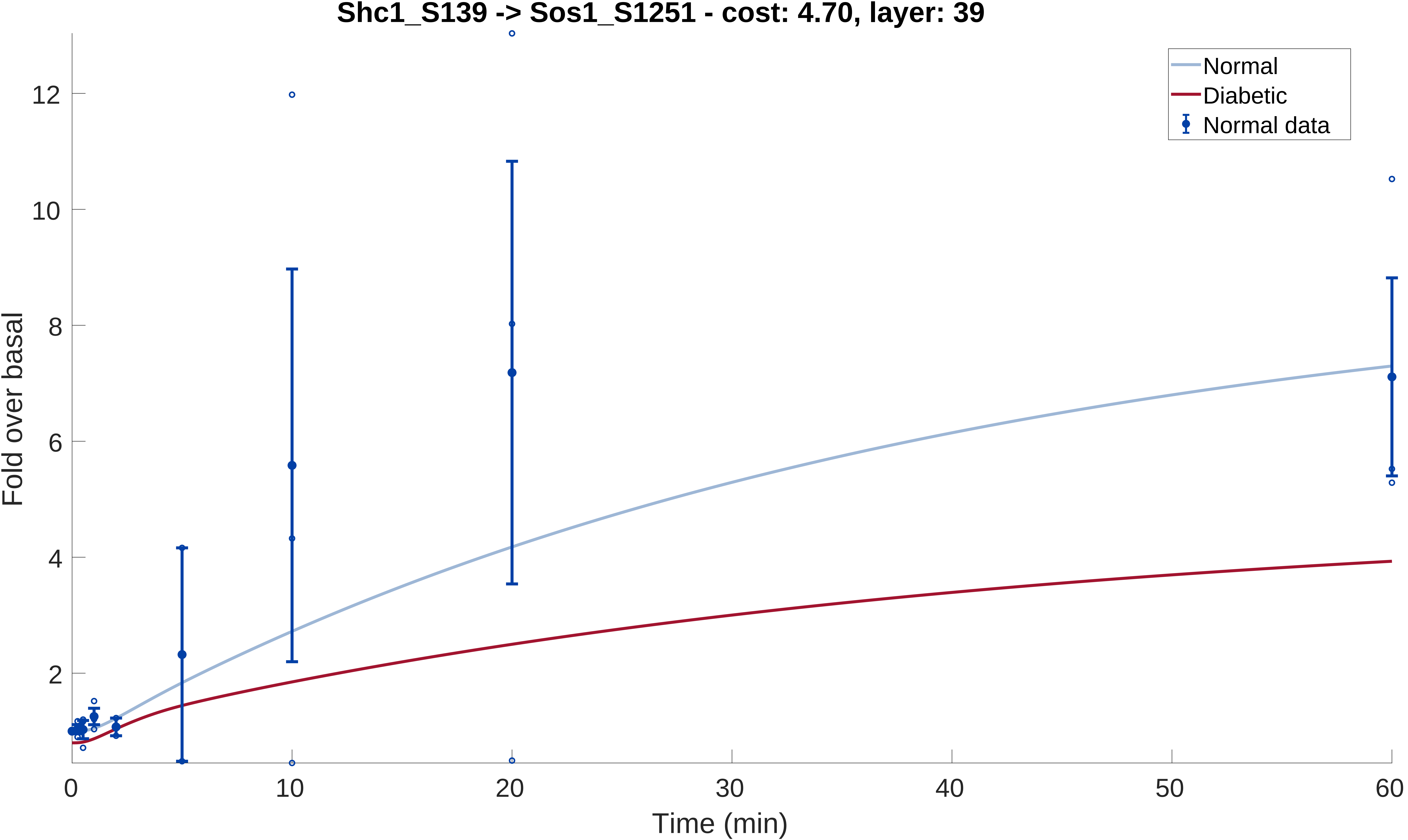

Shc1_S139 -> Sos1_S1251 - cost: 4.70, layer: 39
Normal
Diabetic
Normal data
Fold over basal
Time (min)
0
10
20
30
40
50
60
2
4
6
8
10
12

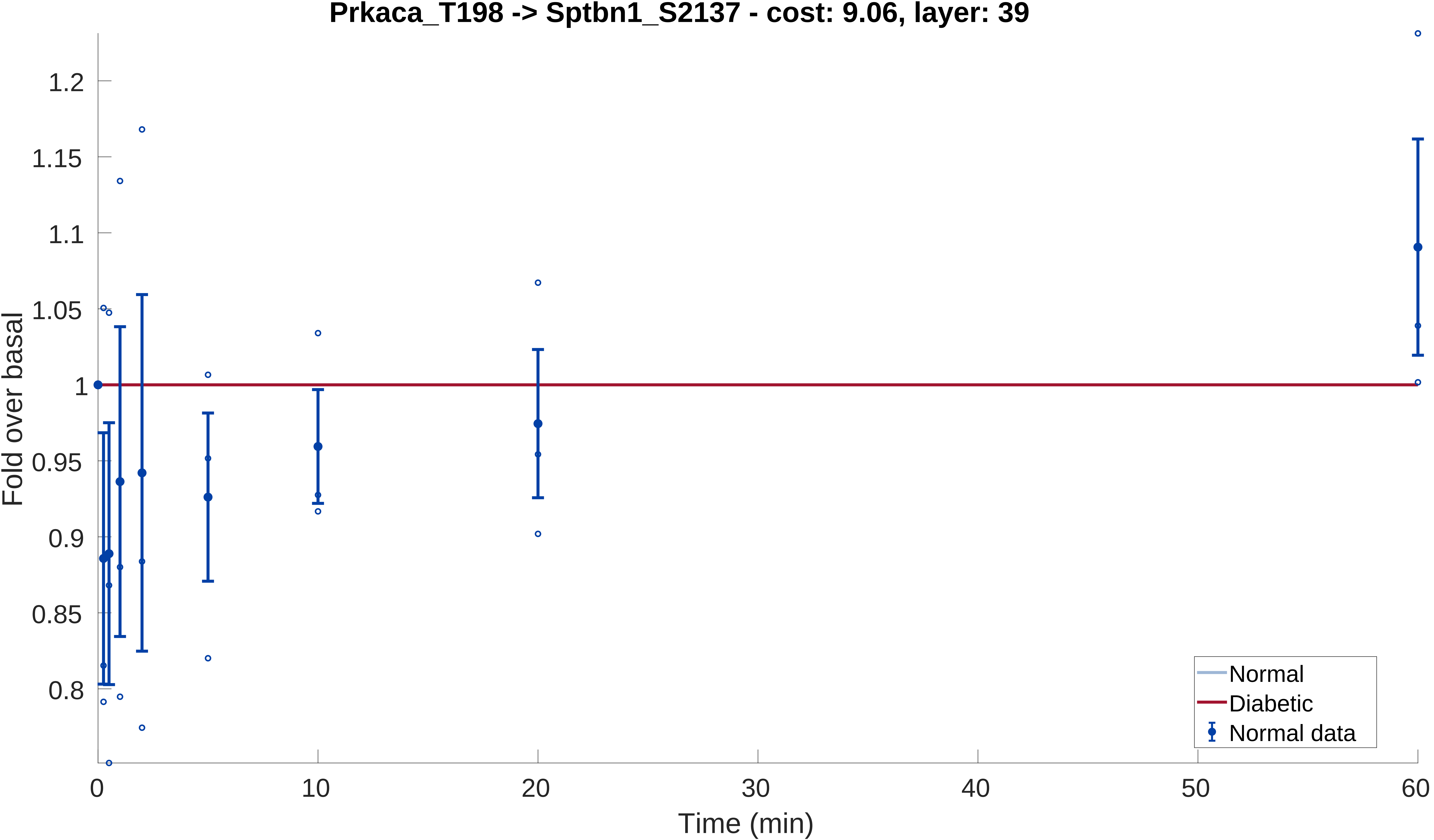

Prkaca_T198 -> Sptbn1_S2137 - cost: 9.06, layer: 39
Fold over basal
1.2
1.15
1.1
1.05
1
0.95
0.9
0.85
0.8
0
10
20
30
40
50
60
Time (min)
Normal
Diabetic
Normal data

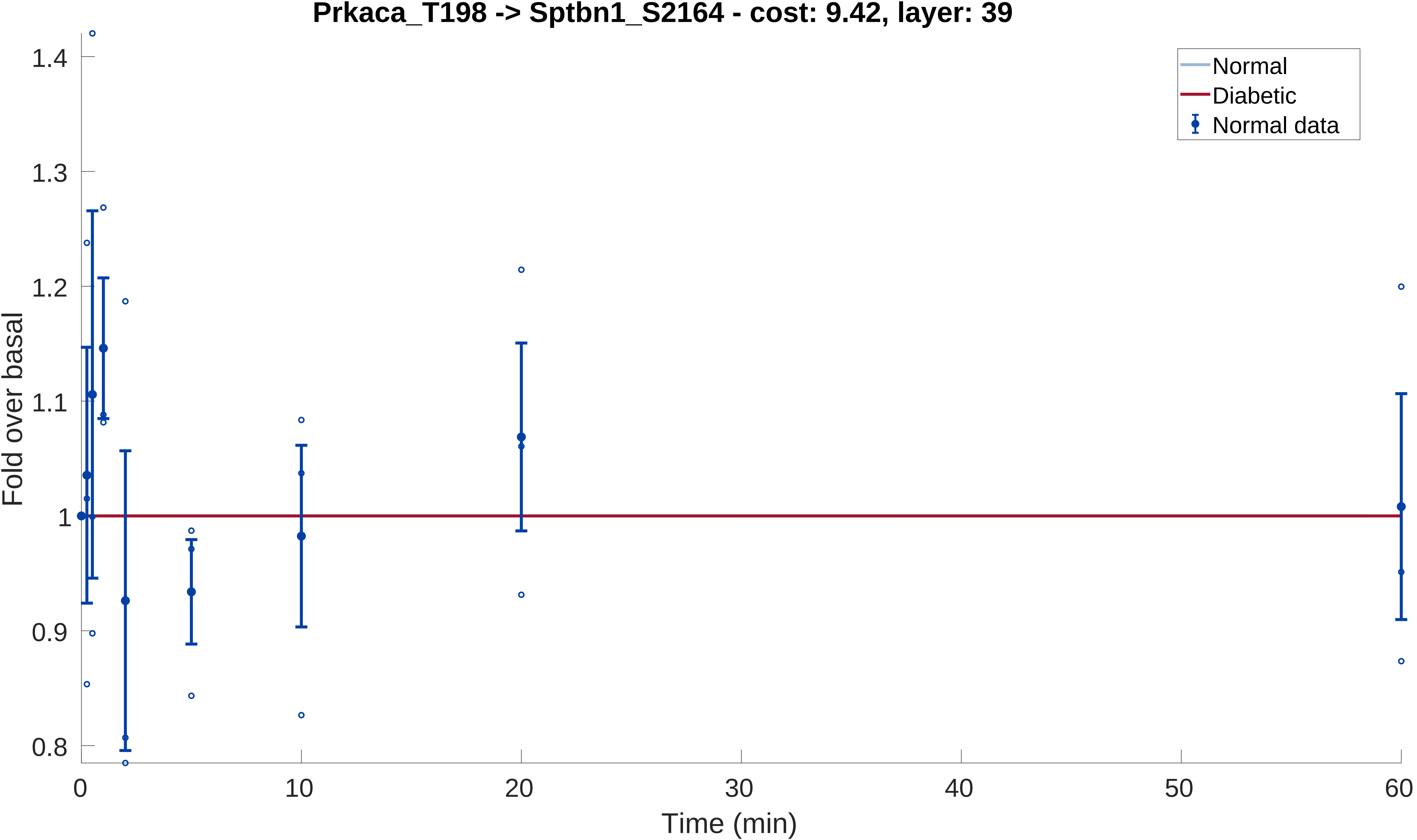

Prkaca_T198 -> Sptbn1_S2164 - cost: 9.42, layer: 39
Normal
Diabetic
Normal data
Fold over basal
Time (min)
1.4
1.3
1.2
1.1
1
0.9
0.8
0
10
20
30
40
50
60

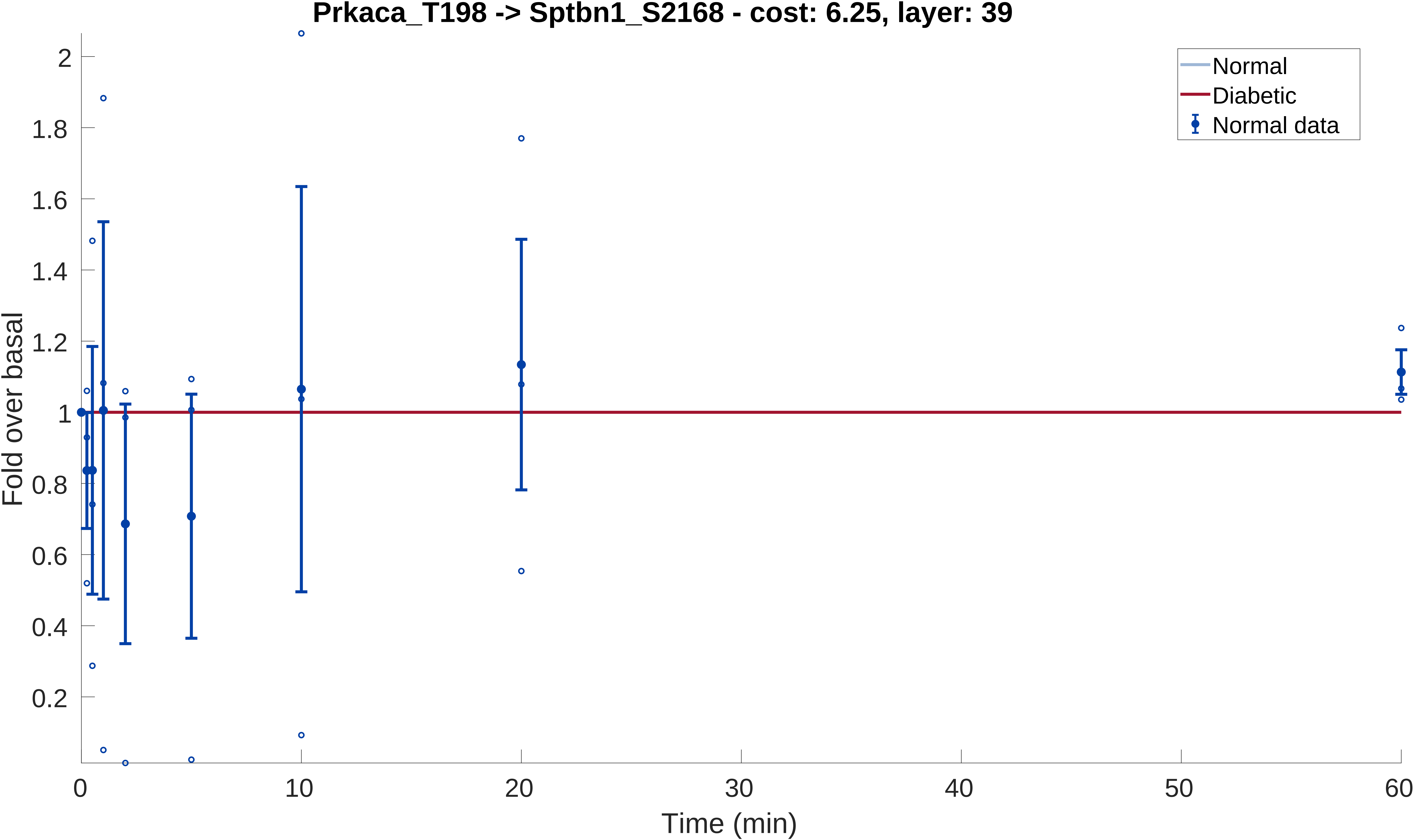
Prkaca_T198 -> Sptbn1_S2168 - cost: 6.25, layer: 39
Fold over basal
Time (min)
Normal
Diabetic
Normal data
0
10
20
30
40
50
60
0.2
0.4
0.6
0.8
1
1.2
1.4
1.6
1.8
2

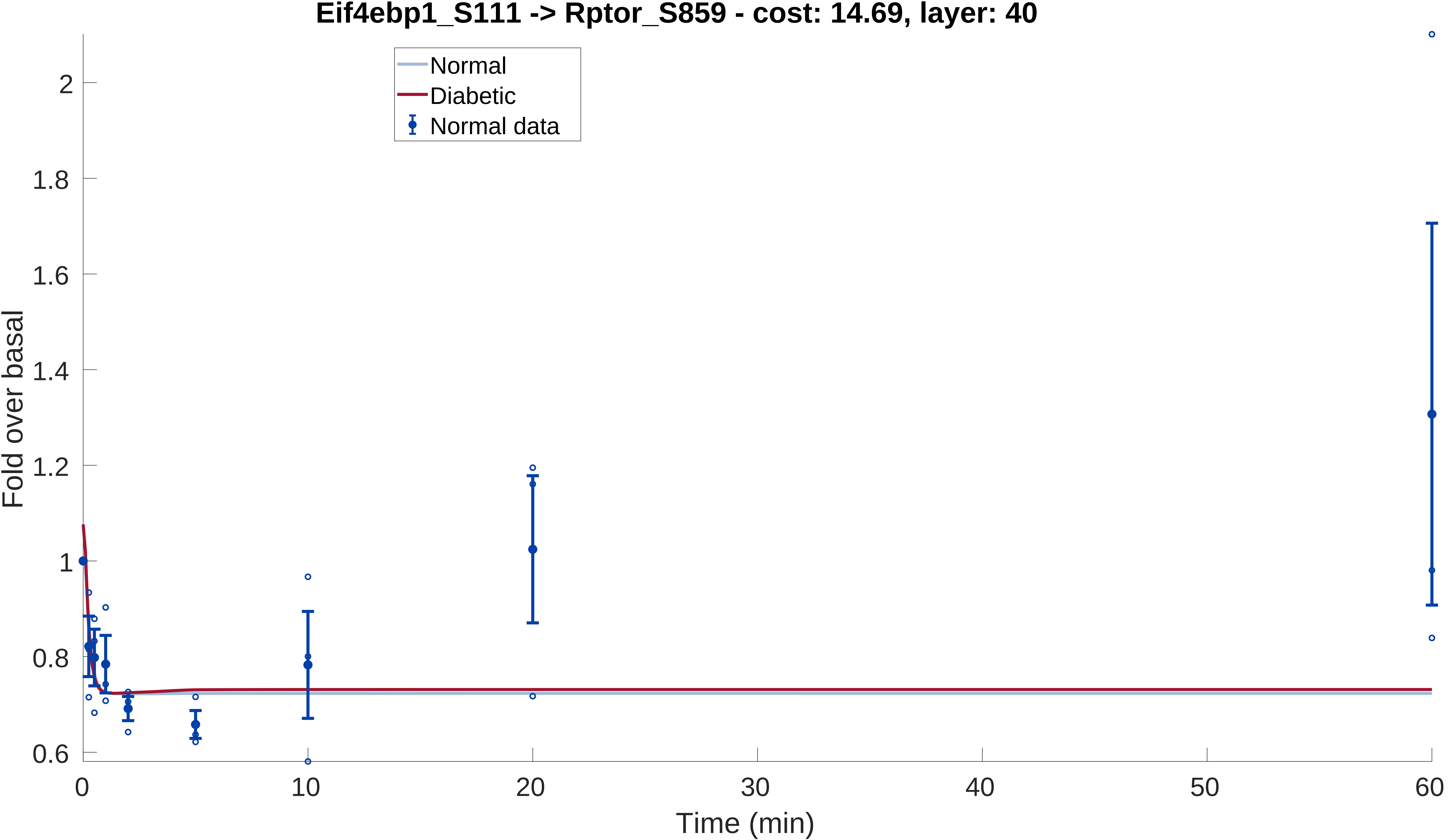

Eif4ebp1_S111 -> Rptor_S859 - cost: 14.69, layer: 40
Normal
Diabetic
Normal data
Fold over basal
0.6
0.8
1
1.2
1.4
1.6
1.8
2
0
10
20
30
40
50
60
Time (min)

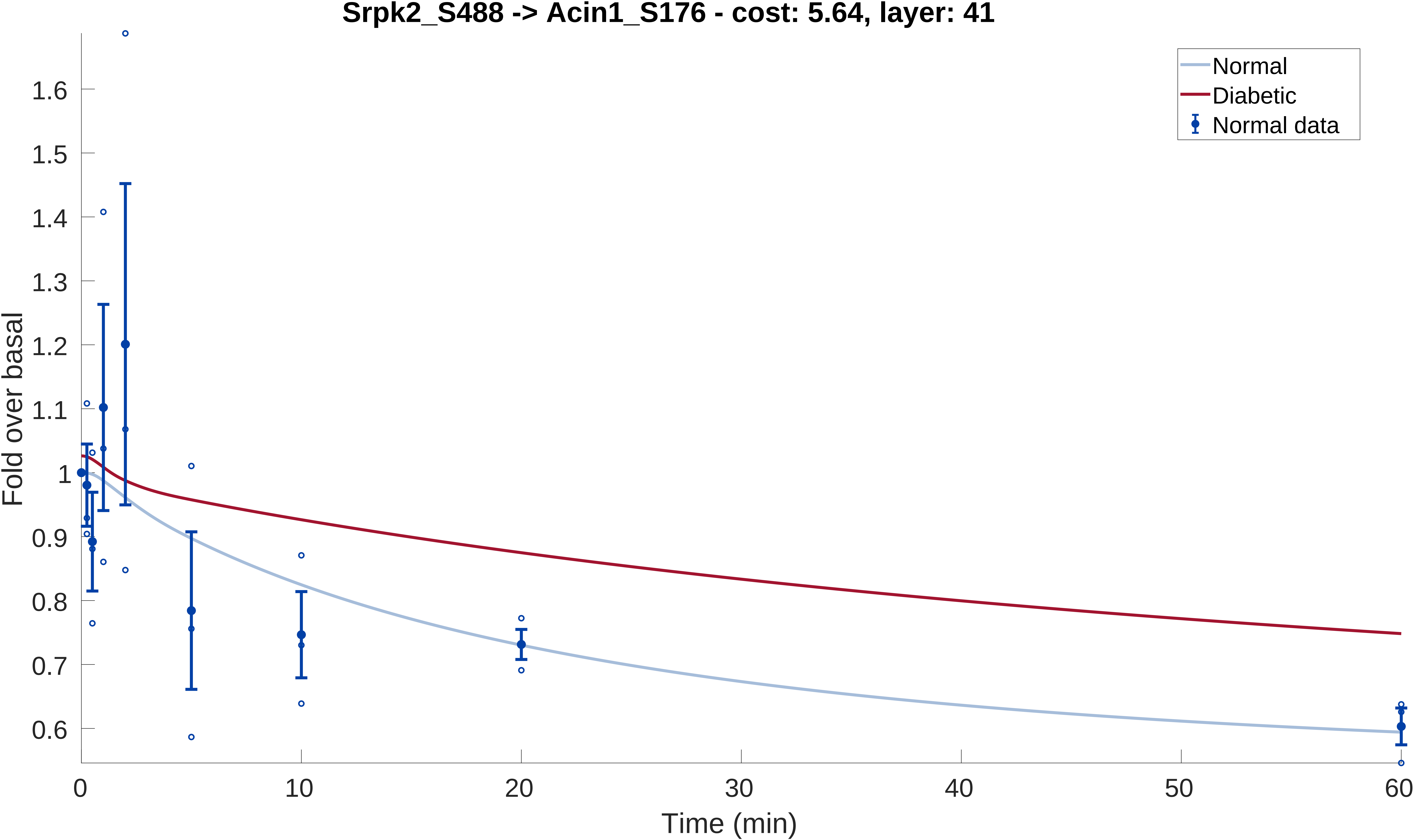

Srpk2_S488 -> Acin1_S176 - cost: 5.64, layer: 41
Normal
Diabetic
Normal data
Fold over basal
1.6
1.5
1.4
1.3
1.2
1.1
1
0.9
0.8
0.7
0.6
0
10
20
30
40
50
60
Time (min)

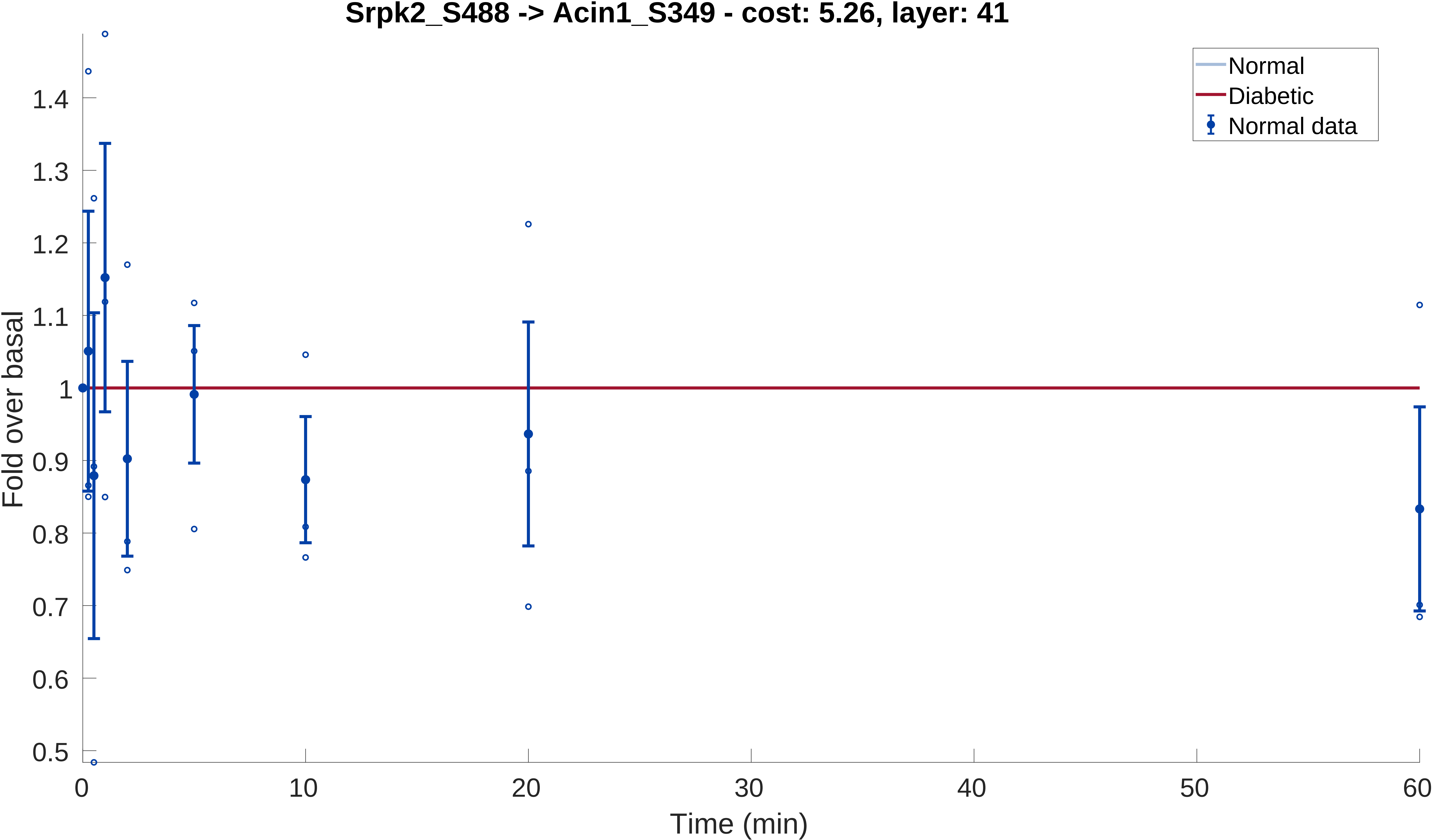

Srpk2_S488 -> Acin1_S349 - cost: 5.26, layer: 41
Normal
Diabetic
Normal data
Fold over basal
1.4
1.3
1.2
1.1
1
0.9
0.8
0.7
0.6
0.5
0
10
20
30
40
50
60
Time (min)

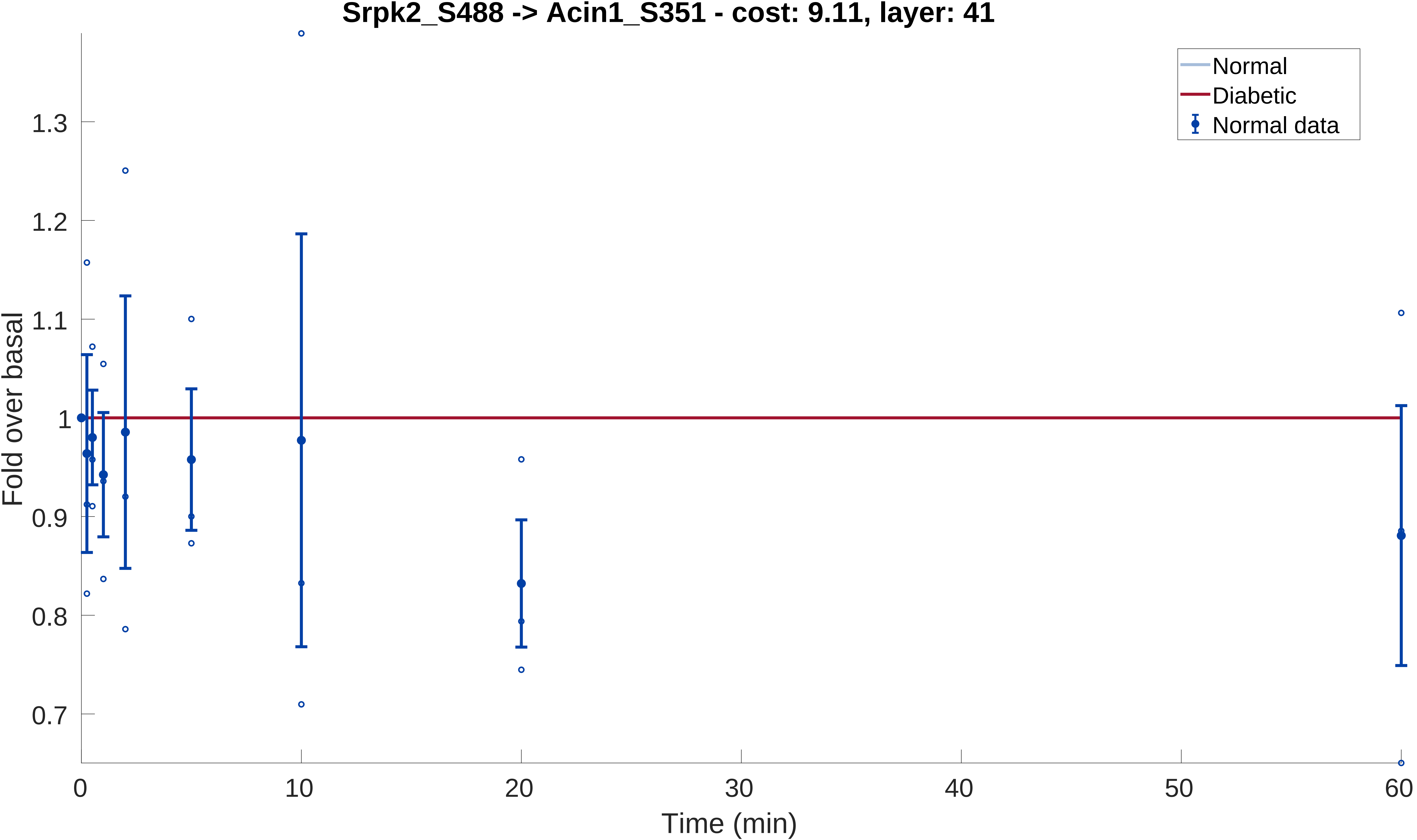

Srpk2_S488 -> Acin1_S351 - cost: 9.11, layer: 41
Normal
Diabetic
Normal data
Fold over basal
1.3
1.2
1.1
1
0.9
0.8
0.7
0
10
20
30
40
50
60
Time (min)

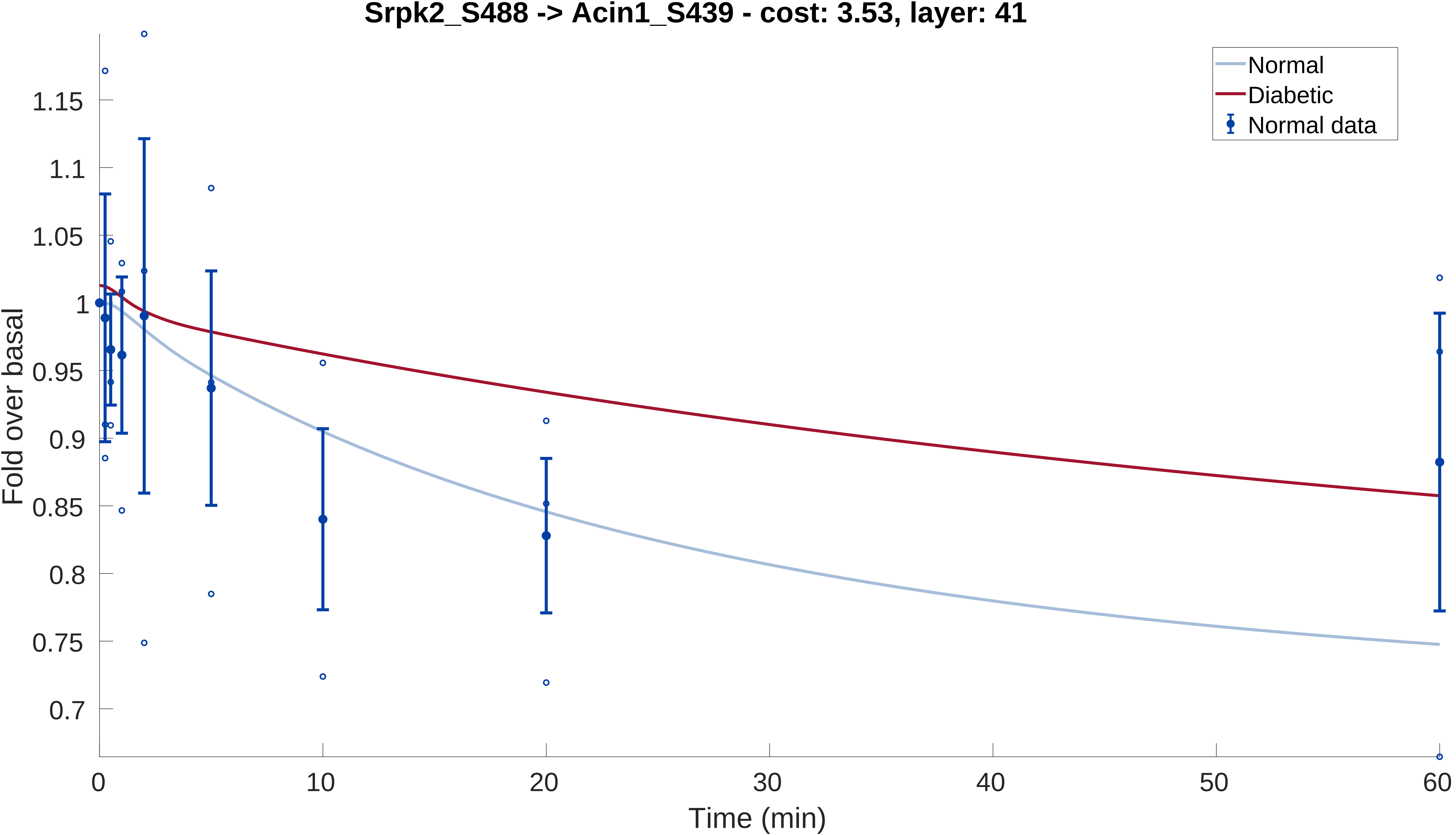

Srpk2_S488 -> Acin1_S439 - cost: 3.53, layer: 41
Normal
Diabetic
Normal data
Fold over basal
1.15
1.1
1.05
1
0.95
0.9
0.85
0.8
0.75
0.7
0
10
20
30
40
50
60
Time (min)

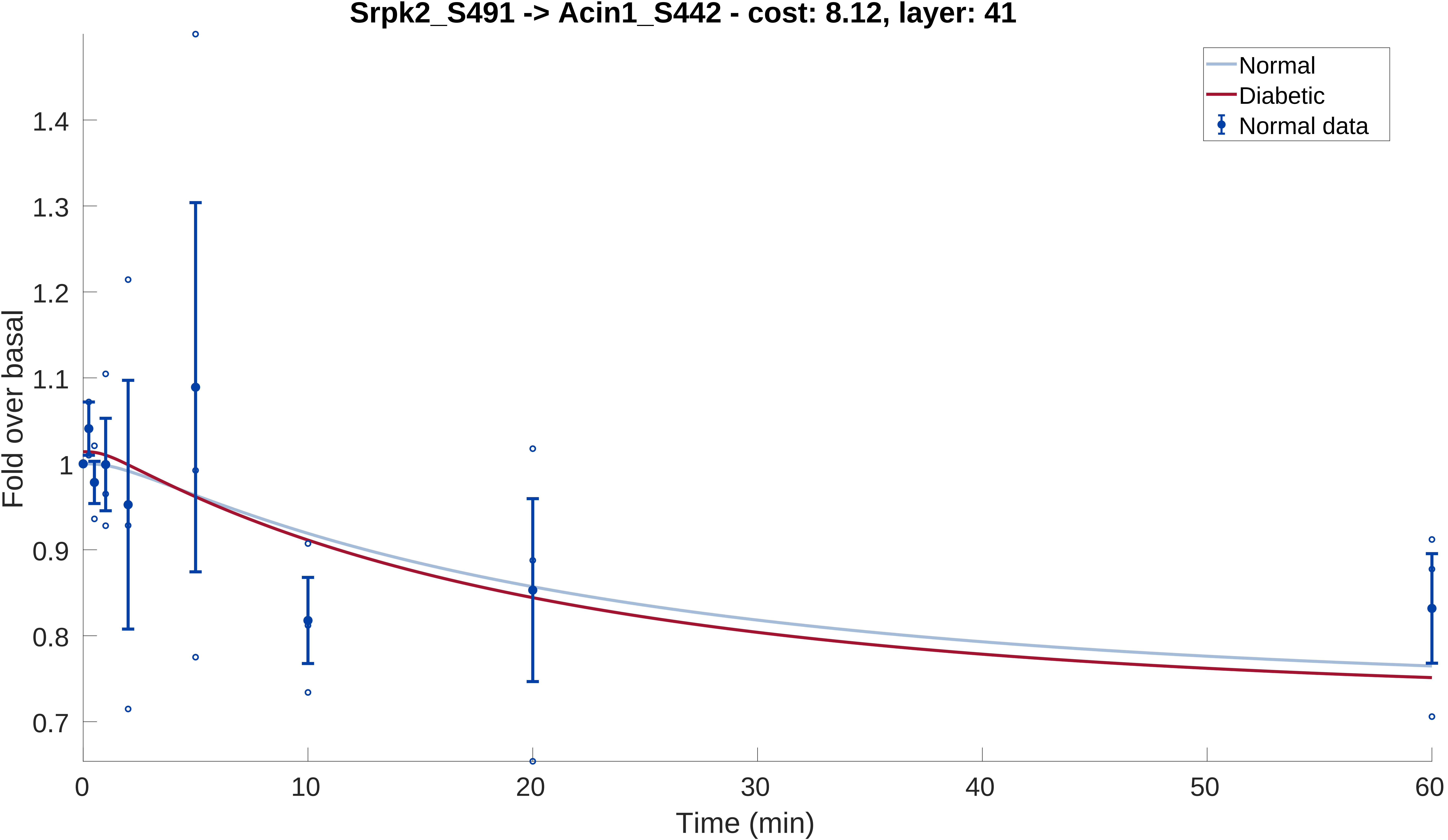

Srpk2_S491 -> Acin1_S442 - cost: 8.12, layer: 41
Normal
Diabetic
Normal data
Fold over basal
1.4
1.3
1.2
1.1
1
0.9
0.8
0.7
0
10
20
30
40
50
60
Time (min)

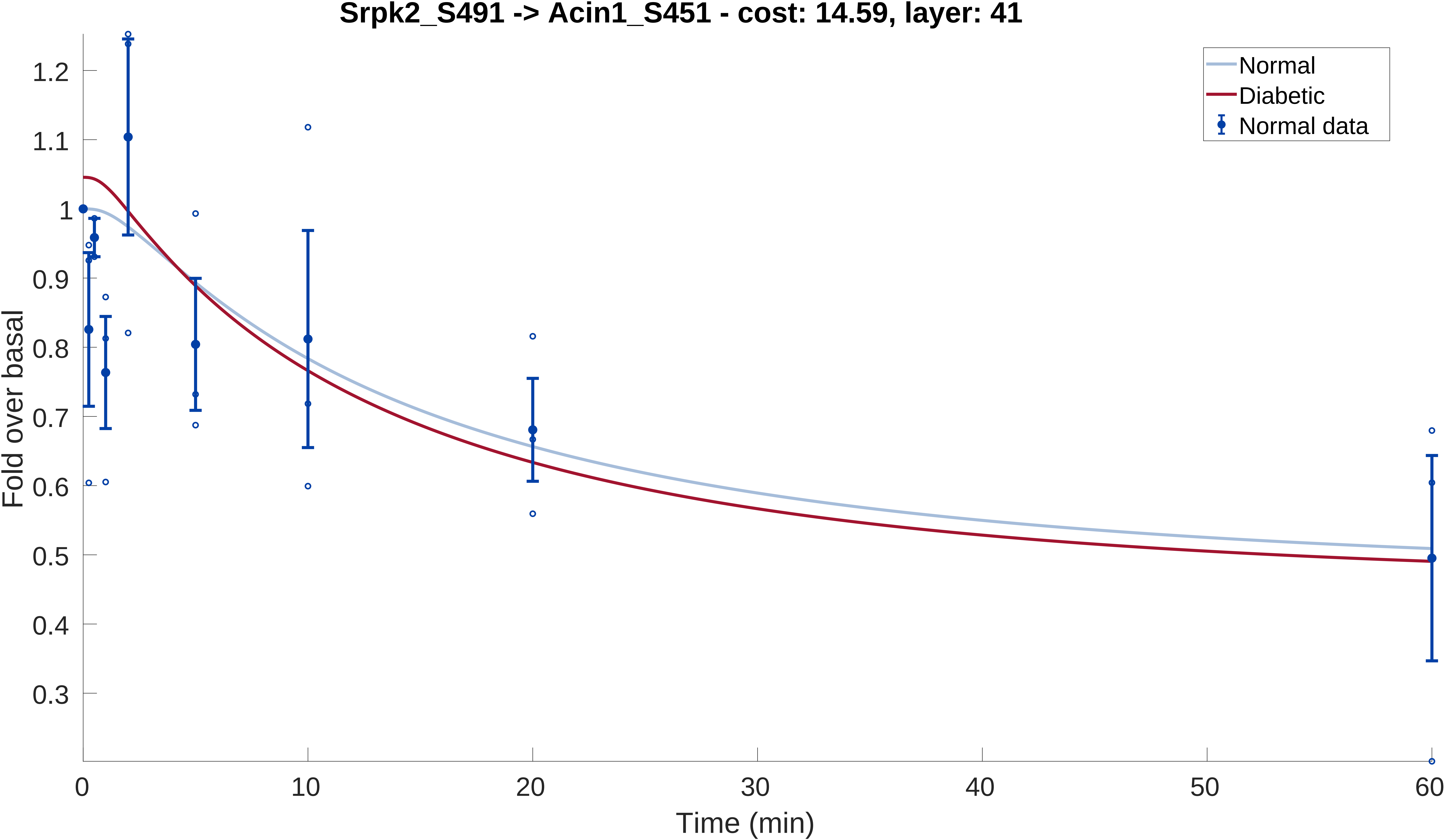

Srpk2_S491 -> Acin1_S451 - cost: 14.59, layer: 41
Normal
Diabetic
Normal data
Fold over basal
1.2
1.1
1
0.9
0.8
0.7
0.6
0.5
0.4
0.3
0
10
20
30
40
50
60
Time (min)

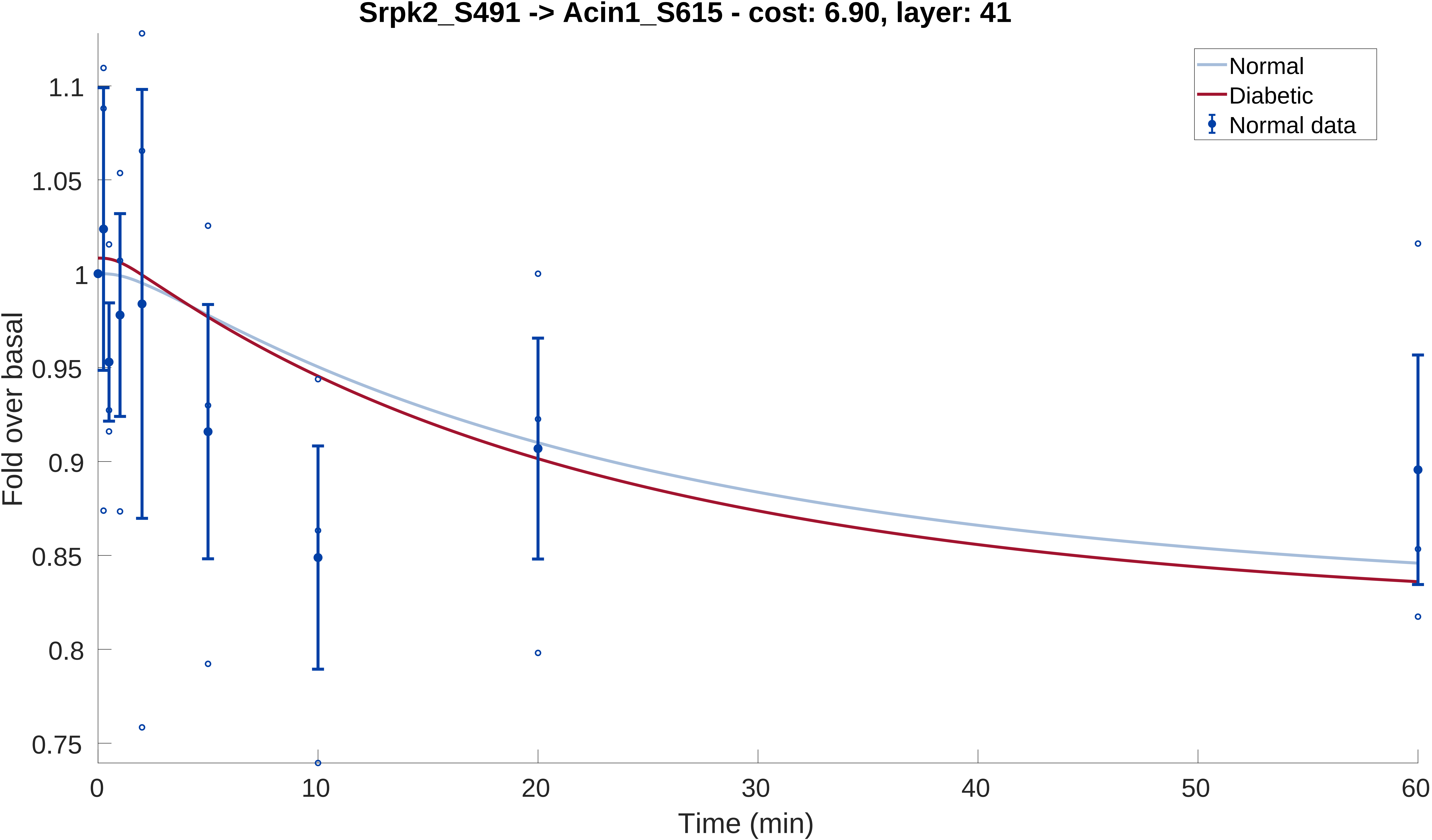

Srpk2_S491 -> Acin1_S615 - cost: 6.90, layer: 41
Normal
Diabetic
Normal data
Fold over basal
1.1
1.05
1
0.95
0.9
0.85
0.8
0.75
0
10
20
30
40
50
60
Time (min)

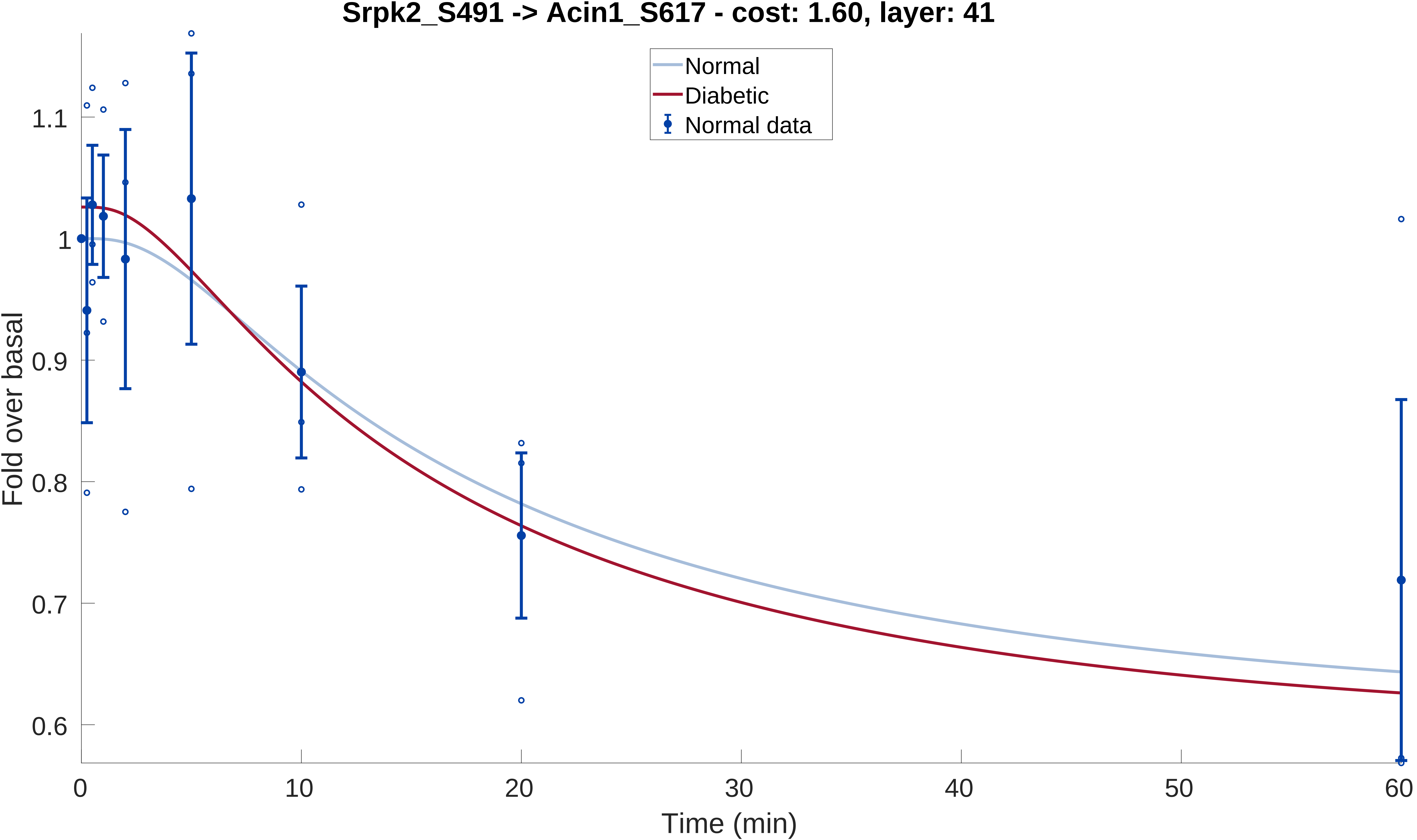
Srpk2_S491 -> Acin1_S617 - cost: 1.60, layer: 41
Normal
Diabetic
Normal data
Fold over basal
1.1
1
0.9
0.8
0.7
0.6
0
10
20
30
40
50
60
Time (min)

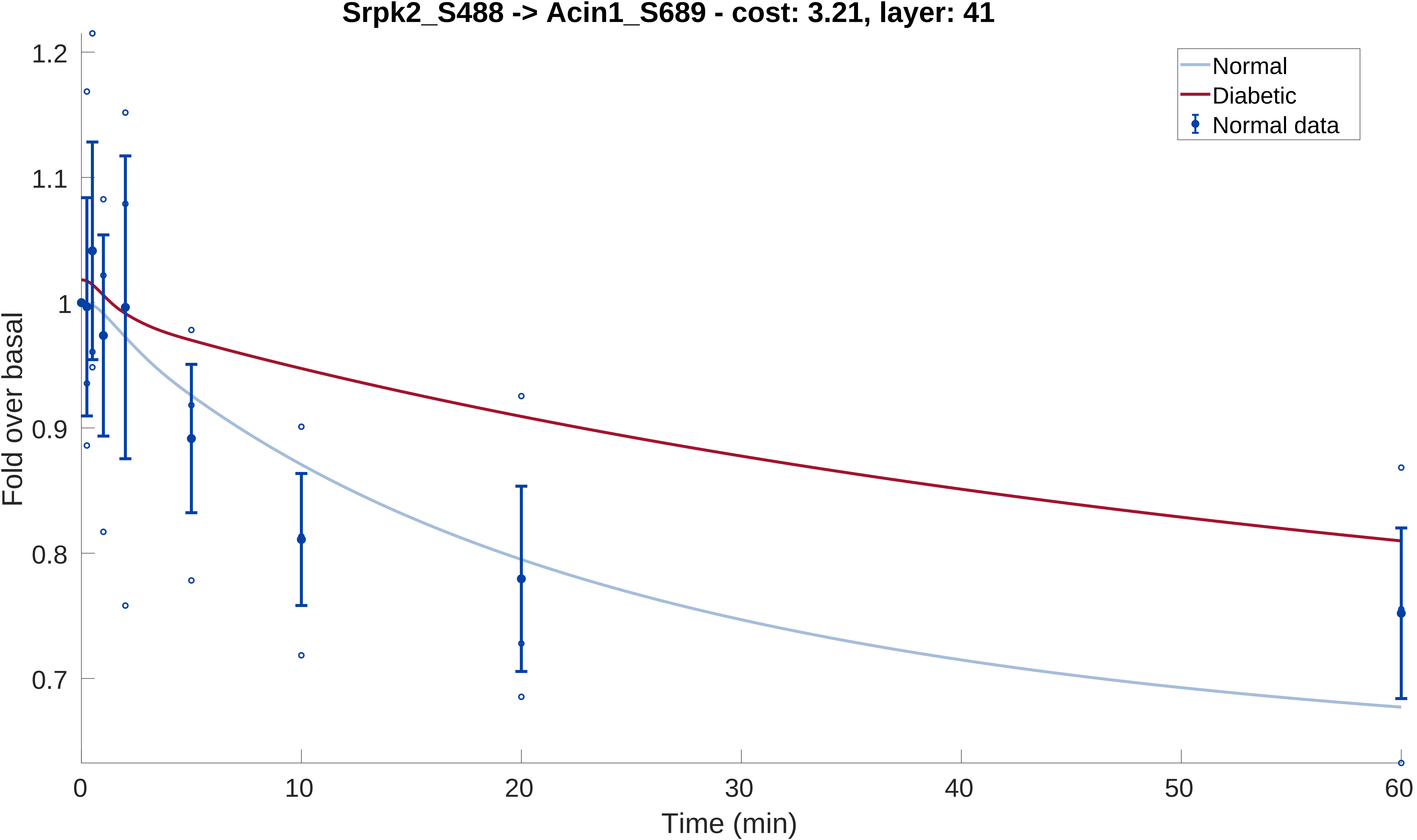

Srpk2_S488 -> Acin1_S689 - cost: 3.21, layer: 41
Fold over basal
Time (min)
Normal
Diabetic
Normal data
1.2
1.1
1
0.9
0.8
0.7
0
10
20
30
40
50
60

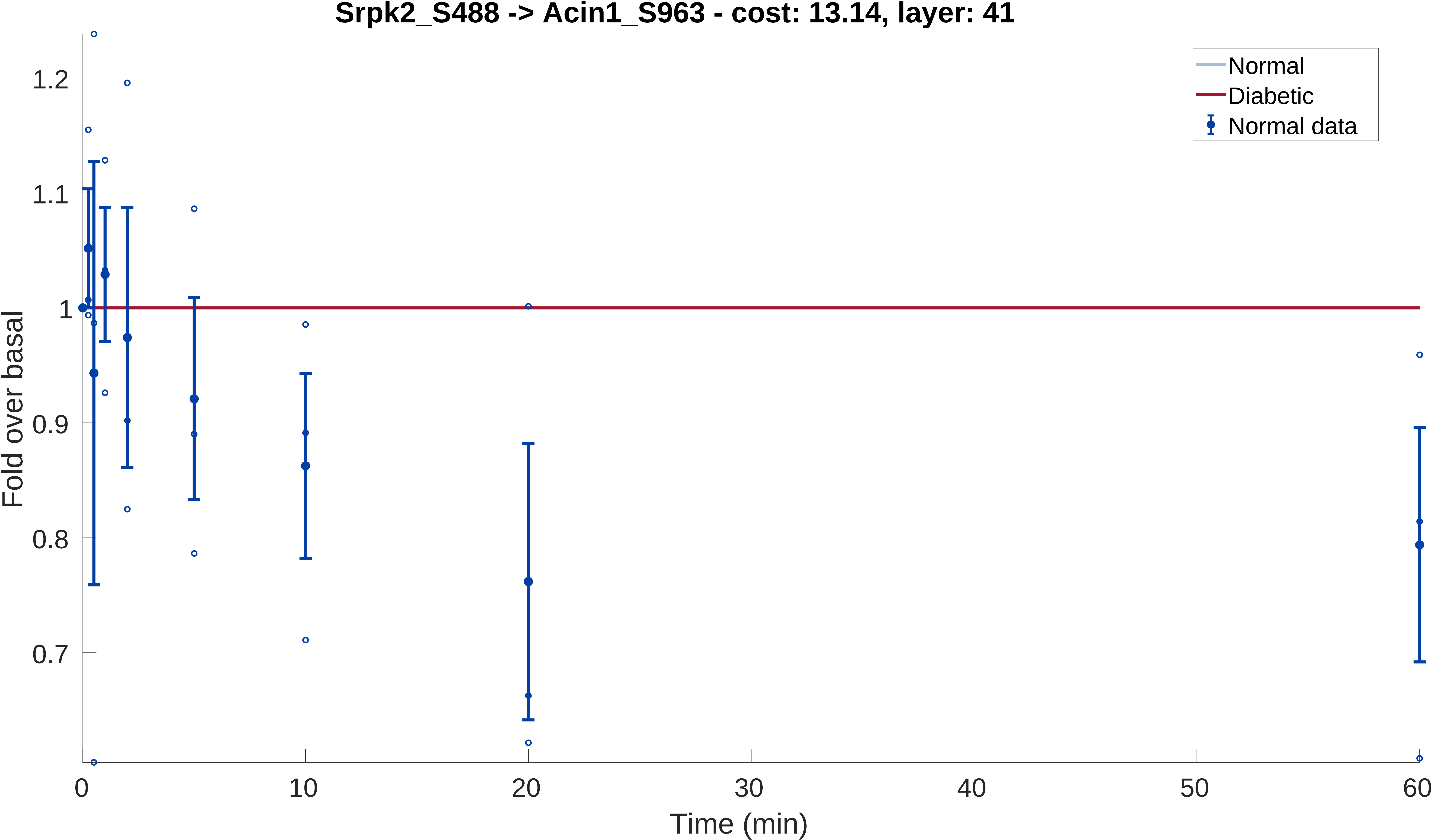

Srpk2_S488 -> Acin1_S963 - cost: 13.14, layer: 41
Normal
Diabetic
Normal data
Fold over basal
1.2
1.1
1
0.9
0.8
0.7
0
10
20
30
40
50
60
Time (min)

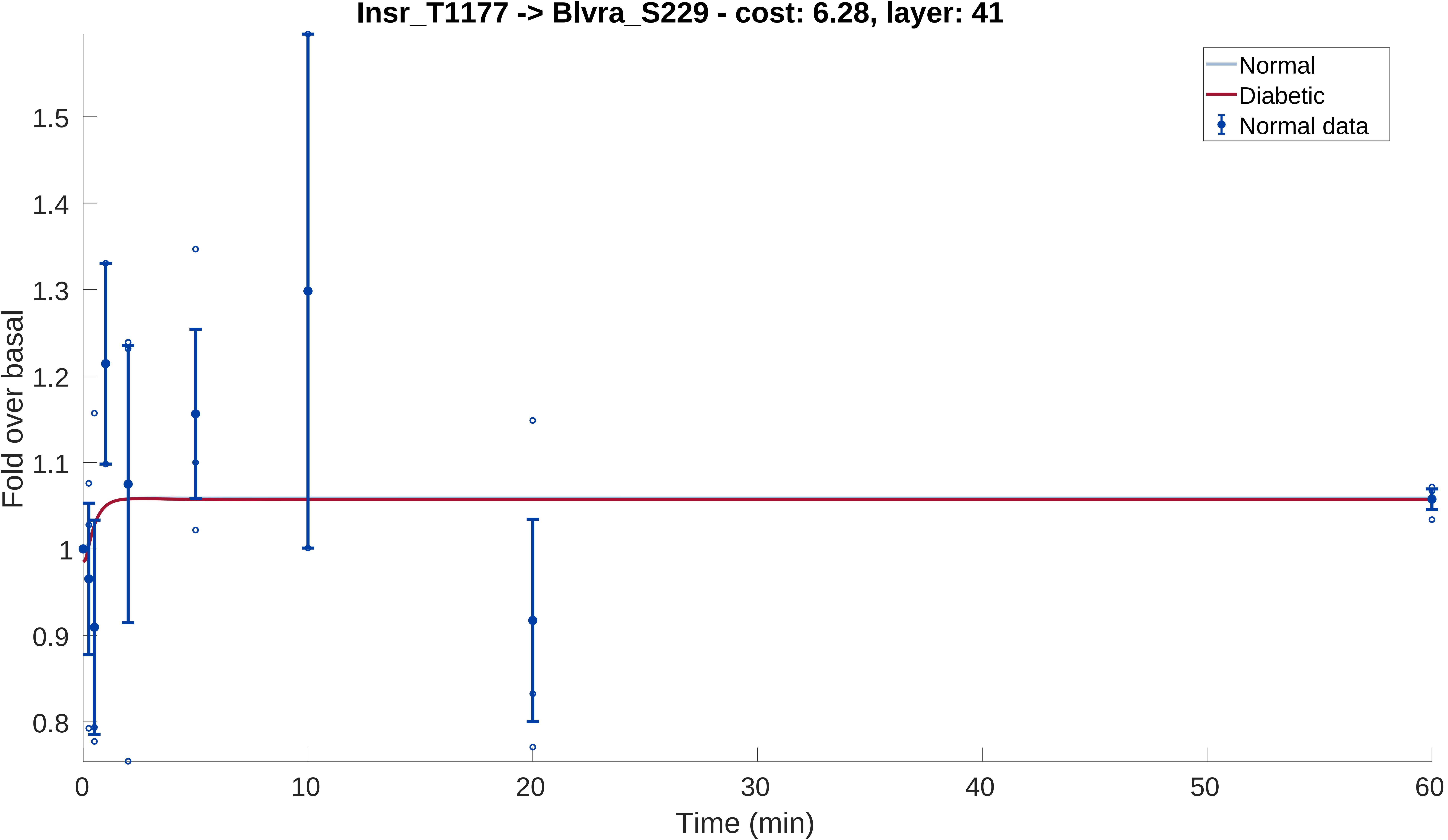

Insr_T1177 -> Blvra_S229 - cost: 6.28, layer: 41
Normal
Diabetic
Normal data
Fold over basal
Time (min)
0.8
0.9
1
1.1
1.2
1.3
1.4
1.5
0
10
20
30
40
50
60

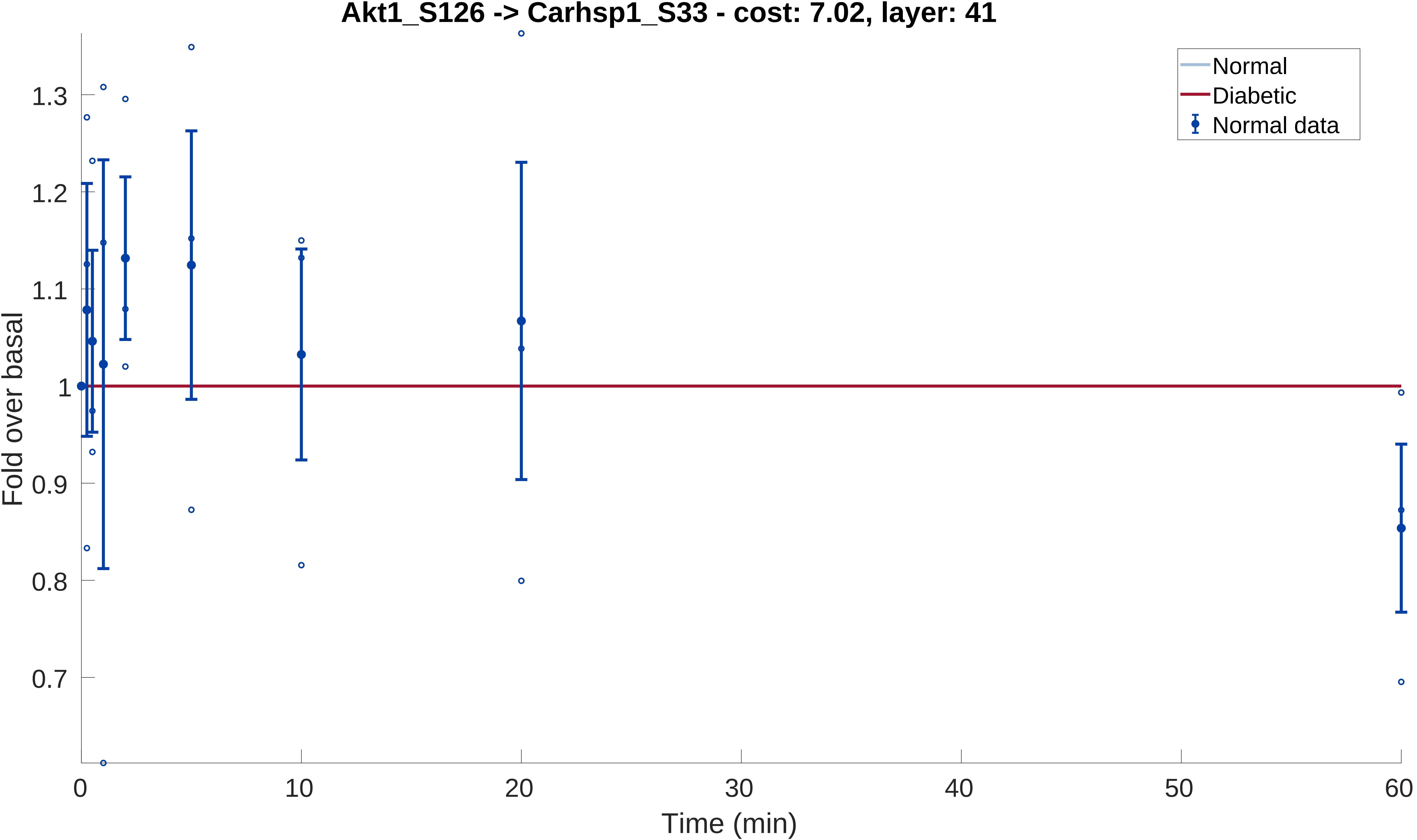
Akt1_S126 -> Carhsp1_S33 - cost: 7.02, layer: 41
Normal
Diabetic
Normal data
Fold over basal
1.3
1.2
1.1
1
0.9
0.8
0.7
0
10
20
30
40
50
60
Time (min)

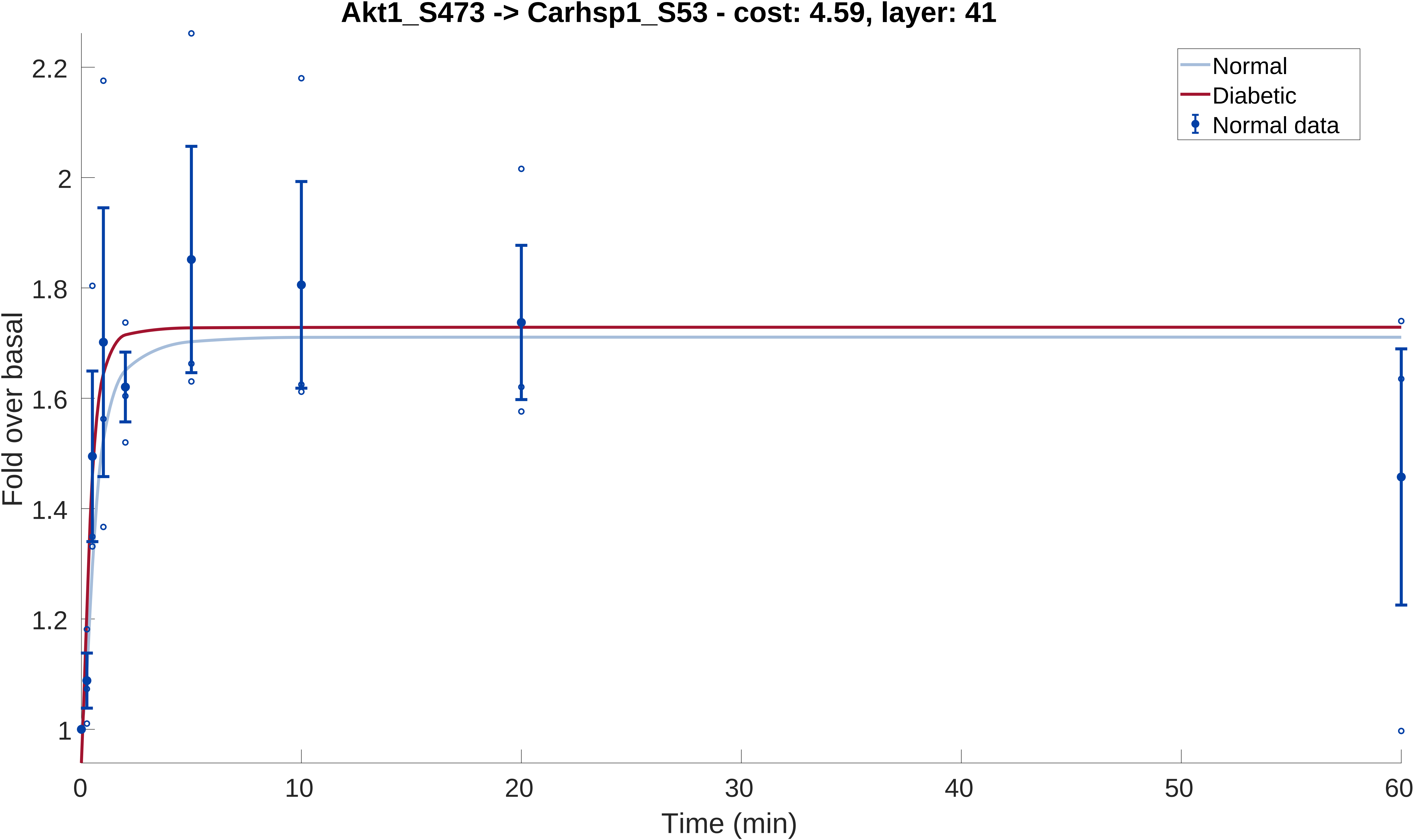

Akt1_S473 -> Carhsp1_S53 - cost: 4.59, layer: 41
Normal
Diabetic
Normal data
Fold over basal
Time (min)
1
1.2
1.4
1.6
1.8
2
2.2
0
10
20
30
40
50
60

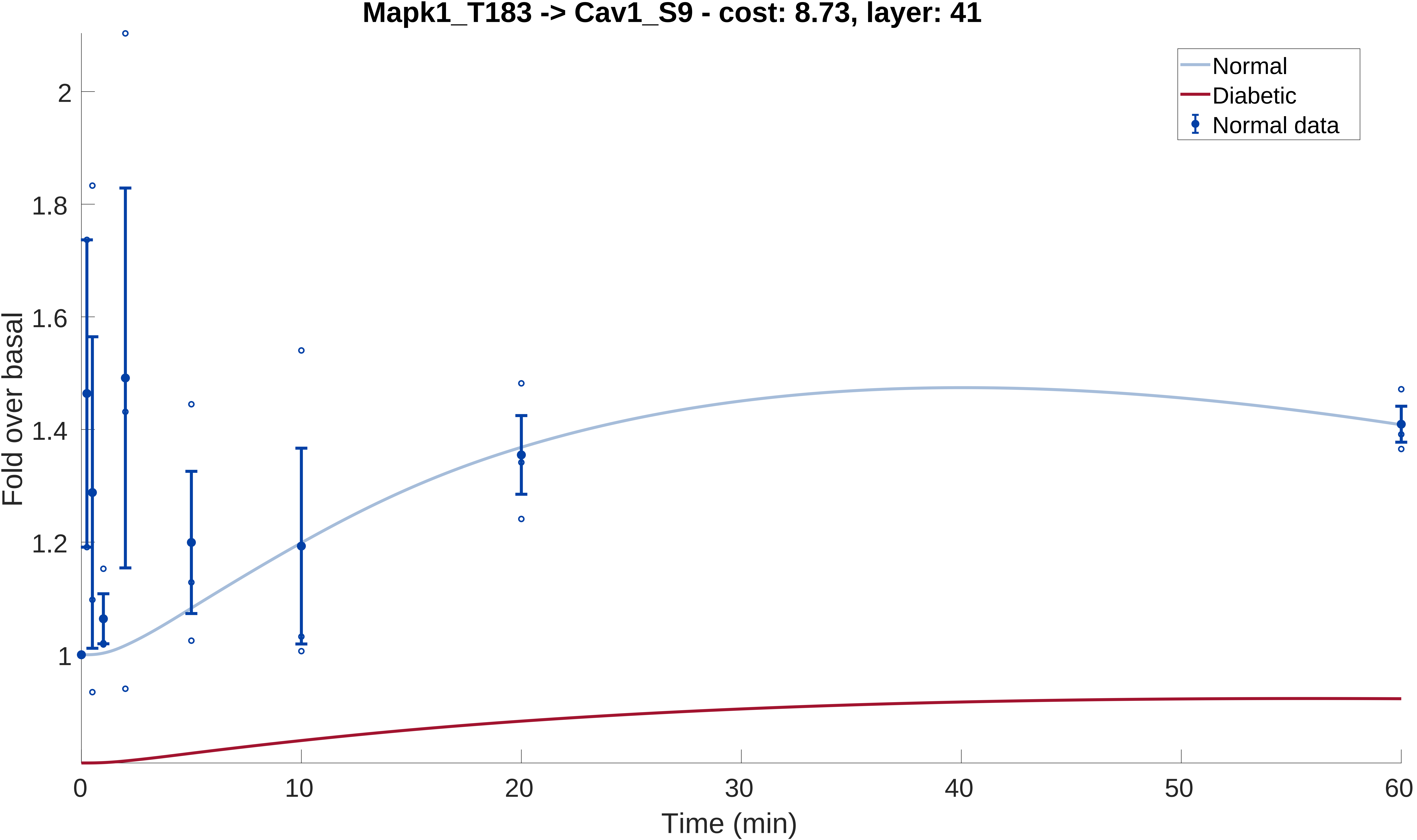

Mapk1_T183 -> Cav1_S9 - cost: 8.73, layer: 41
Normal
Diabetic
Normal data
Fold over basal
2
1.8
1.6
1.4
1.2
1
0
10
20
30
40
50
60
Time (min)

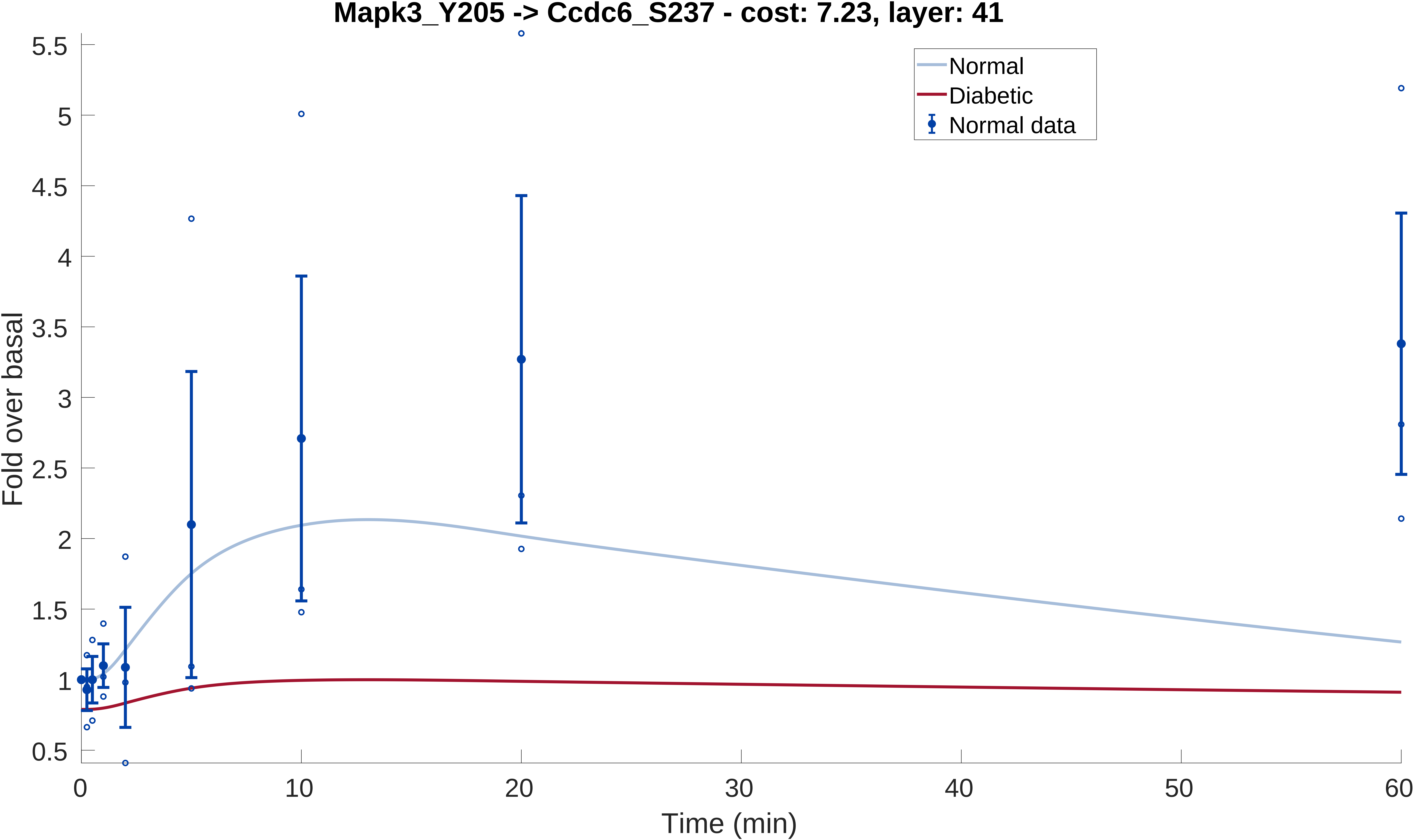

Mapk3_Y205 -> Ccdc6_S237 - cost: 7.23, layer: 41
Normal
Diabetic
Normal data
Fold over basal
Time (min)
0.5
1
1.5
2
2.5
3
3.5
4
4.5
5
5.5
0
10
20
30
40
50
60

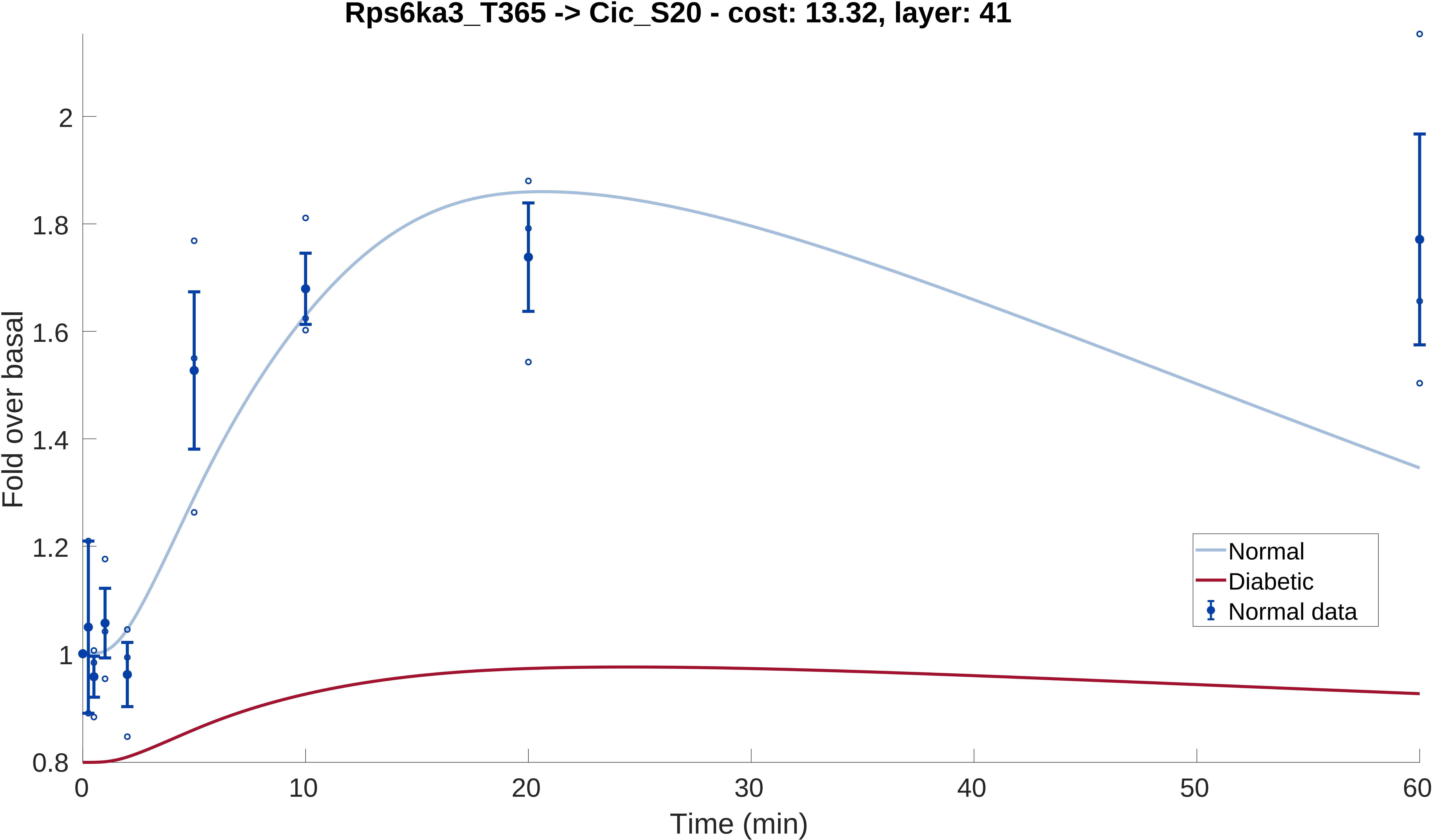

Rps6ka3_T365 -> Cic_S20 - cost: 13.32, layer: 41
Fold over basal
2
1.8
1.6
1.4
1.2
1
0.8
0
10
20
30
40
50
60
Time (min)
Normal
Diabetic
Normal data

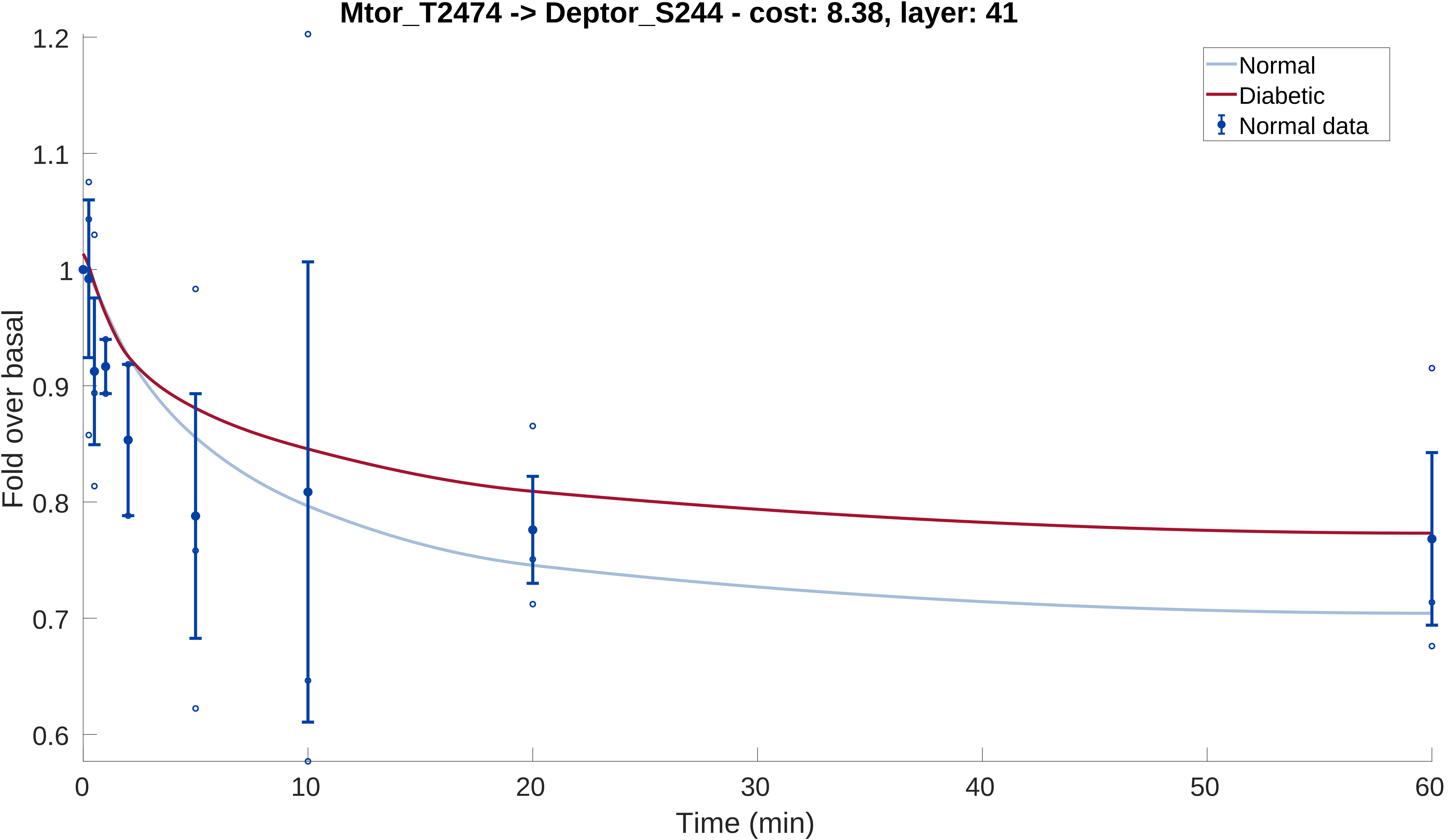

Mtor_T2474 -> Deptor_S244 - cost: 8.38, layer: 41
Normal
Diabetic
Normal data
Fold over basal
Time (min)
1.2
1.1
1
0.9
0.8
0.7
0.6
0
10
20
30
40
50
60

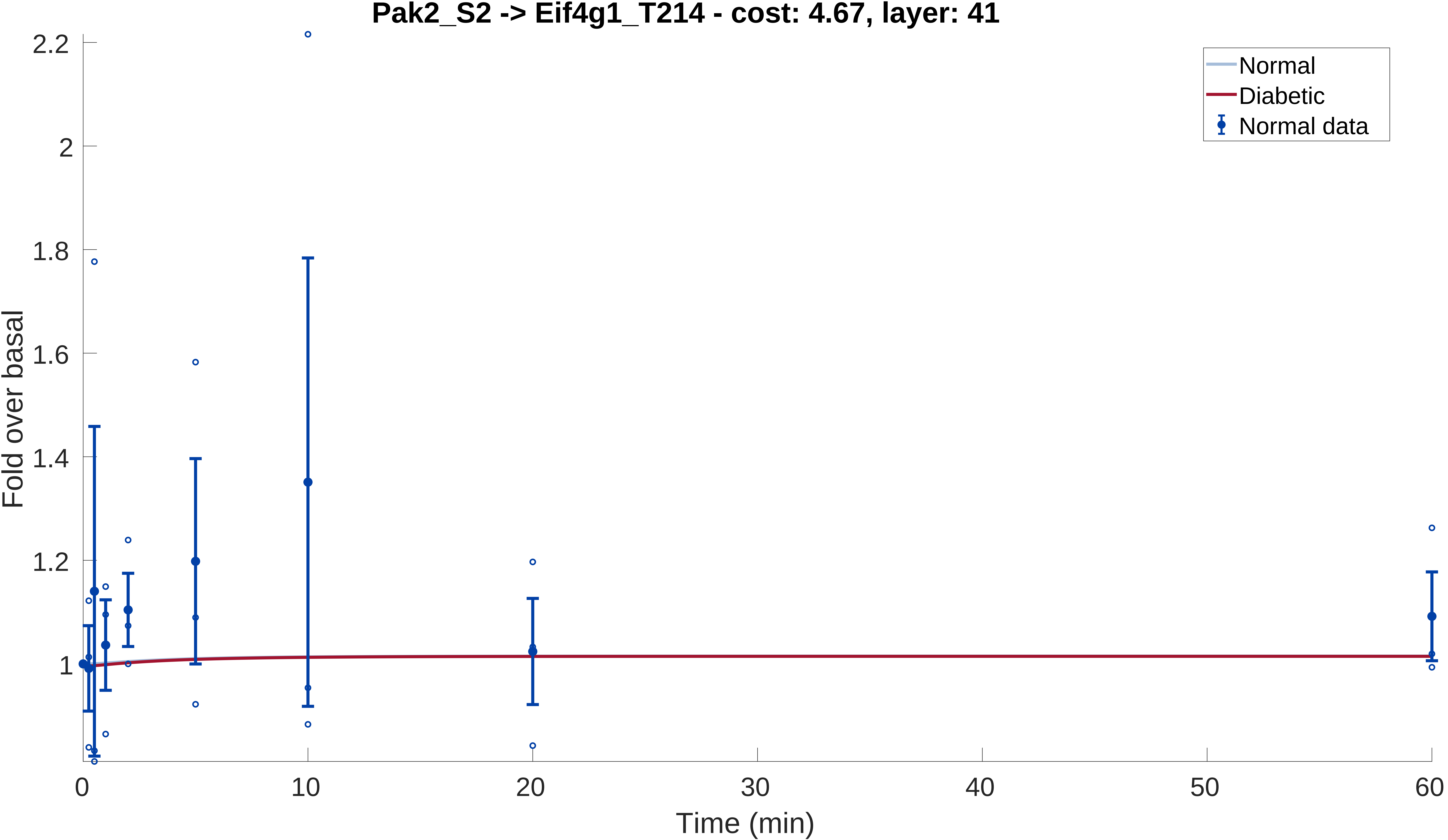
Pak2_S2 -> Eif4g1_T214 - cost: 4.67, layer: 41
Normal
Diabetic
Normal data
Fold over basal
2.2
2
1.8
1.6
1.4
1.2
1
0
10
20
30
40
50
60
Time (min)

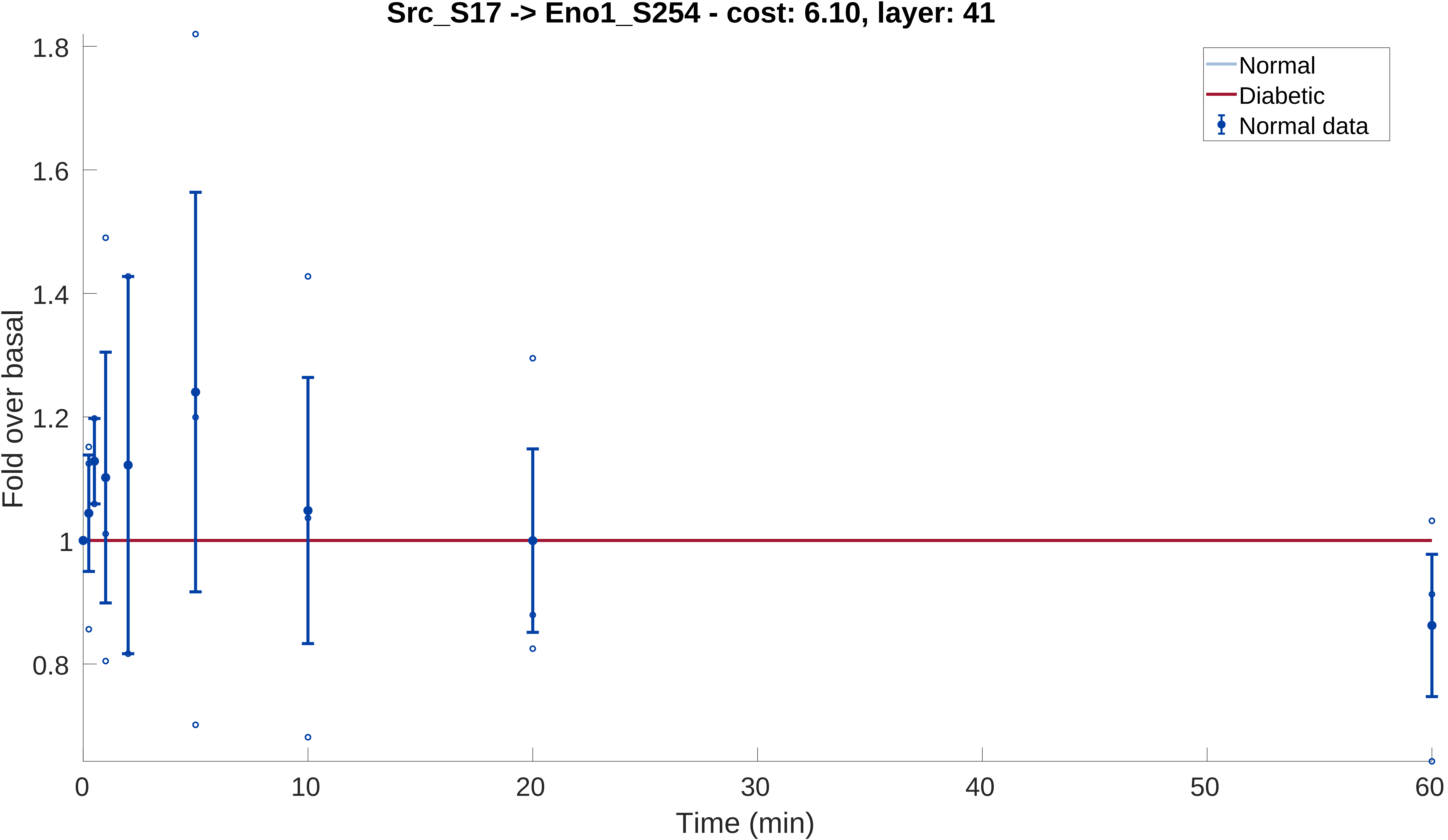

Src_S17 -> Eno1_S254 - cost: 6.10, layer: 41
Normal
Diabetic
Normal data
Fold over basal
1.8
1.6
1.4
1.2
1
0.8
0
10
20
30
40
50
60
Time (min)

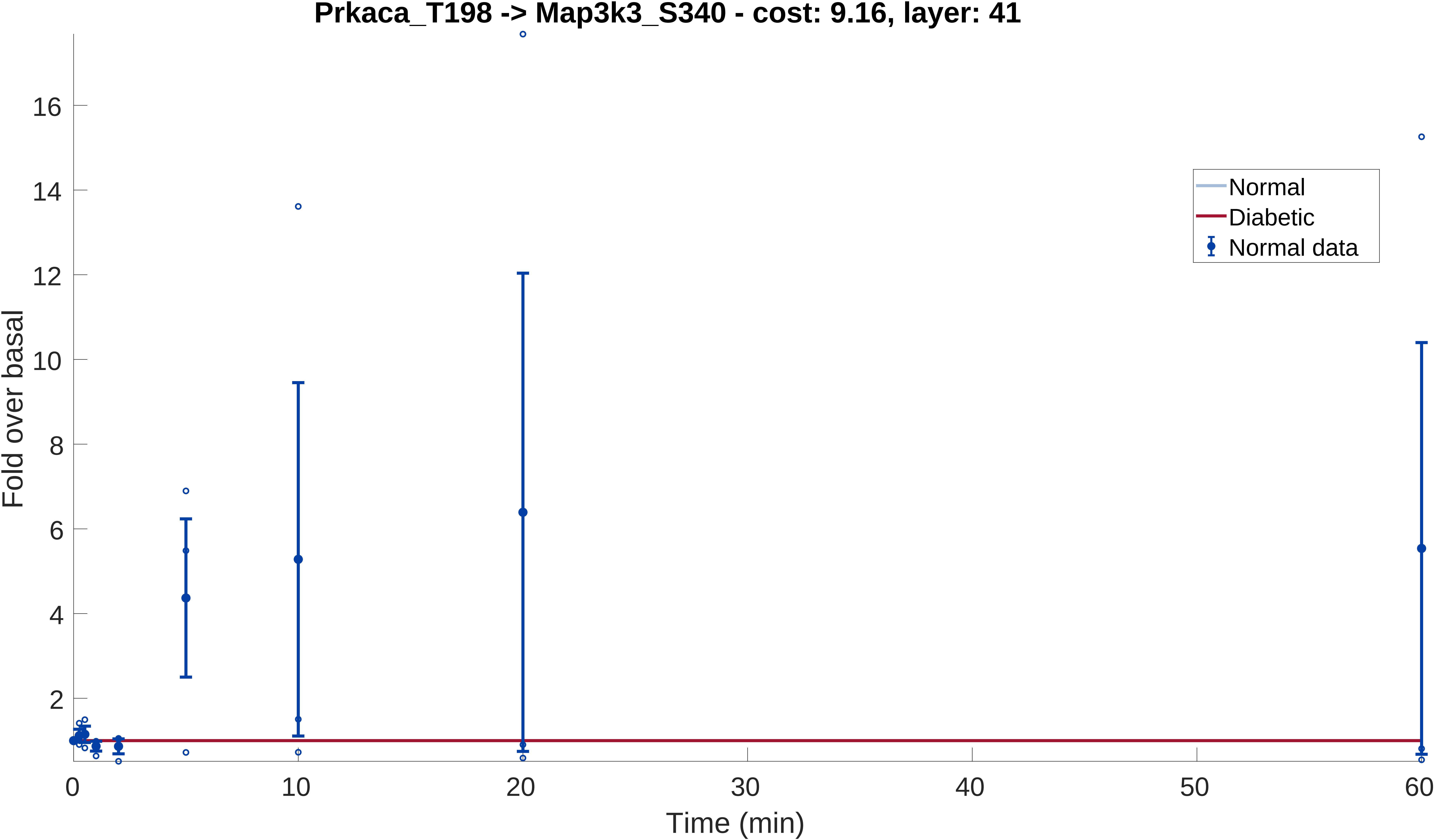
Prkaca_T198 -> Map3k3_S340 - cost: 9.16, layer: 41
Fold over basal
16
14
12
10
8
6
4
2
0
10
20
30
40
50
60
Time (min)
Normal
Diabetic
Normal data

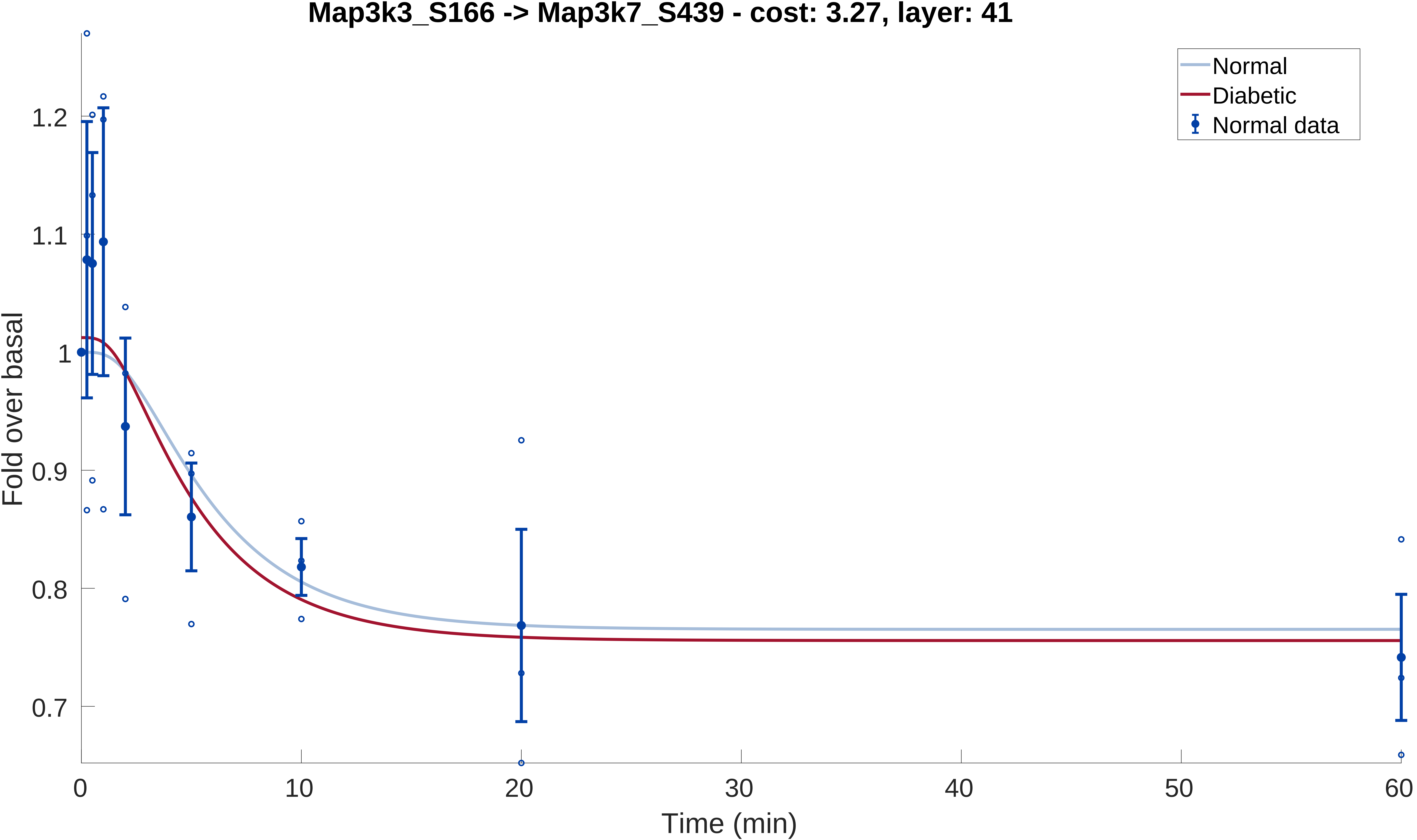

Map3k3_S166 -> Map3k7_S439 - cost: 3.27, layer: 41
Normal
Diabetic
Normal data
Fold over basal
1.2
1.1
1
0.9
0.8
0.7
0
10
20
30
40
50
60
Time (min)

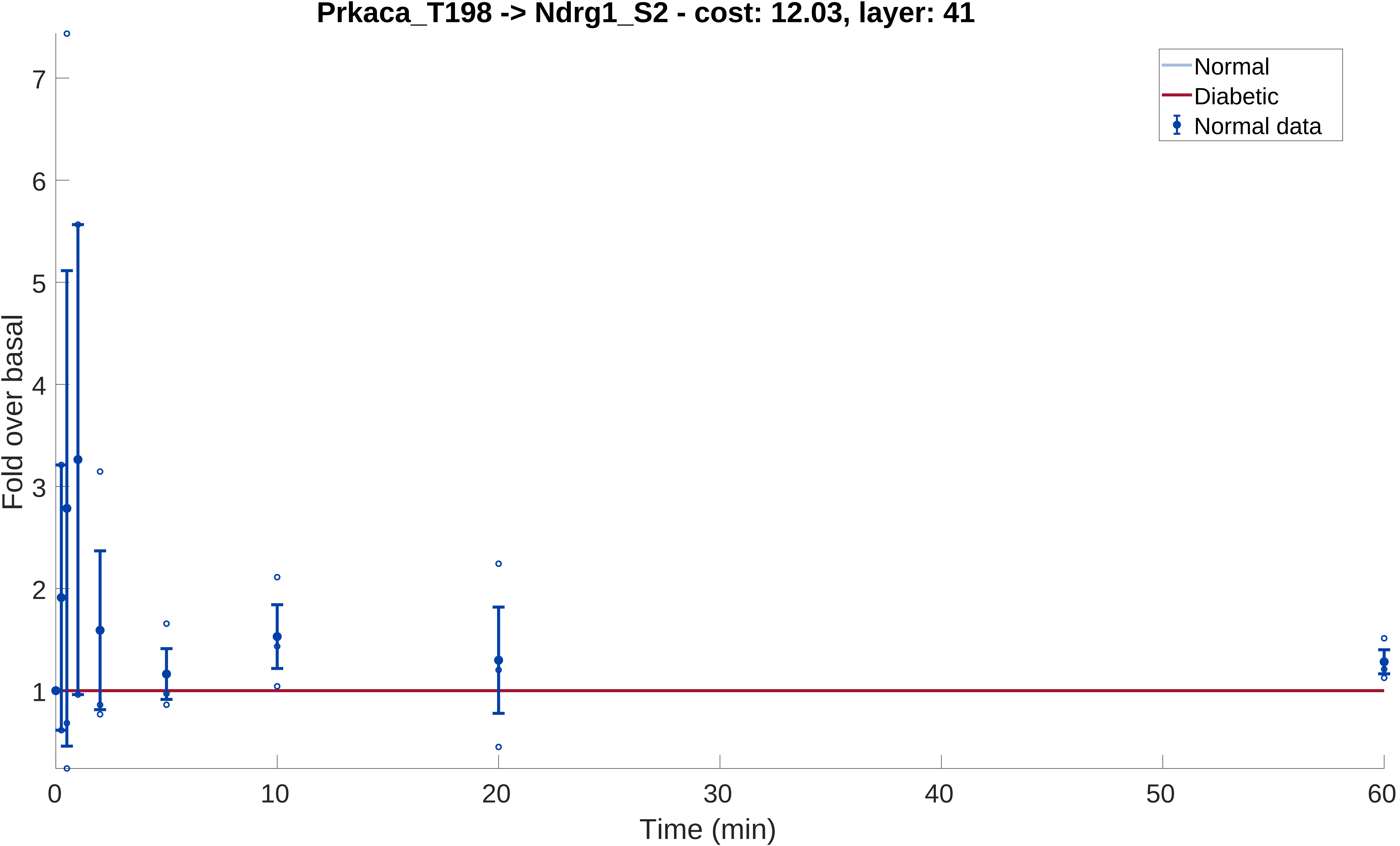

Prrkaca_T198 -> Ndrg1_S2 - cost: 12.03, layer: 41
Normal
Diabetic
Normal data
Fold over basal
Time (min)
0
10
20
30
40
50
60
1
2
3
4
5
6
7

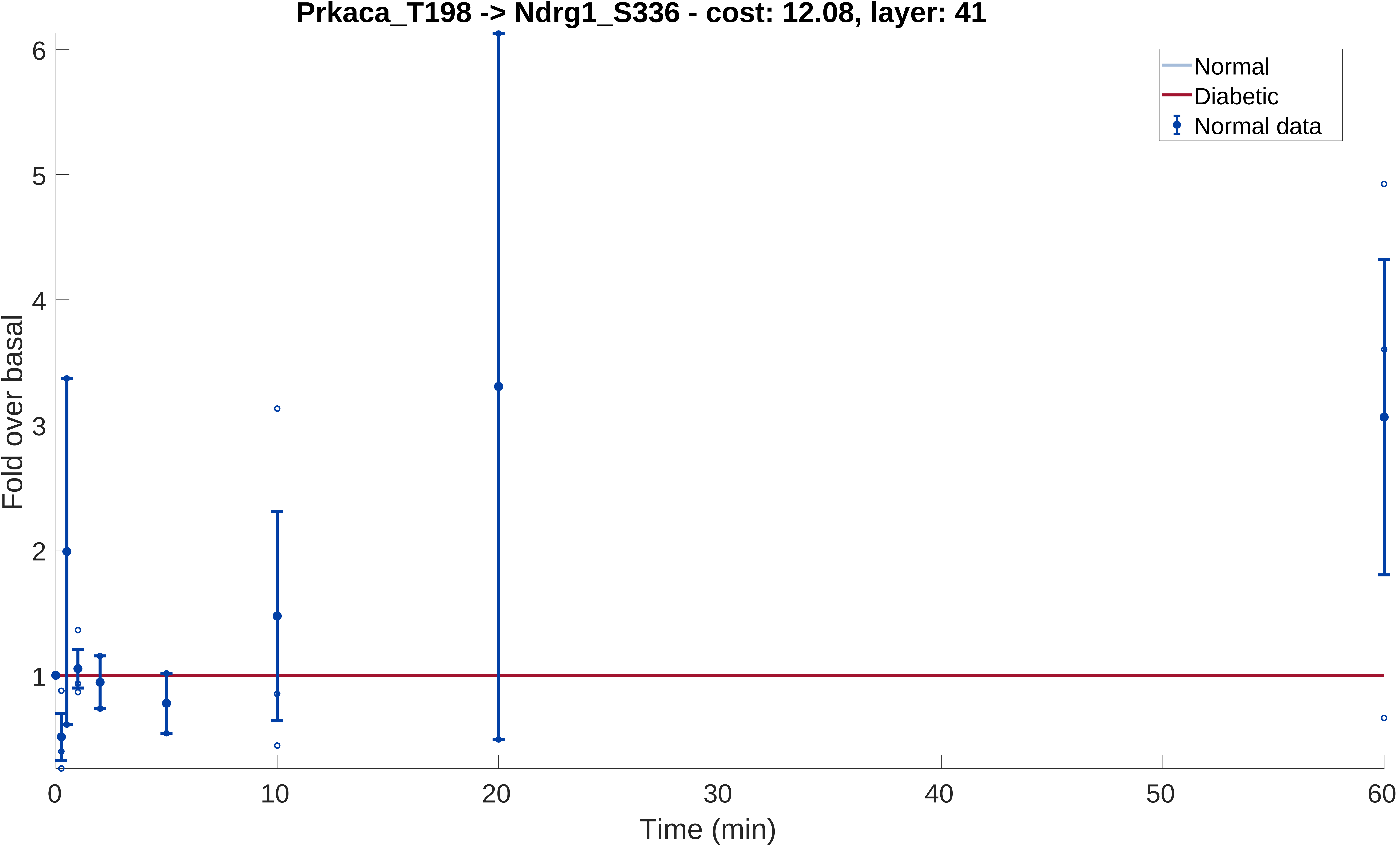

Prrkaca_T198 -> Ndrg1_S336 - cost: 12.08, layer: 41
Normal
Diabetic
Normal data
Fold over basal
Time (min)
0
10
20
30
40
50
60
1
2
3
4
5
6

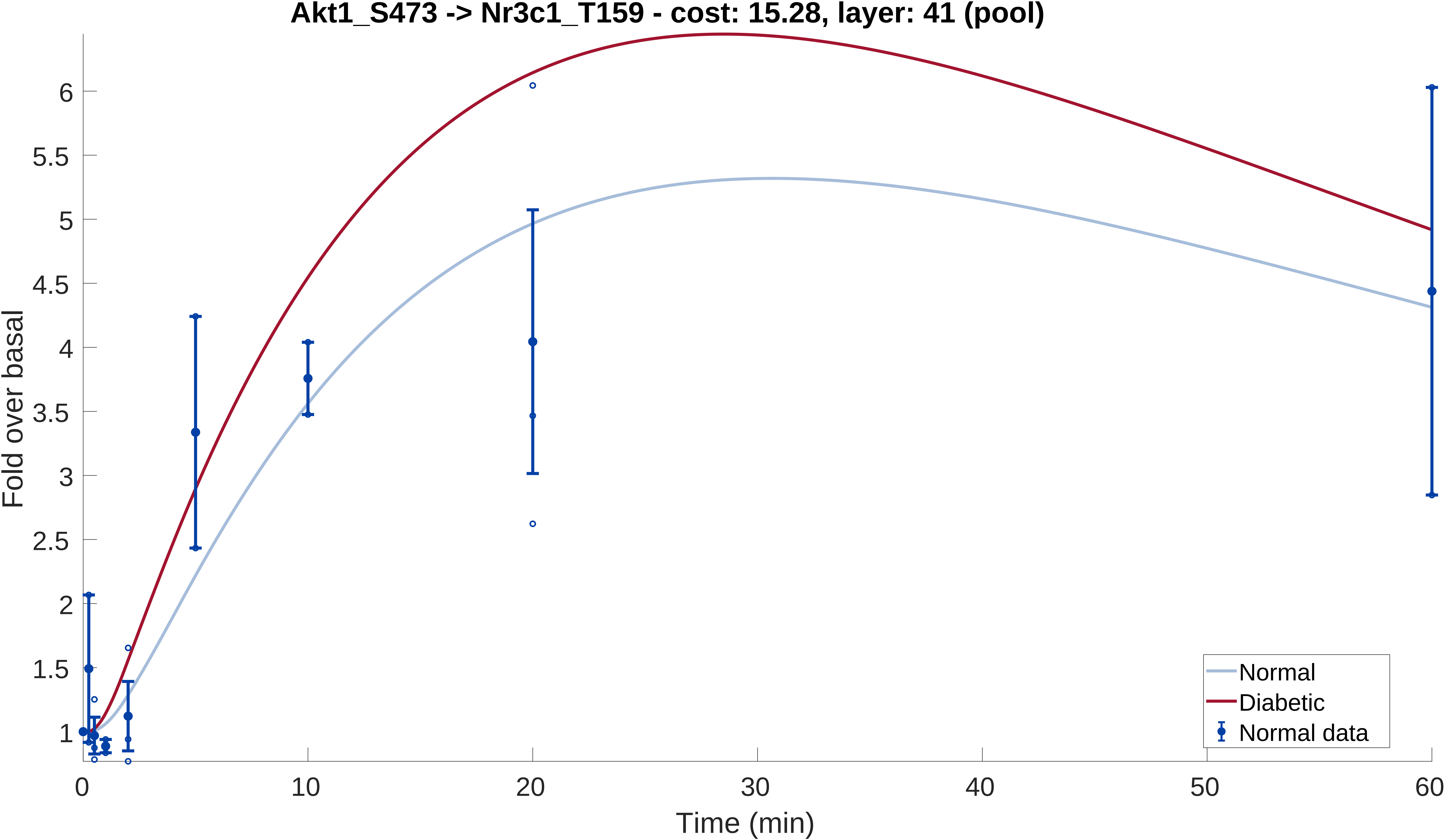
Akt1_S473 -> Nr3c1_T159 - cost: 15.28, layer: 41 (pool)
Fold over basal
Time (min)
0
10
20
30
40
50
60
1
1.5
2
2.5
3
3.5
4
4.5
5
5.5
6
Normal
Diabetic
Normal data

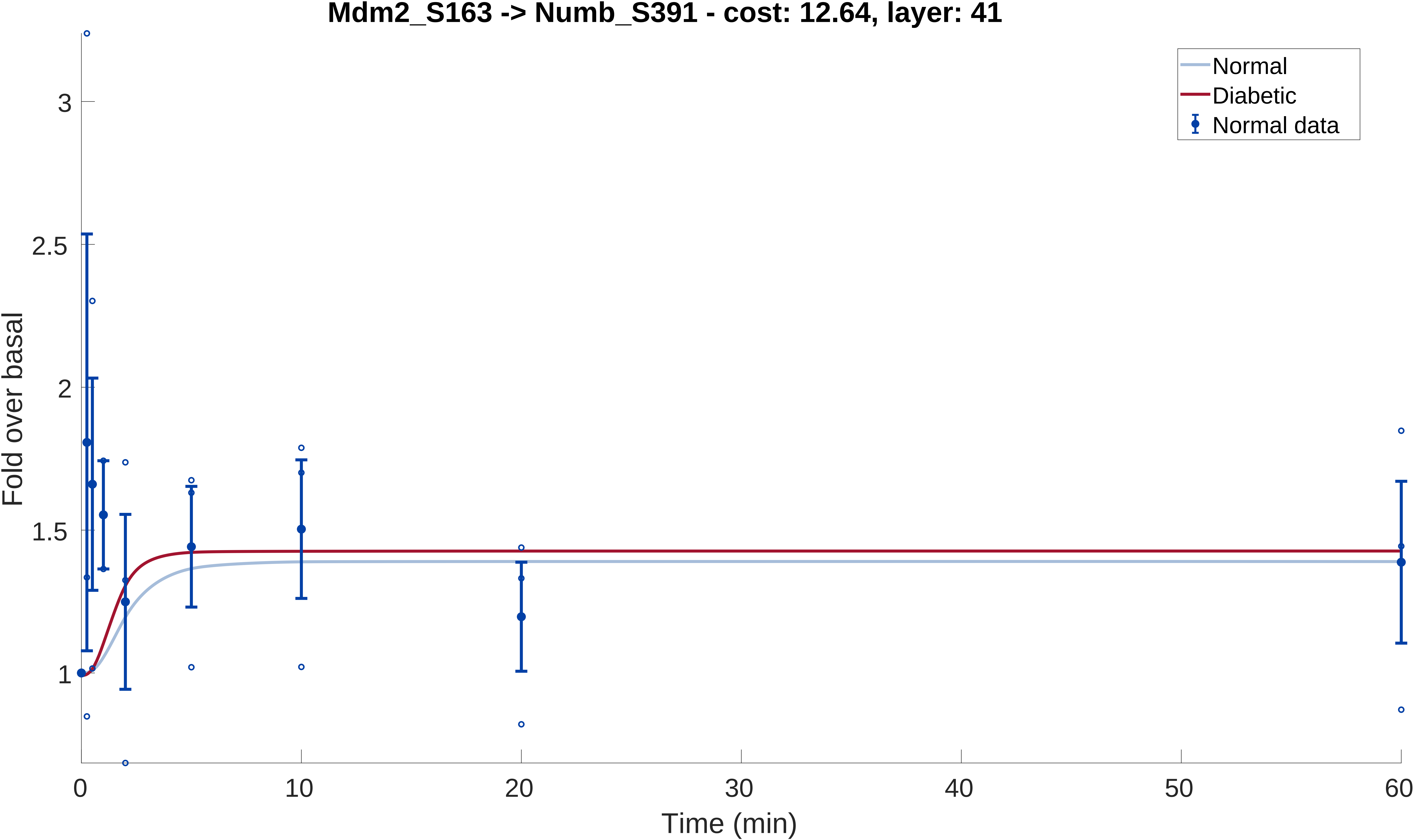

Mdm2_S163 -> Numb_S391 - cost: 12.64, layer: 41
Normal
Diabetic
Normal data
Fold over basal
Time (min)
3
2.5
2
1.5
1
0
10
20
30
40
50
60

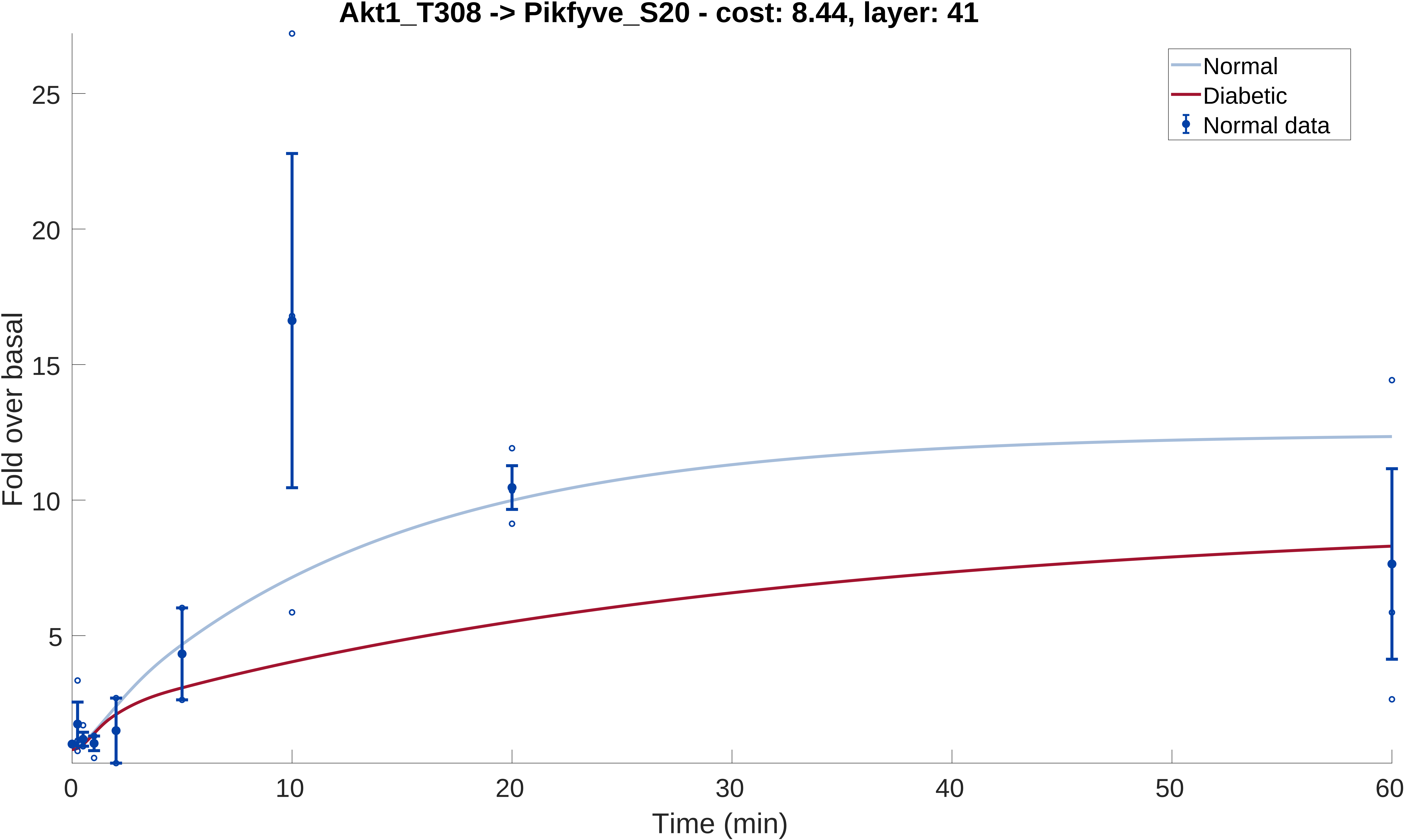
Akt1_T308 -> Pikfyve_S20 - cost: 8.44, layer: 41
Normal
Diabetic
Normal data
Fold over basal
Time (min)
0
10
20
30
40
50
60
5
10
15
20
25

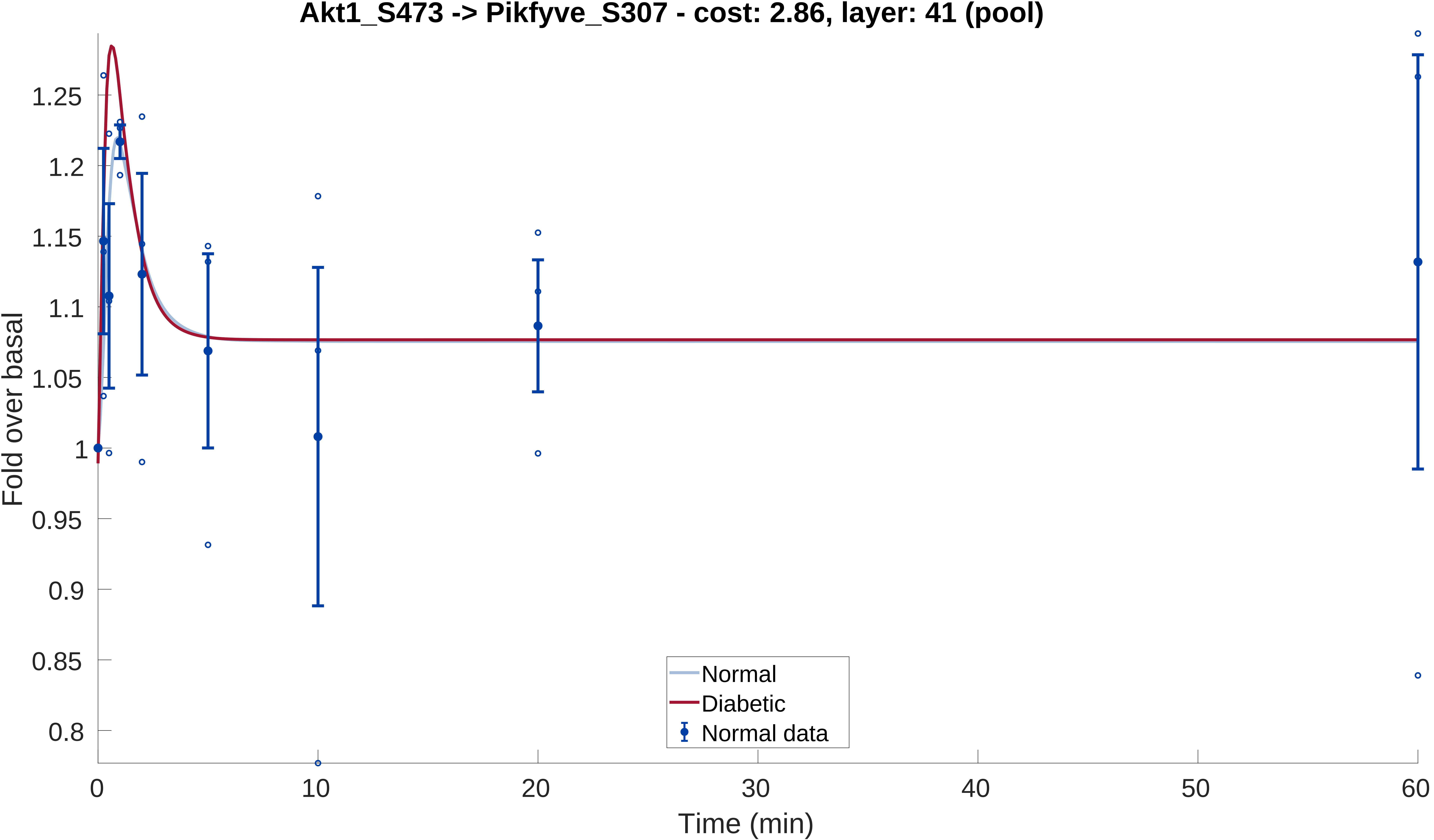

Akt1_S473 -> Pikfyve_S307 - cost: 2.86, layer: 41 (pool)
Fold over basal
1.25
1.2
1.15
1.1
1.05
1
0.95
0.9
0.85
0.8
0
10
20
30
40
50
60
Time (min)
Normal
Diabetic
Normal data

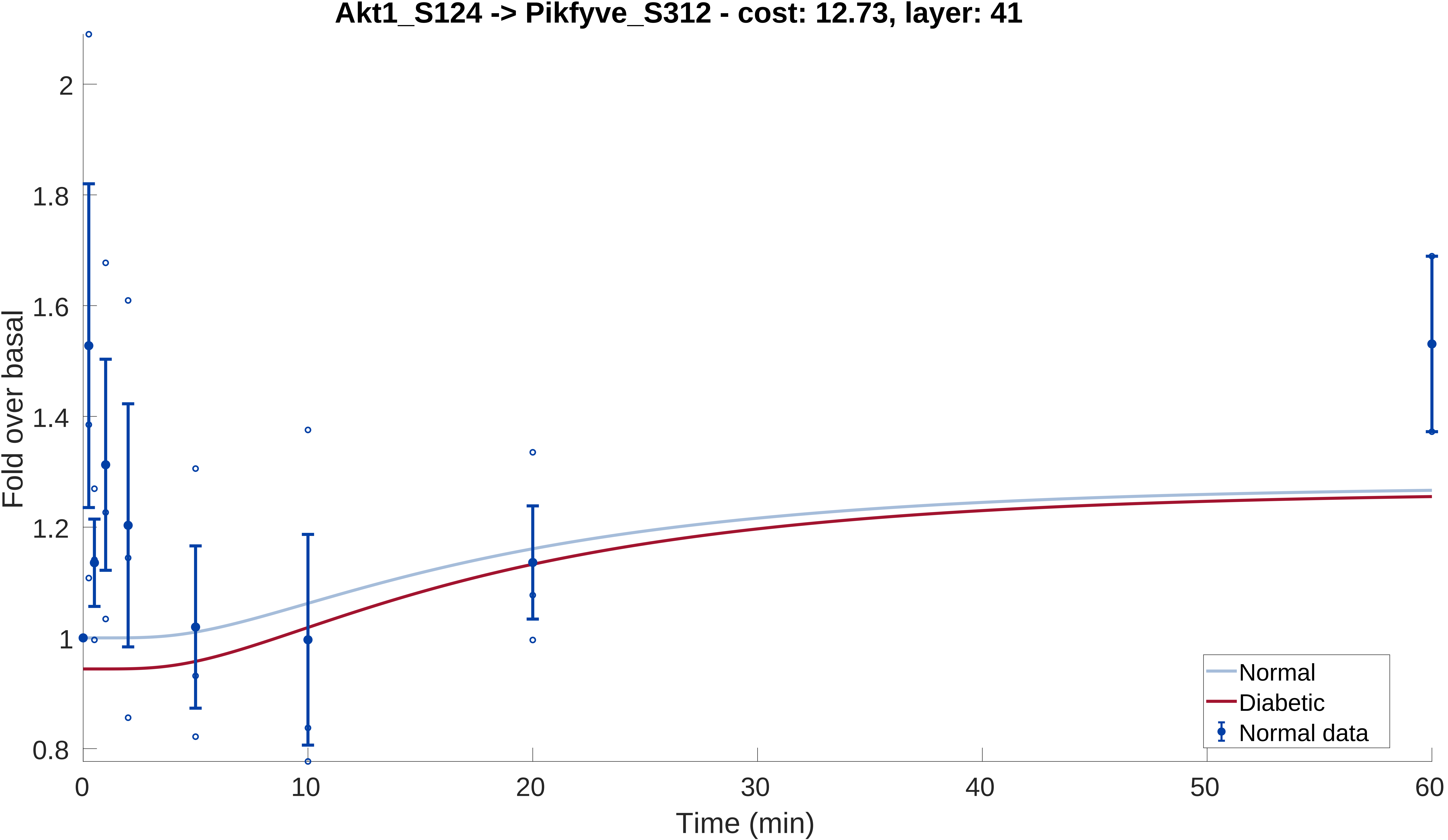

Akt1_S124 -> Pikfyve_S312 - cost: 12.73, layer: 41
Fold over basal
2
1.8
1.6
1.4
1.2
1
0.8
0
10
20
30
40
50
60
Time (min)
Normal
Diabetic
Normal data

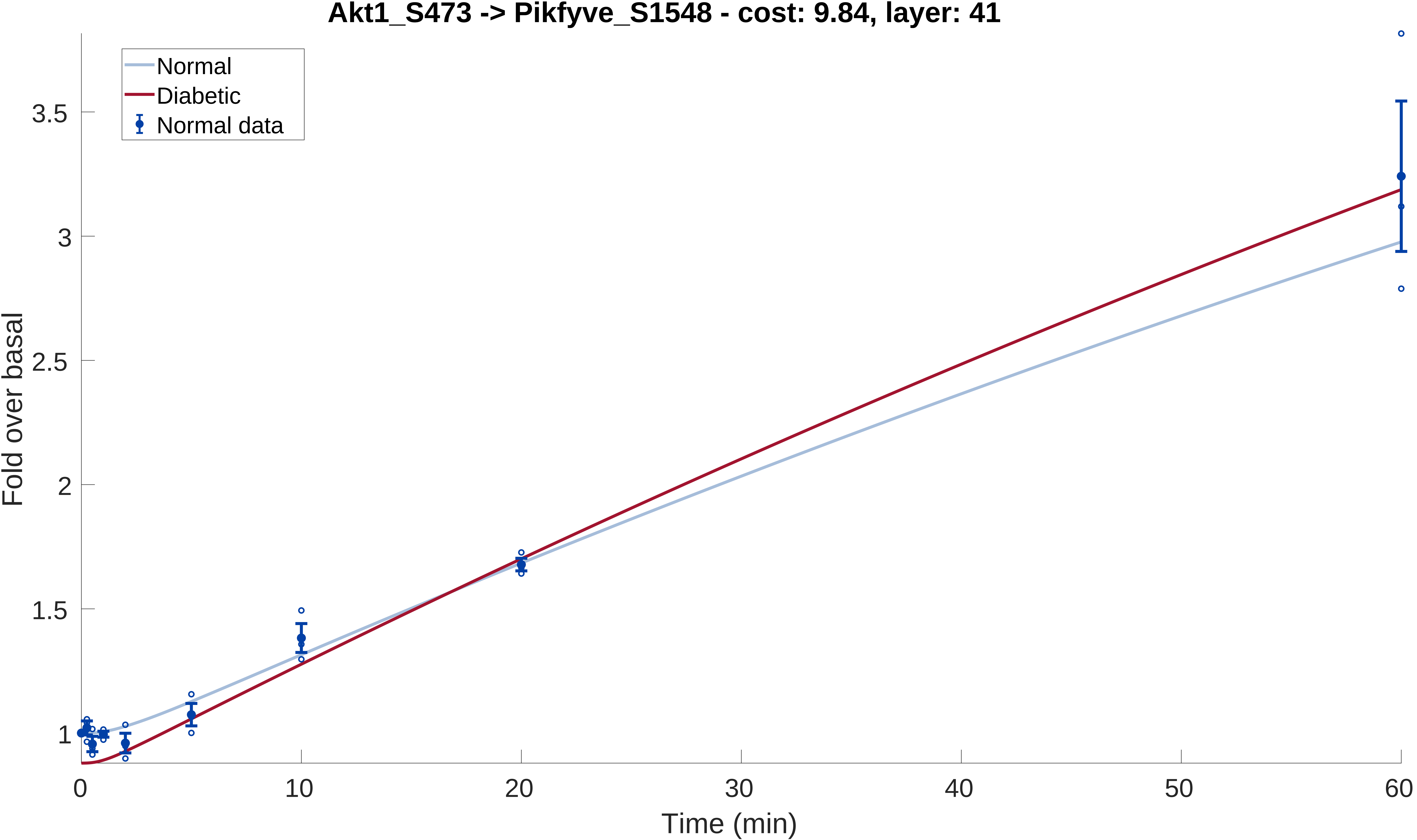

Akt1_S473 -> Pikfyve_S1548 - cost: 9.84, layer: 41
Normal
Diabetic
Normal data
3.5
3
2.5
2
1.5
1
Fold over basal
0
10
20
30
40
50
60
Time (min)

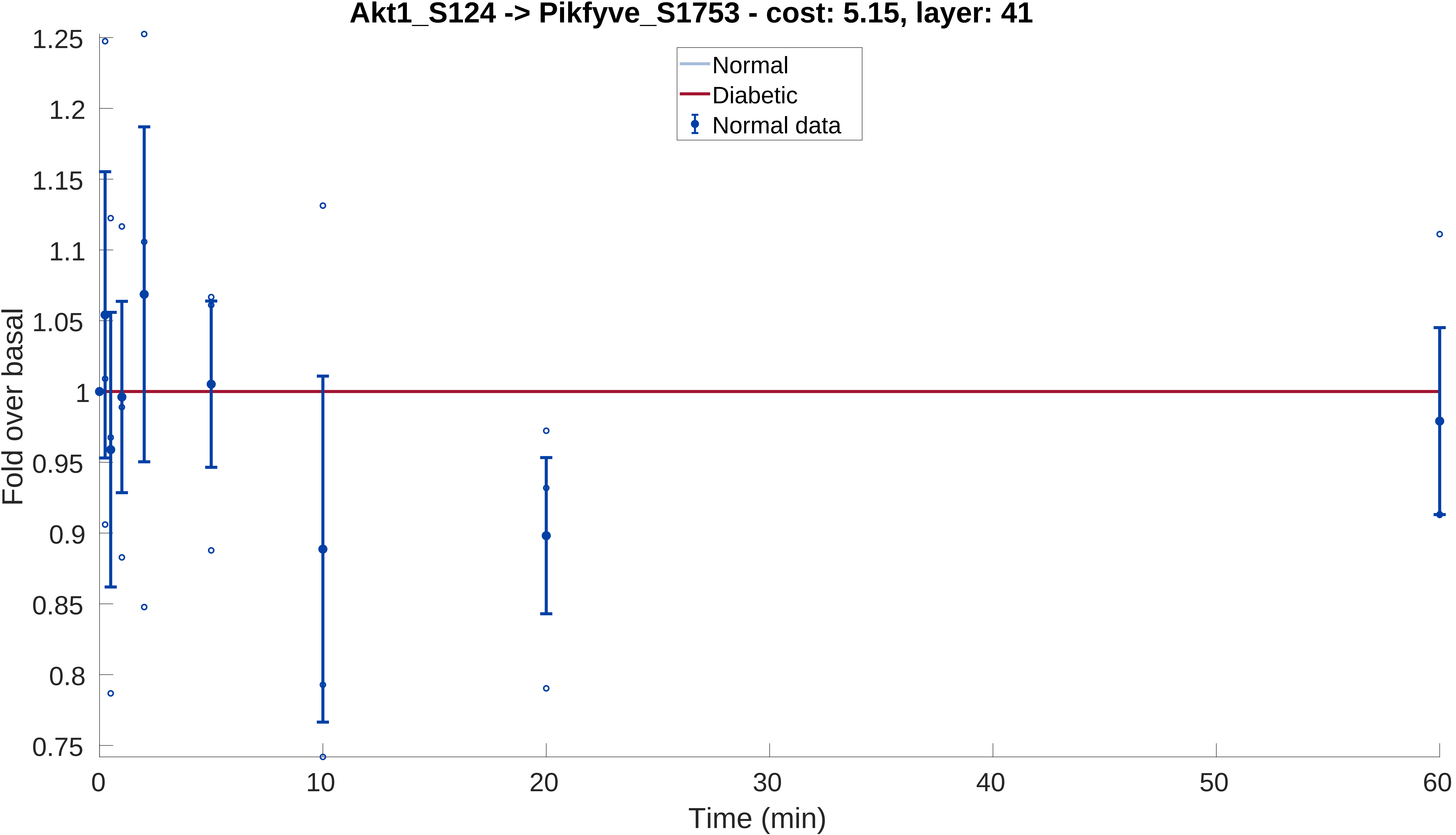

Akt1_S124 -> Pikfyve_S1753 - cost: 5.15, layer: 41
Normal
Diabetic
Normal data
Fold over basal
1.25
1.2
1.15
1.1
1.05
1
0.95
0.9
0.85
0.8
0.75
0
10
20
30
40
50
60
Time (min)

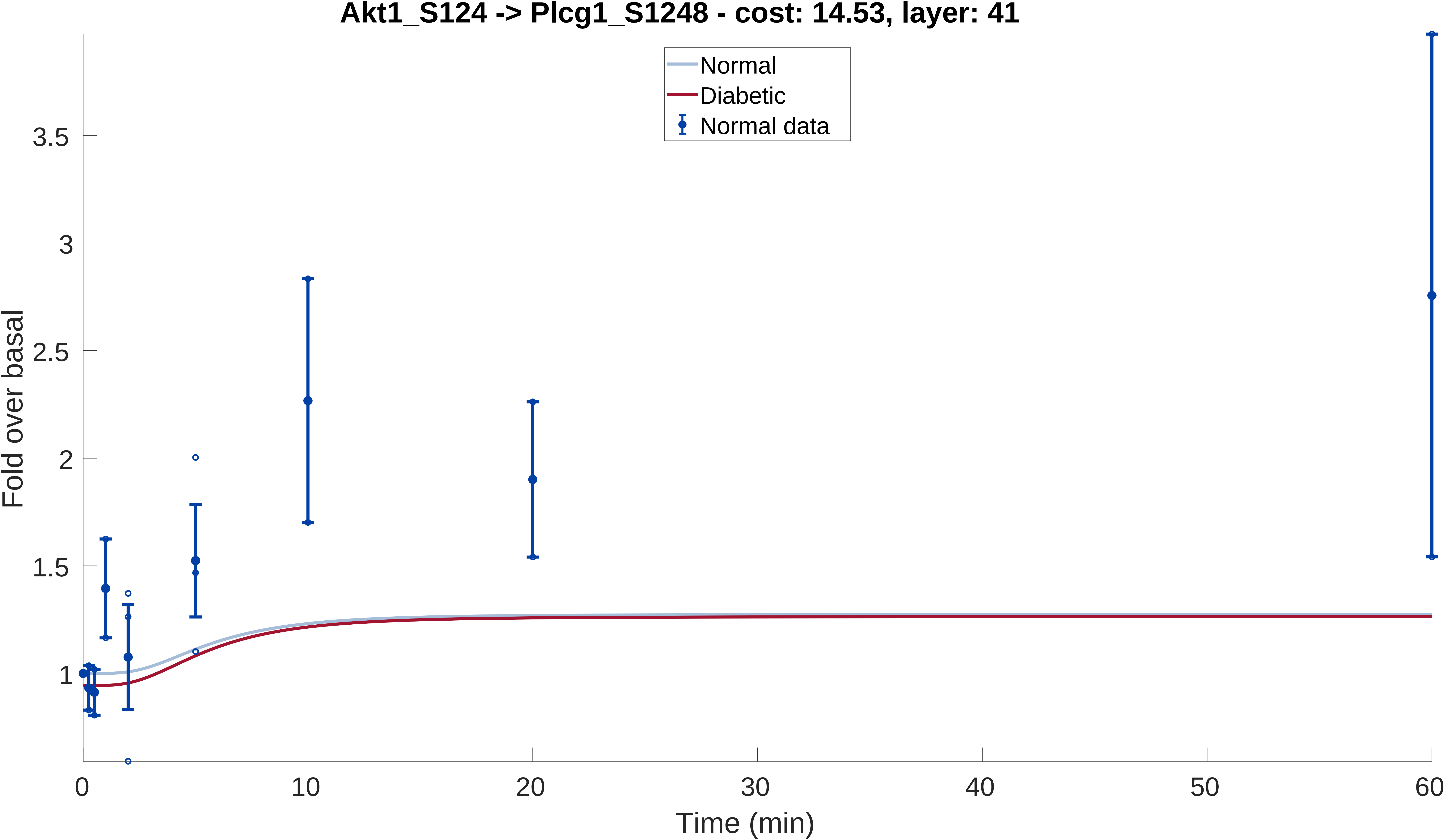

Akt1_S124 -> Plcg1_S1248 - cost: 14.53, layer: 41
Normal
Diabetic
Normal data
Fold over basal
3.5
3
2.5
2
1.5
1
0
10
20
30
40
50
60
Time (min)

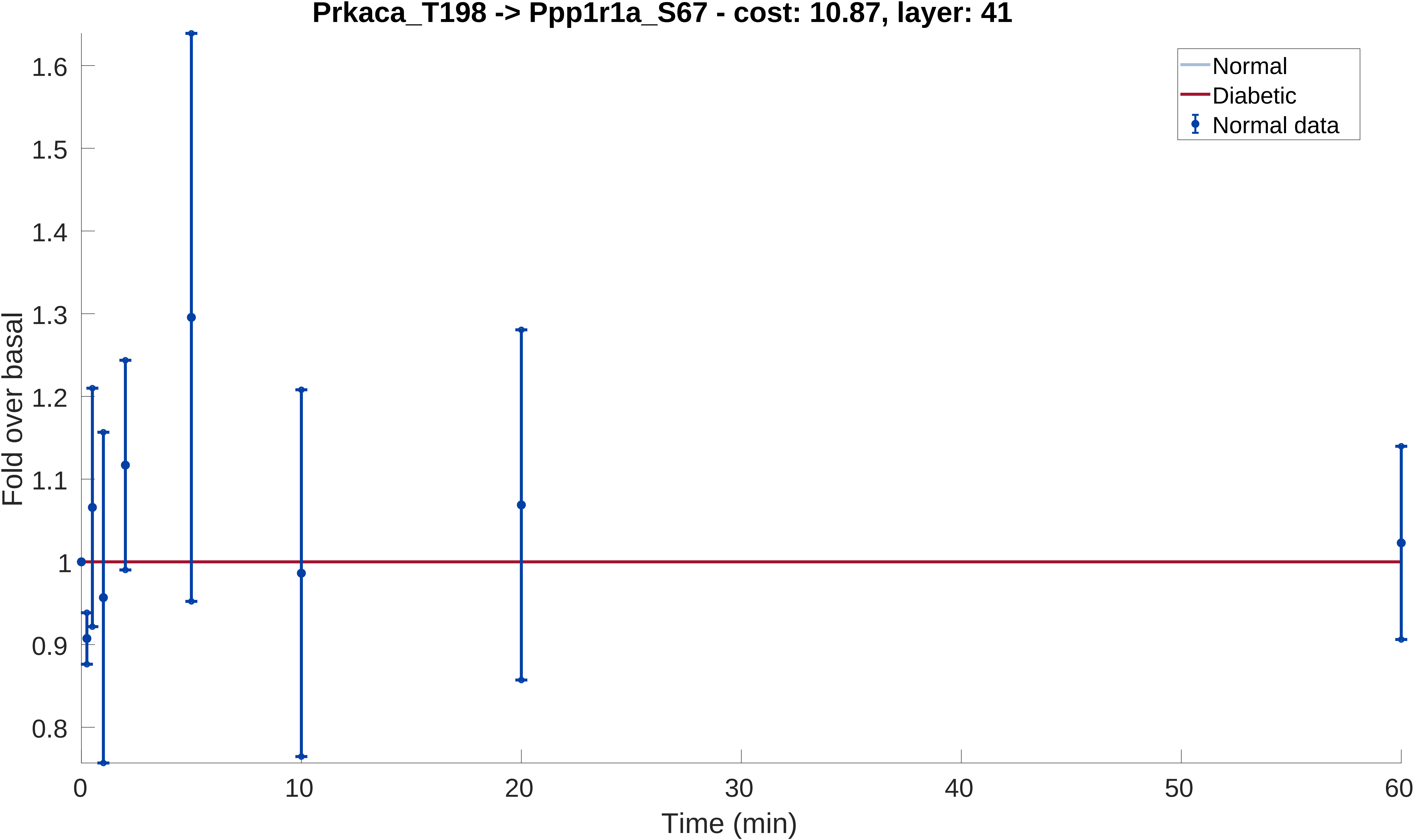
Prkaca_T198 -> Ppp1r1a_S67 - cost: 10.87, layer: 41
Normal
Diabetic
Normal data
Fold over basal
Time (min)
0.8
0.9
1
1.1
1.2
1.3
1.4
1.5
1.6
0
10
20
30
40
50
60

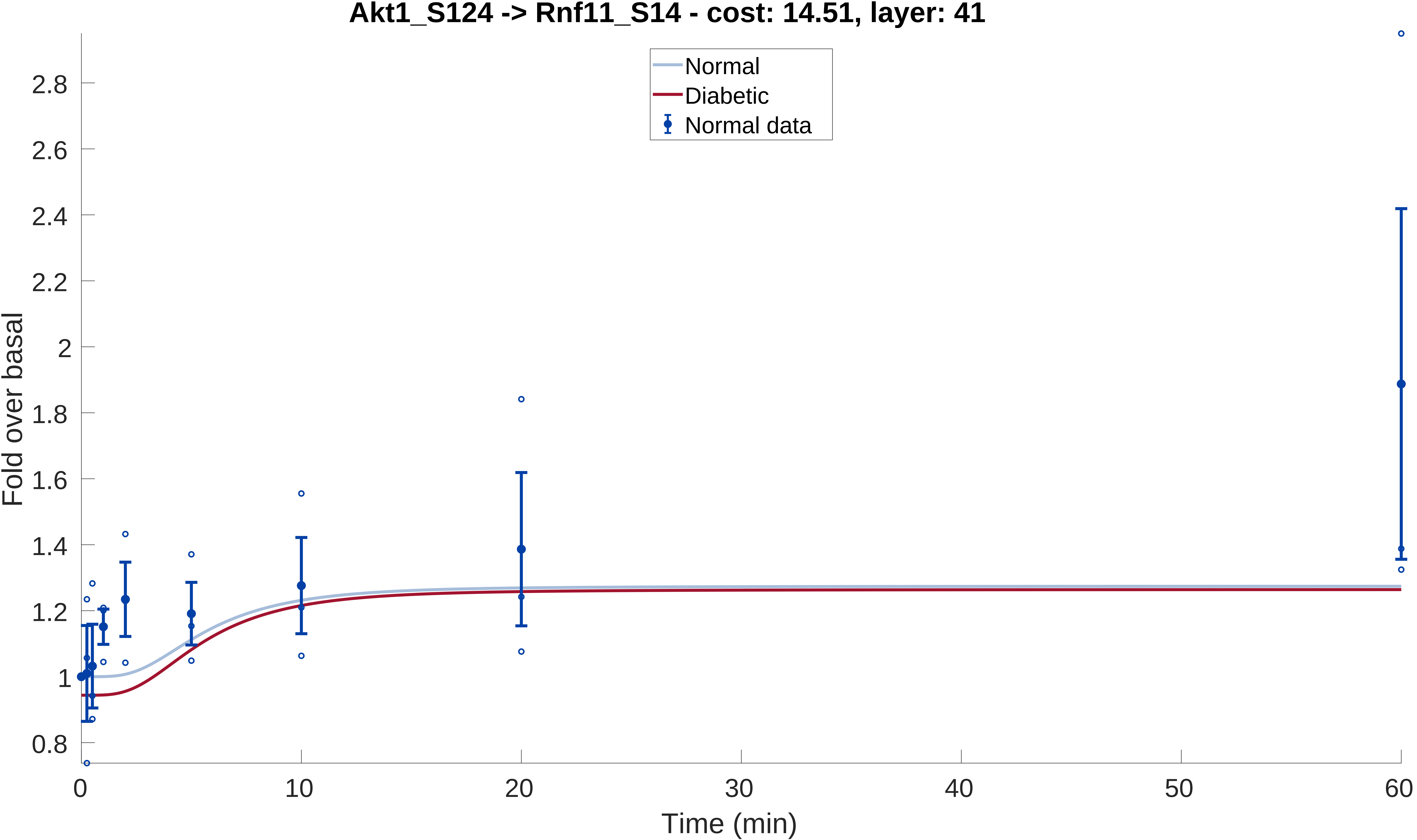

Akt1_S124 -> Rnf11_S14 - cost: 14.51, layer: 41
Normal
Diabetic
Normal data
Fold over basal
Time (min)
0.8
1
1.2
1.4
1.6
1.8
2
2.2
2.4
2.6
2.8
0
10
20
30
40
50
60

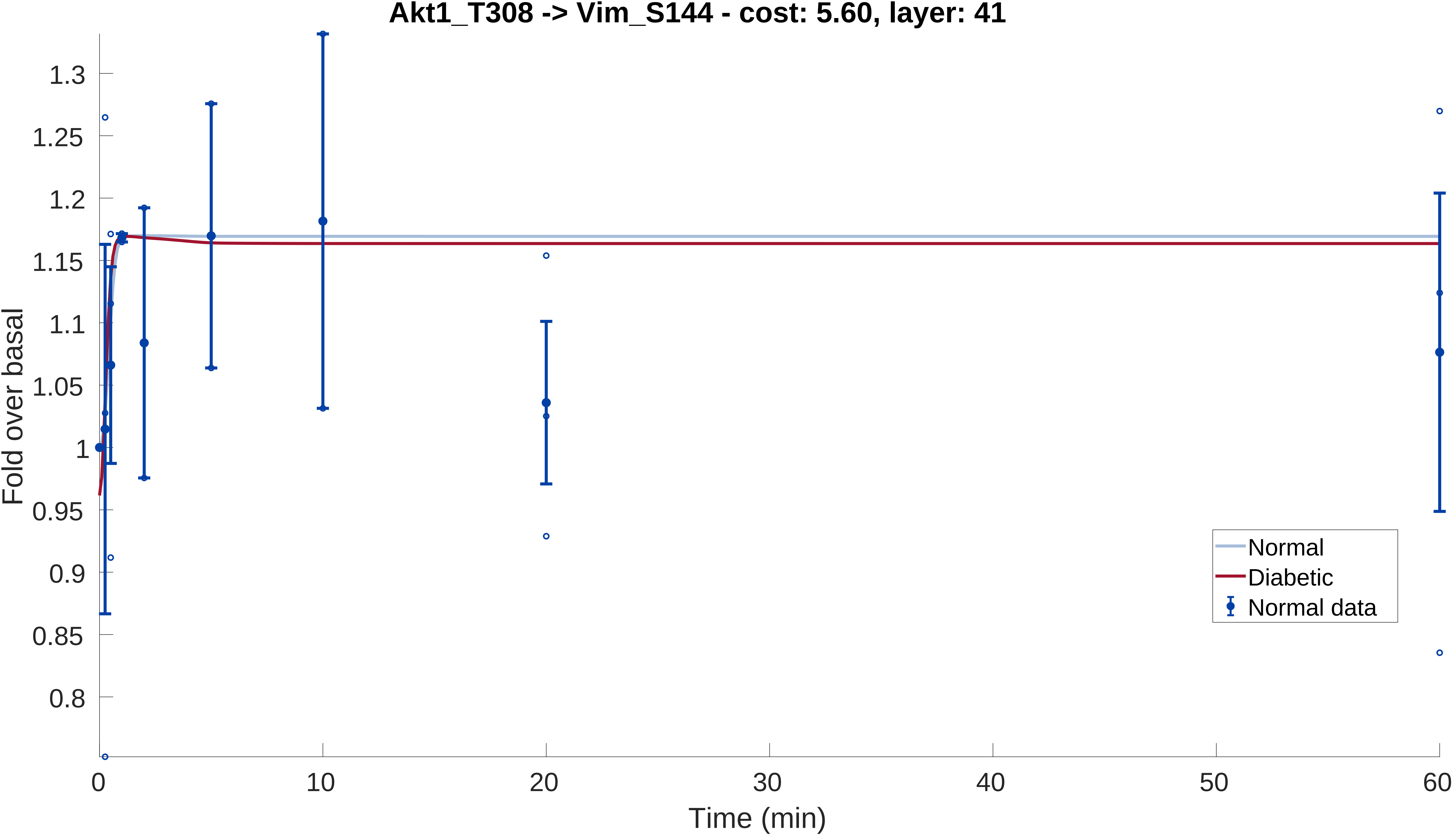
Akt1_T308 -> Vim_S144 - cost: 5.60, layer: 41
Fold over basal
1.3
1.25
1.2
1.15
1.1
1.05
1
0.95
0.9
0.85
0.8
0
10
20
30
40
50
60
Time (min)
Normal
Diabetic
Normal data

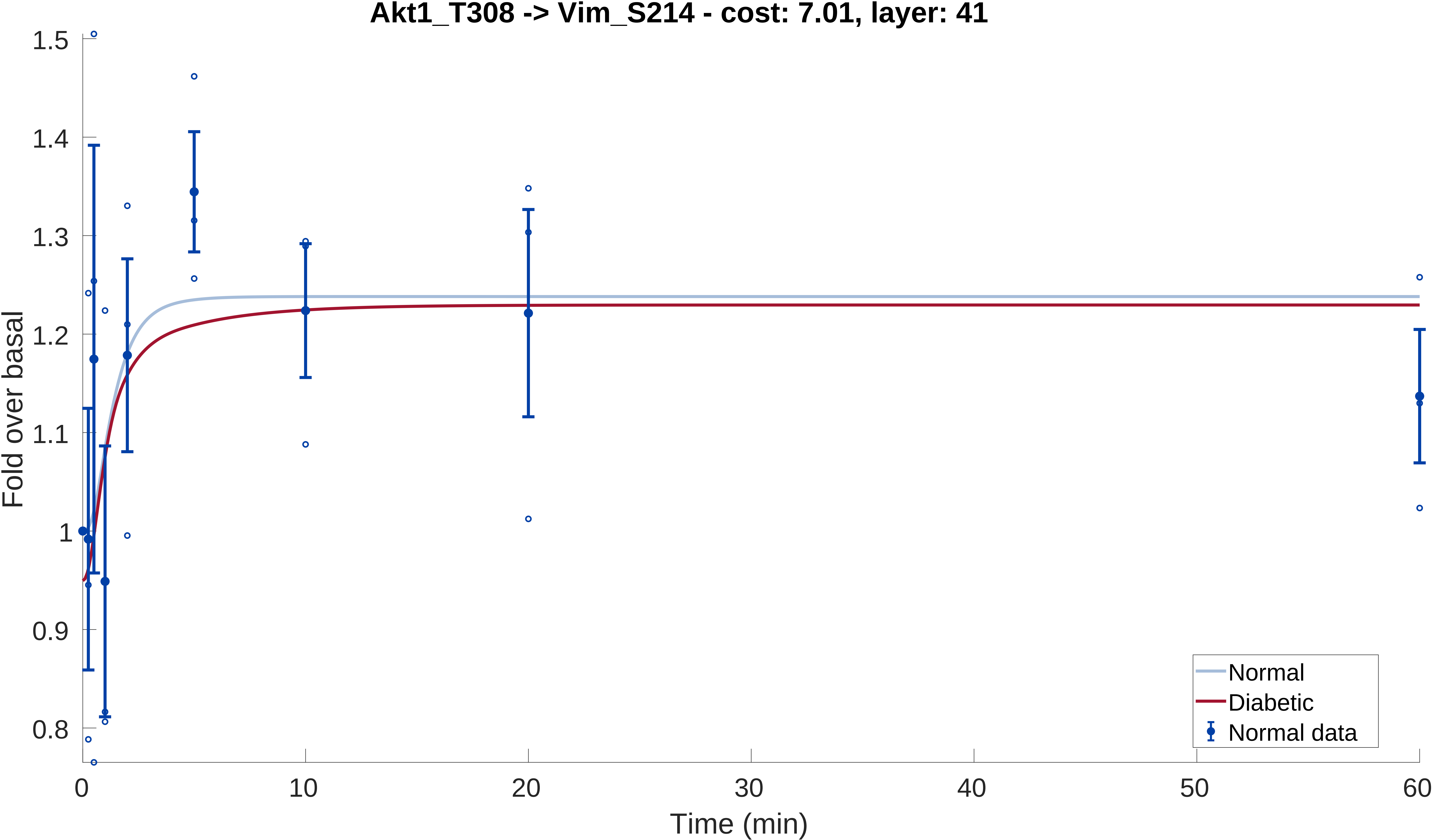

Akt1_T308 -> Vim_S214 - cost: 7.01, layer: 41
Fold over basal
1.5
1.4
1.3
1.2
1.1
1
0.9
0.8
0
10
20
30
40
50
60
Time (min)
Normal
Diabetic
Normal data

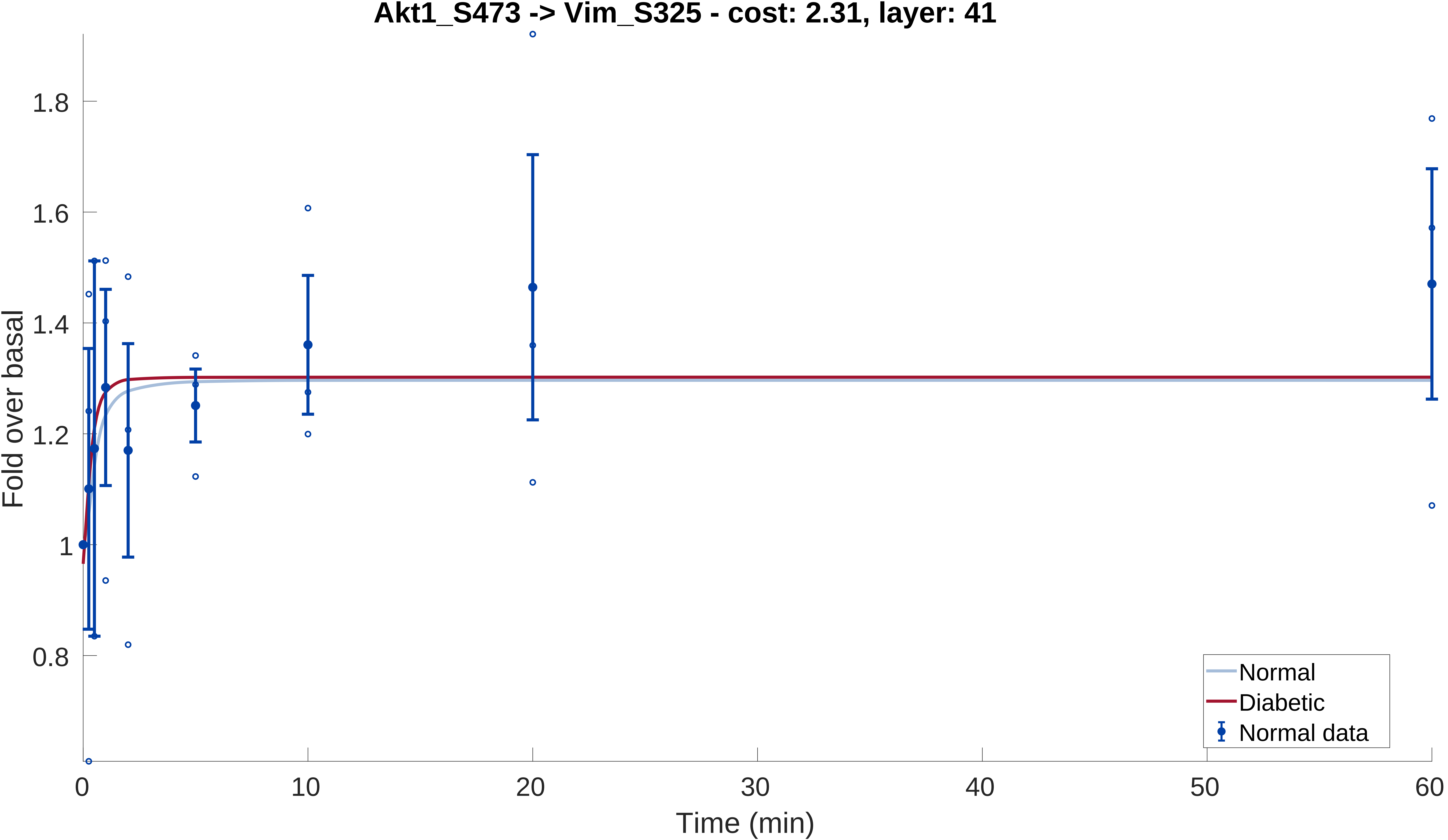

Akt1_S473 -> Vim_S325 - cost: 2.31, layer: 41
Fold over basal
1.8
1.6
1.4
1.2
1
0.8
0
10
20
30
40
50
60
Time (min)
Normal
Diabetic
Normal data

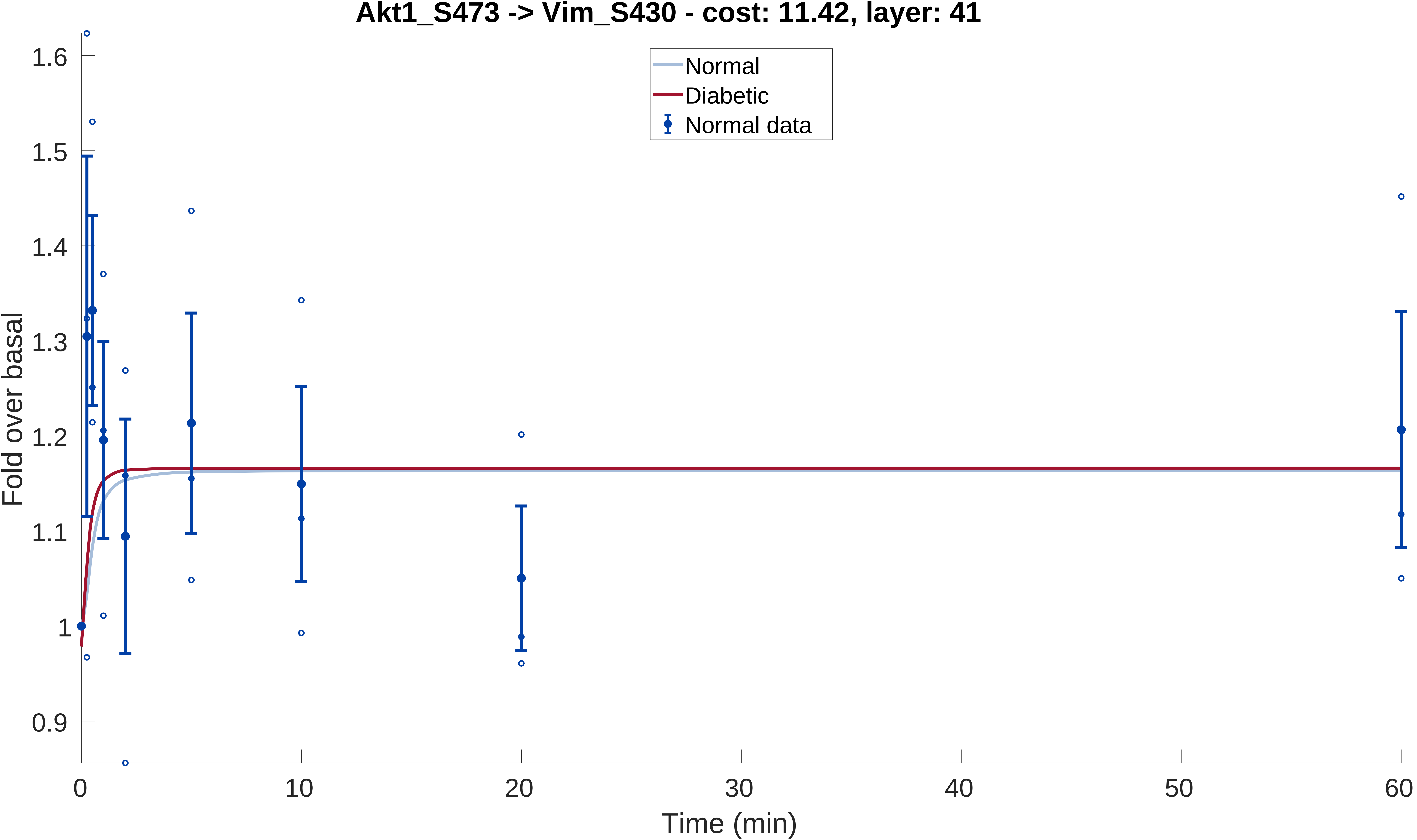
Akt1_S473 -> Vim_S430 - cost: 11.42, layer: 41
Normal
Diabetic
Normal data
Fold over basal
Time (min)
0.9
1
1.1
1.2
1.3
1.4
1.5
1.6
0
10
20
30
40
50
60

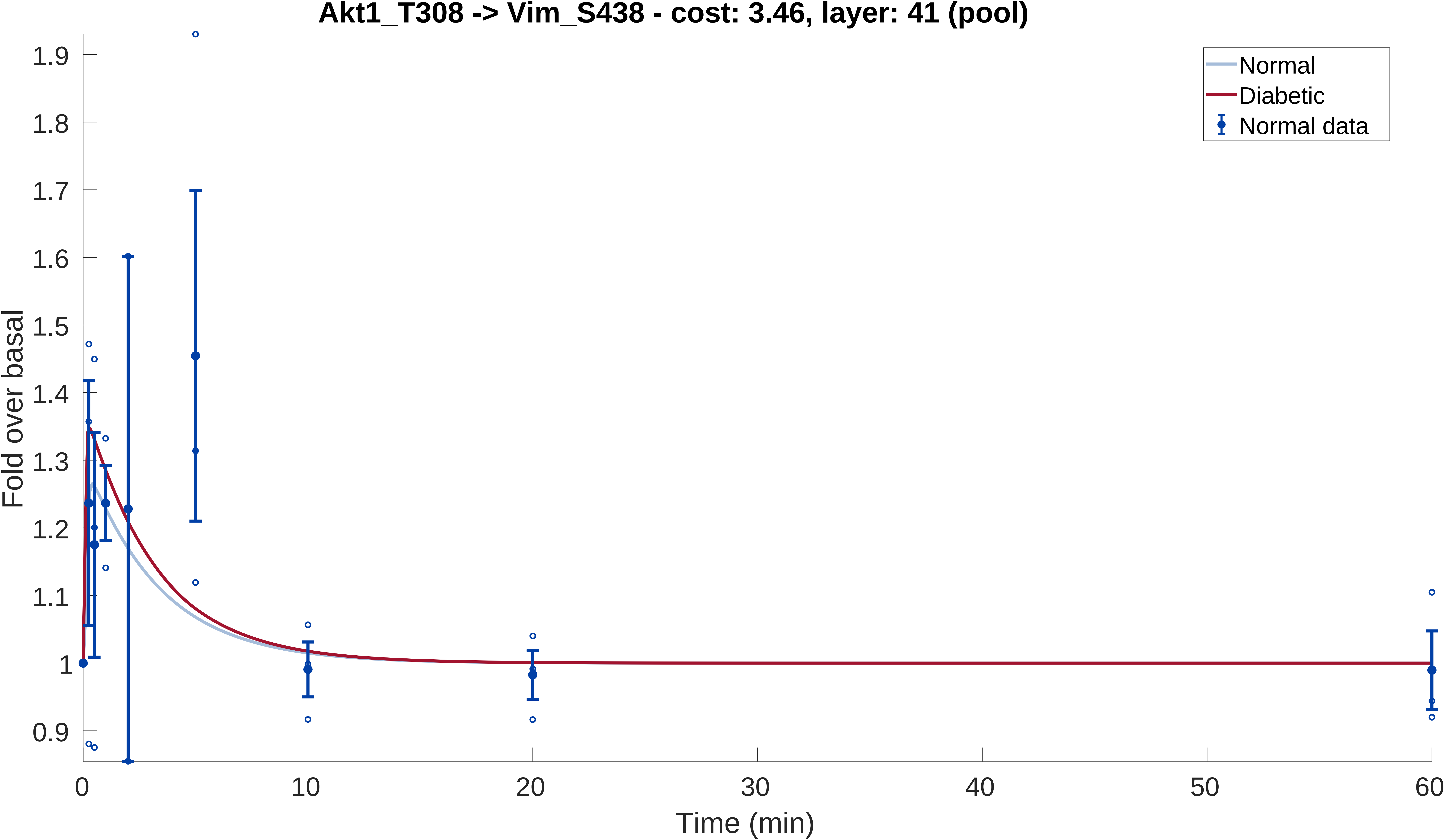

Akt1_T308 -> Vim_S438 - cost: 3.46, layer: 41 (pool)
Normal
Diabetic
Normal data
Fold over basal
Time (min)
1.9
1.8
1.7
1.6
1.5
1.4
1.3
1.2
1.1
1
0.9
0
10
20
30
40
50
60

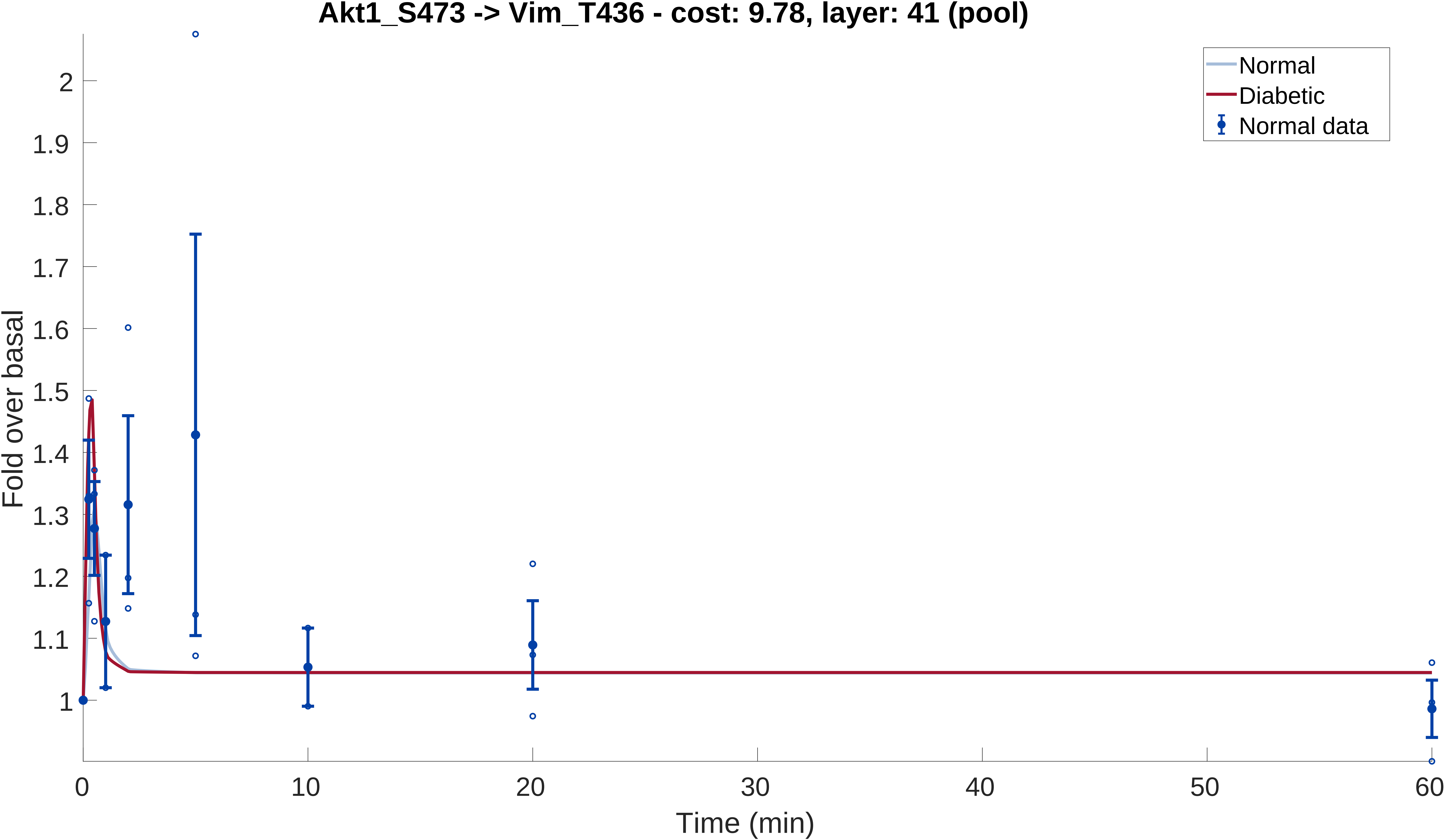

Akt1_S473 -> Vim_T436 - cost: 9.78, layer: 41 (pool)
Normal
Diabetic
Normal data
Fold over basal
2
1.9
1.8
1.7
1.6
1.5
1.4
1.3
1.2
1.1
1
0
10
20
30
40
50
60
Time (min)

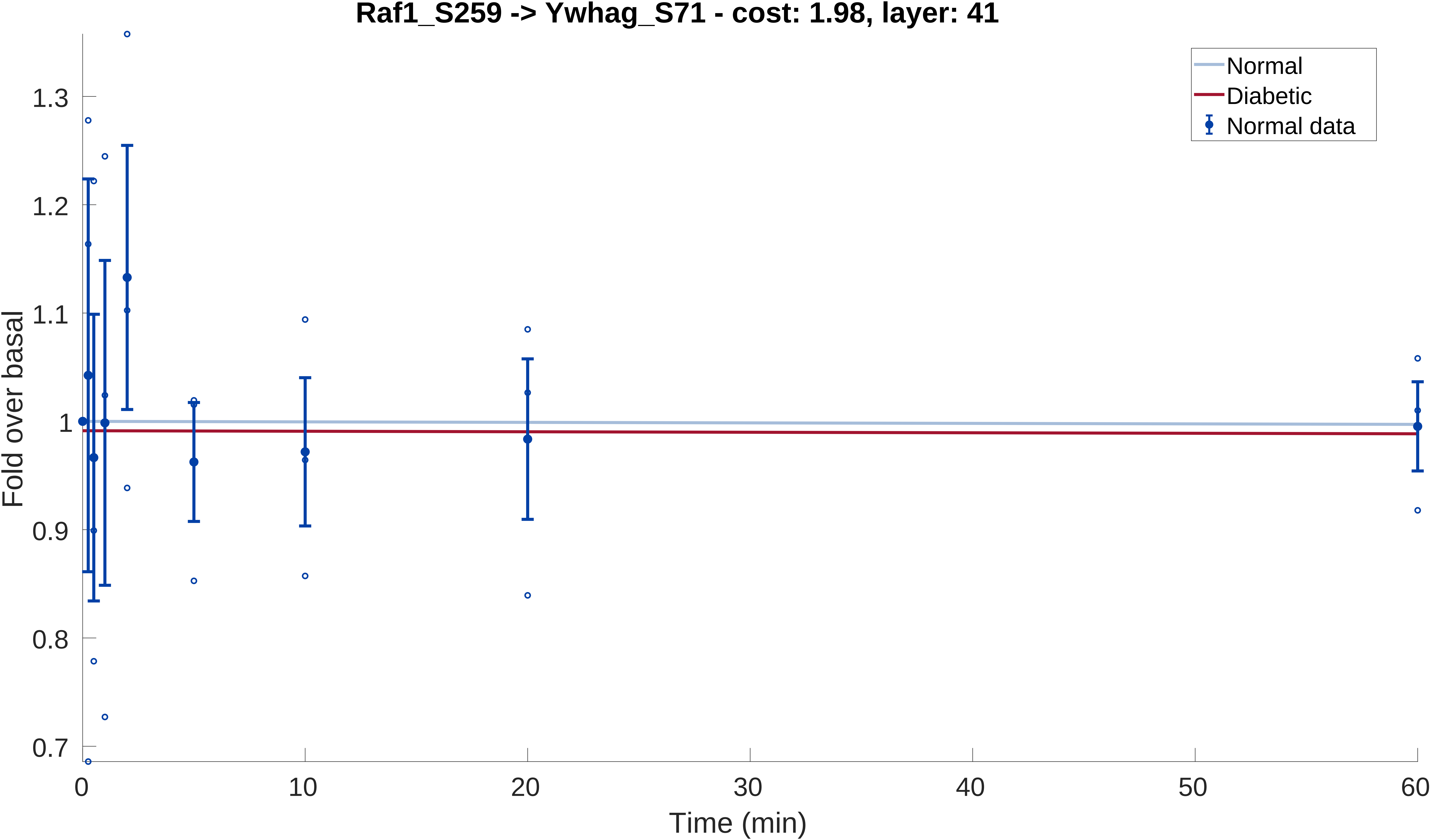

Raf1_S259 -> Ywhag_S71 - cost: 1.98, layer: 41
Normal
Diabetic
Normal data
Fold over basal
1.3
1.2
1.1
1
0.9
0.8
0.7
0
10
20
30
40
50
60
Time (min)

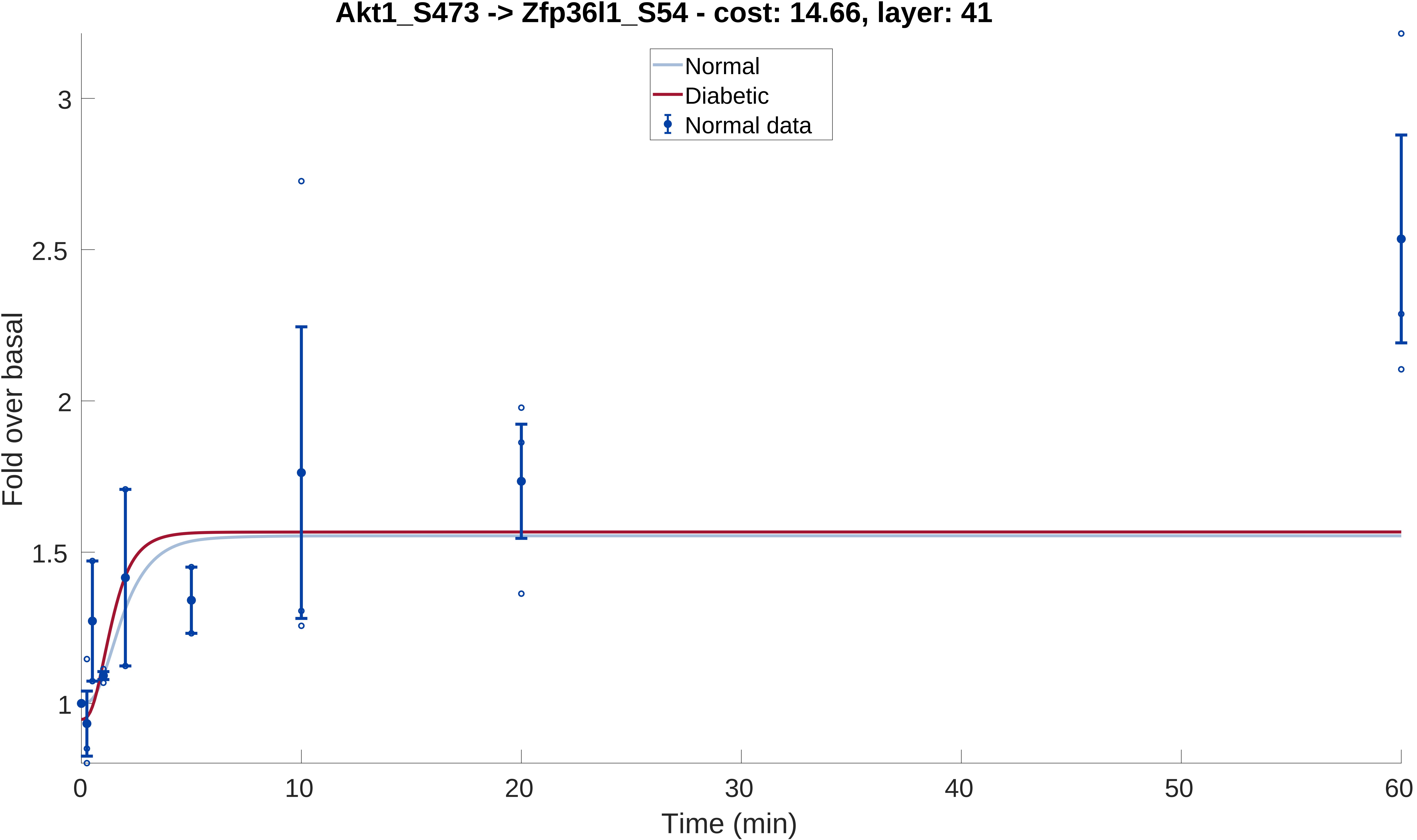

Akt1_S473 -> Zfp36l1_S54 - cost: 14.66, layer: 41
Normal
Diabetic
Normal data
Fold over basal
Time (min)
3
2.5
2
1.5
1
0
10
20
30
40
50
60

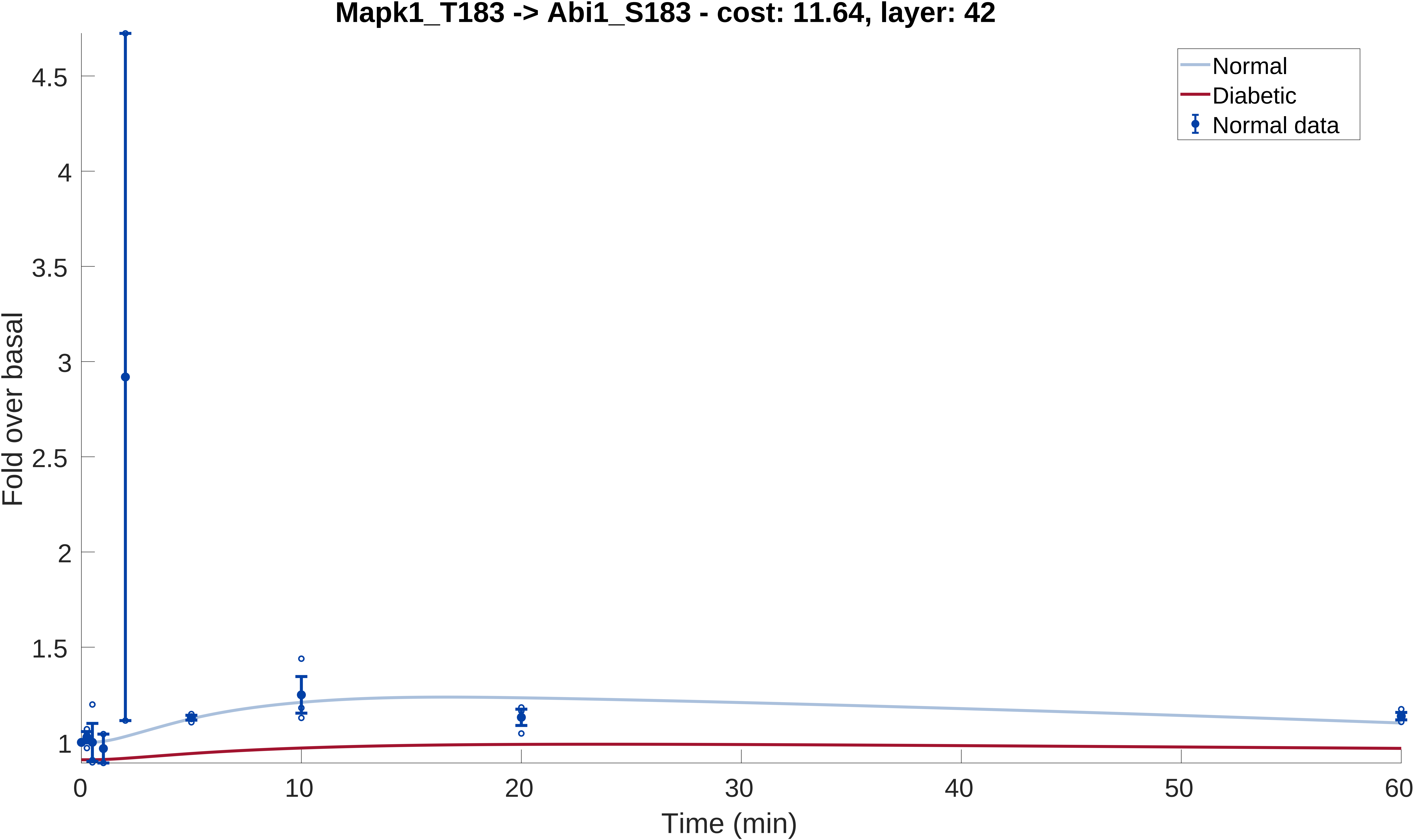

Mapk1_T183 -> Abi1_S183 - cost: 11.64, layer: 42
Fold over basal
Time (min)
Normal
Diabetic
Normal data
4.5
4
3.5
3
2.5
2
1.5
1
0
10
20
30
40
50
60

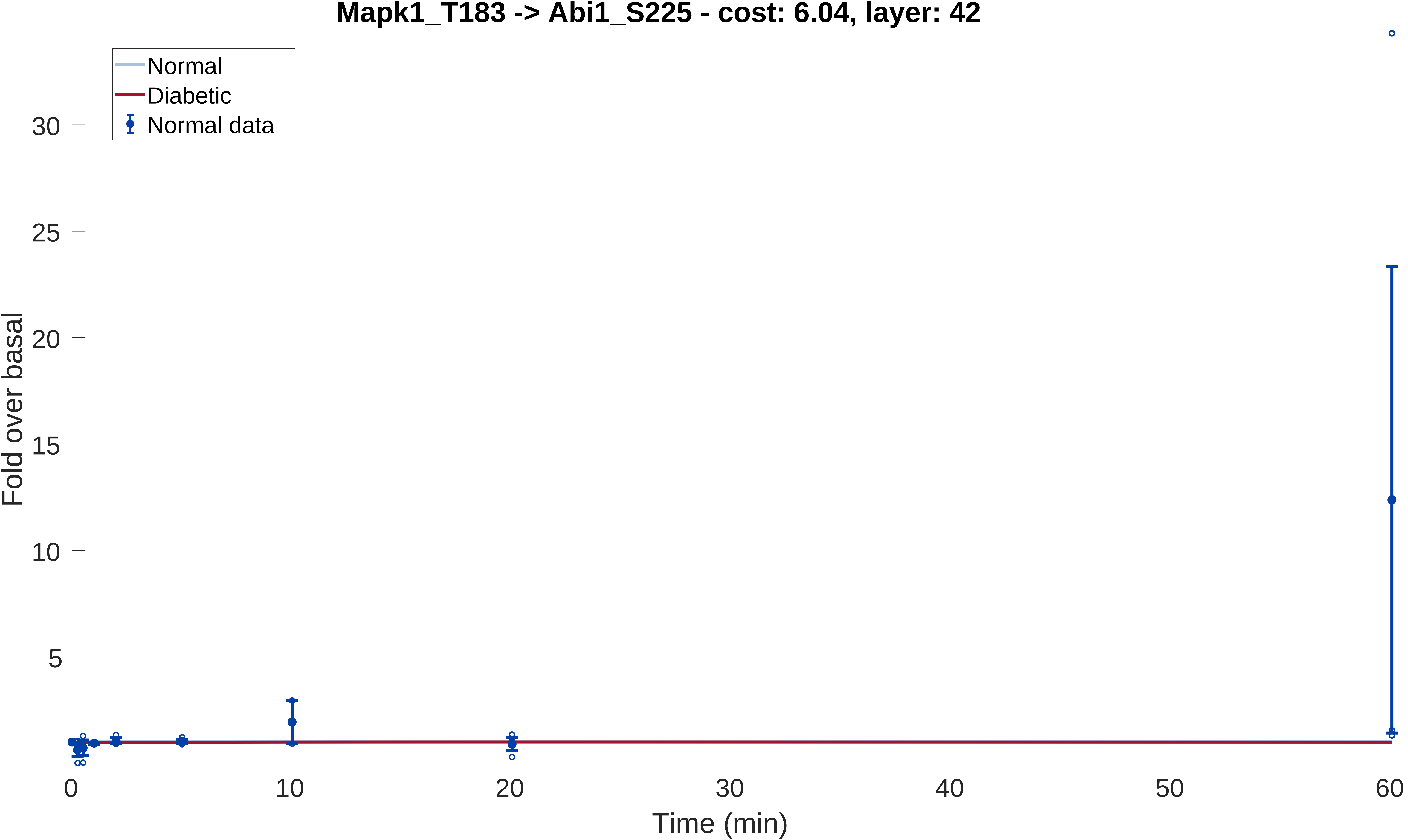

Mapk1_T183 -> Abi1_S225 - cost: 6.04, layer: 42
Normal
Diabetic
Normal data
Fold over basal
Time (min)
30
25
20
15
10
5
0
10
20
30
40
50
60

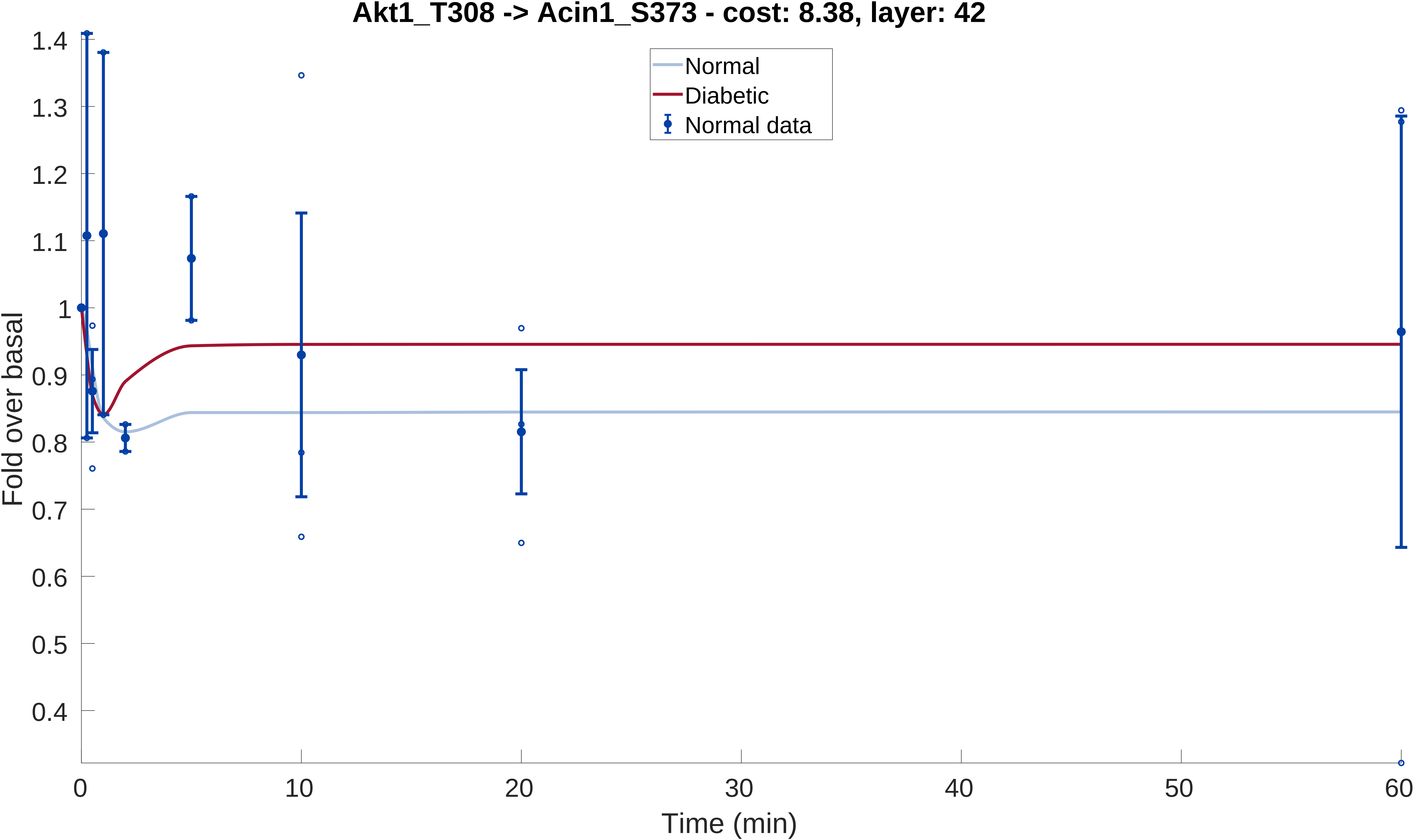

Akt1_T308 -> Acin1_S373 - cost: 8.38, layer: 42
Normal
Diabetic
Normal data
Fold over basal
Time (min)
1.4
1.3
1.2
1.1
1
0.9
0.8
0.7
0.6
0.5
0.4
0
10
20
30
40
50
60

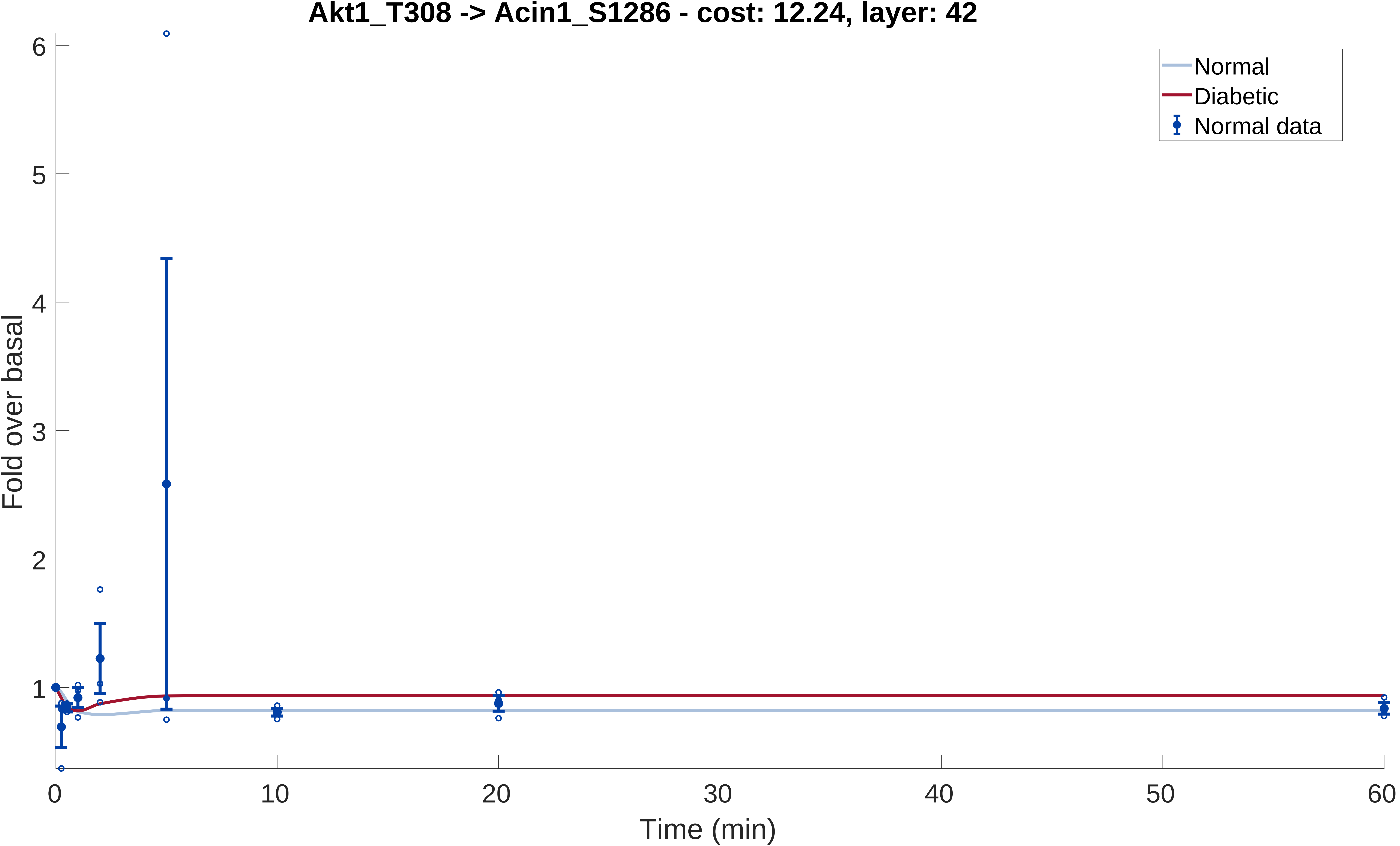

Akt1_T308 -> Acin1_S1286 - cost: 12.24, layer: 42
Normal
Diabetic
Normal data
Fold over basal
Time (min)
0
10
20
30
40
50
60
1
2
3
4
5
6

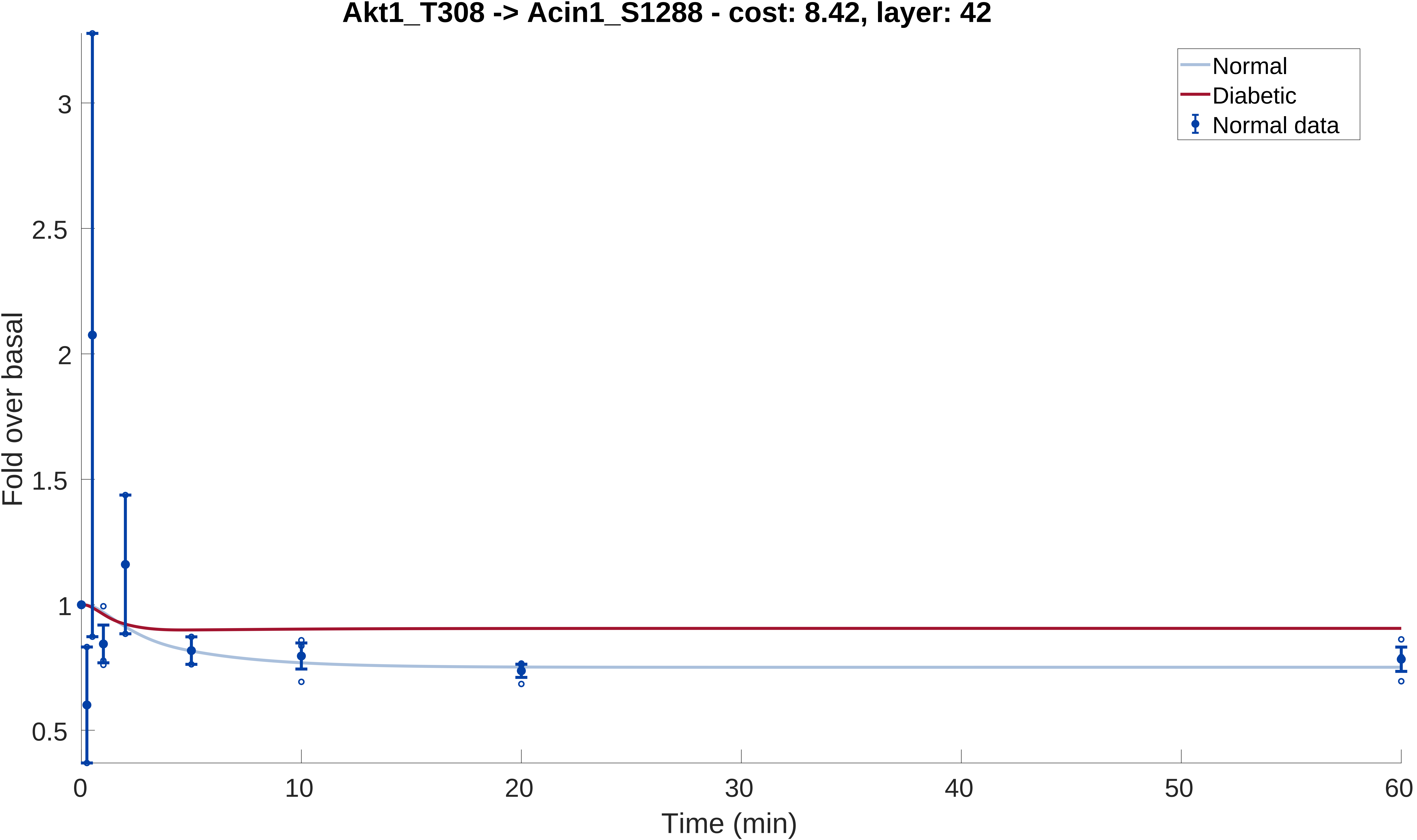

Akt1_T308 -> Acin1_S1288 - cost: 8.42, layer: 42
Normal
Diabetic
Normal data
Fold over basal
3
2.5
2
1.5
1
0.5
0
10
20
30
40
50
60
Time (min)

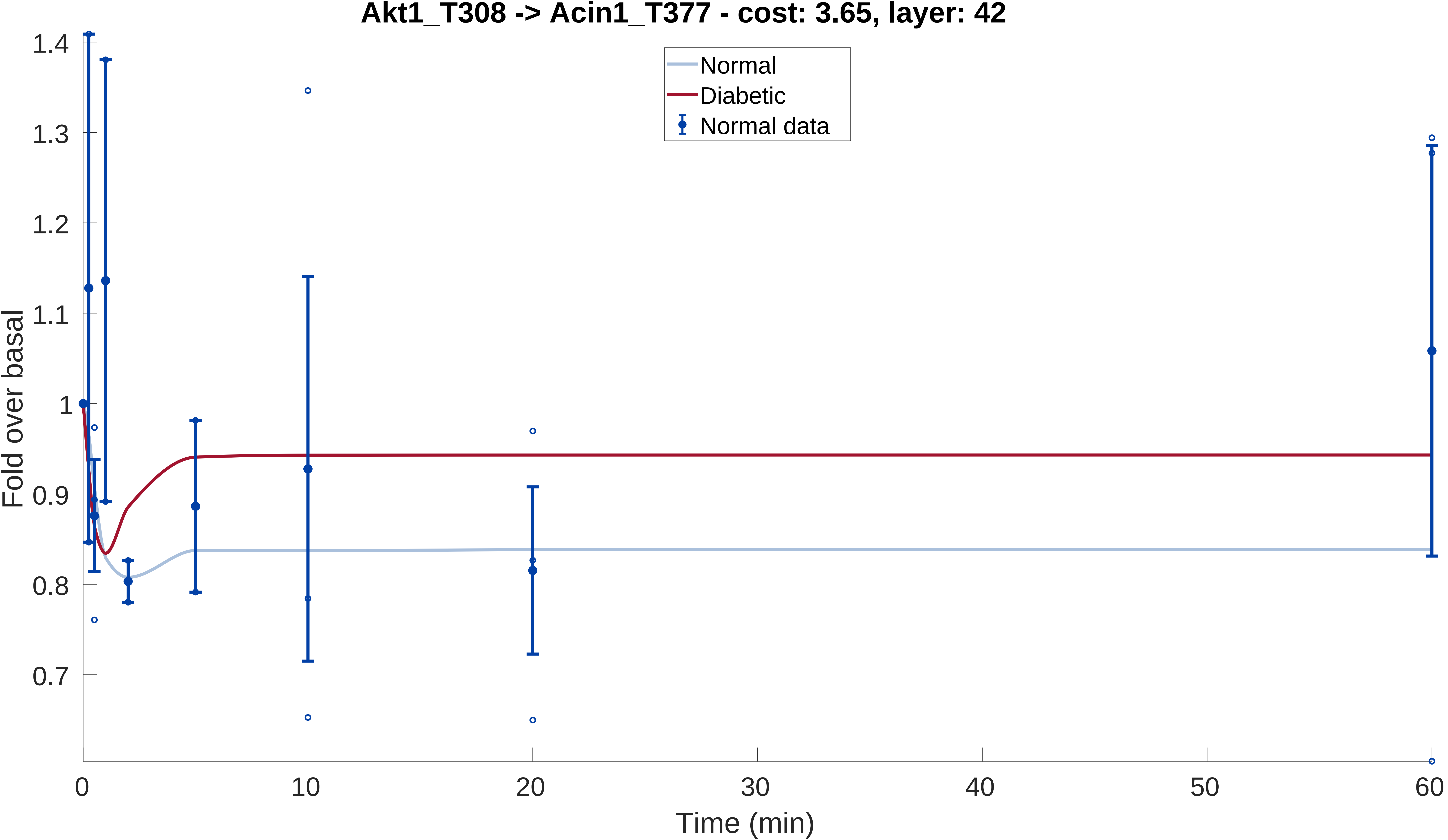

Akt1_T308 -> Acin1_T377 - cost: 3.65, layer: 42
Normal
Diabetic
Normal data
Fold over basal
1.4
1.3
1.2
1.1
1
0.9
0.8
0.7
0
10
20
30
40
50
60
Time (min)

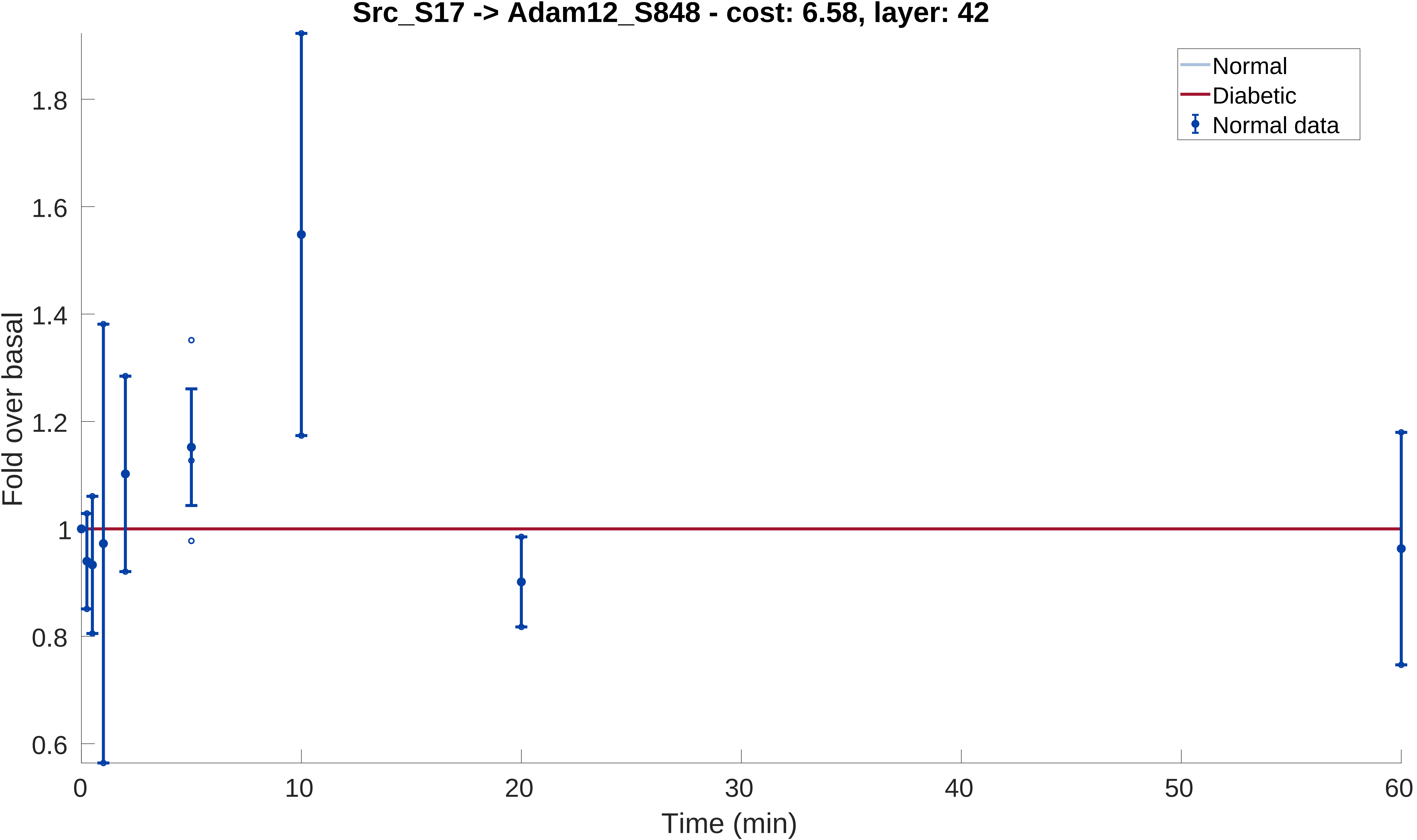

Src_S17 -> Adam12_S848 - cost: 6.58, layer: 42
Normal
Diabetic
Normal data
Fold over basal
1.8
1.6
1.4
1.2
1
0.8
0.6
0
10
20
30
40
50
60
Time (min)

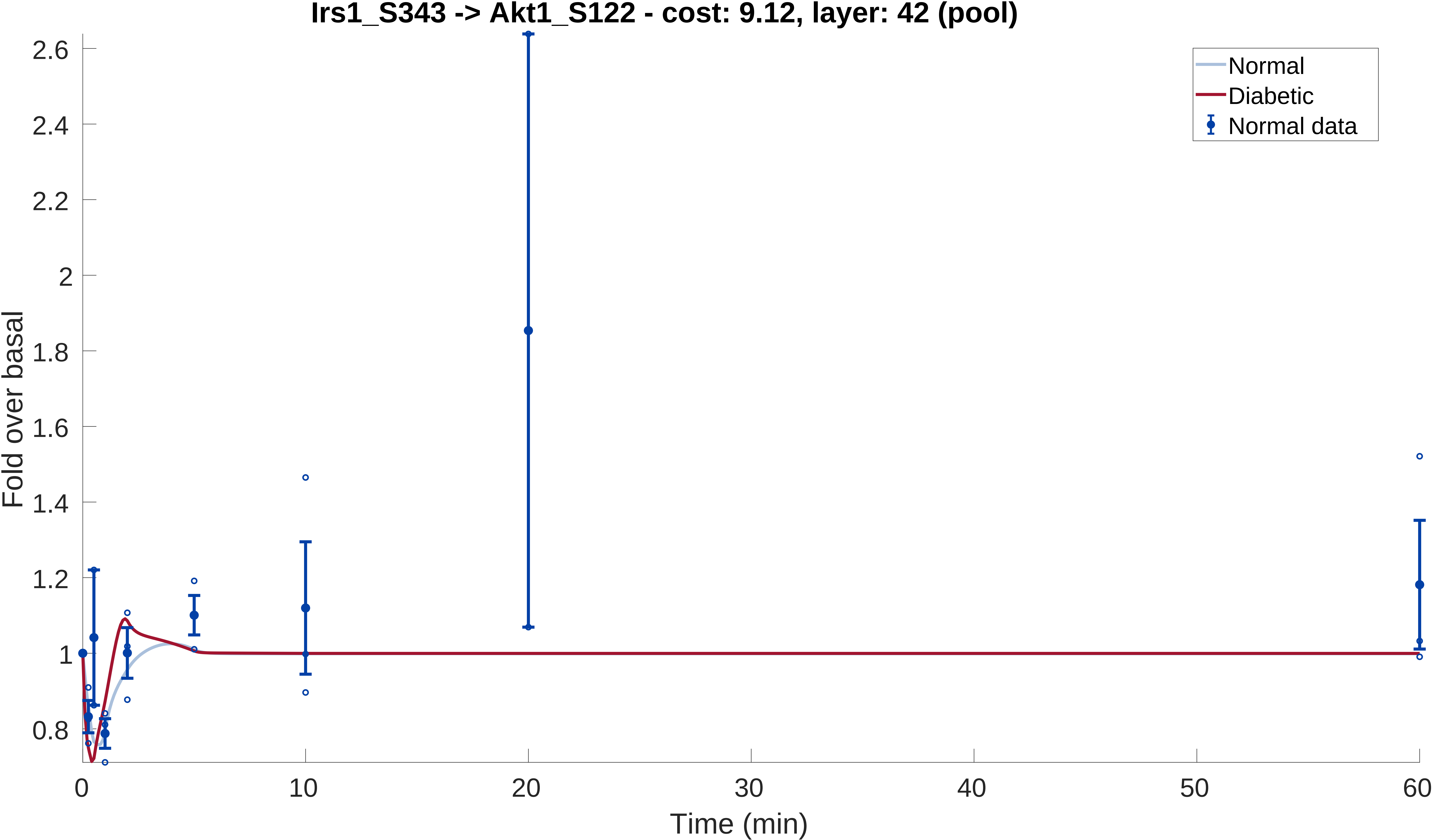

Irs1_S343 -> Akt1_S122 - cost: 9.12, layer: 42 (pool)
Normal
Diabetic
Normal data
Fold over basal
2.6
2.4
2.2
2
1.8
1.6
1.4
1.2
1
0.8
0
10
20
30
40
50
60
Time (min)

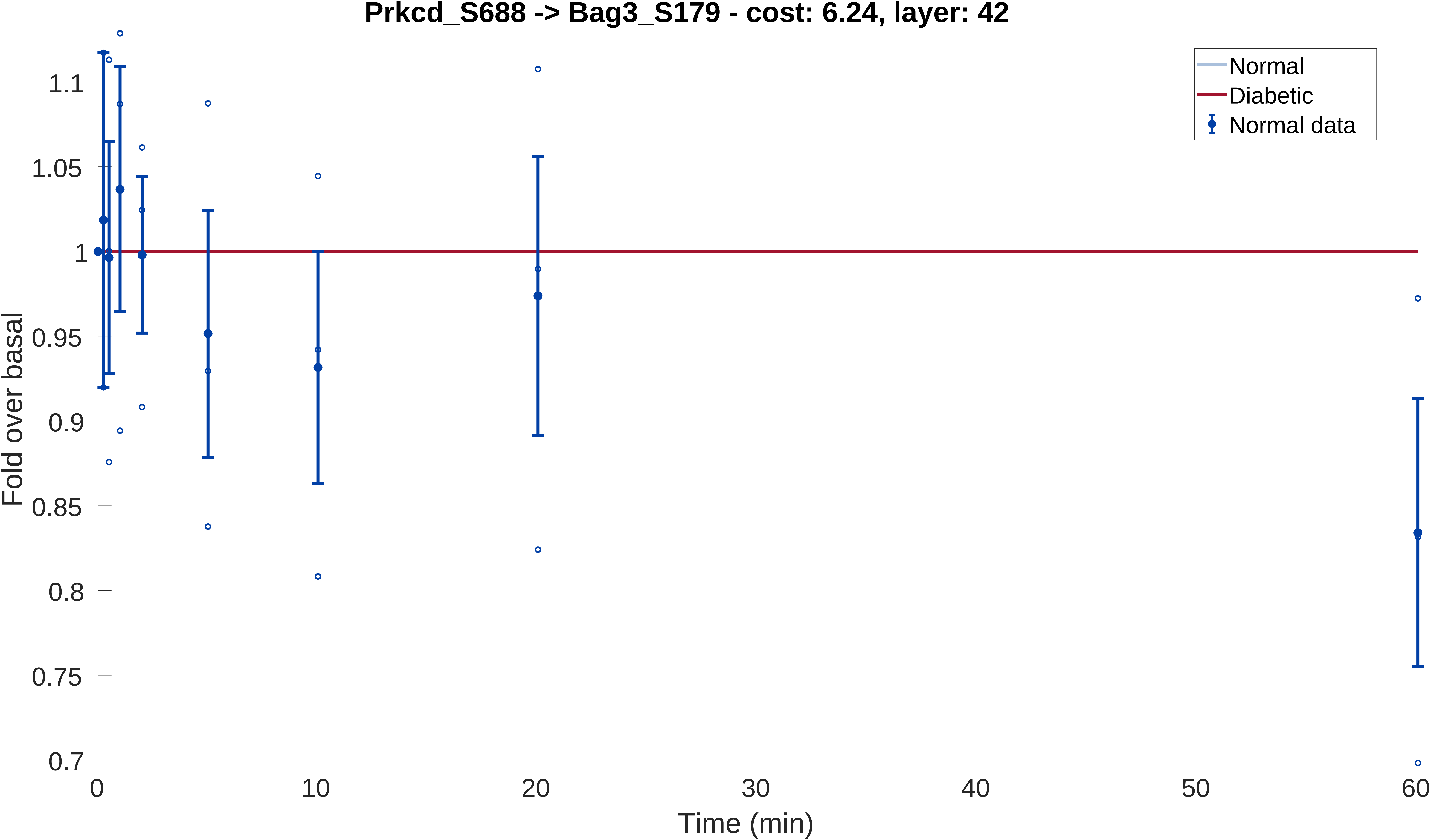

Prkcd_S688 -> Bag3_S179 - cost: 6.24, layer: 42
Normal
Diabetic
Normal data
Fold over basal
Time (min)
1.1
1.05
1
0.95
0.9
0.85
0.8
0.75
0.7
0
10
20
30
40
50
60

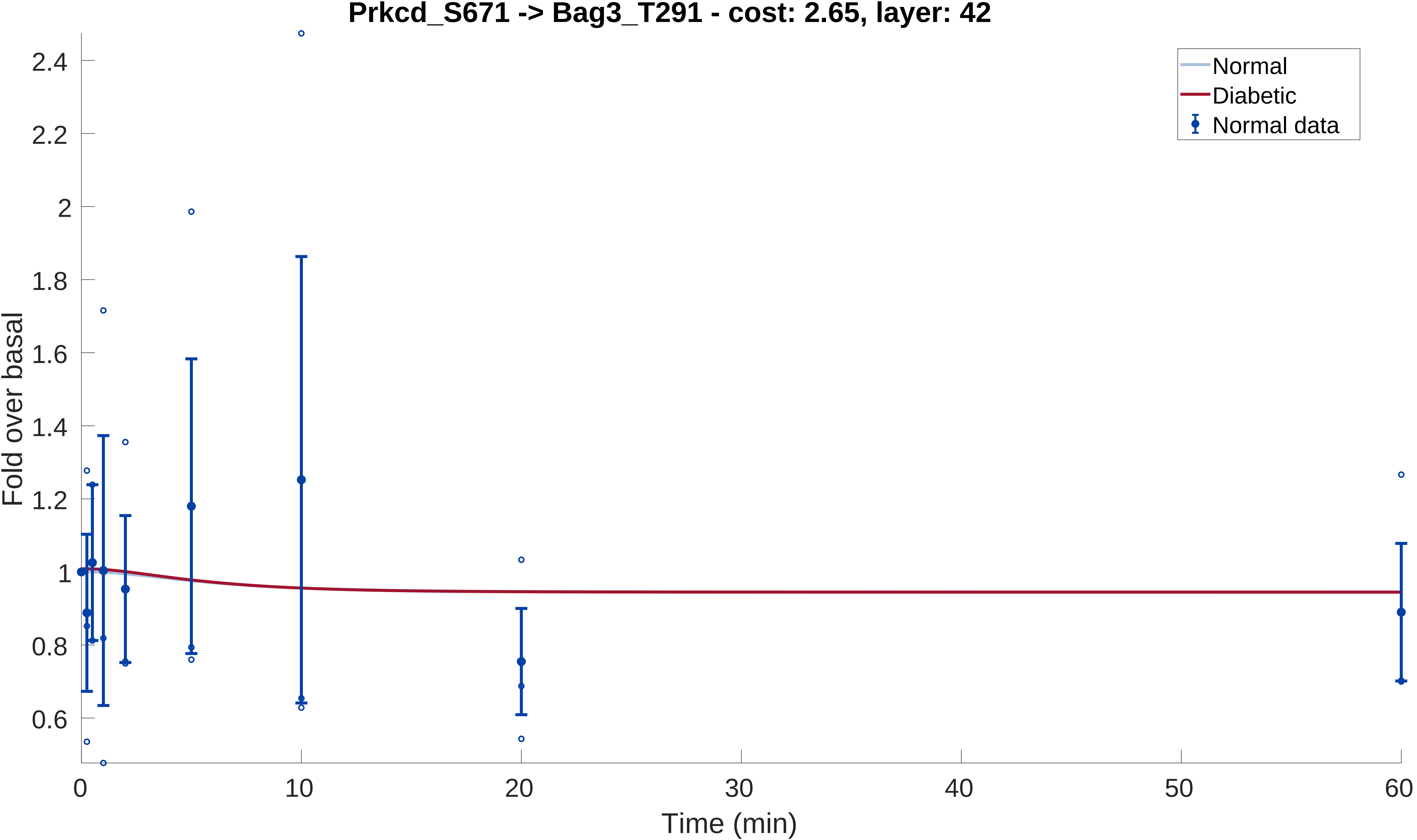
Prkcd_S671 -> Bag3_T291 - cost: 2.65, layer: 42
Normal
Diabetic
Normal data
Fold over basal
Time (min)
2.4
2.2
2
1.8
1.6
1.4
1.2
1
0.8
0.6
0
10
20
30
40
50
60

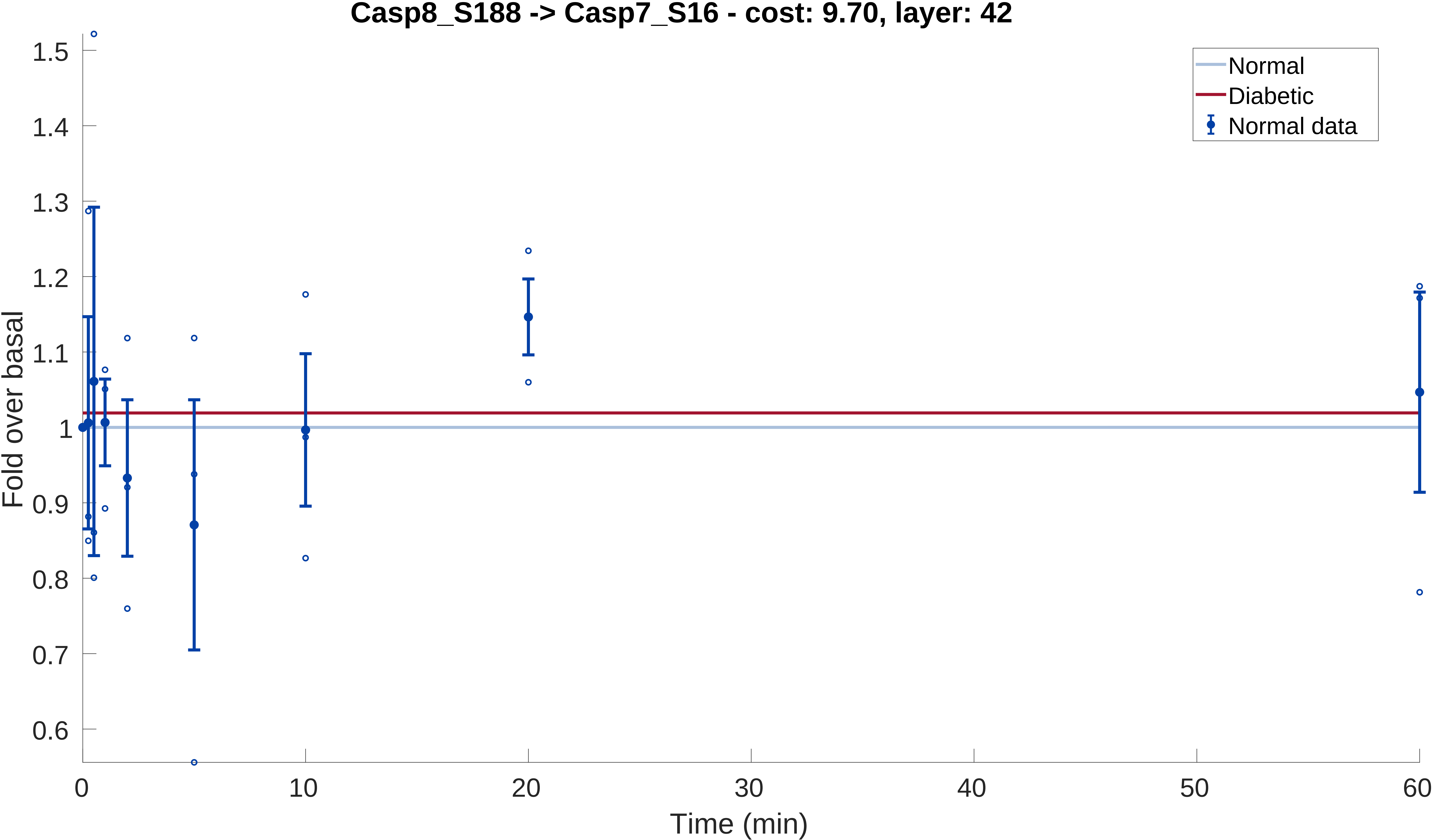

Casp8_S188 -> Casp7_S16 - cost: 9.70, layer: 42
Normal
Diabetic
Normal data
Fold over basal
1.5
1.4
1.3
1.2
1.1
1
0.9
0.8
0.7
0.6
0
10
20
30
40
50
60
Time (min)

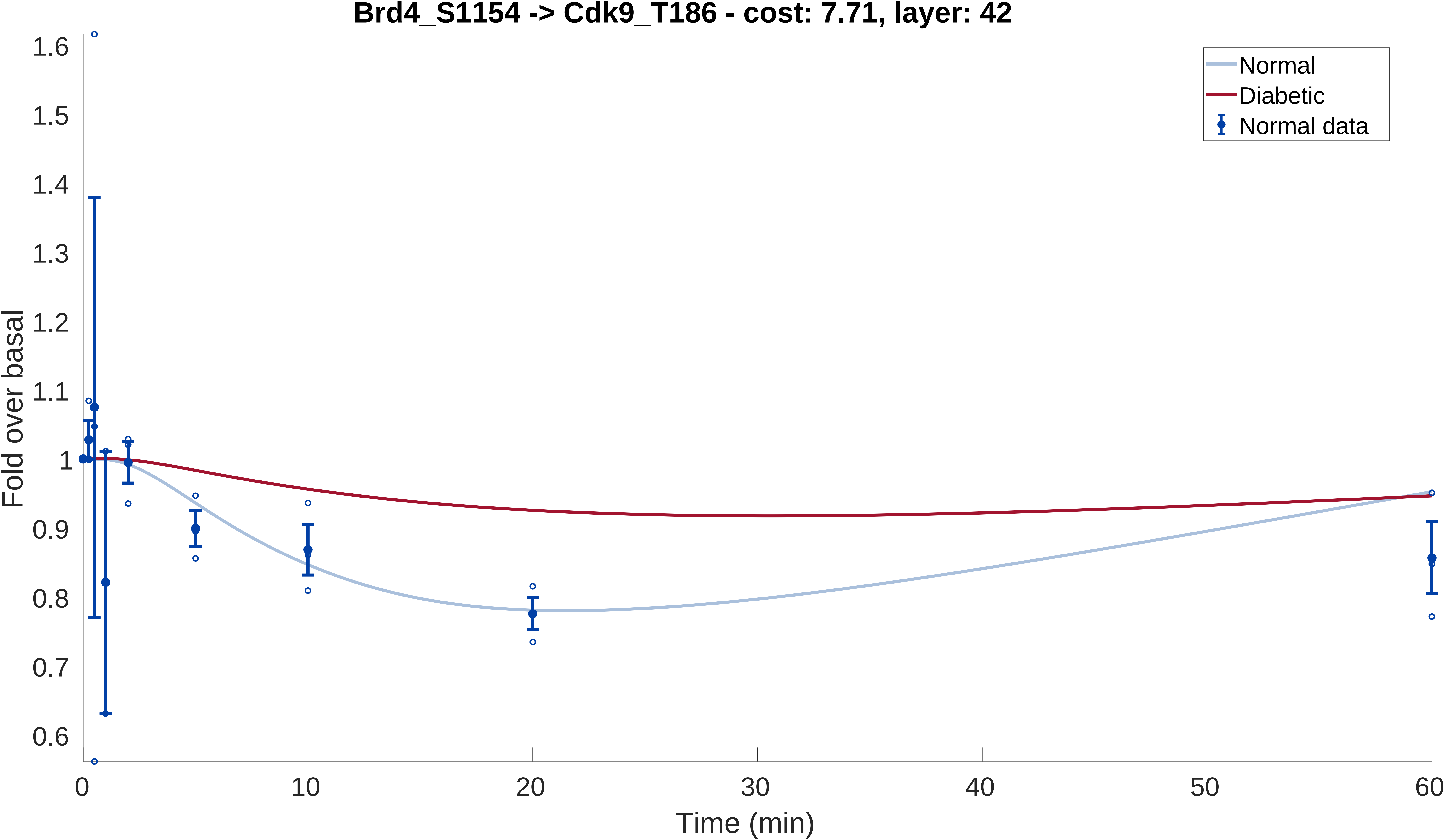

Brd4_S1154 -> Cdk9_T186 - cost: 7.71, layer: 42
Normal
Diabetic
Normal data
Fold over basal
1.6
1.5
1.4
1.3
1.2
1.1
1
0.9
0.8
0.7
0.6
0
10
20
30
40
50
60
Time (min)

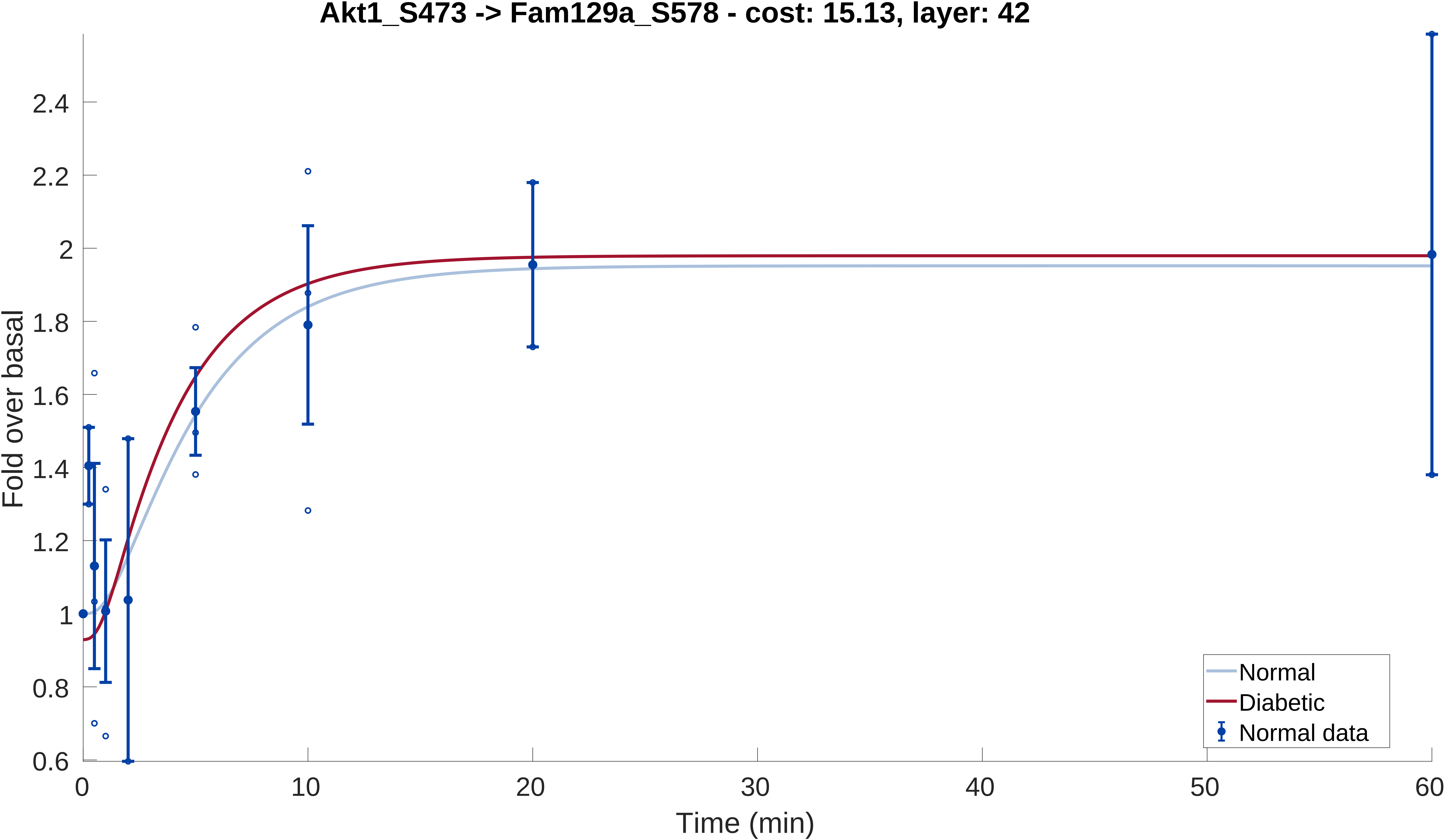

Akt1_S473 -> Fam129a_S578 - cost: 15.13, layer: 42
Fold over basal
2.4
2.2
2
1.8
1.6
1.4
1.2
1
0.8
0.6
0
10
20
30
40
50
60
Time (min)
Normal
Diabetic
Normal data

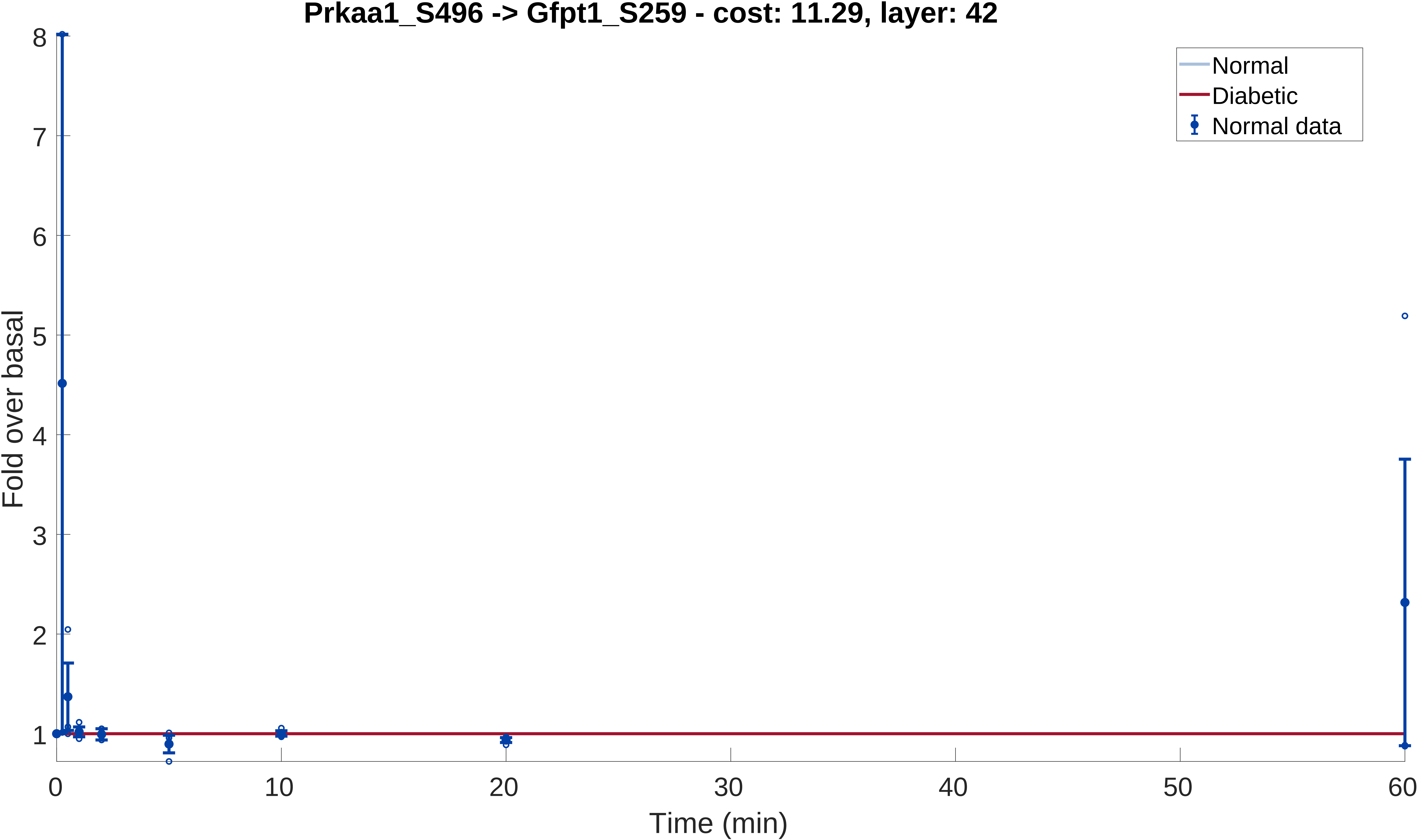

Prkaa1_S496 -> Gfpt1_S259 - cost: 11.29, layer: 42
Fold over basal
Time (min)
Normal
Diabetic
Normal data
0
10
20
30
40
50
60
1
2
3
4
5
6
7
8

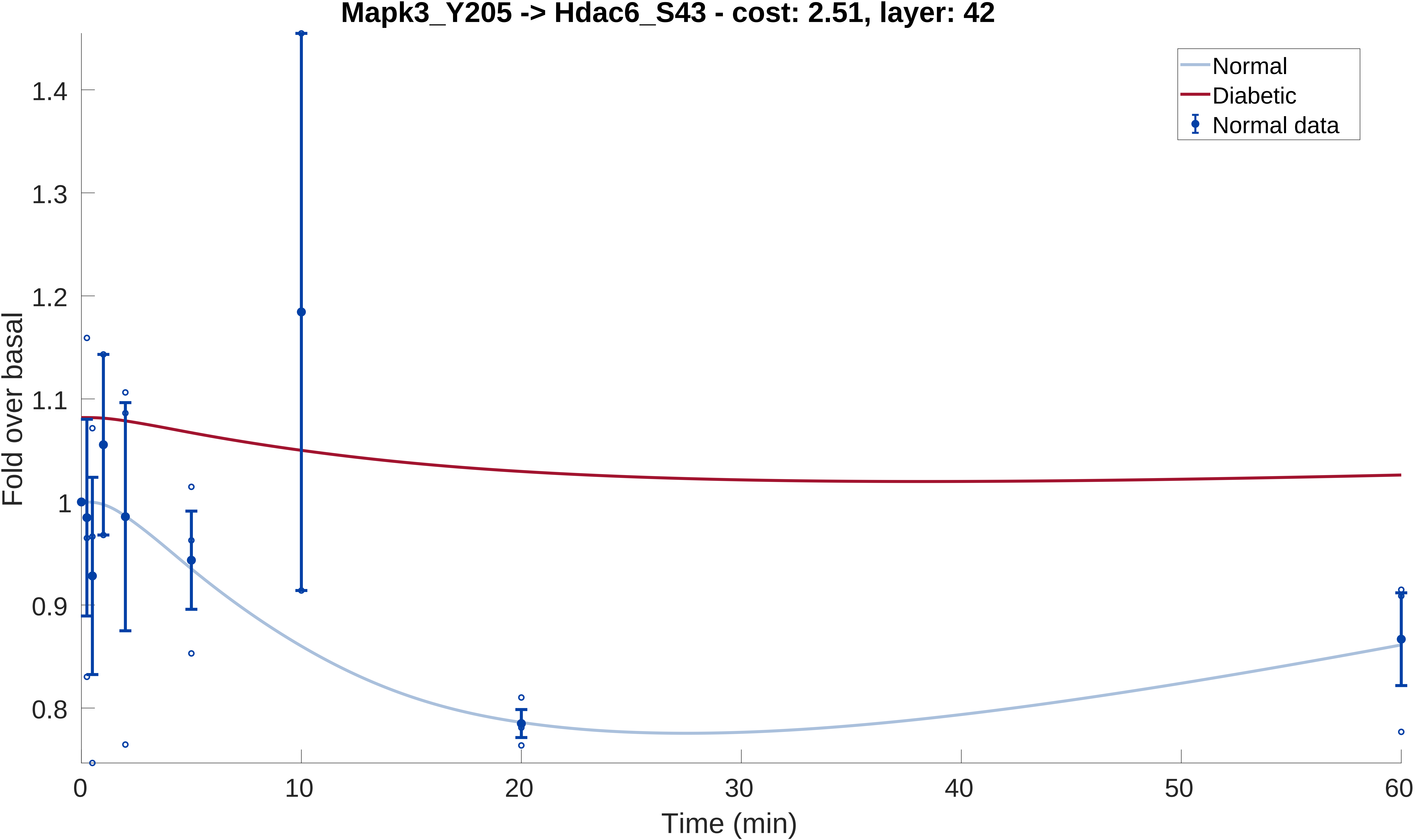
Mapk3_Y205 -> Hdac6_S43 - cost: 2.51, layer: 42
Normal
Diabetic
Normal data
Fold over basal
1.4
1.3
1.2
1.1
1
0.9
0.8
0
10
20
30
40
50
60
Time (min)

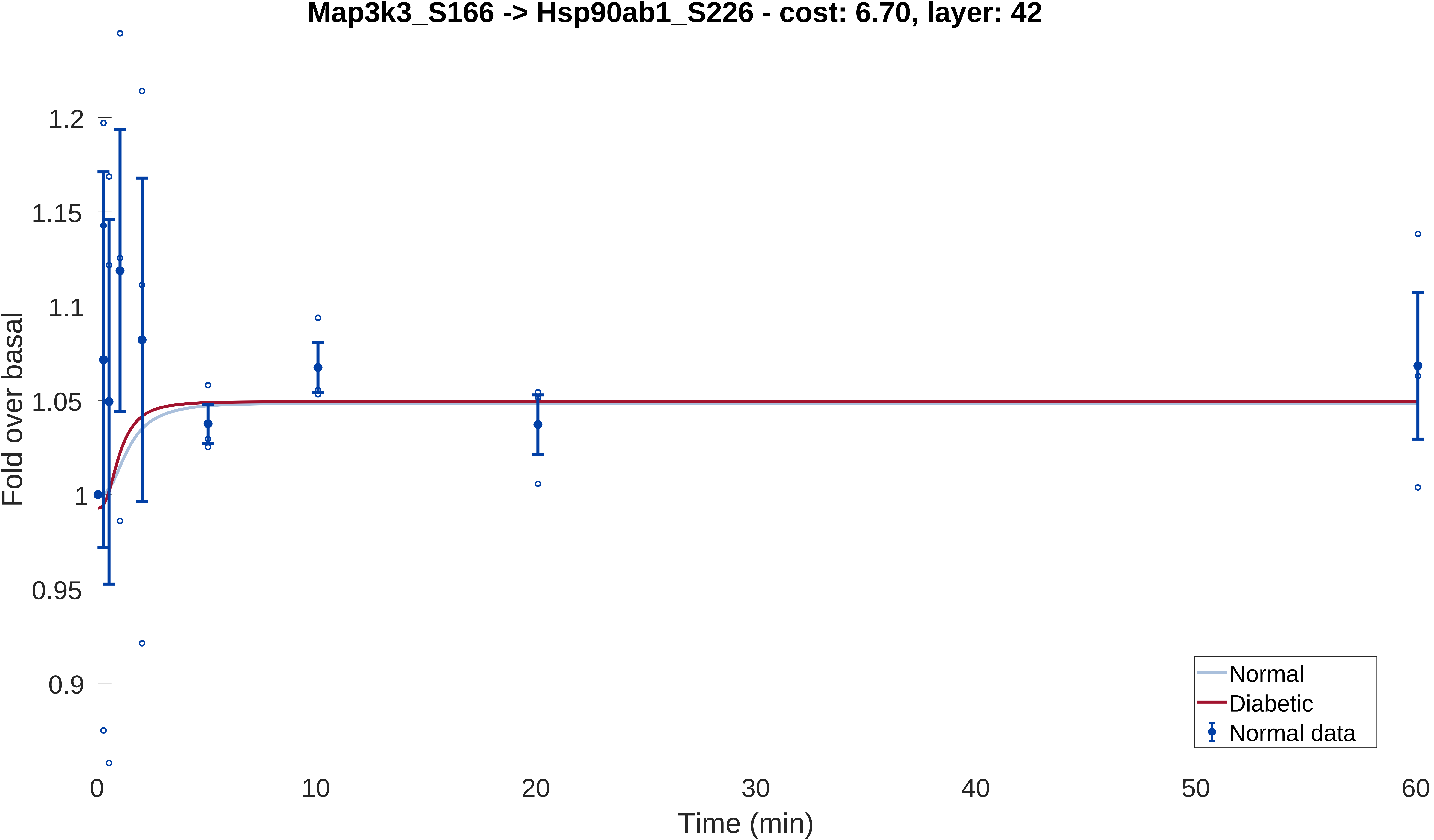

Map3k3_S166 -> Hsp90ab1_S226 - cost: 6.70, layer: 42
Fold over basal
1.2
1.15
1.1
1.05
1
0.95
0.9
0
10
20
30
40
50
60
Time (min)
Normal
Diabetic
Normal data

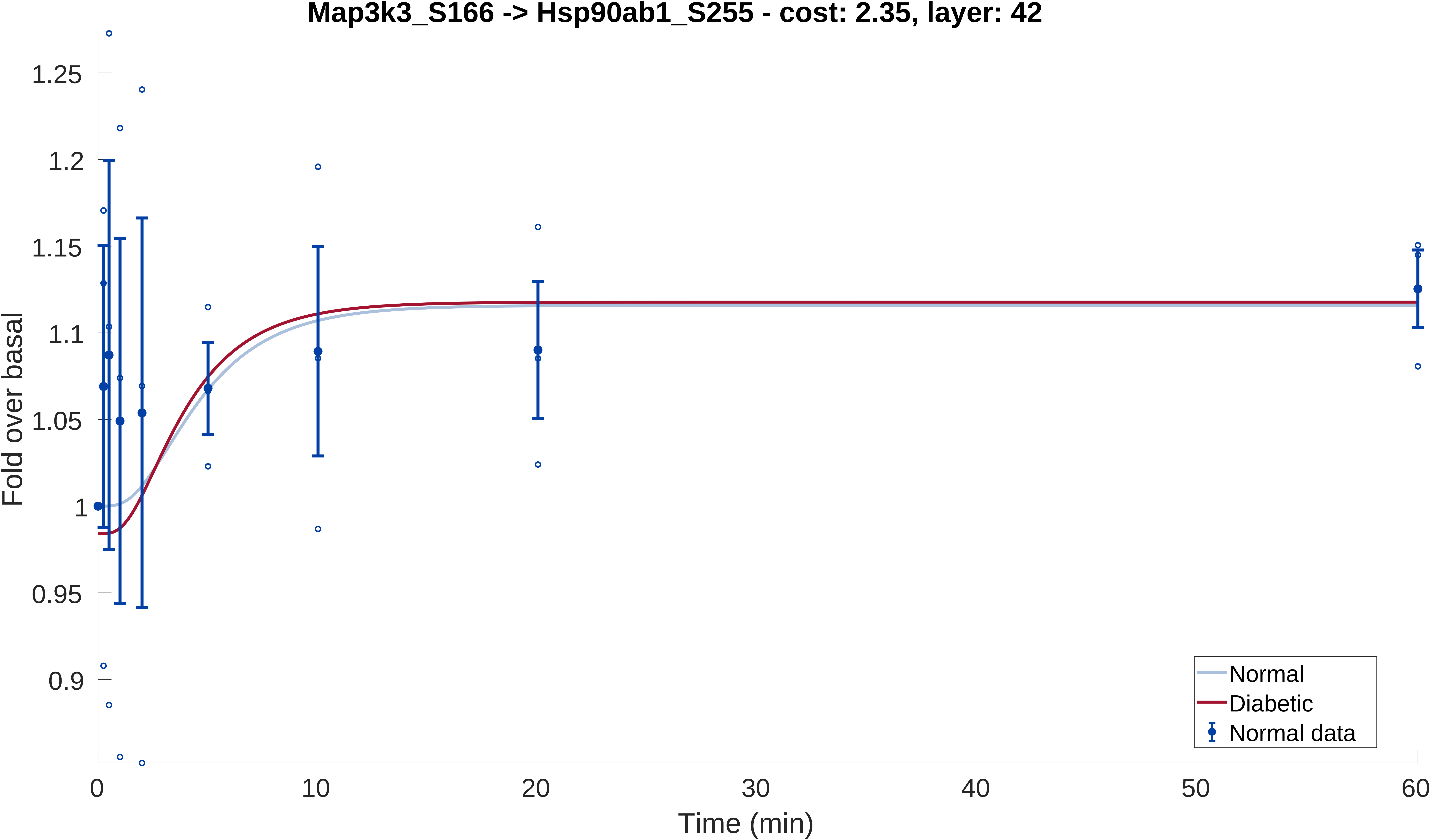
Map3k3_S166 -> Hsp90ab1_S255 - cost: 2.35, layer: 42
Fold over basal
1.25
1.2
1.15
1.1
1.05
1
0.95
0.9
0
10
20
30
40
50
60
Time (min)
Normal
Diabetic
Normal data

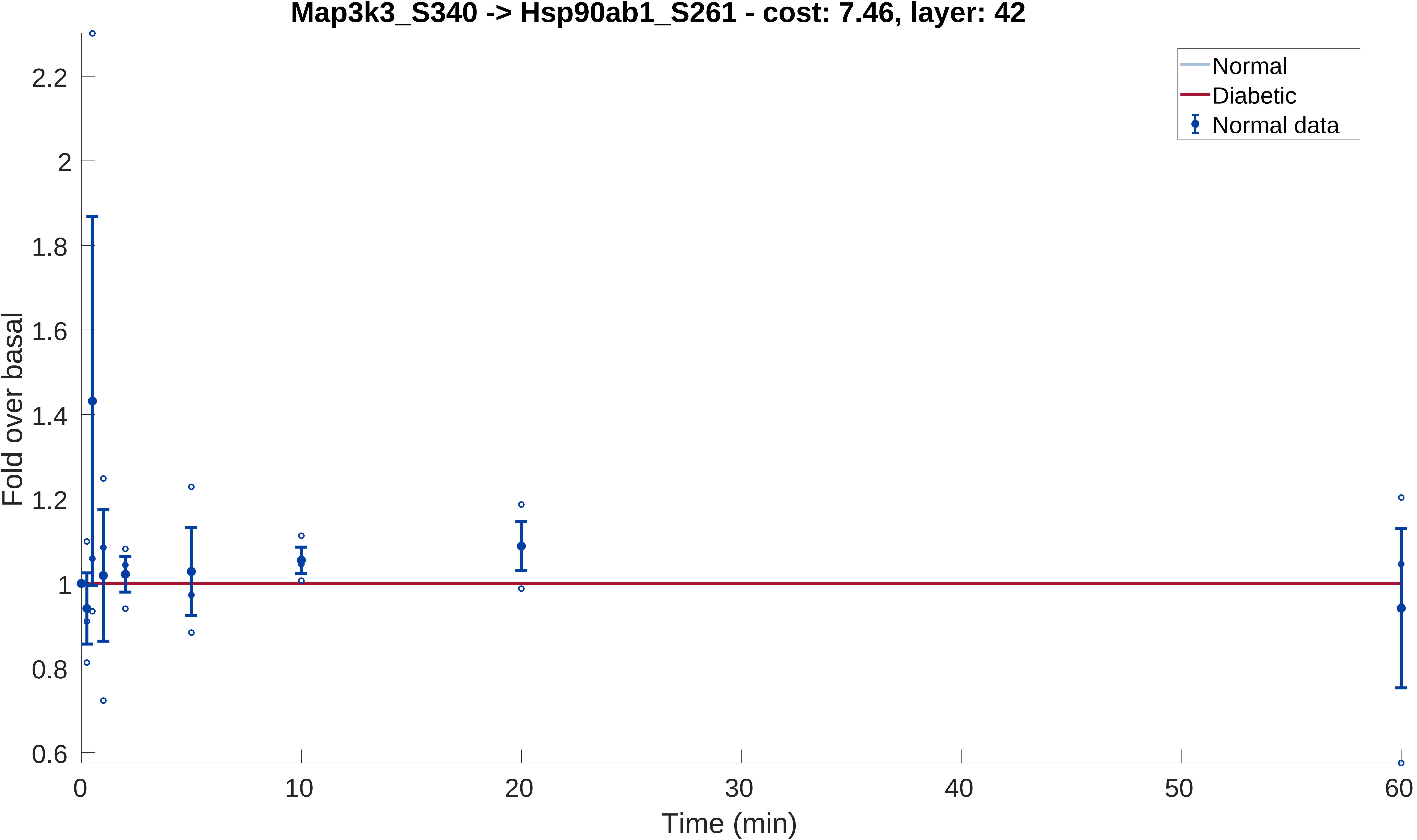
Map3k3_S340 -> Hsp90ab1_S261 - cost: 7.46, layer: 42
Fold over basal
Time (min)
Normal
Diabetic
Normal data
0.6
0.8
1
1.2
1.4
1.6
1.8
2
2.2
0
10
20
30
40
50
60

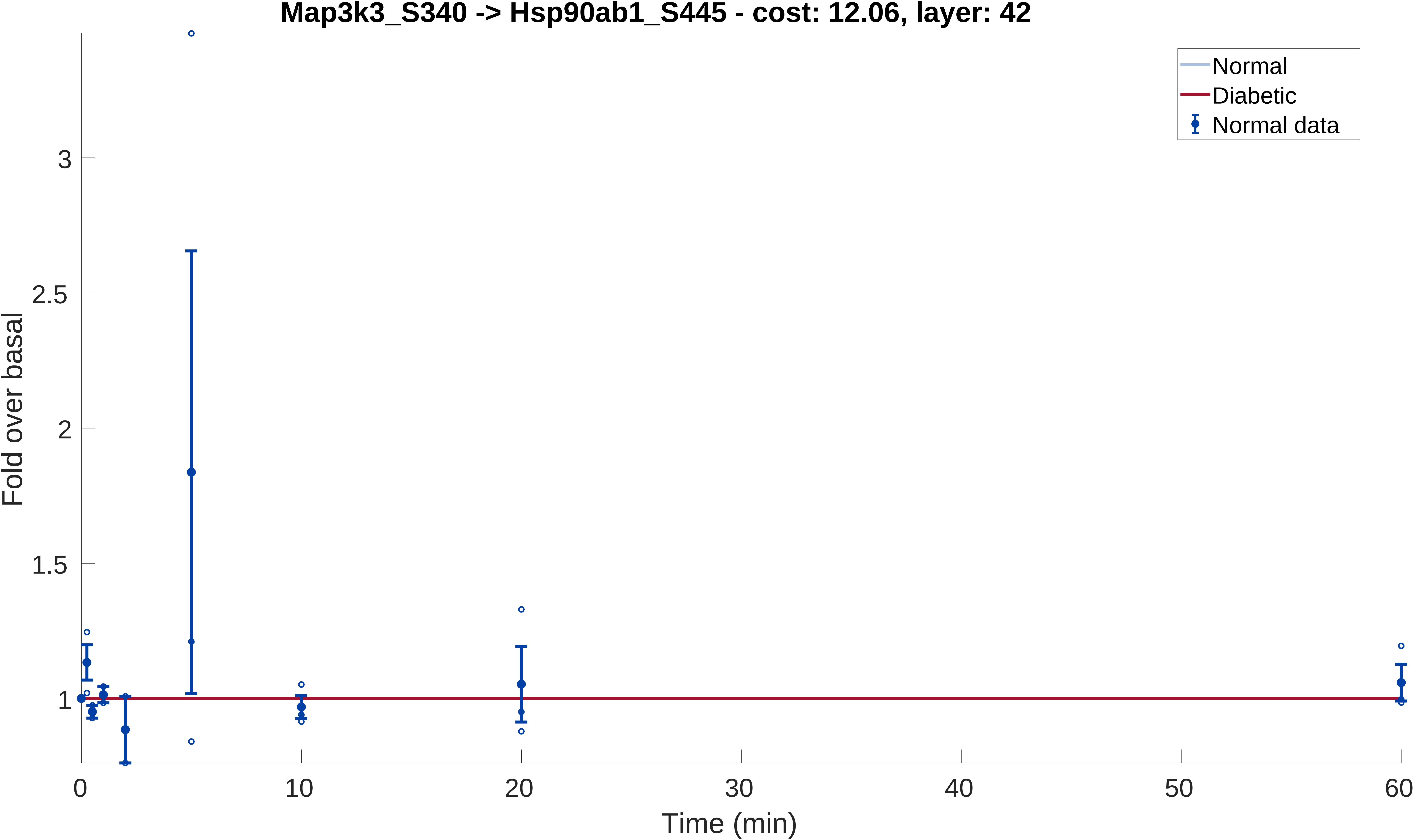

Map3k3_S340 -> Hsp90ab1_S445 - cost: 12.06, layer: 42
Normal
Diabetic
Normal data
Fold over basal
3
2.5
2
1.5
1
0
10
20
30
40
50
60
Time (min)

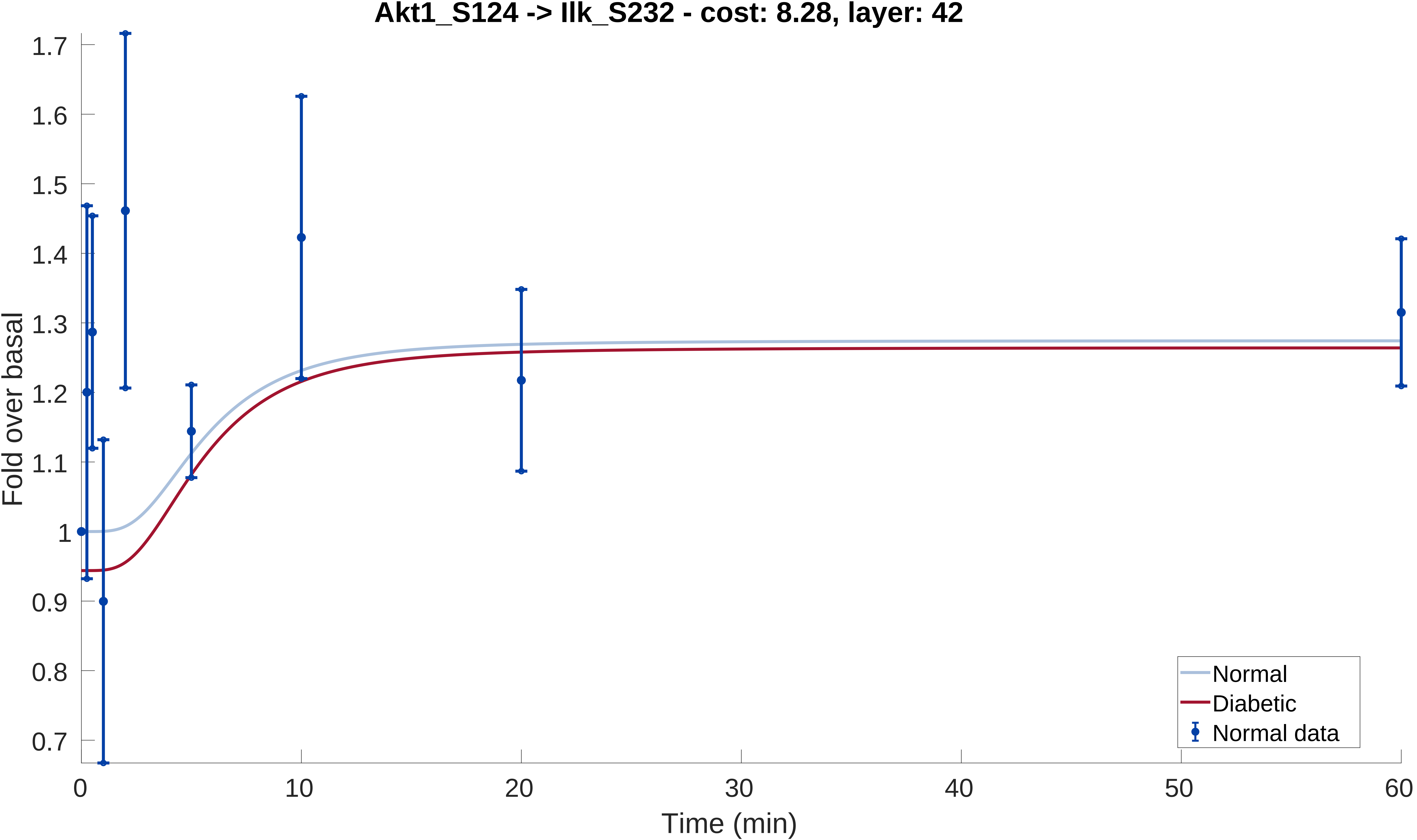

Akt1_S124 -> Ilk_S232 - cost: 8.28, layer: 42
Fold over basal
1.7
1.6
1.5
1.4
1.3
1.2
1.1
1
0.9
0.8
0.7
0
10
20
30
40
50
60
Time (min)
Normal
Diabetic
Normal data

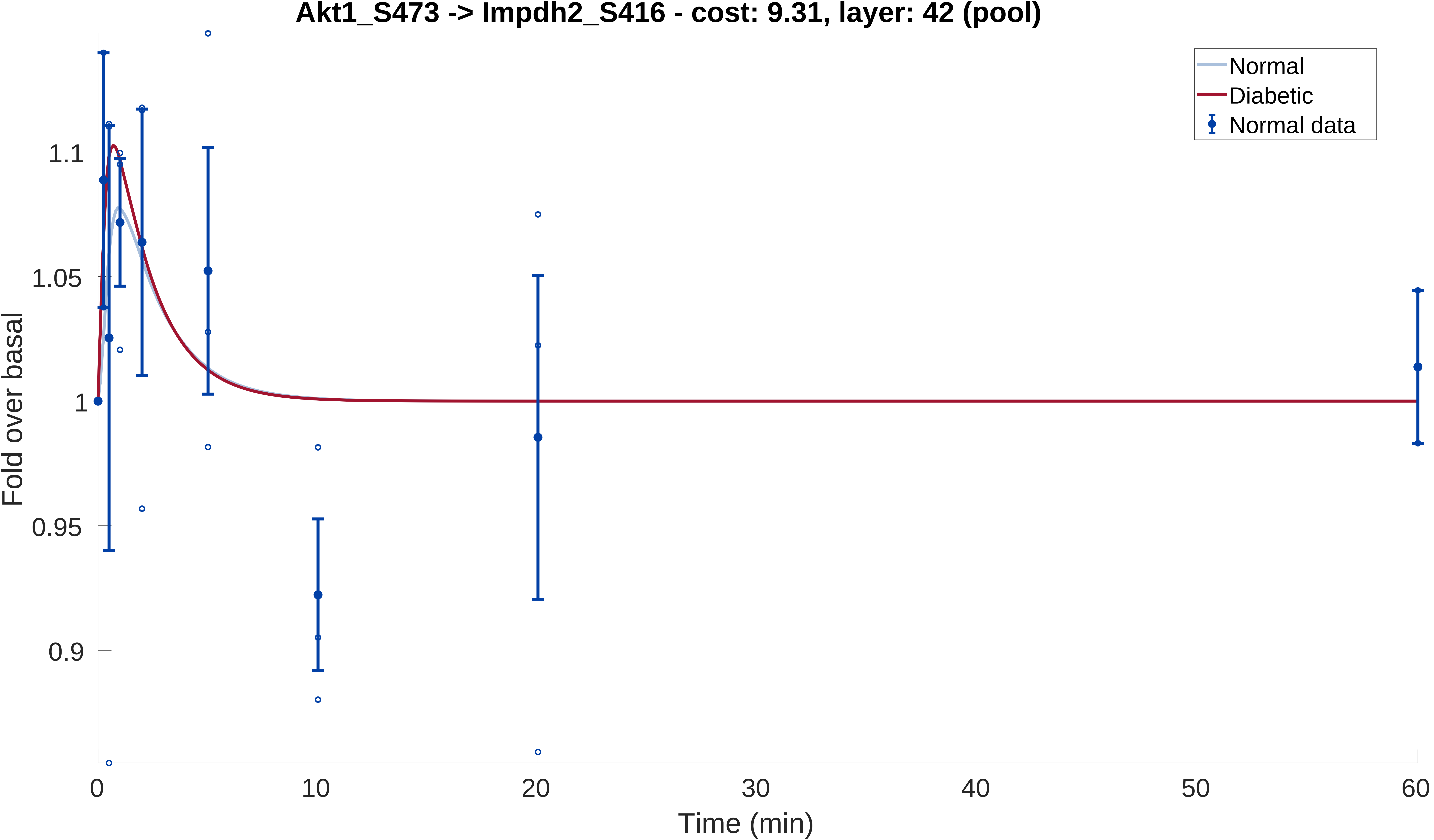

Akt1_S473 -> Impdh2_S416 - cost: 9.31, layer: 42 (pool)
Normal
Diabetic
Normal data
Fold over basal
1.1
1.05
1
0.95
0.9
0
10
20
30
40
50
60
Time (min)

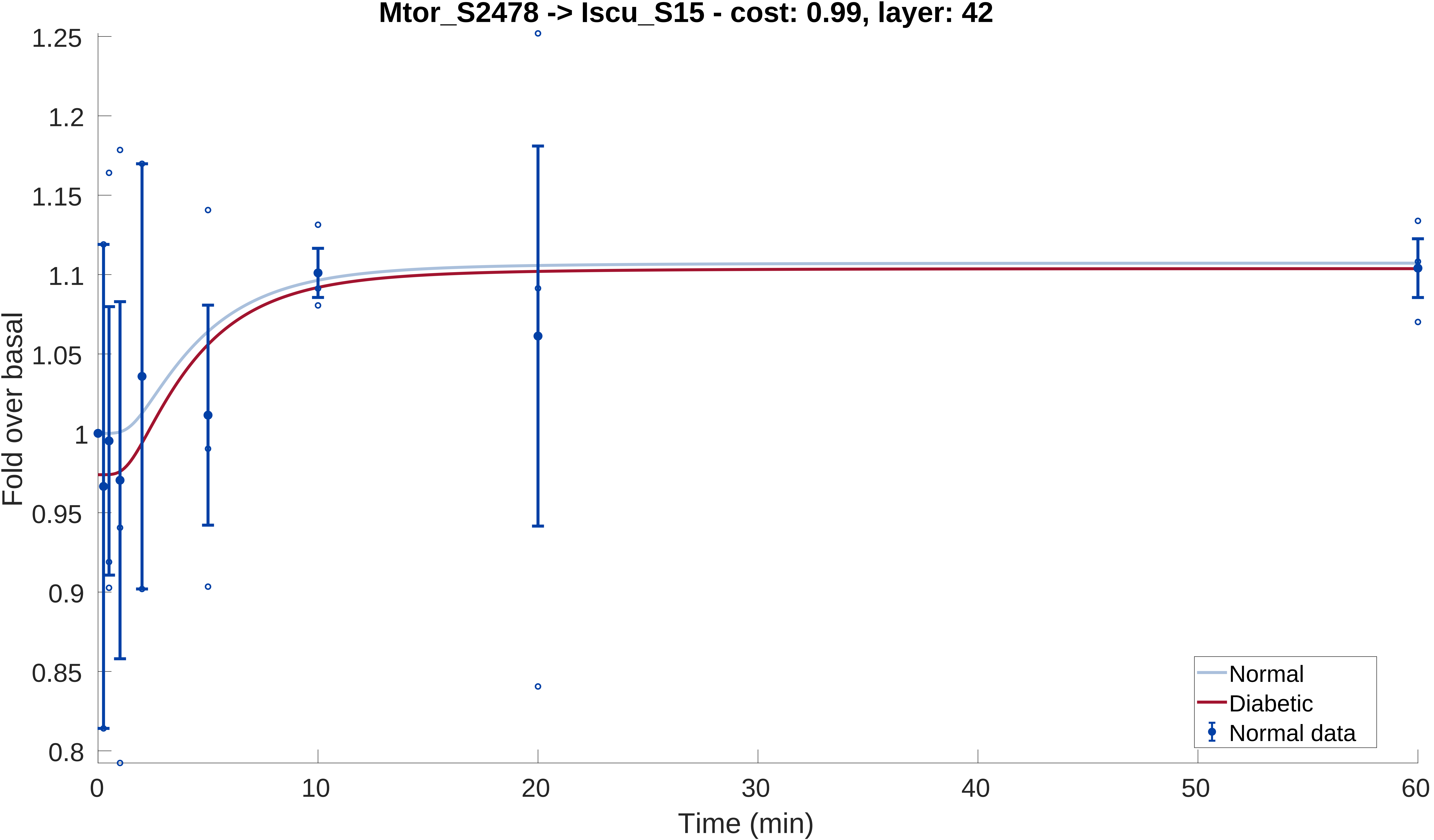

Mtor_S2478 -> Iscu_S15 - cost: 0.99, layer: 42
1.25
1.2
1.15
1.1
1.05
1
0.95
0.9
0.85
0.8
Fold over basal
0
10
20
30
40
50
60
Time (min)
Normal
Diabetic
Normal data

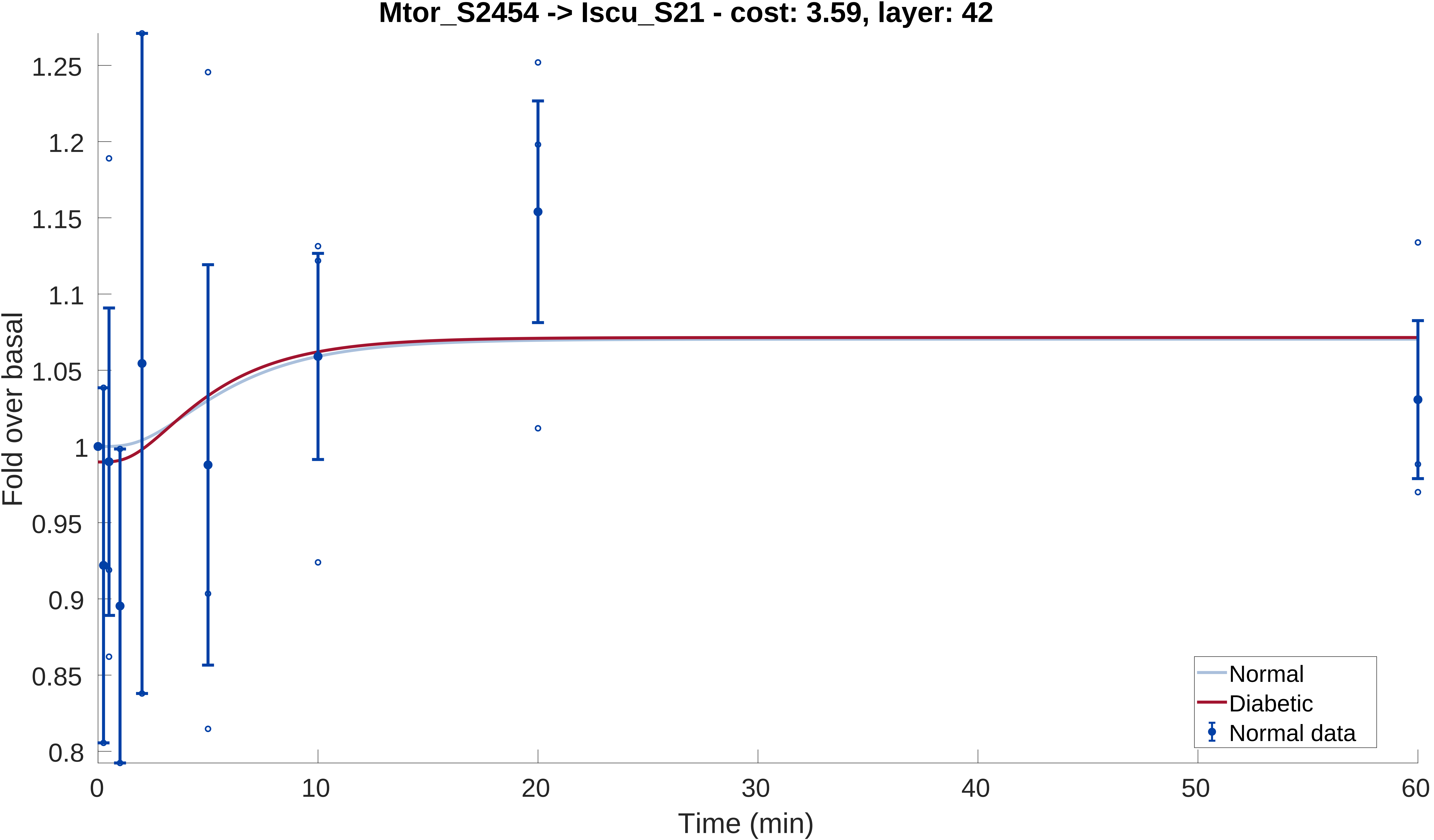

Mtor_S2454 -> Iscu_S21 - cost: 3.59, layer: 42
Fold over basal
1.25
1.2
1.15
1.1
1.05
1
0.95
0.9
0.85
0.8
0
10
20
30
40
50
60
Time (min)
Normal
Diabetic
Normal data

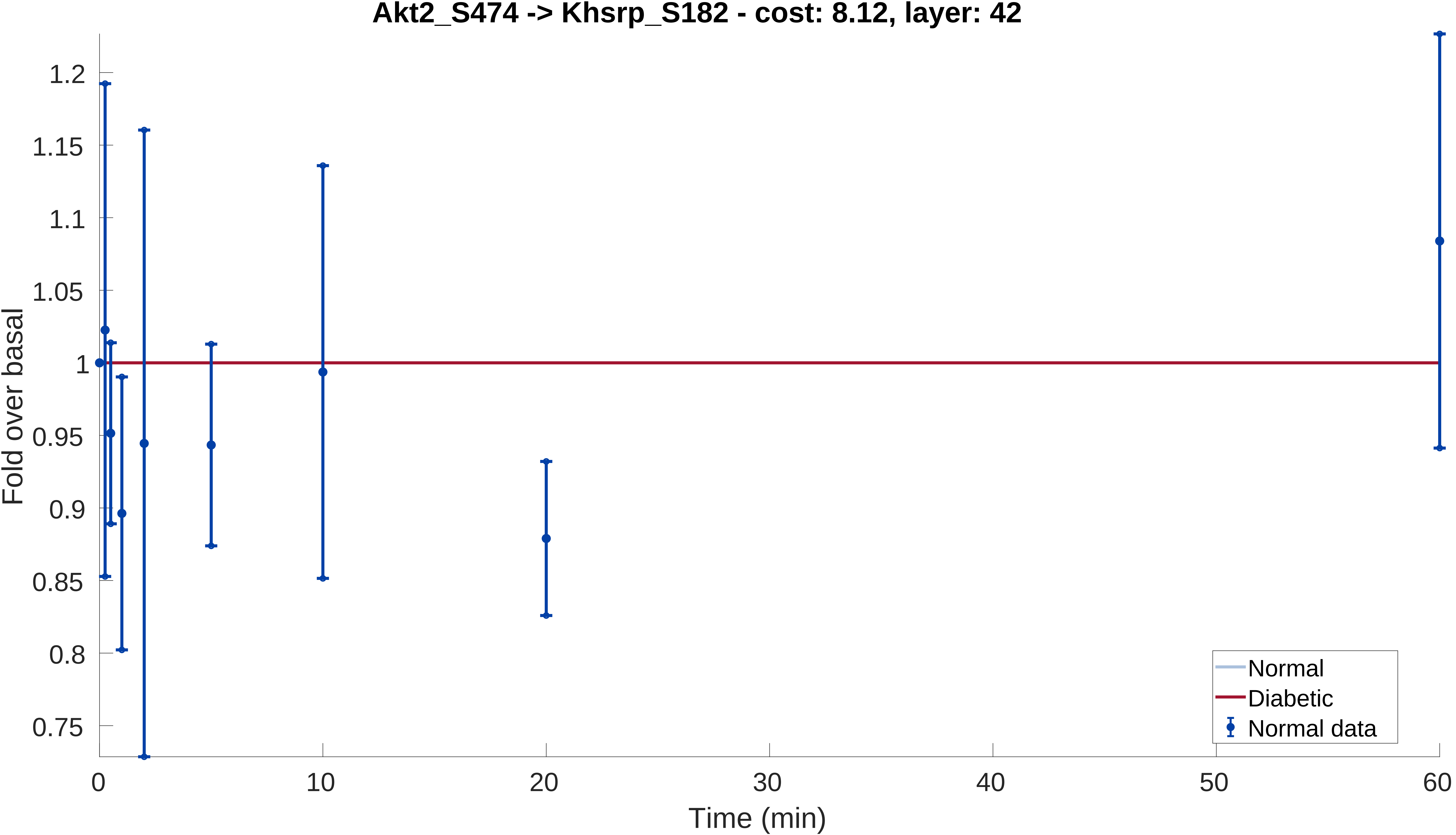
Akt2_S474 -> Khsrp_S182 - cost: 8.12, layer: 42
Fold over basal
Time (min)
1.2
1.15
1.1
1.05
1
0.95
0.9
0.85
0.8
0.75
0
10
20
30
40
50
60
Normal
Diabetic
Normal data

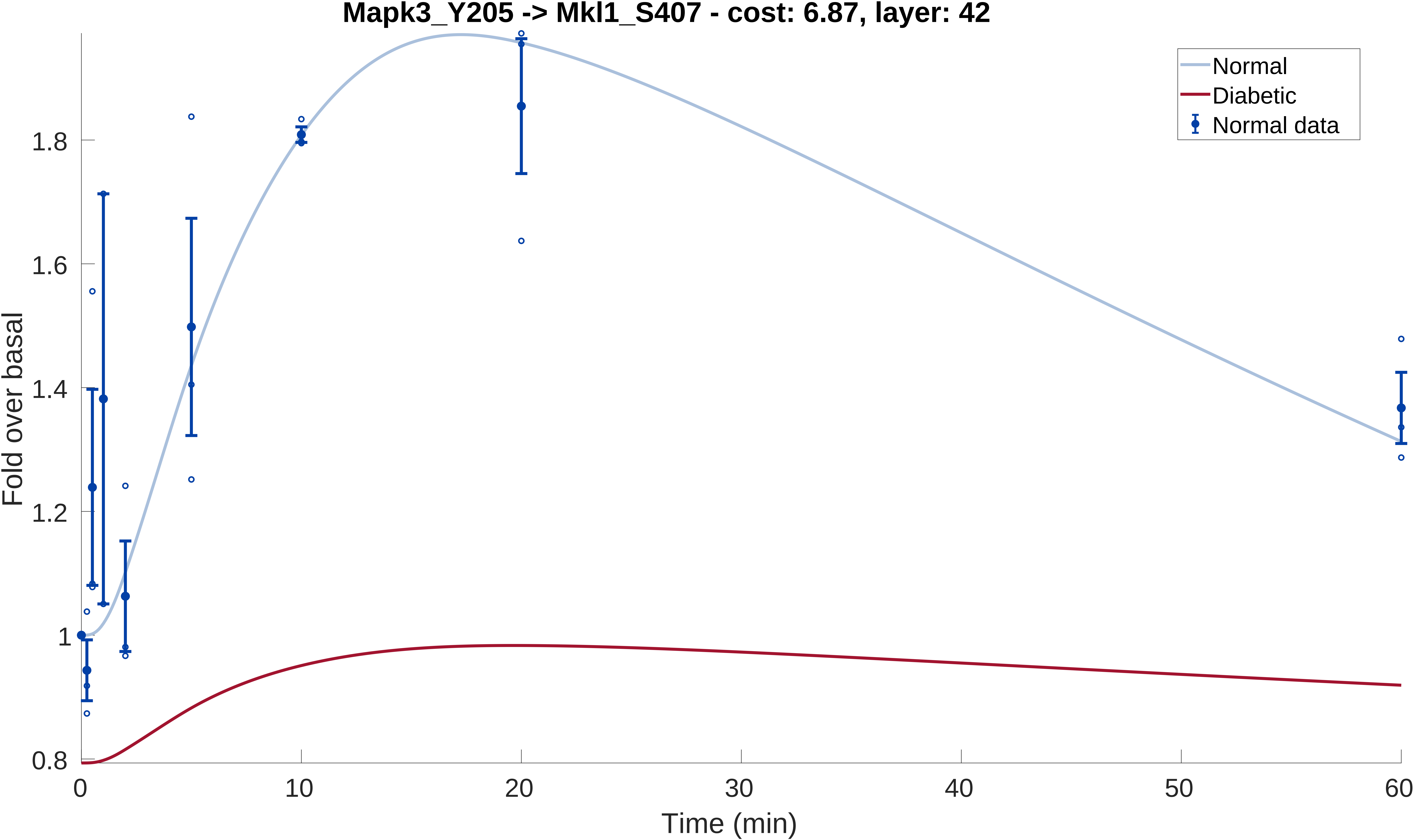

Mapk3_Y205 -> Mkl1_S407 - cost: 6.87, layer: 42
Normal
Diabetic
Normal data
Fold over basal
Time (min)
0.8
1
1.2
1.4
1.6
1.8
0
10
20
30
40
50
60

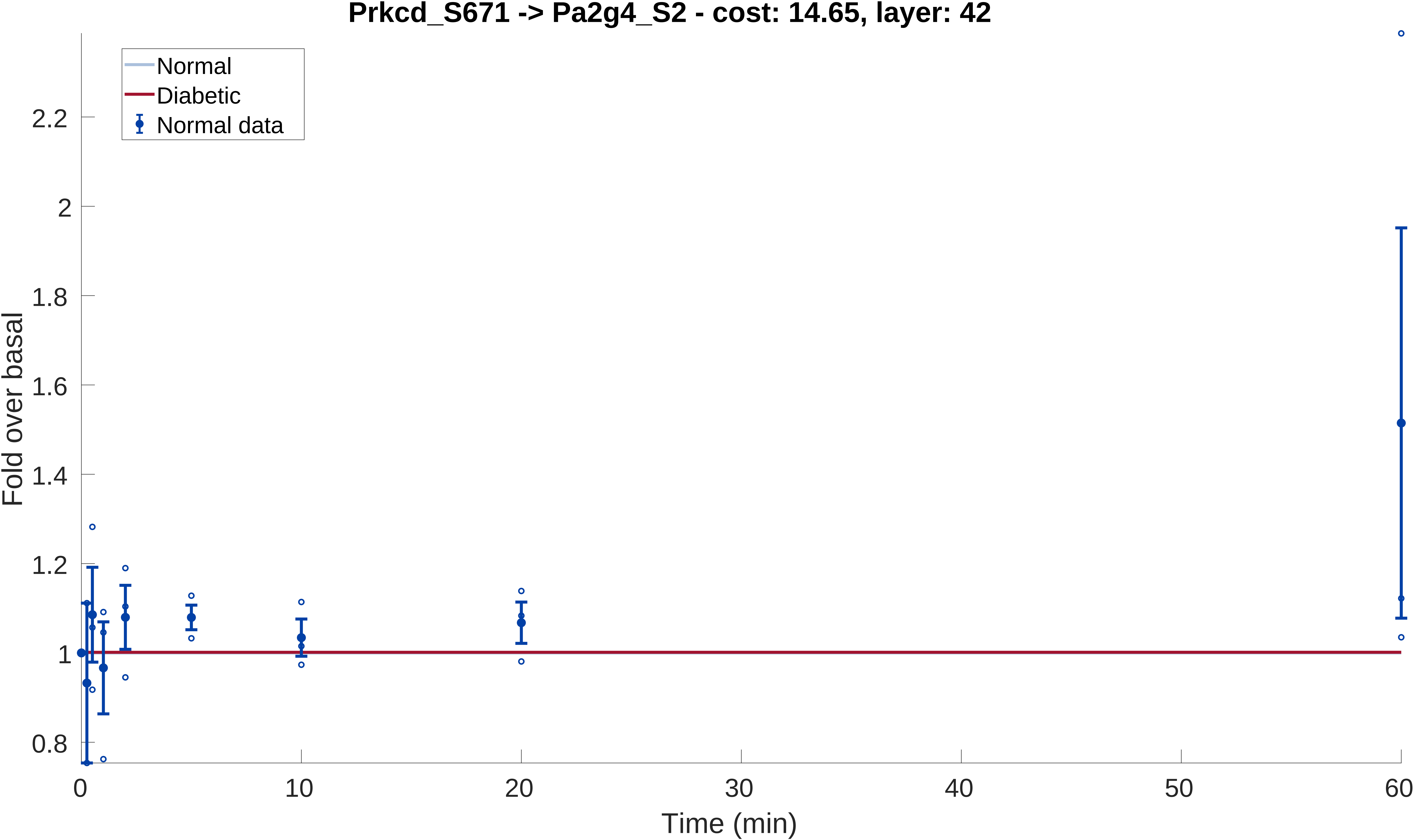

Prkcd_S671 -> Pa2g4_S2 - cost: 14.65, layer: 42
Normal
Diabetic
Normal data
Fold over basal
2.2
2
1.8
1.6
1.4
1.2
1
0.8
0
10
20
30
40
50
60
Time (min)

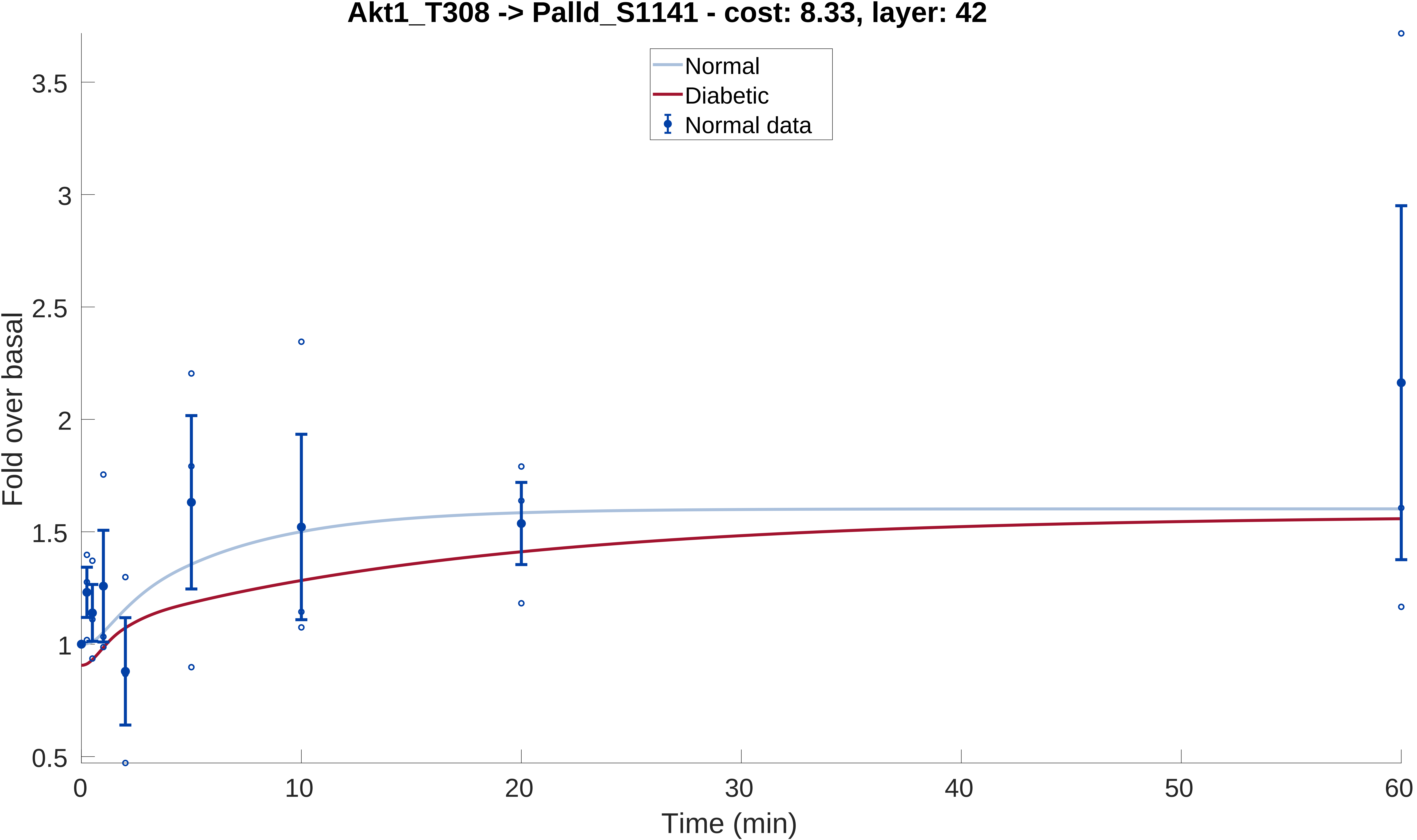

Akt1_T308 -> Palld_S1141 - cost: 8.33, layer: 42
Normal
Diabetic
Normal data
Fold over basal
3.5
3
2.5
2
1.5
1
0.5
0
10
20
30
40
50
60
Time (min)

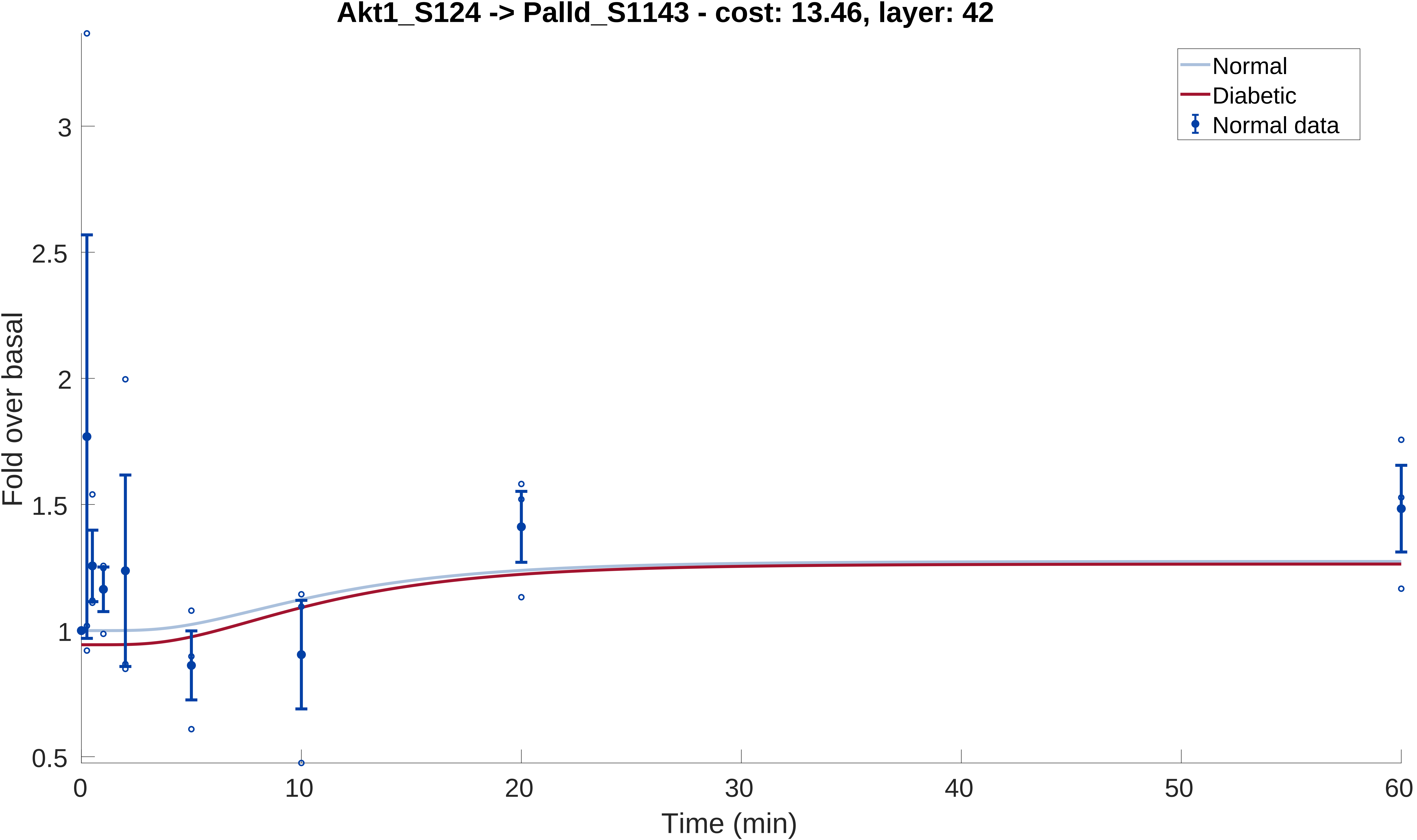
Akt1_S124 -> Palld_S1143 - cost: 13.46, layer: 42
Normal
Diabetic
Normal data
Fold over basal
Time (min)
0.5
1
1.5
2
2.5
3
0
10
20
30
40
50
60

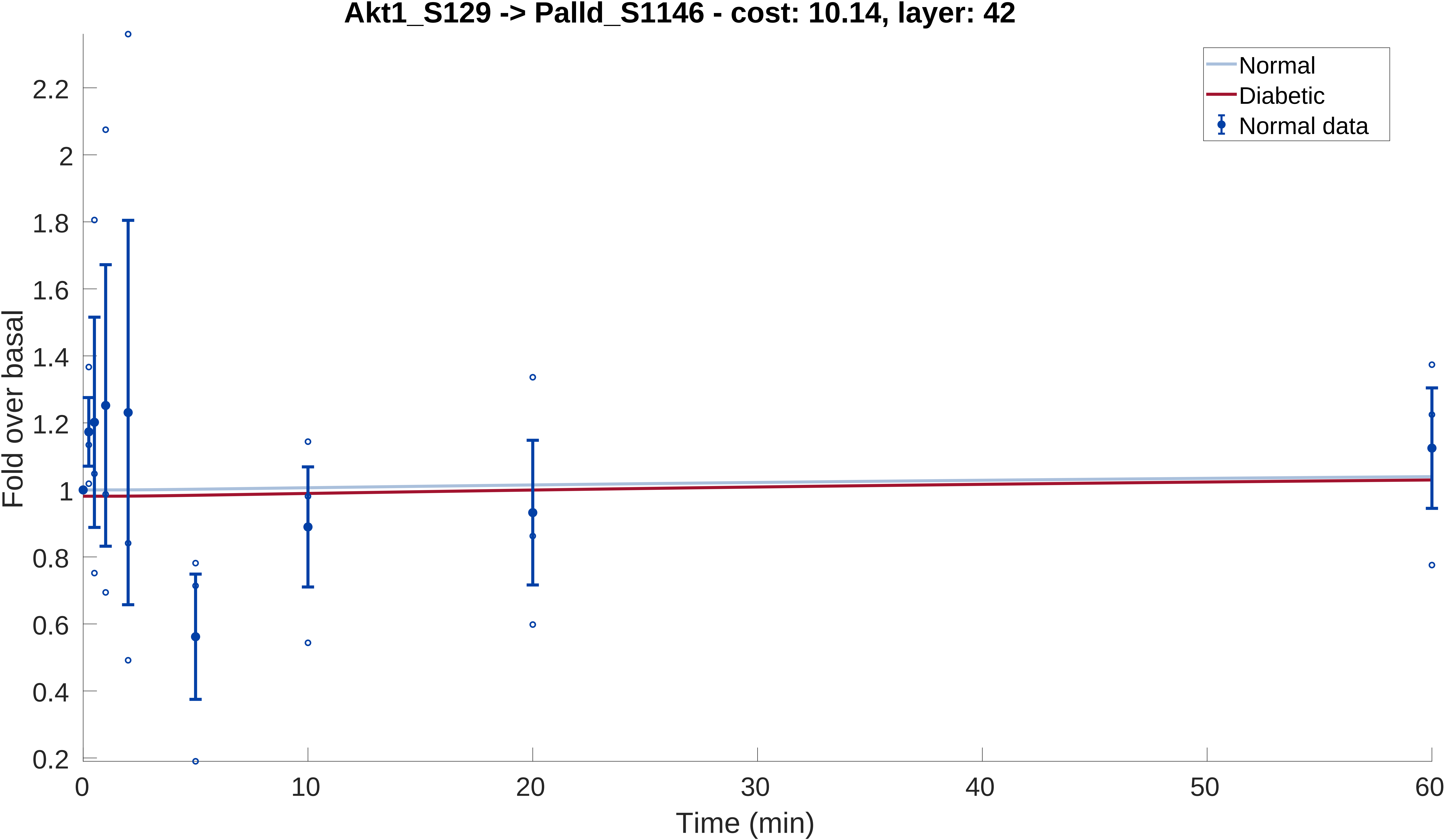

Akt1_S129 -> Palld_S1146 - cost: 10.14, layer: 42
Normal
Diabetic
Normal data
Fold over basal
Time (min)
0.2
0.4
0.6
0.8
1
1.2
1.4
1.6
1.8
2
2.2
0
10
20
30
40
50
60

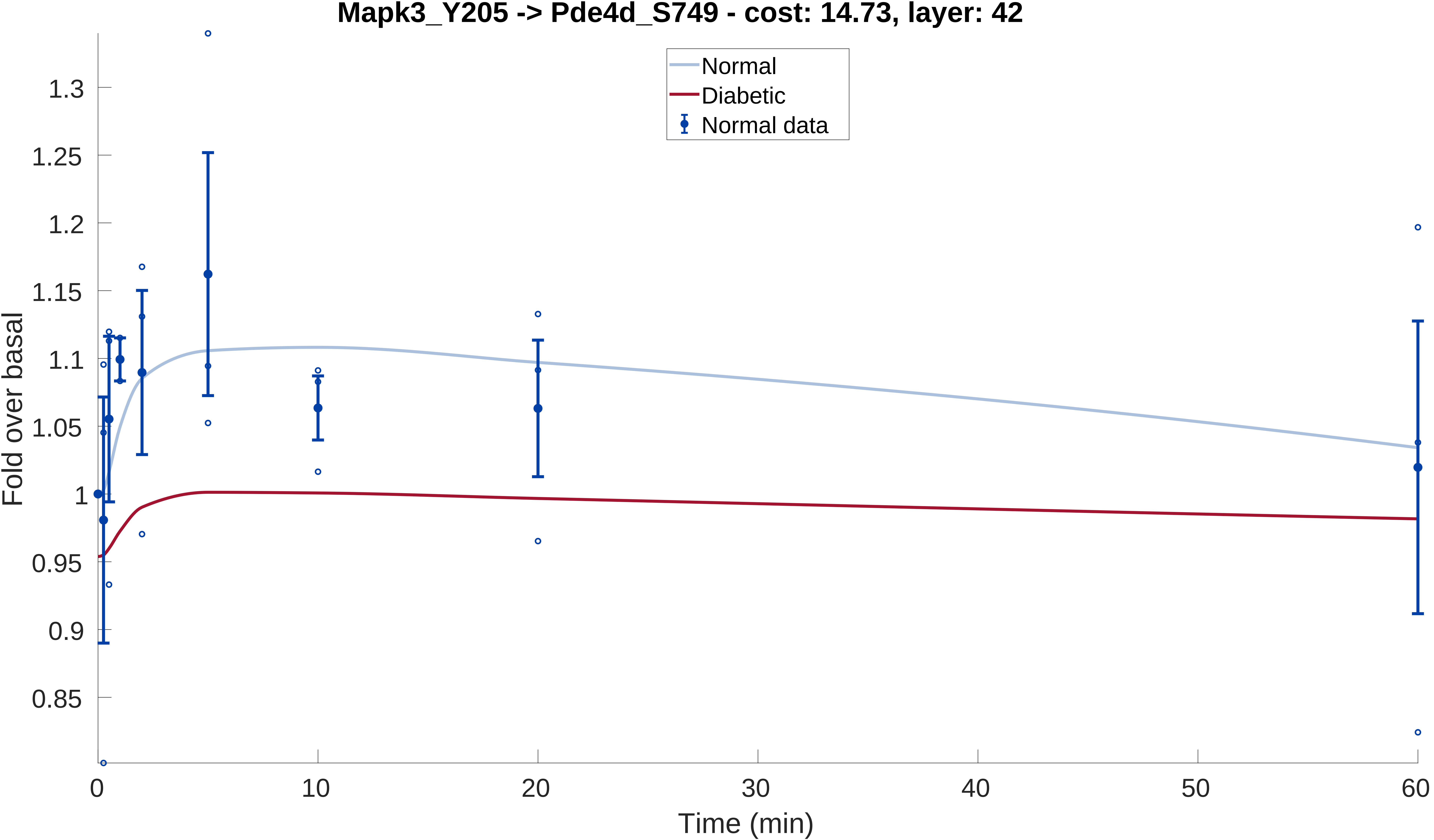

Mapk3_Y205 -> Pde4d_S749 - cost: 14.73, layer: 42
Normal
Diabetic
Normal data
1.3
1.25
1.2
1.15
1.1
1.05
1
0.95
0.9
0.85
Fold over basal
0
10
20
30
40
50
60
Time (min)

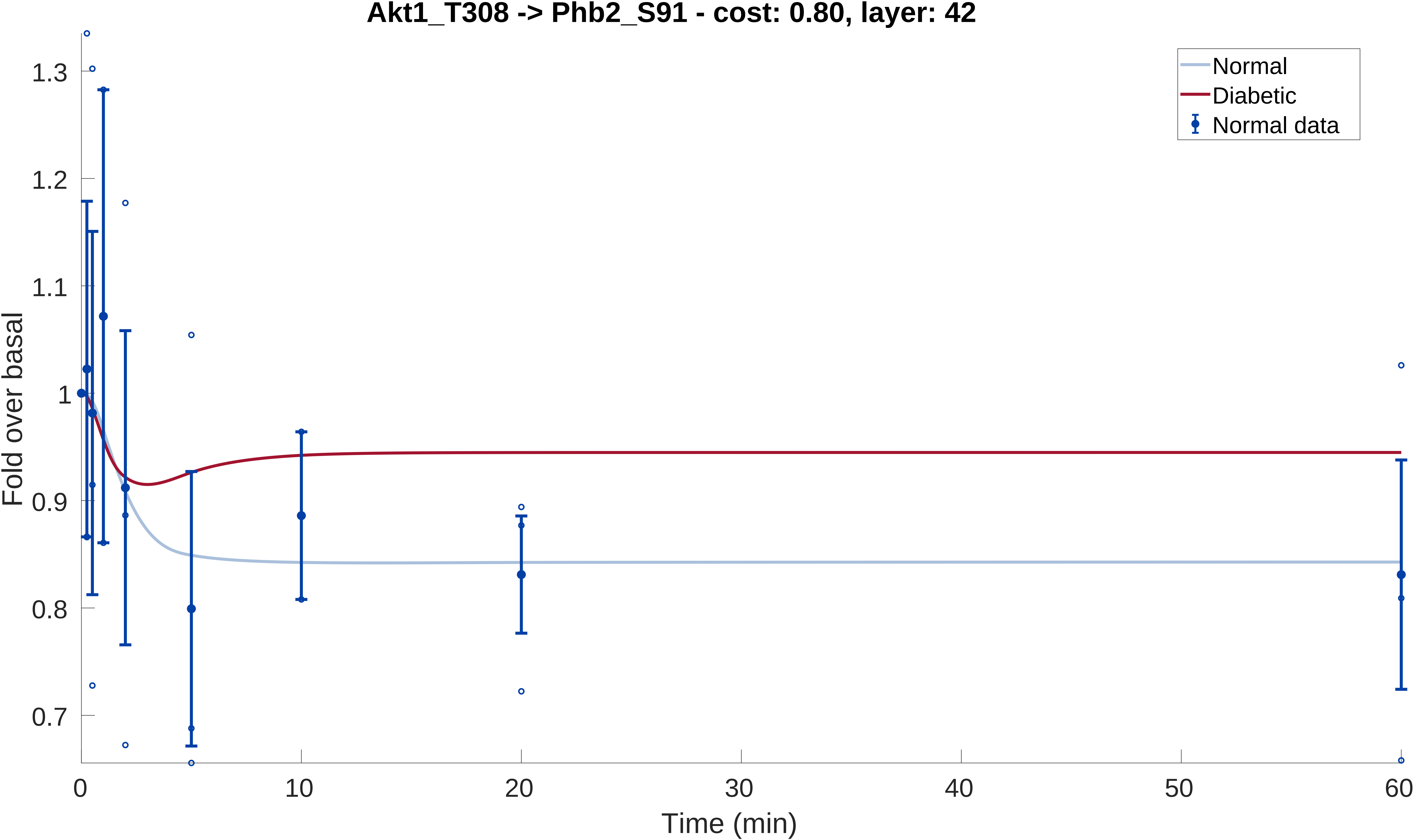

Akt1_T308 -> Phb2_S91 - cost: 0.80, layer: 42
Normal
Diabetic
Normal data
Fold over basal
Time (min)
1.3
1.2
1.1
1
0.9
0.8
0.7
0
10
20
30
40
50
60

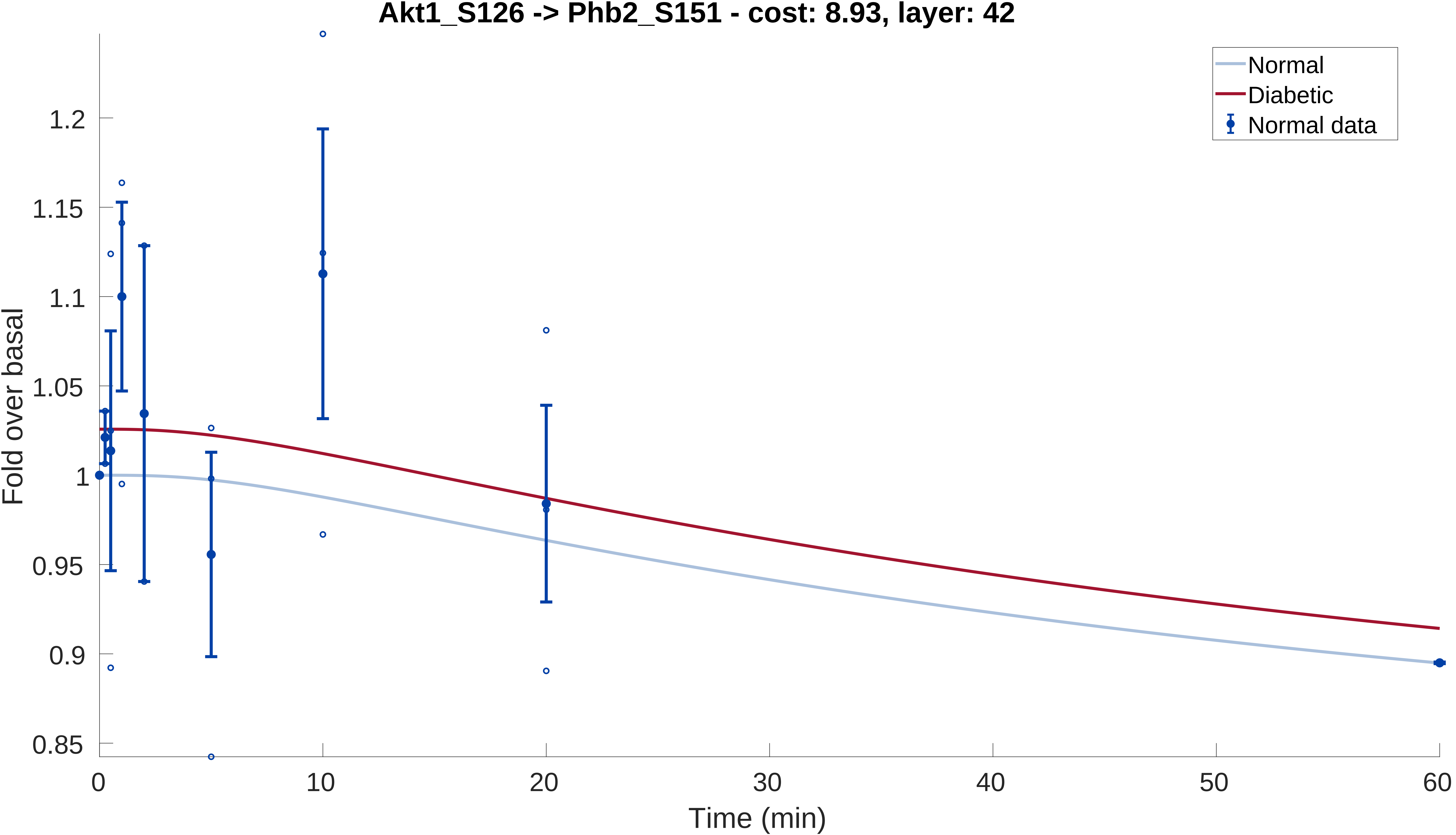
Akt1_S126 -> Phb2_S151 - cost: 8.93, layer: 42
Normal
Diabetic
Normal data
Fold over basal
Time (min)
0
10
20
30
40
50
60
0.85
0.9
0.95
1
1.05
1.1
1.15
1.2

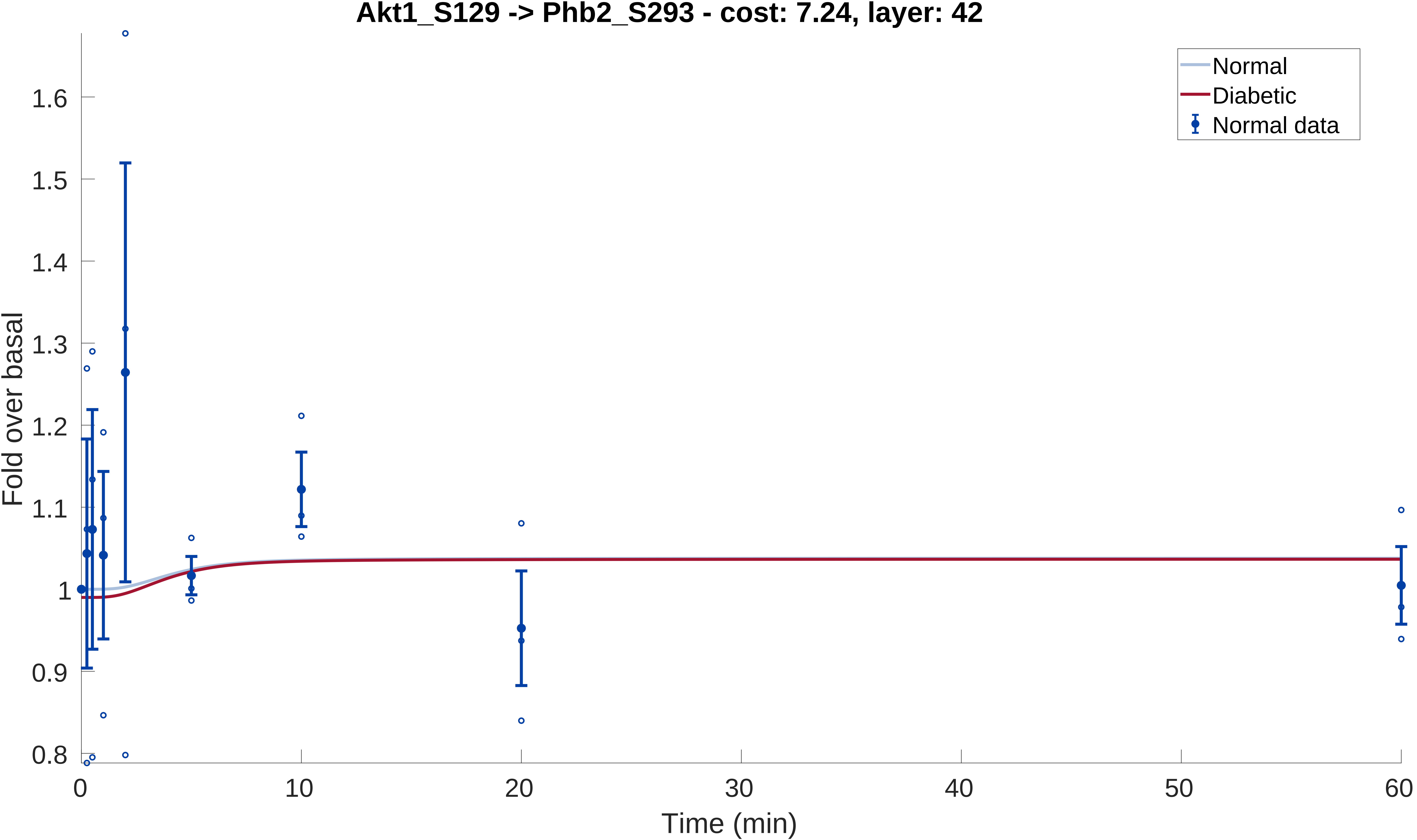

Akt1_S129 -> Phb2_S293 - cost: 7.24, layer: 42
Normal
Diabetic
Normal data
Fold over basal
1.6
1.5
1.4
1.3
1.2
1.1
1
0.9
0.8
0
10
20
30
40
50
60
Time (min)

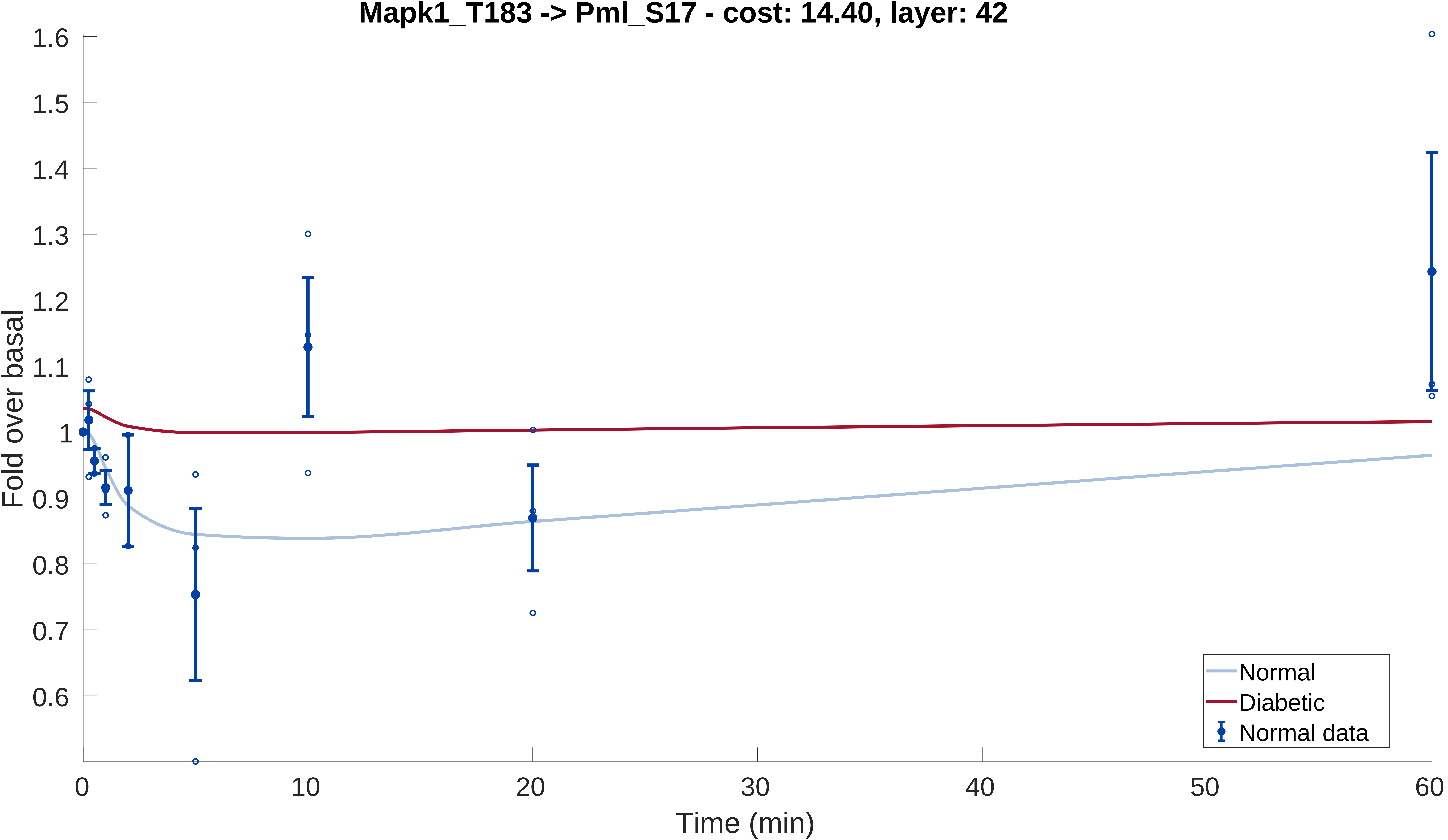
Mapk1_T183 -> Pml_S17 - cost: 14.40, layer: 42
Fold over basal
1.6
1.5
1.4
1.3
1.2
1.1
1
0.9
0.8
0.7
0.6
0
10
20
30
40
50
60
Time (min)
Normal
Diabetic
Normal data

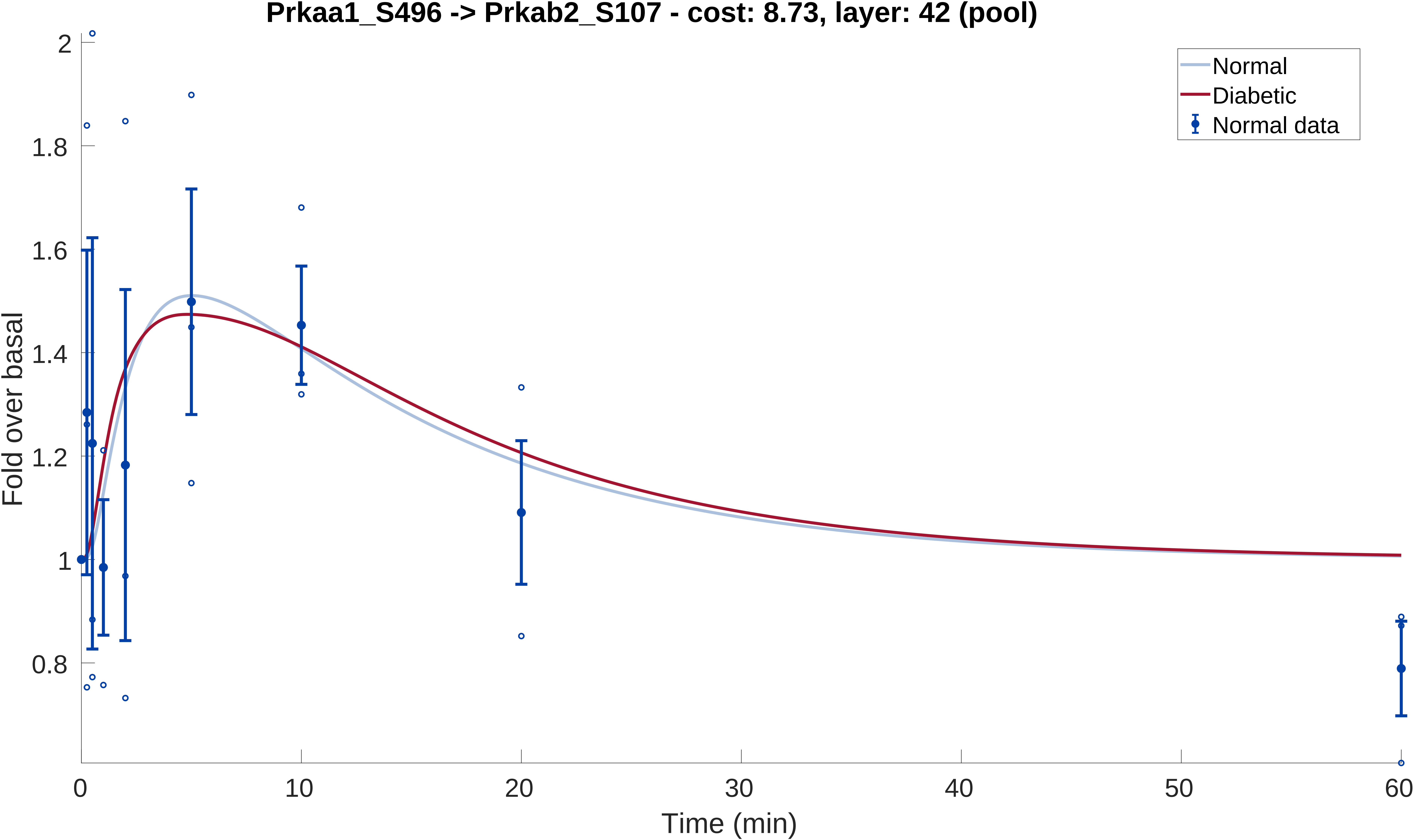

Prrkaa1_S496 -> Prkab2_S107 - cost: 8.73, layer: 42 (pool)
Normal
Diabetic
Normal data
Fold over basal
Time (min)
2
1.8
1.6
1.4
1.2
1
0.8
0
10
20
30
40
50
60

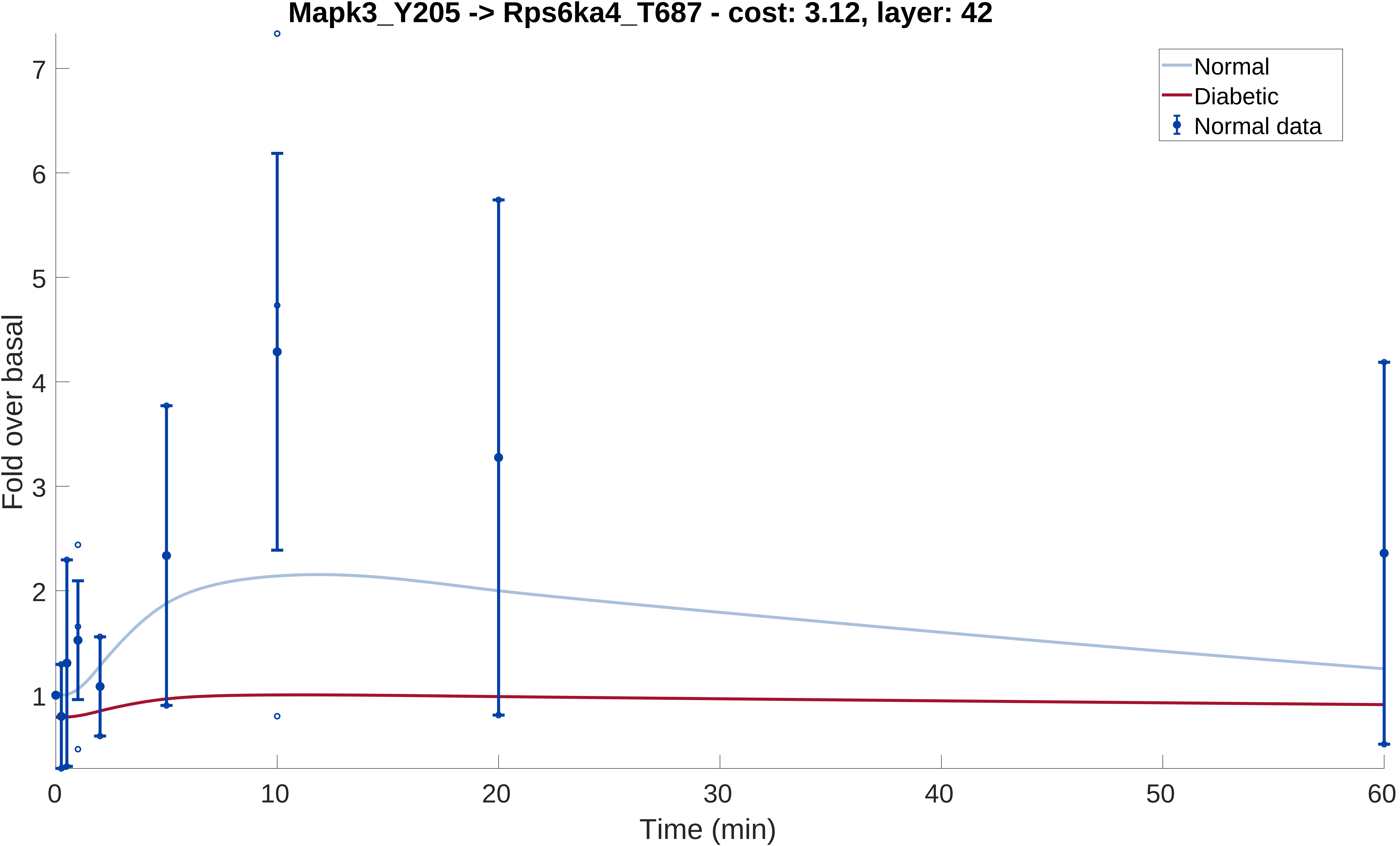

Mapk3_Y205 -> Rps6ka4_T687 - cost: 3.12, layer: 42
Normal
Diabetic
Normal data
Fold over basal
Time (min)
0
10
20
30
40
50
60
1
2
3
4
5
6
7

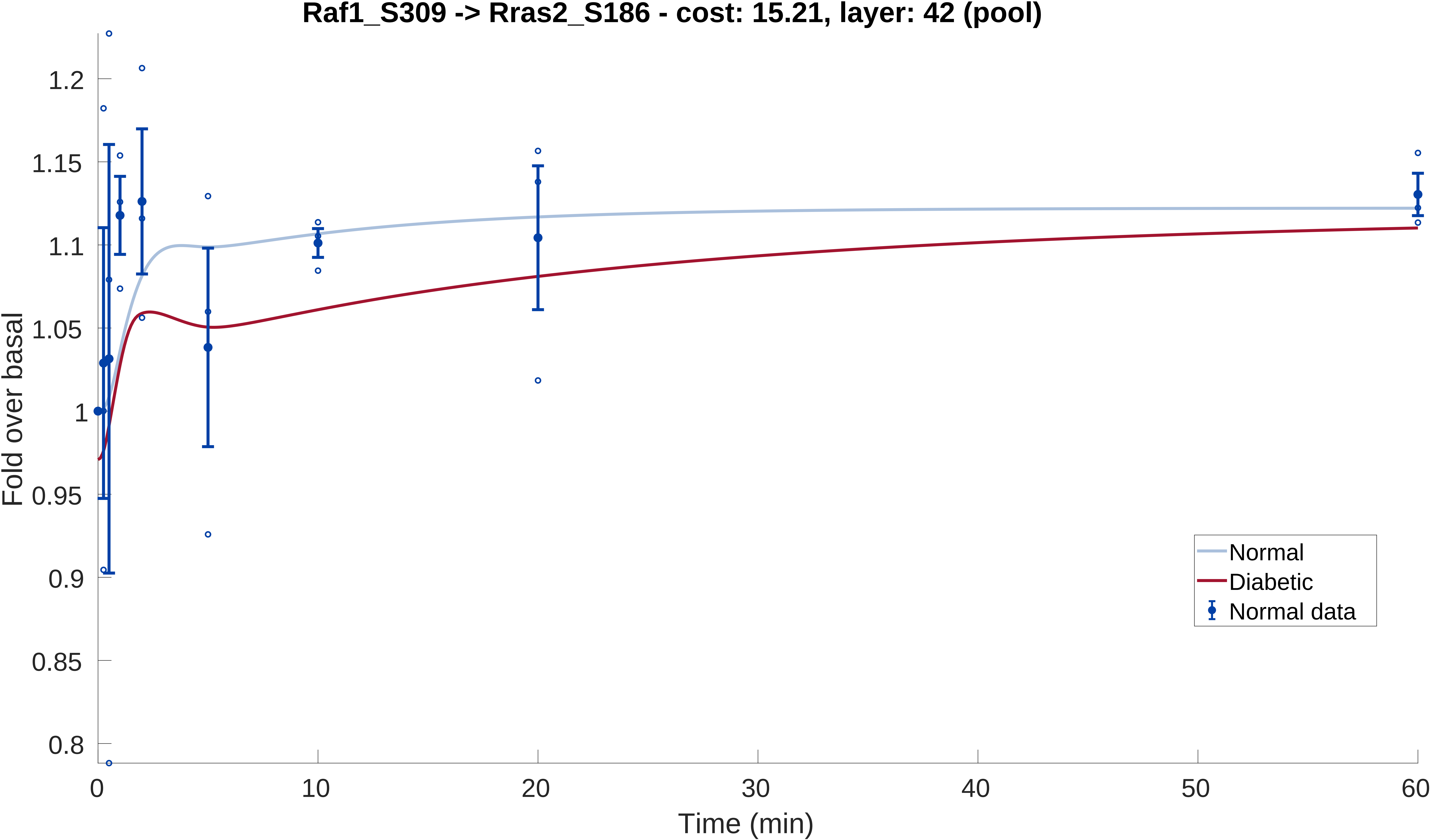
Raf1_S309 -> Rras2_S186 - cost: 15.21, layer: 42 (pool)
Fold over basal
1.2
1.15
1.1
1.05
1
0.95
0.9
0.85
0.8
0
10
20
30
40
50
60
Time (min)
Normal
Diabetic
Normal data

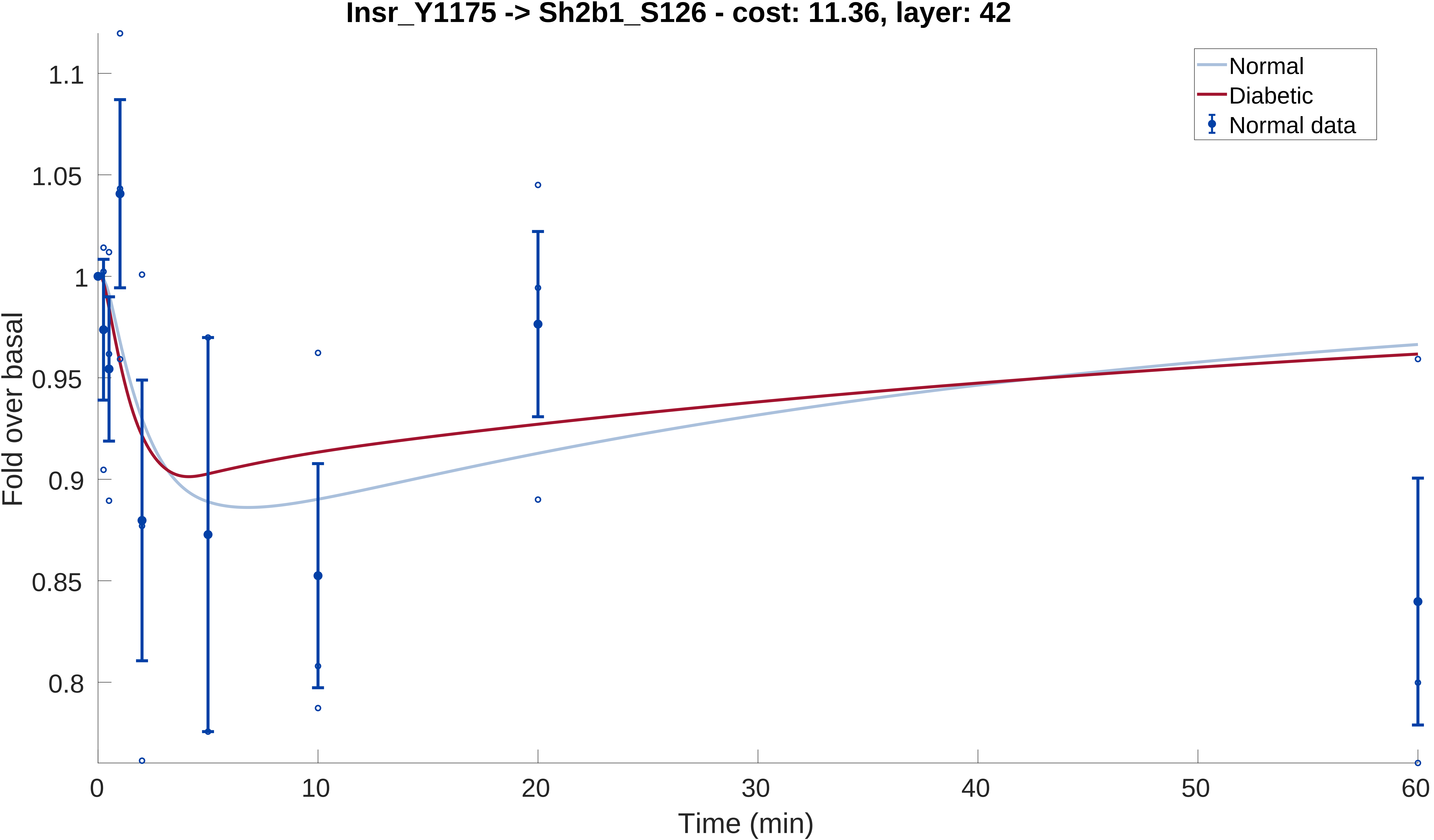

Insr_Y1175 -> Sh2b1_S126 - cost: 11.36, layer: 42
Normal
Diabetic
Normal data
Fold over basal
1.1
1.05
1
0.95
0.9
0.85
0.8
0
10
20
30
40
50
60
Time (min)

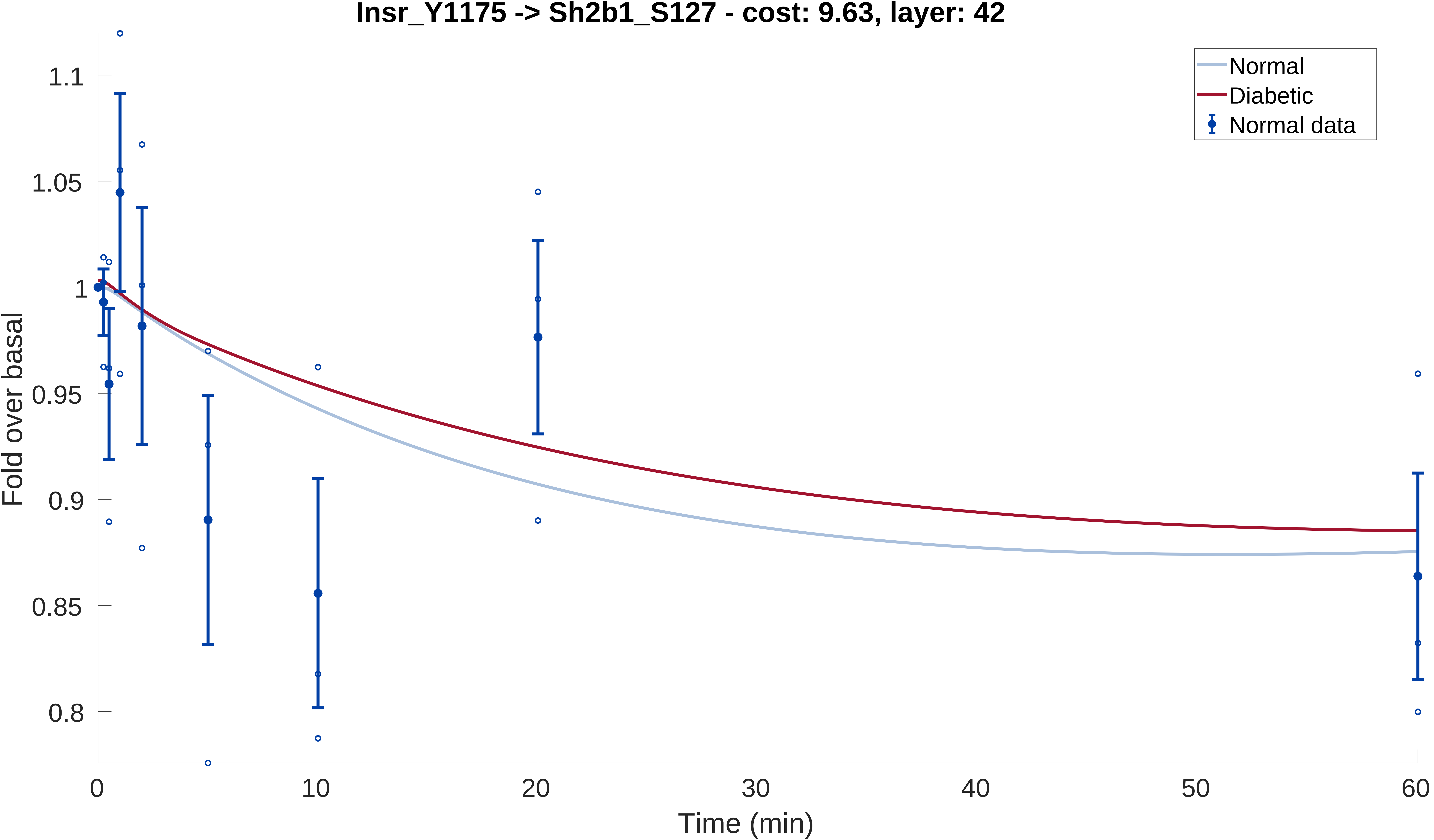
Insr_Y1175 -> Sh2b1_S127 - cost: 9.63, layer: 42
Normal
Diabetic
Normal data
Fold over basal
1.1
1.05
1
0.95
0.9
0.85
0.8
0
10
20
30
40
50
60
Time (min)

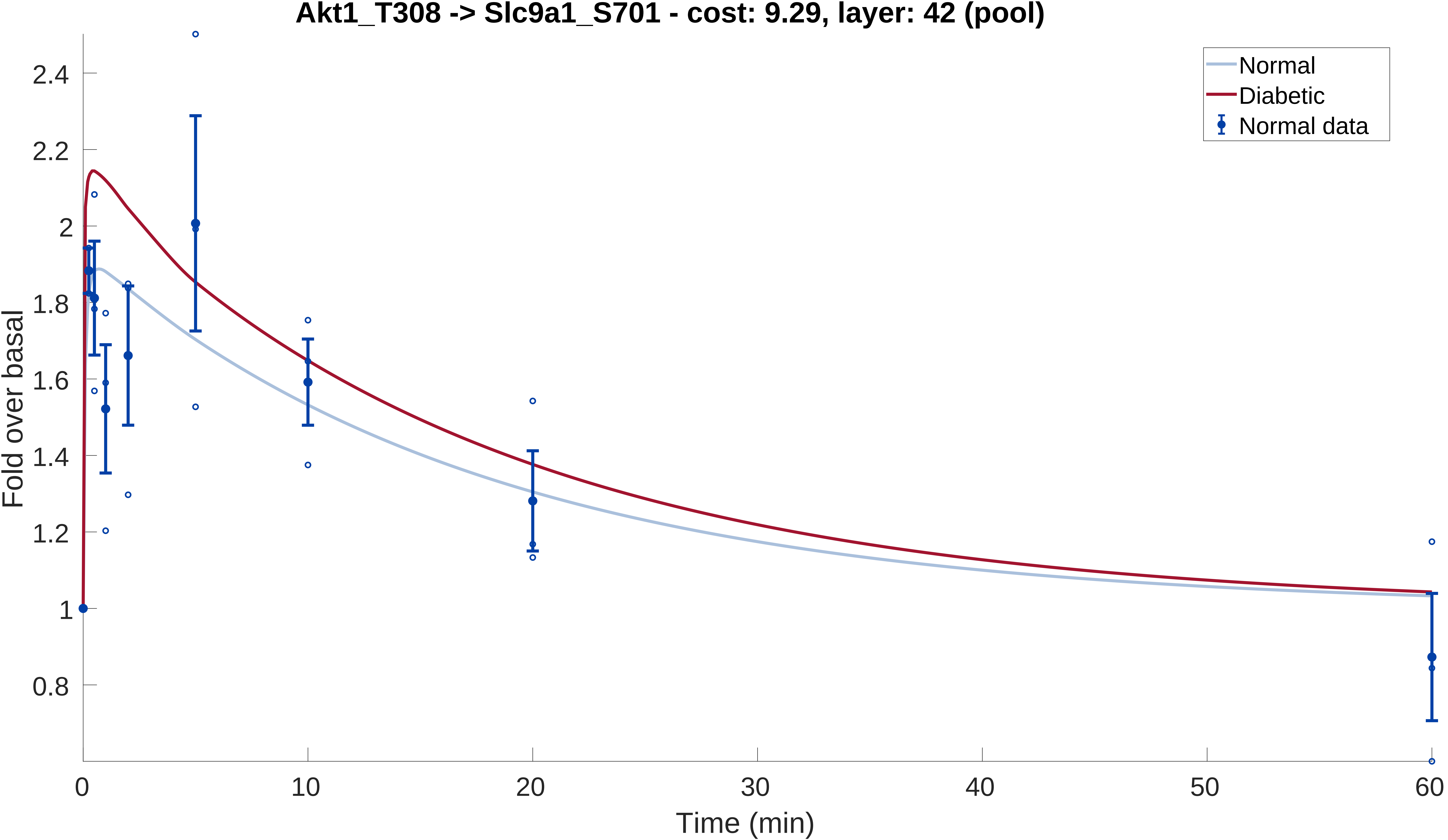

Akt1_T308 -> Slc9a1_S701 - cost: 9.29, layer: 42 (pool)
Normal
Diabetic
Normal data
Fold over basal
Time (min)
2.4
2.2
2
1.8
1.6
1.4
1.2
1
0.8
0
10
20
30
40
50
60

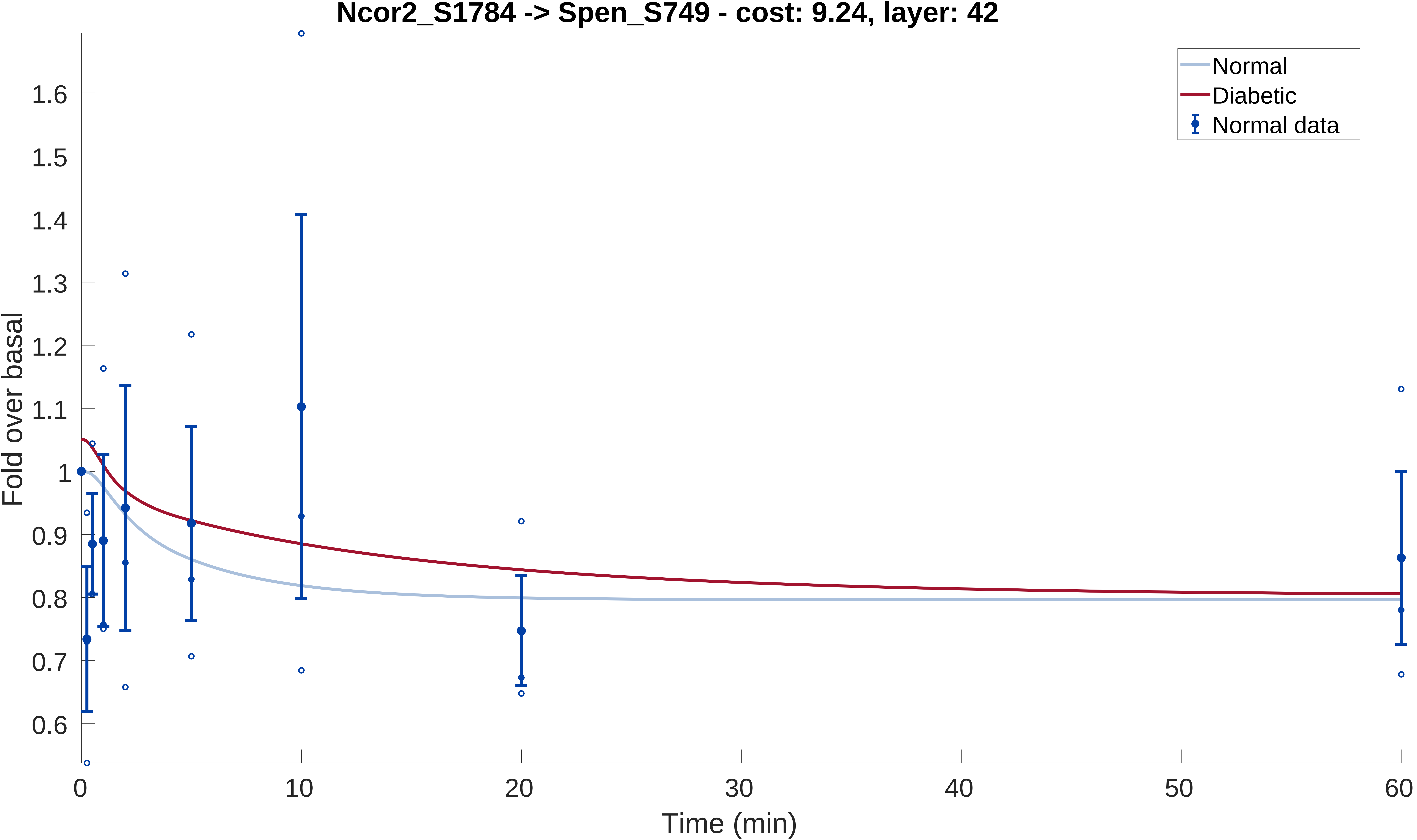
Ncor2_S1784 -> Spen_S749 - cost: 9.24, layer: 42
Normal
Diabetic
Normal data
Fold over basal
1.6
1.5
1.4
1.3
1.2
1.1
1
0.9
0.8
0.7
0.6
0
10
20
30
40
50
60
Time (min)

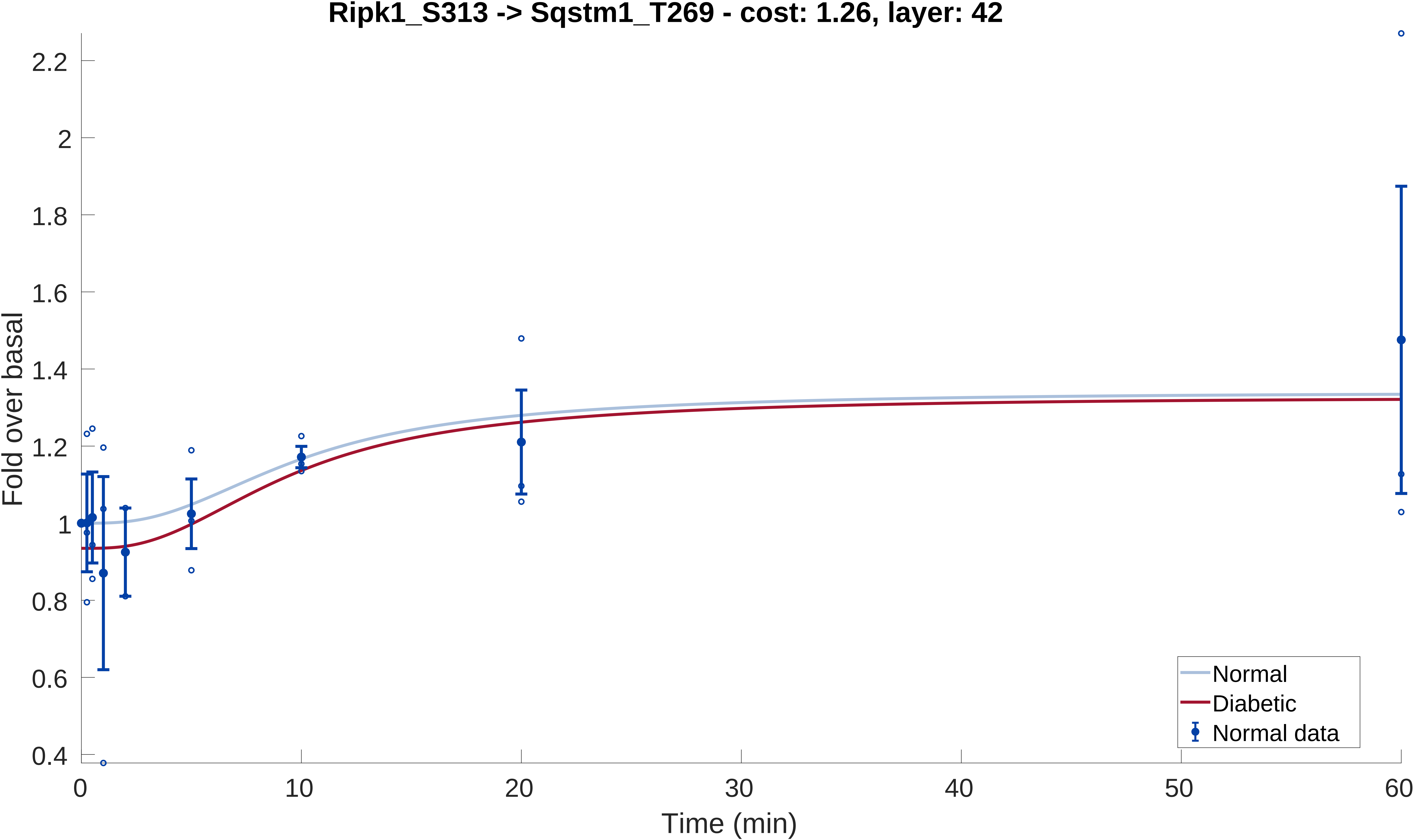

Ripk1_S313 -> Sqstm1_T269 - cost: 1.26, layer: 42
Fold over basal
Time (min)
0.4
0.6
0.8
1
1.2
1.4
1.6
1.8
2
2.2
0
10
20
30
40
50
60
Normal
Diabetic
Normal data

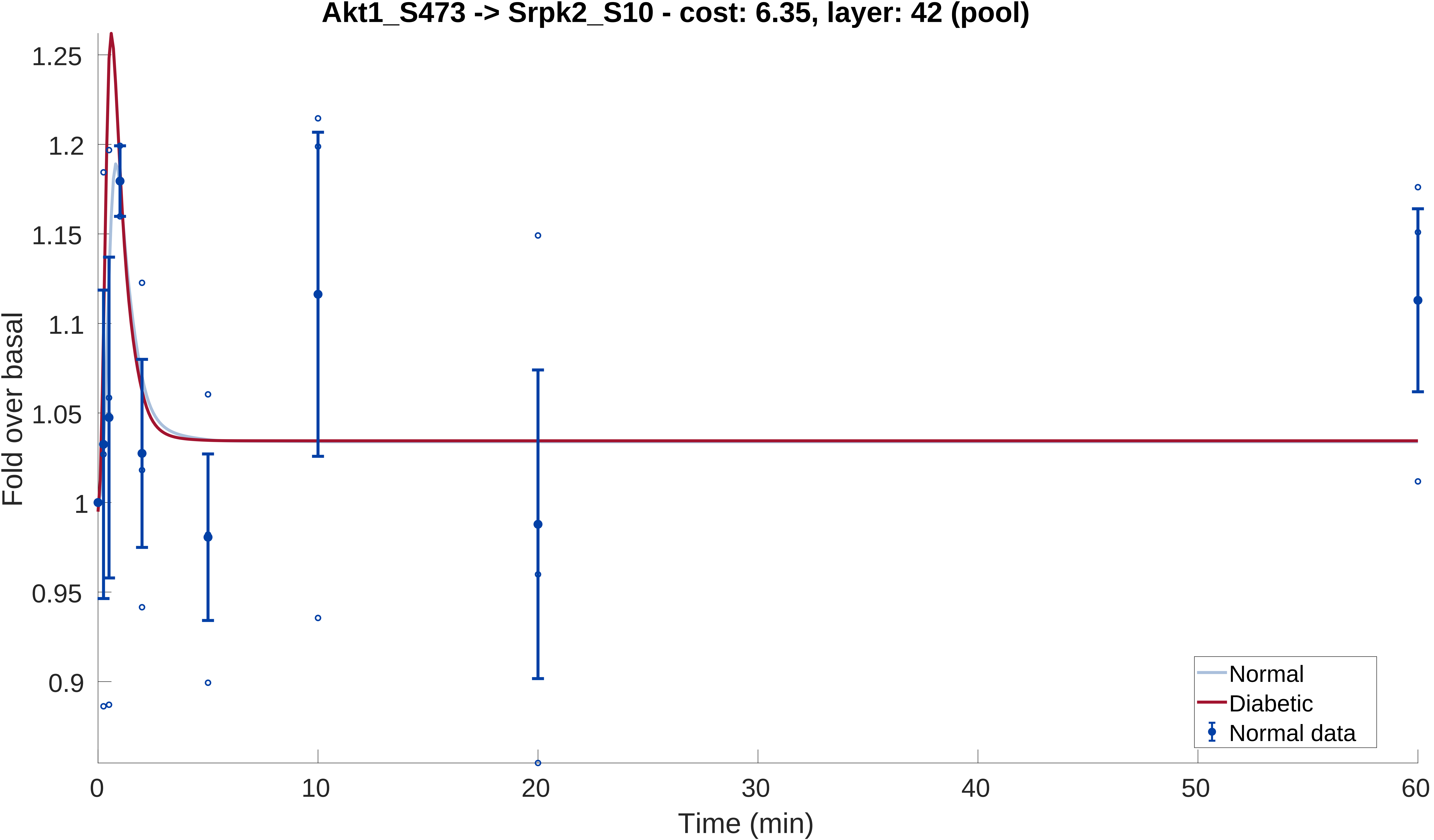
Akt1_S473 -> Srpk2_S10 - cost: 6.35, layer: 42 (pool)
Fold over basal
1.25
1.2
1.15
1.1
1.05
1
0.95
0.9
0
10
20
30
40
50
60
Time (min)
Normal
Diabetic
Normal data

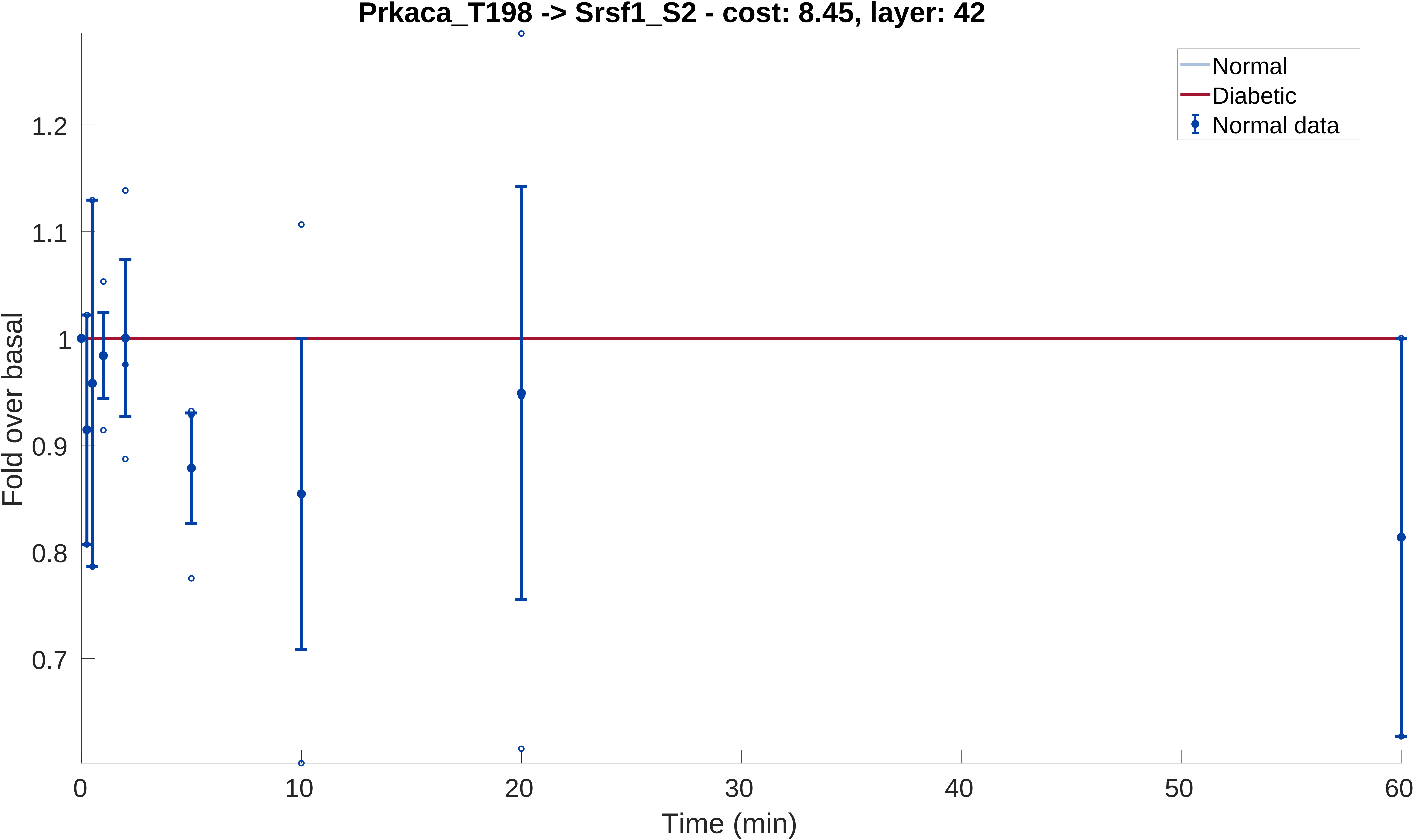
Prkaca_T198 -> Srsf1_S2 - cost: 8.45, layer: 42
Normal
Diabetic
Normal data
Fold over basal
1.2
1.1
1
0.9
0.8
0.7
0
10
20
30
40
50
60
Time (min)

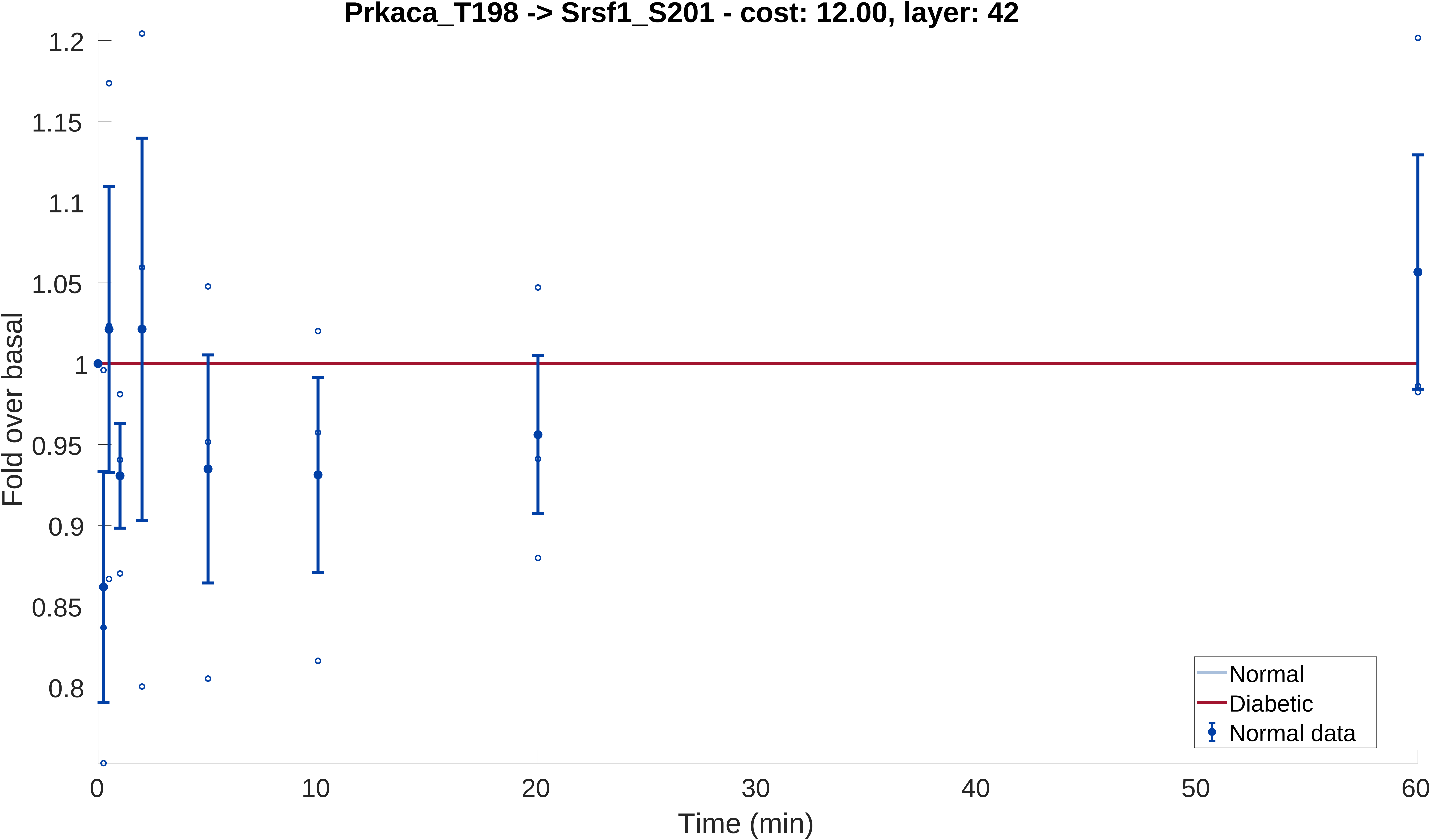

Prkaca_T198 -> Srsf1_S201 - cost: 12.00, layer: 42
Fold over basal
1.2
1.15
1.1
1.05
1
0.95
0.9
0.85
0.8
0
10
20
30
40
50
60
Time (min)
Normal
Diabetic
Normal data

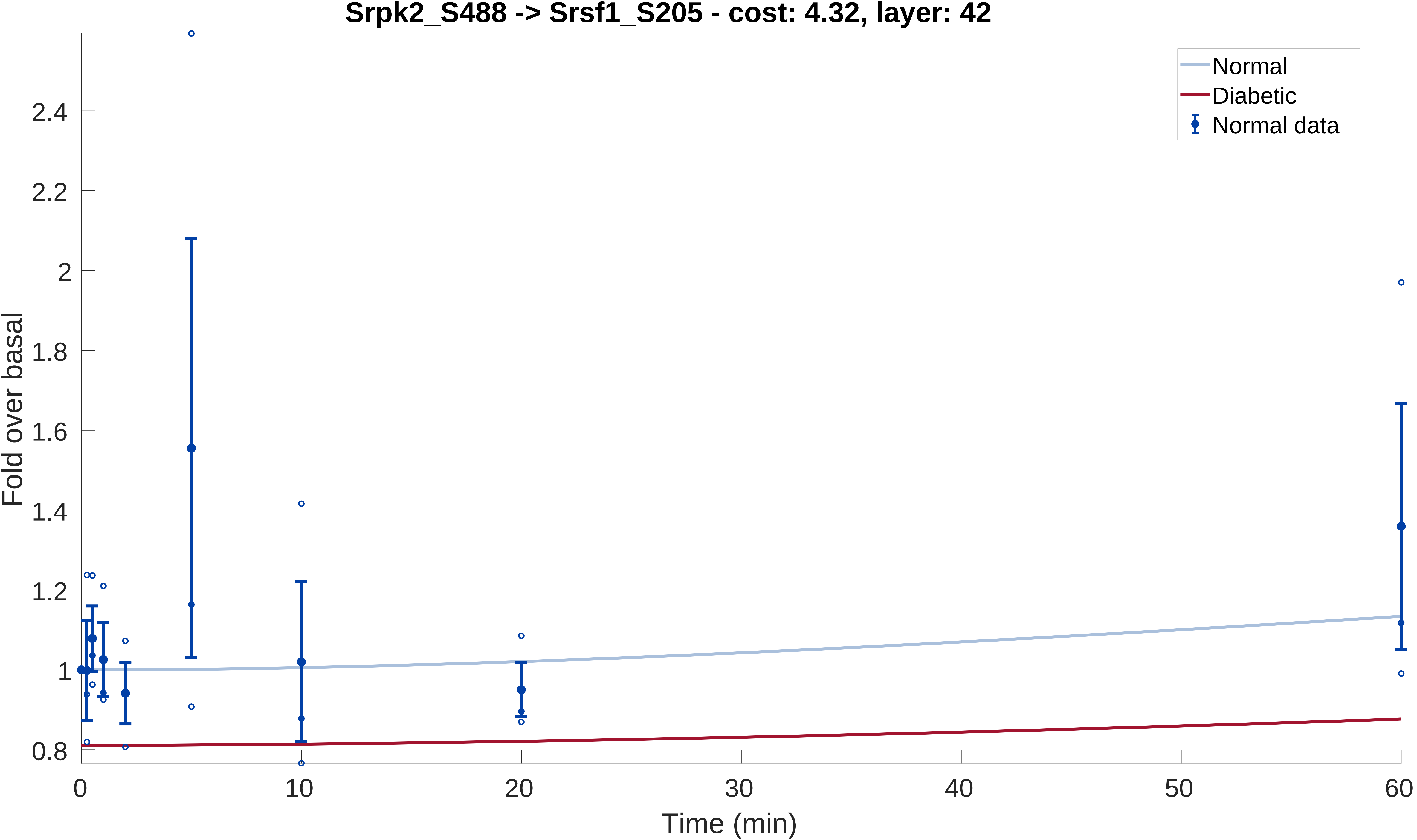
Srpk2_S488 -> Srsf1_S205 - cost: 4.32, layer: 42
Normal
Diabetic
Normal data
Fold over basal
2.4
2.2
2
1.8
1.6
1.4
1.2
1
0.8
0
10
20
30
40
50
60
Time (min)

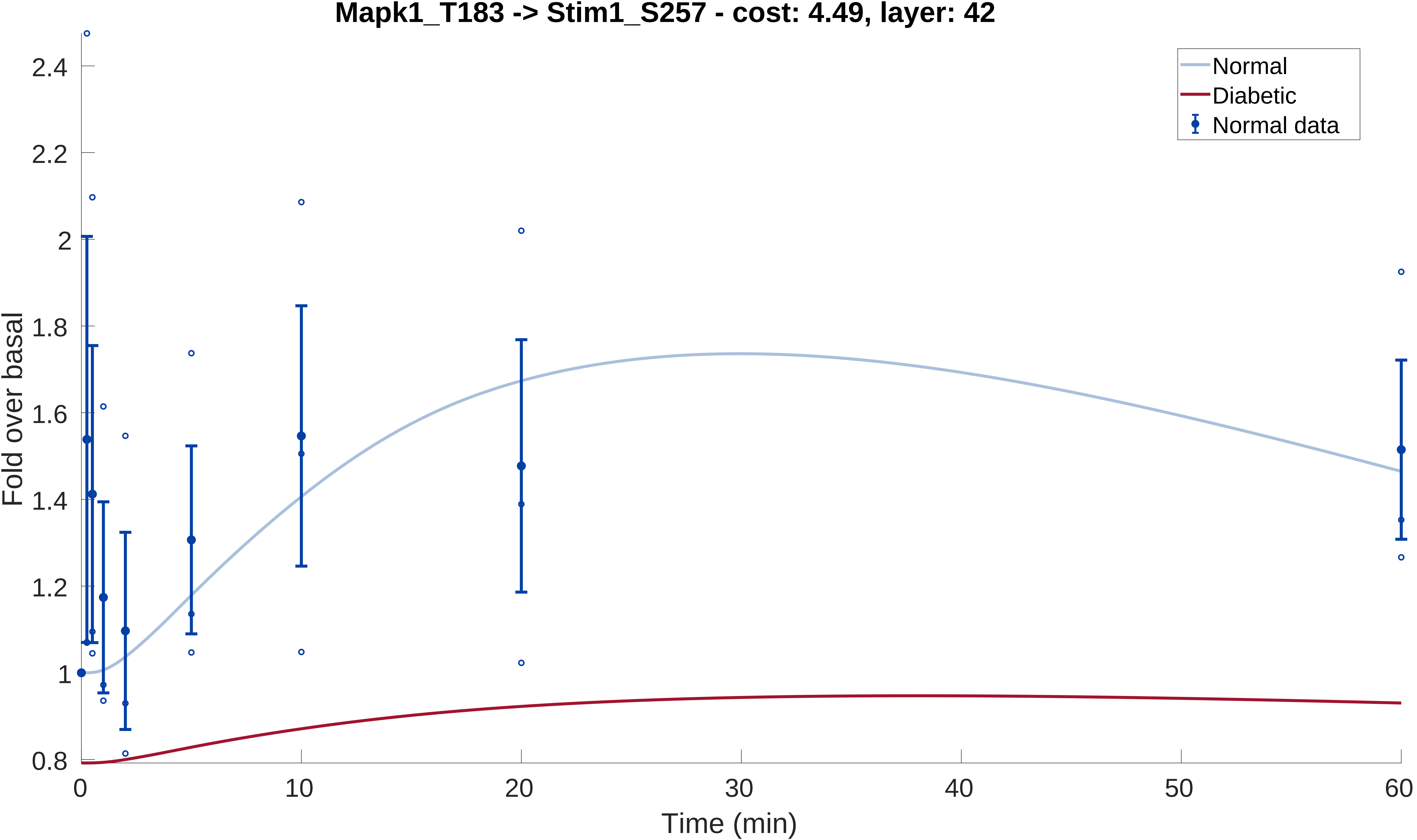

Mapk1_T183 -> Stim1_S257 - cost: 4.49, layer: 42
Normal
Diabetic
Normal data
Fold over basal
Time (min)
0.8
1
1.2
1.4
1.6
1.8
2
2.2
2.4
0
10
20
30
40
50
60

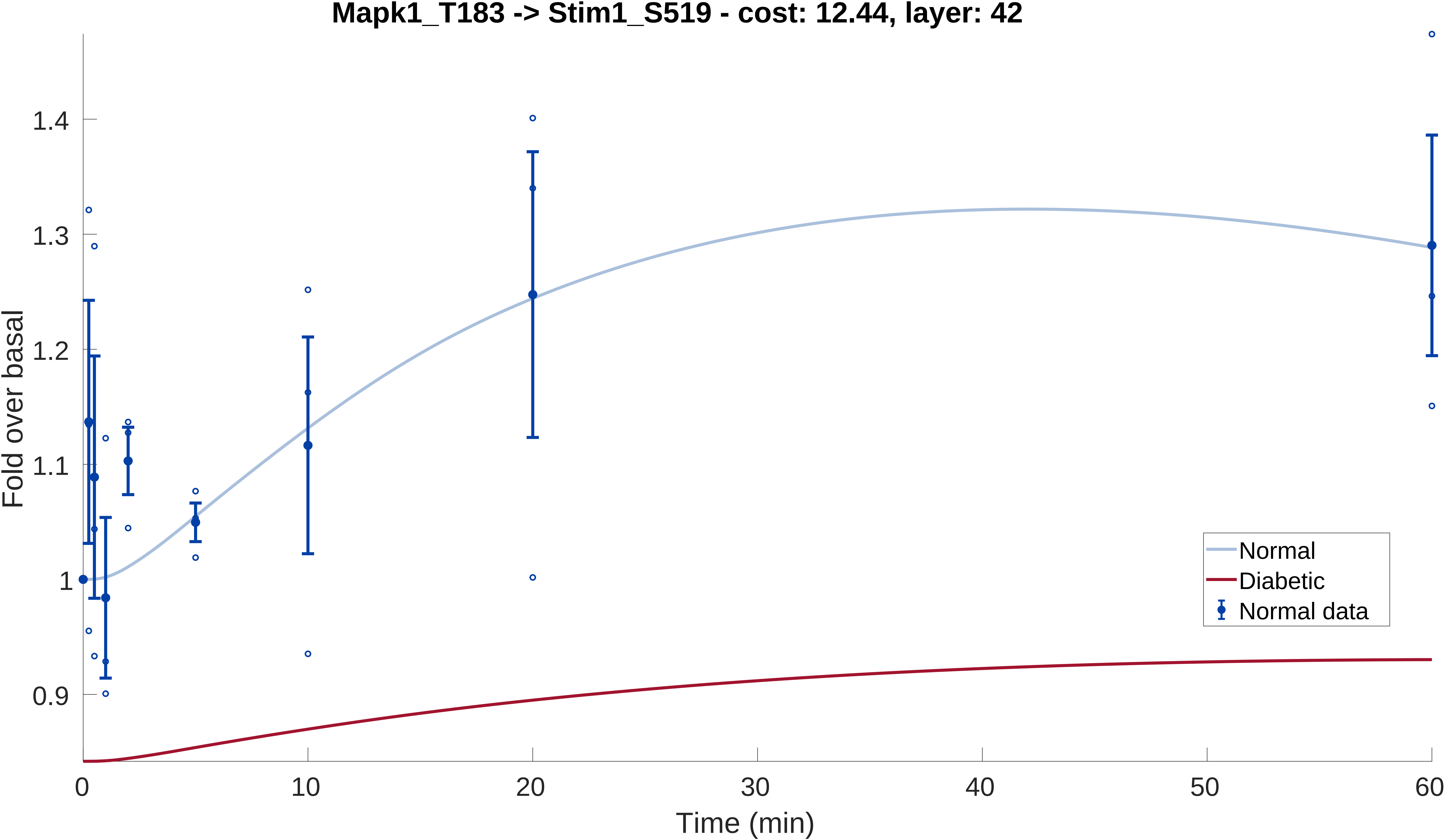

Mapk1_T183 -> Stim1_S519 - cost: 12.44, layer: 42
Fold over basal
Time (min)
0
10
20
30
40
50
60
0.9
1
1.1
1.2
1.3
1.4
Normal
Diabetic
Normal data

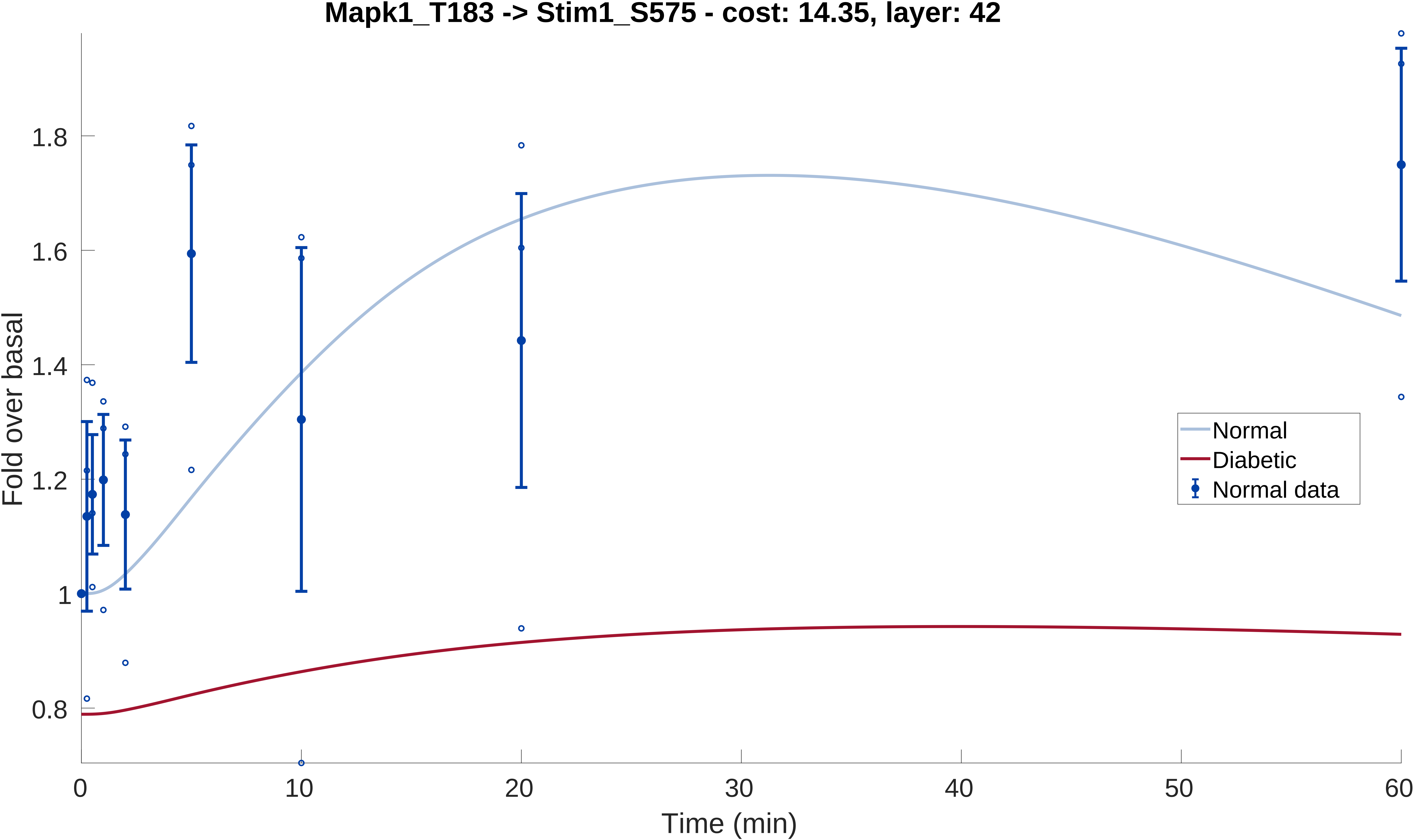
Mapk1_T183 -> Stim1_S575 - cost: 14.35, layer: 42
Fold over basal
1.8
1.6
1.4
1.2
1
0.8
0
10
20
30
40
50
60
Time (min)
Normal
Diabetic
Normal data

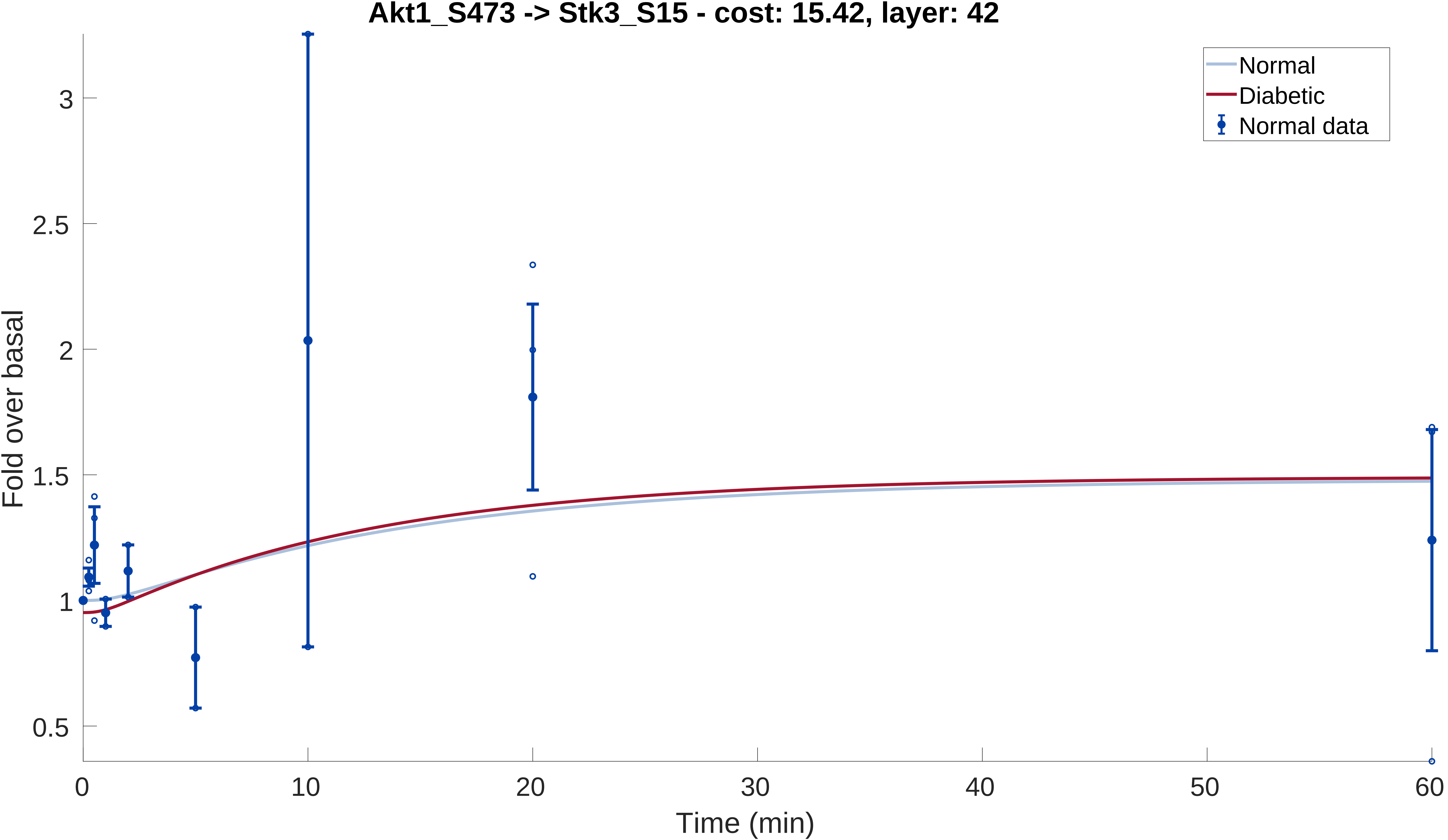

Akt1_S473 -> Stk3_S15 - cost: 15.42, layer: 42
Normal
Diabetic
Normal data
Fold over basal
3
2.5
2
1.5
1
0.5
0
10
20
30
40
50
60
Time (min)

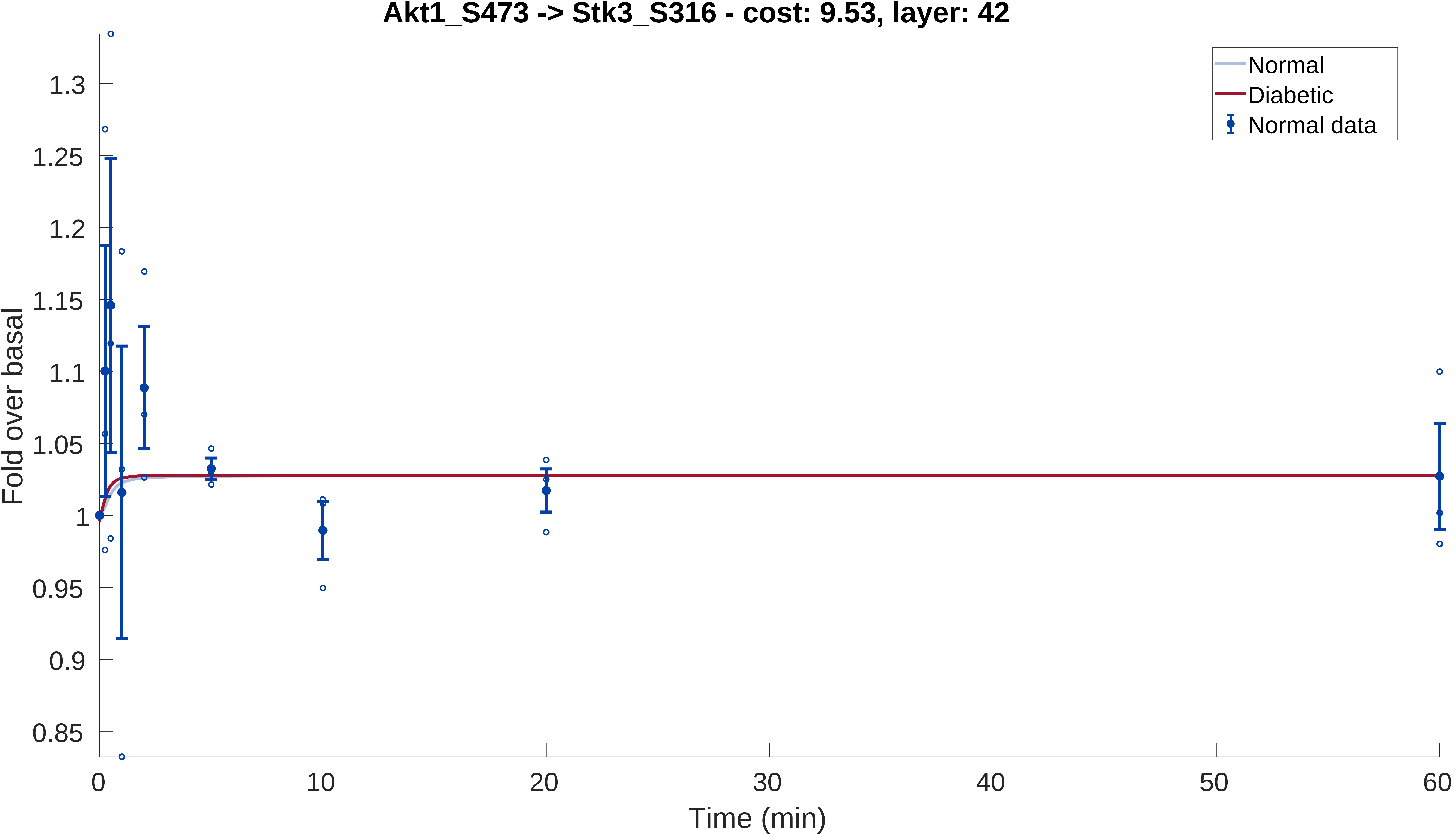
Akt1_S473 -> Stk3_S316 - cost: 9.53, layer: 42
Normal
Diabetic
Normal data
Fold over basal
1.3
1.25
1.2
1.15
1.1
1.05
1
0.95
0.9
0.85
0
10
20
30
40
50
60
Time (min)

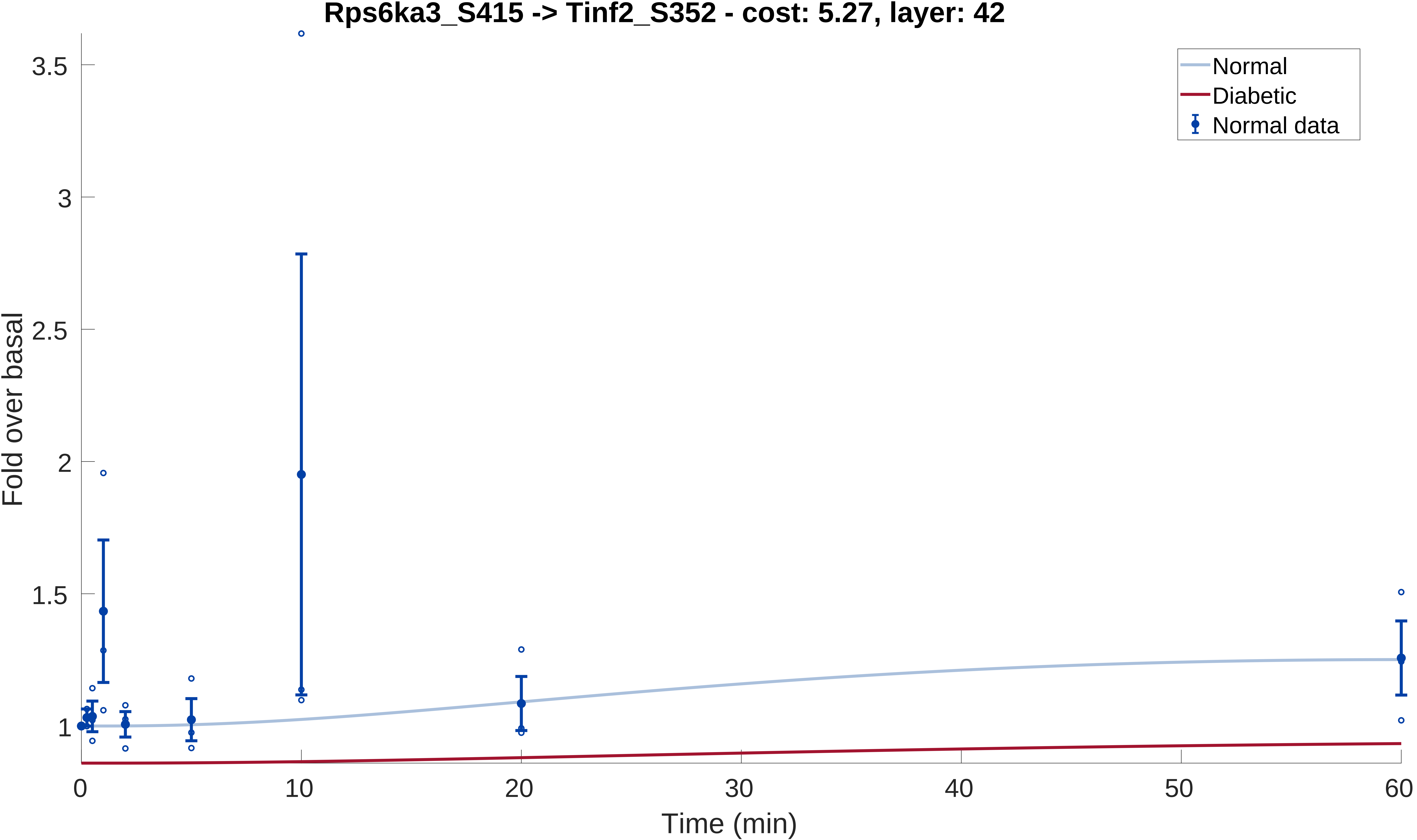

Rps6ka3_S415 -> Tinf2_S352 - cost: 5.27, layer: 42
Normal
Diabetic
Normal data
Fold over basal
3.5
3
2.5
2
1.5
1
0
10
20
30
40
50
60
Time (min)

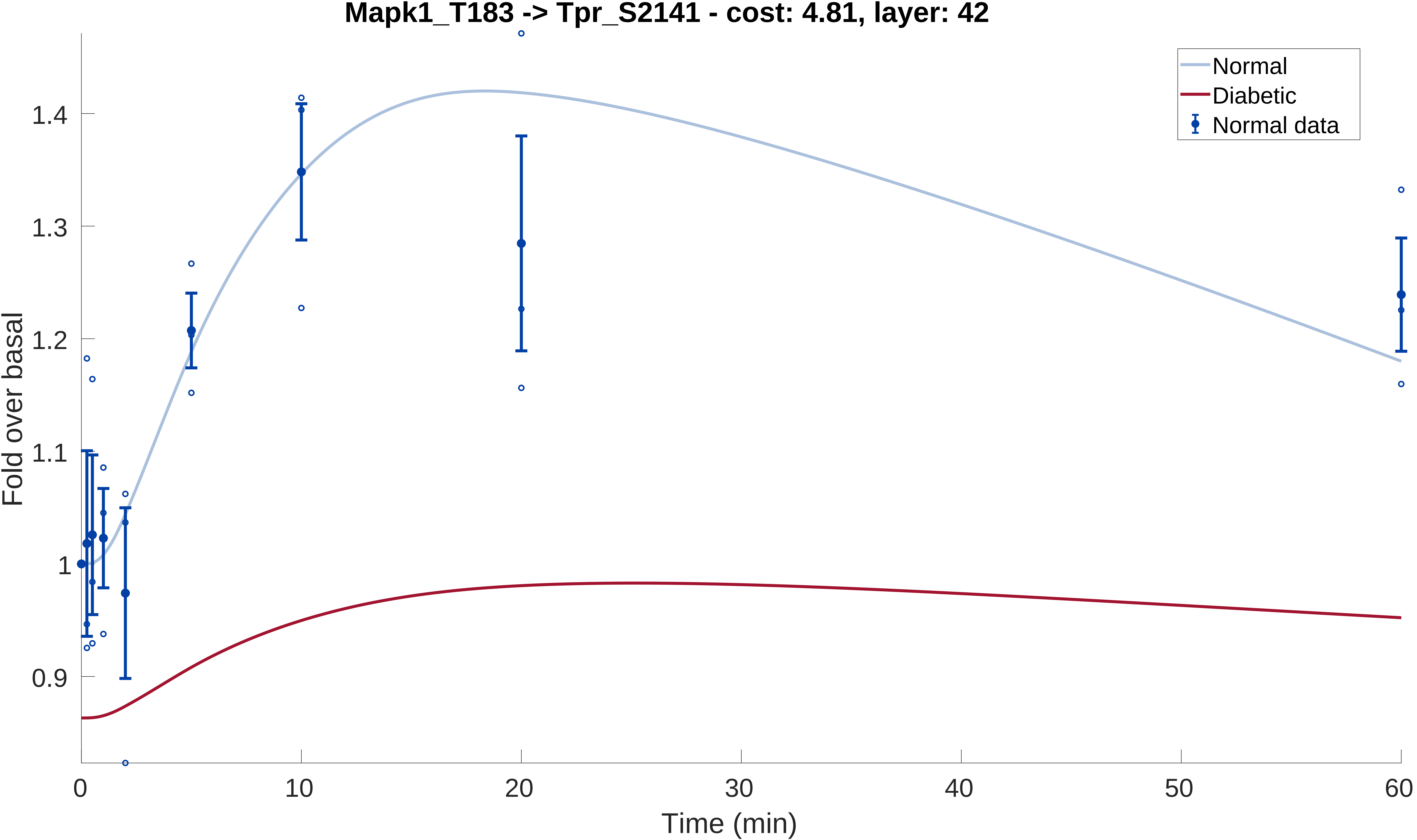

Mapk1_T183 -> Tpr_S2141 - cost: 4.81, layer: 42
Normal
Diabetic
Normal data
Fold over basal
Time (min)
0.9
1
1.1
1.2
1.3
1.4
0
10
20
30
40
50
60

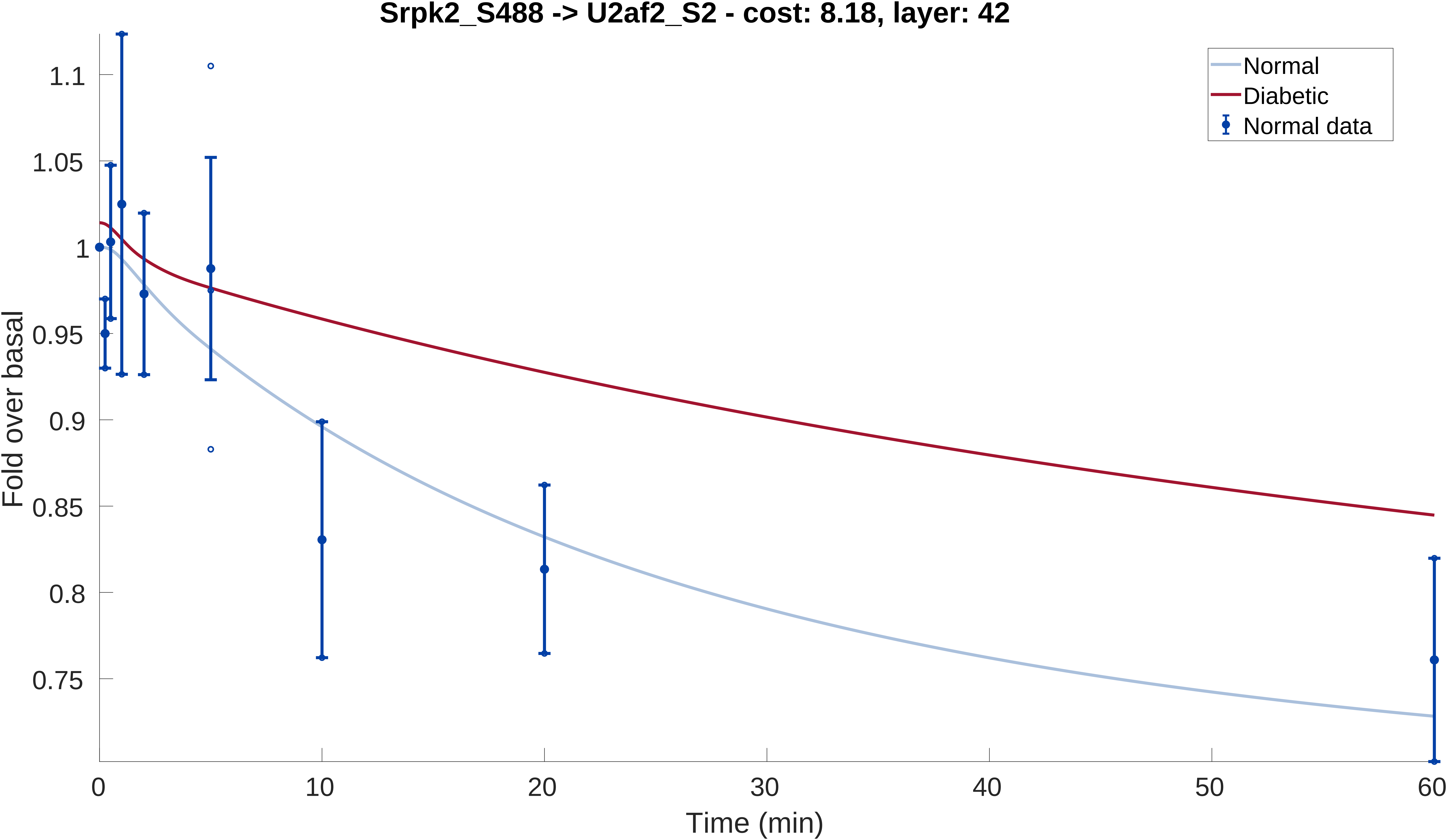

Srpk2_S488 -> U2af2_S2 - cost: 8.18, layer: 42
Normal
Diabetic
Normal data
Fold over basal
1.1
1.05
1
0.95
0.9
0.85
0.8
0.75
0
10
20
30
40
50
60
Time (min)

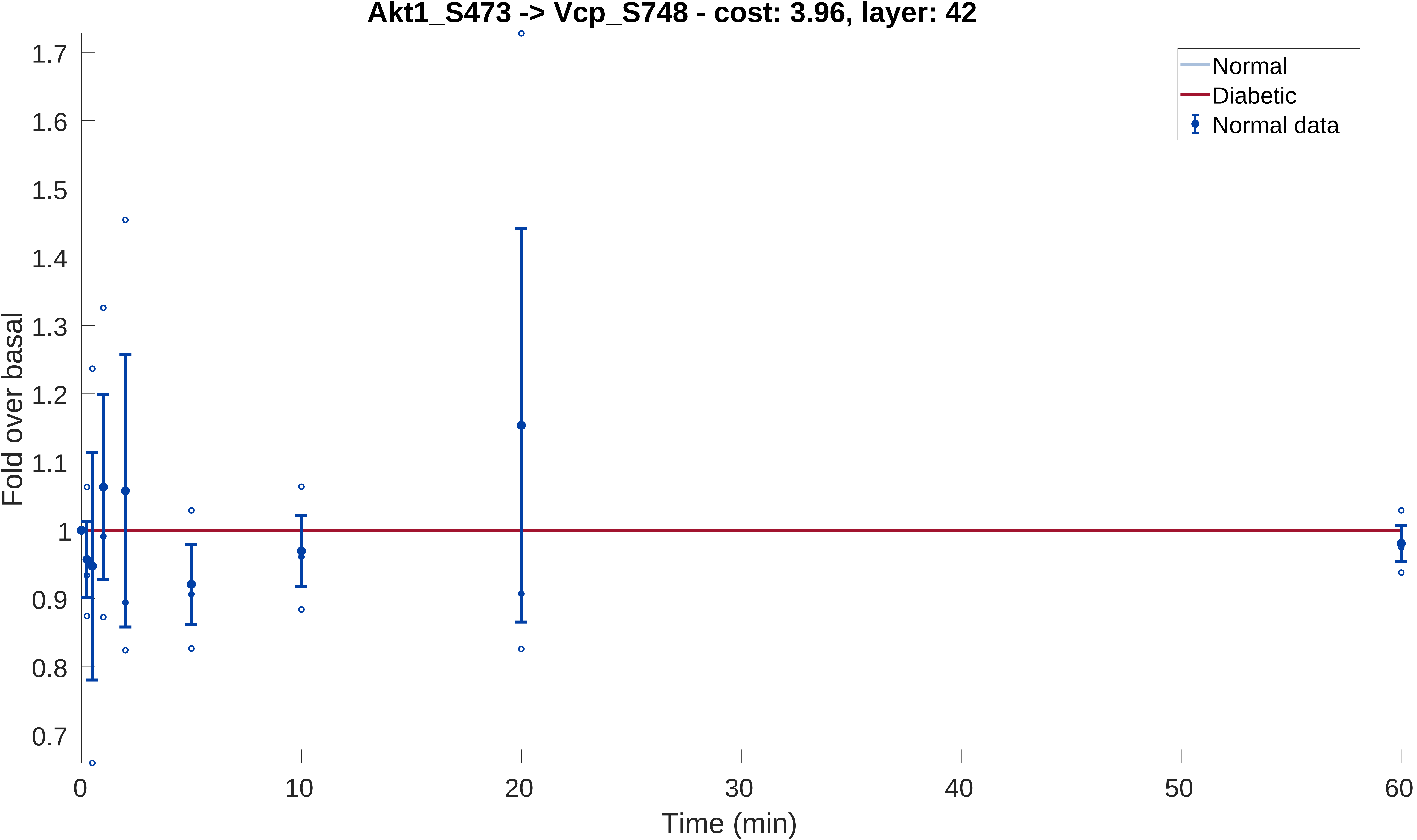

Akt1_S473 -> Vcp_S748 - cost: 3.96, layer: 42
Normal
Diabetic
Normal data
Fold over basal
Time (min)
1.7
1.6
1.5
1.4
1.3
1.2
1.1
1
0.9
0.8
0.7
0
10
20
30
40
50
60

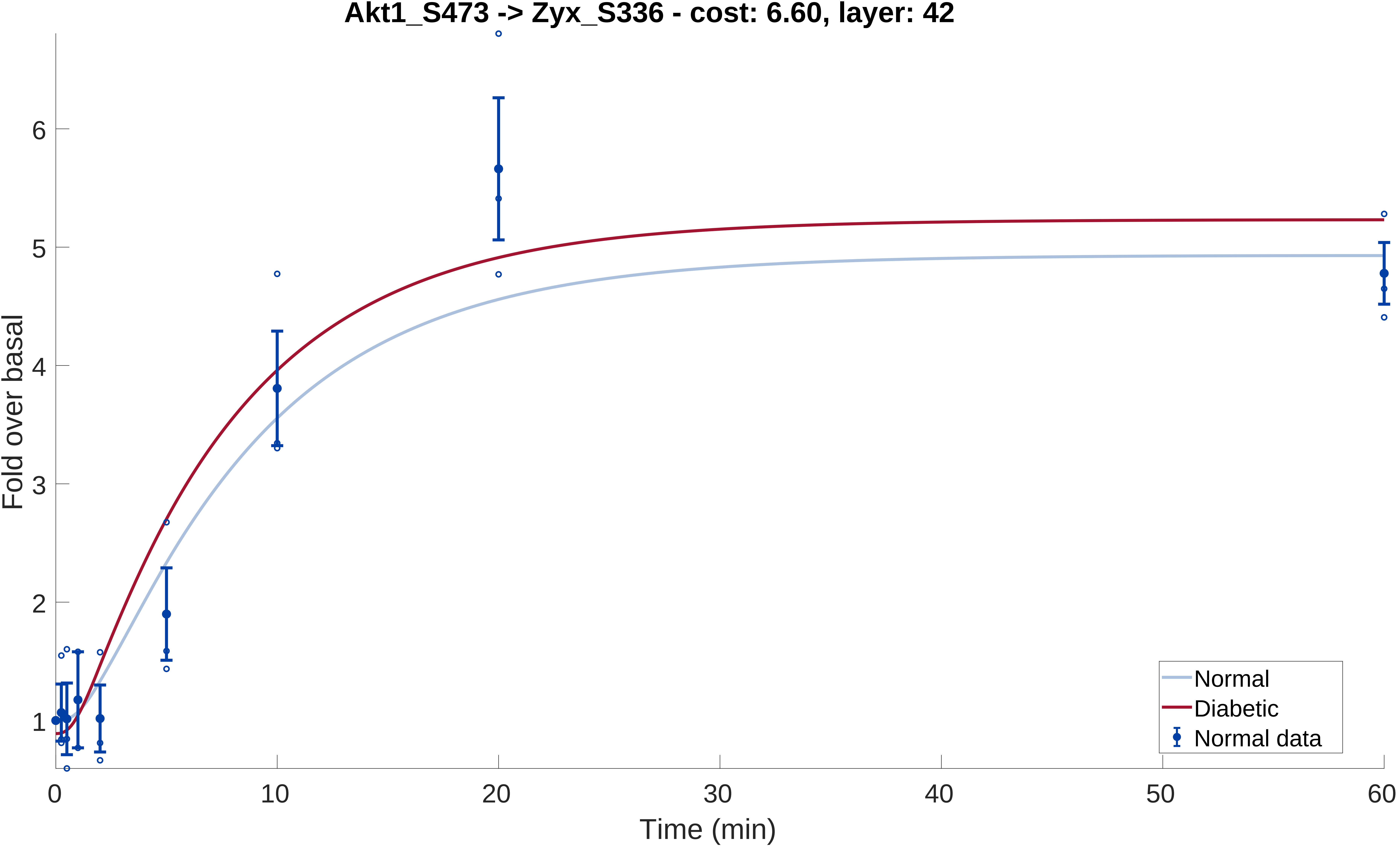

Akt1_S473 -> Zyx_S336 - cost: 6.60, layer: 42
Fold over basal
1
2
3
4
5
6
0
10
20
30
40
50
60
Time (min)
Normal
Diabetic
Normal data

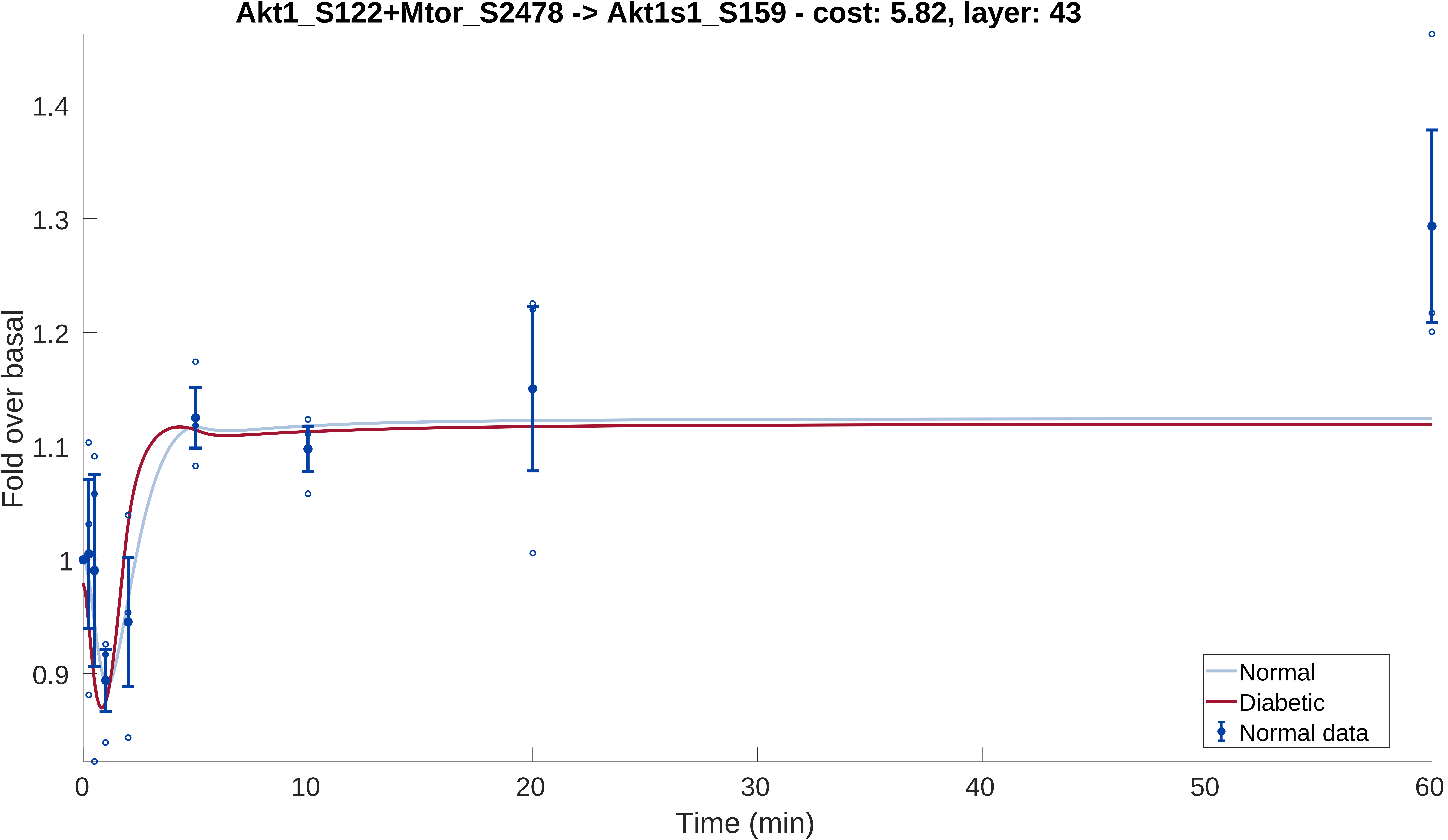

Akt1_S122+Mtor_S2478 -> Akt1s1_S159 - cost: 5.82, layer: 43
Fold over basal
1.4
1.3
1.2
1.1
1
0.9
0
10
20
30
40
50
60
Time (min)
Normal
Diabetic
Normal data

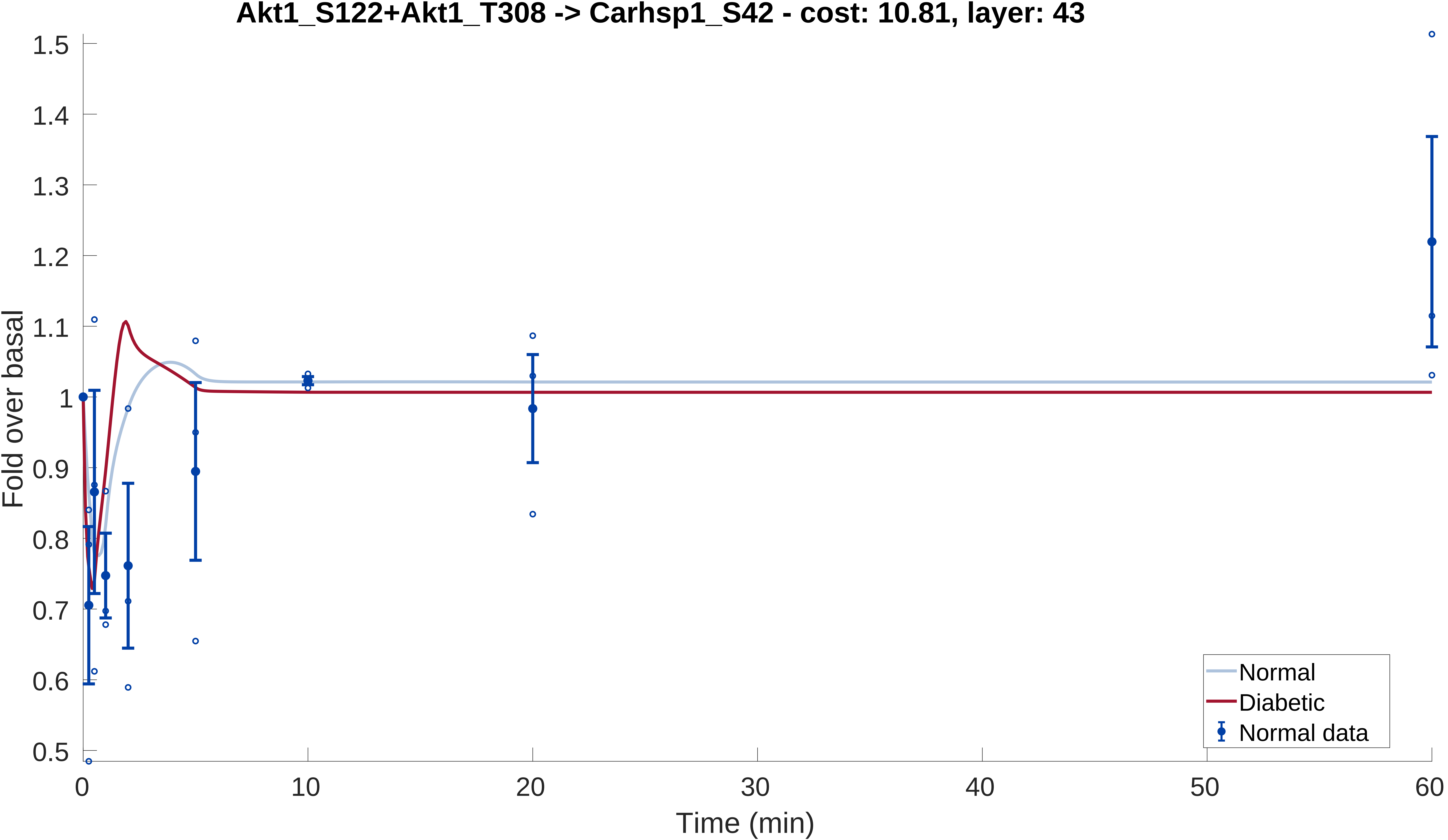
Akt1_S122+Akt1_T308 -> Carhsp1_S42 - cost: 10.81, layer: 43
Fold over basal
1.5
1.4
1.3
1.2
1.1
1
0.9
0.8
0.7
0.6
0.5
0
10
20
30
40
50
60
Time (min)
Normal
Diabetic
Normal data

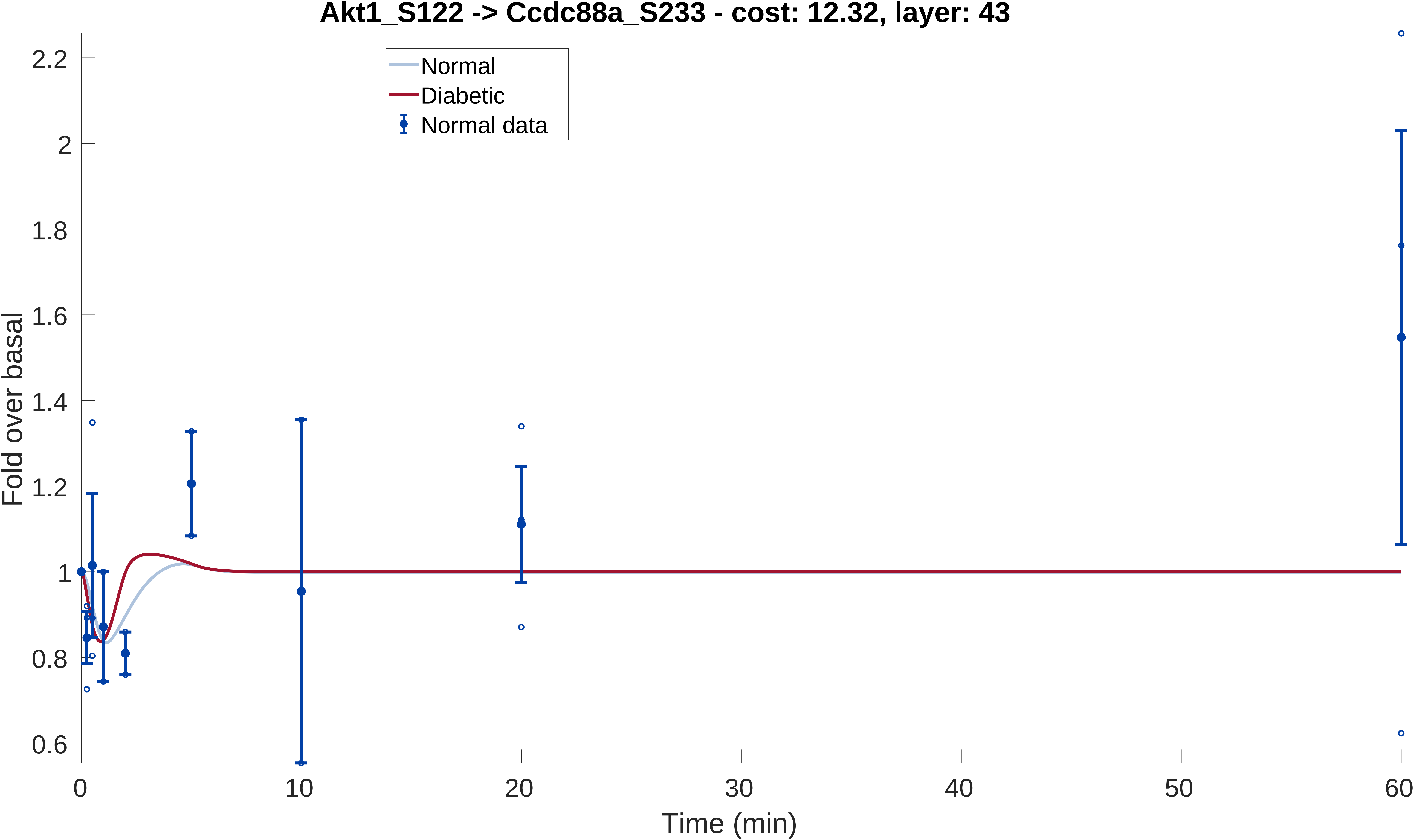
Akt1_S122 -> Ccdc88a_S233 - cost: 12.32, layer: 43
Normal
Diabetic
Normal data
Fold over basal
Time (min)
0.6
0.8
1
1.2
1.4
1.6
1.8
2
2.2
0
10
20
30
40
50
60

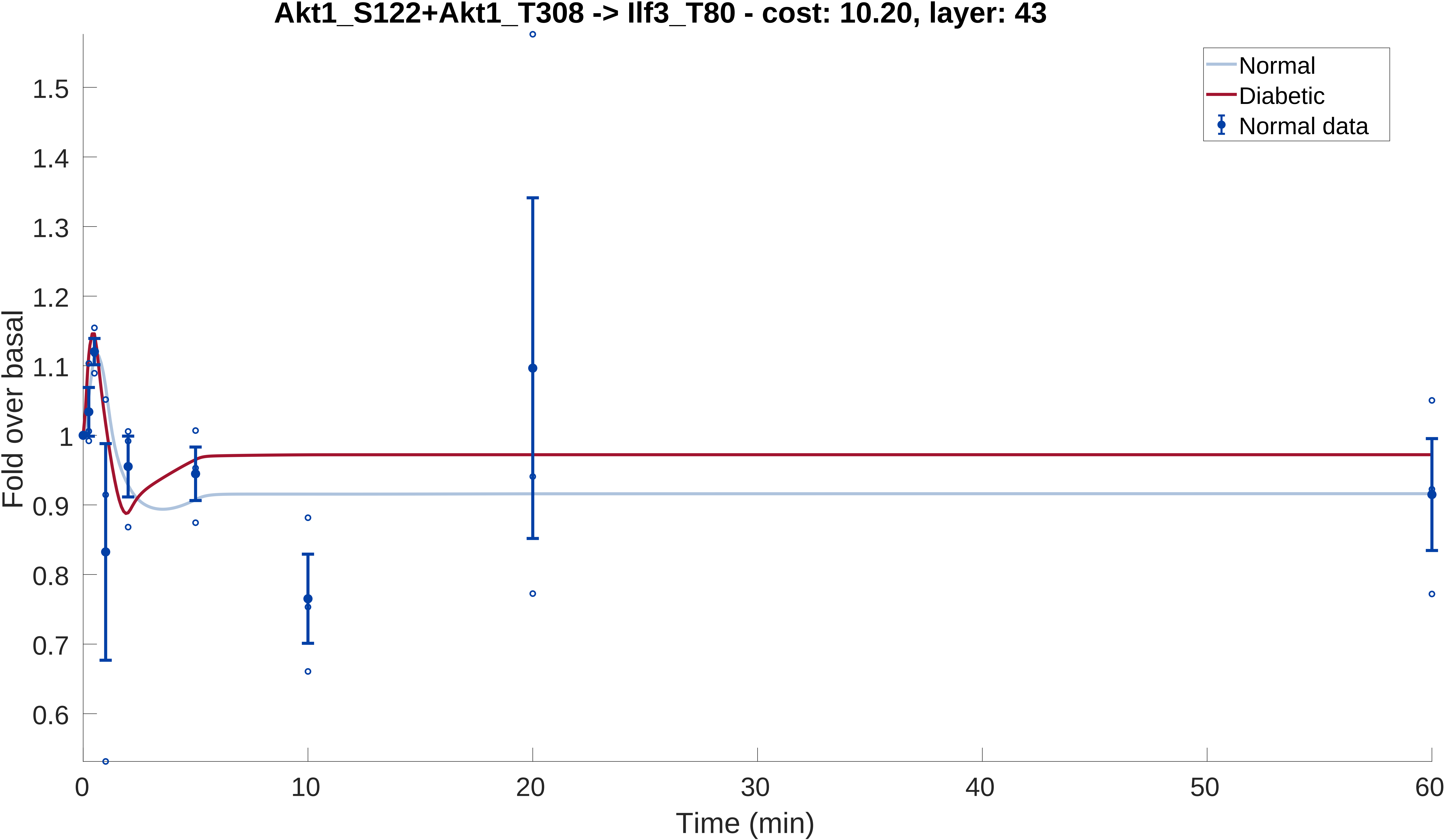

Akt1_S122+Akt1_T308 -> Ilf3_T80 - cost: 10.20, layer: 43
Normal
Diabetic
Normal data
Fold over basal
1.5
1.4
1.3
1.2
1.1
1
0.9
0.8
0.7
0.6
0
10
20
30
40
50
60
Time (min)

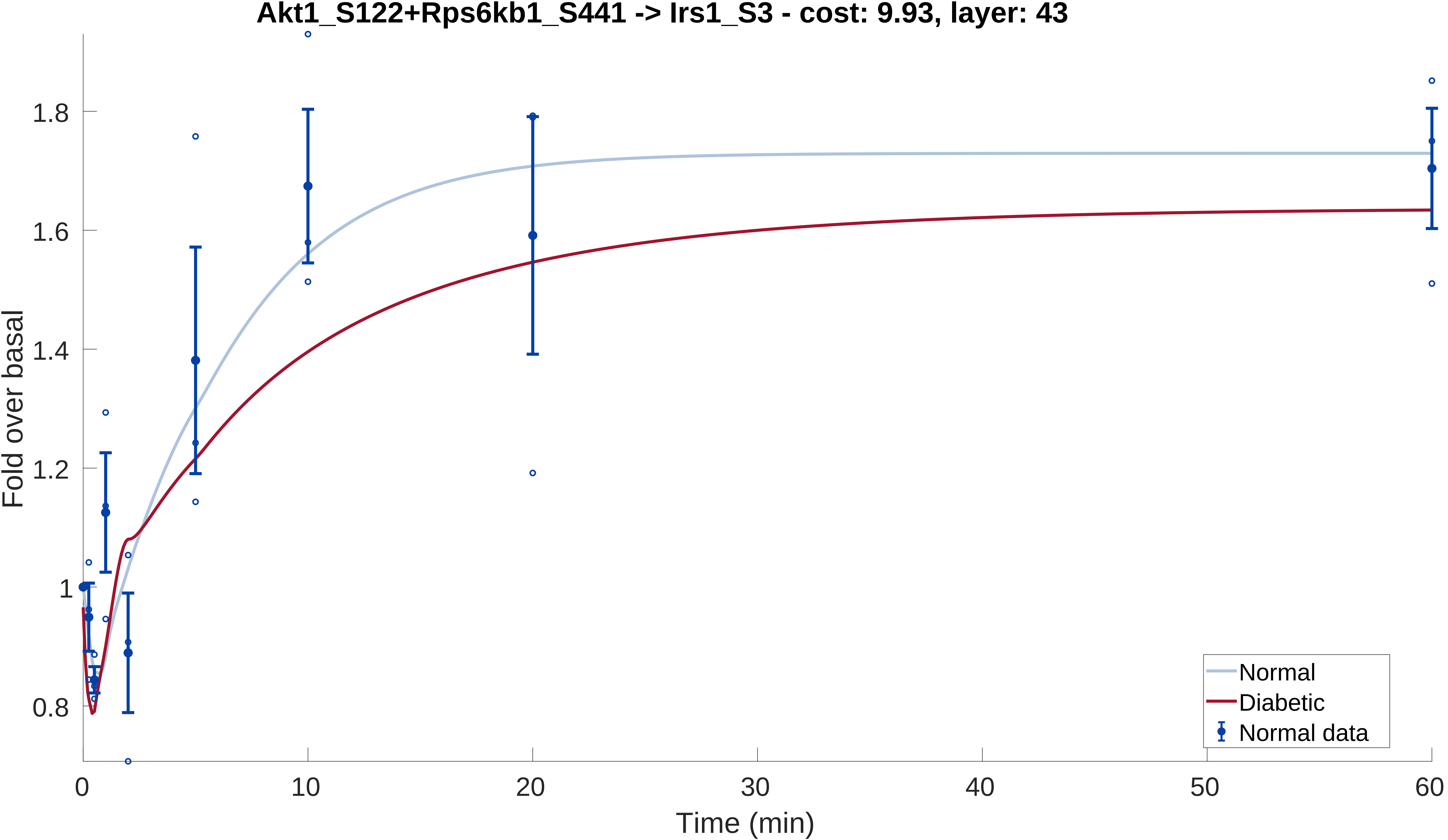
Akt1_S122+Rps6kb1_S441 -> Irs1_S3 - cost: 9.93, layer: 43
Fold over basal
Time (min)
0.8
1
1.2
1.4
1.6
1.8
0
10
20
30
40
50
60
Normal
Diabetic
Normal data

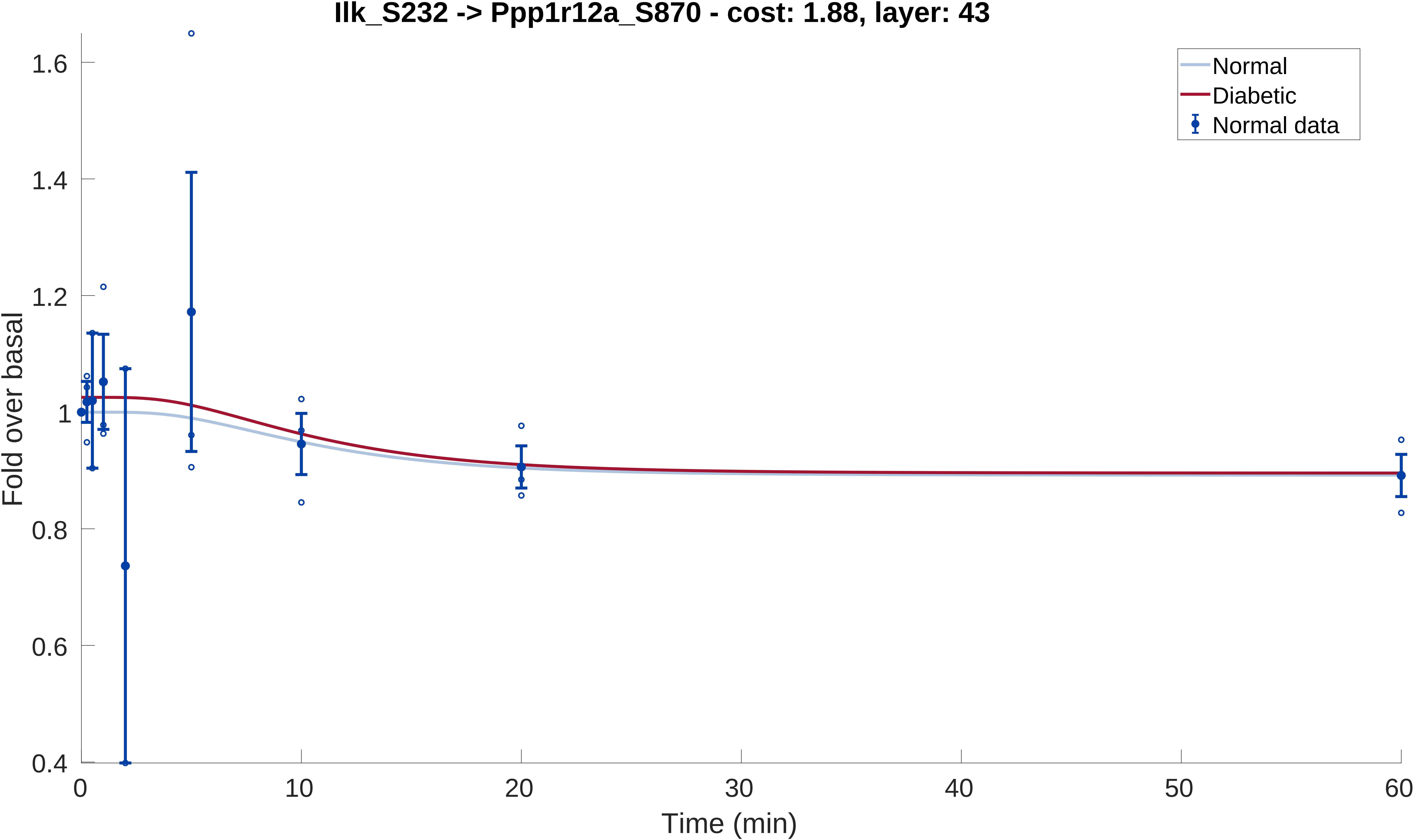
Ilk_S232 -> Ppp1r12a_S870 - cost: 1.88, layer: 43
Normal
Diabetic
Normal data
Fold over basal
1.6
1.4
1.2
1
0.8
0.6
0.4
0
10
20
30
40
50
60
Time (min)

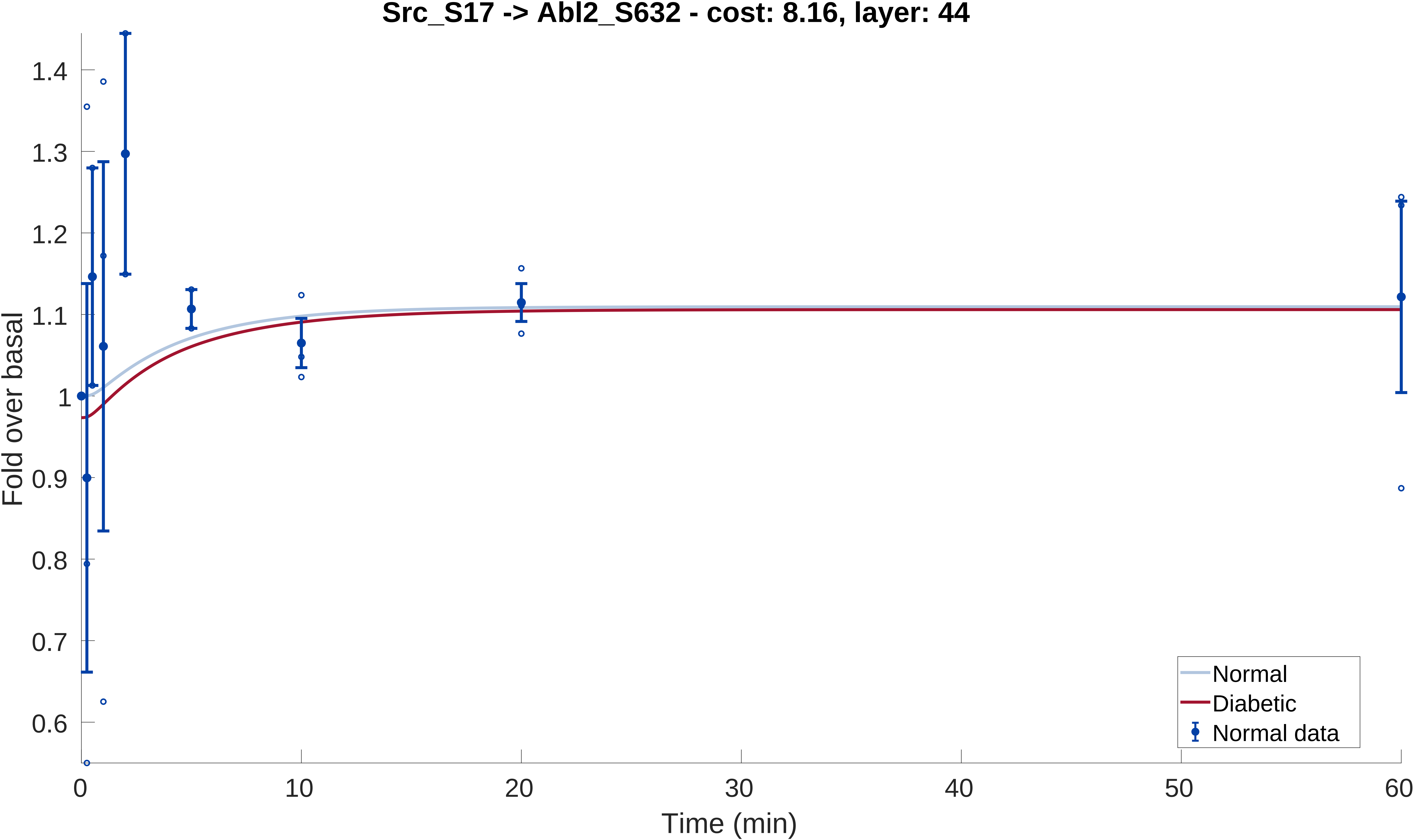

Src_S17 -> Abl2_S632 - cost: 8.16, layer: 44
Fold over basal
Time (min)
0.6
0.7
0.8
0.9
1
1.1
1.2
1.3
1.4
0
10
20
30
40
50
60
Normal
Diabetic
Normal data

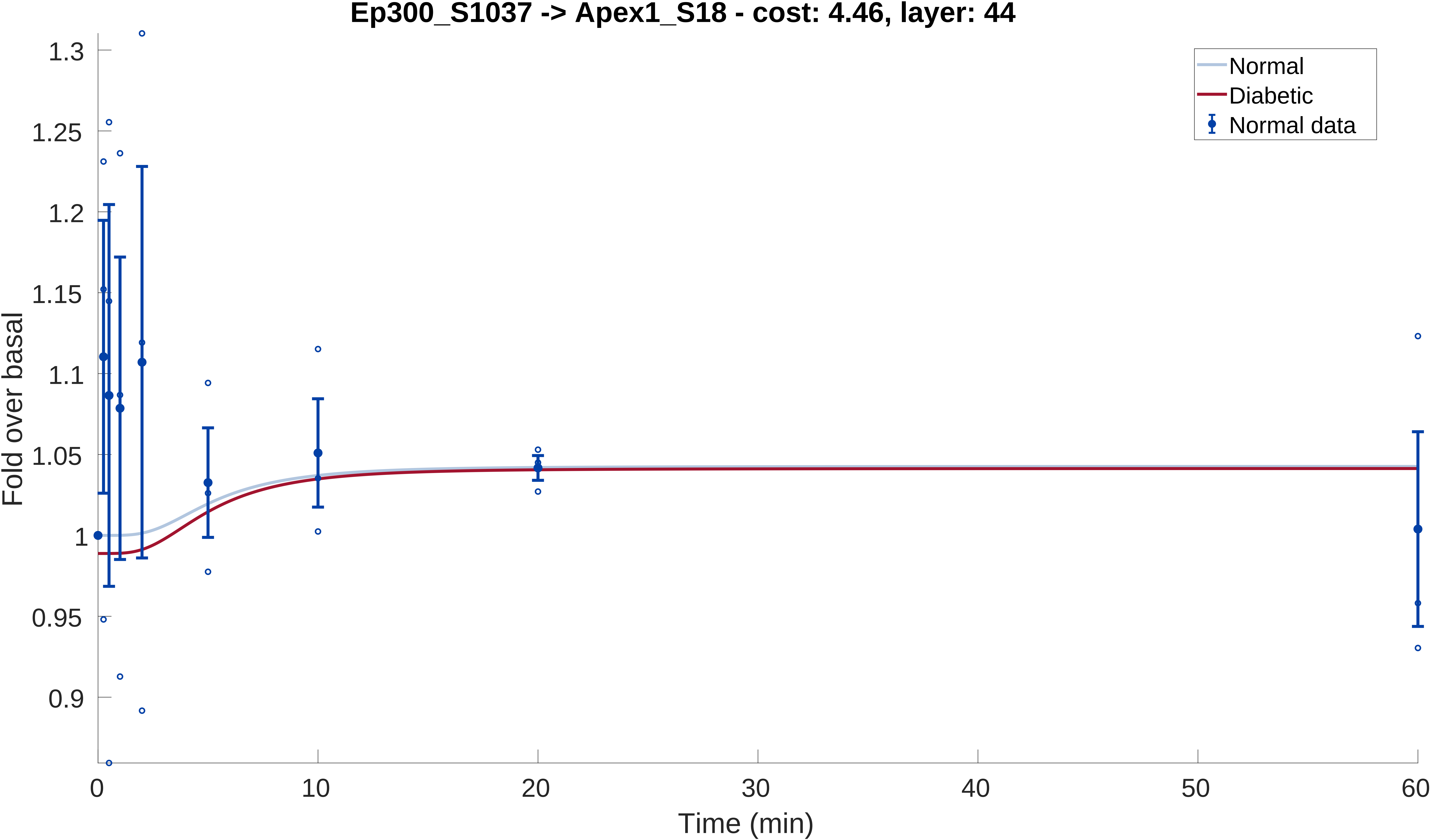

Ep300_S1037 -> Apex1_S18 - cost: 4.46, layer: 44
Normal
Diabetic
Normal data
Fold over basal
Time (min)
1.3
1.25
1.2
1.15
1.1
1.05
1
0.95
0.9
0
10
20
30
40
50
60

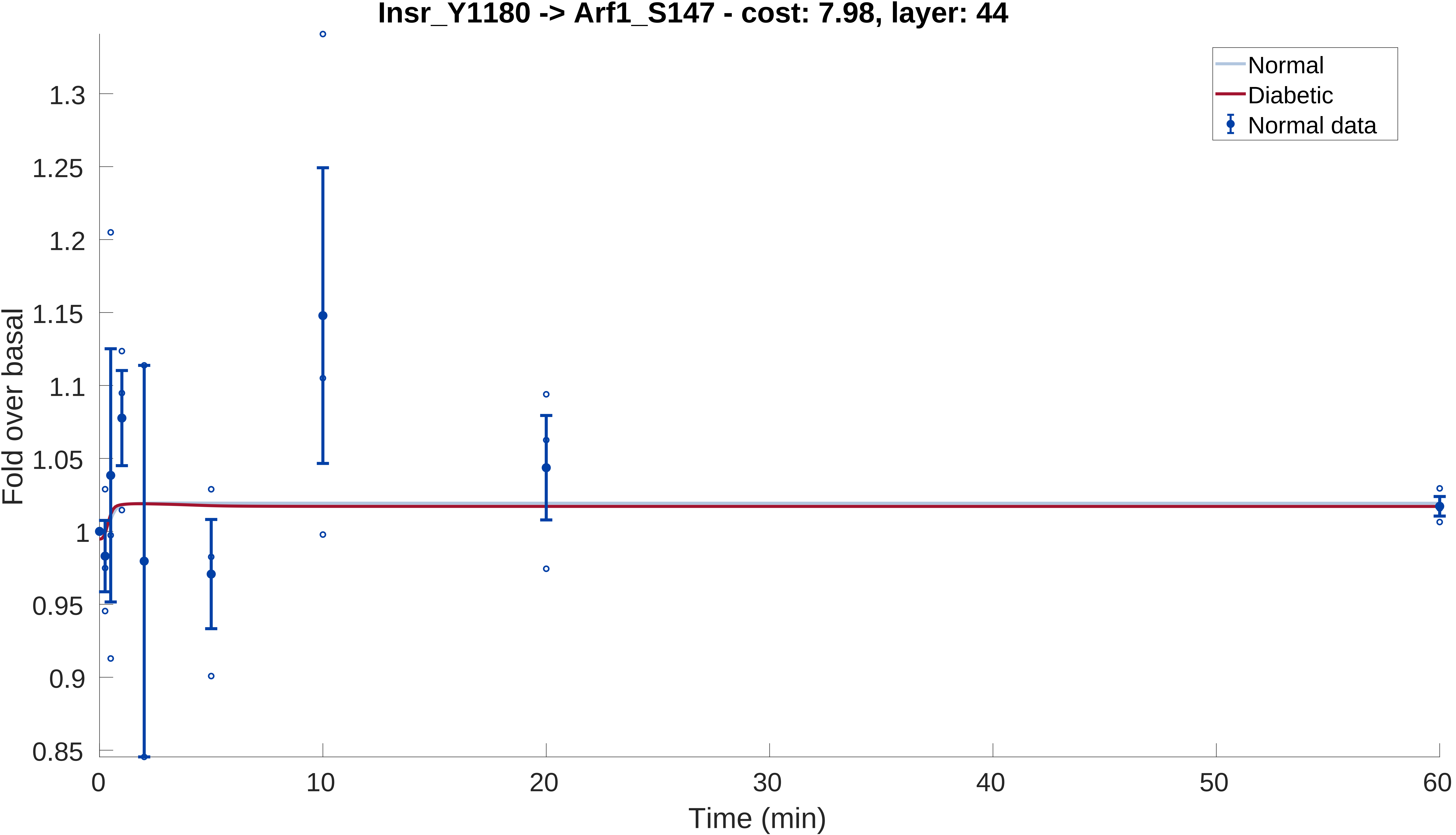

Insr_Y1180 -> Arf1_S147 - cost: 7.98, layer: 44
Normal
Diabetic
Normal data
Fold over basal
Time (min)
1.3
1.25
1.2
1.15
1.1
1.05
1
0.95
0.9
0.85
0
10
20
30
40
50
60

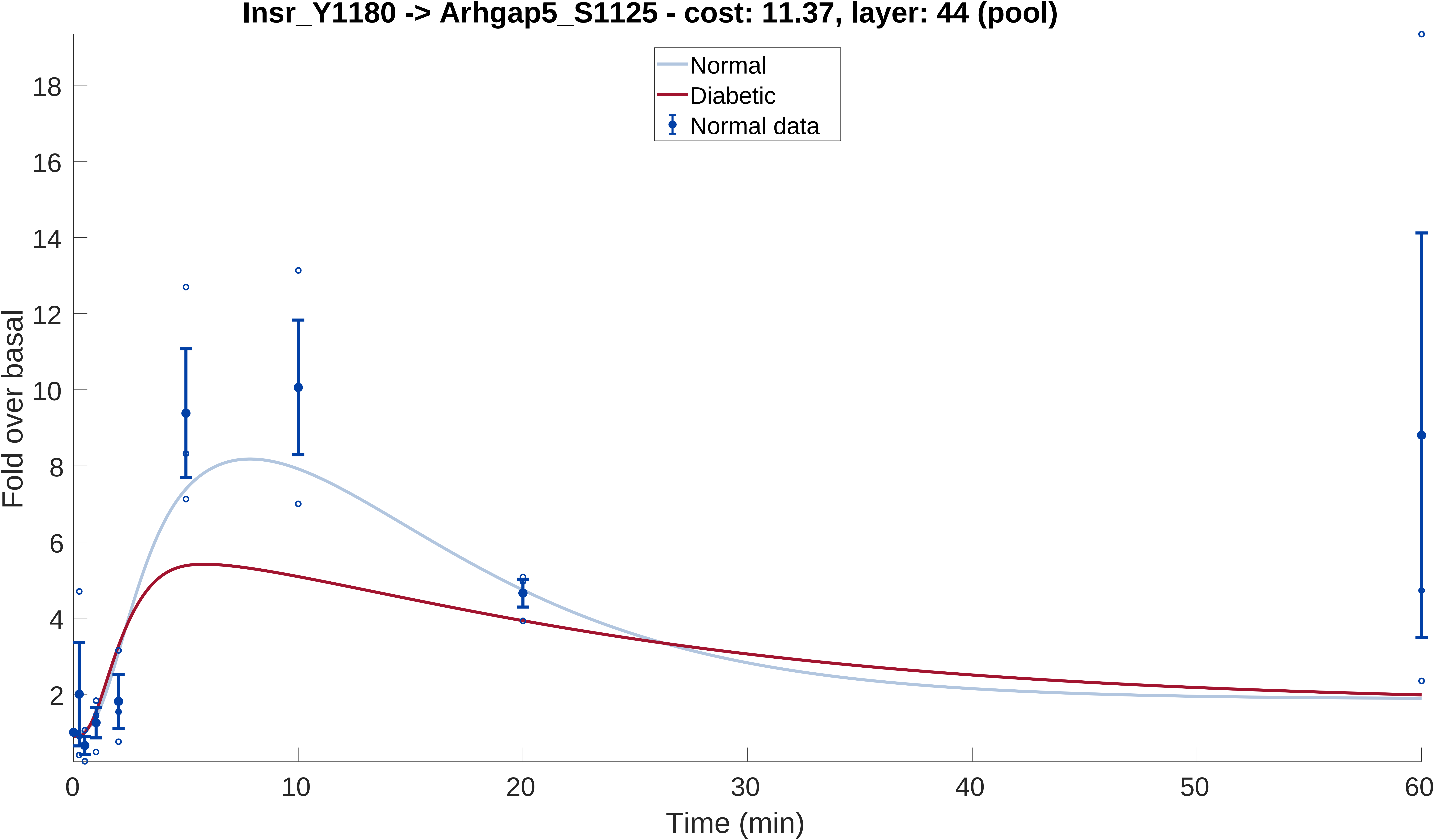

Insr_Y1180 -> Arhgap5_S1125 - cost: 11.37, layer: 44 (pool)
Normal
Diabetic
Normal data
Fold over basal
Time (min)
0
10
20
30
40
50
60
2
4
6
8
10
12
14
16
18

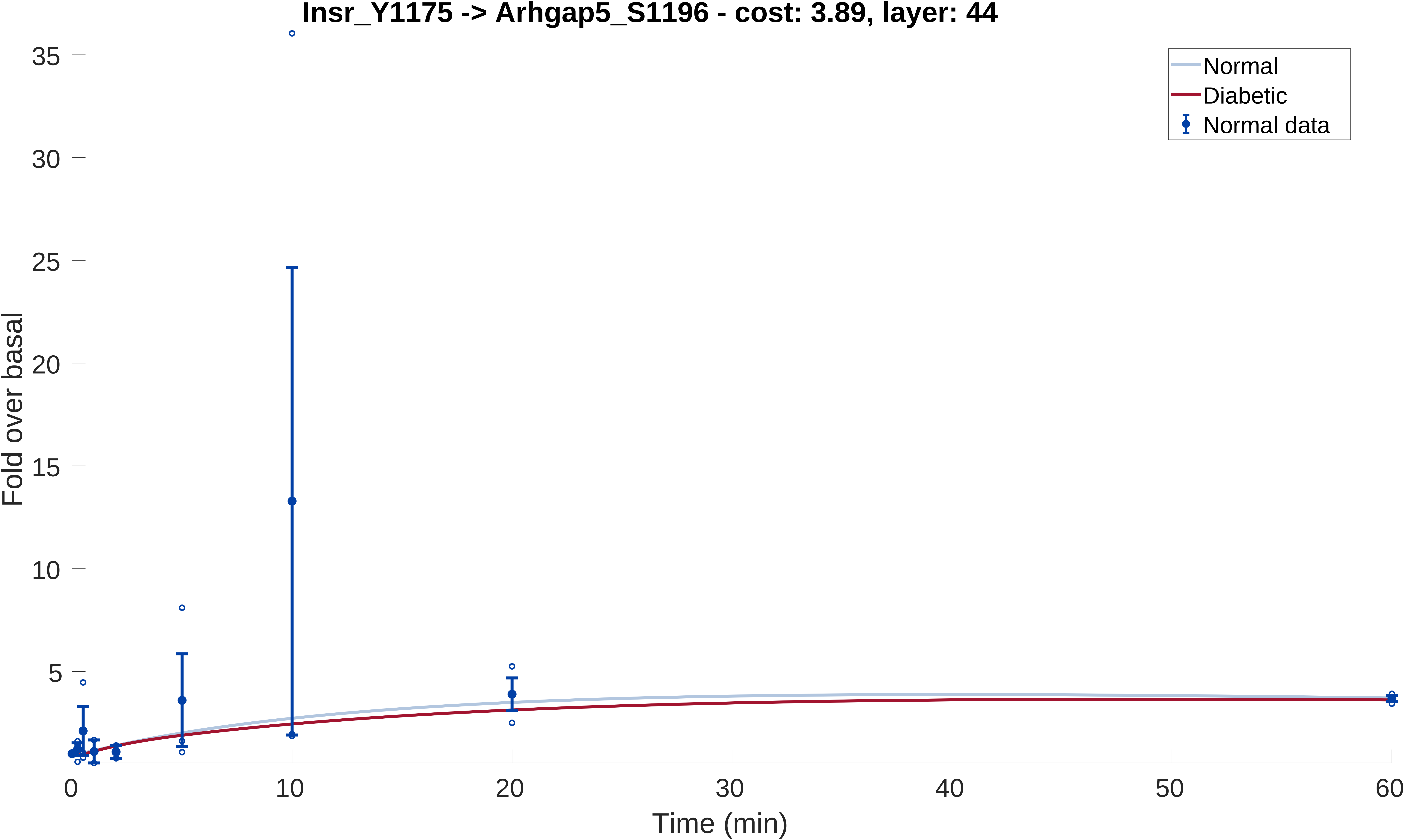

Insr_Y1175 -> Arhgap5_S1196 - cost: 3.89, layer: 44
Normal
Diabetic
Normal data
Fold over basal
Time (min)
35
30
25
20
15
10
5
0
10
20
30
40
50
60

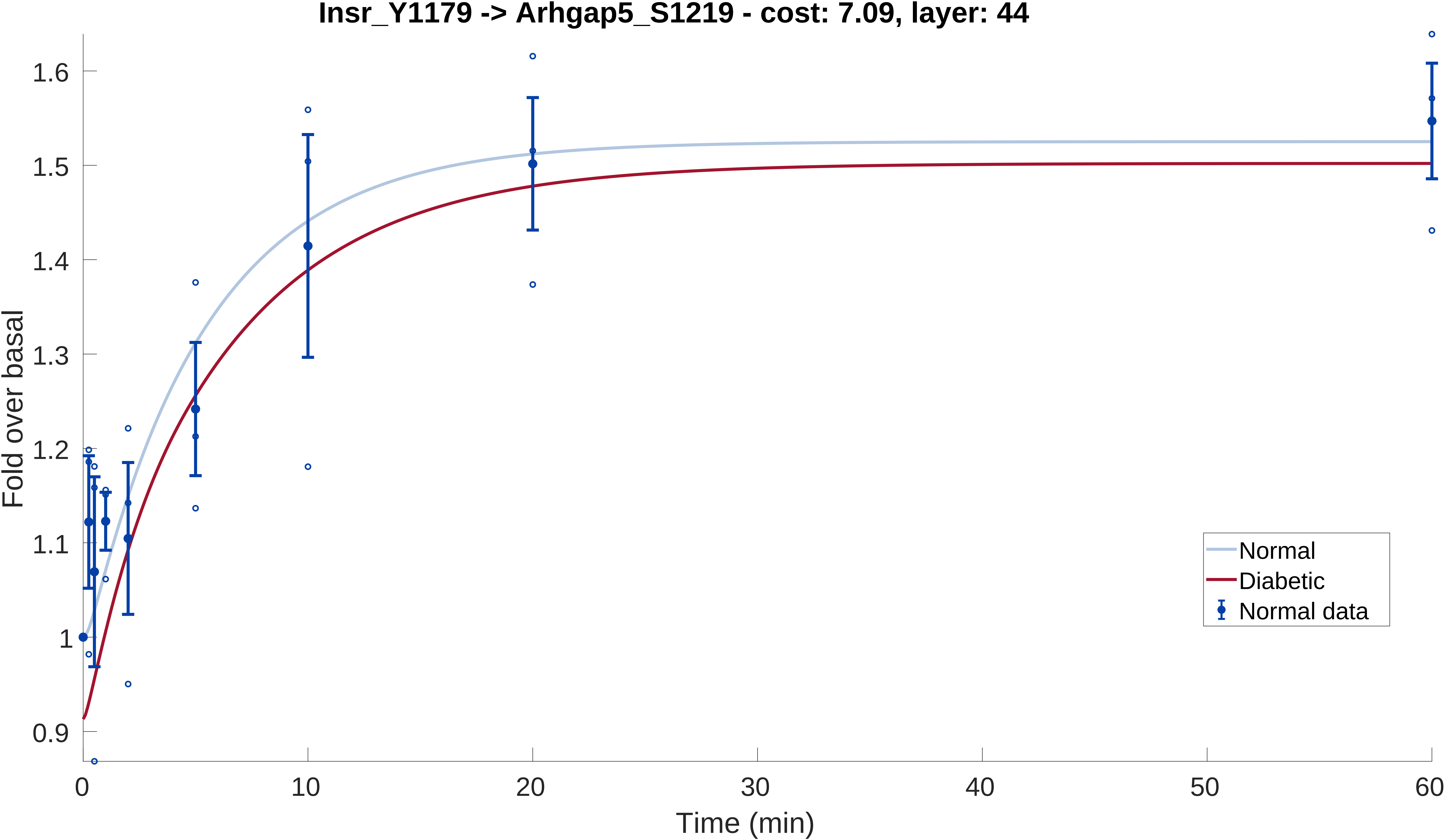
Insr_Y1179 -> Arhgap5_S1219 - cost: 7.09, layer: 44
Fold over basal
1.6
1.5
1.4
1.3
1.2
1.1
1
0.9
0
10
20
30
40
50
60
Time (min)
Normal
Diabetic
Normal data

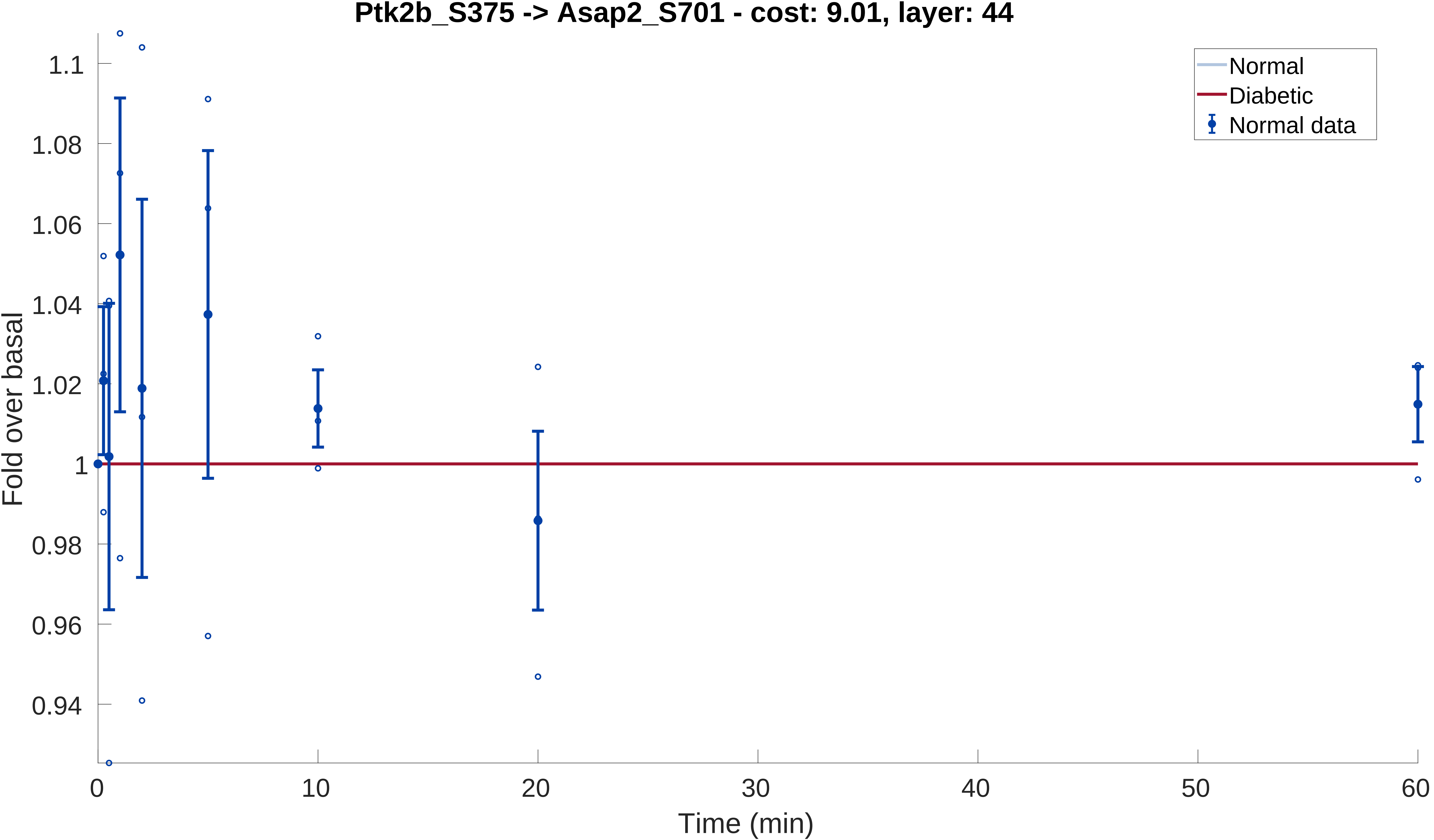

Ptk2b_S375 -> Asap2_S701 - cost: 9.01, layer: 44
Normal
Diabetic
Normal data
Fold over basal
Time (min)
0.94
0.96
0.98
1
1.02
1.04
1.06
1.08
1.1
0
10
20
30
40
50
60

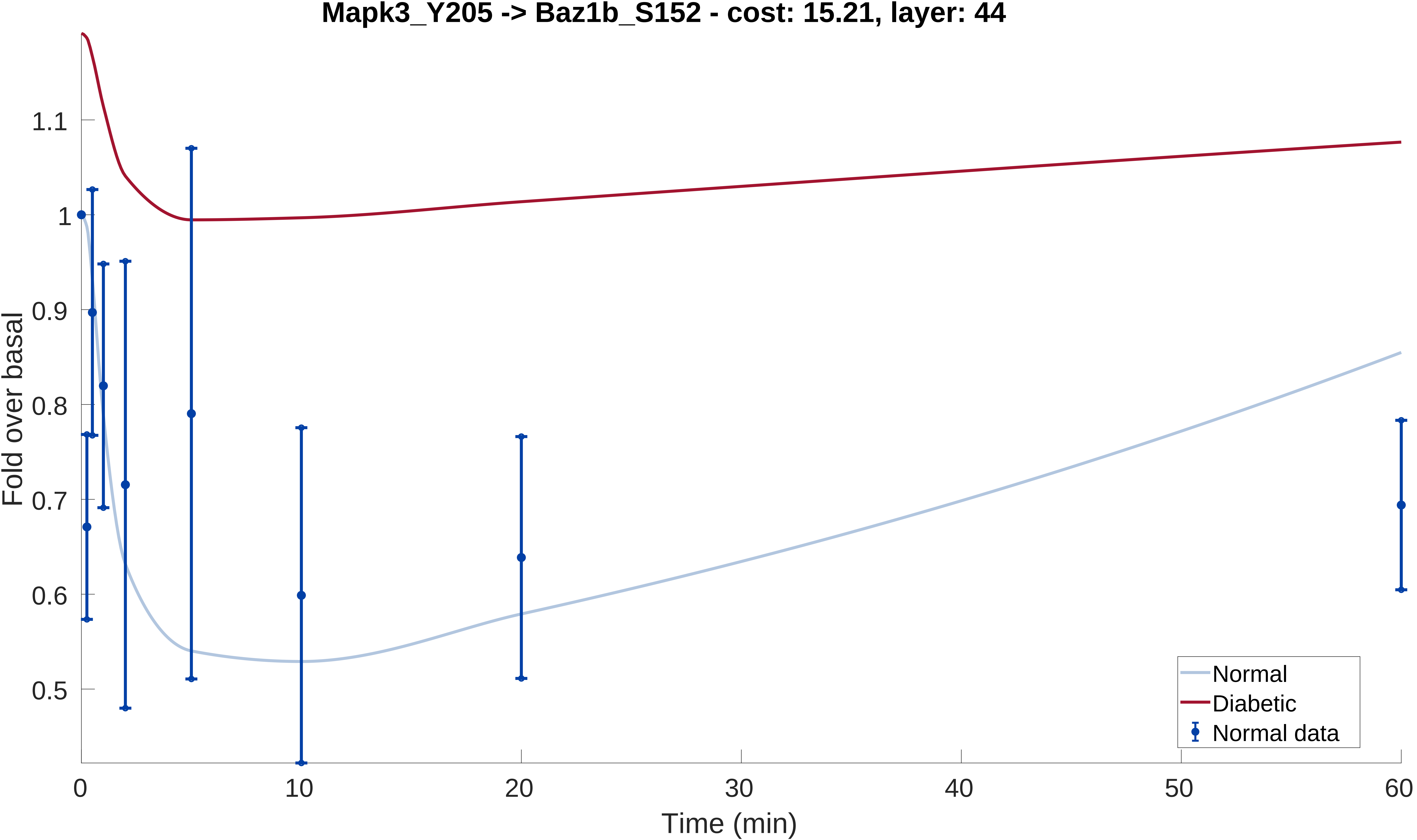

Mapk3_Y205 -> Baz1b_S152 - cost: 15.21, layer: 44
Fold over basal
1.1
1
0.9
0.8
0.7
0.6
0.5
0
10
20
30
40
50
60
Time (min)
Normal
Diabetic
Normal data

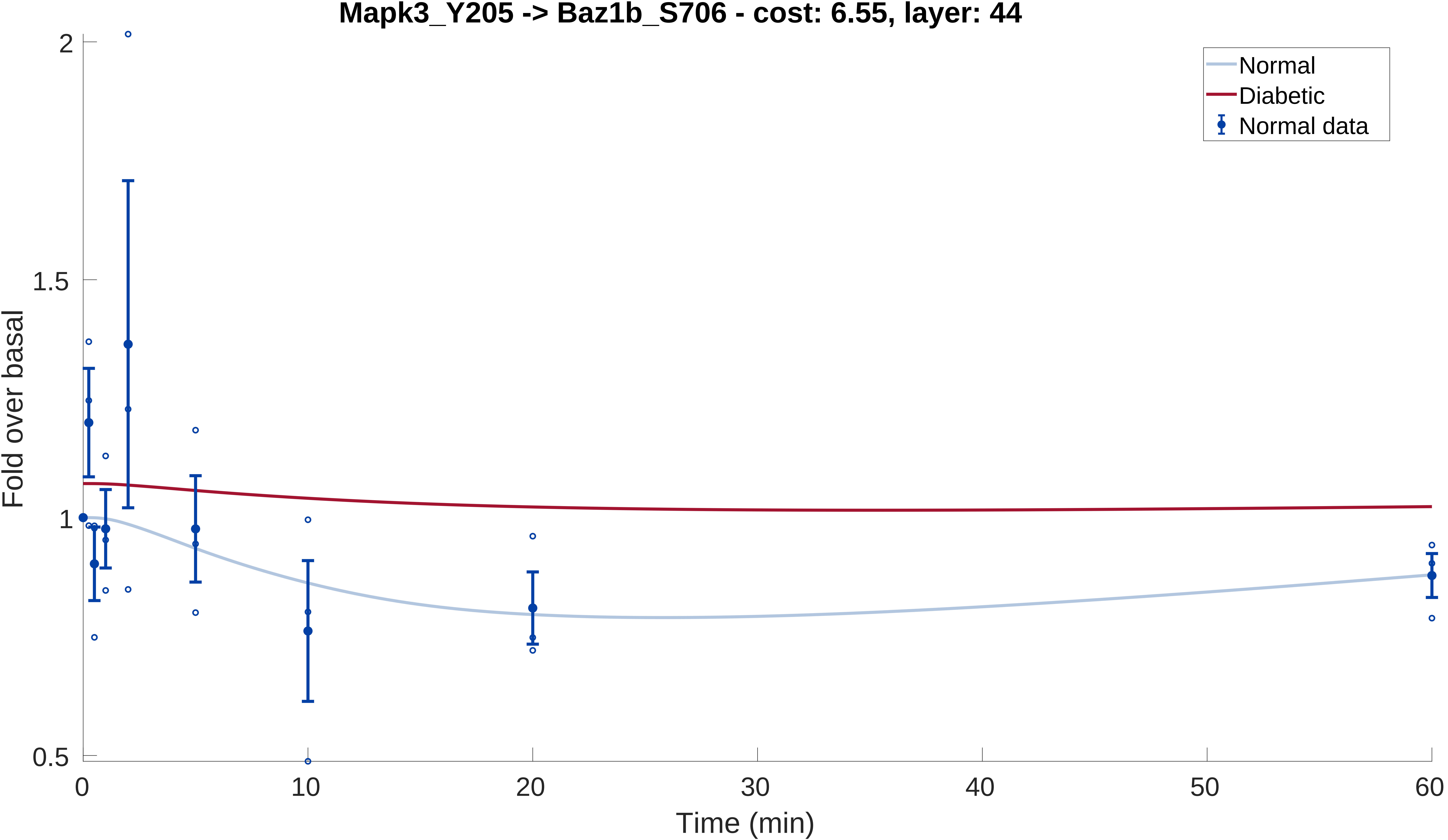
Mapk3_Y205 -> Baz1b_S706 - cost: 6.55, layer: 44
Normal
Diabetic
Normal data
Fold over basal
Time (min)
2
1.5
1
0.5
0
10
20
30
40
50
60

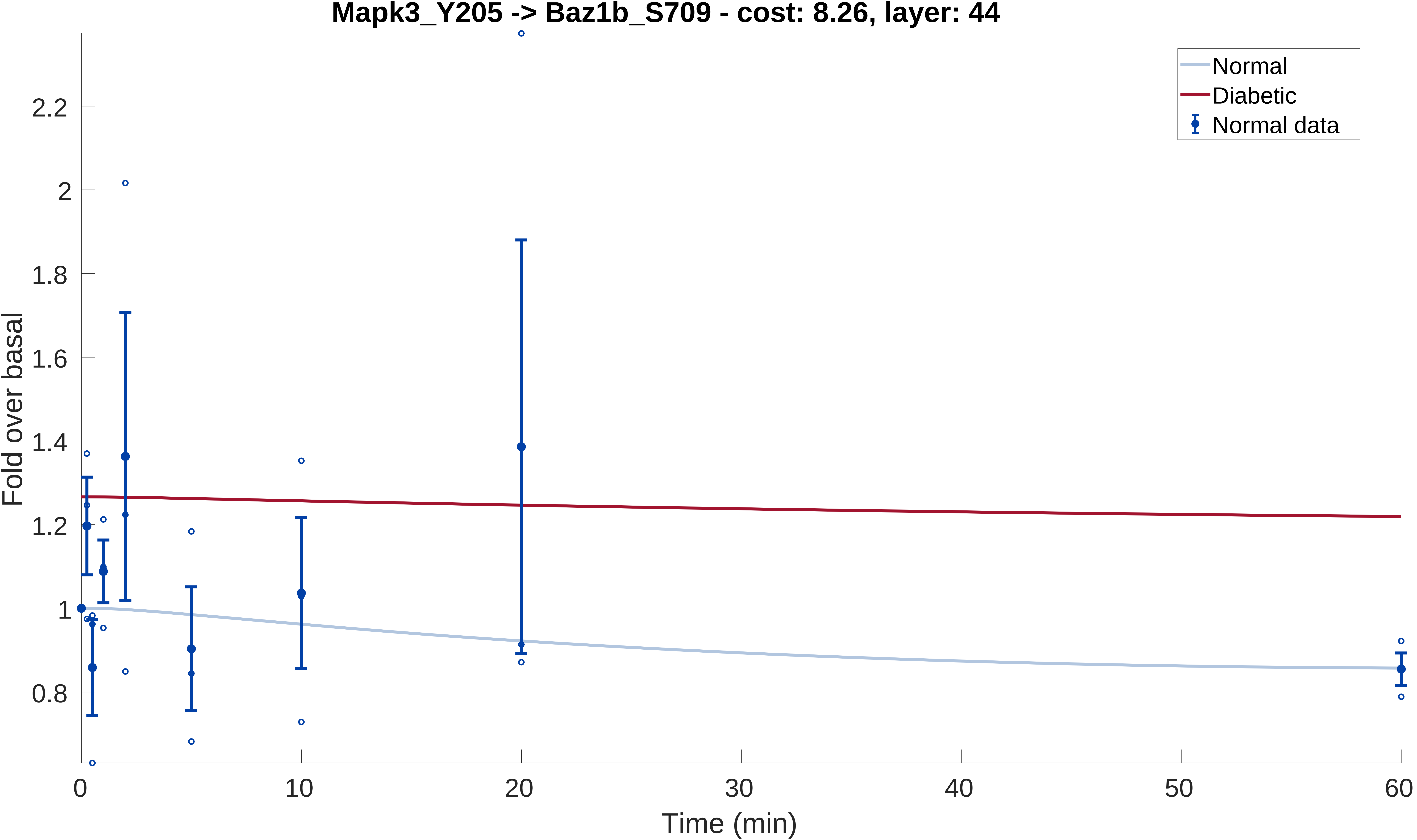
Mapk3_Y205 -> Baz1b_S709 - cost: 8.26, layer: 44
Normal
Diabetic
Normal data
Fold over basal
Time (min)
2.2
2
1.8
1.6
1.4
1.2
1
0.8
0
10
20
30
40
50
60

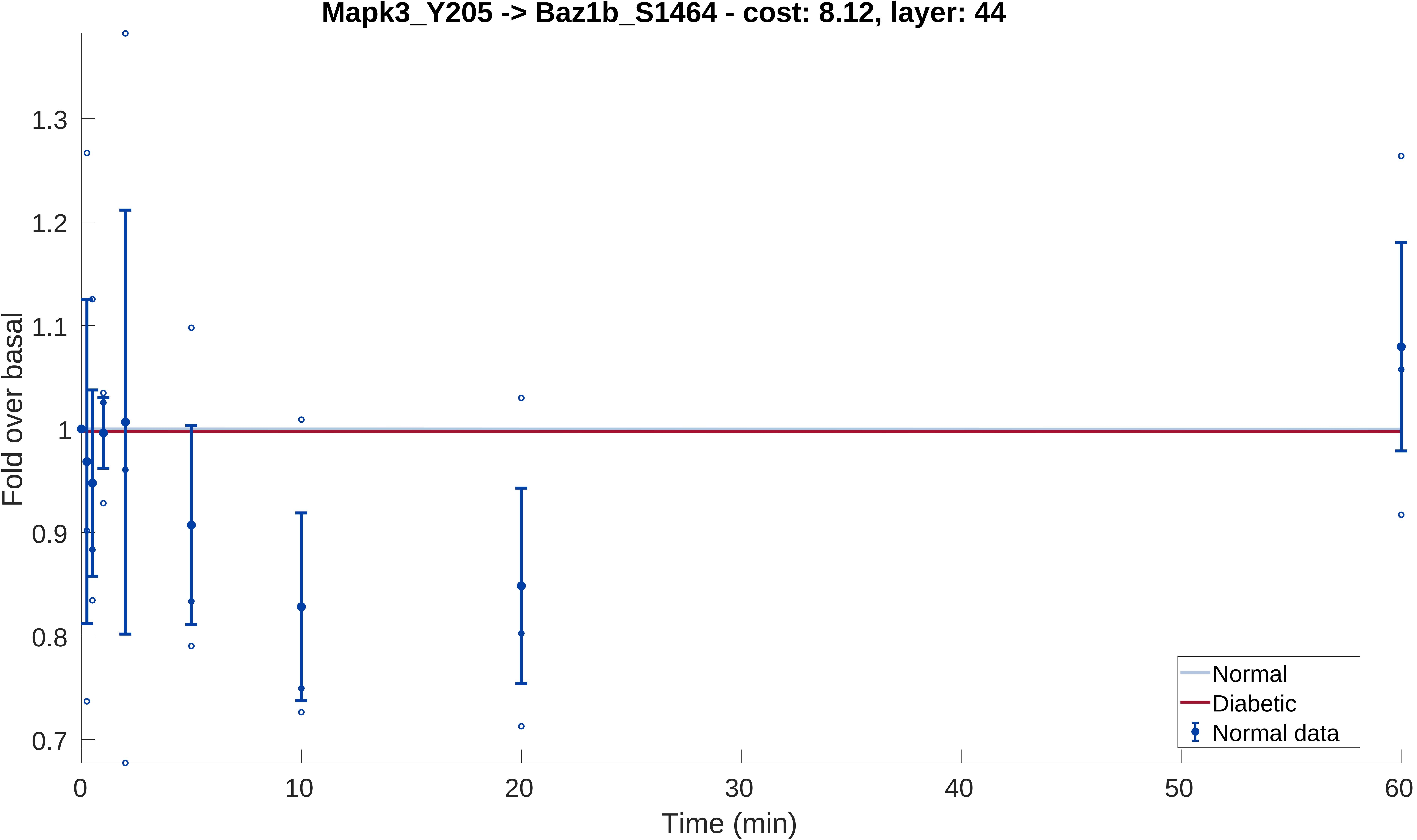

Mapk3_Y205 -> Baz1b_S1464 - cost: 8.12, layer: 44
Fold over basal
1.3
1.2
1.1
1
0.9
0.8
0.7
0
10
20
30
40
50
60
Time (min)
Normal
Diabetic
Normal data

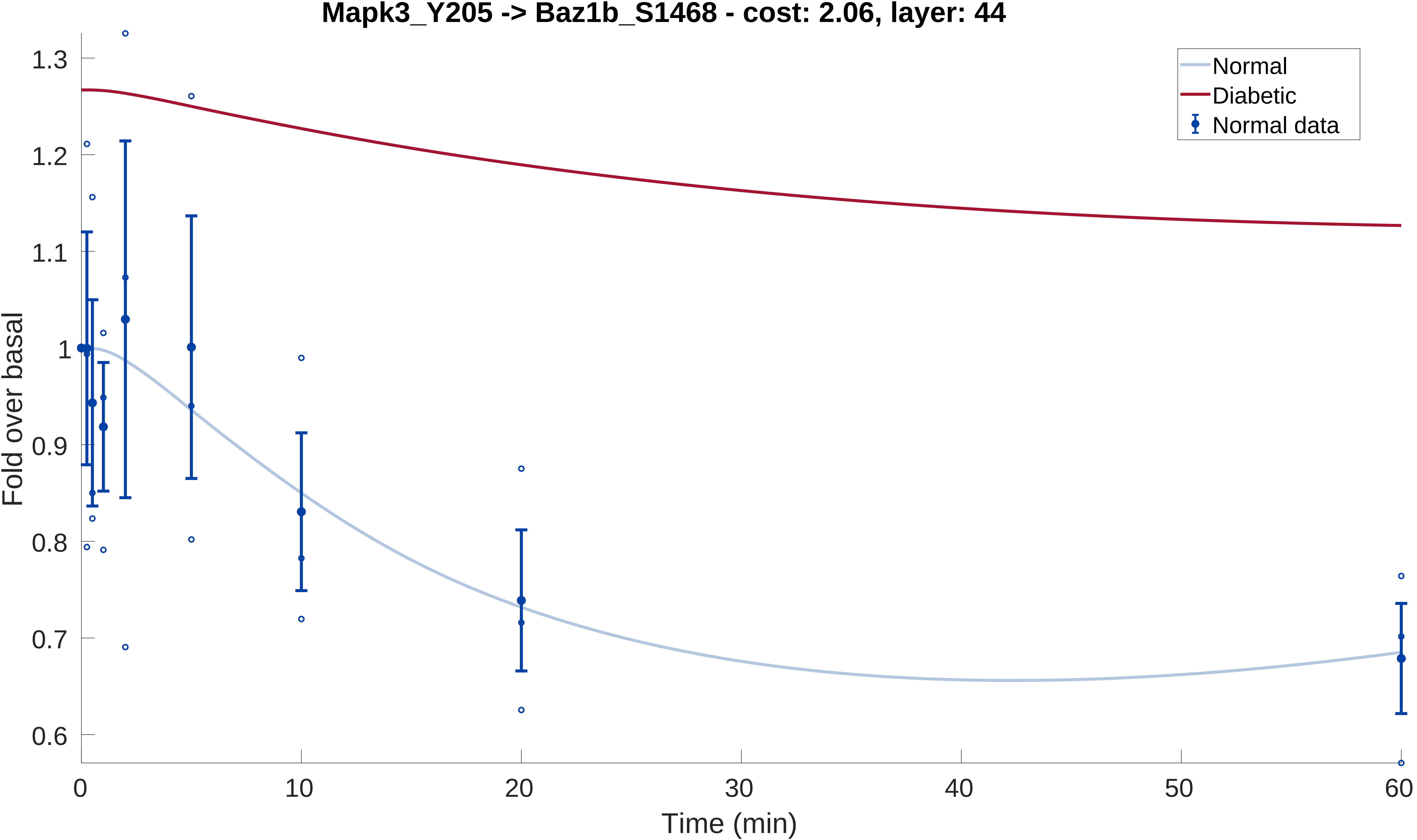

Mapk3_Y205 -> Baz1b_S1468 - cost: 2.06, layer: 44
Normal
Diabetic
Normal data
Fold over basal
Time (min)
1.3
1.2
1.1
1
0.9
0.8
0.7
0.6
0
10
20
30
40
50
60

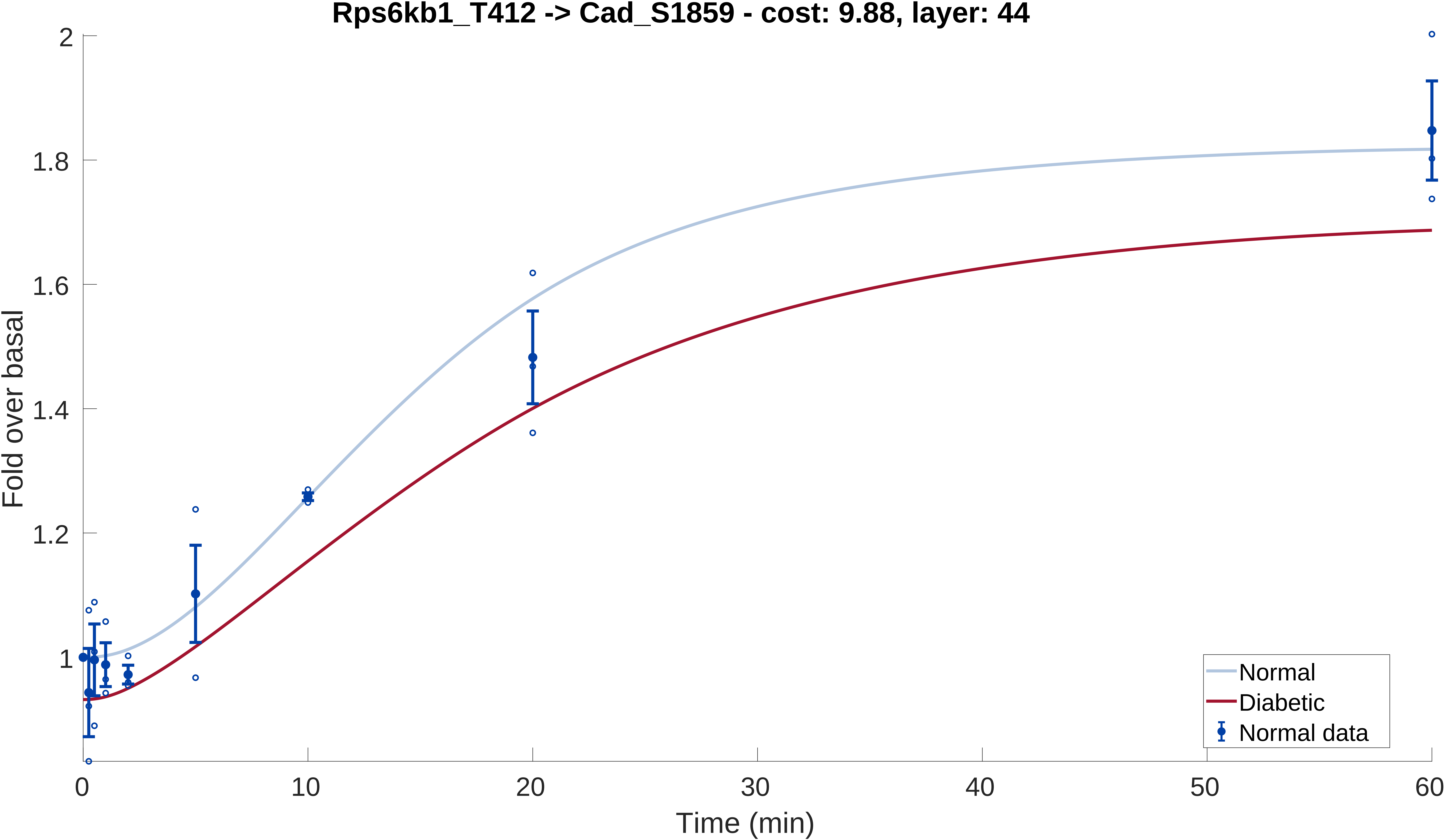

Rps6kb1_T412 -> Cad_S1859 - cost: 9.88, layer: 44
Fold over basal
2
1.8
1.6
1.4
1.2
1
0
10
20
30
40
50
60
Time (min)
Normal
Diabetic
Normal data

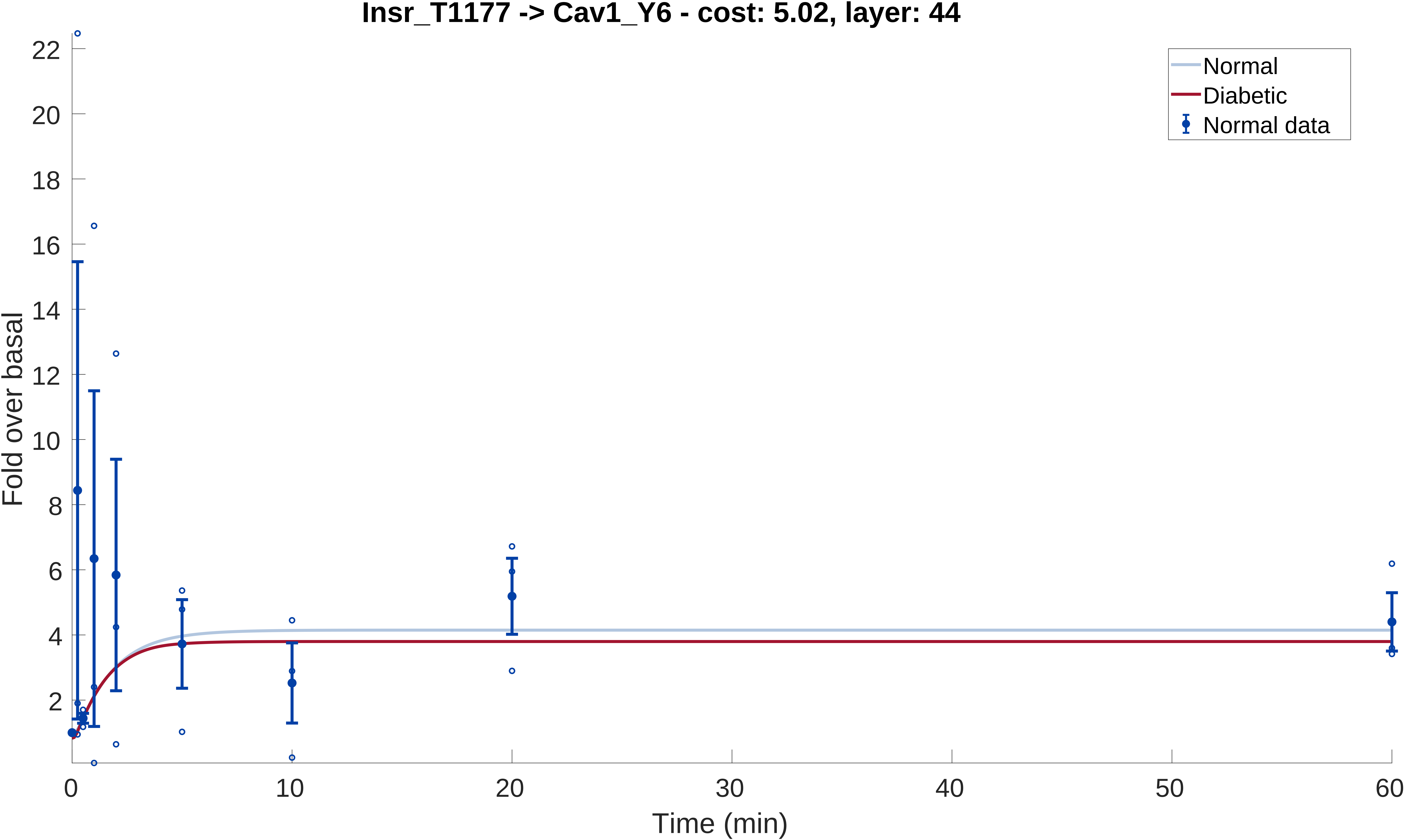
Insr_T1177 -> Cav1_Y6 - cost: 5.02, layer: 44
Normal
Diabetic
Normal data
Fold over basal
Time (min)
0
10
20
30
40
50
60
2
4
6
8
10
12
14
16
18
20
22

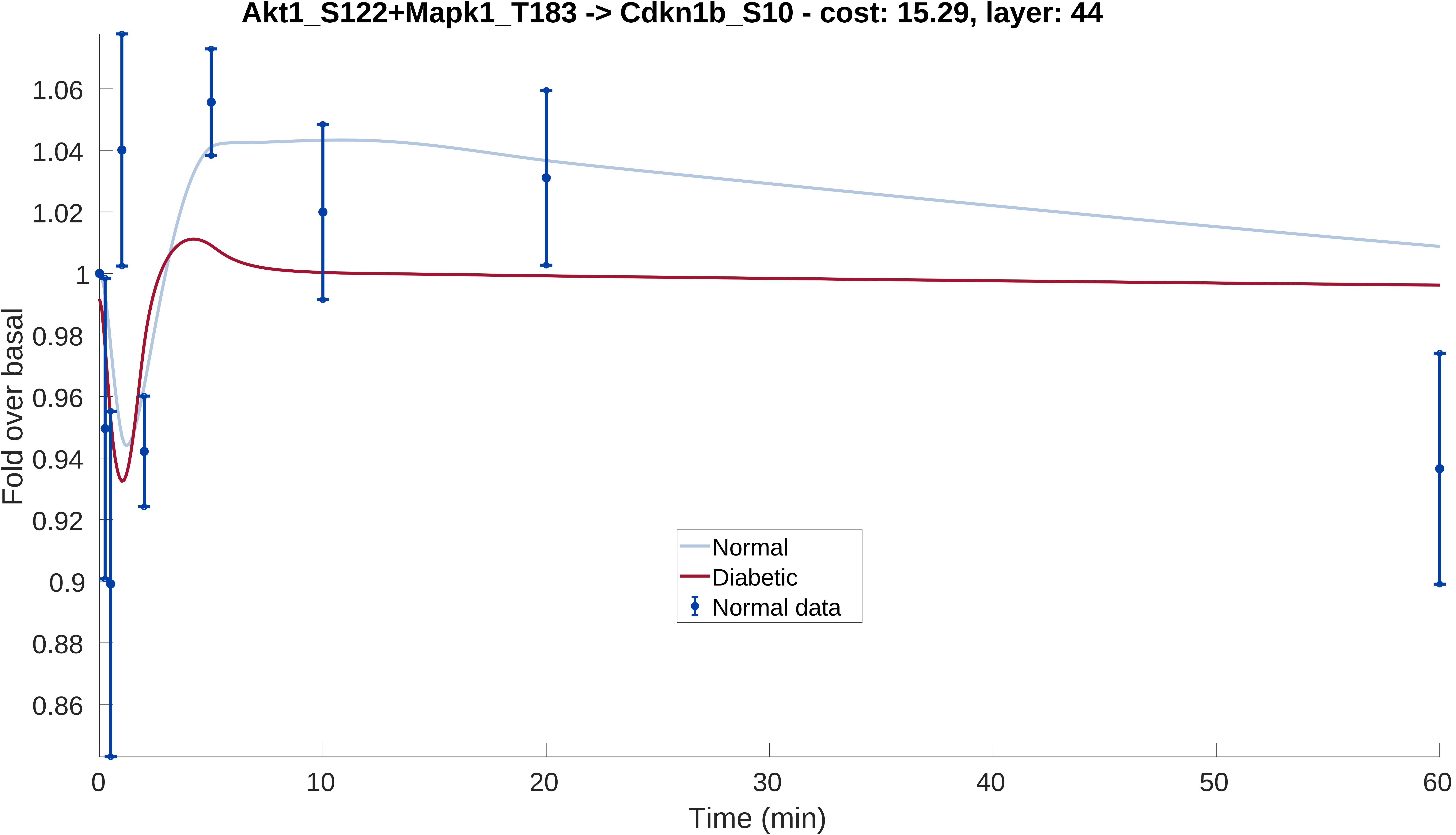

Akt1_S122+Mapk1_T183 -> Cdkn1b_S10 - cost: 15.29, layer: 44
Fold over basal
1.06
1.04
1.02
1
0.98
0.96
0.94
0.92
0.9
0.88
0.86
0
10
20
30
40
50
60
Time (min)
Normal
Diabetic
Normal data

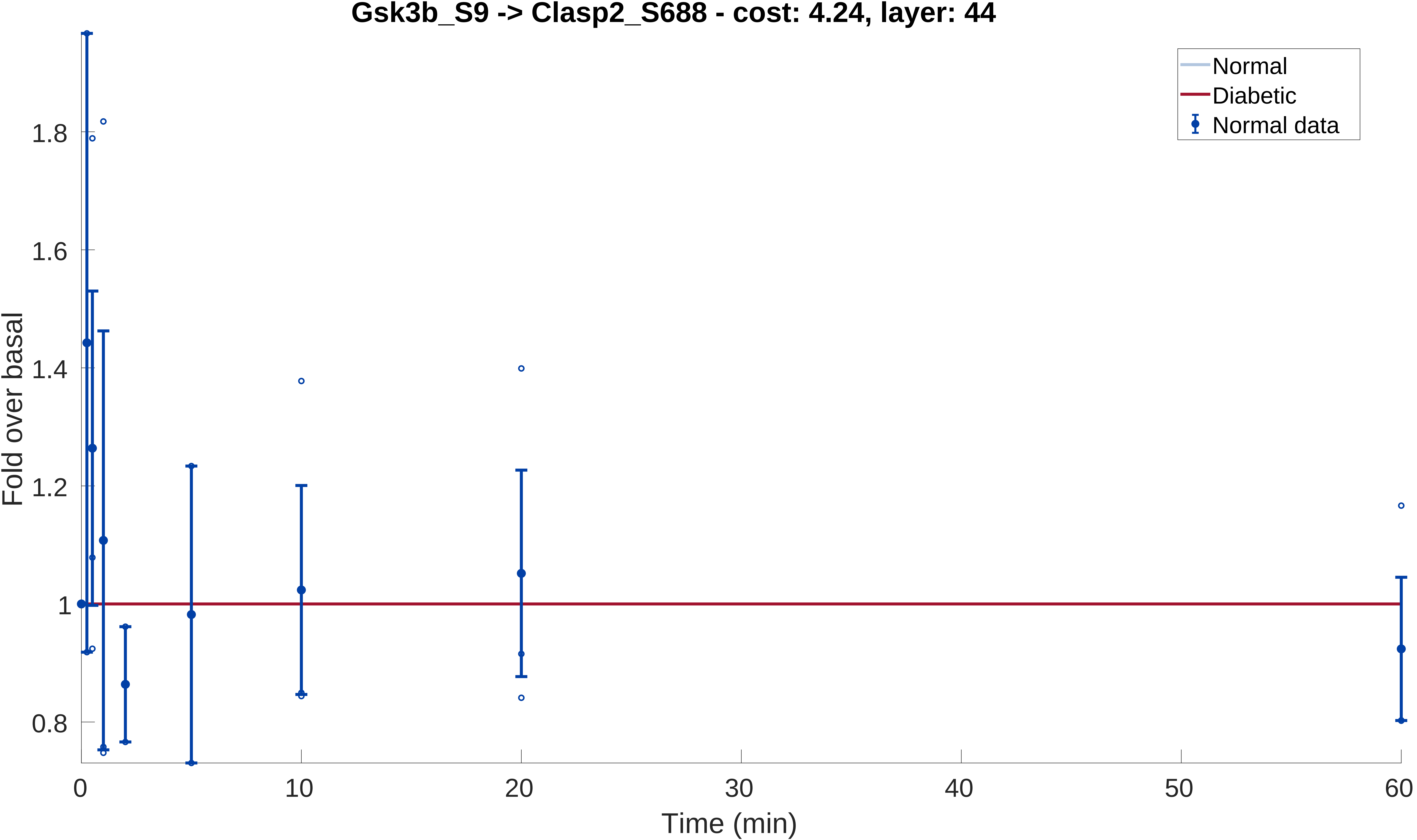

Gsk3b_S9 -> Clasp2_S688 - cost: 4.24, layer: 44
Normal
Diabetic
Normal data
Fold over basal
1.8
1.6
1.4
1.2
1
0.8
0
10
20
30
40
50
60
Time (min)

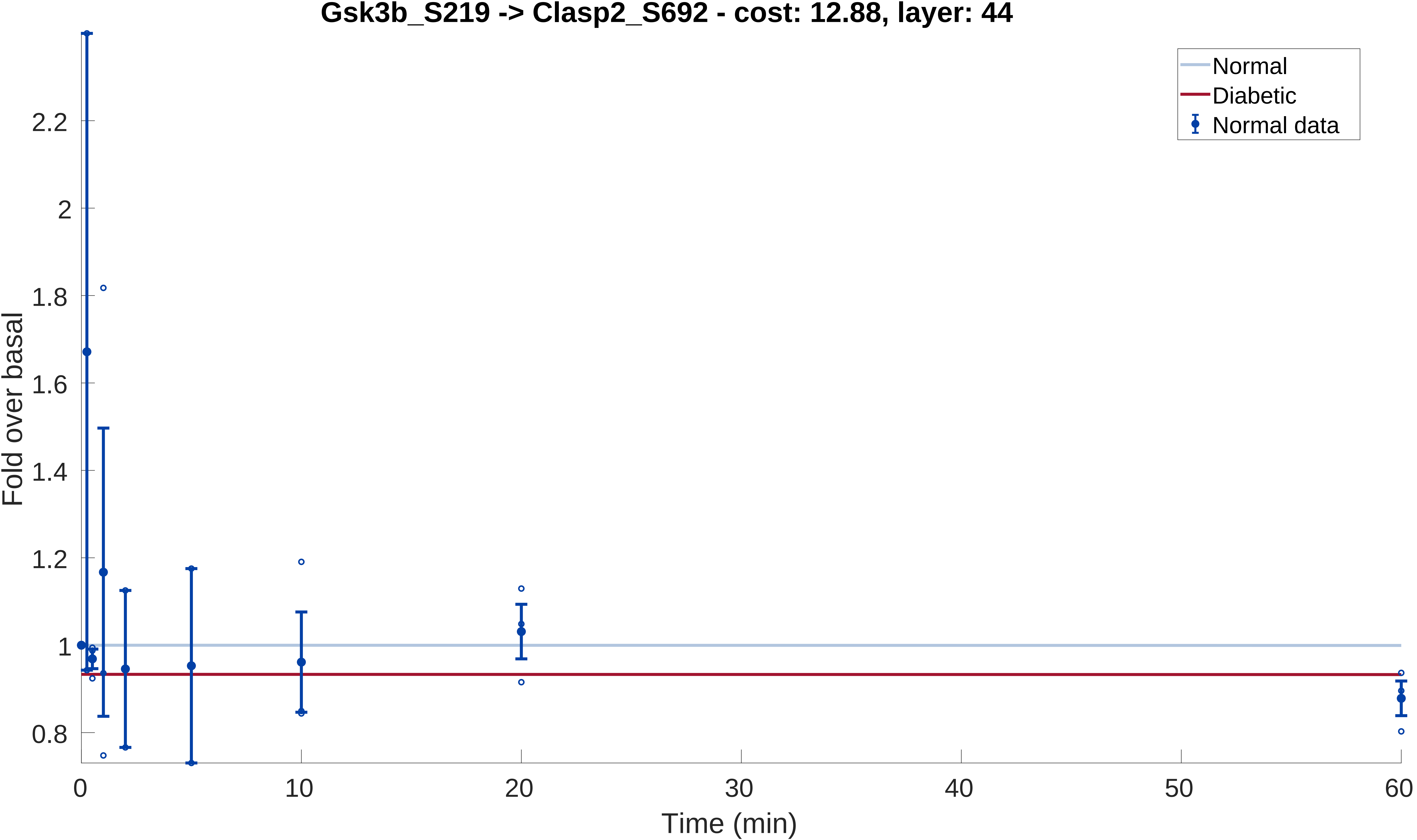

Gsk3b_S219 -> Clasp2_S692 - cost: 12.88, layer: 44
Normal
Diabetic
Normal data
Fold over basal
Time (min)
0.8
1
1.2
1.4
1.6
1.8
2
2.2
0
10
20
30
40
50
60

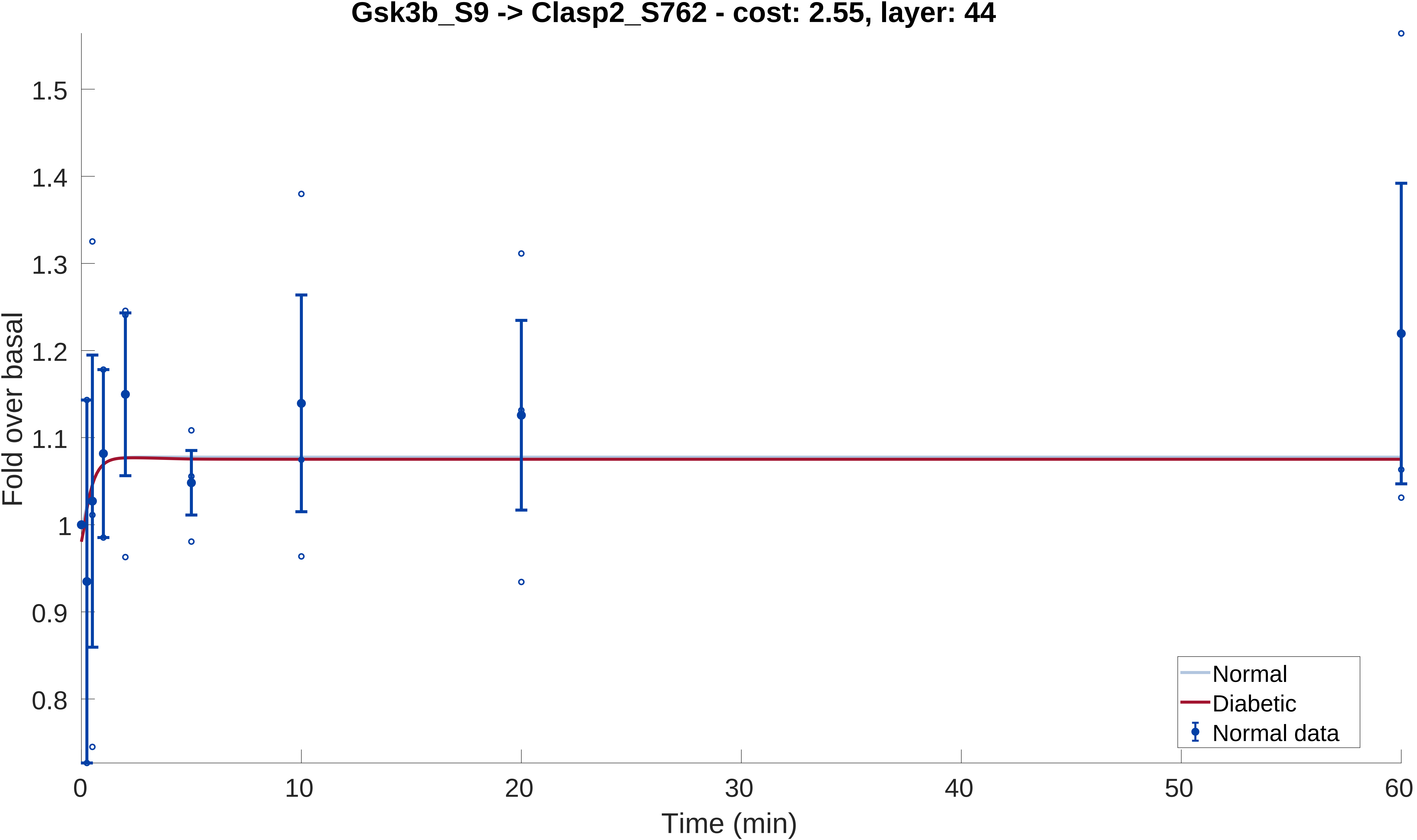
Gsk3b_S9 -> Clasp2_S762 - cost: 2.55, layer: 44
Fold over basal
1.5
1.4
1.3
1.2
1.1
1
0.9
0.8
0
10
20
30
40
50
60
Time (min)
Normal
Diabetic
Normal data

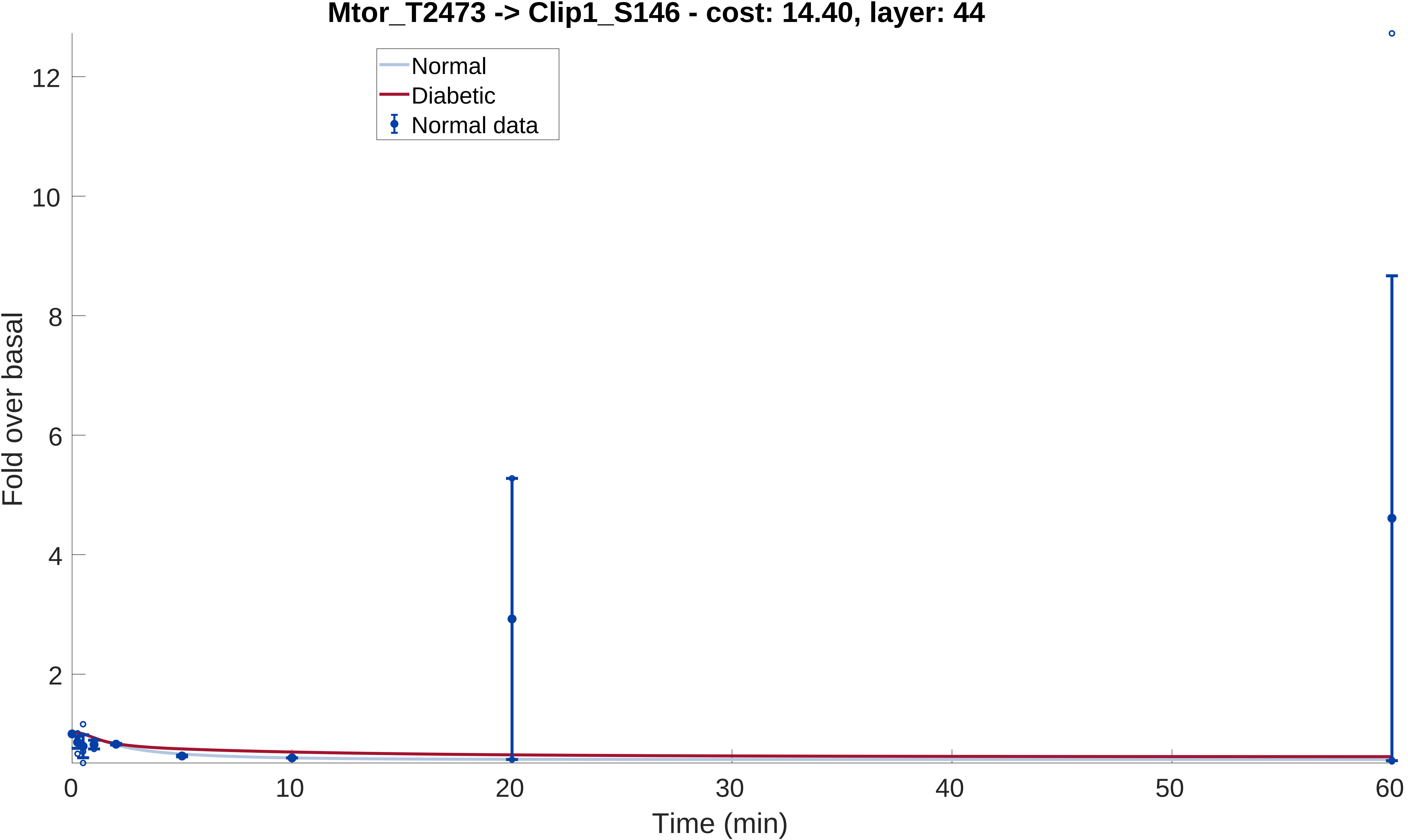

Mtor_T2473 -> Clip1_S146 - cost: 14.40, layer: 44
Normal
Diabetic
Normal data
Fold over basal
Time (min)
0
10
20
30
40
50
60
2
4
6
8
10
12

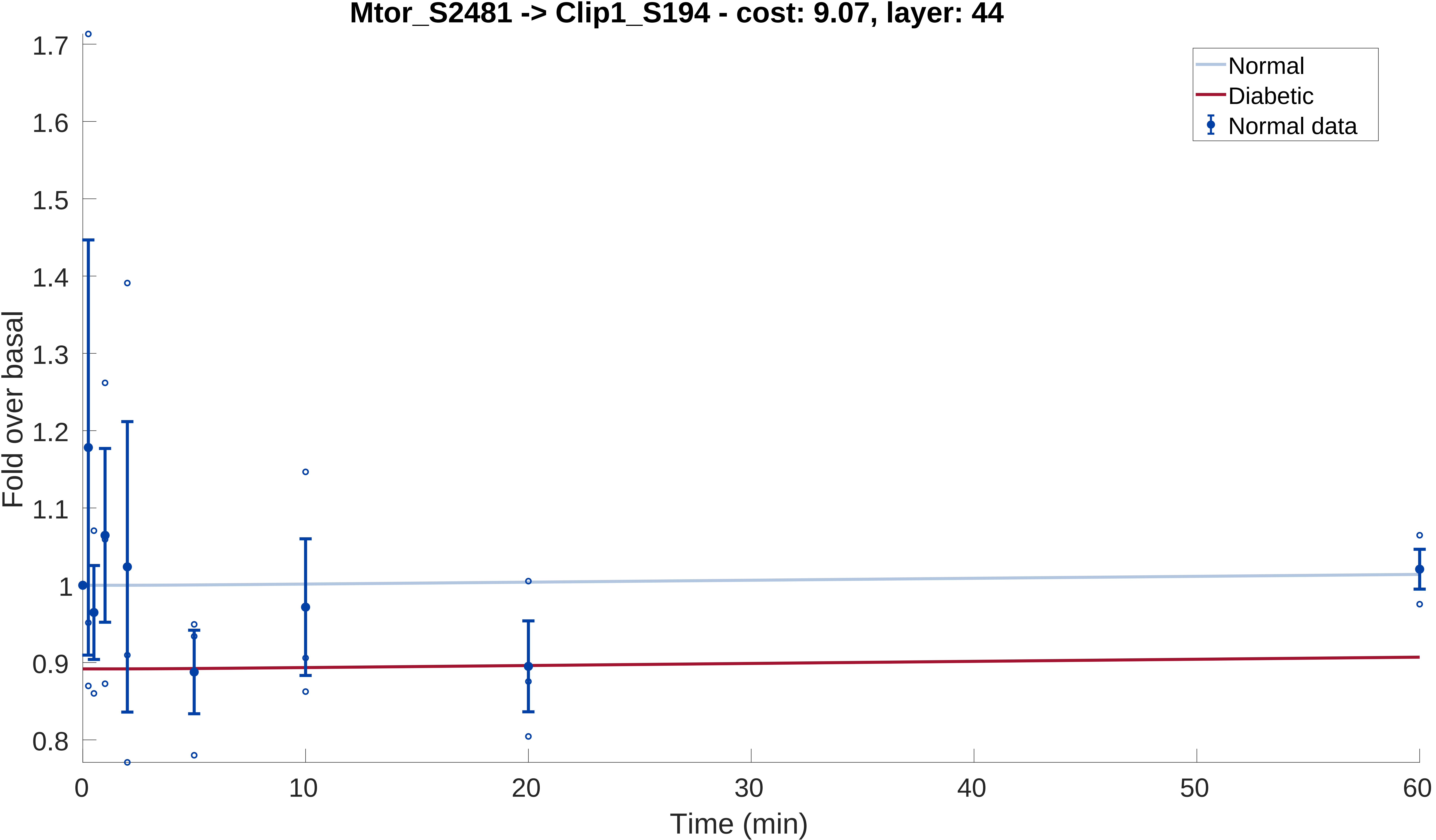

Mtor_S2481 -> Clip1_S194 - cost: 9.07, layer: 44
Normal
Diabetic
Normal data
Fold over basal
Time (min)
0.8
0.9
1
1.1
1.2
1.3
1.4
1.5
1.6
1.7
0
10
20
30
40
50
60

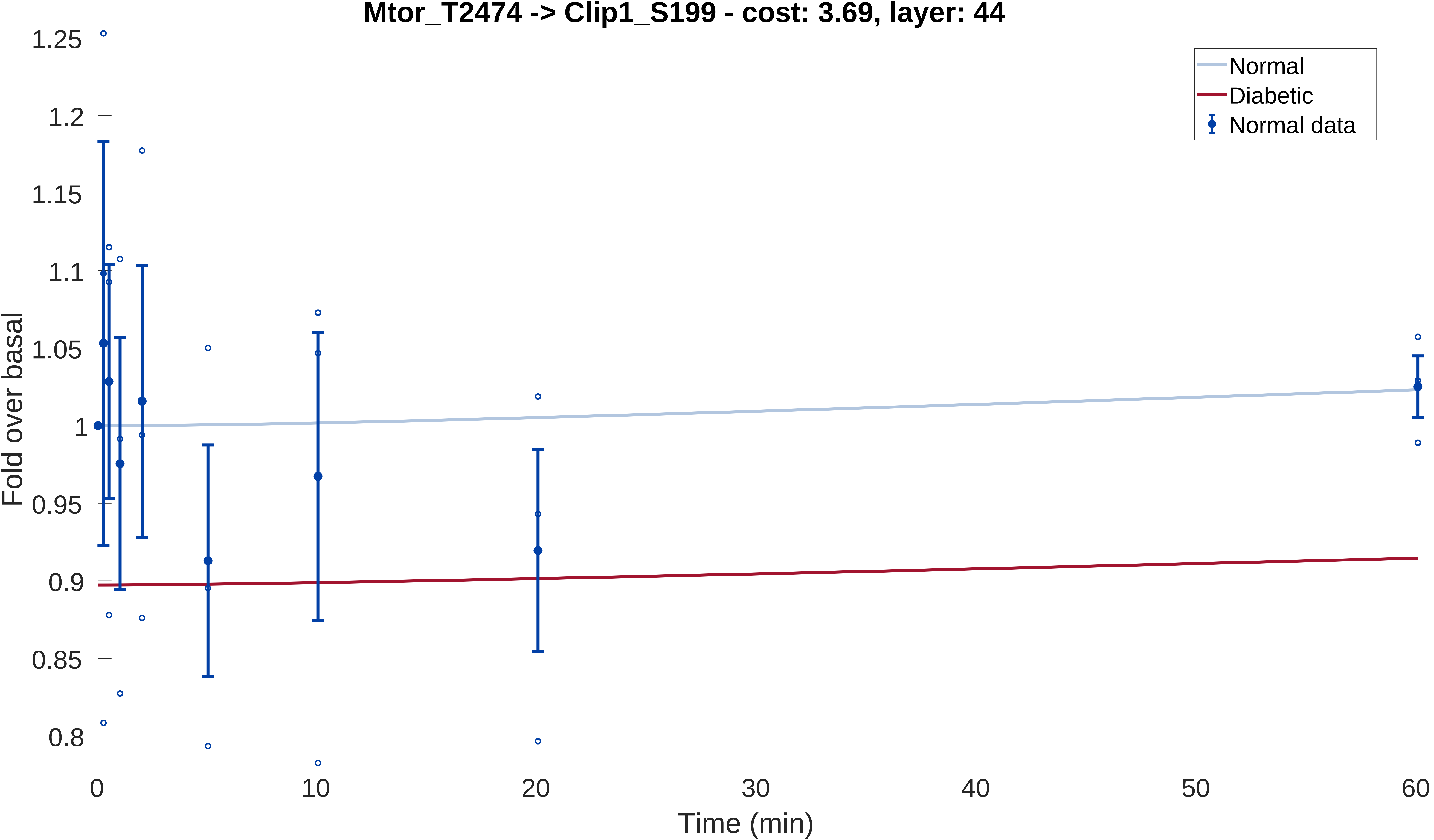
Mtor_T2474 -> Clip1_S199 - cost: 3.69, layer: 44
Normal
Diabetic
Normal data
Fold over basal
Time (min)
1.25
1.2
1.15
1.1
1.05
1
0.95
0.9
0.85
0.8
0
10
20
30
40
50
60

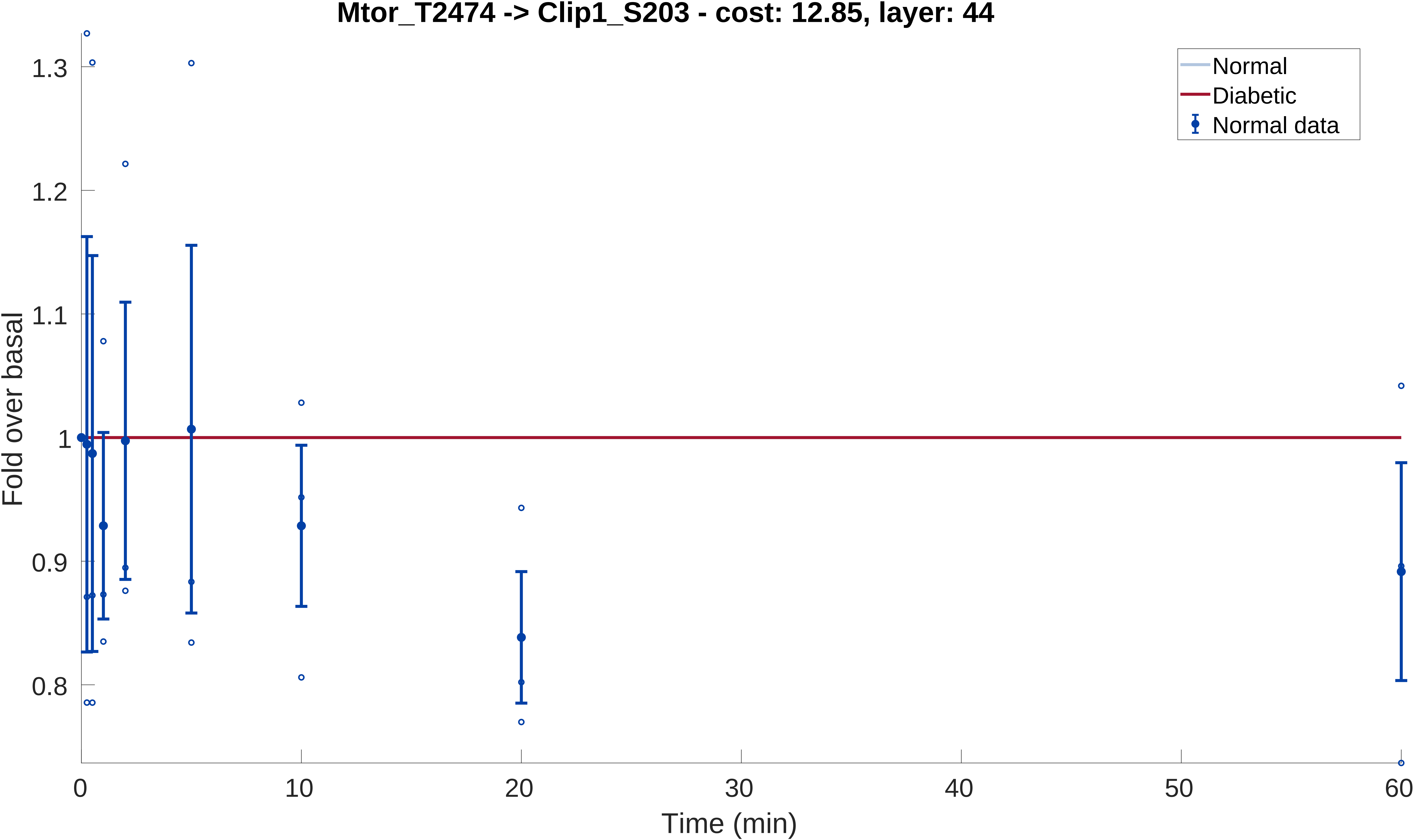
Mtor_T2474 -> Clip1_S203 - cost: 12.85, layer: 44
Normal
Diabetic
Normal data
Fold over basal
1.3
1.2
1.1
1
0.9
0.8
0
10
20
30
40
50
60
Time (min)

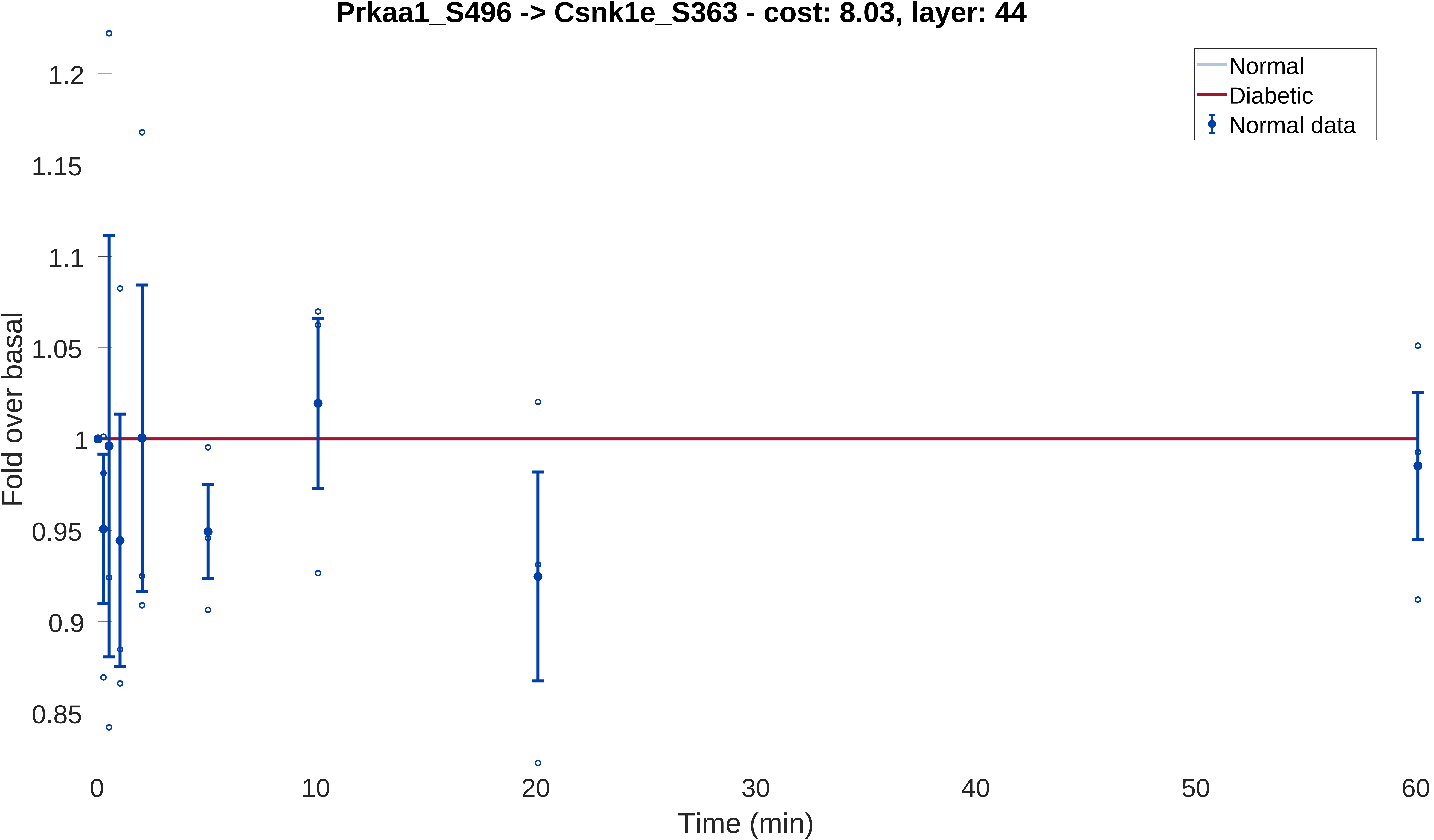

Prkaa1_S496 -> Csnk1e_S363 - cost: 8.03, layer: 44
Normal
Diabetic
Normal data
Fold over basal
1.2
1.15
1.1
1.05
1
0.95
0.9
0.85
0
10
20
30
40
50
60
Time (min)

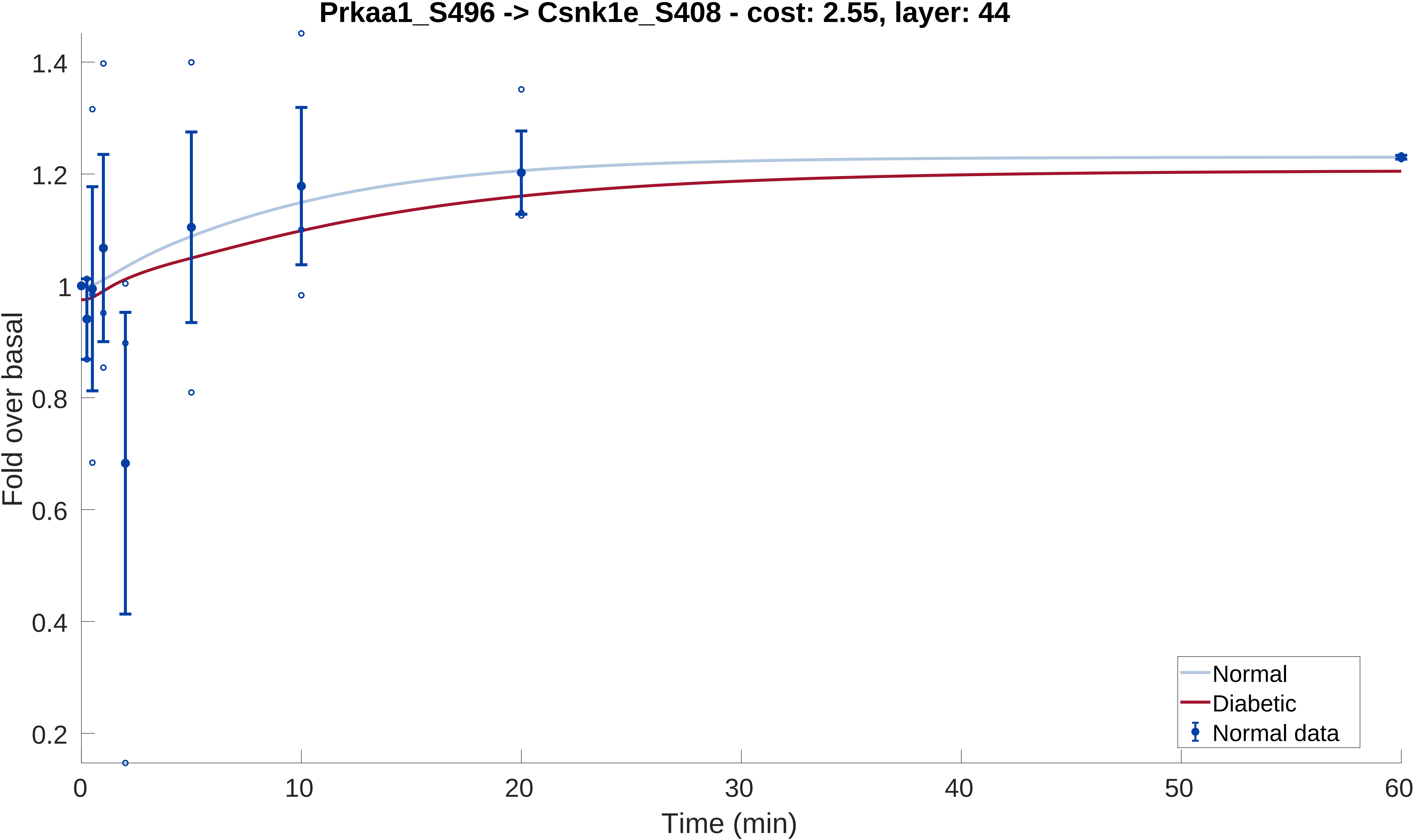

Prkaa1_S496 -> Csnk1e_S408 - cost: 2.55, layer: 44
Fold over basal
1.4
1.2
1
0.8
0.6
0.4
0.2
0
10
20
30
40
50
60
Time (min)
Normal
Diabetic
Normal data

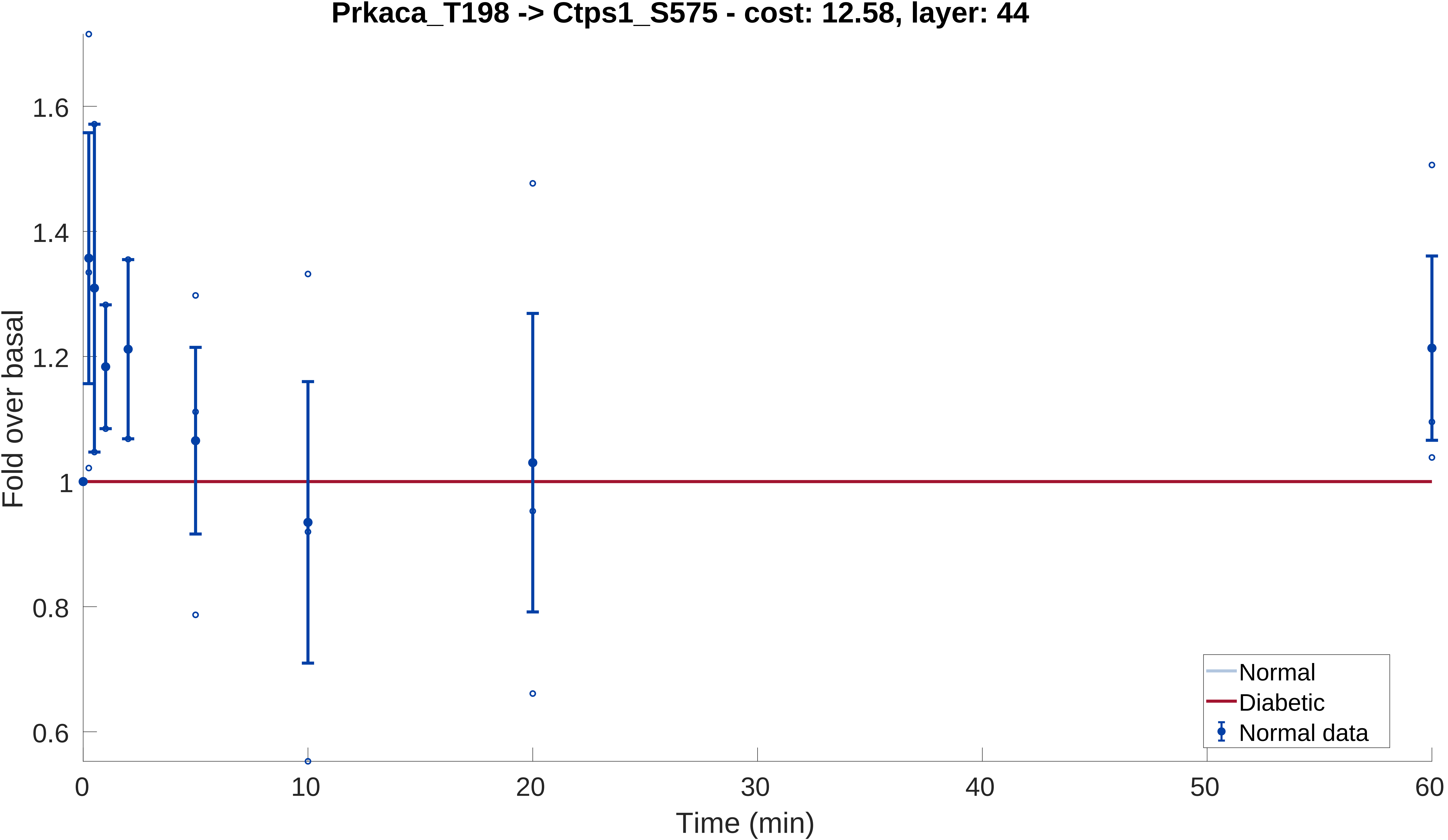

Prkaca_T198 -> Ctps1_S575 - cost: 12.58, layer: 44
Fold over basal
1.6
1.4
1.2
1
0.8
0.6
0
10
20
30
40
50
60
Time (min)
Normal
Diabetic
Normal data

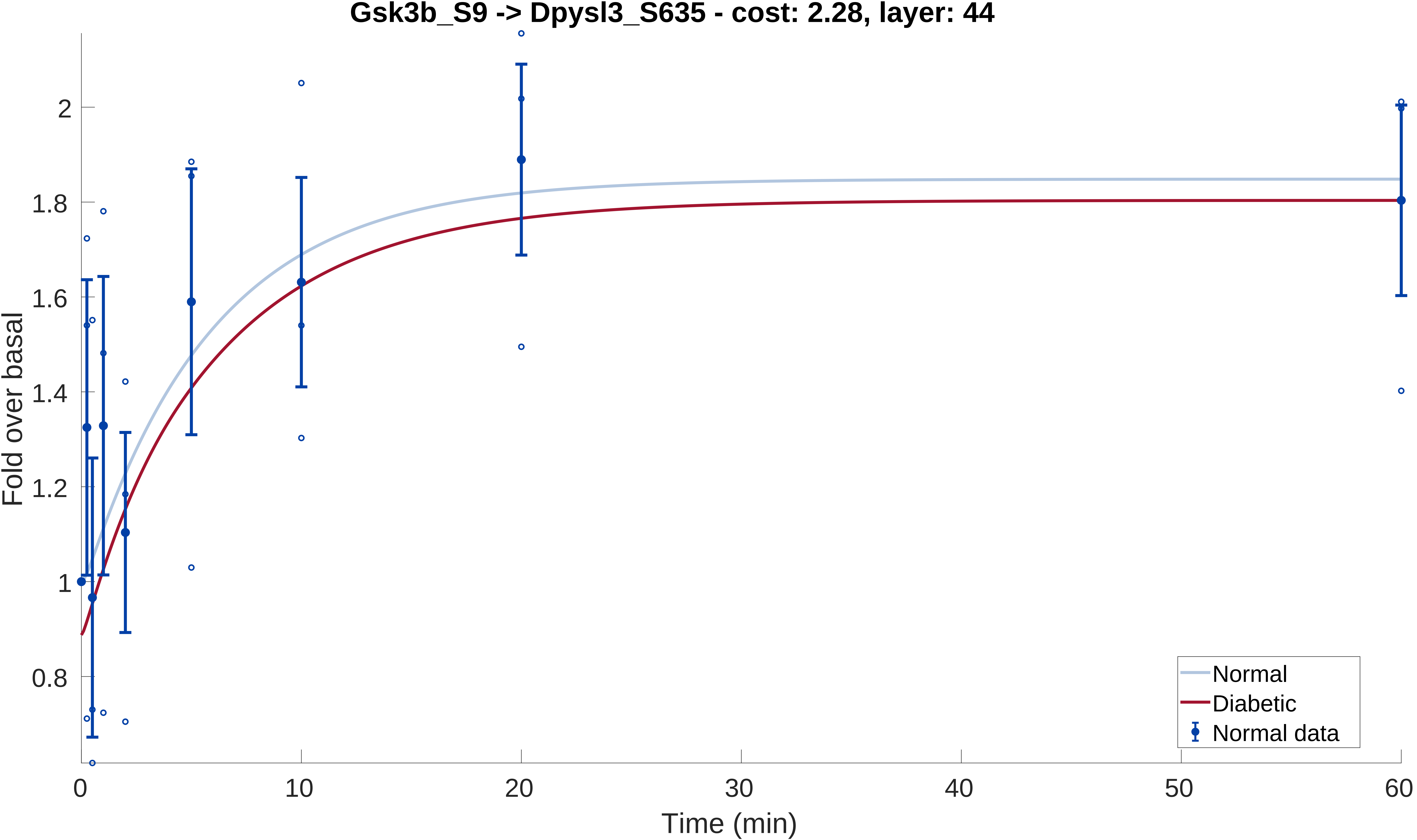
Gsk3b_S9 -> Dpysl3_S635 - cost: 2.28, layer: 44
Fold over basal
2
1.8
1.6
1.4
1.2
1
0.8
0
10
20
30
40
50
60
Time (min)
Normal
Diabetic
Normal data

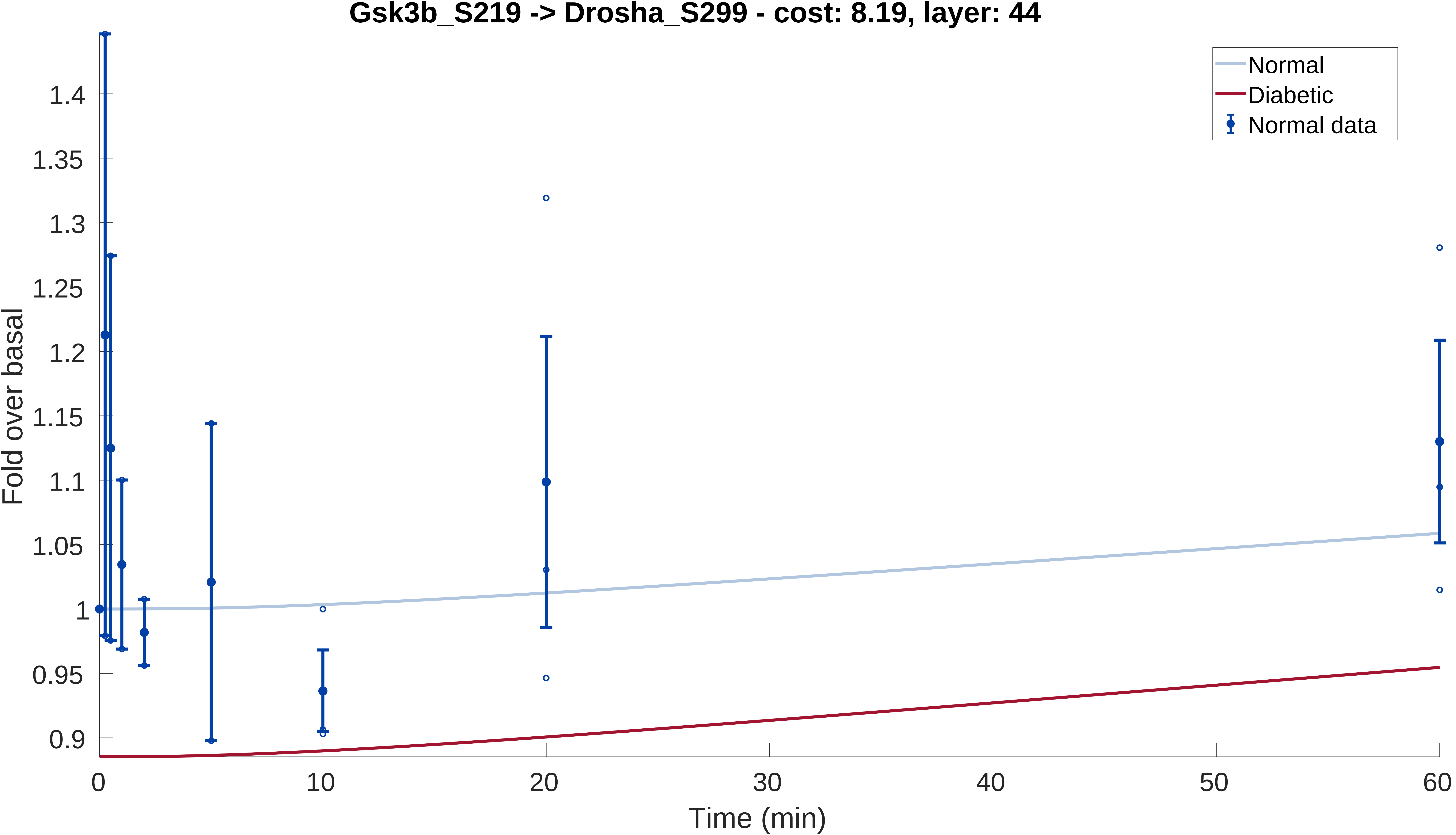
Gsk3b_S219 -> Drosha_S299 - cost: 8.19, layer: 44
Normal
Diabetic
Normal data
Fold over basal
1.4
1.35
1.3
1.25
1.2
1.15
1.1
1.05
1
0.95
0.9
0
10
20
30
40
50
60
Time (min)

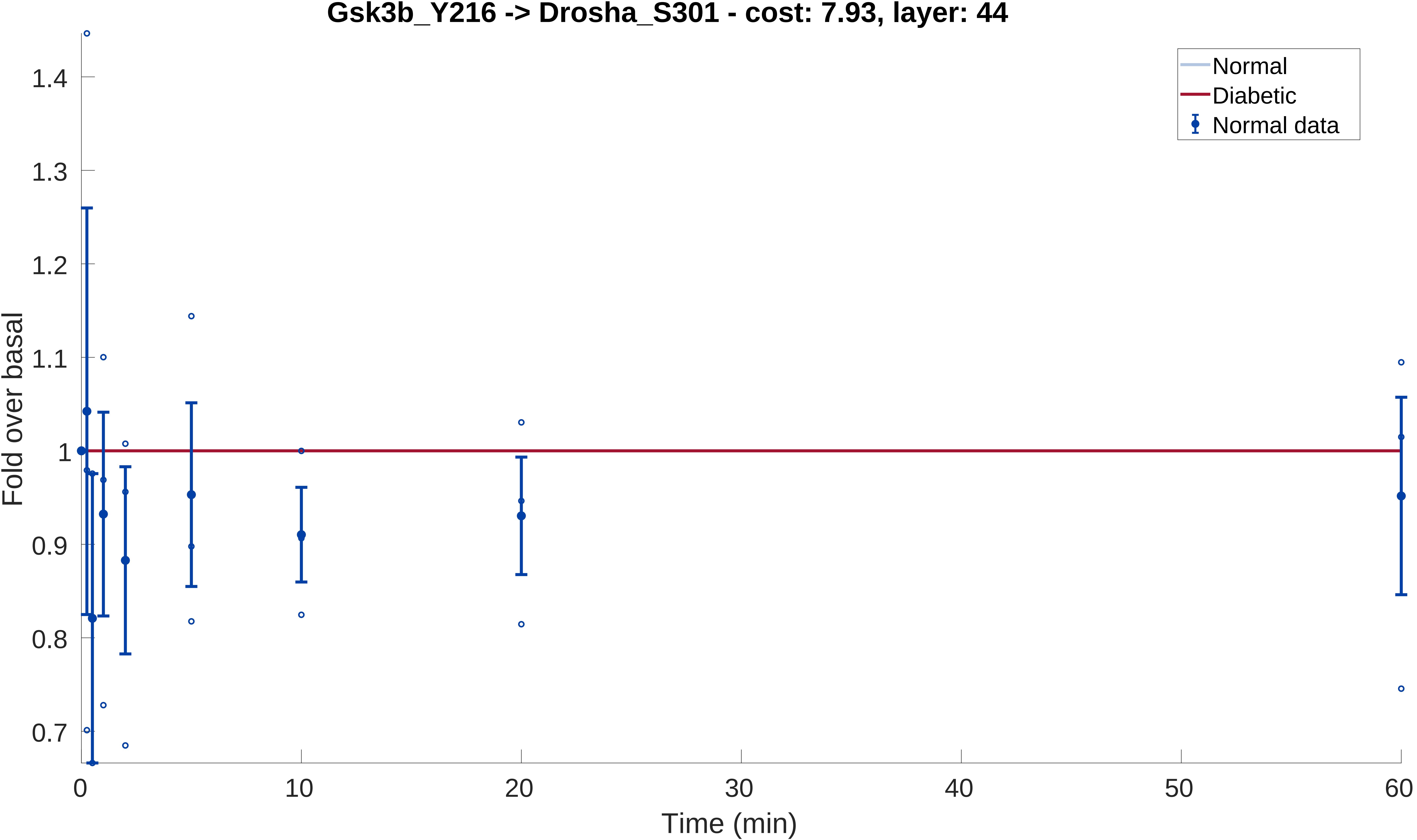

Gsk3b_Y216 -> Drosha_S301 - cost: 7.93, layer: 44
Normal
Diabetic
Normal data
Fold over basal
Time (min)
1.4
1.3
1.2
1.1
1
0.9
0.8
0.7
0
10
20
30
40
50
60

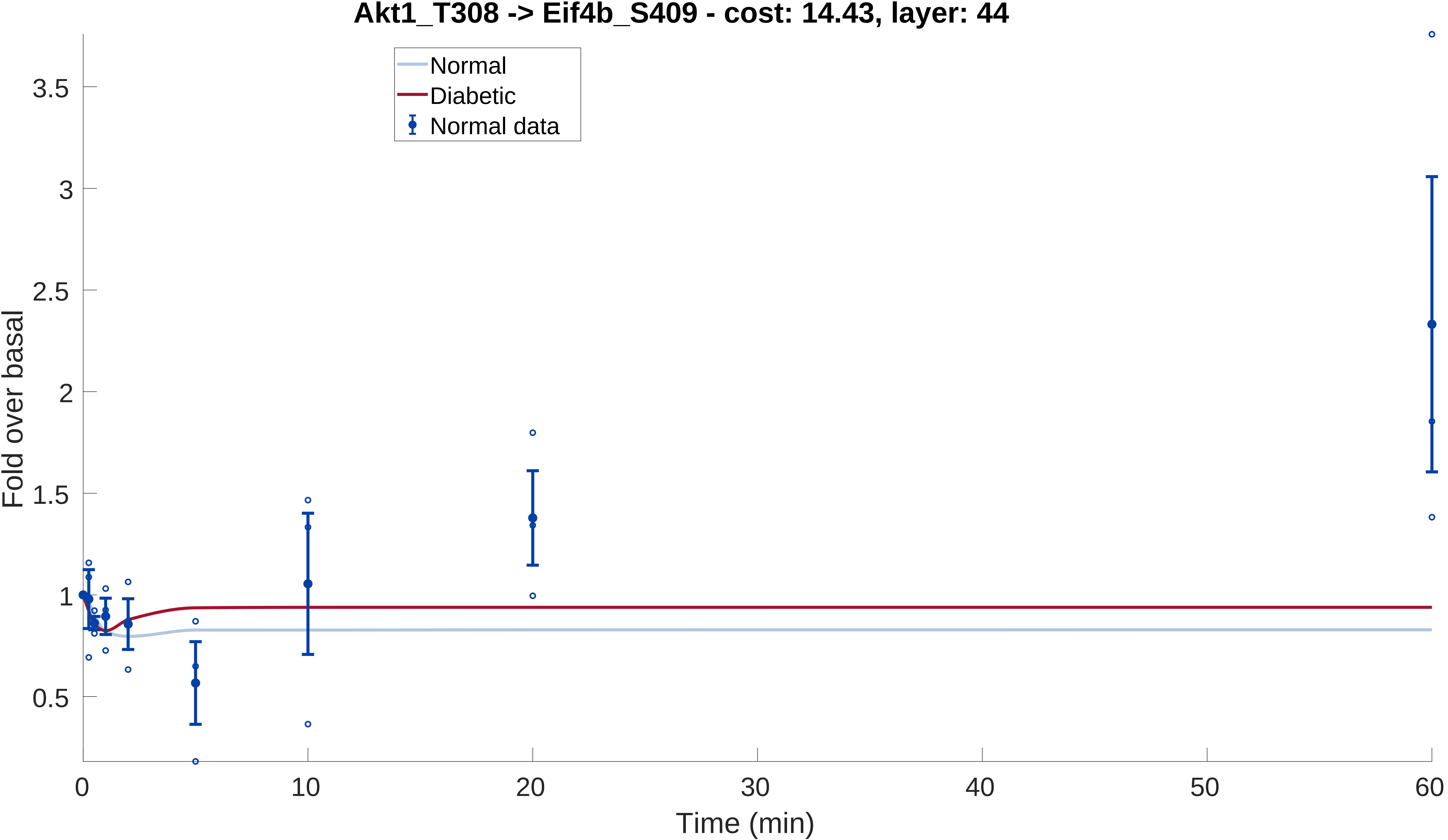
Akt1_T308 -> Eif4b_S409 - cost: 14.43, layer: 44
Normal
Diabetic
Normal data
Fold over basal
Time (min)
0.5
1
1.5
2
2.5
3
3.5
0
10
20
30
40
50
60

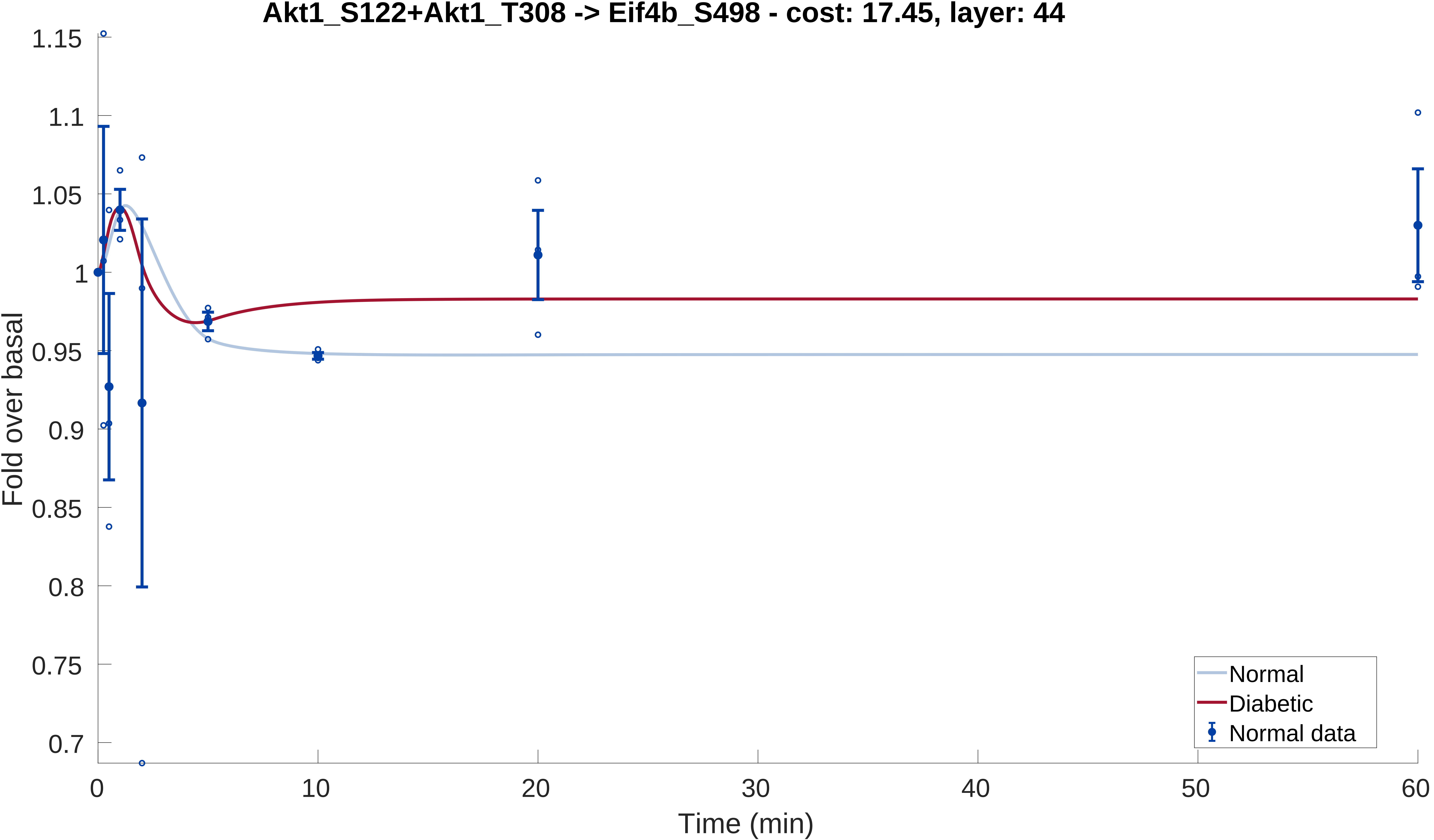
Akt1_S122+Akt1_T308 -> Eif4b_S498 - cost: 17.45, layer: 44
Fold over basal
1.15
1.1
1.05
1
0.95
0.9
0.85
0.8
0.75
0.7
0
10
20
30
40
50
60
Time (min)
Normal
Diabetic
Normal data

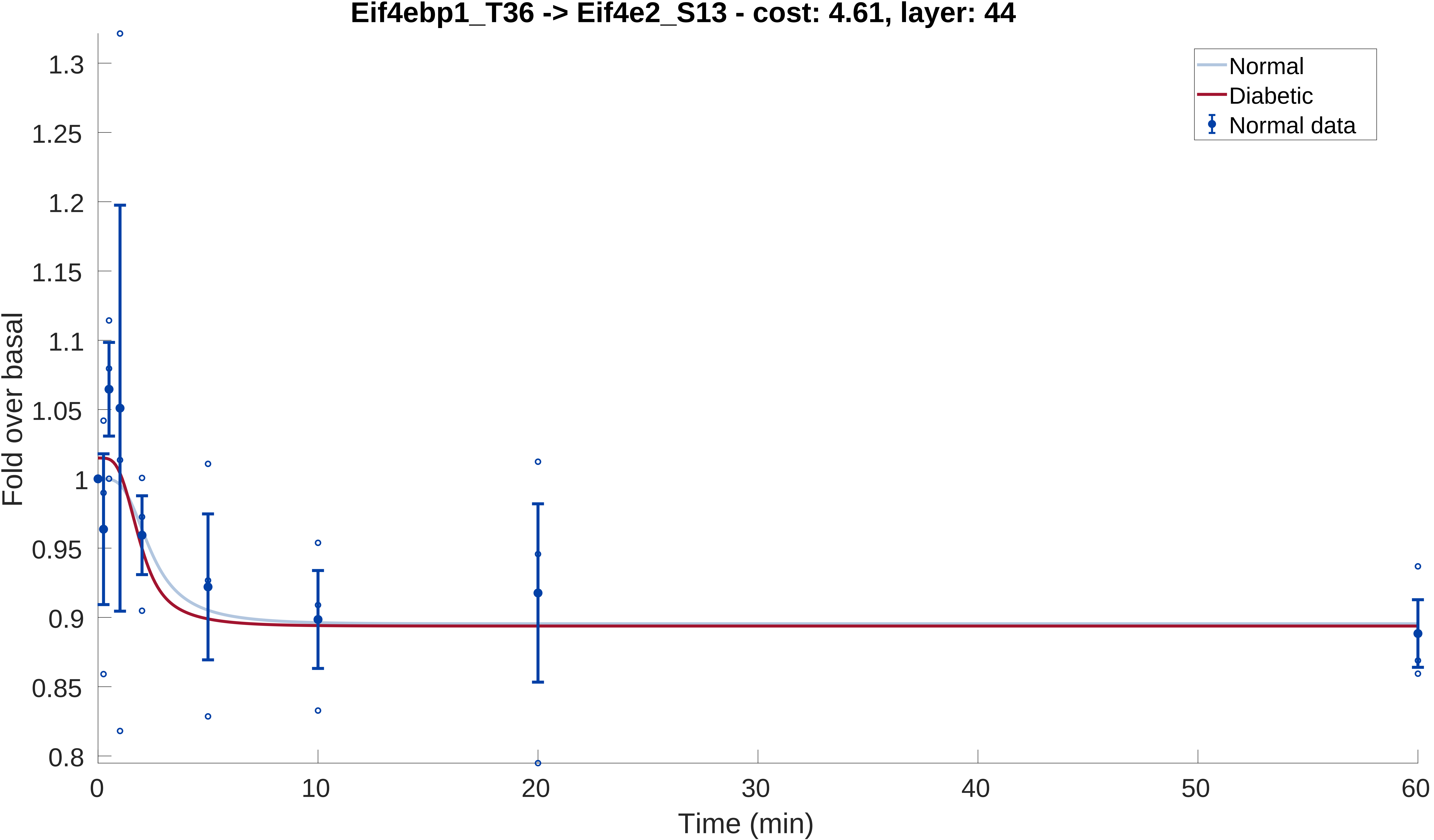

Eif4ebp1_T36 -> Eif4e2_S13 - cost: 4.61, layer: 44
Normal
Diabetic
Normal data
Fold over basal
1.3
1.25
1.2
1.15
1.1
1.05
1
0.95
0.9
0.85
0.8
0
10
20
30
40
50
60
Time (min)

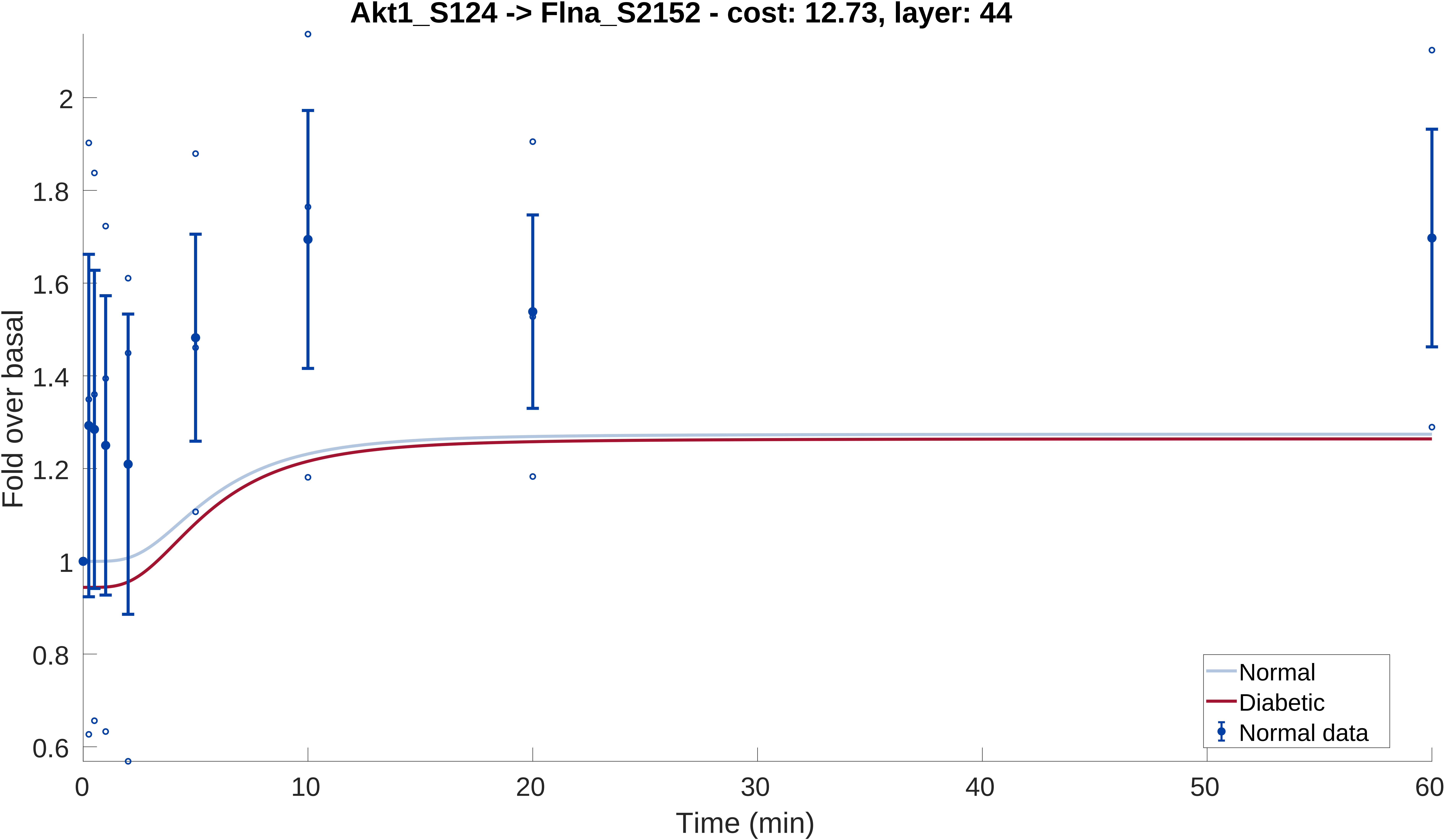
Akt1_S124 -> Flna_S2152 - cost: 12.73, layer: 44
Fold over basal
Time (min)
0
10
20
30
40
50
60
0.6
0.8
1
1.2
1.4
1.6
1.8
2
Normal
Diabetic
Normal data

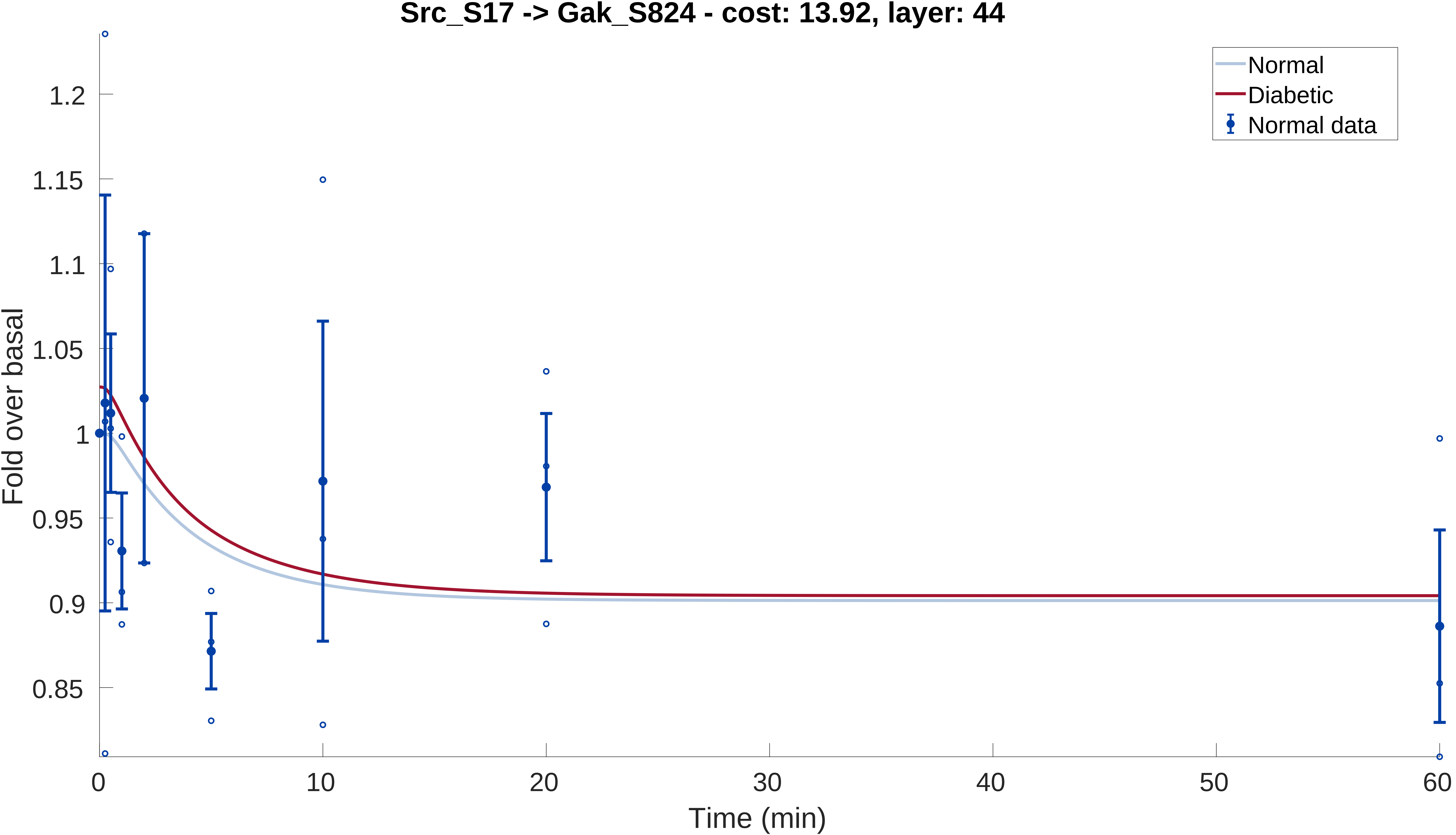

Src_S17 -> Gak_S824 - cost: 13.92, layer: 44
Normal
Diabetic
Normal data
Fold over basal
1.2
1.15
1.1
1.05
1
0.95
0.9
0.85
0
10
20
30
40
50
60
Time (min)

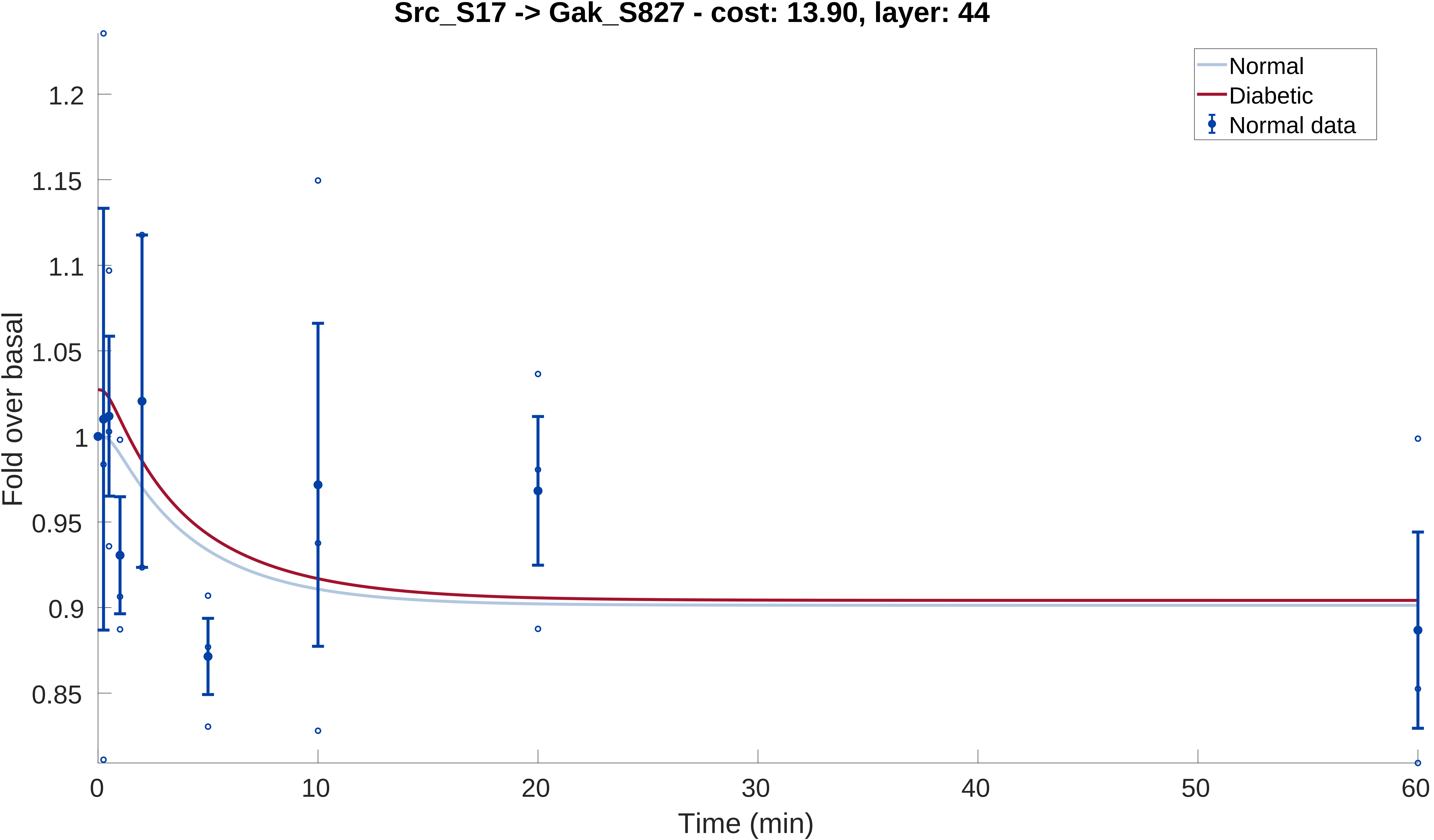

Src_S17 -> Gak_S827 - cost: 13.90, layer: 44
Normal
Diabetic
Normal data
Fold over basal
1.2
1.15
1.1
1.05
1
0.95
0.9
0.85
0
10
20
30
40
50
60
Time (min)

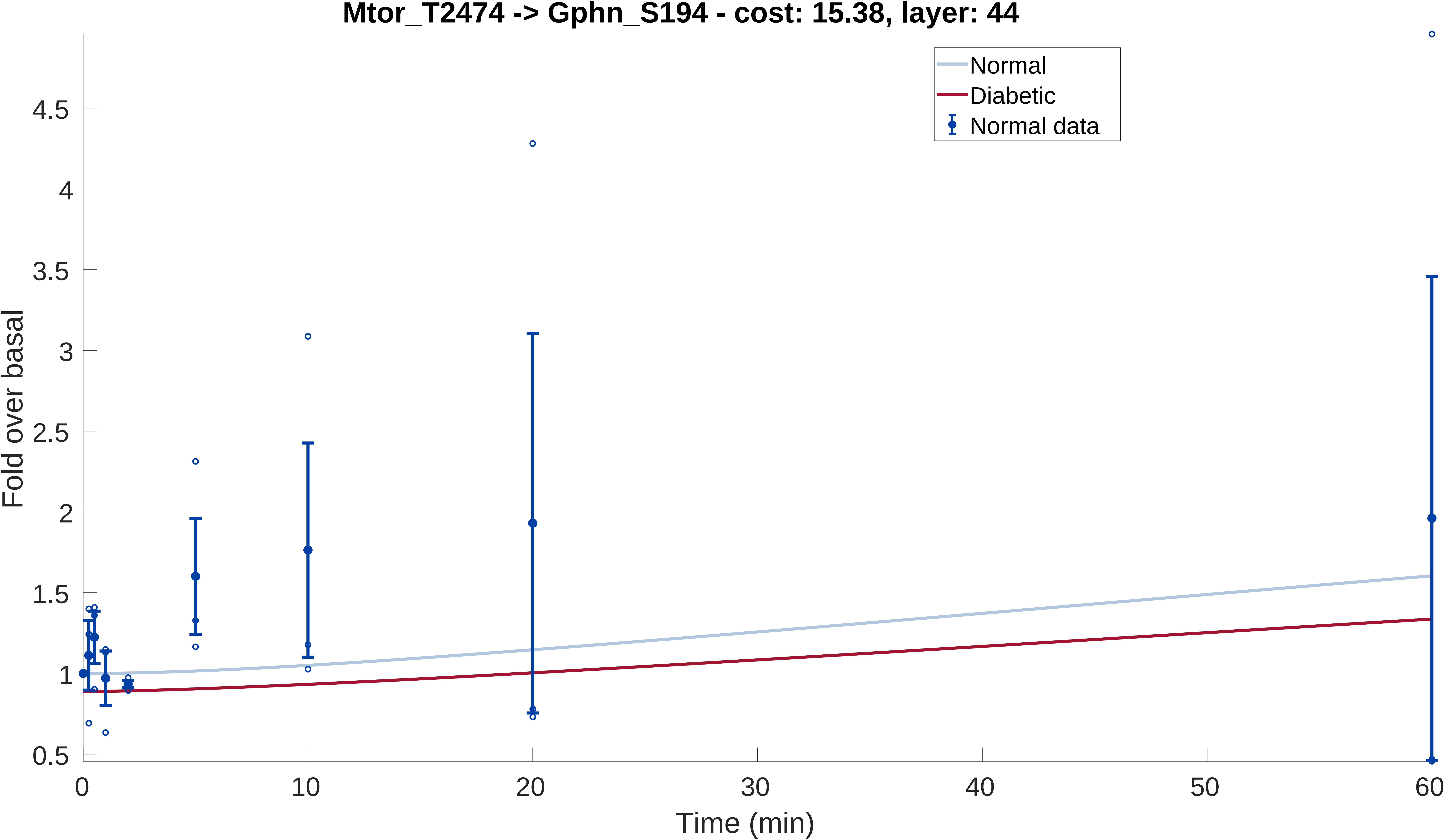

Mtor_T2474 -> Gphn_S194 - cost: 15.38, layer: 44
Normal
Diabetic
Normal data
Fold over basal
Time (min)
0.5
1
1.5
2
2.5
3
3.5
4
4.5
0
10
20
30
40
50
60

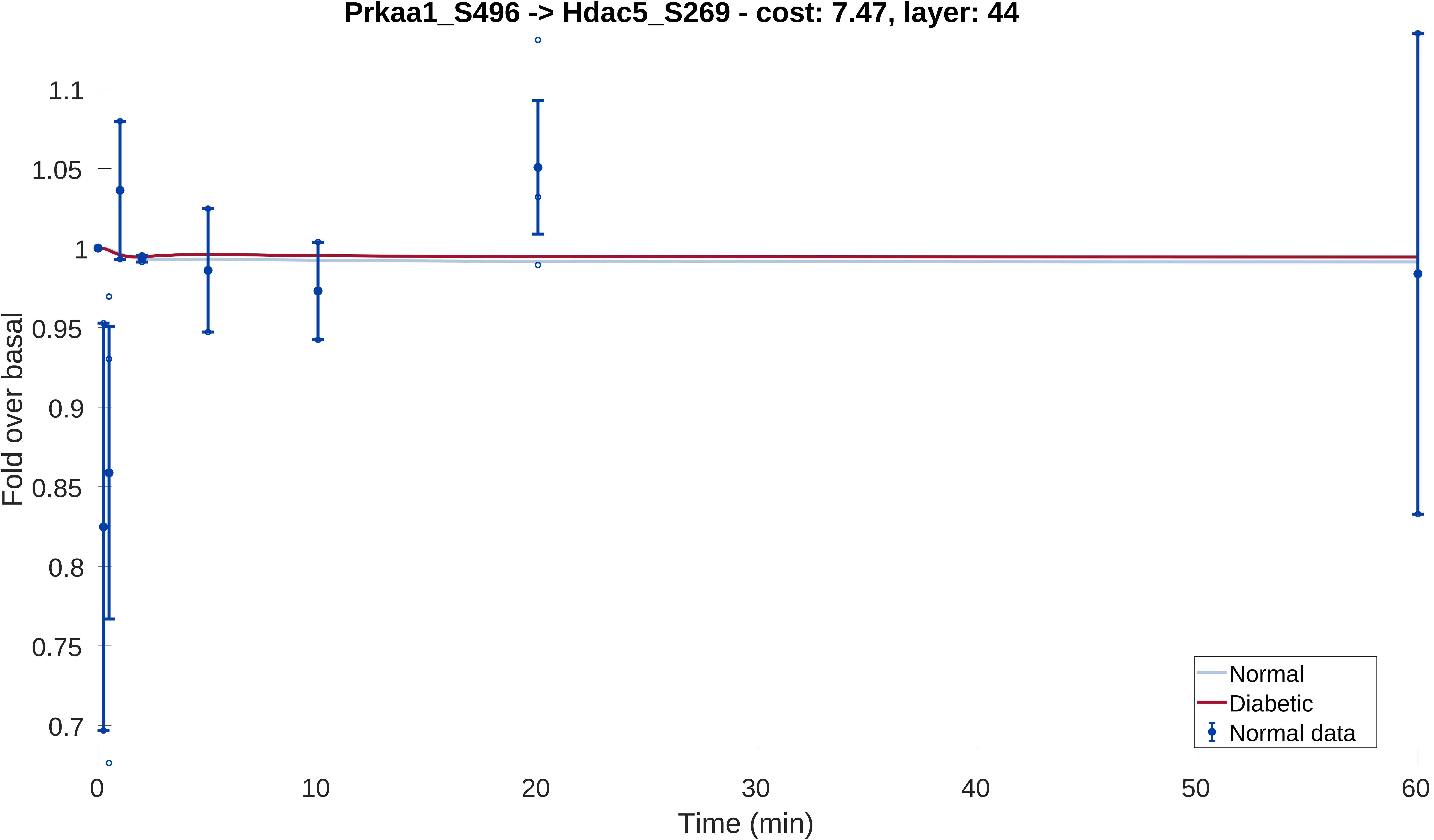

Prkaa1_S496 -> Hdac5_S269 - cost: 7.47, layer: 44
Fold over basal
1.1
1.05
1
0.95
0.9
0.85
0.8
0.75
0.7
0
10
20
30
40
50
60
Time (min)
Normal
Diabetic
Normal data

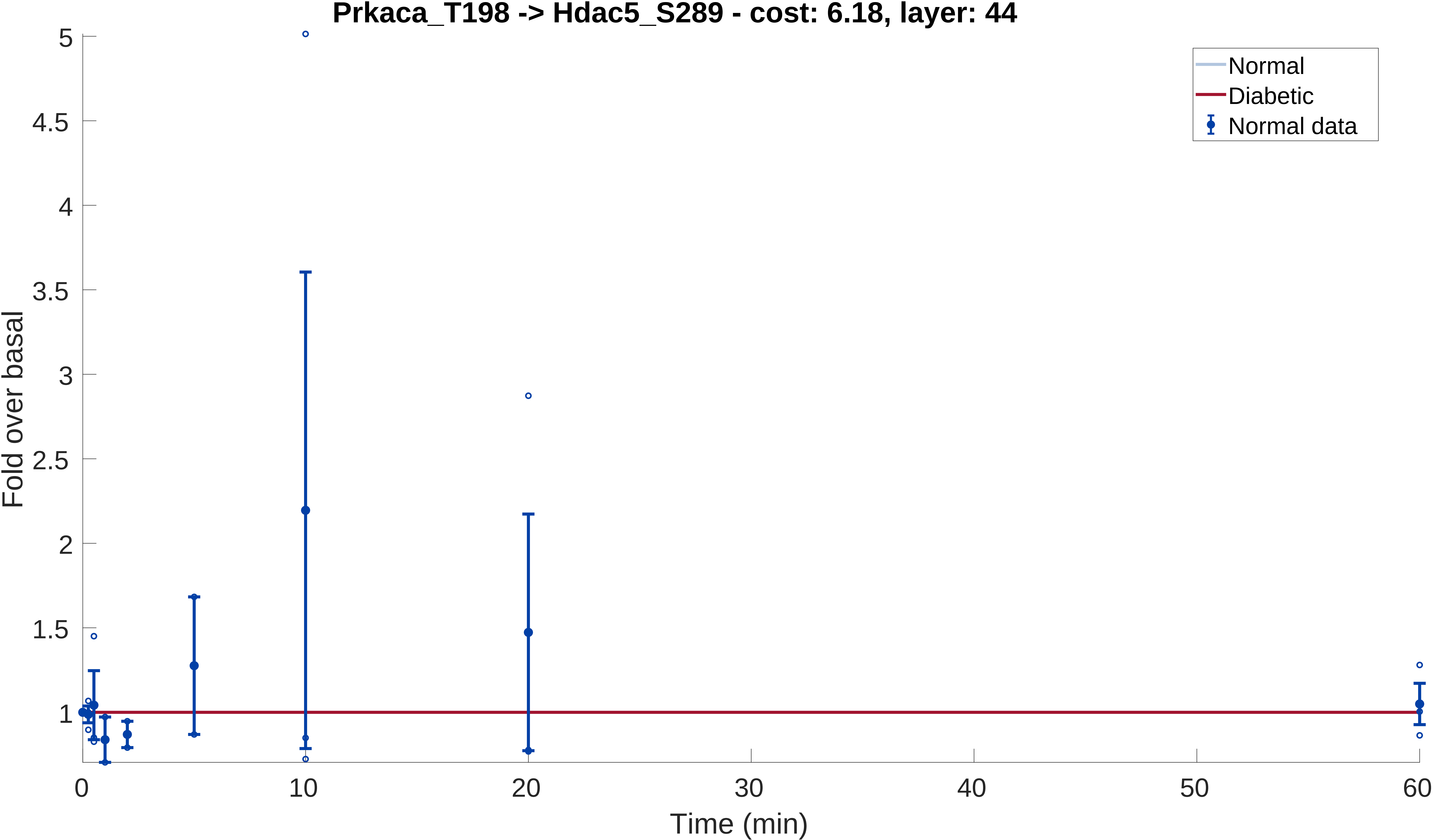

Prkaca_T198 -> Hdac5_S289 - cost: 6.18, layer: 44
Fold over basal
Time (min)
Normal
Diabetic
Normal data
5
4.5
4
3.5
3
2.5
2
1.5
1
0
10
20
30
40
50
60

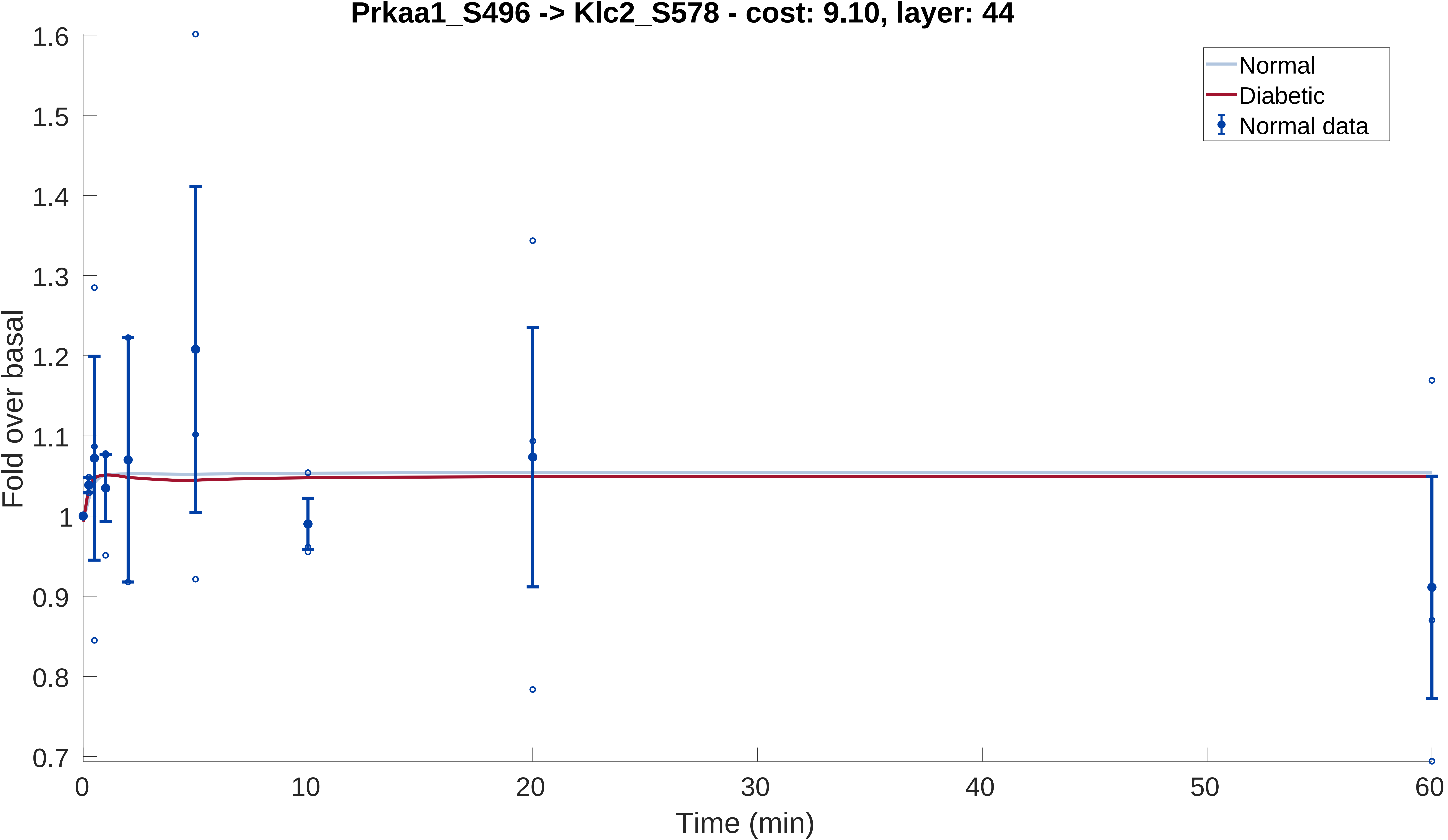

Prrkaa1_S496 -> Klc2_S578 - cost: 9.10, layer: 44
Normal
Diabetic
Normal data
Fold over basal
Time (min)
1.6
1.5
1.4
1.3
1.2
1.1
1
0.9
0.8
0.7
0
10
20
30
40
50
60

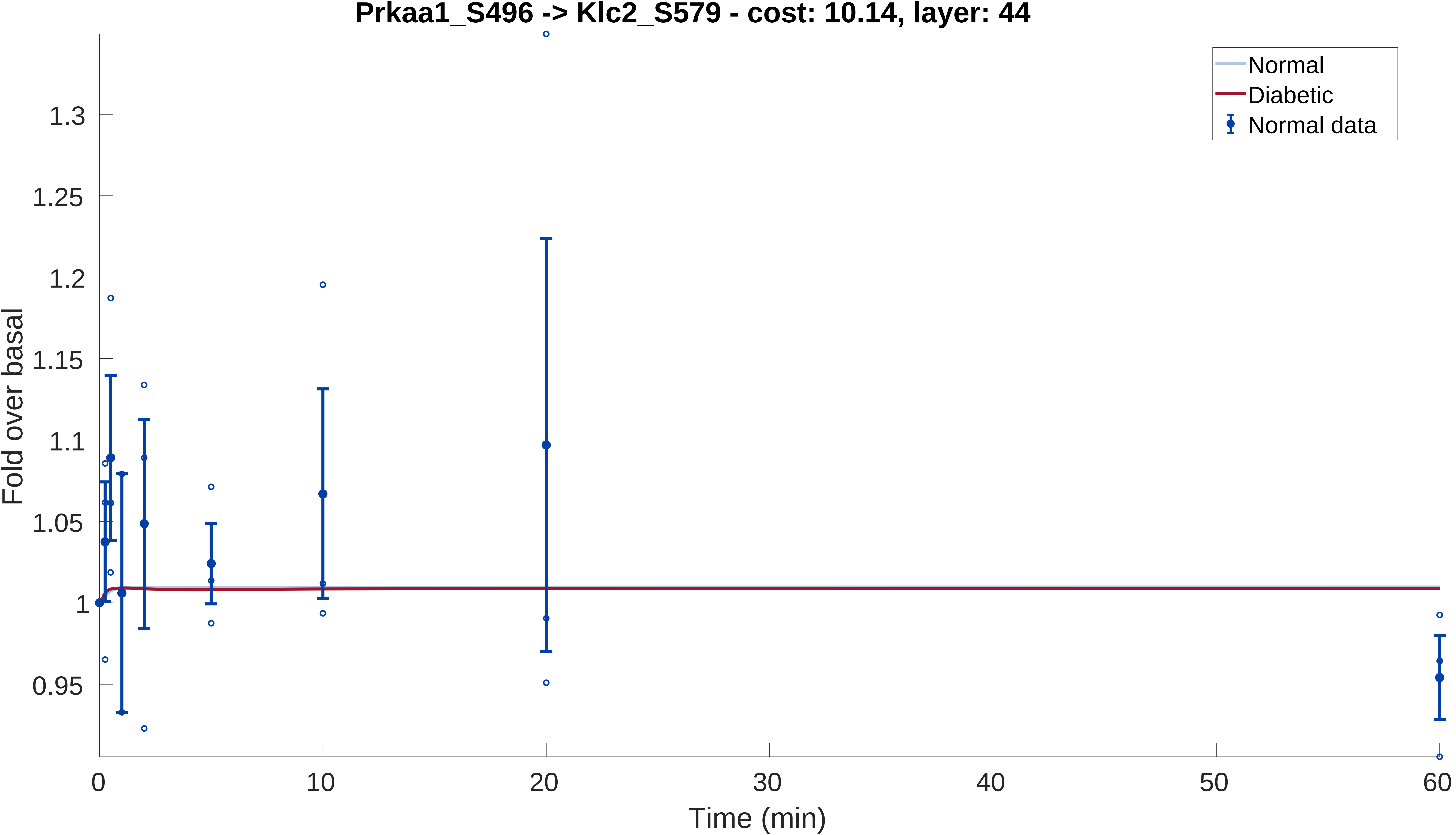
Prkaa1_S496 -> Klc2_S579 - cost: 10.14, layer: 44
Normal
Diabetic
Normal data
Fold over basal
Time (min)
1.3
1.25
1.2
1.15
1.1
1.05
1
0.95
0
10
20
30
40
50
60

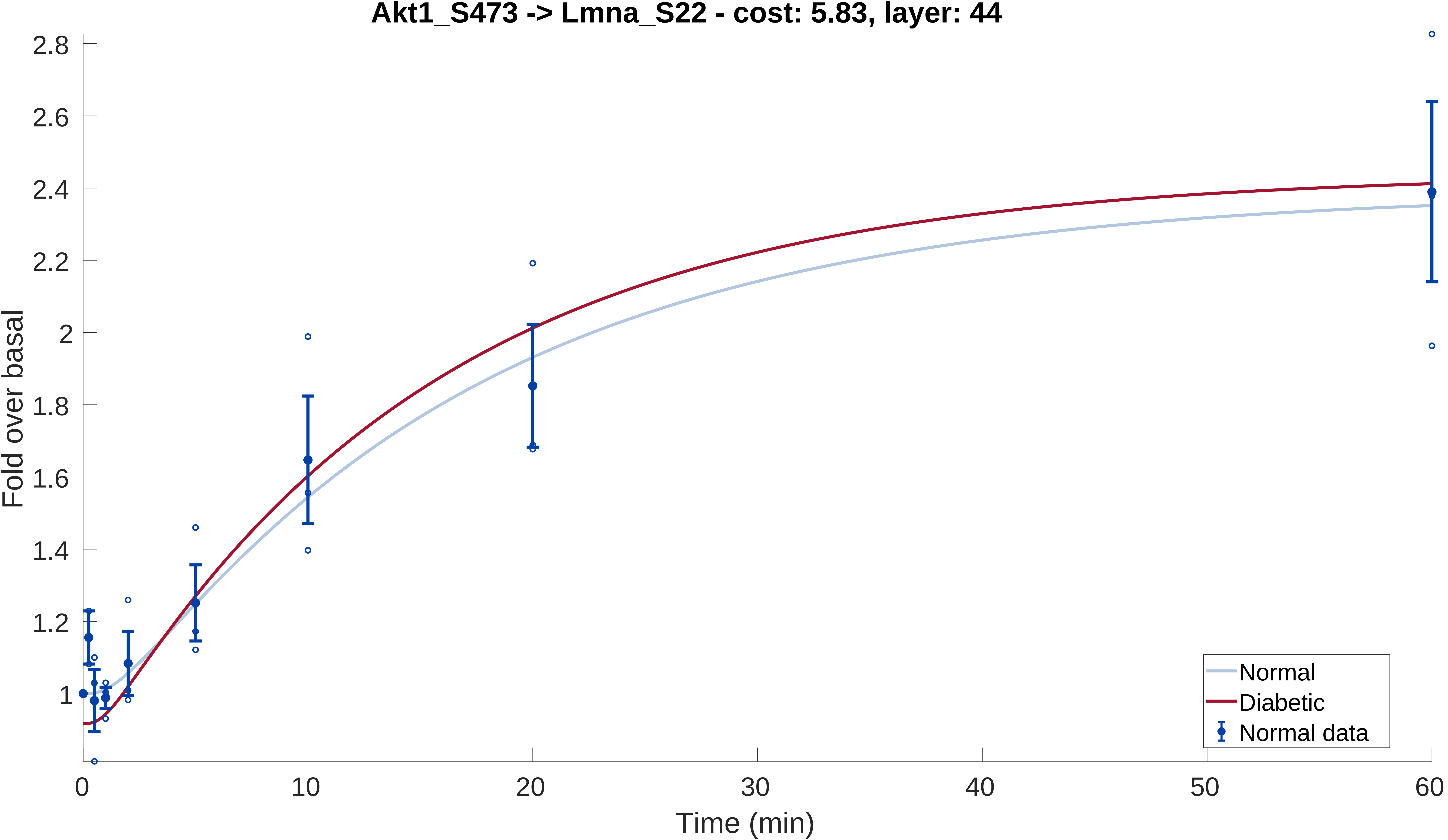

Akt1_S473 -> Lmna_S22 - cost: 5.83, layer: 44
Fold over basal
2.8
2.6
2.4
2.2
2
1.8
1.6
1.4
1.2
1
0
10
20
30
40
50
60
Time (min)
Normal
Diabetic
Normal data

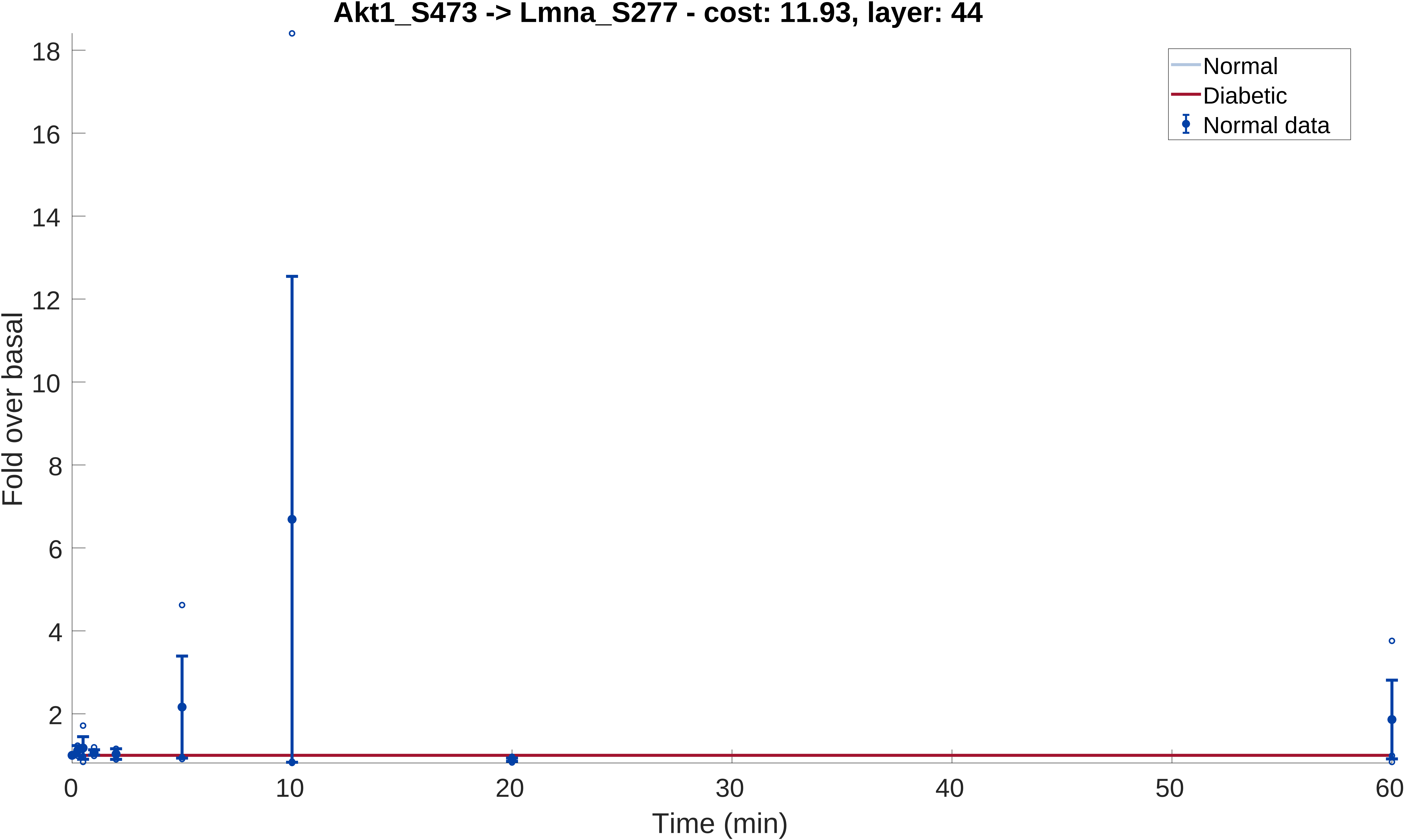
Akt1_S473 -> Lmna_S277 - cost: 11.93, layer: 44
Normal
Diabetic
Normal data
Fold over basal
Time (min)
0
10
20
30
40
50
60
2
4
6
8
10
12
14
16
18

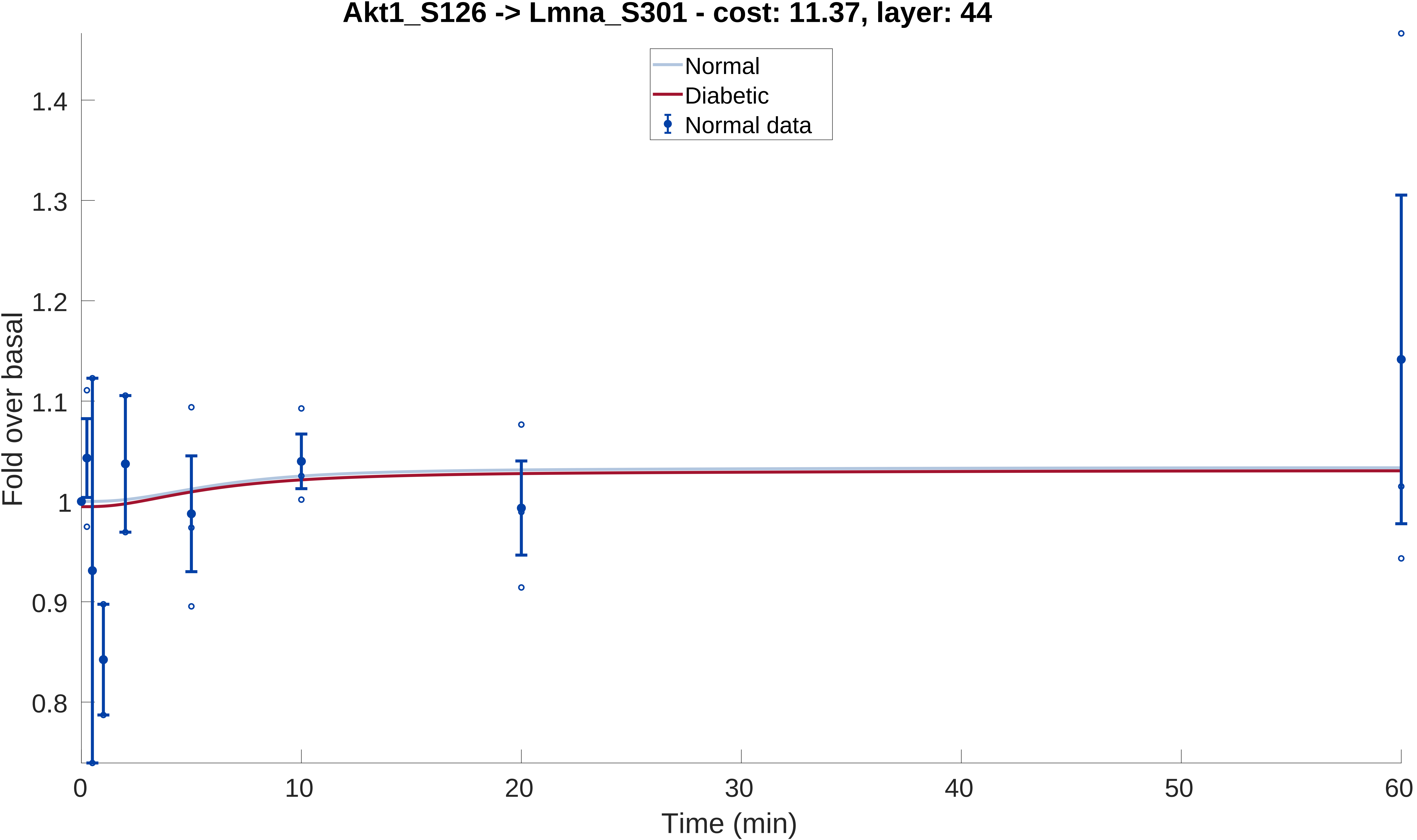

Akt1_S126 -> Lmna_S301 - cost: 11.37, layer: 44
Normal
Diabetic
Normal data
1.4
1.3
1.2
1.1
1
0.9
0.8
Fold over basal
0
10
20
30
40
50
60
Time (min)

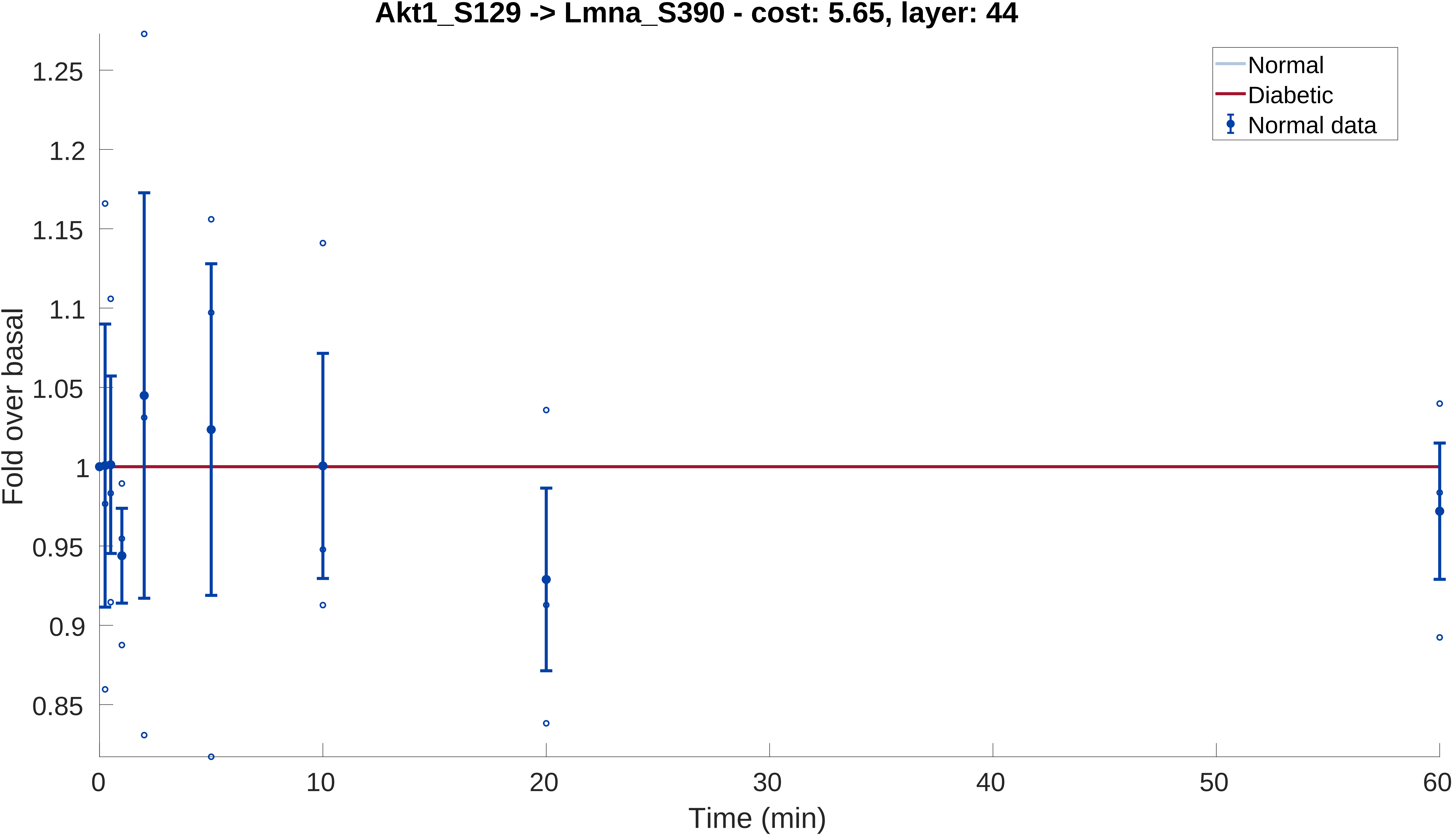

Akt1_S129 -> Lmna_S390 - cost: 5.65, layer: 44
Normal
Diabetic
Normal data
Fold over basal
1.25
1.2
1.15
1.1
1.05
1
0.95
0.9
0.85
0
10
20
30
40
50
60
Time (min)

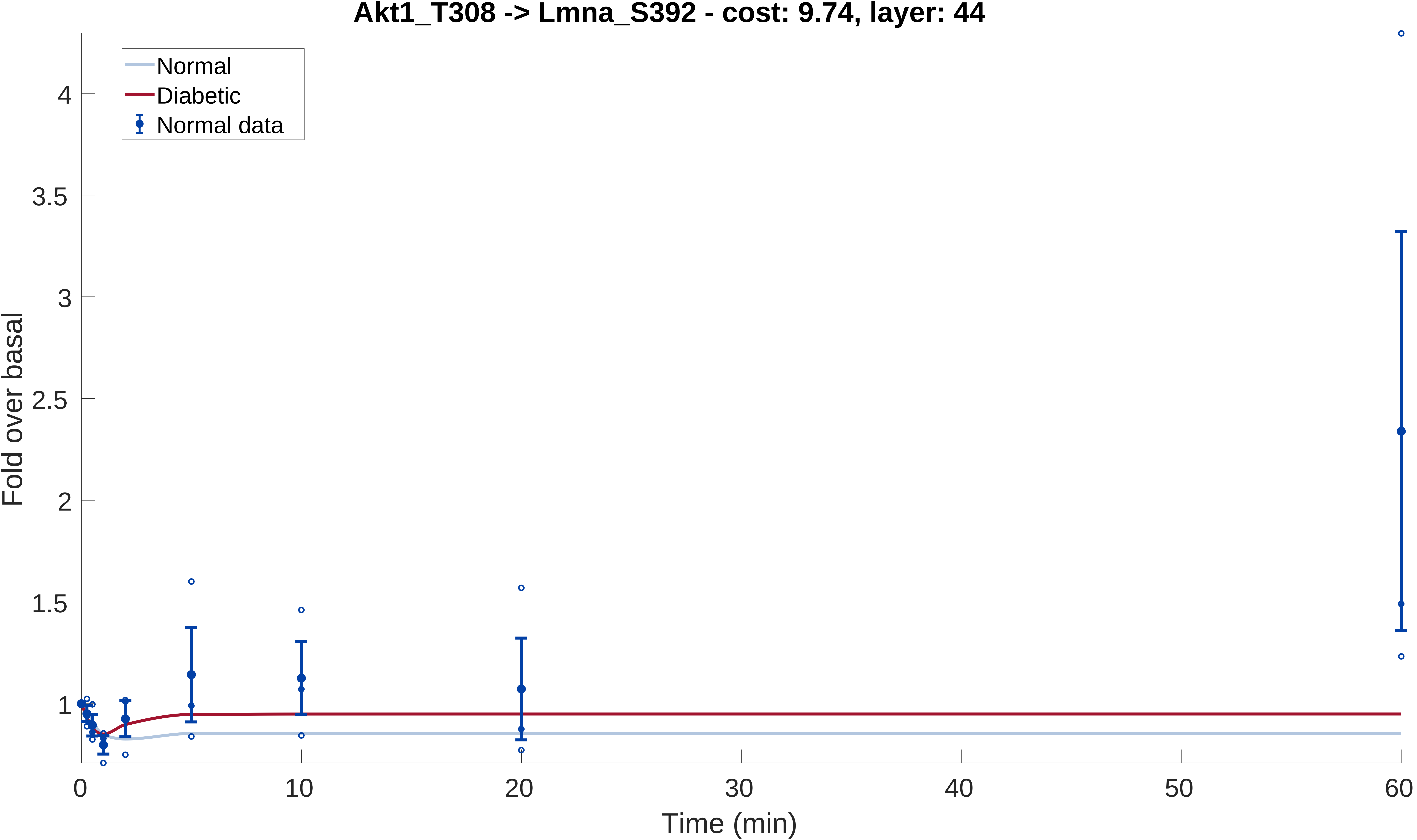

Akt1_T308 -> Lmna_S392 - cost: 9.74, layer: 44
Normal
Diabetic
Normal data
Fold over basal
4
3.5
3
2.5
2
1.5
1
0
10
20
30
40
50
60
Time (min)

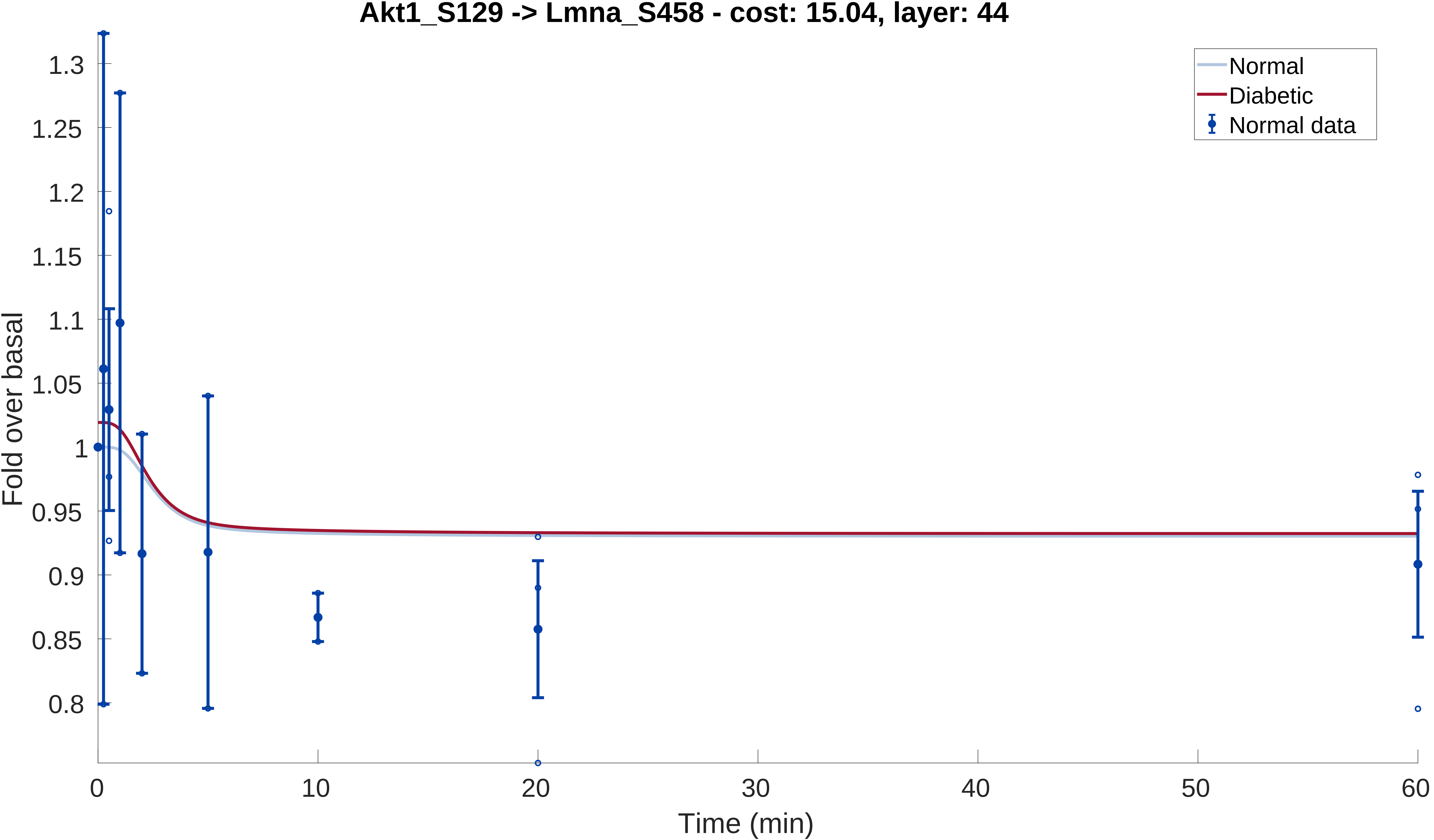

Akt1_S129 -> Lmna_S458 - cost: 15.04, layer: 44
Normal
Diabetic
Normal data
Fold over basal
1.3
1.25
1.2
1.15
1.1
1.05
1
0.95
0.9
0.85
0.8
0
10
20
30
40
50
60
Time (min)

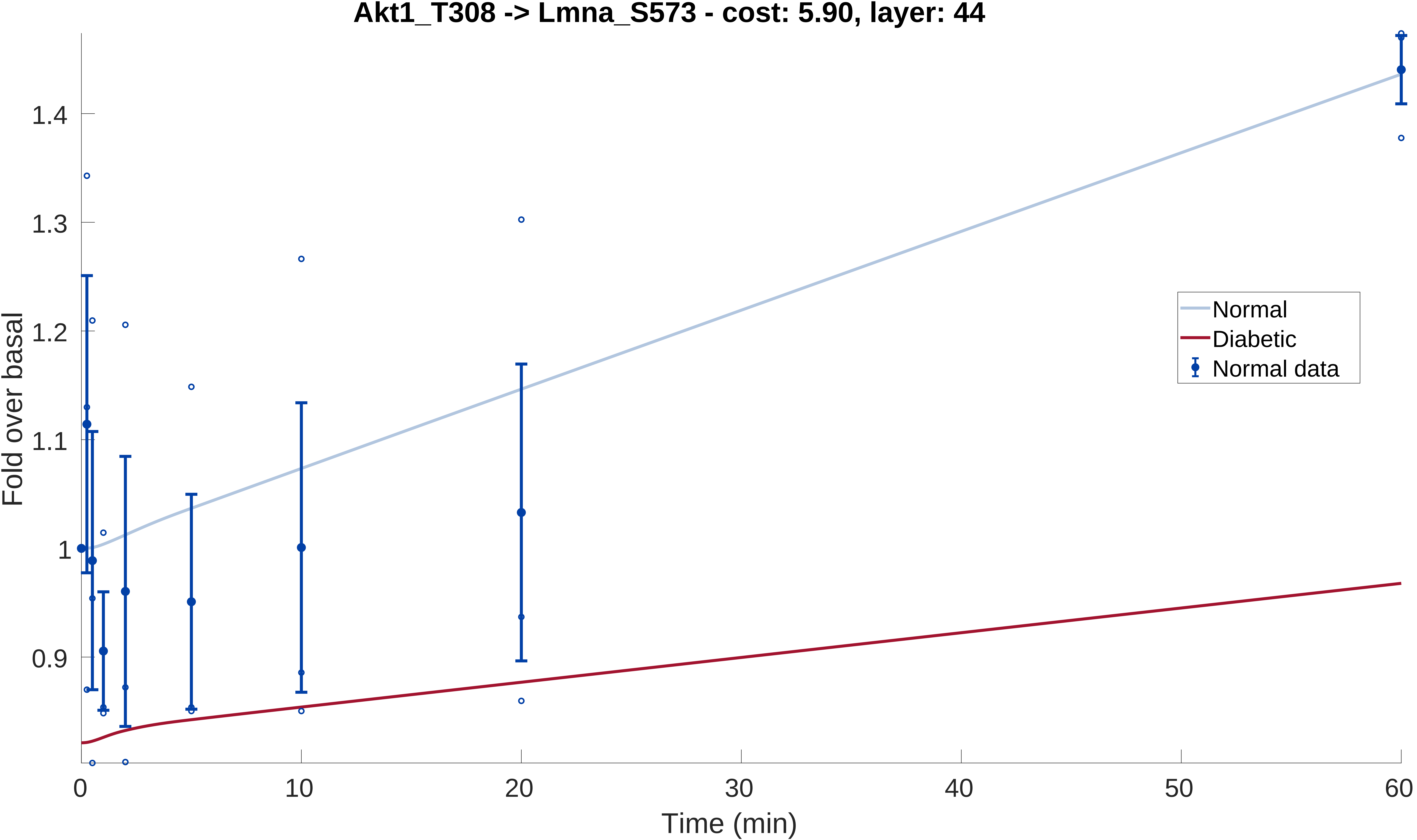
Akt1_T308 -> Lmna_S573 - cost: 5.90, layer: 44
Fold over basal
Time (min)
1.4
1.3
1.2
1.1
1
0.9
0
10
20
30
40
50
60
Normal
Diabetic
Normal data

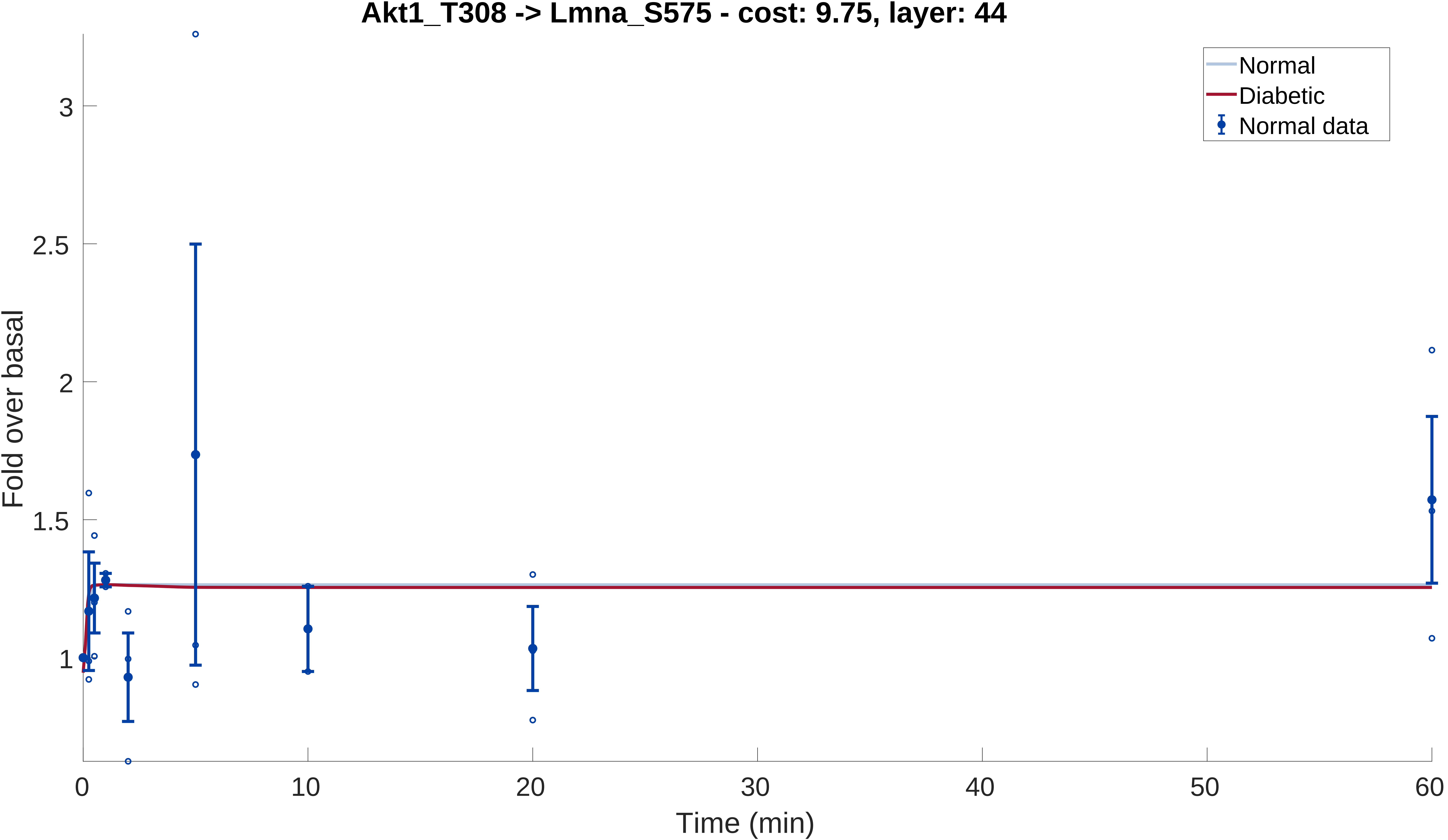
Akt1_T308 -> Lmna_S575 - cost: 9.75, layer: 44
Normal
Diabetic
Normal data
Fold over basal
3
2.5
2
1.5
1
0
10
20
30
40
50
60
Time (min)

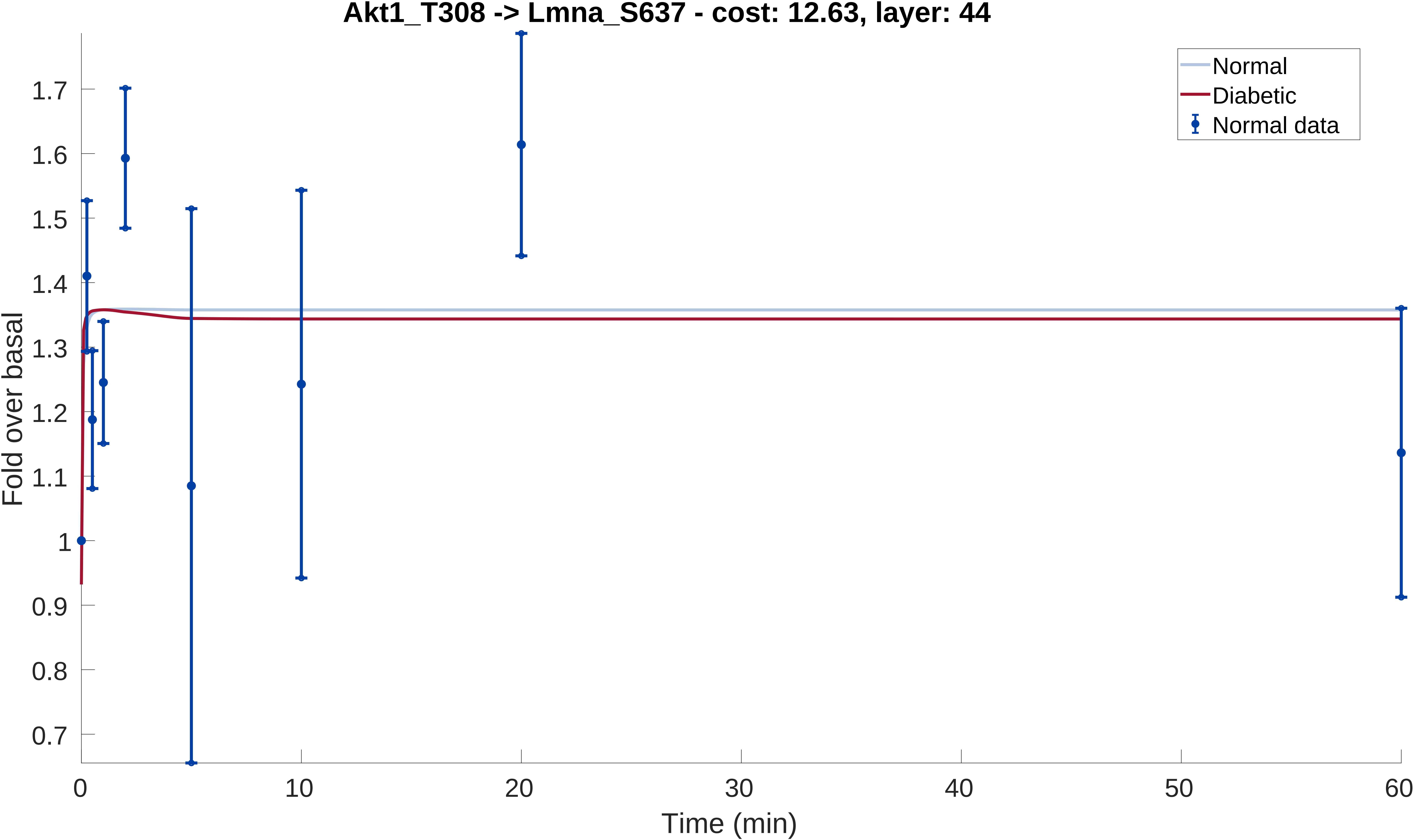

Akt1_T308 -> Lmna_S637 - cost: 12.63, layer: 44
Normal
Diabetic
Normal data
Fold over basal
0.7
0.8
0.9
1
1.1
1.2
1.3
1.4
1.5
1.6
1.7
0
10
20
30
40
50
60
Time (min)

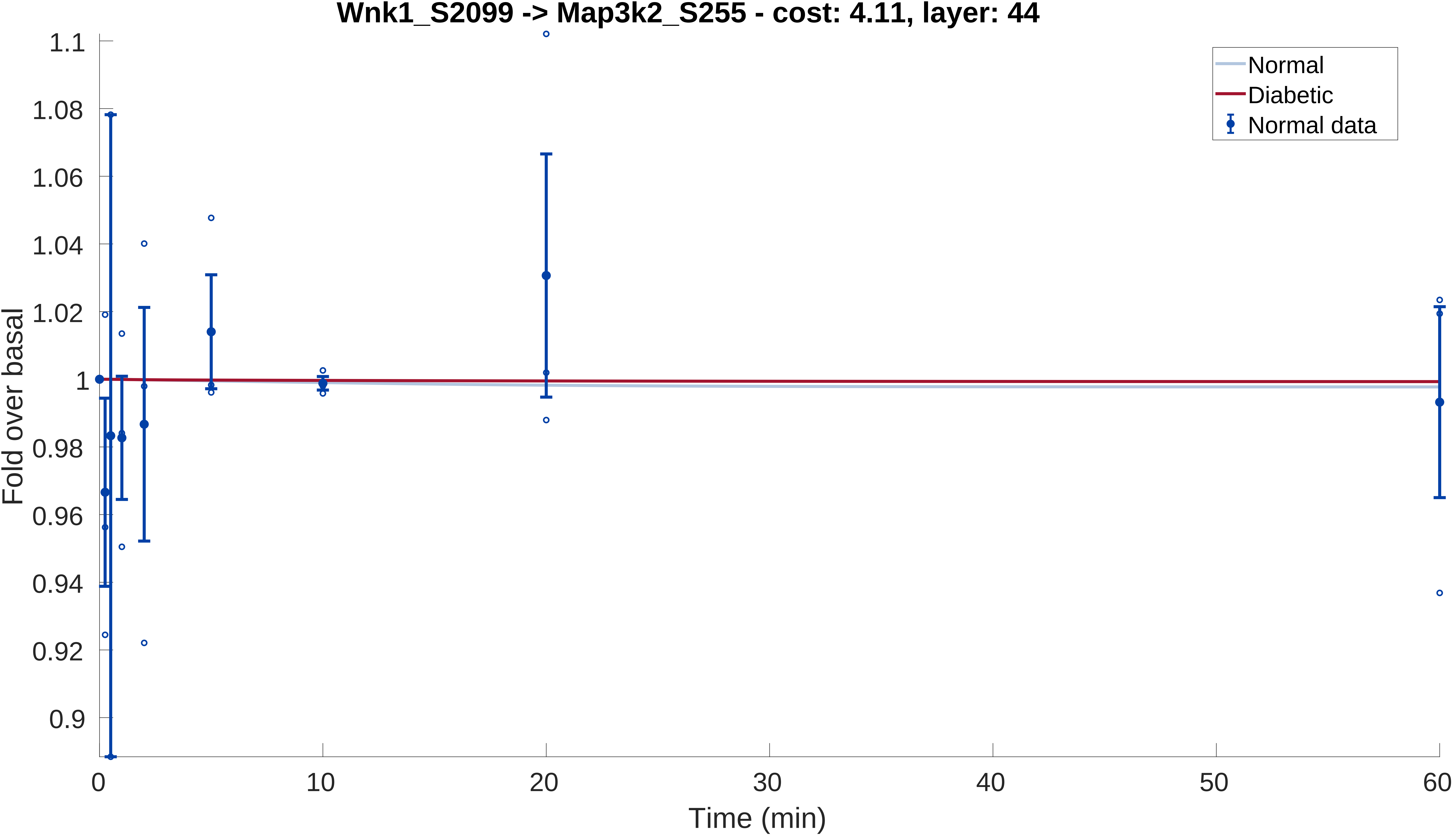

Wnk1_S2099 -> Map3k2_S255 - cost: 4.11, layer: 44
Fold over basal
Time (min)
Normal
Diabetic
Normal data
1.1
1.08
1.06
1.04
1.02
1
0.98
0.96
0.94
0.92
0.9
0
10
20
30
40
50
60

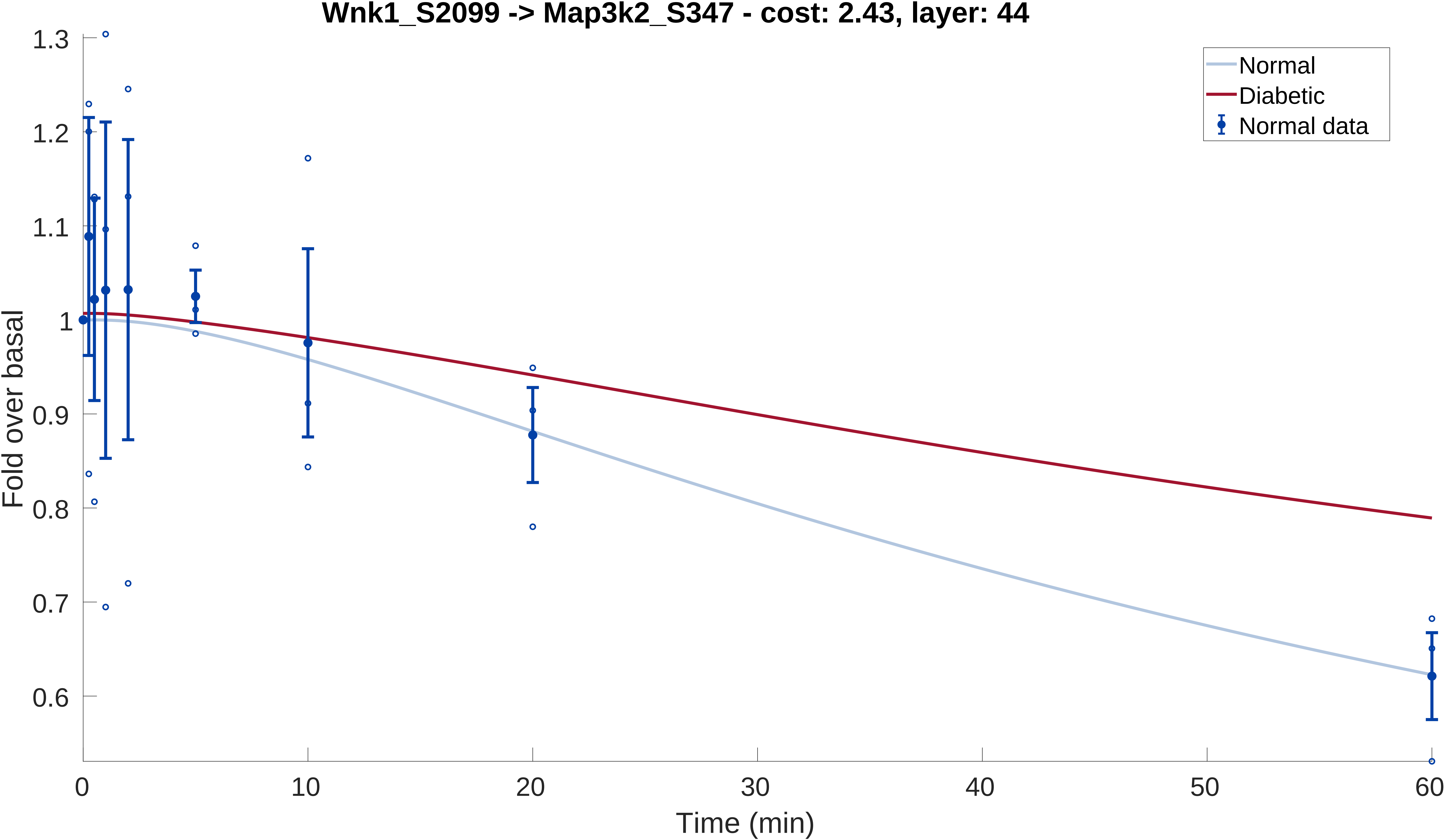
Wnk1_S2099 -> Map3k2_S347 - cost: 2.43, layer: 44
Fold over basal
1.3
1.2
1.1
1
0.9
0.8
0.7
0.6
0
10
20
30
40
50
60
Time (min)
Normal
Diabetic
Normal data

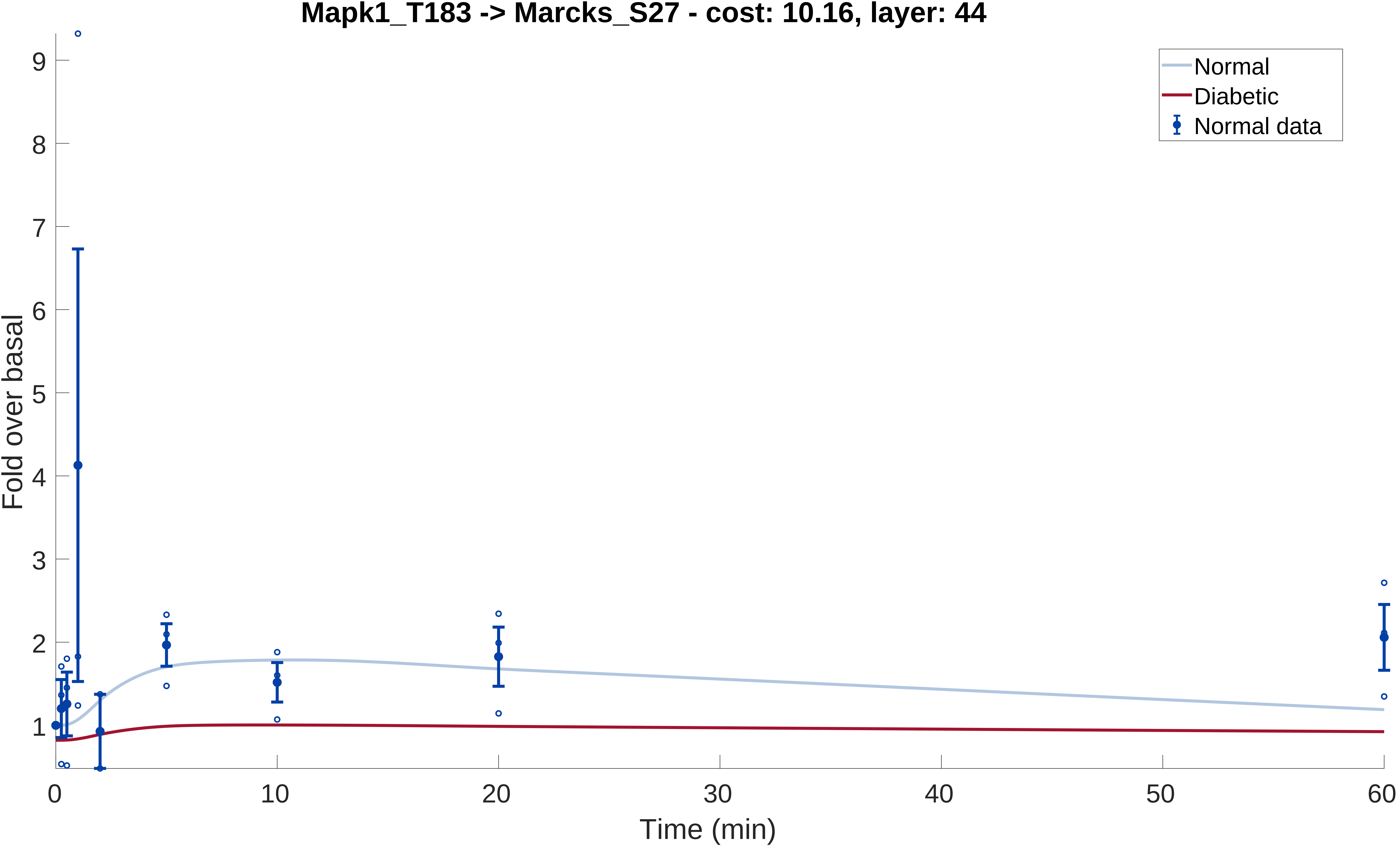

Mapk1_T183 -> Marcks_S27 - cost: 10.16, layer: 44
Normal
Diabetic
Normal data
Fold over basal
9
8
7
6
5
4
3
2
1
0
10
20
30
40
50
60
Time (min)

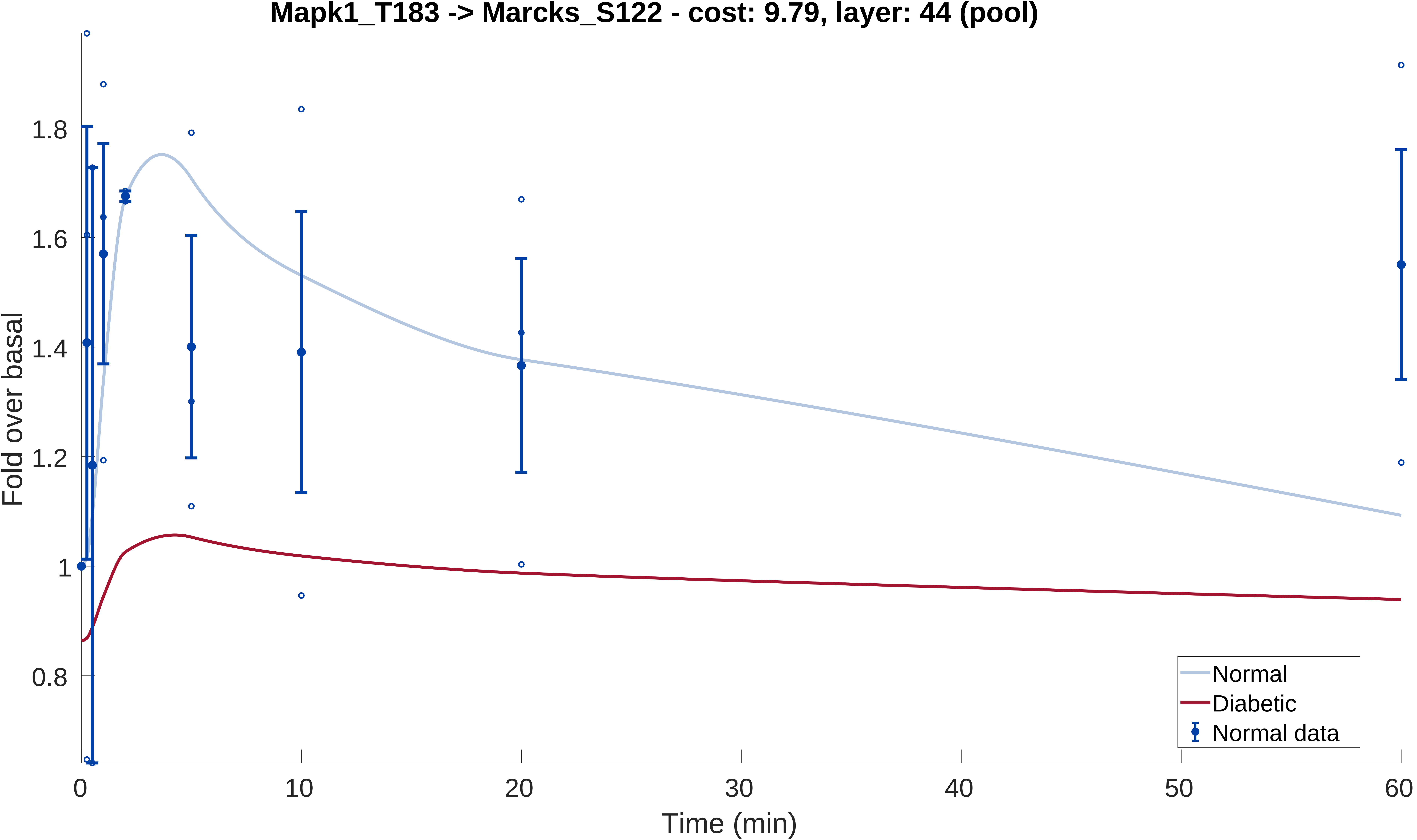

Mapk1_T183 -> Marcks_S122 - cost: 9.79, layer: 44 (pool)
Fold over basal
Time (min)
0.8
1
1.2
1.4
1.6
1.8
0
10
20
30
40
50
60
Normal
Diabetic
Normal data

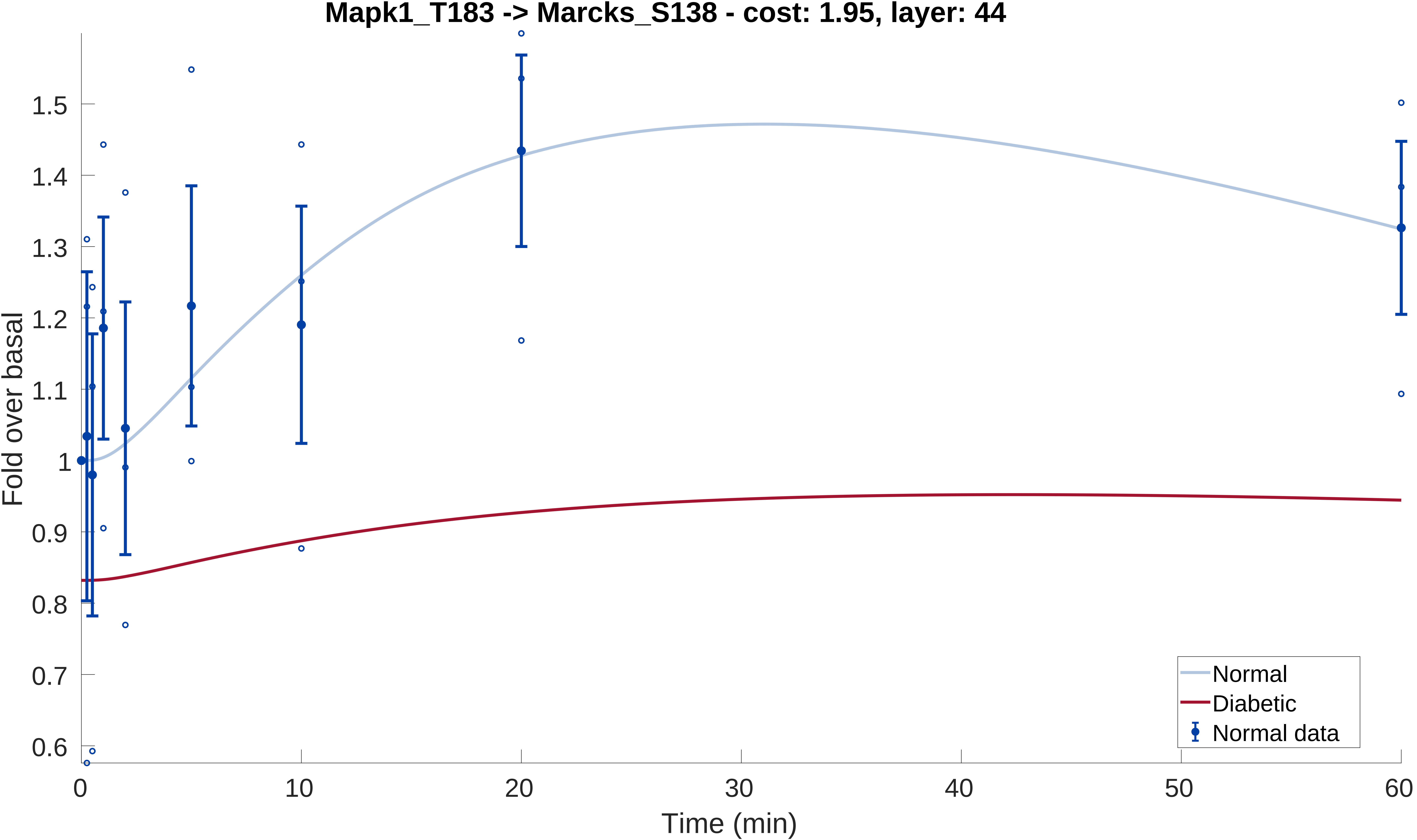
Mapk1_T183 -> Marcks_S138 - cost: 1.95, layer: 44
Fold over basal
Time (min)
0.6
0.7
0.8
0.9
1
1.1
1.2
1.3
1.4
1.5
0
10
20
30
40
50
60
Normal
Diabetic
Normal data

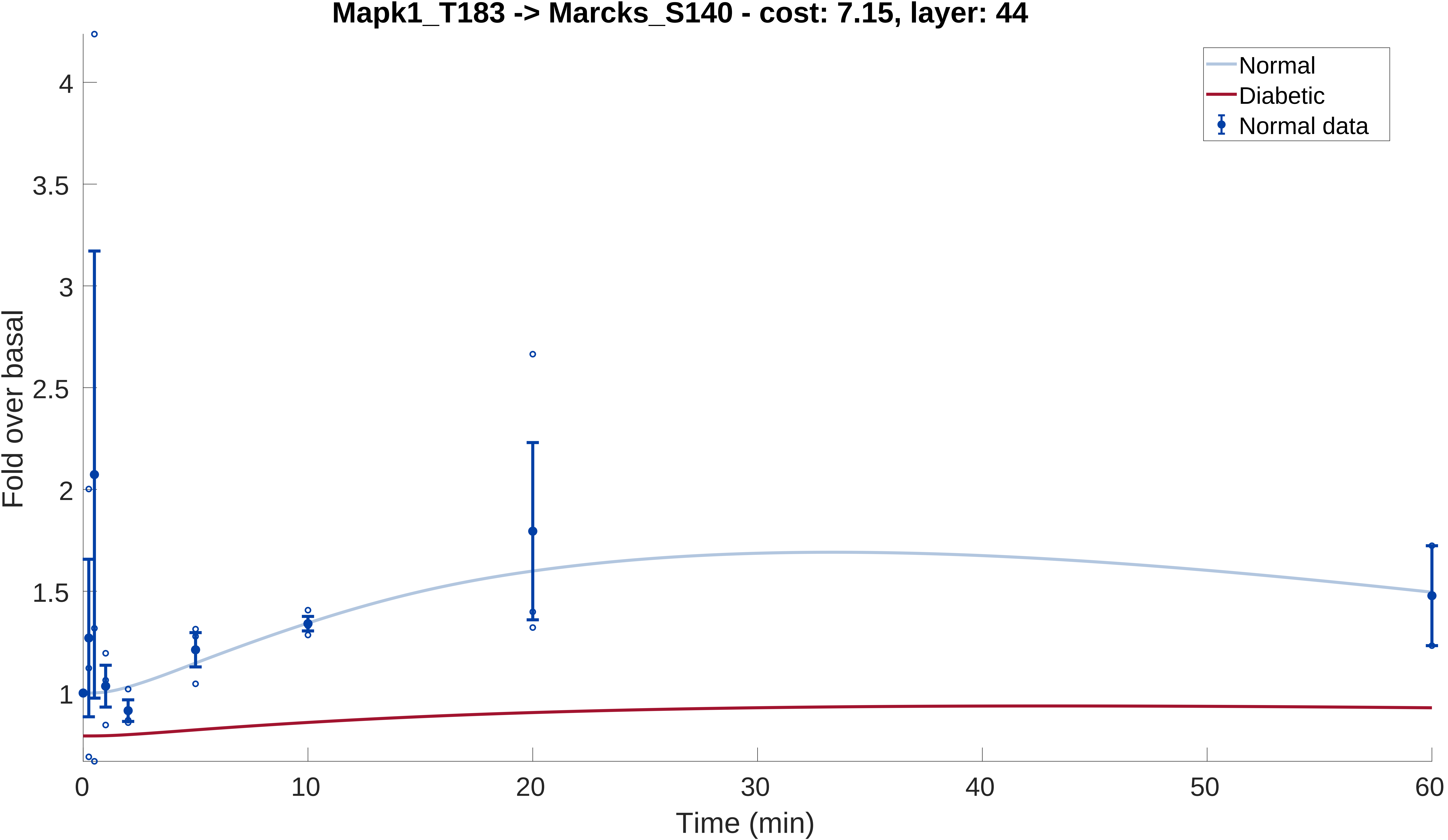
Mapk1_T183 -> Marcks_S140 - cost: 7.15, layer: 44
Fold over basal
Time (min)
Normal
Diabetic
Normal data
0
10
20
30
40
50
60
1
1.5
2
2.5
3
3.5
4

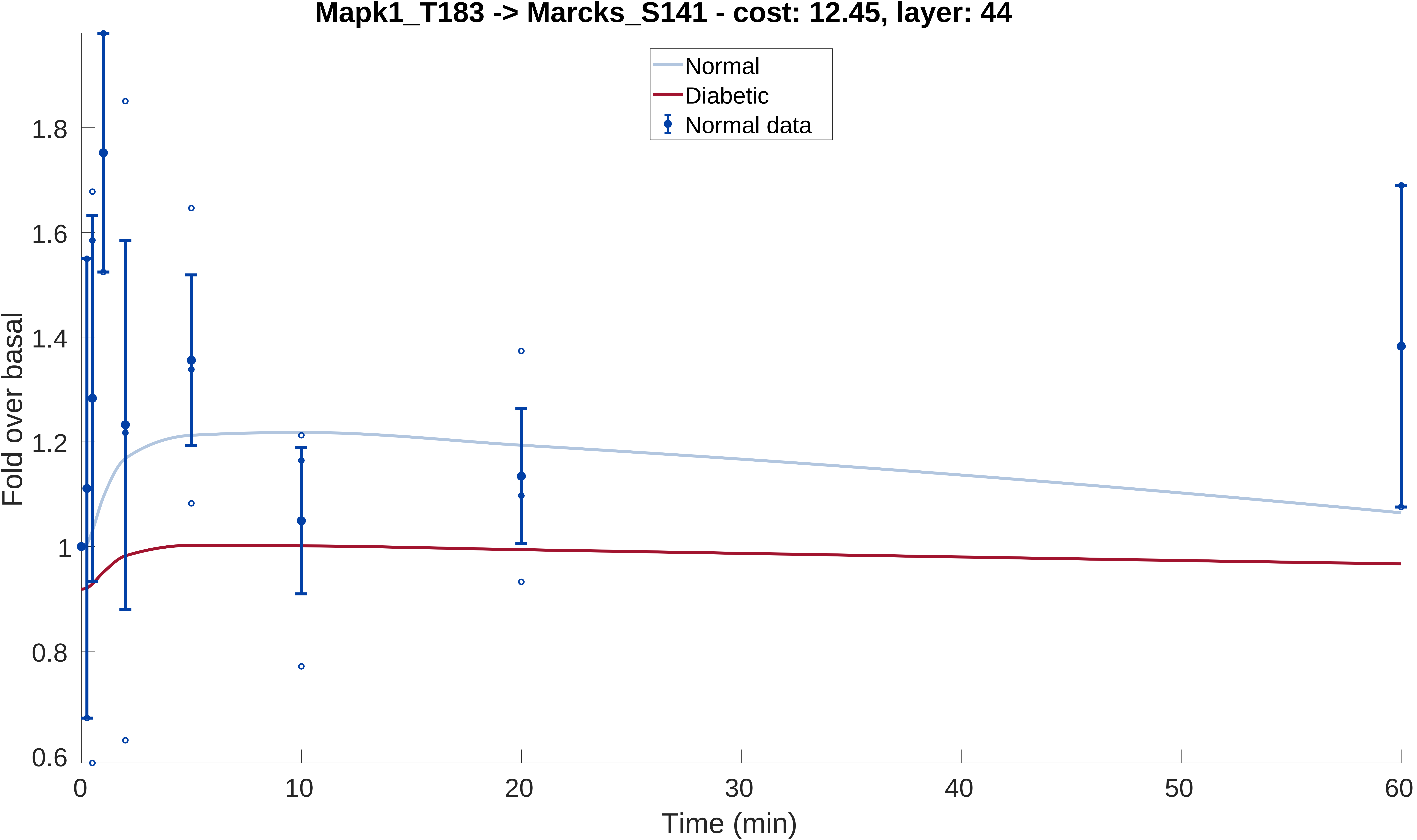
Mapk1_T183 -> Marcks_S141 - cost: 12.45, layer: 44
Normal
Diabetic
Normal data
Fold over basal
1.8
1.6
1.4
1.2
1
0.8
0.6
0
10
20
30
40
50
60
Time (min)

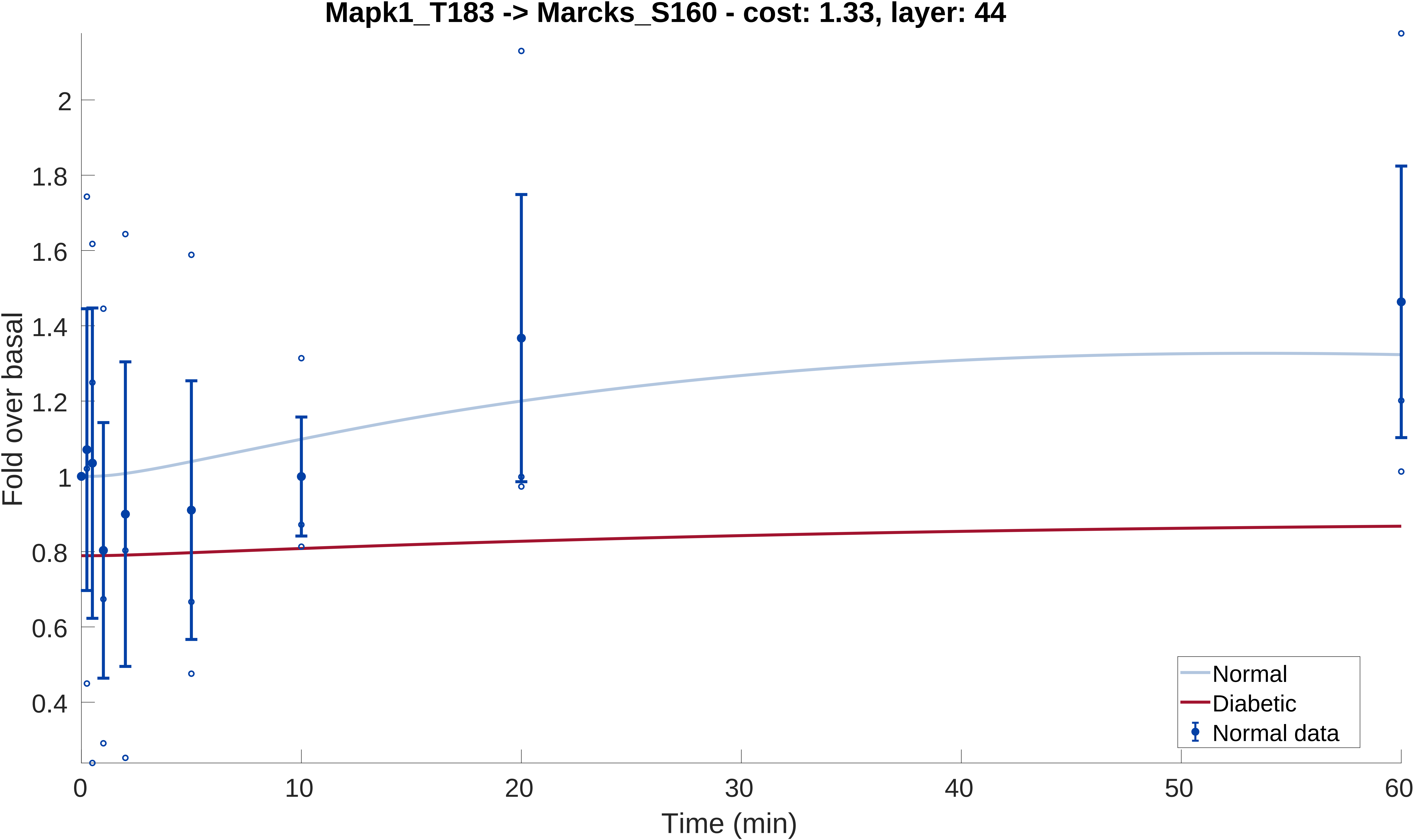

Mapk1_T183 -> Marcks_S160 - cost: 1.33, layer: 44
Fold over basal
Time (min)
0.4
0.6
0.8
1
1.2
1.4
1.6
1.8
2
0
10
20
30
40
50
60
Normal
Diabetic
Normal data

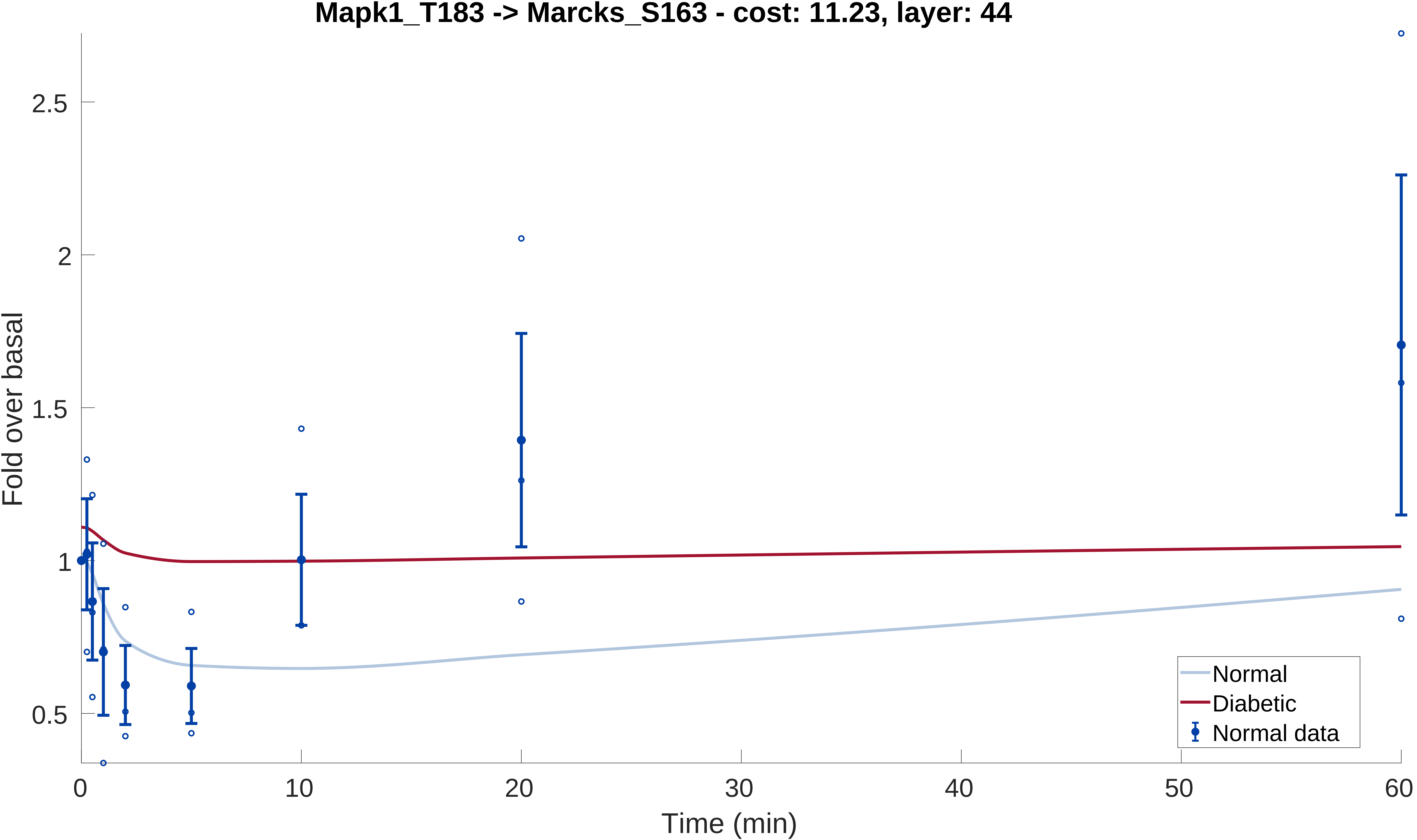

Mapk1_T183 -> Marcks_S163 - cost: 11.23, layer: 44
Fold over basal
2.5
2
1.5
1
0.5
0
10
20
30
40
50
60
Time (min)
Normal
Diabetic
Normal data

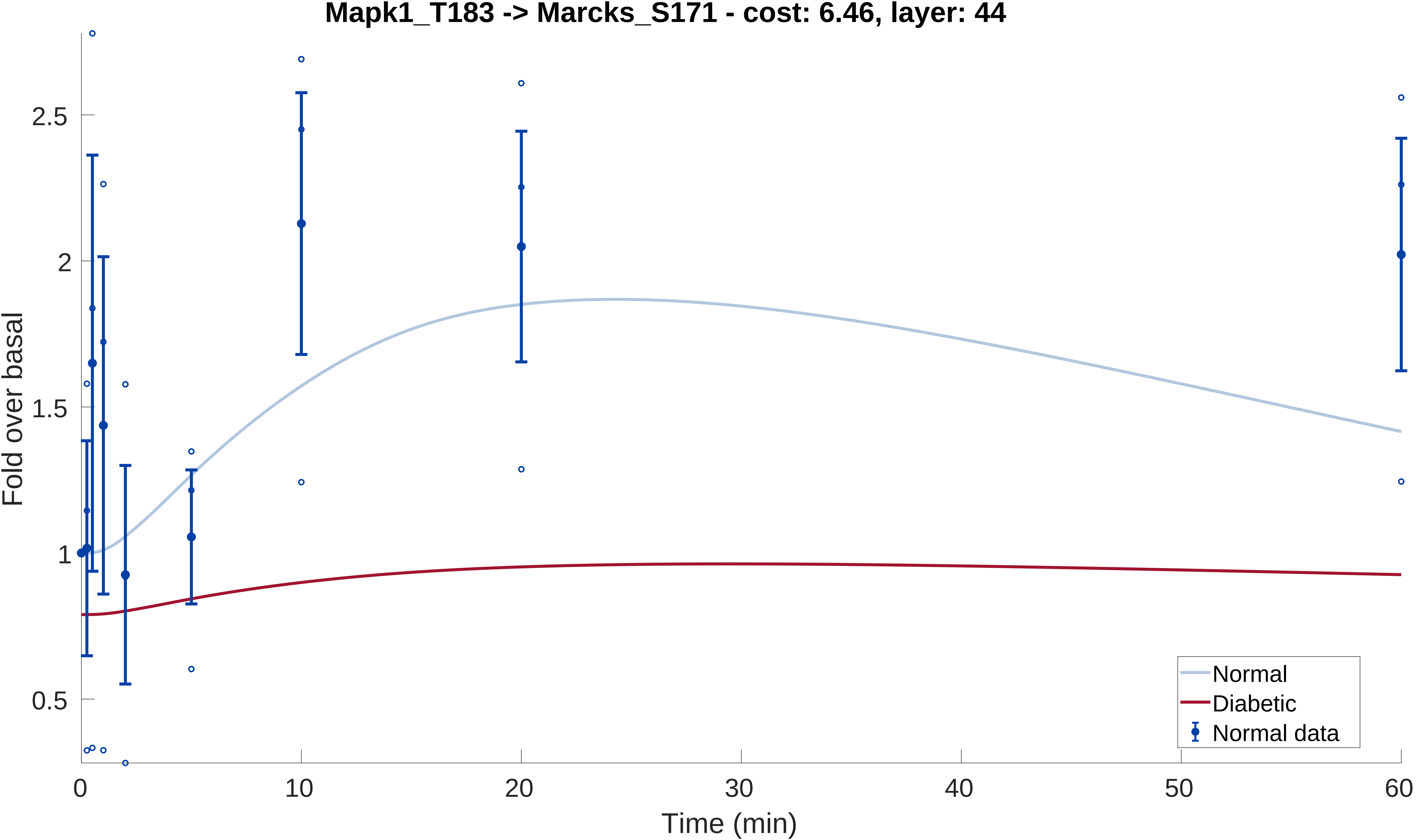

Mapk1_T183 -> Marcks_S171 - cost: 6.46, layer: 44
Fold over basal
2.5
2
1.5
1
0.5
0
10
20
30
40
50
60
Time (min)
Normal
Diabetic
Normal data

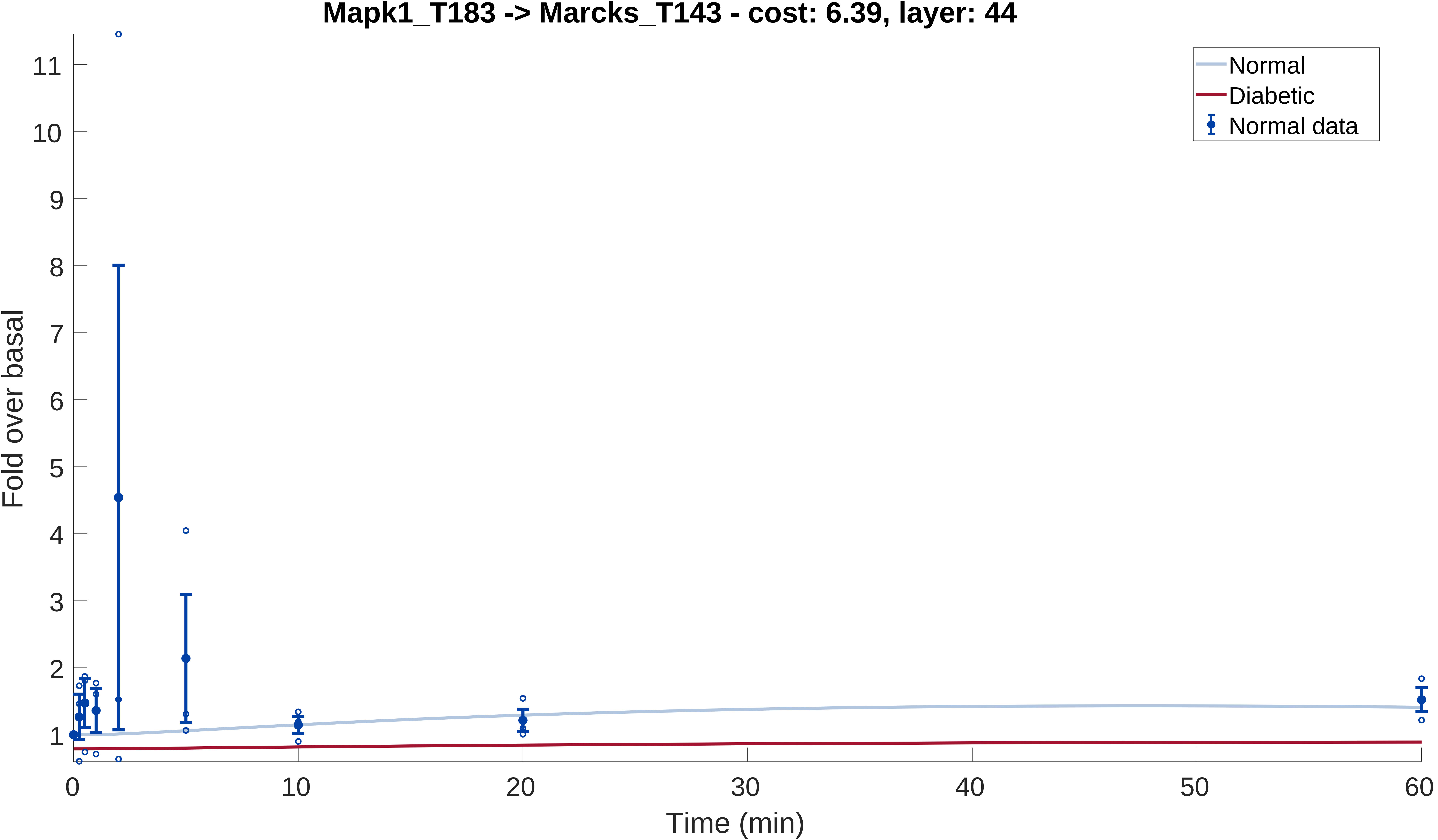

Mapk1_T183 -> Marcks_T143 - cost: 6.39, layer: 44
Normal
Diabetic
Normal data
Fold over basal
1
2
3
4
5
6
7
8
9
10
11
0
10
20
30
40
50
60
Time (min)

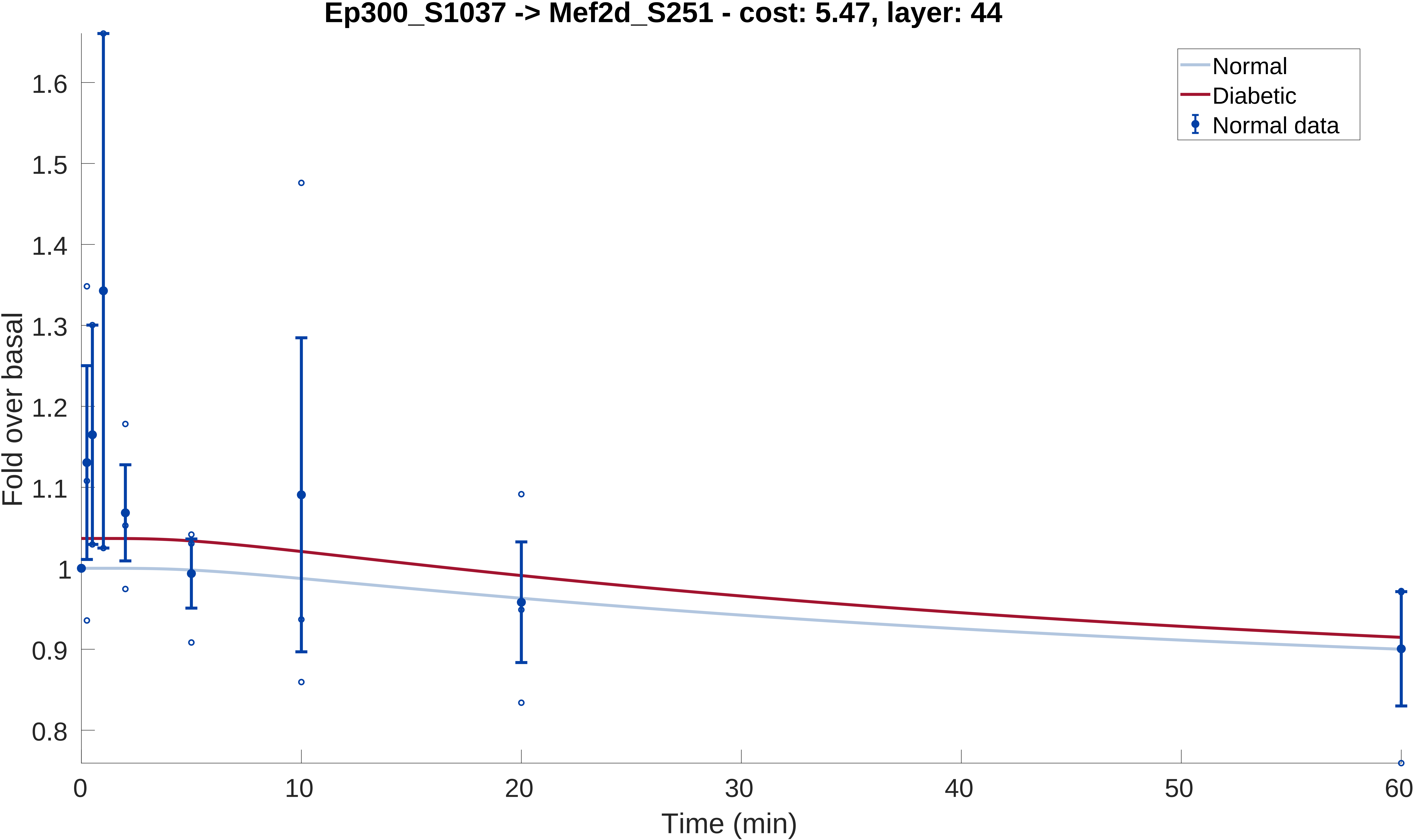

Ep300_S1037 -> Mef2d_S251 - cost: 5.47, layer: 44
Normal
Diabetic
Normal data
Fold over basal
Time (min)
0.8
0.9
1
1.1
1.2
1.3
1.4
1.5
1.6
0
10
20
30
40
50
60

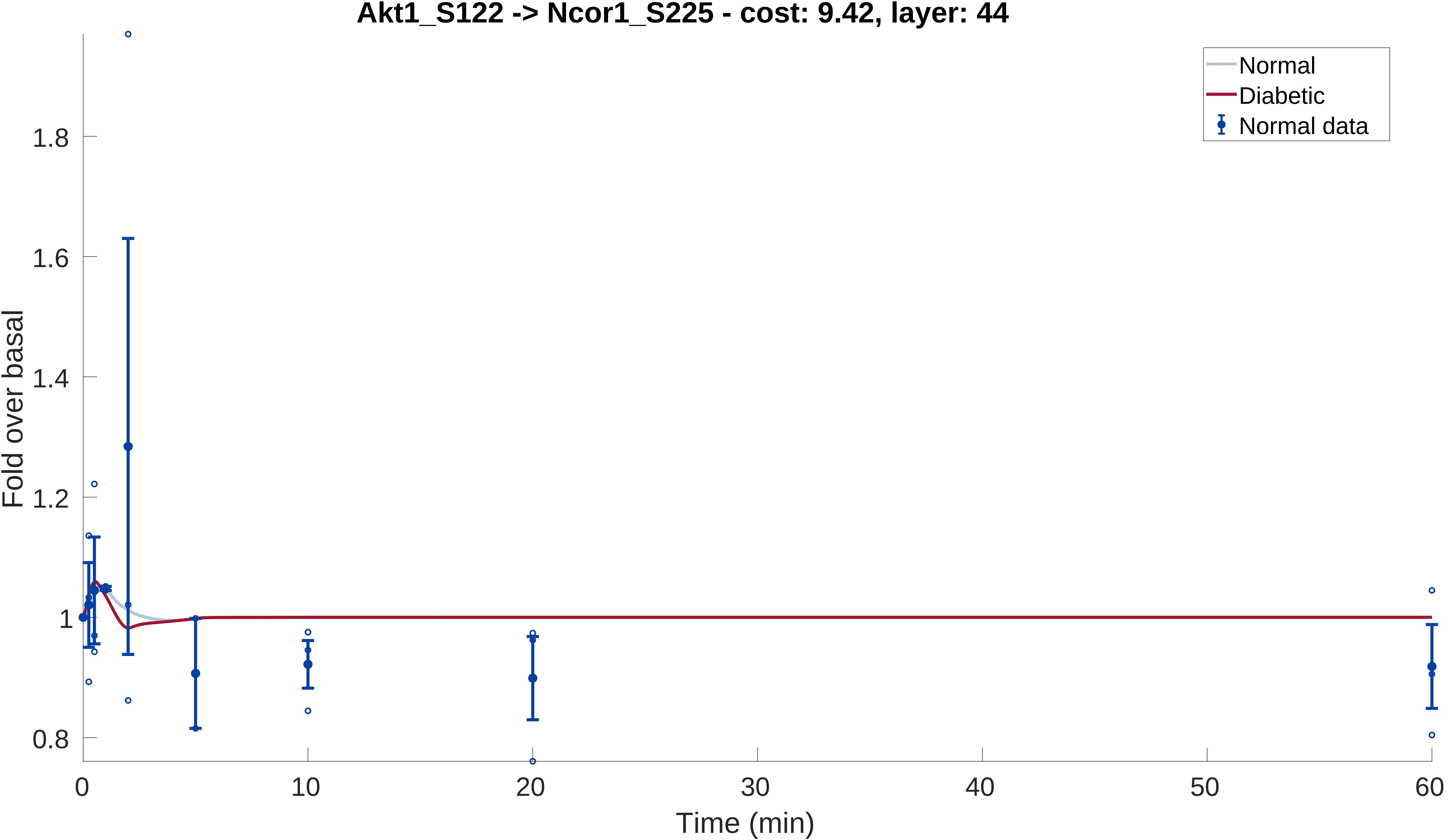

Akt1_S122 -> Ncor1_S225 - cost: 9.42, layer: 44
Normal
Diabetic
Normal data
Fold over basal
1.8
1.6
1.4
1.2
1
0.8
0
10
20
30
40
50
60
Time (min)

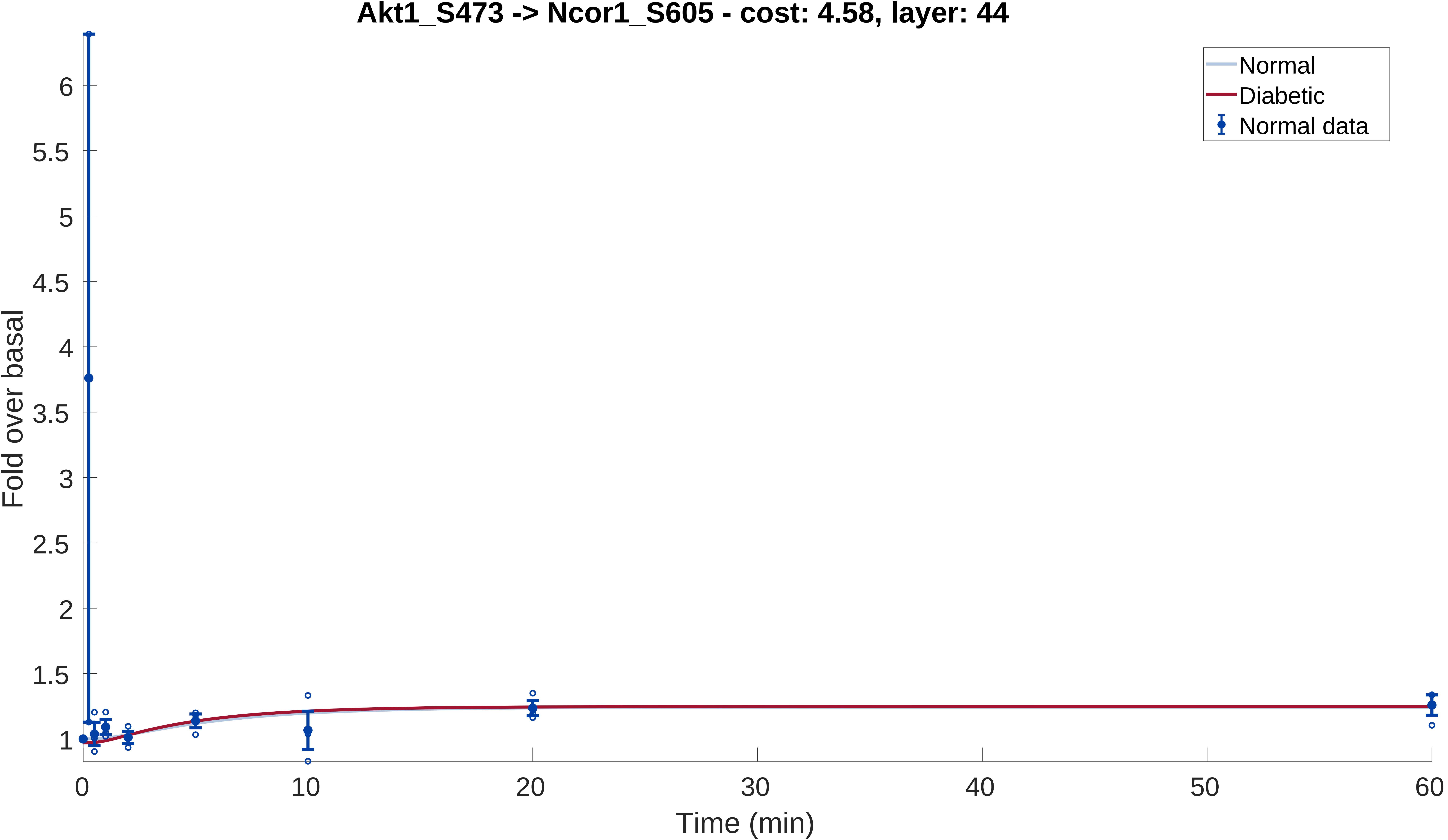

Akt1_S473 -> Ncor1_S605 - cost: 4.58, layer: 44
Normal
Diabetic
Normal data
Fold over basal
Time (min)
6
5.5
5
4.5
4
3.5
3
2.5
2
1.5
1
0
10
20
30
40
50
60

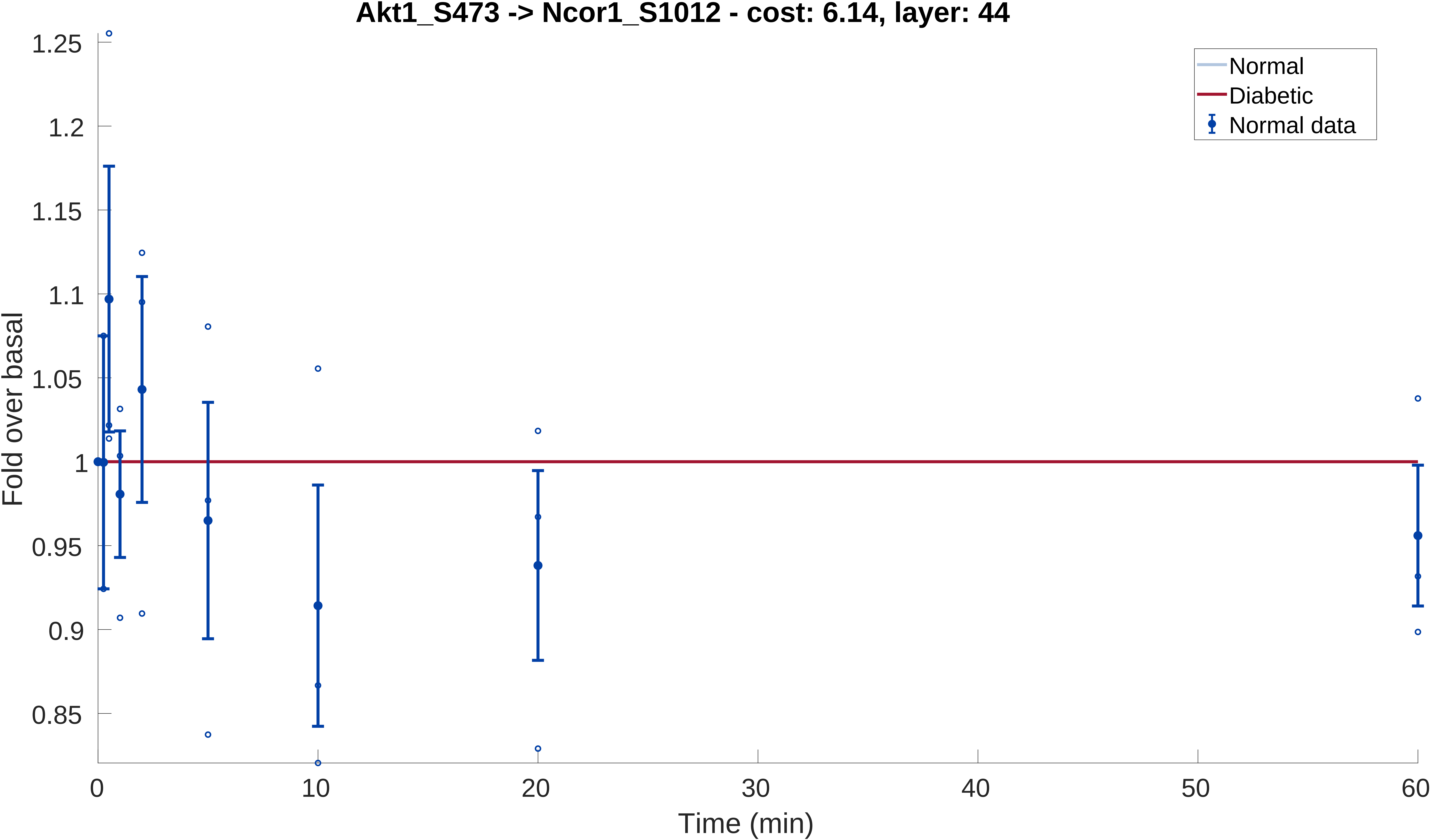

Akt1_S473 -> Ncor1_S1012 - cost: 6.14, layer: 44
Normal
Diabetic
Normal data
Fold over basal
1.25
1.2
1.15
1.1
1.05
1
0.95
0.9
0.85
0
10
20
30
40
50
60
Time (min)

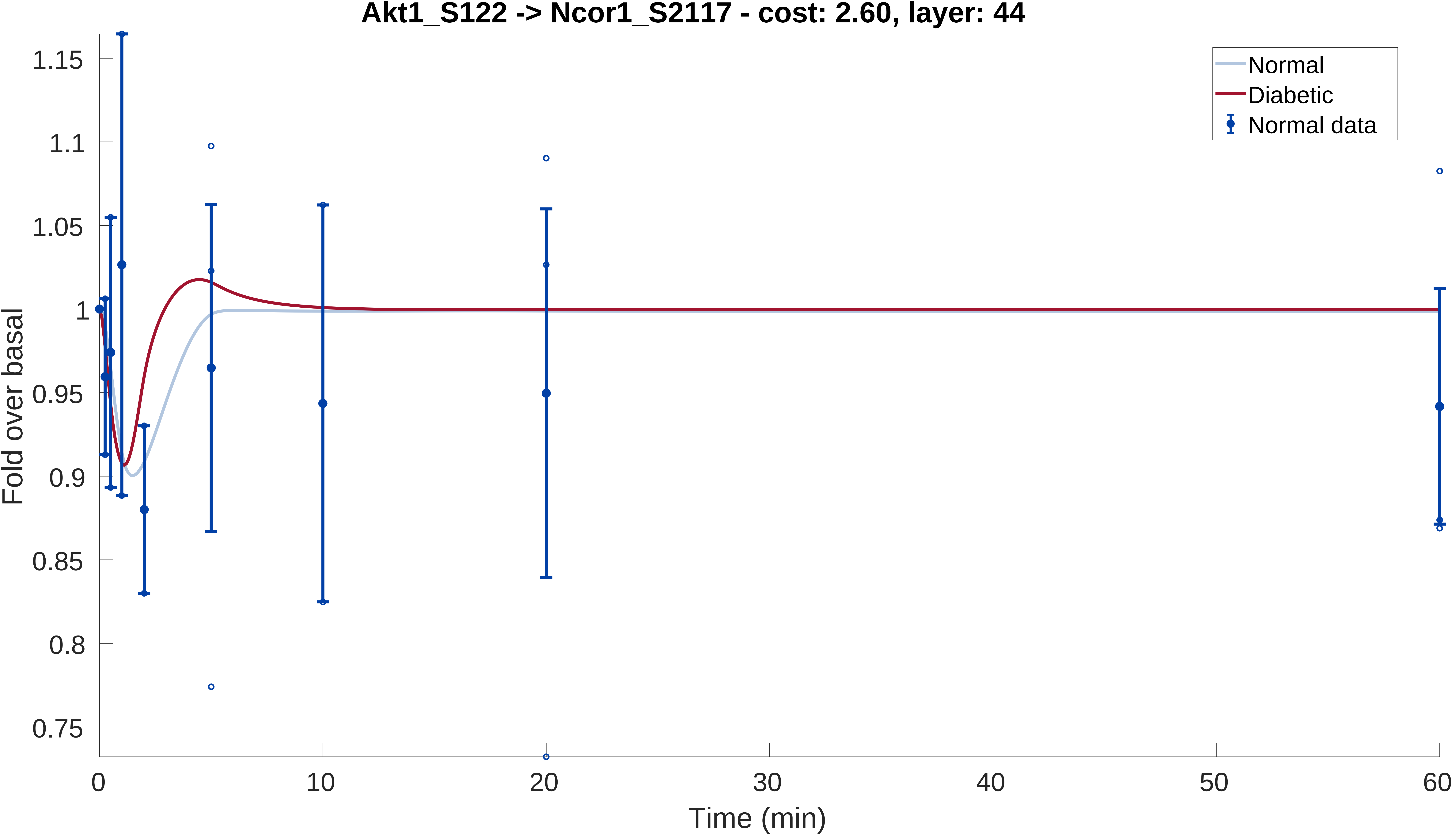
Akt1_S122 -> Ncor1_S2117 - cost: 2.60, layer: 44
Normal
Diabetic
Normal data
Fold over basal
Time (min)
1.15
1.1
1.05
1
0.95
0.9
0.85
0.8
0.75
0
10
20
30
40
50
60

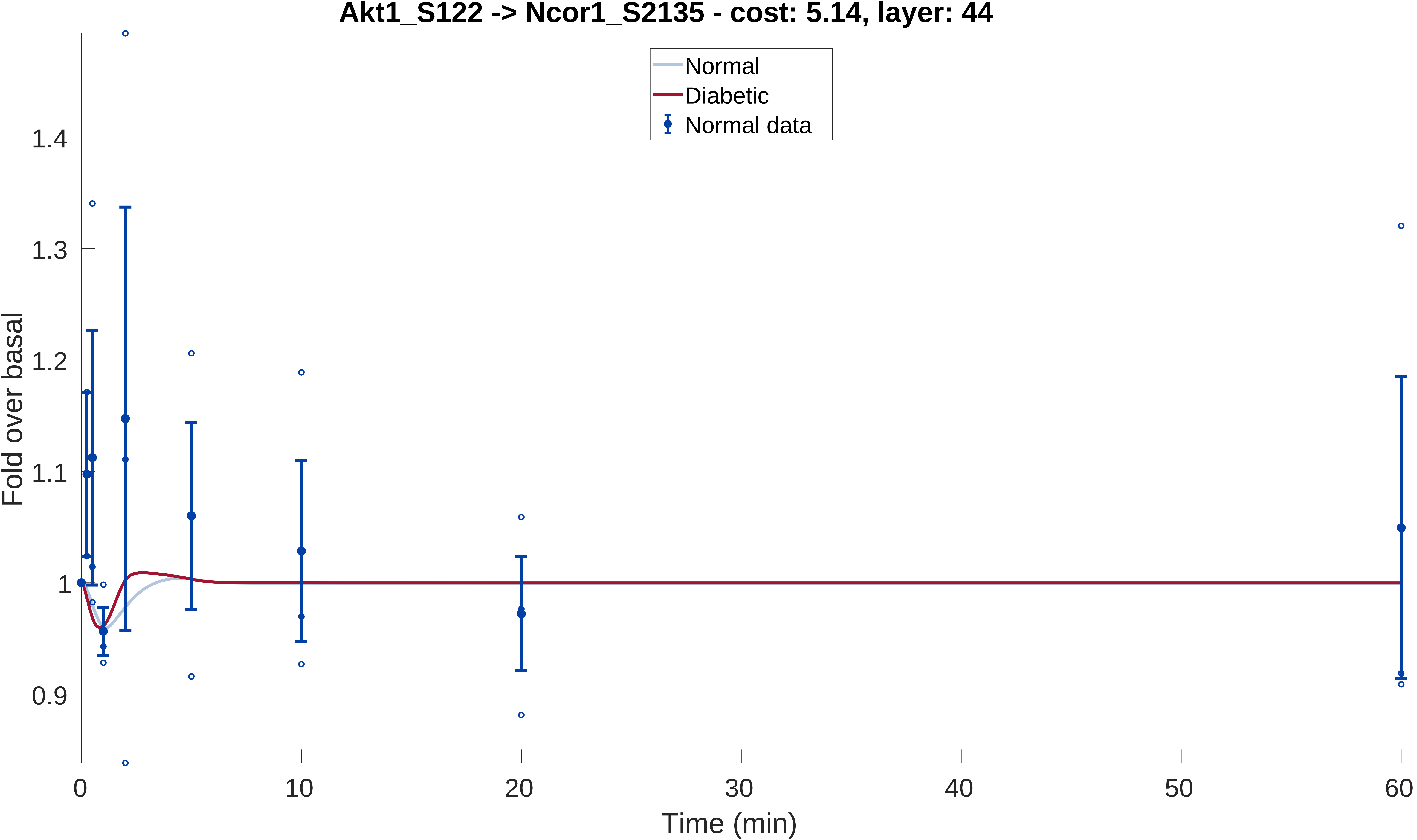

Akt1_S122 -> Ncor1_S2135 - cost: 5.14, layer: 44
Normal
Diabetic
Normal data
Fold over basal
1.4
1.3
1.2
1.1
1
0.9
0
10
20
30
40
50
60
Time (min)

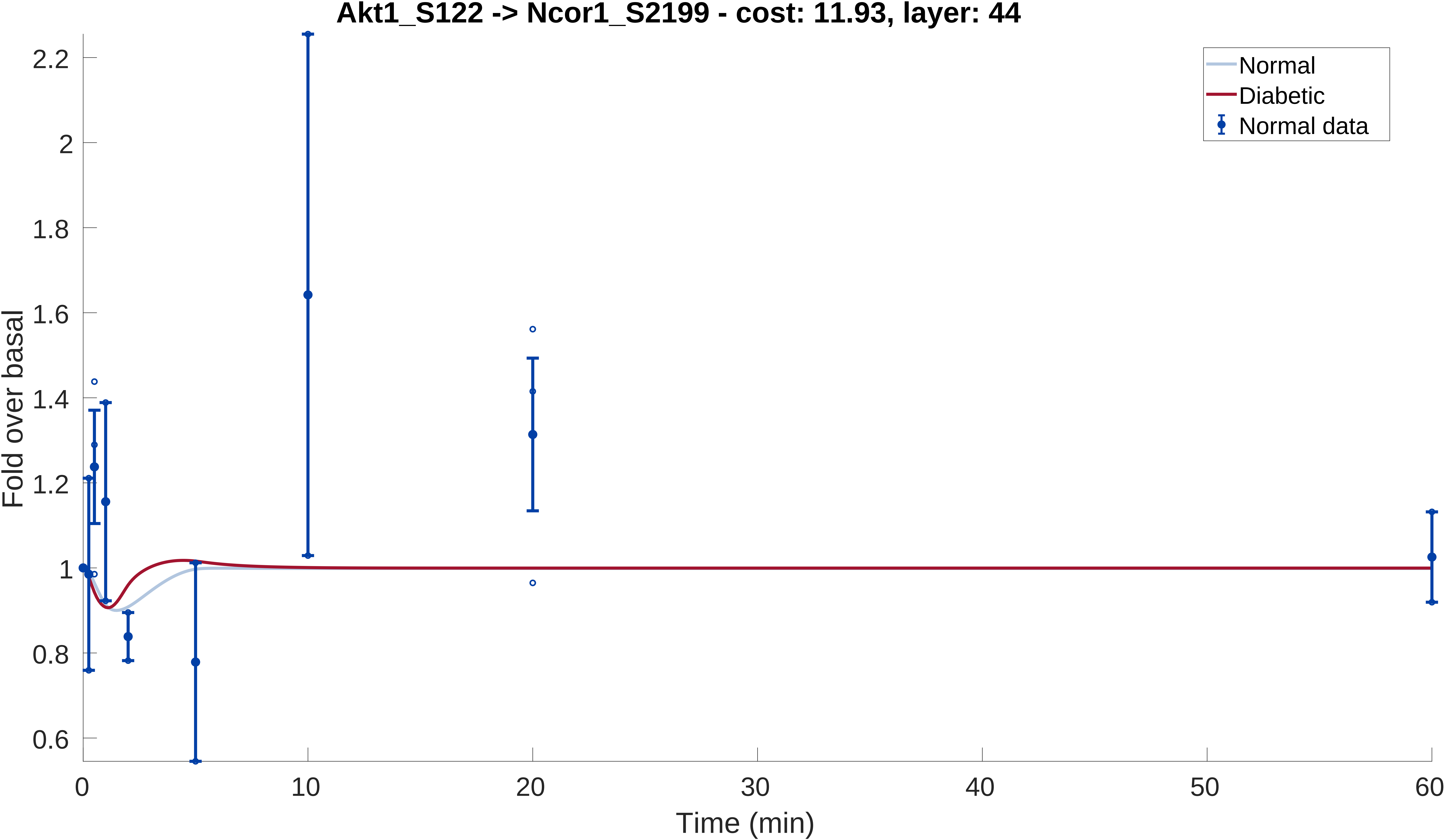

Akt1_S122 -> Ncor1_S2199 - cost: 11.93, layer: 44
Normal
Diabetic
Normal data
Fold over basal
Time (min)
0.6
0.8
1
1.2
1.4
1.6
1.8
2
2.2
0
10
20
30
40
50
60

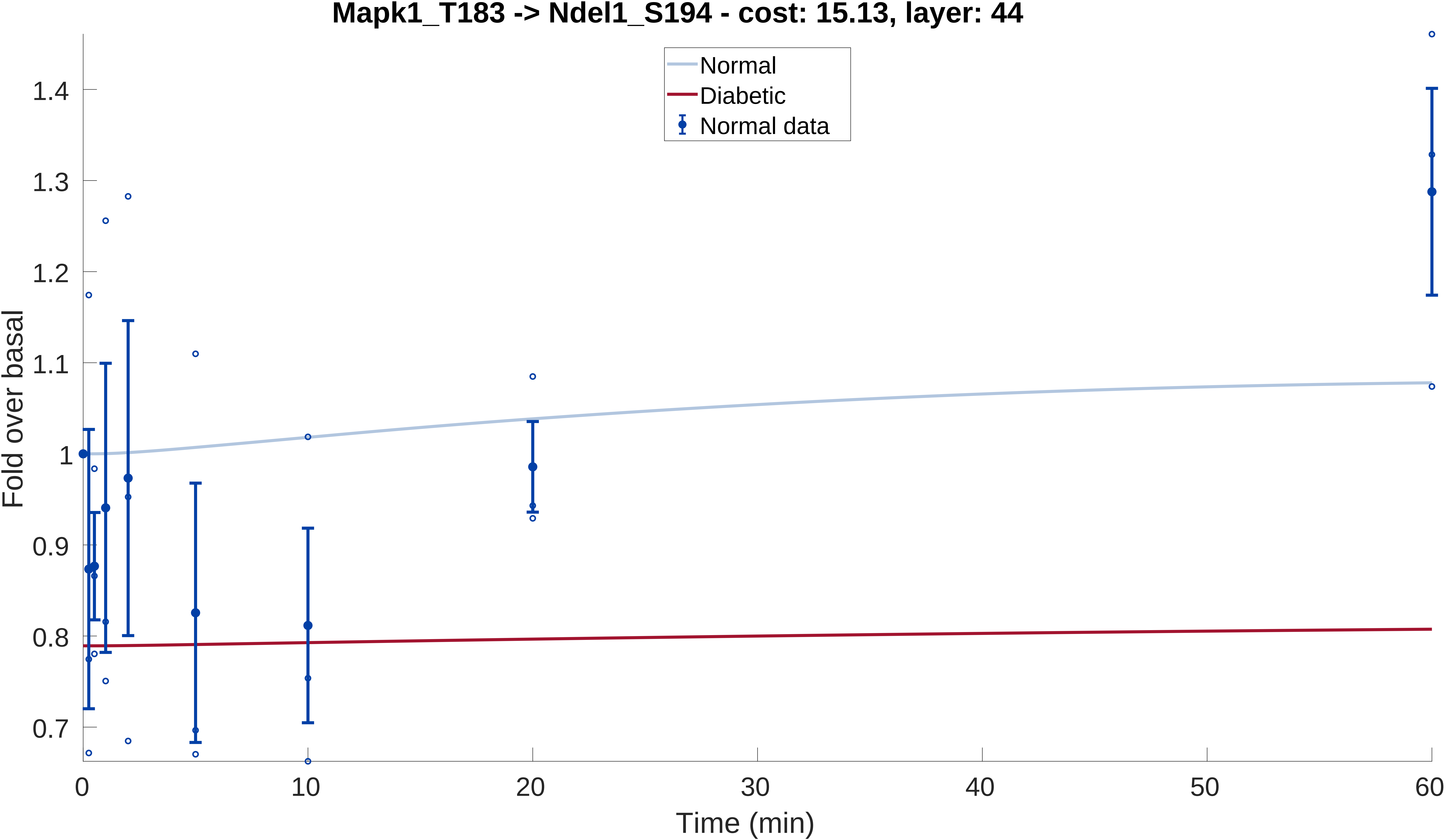

Mapk1_T183 -> Ndel1_S194 - cost: 15.13, layer: 44
Normal
Diabetic
Normal data
1.4
1.3
1.2
1.1
1
0.9
0.8
0.7
Fold over basal
0
10
20
30
40
50
60
Time (min)

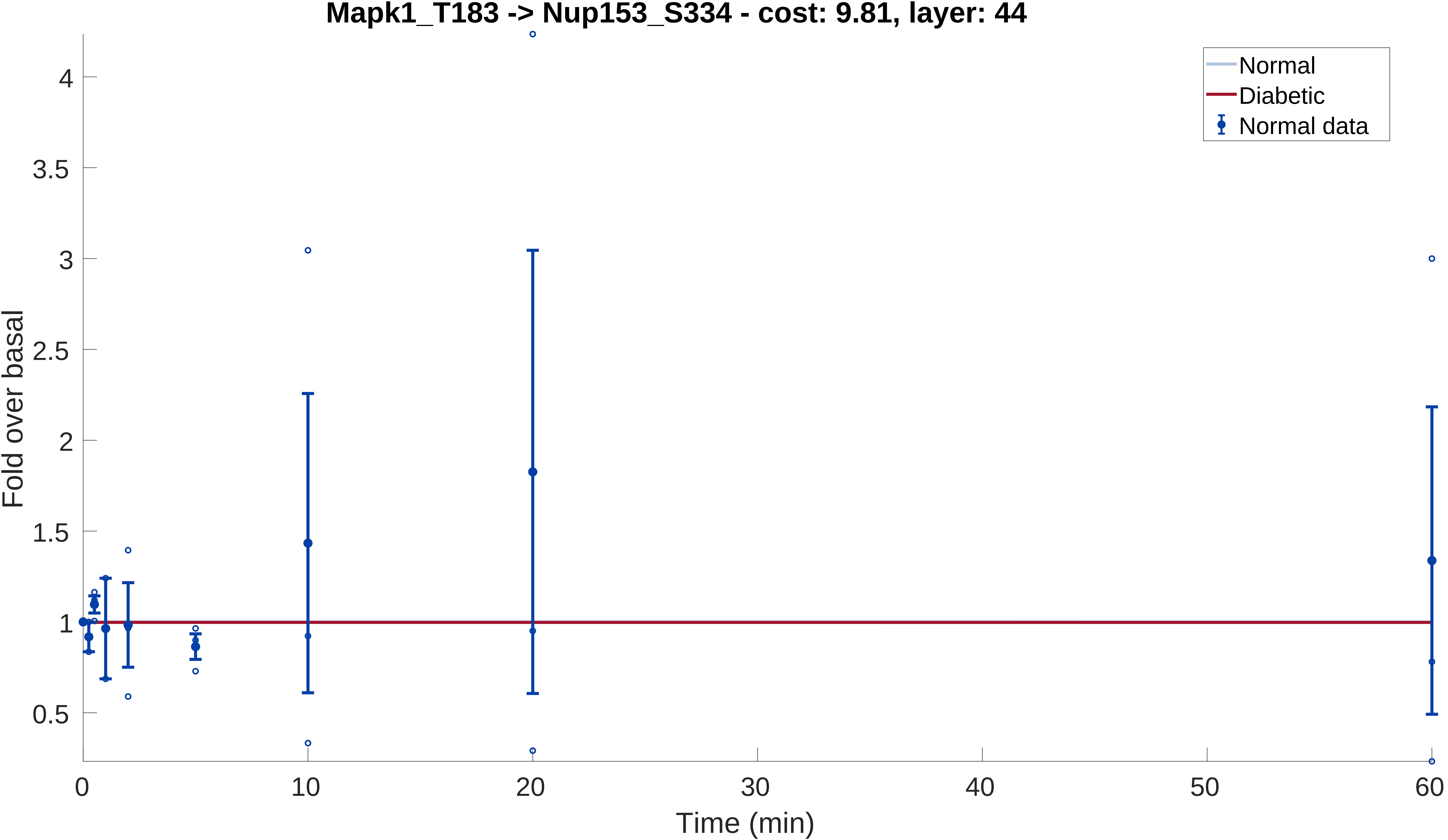

Mapk1_T183 -> Nup153_S334 - cost: 9.81, layer: 44
Normal
Diabetic
Normal data
Fold over basal
0.5
1
1.5
2
2.5
3
3.5
4
0
10
20
30
40
50
60
Time (min)

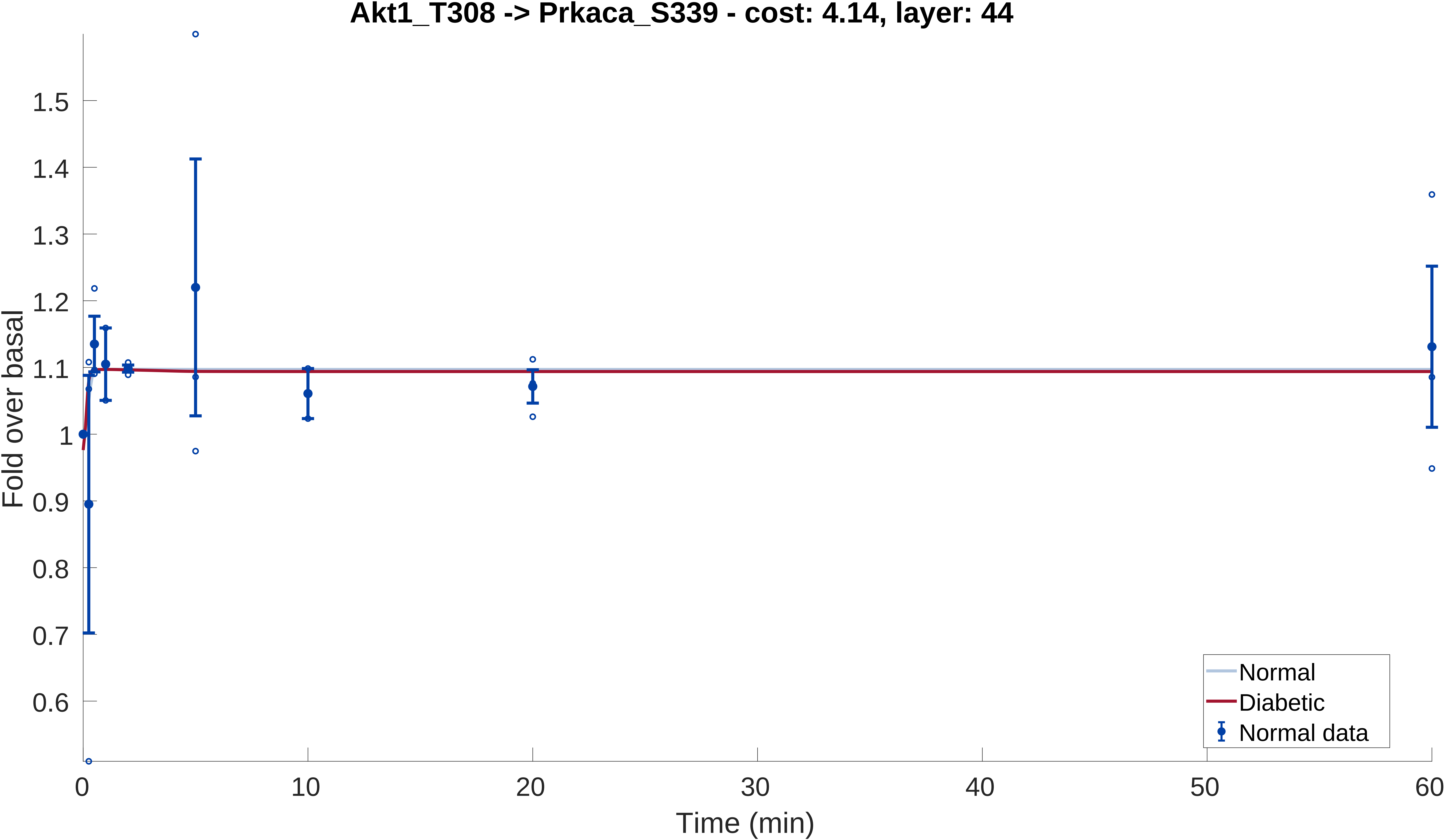

Akt1_T308 -> Prkaca_S339 - cost: 4.14, layer: 44
Fold over basal
1.5
1.4
1.3
1.2
1.1
1
0.9
0.8
0.7
0.6
0
10
20
30
40
50
60
Time (min)
Normal
Diabetic
Normal data

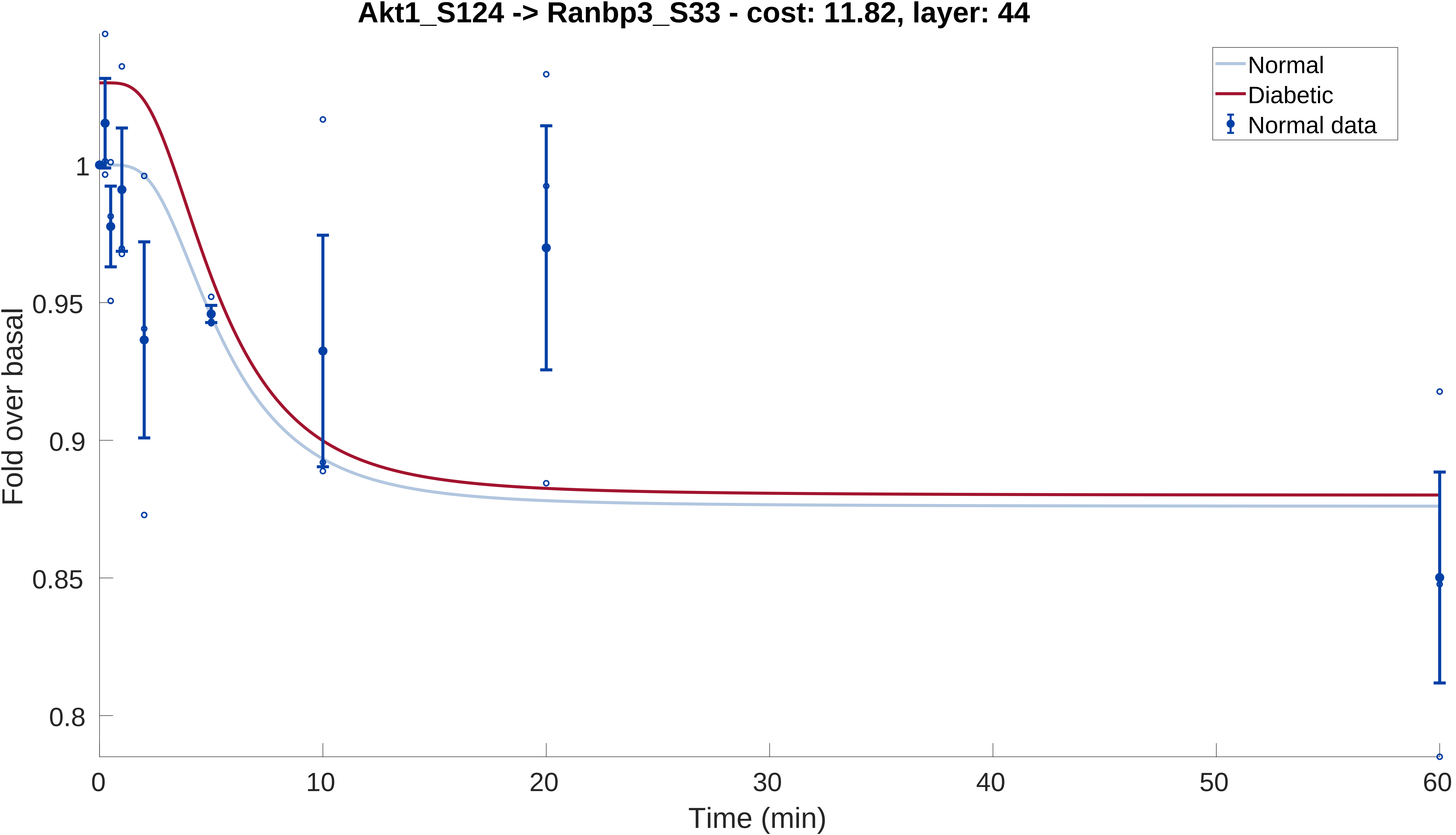

Akt1_S124 -> Ranbp3_S33 - cost: 11.82, layer: 44
Normal
Diabetic
Normal data
Fold over basal
1
0.95
0.9
0.85
0.8
0
10
20
30
40
50
60
Time (min)

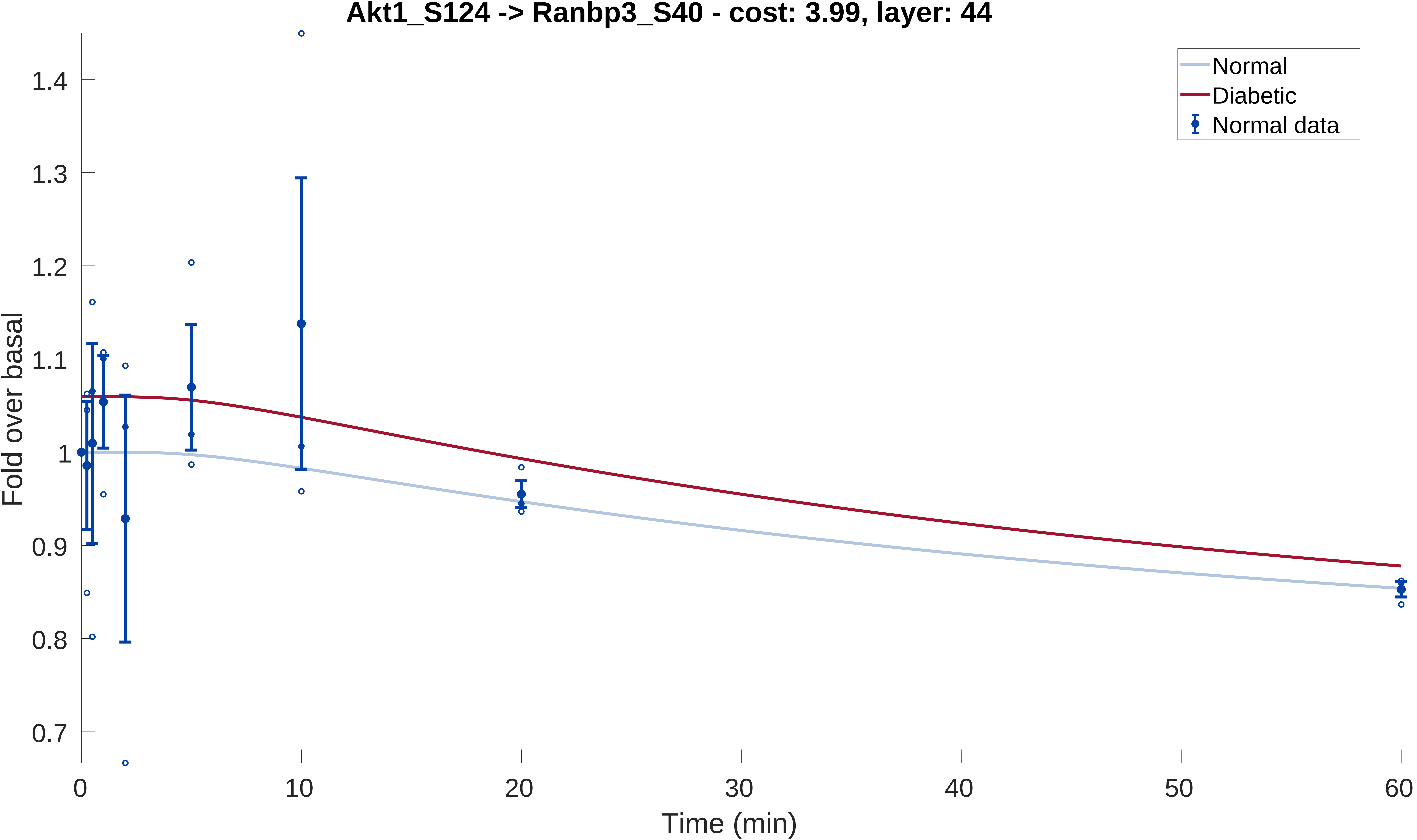
Akt1_S124 -> Ranbp3_S40 - cost: 3.99, layer: 44
Normal
Diabetic
Normal data
Fold over basal
1.4
1.3
1.2
1.1
1
0.9
0.8
0.7
0
10
20
30
40
50
60
Time (min)

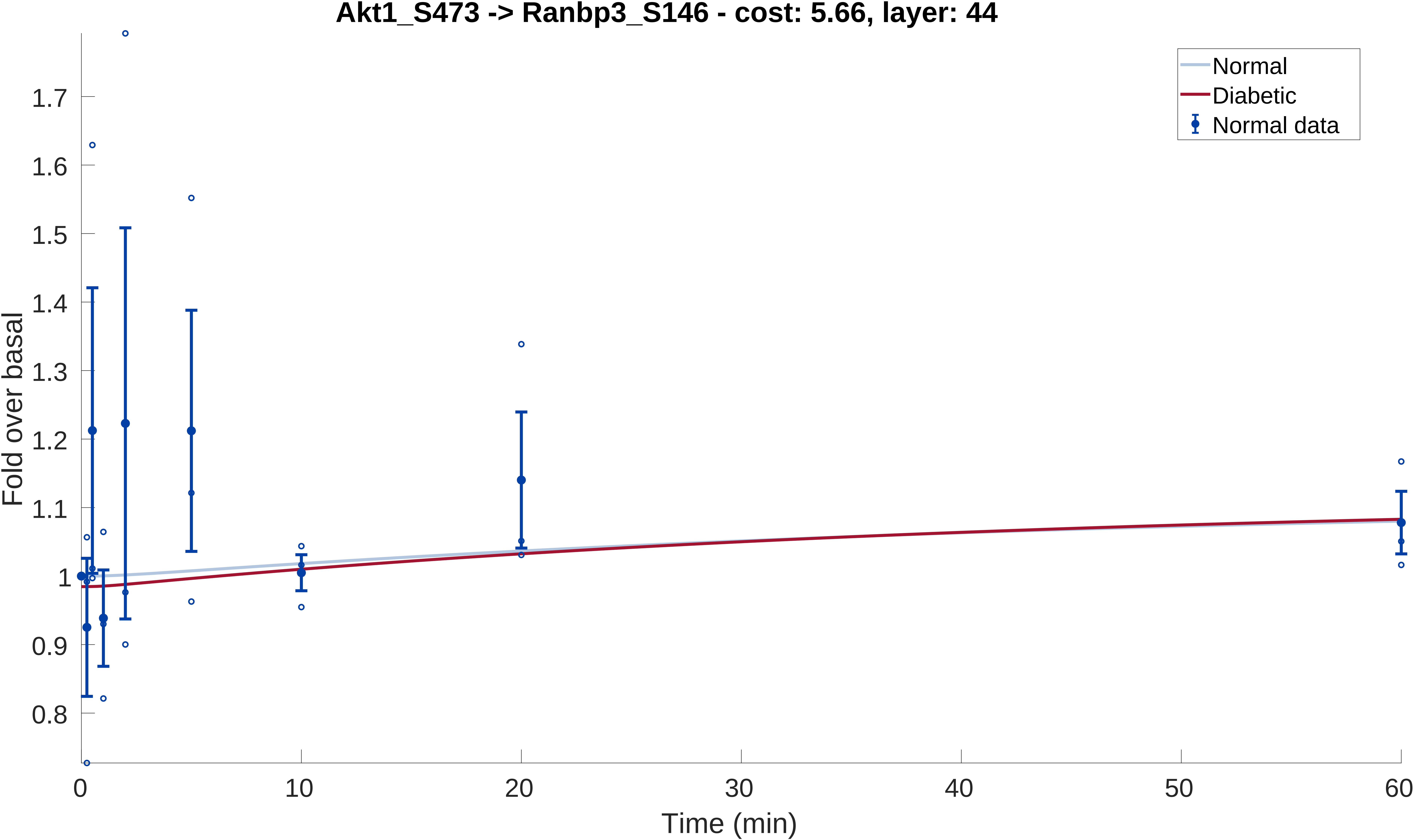
Akt1_S473 -> Ranbp3_S146 - cost: 5.66, layer: 44
Normal
Diabetic
Normal data
Fold over basal
1.7
1.6
1.5
1.4
1.3
1.2
1.1
1
0.9
0.8
0
10
20
30
40
50
60
Time (min)

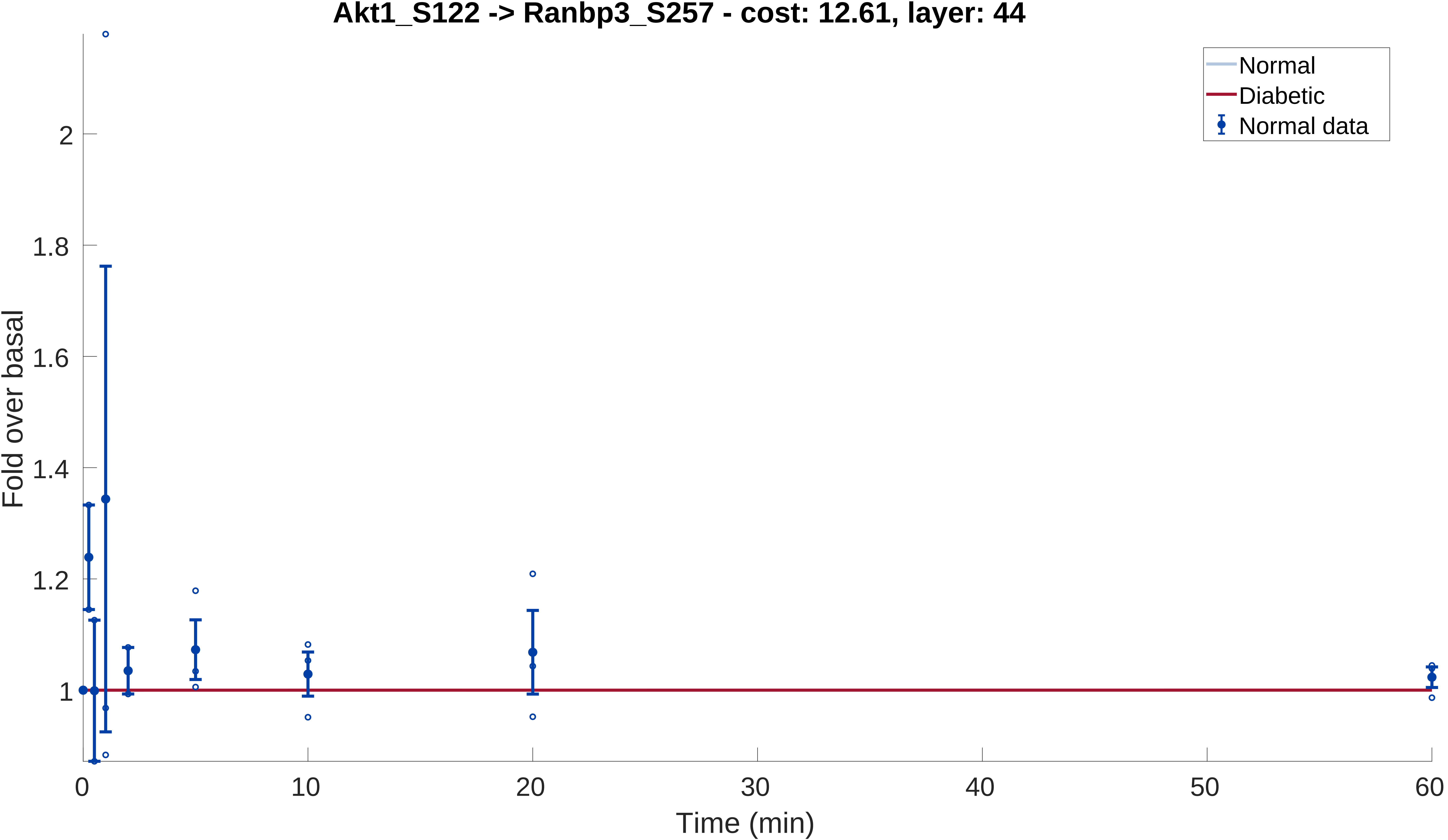
Akt1_S122 -> Ranbp3_S257 - cost: 12.61, layer: 44
Normal
Diabetic
Normal data
Fold over basal
2
1.8
1.6
1.4
1.2
1
0
10
20
30
40
50
60
Time (min)

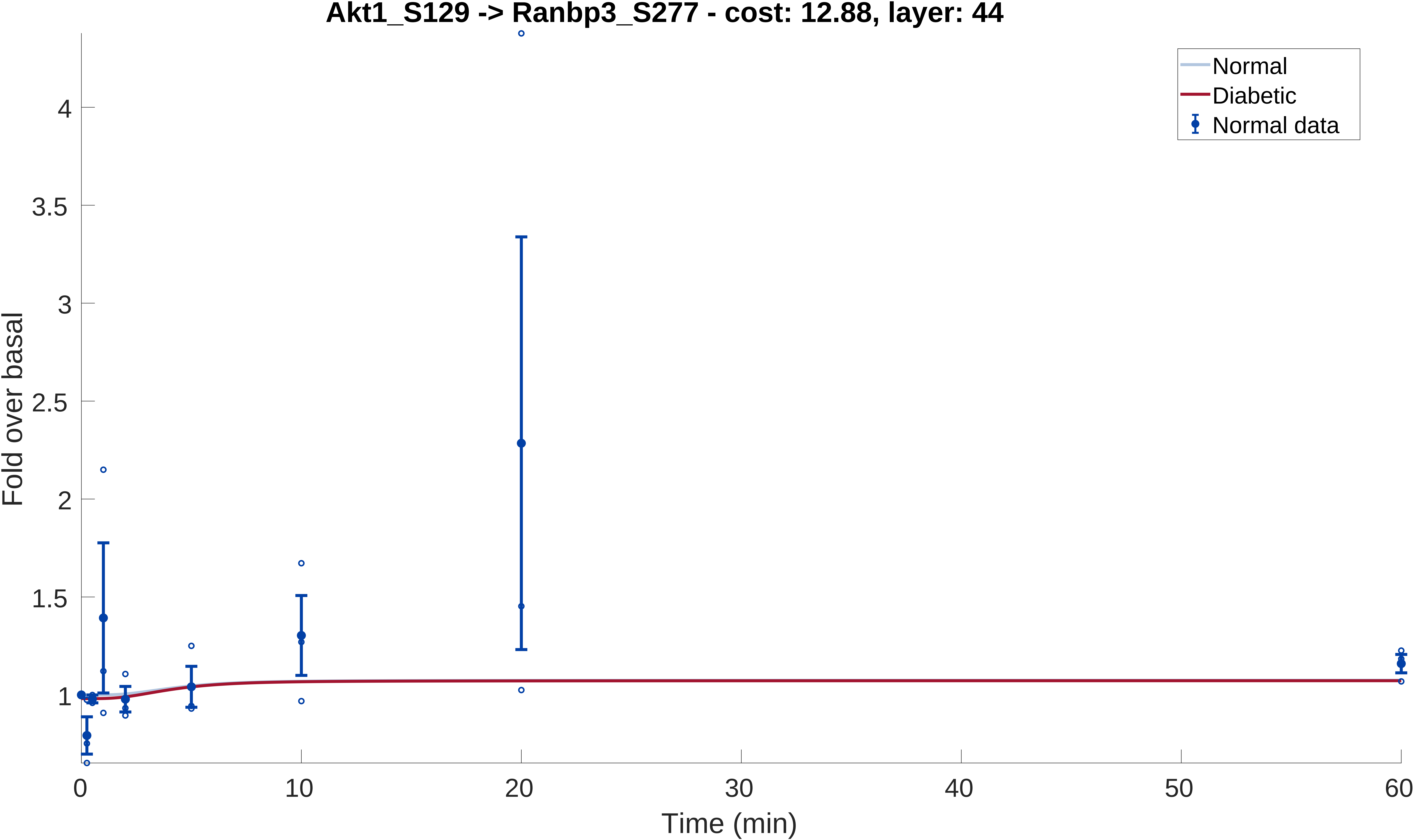
Akt1_S129 -> Ranbp3_S277 - cost: 12.88, layer: 44
Normal
Diabetic
Normal data
Fold over basal
Time (min)
4
3.5
3
2.5
2
1.5
1
0
10
20
30
40
50
60

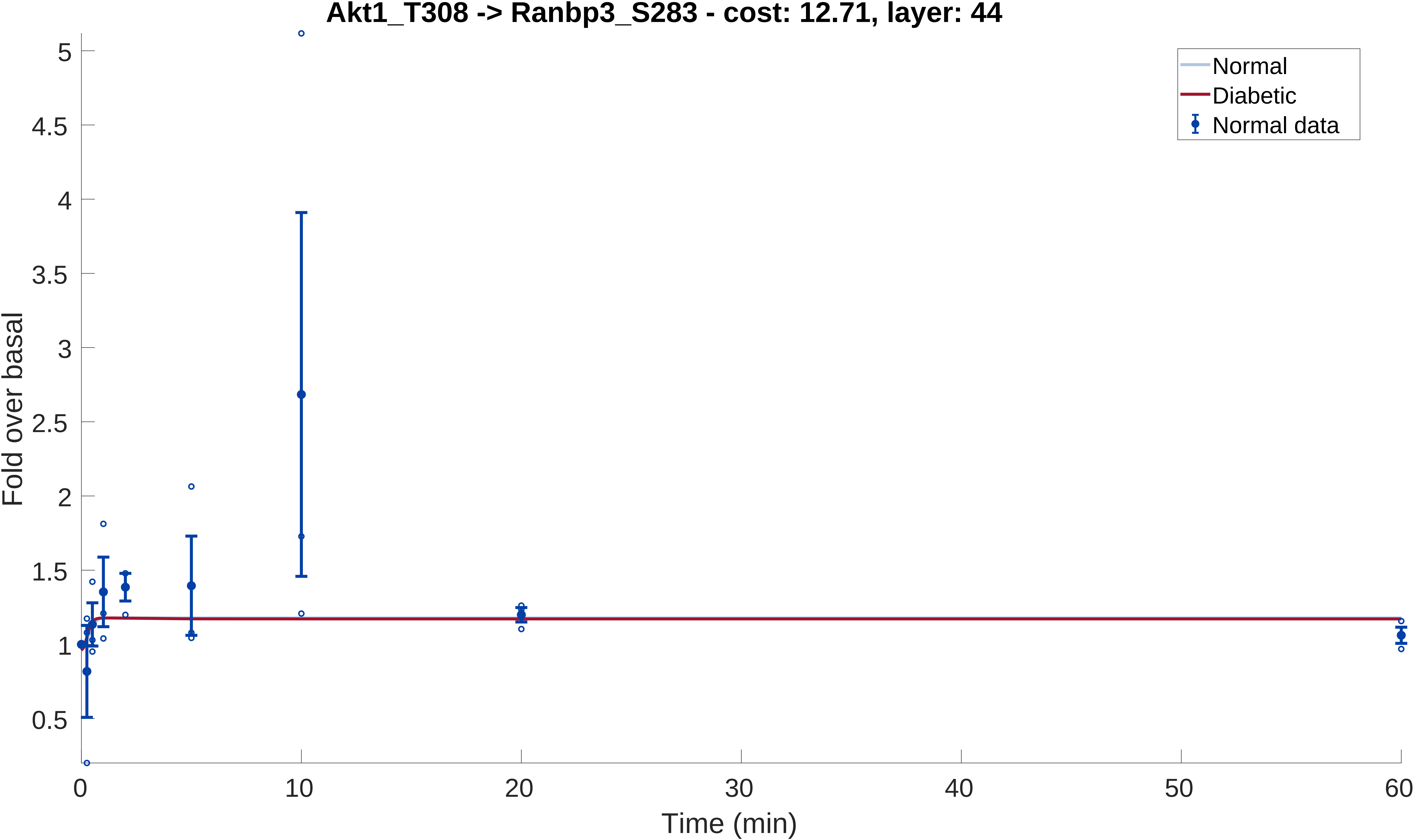
Akt1_T308 -> Ranbp3_S283 - cost: 12.71, layer: 44
Normal
Diabetic
Normal data
Fold over basal
Time (min)
0
10
20
30
40
50
60
0.5
1
1.5
2
2.5
3
3.5
4
4.5
5

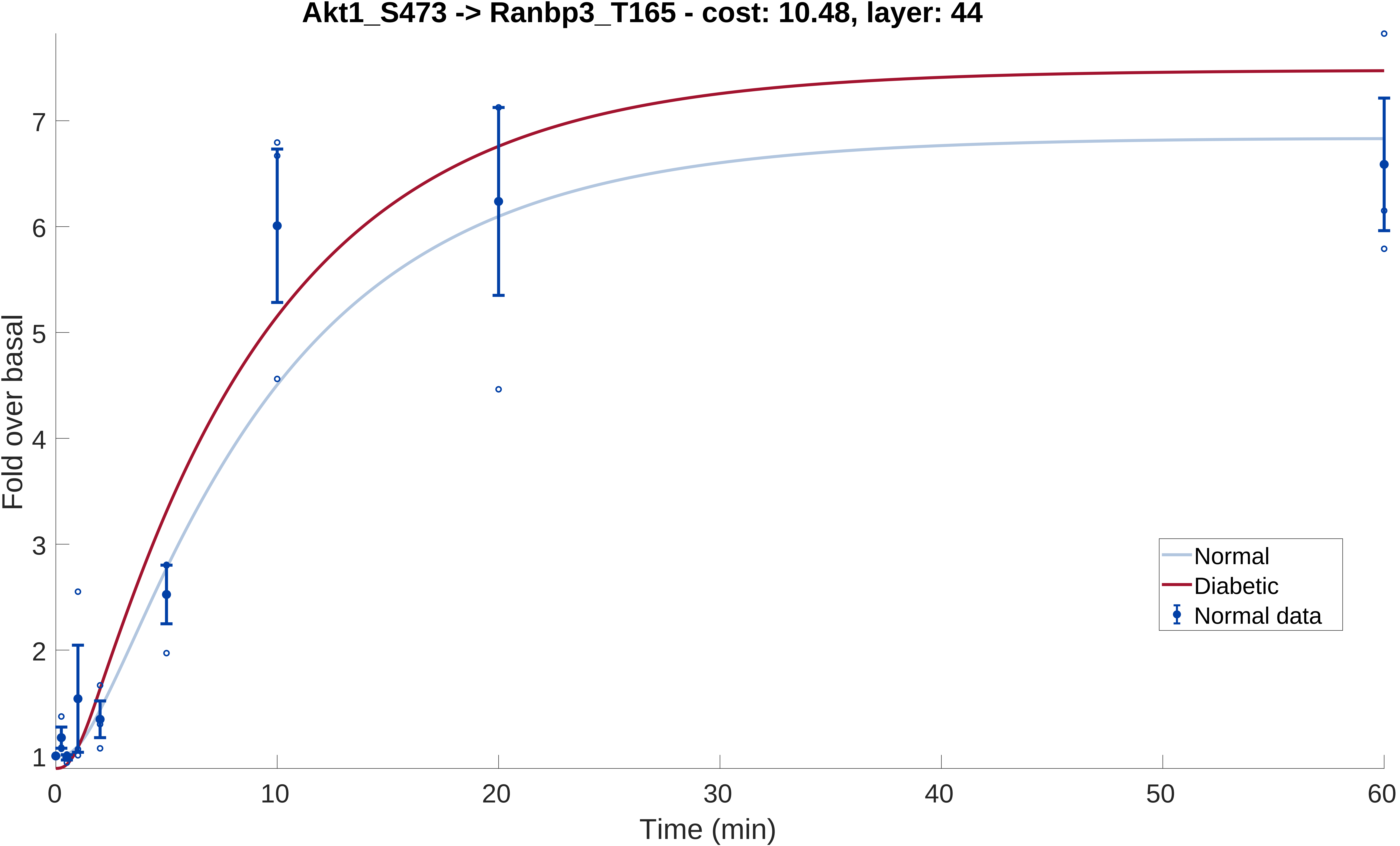
Akt1_S473 -> Ranbp3_T165 - cost: 10.48, layer: 44
Fold over basal
Time (min)
1
2
3
4
5
6
7
0
10
20
30
40
50
60
Normal
Diabetic
Normal data

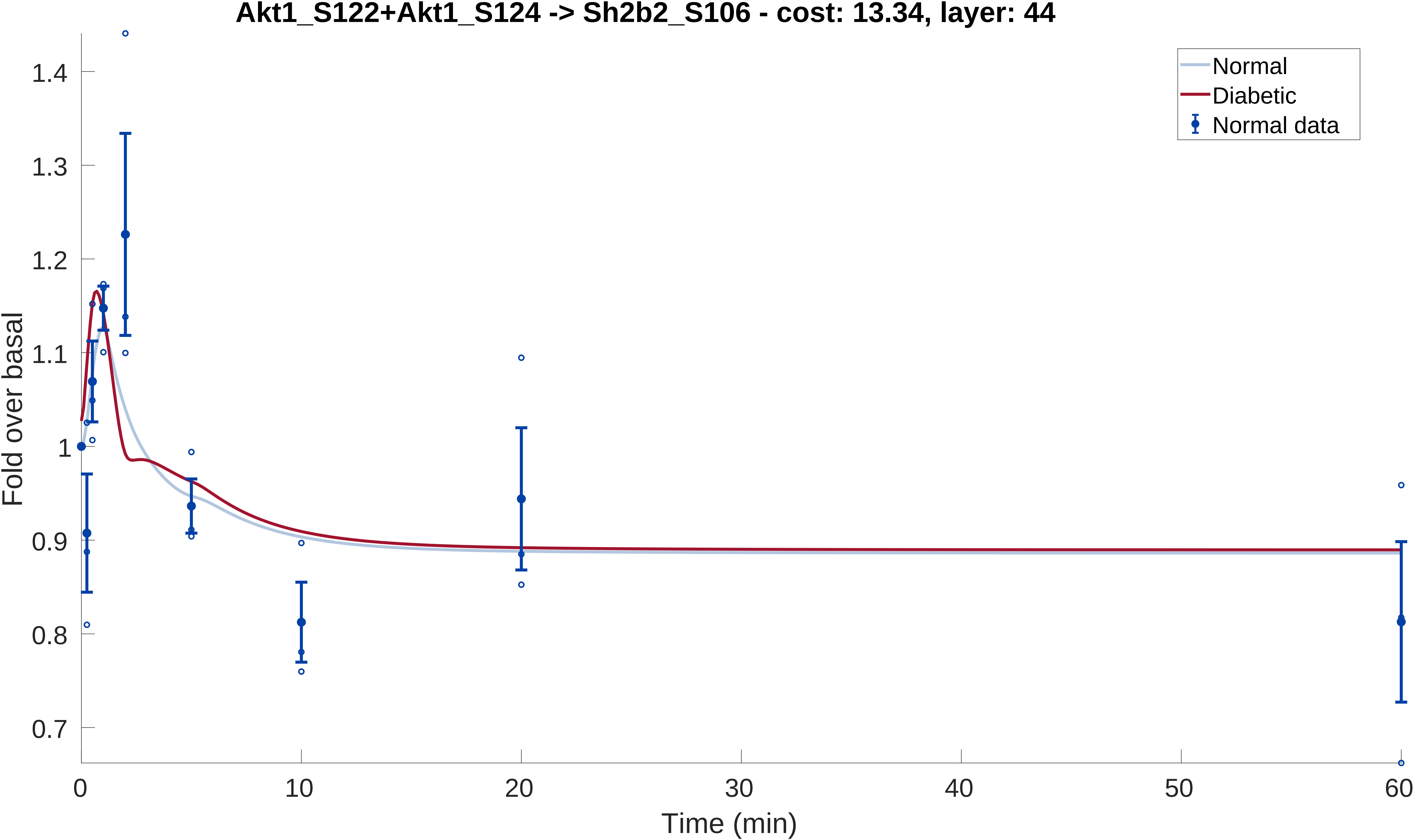

Akt1_S122+Akt1_S124 -> Sh2b2_S106 - cost: 13.34, layer: 44
Normal
Diabetic
Normal data
Fold over basal
Time (min)
1.4
1.3
1.2
1.1
1
0.9
0.8
0.7
0
10
20
30
40
50
60

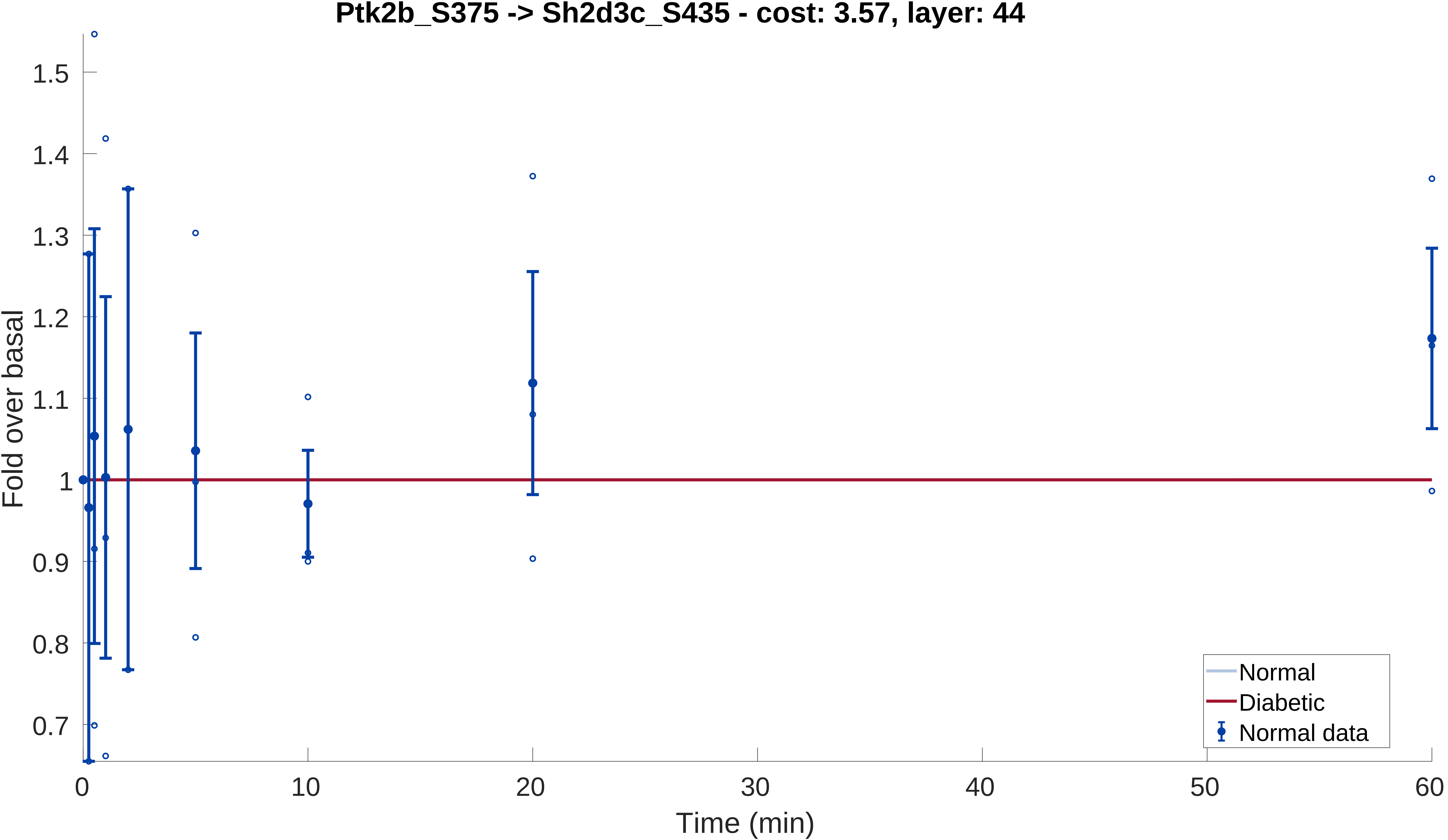

Ptk2b_S375 -> Sh2d3c_S435 - cost: 3.57, layer: 44
Fold over basal
1.5
1.4
1.3
1.2
1.1
1
0.9
0.8
0.7
0
10
20
30
40
50
60
Time (min)
Normal
Diabetic
Normal data

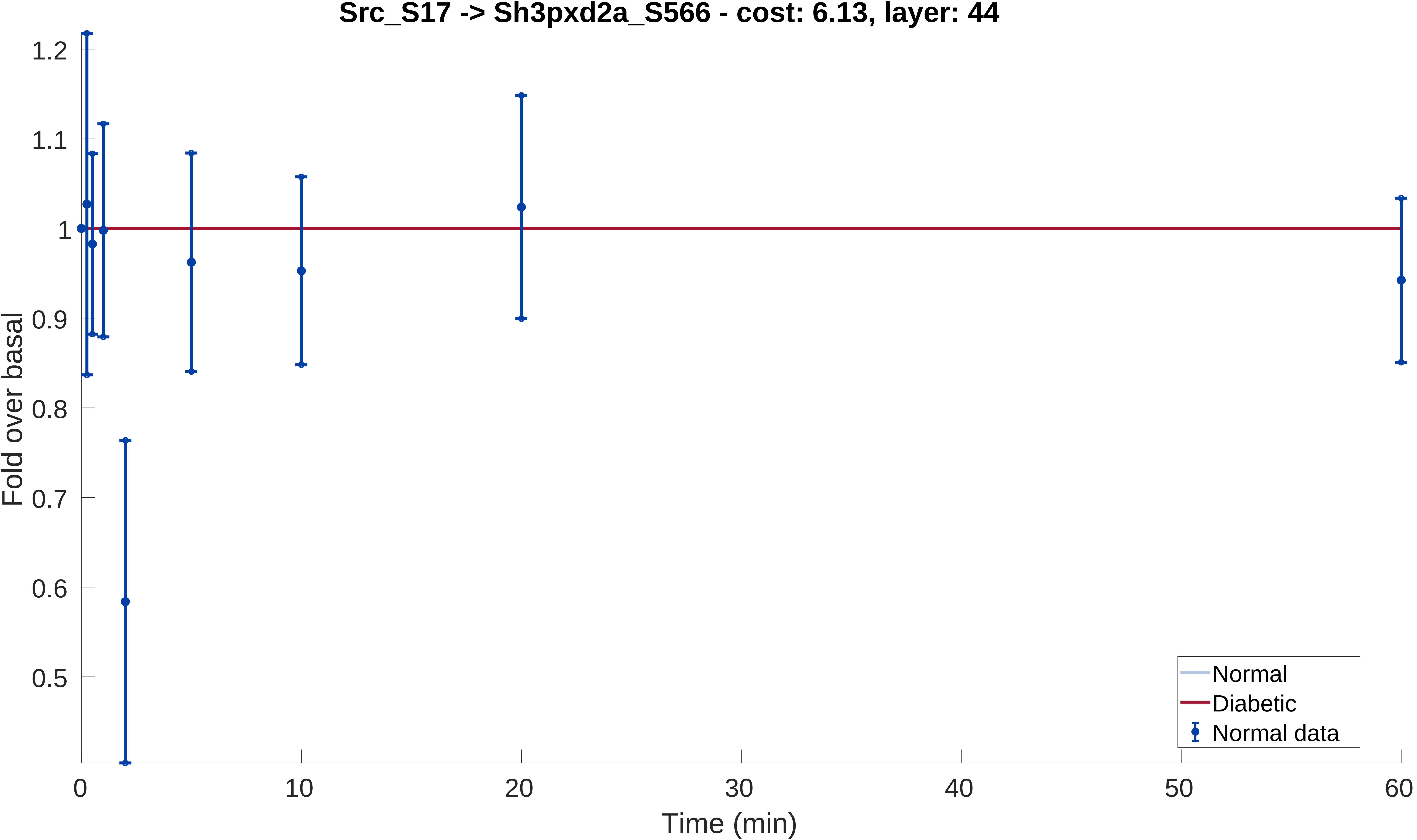

Src_S17 -> Sh3pxd2a_S566 - cost: 6.13, layer: 44
Fold over basal
1.2
1.1
1
0.9
0.8
0.7
0.6
0.5
0
10
20
30
40
50
60
Time (min)
Normal
Diabetic
Normal data

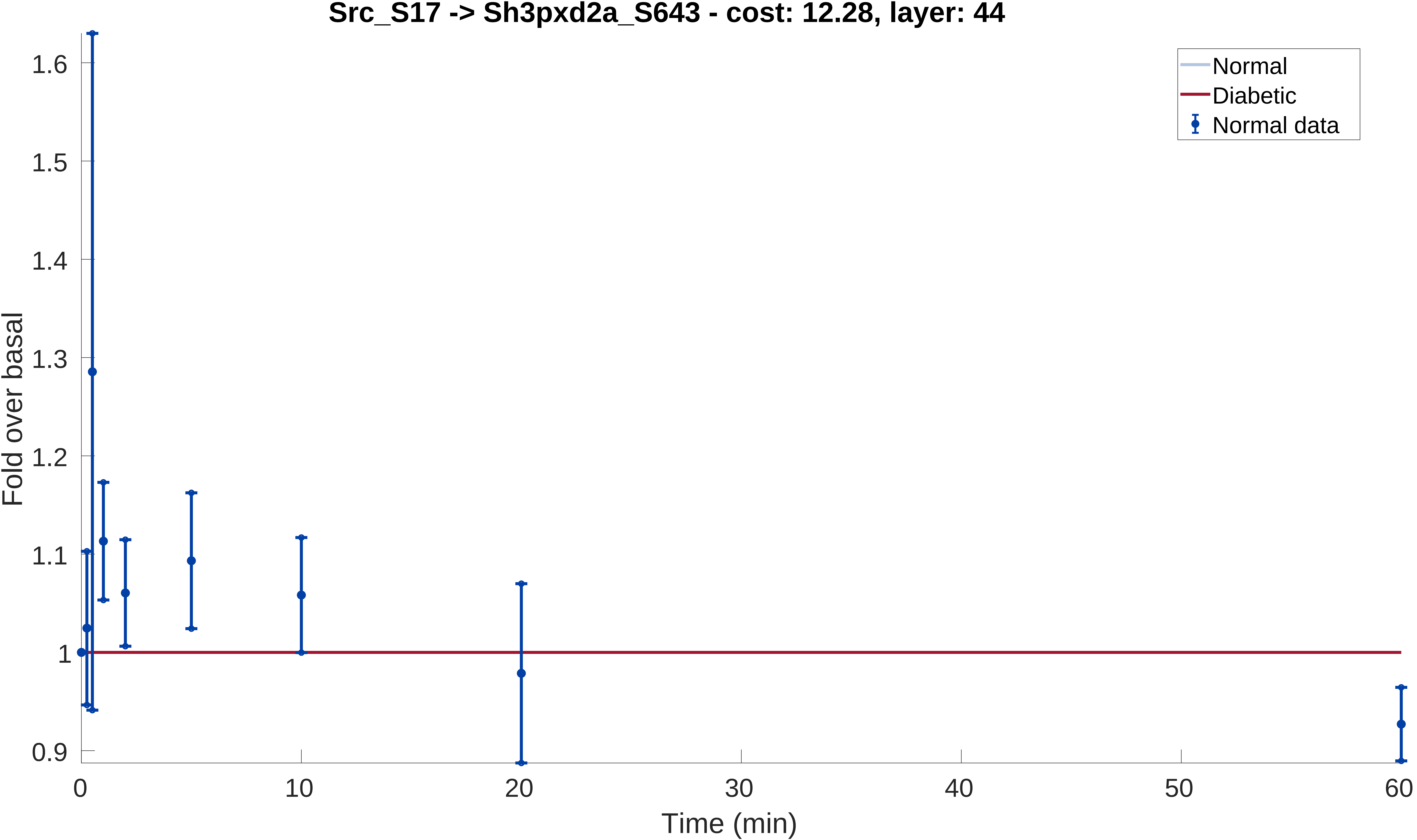

Src_S17 -> Sh3pxd2a_S643 - cost: 12.28, layer: 44
Normal
Diabetic
Normal data
Fold over basal
1.6
1.5
1.4
1.3
1.2
1.1
1
0.9
0
10
20
30
40
50
60
Time (min)

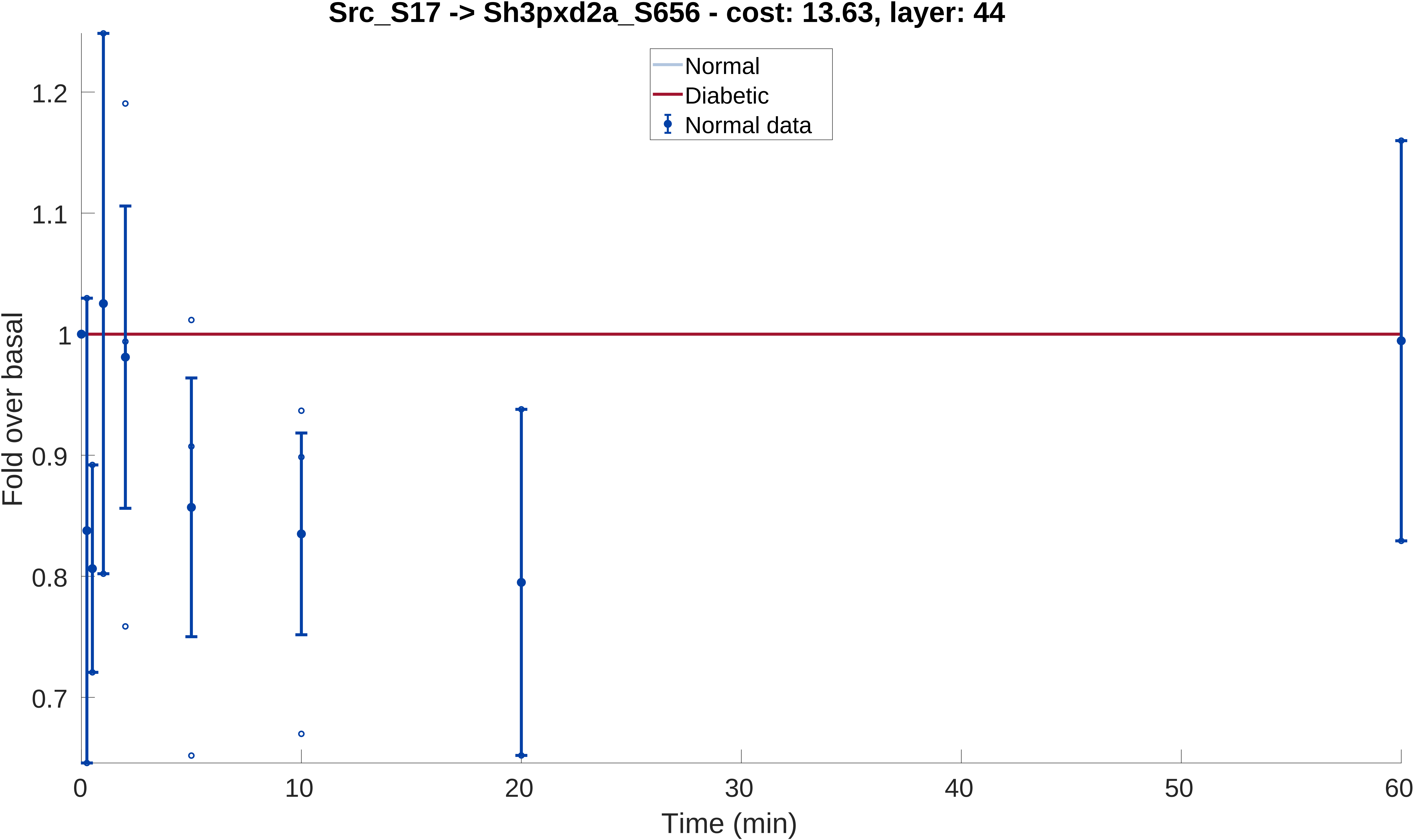

Src_S17 -> Sh3pxd2a_S656 - cost: 13.63, layer: 44
Normal
Diabetic
Normal data
Fold over basal
1.2
1.1
1
0.9
0.8
0.7
0
10
20
30
40
50
60
Time (min)

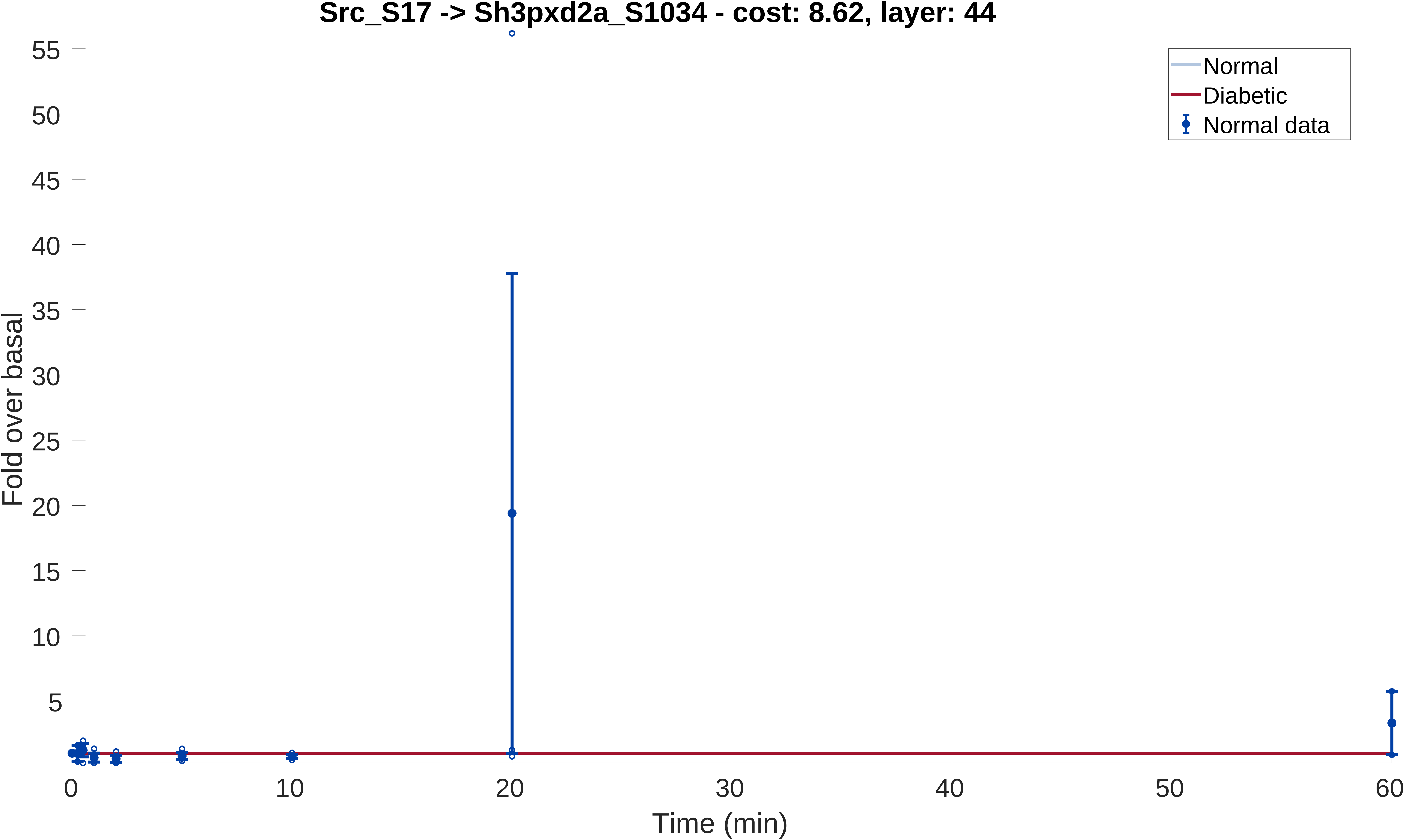

Src_S17 -> Sh3pxd2a_S1034 - cost: 8.62, layer: 44
Normal
Diabetic
Normal data
Fold over basal
55
50
45
40
35
30
25
20
15
10
5
0
10
20
30
40
50
60
Time (min)

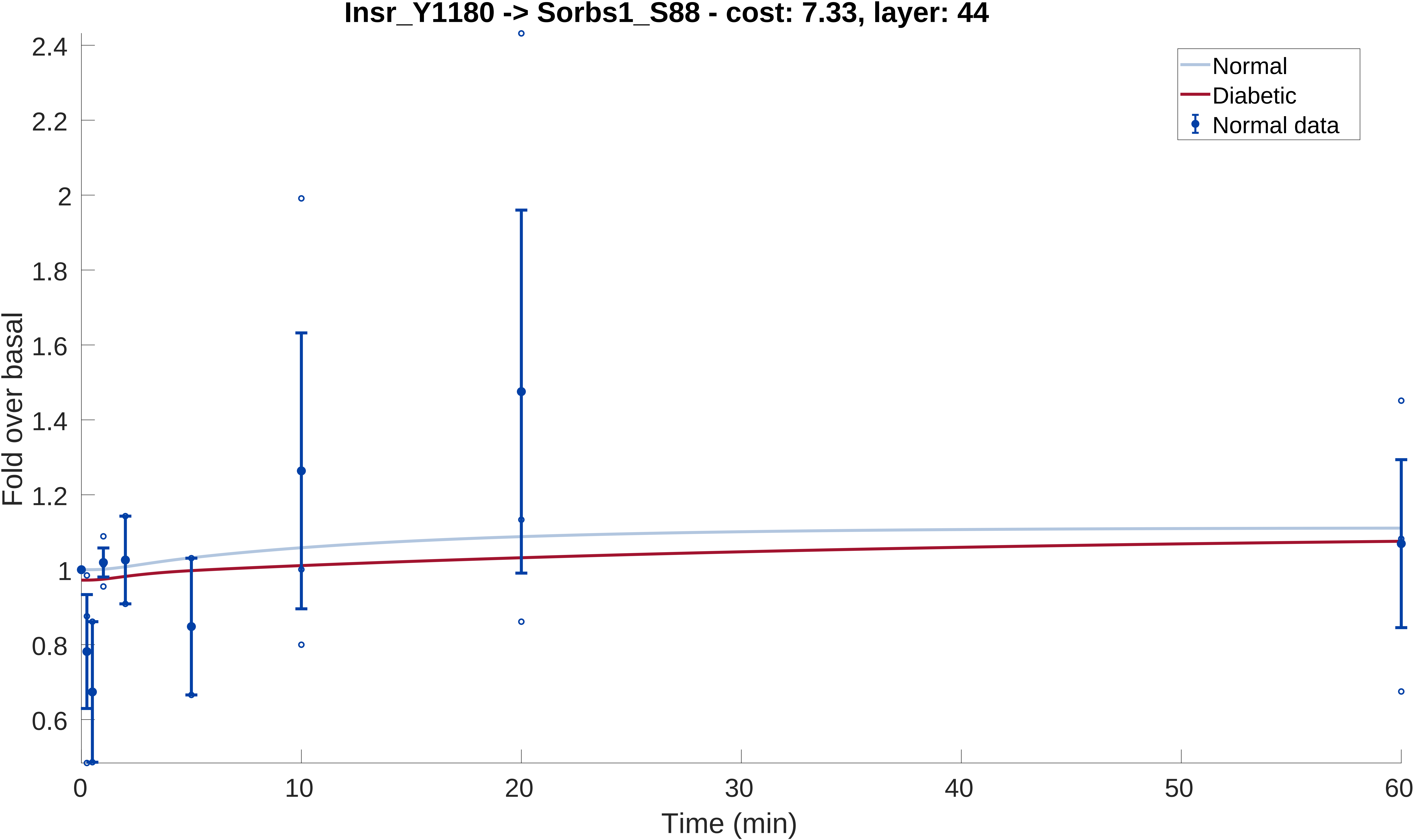
Insr_Y1180 -> Sorbs1_S88 - cost: 7.33, layer: 44
Normal
Diabetic
Normal data
Fold over basal
Time (min)
2.4
2.2
2
1.8
1.6
1.4
1.2
1
0.8
0.6
0
10
20
30
40
50
60

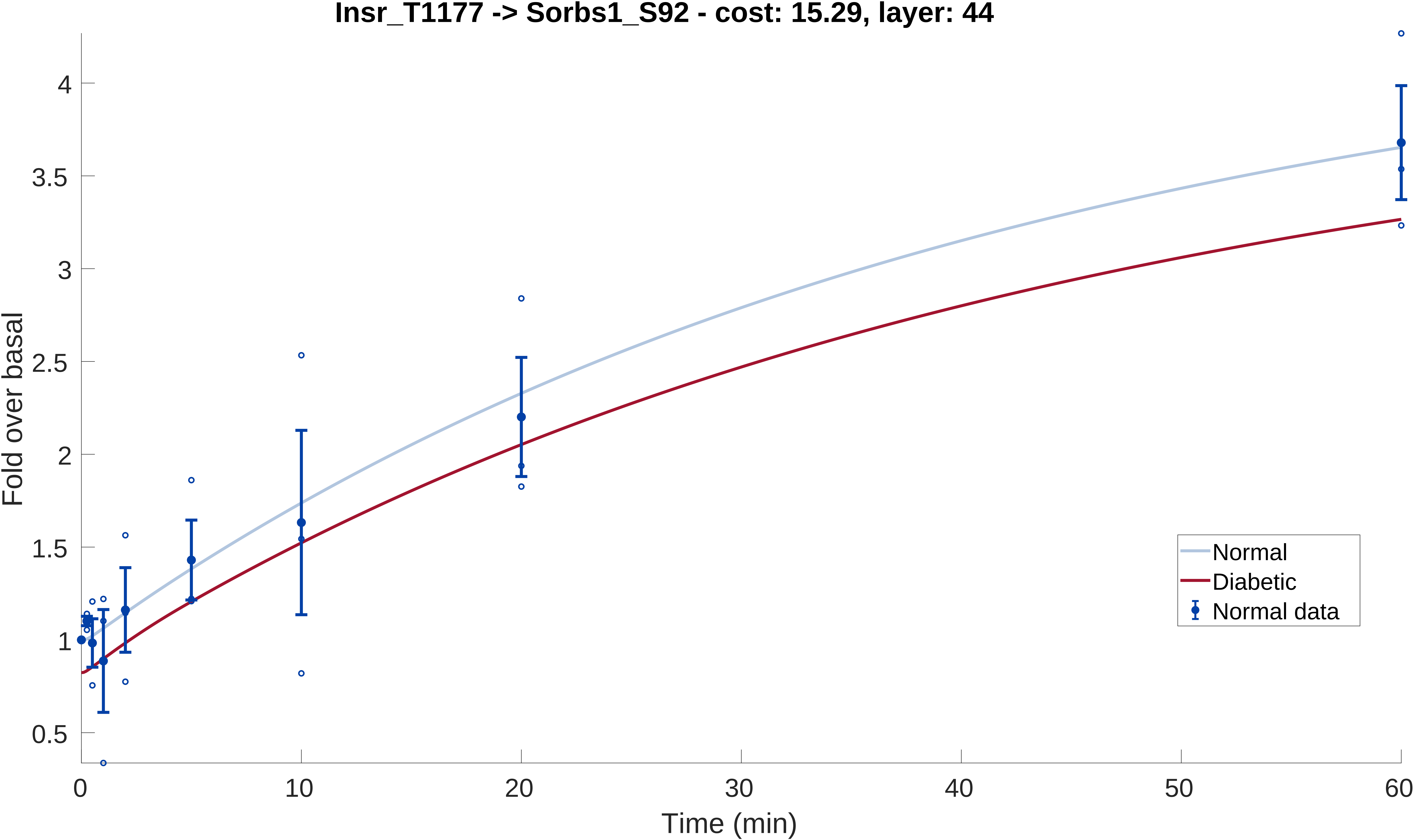
Insr_T1177 -> Sorbs1_S92 - cost: 15.29, layer: 44
Fold over basal
4
3.5
3
2.5
2
1.5
1
0.5
0
10
20
30
40
50
60
Time (min)
Normal
Diabetic
Normal data

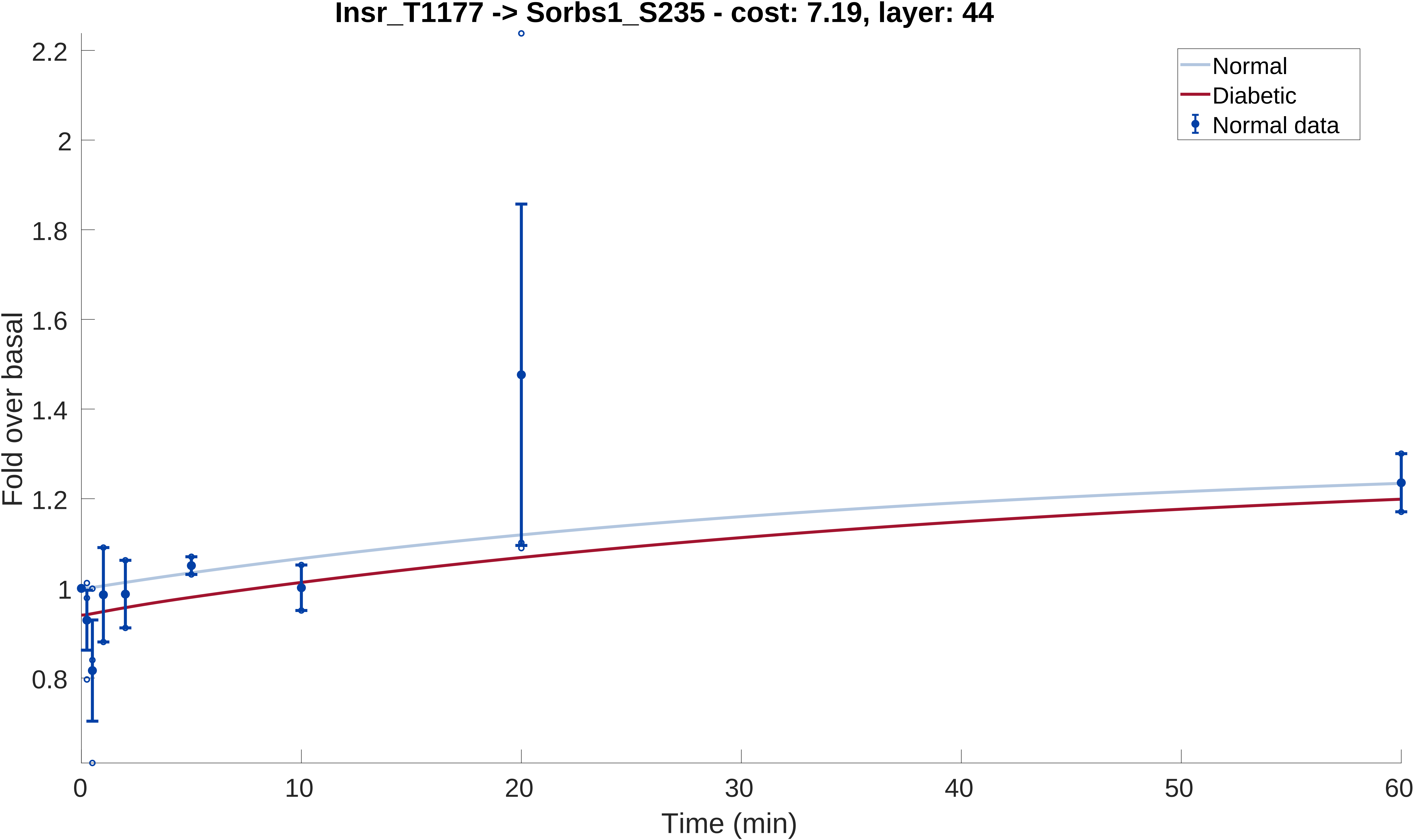

Insr_T1177 -> Sorbs1_S235 - cost: 7.19, layer: 44
Normal
Diabetic
Normal data
Fold over basal
2.2
2
1.8
1.6
1.4
1.2
1
0.8
0
10
20
30
40
50
60
Time (min)

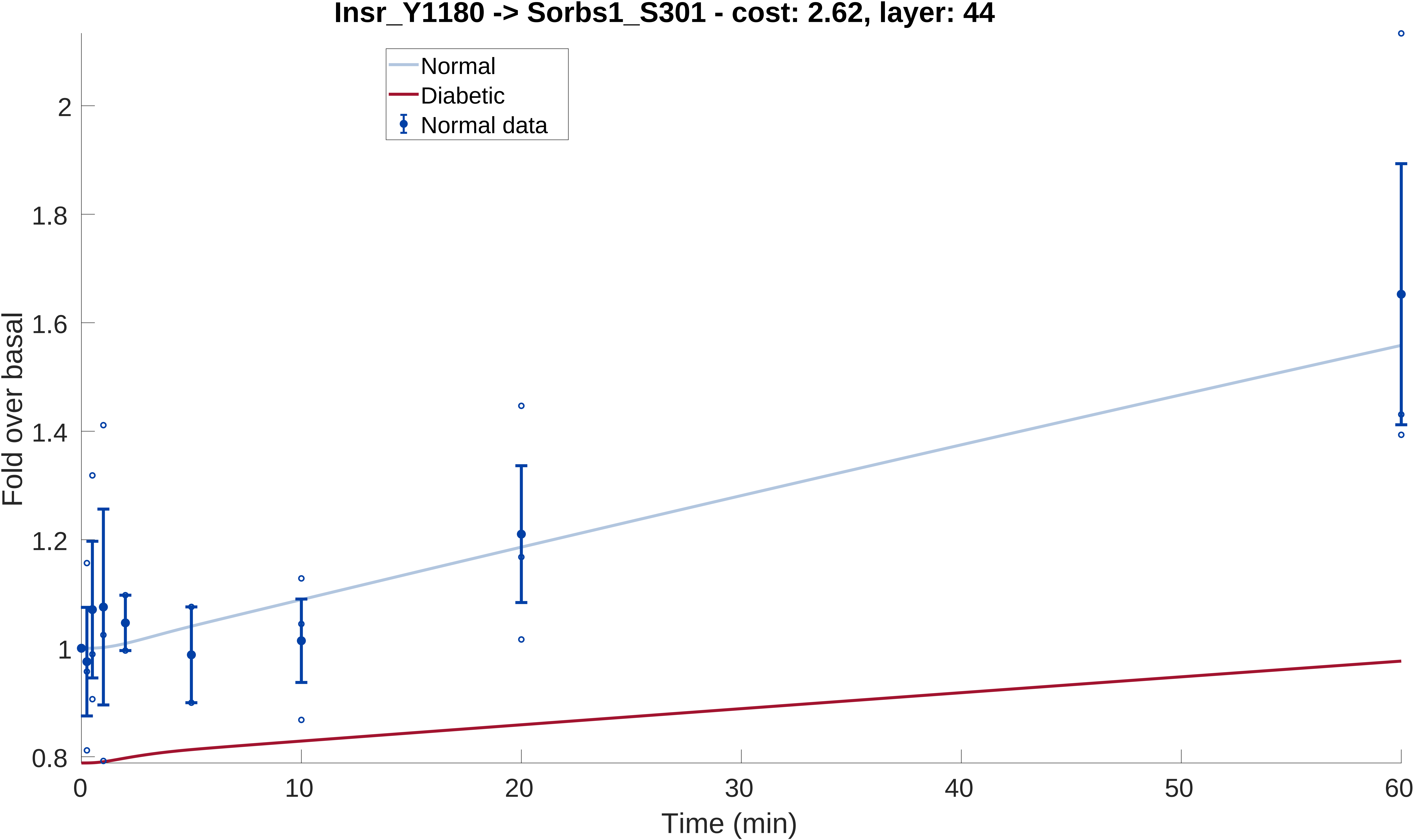

Insr_Y1180 -> Sorbs1_S301 - cost: 2.62, layer: 44
Normal
Diabetic
Normal data
Fold over basal
Time (min)
0.8
1
1.2
1.4
1.6
1.8
2
0
10
20
30
40
50
60

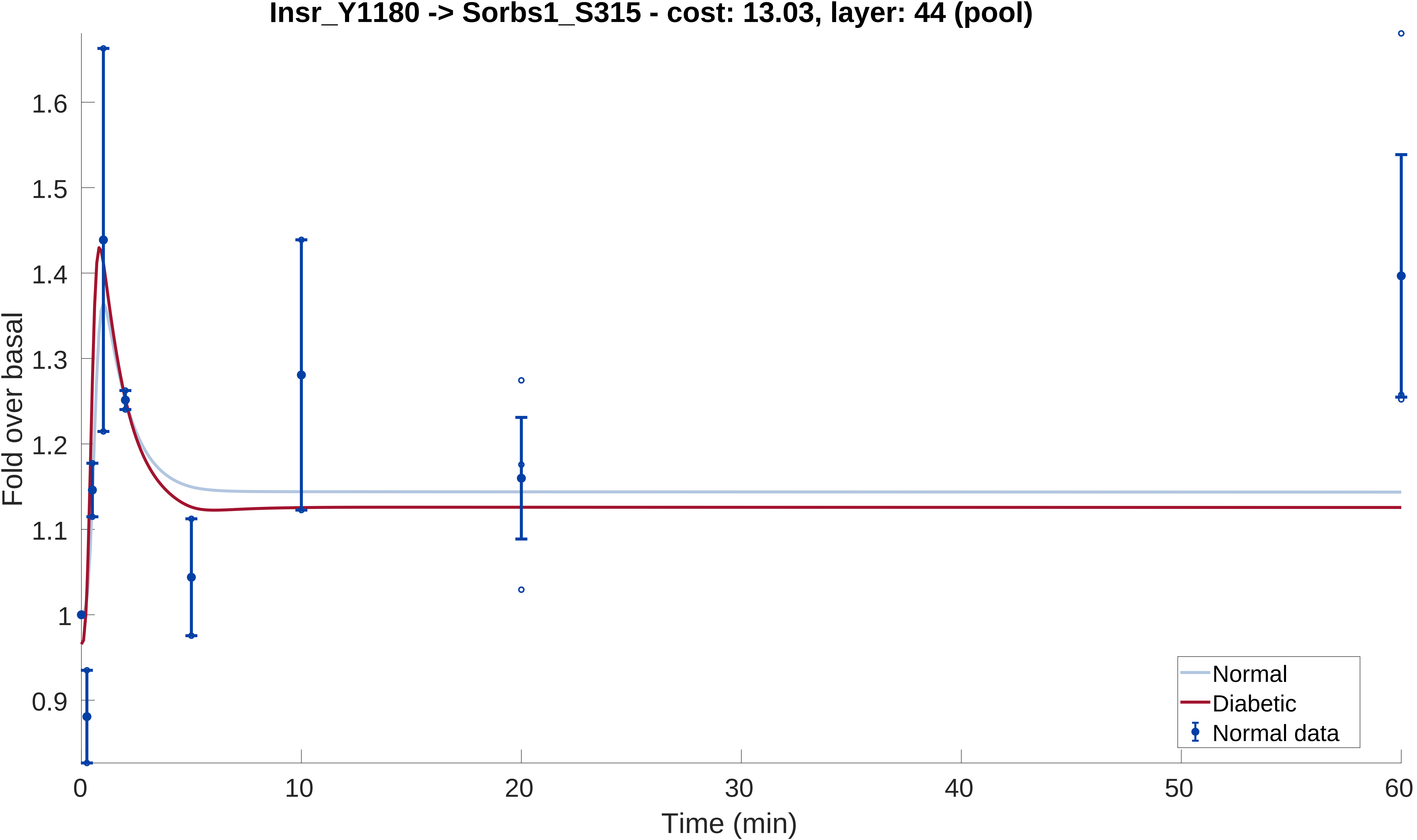

Insr_Y1180 -> Sorbs1_S315 - cost: 13.03, layer: 44 (pool)
Fold over basal
1.6
1.5
1.4
1.3
1.2
1.1
1
0.9
0
10
20
30
40
50
60
Time (min)
Normal
Diabetic
Normal data

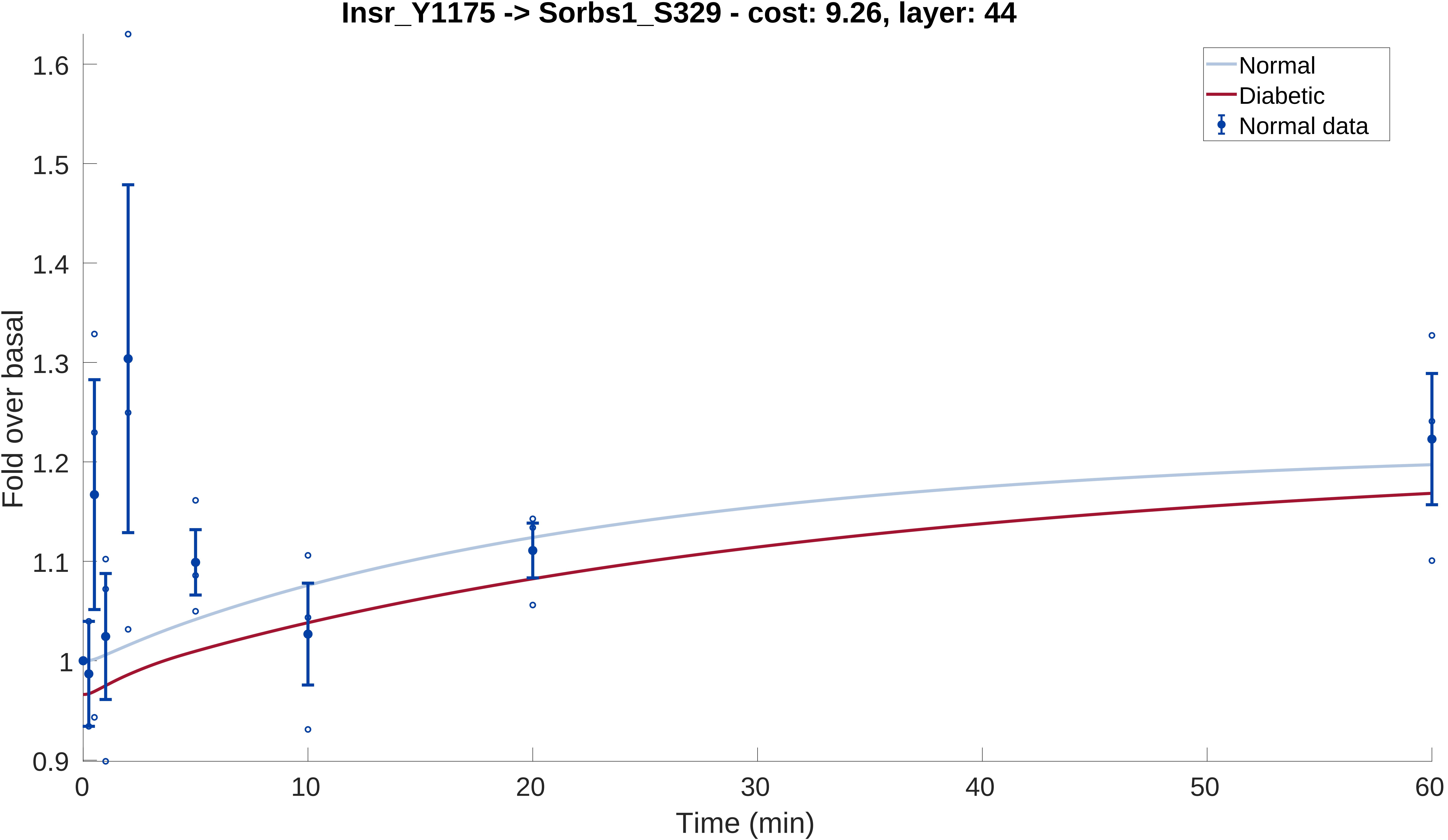

Insr_Y1175 -> Sorbs1_S329 - cost: 9.26, layer: 44
Normal
Diabetic
Normal data
Fold over basal
Time (min)
0.9
1
1.1
1.2
1.3
1.4
1.5
1.6
0
10
20
30
40
50
60

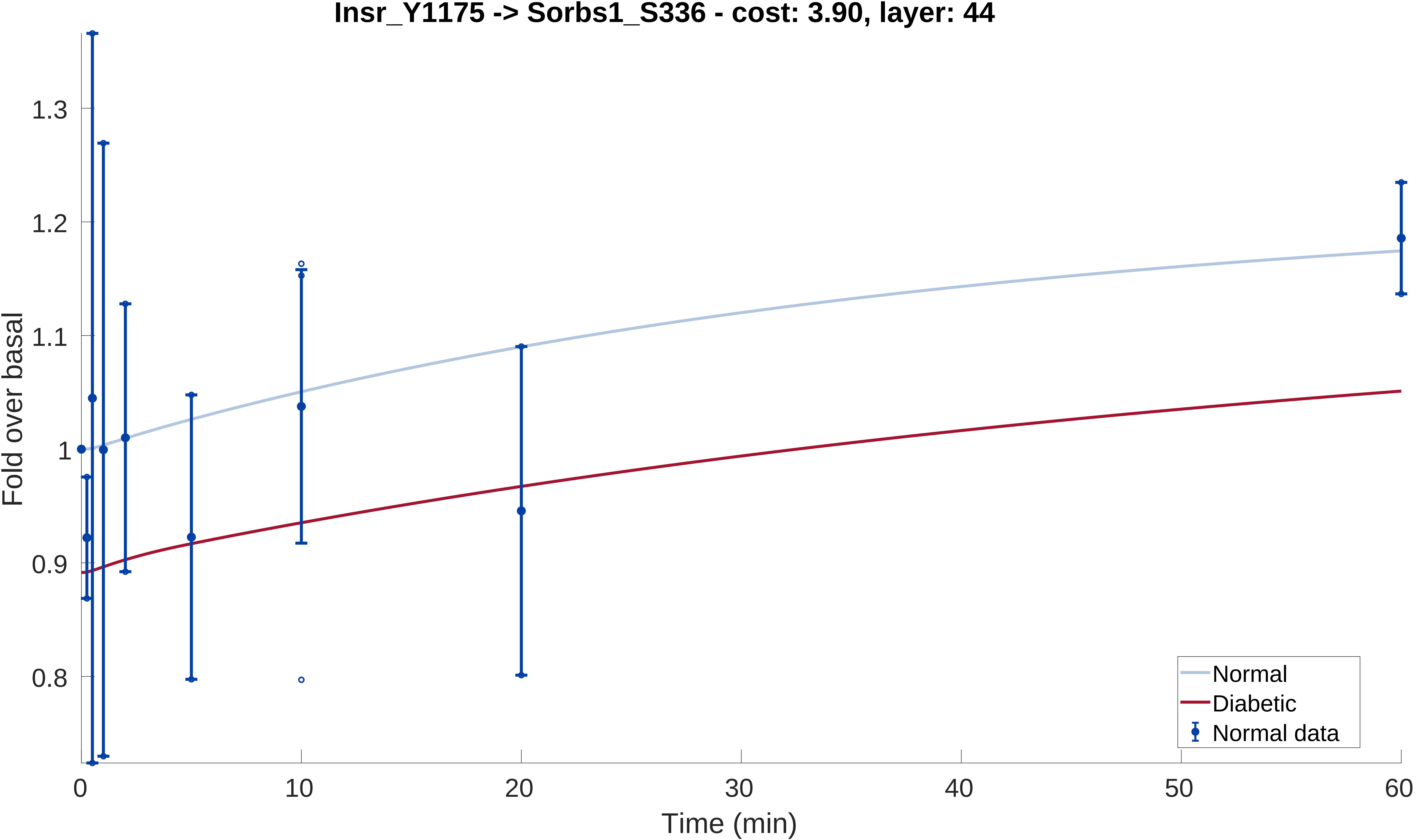

Insr_Y1175 -> Sorbs1_S336 - cost: 3.90, layer: 44
Fold over basal
1.3
1.2
1.1
1
0.9
0.8
0
10
20
30
40
50
60
Time (min)
Normal
Diabetic
Normal data

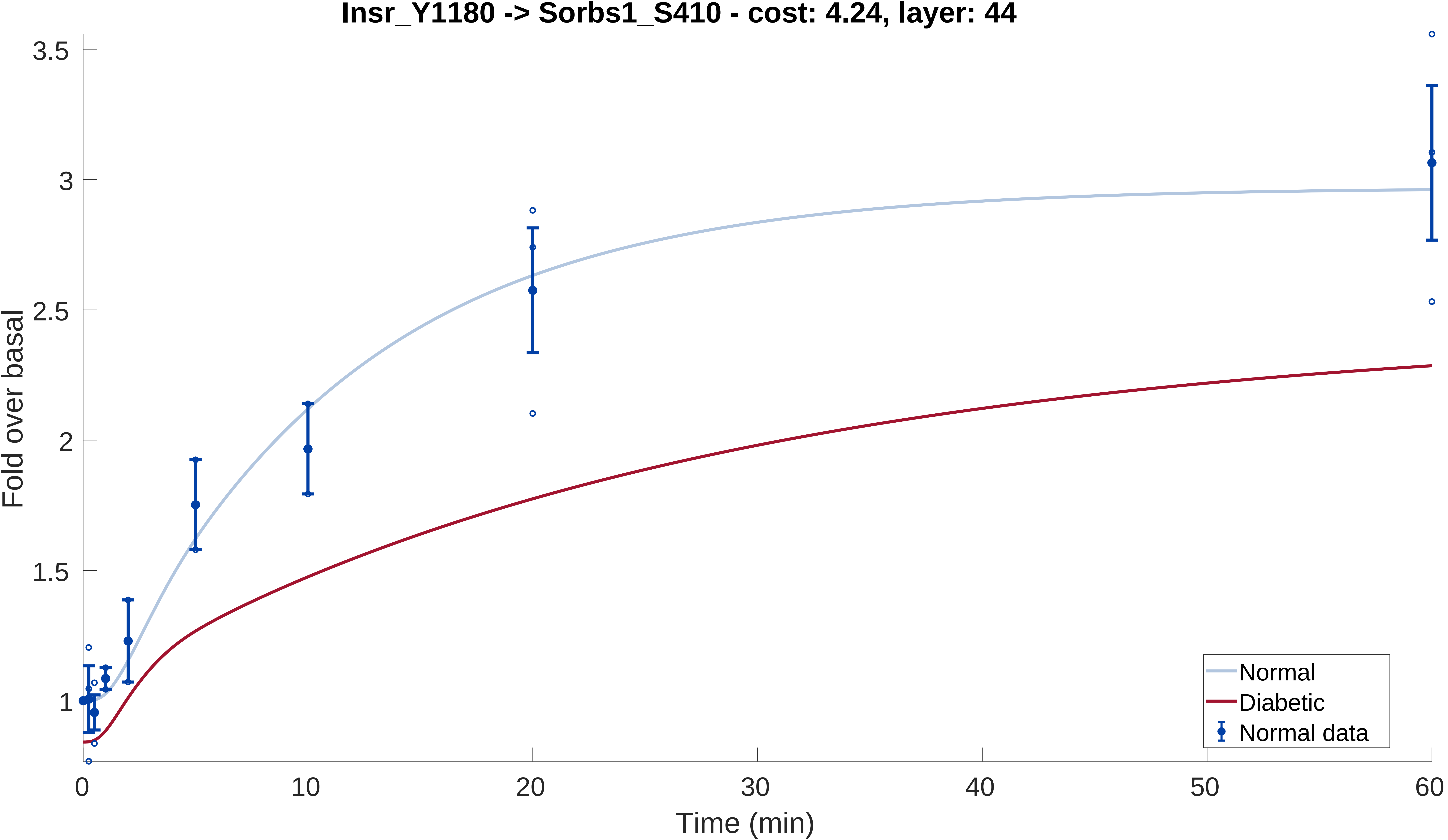

Insr_Y1180 -> Sorbs1_S410 - cost: 4.24, layer: 44
Fold over basal
3.5
3
2.5
2
1.5
1
0
10
20
30
40
50
60
Time (min)
Normal
Diabetic
Normal data

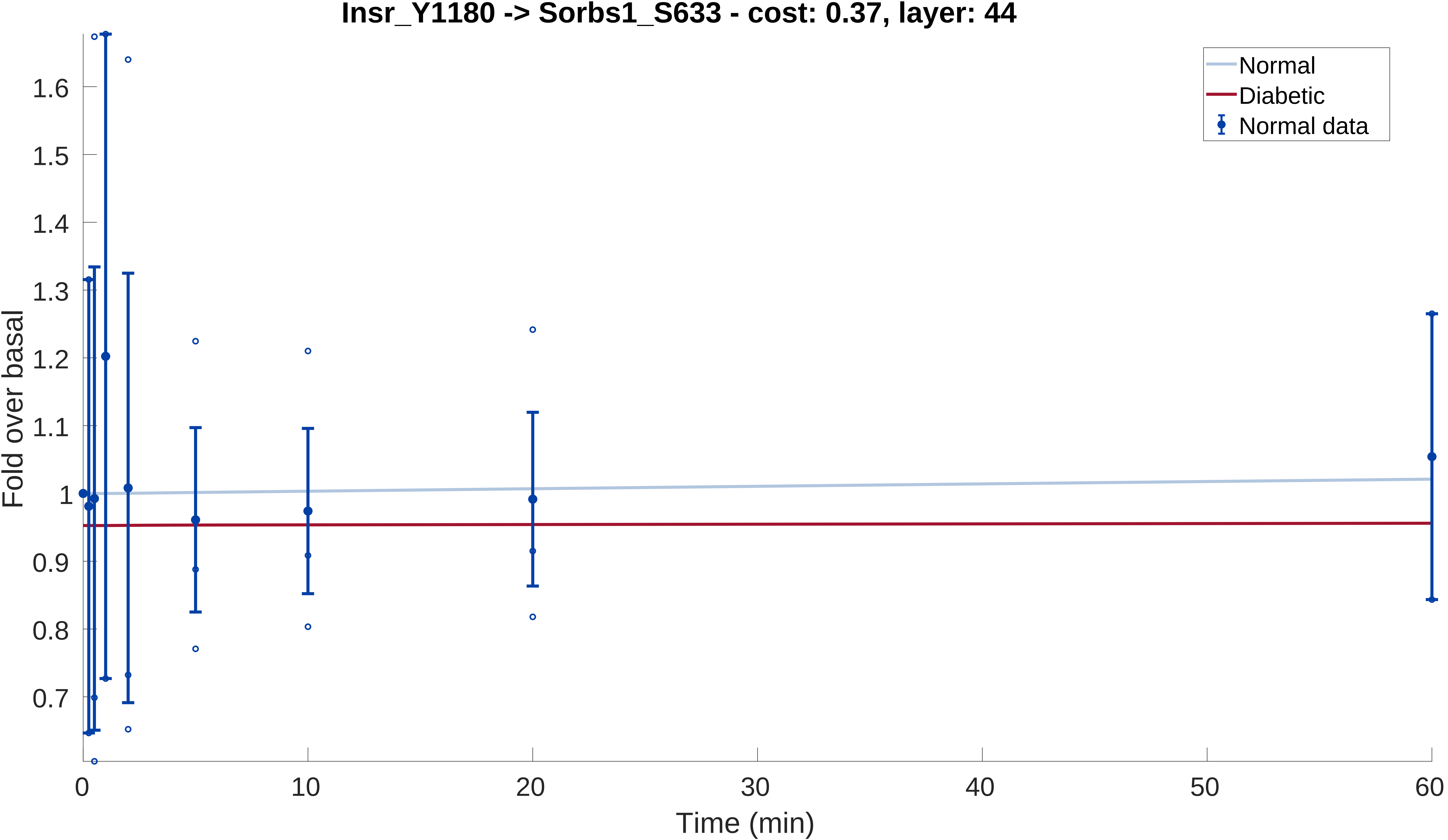
Insr_Y1180 -> Sorbs1_S633 - cost: 0.37, layer: 44
Normal
Diabetic
Normal data
Fold over basal
1.6
1.5
1.4
1.3
1.2
1.1
1
0.9
0.8
0.7
0
10
20
30
40
50
60
Time (min)

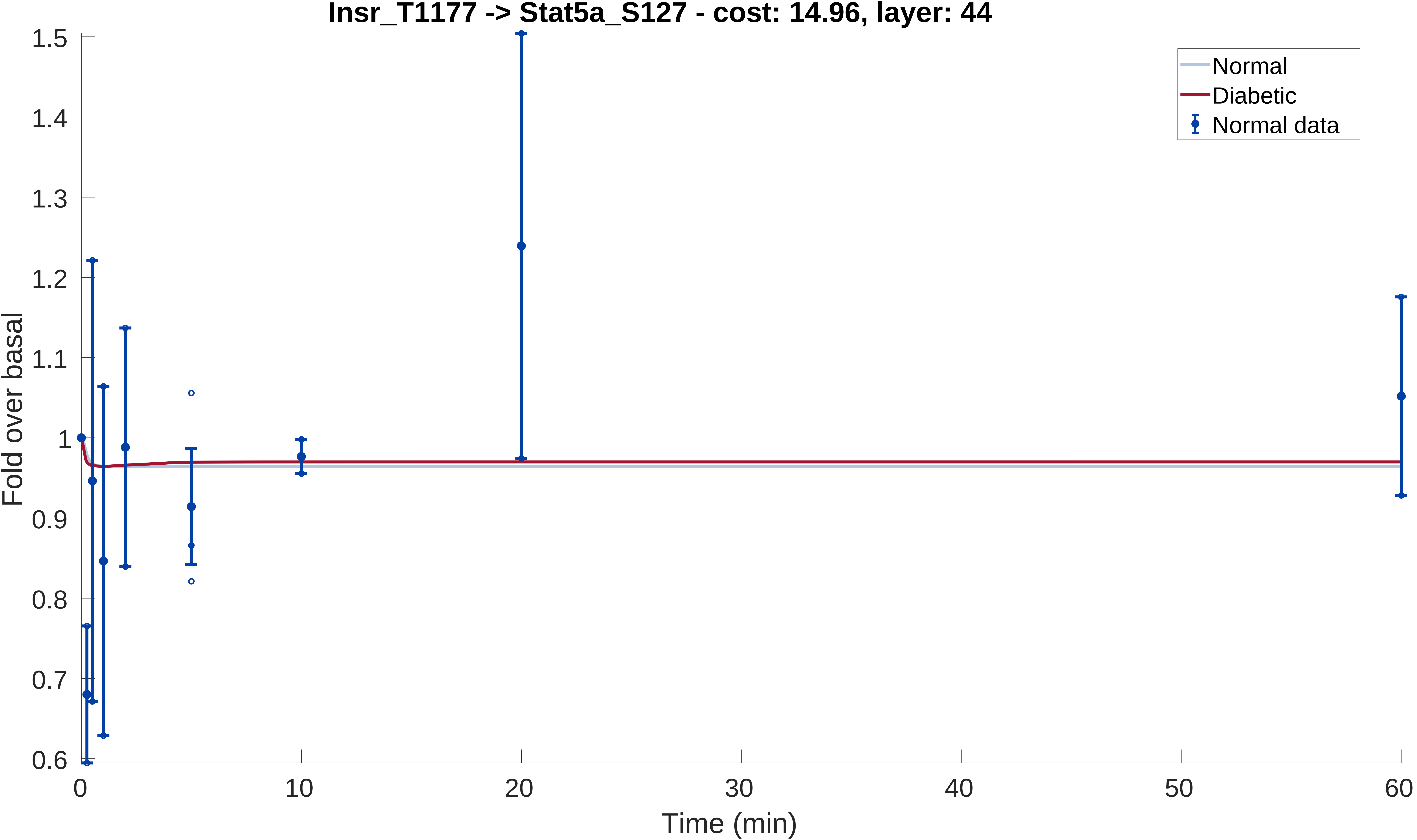
Insr_T1177 -> Stat5a_S127 - cost: 14.96, layer: 44
Fold over basal
Time (min)
Normal
Diabetic
Normal data
1.5
1.4
1.3
1.2
1.1
1
0.9
0.8
0.7
0.6
0
10
20
30
40
50
60

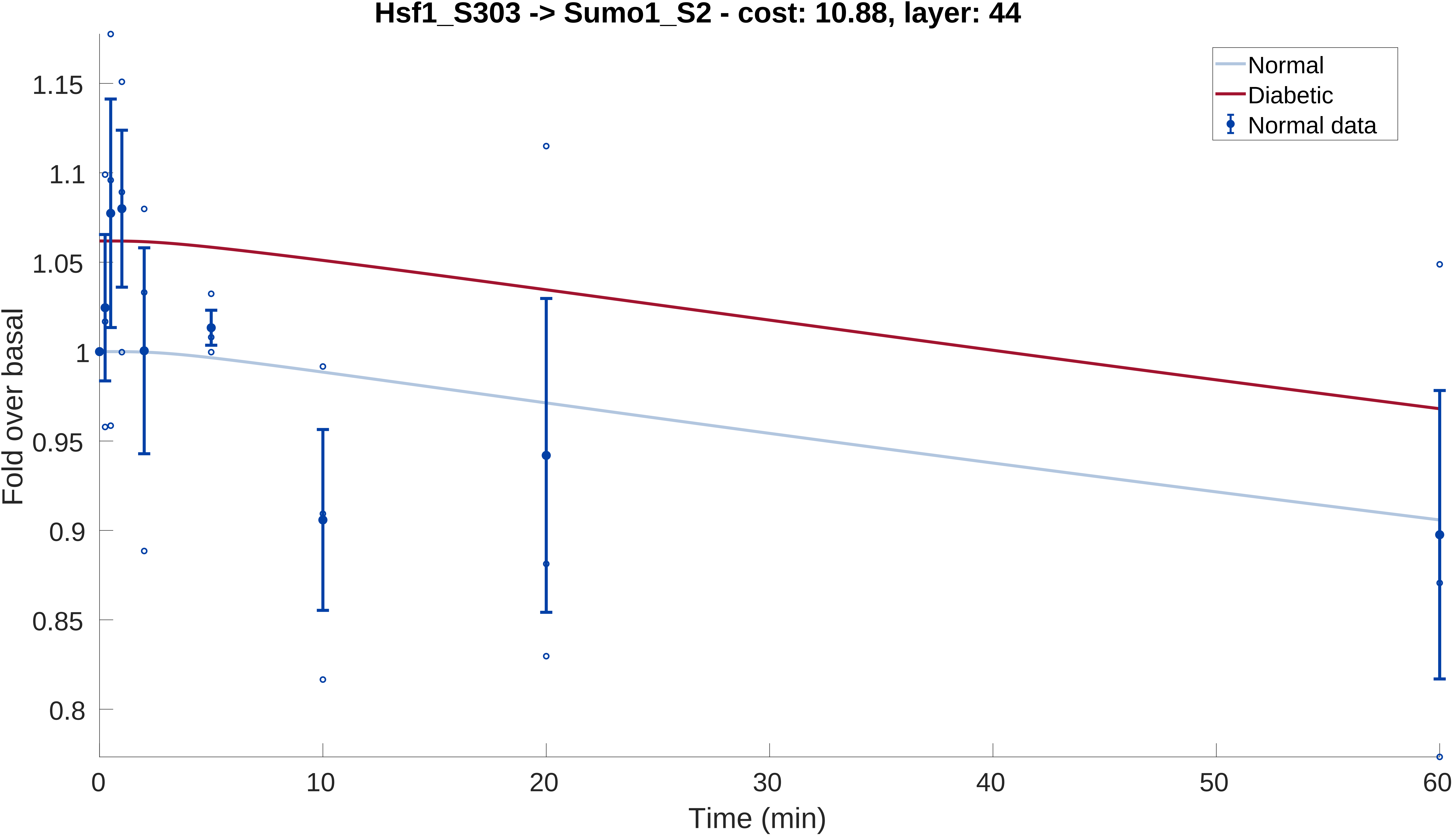

Hsf1_S303 -> Sumo1_S2 - cost: 10.88, layer: 44
Normal
Diabetic
Normal data
Fold over basal
1.15
1.1
1.05
1
0.95
0.9
0.85
0.8
0
10
20
30
40
50
60
Time (min)

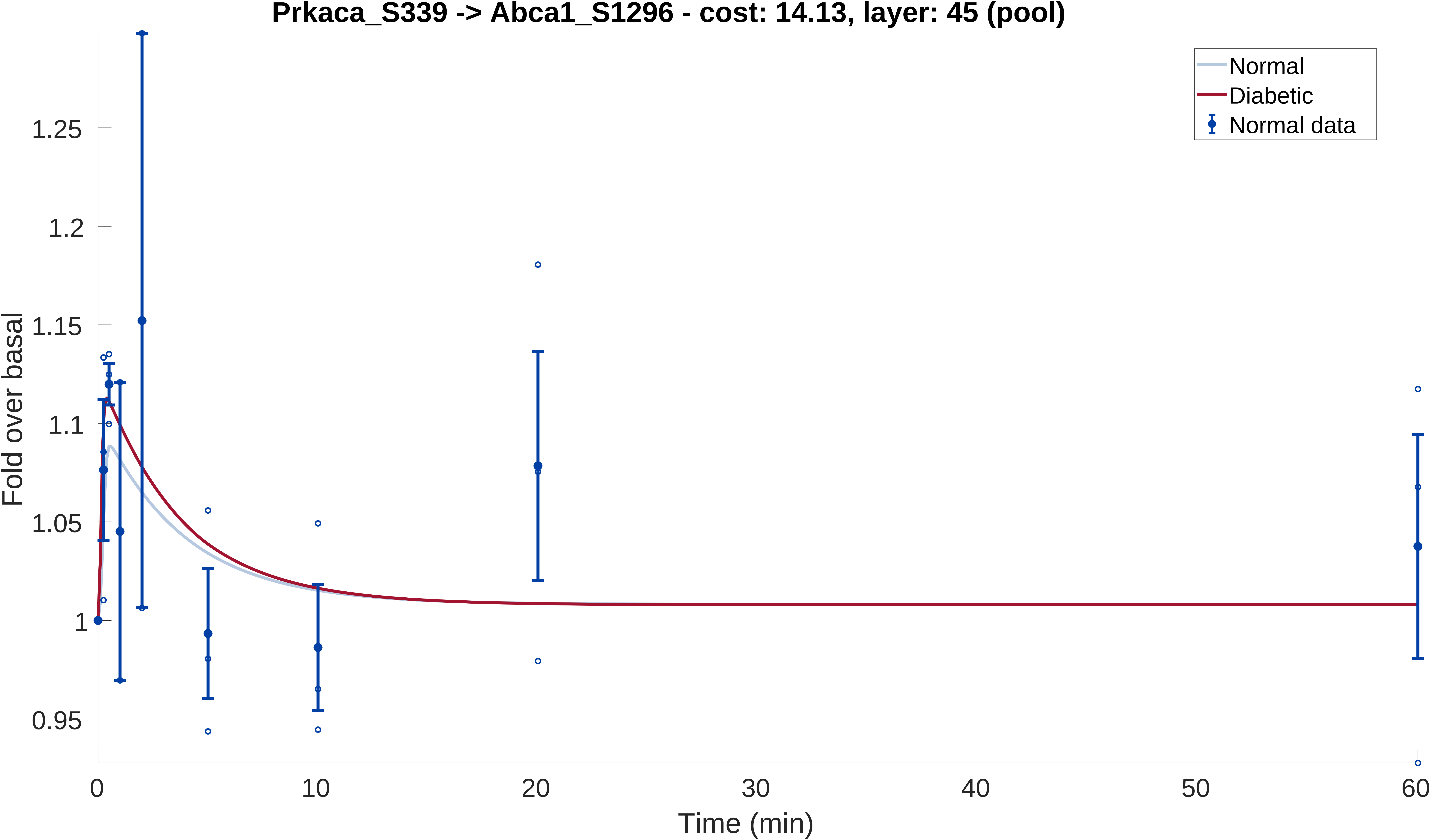

Prkaca_S339 -> Abca1_S1296 - cost: 14.13, layer: 45 (pool)
Normal
Diabetic
Normal data
Fold over basal
1.25
1.2
1.15
1.1
1.05
1
0.95
0
10
20
30
40
50
60
Time (min)

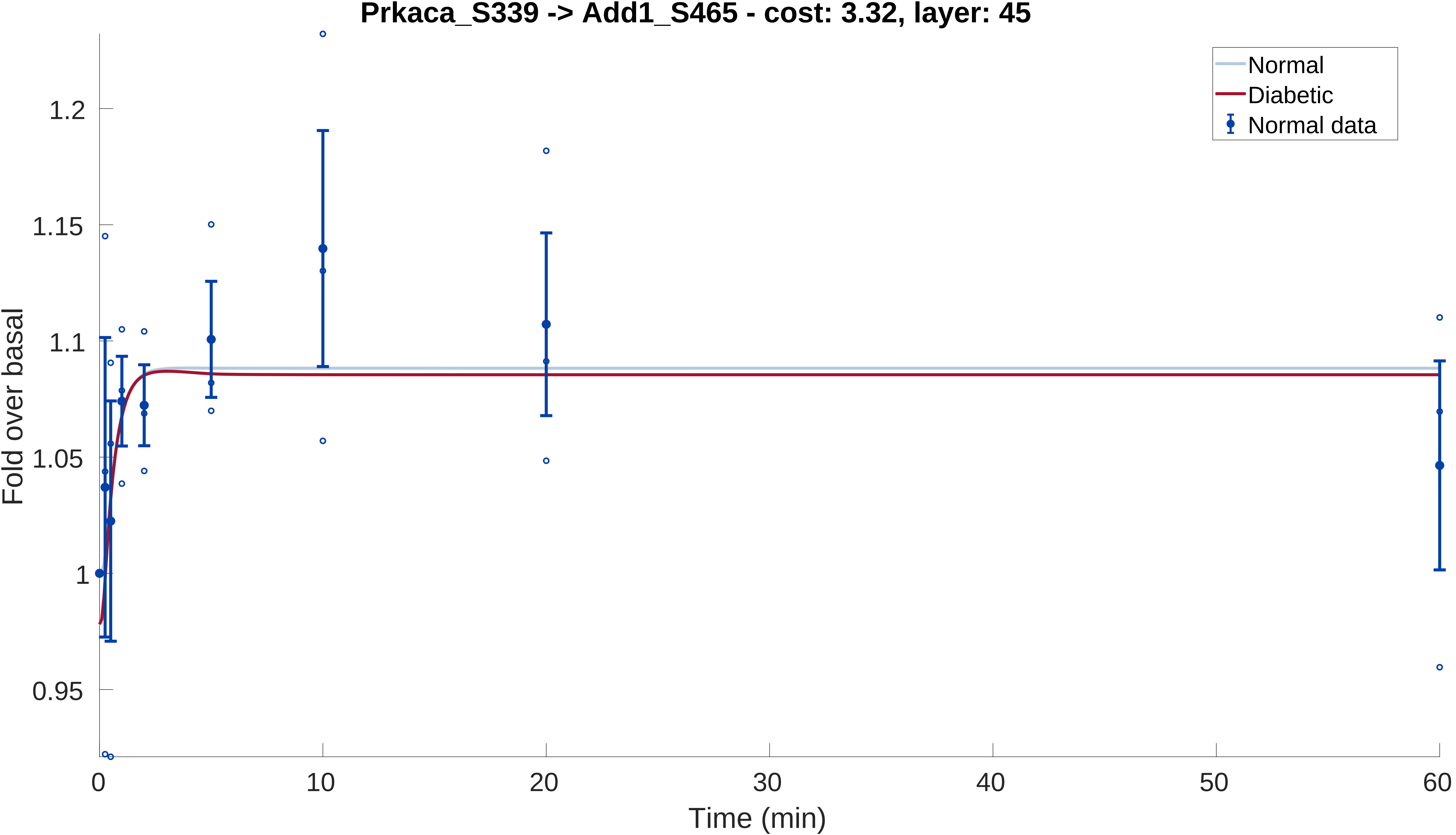
Prkaca_S339 -> Add1_S465 - cost: 3.32, layer: 45
Normal
Diabetic
Normal data
Fold over basal
Time (min)
0.95
1
1.05
1.1
1.15
1.2
0
10
20
30
40
50
60

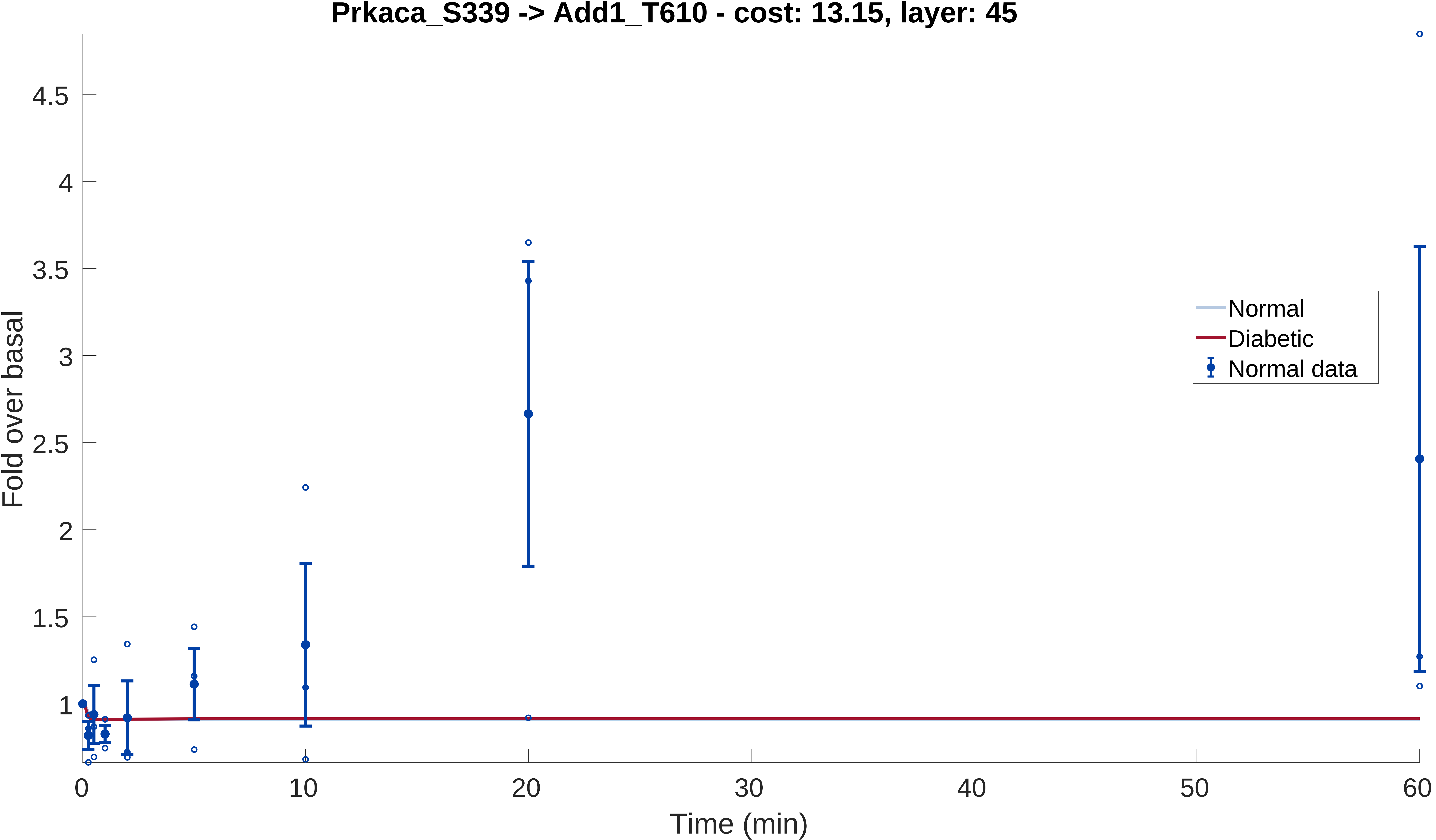

Prkaca_S339 -> Add1_T610 - cost: 13.15, layer: 45
Fold over basal
Time (min)
4.5
4
3.5
3
2.5
2
1.5
1
0
10
20
30
40
50
60
Normal
Diabetic
Normal data

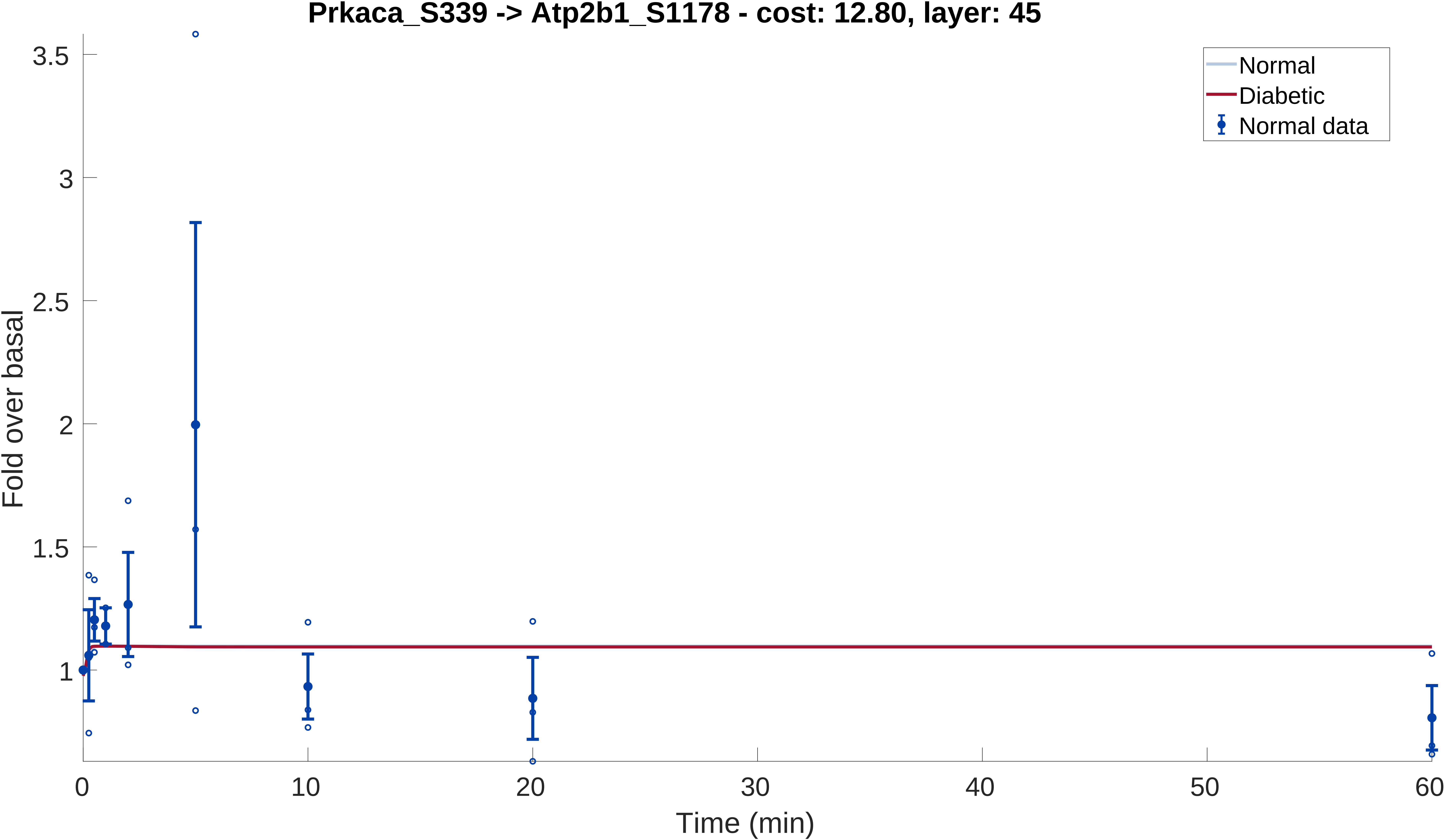

Prkaca_S339 -> Atp2b1_S1178 - cost: 12.80, layer: 45
Fold over basal
3.5
3
2.5
2
1.5
1
0
10
20
30
40
50
60
Time (min)
Normal
Diabetic
Normal data

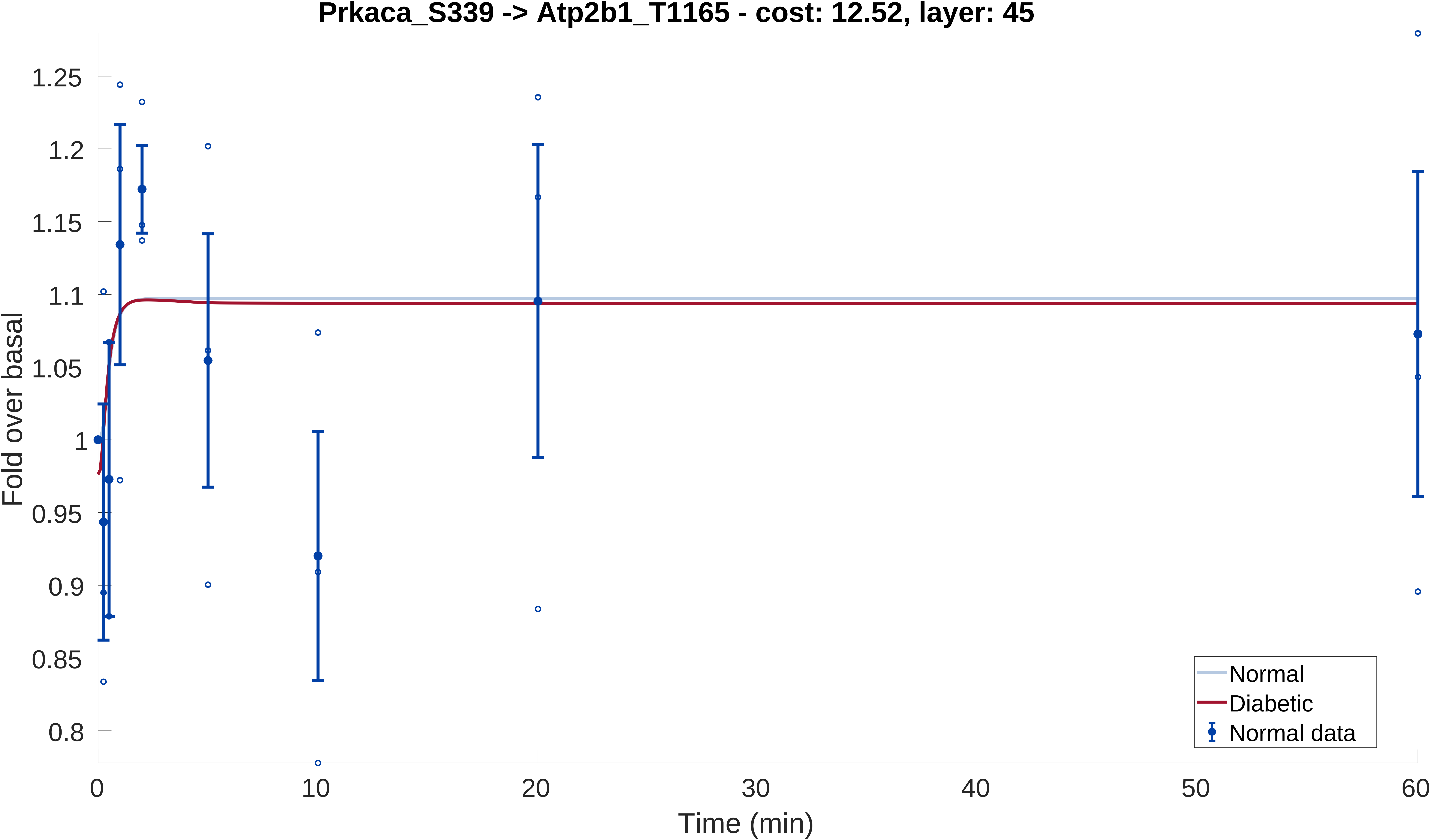

Prkaca_S339 -> Atp2b1_T1165 - cost: 12.52, layer: 45
Fold over basal
1.25
1.2
1.15
1.1
1.05
1
0.95
0.9
0.85
0.8
0
10
20
30
40
50
60
Time (min)
Normal
Diabetic
Normal data

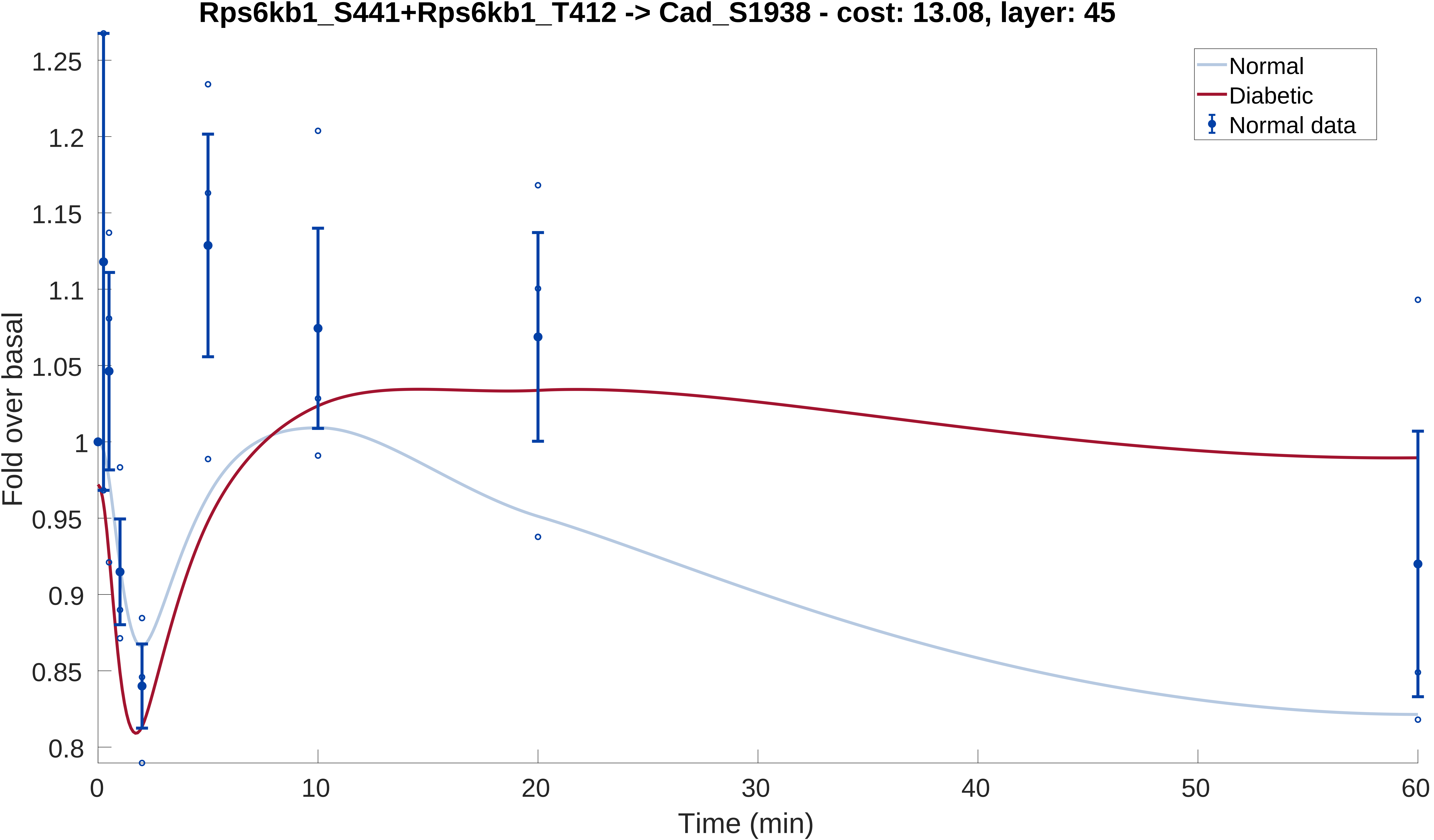

Rps6kb1_S441+Rps6kb1_T412 -> Cad_S1938 - cost: 13.08, layer: 45
Normal
Diabetic
Normal data
Fold over basal
1.25
1.2
1.15
1.1
1.05
1
0.95
0.9
0.85
0.8
0
10
20
30
40
50
60
Time (min)

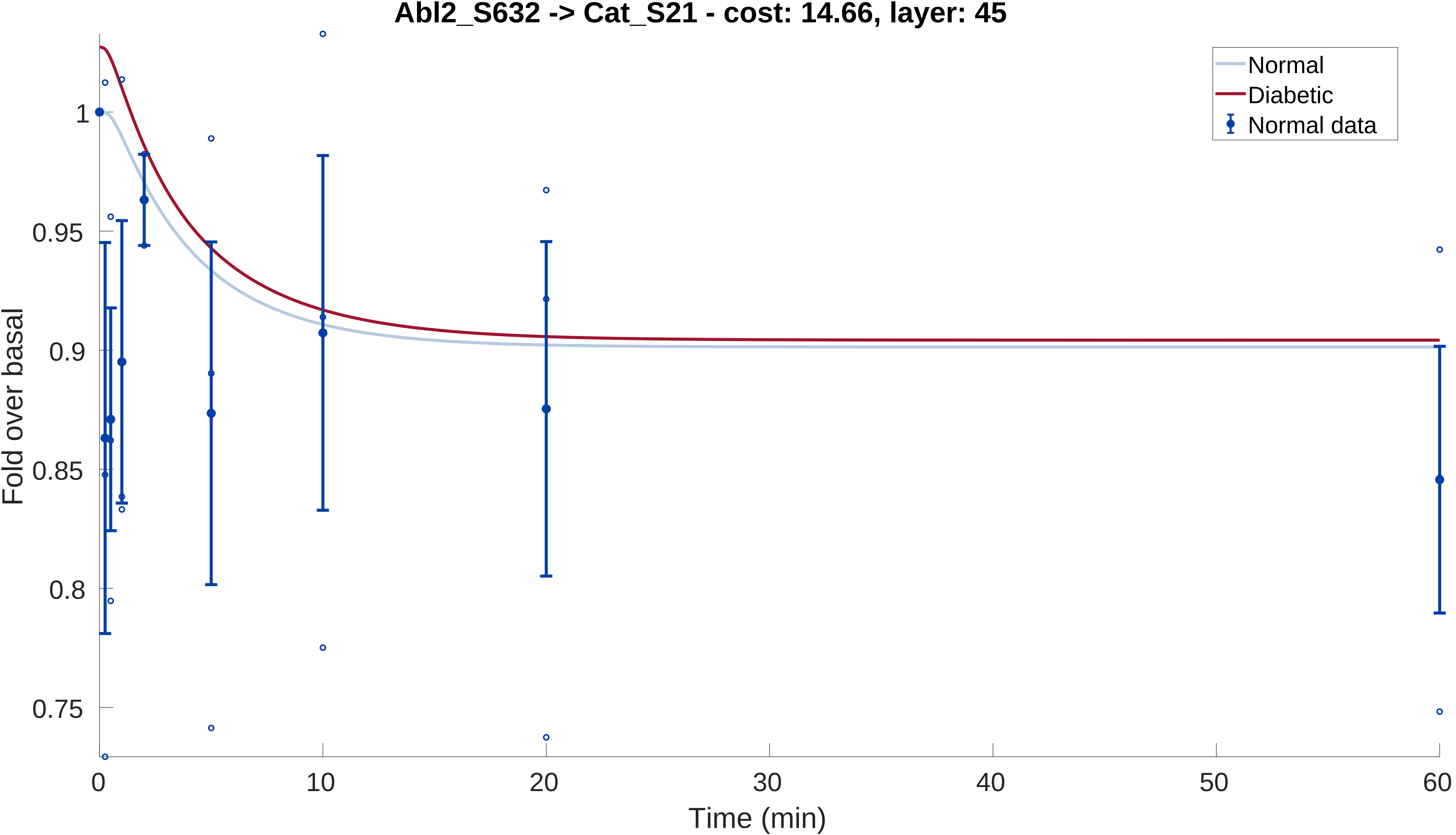

Abl2_S632 -> Cat_S21 - cost: 14.66, layer: 45
Normal
Diabetic
Normal data
Fold over basal
1
0.95
0.9
0.85
0.8
0.75
0
10
20
30
40
50
60
Time (min)

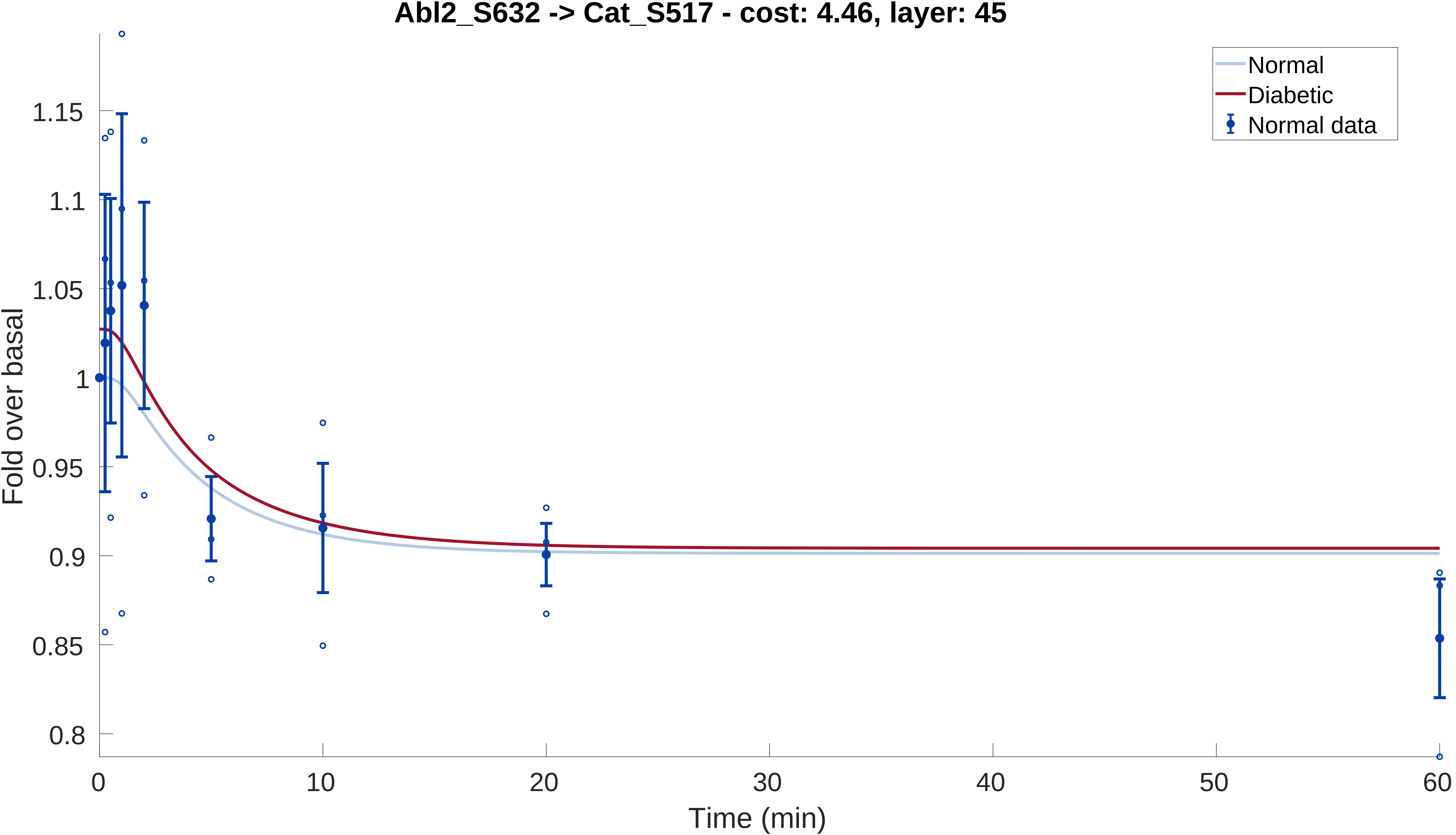
Abl2_S632 -> Cat_S517 - cost: 4.46, layer: 45
Normal
Diabetic
Normal data
Fold over basal
1.15
1.1
1.05
1
0.95
0.9
0.85
0.8
0
10
20
30
40
50
60
Time (min)

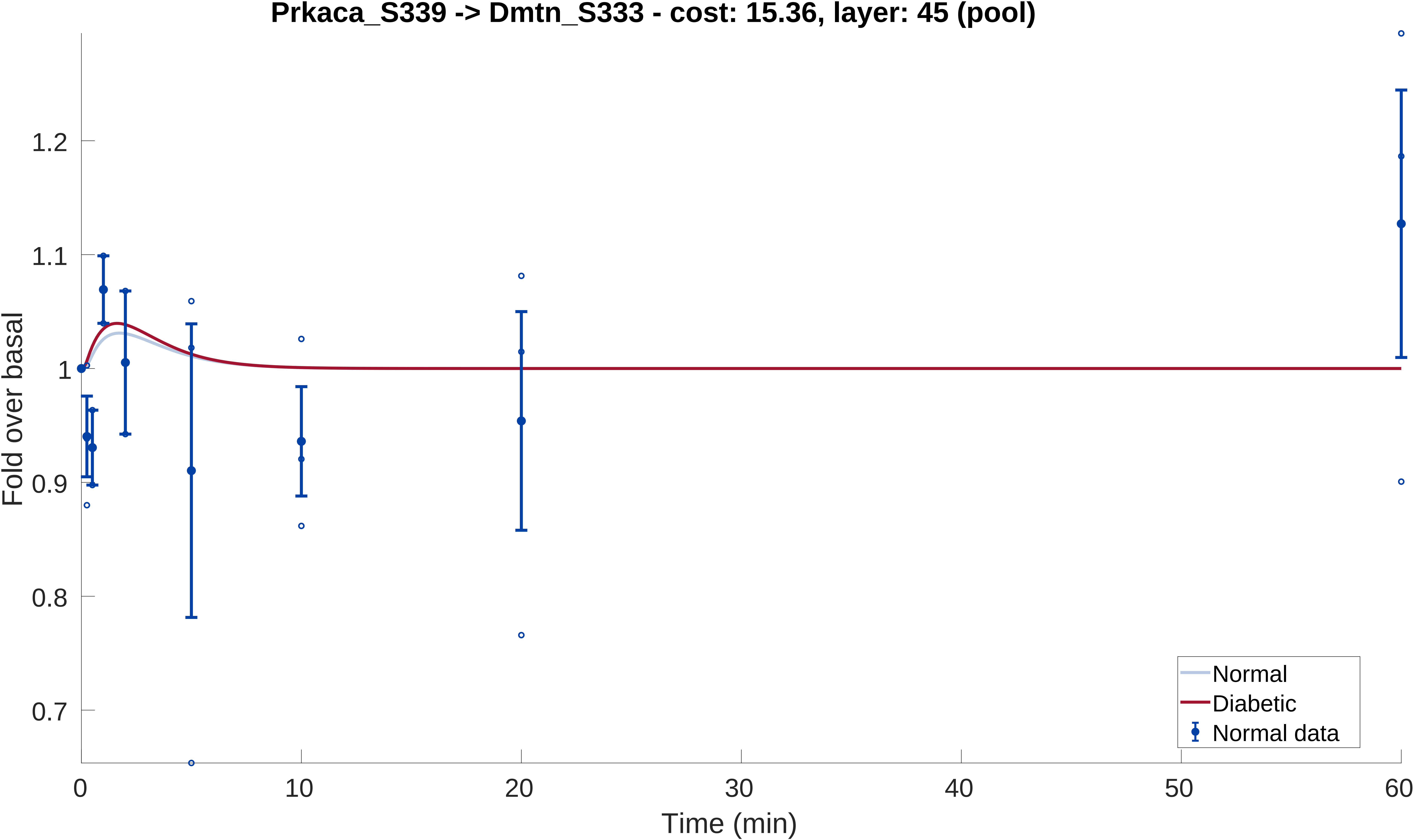

Prkaca_S339 -> Dmtn_S333 - cost: 15.36, layer: 45 (pool)
Fold over basal
1.2
1.1
1
0.9
0.8
0.7
0
10
20
30
40
50
60
Time (min)
Normal
Diabetic
Normal data

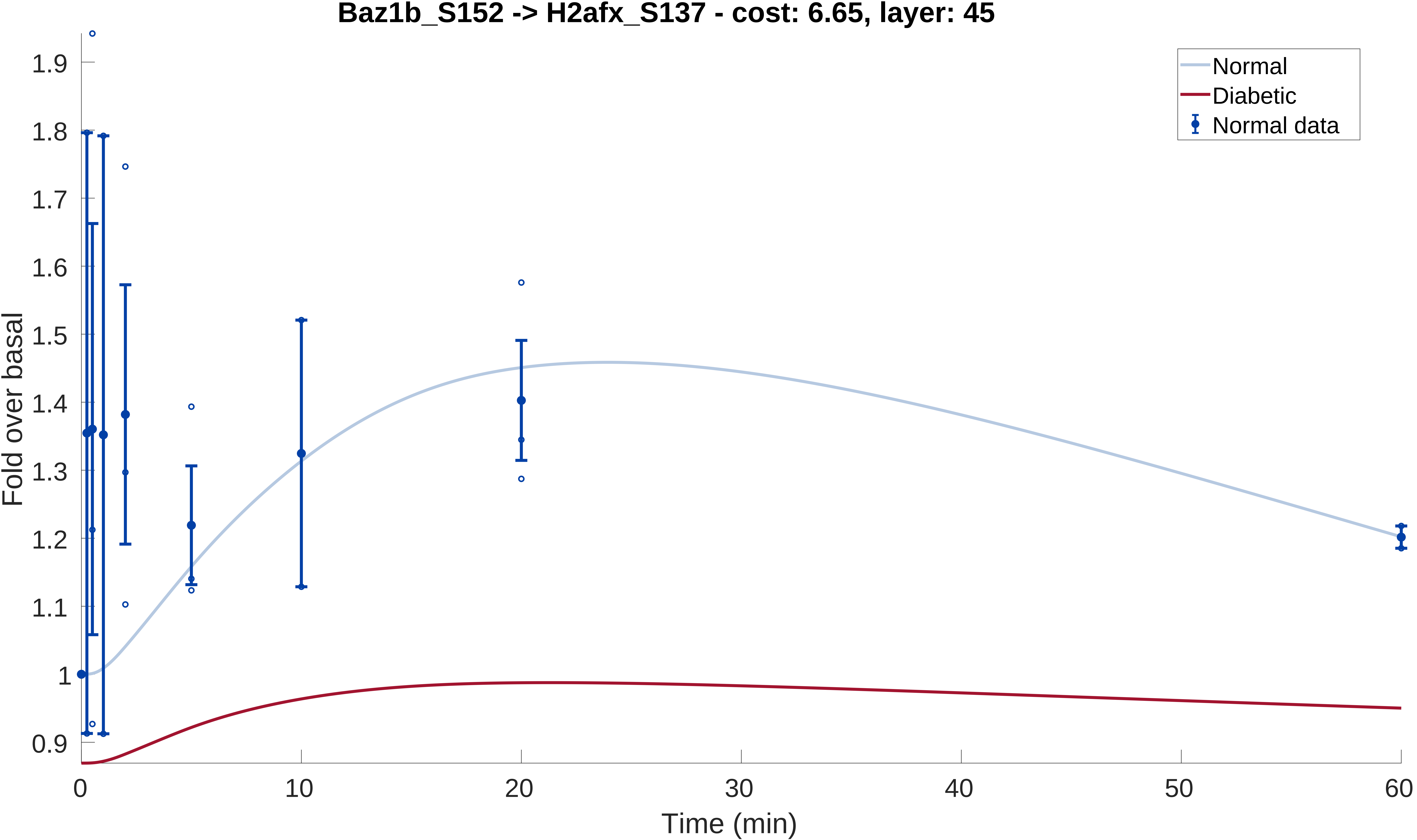

Baz1b_S152 -> H2afx_S137 - cost: 6.65, layer: 45
Normal
Diabetic
Normal data
Fold over basal
Time (min)
0.9
1
1.1
1.2
1.3
1.4
1.5
1.6
1.7
1.8
1.9
0
10
20
30
40
50
60

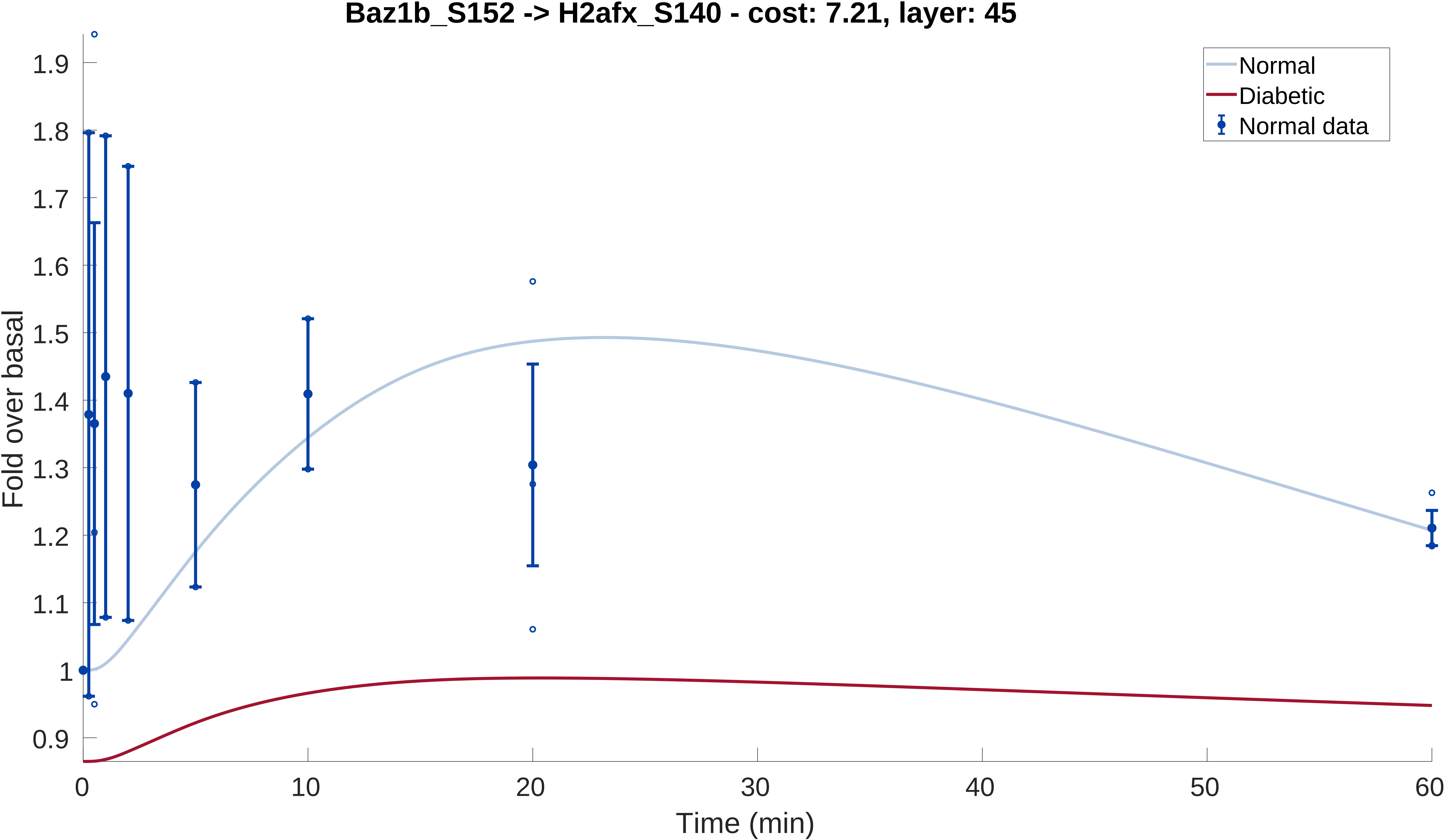

Baz1b_S152 -> H2afx_S140 - cost: 7.21, layer: 45
Normal
Diabetic
Normal data
Fold over basal
Time (min)
0.9
1
1.1
1.2
1.3
1.4
1.5
1.6
1.7
1.8
1.9
0
10
20
30
40
50
60

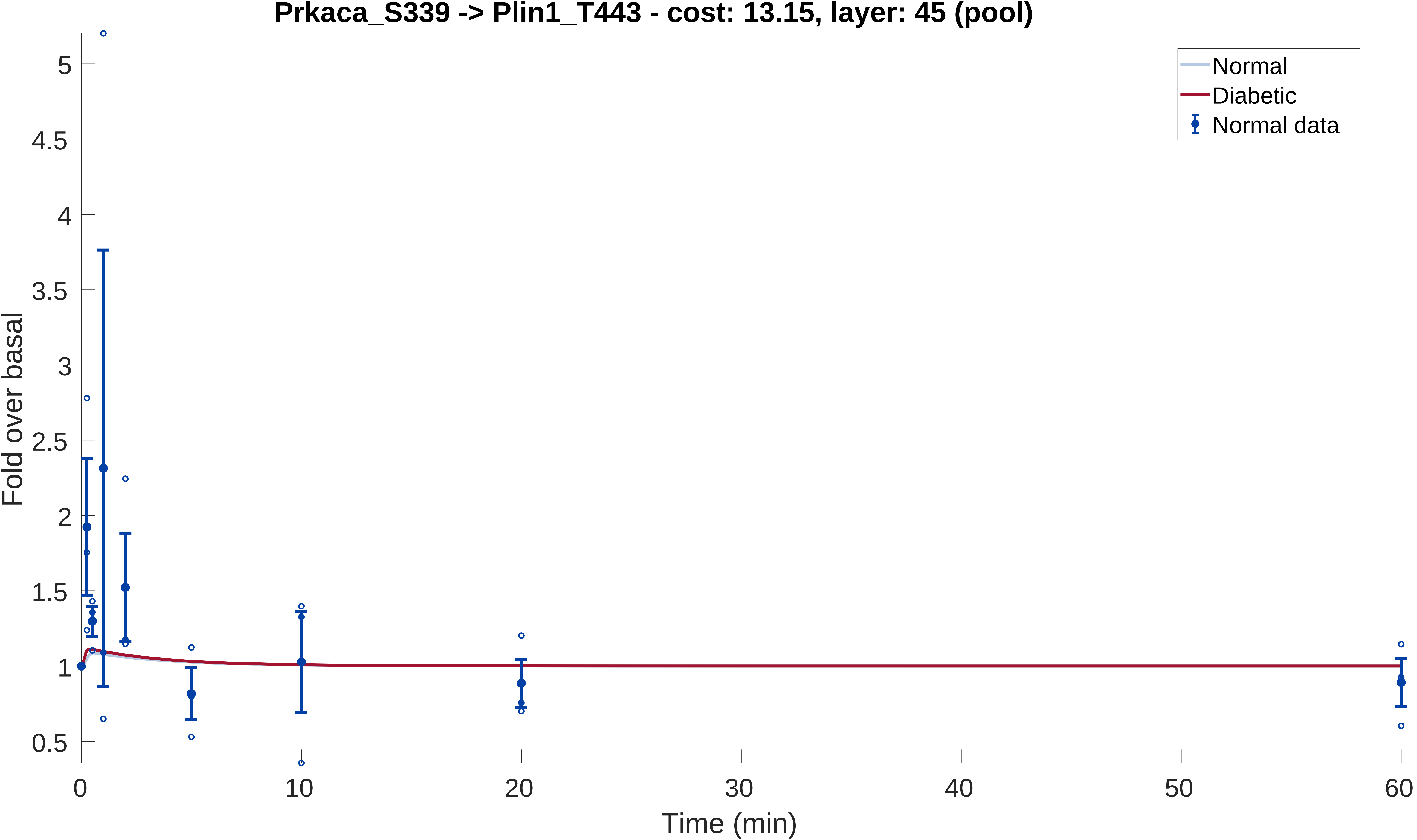
Prkaca_S339 -> Plin1_T443 - cost: 13.15, layer: 45 (pool)
Normal
Diabetic
Normal data
Fold over basal
Time (min)
0.5
1
1.5
2
2.5
3
3.5
4
4.5
5
0
10
20
30
40
50
60

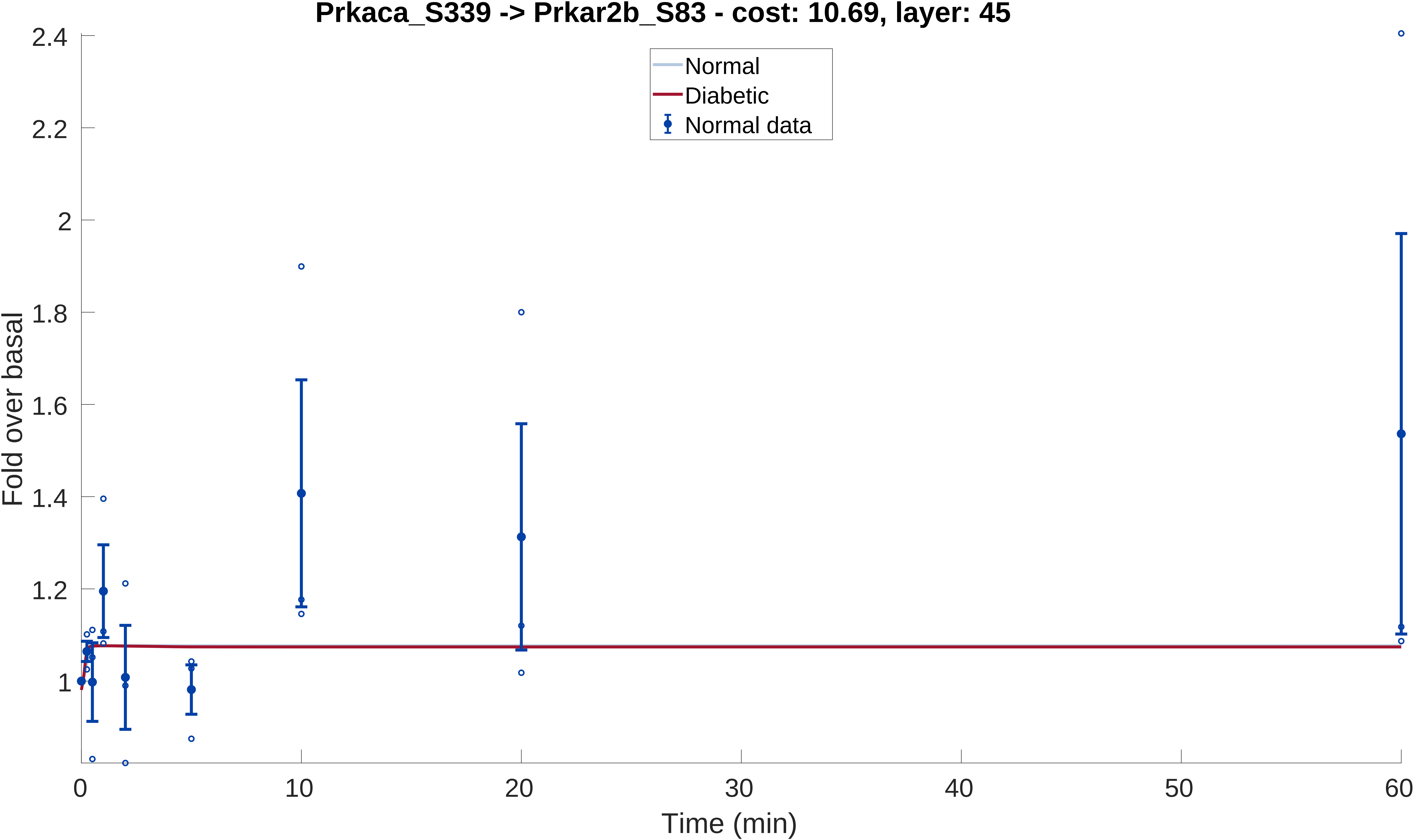
Prkaca_S339 -> Prkar2b_S83 - cost: 10.69, layer: 45
Normal
Diabetic
Normal data
Fold over basal
Time (min)
2.4
2.2
2
1.8
1.6
1.4
1.2
1
0
10
20
30
40
50
60

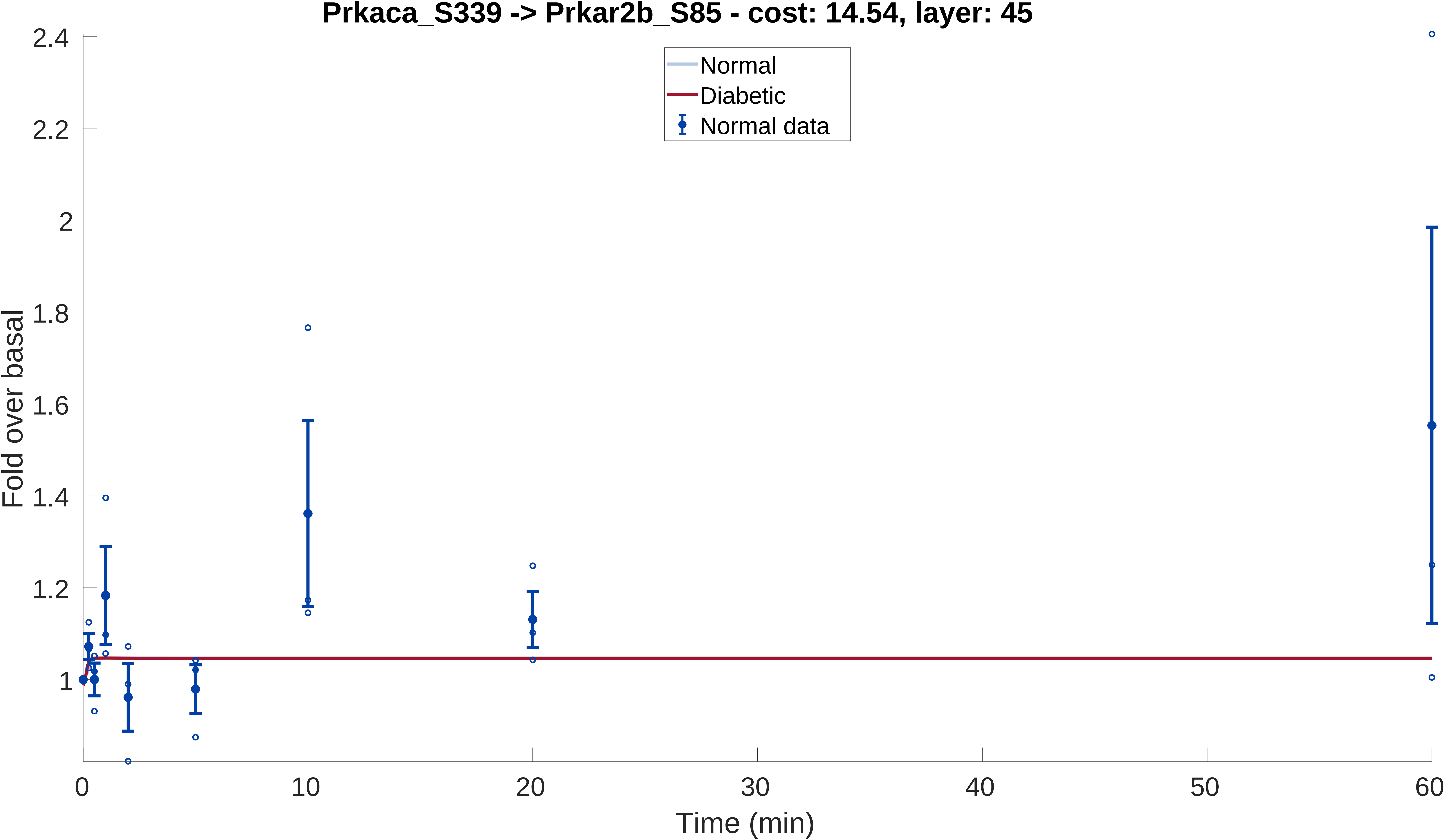

Prkaca_S339 -> Prkar2b_S85 - cost: 14.54, layer: 45
Normal
Diabetic
Normal data
Fold over basal
2.4
2.2
2
1.8
1.6
1.4
1.2
1
0
10
20
30
40
50
60
Time (min)

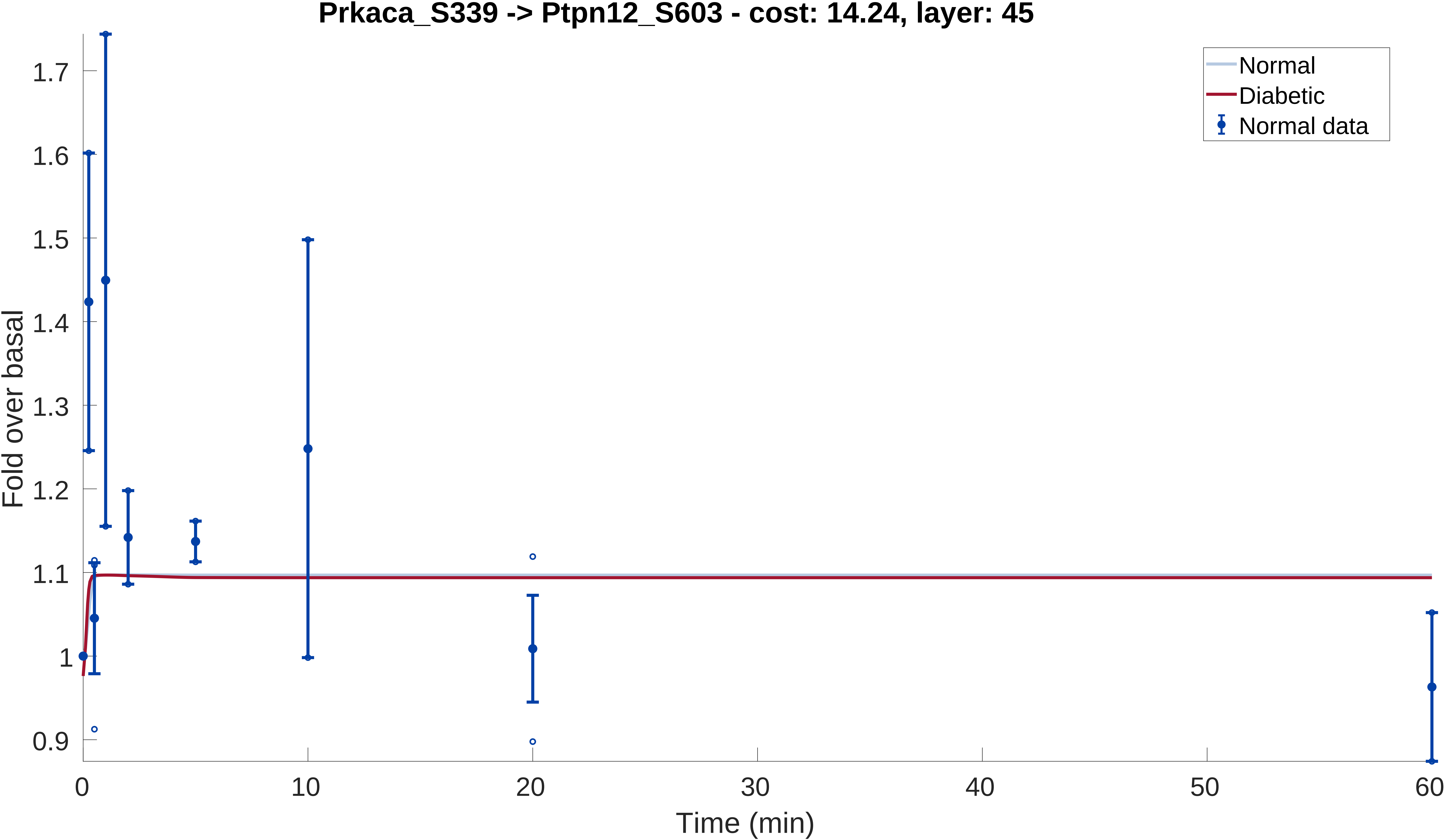
Prkaca_S339 -> Ptpn12_S603 - cost: 14.24, layer: 45
Normal
Diabetic
Normal data
Fold over basal
Time (min)
1.7
1.6
1.5
1.4
1.3
1.2
1.1
1
0.9
0
10
20
30
40
50
60

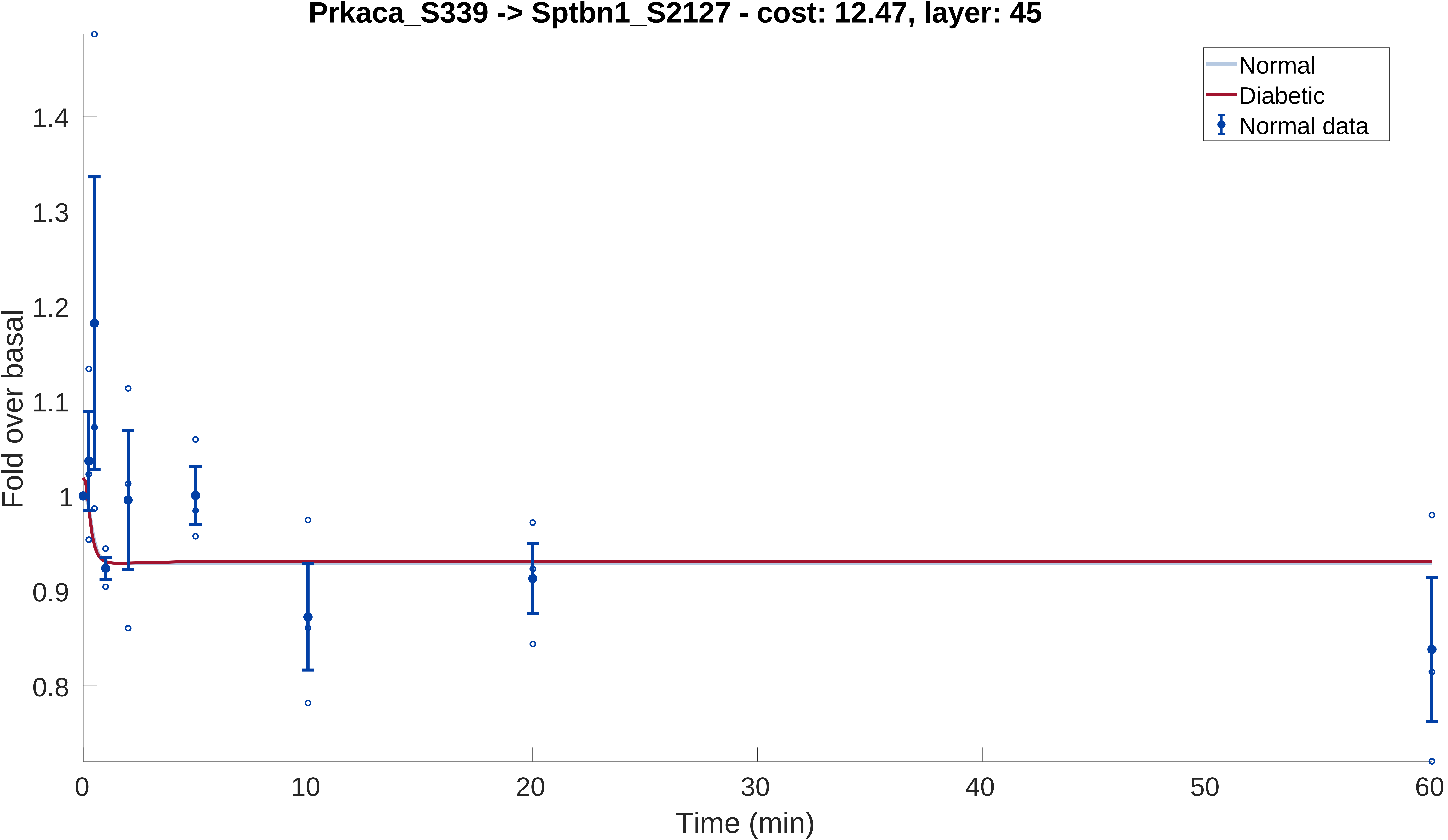

Prkaca_S339 -> Sptbn1_S2127 - cost: 12.47, layer: 45
Normal
Diabetic
Normal data
Fold over basal
1.4
1.3
1.2
1.1
1
0.9
0.8
0
10
20
30
40
50
60
Time (min)

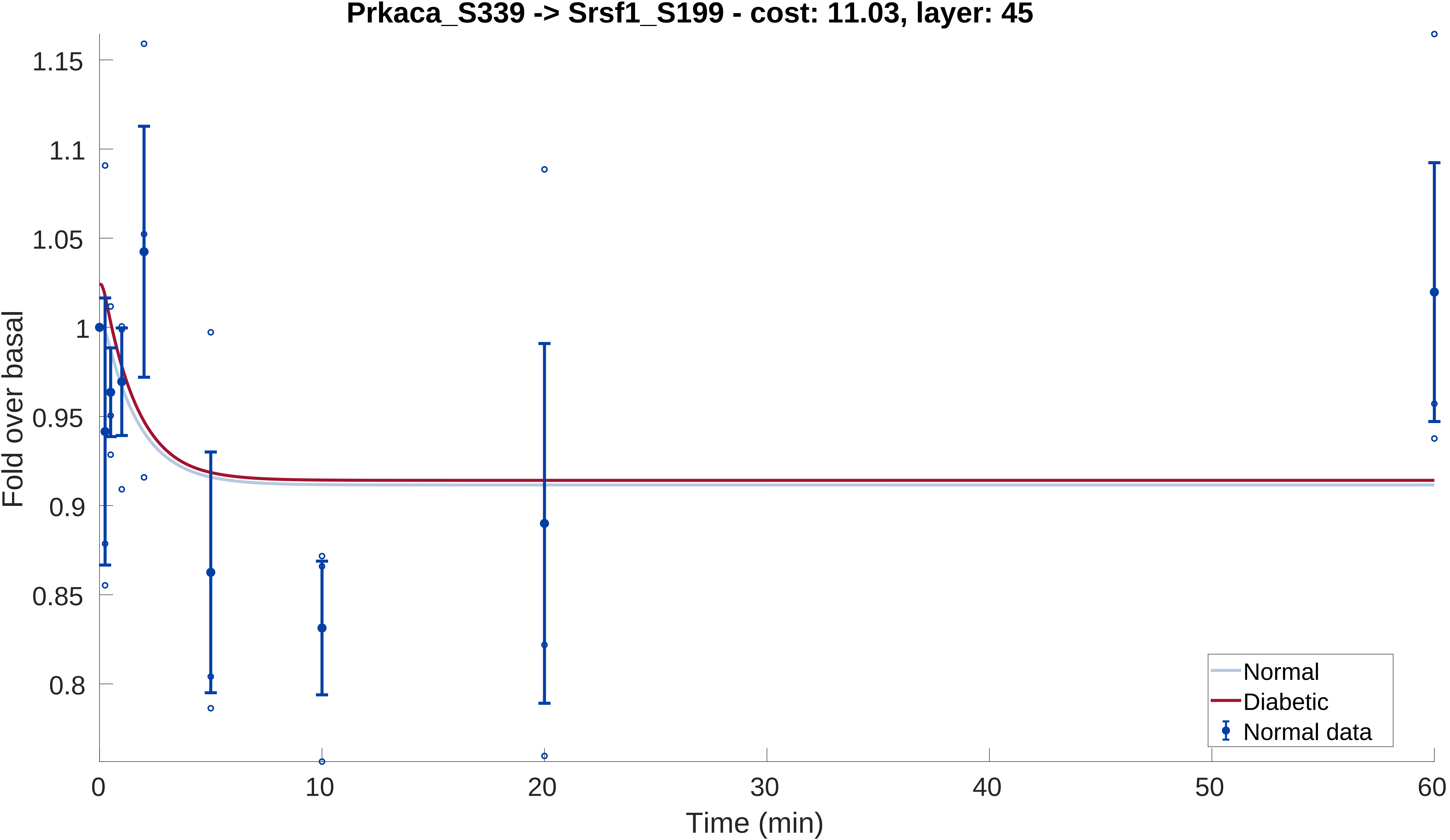

Prrkaca_S339 -> Srsf1_S199 - cost: 11.03, layer: 45
Fold over basal
1.15
1.1
1.05
1
0.95
0.9
0.85
0.8
0
10
20
30
40
50
60
Time (min)
Normal
Diabetic
Normal data

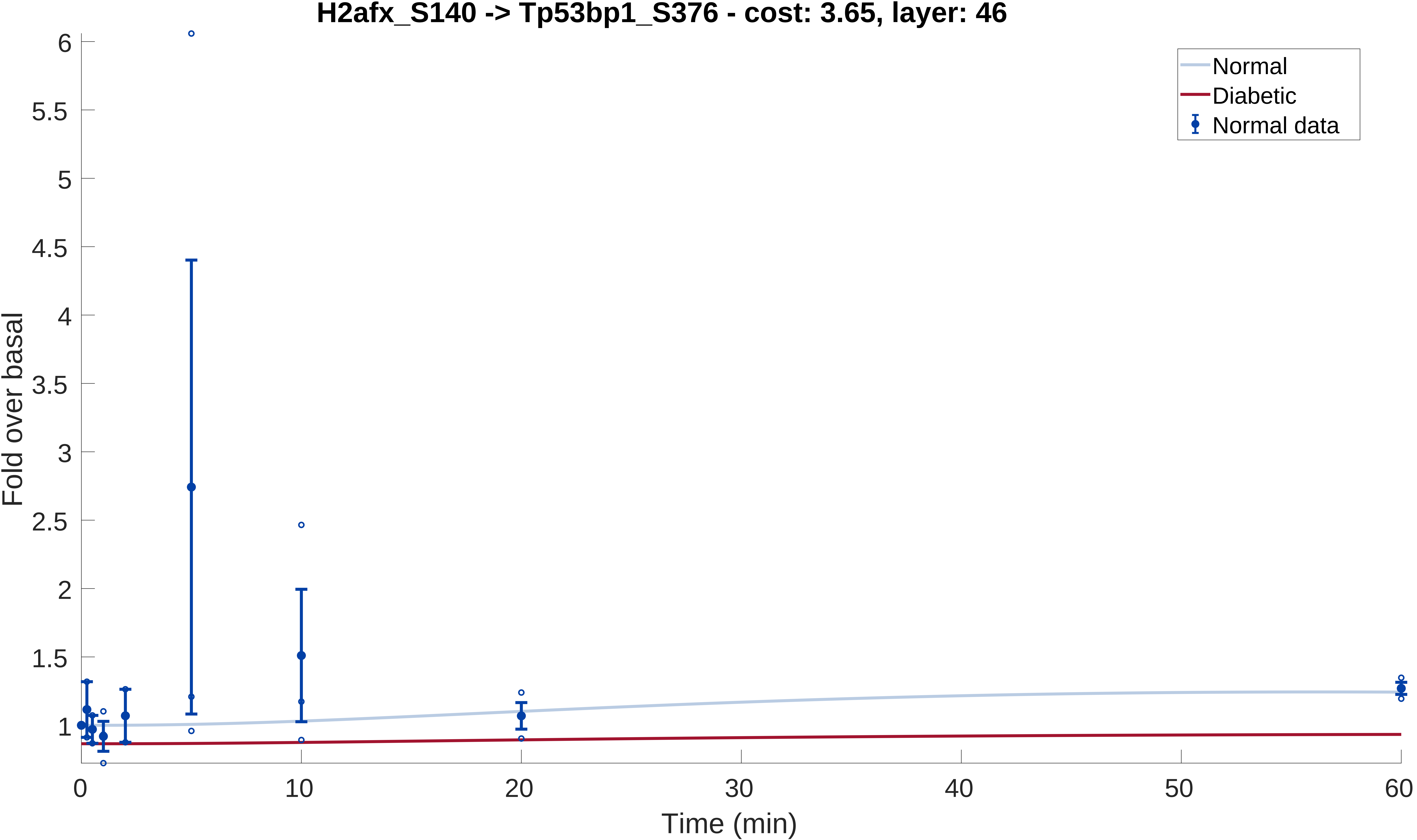

H2afx_S140 -> Tp53bp1_S376 - cost: 3.65, layer: 46
Normal
Diabetic
Normal data
Fold over basal
6
5.5
5
4.5
4
3.5
3
2.5
2
1.5
1
0
10
20
30
40
50
60
Time (min)

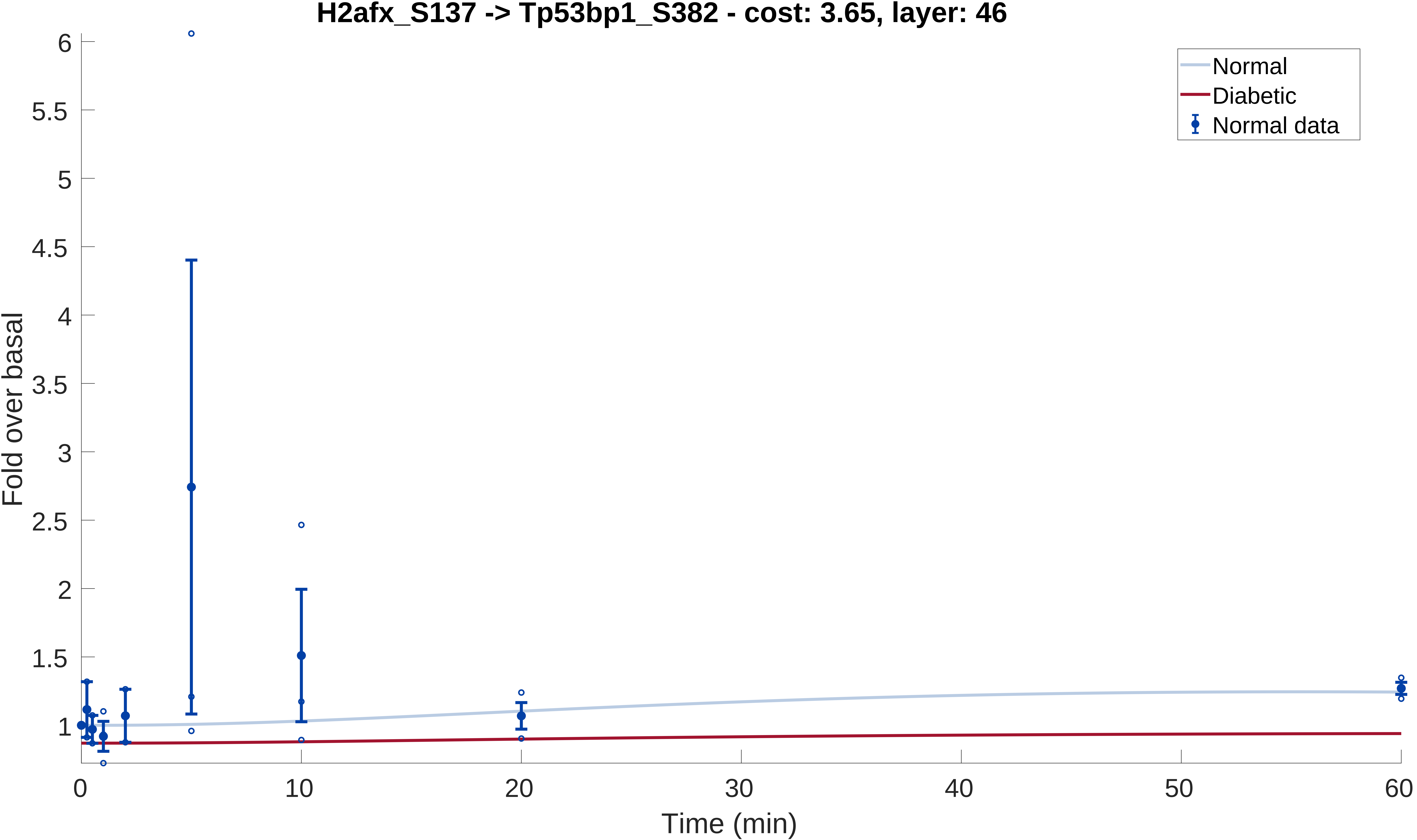

H2afx_S137 -> Tp53bp1_S382 - cost: 3.65, layer: 46
Normal
Diabetic
Normal data
Fold over basal
Time (min)
6
5.5
5
4.5
4
3.5
3
2.5
2
1.5
1
0
10
20
30
40
50
60

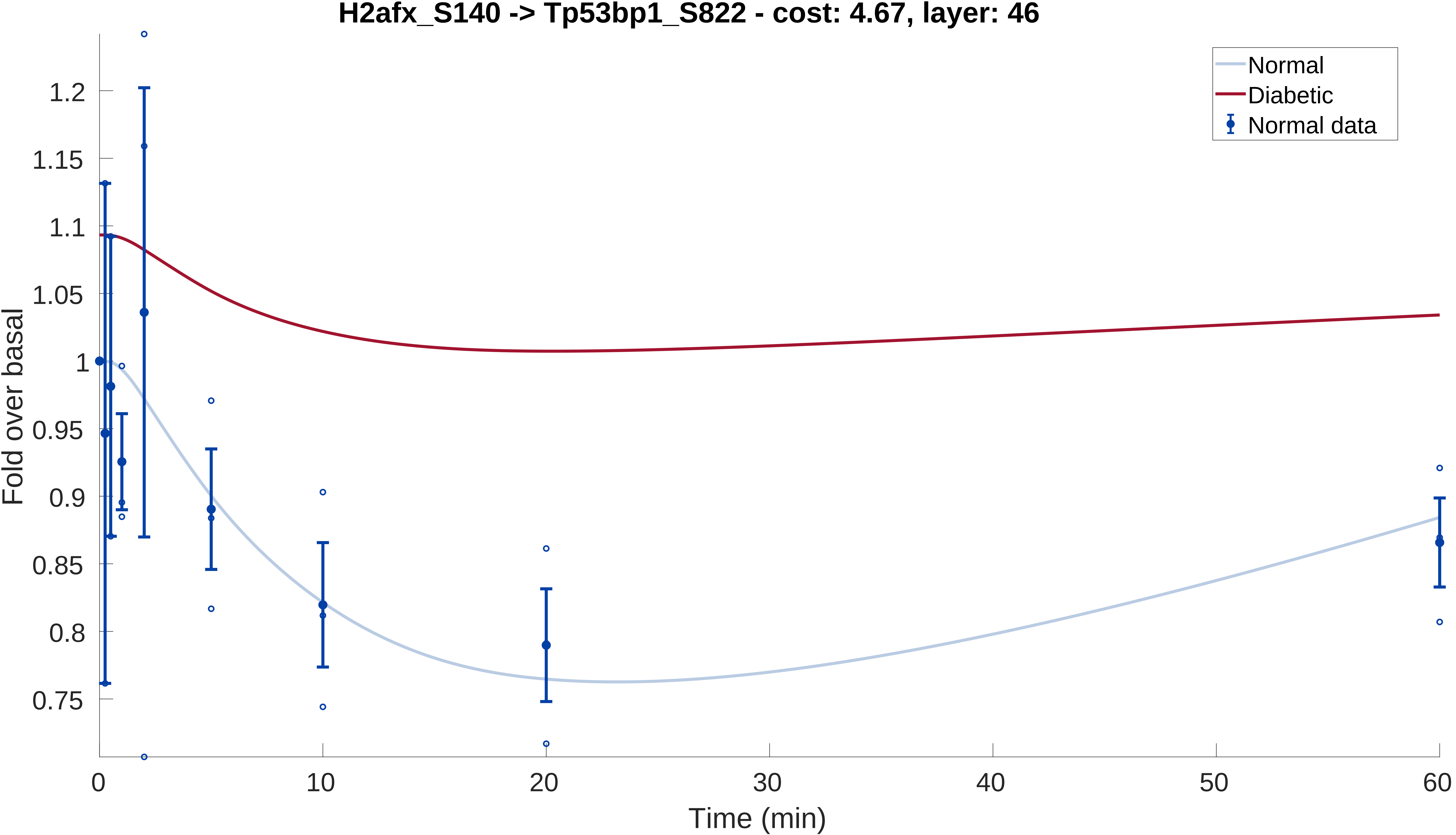

H2afx_S140 -> Tp53bp1_S822 - cost: 4.67, layer: 46
Normal
Diabetic
Normal data
Fold over basal
1.2
1.15
1.1
1.05
1
0.95
0.9
0.85
0.8
0.75
0
10
20
30
40
50
60
Time (min)

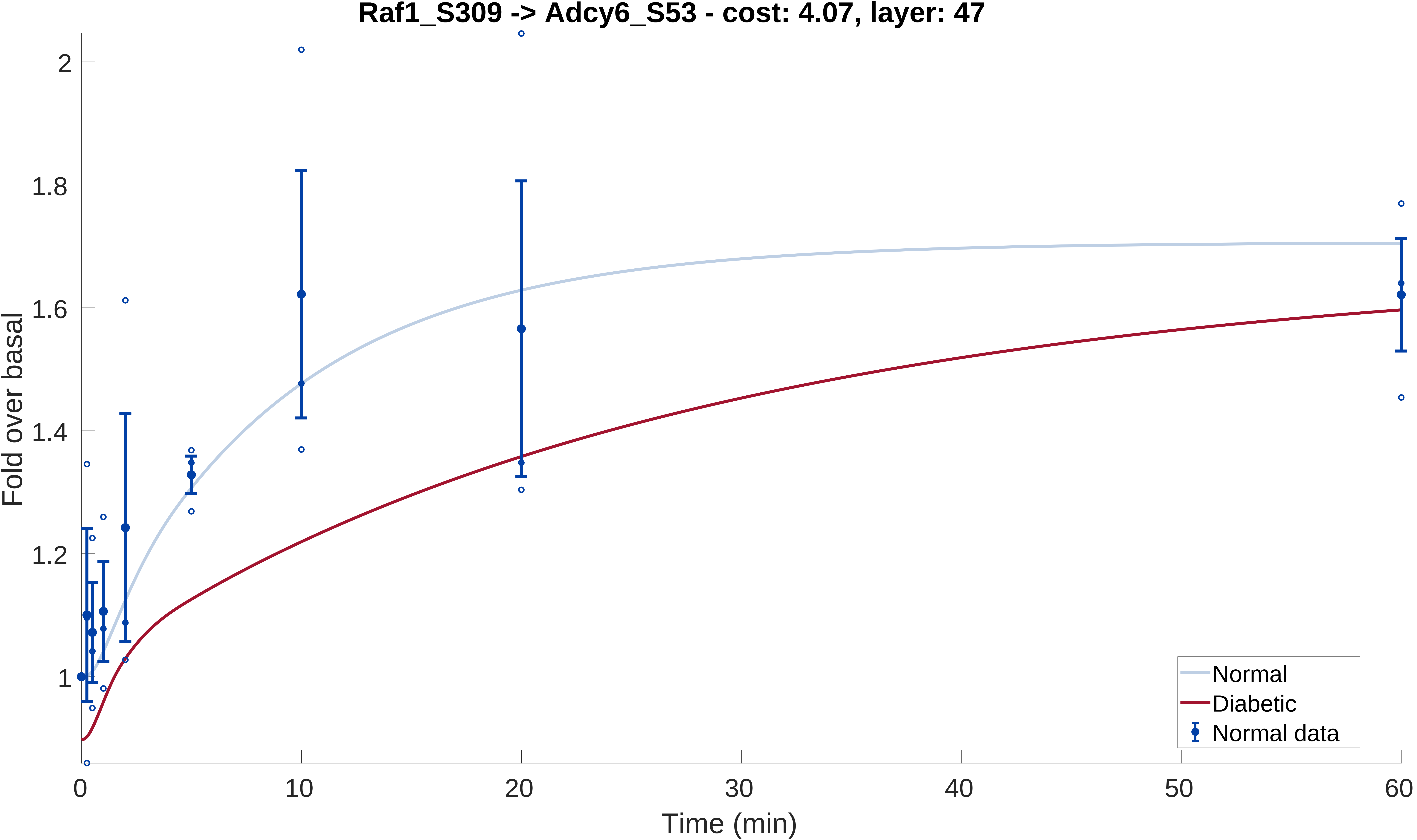

Raf1_S309 -> Adcy6_S53 - cost: 4.07, layer: 47
Fold over basal
Time (min)
0
10
20
30
40
50
60
1
1.2
1.4
1.6
1.8
2
Normal
Diabetic
Normal data

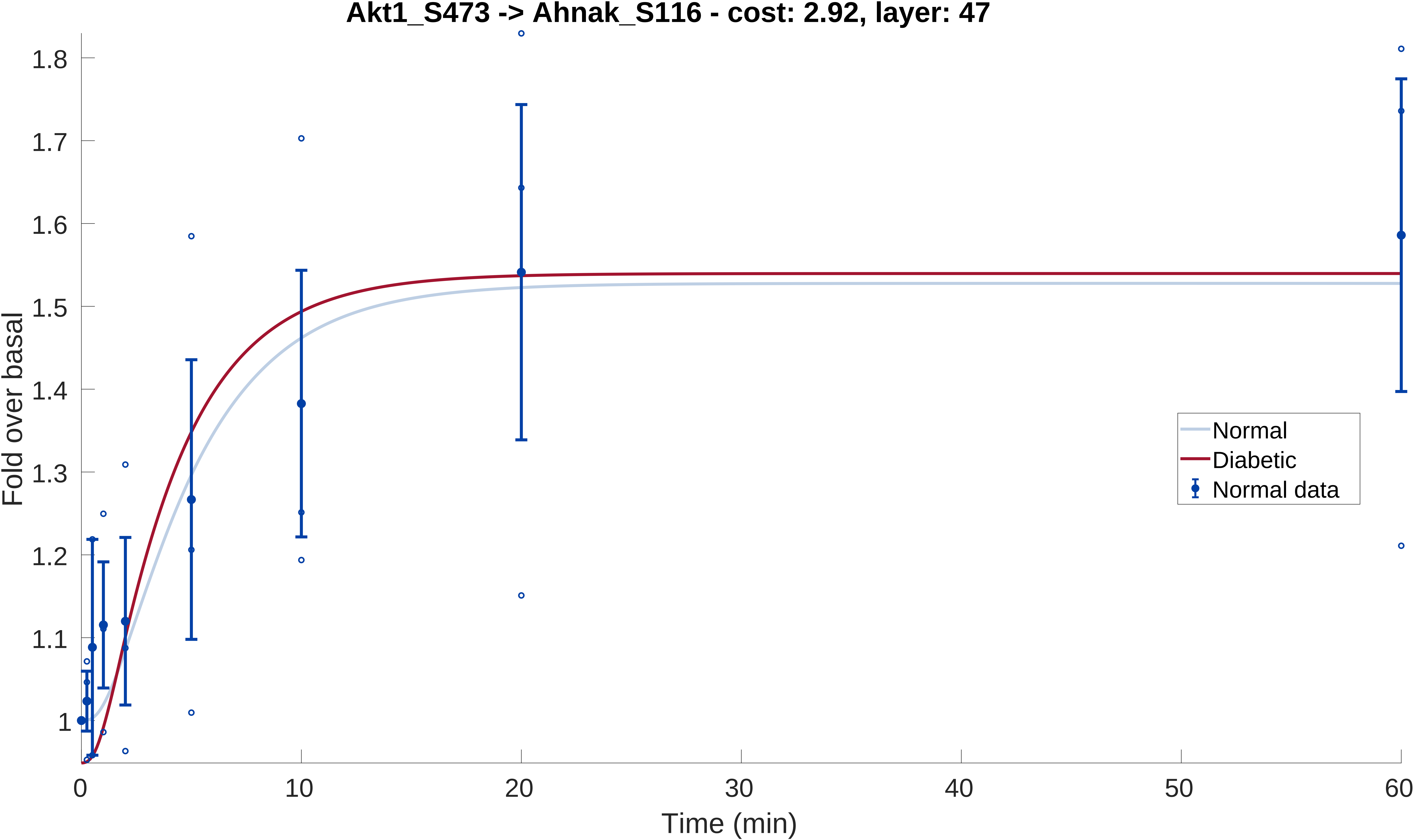

Akt1_S473 -> Ahnak_S116 - cost: 2.92, layer: 47
Fold over basal
Time (min)
Normal
Diabetic
Normal data
1.8
1.7
1.6
1.5
1.4
1.3
1.2
1.1
1
0
10
20
30
40
50
60

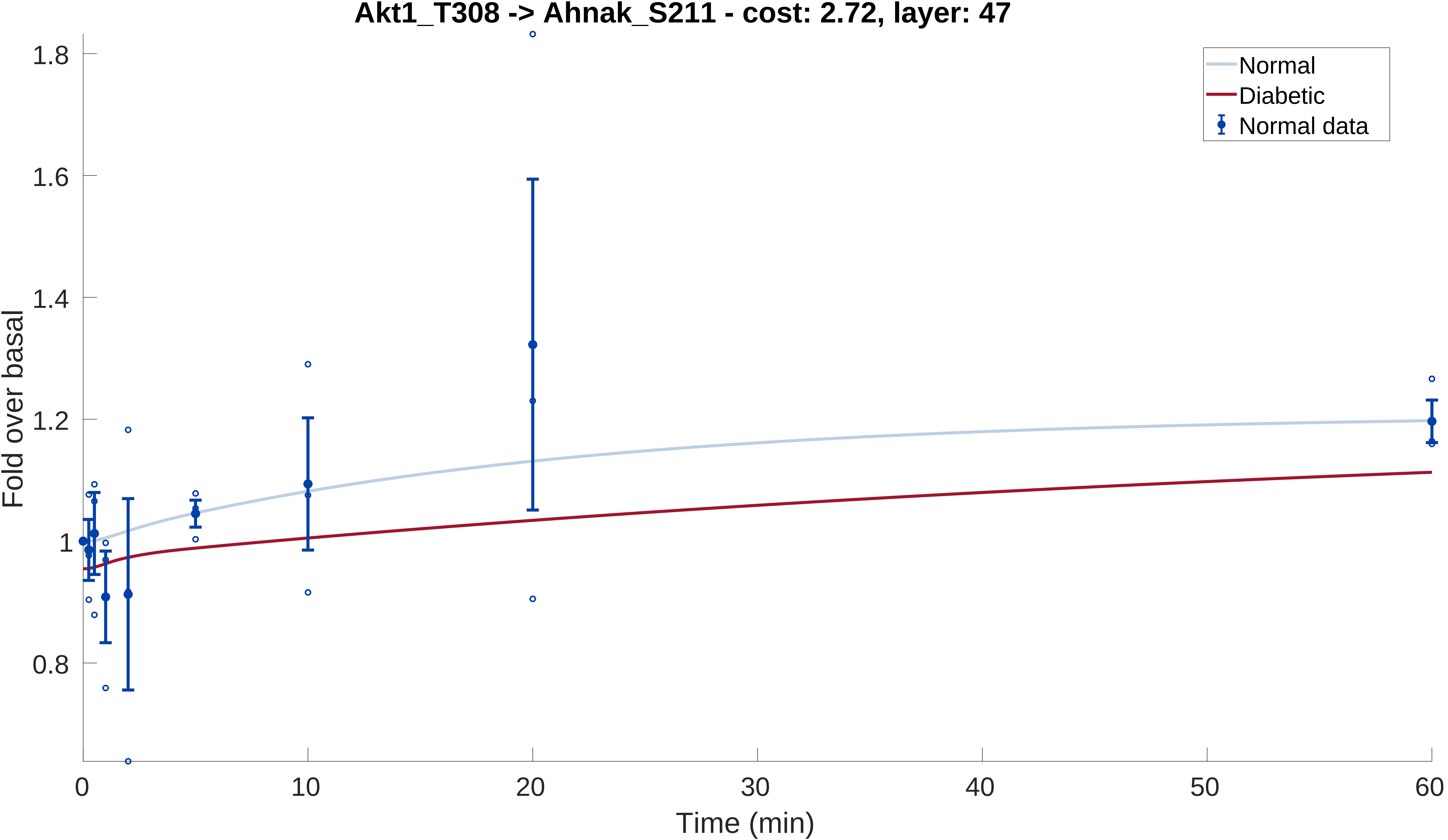

Akt1_T308 -> Ahnak_S211 - cost: 2.72, layer: 47
Normal
Diabetic
Normal data
Fold over basal
1.8
1.6
1.4
1.2
1
0.8
0
10
20
30
40
50
60
Time (min)

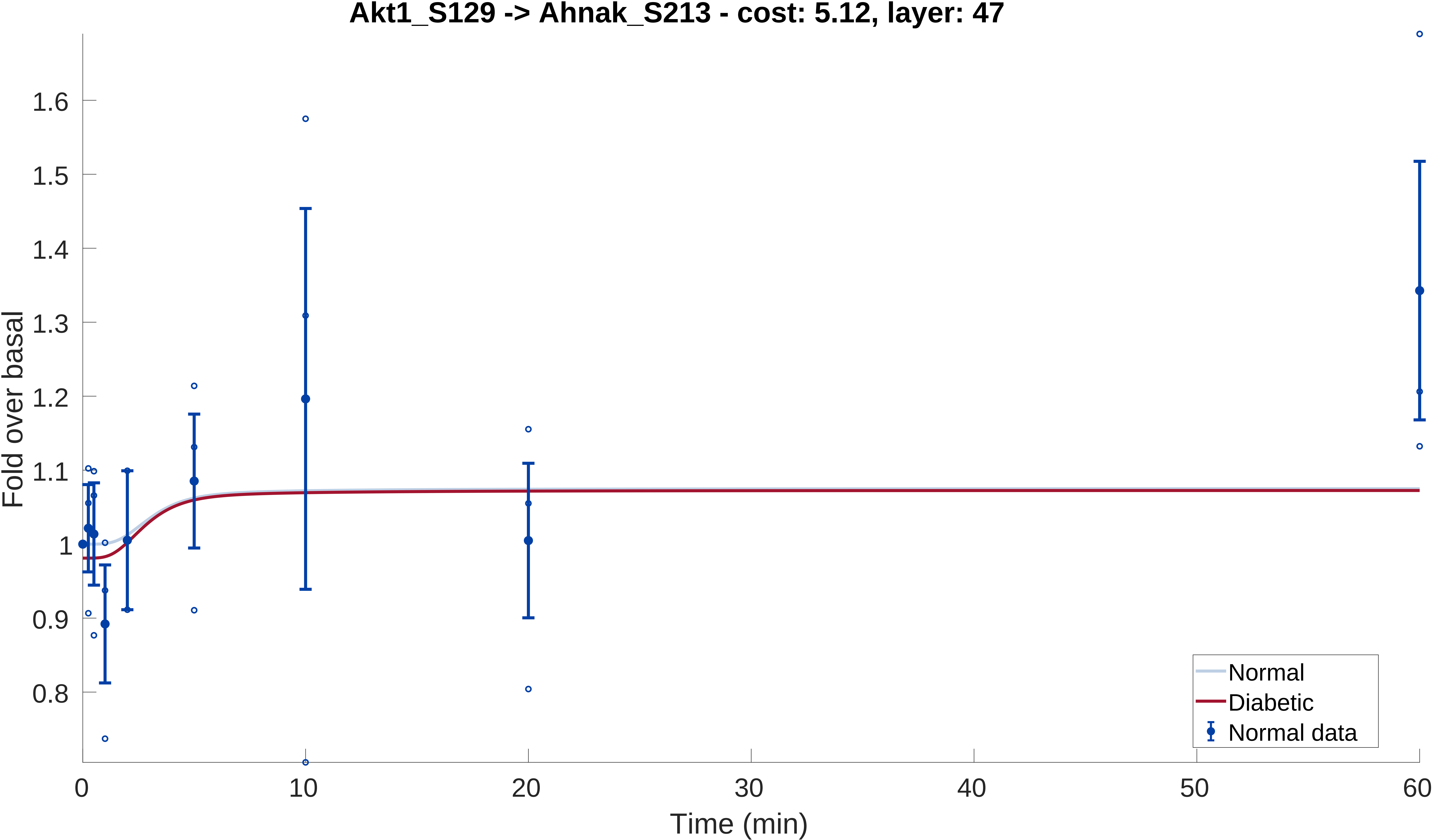

Akt1_S129 -> Ahnak_S213 - cost: 5.12, layer: 47
Fold over basal
1.6
1.5
1.4
1.3
1.2
1.1
1
0.9
0.8
0
10
20
30
40
50
60
Time (min)
Normal
Diabetic
Normal data

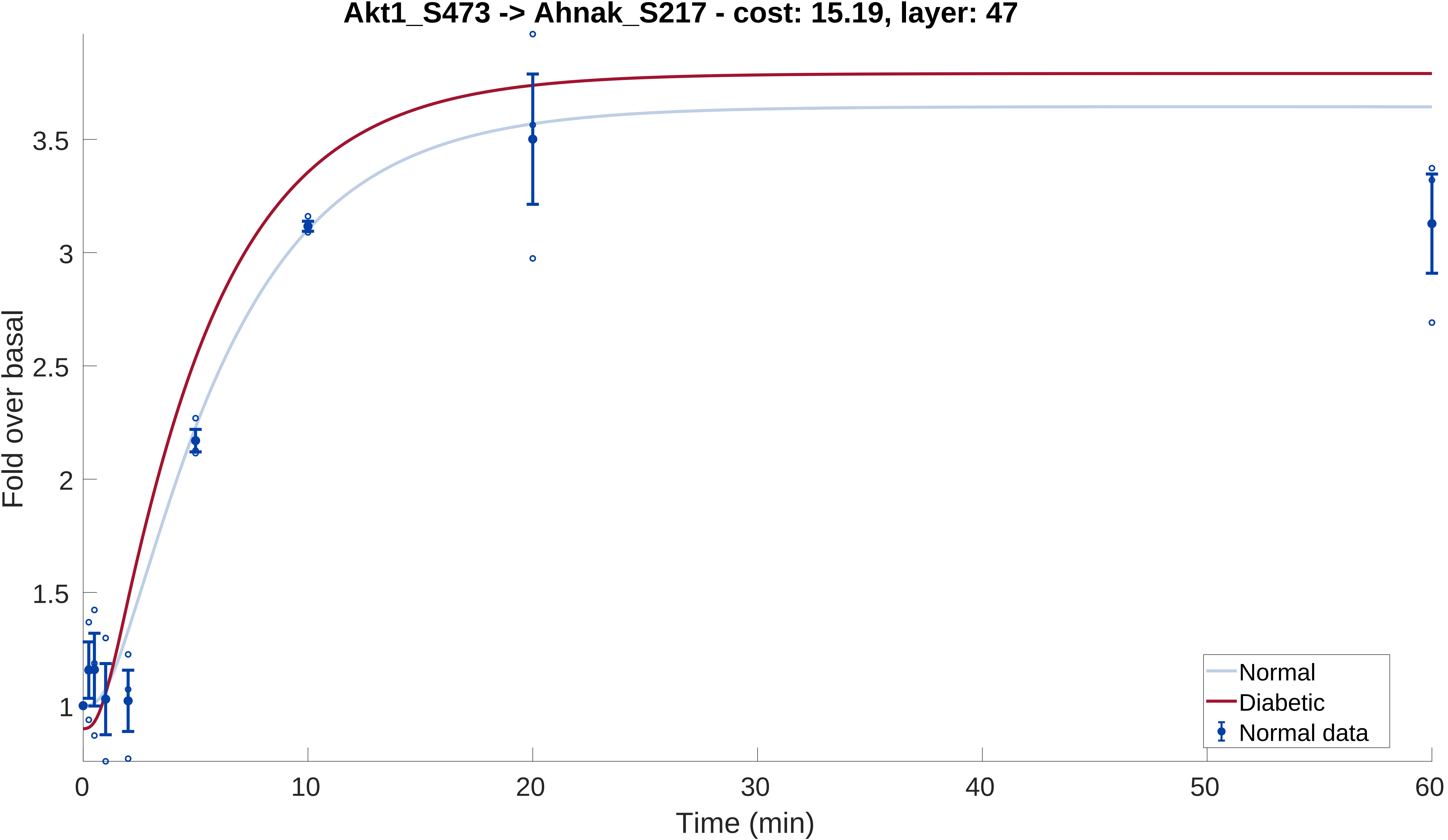

Akt1_S473 -> Ahnak_S217 - cost: 15.19, layer: 47
Fold over basal
3.5
3
2.5
2
1.5
1
0
10
20
30
40
50
60
Time (min)
Normal
Diabetic
Normal data

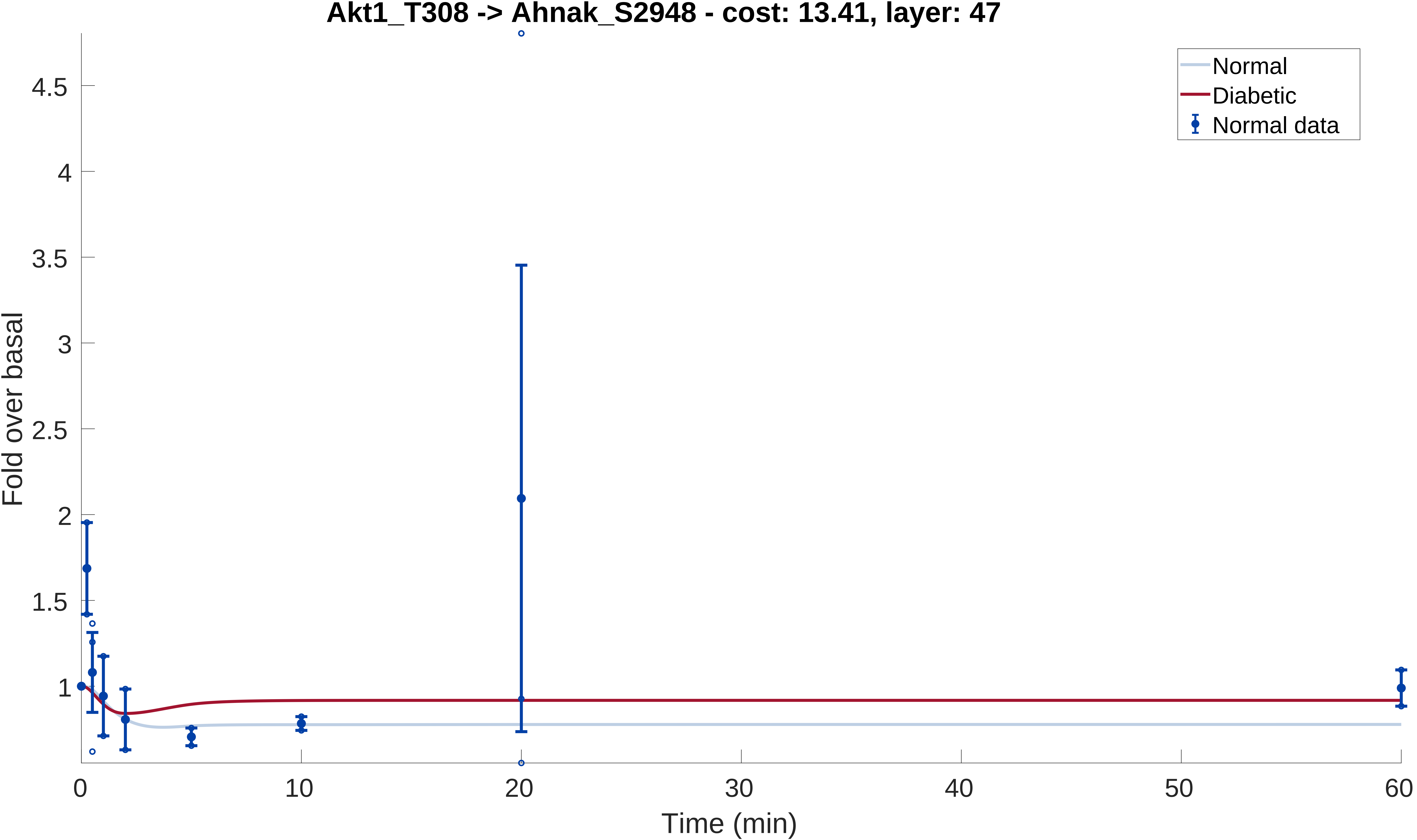

Akt1_T308 -> Ahnak_S2948 - cost: 13.41, layer: 47
Normal
Diabetic
Normal data
Fold over basal
4.5
4
3.5
3
2.5
2
1.5
1
0
10
20
30
40
50
60
Time (min)

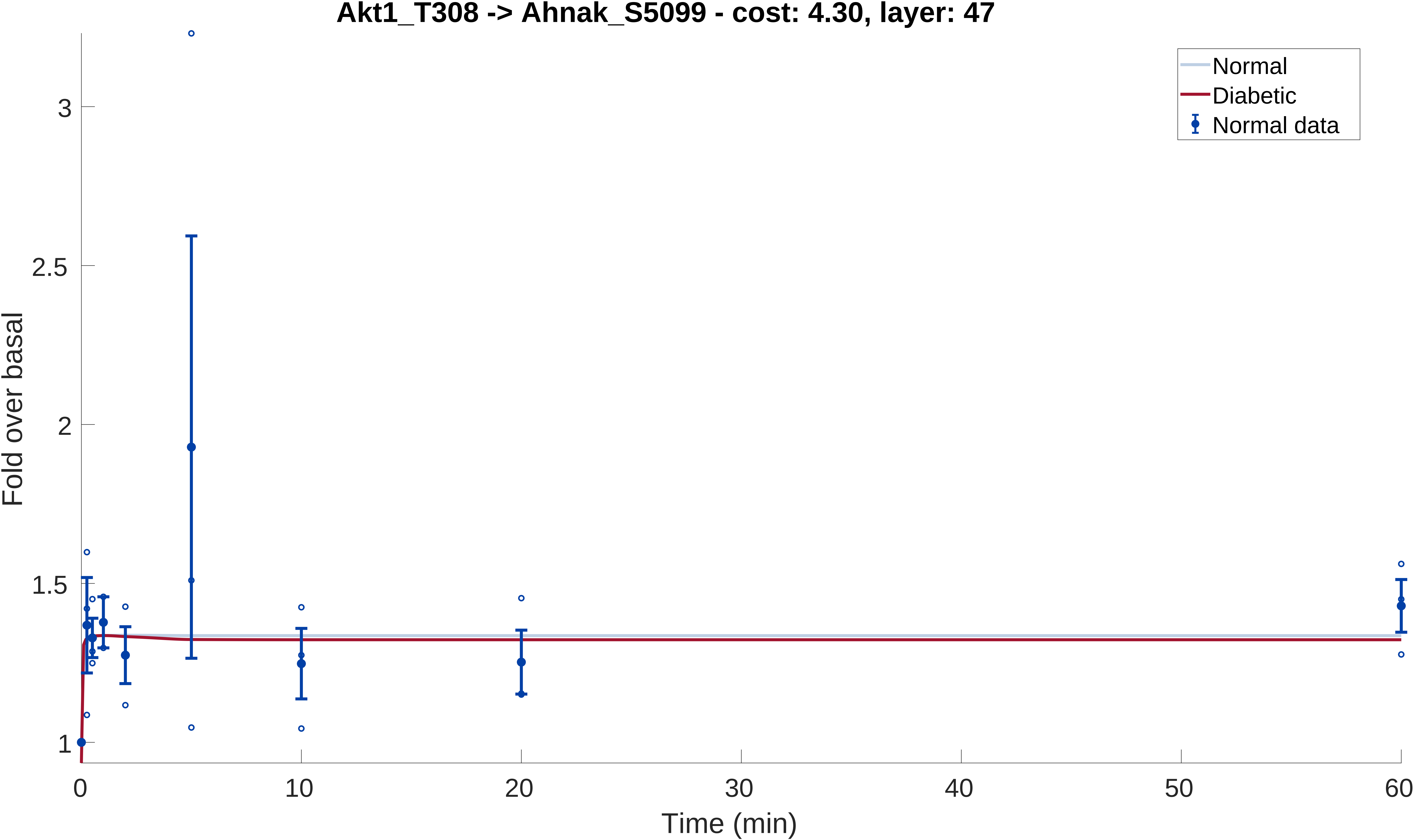

Akt1_T308 -> Ahnak_S5099 - cost: 4.30, layer: 47
Normal
Diabetic
Normal data
Fold over basal
Time (min)
1
1.5
2
2.5
3
0
10
20
30
40
50
60

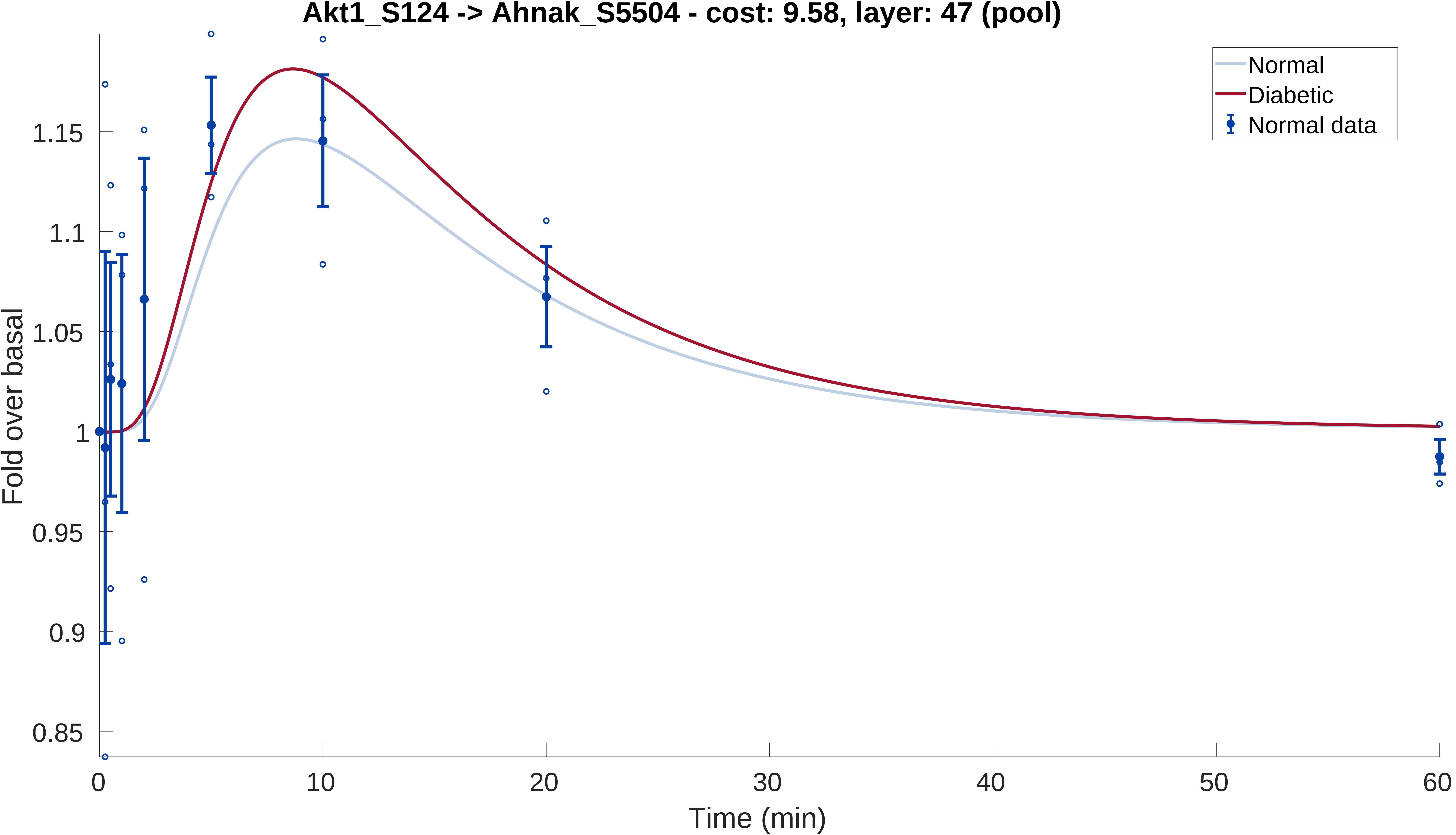

Akt1_S124 -> Ahnak_S5504 - cost: 9.58, layer: 47 (pool)
Normal
Diabetic
Normal data
Fold over basal
1.15
1.1
1.05
1
0.95
0.9
0.85
0
10
20
30
40
50
60
Time (min)

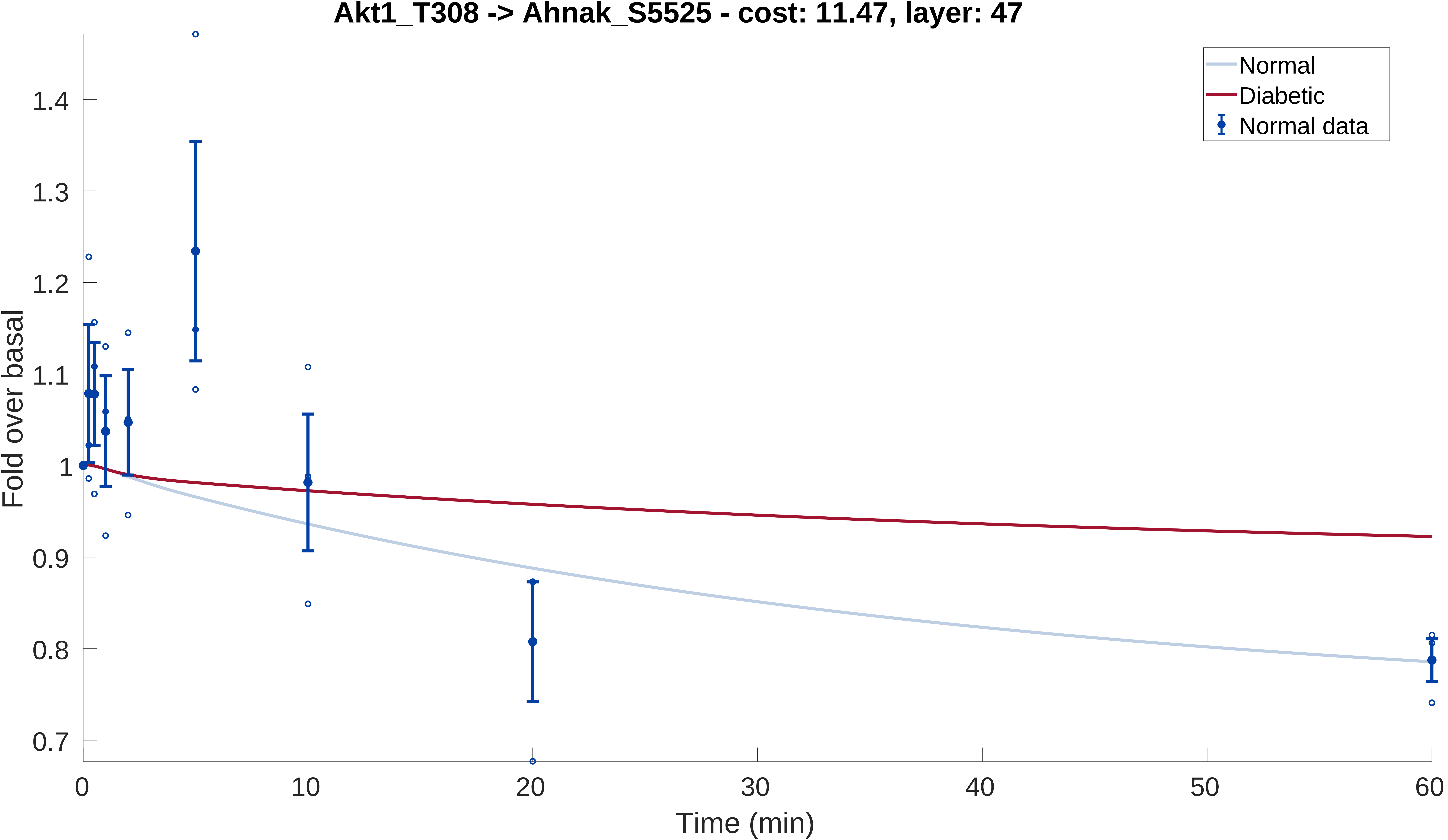

Akt1_T308 -> Ahnak_S5525 - cost: 11.47, layer: 47
Normal
Diabetic
Normal data
Fold over basal
1.4
1.3
1.2
1.1
1
0.9
0.8
0.7
0
10
20
30
40
50
60
Time (min)

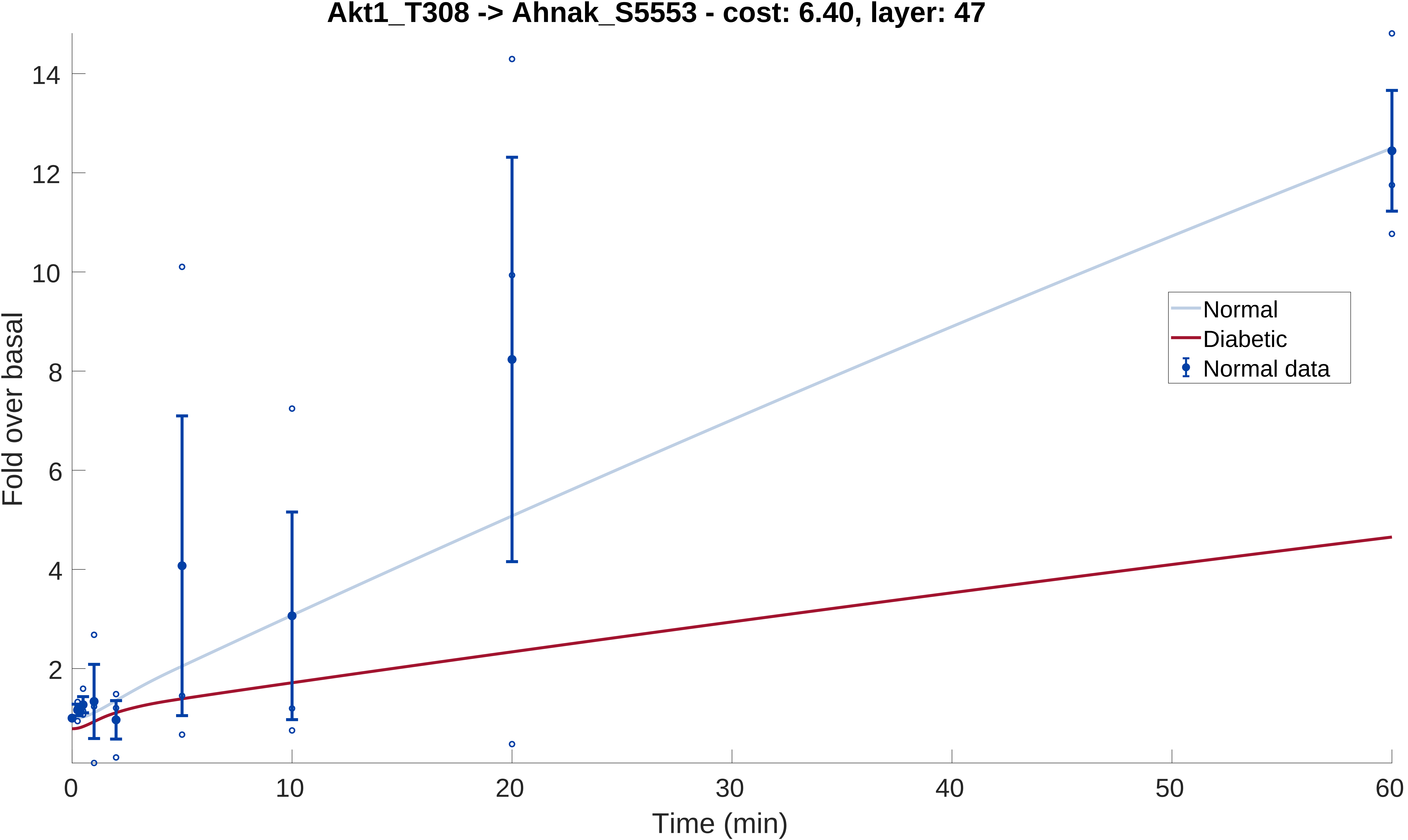
Akt1_T308 -> Ahnak_S5553 - cost: 6.40, layer: 47
Fold over basal
Time (min)
Normal
Diabetic
Normal data
0
2
4
6
8
10
12
14
0
10
20
30
40
50
60

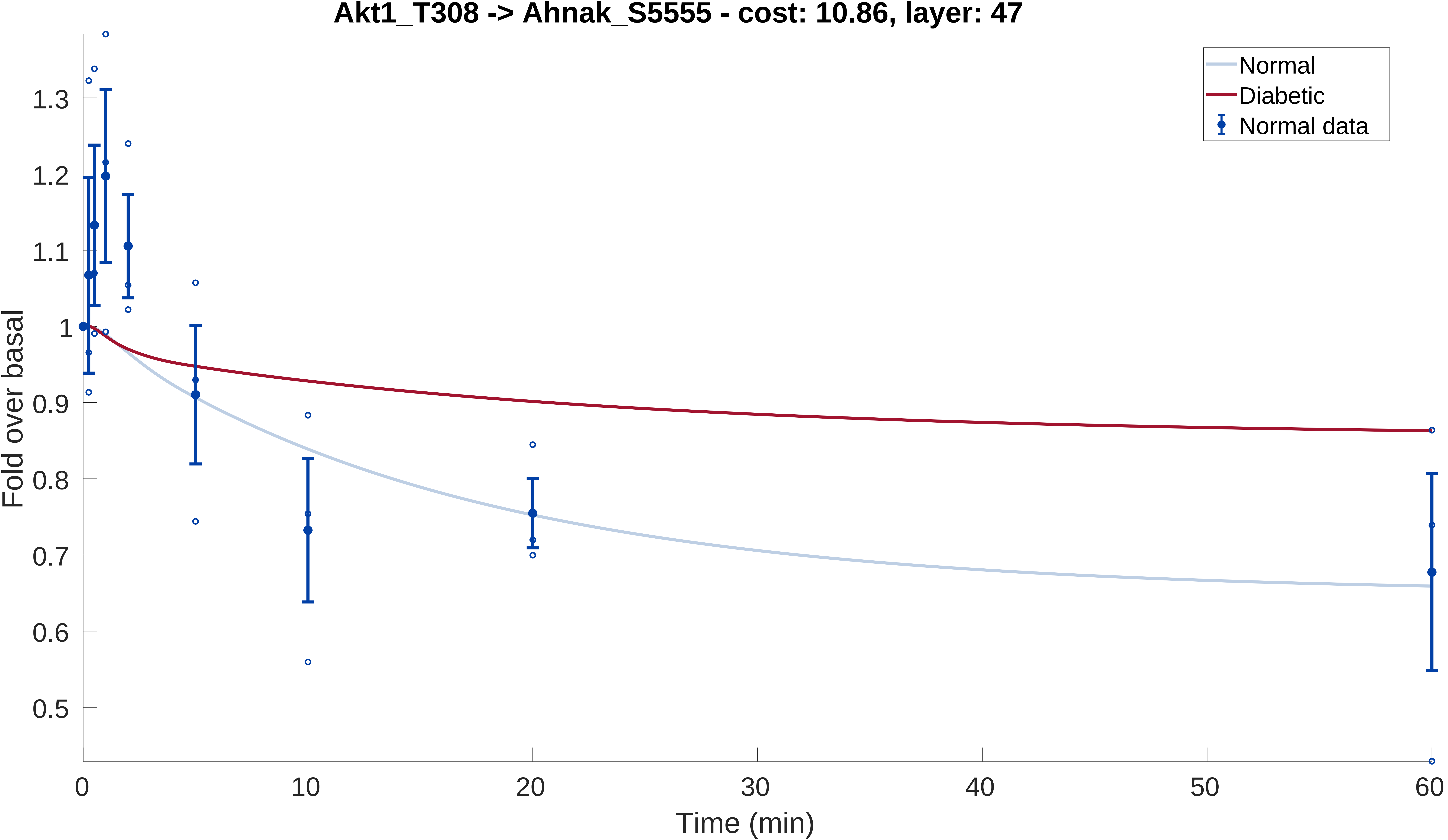
Akt1_T308 -> Ahnak_S5555 - cost: 10.86, layer: 47
Normal
Diabetic
Normal data
Fold over basal
Time (min)
1.3
1.2
1.1
1
0.9
0.8
0.7
0.6
0.5
0
10
20
30
40
50
60

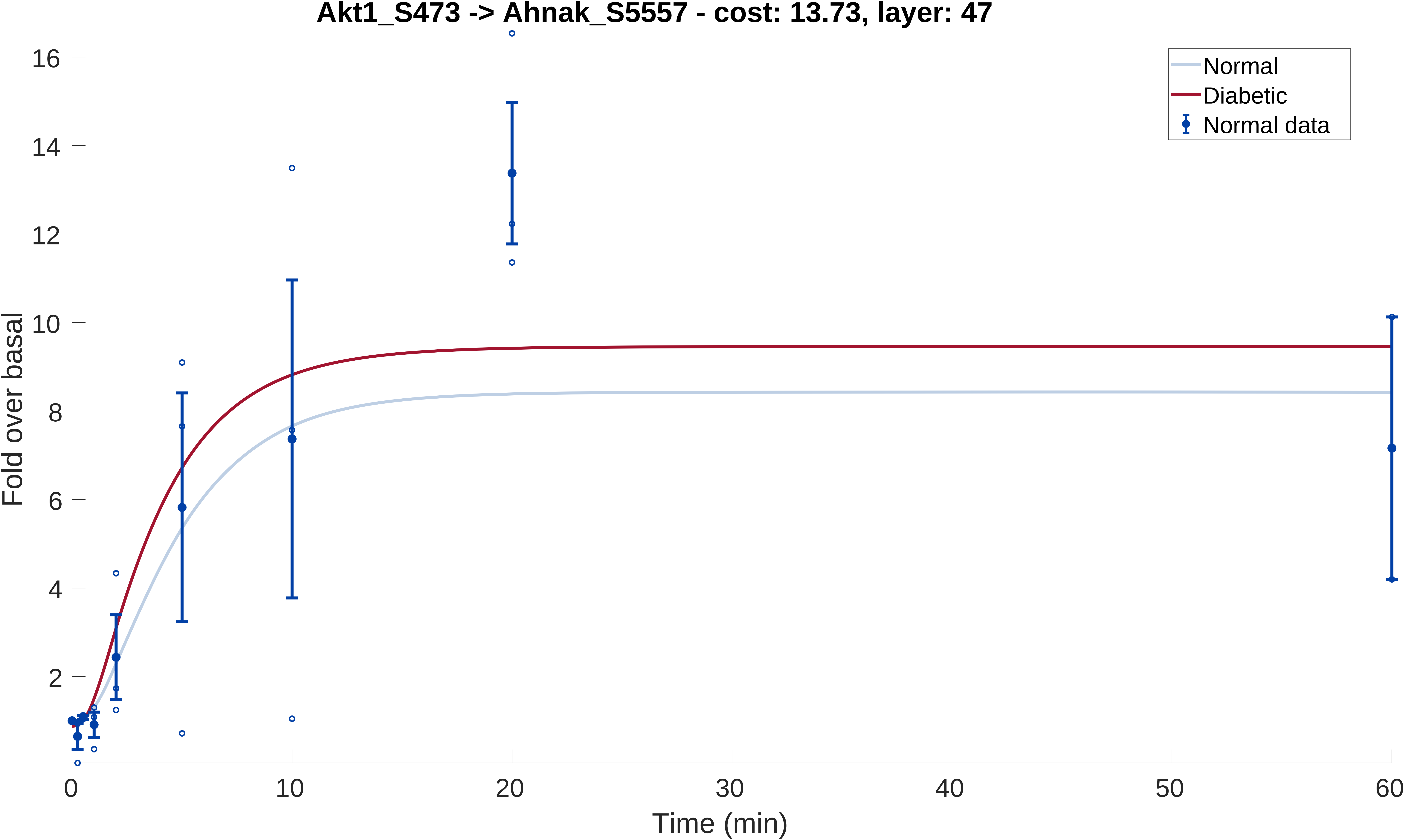

Akt1_S473 -> Ahnak_S5557 - cost: 13.73, layer: 47
Normal
Diabetic
Normal data
Fold over basal
Time (min)
0
10
20
30
40
50
60
2
4
6
8
10
12
14
16

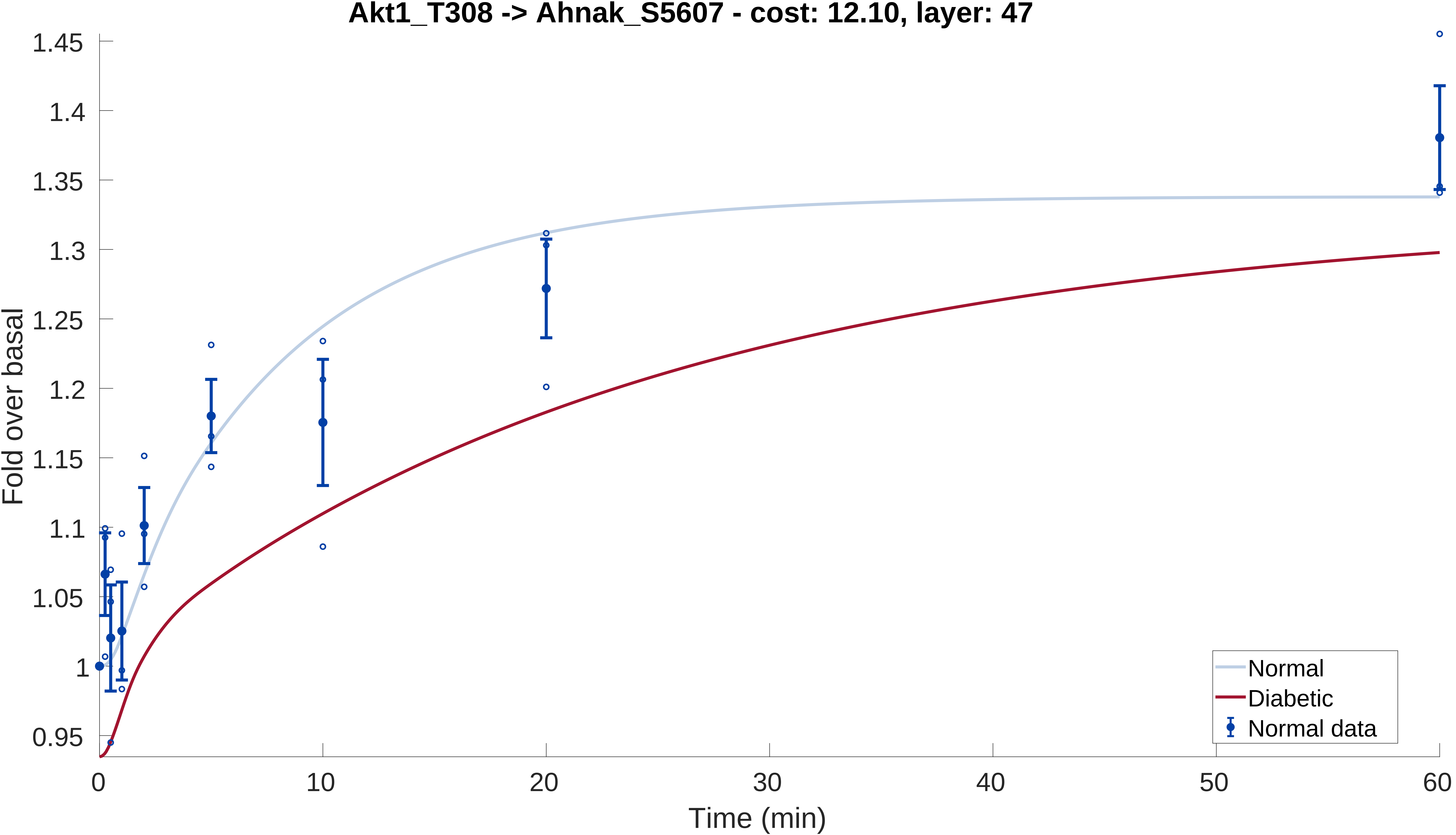
Akt1_T308 -> Ahnak_S5607 - cost: 12.10, layer: 47
Fold over basal
1.45
1.4
1.35
1.3
1.25
1.2
1.15
1.1
1.05
1
0.95
0
10
20
30
40
50
60
Time (min)
Normal
Diabetic
Normal data

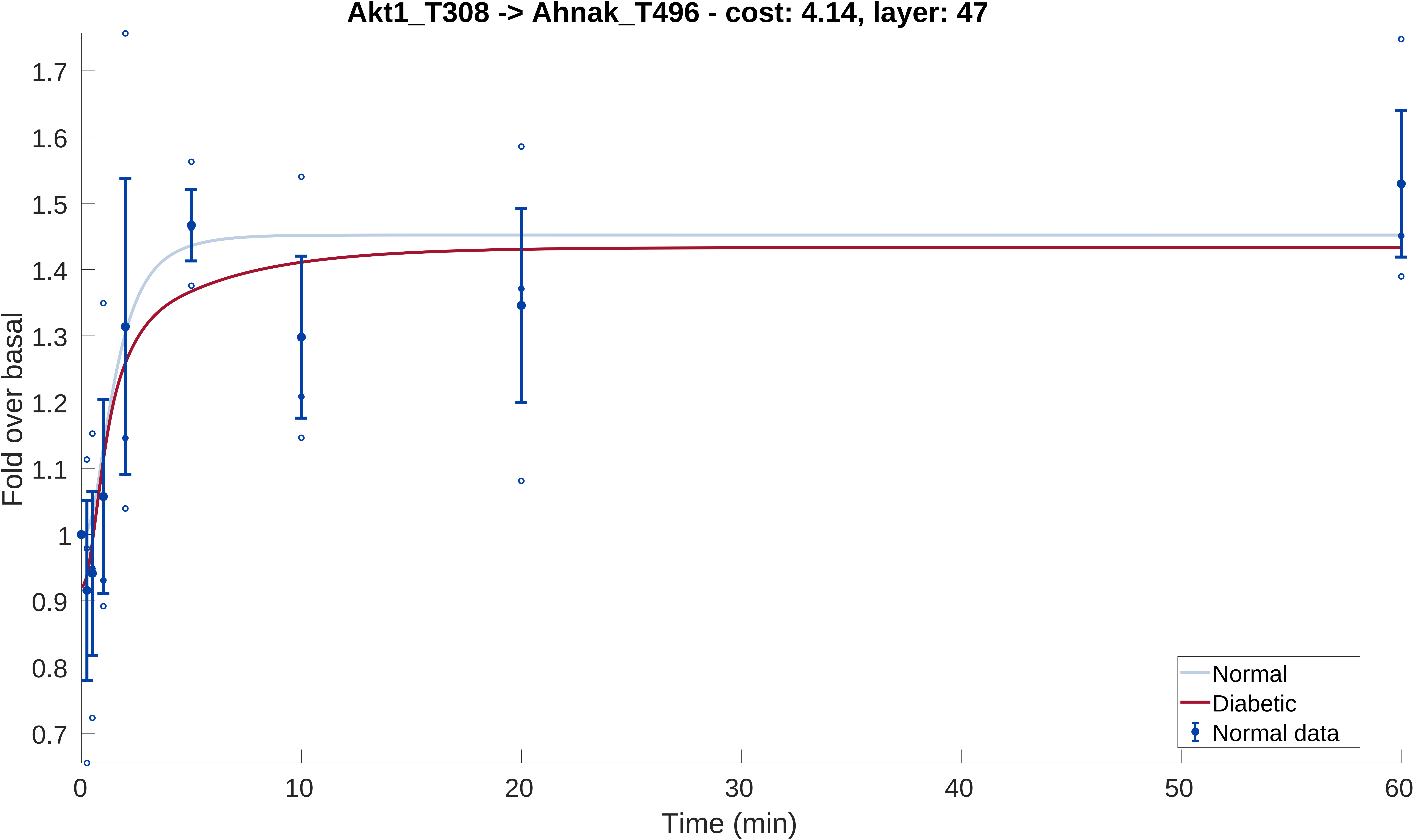

Akt1_T308 -> Ahnak_T496 - cost: 4.14, layer: 47
Fold over basal
1.7
1.6
1.5
1.4
1.3
1.2
1.1
1
0.9
0.8
0.7
0
10
20
30
40
50
60
Time (min)
Normal
Diabetic
Normal data

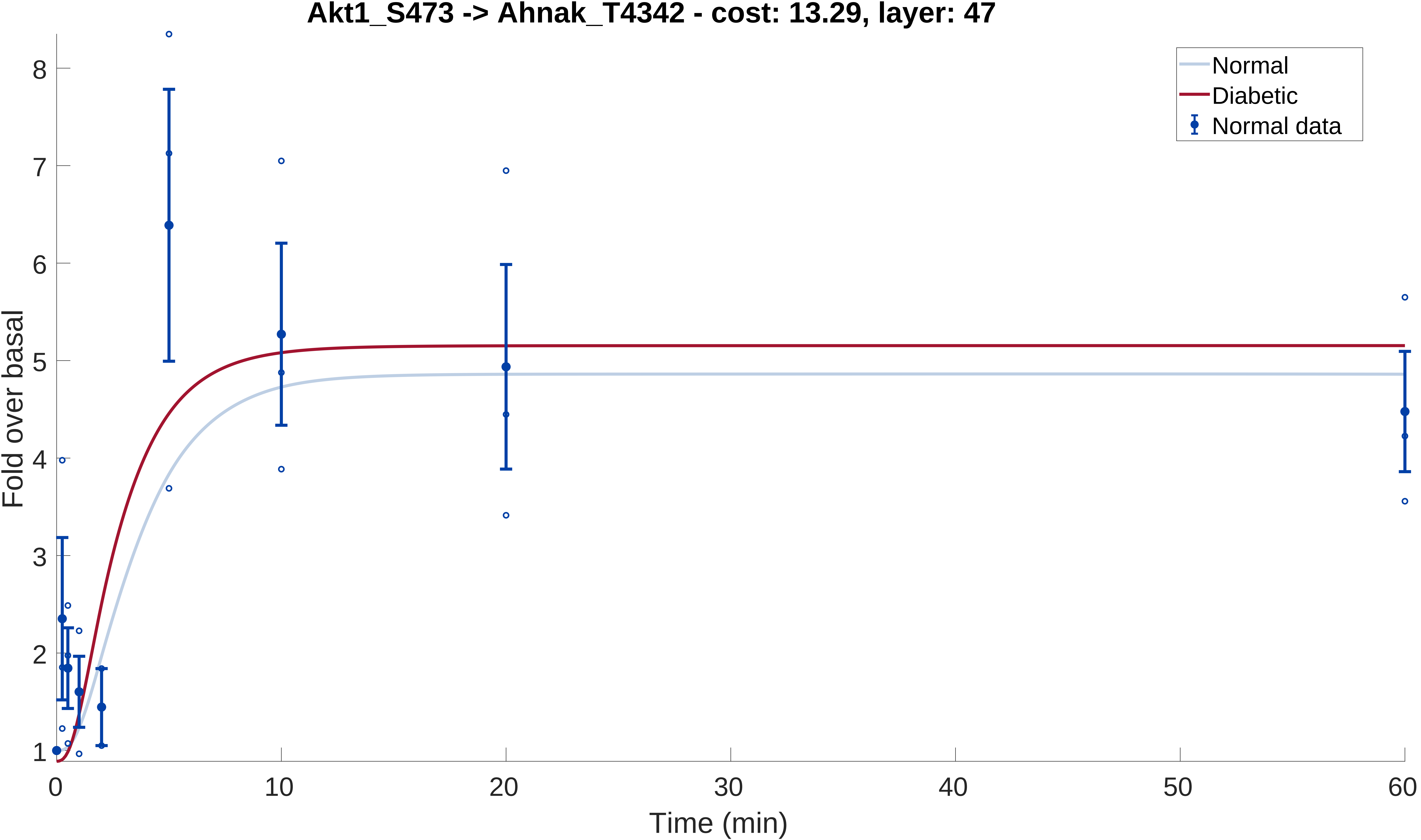

Akt1_S473 -> Ahnak_T4342 - cost: 13.29, layer: 47
Normal
Diabetic
Normal data
Fold over basal
Time (min)
1
2
3
4
5
6
7
8
0
10
20
30
40
50
60

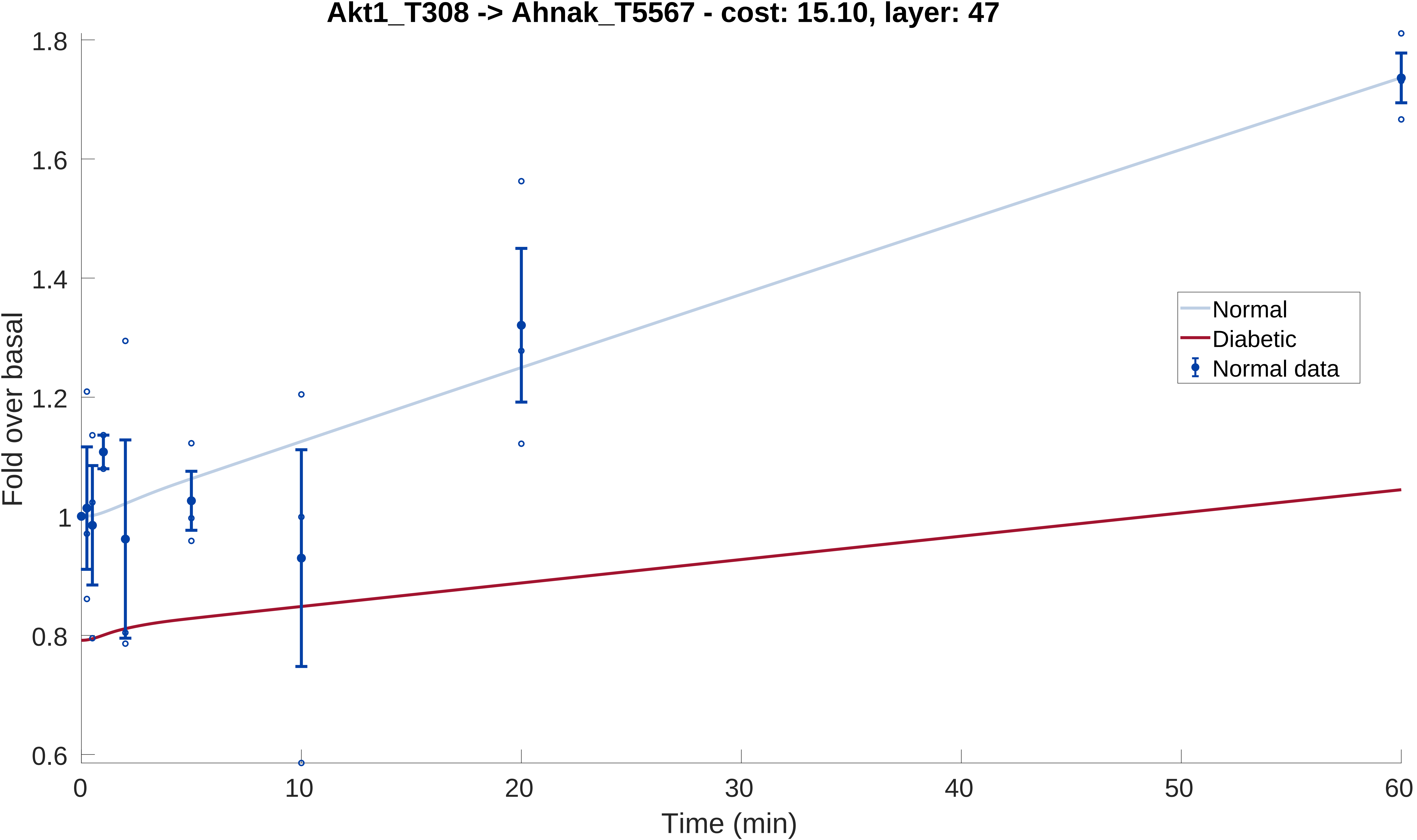

Akt1_T308 -> Ahnak_T5567 - cost: 15.10, layer: 47
Fold over basal
Time (min)
Normal
Diabetic
Normal data
1.8
1.6
1.4
1.2
1
0.8
0.6
0
10
20
30
40
50
60

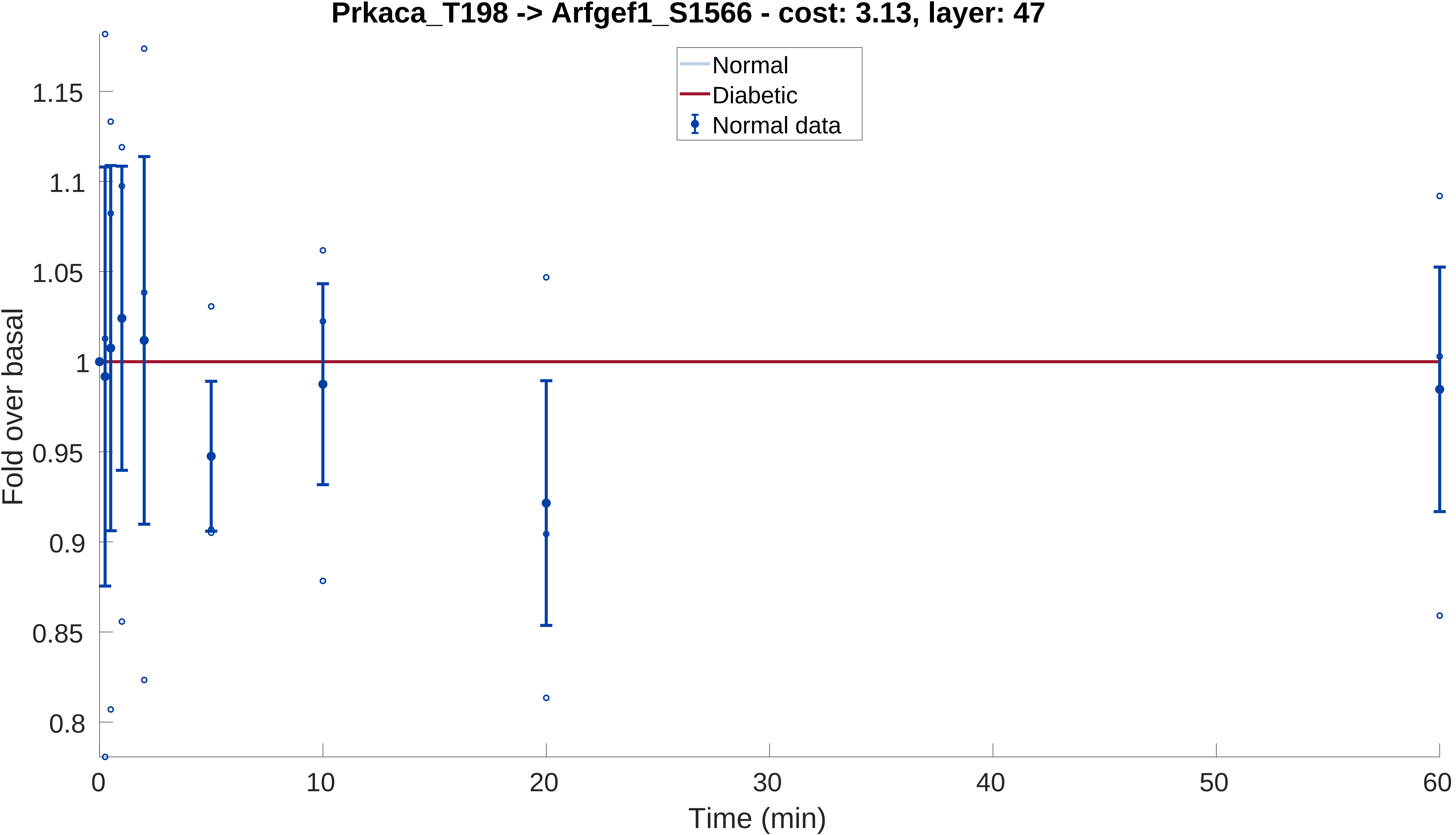

Prkaca_T198 -> Arfgef1_S1566 - cost: 3.13, layer: 47
Normal
Diabetic
Normal data
Fold over basal
Time (min)
1.15
1.1
1.05
1
0.95
0.9
0.85
0.8
0
10
20
30
40
50
60

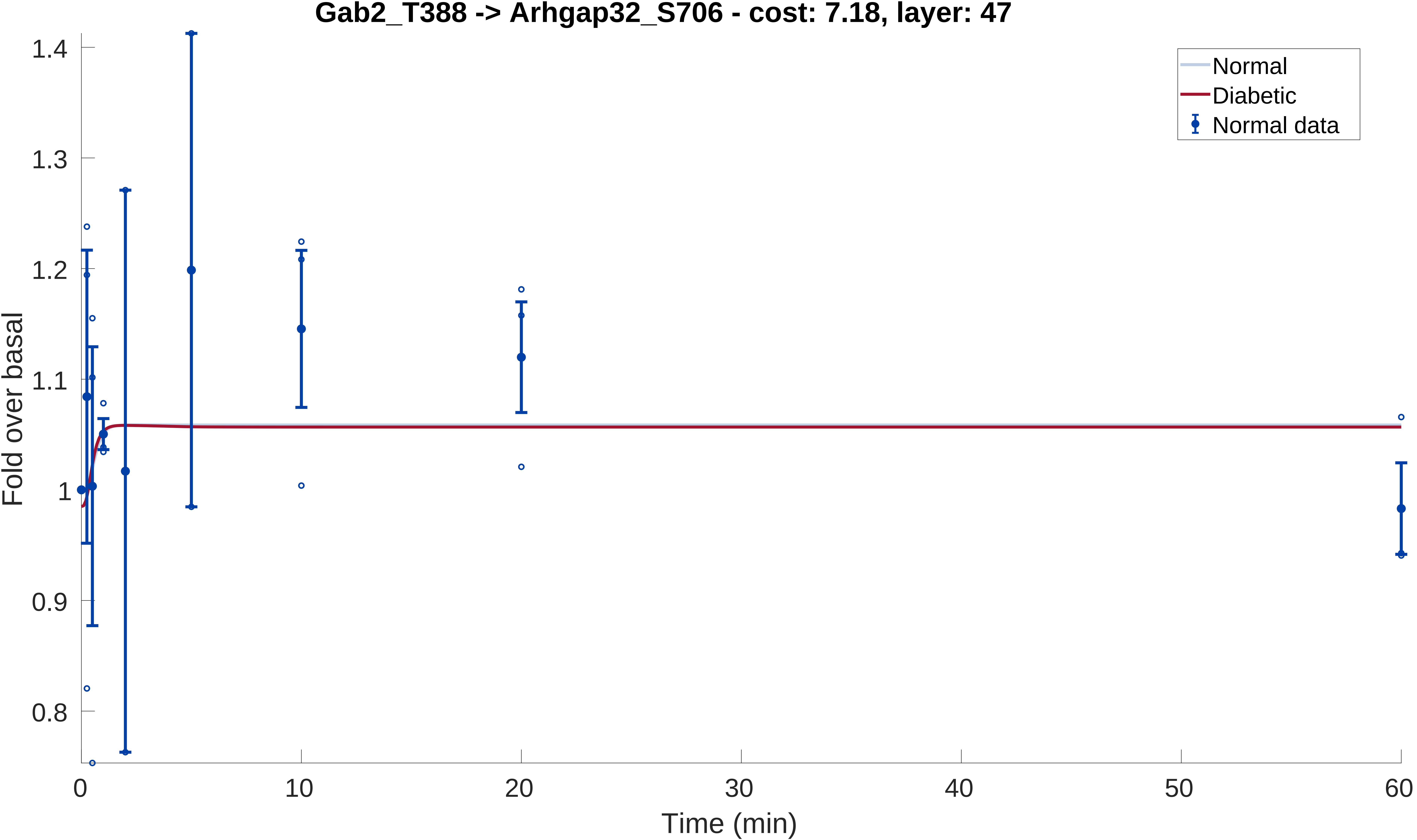

Gab2_T388 -> Arhgap32_S706 - cost: 7.18, layer: 47
Fold over basal
Time (min)
Normal
Diabetic
Normal data
1.4
1.3
1.2
1.1
1
0.9
0.8
0
10
20
30
40
50
60

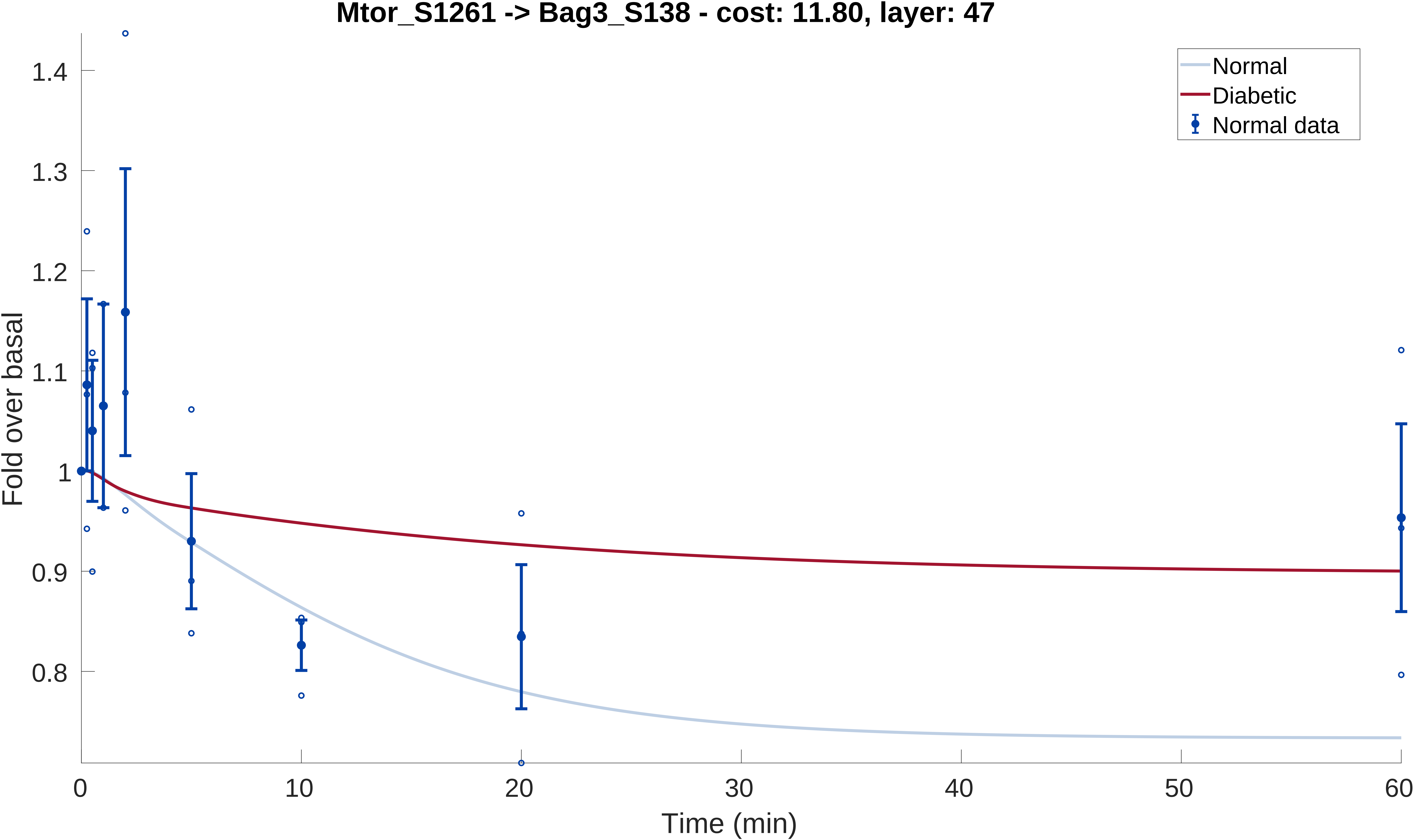

Mtor_S1261 -> Bag3_S138 - cost: 11.80, layer: 47
Normal
Diabetic
Normal data
Fold over basal
Time (min)
1.4
1.3
1.2
1.1
1
0.9
0.8
0
10
20
30
40
50
60

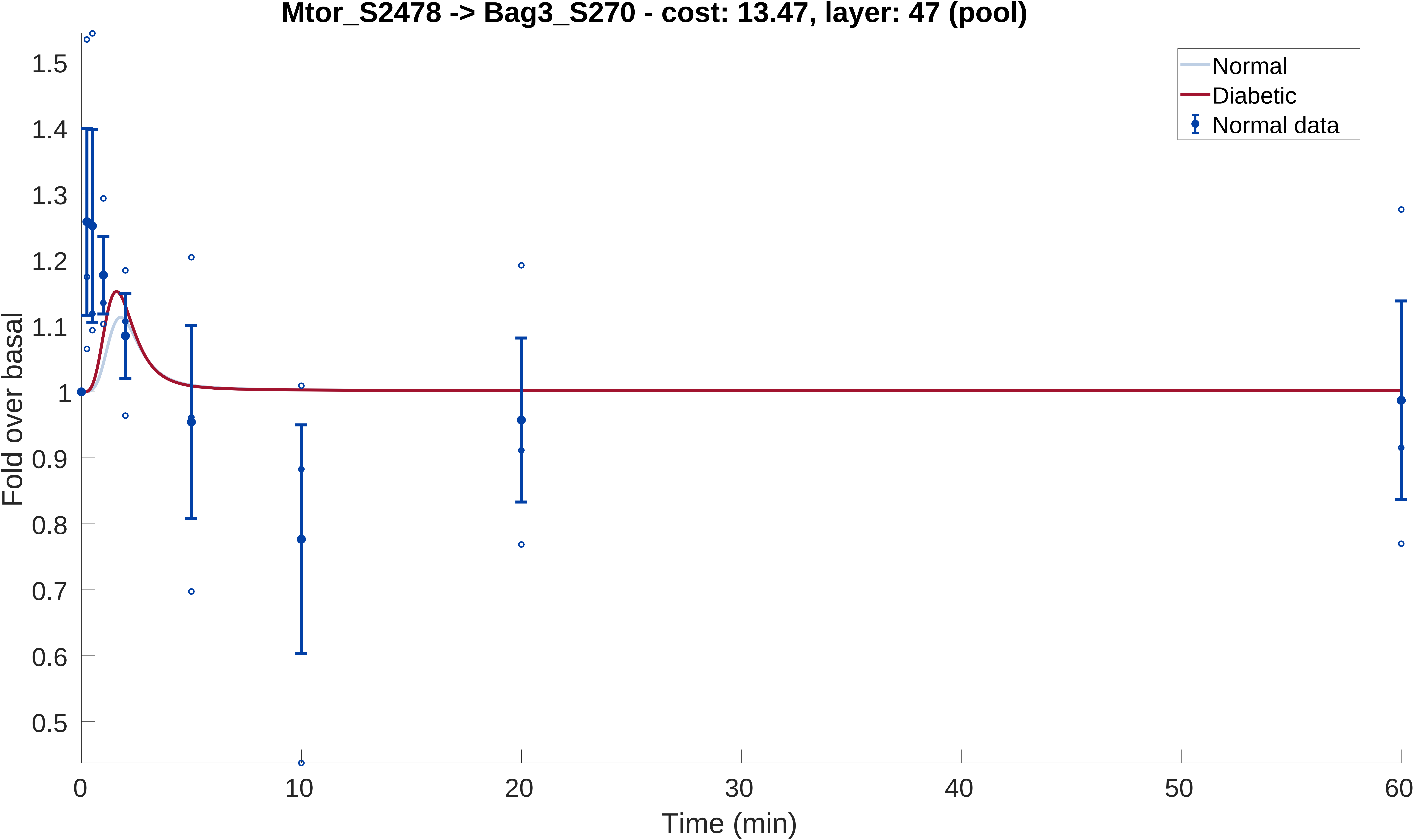
Mtor_S2478 -> Bag3_S270 - cost: 13.47, layer: 47 (pool)
Normal
Diabetic
Normal data
Fold over basal
Time (min)
1.5
1.4
1.3
1.2
1.1
1
0.9
0.8
0.7
0.6
0.5
0
10
20
30
40
50
60

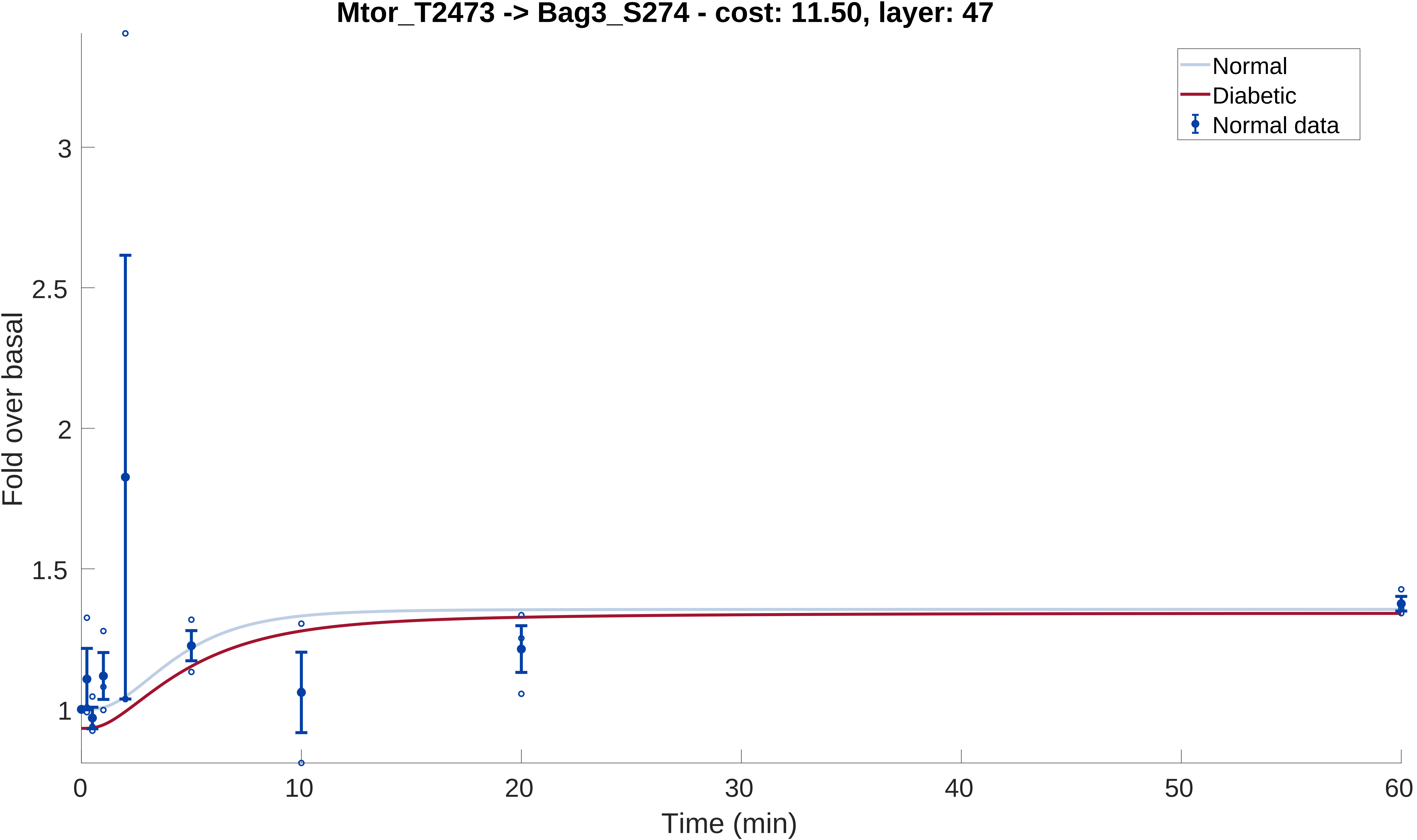
Mtor_T2473 -> Bag3_S274 - cost: 11.50, layer: 47
Normal
Diabetic
Normal data
Fold over basal
Time (min)
1
1.5
2
2.5
3
0
10
20
30
40
50
60

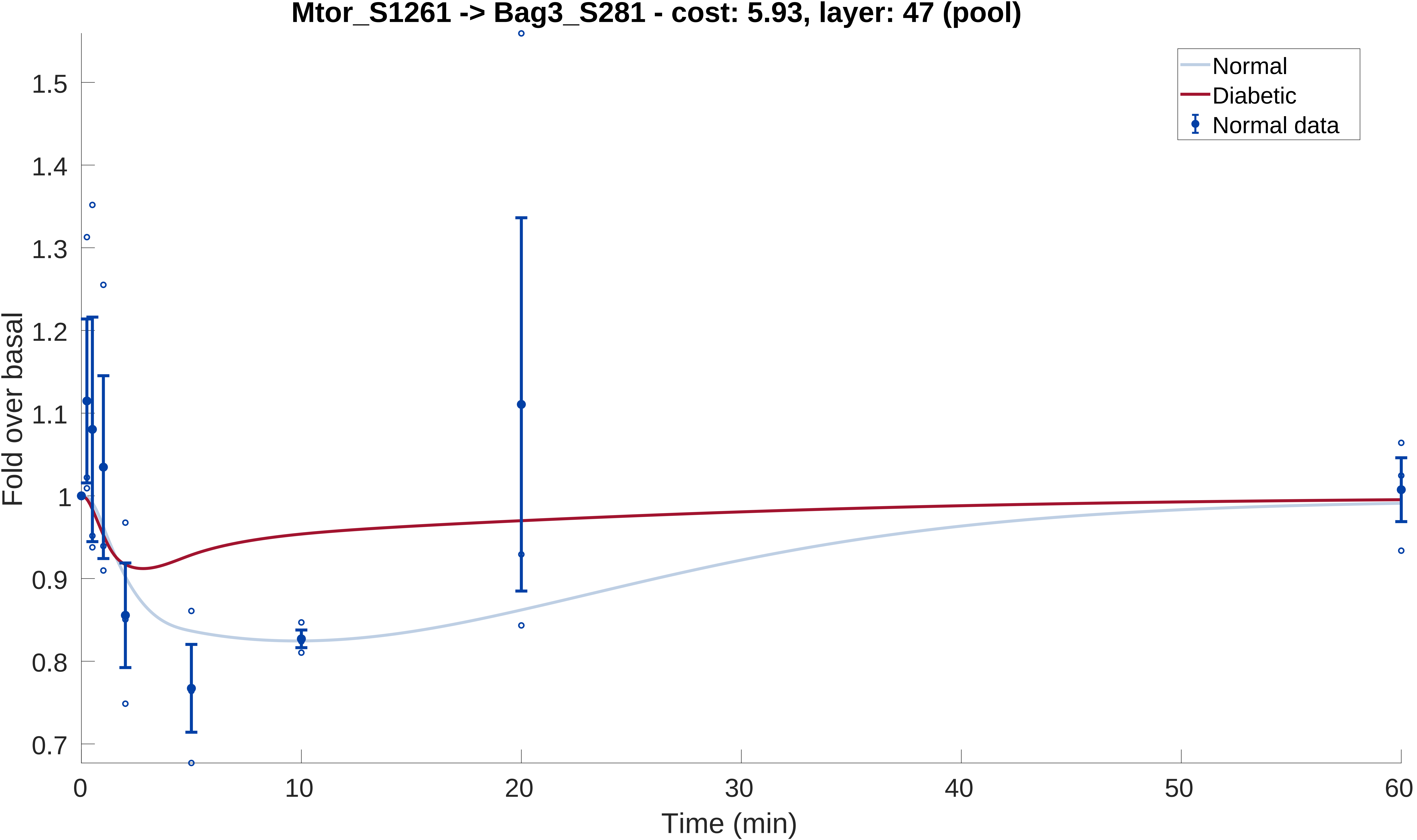

Mtor_S1261 -> Bag3_S281 - cost: 5.93, layer: 47 (pool)
Normal
Diabetic
Normal data
Fold over basal
1.5
1.4
1.3
1.2
1.1
1
0.9
0.8
0.7
0
10
20
30
40
50
60
Time (min)

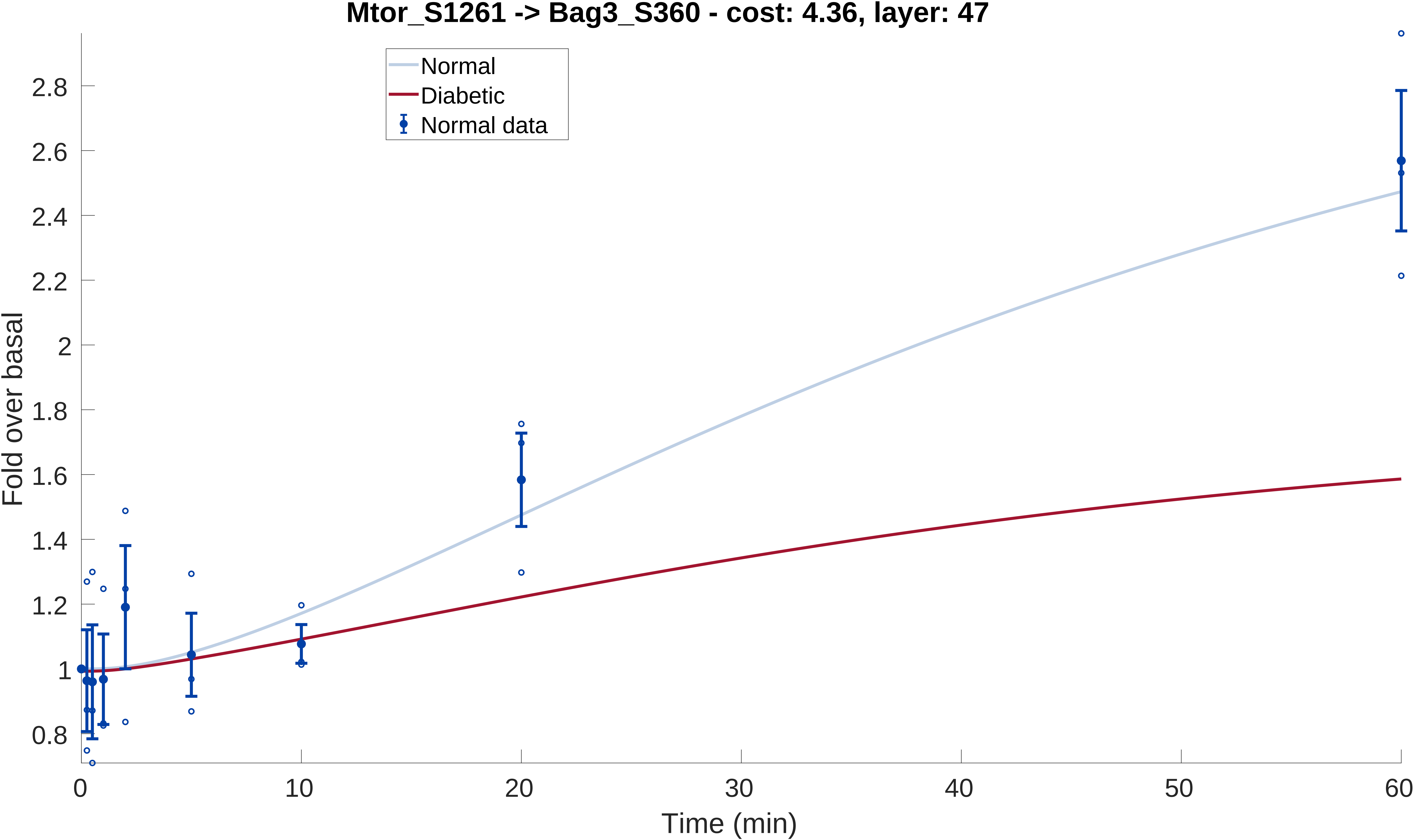
Mtor_S1261 -> Bag3_S360 - cost: 4.36, layer: 47
Normal
Diabetic
Normal data
Fold over basal
Time (min)
0.8
1
1.2
1.4
1.6
1.8
2
2.2
2.4
2.6
2.8
0
10
20
30
40
50
60

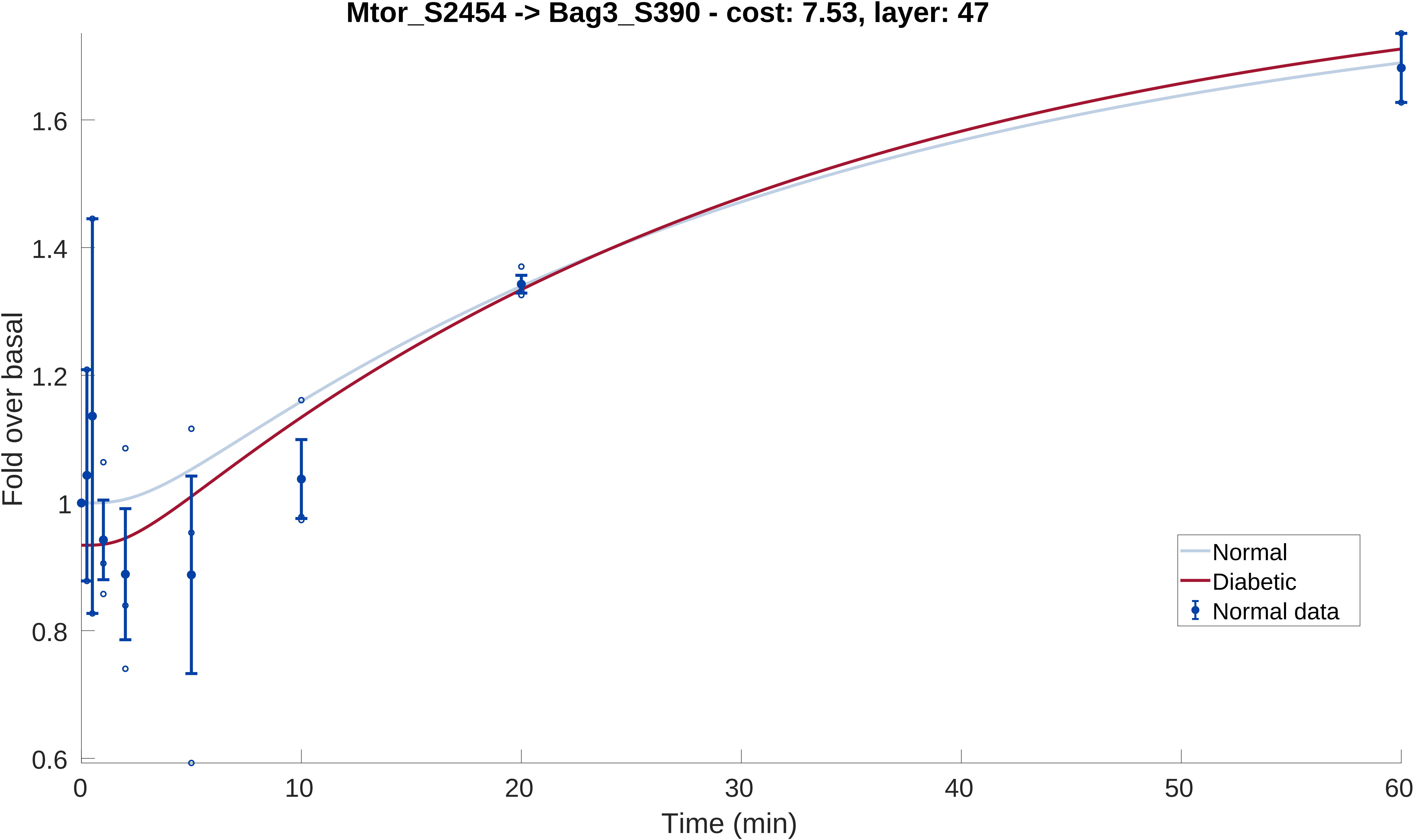
Mtor_S2454 -> Bag3_S390 - cost: 7.53, layer: 47
Fold over basal
Time (min)
0.6
0.8
1
1.2
1.4
1.6
0
10
20
30
40
50
60
Normal
Diabetic
Normal data

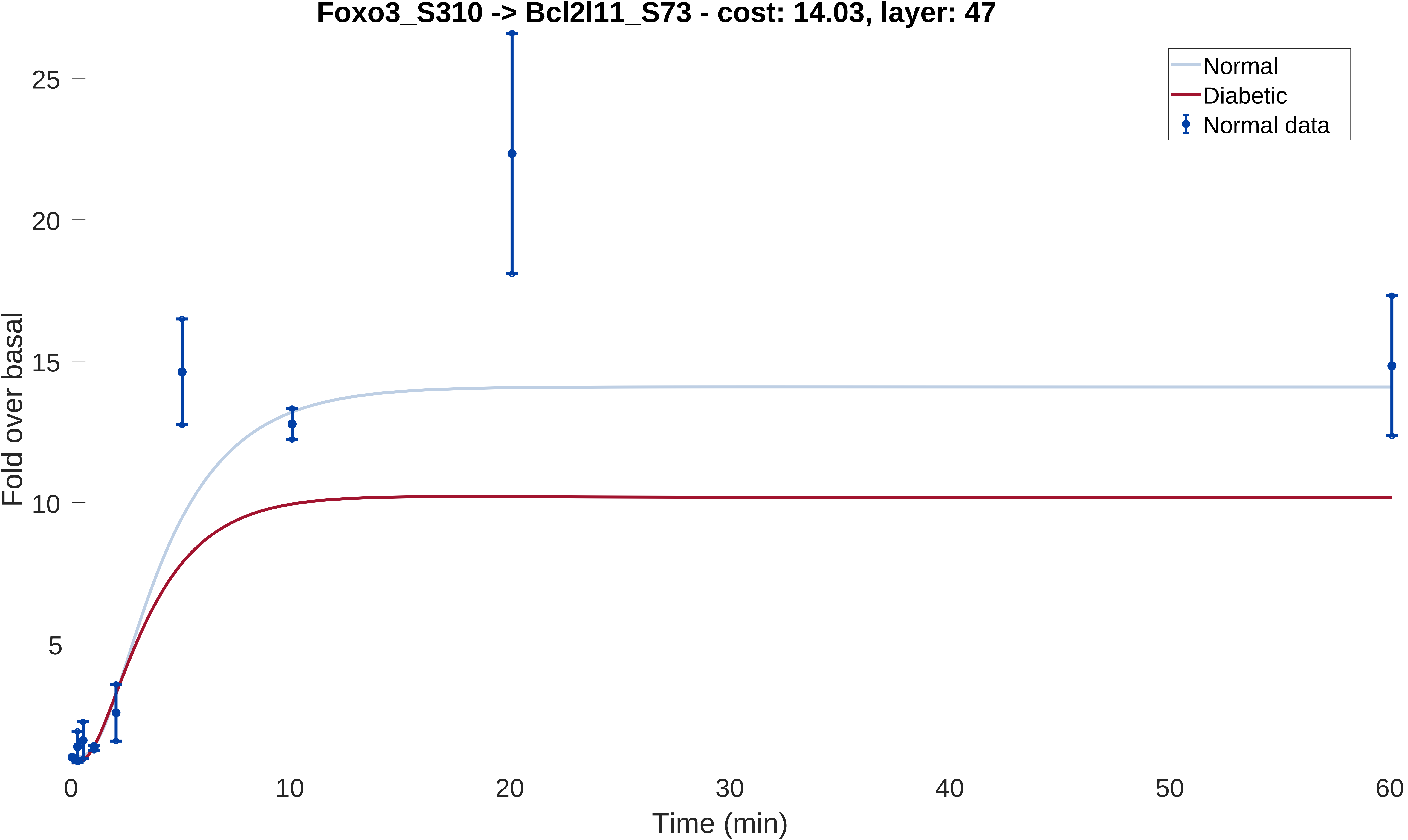
Foxo3_S310 -> Bcl2l11_S73 - cost: 14.03, layer: 47
Normal
Diabetic
Normal data
Fold over basal
25
20
15
10
5
0
10
20
30
40
50
60
Time (min)

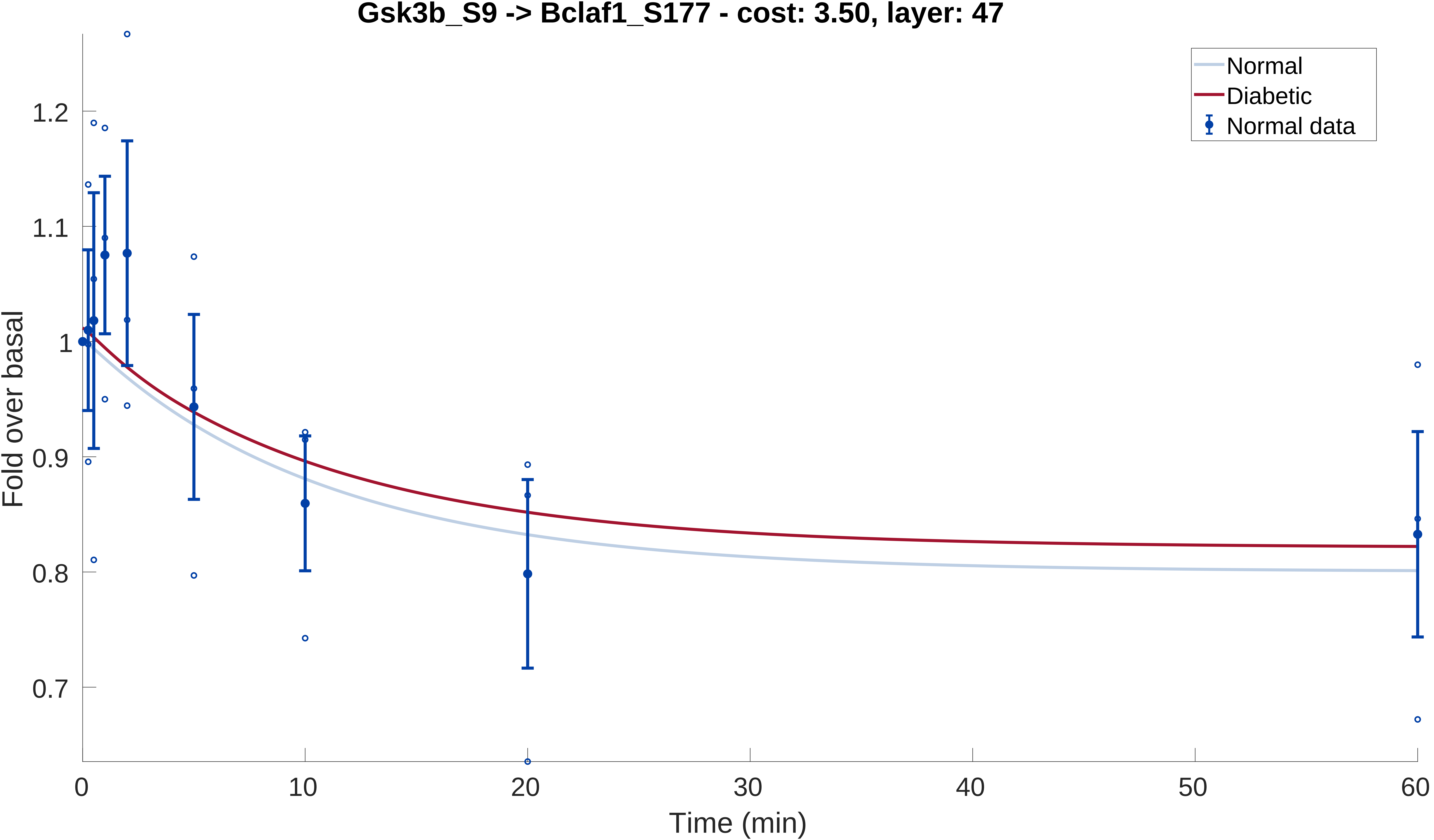
Gsk3b_S9 -> Bclaf1_S177 - cost: 3.50, layer: 47
Normal
Diabetic
Normal data
Fold over basal
1.2
1.1
1
0.9
0.8
0.7
0
10
20
30
40
50
60
Time (min)

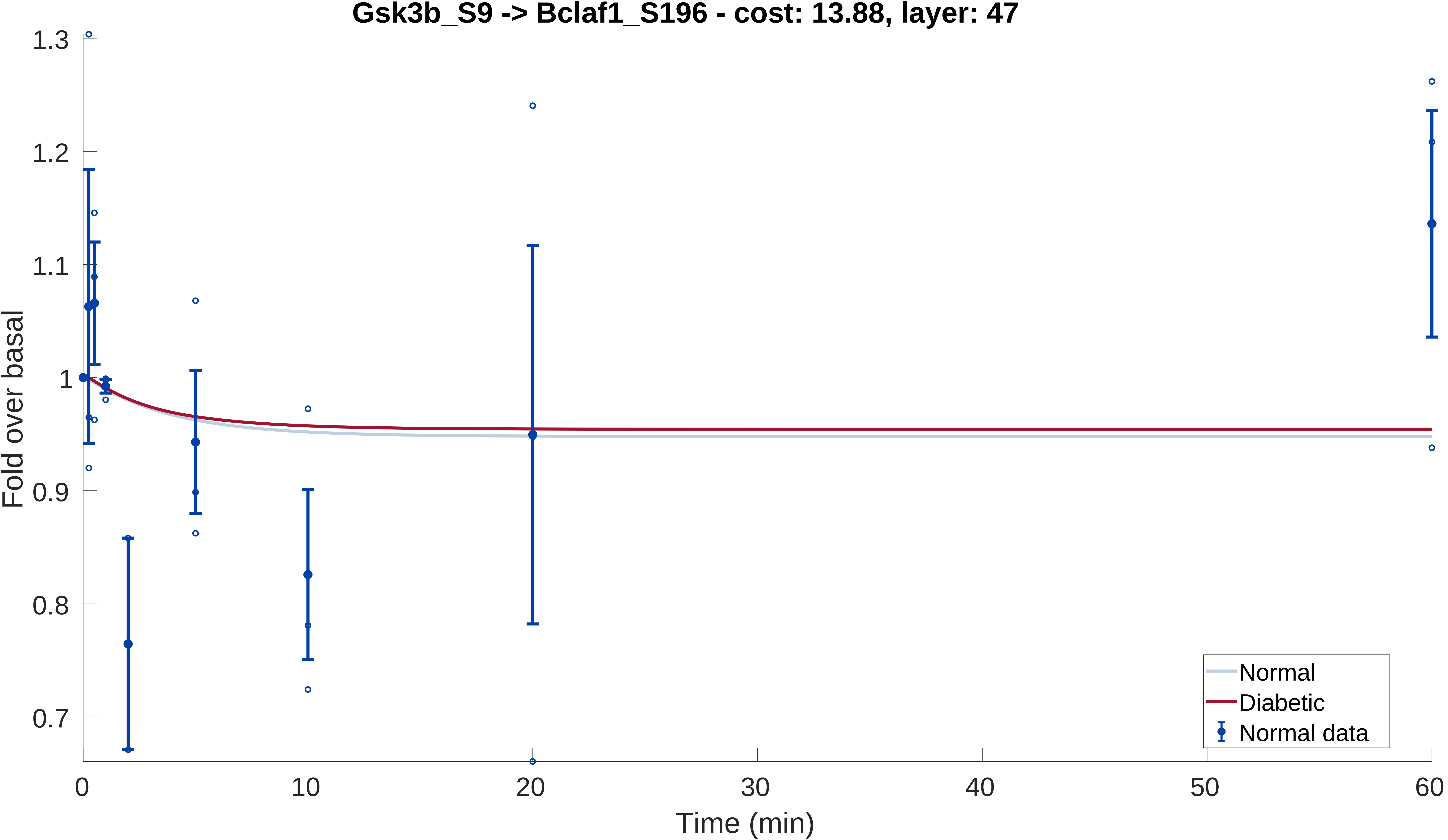

Gsk3b_S9 -> Bclaf1_S196 - cost: 13.88, layer: 47
Fold over basal
1.3
1.2
1.1
1
0.9
0.8
0.7
0
10
20
30
40
50
60
Time (min)
Normal
Diabetic
Normal data

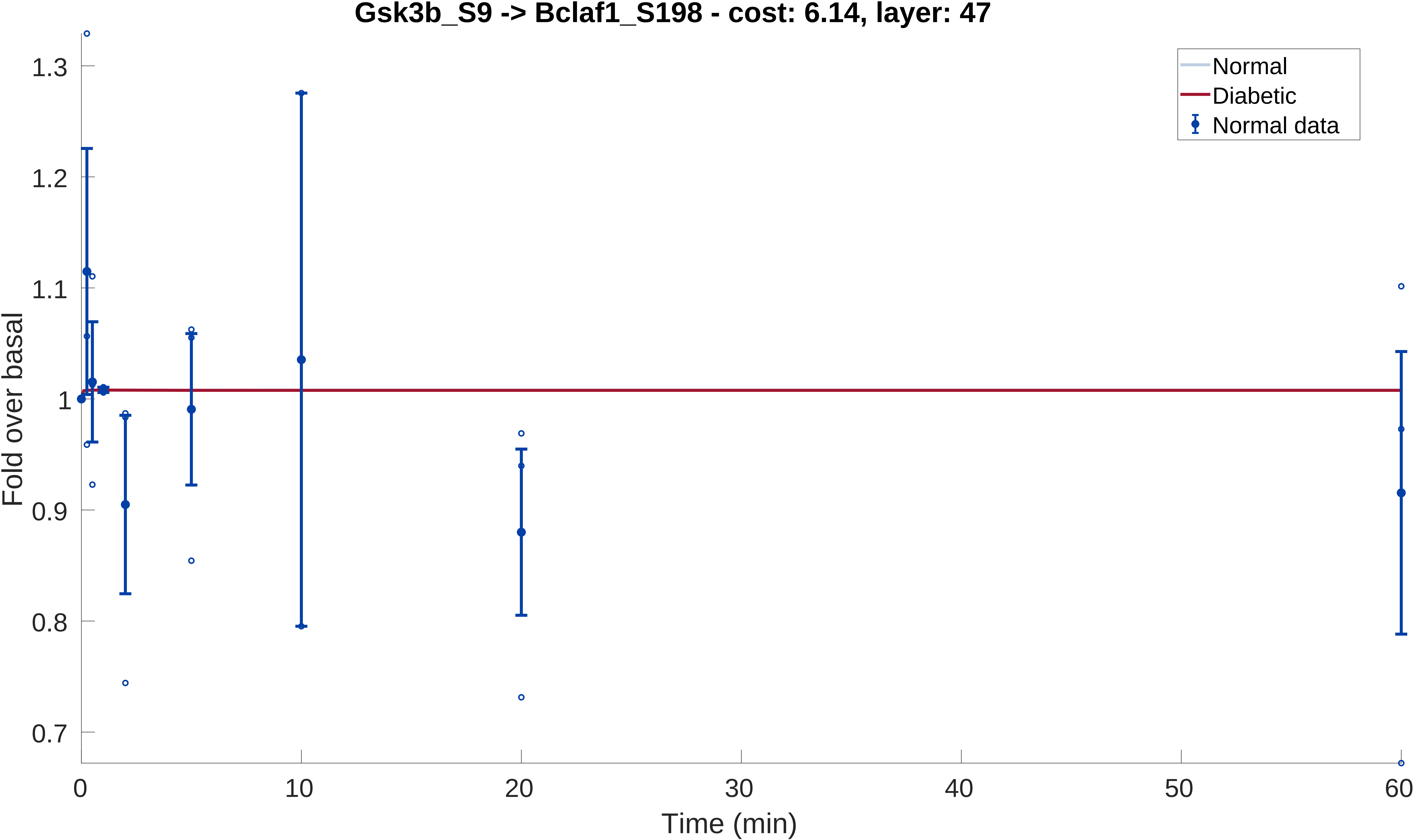

Gsk3b_S9 -> Bclaf1_S198 - cost: 6.14, layer: 47
Normal
Diabetic
Normal data
Fold over basal
Time (min)
0.7
0.8
0.9
1
1.1
1.2
1.3
0
10
20
30
40
50
60

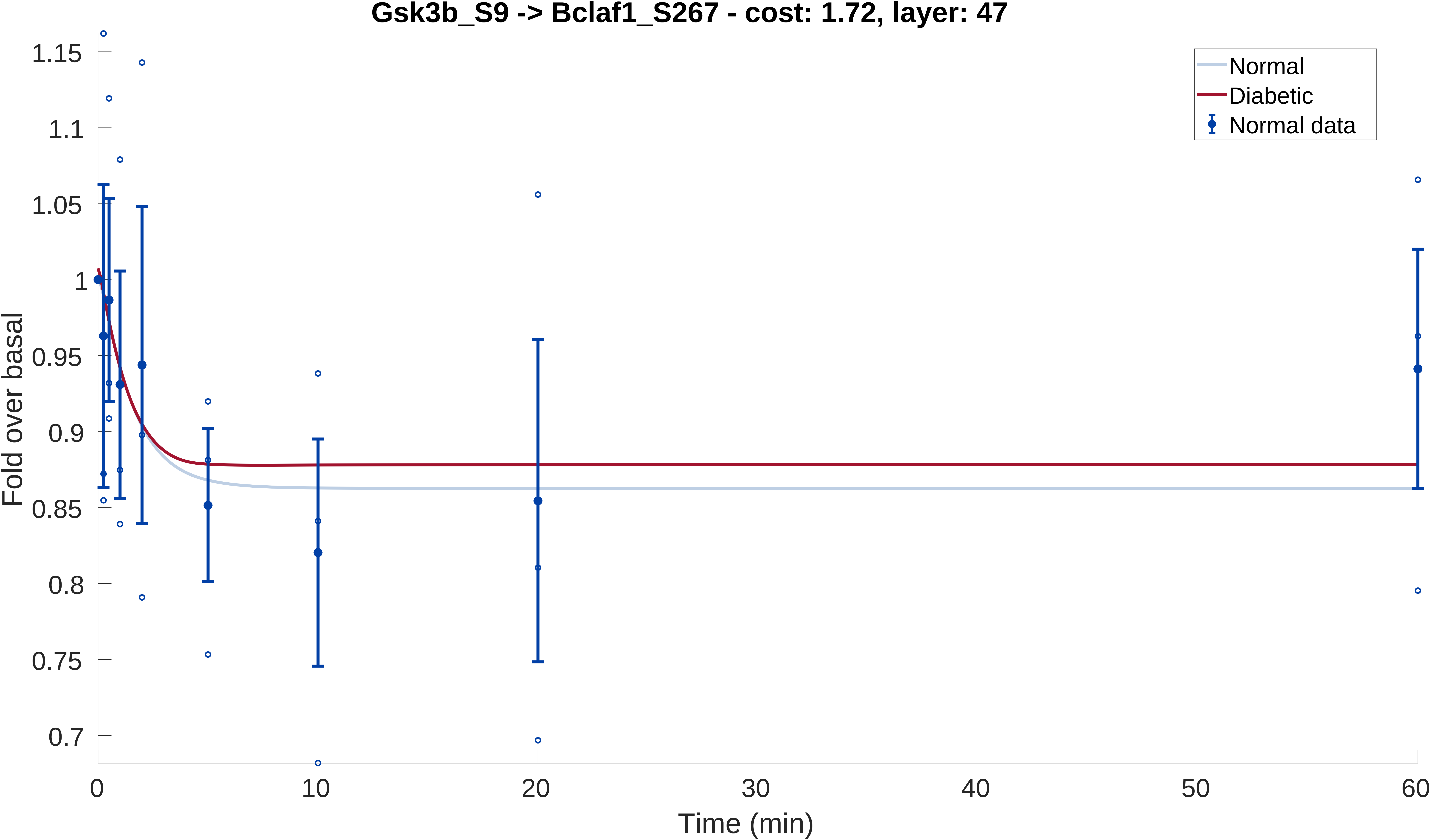

Gsk3b_S9 -> Bclaf1_S267 - cost: 1.72, layer: 47
Normal
Diabetic
Normal data
Fold over basal
Time (min)
1.15
1.1
1.05
1
0.95
0.9
0.85
0.8
0.75
0.7
0
10
20
30
40
50
60

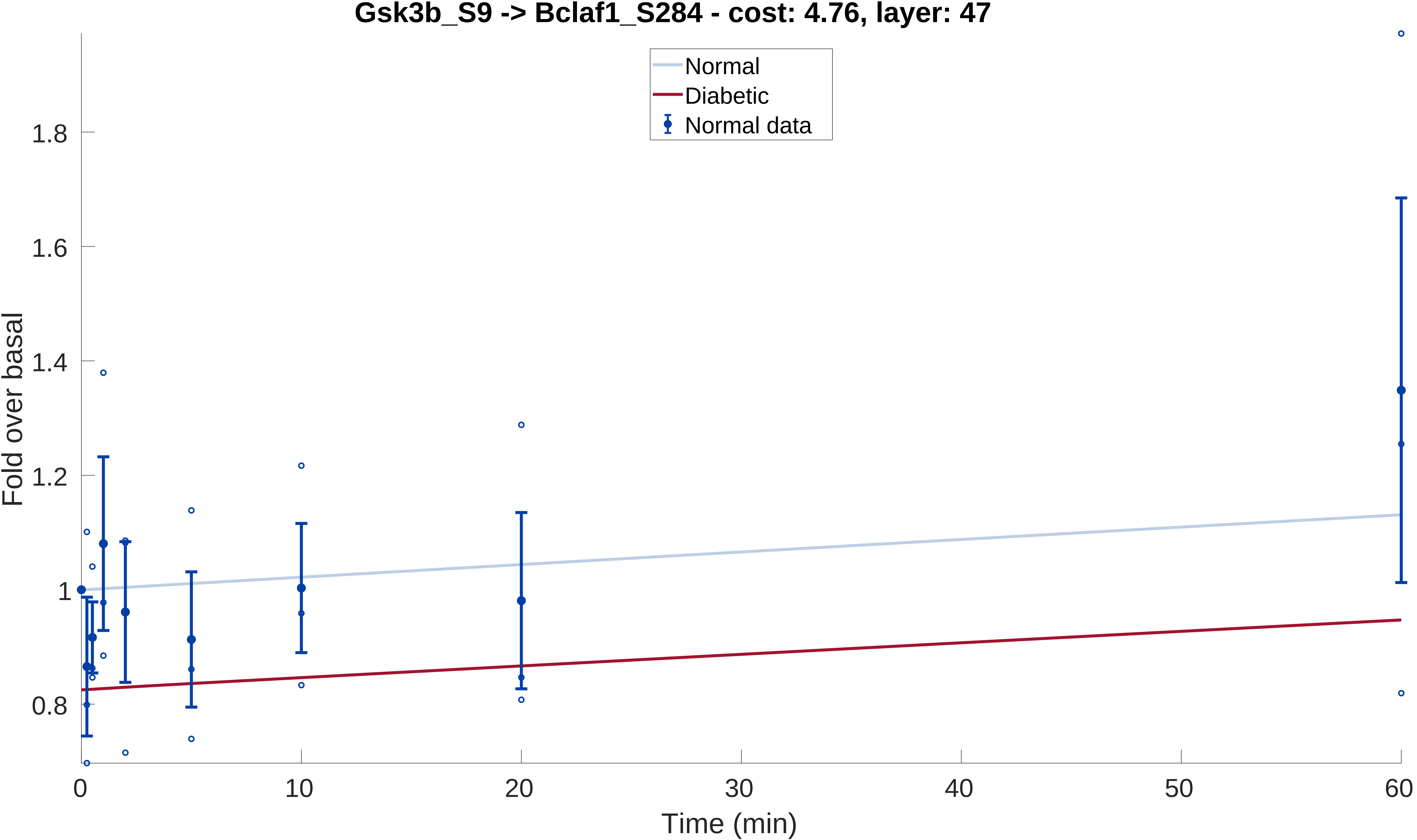

Gsk3b_S9 -> Bclaf1_S284 - cost: 4.76, layer: 47
Normal
Diabetic
Normal data
Fold over basal
1.8
1.6
1.4
1.2
1
0.8
0
10
20
30
40
50
60
Time (min)

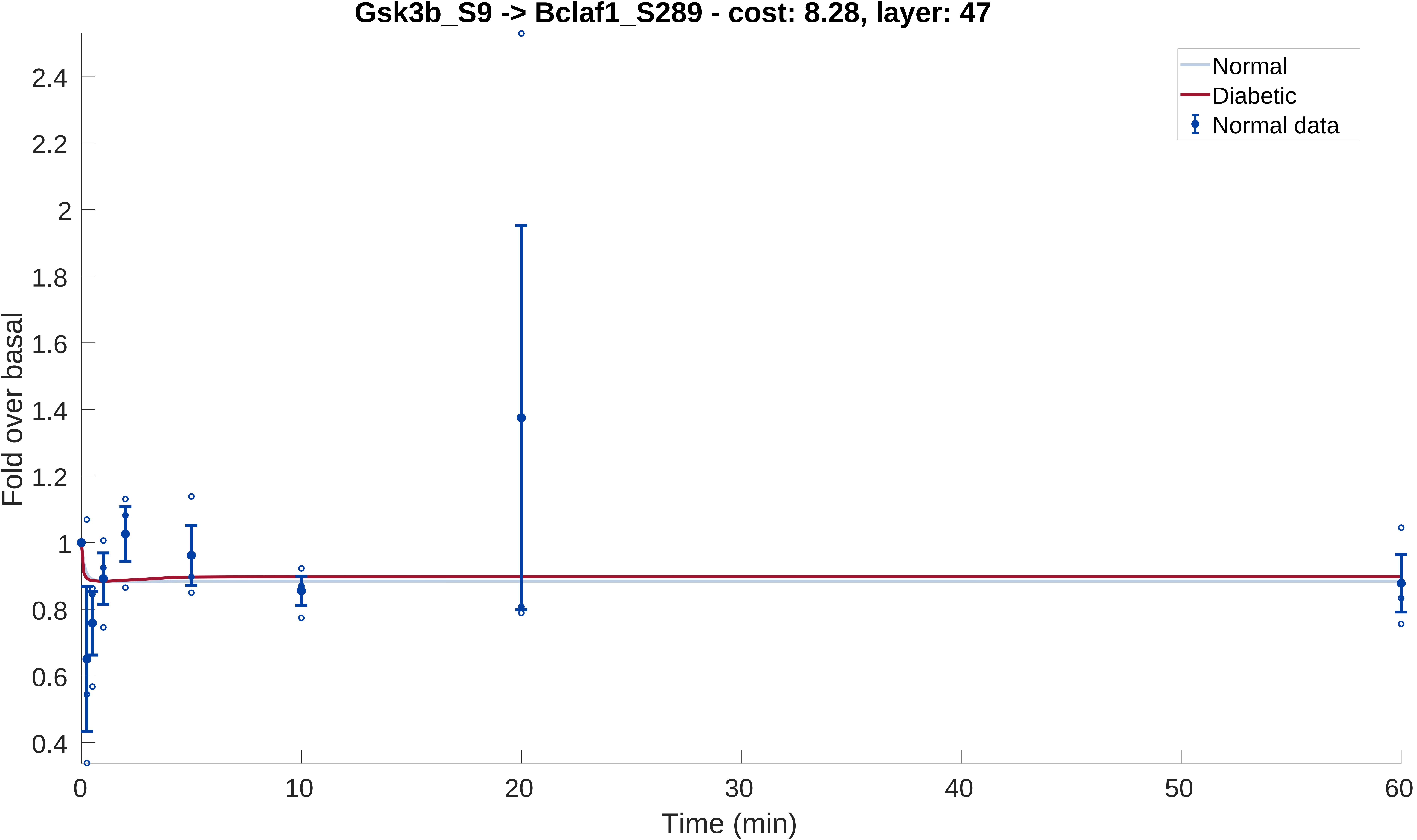

Gsk3b_S9 -> Bclaf1_S289 - cost: 8.28, layer: 47
Normal
Diabetic
Normal data
Fold over basal
Time (min)
2.4
2.2
2
1.8
1.6
1.4
1.2
1
0.8
0.6
0.4
0
10
20
30
40
50
60

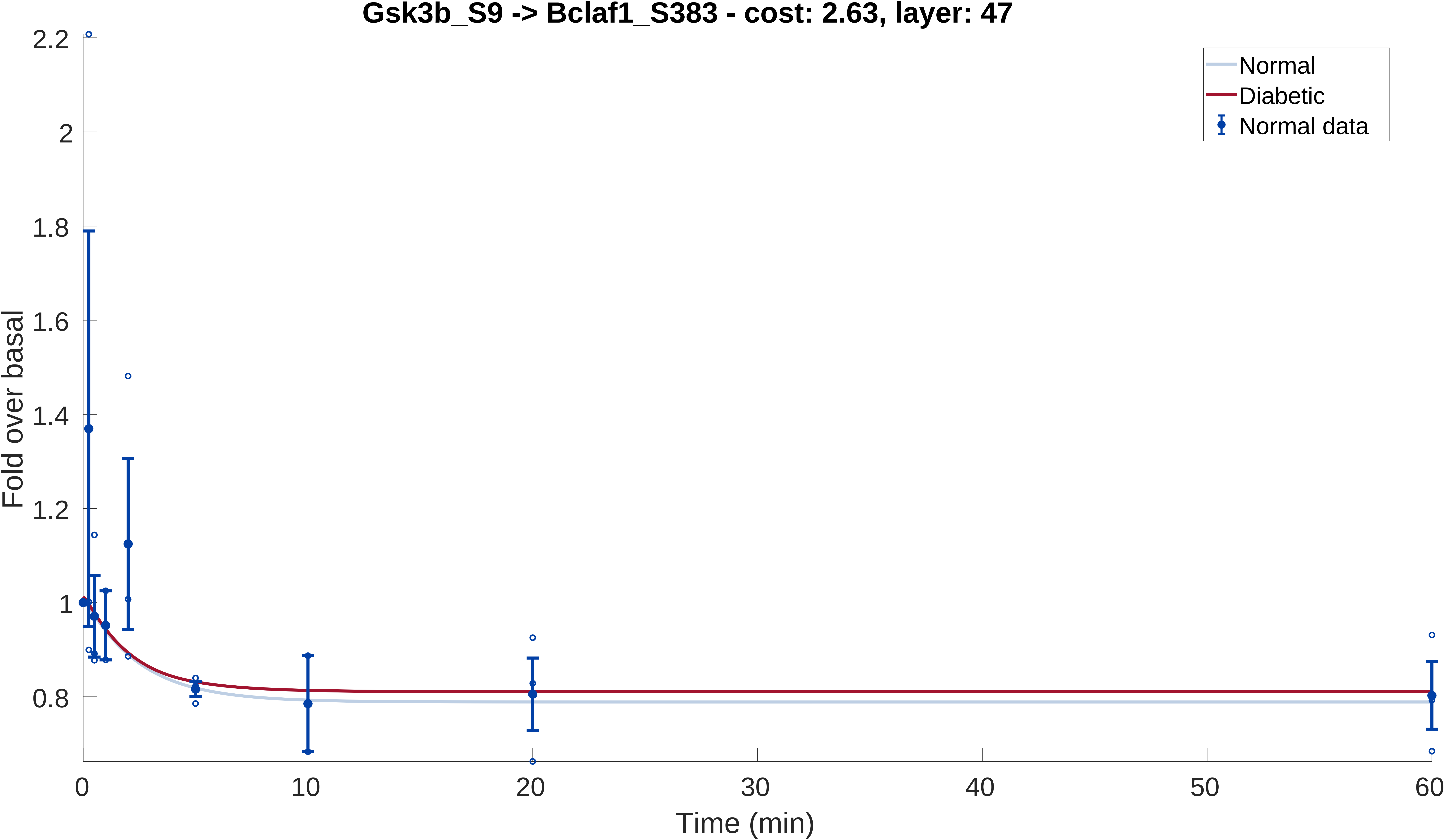
Gsk3b_S9 -> Bclaf1_S383 - cost: 2.63, layer: 47
Fold over basal
Time (min)
Normal
Diabetic
Normal data
2.2
2
1.8
1.6
1.4
1.2
1
0.8
0
10
20
30
40
50
60

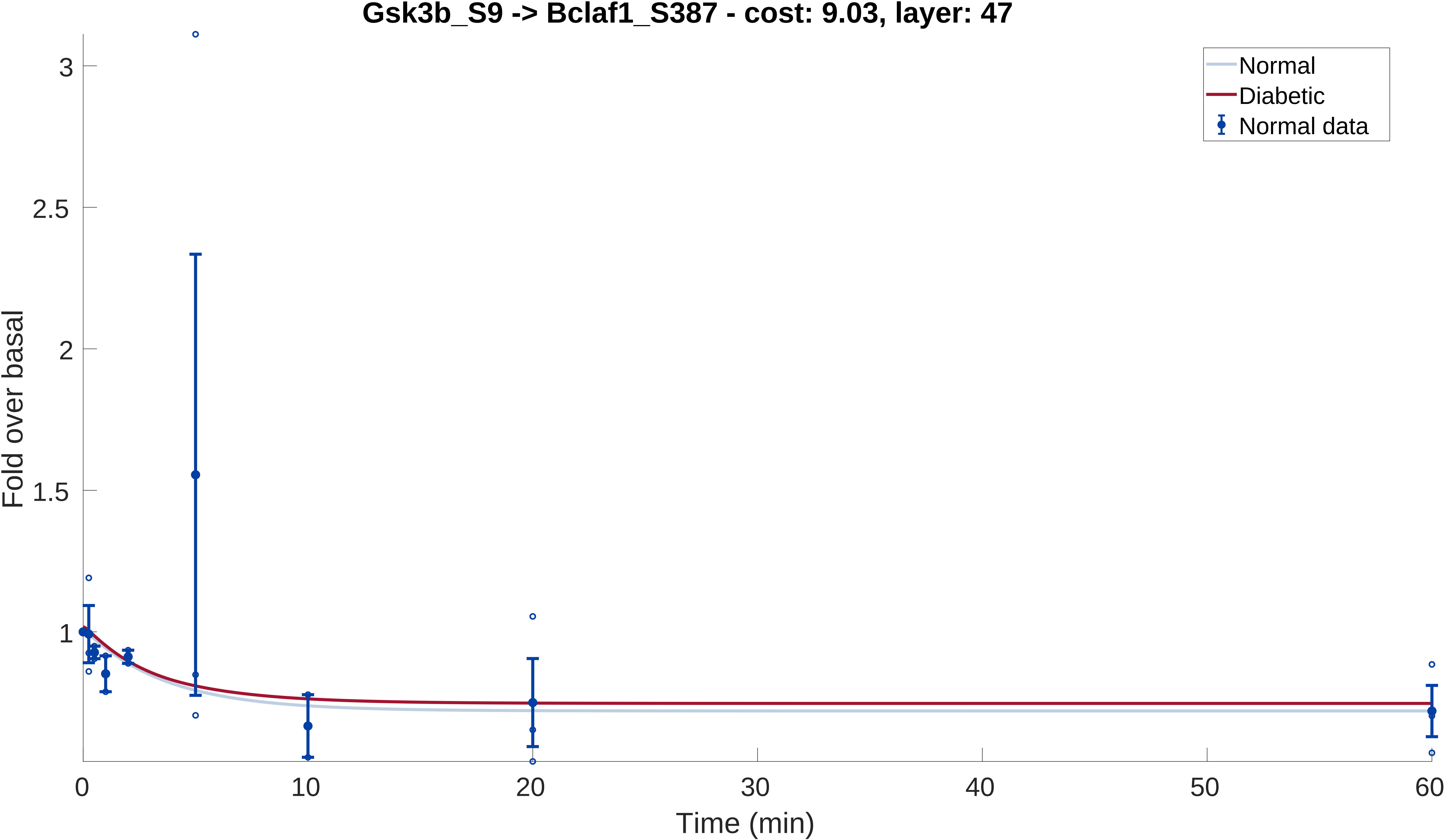

Gsk3b_S9 -> Bclaf1_S387 - cost: 9.03, layer: 47
Normal
Diabetic
Normal data
Fold over basal
Time (min)
3
2.5
2
1.5
1
0
10
20
30
40
50
60

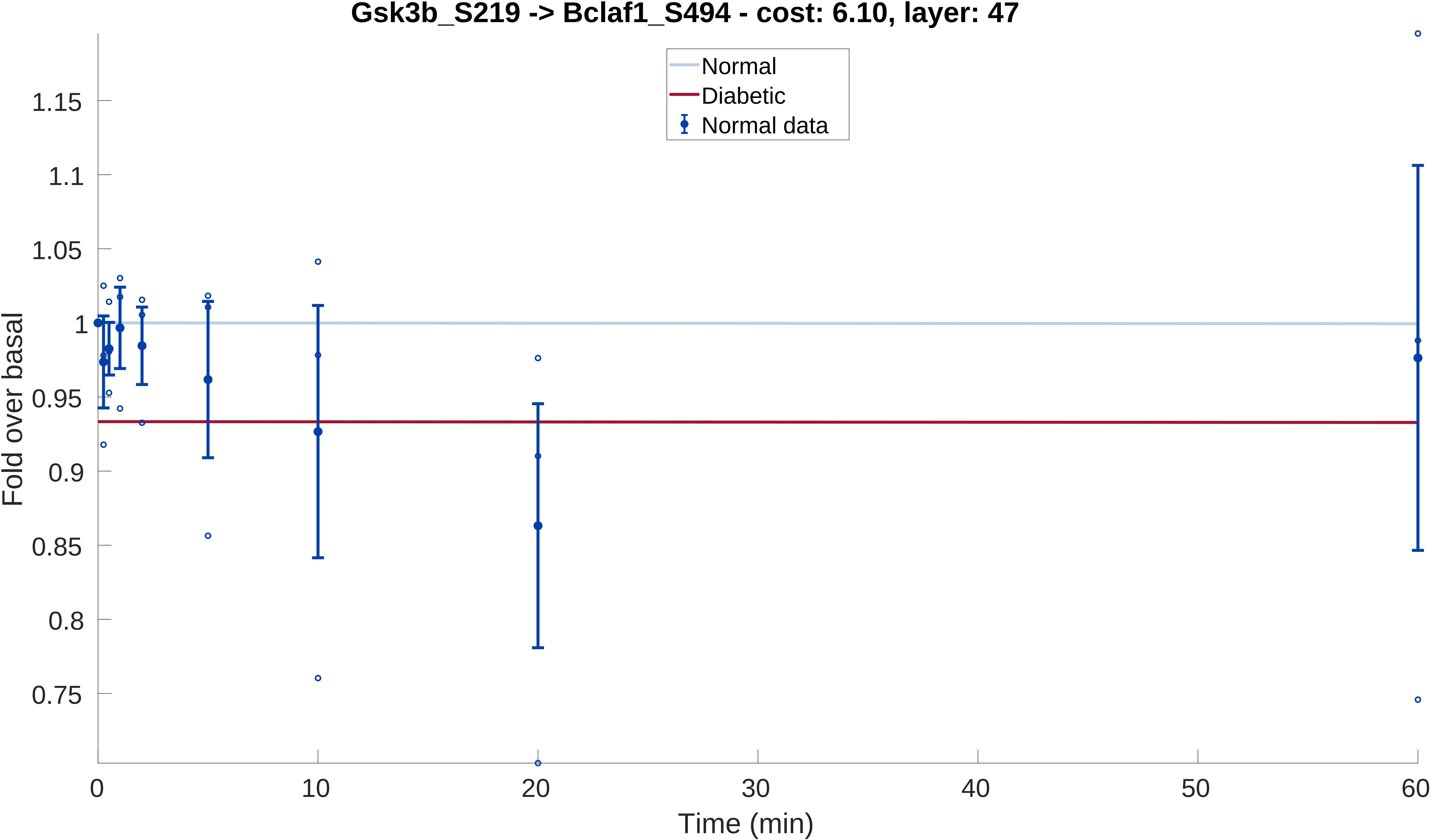

Gsk3b_S219 -> Bclaf1_S494 - cost: 6.10, layer: 47
Normal
Diabetic
Normal data
Fold over basal
1.15
1.1
1.05
1
0.95
0.9
0.85
0.8
0.75
0
10
20
30
40
50
60
Time (min)

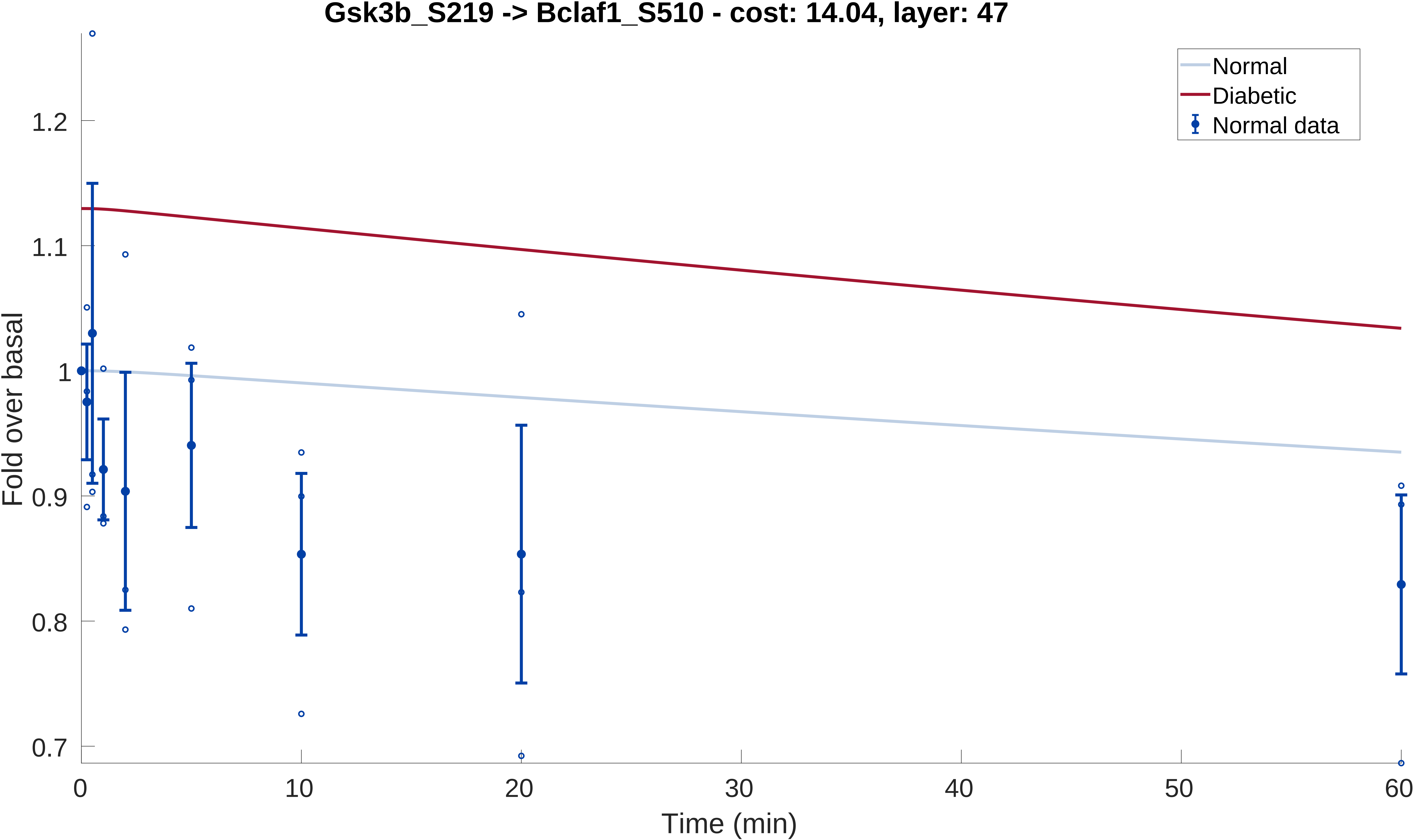

Gsk3b_S219 -> Bclaf1_S510 - cost: 14.04, layer: 47
Normal
Diabetic
Normal data
Fold over basal
1.2
1.1
1
0.9
0.8
0.7
0
10
20
30
40
50
60
Time (min)

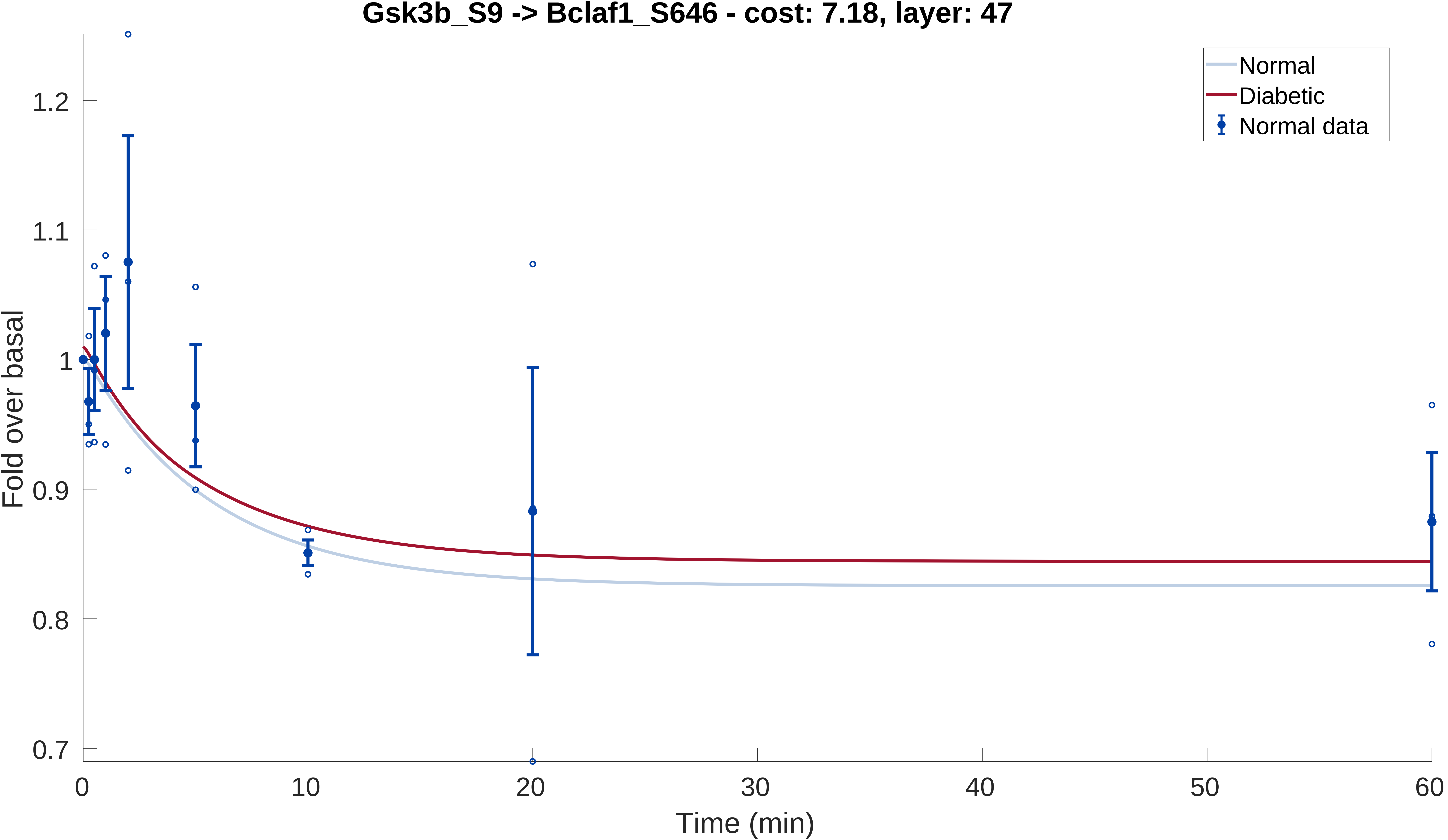
Gsk3b_S9 -> Bclaf1_S646 - cost: 7.18, layer: 47
Normal
Diabetic
Normal data
1.2
1.1
1
0.9
0.8
0.7
Fold over basal
0
10
20
30
40
50
60
Time (min)

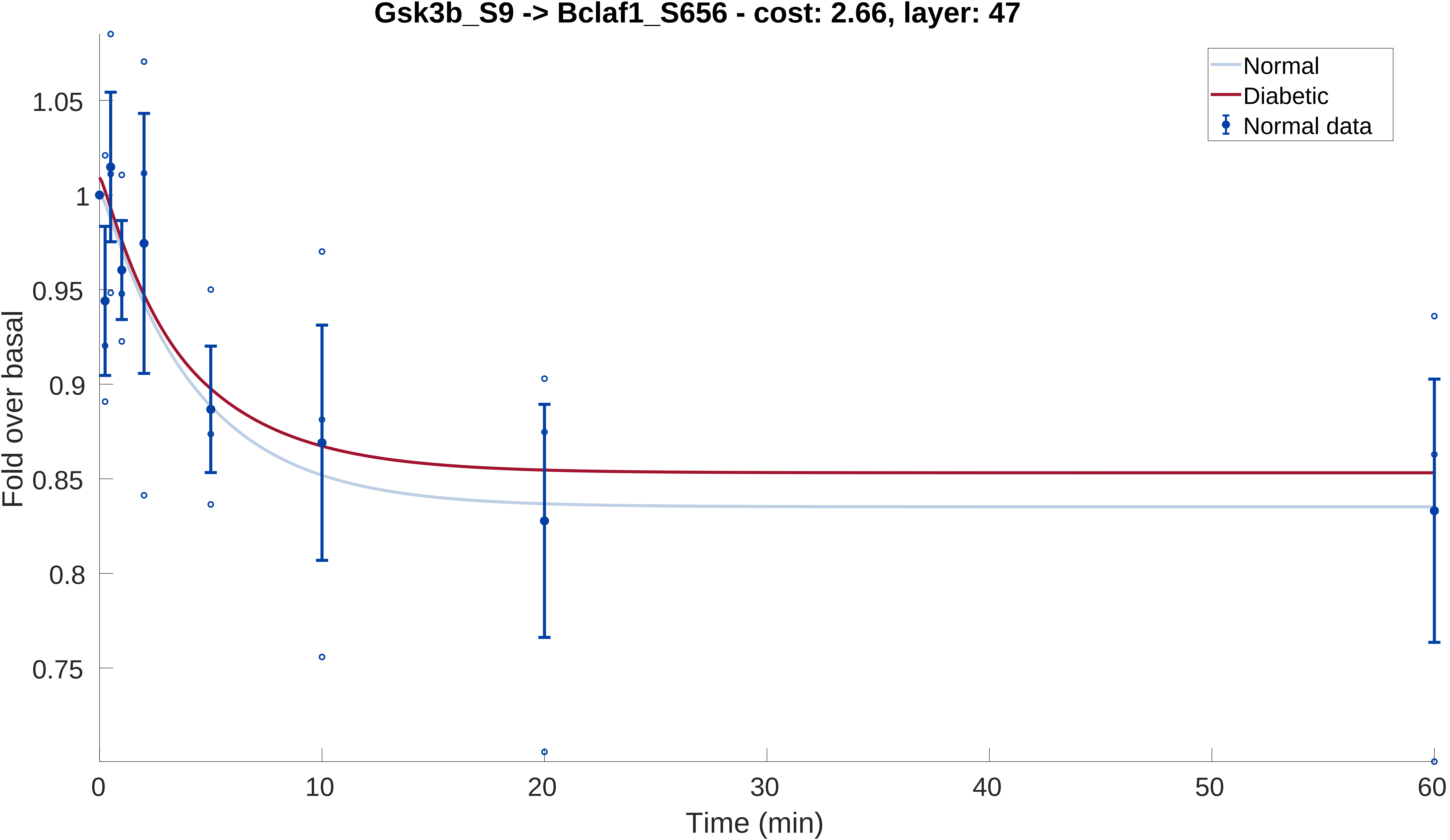

Gsk3b_S9 -> Bclaf1_S656 - cost: 2.66, layer: 47
Normal
Diabetic
Normal data
1.05
1
0.95
0.9
0.85
0.8
0.75
Fold over basal
0
10
20
30
40
50
60
Time (min)

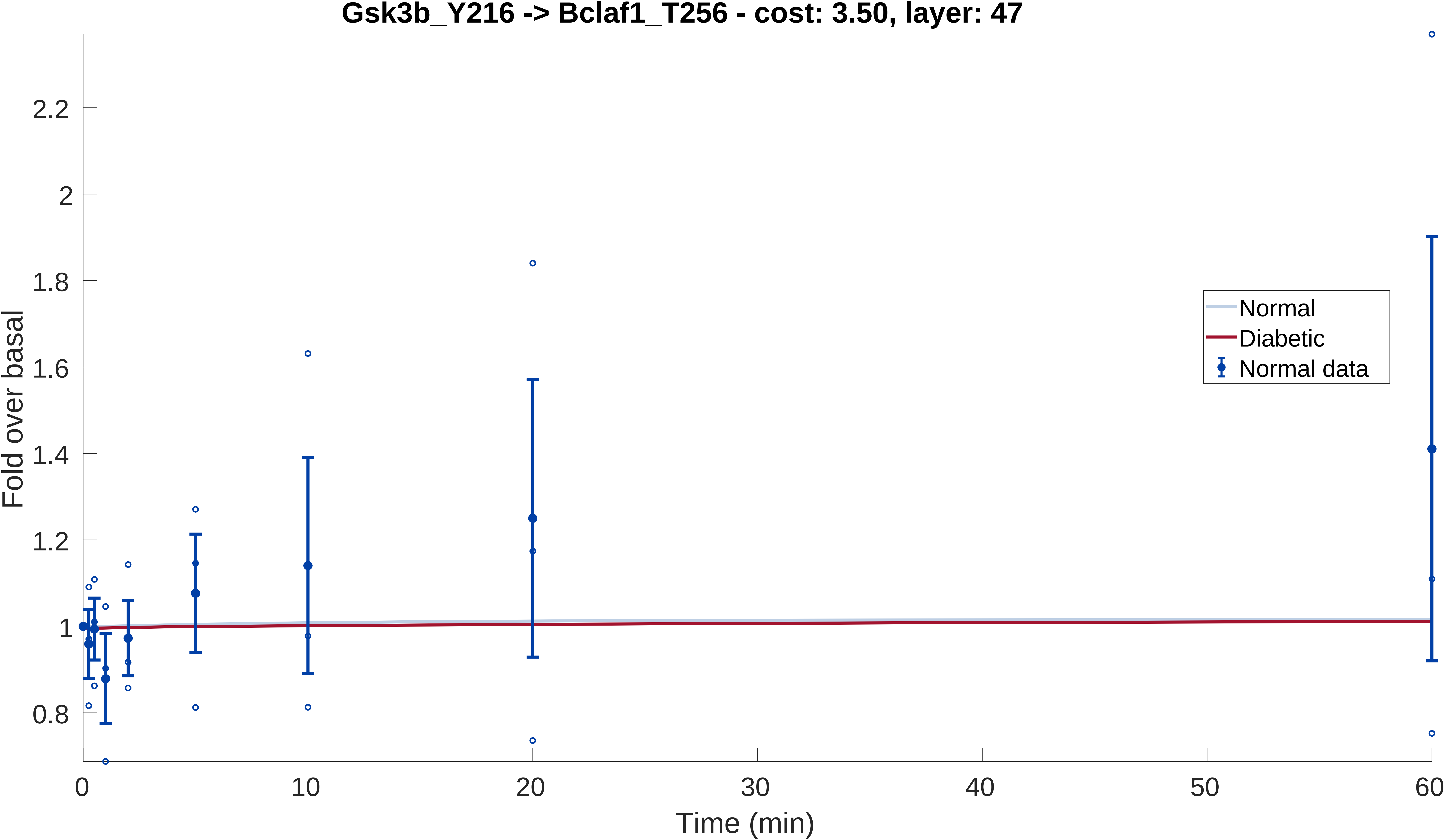

Gsk3b_Y216 -> Bclaf1_T256 - cost: 3.50, layer: 47
Fold over basal
2.2
2
1.8
1.6
1.4
1.2
1
0.8
0
10
20
30
40
50
60
Time (min)
Normal
Diabetic
Normal data

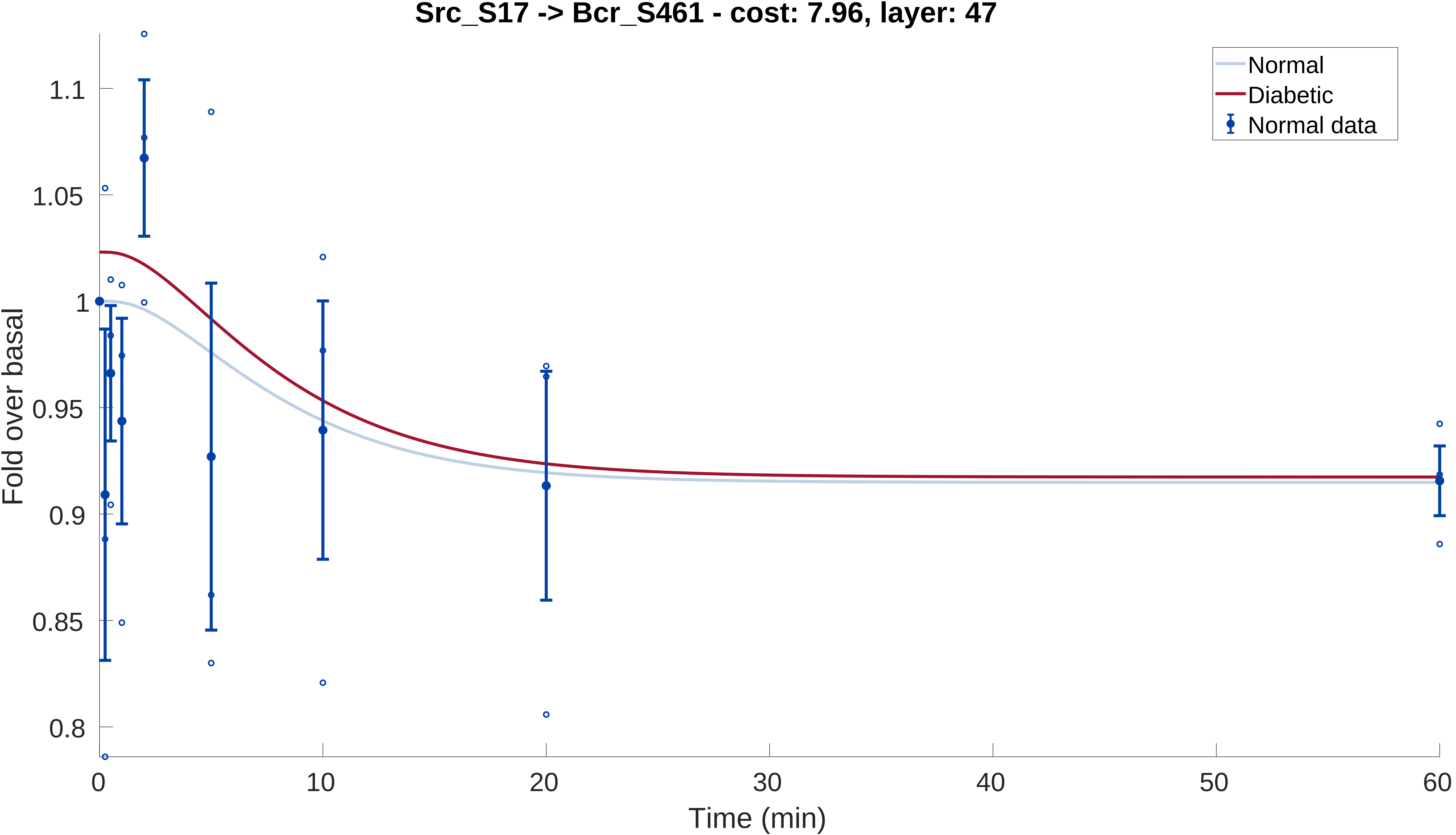

Src_S17 -> Bcr_S461 - cost: 7.96, layer: 47
Normal
Diabetic
Normal data
Fold over basal
1.1
1.05
1
0.95
0.9
0.85
0.8
0
10
20
30
40
50
60
Time (min)

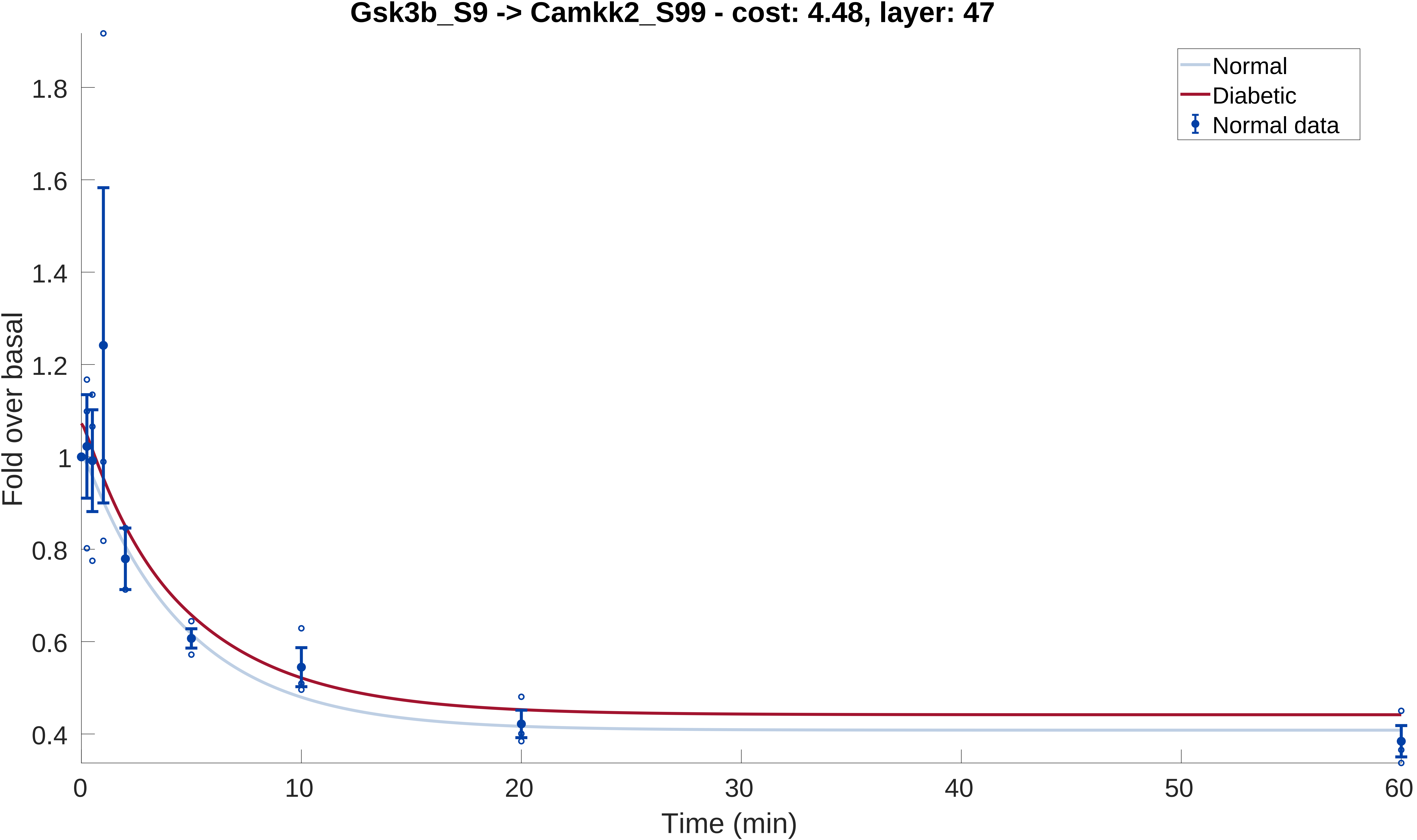

Gsk3b_S9 -> Camkk2_S99 - cost: 4.48, layer: 47
Normal
Diabetic
Normal data
Fold over basal
1.8
1.6
1.4
1.2
1
0.8
0.6
0.4
0
10
20
30
40
50
60
Time (min)

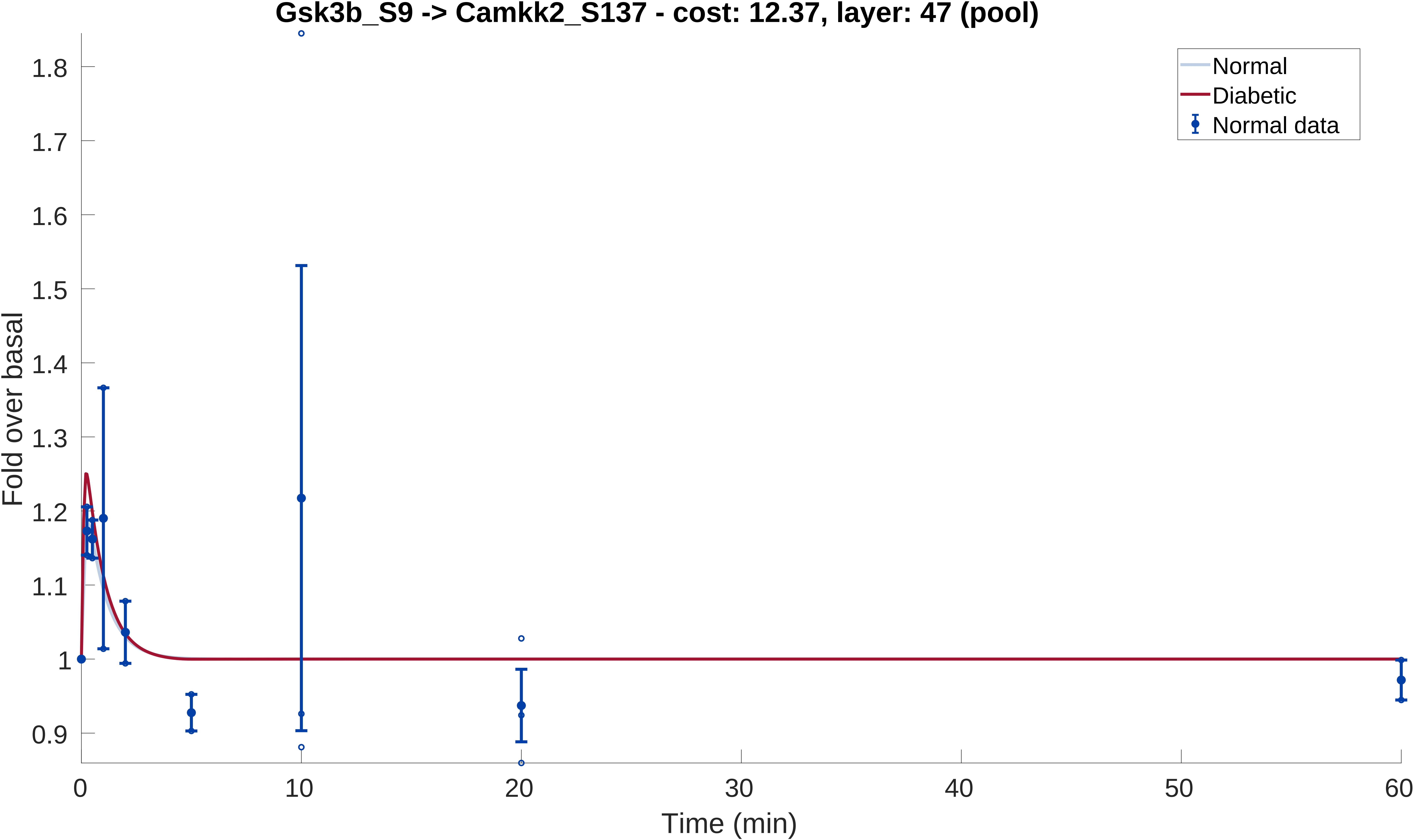

Gsk3b_S9 -> Camkk2_S137 - cost: 12.37, layer: 47 (pool)
Normal
Diabetic
Normal data
Fold over basal
1.8
1.7
1.6
1.5
1.4
1.3
1.2
1.1
1
0.9
0
10
20
30
40
50
60
Time (min)

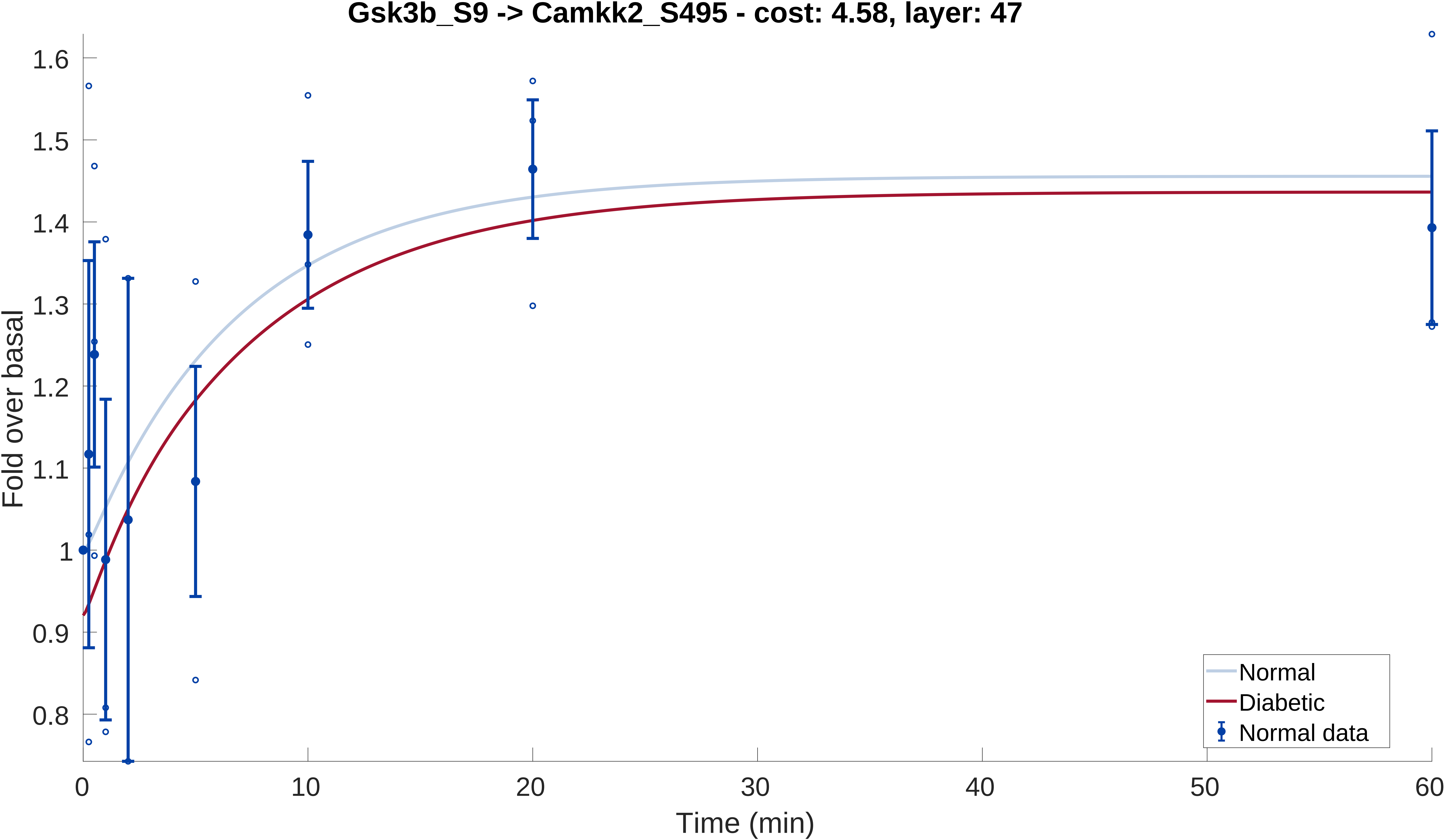

Gsk3b_S9 -> Camkk2_S495 - cost: 4.58, layer: 47
Fold over basal
Time (min)
0
10
20
30
40
50
60
0.8
0.9
1
1.1
1.2
1.3
1.4
1.5
1.6
Normal
Diabetic
Normal data

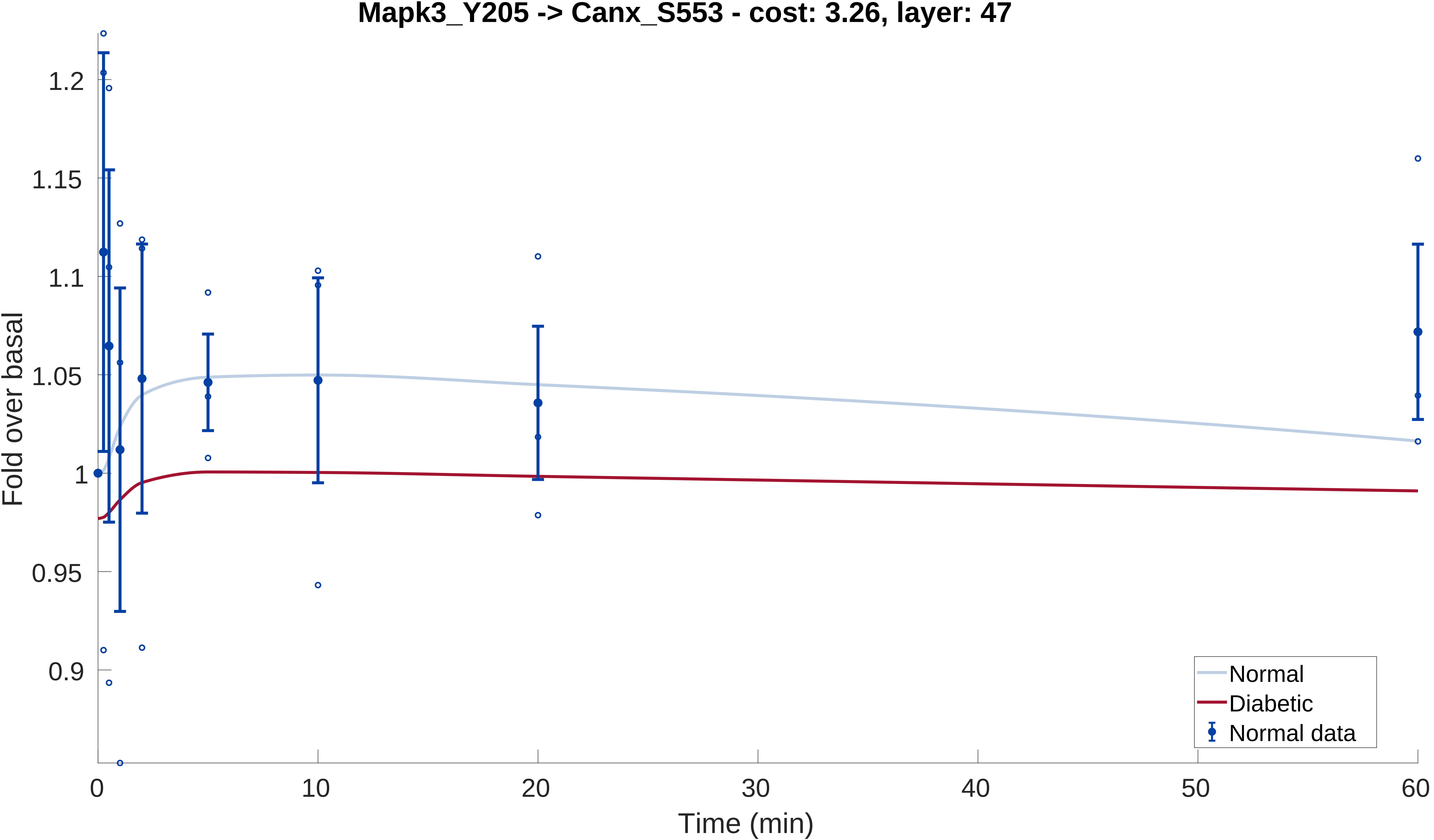

Mapk3_Y205 -> Canx_S553 - cost: 3.26, layer: 47
Fold over basal
1.2
1.15
1.1
1.05
1
0.95
0.9
0
10
20
30
40
50
60
Time (min)
Normal
Diabetic
Normal data

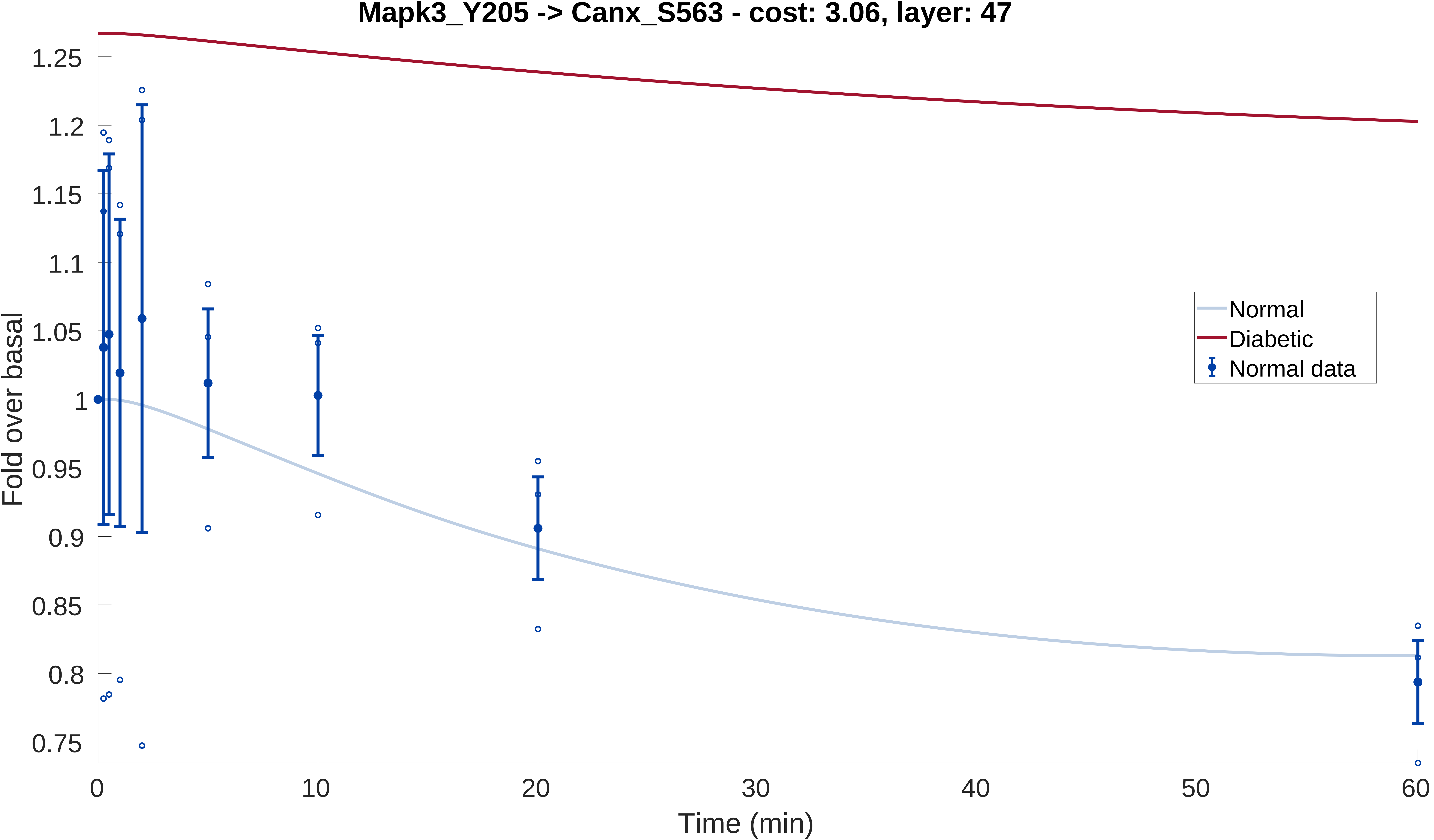

Mapk3_Y205 -> Canx_S563 - cost: 3.06, layer: 47
Fold over basal
1.25
1.2
1.15
1.1
1.05
1
0.95
0.9
0.85
0.8
0.75
0
10
20
30
40
50
60
Time (min)
Normal
Diabetic
Normal data

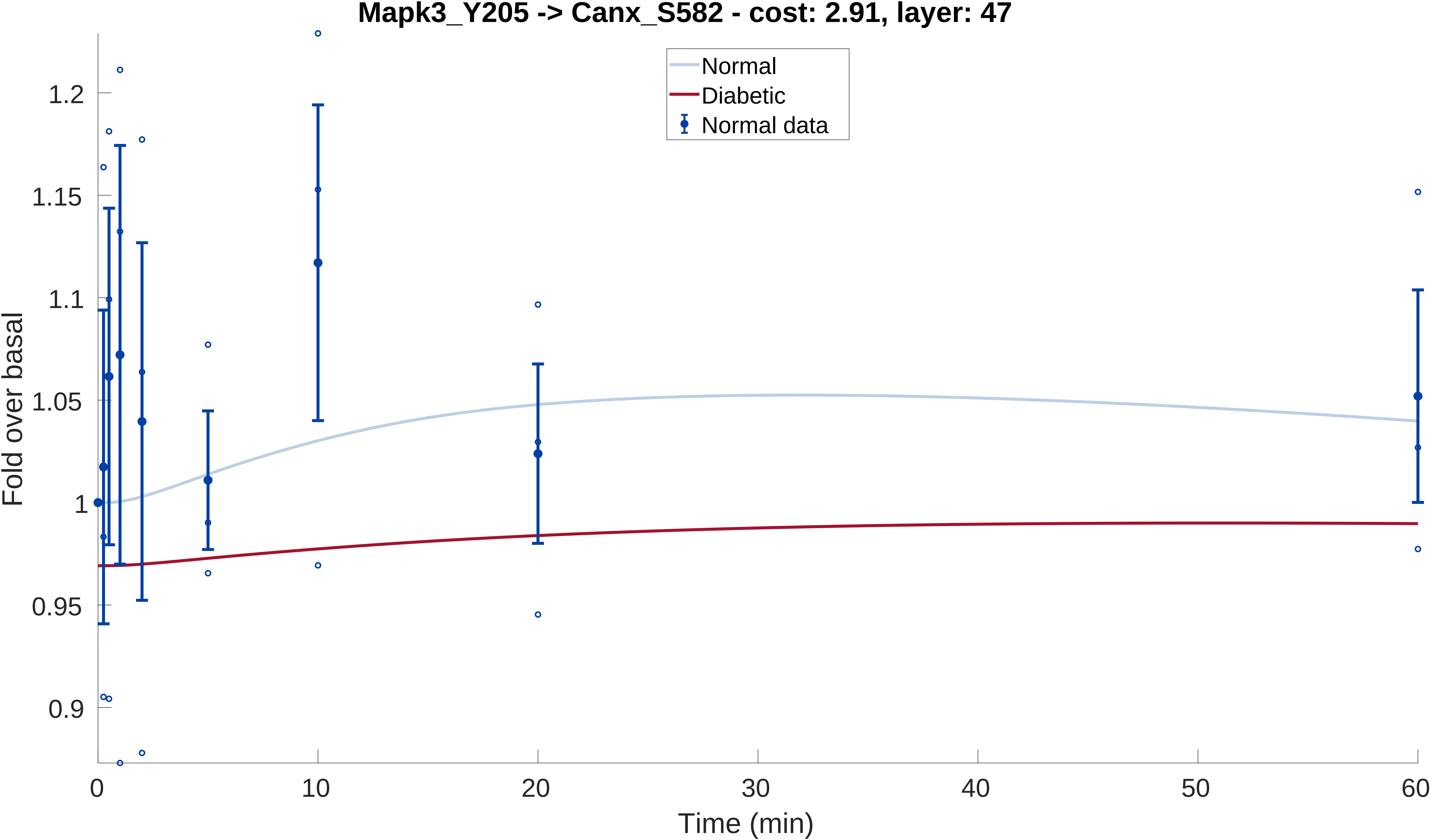

Mapk3_Y205 -> Canx_S582 - cost: 2.91, layer: 47
Normal
Diabetic
Normal data
Fold over basal
1.2
1.15
1.1
1.05
1
0.95
0.9
0
10
20
30
40
50
60
Time (min)

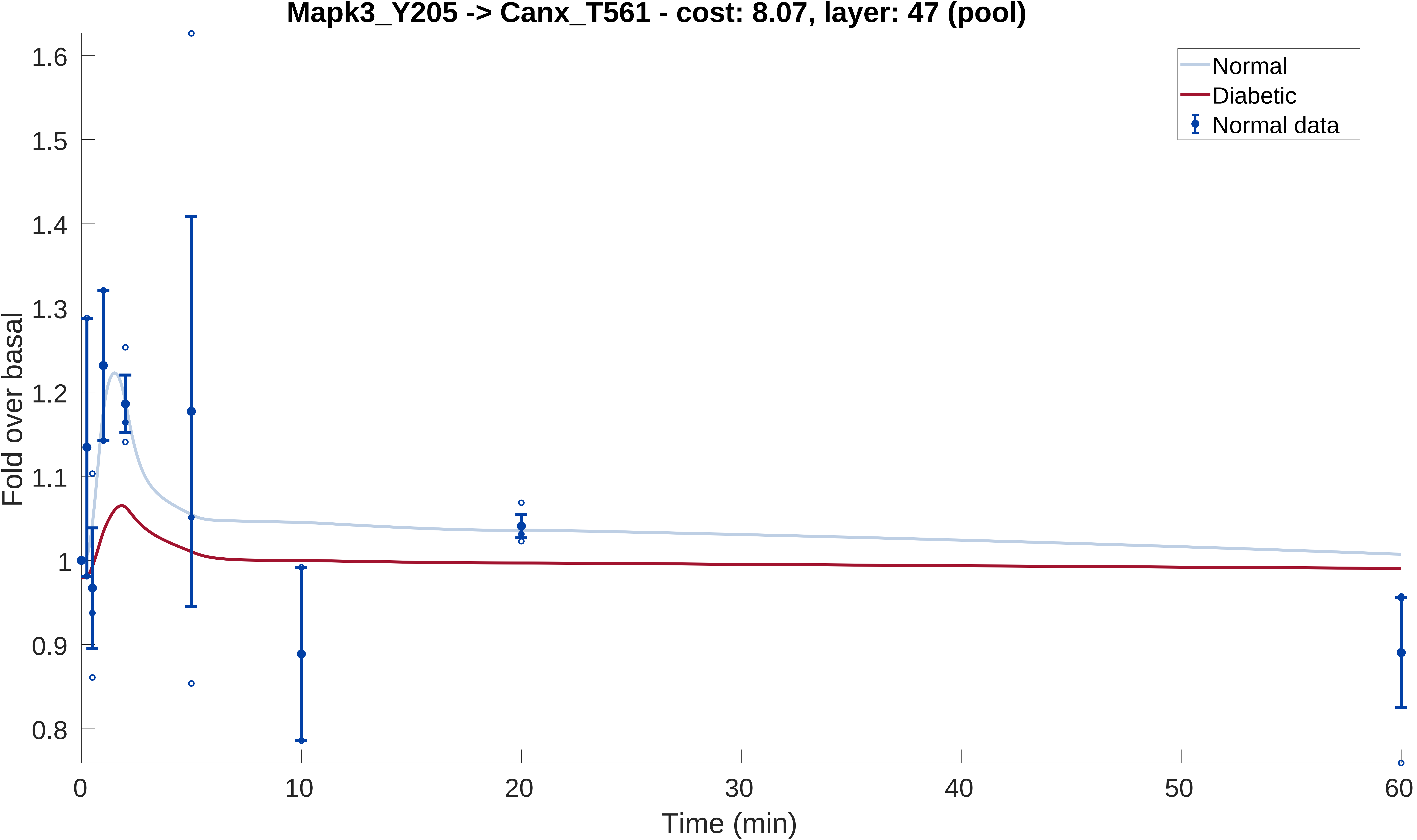

Mapk3_Y205 -> Canx_T561 - cost: 8.07, layer: 47 (pool)
Normal
Diabetic
Normal data
Fold over basal
Time (min)
0.8
0.9
1
1.1
1.2
1.3
1.4
1.5
1.6
0
10
20
30
40
50
60

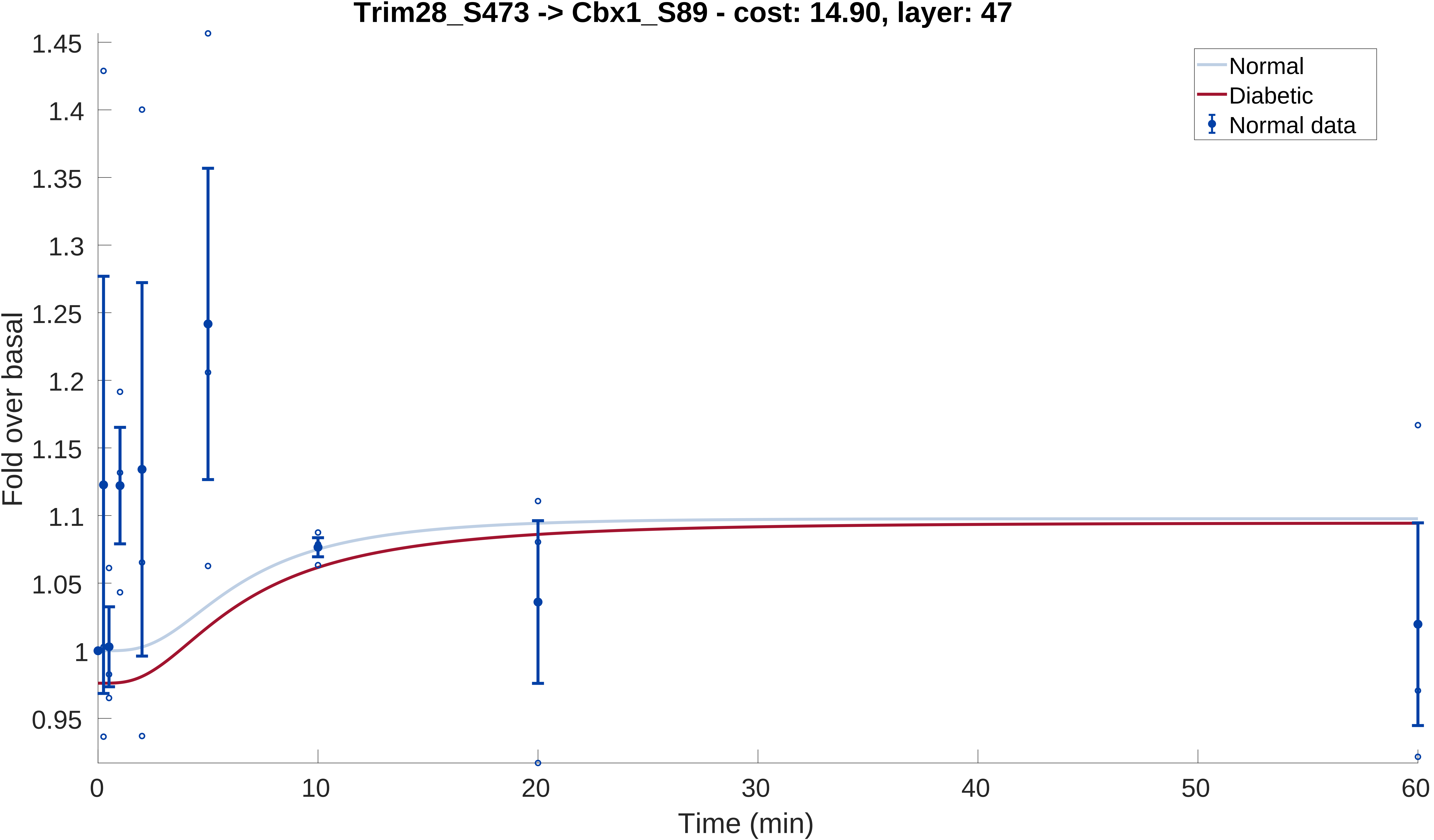

Trim28_S473 -> Cbx1_S89 - cost: 14.90, layer: 47
Normal
Diabetic
Normal data
Fold over basal
Time (min)
1.45
1.4
1.35
1.3
1.25
1.2
1.15
1.1
1.05
1
0.95
0
10
20
30
40
50
60

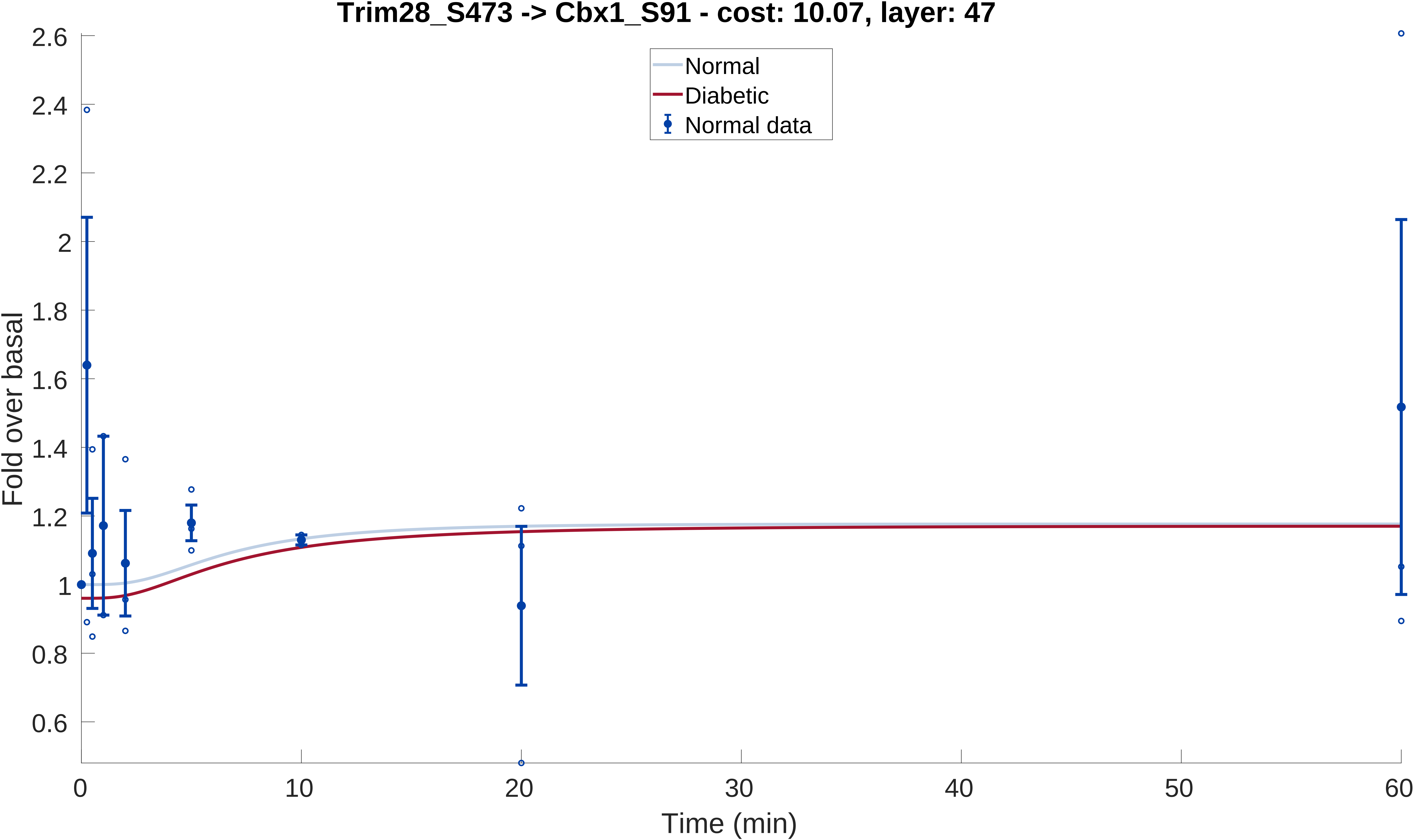

Trim28_S473 -> Cbx1_S91 - cost: 10.07, layer: 47
Normal
Diabetic
Normal data
Fold over basal
Time (min)
0.6
0.8
1
1.2
1.4
1.6
1.8
2
2.2
2.4
2.6
0
10
20
30
40
50
60

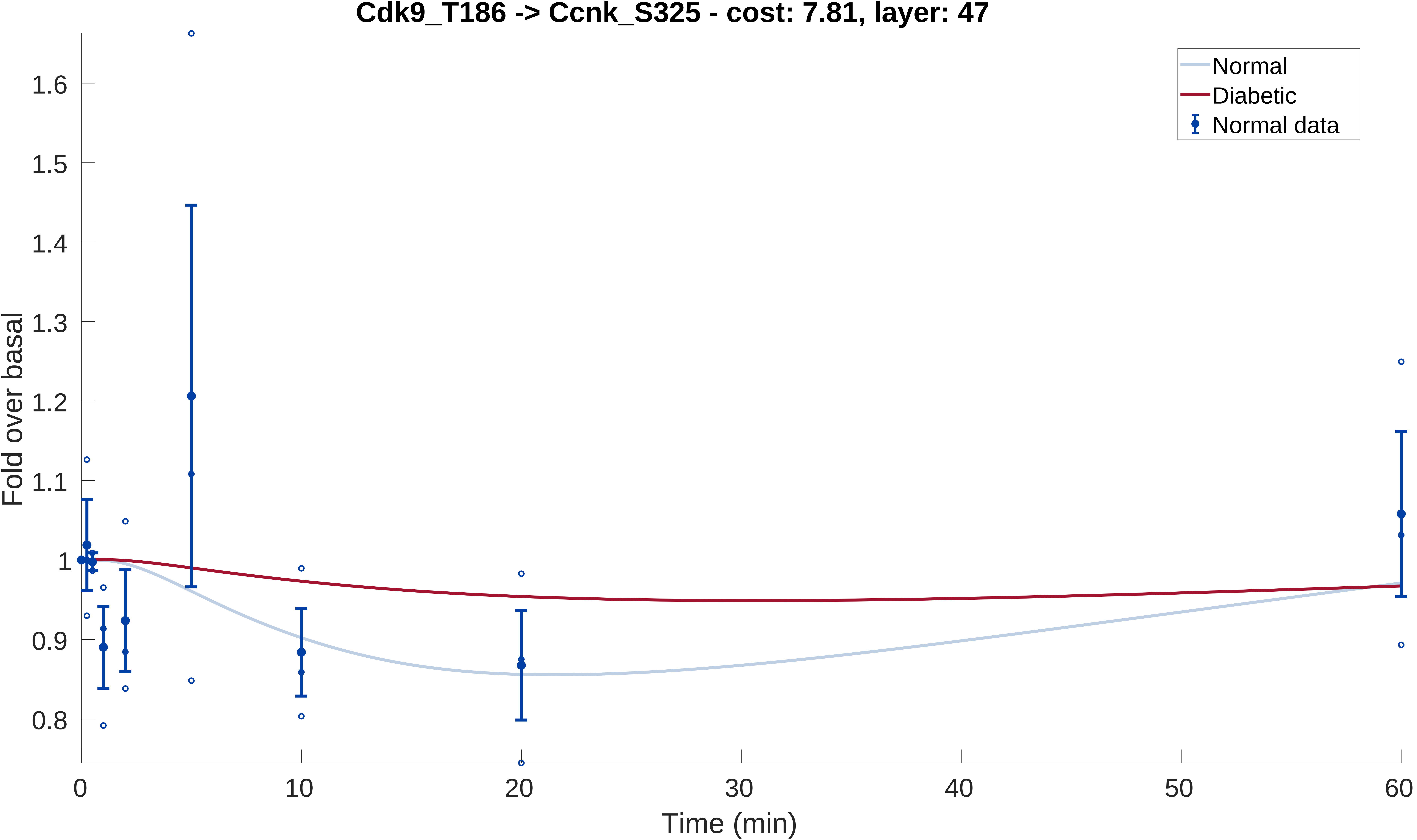
Cdk9_T186 -> Ccnk_S325 - cost: 7.81, layer: 47
Normal
Diabetic
Normal data
Fold over basal
1.6
1.5
1.4
1.3
1.2
1.1
1
0.9
0.8
0
10
20
30
40
50
60
Time (min)

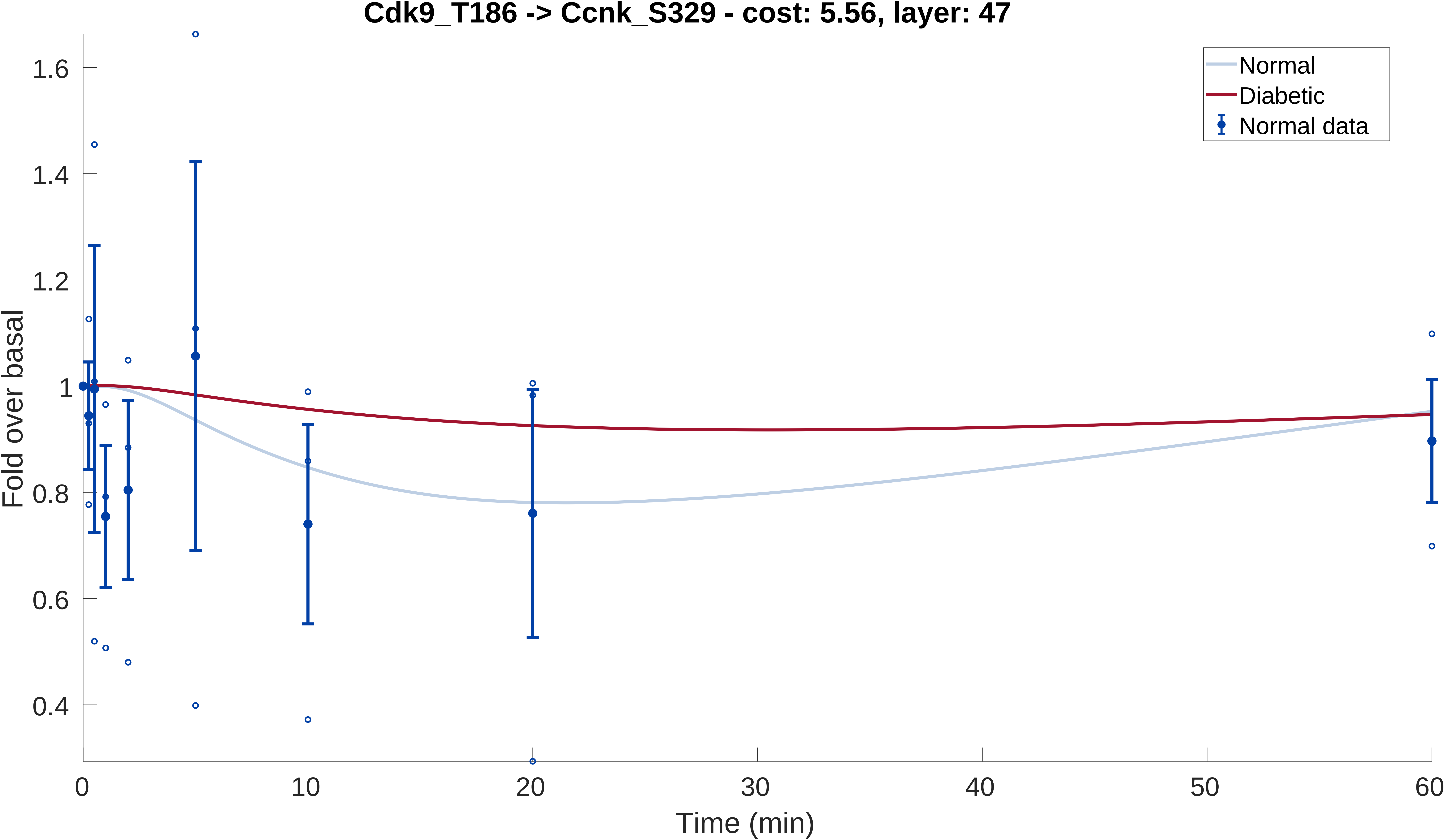

Cdk9_T186 -> Ccnk_S329 - cost: 5.56, layer: 47
Normal
Diabetic
Normal data
Fold over basal
Time (min)
1.6
1.4
1.2
1
0.8
0.6
0.4
0
10
20
30
40
50
60

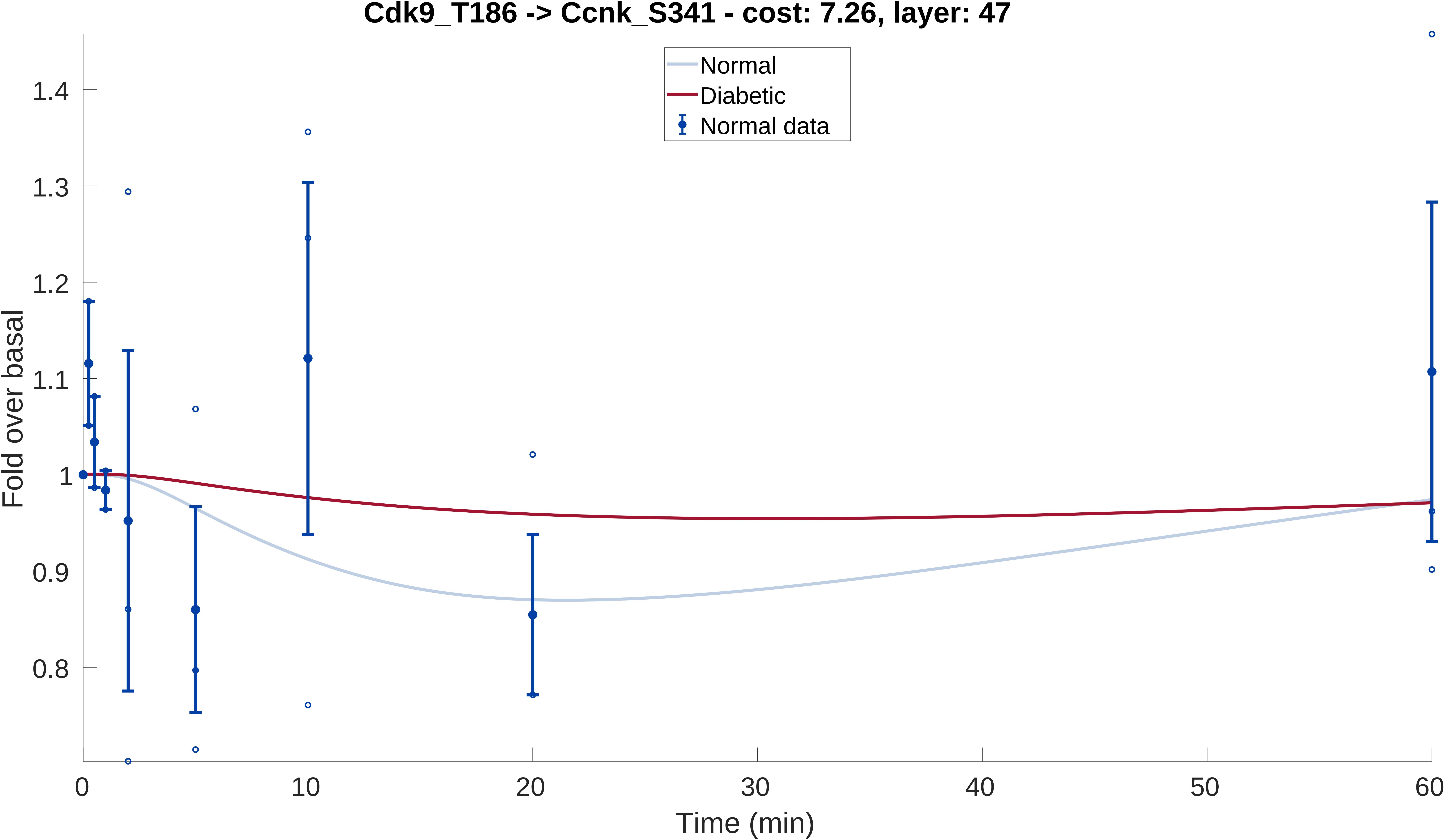

Cdk9_T186 -> Ccnk_S341 - cost: 7.26, layer: 47
Normal
Diabetic
Normal data
Fold over basal
1.4
1.3
1.2
1.1
1
0.9
0.8
0
10
20
30
40
50
60
Time (min)

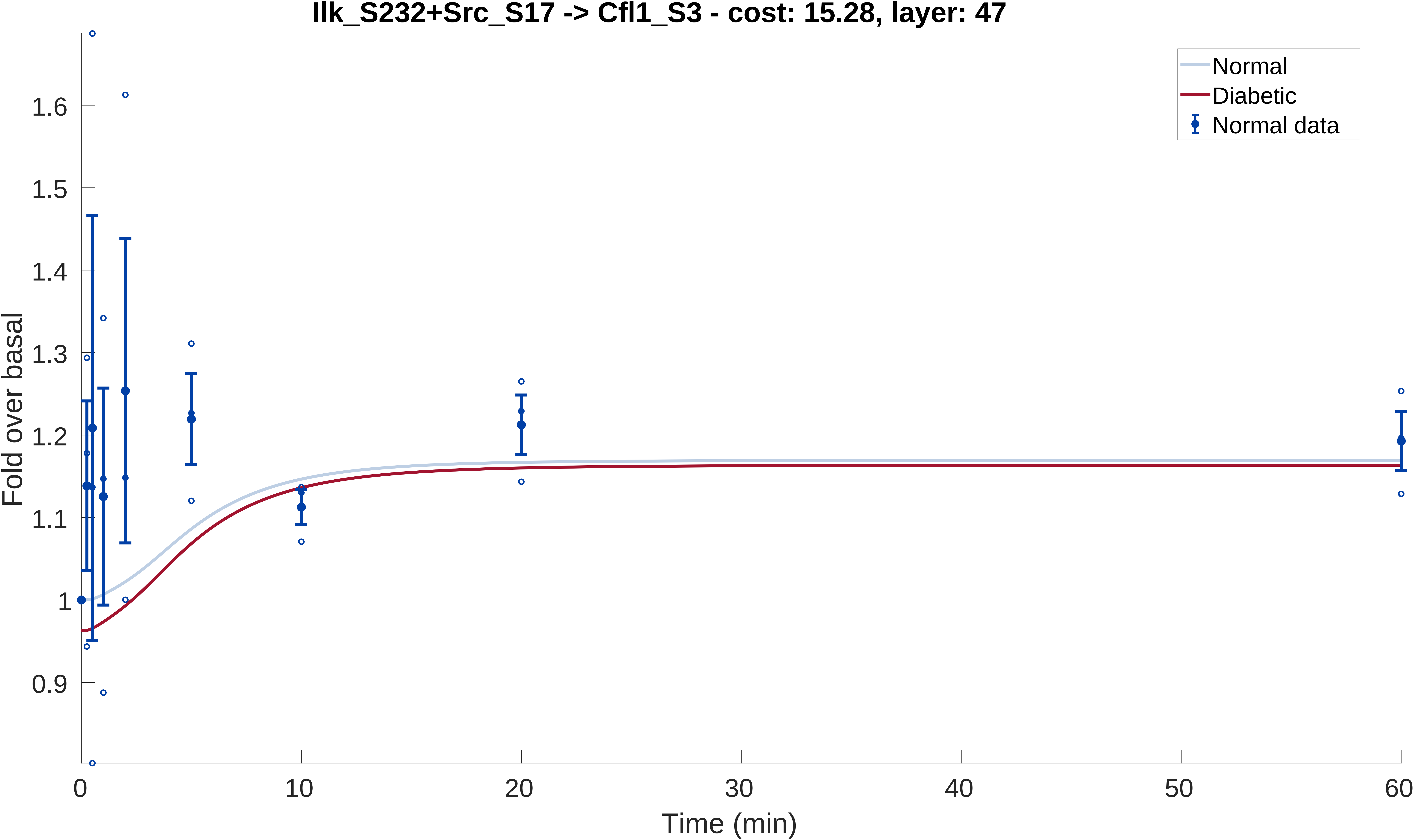

Ilk_S232+Src_S17 -> Cfl1_S3 - cost: 15.28, layer: 47
Normal
Diabetic
Normal data
Fold over basal
1.6
1.5
1.4
1.3
1.2
1.1
1
0.9
0
10
20
30
40
50
60
Time (min)

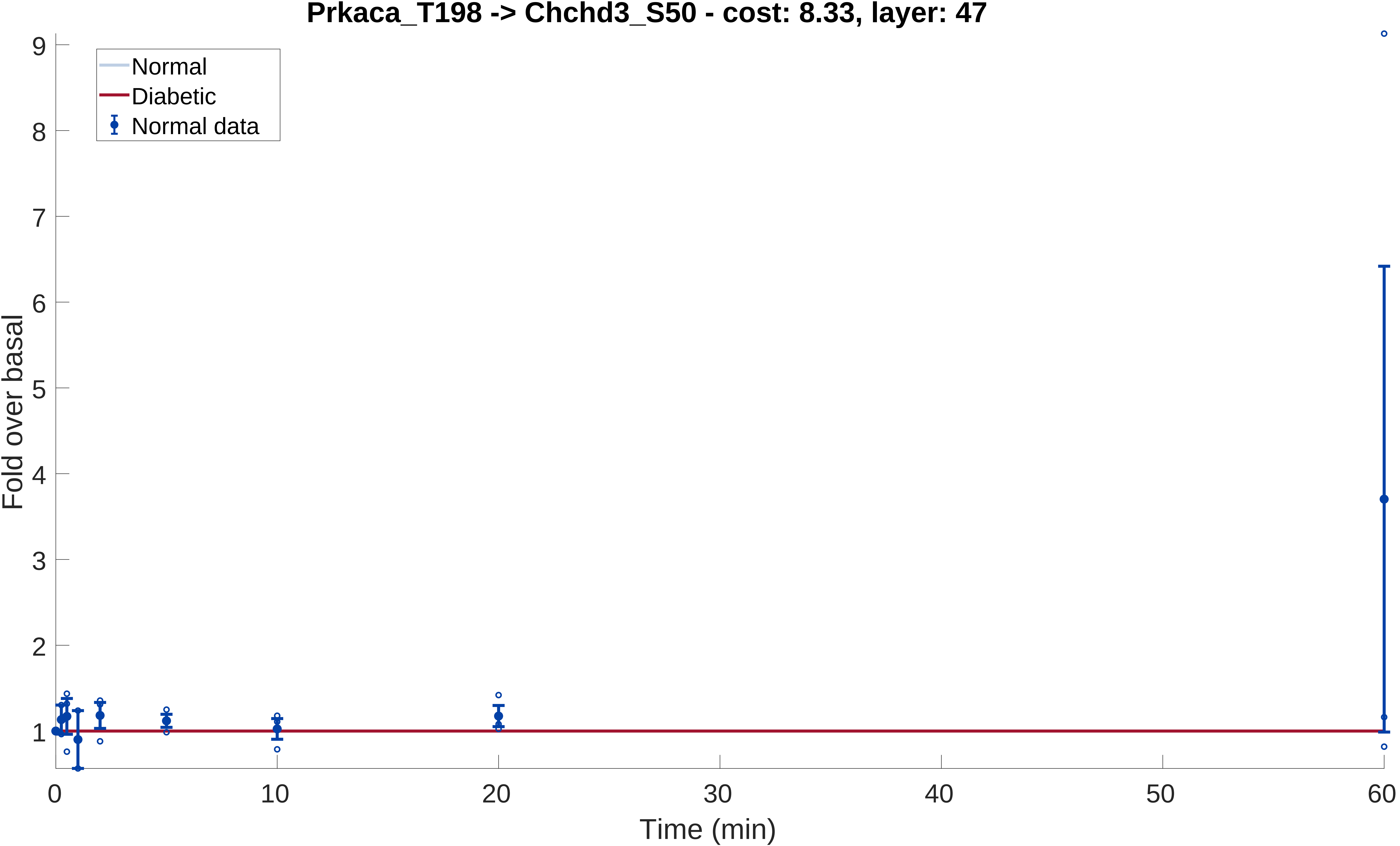
Prkaca_T198 -> Chchd3_S50 - cost: 8.33, layer: 47
Normal
Diabetic
Normal data
Fold over basal
Time (min)
1
2
3
4
5
6
7
8
9
0
10
20
30
40
50
60

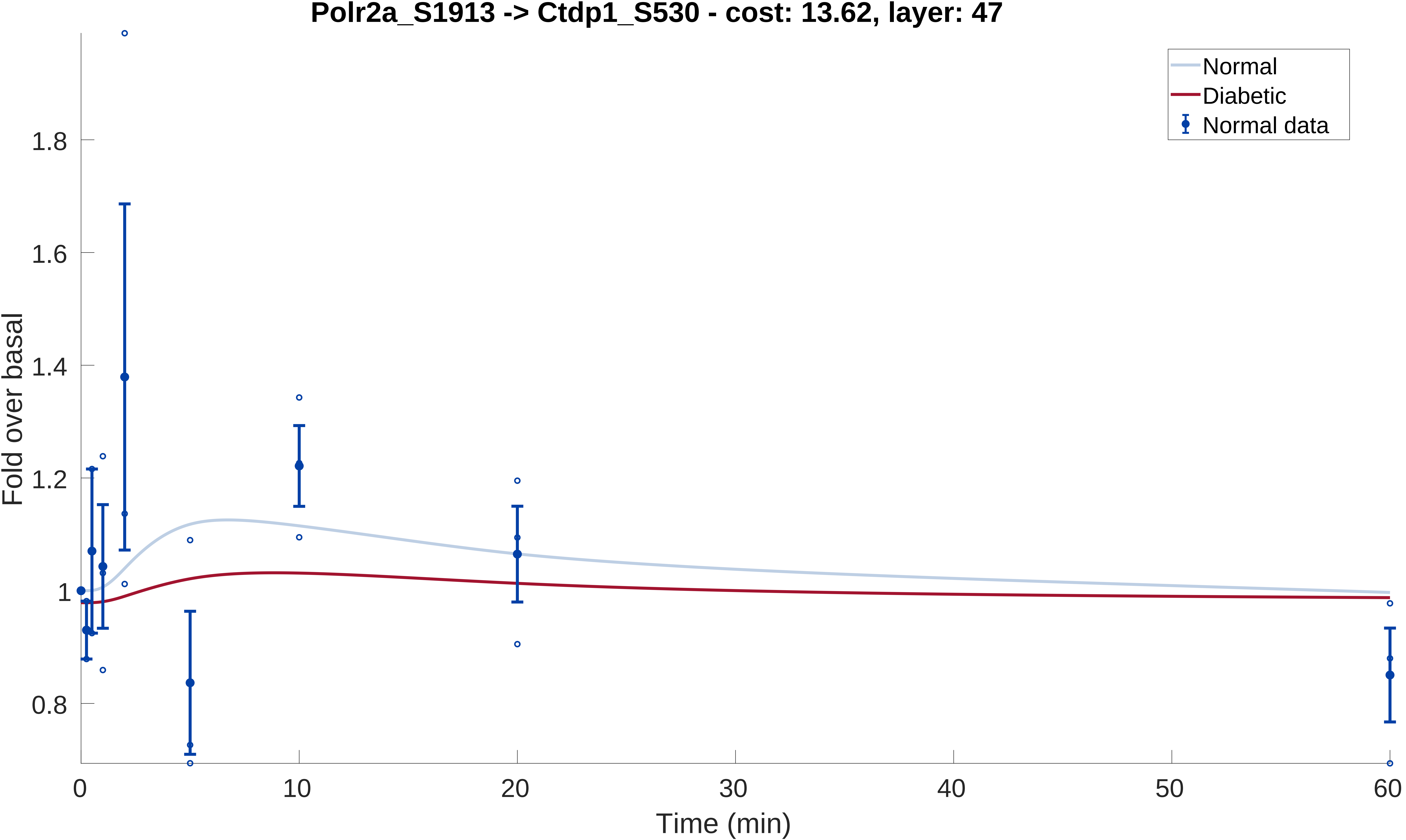
Polr2a_S1913 -> Ctdp1_S530 - cost: 13.62, layer: 47
Normal
Diabetic
Normal data
Fold over basal
1.8
1.6
1.4
1.2
1
0.8
0
10
20
30
40
50
60
Time (min)

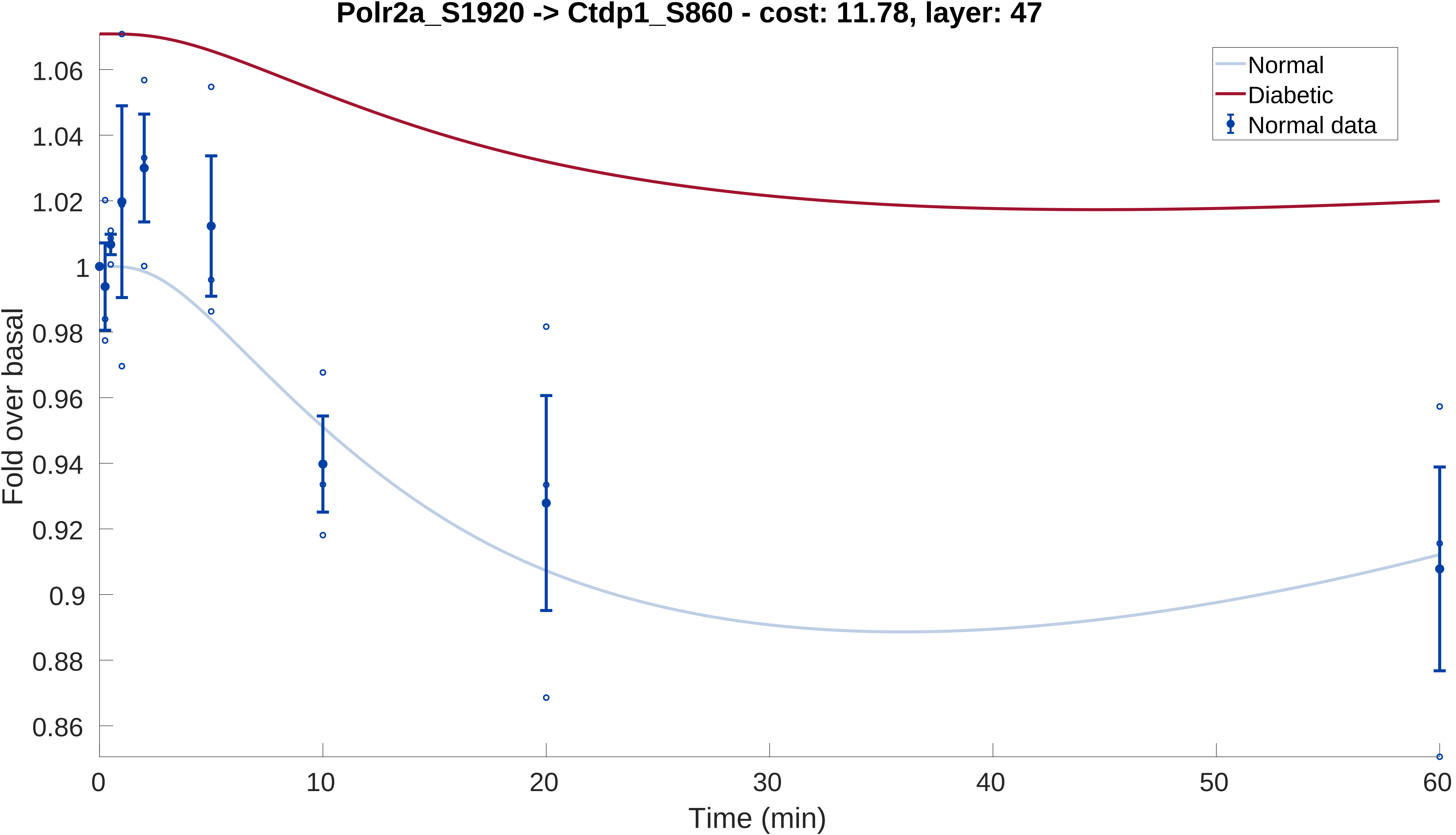
Polr2a_S1920 -> Ctdp1_S860 - cost: 11.78, layer: 47
Normal
Diabetic
Normal data
Fold over basal
Time (min)
1.06
1.04
1.02
1
0.98
0.96
0.94
0.92
0.9
0.88
0.86
0
10
20
30
40
50
60

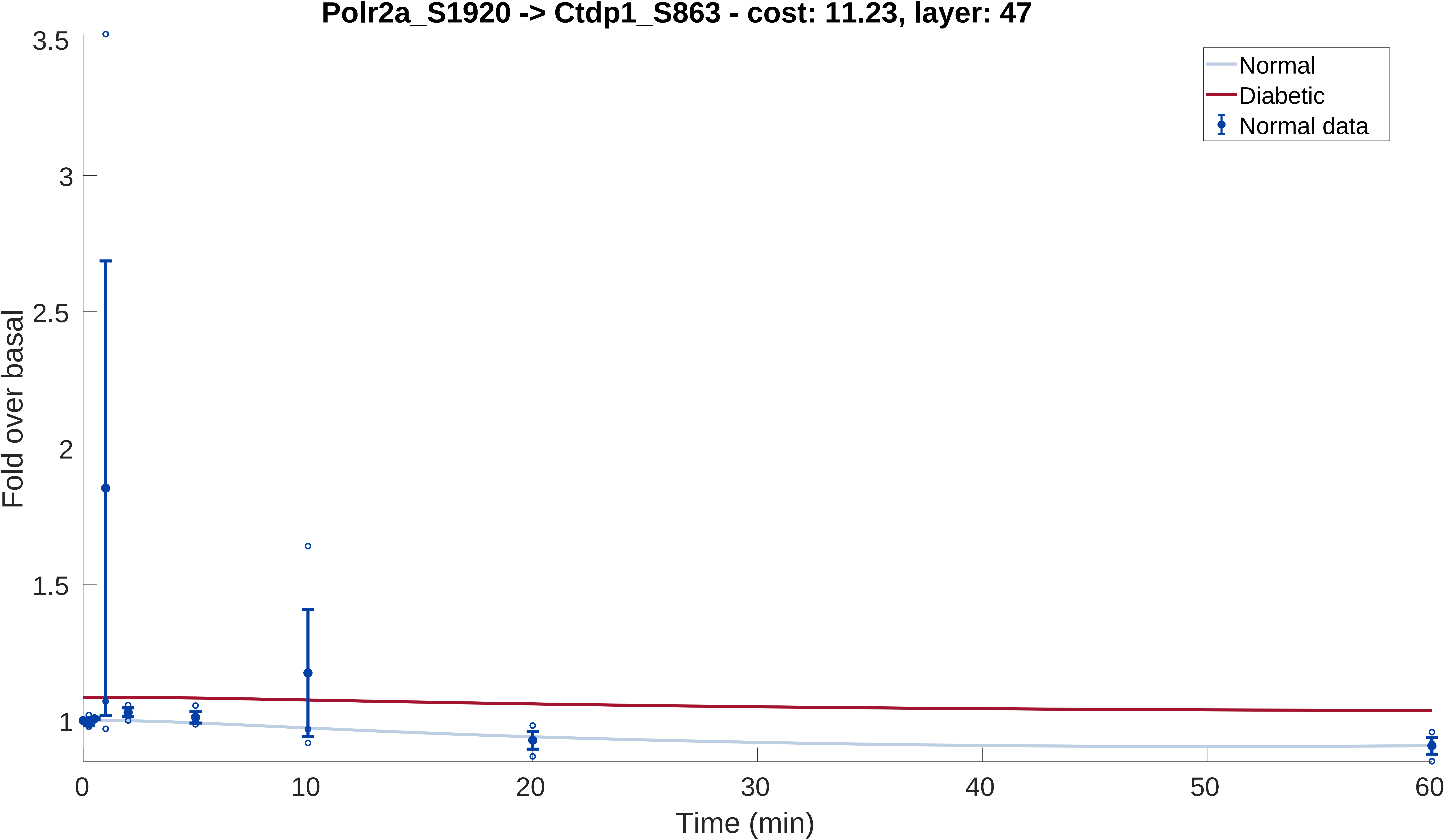
Polr2a_S1920 -> Ctdp1_S863 - cost: 11.23, layer: 47
Fold over basal
3.5
3
2.5
2
1.5
1
Time (min)
0
10
20
30
40
50
60
Normal
Diabetic
Normal data

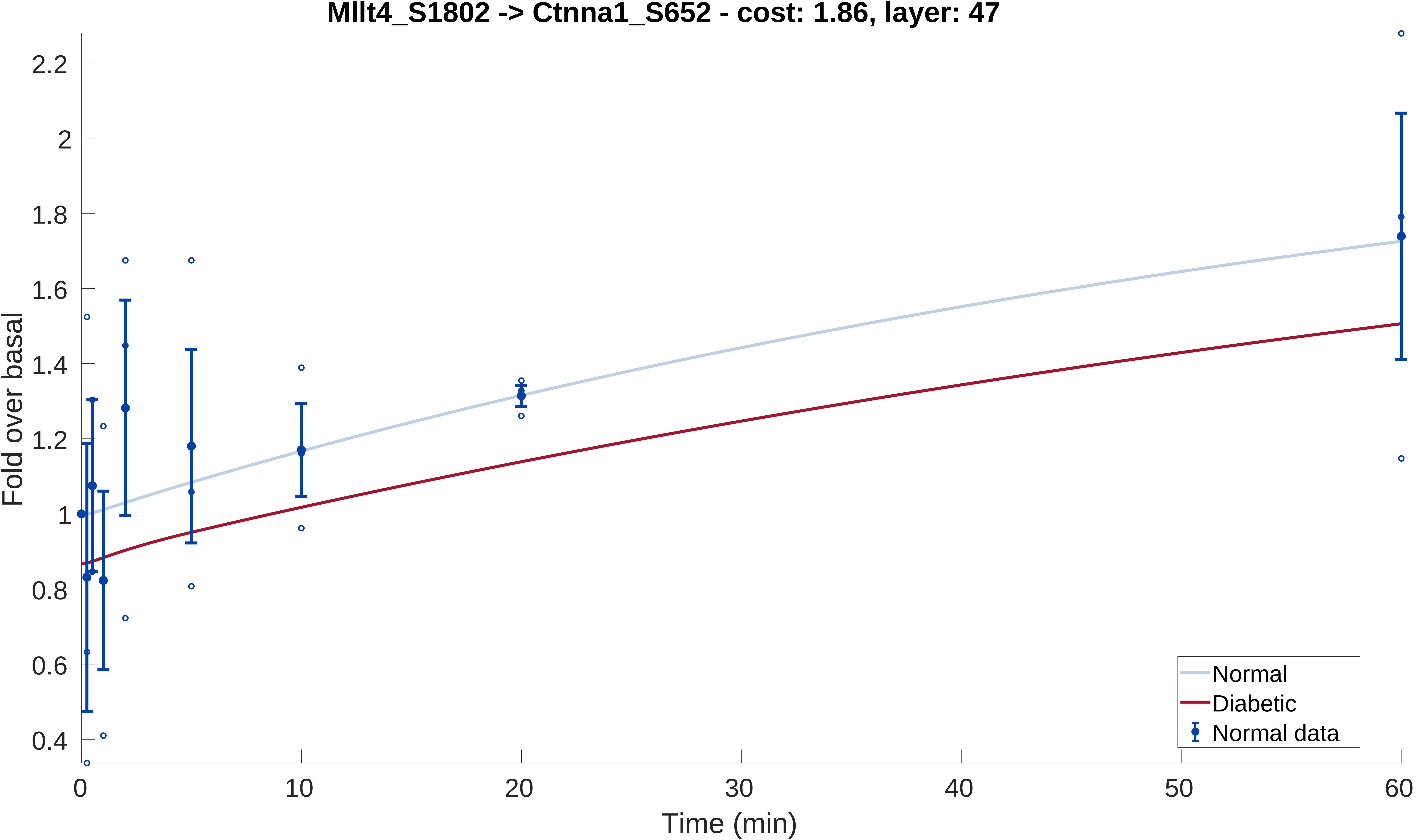
Mllt4_S1802 -> Ctnna1_S652 - cost: 1.86, layer: 47
Fold over basal
Time (min)
0.4
0.6
0.8
1
1.2
1.4
1.6
1.8
2
2.2
0
10
20
30
40
50
60
Normal
Diabetic
Normal data

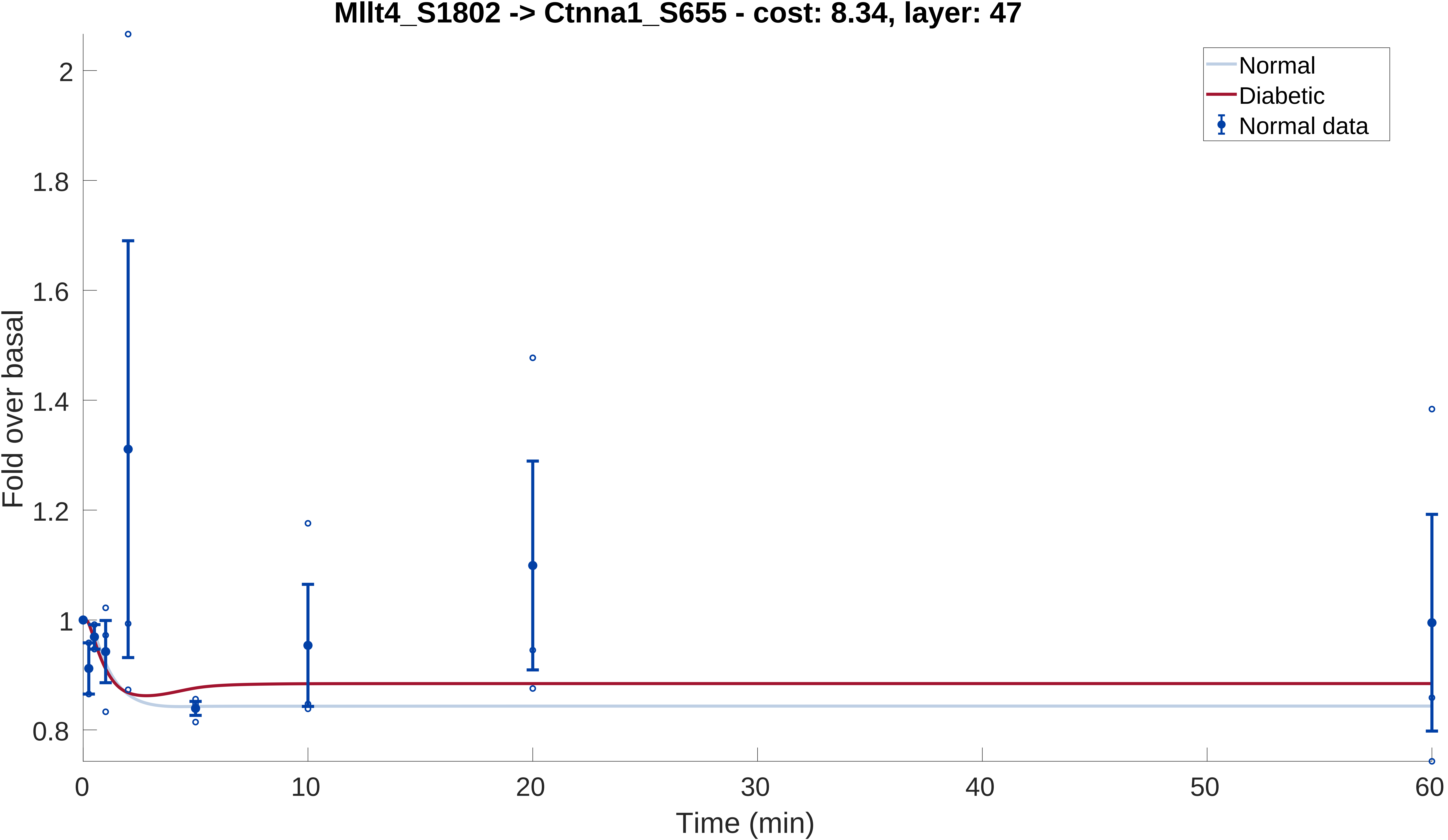

Mllt4_S1802 -> Ctnna1_S655 - cost: 8.34, layer: 47
Normal
Diabetic
Normal data
Fold over basal
2
1.8
1.6
1.4
1.2
1
0.8
0
10
20
30
40
50
60
Time (min)

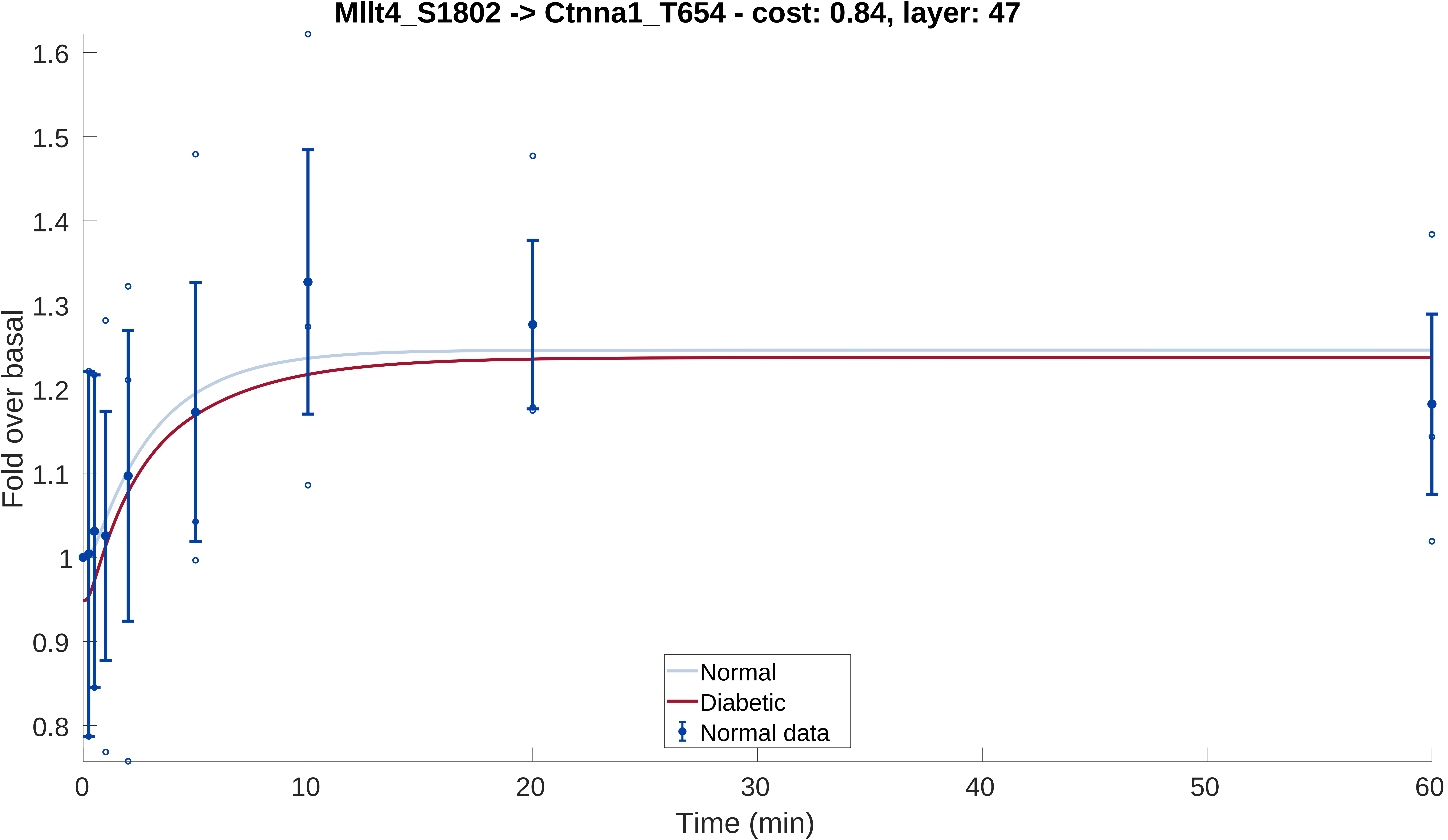

Mllt4_S1802 -> Ctnna1_T654 - cost: 0.84, layer: 47
Fold over basal
1.6
1.5
1.4
1.3
1.2
1.1
1
0.9
0.8
0
10
20
30
40
50
60
Time (min)
Normal
Diabetic
Normal data

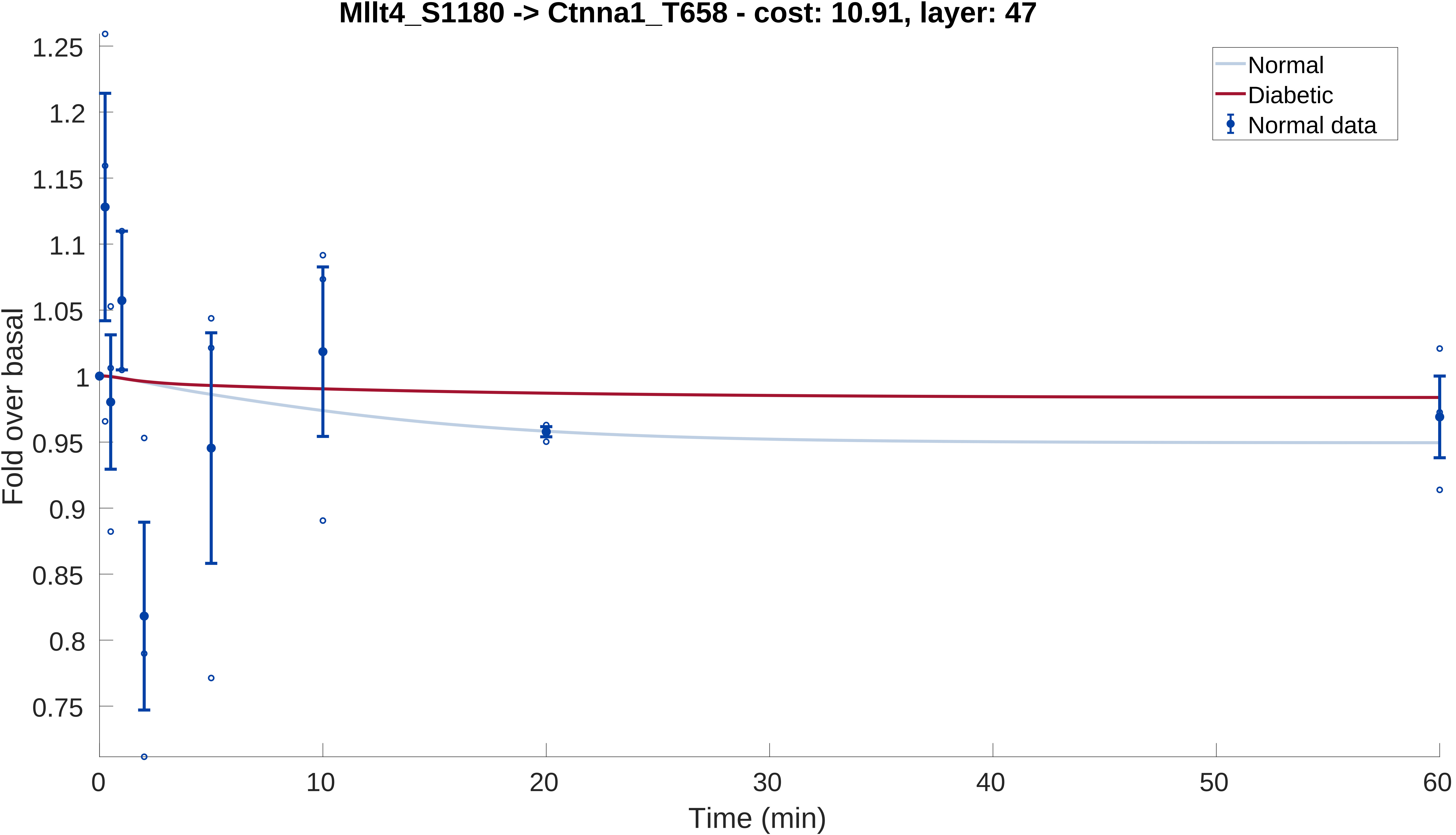
Mllt4_S1180 -> Ctnna1_T658 - cost: 10.91, layer: 47
Normal
Diabetic
Normal data
Fold over basal
Time (min)
1.25
1.2
1.15
1.1
1.05
1
0.95
0.9
0.85
0.8
0.75
0
10
20
30
40
50
60

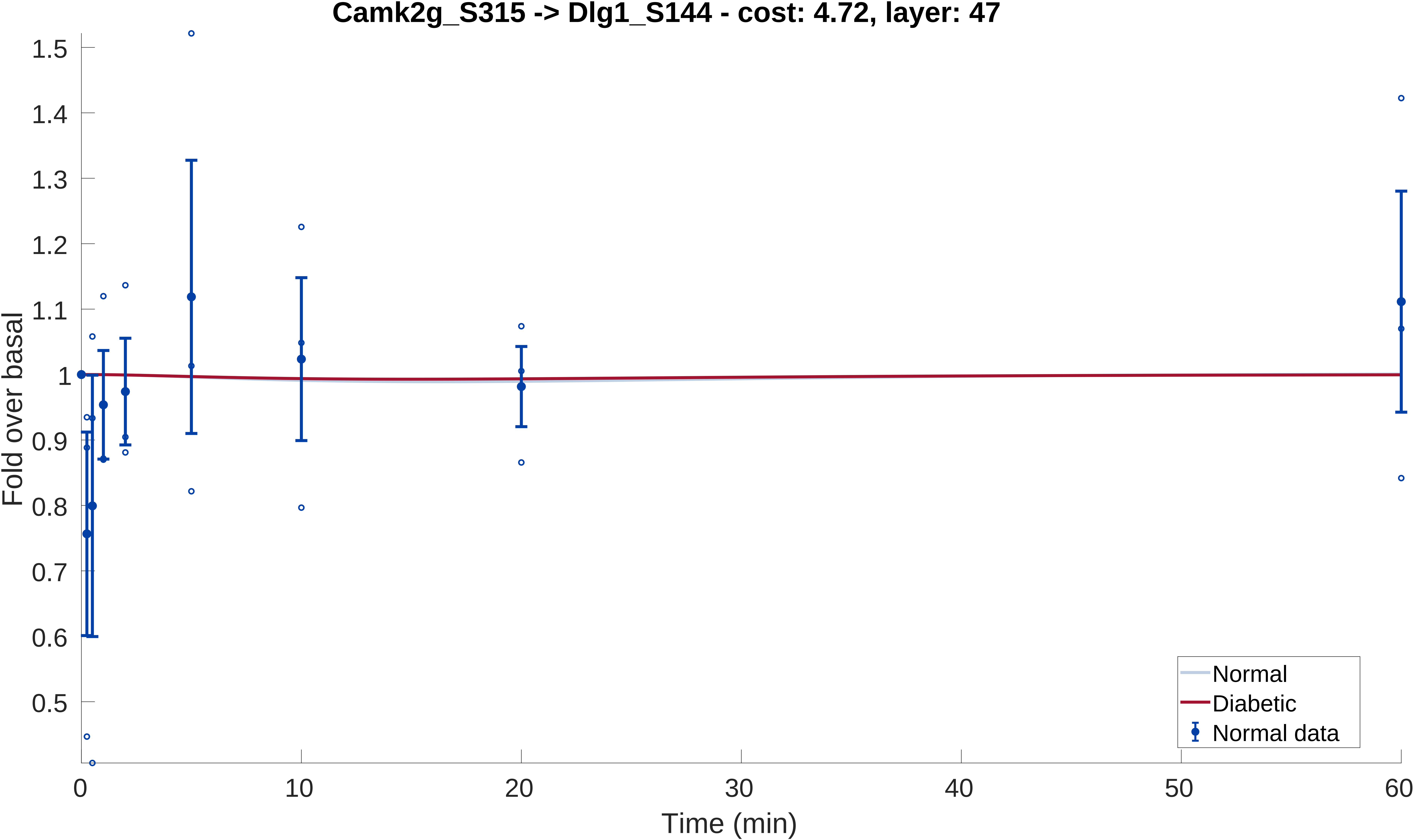

Camk2g_S315 -> Dlg1_S144 - cost: 4.72, layer: 47
Fold over basal
1.5
1.4
1.3
1.2
1.1
1
0.9
0.8
0.7
0.6
0.5
0
10
20
30
40
50
60
Time (min)
Normal
Diabetic
Normal data

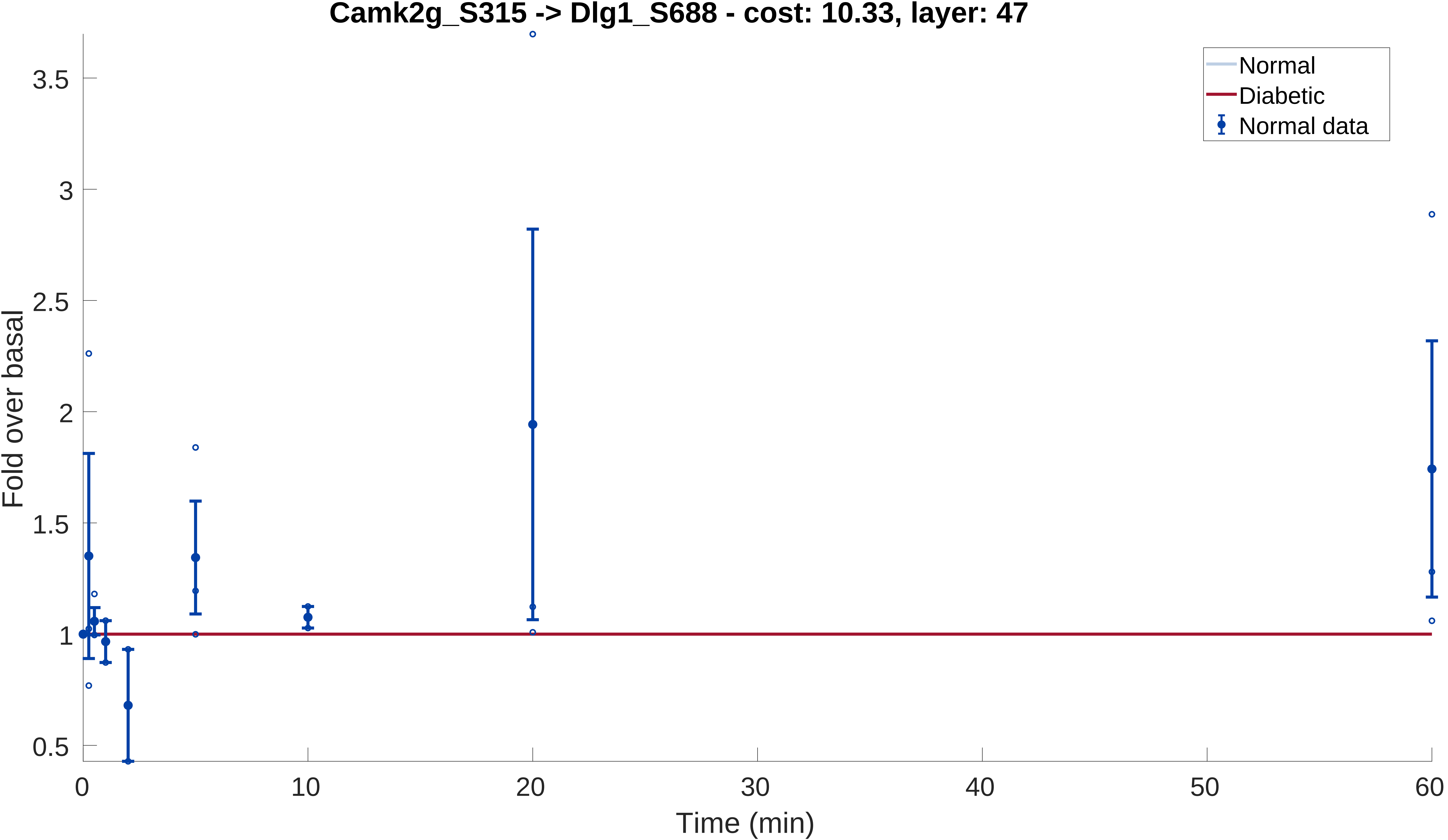

Camk2g_S315 -> Dlg1_S688 - cost: 10.33, layer: 47
Normal
Diabetic
Normal data
Fold over basal
Time (min)
0.5
1
1.5
2
2.5
3
3.5
0
10
20
30
40
50
60

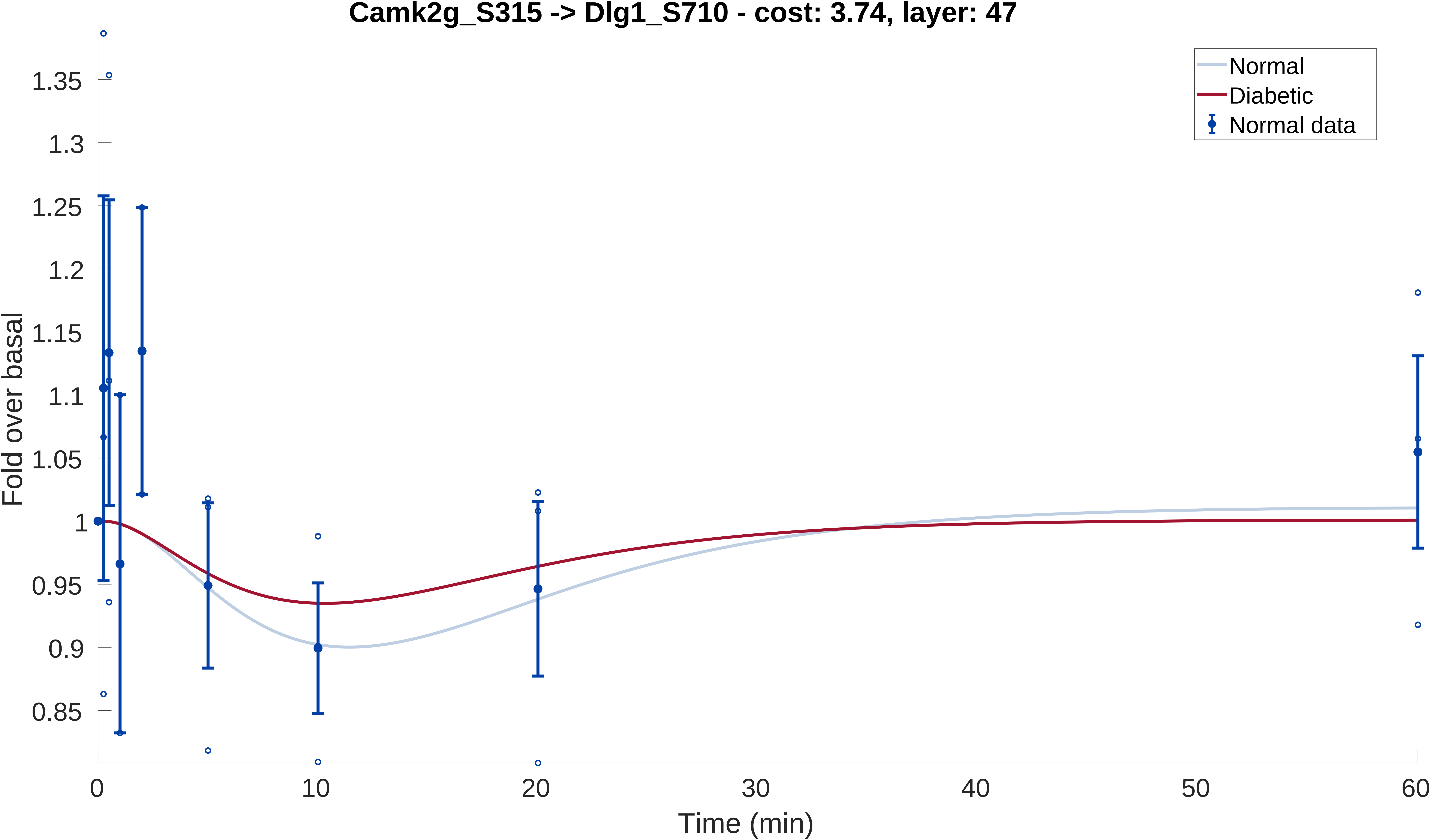

Camk2g_S315 -> Dlg1_S710 - cost: 3.74, layer: 47
Normal
Diabetic
Normal data
Fold over basal
Time (min)
1.35
1.3
1.25
1.2
1.15
1.1
1.05
1
0.95
0.9
0.85
0
10
20
30
40
50
60

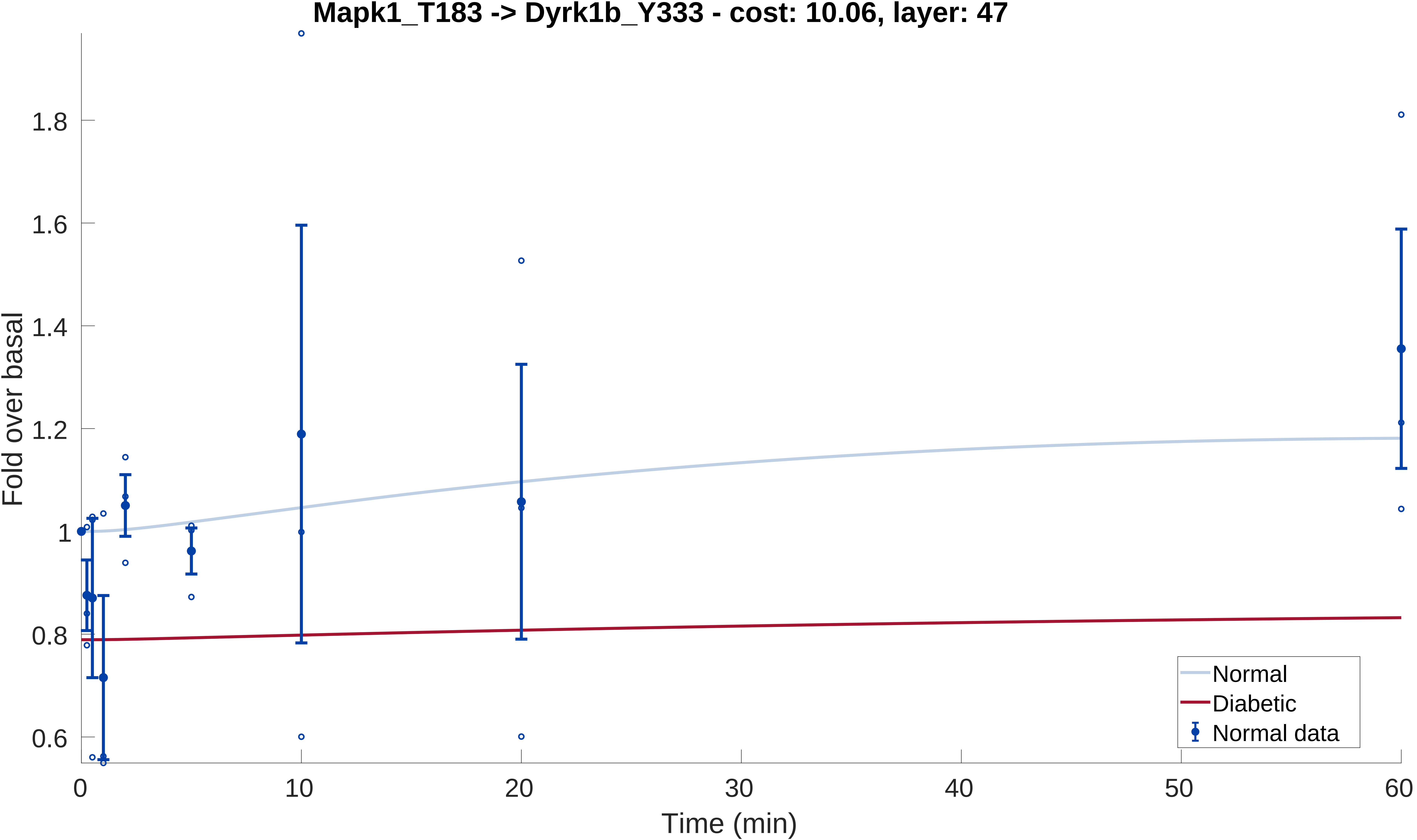

Mapk1_T183 -> Dyrk1b_Y333 - cost: 10.06, layer: 47
Fold over basal
1.8
1.6
1.4
1.2
1
0.8
0.6
0
10
20
30
40
50
60
Time (min)
Normal
Diabetic
Normal data

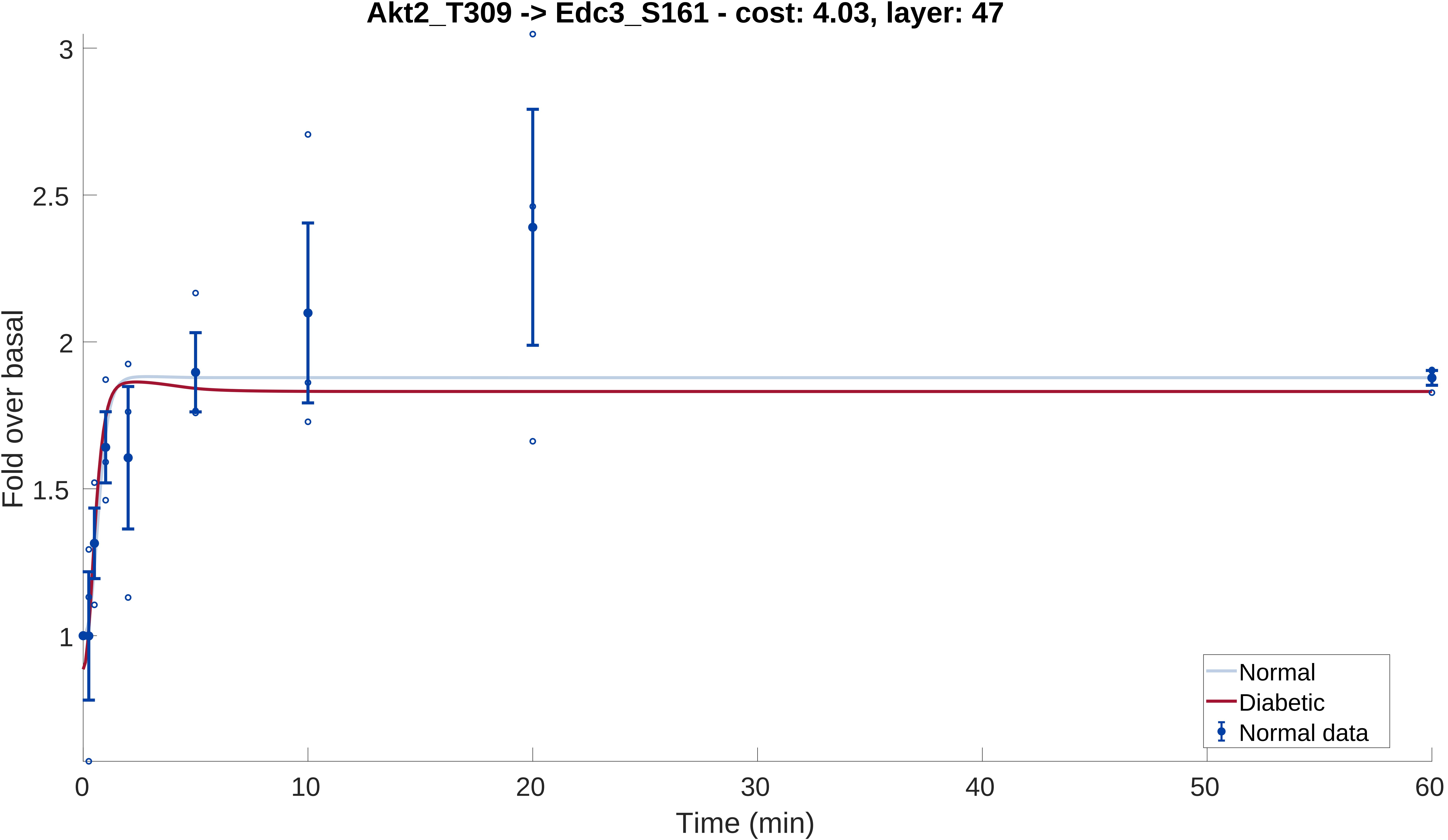
Akt2_T309 -> Edc3_S161 - cost: 4.03, layer: 47
Fold over basal
3
2.5
2
1.5
1
0
10
20
30
40
50
60
Time (min)
Normal
Diabetic
Normal data

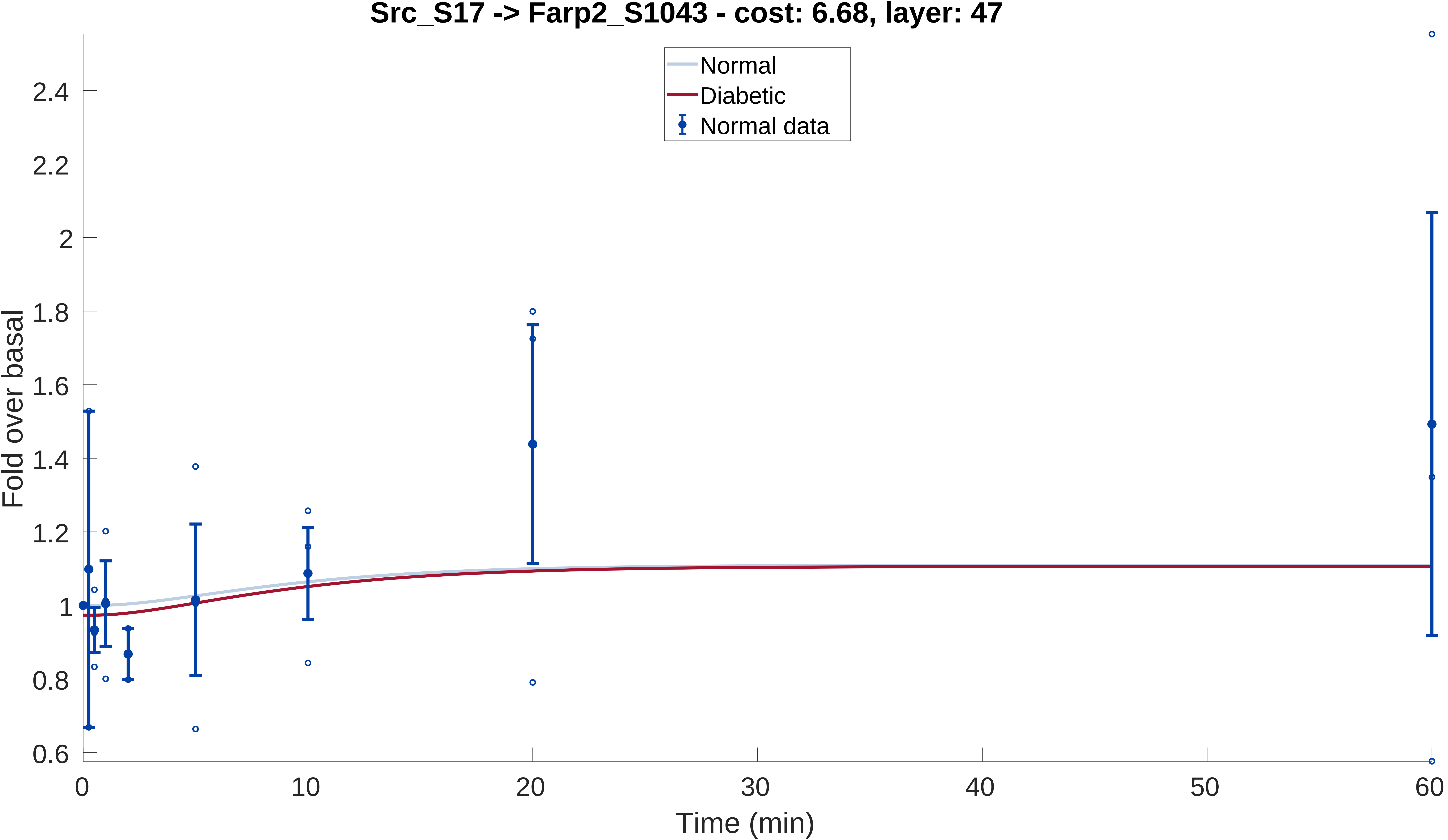

Src_S17 -> Farp2_S1043 - cost: 6.68, layer: 47
Normal
Diabetic
Normal data
Fold over basal
Time (min)
0.6
0.8
1
1.2
1.4
1.6
1.8
2
2.2
2.4
0
10
20
30
40
50
60

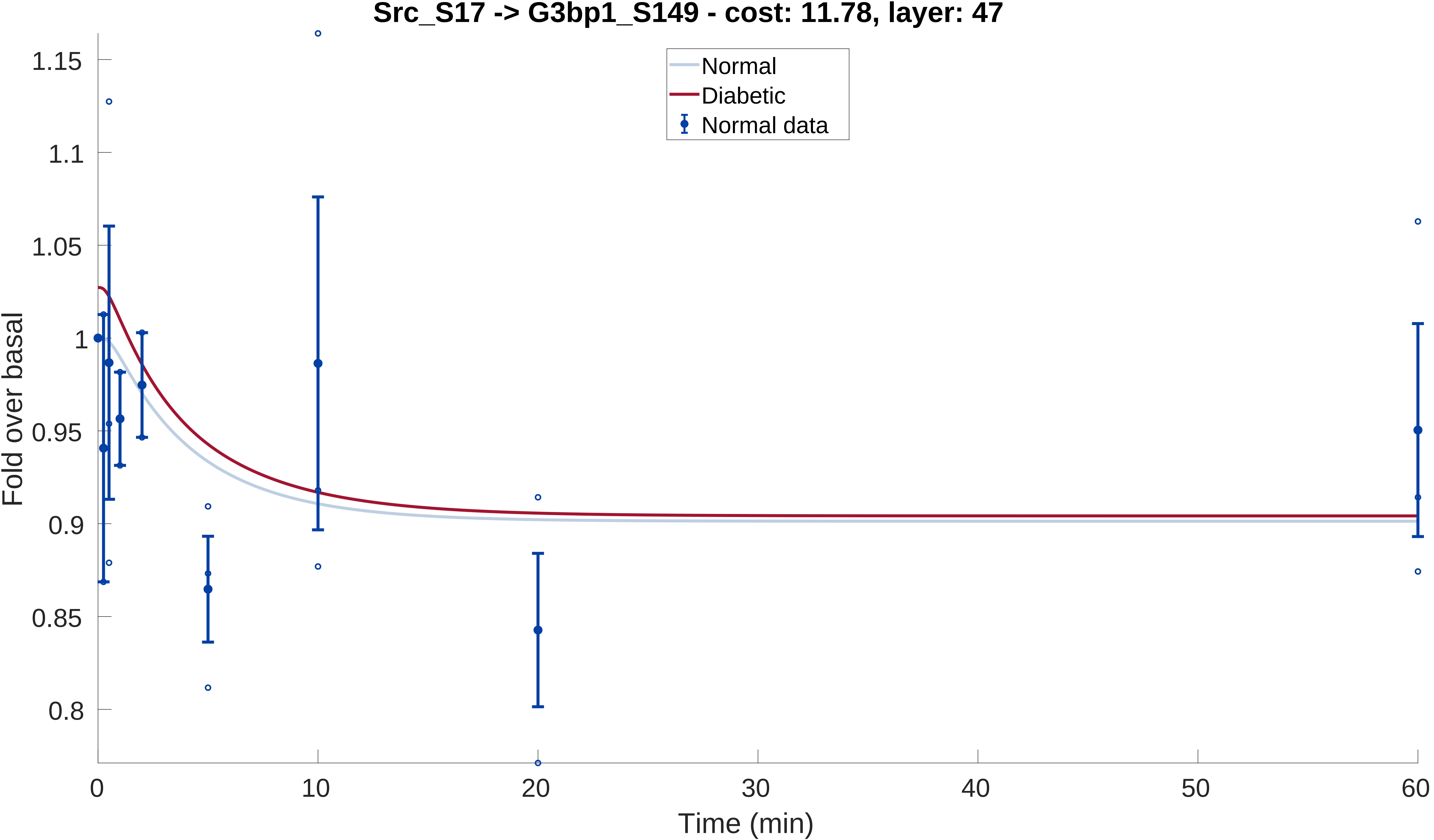
Src_S17 -> G3bp1_S149 - cost: 11.78, layer: 47
Normal
Diabetic
Normal data
Fold over basal
1.15
1.1
1.05
1
0.95
0.9
0.85
0.8
0
10
20
30
40
50
60
Time (min)

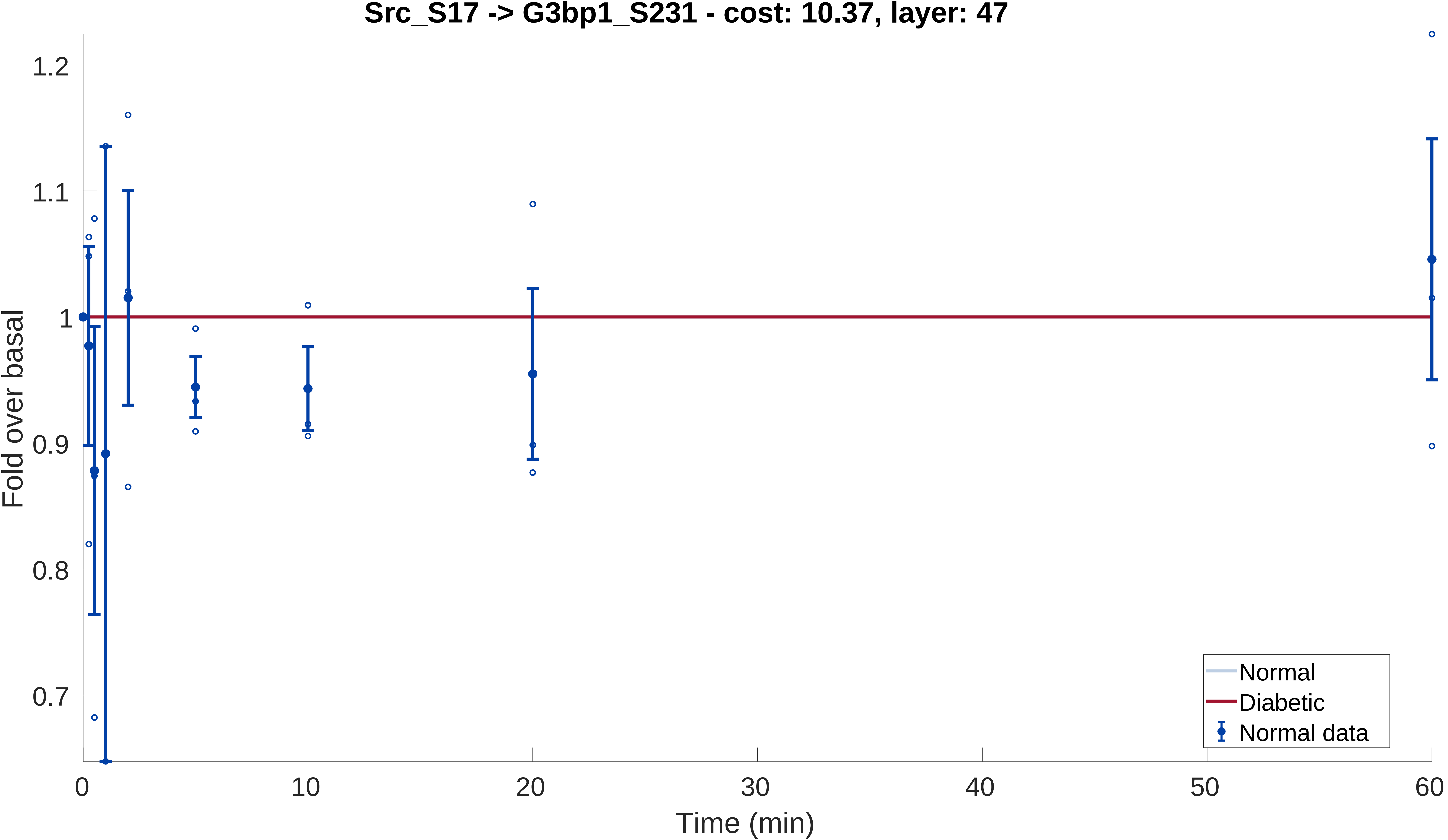

Src_S17 -> G3bp1_S231 - cost: 10.37, layer: 47
Fold over basal
1.2
1.1
1
0.9
0.8
0.7
0
10
20
30
40
50
60
Time (min)
Normal
Diabetic
Normal data

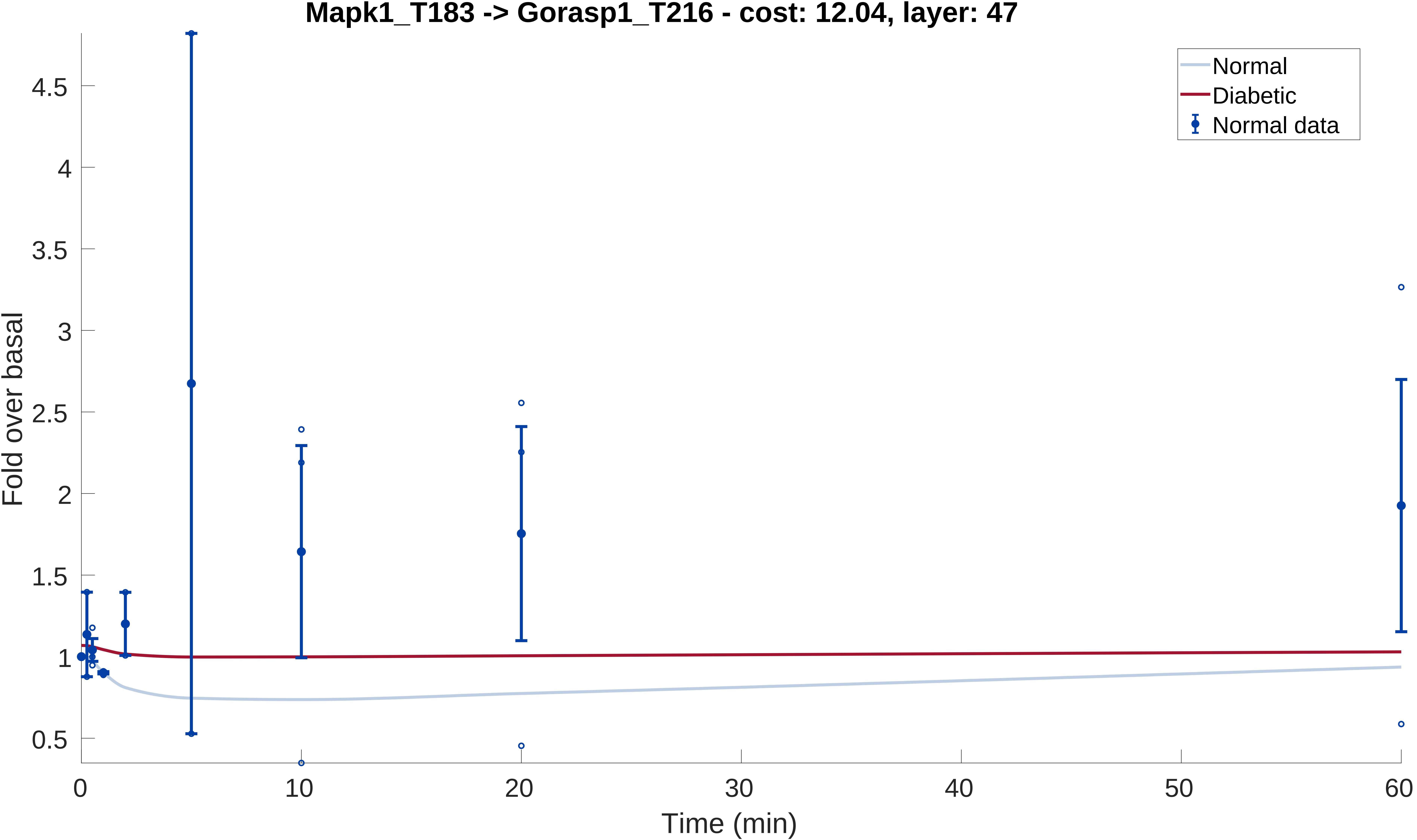

Mapk1_T183 -> Gorasp1_T216 - cost: 12.04, layer: 47
Normal
Diabetic
Normal data
Fold over basal
4.5
4
3.5
3
2.5
2
1.5
1
0.5
0
10
20
30
40
50
60
Time (min)

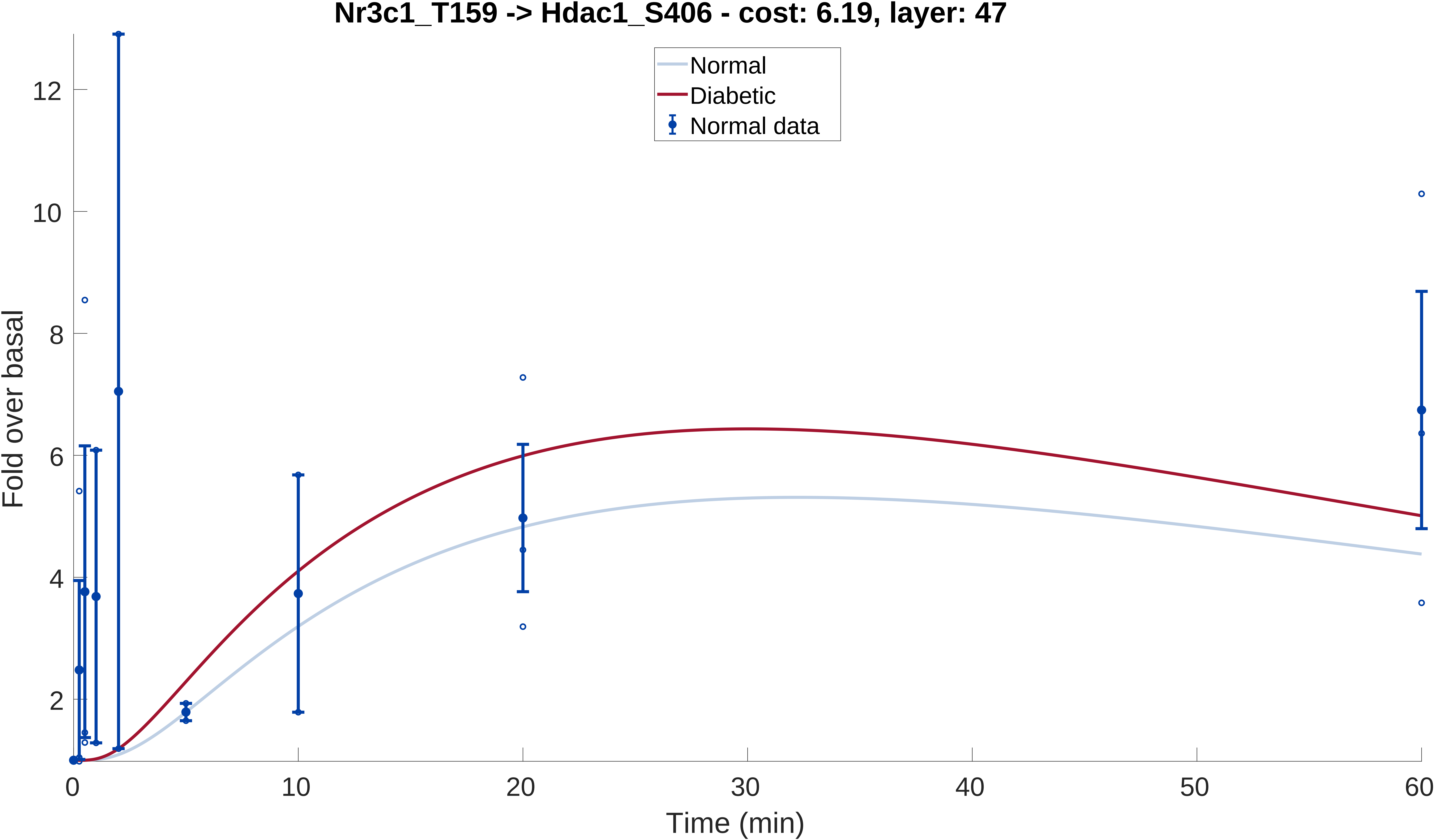

Nr3c1_T159 -> Hdac1_S406 - cost: 6.19, layer: 47
Normal
Diabetic
Normal data
Fold over basal
12
10
8
6
4
2
0
10
20
30
40
50
60
Time (min)

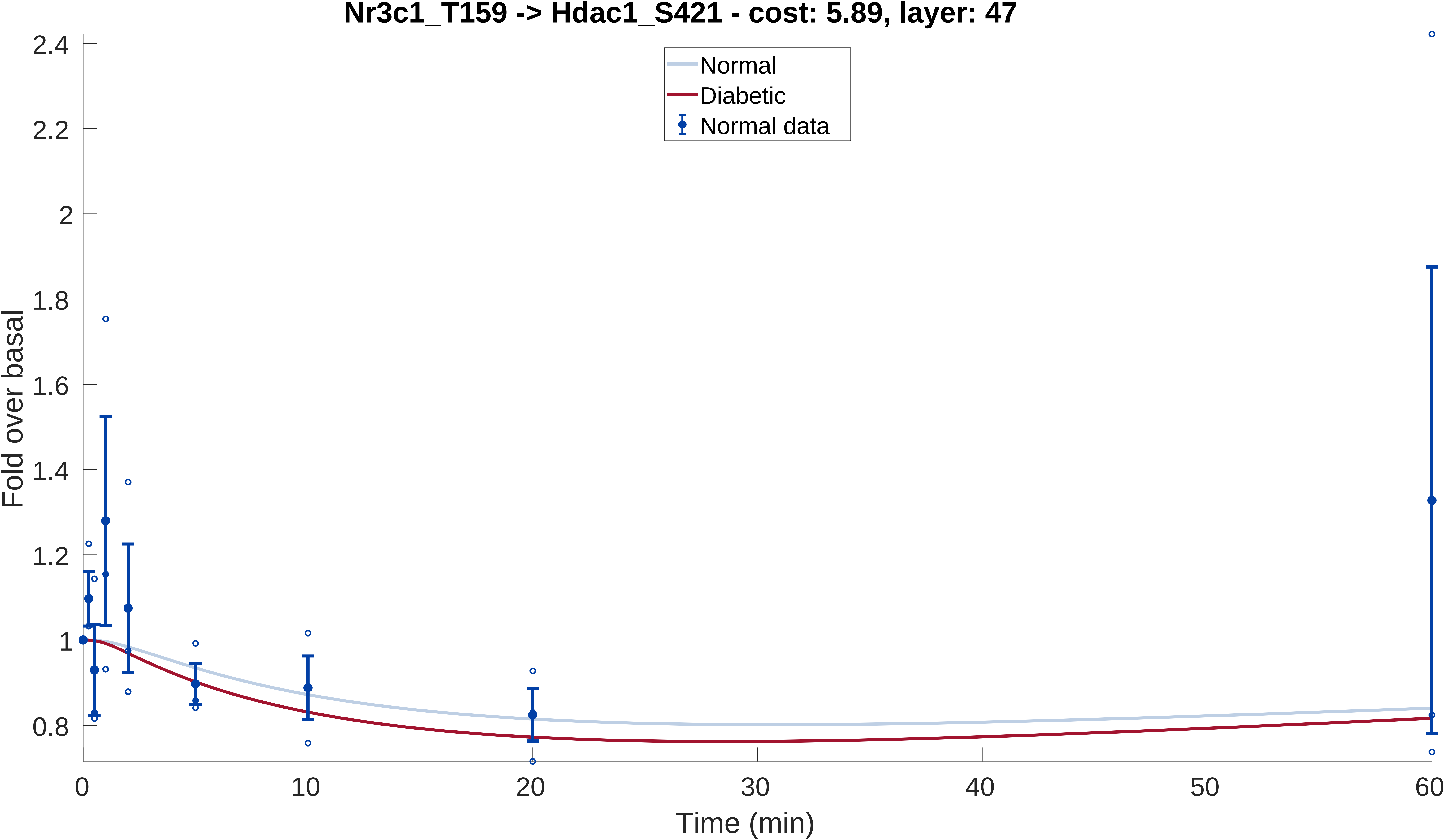

Nr3c1_T159 -> Hdac1_S421 - cost: 5.89, layer: 47
Normal
Diabetic
Normal data
Fold over basal
Time (min)
2.4
2.2
2
1.8
1.6
1.4
1.2
1
0.8
0
10
20
30
40
50
60

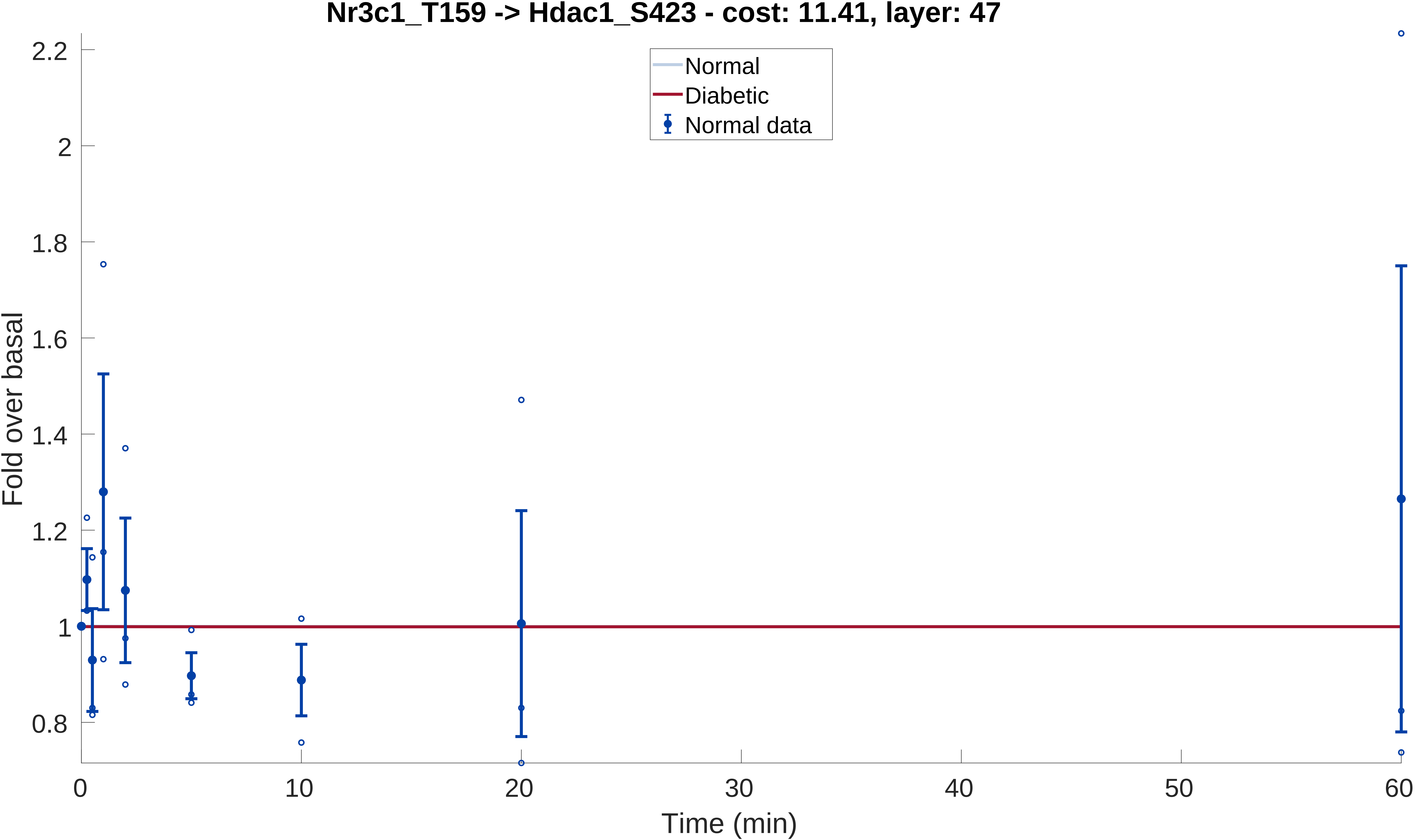

Nr3c1_T159 -> Hdac1_S423 - cost: 11.41, layer: 47
Normal
Diabetic
Normal data
Fold over basal
Time (min)
2.2
2
1.8
1.6
1.4
1.2
1
0.8
0
10
20
30
40
50
60

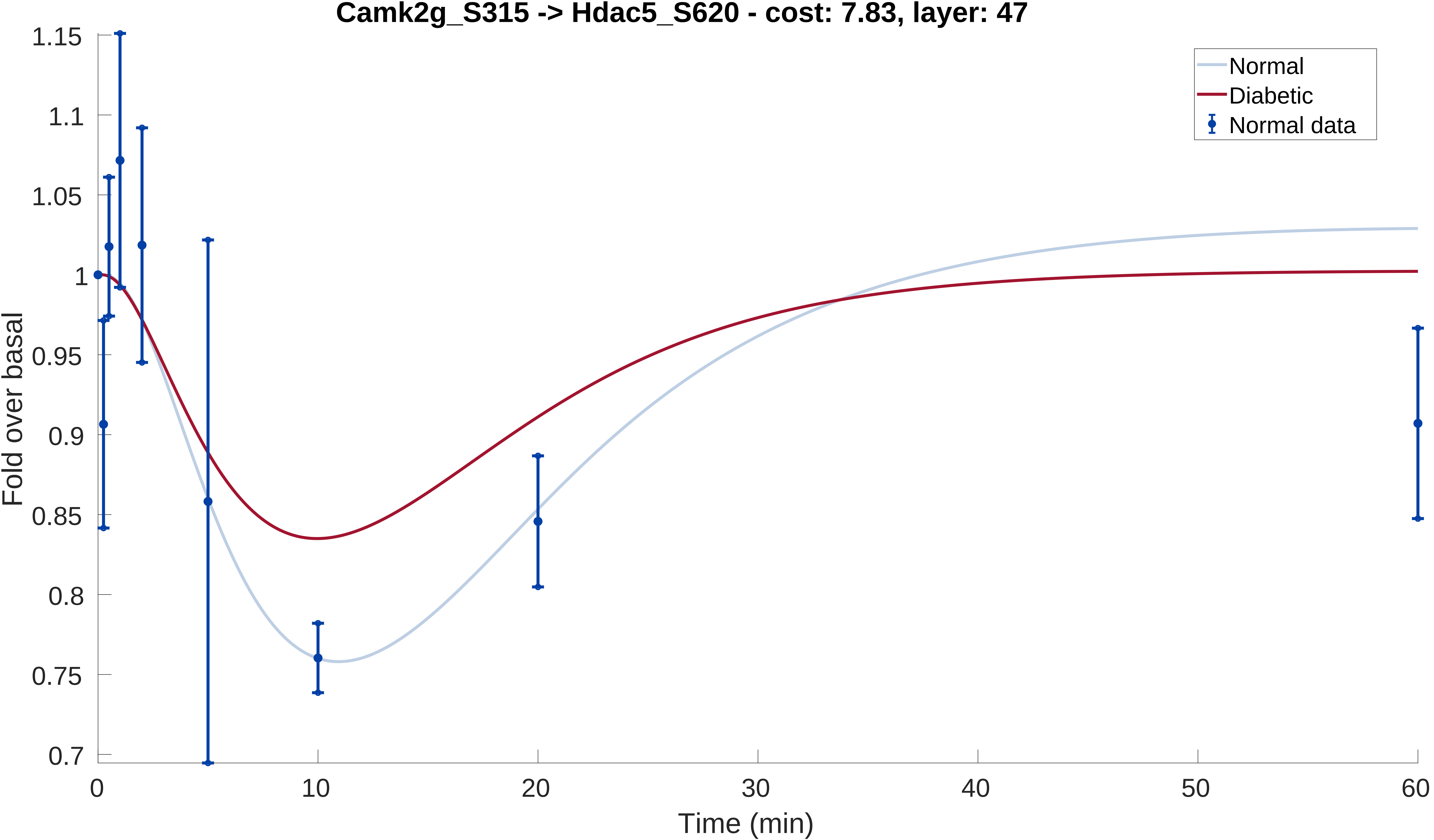
Camk2g_S315 -> Hdac5_S620 - cost: 7.83, layer: 47
Normal
Diabetic
Normal data
Fold over basal
1.15
1.1
1.05
1
0.95
0.9
0.85
0.8
0.75
0.7
0
10
20
30
40
50
60
Time (min)

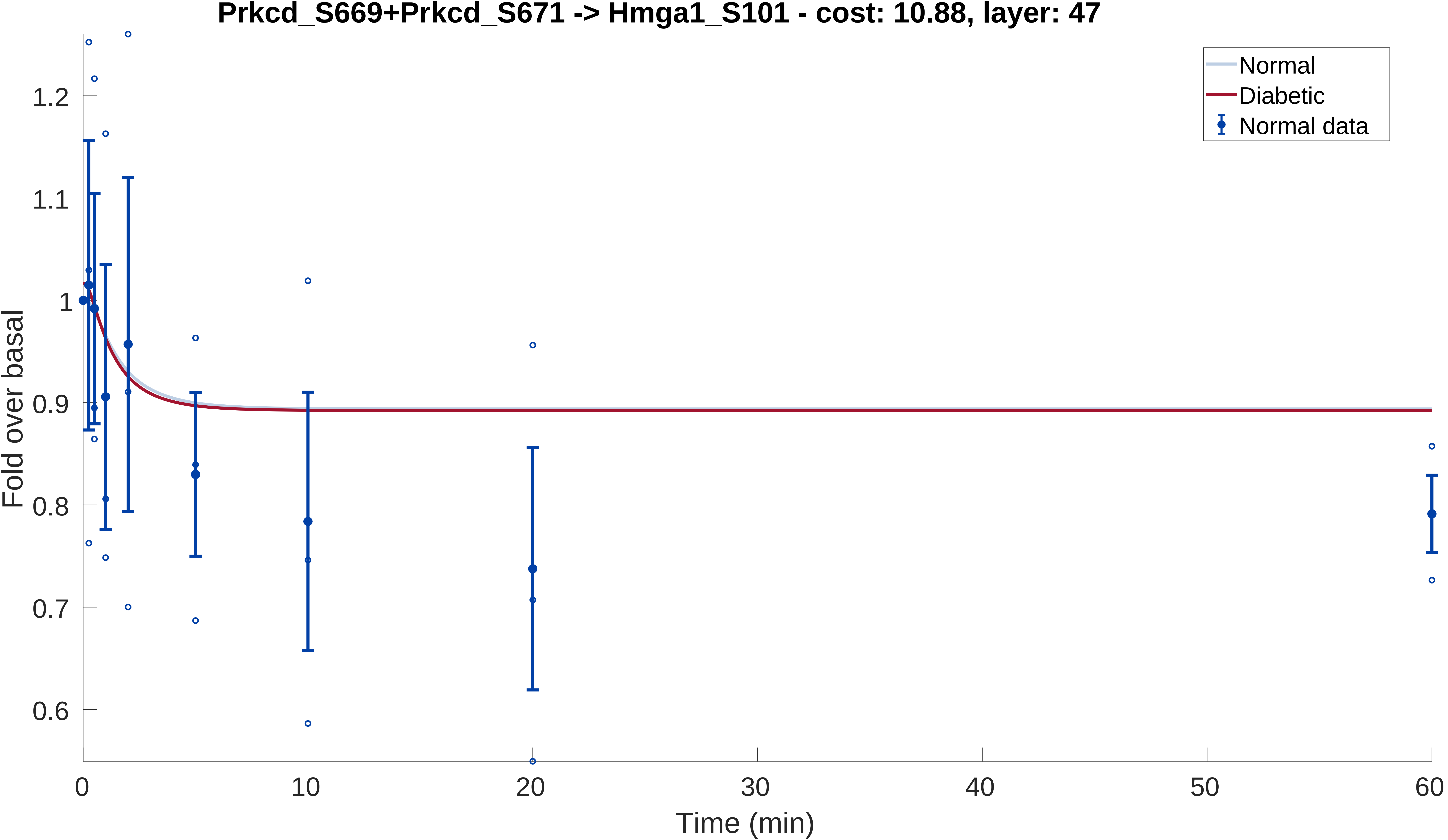

Prkcd_S669+Prkcd_S671 -> Hmga1_S101 - cost: 10.88, layer: 47
Normal
Diabetic
Normal data
Fold over basal
Time (min)
0.6
0.7
0.8
0.9
1
1.1
1.2
0
10
20
30
40
50
60

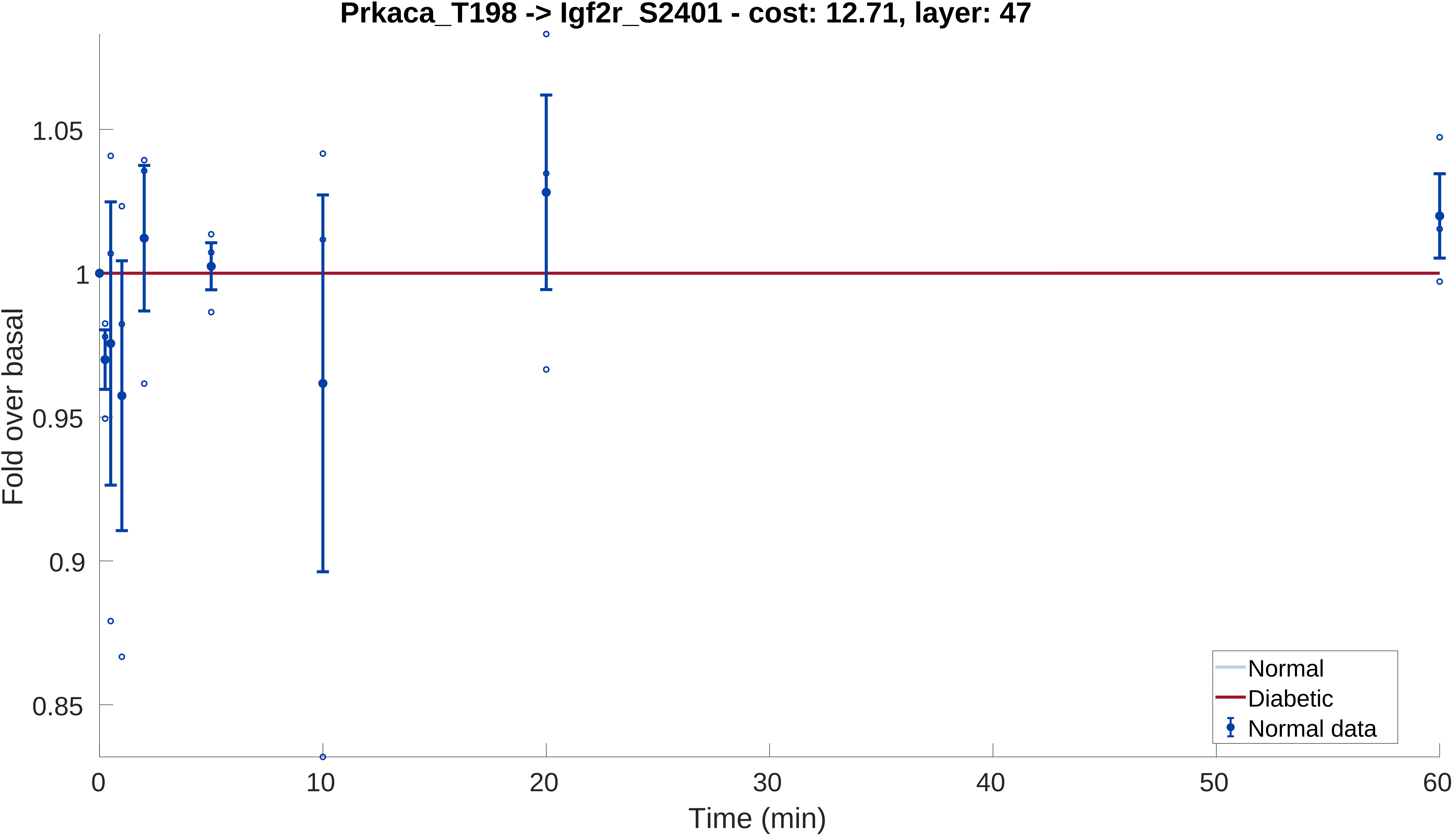

Prkaca_T198 -> Igf2r_S2401 - cost: 12.71, layer: 47
1.05
1
0.95
0.9
0.85
Fold over basal
0
10
20
30
40
50
60
Time (min)
Normal
Diabetic
Normal data

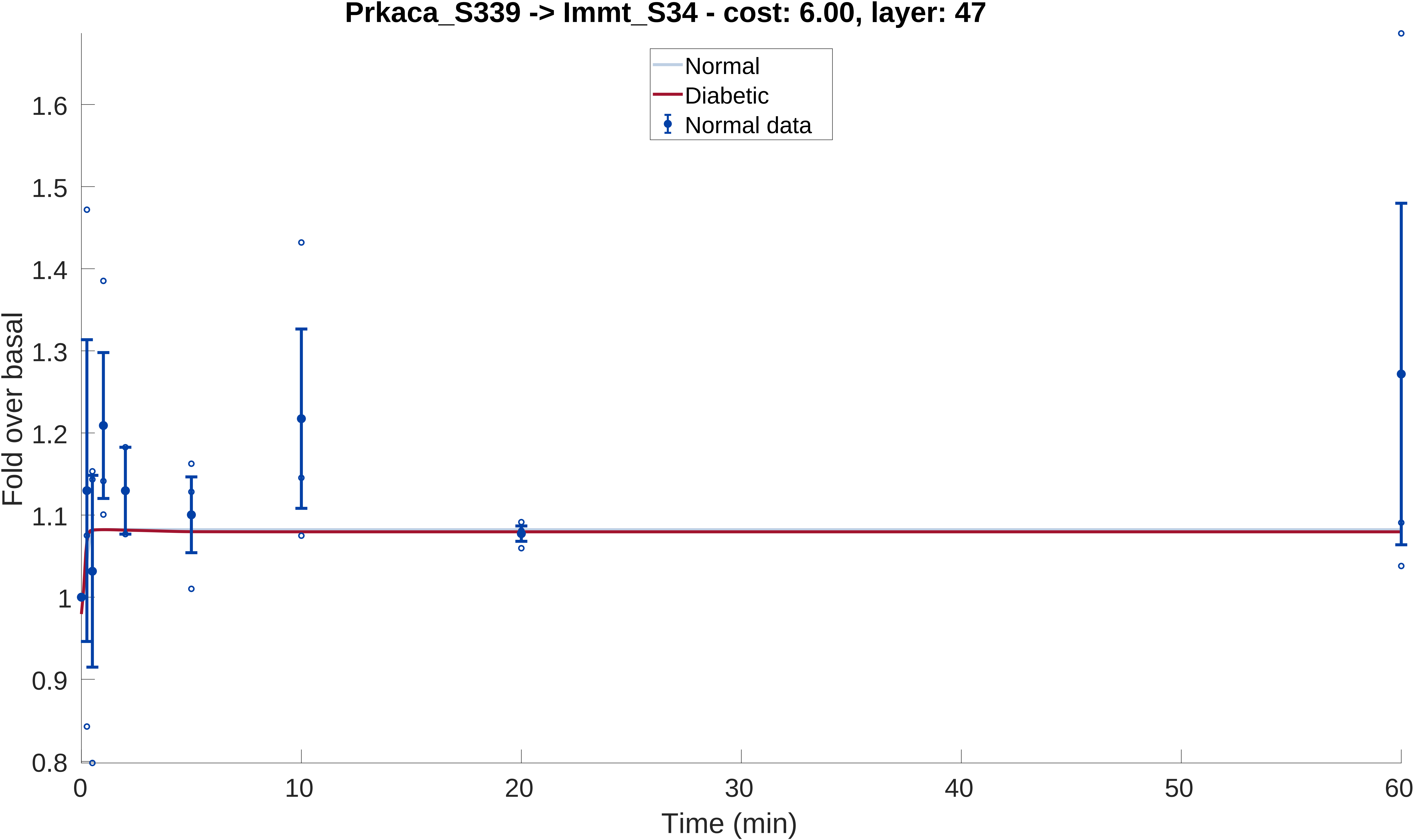

Prkaca_S339 -> Immt_S34 - cost: 6.00, layer: 47
Normal
Diabetic
Normal data
Fold over basal
Time (min)
0.8
0.9
1
1.1
1.2
1.3
1.4
1.5
1.6
0
10
20
30
40
50
60

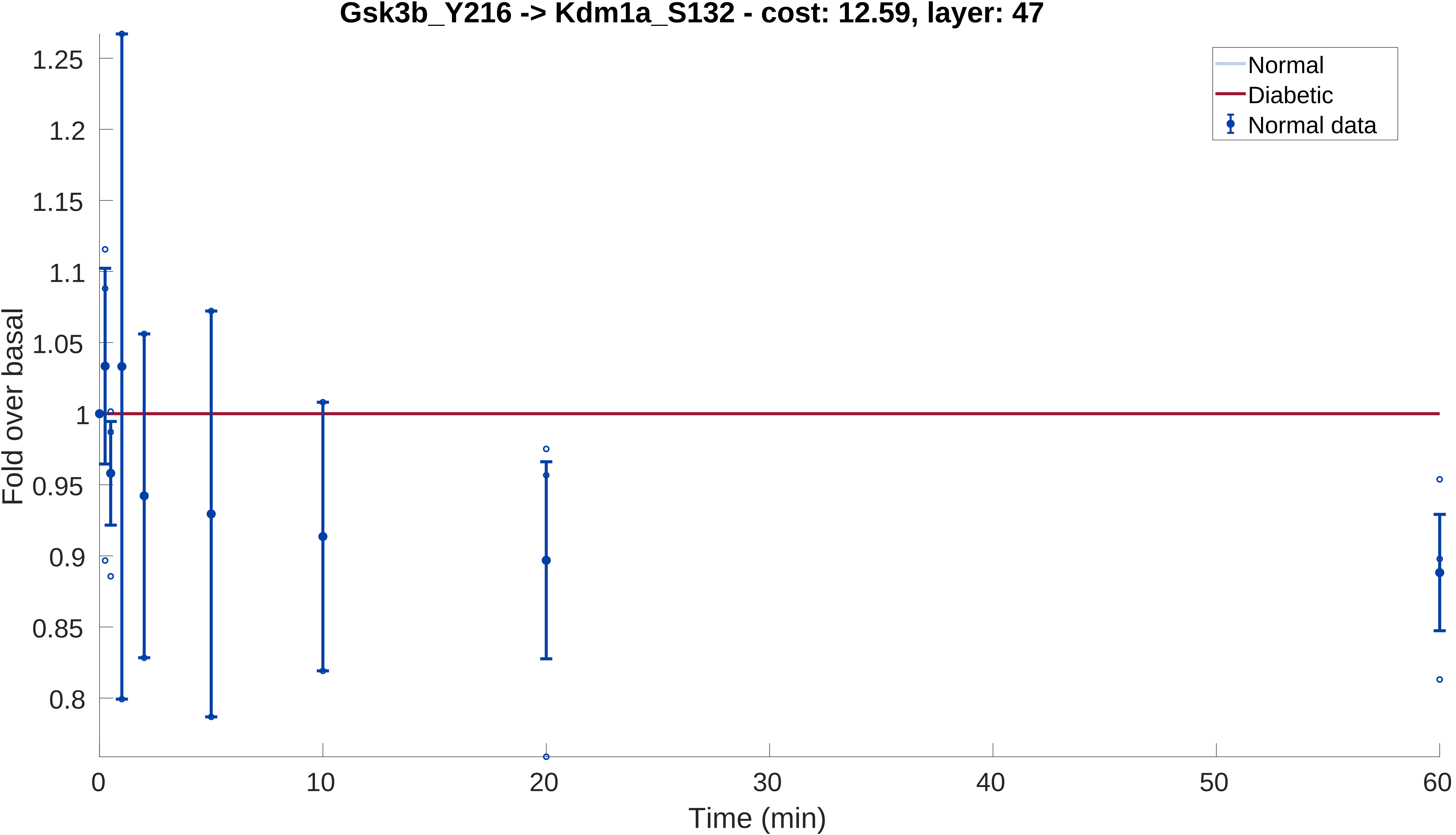

Gsk3b_Y216 -> Kdm1a_S132 - cost: 12.59, layer: 47
Normal
Diabetic
Normal data
Fold over basal
Time (min)
1.25
1.2
1.15
1.1
1.05
1
0.95
0.9
0.85
0.8
0
10
20
30
40
50
60

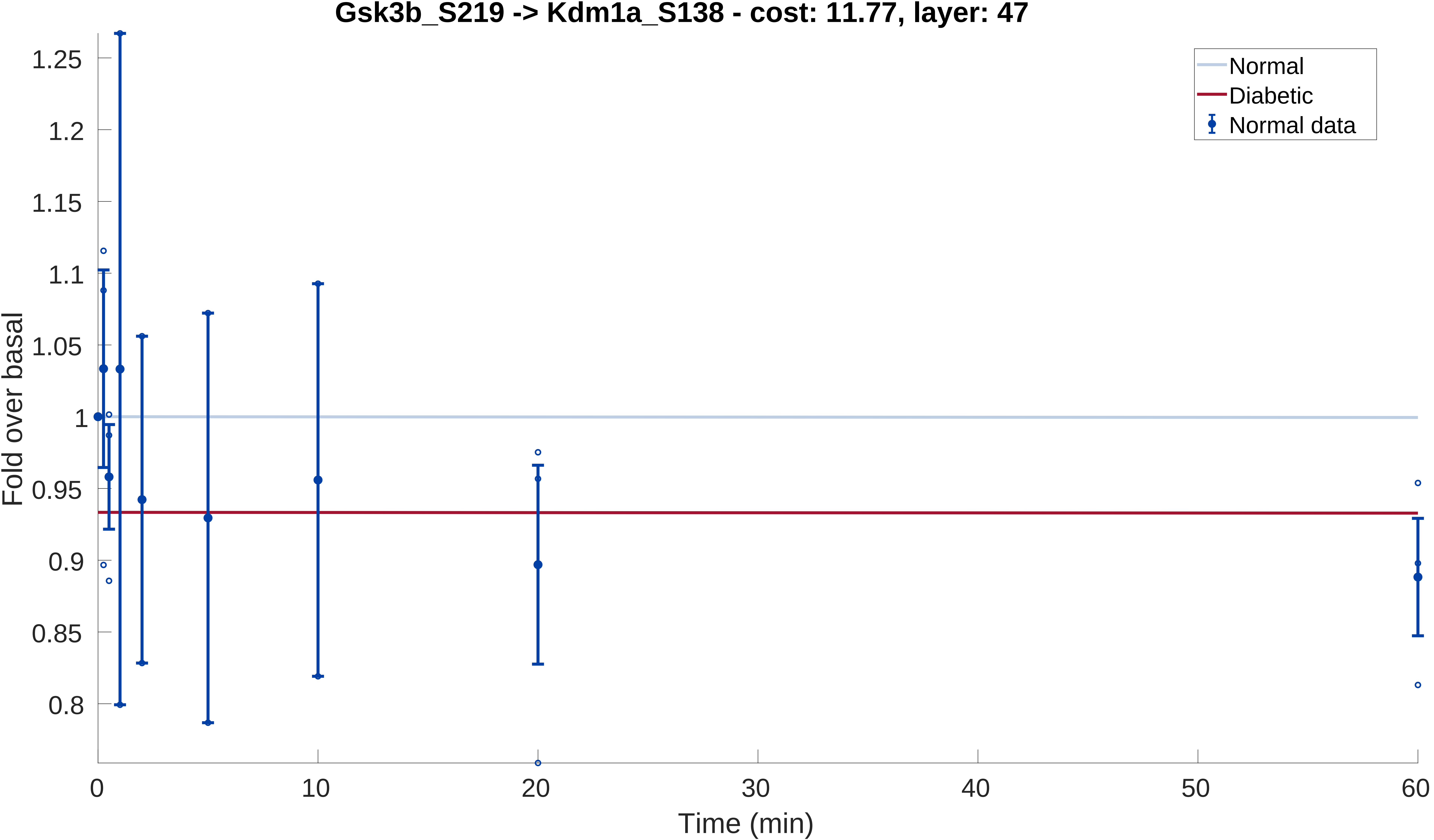

Gsk3b_S219 -> Kdm1a_S138 - cost: 11.77, layer: 47
Normal
Diabetic
Normal data
Fold over basal
1.25
1.2
1.15
1.1
1.05
1
0.95
0.9
0.85
0.8
0
10
20
30
40
50
60
Time (min)

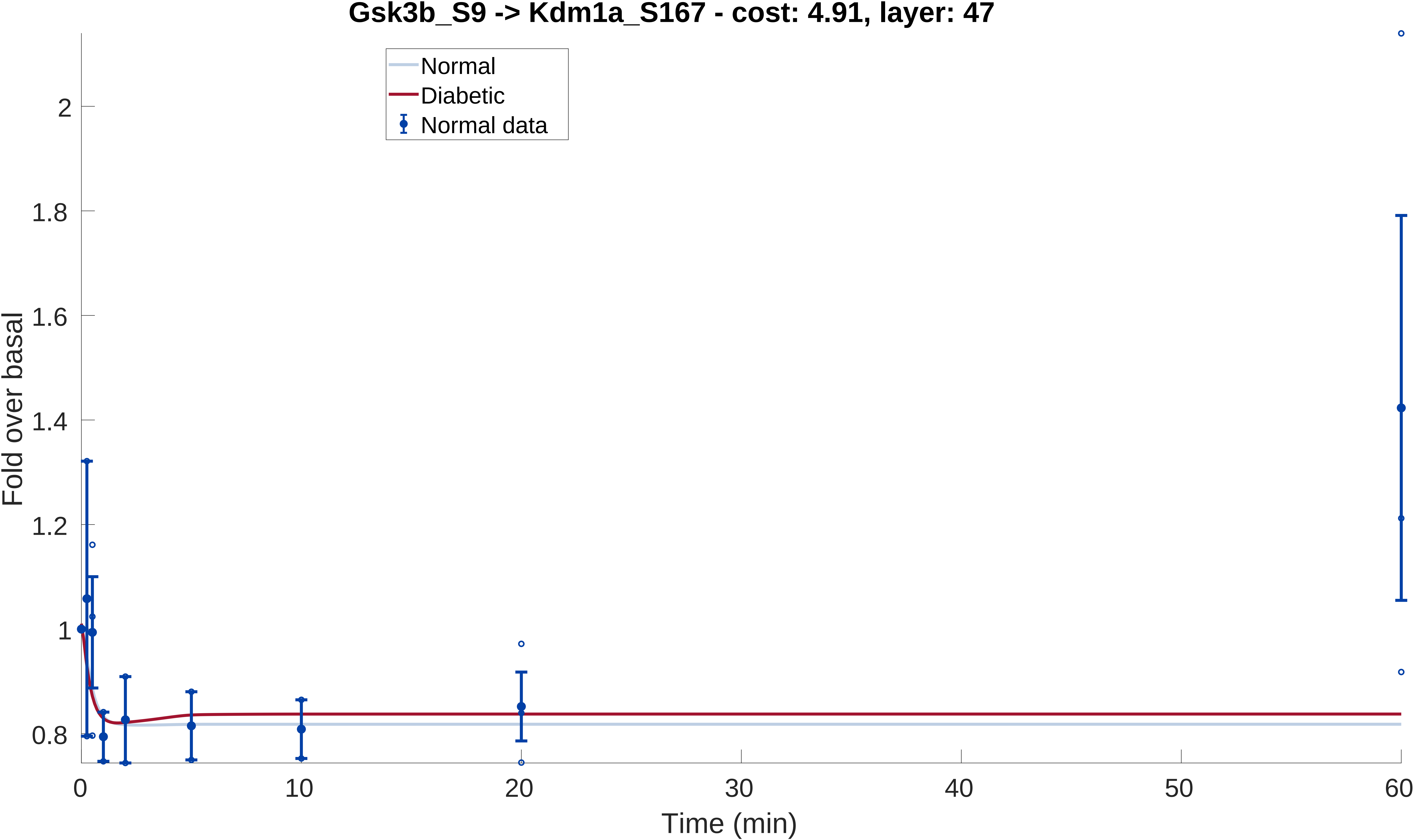

Gsk3b_S9 -> Kdm1a_S167 - cost: 4.91, layer: 47
Normal
Diabetic
Normal data
Fold over basal
2
1.8
1.6
1.4
1.2
1
0.8
0
10
20
30
40
50
60
Time (min)

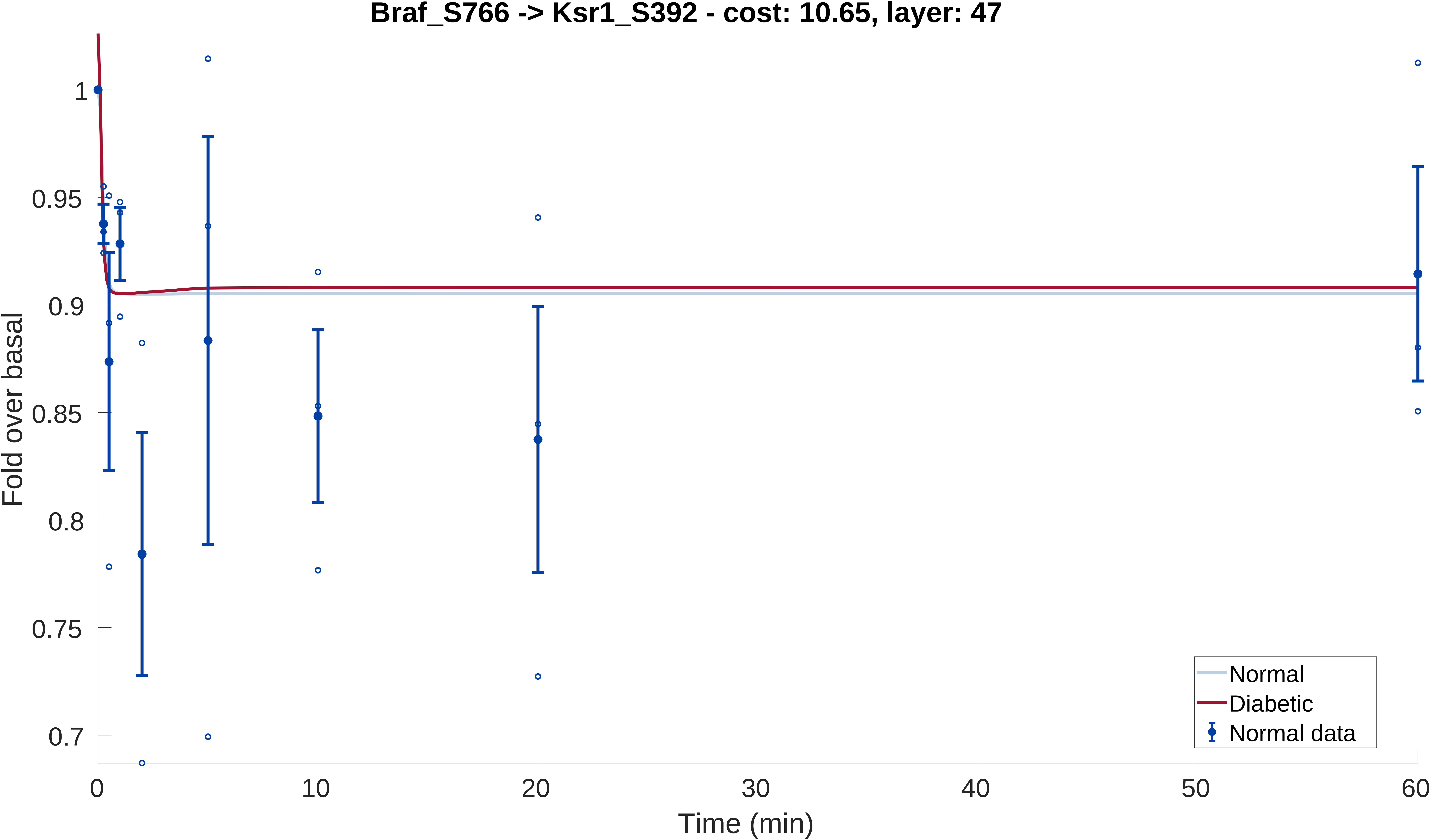
Braf_S766 -> Ksr1_S392 - cost: 10.65, layer: 47
Fold over basal
Time (min)
Normal
Diabetic
Normal data
1
0.95
0.9
0.85
0.8
0.75
0.7
0
10
20
30
40
50
60

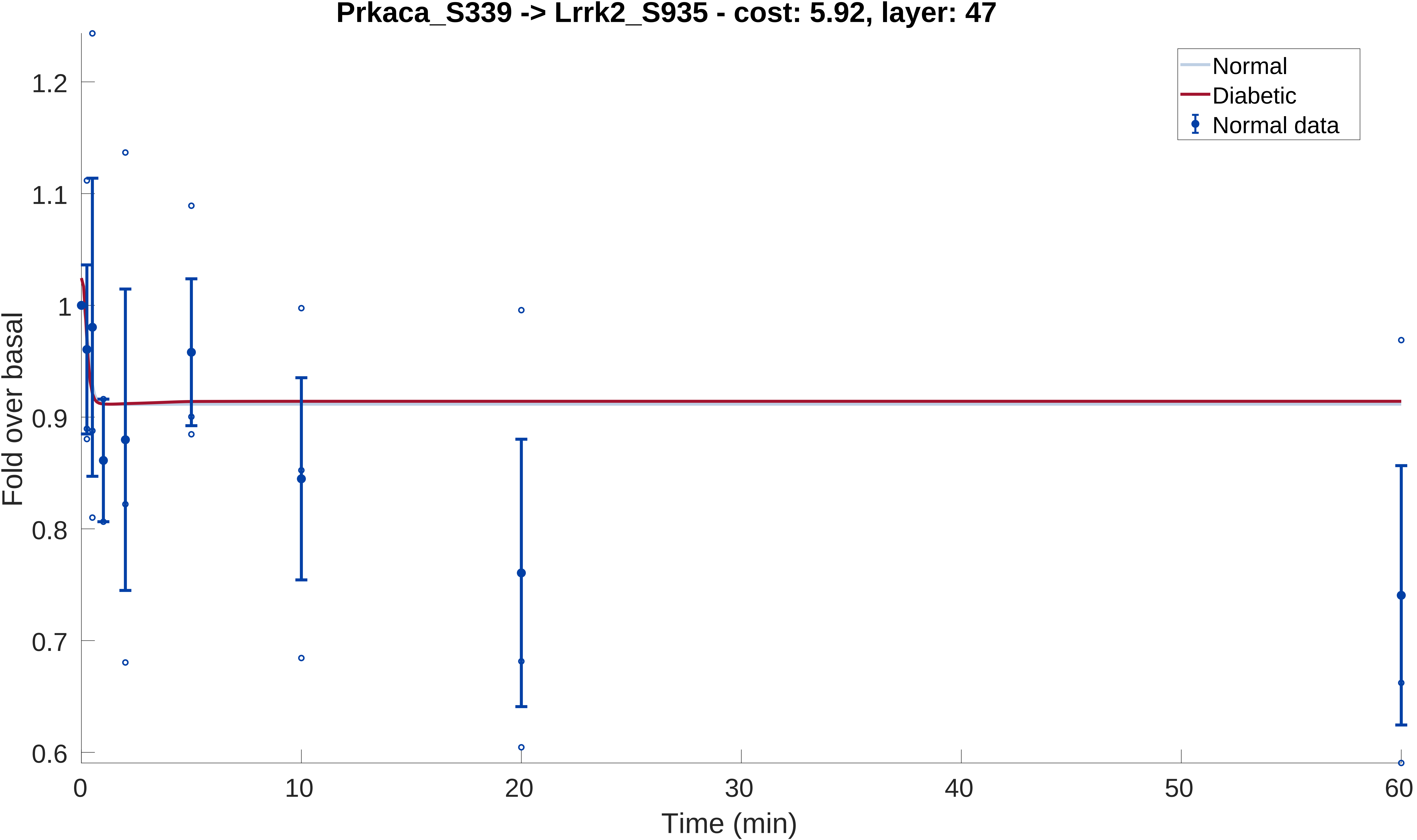

Prkaca_S339 -> Lrrk2_S935 - cost: 5.92, layer: 47
Normal
Diabetic
Normal data
Fold over basal
1.2
1.1
1
0.9
0.8
0.7
0.6
0
10
20
30
40
50
60
Time (min)

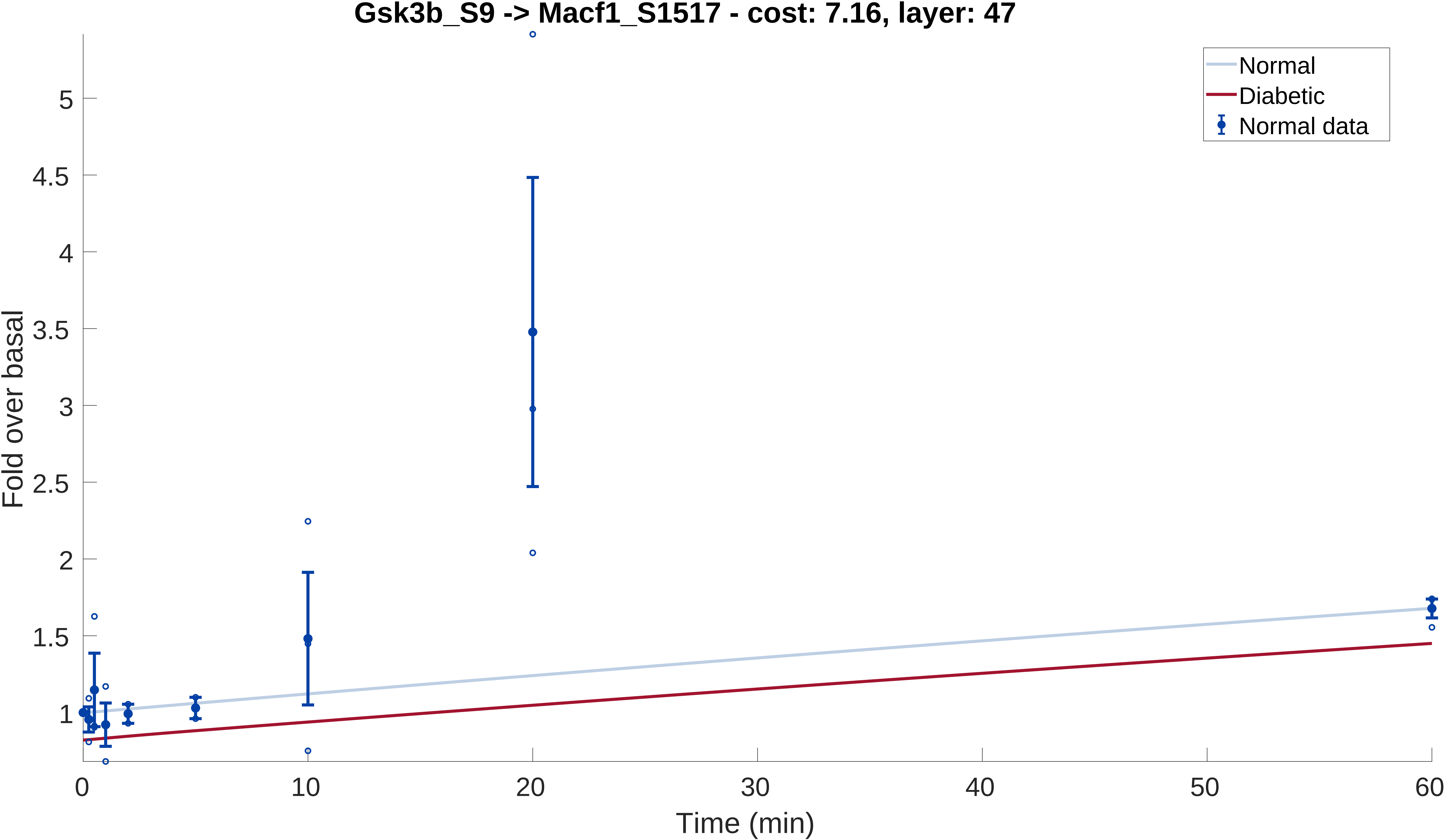

Gsk3b_S9 -> Macf1_S1517 - cost: 7.16, layer: 47
Normal
Diabetic
Normal data
Fold over basal
5
4.5
4
3.5
3
2.5
2
1.5
1
0
10
20
30
40
50
60
Time (min)

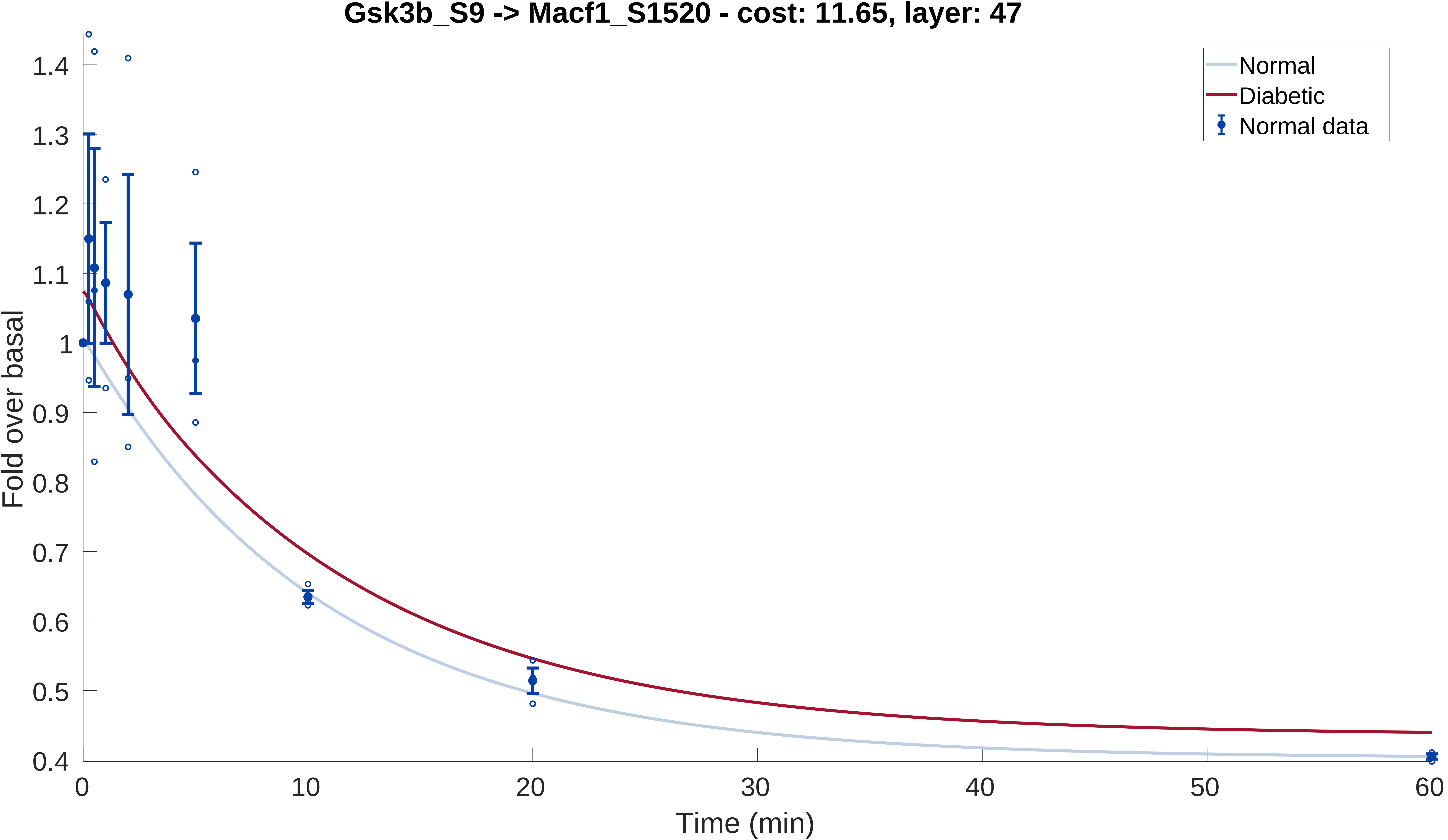

Gsk3b_S9 -> Macf1_S1520 - cost: 11.65, layer: 47
Normal
Diabetic
Normal data
Fold over basal
1.4
1.3
1.2
1.1
1
0.9
0.8
0.7
0.6
0.5
0.4
0
10
20
30
40
50
60
Time (min)

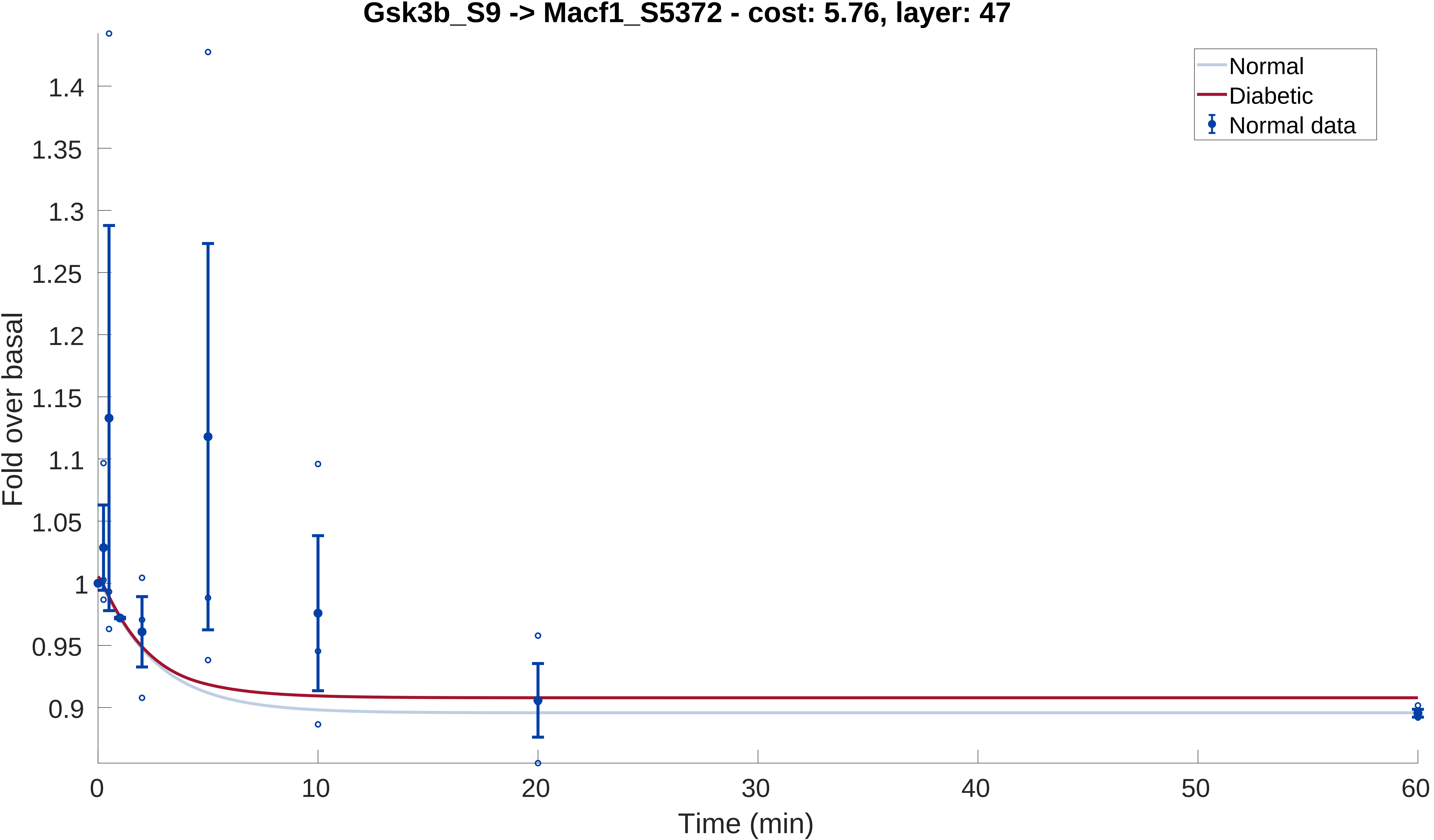

Gsk3b_S9 -> Macf1_S5372 - cost: 5.76, layer: 47
Normal
Diabetic
Normal data
Fold over basal
Time (min)
1.4
1.35
1.3
1.25
1.2
1.15
1.1
1.05
1
0.95
0.9
0
10
20
30
40
50
60

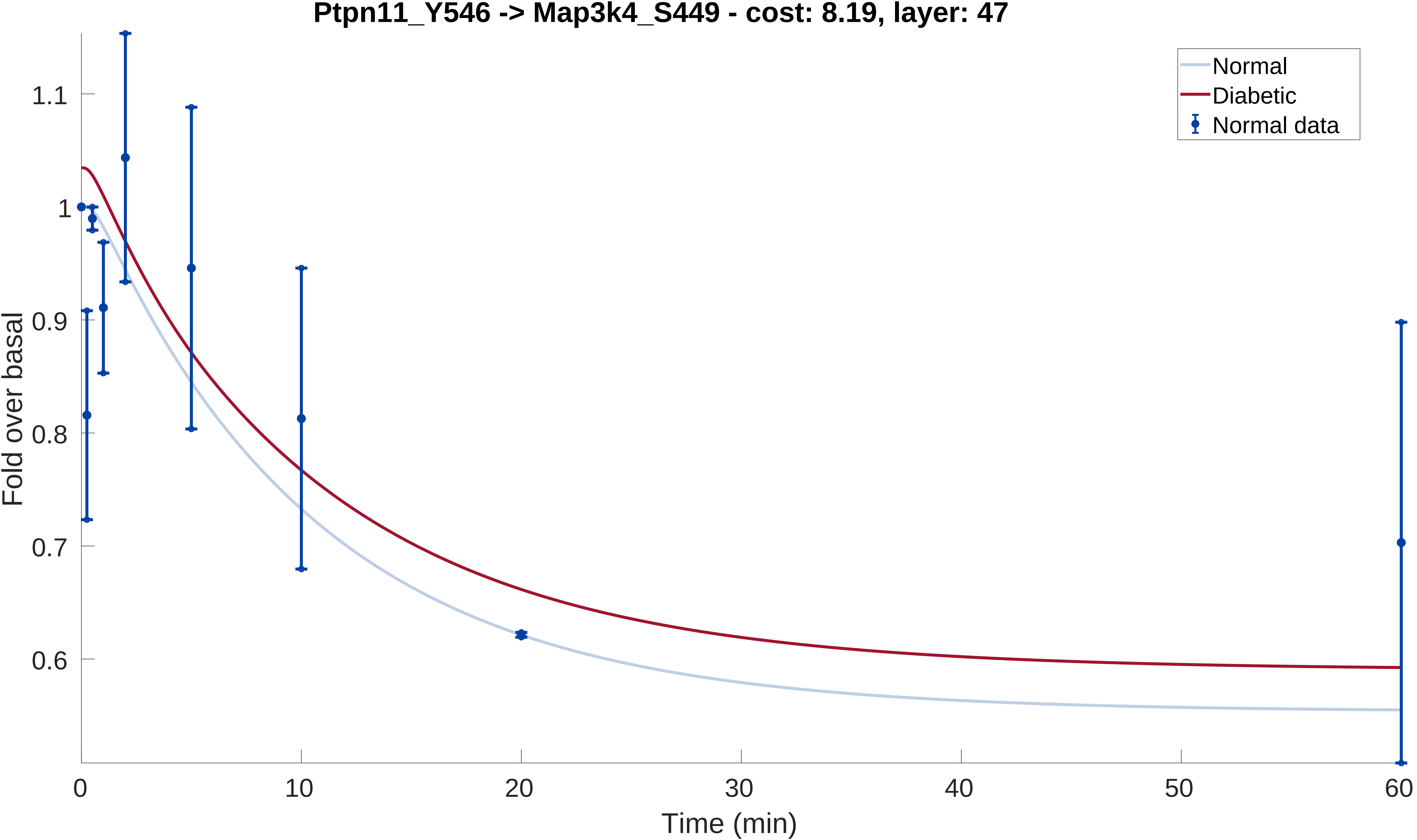

Ptpn11_Y546 -> Map3k4_S449 - cost: 8.19, layer: 47
Normal
Diabetic
Normal data
Fold over basal
1.1
1
0.9
0.8
0.7
0.6
0
10
20
30
40
50
60
Time (min)

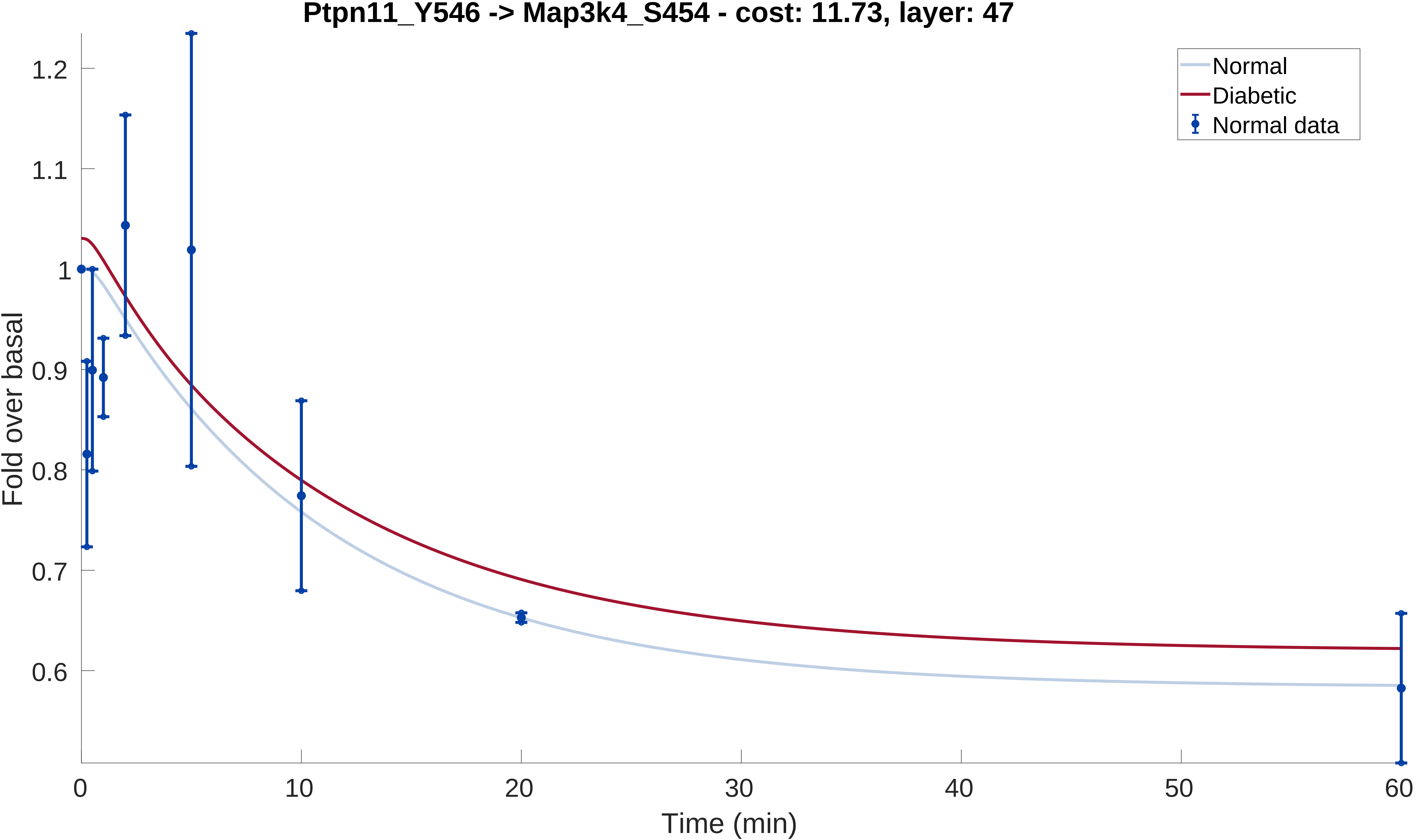

Ptpn11_Y546 -> Map3k4_S454 - cost: 11.73, layer: 47
Normal
Diabetic
Normal data
Fold over basal
1.2
1.1
1
0.9
0.8
0.7
0.6
0
10
20
30
40
50
60
Time (min)

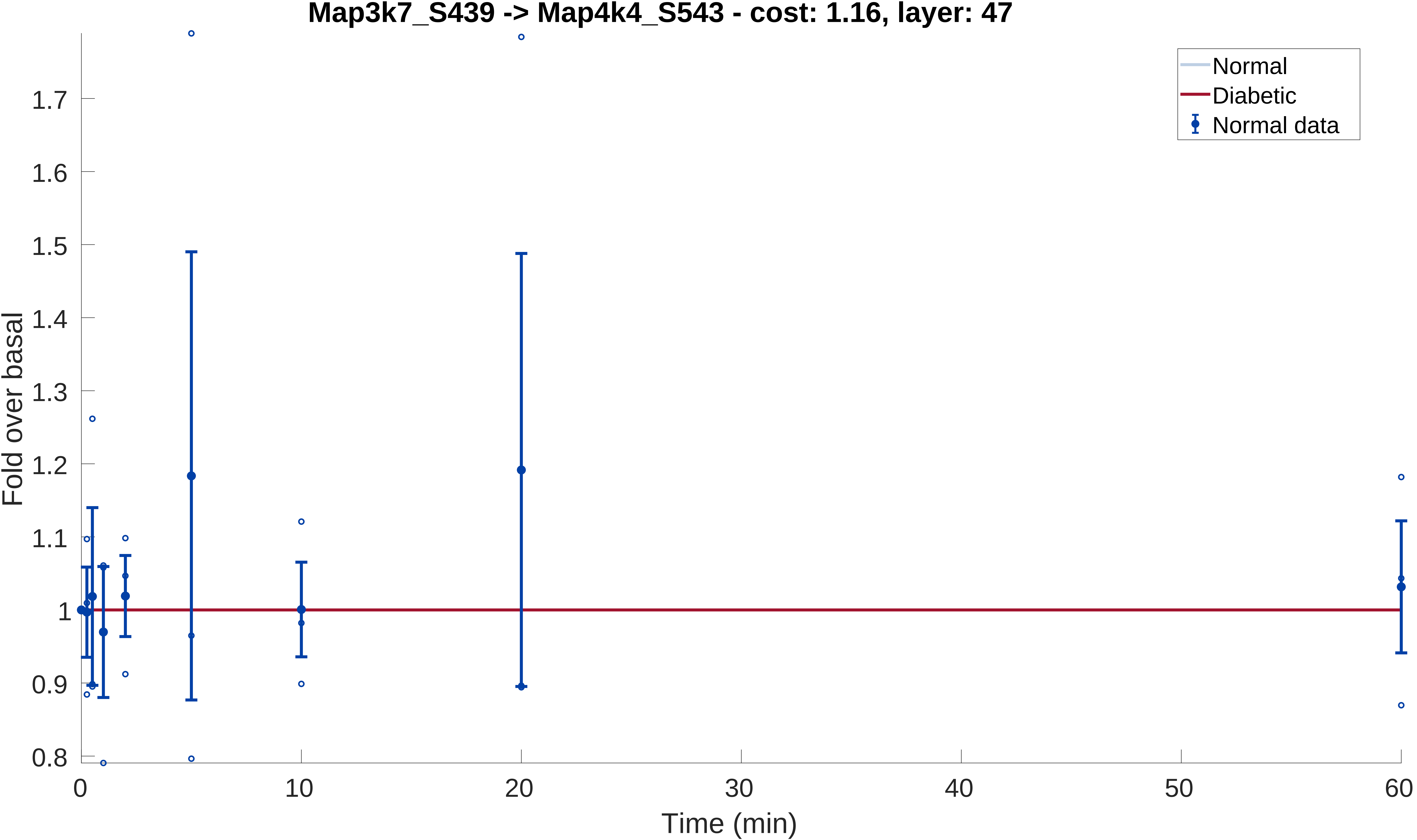

Map3k7_S439 -> Map4k4_S543 - cost: 1.16, layer: 47
Normal
Diabetic
Normal data
Fold over basal
Time (min)
0.8
0.9
1
1.1
1.2
1.3
1.4
1.5
1.6
1.7
0
10
20
30
40
50
60

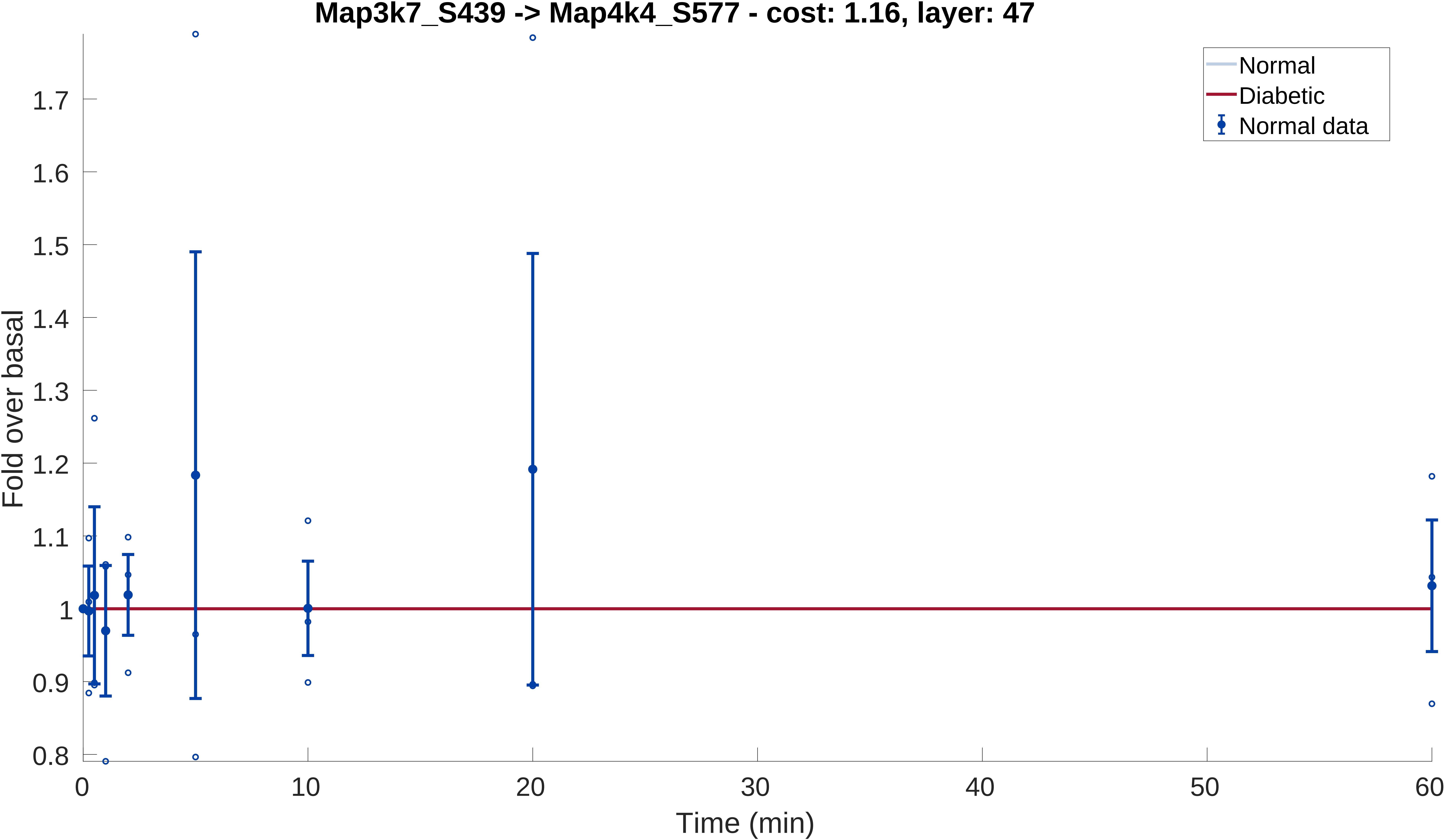

Map3k7_S439 -> Map4k4_S577 - cost: 1.16, layer: 47
Normal
Diabetic
Normal data
Fold over basal
1.7
1.6
1.5
1.4
1.3
1.2
1.1
1
0.9
0.8
0
10
20
30
40
50
60
Time (min)

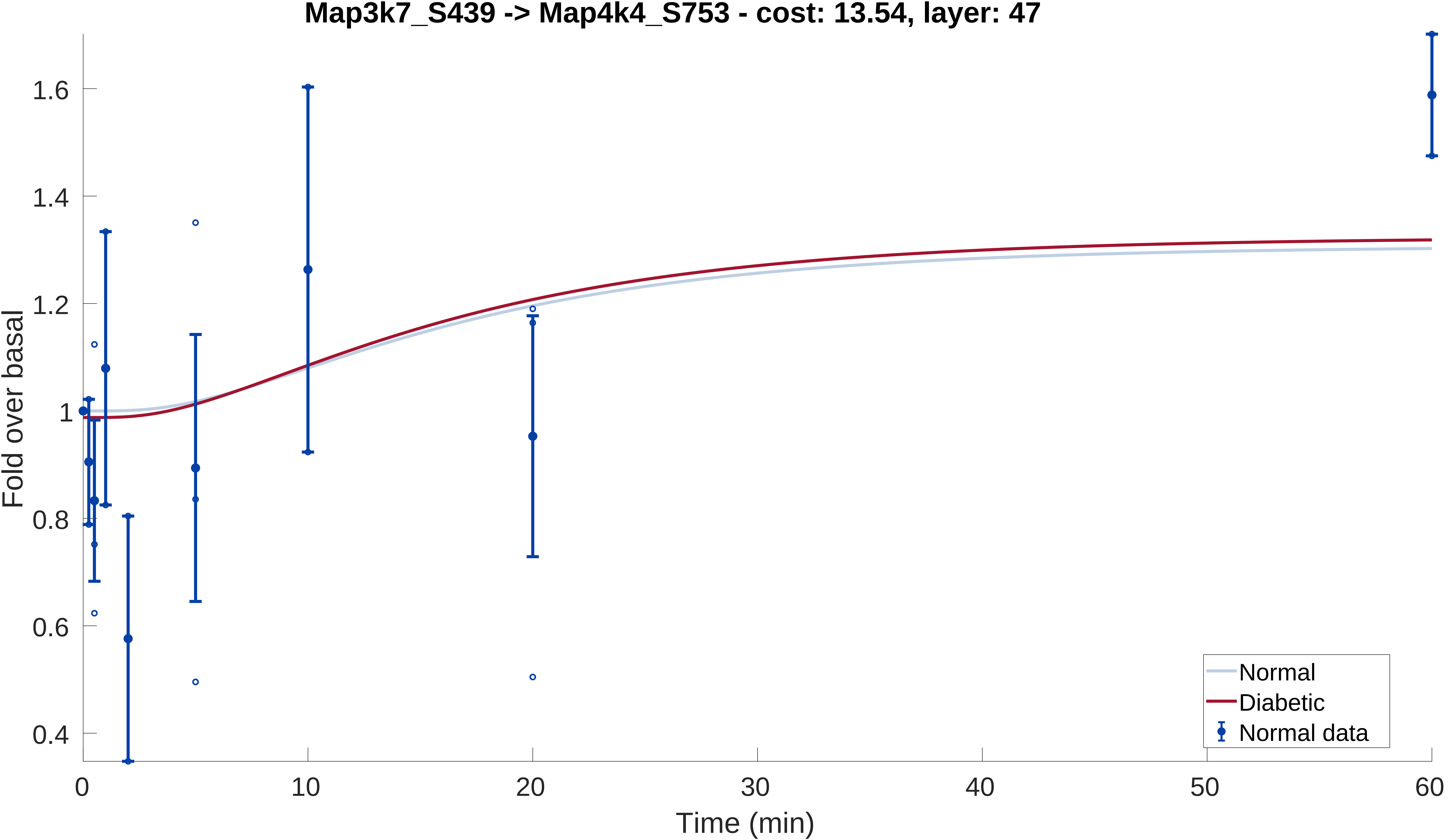
Map3k7_S439 -> Map4k4_S753 - cost: 13.54, layer: 47
Fold over basal
1.6
1.4
1.2
1
0.8
0.6
0.4
0
10
20
30
40
50
60
Time (min)
Normal
Diabetic
Normal data

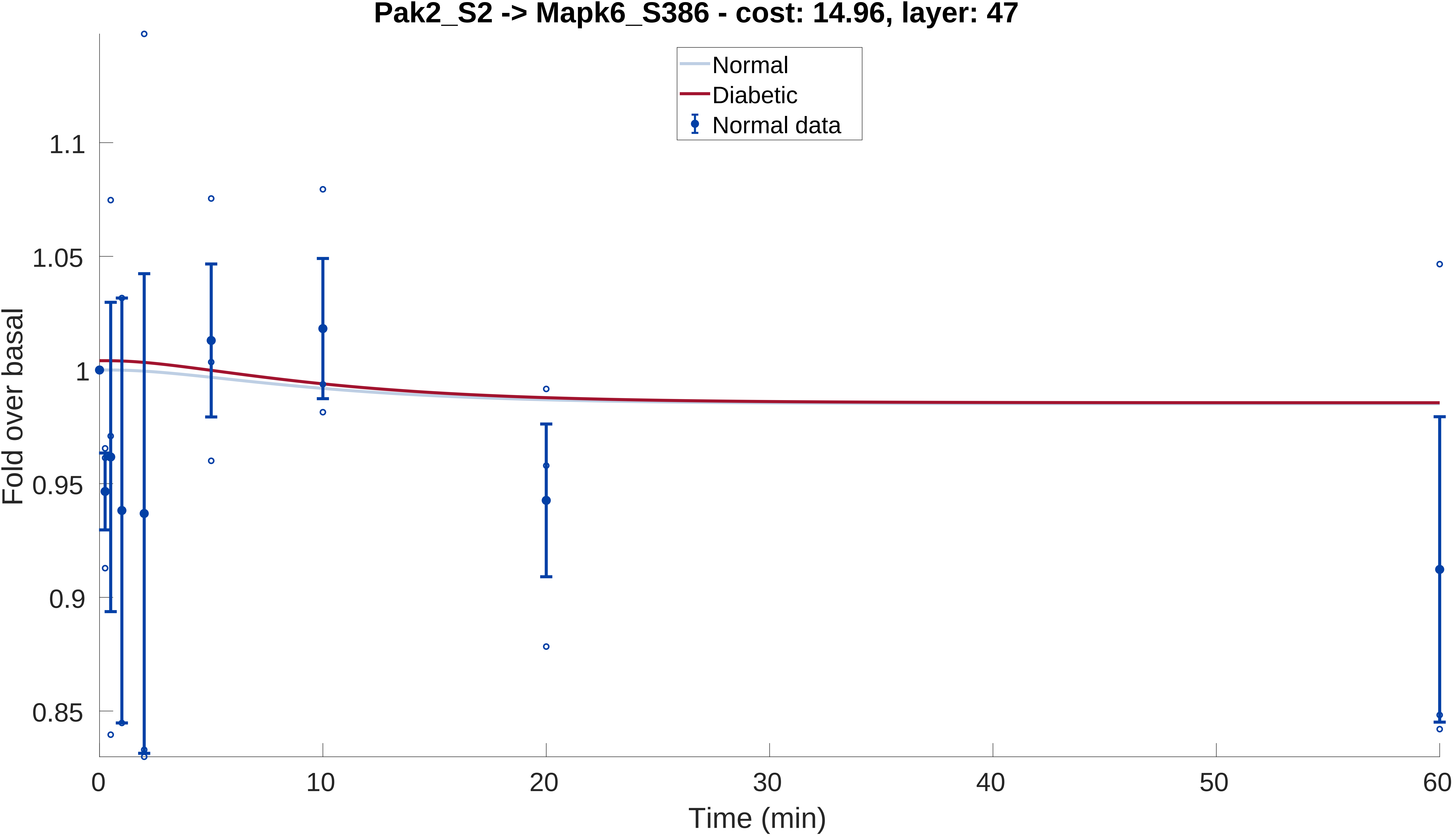
Pak2_S2 -> Mapk6_S386 - cost: 14.96, layer: 47
Normal
Diabetic
Normal data
Fold over basal
1.1
1.05
1
0.95
0.9
0.85
0
10
20
30
40
50
60
Time (min)

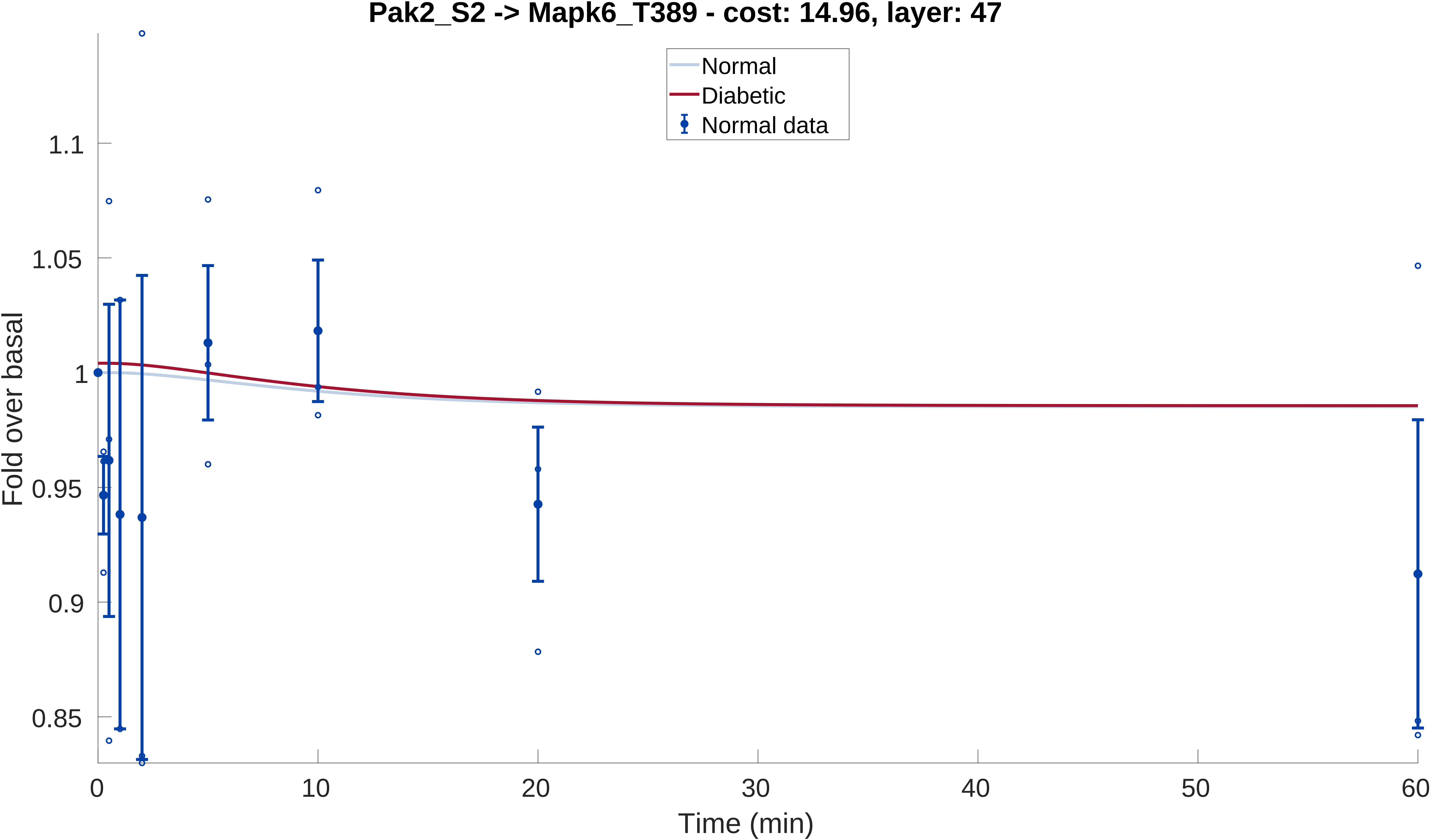

Pak2_S2 -> Mapk6_T389 - cost: 14.96, layer: 47
Normal
Diabetic
Normal data
Fold over basal
1.1
1.05
1
0.95
0.9
0.85
0
10
20
30
40
50
60
Time (min)

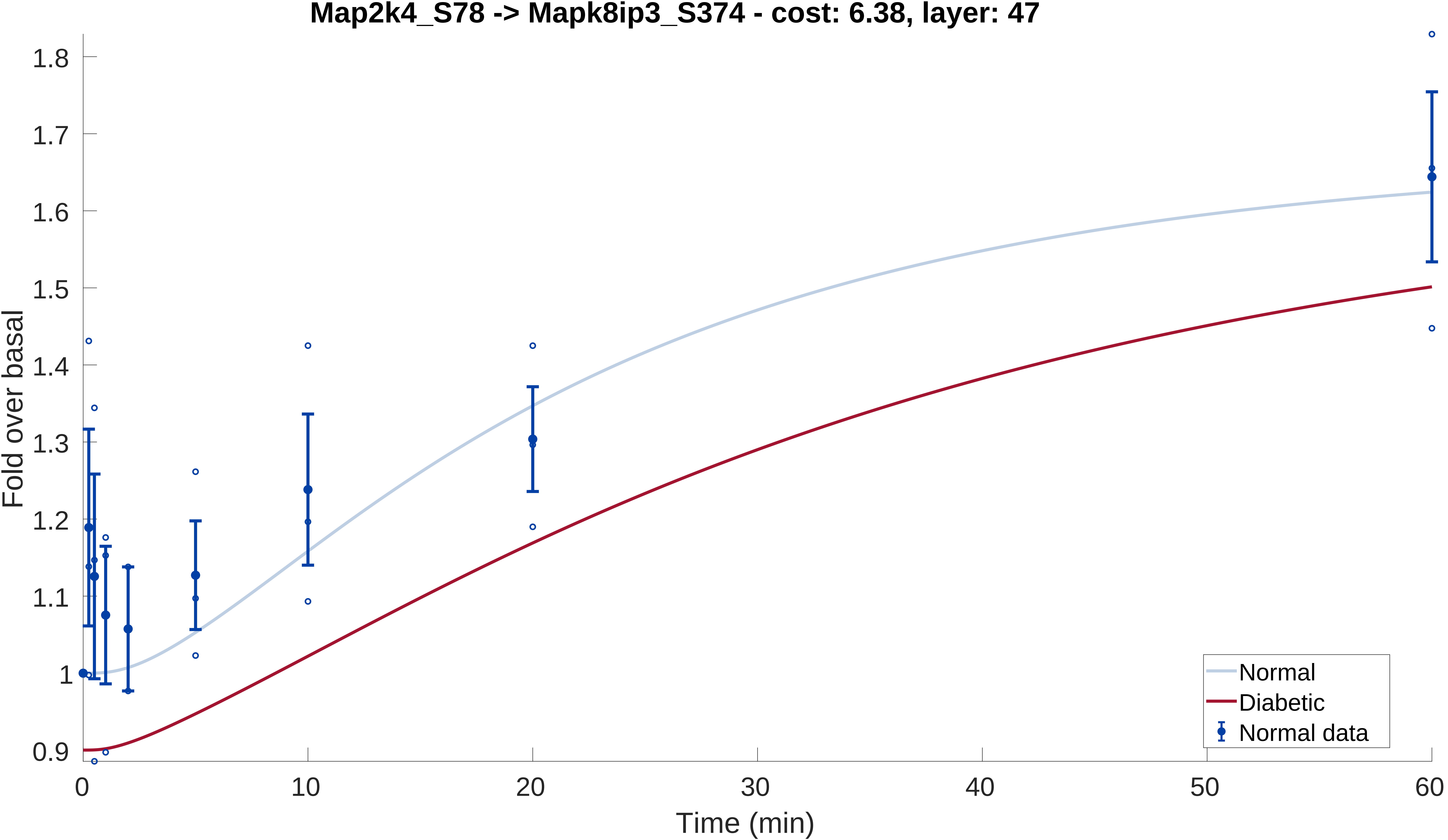
Map2k4_S78 -> Mapk8ip3_S374 - cost: 6.38, layer: 47
Fold over basal
1.8
1.7
1.6
1.5
1.4
1.3
1.2
1.1
1
0.9
0
10
20
30
40
50
60
Time (min)
Normal
Diabetic
Normal data

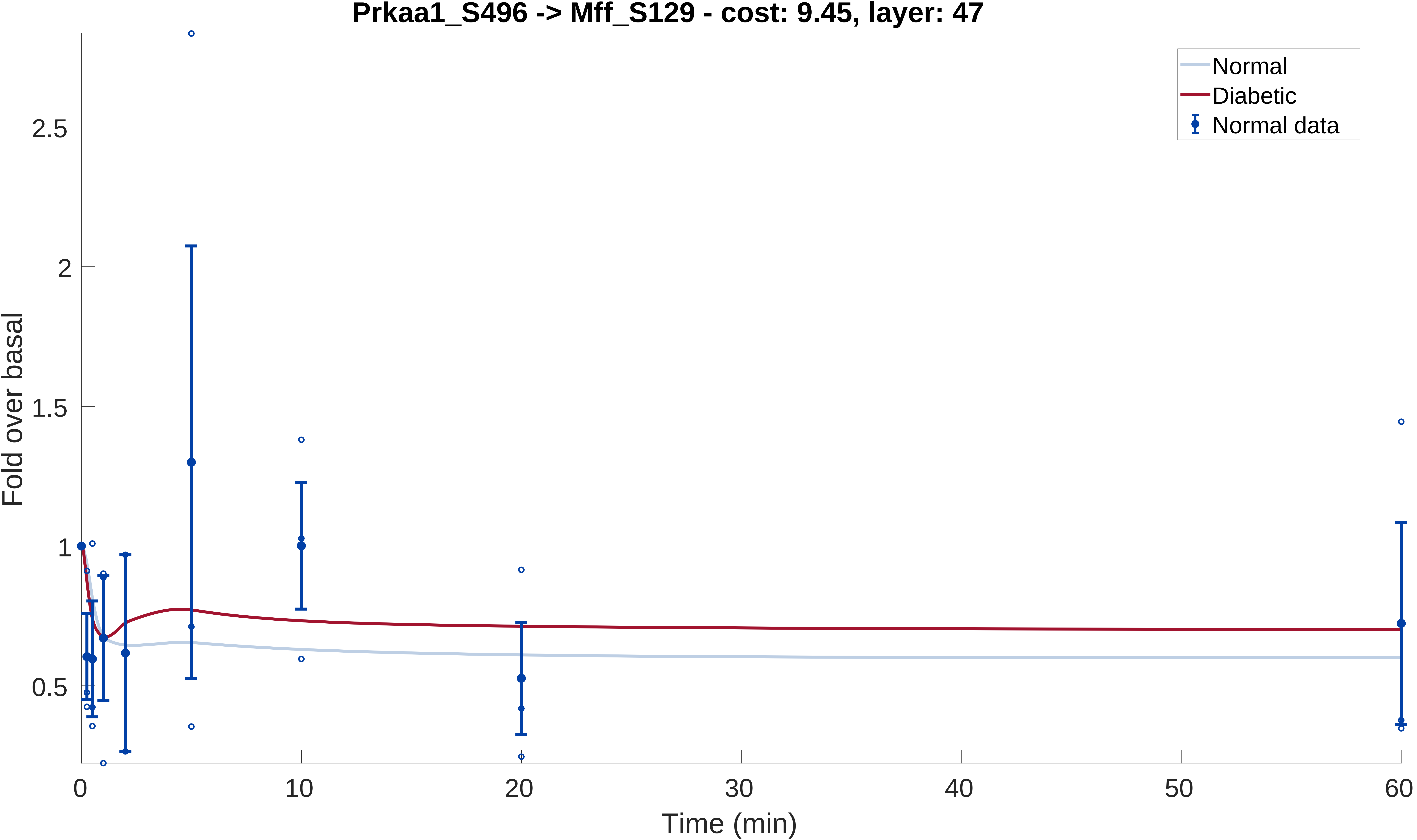

Prkaa1_S496 -> Mff_S129 - cost: 9.45, layer: 47
Normal
Diabetic
Normal data
Fold over basal
Time (min)
0.5
1
1.5
2
2.5
0
10
20
30
40
50
60

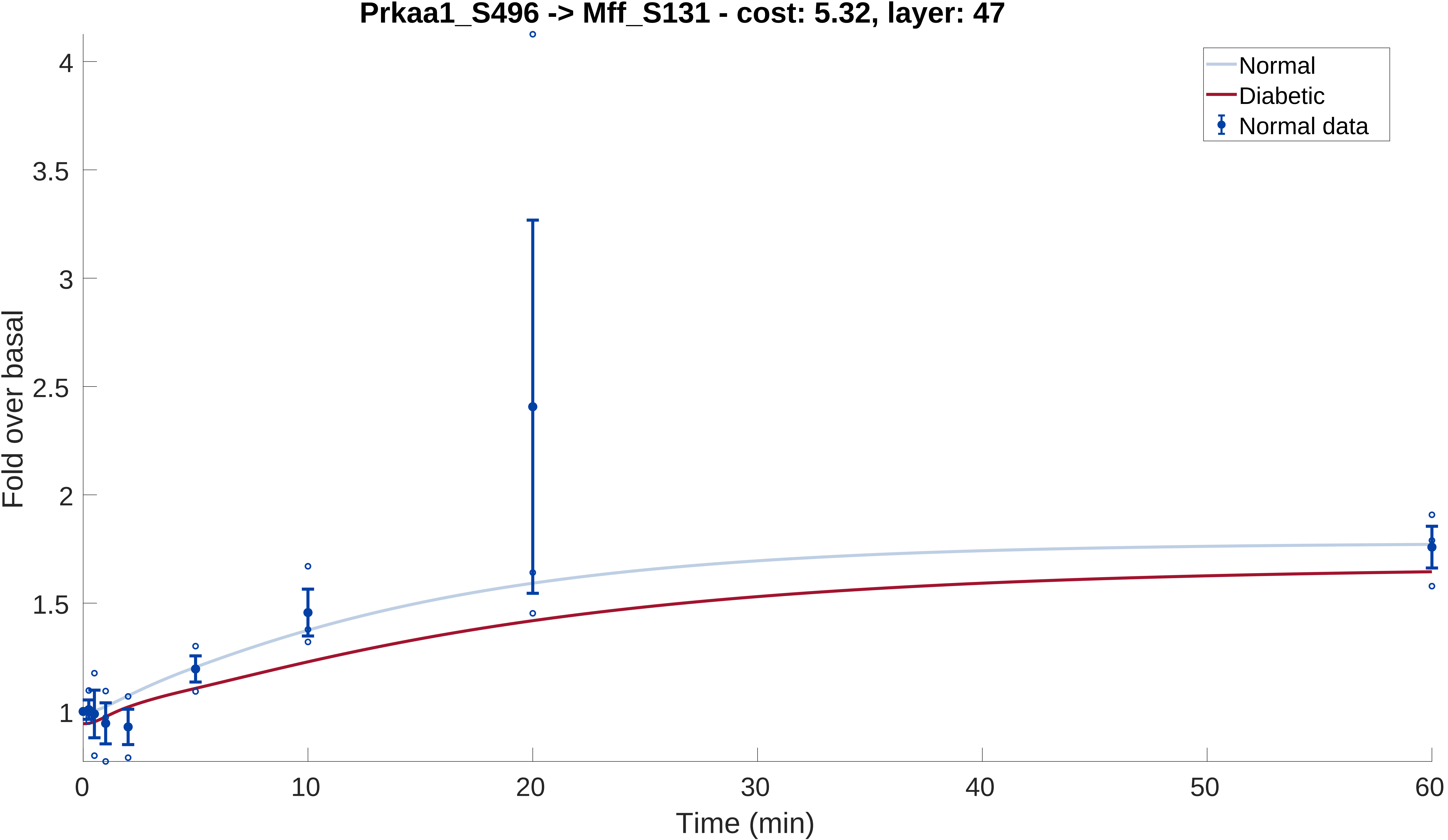

Prkaa1_S496 -> Mff_S131 - cost: 5.32, layer: 47
Normal
Diabetic
Normal data
Fold over basal
4
3.5
3
2.5
2
1.5
1
0
10
20
30
40
50
60
Time (min)

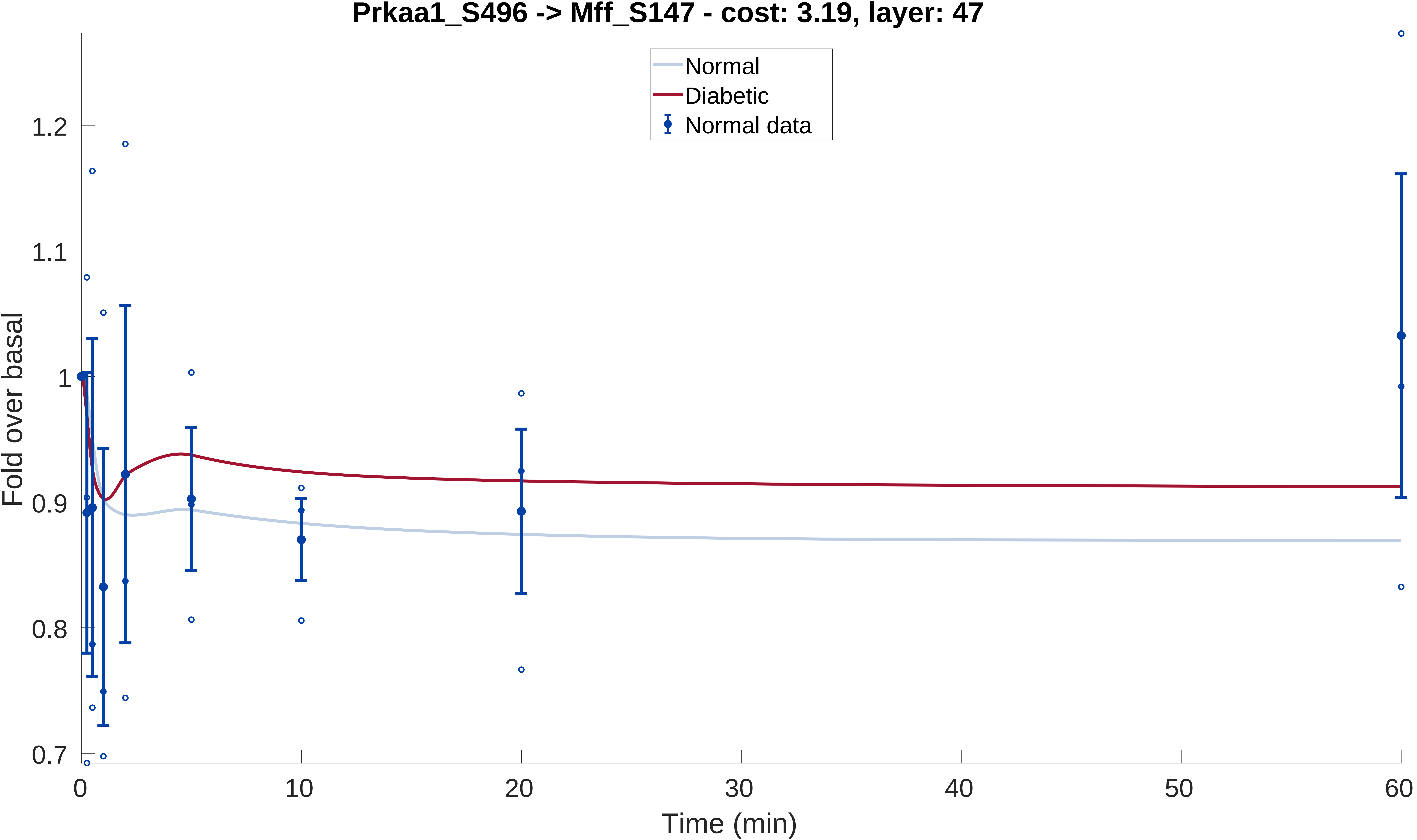

Prkaa1_S496 -> Mff_S147 - cost: 3.19, layer: 47
Normal
Diabetic
Normal data
Fold over basal
1.2
1.1
1
0.9
0.8
0.7
0
10
20
30
40
50
60
Time (min)

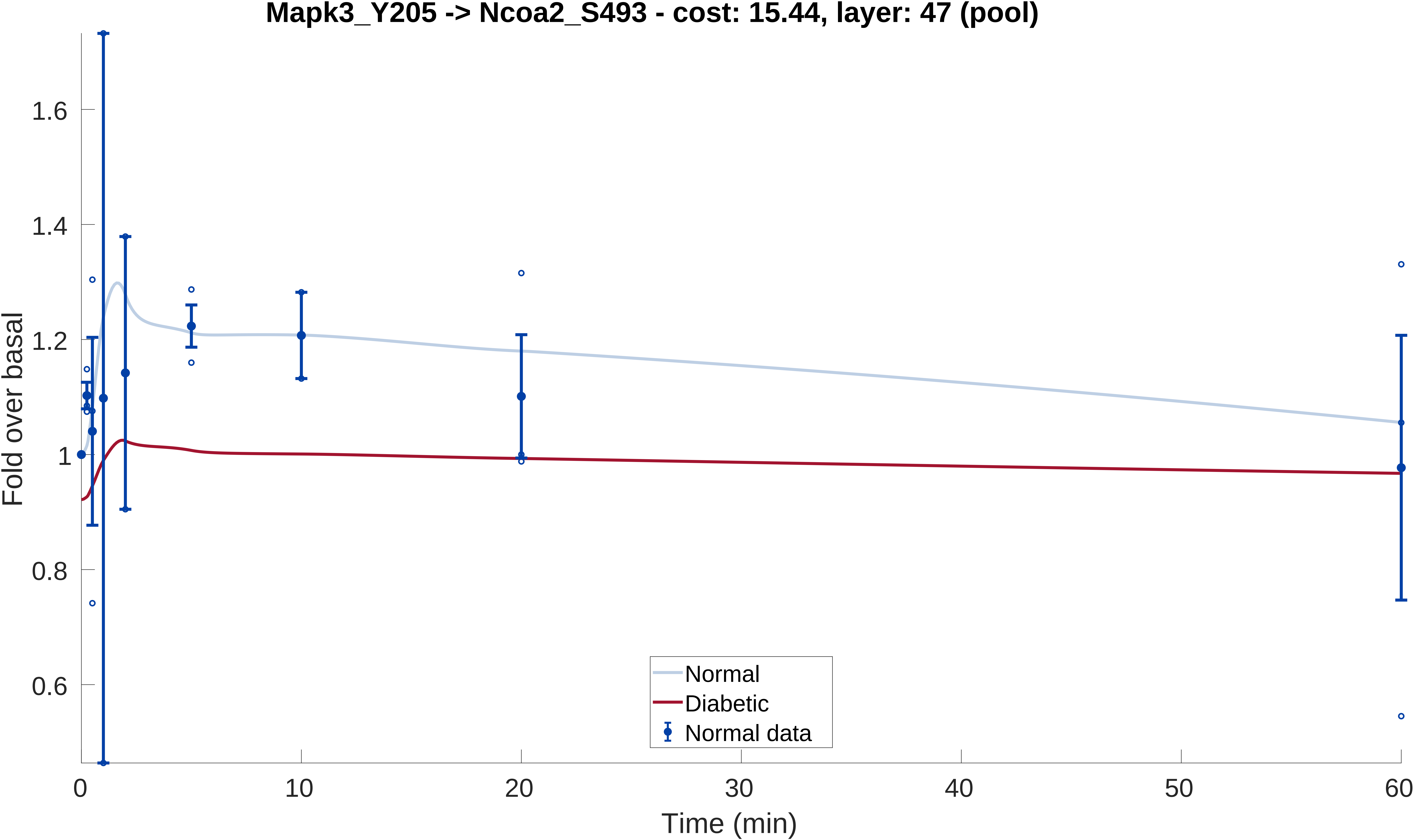

Mapk3_Y205 -> Ncoa2_S493 - cost: 15.44, layer: 47 (pool)
Fold over basal
Time (min)
1.6
1.4
1.2
1
0.8
0.6
0
10
20
30
40
50
60
Normal
Diabetic
Normal data

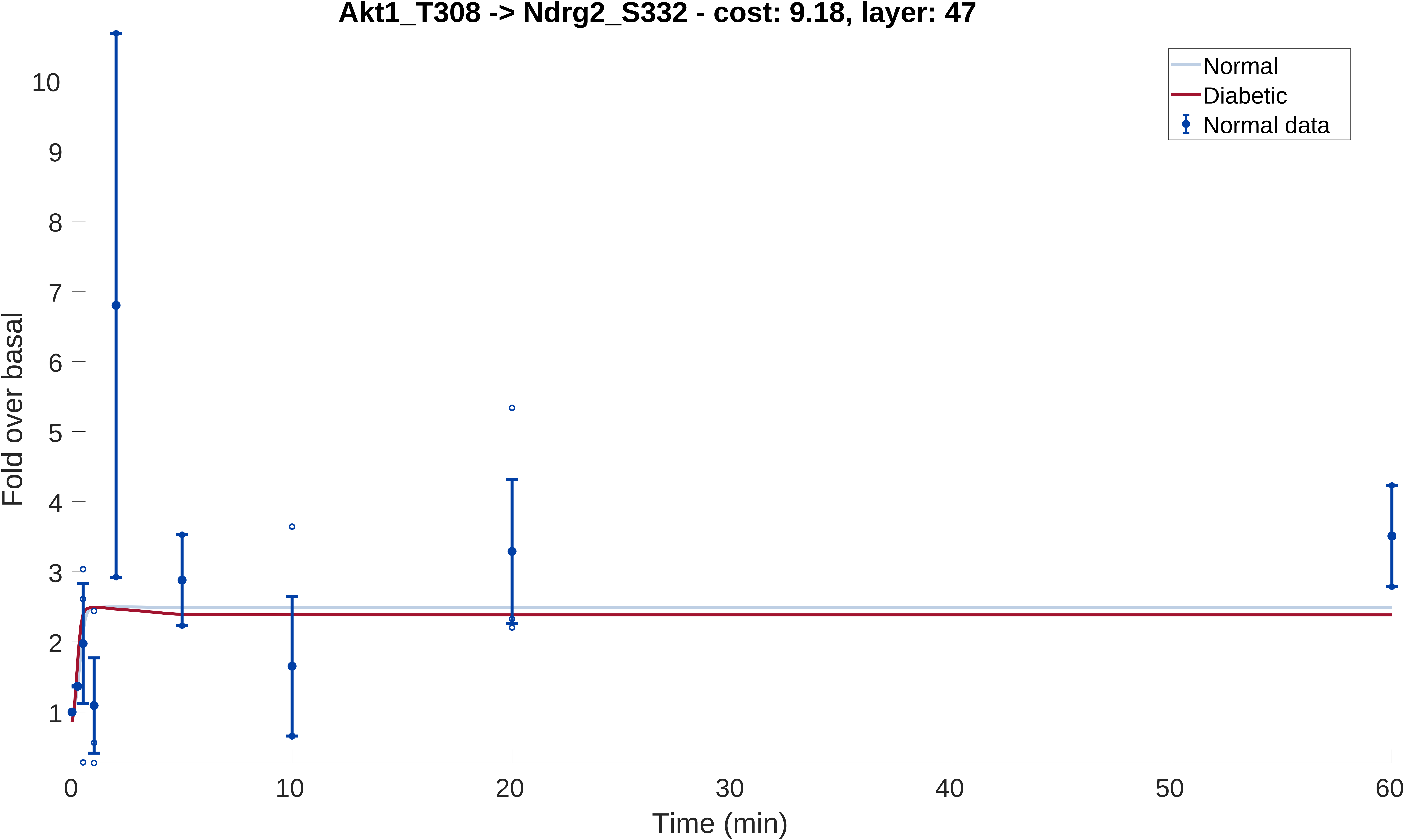

Akt1_T308 -> Ndrg2_S332 - cost: 9.18, layer: 47
Normal
Diabetic
Normal data
Fold over basal
Time (min)
0
10
20
30
40
50
60
1
2
3
4
5
6
7
8
9
10

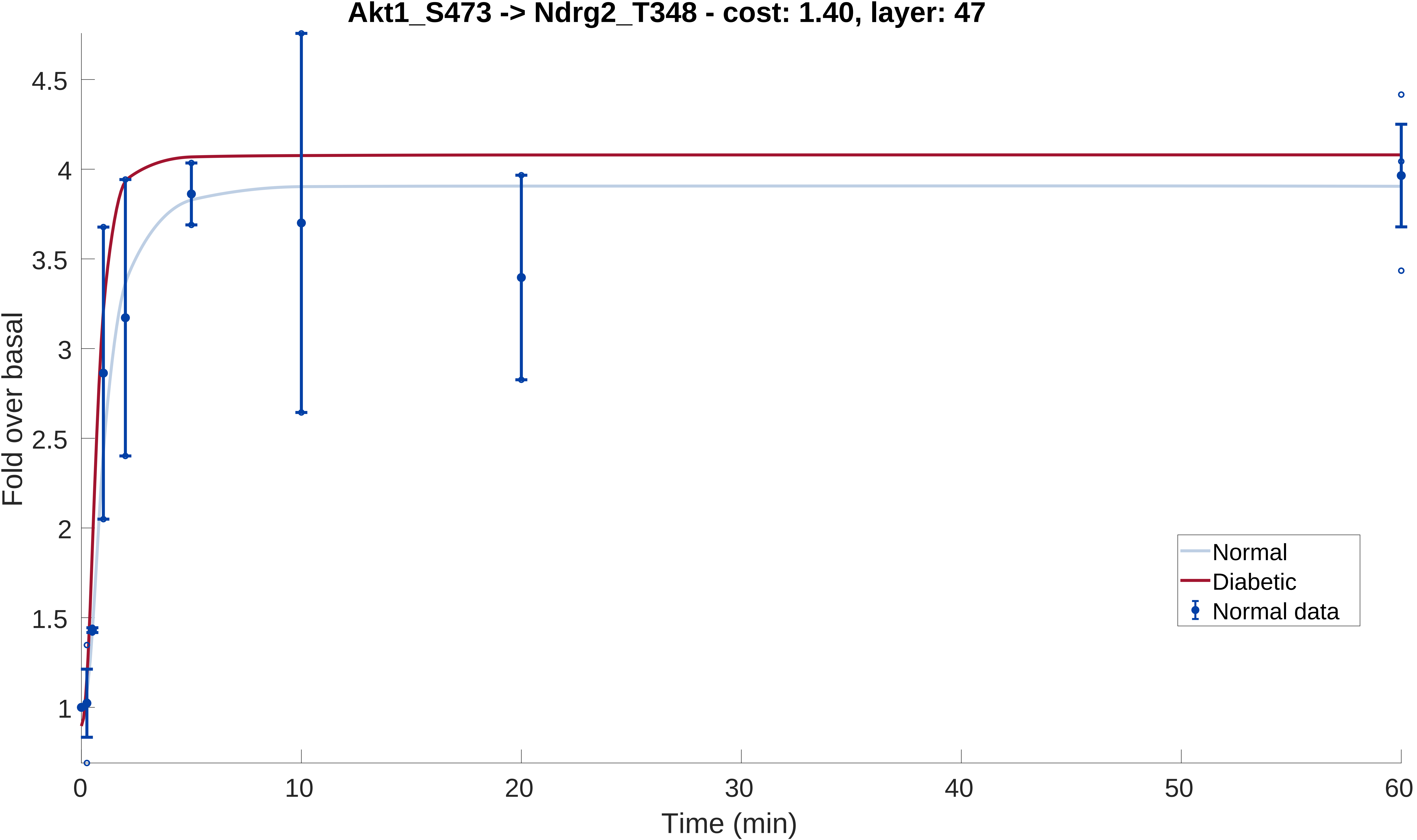

Akt1_S473 -> Ndrg2_T348 - cost: 1.40, layer: 47
Fold over basal
Time (min)
4.5
4
3.5
3
2.5
2
1.5
1
0
10
20
30
40
50
60
Normal
Diabetic
Normal data

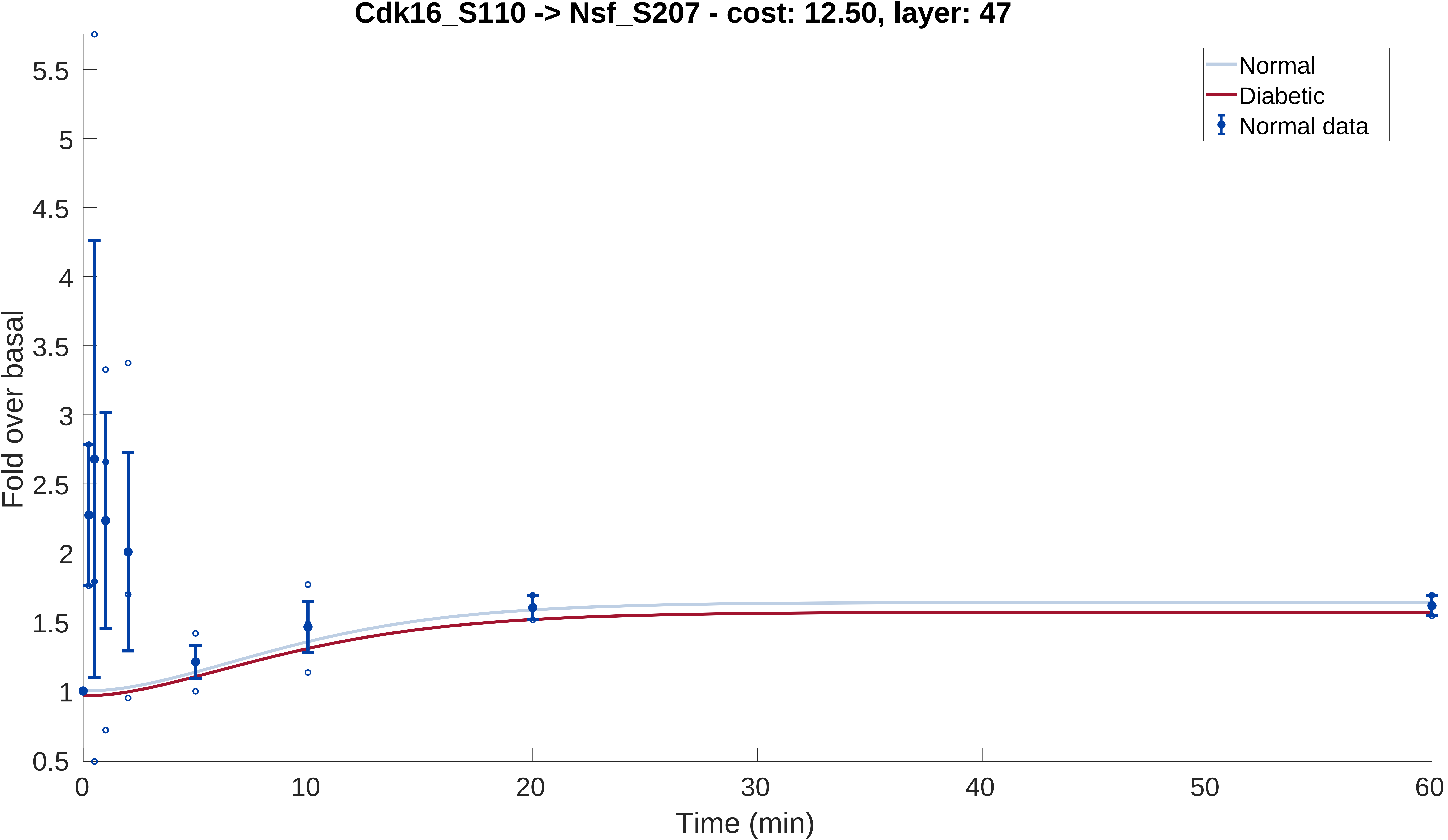
Cdk16_S110 -> Nsf_S207 - cost: 12.50, layer: 47
Normal
Diabetic
Normal data
Fold over basal
5.5
5
4.5
4
3.5
3
2.5
2
1.5
1
0.5
0
10
20
30
40
50
60
Time (min)

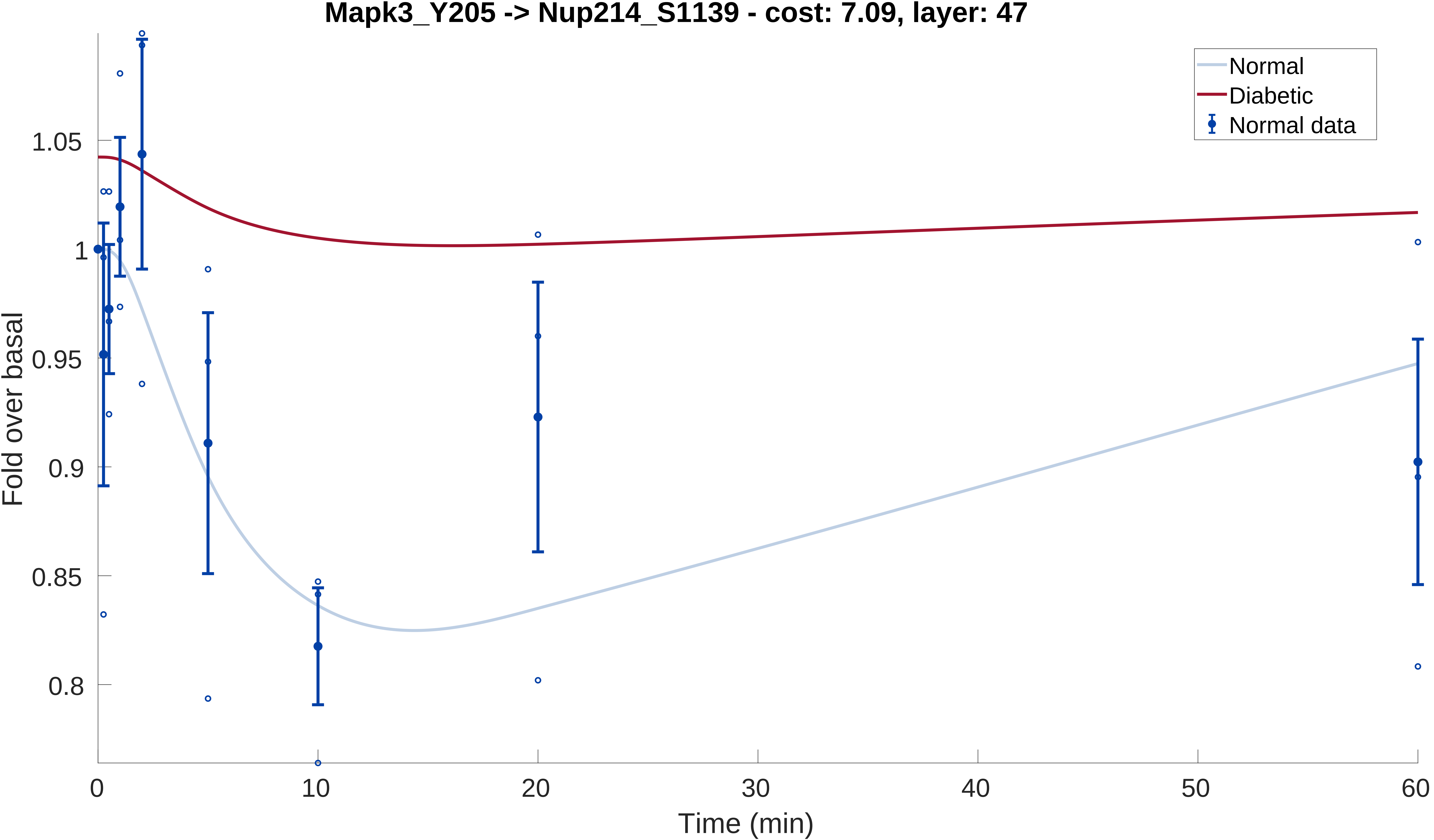

Mapk3_Y205 -> Nup214_S1139 - cost: 7.09, layer: 47
Normal
Diabetic
Normal data
1.05
1
0.95
0.9
0.85
0.8
Fold over basal
0
10
20
30
40
50
60
Time (min)

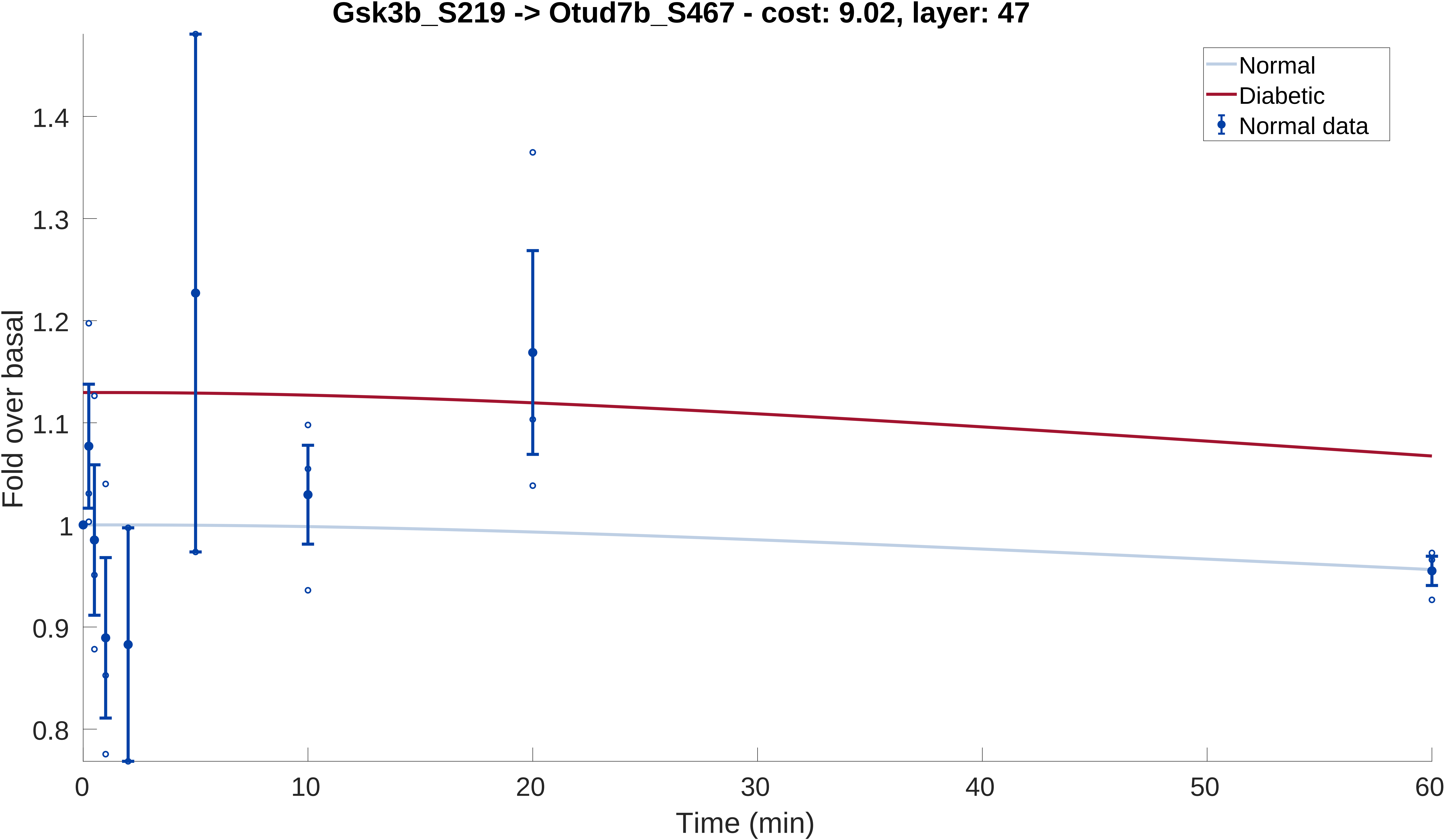
Gsk3b_S219 -> Otud7b_S467 - cost: 9.02, layer: 47
Normal
Diabetic
Normal data
Fold over basal
1.4
1.3
1.2
1.1
1
0.9
0.8
0
10
20
30
40
50
60
Time (min)

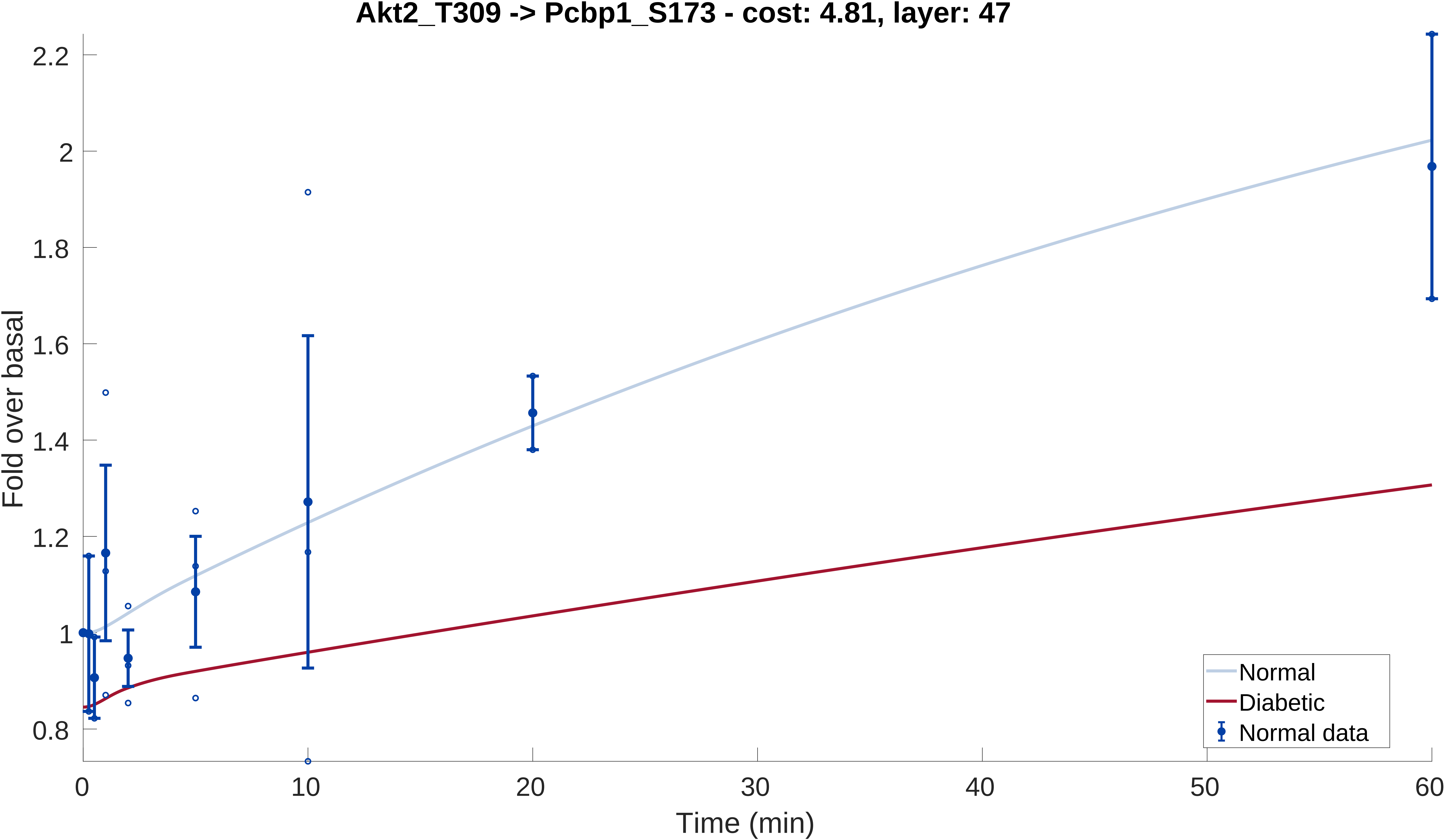

Akt2_T309 -> Pcbp1_S173 - cost: 4.81, layer: 47
Fold over basal
2.2
2
1.8
1.6
1.4
1.2
1
0.8
0
10
20
30
40
50
60
Time (min)
Normal
Diabetic
Normal data

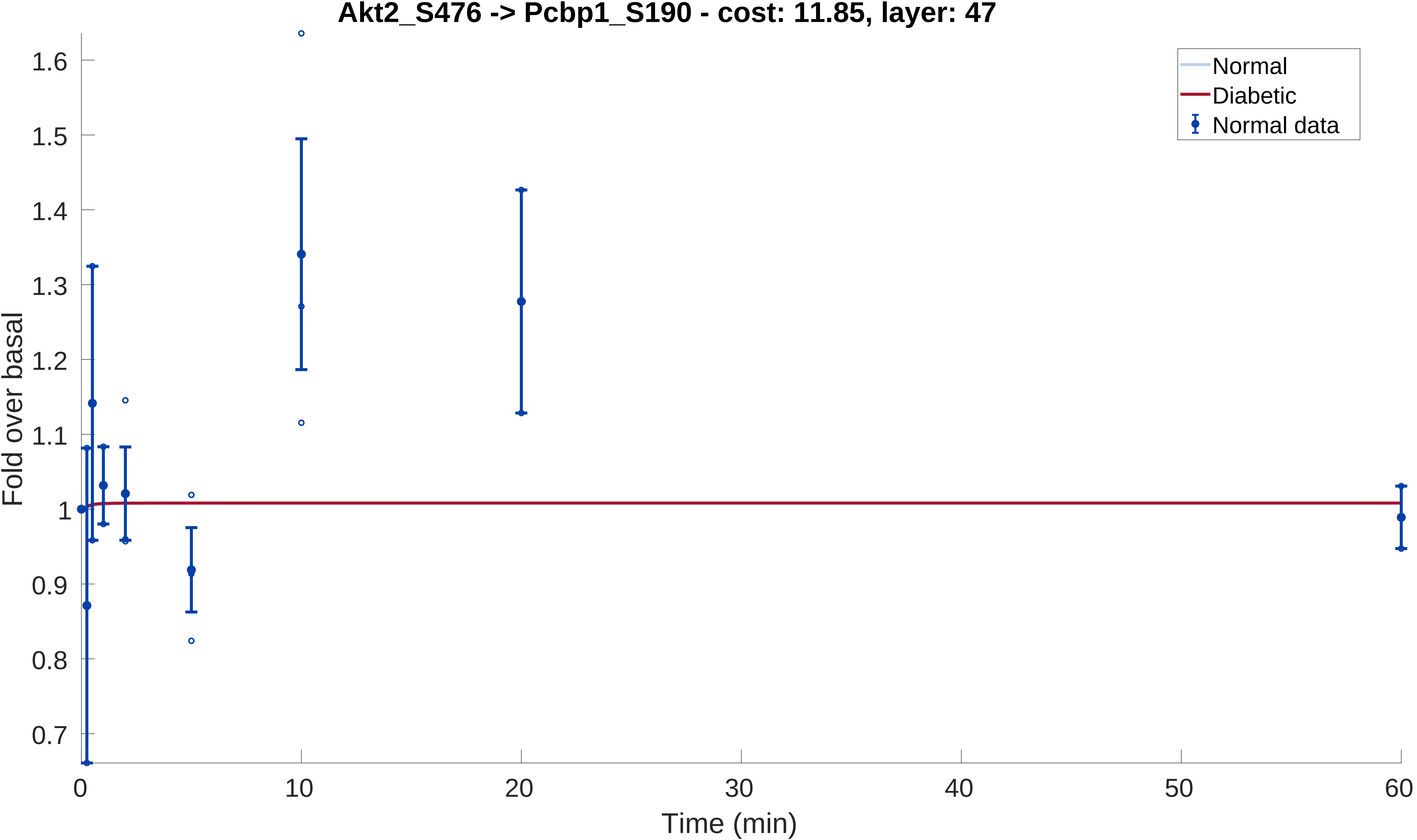
Akt2_S476 -> Pcbp1_S190 - cost: 11.85, layer: 47
Fold over basal
1.6
1.5
1.4
1.3
1.2
1.1
1
0.9
0.8
0.7
0
10
20
30
40
50
60
Time (min)
Normal
Diabetic
Normal data

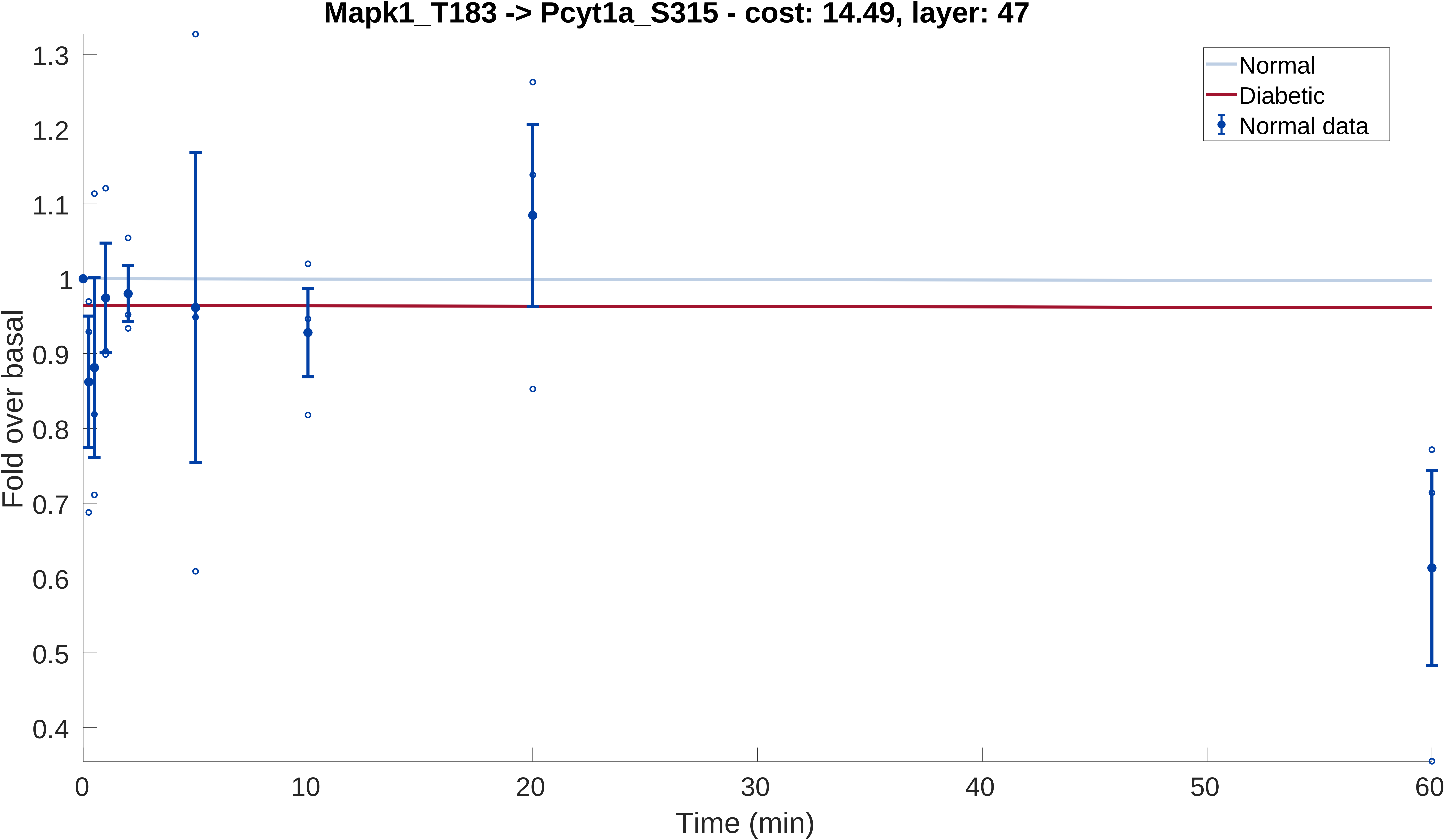

Mapk1_T183 -> Pcyt1a_S315 - cost: 14.49, layer: 47
Normal
Diabetic
Normal data
Fold over basal
Time (min)
1.3
1.2
1.1
1
0.9
0.8
0.7
0.6
0.5
0.4
0
10
20
30
40
50
60

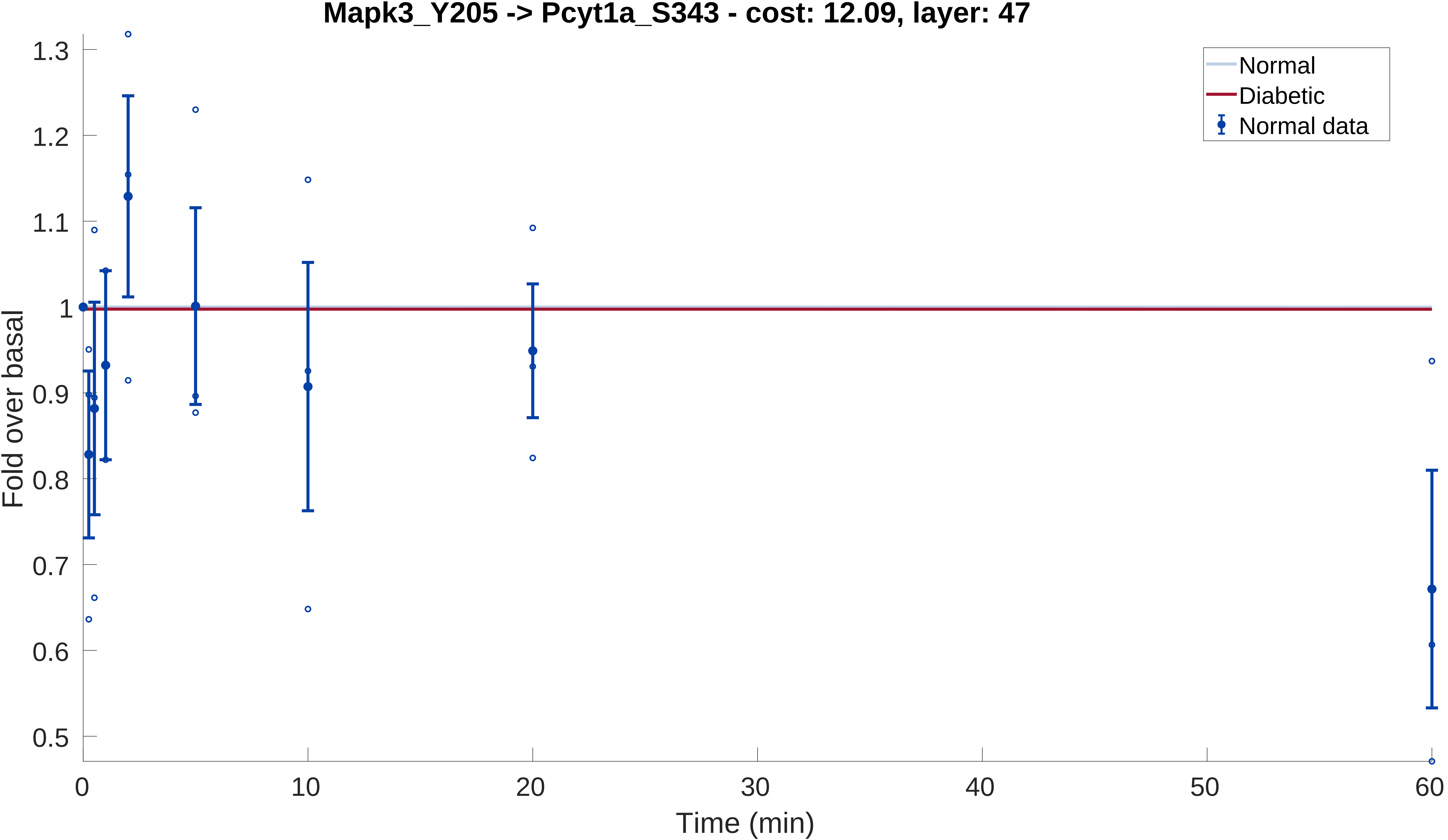

Mapk3_Y205 -> Pcyt1a_S343 - cost: 12.09, layer: 47
Normal
Diabetic
Normal data
Fold over basal
Time (min)
1.3
1.2
1.1
1
0.9
0.8
0.7
0.6
0.5
0
10
20
30
40
50
60

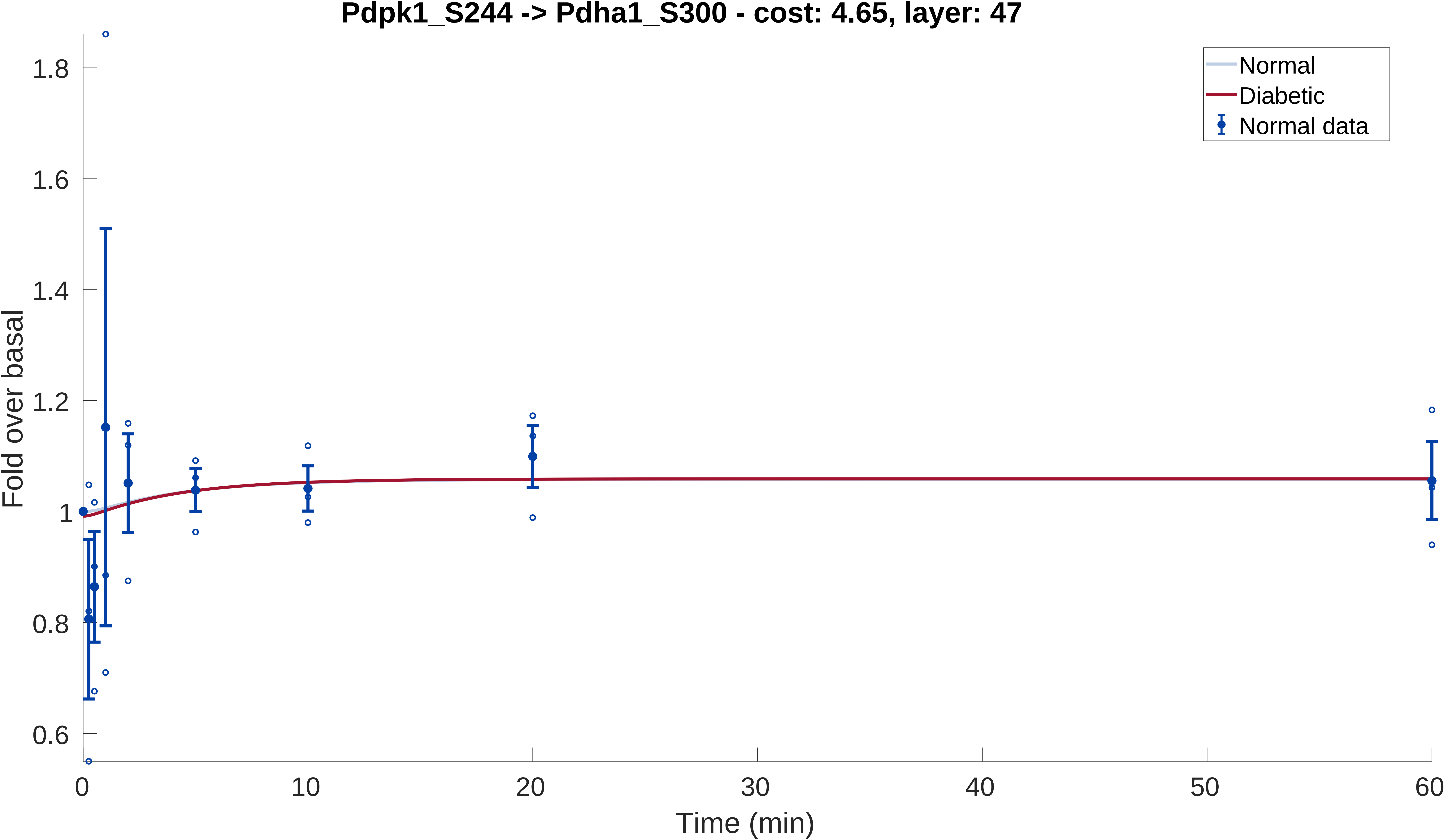
Pdpk1_S244 -> Pdha1_S300 - cost: 4.65, layer: 47
Normal
Diabetic
Normal data
Fold over basal
Time (min)
0.6
0.8
1
1.2
1.4
1.6
1.8
0
10
20
30
40
50
60

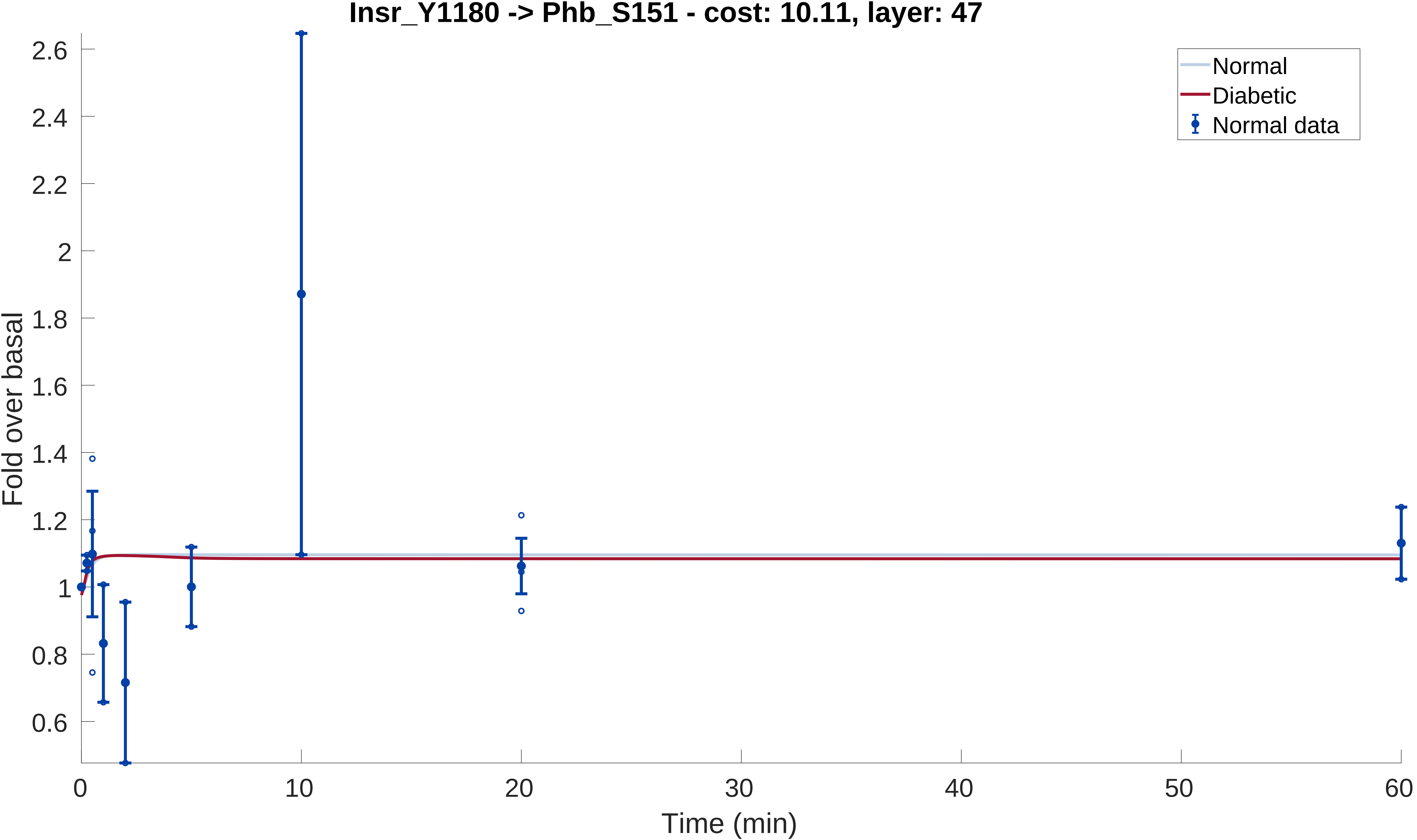

Insr_Y1180 -> Phb_S151 - cost: 10.11, layer: 47
Normal
Diabetic
Normal data
Fold over basal
Time (min)
2.6
2.4
2.2
2
1.8
1.6
1.4
1.2
1
0.8
0.6
0
10
20
30
40
50
60

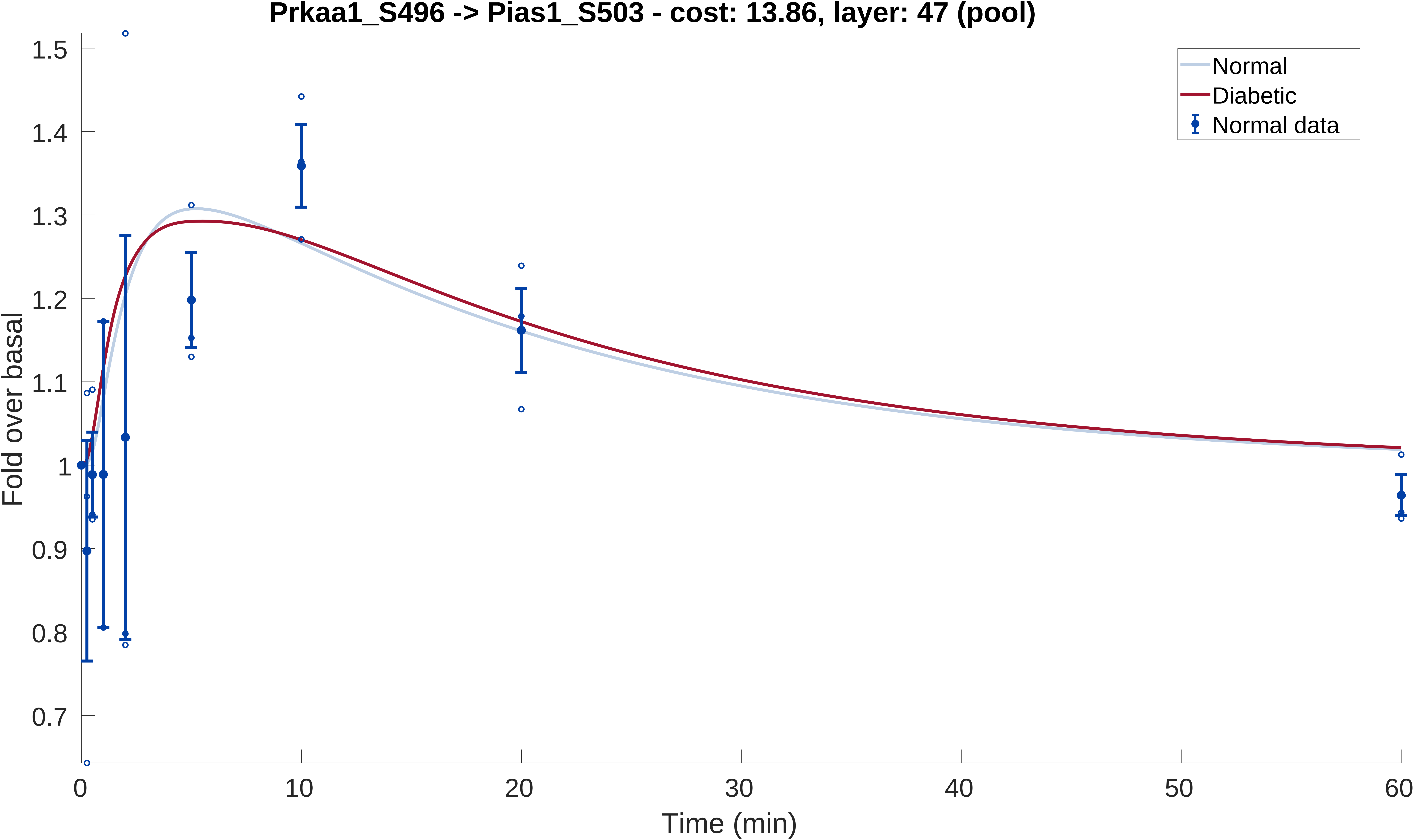
Prkaa1_S496 -> Pias1_S503 - cost: 13.86, layer: 47 (pool)
Normal
Diabetic
Normal data
Fold over basal
1.5
1.4
1.3
1.2
1.1
1
0.9
0.8
0.7
0
10
20
30
40
50
60
Time (min)

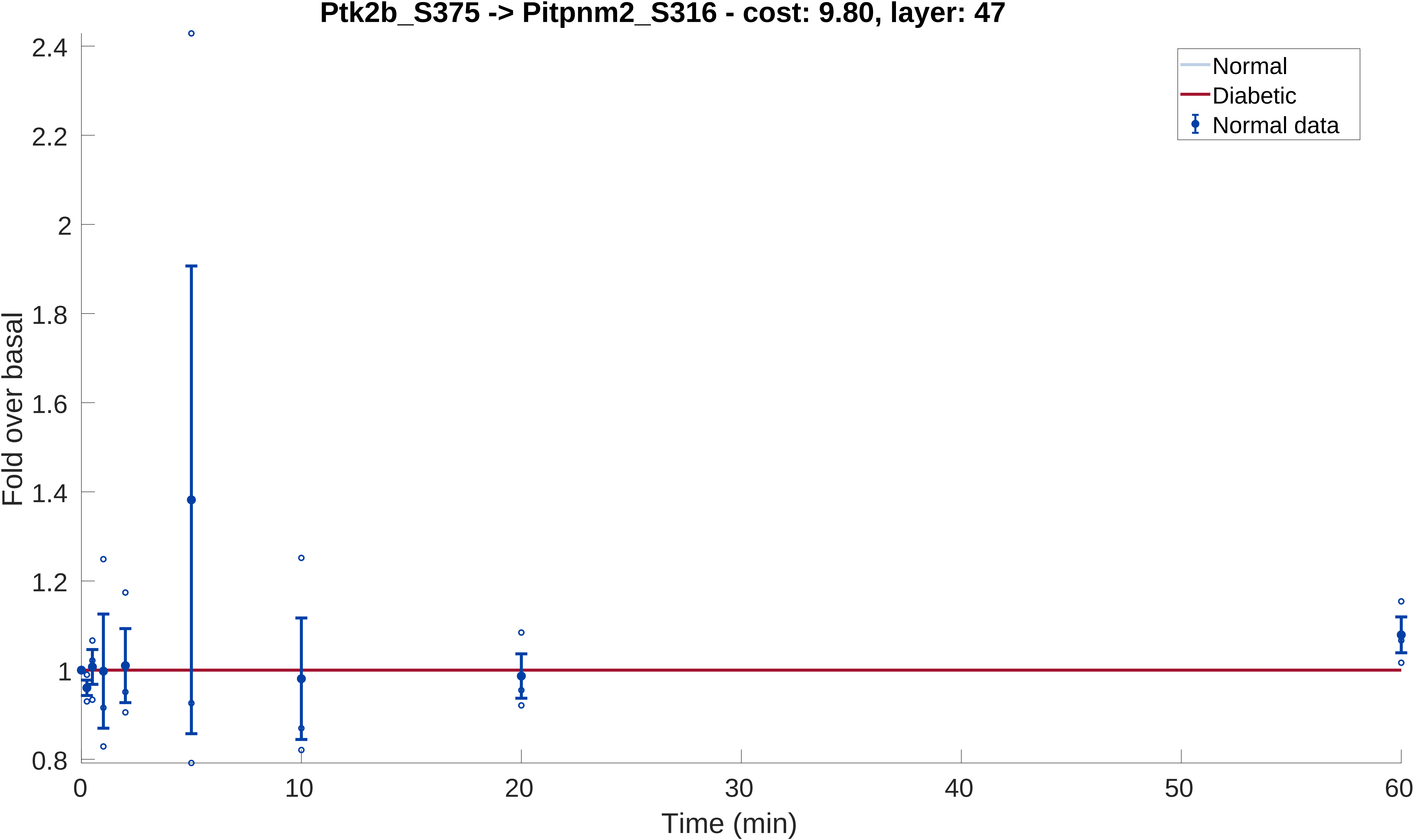
Ptk2b_S375 -> Pitpnm2_S316 - cost: 9.80, layer: 47
Normal
Diabetic
Normal data
Fold over basal
Time (min)
0.8
1
1.2
1.4
1.6
1.8
2
2.2
2.4
0
10
20
30
40
50
60

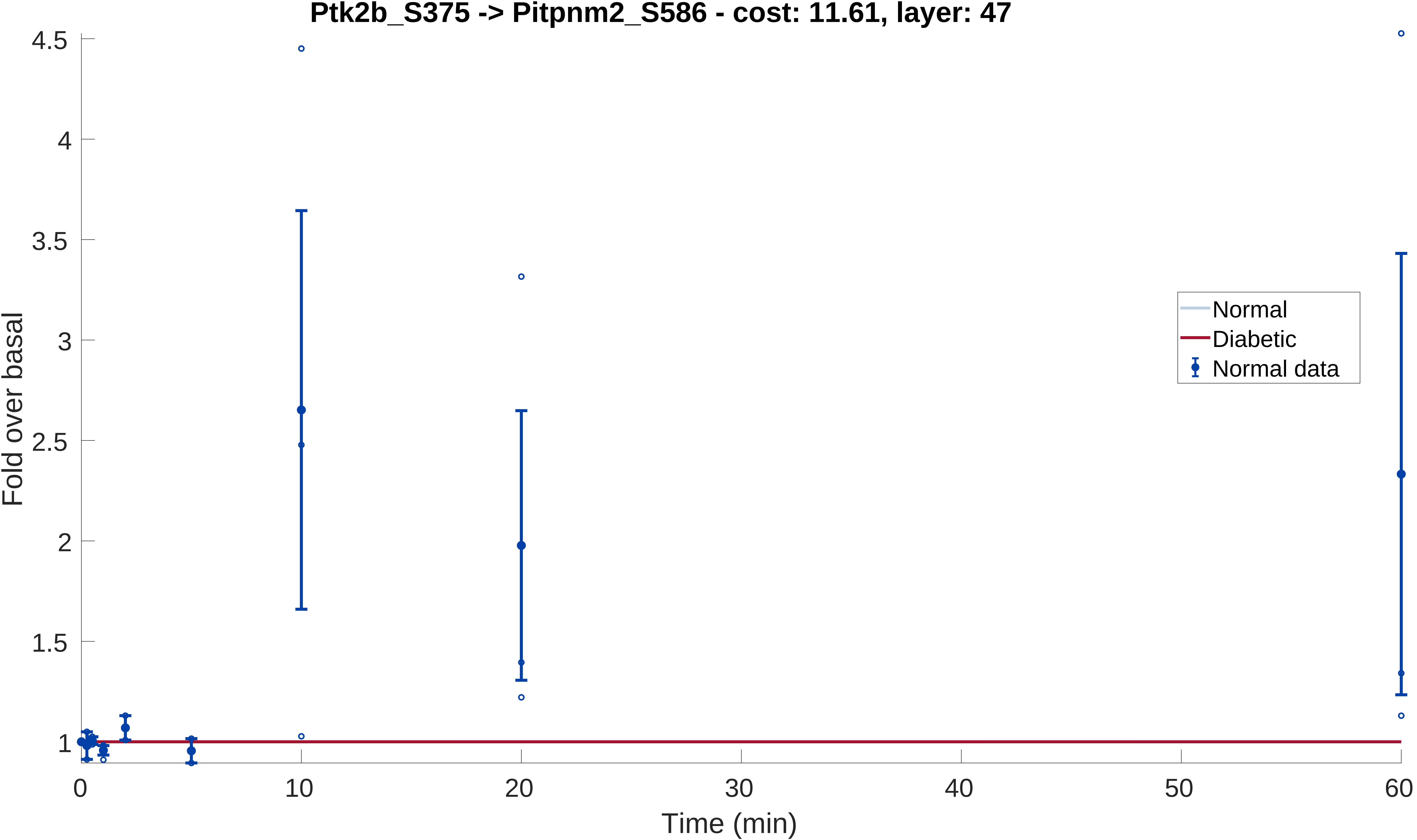

Ptk2b_S375 -> Pitpnm2_S586 - cost: 11.61, layer: 47
Fold over basal
4.5
4
3.5
3
2.5
2
1.5
1
0
10
20
30
40
50
60
Time (min)
Normal
Diabetic
Normal data

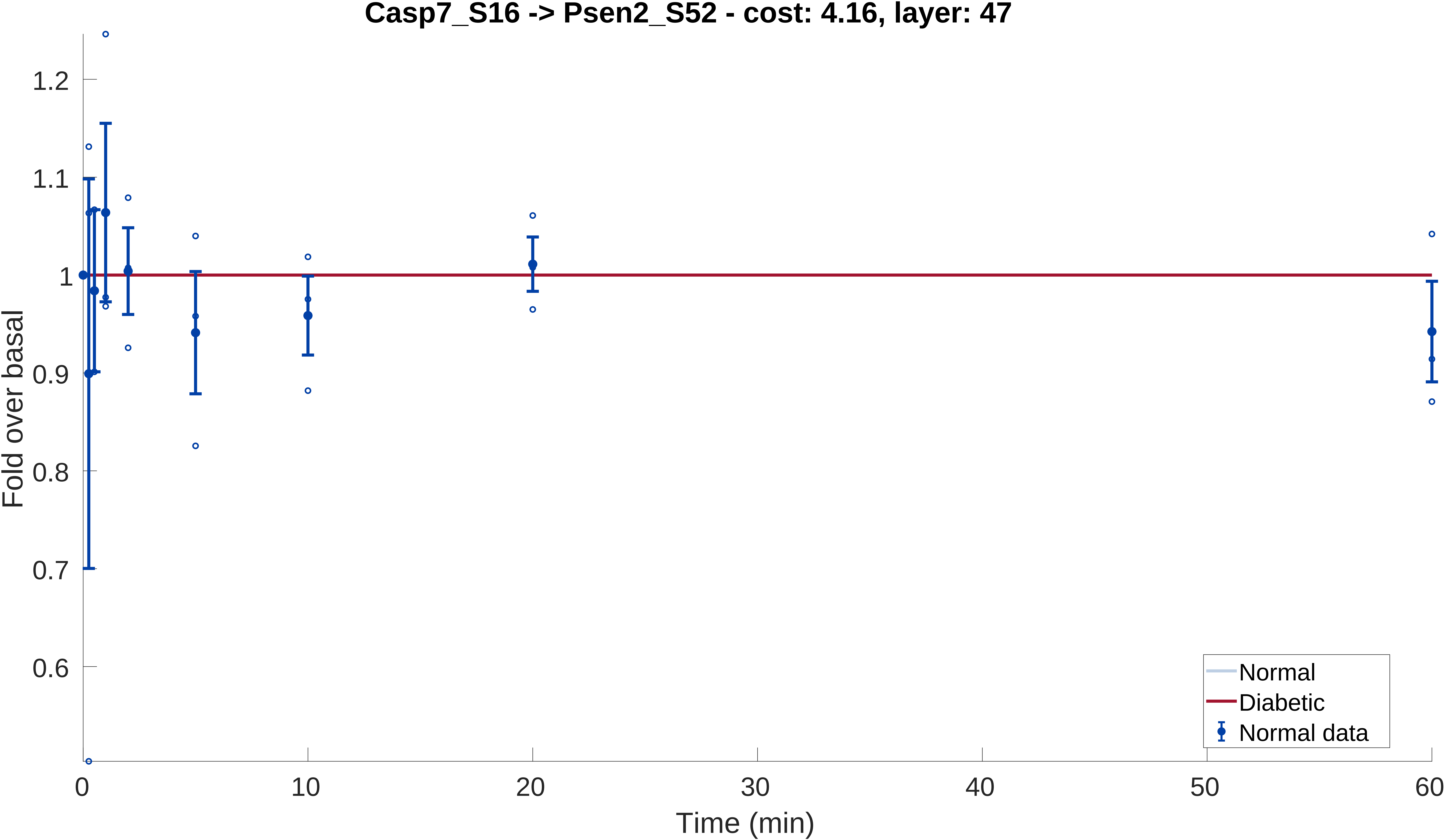

Casp7_S16 -> Psen2_S52 - cost: 4.16, layer: 47
Fold over basal
1.2
1.1
1
0.9
0.8
0.7
0.6
0
10
20
30
40
50
60
Time (min)
Normal
Diabetic
Normal data

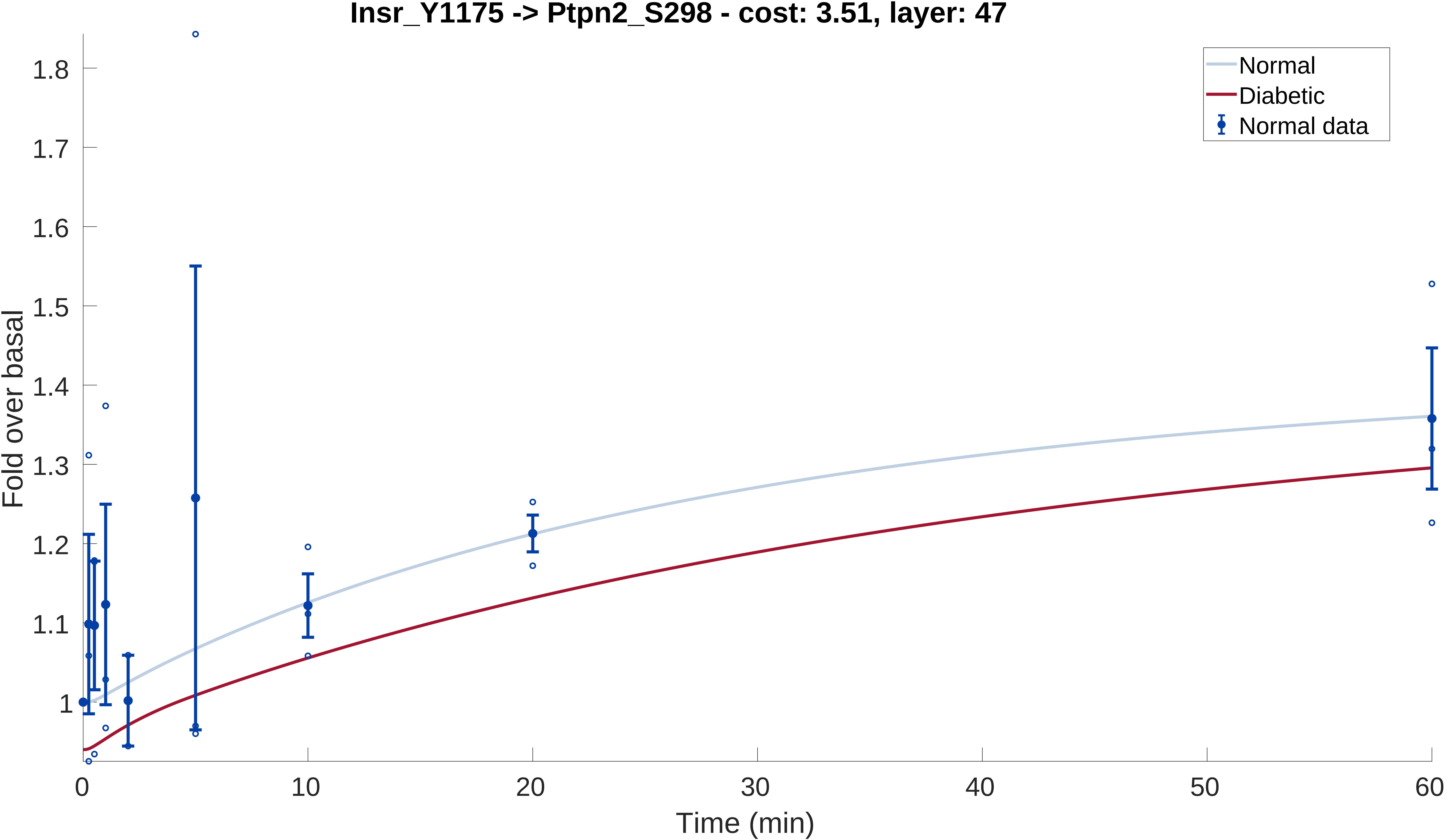

Insr_Y1175 -> Ptpn2_S298 - cost: 3.51, layer: 47
Normal
Diabetic
Normal data
Fold over basal
1.8
1.7
1.6
1.5
1.4
1.3
1.2
1.1
1
0
10
20
30
40
50
60
Time (min)

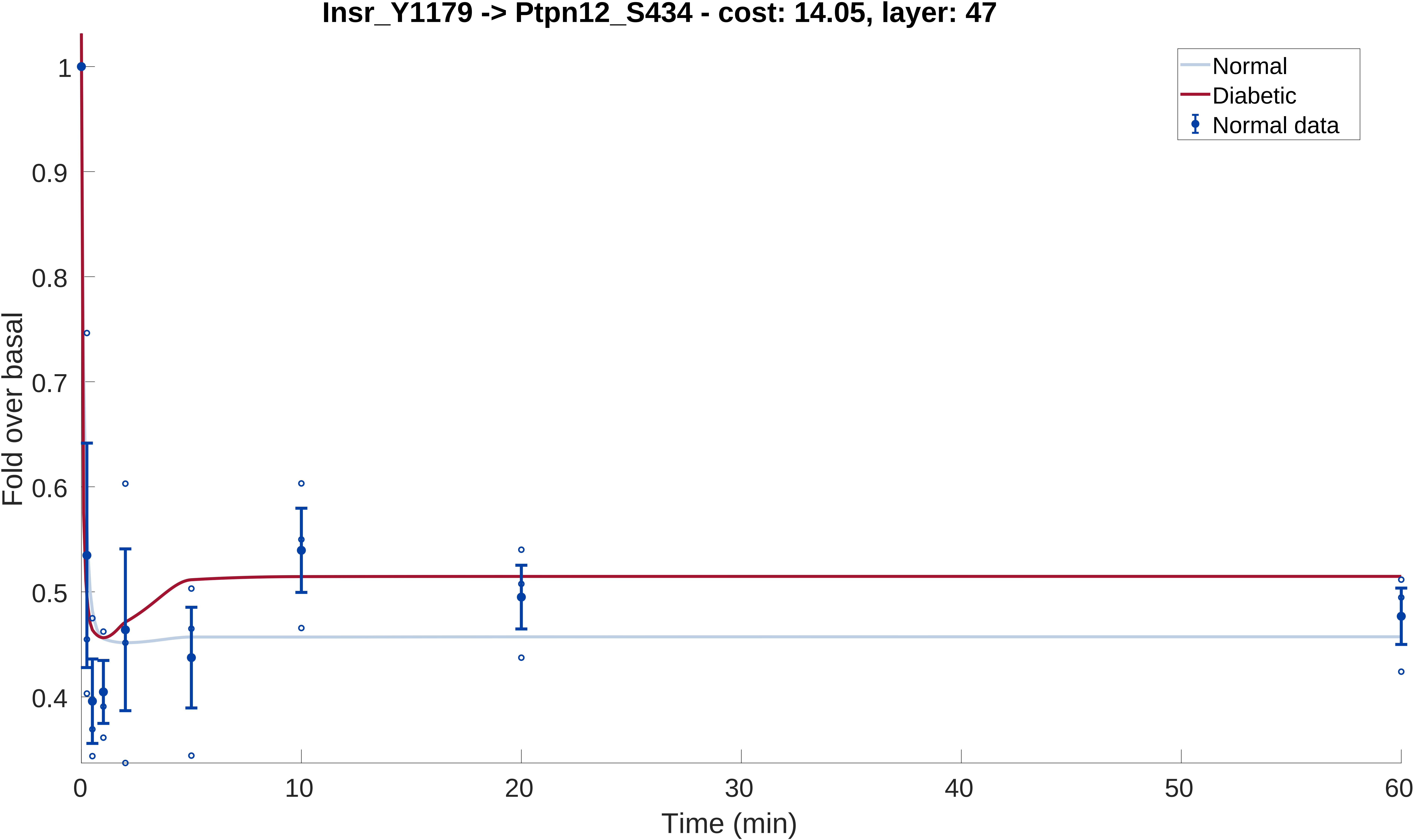

Insr_Y1179 -> Ptpn12_S434 - cost: 14.05, layer: 47
Normal
Diabetic
Normal data
Fold over basal
1
0.9
0.8
0.7
0.6
0.5
0.4
0
10
20
30
40
50
60
Time (min)

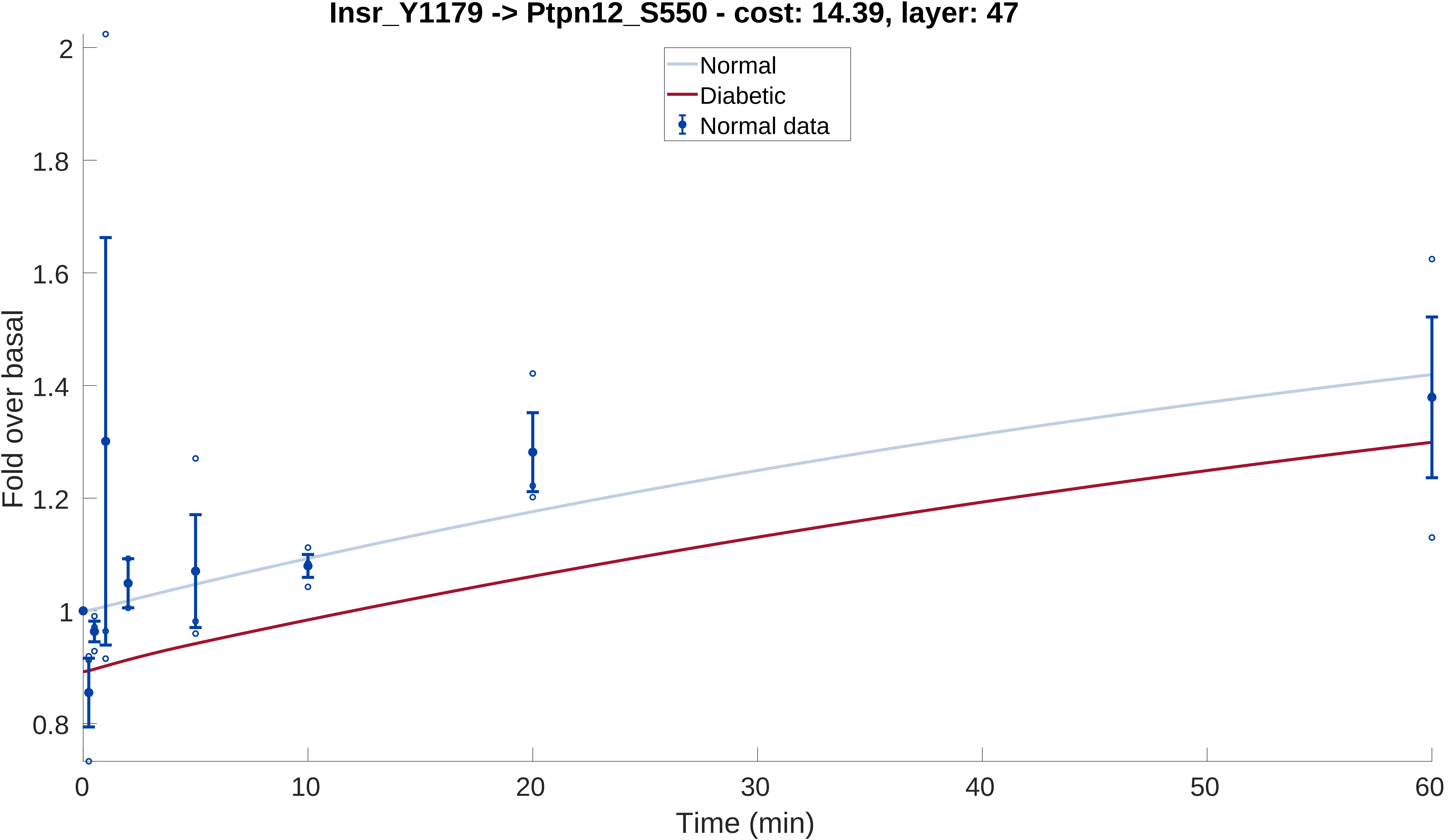

Insr_Y1179 -> Ptpn12_S550 - cost: 14.39, layer: 47
Normal
Diabetic
Normal data
Fold over basal
Time (min)
0.8
1
1.2
1.4
1.6
1.8
2
0
10
20
30
40
50
60

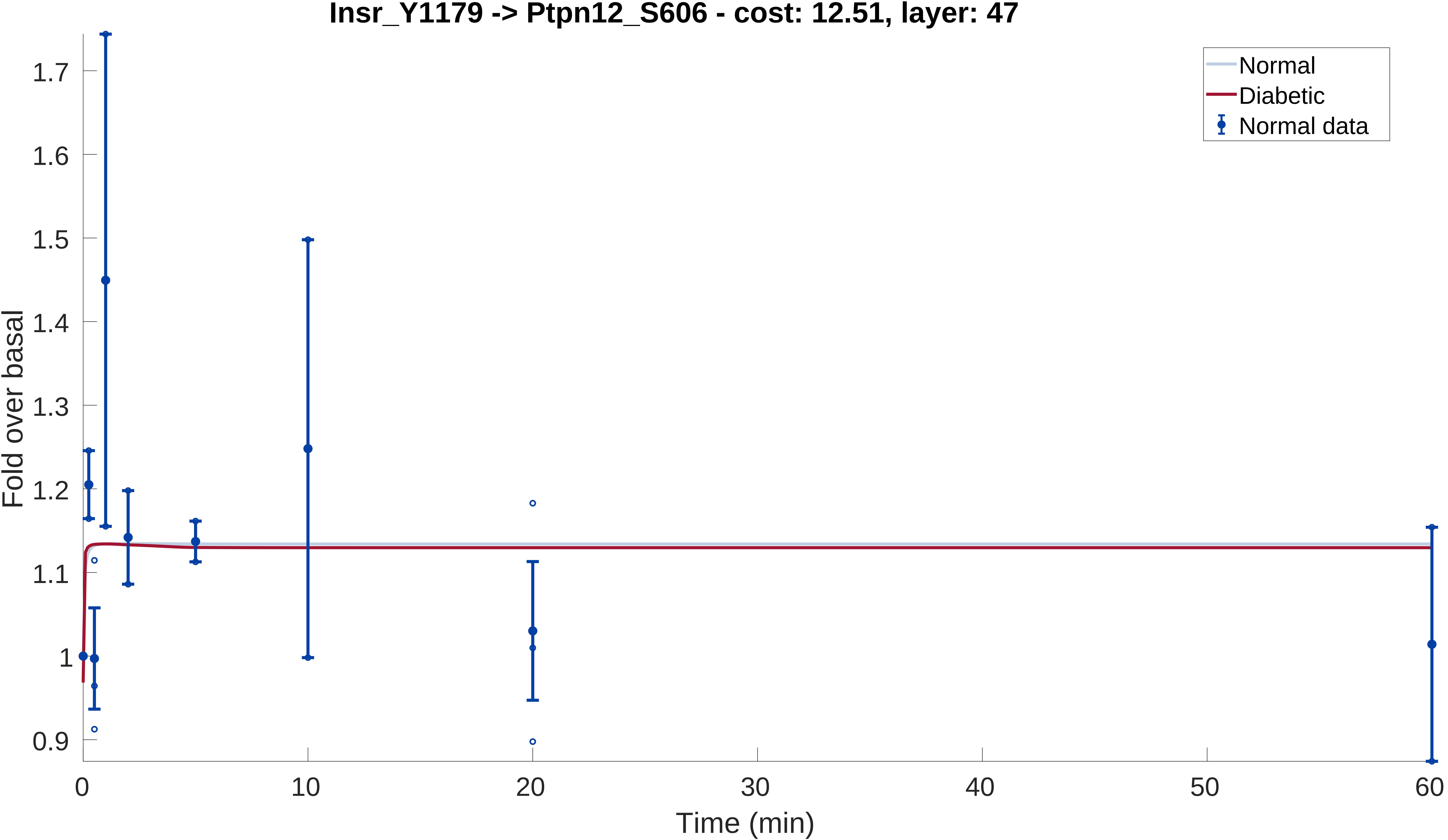

Insr_Y1179 -> Ptpn12_S606 - cost: 12.51, layer: 47
Normal
Diabetic
Normal data
Fold over basal
Time (min)
0.9
1
1.1
1.2
1.3
1.4
1.5
1.6
1.7
0
10
20
30
40
50
60

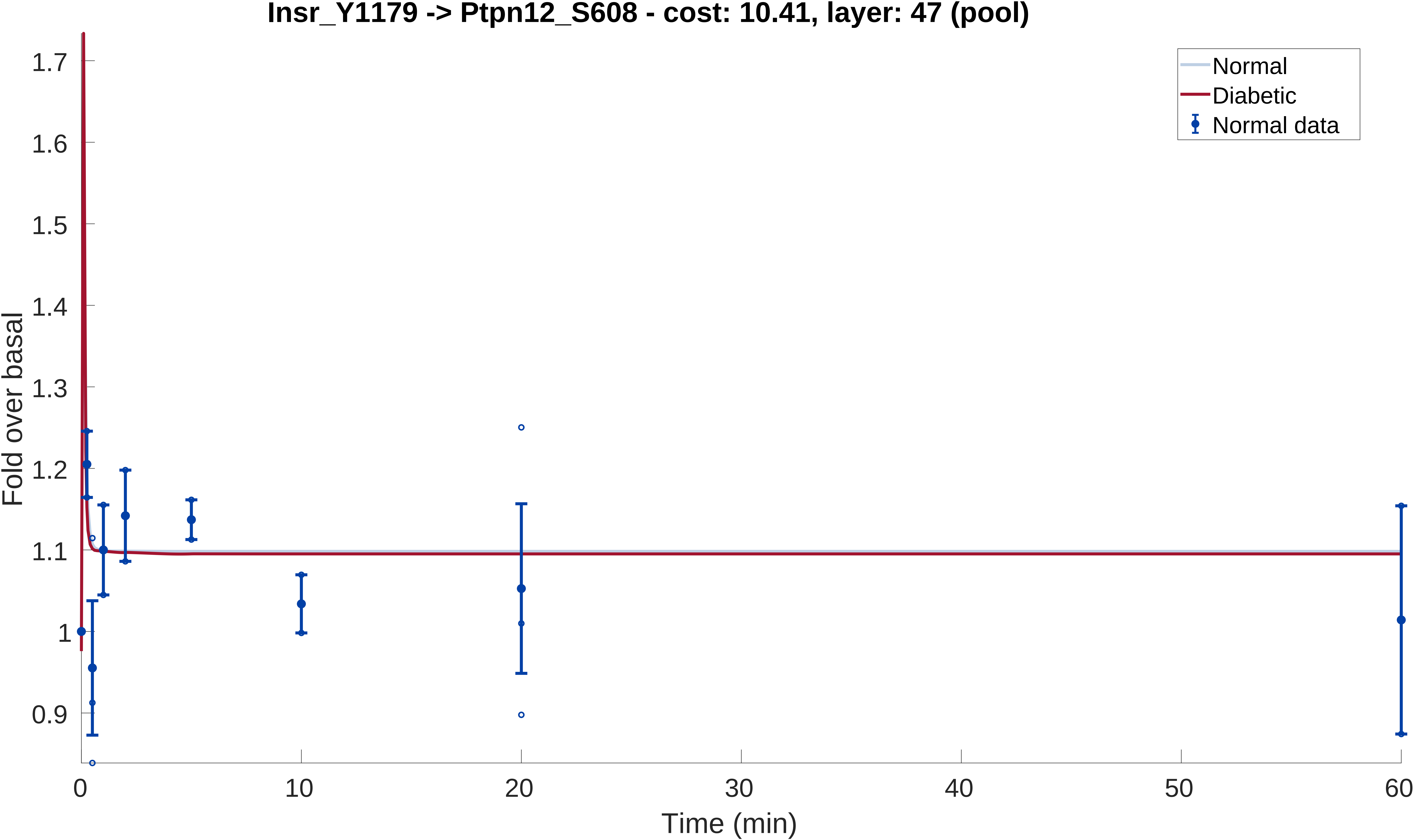
Insr_Y1179 -> Ptpn12_S608 - cost: 10.41, layer: 47 (pool)
Normal
Diabetic
Normal data
Fold over basal
1.7
1.6
1.5
1.4
1.3
1.2
1.1
1
0.9
0
10
20
30
40
50
60
Time (min)

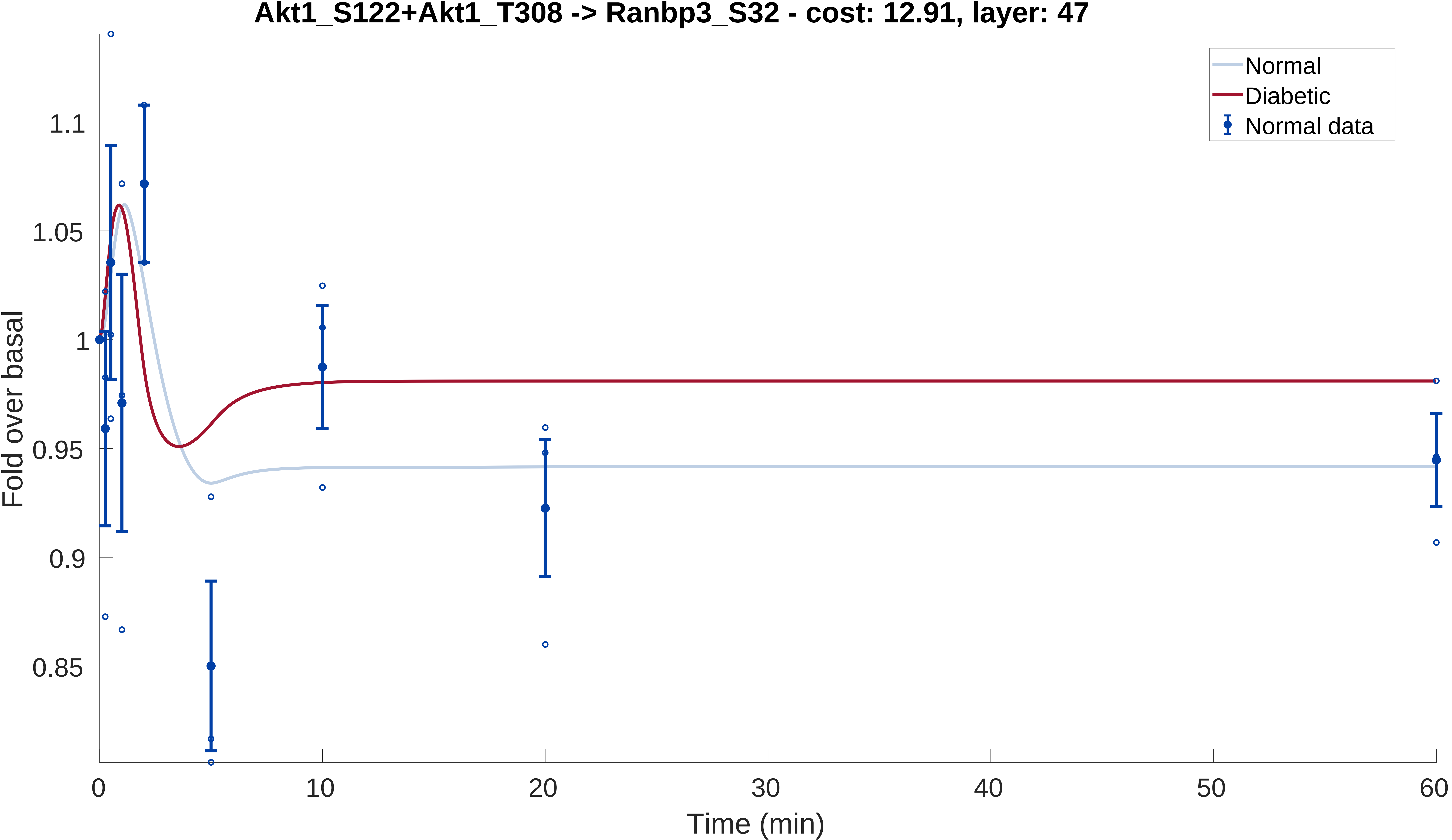

Akt1_S122+Akt1_T308 -> Ranbp3_S32 - cost: 12.91, layer: 47
Normal
Diabetic
Normal data
1.1
1.05
1
0.95
0.9
0.85
Fold over basal
0
10
20
30
40
50
60
Time (min)

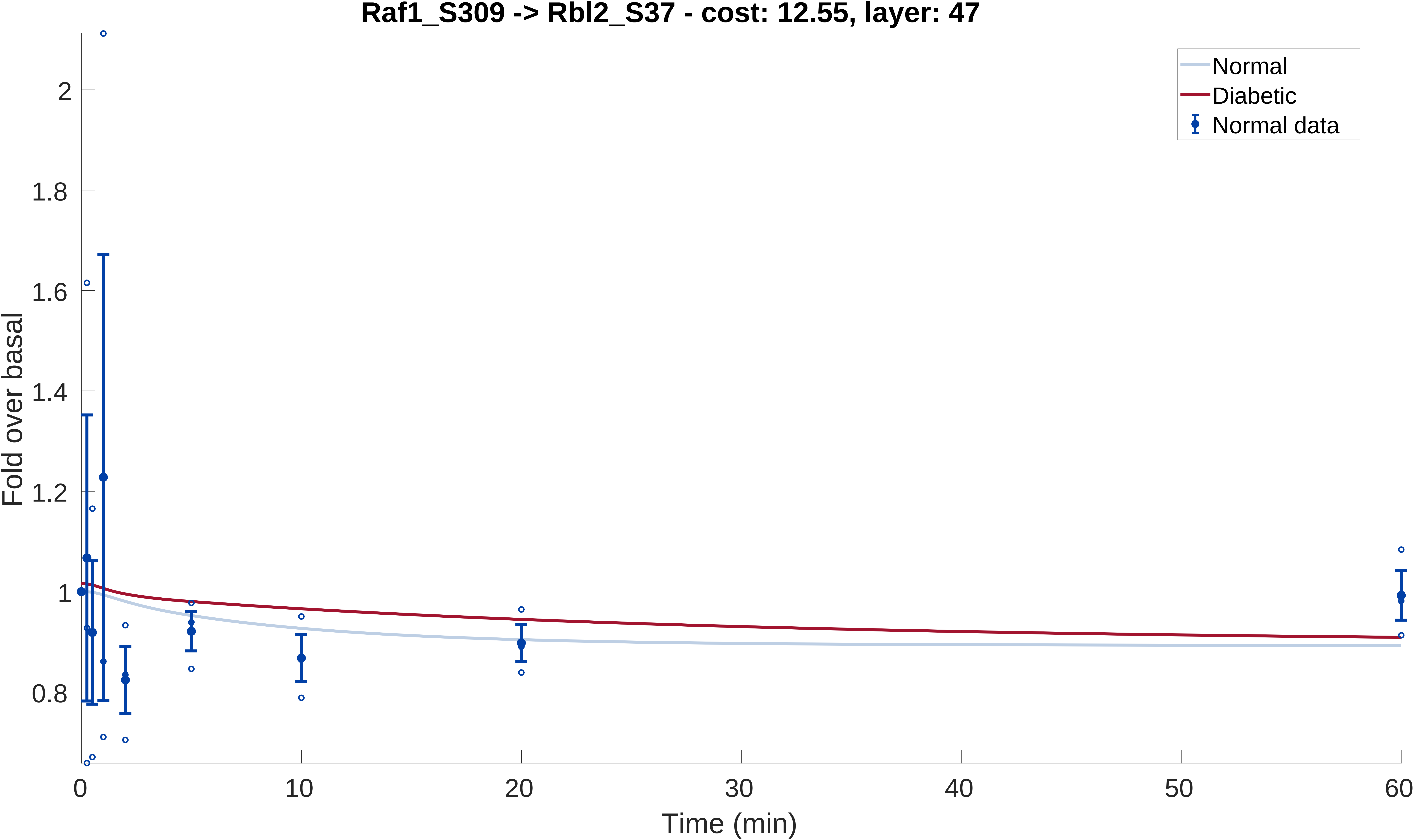
Raf1_S309 -> Rbl2_S37 - cost: 12.55, layer: 47
Normal
Diabetic
Normal data
Fold over basal
2
1.8
1.6
1.4
1.2
1
0.8
0
10
20
30
40
50
60
Time (min)

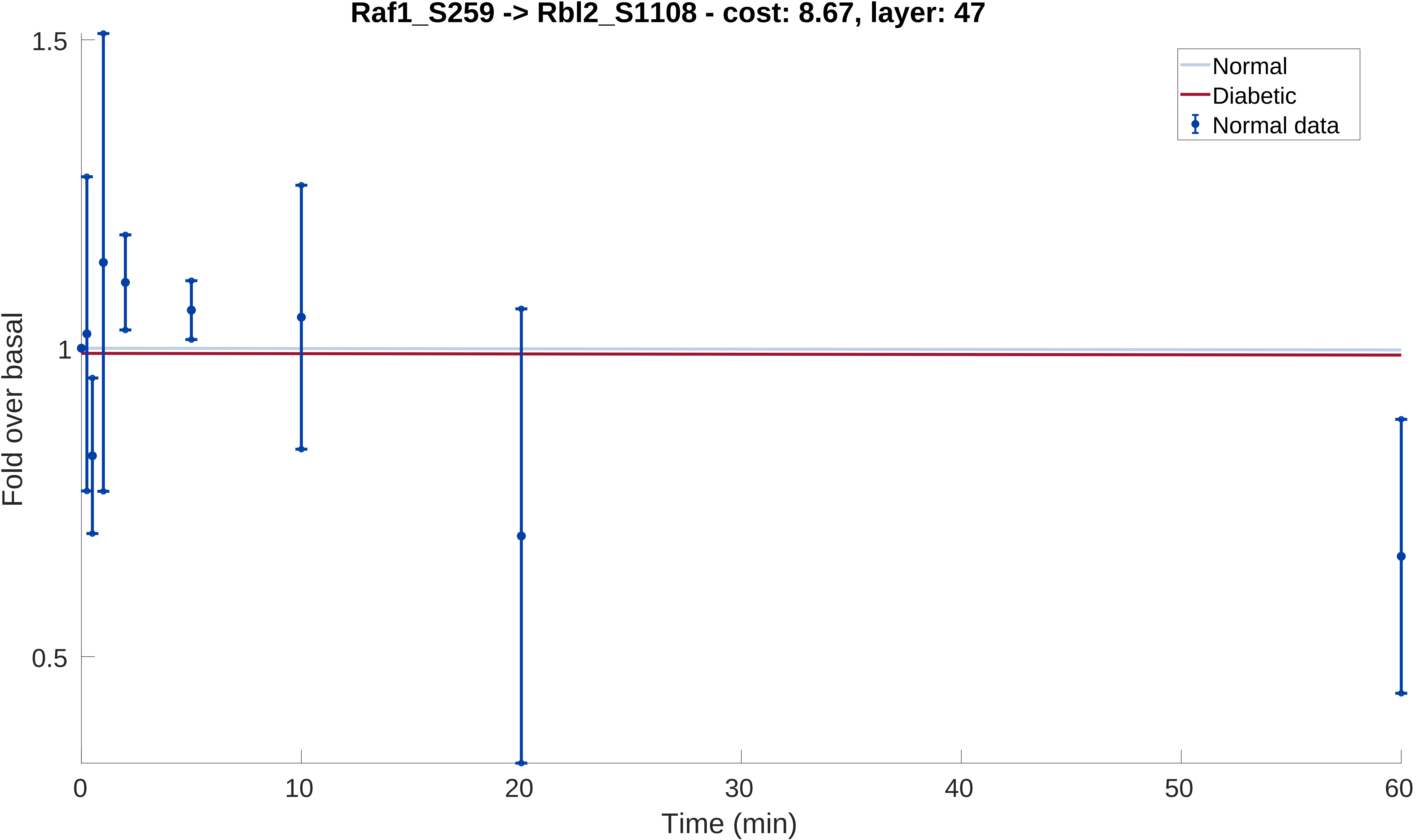

Raf1_S259 -> Rbl2_S1108 - cost: 8.67, layer: 47
Normal
Diabetic
Normal data
Fold over basal
1.5
1
0.5
0
10
20
30
40
50
60
Time (min)

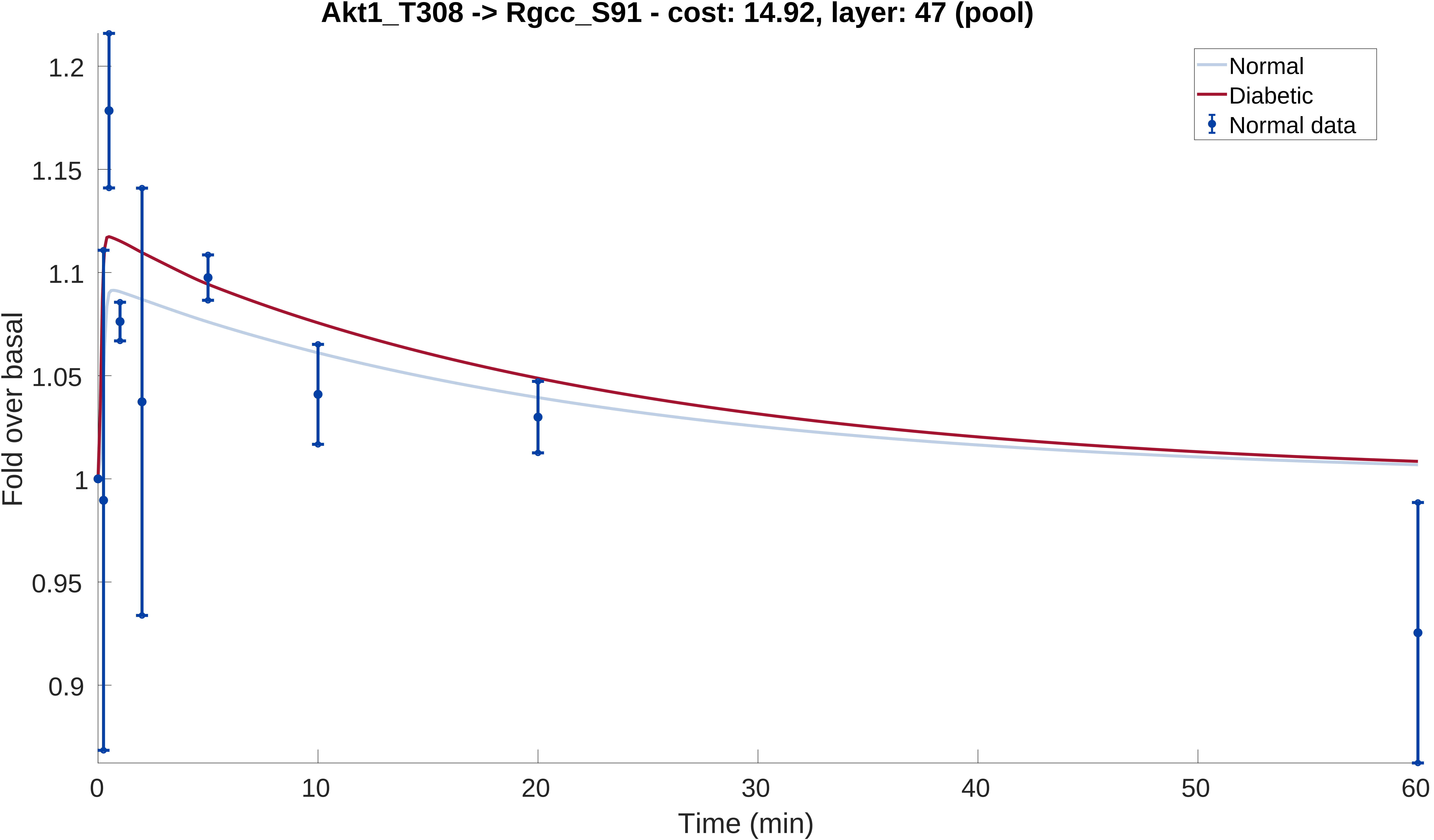

Akt1_T308 -> Rgcc_S91 - cost: 14.92, layer: 47 (pool)
Normal
Diabetic
Normal data
Fold over basal
1.2
1.15
1.1
1.05
1
0.95
0.9
0
10
20
30
40
50
60
Time (min)

Rps6ka3_S375 -> Rps6ka1_S363 - cost: 11.66, layer: 47

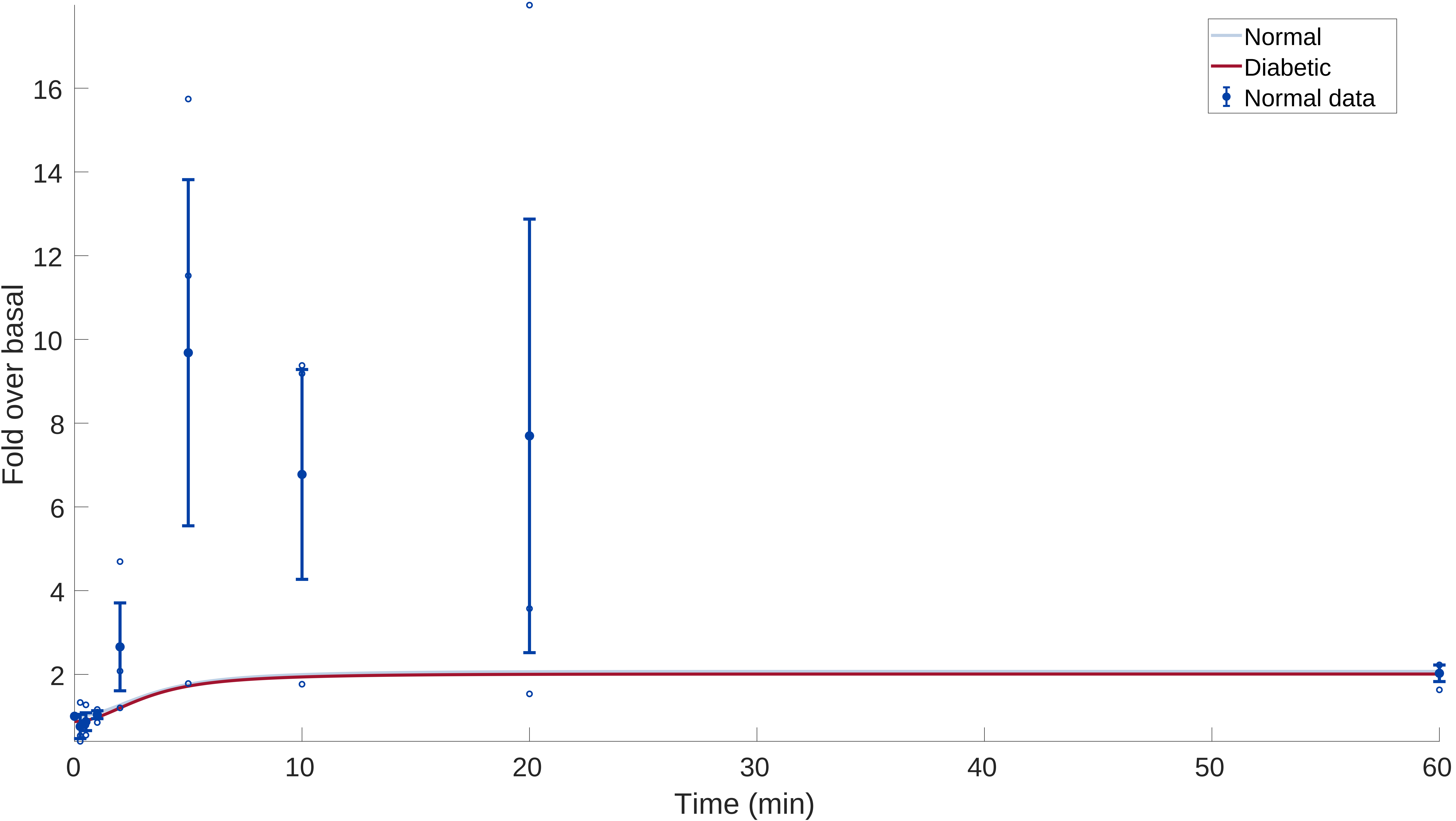

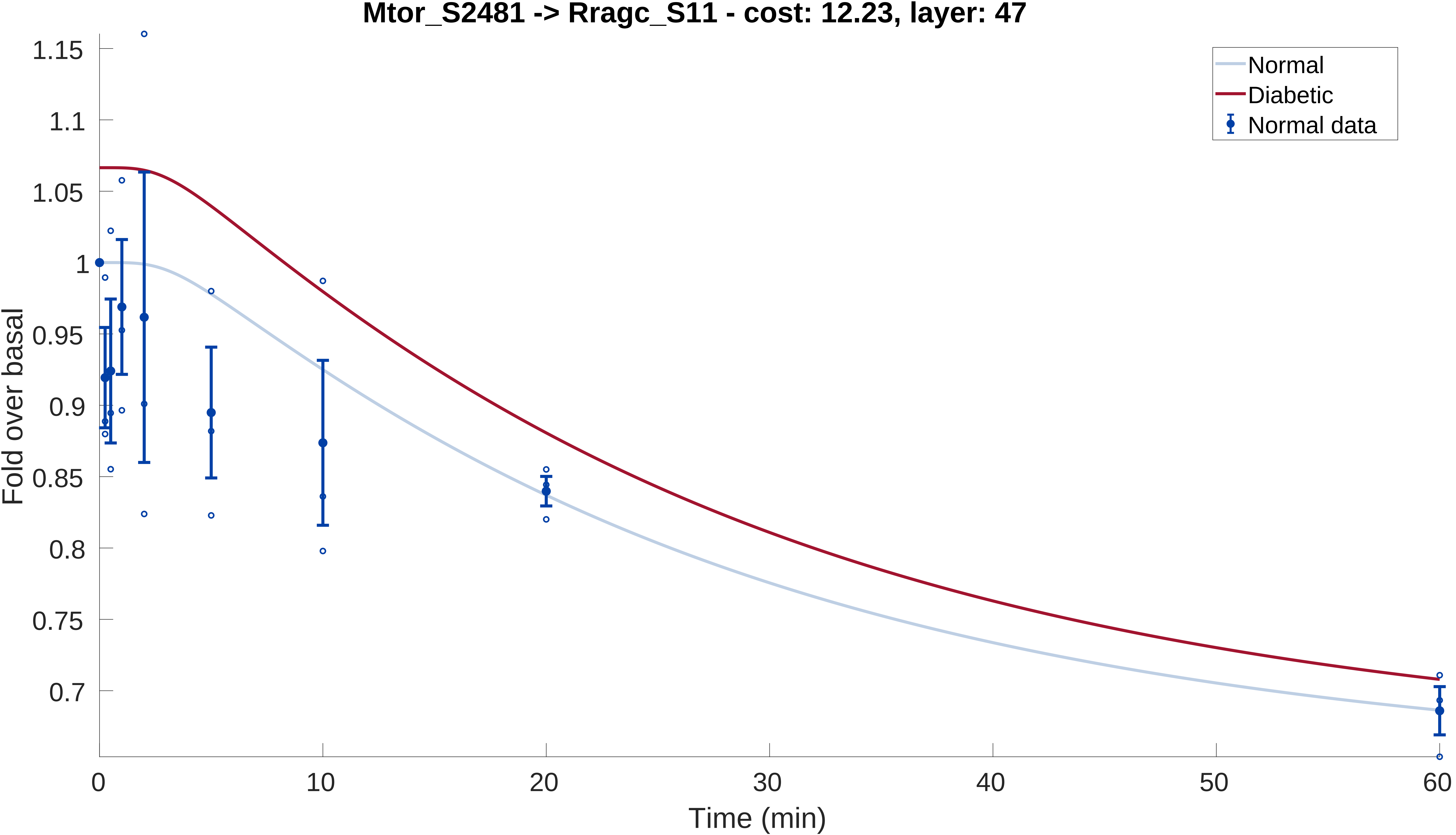
Mtor_S2481 -> Rragc_S11 - cost: 12.23, layer: 47
Normal
Diabetic
Normal data
Fold over basal
1.15
1.1
1.05
1
0.95
0.9
0.85
0.8
0.75
0.7
0
10
20
30
40
50
60
Time (min)

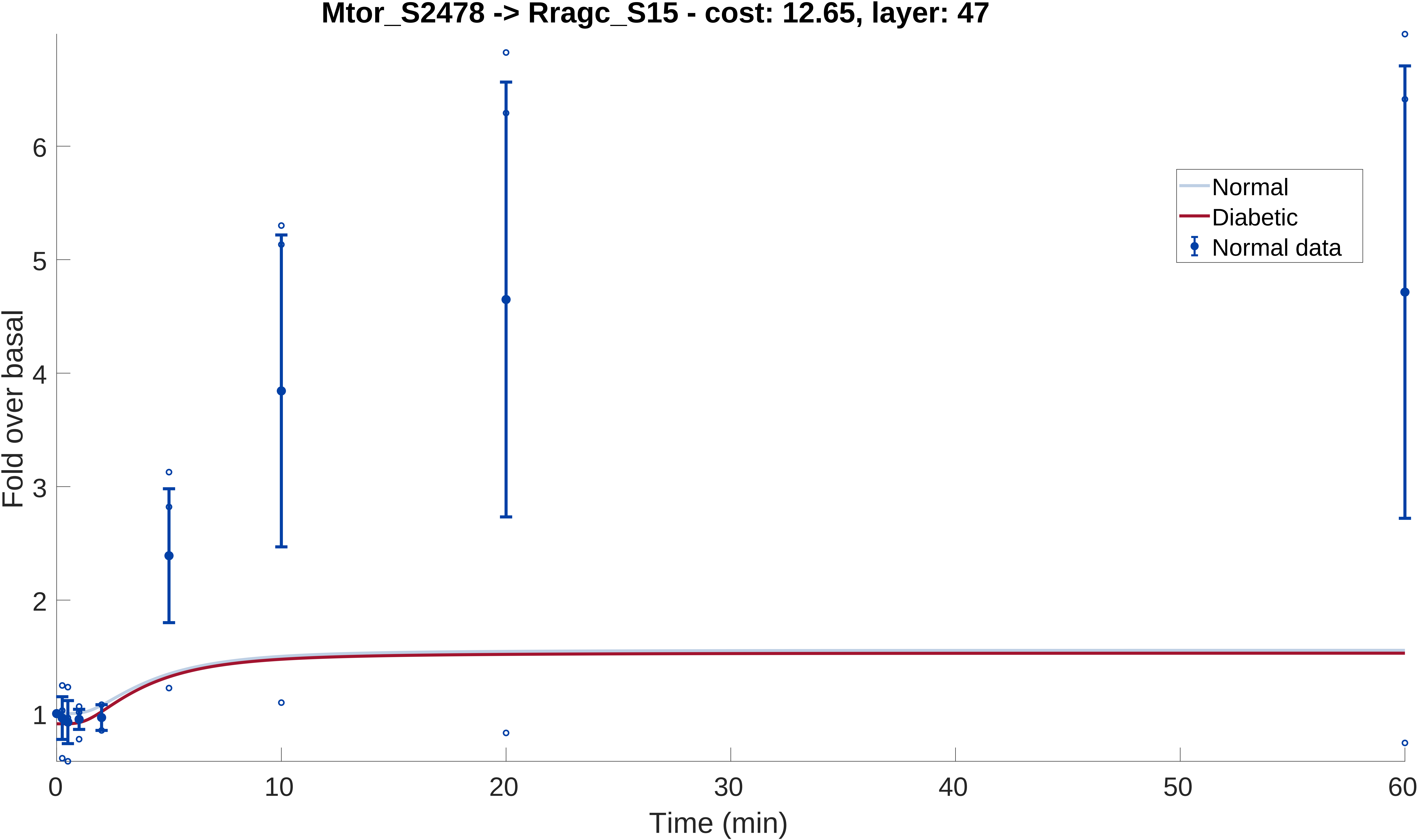

Mtor_S2478 -> Rragc_S15 - cost: 12.65, layer: 47
Fold over basal
1
2
3
4
5
6
0
10
20
30
40
50
60
Time (min)
Normal
Diabetic
Normal data

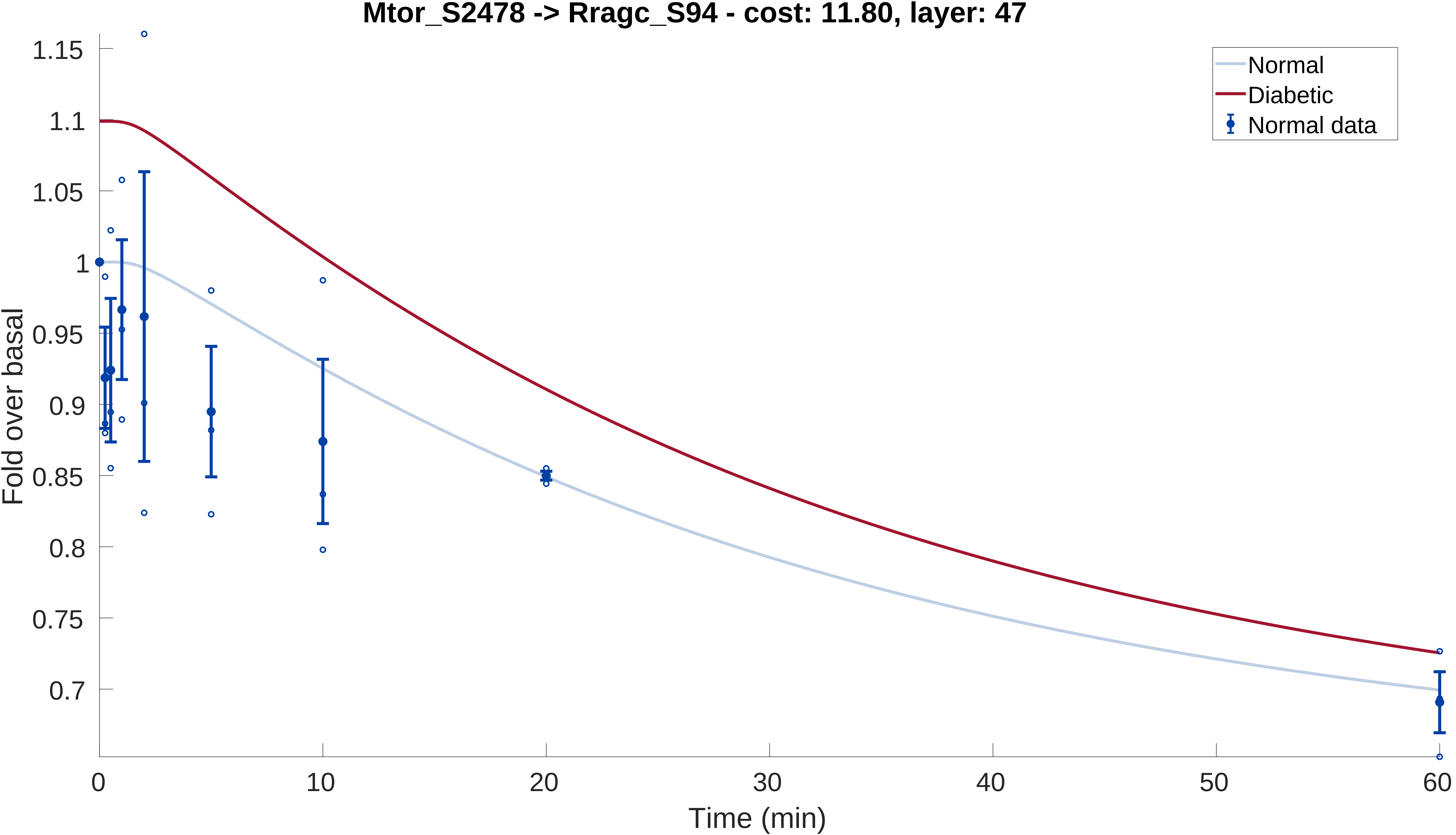
Mtor_S2478 -> Rragc_S94 - cost: 11.80, layer: 47
Normal
Diabetic
Normal data
Fold over basal
1.15
1.1
1.05
1
0.95
0.9
0.85
0.8
0.75
0.7
0
10
20
30
40
50
60
Time (min)

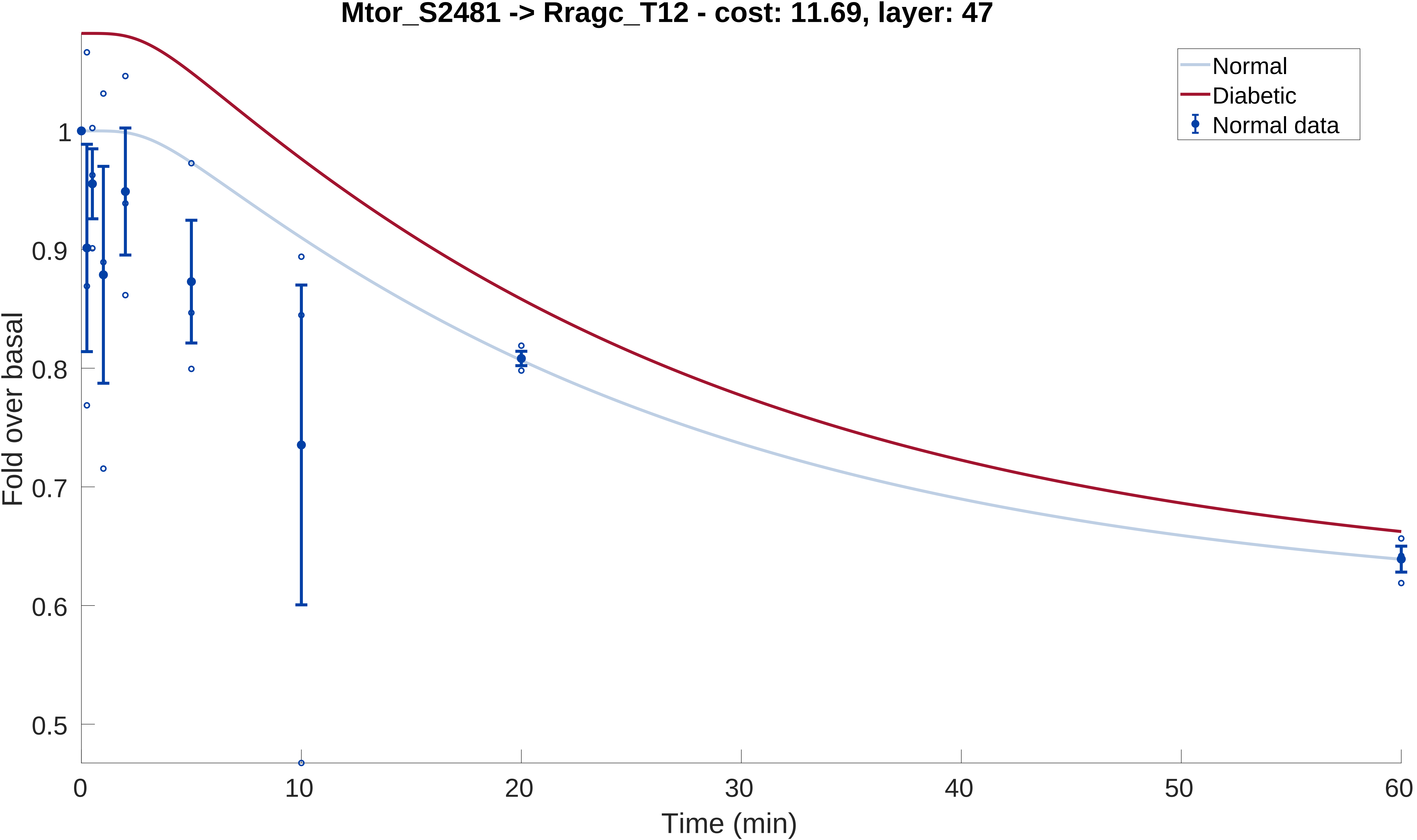

Mtor_S2481 -> Rragc_T12 - cost: 11.69, layer: 47
Normal
Diabetic
Normal data
Fold over basal
1
0.9
0.8
0.7
0.6
0.5
0
10
20
30
40
50
60
Time (min)

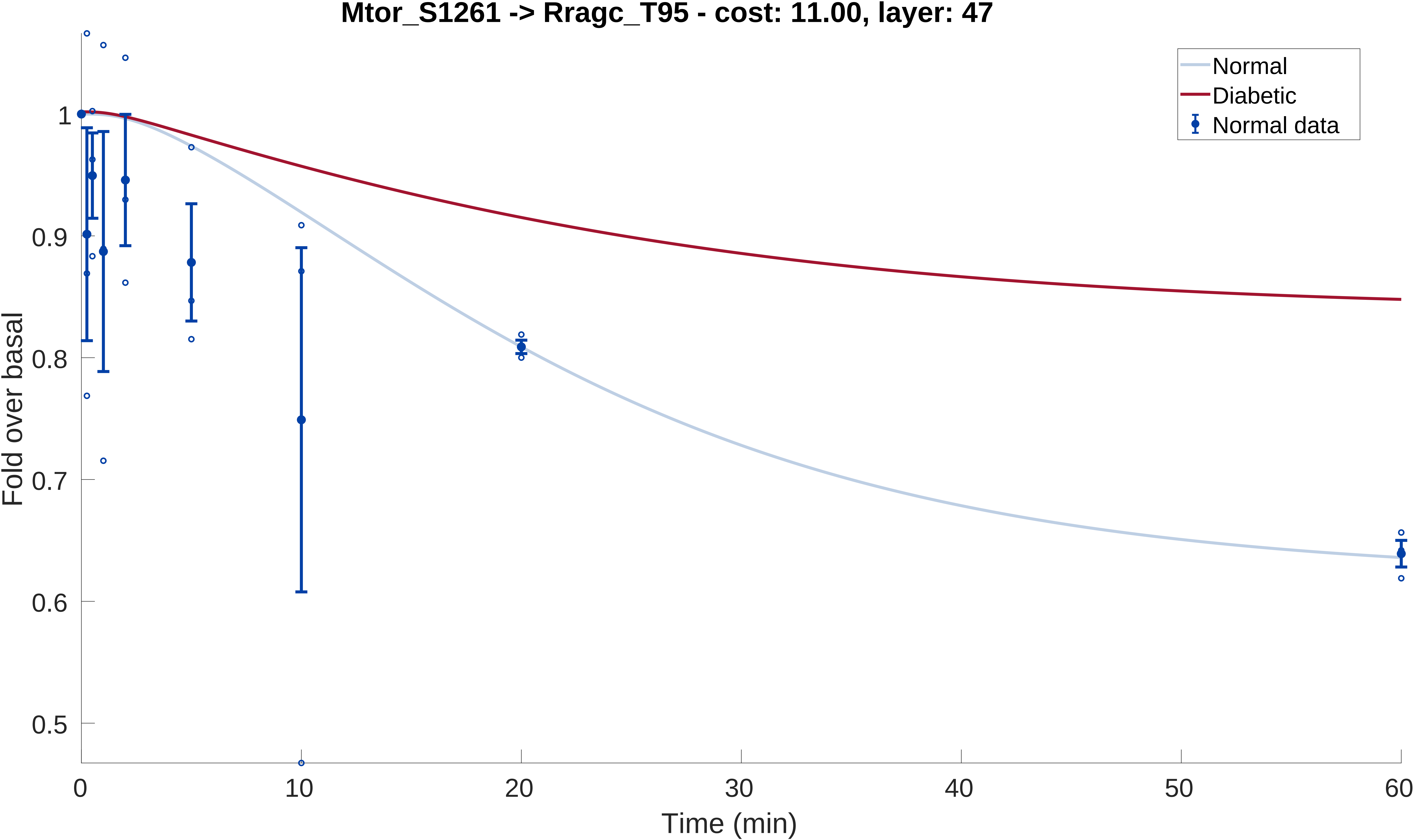
Mtor_S1261 -> Rragc_T95 - cost: 11.00, layer: 47
Normal
Diabetic
Normal data
Fold over basal
1
0.9
0.8
0.7
0.6
0.5
0
10
20
30
40
50
60
Time (min)

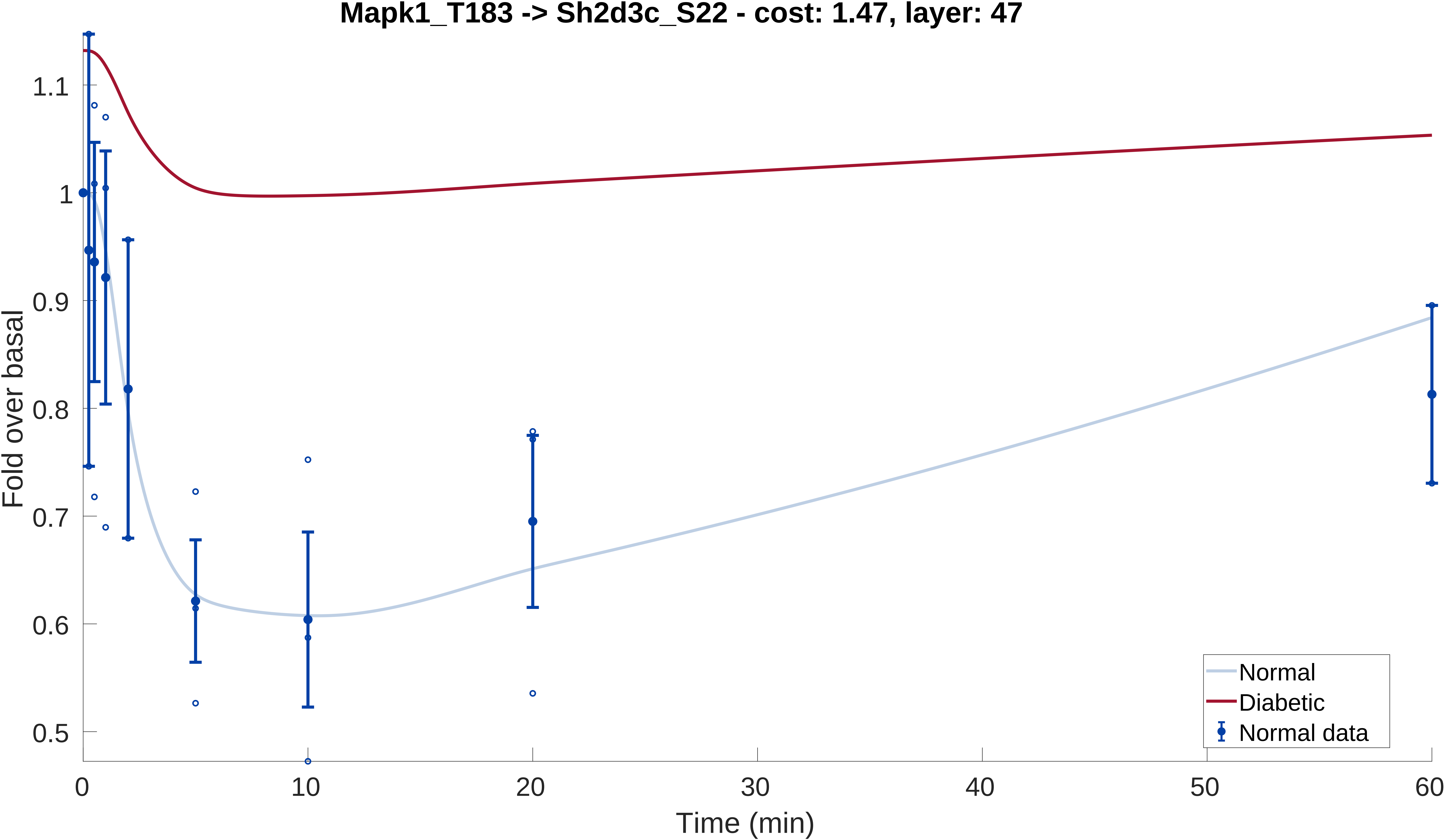

Mapk1_T183 -> Sh2d3c_S22 - cost: 1.47, layer: 47
Fold over basal
1.1
1
0.9
0.8
0.7
0.6
0.5
0
10
20
30
40
50
60
Time (min)
Normal
Diabetic
Normal data

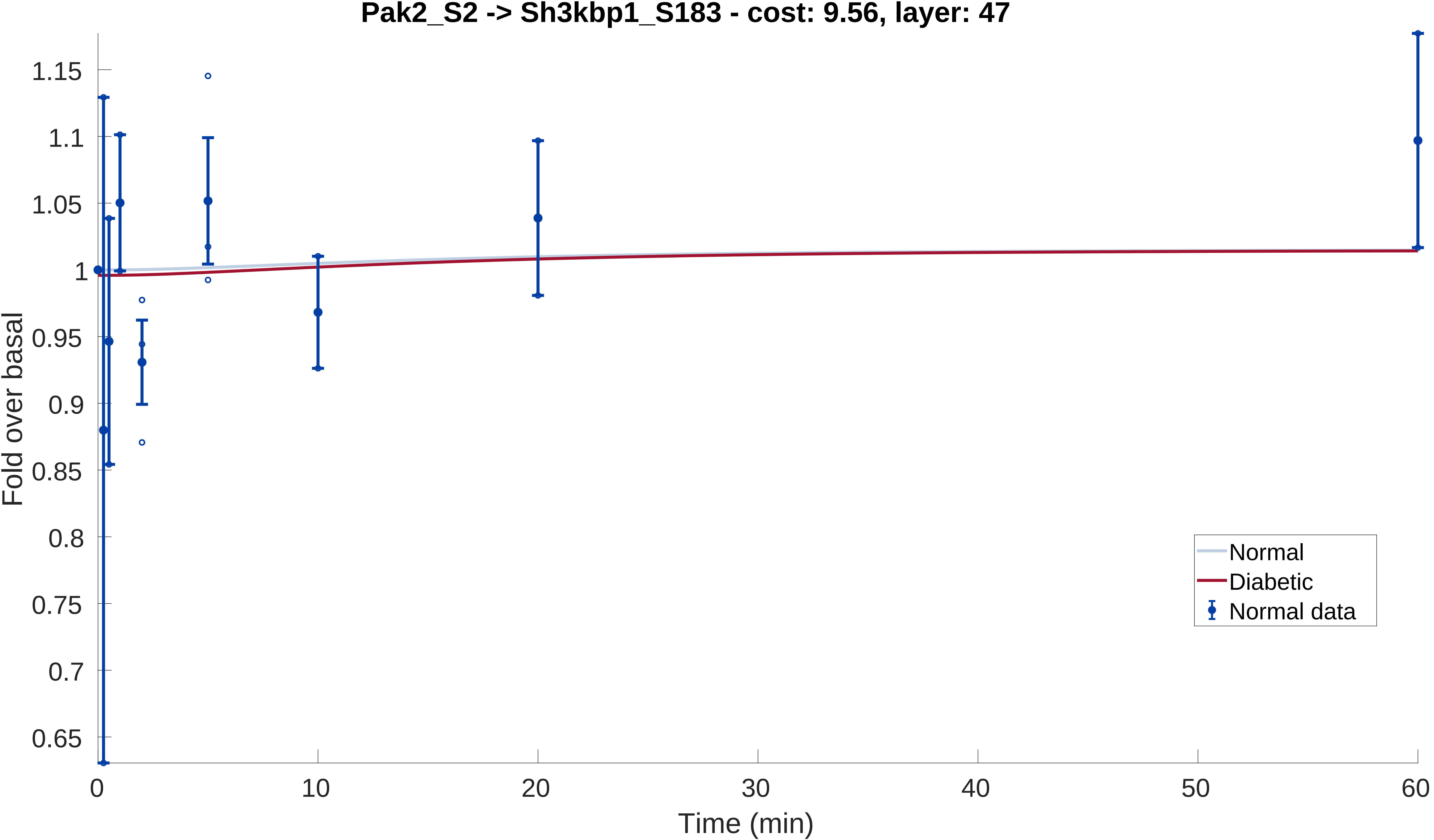

Pak2_S2 -> Sh3kbp1_S183 - cost: 9.56, layer: 47
Fold over basal
Time (min)
1.15
1.1
1.05
1
0.95
0.9
0.85
0.8
0.75
0.7
0.65
0
10
20
30
40
50
60
Normal
Diabetic
Normal data

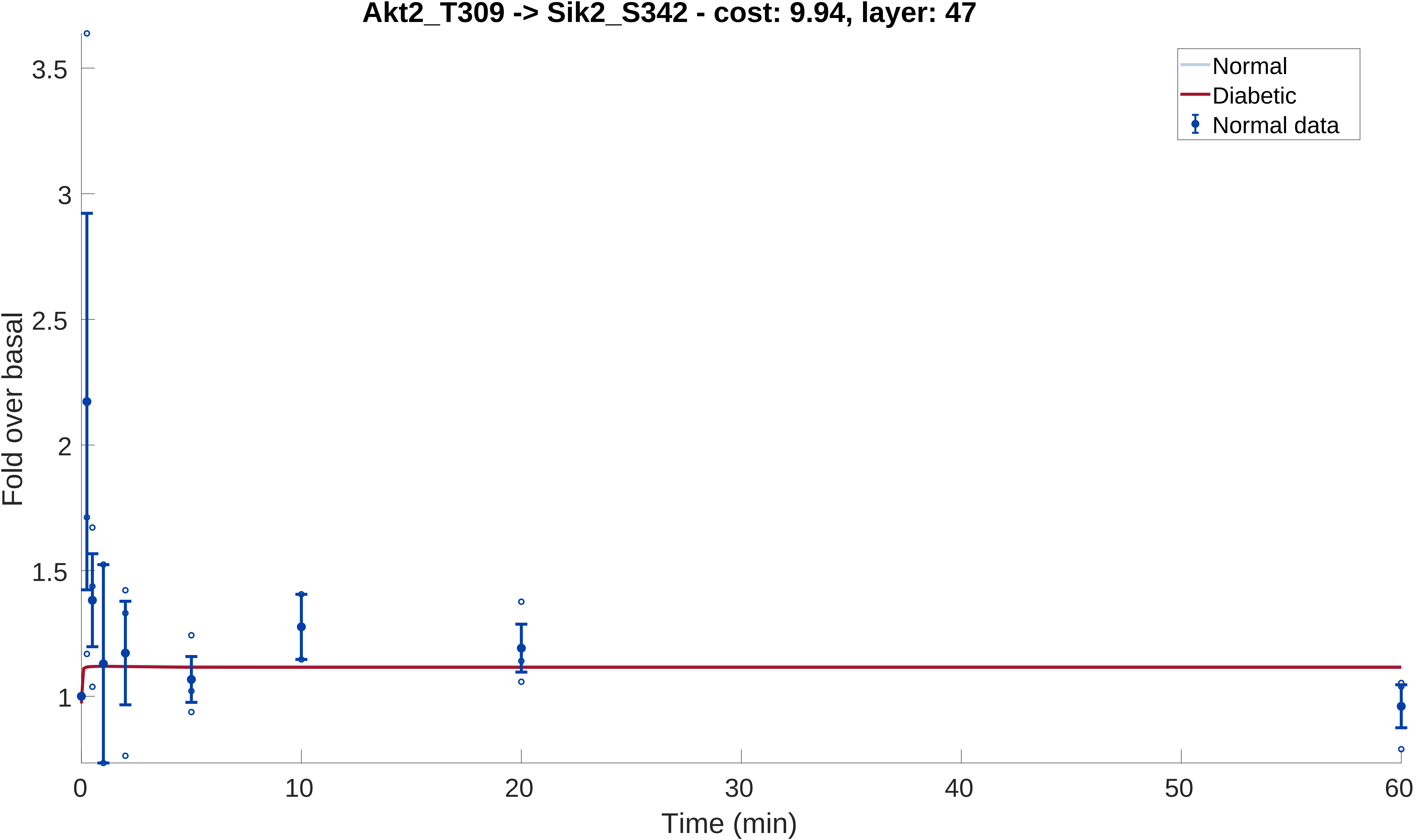

Akt2_T309 -> Sik2_S342 - cost: 9.94, layer: 47
Normal
Diabetic
Normal data
Fold over basal
3.5
3
2.5
2
1.5
1
0
10
20
30
40
50
60
Time (min)

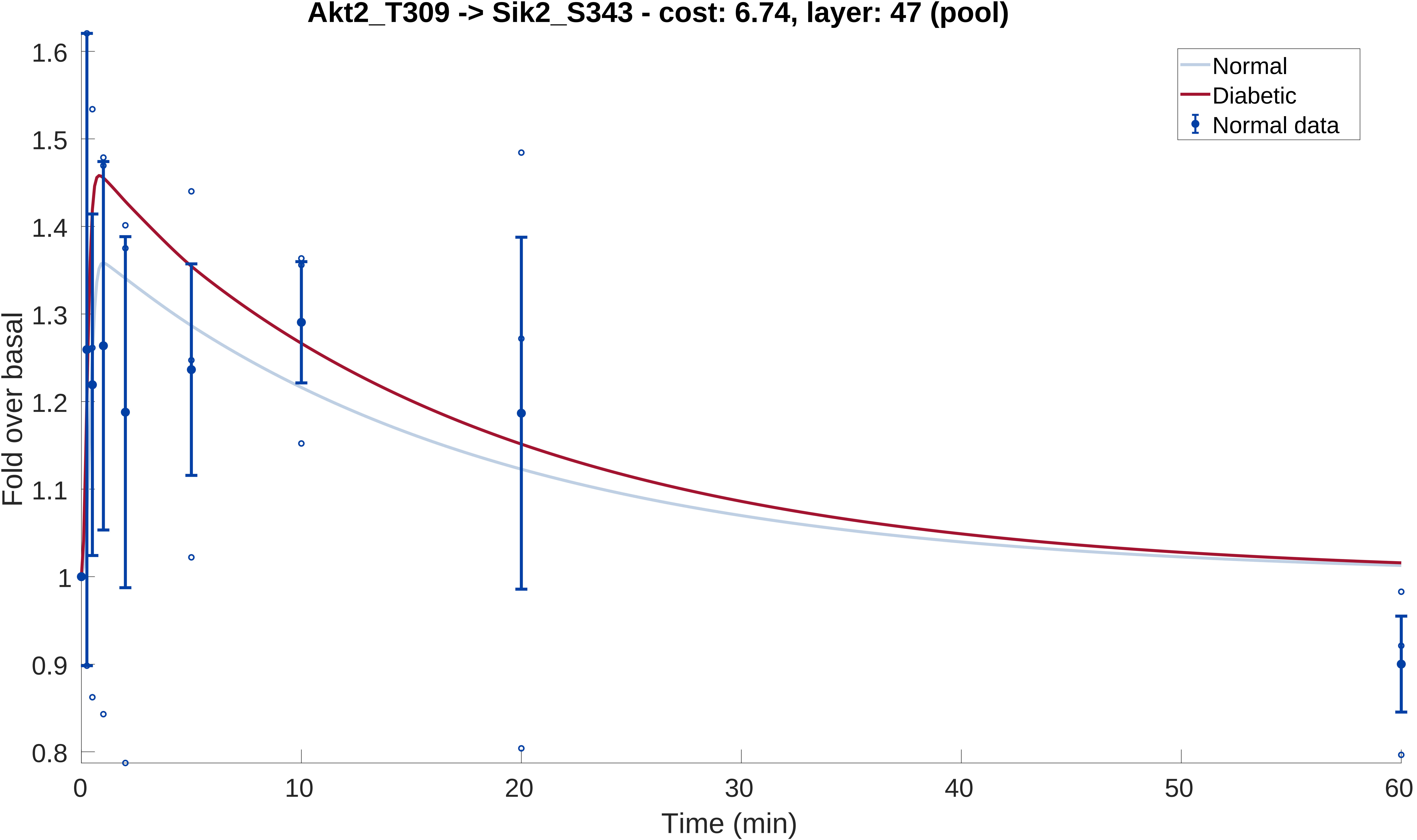

Akt2_T309 -> Sik2_S343 - cost: 6.74, layer: 47 (pool)
Normal
Diabetic
Normal data
Fold over basal
Time (min)
0.8
0.9
1
1.1
1.2
1.3
1.4
1.5
1.6
0
10
20
30
40
50
60

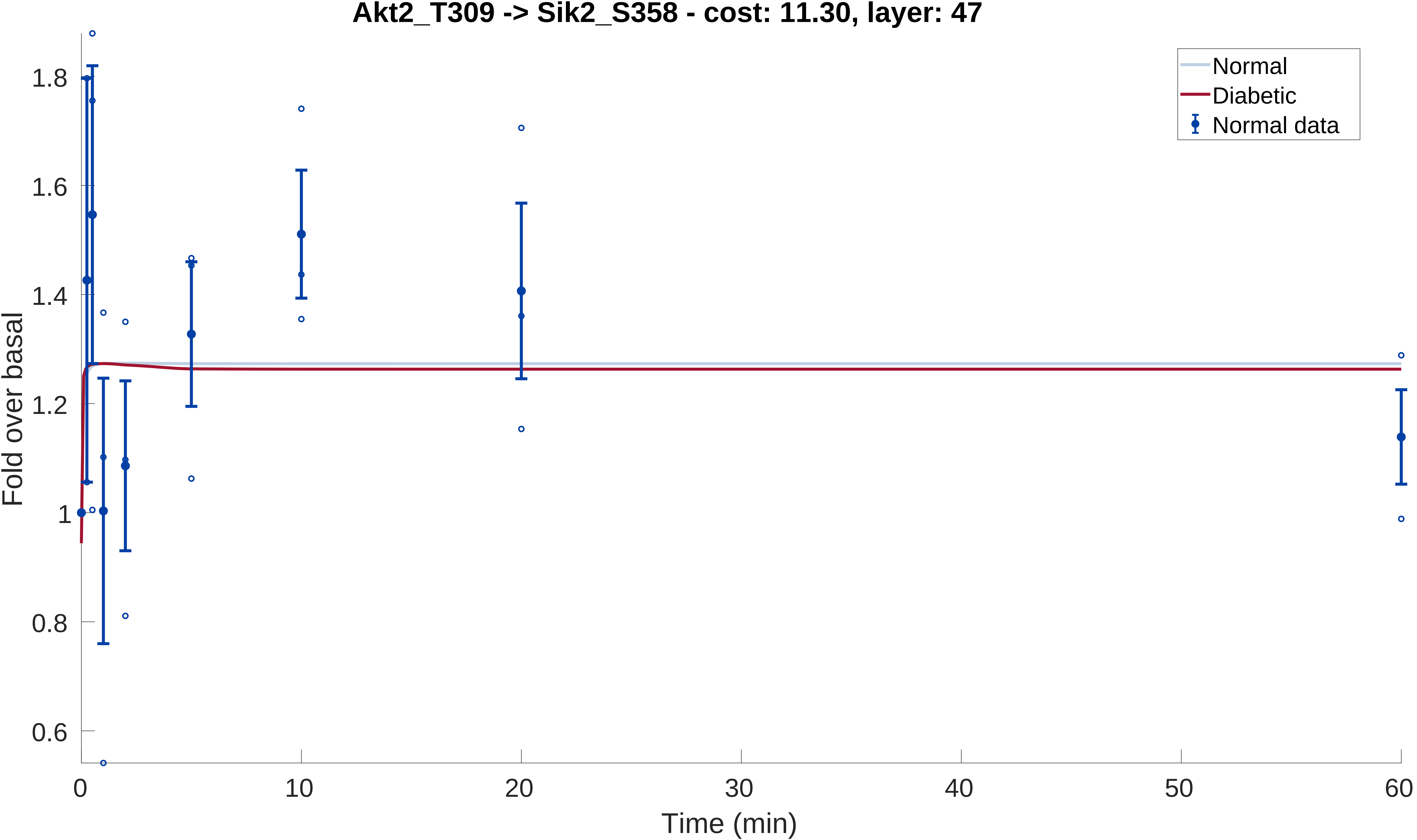

Akt2_T309 -> Sik2_S358 - cost: 11.30, layer: 47
Normal
Diabetic
Normal data
Fold over basal
Time (min)
1.8
1.6
1.4
1.2
1
0.8
0.6
0
10
20
30
40
50
60

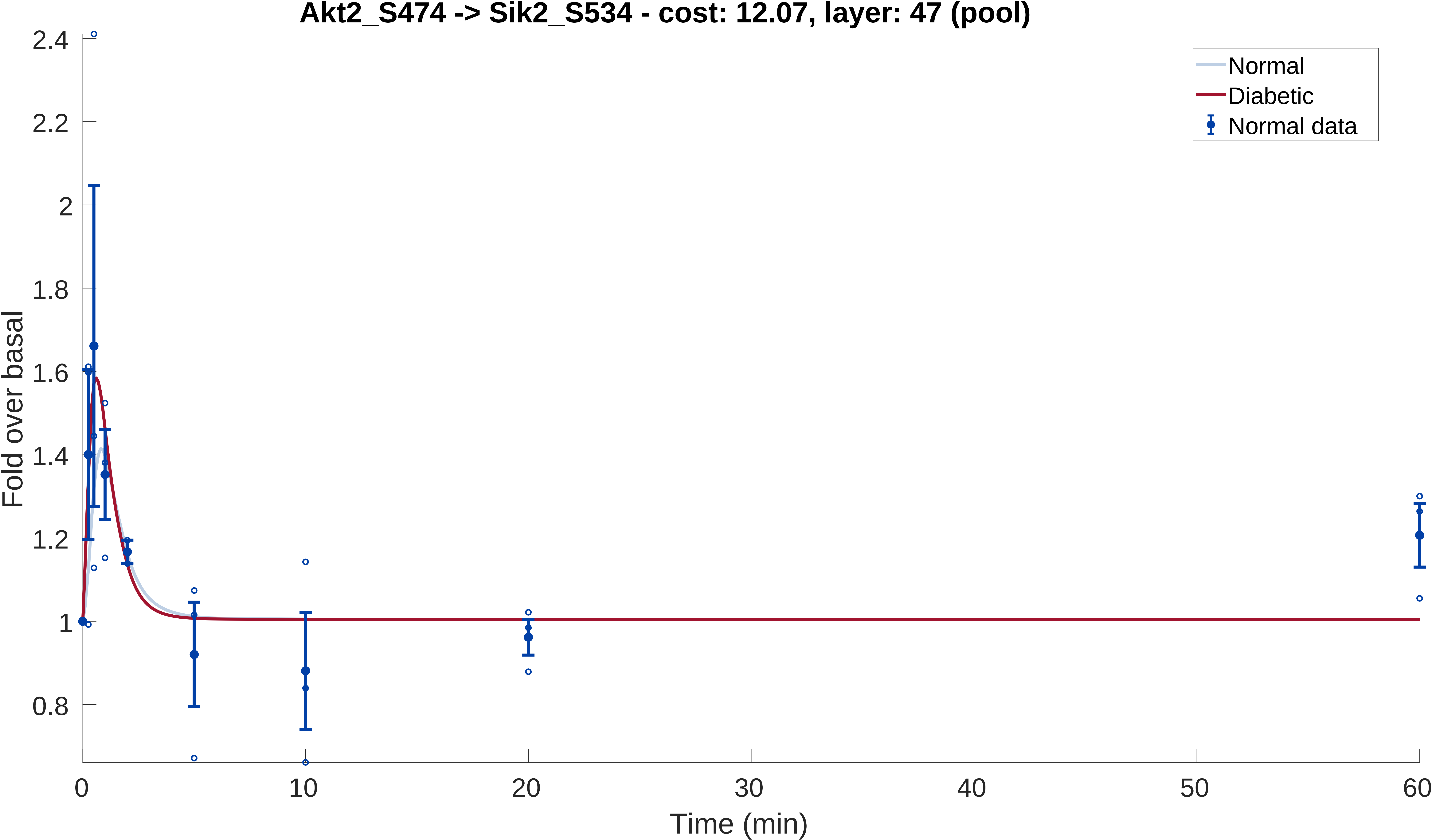
Akt2_S474 -> Sik2_S534 - cost: 12.07, layer: 47 (pool)
Normal
Diabetic
Normal data
Fold over basal
Time (min)
2.4
2.2
2
1.8
1.6
1.4
1.2
1
0.8
0
10
20
30
40
50
60

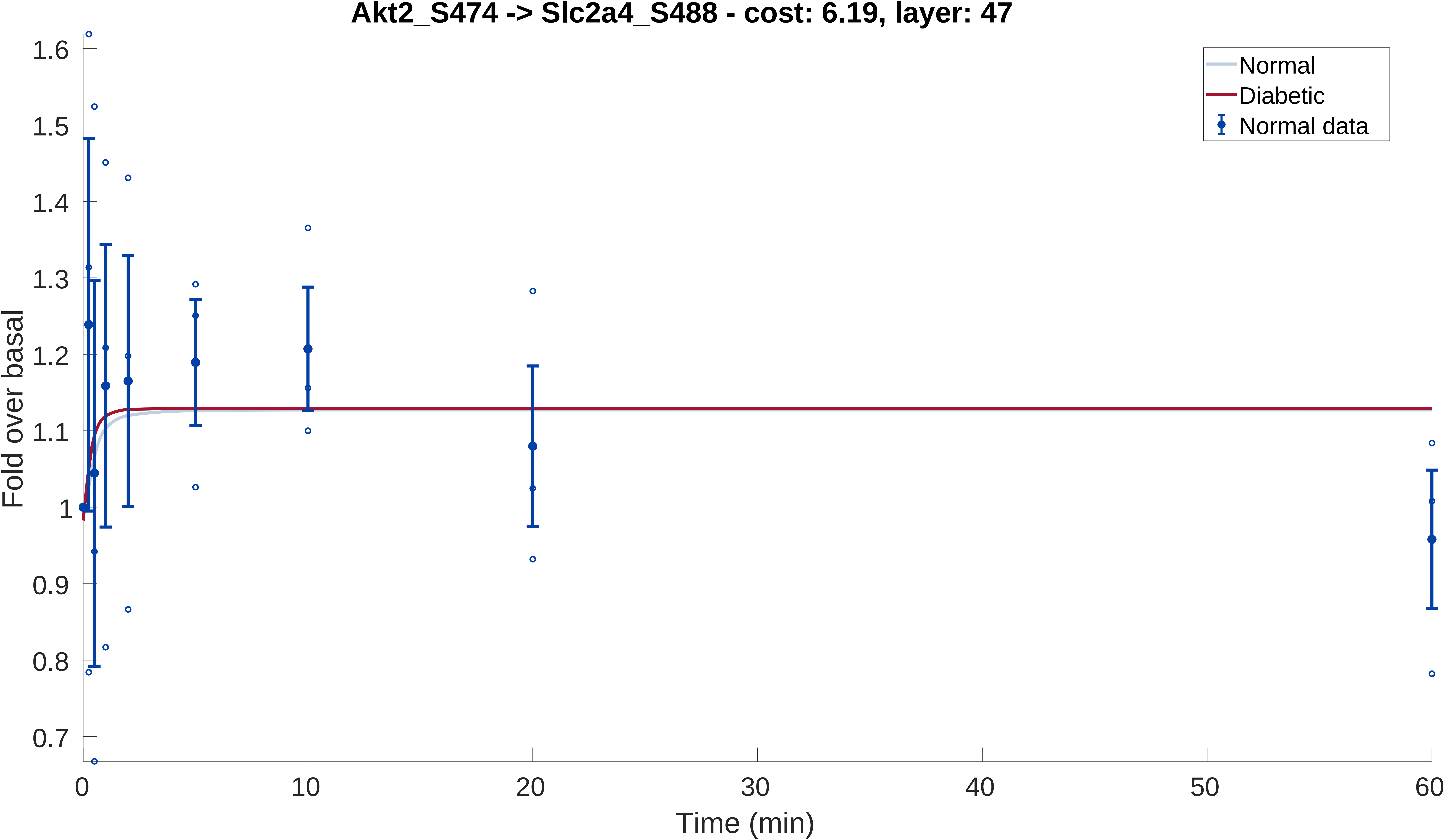

Akt2_S474 -> Slc2a4_S488 - cost: 6.19, layer: 47
Fold over basal
1.6
1.5
1.4
1.3
1.2
1.1
1
0.9
0.8
0.7
0
10
20
30
40
50
60
Time (min)
Normal
Diabetic
Normal data

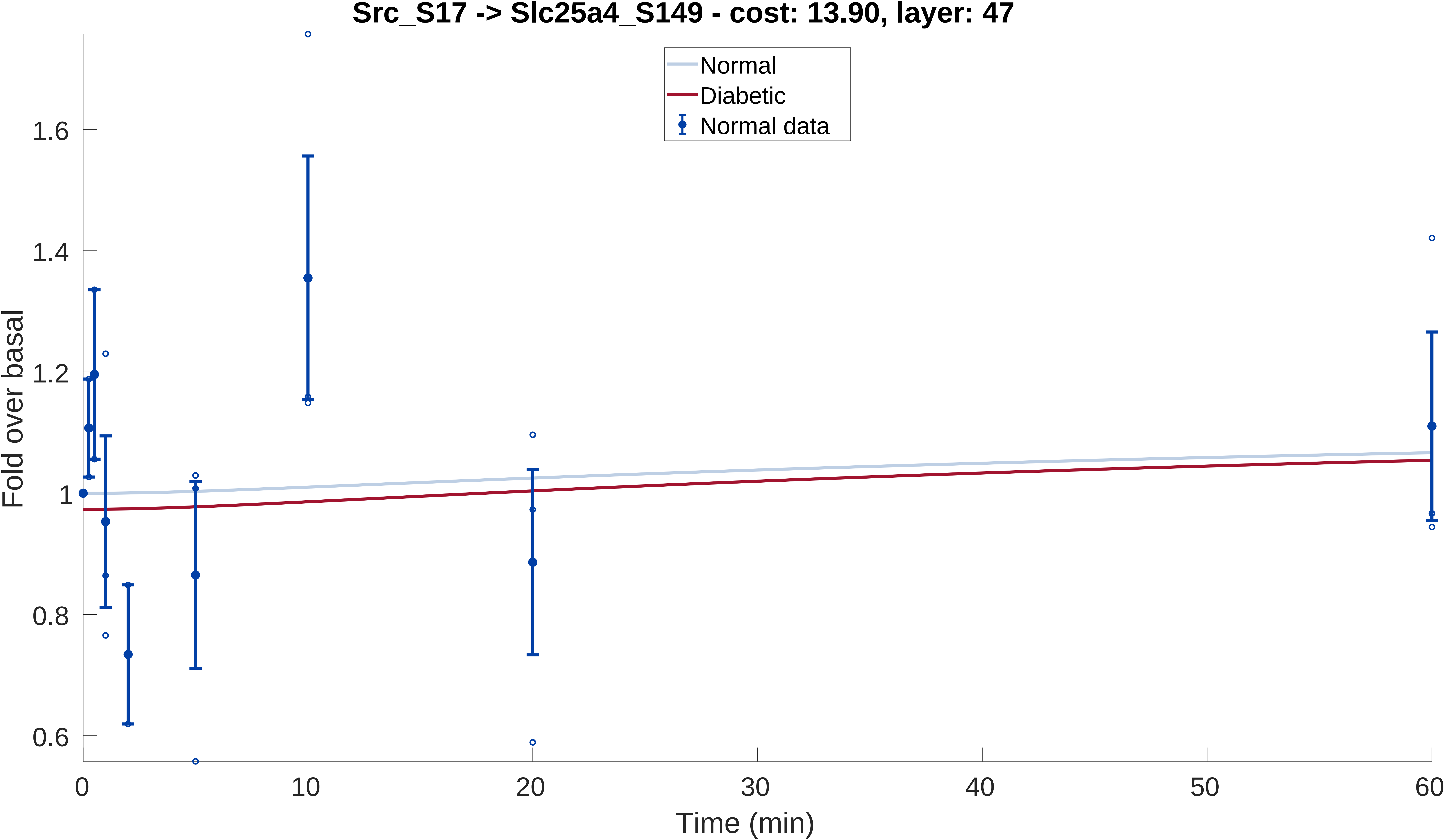

Src_S17 -> Slc25a4_S149 - cost: 13.90, layer: 47
Normal
Diabetic
Normal data
1.6
1.4
1.2
1
0.8
0.6
Fold over basal
0
10
20
30
40
50
60
Time (min)

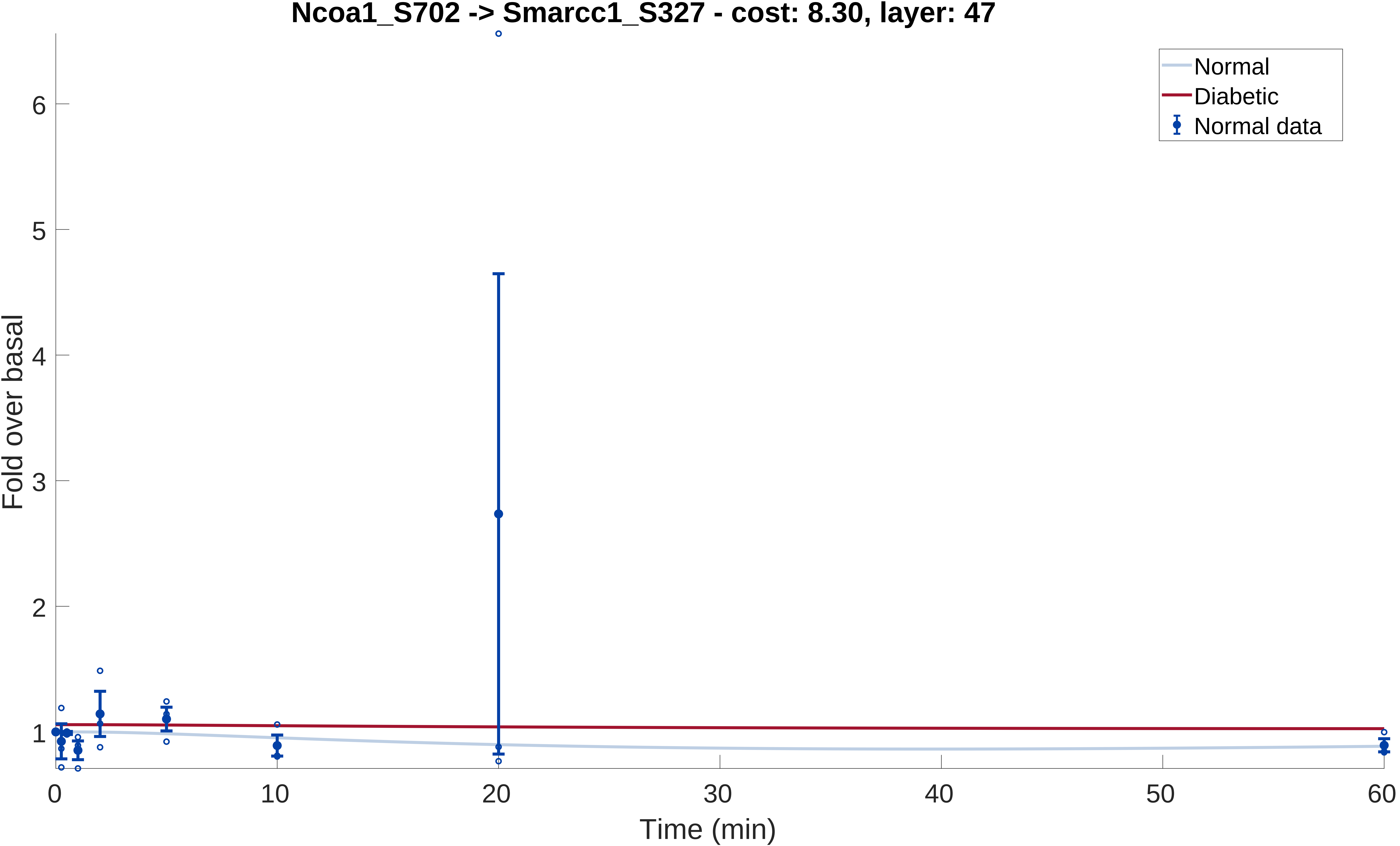

Ncoa1_S702 -> Smarcc1_S327 - cost: 8.30, layer: 47
Normal
Diabetic
Normal data
Fold over basal
Time (min)
1
2
3
4
5
6
0
10
20
30
40
50
60

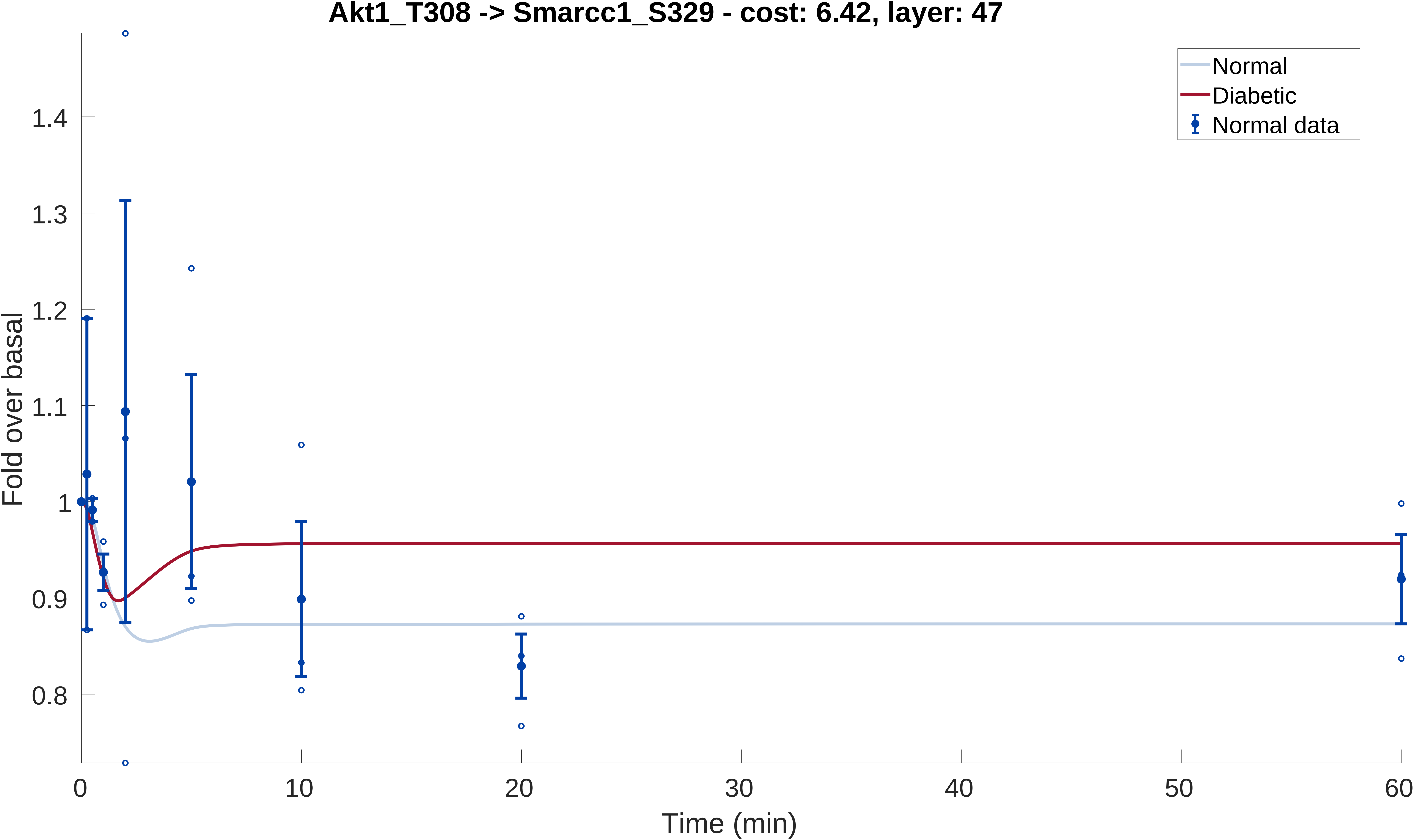

Akt1_T308 -> Smarcc1_S329 - cost: 6.42, layer: 47
Normal
Diabetic
Normal data
Fold over basal
Time (min)
0.8
0.9
1
1.1
1.2
1.3
1.4
0
10
20
30
40
50
60

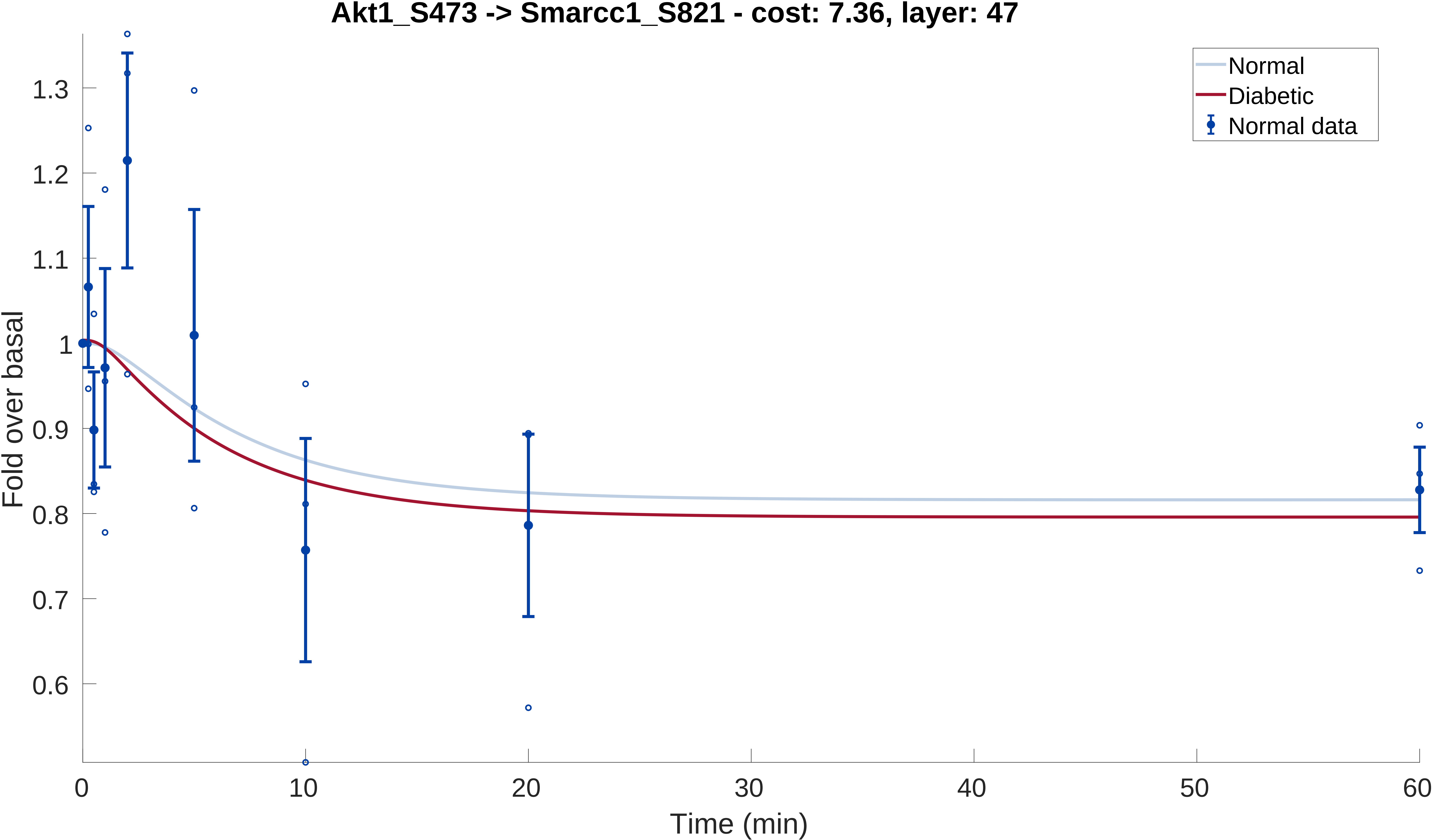

Akt1_S473 -> Smarcc1_S821 - cost: 7.36, layer: 47
Normal
Diabetic
Normal data
Fold over basal
Time (min)
1.3
1.2
1.1
1
0.9
0.8
0.7
0.6
0
10
20
30
40
50
60

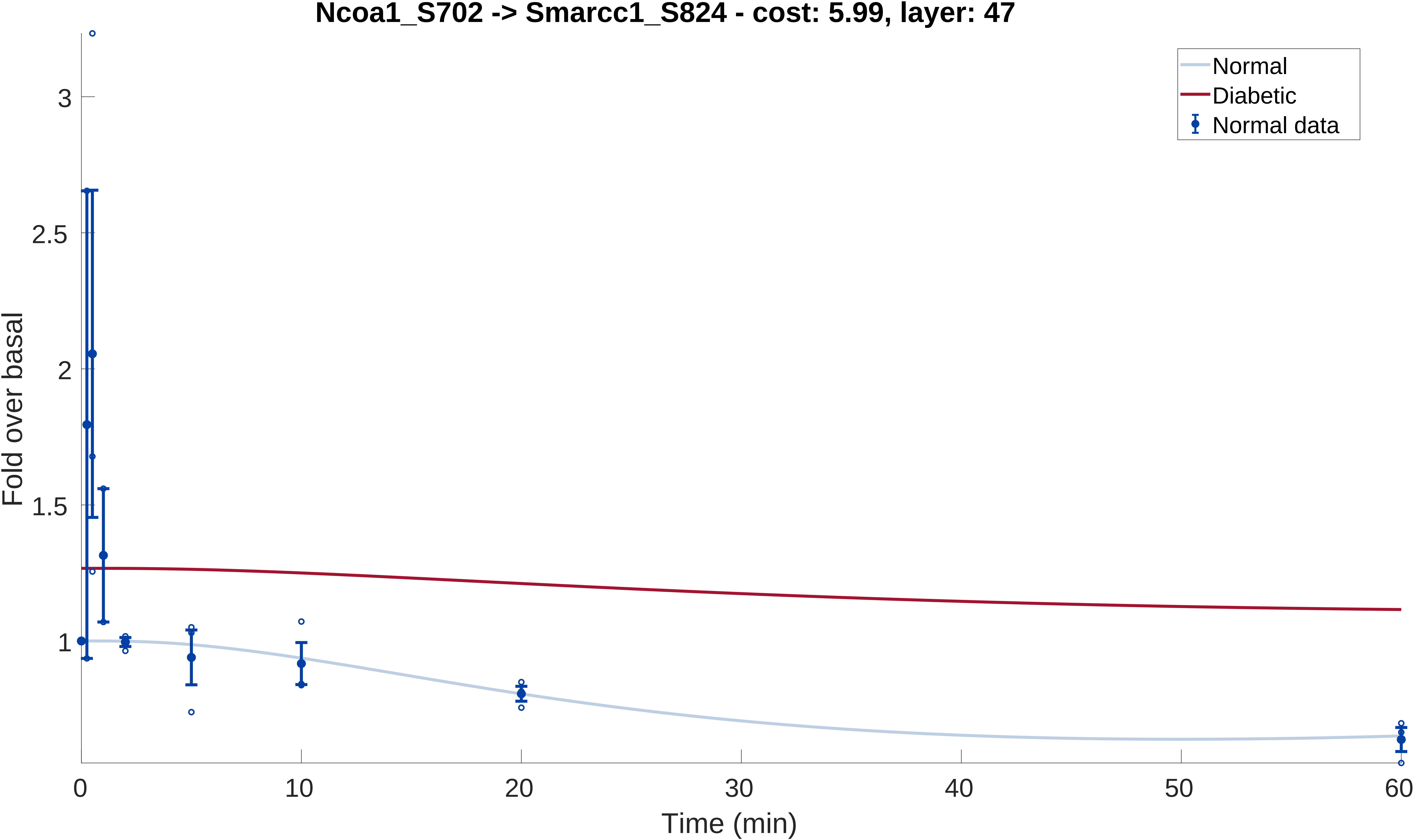

Ncoa1_S702 -> Smarcc1_S824 - cost: 5.99, layer: 47
Normal
Diabetic
Normal data
Fold over basal
Time (min)
3
2.5
2
1.5
1
0
10
20
30
40
50
60

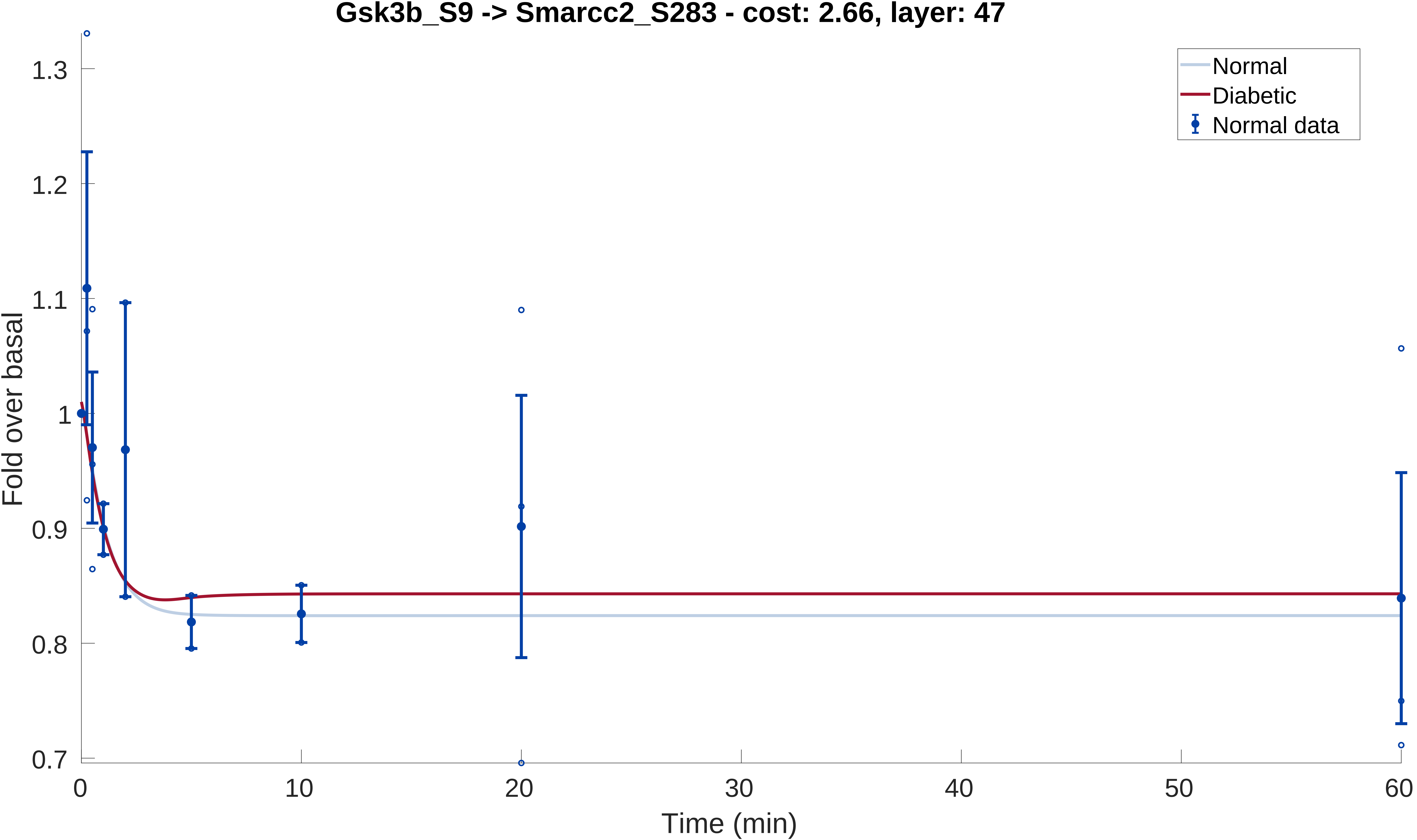
Gsk3b_S9 -> Smarcc2_S283 - cost: 2.66, layer: 47
Normal
Diabetic
Normal data
Fold over basal
1.3
1.2
1.1
1
0.9
0.8
0.7
0
10
20
30
40
50
60
Time (min)

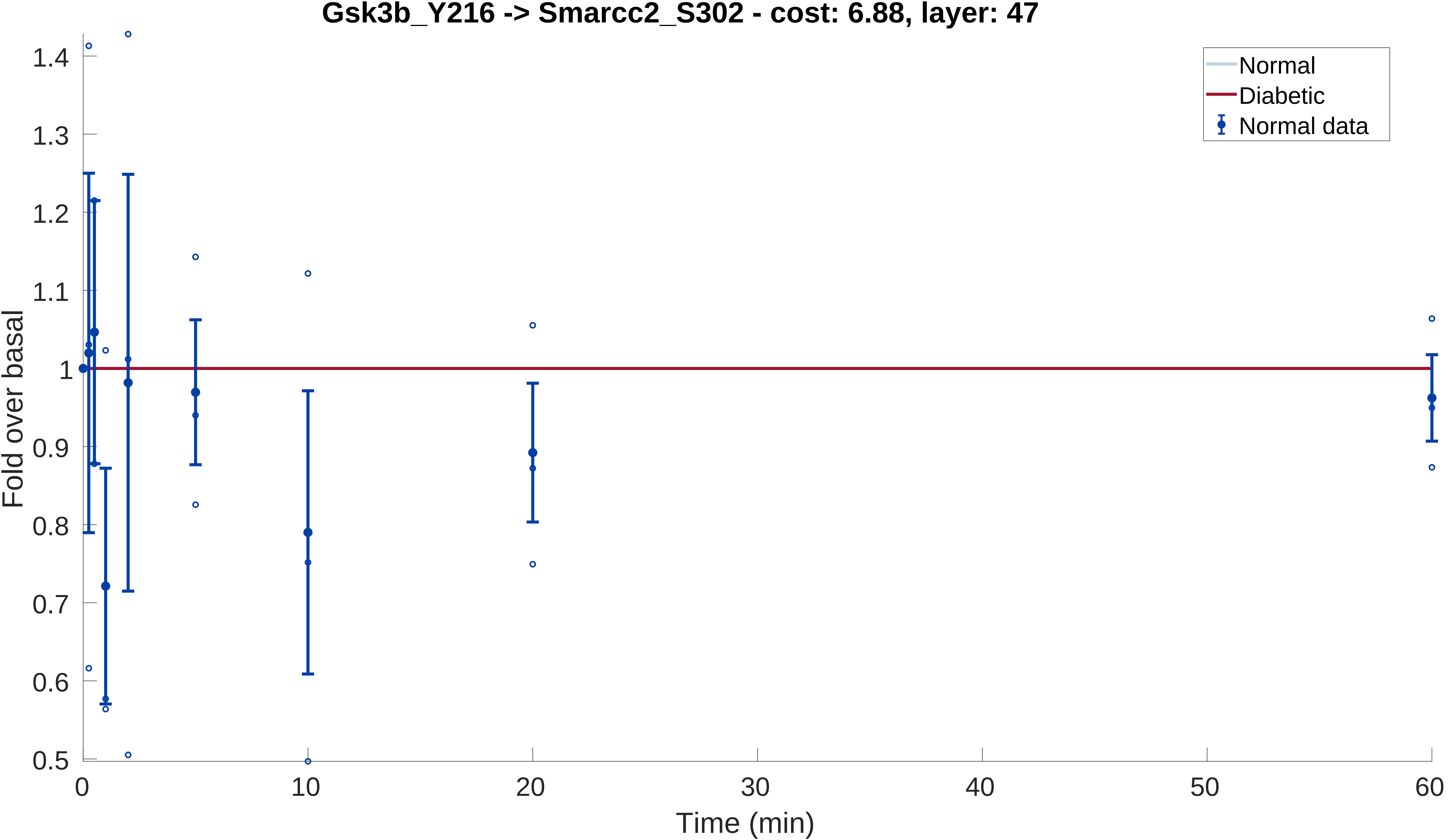

Gsk3b_Y216 -> Smarcc2_S302 - cost: 6.88, layer: 47
Normal
Diabetic
Normal data
Fold over basal
Time (min)
1.4
1.3
1.2
1.1
1
0.9
0.8
0.7
0.6
0.5
0
10
20
30
40
50
60

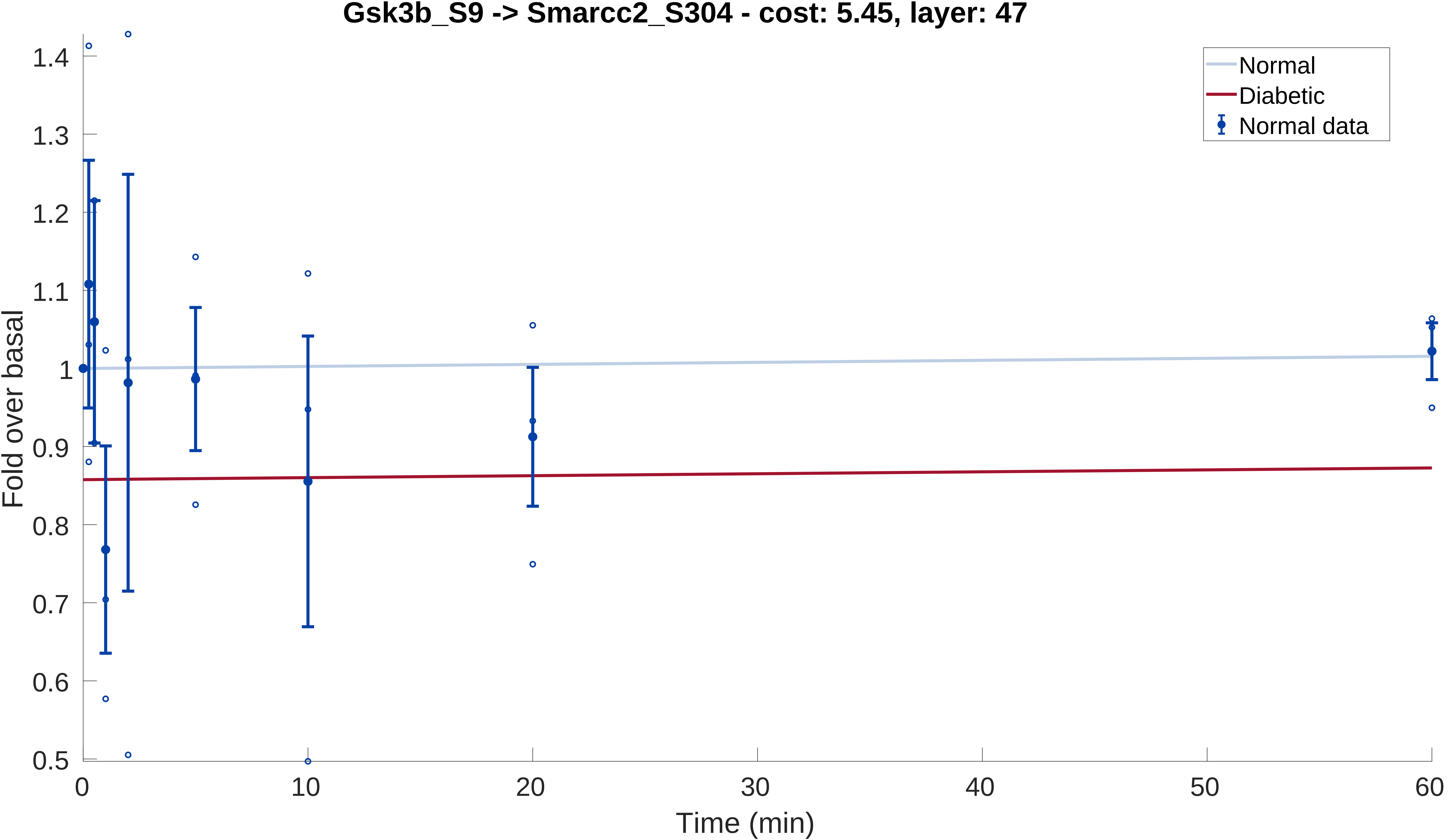
Gsk3b_S9 -> Smarcc2_S304 - cost: 5.45, layer: 47
Normal
Diabetic
Normal data
Fold over basal
1.4
1.3
1.2
1.1
1
0.9
0.8
0.7
0.6
0.5
0
10
20
30
40
50
60
Time (min)

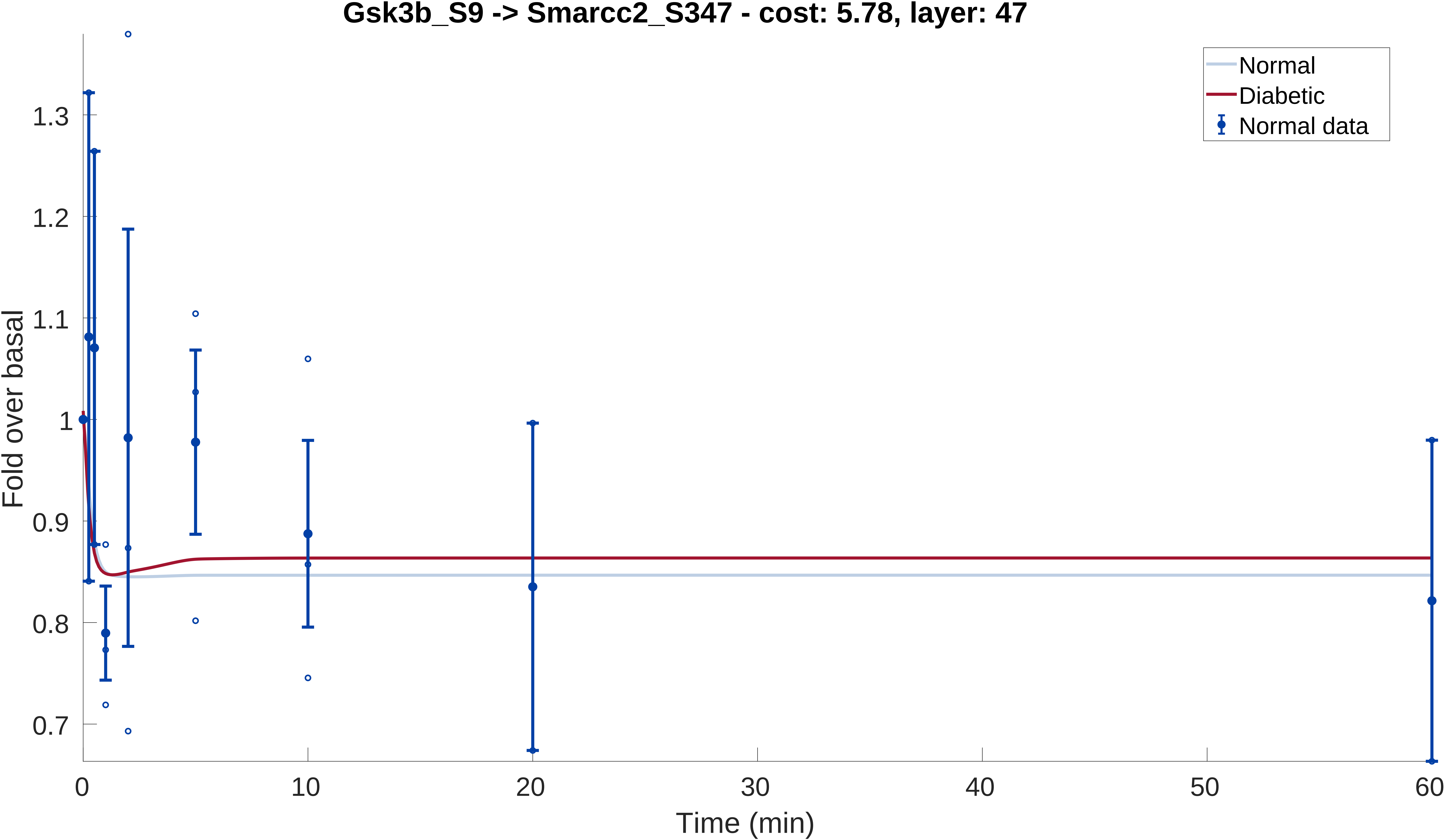
Gsk3b_S9 -> Smarcc2_S347 - cost: 5.78, layer: 47
Normal
Diabetic
Normal data
1.3
1.2
1.1
1
0.9
0.8
0.7
Fold over basal
0
10
20
30
40
50
60
Time (min)

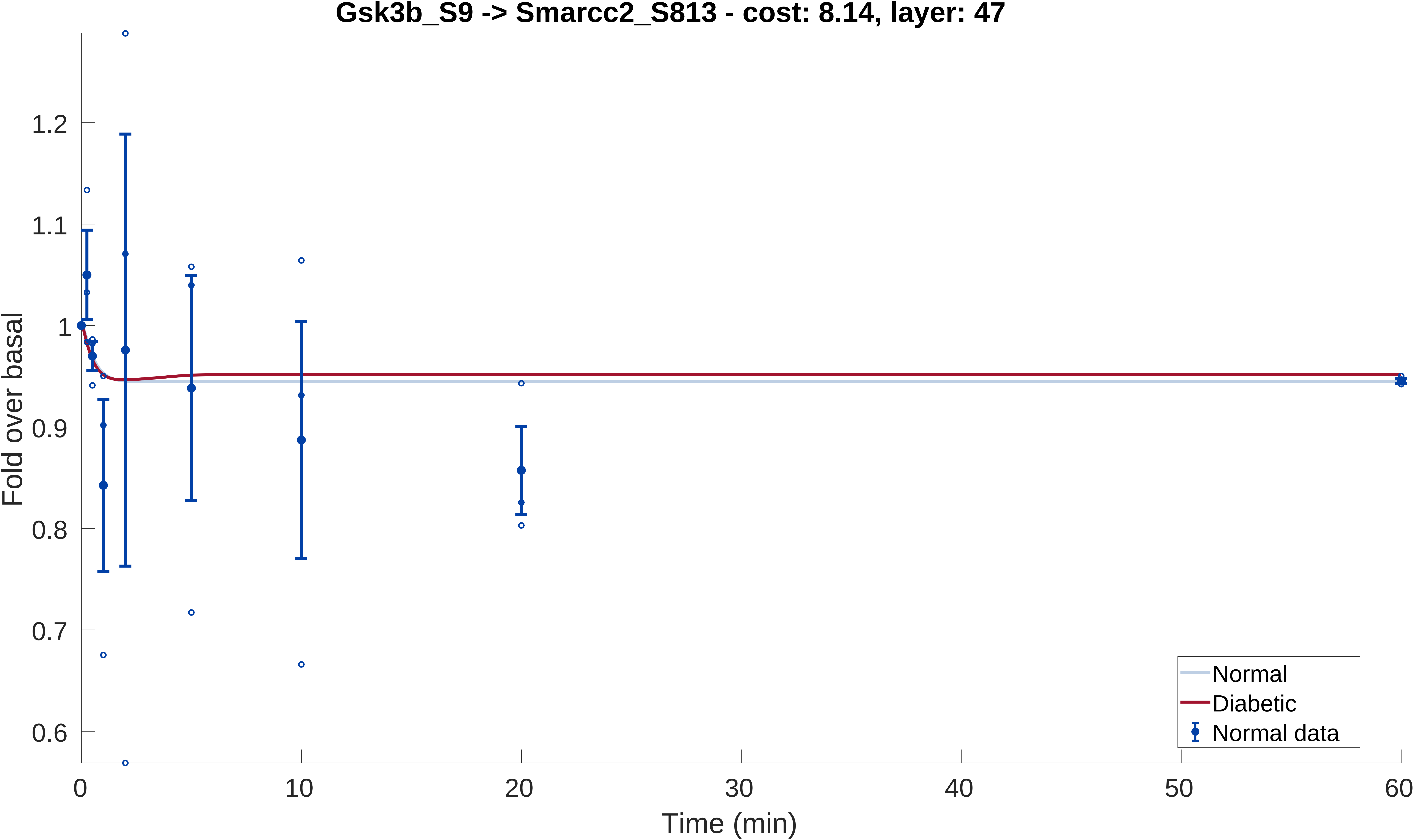

Gsk3b_S9 -> Smarcc2_S813 - cost: 8.14, layer: 47
Fold over basal
1.2
1.1
1
0.9
0.8
0.7
0.6
0
10
20
30
40
50
60
Time (min)
Normal
Diabetic
Normal data

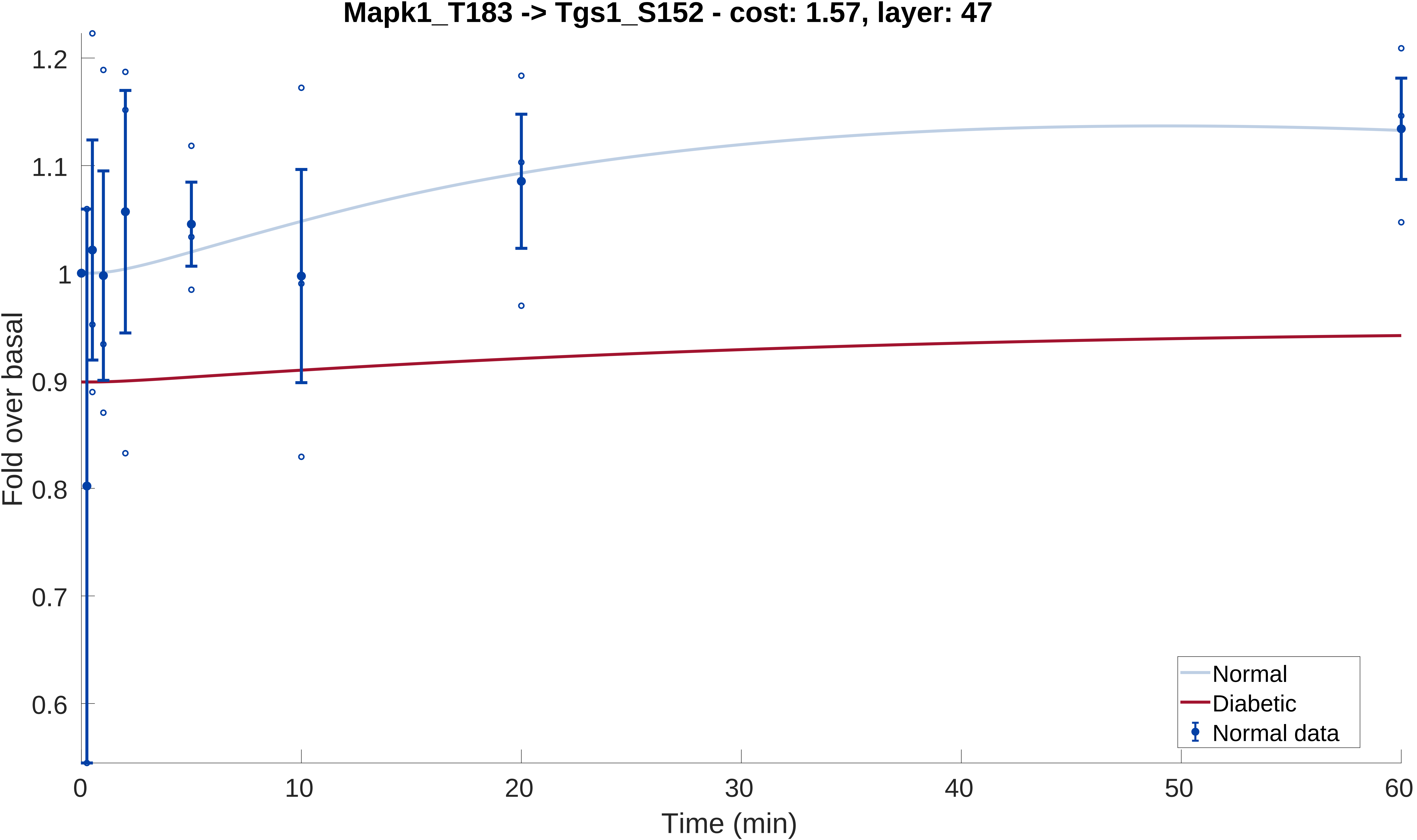

Mapk1_T183 -> Tgs1_S152 - cost: 1.57, layer: 47
Fold over basal
Time (min)
1.2
1.1
1
0.9
0.8
0.7
0.6
0
10
20
30
40
50
60
Normal
Diabetic
Normal data

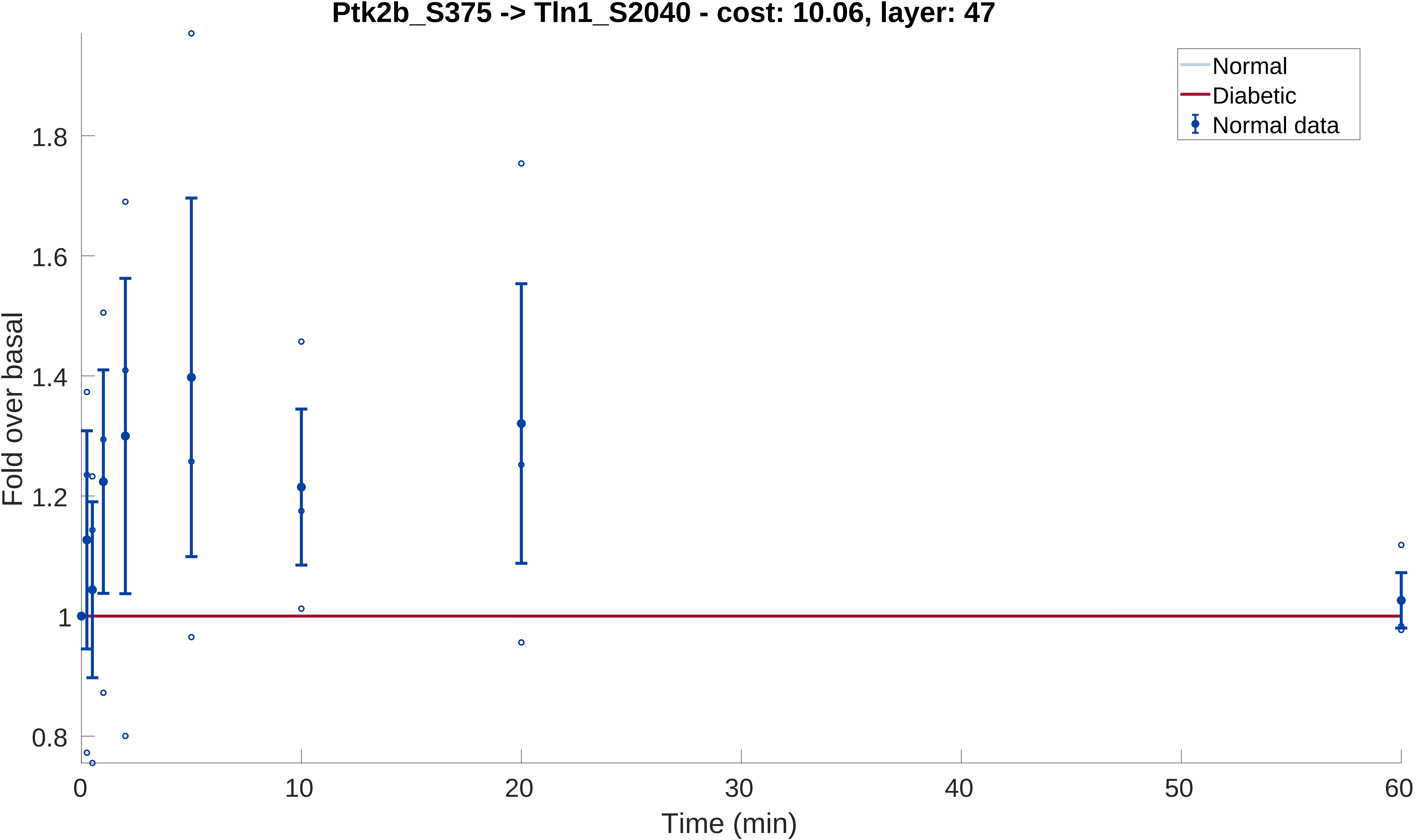

Ptk2b_S375 -> Tln1_S2040 - cost: 10.06, layer: 47
Normal
Diabetic
Normal data
Fold over basal
1.8
1.6
1.4
1.2
1
0.8
0
10
20
30
40
50
60
Time (min)

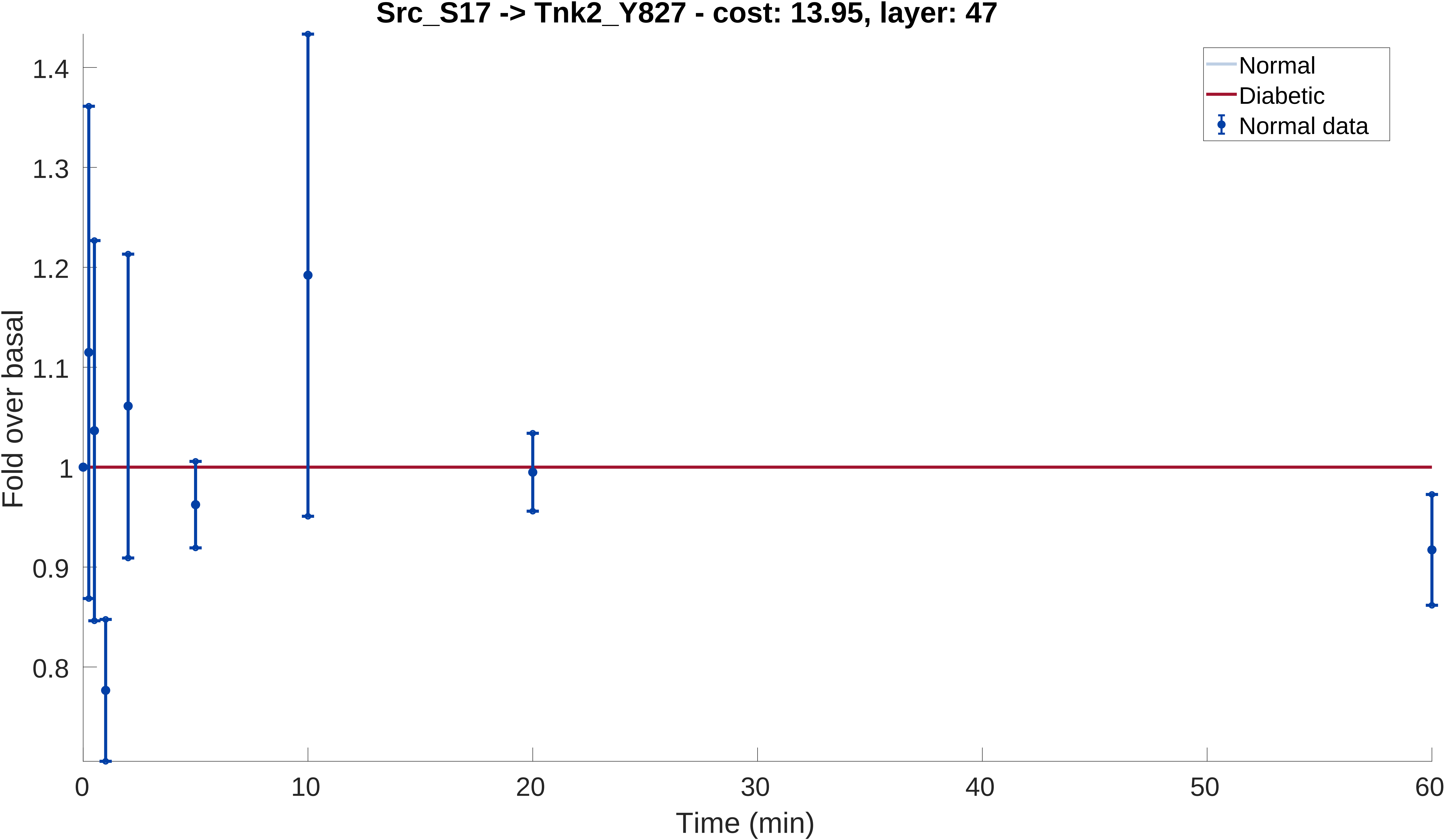

Src_S17 -> Tnk2_Y827 - cost: 13.95, layer: 47
Normal
Diabetic
Normal data
Fold over basal
1.4
1.3
1.2
1.1
1
0.9
0.8
0
10
20
30
40
50
60
Time (min)

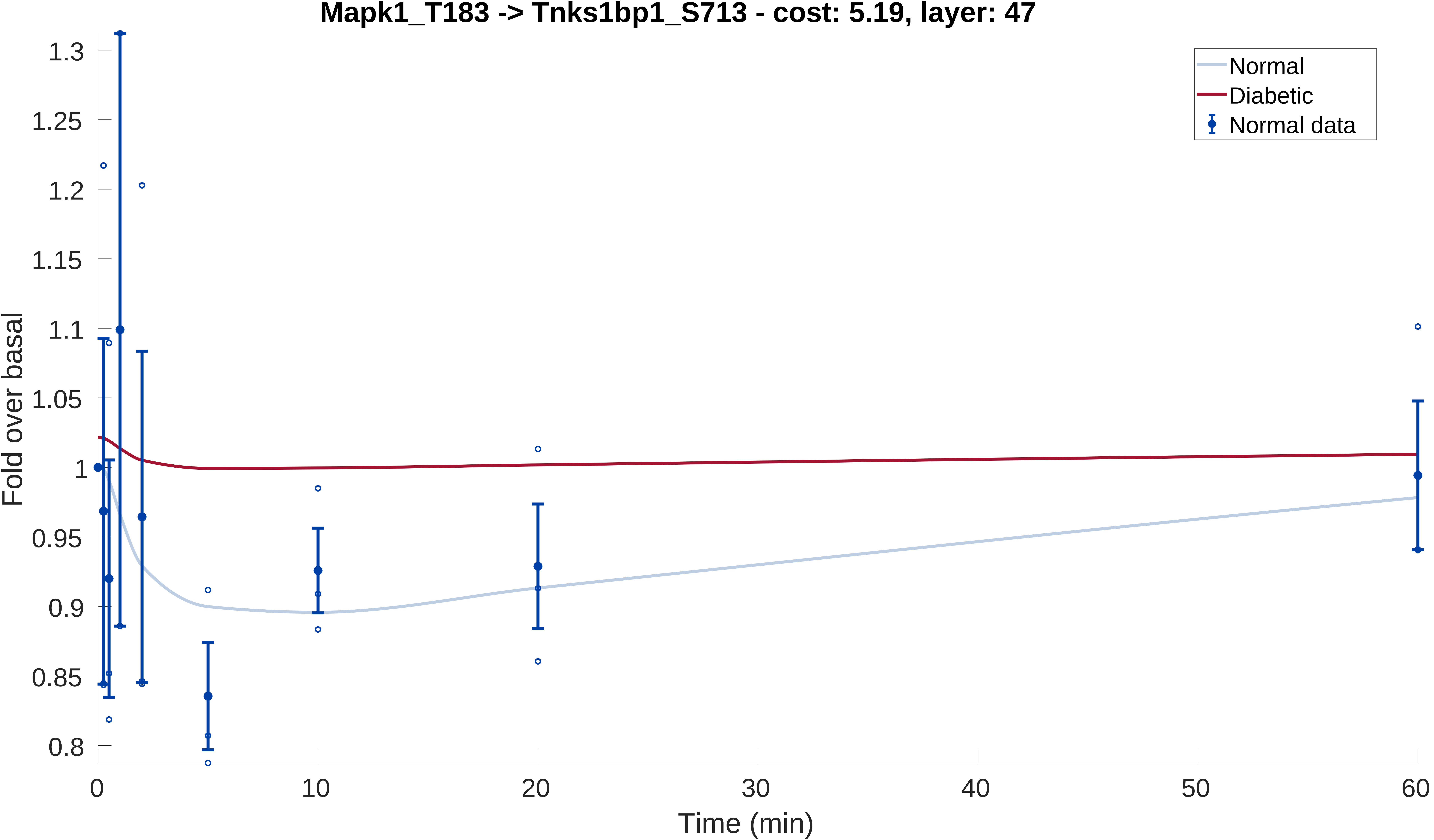

Mapk1_T183 -> Tnks1bp1_S713 - cost: 5.19, layer: 47
Normal
Diabetic
Normal data
Fold over basal
1.3
1.25
1.2
1.15
1.1
1.05
1
0.95
0.9
0.85
0.8
0
10
20
30
40
50
60
Time (min)

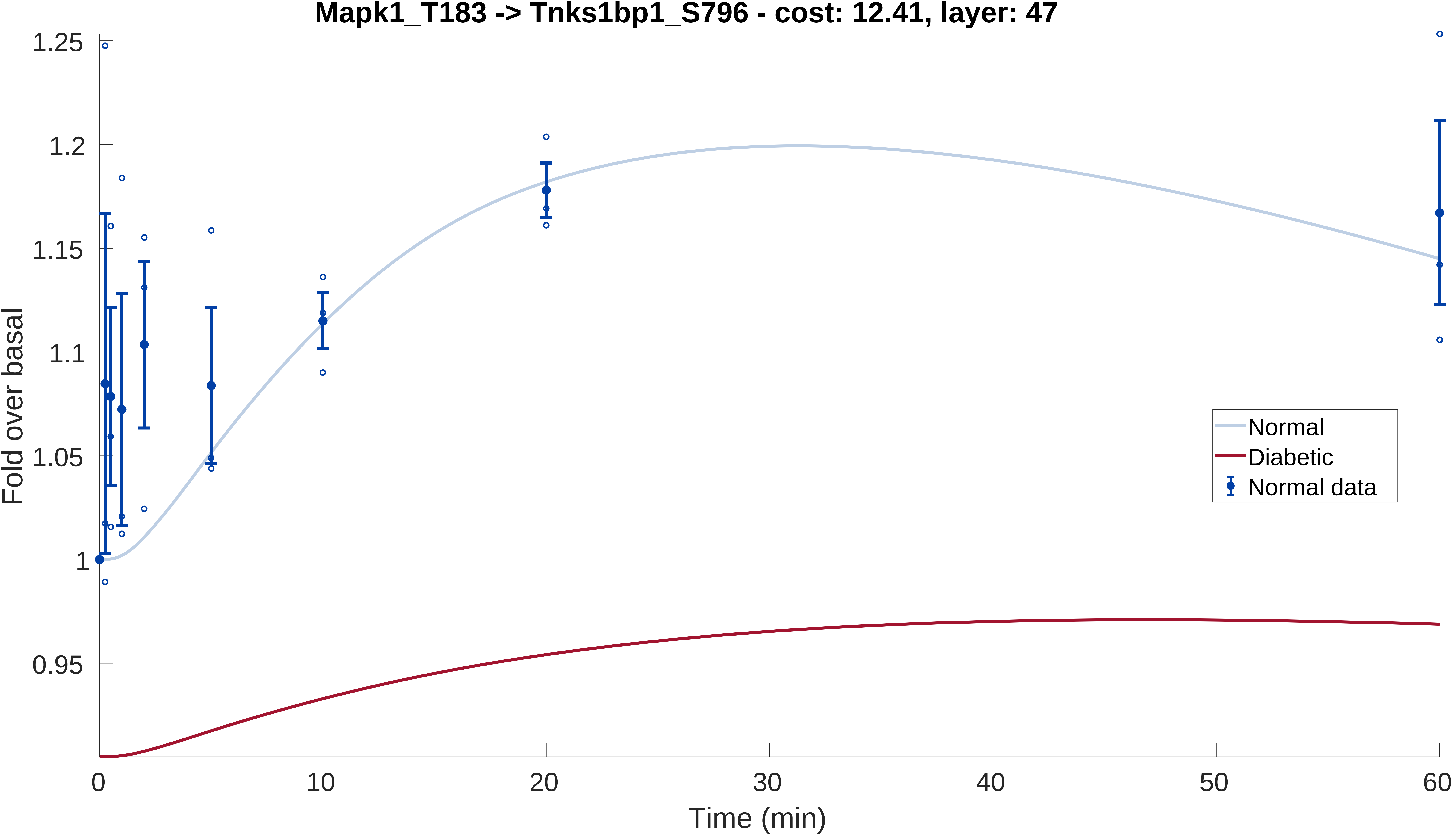
Mapk1_T183 -> Tnks1bp1_S796 - cost: 12.41, layer: 47
Fold over basal
1.25
1.2
1.15
1.1
1.05
1
0.95
0
10
20
30
40
50
60
Time (min)
Normal
Diabetic
Normal data

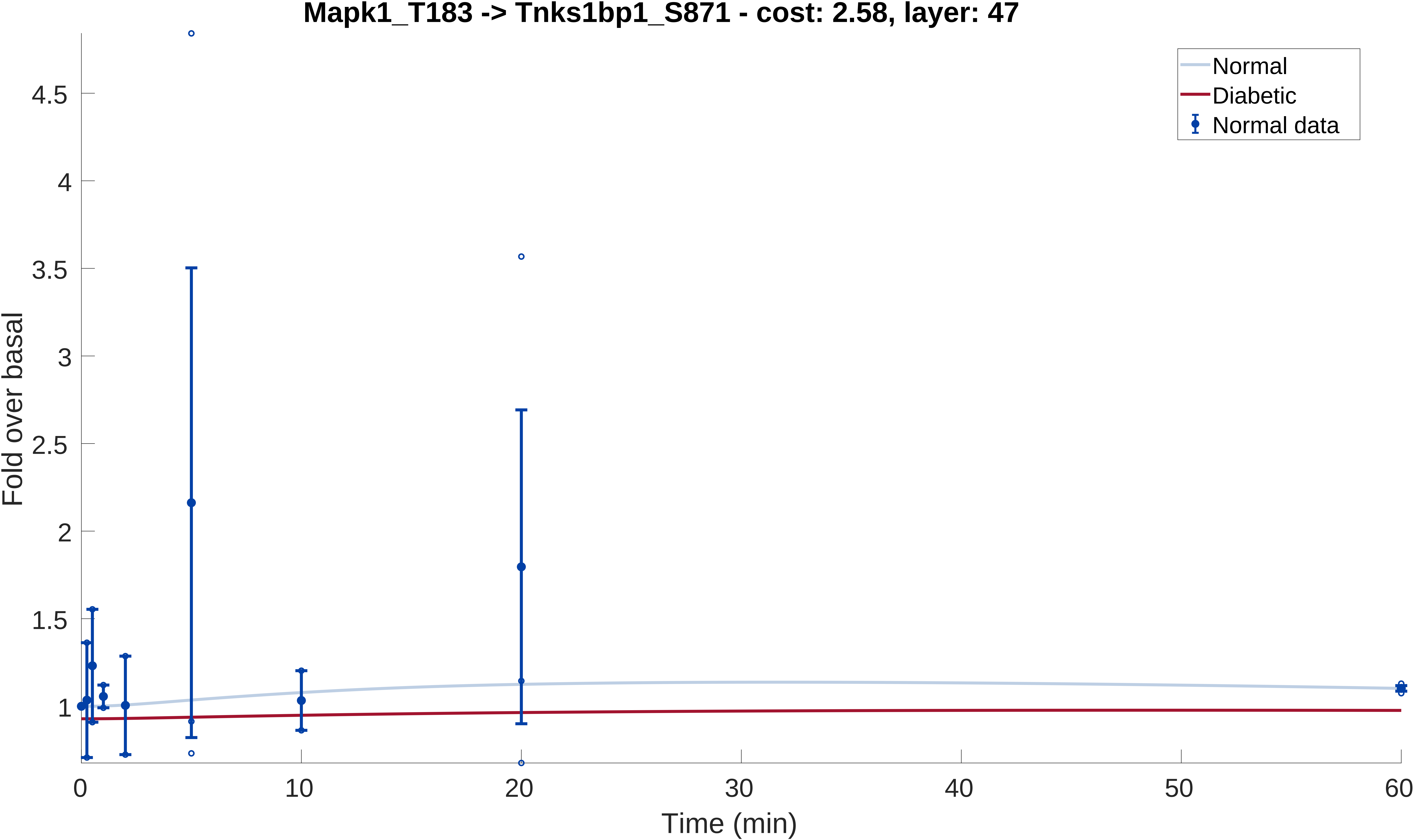

Mapk1_T183 -> Tnks1bp1_S871 - cost: 2.58, layer: 47
Normal
Diabetic
Normal data
Fold over basal
4.5
4
3.5
3
2.5
2
1.5
1
0
10
20
30
40
50
60
Time (min)

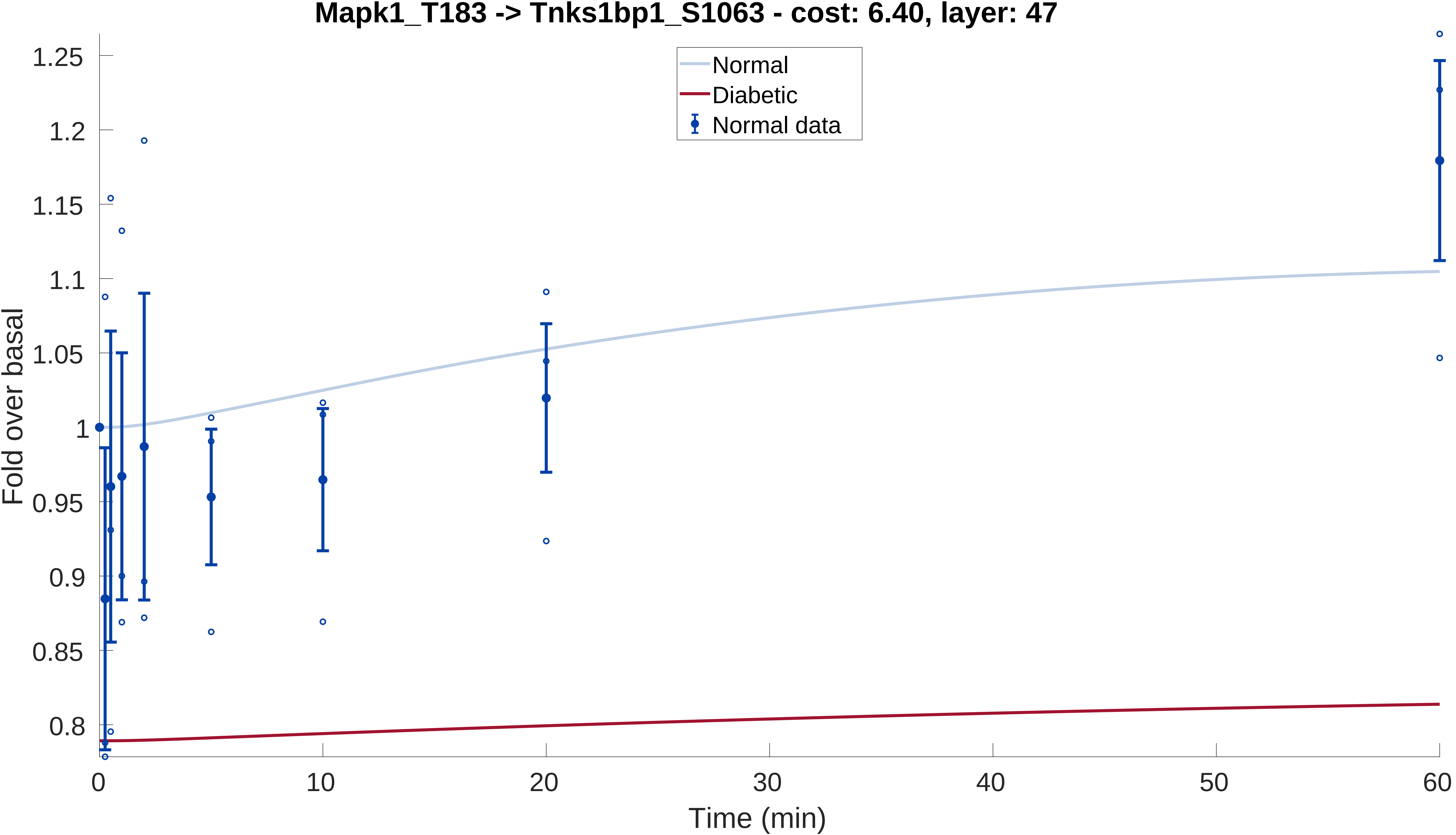
Mapk1_T183 -> Tnks1bp1_S1063 - cost: 6.40, layer: 47
Normal
Diabetic
Normal data
Fold over basal
1.25
1.2
1.15
1.1
1.05
1
0.95
0.9
0.85
0.8
0
10
20
30
40
50
60
Time (min)

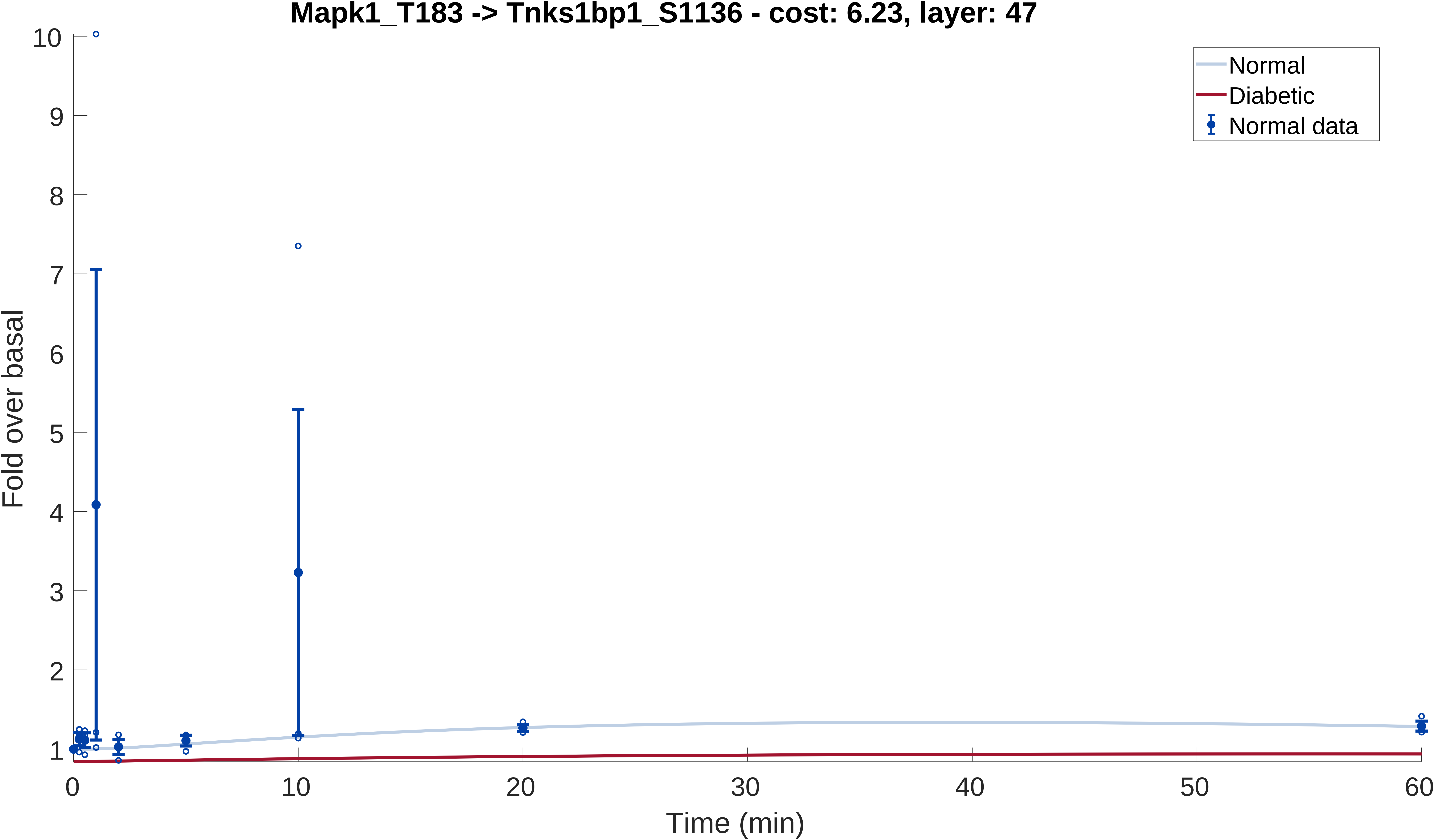

Mapk1_T183 -> Tnks1bp1_S1136 - cost: 6.23, layer: 47
Normal
Diabetic
Normal data
Fold over basal
Time (min)
1
2
3
4
5
6
7
8
9
10
0
10
20
30
40
50
60

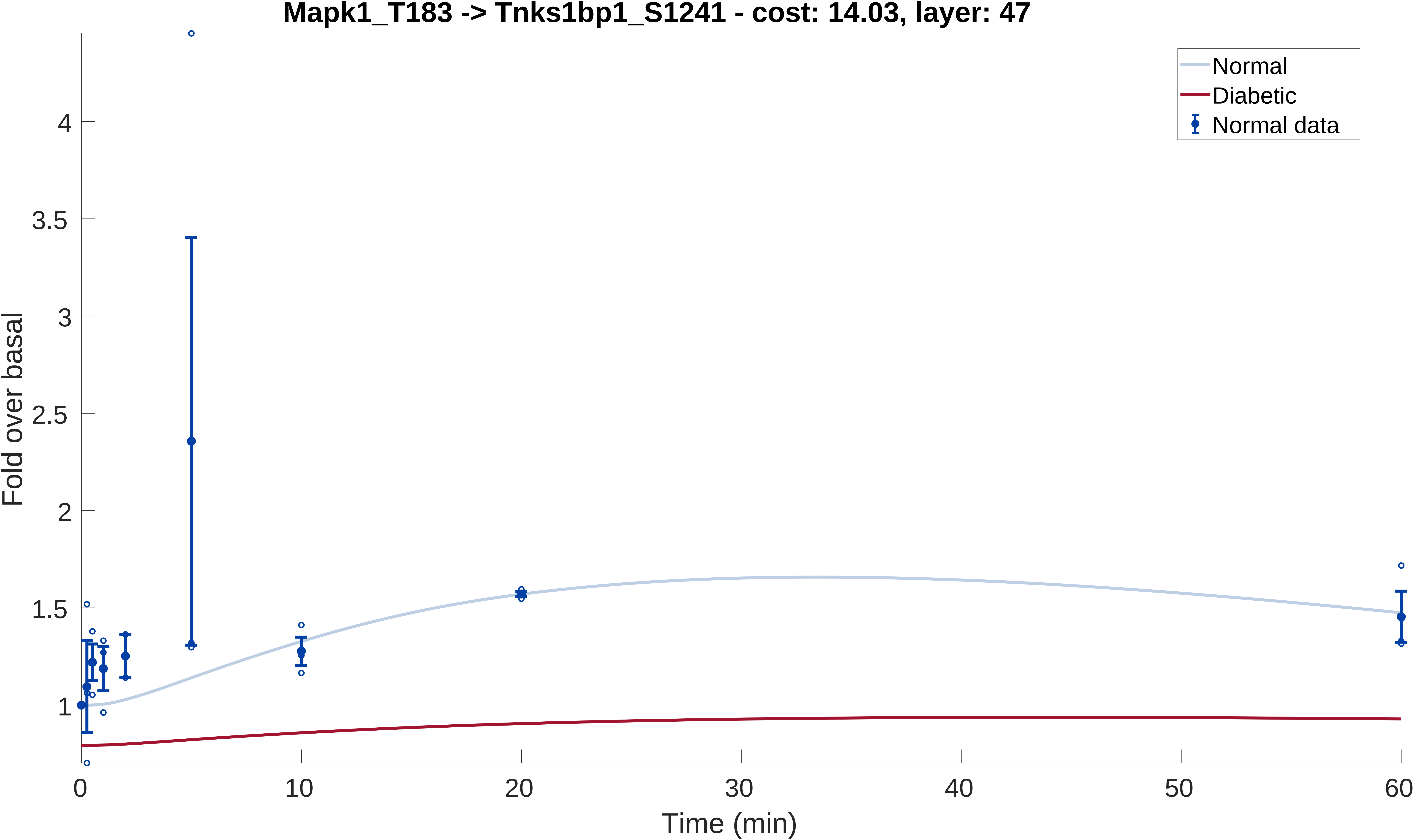

Mapk1_T183 -> Tnks1bp1_S1241 - cost: 14.03, layer: 47
Normal
Diabetic
Normal data
Fold over basal
Time (min)
1
1.5
2
2.5
3
3.5
4
0
10
20
30
40
50
60

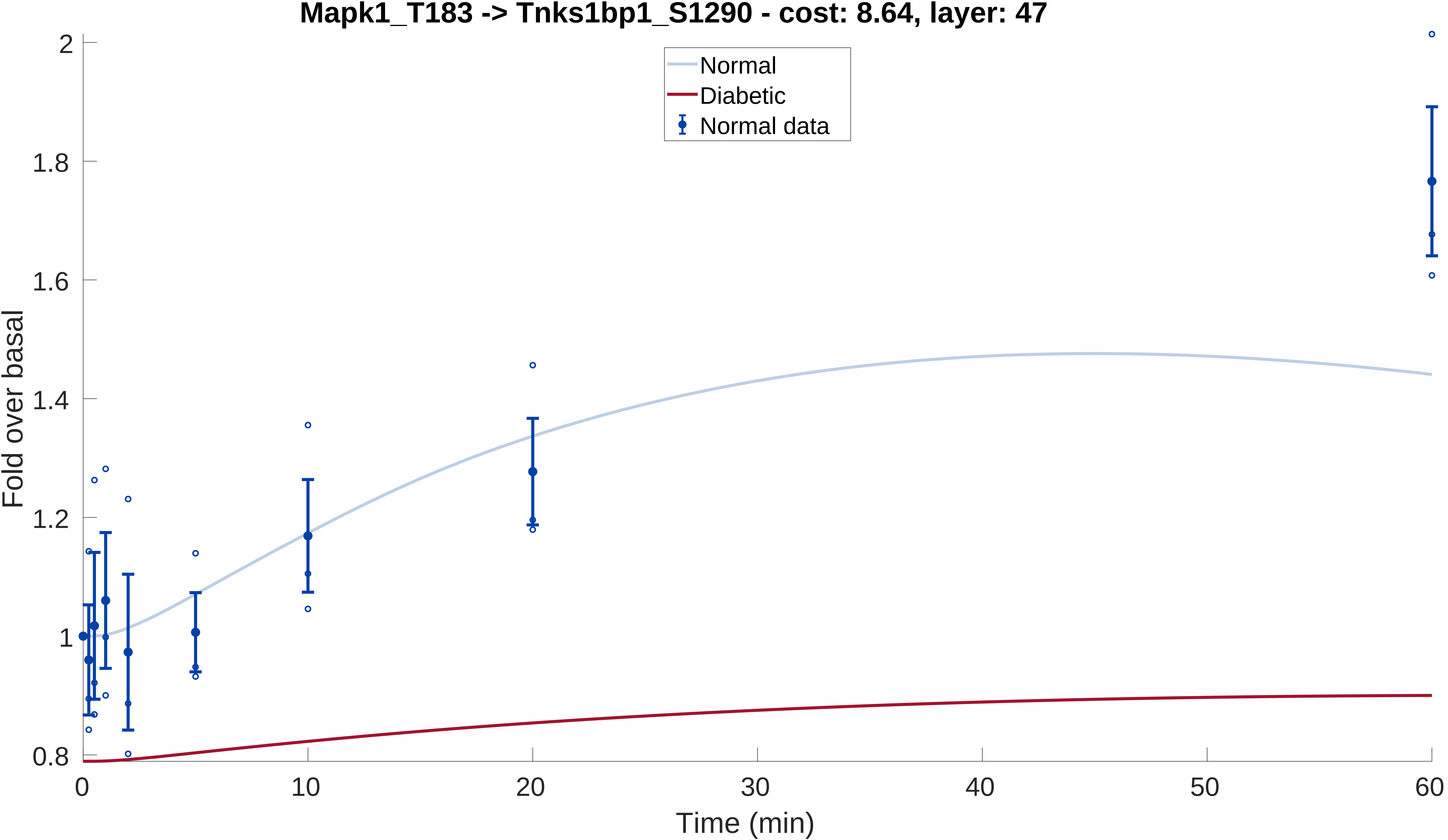

Mapk1_T183 -> Tnks1bp1_S1290 - cost: 8.64, layer: 47
Normal
Diabetic
Normal data
Fold over basal
Time (min)
0.8
1
1.2
1.4
1.6
1.8
2
0
10
20
30
40
50
60

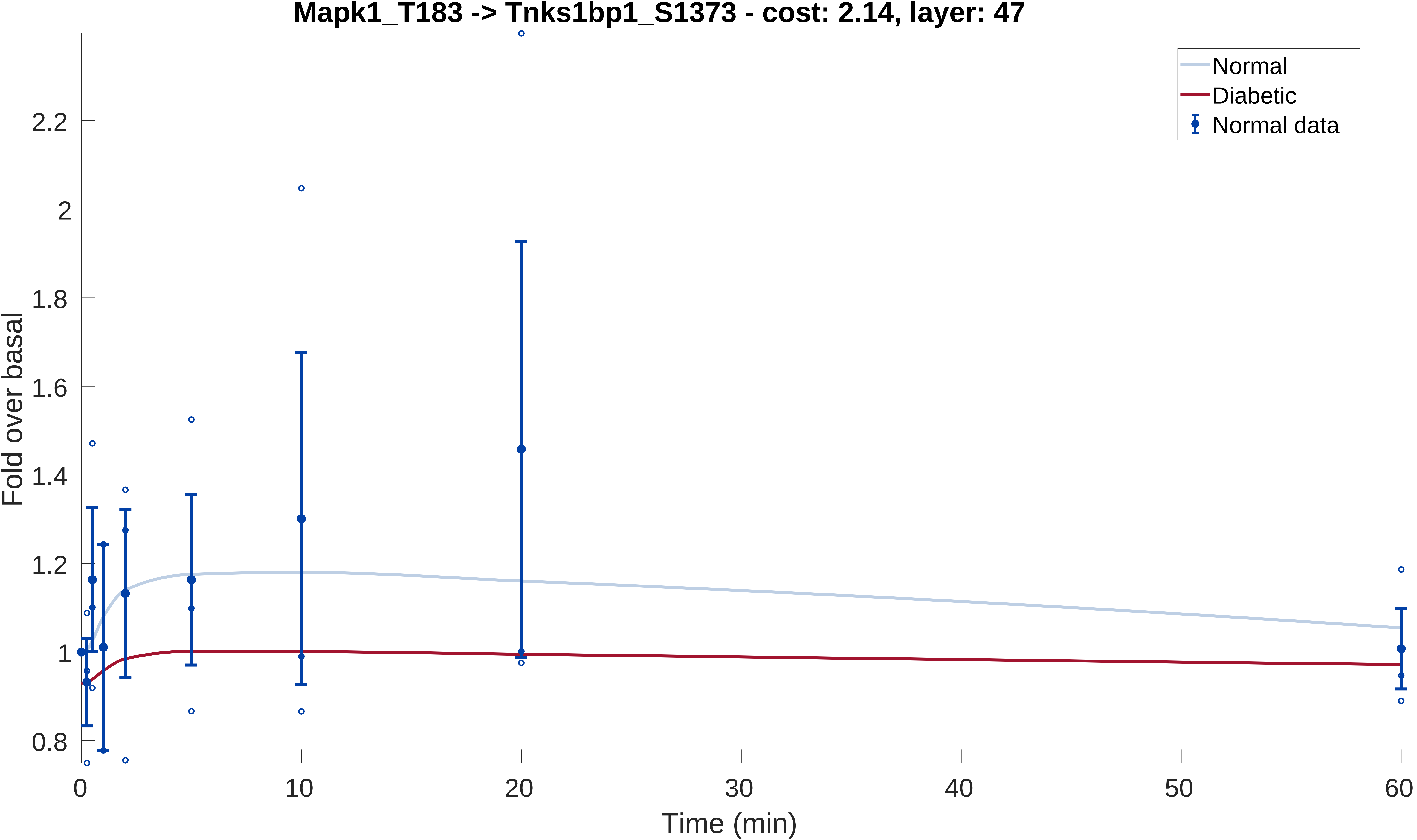

Mapk1_T183 -> Tnks1bp1_S1373 - cost: 2.14, layer: 47
Normal
Diabetic
Normal data
Fold over basal
Time (min)
0.8
1
1.2
1.4
1.6
1.8
2
2.2
0
10
20
30
40
50
60

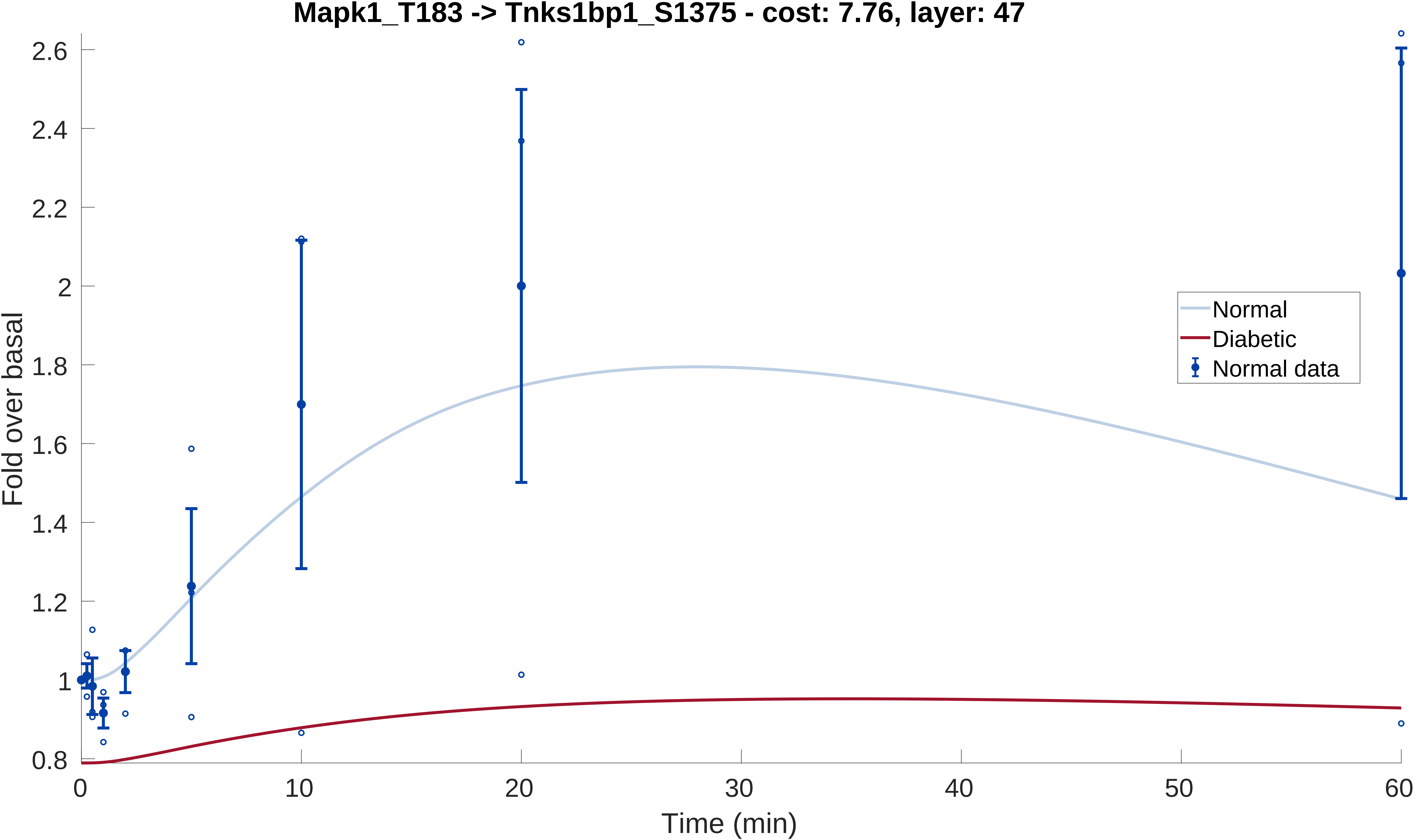

Mapk1_T183 -> Tnks1bp1_S1375 - cost: 7.76, layer: 47
Fold over basal
Time (min)
0.8
1
1.2
1.4
1.6
1.8
2
2.2
2.4
2.6
0
10
20
30
40
50
60
Normal
Diabetic
Normal data

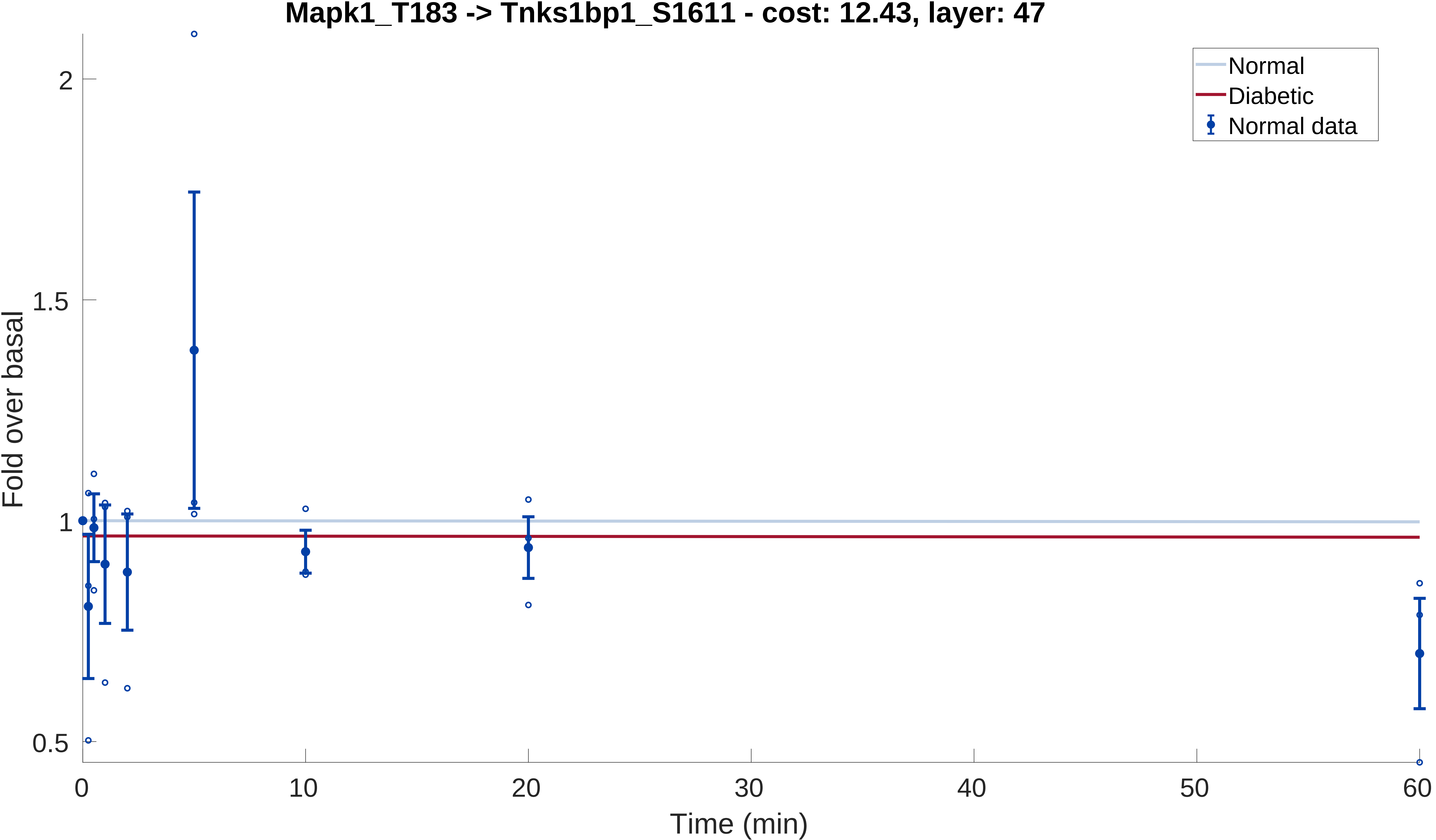

Mapk1_T183 -> Tnks1bp1_S1611 - cost: 12.43, layer: 47
Normal
Diabetic
Normal data
Fold over basal
2
1.5
1
0.5
0
10
20
30
40
50
60
Time (min)

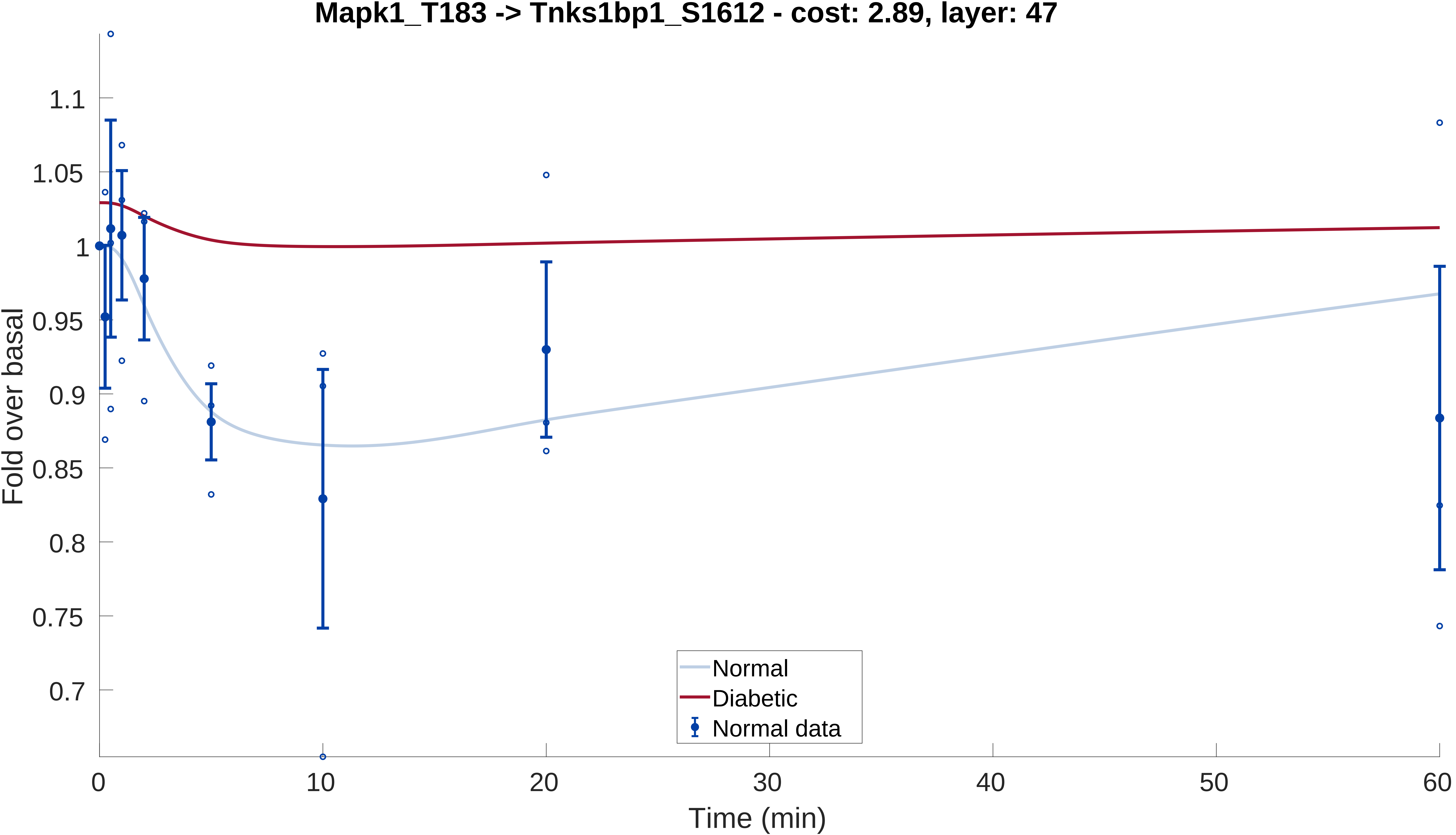
Mapk1_T183 -> Tnks1bp1_S1612 - cost: 2.89, layer: 47
Fold over basal
1.1
1.05
1
0.95
0.9
0.85
0.8
0.75
0.7
0
10
20
30
40
50
60
Time (min)
Normal
Diabetic
Normal data

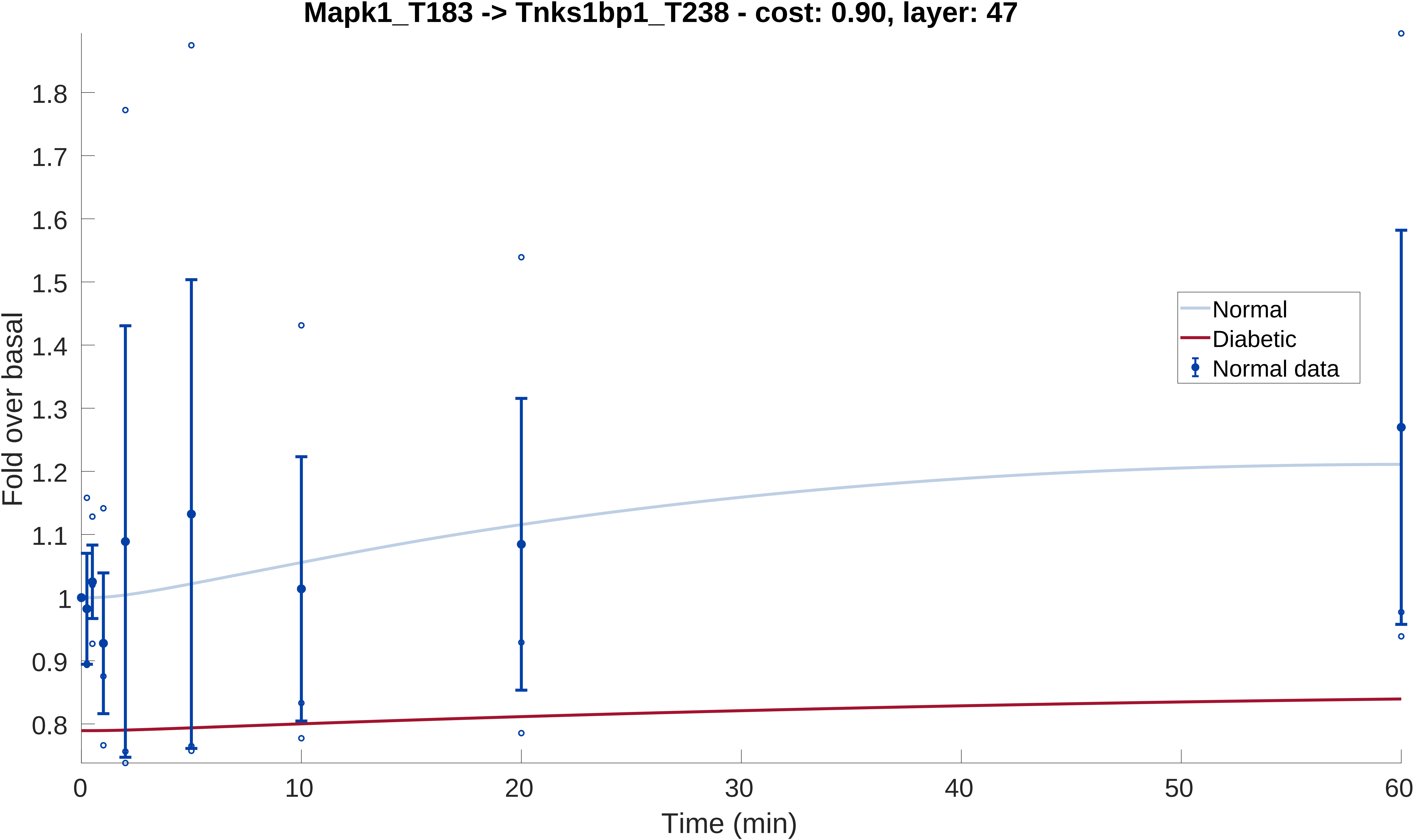

Mapk1_T183 -> Tnks1bp1_T238 - cost: 0.90, layer: 47
Fold over basal
Time (min)
Normal
Diabetic
Normal data
0.8
0.9
1
1.1
1.2
1.3
1.4
1.5
1.6
1.7
1.8
0
10
20
30
40
50
60

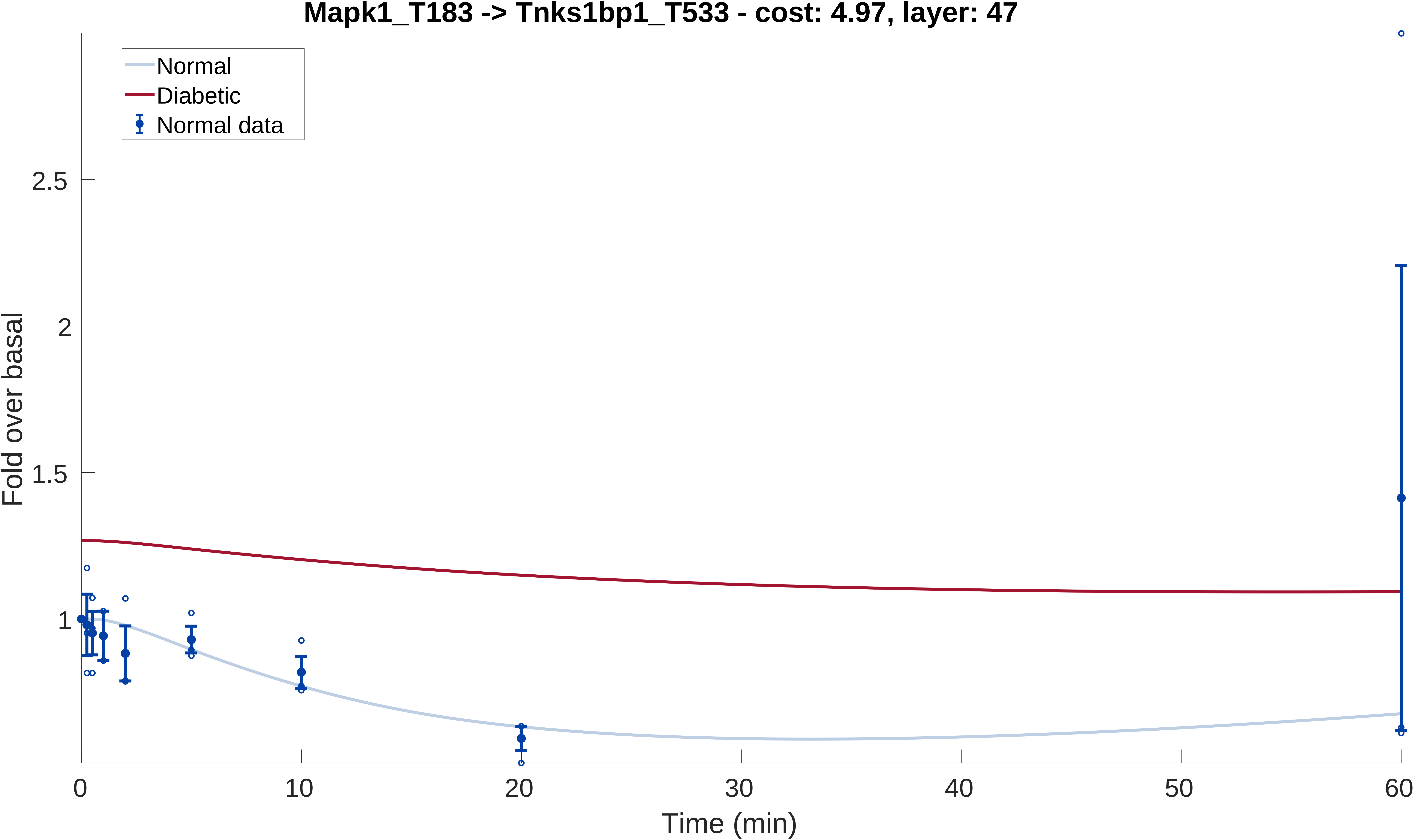

Mapk1_T183 -> Tnks1bp1_T533 - cost: 4.97, layer: 47
Normal
Diabetic
Normal data
Fold over basal
2.5
2
1.5
1
0
10
20
30
40
50
60
Time (min)

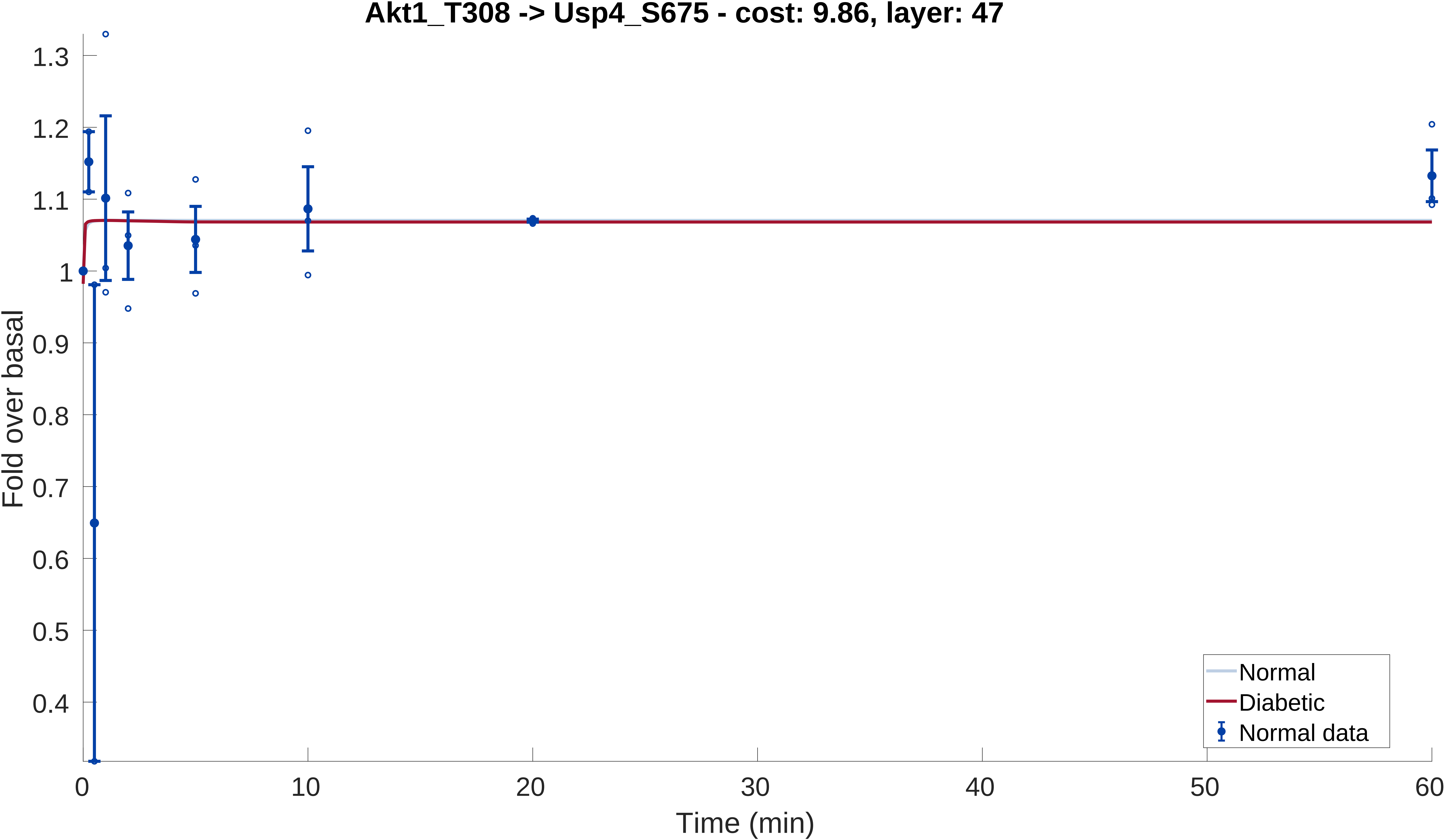
Akt1_T308 -> Usp4_S675 - cost: 9.86, layer: 47
Fold over basal
1.3
1.2
1.1
1
0.9
0.8
0.7
0.6
0.5
0.4
0
10
20
30
40
50
60
Time (min)
Normal
Diabetic
Normal data

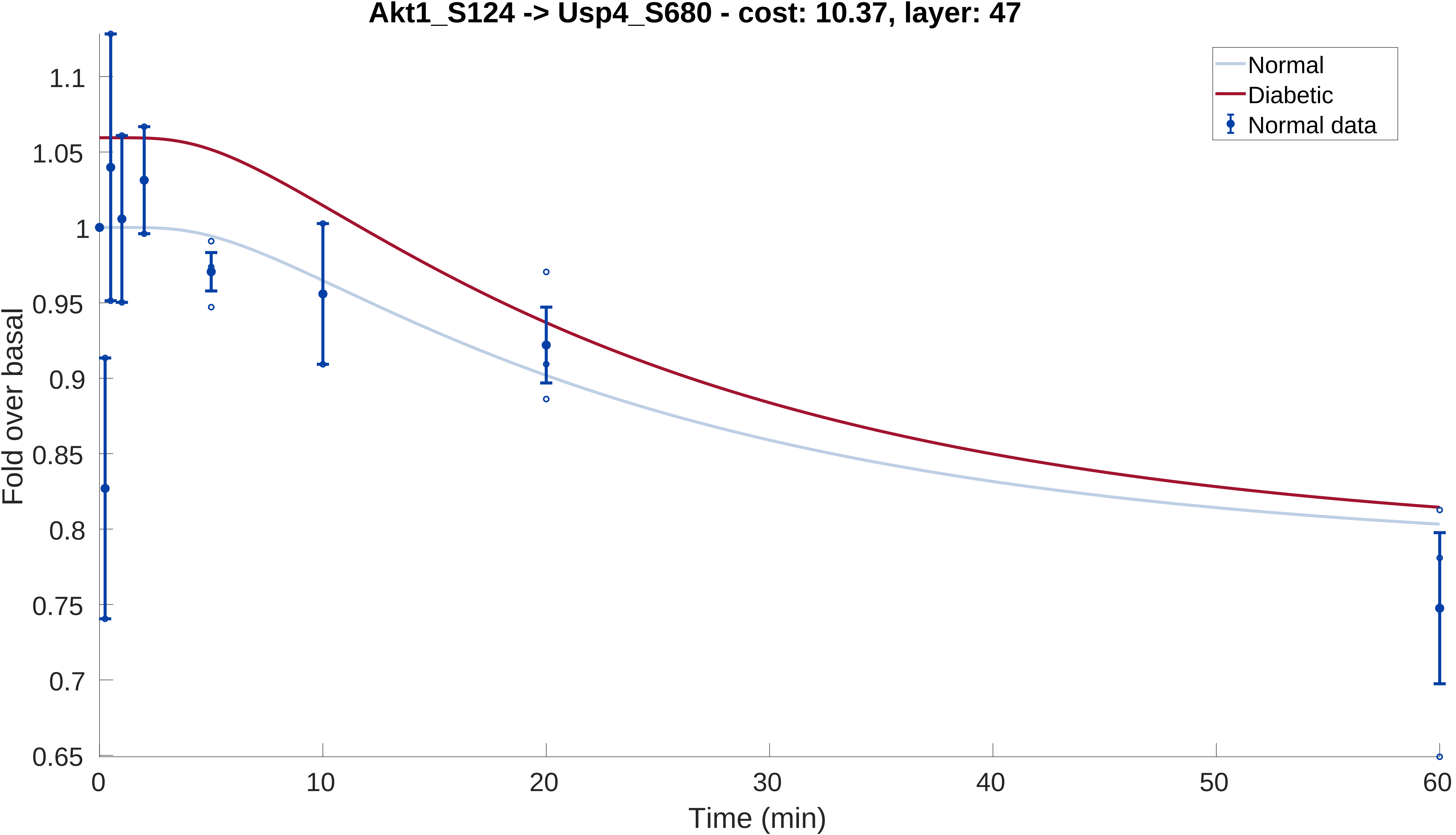

Akt1_S124 -> Usp4_S680 - cost: 10.37, layer: 47
Normal
Diabetic
Normal data
Fold over basal
Time (min)
1.1
1.05
1
0.95
0.9
0.85
0.8
0.75
0.7
0.65
0
10
20
30
40
50
60

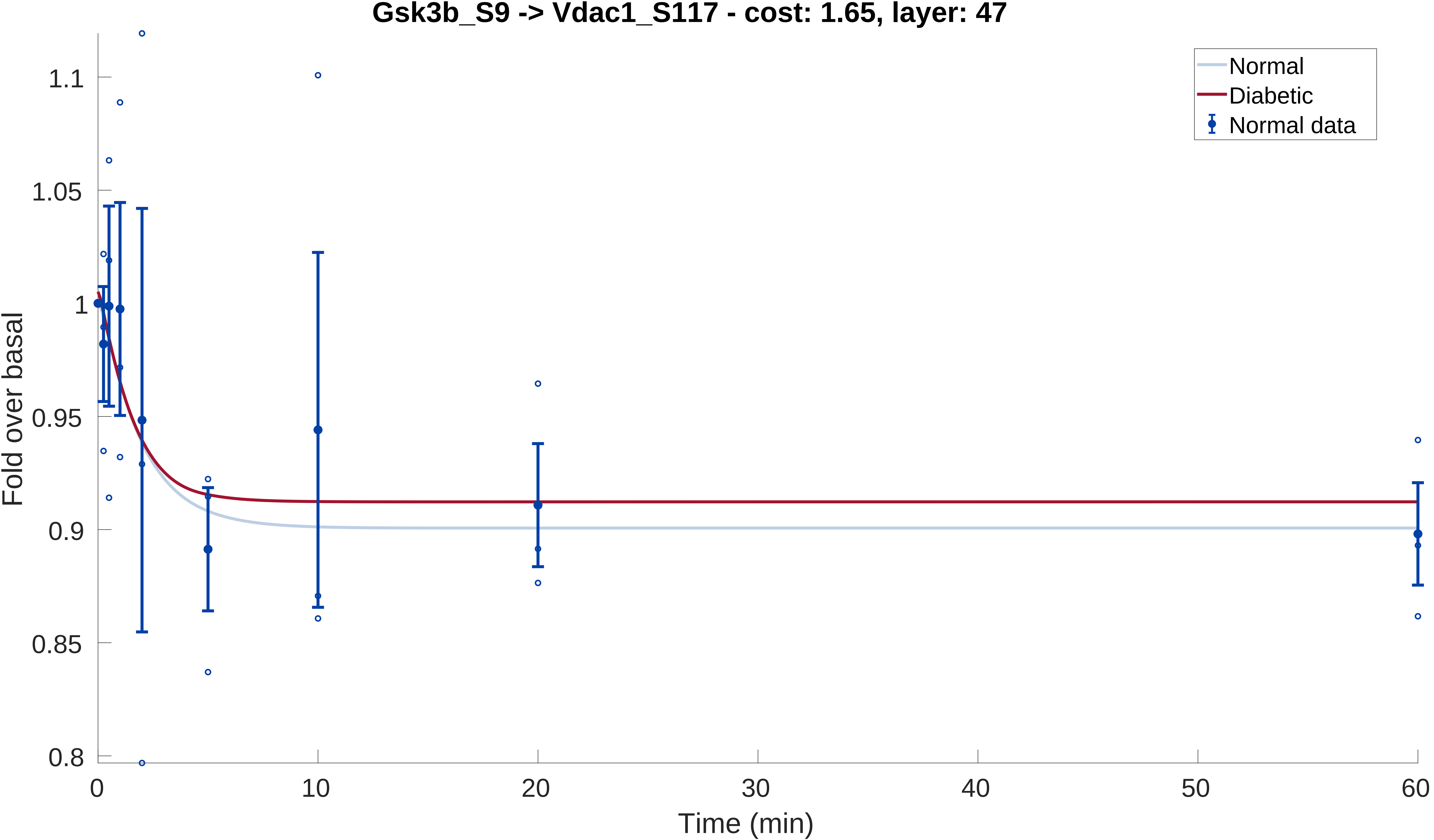
Gsk3b_S9 -> Vdac1_S117 - cost: 1.65, layer: 47
Normal
Diabetic
Normal data
Fold over basal
Time (min)
0.8
0.85
0.9
0.95
1
1.05
1.1
0
10
20
30
40
50
60

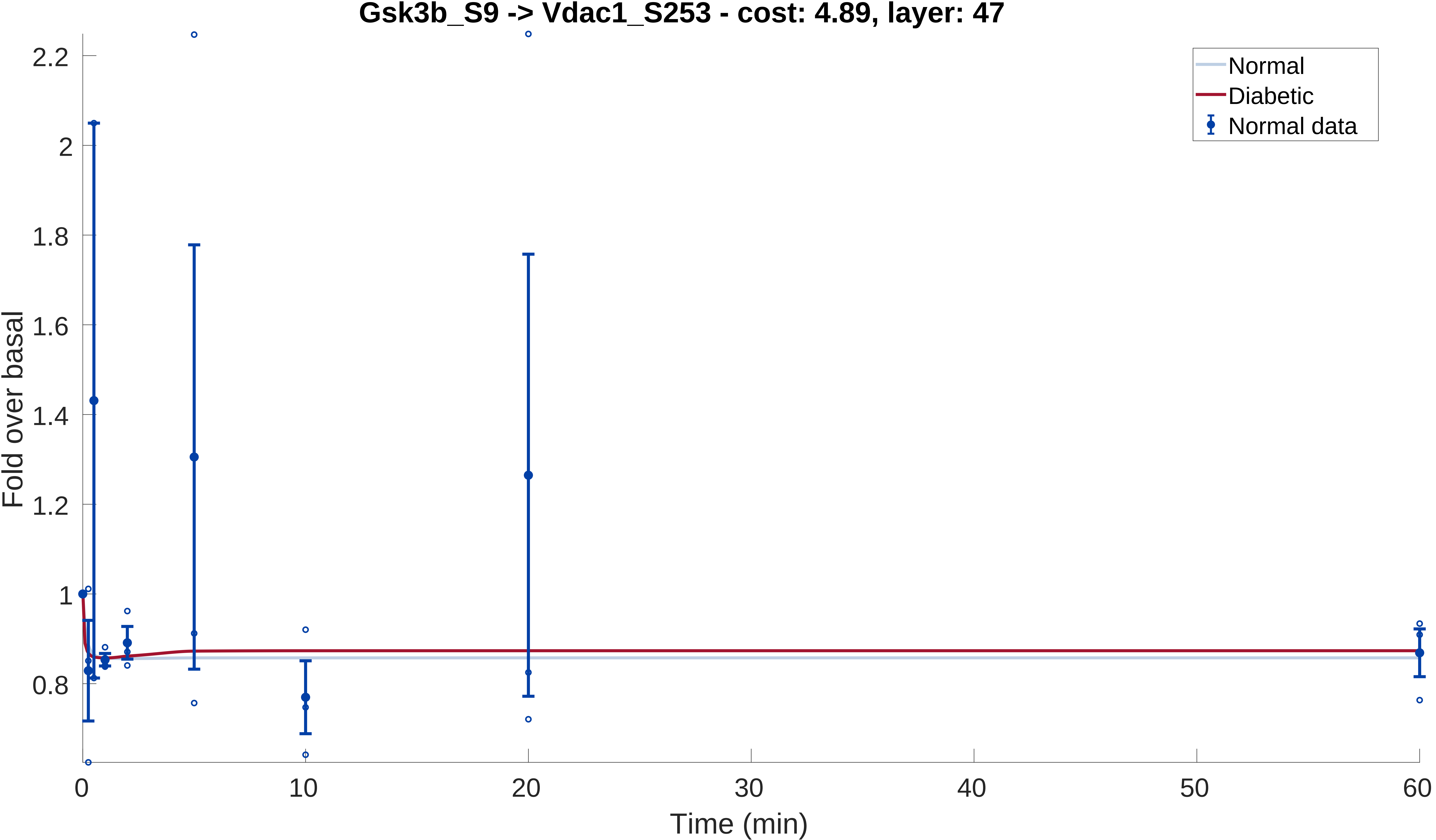
Gsk3b_S9 -> Vdac1_S253 - cost: 4.89, layer: 47
Normal
Diabetic
Normal data
Fold over basal
0.8
1
1.2
1.4
1.6
1.8
2
2.2
0
10
20
30
40
50
60
Time (min)

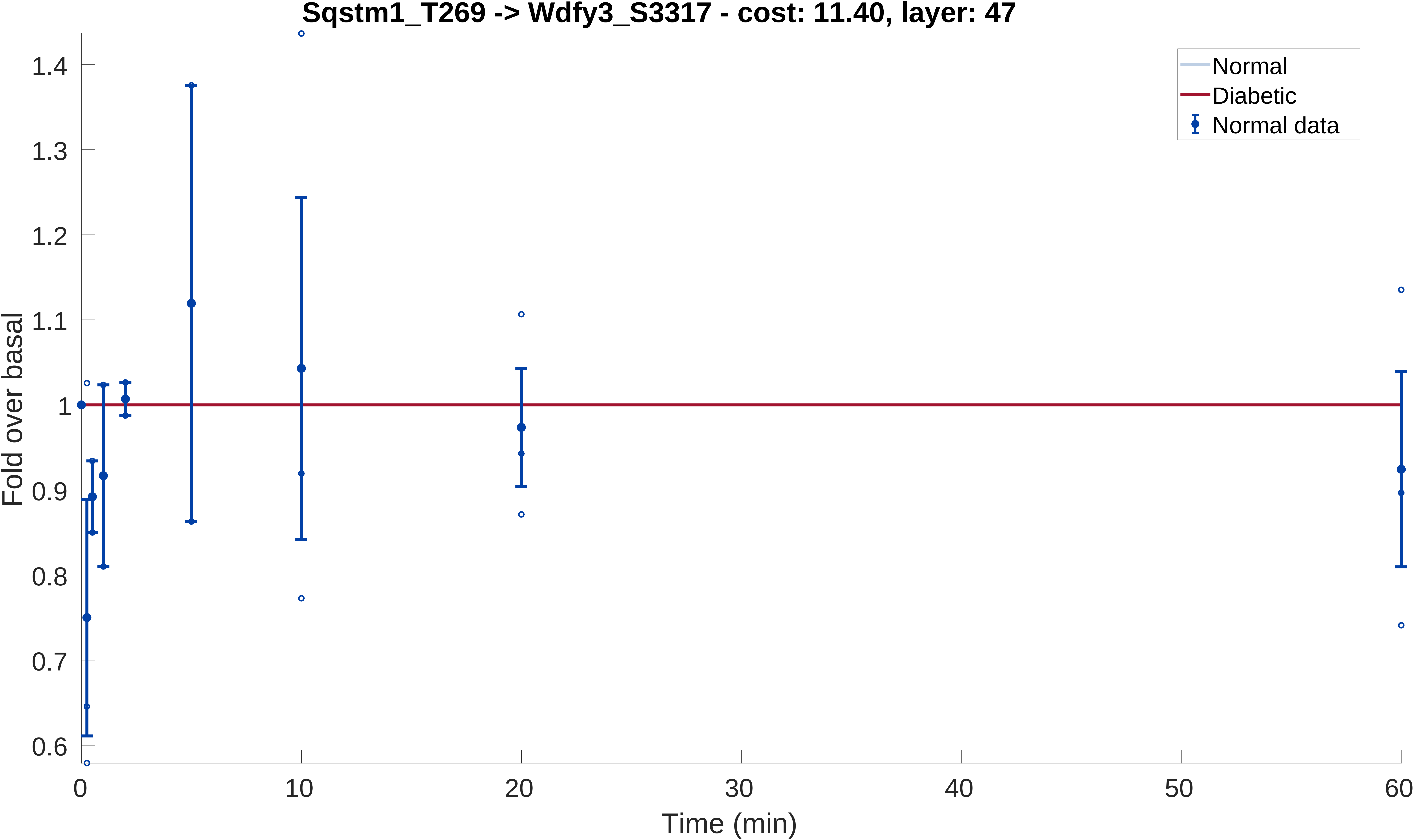

Sqstm1_T269 -> Wdfy3_S3317 - cost: 11.40, layer: 47
Normal
Diabetic
Normal data
Fold over basal
1.4
1.3
1.2
1.1
1
0.9
0.8
0.7
0.6
0
10
20
30
40
50
60
Time (min)

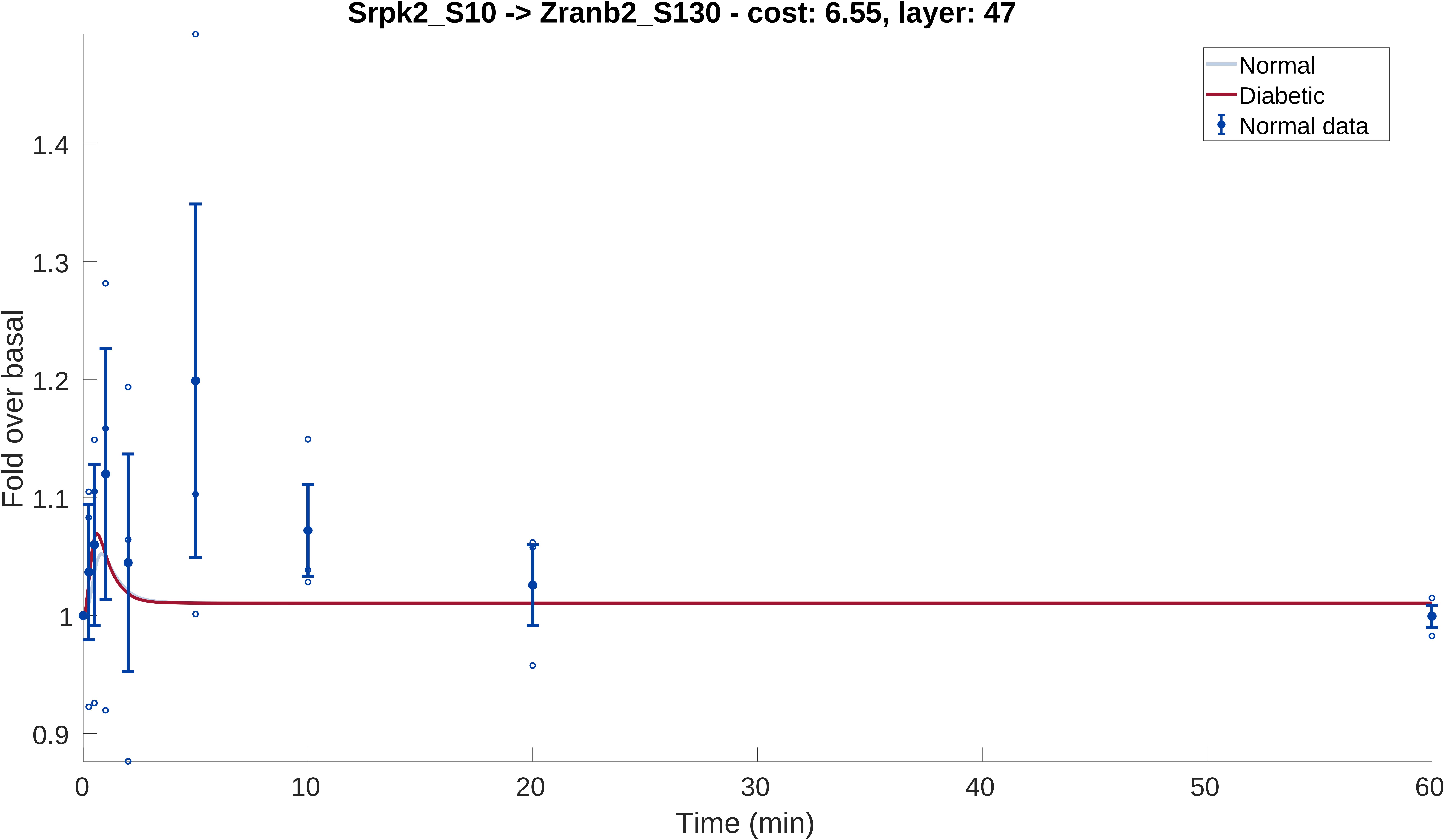

Srpk2_S10 -> Zranb2_S130 - cost: 6.55, layer: 47
Normal
Diabetic
Normal data
Fold over basal
1.4
1.3
1.2
1.1
1
0.9
0
10
20
30
40
50
60
Time (min)

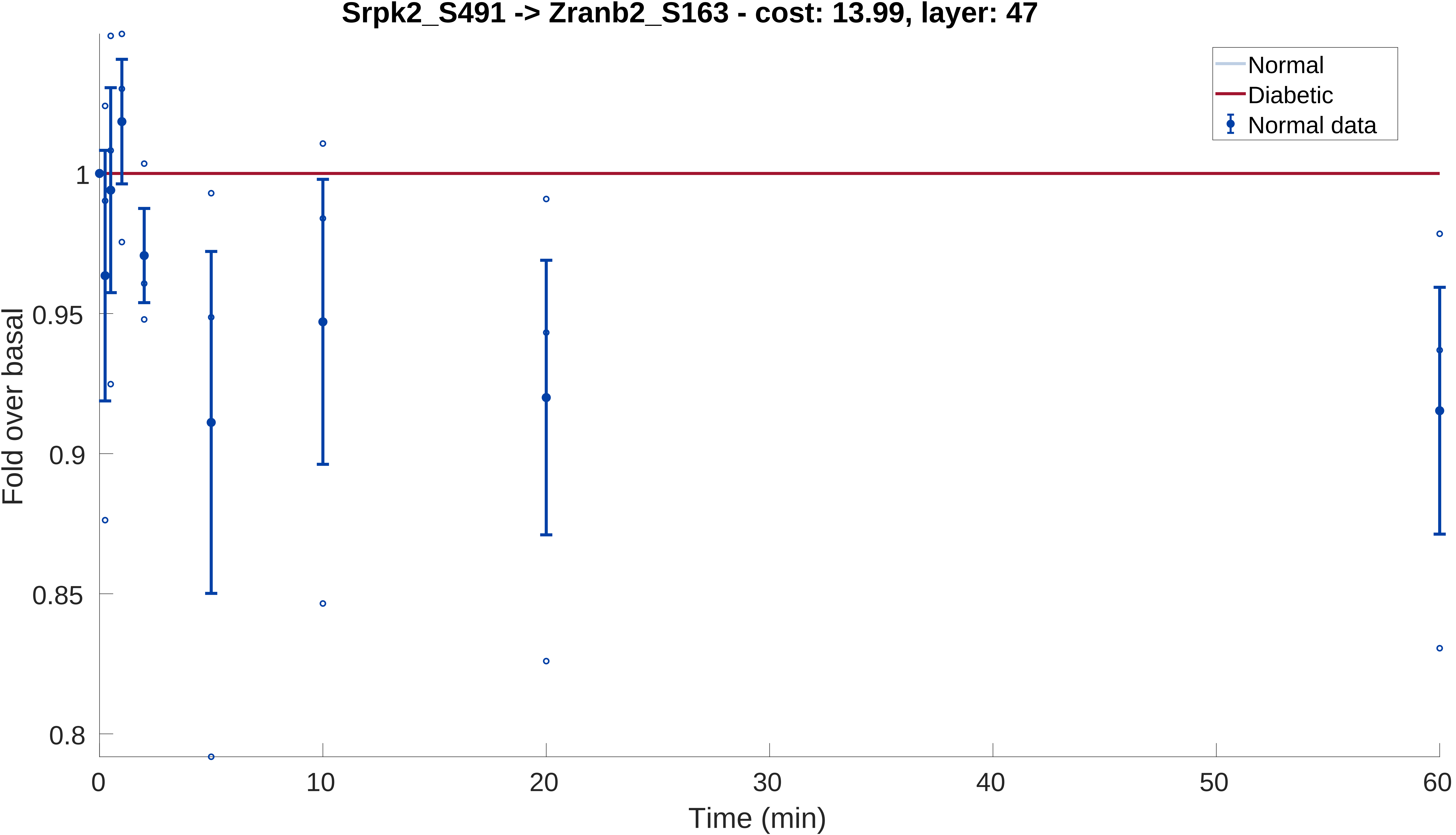

Srpk2_S491 -> Zranb2_S163 - cost: 13.99, layer: 47
Normal
Diabetic
Normal data
Fold over basal
1
0.95
0.9
0.85
0.8
0
10
20
30
40
50
60
Time (min)

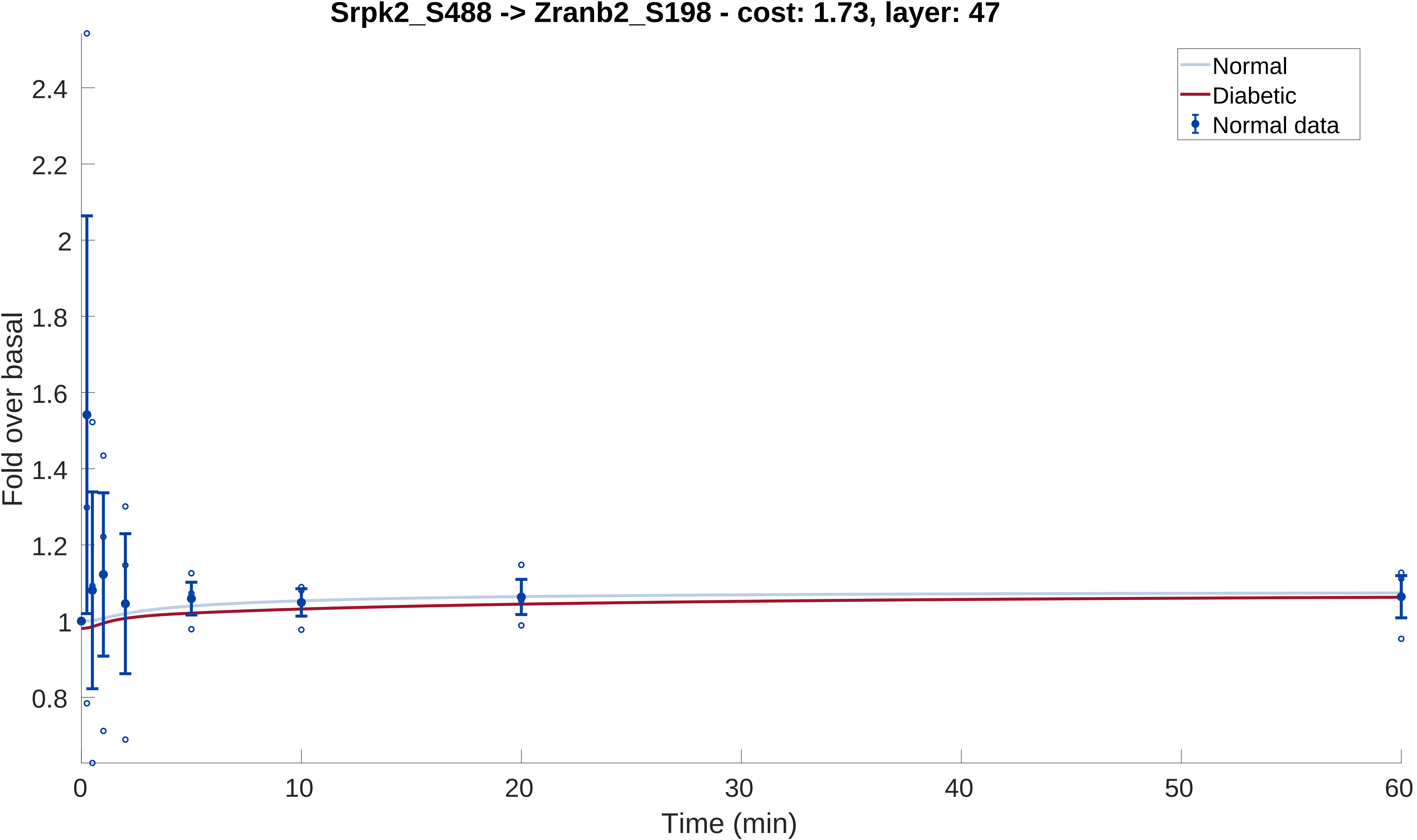
Srpk2_S488 -> Zranb2_S198 - cost: 1.73, layer: 47
Normal
Diabetic
Normal data
Fold over basal
2.4
2.2
2
1.8
1.6
1.4
1.2
1
0.8
0
10
20
30
40
50
60
Time (min)

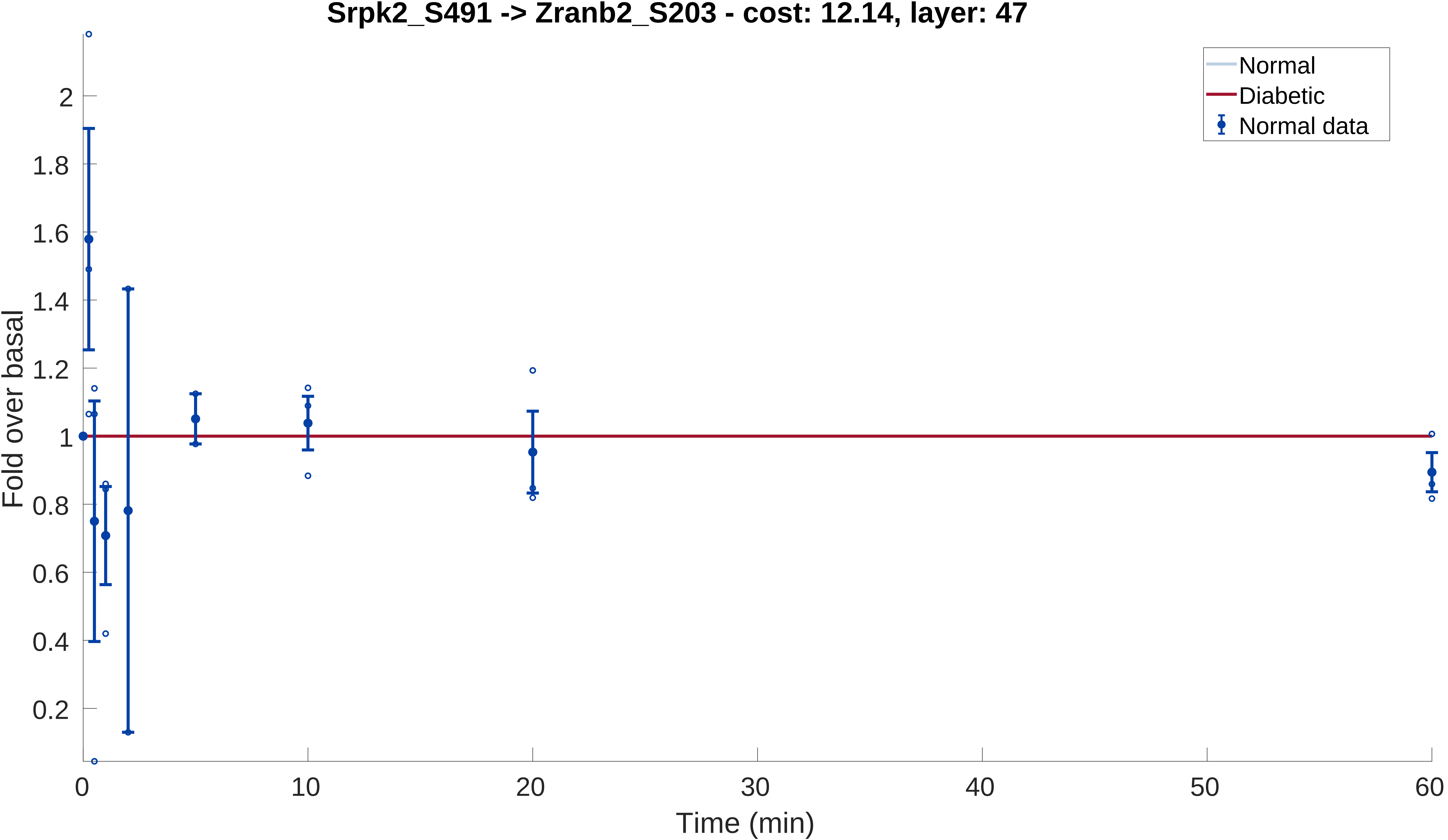

Srpk2_S491 -> Zranb2_S203 - cost: 12.14, layer: 47
Normal
Diabetic
Normal data
Fold over basal
2
1.8
1.6
1.4
1.2
1
0.8
0.6
0.4
0.2
0
10
20
30
40
50
60
Time (min)

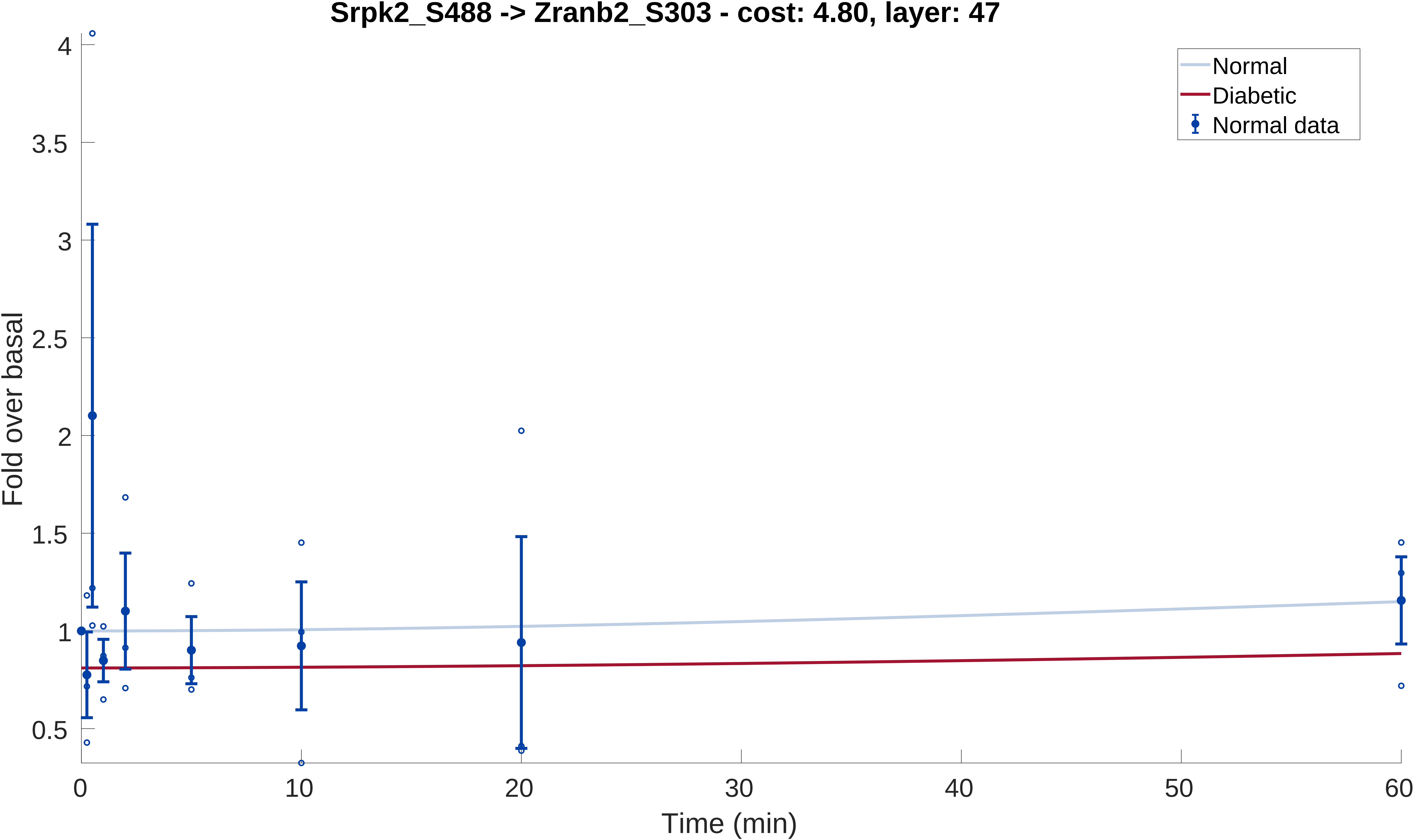
Srpk2_S488 -> Zranb2_S303 - cost: 4.80, layer: 47
Normal
Diabetic
Normal data
Fold over basal
Time (min)
0.5
1
1.5
2
2.5
3
3.5
4
0
10
20
30
40
50
60

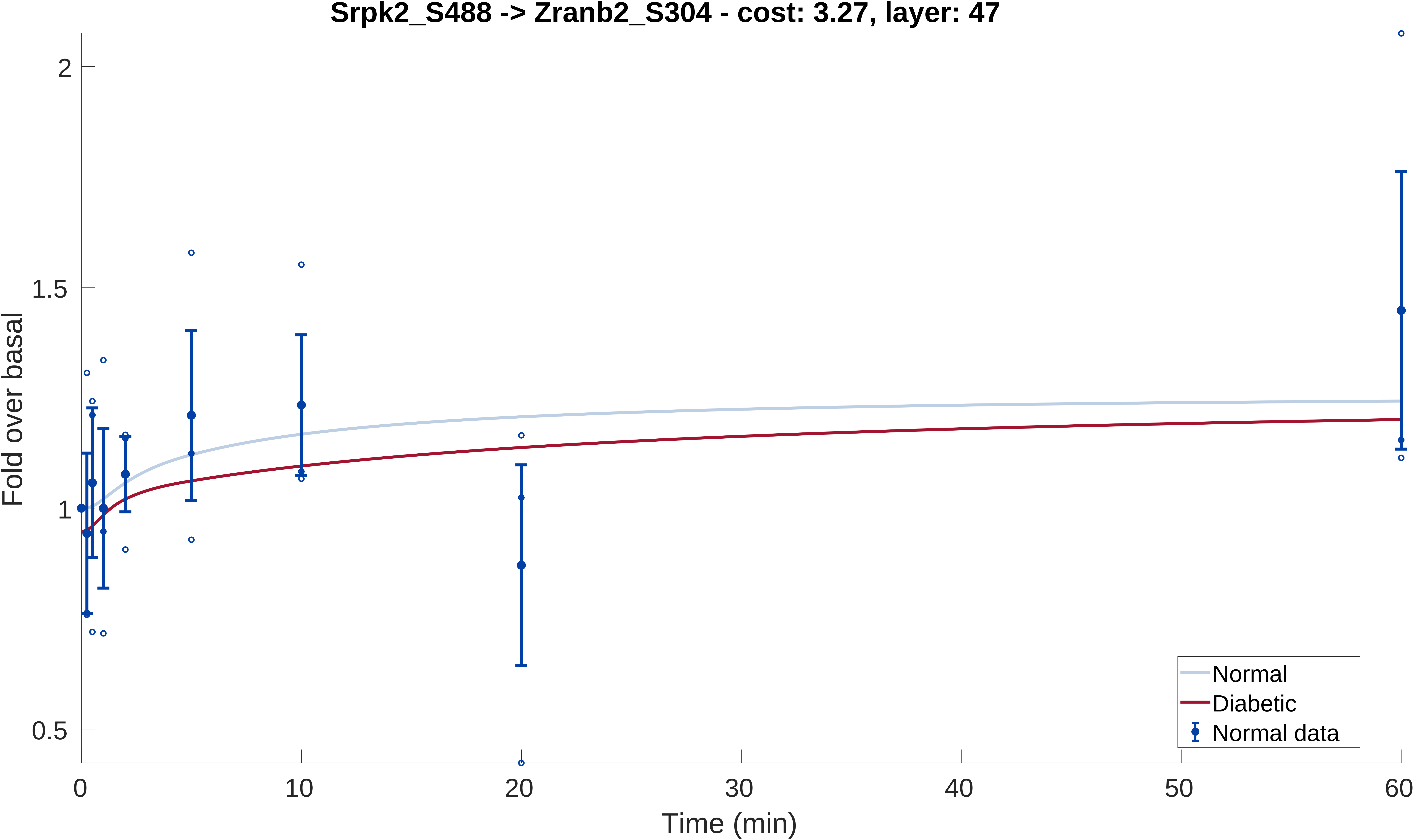
Srpk2_S488 -> Zranb2_S304 - cost: 3.27, layer: 47
Fold over basal
2
1.5
1
0.5
0
10
20
30
40
50
60
Time (min)
Normal
Diabetic
Normal data

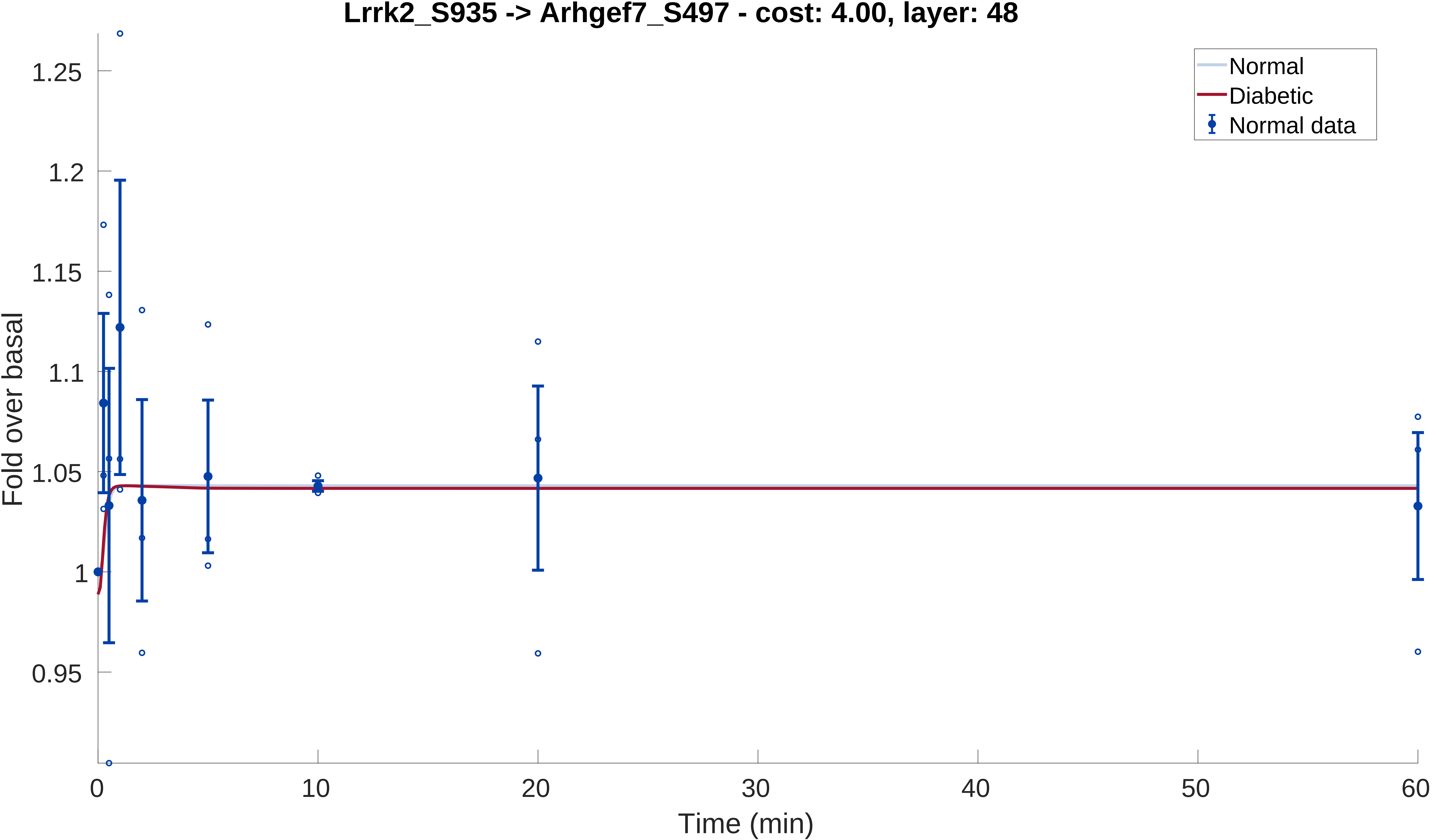

Lrrk2_S935 -> Arhgef7_S497 - cost: 4.00, layer: 48
Normal
Diabetic
Normal data
Fold over basal
1.25
1.2
1.15
1.1
1.05
1
0.95
0
10
20
30
40
50
60
Time (min)

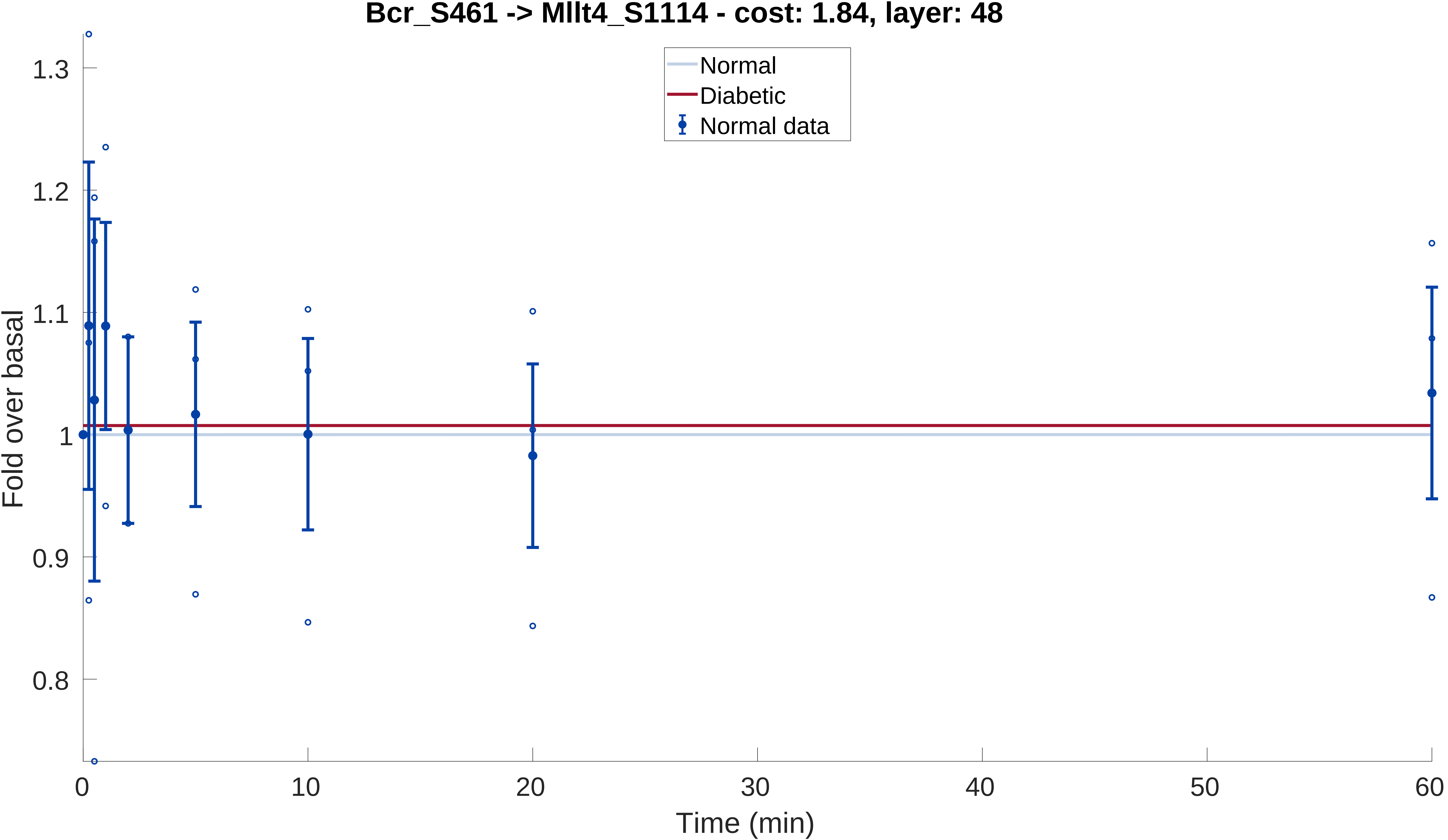

Bcr_S461 -> Mllt4_S1114 - cost: 1.84, layer: 48
Normal
Diabetic
Normal data
Fold over basal
Time (min)
1.3
1.2
1.1
1
0.9
0.8
0
10
20
30
40
50
60

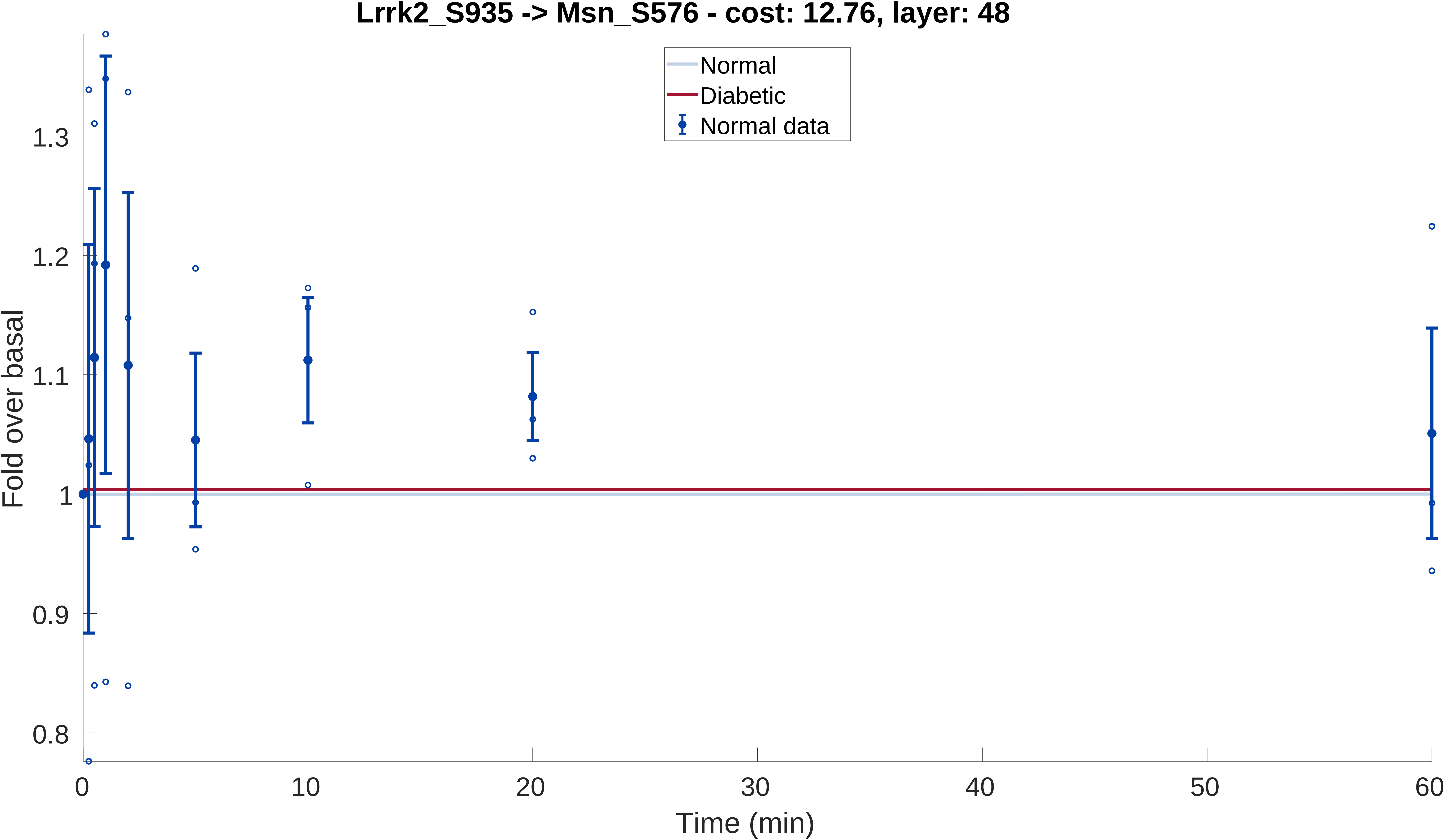

Lrrk2_S935 -> Msn_S576 - cost: 12.76, layer: 48
Normal
Diabetic
Normal data
Fold over basal
1.3
1.2
1.1
1
0.9
0.8
0
10
20
30
40
50
60
Time (min)

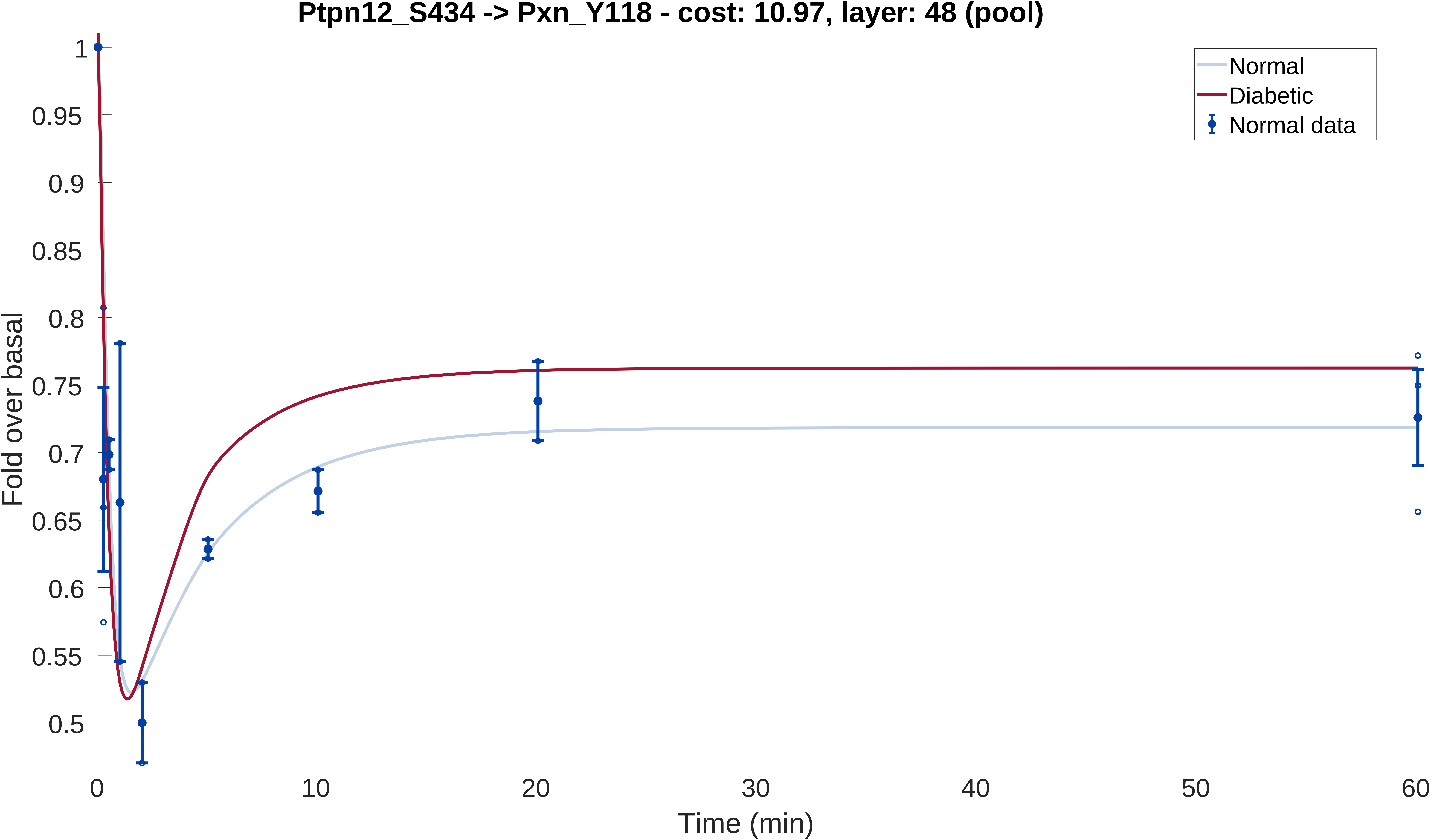
Ptpn12_S434 -> Pxn_Y118 - cost: 10.97, layer: 48 (pool)
Normal
Diabetic
Normal data
Fold over basal
Time (min)
1
0.95
0.9
0.85
0.8
0.75
0.7
0.65
0.6
0.55
0.5
0
10
20
30
40
50
60

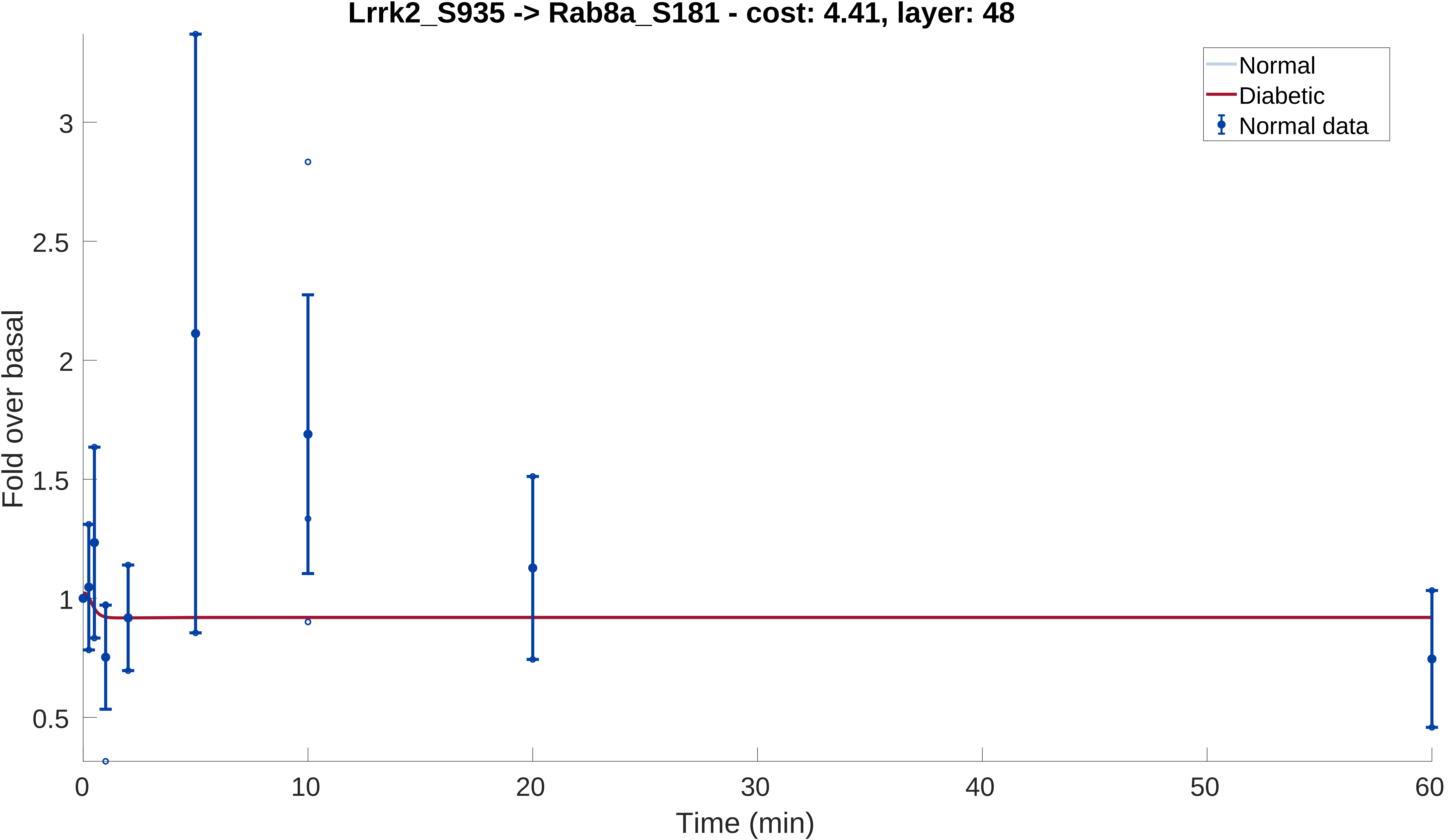
Lrrk2_S935 -> Rab8a_S181 - cost: 4.41, layer: 48
Normal
Diabetic
Normal data
Fold over basal
3
2.5
2
1.5
1
0.5
0
10
20
30
40
50
60
Time (min)

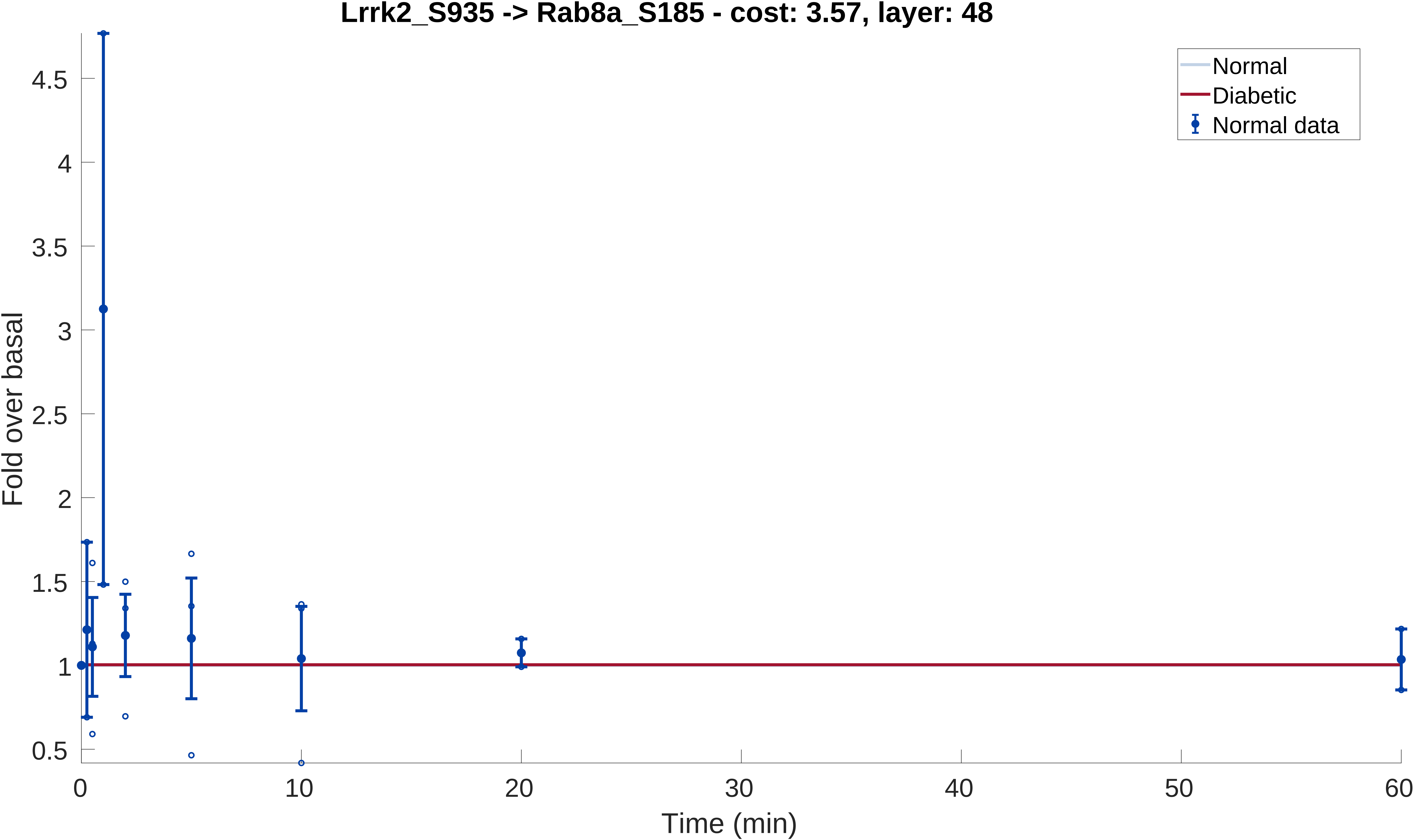

Lrrk2_S935 -> Rab8a_S185 - cost: 3.57, layer: 48
Normal
Diabetic
Normal data
Fold over basal
Time (min)
0.5
1
1.5
2
2.5
3
3.5
4
4.5
0
10
20
30
40
50
60

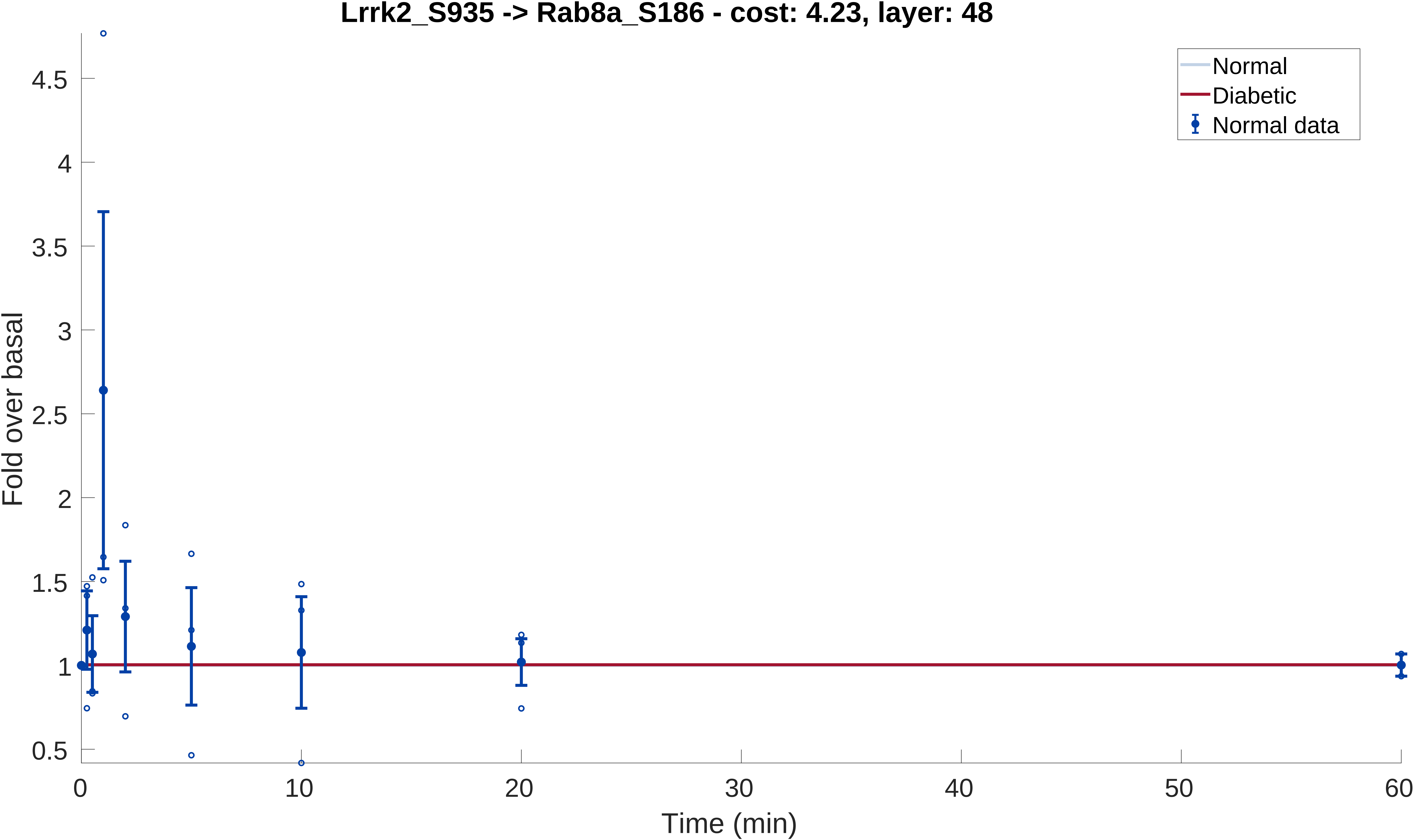
Lrrk2_S935 -> Rab8a_S186 - cost: 4.23, layer: 48
Normal
Diabetic
Normal data
Fold over basal
Time (min)
0.5
1
1.5
2
2.5
3
3.5
4
4.5
0
10
20
30
40
50
60

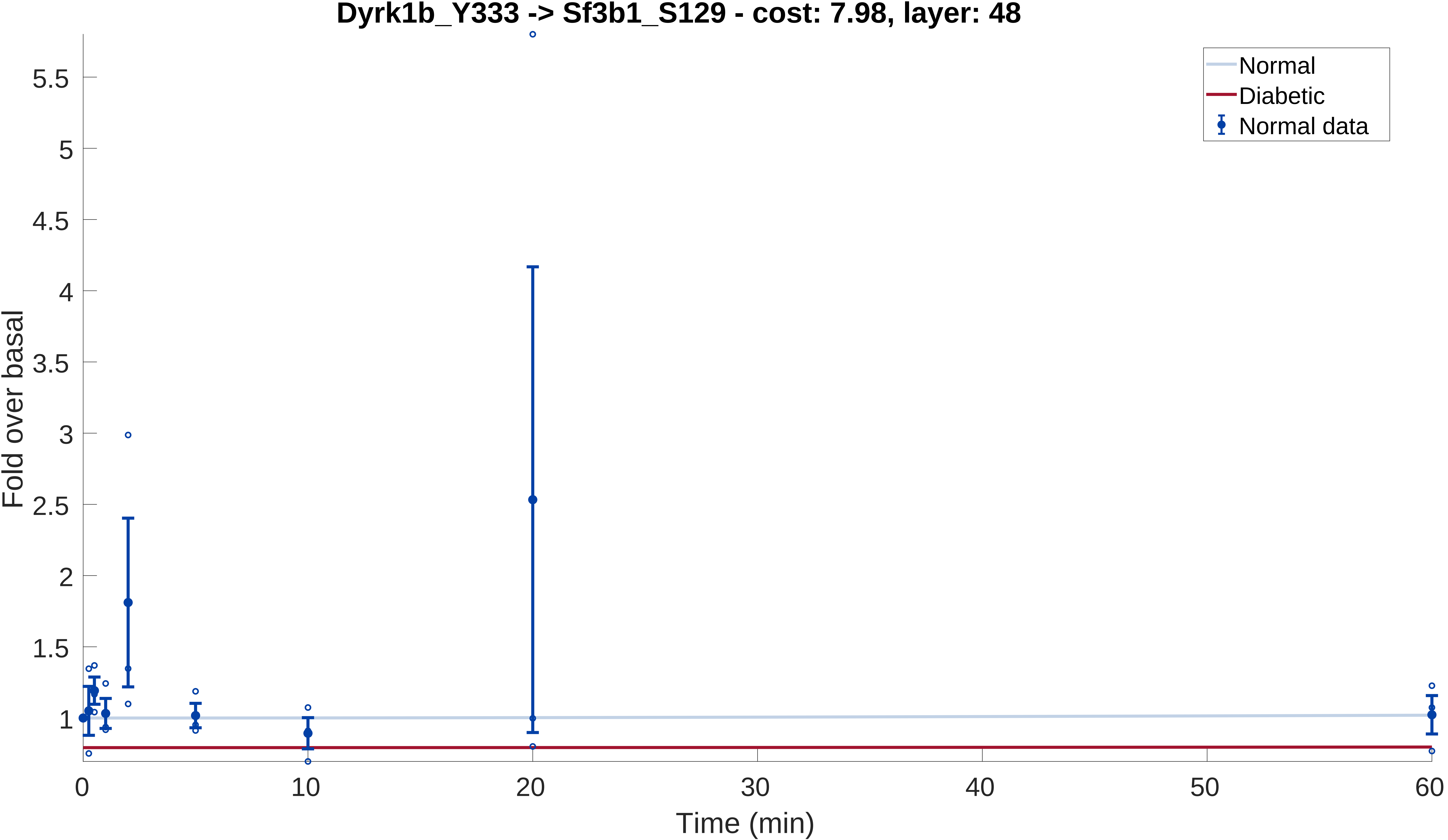
Dyrk1b_Y333 -> Sf3b1_S129 - cost: 7.98, layer: 48
Normal
Diabetic
Normal data
Fold over basal
5.5
5
4.5
4
3.5
3
2.5
2
1.5
1
0
10
20
30
40
50
60
Time (min)

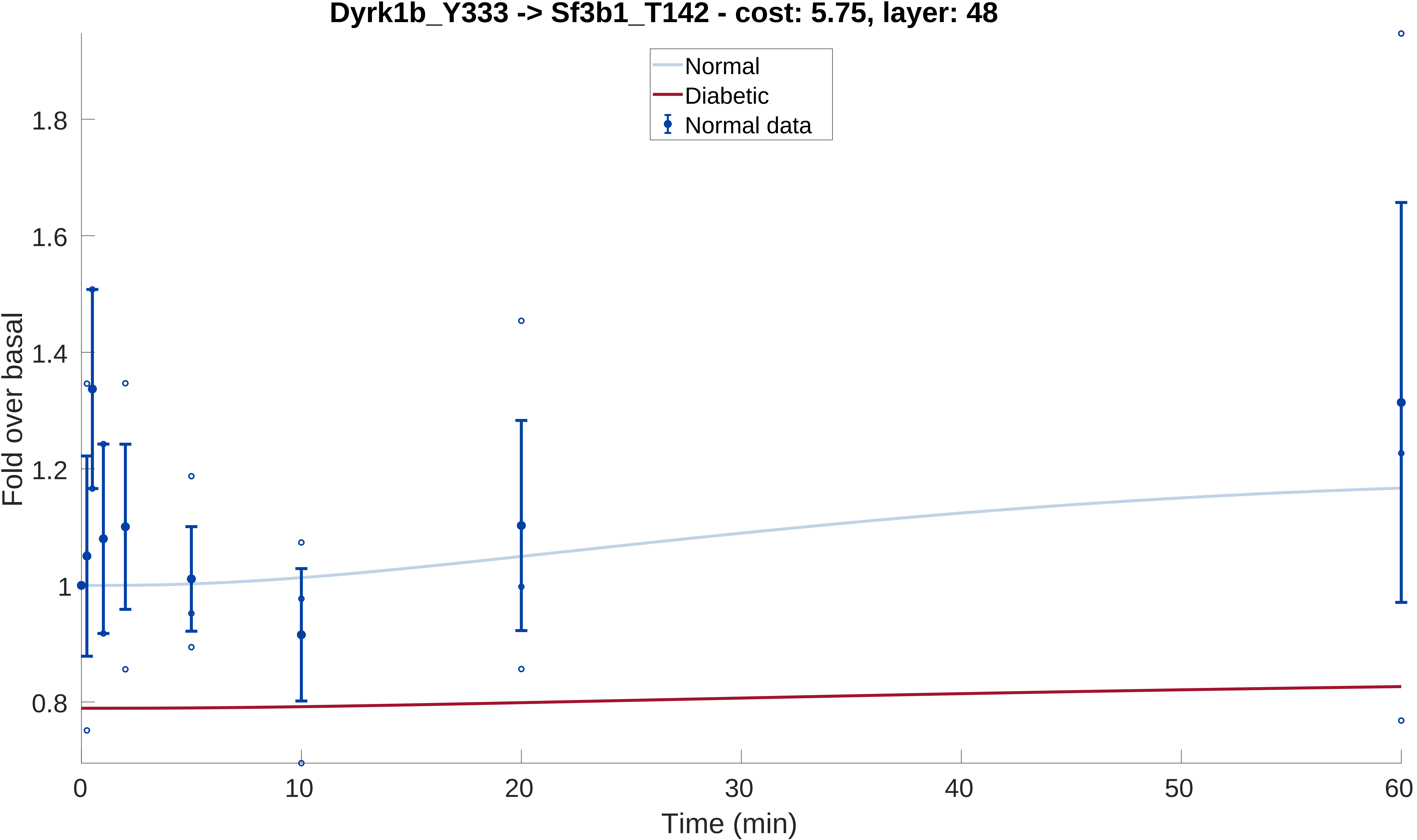

Dyrk1b_Y333 -> Sf3b1_T142 - cost: 5.75, layer: 48
Normal
Diabetic
Normal data
Fold over basal
1.8
1.6
1.4
1.2
1
0.8
0
10
20
30
40
50
60
Time (min)

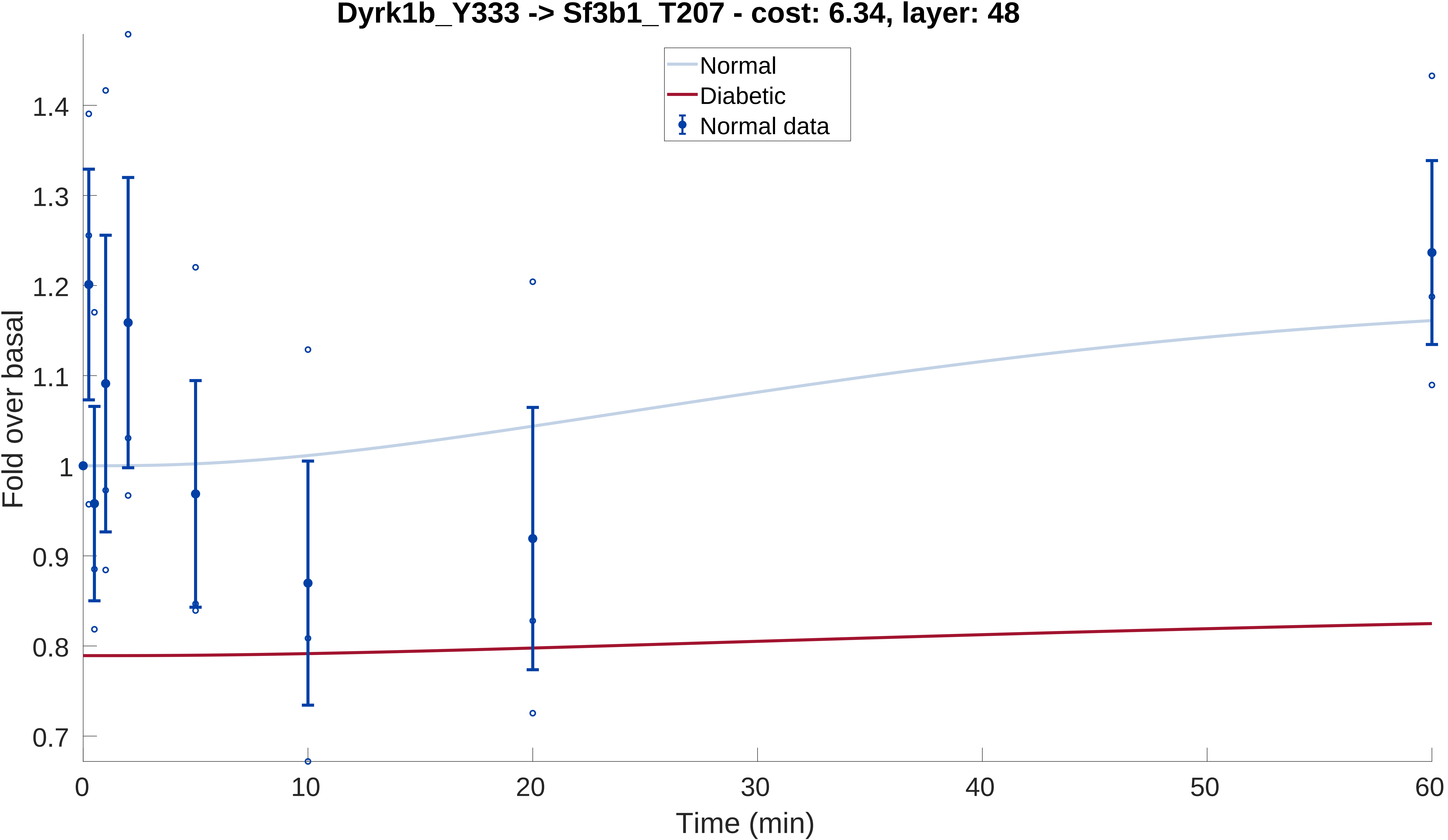

Dyrk1b_Y333 -> Sf3b1_T207 - cost: 6.34, layer: 48
Normal
Diabetic
Normal data
Fold over basal
Time (min)
0.7
0.8
0.9
1
1.1
1.2
1.3
1.4
0
10
20
30
40
50
60

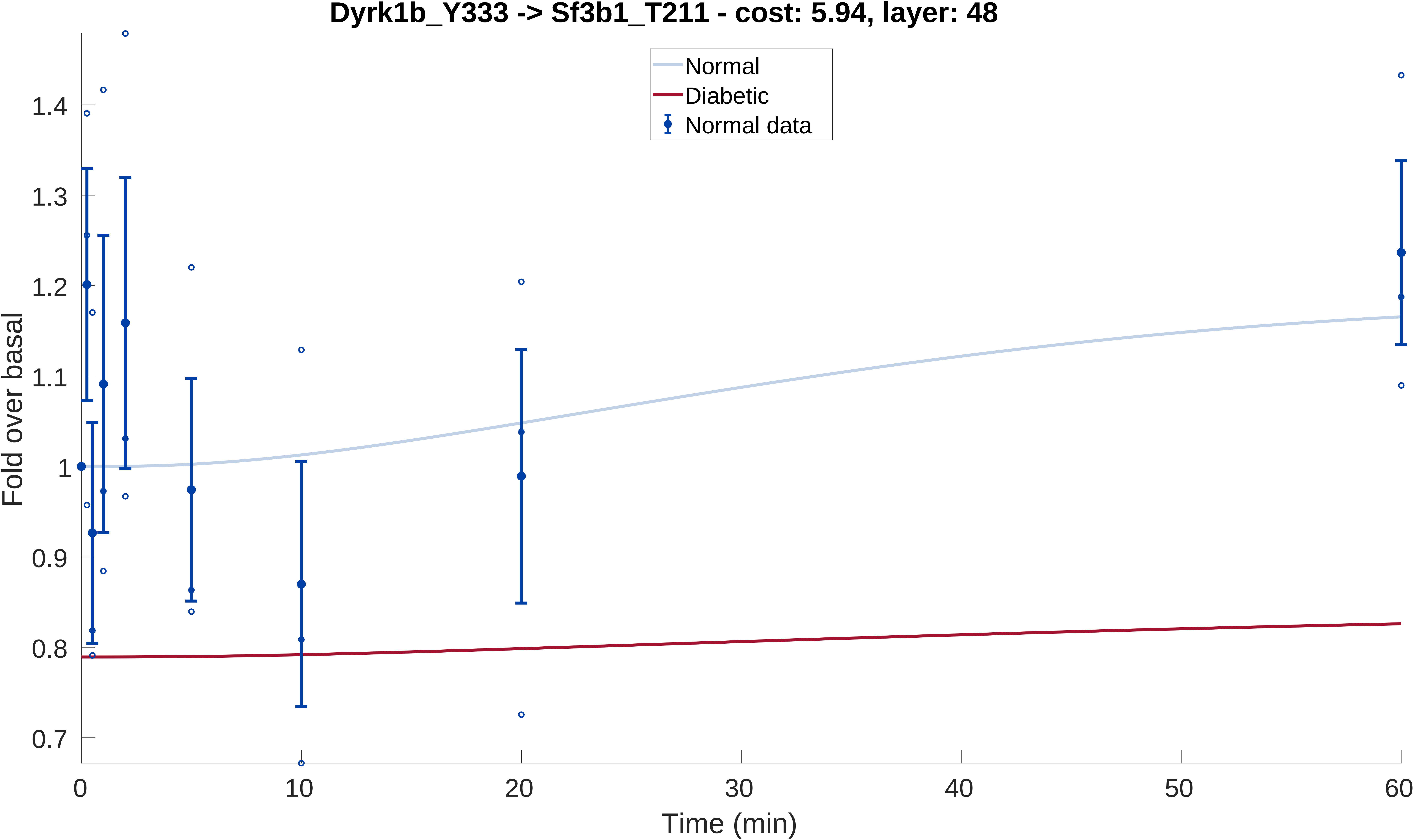
Dyrk1b_Y333 -> Sf3b1_T211 - cost: 5.94, layer: 48
Normal
Diabetic
Normal data
Fold over basal
0.7
0.8
0.9
1
1.1
1.2
1.3
1.4
0
10
20
30
40
50
60
Time (min)

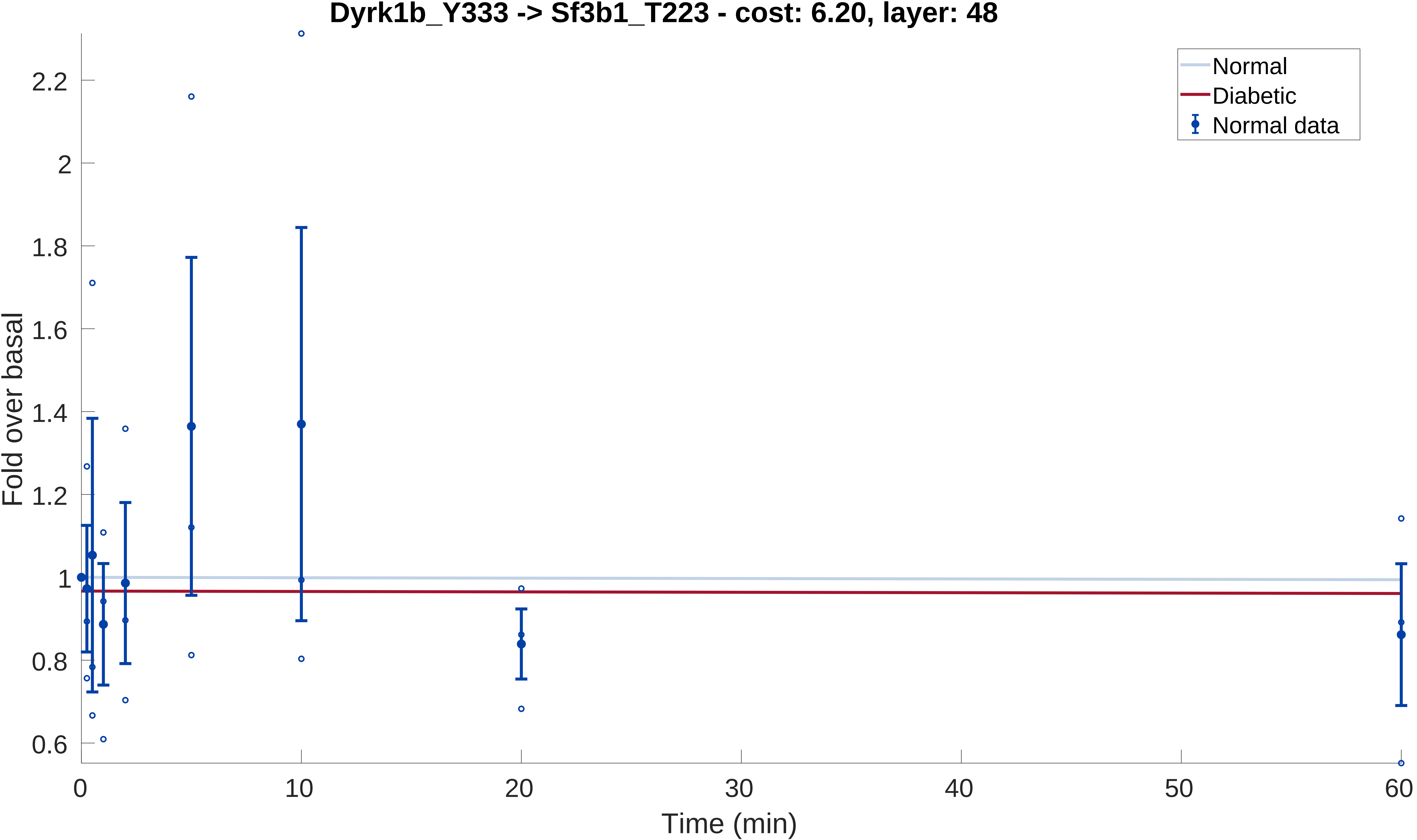

Dyrk1b_Y333 -> Sf3b1_T223 - cost: 6.20, layer: 48
Normal
Diabetic
Normal data
Fold over basal
2.2
2
1.8
1.6
1.4
1.2
1
0.8
0.6
0
10
20
30
40
50
60
Time (min)

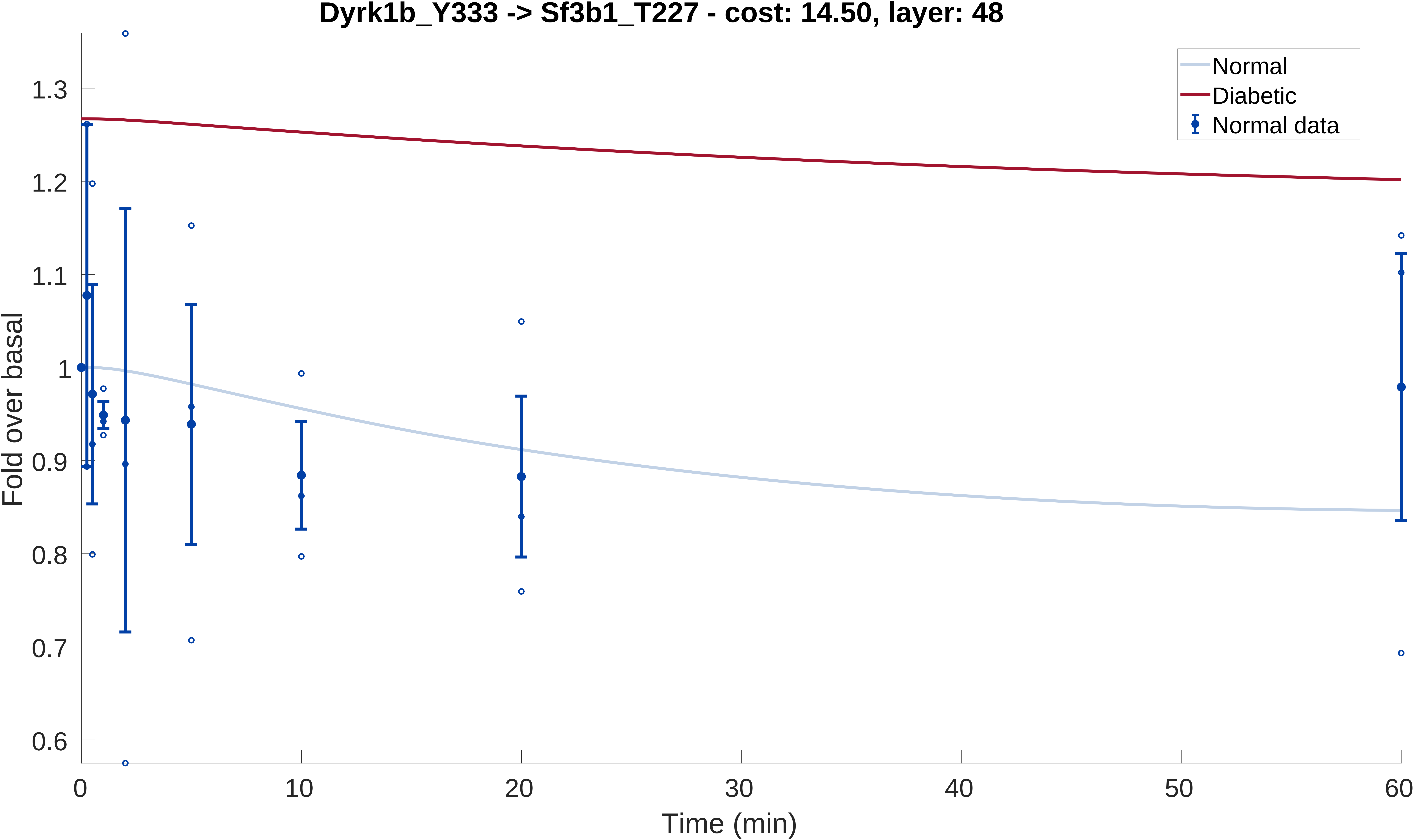

Dyrk1b_Y333 -> Sf3b1_T227 - cost: 14.50, layer: 48
Normal
Diabetic
Normal data
Fold over basal
1.3
1.2
1.1
1
0.9
0.8
0.7
0.6
0
10
20
30
40
50
60
Time (min)

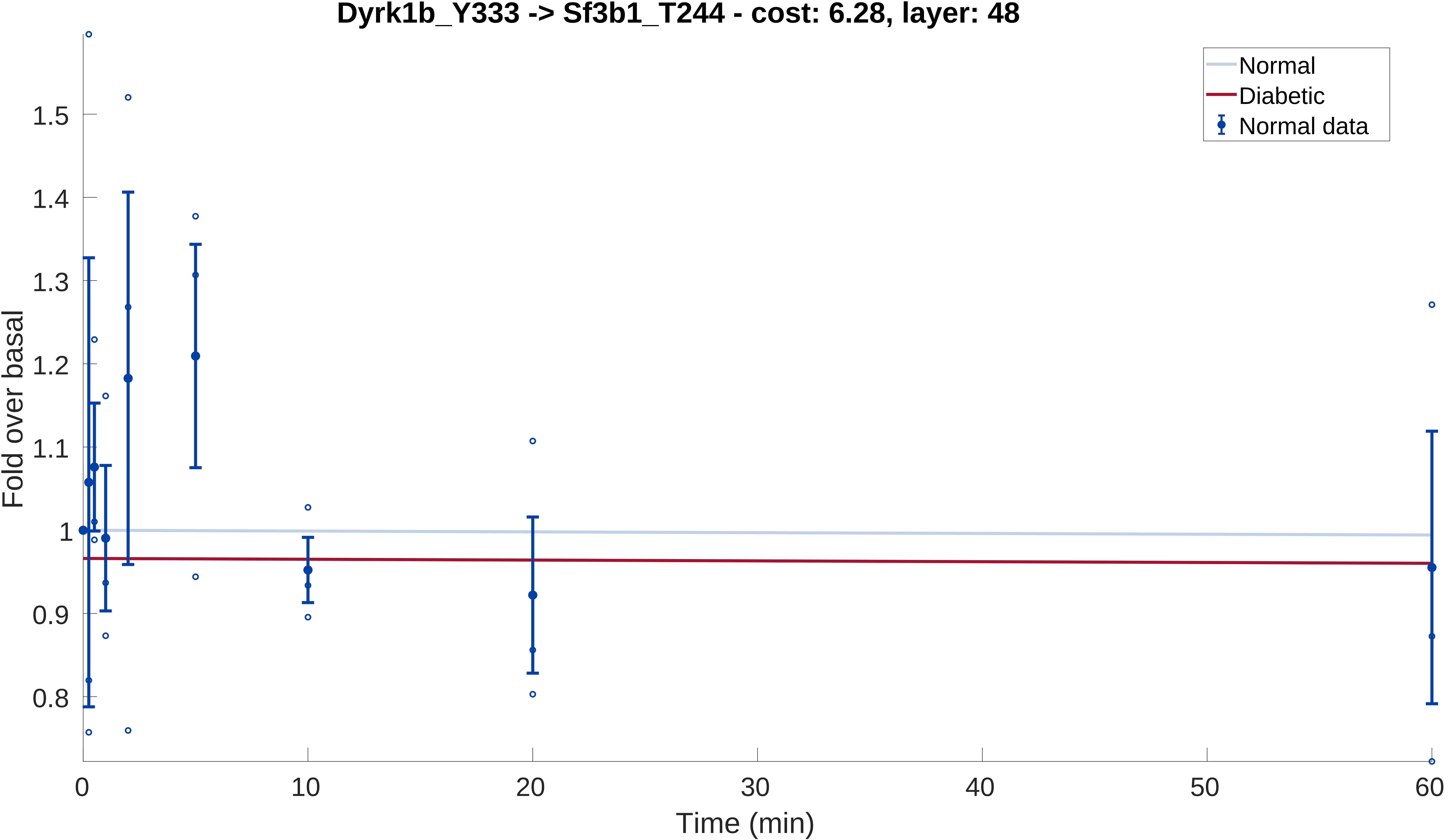

Dyrk1b_Y333 -> Sf3b1_T244 - cost: 6.28, layer: 48
Normal
Diabetic
Normal data
Fold over basal
1.5
1.4
1.3
1.2
1.1
1
0.9
0.8
0
10
20
30
40
50
60
Time (min)

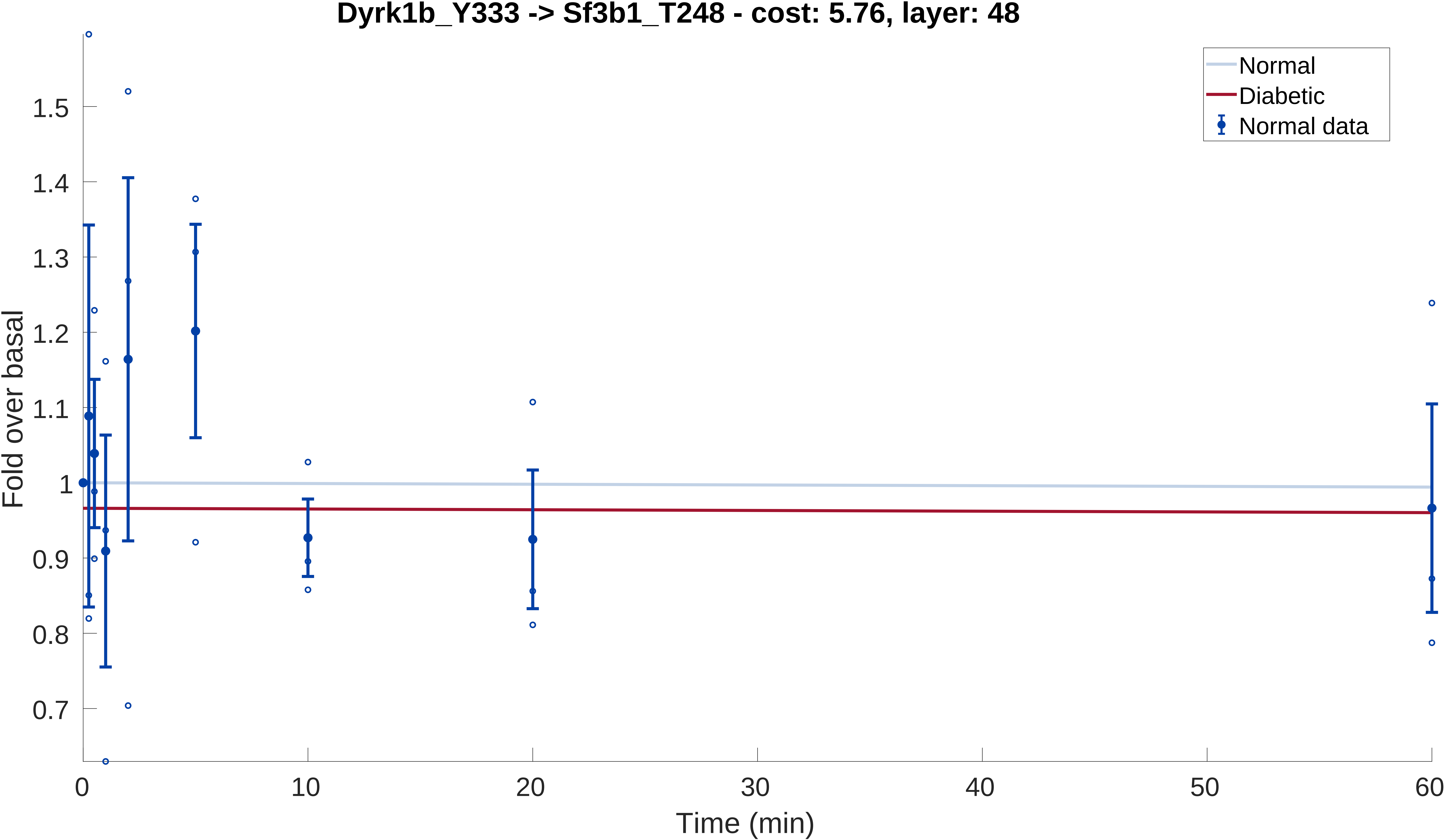

Dyrk1b_Y333 -> Sf3b1_T248 - cost: 5.76, layer: 48
Normal
Diabetic
Normal data
Fold over basal
1.5
1.4
1.3
1.2
1.1
1
0.9
0.8
0.7
0
10
20
30
40
50
60
Time (min)

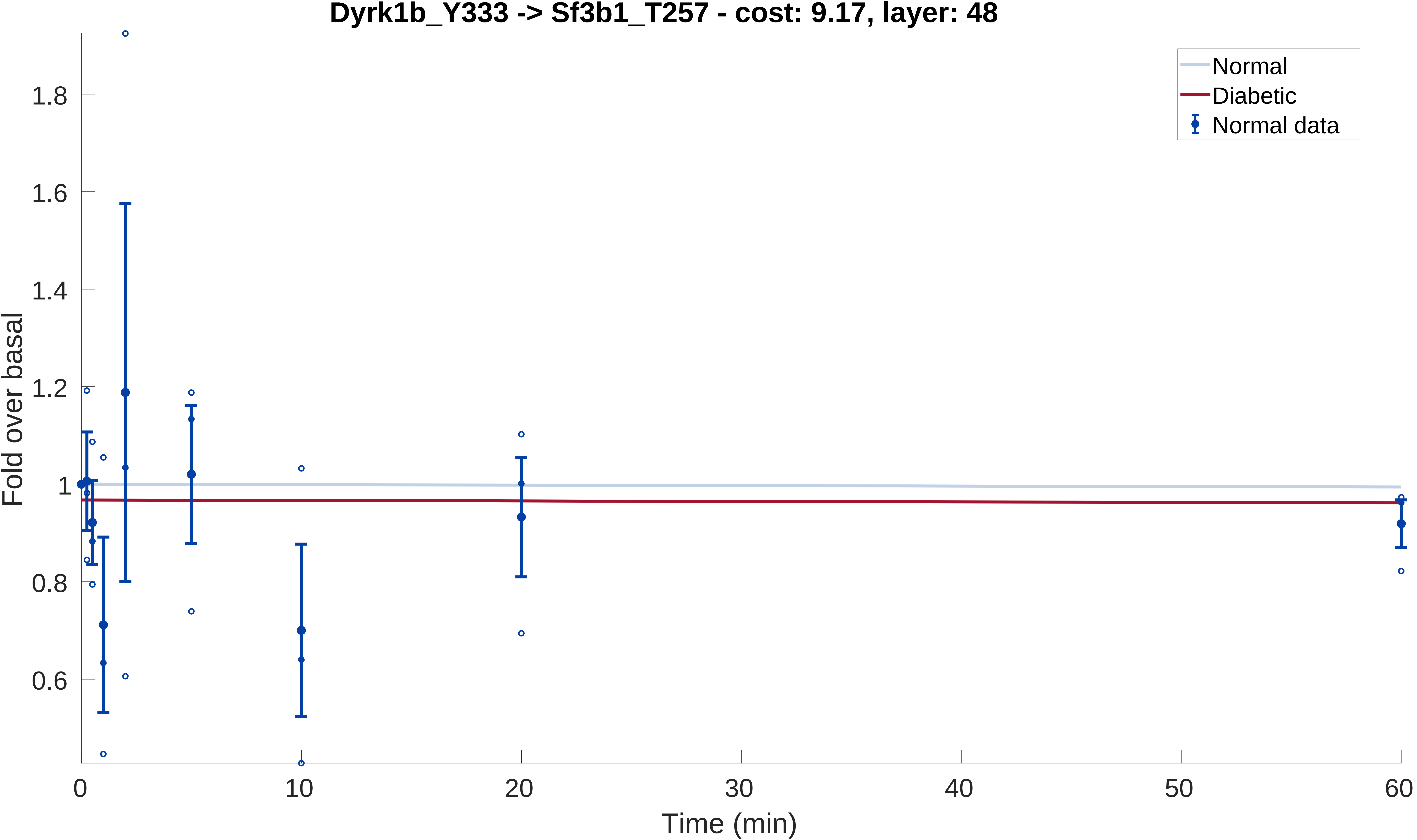

Dyrk1b_Y333 -> Sf3b1_T257 - cost: 9.17, layer: 48
Normal
Diabetic
Normal data
Fold over basal
Time (min)
1.8
1.6
1.4
1.2
1
0.8
0.6
0
10
20
30
40
50
60

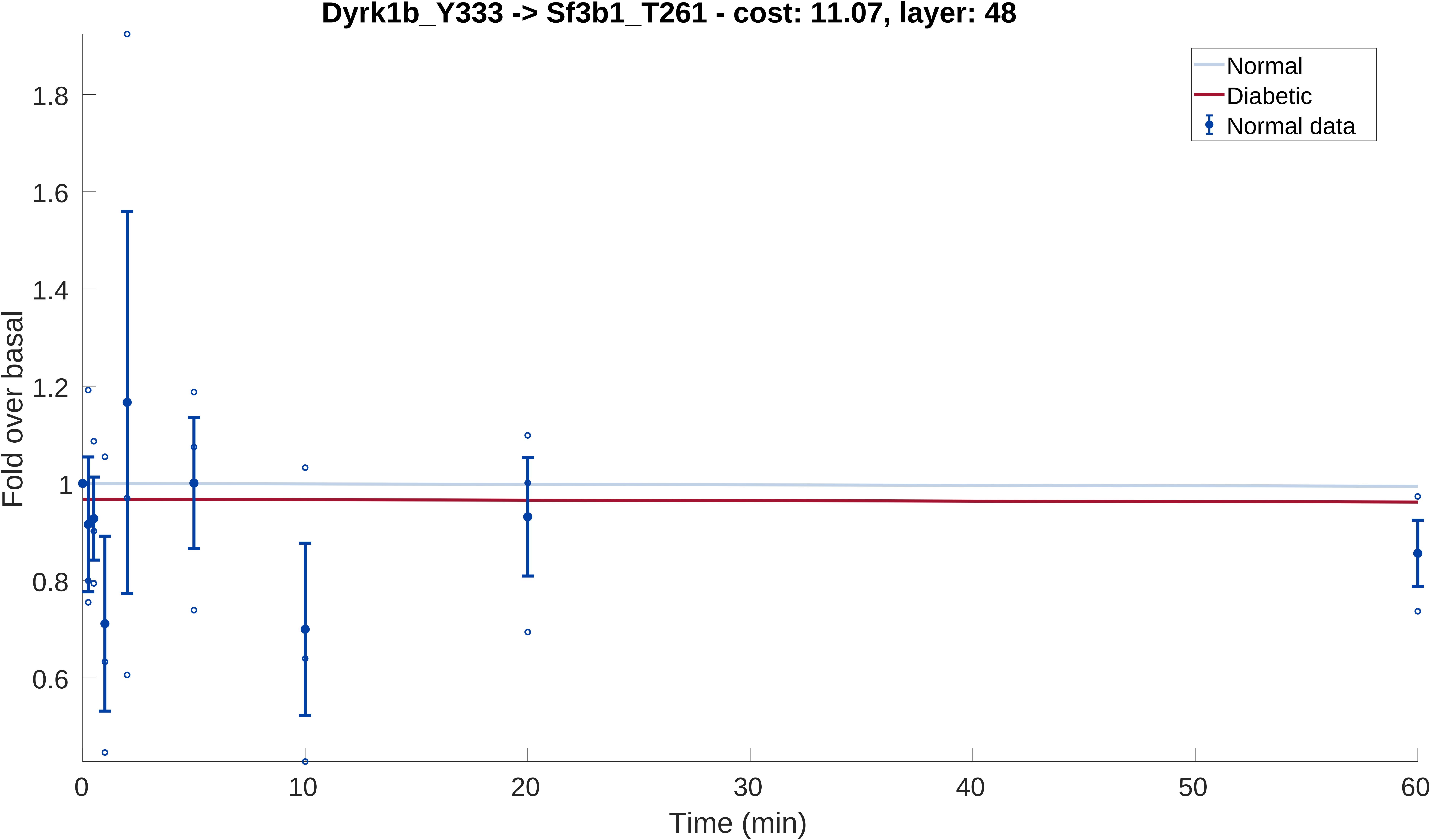

Dyrk1b_Y333 -> Sf3b1_T261 - cost: 11.07, layer: 48
Normal
Diabetic
Normal data
Fold over basal
1.8
1.6
1.4
1.2
1
0.8
0.6
0
10
20
30
40
50
60
Time (min)

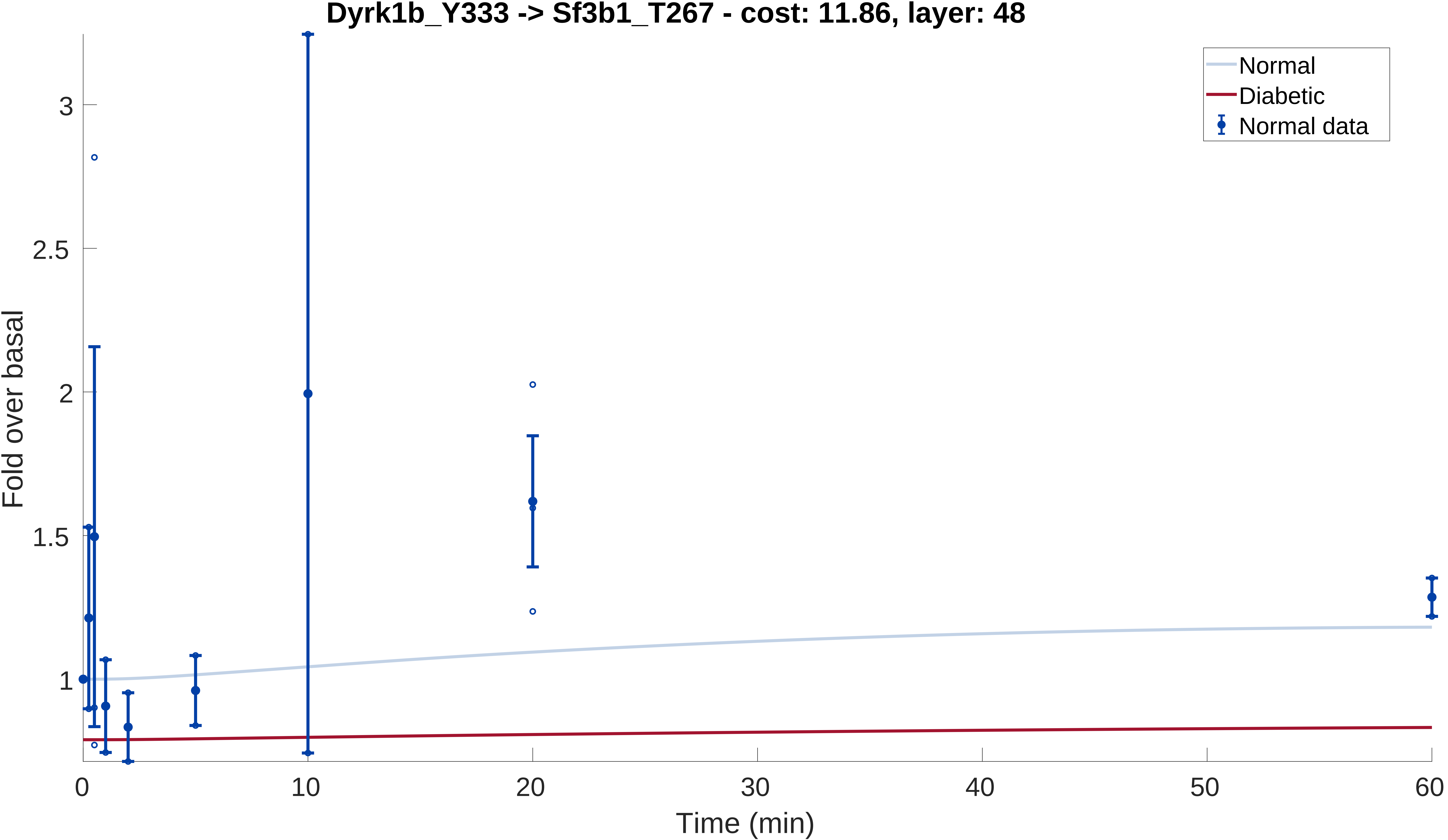

Dyrk1b_Y333 -> Sf3b1_T267 - cost: 11.86, layer: 48
Normal
Diabetic
Normal data
Fold over basal
3
2.5
2
1.5
1
0
10
20
30
40
50
60
Time (min)

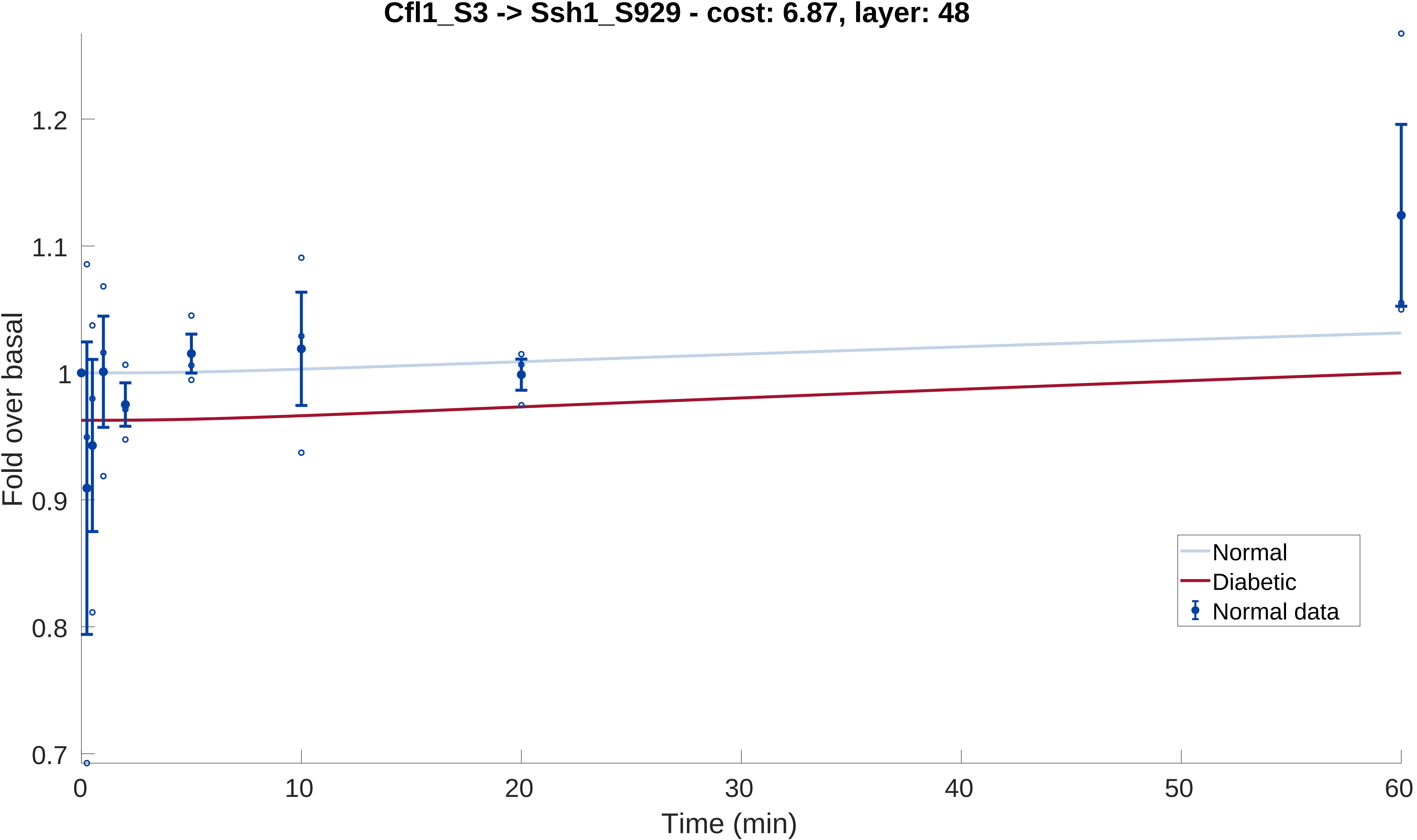
Cfl1_S3 -> Ssh1_S929 - cost: 6.87, layer: 48
Fold over basal
1.2
1.1
1
0.9
0.8
0.7
0
10
20
30
40
50
60
Time (min)
Normal
Diabetic
Normal data

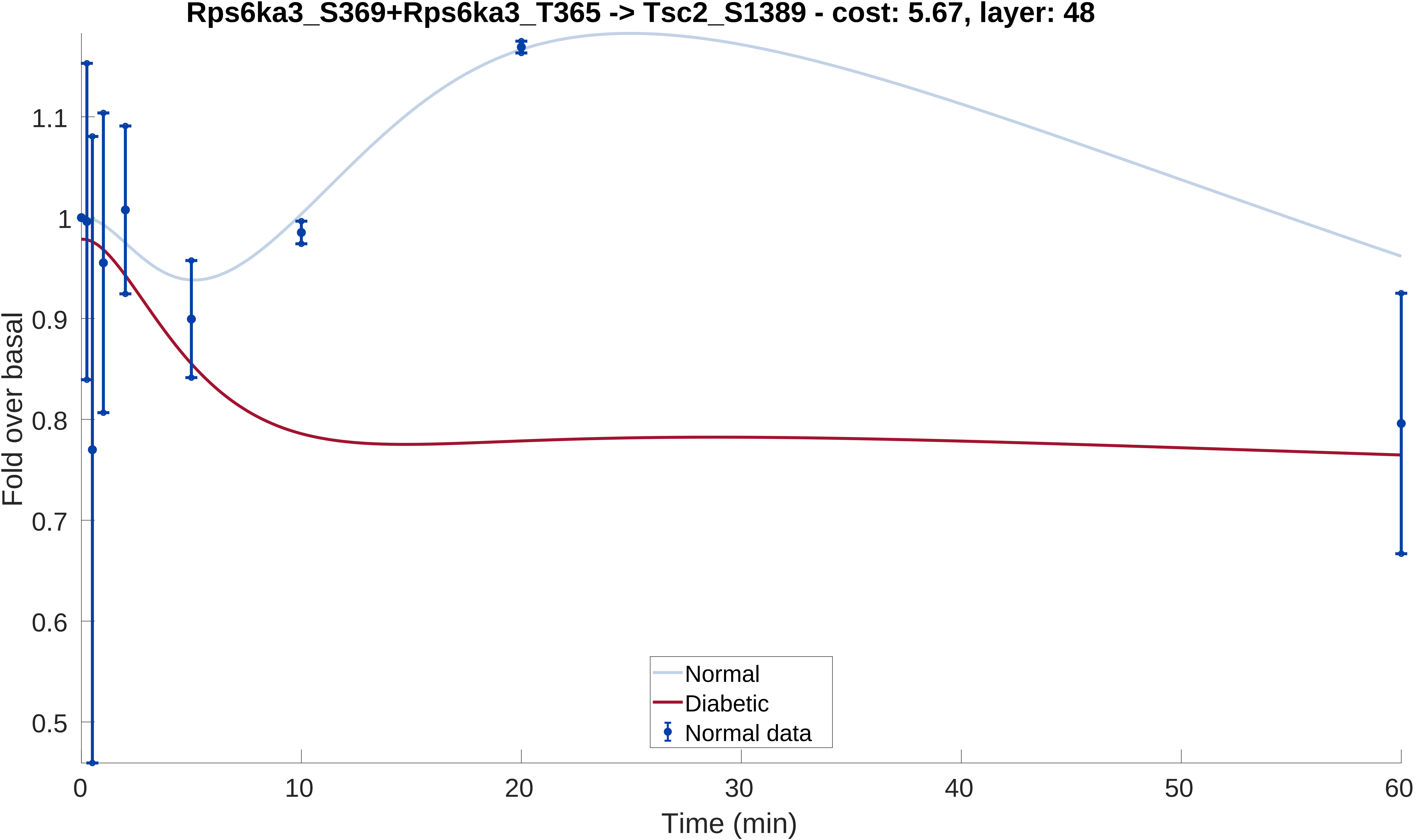

Rps6ka3_S369+Rps6ka3_T365 -> Tsc2_S1389 - cost: 5.67, layer: 48
Fold over basal
1.1
1
0.9
0.8
0.7
0.6
0.5
0
10
20
30
40
50
60
Time (min)
Normal
Diabetic
Normal data

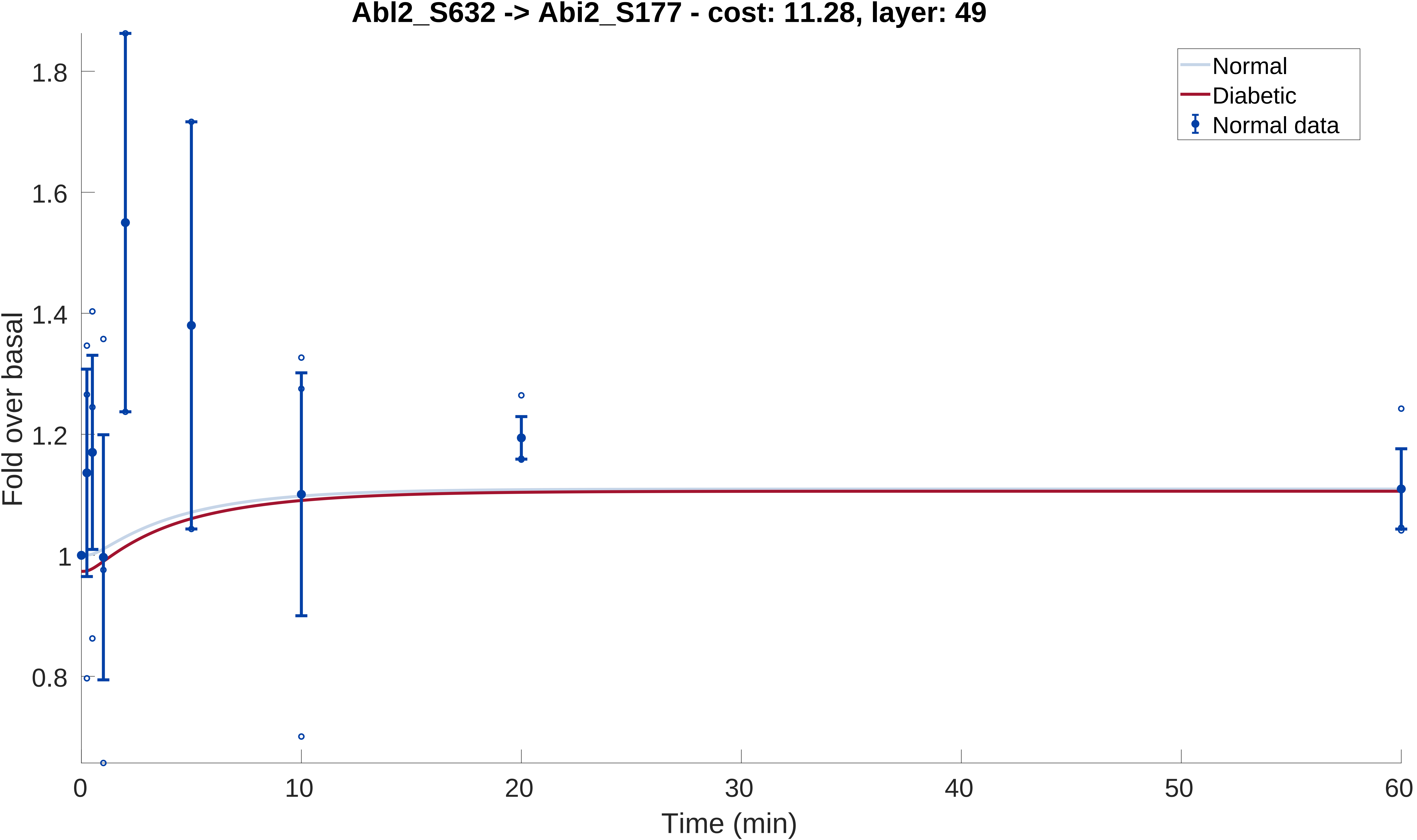
Abl2_S632 -> Abi2_S177 - cost: 11.28, layer: 49
Fold over basal
1.8
1.6
1.4
1.2
1
0.8
0
10
20
30
40
50
60
Time (min)
Normal
Diabetic
Normal data

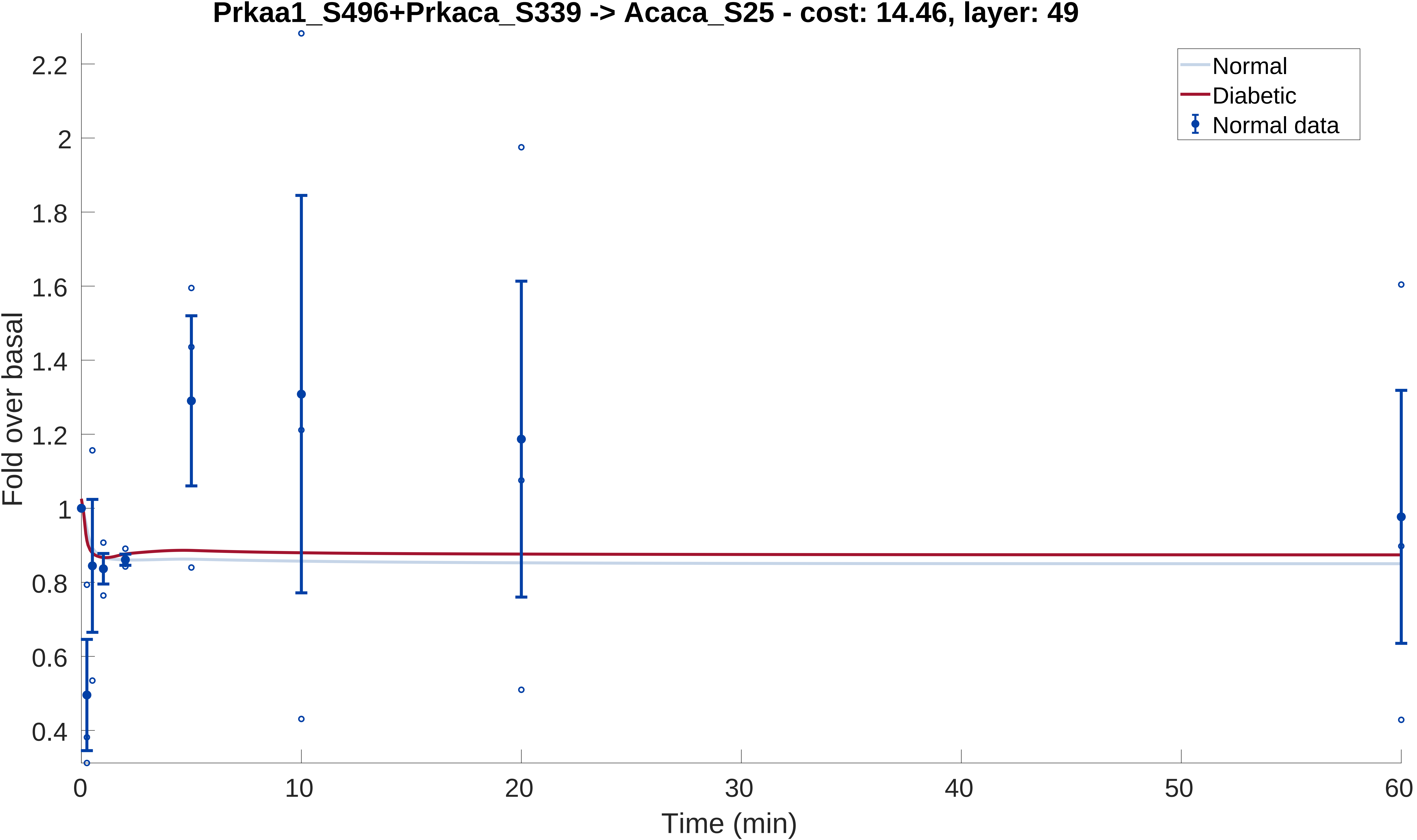
Prkaa1_S496+Prkaca_S339 -> Acaca_S25 - cost: 14.46, layer: 49
Normal
Diabetic
Normal data
Fold over basal
Time (min)
2.2
2
1.8
1.6
1.4
1.2
1
0.8
0.6
0.4
0
10
20
30
40
50
60

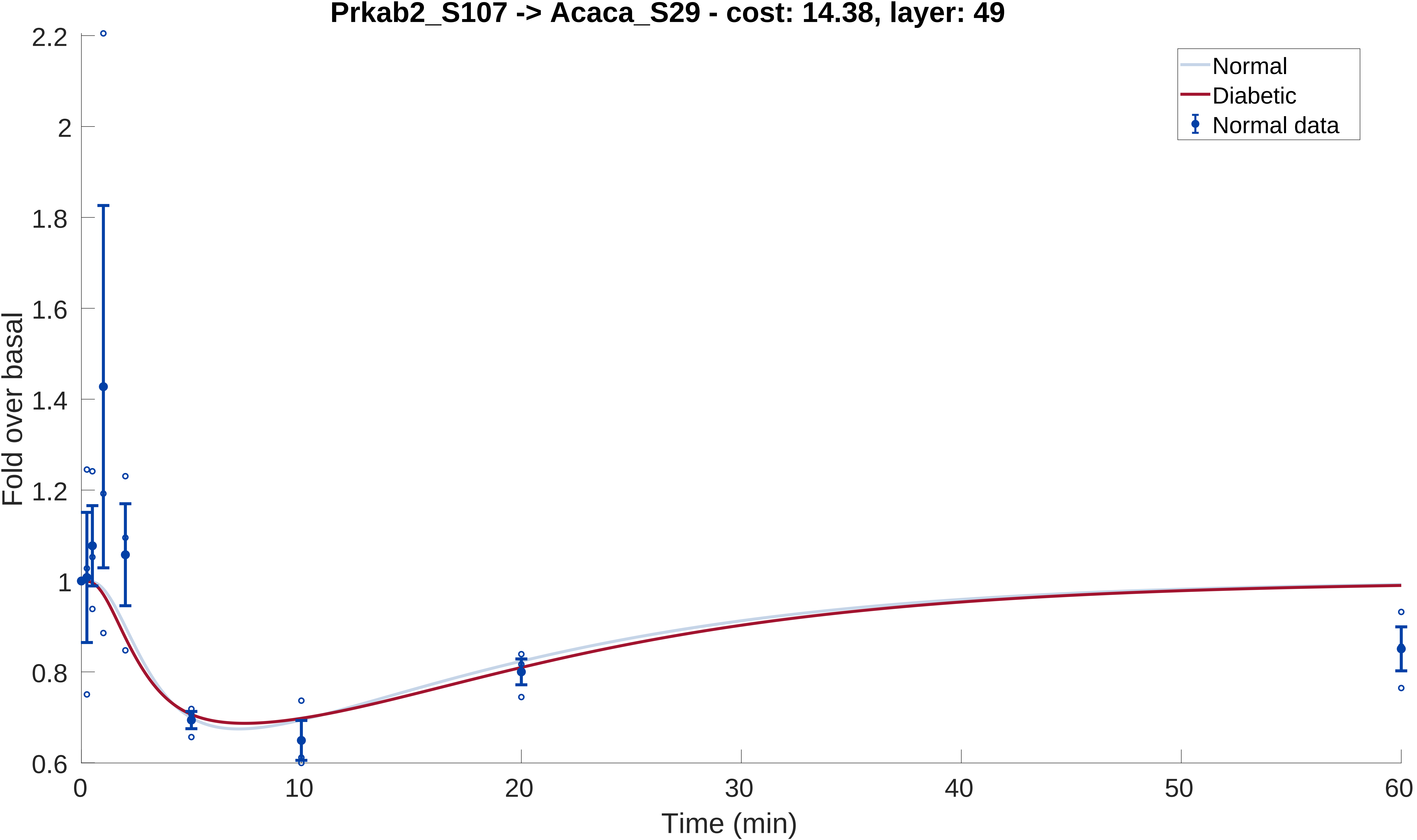

Prkab2_S107 -> Acaca_S29 - cost: 14.38, layer: 49
Normal
Diabetic
Normal data
Fold over basal
Time (min)
2.2
2
1.8
1.6
1.4
1.2
1
0.8
0.6
0
10
20
30
40
50
60

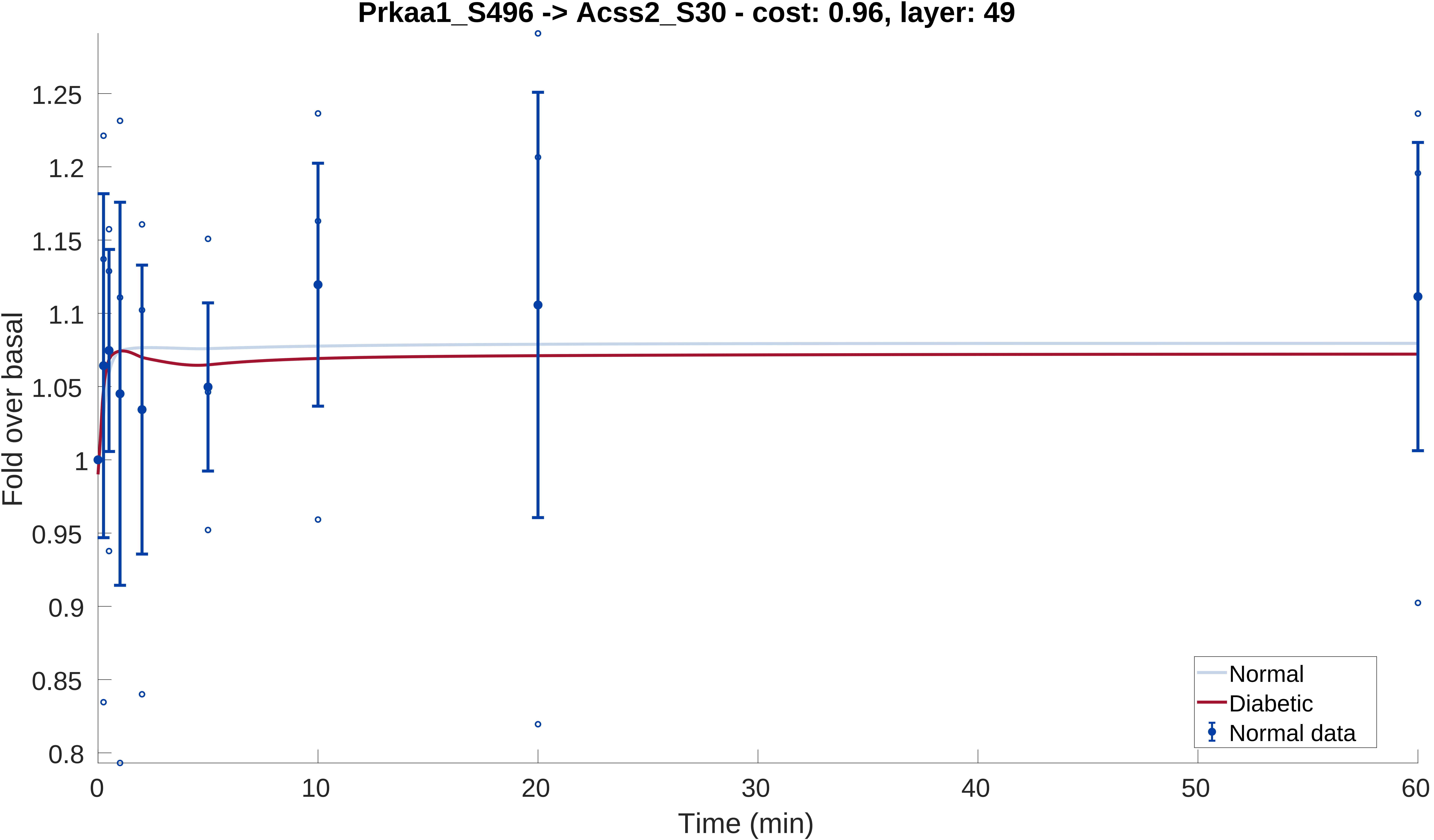

Prkaa1_S496 -> Acss2_S30 - cost: 0.96, layer: 49
Fold over basal
1.25
1.2
1.15
1.1
1.05
1
0.95
0.9
0.85
0.8
0
10
20
30
40
50
60
Time (min)
Normal
Diabetic
Normal data

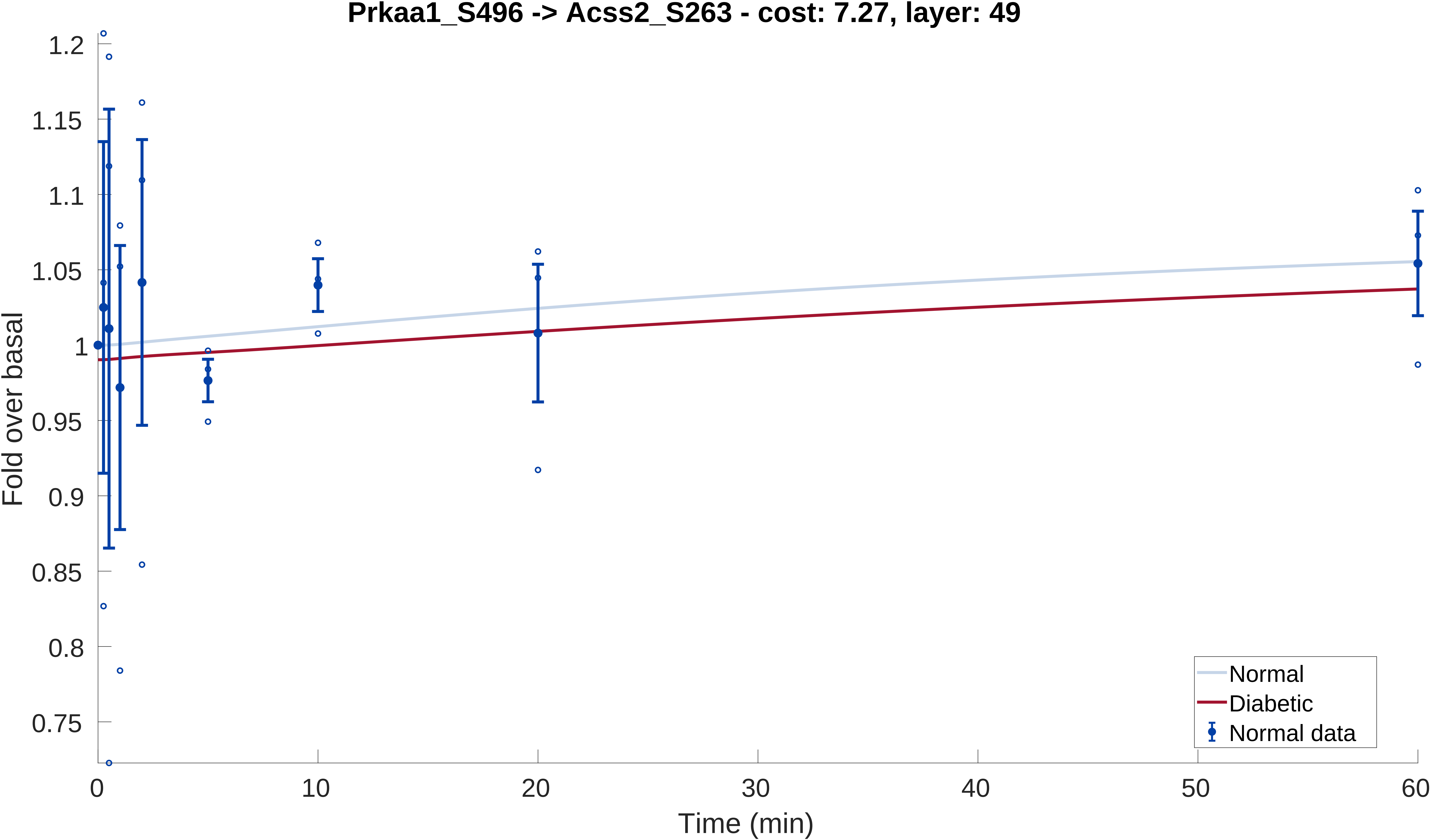

Prkaa1_S496 -> Acss2_S263 - cost: 7.27, layer: 49
Fold over basal
Time (min)
1.2
1.15
1.1
1.05
1
0.95
0.9
0.85
0.8
0.75
0
10
20
30
40
50
60
Normal
Diabetic
Normal data

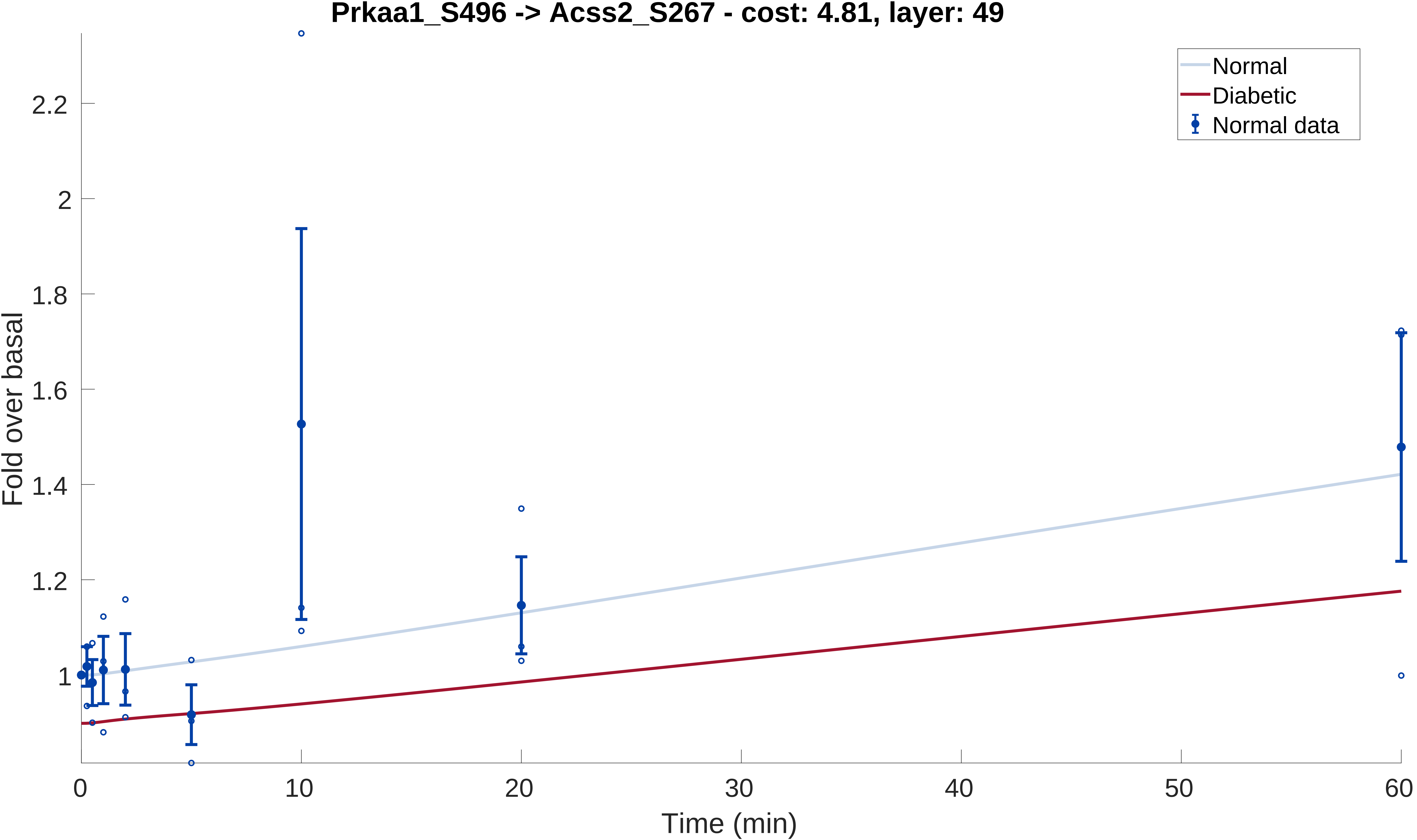
Prkaa1_S496 -> Acss2_S267 - cost: 4.81, layer: 49
Normal
Diabetic
Normal data
Fold over basal
Time (min)
2.2
2
1.8
1.6
1.4
1.2
1
0
10
20
30
40
50
60

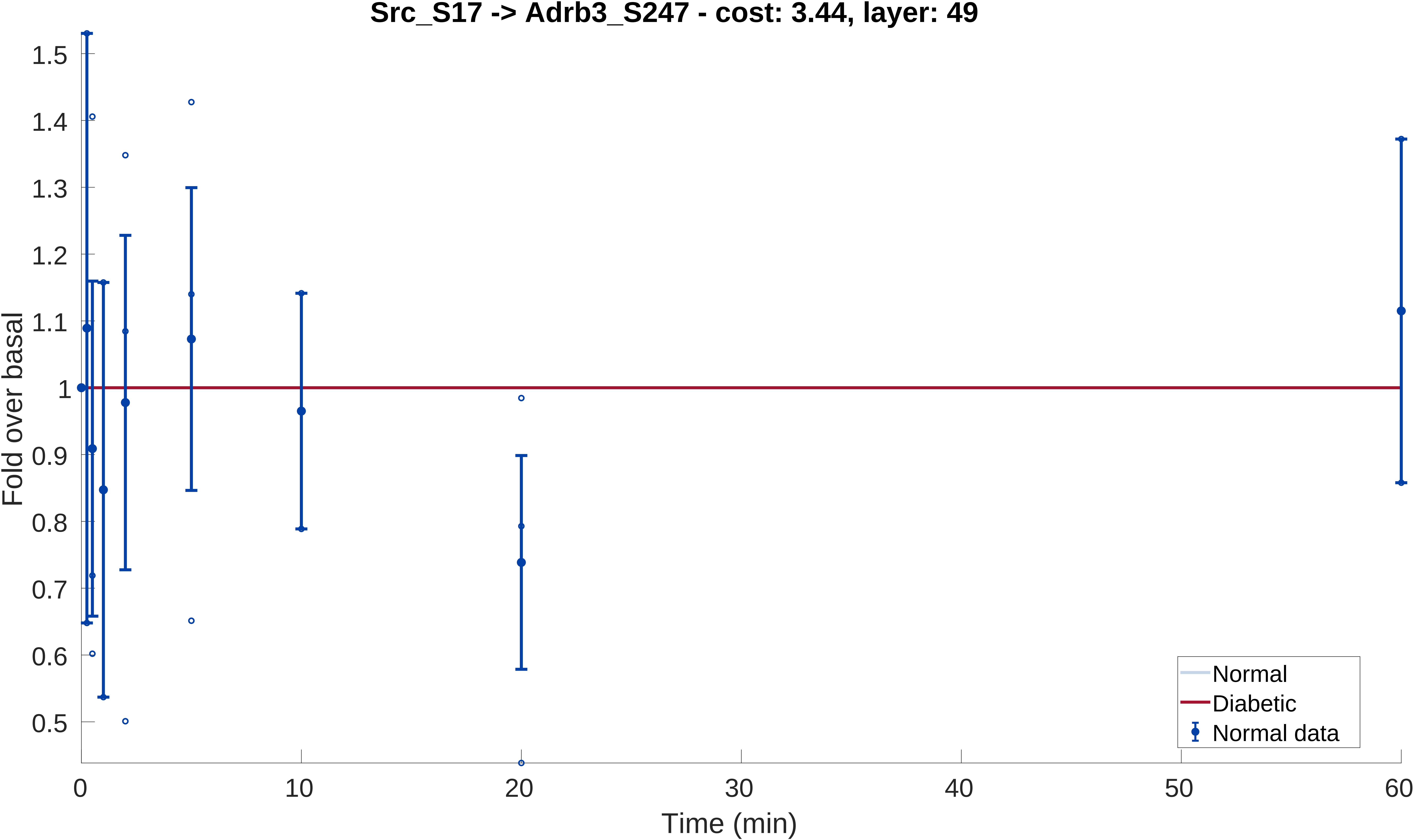

Src_S17 -> Adrb3_S247 - cost: 3.44, layer: 49
Fold over basal
1.5
1.4
1.3
1.2
1.1
1
0.9
0.8
0.7
0.6
0.5
0
10
20
30
40
50
60
Time (min)
Normal
Diabetic
Normal data

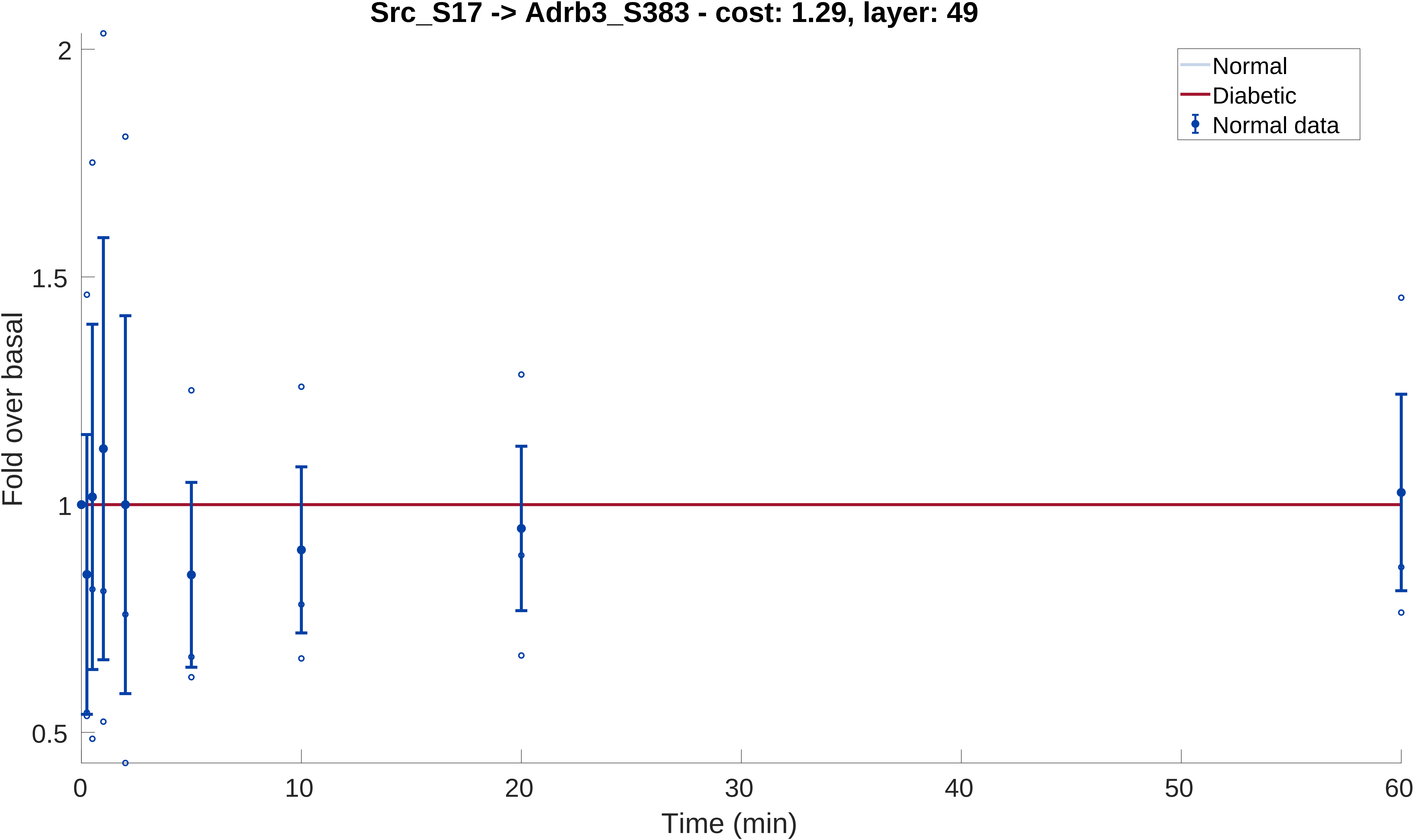

Src_S17 -> Adrb3_S383 - cost: 1.29, layer: 49
Normal
Diabetic
Normal data
Fold over basal
2
1.5
1
0.5
0
10
20
30
40
50
60
Time (min)

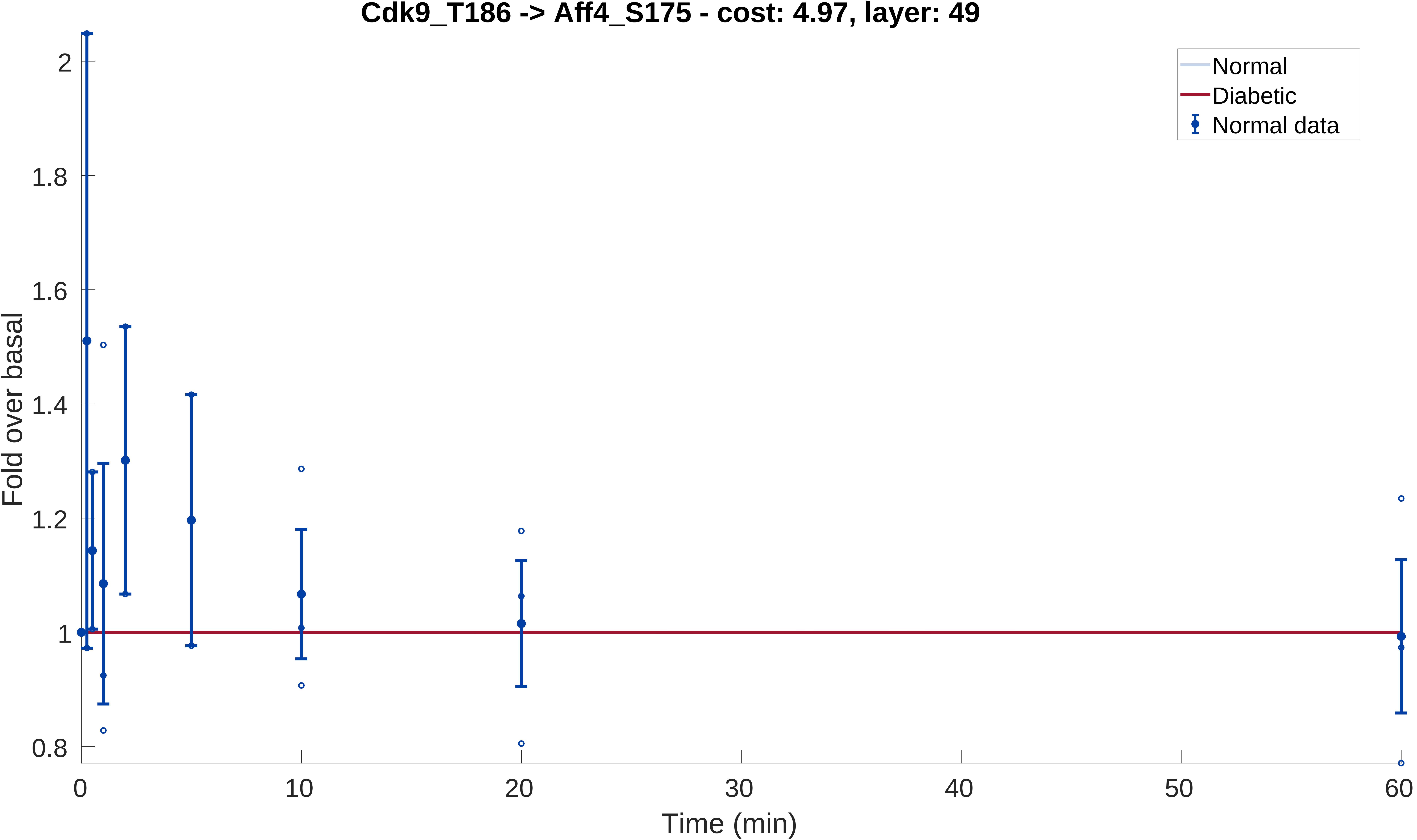

Cdk9_T186 -> Aff4_S175 - cost: 4.97, layer: 49
Normal
Diabetic
Normal data
Fold over basal
Time (min)
0.8
1
1.2
1.4
1.6
1.8
2
0
10
20
30
40
50
60

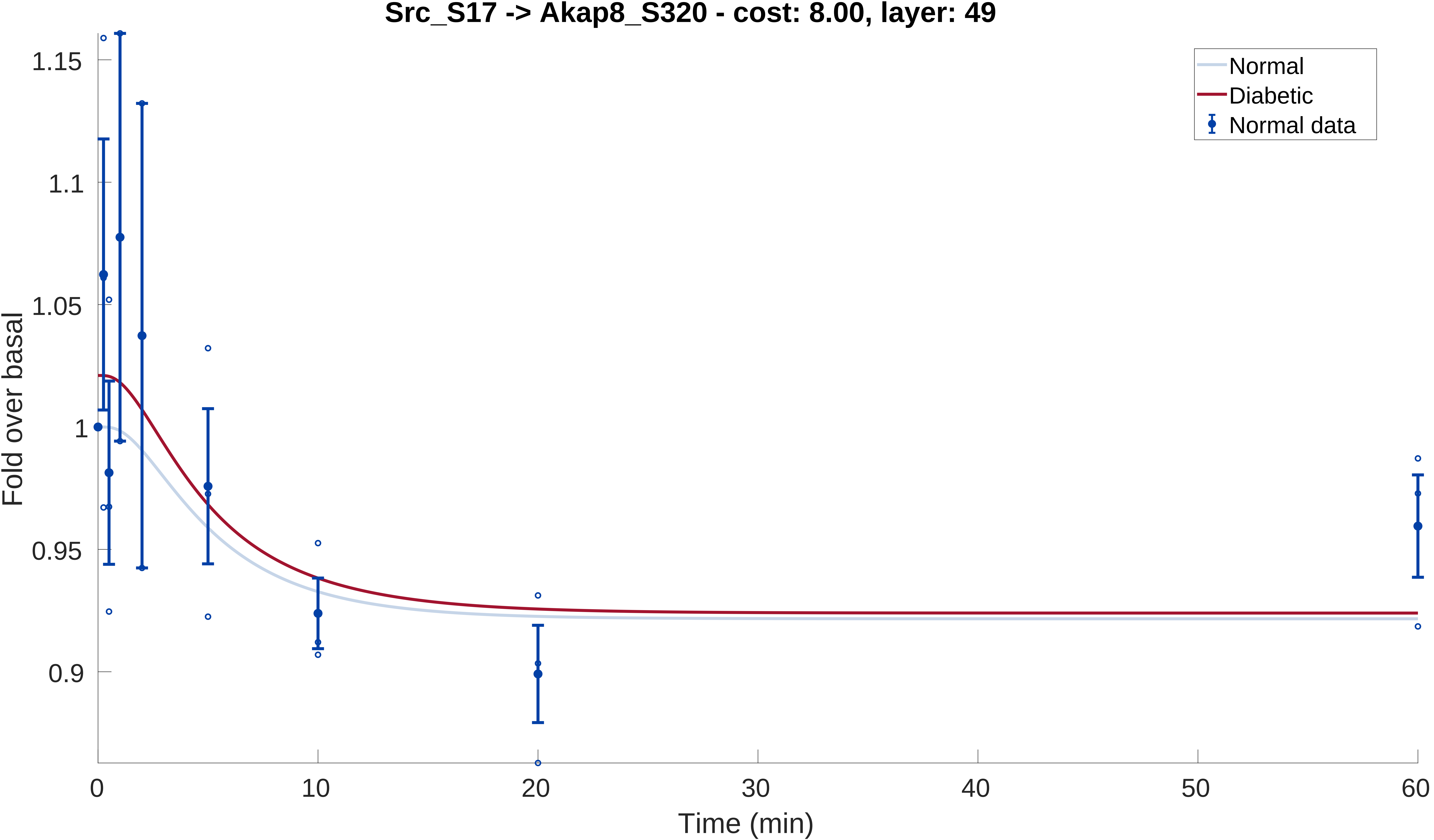

Src_S17 -> Akap8_S320 - cost: 8.00, layer: 49
Normal
Diabetic
Normal data
Fold over basal
1.15
1.1
1.05
1
0.95
0.9
0
10
20
30
40
50
60
Time (min)

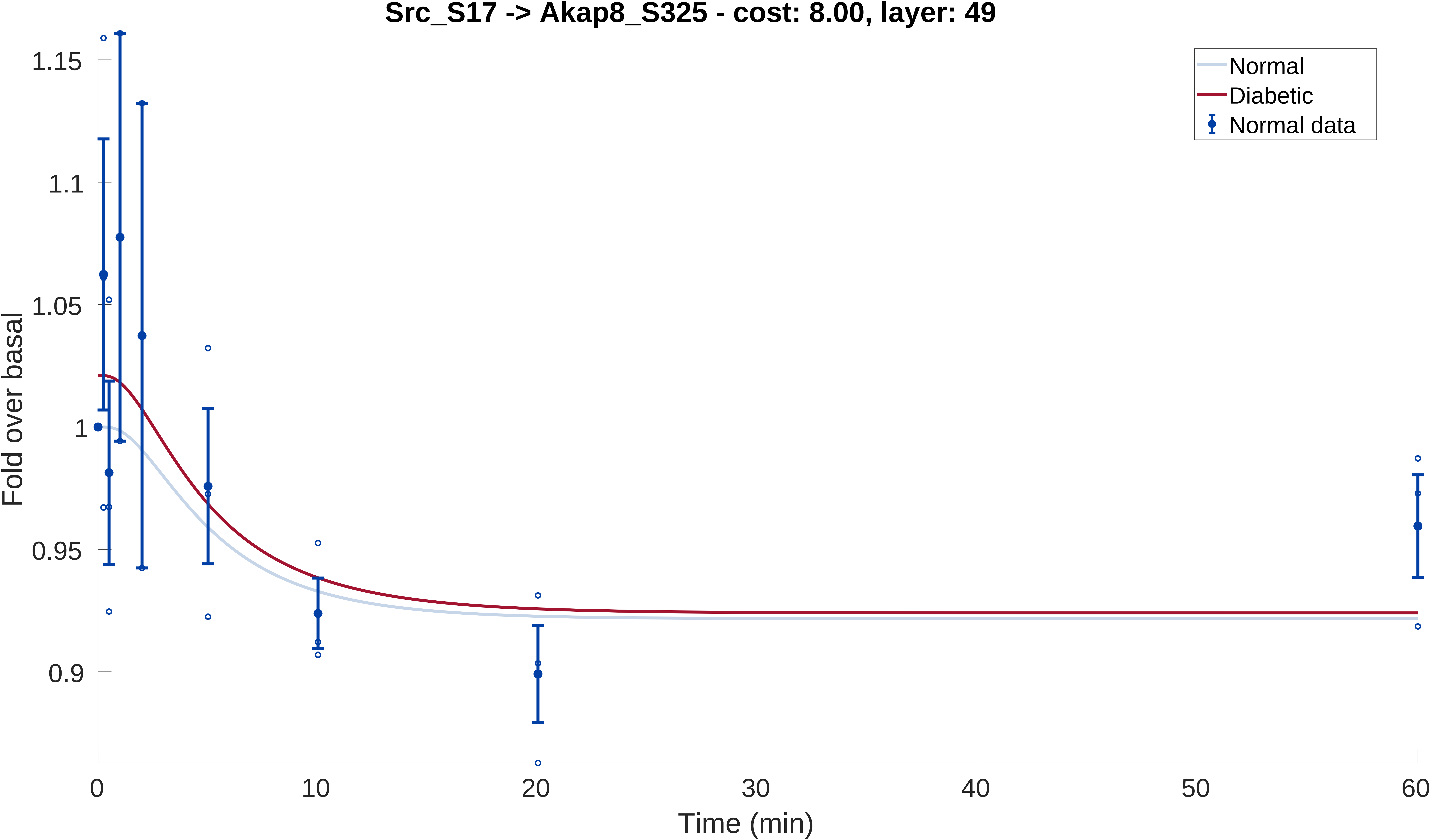

Src_S17 -> Akap8_S325 - cost: 8.00, layer: 49
Normal
Diabetic
Normal data
Fold over basal
1.15
1.1
1.05
1
0.95
0.9
0
10
20
30
40
50
60
Time (min)

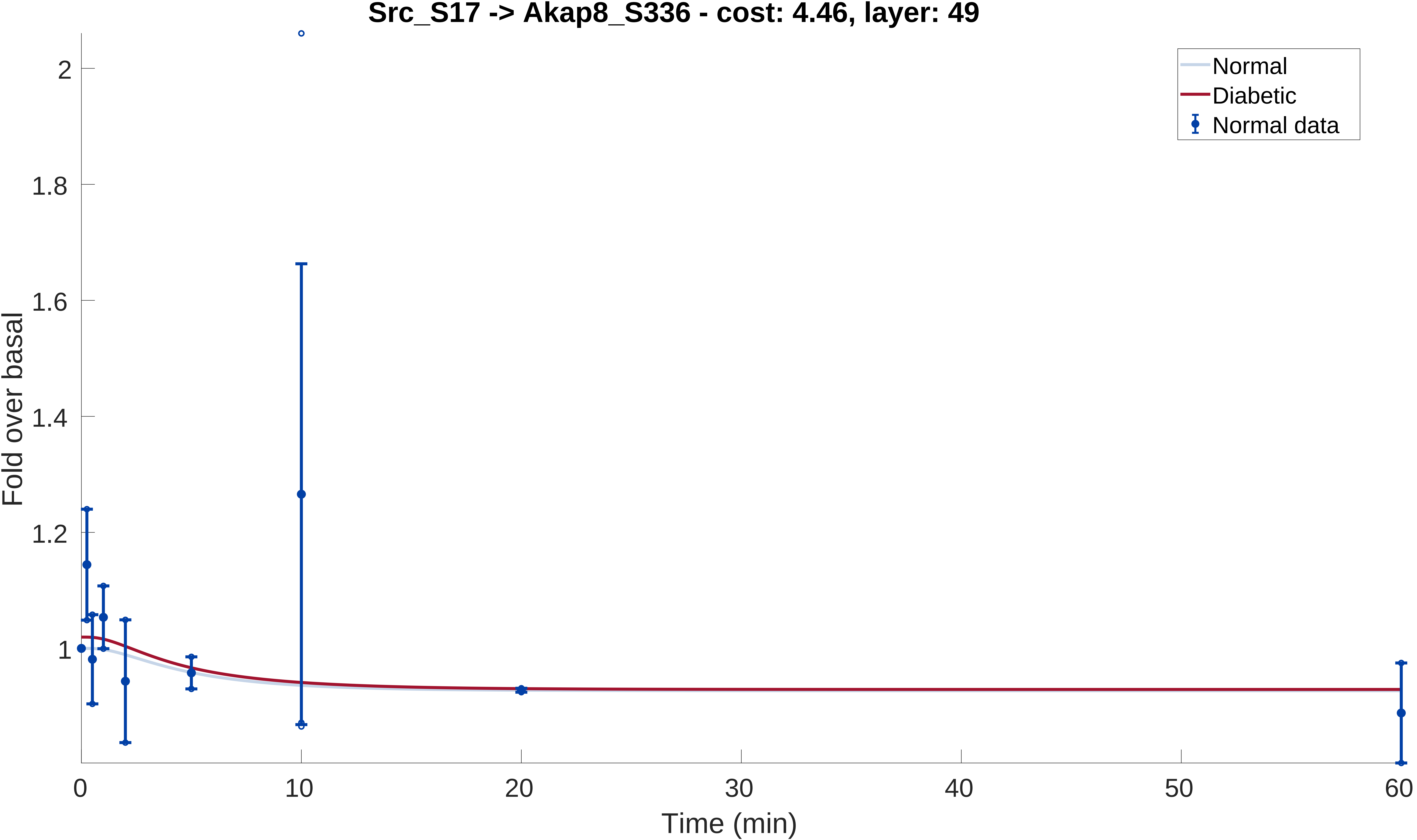

Src_S17 -> Akap8_S336 - cost: 4.46, layer: 49
Normal
Diabetic
Normal data
Fold over basal
2
1.8
1.6
1.4
1.2
1
0
10
20
30
40
50
60
Time (min)

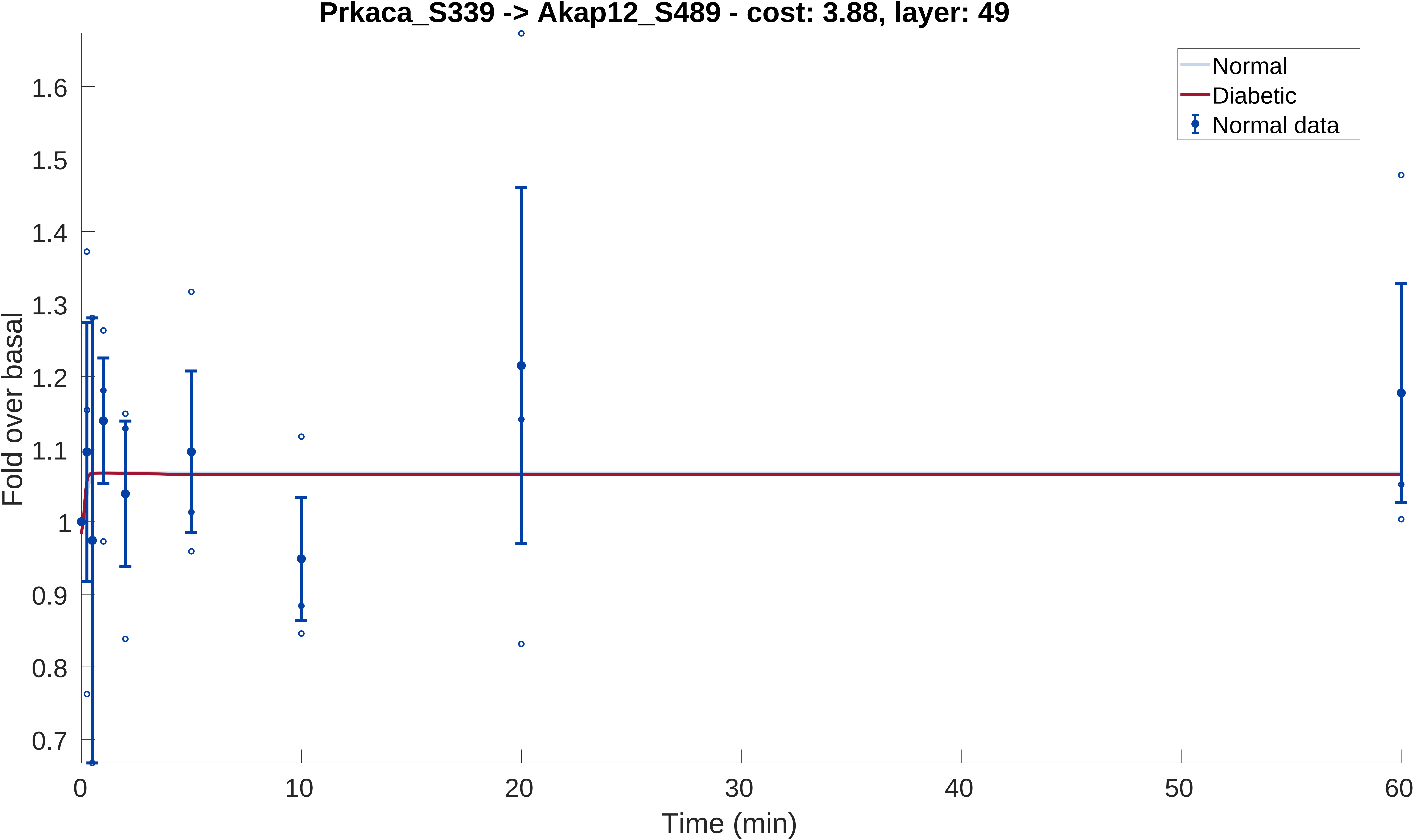

Prkaca_S339 -> Akap12_S489 - cost: 3.88, layer: 49
Normal
Diabetic
Normal data
Fold over basal
Time (min)
1.6
1.5
1.4
1.3
1.2
1.1
1
0.9
0.8
0.7
0
10
20
30
40
50
60

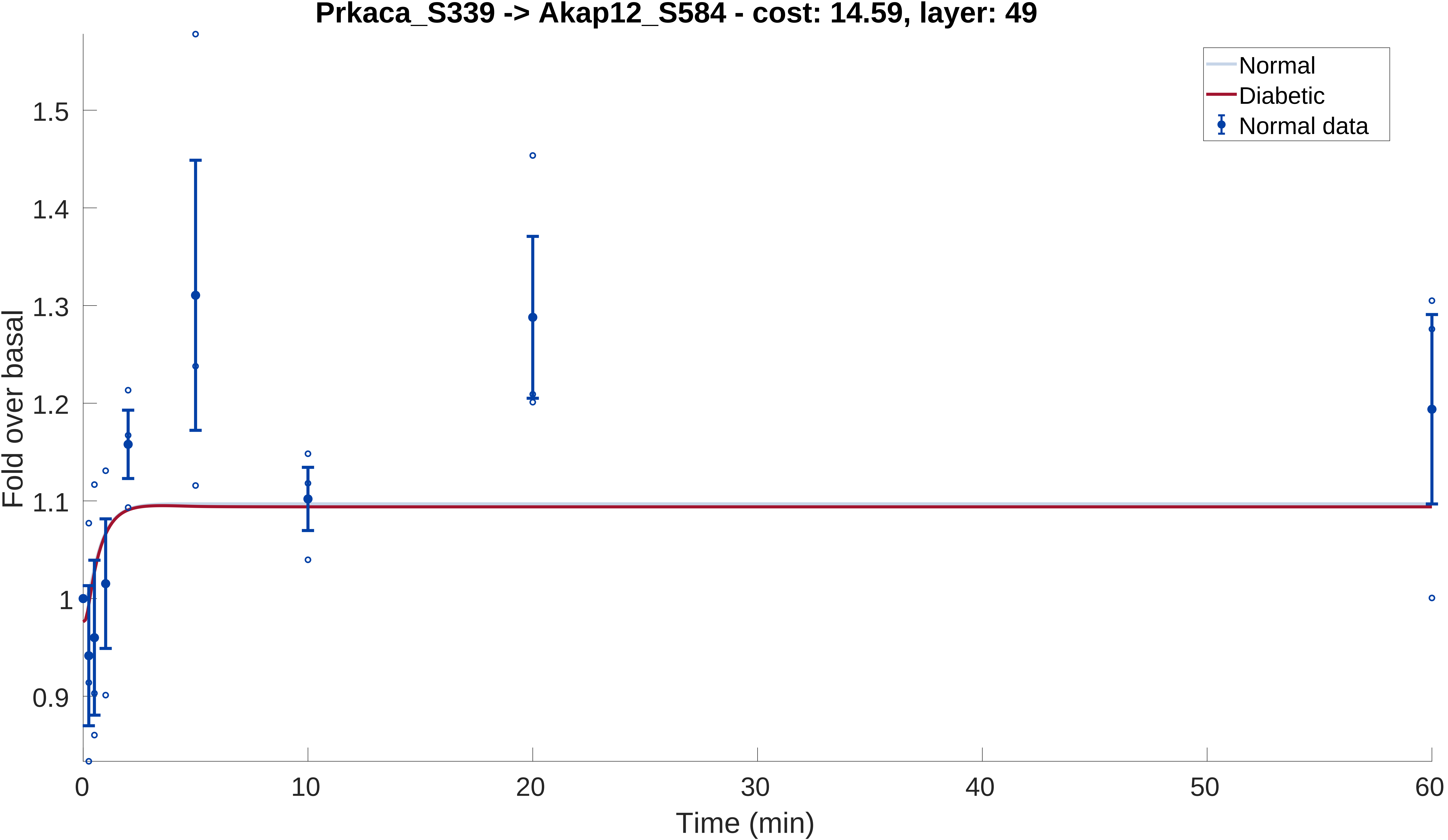
Prkaca_S339 -> Akap12_S584 - cost: 14.59, layer: 49
Normal
Diabetic
Normal data
Fold over basal
1.5
1.4
1.3
1.2
1.1
1
0.9
0
10
20
30
40
50
60
Time (min)

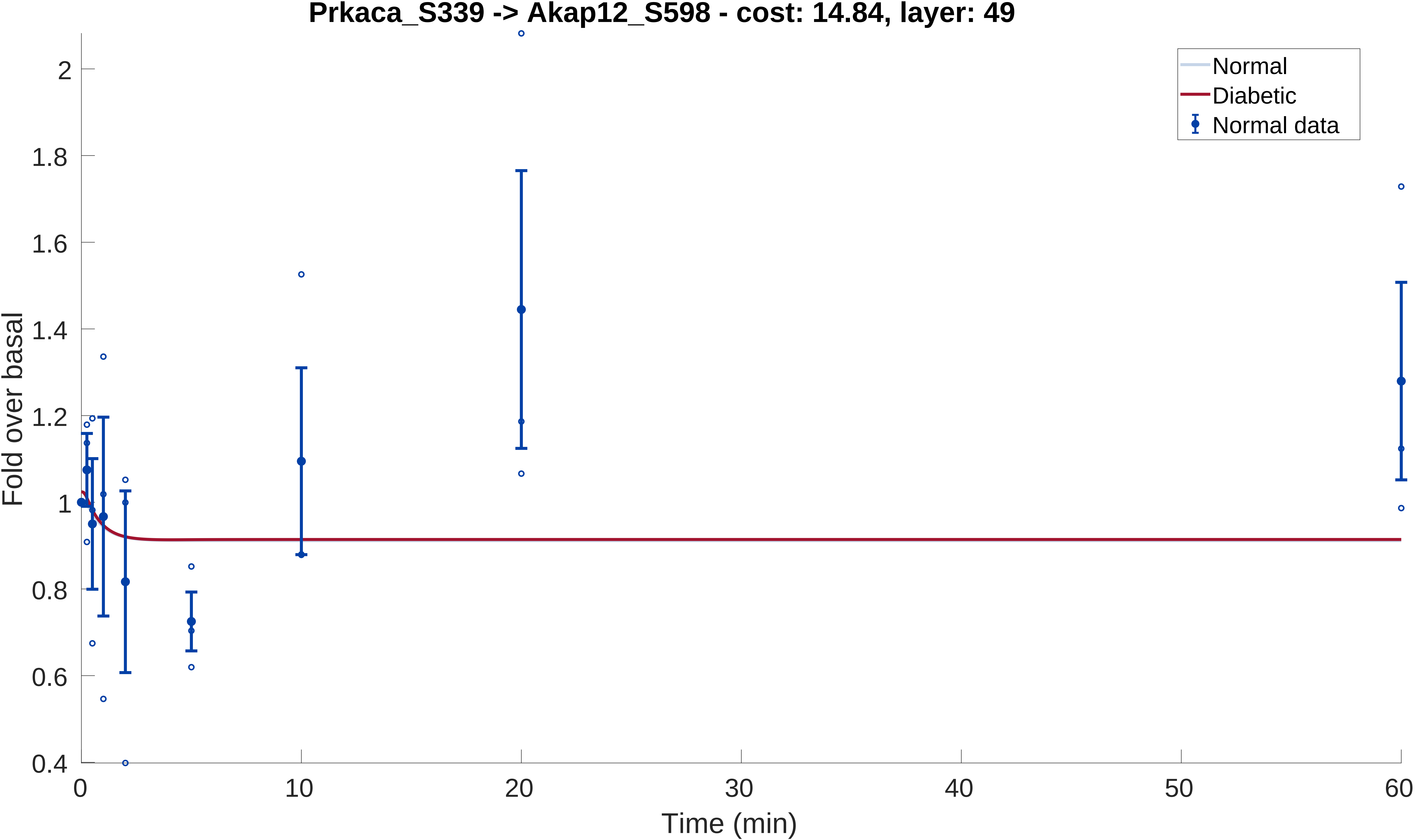
Prkaca_S339 -> Akap12_S598 - cost: 14.84, layer: 49
Normal
Diabetic
Normal data
Fold over basal
Time (min)
0.4
0.6
0.8
1
1.2
1.4
1.6
1.8
2
0
10
20
30
40
50
60

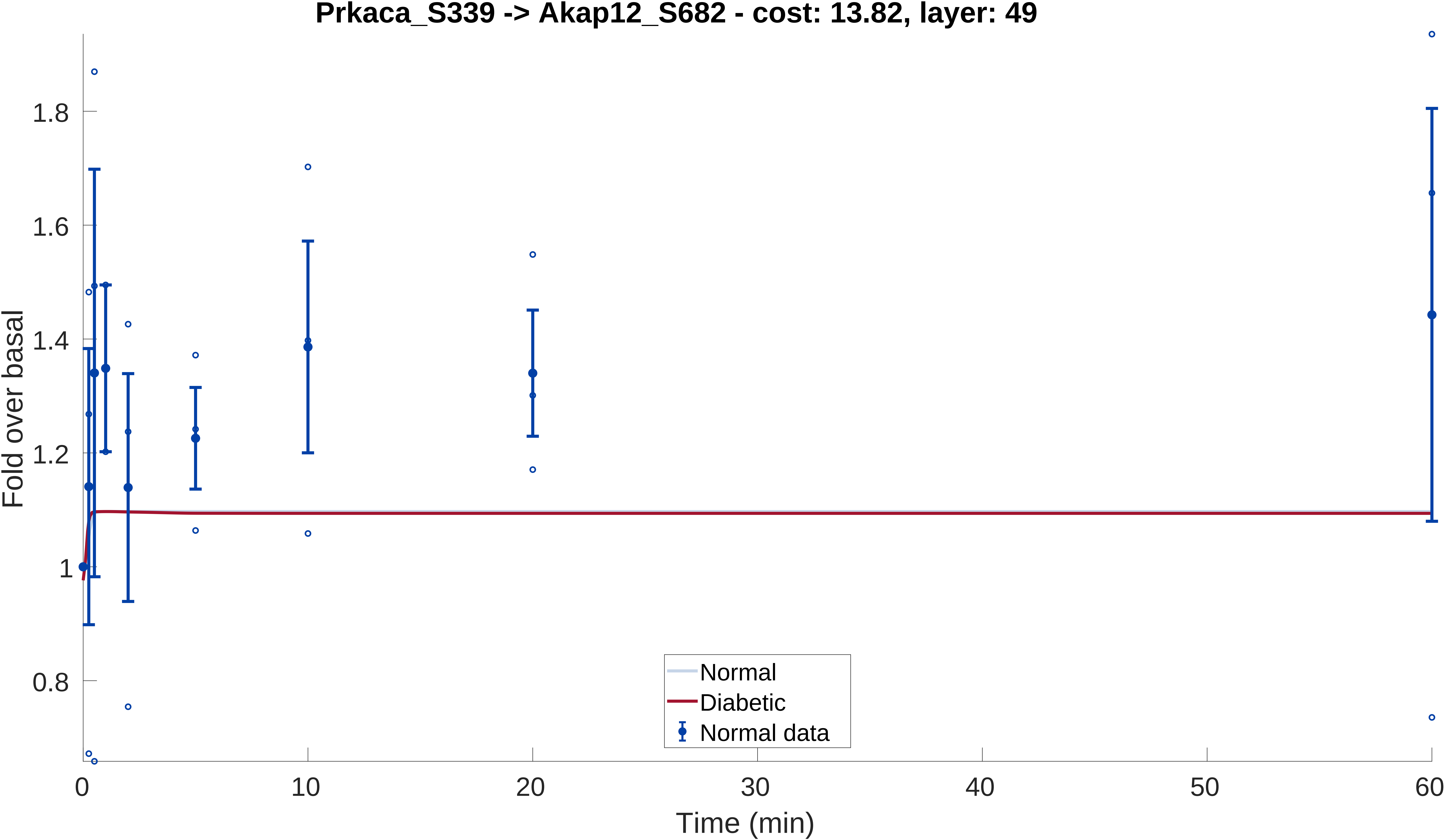

Prrkaca_S339 -> Akap12_S682 - cost: 13.82, layer: 49
Fold over basal
0.8
1
1.2
1.4
1.6
1.8
0
10
20
30
40
50
60
Time (min)
Normal
Diabetic
Normal data

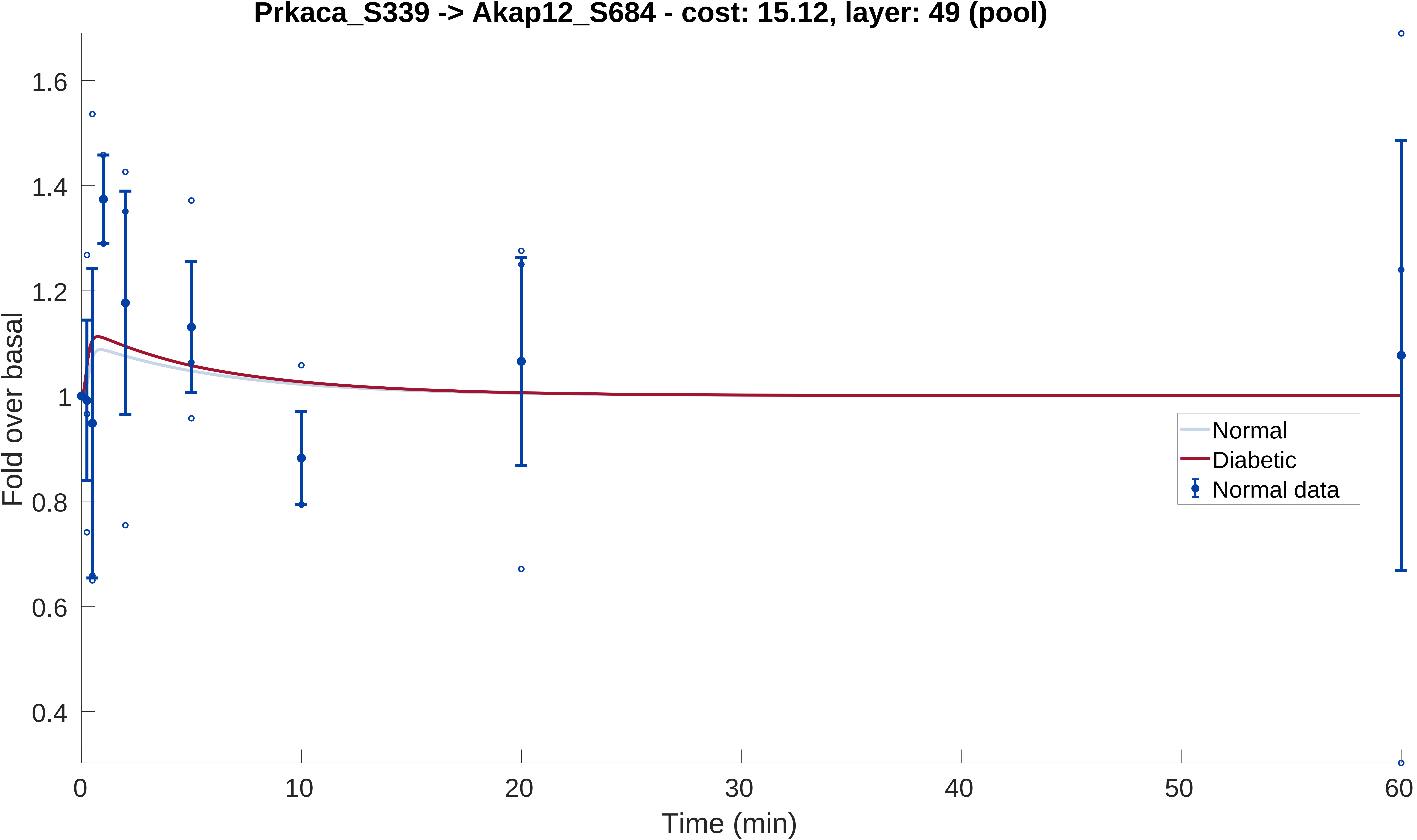

Prkaca_S339 -> Akap12_S684 - cost: 15.12, layer: 49 (pool)
Fold over basal
1.6
1.4
1.2
1
0.8
0.6
0.4
0
10
20
30
40
50
60
Time (min)
Normal
Diabetic
Normal data

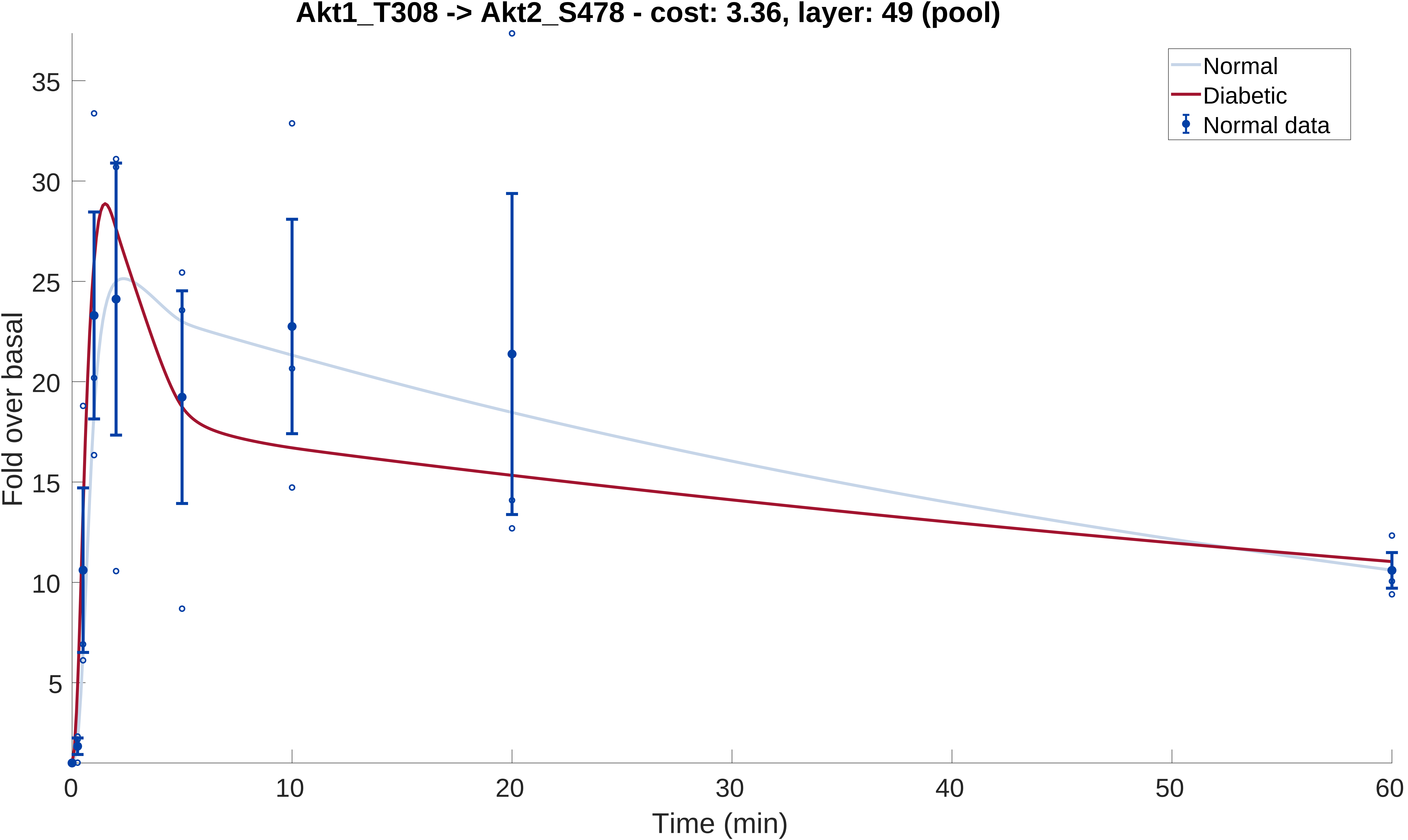

Akt1_T308 -> Akt2_S478 - cost: 3.36, layer: 49 (pool)
Normal
Diabetic
Normal data
Fold over basal
Time (min)
0
10
20
30
40
50
60
5
10
15
20
25
30
35

Akt1_T308 -> Akt2_T313 - cost: 5.38, layer: 49

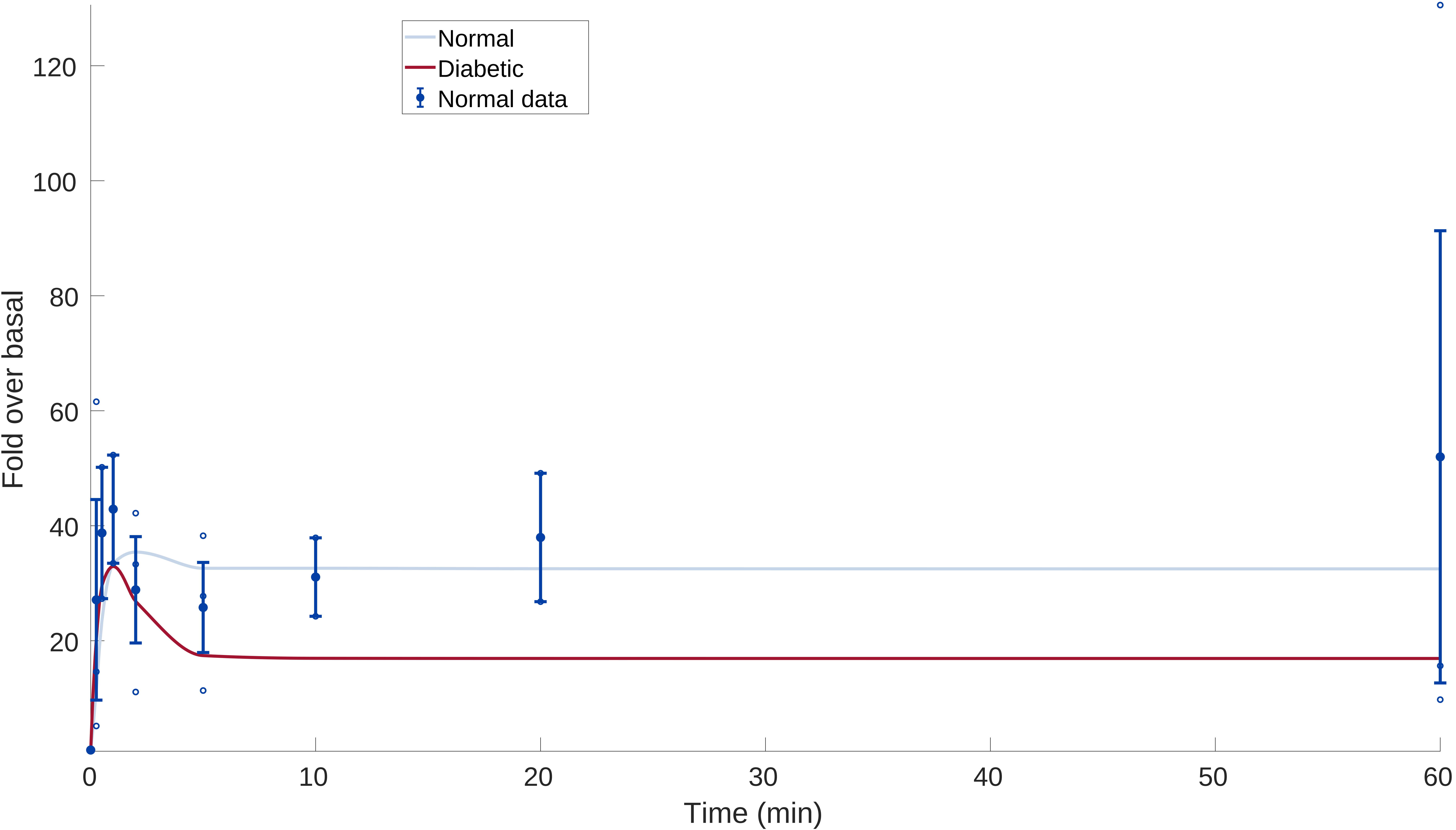

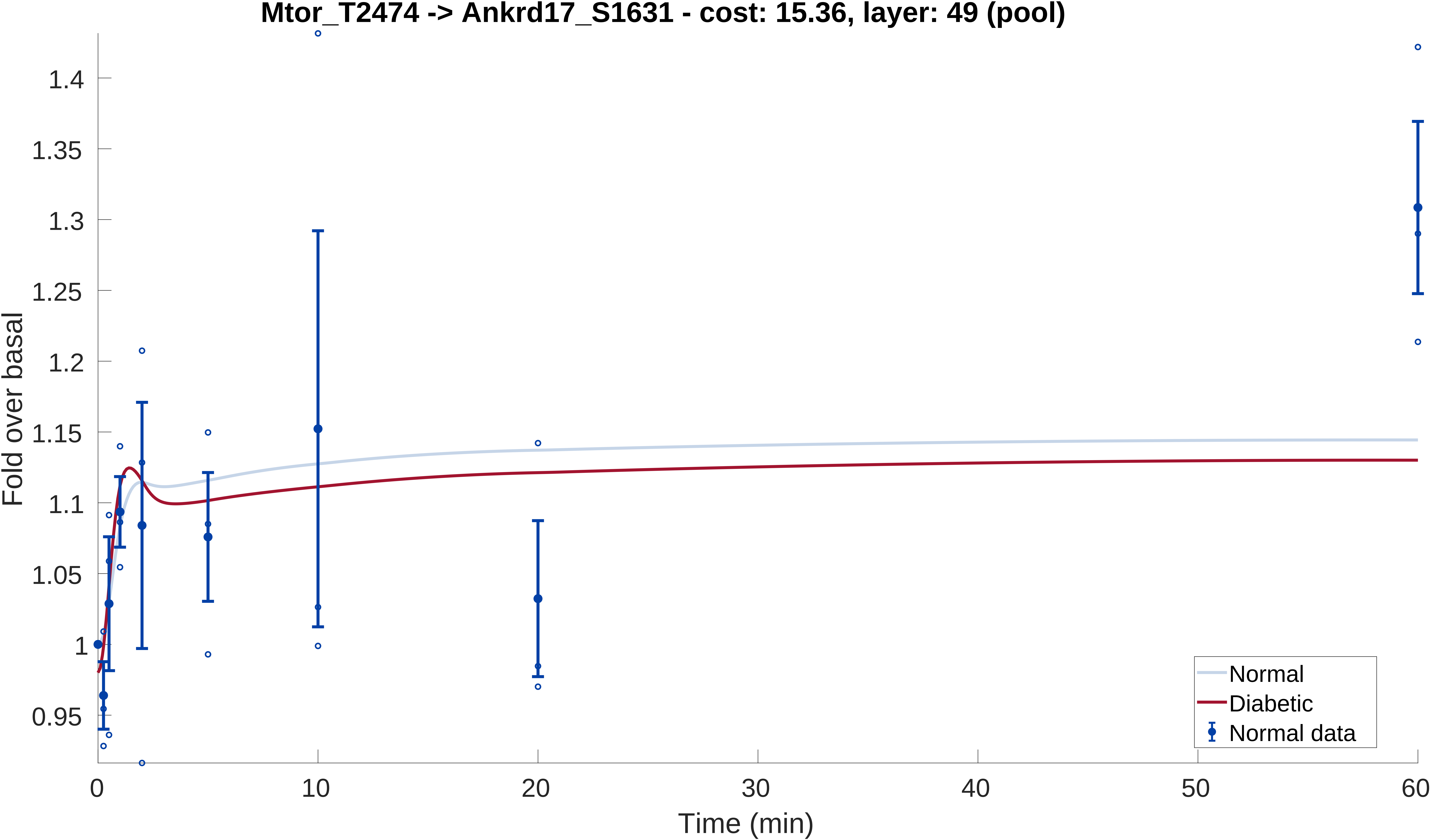

Mtor_T2474 -> Ankrd17_S1631 - cost: 15.36, layer: 49 (pool)
Fold over basal
1.4
1.35
1.3
1.25
1.2
1.15
1.1
1.05
1
0.95
0
10
20
30
40
50
60
Time (min)
Normal
Diabetic
Normal data

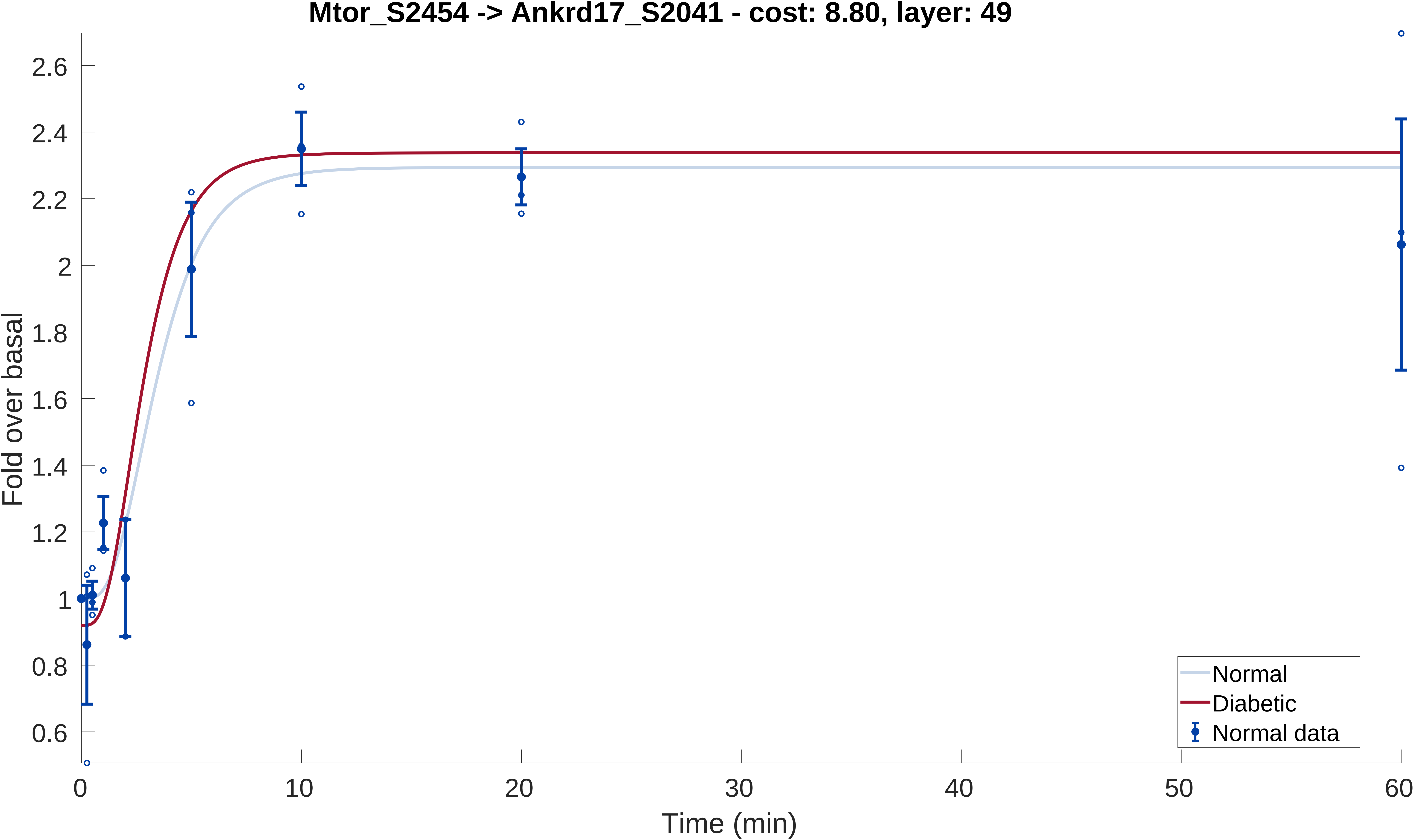
Mtor_S2454 -> Ankrd17_S2041 - cost: 8.80, layer: 49
Fold over basal
Time (min)
0.6
0.8
1
1.2
1.4
1.6
1.8
2
2.2
2.4
2.6
0
10
20
30
40
50
60
Normal
Diabetic
Normal data

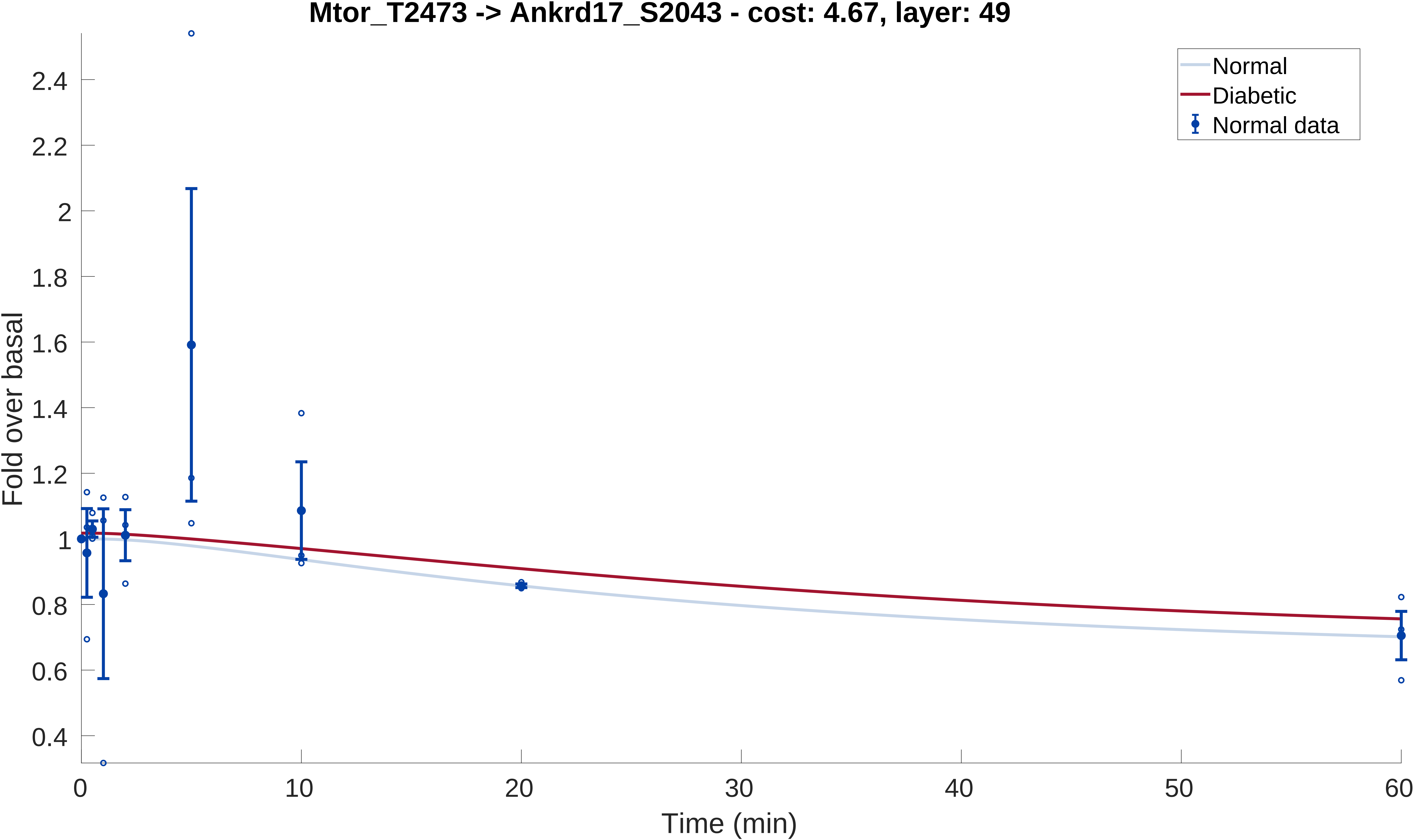

Mtor_T2473 -> Ankrd17_S2043 - cost: 4.67, layer: 49
Normal
Diabetic
Normal data
Fold over basal
2.4
2.2
2
1.8
1.6
1.4
1.2
1
0.8
0.6
0.4
0
10
20
30
40
50
60
Time (min)

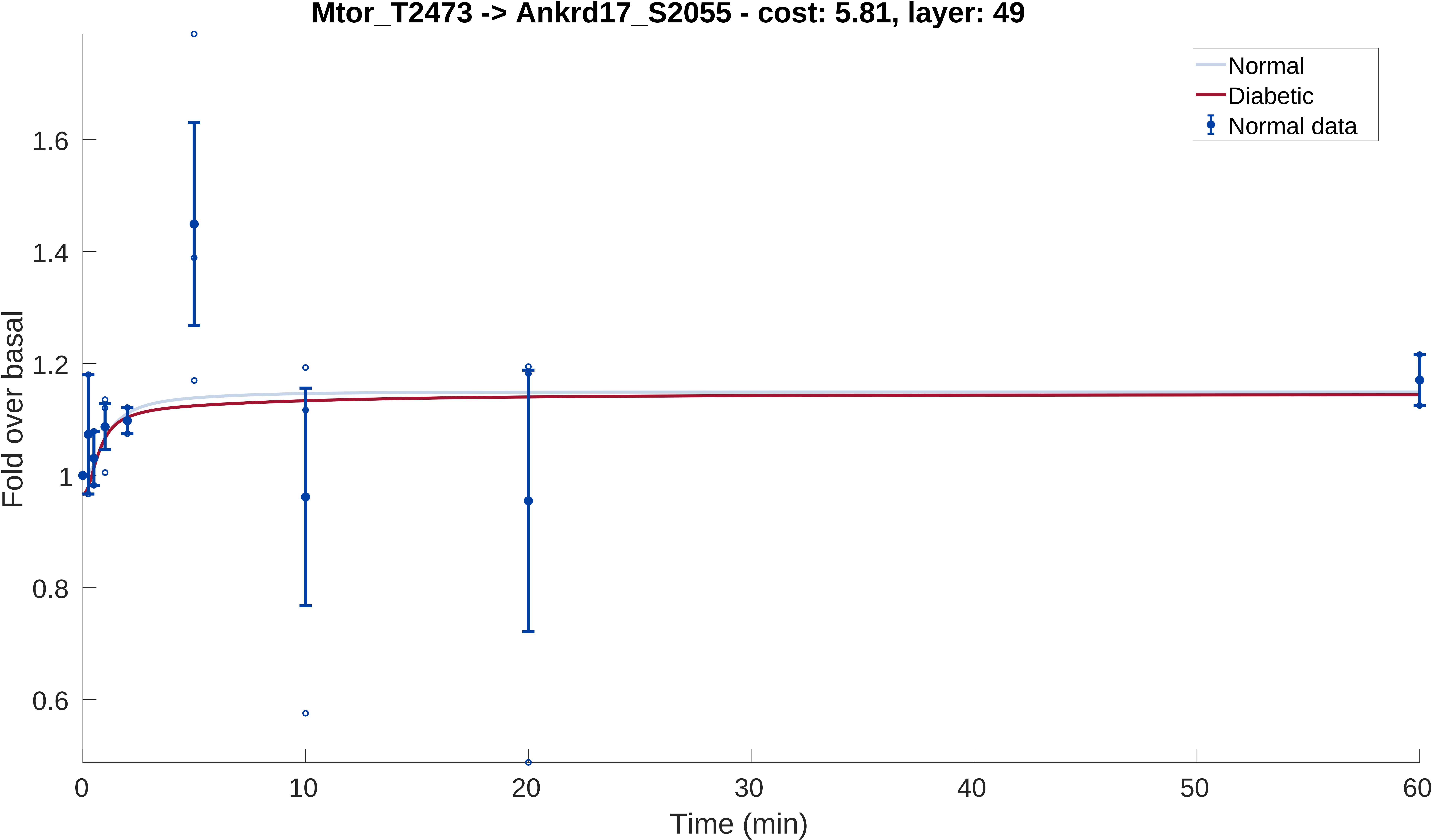

Mtor_T2473 -> Ankrd17_S2055 - cost: 5.81, layer: 49
Normal
Diabetic
Normal data
1.6
1.4
1.2
1
0.8
0.6
Fold over basal
0
10
20
30
40
50
60
Time (min)

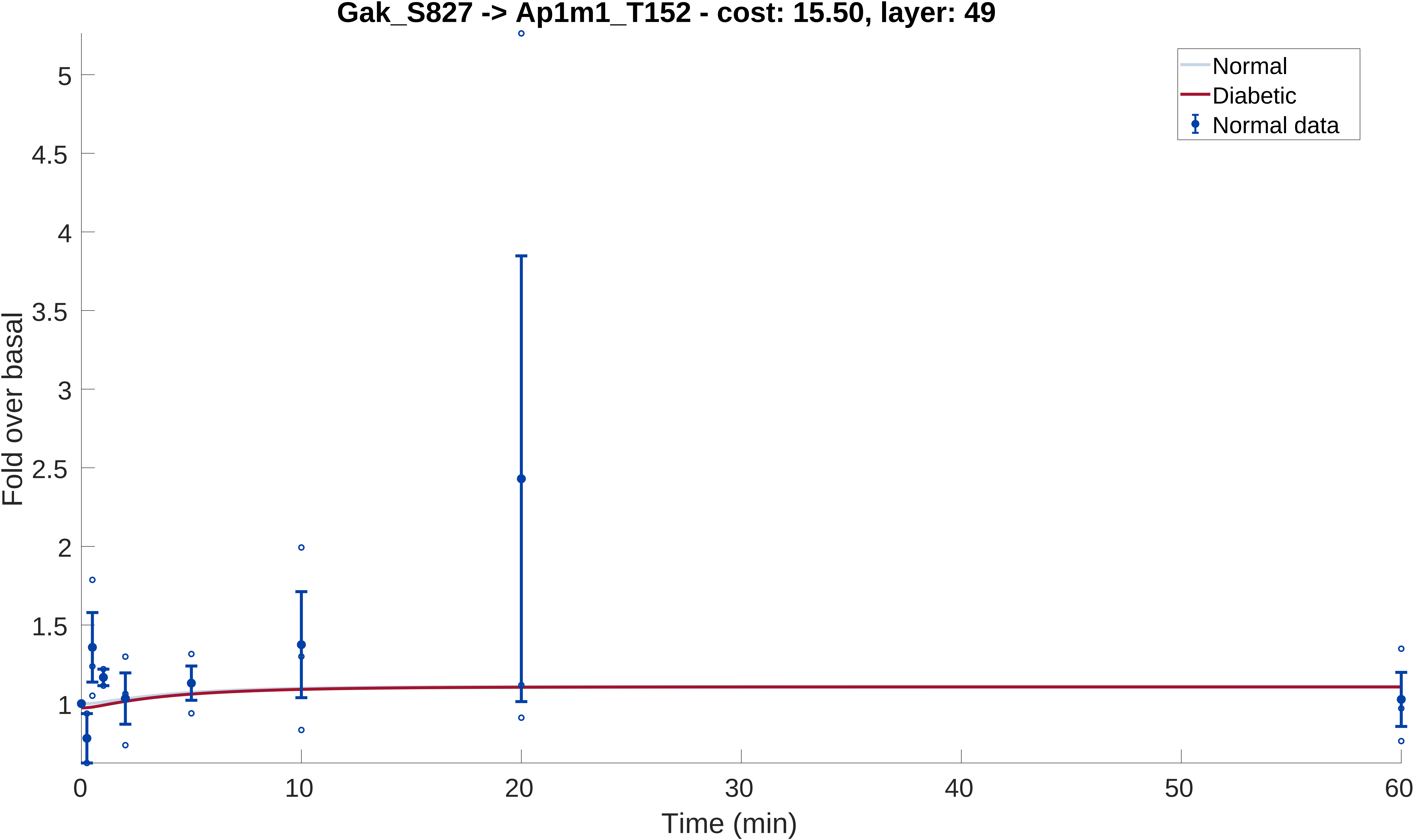

Gak_S827 -> Ap1m1_T152 - cost: 15.50, layer: 49
Normal
Diabetic
Normal data
Fold over basal
5
4.5
4
3.5
3
2.5
2
1.5
1
0
10
20
30
40
50
60
Time (min)

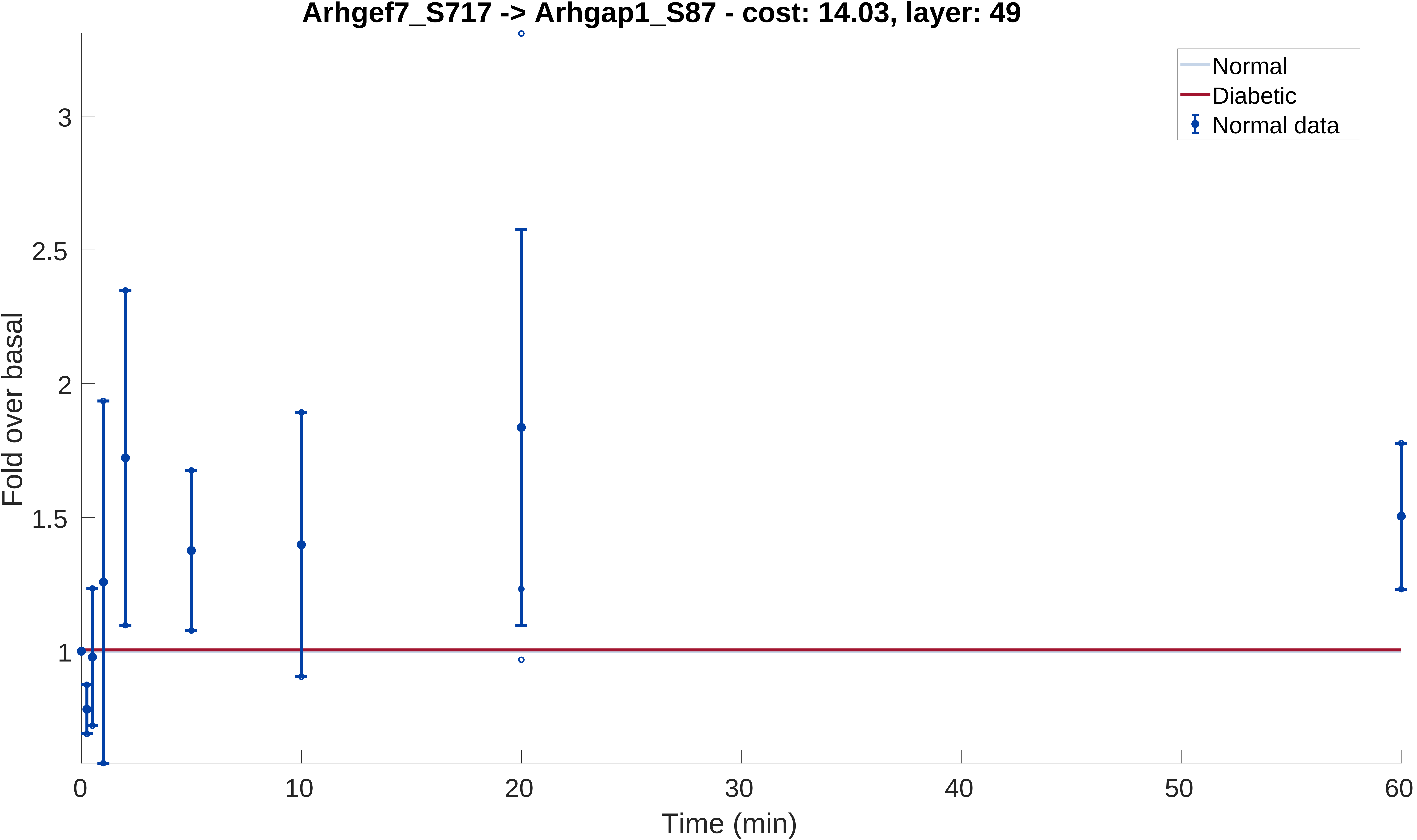

Arhgef7_S717 -> Arhgap1_S87 - cost: 14.03, layer: 49
Fold over basal
3
2.5
2
1.5
1
Time (min)
0
10
20
30
40
50
60
Normal
Diabetic
Normal data

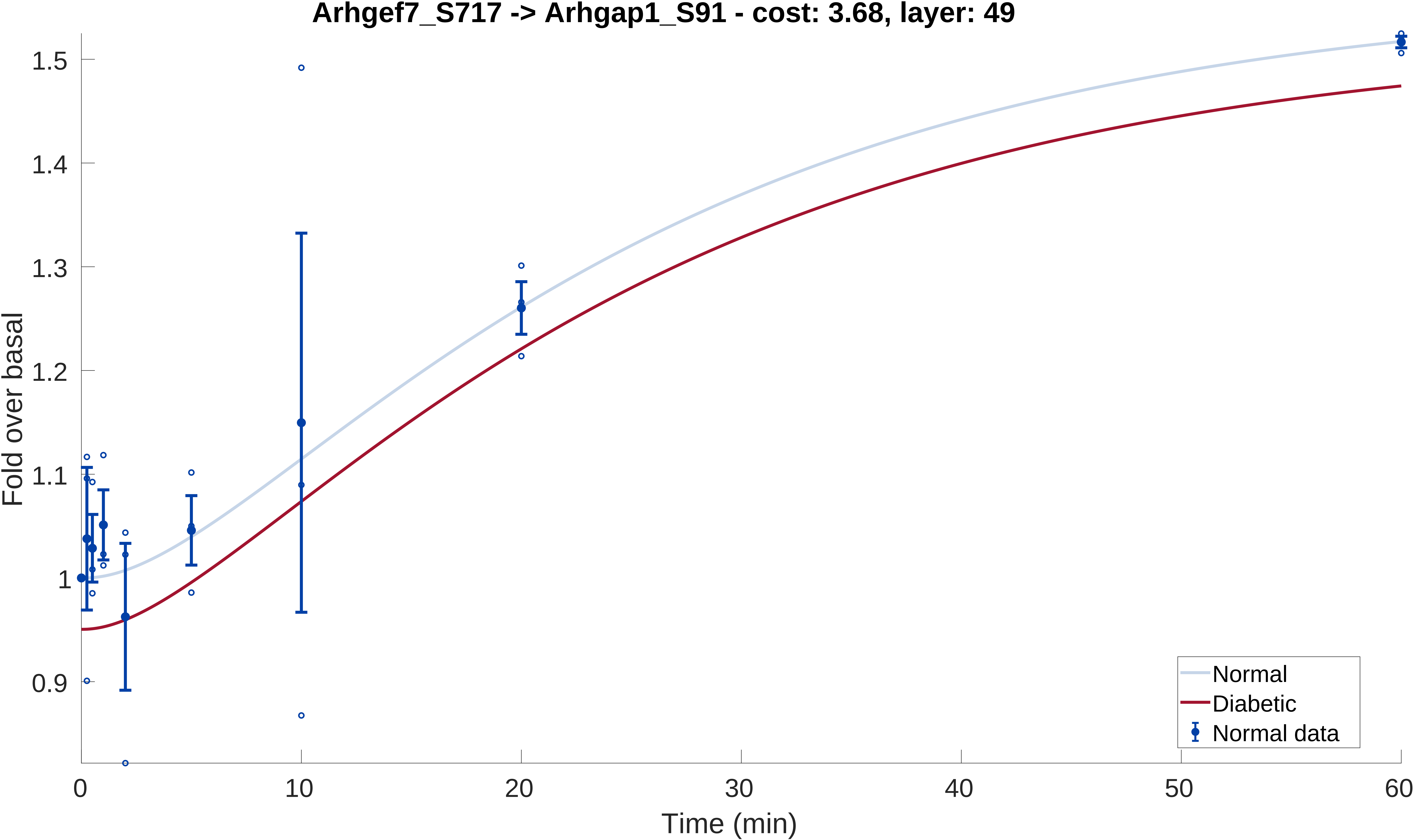

Arhgef7_S717 -> Arhgap1_S91 - cost: 3.68, layer: 49
Fold over basal
1.5
1.4
1.3
1.2
1.1
1
0.9
0
10
20
30
40
50
60
Time (min)
Normal
Diabetic
Normal data

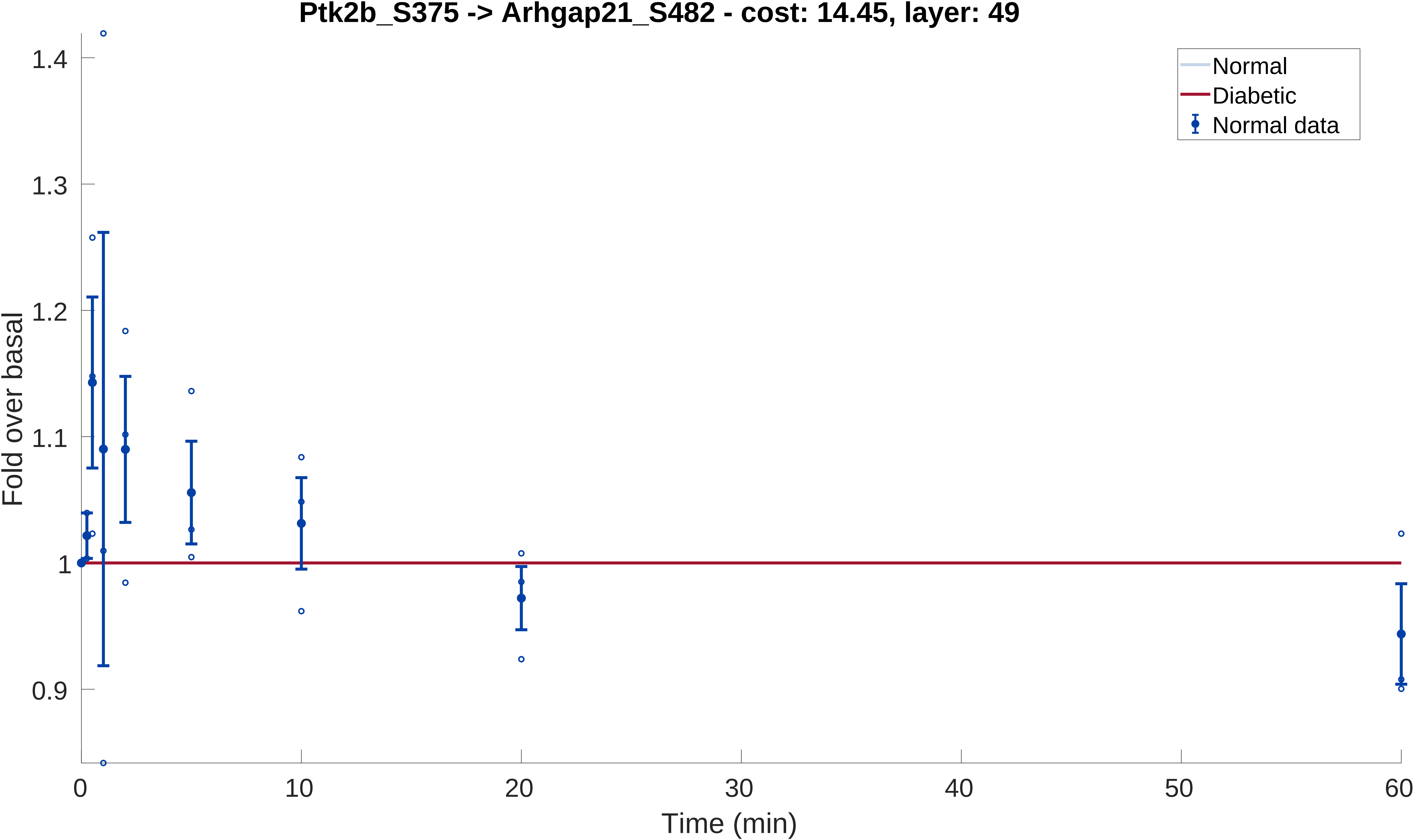
Ptk2b_S375 -> Arhgap21_S482 - cost: 14.45, layer: 49
Normal
Diabetic
Normal data
Fold over basal
1.4
1.3
1.2
1.1
1
0.9
Time (min)
0
10
20
30
40
50
60

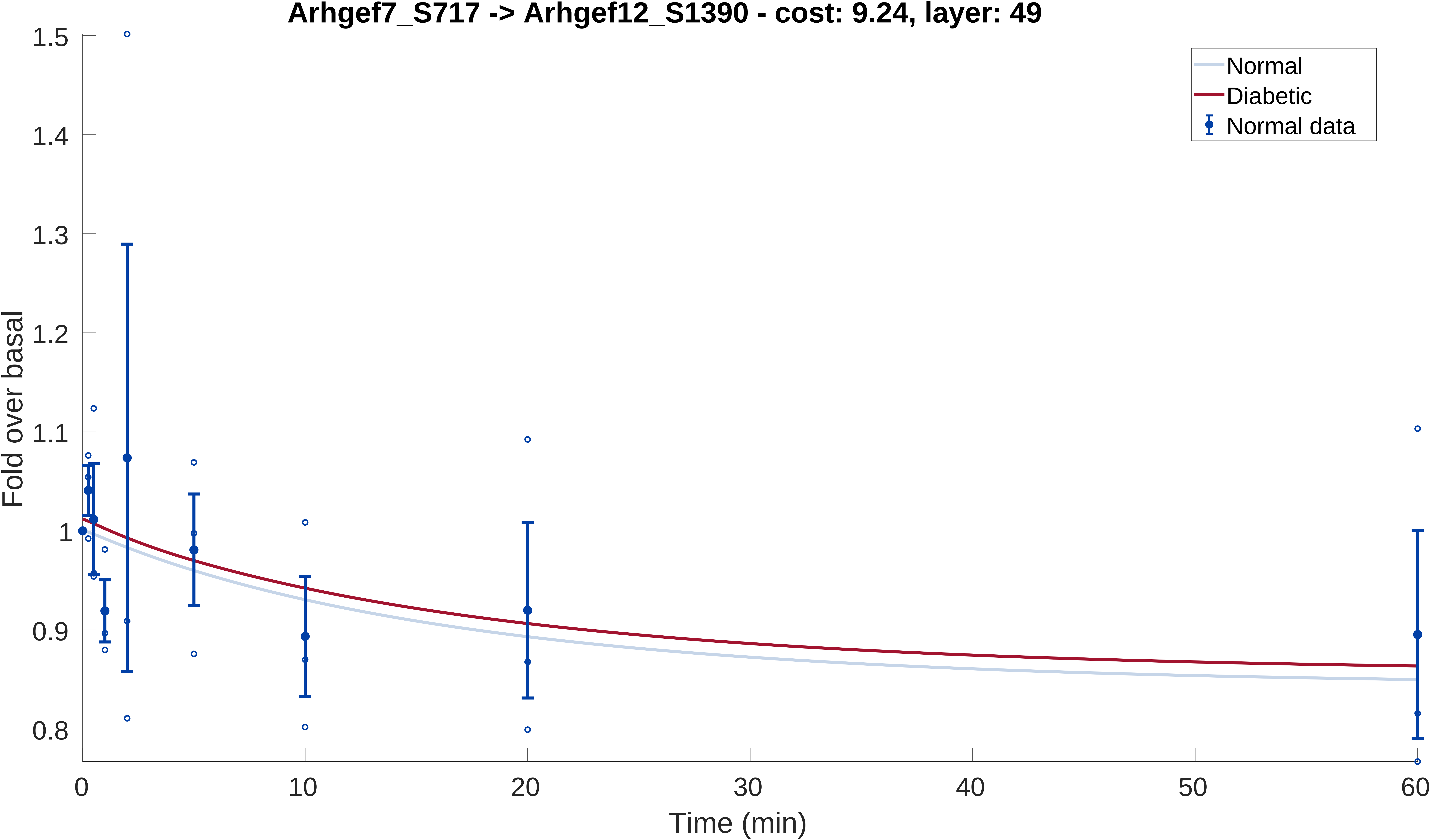

Arhgef7_S717 -> Arhgef12_S1390 - cost: 9.24, layer: 49
Normal
Diabetic
Normal data
Fold over basal
Time (min)
1.5
1.4
1.3
1.2
1.1
1
0.9
0.8
0
10
20
30
40
50
60

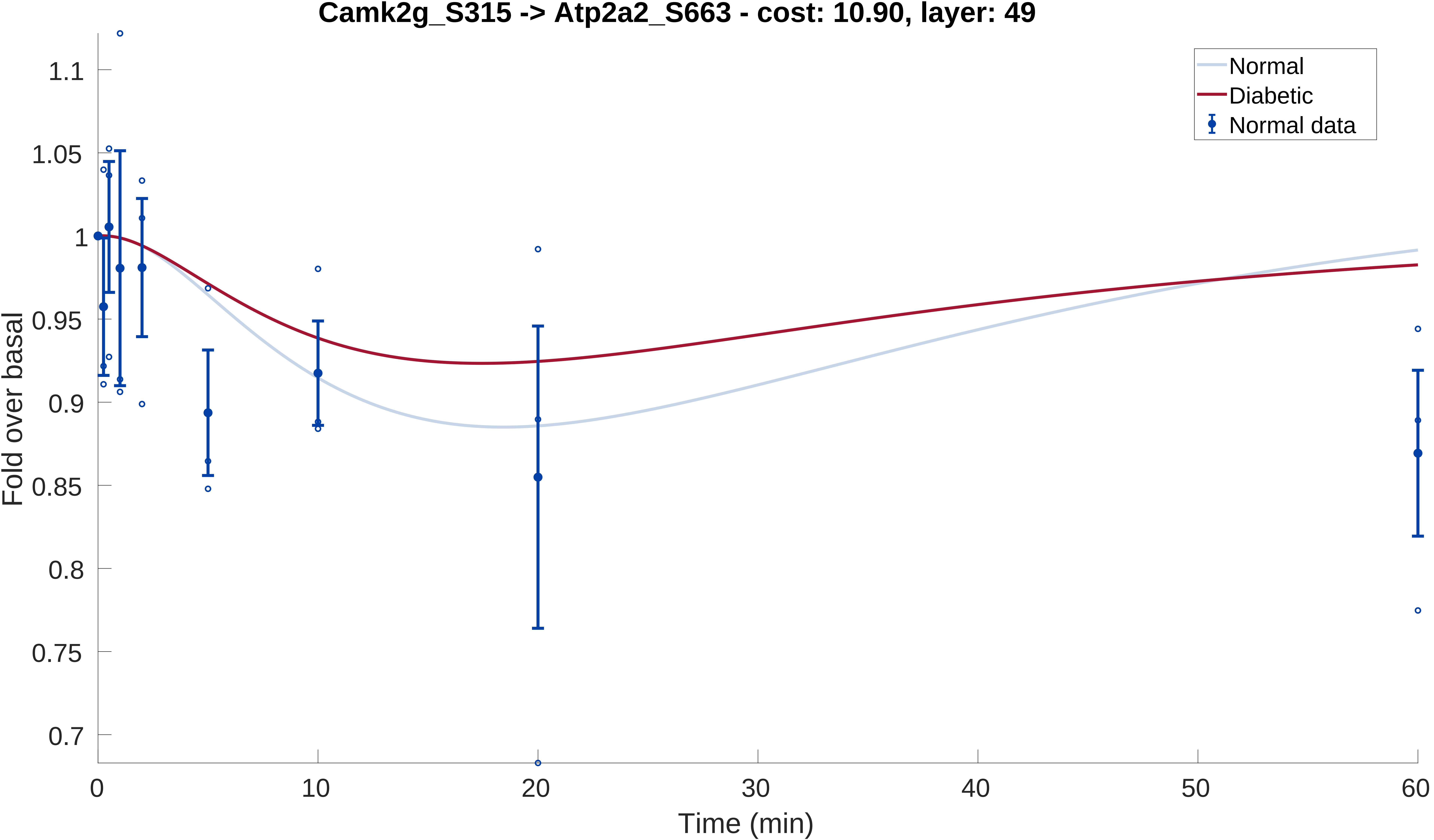
Camk2g_S315 -> Atp2a2_S663 - cost: 10.90, layer: 49
Normal
Diabetic
Normal data
Fold over basal
Time (min)
1.1
1.05
1
0.95
0.9
0.85
0.8
0.75
0.7
0
10
20
30
40
50
60

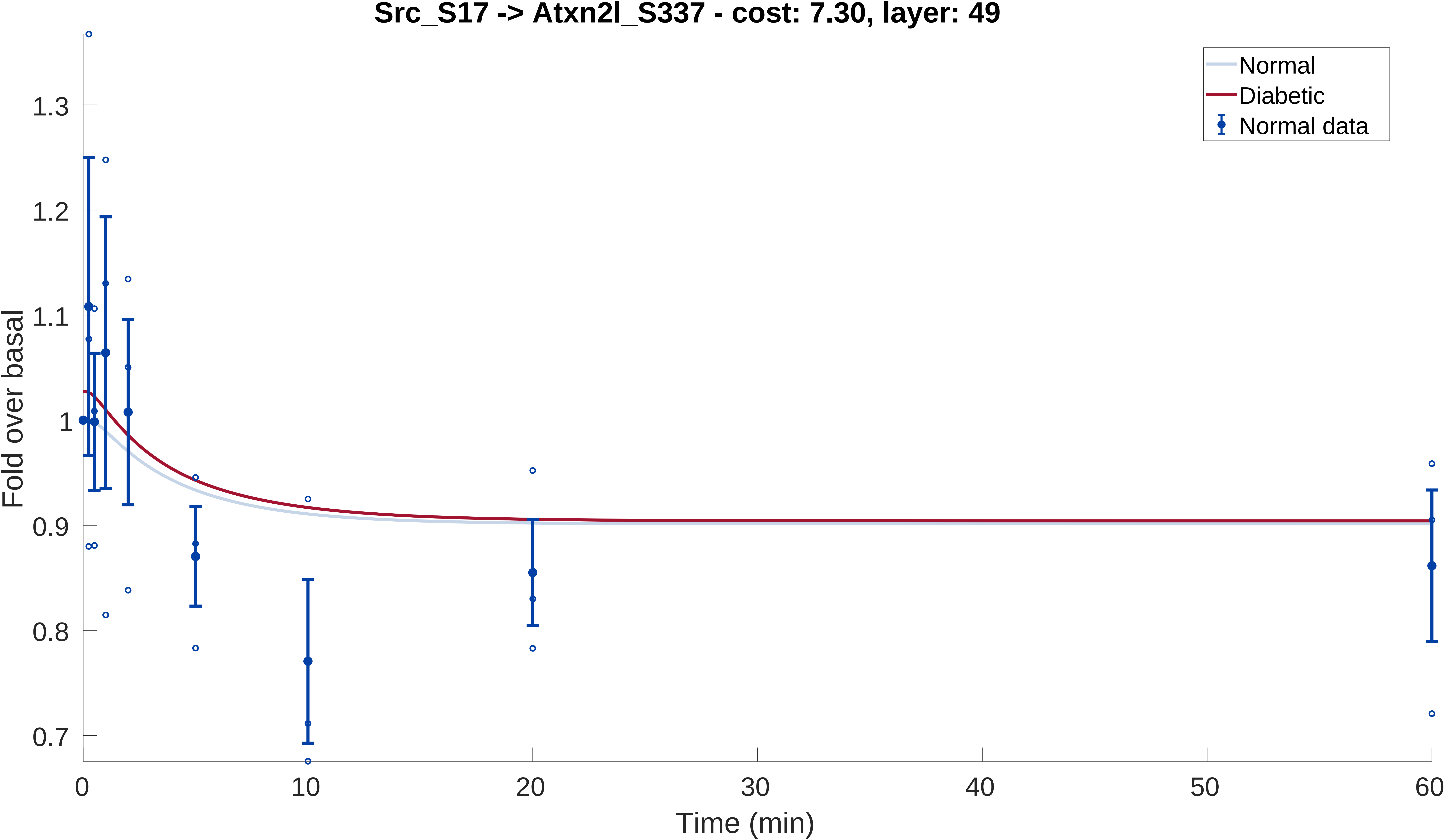

Src_S17 -> Atxn2l_S337 - cost: 7.30, layer: 49
Normal
Diabetic
Normal data
Fold over basal
1.3
1.2
1.1
1
0.9
0.8
0.7
0
10
20
30
40
50
60
Time (min)

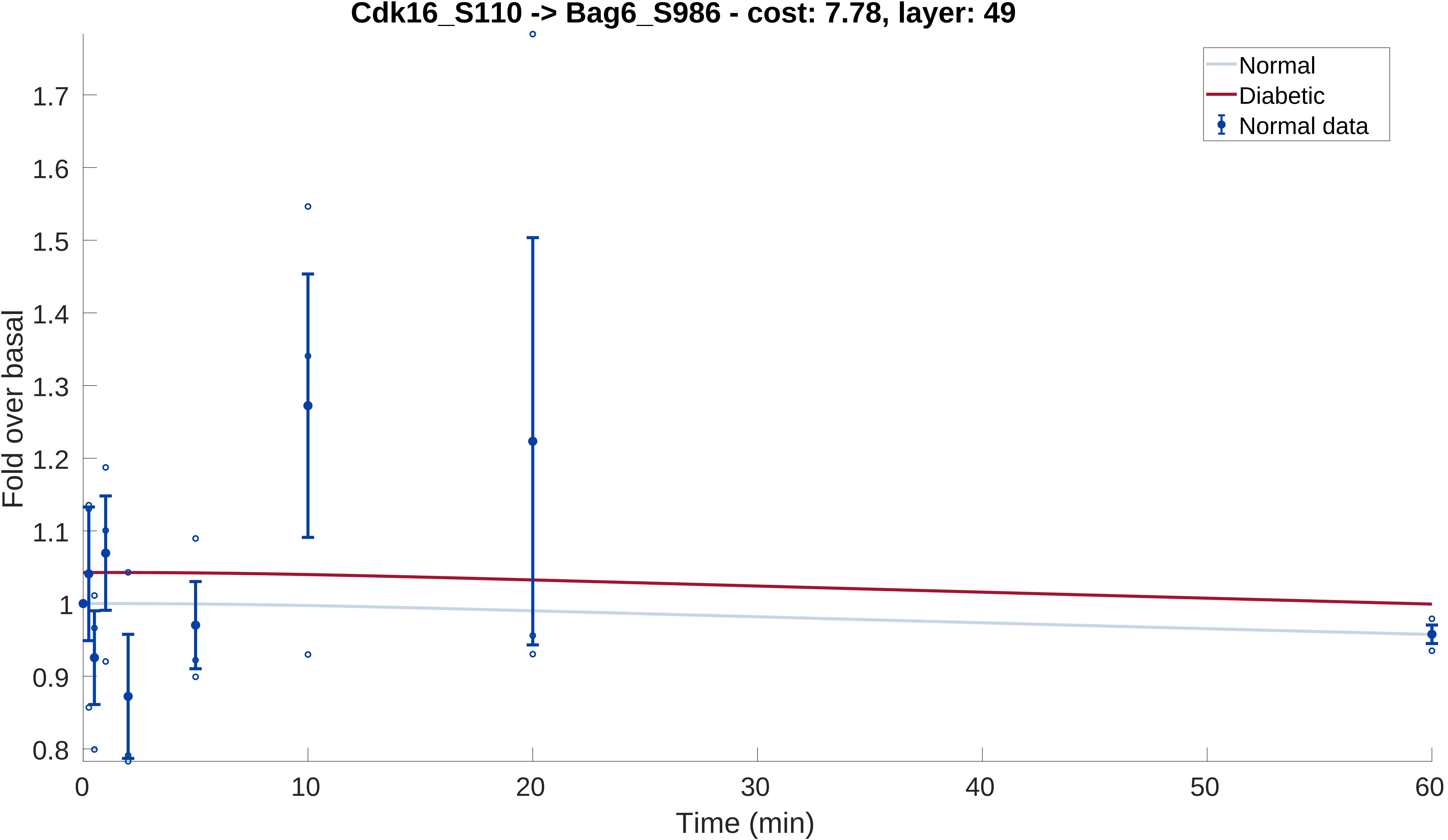

Cdk16_S110 -> Bag6_S986 - cost: 7.78, layer: 49
Normal
Diabetic
Normal data
Fold over basal
1.7
1.6
1.5
1.4
1.3
1.2
1.1
1
0.9
0.8
0
10
20
30
40
50
60
Time (min)

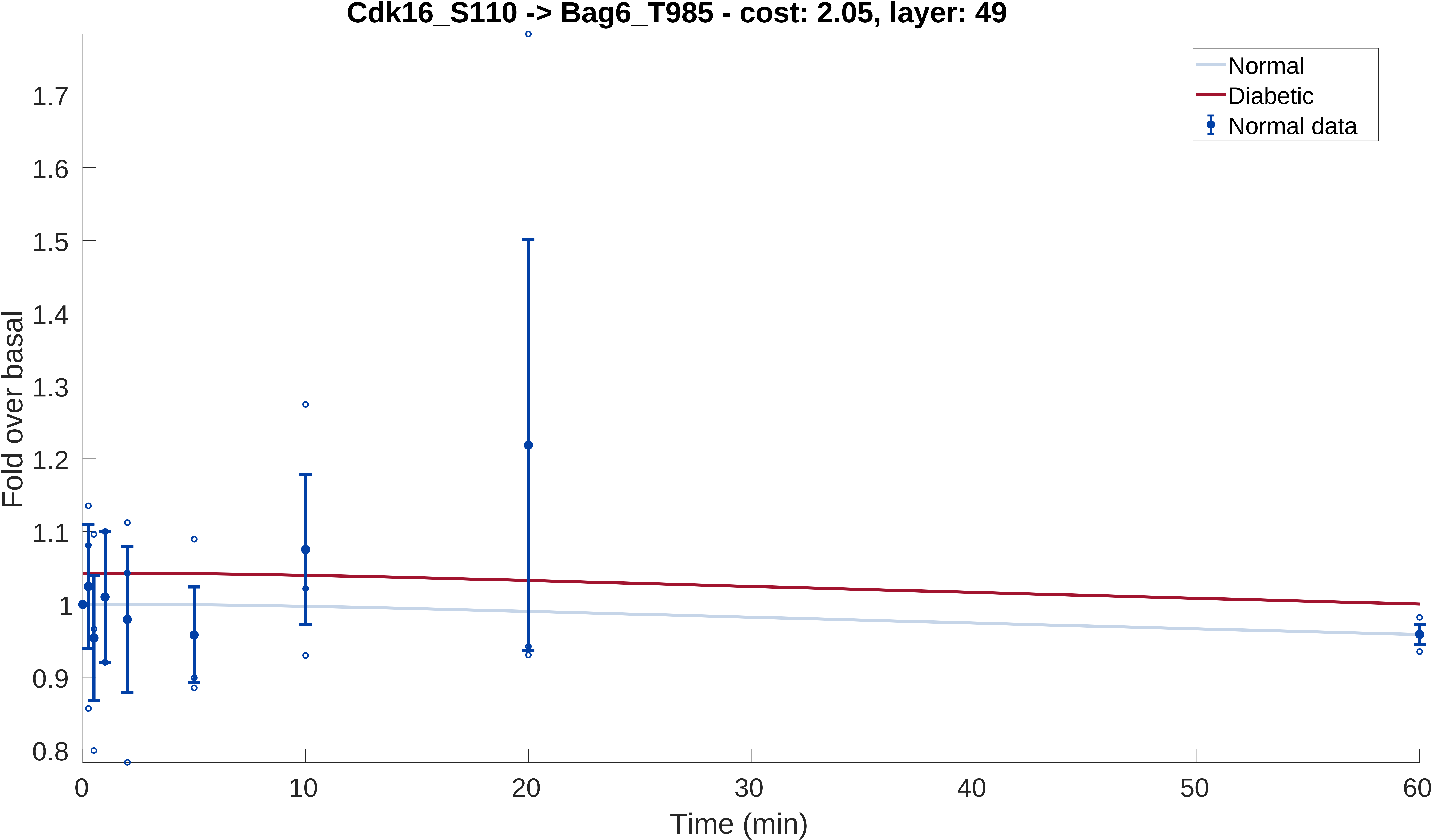
Cdk16_S110 -> Bag6_T985 - cost: 2.05, layer: 49
Normal
Diabetic
Normal data
Fold over basal
1.7
1.6
1.5
1.4
1.3
1.2
1.1
1
0.9
0.8
0
10
20
30
40
50
60
Time (min)

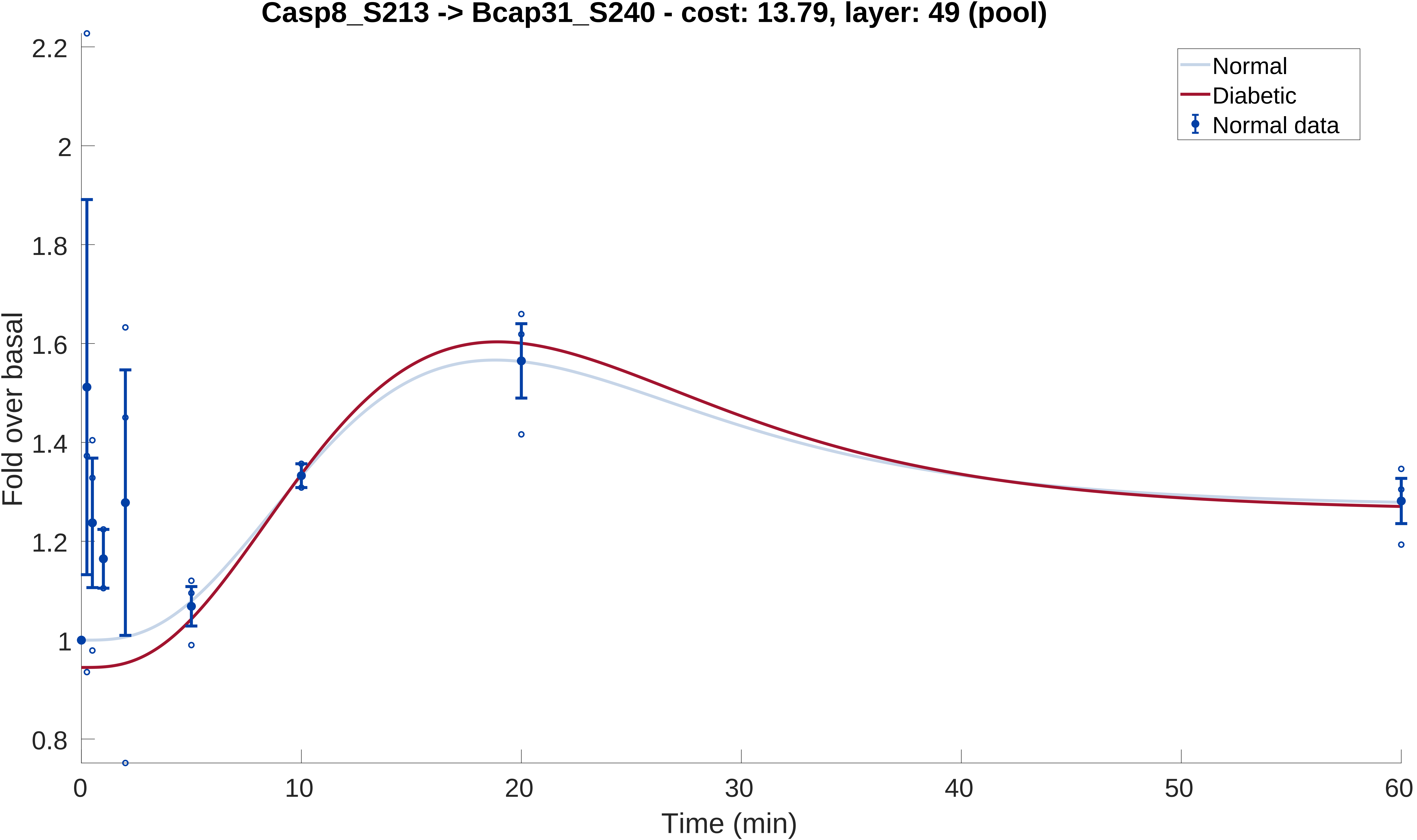

Casp8_S213 -> Bcap31_S240 - cost: 13.79, layer: 49 (pool)
Normal
Diabetic
Normal data
Fold over basal
Time (min)
2.2
2
1.8
1.6
1.4
1.2
1
0.8
0
10
20
30
40
50
60

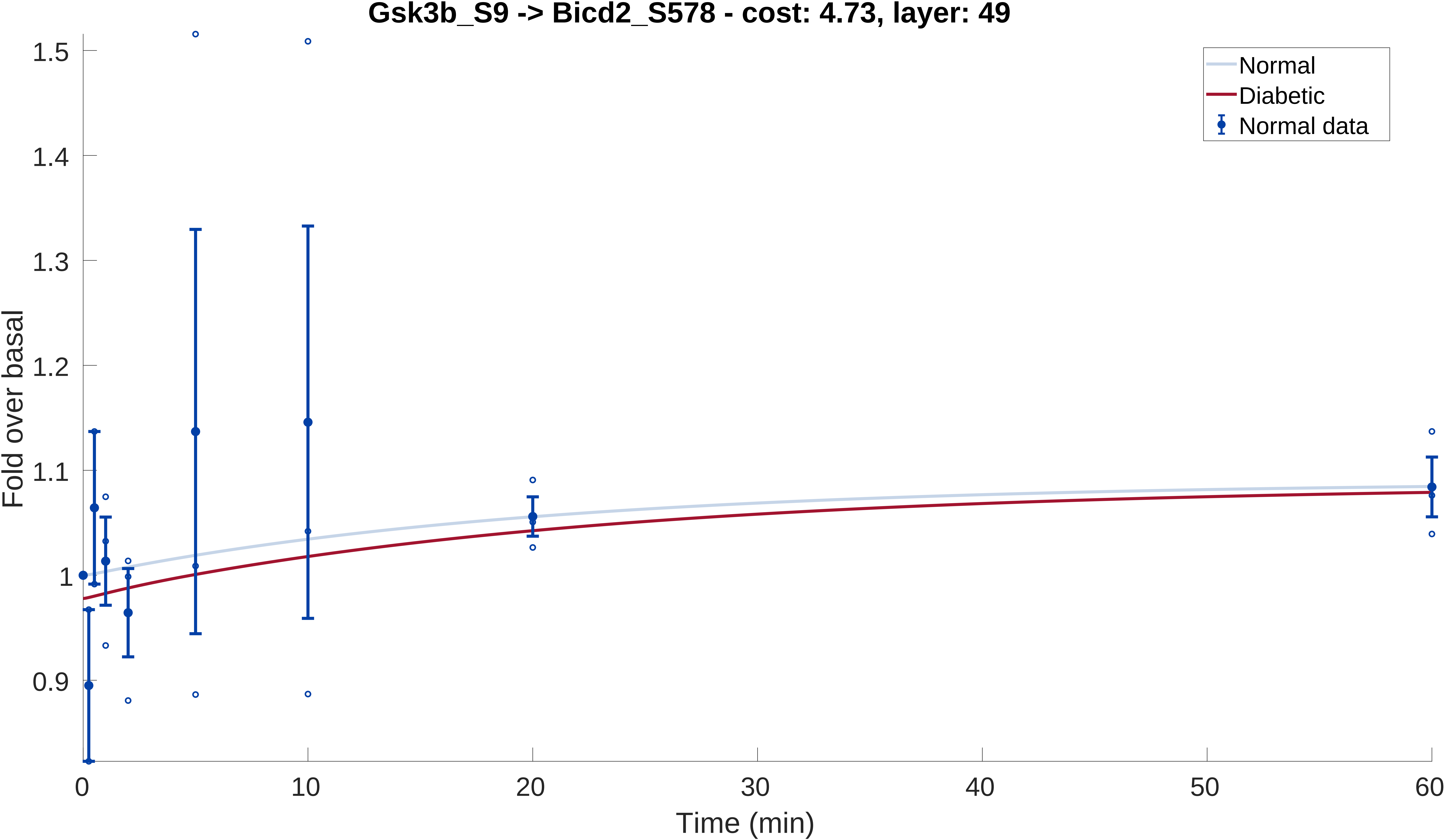
Gsk3b_S9 -> Bicd2_S578 - cost: 4.73, layer: 49
Normal
Diabetic
Normal data
Fold over basal
Time (min)
0
10
20
30
40
50
60
0.9
1
1.1
1.2
1.3
1.4
1.5

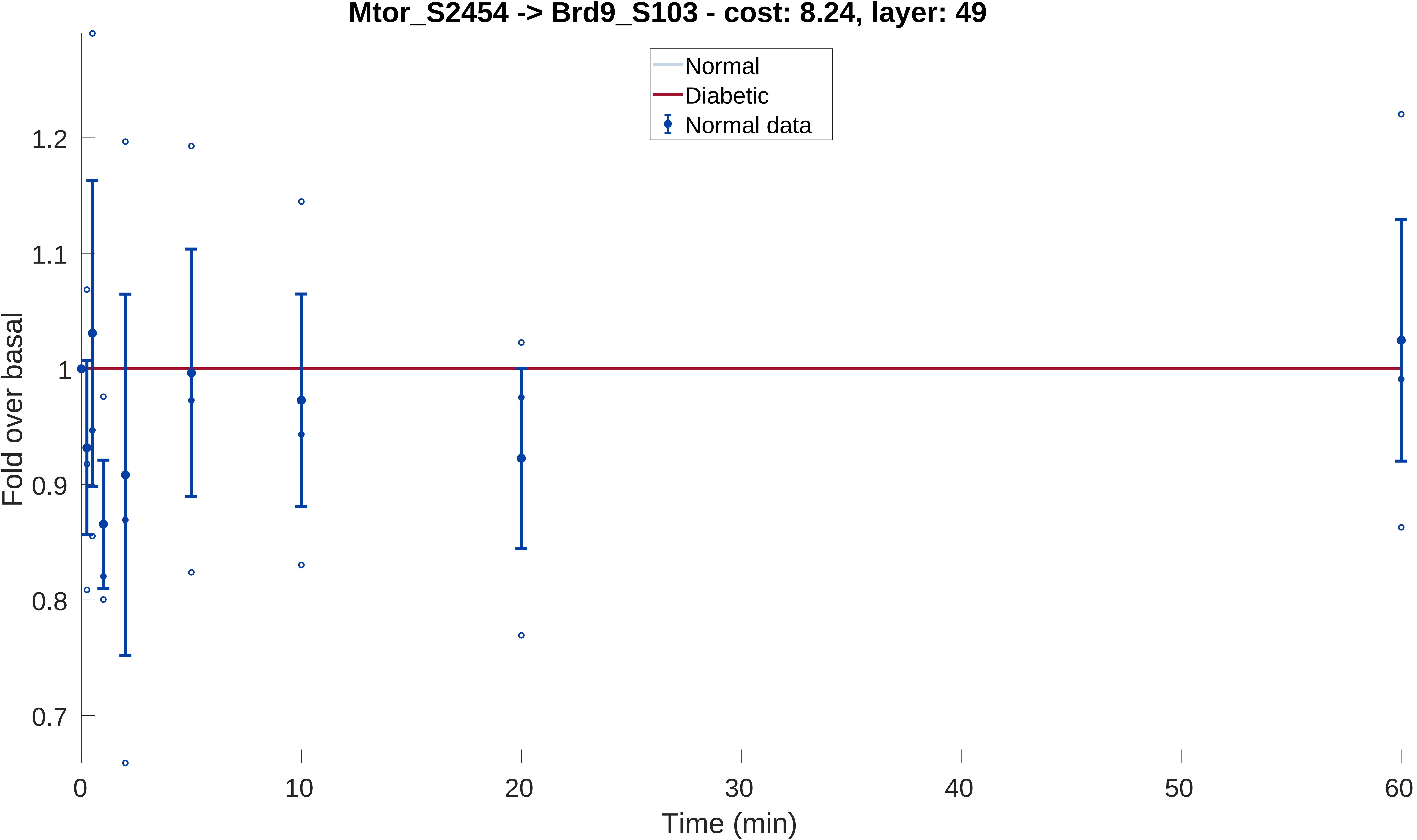
Mtor_S2454 -> Brd9_S103 - cost: 8.24, layer: 49
Normal
Diabetic
Normal data
Fold over basal
Time (min)
1.2
1.1
1
0.9
0.8
0.7
0
10
20
30
40
50
60

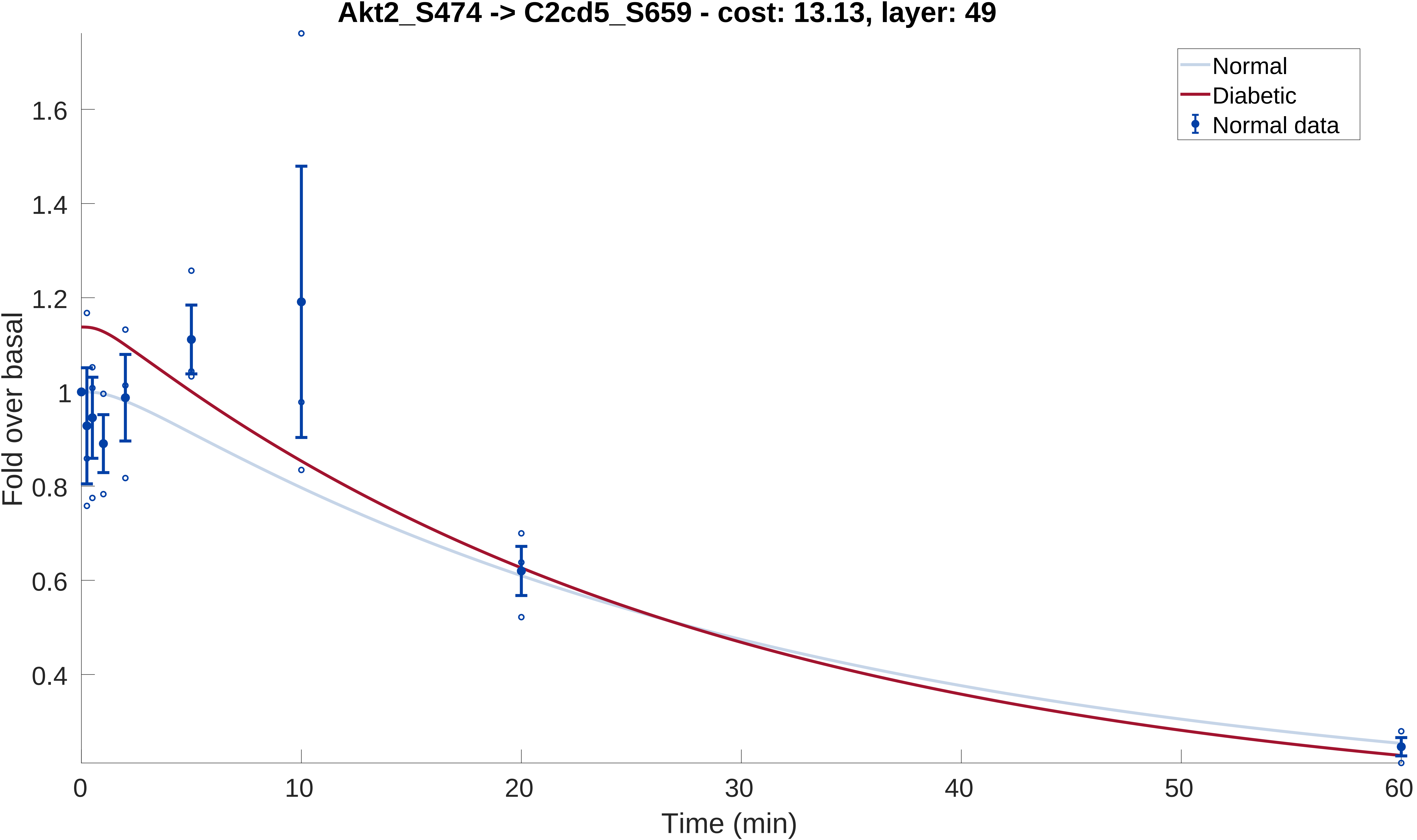
Akt2_S474 -> C2cd5_S659 - cost: 13.13, layer: 49
Normal
Diabetic
Normal data
1.6
1.4
1.2
1
0.8
0.6
0.4
Fold over basal
0
10
20
30
40
50
60
Time (min)

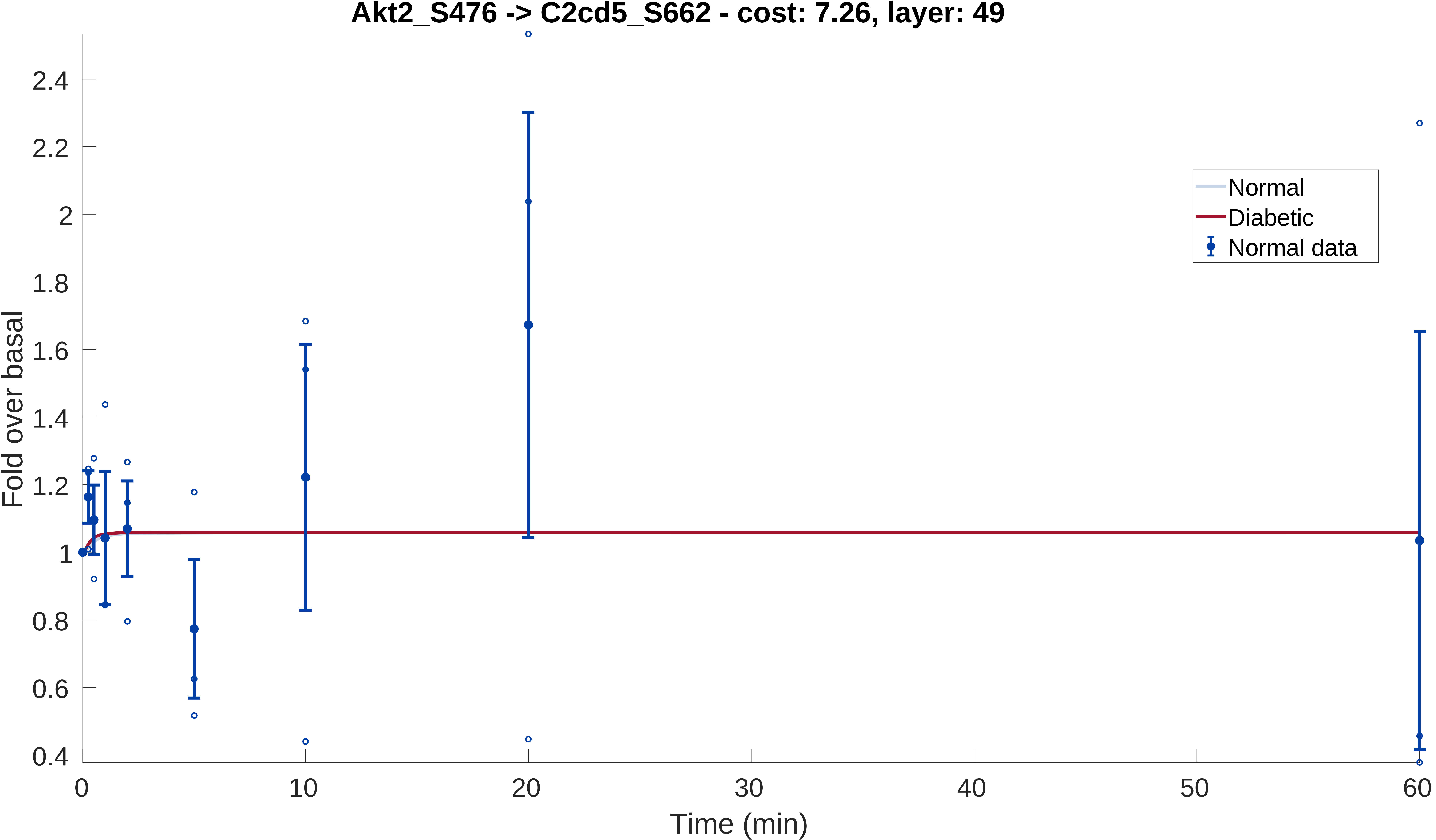

Akt2_S476 -> C2cd5_S662 - cost: 7.26, layer: 49
Fold over basal
2.4
2.2
2
1.8
1.6
1.4
1.2
1
0.8
0.6
0.4
0
10
20
30
40
50
60
Time (min)
Normal
Diabetic
Normal data

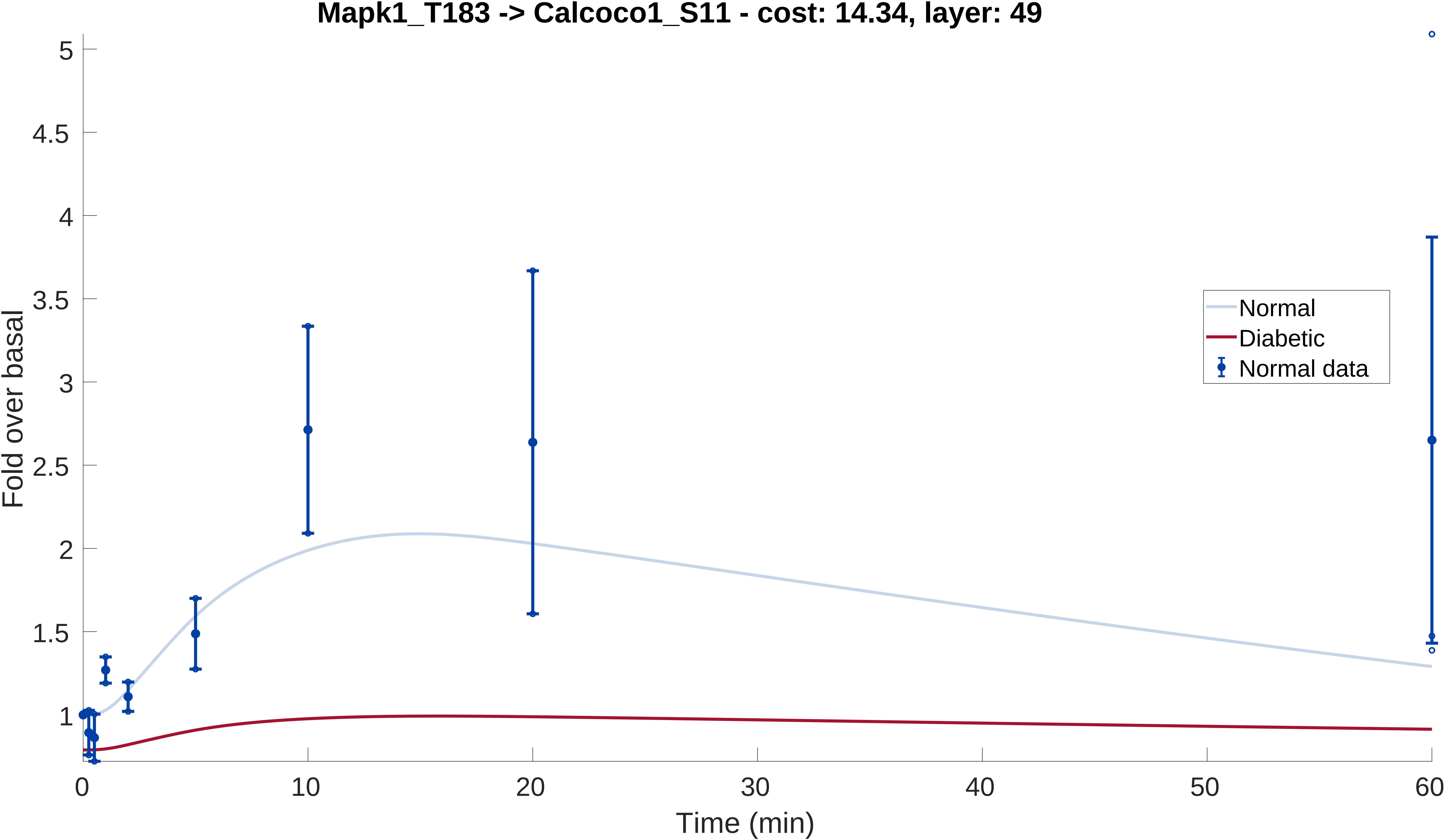
Mapk1_T183 -> Calcoco1_S11 - cost: 14.34, layer: 49
Fold over basal
Time (min)
Normal
Diabetic
Normal data
5
4.5
4
3.5
3
2.5
2
1.5
1
0
10
20
30
40
50
60

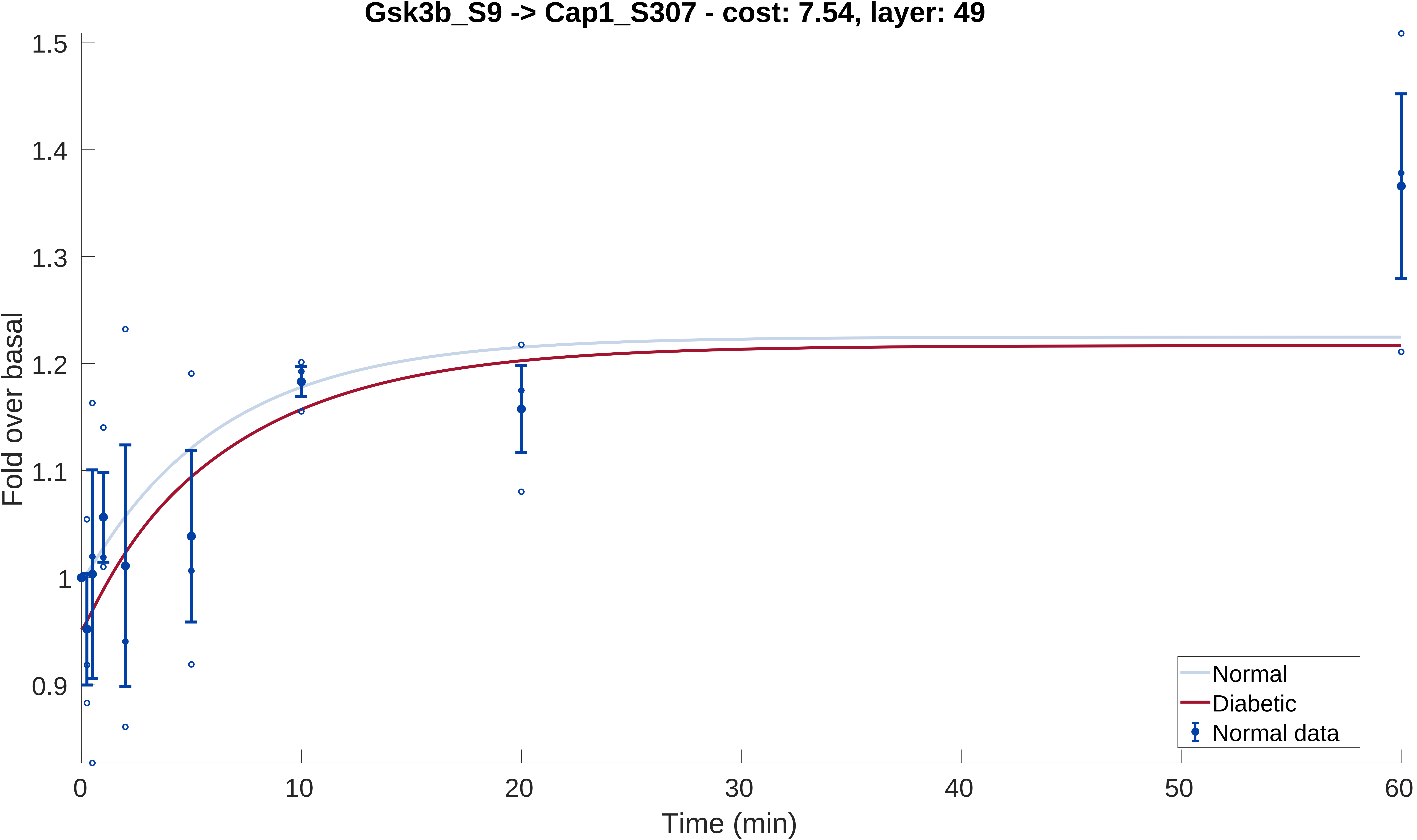
Gsk3b_S9 -> Cap1_S307 - cost: 7.54, layer: 49
Fold over basal
1.5
1.4
1.3
1.2
1.1
1
0.9
0
10
20
30
40
50
60
Time (min)
Normal
Diabetic
Normal data

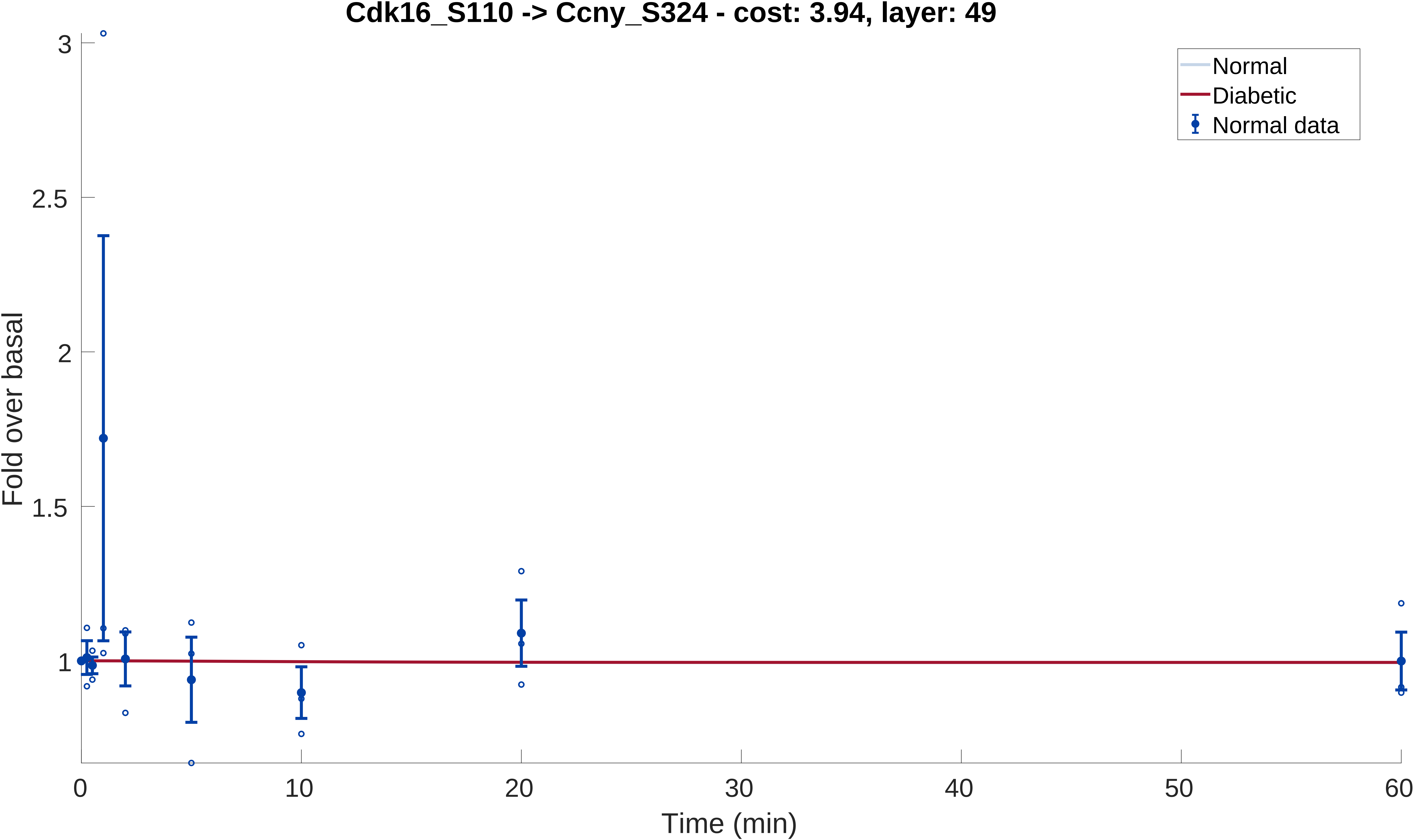

Cdk16_S110 -> Ccny_S324 - cost: 3.94, layer: 49
Normal
Diabetic
Normal data
Fold over basal
Time (min)
3
2.5
2
1.5
1
0
10
20
30
40
50
60

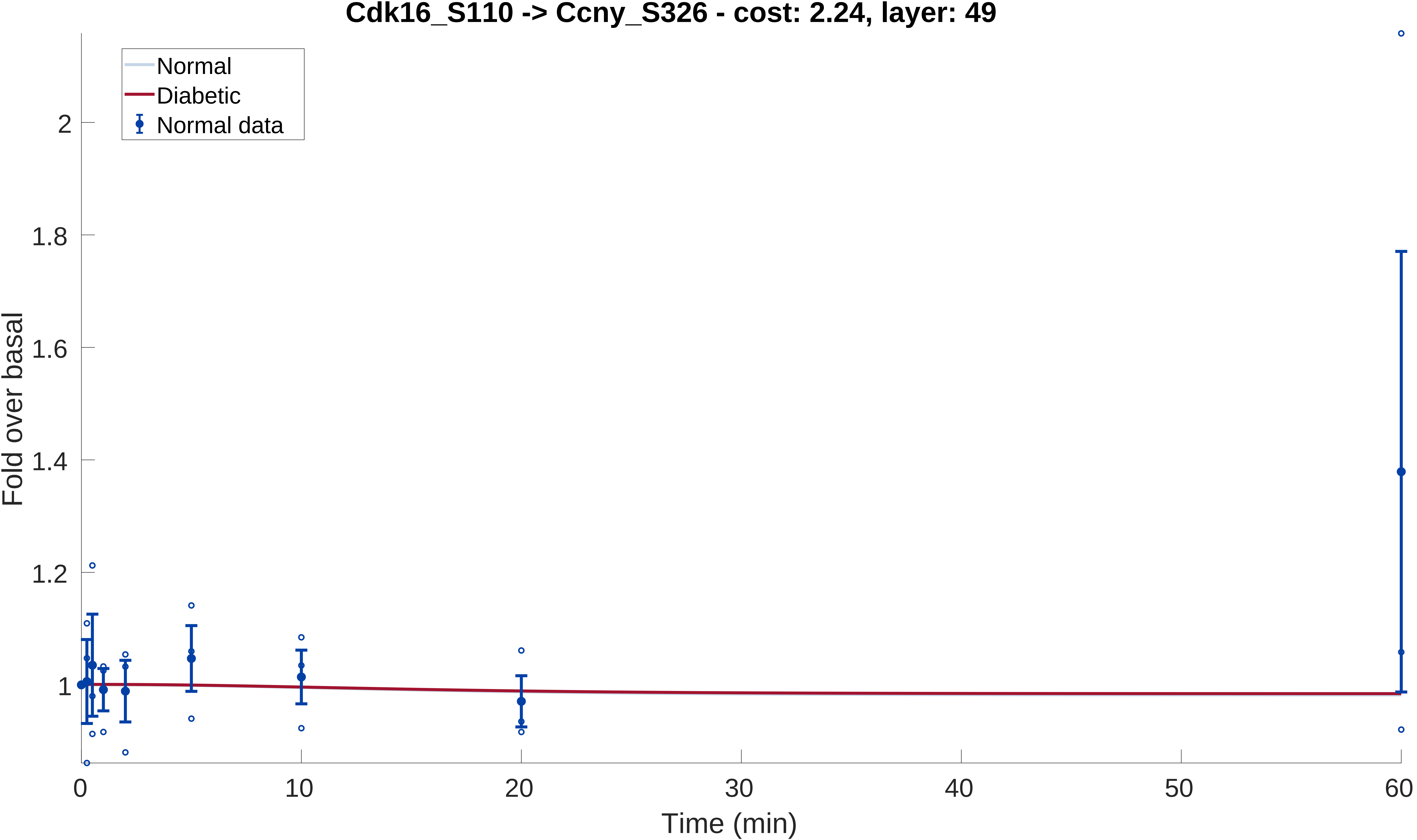

Cdk16_S110 -> Ccny_S326 - cost: 2.24, layer: 49
Normal
Diabetic
Normal data
Fold over basal
2
1.8
1.6
1.4
1.2
1
0
10
20
30
40
50
60
Time (min)

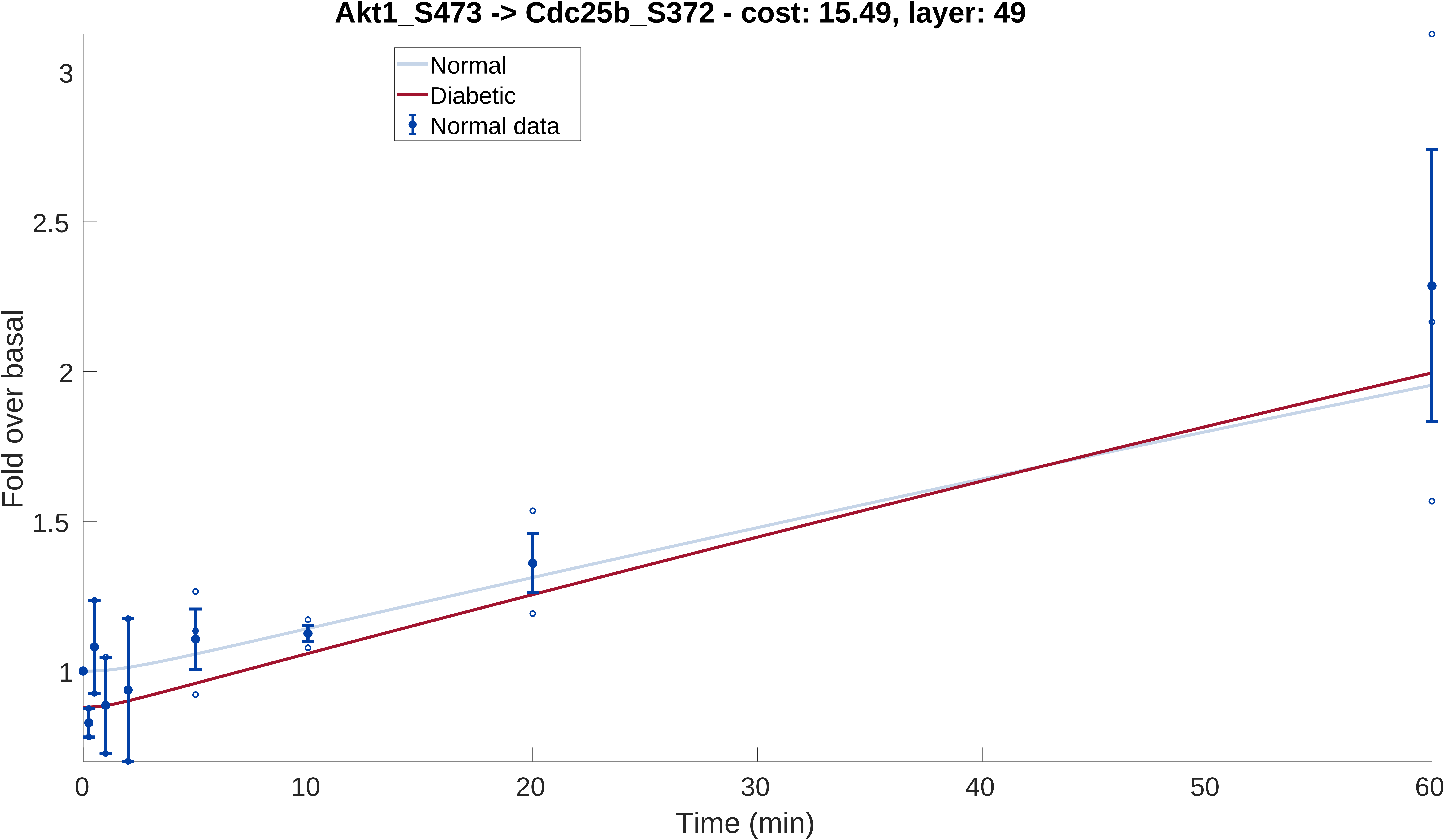

Akt1_S473 -> Cdc25b_S372 - cost: 15.49, layer: 49
Normal
Diabetic
Normal data
Fold over basal
3
2.5
2
1.5
1
0
10
20
30
40
50
60
Time (min)

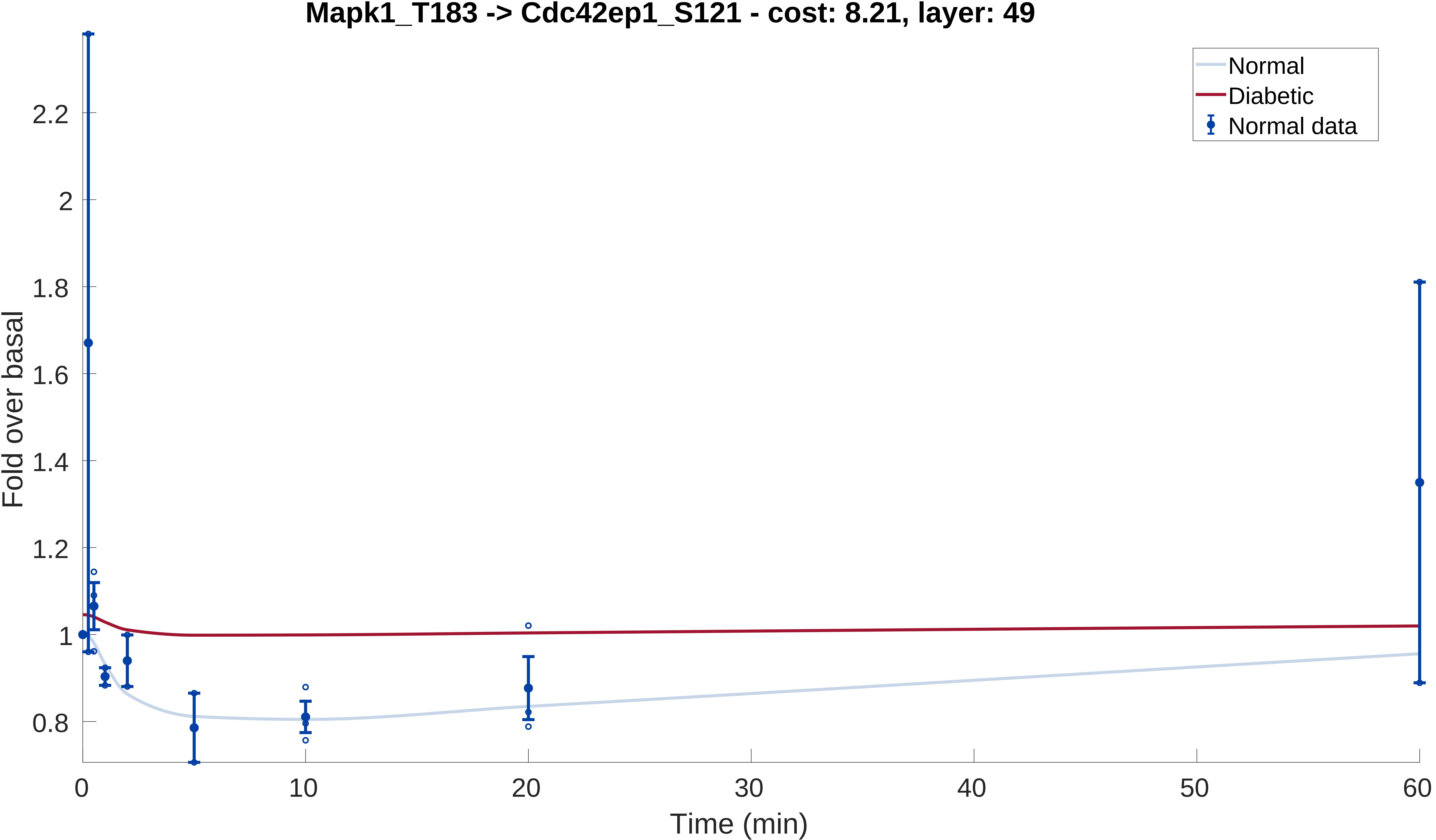

Mapk1_T183 -> Cdc42ep1_S121 - cost: 8.21, layer: 49
Normal
Diabetic
Normal data
2.2
2
1.8
1.6
1.4
1.2
1
0.8
Fold over basal
0
10
20
30
40
50
60
Time (min)

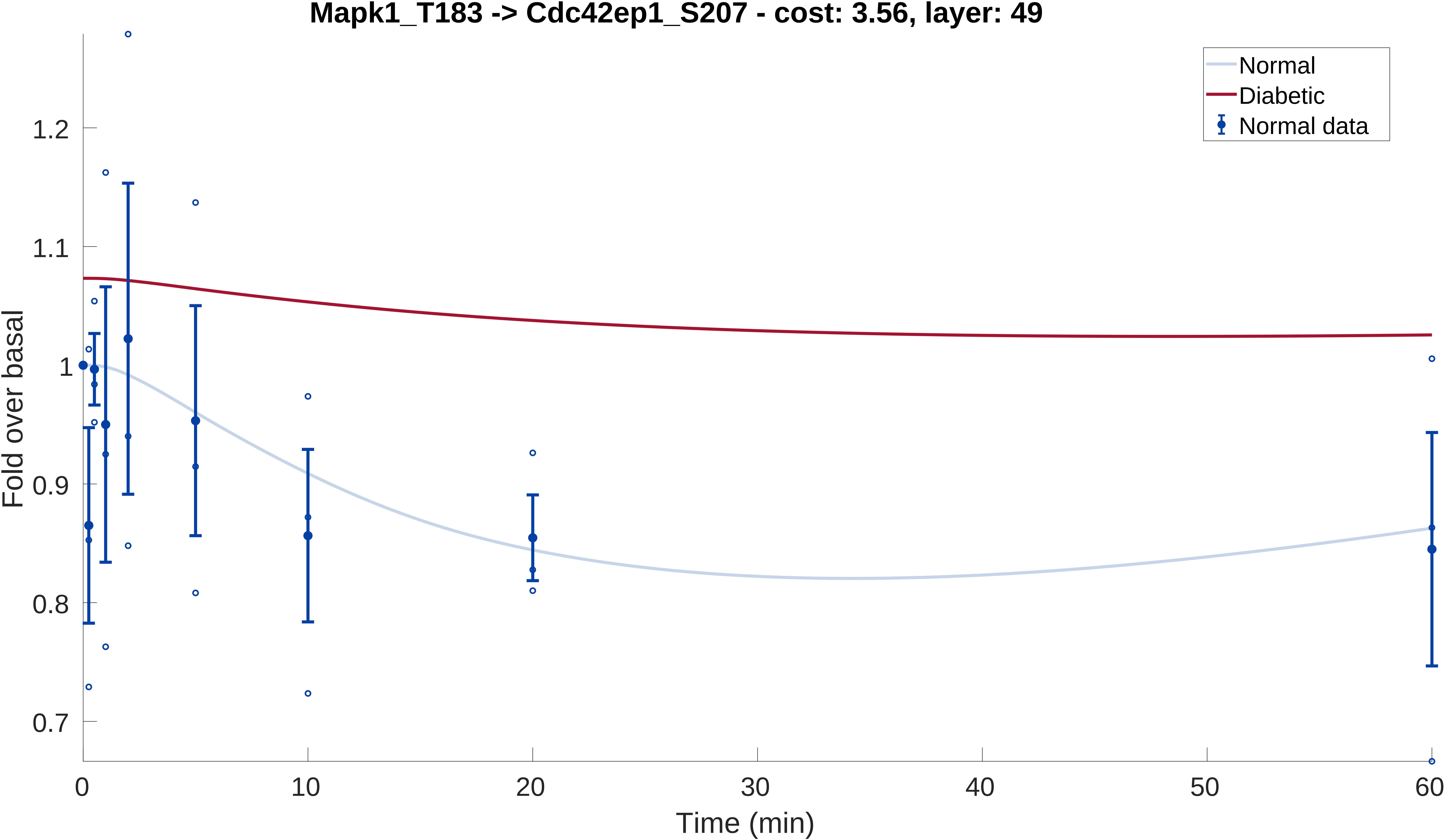

Mapk1_T183 -> Cdc42ep1_S207 - cost: 3.56, layer: 49
Normal
Diabetic
Normal data
Fold over basal
1.2
1.1
1
0.9
0.8
0.7
0
10
20
30
40
50
60
Time (min)

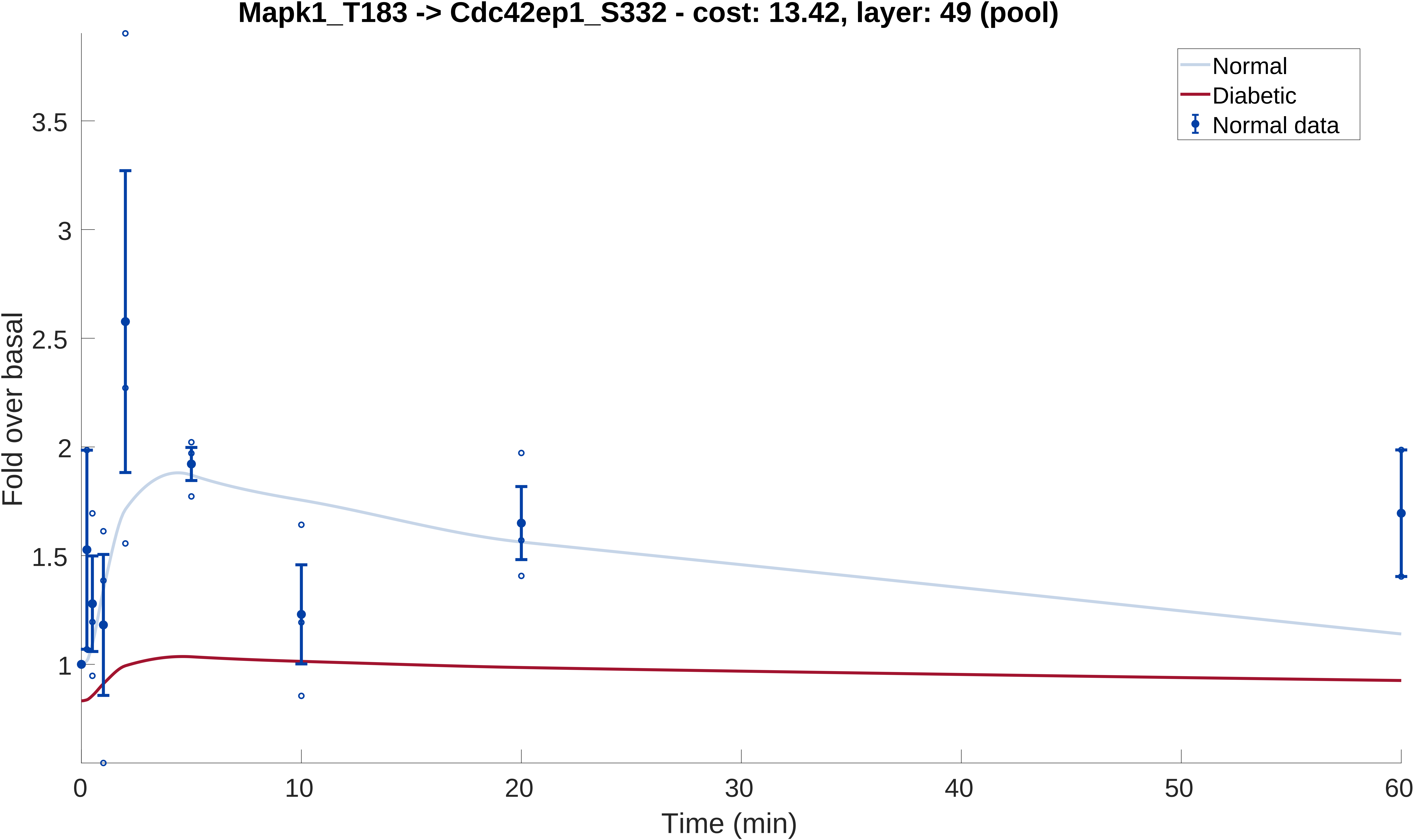
Mapk1_T183 -> Cdc42ep1_S332 - cost: 13.42, layer: 49 (pool)
Normal
Diabetic
Normal data
Fold over basal
3.5
3
2.5
2
1.5
1
0
10
20
30
40
50
60
Time (min)

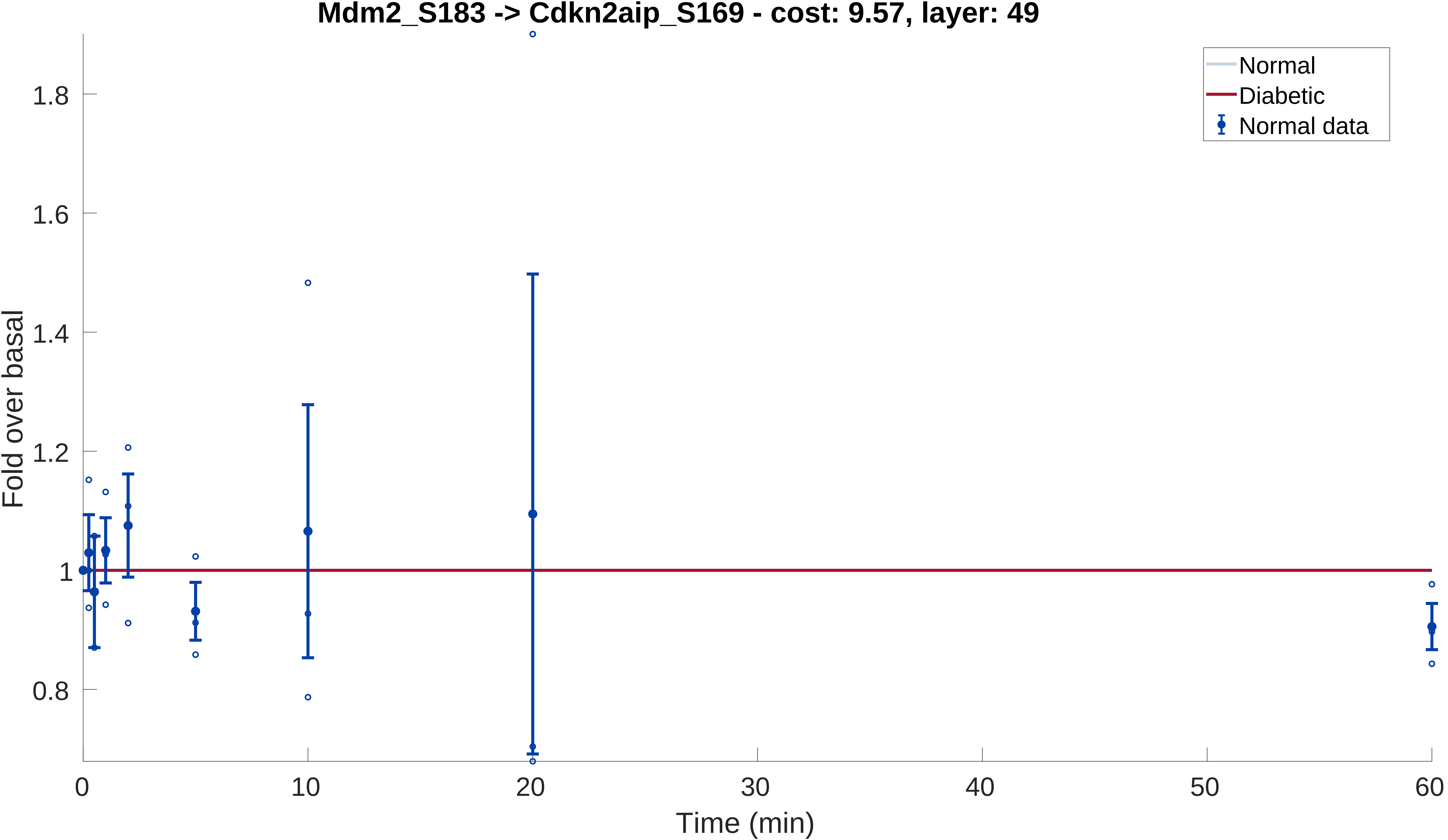

Mdm2_S183 -> Cdkn2aip_S169 - cost: 9.57, layer: 49
Normal
Diabetic
Normal data
Fold over basal
1.8
1.6
1.4
1.2
1
0.8
0
10
20
30
40
50
60
Time (min)

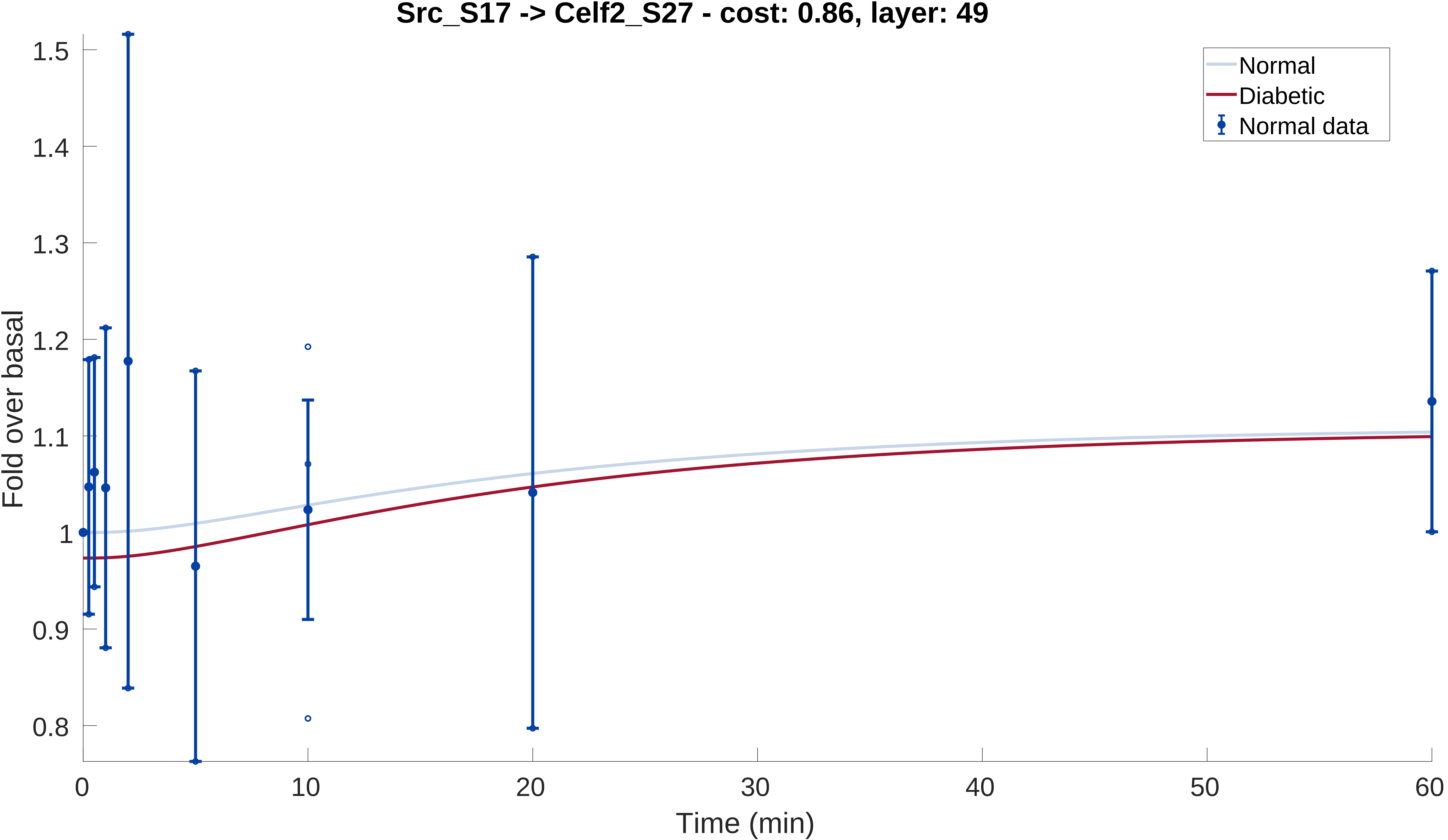

Src_S17 -> Celf2_S27 - cost: 0.86, layer: 49
Normal
Diabetic
Normal data
Fold over basal
1.5
1.4
1.3
1.2
1.1
1
0.9
0.8
0
10
20
30
40
50
60
Time (min)

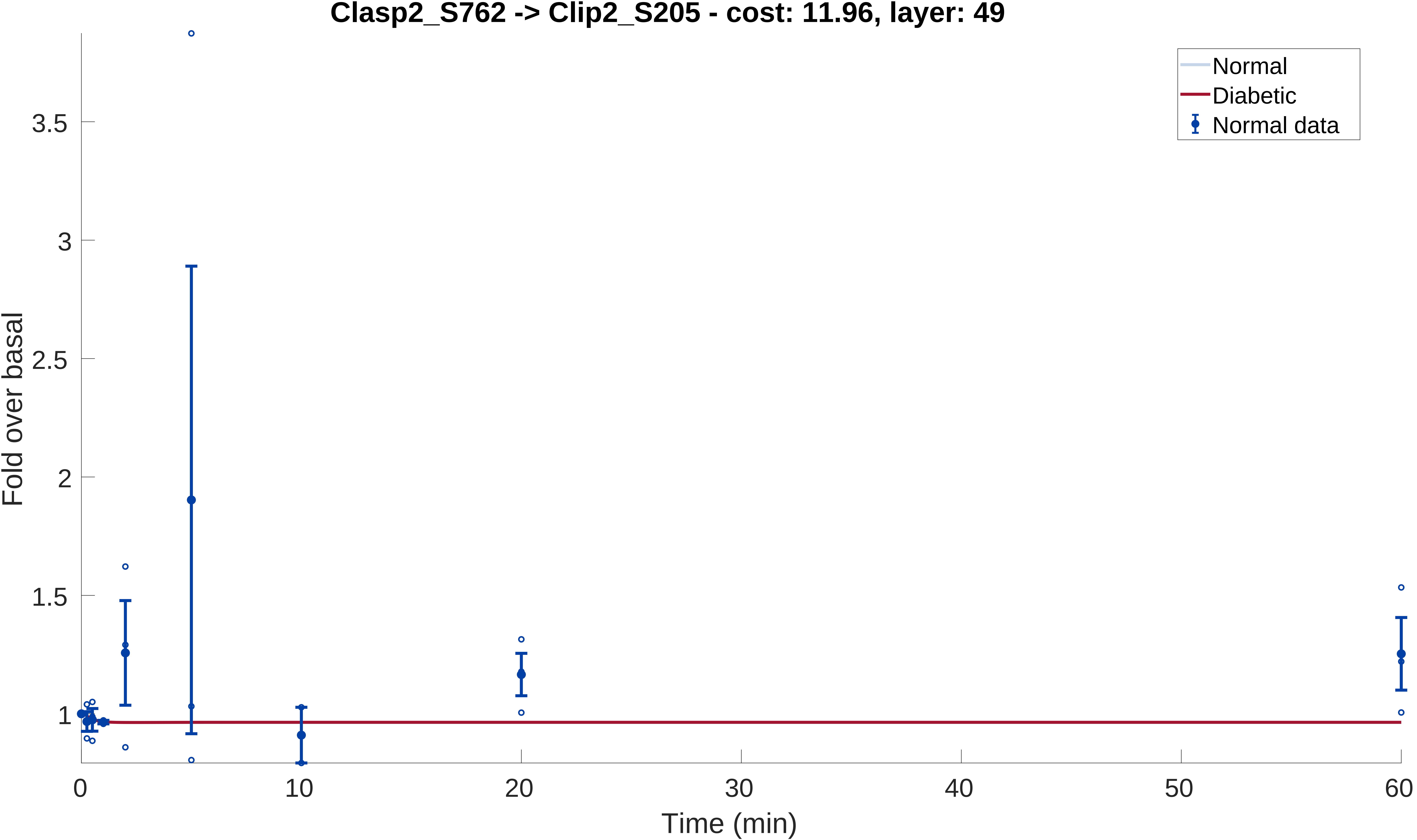
Clasp2_S762 -> Clip2_S205 - cost: 11.96, layer: 49
Normal
Diabetic
Normal data
3.5
3
2.5
2
1.5
1
Fold over basal
0
10
20
30
40
50
60
Time (min)

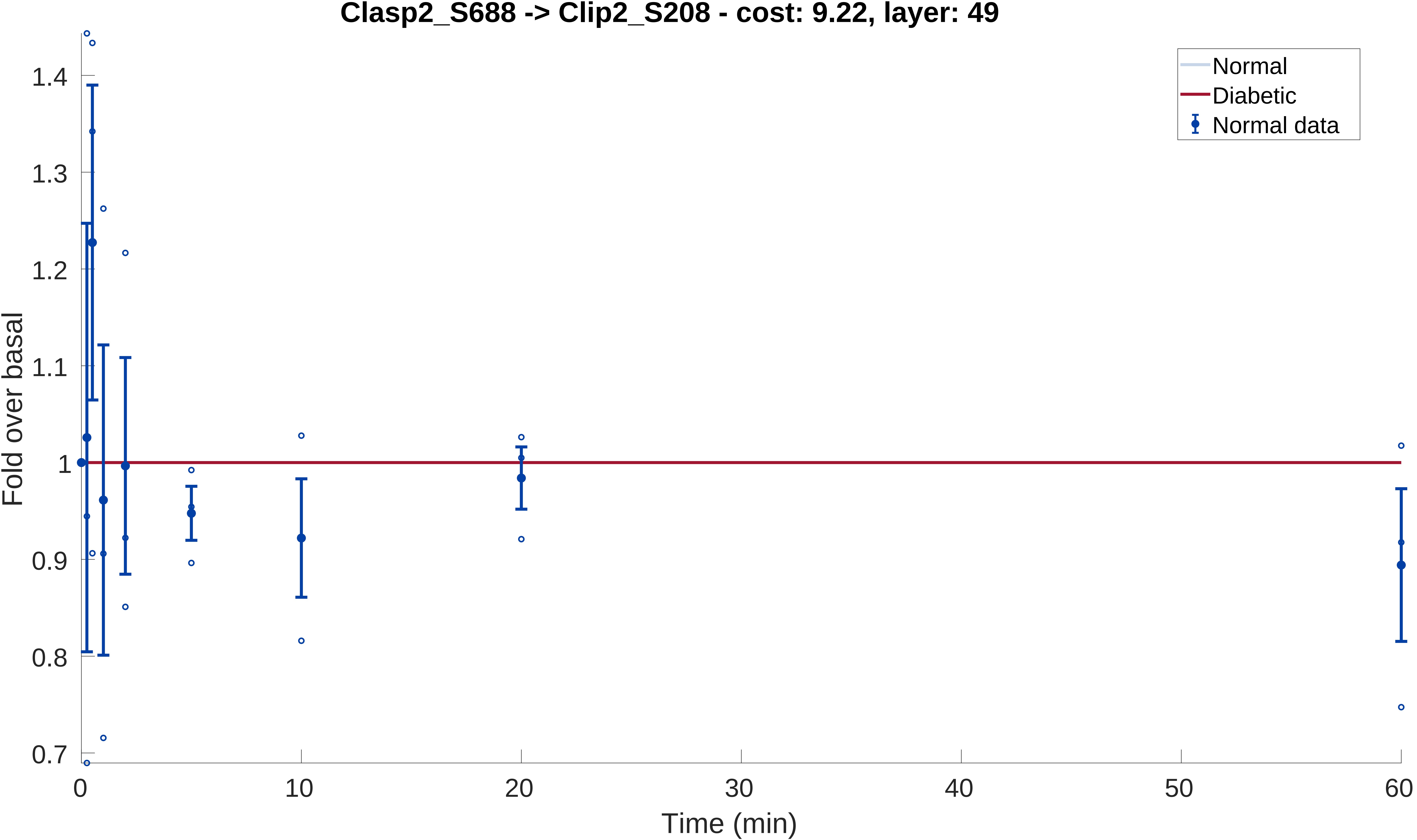

Clasp2_S688 -> Clip2_S208 - cost: 9.22, layer: 49
Normal
Diabetic
Normal data
Fold over basal
Time (min)
1.4
1.3
1.2
1.1
1
0.9
0.8
0.7
0
10
20
30
40
50
60

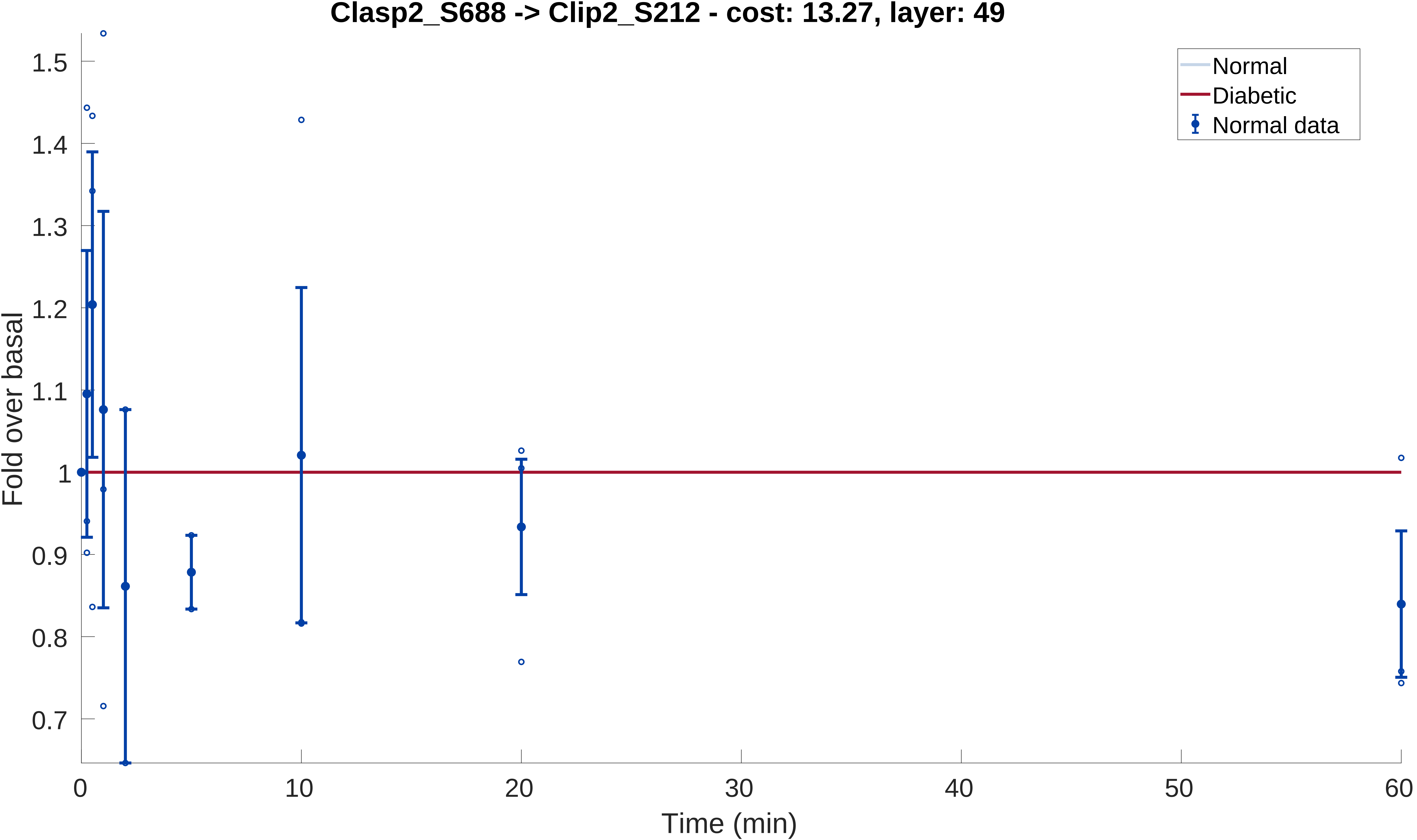
Clasp2_S688 -> Clip2_S212 - cost: 13.27, layer: 49
Normal
Diabetic
Normal data
Fold over basal
1.5
1.4
1.3
1.2
1.1
1
0.9
0.8
0.7
0
10
20
30
40
50
60
Time (min)

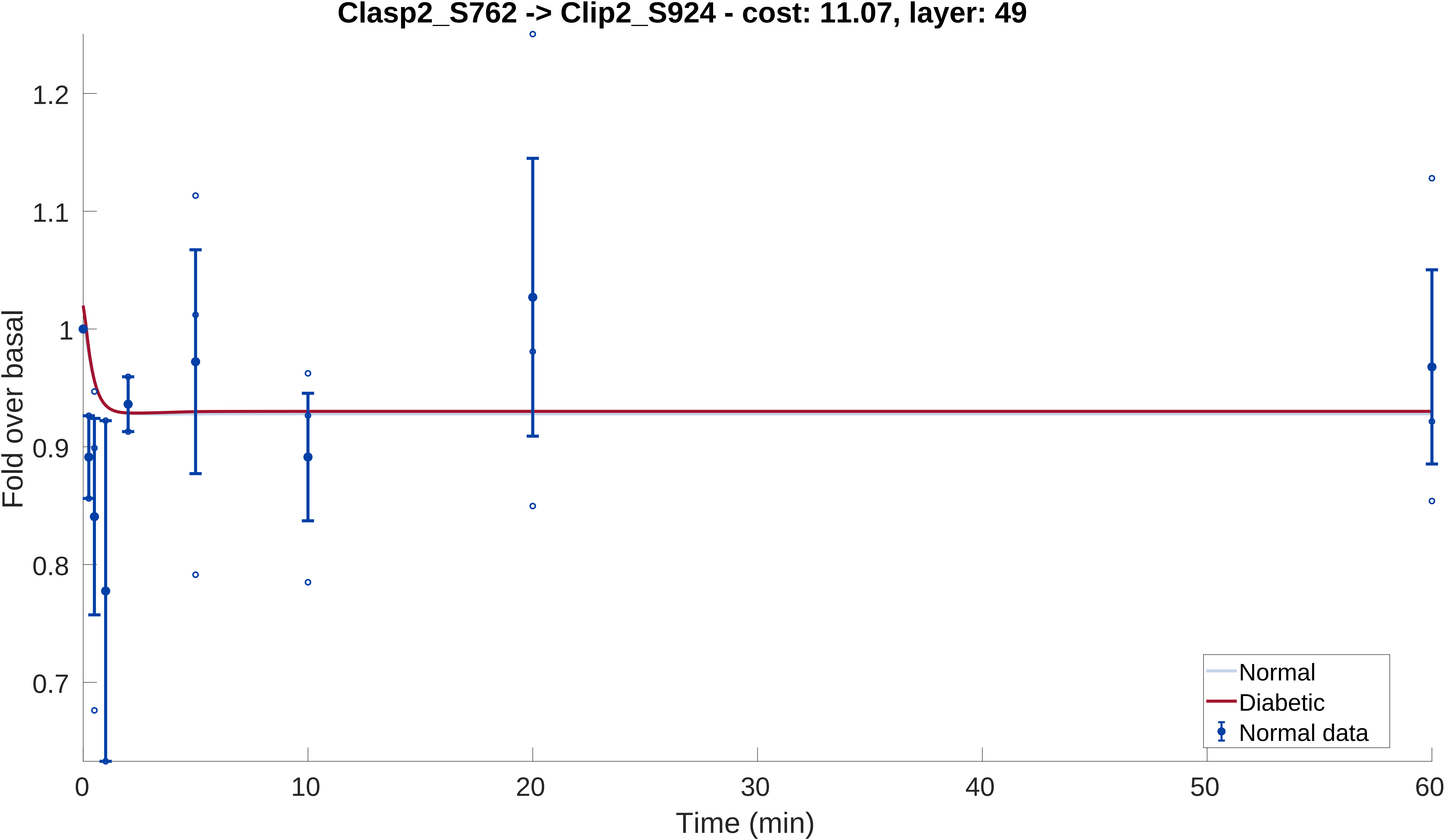

Clasp2_S762 -> Clip2_S924 - cost: 11.07, layer: 49
Fold over basal
1.2
1.1
1
0.9
0.8
0.7
0
10
20
30
40
50
60
Time (min)
Normal
Diabetic
Normal data

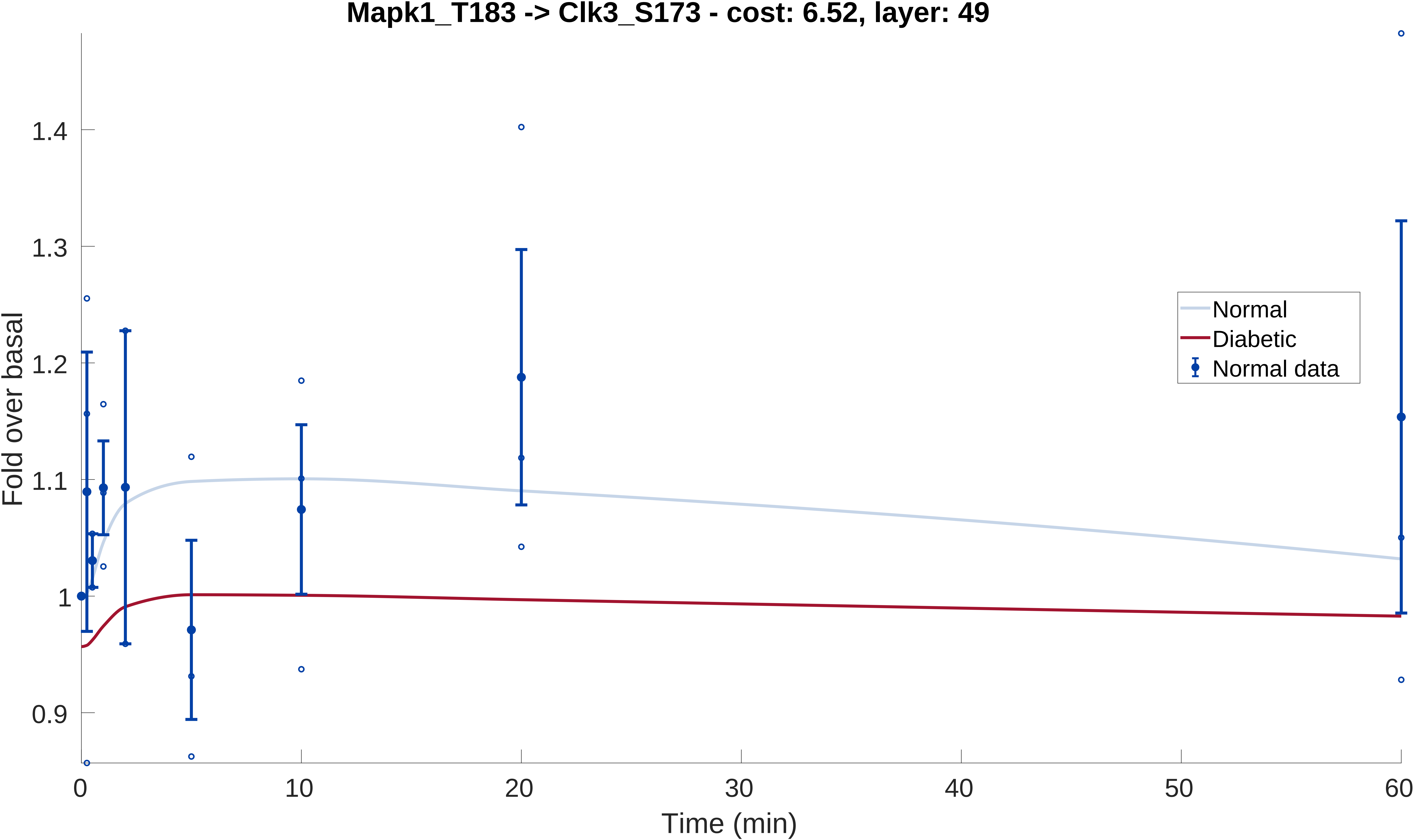

Mapk1_T183 -> Clk3_S173 - cost: 6.52, layer: 49
Fold over basal
1.4
1.3
1.2
1.1
1
0.9
0
10
20
30
40
50
60
Time (min)
Normal
Diabetic
Normal data

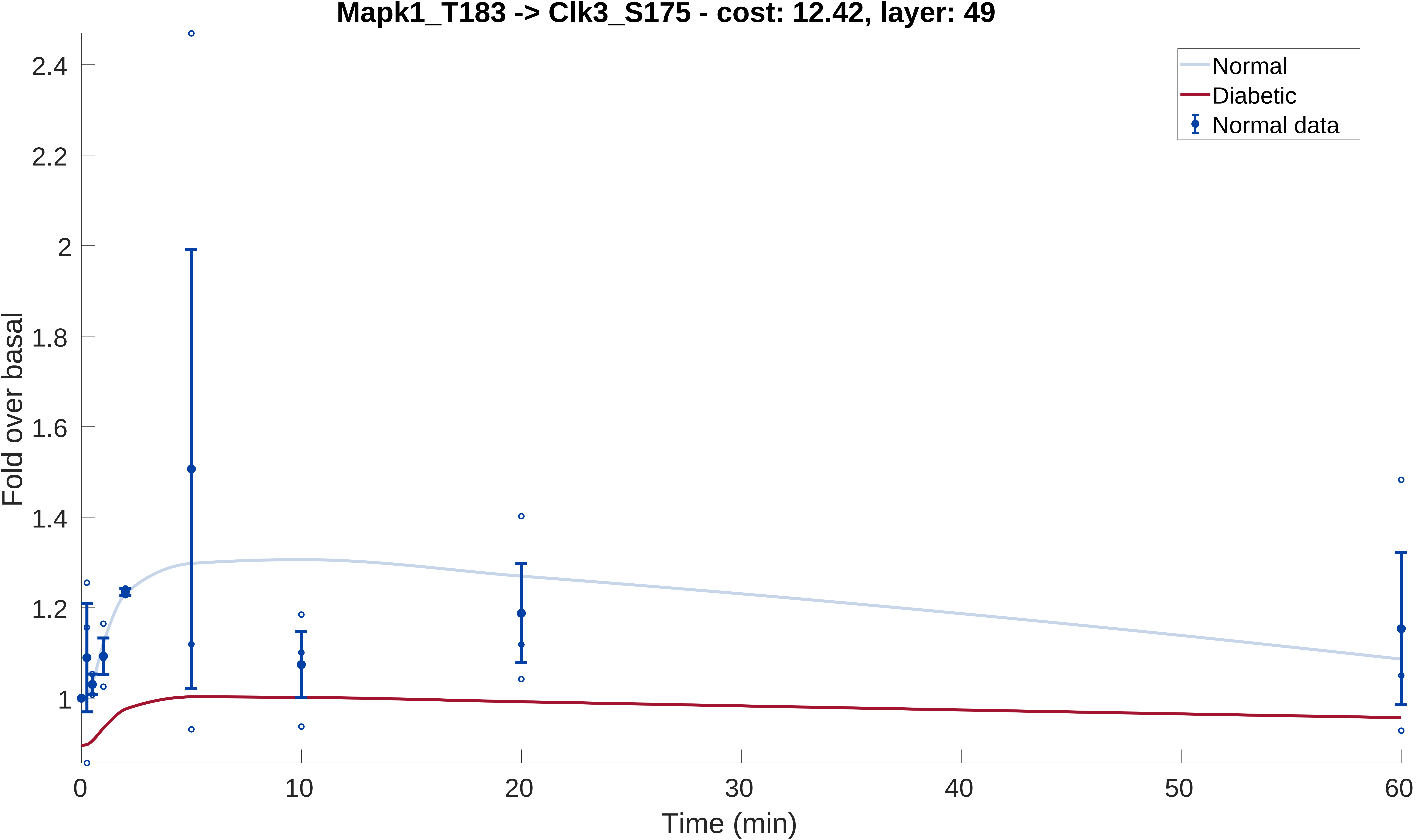

Mapk1_T183 -> Clk3_S175 - cost: 12.42, layer: 49
Normal
Diabetic
Normal data
Fold over basal
2.4
2.2
2
1.8
1.6
1.4
1.2
1
0
10
20
30
40
50
60
Time (min)

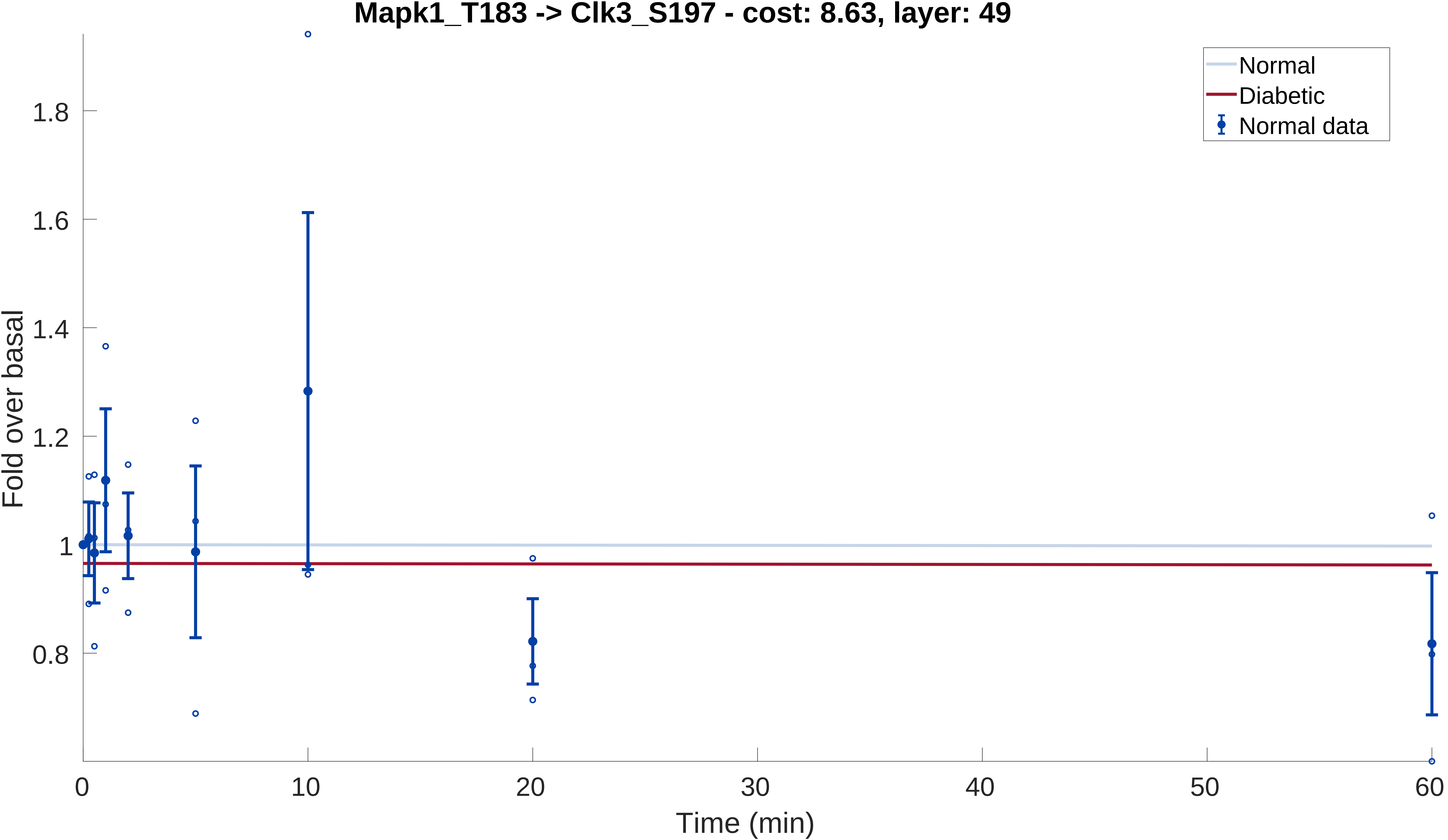

Mapk1_T183 -> Clk3_S197 - cost: 8.63, layer: 49
Normal
Diabetic
Normal data
Fold over basal
1.8
1.6
1.4
1.2
1
0.8
0
10
20
30
40
50
60
Time (min)

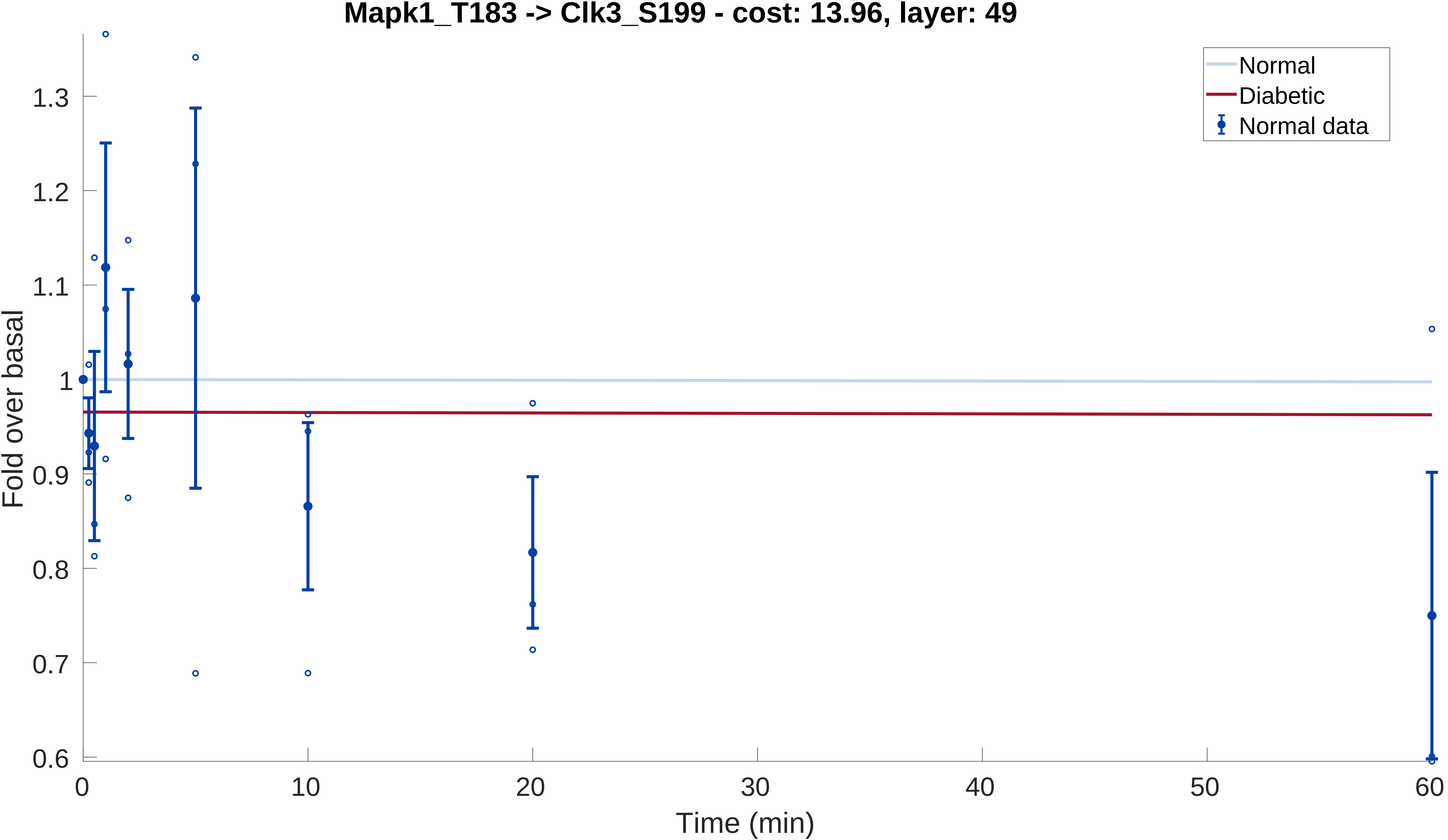

Mapk1_T183 -> Clk3_S199 - cost: 13.96, layer: 49
Normal
Diabetic
Normal data
Fold over basal
Time (min)
1.3
1.2
1.1
1
0.9
0.8
0.7
0.6
0
10
20
30
40
50
60

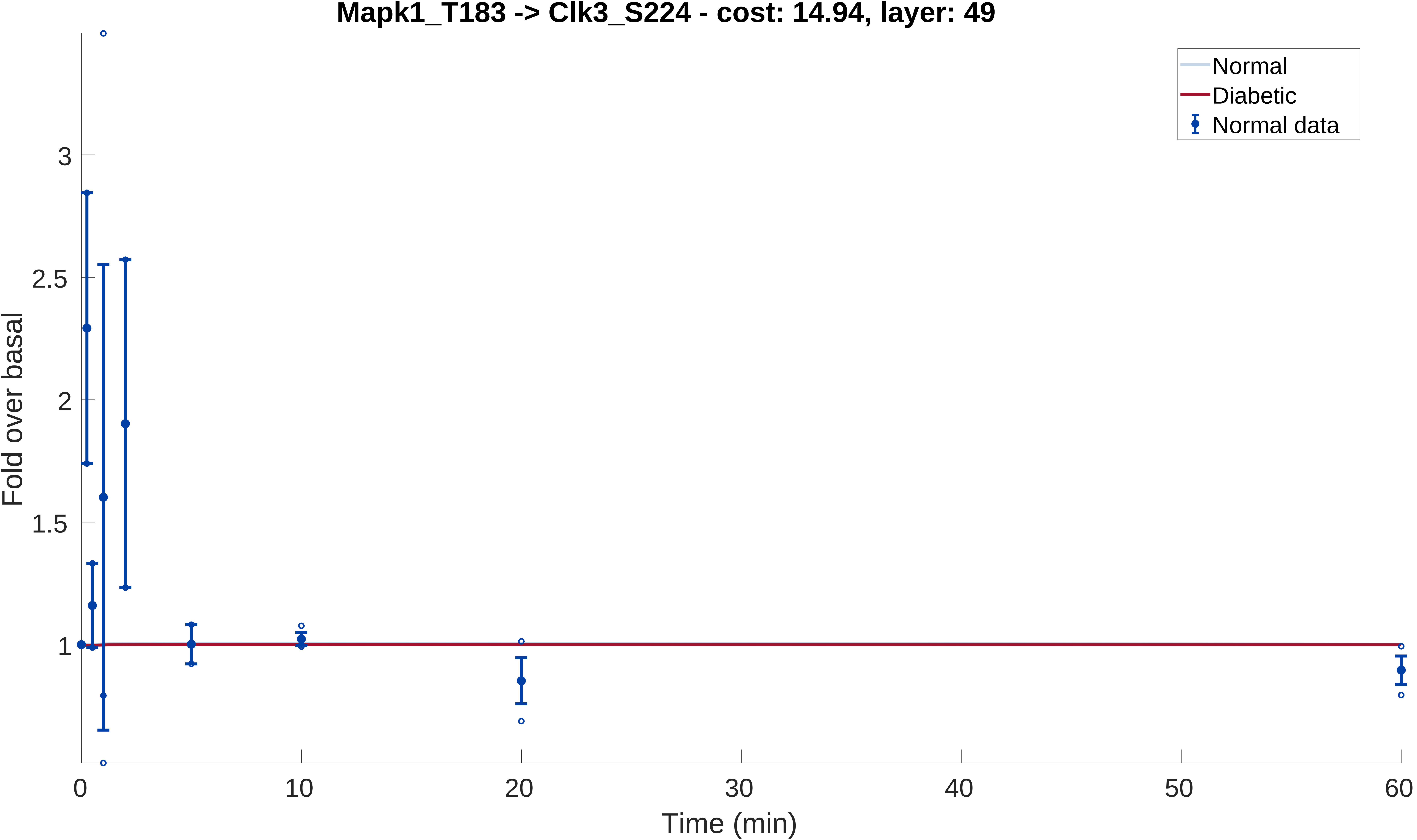

Mapk1_T183 -> Clk3_S224 - cost: 14.94, layer: 49
Normal
Diabetic
Normal data
Fold over basal
3
2.5
2
1.5
1
0
10
20
30
40
50
60
Time (min)

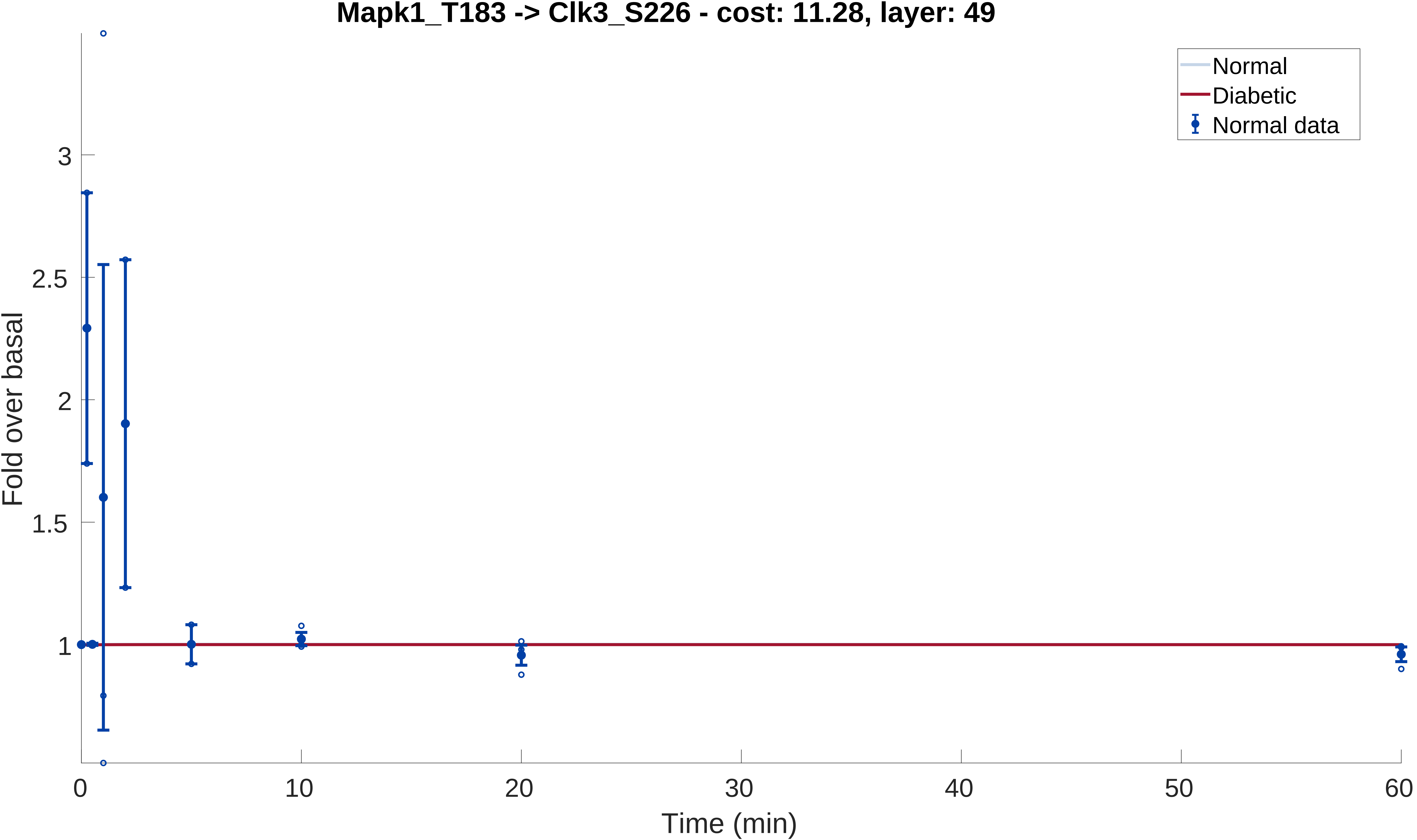

Mapk1_T183 -> Clk3_S226 - cost: 11.28, layer: 49
Normal
Diabetic
Normal data
Fold over basal
3
2.5
2
1.5
1
0
10
20
30
40
50
60
Time (min)

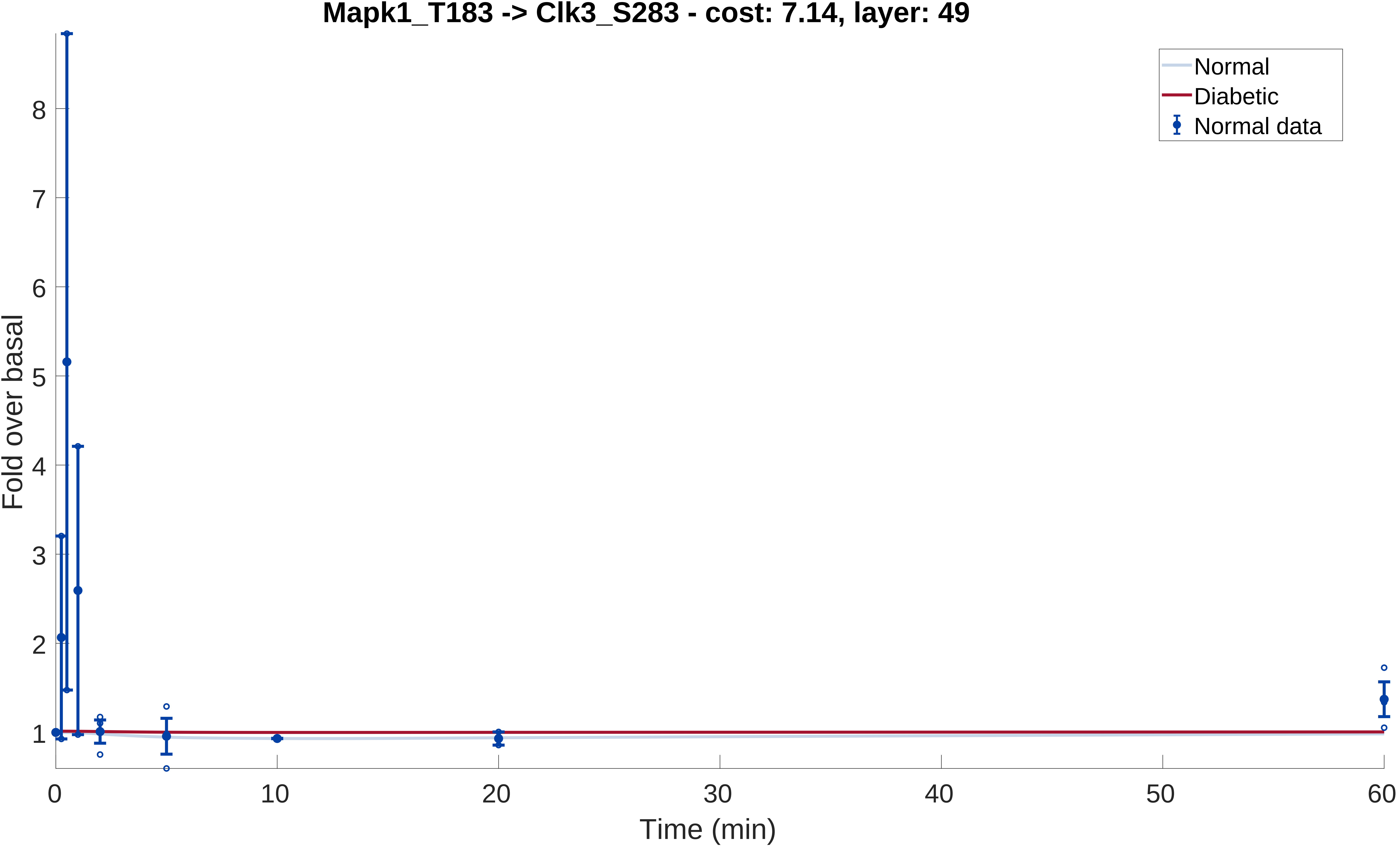
Mapk1_T183 -> Clk3_S283 - cost: 7.14, layer: 49
Normal
Diabetic
Normal data
Fold over basal
Time (min)
1
2
3
4
5
6
7
8
0
10
20
30
40
50
60

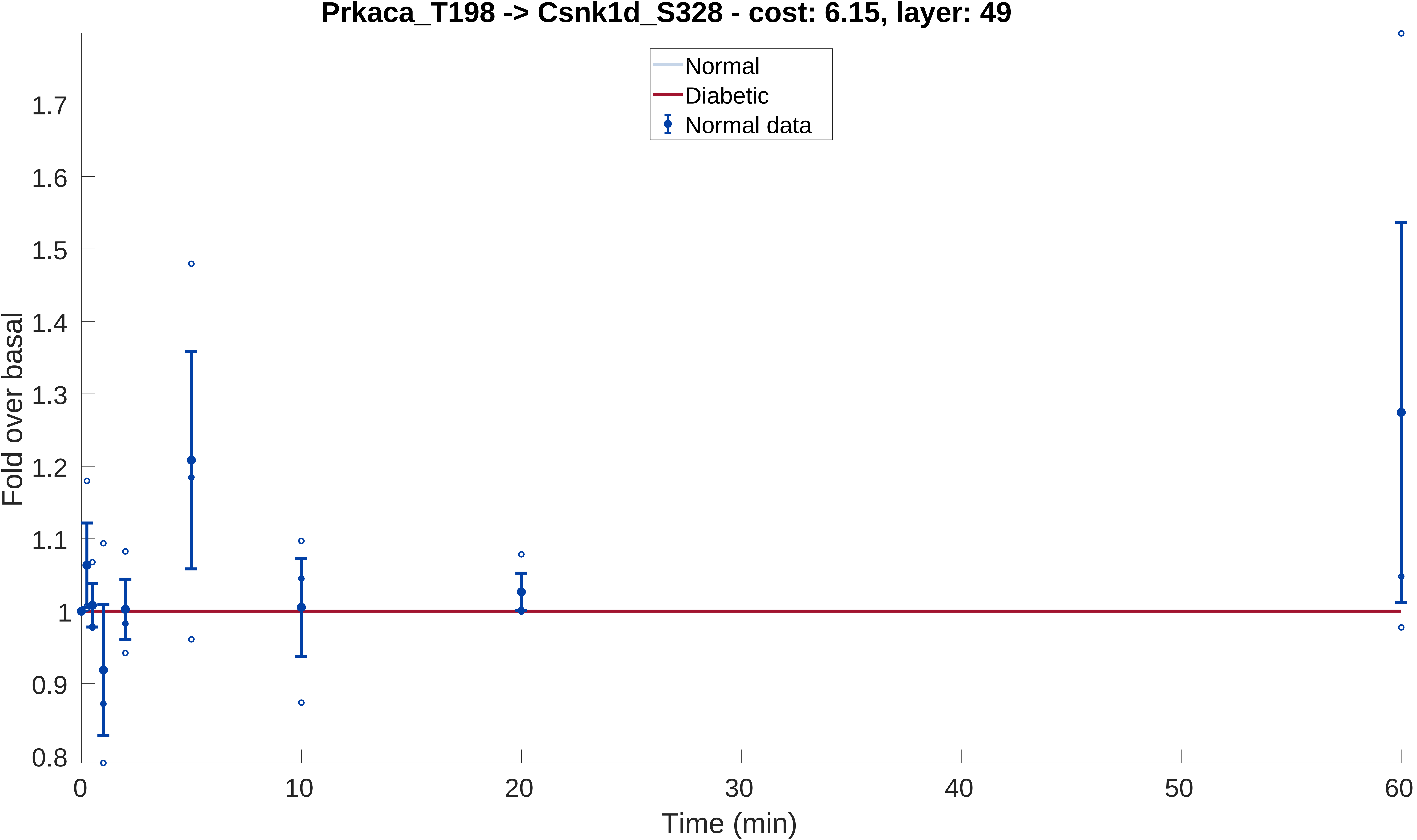

Prkaca_T198 -> Csnk1d_S328 - cost: 6.15, layer: 49
Normal
Diabetic
Normal data
Fold over basal
Time (min)
0.8
0.9
1
1.1
1.2
1.3
1.4
1.5
1.6
1.7
0
10
20
30
40
50
60

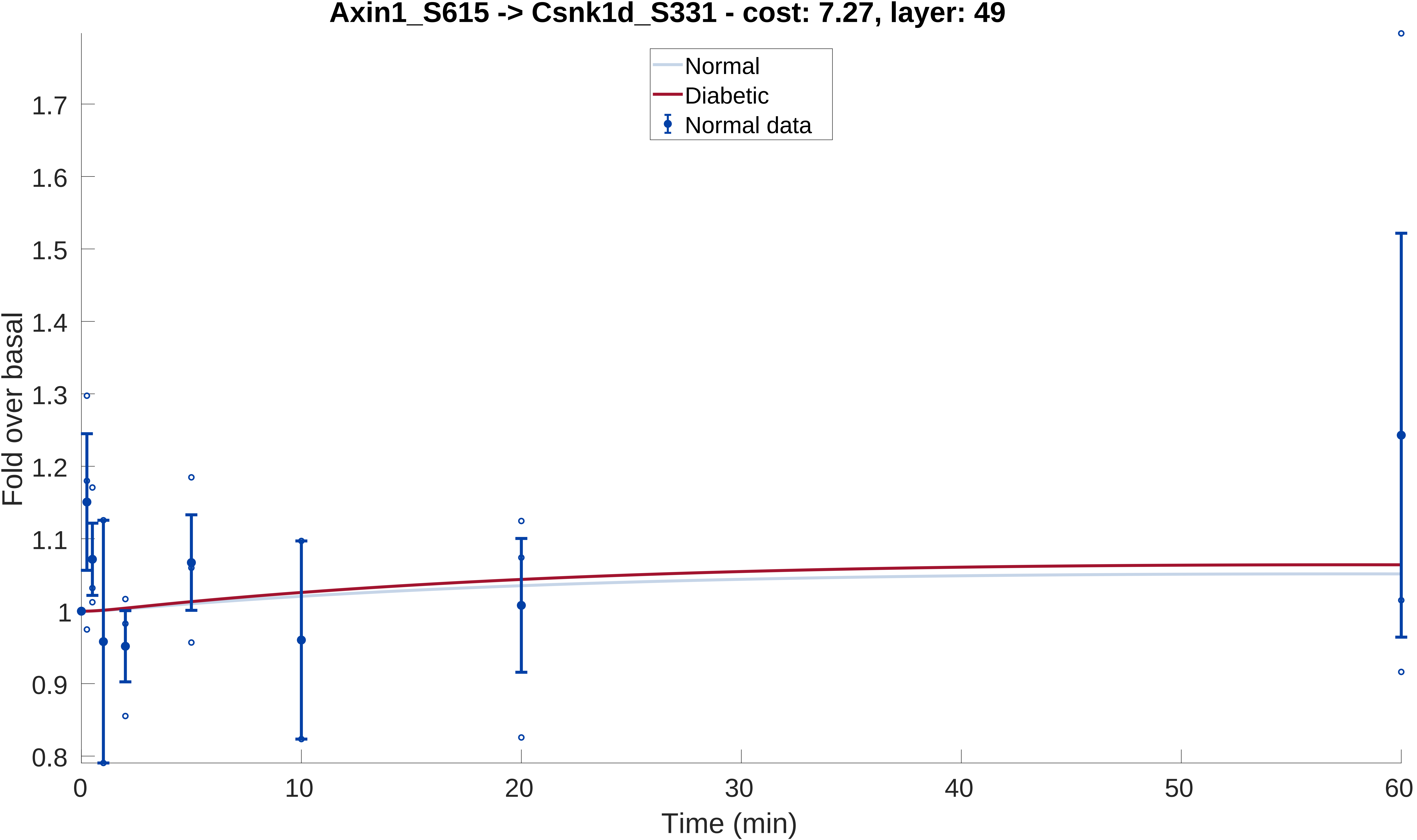

Axin1_S615 -> Csnk1d_S331 - cost: 7.27, layer: 49
Normal
Diabetic
Normal data
Fold over basal
1.7
1.6
1.5
1.4
1.3
1.2
1.1
1
0.9
0.8
0
10
20
30
40
50
60
Time (min)

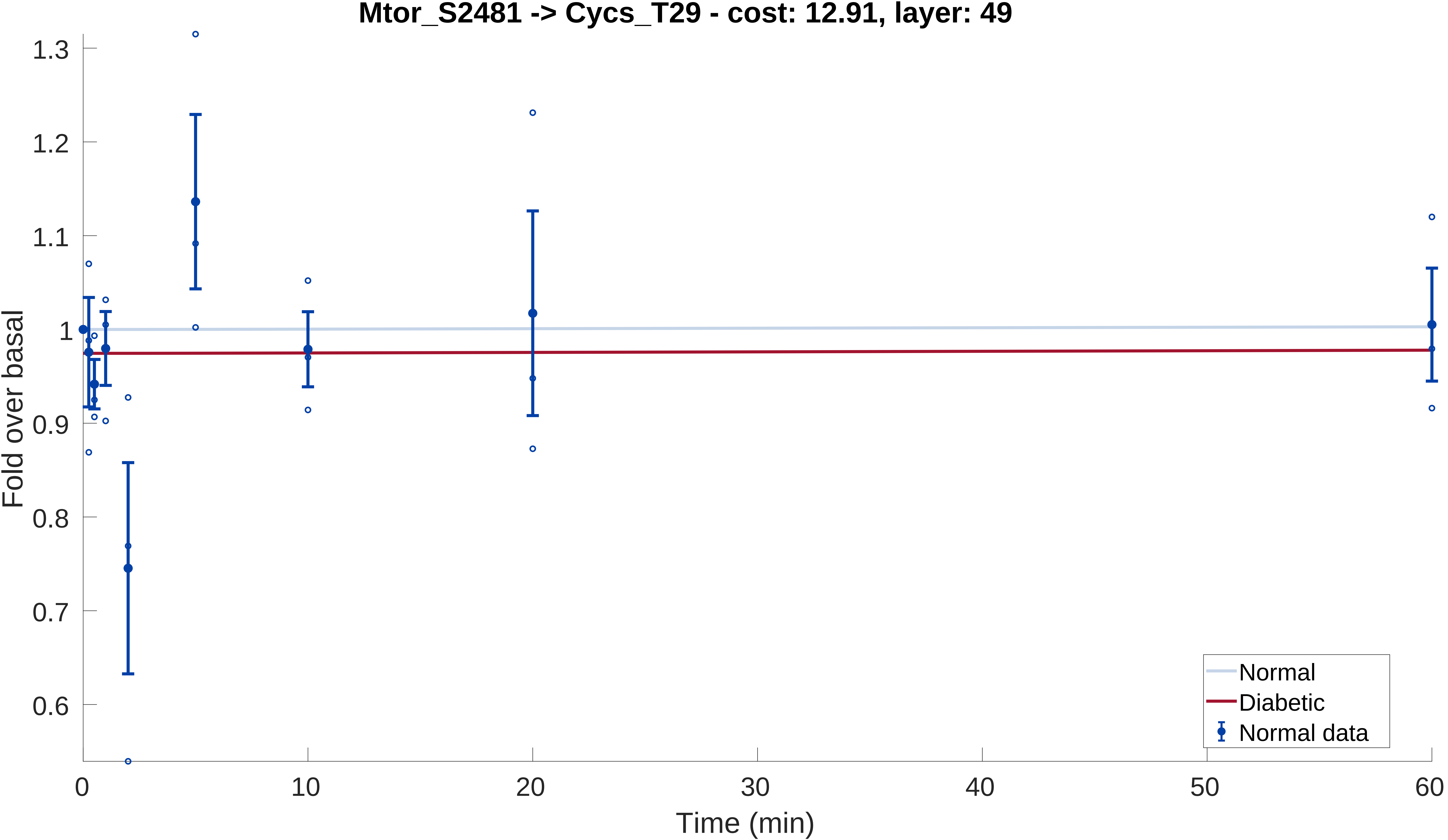

Mtor_S2481 -> Cycs_T29 - cost: 12.91, layer: 49
Fold over basal
1.3
1.2
1.1
1
0.9
0.8
0.7
0.6
0
10
20
30
40
50
60
Time (min)
Normal
Diabetic
Normal data

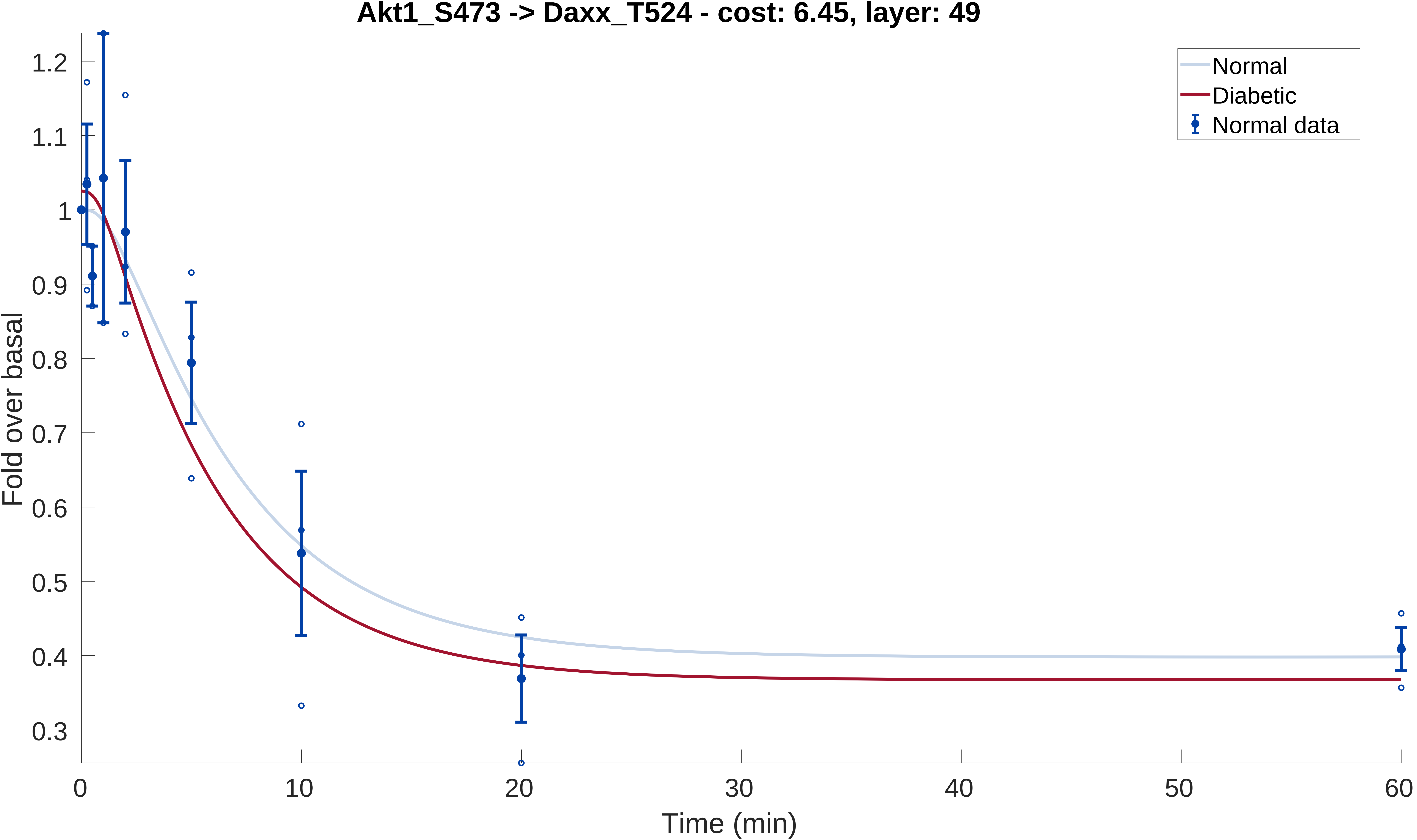

Akt1_S473 -> Daxx_T524 - cost: 6.45, layer: 49
Normal
Diabetic
Normal data
Fold over basal
1.2
1.1
1
0.9
0.8
0.7
0.6
0.5
0.4
0.3
0
10
20
30
40
50
60
Time (min)

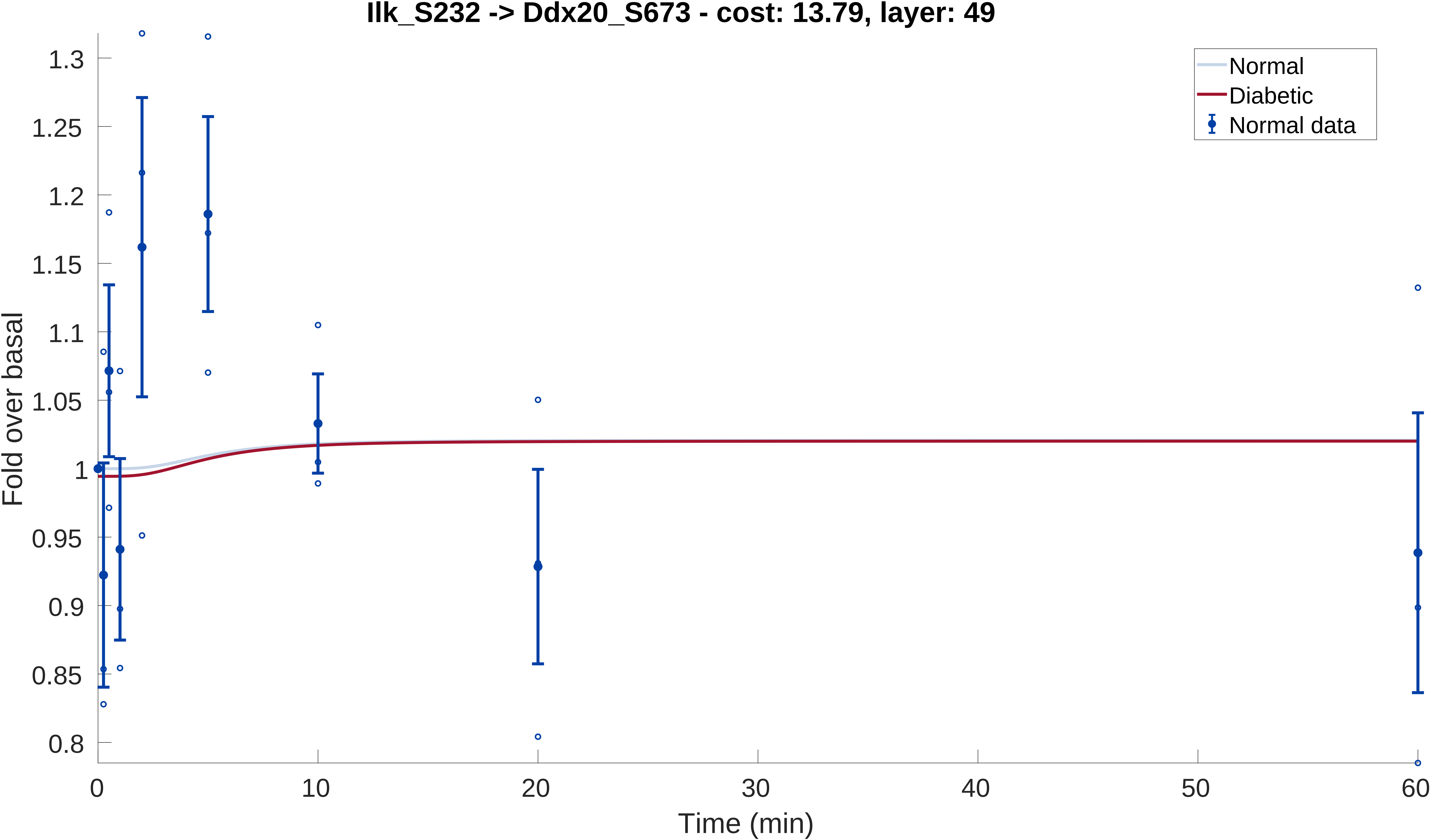

Ilk_S232 -> Ddx20_S673 - cost: 13.79, layer: 49
Normal
Diabetic
Normal data
Fold over basal
1.3
1.25
1.2
1.15
1.1
1.05
1
0.95
0.9
0.85
0.8
0
10
20
30
40
50
60
Time (min)

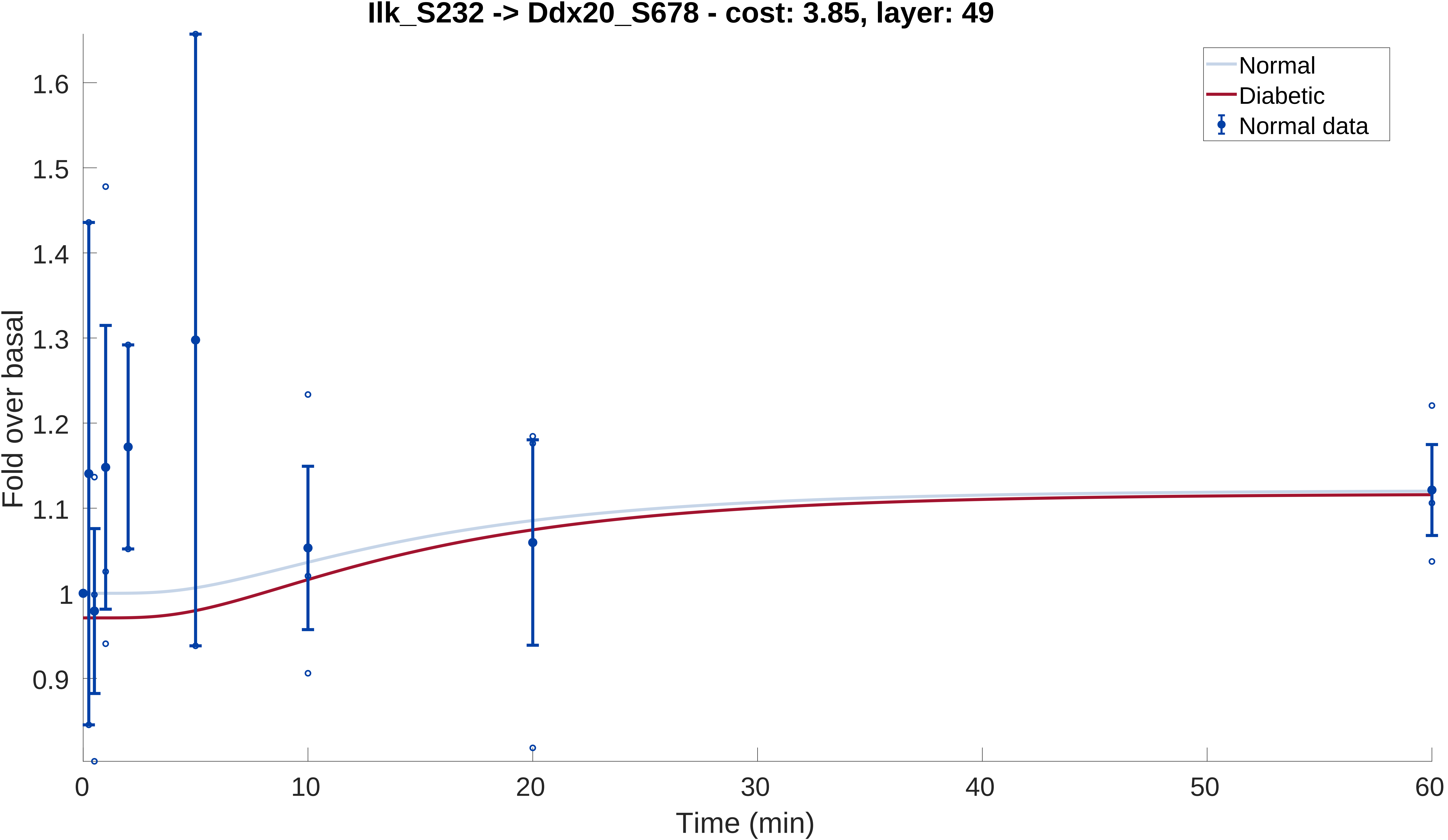

Ilk_S232 -> Ddx20_S678 - cost: 3.85, layer: 49
Normal
Diabetic
Normal data
Fold over basal
1.6
1.5
1.4
1.3
1.2
1.1
1
0.9
0
10
20
30
40
50
60
Time (min)

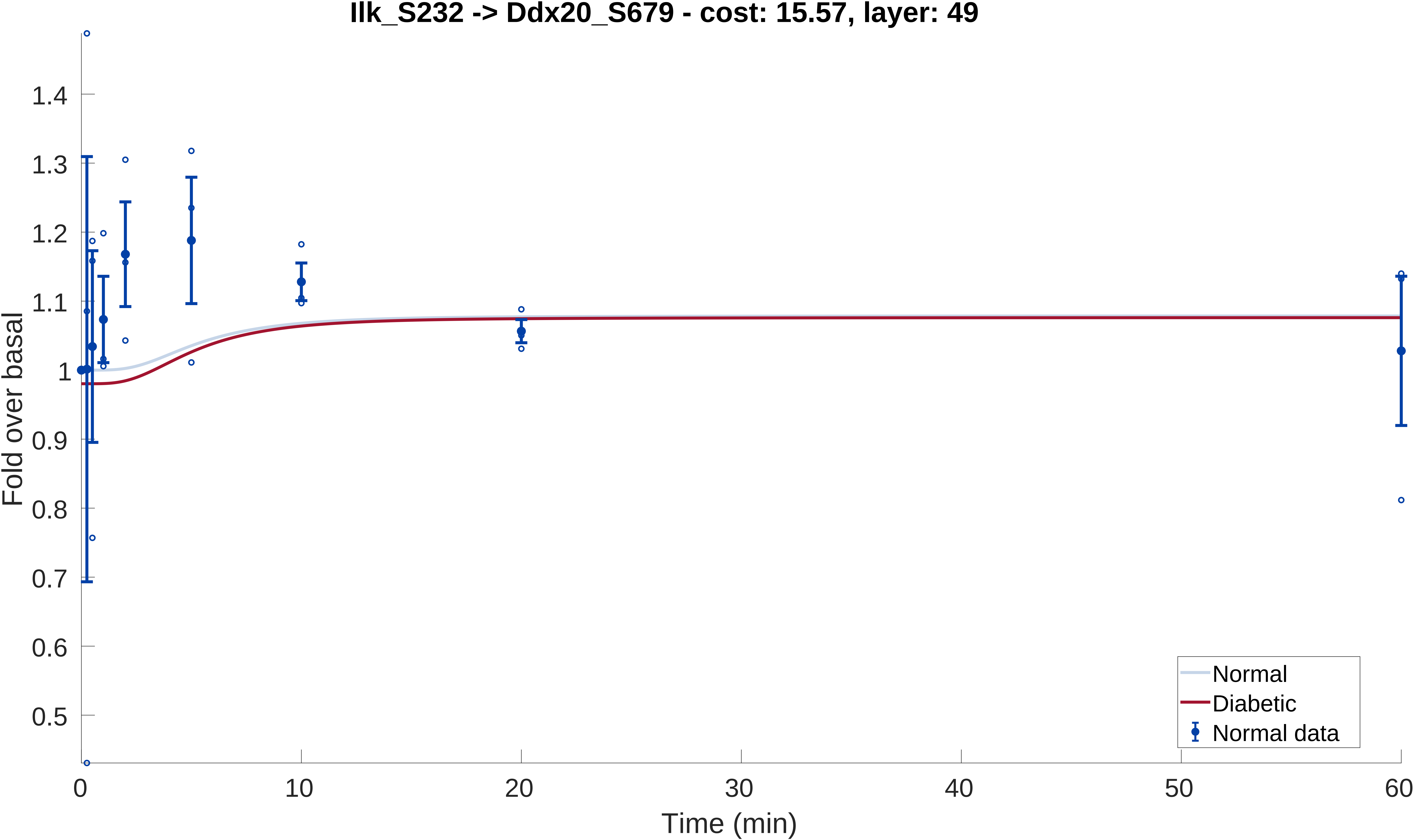

Ilk_S232 -> Ddx20_S679 - cost: 15.57, layer: 49
Fold over basal
Time (min)
1.4
1.3
1.2
1.1
1
0.9
0.8
0.7
0.6
0.5
0
10
20
30
40
50
60
Normal
Diabetic
Normal data

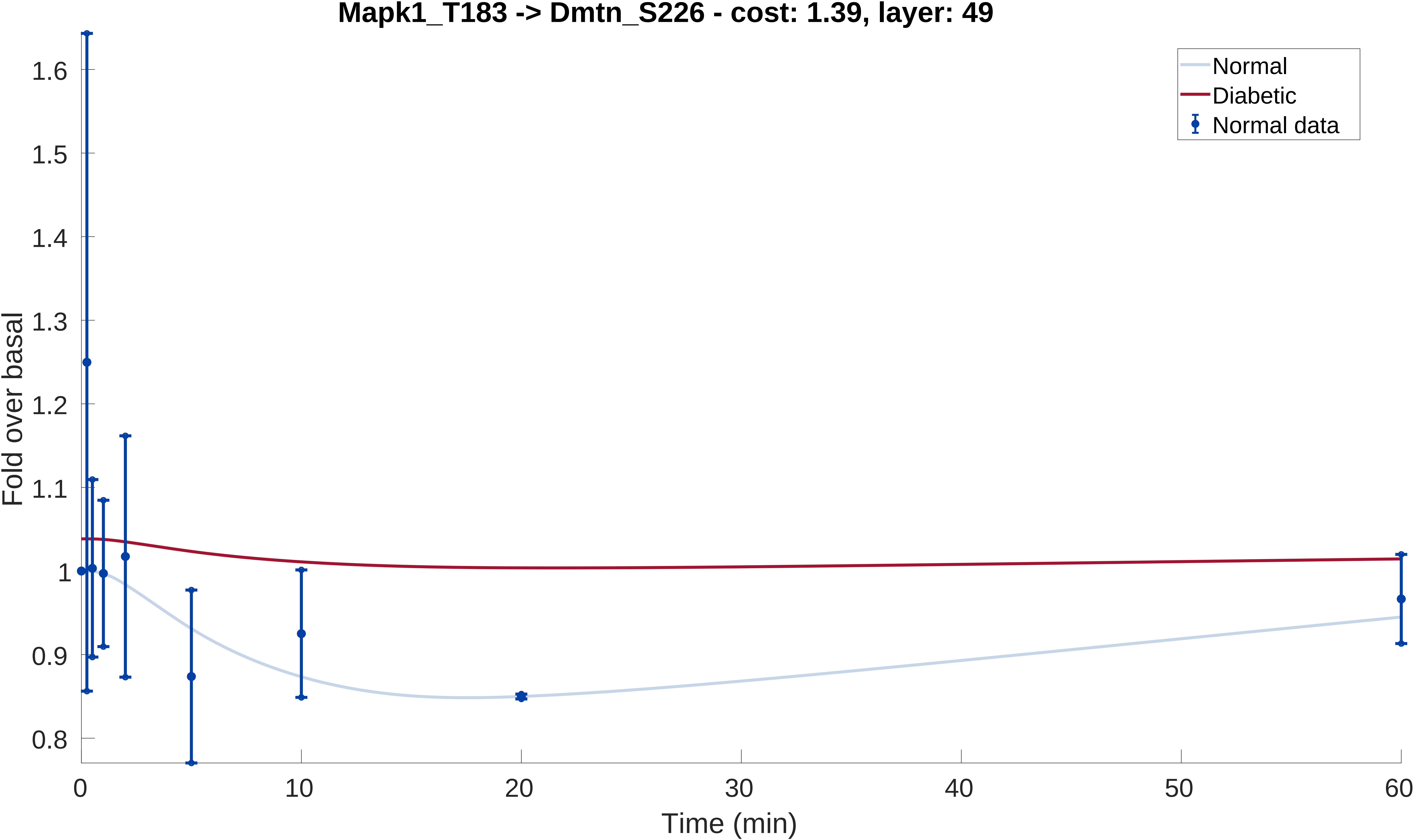

Mapk1_T183 -> Dmtn_S226 - cost: 1.39, layer: 49
Normal
Diabetic
Normal data
Fold over basal
1.6
1.5
1.4
1.3
1.2
1.1
1
0.9
0.8
0
10
20
30
40
50
60
Time (min)

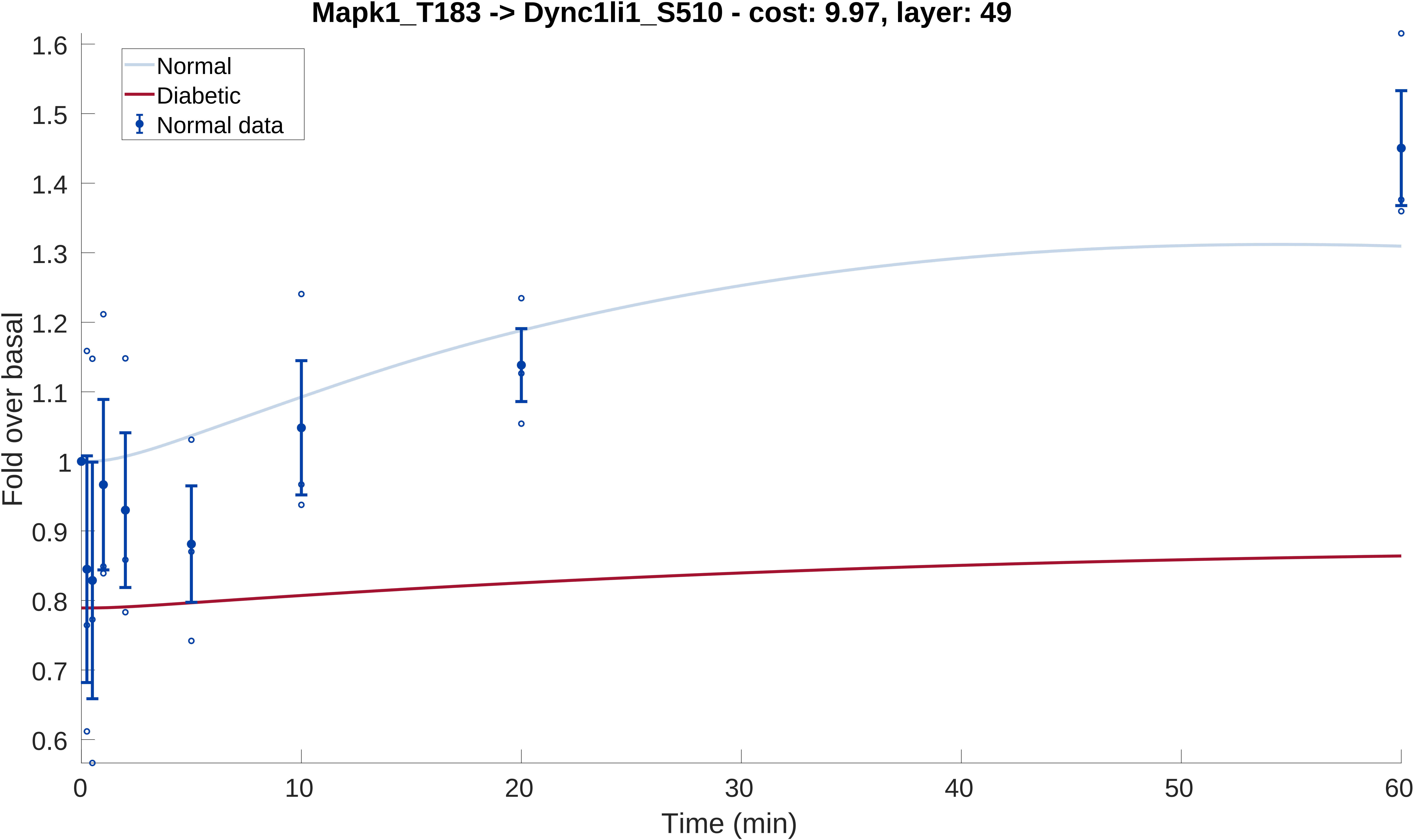

Mapk1_T183 -> Dync1li1_S510 - cost: 9.97, layer: 49
Normal
Diabetic
Normal data
Fold over basal
Time (min)
1.6
1.5
1.4
1.3
1.2
1.1
1
0.9
0.8
0.7
0.6
0
10
20
30
40
50
60

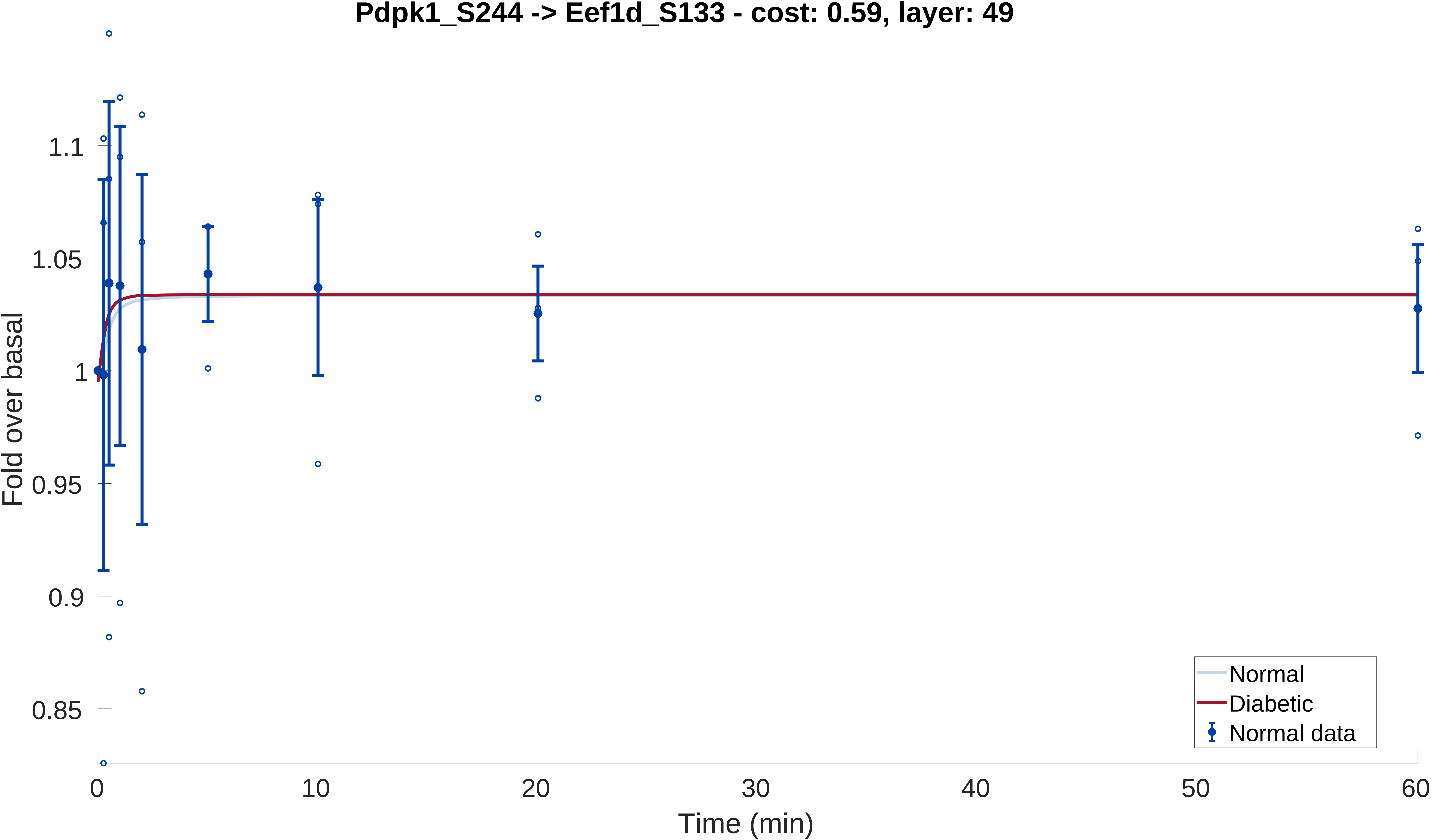

Pdpk1_S244 -> Eef1d_S133 - cost: 0.59, layer: 49
Fold over basal
1.1
1.05
1
0.95
0.9
0.85
0
10
20
30
40
50
60
Time (min)
Normal
Diabetic
Normal data

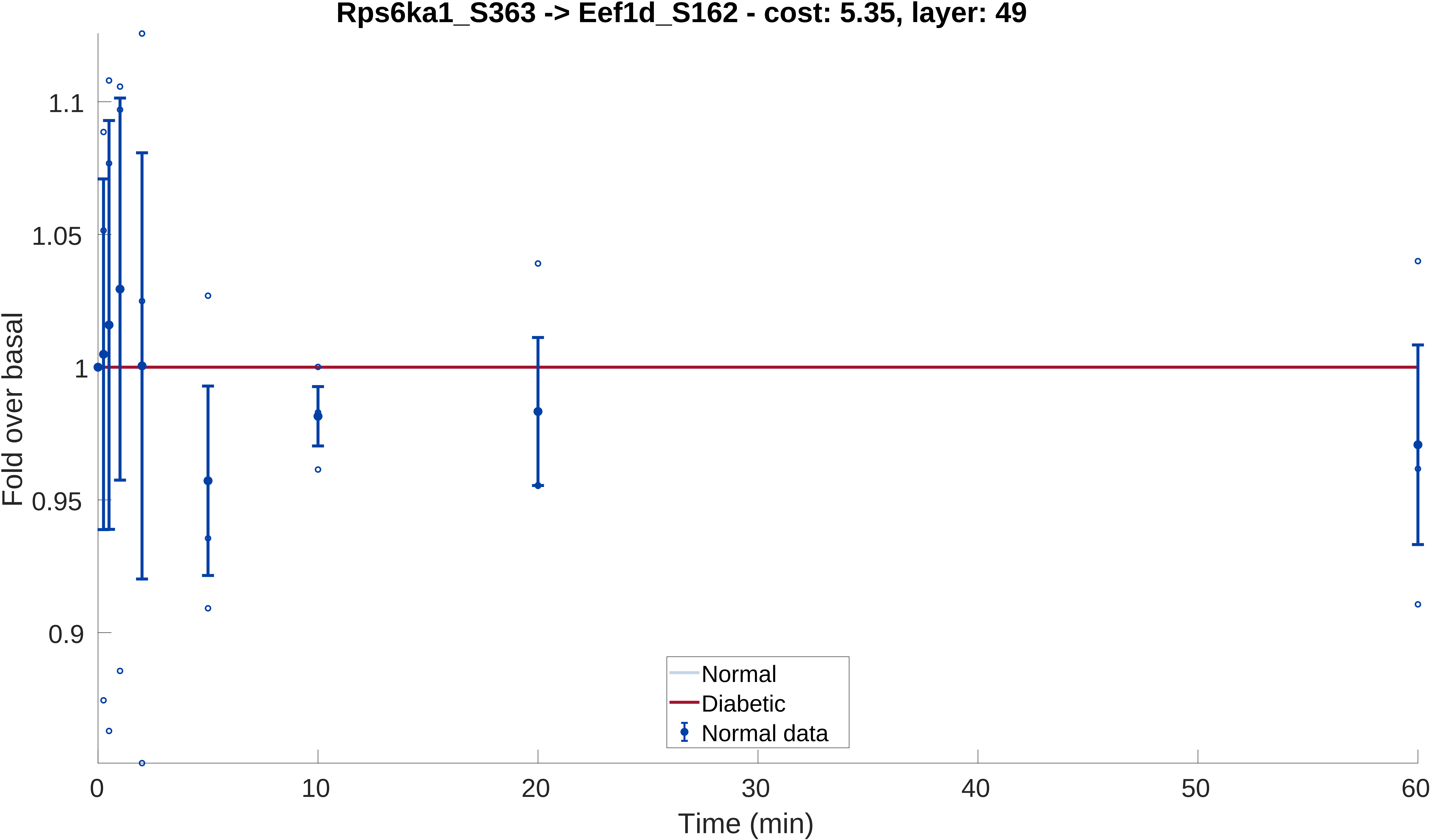

Rps6ka1_S363 -> Eef1d_S162 - cost: 5.35, layer: 49
Fold over basal
1.1
1.05
1
0.95
0.9
0
10
20
30
40
50
60
Time (min)
Normal
Diabetic
Normal data

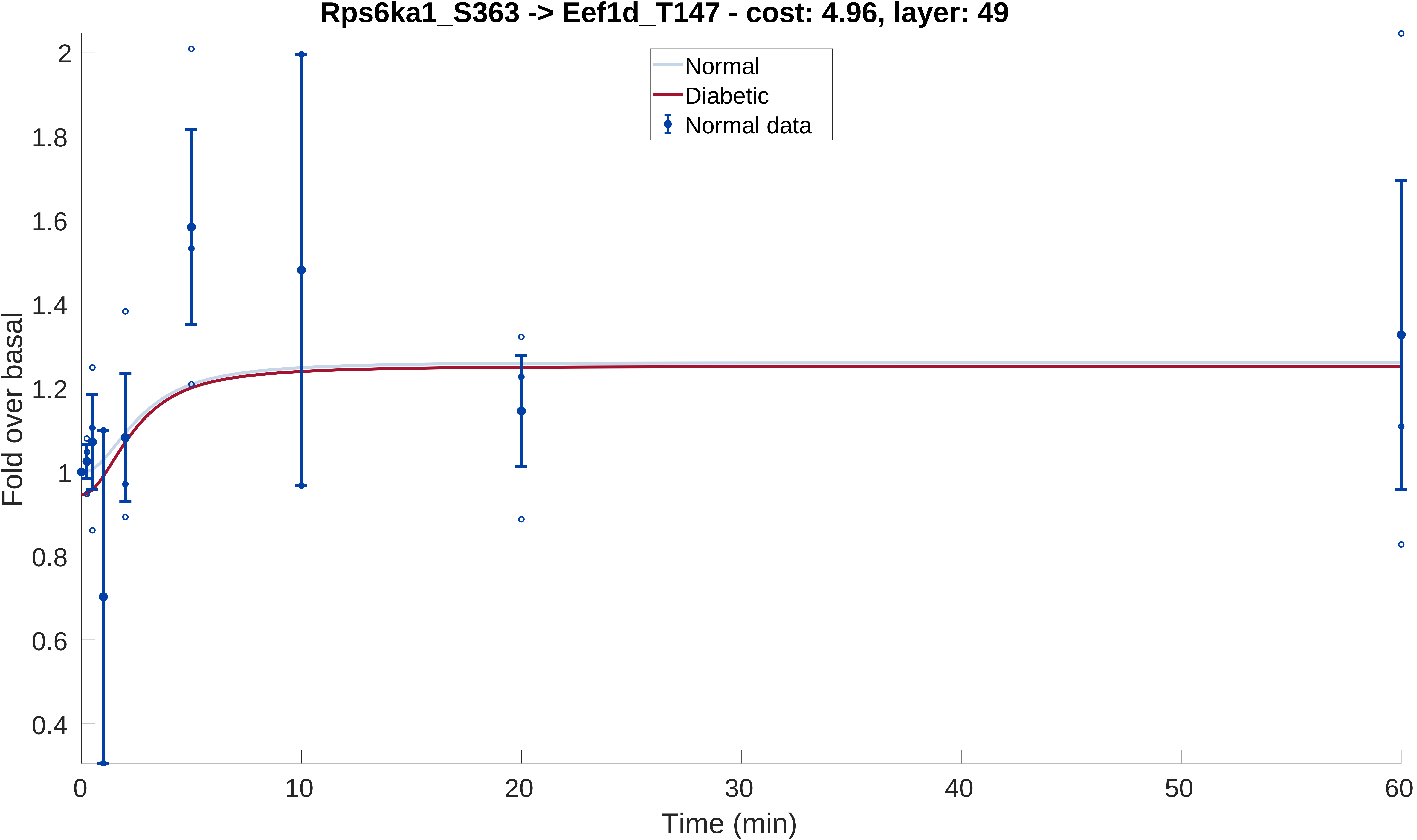
Rps6ka1_S363 -> Eef1d_T147 - cost: 4.96, layer: 49
Normal
Diabetic
Normal data
Fold over basal
Time (min)
0.4
0.6
0.8
1
1.2
1.4
1.6
1.8
2
0
10
20
30
40
50
60

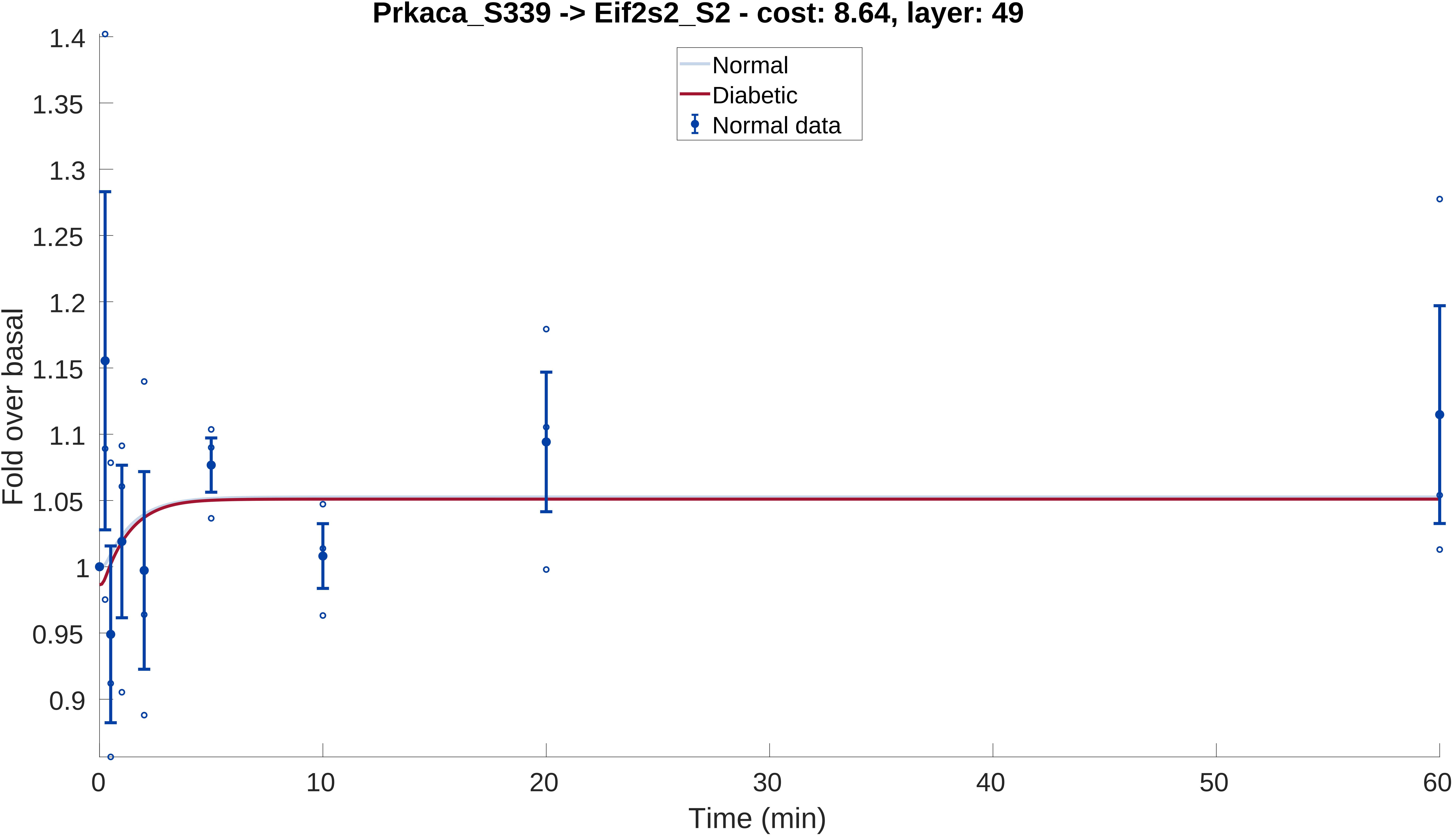

Prrkaca_S339 -> Eif2s2_S2 - cost: 8.64, layer: 49
Normal
Diabetic
Normal data
Fold over basal
Time (min)
1.4
1.35
1.3
1.25
1.2
1.15
1.1
1.05
1
0.95
0.9
0
10
20
30
40
50
60

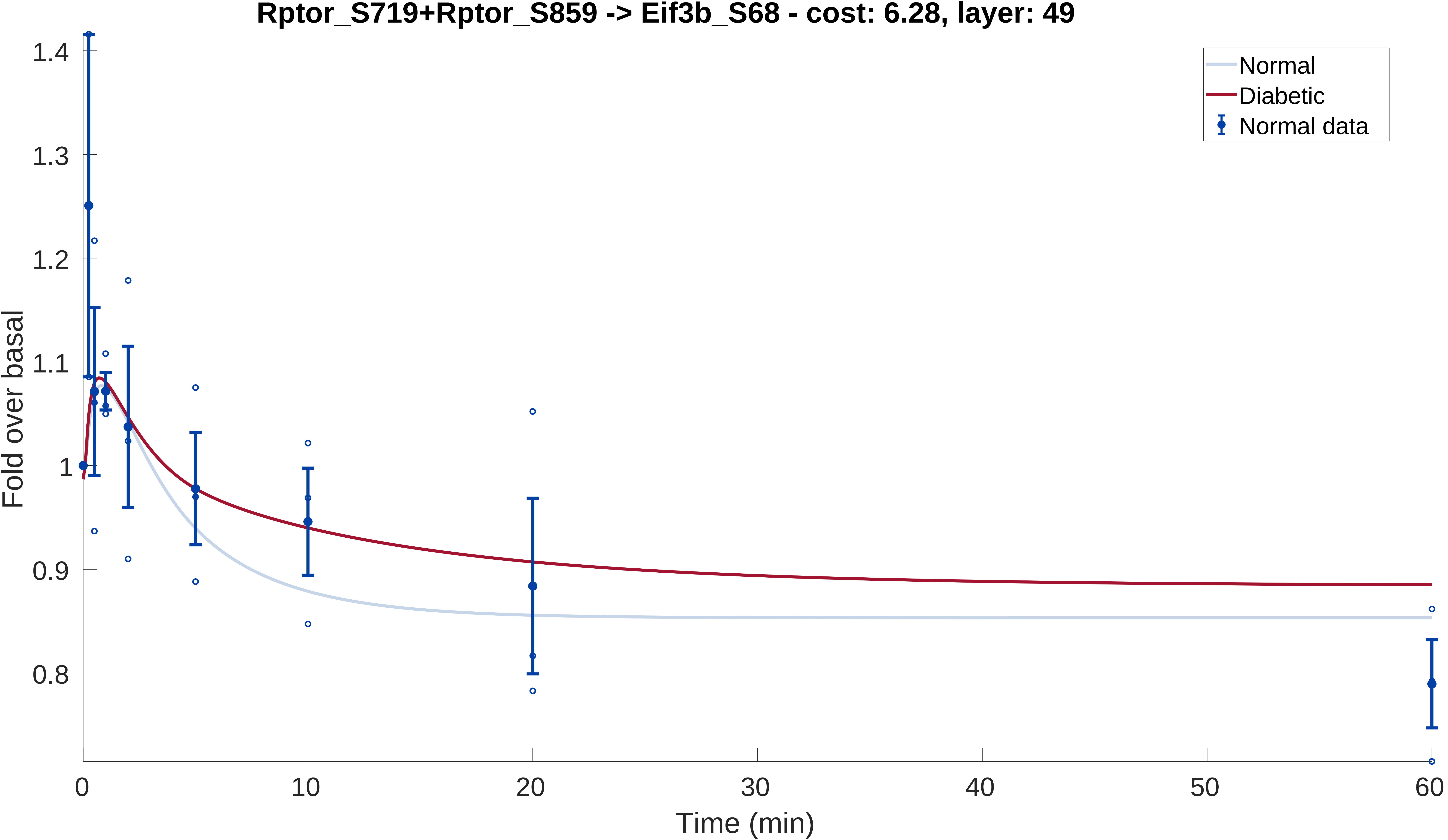

Rptor_S719+Rptor_S859 -> Eif3b_S68 - cost: 6.28, layer: 49
Fold over basal
Time (min)
Normal
Diabetic
Normal data
1.4
1.3
1.2
1.1
1
0.9
0.8
0
10
20
30
40
50
60

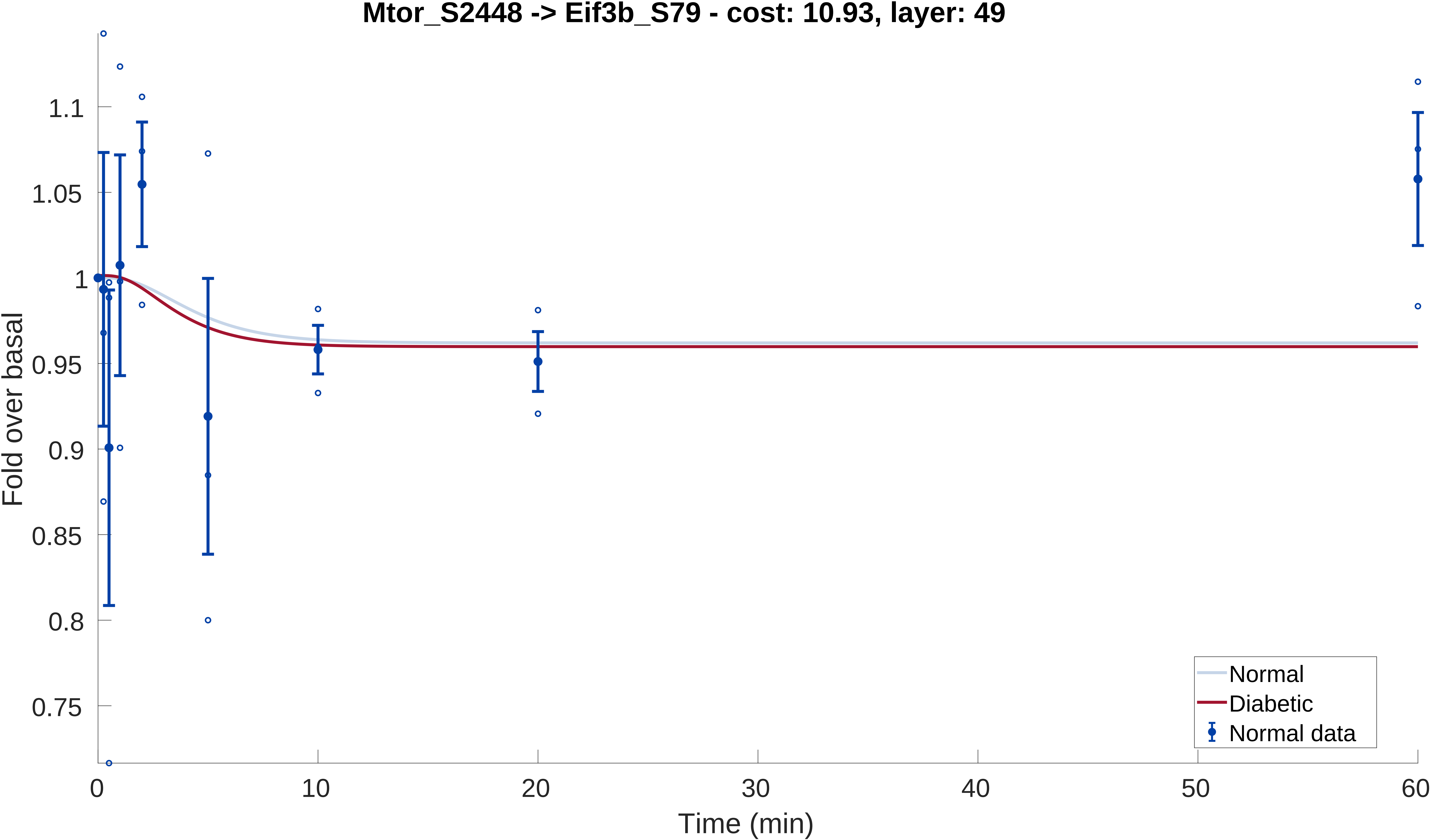

Mtor_S2448 -> Eif3b_S79 - cost: 10.93, layer: 49
Fold over basal
1.1
1.05
1
0.95
0.9
0.85
0.8
0.75
0
10
20
30
40
50
60
Time (min)
Normal
Diabetic
Normal data

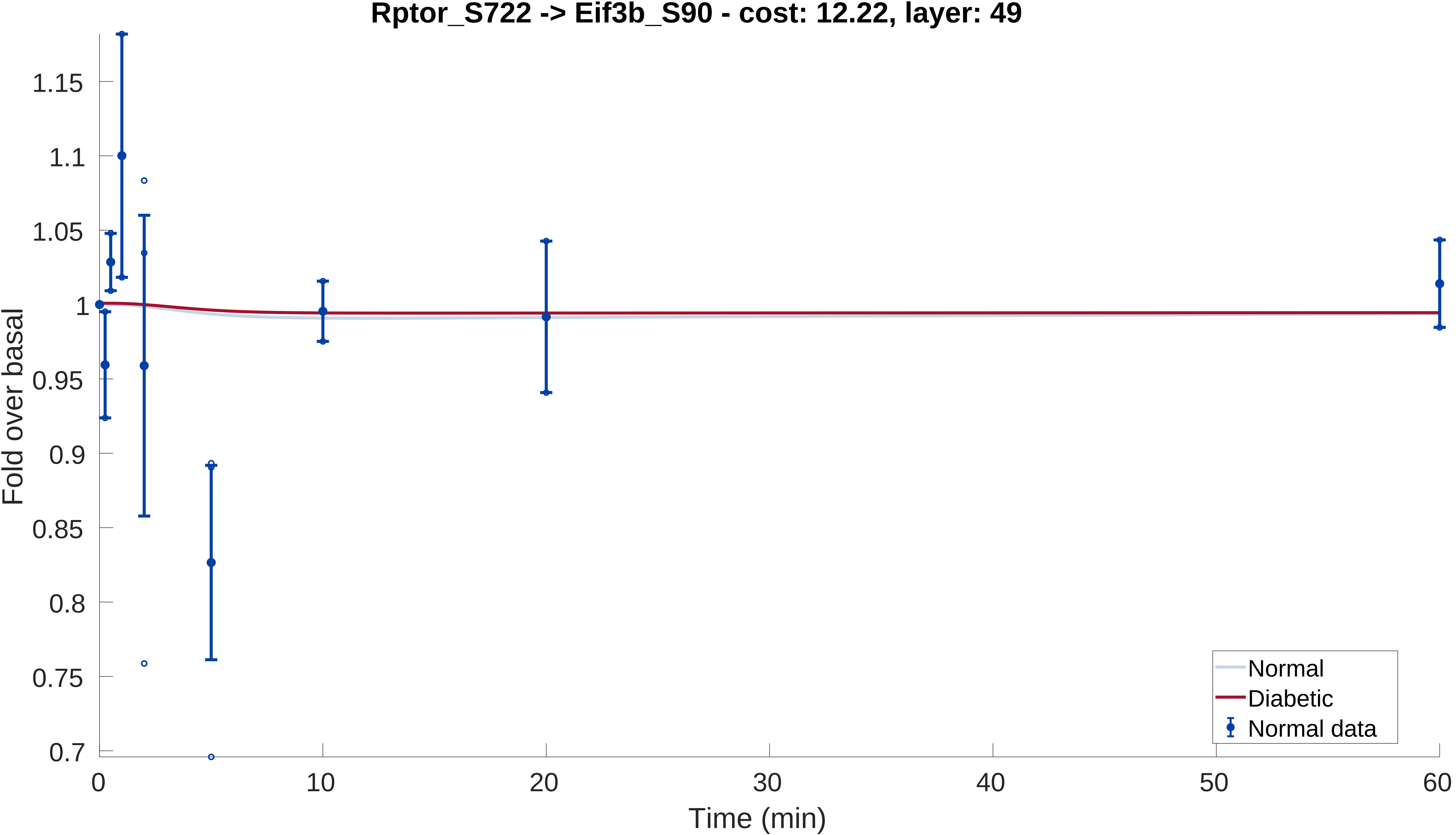
Rptor_S722 -> Eif3b_S90 - cost: 12.22, layer: 49
Fold over basal
Time (min)
0
10
20
30
40
50
60
0.7
0.75
0.8
0.85
0.9
0.95
1
1.05
1.1
1.15
Normal
Diabetic
Normal data

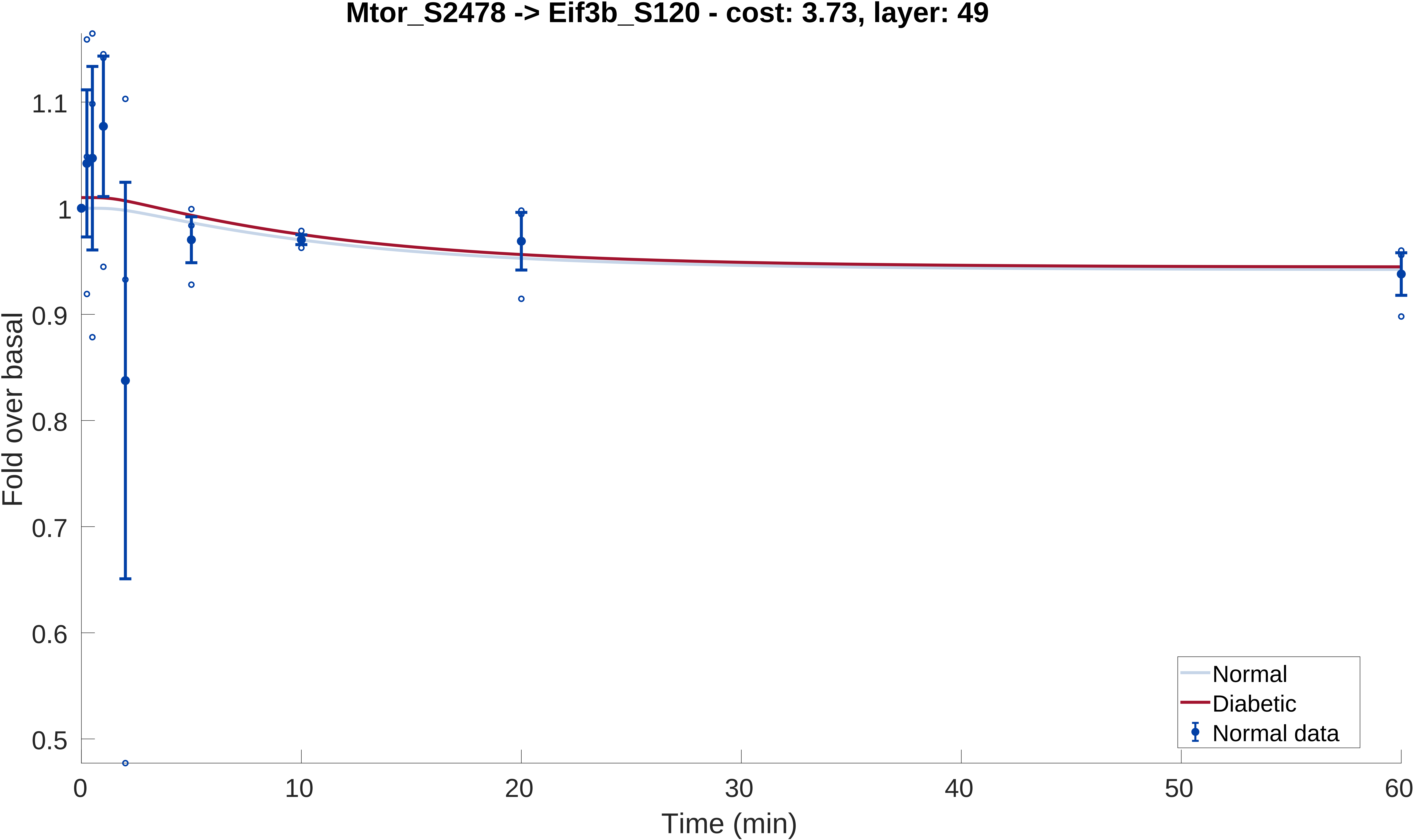

Mtor_S2478 -> Eif3b_S120 - cost: 3.73, layer: 49
Fold over basal
1.1
1
0.9
0.8
0.7
0.6
0.5
0
10
20
30
40
50
60
Time (min)
Normal
Diabetic
Normal data

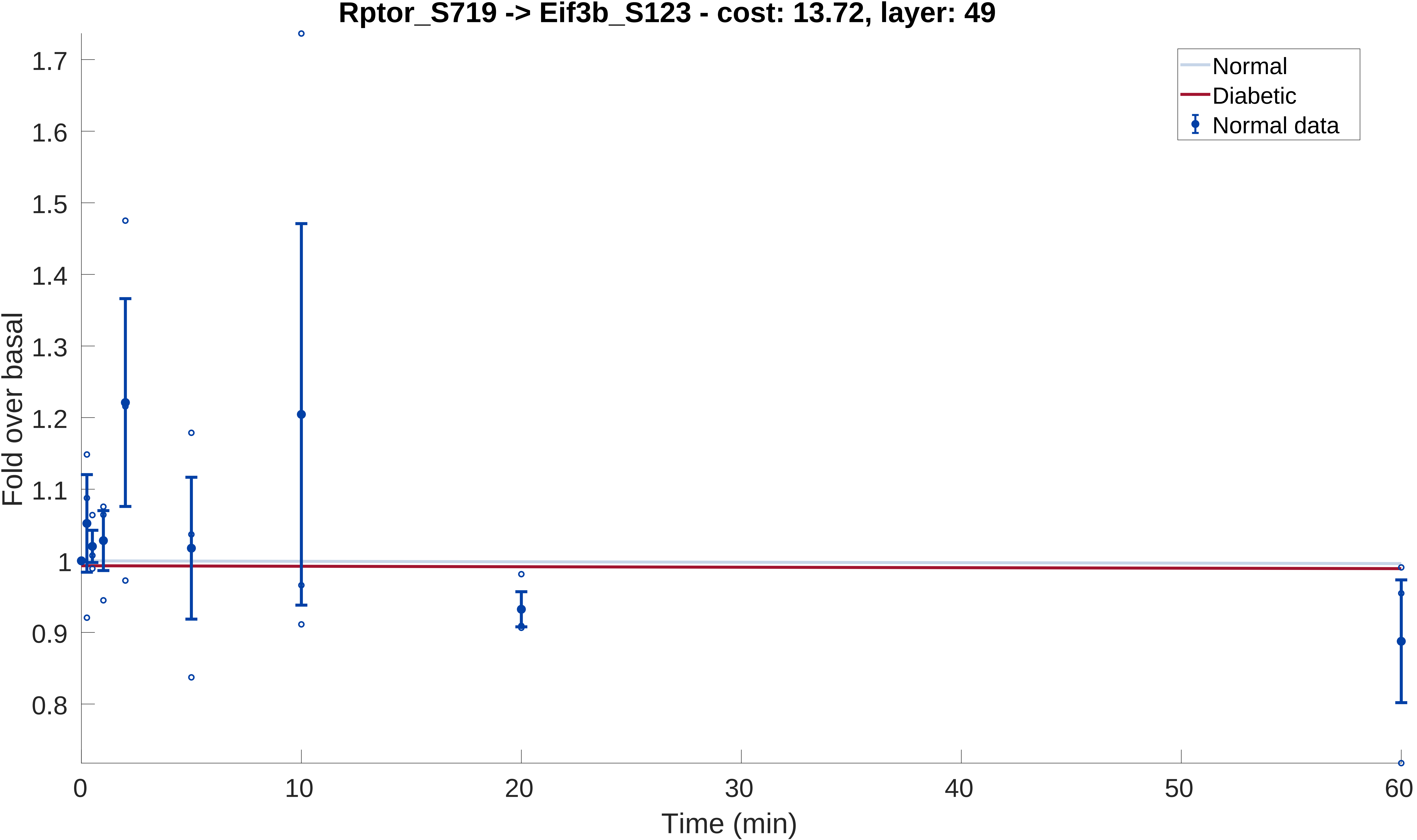

Rptor_S719 -> Eif3b_S123 - cost: 13.72, layer: 49
Normal
Diabetic
Normal data
Fold over basal
Time (min)
0.8
0.9
1
1.1
1.2
1.3
1.4
1.5
1.6
1.7
0
10
20
30
40
50
60

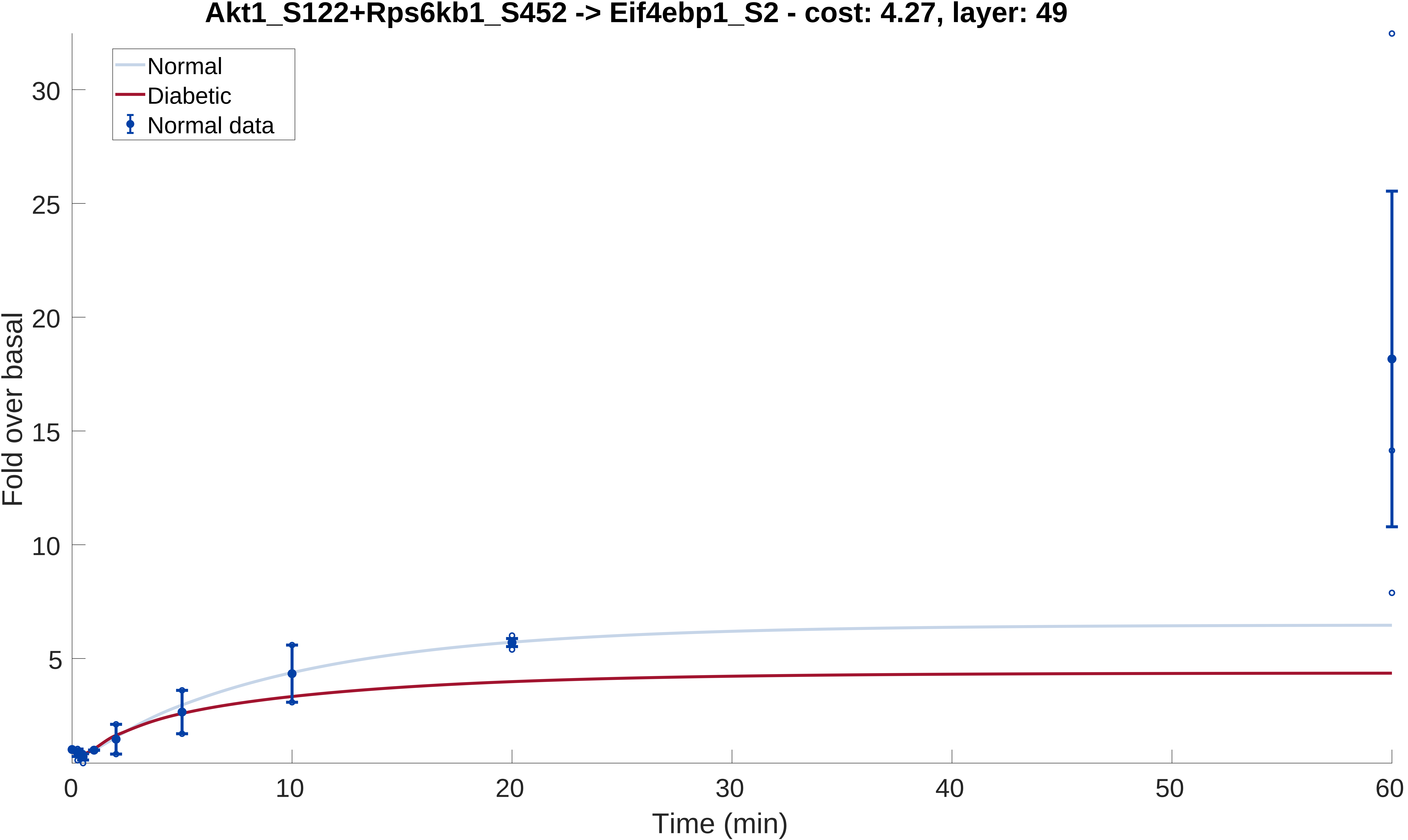

Akt1_S122+Rps6kb1_S452 -> Eif4ebp1_S2 - cost: 4.27, layer: 49
Normal
Diabetic
Normal data
Fold over basal
Time (min)
0
5
10
15
20
25
30
0
10
20
30
40
50
60

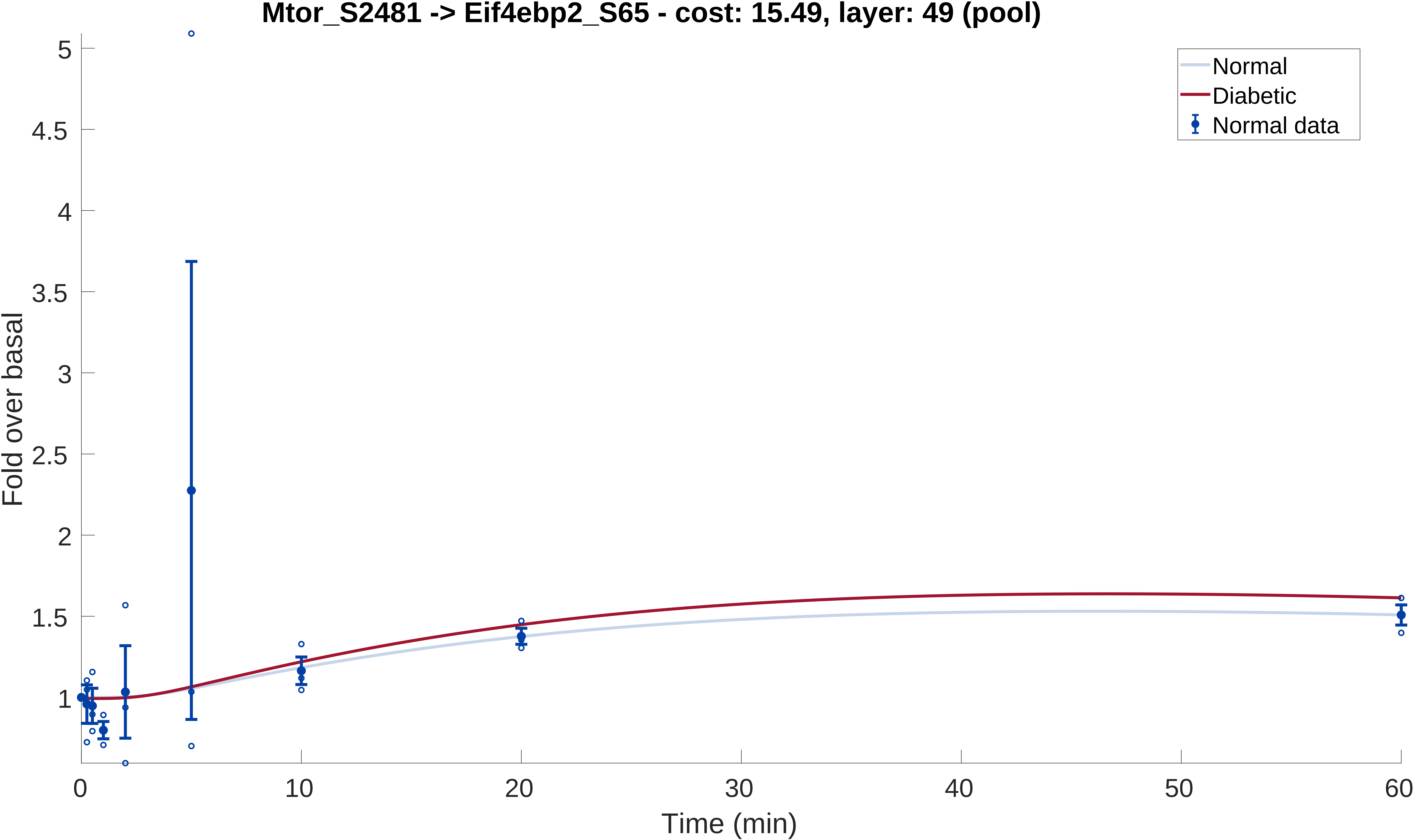

Mtor_S2481 -> Eif4ebp2_S65 - cost: 15.49, layer: 49 (pool)
Normal
Diabetic
Normal data
Fold over basal
Time (min)
0
10
20
30
40
50
60
1
1.5
2
2.5
3
3.5
4
4.5
5

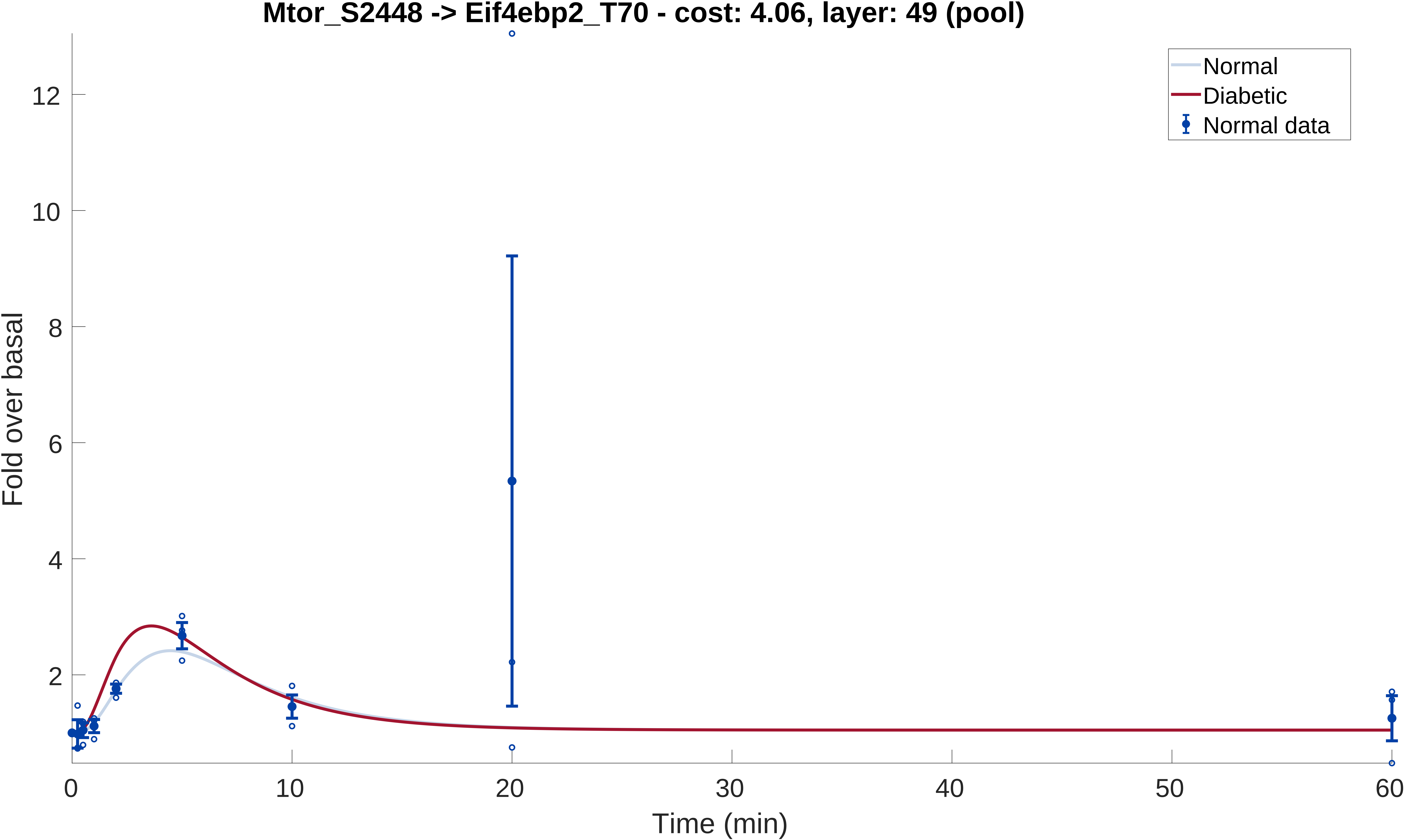

Mtor_S2448 -> Eif4ebp2_T70 - cost: 4.06, layer: 49 (pool)
Normal
Diabetic
Normal data
Fold over basal
Time (min)
0
2
4
6
8
10
12
0
10
20
30
40
50
60

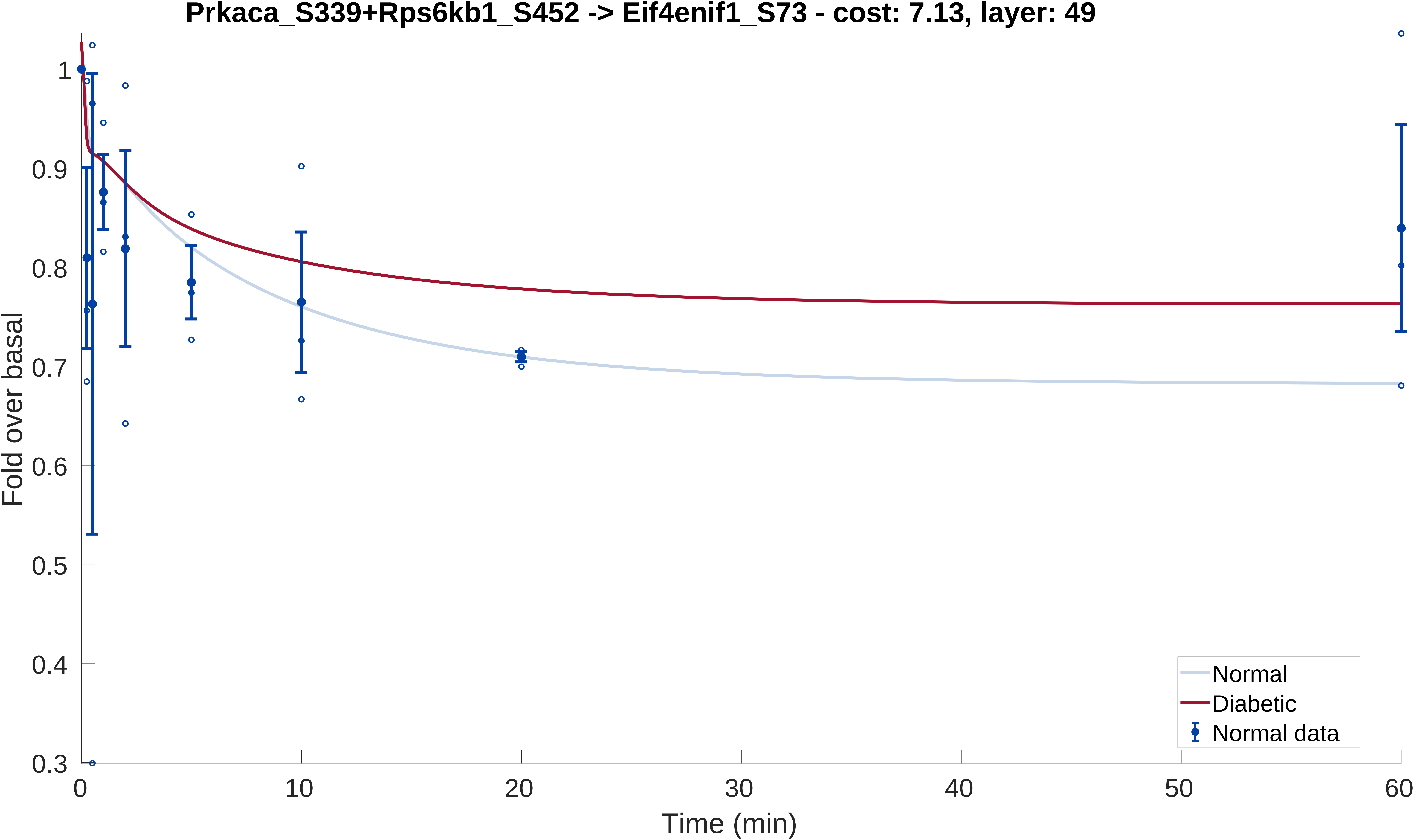

Prkaca_S339+Rps6kb1_S452 -> Eif4enif1_S73 - cost: 7.13, layer: 49
Fold over basal
Time (min)
0.3
0.4
0.5
0.6
0.7
0.8
0.9
1
0
10
20
30
40
50
60
Normal
Diabetic
Normal data

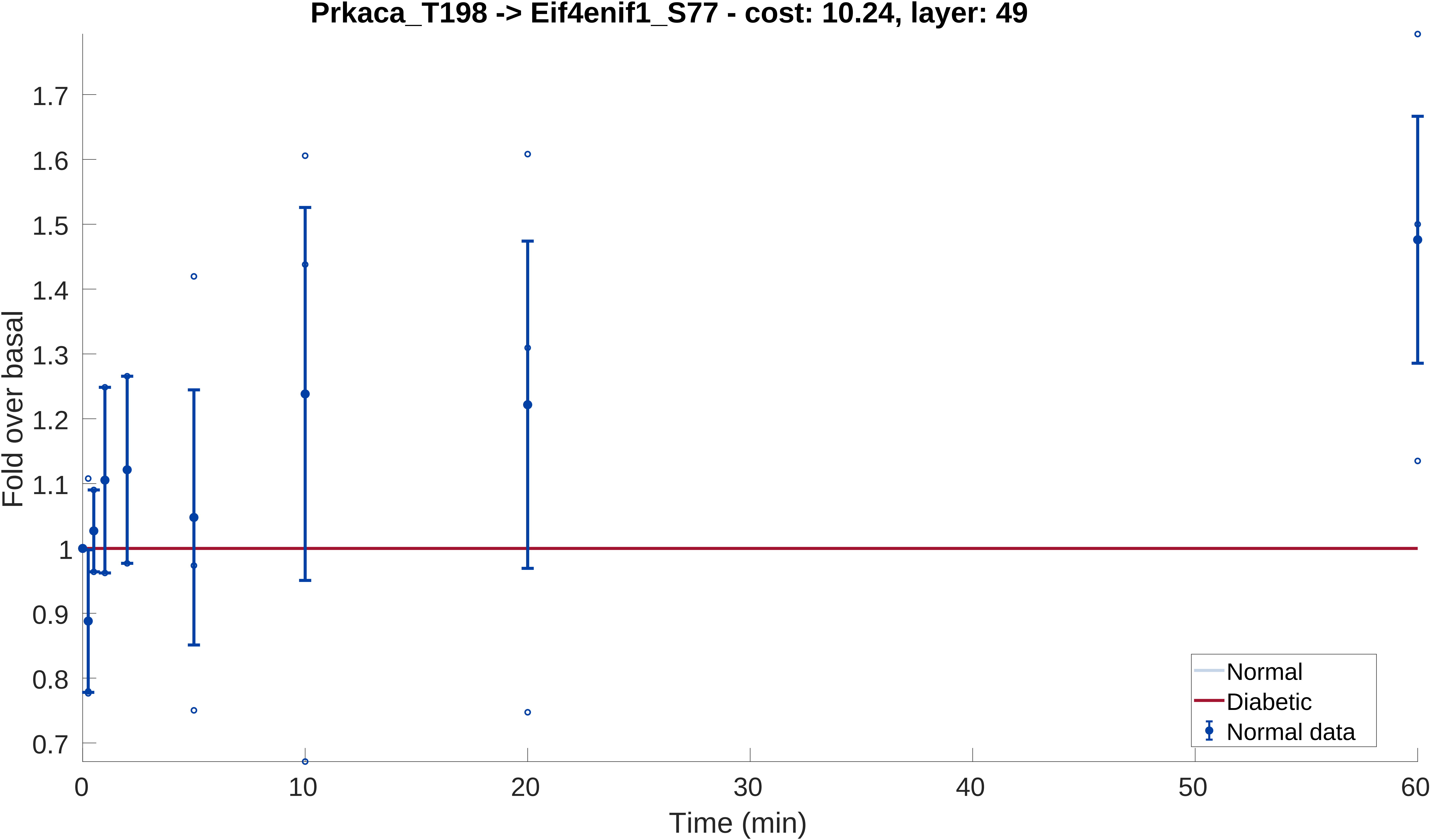

Prrkaca_T198 -> Eif4enif1_S77 - cost: 10.24, layer: 49
Fold over basal
Time (min)
Normal
Diabetic
Normal data
1.7
1.6
1.5
1.4
1.3
1.2
1.1
1
0.9
0.8
0.7
0
10
20
30
40
50
60

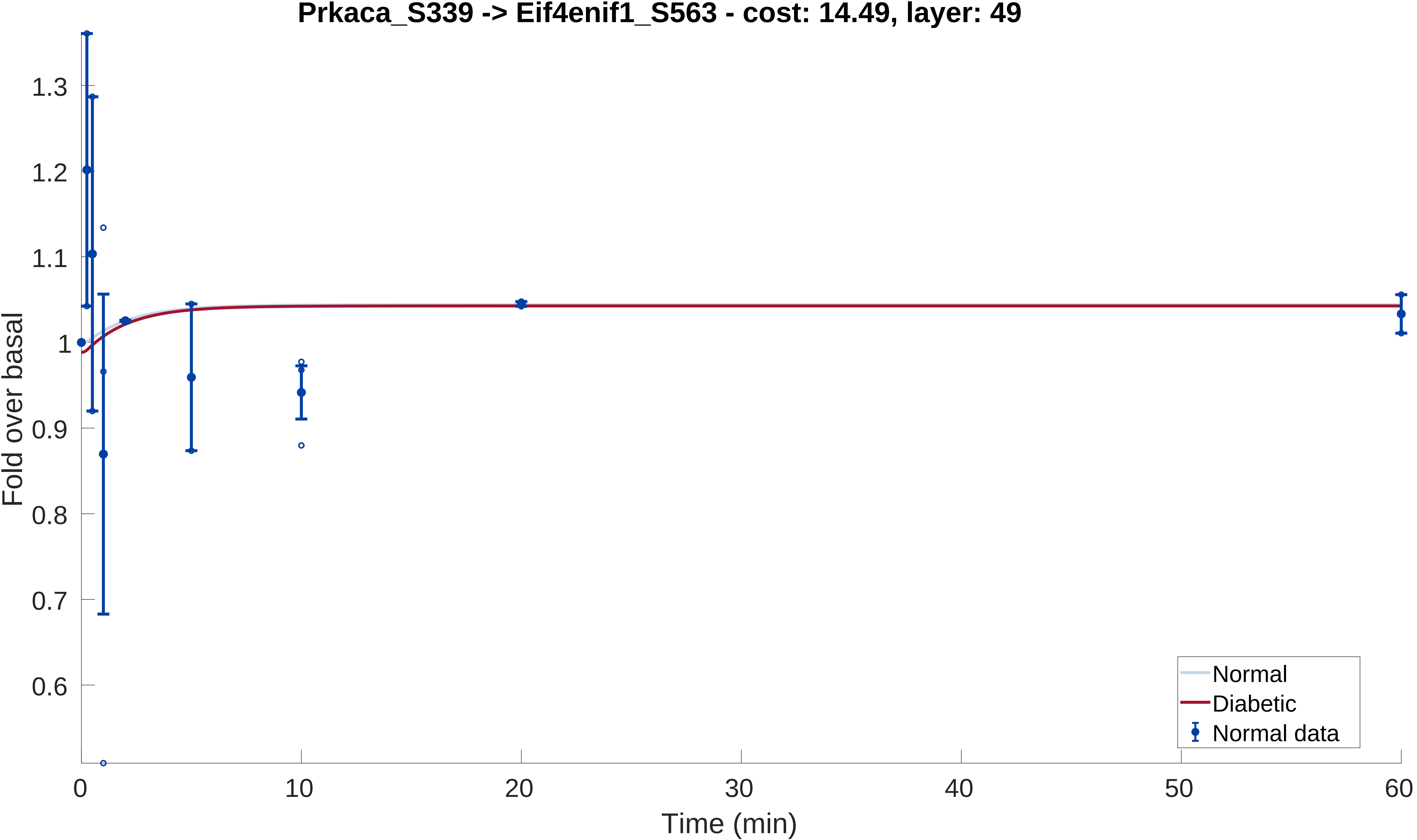

Prkaca_S339 -> Eif4enif1_S563 - cost: 14.49, layer: 49
Fold over basal
1.3
1.2
1.1
1
0.9
0.8
0.7
0.6
0
10
20
30
40
50
60
Time (min)
Normal
Diabetic
Normal data

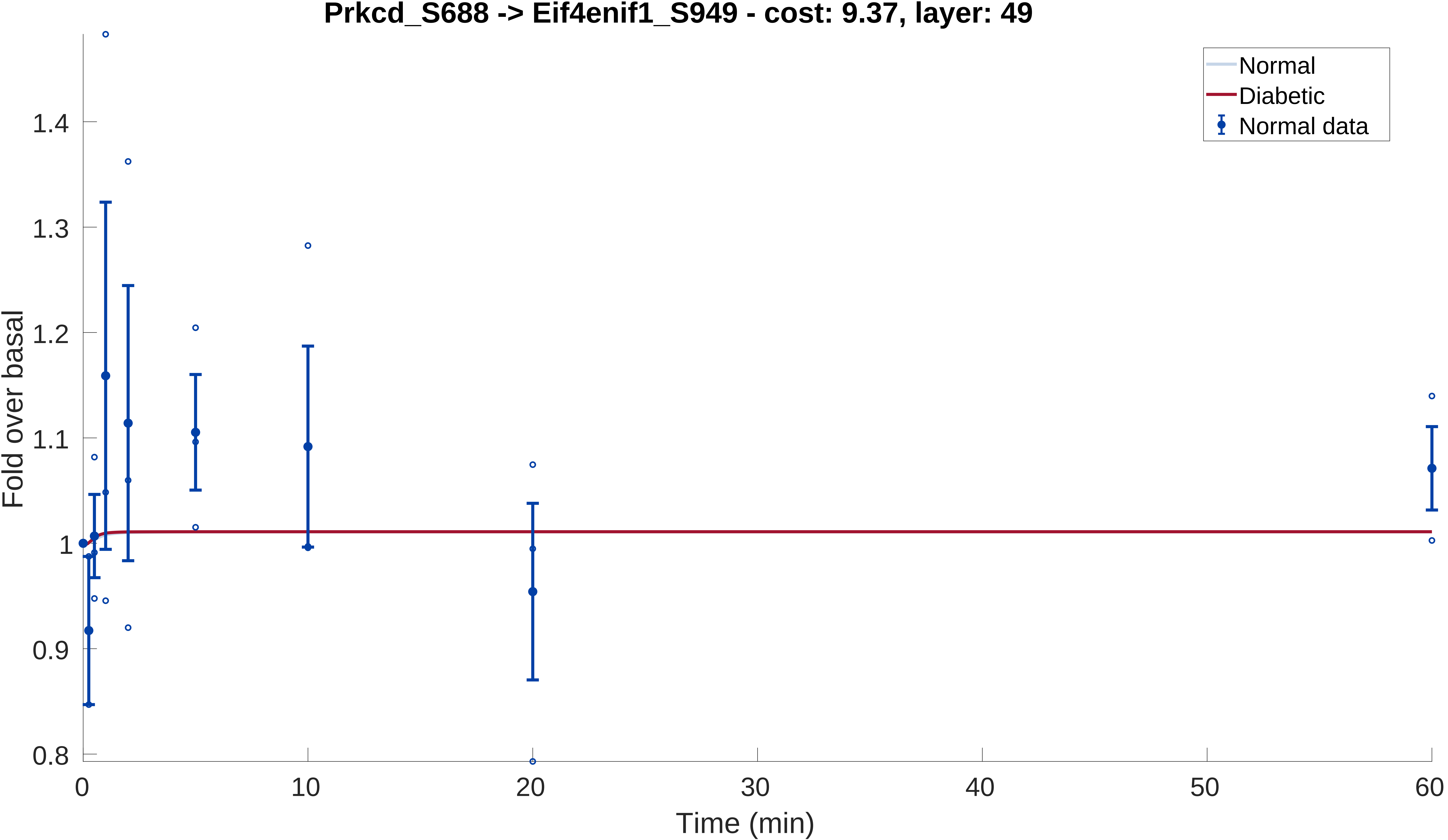
Prkcd_S688 -> Eif4enif1_S949 - cost: 9.37, layer: 49
Normal
Diabetic
Normal data
Fold over basal
Time (min)
0.8
0.9
1
1.1
1.2
1.3
1.4
0
10
20
30
40
50
60

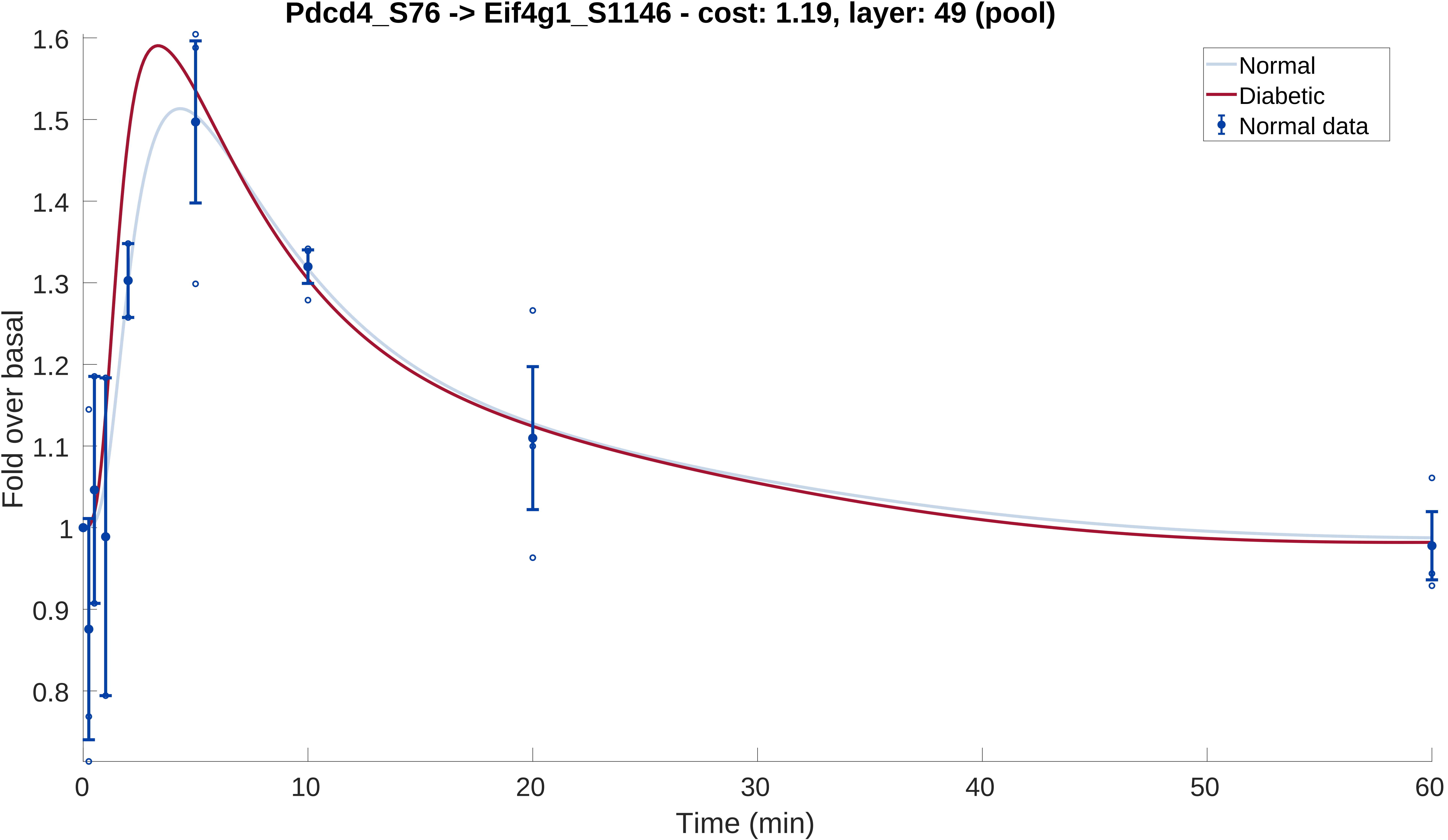

Pdcd4_S76 -> Eif4g1_S1146 - cost: 1.19, layer: 49 (pool)
Fold over basal
1.6
1.5
1.4
1.3
1.2
1.1
1
0.9
0.8
0
10
20
30
40
50
60
Time (min)
Normal
Diabetic
Normal data

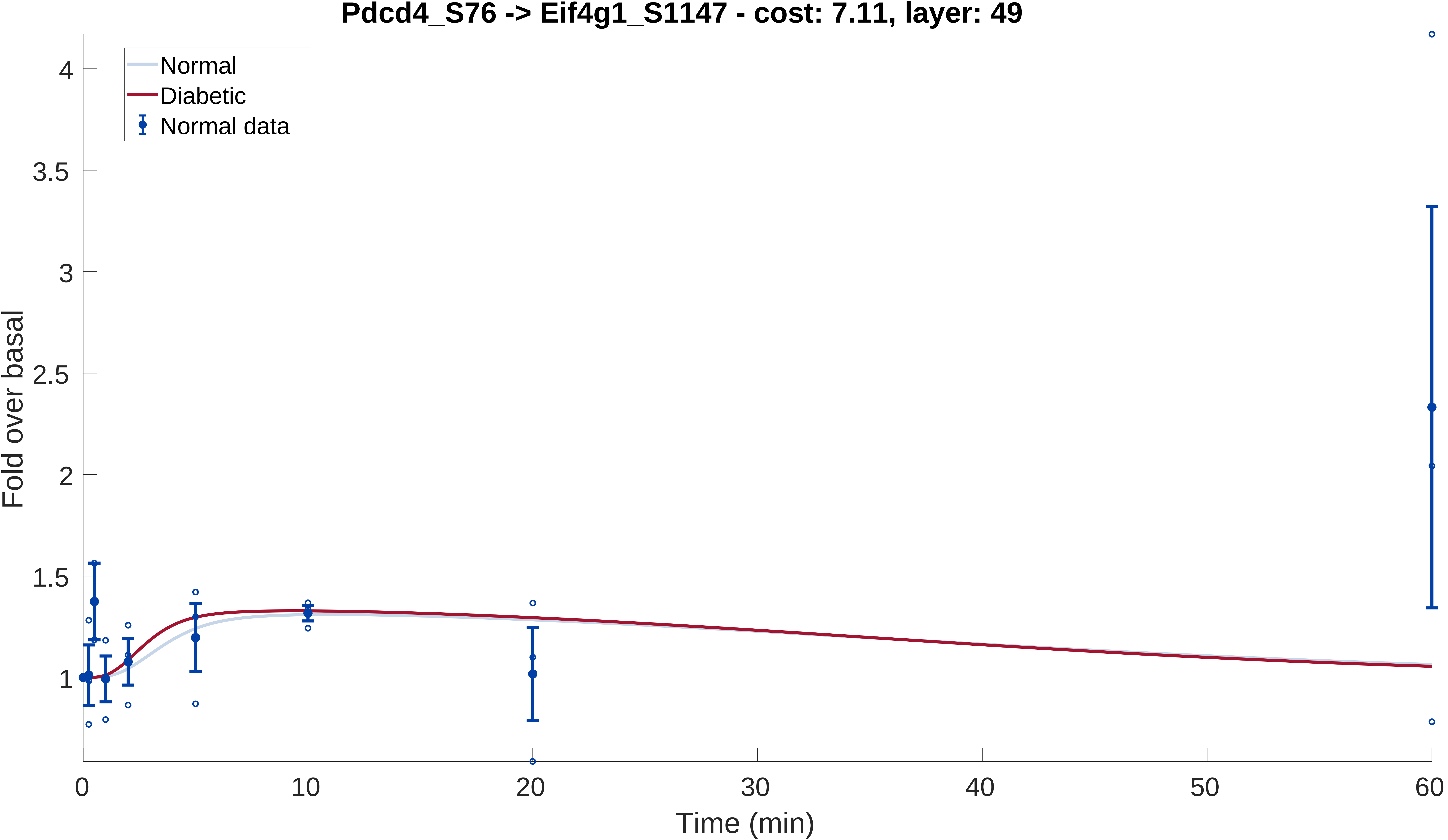

Pdcd4_S76 -> Eif4g1_S1147 - cost: 7.11, layer: 49
Normal
Diabetic
Normal data
Fold over basal
4
3.5
3
2.5
2
1.5
1
0
10
20
30
40
50
60
Time (min)

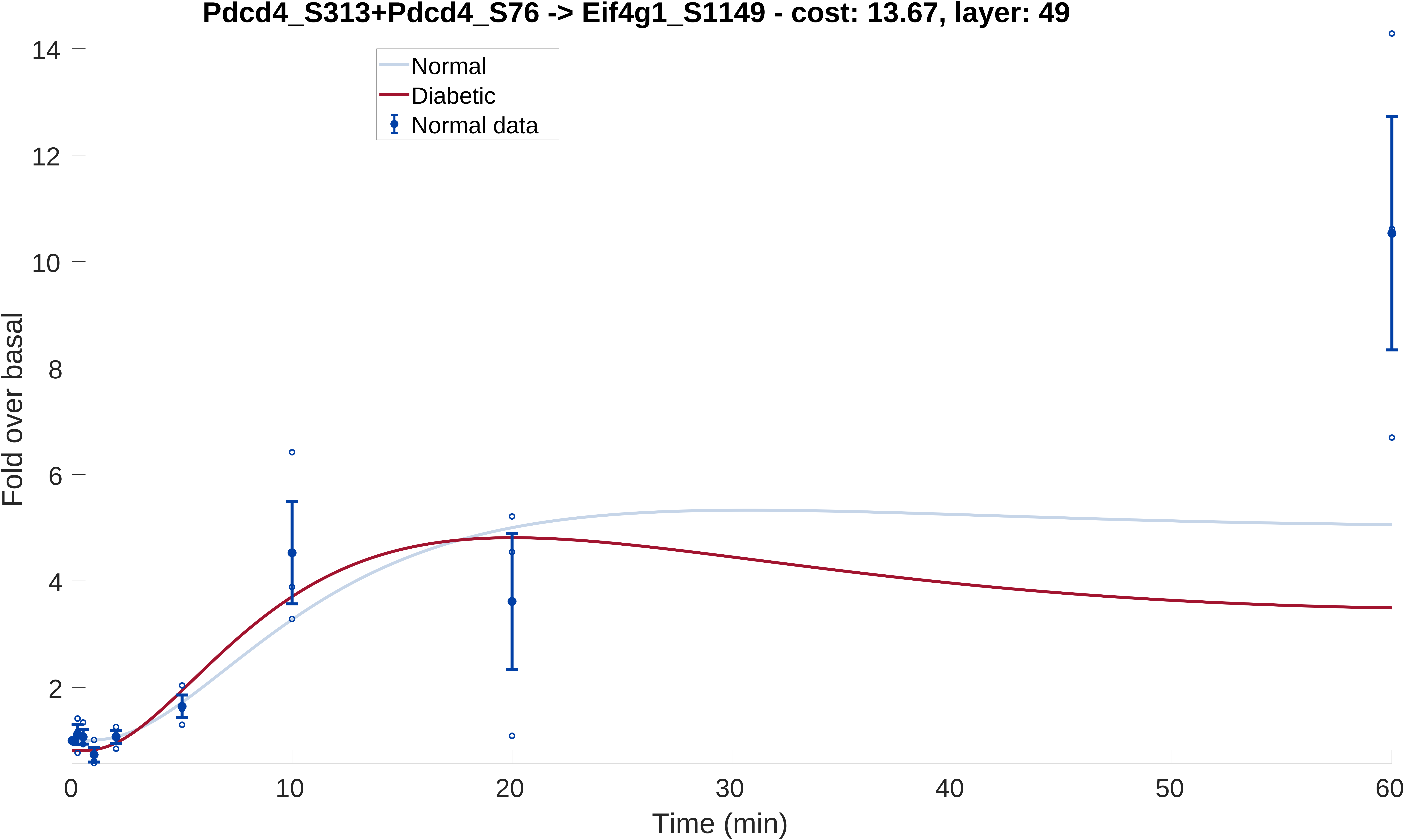

Pdcd4_S313+Pdcd4_S76 -> Eif4g1_S1149 - cost: 13.67, layer: 49
Normal
Diabetic
Normal data
Fold over basal
Time (min)
0
10
20
30
40
50
60
2
4
6
8
10
12
14

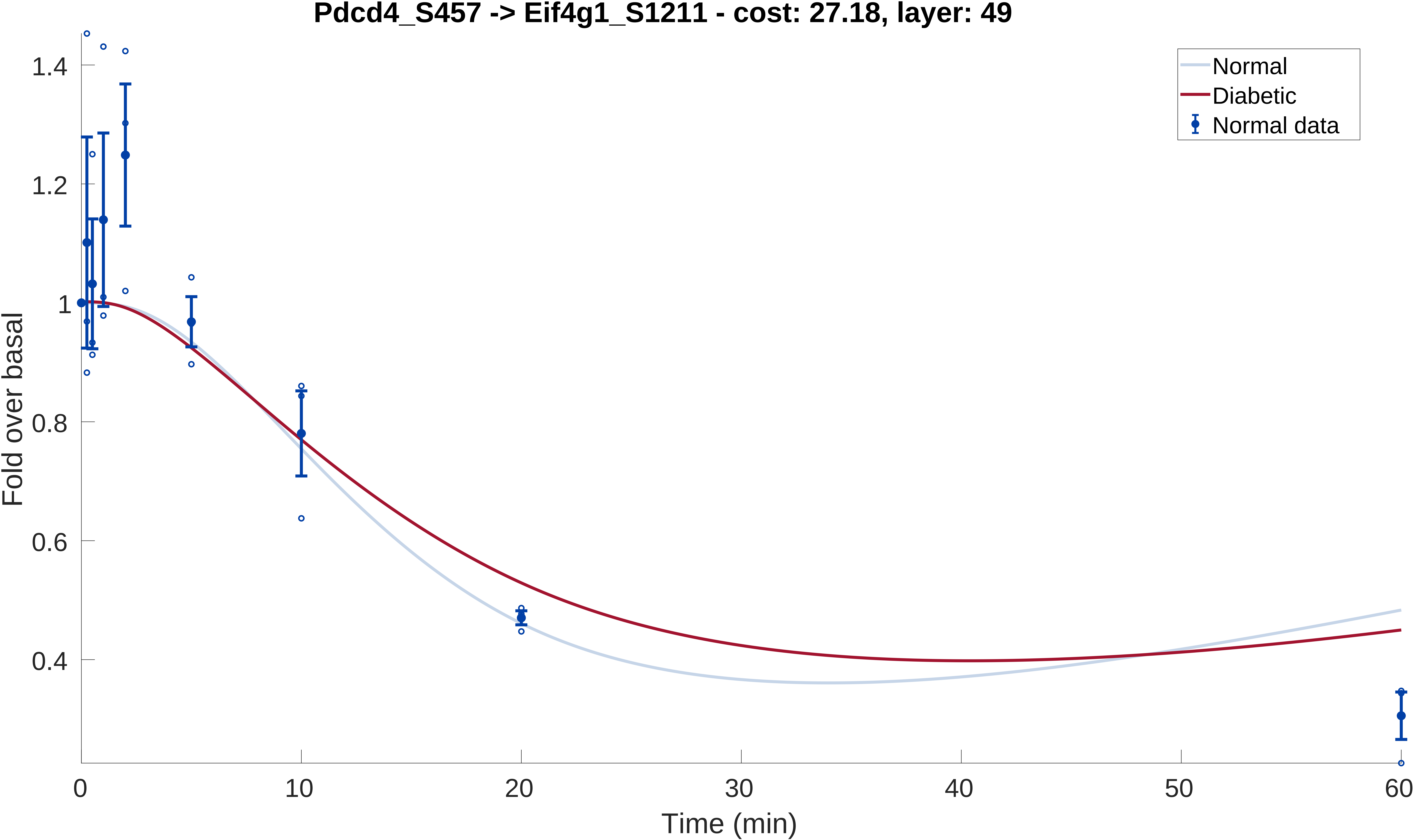

Pdcd4_S457 -> Eif4g1_S1211 - cost: 27.18, layer: 49
Normal
Diabetic
Normal data
Fold over basal
1.4
1.2
1
0.8
0.6
0.4
0
10
20
30
40
50
60
Time (min)

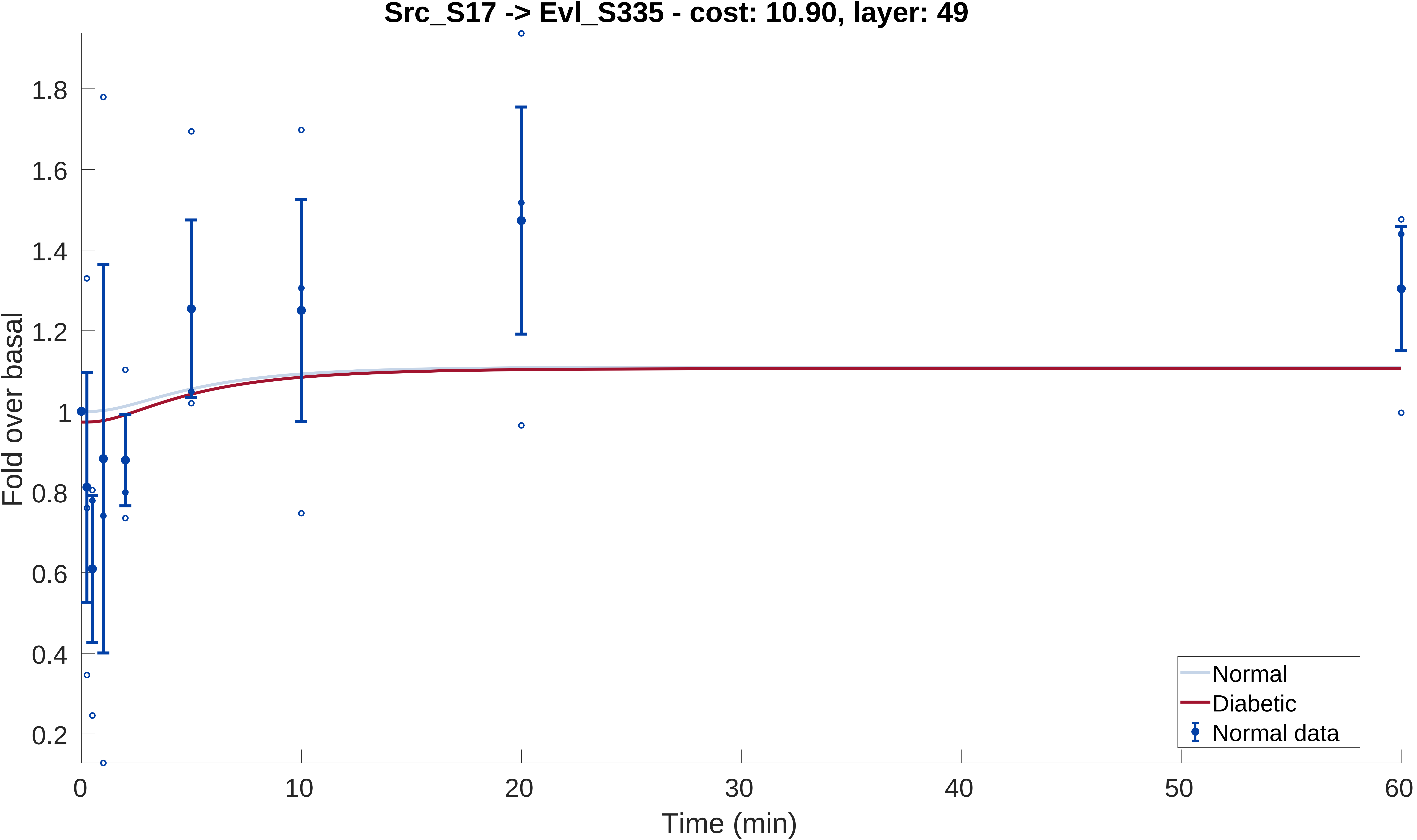
Src_S17 -> Evl_S335 - cost: 10.90, layer: 49
Fold over basal
1.8
1.6
1.4
1.2
1
0.8
0.6
0.4
0.2
0
10
20
30
40
50
60
Time (min)
Normal
Diabetic
Normal data

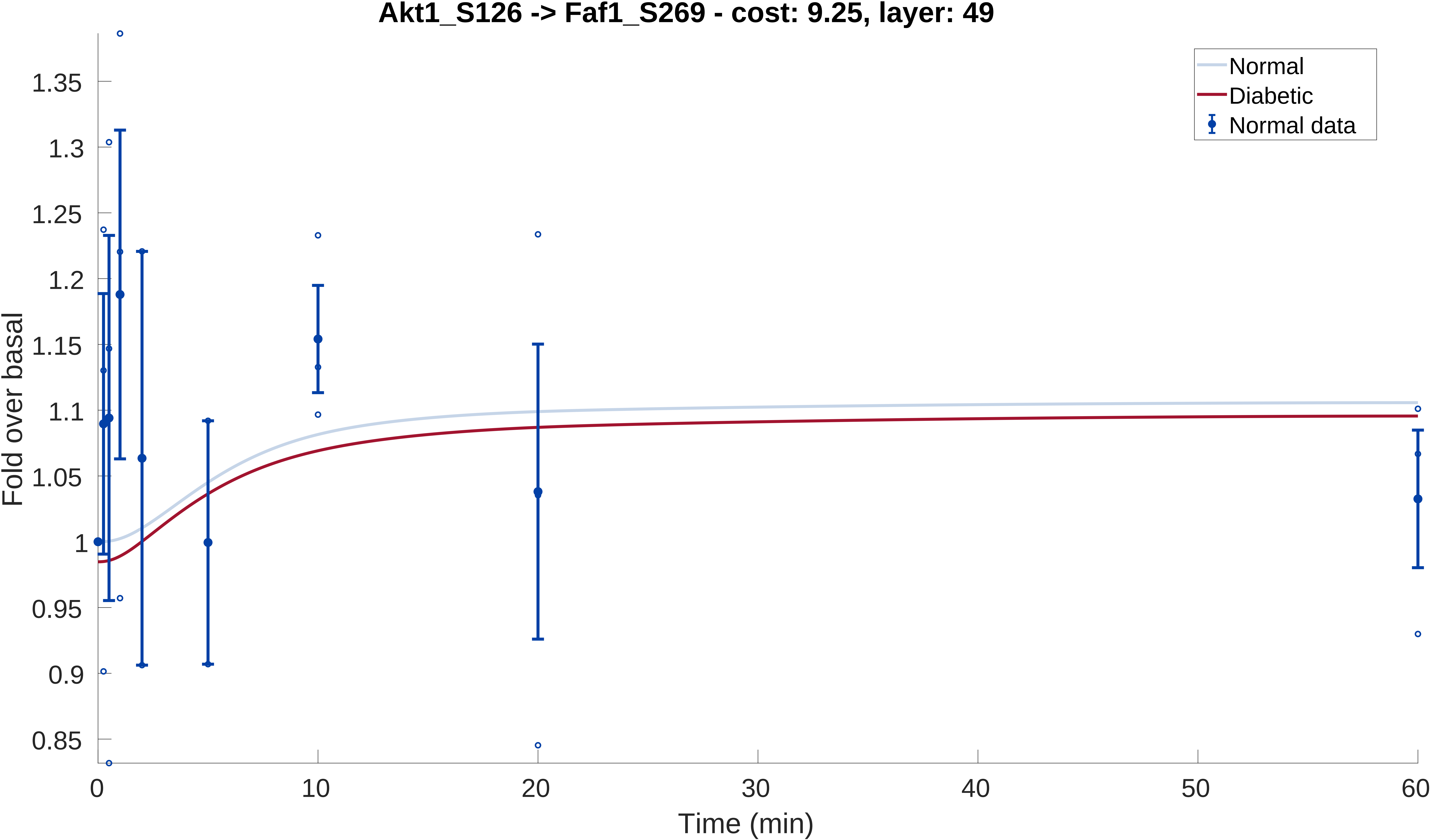

Akt1_S126 -> Faf1_S269 - cost: 9.25, layer: 49
Normal
Diabetic
Normal data
Fold over basal
Time (min)
1.35
1.3
1.25
1.2
1.15
1.1
1.05
1
0.95
0.9
0.85
0
10
20
30
40
50
60

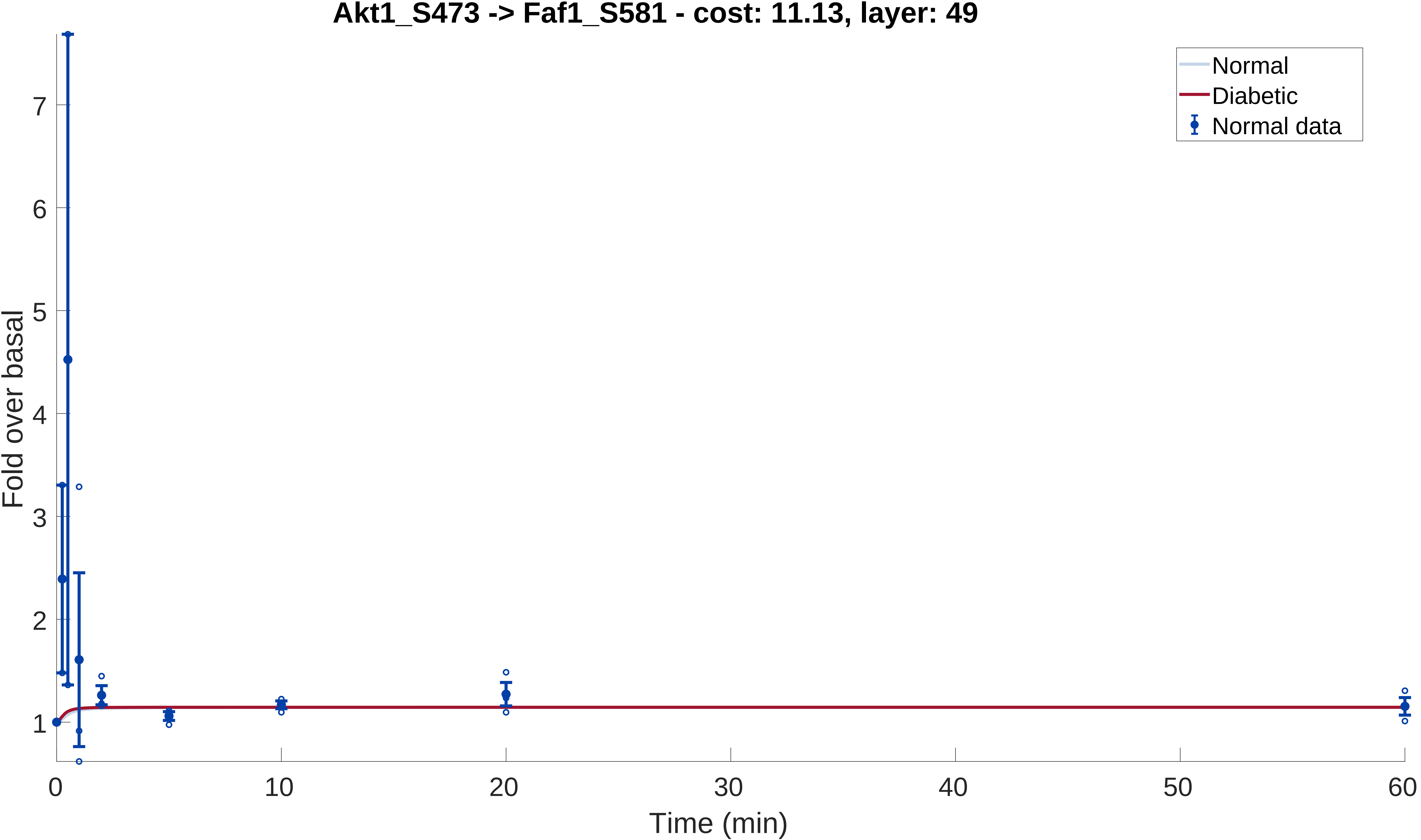

Akt1_S473 -> Faf1_S581 - cost: 11.13, layer: 49
Normal
Diabetic
Normal data
Fold over basal
Time (min)
1
2
3
4
5
6
7
0
10
20
30
40
50
60

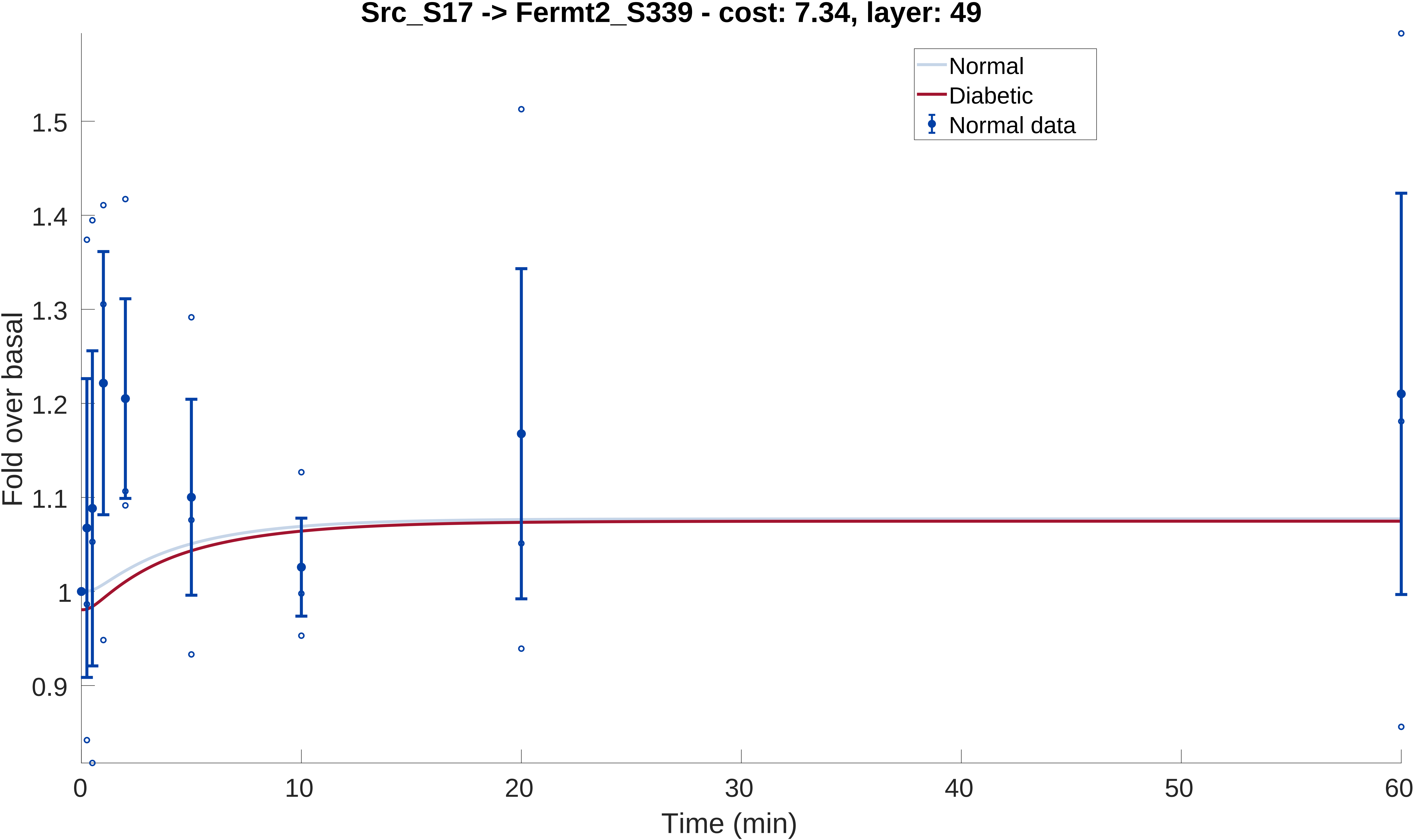

Src_S17 -> Fermt2_S339 - cost: 7.34, layer: 49
Normal
Diabetic
Normal data
Fold over basal
1.5
1.4
1.3
1.2
1.1
1
0.9
0
10
20
30
40
50
60
Time (min)

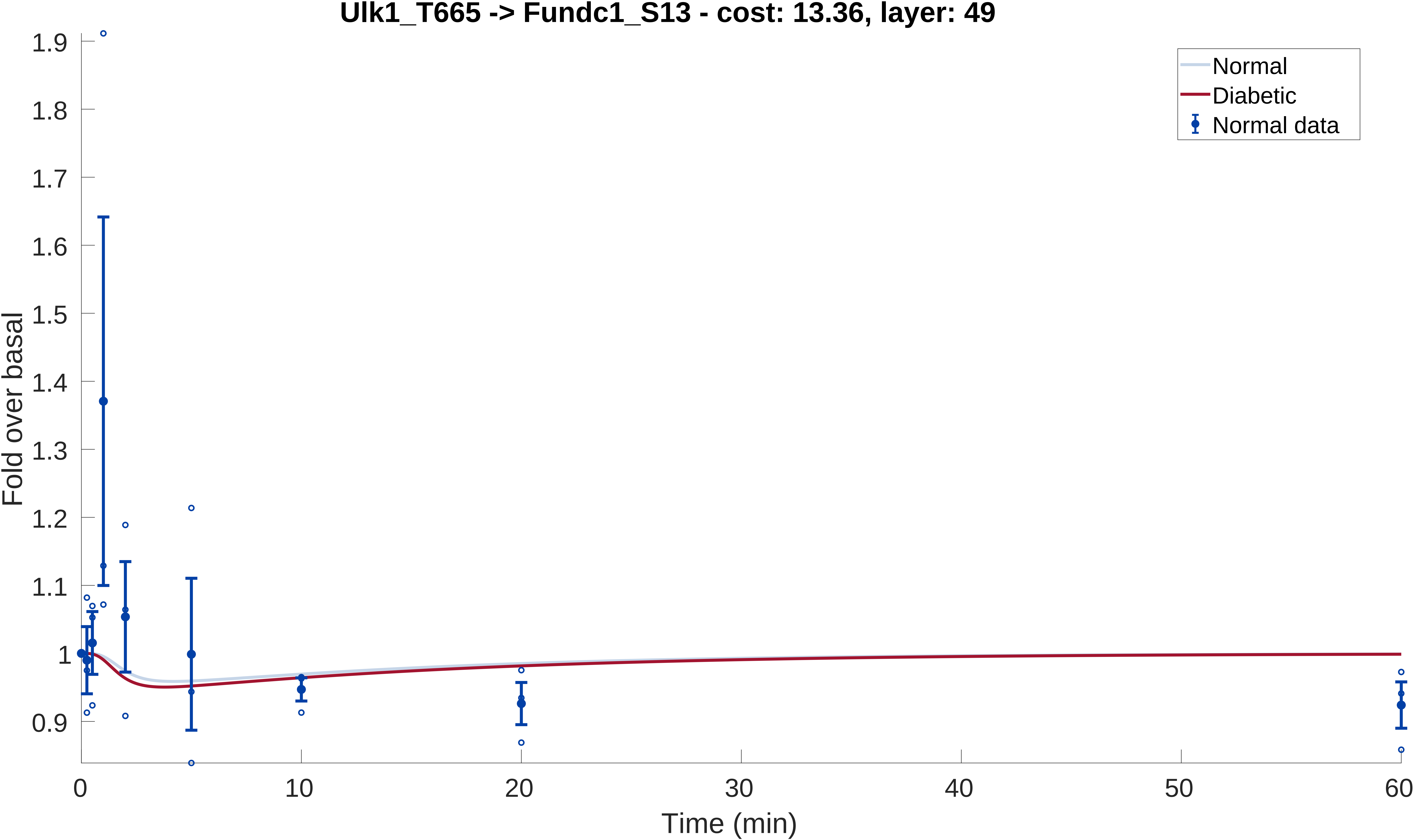
Ulk1_T665 -> Fundc1_S13 - cost: 13.36, layer: 49
Normal
Diabetic
Normal data
Fold over basal
Time (min)
1.9
1.8
1.7
1.6
1.5
1.4
1.3
1.2
1.1
1
0.9
0
10
20
30
40
50
60

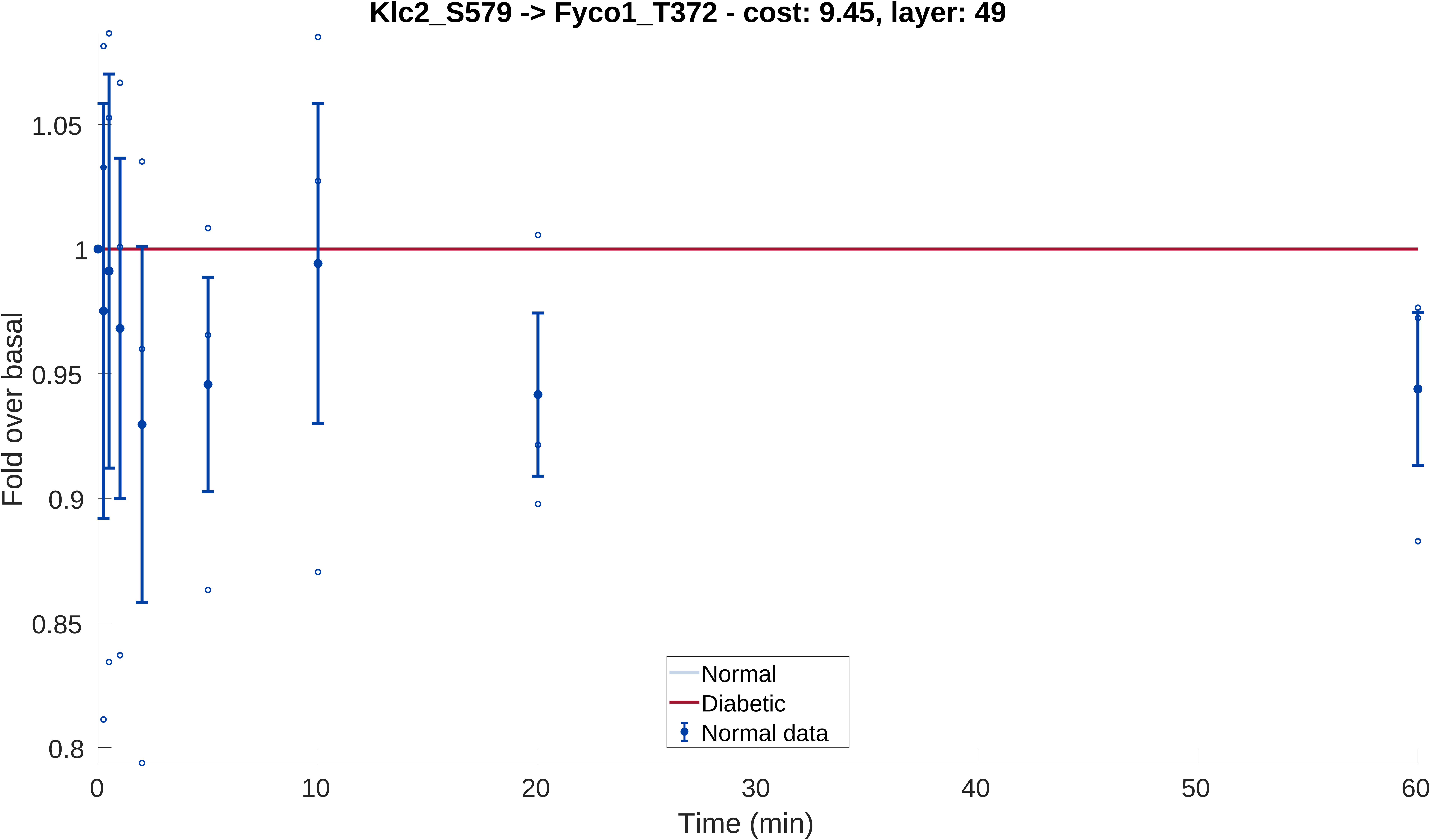

Klc2_S579 -> Fyco1_T372 - cost: 9.45, layer: 49
Fold over basal
1.05
1
0.95
0.9
0.85
0.8
0
10
20
30
40
50
60
Time (min)
Normal
Diabetic
Normal data

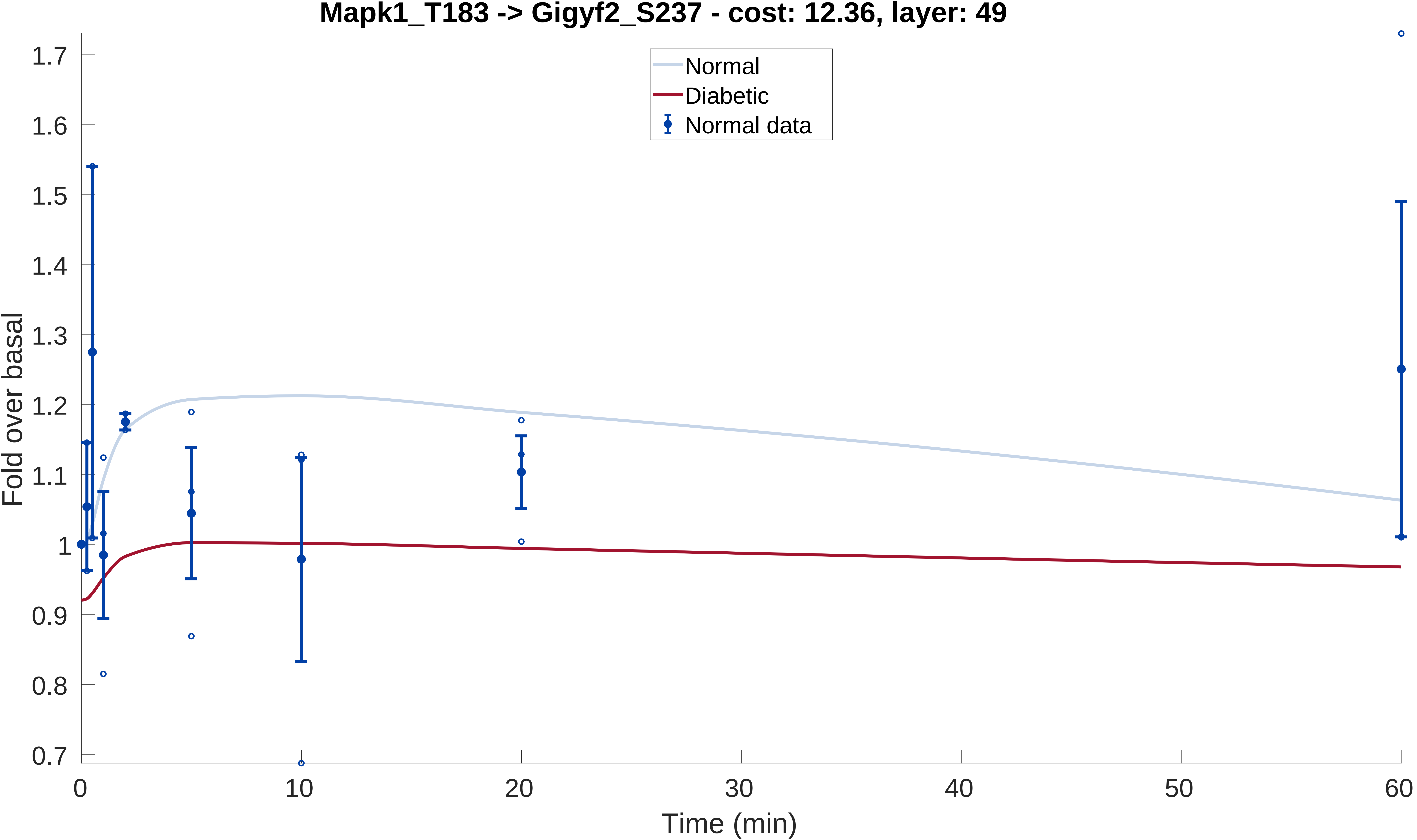

Mapk1_T183 -> Gigyf2_S237 - cost: 12.36, layer: 49
Normal
Diabetic
Normal data
Fold over basal
Time (min)
1.7
1.6
1.5
1.4
1.3
1.2
1.1
1
0.9
0.8
0.7
0
10
20
30
40
50
60

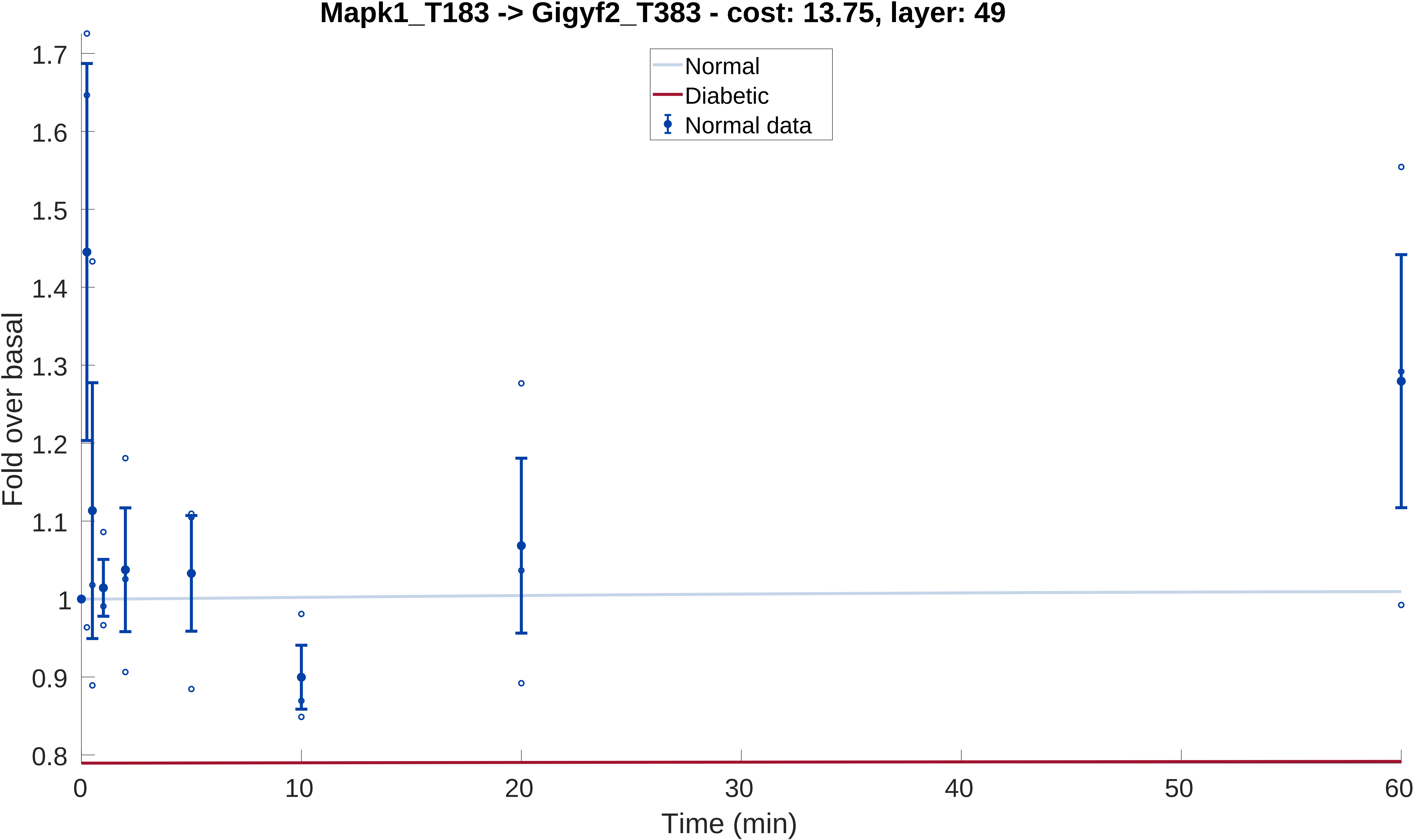

Mapk1_T183 -> Gigyf2_T383 - cost: 13.75, layer: 49
Normal
Diabetic
Normal data
Fold over basal
1.7
1.6
1.5
1.4
1.3
1.2
1.1
1
0.9
0.8
0
10
20
30
40
50
60
Time (min)

Ptpn12_S434 -> Git2_S359 - cost: 4.88, layer: 49

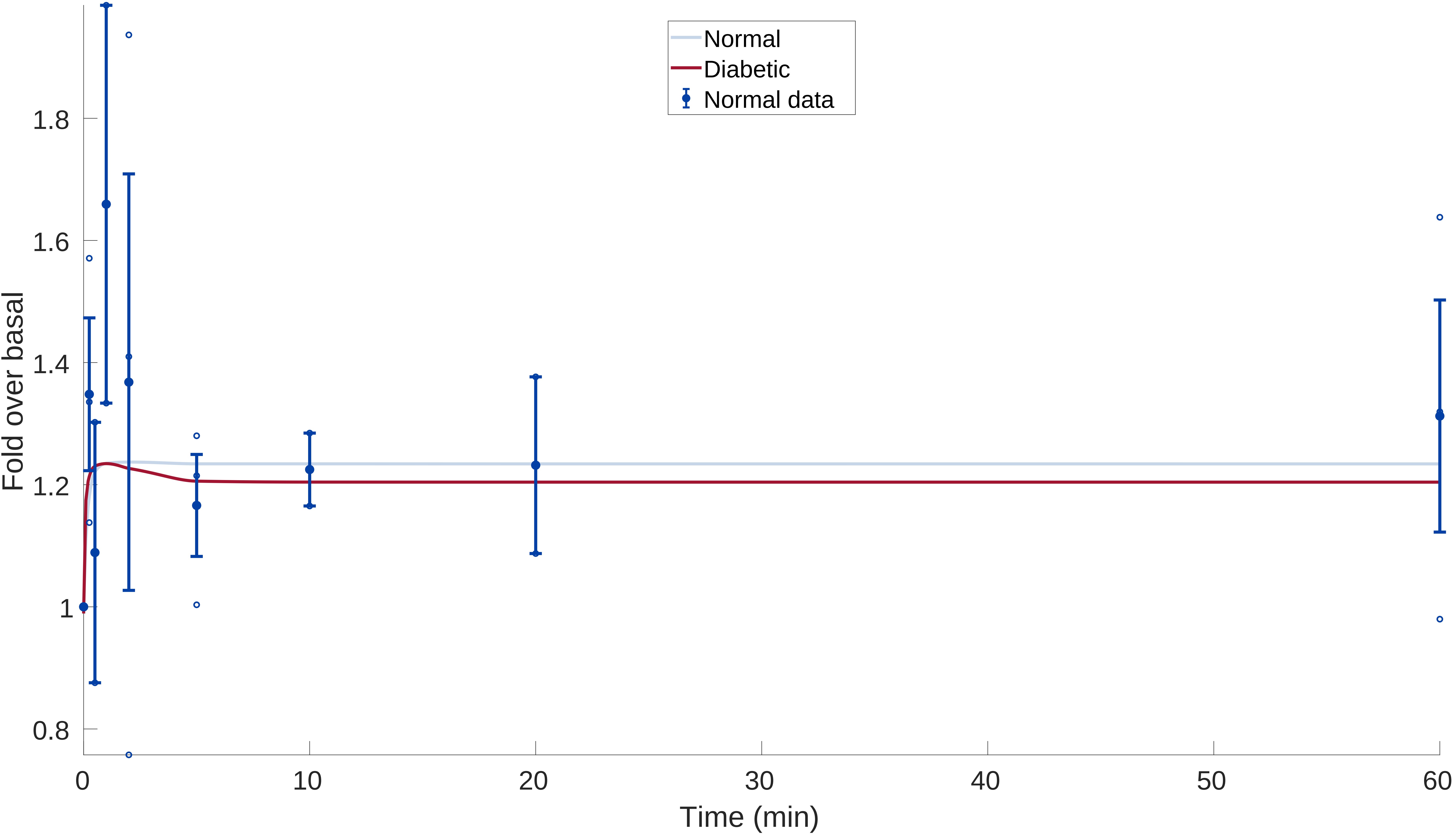

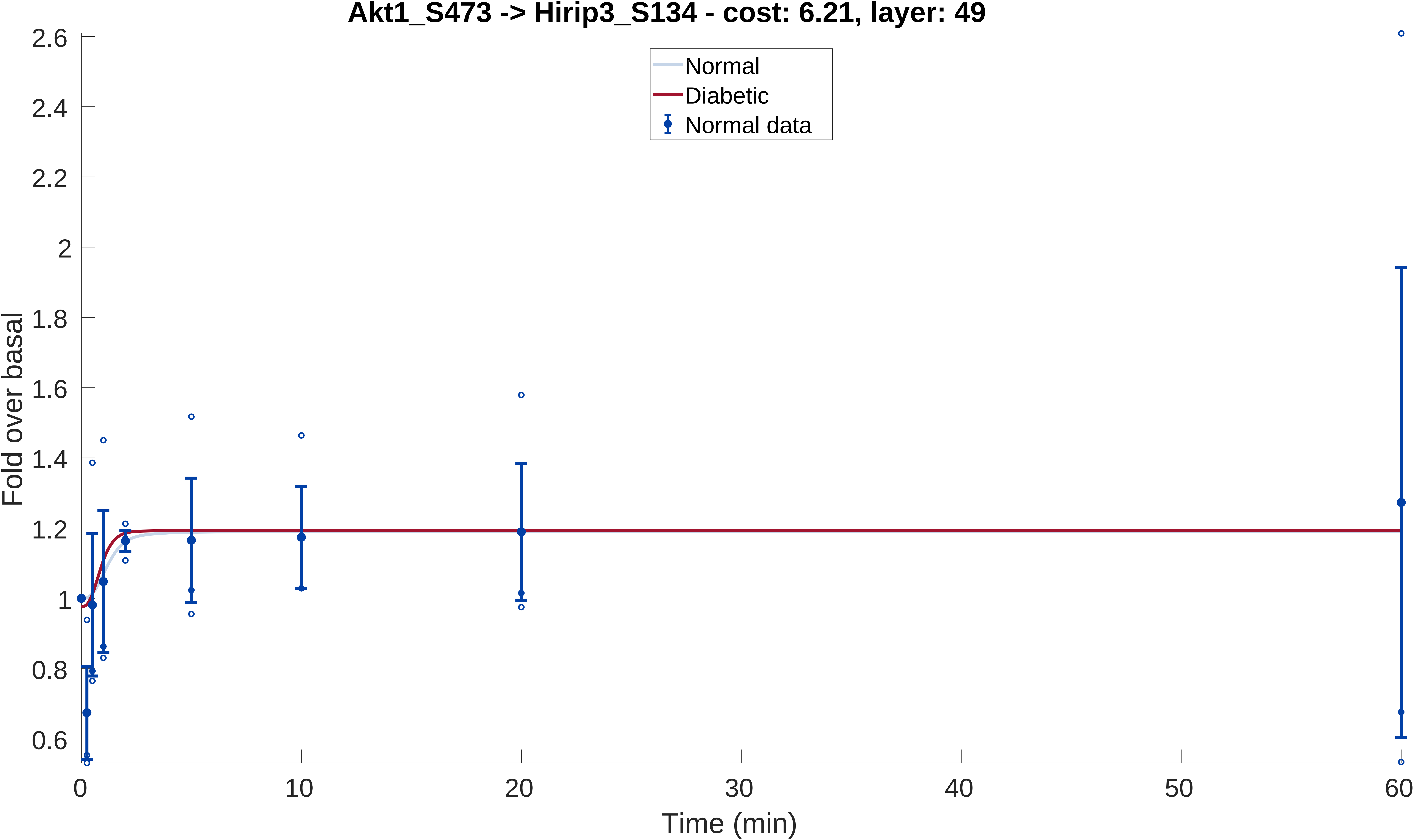

Akt1_S473 -> Hirip3_S134 - cost: 6.21, layer: 49
Normal
Diabetic
Normal data
Fold over basal
Time (min)
0.6
0.8
1
1.2
1.4
1.6
1.8
2
2.2
2.4
2.6
0
10
20
30
40
50
60

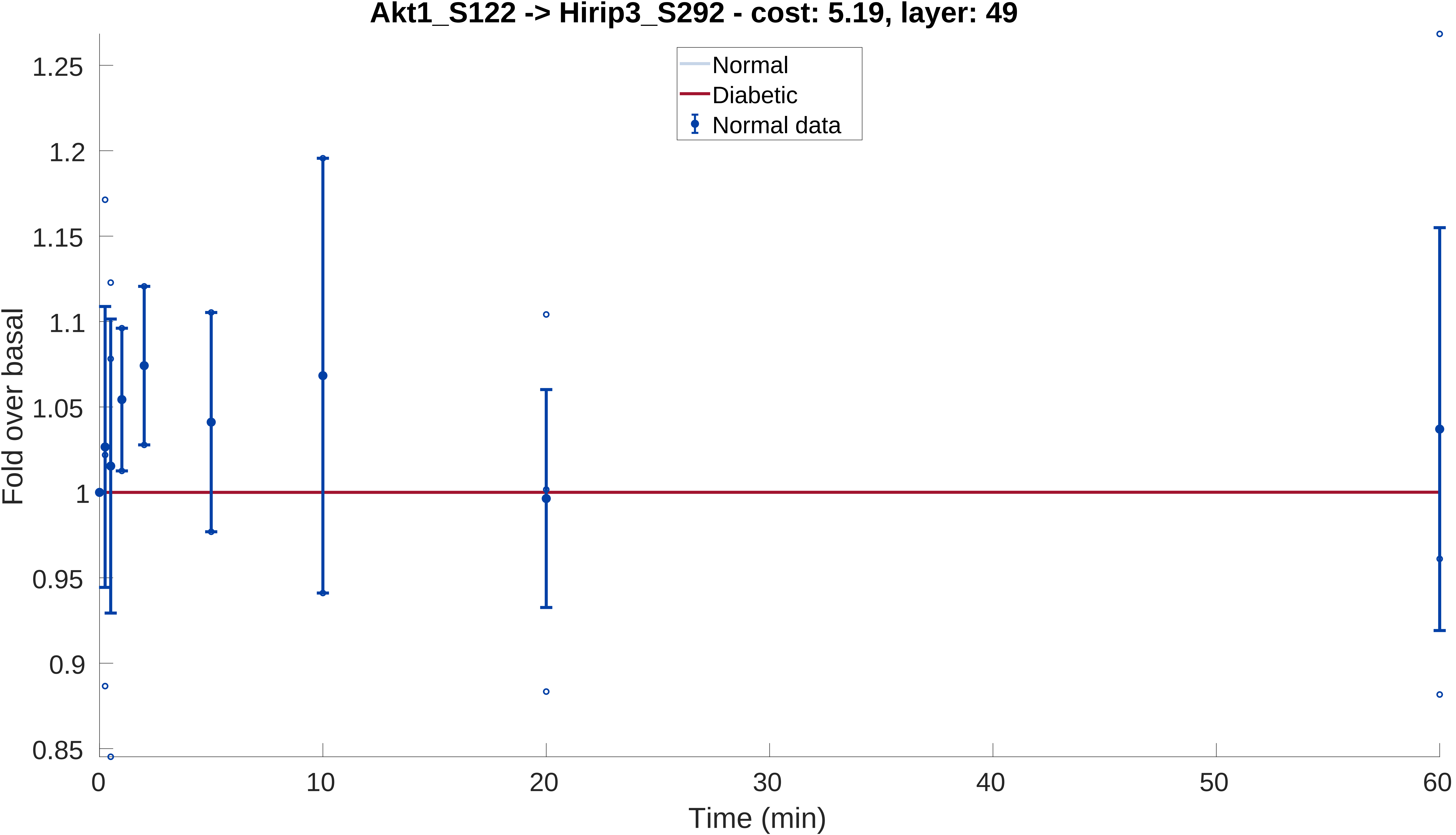

Akt1_S122 -> Hirip3_S292 - cost: 5.19, layer: 49
Normal
Diabetic
Normal data
Fold over basal
1.25
1.2
1.15
1.1
1.05
1
0.95
0.9
0.85
0
10
20
30
40
50
60
Time (min)

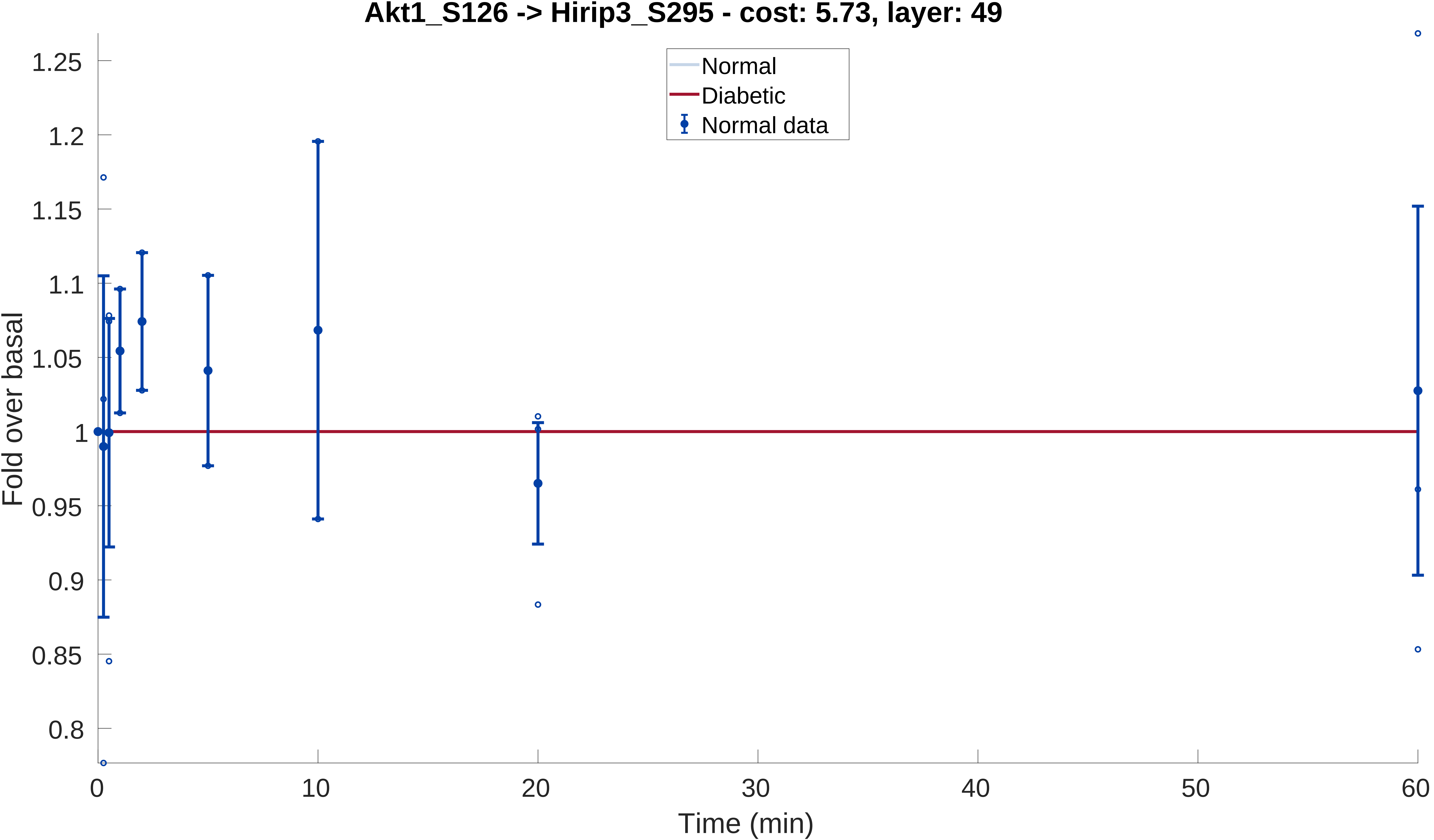

Akt1_S126 -> Hirip3_S295 - cost: 5.73, layer: 49
Normal
Diabetic
Normal data
Fold over basal
Time (min)
1.25
1.2
1.15
1.1
1.05
1
0.95
0.9
0.85
0.8
0
10
20
30
40
50
60

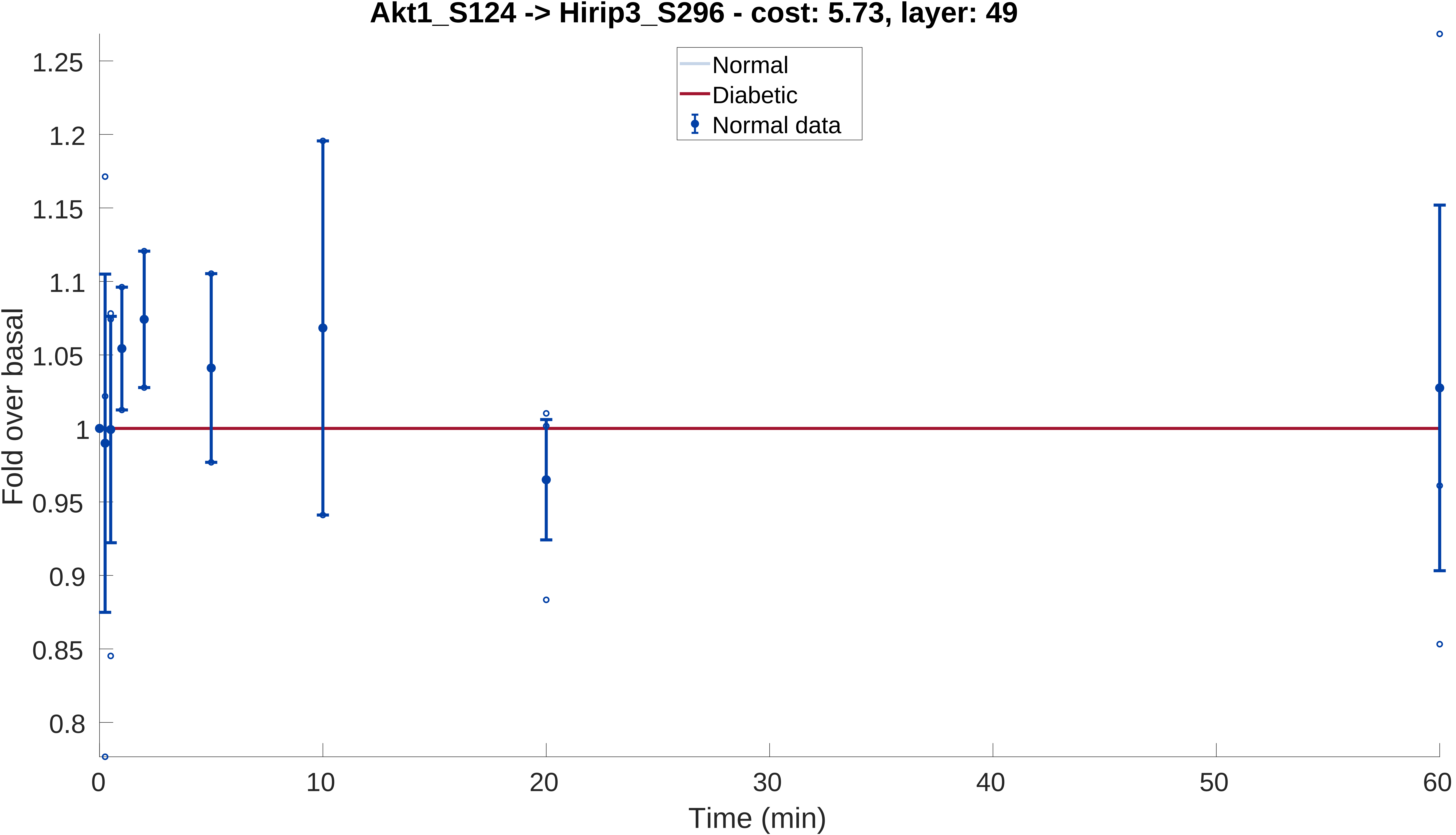

Akt1_S124 -> Hirip3_S296 - cost: 5.73, layer: 49
Normal
Diabetic
Normal data
Fold over basal
Time (min)
1.25
1.2
1.15
1.1
1.05
1
0.95
0.9
0.85
0.8
0
10
20
30
40
50
60

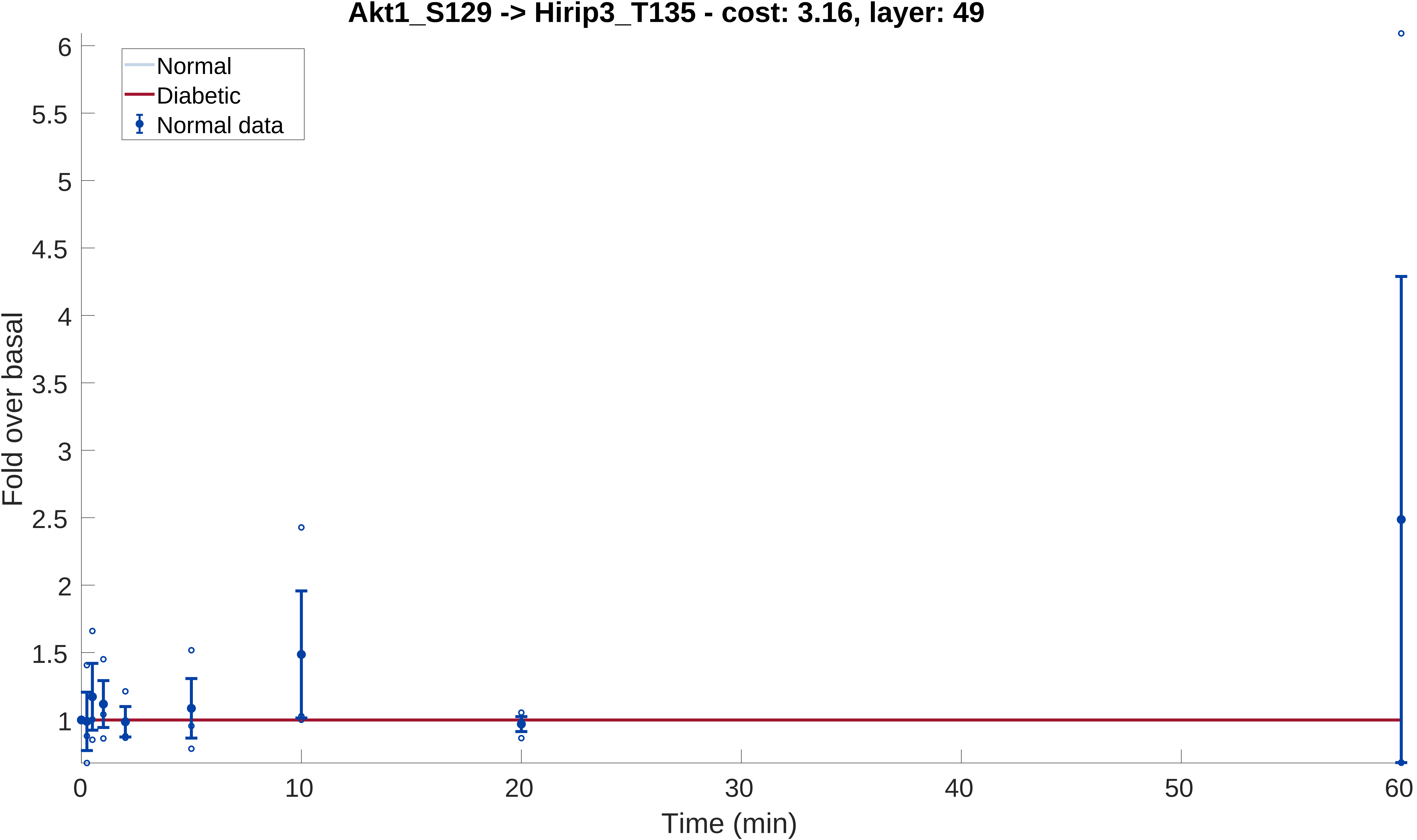

Akt1_S129 -> Hirip3_T135 - cost: 3.16, layer: 49
Normal
Diabetic
Normal data
Fold over basal
Time (min)
0
10
20
30
40
50
60
1
1.5
2
2.5
3
3.5
4
4.5
5
5.5
6

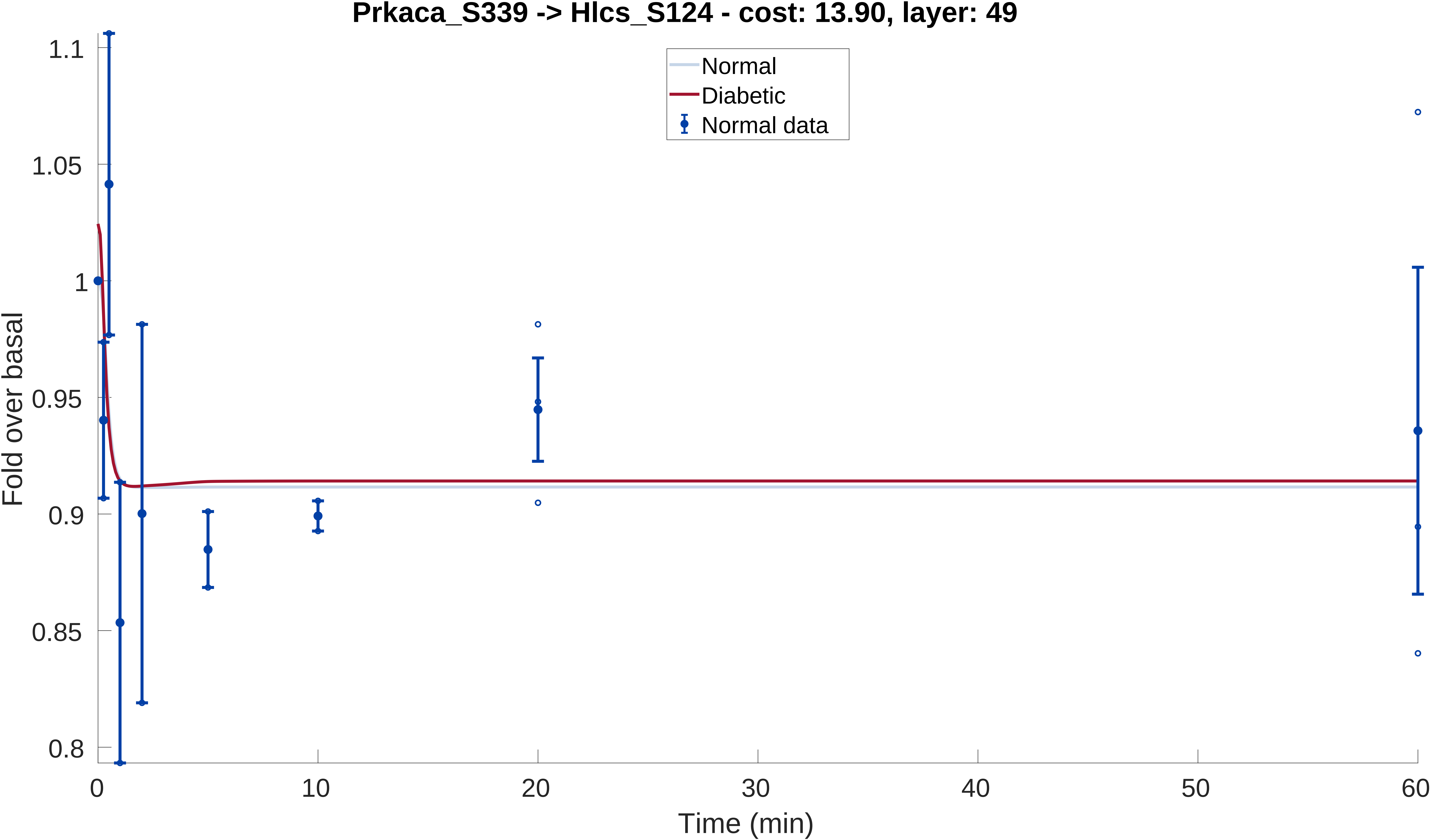

Prkaca_S339 -> Hlcs_S124 - cost: 13.90, layer: 49
Normal
Diabetic
Normal data
Fold over basal
1.1
1.05
1
0.95
0.9
0.85
0.8
0
10
20
30
40
50
60
Time (min)

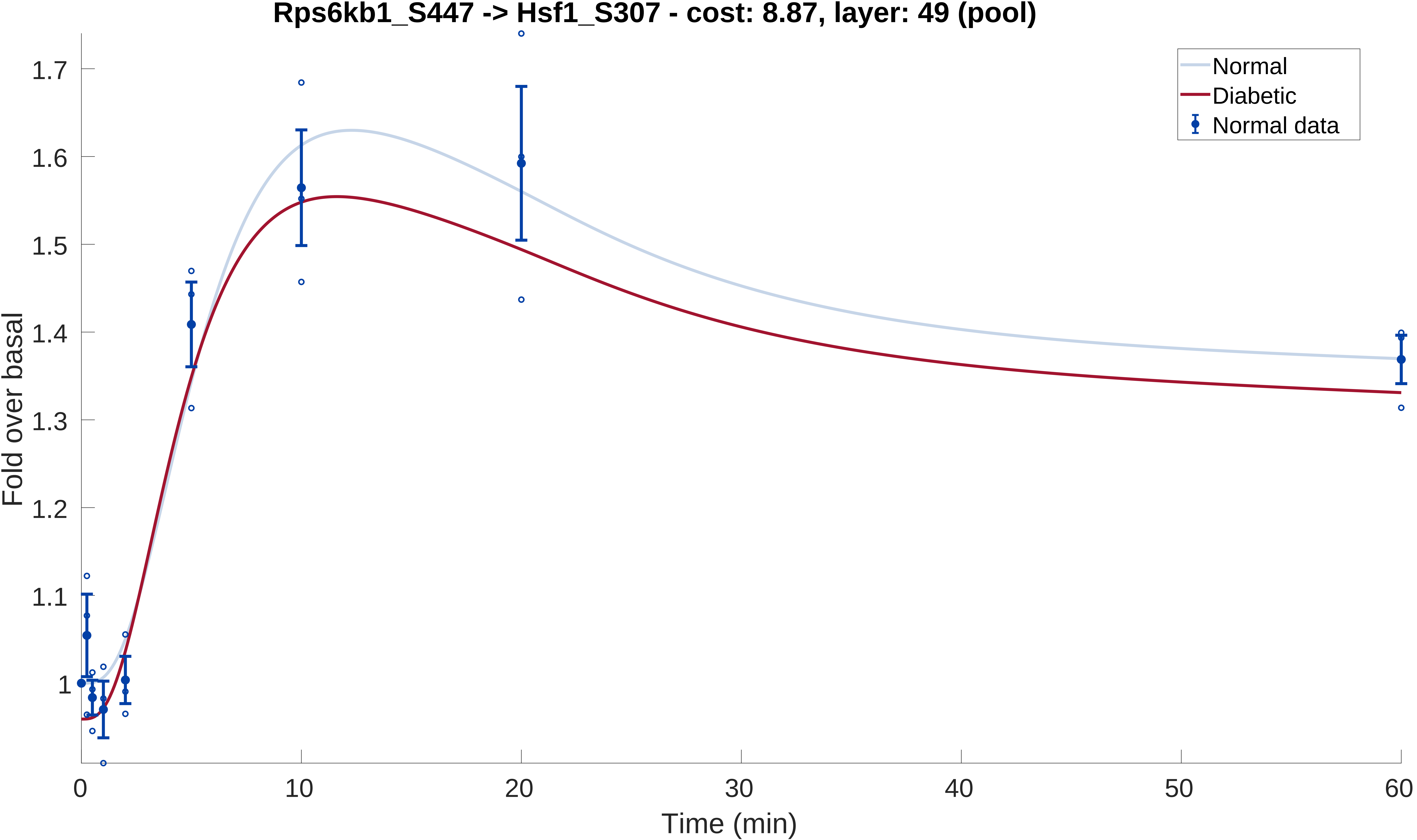
Rps6kb1_S447 -> Hsf1_S307 - cost: 8.87, layer: 49 (pool)
Normal
Diabetic
Normal data
Fold over basal
Time (min)
1
1.1
1.2
1.3
1.4
1.5
1.6
1.7
0
10
20
30
40
50
60

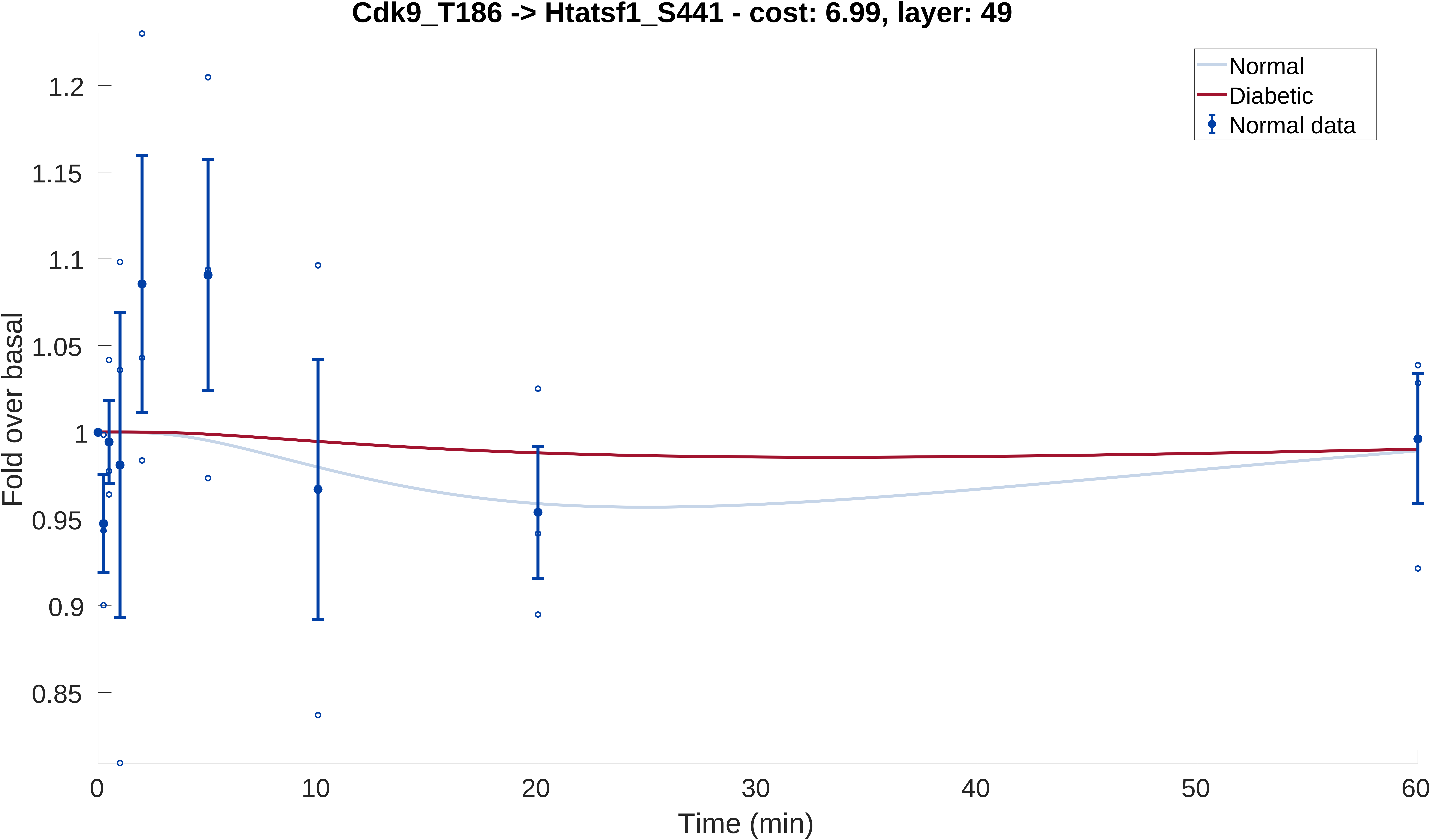

Cdk9_T186 -> Htatsf1_S441 - cost: 6.99, layer: 49
Normal
Diabetic
Normal data
Fold over basal
1.2
1.15
1.1
1.05
1
0.95
0.9
0.85
0
10
20
30
40
50
60
Time (min)

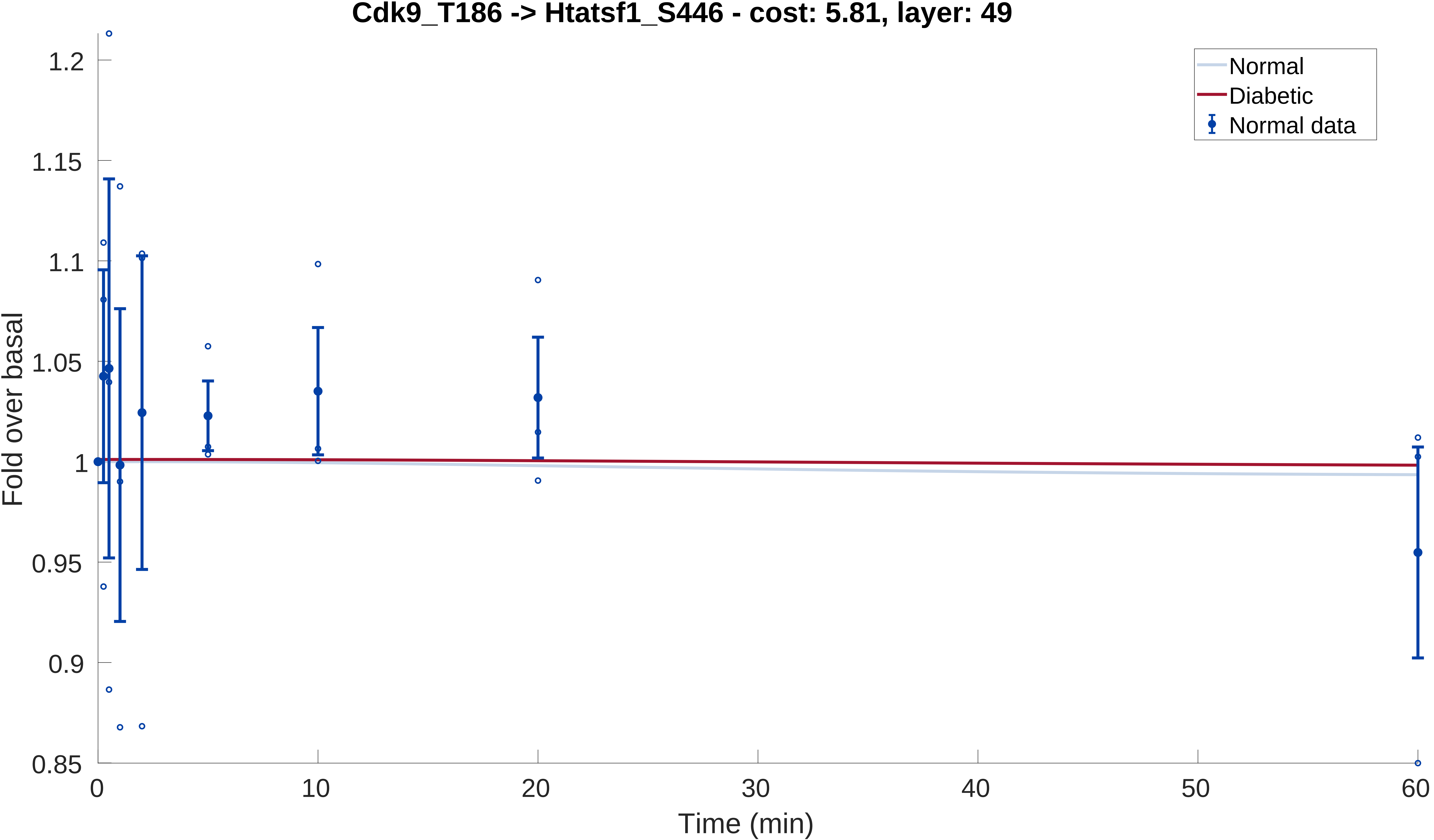

Cdk9_T186 -> Htatsf1_S446 - cost: 5.81, layer: 49
Normal
Diabetic
Normal data
Fold over basal
1.2
1.15
1.1
1.05
1
0.95
0.9
0.85
0
10
20
30
40
50
60
Time (min)

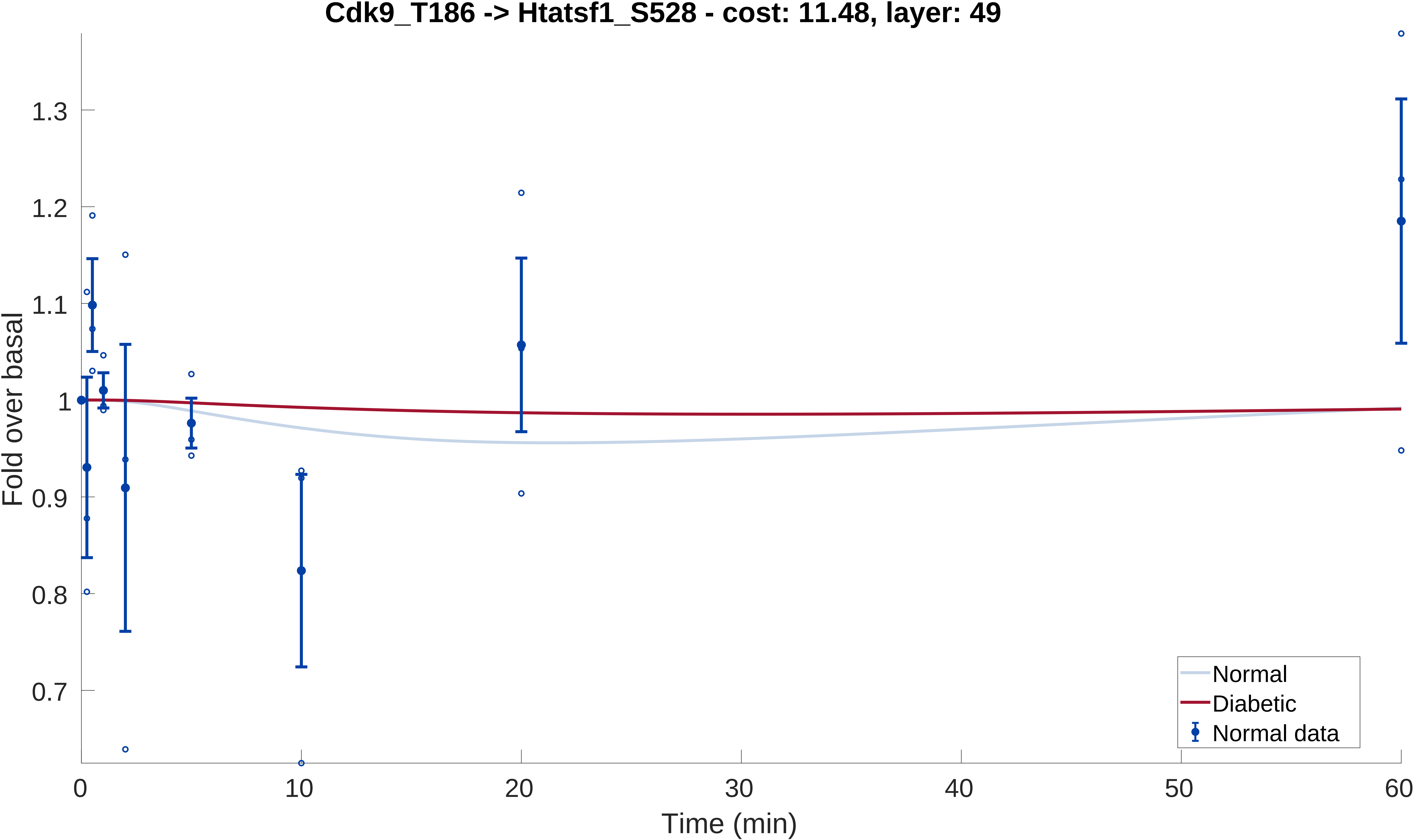
Cdk9_T186 -> Htatsf1_S528 - cost: 11.48, layer: 49
Fold over basal
Time (min)
0.7
0.8
0.9
1
1.1
1.2
1.3
0
10
20
30
40
50
60
Normal
Diabetic
Normal data

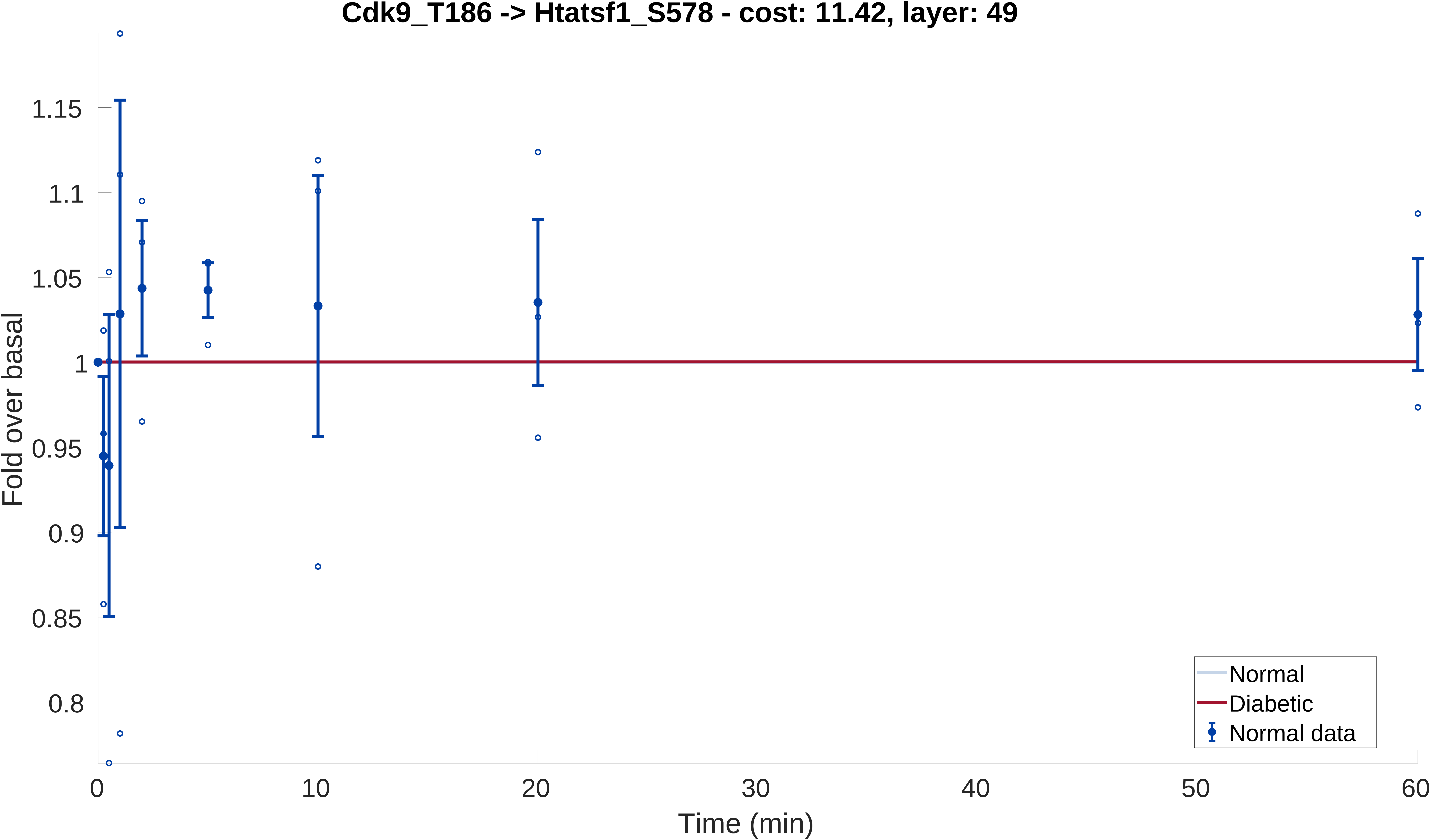
Cdk9_T186 -> Htatsf1_S578 - cost: 11.42, layer: 49
Fold over basal
1.15
1.1
1.05
1
0.95
0.9
0.85
0.8
0
10
20
30
40
50
60
Time (min)
Normal
Diabetic
Normal data

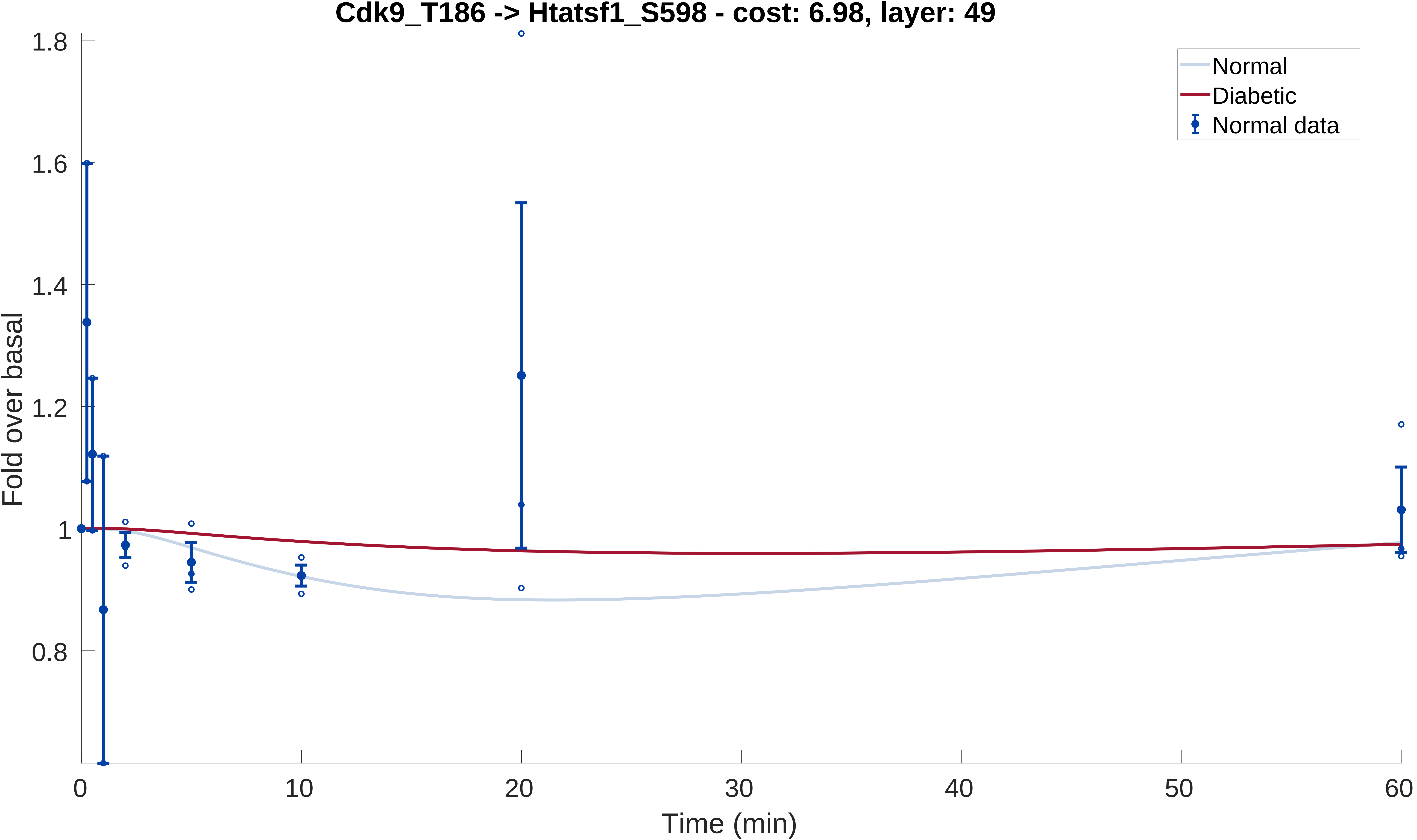

Cdk9_T186 -> Htatsf1_S598 - cost: 6.98, layer: 49
Normal
Diabetic
Normal data
Fold over basal
1.8
1.6
1.4
1.2
1
0.8
0
10
20
30
40
50
60
Time (min)

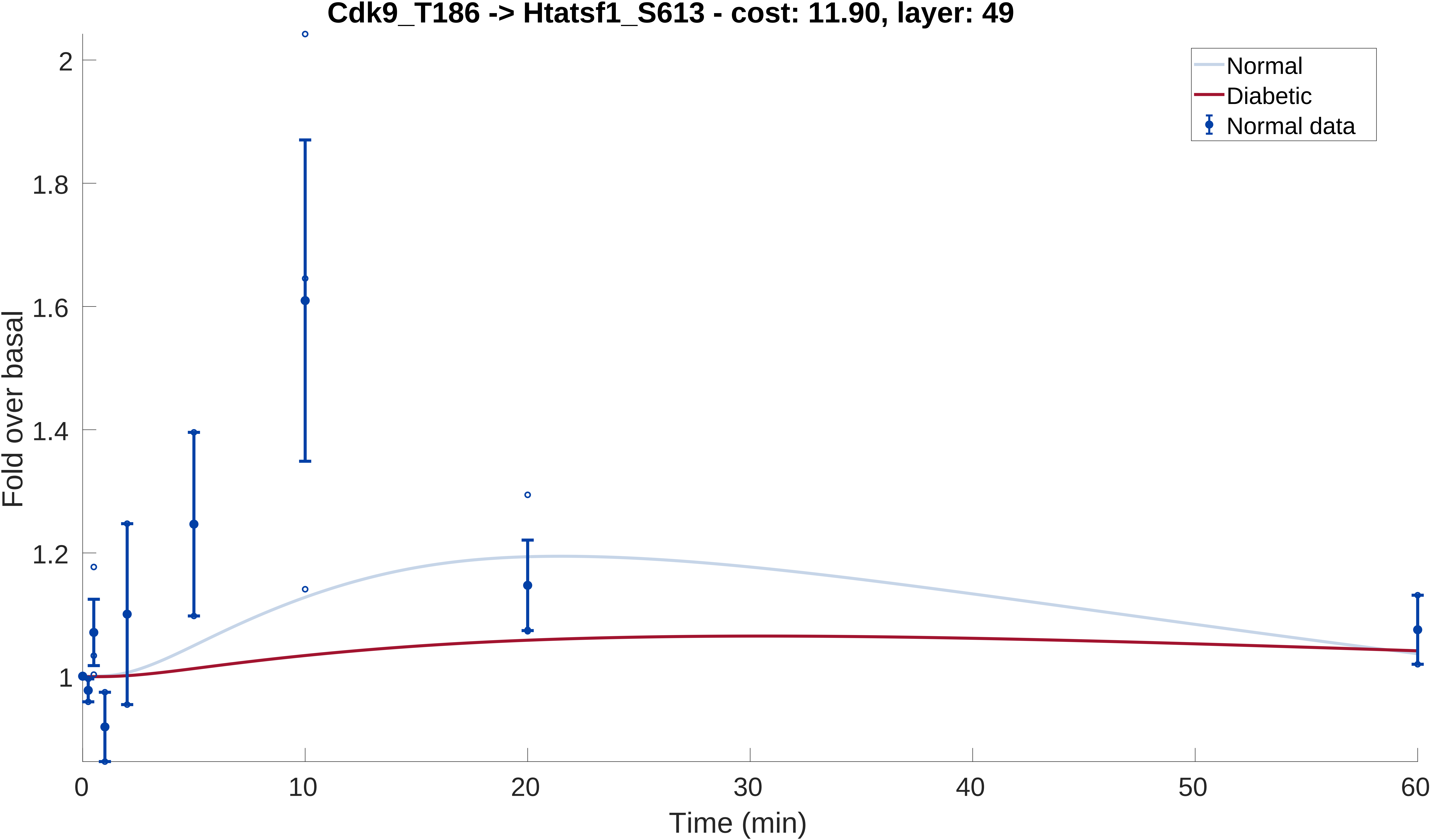
Cdk9_T186 -> Htatsf1_S613 - cost: 11.90, layer: 49
Normal
Diabetic
Normal data
Fold over basal
Time (min)
2
1.8
1.6
1.4
1.2
1
0
10
20
30
40
50
60

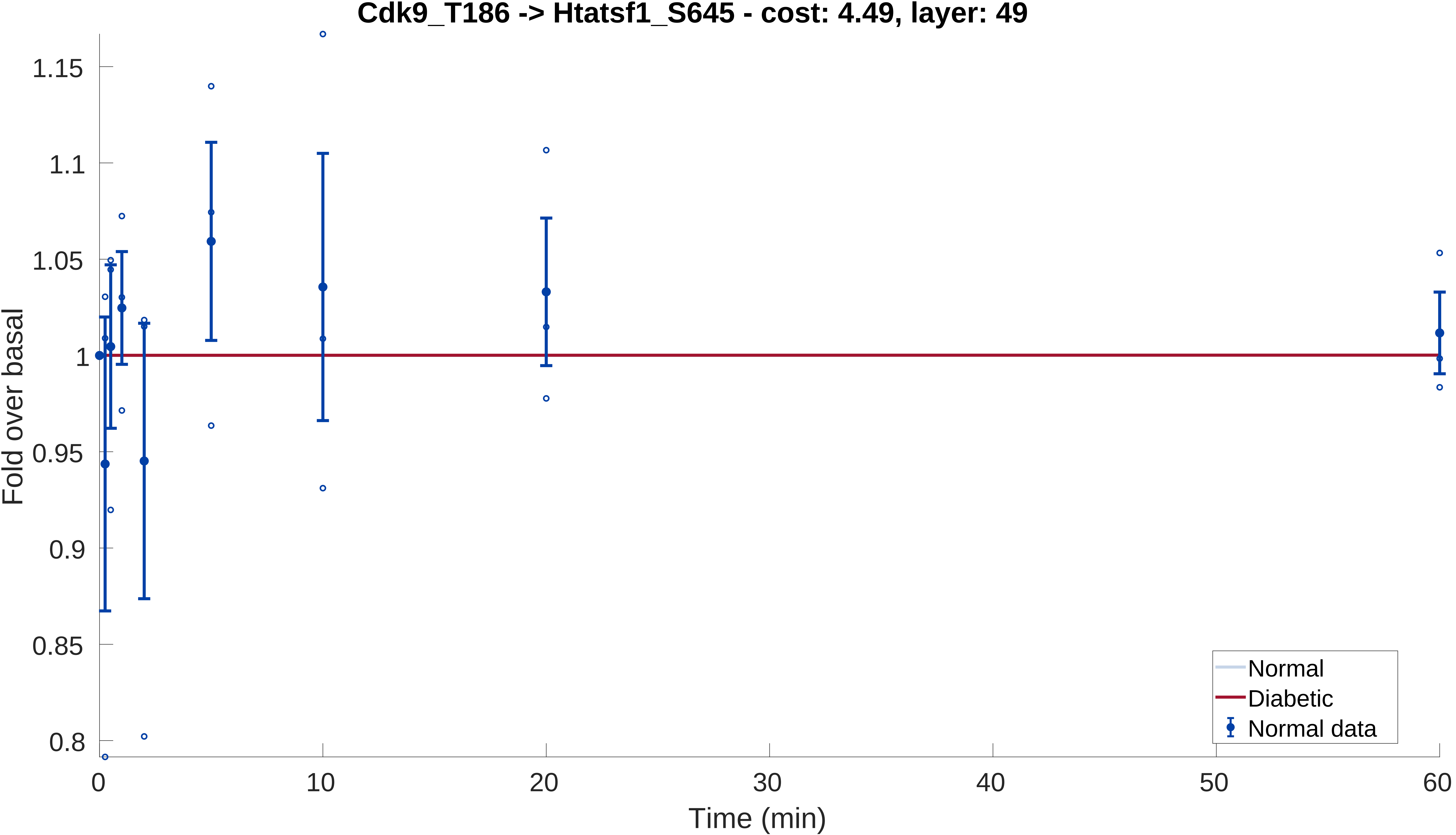

Cdk9_T186 -> Htatsf1_S645 - cost: 4.49, layer: 49
Fold over basal
1.15
1.1
1.05
1
0.95
0.9
0.85
0.8
0
10
20
30
40
50
60
Time (min)
Normal
Diabetic
Normal data

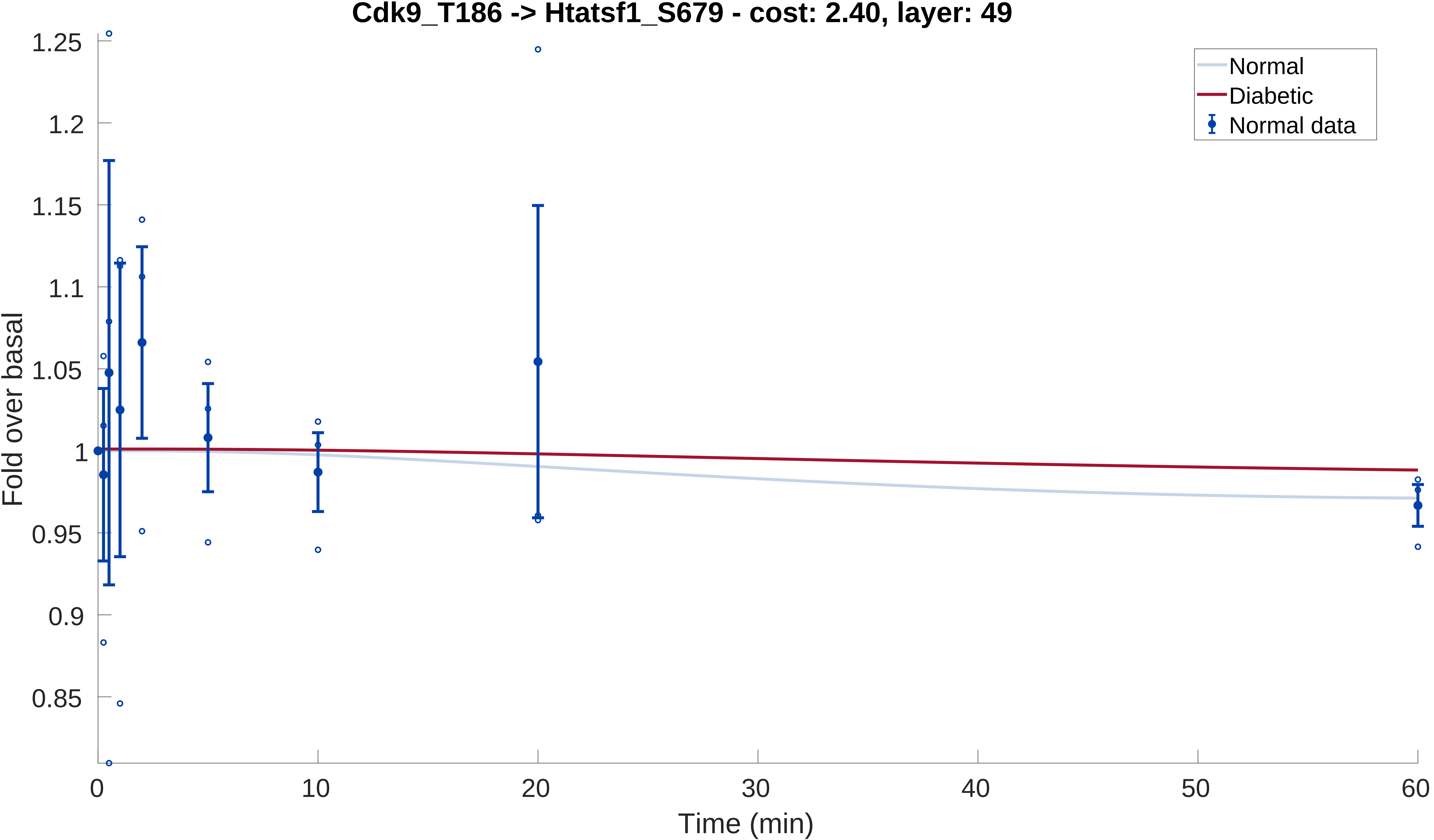
Cdk9_T186 -> Htatsf1_S679 - cost: 2.40, layer: 49
Fold over basal
Time (min)
Normal
Diabetic
Normal data
1.25
1.2
1.15
1.1
1.05
1
0.95
0.9
0.85
0
10
20
30
40
50
60

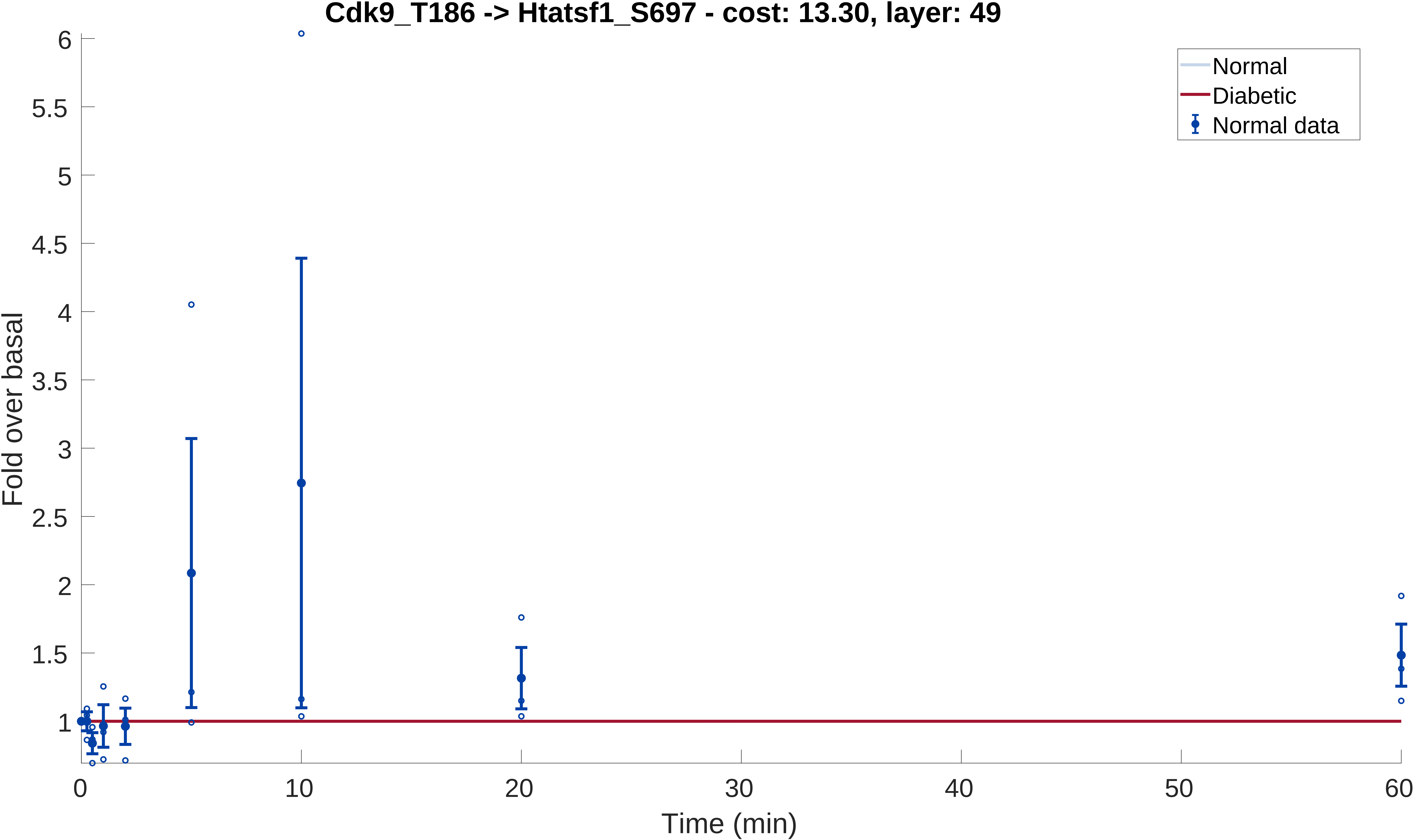

Cdk9_T186 -> Htatsf1_S697 - cost: 13.30, layer: 49
Normal
Diabetic
Normal data
Fold over basal
Time (min)
6
5.5
5
4.5
4
3.5
3
2.5
2
1.5
1
0
10
20
30
40
50
60

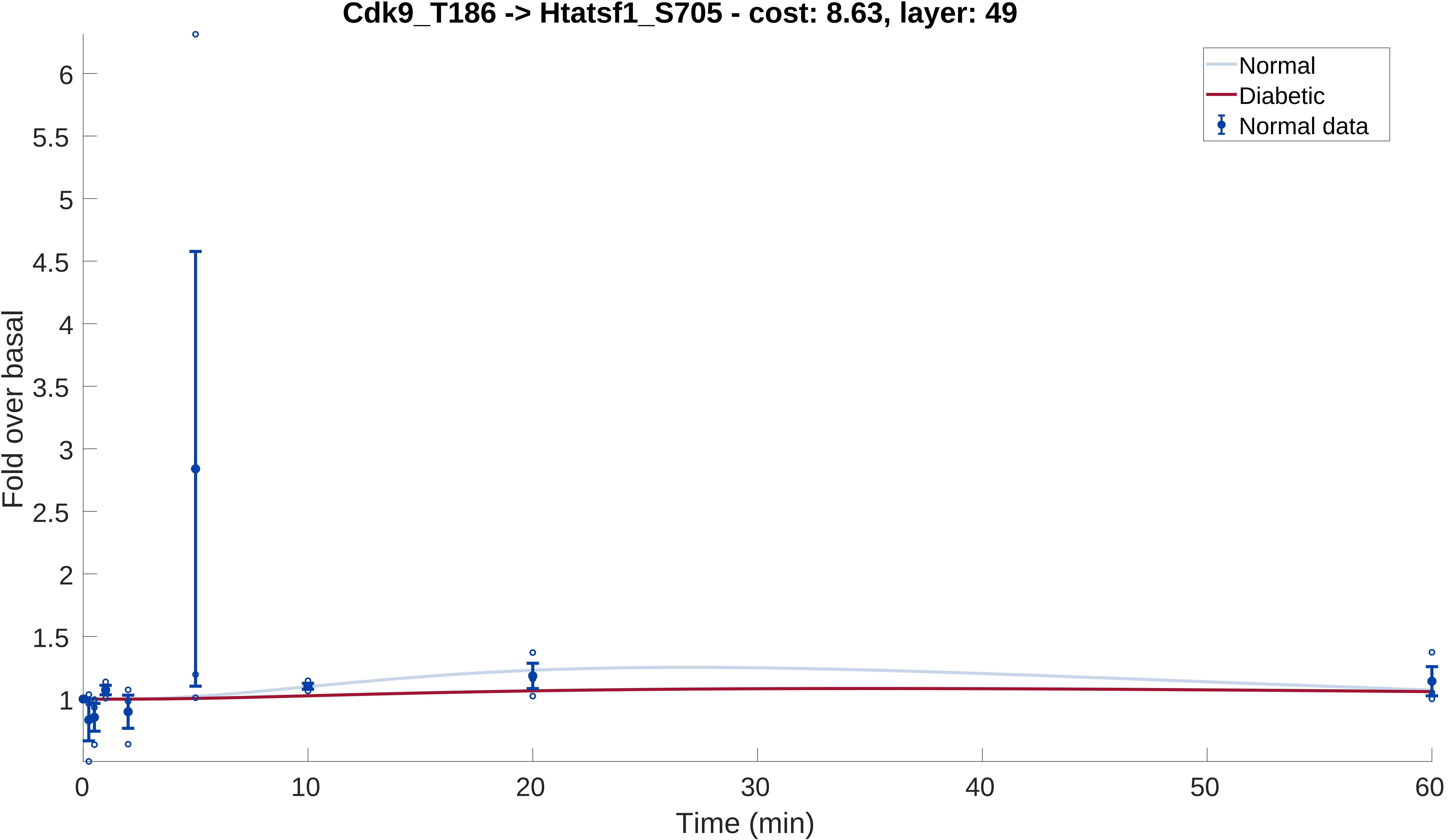

Cdk9_T186 -> Htatsf1_S705 - cost: 8.63, layer: 49
Normal
Diabetic
Normal data
Fold over basal
Time (min)
0
10
20
30
40
50
60
1
1.5
2
2.5
3
3.5
4
4.5
5
5.5
6

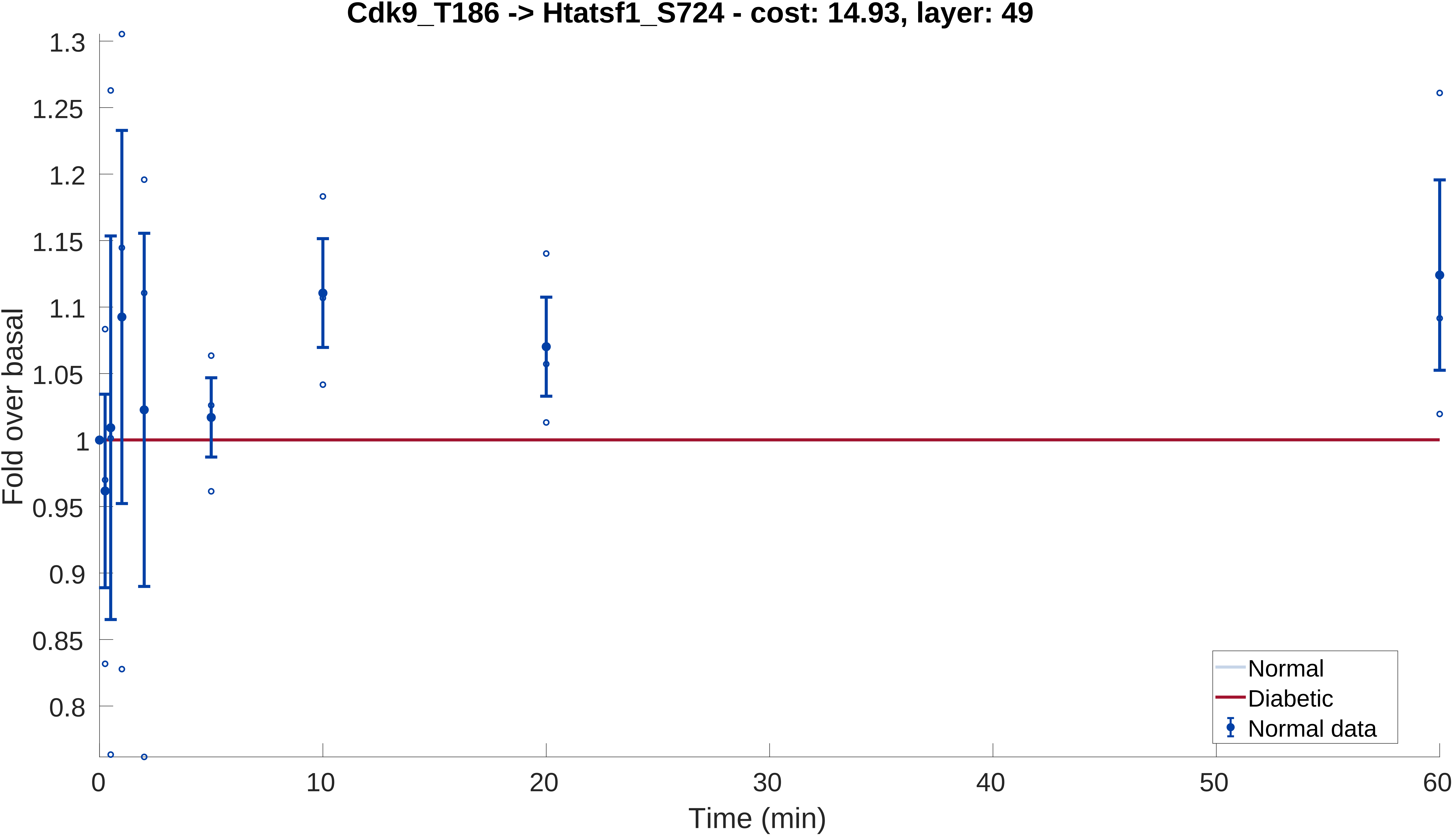
Cdk9_T186 -> Htatsf1_S724 - cost: 14.93, layer: 49
Fold over basal
Time (min)
1.3
1.25
1.2
1.15
1.1
1.05
1
0.95
0.9
0.85
0.8
0
10
20
30
40
50
60
Normal
Diabetic
Normal data

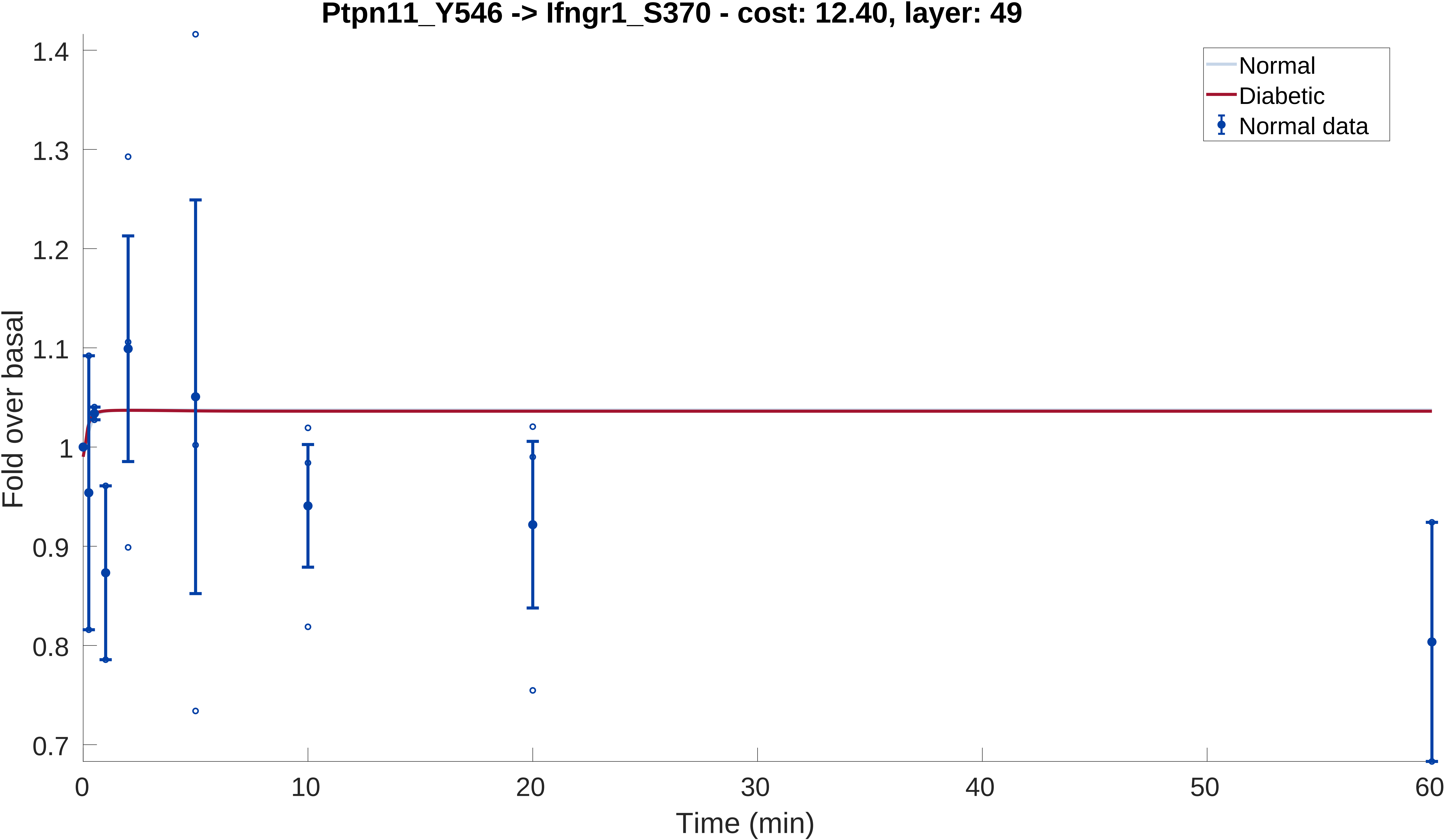
Ptpn11_Y546 -> Ifngr1_S370 - cost: 12.40, layer: 49
Normal
Diabetic
Normal data
Fold over basal
1.4
1.3
1.2
1.1
1
0.9
0.8
0.7
0
10
20
30
40
50
60
Time (min)

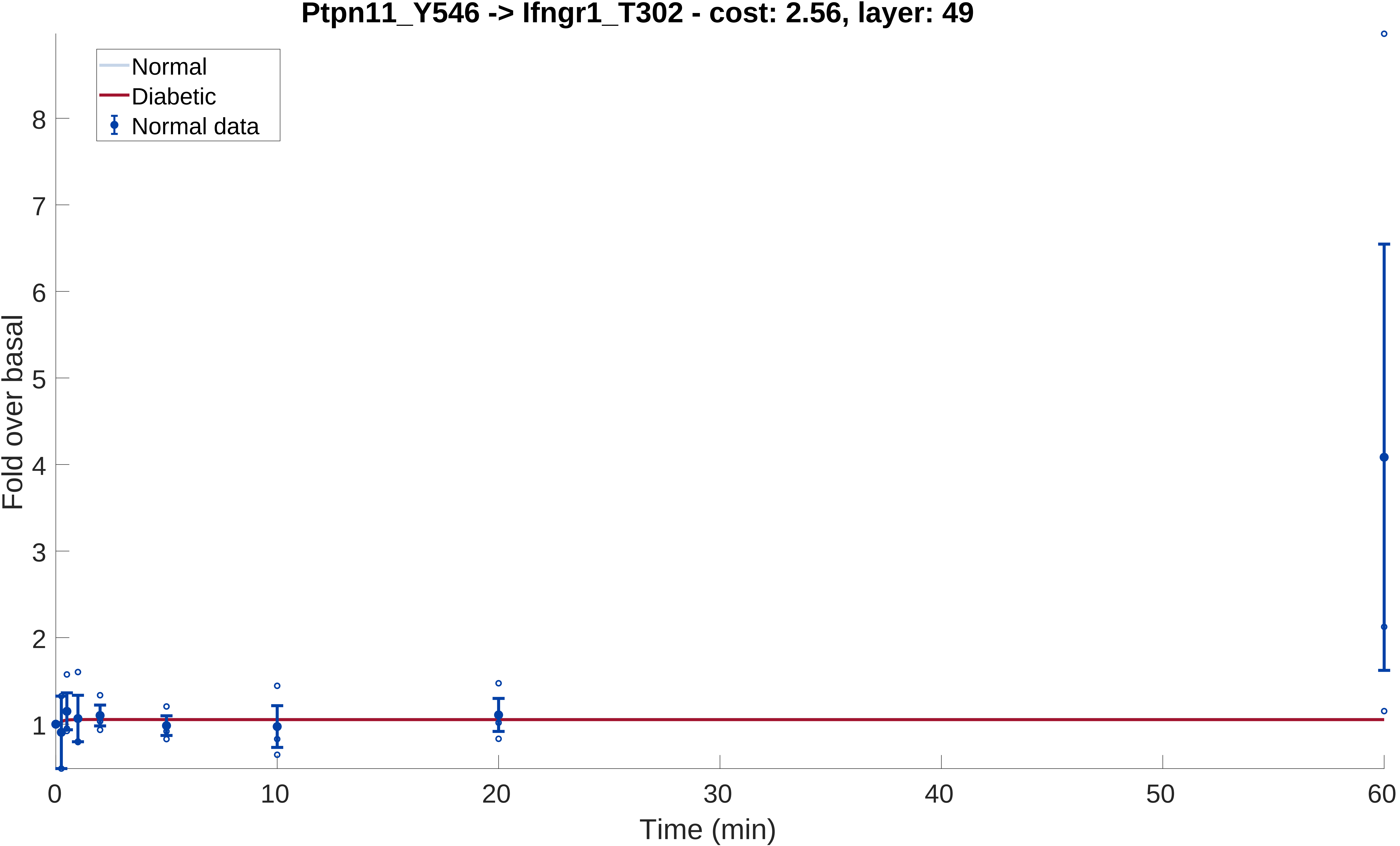

Ptpn11_Y546 -> Ifngr1_T302 - cost: 2.56, layer: 49
Normal
Diabetic
Normal data
Fold over basal
1
2
3
4
5
6
7
8
0
10
20
30
40
50
60
Time (min)

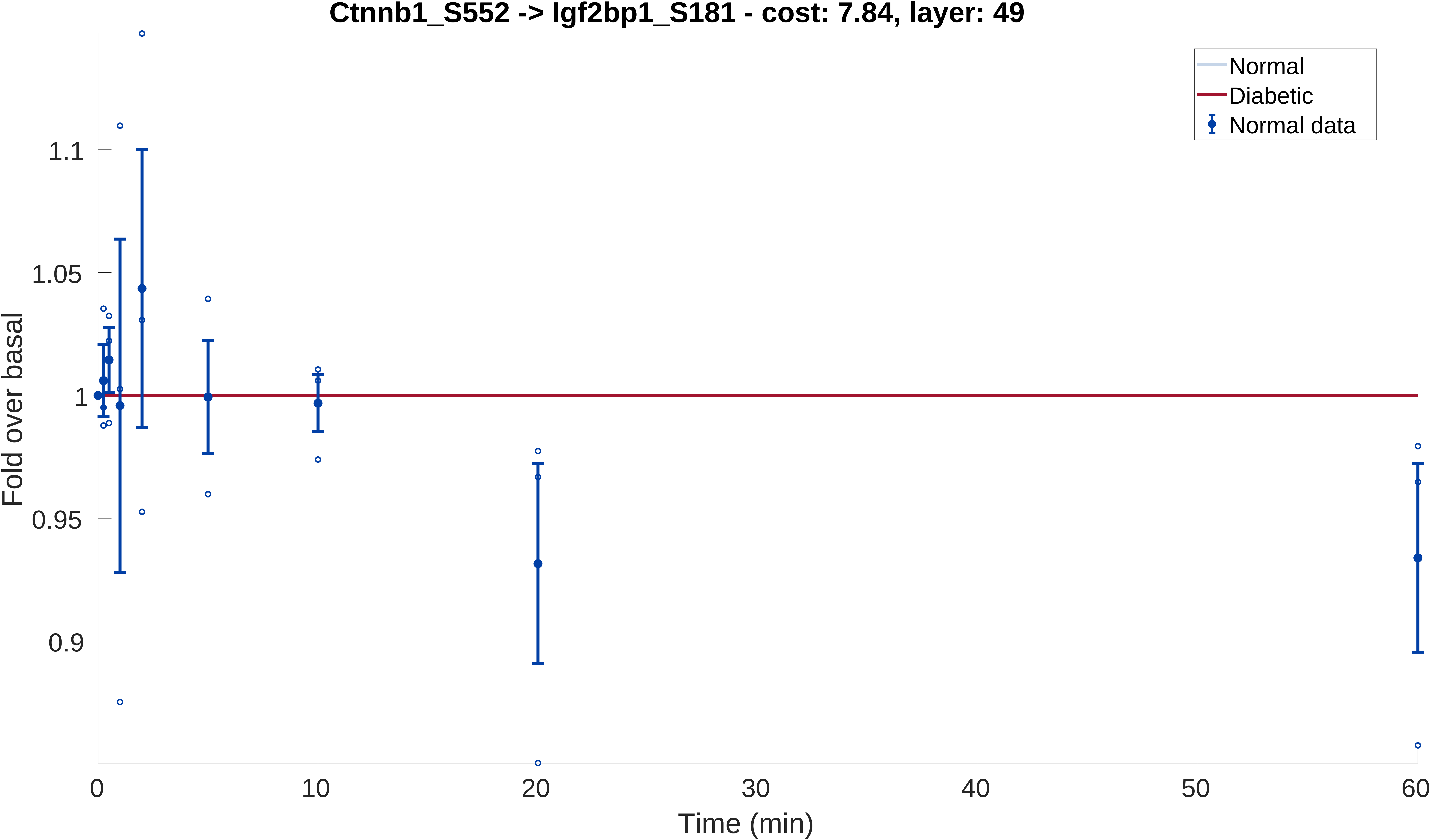

Ctnnb1_S552 -> Igf2bp1_S181 - cost: 7.84, layer: 49
Normal
Diabetic
Normal data
Fold over basal
1.1
1.05
1
0.95
0.9
0
10
20
30
40
50
60
Time (min)

Rps6ka1_S363 -> Irf3_S130 - cost: 8.59, layer: 49

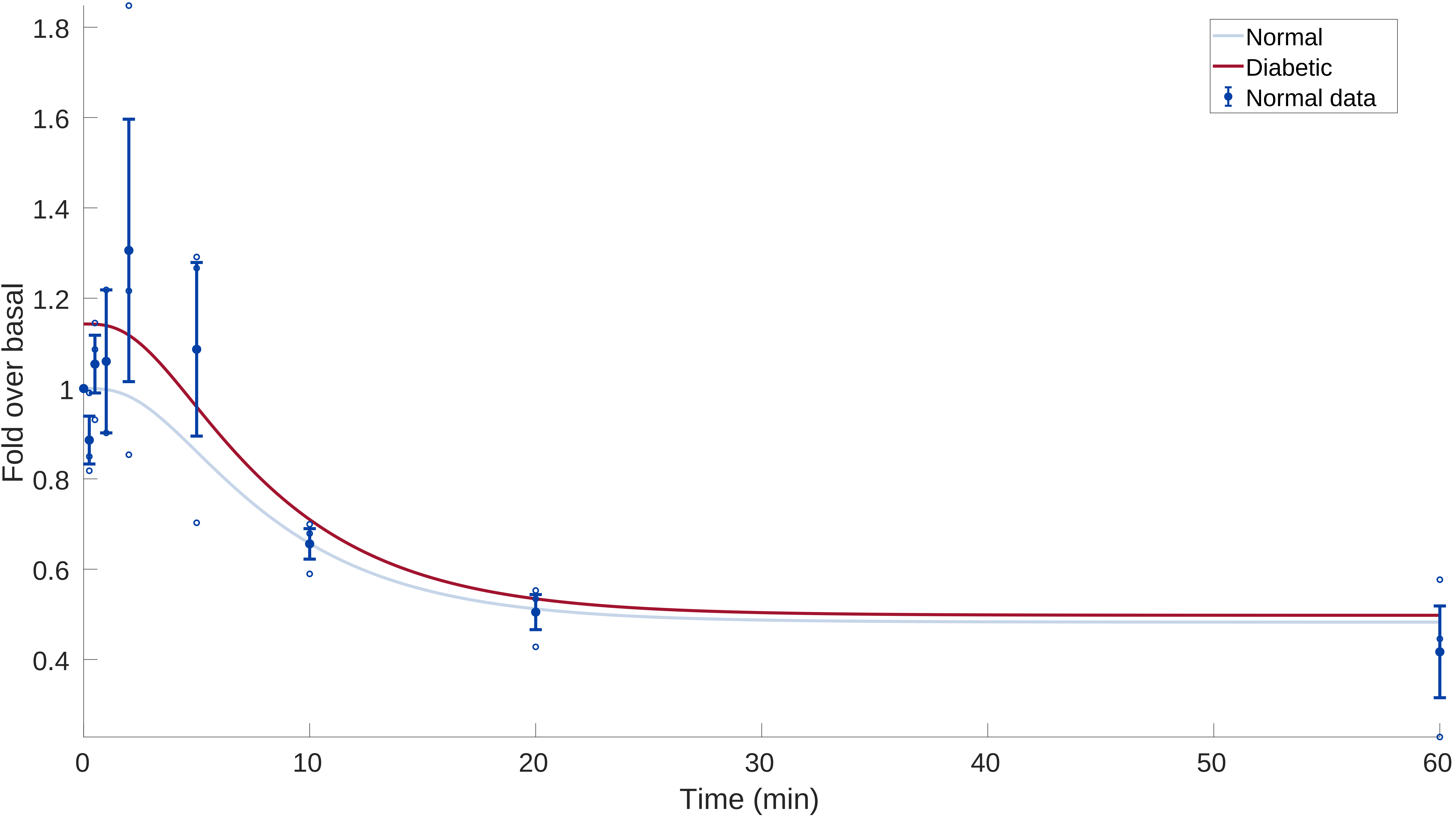

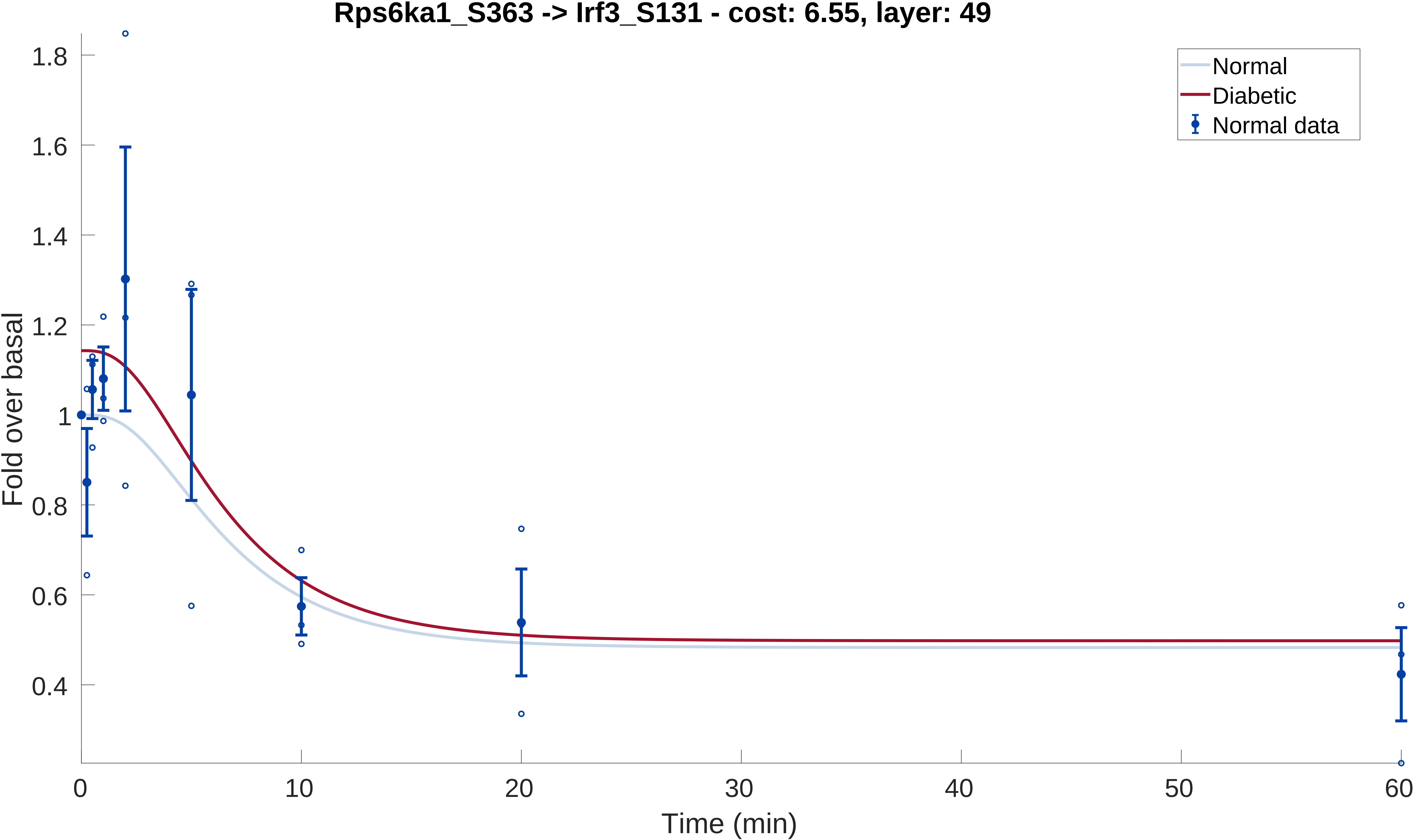

Rps6ka1_S363 -> Irf3_S131 - cost: 6.55, layer: 49
Normal
Diabetic
Normal data
Fold over basal
Time (min)
1.8
1.6
1.4
1.2
1
0.8
0.6
0.4
0
10
20
30
40
50
60

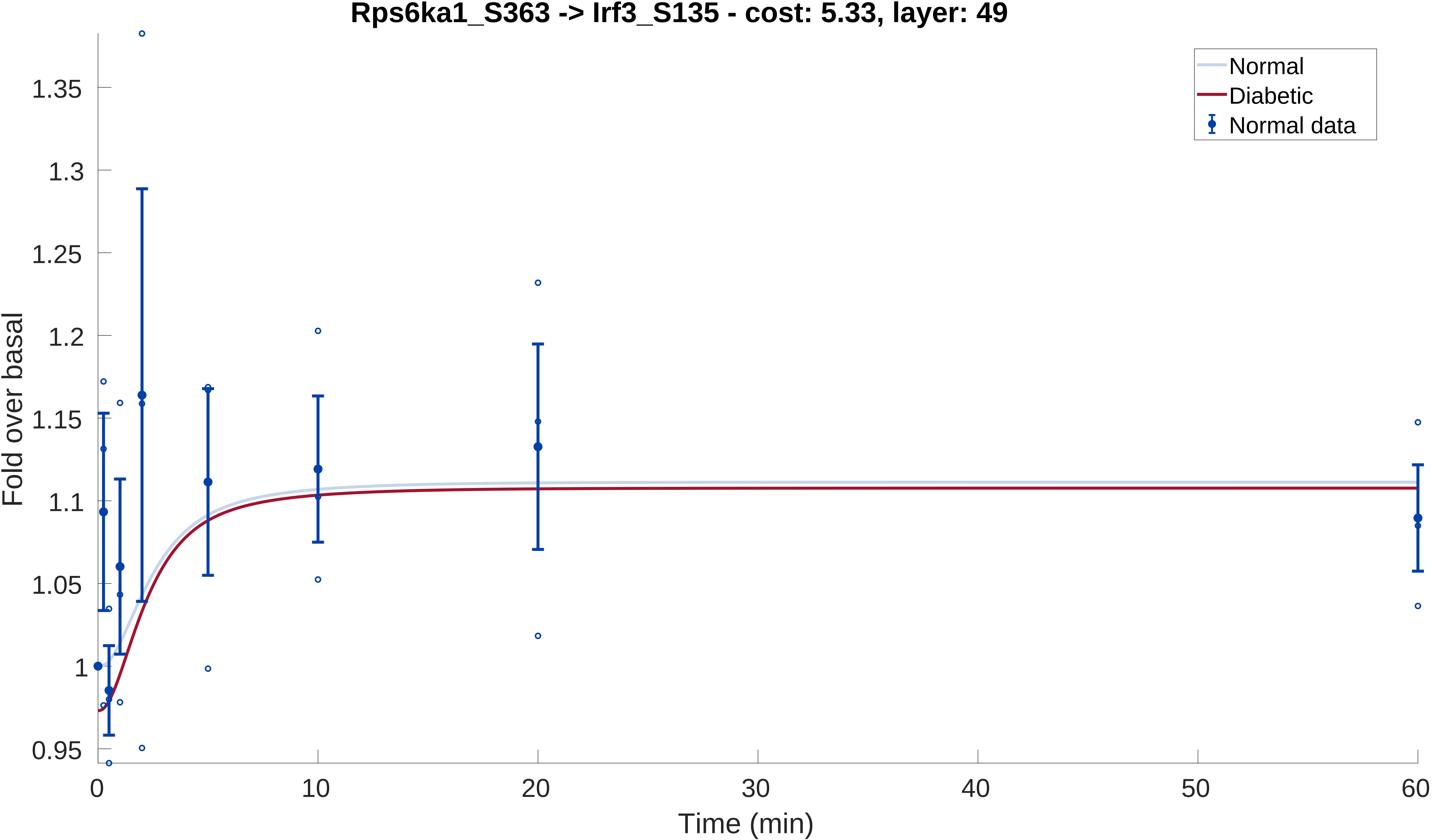
Rps6ka1_S363 -> Irf3_S135 - cost: 5.33, layer: 49
Normal
Diabetic
Normal data
Fold over basal
1.35
1.3
1.25
1.2
1.15
1.1
1.05
1
0.95
0
10
20
30
40
50
60
Time (min)

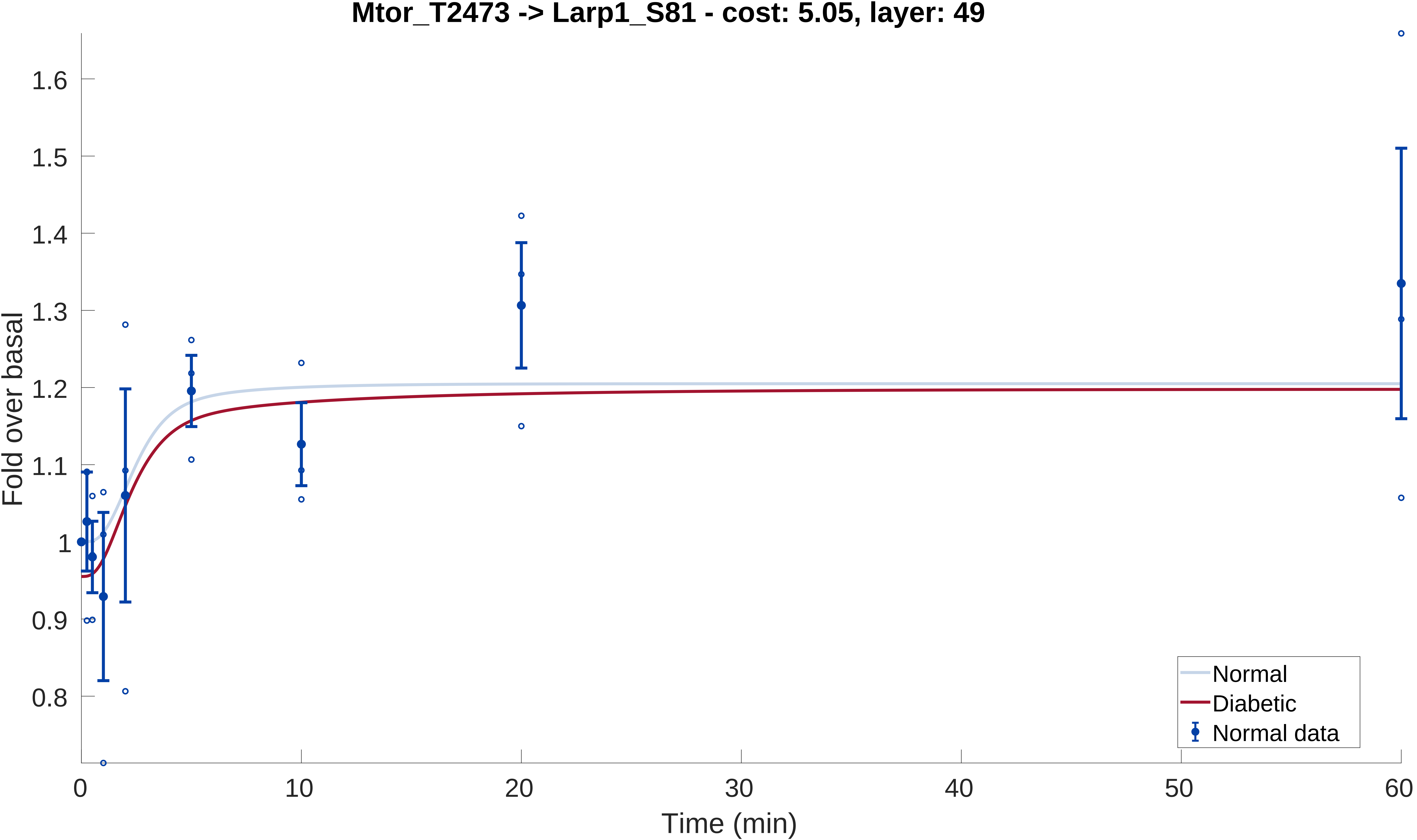

Mtor_T2473 -> Larp1_S81 - cost: 5.05, layer: 49
Fold over basal
1.6
1.5
1.4
1.3
1.2
1.1
1
0.9
0.8
0
10
20
30
40
50
60
Time (min)
Normal
Diabetic
Normal data

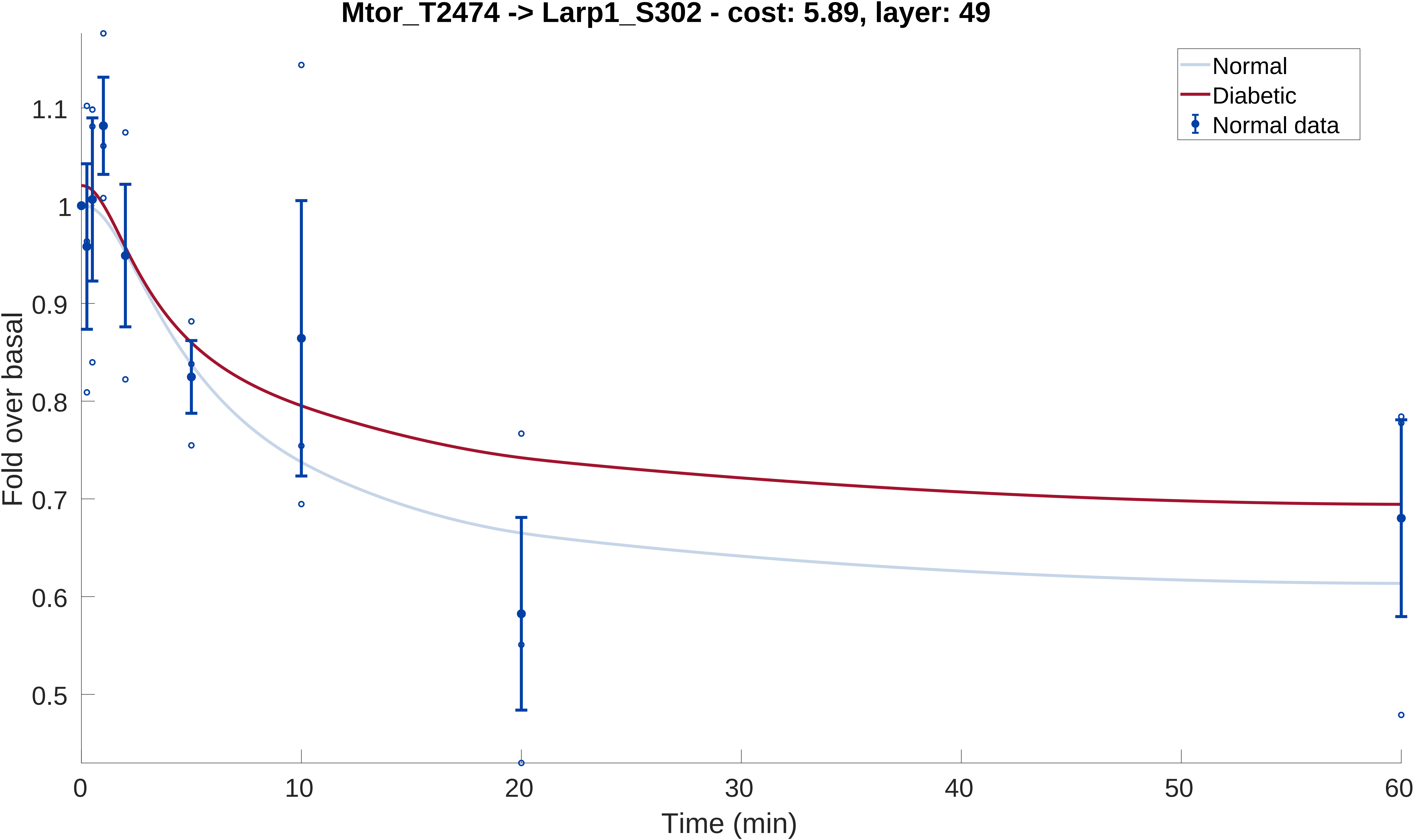

Mtor_T2474 -> Larp1_S302 - cost: 5.89, layer: 49
Normal
Diabetic
Normal data
1.1
1
0.9
0.8
0.7
0.6
0.5
Fold over basal
0
10
20
30
40
50
60
Time (min)

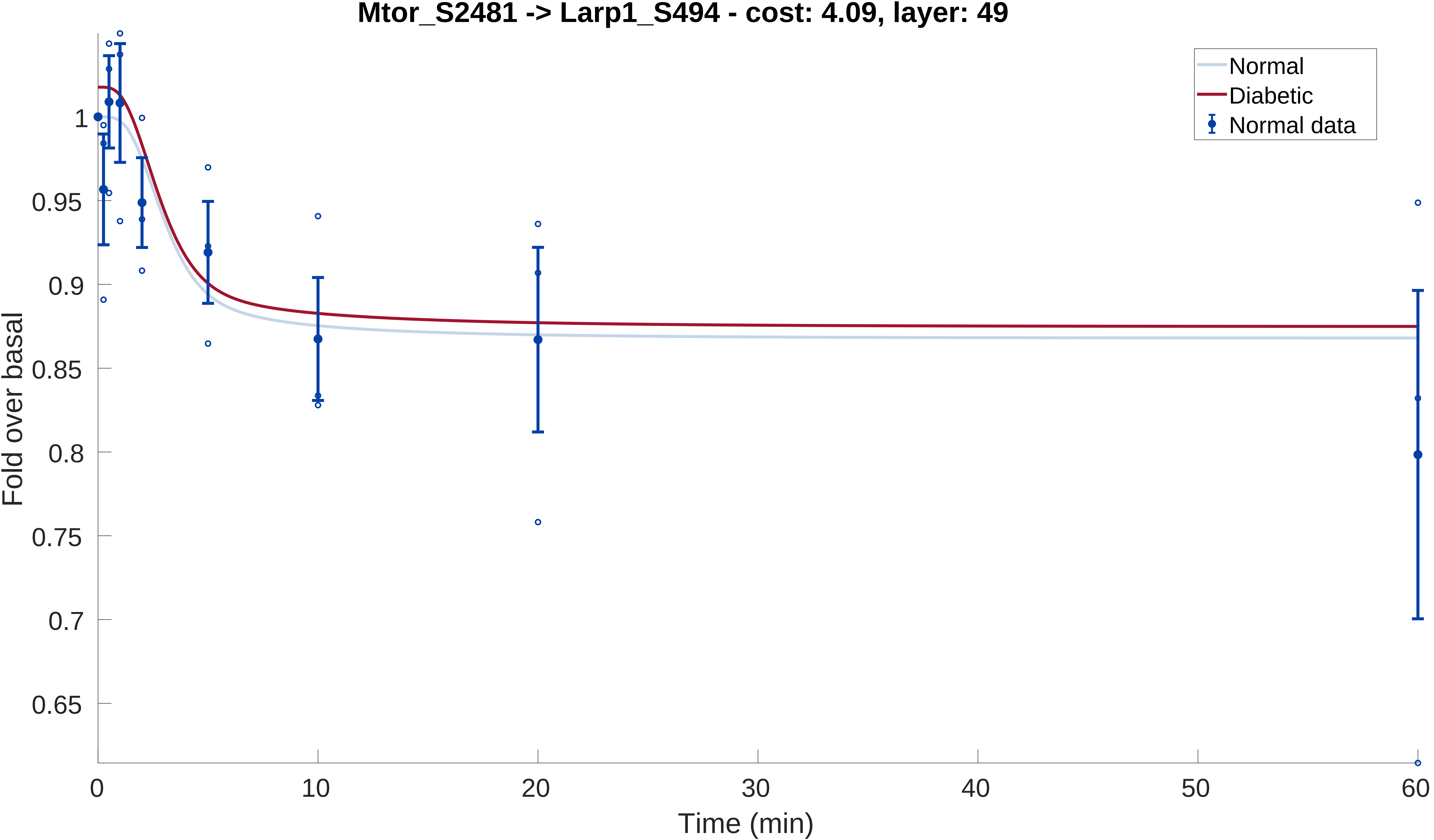

Mtor_S2481 -> Larp1_S494 - cost: 4.09, layer: 49
Normal
Diabetic
Normal data
Fold over basal
1
0.95
0.9
0.85
0.8
0.75
0.7
0.65
0
10
20
30
40
50
60
Time (min)

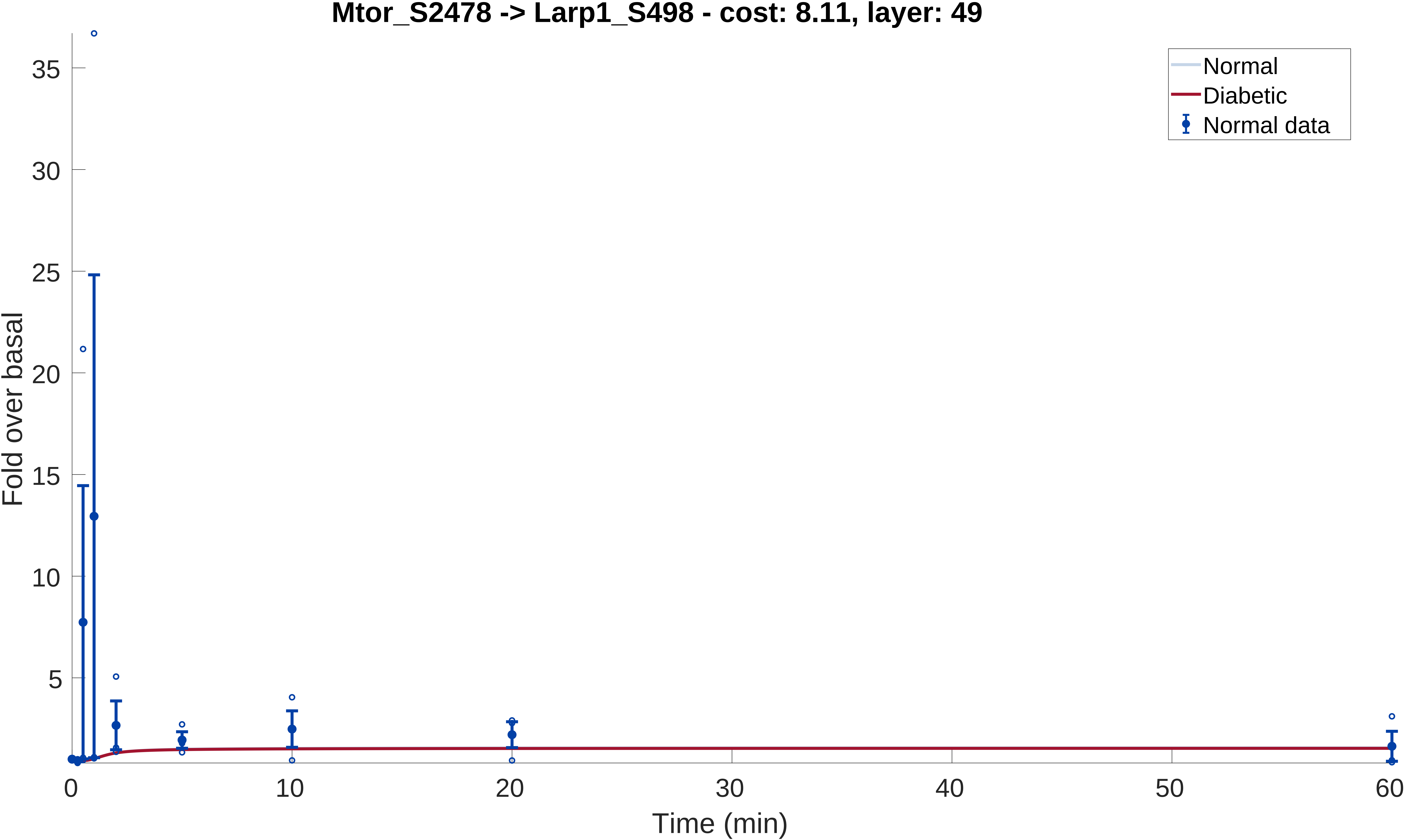

Mtor_S2478 -> Larp1_S498 - cost: 8.11, layer: 49
Normal
Diabetic
Normal data
Fold over basal
Time (min)
0
10
20
30
40
50
60
5
10
15
20
25
30
35

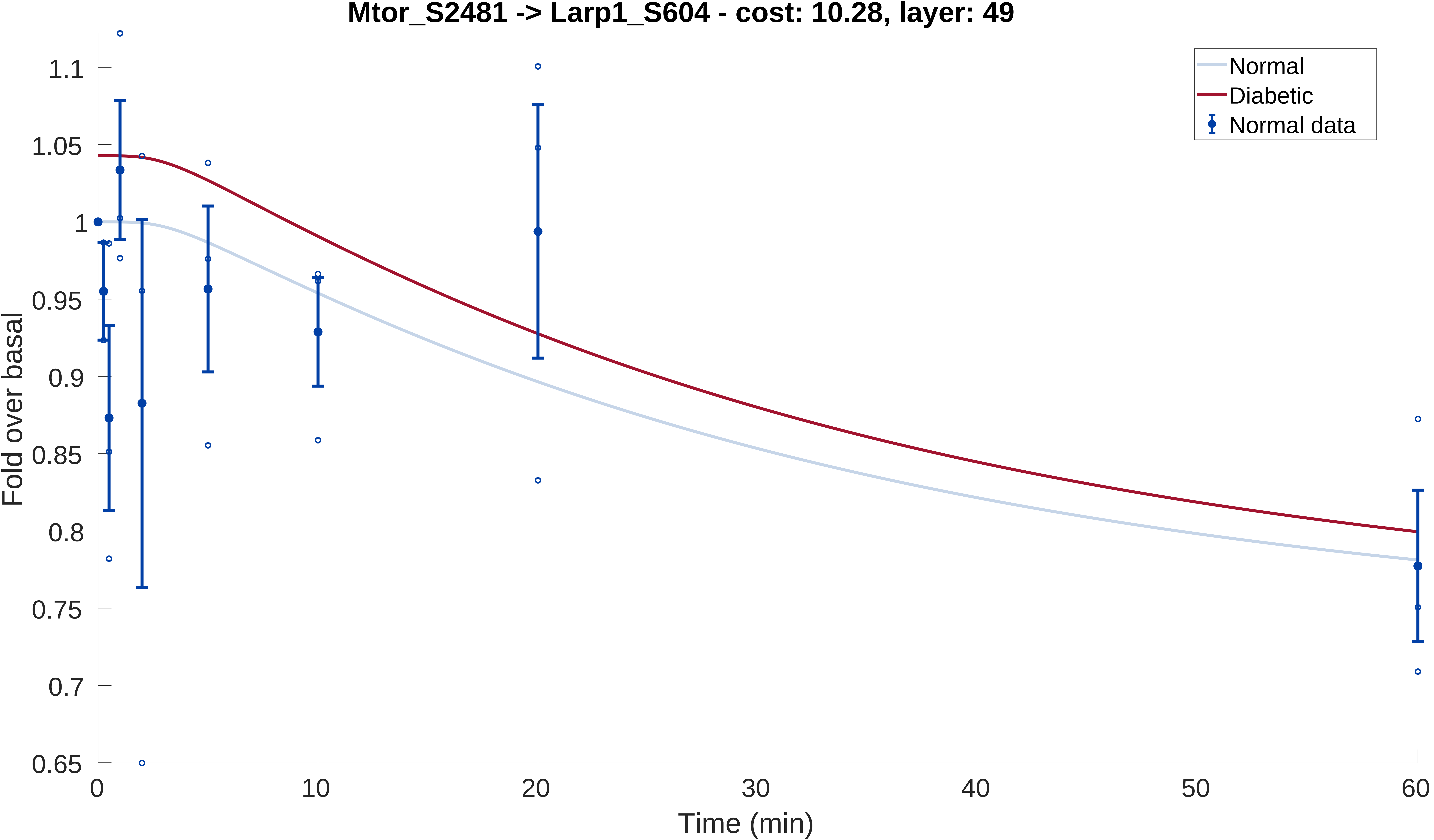
Mtor_S2481 -> Larp1_S604 - cost: 10.28, layer: 49
Fold over basal
Time (min)
Normal
Diabetic
Normal data
1.1
1.05
1
0.95
0.9
0.85
0.8
0.75
0.7
0.65
0
10
20
30
40
50
60

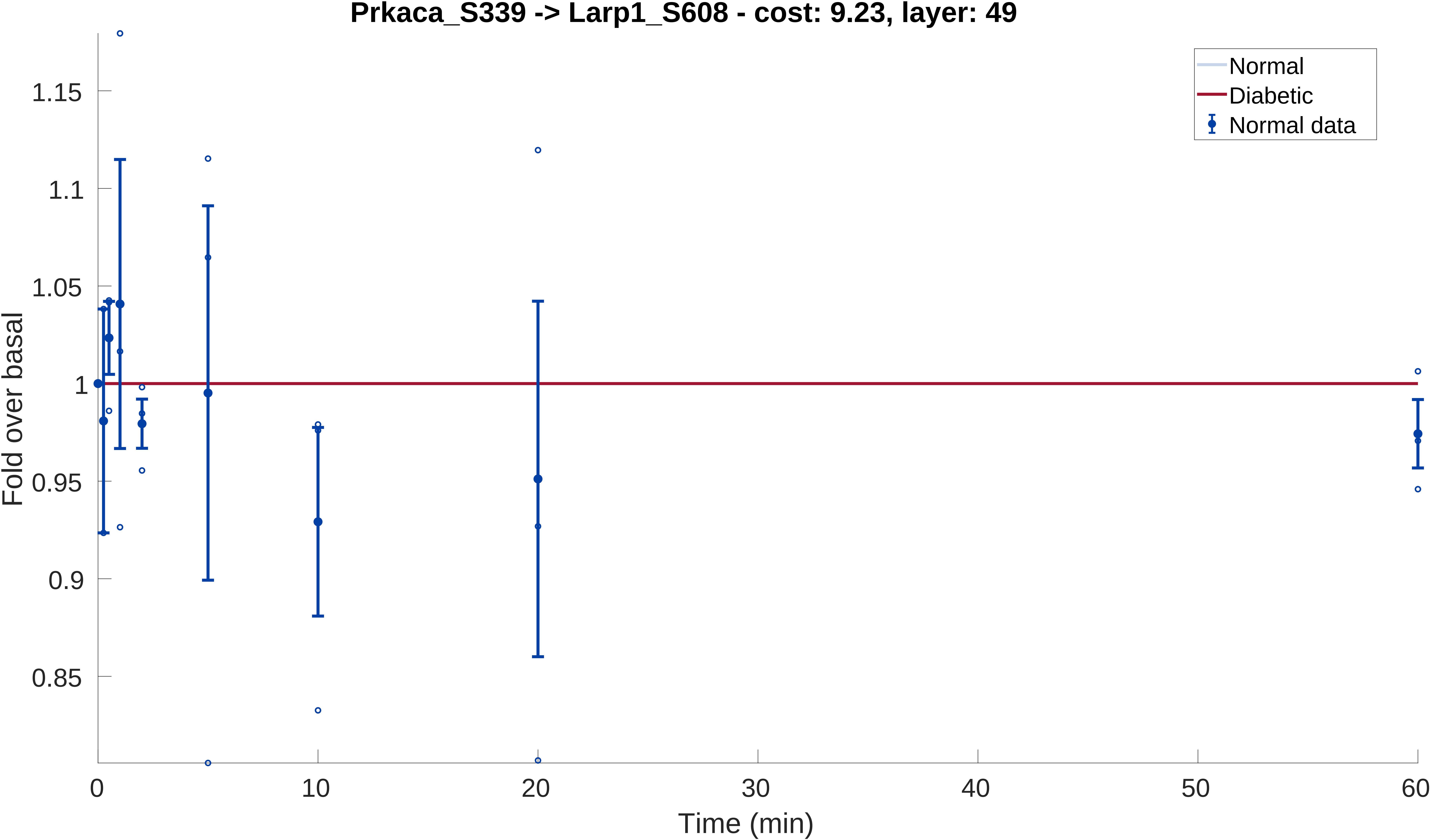

Prkaca_S339 -> Larp1_S608 - cost: 9.23, layer: 49
Normal
Diabetic
Normal data
Fold over basal
1.15
1.1
1.05
1
0.95
0.9
0.85
0
10
20
30
40
50
60
Time (min)

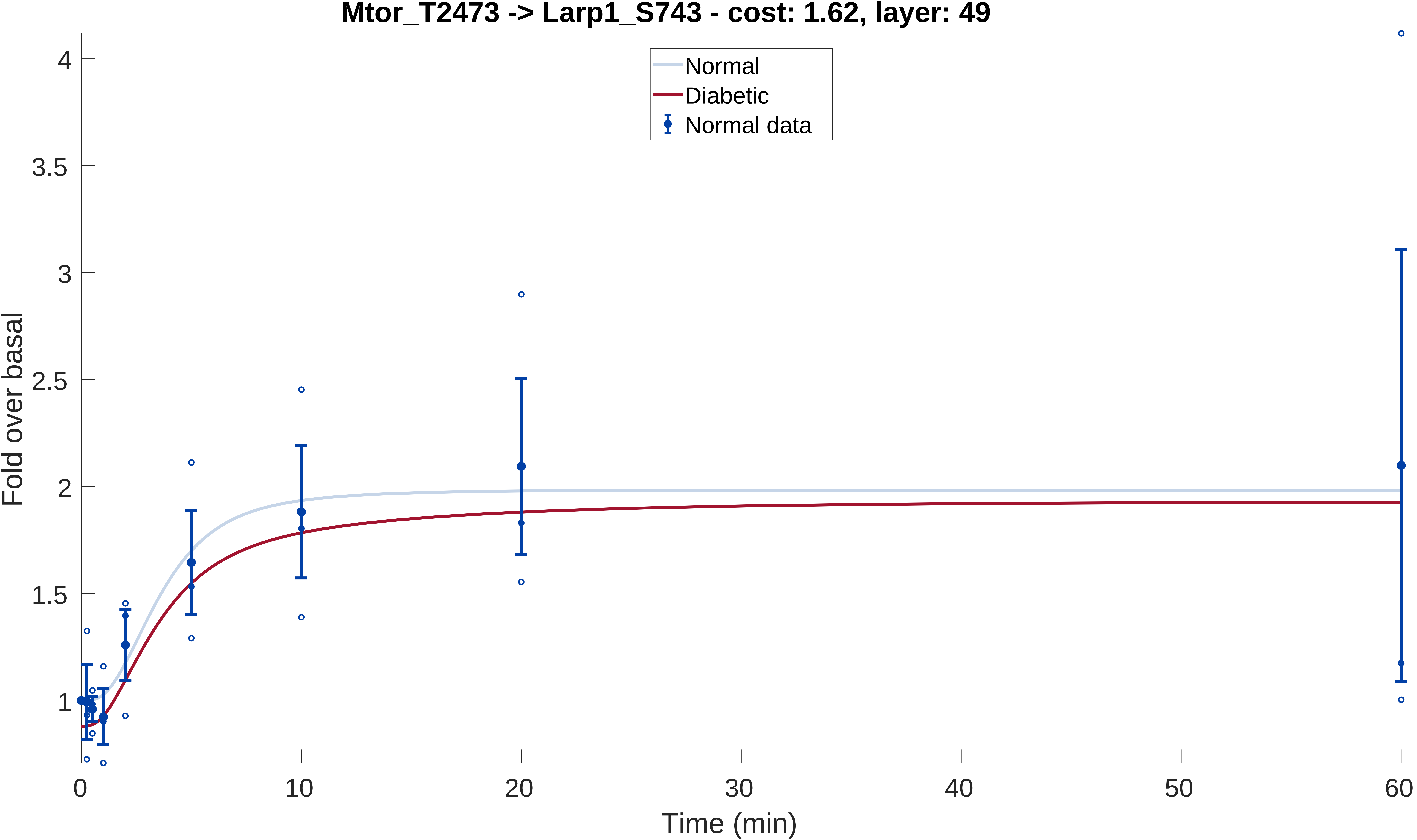

Mtor_T2473 -> Larp1_S743 - cost: 1.62, layer: 49
Normal
Diabetic
Normal data
Fold over basal
Time (min)
4
3.5
3
2.5
2
1.5
1
0
10
20
30
40
50
60

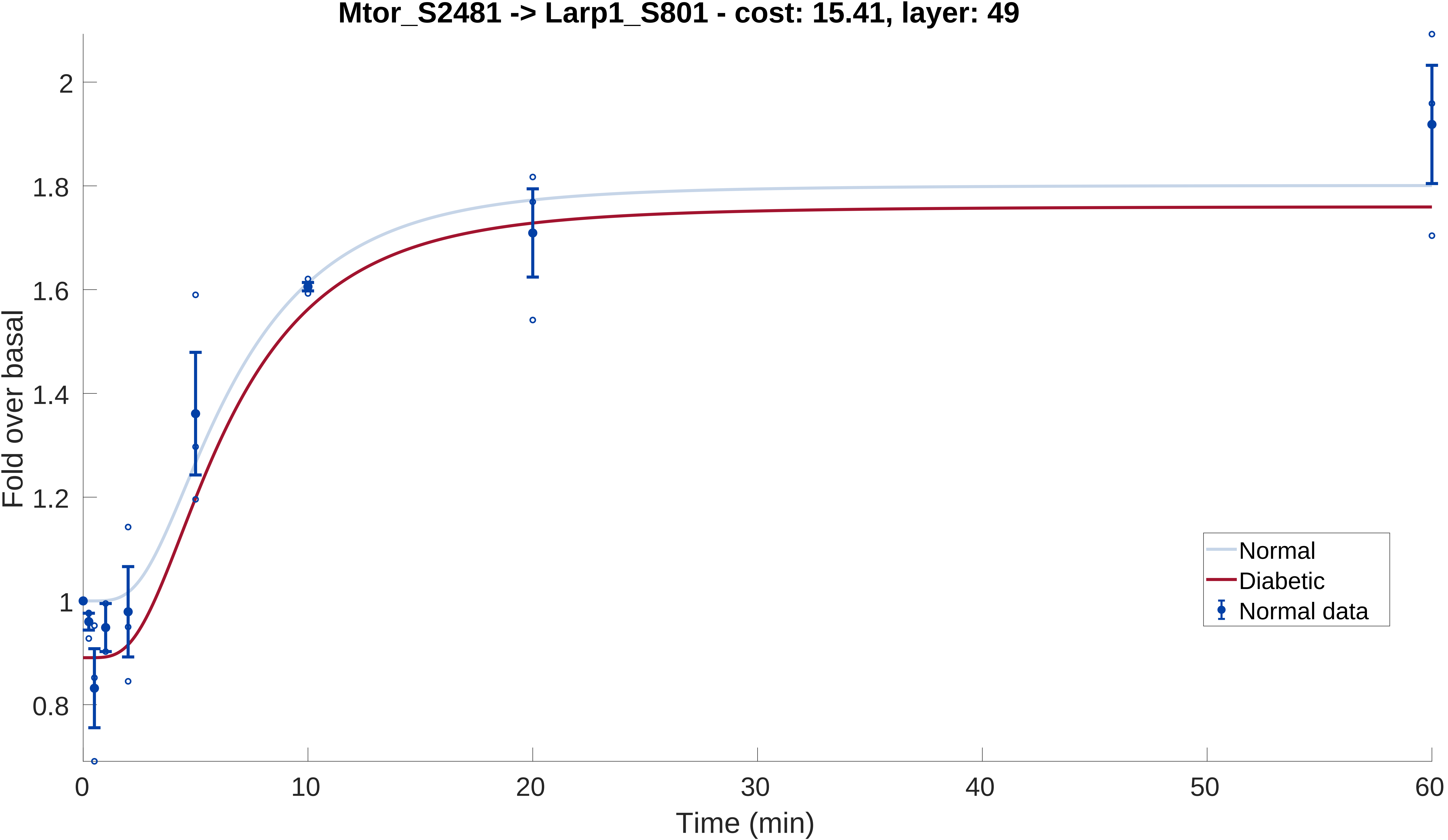

Mtor_S2481 -> Larp1_S801 - cost: 15.41, layer: 49
Fold over basal
Time (min)
0.8
1
1.2
1.4
1.6
1.8
2
0
10
20
30
40
50
60
Normal
Diabetic
Normal data

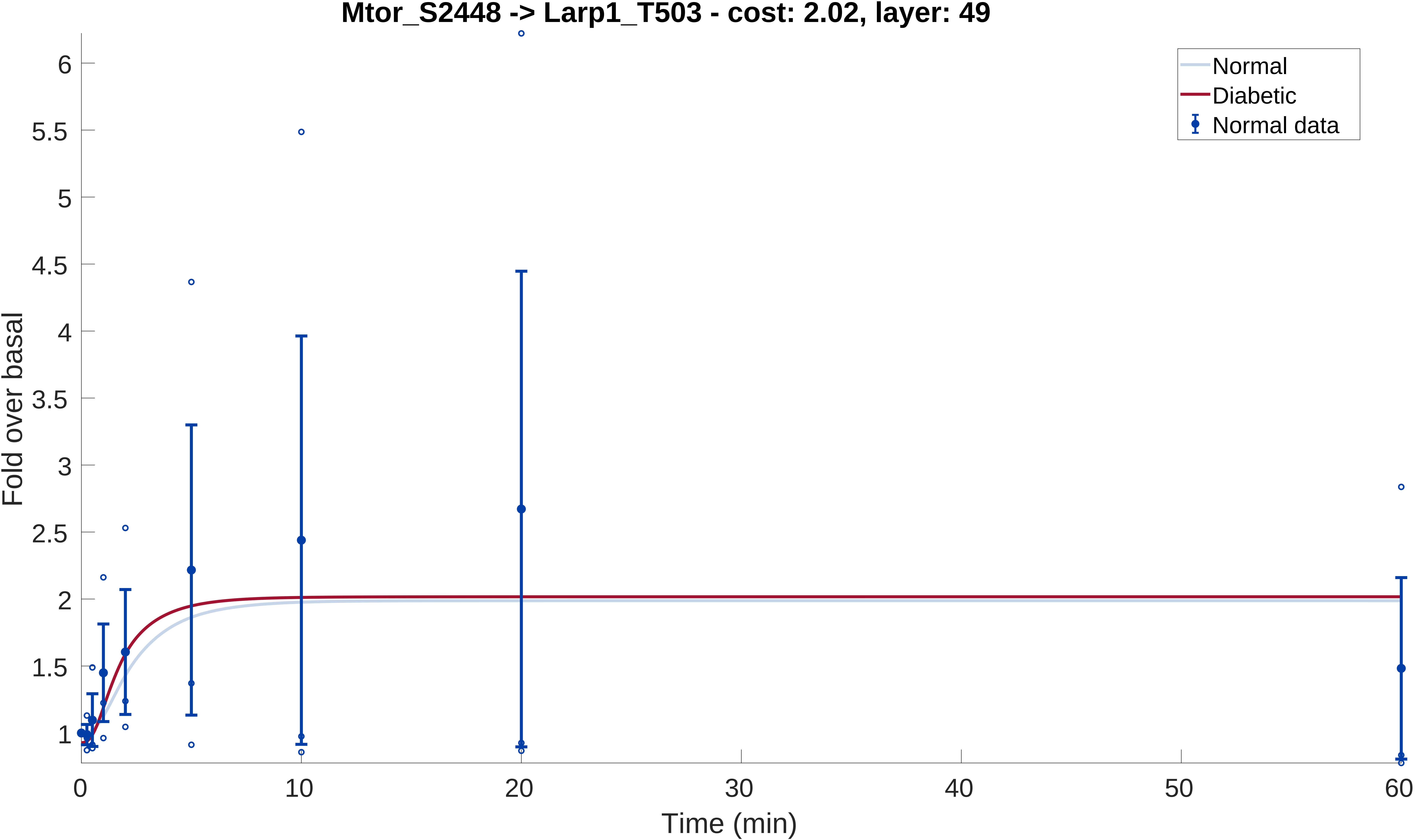

Mtor_S2448 -> Larp1_T503 - cost: 2.02, layer: 49
Normal
Diabetic
Normal data
Fold over basal
Time (min)
6
5.5
5
4.5
4
3.5
3
2.5
2
1.5
1
0
10
20
30
40
50
60

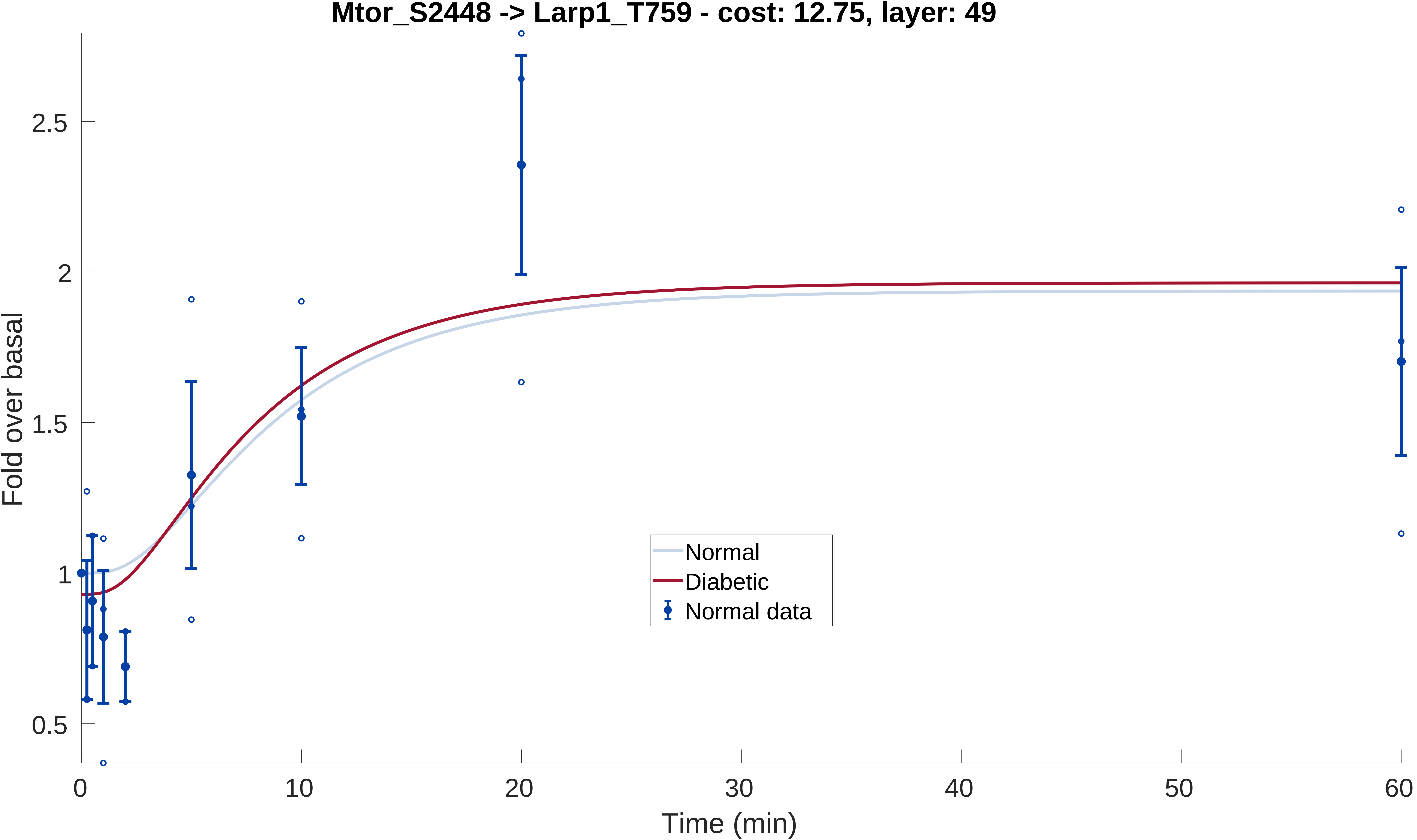

Mtor_S2448 -> Larp1_T759 - cost: 12.75, layer: 49
Fold over basal
0.5
1
1.5
2
2.5
0
10
20
30
40
50
60
Time (min)
Normal
Diabetic
Normal data

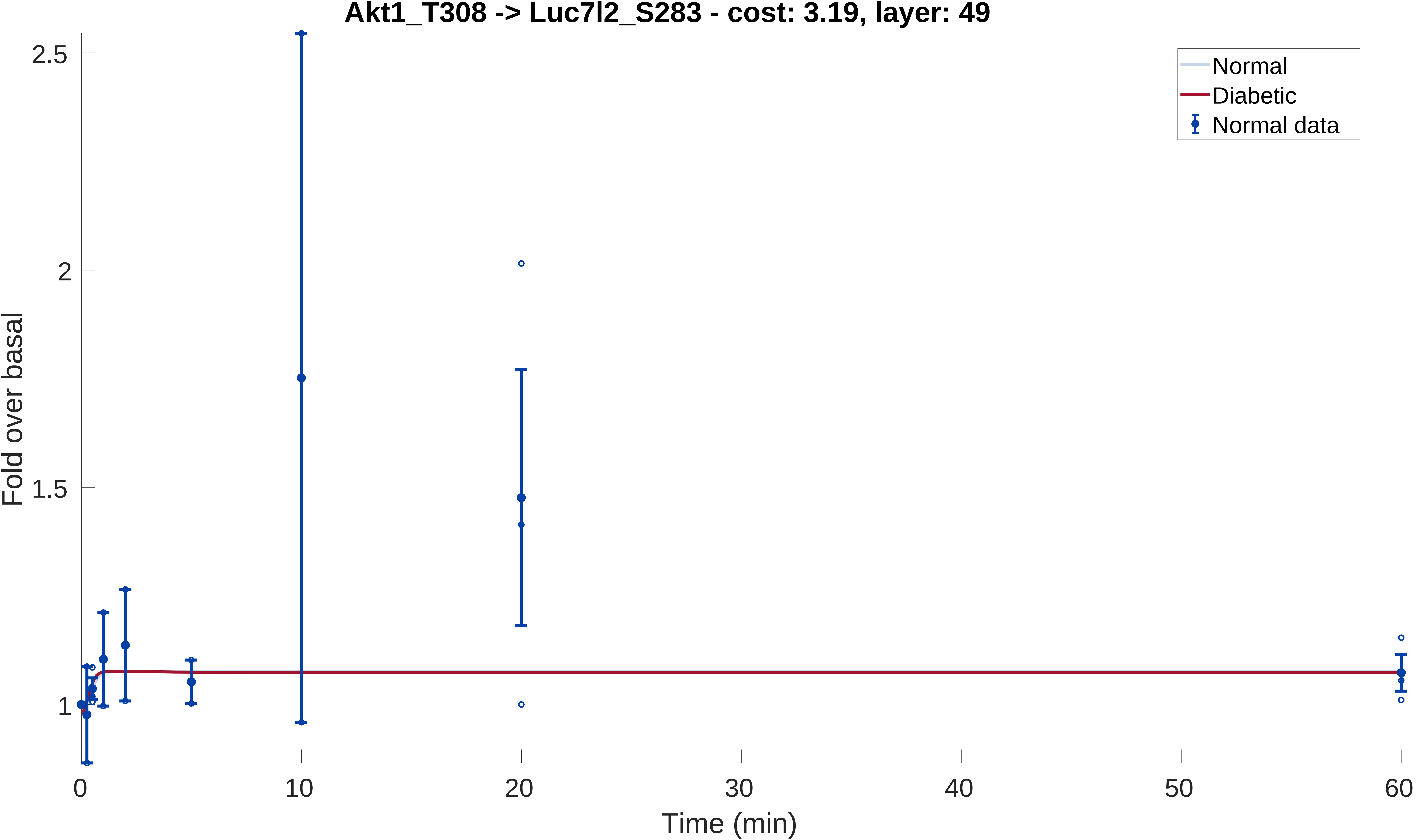
Akt1_T308 -> Luc7l2_S283 - cost: 3.19, layer: 49
Fold over basal
2.5
2
1.5
1
0
10
20
30
40
50
60
Time (min)
Normal
Diabetic
Normal data

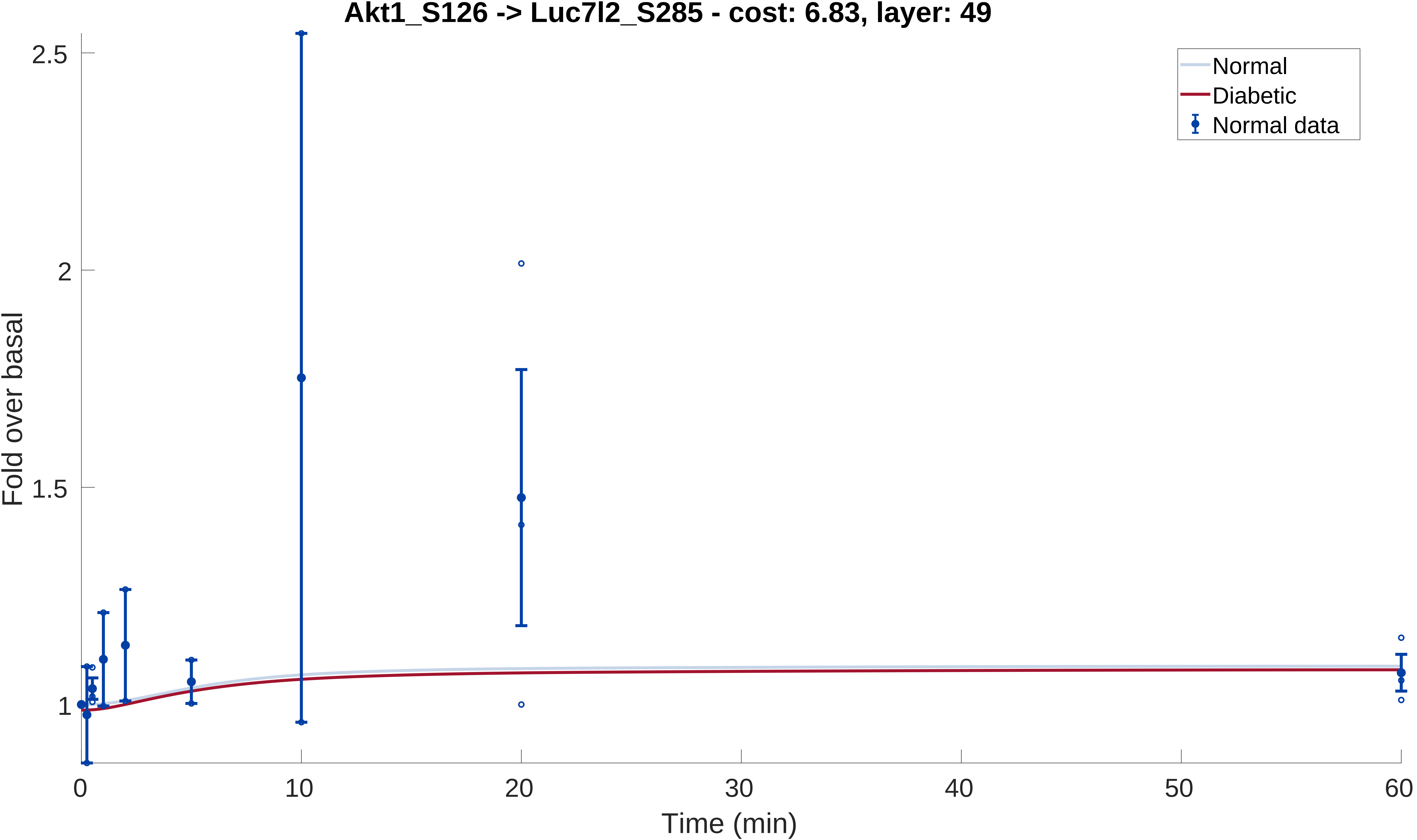

Akt1_S126 -> Luc7l2_S285 - cost: 6.83, layer: 49
Fold over basal
Time (min)
2.5
2
1.5
1
0
10
20
30
40
50
60
Normal
Diabetic
Normal data

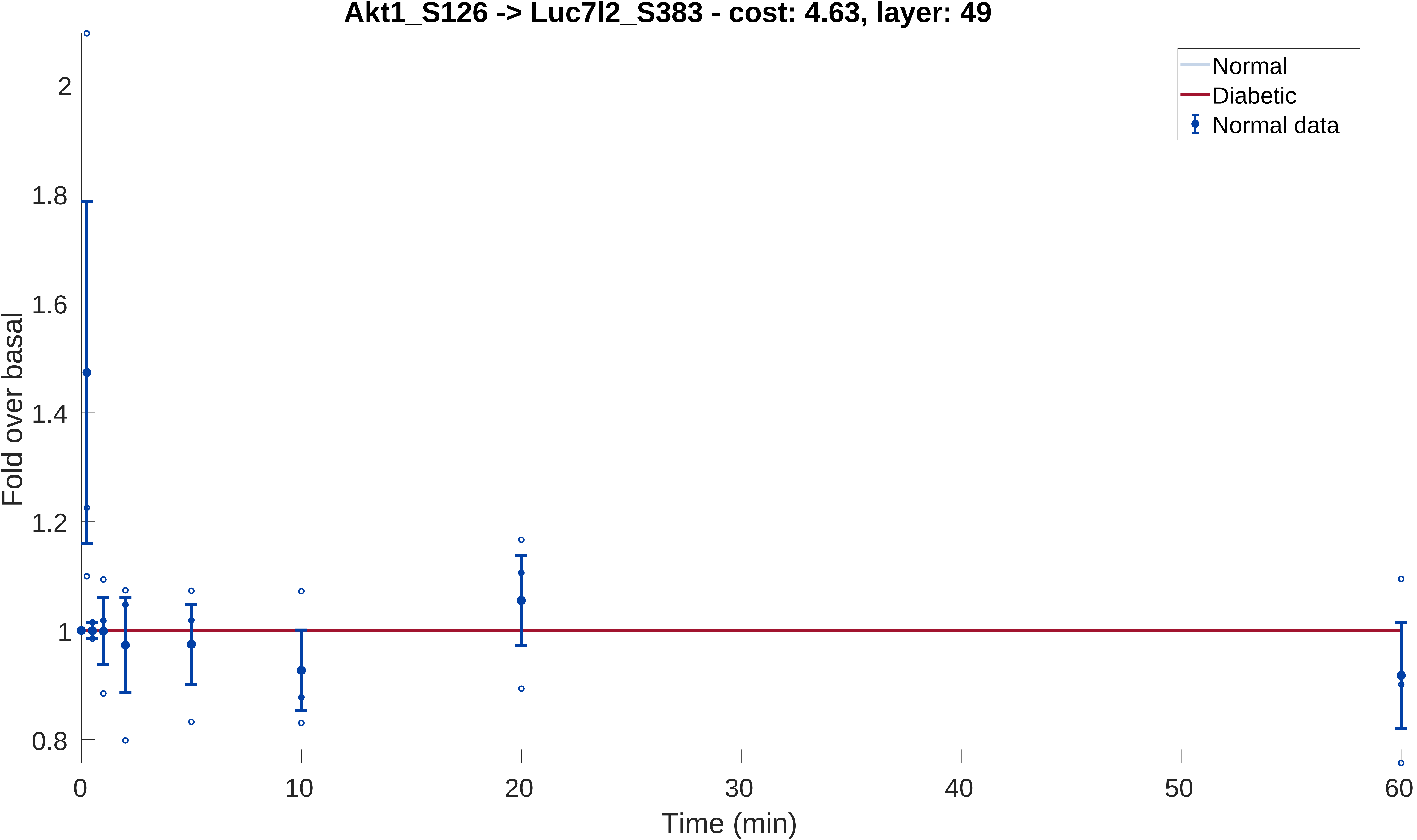
Akt1_S126 -> Luc7l2_S383 - cost: 4.63, layer: 49
Normal
Diabetic
Normal data
Fold over basal
2
1.8
1.6
1.4
1.2
1
0.8
0
10
20
30
40
50
60
Time (min)

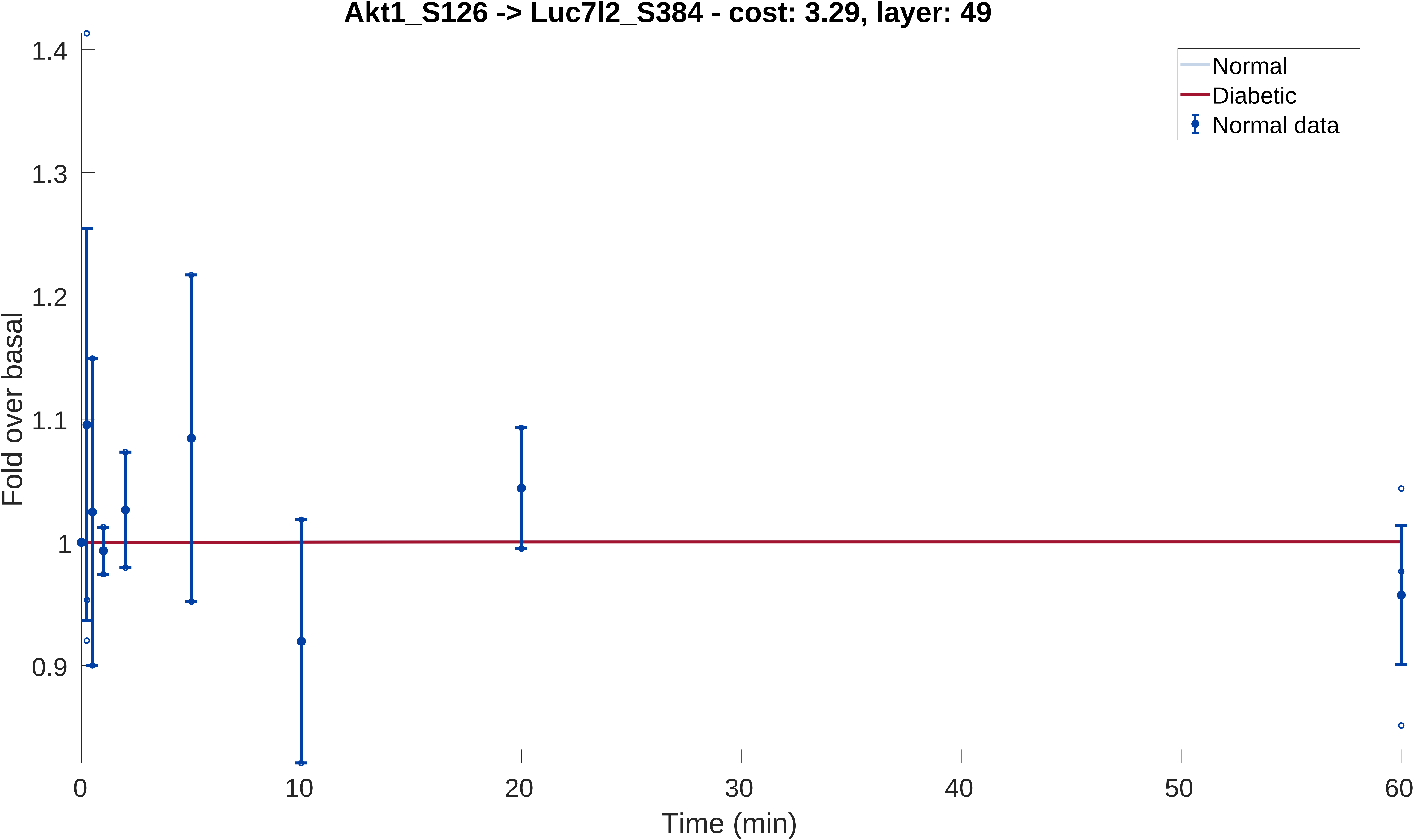

Akt1_S126 -> Luc7l2_S384 - cost: 3.29, layer: 49
Normal
Diabetic
Normal data
Fold over basal
1.4
1.3
1.2
1.1
1
0.9
0
10
20
30
40
50
60
Time (min)

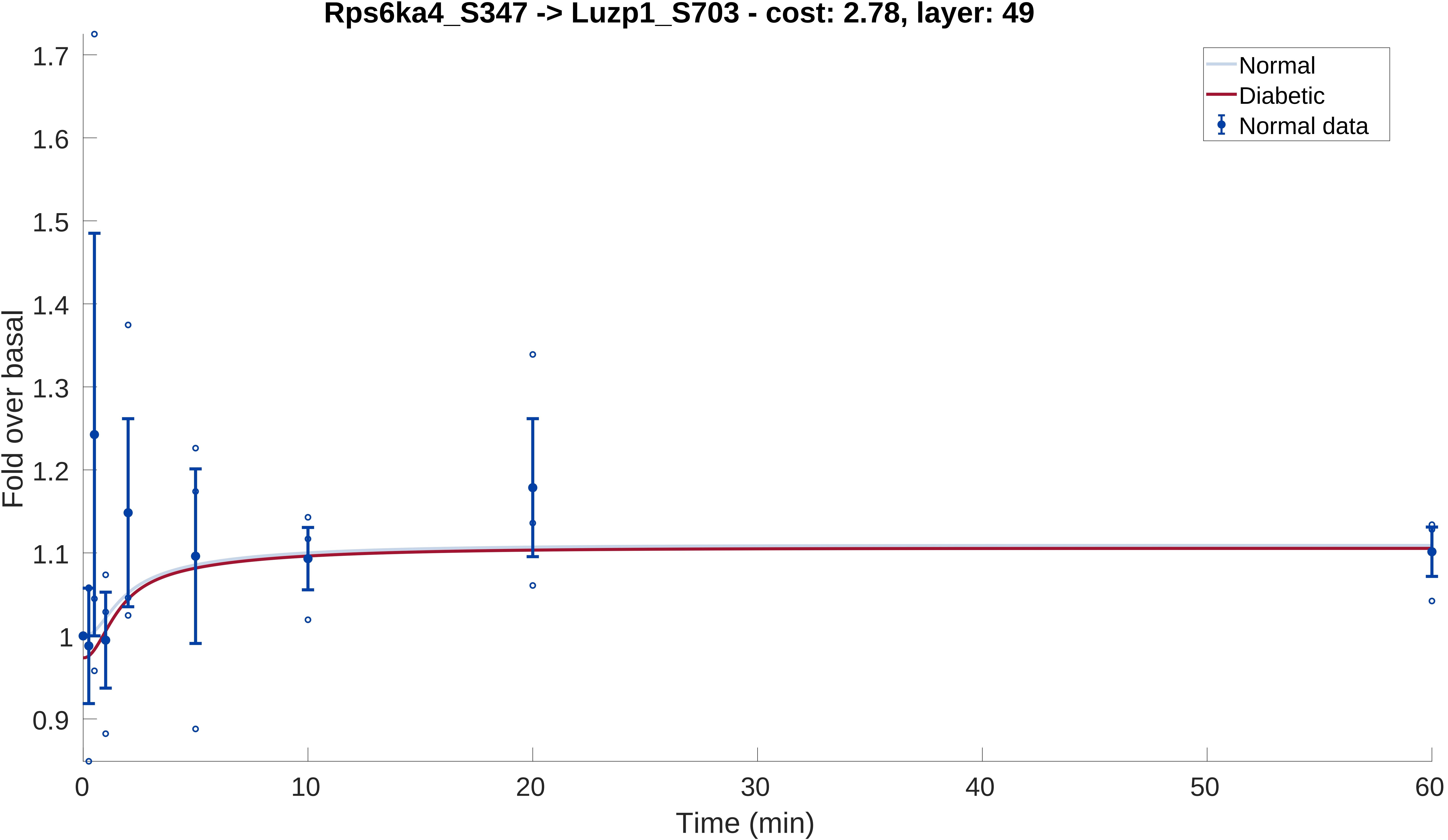

Rps6ka4_S347 -> Luzp1_S703 - cost: 2.78, layer: 49
Normal
Diabetic
Normal data
Fold over basal
1.7
1.6
1.5
1.4
1.3
1.2
1.1
1
0.9
0
10
20
30
40
50
60
Time (min)

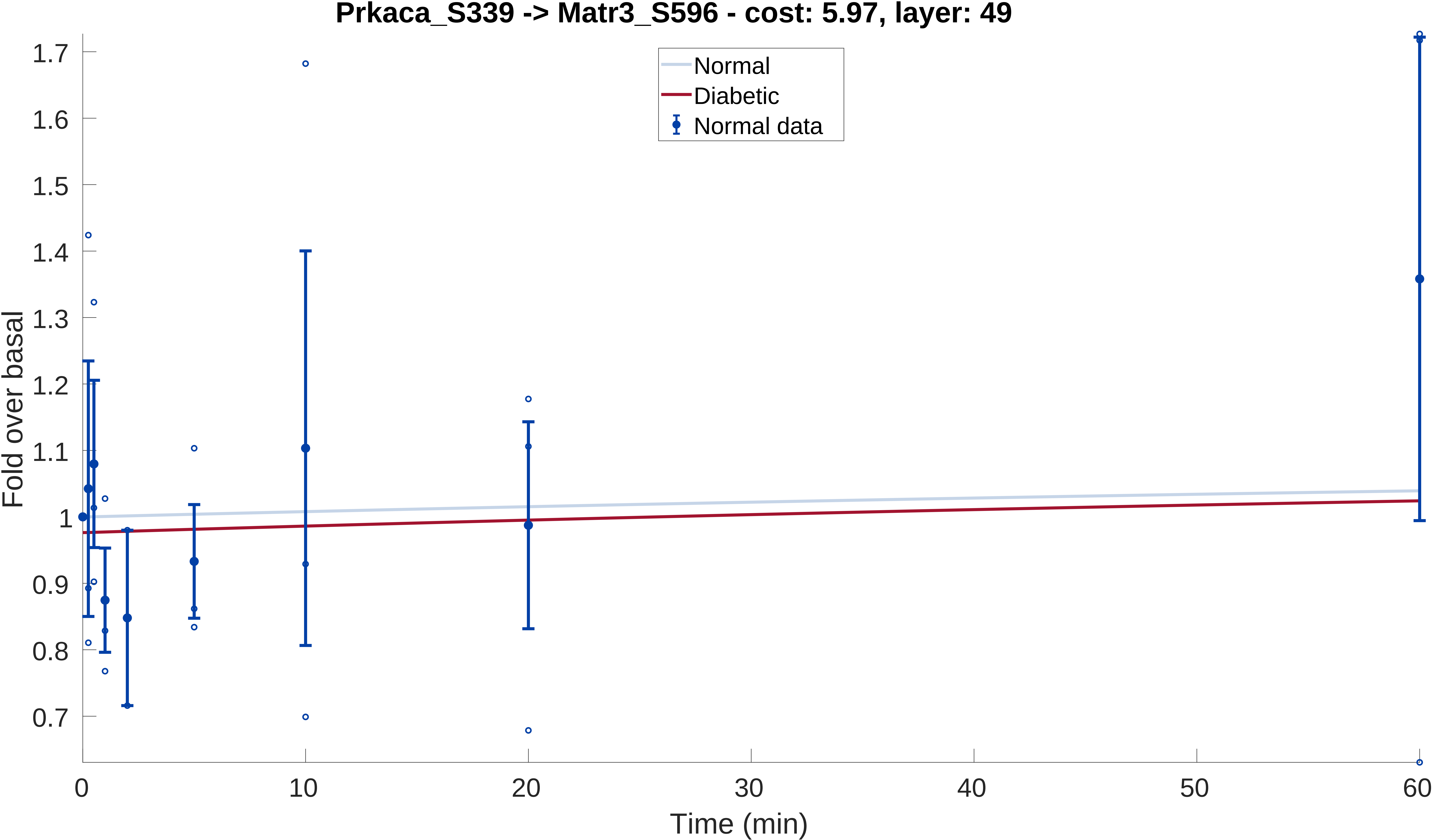

Prkaca_S339 -> Matr3_S596 - cost: 5.97, layer: 49
Normal
Diabetic
Normal data
Fold over basal
1.7
1.6
1.5
1.4
1.3
1.2
1.1
1
0.9
0.8
0.7
0
10
20
30
40
50
60
Time (min)

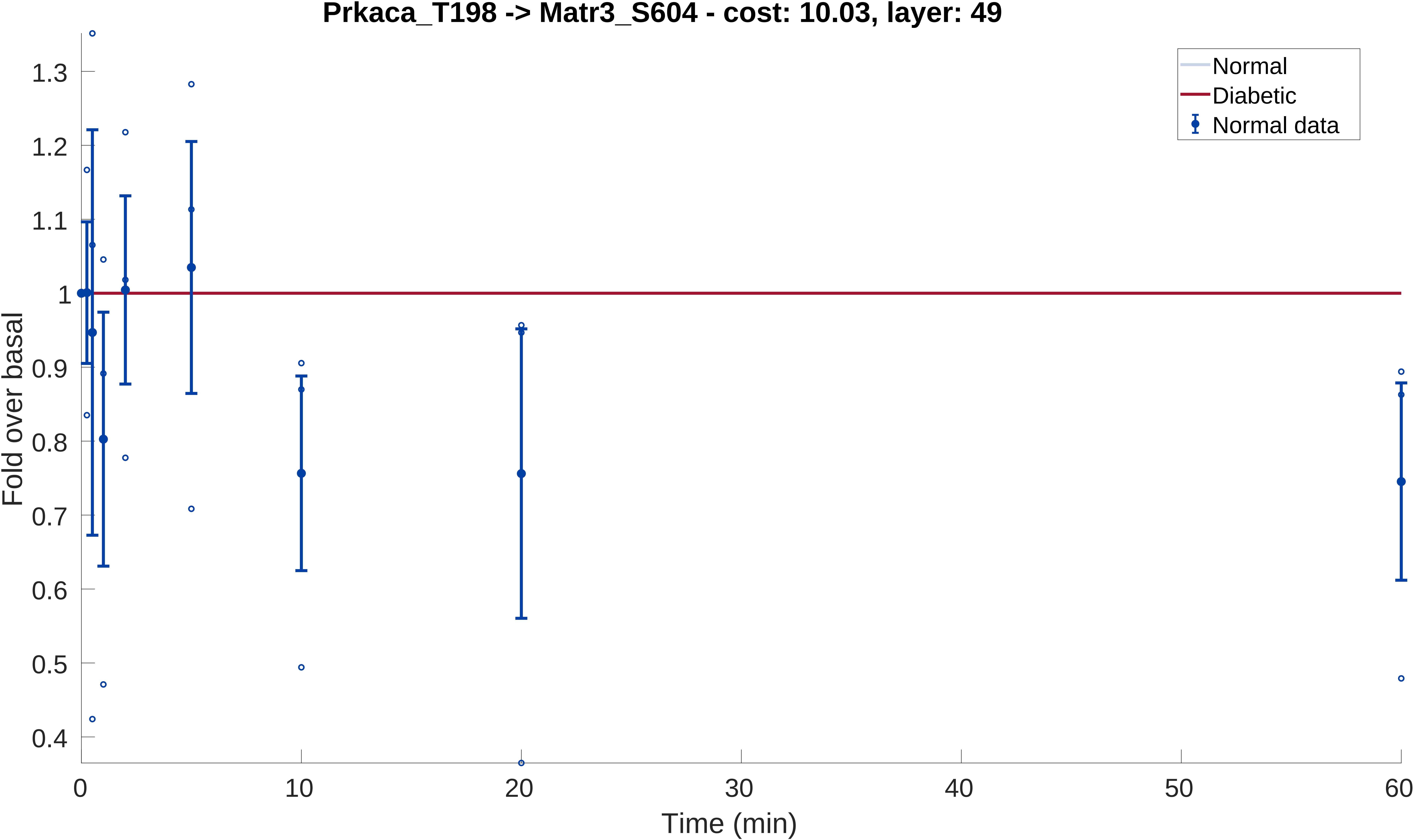

Prkaca_T198 -> Matr3_S604 - cost: 10.03, layer: 49
Normal
Diabetic
Normal data
Fold over basal
Time (min)
0.4
0.5
0.6
0.7
0.8
0.9
1
1.1
1.2
1.3
0
10
20
30
40
50
60

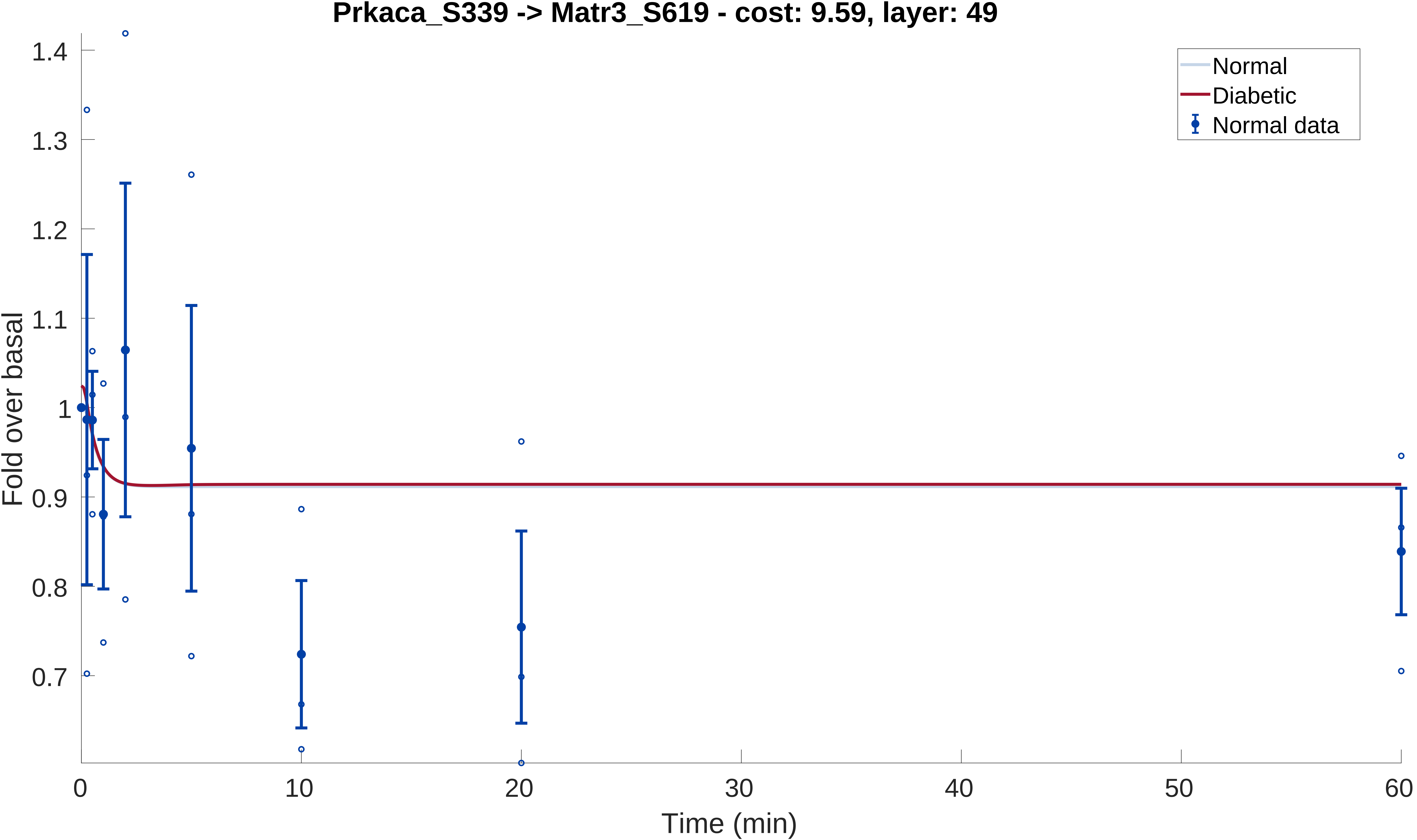

Prkaca_S339 -> Matr3_S619 - cost: 9.59, layer: 49
Fold over basal
1.4
1.3
1.2
1.1
1
0.9
0.8
0.7
0
10
20
30
40
50
60
Time (min)
Normal
Diabetic
Normal data

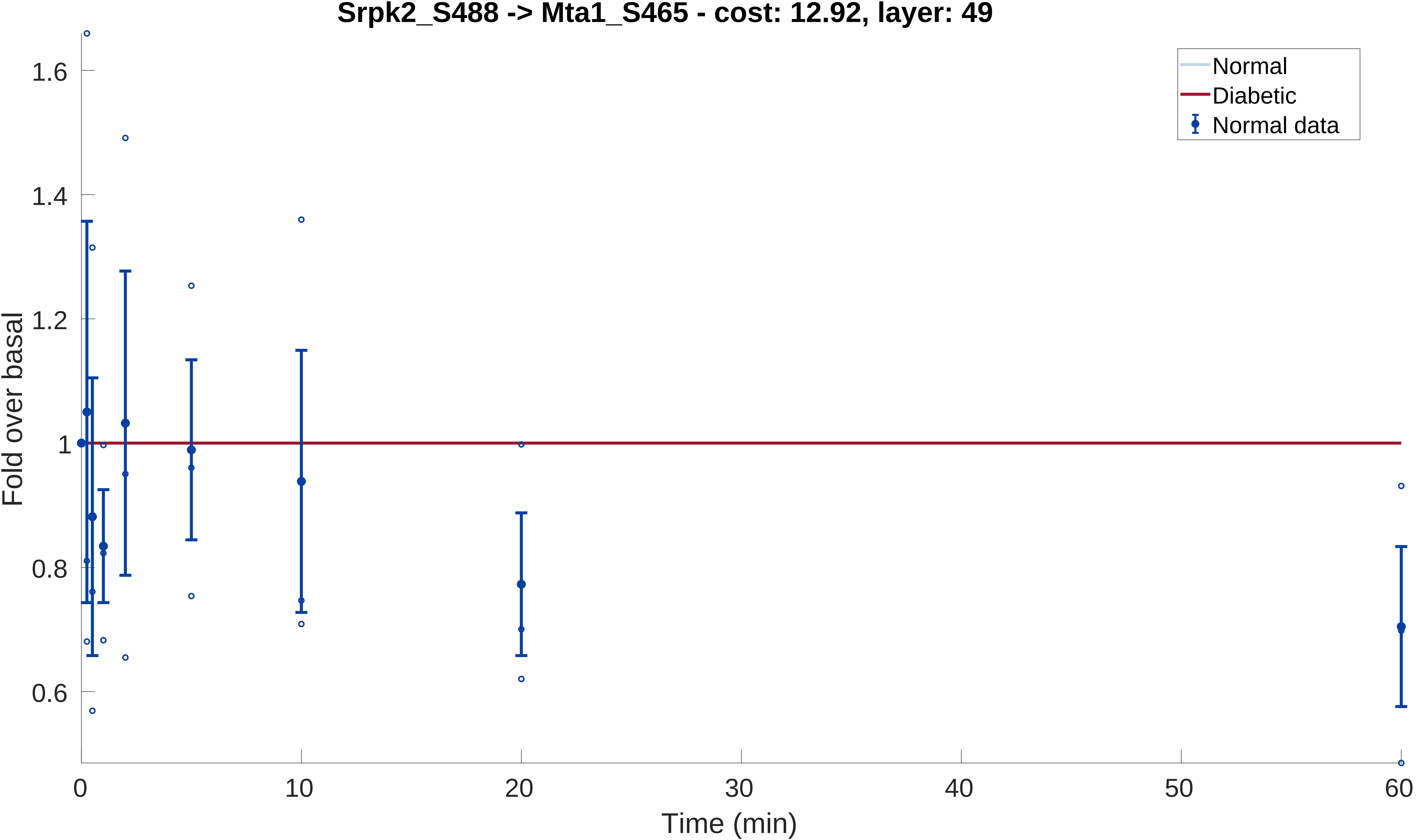

Srpk2_S488 -> Mta1_S465 - cost: 12.92, layer: 49
Normal
Diabetic
Normal data
Fold over basal
Time (min)
1.6
1.4
1.2
1
0.8
0.6
0
10
20
30
40
50
60

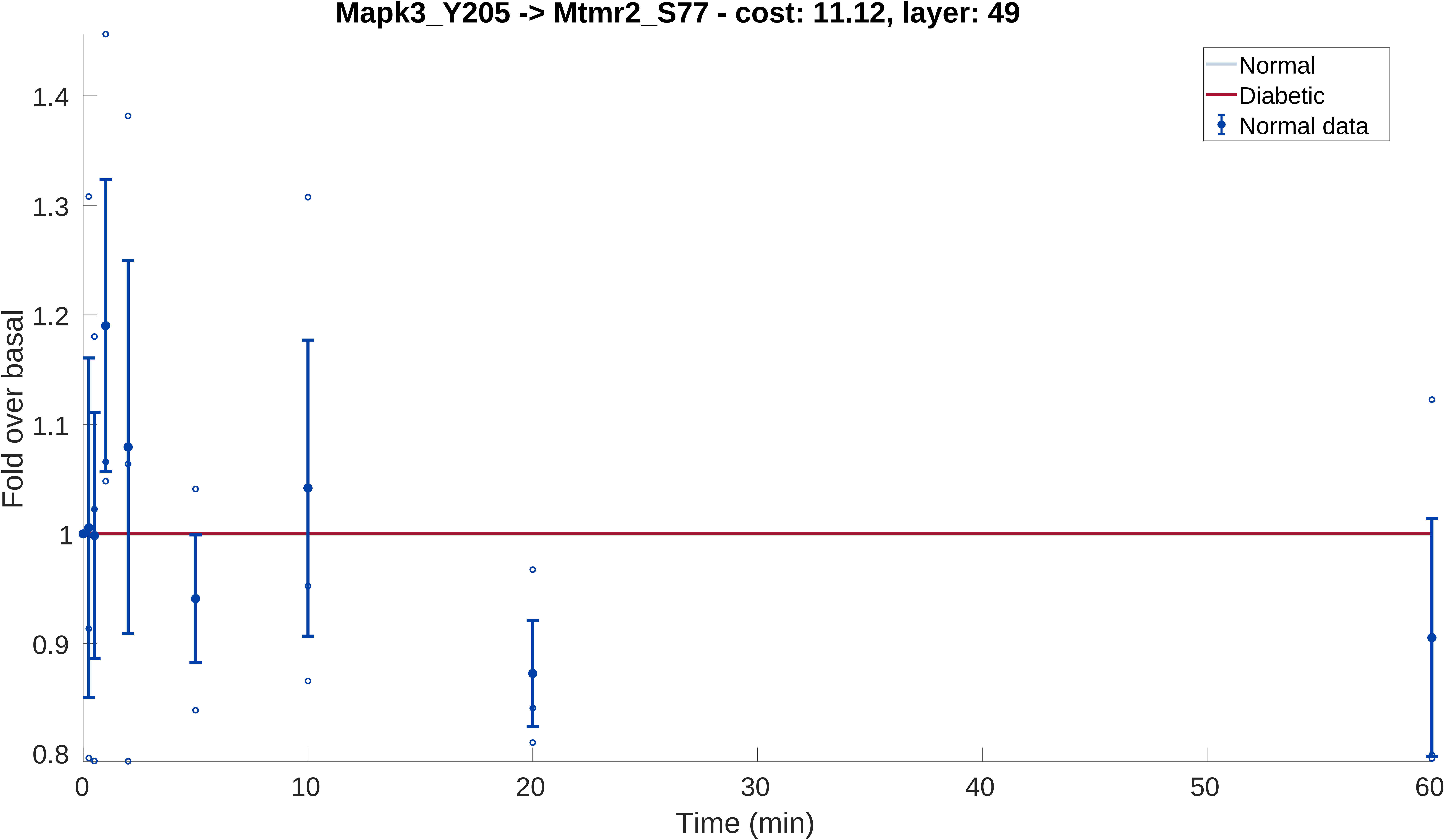

Mapk3_Y205 -> Mtmr2_S77 - cost: 11.12, layer: 49
Normal
Diabetic
Normal data
Fold over basal
1.4
1.3
1.2
1.1
1
0.9
0.8
0
10
20
30
40
50
60
Time (min)

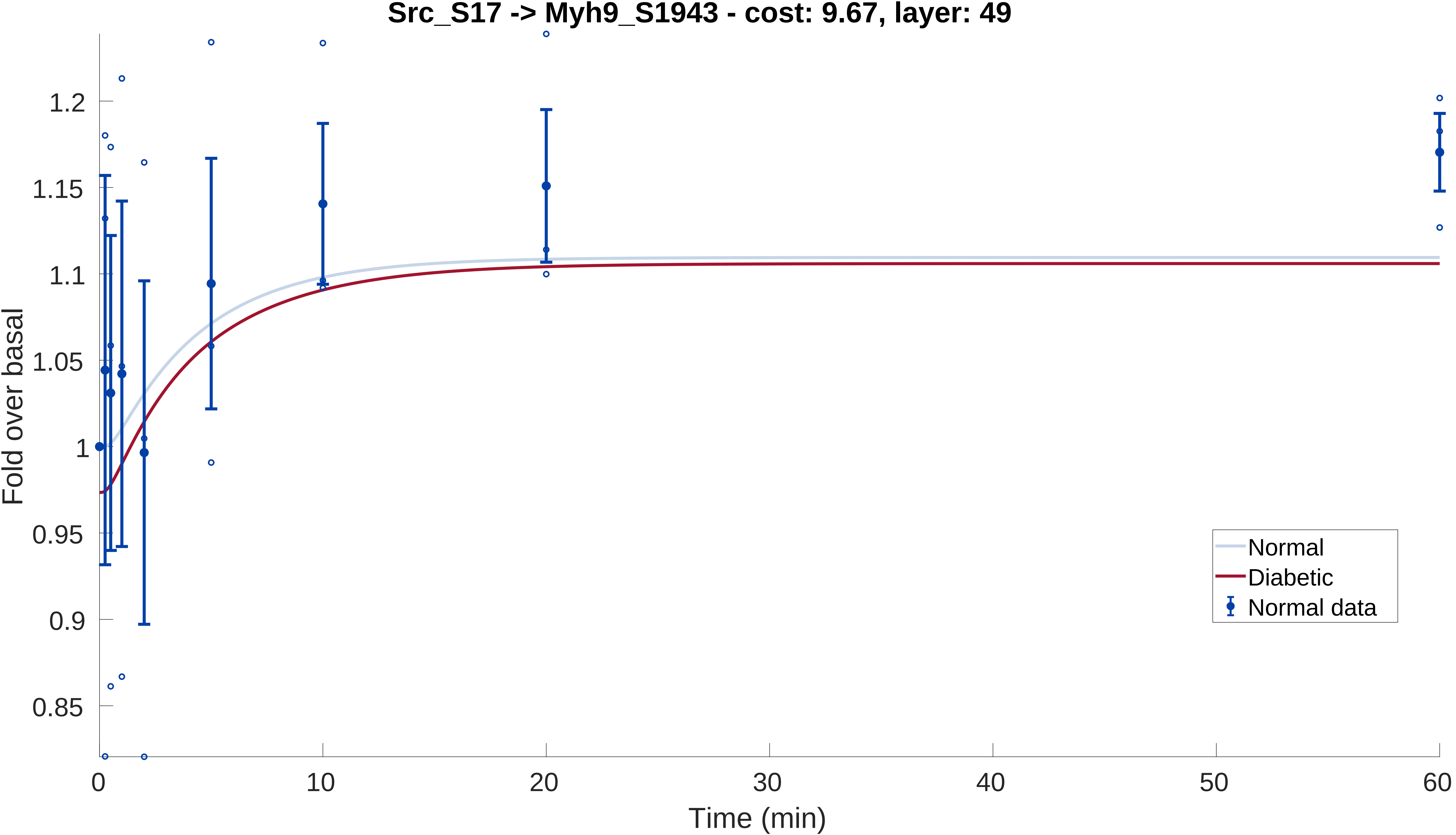
Src_S17 -> Myh9_S1943 - cost: 9.67, layer: 49
Fold over basal
1.2
1.15
1.1
1.05
1
0.95
0.9
0.85
0
10
20
30
40
50
60
Time (min)
Normal
Diabetic
Normal data

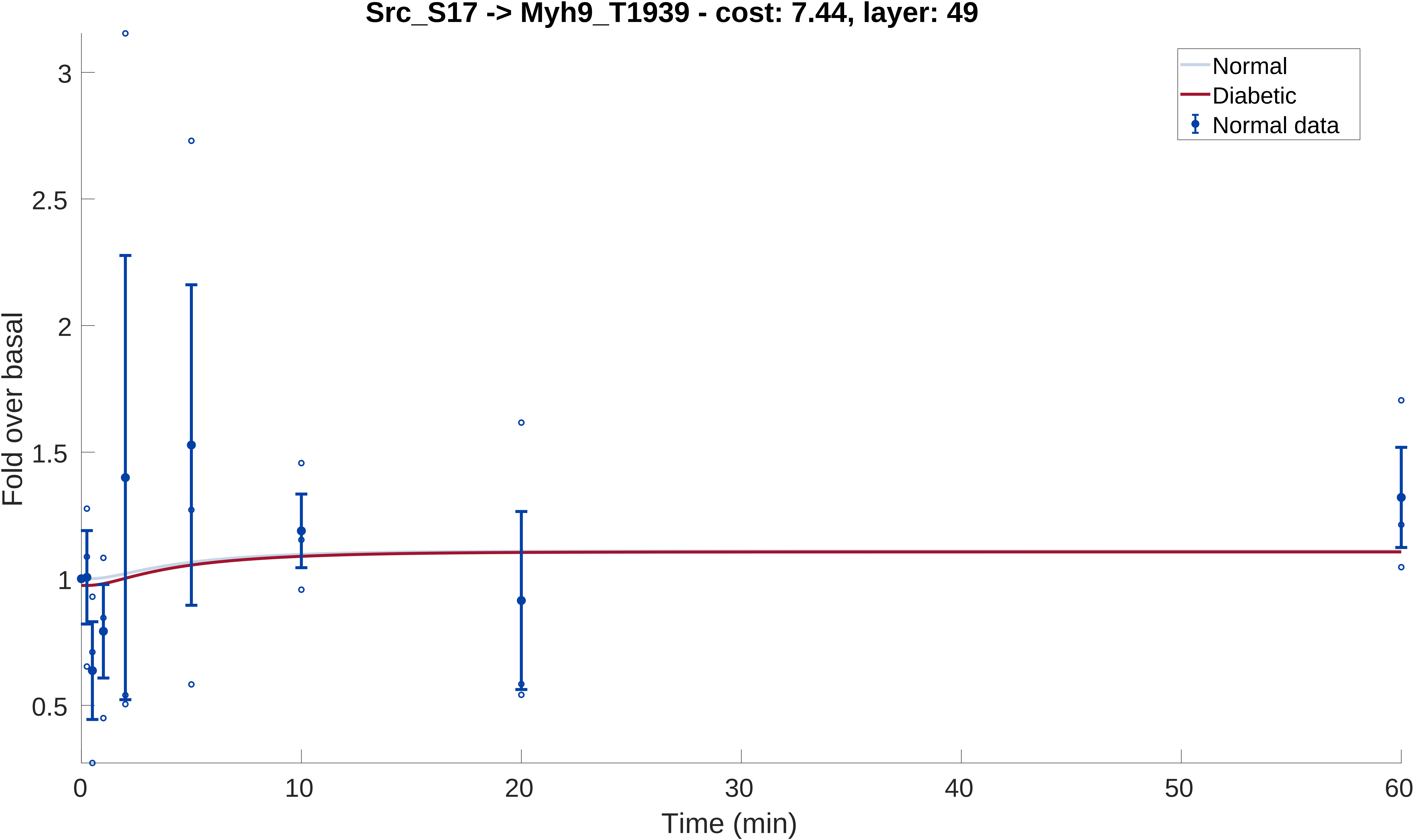

Src_S17 -> Myh9_T1939 - cost: 7.44, layer: 49
Normal
Diabetic
Normal data
Fold over basal
Time (min)
0.5
1
1.5
2
2.5
3
0
10
20
30
40
50
60

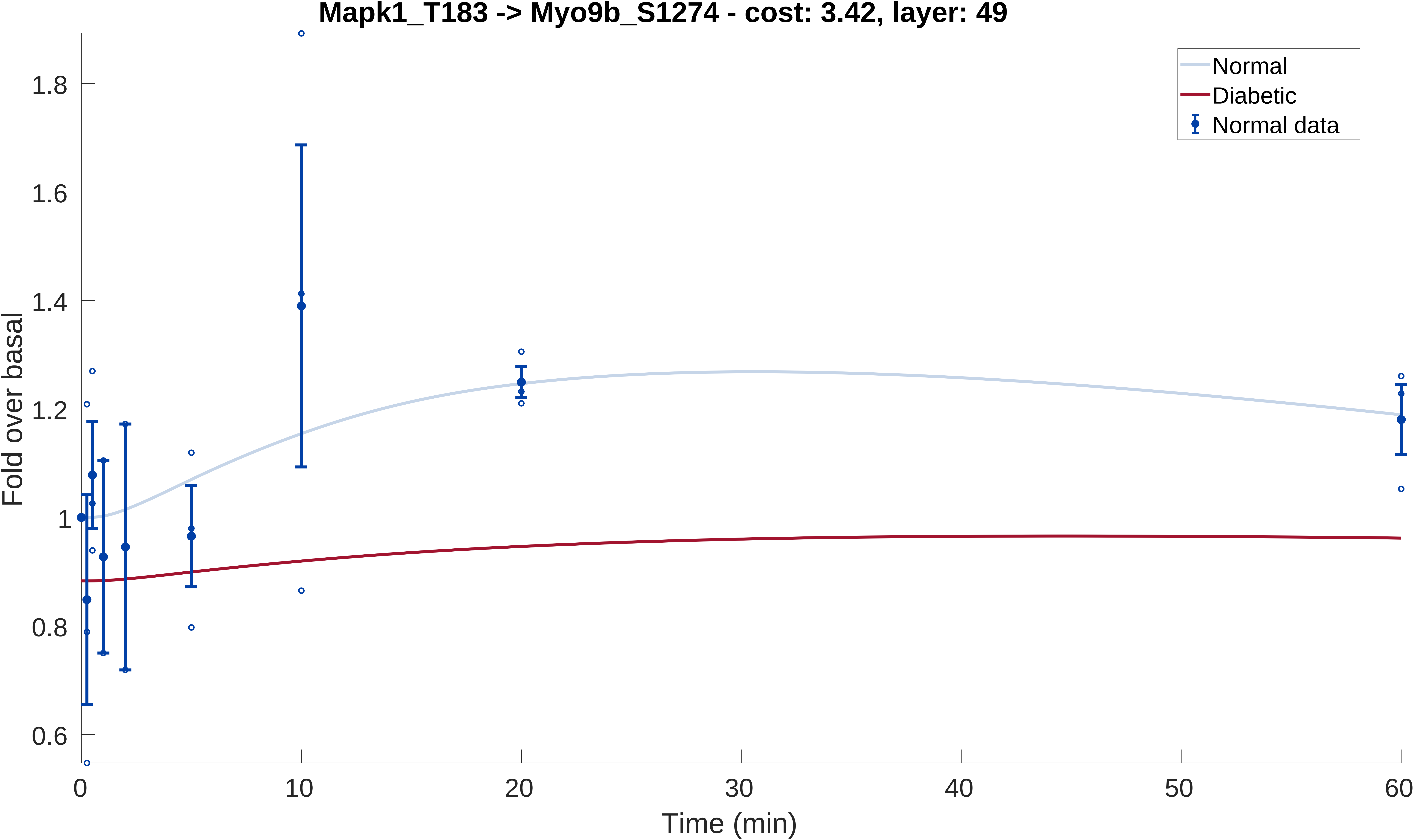

Mapk1_T183 -> Myo9b_S1274 - cost: 3.42, layer: 49
Normal
Diabetic
Normal data
Fold over basal
1.8
1.6
1.4
1.2
1
0.8
0.6
0
10
20
30
40
50
60
Time (min)

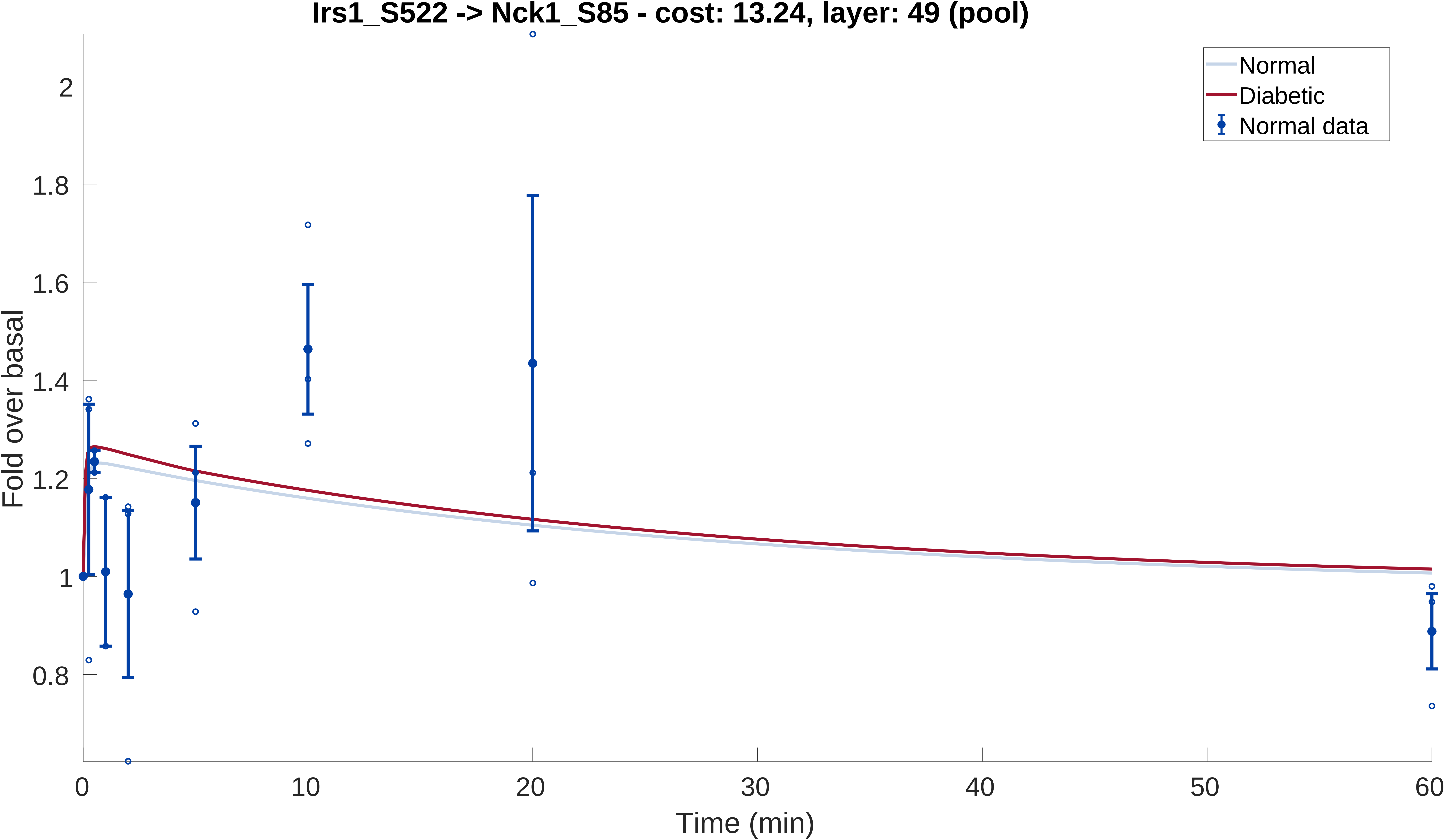
Irs1_S522 -> Nck1_S85 - cost: 13.24, layer: 49 (pool)
Normal
Diabetic
Normal data
Fold over basal
Time (min)
0.8
1
1.2
1.4
1.6
1.8
2
0
10
20
30
40
50
60

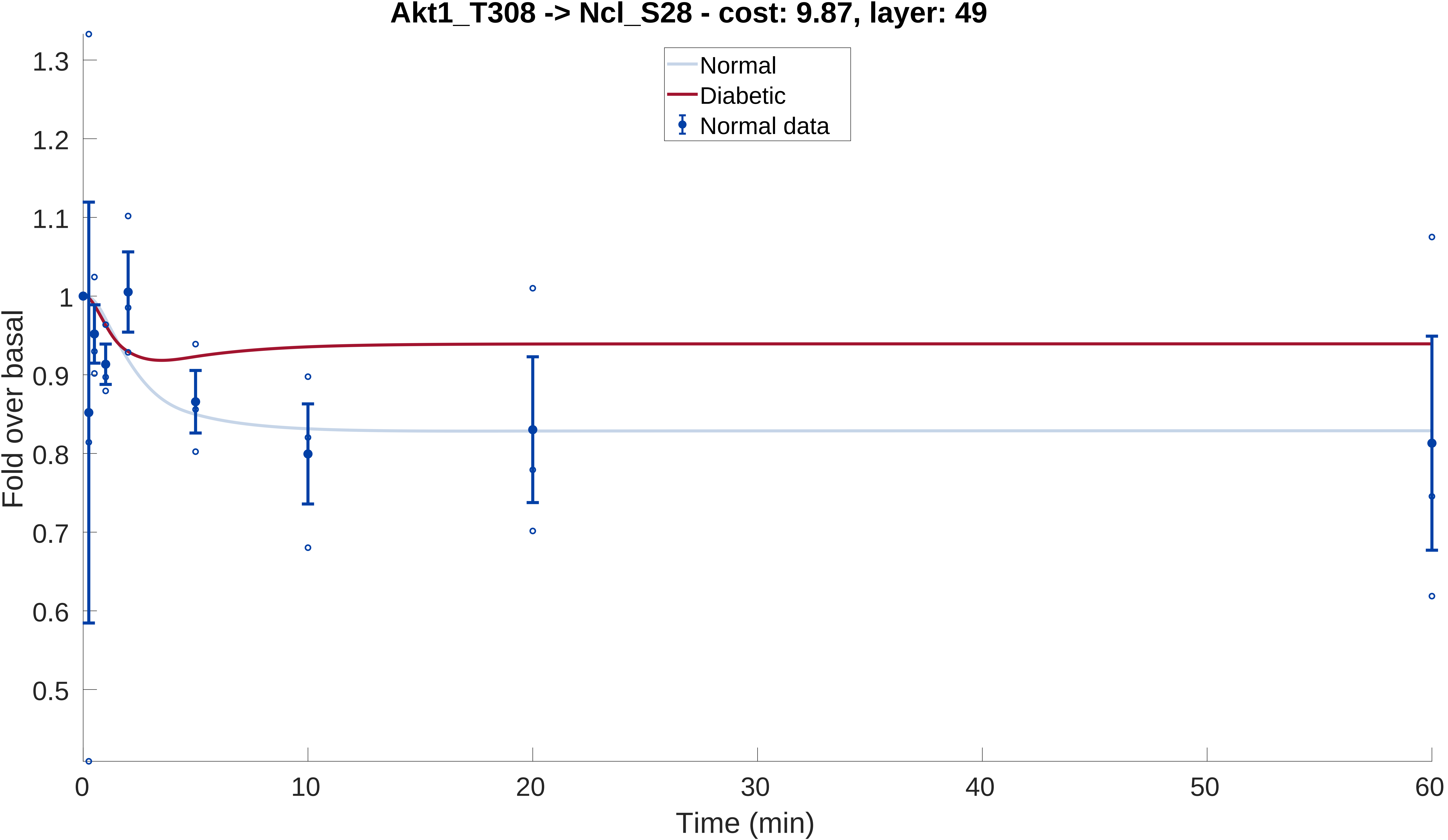

Akt1_T308 -> Ncl_S28 - cost: 9.87, layer: 49
Normal
Diabetic
Normal data
Fold over basal
1.3
1.2
1.1
1
0.9
0.8
0.7
0.6
0.5
0
10
20
30
40
50
60
Time (min)

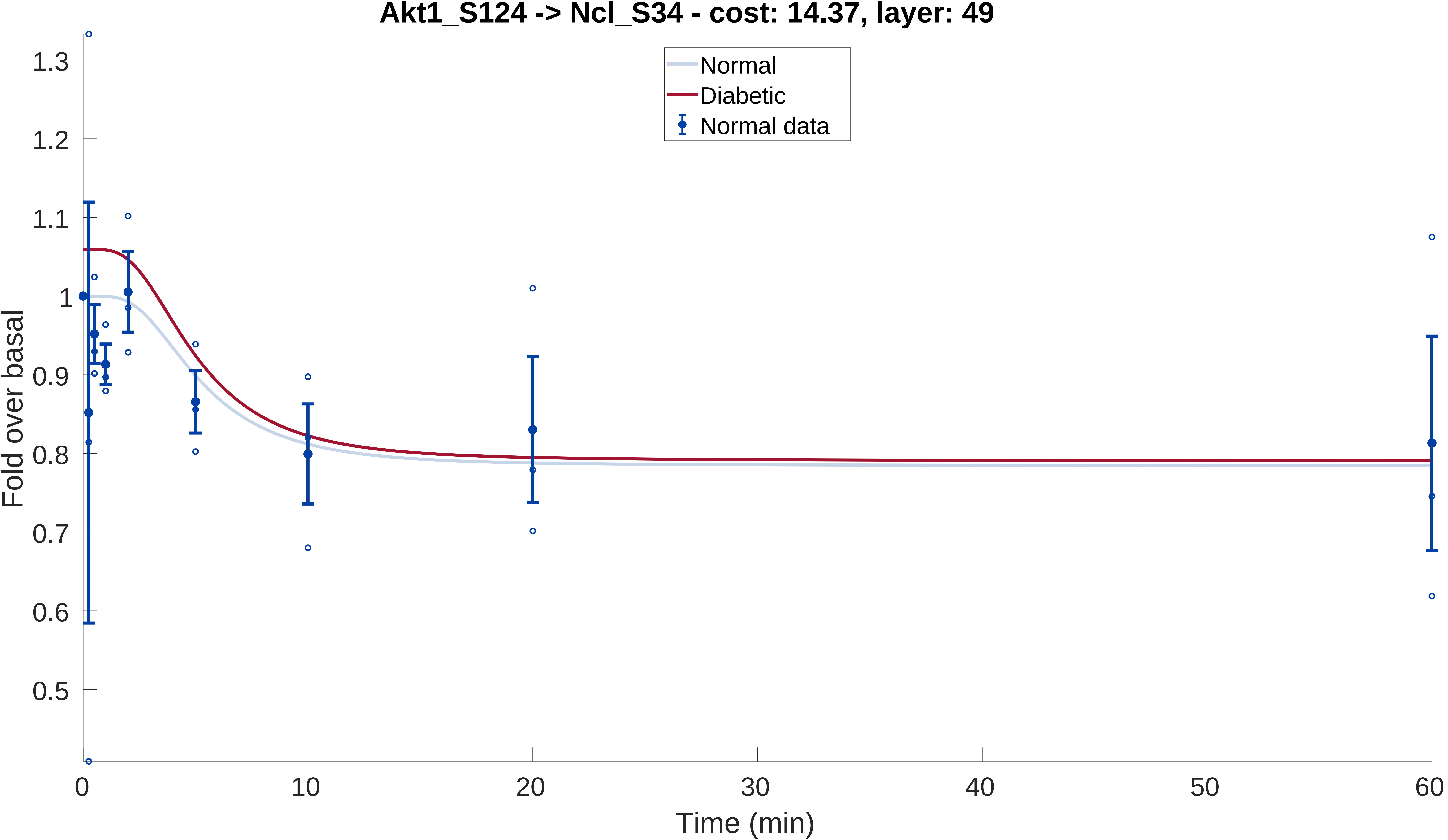

Akt1_S124 -> Ncl_S34 - cost: 14.37, layer: 49
Normal
Diabetic
Normal data
Fold over basal
1.3
1.2
1.1
1
0.9
0.8
0.7
0.6
0.5
0
10
20
30
40
50
60
Time (min)

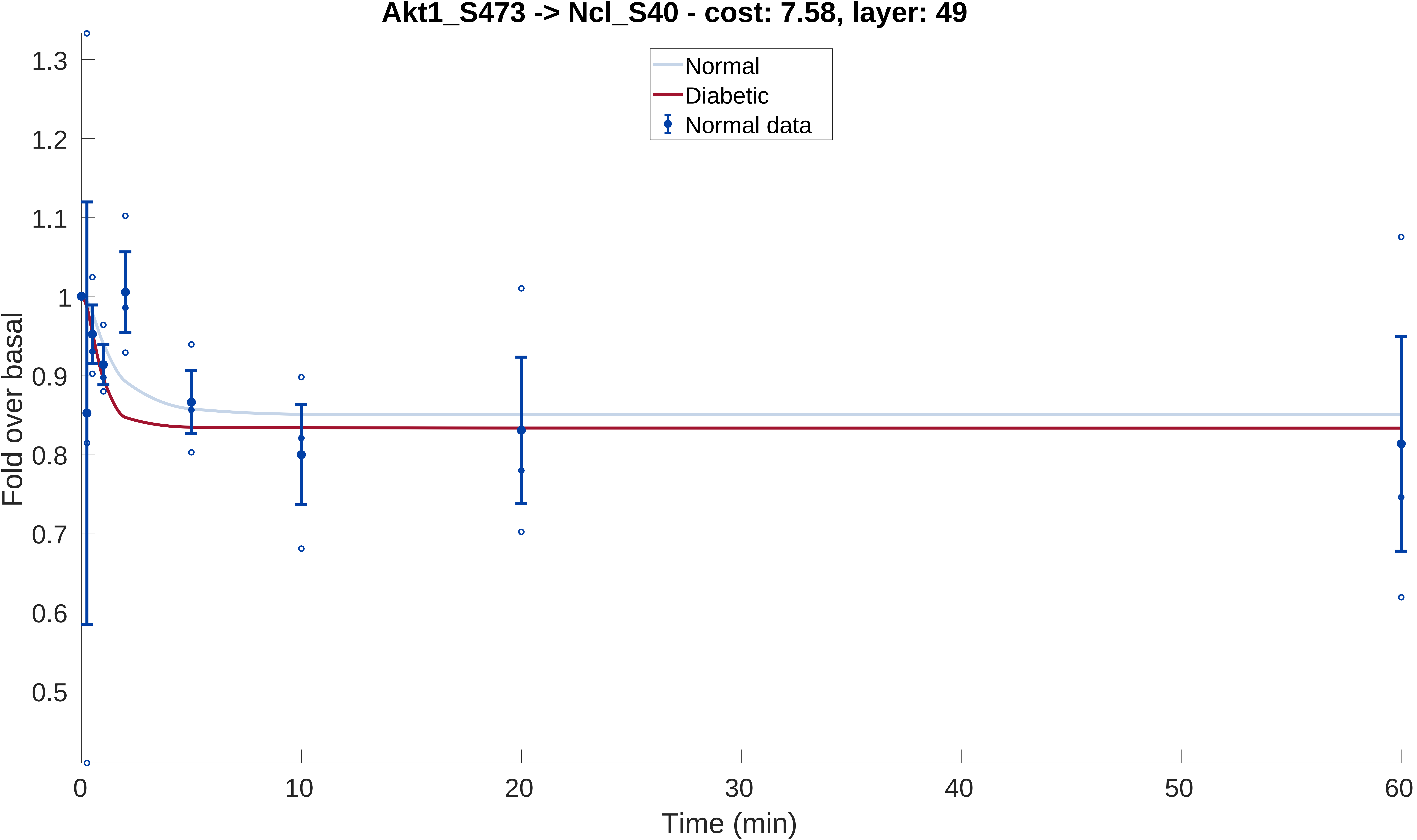

Akt1_S473 -> Ncl_S40 - cost: 7.58, layer: 49
Normal
Diabetic
Normal data
Fold over basal
Time (min)
1.3
1.2
1.1
1
0.9
0.8
0.7
0.6
0.5
0
10
20
30
40
50
60

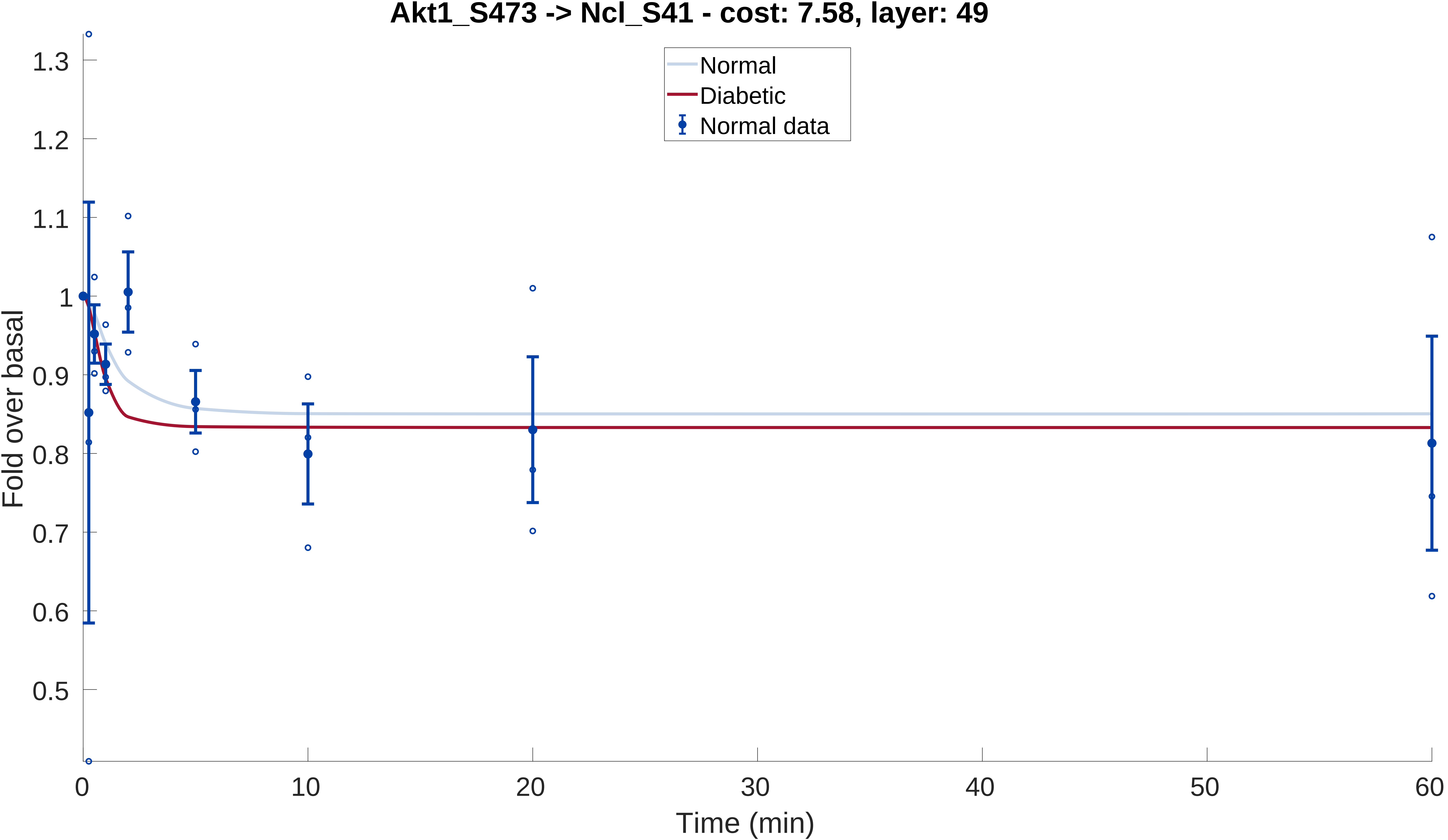

Akt1_S473 -> Ncl_S41 - cost: 7.58, layer: 49
Normal
Diabetic
Normal data
Fold over basal
Time (min)
1.3
1.2
1.1
1
0.9
0.8
0.7
0.6
0.5
0
10
20
30
40
50
60

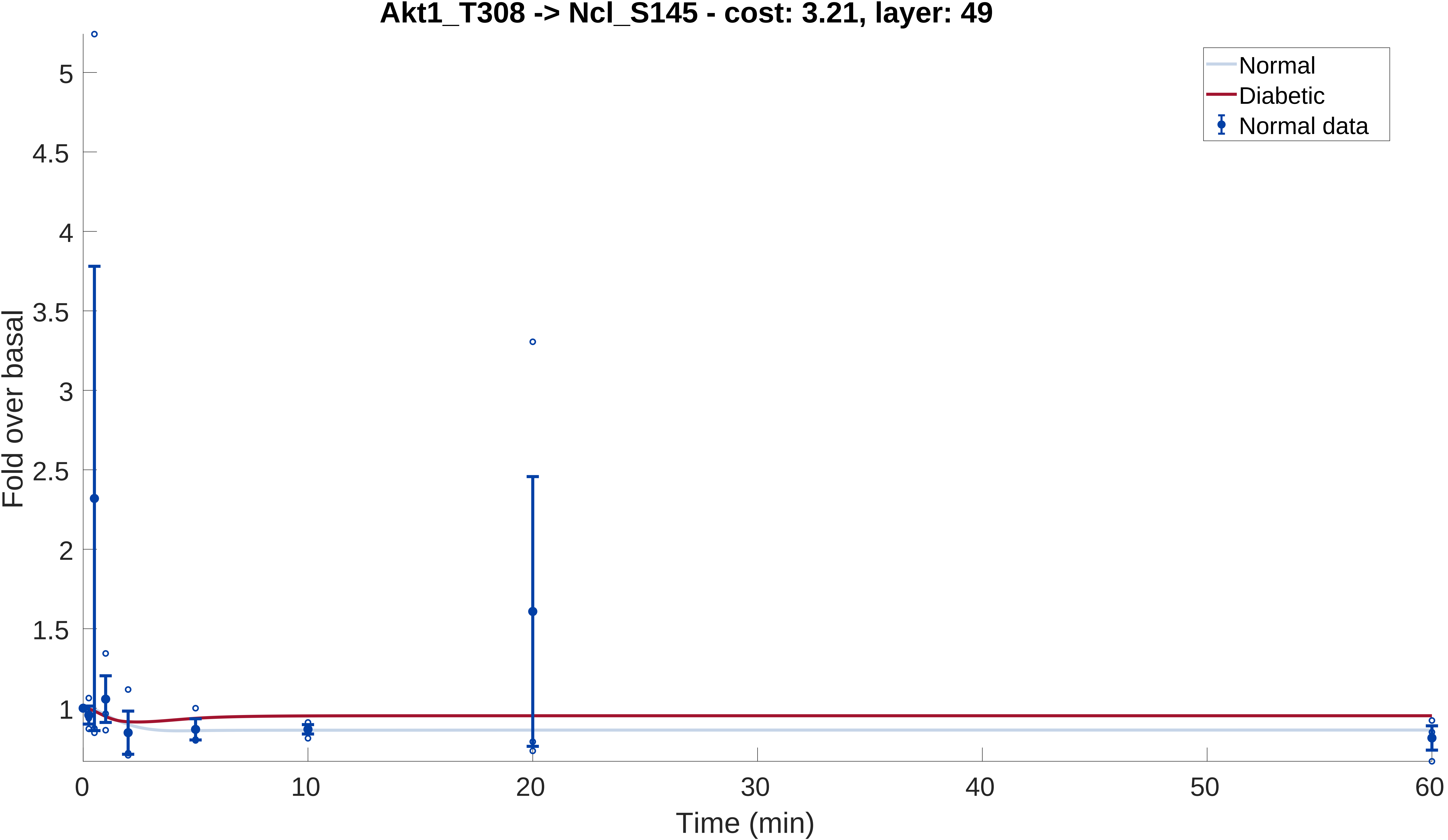

Akt1_T308 -> Ncl_S145 - cost: 3.21, layer: 49
Normal
Diabetic
Normal data
Fold over basal
5
4.5
4
3.5
3
2.5
2
1.5
1
0
10
20
30
40
50
60
Time (min)

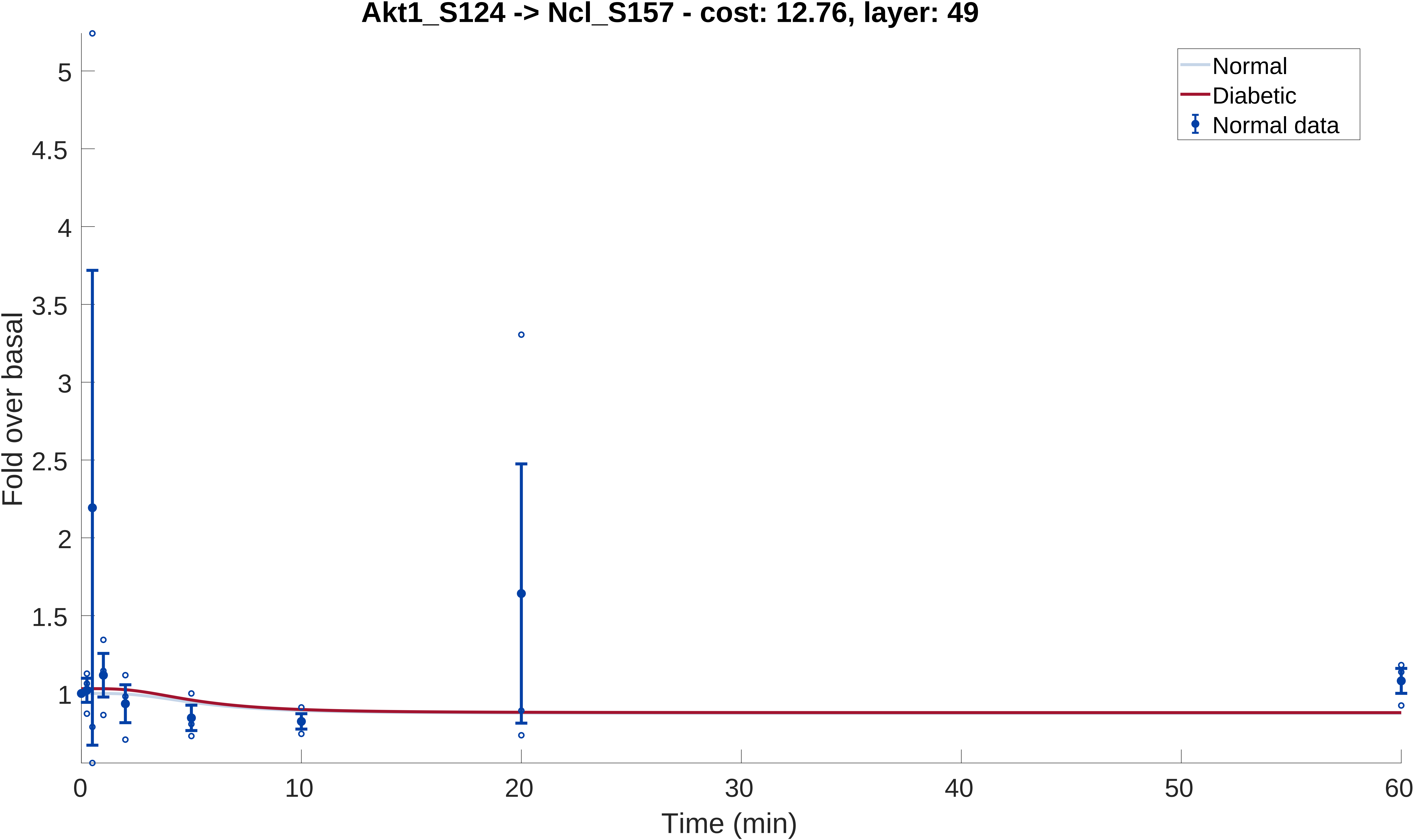
Akt1_S124 -> Ncl_S157 - cost: 12.76, layer: 49
Normal
Diabetic
Normal data
Fold over basal
Time (min)
5
4.5
4
3.5
3
2.5
2
1.5
1
0
10
20
30
40
50
60

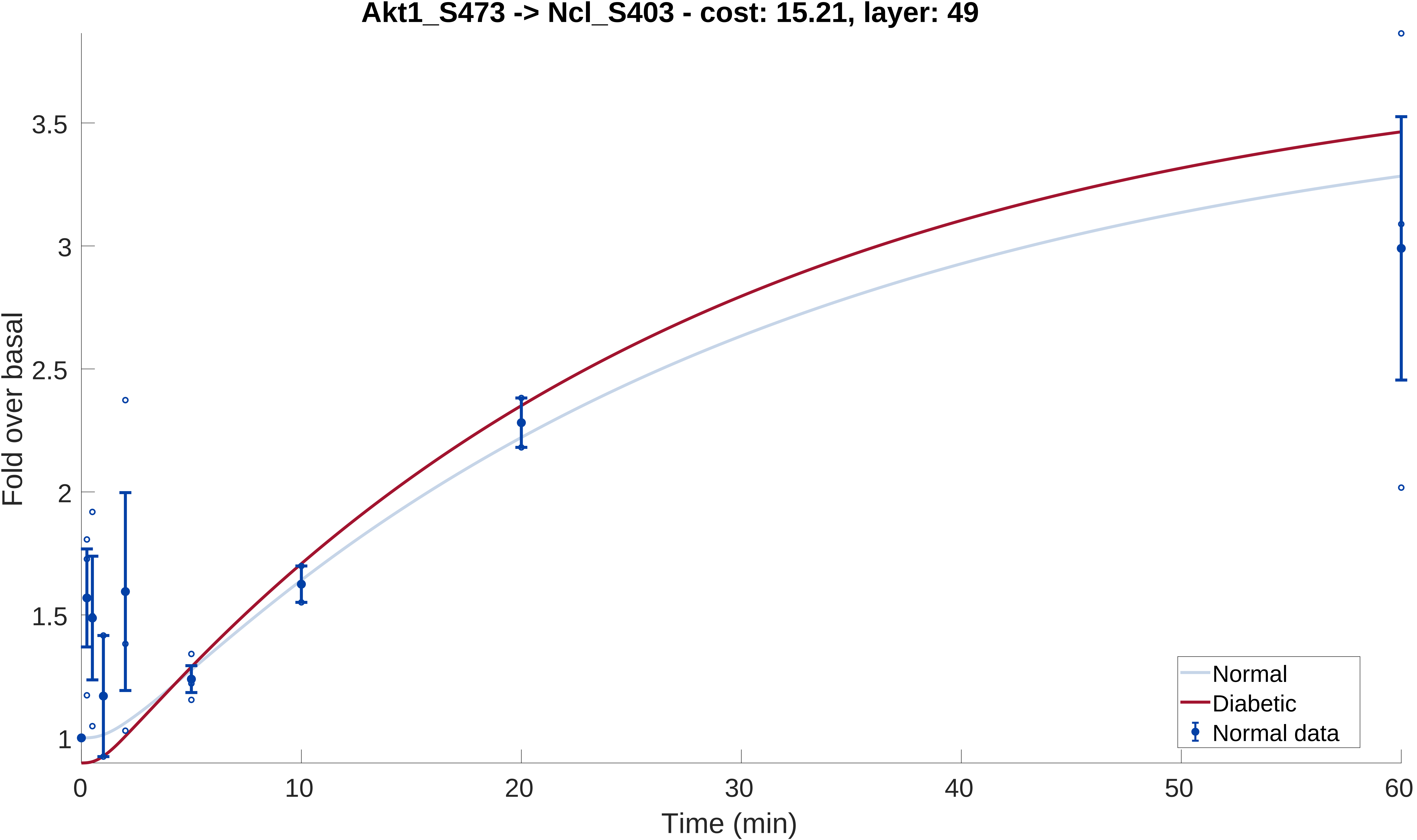
Akt1_S473 -> Ncl_S403 - cost: 15.21, layer: 49
Fold over basal
3.5
3
2.5
2
1.5
1
0
10
20
30
40
50
60
Time (min)
Normal
Diabetic
Normal data

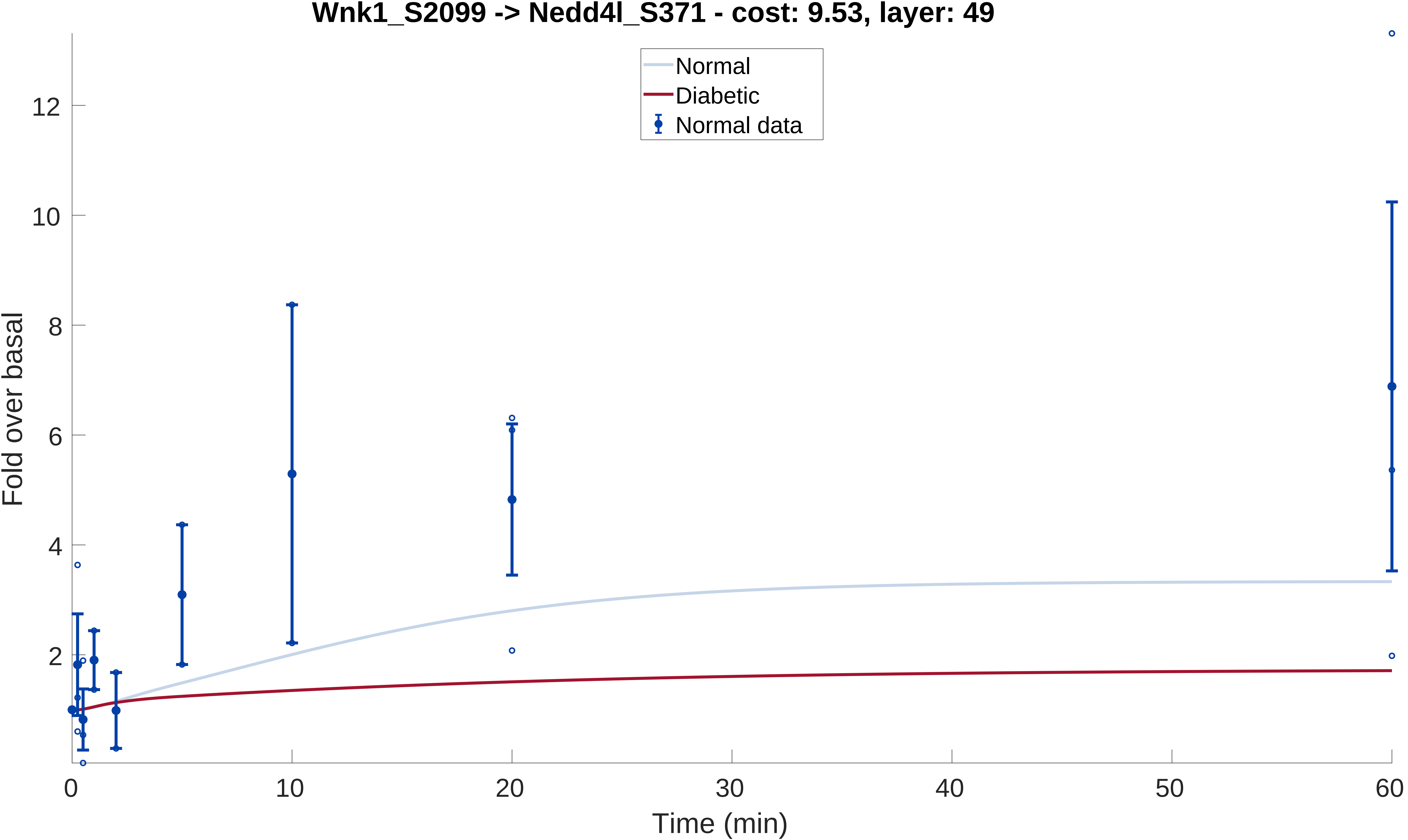

Wnk1_S2099 -> Nedd4l_S371 - cost: 9.53, layer: 49
Normal
Diabetic
Normal data
Fold over basal
Time (min)
0
10
20
30
40
50
60
2
4
6
8
10
12

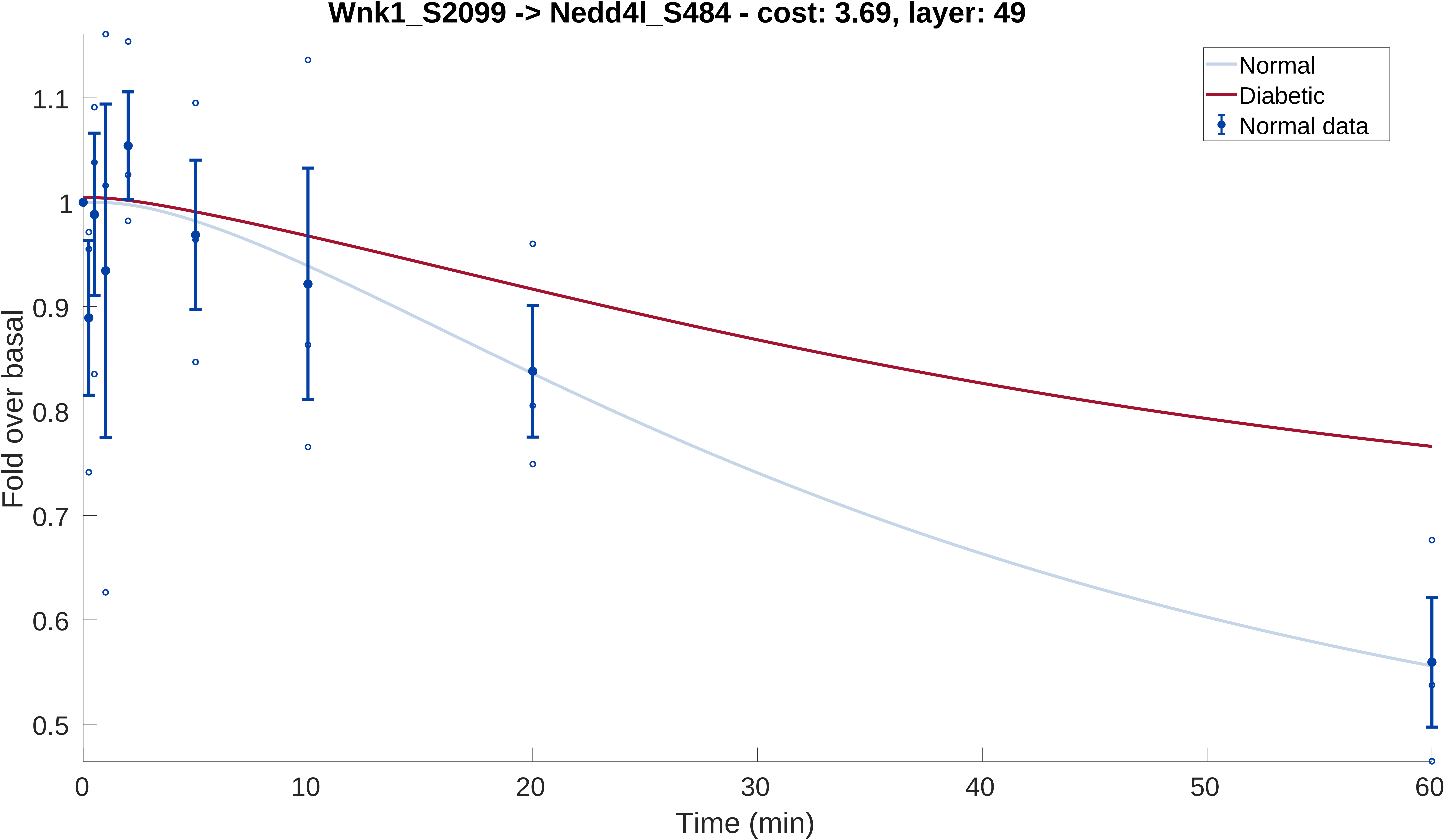

Wnk1_S2099 -> Nedd4l_S484 - cost: 3.69, layer: 49
Normal
Diabetic
Normal data
Fold over basal
Time (min)
1.1
1
0.9
0.8
0.7
0.6
0.5
0
10
20
30
40
50
60

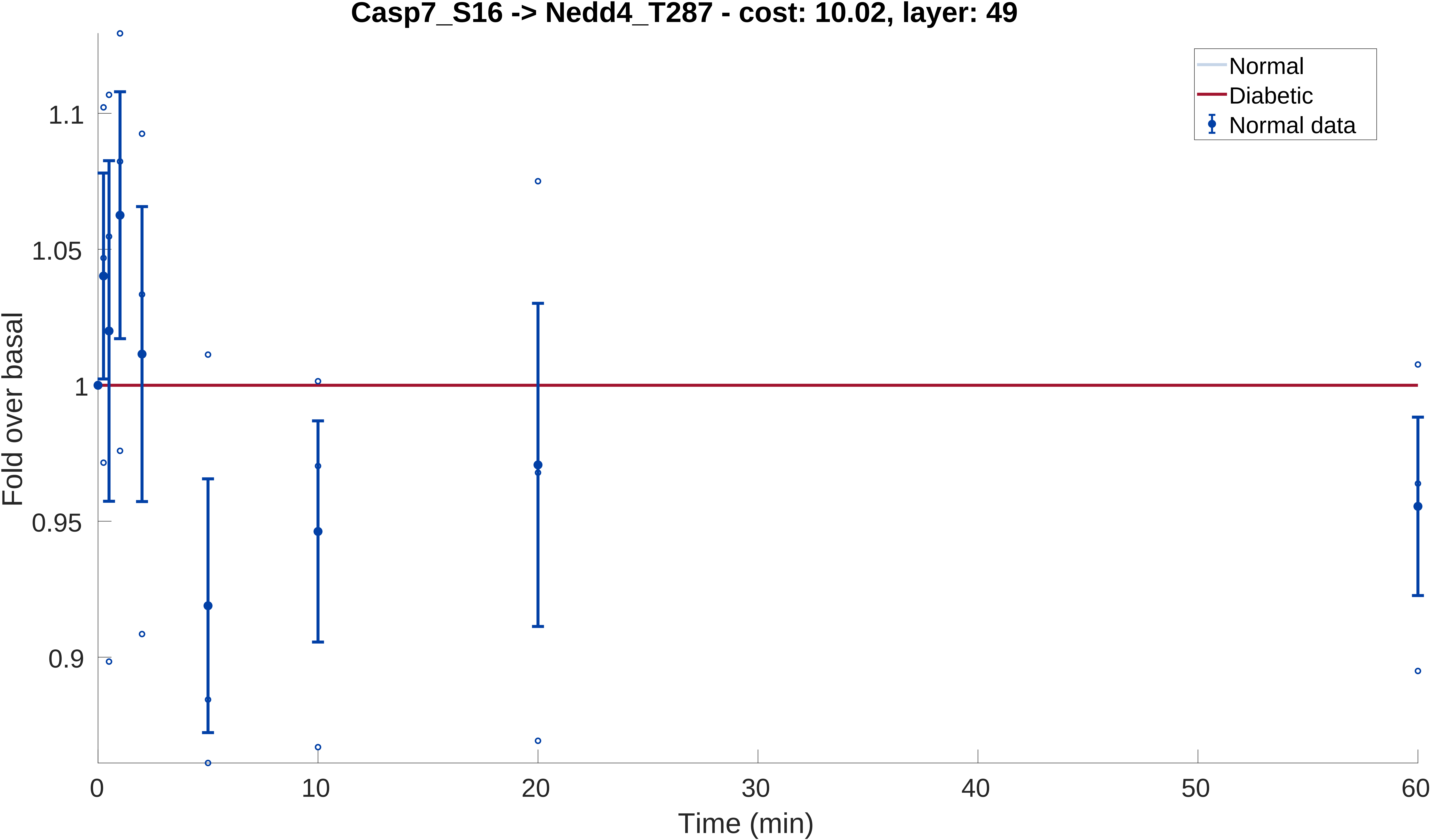
Casp7_S16 -> Nedd4_T287 - cost: 10.02, layer: 49
Normal
Diabetic
Normal data
Fold over basal
1.1
1.05
1
0.95
0.9
0
10
20
30
40
50
60
Time (min)

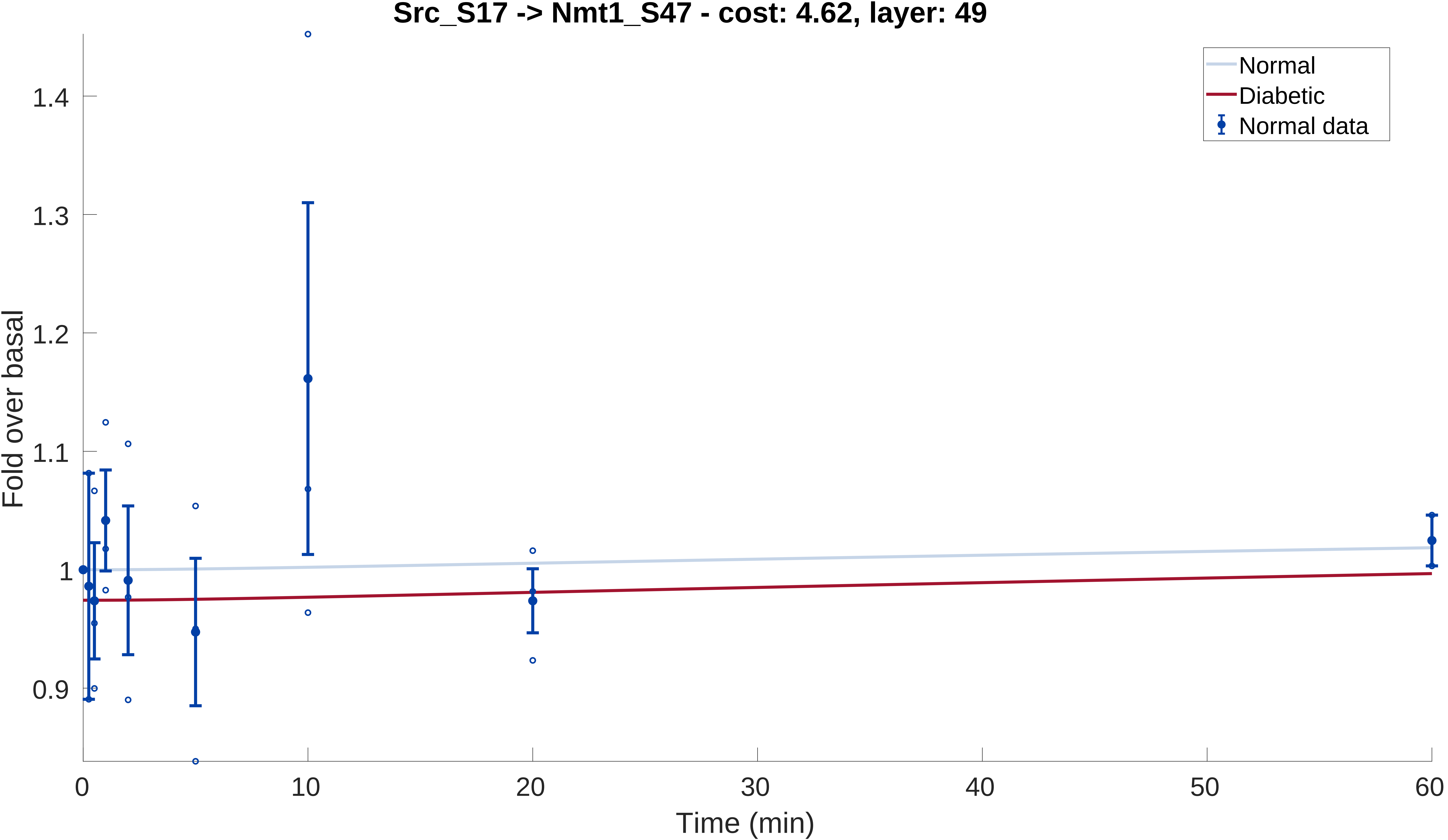

Src_S17 -> Nmt1_S47 - cost: 4.62, layer: 49
Normal
Diabetic
Normal data
Fold over basal
1.4
1.3
1.2
1.1
1
0.9
0
10
20
30
40
50
60
Time (min)

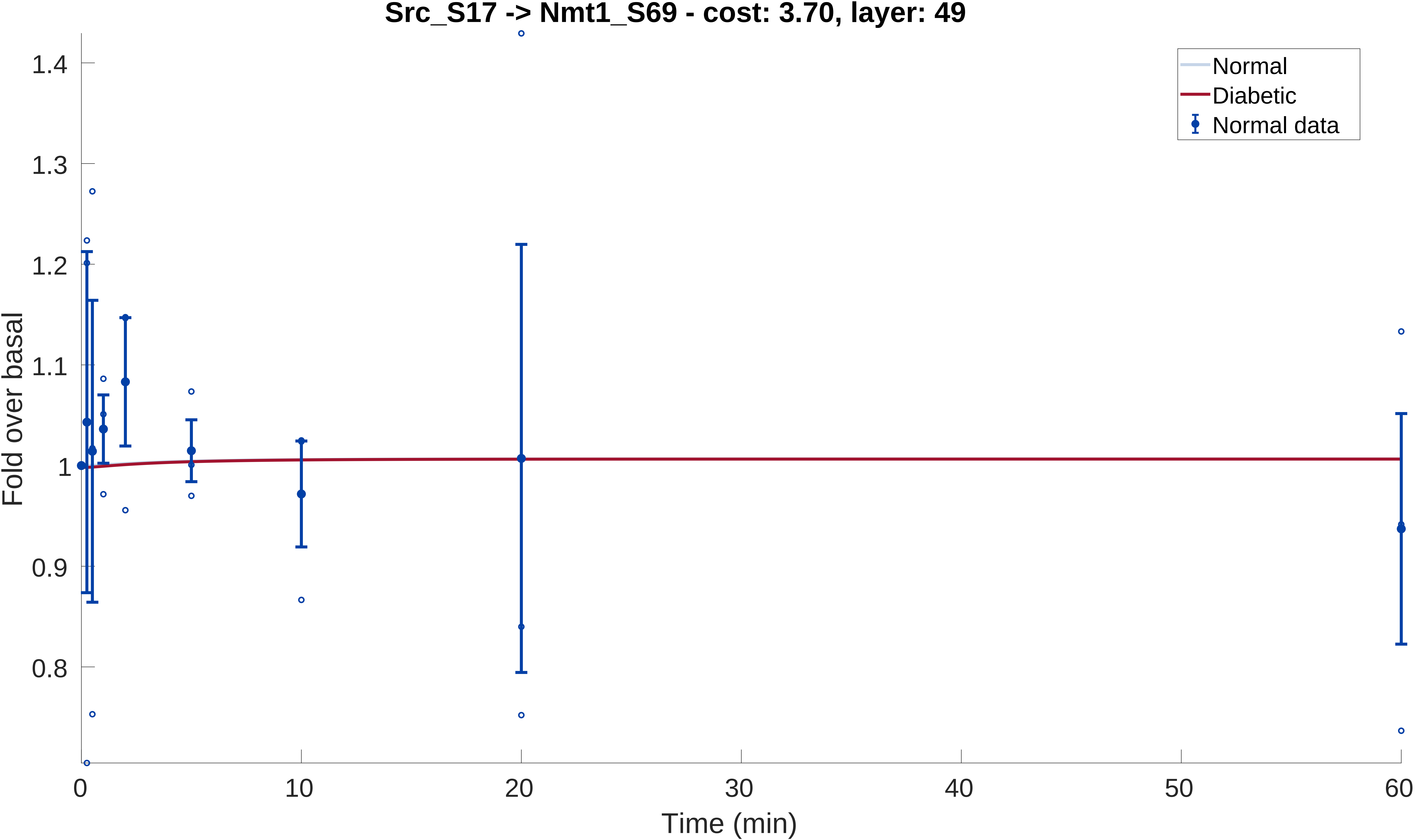

Src_S17 -> Nmt1_S69 - cost: 3.70, layer: 49
Normal
Diabetic
Normal data
Fold over basal
Time (min)
1.4
1.3
1.2
1.1
1
0.9
0.8
0
10
20
30
40
50
60

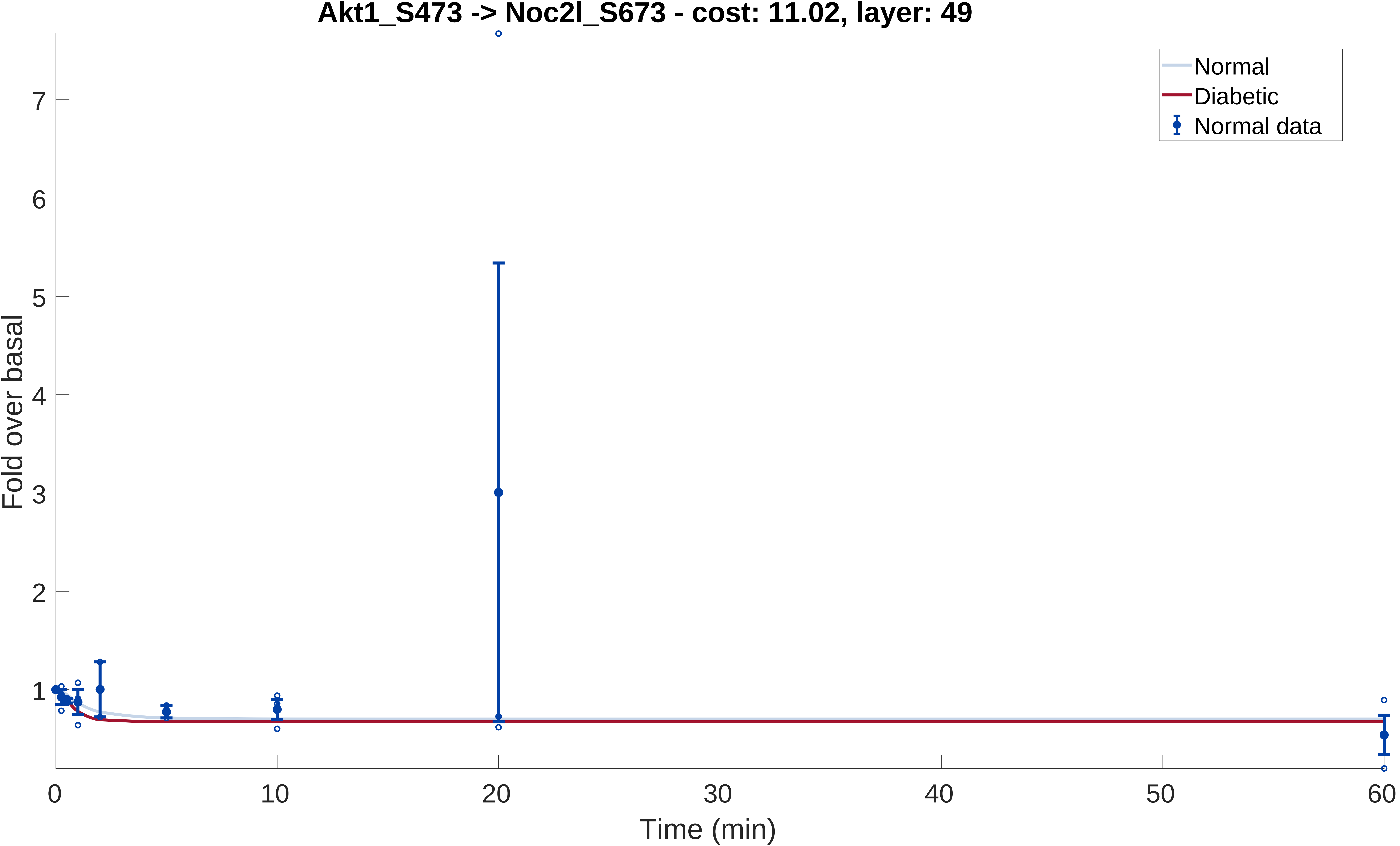

Akt1_S473 -> Noc2l_S673 - cost: 11.02, layer: 49
Normal
Diabetic
Normal data
Fold over basal
Time (min)
0
10
20
30
40
50
60
1
2
3
4
5
6
7

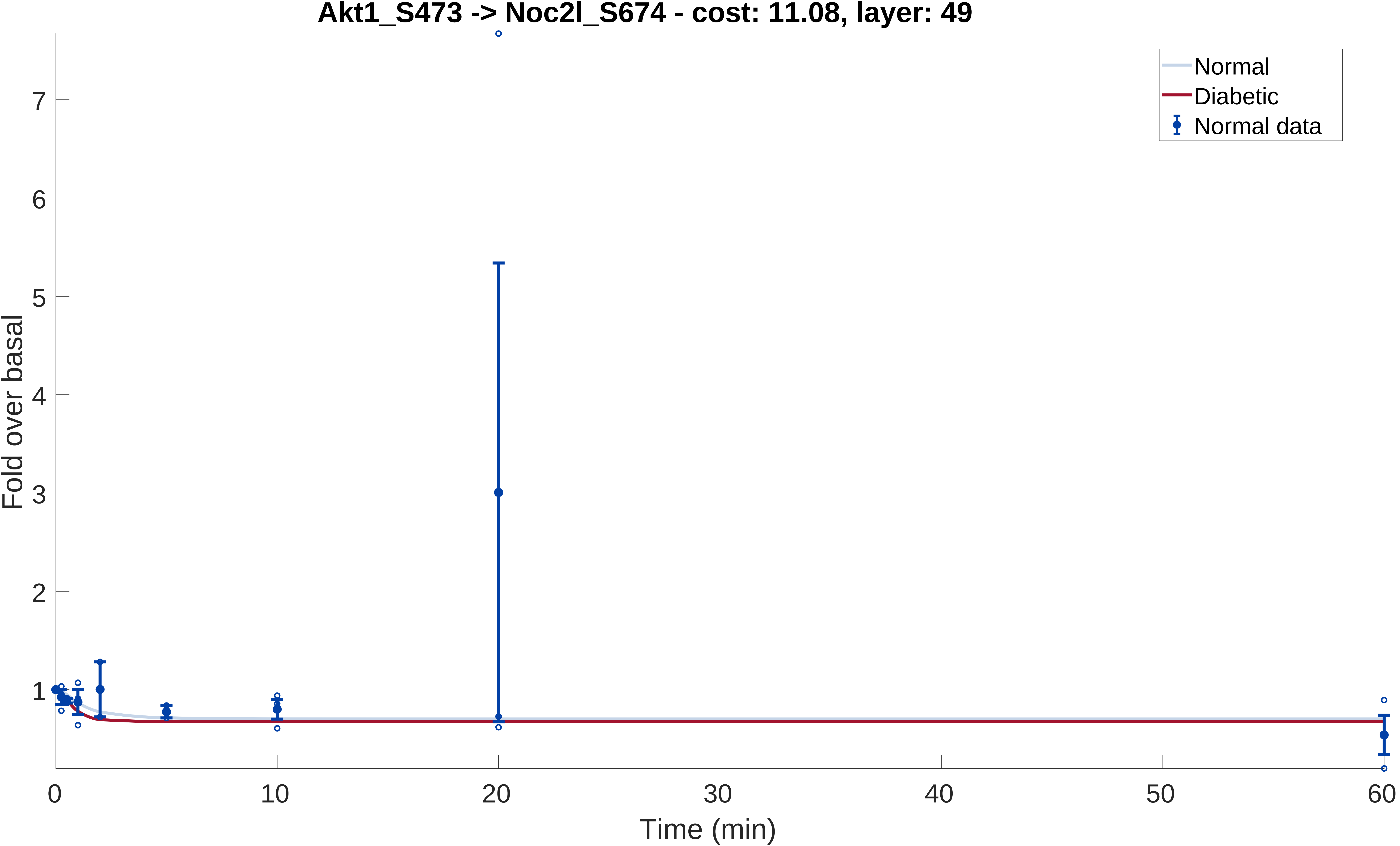
Akt1_S473 -> Noc2l_S674 - cost: 11.08, layer: 49
Normal
Diabetic
Normal data
Fold over basal
Time (min)
1
2
3
4
5
6
7
0
10
20
30
40
50
60

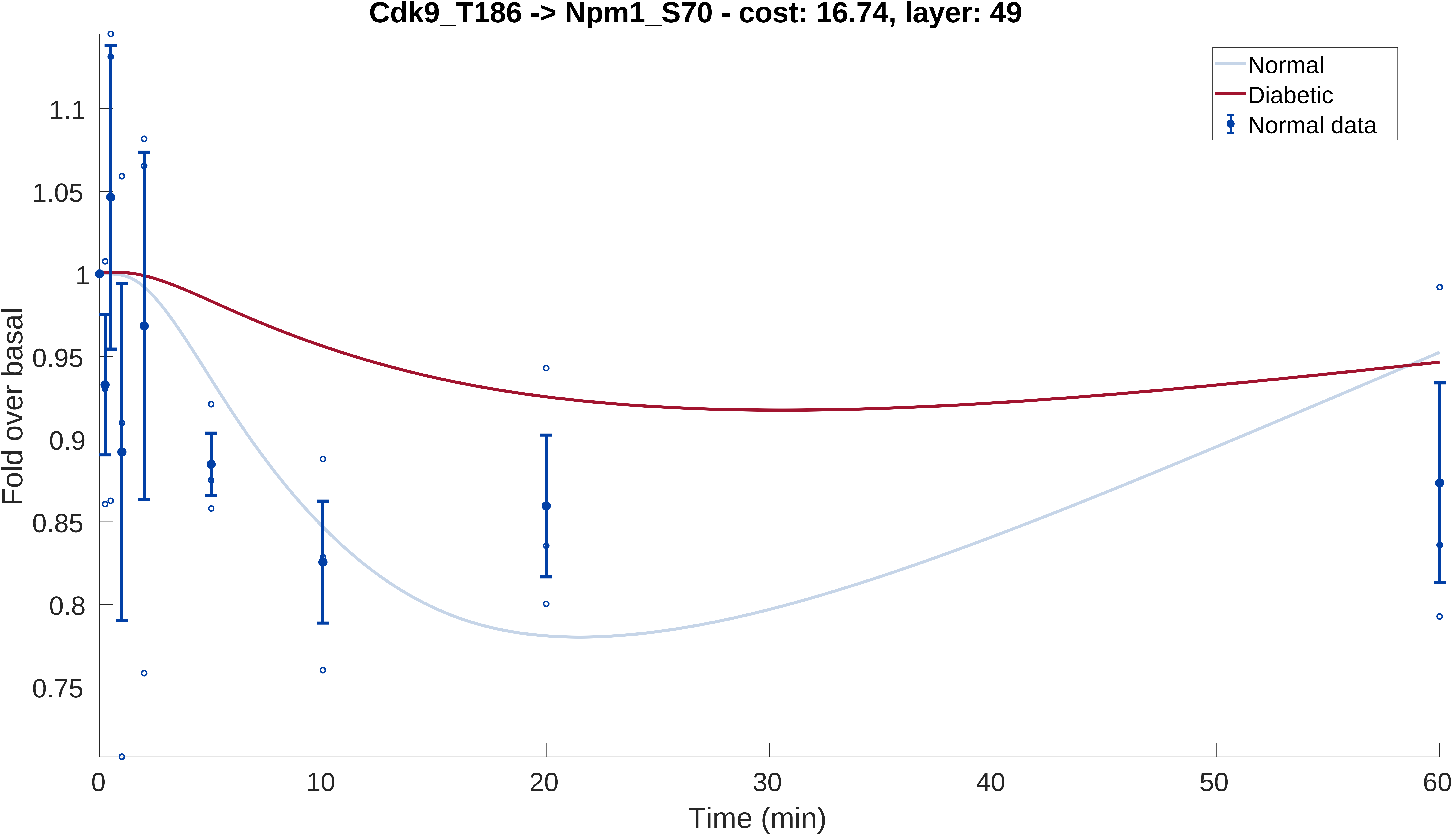

Cdk9_T186 -> Npm1_S70 - cost: 16.74, layer: 49
Normal
Diabetic
Normal data
Fold over basal
Time (min)
1.1
1.05
1
0.95
0.9
0.85
0.8
0.75
0
10
20
30
40
50
60

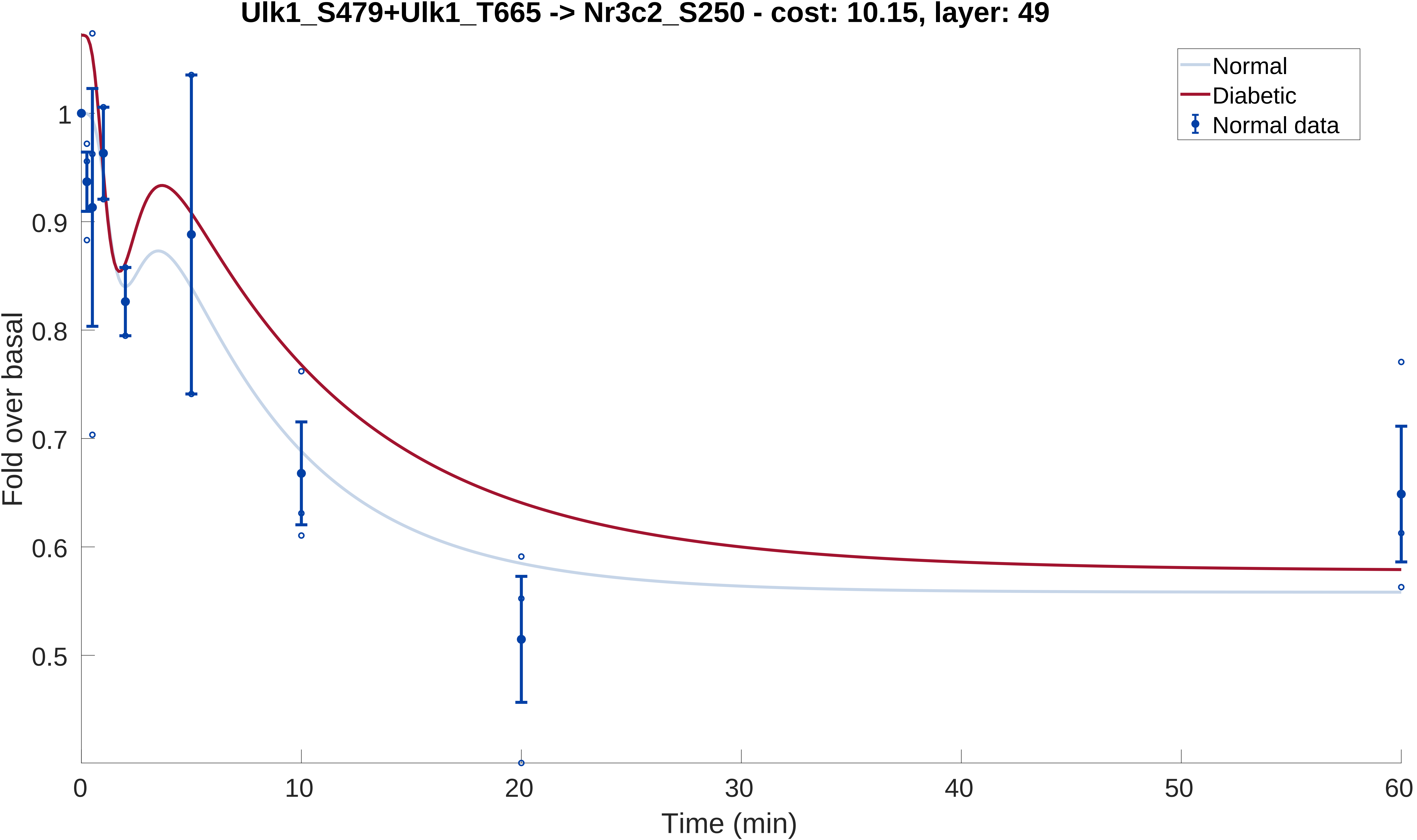

Ulk1_S479+Ulk1_T665 -> Nr3c2_S250 - cost: 10.15, layer: 49
Normal
Diabetic
Normal data
Fold over basal
1
0.9
0.8
0.7
0.6
0.5
0
10
20
30
40
50
60
Time (min)

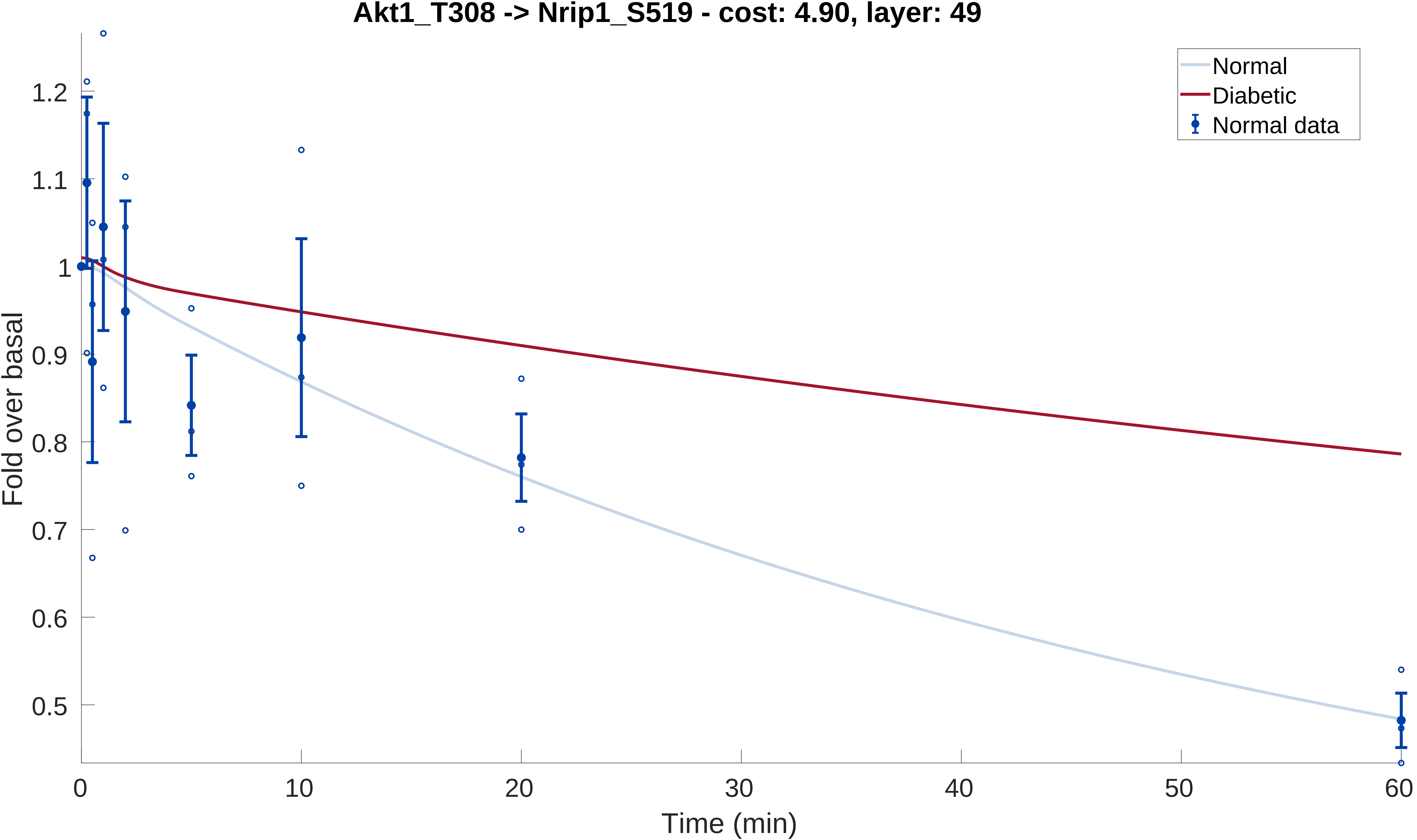

Akt1_T308 -> Nrip1_S519 - cost: 4.90, layer: 49
Normal
Diabetic
Normal data
Fold over basal
Time (min)
1.2
1.1
1
0.9
0.8
0.7
0.6
0.5
0
10
20
30
40
50
60

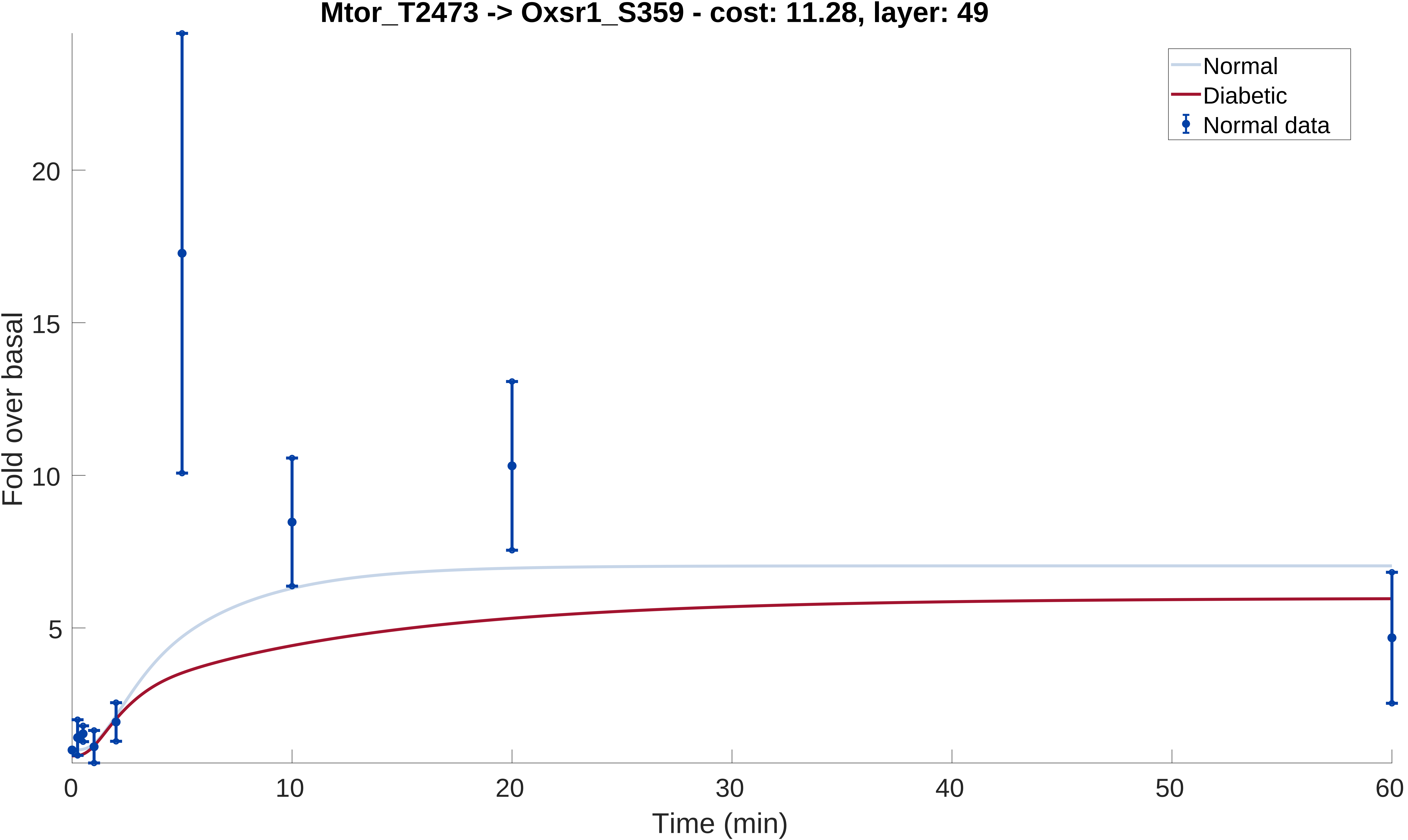

Mtor_T2473 -> Oxsr1_S359 - cost: 11.28, layer: 49
Normal
Diabetic
Normal data
Fold over basal
Time (min)
0
10
20
30
40
50
60
5
10
15
20

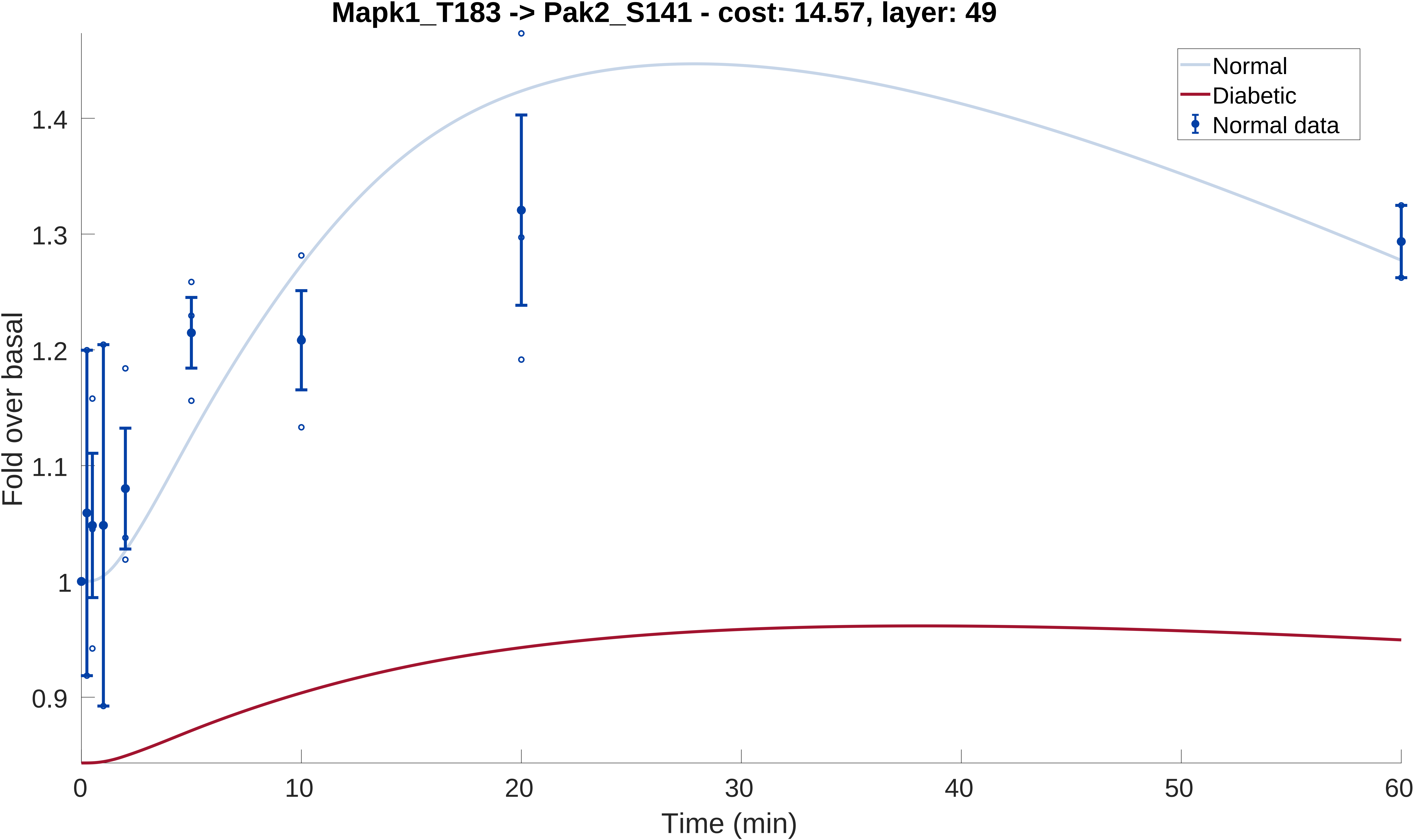

Mapk1_T183 -> Pak2_S141 - cost: 14.57, layer: 49
Normal
Diabetic
Normal data
Fold over basal
0.9
1
1.1
1.2
1.3
1.4
0
10
20
30
40
50
60
Time (min)

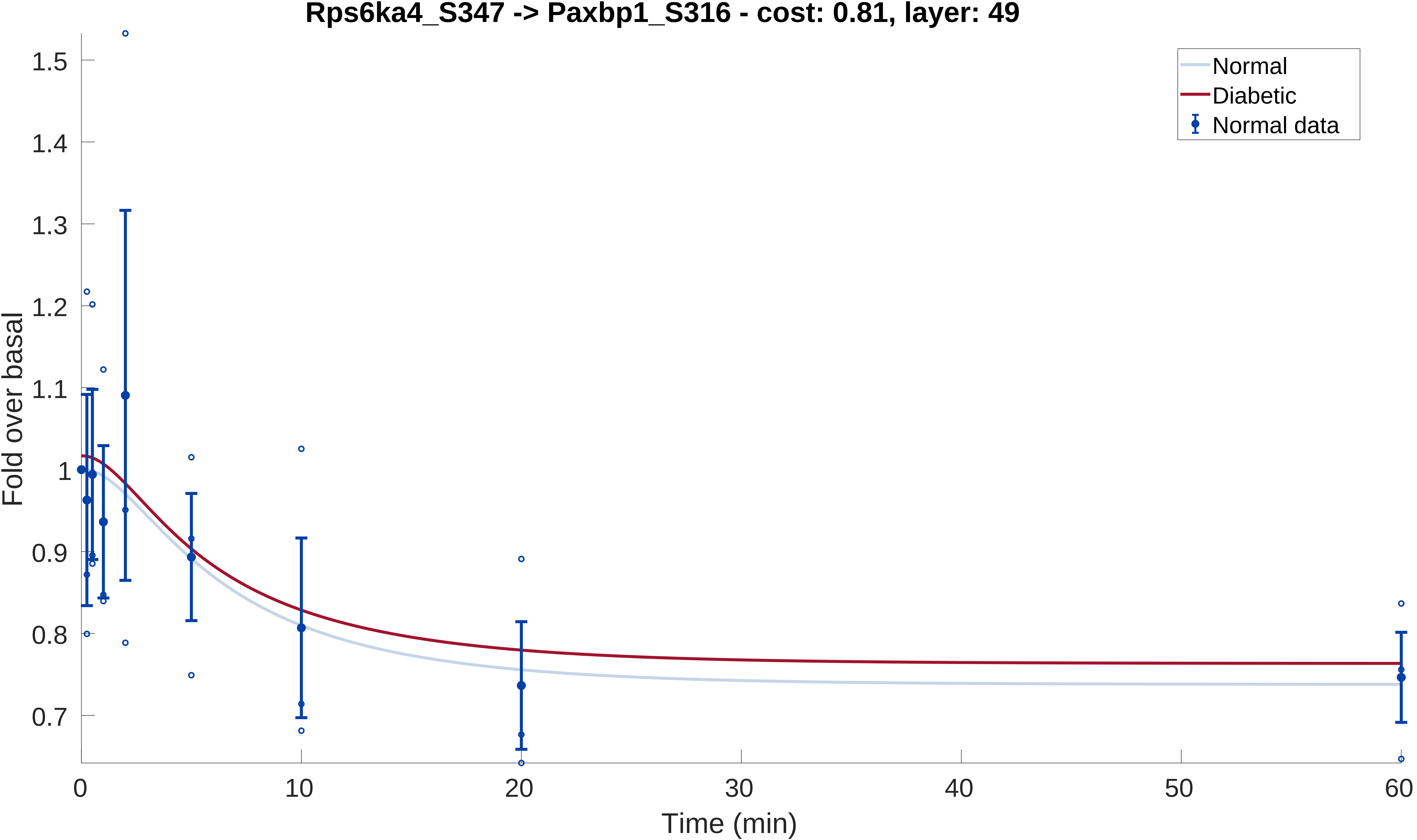

Rps6ka4_S347 -> Paxbp1_S316 - cost: 0.81, layer: 49
Normal
Diabetic
Normal data
Fold over basal
1.5
1.4
1.3
1.2
1.1
1
0.9
0.8
0.7
0
10
20
30
40
50
60
Time (min)

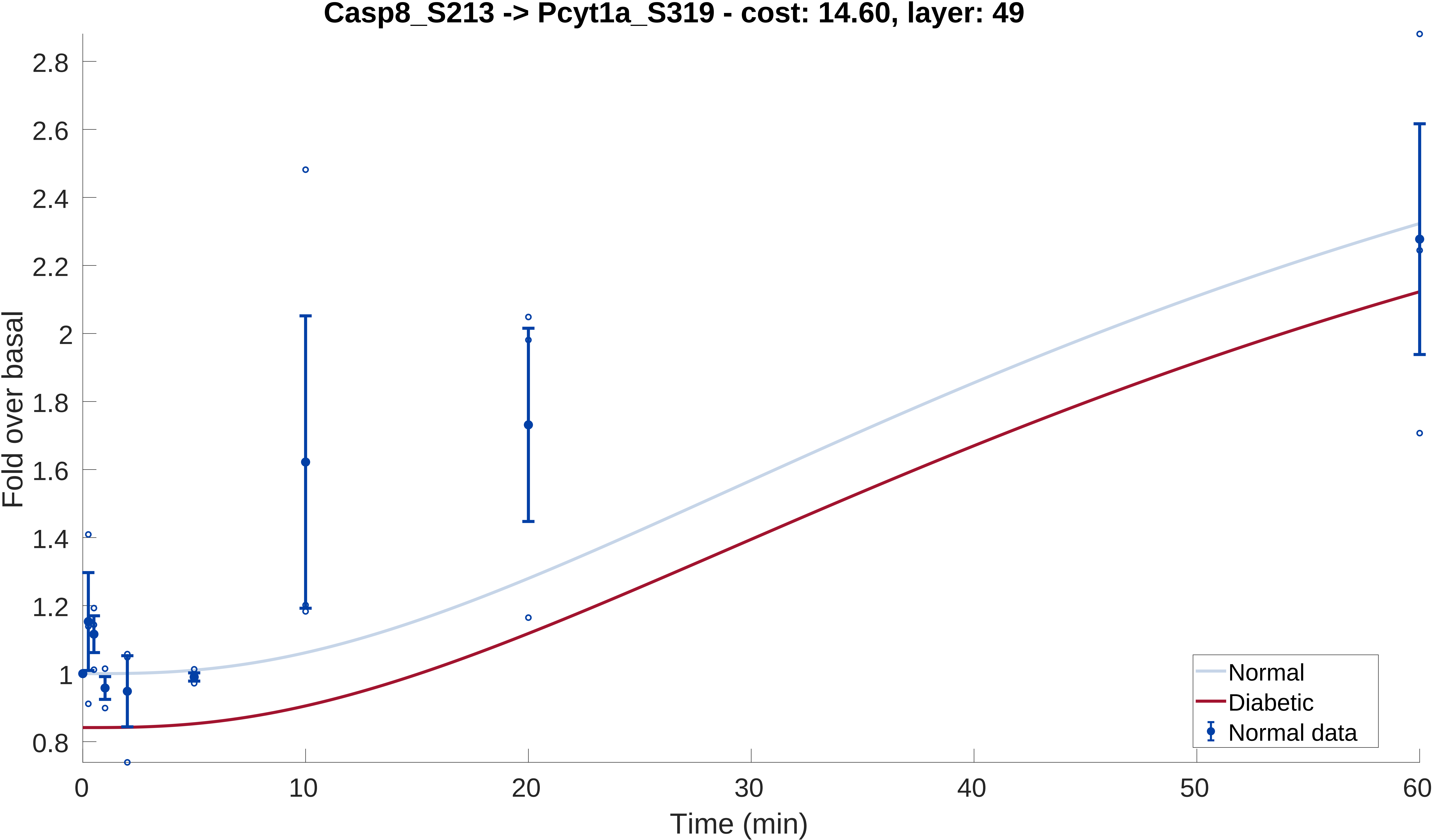

Casp8_S213 -> Pcyt1a_S319 - cost: 14.60, layer: 49
Fold over basal
2.8
2.6
2.4
2.2
2
1.8
1.6
1.4
1.2
1
0.8
0
10
20
30
40
50
60
Time (min)
Normal
Diabetic
Normal data

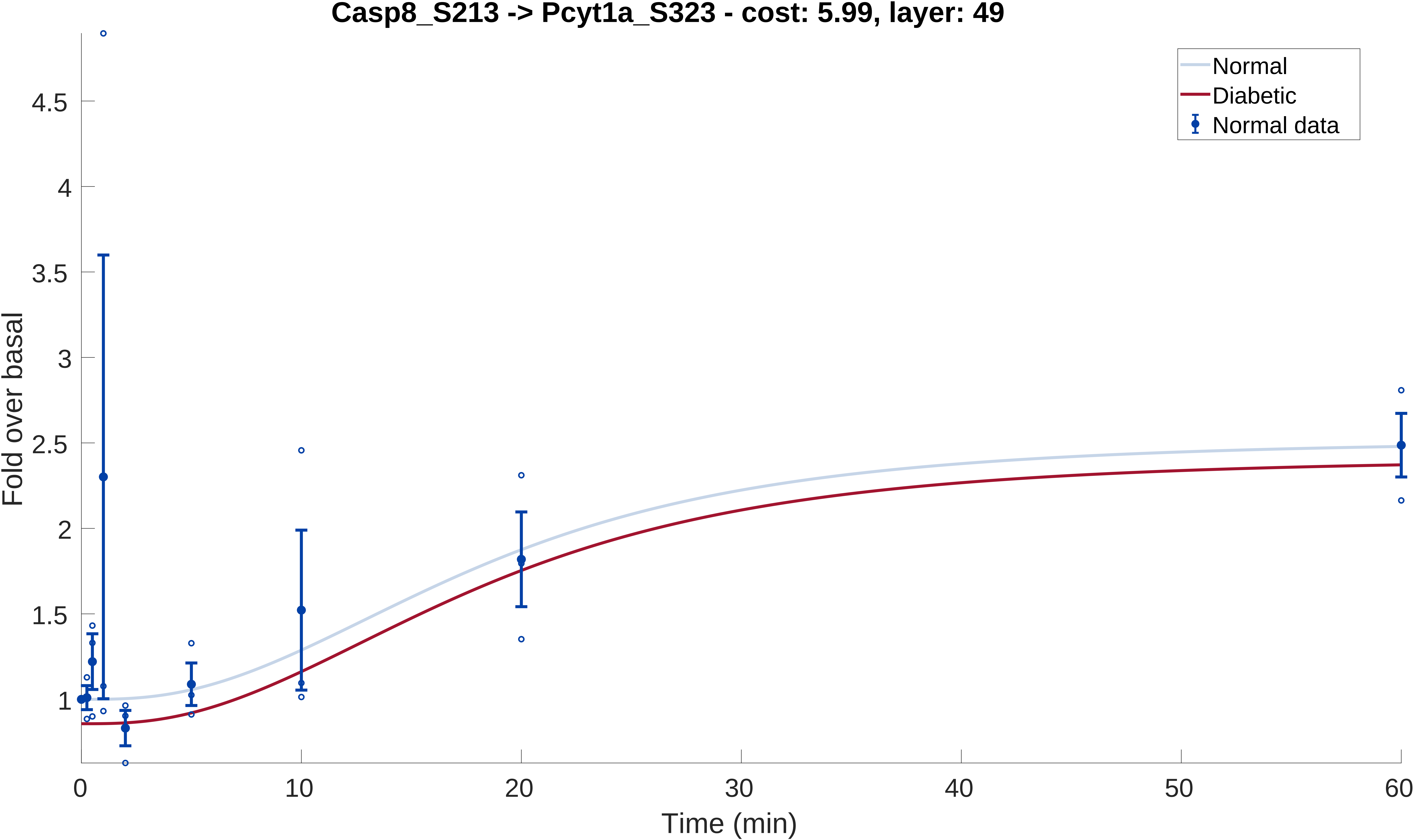

Casp8_S213 -> Pcyt1a_S323 - cost: 5.99, layer: 49
Normal
Diabetic
Normal data
Fold over basal
Time (min)
4.5
4
3.5
3
2.5
2
1.5
1
0
10
20
30
40
50
60

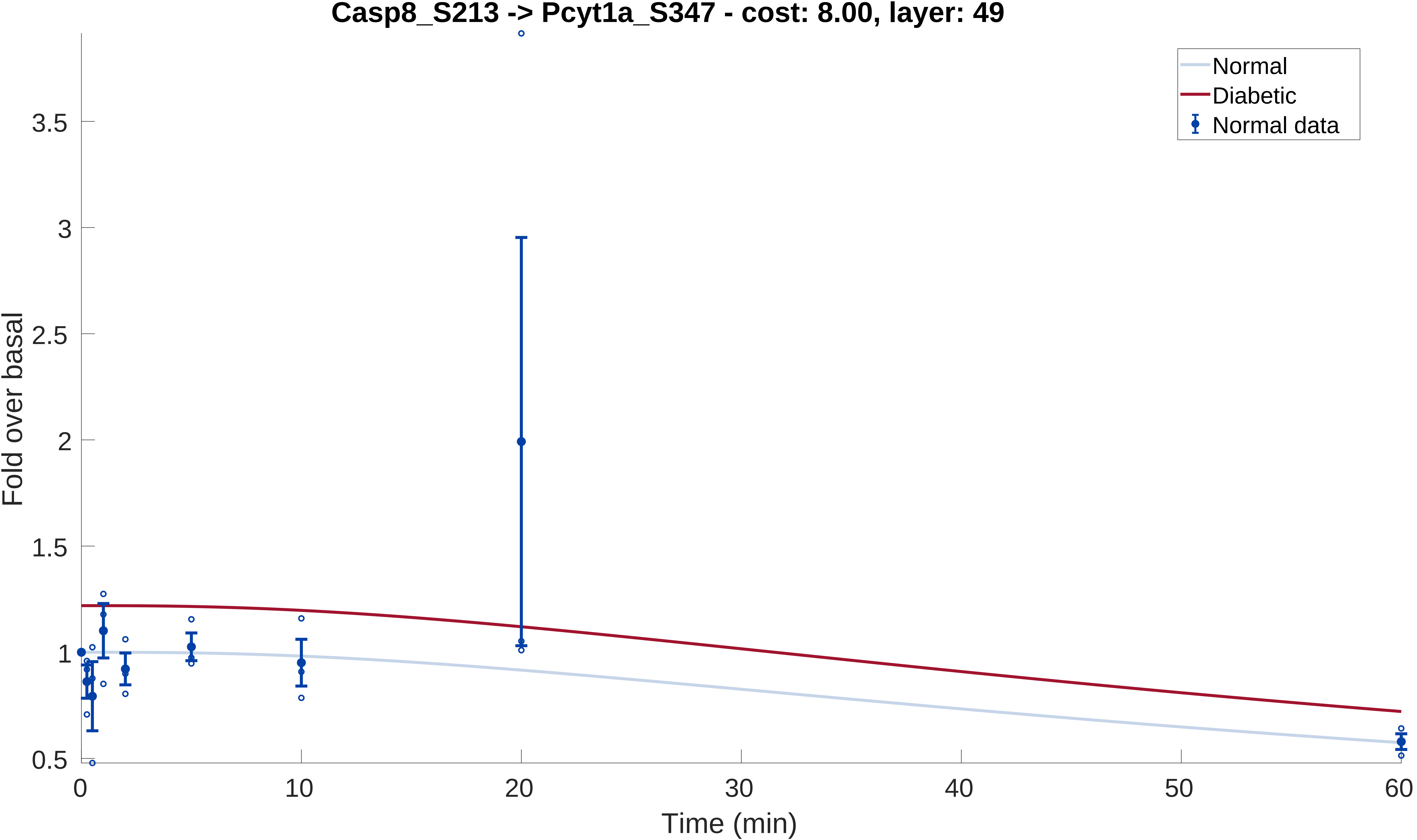

Casp8_S213 -> Pcyt1a_S347 - cost: 8.00, layer: 49
Normal
Diabetic
Normal data
Fold over basal
Time (min)
0.5
1
1.5
2
2.5
3
3.5
0
10
20
30
40
50
60

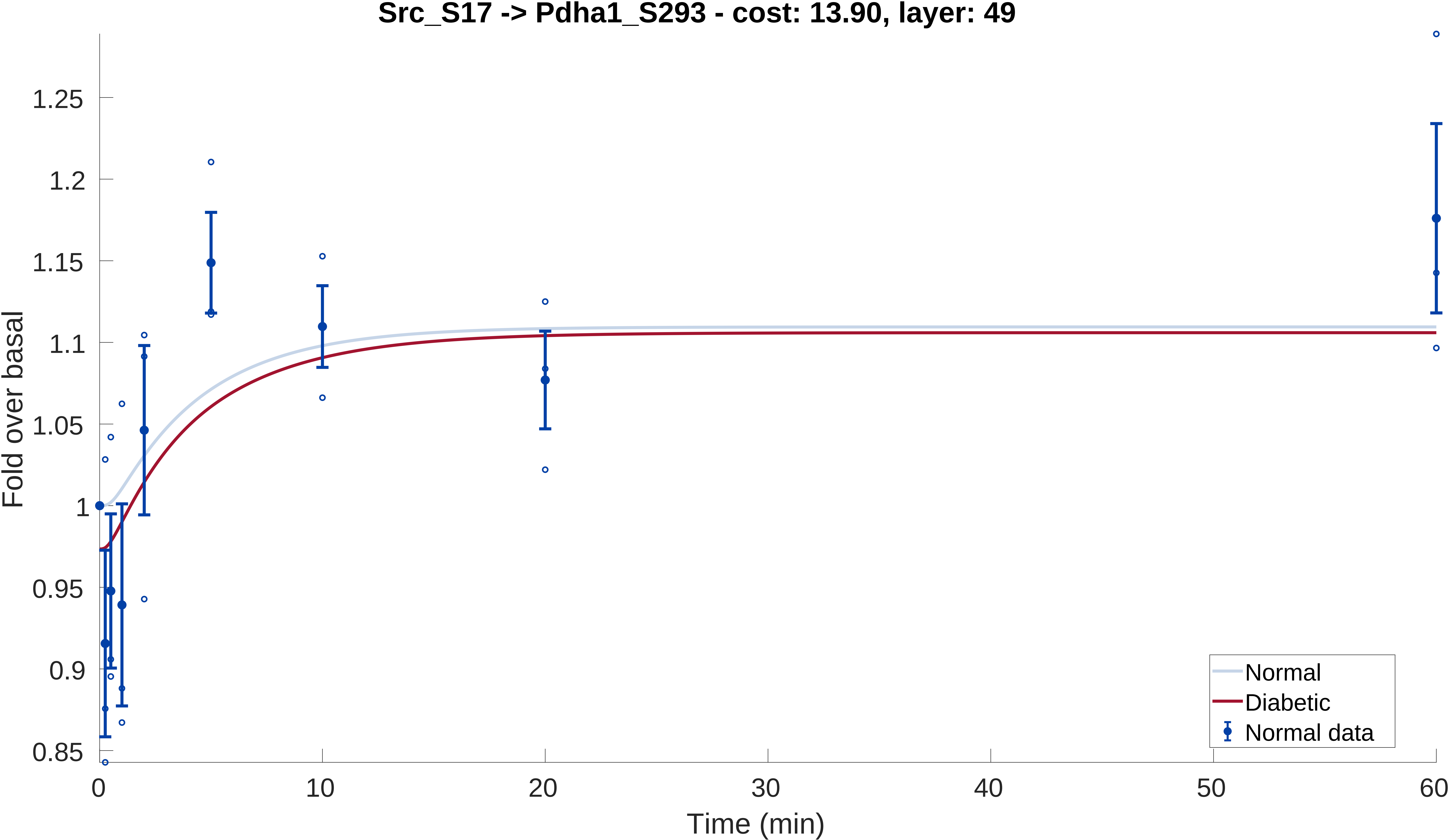

Src_S17 -> Pdha1_S293 - cost: 13.90, layer: 49
Fold over basal
1.25
1.2
1.15
1.1
1.05
1
0.95
0.9
0.85
0
10
20
30
40
50
60
Time (min)
Normal
Diabetic
Normal data

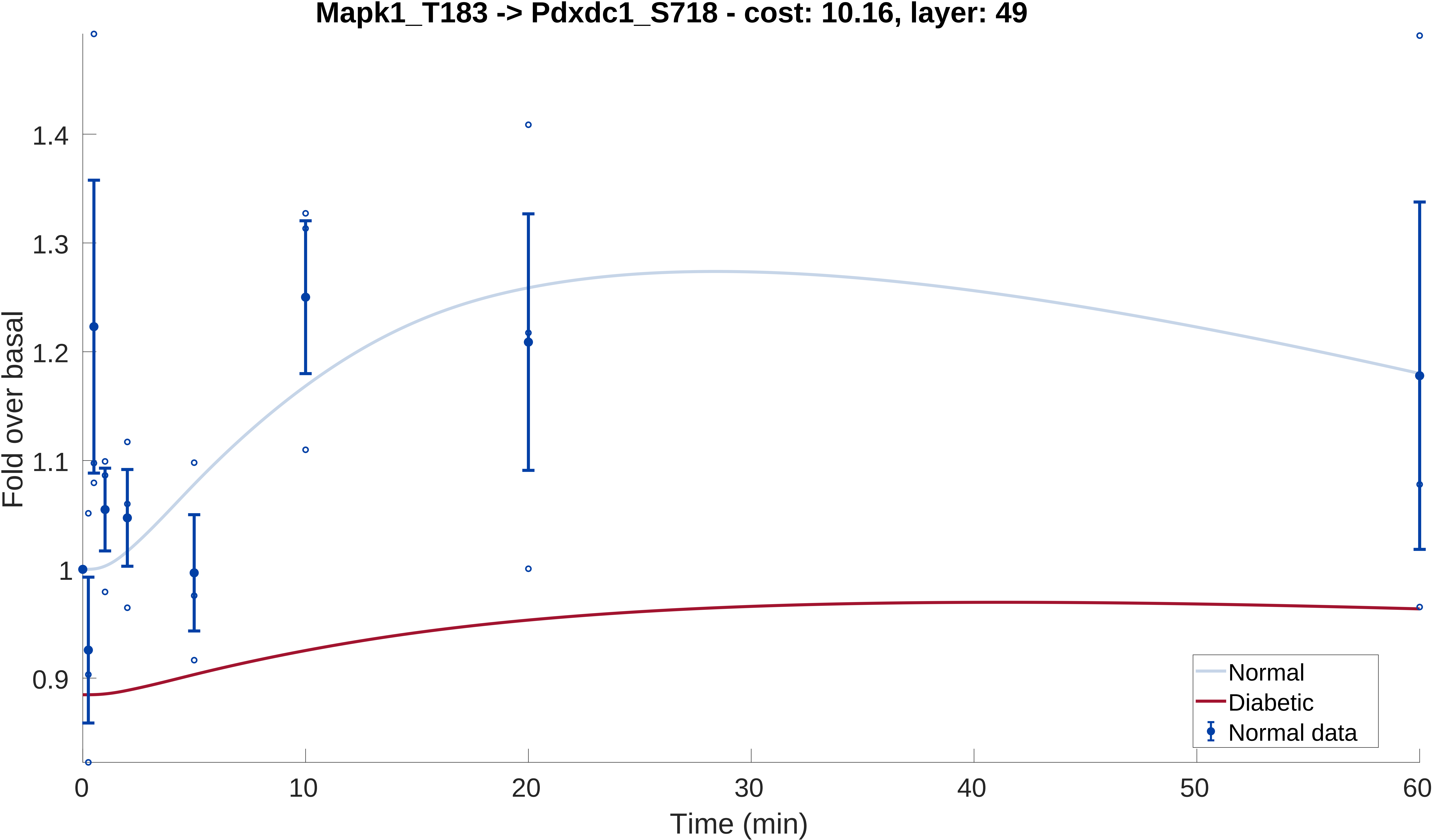
Mapk1_T183 -> Pdxdc1_S718 - cost: 10.16, layer: 49
Fold over basal
1.4
1.3
1.2
1.1
1
0.9
0
10
20
30
40
50
60
Time (min)
Normal
Diabetic
Normal data

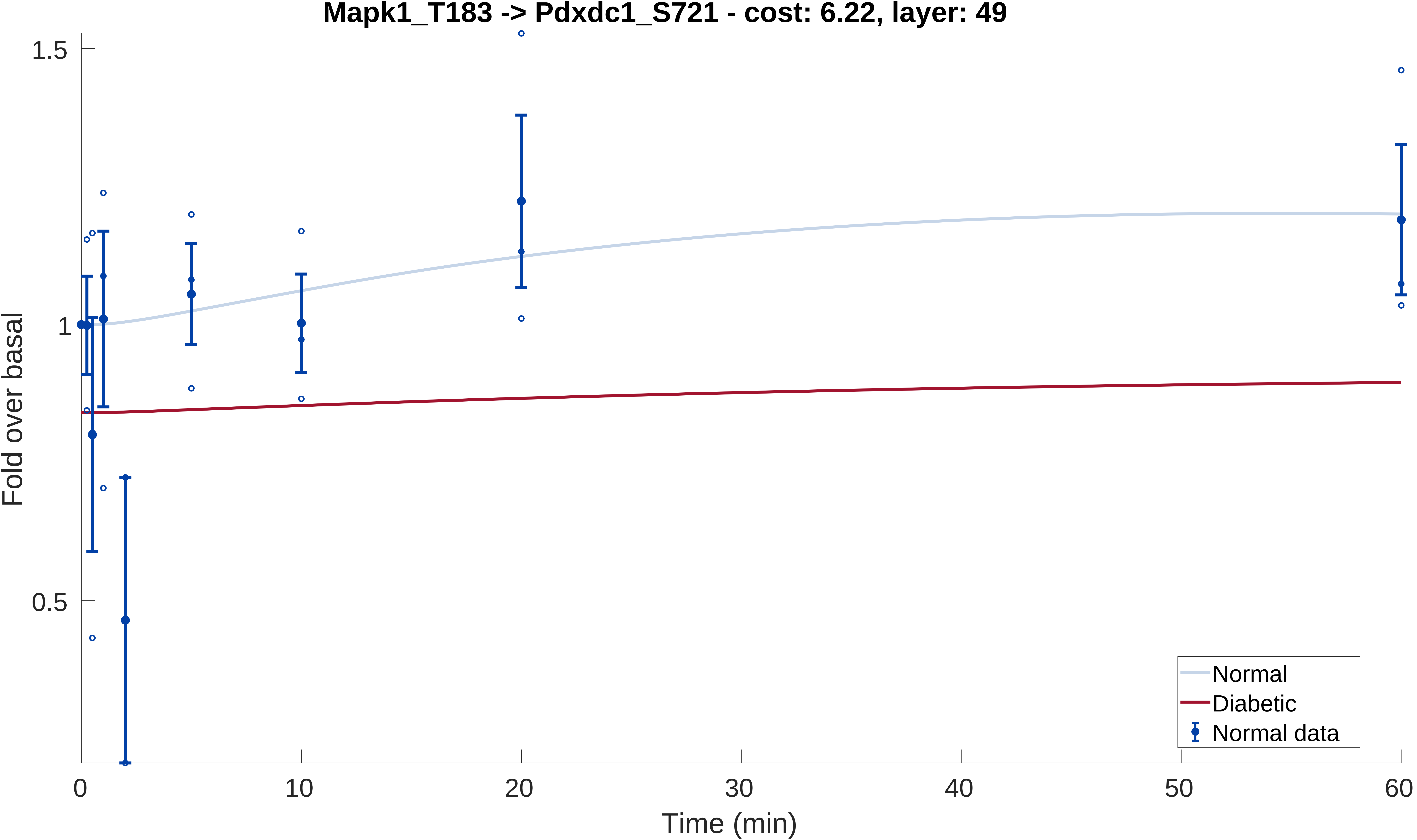

Mapk1_T183 -> Pdxdc1_S721 - cost: 6.22, layer: 49
Fold over basal
1.5
1
0.5
0
10
20
30
40
50
60
Time (min)
Normal
Diabetic
Normal data

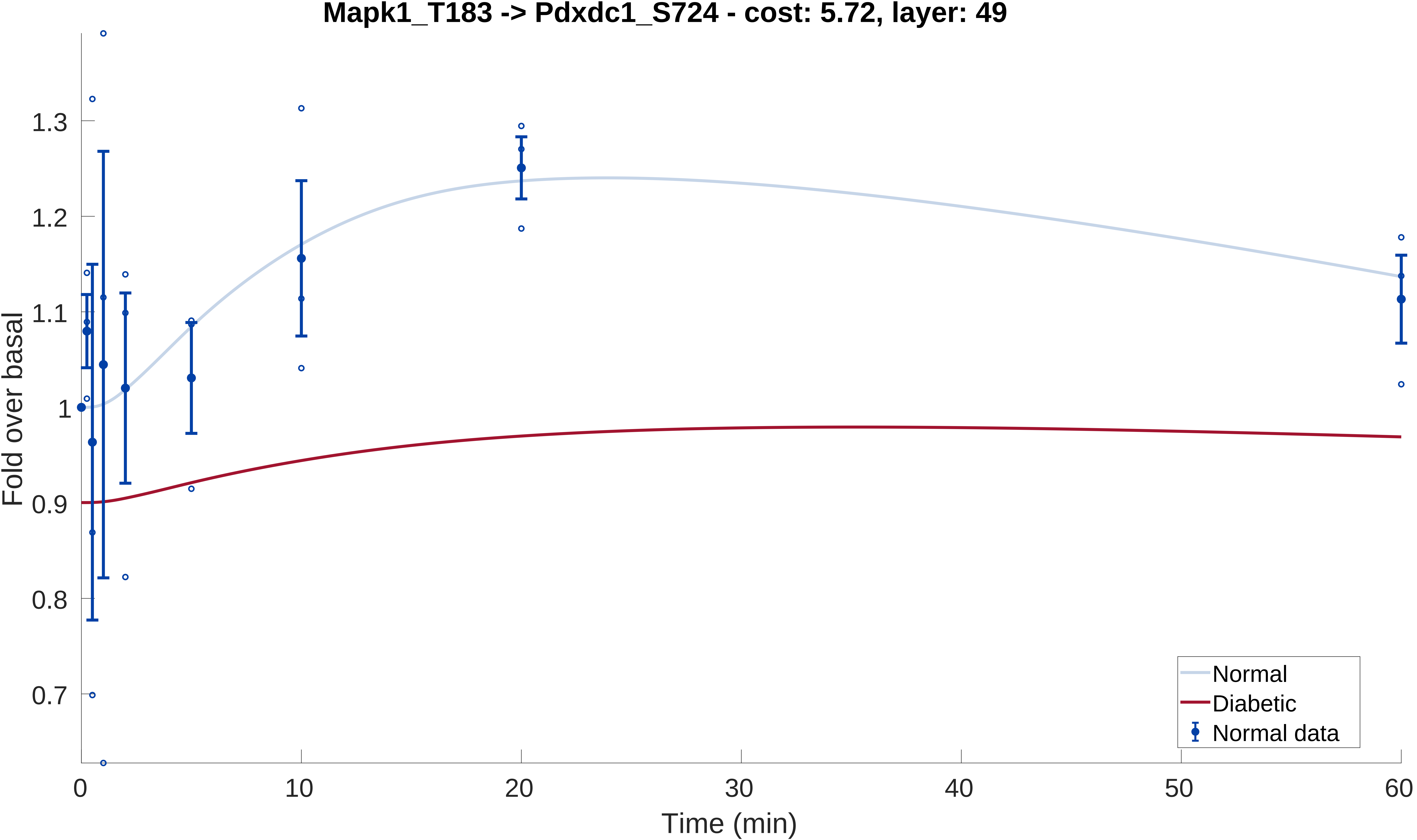

Mapk1_T183 -> Pdxdc1_S724 - cost: 5.72, layer: 49
Fold over basal
Time (min)
1.3
1.2
1.1
1
0.9
0.8
0.7
0
10
20
30
40
50
60
Normal
Diabetic
Normal data

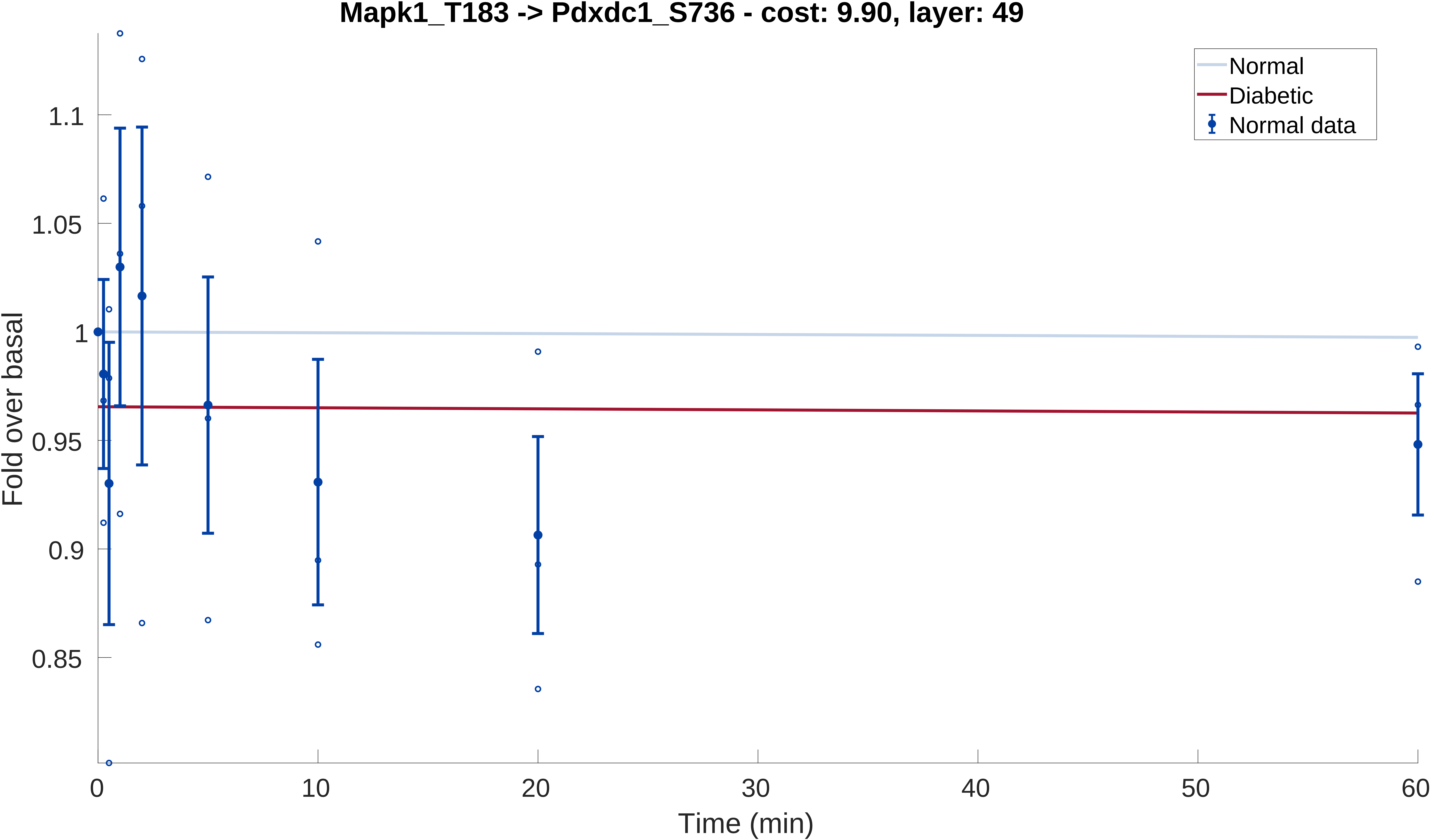
Mapk1_T183 -> Pdxdc1_S736 - cost: 9.90, layer: 49
Normal
Diabetic
Normal data
Fold over basal
1.1
1.05
1
0.95
0.9
0.85
0
10
20
30
40
50
60
Time (min)

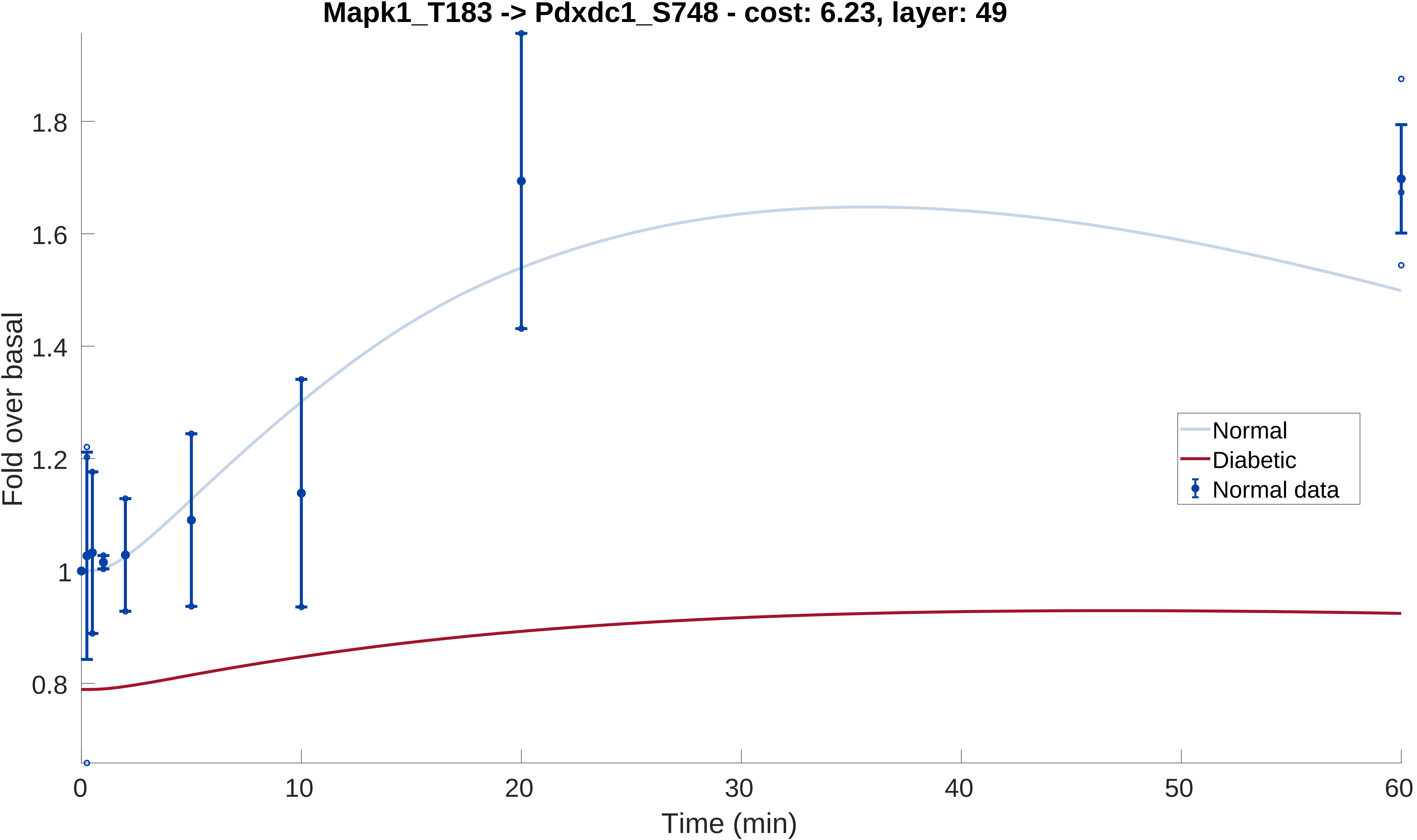
Mapk1_T183 -> Pdxdc1_S748 - cost: 6.23, layer: 49
Fold over basal
1.8
1.6
1.4
1.2
1
0.8
0
10
20
30
40
50
60
Time (min)
Normal
Diabetic
Normal data

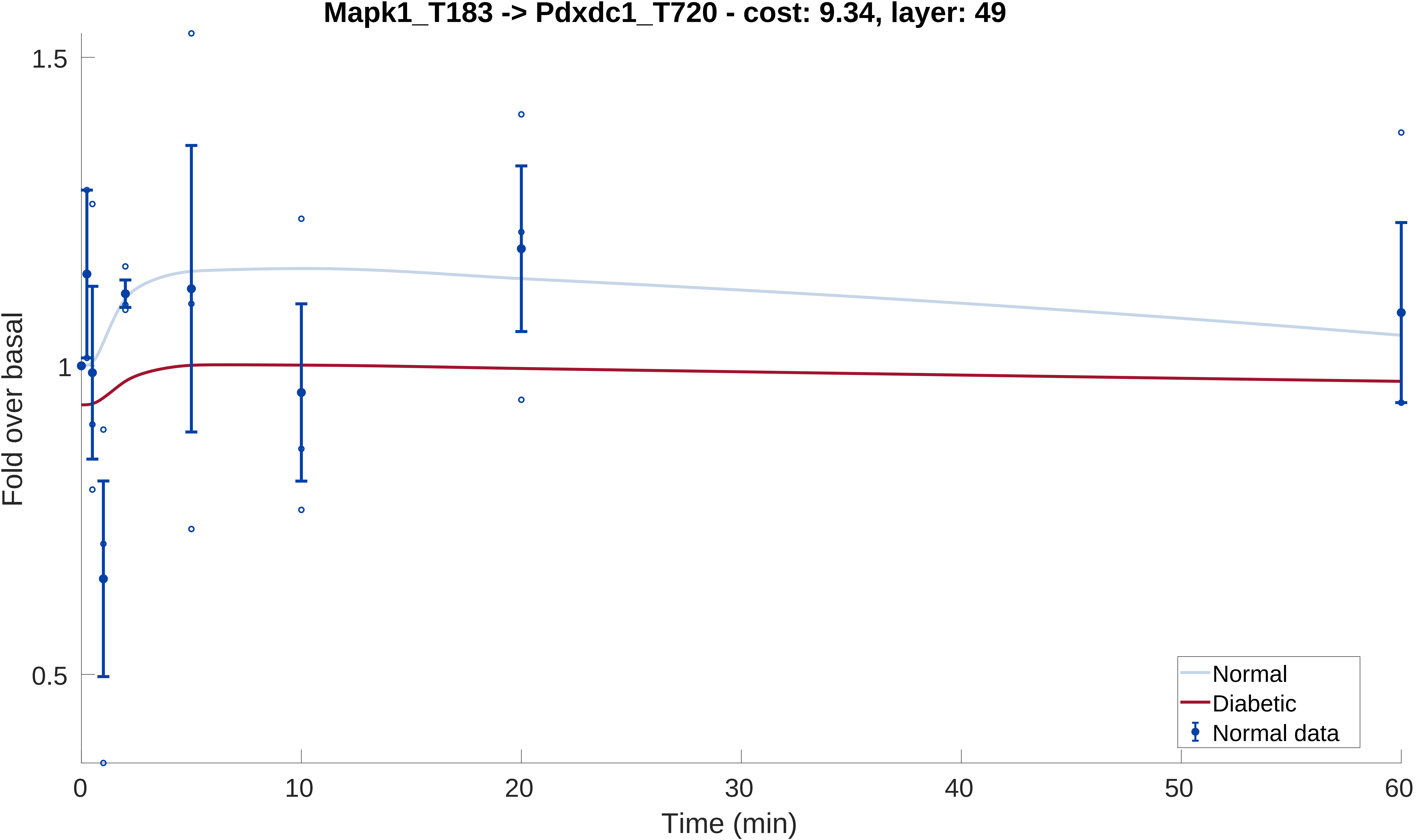
Mapk1_T183 -> Pdxdc1_T720 - cost: 9.34, layer: 49
Fold over basal
1.5
1
0.5
0
10
20
30
40
50
60
Time (min)
Normal
Diabetic
Normal data

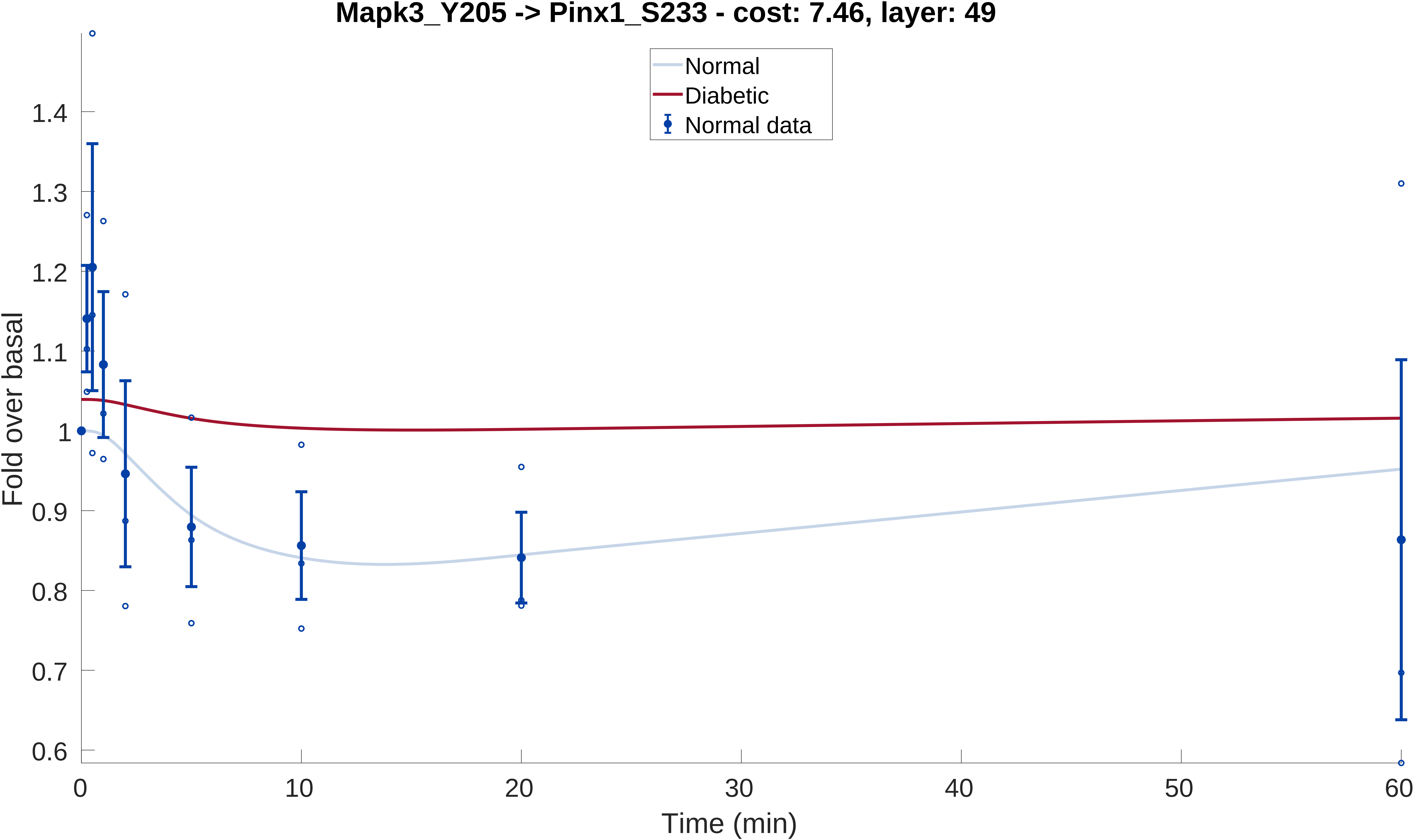

Mapk3_Y205 -> Pinx1_S233 - cost: 7.46, layer: 49
Normal
Diabetic
Normal data
Fold over basal
Time (min)
1.4
1.3
1.2
1.1
1
0.9
0.8
0.7
0.6
0
10
20
30
40
50
60

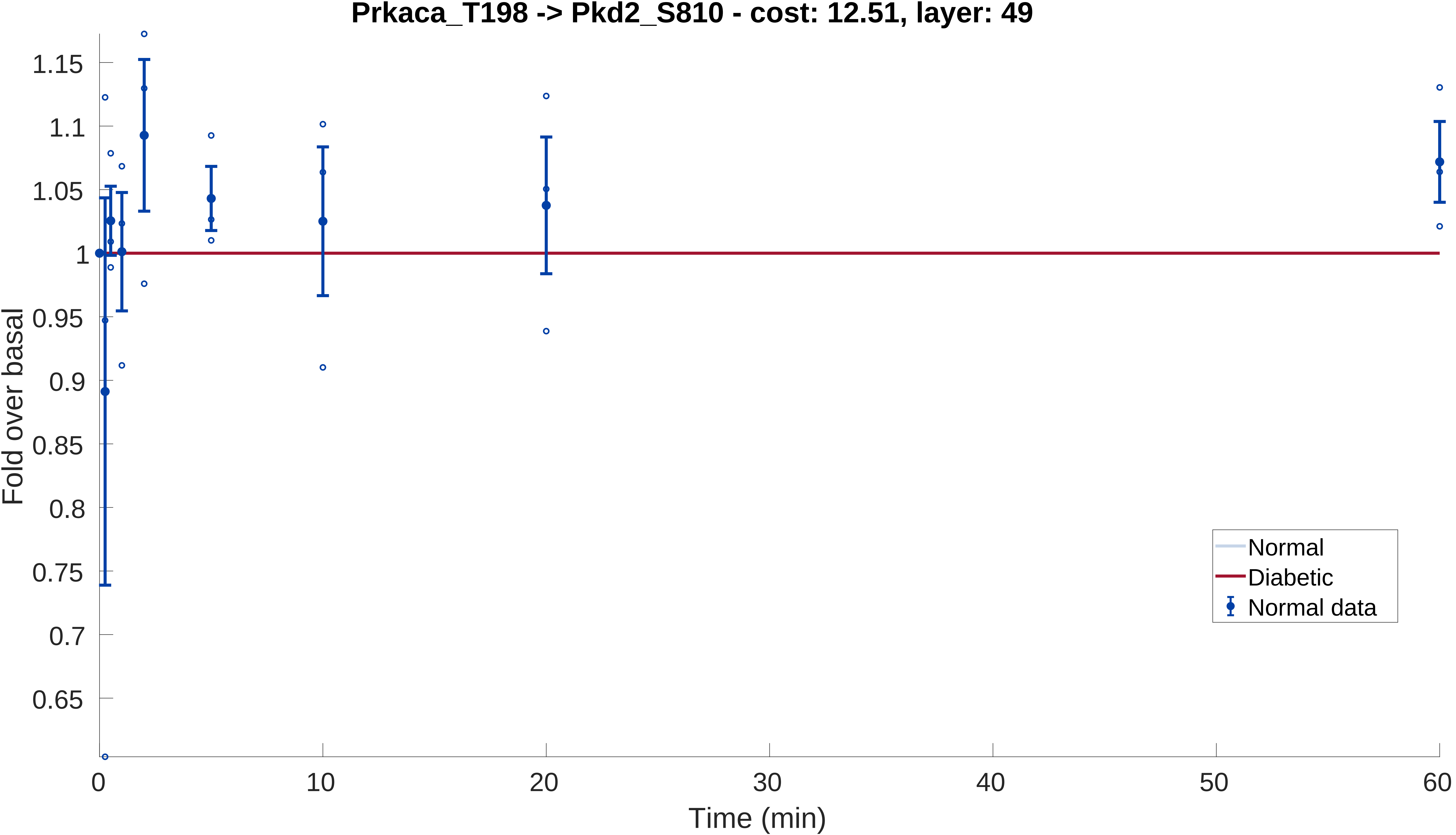

Prkaca_T198 -> Pkd2_S810 - cost: 12.51, layer: 49
Fold over basal
1.15
1.1
1.05
1
0.95
0.9
0.85
0.8
0.75
0.7
0.65
0
10
20
30
40
50
60
Time (min)
Normal
Diabetic
Normal data

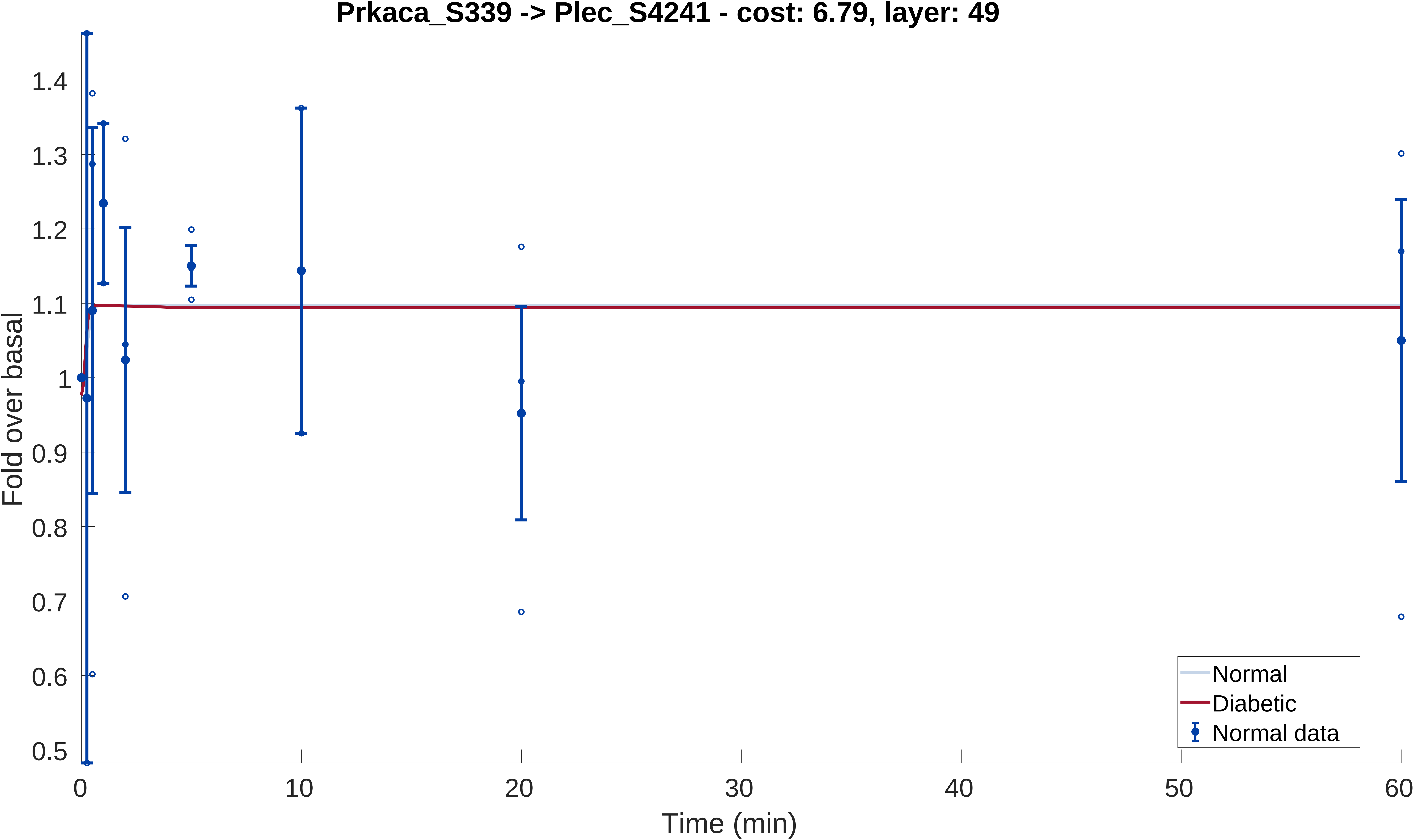

Prkaca_S339 -> Plec_S4241 - cost: 6.79, layer: 49
Fold over basal
1.4
1.3
1.2
1.1
1
0.9
0.8
0.7
0.6
0.5
0
10
20
30
40
50
60
Time (min)
Normal
Diabetic
Normal data

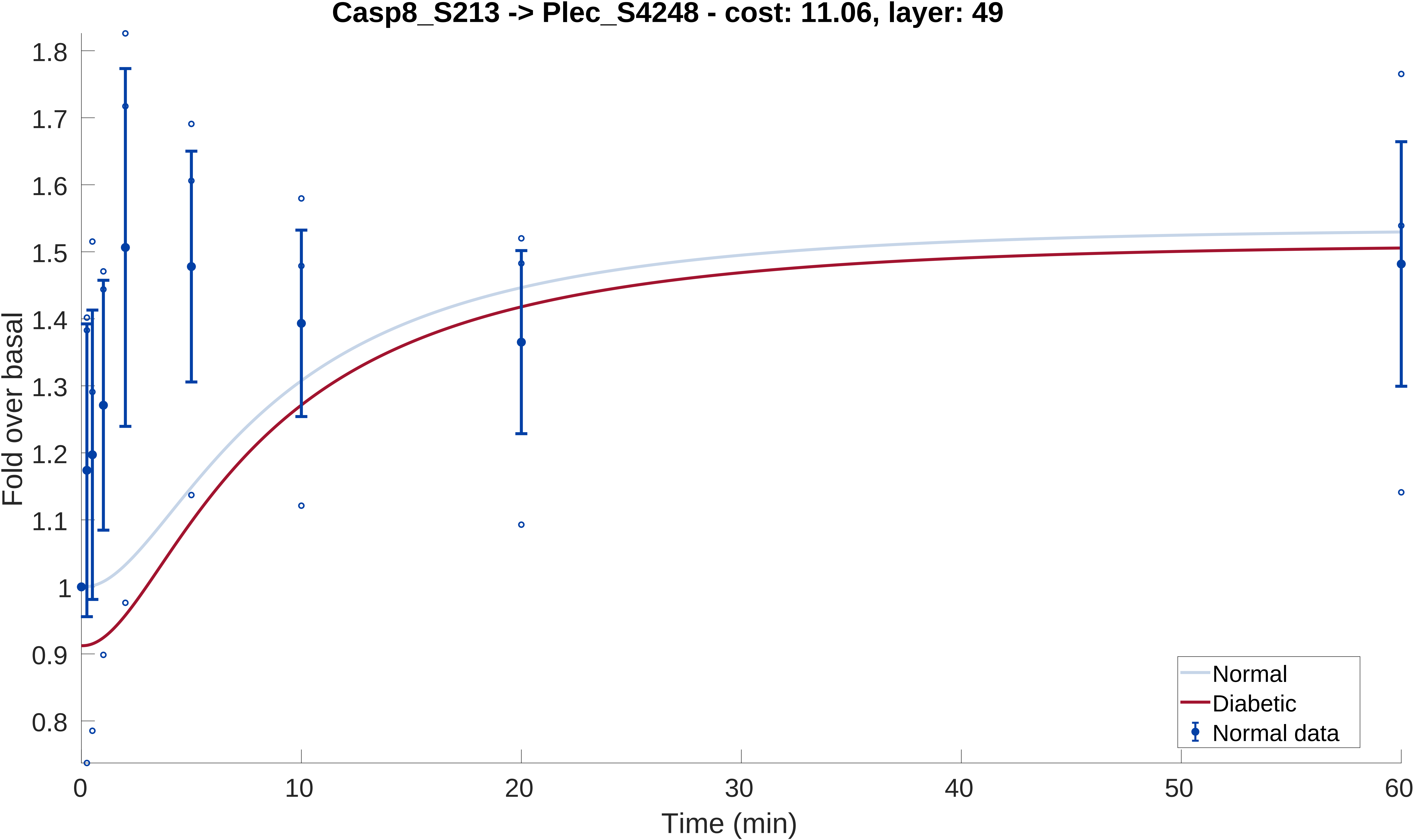

Casp8_S213 -> Plec_S4248 - cost: 11.06, layer: 49
Fold over basal
Time (min)
1.8
1.7
1.6
1.5
1.4
1.3
1.2
1.1
1
0.9
0.8
0
10
20
30
40
50
60
Normal
Diabetic
Normal data

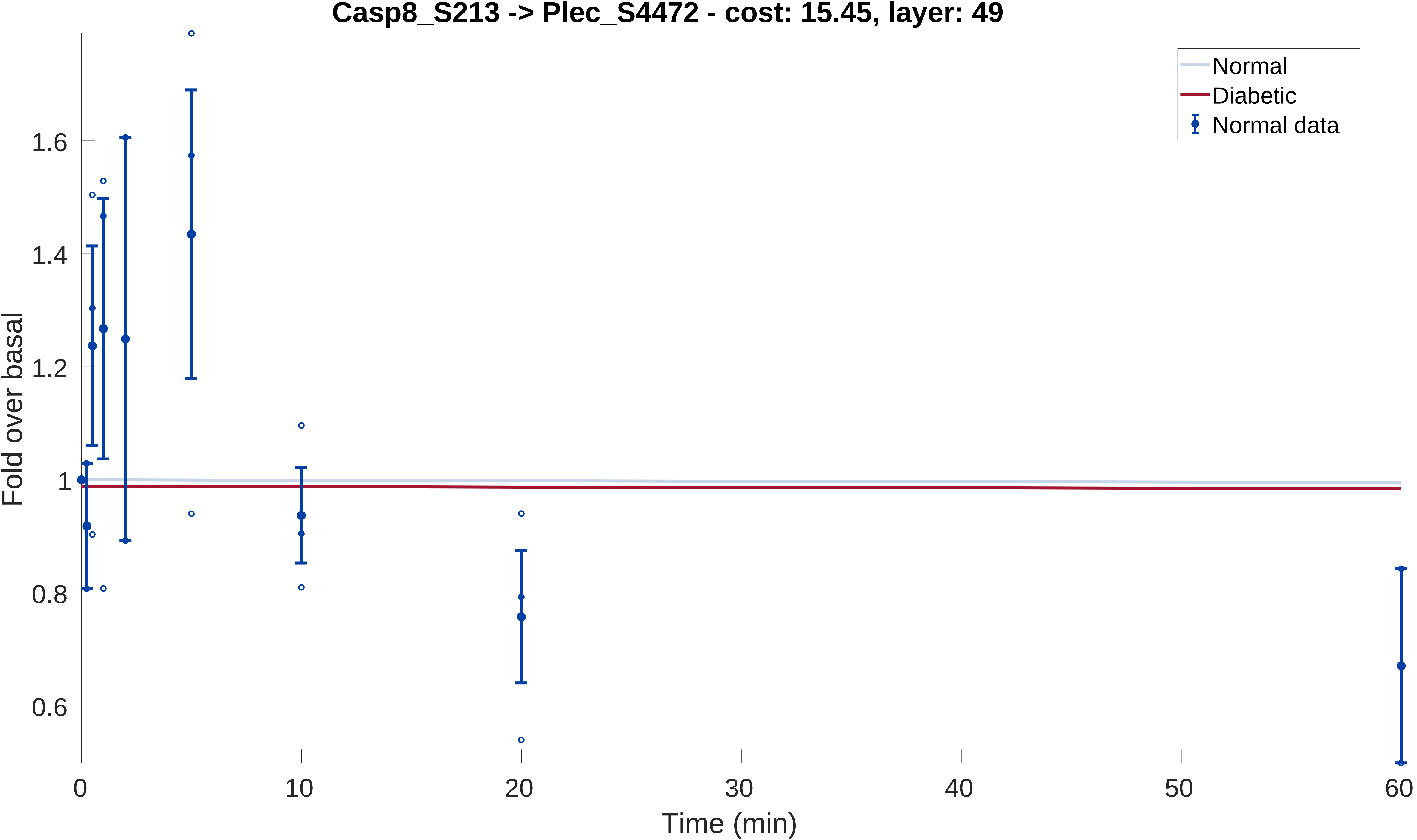

Casp8_S213 -> Plec_S4472 - cost: 15.45, layer: 49
Normal
Diabetic
Normal data
Fold over basal
1.6
1.4
1.2
1
0.8
0.6
0
10
20
30
40
50
60
Time (min)

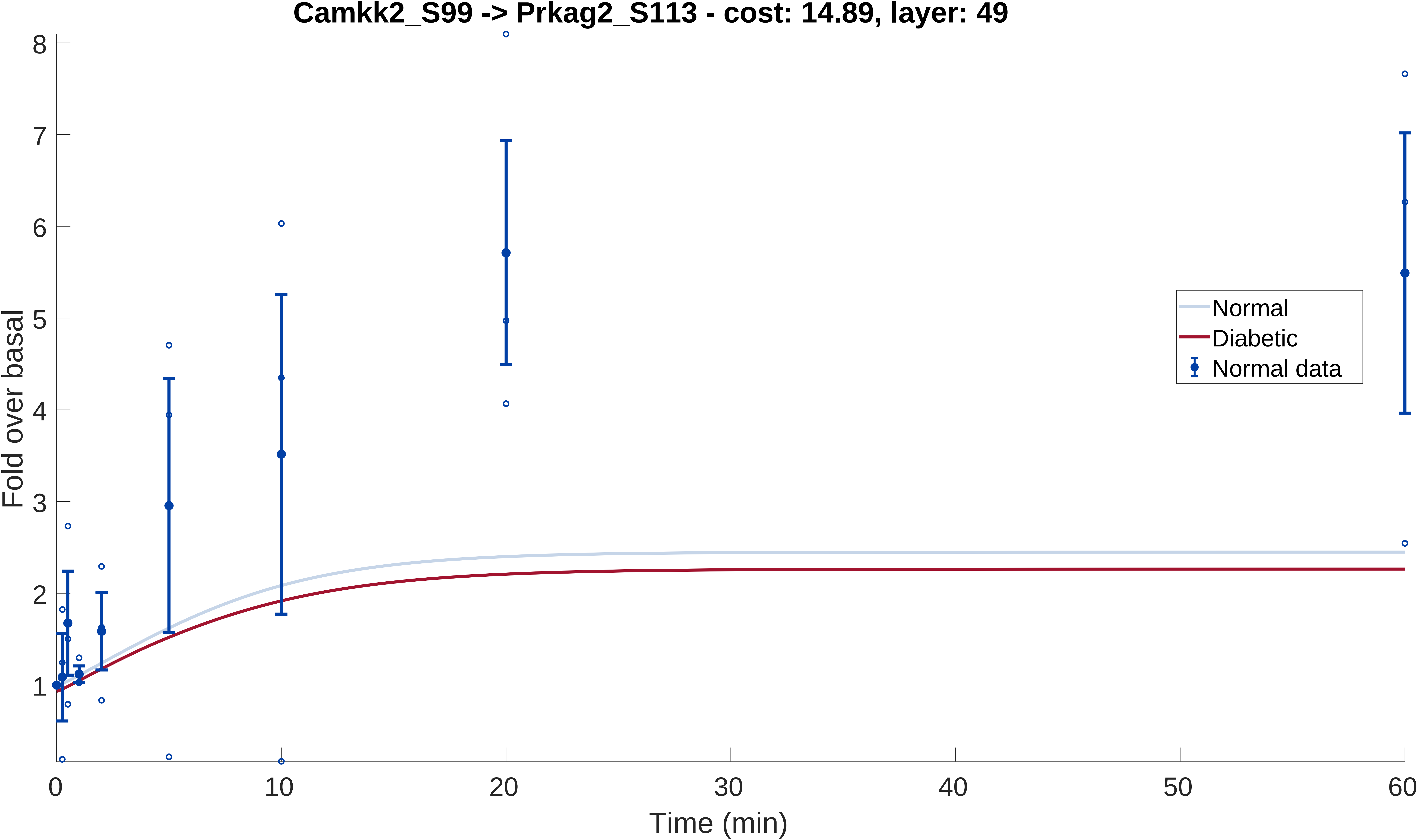

Camkk2_S99 -> Prkag2_S113 - cost: 14.89, layer: 49
Fold over basal
Time (min)
Normal
Diabetic
Normal data
0
10
20
30
40
50
60
1
2
3
4
5
6
7
8

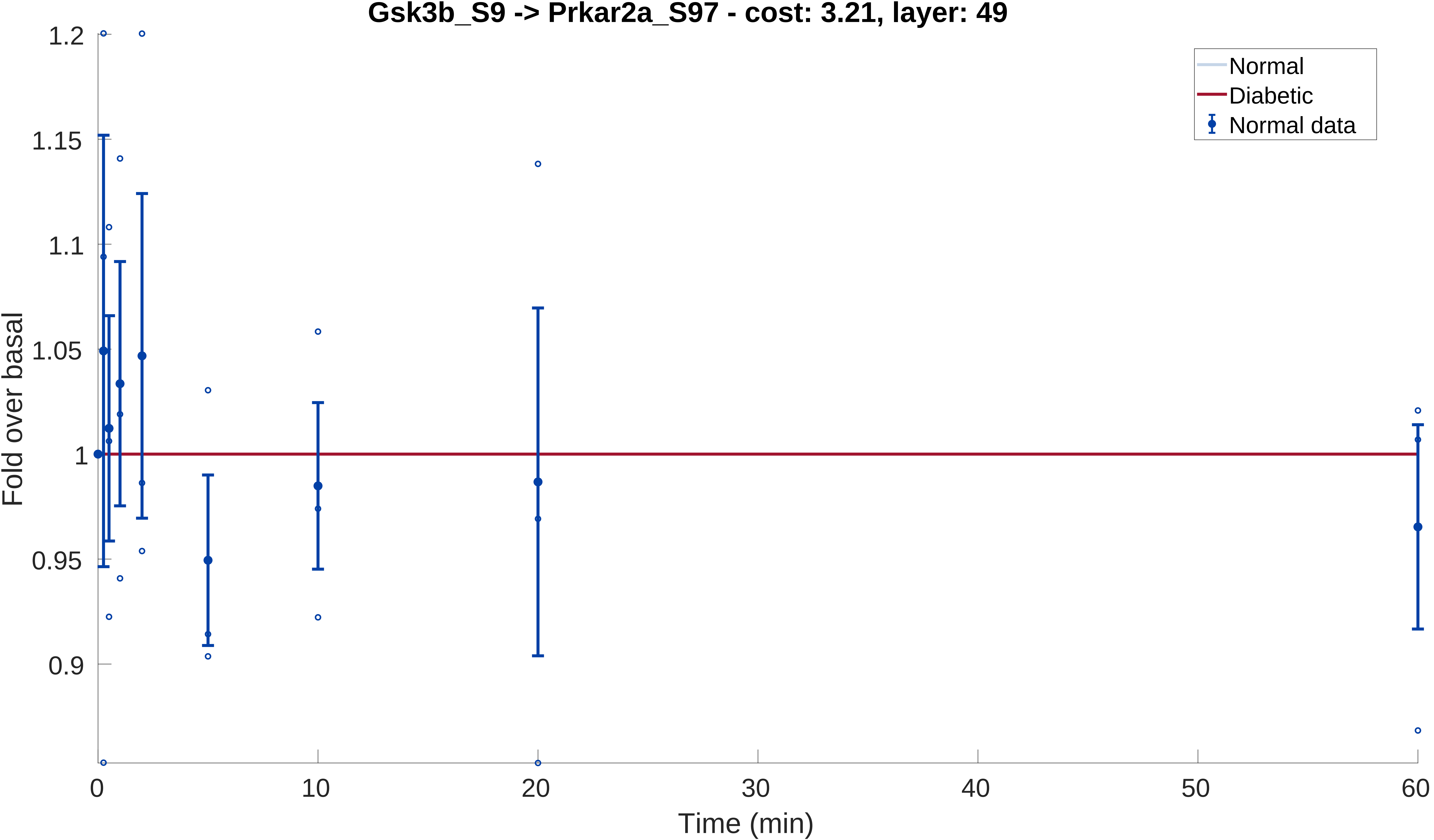
Gsk3b_S9 -> Prkar2a_S97 - cost: 3.21, layer: 49
Normal
Diabetic
Normal data
Fold over basal
Time (min)
1.2
1.15
1.1
1.05
1
0.95
0.9
0
10
20
30
40
50
60

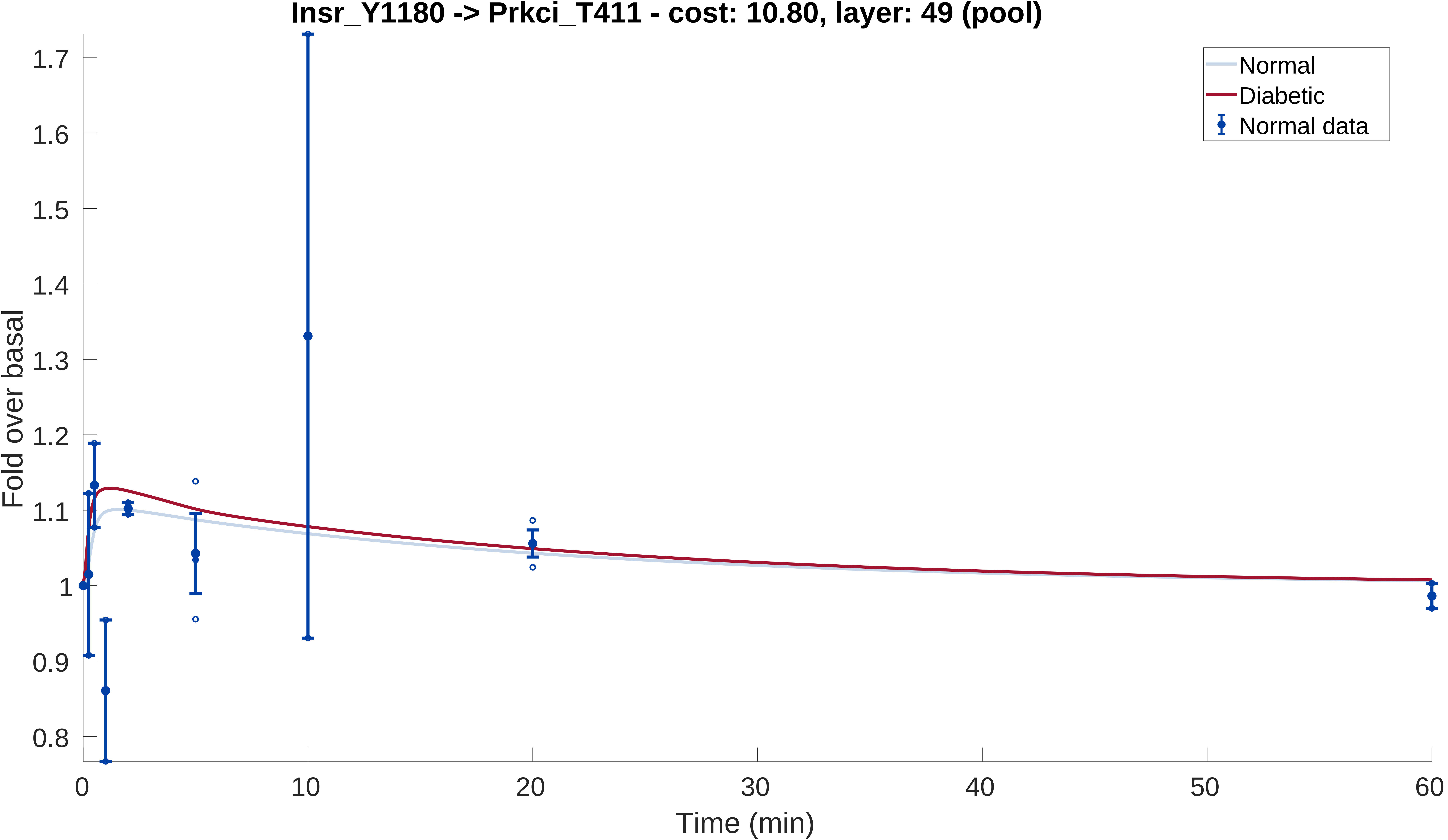

Insr_Y1180 -> Prkci_T411 - cost: 10.80, layer: 49 (pool)
Fold over basal
Time (min)
Normal
Diabetic
Normal data
1.7
1.6
1.5
1.4
1.3
1.2
1.1
1
0.9
0.8
0
10
20
30
40
50
60

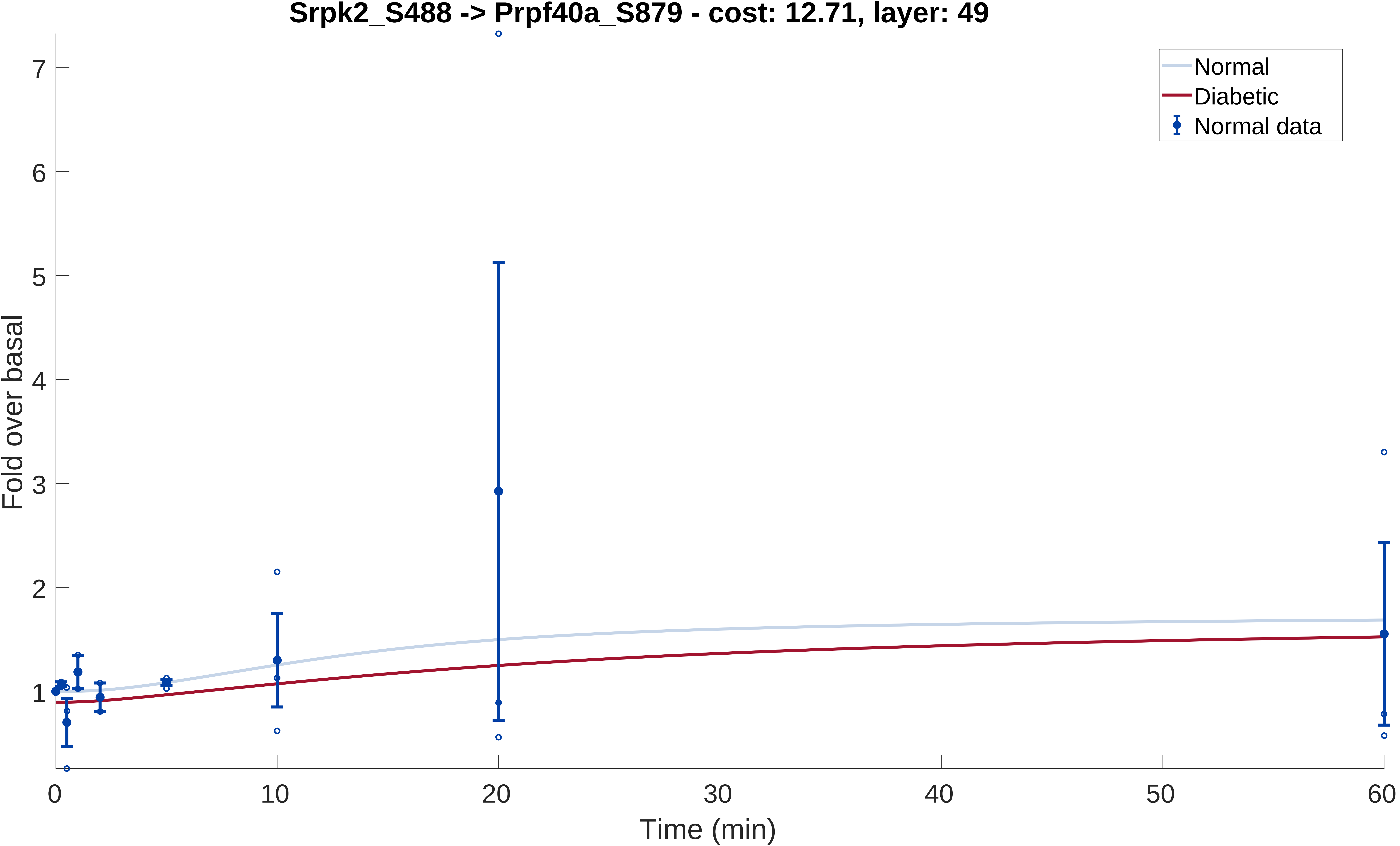

Srpk2_S488 -> Prpf40a_S879 - cost: 12.71, layer: 49
Normal
Diabetic
Normal data
Fold over basal
Time (min)
0
10
20
30
40
50
60
1
2
3
4
5
6
7

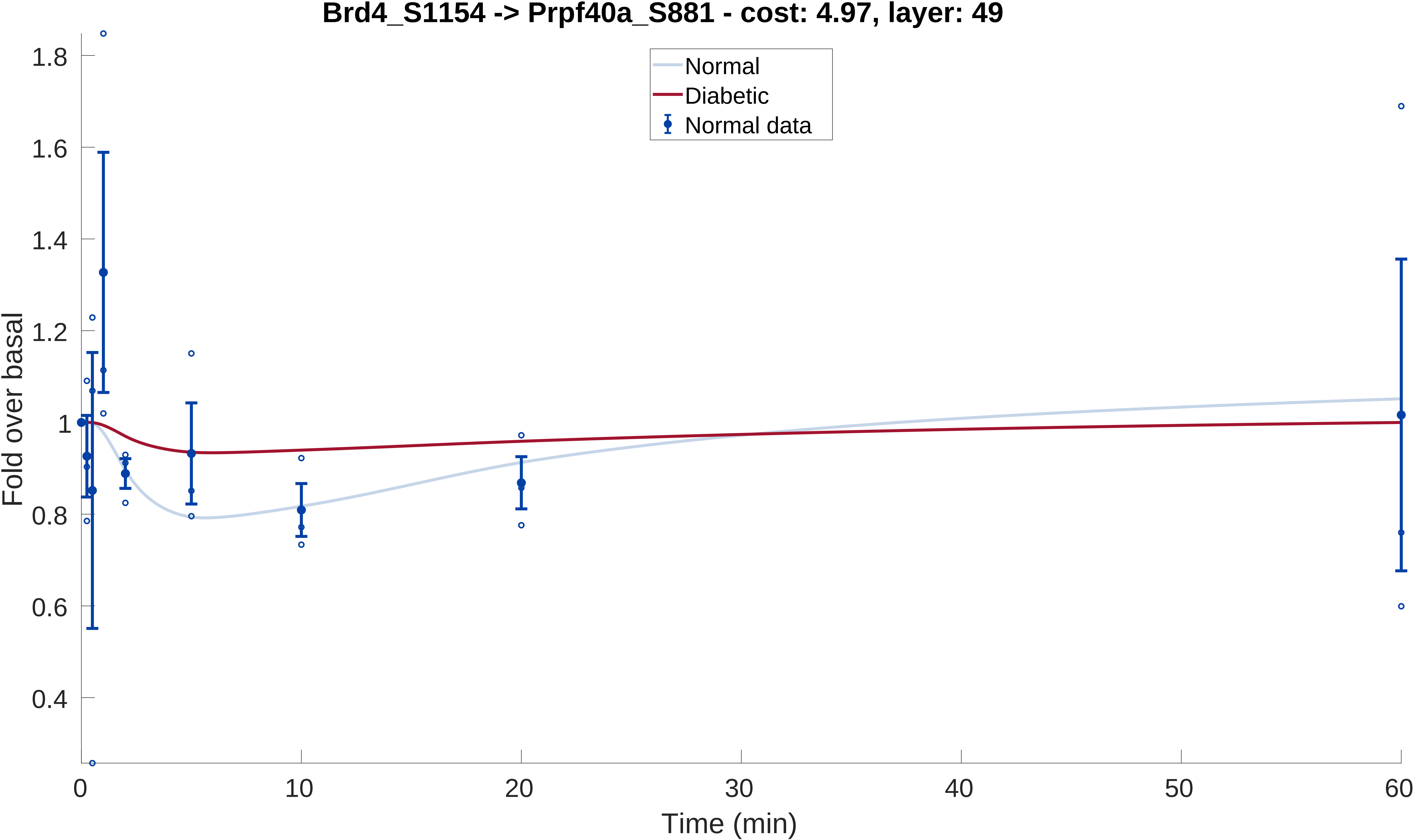
Brd4_S1154 -> Prpf40a_S881 - cost: 4.97, layer: 49
Normal
Diabetic
Normal data
Fold over basal
Time (min)
1.8
1.6
1.4
1.2
1
0.8
0.6
0.4
0
10
20
30
40
50
60

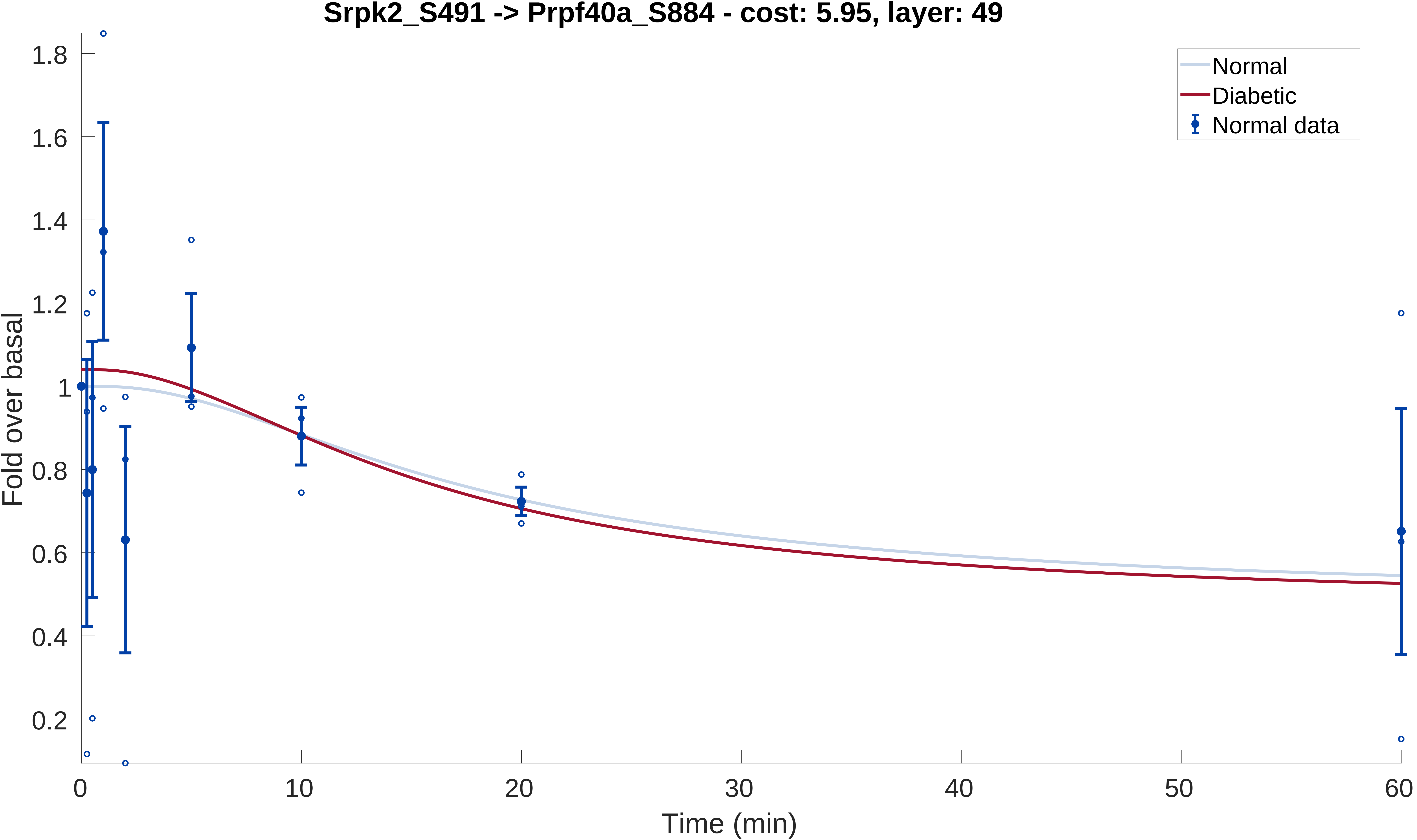
Srpk2_S491 -> Prpf40a_S884 - cost: 5.95, layer: 49
Normal
Diabetic
Normal data
Fold over basal
Time (min)
1.8
1.6
1.4
1.2
1
0.8
0.6
0.4
0.2
0
10
20
30
40
50
60

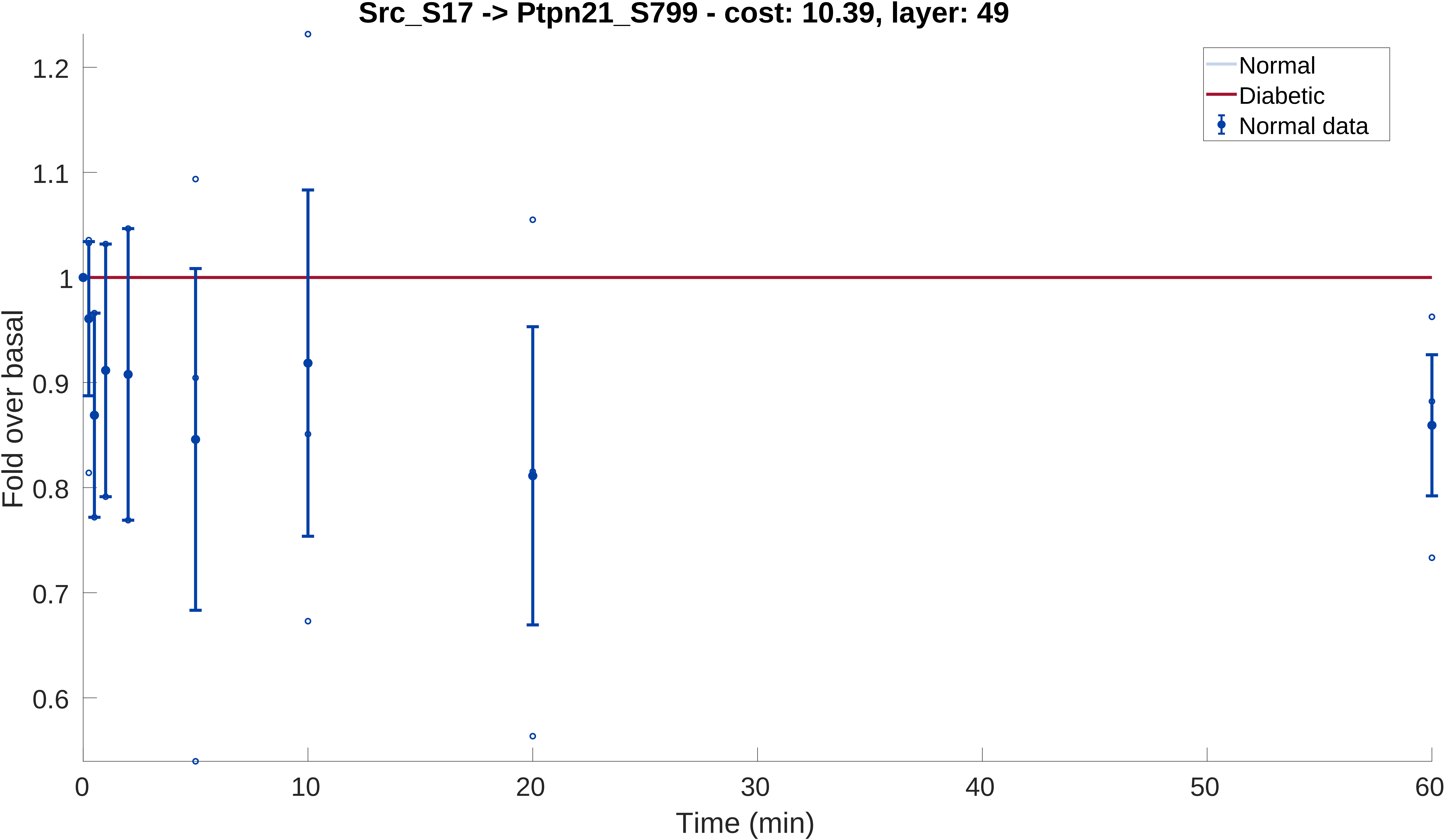

Src_S17 -> Ptpn21_S799 - cost: 10.39, layer: 49
Normal
Diabetic
Normal data
Fold over basal
1.2
1.1
1
0.9
0.8
0.7
0.6
0
10
20
30
40
50
60
Time (min)

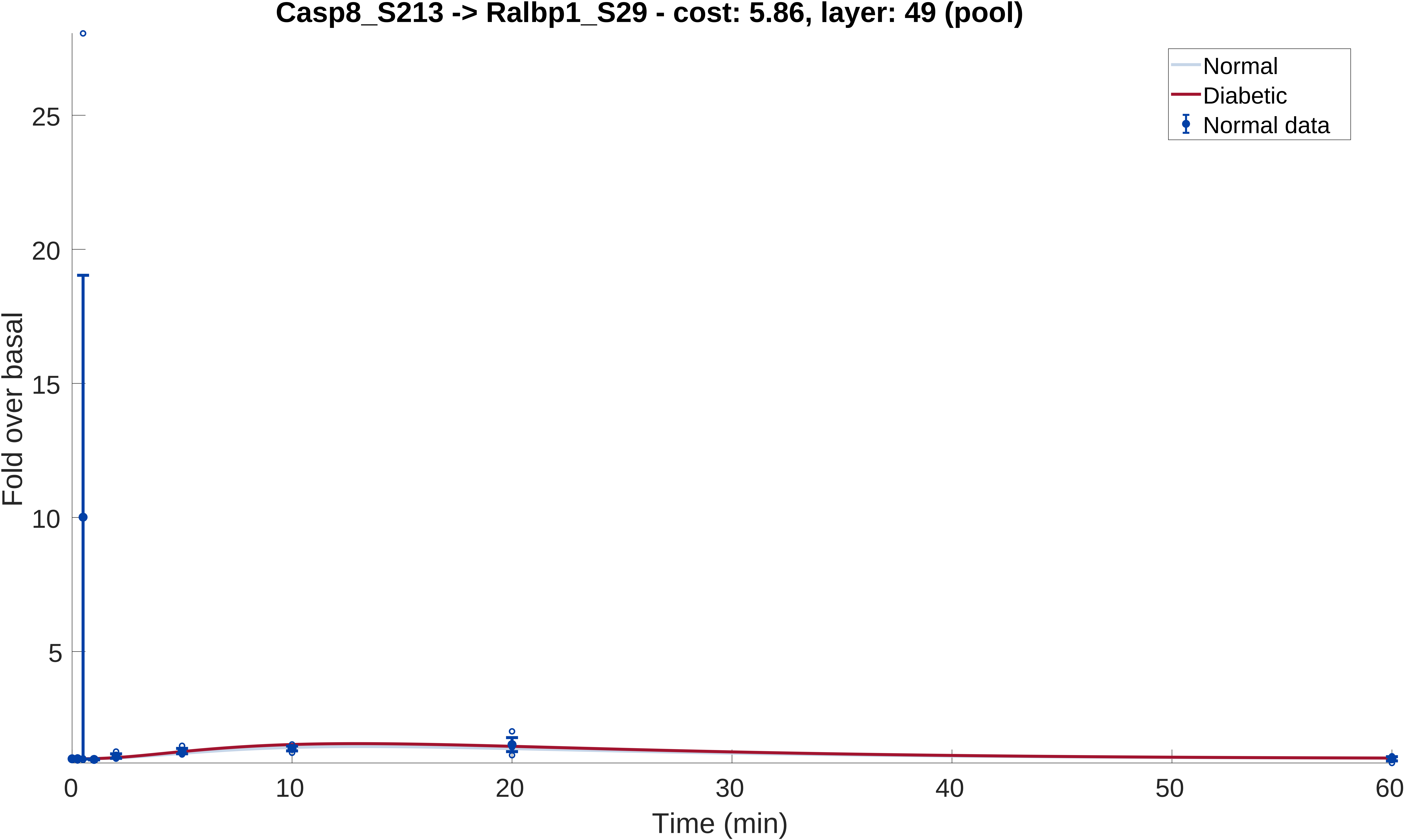

Casp8_S213 -> Ralbp1_S29 - cost: 5.86, layer: 49 (pool)
Normal
Diabetic
Normal data
Fold over basal
25
20
15
10
5
0
10
20
30
40
50
60
Time (min)

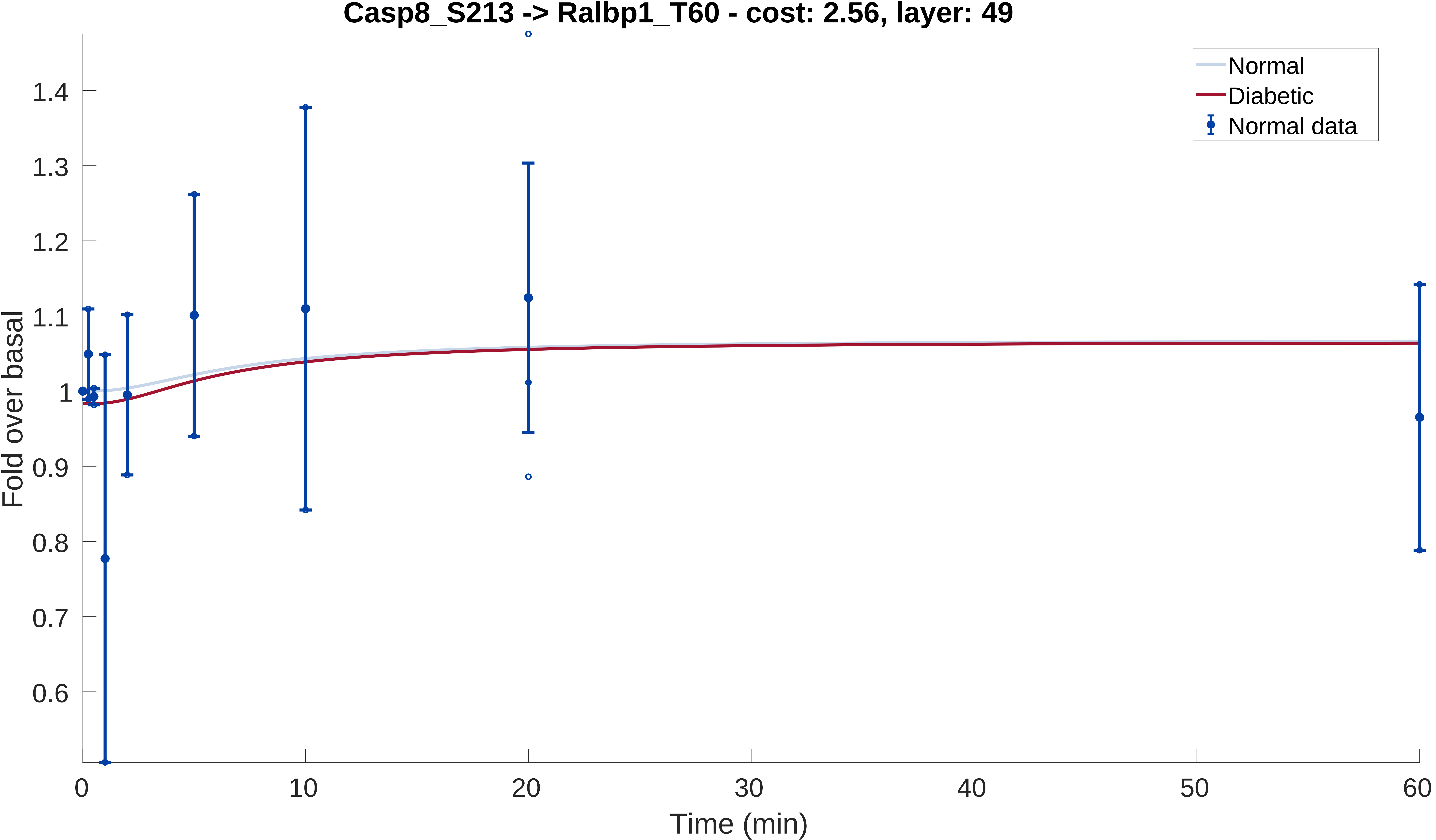
Casp8_S213 -> Ralbp1_T60 - cost: 2.56, layer: 49
Normal
Diabetic
Normal data
Fold over basal
1.4
1.3
1.2
1.1
1
0.9
0.8
0.7
0.6
0
10
20
30
40
50
60
Time (min)

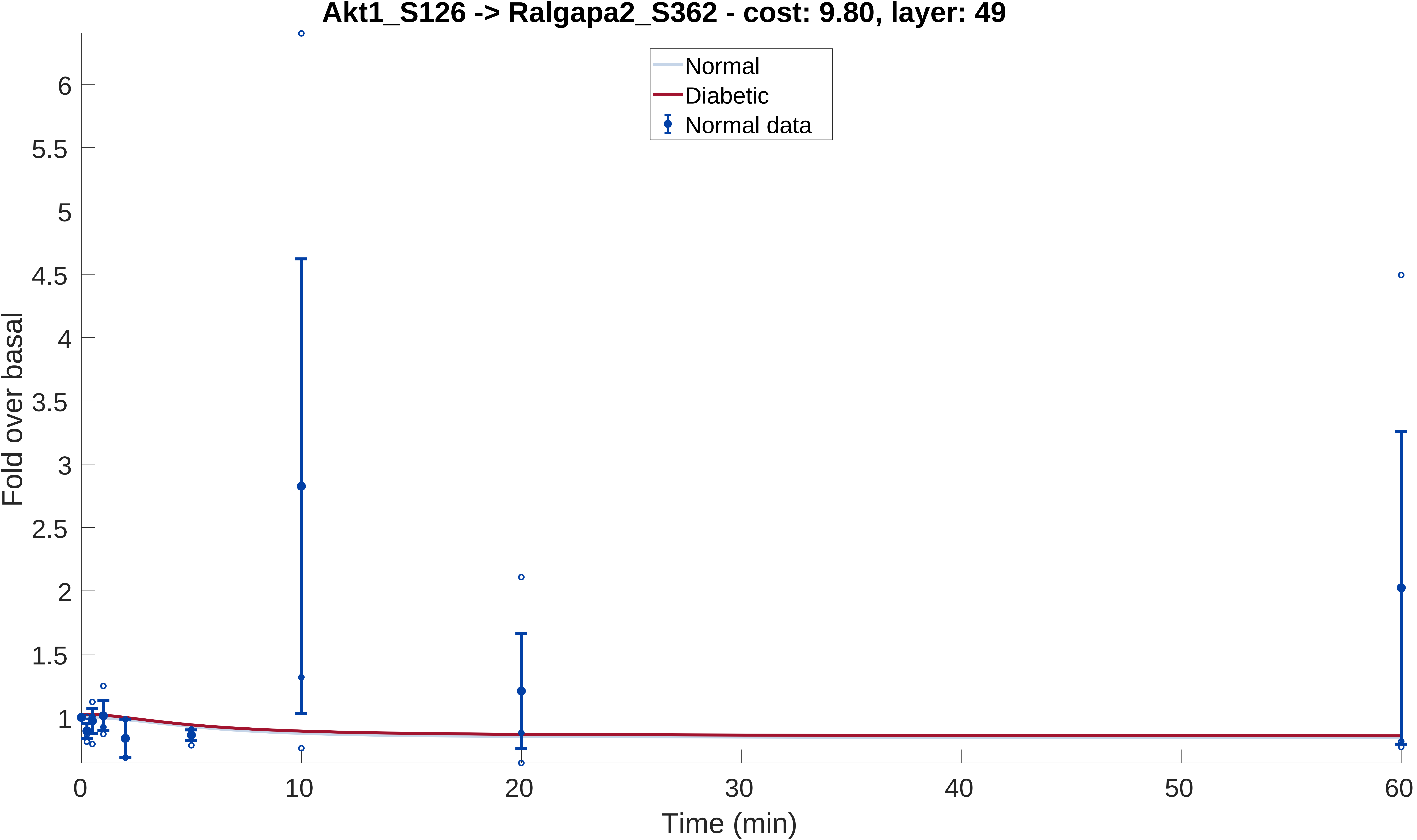

Akt1_S126 -> Ralgapa2_S362 - cost: 9.80, layer: 49
Normal
Diabetic
Normal data
Fold over basal
Time (min)
6
5.5
5
4.5
4
3.5
3
2.5
2
1.5
1
0
10
20
30
40
50
60

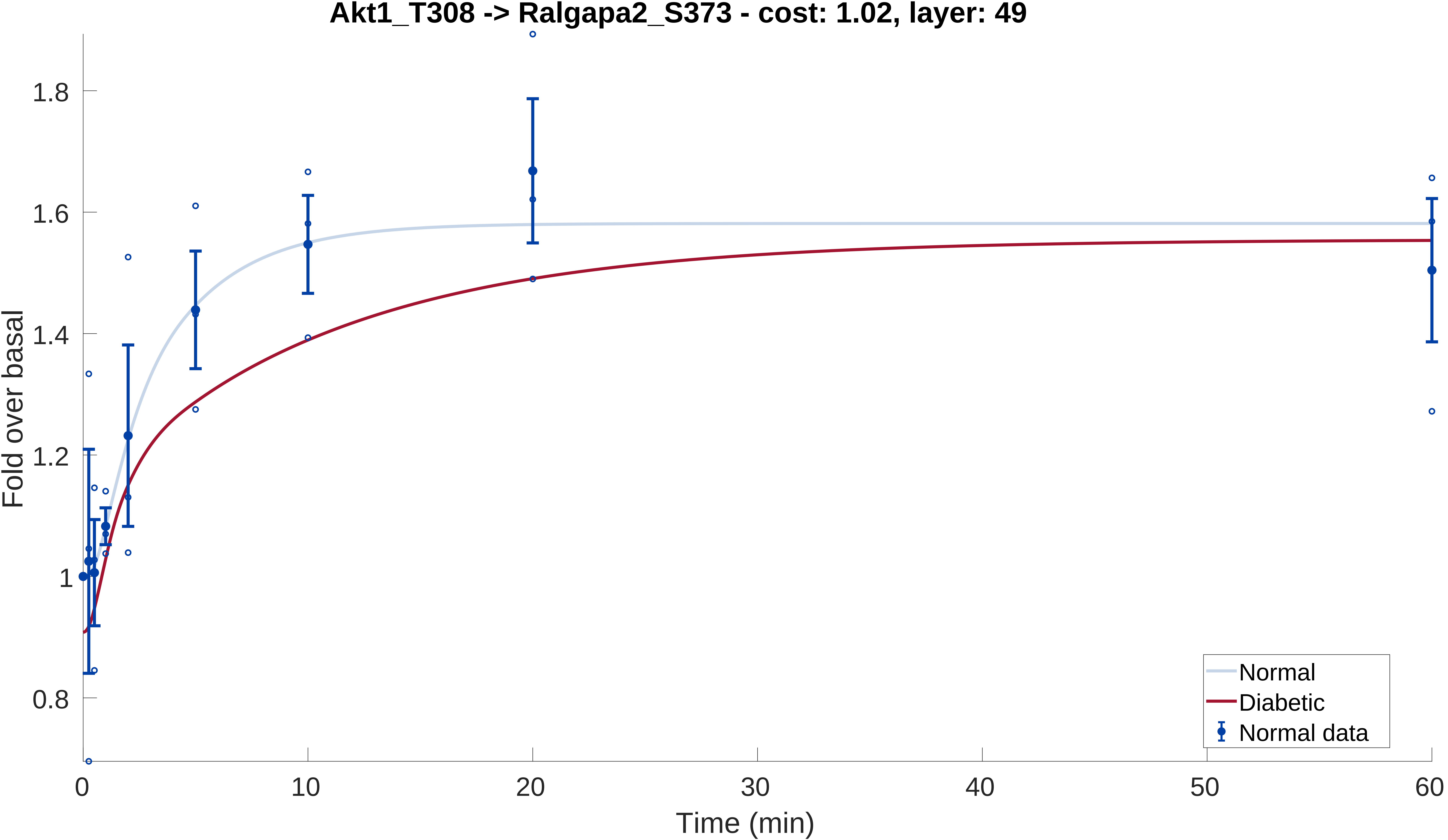

Akt1_T308 -> Ralgapa2_S373 - cost: 1.02, layer: 49
1.8
1.6
1.4
1.2
1
0.8
Fold over basal
0
10
20
30
40
50
60
Time (min)
Normal
Diabetic
Normal data

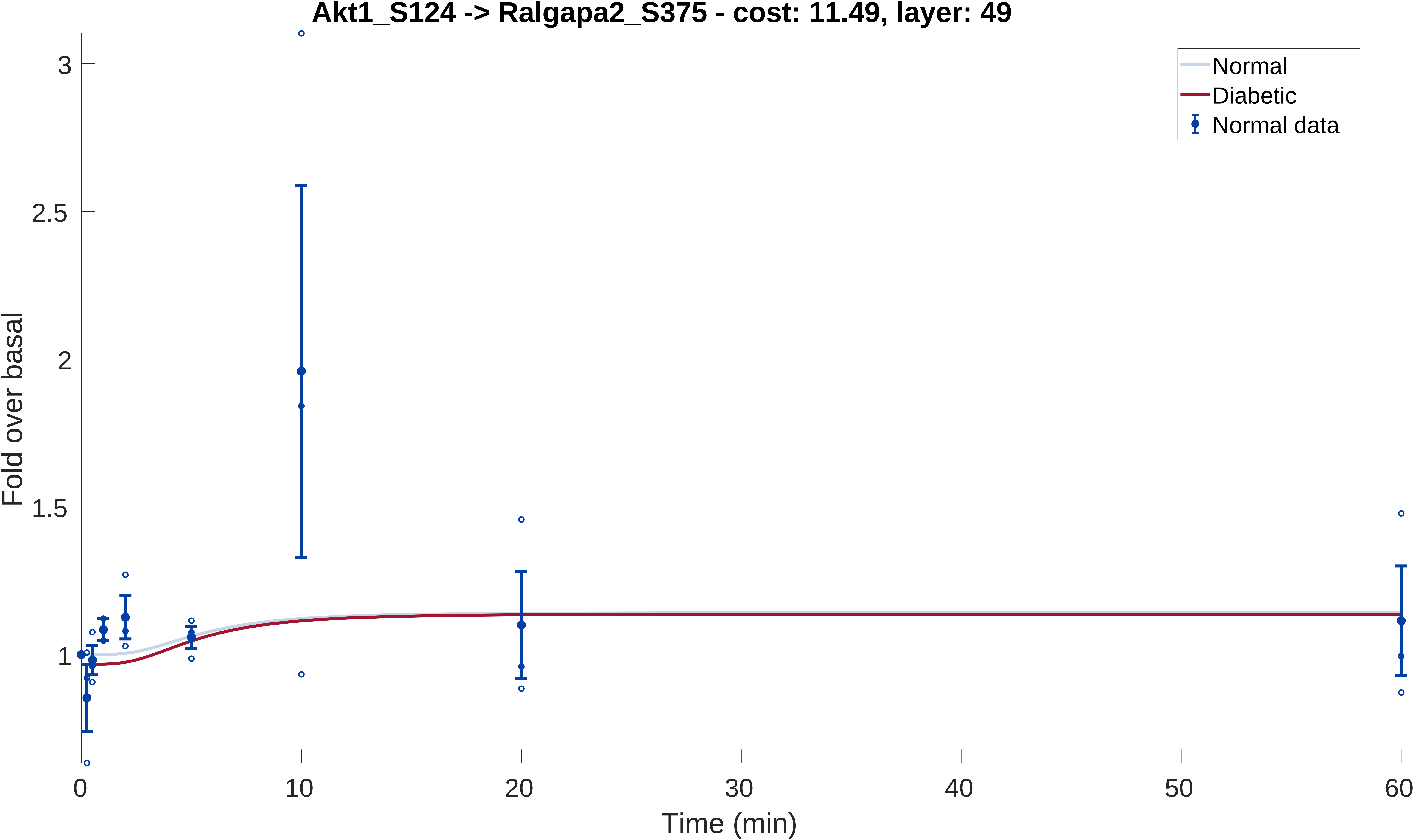
Akt1_S124 -> Ralgapa2_S375 - cost: 11.49, layer: 49
Normal
Diabetic
Normal data
Fold over basal
Time (min)
3
2.5
2
1.5
1
0
10
20
30
40
50
60

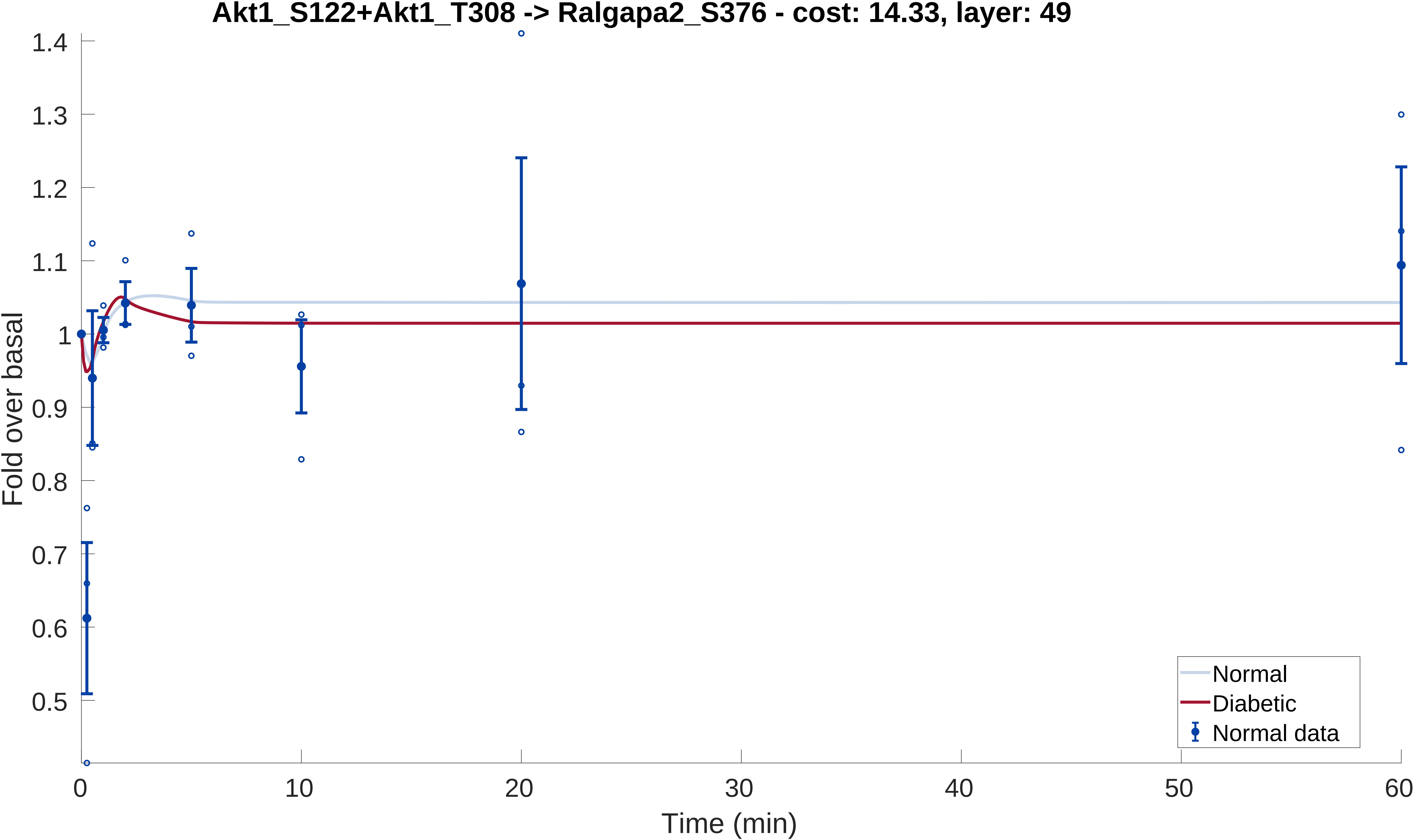

Akt1_S122+Akt1_T308 -> Ralgapa2_S376 - cost: 14.33, layer: 49
Fold over basal
1.4
1.3
1.2
1.1
1
0.9
0.8
0.7
0.6
0.5
0
10
20
30
40
50
60
Time (min)
Normal
Diabetic
Normal data

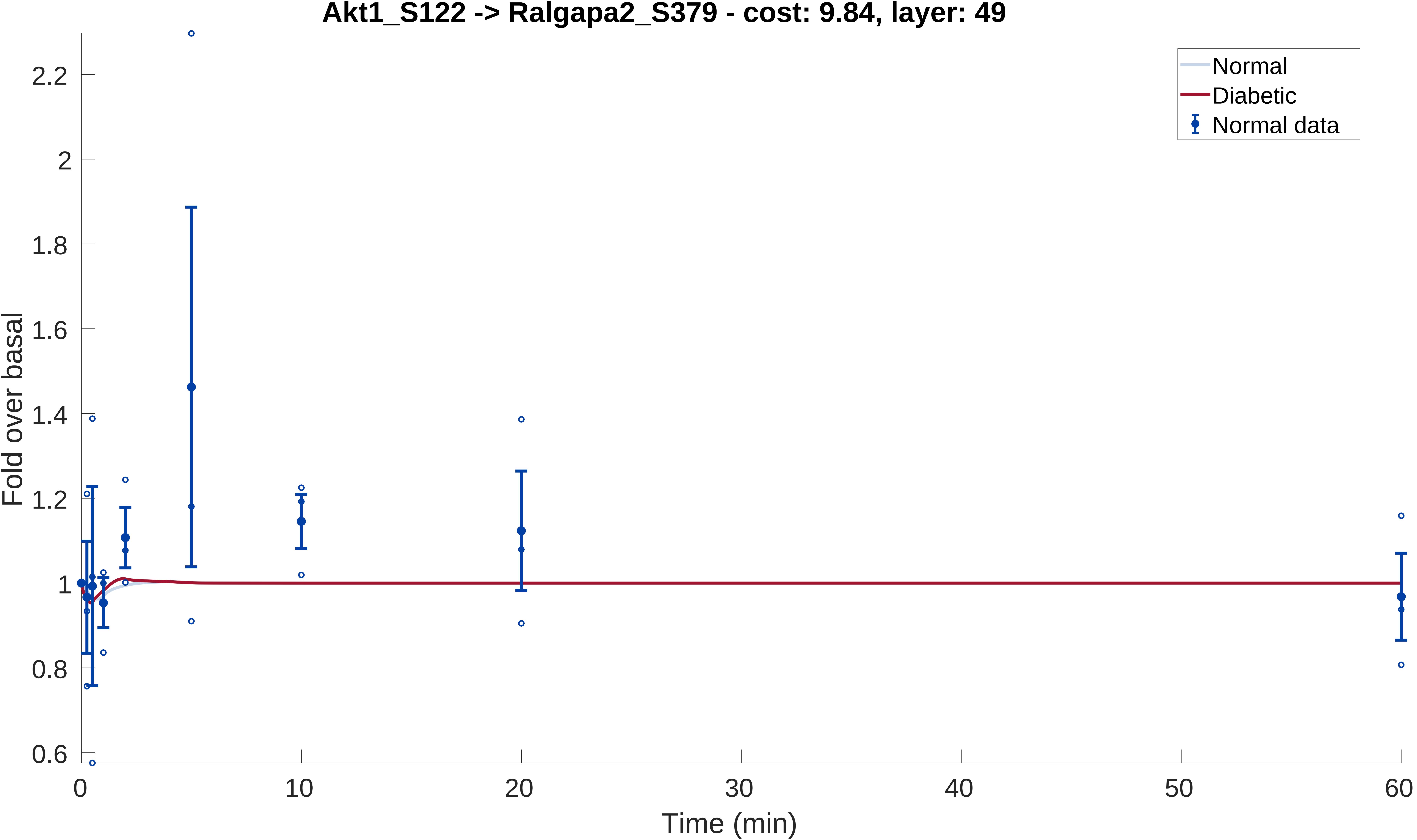

Akt1_S122 -> Ralgapa2_S379 - cost: 9.84, layer: 49
Normal
Diabetic
Normal data
Fold over basal
Time (min)
0.6
0.8
1
1.2
1.4
1.6
1.8
2
2.2
0
10
20
30
40
50
60

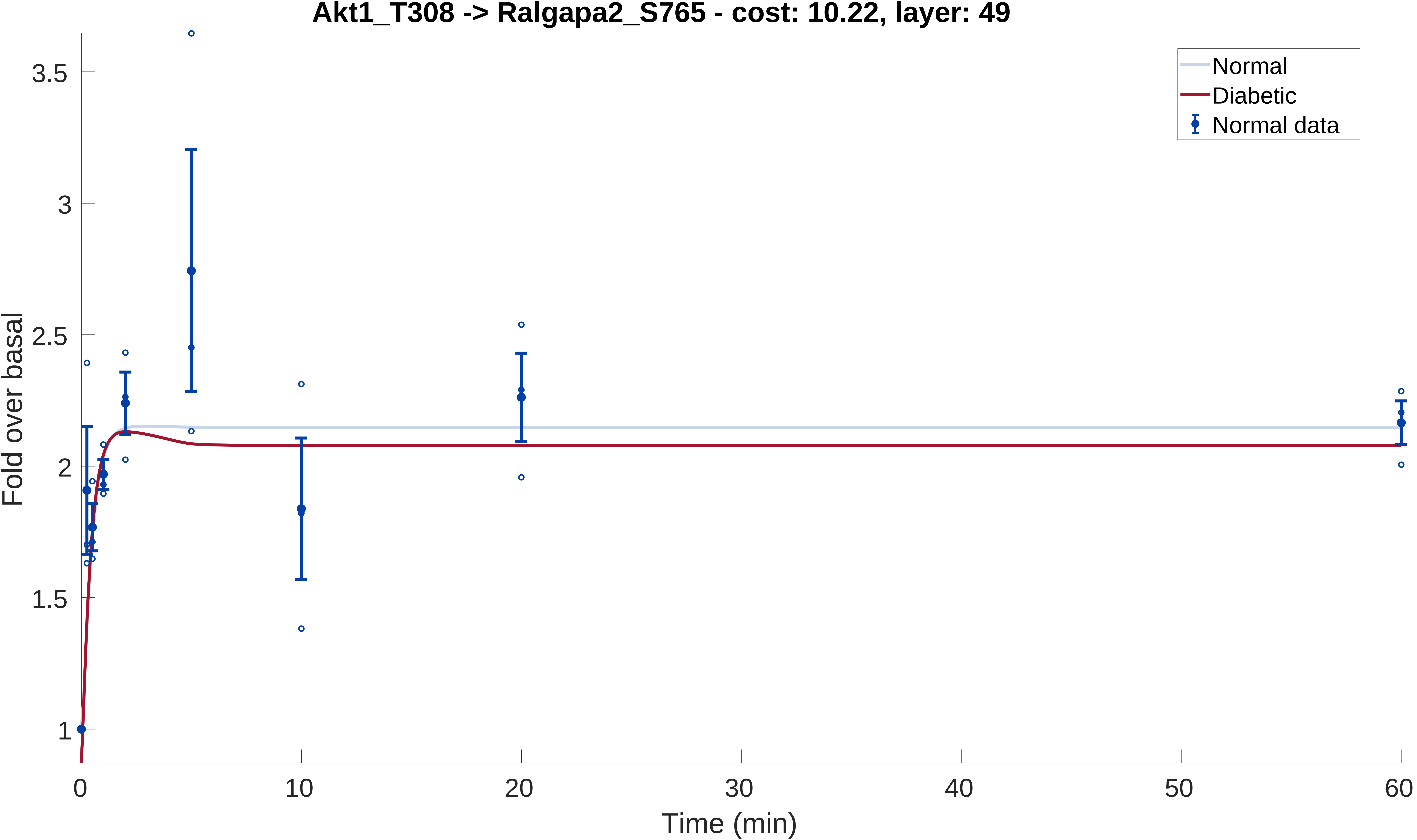

Akt1_T308 -> Ralgapa2_S765 - cost: 10.22, layer: 49
Normal
Diabetic
Normal data
Fold over basal
Time (min)
3.5
3
2.5
2
1.5
1
0
10
20
30
40
50
60

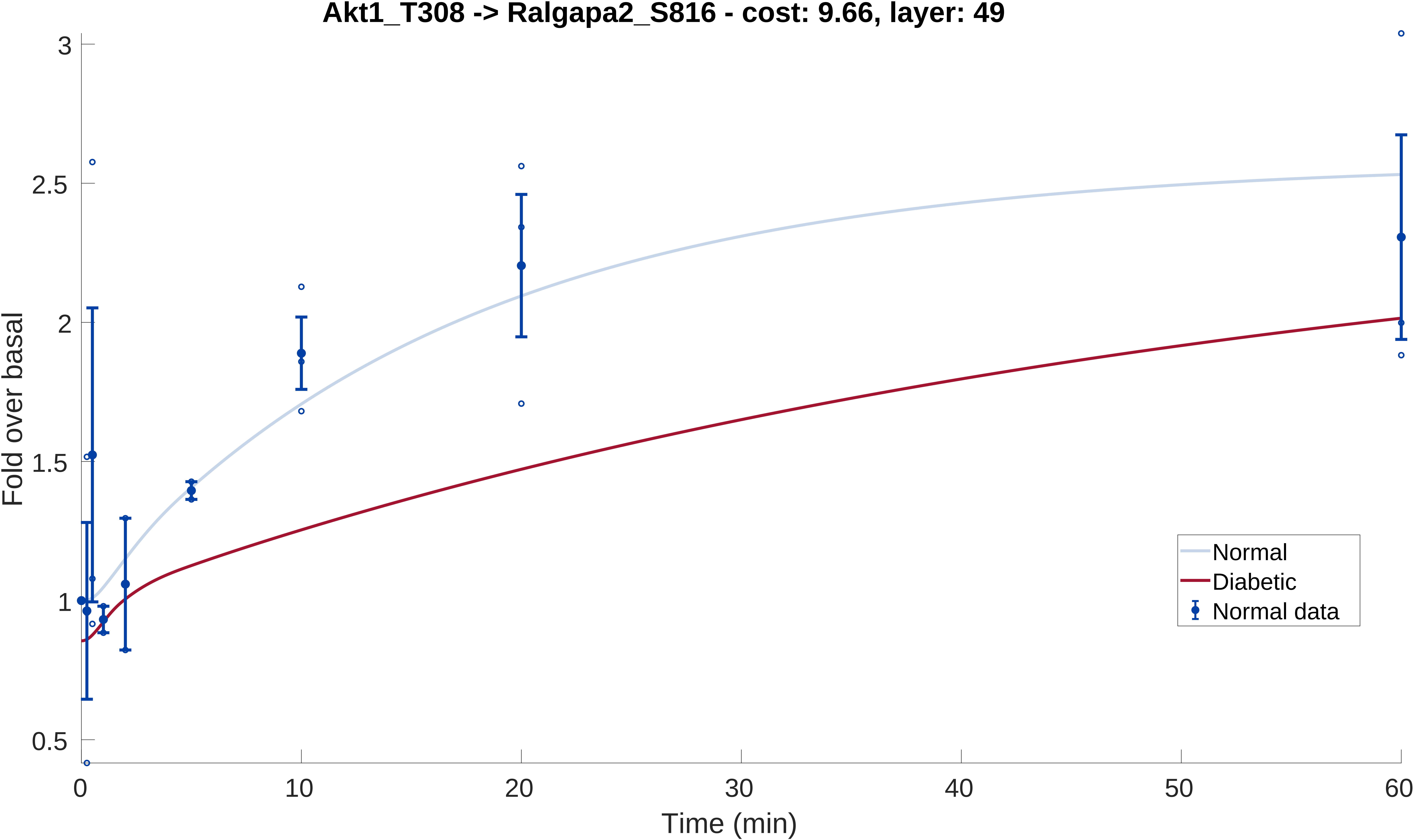

Akt1_T308 -> Ralgapa2_S816 - cost: 9.66, layer: 49
Fold over basal
Time (min)
0.5
1
1.5
2
2.5
3
0
10
20
30
40
50
60
Normal
Diabetic
Normal data

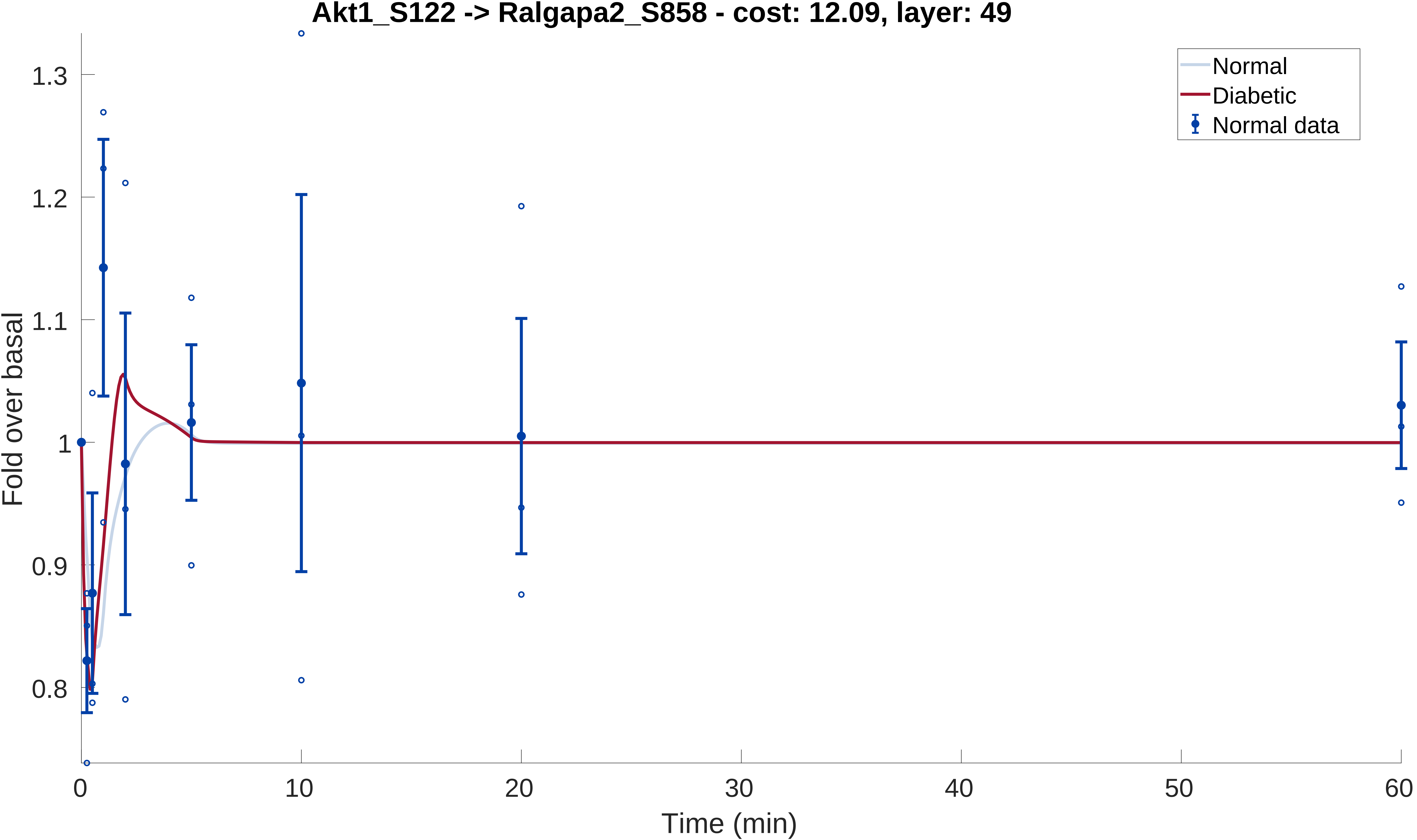

Akt1_S122 -> Ralgapa2_S858 - cost: 12.09, layer: 49
Normal
Diabetic
Normal data
Fold over basal
1.3
1.2
1.1
1
0.9
0.8
0
10
20
30
40
50
60
Time (min)

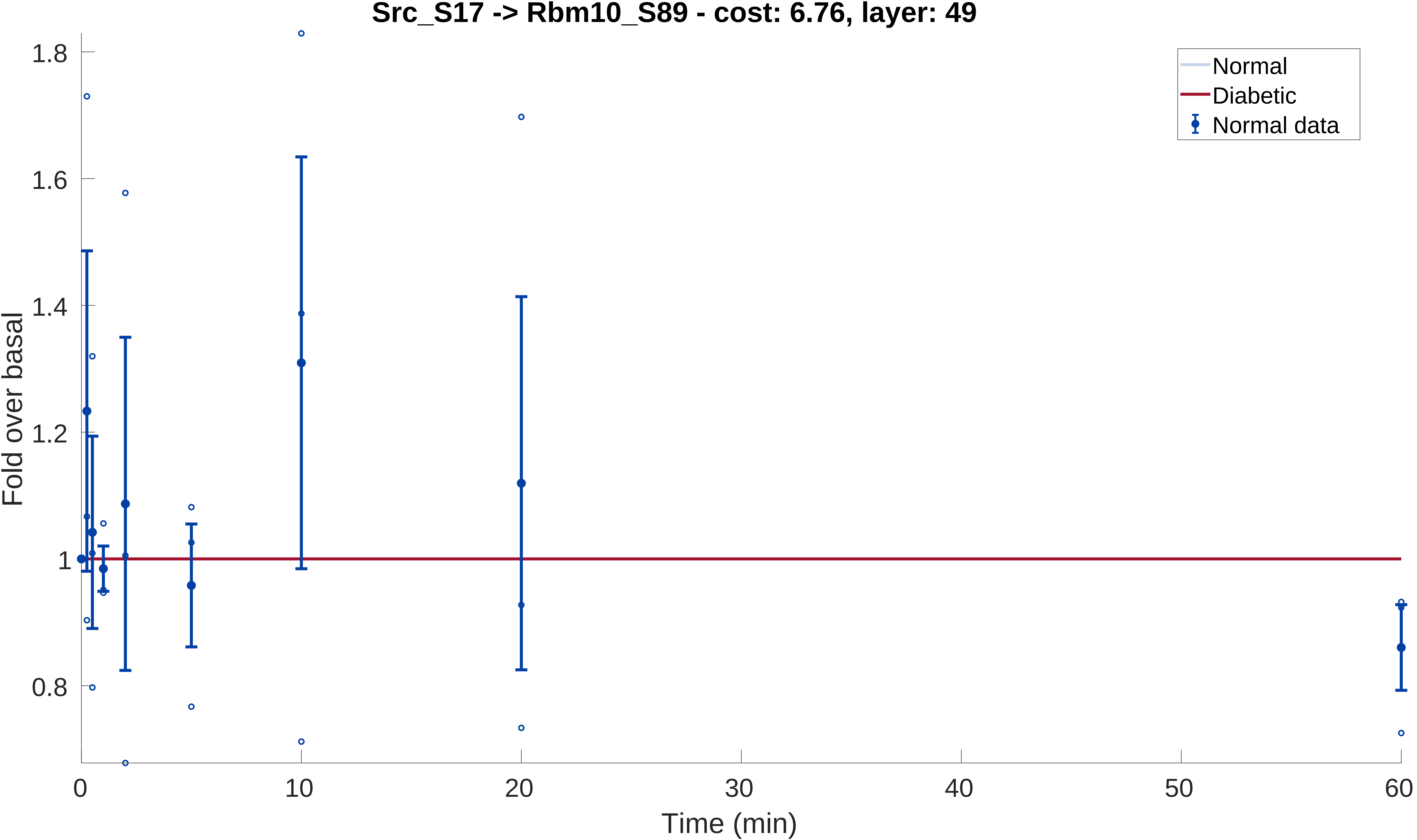
Src_S17 -> Rbm10_S89 - cost: 6.76, layer: 49
Fold over basal
Time (min)
Normal
Diabetic
Normal data
1.8
1.6
1.4
1.2
1
0.8
0
10
20
30
40
50
60

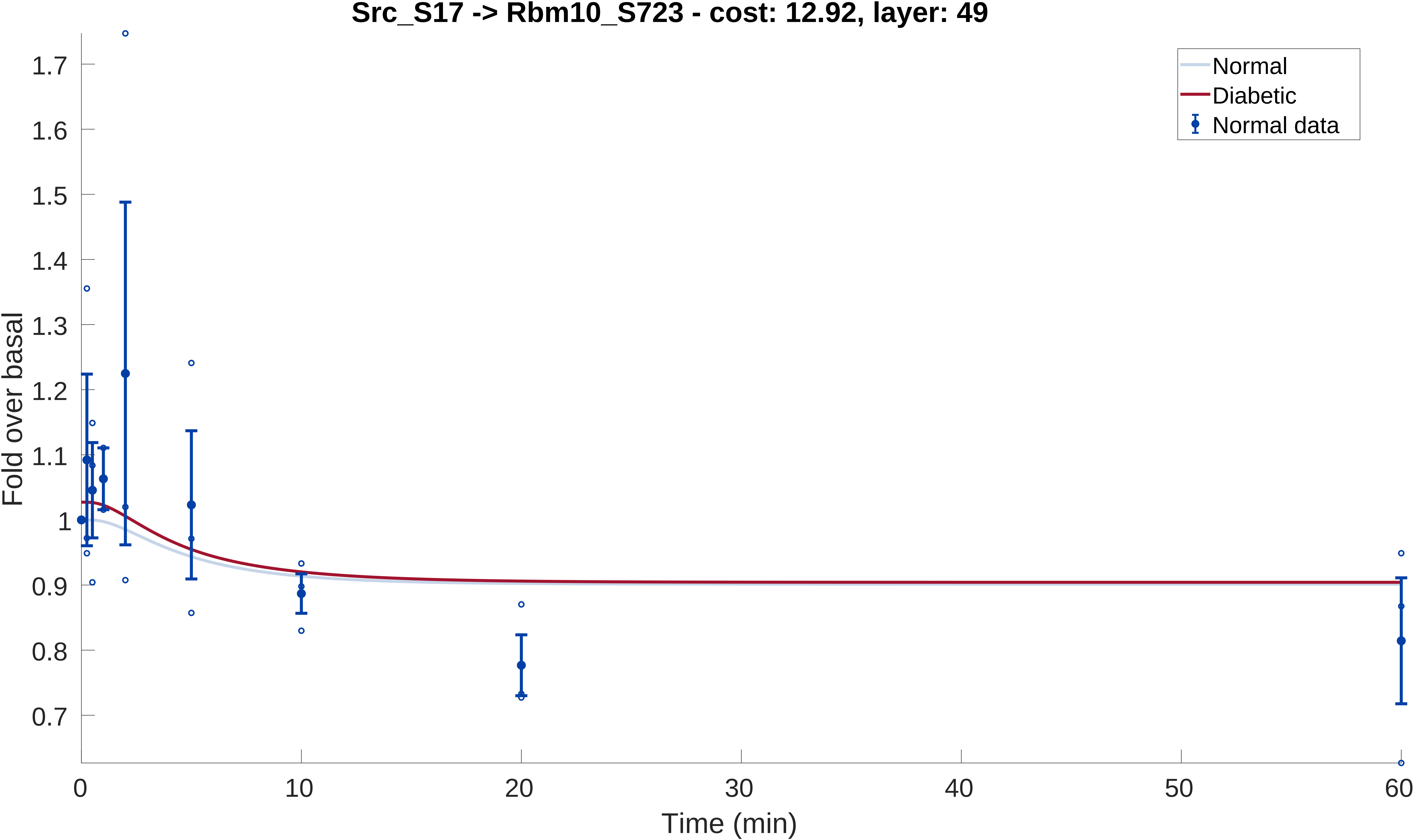
Src_S17 -> Rbm10_S723 - cost: 12.92, layer: 49
Normal
Diabetic
Normal data
Fold over basal
Time (min)
0.7
0.8
0.9
1
1.1
1.2
1.3
1.4
1.5
1.6
1.7
0
10
20
30
40
50
60

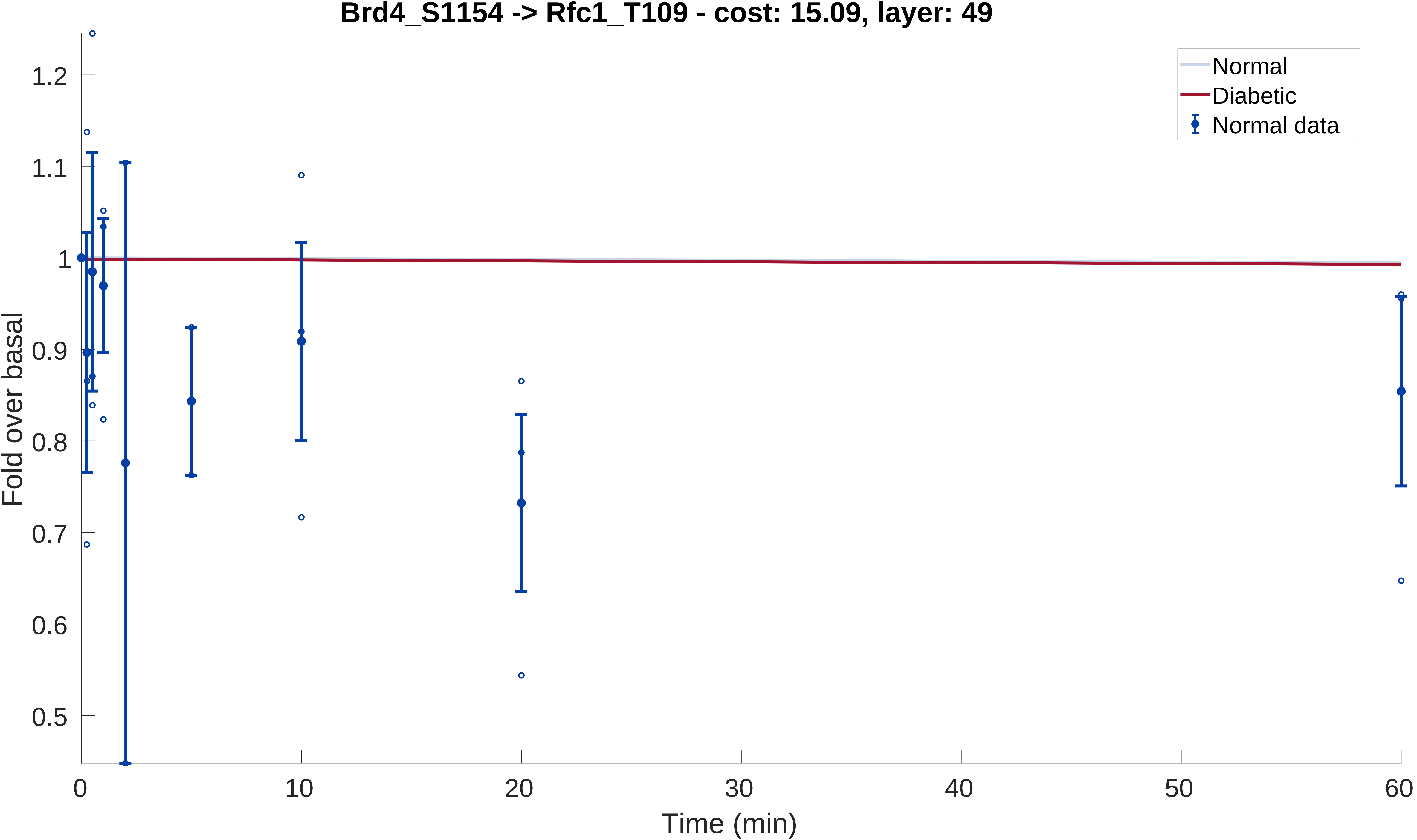

Brd4_S1154 -> Rfc1_T109 - cost: 15.09, layer: 49
Normal
Diabetic
Normal data
Fold over basal
1.2
1.1
1
0.9
0.8
0.7
0.6
0.5
0
10
20
30
40
50
60
Time (min)

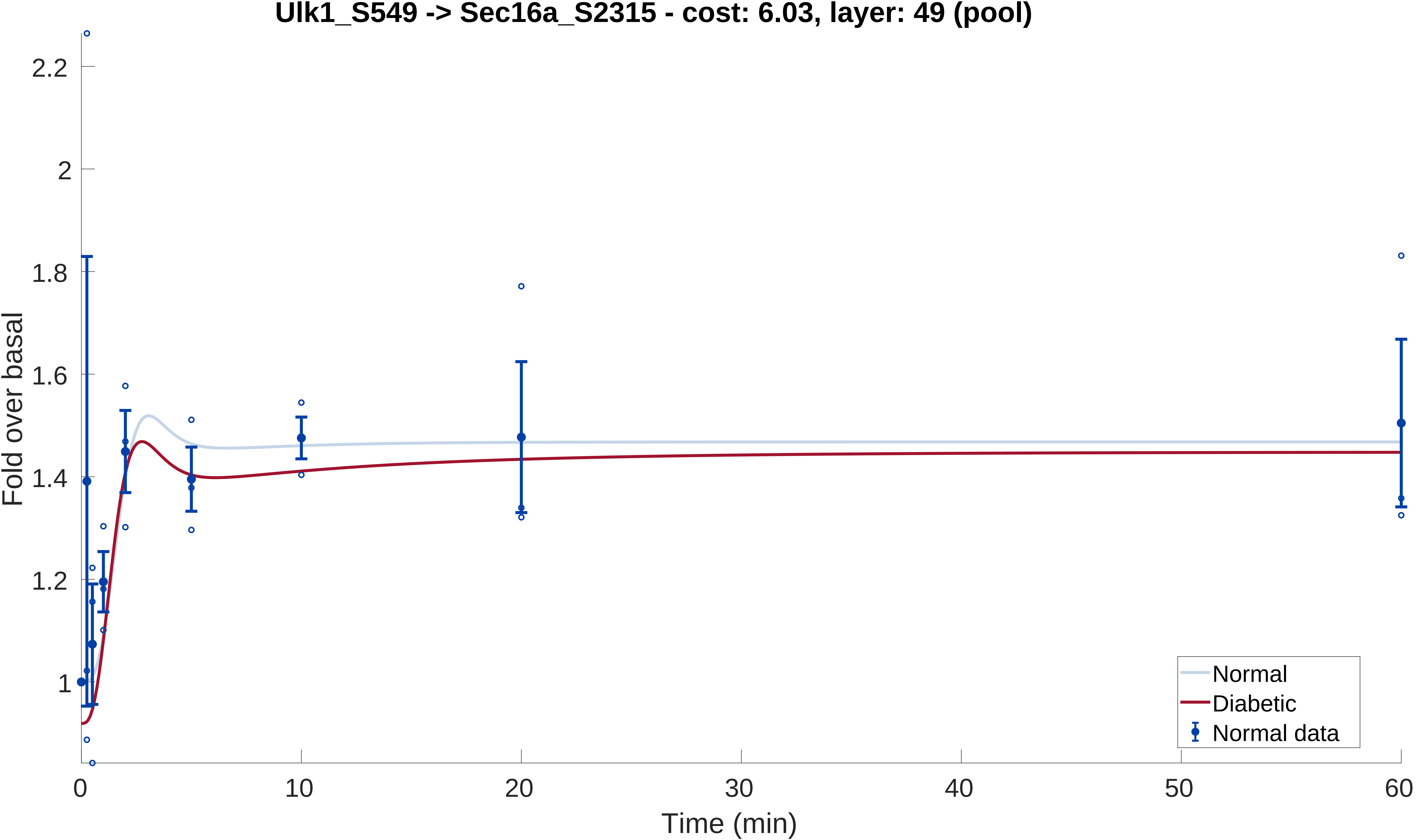
Ulk1_S549 -> Sec16a_S2315 - cost: 6.03, layer: 49 (pool)
Fold over basal
2.2
2
1.8
1.6
1.4
1.2
1
0
10
20
30
40
50
60
Time (min)
Normal
Diabetic
Normal data

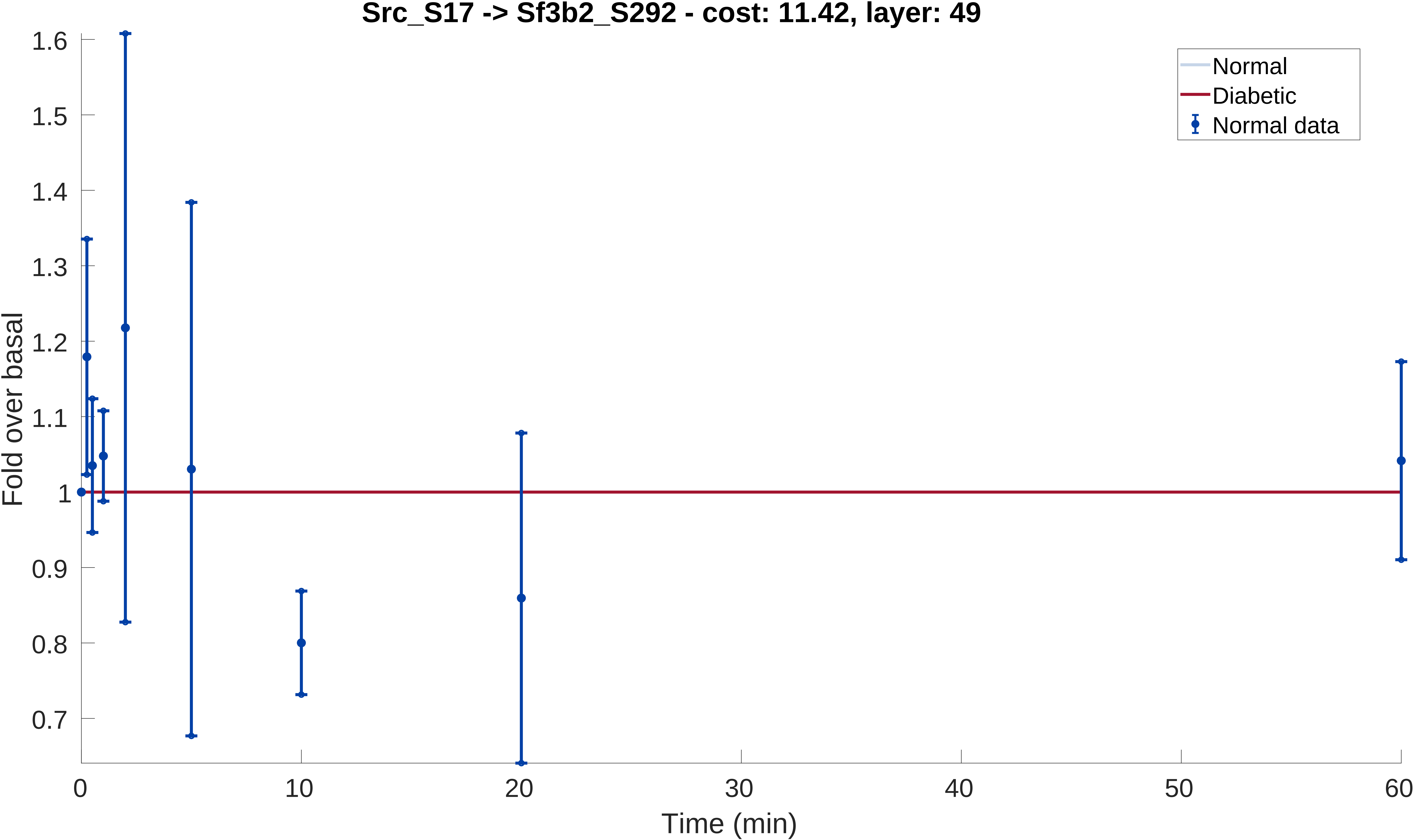

Src_S17 -> Sf3b2_S292 - cost: 11.42, layer: 49
Normal
Diabetic
Normal data
Fold over basal
1.6
1.5
1.4
1.3
1.2
1.1
1
0.9
0.8
0.7
0
10
20
30
40
50
60
Time (min)

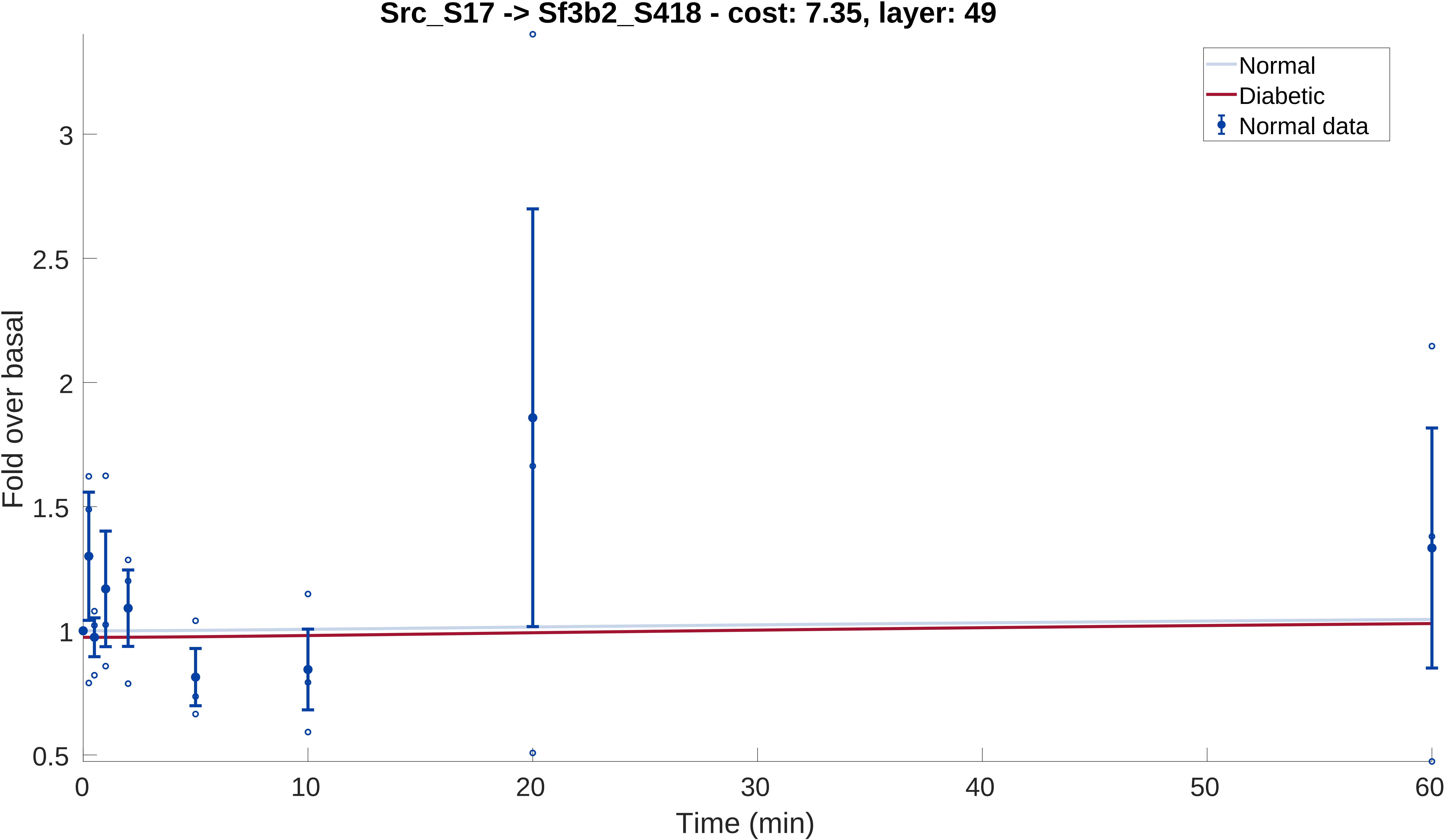

Src_S17 -> Sf3b2_S418 - cost: 7.35, layer: 49
Normal
Diabetic
Normal data
Fold over basal
0.5
1
1.5
2
2.5
3
0
10
20
30
40
50
60
Time (min)

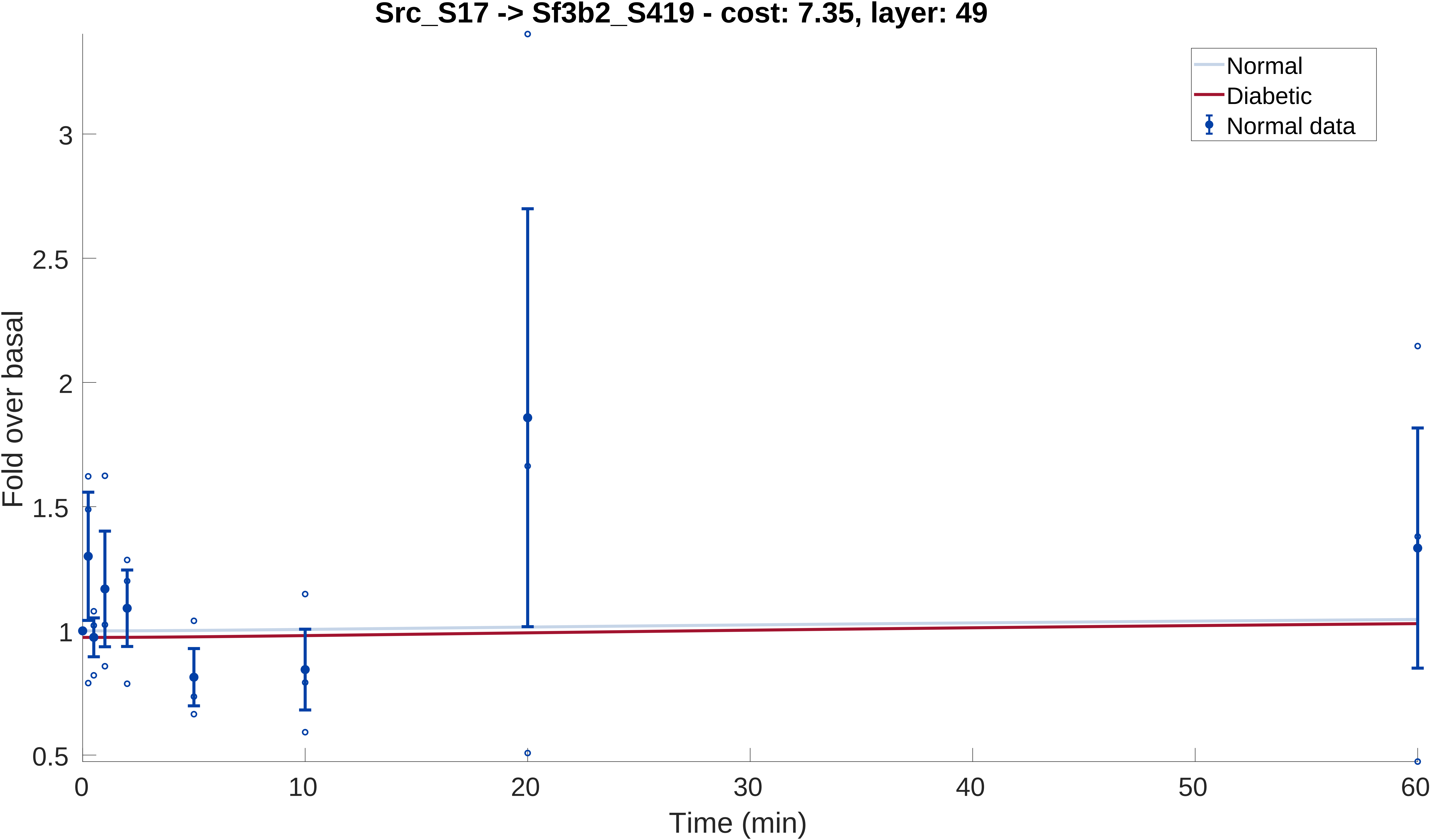
Src_S17 -> Sf3b2_S419 - cost: 7.35, layer: 49
Normal
Diabetic
Normal data
Fold over basal
Time (min)
0.5
1
1.5
2
2.5
3
0
10
20
30
40
50
60

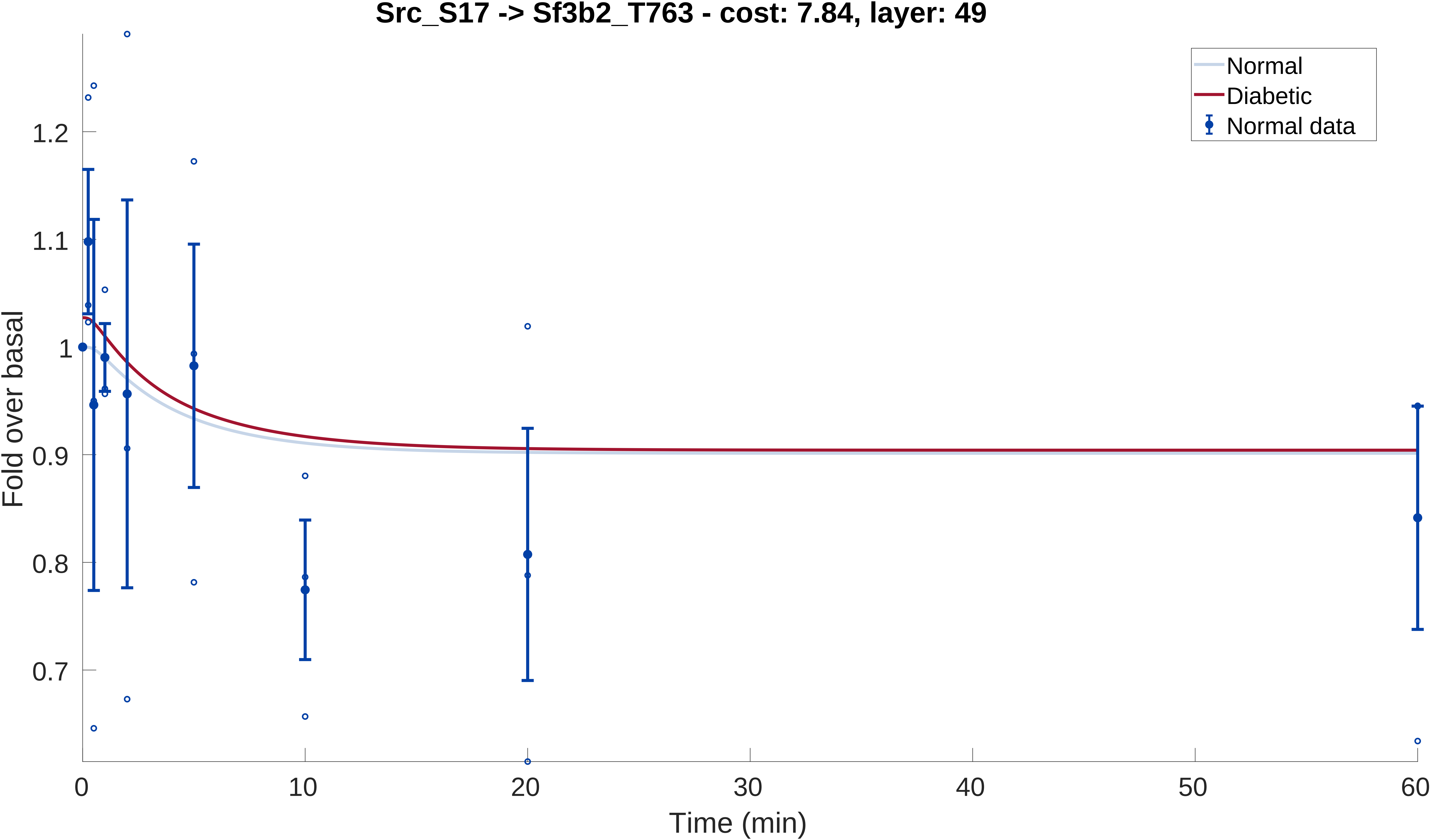

Src_S17 -> Sf3b2_T763 - cost: 7.84, layer: 49
Normal
Diabetic
Normal data
Fold over basal
1.2
1.1
1
0.9
0.8
0.7
0
10
20
30
40
50
60
Time (min)

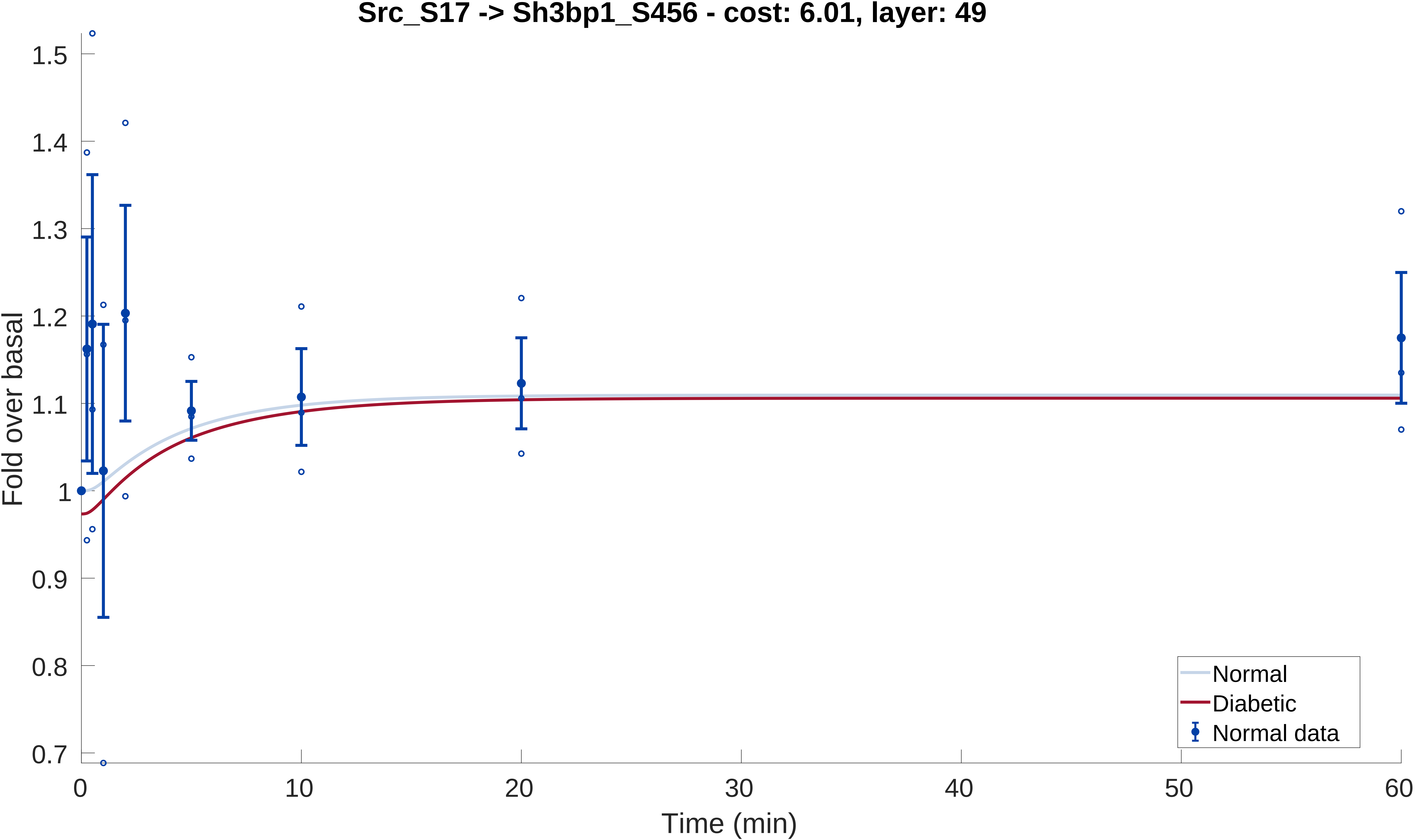

Src_S17 -> Sh3bp1_S456 - cost: 6.01, layer: 49
Fold over basal
Time (min)
1.5
1.4
1.3
1.2
1.1
1
0.9
0.8
0.7
0
10
20
30
40
50
60
Normal
Diabetic
Normal data

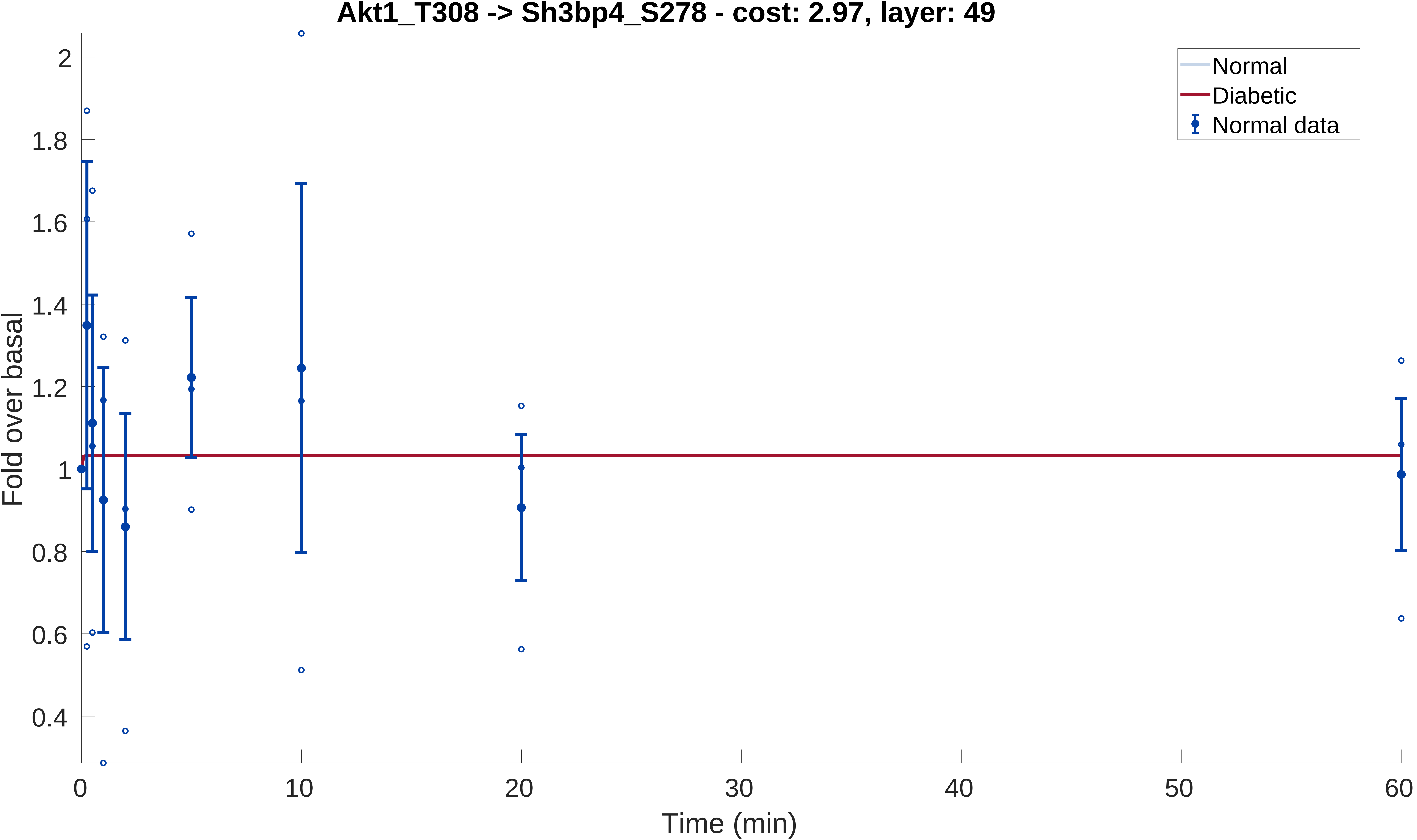

Akt1_T308 -> Sh3bp4_S278 - cost: 2.97, layer: 49
Normal
Diabetic
Normal data
Fold over basal
Time (min)
0
10
20
30
40
50
60
0.4
0.6
0.8
1
1.2
1.4
1.6
1.8
2

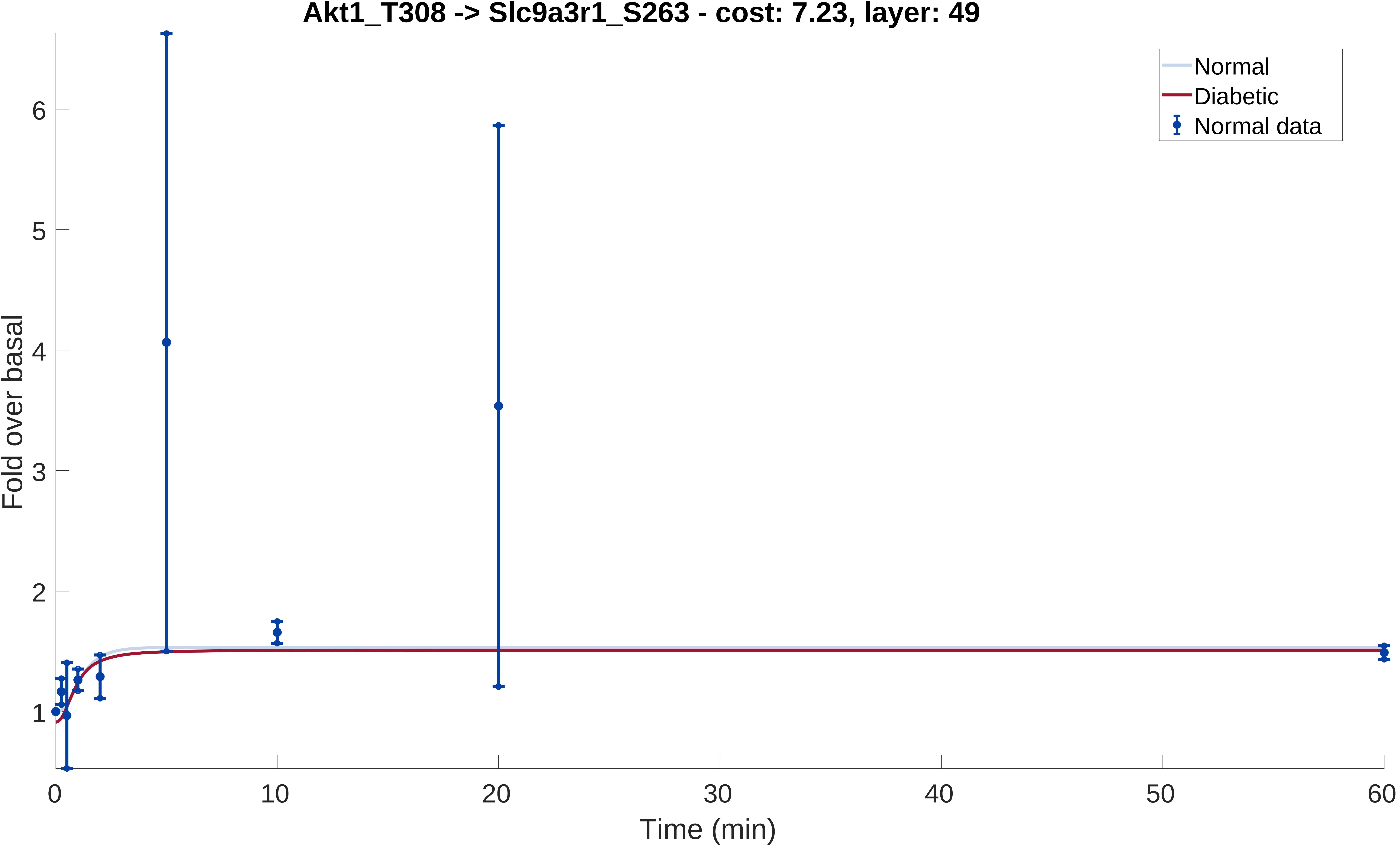

Akt1_T308 -> Slc9a3r1_S263 - cost: 7.23, layer: 49
Normal
Diabetic
Normal data
Fold over basal
Time (min)
1
2
3
4
5
6
0
10
20
30
40
50
60

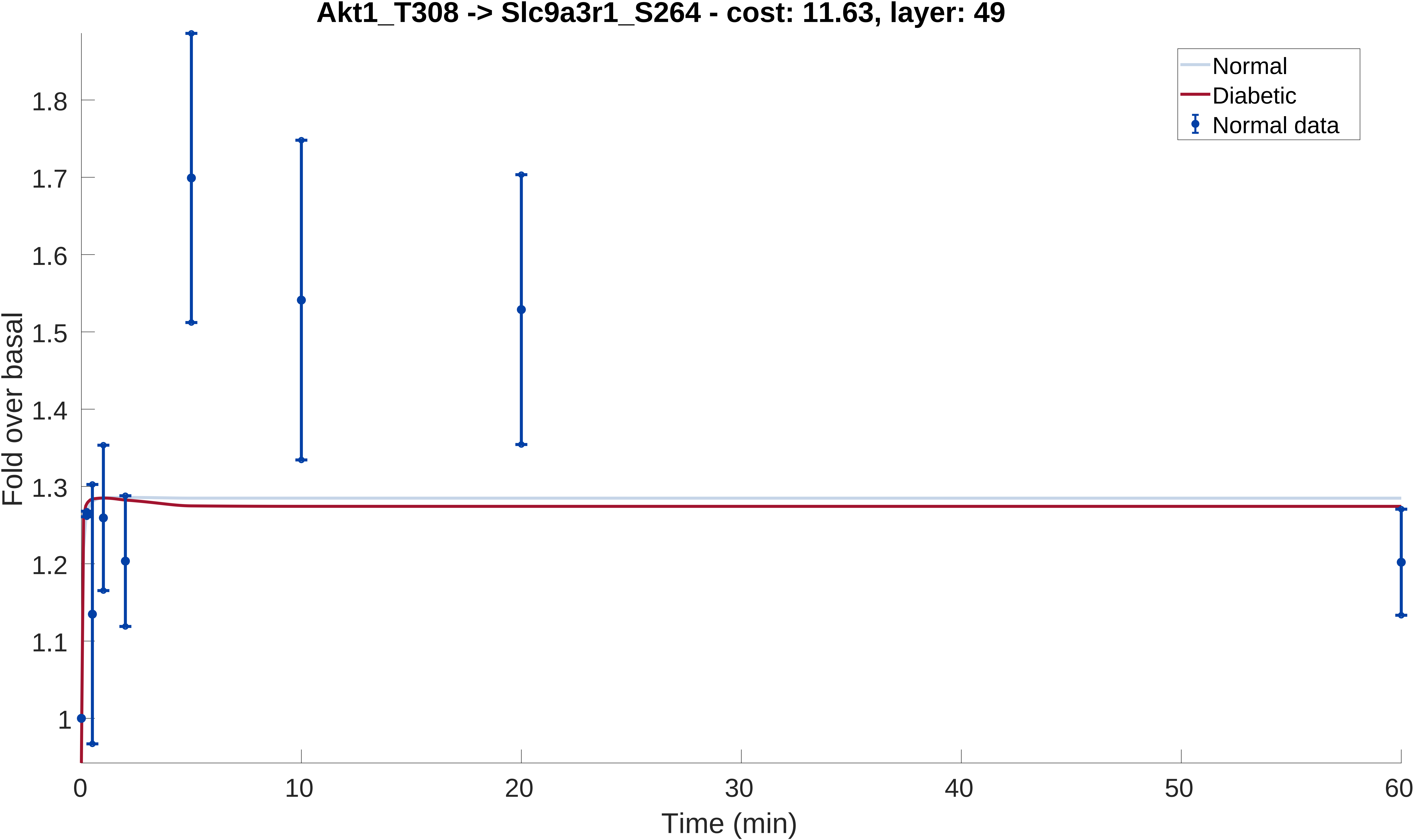

Akt1_T308 -> Slc9a3r1_S264 - cost: 11.63, layer: 49
Normal
Diabetic
Normal data
Fold over basal
1
1.1
1.2
1.3
1.4
1.5
1.6
1.7
1.8
0
10
20
30
40
50
60
Time (min)

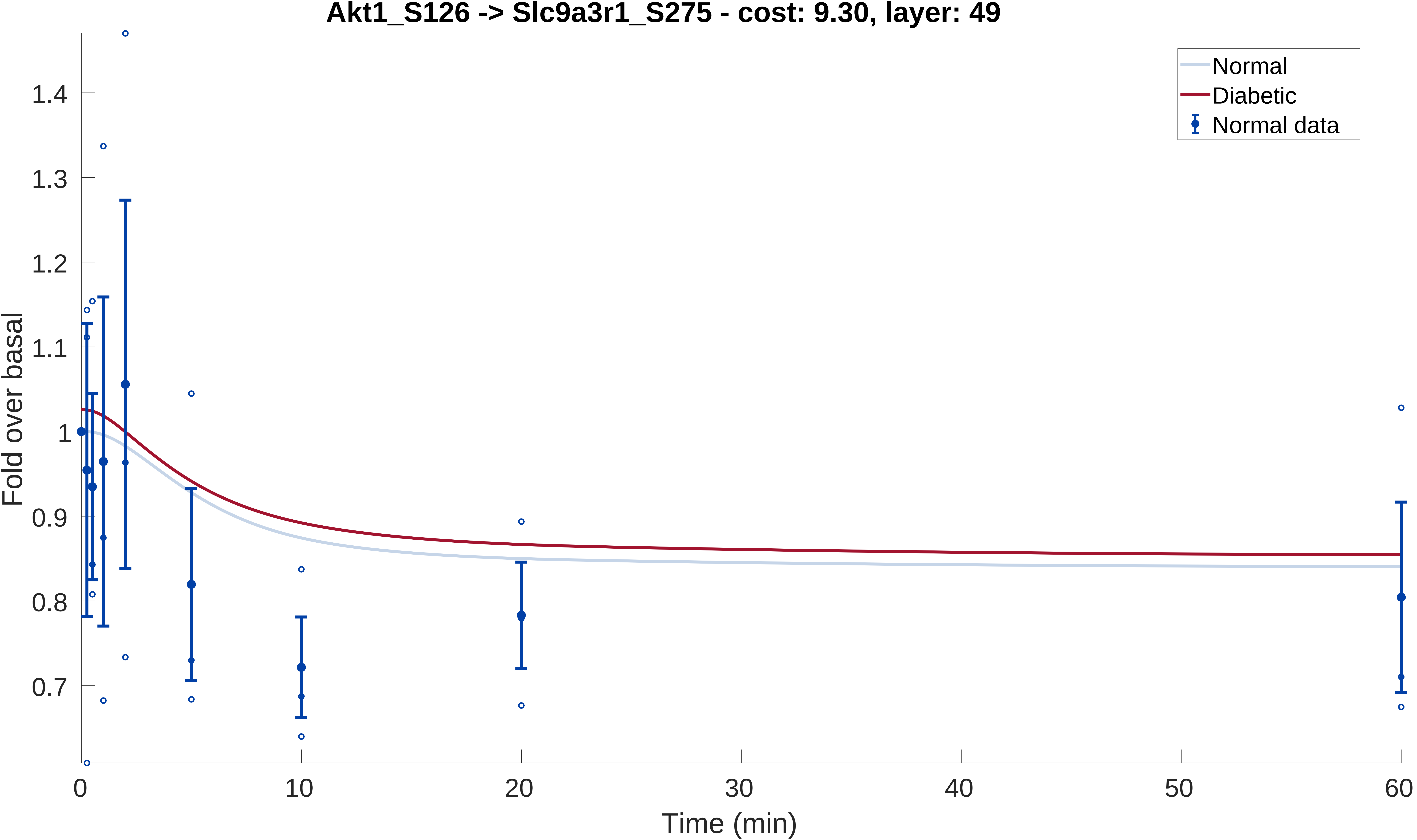

Akt1_S126 -> Slc9a3r1_S275 - cost: 9.30, layer: 49
Normal
Diabetic
Normal data
Fold over basal
1.4
1.3
1.2
1.1
1
0.9
0.8
0.7
0
10
20
30
40
50
60
Time (min)

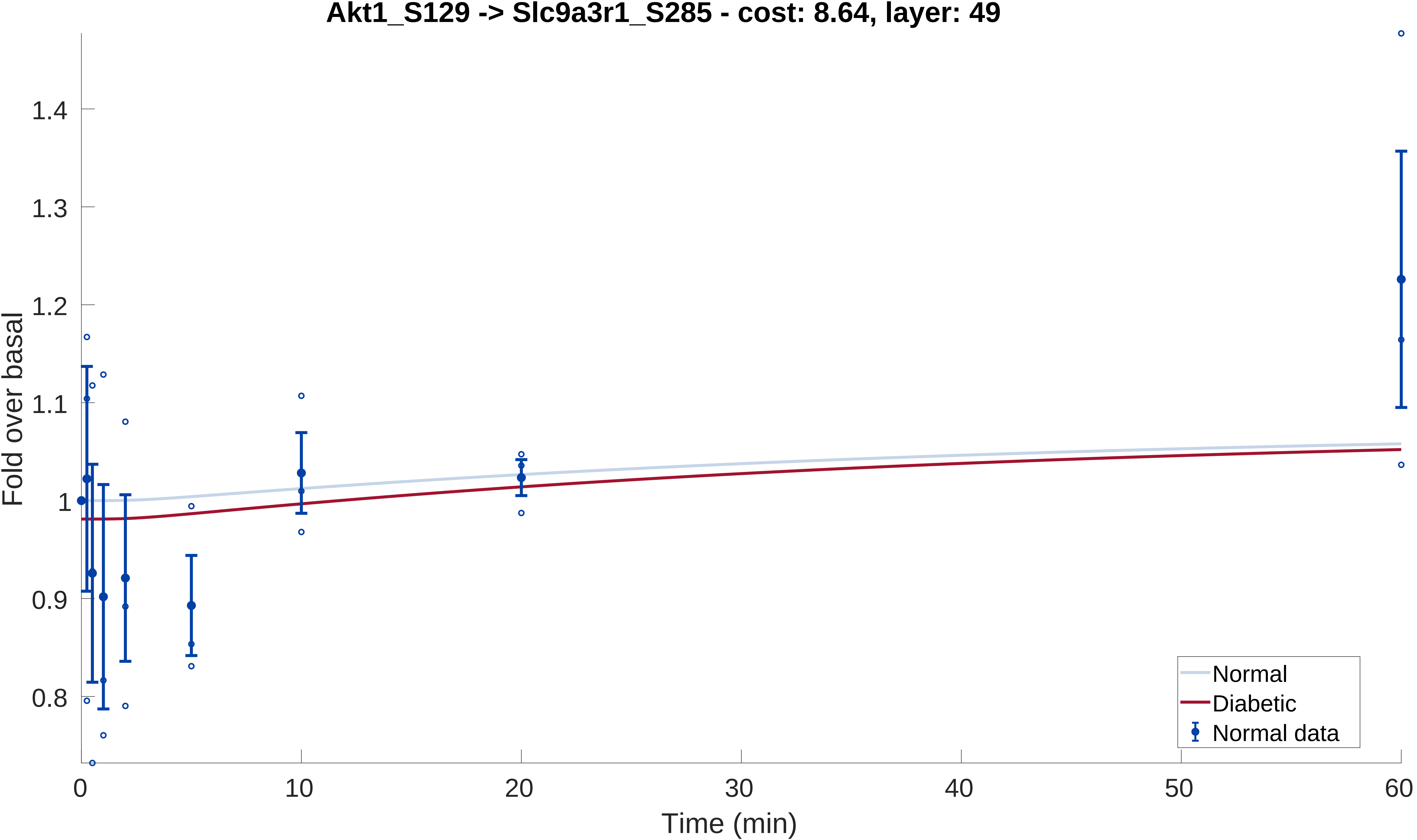
Akt1_S129 -> Slc9a3r1_S285 - cost: 8.64, layer: 49
Fold over basal
1.4
1.3
1.2
1.1
1
0.9
0.8
0
10
20
30
40
50
60
Time (min)
Normal
Diabetic
Normal data

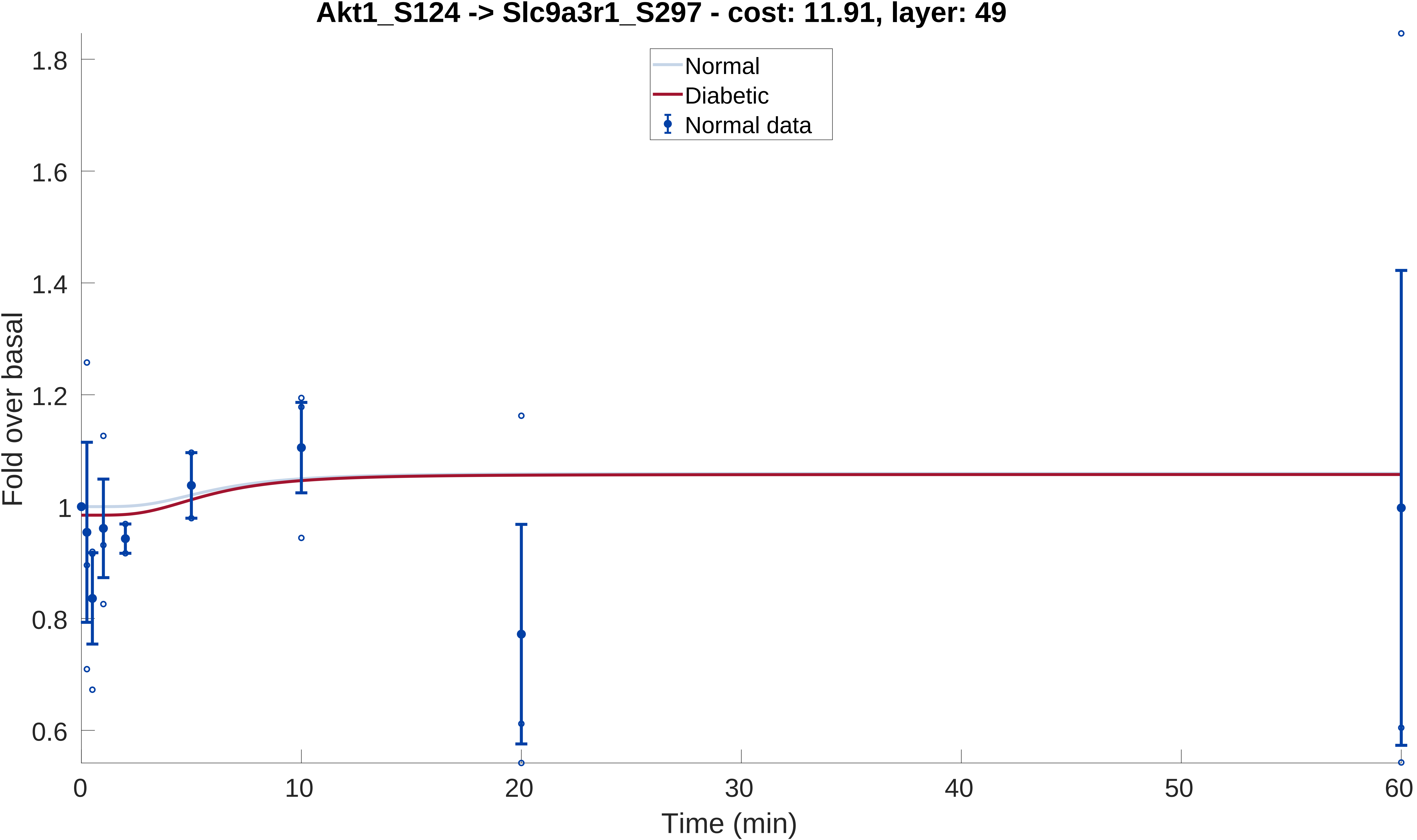

Akt1_S124 -> Slc9a3r1_S297 - cost: 11.91, layer: 49
Normal
Diabetic
Normal data
Fold over basal
1.8
1.6
1.4
1.2
1
0.8
0.6
0
10
20
30
40
50
60
Time (min)

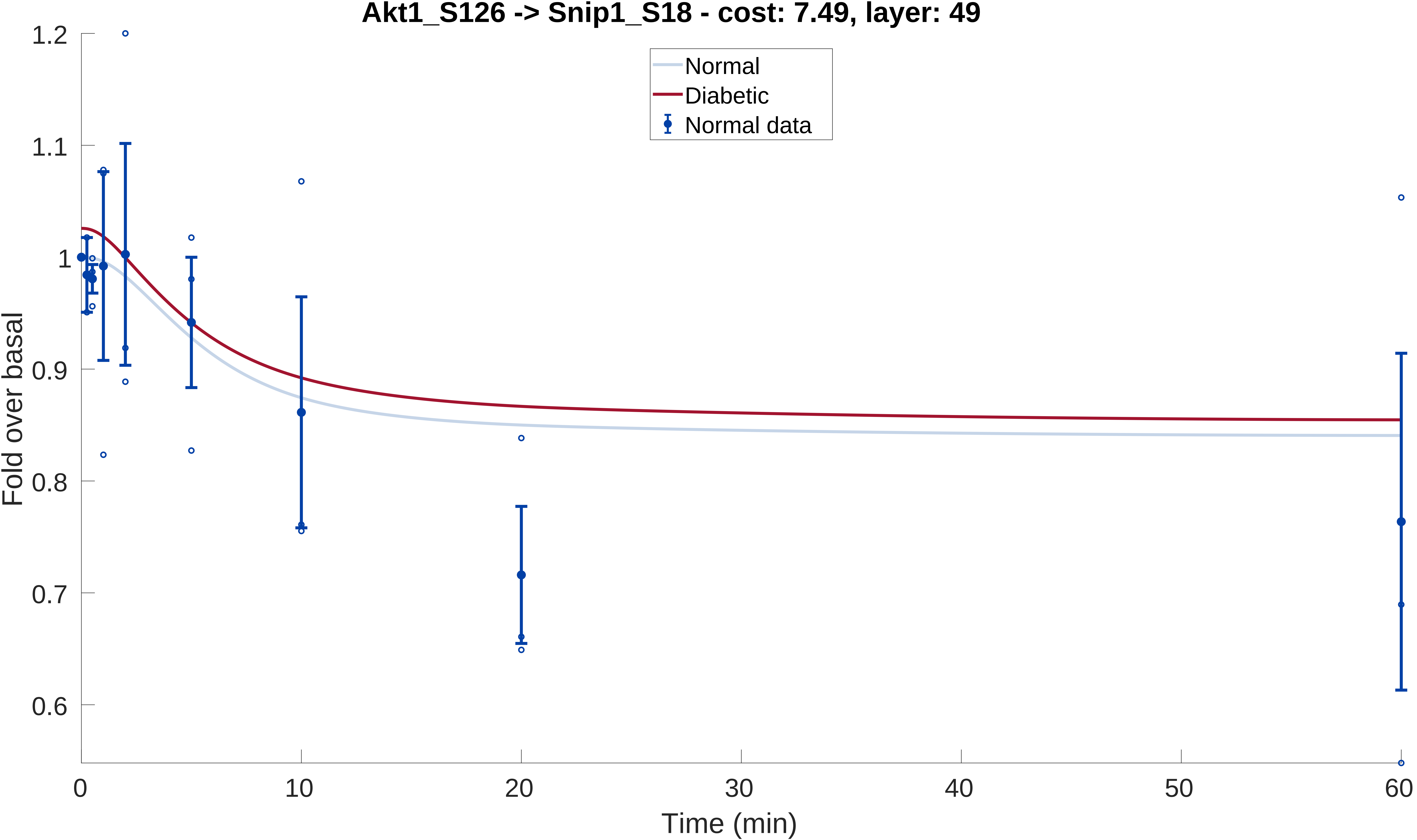
Akt1_S126 -> Snip1_S18 - cost: 7.49, layer: 49
Normal
Diabetic
Normal data
Fold over basal
Time (min)
1.2
1.1
1
0.9
0.8
0.7
0.6
0
10
20
30
40
50
60

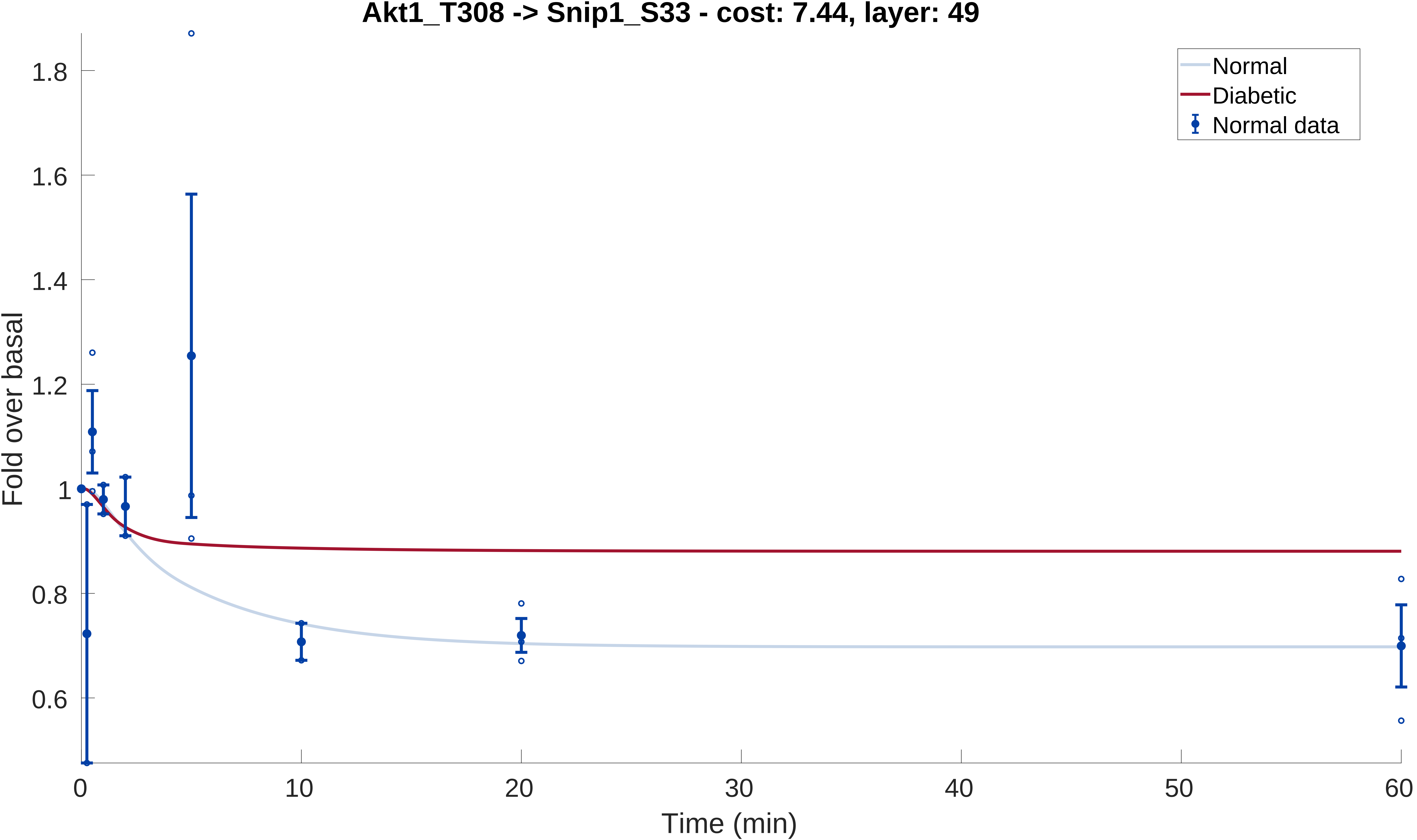

Akt1_T308 -> Snip1_S33 - cost: 7.44, layer: 49
Normal
Diabetic
Normal data
Fold over basal
1.8
1.6
1.4
1.2
1
0.8
0.6
0
10
20
30
40
50
60
Time (min)

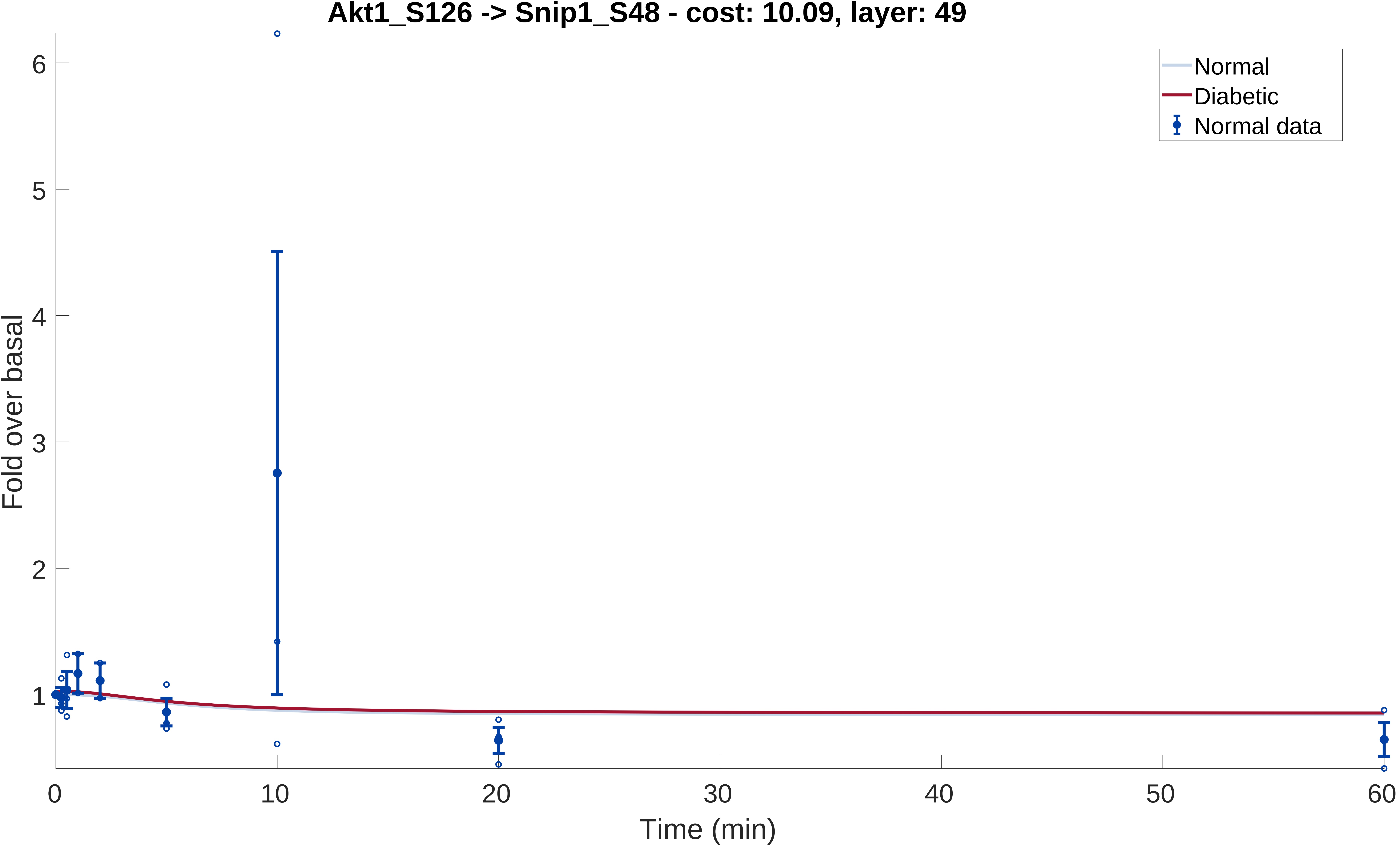
Akt1_S126 -> Snip1_S48 - cost: 10.09, layer: 49
Normal
Diabetic
Normal data
Fold over basal
1
2
3
4
5
6
0
10
20
30
40
50
60
Time (min)

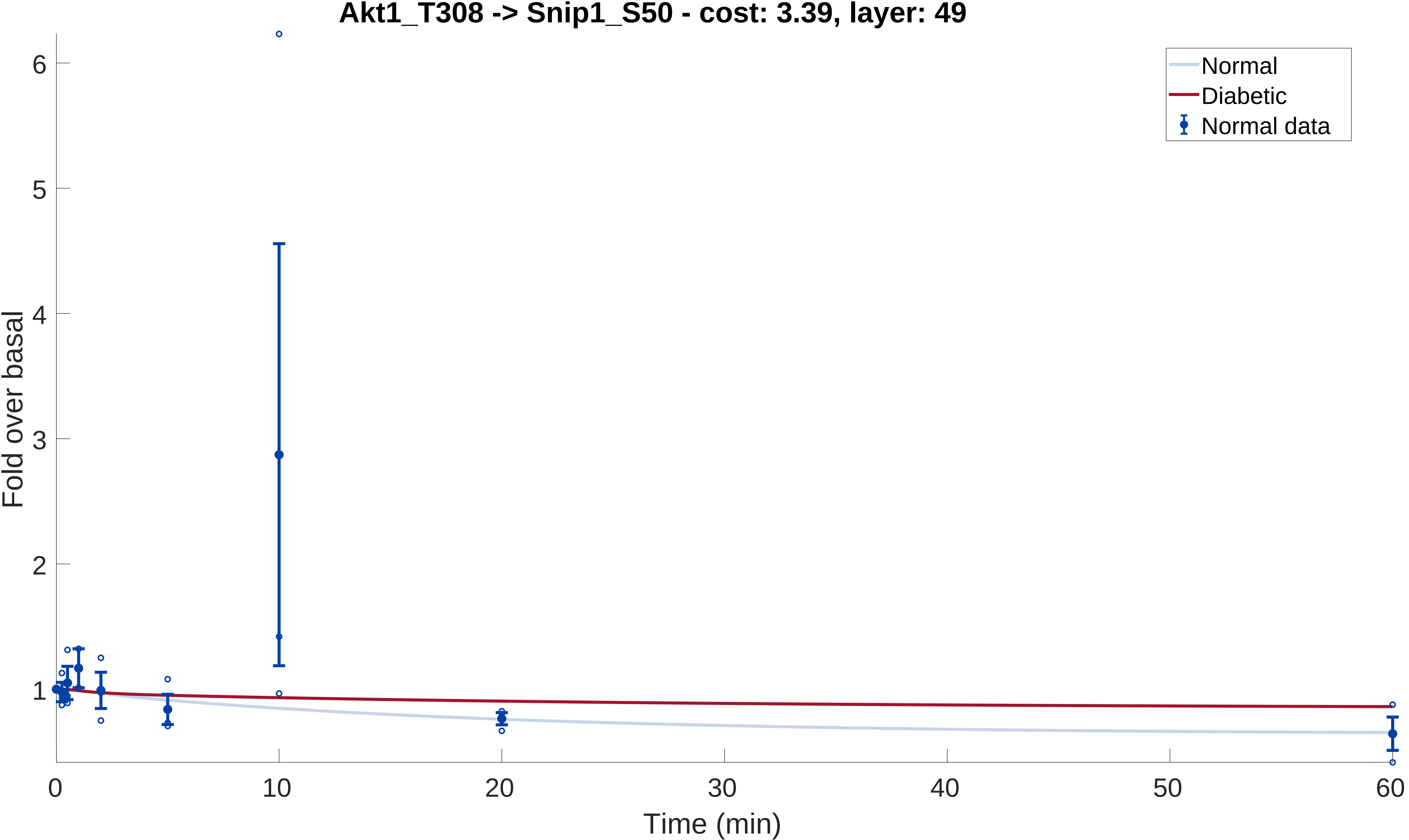
Akt1_T308 -> Snip1_S50 - cost: 3.39, layer: 49
Normal
Diabetic
Normal data
Fold over basal
Time (min)
1
2
3
4
5
6
0
10
20
30
40
50
60

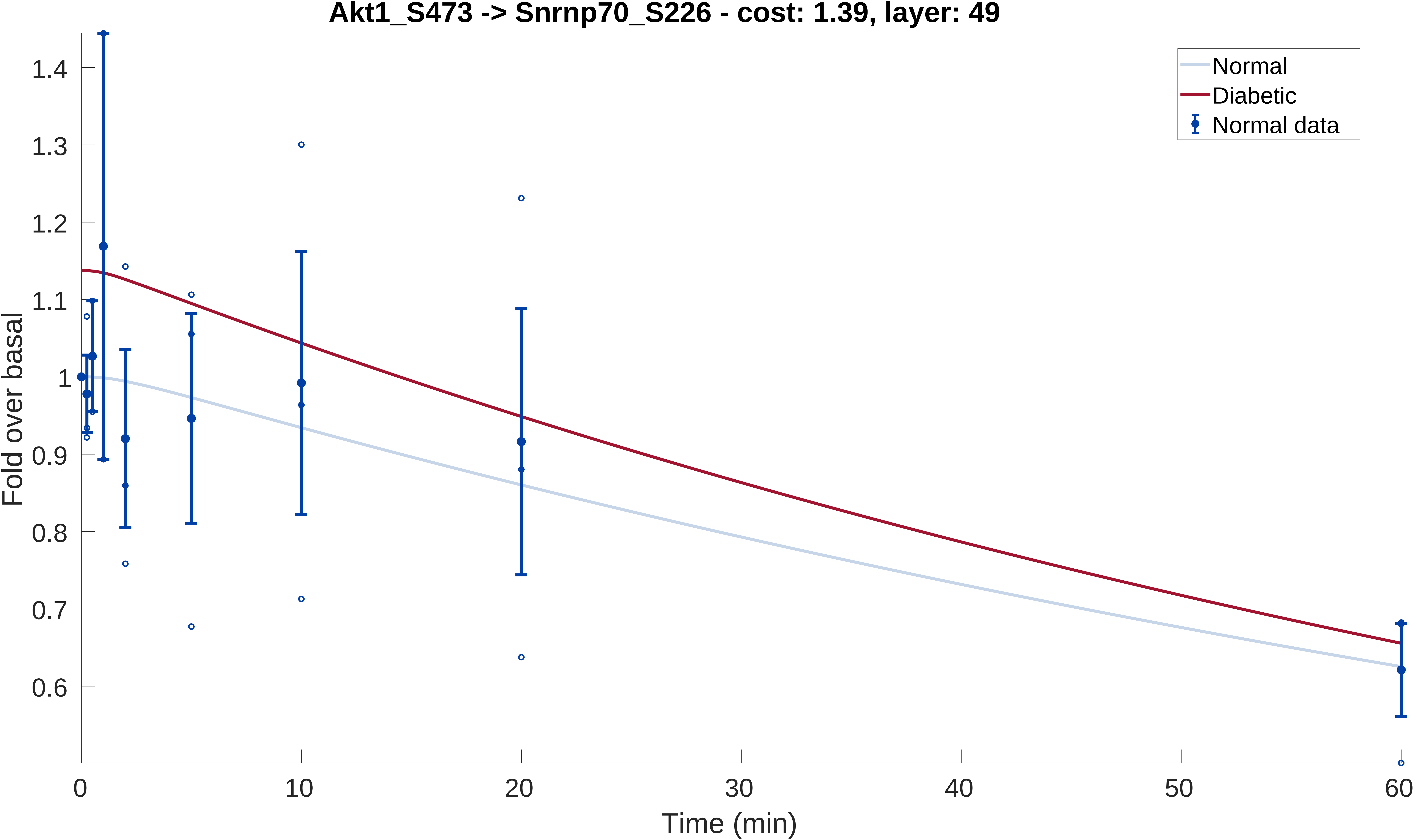

Akt1_S473 -> Snrnp70_S226 - cost: 1.39, layer: 49
Normal
Diabetic
Normal data
Fold over basal
1.4
1.3
1.2
1.1
1
0.9
0.8
0.7
0.6
0
10
20
30
40
50
60
Time (min)

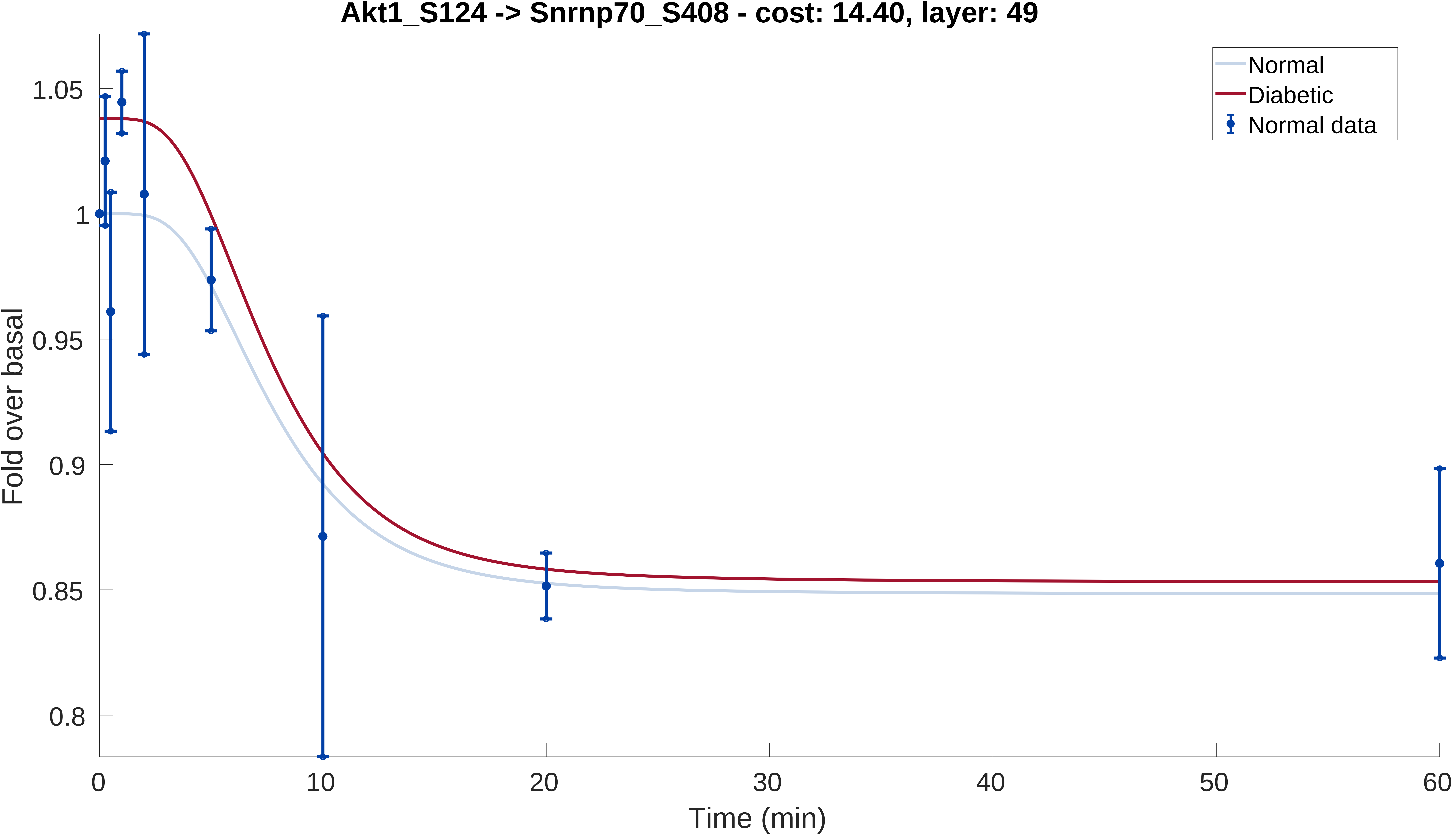

Akt1_S124 -> Snrnp70_S408 - cost: 14.40, layer: 49
Normal
Diabetic
Normal data
Fold over basal
1.05
1
0.95
0.9
0.85
0.8
0
10
20
30
40
50
60
Time (min)

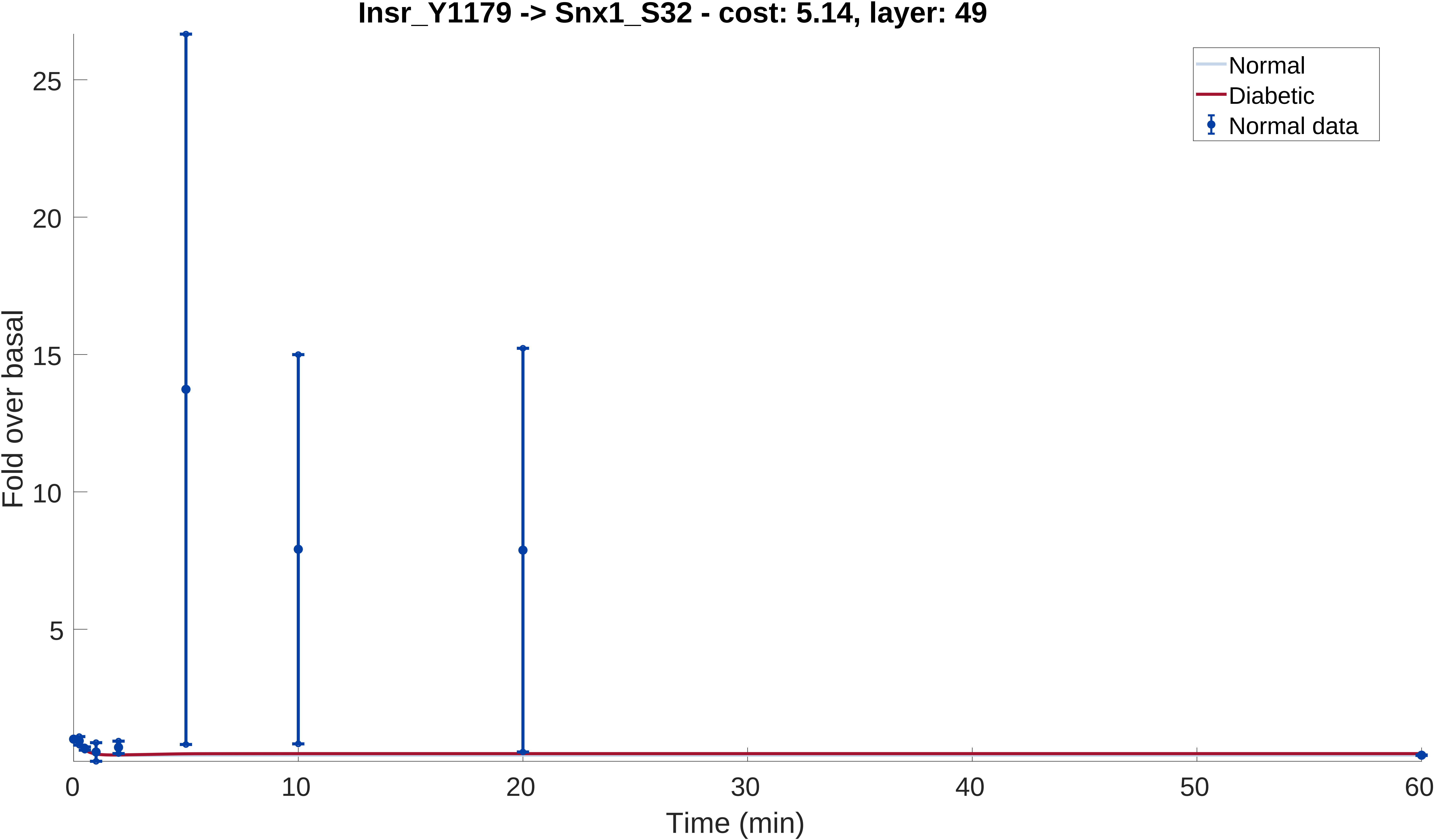
Insr_Y1179 -> Snx1_S32 - cost: 5.14, layer: 49
Normal
Diabetic
Normal data
Fold over basal
25
20
15
10
5
0
10
20
30
40
50
60
Time (min)

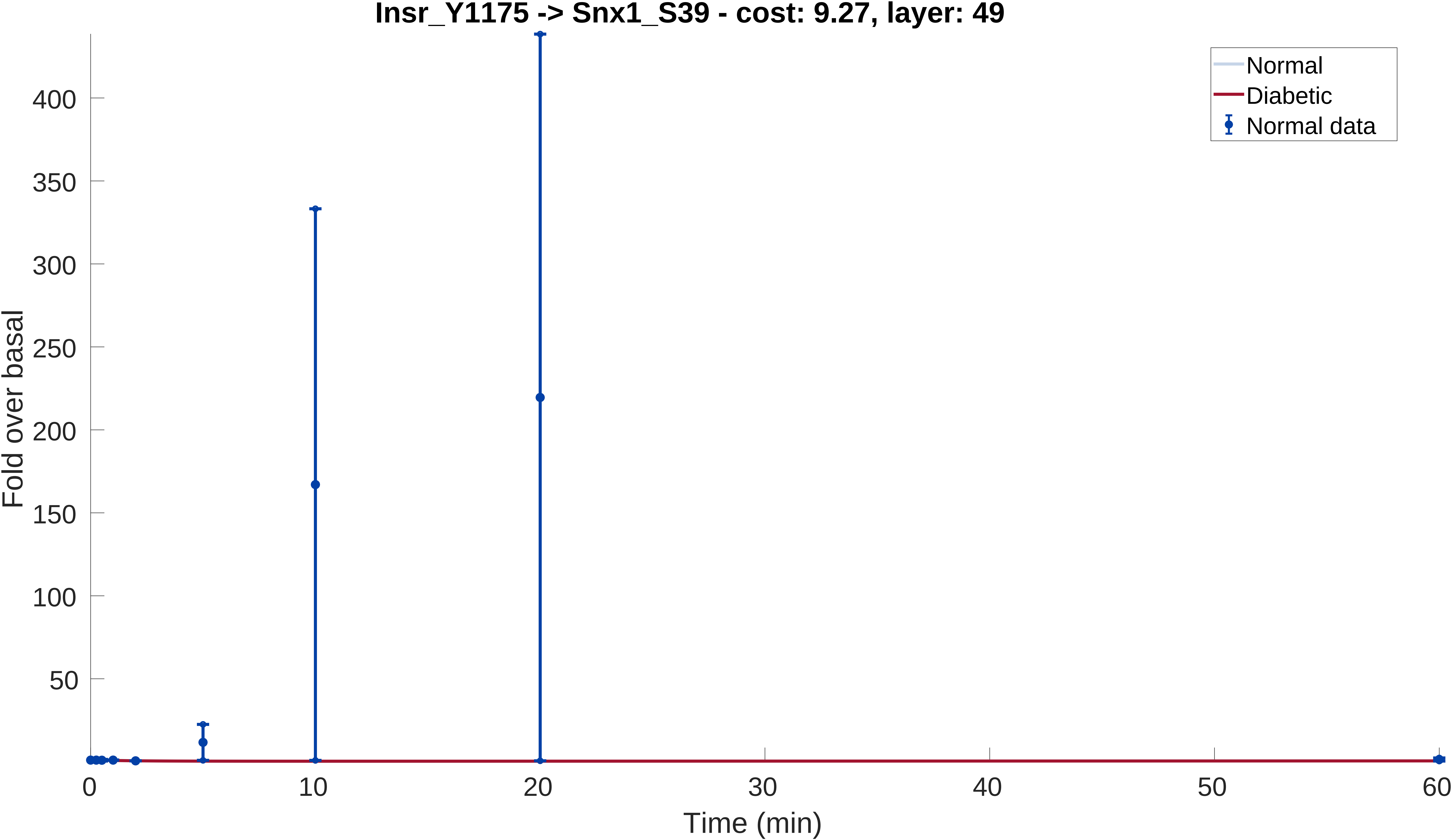
Insr_Y1175 -> Snx1_S39 - cost: 9.27, layer: 49
Normal
Diabetic
Normal data
Fold over basal
400
350
300
250
200
150
100
50
0
10
20
30
40
50
60
Time (min)

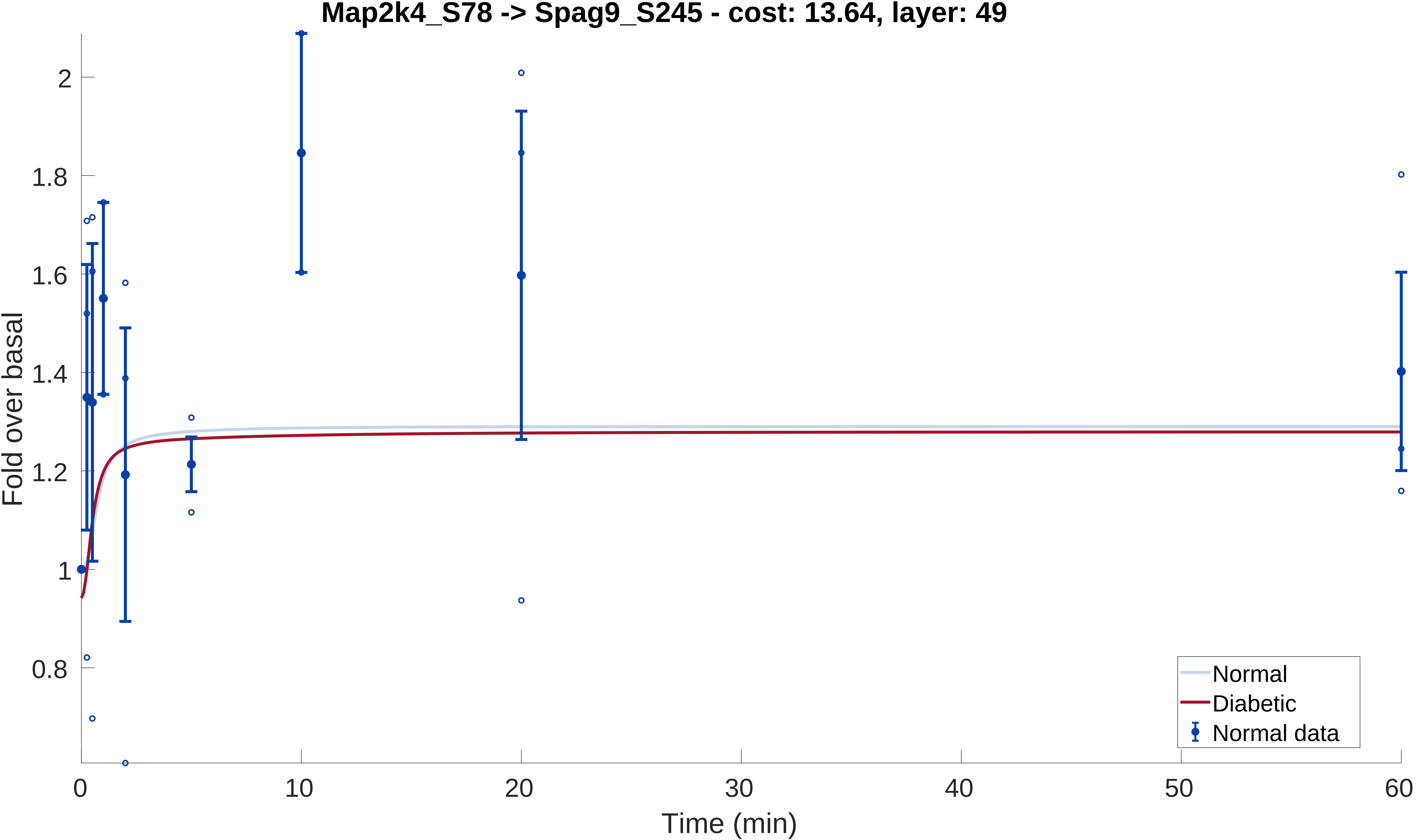

Map2k4_S78 -> Spag9_S245 - cost: 13.64, layer: 49
Fold over basal
0.8
1
1.2
1.4
1.6
1.8
2
0
10
20
30
40
50
60
Time (min)
Normal
Diabetic
Normal data

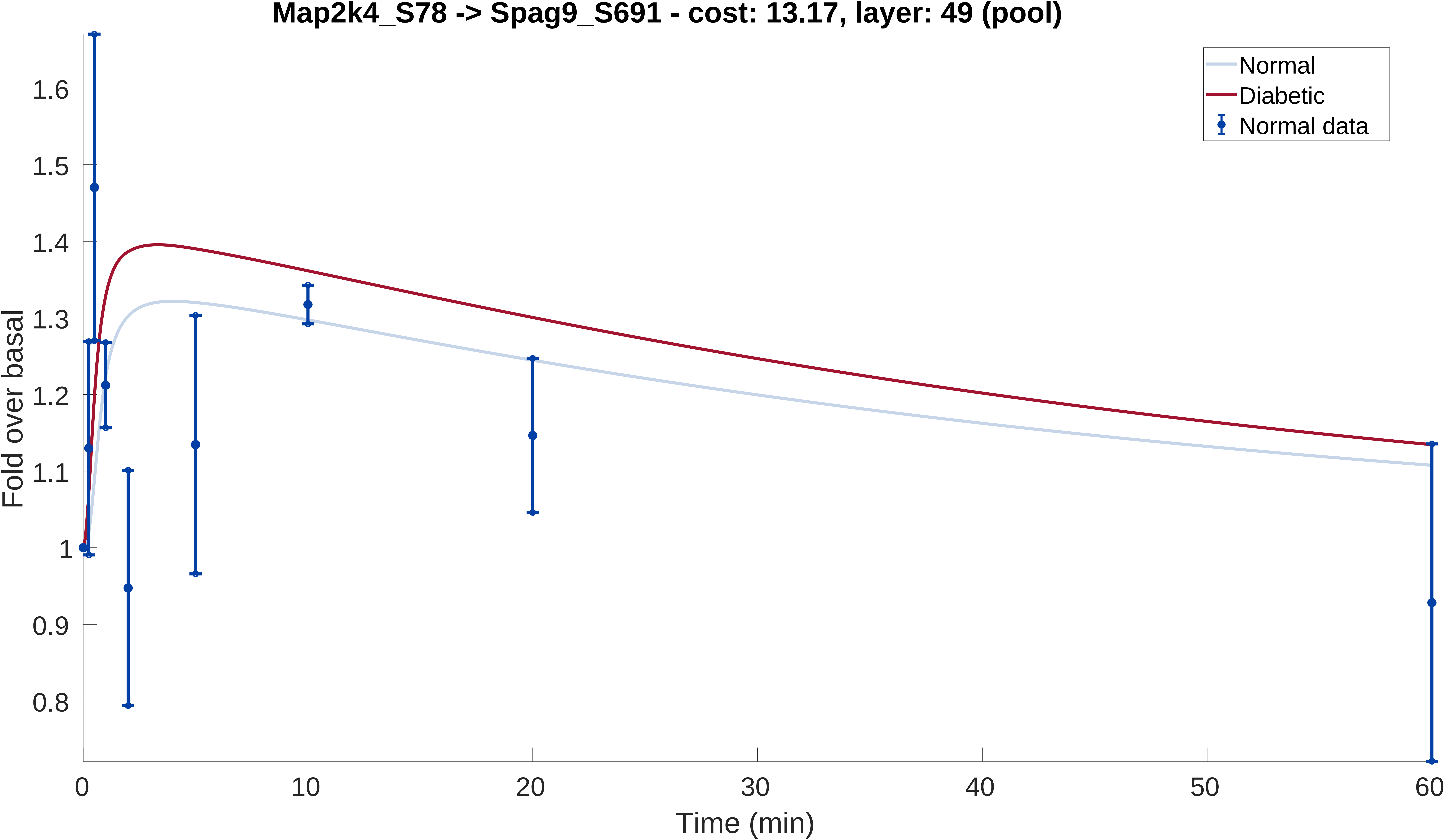

Map2k4_S78 -> Spag9_S691 - cost: 13.17, layer: 49 (pool)
Normal
Diabetic
Normal data
Fold over basal
1.6
1.5
1.4
1.3
1.2
1.1
1
0.9
0.8
0
10
20
30
40
50
60
Time (min)

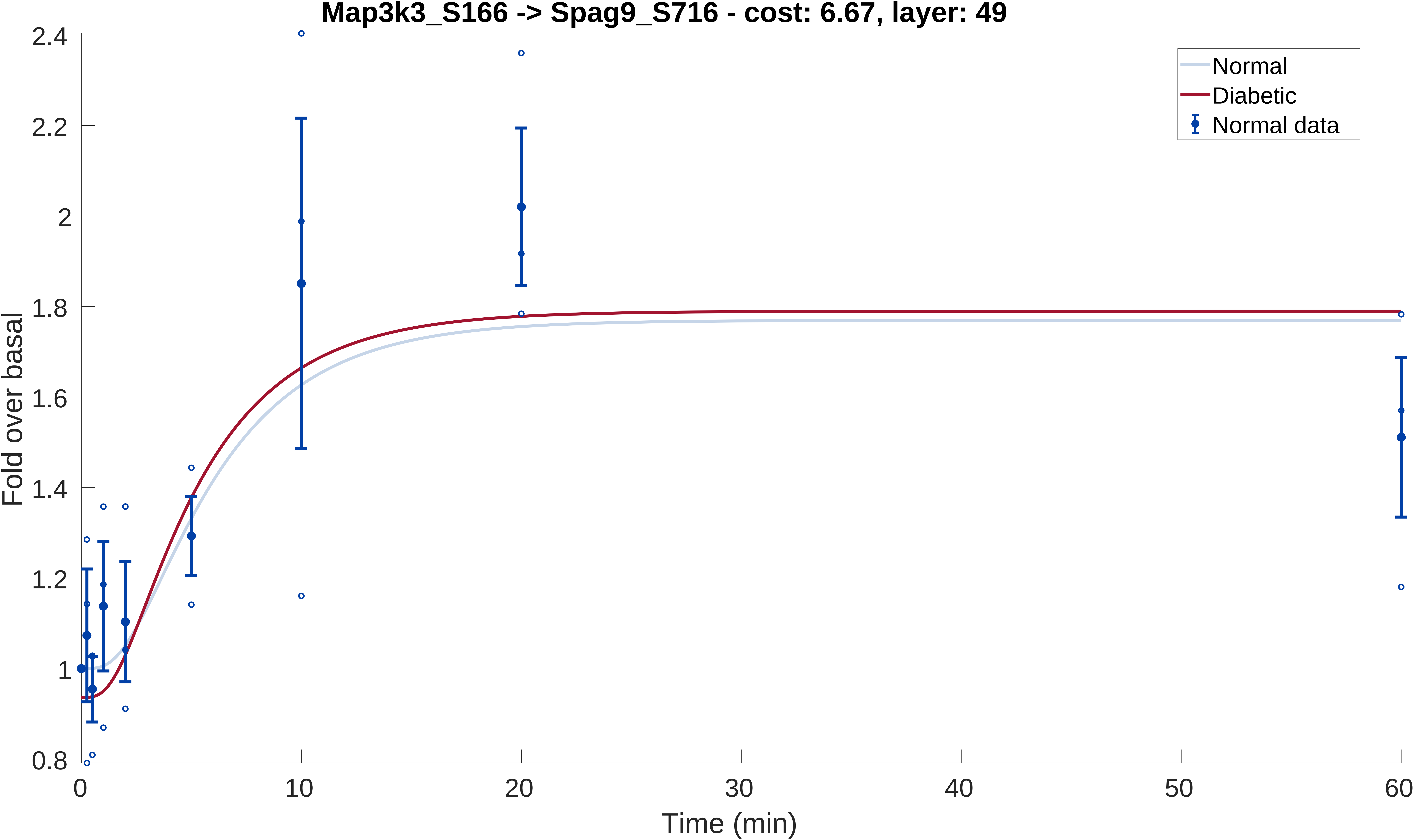

Map3k3_S166 -> Spag9_S716 - cost: 6.67, layer: 49
Normal
Diabetic
Normal data
Fold over basal
Time (min)
0.8
1
1.2
1.4
1.6
1.8
2
2.2
2.4
0
10
20
30
40
50
60

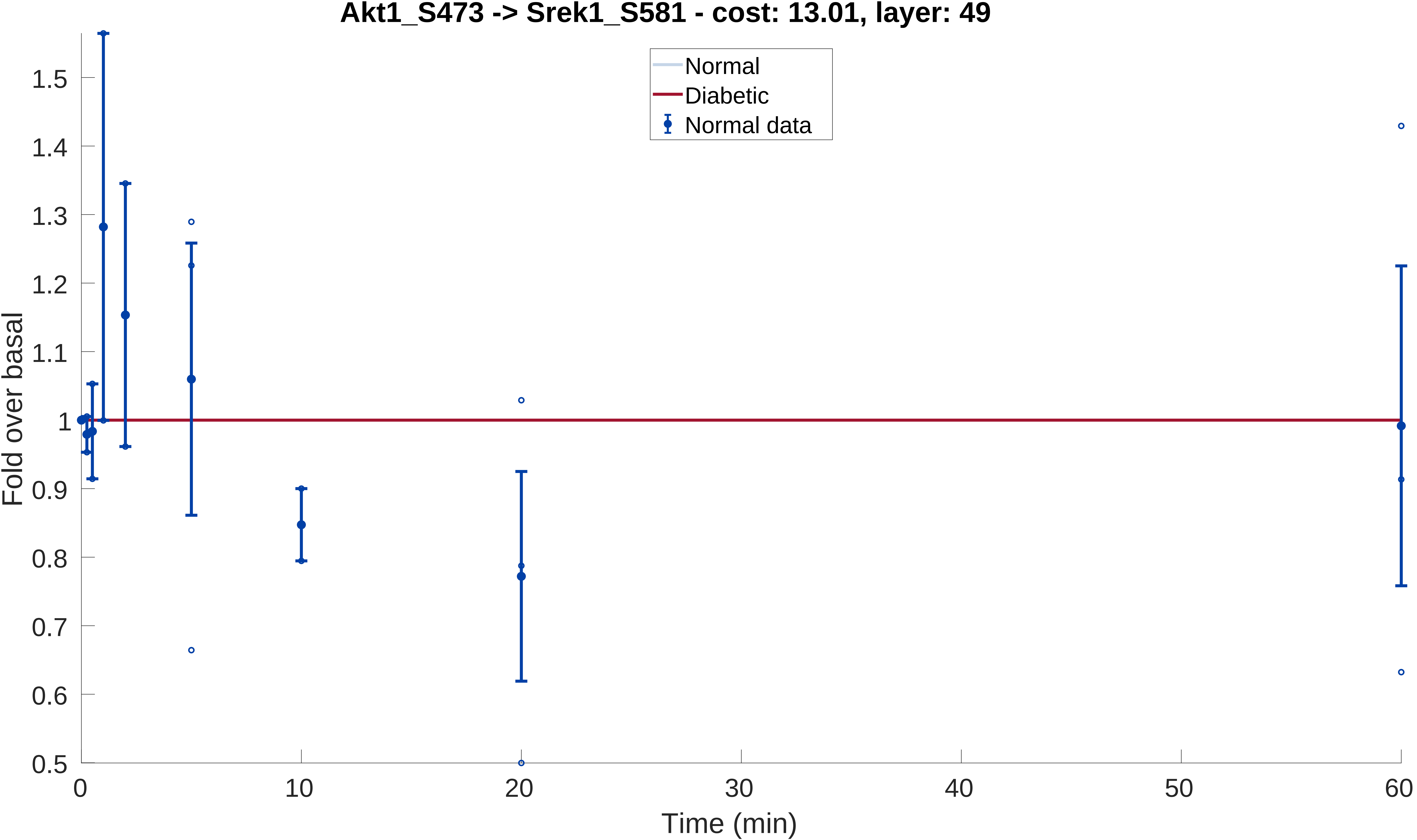

Akt1_S473 -> Srek1_S581 - cost: 13.01, layer: 49
Normal
Diabetic
Normal data
Fold over basal
Time (min)
0.5
0.6
0.7
0.8
0.9
1
1.1
1.2
1.3
1.4
1.5
0
10
20
30
40
50
60

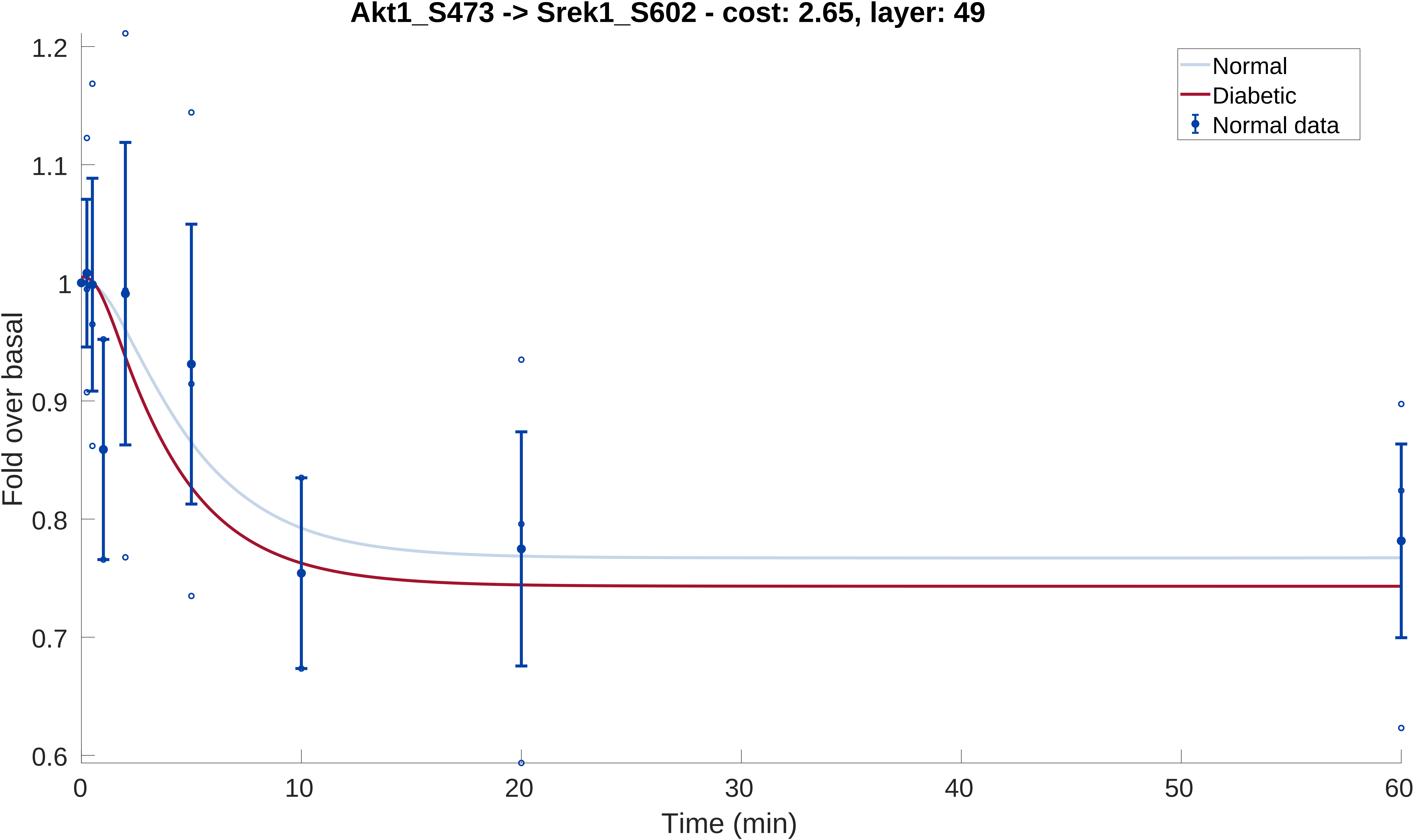

Akt1_S473 -> Srek1_S602 - cost: 2.65, layer: 49
Normal
Diabetic
Normal data
Fold over basal
Time (min)
1.2
1.1
1
0.9
0.8
0.7
0.6
0
10
20
30
40
50
60

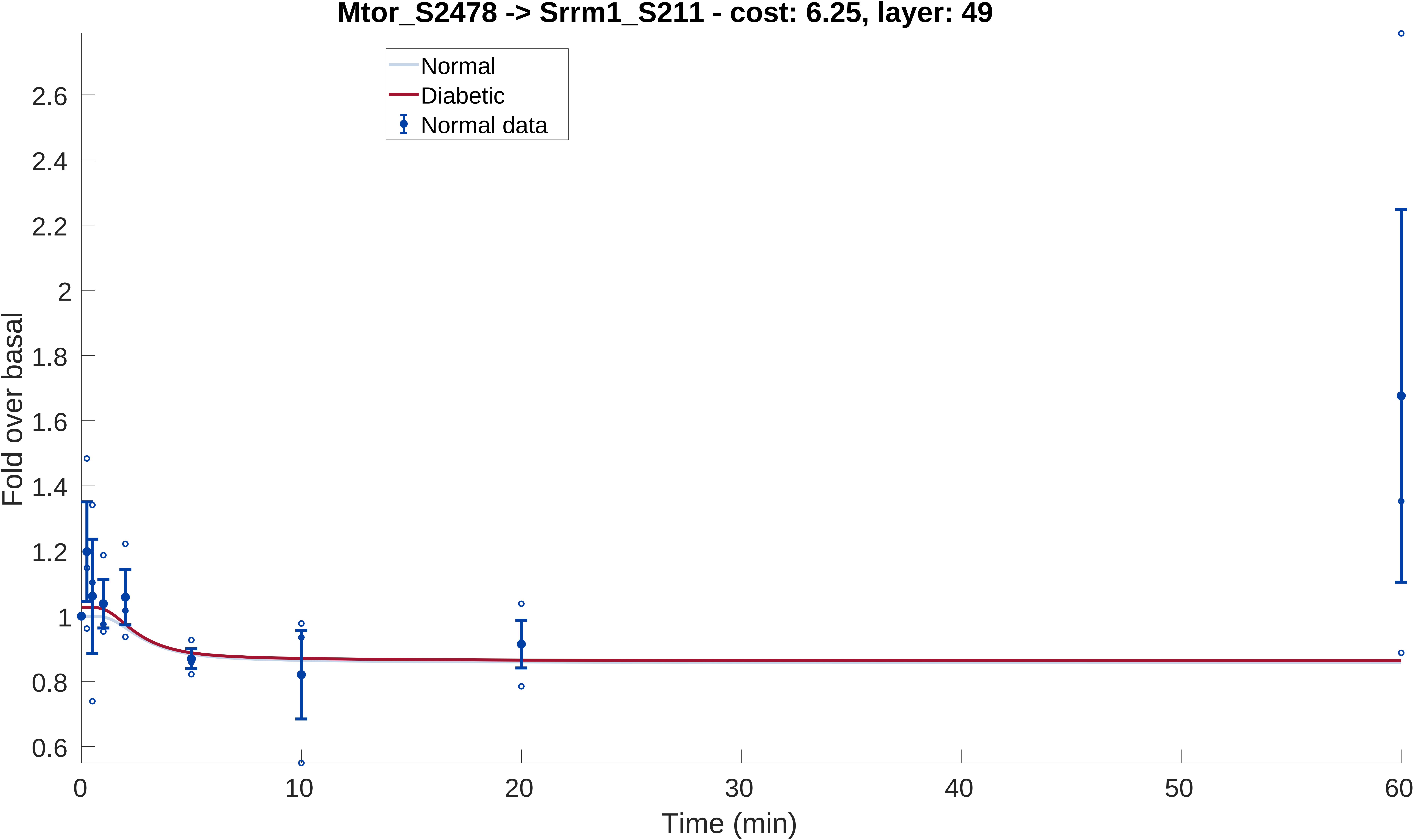

Mtor_S2478 -> Srrm1_S211 - cost: 6.25, layer: 49
Normal
Diabetic
Normal data
Fold over basal
2.6
2.4
2.2
2
1.8
1.6
1.4
1.2
1
0.8
0.6
0
10
20
30
40
50
60
Time (min)

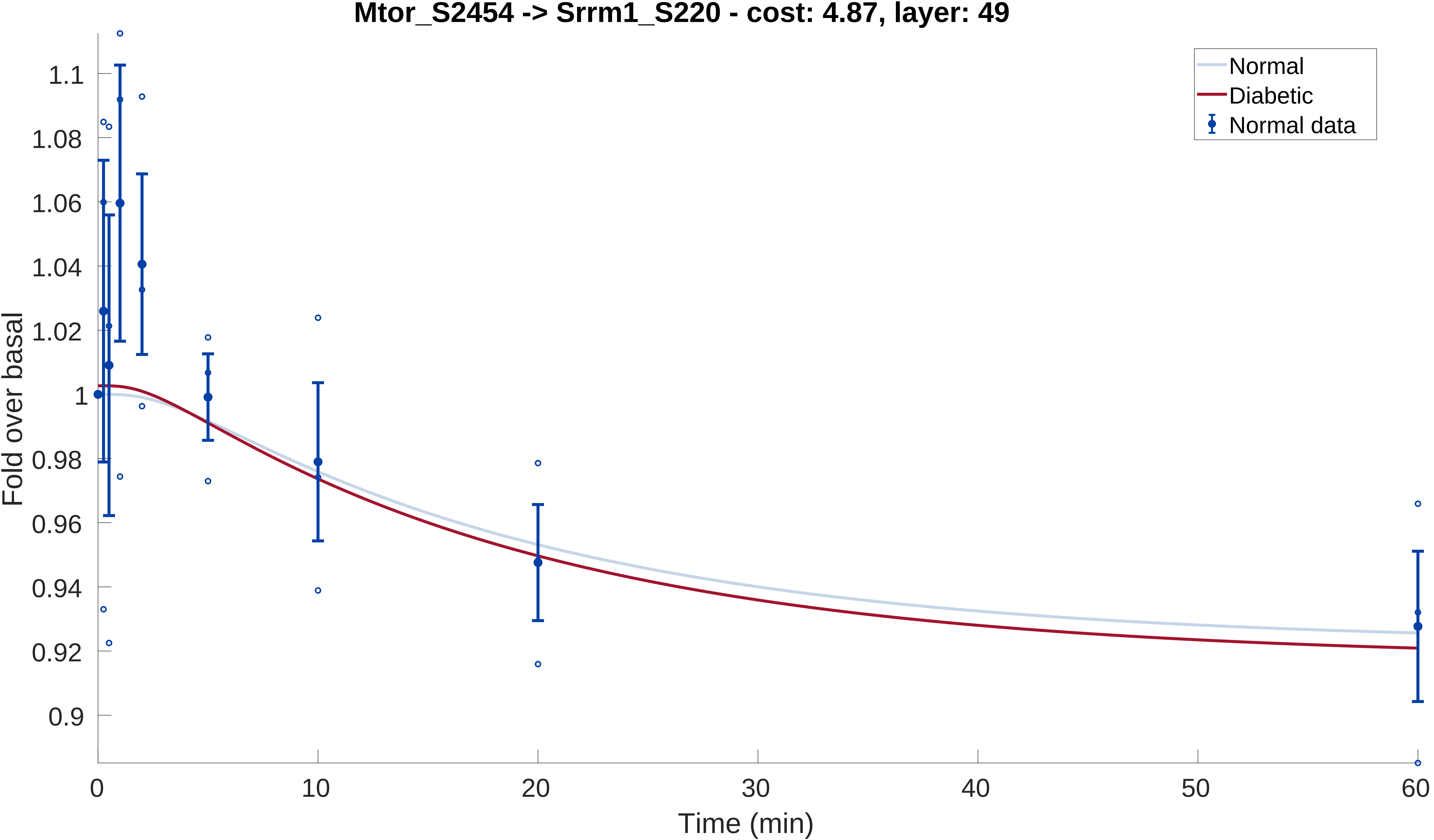

Mtor_S2454 -> Srrm1_S220 - cost: 4.87, layer: 49
Normal
Diabetic
Normal data
Fold over basal
1.1
1.08
1.06
1.04
1.02
1
0.98
0.96
0.94
0.92
0.9
0
10
20
30
40
50
60
Time (min)

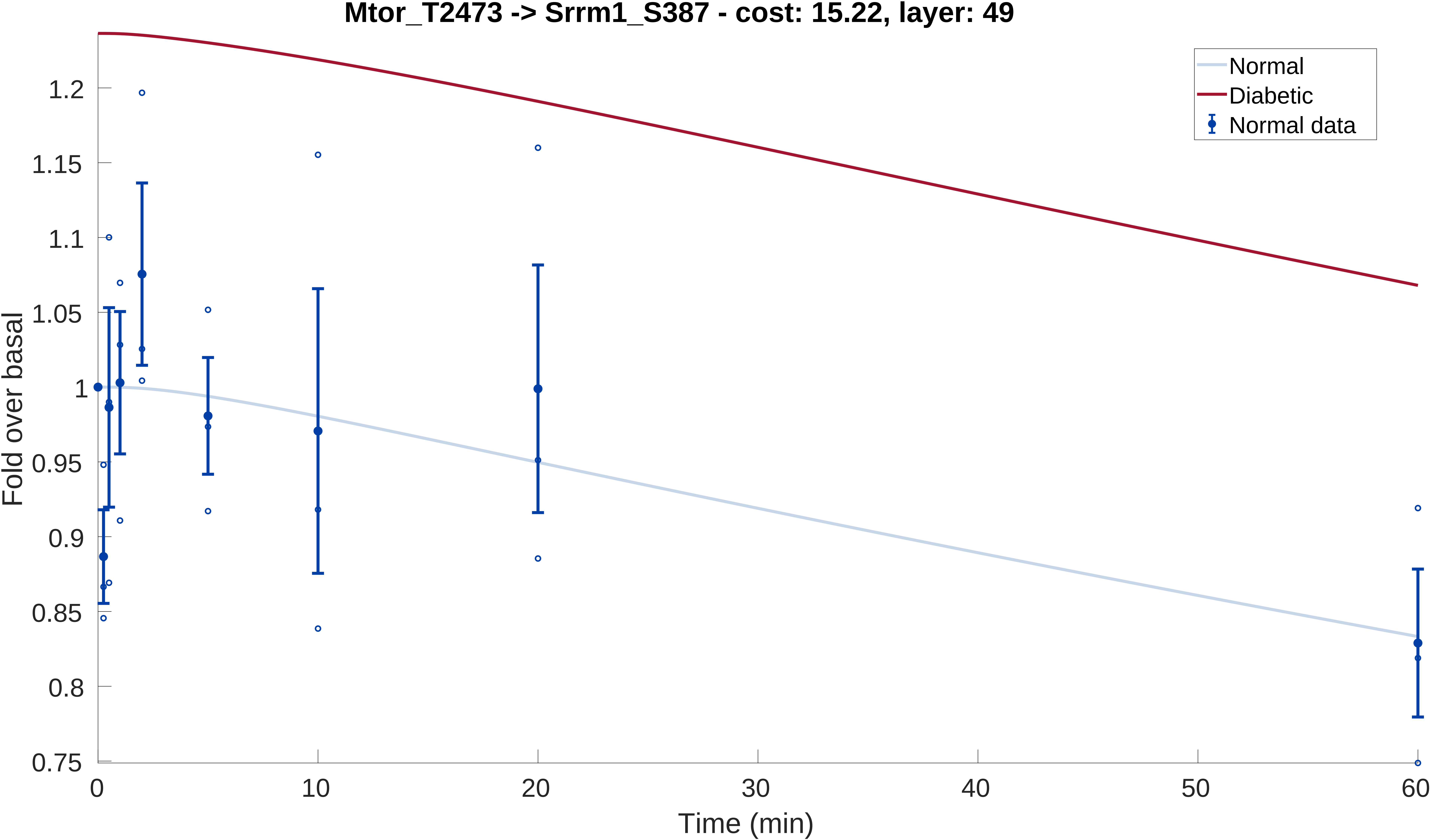
Mtor_T2473 -> Srrm1_S387 - cost: 15.22, layer: 49
Normal
Diabetic
Normal data
Fold over basal
1.2
1.15
1.1
1.05
1
0.95
0.9
0.85
0.8
0.75
0
10
20
30
40
50
60
Time (min)

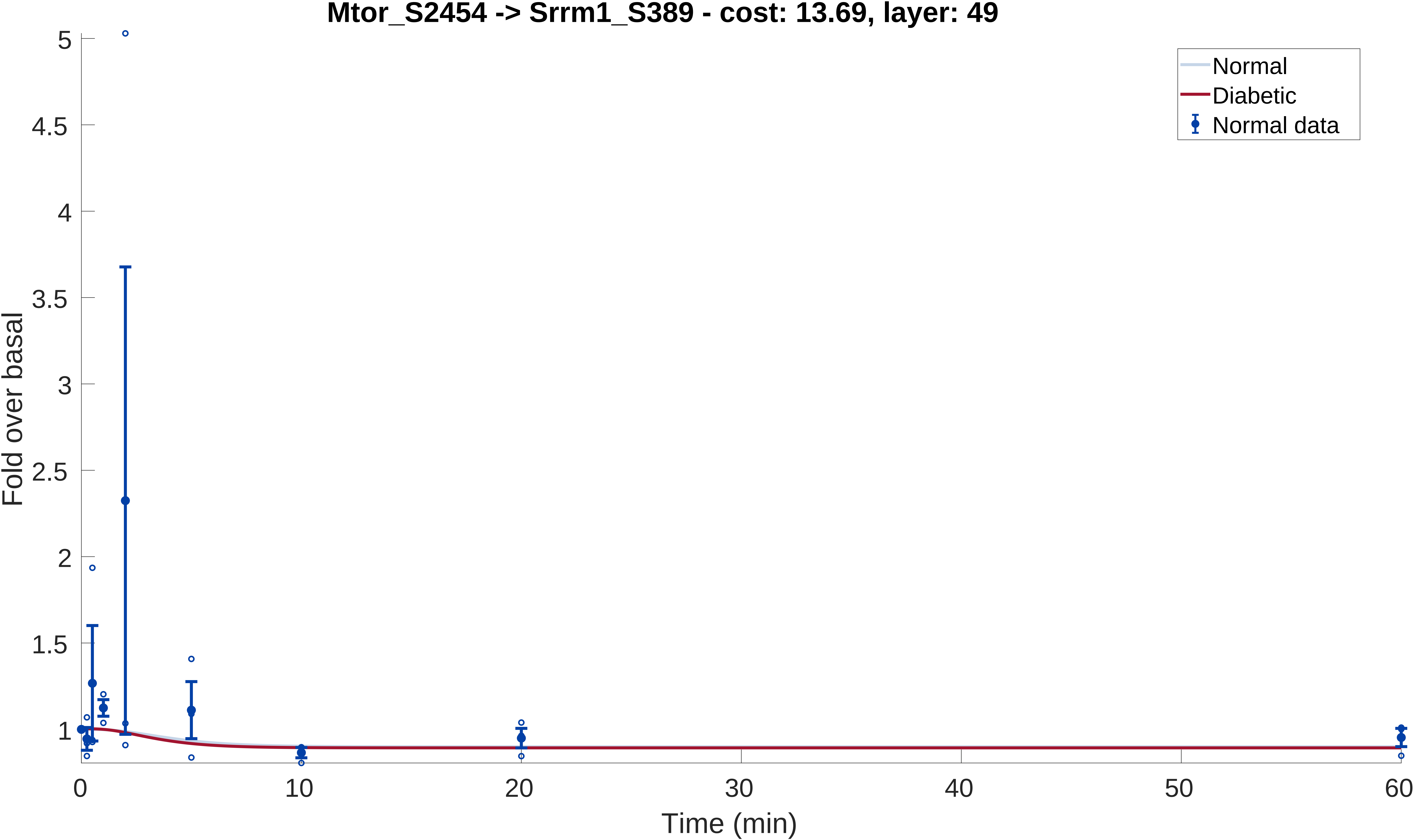

Mtor_S2454 -> Srrm1_S389 - cost: 13.69, layer: 49
Fold over basal
Time (min)
Normal
Diabetic
Normal data
5
4.5
4
3.5
3
2.5
2
1.5
1
0
10
20
30
40
50
60

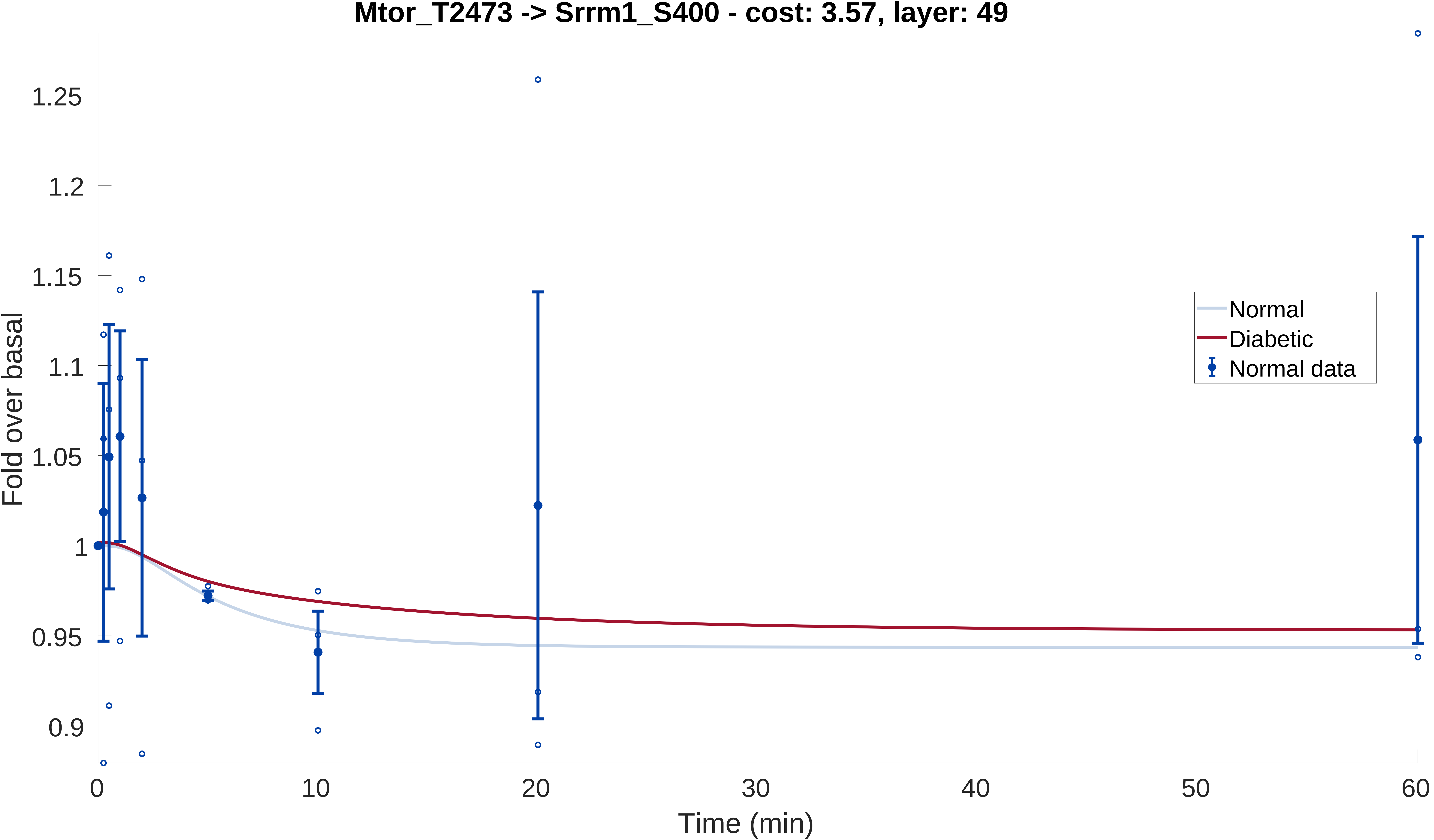
Mtor_T2473 -> Srrm1_S400 - cost: 3.57, layer: 49
Fold over basal
1.25
1.2
1.15
1.1
1.05
1
0.95
0.9
0
10
20
30
40
50
60
Time (min)
Normal
Diabetic
Normal data

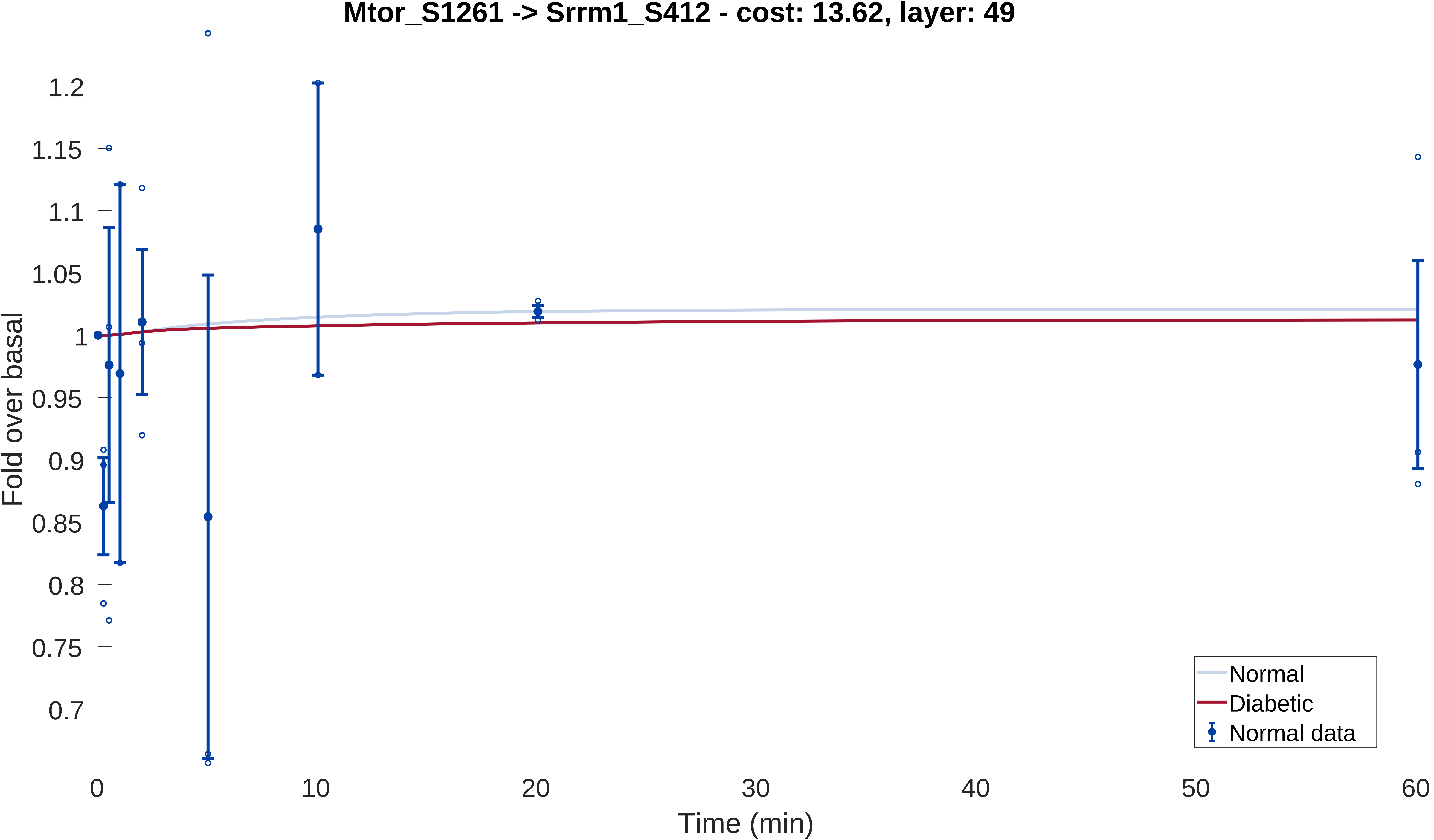

Mtor_S1261 -> Srrm1_S412 - cost: 13.62, layer: 49
Fold over basal
1.2
1.15
1.1
1.05
1
0.95
0.9
0.85
0.8
0.75
0.7
Time (min)
0
10
20
30
40
50
60
Normal
Diabetic
Normal data

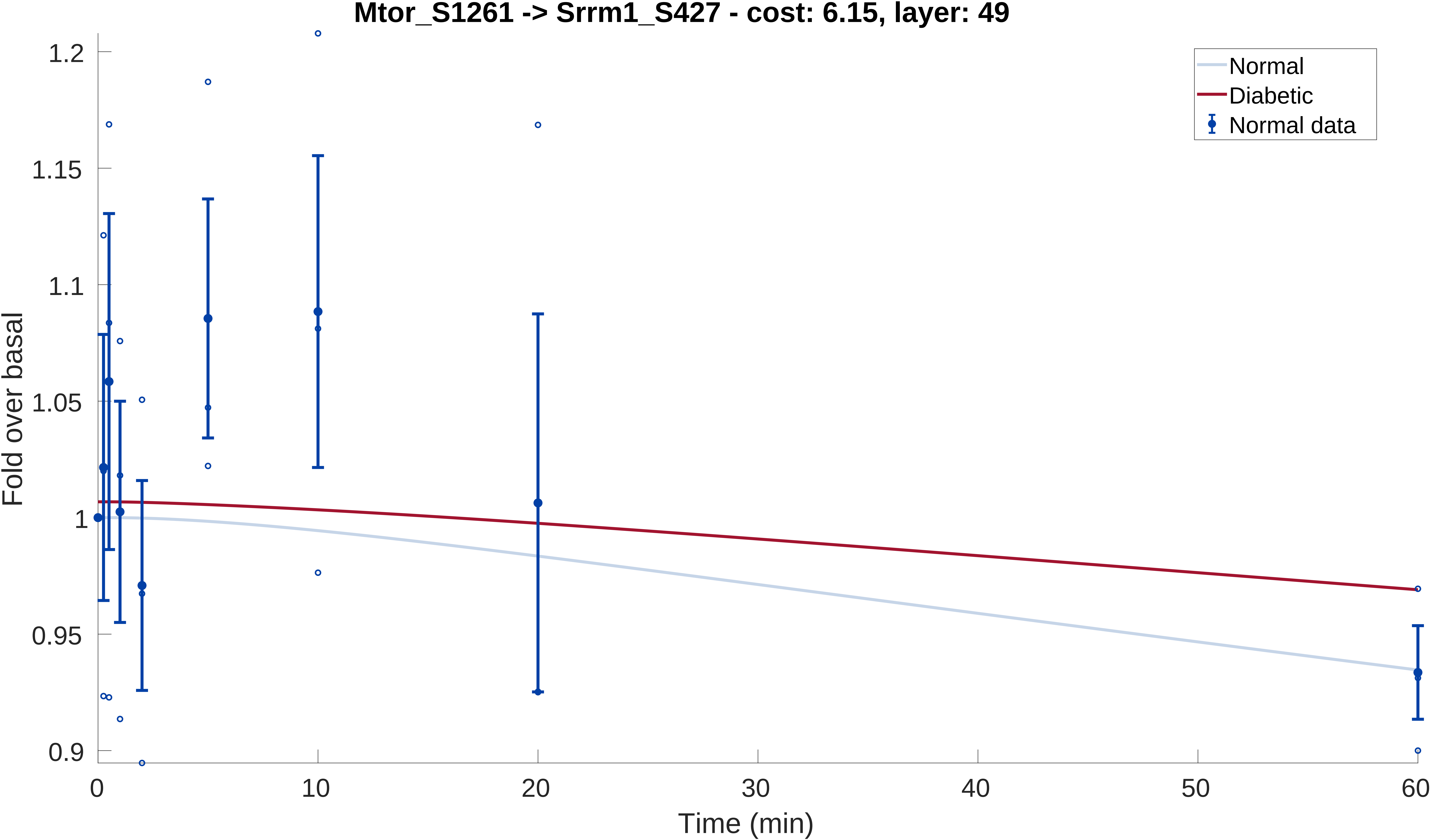

Mtor_S1261 -> Srrm1_S427 - cost: 6.15, layer: 49
Normal
Diabetic
Normal data
Fold over basal
Time (min)
1.2
1.15
1.1
1.05
1
0.95
0.9
0
10
20
30
40
50
60

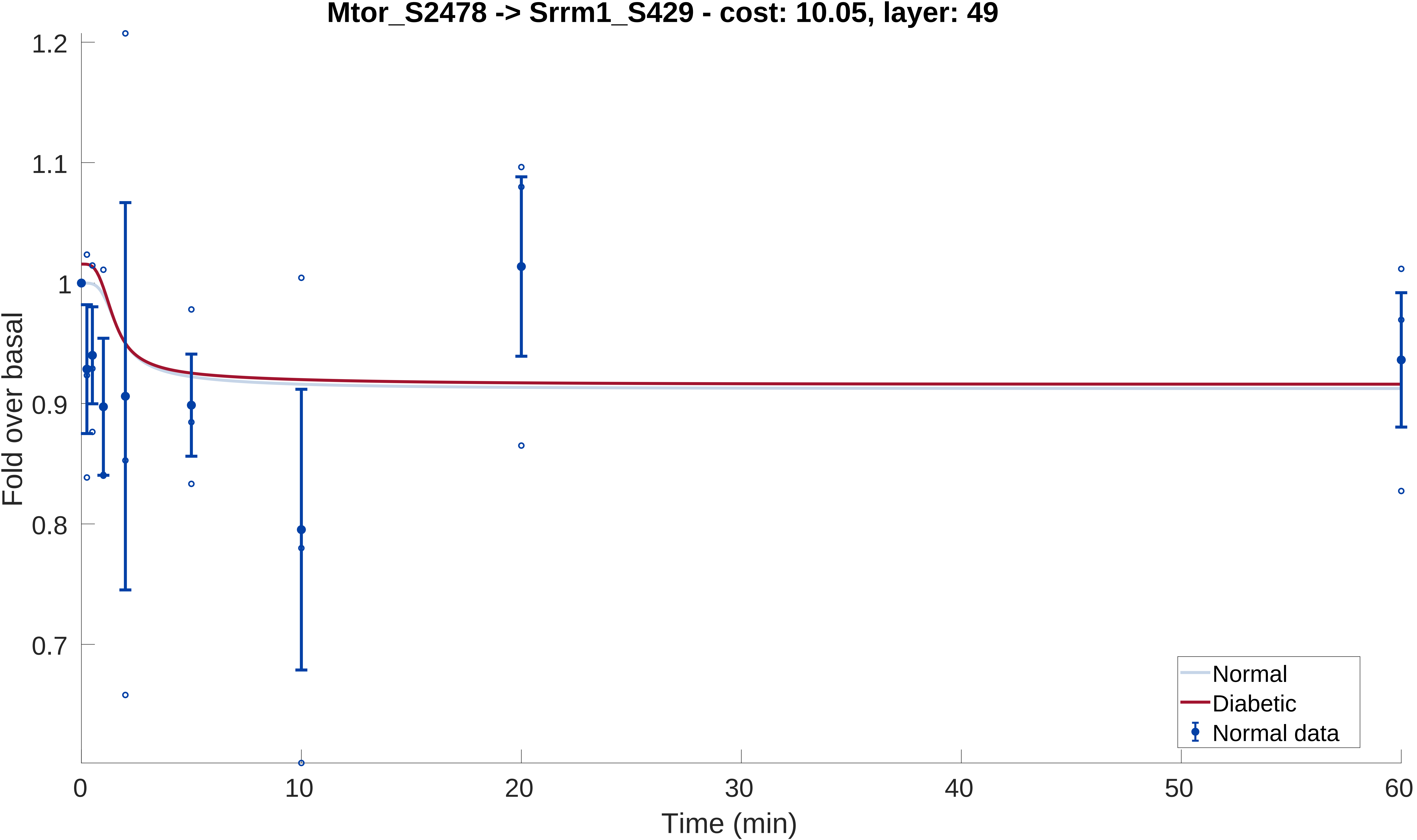

Mtor_S2478 -> Srrm1_S429 - cost: 10.05, layer: 49
Fold over basal
1.2
1.1
1
0.9
0.8
0.7
0
10
20
30
40
50
60
Time (min)
Normal
Diabetic
Normal data

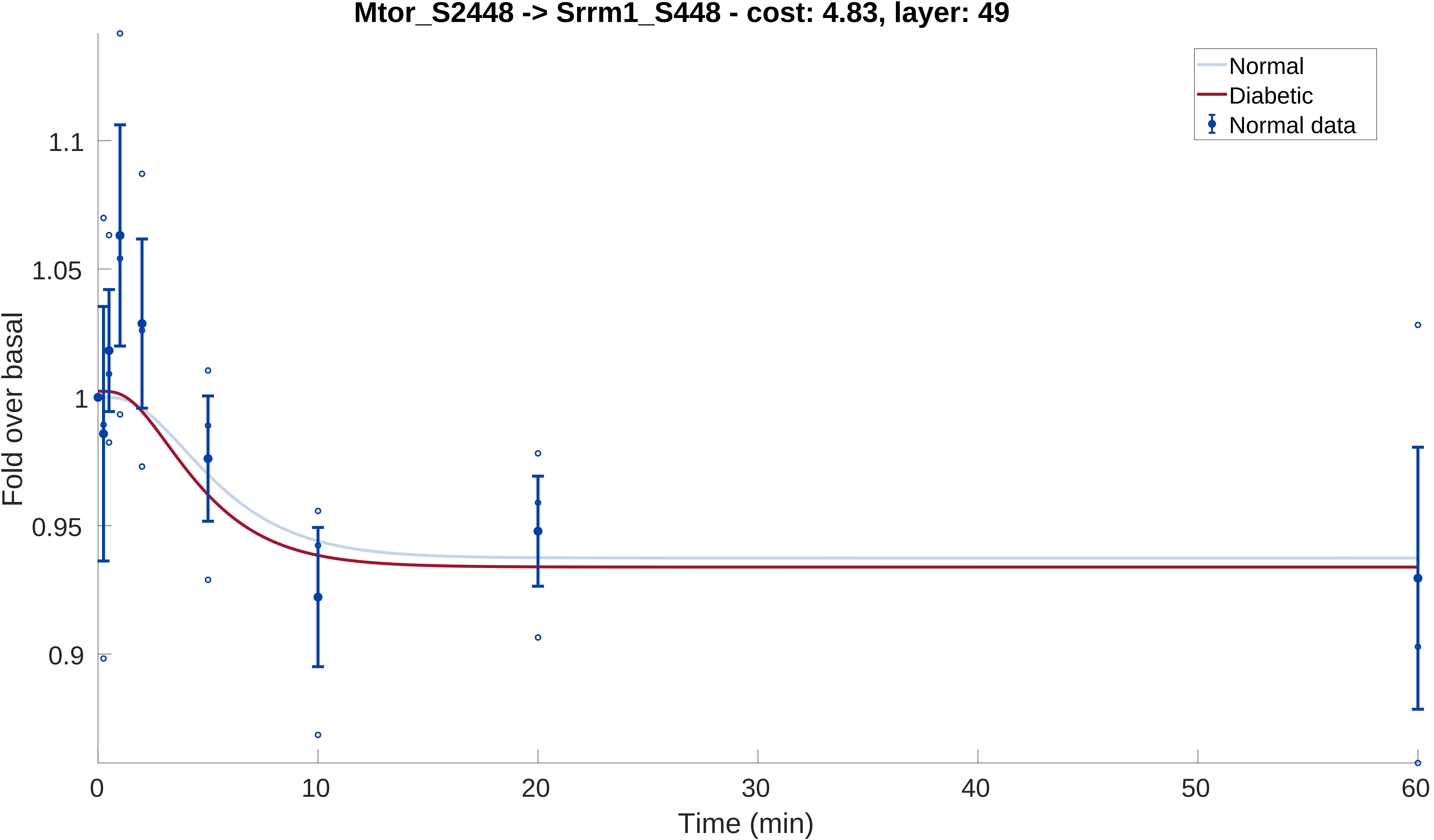
Mtor_S2448 -> Srrm1_S448 - cost: 4.83, layer: 49
Normal
Diabetic
Normal data
Fold over basal
1.1
1.05
1
0.95
0.9
0
10
20
30
40
50
60
Time (min)

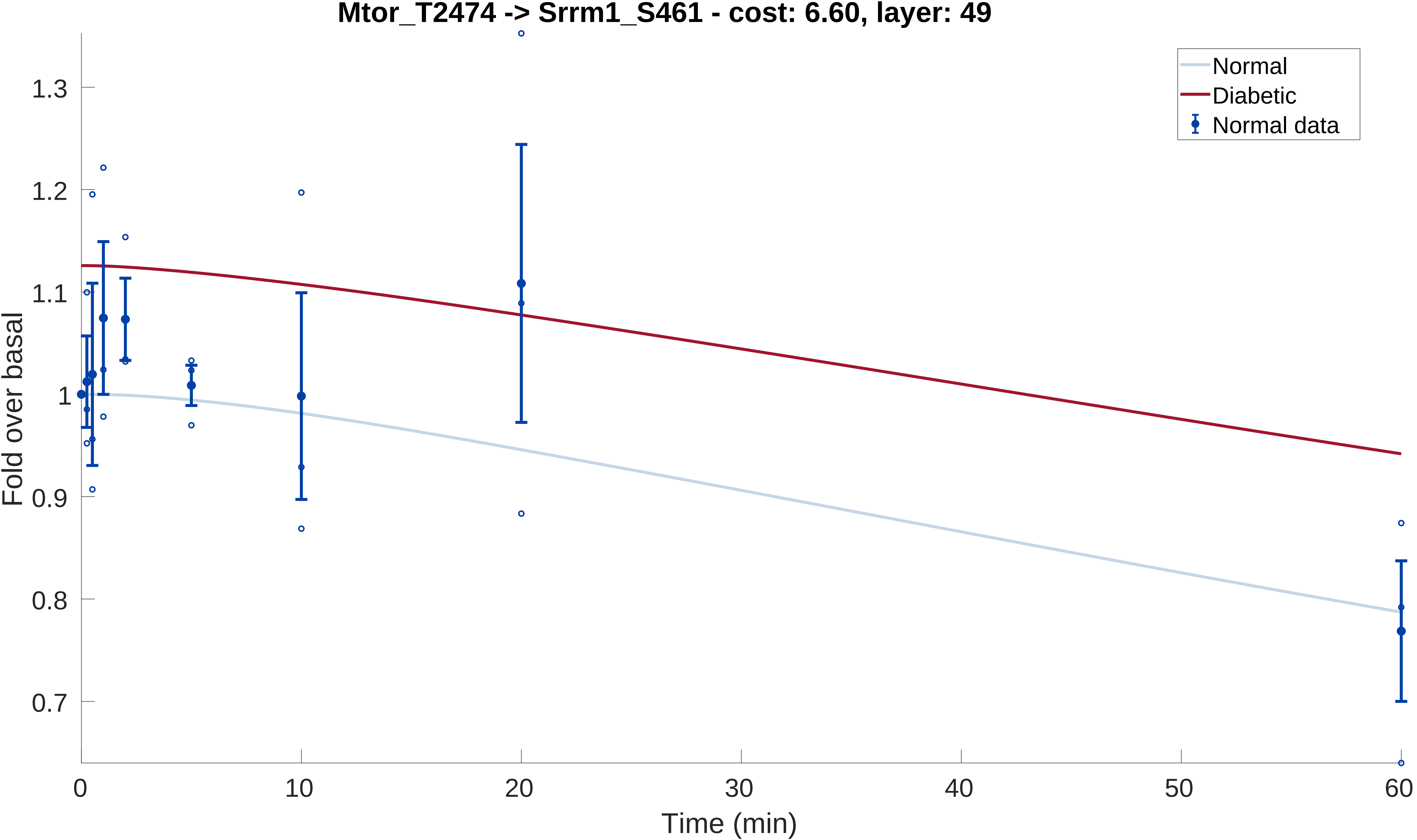
Mtor_T2474 -> Srrm1_S461 - cost: 6.60, layer: 49
Normal
Diabetic
Normal data
Fold over basal
Time (min)
1.3
1.2
1.1
1
0.9
0.8
0.7
0
10
20
30
40
50
60

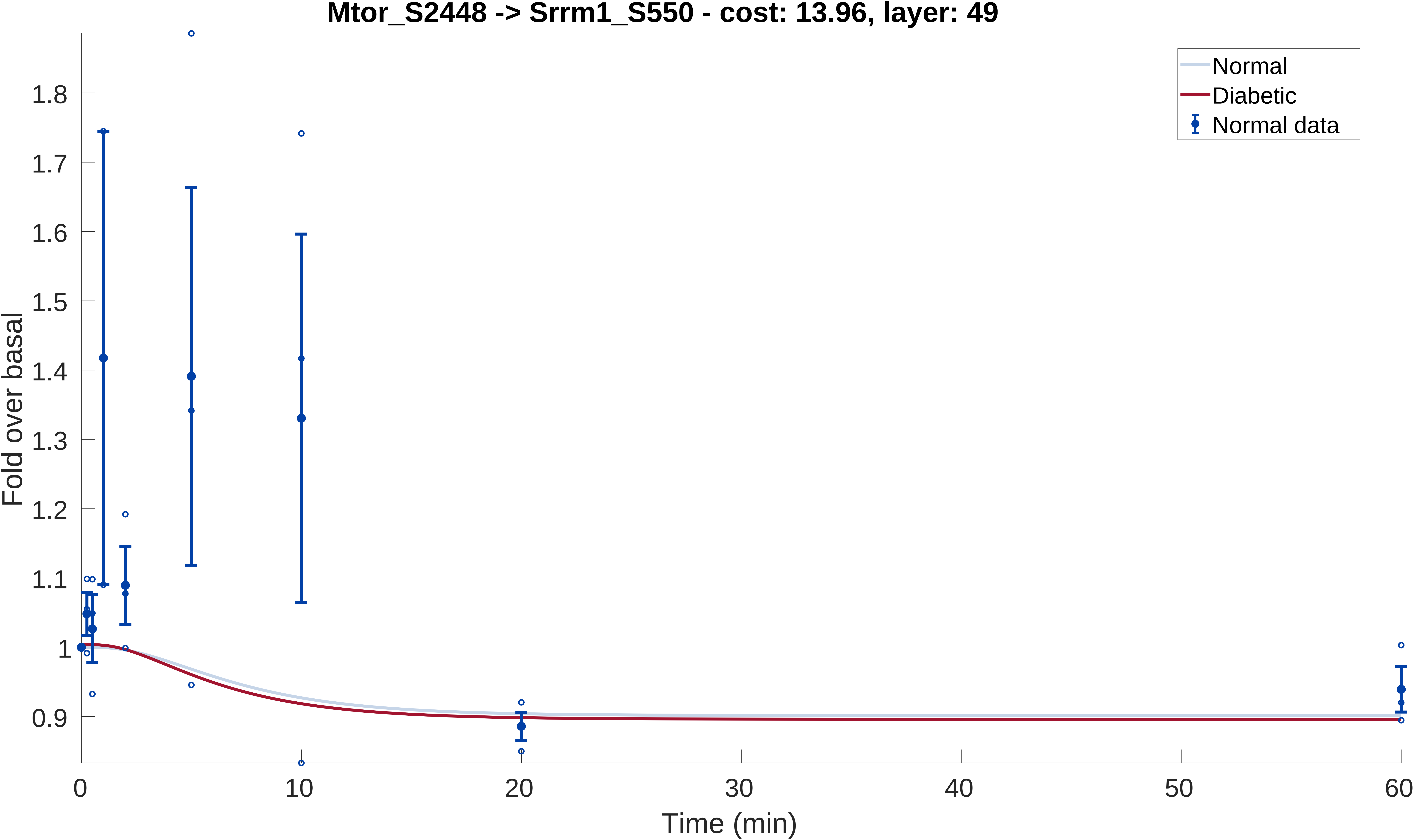

Mtor_S2448 -> Srrm1_S550 - cost: 13.96, layer: 49
Normal
Diabetic
Normal data
1.8
1.7
1.6
1.5
1.4
1.3
1.2
1.1
1
0.9
Fold over basal
0
10
20
30
40
50
60
Time (min)

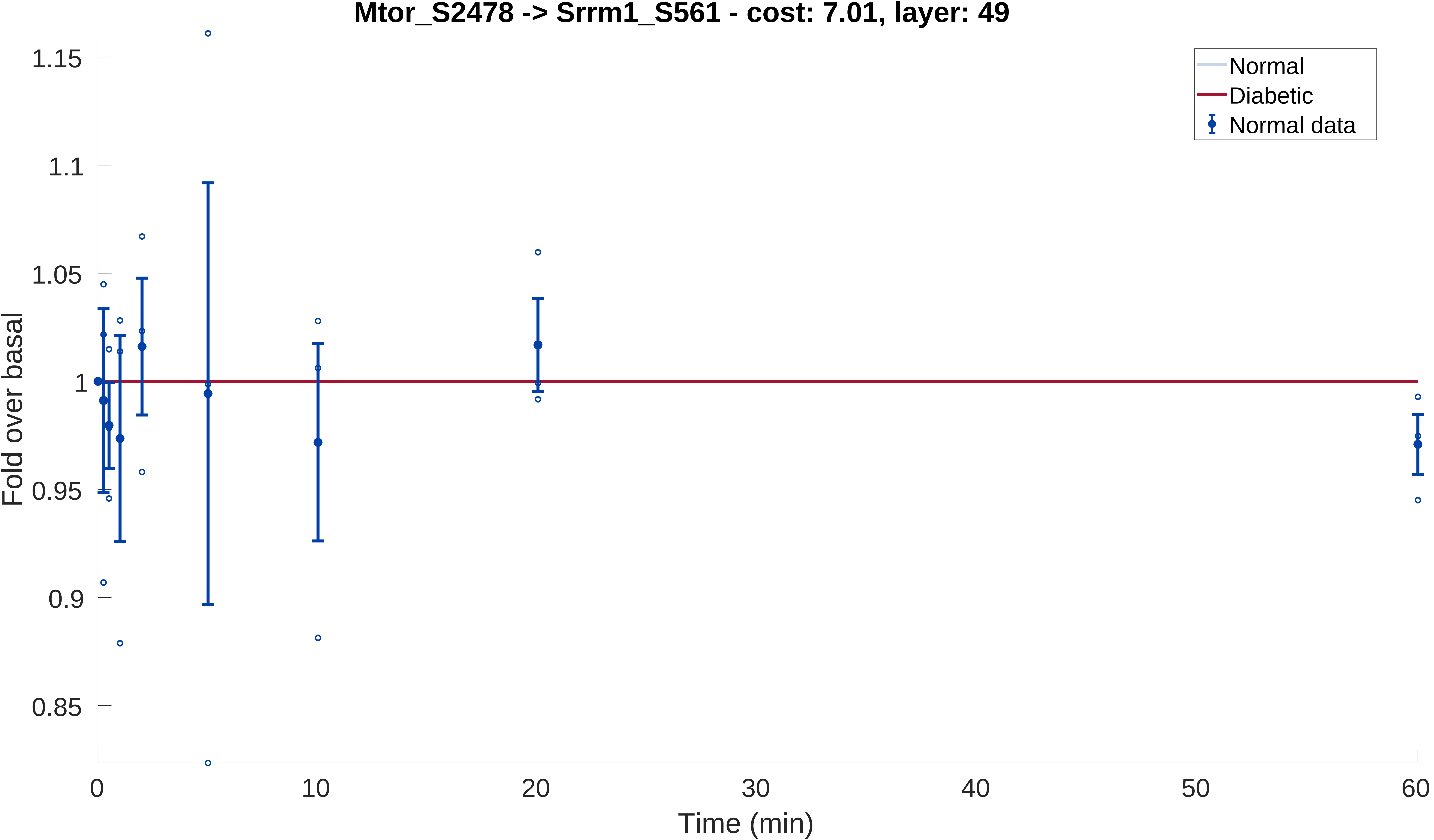
Mtor_S2478 -> Srrm1_S561 - cost: 7.01, layer: 49
Normal
Diabetic
Normal data
Fold over basal
Time (min)
1.15
1.1
1.05
1
0.95
0.9
0.85
0
10
20
30
40
50
60

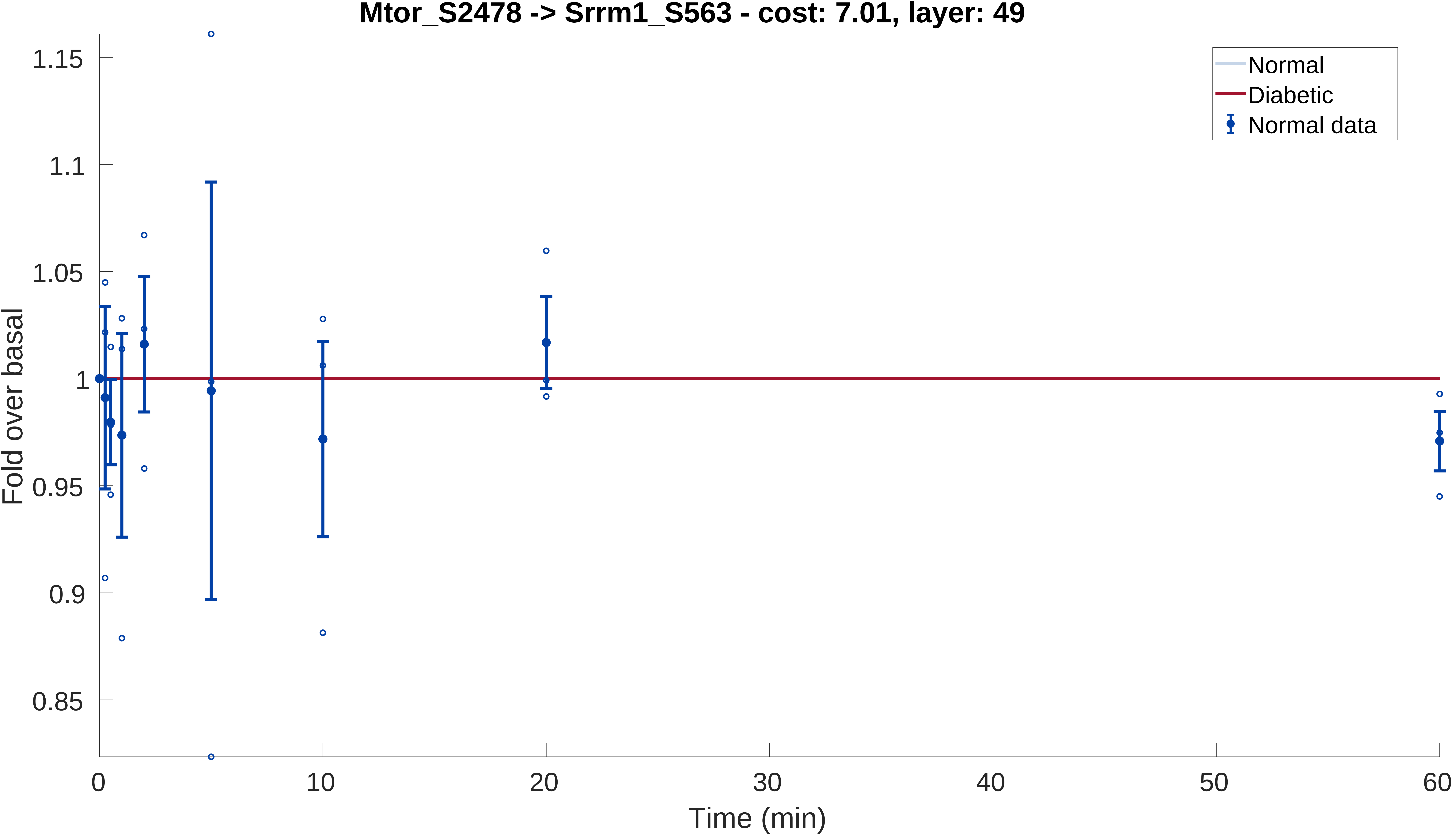
Mtor_S2478 -> Srrm1_S563 - cost: 7.01, layer: 49
Fold over basal
Time (min)
Normal
Diabetic
Normal data
1.15
1.1
1.05
1
0.95
0.9
0.85
0
10
20
30
40
50
60

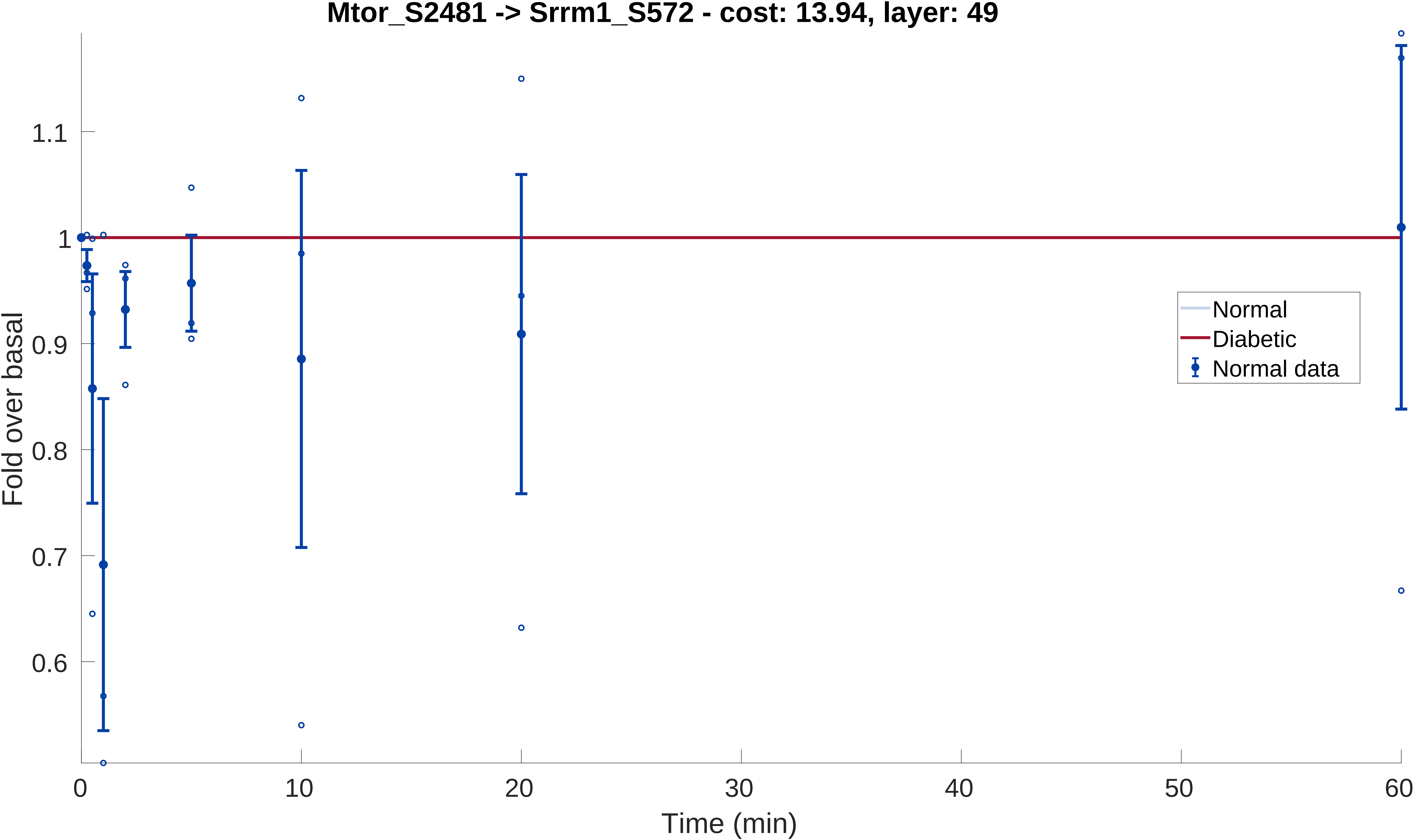

Mtor_S2481 -> Srrm1_S572 - cost: 13.94, layer: 49
Fold over basal
Time (min)
1.1
1
0.9
0.8
0.7
0.6
0
10
20
30
40
50
60
Normal
Diabetic
Normal data

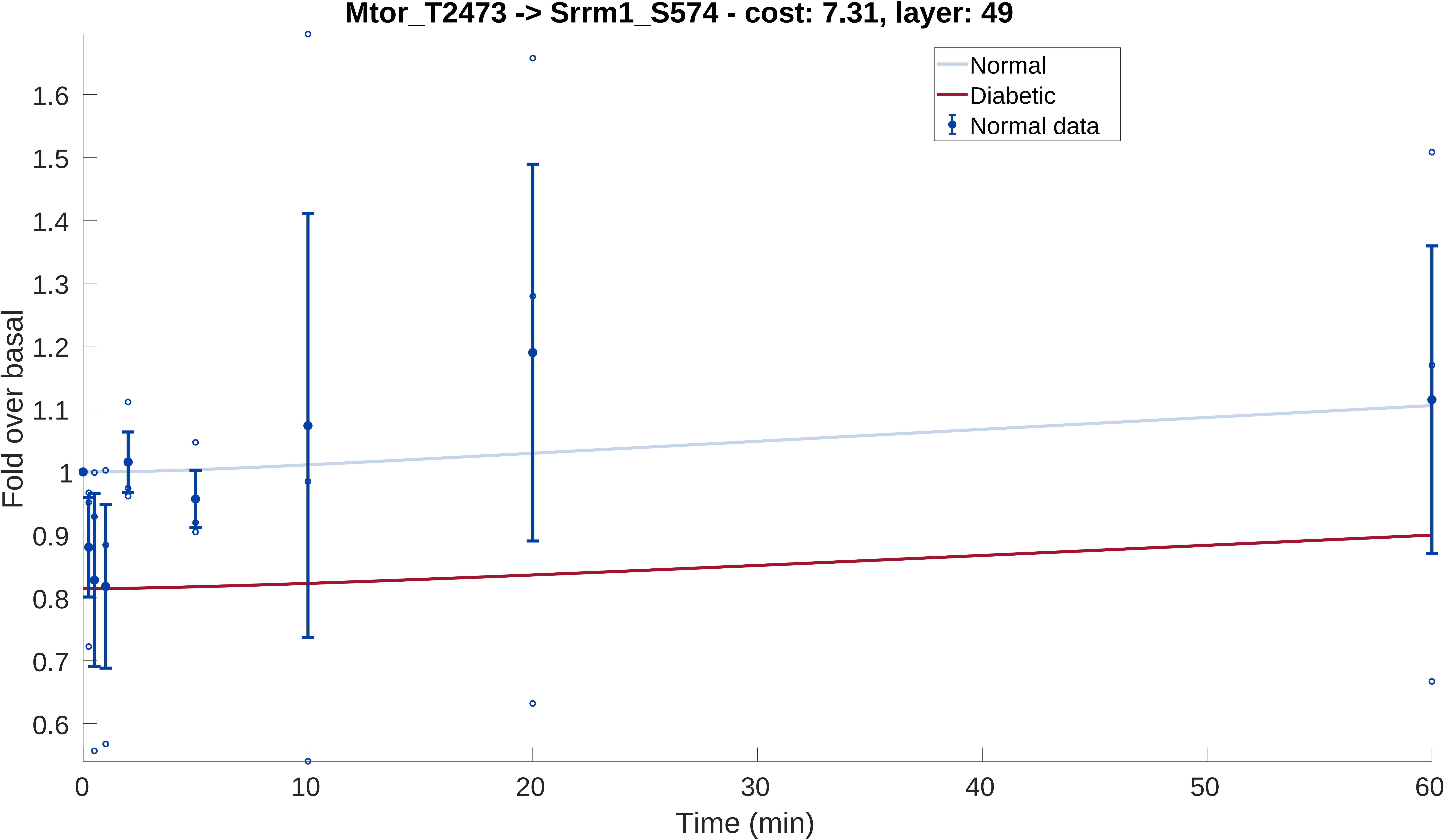

Mtor_T2473 -> Srrm1_S574 - cost: 7.31, layer: 49
Normal
Diabetic
Normal data
Fold over basal
1.6
1.5
1.4
1.3
1.2
1.1
1
0.9
0.8
0.7
0.6
0
10
20
30
40
50
60
Time (min)

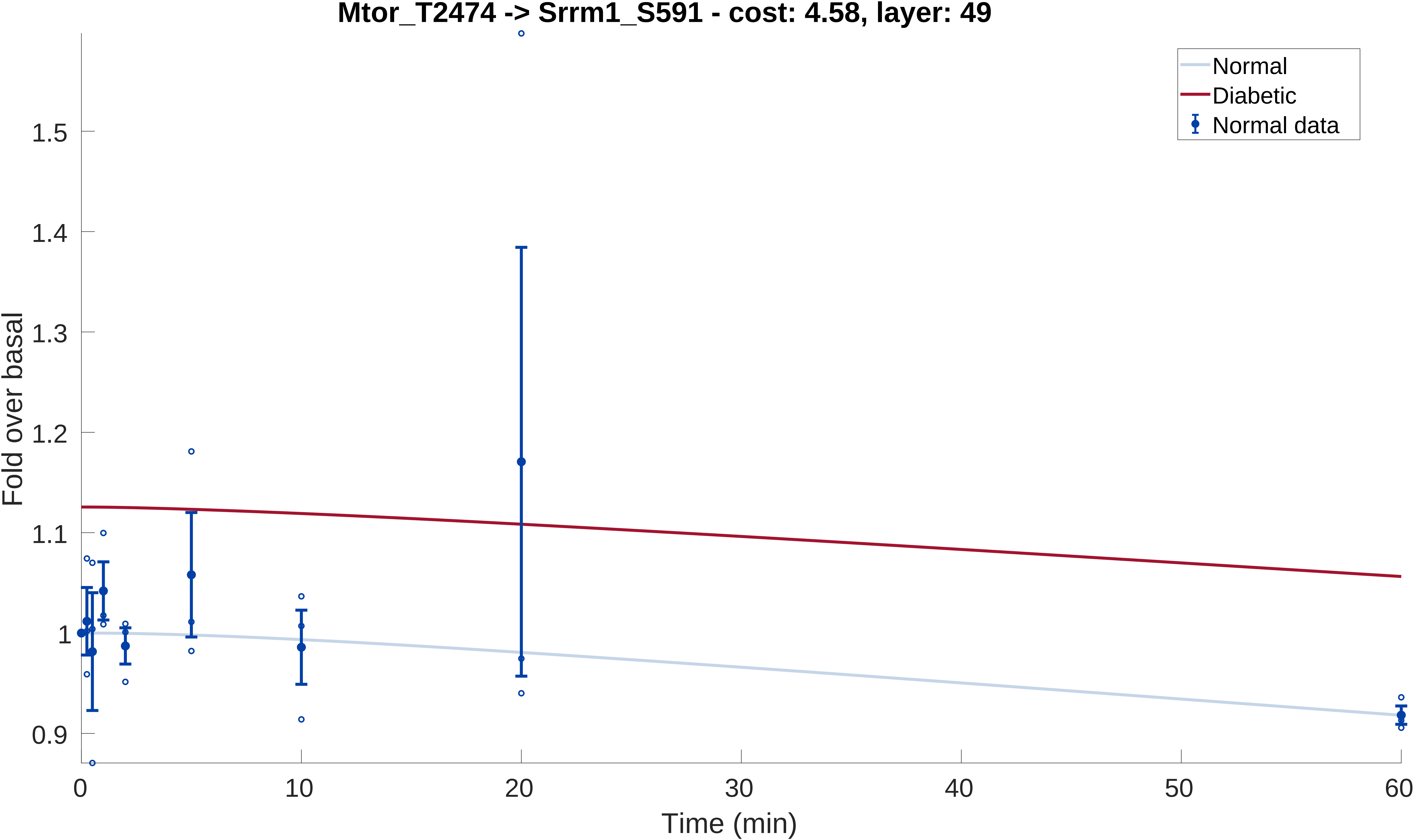
Mtor_T2474 -> Srrm1_S591 - cost: 4.58, layer: 49
Normal
Diabetic
Normal data
Fold over basal
1.5
1.4
1.3
1.2
1.1
1
0.9
0
10
20
30
40
50
60
Time (min)

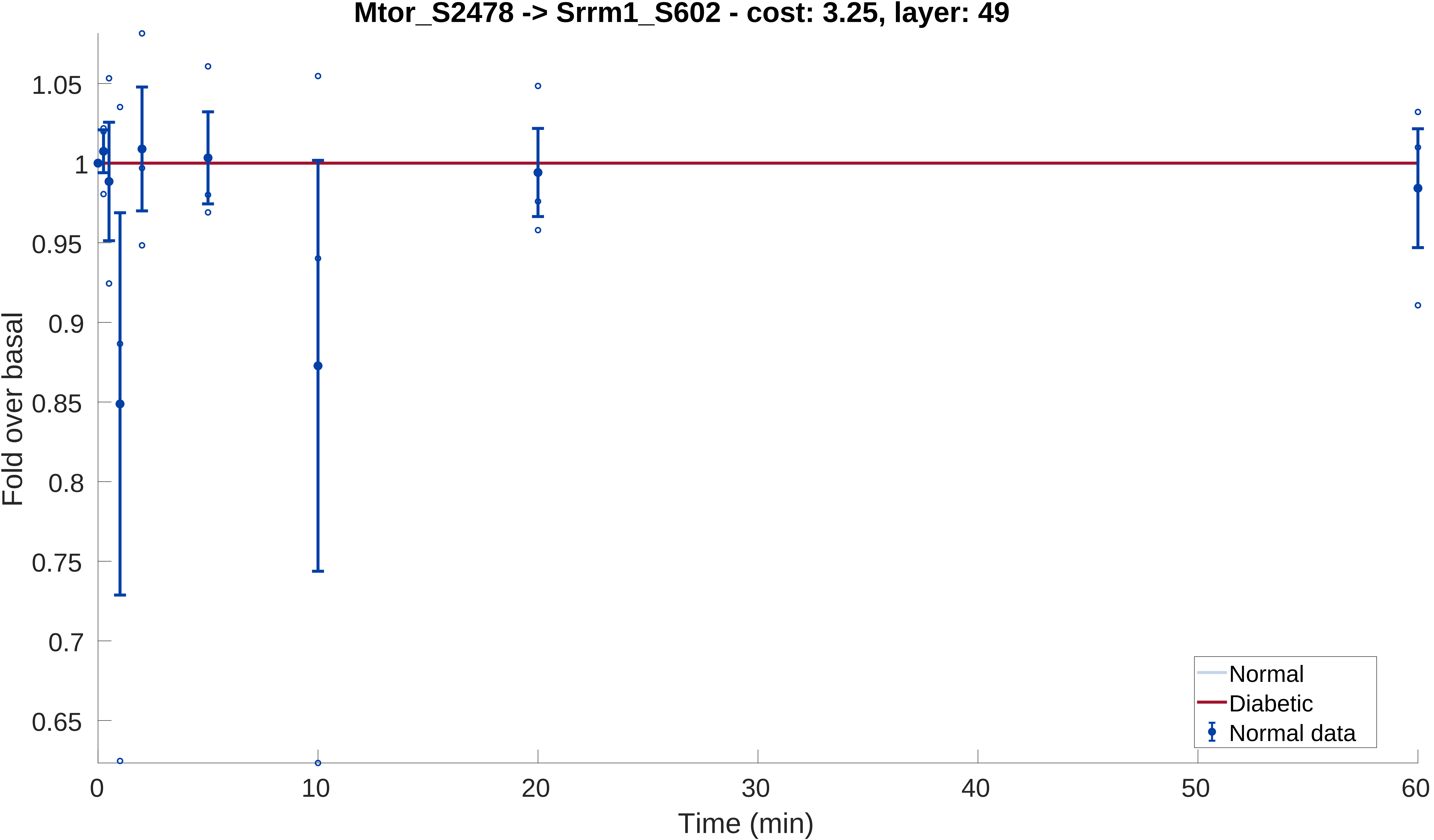
Mtor_S2478 -> Srrm1_S602 - cost: 3.25, layer: 49
Fold over basal
1.05
1
0.95
0.9
0.85
0.8
0.75
0.7
0.65
0
10
20
30
40
50
60
Time (min)
Normal
Diabetic
Normal data

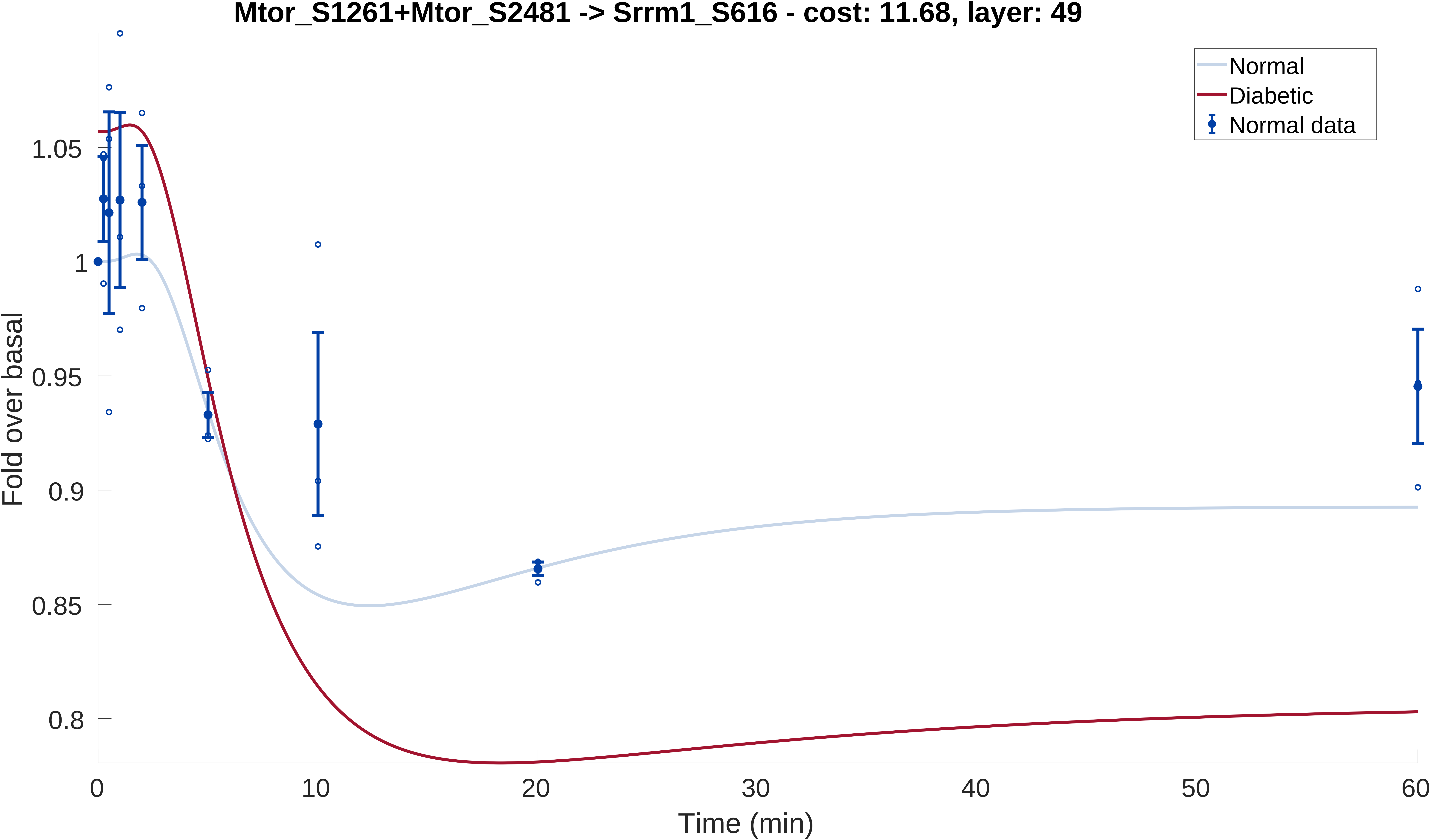
Mtor_S1261+Mtor_S2481 -> Srrm1_S616 - cost: 11.68, layer: 49
Normal
Diabetic
Normal data
Fold over basal
1.05
1
0.95
0.9
0.85
0.8
0
10
20
30
40
50
60
Time (min)

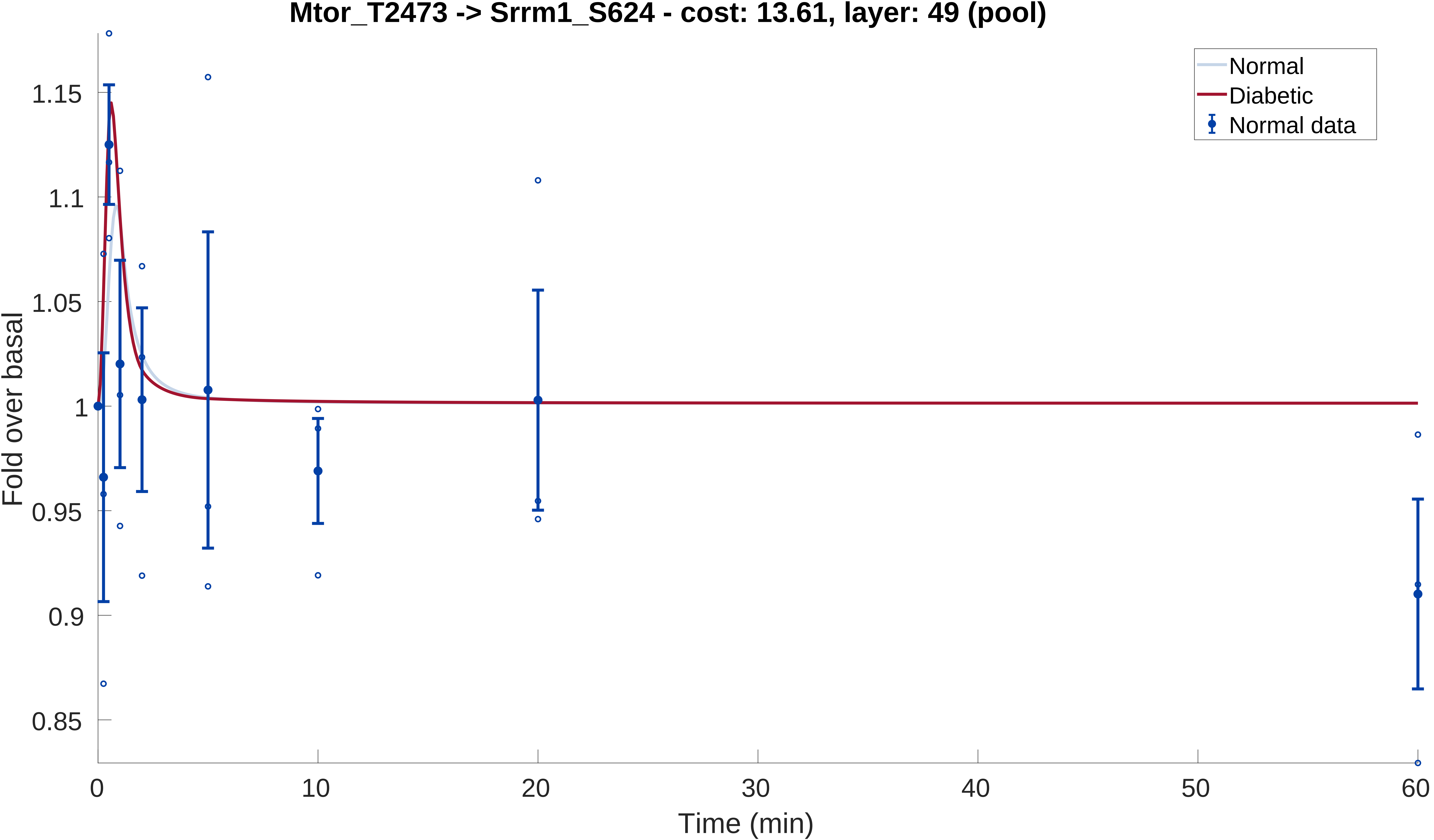

Mtor_T2473 -> Srrm1_S624 - cost: 13.61, layer: 49 (pool)
Normal
Diabetic
Normal data
Fold over basal
Time (min)
1.15
1.1
1.05
1
0.95
0.9
0.85
0
10
20
30
40
50
60

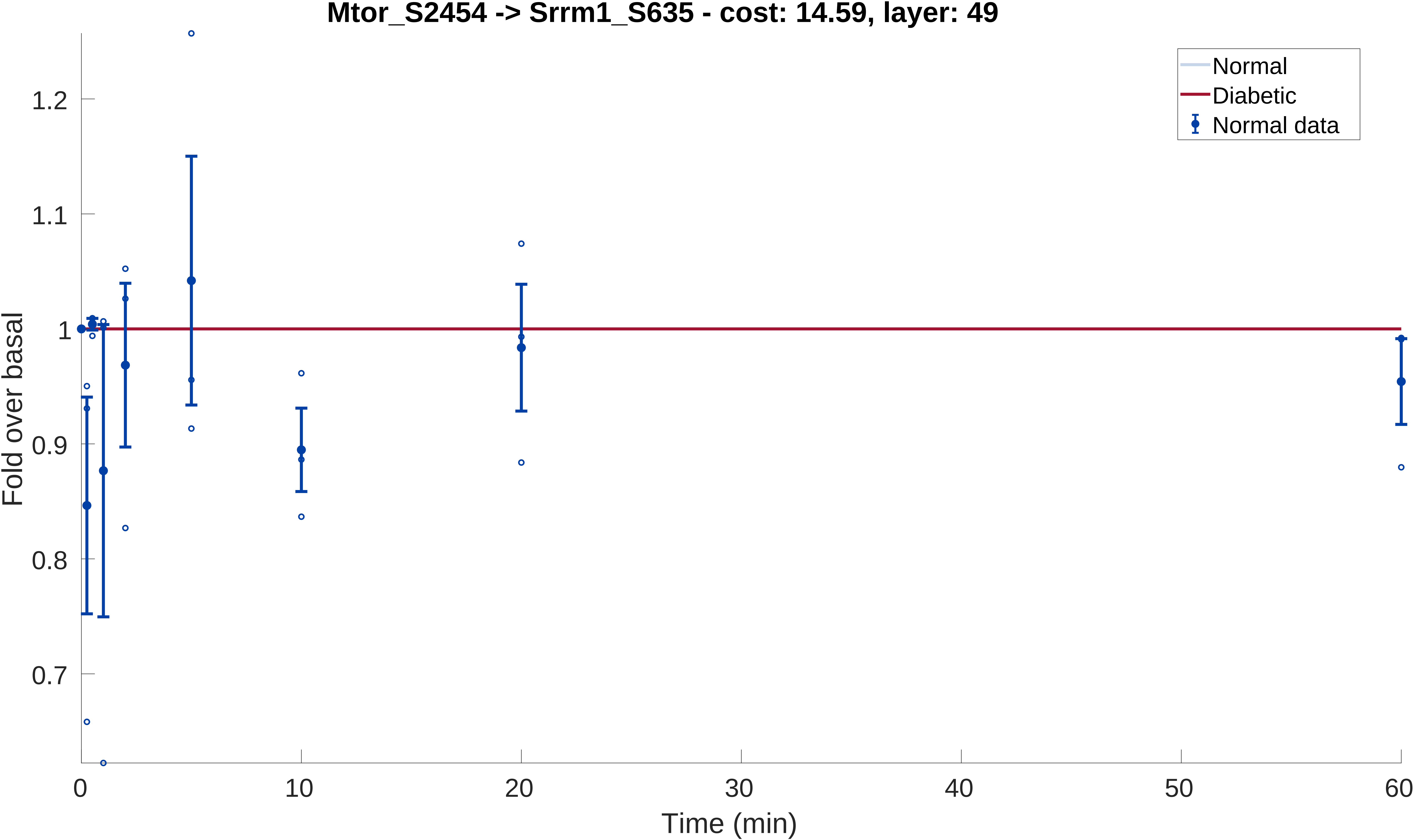

Mtor_S2454 -> Srrm1_S635 - cost: 14.59, layer: 49
Normal
Diabetic
Normal data
Fold over basal
Time (min)
1.2
1.1
1
0.9
0.8
0.7
0
10
20
30
40
50
60

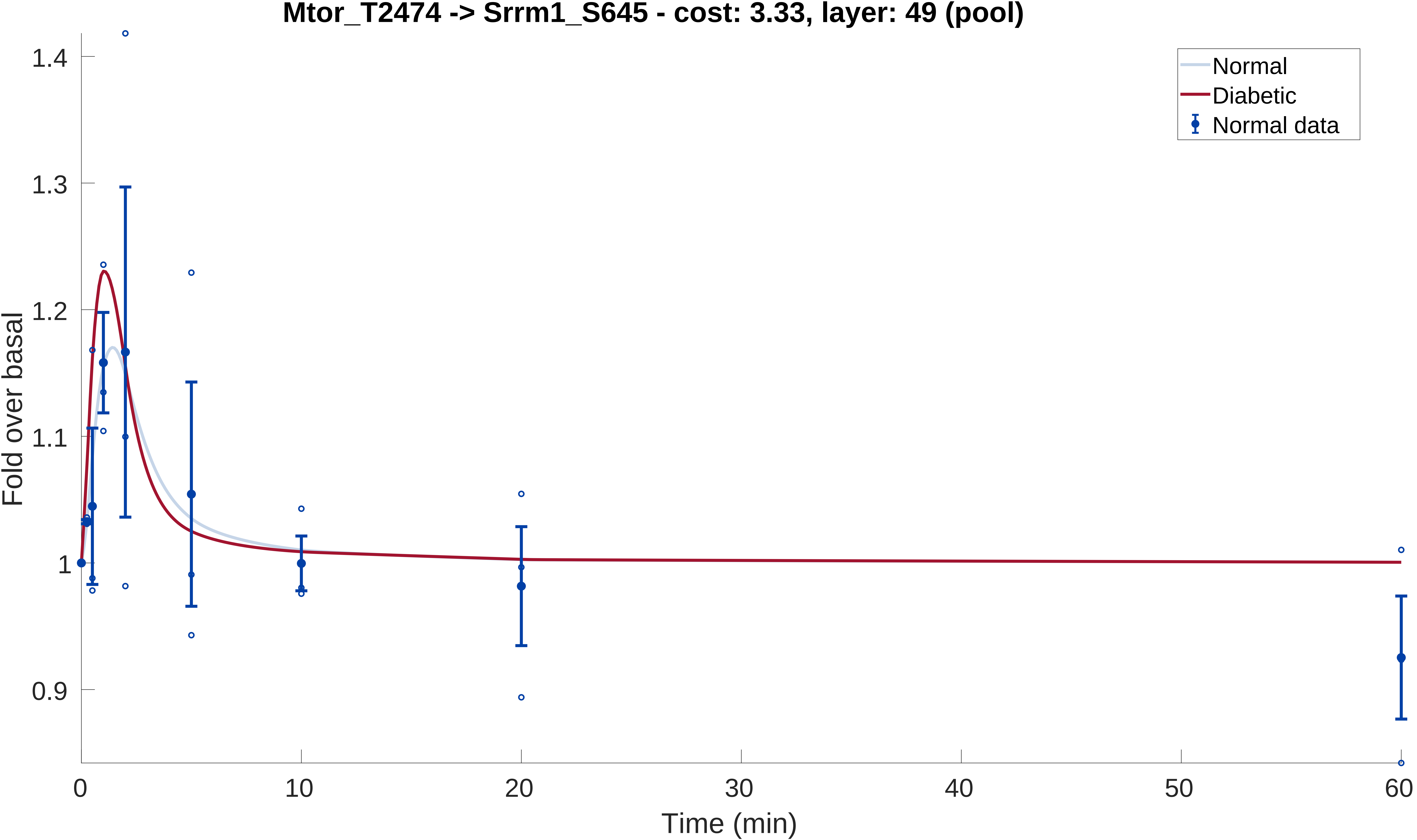

Mtor_T2474 -> Srrm1_S645 - cost: 3.33, layer: 49 (pool)
Normal
Diabetic
Normal data
Fold over basal
Time (min)
1.4
1.3
1.2
1.1
1
0.9
0
10
20
30
40
50
60

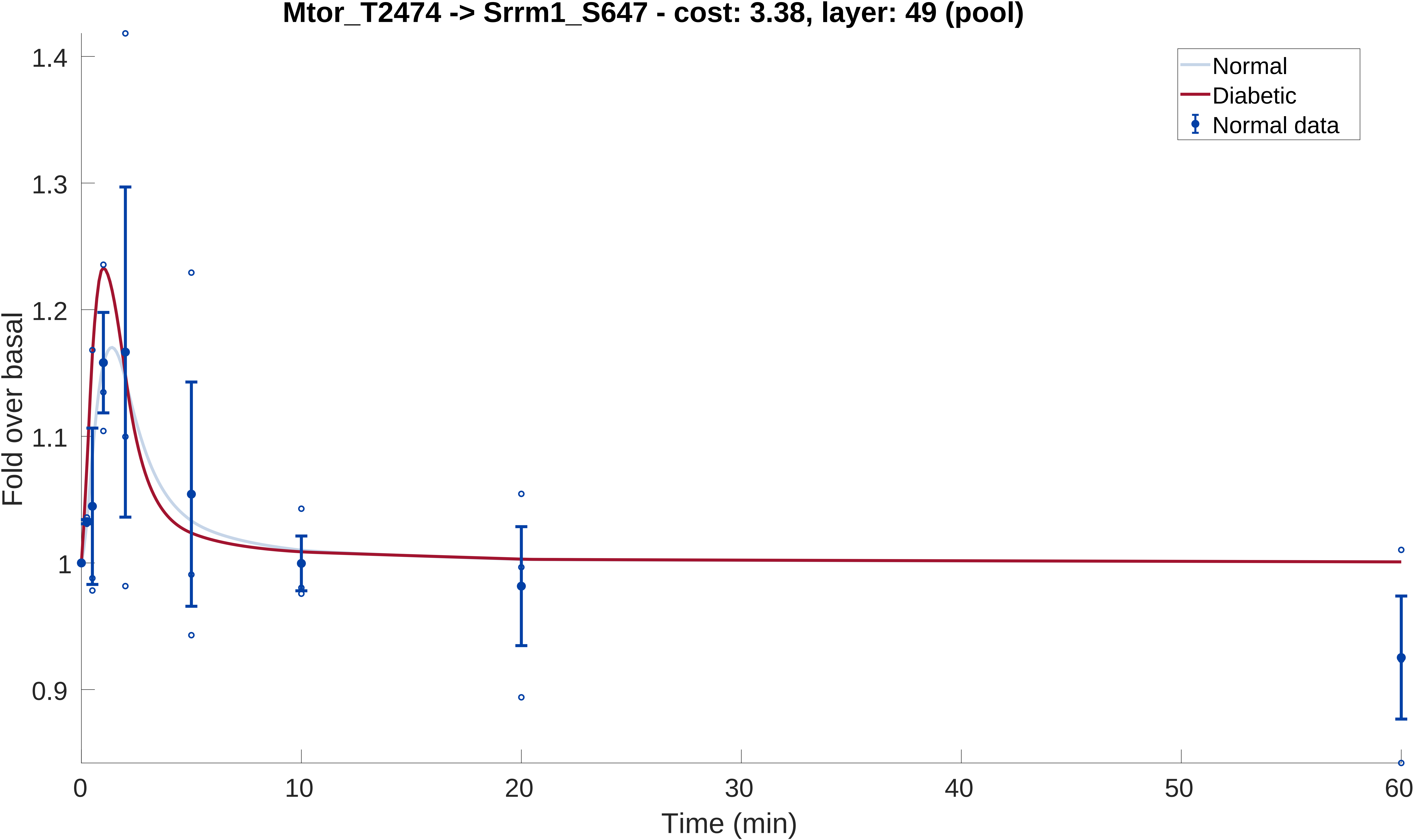

Mtor_T2474 -> Srrm1_S647 - cost: 3.38, layer: 49 (pool)
Fold over basal
Time (min)
Normal
Diabetic
Normal data
1.4
1.3
1.2
1.1
1
0.9
0
10
20
30
40
50
60

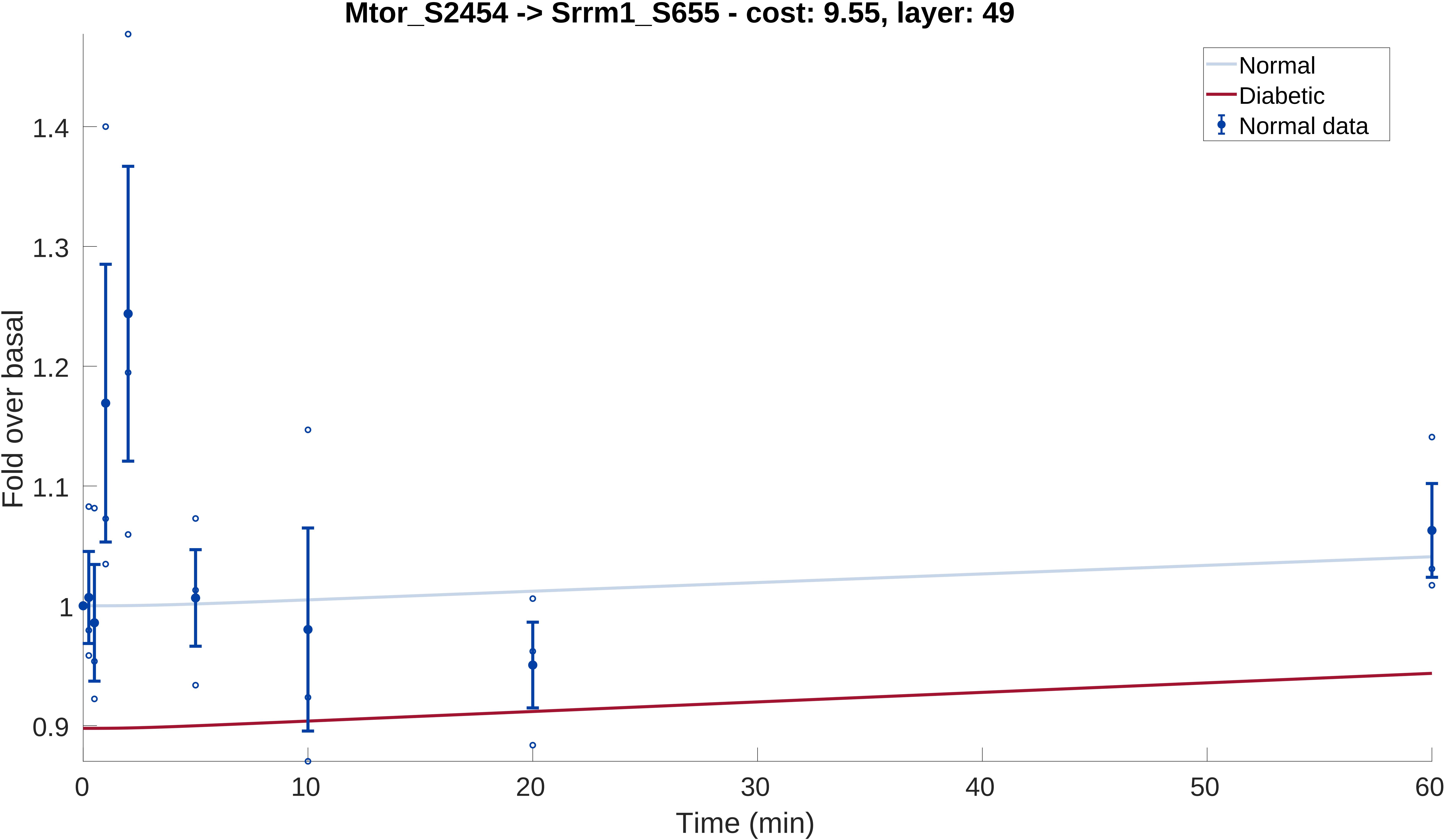

Mtor_S2454 -> Srrm1_S655 - cost: 9.55, layer: 49
Normal
Diabetic
Normal data
Fold over basal
1.4
1.3
1.2
1.1
1
0.9
0
10
20
30
40
50
60
Time (min)

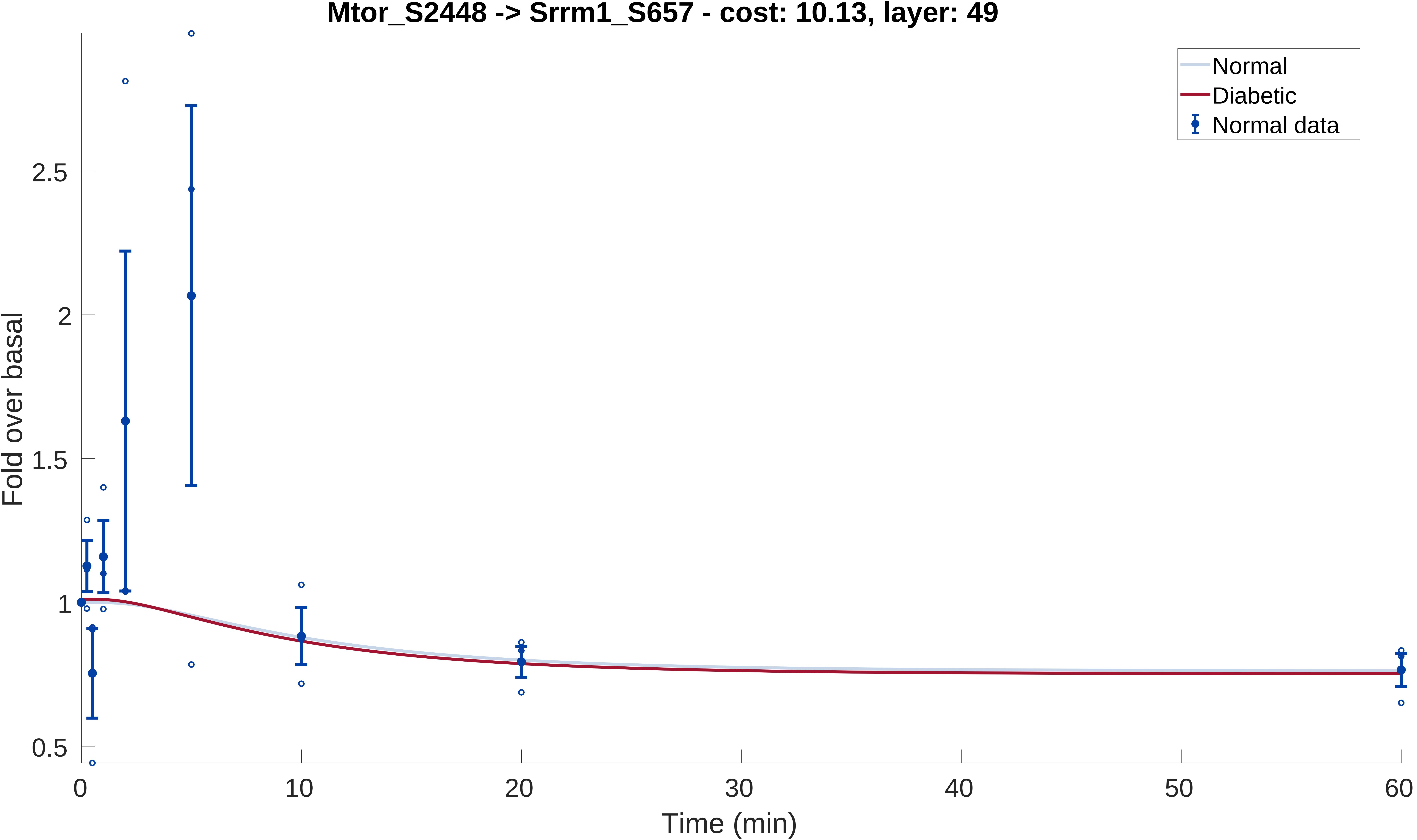
Mtor_S2448 -> Srrm1_S657 - cost: 10.13, layer: 49
Normal
Diabetic
Normal data
Fold over basal
Time (min)
0.5
1
1.5
2
2.5
0
10
20
30
40
50
60

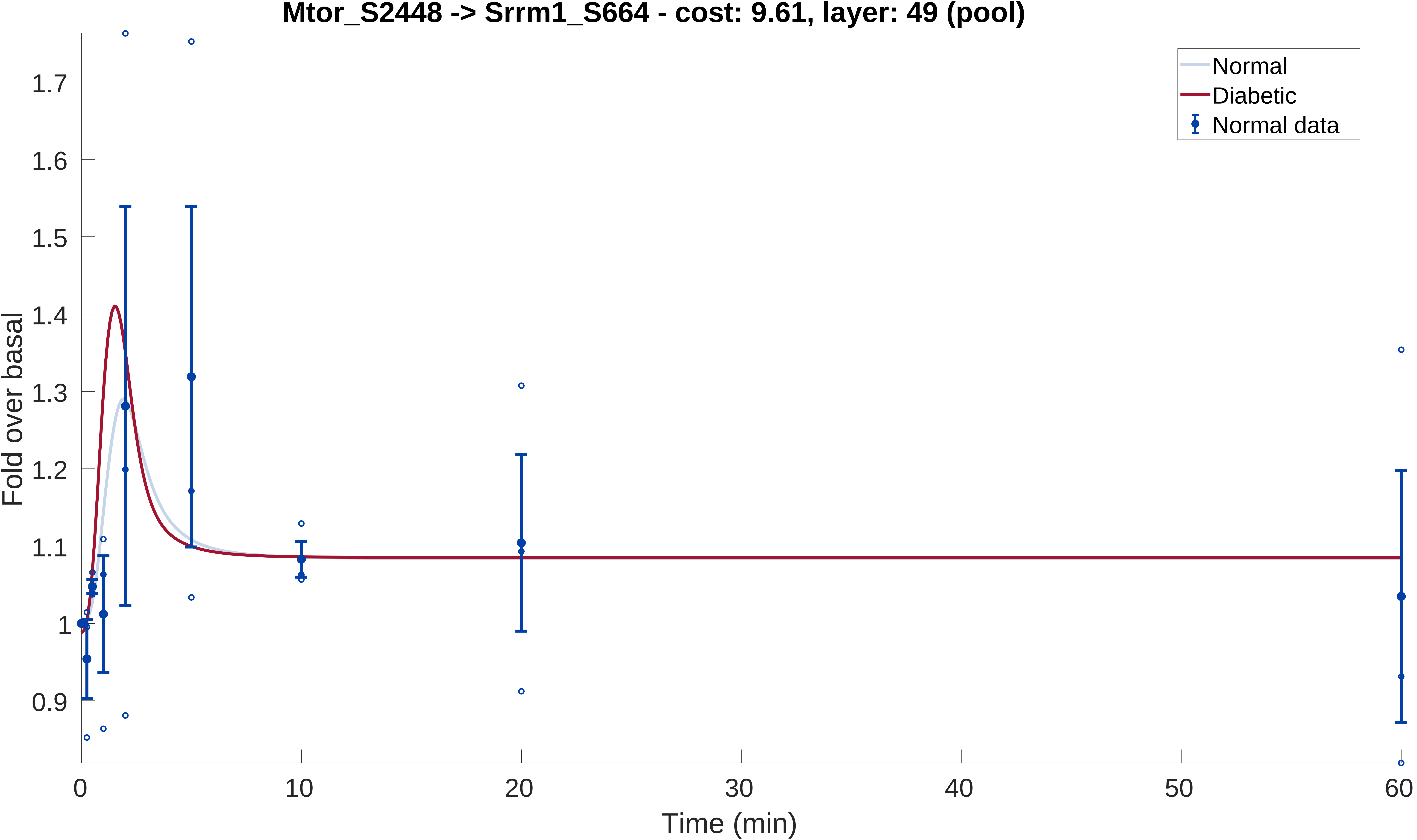

Mtor_S2448 -> Srrm1_S664 - cost: 9.61, layer: 49 (pool)
Normal
Diabetic
Normal data
Fold over basal
Time (min)
1.7
1.6
1.5
1.4
1.3
1.2
1.1
1
0.9
0
10
20
30
40
50
60

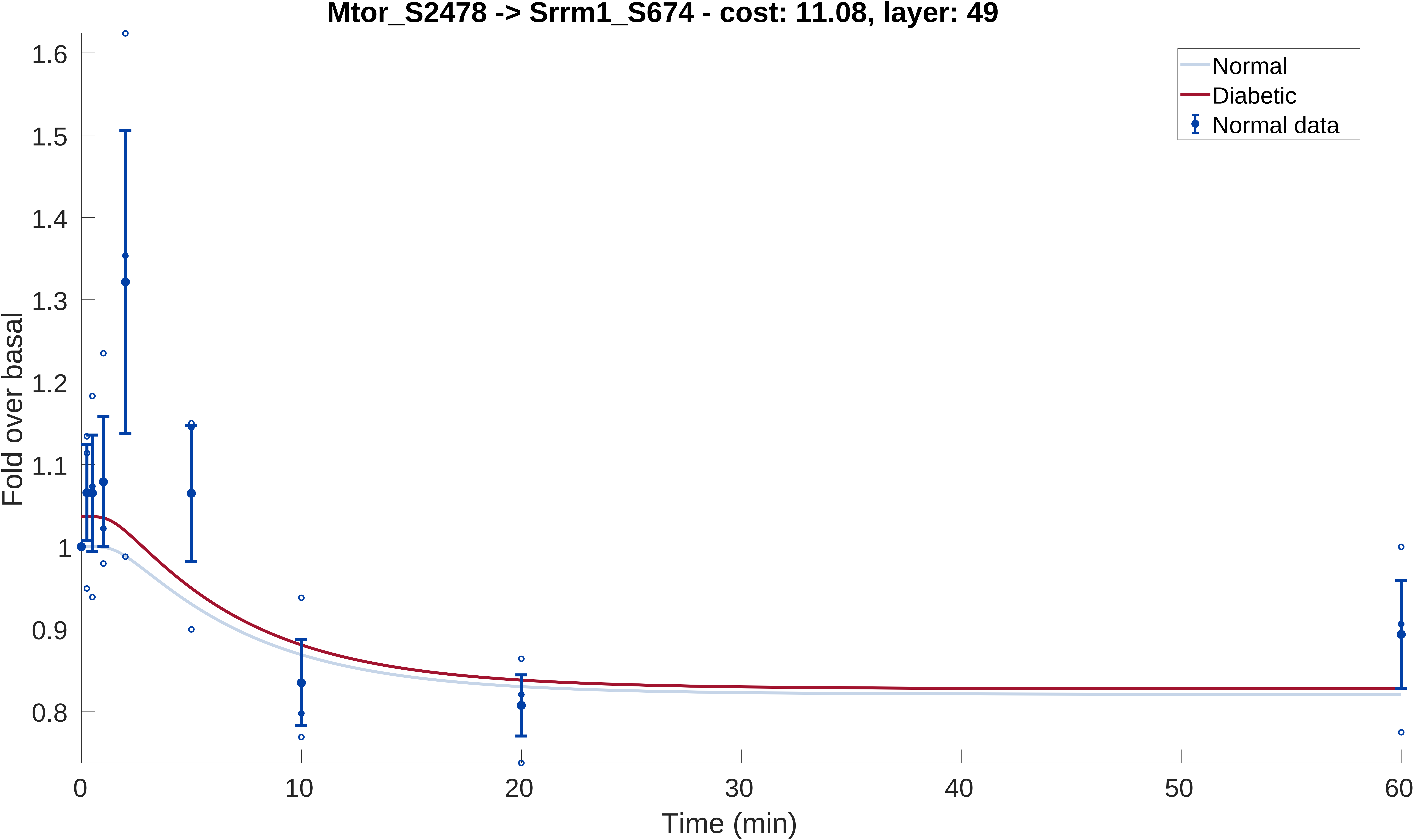
Mtor_S2478 -> Srrm1_S674 - cost: 11.08, layer: 49
Normal
Diabetic
Normal data
Fold over basal
1.6
1.5
1.4
1.3
1.2
1.1
1
0.9
0.8
0
10
20
30
40
50
60
Time (min)

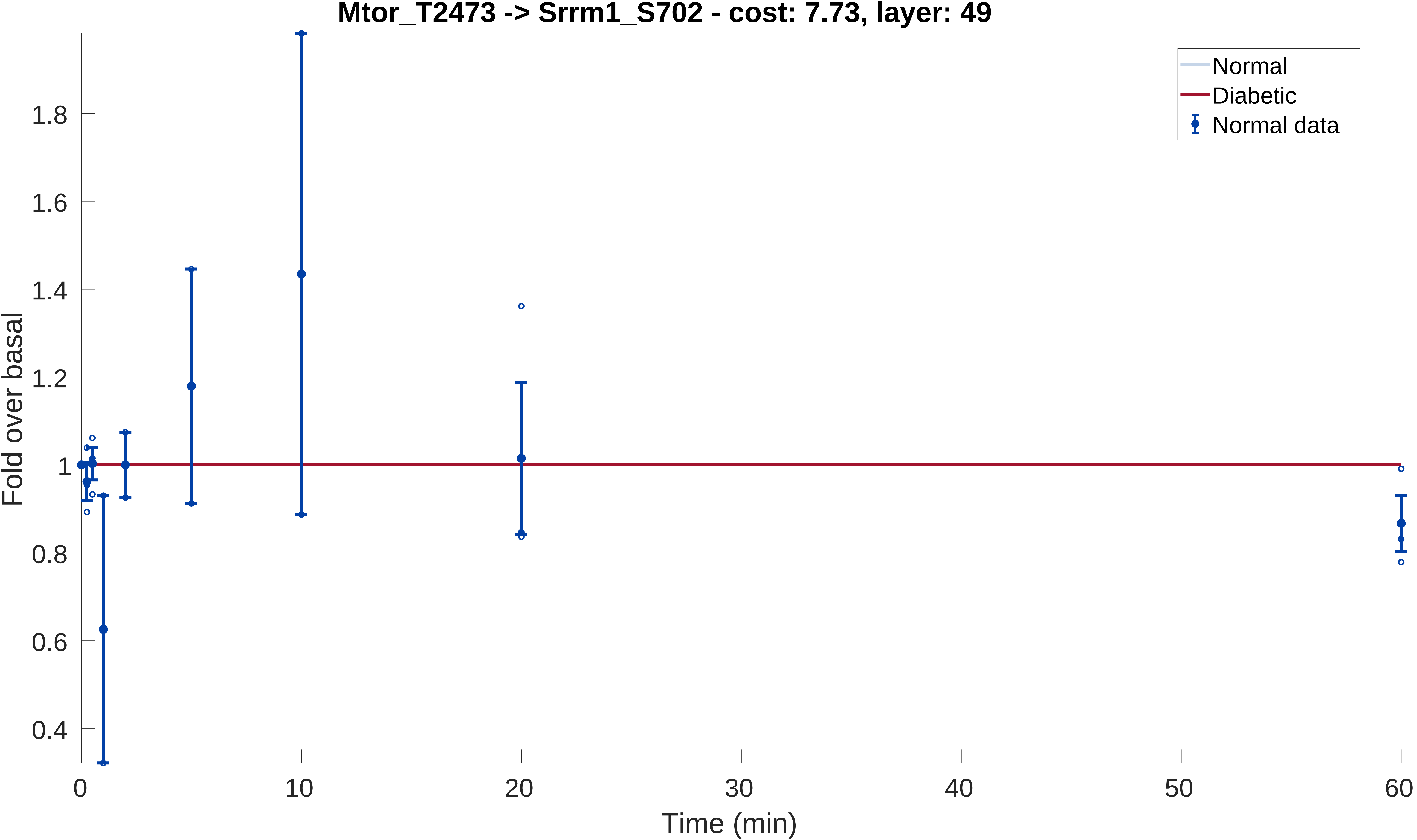

Mtor_T2473 -> Srrm1_S702 - cost: 7.73, layer: 49
Normal
Diabetic
Normal data
Fold over basal
0.4
0.6
0.8
1
1.2
1.4
1.6
1.8
0
10
20
30
40
50
60
Time (min)

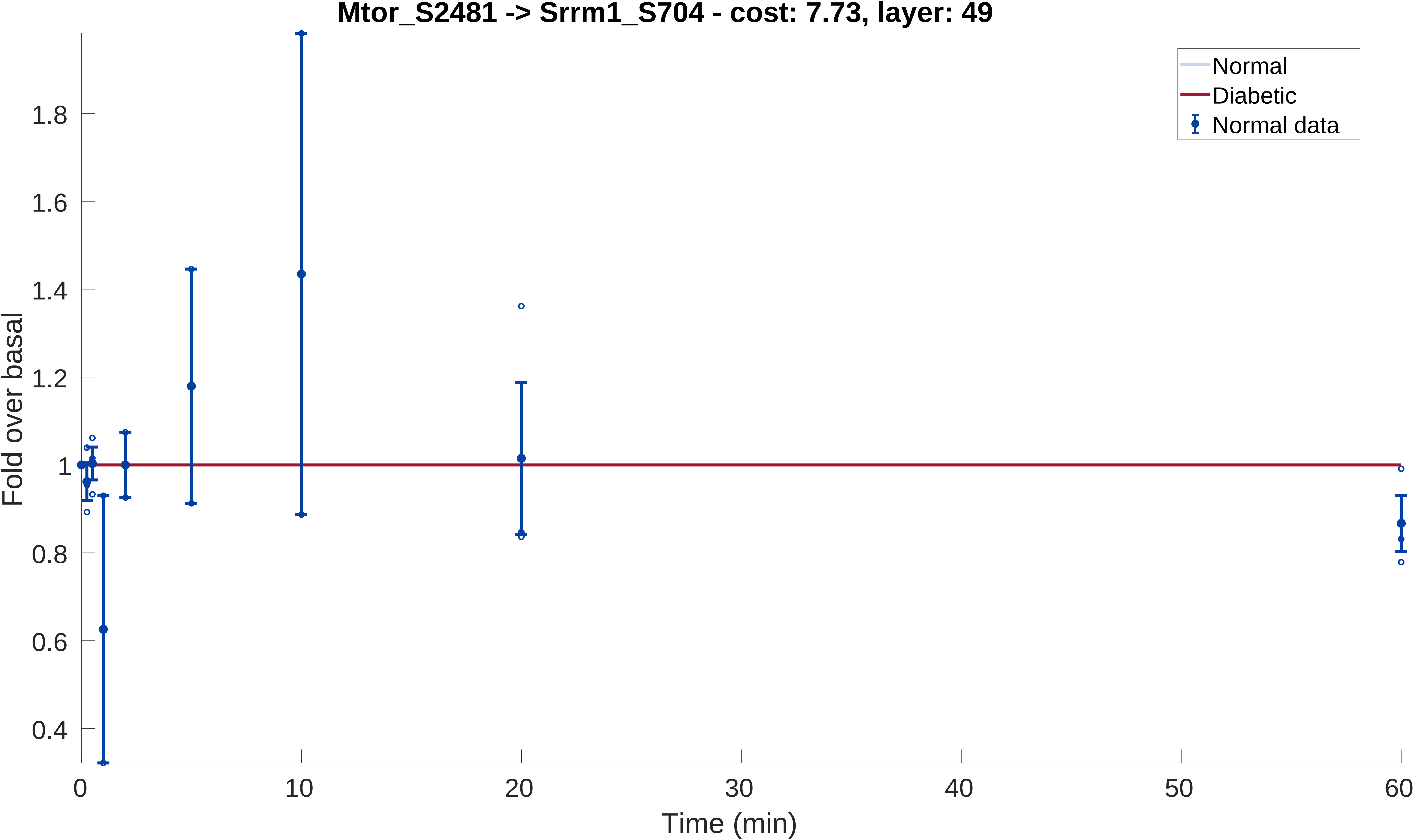

Mtor_S2481 -> Srrm1_S704 - cost: 7.73, layer: 49
Normal
Diabetic
Normal data
Fold over basal
1.8
1.6
1.4
1.2
1
0.8
0.6
0.4
0
10
20
30
40
50
60
Time (min)

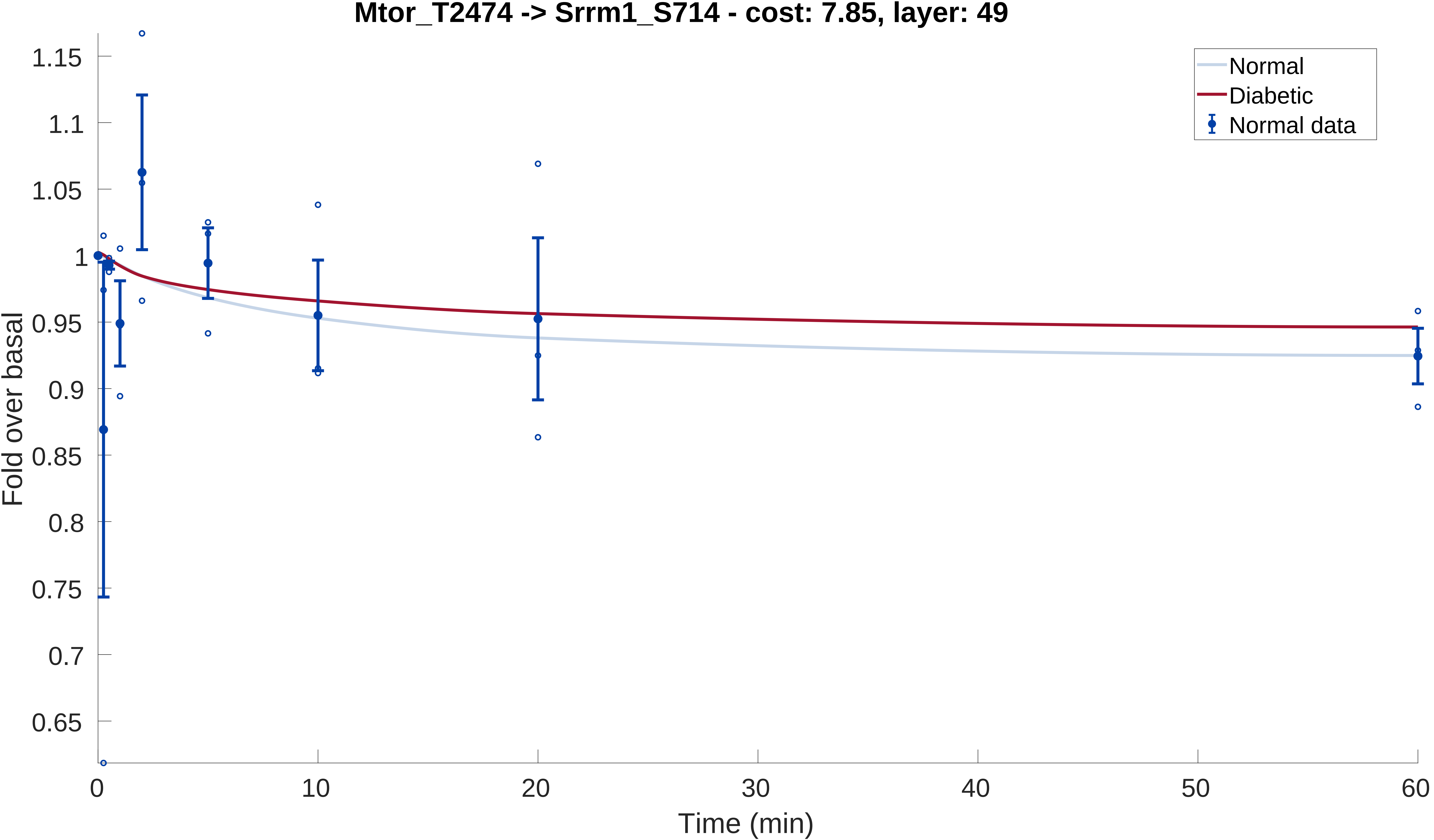

Mtor_T2474 -> Srrm1_S714 - cost: 7.85, layer: 49
Normal
Diabetic
Normal data
Fold over basal
Time (min)
1.15
1.1
1.05
1
0.95
0.9
0.85
0.8
0.75
0.7
0.65
0
10
20
30
40
50
60

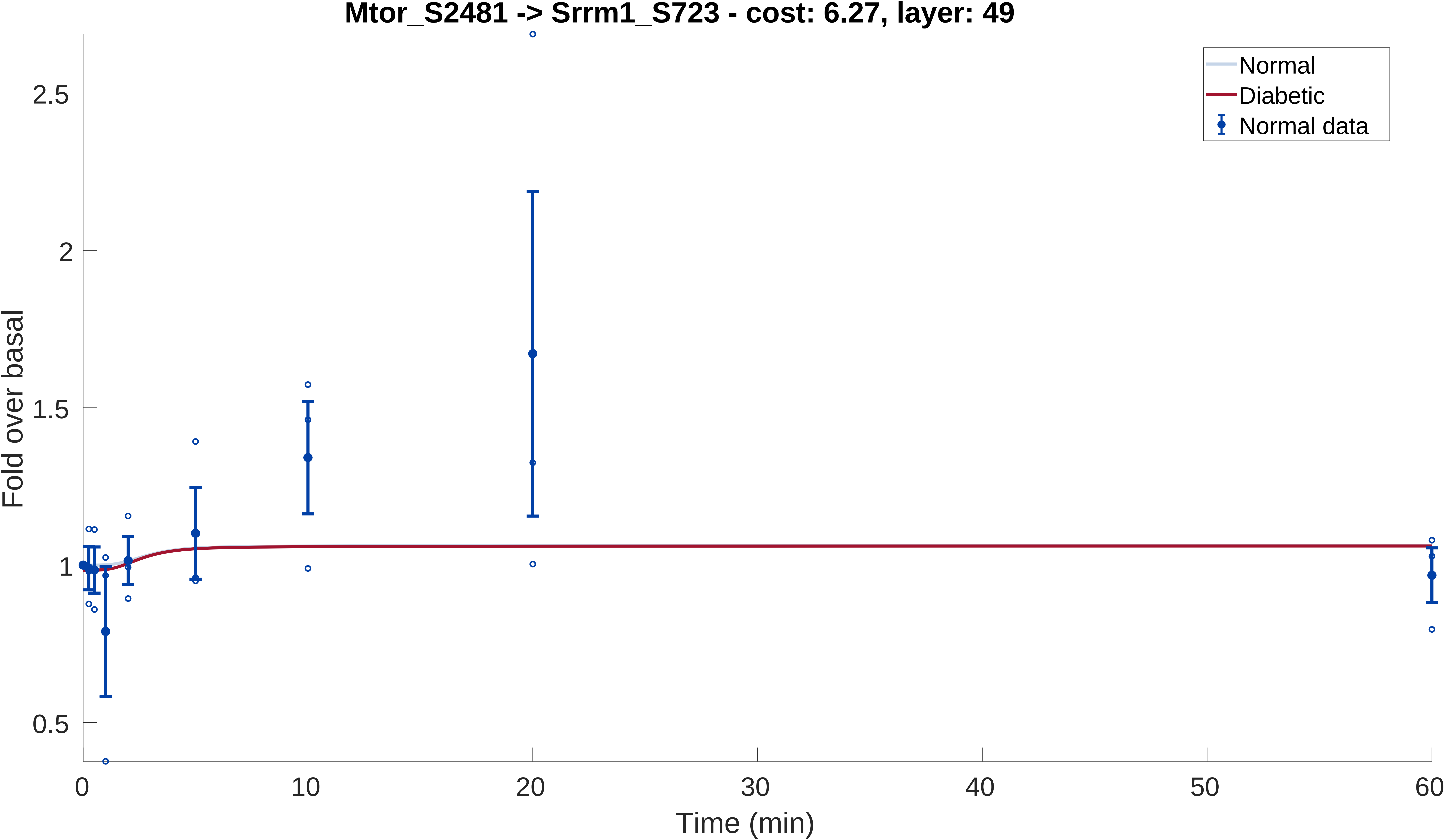

Mtor_S2481 -> Srrm1_S723 - cost: 6.27, layer: 49
Normal
Diabetic
Normal data
Fold over basal
2.5
2
1.5
1
0.5
0
10
20
30
40
50
60
Time (min)

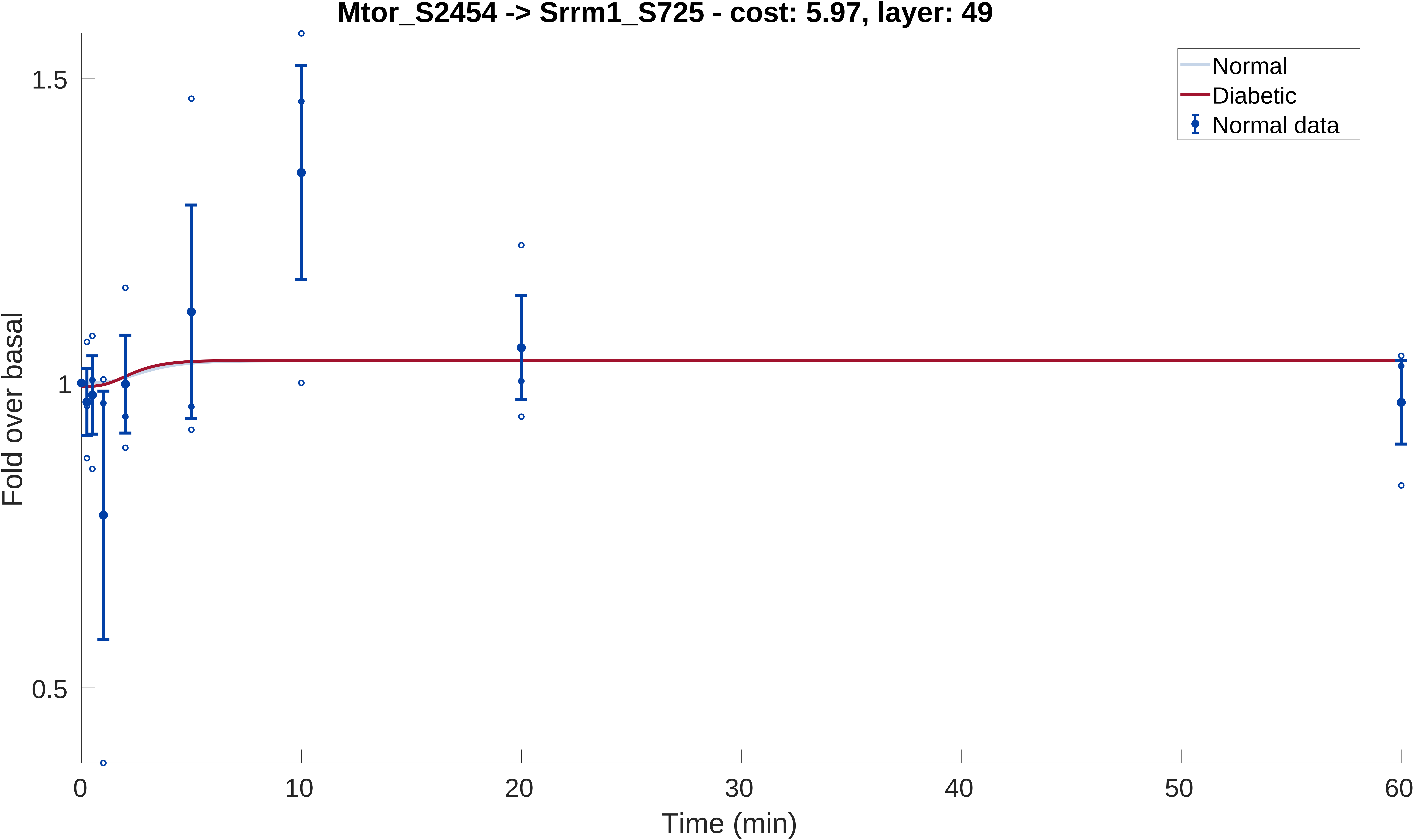
Mtor_S2454 -> Srrm1_S725 - cost: 5.97, layer: 49
Normal
Diabetic
Normal data
1.5
1
0.5
Fold over basal
0
10
20
30
40
50
60
Time (min)

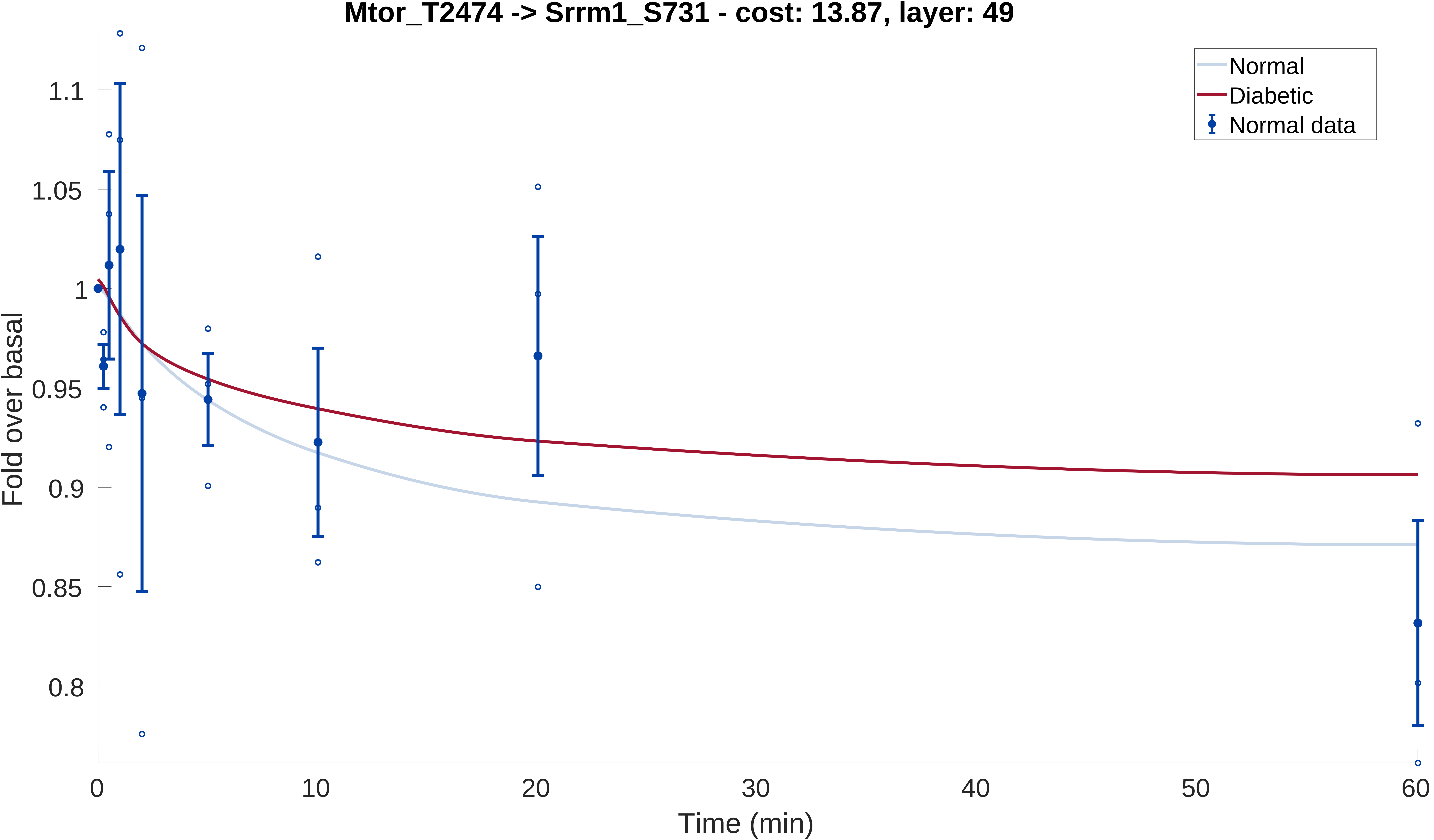
Mtor_T2474 -> Srrm1_S731 - cost: 13.87, layer: 49
Normal
Diabetic
Normal data
Fold over basal
1.1
1.05
1
0.95
0.9
0.85
0.8
0
10
20
30
40
50
60
Time (min)

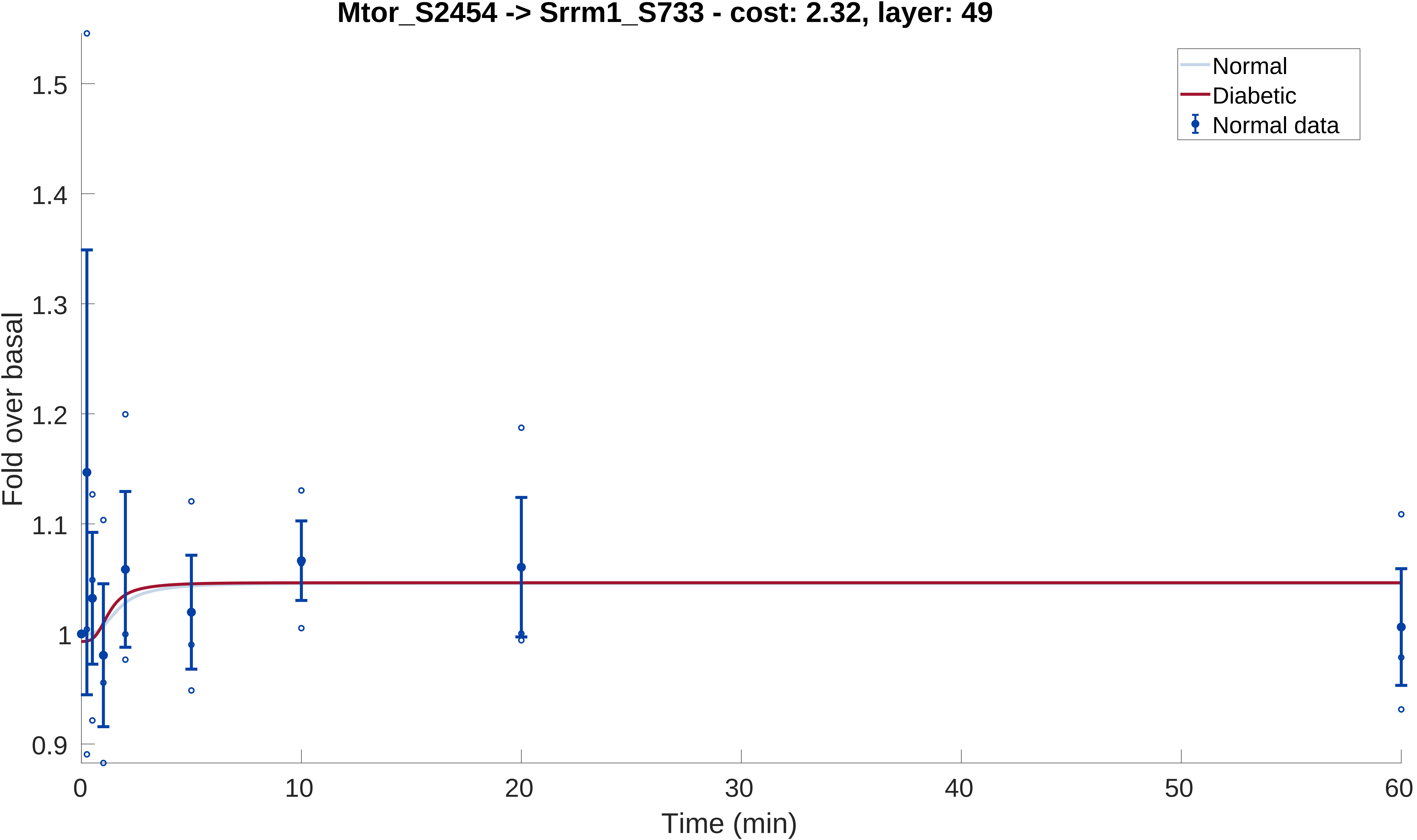

Mtor_S2454 -> Srrm1_S733 - cost: 2.32, layer: 49
Fold over basal
1.5
1.4
1.3
1.2
1.1
1
0.9
0
10
20
30
40
50
60
Time (min)
Normal
Diabetic
Normal data

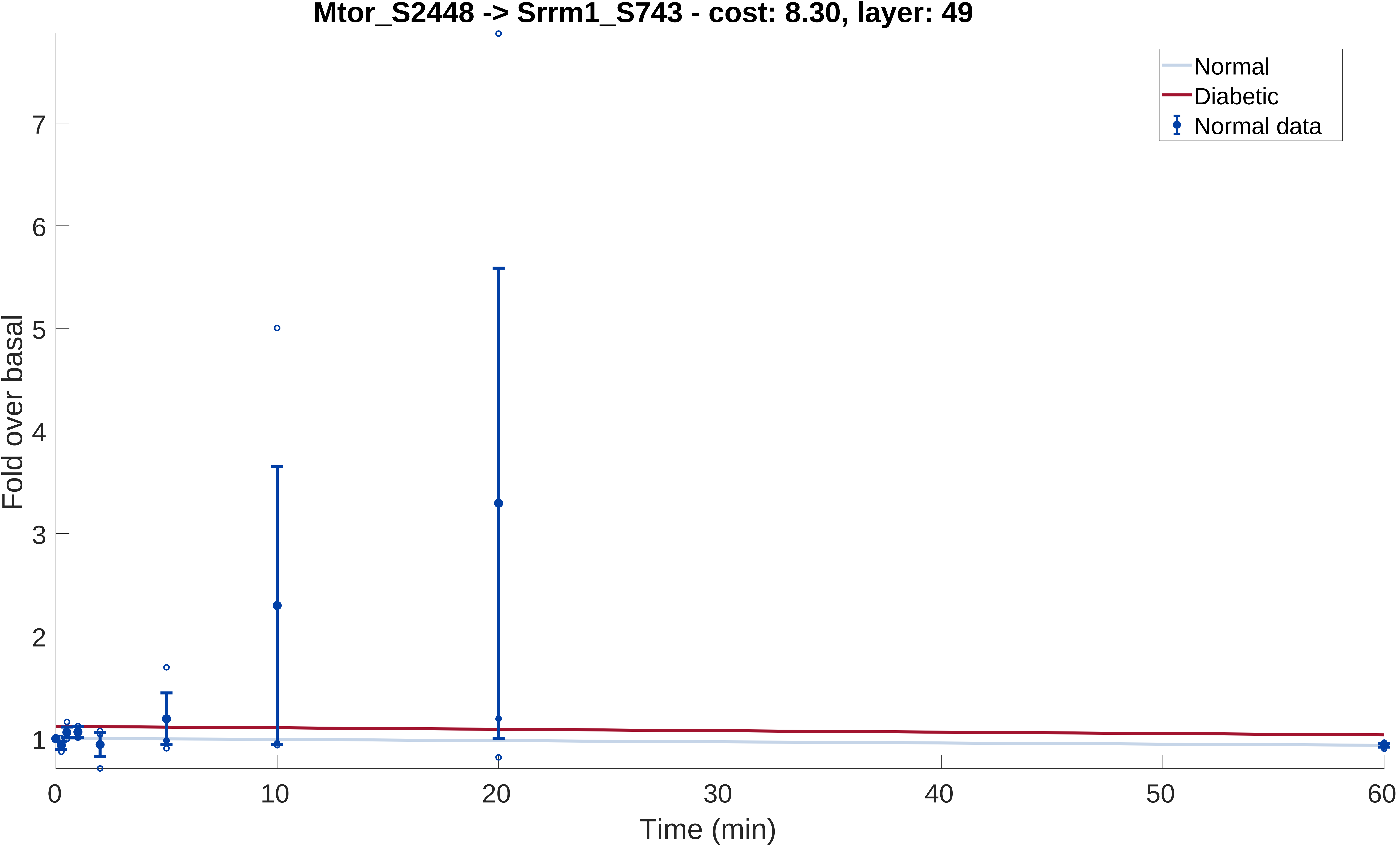

Mtor_S2448 -> Srrm1_S743 - cost: 8.30, layer: 49
Normal
Diabetic
Normal data
Fold over basal
Time (min)
1
2
3
4
5
6
7
0
10
20
30
40
50
60

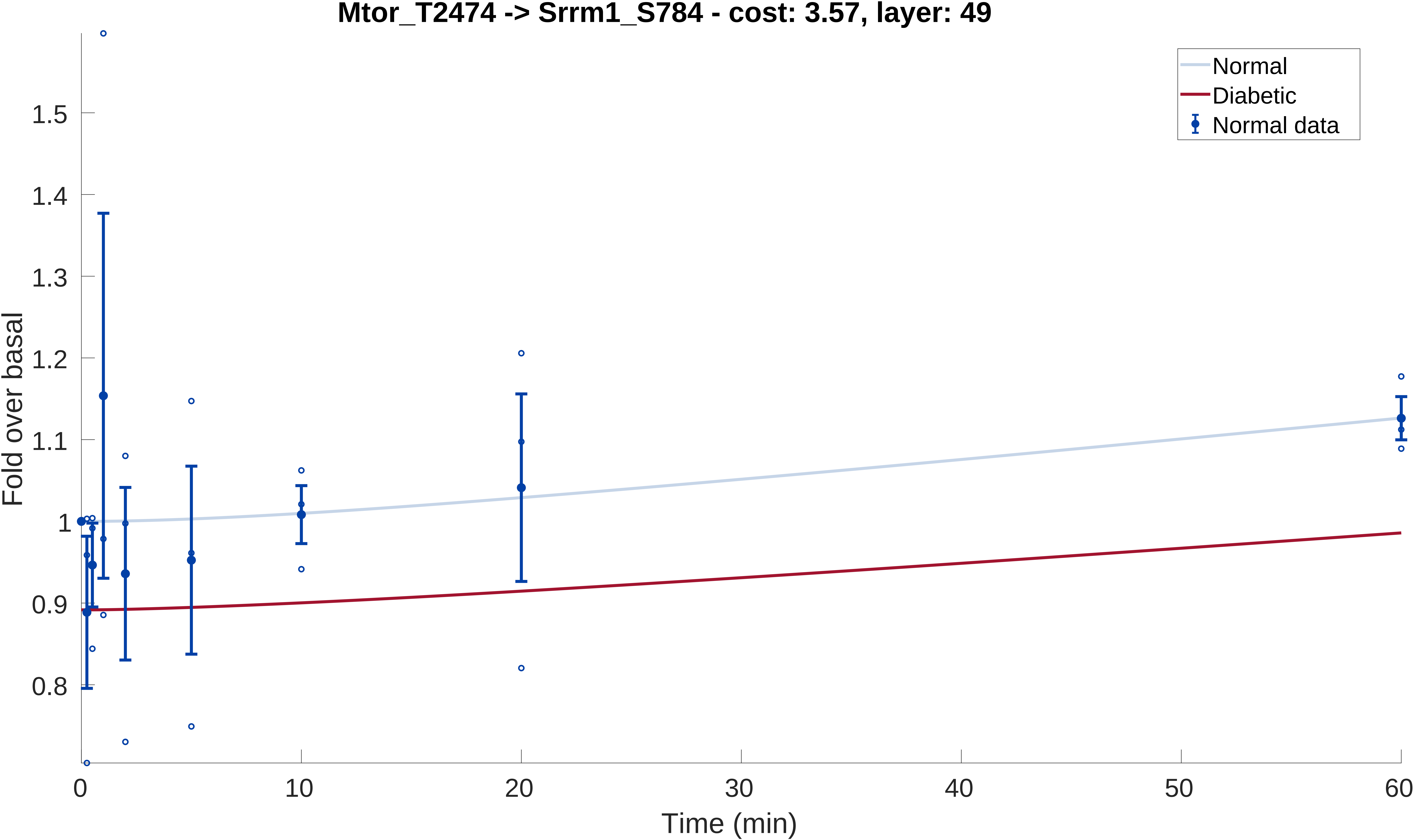

Mtor_T2474 -> Srrm1_S784 - cost: 3.57, layer: 49
Normal
Diabetic
Normal data
1.5
1.4
1.3
1.2
1.1
1
0.9
0.8
Fold over basal
0
10
20
30
40
50
60
Time (min)

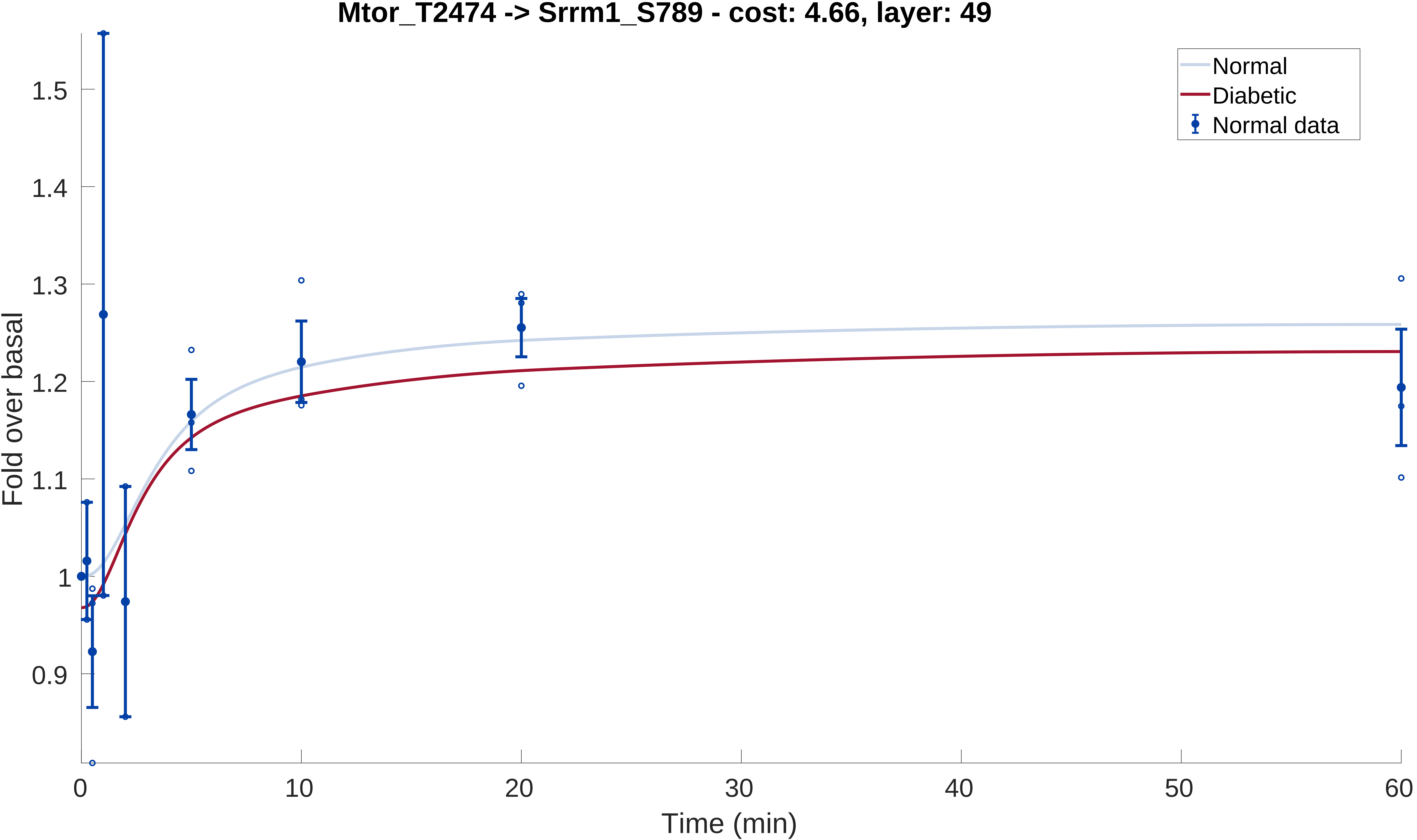
Mtor_T2474 -> Srrm1_S789 - cost: 4.66, layer: 49
Fold over basal
Time (min)
Normal
Diabetic
Normal data
0.9
1
1.1
1.2
1.3
1.4
1.5
0
10
20
30
40
50
60

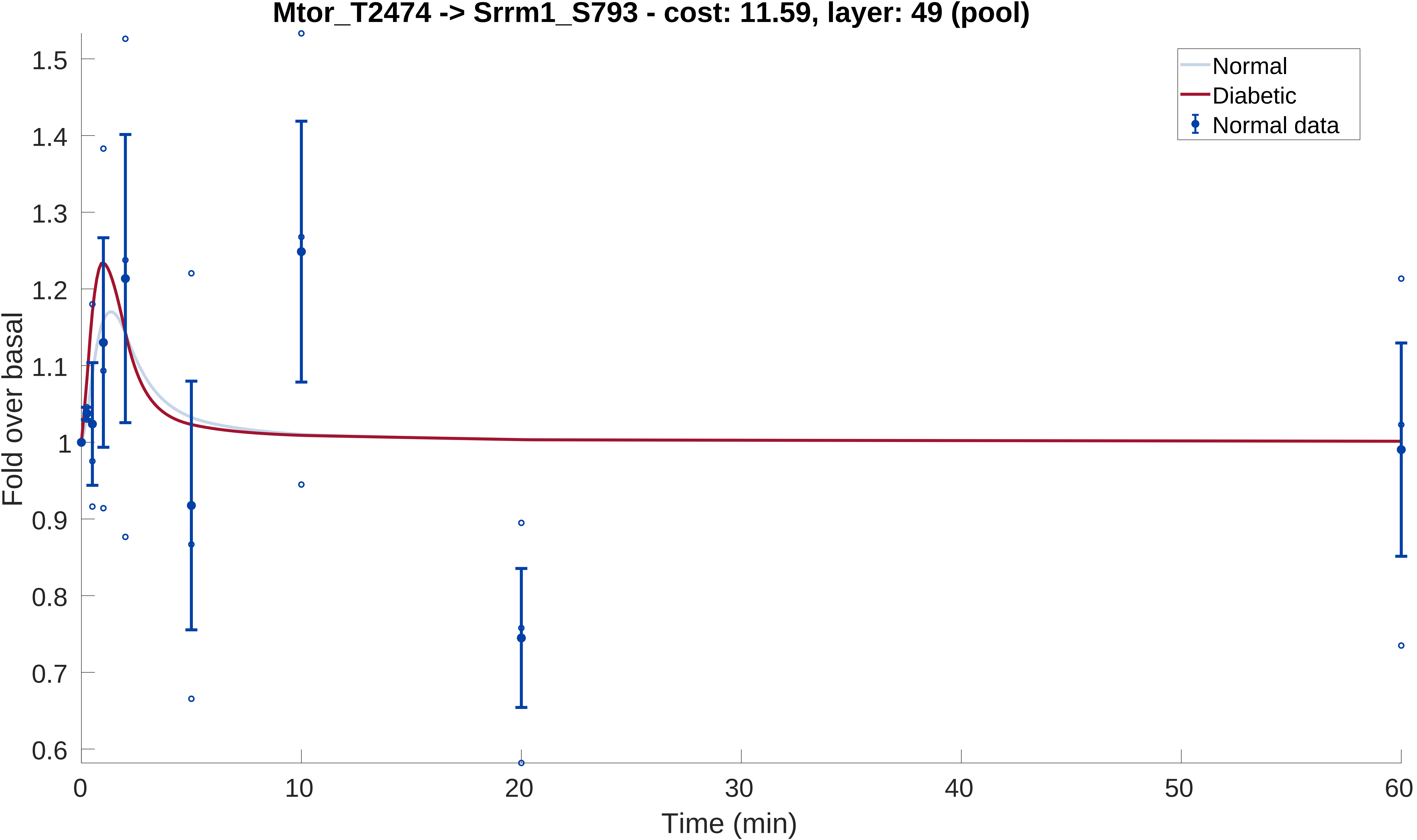
Mtor_T2474 -> Srrm1_S793 - cost: 11.59, layer: 49 (pool)
Fold over basal
Time (min)
Normal
Diabetic
Normal data
0.6
0.7
0.8
0.9
1
1.1
1.2
1.3
1.4
1.5
0
10
20
30
40
50
60

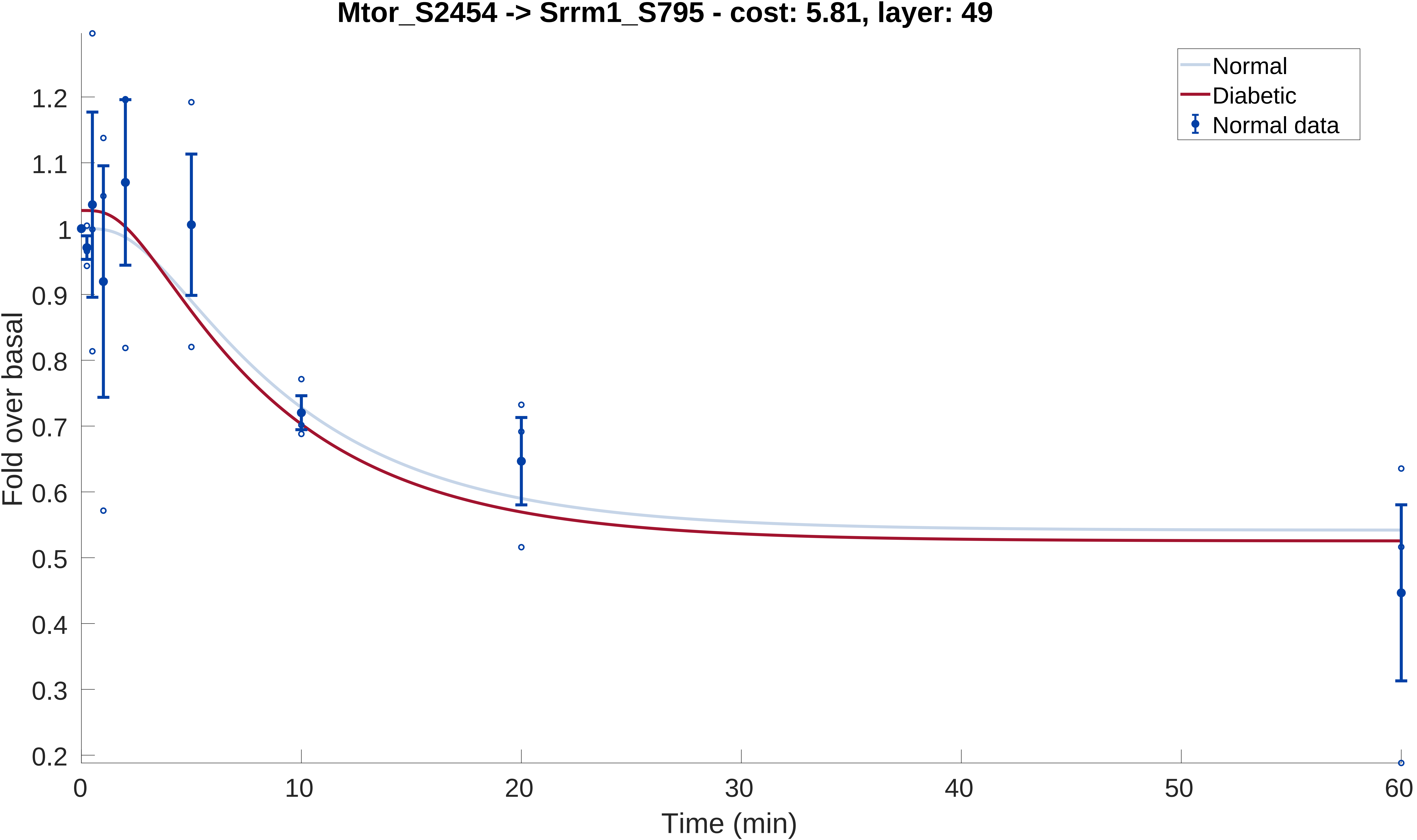

Mtor_S2454 -> Srrm1_S795 - cost: 5.81, layer: 49
Normal
Diabetic
Normal data
Fold over basal
Time (min)
0.2
0.3
0.4
0.5
0.6
0.7
0.8
0.9
1
1.1
1.2
0
10
20
30
40
50
60

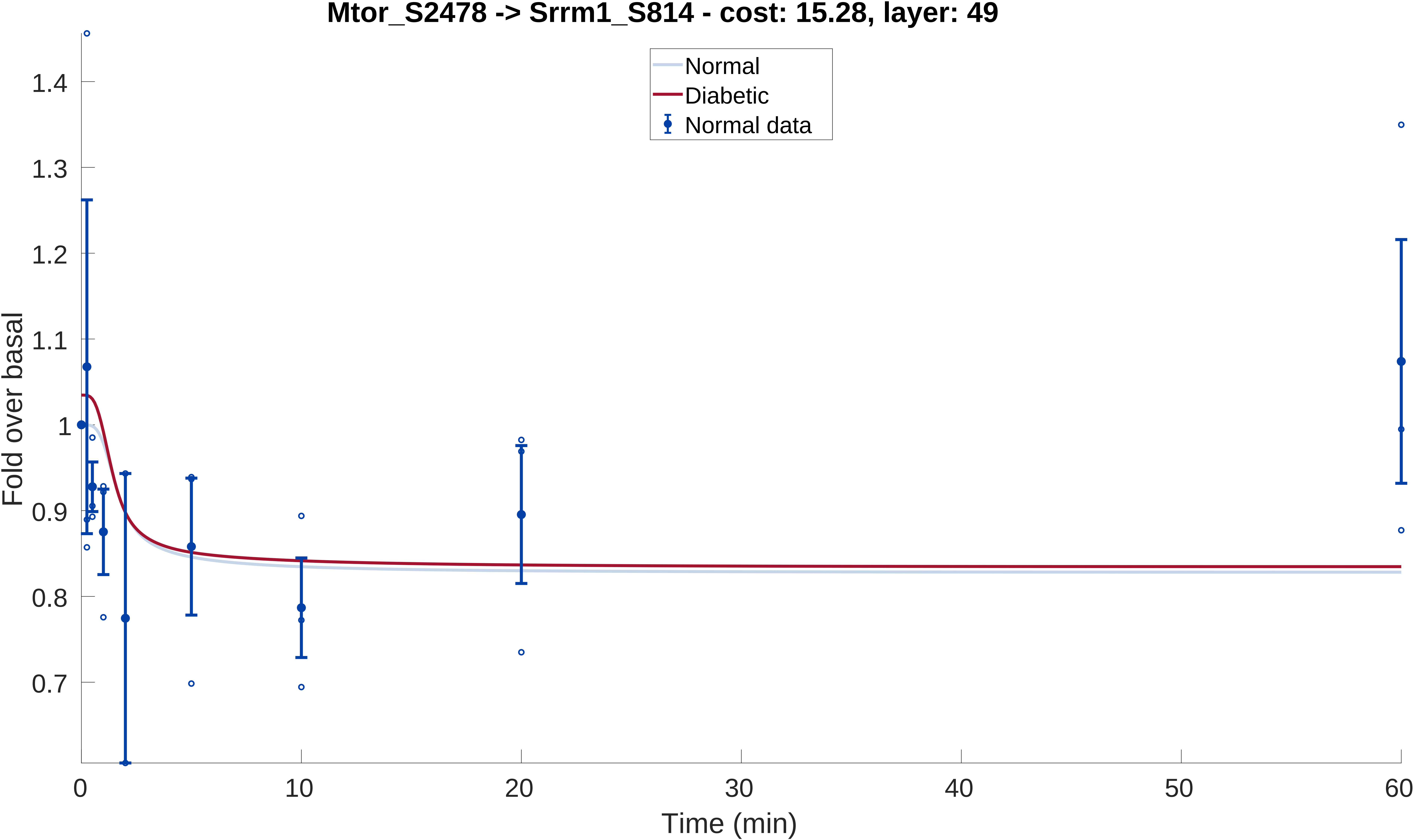

Mtor_S2478 -> Srrm1_S814 - cost: 15.28, layer: 49
Normal
Diabetic
Normal data
Fold over basal
1.4
1.3
1.2
1.1
1
0.9
0.8
0.7
0
10
20
30
40
50
60
Time (min)

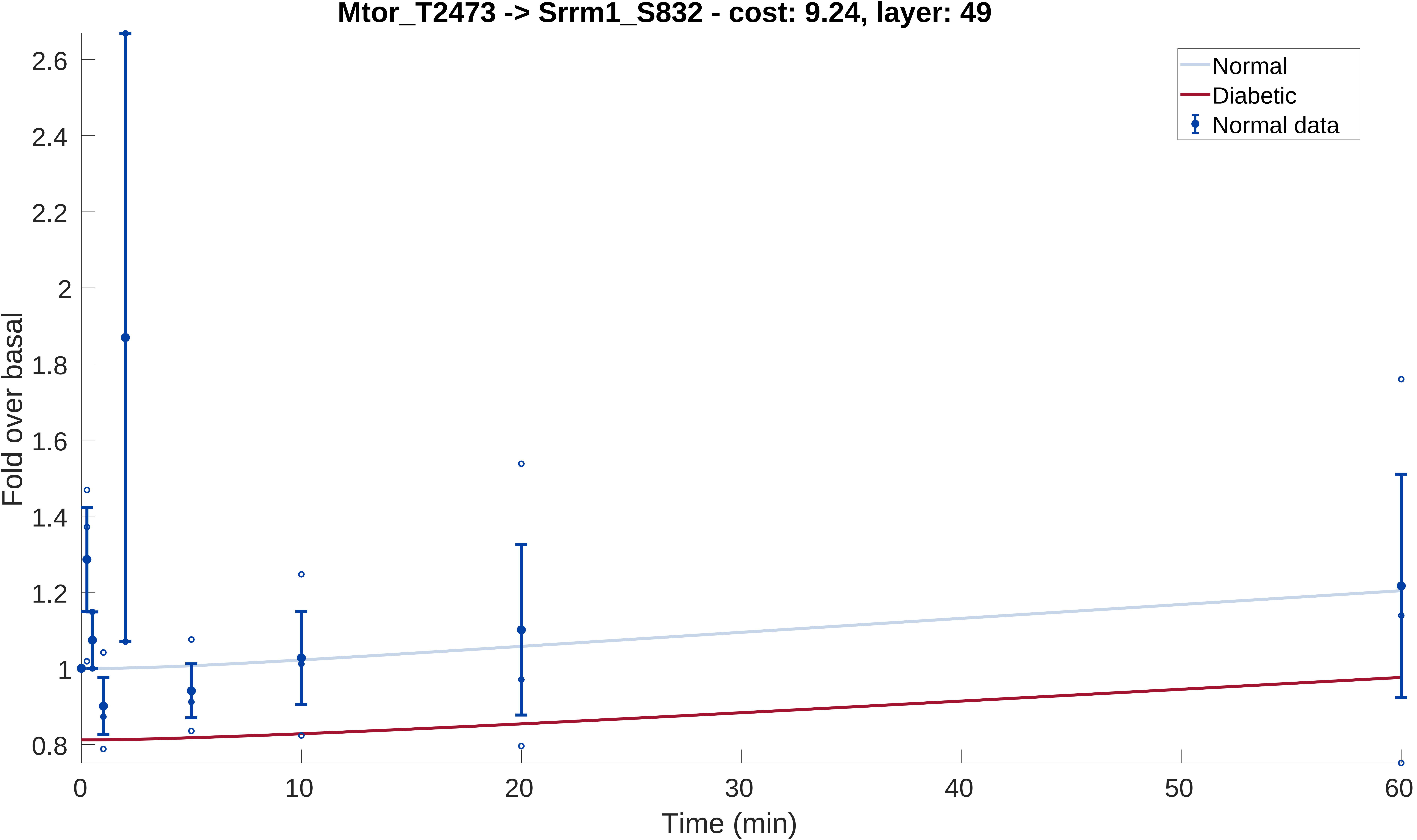

Mtor_T2473 -> Srrm1_S832 - cost: 9.24, layer: 49
Normal
Diabetic
Normal data
Fold over basal
2.6
2.4
2.2
2
1.8
1.6
1.4
1.2
1
0.8
0
10
20
30
40
50
60
Time (min)

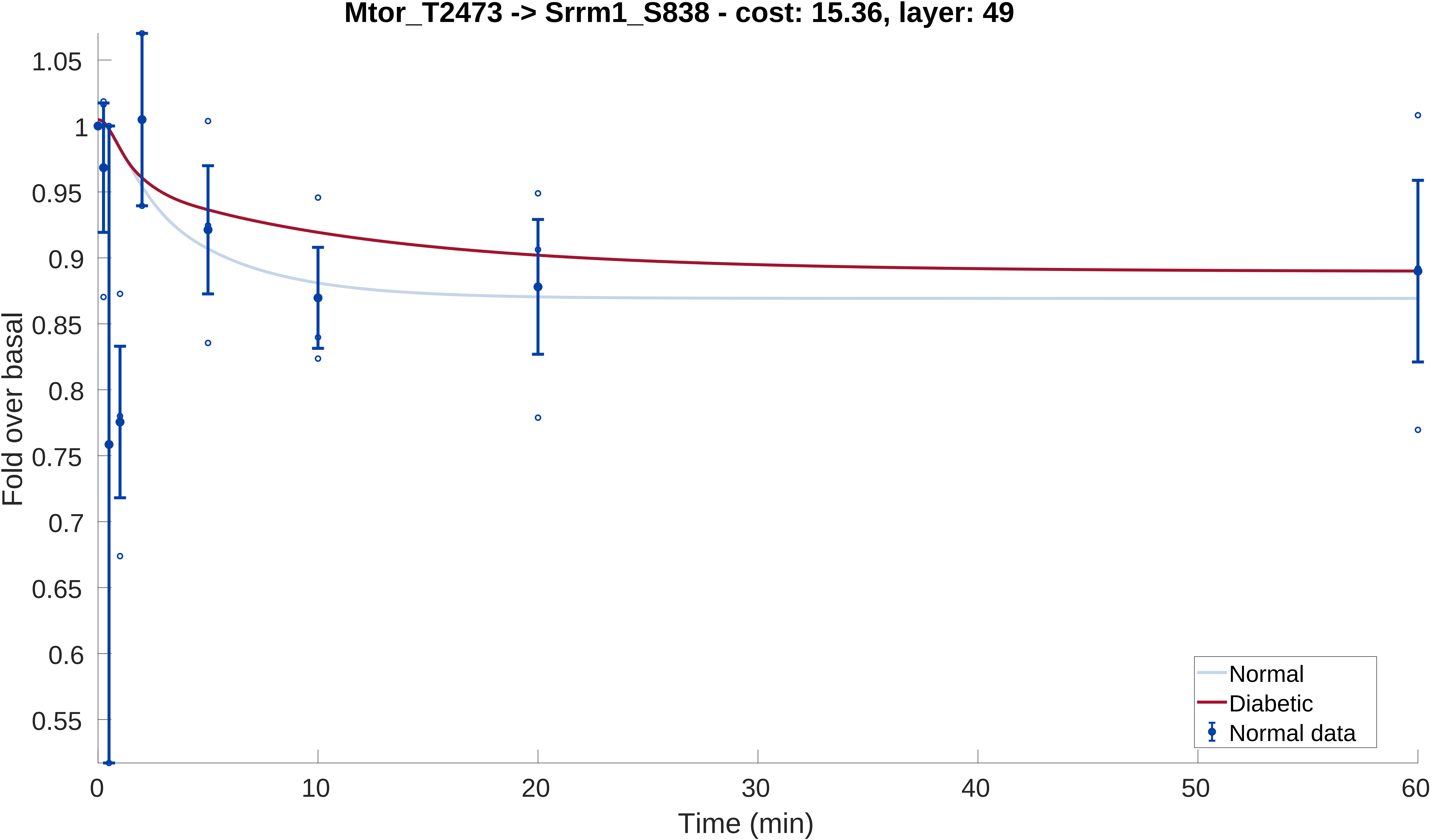

Mtor_T2473 -> Srrm1_S838 - cost: 15.36, layer: 49
Fold over basal
1.05
1
0.95
0.9
0.85
0.8
0.75
0.7
0.65
0.6
0.55
0
10
20
30
40
50
60
Time (min)
Normal
Diabetic
Normal data

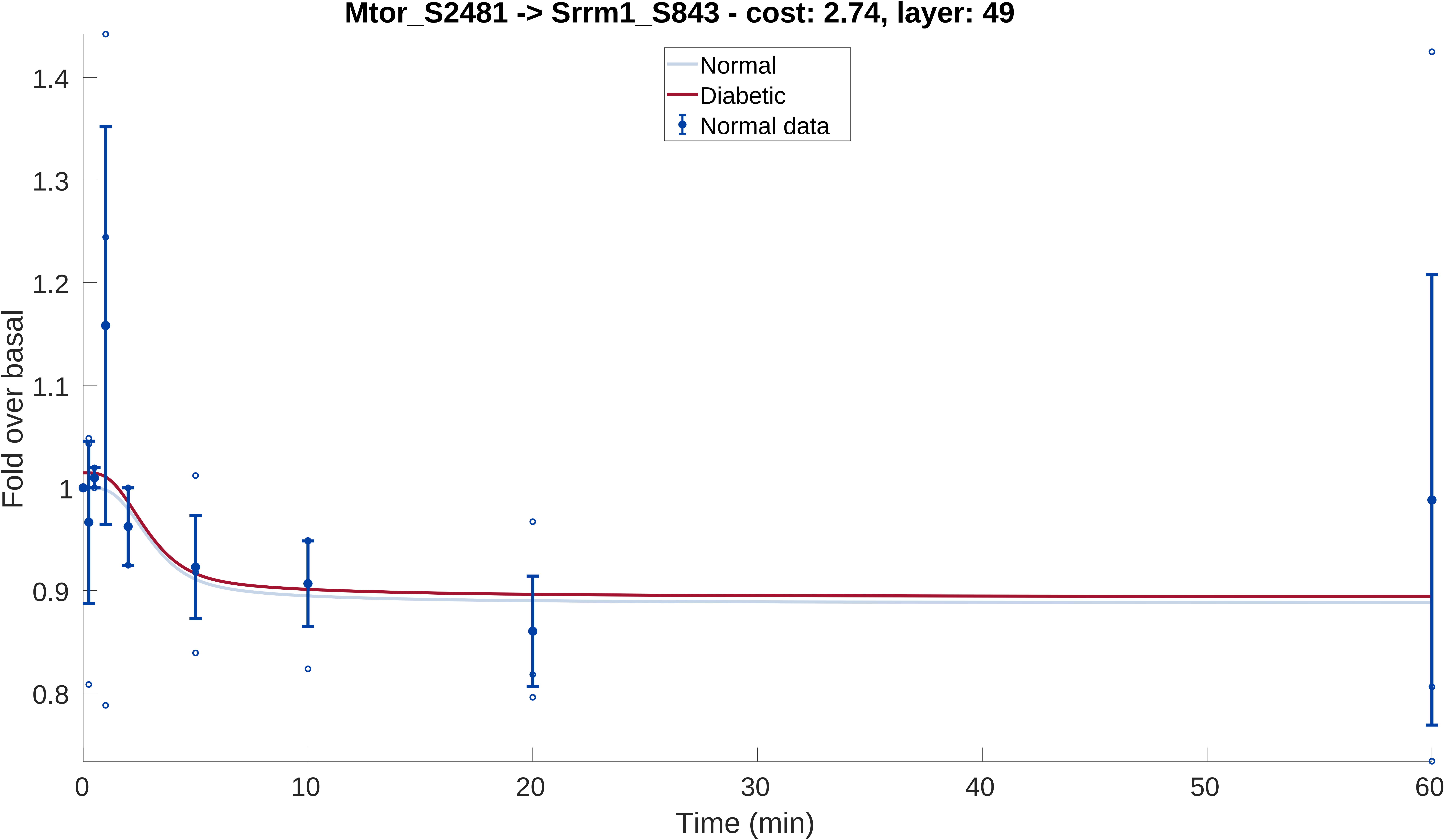

Mtor_S2481 -> Srrm1_S843 - cost: 2.74, layer: 49
Normal
Diabetic
Normal data
Fold over basal
1.4
1.3
1.2
1.1
1
0.9
0.8
0
10
20
30
40
50
60
Time (min)

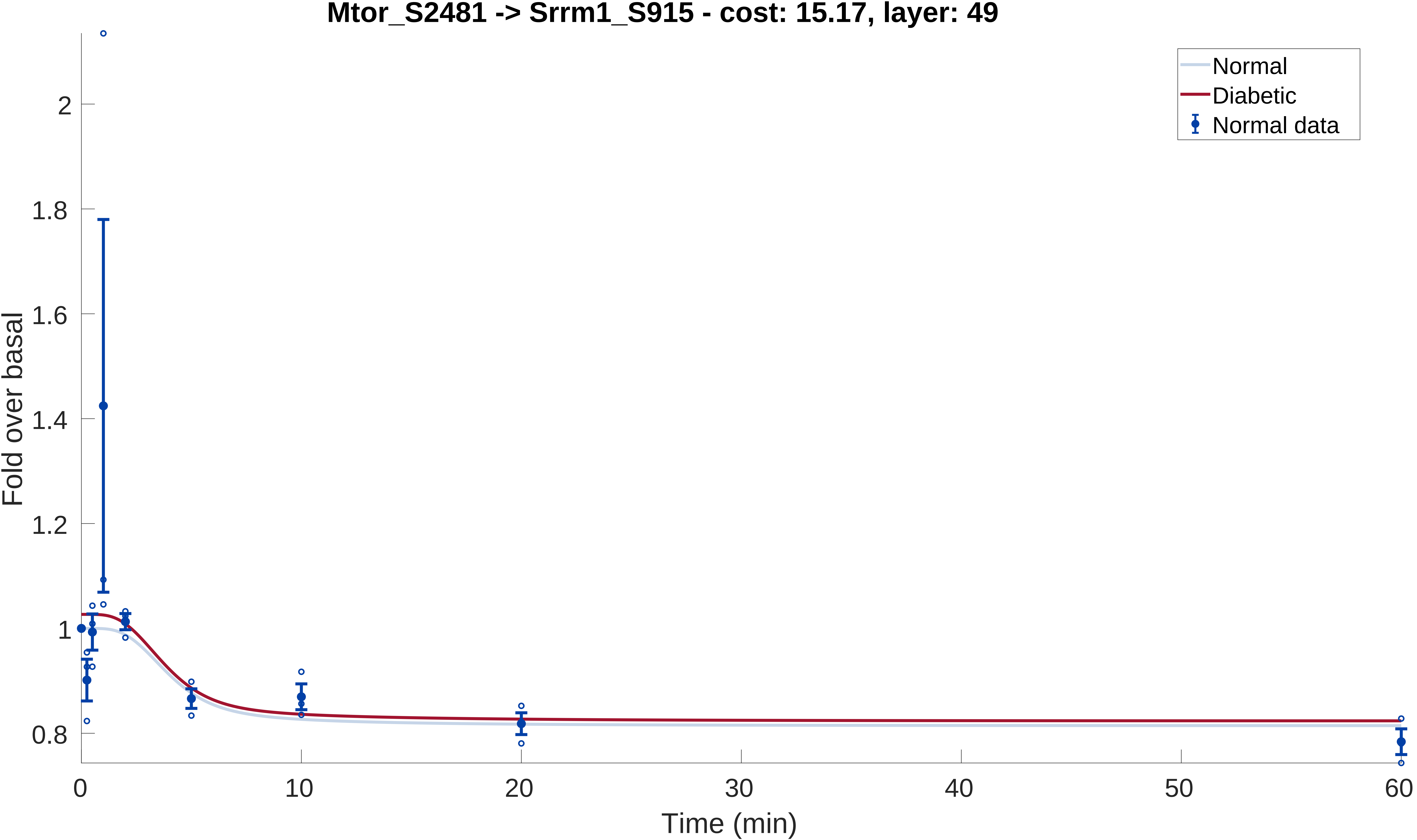

Mtor_S2481 -> Srrm1_S915 - cost: 15.17, layer: 49
Normal
Diabetic
Normal data
Fold over basal
Time (min)
0.8
1
1.2
1.4
1.6
1.8
2
0
10
20
30
40
50
60

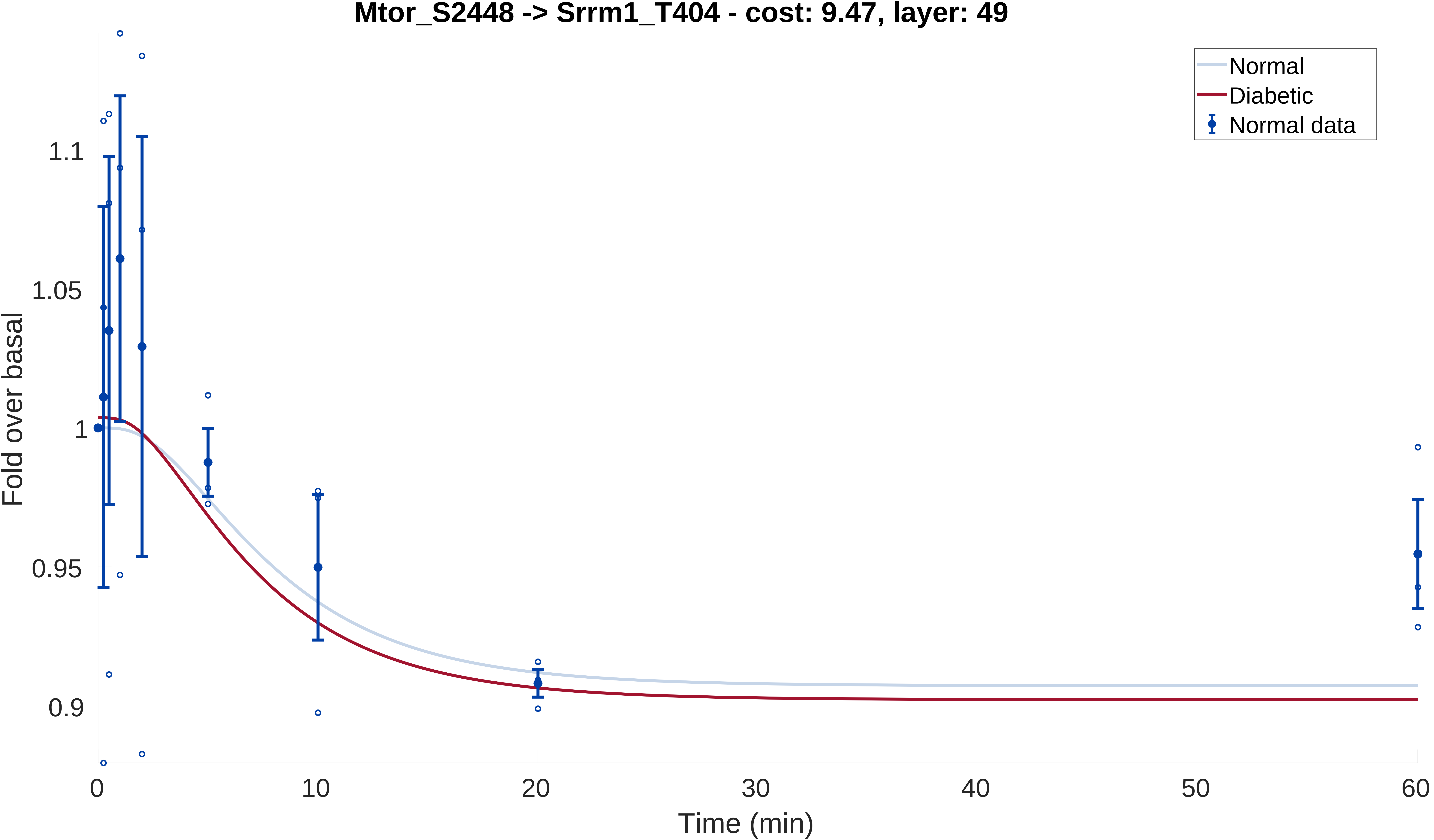

Mtor_S2448 -> Srrm1_T404 - cost: 9.47, layer: 49
Normal
Diabetic
Normal data
Fold over basal
1.1
1.05
1
0.95
0.9
0
10
20
30
40
50
60
Time (min)

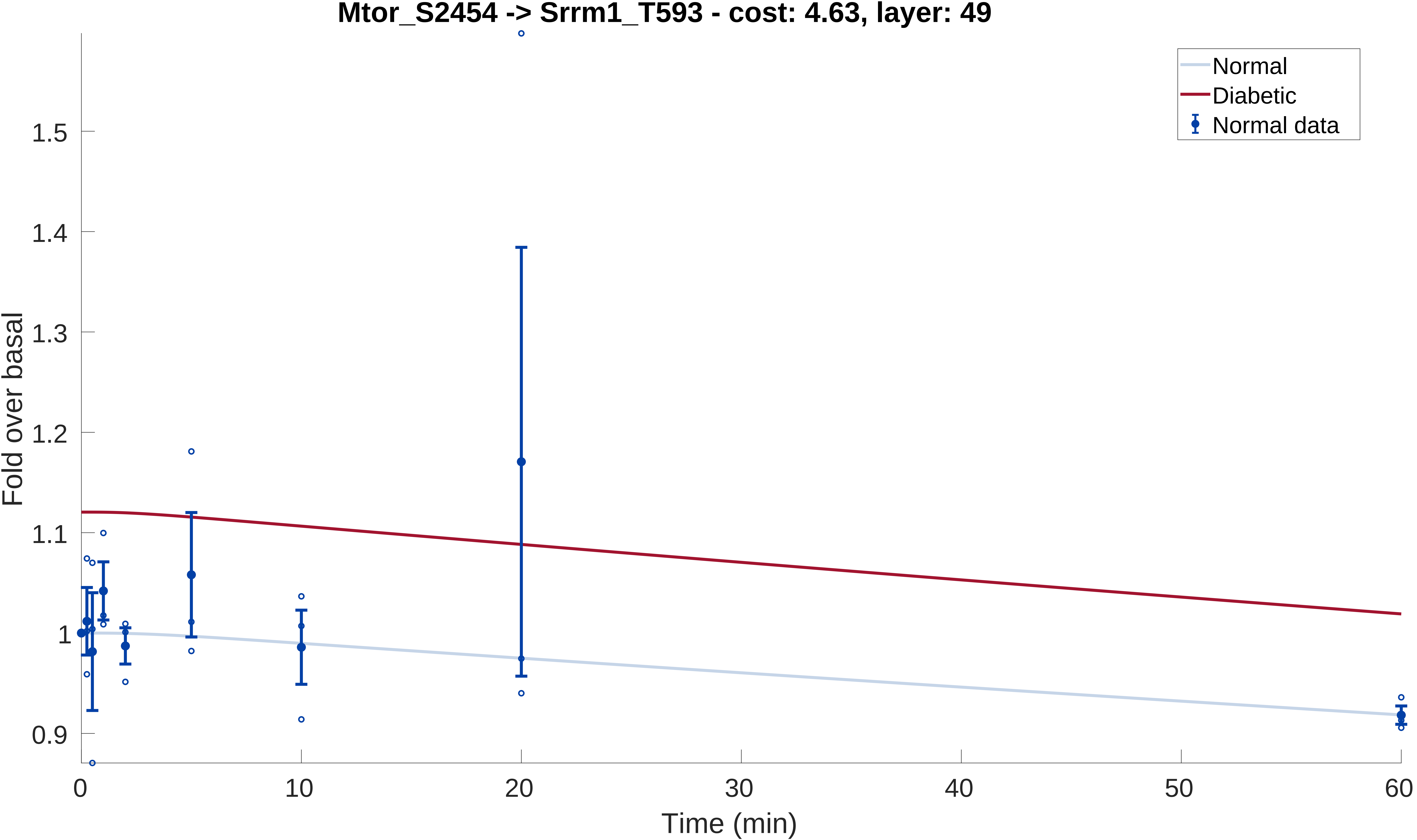

Mtor_S2454 -> Srrm1_T593 - cost: 4.63, layer: 49
Normal
Diabetic
Normal data
Fold over basal
Time (min)
0.9
1
1.1
1.2
1.3
1.4
1.5
0
10
20
30
40
50
60

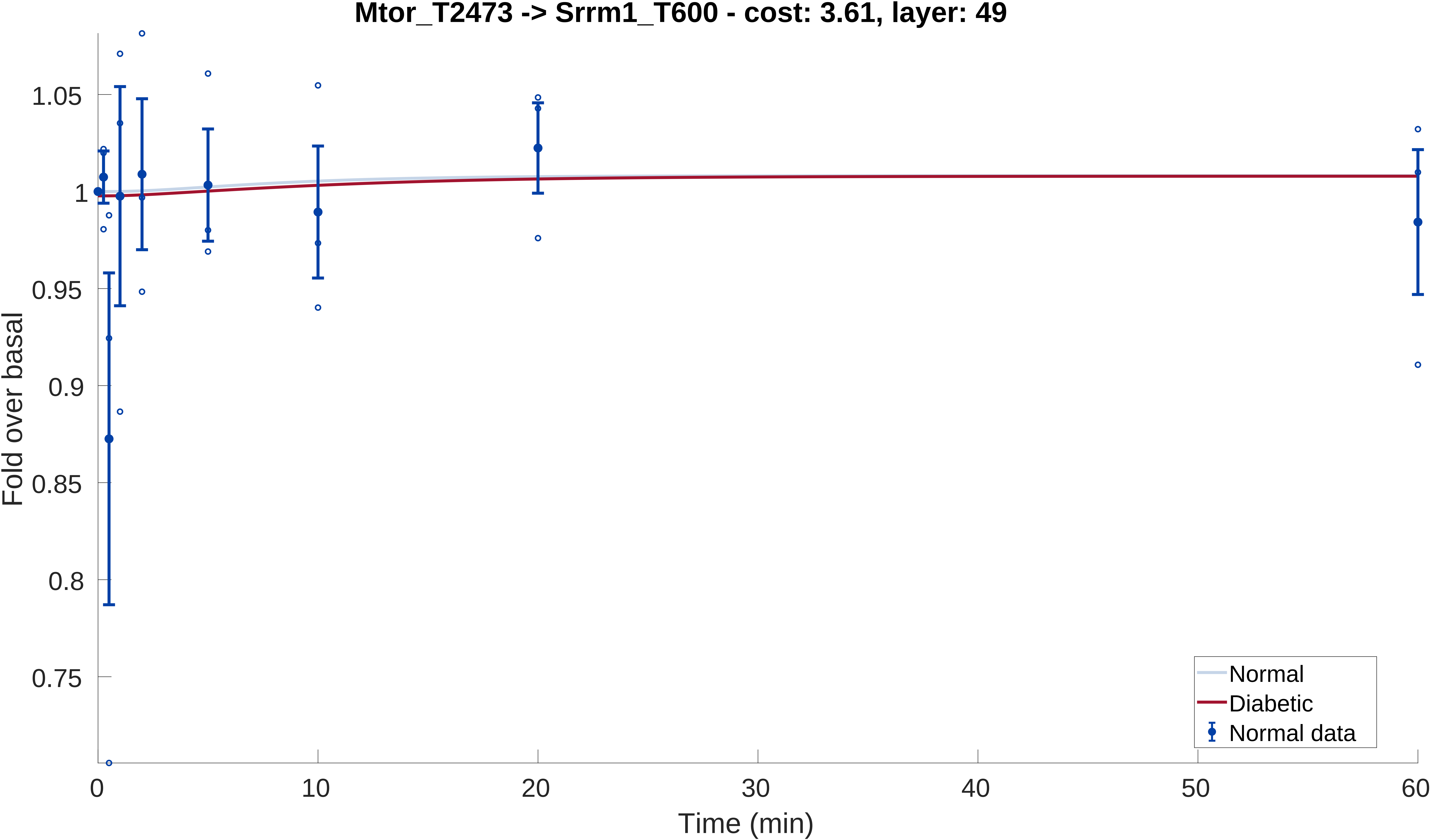

Mtor_T2473 -> Srrm1_T600 - cost: 3.61, layer: 49
1.05
1
0.95
0.9
0.85
0.8
0.75
Fold over basal
0
10
20
30
40
50
60
Time (min)
Normal
Diabetic
Normal data

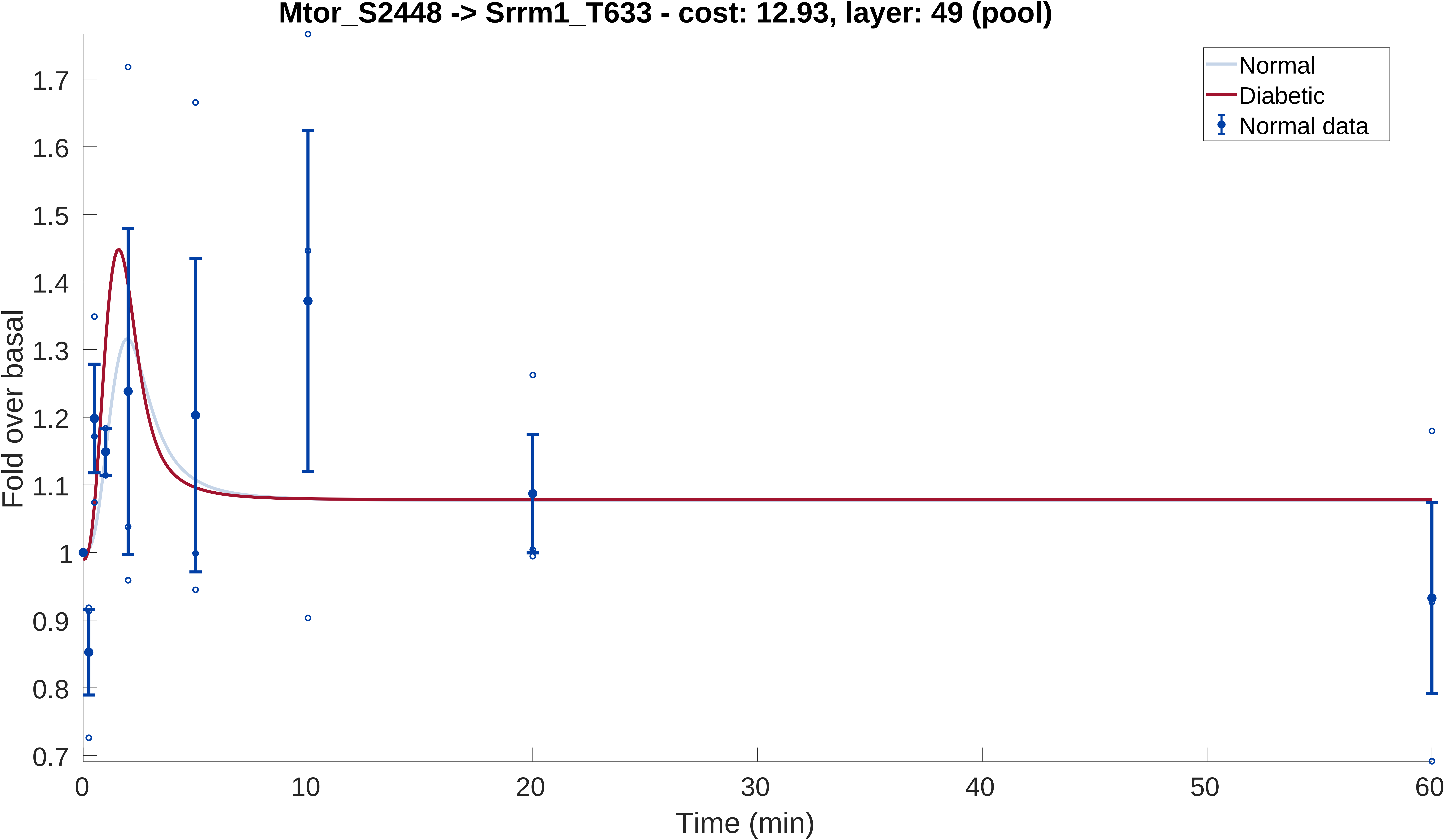

Mtor_S2448 -> Srrm1_T633 - cost: 12.93, layer: 49 (pool)
Normal
Diabetic
Normal data
Fold over basal
1.7
1.6
1.5
1.4
1.3
1.2
1.1
1
0.9
0.8
0.7
0
10
20
30
40
50
60
Time (min)

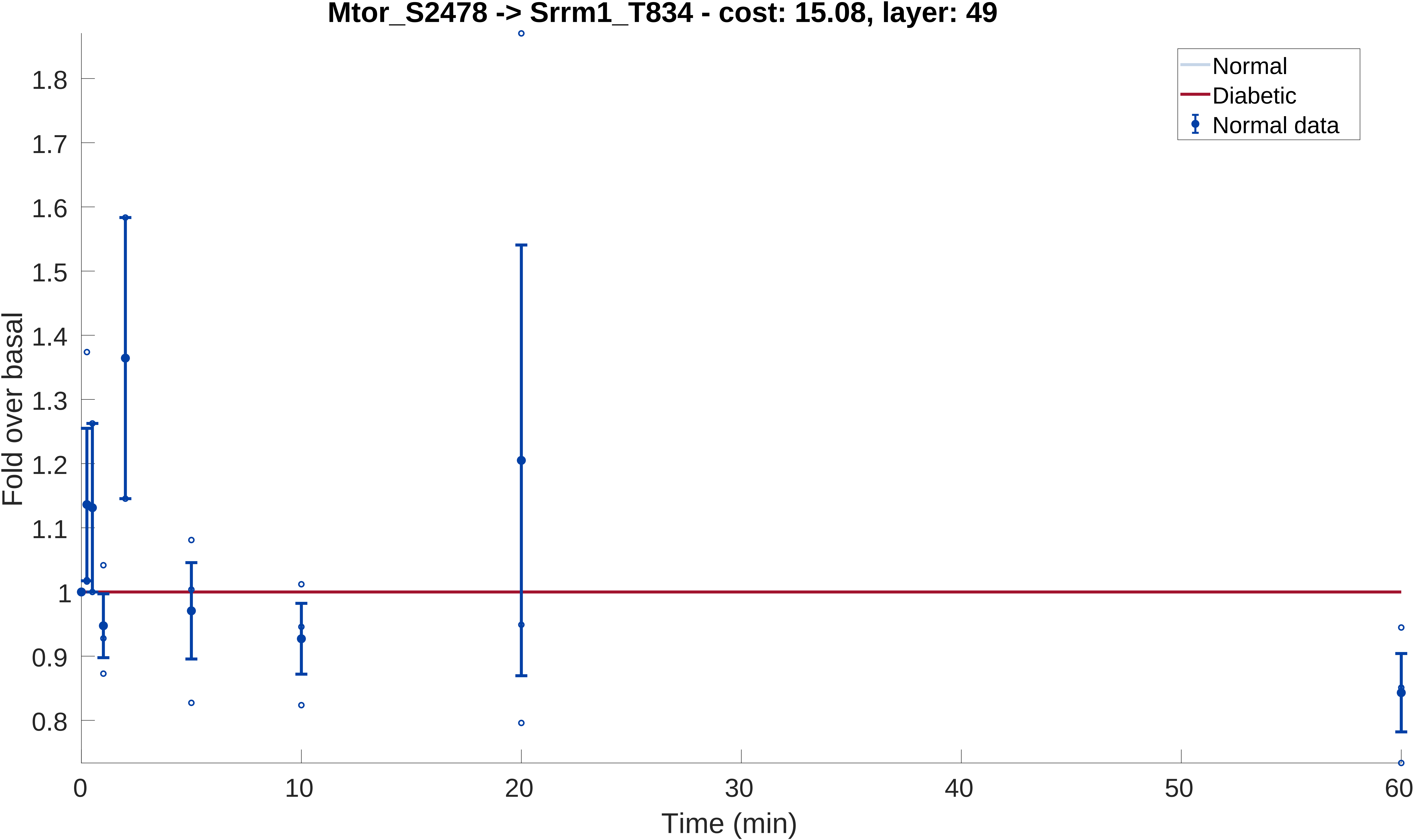
Mtor_S2478 -> Srrm1_T834 - cost: 15.08, layer: 49
Normal
Diabetic
Normal data
Fold over basal
Time (min)
1.8
1.7
1.6
1.5
1.4
1.3
1.2
1.1
1
0.9
0.8
0
10
20
30
40
50
60

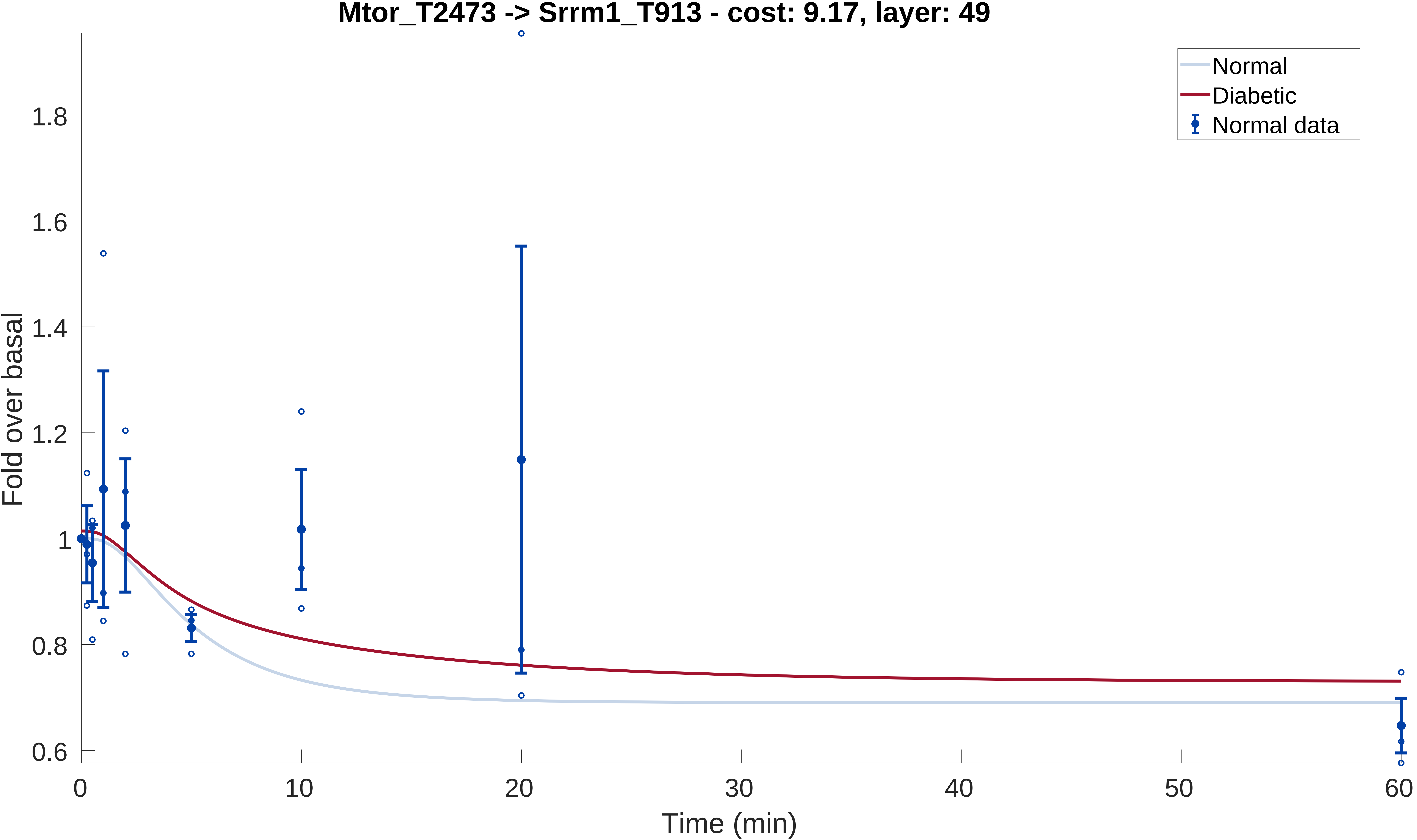

Mtor_T2473 -> Srrm1_T913 - cost: 9.17, layer: 49
Normal
Diabetic
Normal data
1.8
1.6
1.4
1.2
1
0.8
0.6
Fold over basal
0
10
20
30
40
50
60
Time (min)

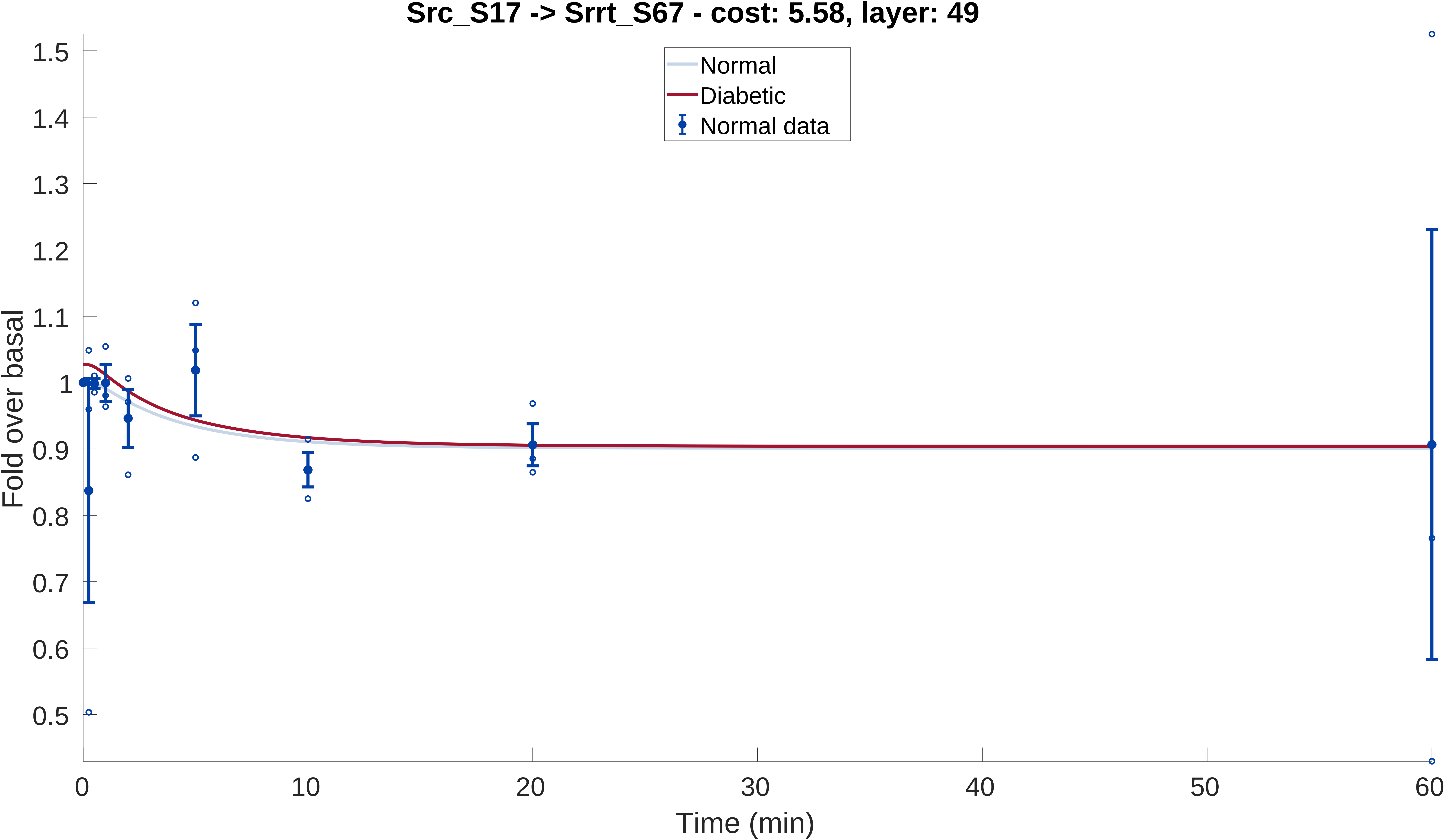

Src_S17 -> Srrt_S67 - cost: 5.58, layer: 49
Normal
Diabetic
Normal data
Fold over basal
1.5
1.4
1.3
1.2
1.1
1
0.9
0.8
0.7
0.6
0.5
0
10
20
30
40
50
60
Time (min)

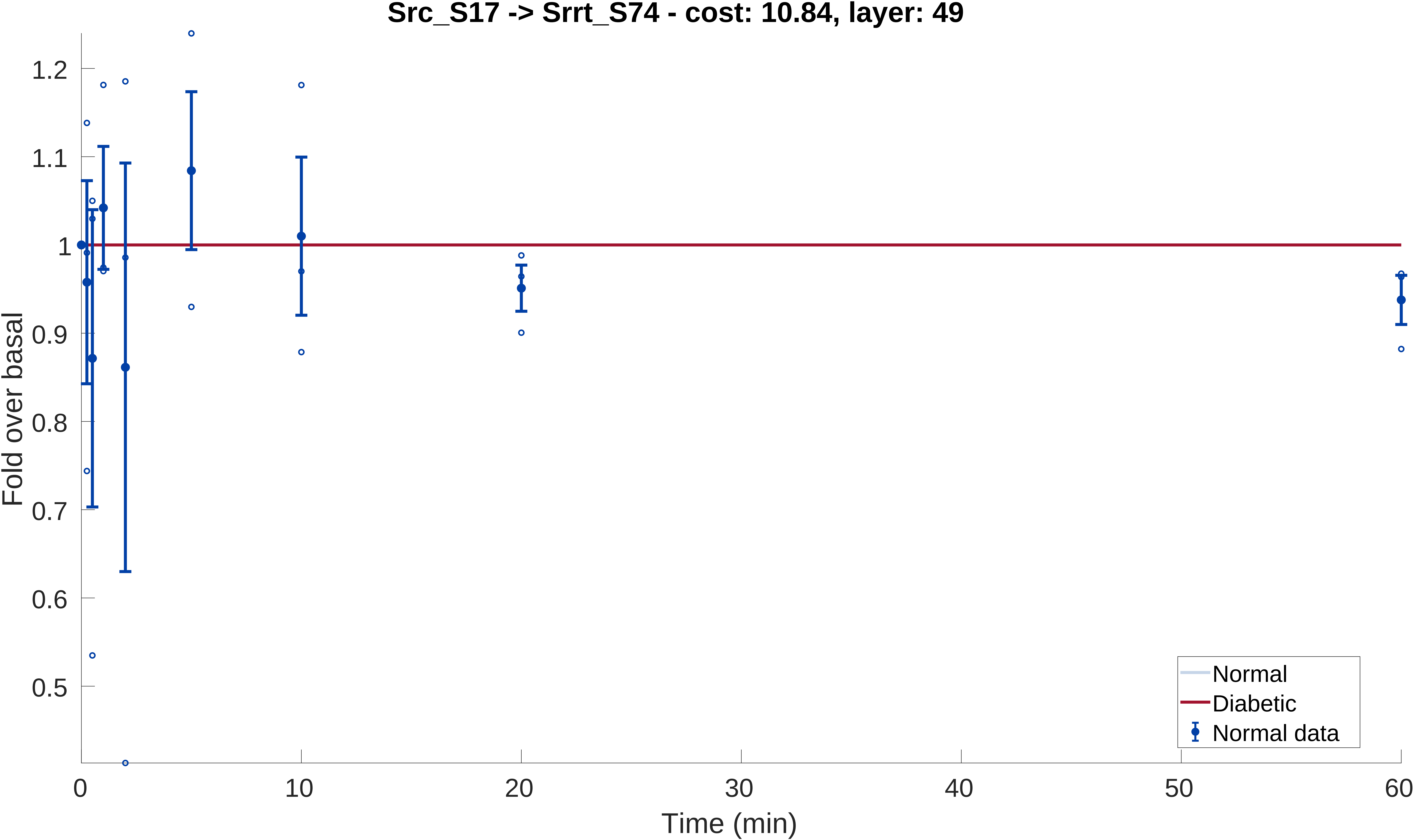

Src_S17 -> Srrt_S74 - cost: 10.84, layer: 49
Fold over basal
1.2
1.1
1
0.9
0.8
0.7
0.6
0.5
0
10
20
30
40
50
60
Time (min)
Normal
Diabetic
Normal data

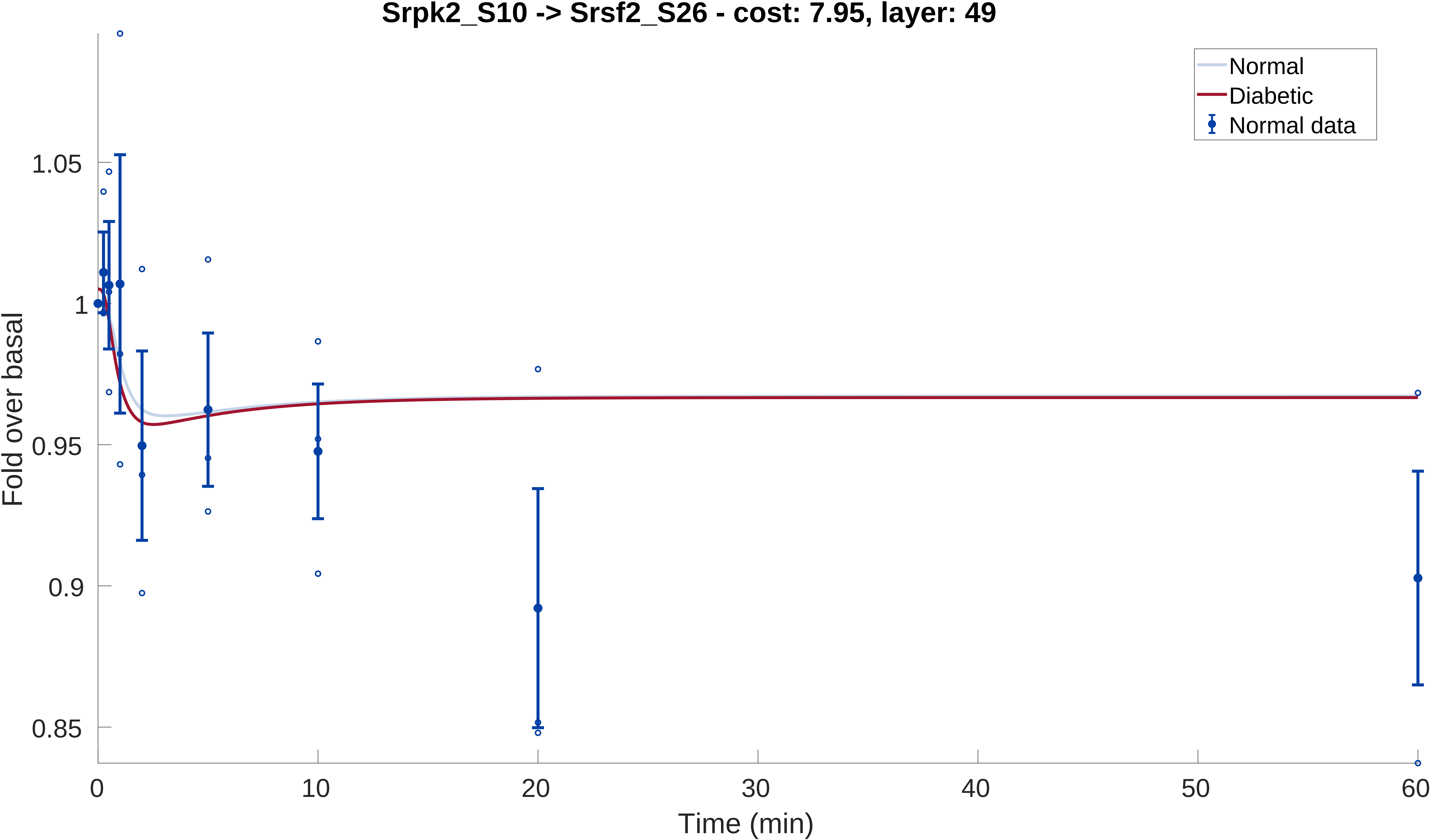

Srpk2_S10 -> Srsf2_S26 - cost: 7.95, layer: 49
Normal
Diabetic
Normal data
Fold over basal
1.05
1
0.95
0.9
0.85
0
10
20
30
40
50
60
Time (min)

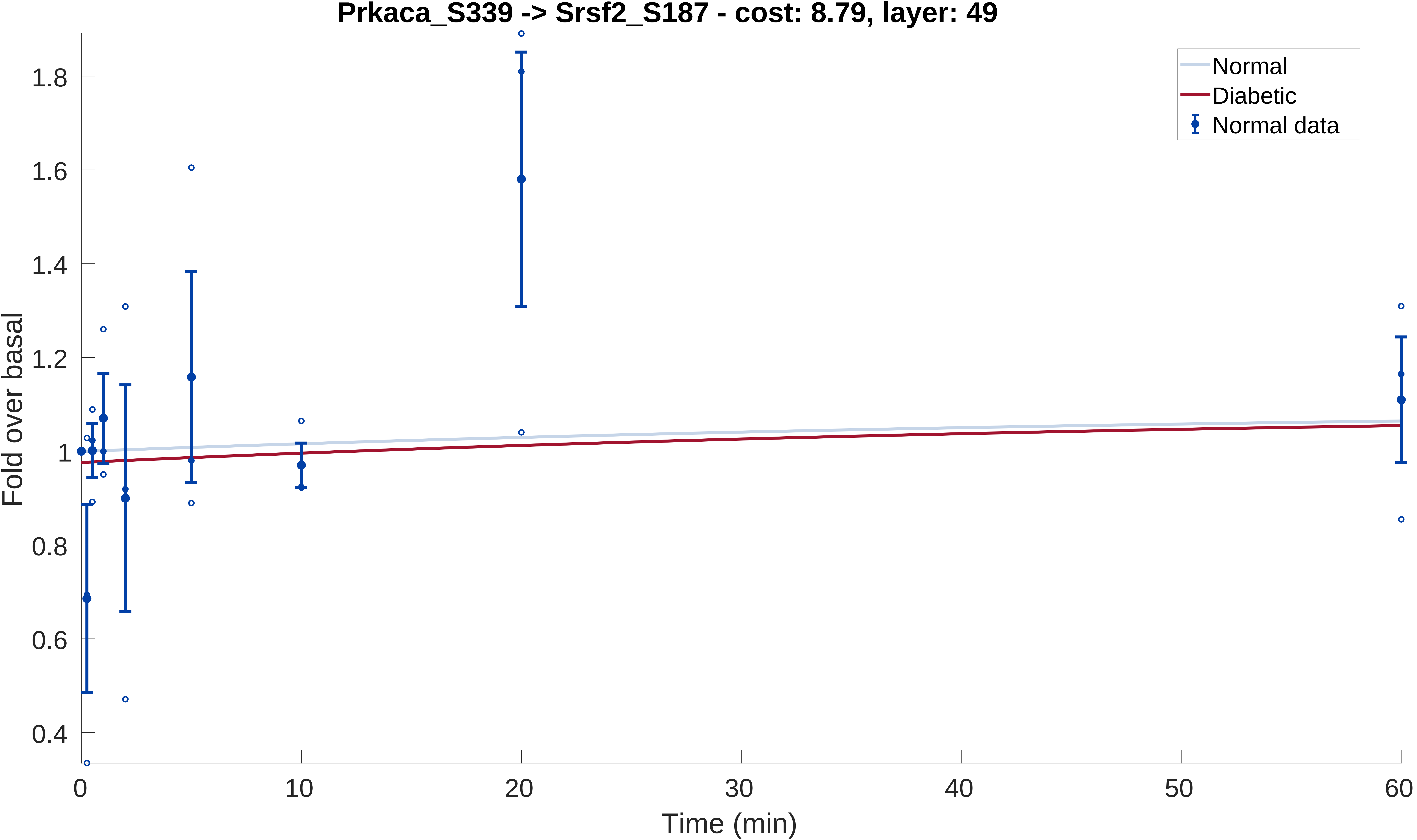
Prkaca_S339 -> Srsf2_S187 - cost: 8.79, layer: 49
Normal
Diabetic
Normal data
Fold over basal
1.8
1.6
1.4
1.2
1
0.8
0.6
0.4
0
10
20
30
40
50
60
Time (min)

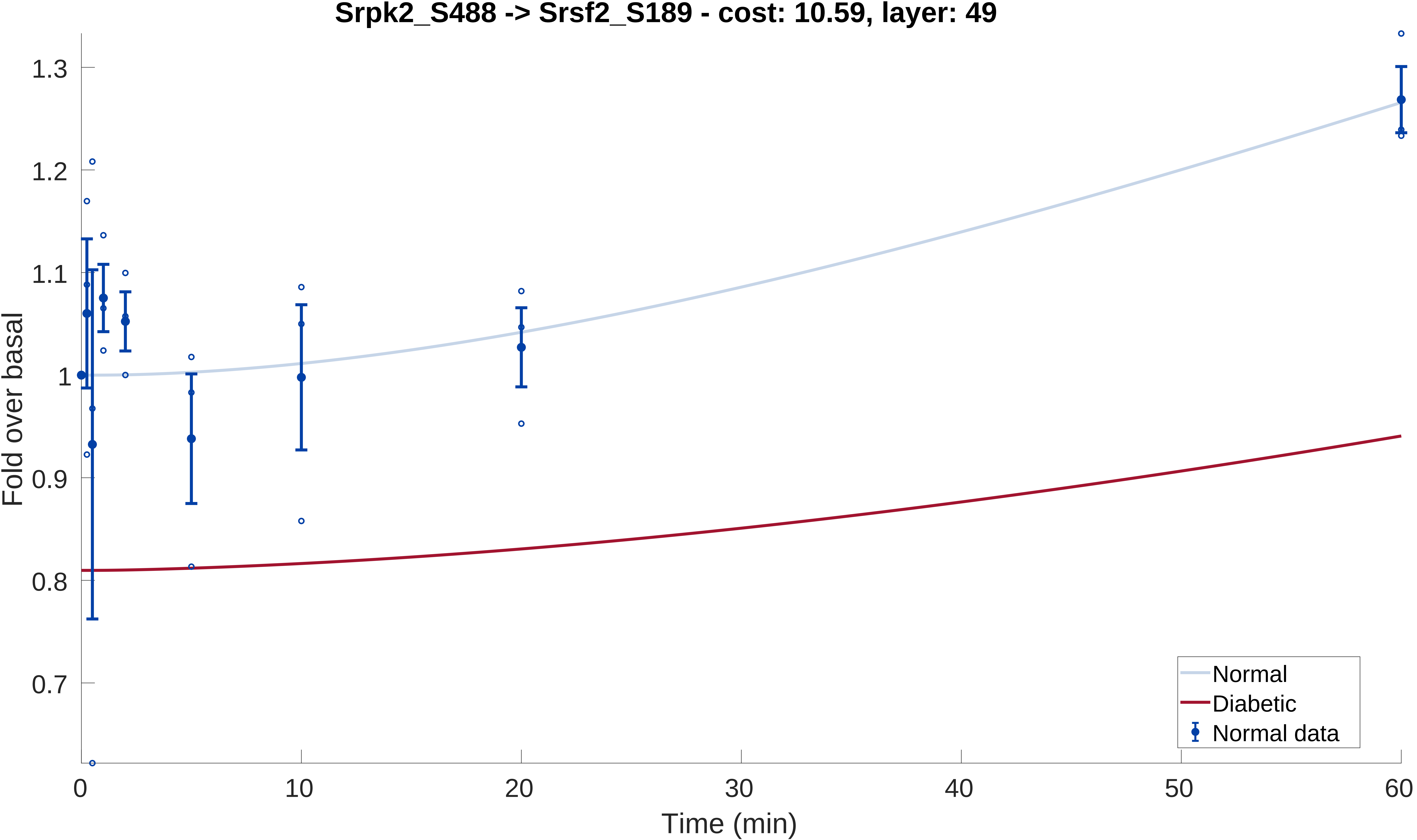

Srpk2_S488 -> Srsf2_S189 - cost: 10.59, layer: 49
Fold over basal
1.3
1.2
1.1
1
0.9
0.8
0.7
0
10
20
30
40
50
60
Time (min)
Normal
Diabetic
Normal data

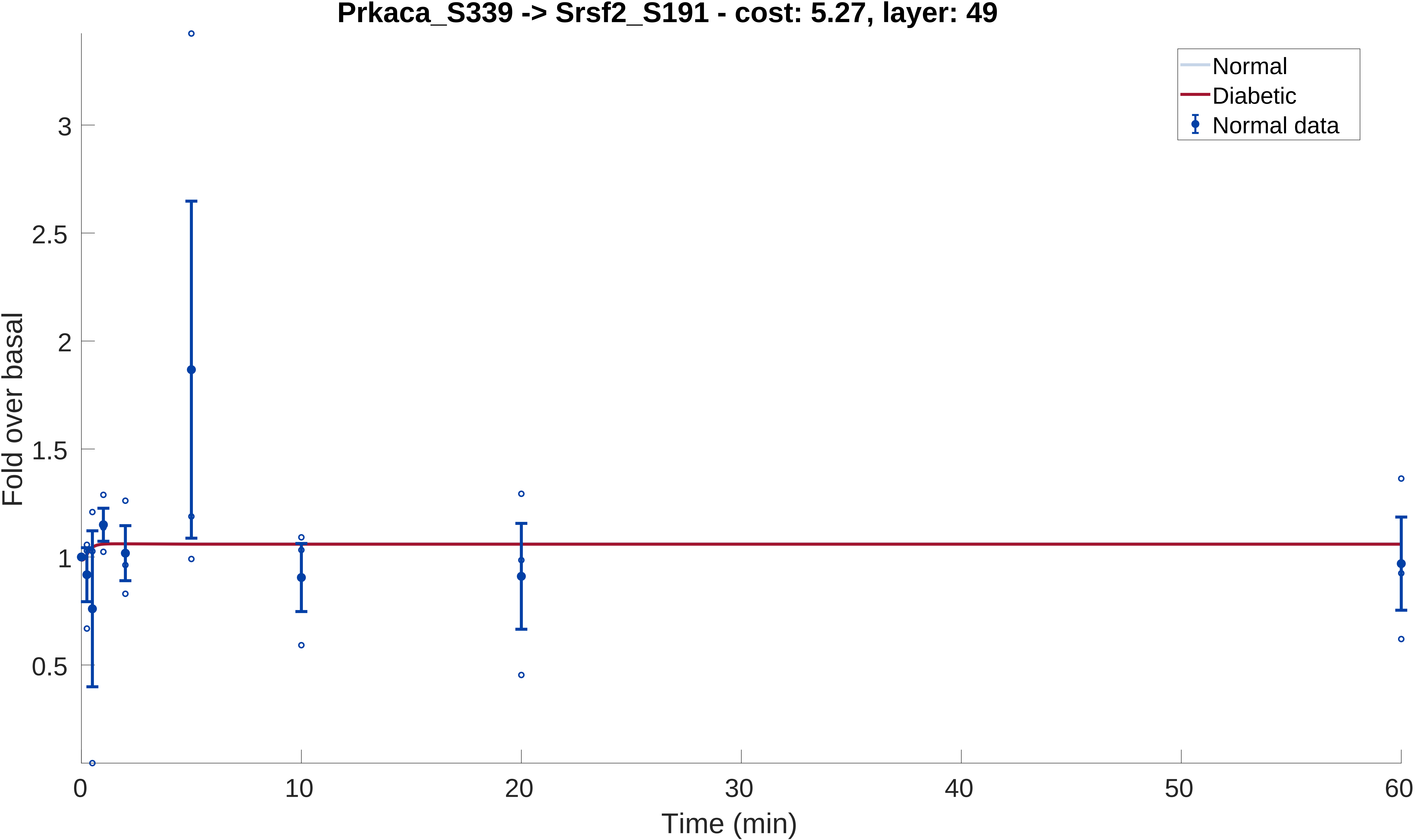

Prrkaca_S339 -> Srsf2_S191 - cost: 5.27, layer: 49
Normal
Diabetic
Normal data
Fold over basal
Time (min)
0.5
1
1.5
2
2.5
3
0
10
20
30
40
50
60

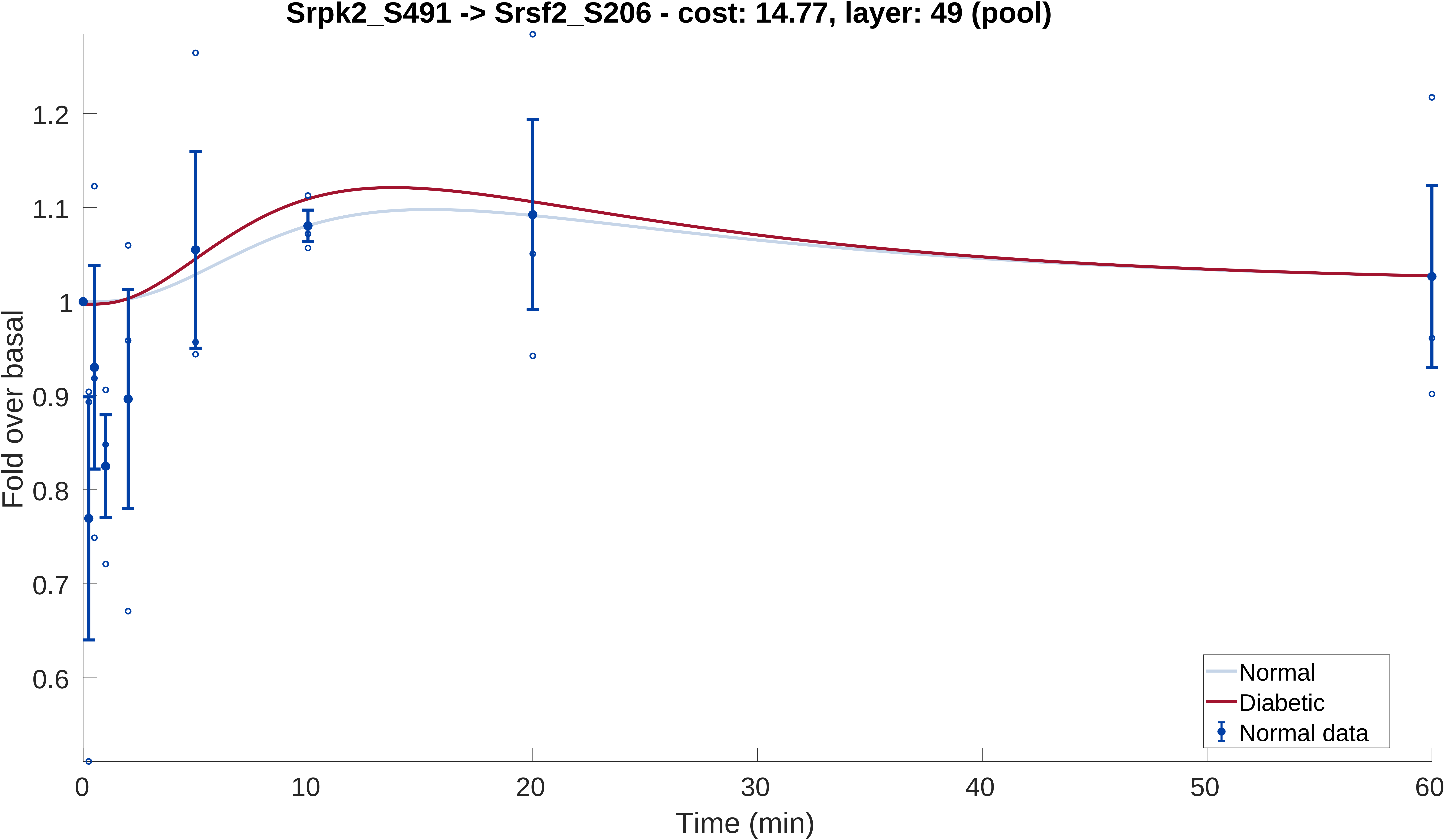

Srpk2_S491 -> Srsf2_S206 - cost: 14.77, layer: 49 (pool)
Fold over basal
0.6
0.7
0.8
0.9
1
1.1
1.2
Time (min)
0
10
20
30
40
50
60
Normal
Diabetic
Normal data

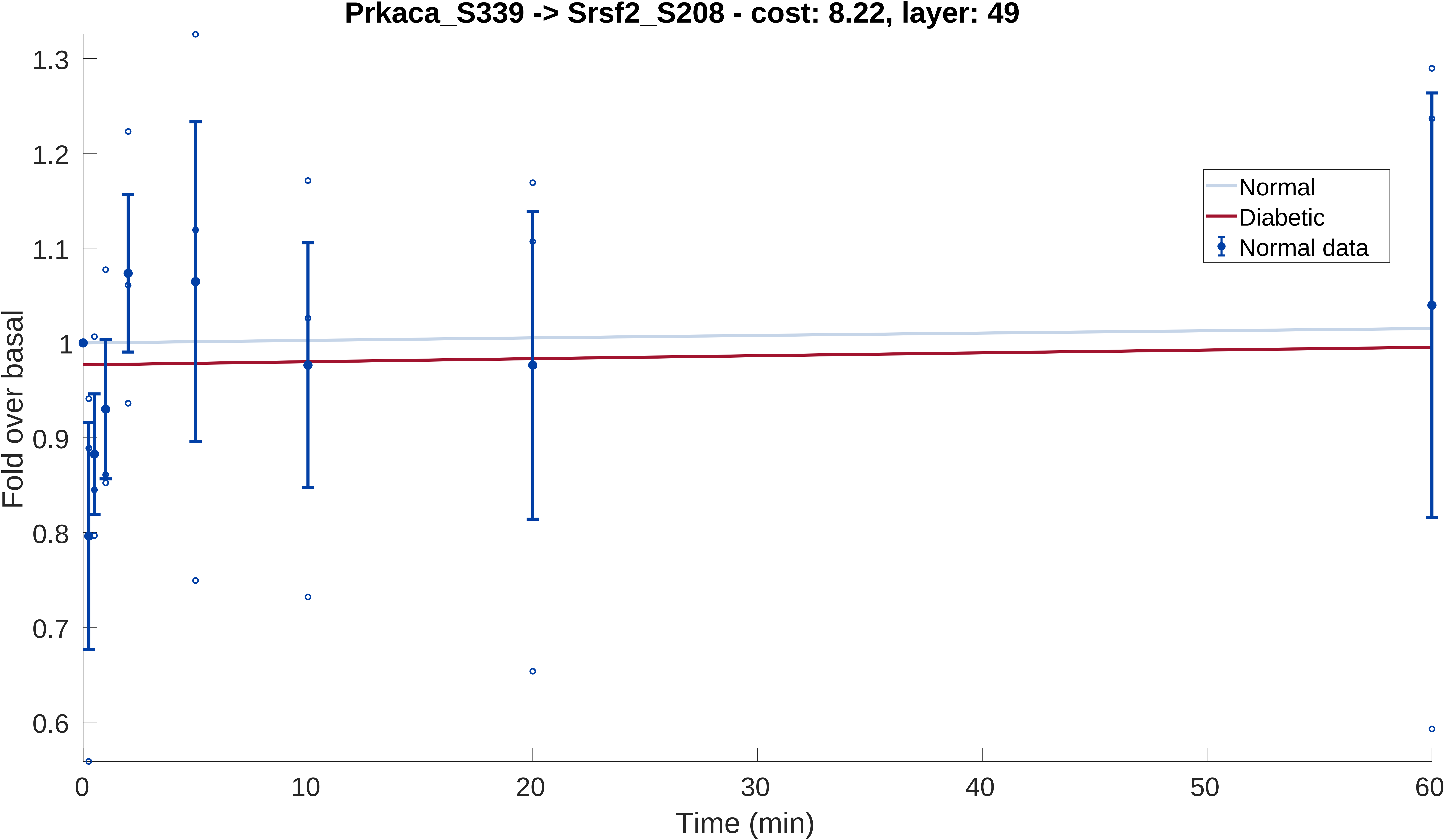

Prkaca_S339 -> Srsf2_S208 - cost: 8.22, layer: 49
Normal
Diabetic
Normal data
Fold over basal
Time (min)
1.3
1.2
1.1
1
0.9
0.8
0.7
0.6
0
10
20
30
40
50
60

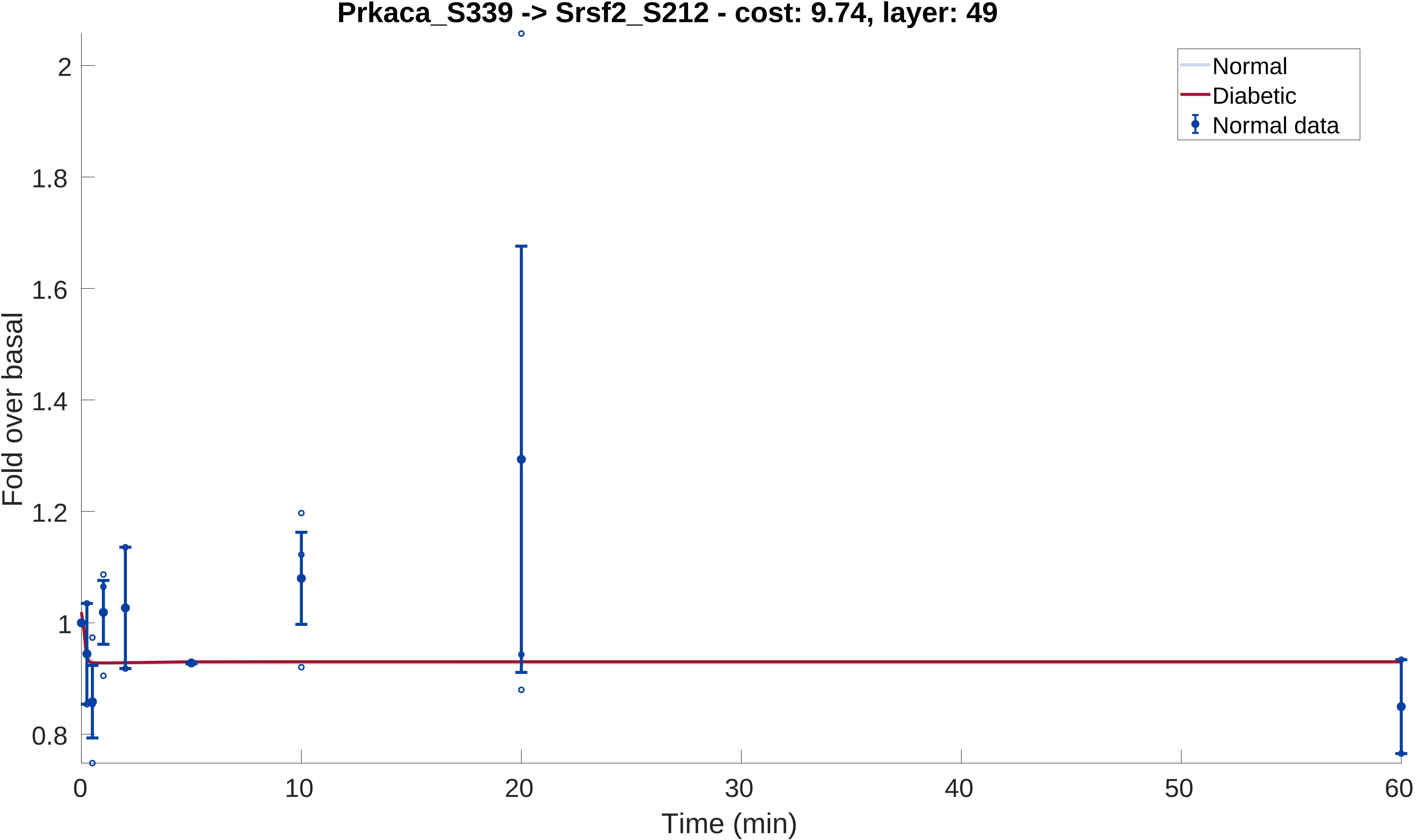

Prkaca_S339 -> Srsf2_S212 - cost: 9.74, layer: 49
Normal
Diabetic
Normal data
Fold over basal
Time (min)
2
1.8
1.6
1.4
1.2
1
0.8
0
10
20
30
40
50
60

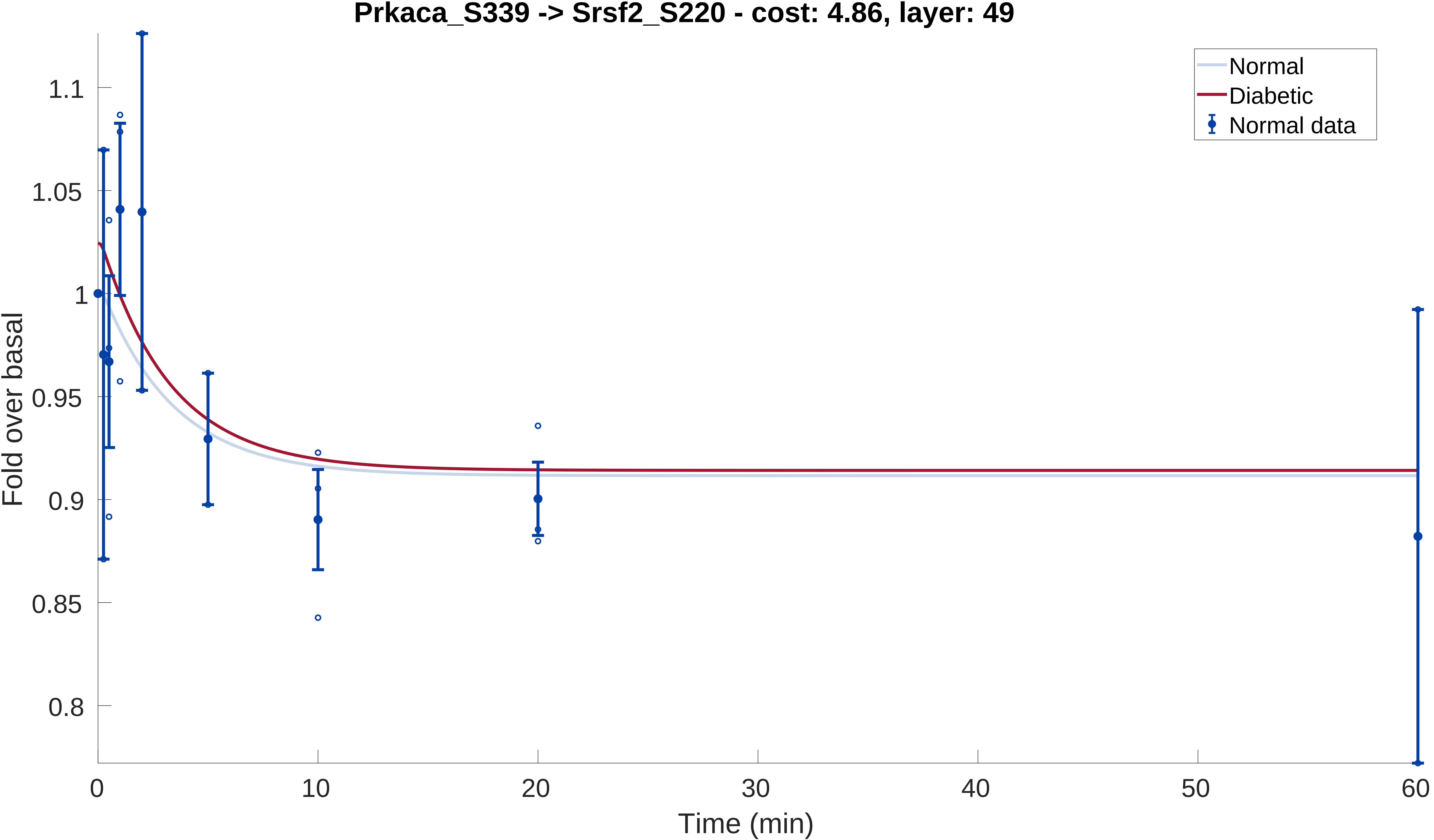
Prkaca_S339 -> Srsf2_S220 - cost: 4.86, layer: 49
Normal
Diabetic
Normal data
Fold over basal
1.1
1.05
1
0.95
0.9
0.85
0.8
0
10
20
30
40
50
60
Time (min)

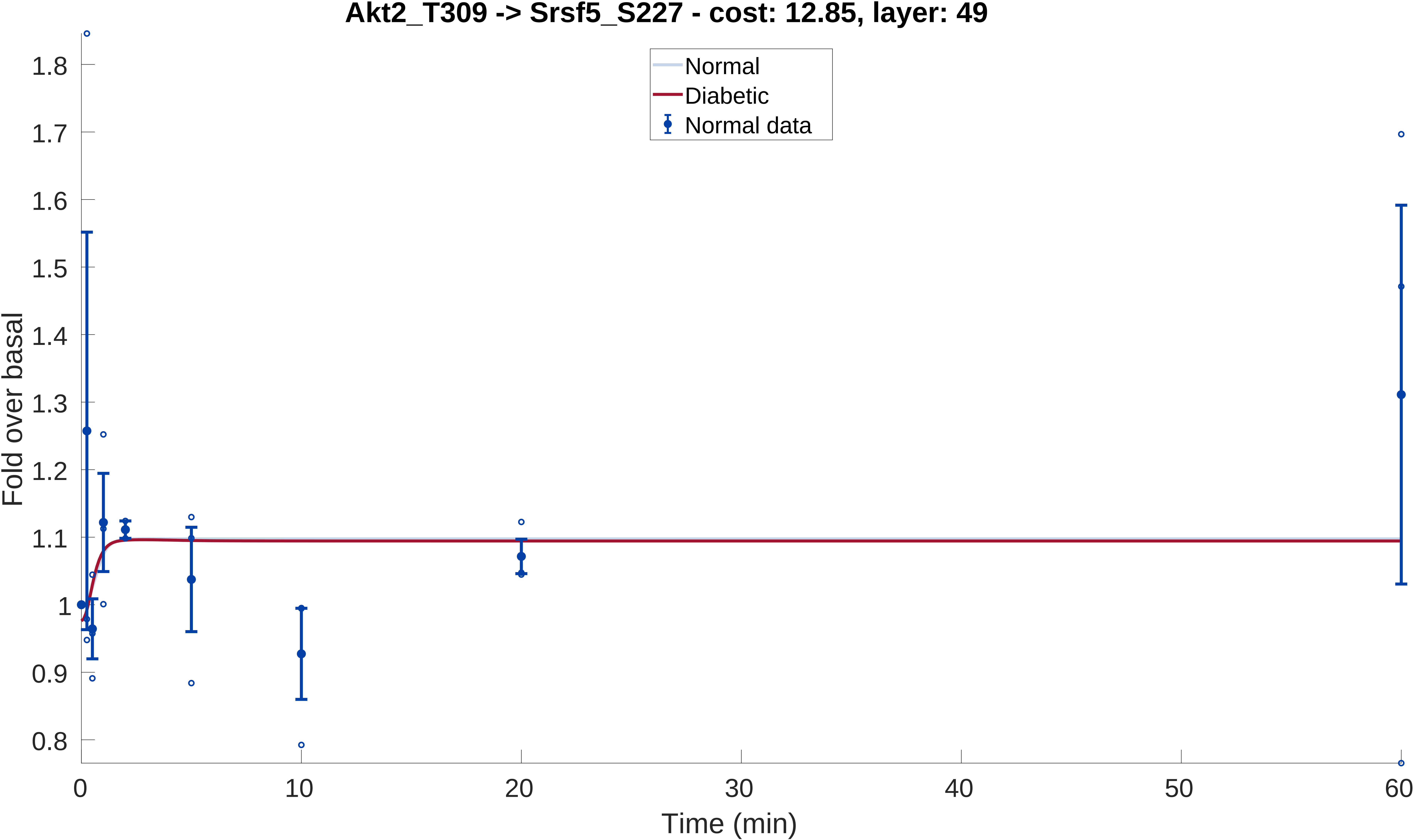

Akt2_T309 -> Srsf5_S227 - cost: 12.85, layer: 49
Normal
Diabetic
Normal data
Fold over basal
1.8
1.7
1.6
1.5
1.4
1.3
1.2
1.1
1
0.9
0.8
0
10
20
30
40
50
60
Time (min)

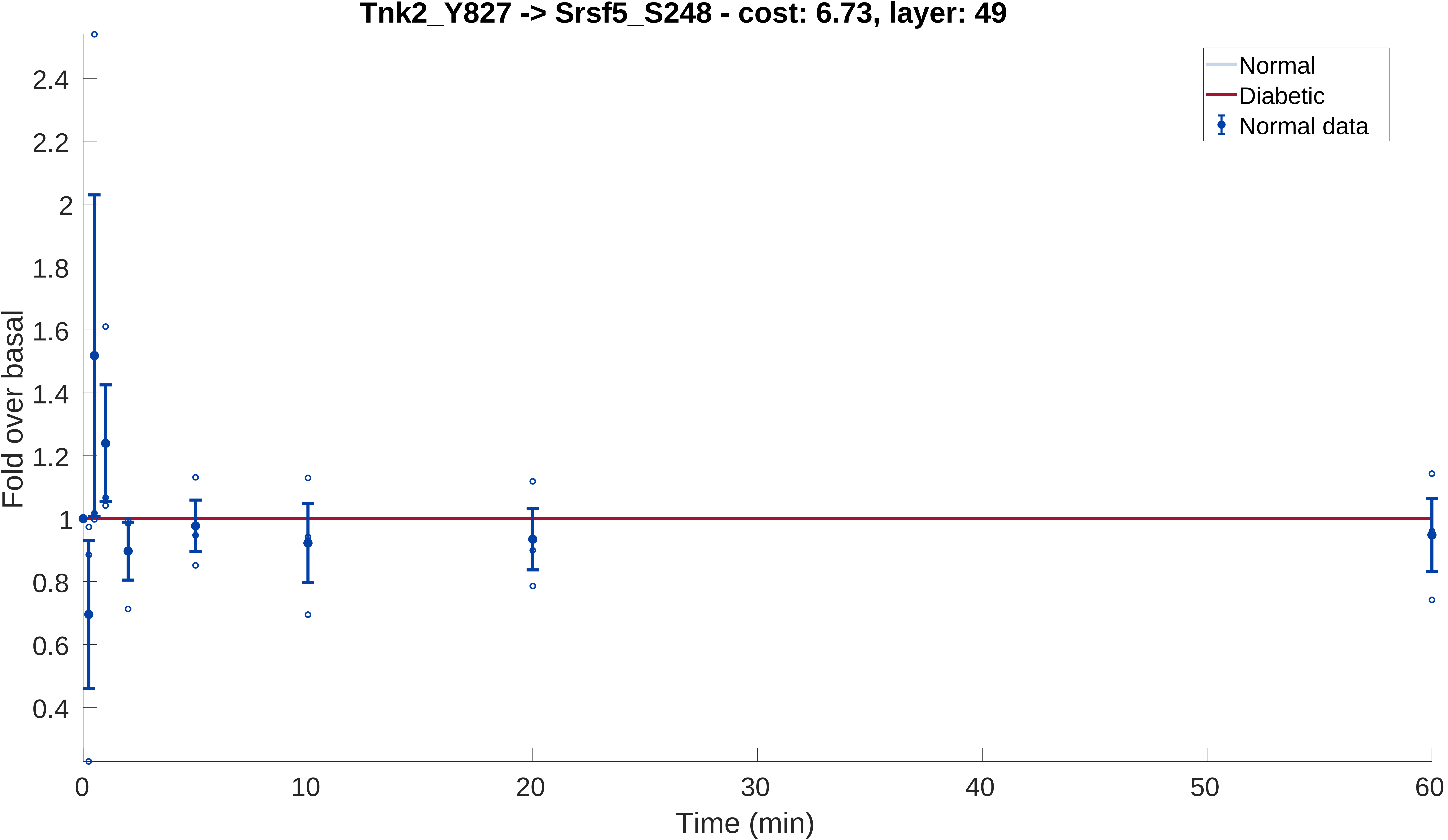

Tnk2_Y827 -> Srsf5_S248 - cost: 6.73, layer: 49
Normal
Diabetic
Normal data
Fold over basal
2.4
2.2
2
1.8
1.6
1.4
1.2
1
0.8
0.6
0.4
0
10
20
30
40
50
60
Time (min)

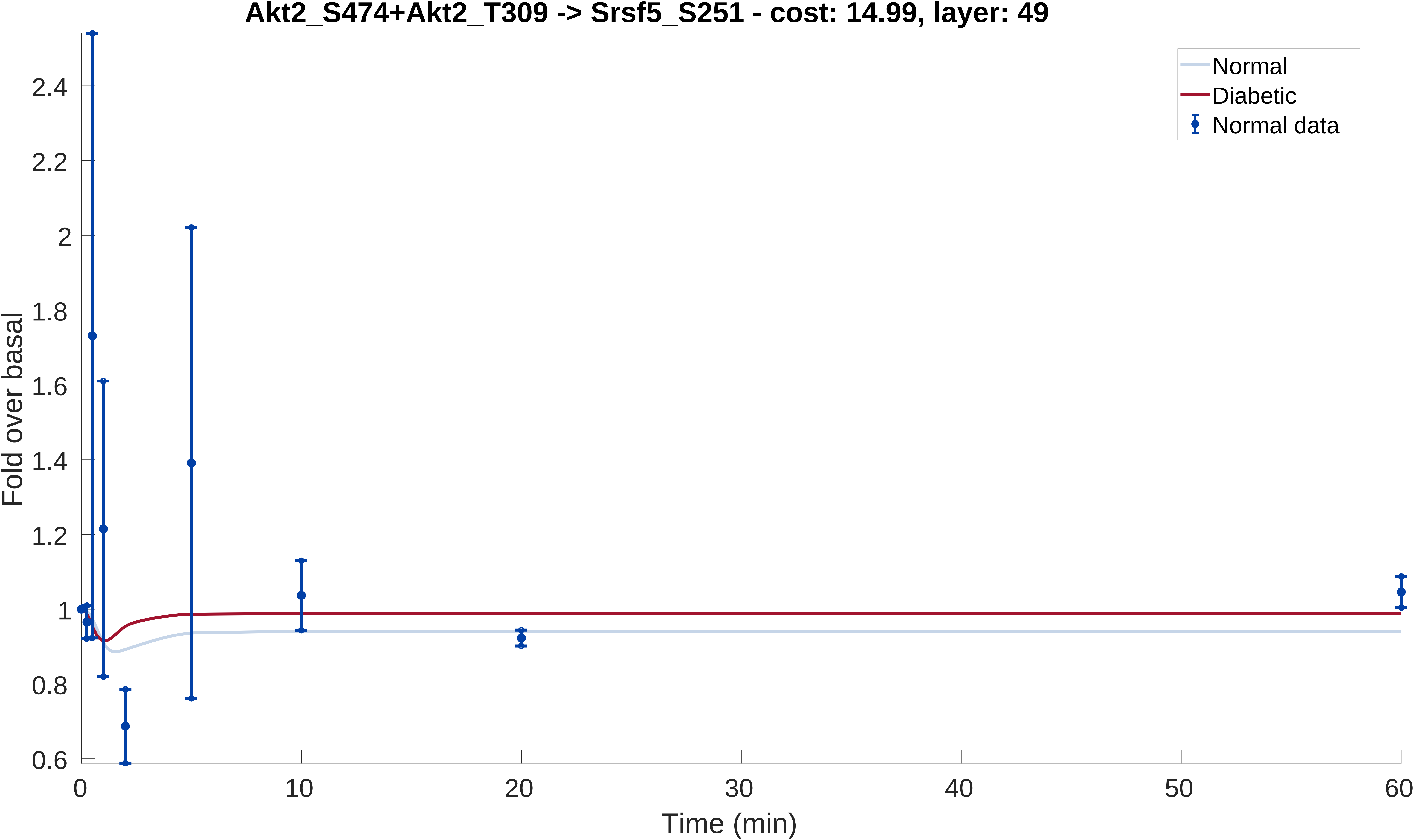

Akt2_S474+Akt2_T309 -> Srsf5_S251 - cost: 14.99, layer: 49
Normal
Diabetic
Normal data
Fold over basal
2.4
2.2
2
1.8
1.6
1.4
1.2
1
0.8
0.6
0
10
20
30
40
50
60
Time (min)

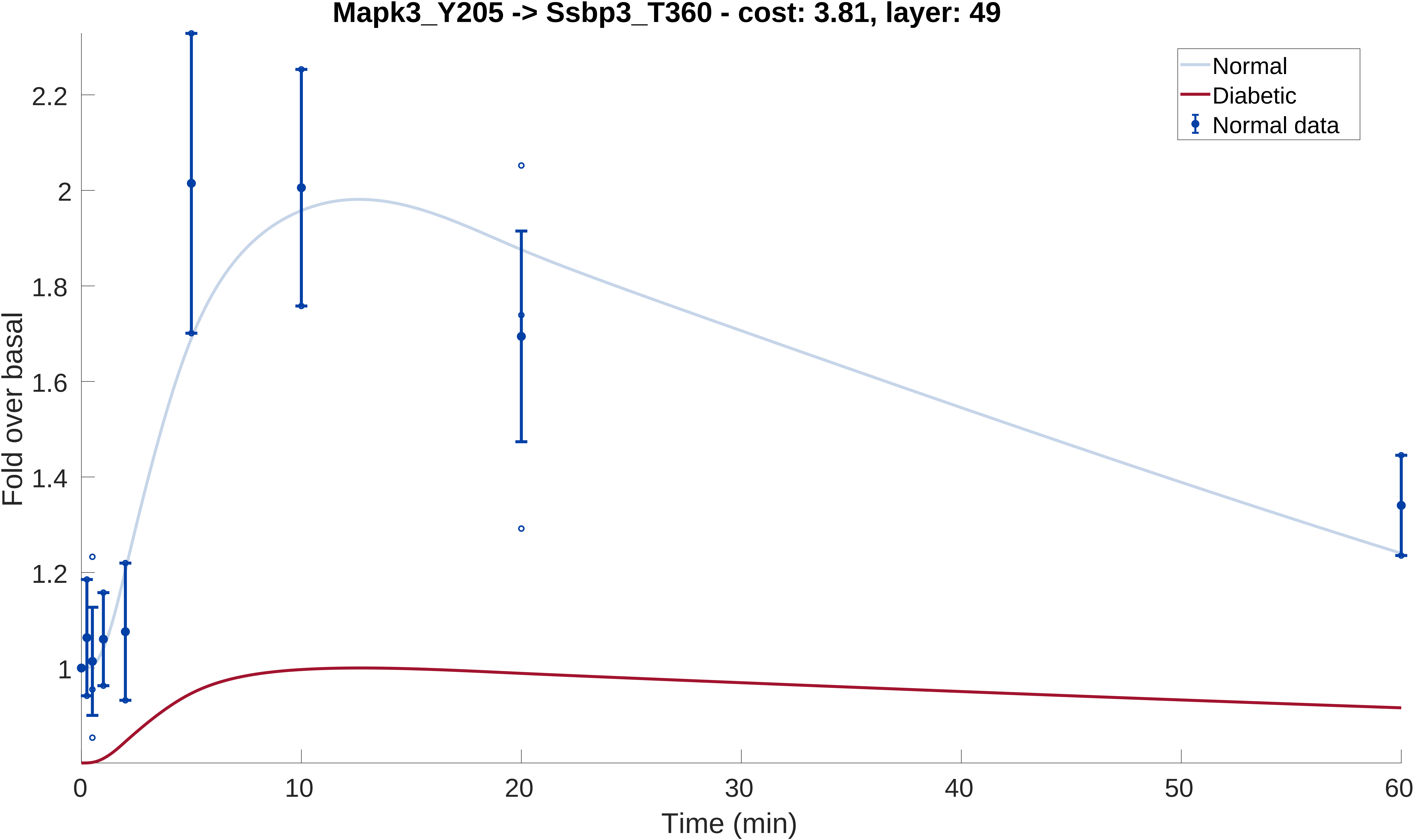

Mapk3_Y205 -> Ssbp3_T360 - cost: 3.81, layer: 49
Fold over basal
Time (min)
Normal
Diabetic
Normal data
2.2
2
1.8
1.6
1.4
1.2
1
0
10
20
30
40
50
60

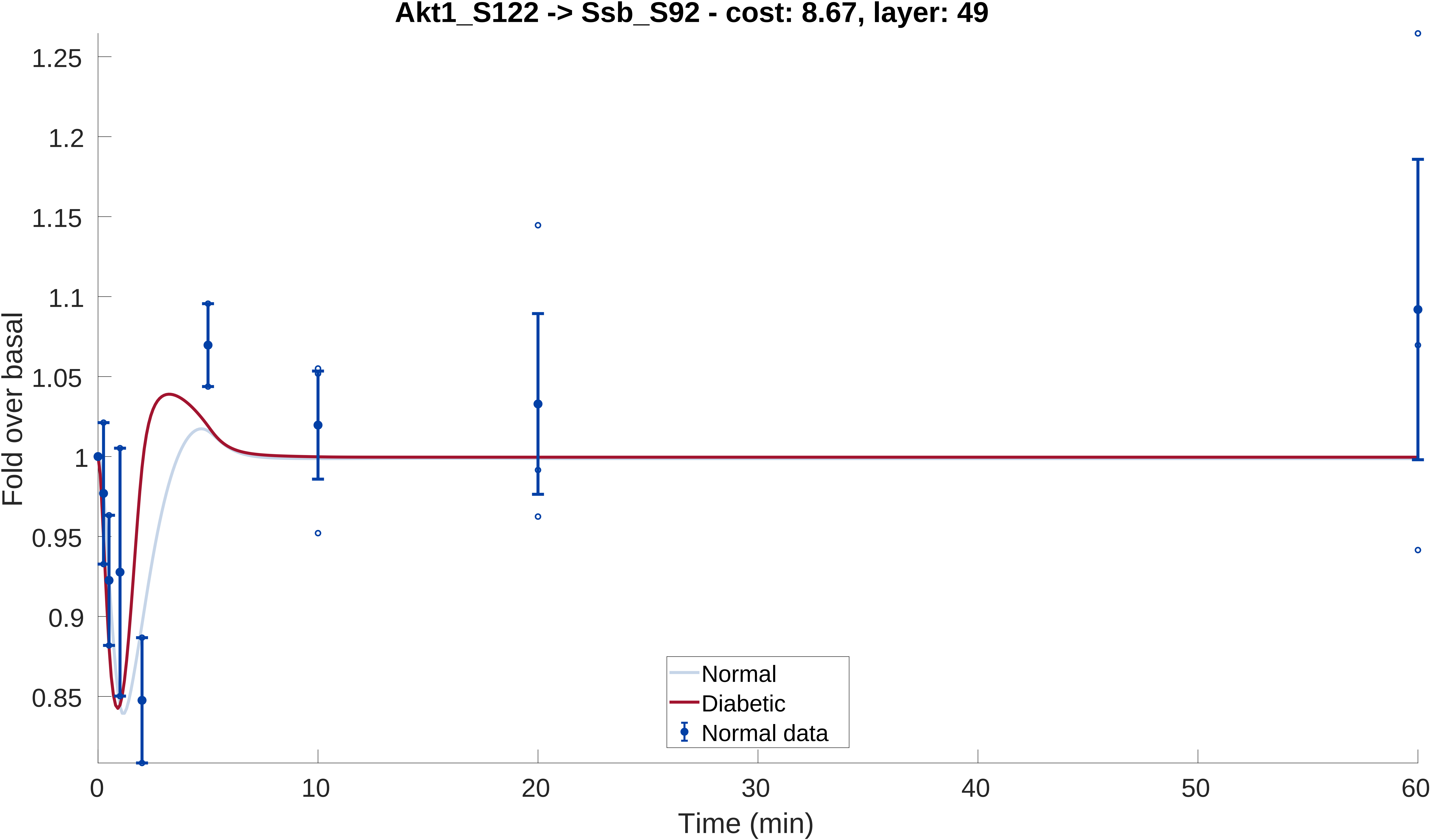

Akt1_S122 -> Ssb_S92 - cost: 8.67, layer: 49
Fold over basal
Time (min)
Normal
Diabetic
Normal data
1.25
1.2
1.15
1.1
1.05
1
0.95
0.9
0.85
0
10
20
30
40
50
60

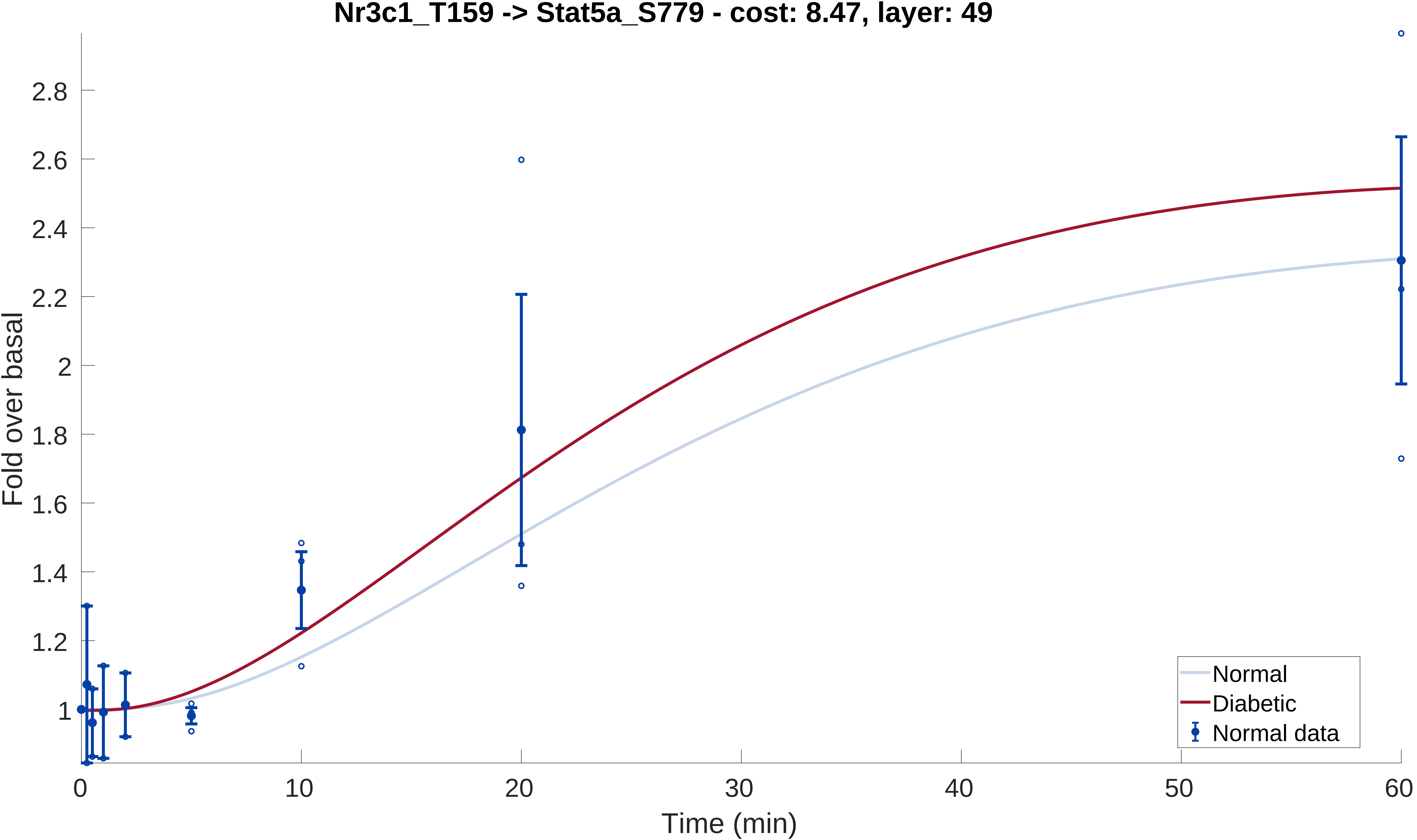

Nr3c1_T159 -> Stat5a_S779 - cost: 8.47, layer: 49
Fold over basal
2.8
2.6
2.4
2.2
2
1.8
1.6
1.4
1.2
1
0
10
20
30
40
50
60
Time (min)
Normal
Diabetic
Normal data

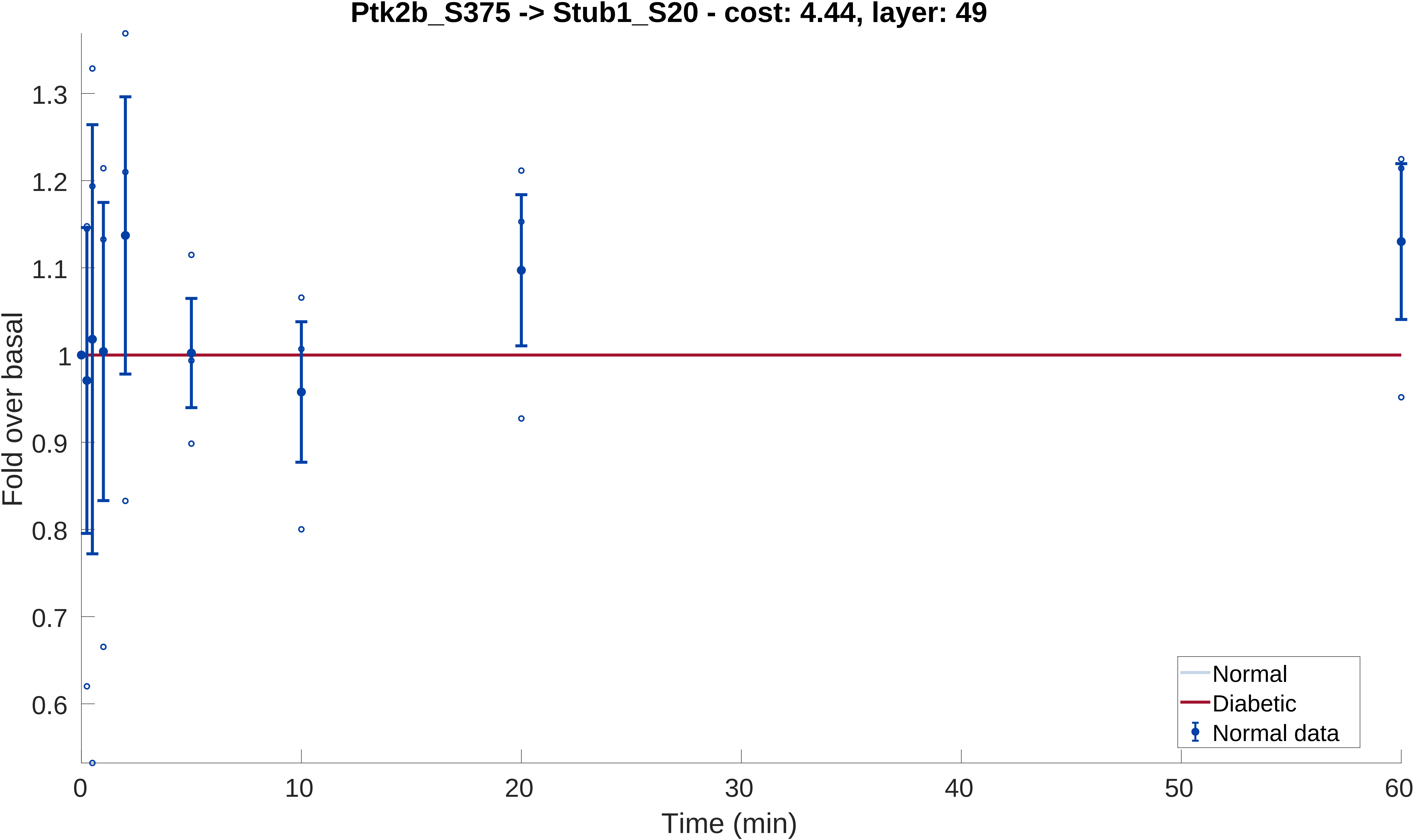

Ptk2b_S375 -> Stub1_S20 - cost: 4.44, layer: 49
Fold over basal
1.3
1.2
1.1
1
0.9
0.8
0.7
0.6
0
10
20
30
40
50
60
Time (min)
Normal
Diabetic
Normal data

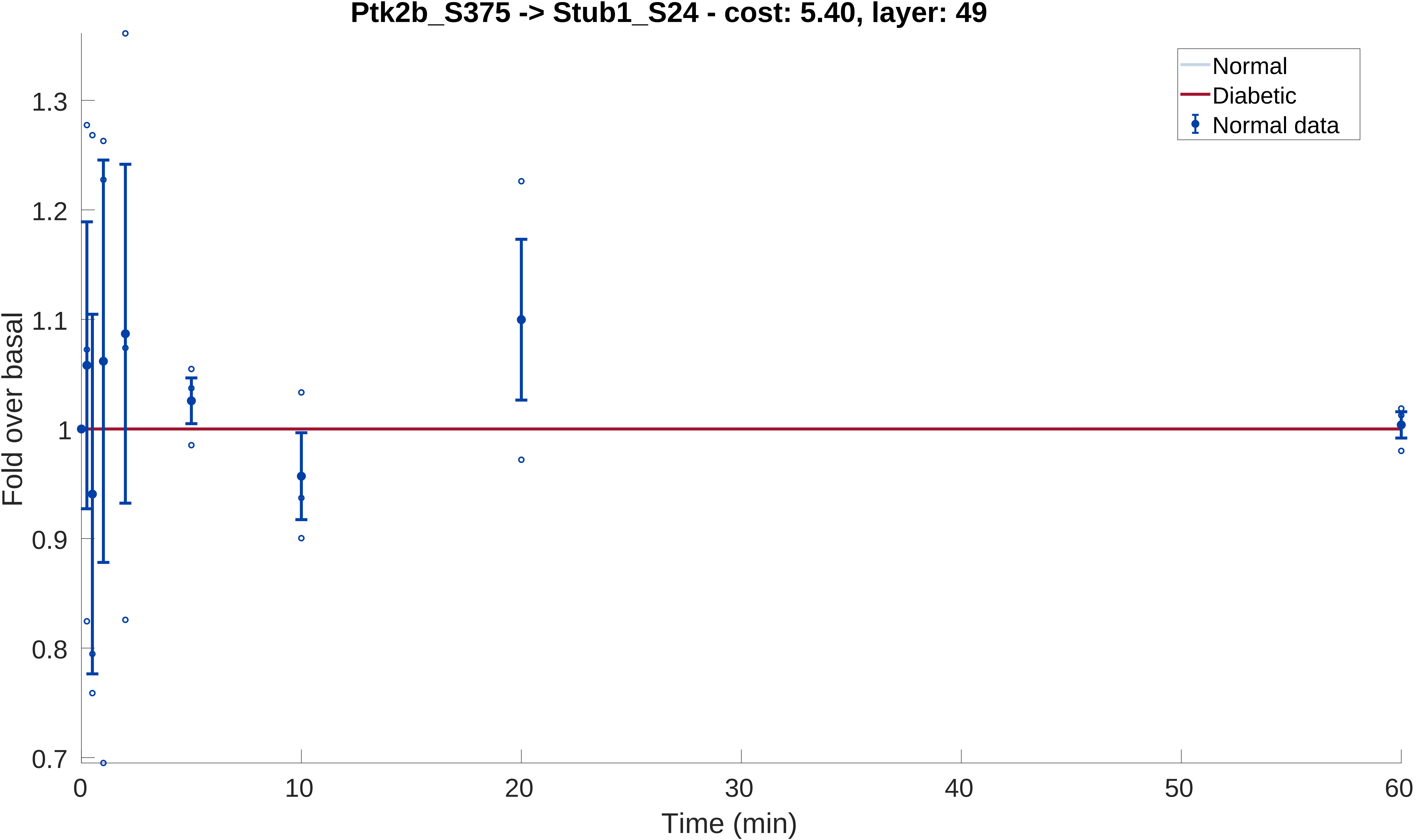

Ptk2b_S375 -> Stub1_S24 - cost: 5.40, layer: 49
Normal
Diabetic
Normal data
Fold over basal
1.3
1.2
1.1
1
0.9
0.8
0.7
0
10
20
30
40
50
60
Time (min)

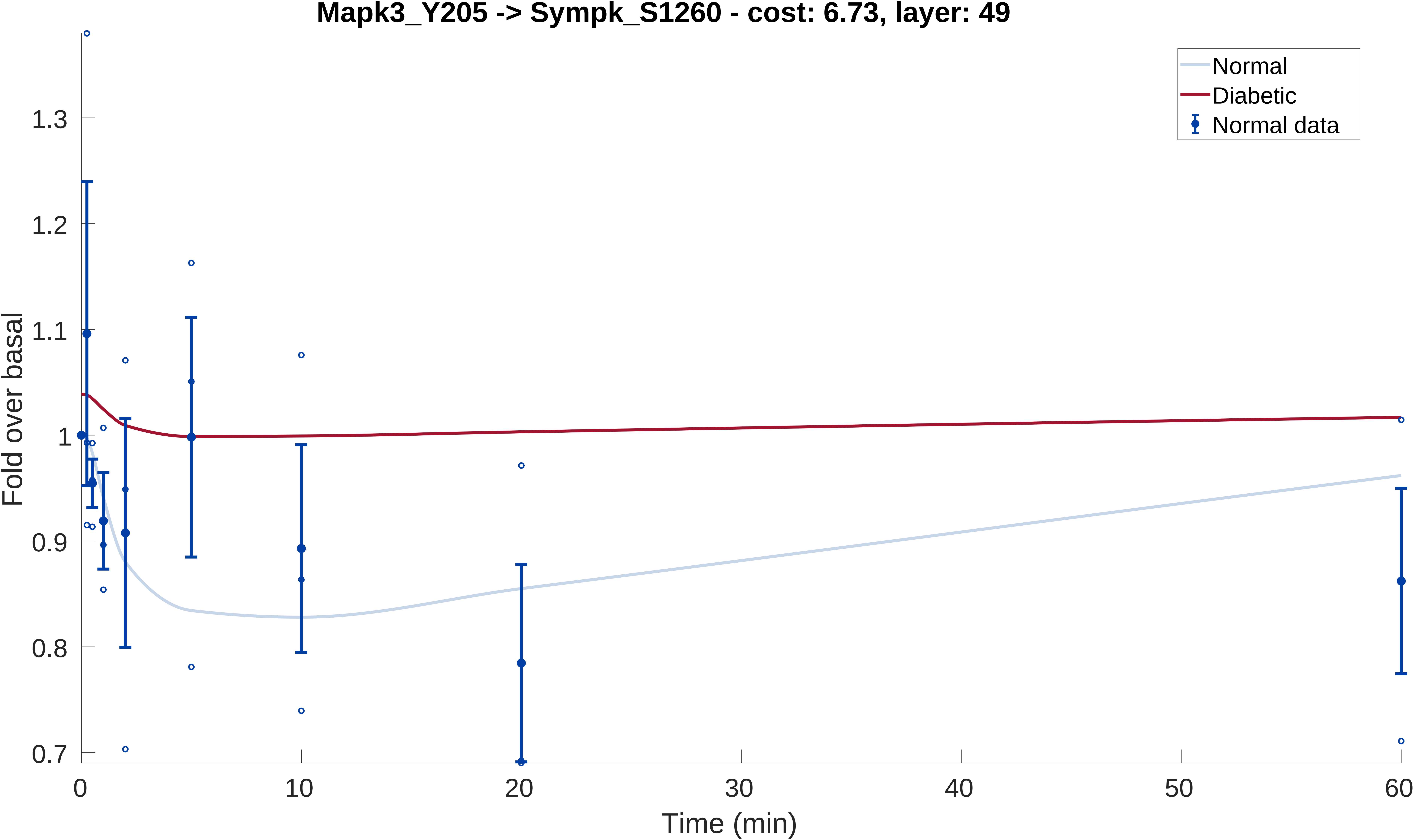

Mapk3_Y205 -> Sympk_S1260 - cost: 6.73, layer: 49
Normal
Diabetic
Normal data
Fold over basal
0.7
0.8
0.9
1
1.1
1.2
1.3
0
10
20
30
40
50
60
Time (min)

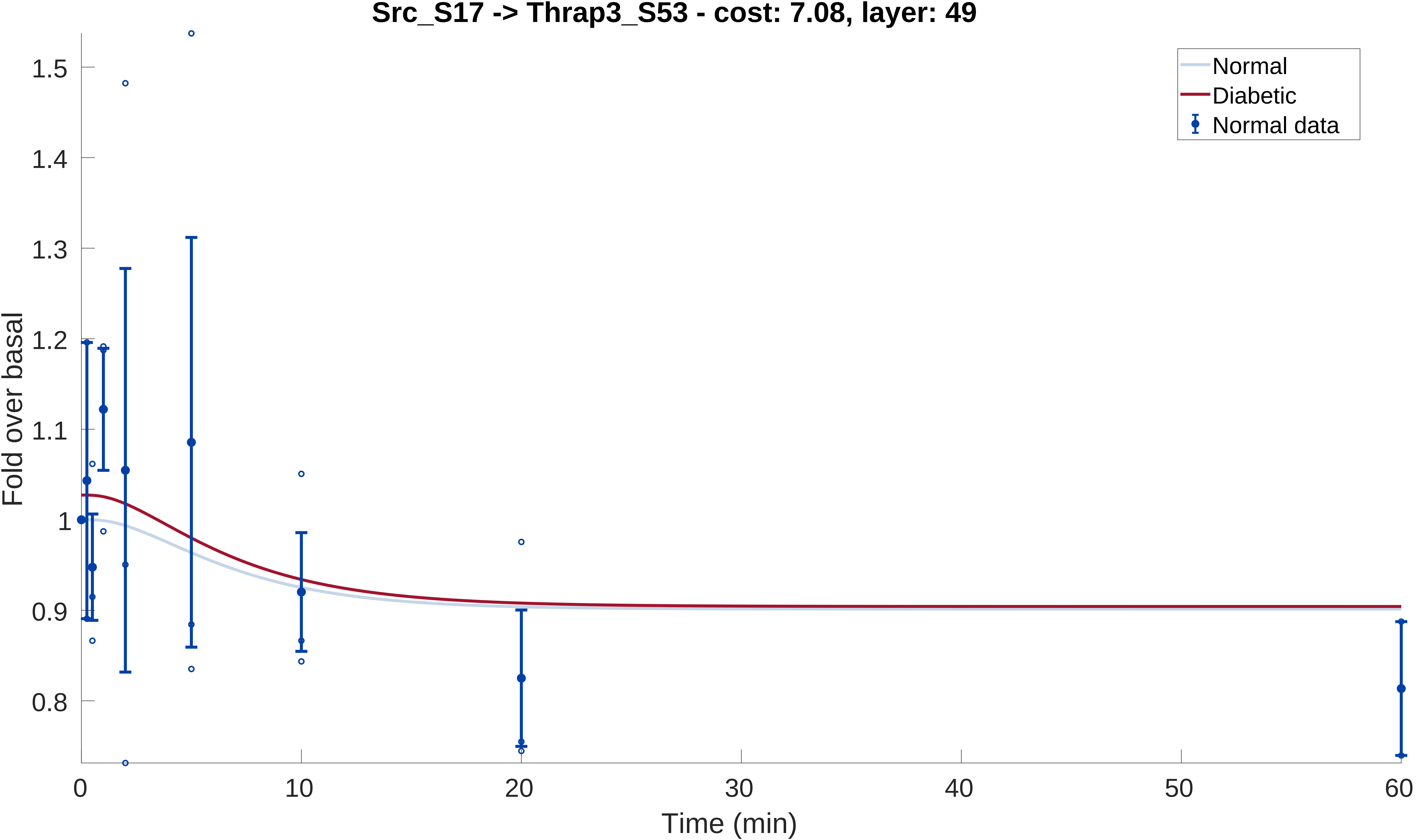
Src_S17 -> Thrap3_S53 - cost: 7.08, layer: 49
Normal
Diabetic
Normal data
Fold over basal
1.5
1.4
1.3
1.2
1.1
1
0.9
0.8
0
10
20
30
40
50
60
Time (min)

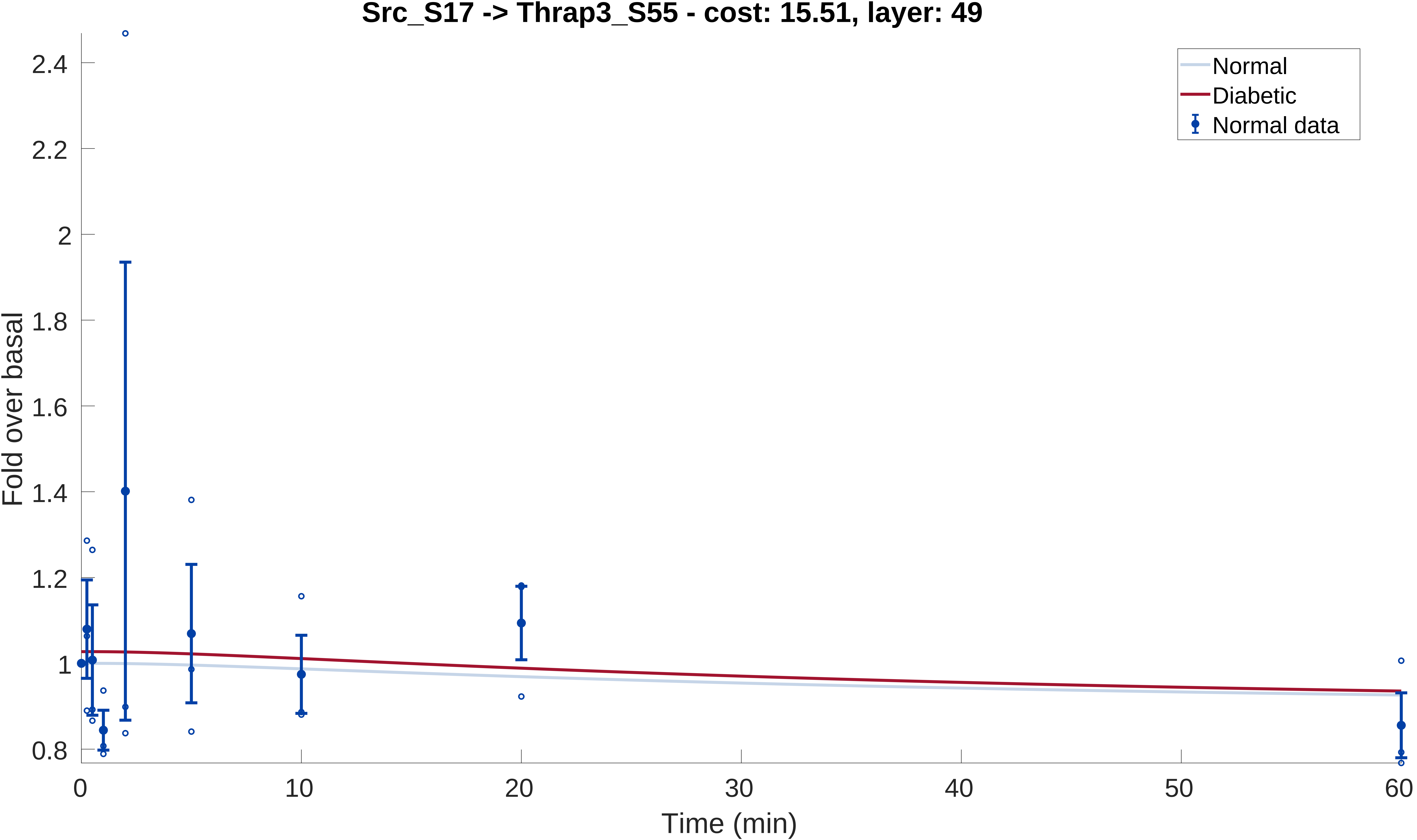

Src_S17 -> Thrap3_S55 - cost: 15.51, layer: 49
Normal
Diabetic
Normal data
Fold over basal
2.4
2.2
2
1.8
1.6
1.4
1.2
1
0.8
0
10
20
30
40
50
60
Time (min)

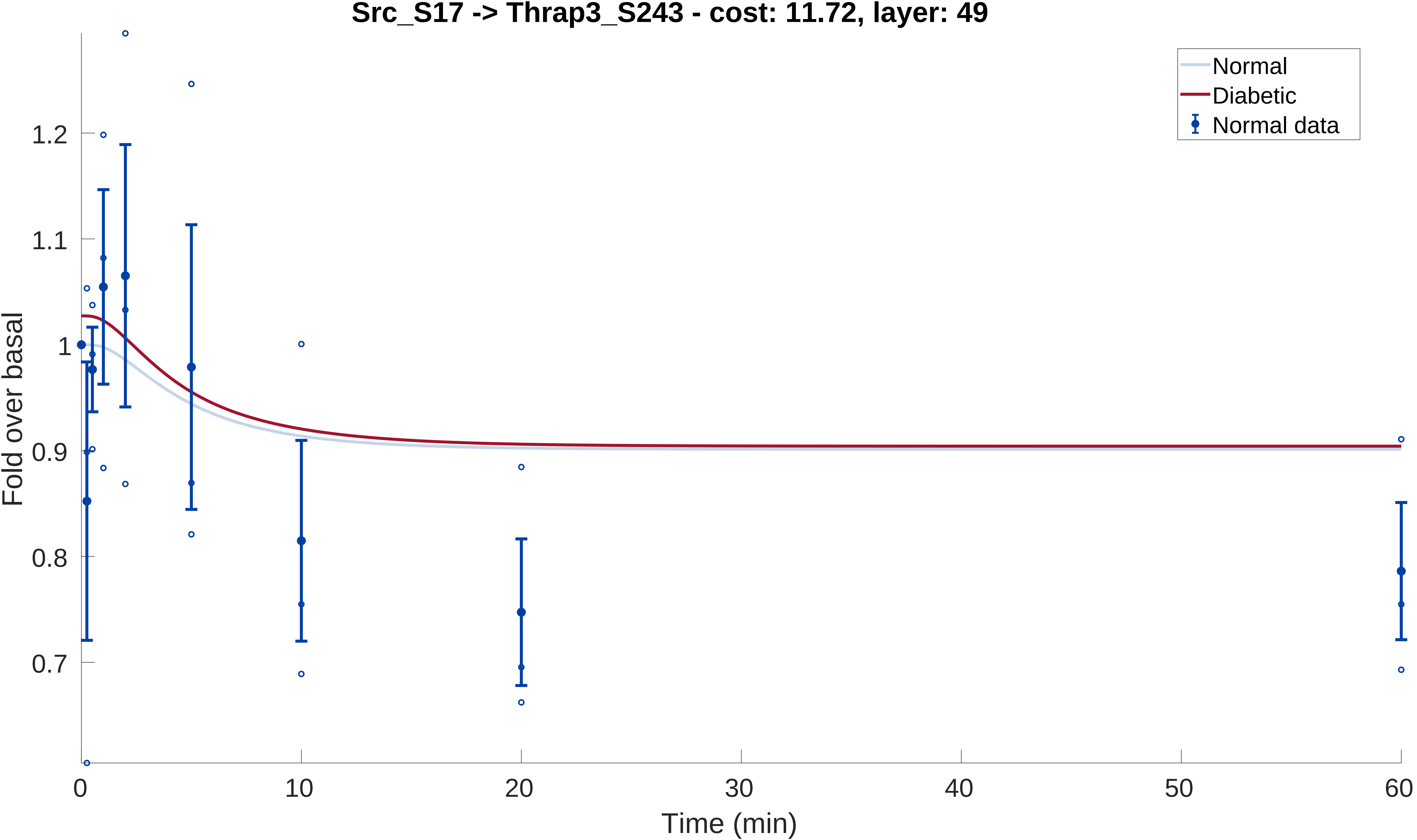
Src_S17 -> Thrap3_S243 - cost: 11.72, layer: 49
Normal
Diabetic
Normal data
1.2
1.1
1
0.9
0.8
0.7
Fold over basal
0
10
20
30
40
50
60
Time (min)

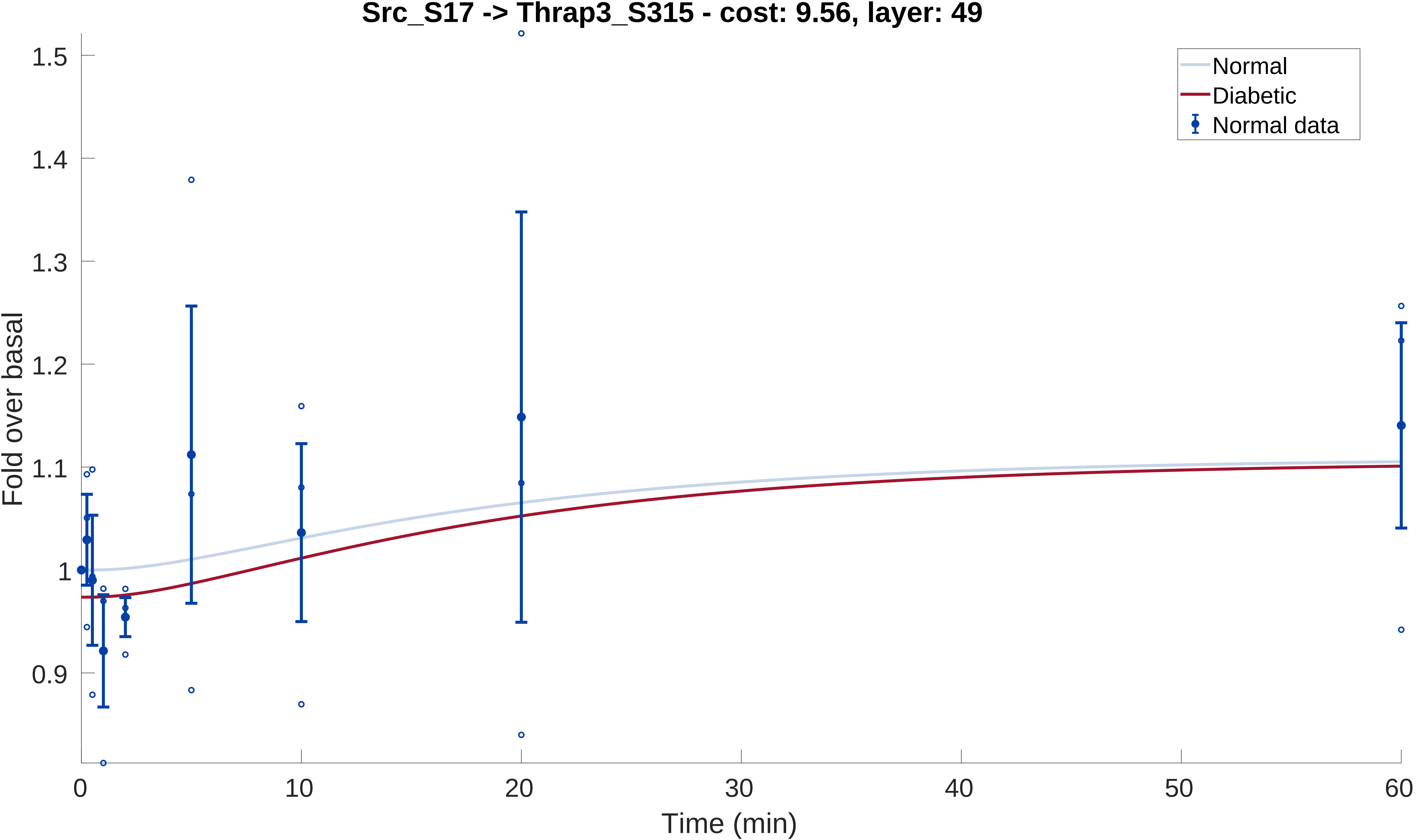
Src_S17 -> Thrap3_S315 - cost: 9.56, layer: 49
Fold over basal
1.5
1.4
1.3
1.2
1.1
1
0.9
Time (min)
0
10
20
30
40
50
60
Normal
Diabetic
Normal data

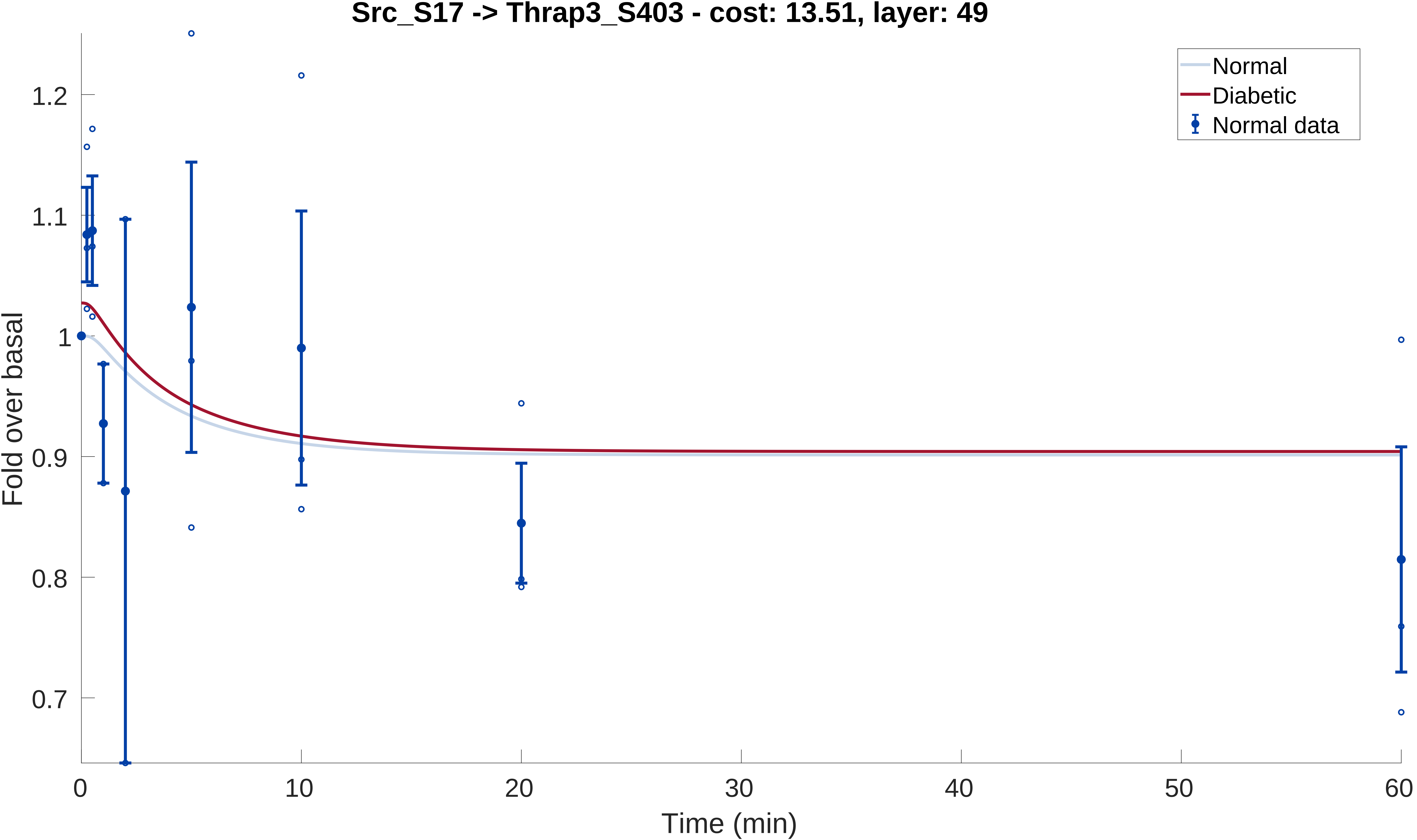
Src_S17 -> Thrap3_S403 - cost: 13.51, layer: 49
Normal
Diabetic
Normal data
1.2
1.1
1
0.9
0.8
0.7
Fold over basal
0
10
20
30
40
50
60
Time (min)

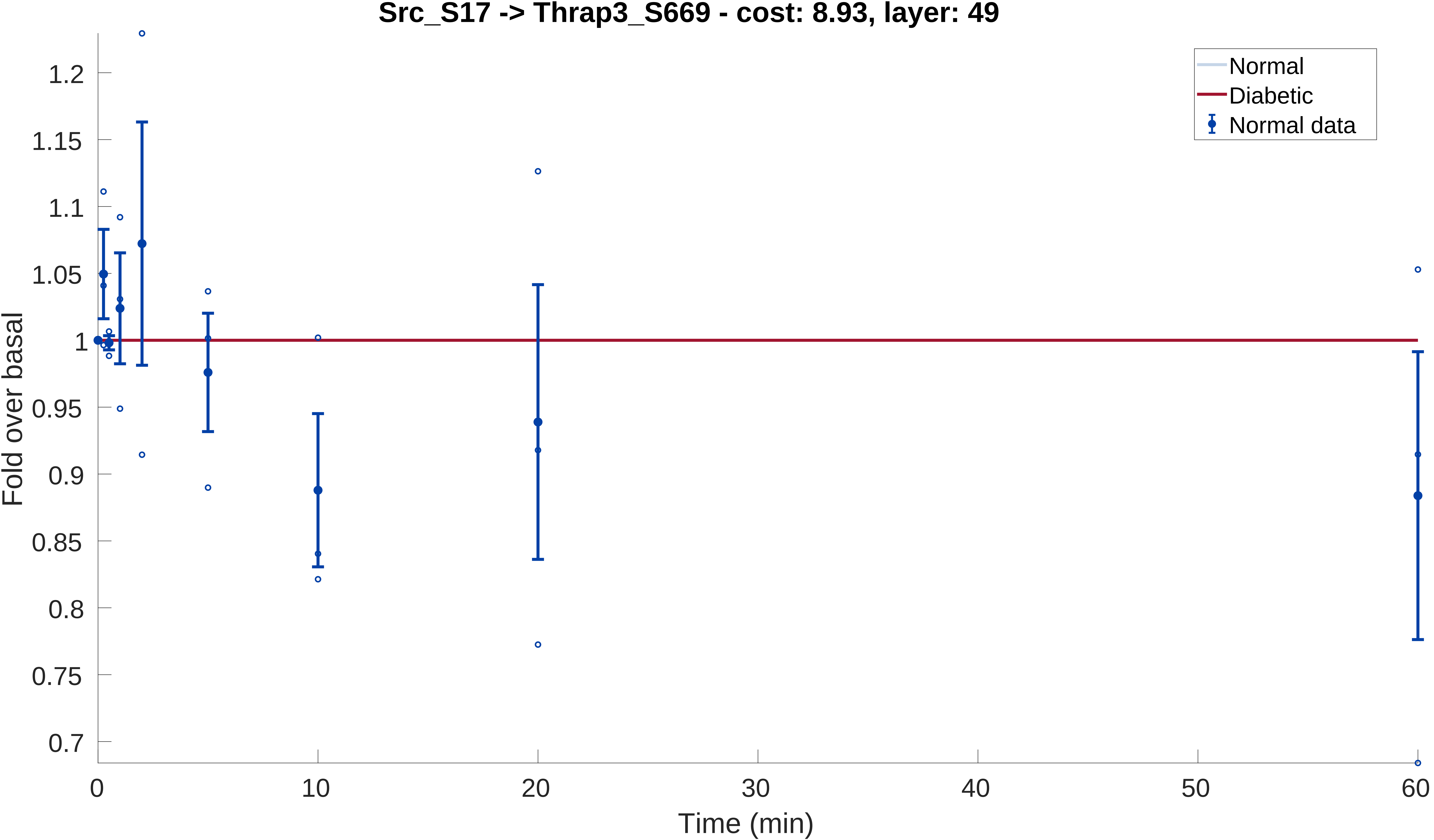

Src_S17 -> Thrap3_S669 - cost: 8.93, layer: 49
Normal
Diabetic
Normal data
Fold over basal
Time (min)
1.2
1.15
1.1
1.05
1
0.95
0.9
0.85
0.8
0.75
0.7
0
10
20
30
40
50
60

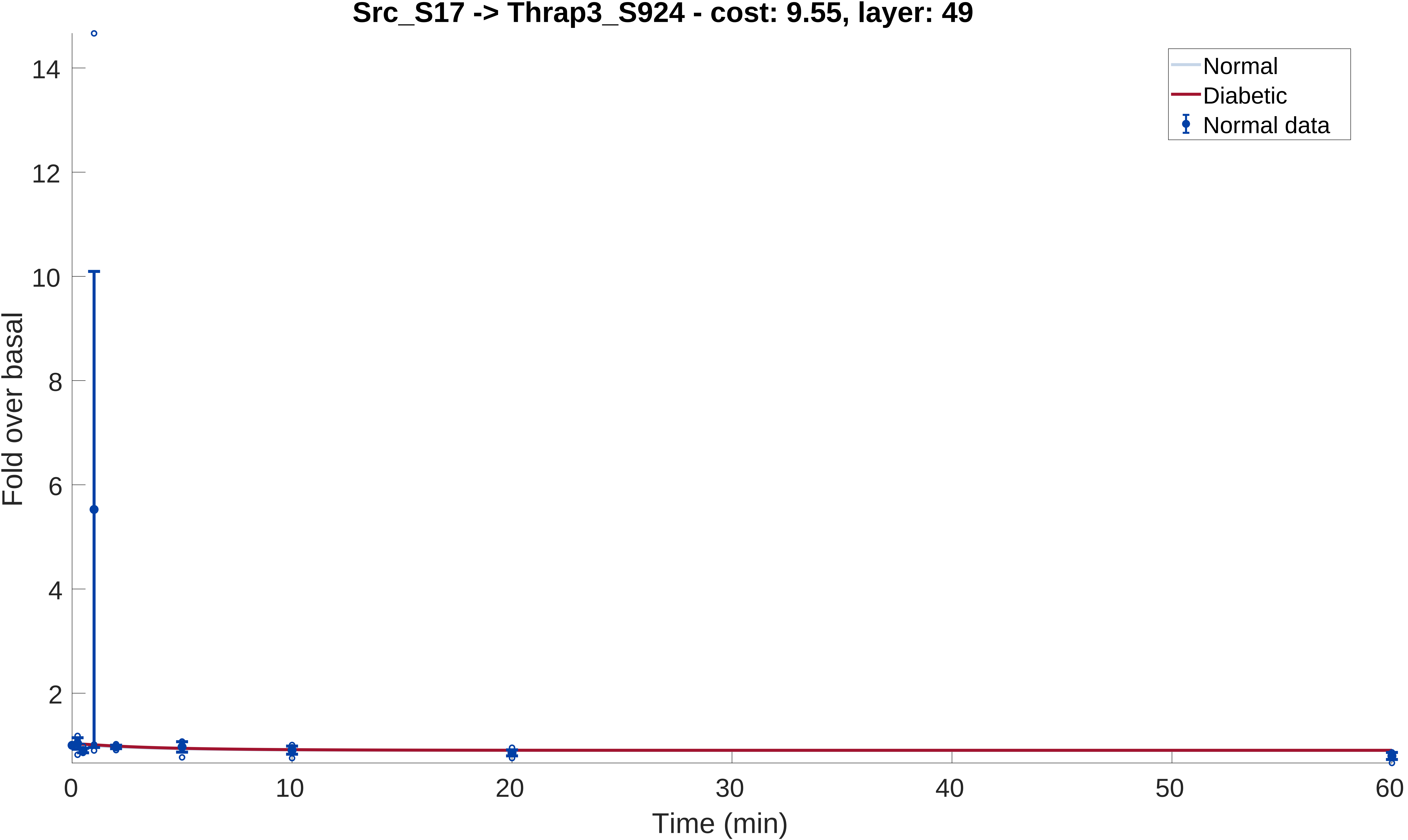

Src_S17 -> Thrap3_S924 - cost: 9.55, layer: 49
Normal
Diabetic
Normal data
Fold over basal
Time (min)
0
10
20
30
40
50
60
2
4
6
8
10
12
14

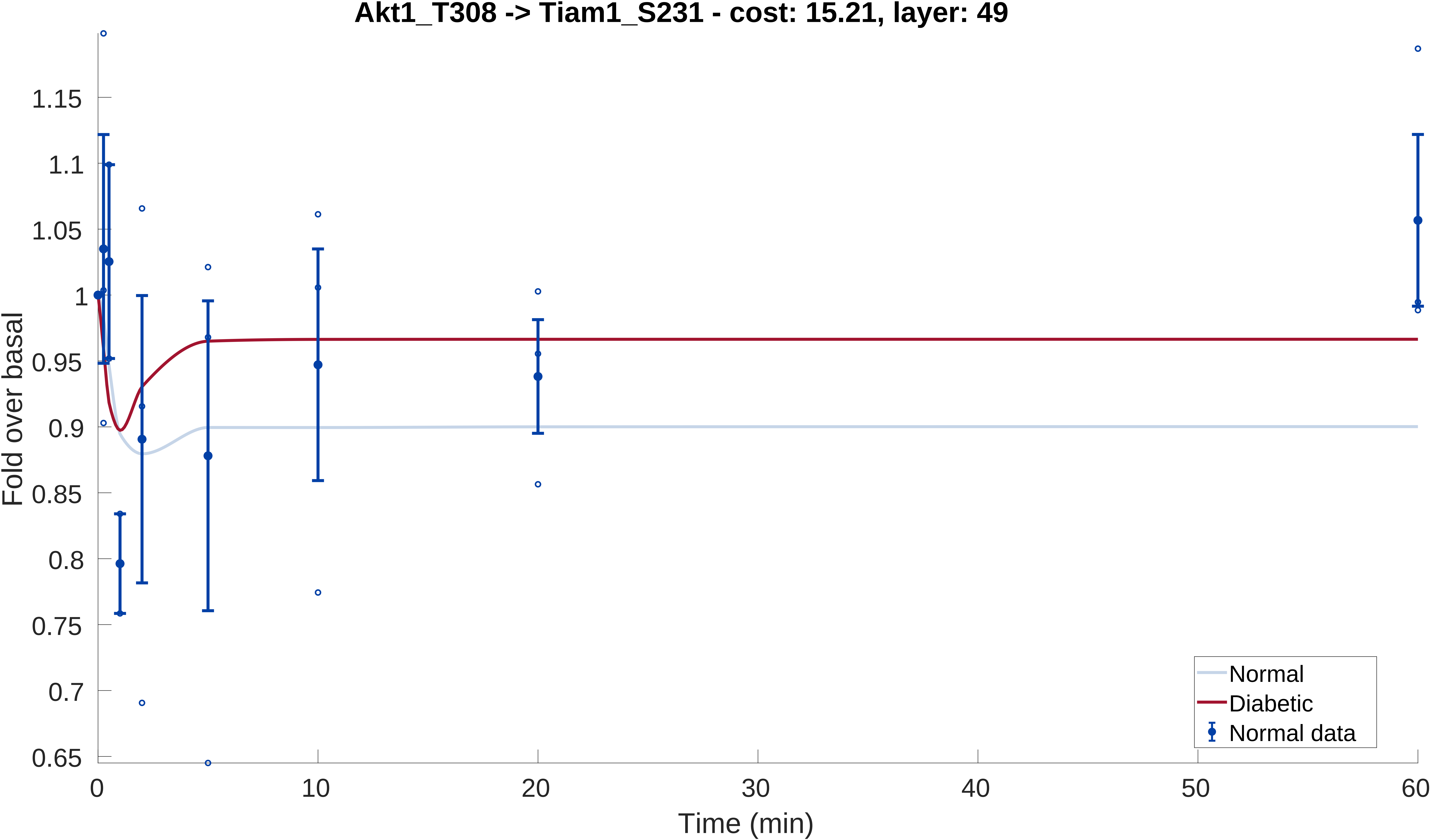

Akt1_T308 -> Tiam1_S231 - cost: 15.21, layer: 49
Fold over basal
1.15
1.1
1.05
1
0.95
0.9
0.85
0.8
0.75
0.7
0.65
0
10
20
30
40
50
60
Time (min)
Normal
Diabetic
Normal data

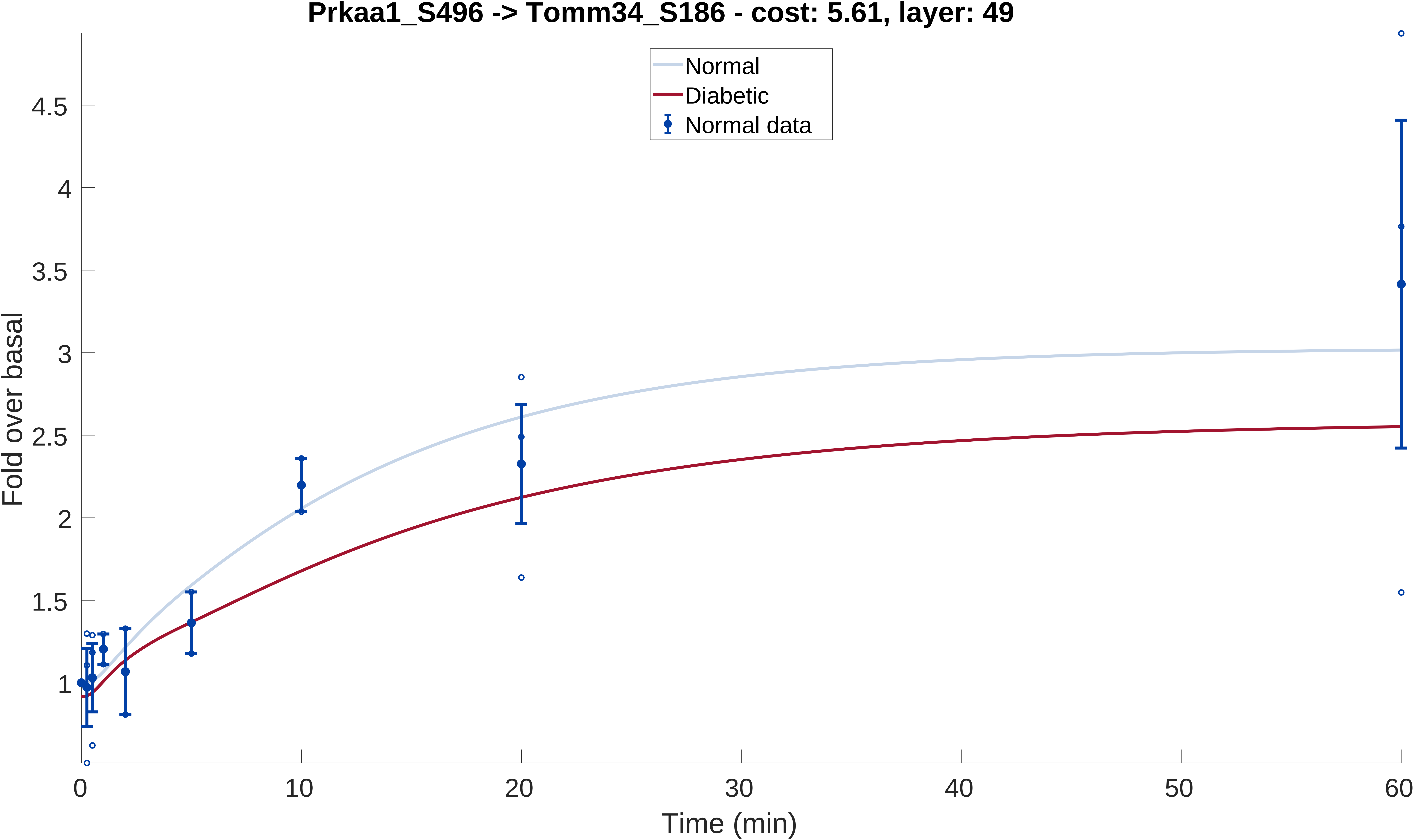

Prkaa1_S496 -> Tomm34_S186 - cost: 5.61, layer: 49
Normal
Diabetic
Normal data
Fold over basal
Time (min)
4.5
4
3.5
3
2.5
2
1.5
1
0
10
20
30
40
50
60

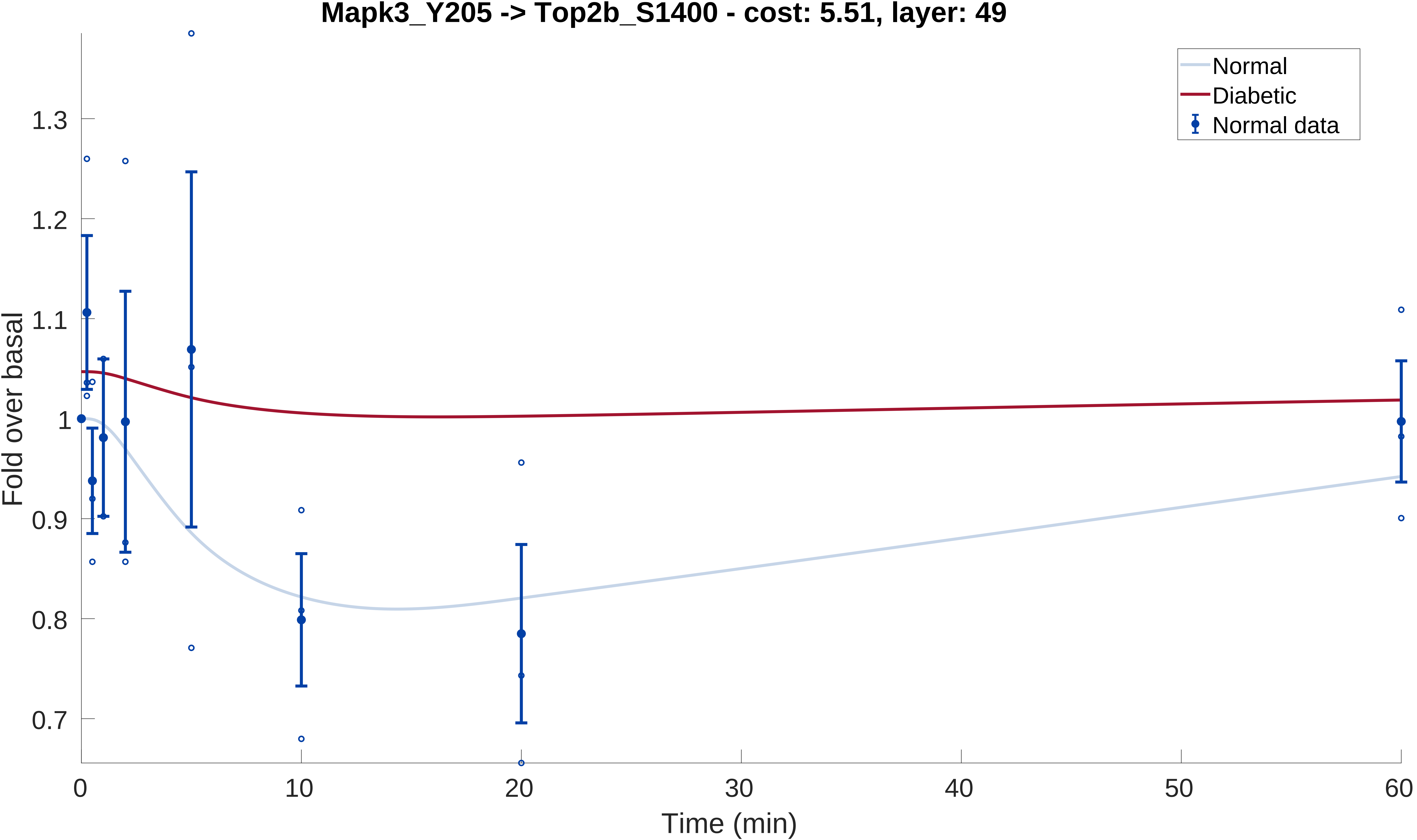

Mapk3_Y205 -> Top2b_S1400 - cost: 5.51, layer: 49
Normal
Diabetic
Normal data
Fold over basal
1.3
1.2
1.1
1
0.9
0.8
0.7
0
10
20
30
40
50
60
Time (min)

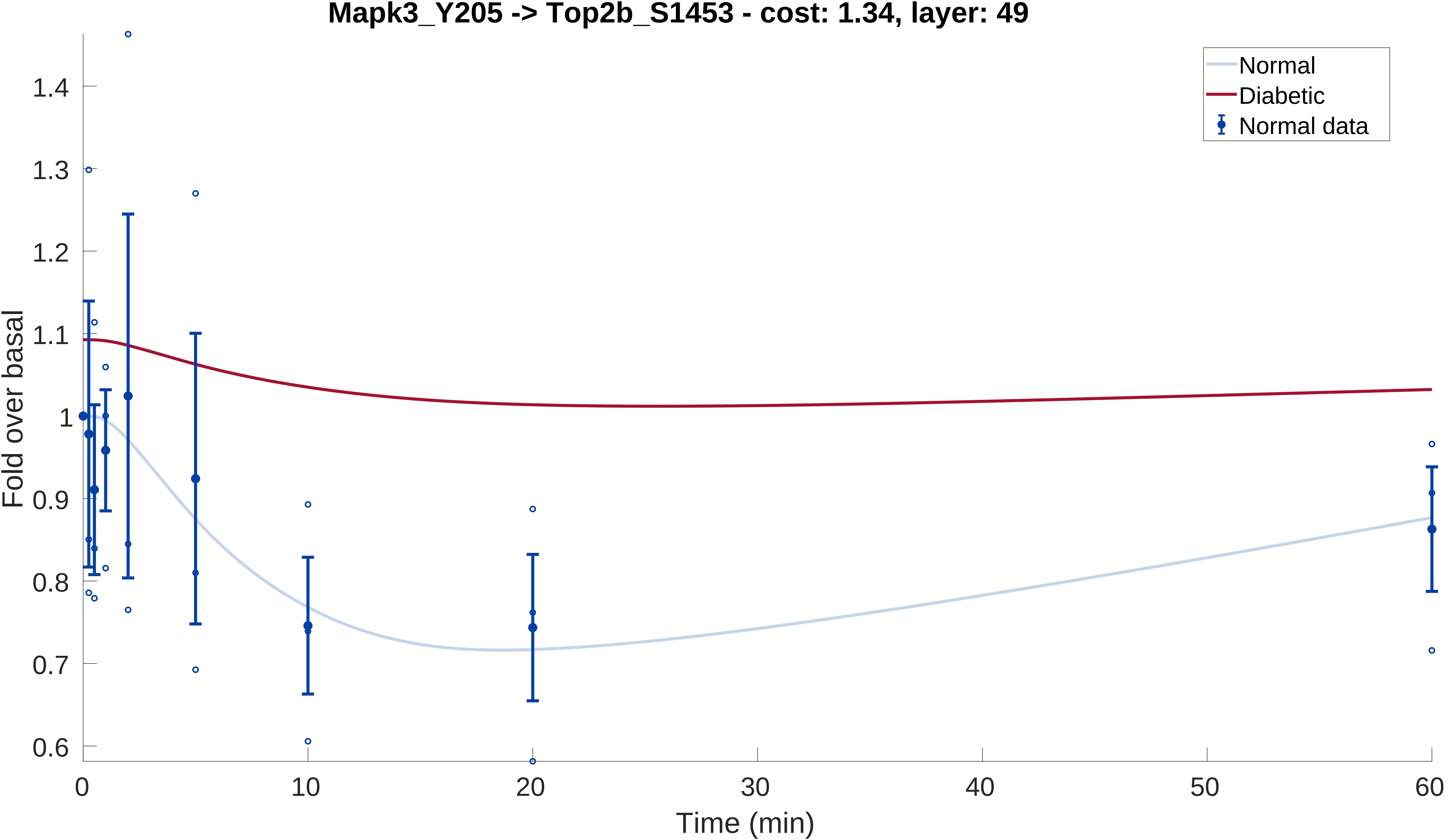

Mapk3_Y205 -> Top2b_S1453 - cost: 1.34, layer: 49
Normal
Diabetic
Normal data
Fold over basal
Time (min)
1.4
1.3
1.2
1.1
1
0.9
0.8
0.7
0.6
0
10
20
30
40
50
60

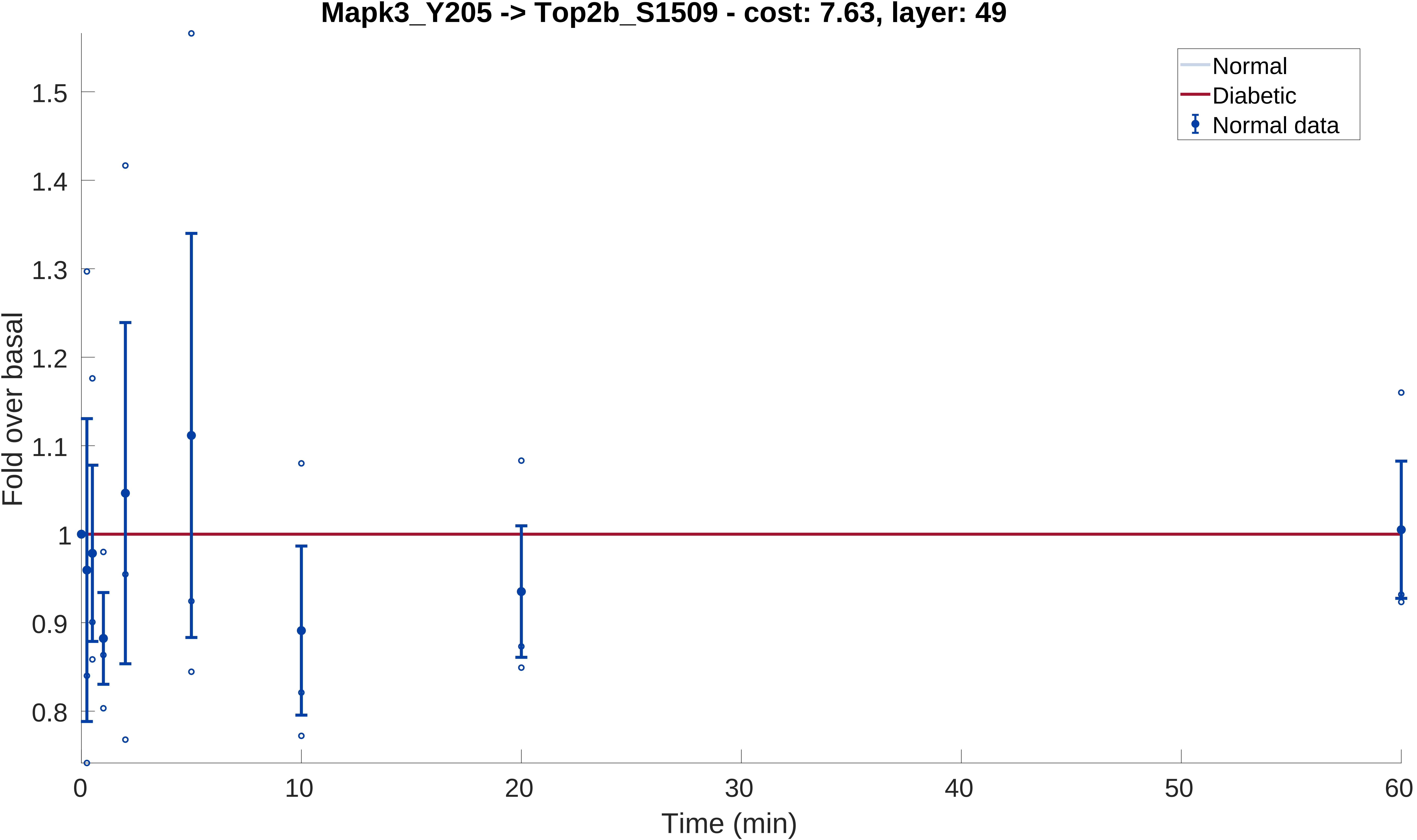

Mapk3_Y205 -> Top2b_S1509 - cost: 7.63, layer: 49
Normal
Diabetic
Normal data
Fold over basal
1.5
1.4
1.3
1.2
1.1
1
0.9
0.8
0
10
20
30
40
50
60
Time (min)

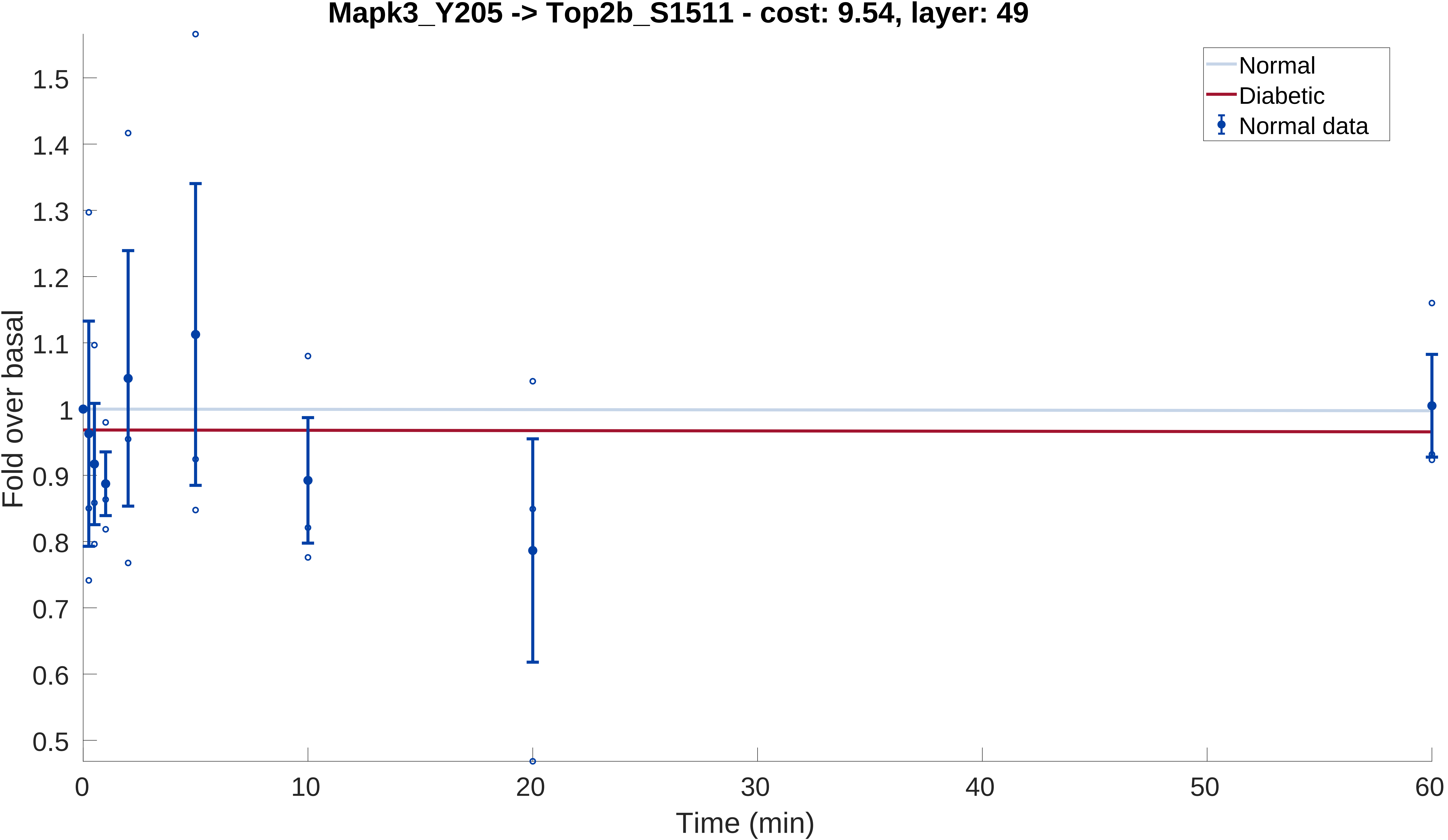

Mapk3_Y205 -> Top2b_S1511 - cost: 9.54, layer: 49
Normal
Diabetic
Normal data
Fold over basal
Time (min)
0
10
20
30
40
50
60
0.5
0.6
0.7
0.8
0.9
1
1.1
1.2
1.3
1.4
1.5

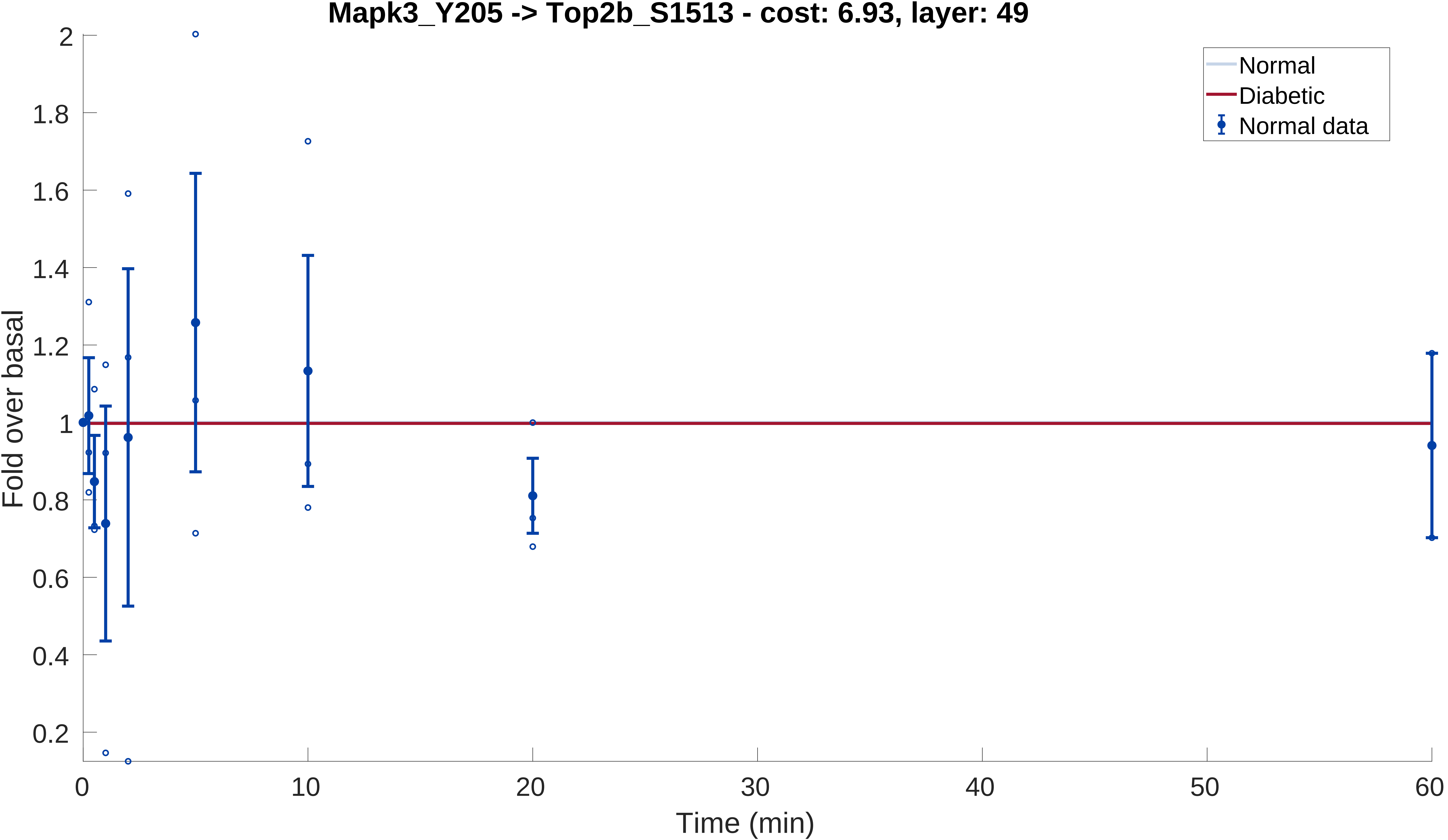

Mapk3_Y205 -> Top2b_S1513 - cost: 6.93, layer: 49
Normal
Diabetic
Normal data
Fold over basal
2
1.8
1.6
1.4
1.2
1
0.8
0.6
0.4
0.2
0
10
20
30
40
50
60
Time (min)

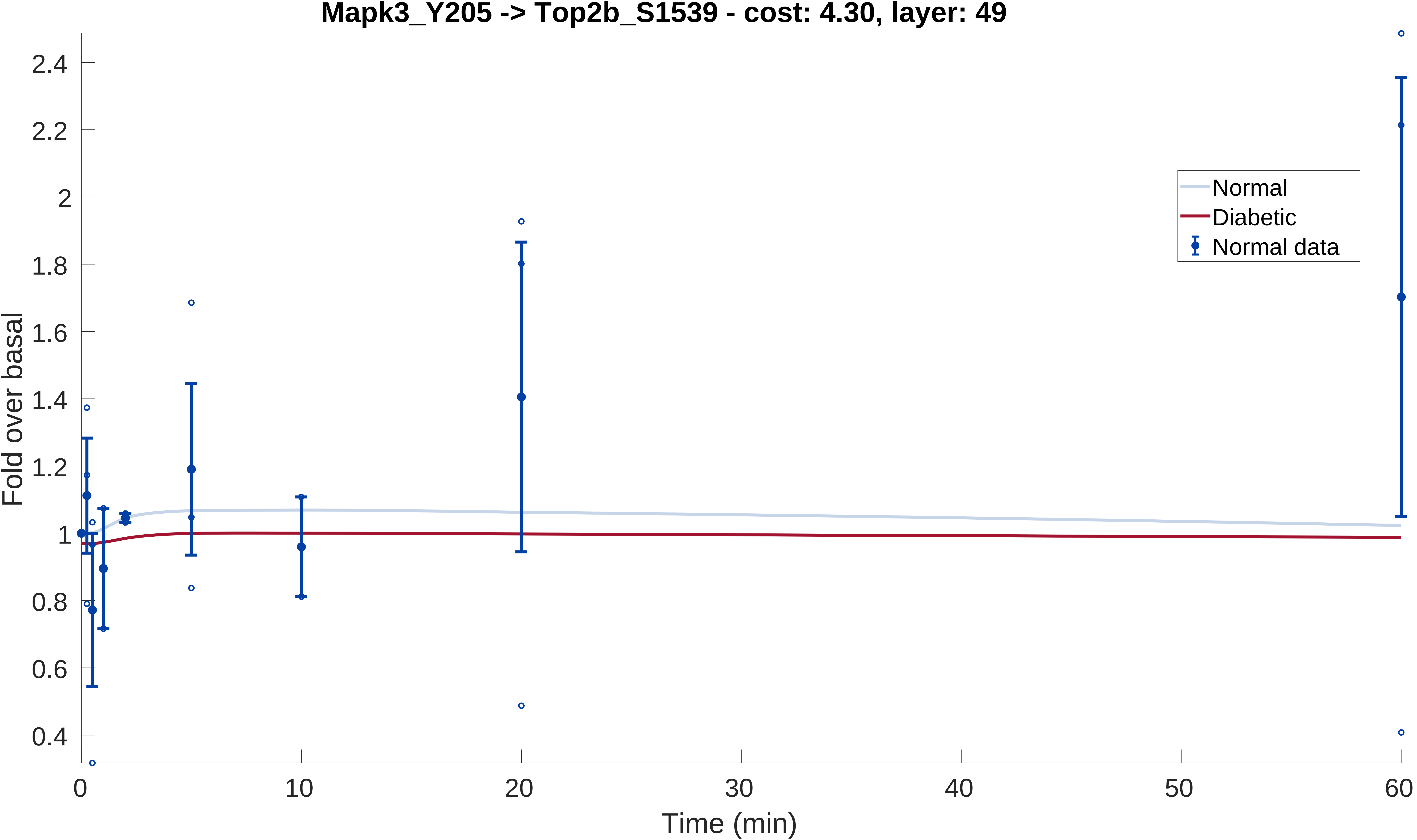

Mapk3_Y205 -> Top2b_S1539 - cost: 4.30, layer: 49
Fold over basal
2.4
2.2
2
1.8
1.6
1.4
1.2
1
0.8
0.6
0.4
0
10
20
30
40
50
60
Time (min)
Normal
Diabetic
Normal data

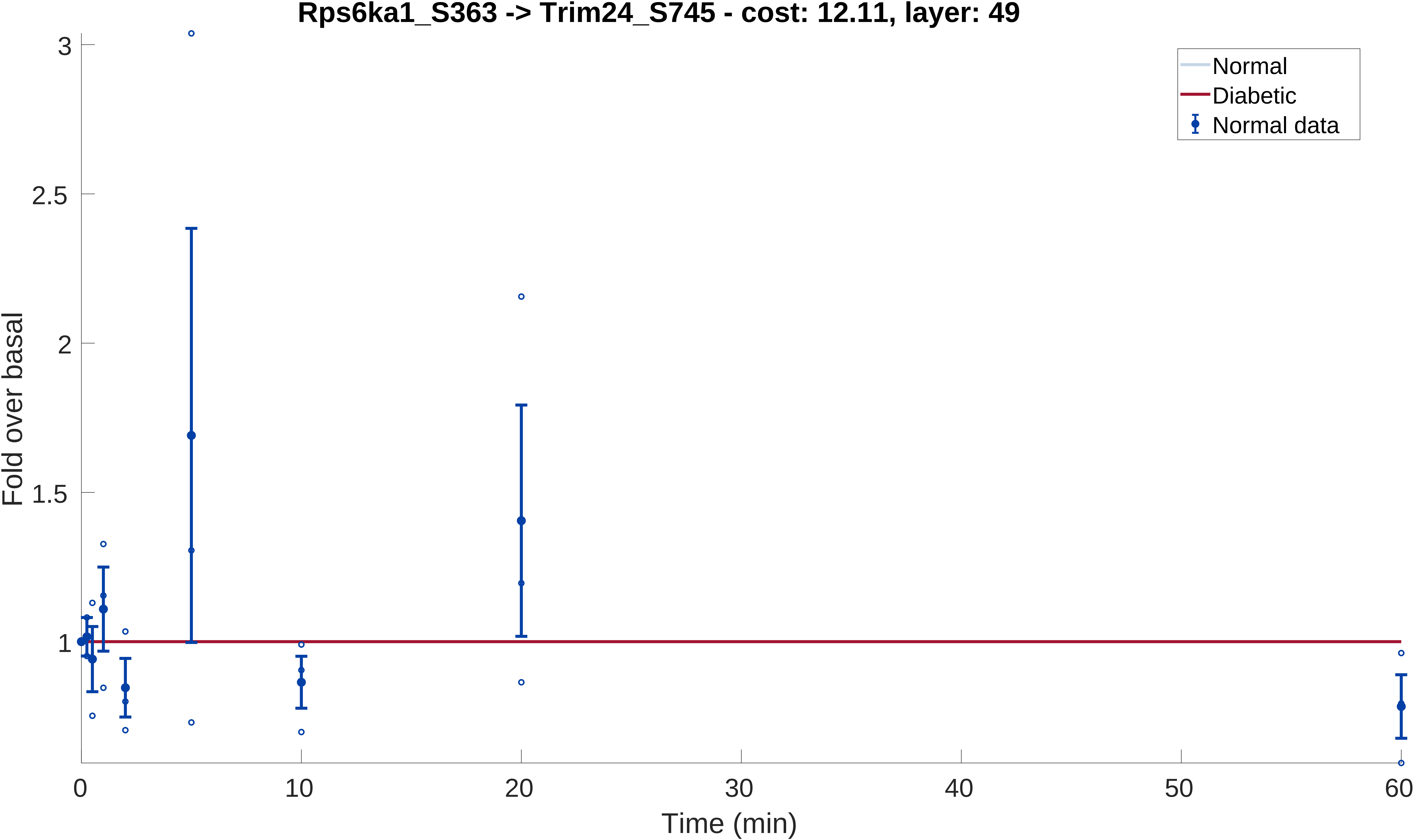

Rps6ka1_S363 -> Trim24_S745 - cost: 12.11, layer: 49
Normal
Diabetic
Normal data
Fold over basal
Time (min)
3
2.5
2
1.5
1
0
10
20
30
40
50
60

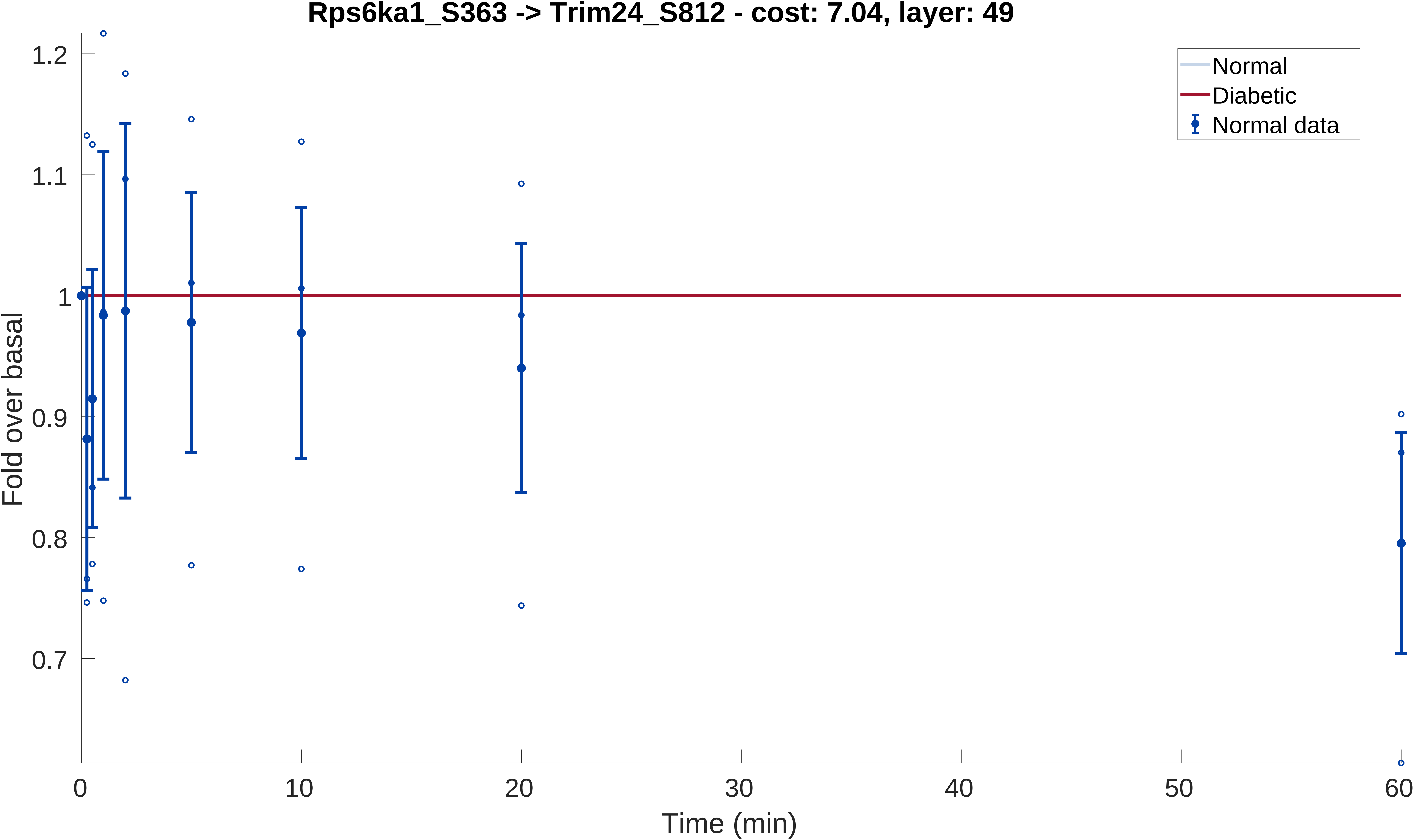

Rps6ka1_S363 -> Trim24_S812 - cost: 7.04, layer: 49
Normal
Diabetic
Normal data
Fold over basal
Time (min)
1.2
1.1
1
0.9
0.8
0.7
0
10
20
30
40
50
60

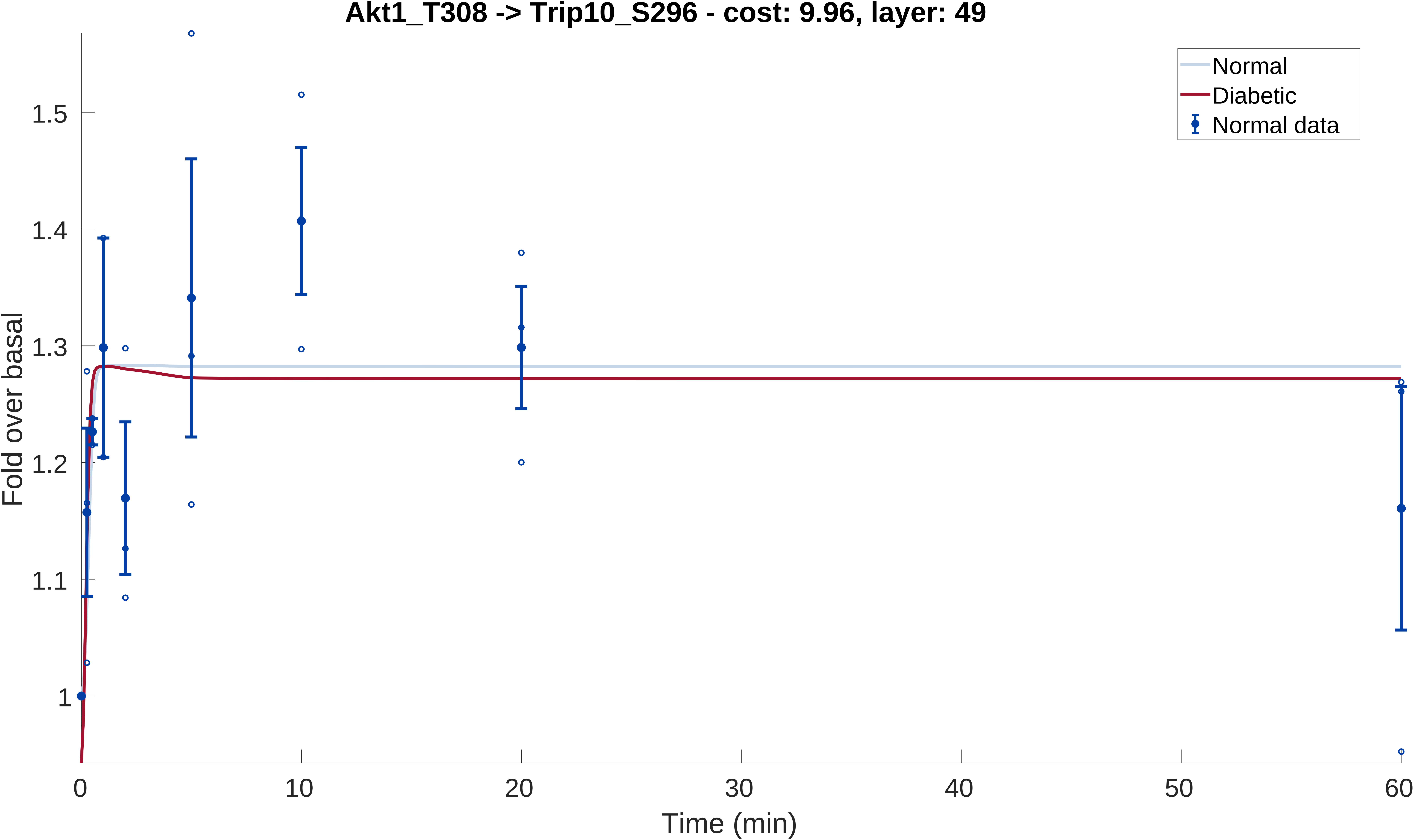

Akt1_T308 -> Trip10_S296 - cost: 9.96, layer: 49
Normal
Diabetic
Normal data
Fold over basal
1
1.1
1.2
1.3
1.4
1.5
0
10
20
30
40
50
60
Time (min)

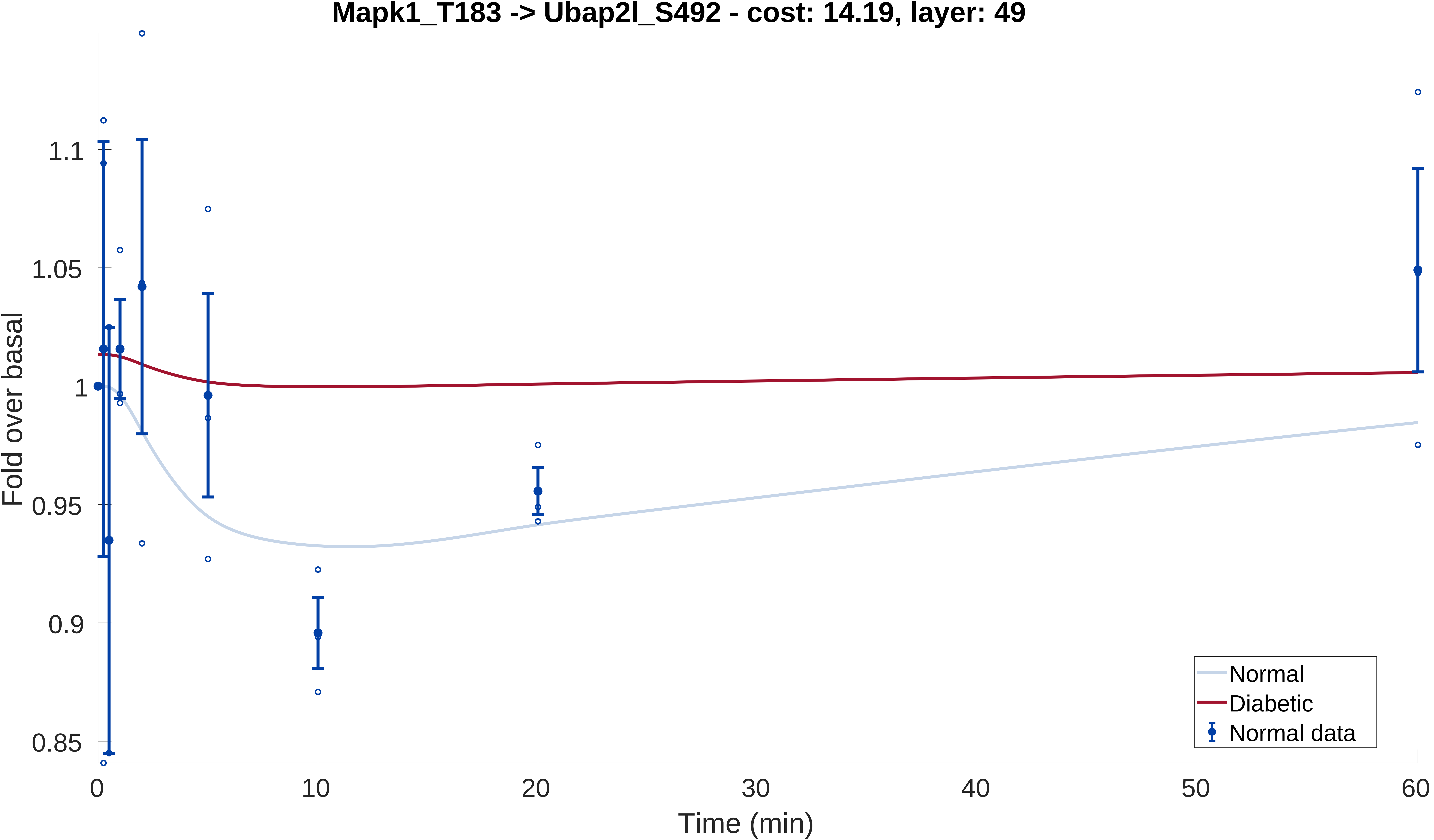

Mapk1_T183 -> Ubap2l_S492 - cost: 14.19, layer: 49
Fold over basal
1.1
1.05
1
0.95
0.9
0.85
0
10
20
30
40
50
60
Time (min)
Normal
Diabetic
Normal data

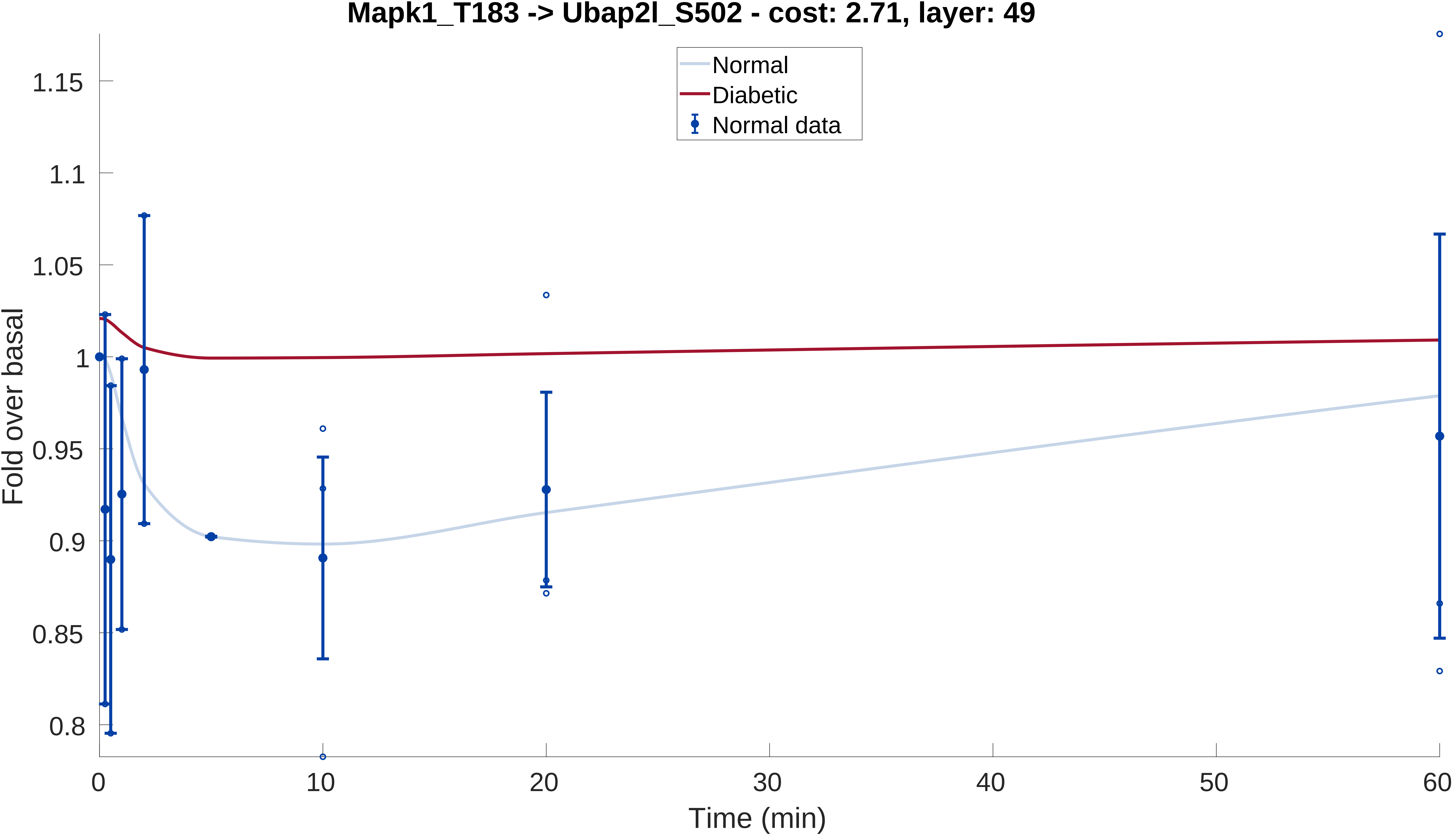

Mapk1_T183 -> Ubap2l_S502 - cost: 2.71, layer: 49
Normal
Diabetic
Normal data
Fold over basal
1.15
1.1
1.05
1
0.95
0.9
0.85
0.8
0
10
20
30
40
50
60
Time (min)

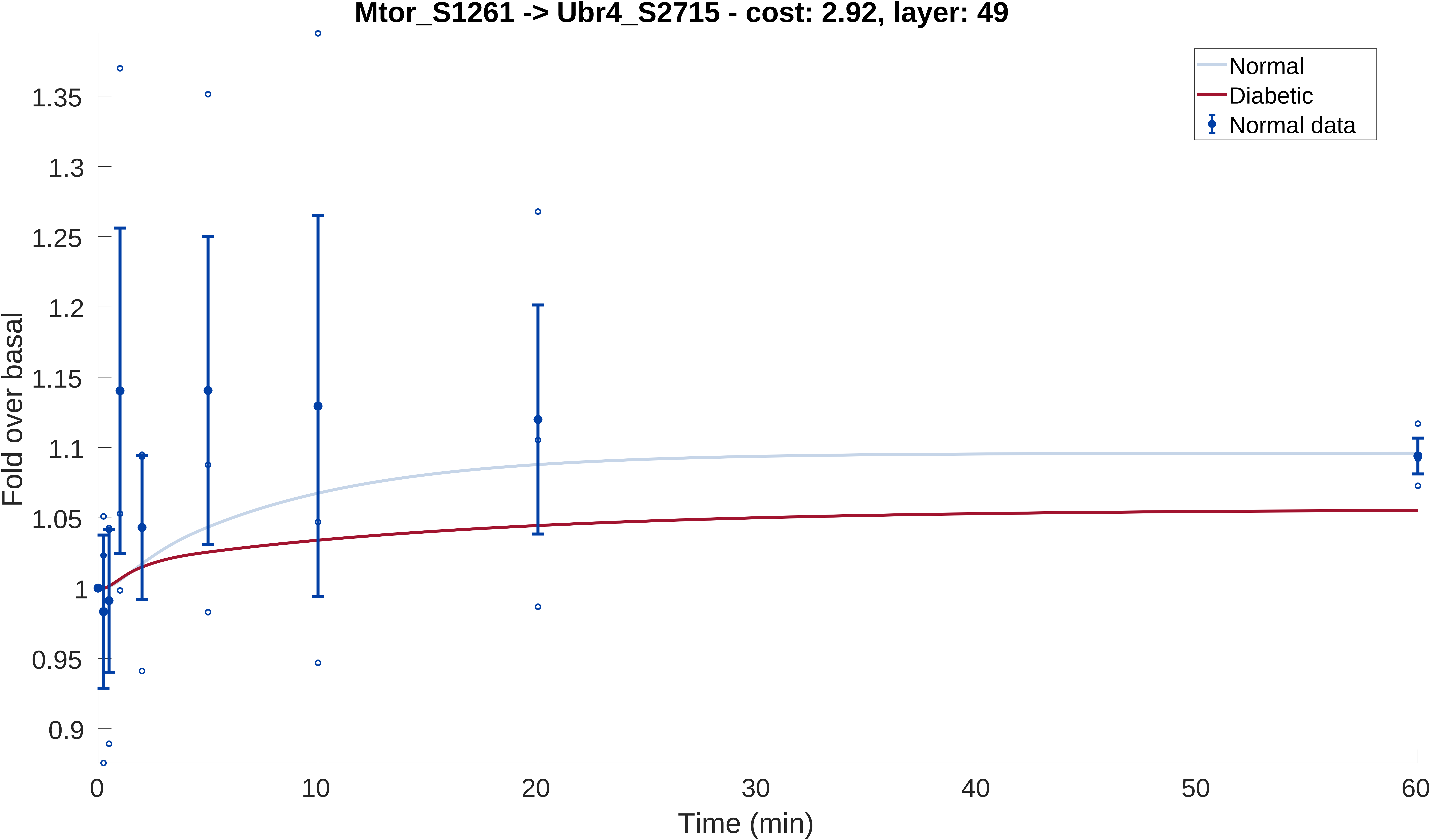

Mtor_S1261 -> Ubr4_S2715 - cost: 2.92, layer: 49
Normal
Diabetic
Normal data
Fold over basal
1.35
1.3
1.25
1.2
1.15
1.1
1.05
1
0.95
0.9
0
10
20
30
40
50
60
Time (min)

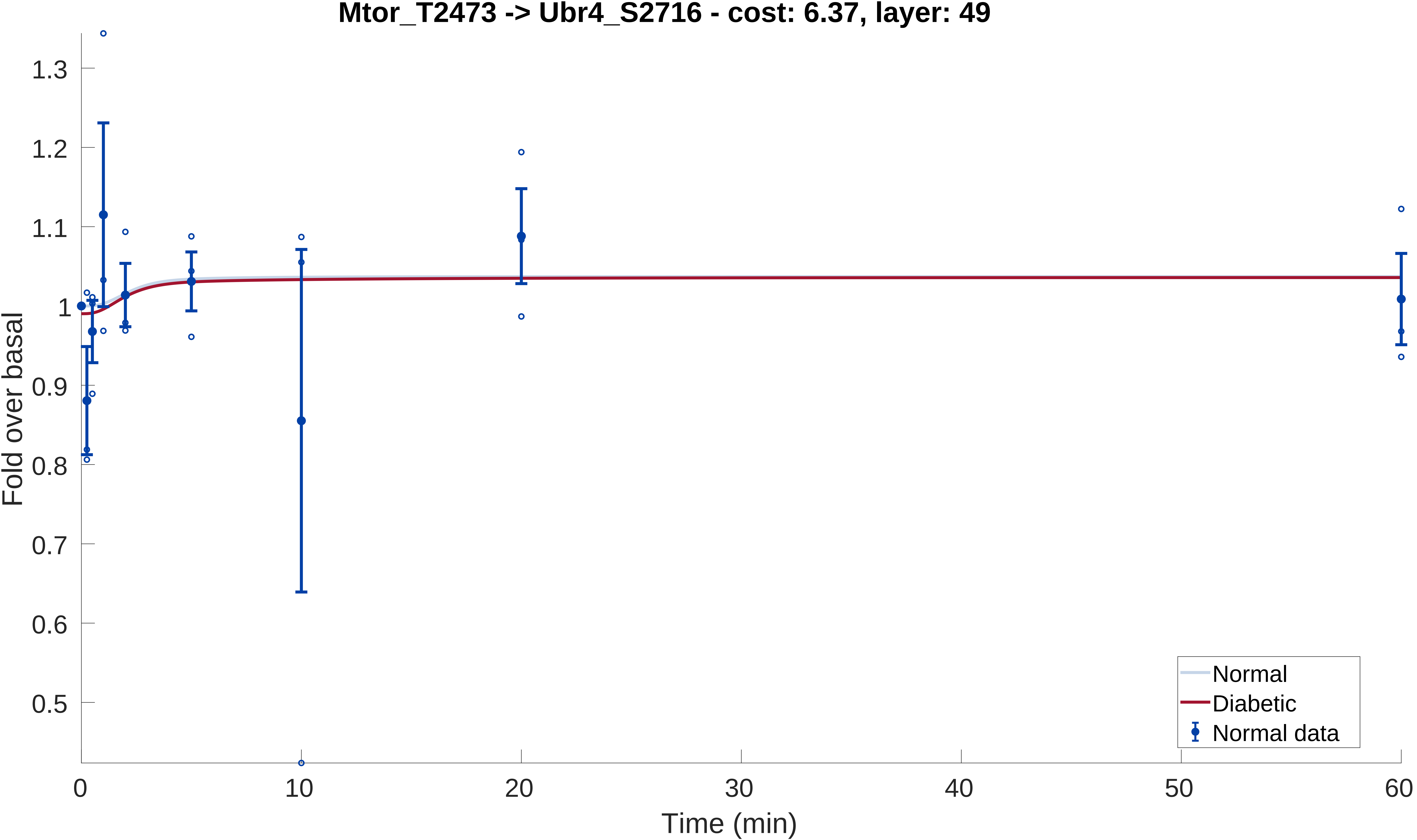

Mtor_T2473 -> Ubr4_S2716 - cost: 6.37, layer: 49
Fold over basal
1.3
1.2
1.1
1
0.9
0.8
0.7
0.6
0.5
0
10
20
30
40
50
60
Time (min)
Normal
Diabetic
Normal data

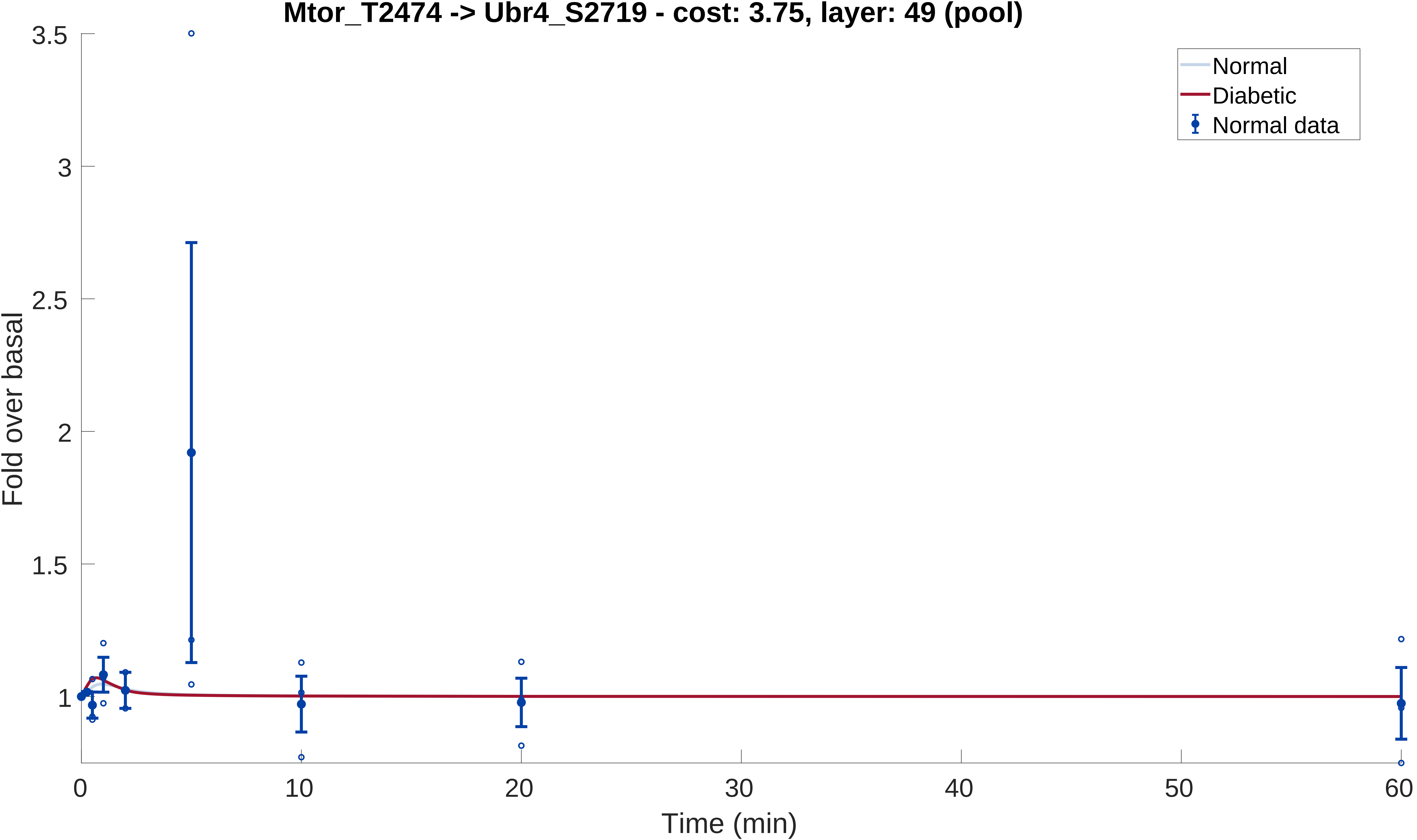
Mtor_T2474 -> Ubr4_S2719 - cost: 3.75, layer: 49 (pool)
Normal
Diabetic
Normal data
Fold over basal
Time (min)
3.5
3
2.5
2
1.5
1
0
10
20
30
40
50
60

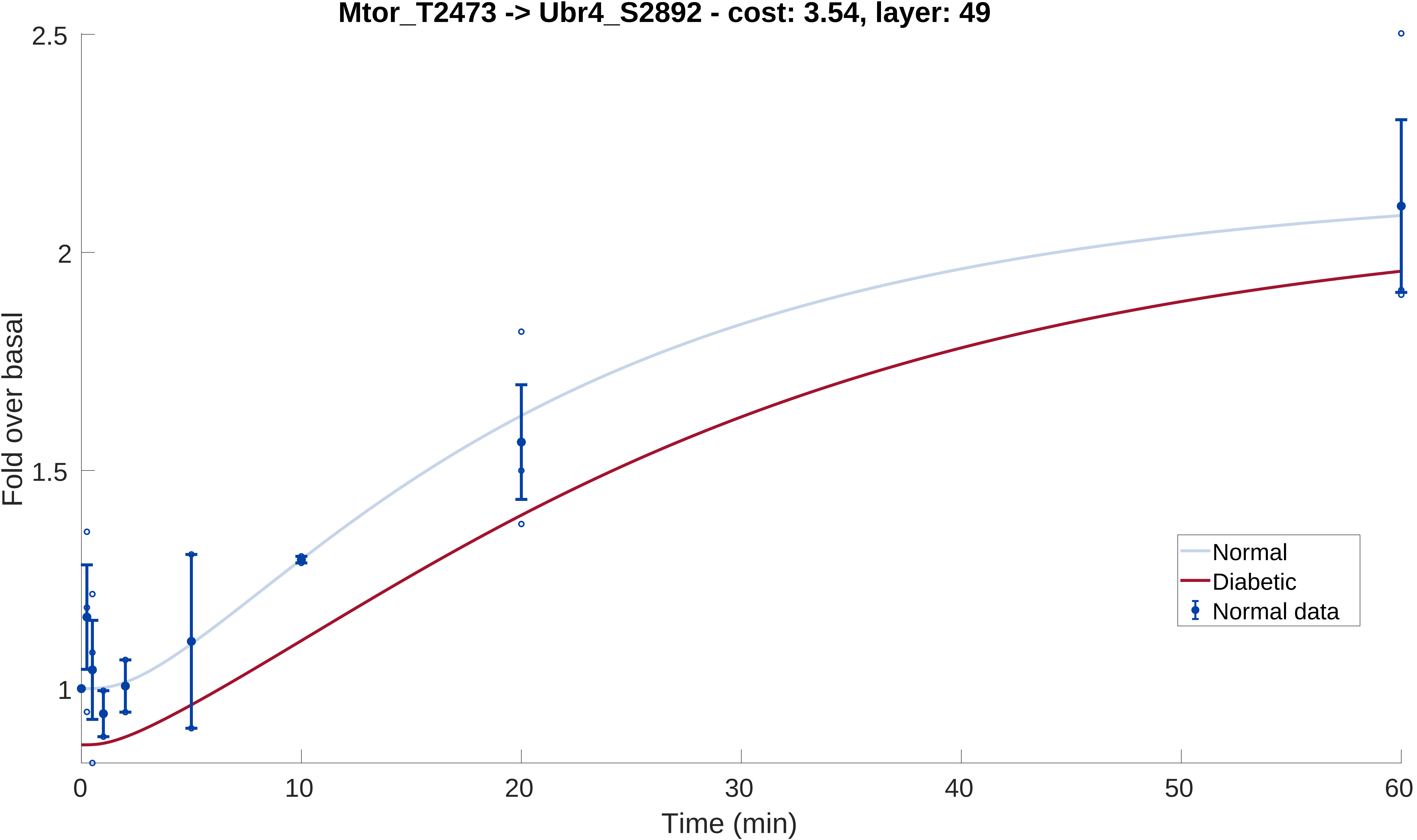

Mtor_T2473 -> Ubr4_S2892 - cost: 3.54, layer: 49
Fold over basal
2.5
2
1.5
1
0
10
20
30
40
50
60
Time (min)
Normal
Diabetic
Normal data

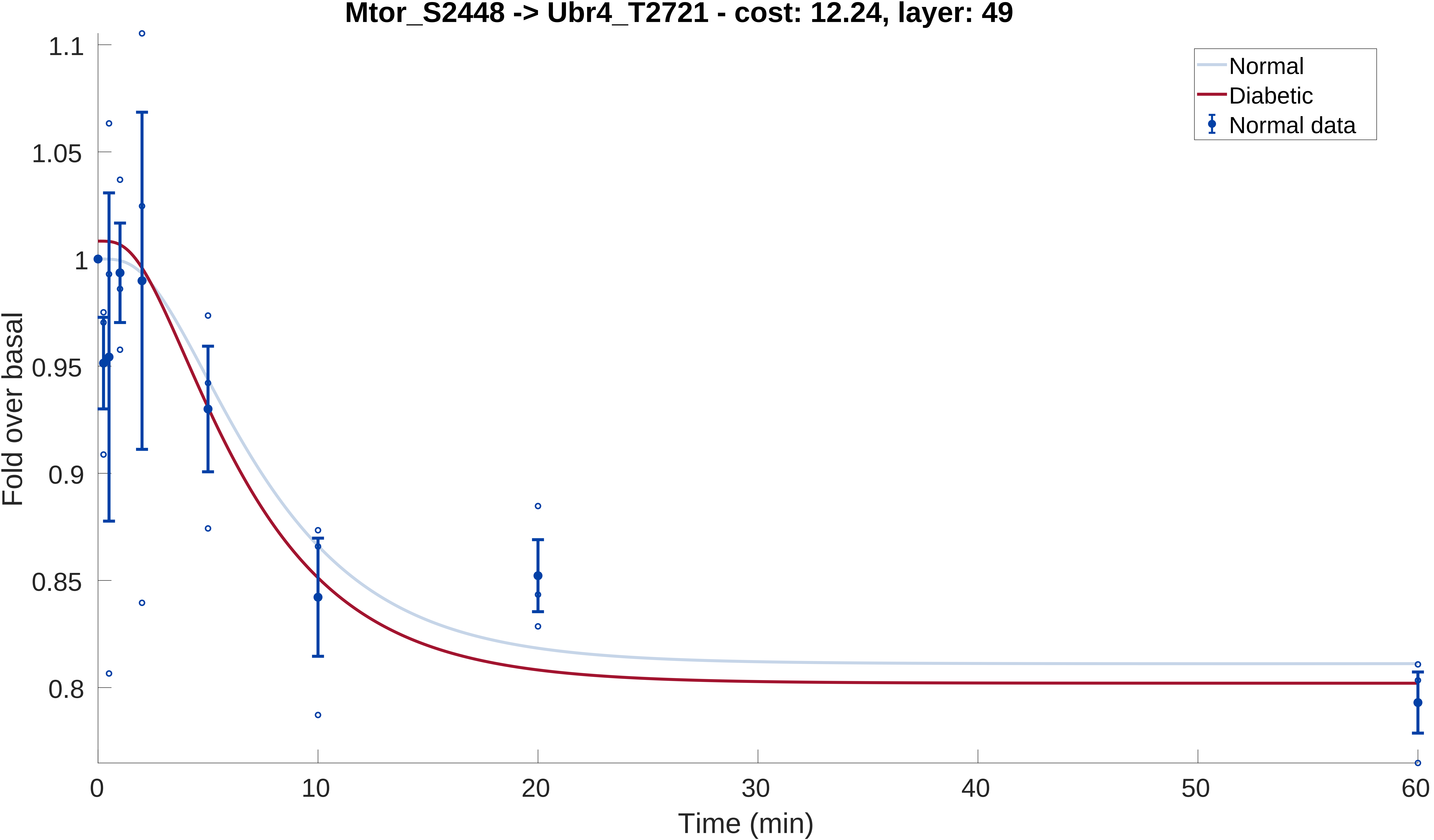

Mtor_S2448 -> Ubr4_T2721 - cost: 12.24, layer: 49
Normal
Diabetic
Normal data
Fold over basal
1.1
1.05
1
0.95
0.9
0.85
0.8
0
10
20
30
40
50
60
Time (min)

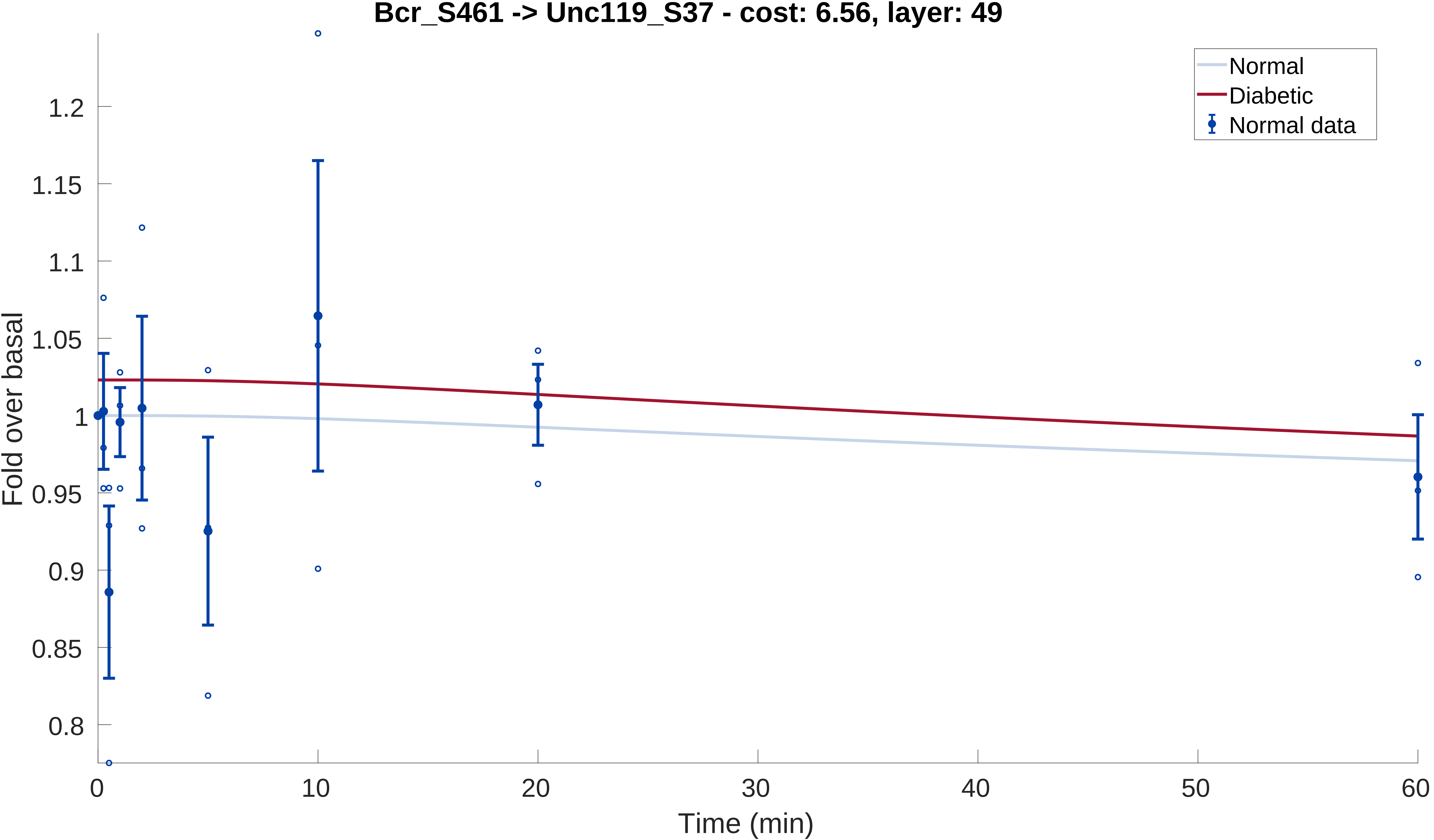

Bcr_S461 -> Unc119_S37 - cost: 6.56, layer: 49
Normal
Diabetic
Normal data
Fold over basal
Time (min)
1.2
1.15
1.1
1.05
1
0.95
0.9
0.85
0.8
0
10
20
30
40
50
60

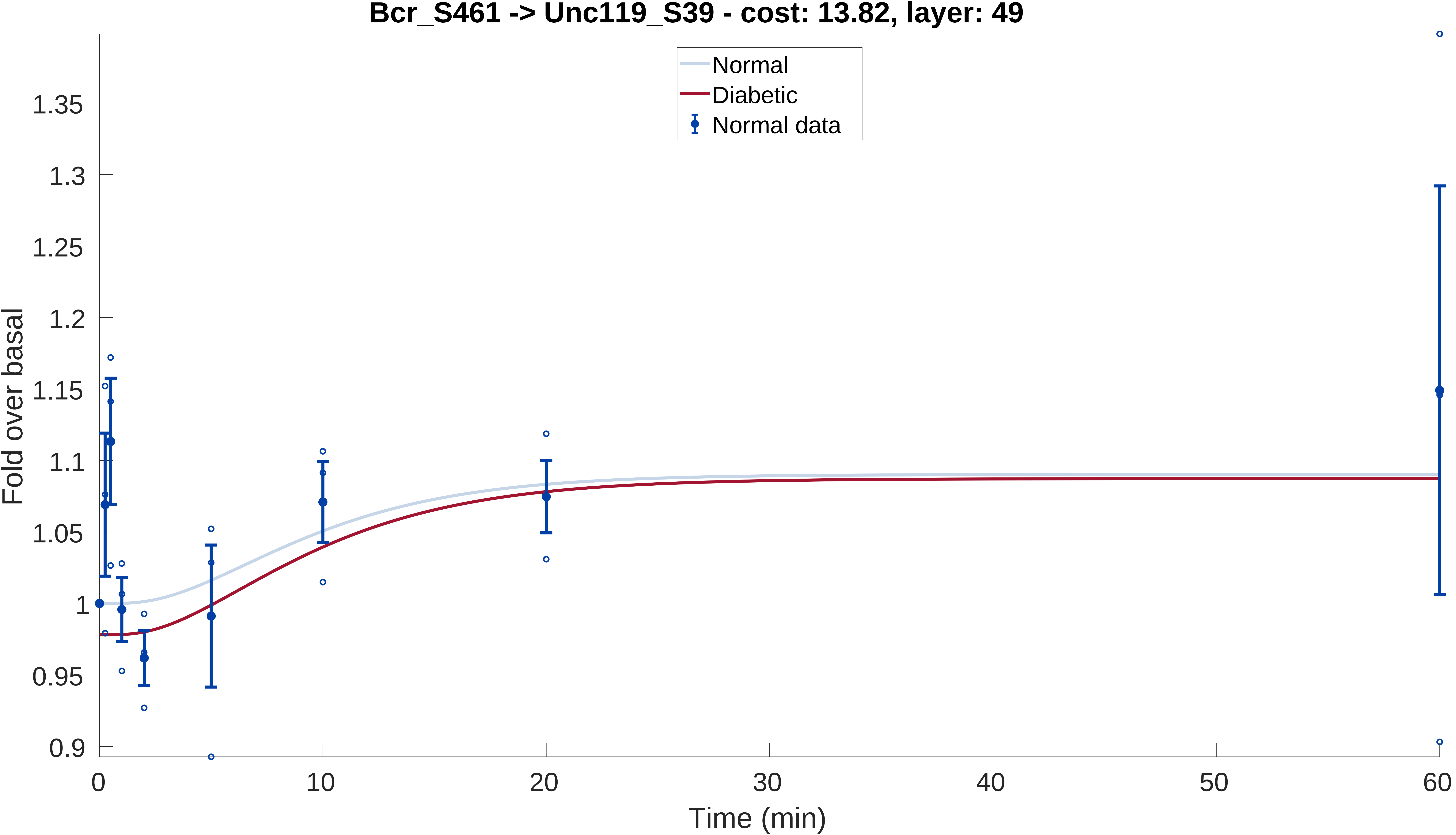
Bcr_S461 -> Unc119_S39 - cost: 13.82, layer: 49
Normal
Diabetic
Normal data
Fold over basal
1.35
1.3
1.25
1.2
1.15
1.1
1.05
1
0.95
0.9
0
10
20
30
40
50
60
Time (min)

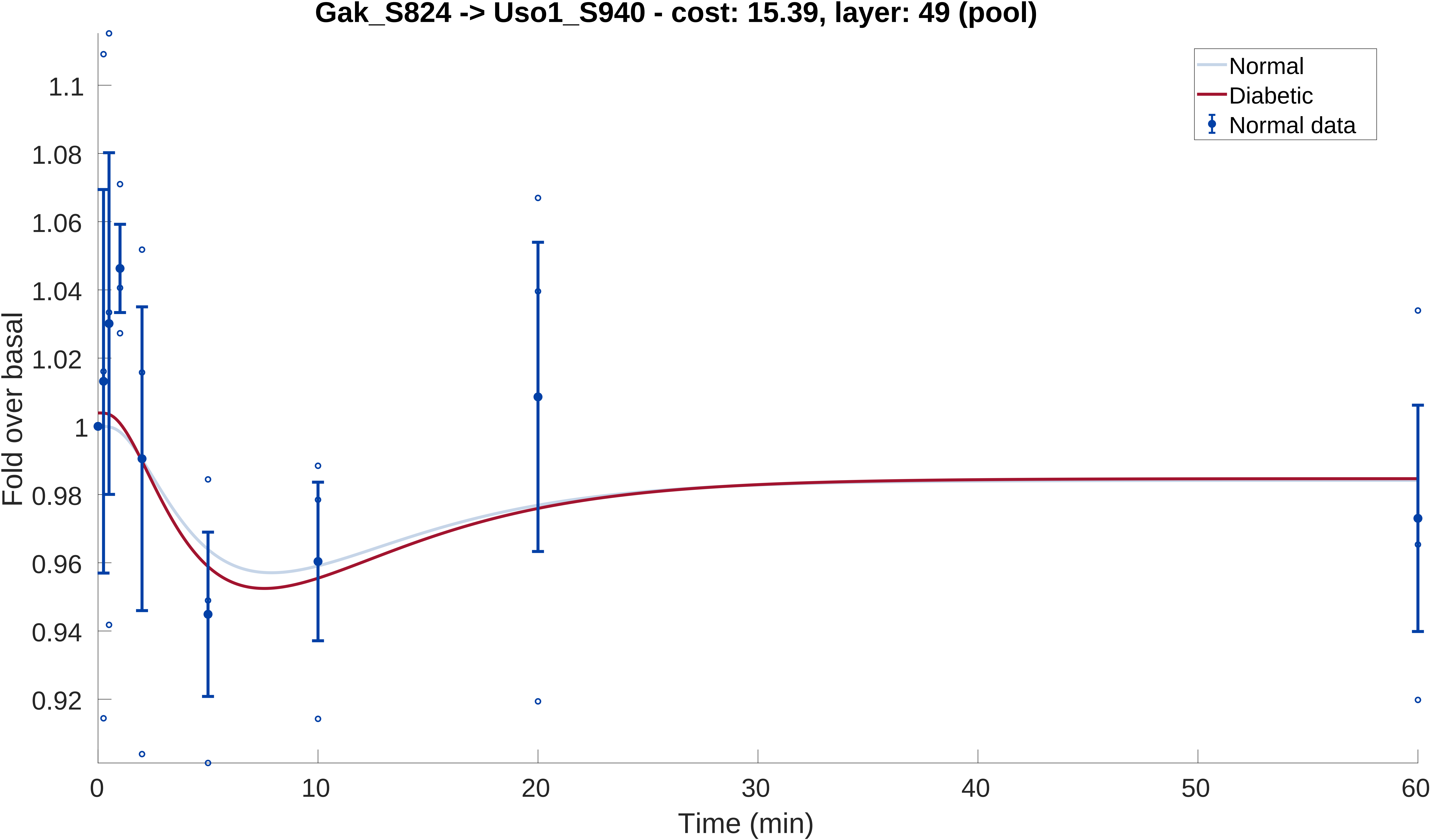

Gak_S824 -> Uso1_S940 - cost: 15.39, layer: 49 (pool)
Normal
Diabetic
Normal data
Fold over basal
1.1
1.08
1.06
1.04
1.02
1
0.98
0.96
0.94
0.92
0
10
20
30
40
50
60
Time (min)

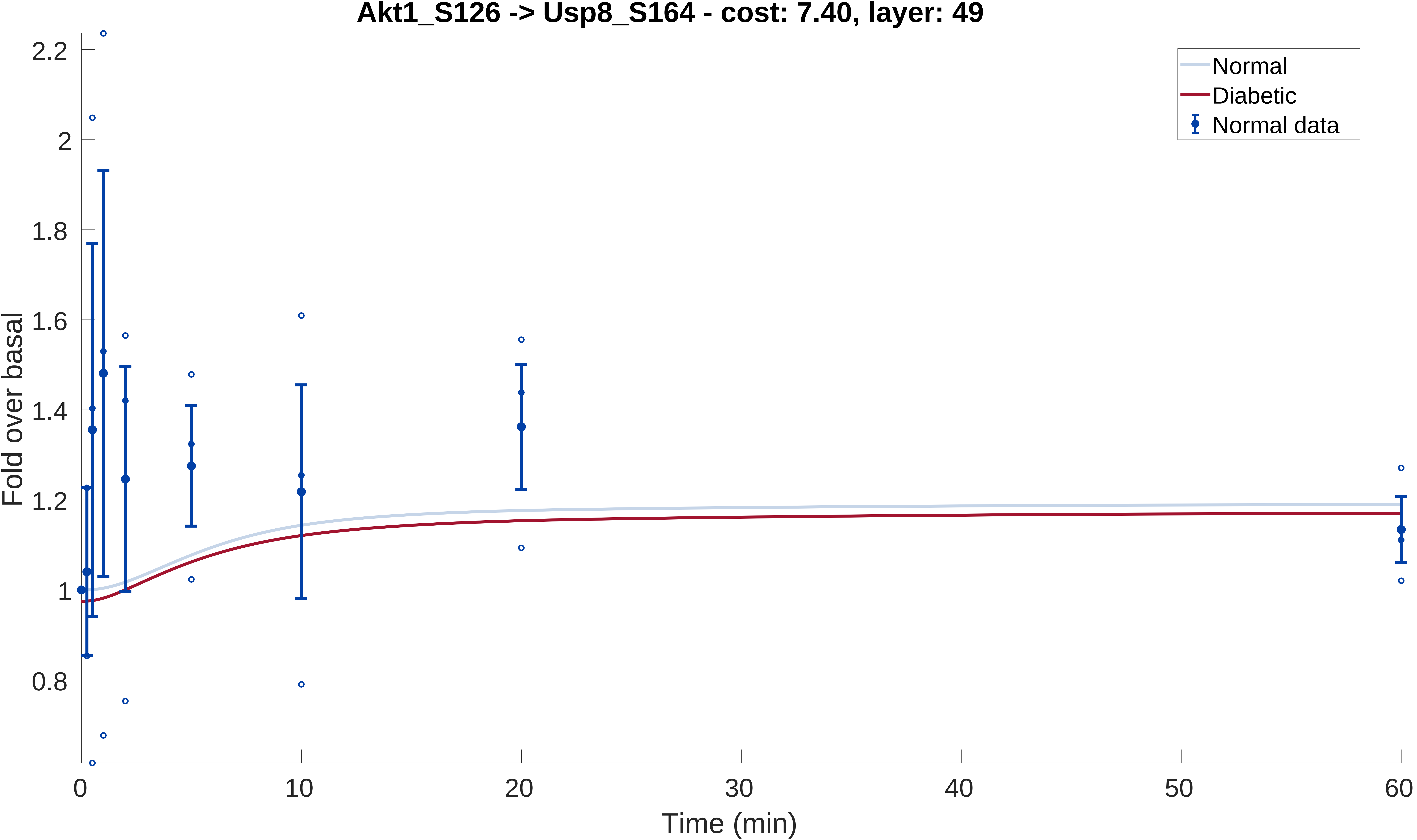

Akt1_S126 -> Usp8_S164 - cost: 7.40, layer: 49
Normal
Diabetic
Normal data
Fold over basal
Time (min)
0.8
1
1.2
1.4
1.6
1.8
2
2.2
0
10
20
30
40
50
60

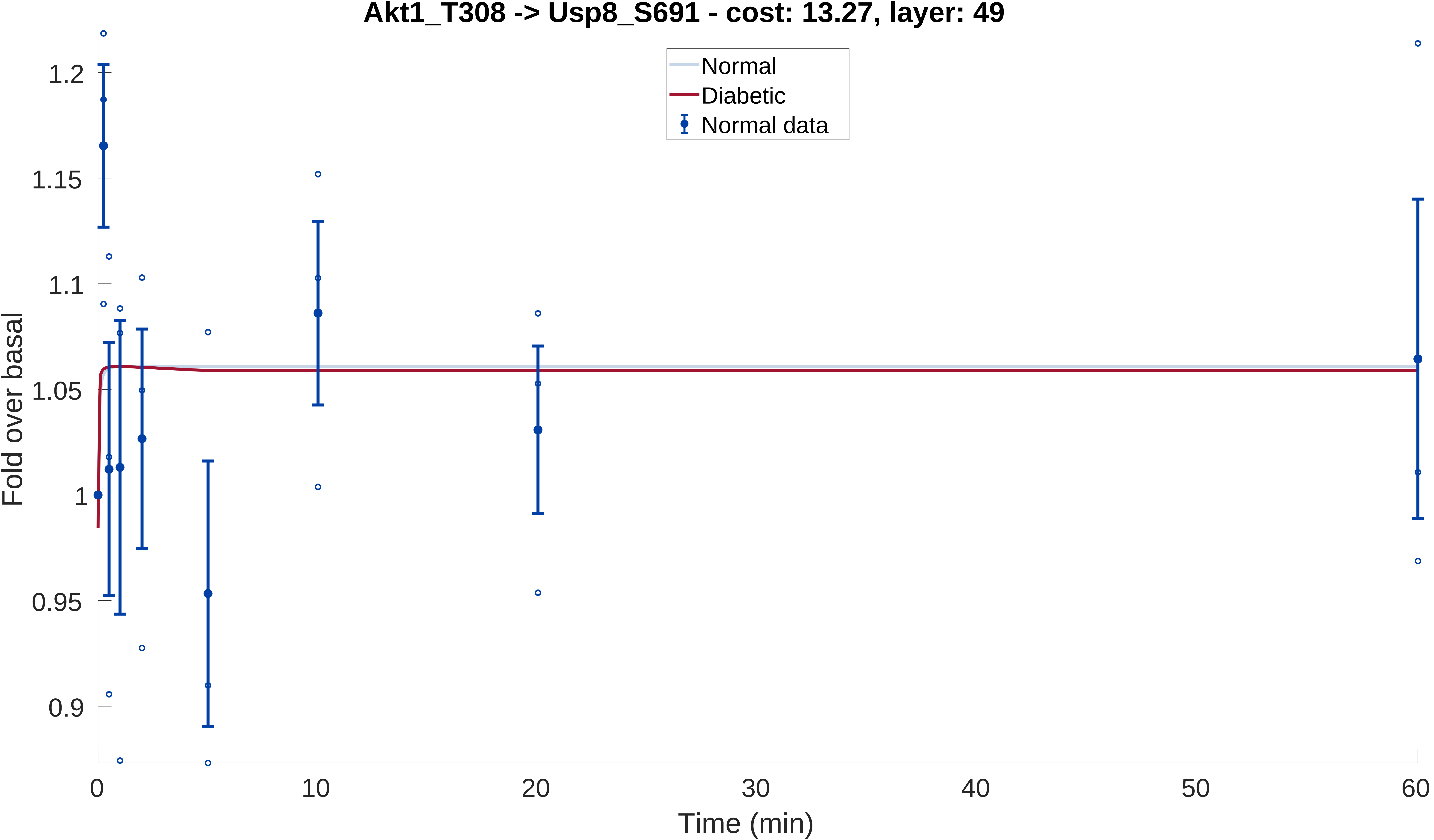
Akt1_T308 -> Usp8_S691 - cost: 13.27, layer: 49
Normal
Diabetic
Normal data
Fold over basal
1.2
1.15
1.1
1.05
1
0.95
0.9
0
10
20
30
40
50
60
Time (min)

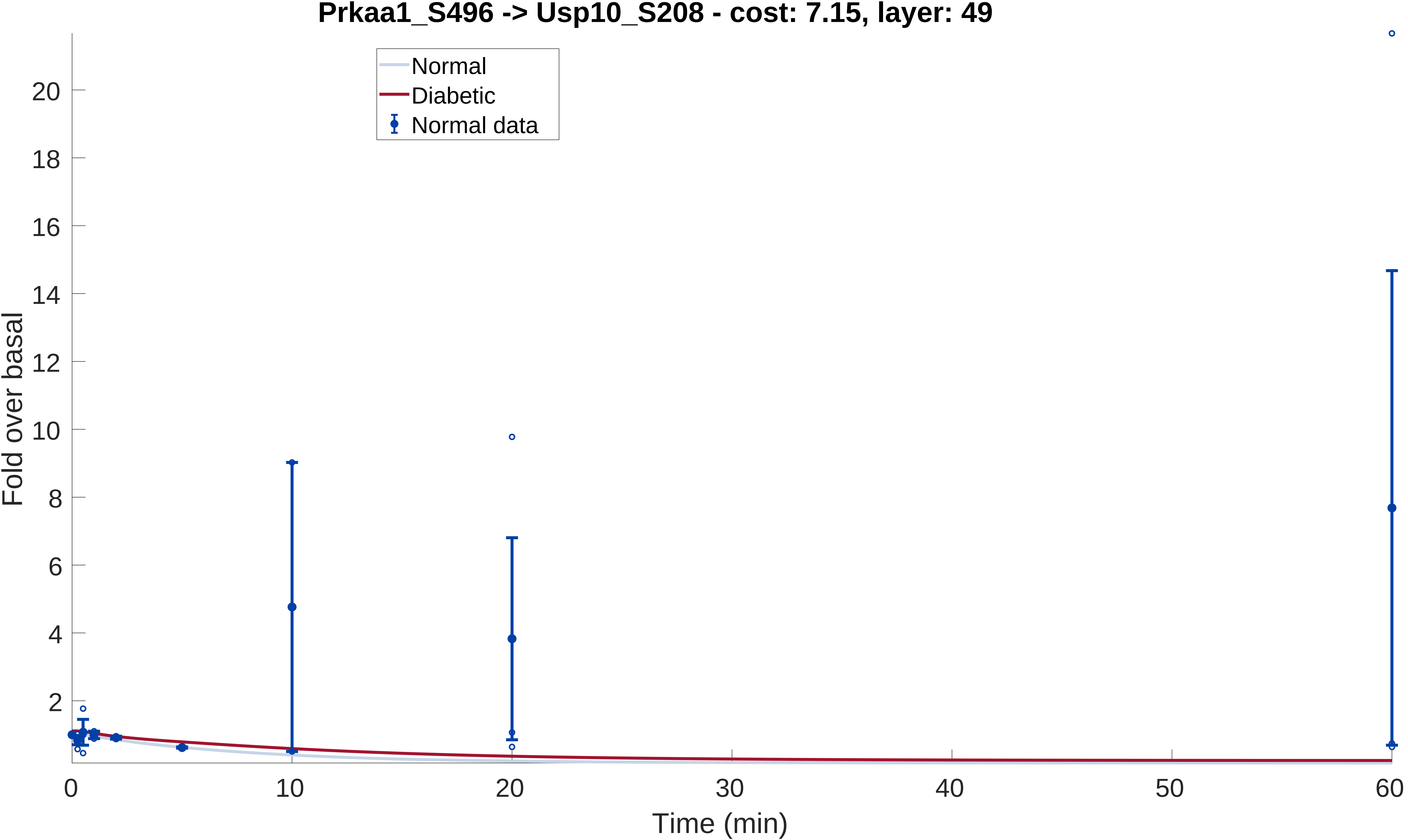

Prkaa1_S496 -> Usp10_S208 - cost: 7.15, layer: 49
Normal
Diabetic
Normal data
Fold over basal
Time (min)
0
10
20
30
40
50
60
2
4
6
8
10
12
14
16
18
20

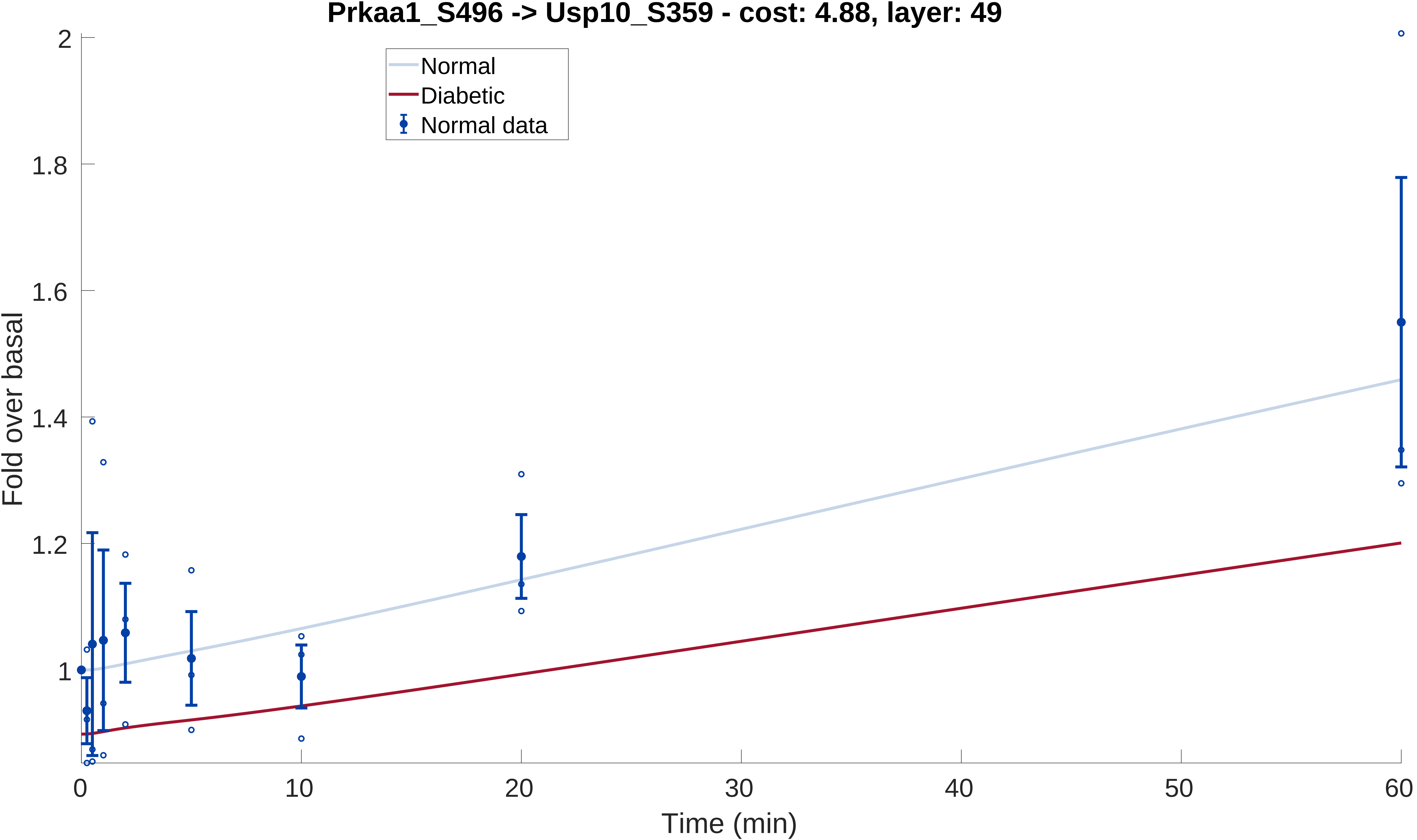

Prkaa1_S496 -> Usp10_S359 - cost: 4.88, layer: 49
Normal
Diabetic
Normal data
Fold over basal
Time (min)
2
1.8
1.6
1.4
1.2
1
0
10
20
30
40
50
60

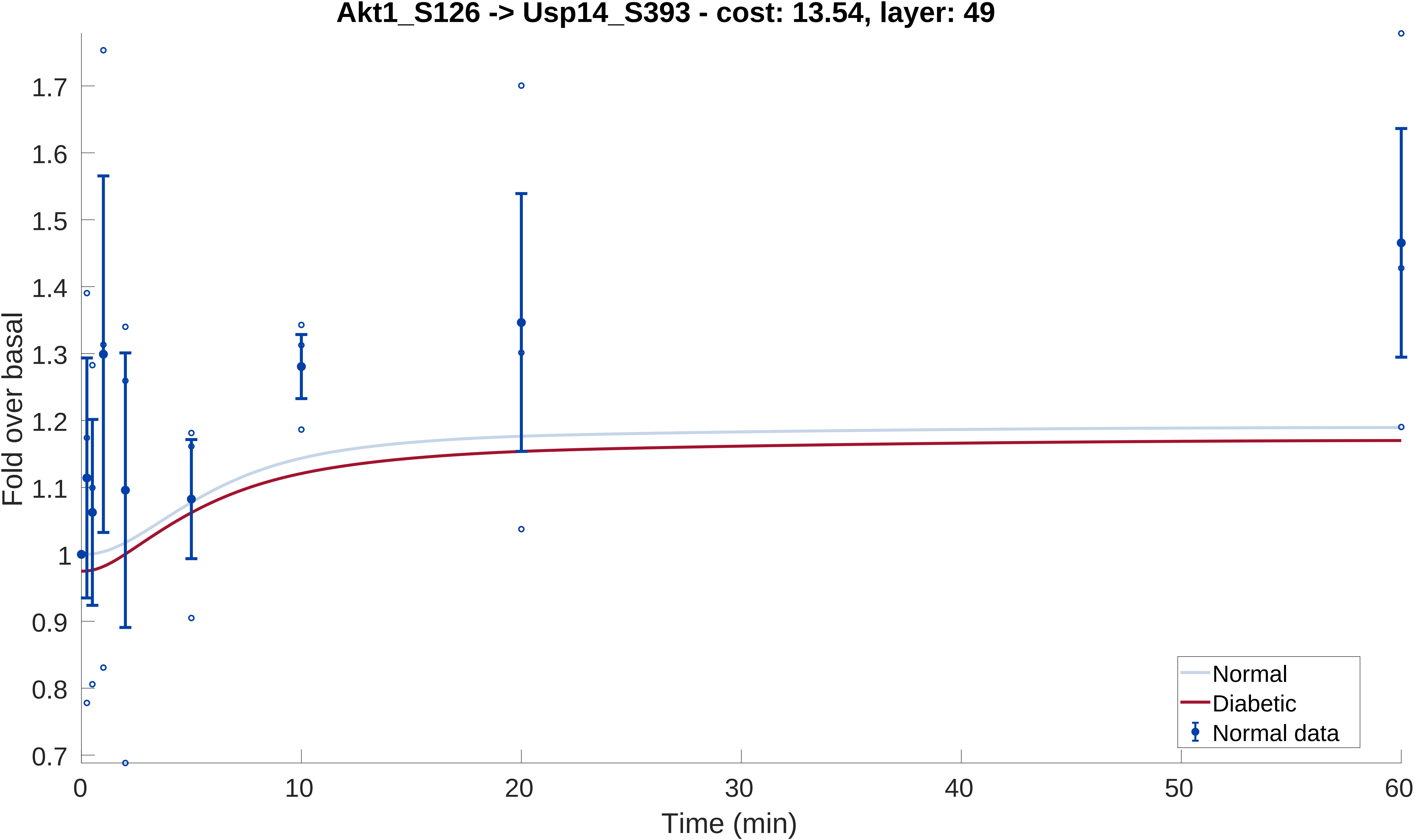

Akt1_S126 -> Usp14_S393 - cost: 13.54, layer: 49
Fold over basal
1.7
1.6
1.5
1.4
1.3
1.2
1.1
1
0.9
0.8
0.7
0
10
20
30
40
50
60
Time (min)
Normal
Diabetic
Normal data

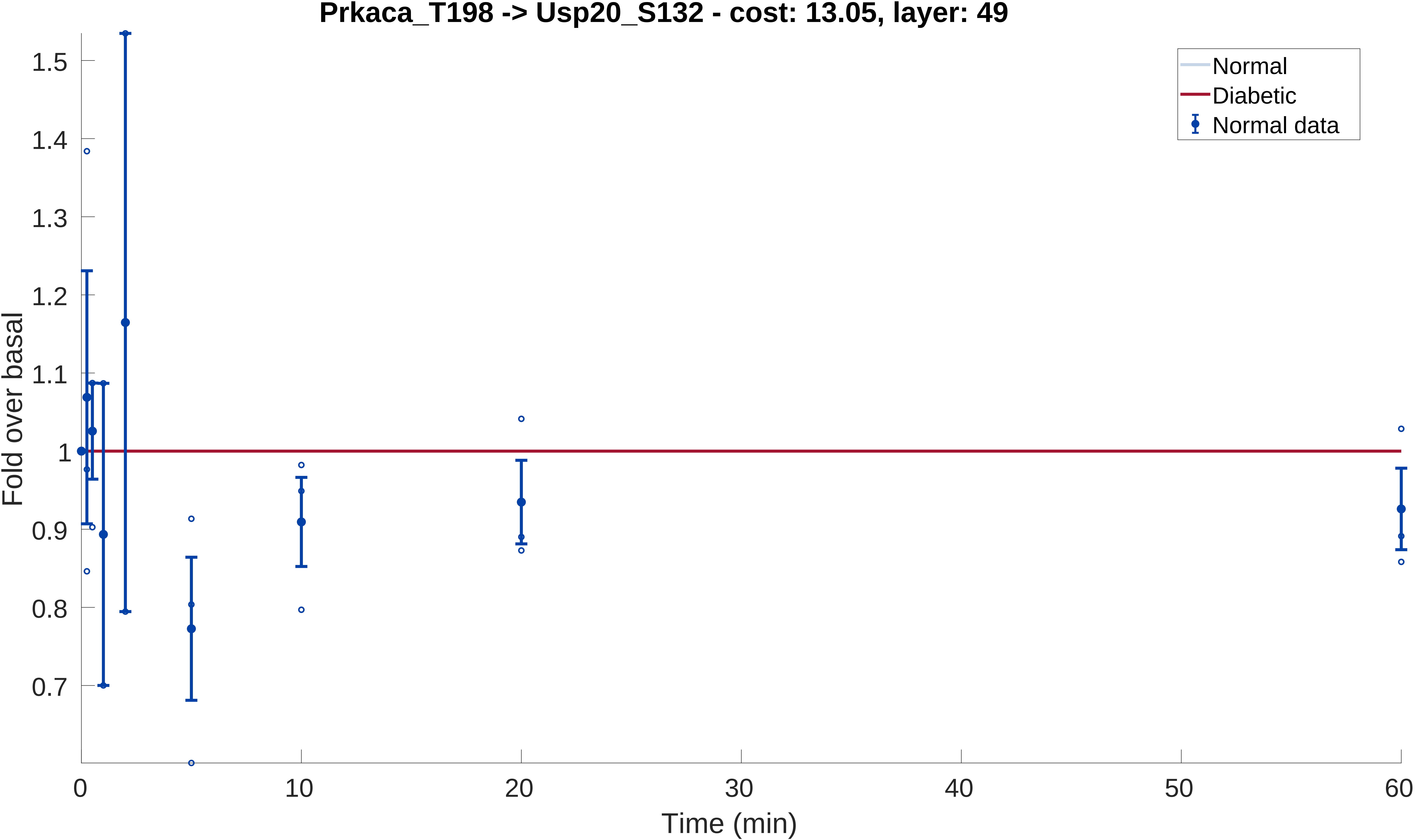

Prkaca_T198 -> Usp20_S132 - cost: 13.05, layer: 49
Normal
Diabetic
Normal data
Fold over basal
Time (min)
1.5
1.4
1.3
1.2
1.1
1
0.9
0.8
0.7
0
10
20
30
40
50
60

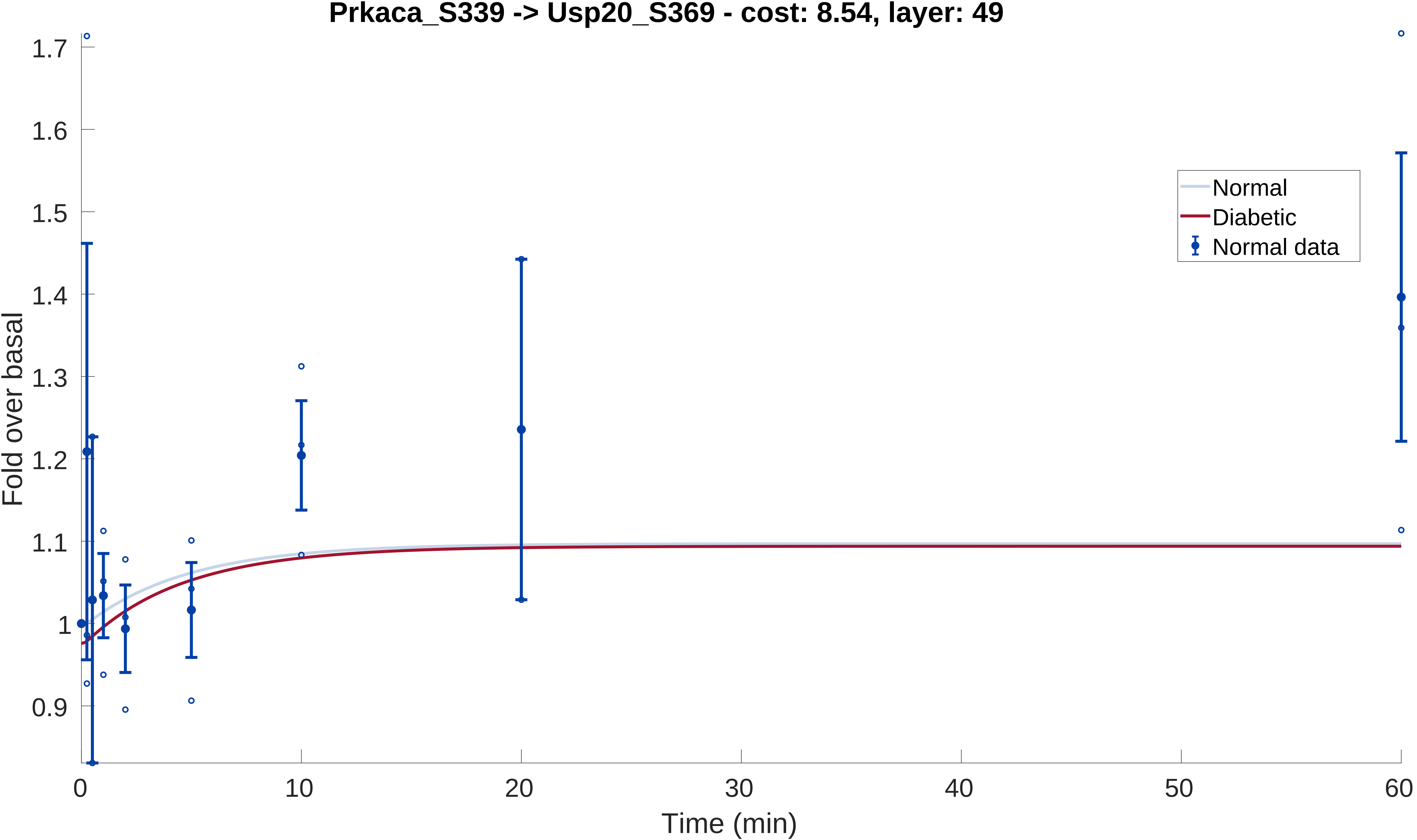

Prkaca_S339 -> Usp20_S369 - cost: 8.54, layer: 49
Fold over basal
1.7
1.6
1.5
1.4
1.3
1.2
1.1
1
0.9
0
10
20
30
40
50
60
Time (min)
Normal
Diabetic
Normal data

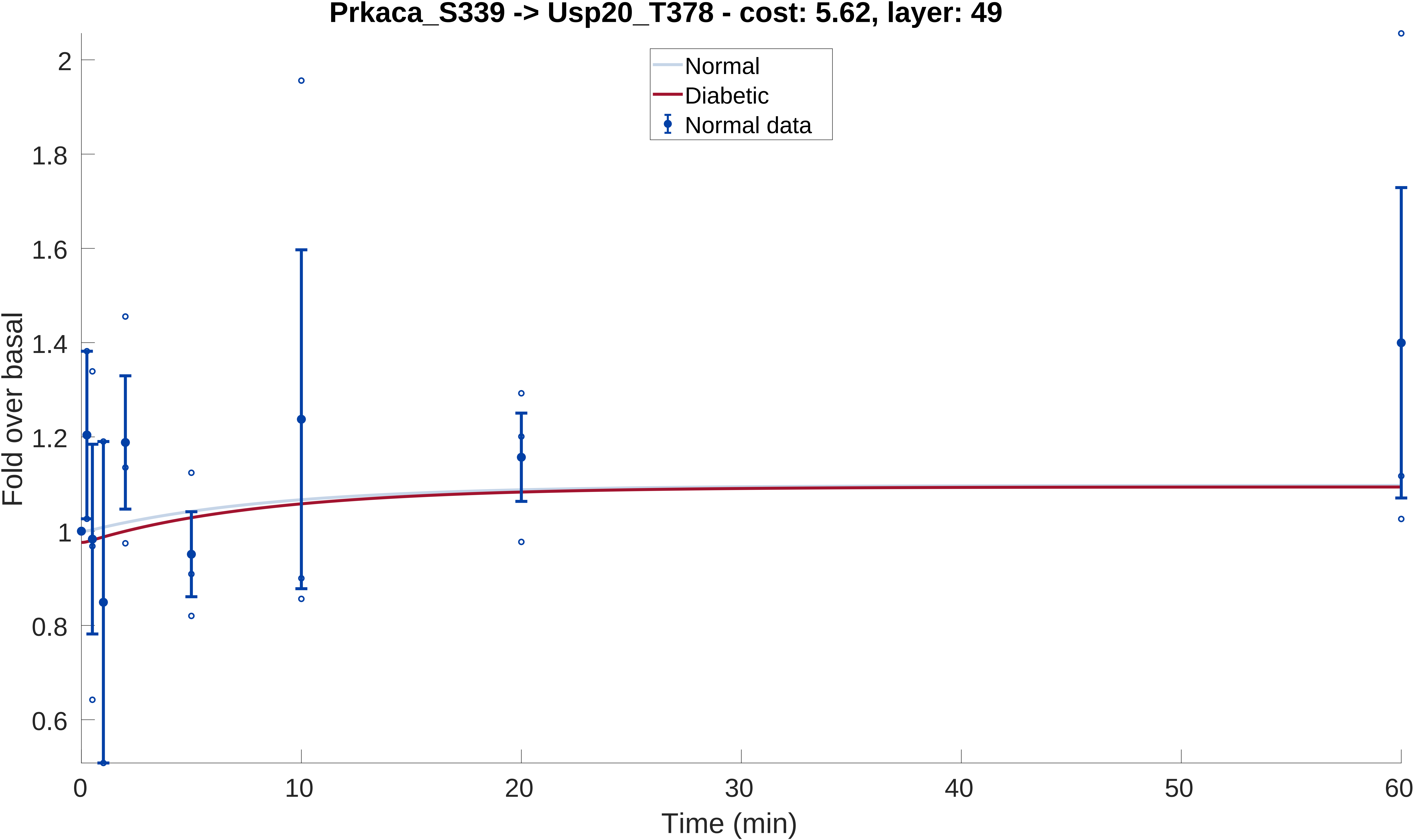

Prkaca_S339 -> Usp20_T378 - cost: 5.62, layer: 49
Normal
Diabetic
Normal data
Fold over basal
Time (min)
0.6
0.8
1
1.2
1.4
1.6
1.8
2
0
10
20
30
40
50
60

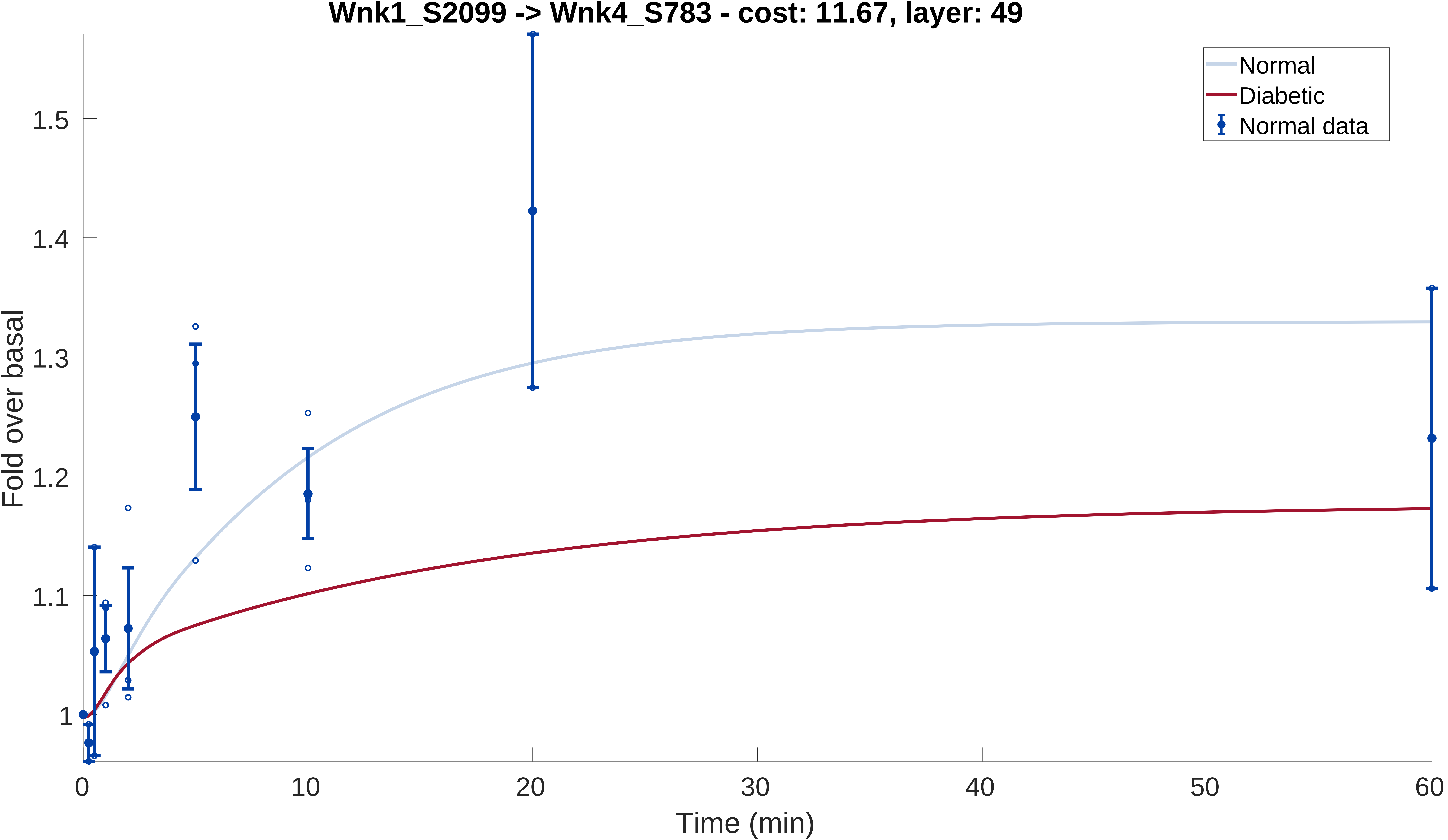
Wnk1_S2099 -> Wnk4_S783 - cost: 11.67, layer: 49
Normal
Diabetic
Normal data
1.5
1.4
1.3
1.2
1.1
1
Fold over basal
0
10
20
30
40
50
60
Time (min)

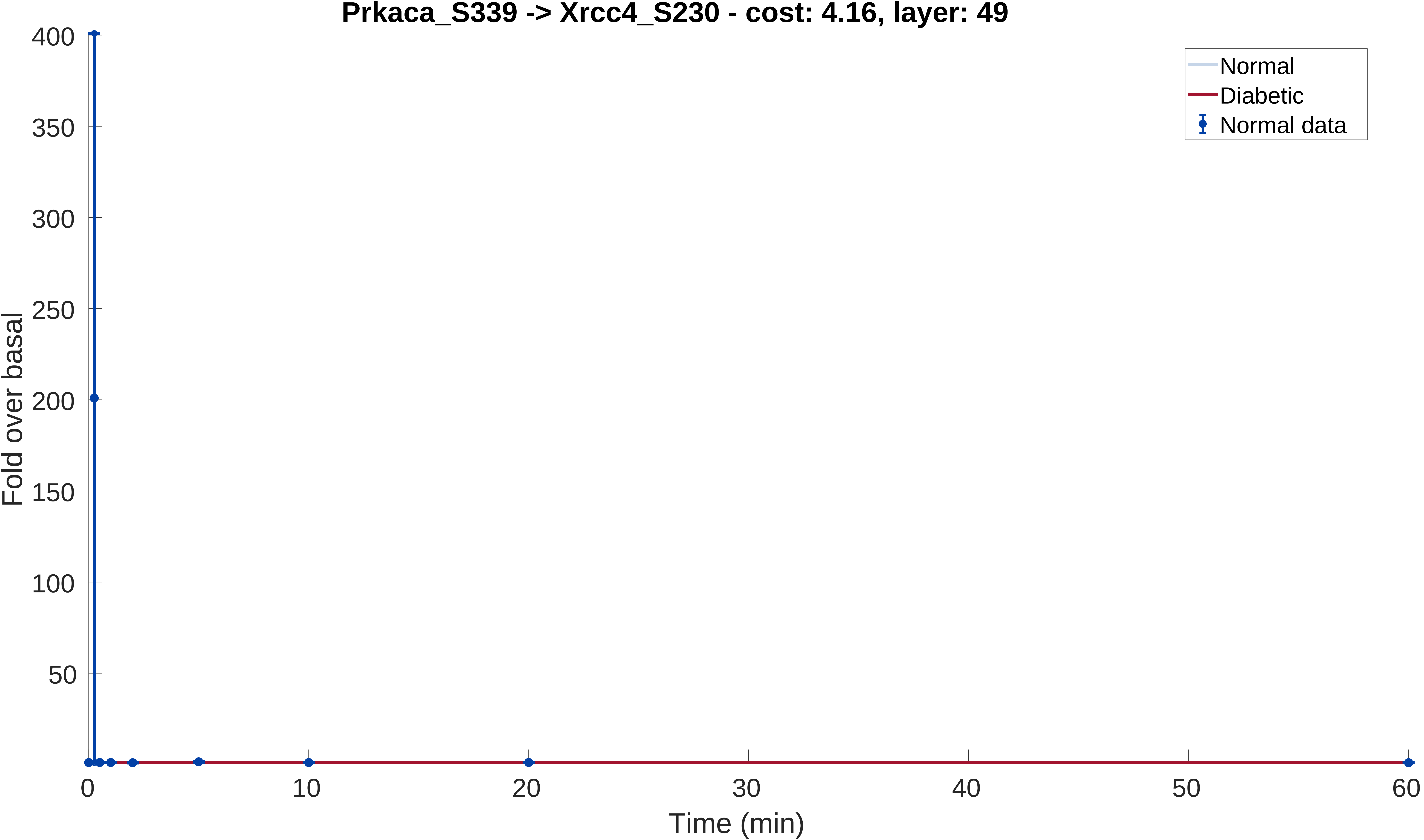
Prkaca_S339 -> Xrcc4_S230 - cost: 4.16, layer: 49
Fold over basal
400
350
300
250
200
150
100
50
0
10
20
30
40
50
60
Time (min)
Normal
Diabetic
Normal data

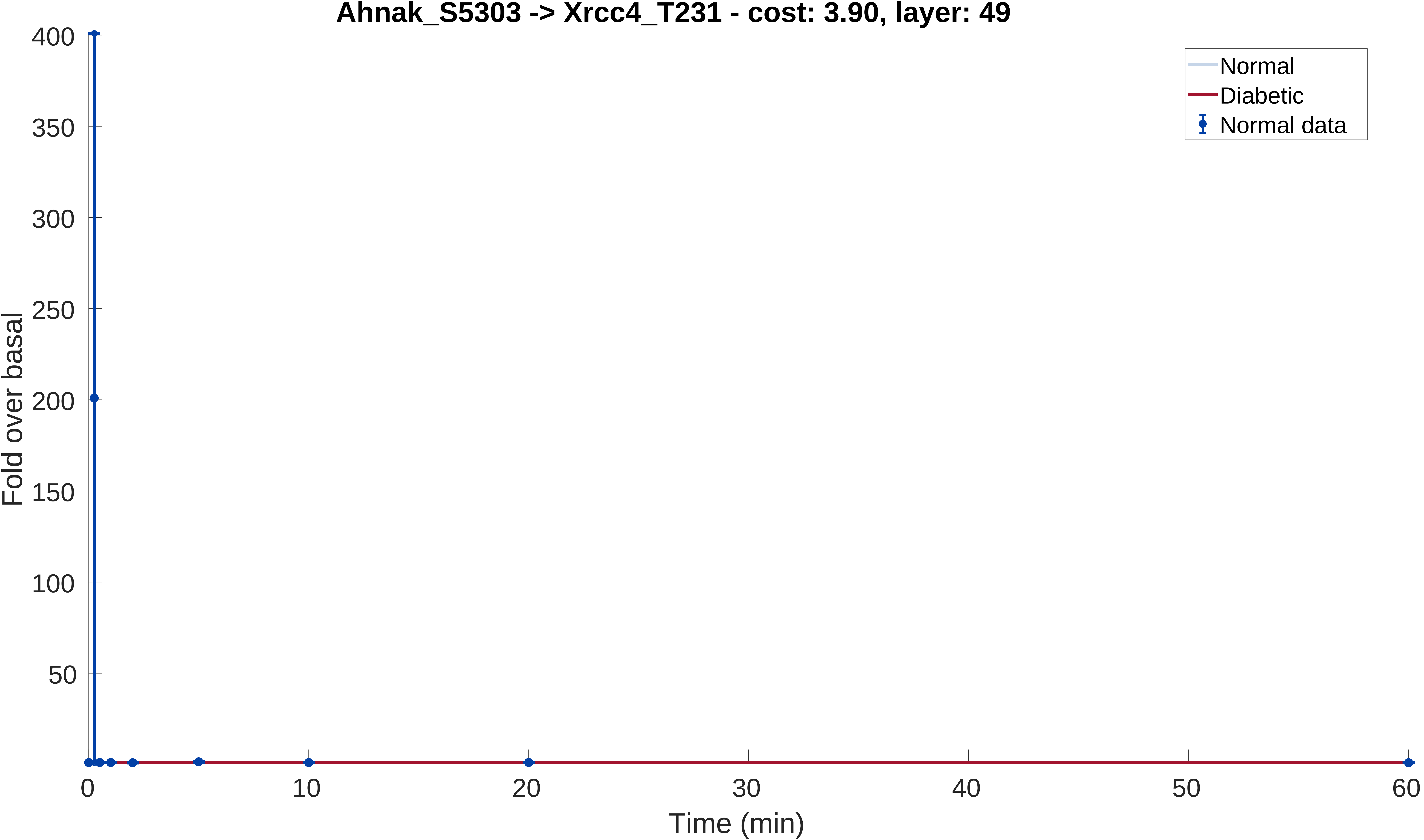

Ahnak_S5303 -> Xrcc4_T231 - cost: 3.90, layer: 49
Normal
Diabetic
Normal data
Fold over basal
Time (min)
400
350
300
250
200
150
100
50
0
10
20
30
40
50
60

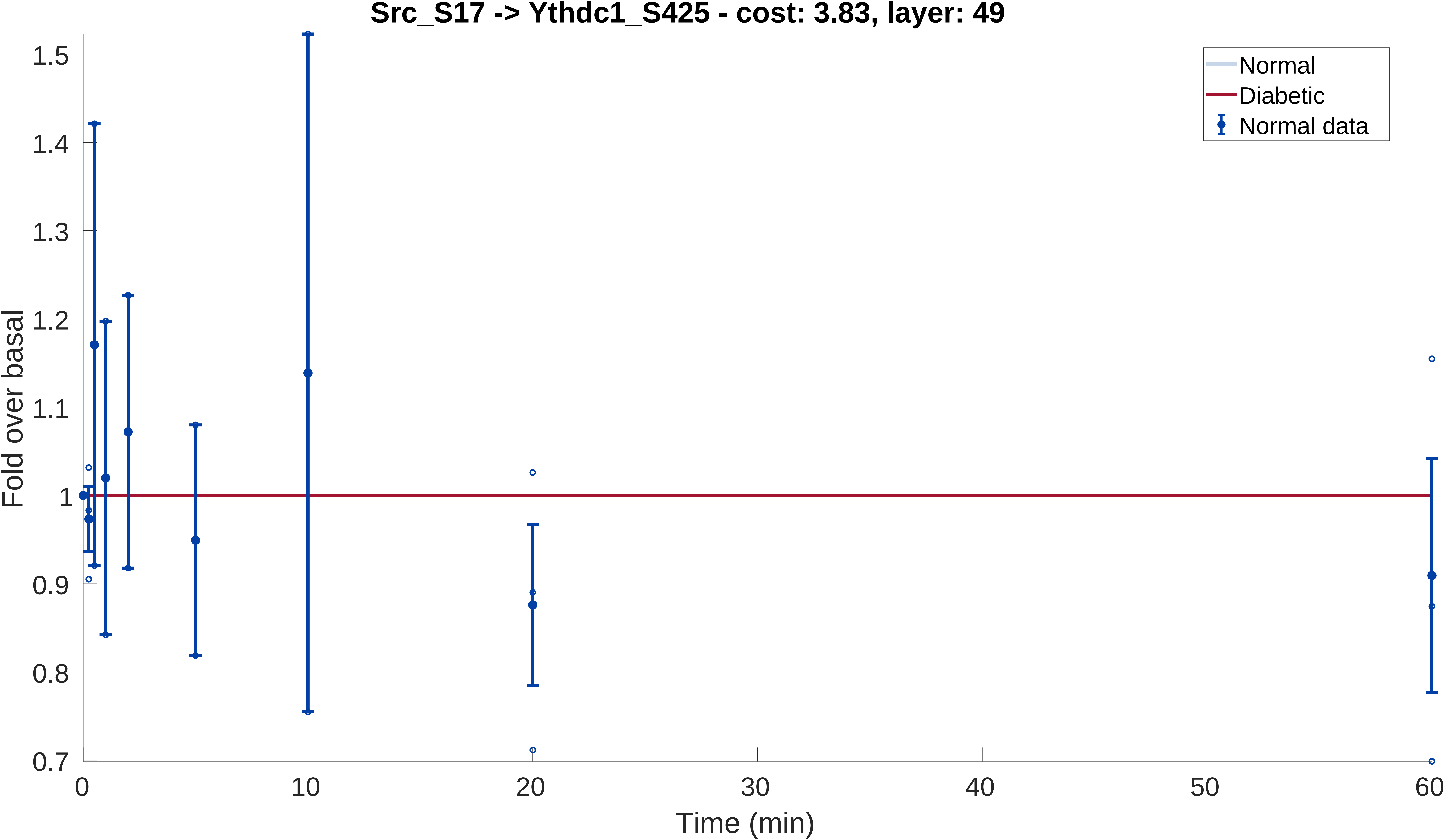
Src_S17 -> Ythdc1_S425 - cost: 3.83, layer: 49
Fold over basal
Time (min)
Normal
Diabetic
Normal data
1.5
1.4
1.3
1.2
1.1
1
0.9
0.8
0.7
0
10
20
30
40
50
60

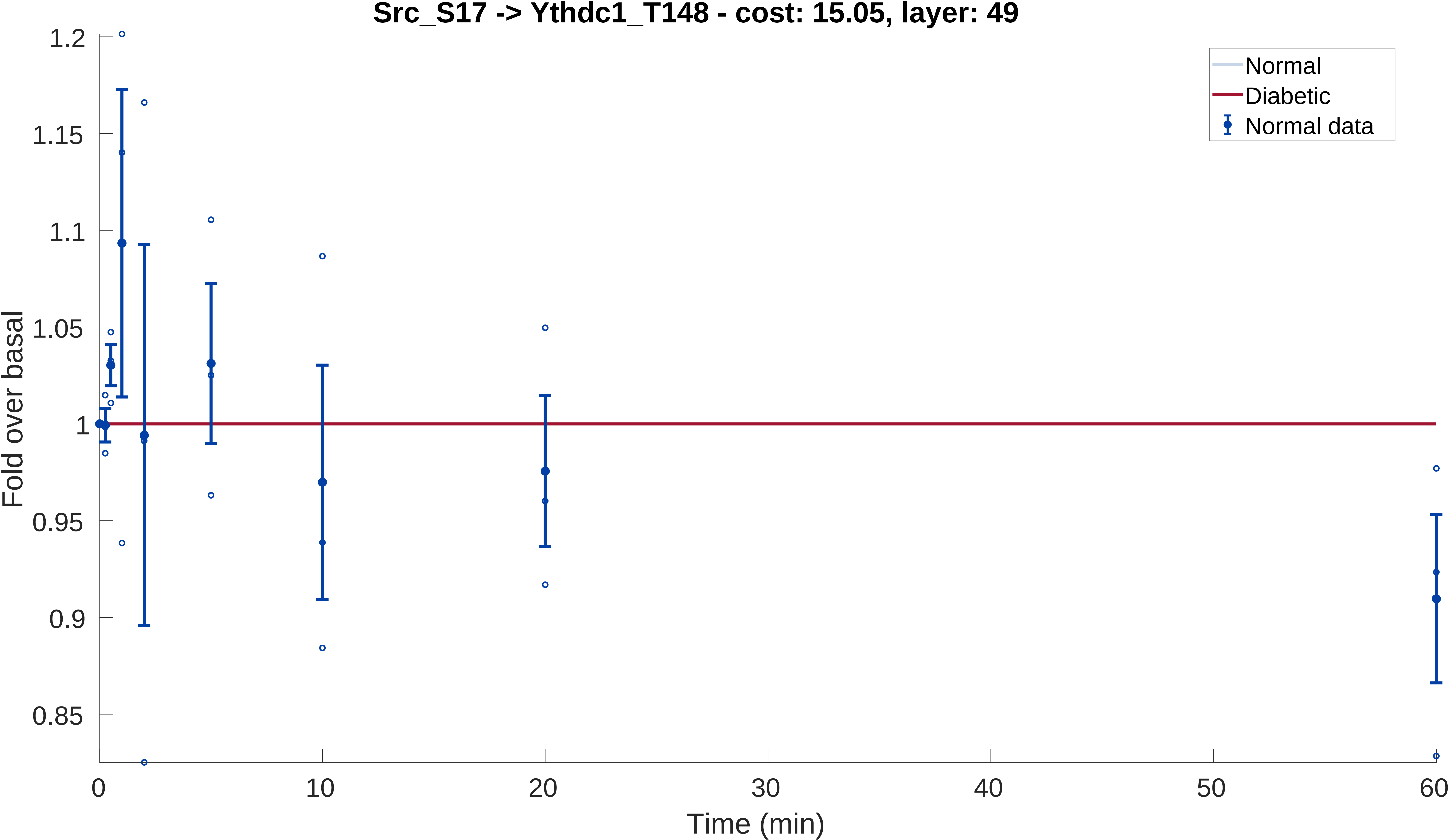

Src_S17 -> Ythdc1_T148 - cost: 15.05, layer: 49
Normal
Diabetic
Normal data
Fold over basal
1.2
1.15
1.1
1.05
1
0.95
0.9
0.85
0
10
20
30
40
50
60
Time (min)

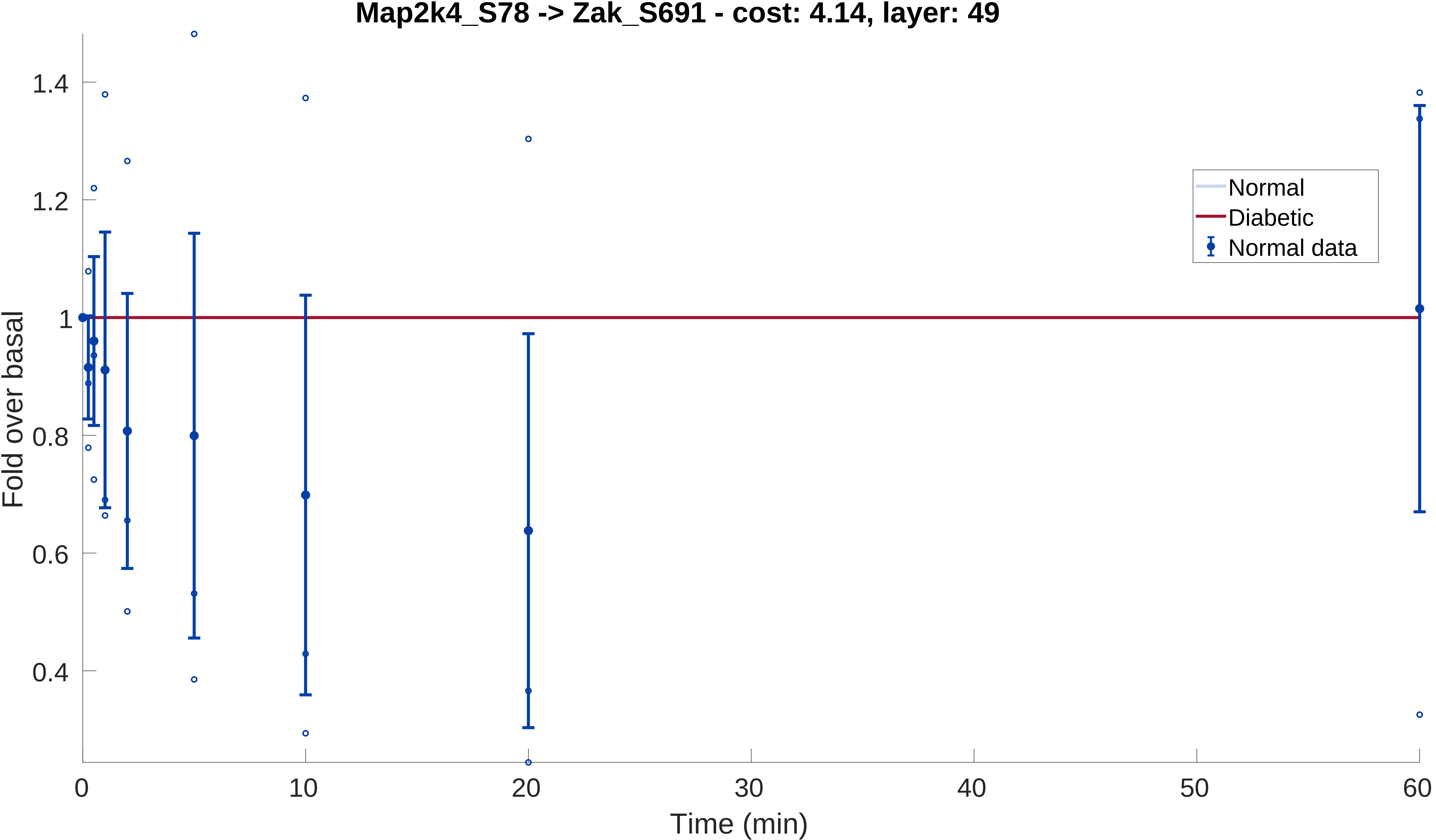

Map2k4_S78 -> Zak_S691 - cost: 4.14, layer: 49
Fold over basal
Time (min)
Normal
Diabetic
Normal data
1.4
1.2
1
0.8
0.6
0.4
0
10
20
30
40
50
60

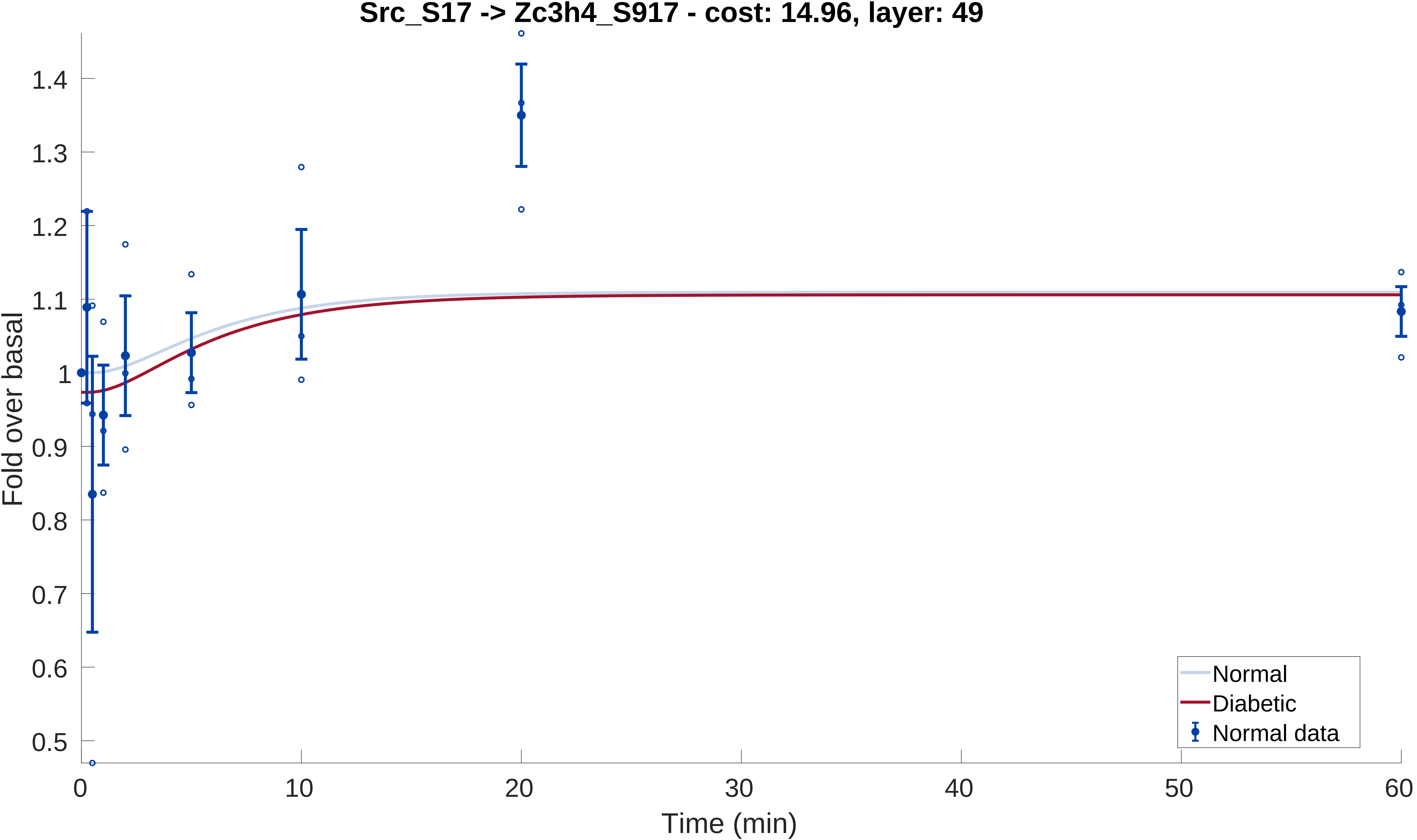

Src_S17 -> Zc3h4_S917 - cost: 14.96, layer: 49
Fold over basal
1.4
1.3
1.2
1.1
1
0.9
0.8
0.7
0.6
0.5
0
10
20
30
40
50
60
Time (min)
Normal
Diabetic
Normal data

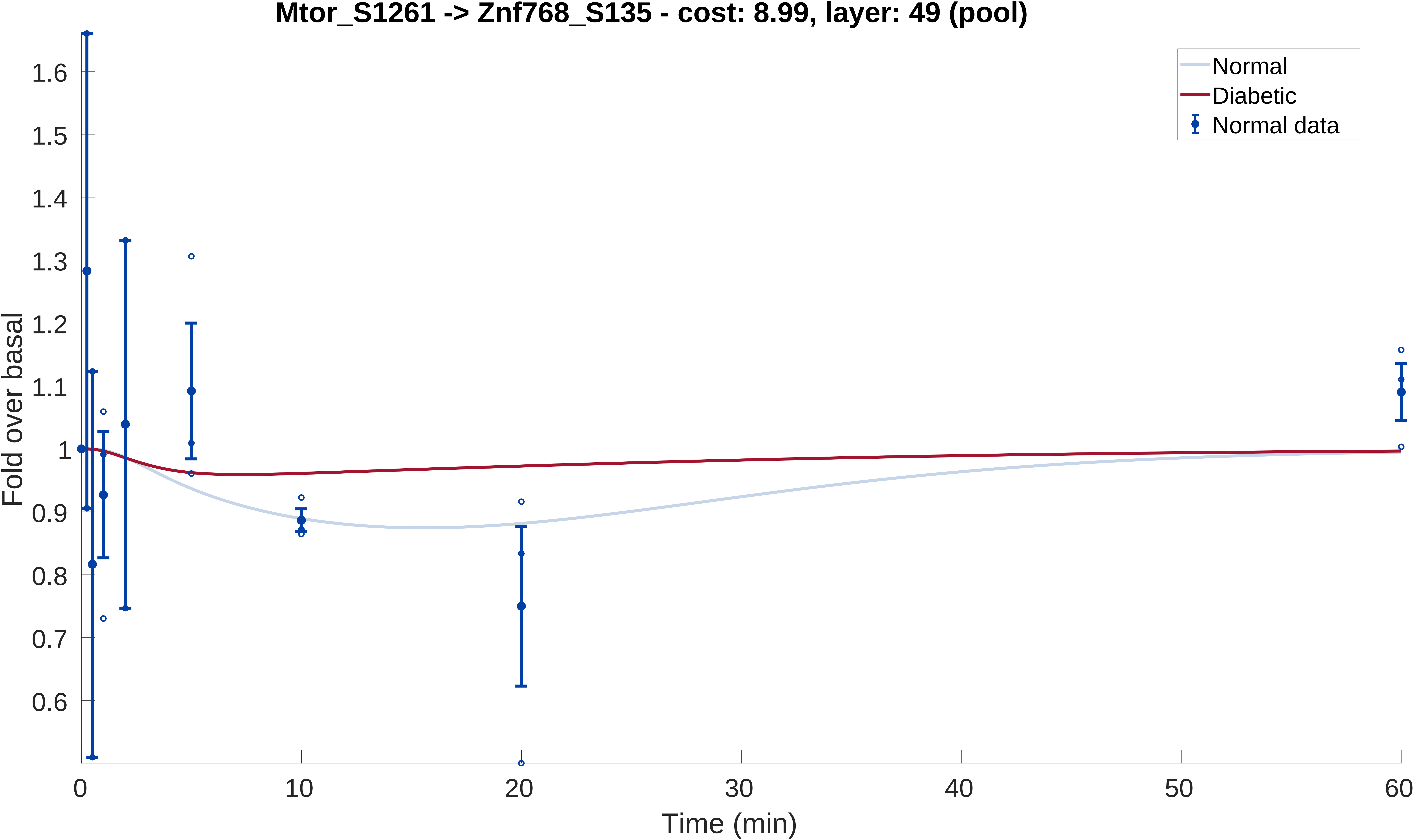
Mtor_S1261 -> Znf768_S135 - cost: 8.99, layer: 49 (pool)
Normal
Diabetic
Normal data
Fold over basal
Time (min)
1.6
1.5
1.4
1.3
1.2
1.1
1
0.9
0.8
0.7
0.6
0
10
20
30
40
50
60

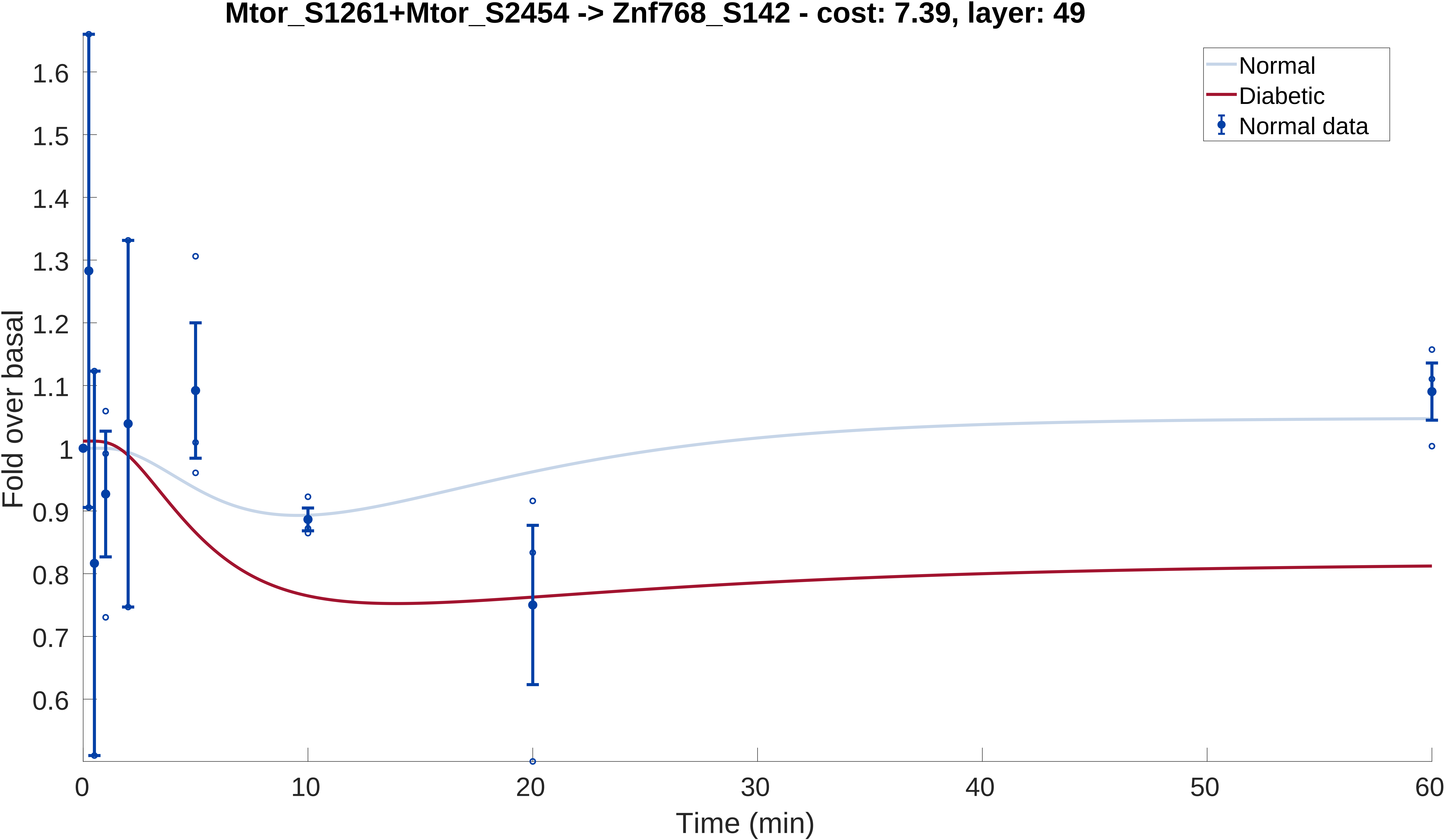
Mtor_S1261+Mtor_S2454 -> Znf768_S142 - cost: 7.39, layer: 49
Normal
Diabetic
Normal data
Fold over basal
1.6
1.5
1.4
1.3
1.2
1.1
1
0.9
0.8
0.7
0.6
0
10
20
30
40
50
60
Time (min)

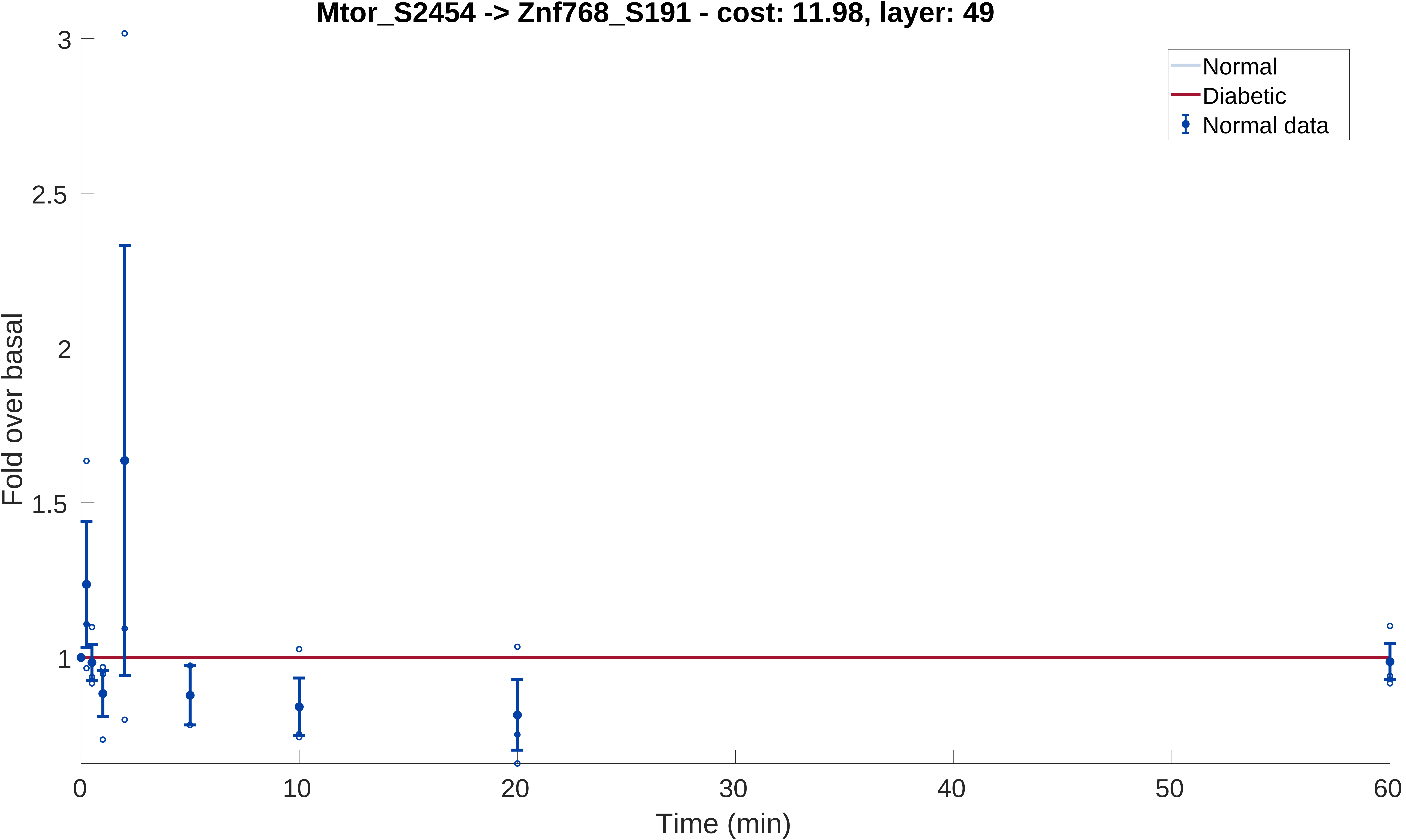

Mtor_S2454 -> Znf768_S191 - cost: 11.98, layer: 49
Normal
Diabetic
Normal data
Fold over basal
Time (min)
3
2.5
2
1.5
1
0
10
20
30
40
50
60

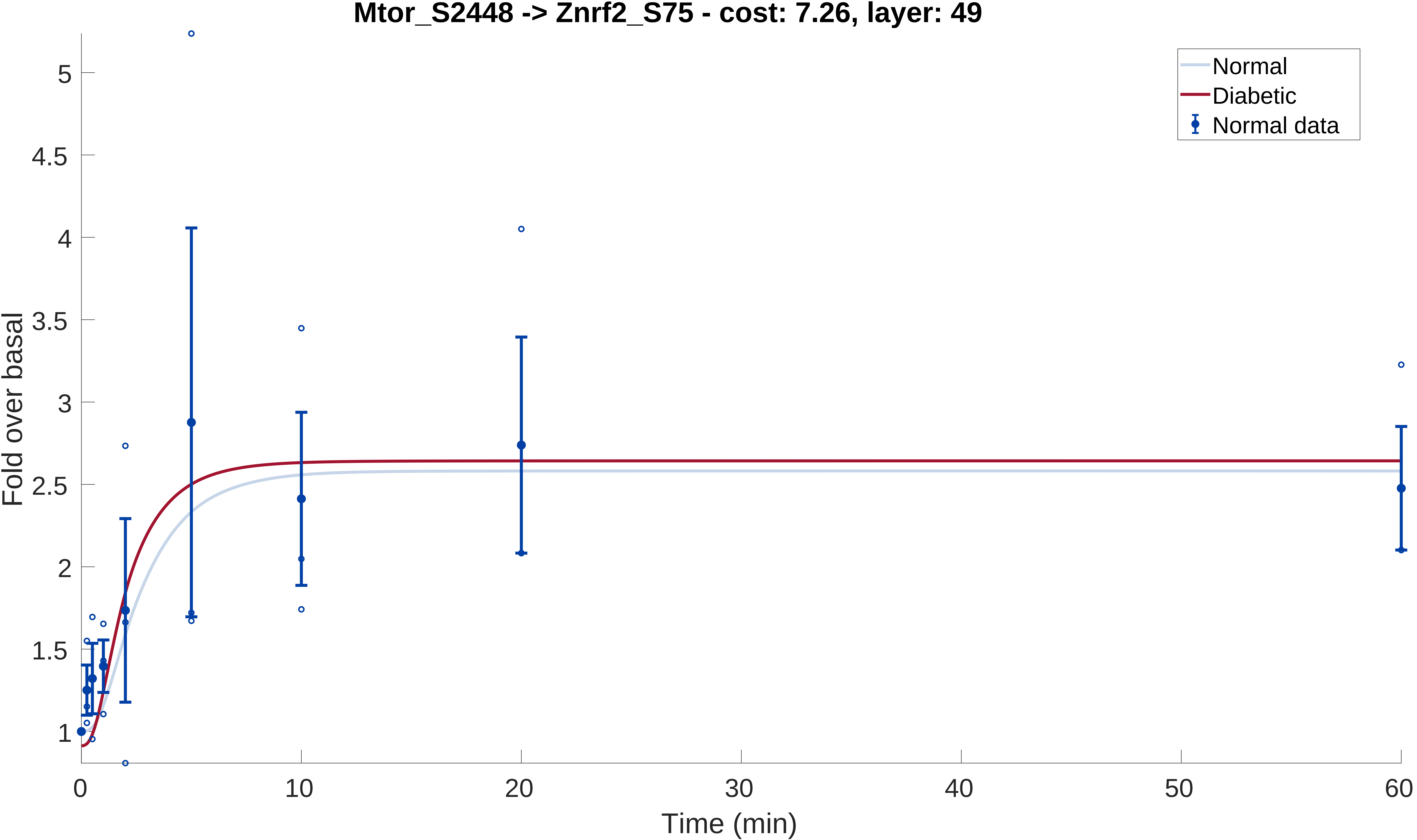
Mtor_S2448 -> Znrf2_S75 - cost: 7.26, layer: 49
Normal
Diabetic
Normal data
Fold over basal
Time (min)
5
4.5
4
3.5
3
2.5
2
1.5
1
0
10
20
30
40
50
60

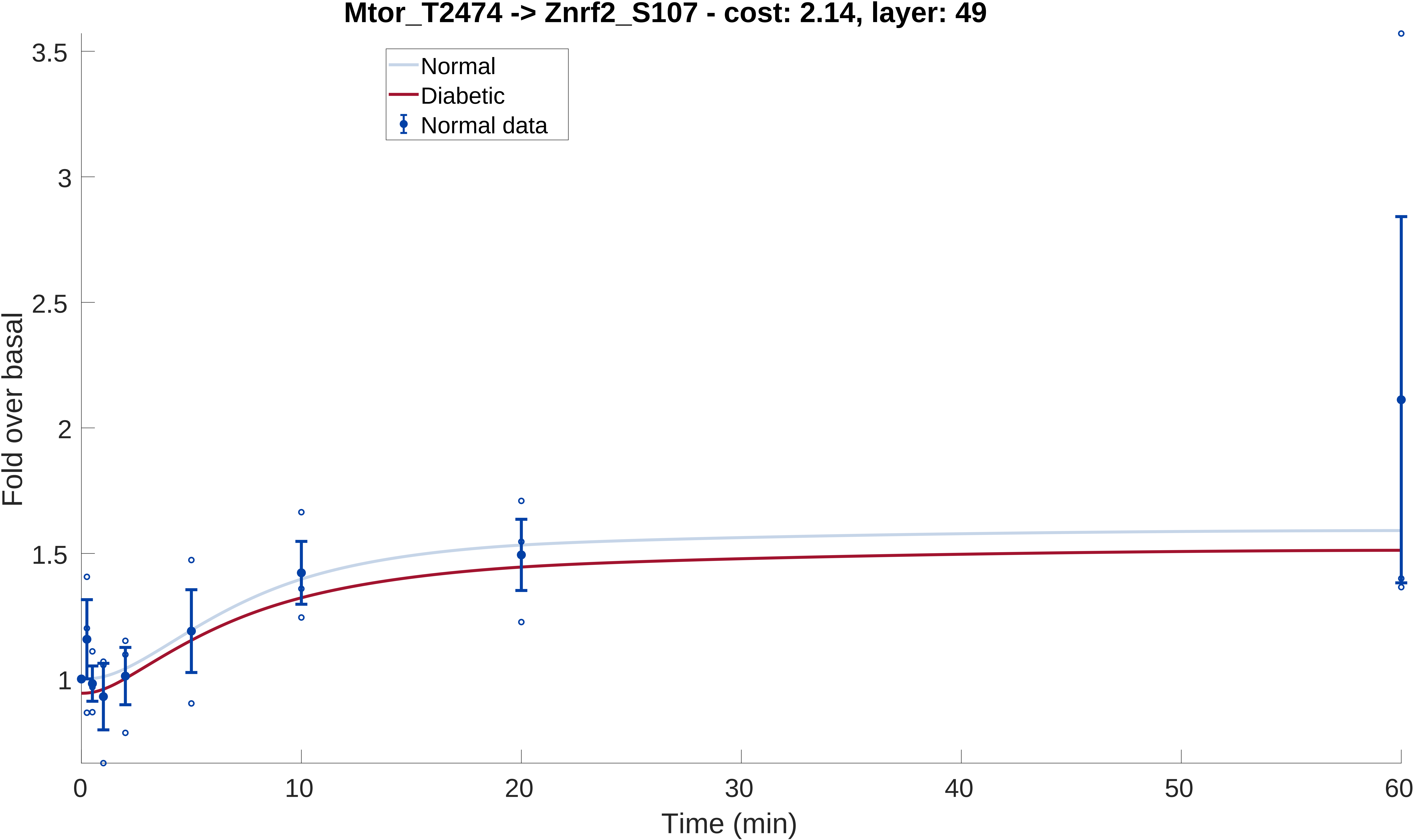
Mtor_T2474 -> Znrf2_S107 - cost: 2.14, layer: 49
Normal
Diabetic
Normal data
Fold over basal
3.5
3
2.5
2
1.5
1
0
10
20
30
40
50
60
Time (min)

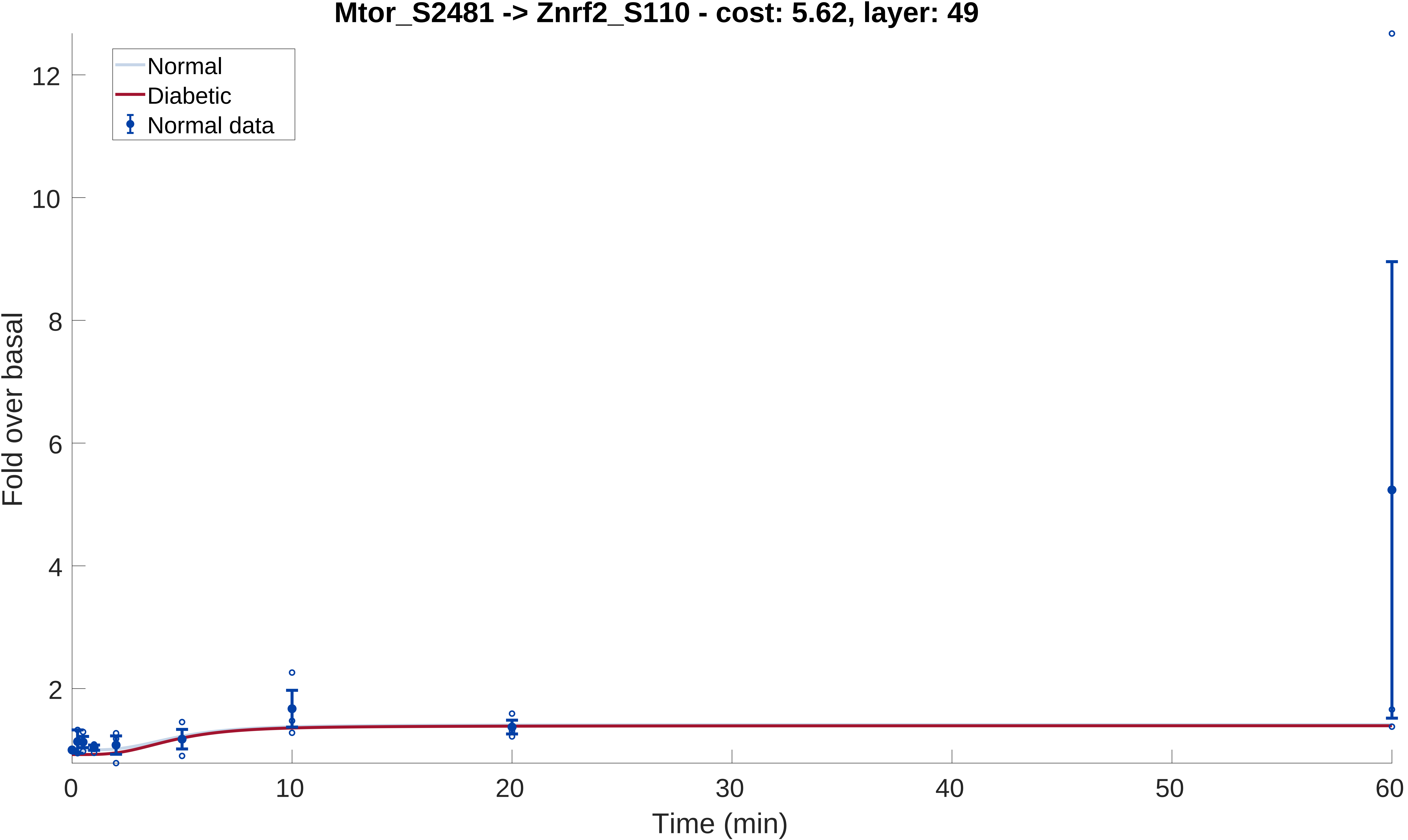
Mtor_S2481 -> Znrf2_S110 - cost: 5.62, layer: 49
Normal
Diabetic
Normal data
Fold over basal
Time (min)
0
2
4
6
8
10
12
0
10
20
30
40
50
60

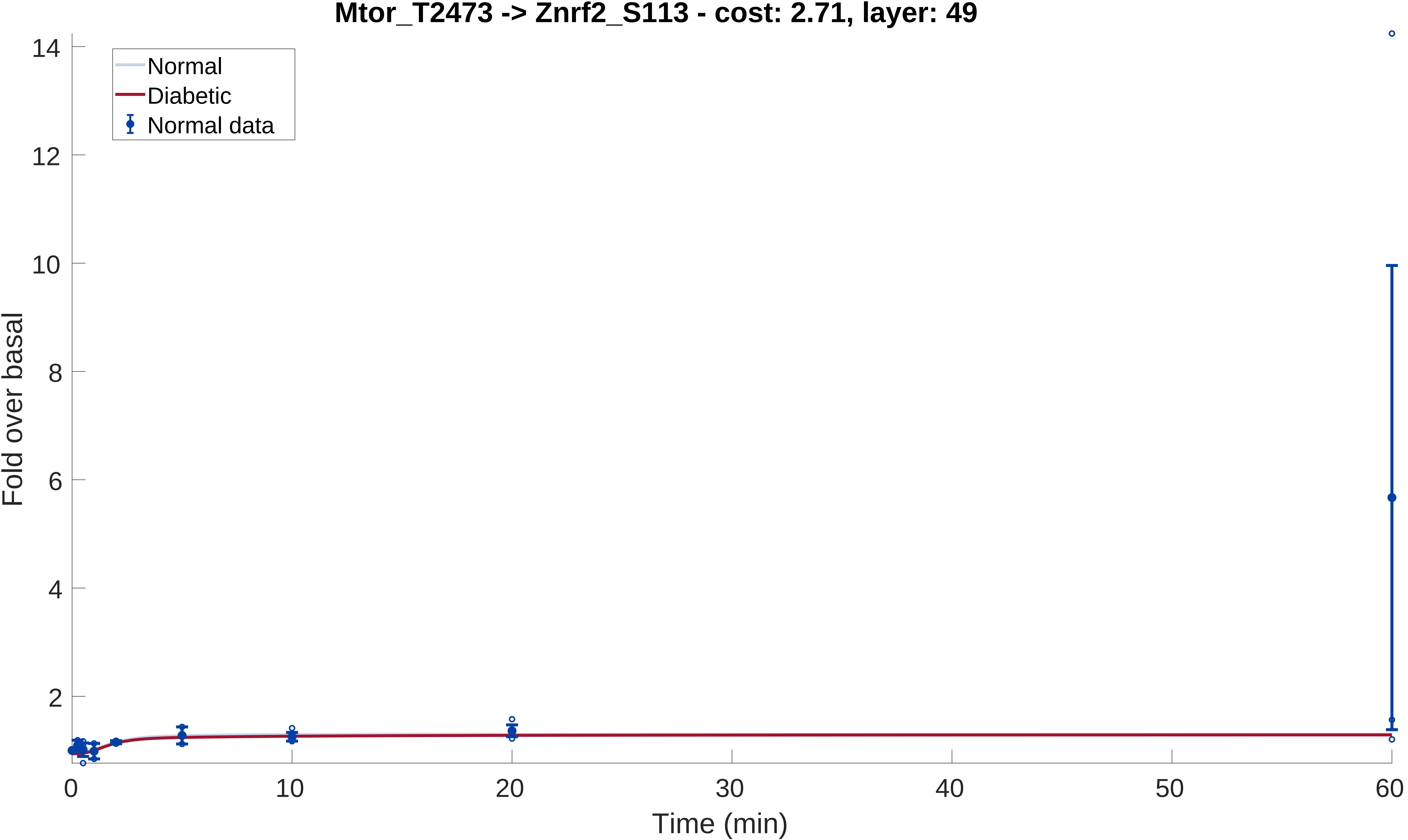

Mtor_T2473 -> Znrf2_S113 - cost: 2.71, layer: 49
Normal
Diabetic
Normal data
Fold over basal
Time (min)
0
10
20
30
40
50
60
2
4
6
8
10
12
14

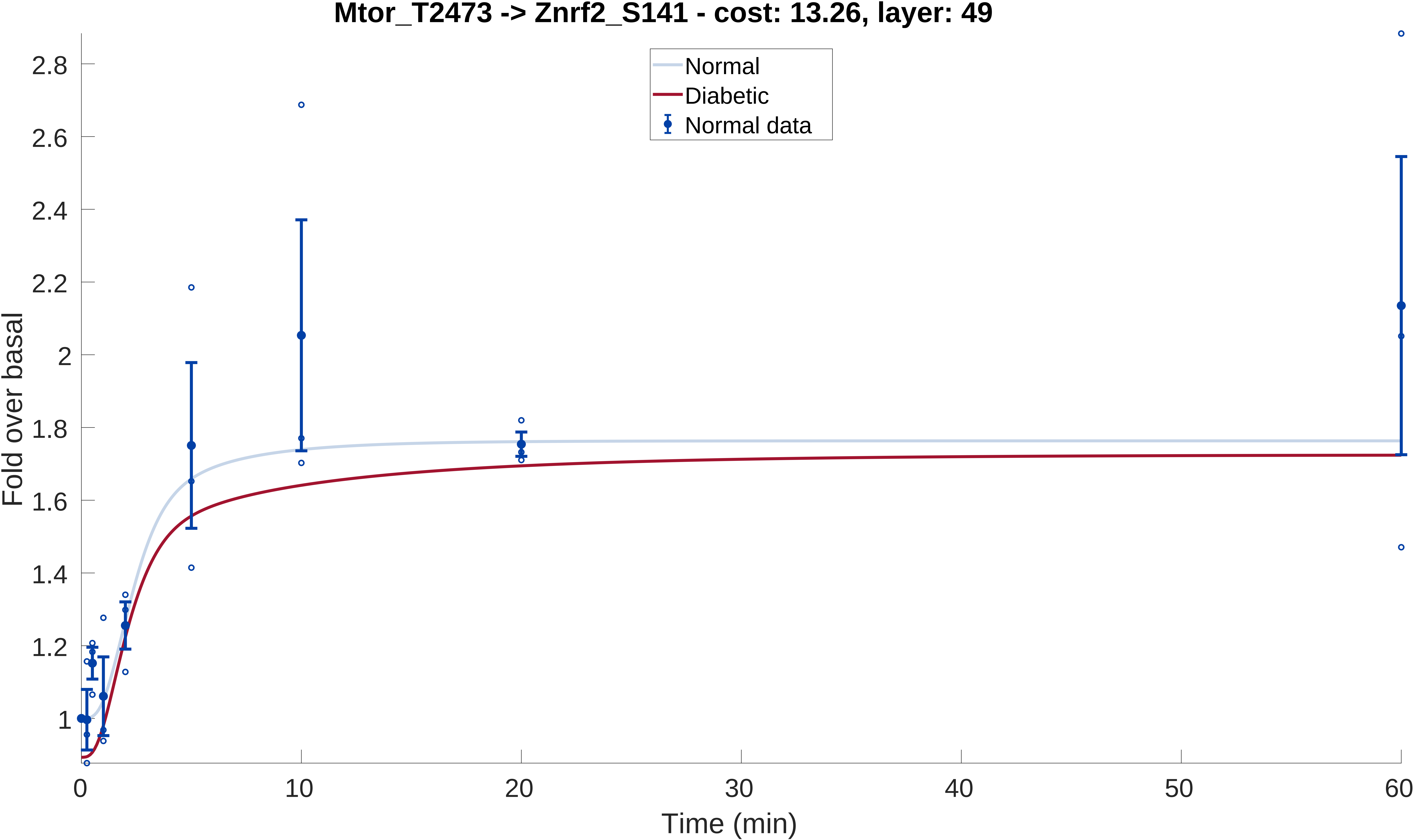

Mtor_T2473 -> Znrf2_S141 - cost: 13.26, layer: 49
Normal
Diabetic
Normal data
Fold over basal
Time (min)
2.8
2.6
2.4
2.2
2
1.8
1.6
1.4
1.2
1
0
10
20
30
40
50
60

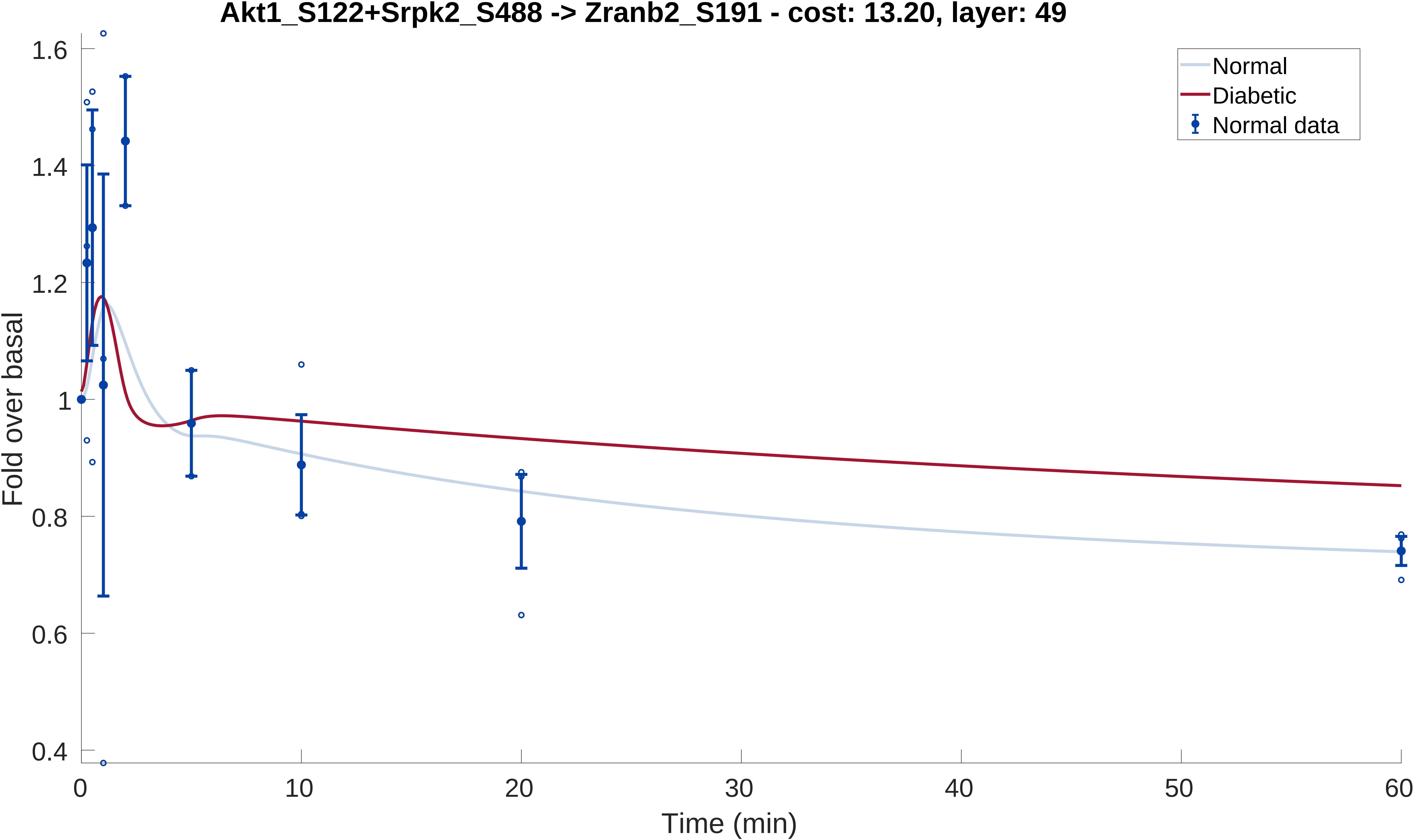

Akt1_S122+Srpk2_S488 -> Zranb2_S191 - cost: 13.20, layer: 49
Normal
Diabetic
Normal data
Fold over basal
1.6
1.4
1.2
1
0.8
0.6
0.4
0
10
20
30
40
50
60
Time (min)

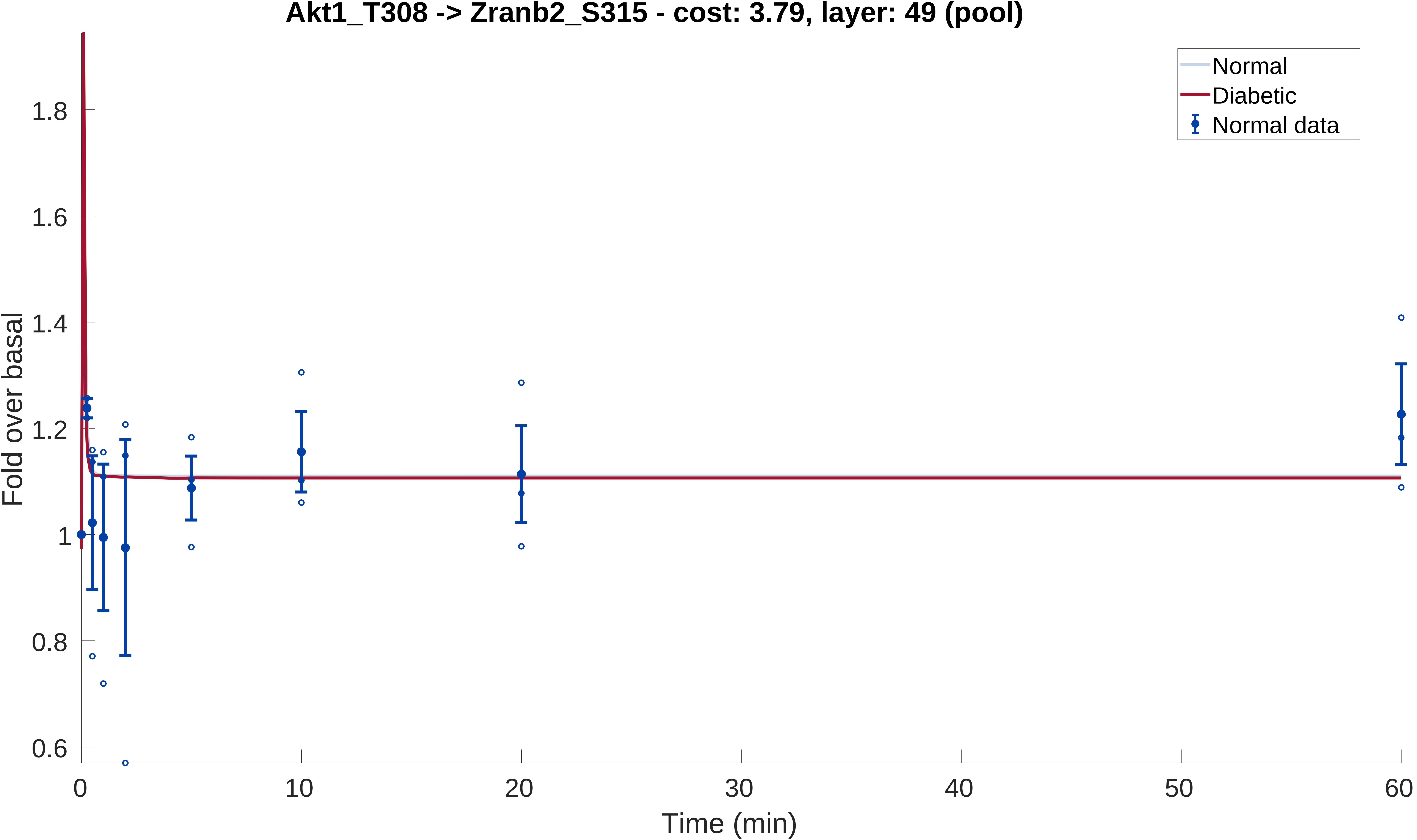

Akt1_T308 -> Zranb2_S315 - cost: 3.79, layer: 49 (pool)
Normal
Diabetic
Normal data
Fold over basal
1.8
1.6
1.4
1.2
1
0.8
0.6
0
10
20
30
40
50
60
Time (min)

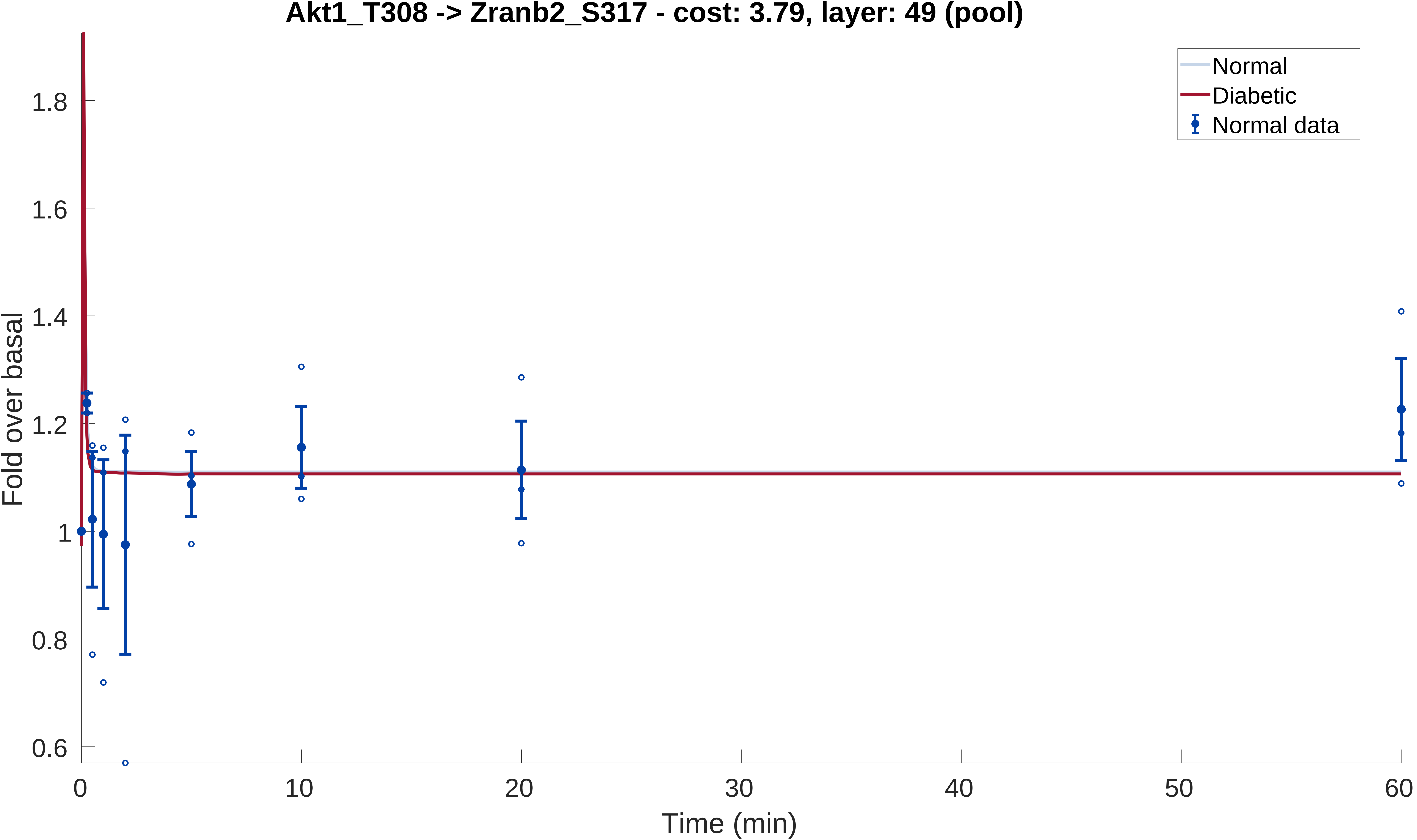
Akt1_T308 -> Zranb2_S317 - cost: 3.79, layer: 49 (pool)
Normal
Diabetic
Normal data
Fold over basal
Time (min)
0.6
0.8
1
1.2
1.4
1.6
1.8
0
10
20
30
40
50
60

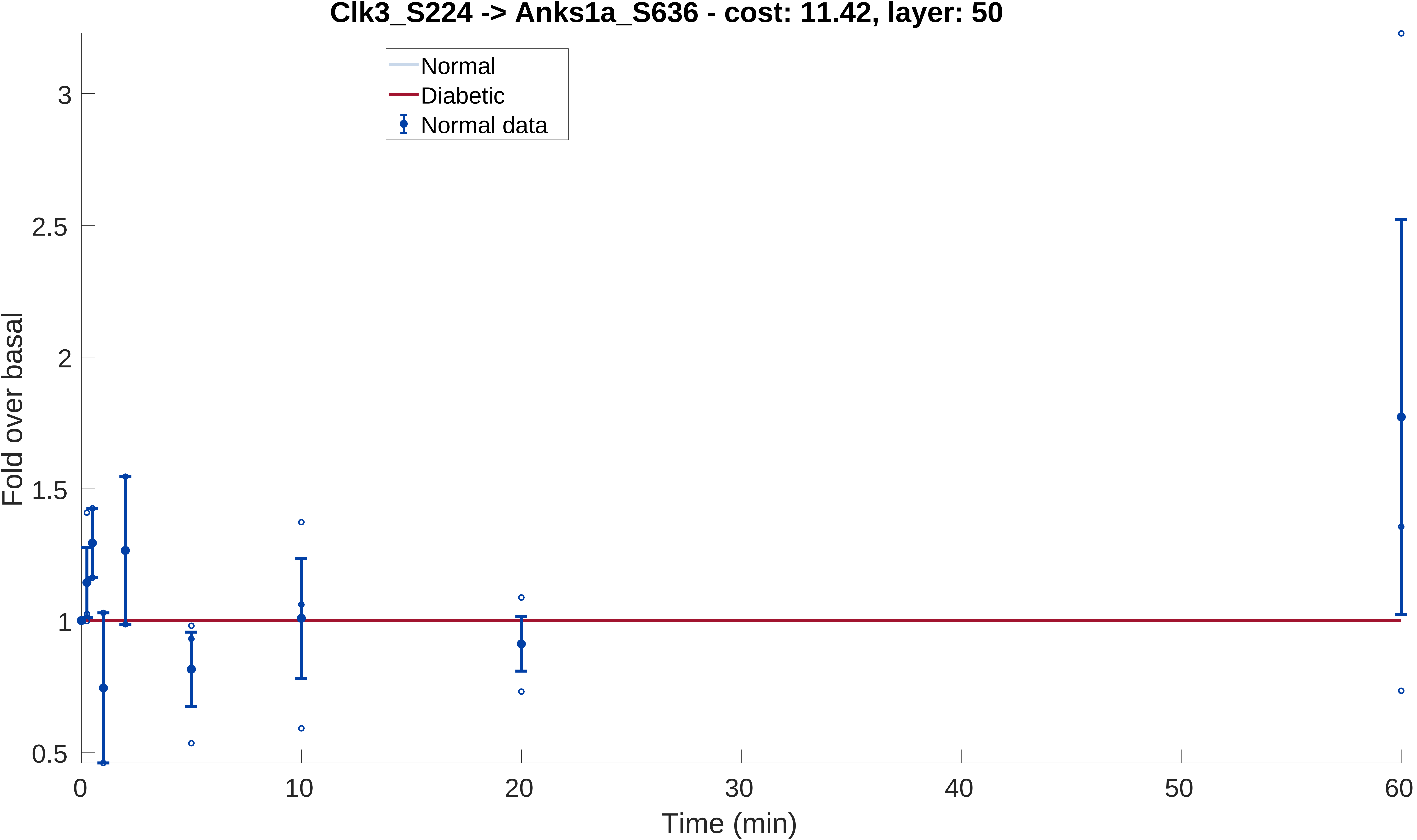

Clk3_S224 -> Anks1a_S636 - cost: 11.42, layer: 50
Normal
Diabetic
Normal data
Fold over basal
Time (min)
0.5
1
1.5
2
2.5
3
0
10
20
30
40
50
60

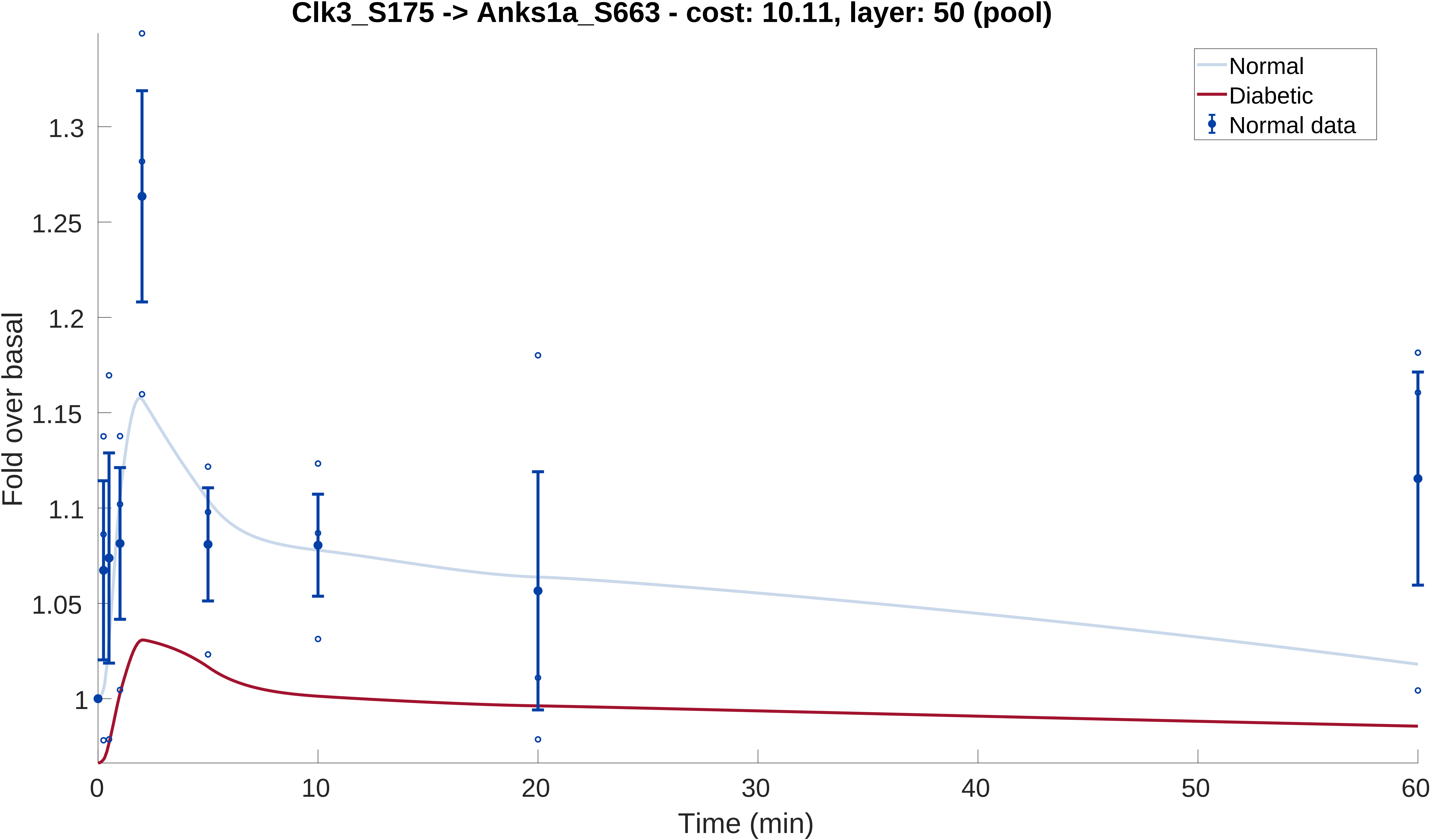

Clk3_S175 -> Anks1a_S663 - cost: 10.11, layer: 50 (pool)
Normal
Diabetic
Normal data
Fold over basal
1.3
1.25
1.2
1.15
1.1
1.05
1
0
10
20
30
40
50
60
Time (min)

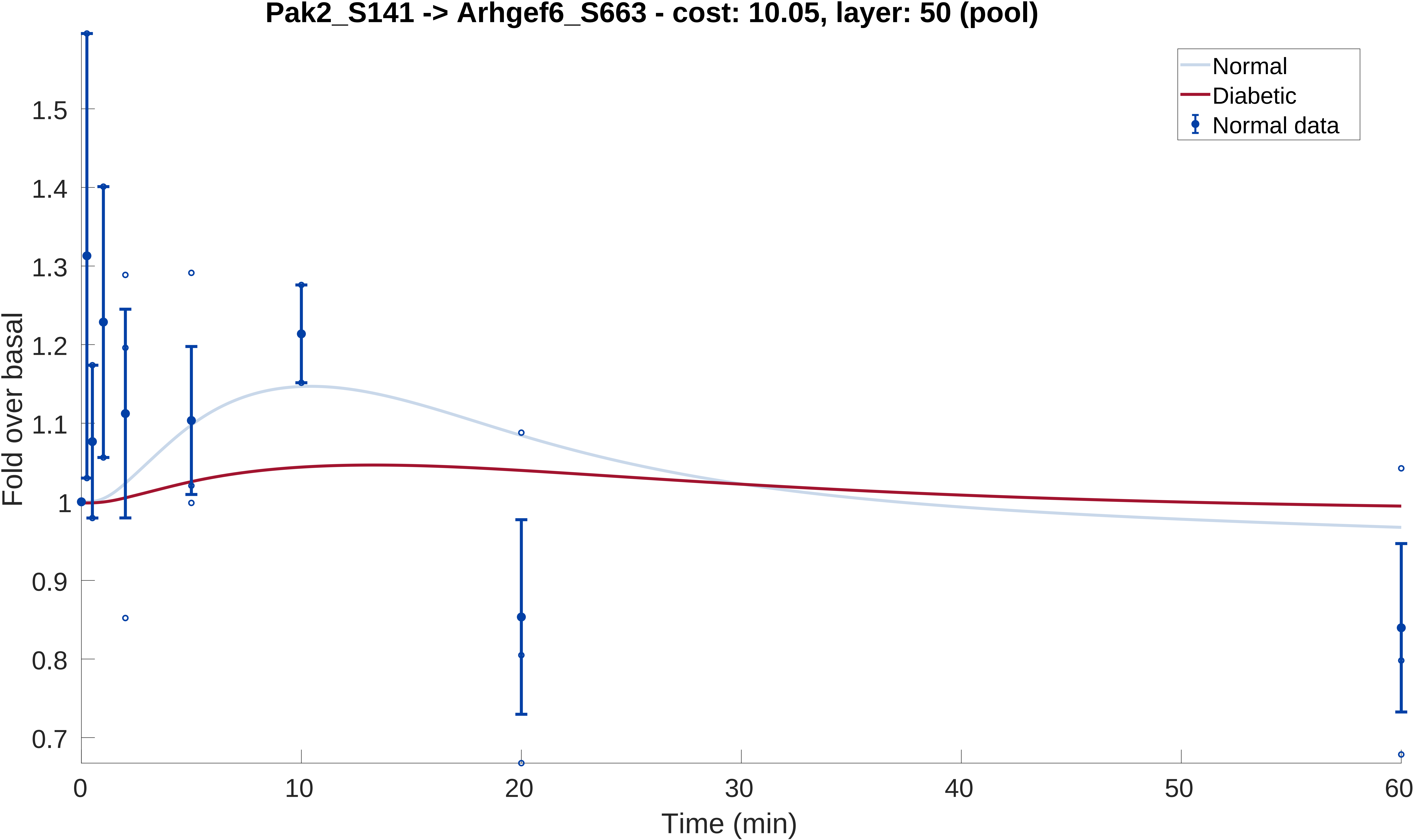

Pak2_S141 -> Arhgef6_S663 - cost: 10.05, layer: 50 (pool)
Normal
Diabetic
Normal data
Fold over basal
1.5
1.4
1.3
1.2
1.1
1
0.9
0.8
0.7
0
10
20
30
40
50
60
Time (min)

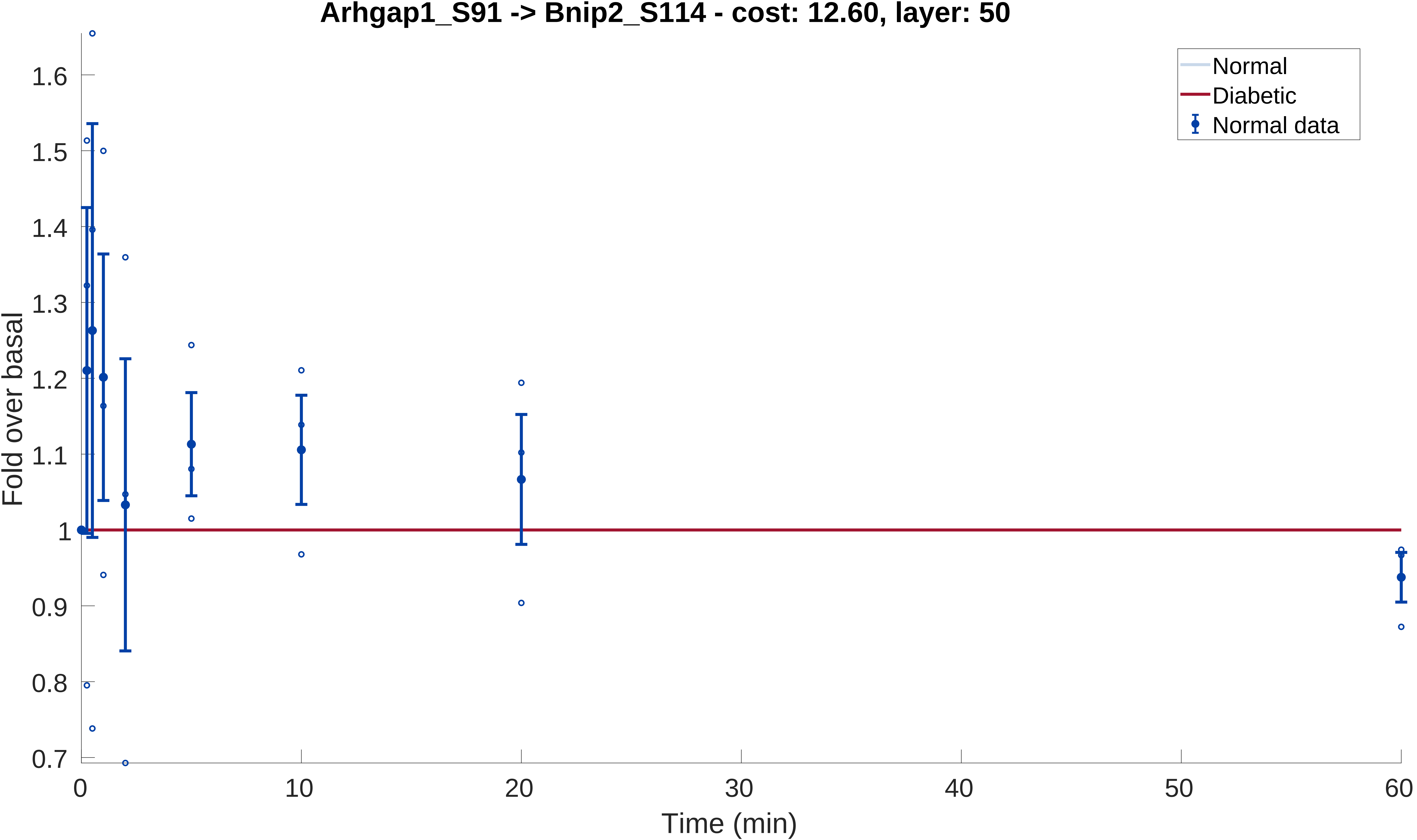

Arhgap1_S91 -> Bnip2_S114 - cost: 12.60, layer: 50
Normal
Diabetic
Normal data
Fold over basal
Time (min)
1.6
1.5
1.4
1.3
1.2
1.1
1
0.9
0.8
0.7
0
10
20
30
40
50
60

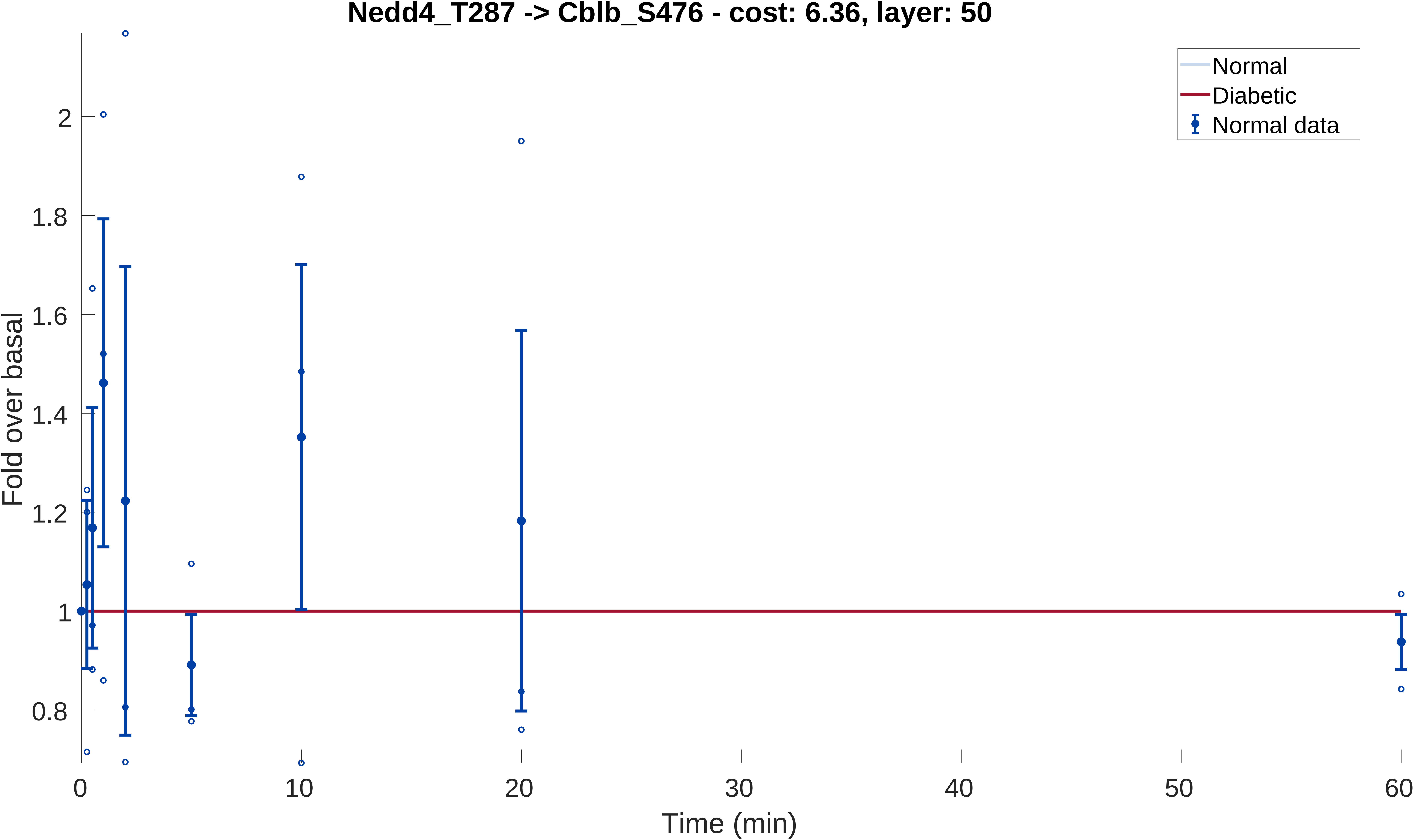

Nedd4_T287 -> Cblb_S476 - cost: 6.36, layer: 50
Normal
Diabetic
Normal data
Fold over basal
Time (min)
0.8
1
1.2
1.4
1.6
1.8
2
0
10
20
30
40
50
60

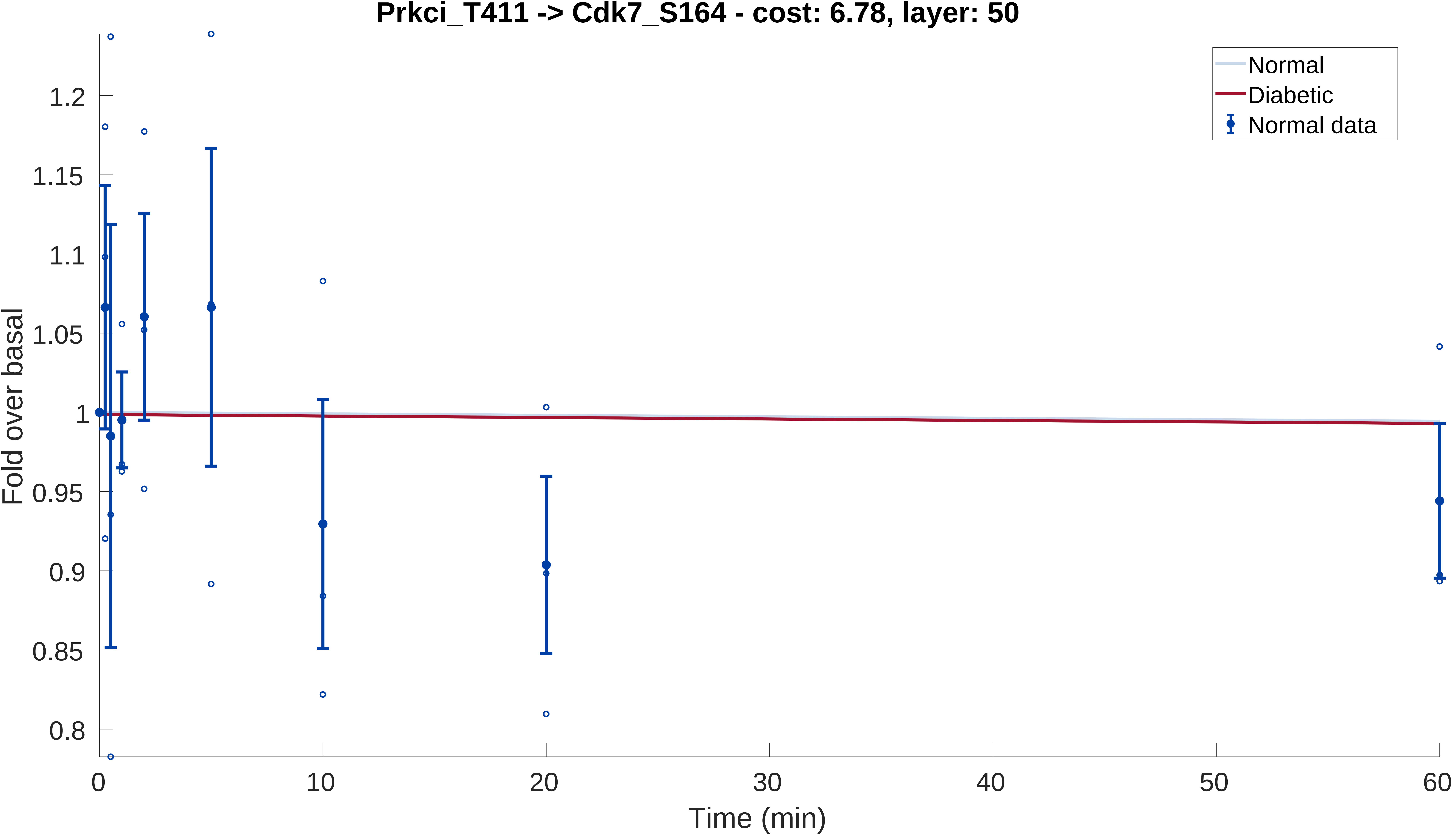

Prkci_T411 -> Cdk7_S164 - cost: 6.78, layer: 50
Normal
Diabetic
Normal data
Fold over basal
Time (min)
1.2
1.15
1.1
1.05
1
0.95
0.9
0.85
0.8
0
10
20
30
40
50
60

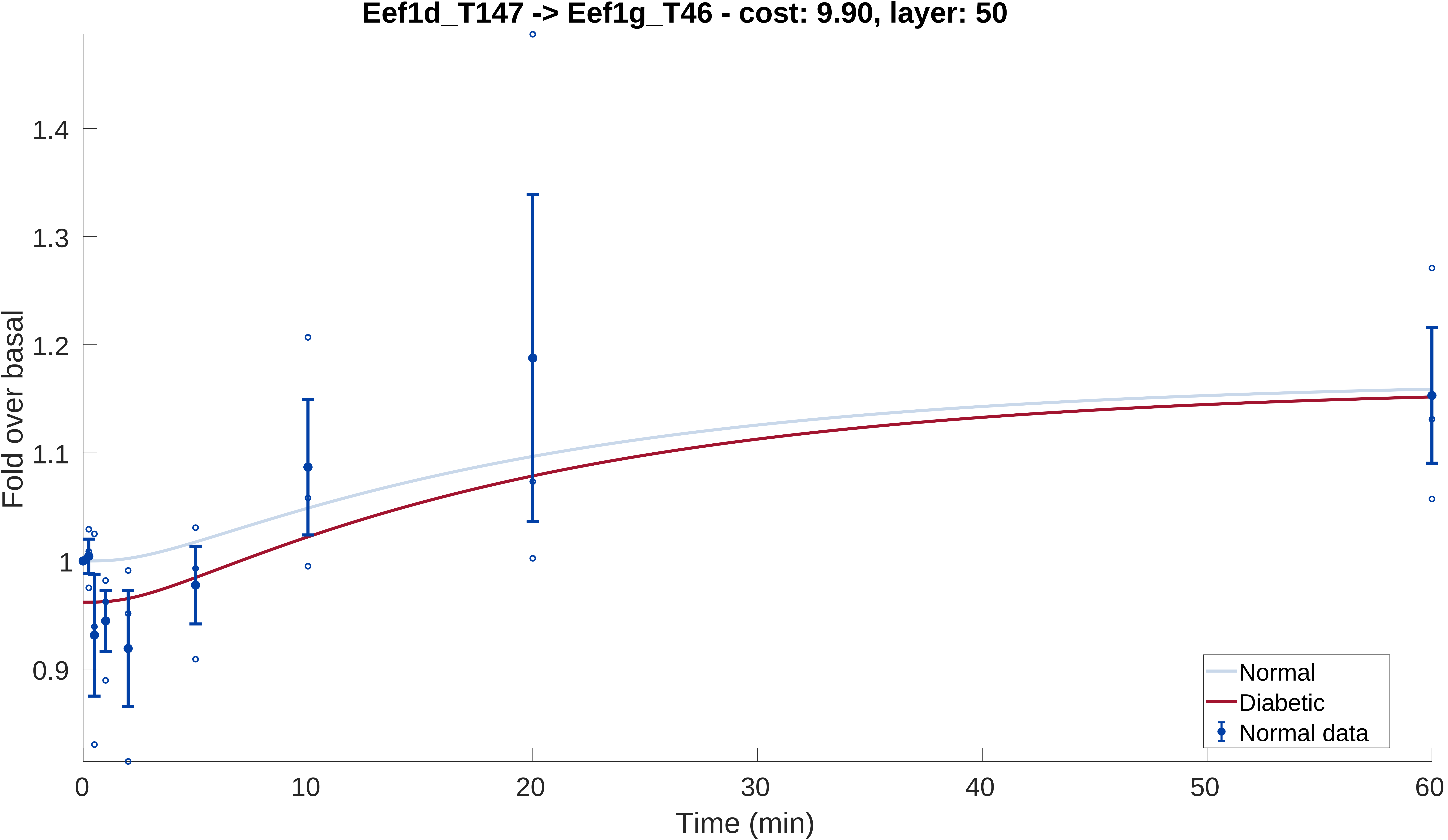

Eef1d_T147 -> Eef1g_T46 - cost: 9.90, layer: 50
Fold over basal
1.4
1.3
1.2
1.1
1
0.9
0
10
20
30
40
50
60
Time (min)
Normal
Diabetic
Normal data

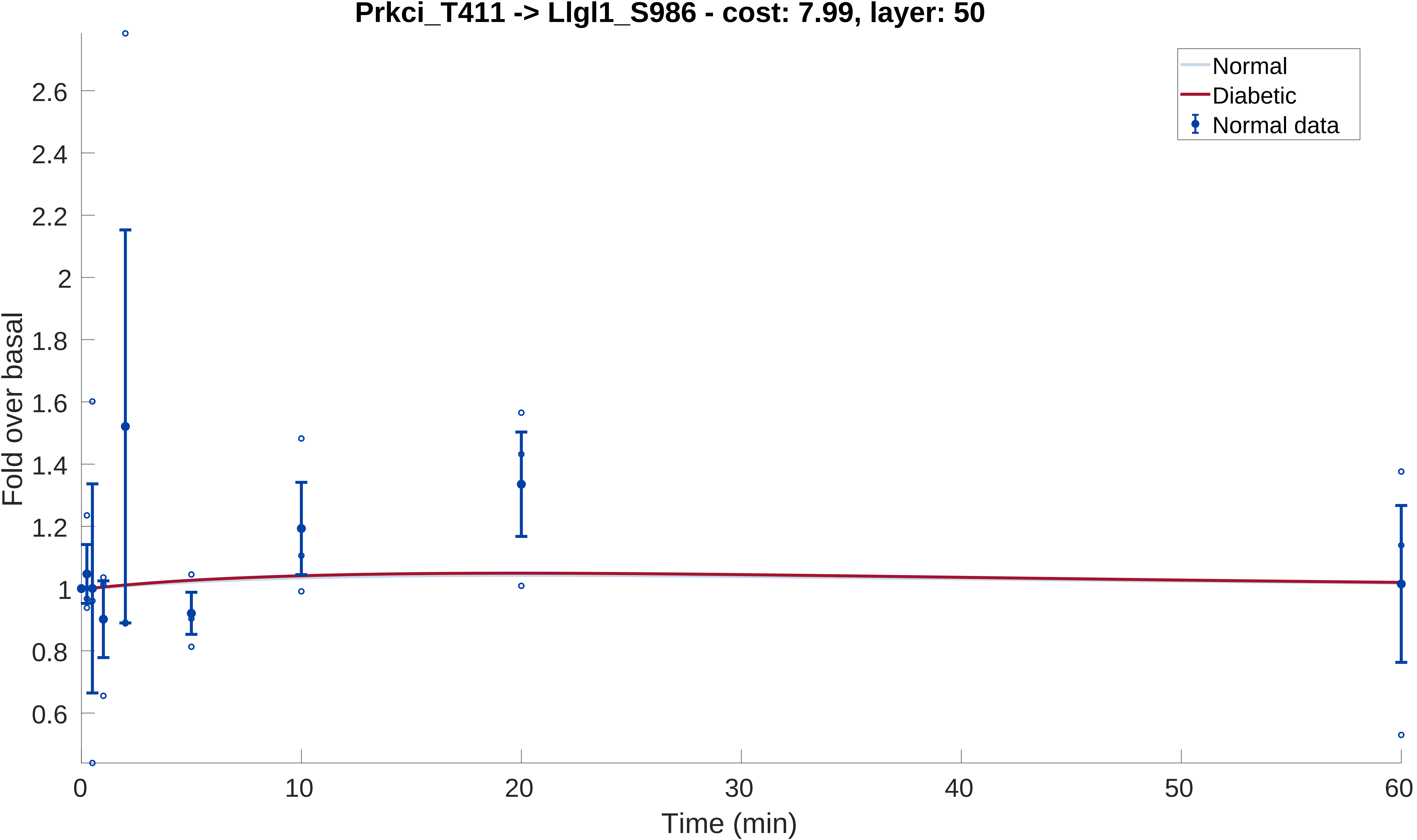
Prrkci_T411 -> Llgl1_S986 - cost: 7.99, layer: 50
Fold over basal
Time (min)
Normal
Diabetic
Normal data
0.6
0.8
1
1.2
1.4
1.6
1.8
2
2.2
2.4
2.6
0
10
20
30
40
50
60

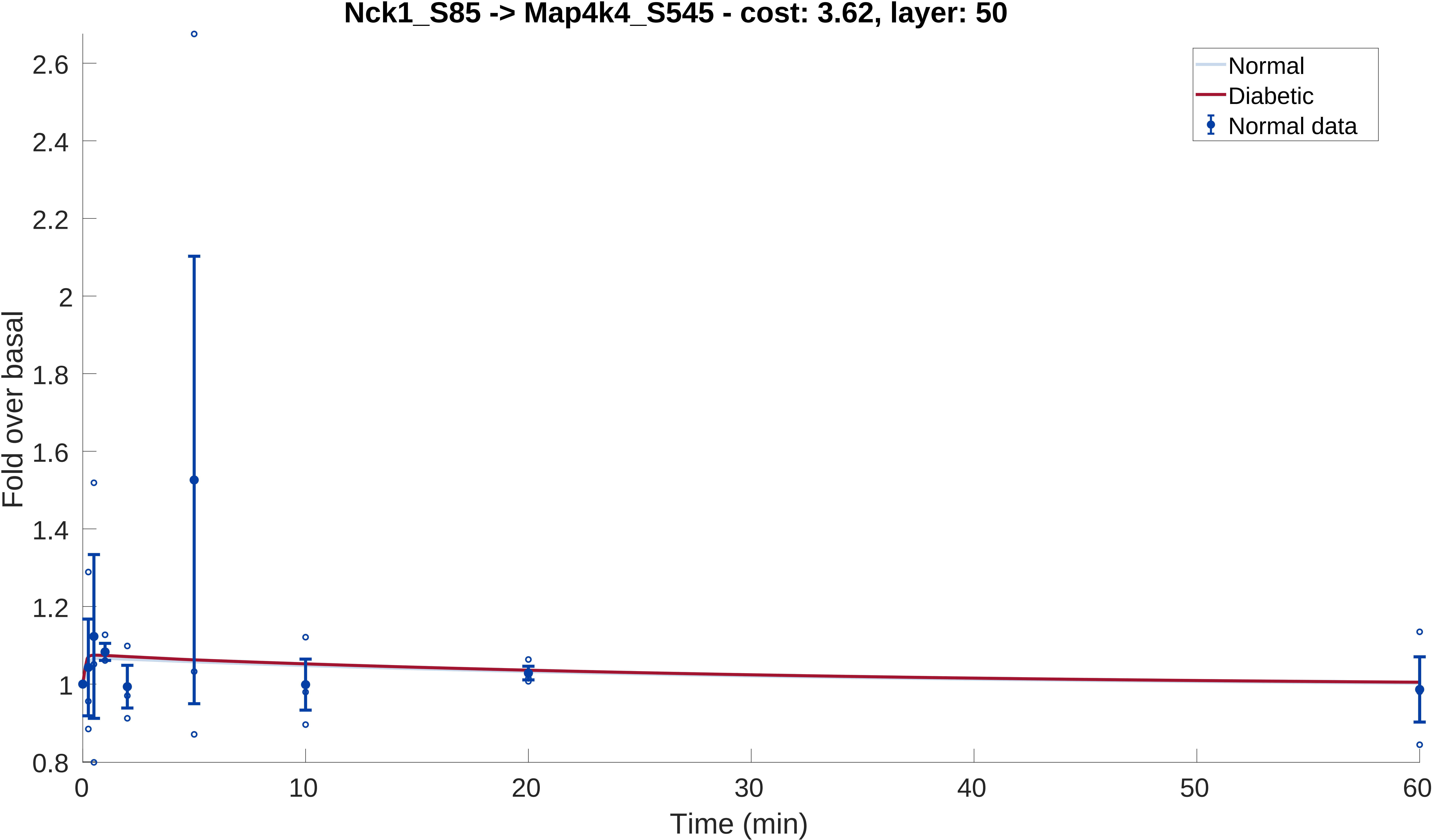

Nck1_S85 -> Map4k4_S545 - cost: 3.62, layer: 50
Normal
Diabetic
Normal data
Fold over basal
Time (min)
0.8
1
1.2
1.4
1.6
1.8
2
2.2
2.4
2.6
0
10
20
30
40
50
60

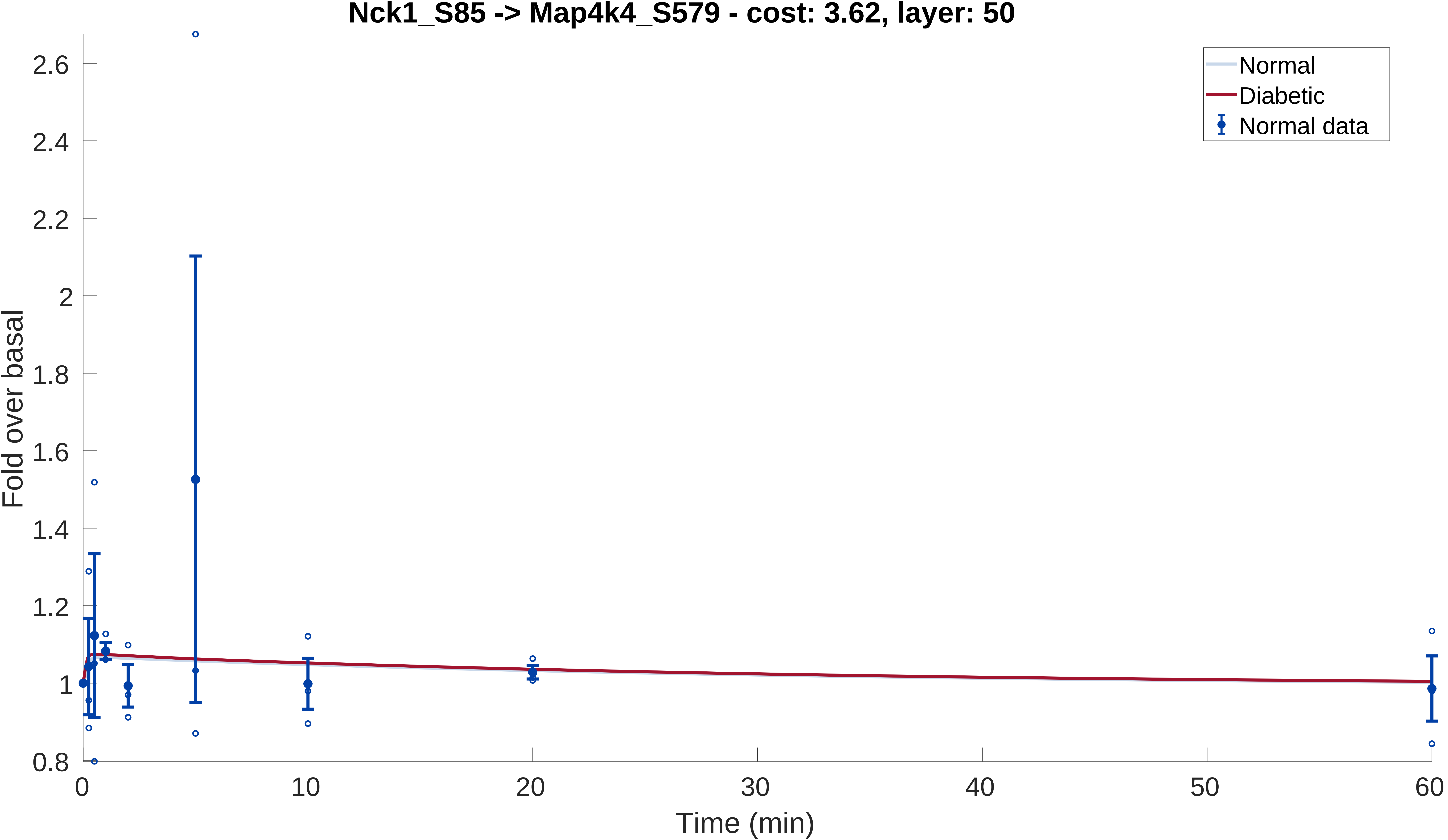

Nck1_S85 -> Map4k4_S579 - cost: 3.62, layer: 50
Normal
Diabetic
Normal data
Fold over basal
Time (min)
2.6
2.4
2.2
2
1.8
1.6
1.4
1.2
1
0.8
0
10
20
30
40
50
60

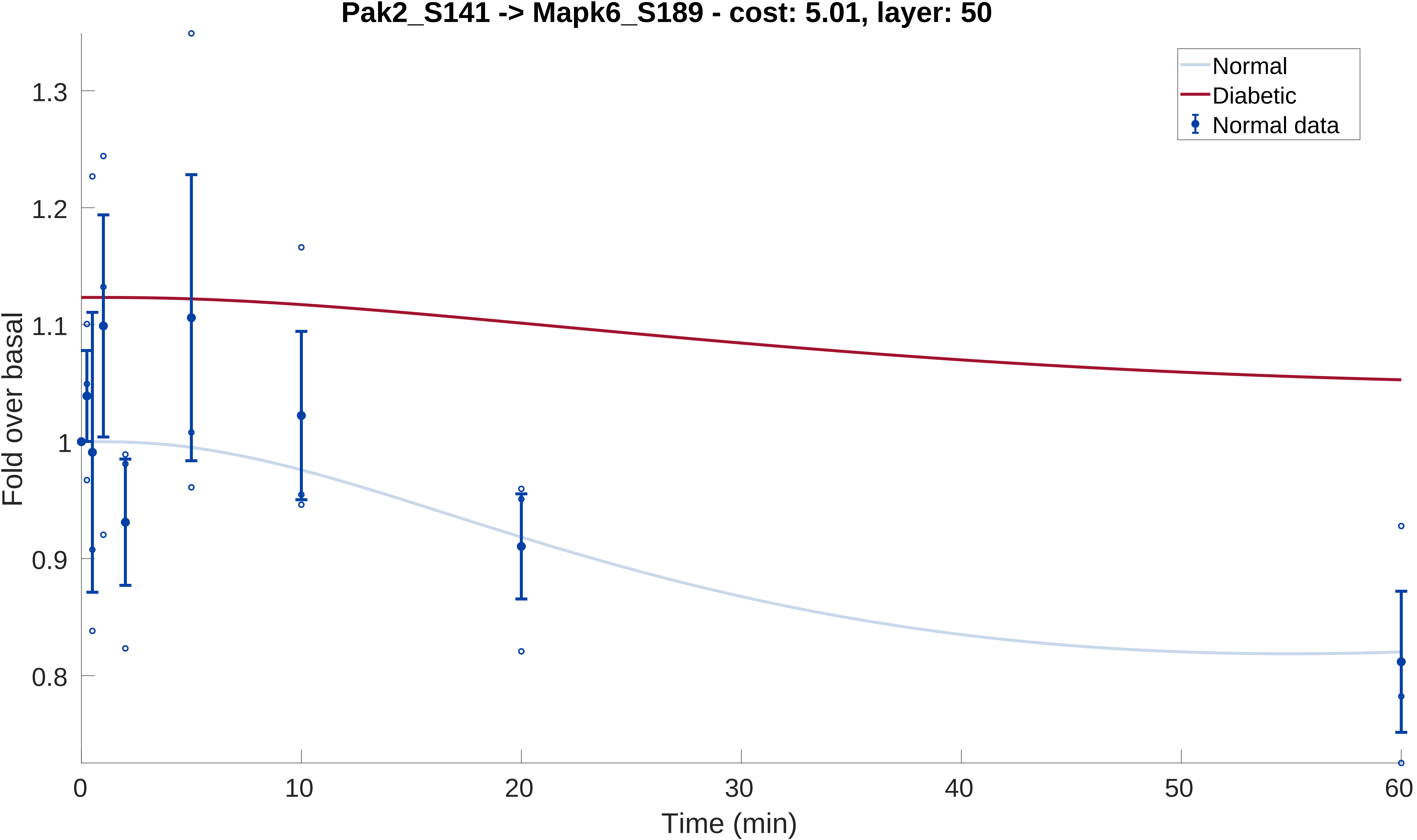

Pak2_S141 -> Mapk6_S189 - cost: 5.01, layer: 50
Normal
Diabetic
Normal data
Fold over basal
1.3
1.2
1.1
1
0.9
0.8
0
10
20
30
40
50
60
Time (min)

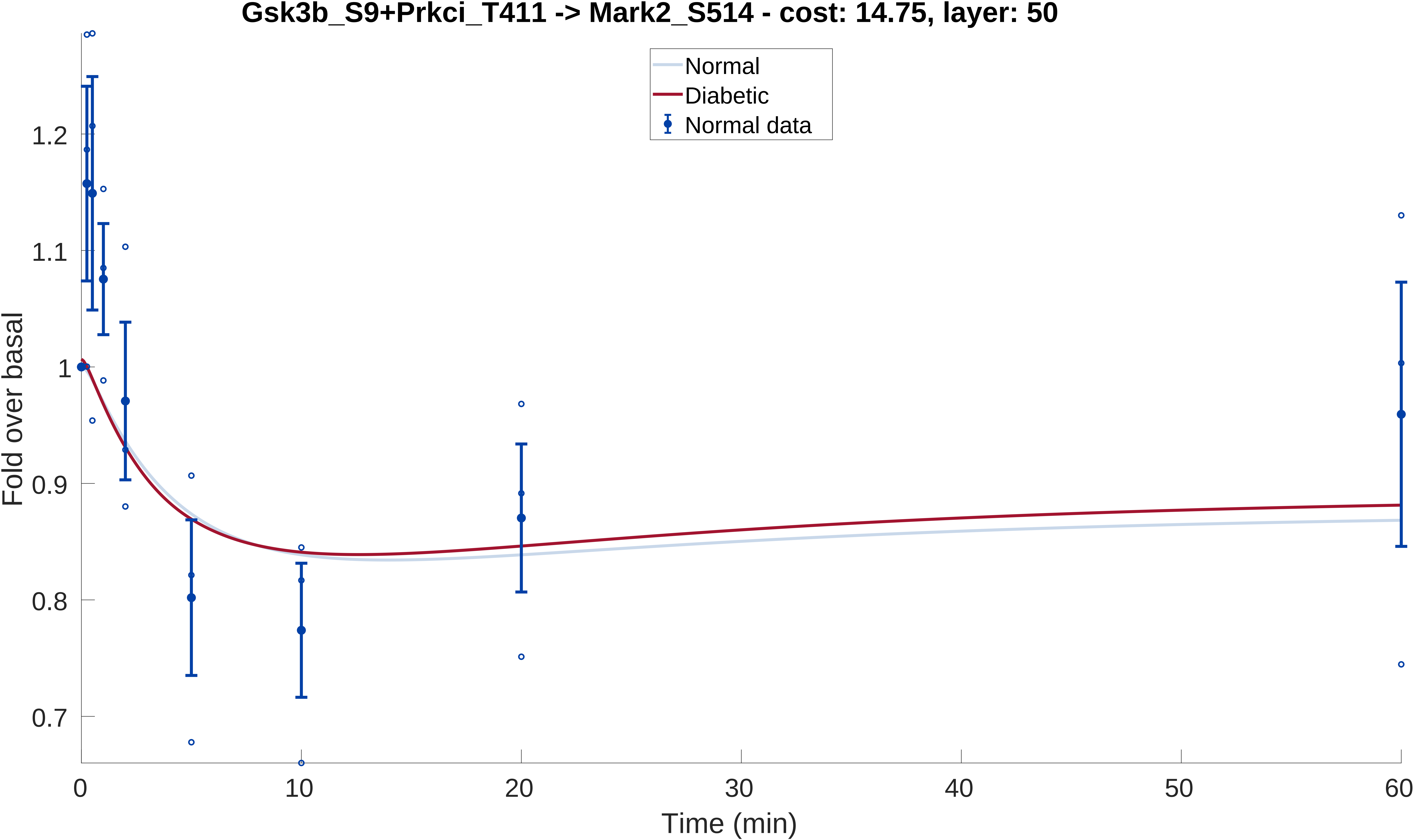

Gsk3b_S9+Prkci_T411 -> Mark2_S514 - cost: 14.75, layer: 50
Normal
Diabetic
Normal data
Fold over basal
1.2
1.1
1
0.9
0.8
0.7
0
10
20
30
40
50
60
Time (min)

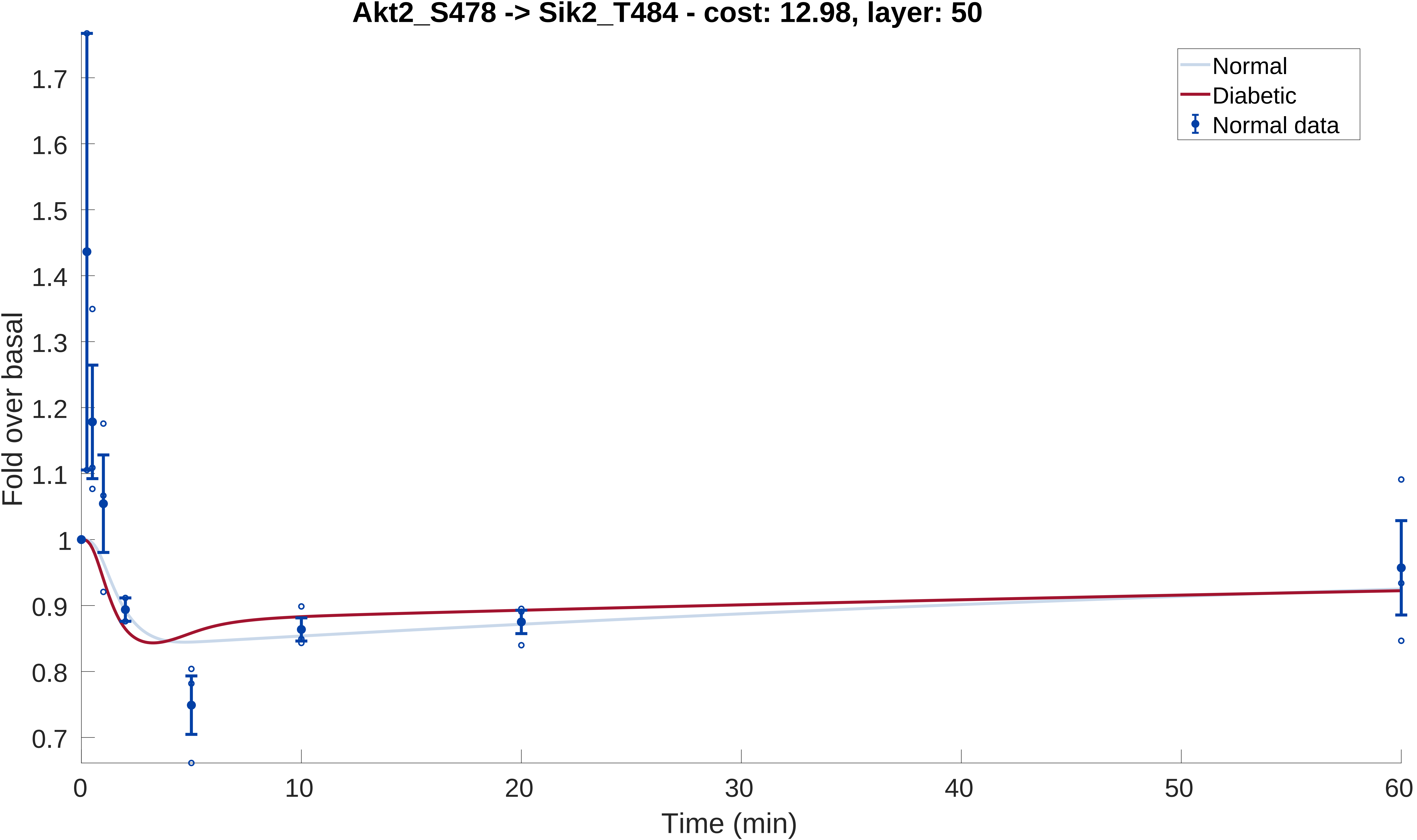

Akt2_S478 -> Sik2_T484 - cost: 12.98, layer: 50
Normal
Diabetic
Normal data
Fold over basal
Time (min)
0.7
0.8
0.9
1
1.1
1.2
1.3
1.4
1.5
1.6
1.7
0
10
20
30
40
50
60

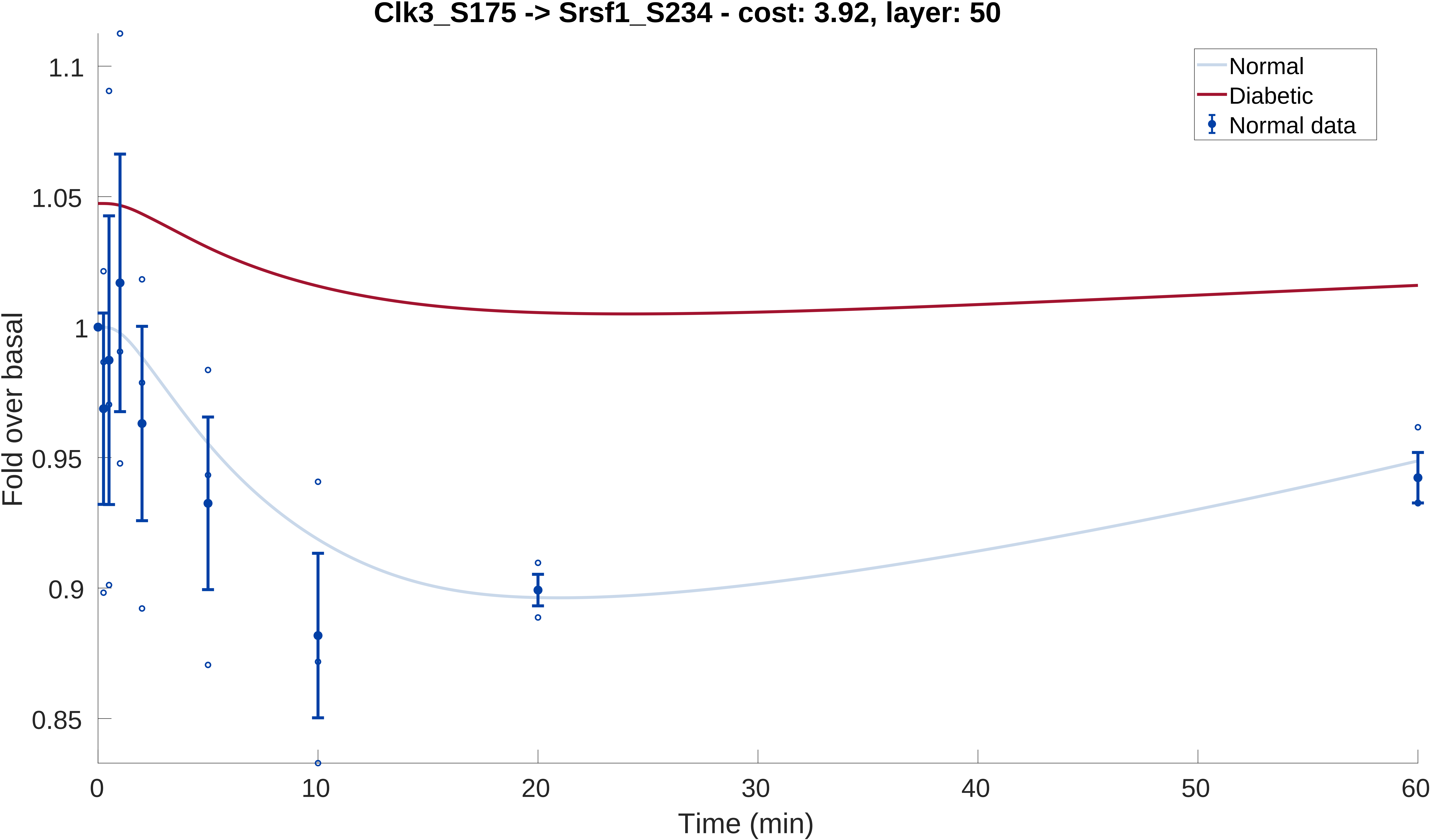

Clk3_S175 -> Srsf1_S234 - cost: 3.92, layer: 50
Normal
Diabetic
Normal data
Fold over basal
1.1
1.05
1
0.95
0.9
0.85
0
10
20
30
40
50
60
Time (min)

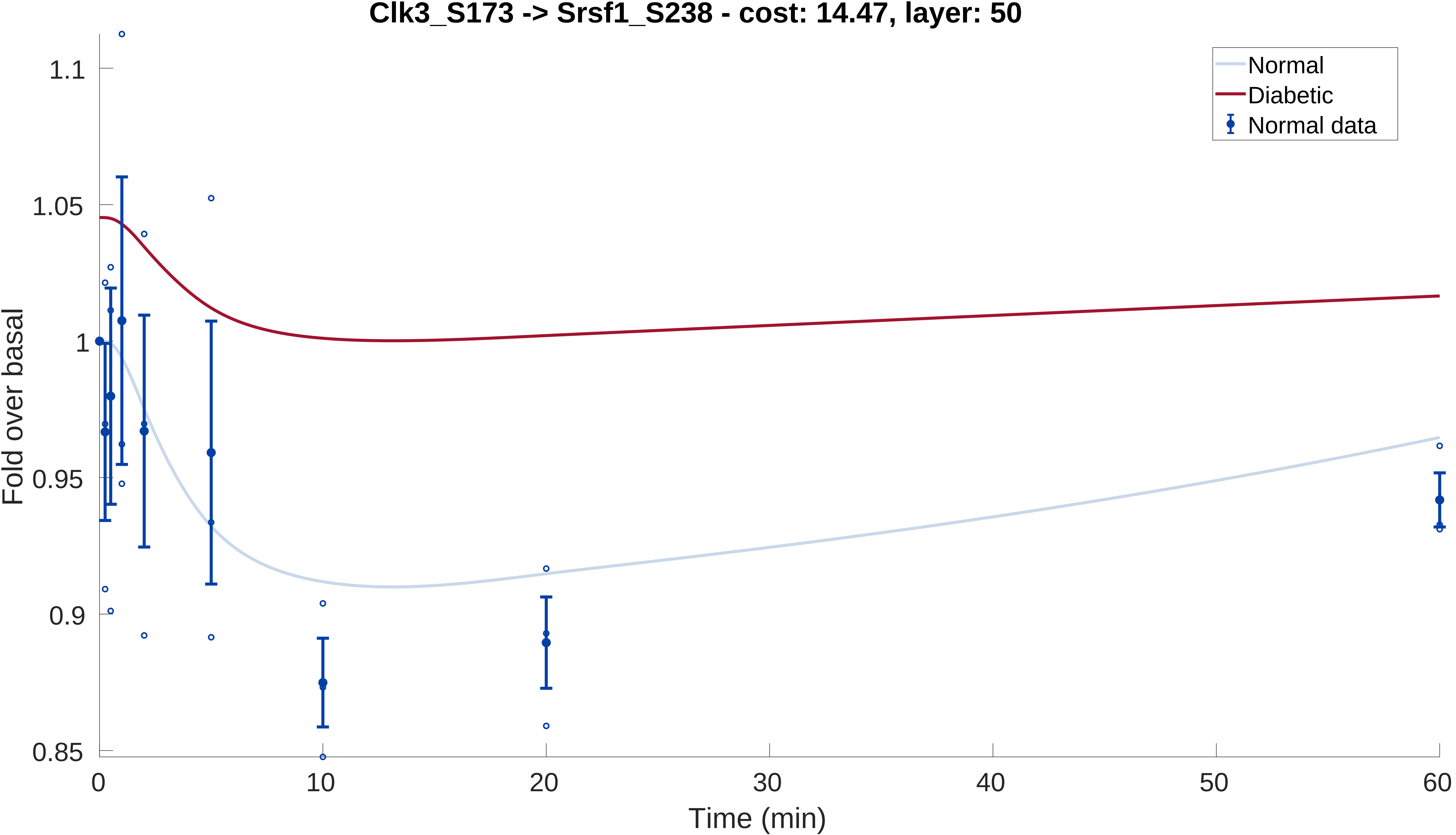
Clk3_S173 -> Srsf1_S238 - cost: 14.47, layer: 50
Normal
Diabetic
Normal data
Fold over basal
Time (min)
1.1
1.05
1
0.95
0.9
0.85
0
10
20
30
40
50
60

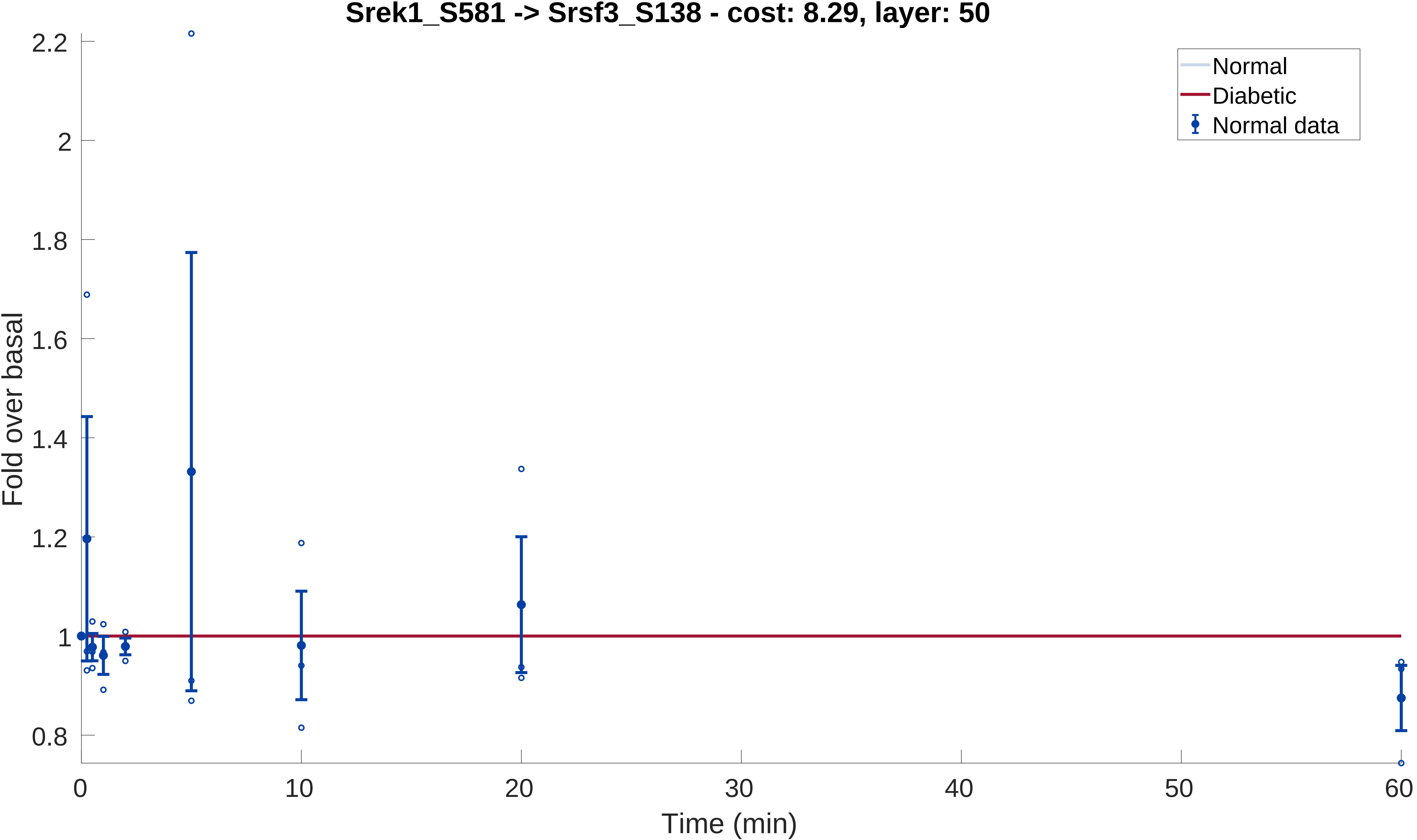

Srek1_S581 -> Srsf3_S138 - cost: 8.29, layer: 50
Normal
Diabetic
Normal data
Fold over basal
2.2
2
1.8
1.6
1.4
1.2
1
0.8
0
10
20
30
40
50
60
Time (min)

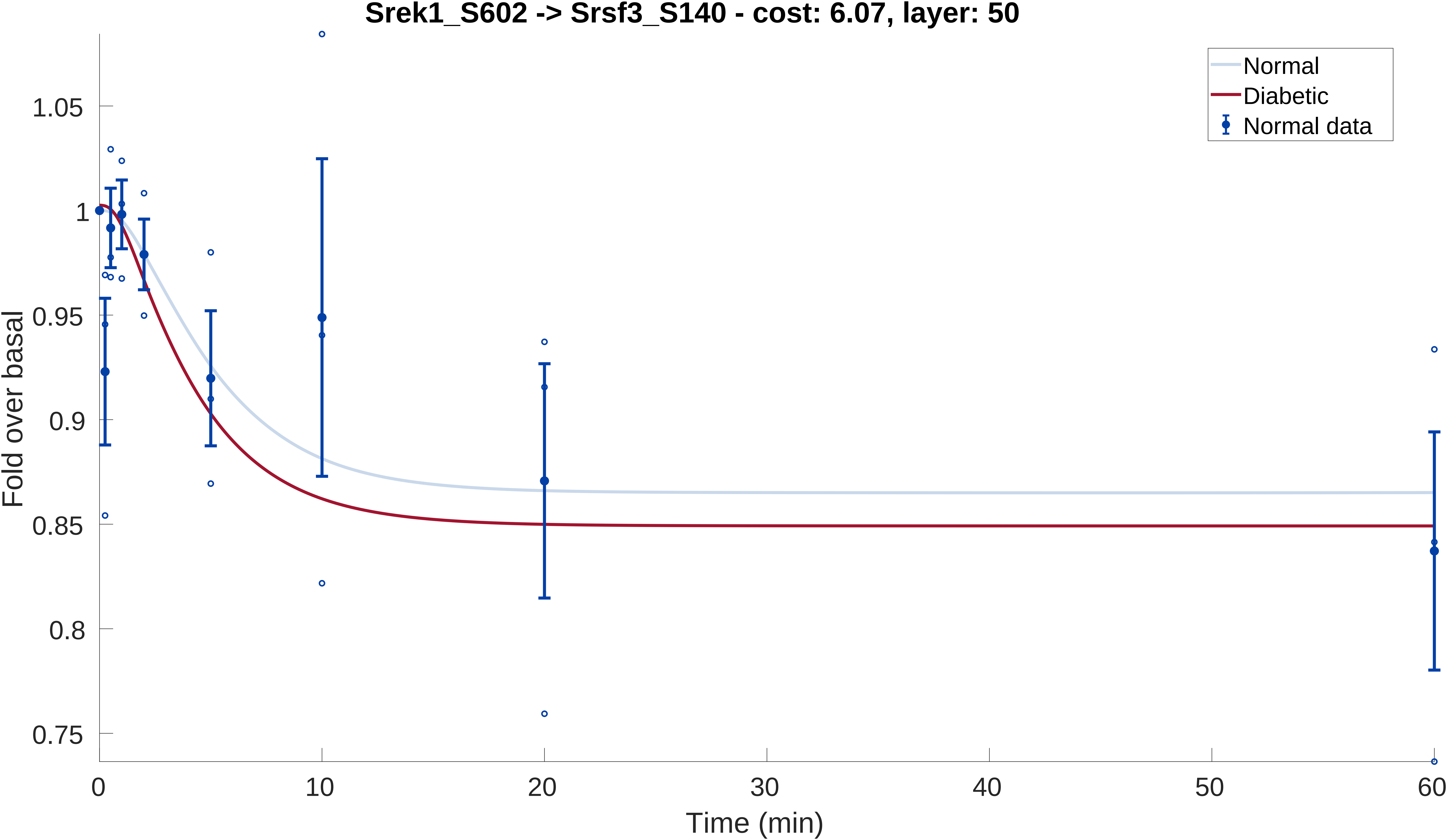
Srek1_S602 -> Srsf3_S140 - cost: 6.07, layer: 50
Normal
Diabetic
Normal data
Fold over basal
1.05
1
0.95
0.9
0.85
0.8
0.75
0
10
20
30
40
50
60
Time (min)

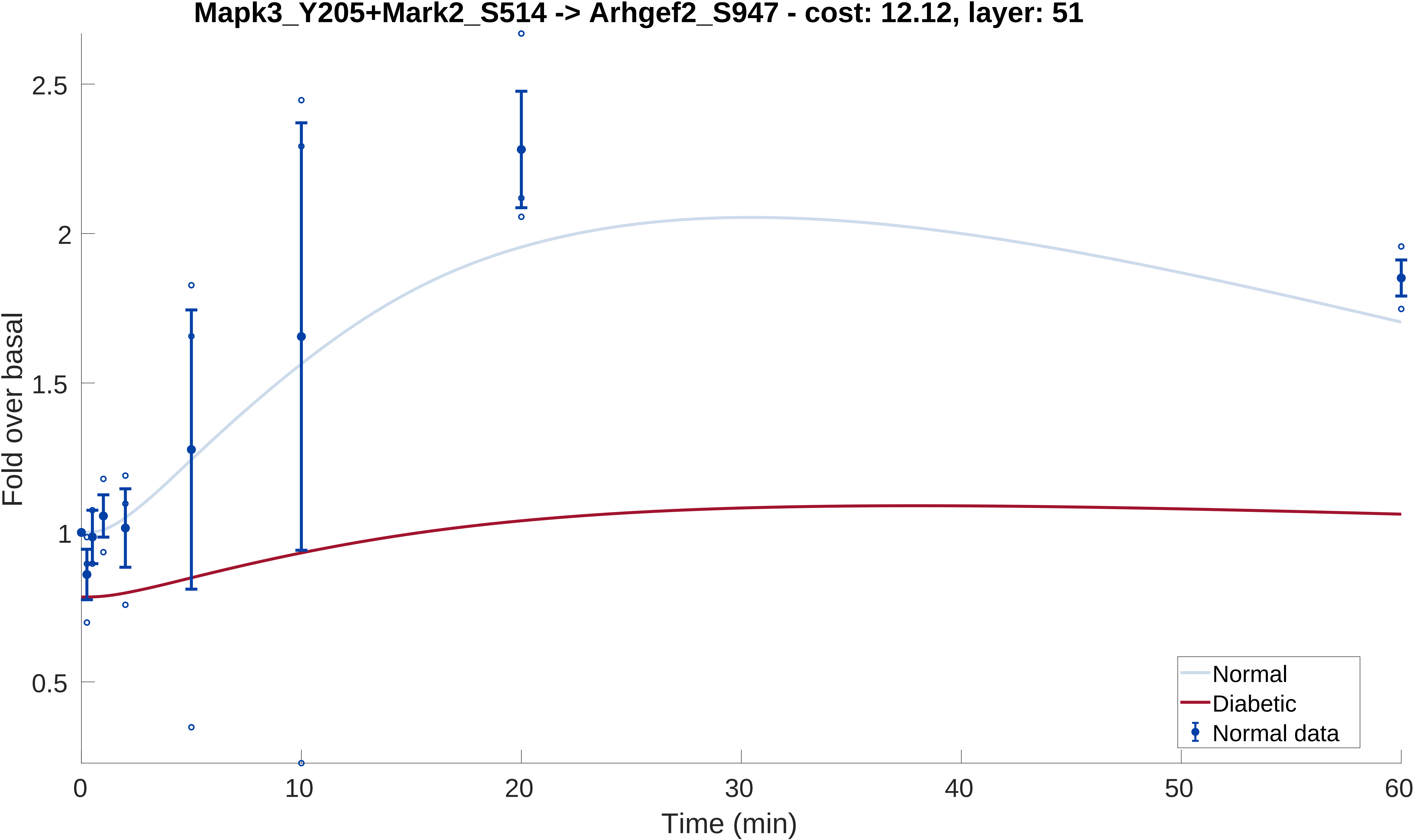

Mapk3_Y205+Mark2_S514 -> Arhgef2_S947 - cost: 12.12, layer: 51
Fold over basal
2.5
2
1.5
1
0.5
0
10
20
30
40
50
60
Time (min)
Normal
Diabetic
Normal data

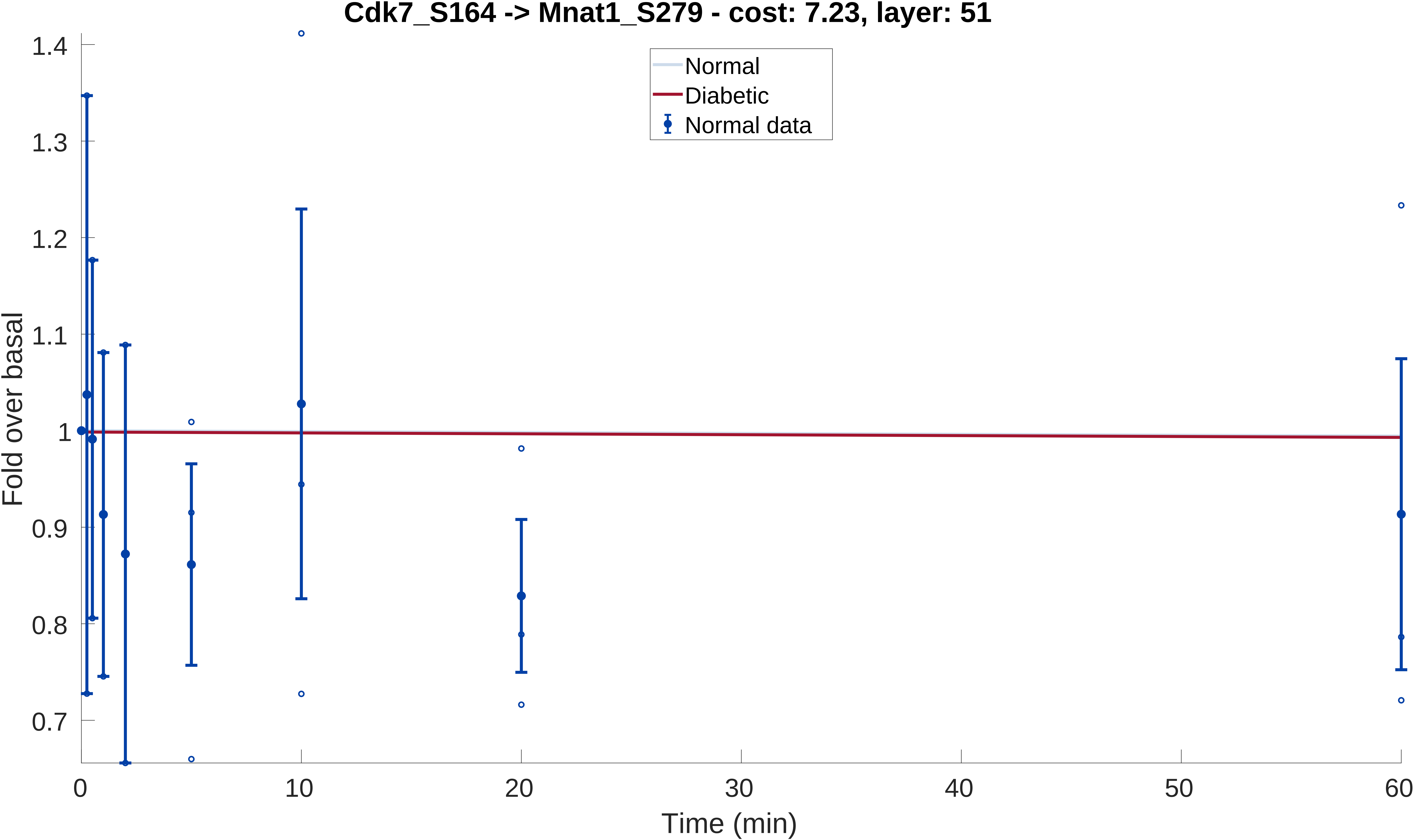

Cdk7_S164 -> Mnat1_S279 - cost: 7.23, layer: 51
Normal
Diabetic
Normal data
Fold over basal
1.4
1.3
1.2
1.1
1
0.9
0.8
0.7
0
10
20
30
40
50
60
Time (min)

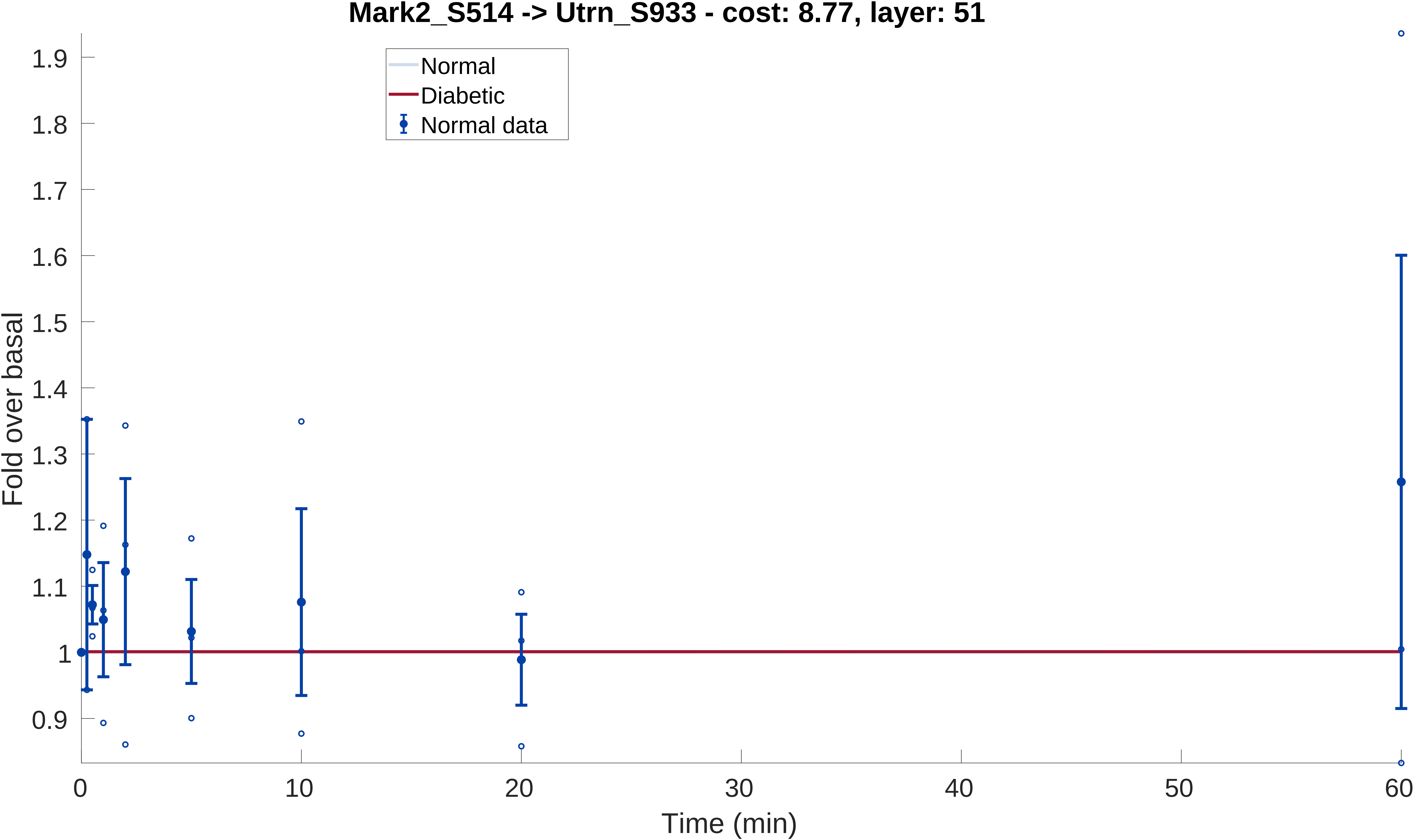

Mark2_S514 -> Utrn_S933 - cost: 8.77, layer: 51
Normal
Diabetic
Normal data
Fold over basal
Time (min)
1.9
1.8
1.7
1.6
1.5
1.4
1.3
1.2
1.1
1
0.9
0
10
20
30
40
50
60

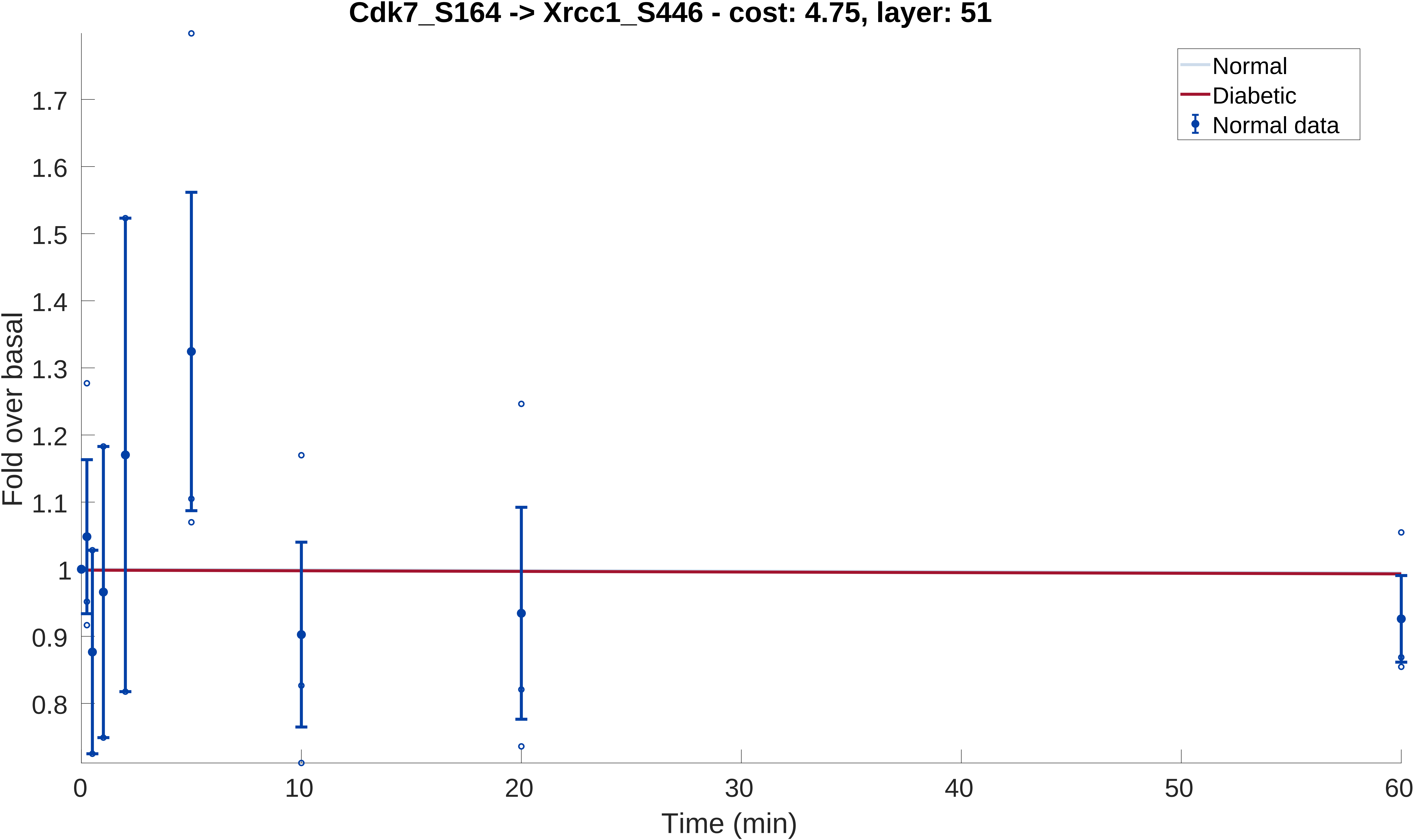

Cdk7_S164 -> Xrcc1_S446 - cost: 4.75, layer: 51
Normal
Diabetic
Normal data
Fold over basal
Time (min)
0.8
0.9
1
1.1
1.2
1.3
1.4
1.5
1.6
1.7
0
10
20
30
40
50
60

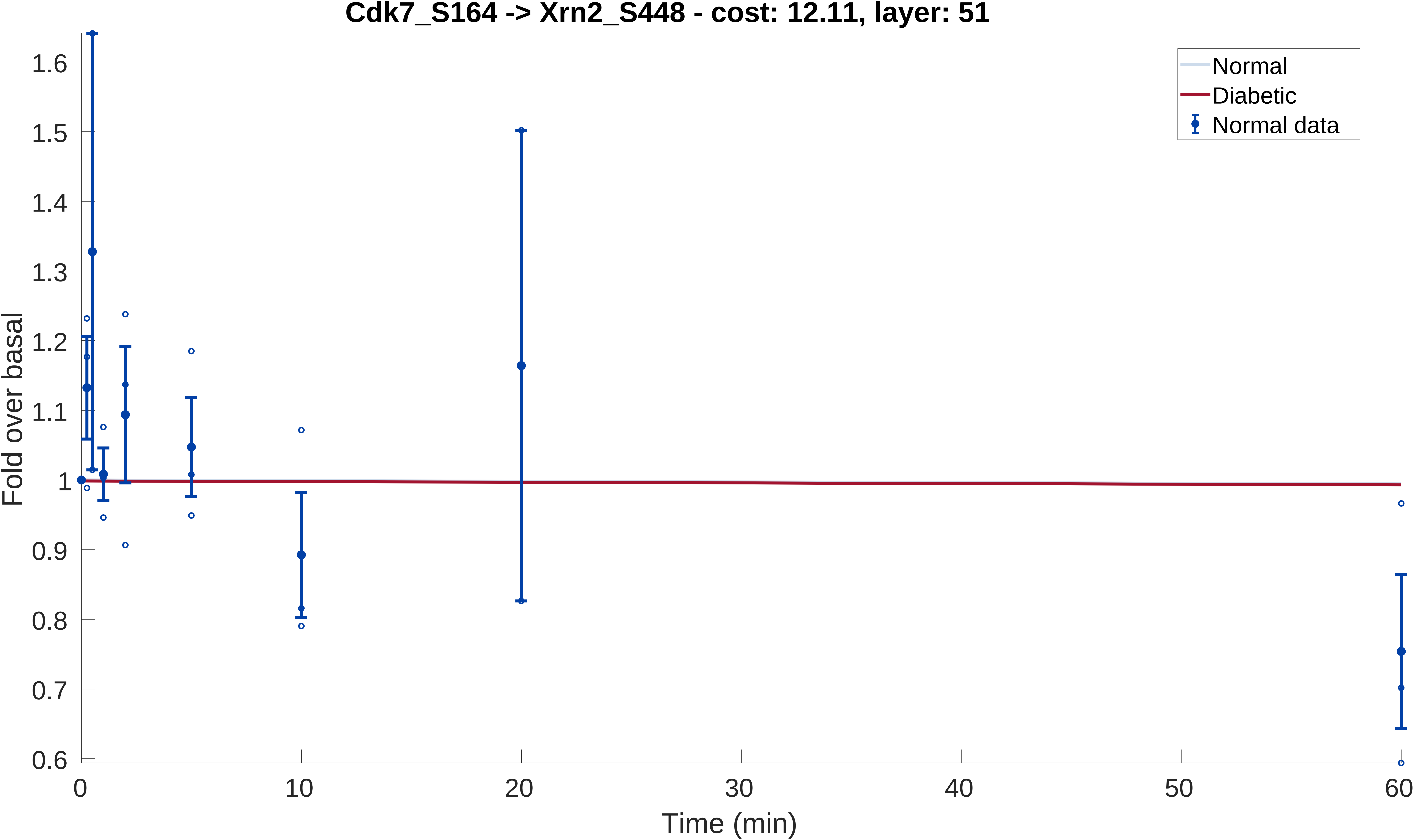

Cdk7_S164 -> Xrn2_S448 - cost: 12.11, layer: 51
Normal
Diabetic
Normal data
Fold over basal
Time (min)
1.6
1.5
1.4
1.3
1.2
1.1
1
0.9
0.8
0.7
0.6
0
10
20
30
40
50
60

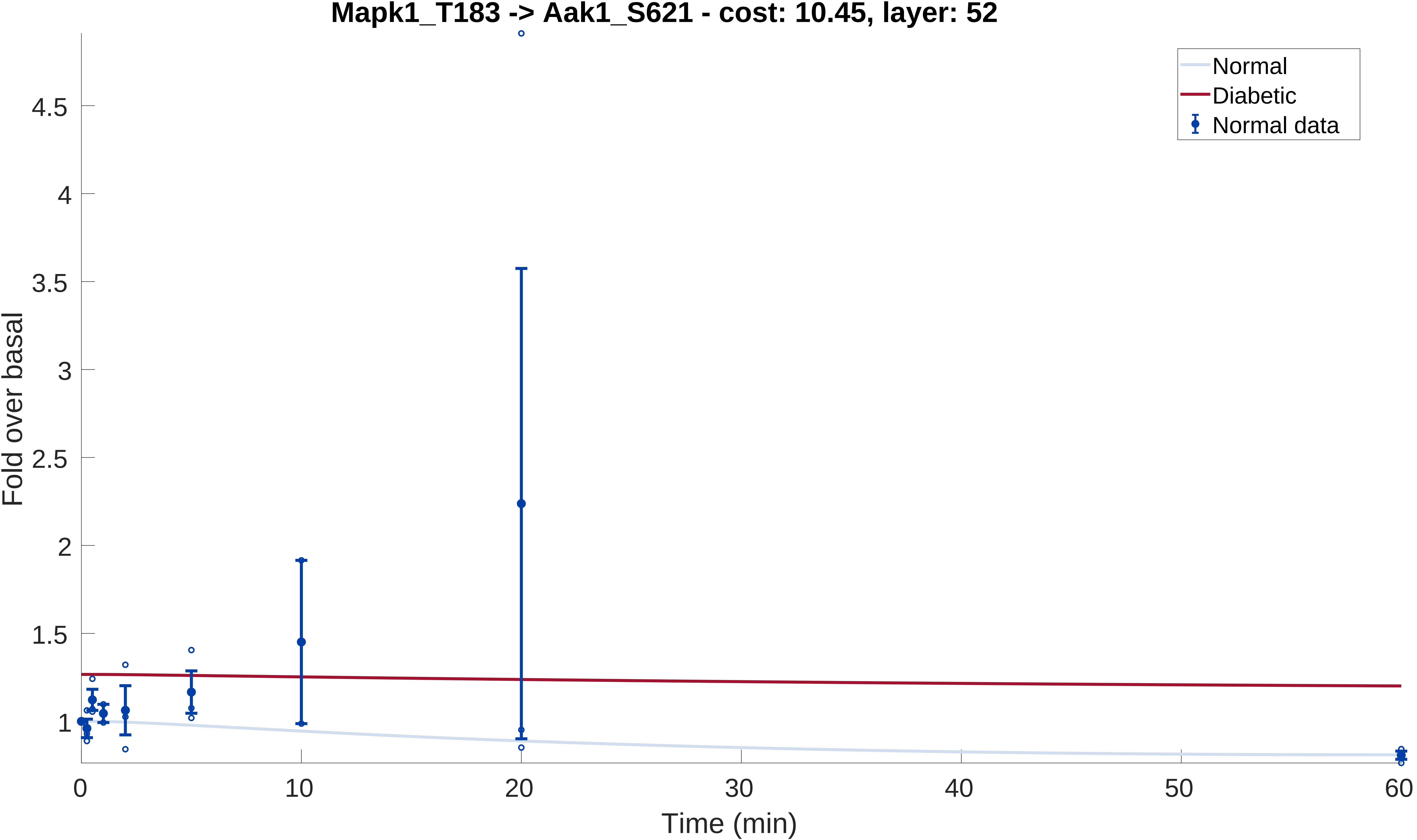

Mapk1_T183 -> Aak1_S621 - cost: 10.45, layer: 52
Normal
Diabetic
Normal data
Fold over basal
4.5
4
3.5
3
2.5
2
1.5
1
0
10
20
30
40
50
60
Time (min)

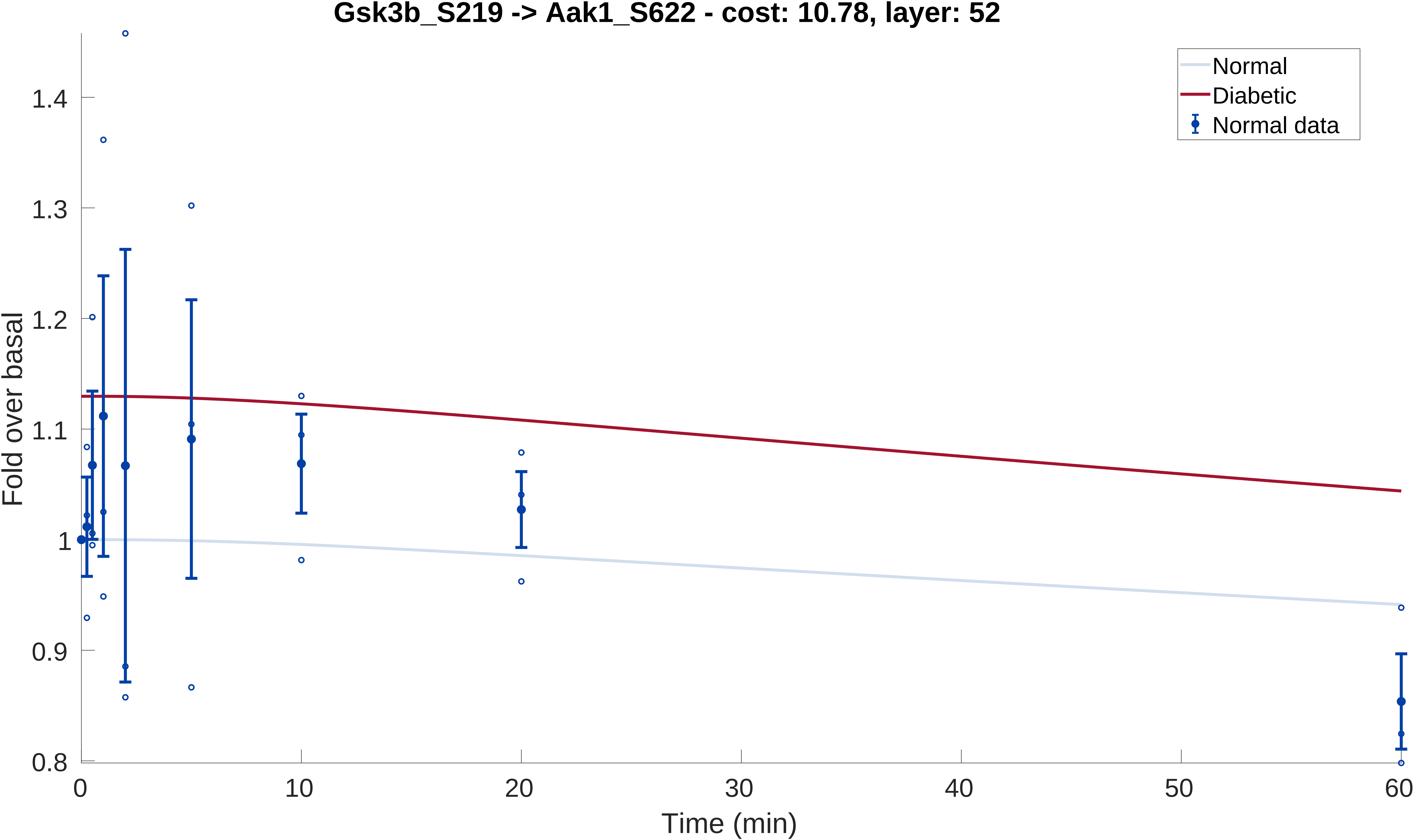

Gsk3b_S219 -> Aak1_S622 - cost: 10.78, layer: 52
Normal
Diabetic
Normal data
Fold over basal
1.4
1.3
1.2
1.1
1
0.9
0.8
0
10
20
30
40
50
60
Time (min)

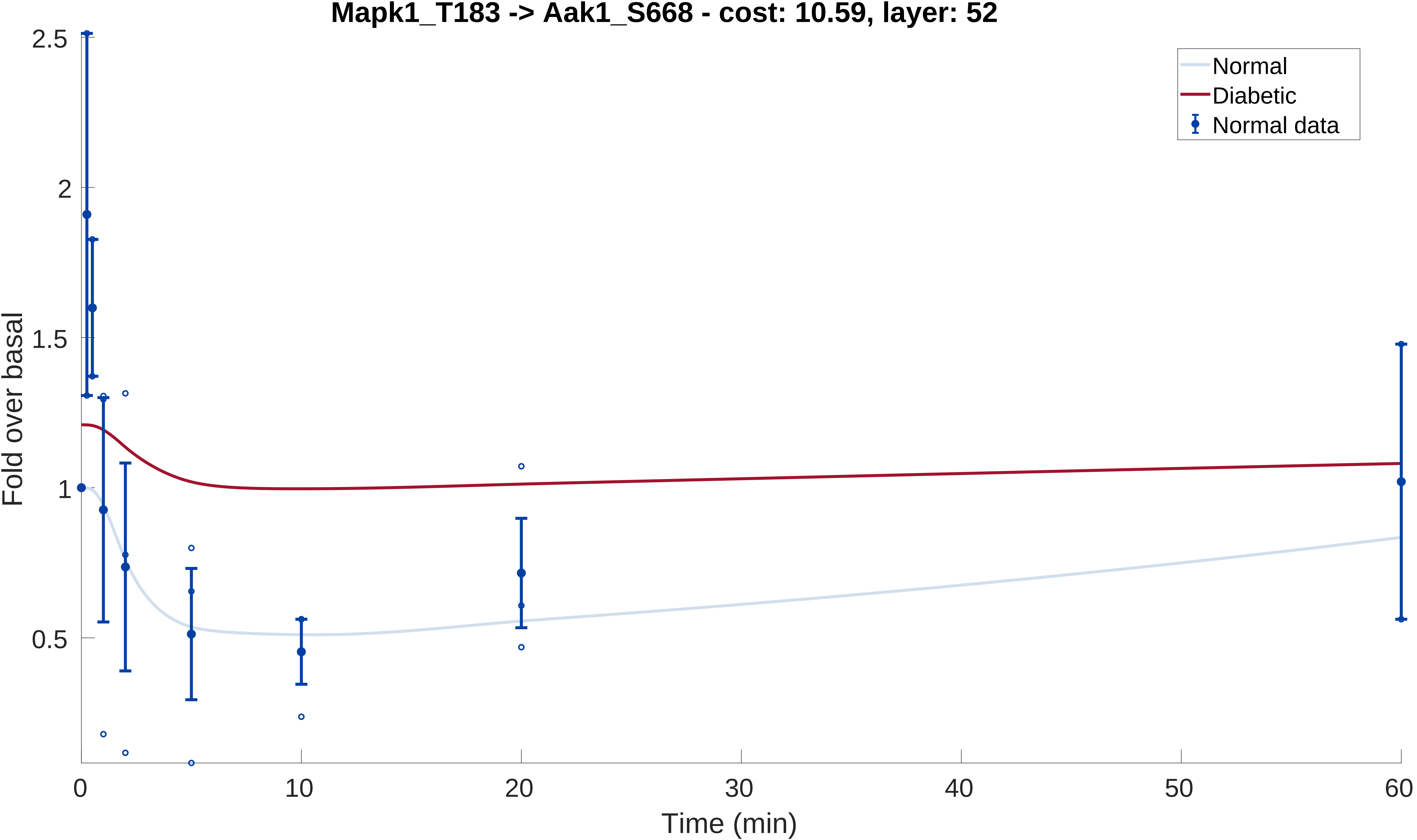

Mapk1_T183 -> Aak1_S668 - cost: 10.59, layer: 52
Fold over basal
Time (min)
Normal
Diabetic
Normal data
2.5
2
1.5
1
0.5
0
10
20
30
40
50
60

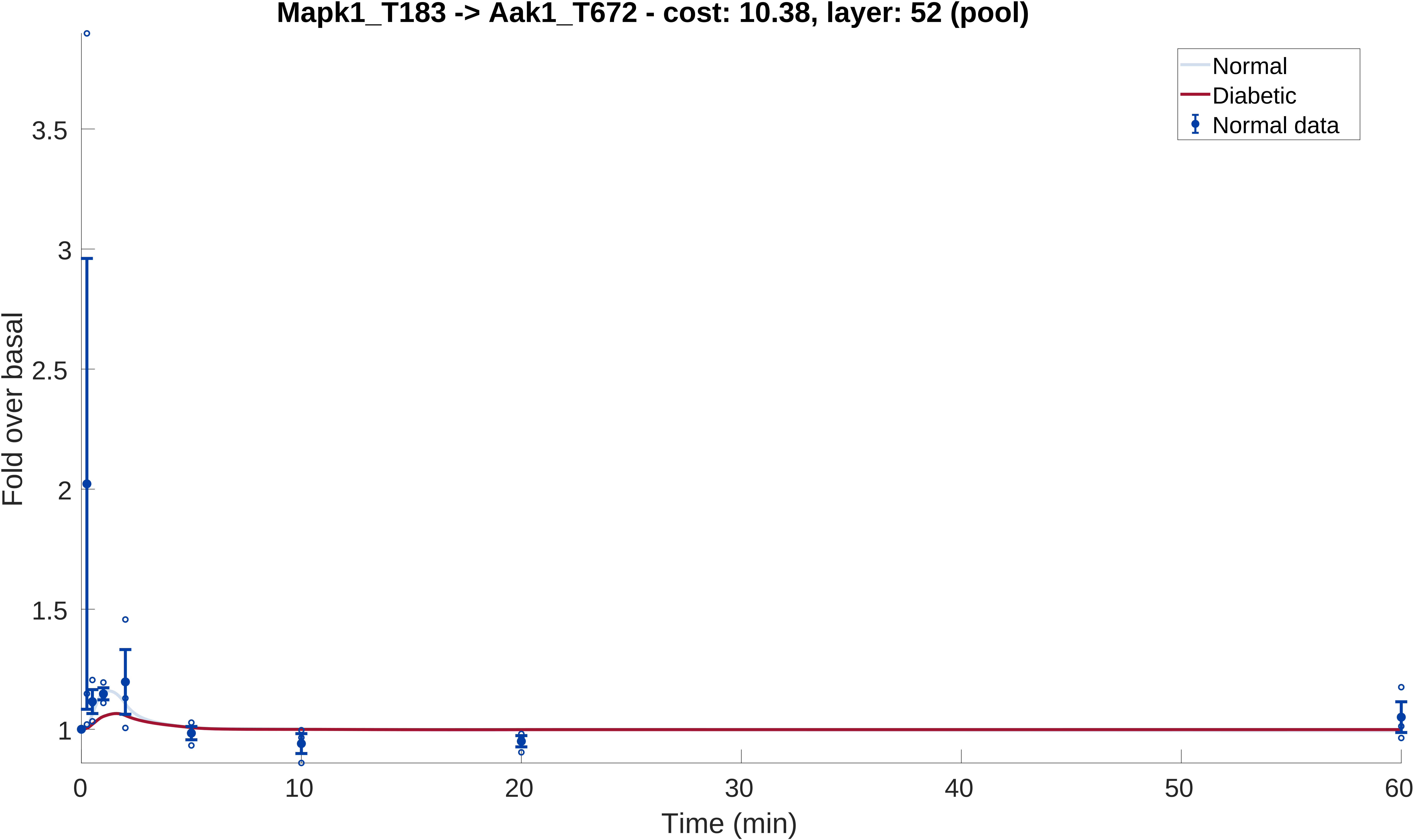

Mapk1_T183 -> Aak1_T672 - cost: 10.38, layer: 52 (pool)
Normal
Diabetic
Normal data
Fold over basal
Time (min)
1
1.5
2
2.5
3
3.5
0
10
20
30
40
50
60

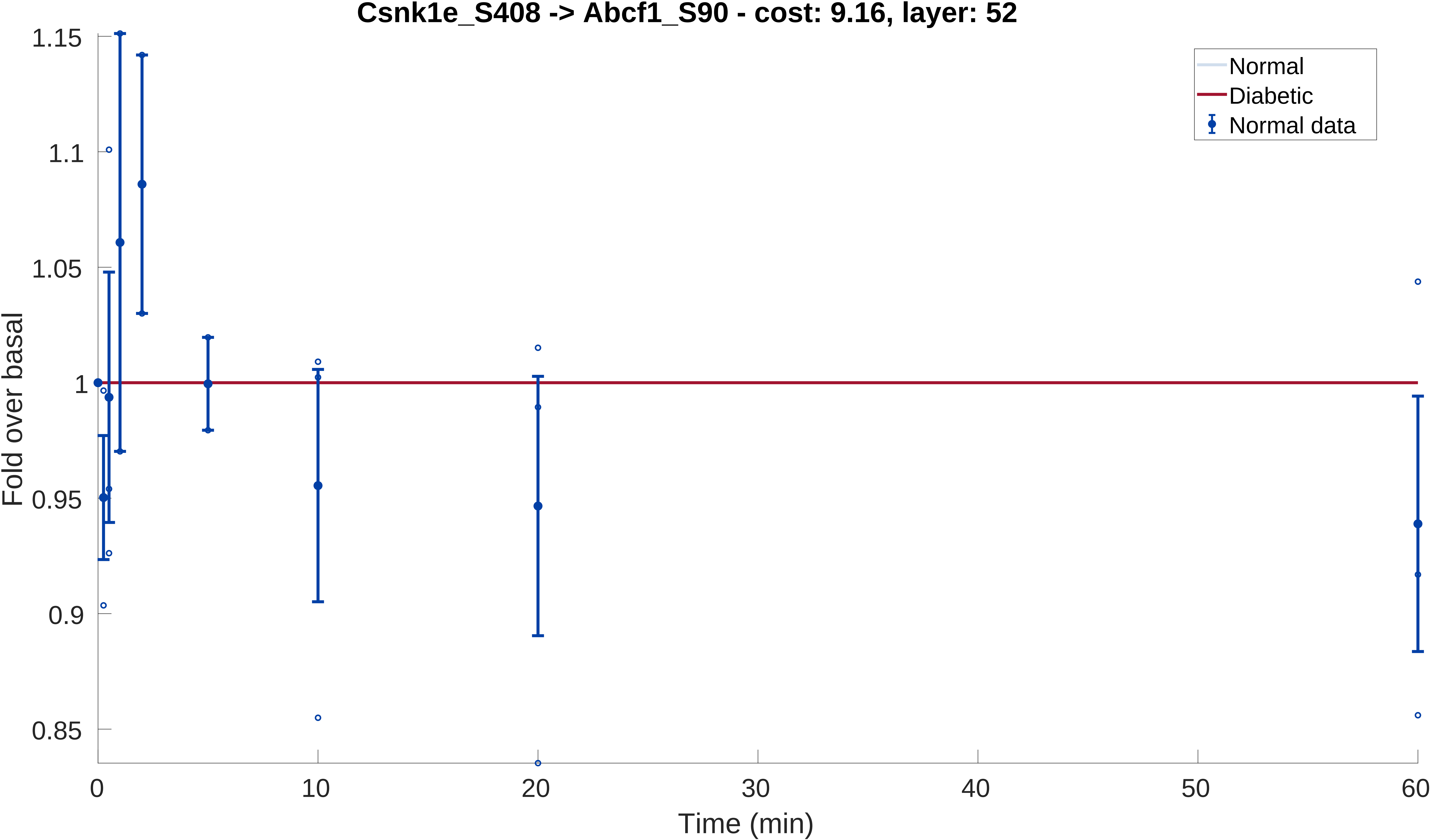
Csnk1e_S408 -> Abcf1_S90 - cost: 9.16, layer: 52
Normal
Diabetic
Normal data
Fold over basal
1.15
1.1
1.05
1
0.95
0.9
0.85
0
10
20
30
40
50
60
Time (min)

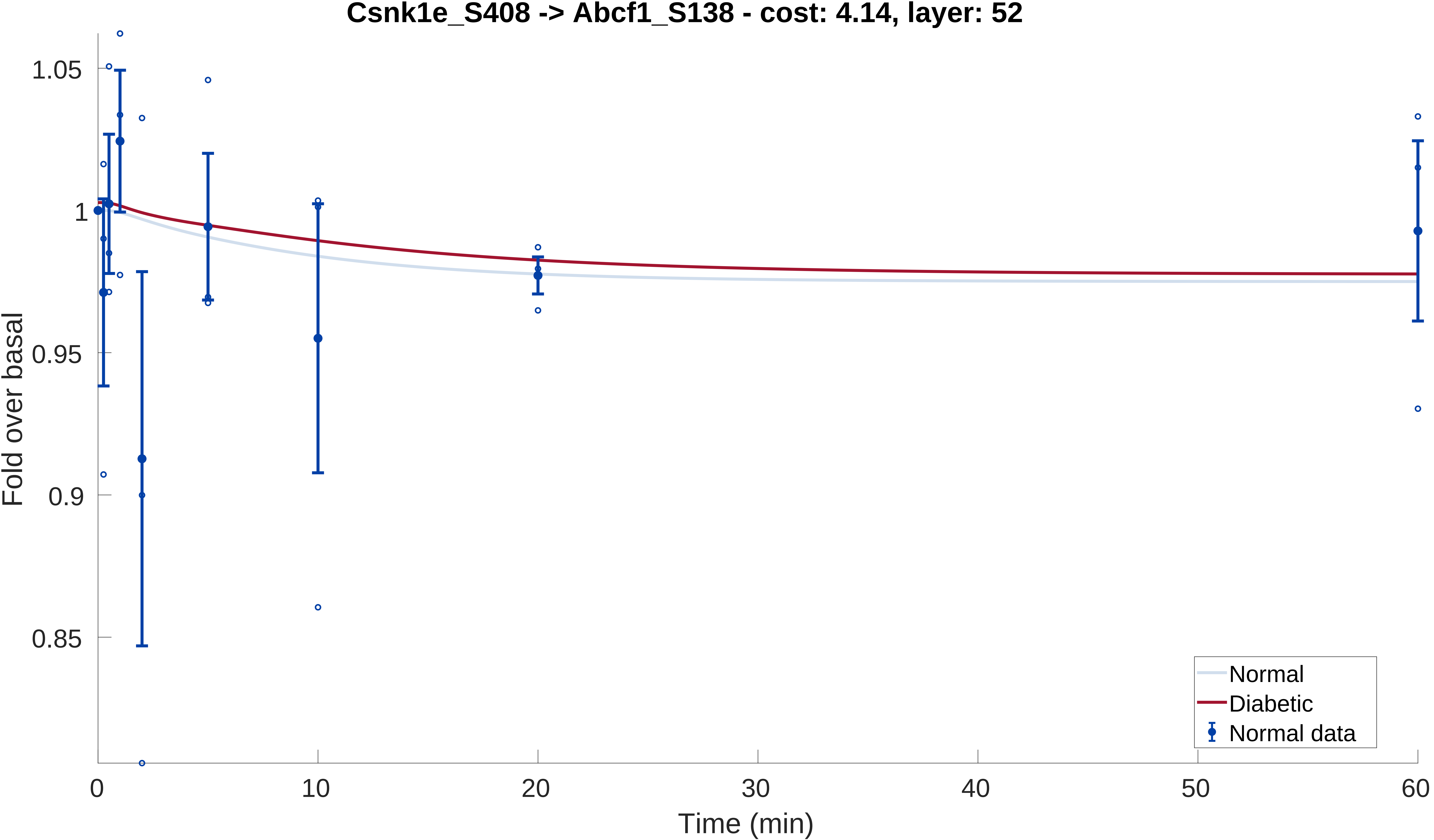
Csnk1e_S408 -> Abcf1_S138 - cost: 4.14, layer: 52
Fold over basal
1.05
1
0.95
0.9
0.85
0
10
20
30
40
50
60
Time (min)
Normal
Diabetic
Normal data

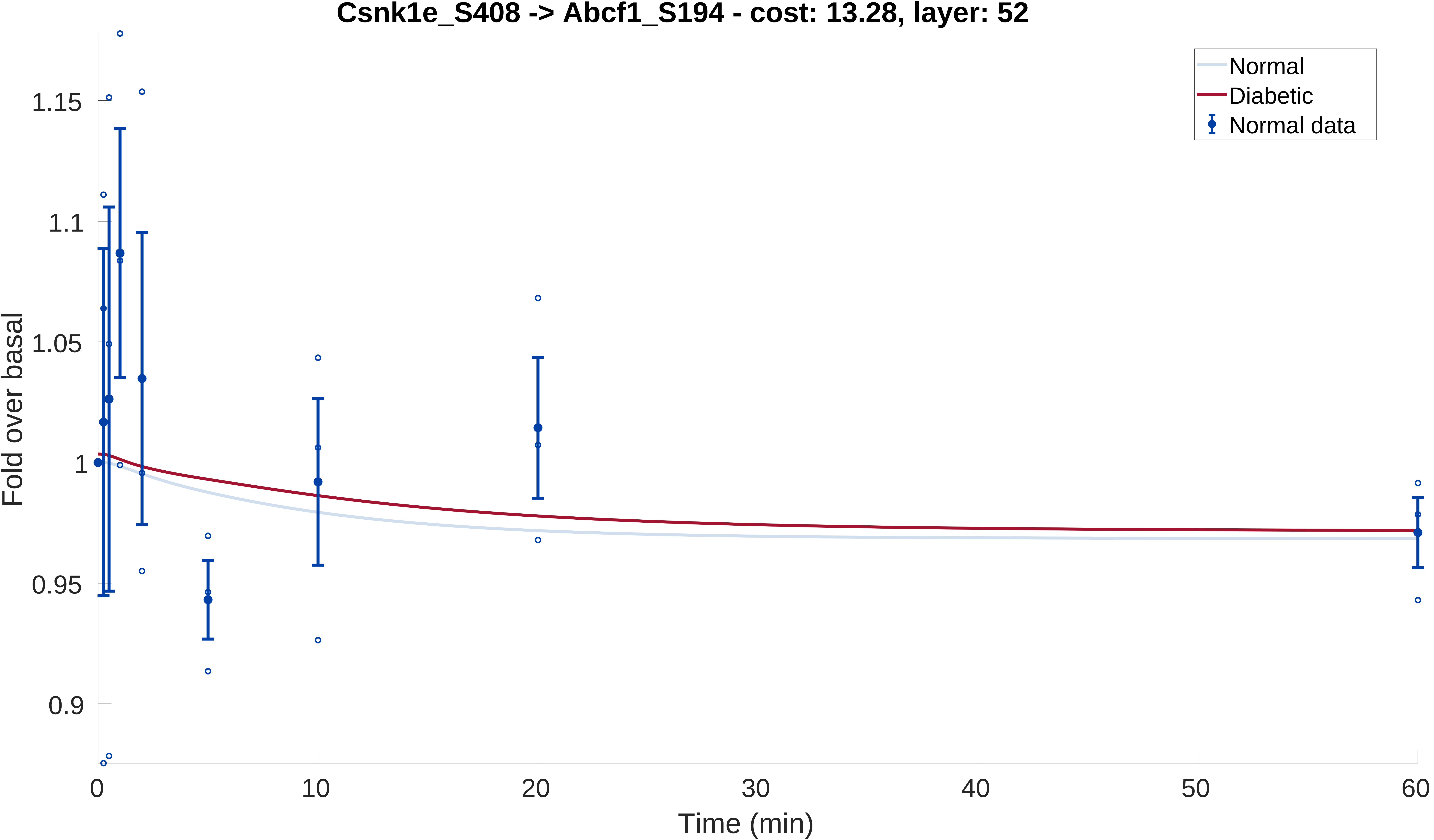

Csnk1e_S408 -> Abcf1_S194 - cost: 13.28, layer: 52
Normal
Diabetic
Normal data
1.15
1.1
1.05
1
0.95
0.9
Fold over basal
0
10
20
30
40
50
60
Time (min)

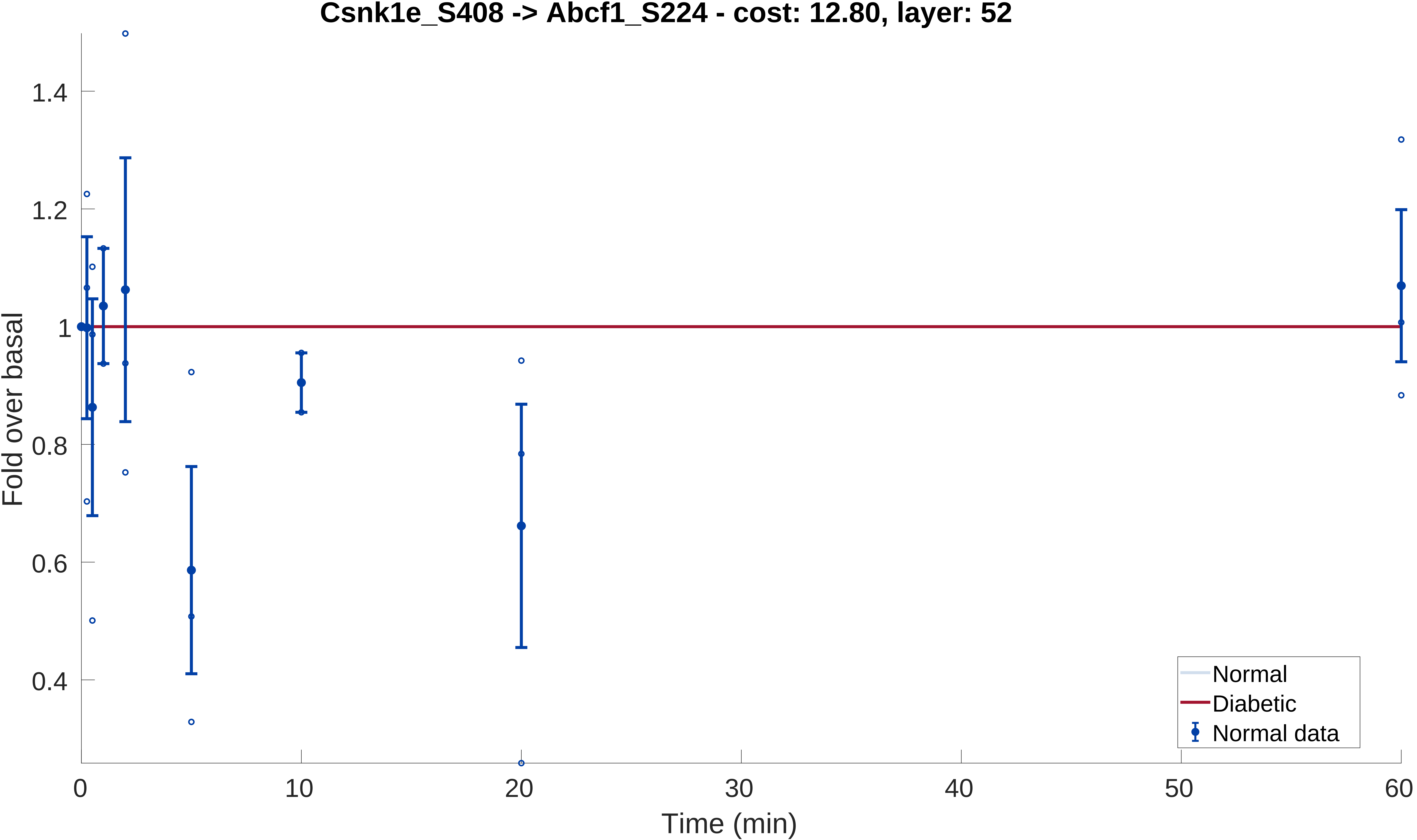
Csnk1e_S408 -> Abcf1_S224 - cost: 12.80, layer: 52
Fold over basal
1.4
1.2
1
0.8
0.6
0.4
Time (min)
0
10
20
30
40
50
60
Normal
Diabetic
Normal data

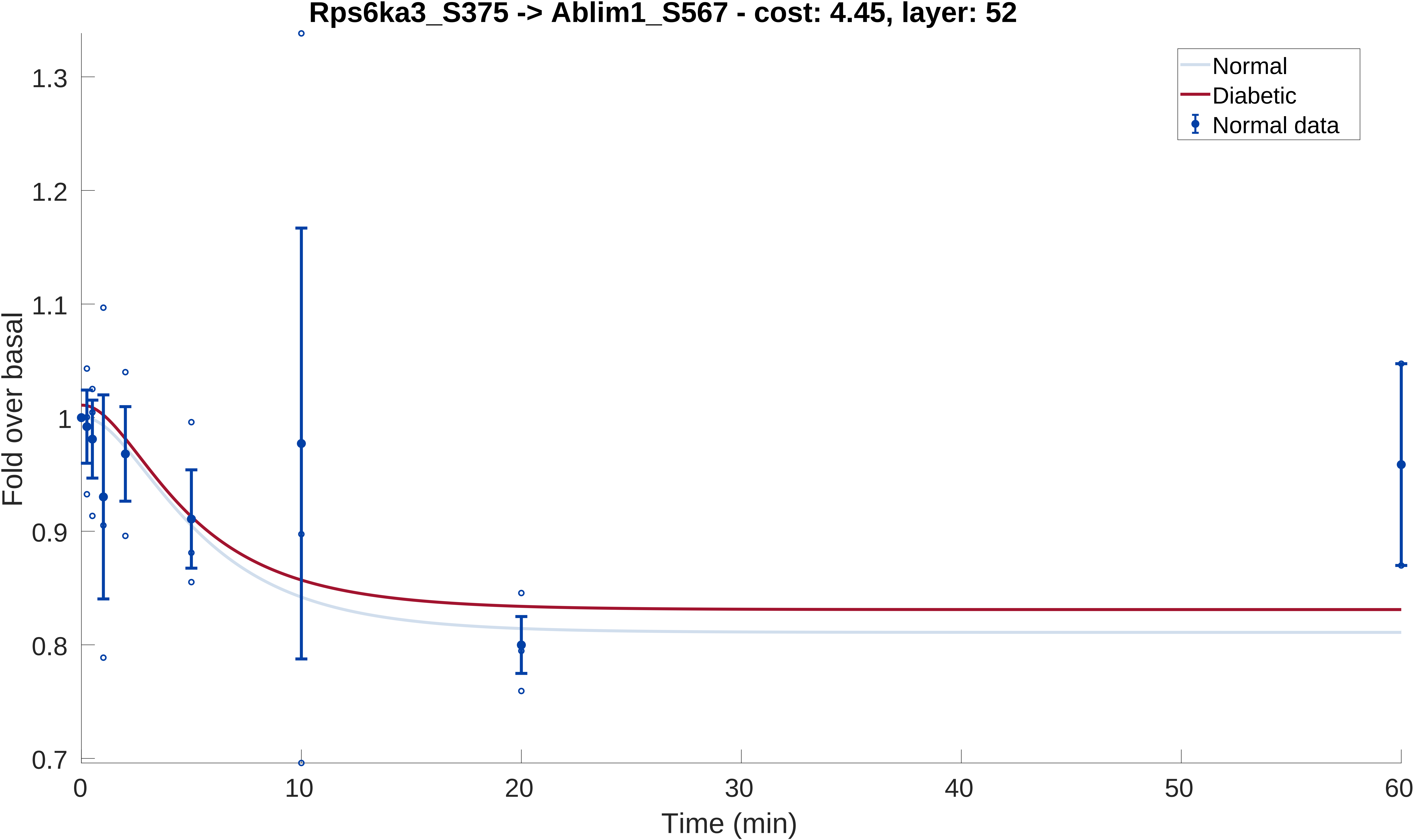

Rps6ka3_S375 -> Ablim1_S567 - cost: 4.45, layer: 52
Normal
Diabetic
Normal data
Fold over basal
Time (min)
1.3
1.2
1.1
1
0.9
0.8
0.7
0
10
20
30
40
50
60

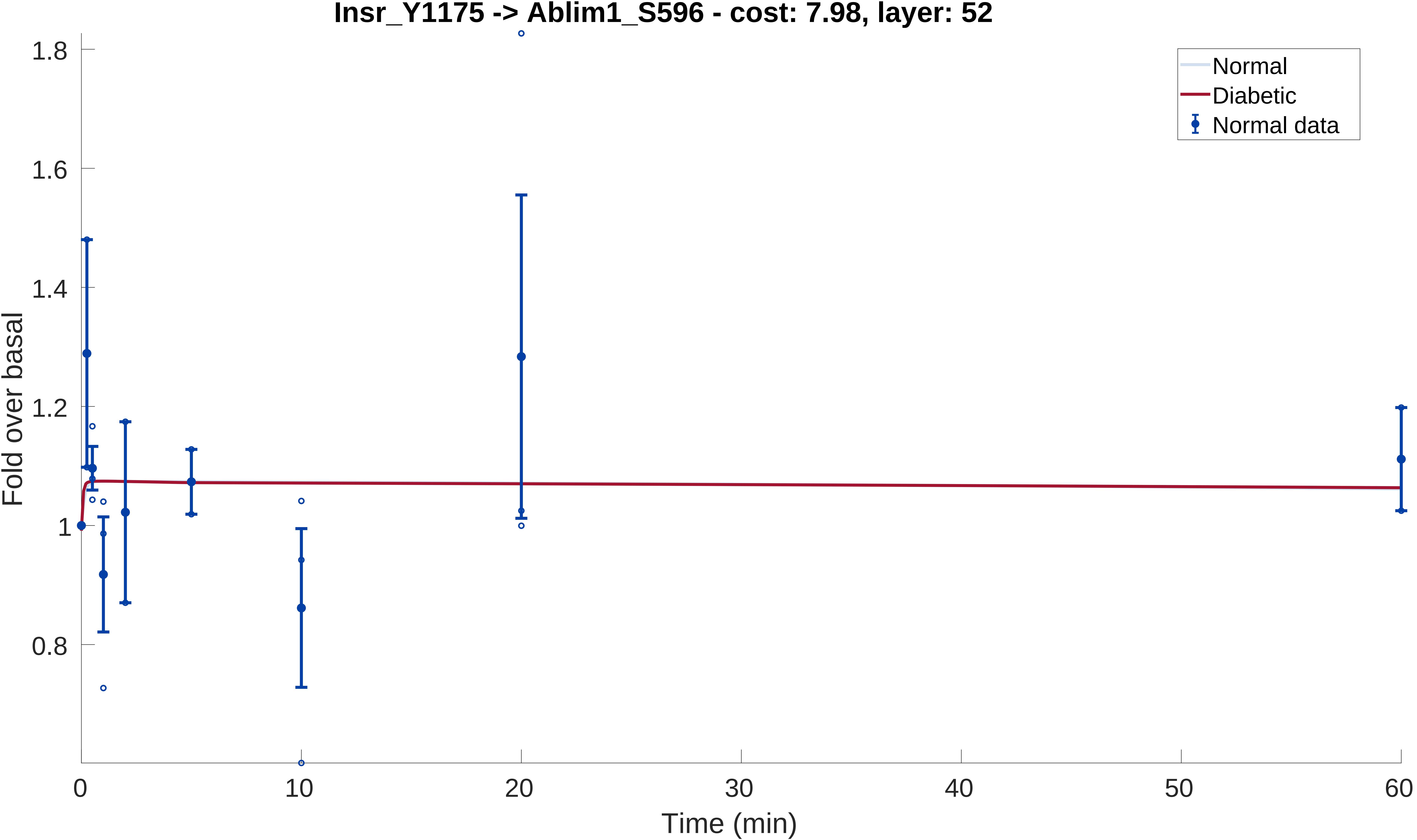

Insr_Y1175 -> Ablim1_S596 - cost: 7.98, layer: 52
Normal
Diabetic
Normal data
Fold over basal
Time (min)
1.8
1.6
1.4
1.2
1
0.8
0
10
20
30
40
50
60

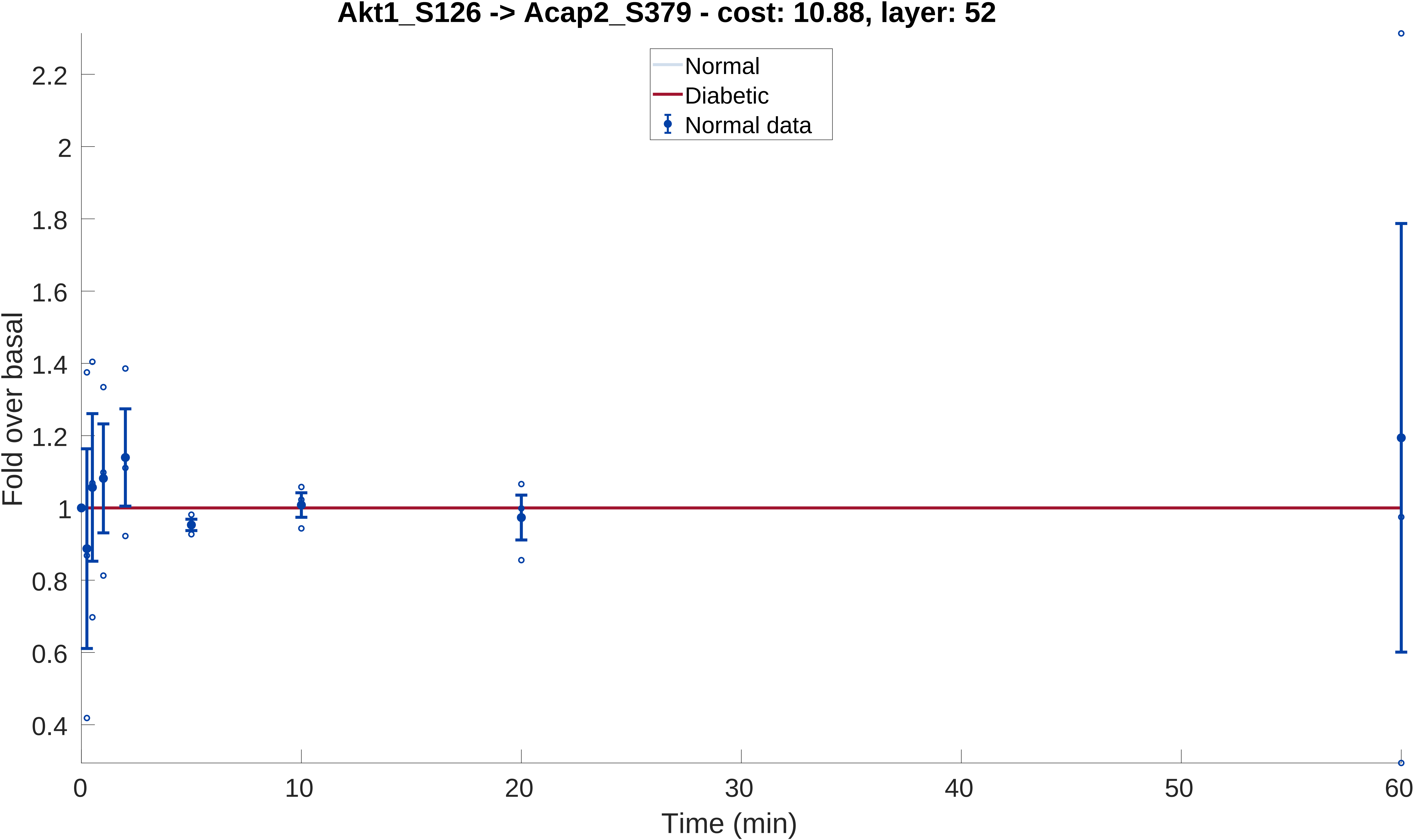
Akt1_S126 -> Acap2_S379 - cost: 10.88, layer: 52
Normal
Diabetic
Normal data
Fold over basal
2.2
2
1.8
1.6
1.4
1.2
1
0.8
0.6
0.4
0
10
20
30
40
50
60
Time (min)

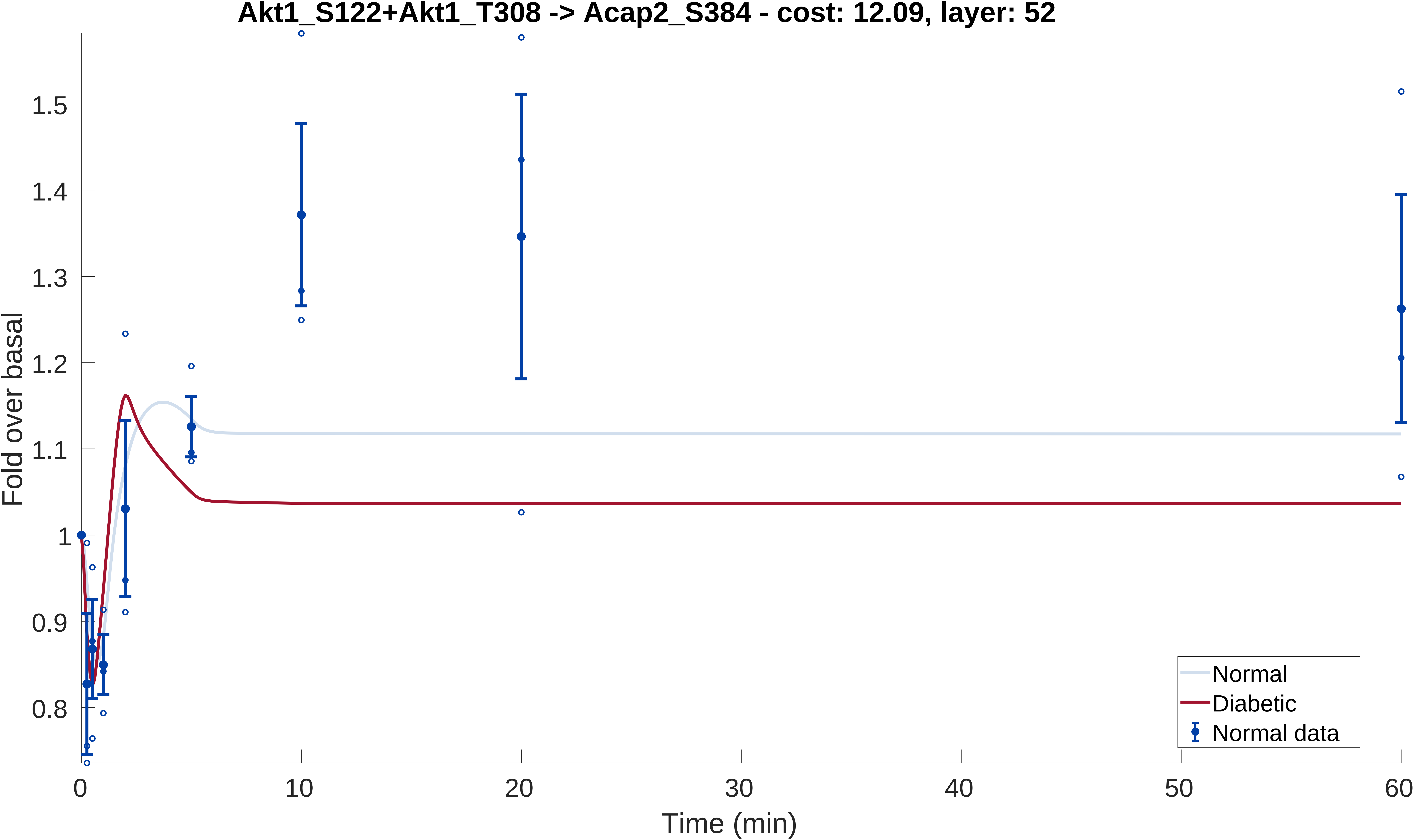

Akt1_S122+Akt1_T308 -> Acap2_S384 - cost: 12.09, layer: 52
Fold over basal
1.5
1.4
1.3
1.2
1.1
1
0.9
0.8
0
10
20
30
40
50
60
Time (min)
Normal
Diabetic
Normal data

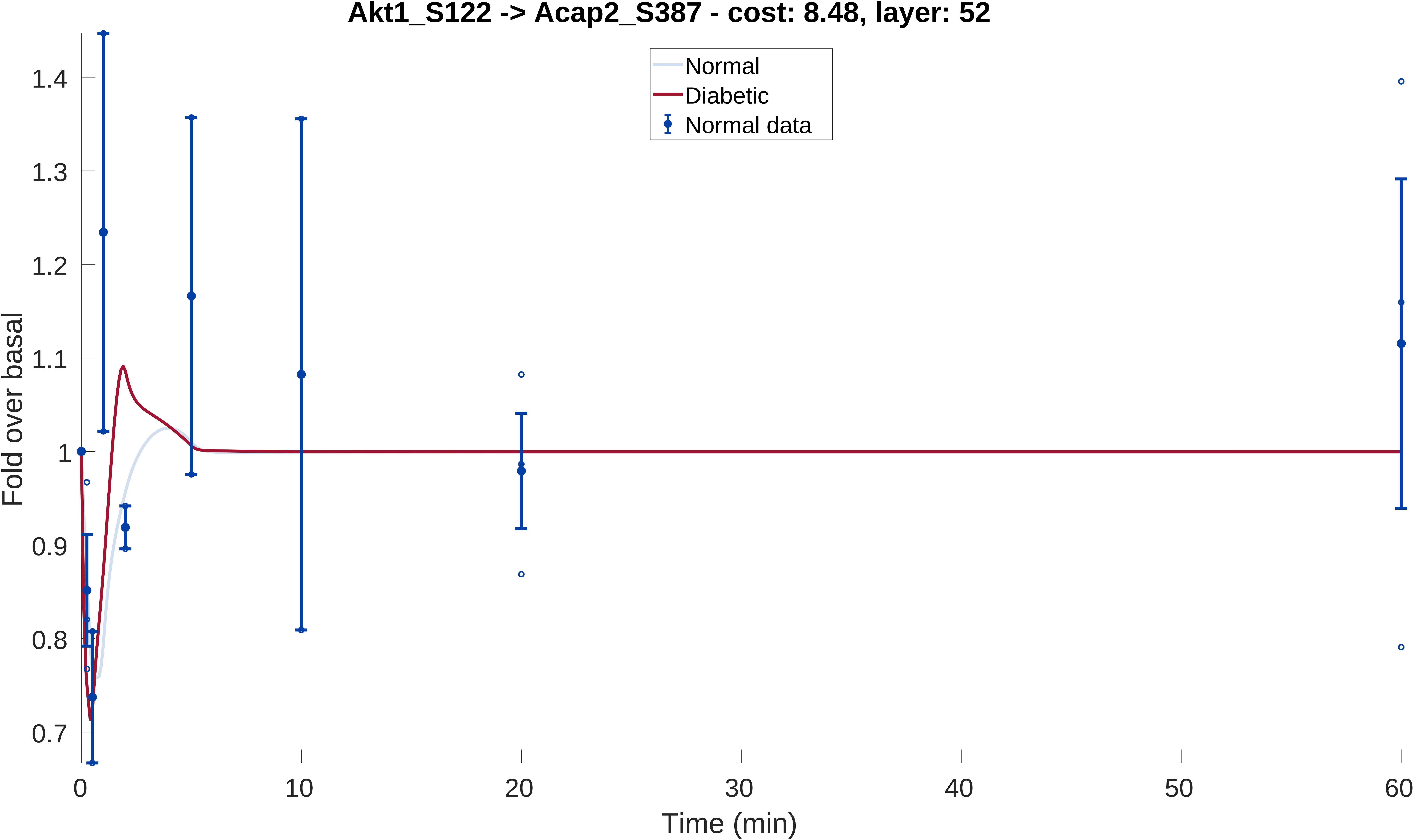

Akt1_S122 -> Acap2_S387 - cost: 8.48, layer: 52
Normal
Diabetic
Normal data
Fold over basal
Time (min)
0.7
0.8
0.9
1
1.1
1.2
1.3
1.4
0
10
20
30
40
50
60

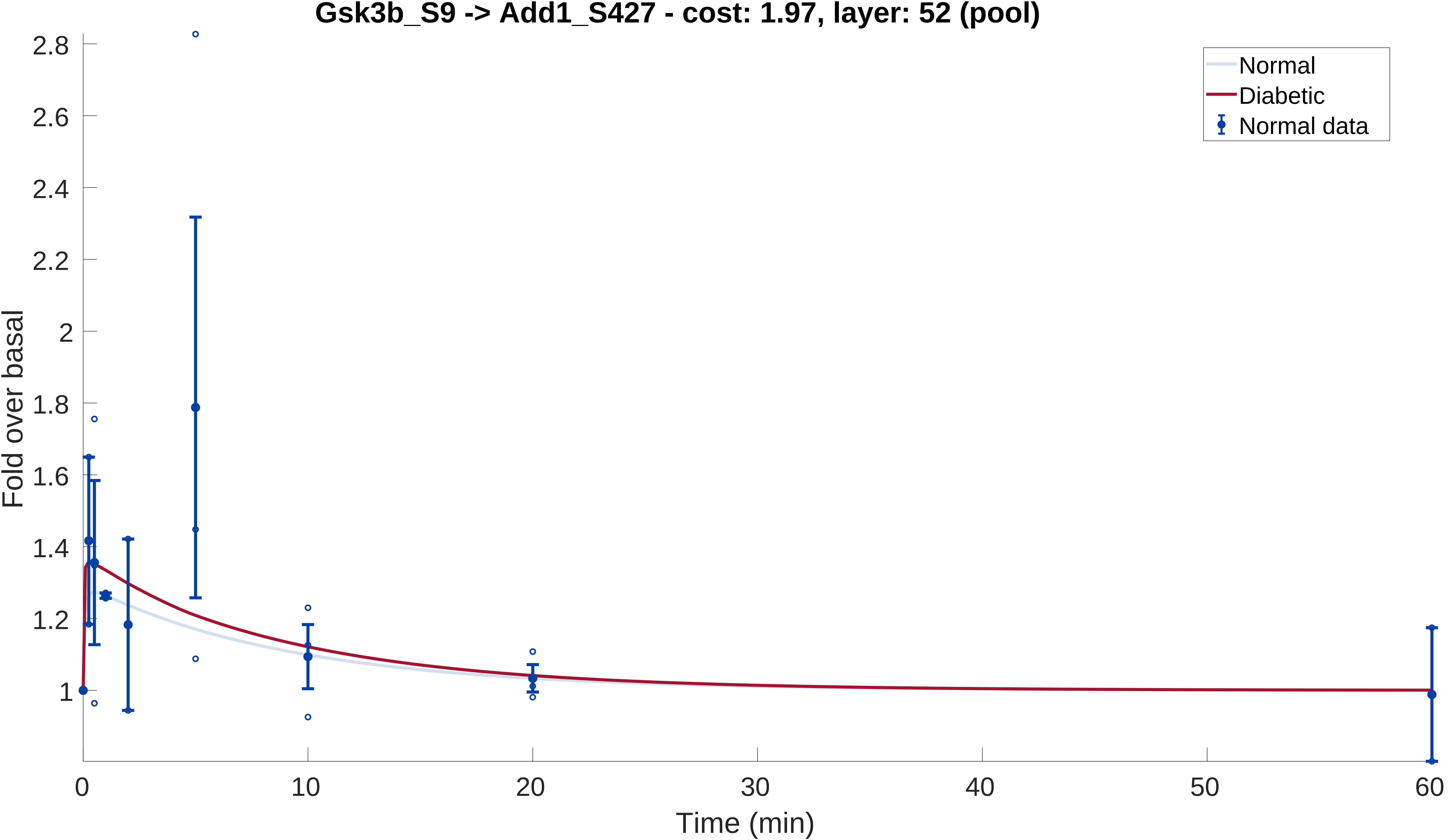
Gsk3b_S9 -> Add1_S427 - cost: 1.97, layer: 52 (pool)
Normal
Diabetic
Normal data
Fold over basal
Time (min)
2.8
2.6
2.4
2.2
2
1.8
1.6
1.4
1.2
1
0
10
20
30
40
50
60

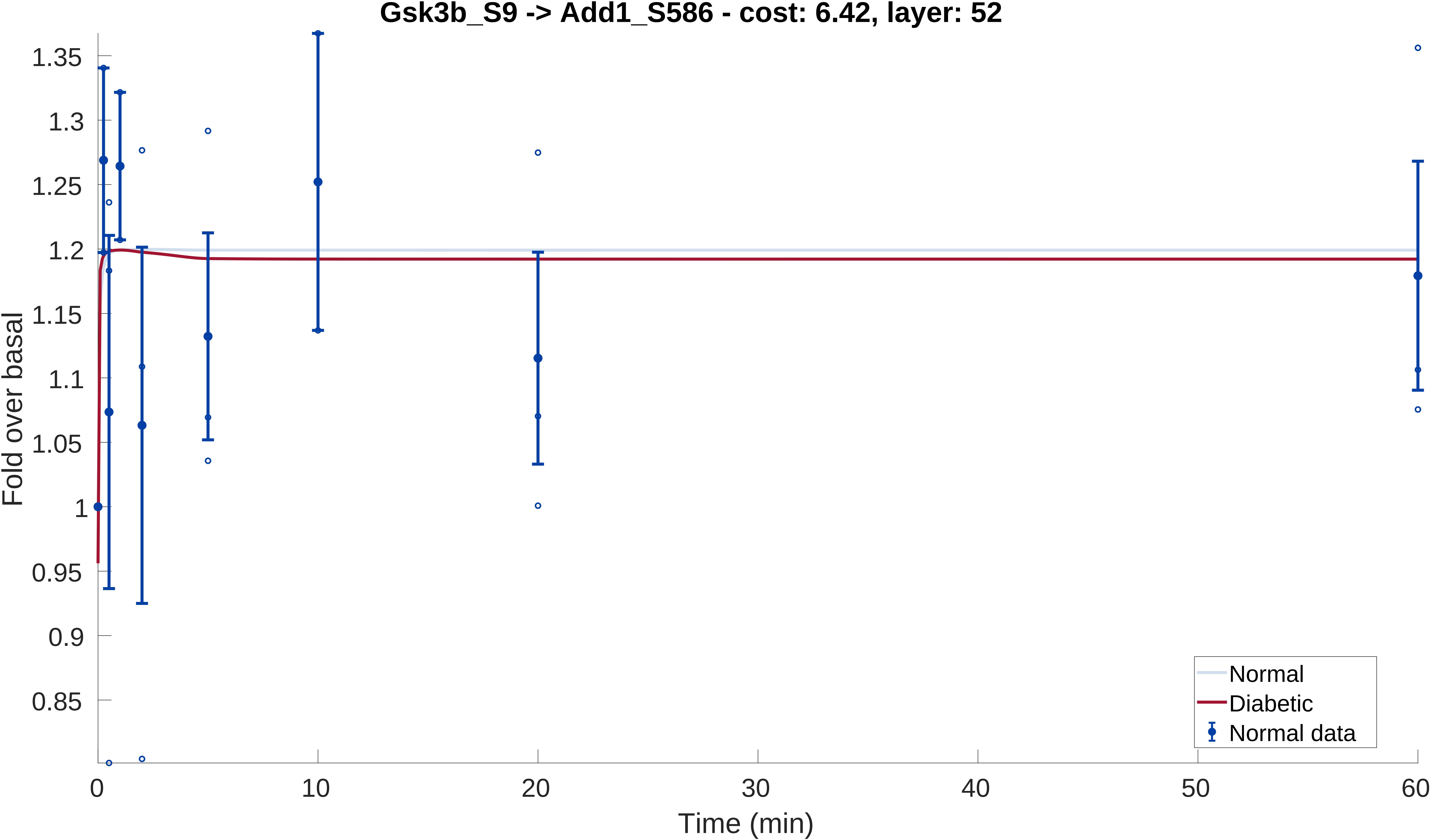

Gsk3b_S9 -> Add1_S586 - cost: 6.42, layer: 52
Fold over basal
1.35
1.3
1.25
1.2
1.15
1.1
1.05
1
0.95
0.9
0.85
0
10
20
30
40
50
60
Time (min)
Normal
Diabetic
Normal data

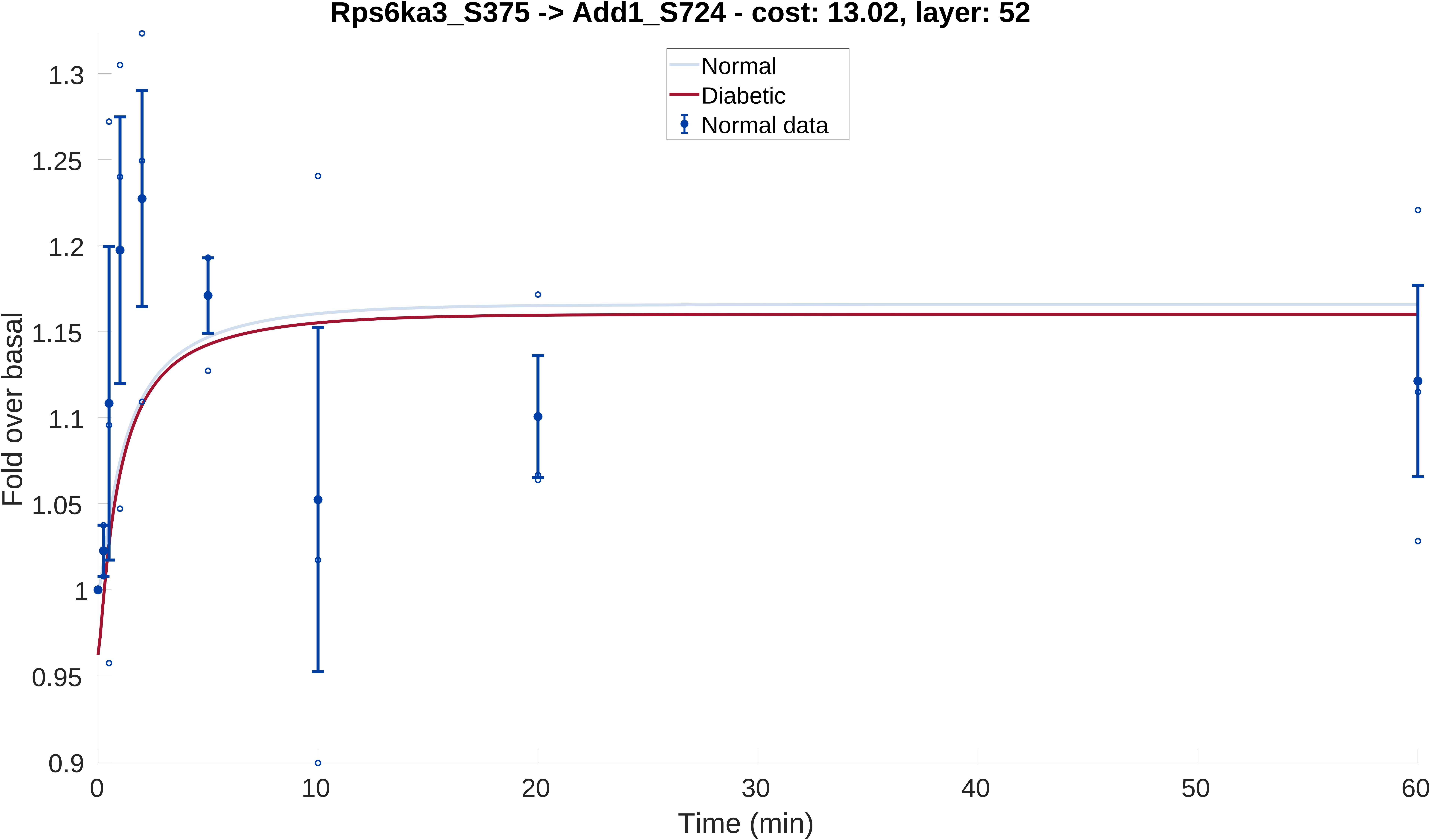

Rps6ka3_S375 -> Add1_S724 - cost: 13.02, layer: 52
Normal
Diabetic
Normal data
Fold over basal
1.3
1.25
1.2
1.15
1.1
1.05
1
0.95
0.9
0
10
20
30
40
50
60
Time (min)

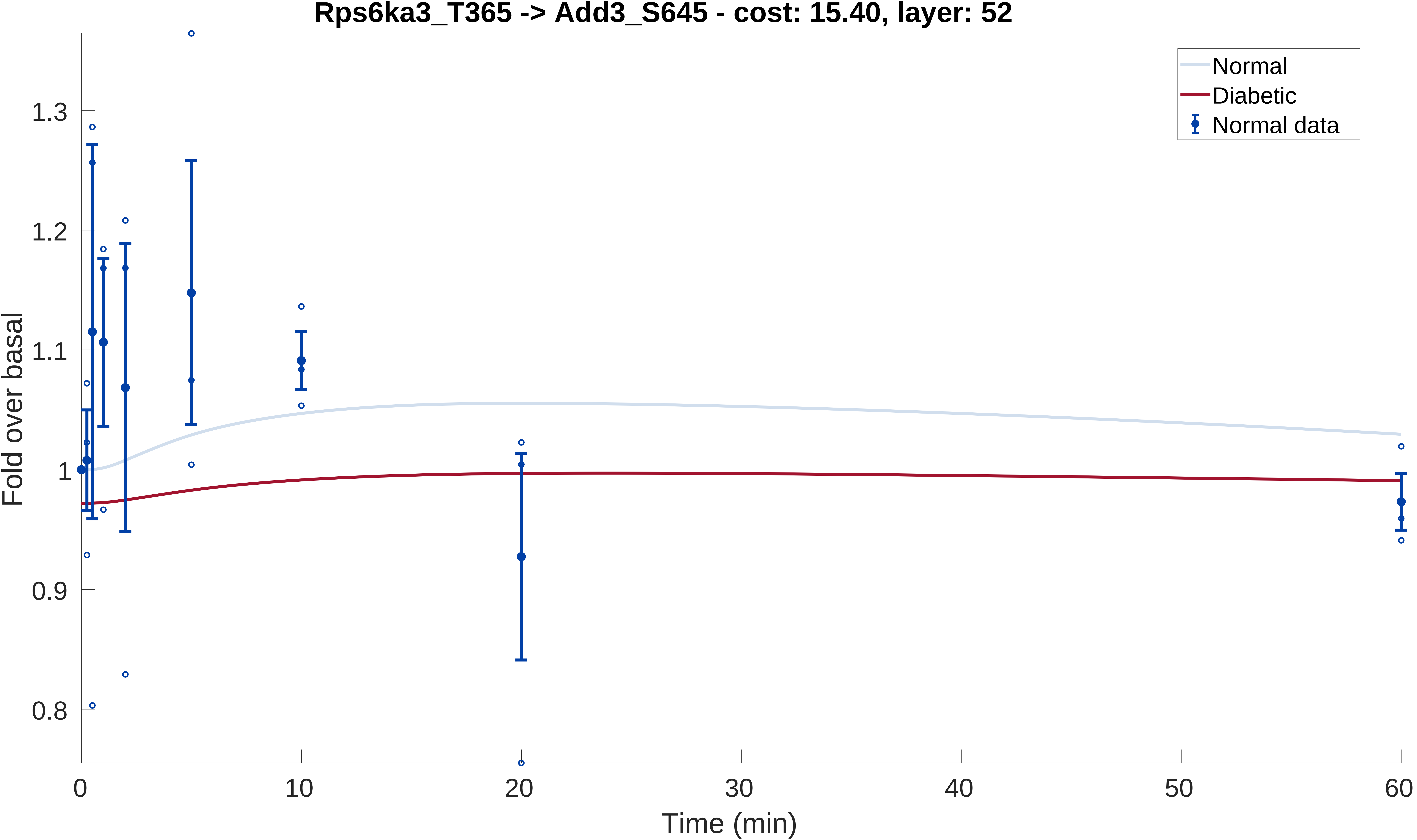
Rps6ka3_T365 -> Add3_S645 - cost: 15.40, layer: 52
Normal
Diabetic
Normal data
Fold over basal
Time (min)
0.8
0.9
1
1.1
1.2
1.3
0
10
20
30
40
50
60

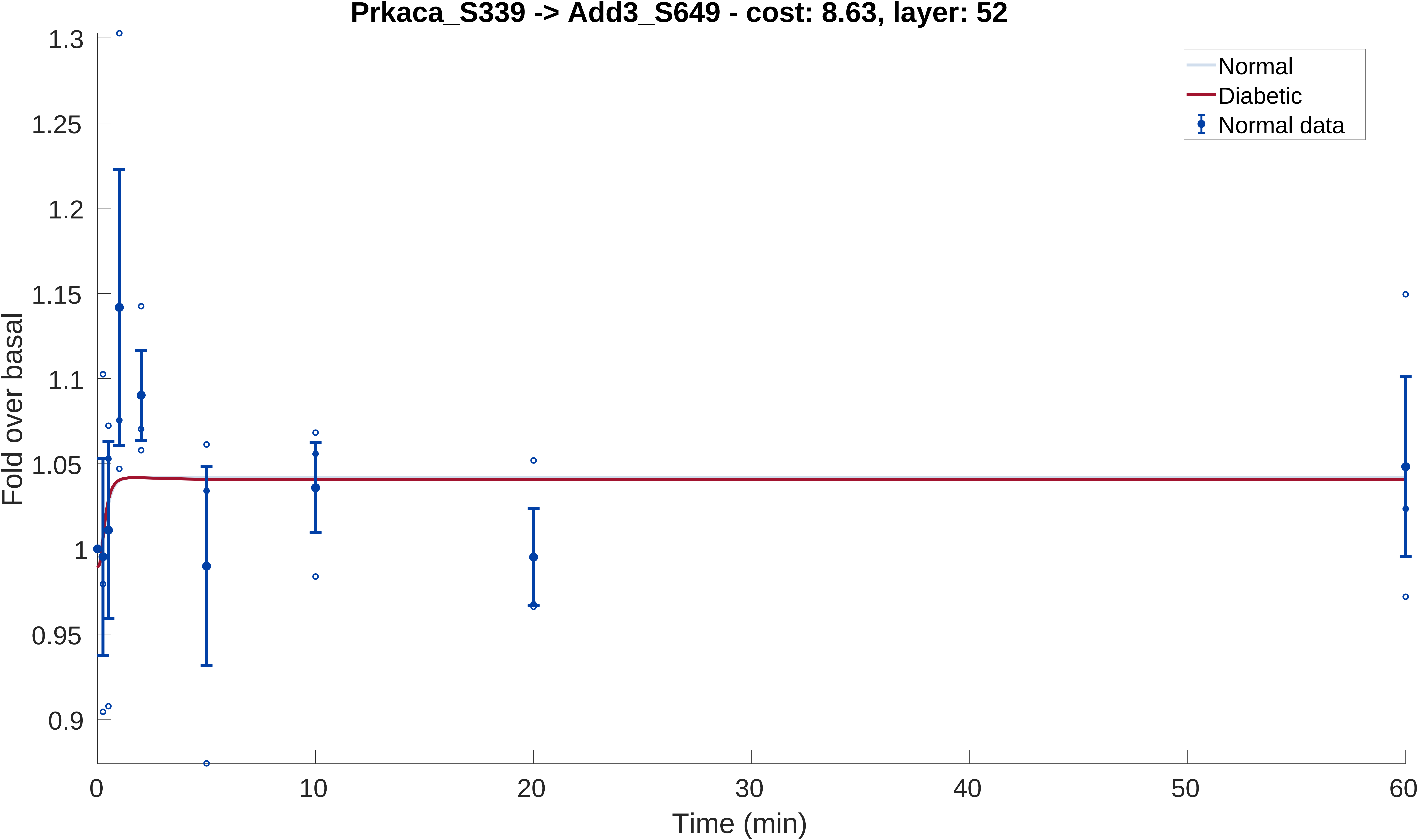
Prkaca_S339 -> Add3_S649 - cost: 8.63, layer: 52
Normal
Diabetic
Normal data
Fold over basal
Time (min)
1.3
1.25
1.2
1.15
1.1
1.05
1
0.95
0.9
0
10
20
30
40
50
60

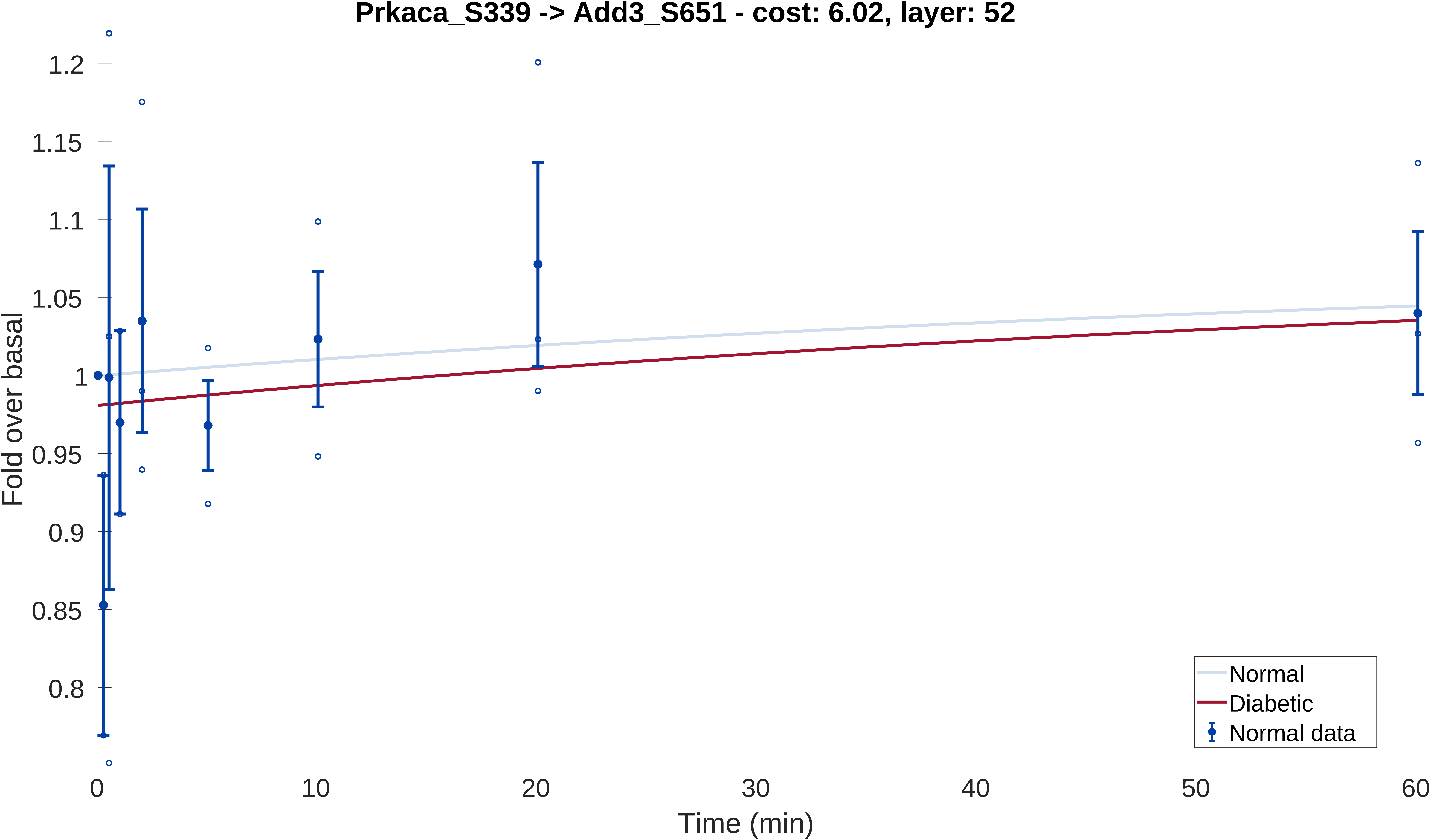

Prkaca_S339 -> Add3_S651 - cost: 6.02, layer: 52
Fold over basal
Time (min)
1.2
1.15
1.1
1.05
1
0.95
0.9
0.85
0.8
0
10
20
30
40
50
60
Normal
Diabetic
Normal data

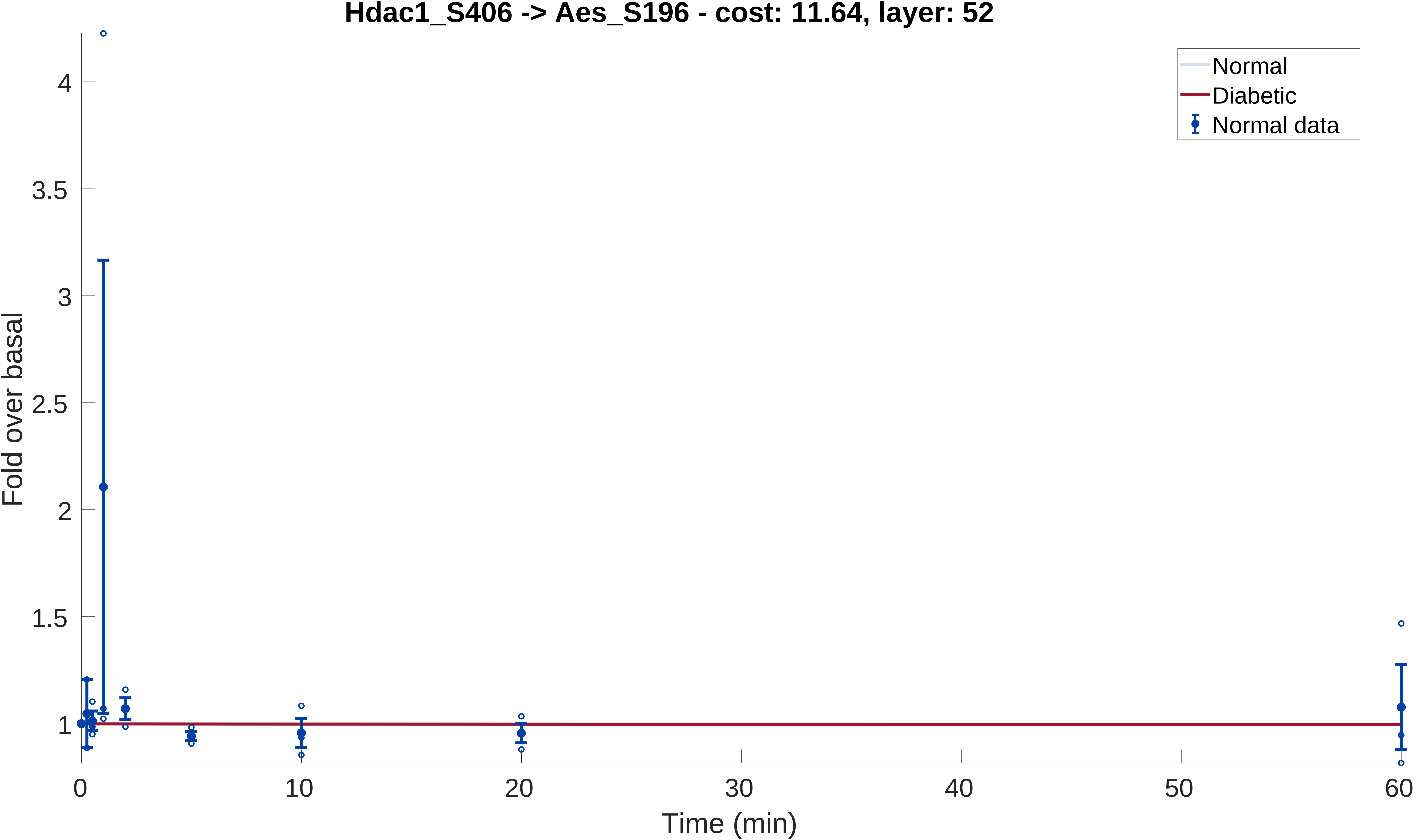
Hdac1_S406 -> Aes_S196 - cost: 11.64, layer: 52
Fold over basal
4
3.5
3
2.5
2
1.5
1
0
10
20
30
40
50
60
Time (min)
Normal
Diabetic
Normal data

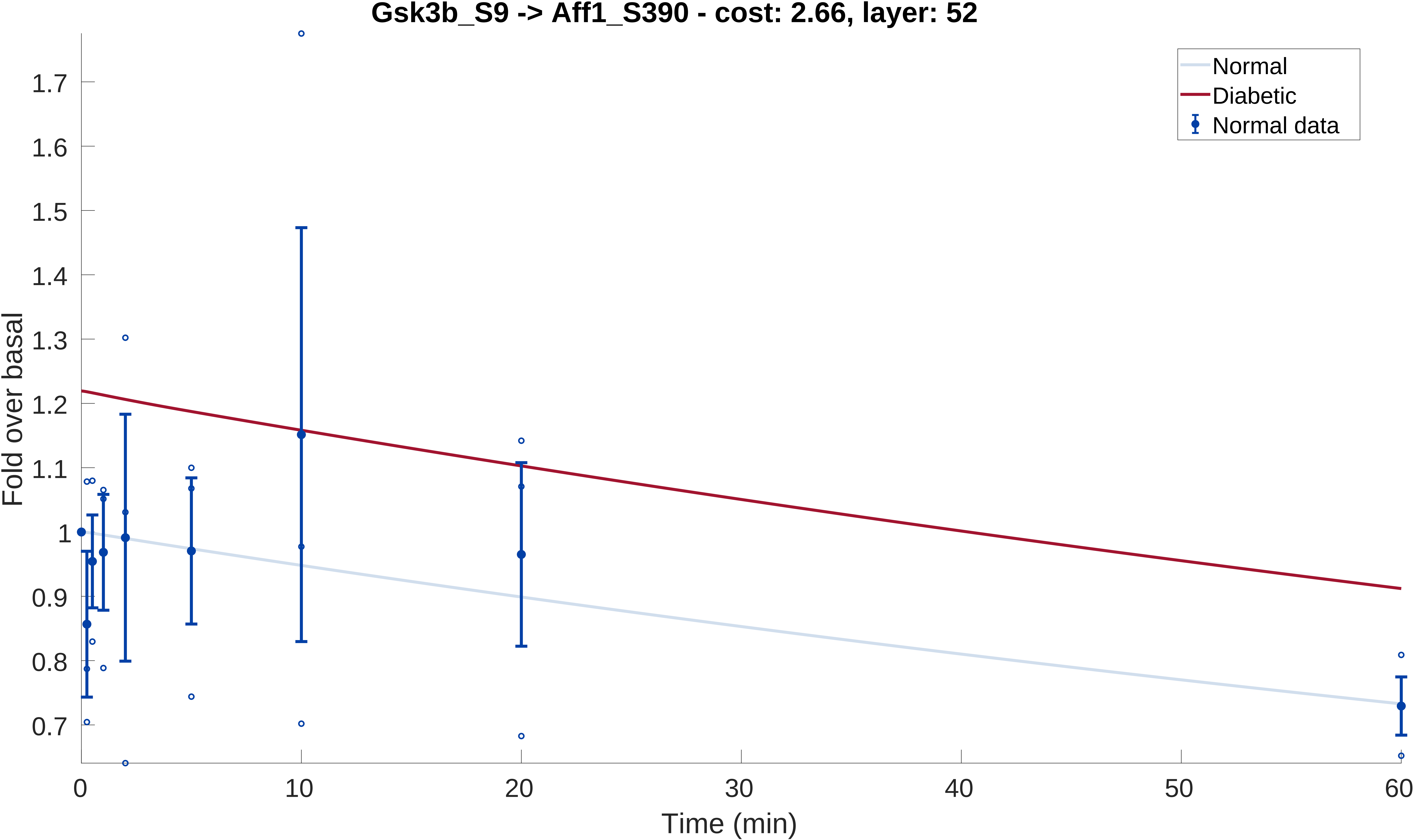

Gsk3b_S9 -> Aff1_S390 - cost: 2.66, layer: 52
Normal
Diabetic
Normal data
Fold over basal
Time (min)
1.7
1.6
1.5
1.4
1.3
1.2
1.1
1
0.9
0.8
0.7
0
10
20
30
40
50
60

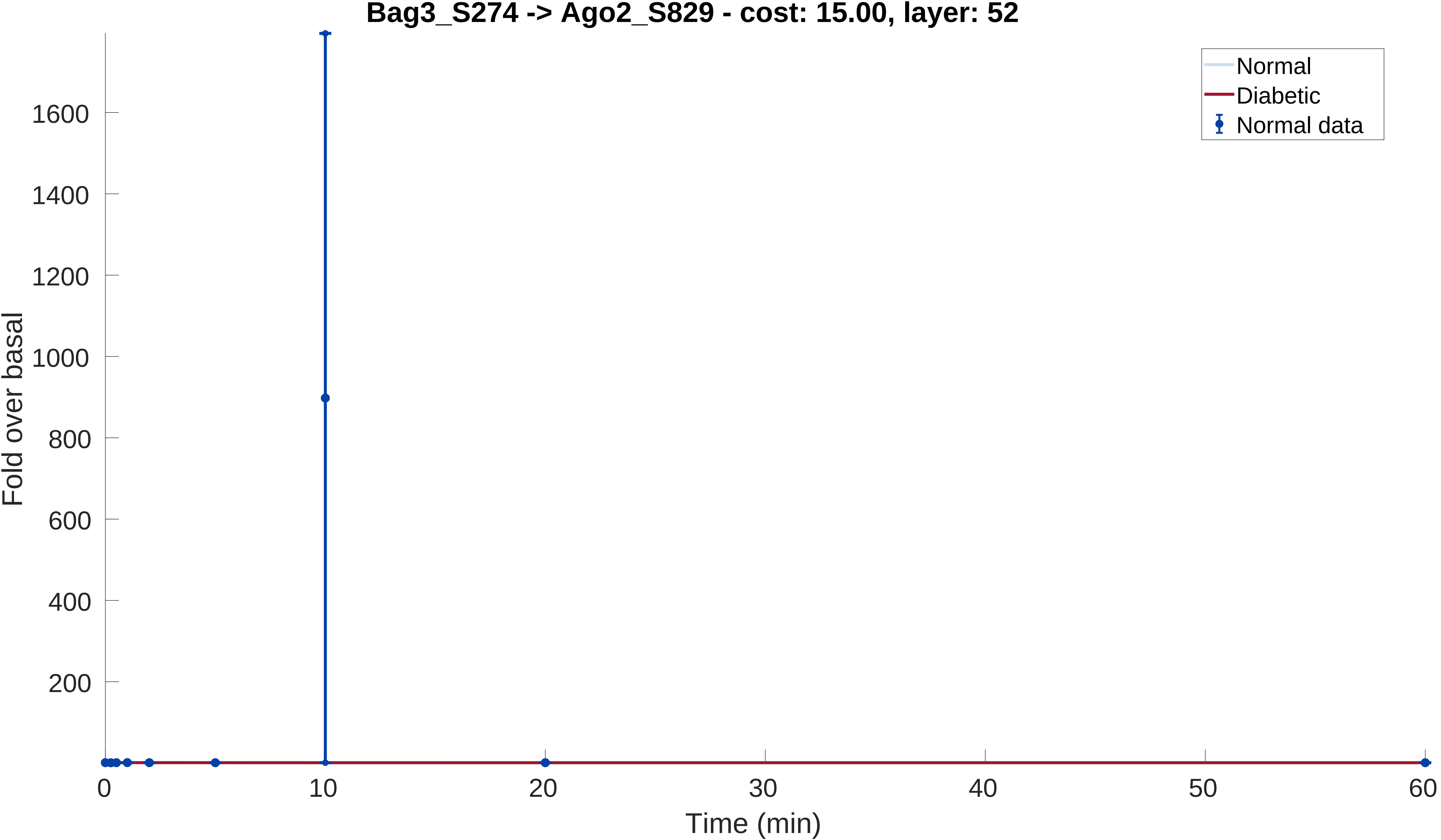
Bag3_S274 -> Ago2_S829 - cost: 15.00, layer: 52
Fold over basal
1600
1400
1200
1000
800
600
400
200
0
10
20
30
40
50
60
Time (min)
Normal
Diabetic
Normal data

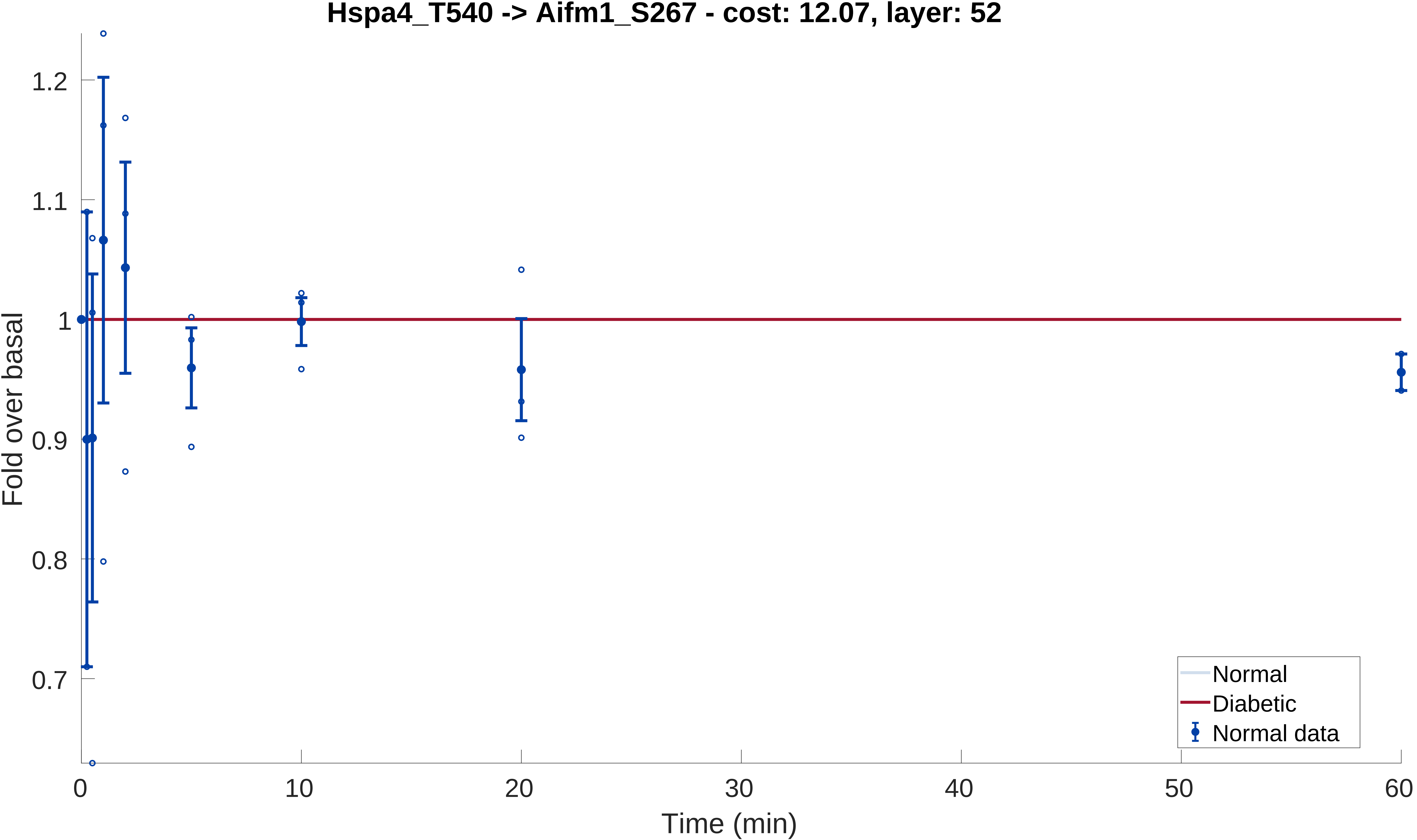

Hspa4_T540 -> Aifm1_S267 - cost: 12.07, layer: 52
Fold over basal
1.2
1.1
1
0.9
0.8
0.7
0
10
20
30
40
50
60
Time (min)
Normal
Diabetic
Normal data

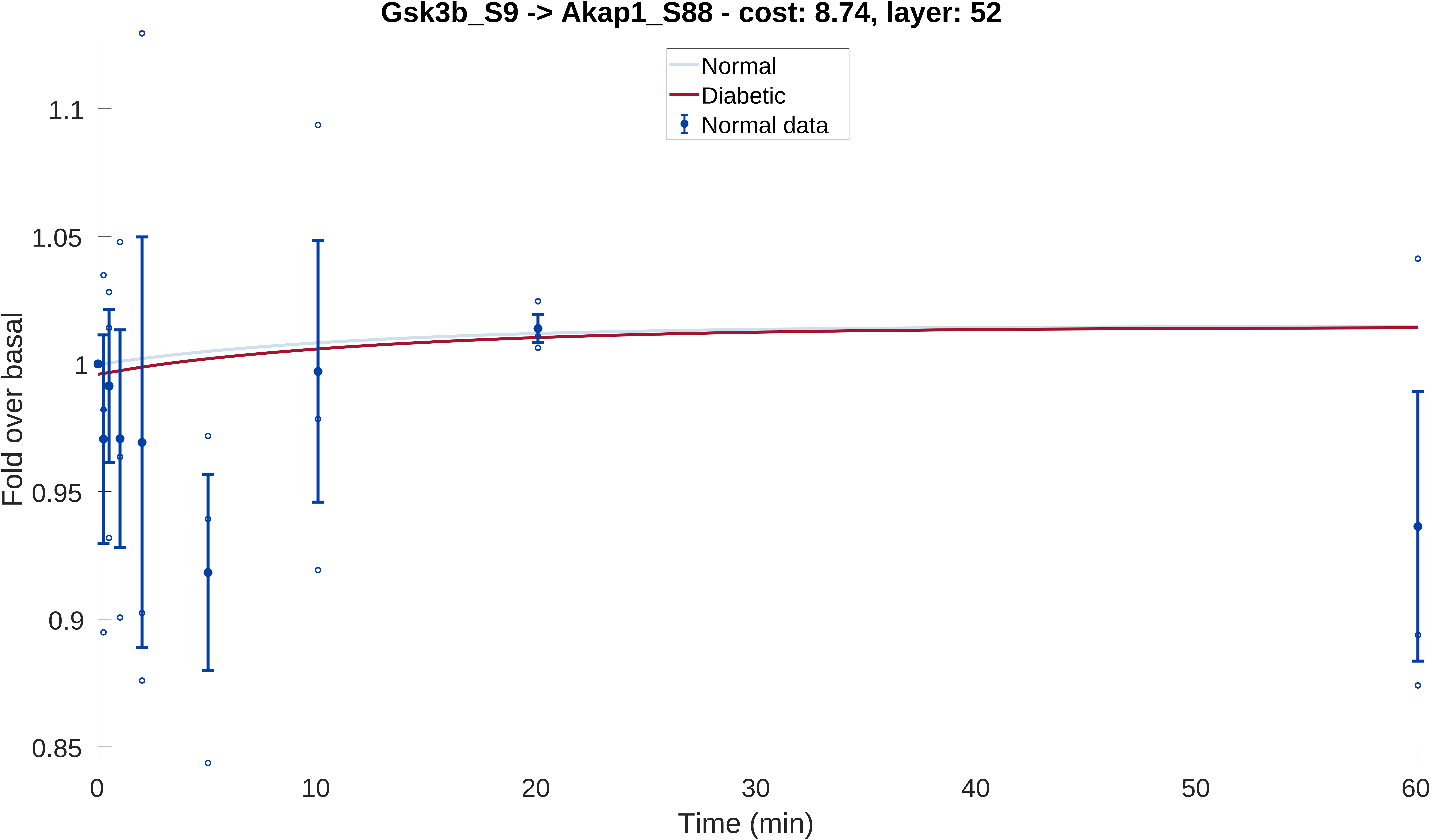
Gsk3b_S9 -> Akap1_S88 - cost: 8.74, layer: 52
Normal
Diabetic
Normal data
1.1
1.05
1
0.95
0.9
0.85
Fold over basal
0
10
20
30
40
50
60
Time (min)

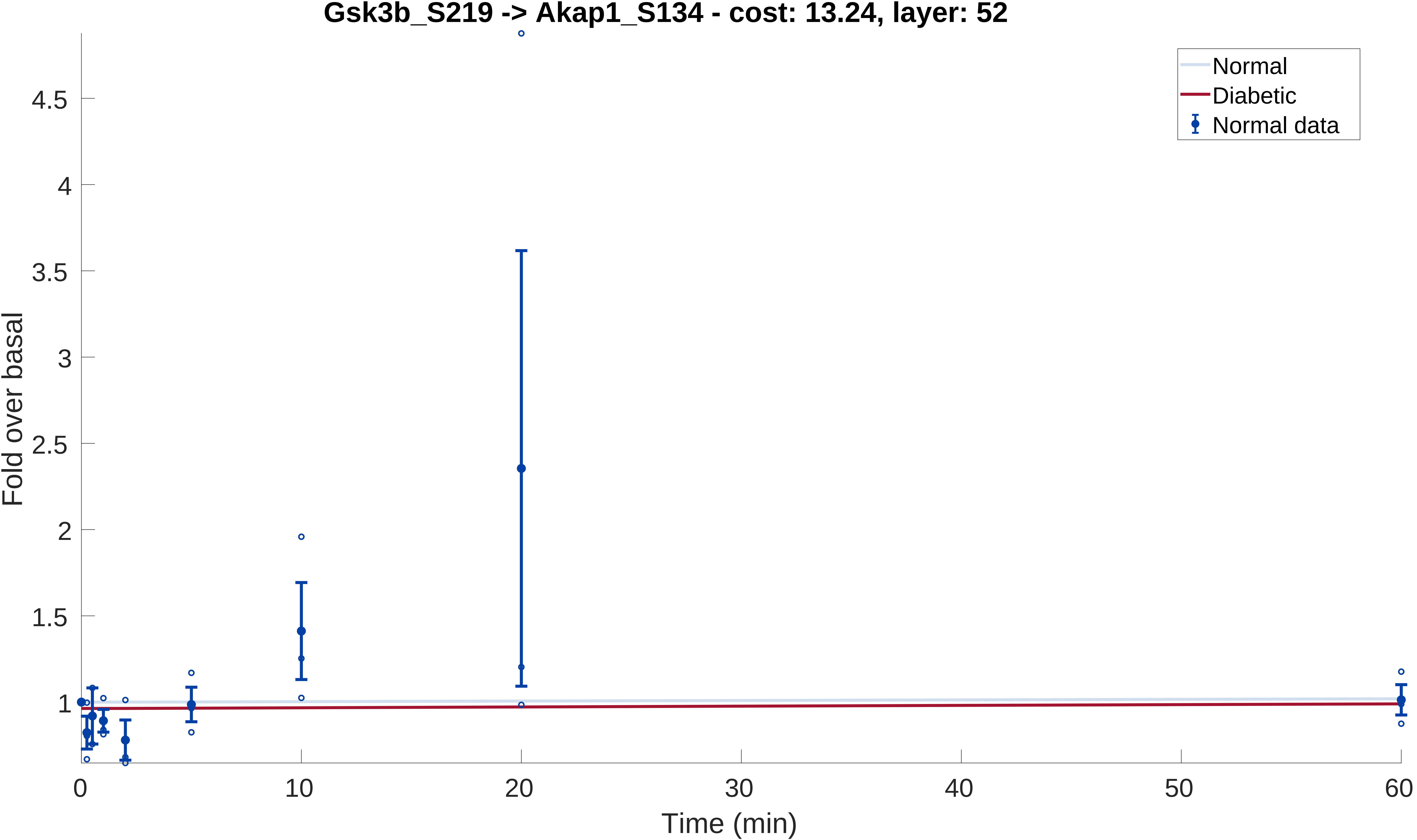
Gsk3b_S219 -> Akap1_S134 - cost: 13.24, layer: 52
Normal
Diabetic
Normal data
Fold over basal
4.5
4
3.5
3
2.5
2
1.5
1
0
10
20
30
40
50
60
Time (min)

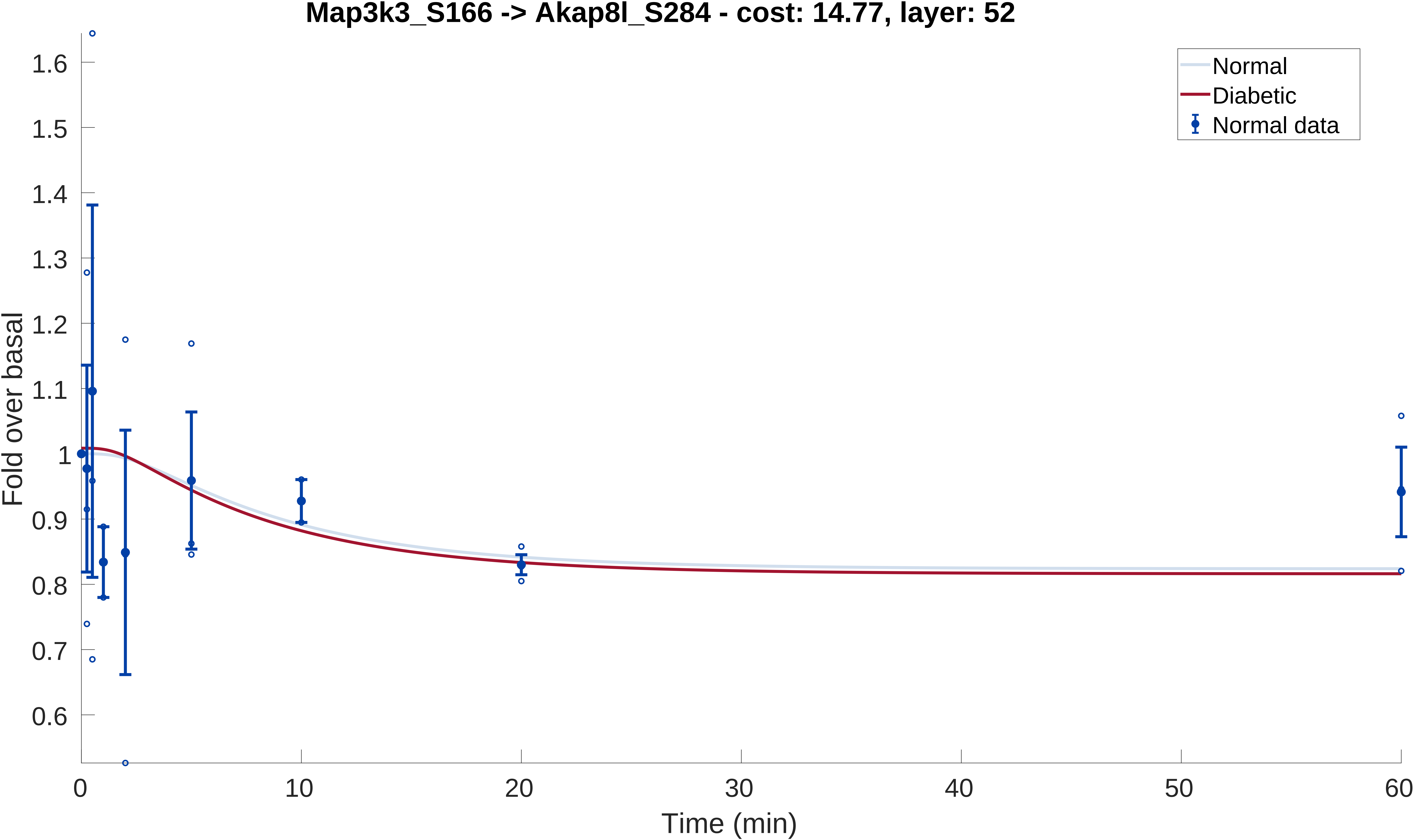

Map3k3_S166 -> Akap8l_S284 - cost: 14.77, layer: 52
Normal
Diabetic
Normal data
Fold over basal
1.6
1.5
1.4
1.3
1.2
1.1
1
0.9
0.8
0.7
0.6
0
10
20
30
40
50
60
Time (min)

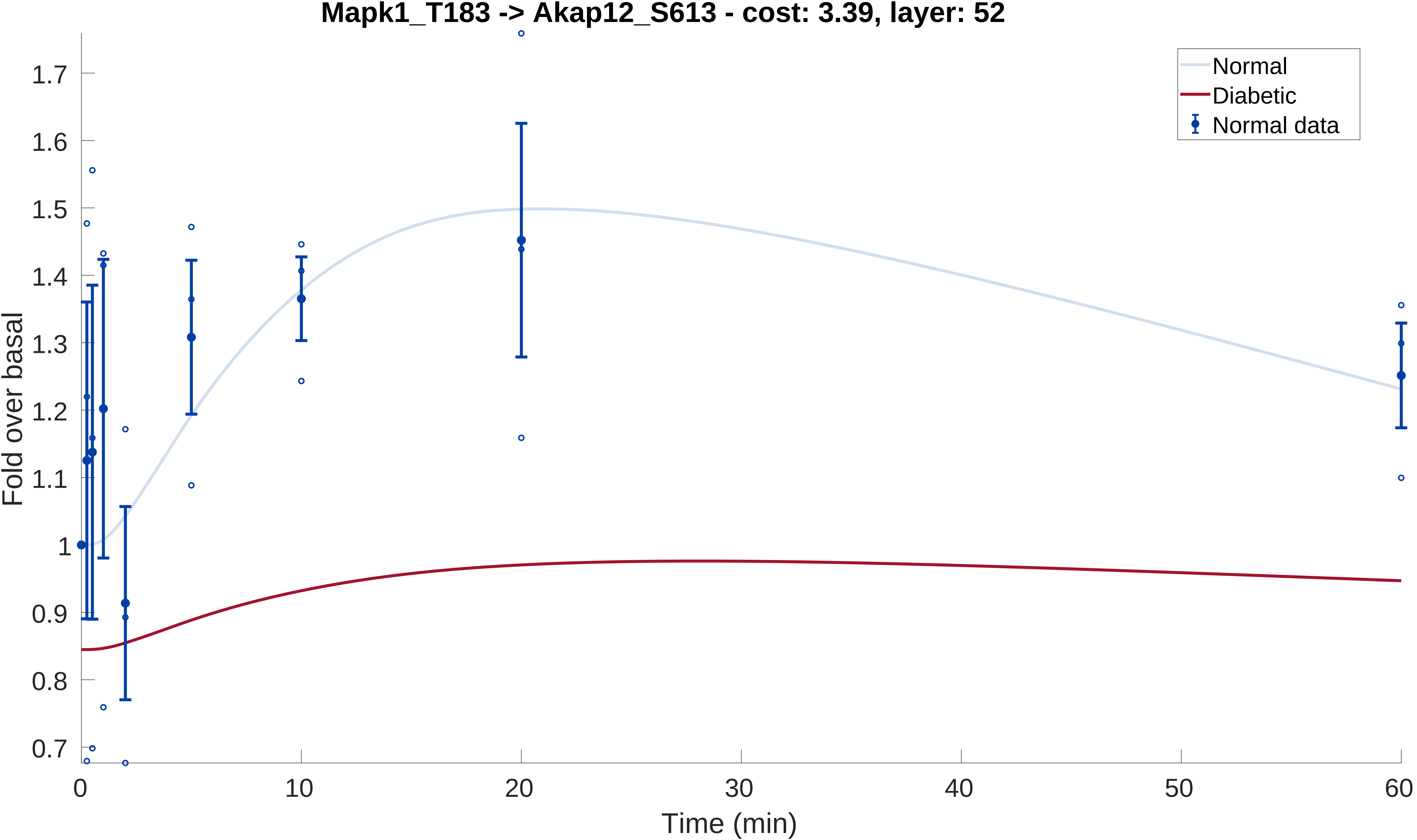
Mapk1_T183 -> Akap12_S613 - cost: 3.39, layer: 52
Normal
Diabetic
Normal data
Fold over basal
Time (min)
0.7
0.8
0.9
1
1.1
1.2
1.3
1.4
1.5
1.6
1.7
0
10
20
30
40
50
60

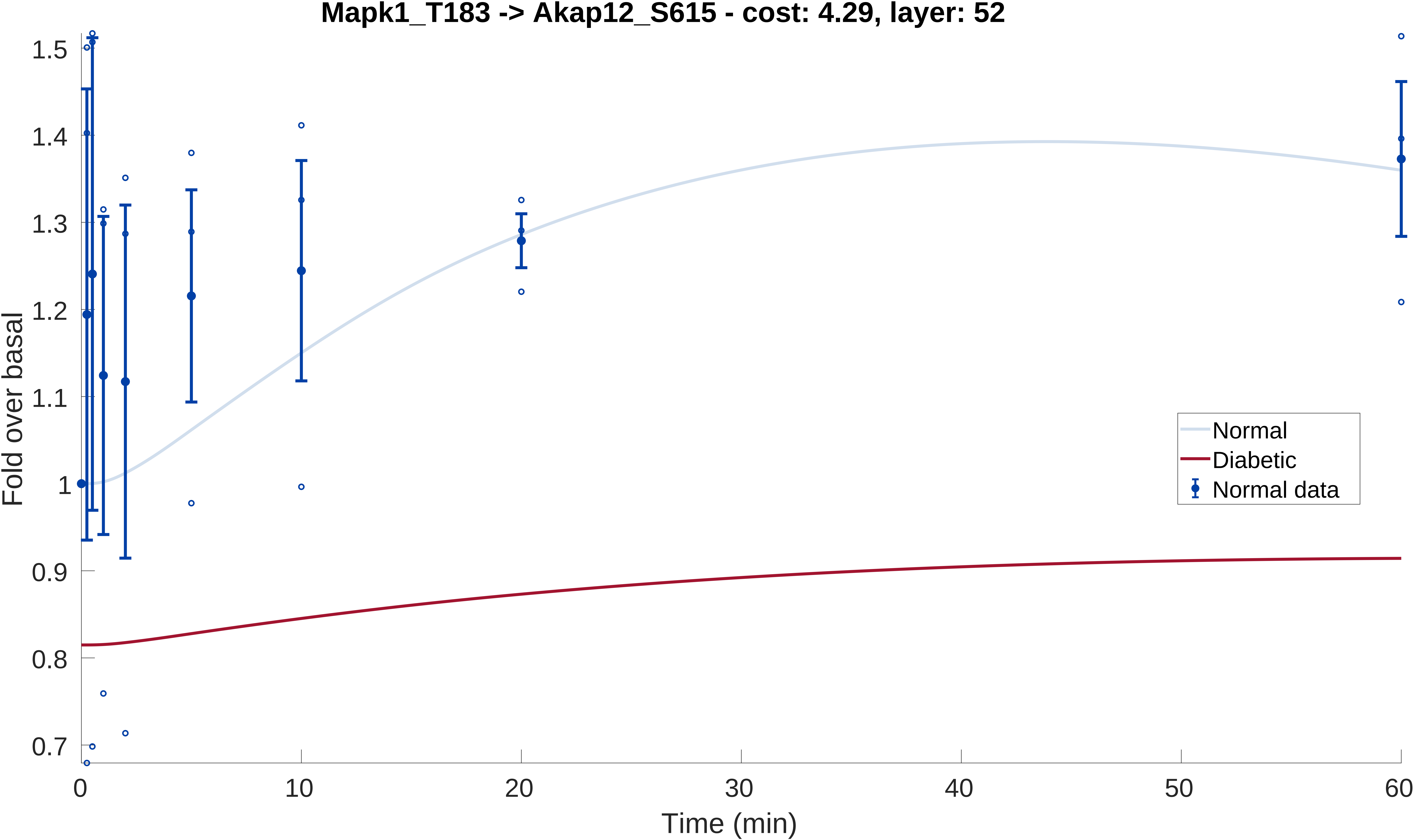
Mapk1_T183 -> Akap12_S615 - cost: 4.29, layer: 52
Fold over basal
1.5
1.4
1.3
1.2
1.1
1
0.9
0.8
0.7
0
10
20
30
40
50
60
Time (min)
Normal
Diabetic
Normal data

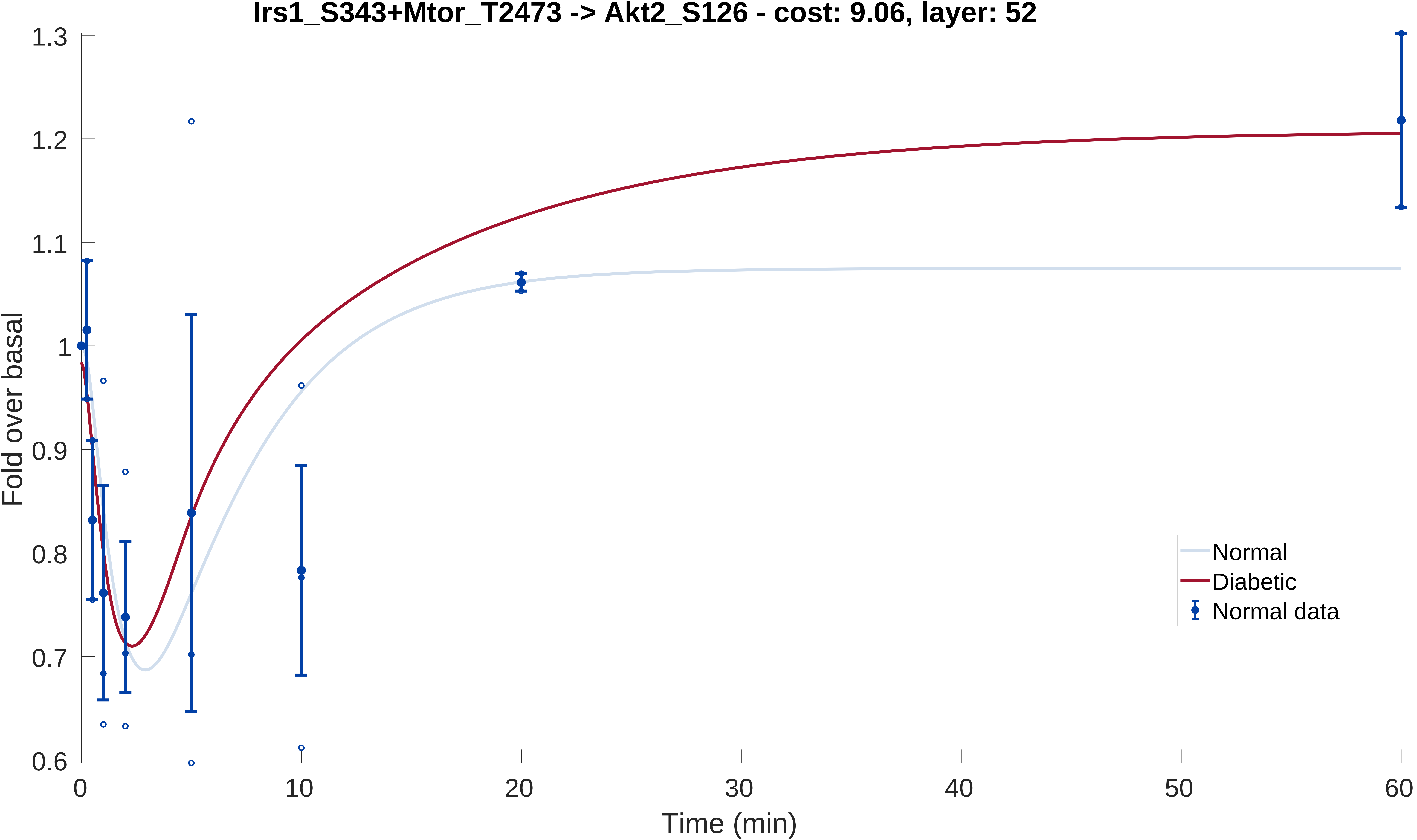

Irs1_S343+Mtor_T2473 -> Akt2_S126 - cost: 9.06, layer: 52
Fold over basal
Time (min)
1.3
1.2
1.1
1
0.9
0.8
0.7
0.6
0
10
20
30
40
50
60
Normal
Diabetic
Normal data

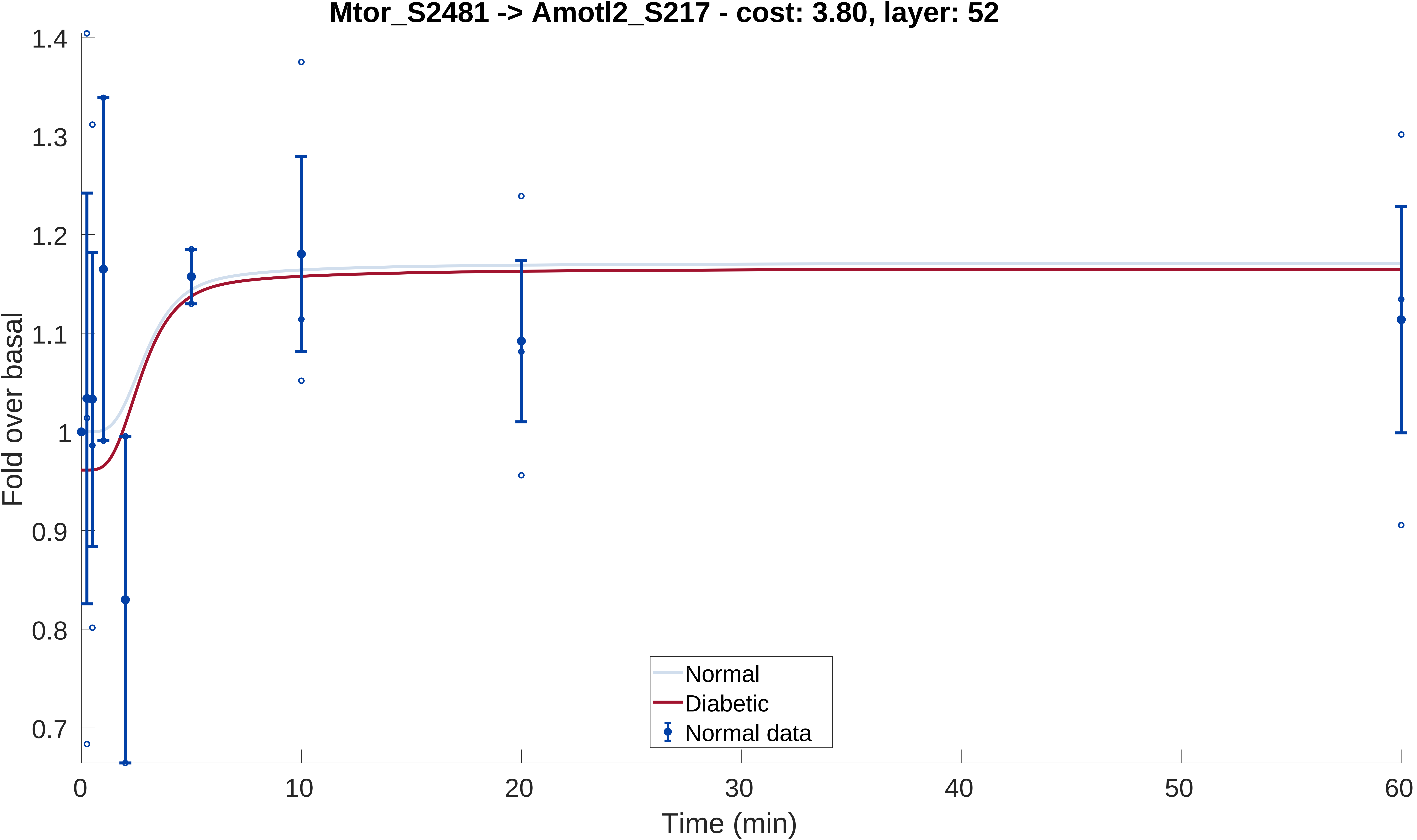

Mtor_S2481 -> Amotl2_S217 - cost: 3.80, layer: 52
Fold over basal
1.4
1.3
1.2
1.1
1
0.9
0.8
0.7
Time (min)
0
10
20
30
40
50
60
Normal
Diabetic
Normal data

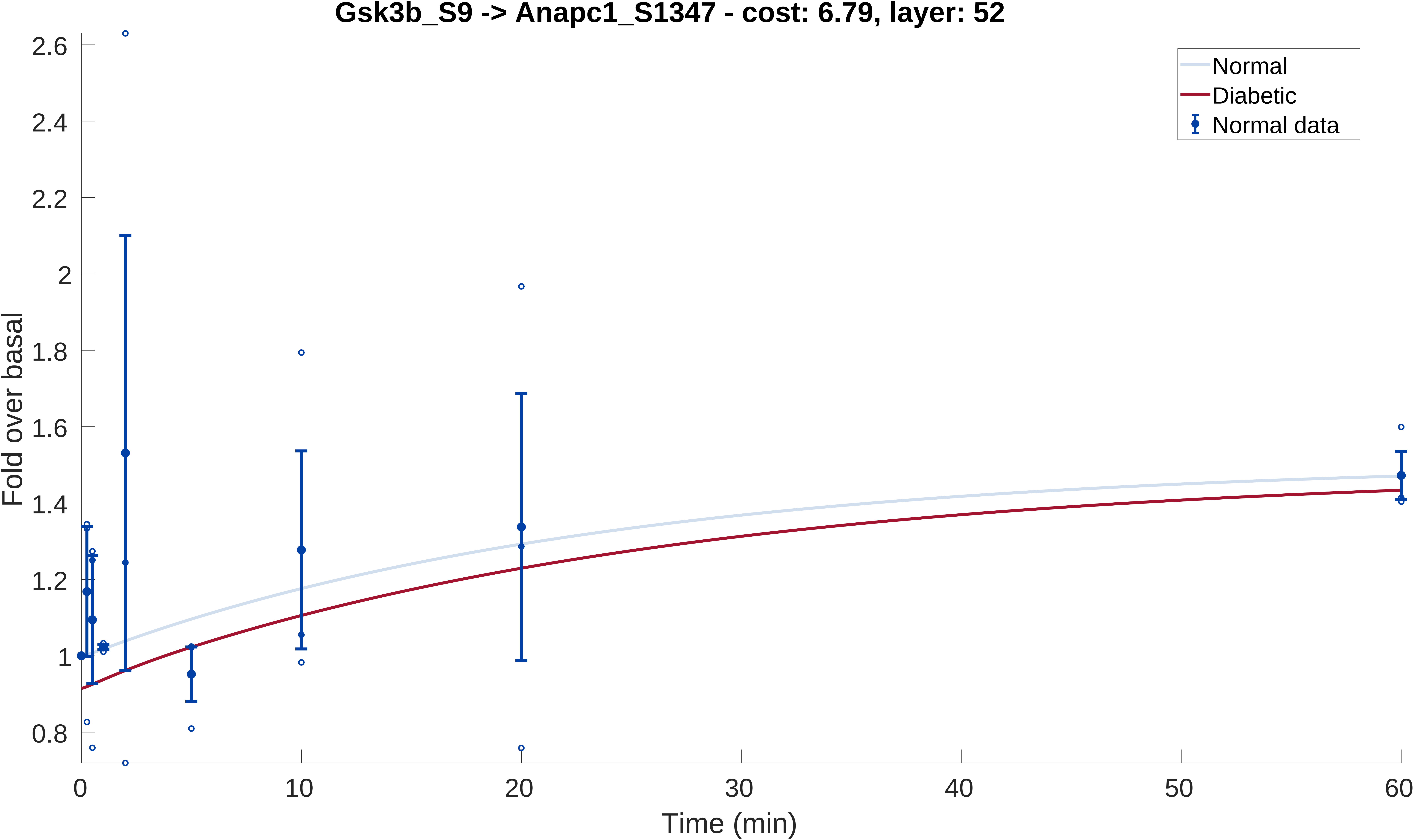

Gsk3b_S9 -> Anapc1_S1347 - cost: 6.79, layer: 52
Fold over basal
Time (min)
Normal
Diabetic
Normal data
2.6
2.4
2.2
2
1.8
1.6
1.4
1.2
1
0.8
0
10
20
30
40
50
60

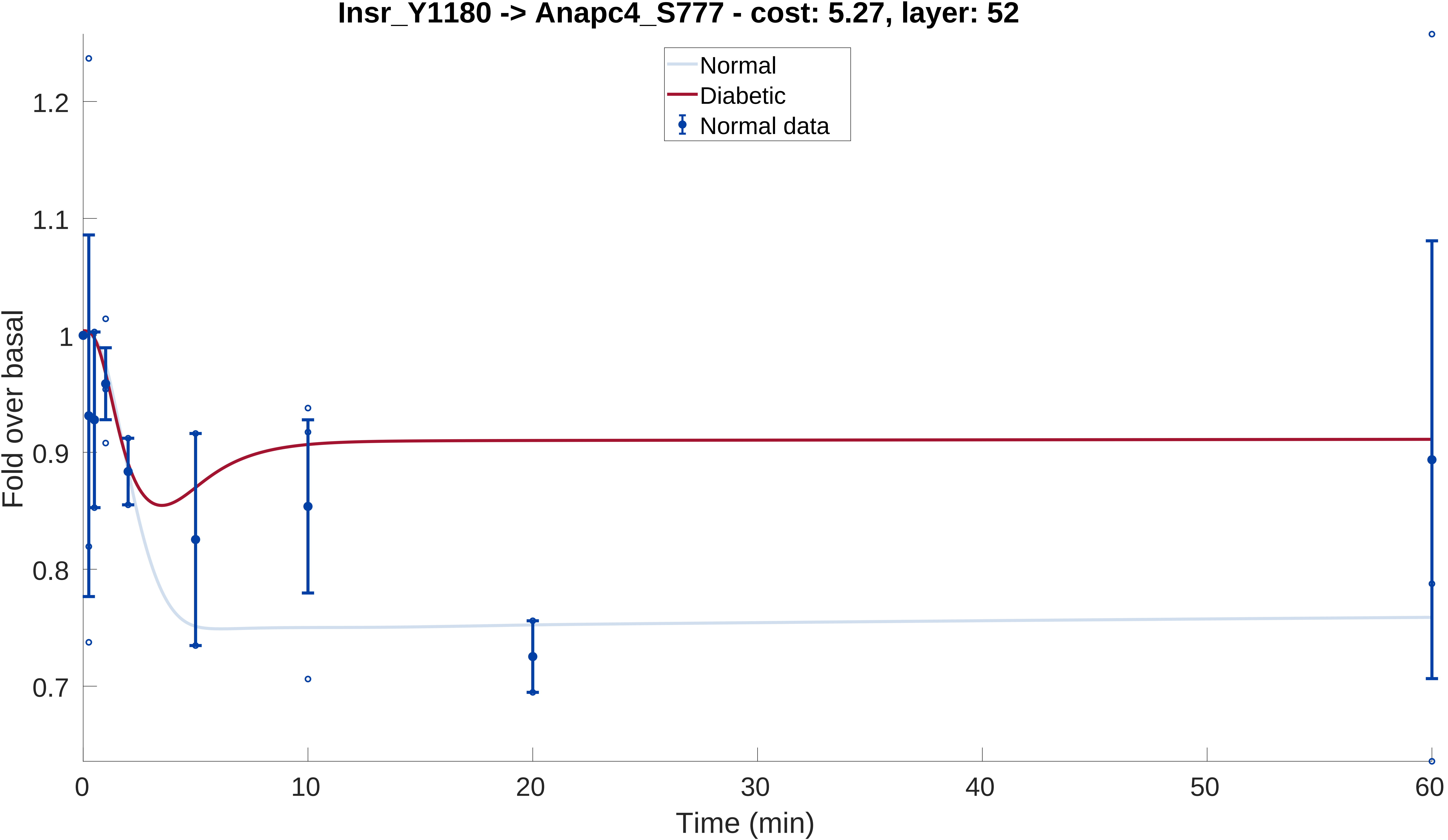
Insr_Y1180 -> Anapc4_S777 - cost: 5.27, layer: 52
Normal
Diabetic
Normal data
Fold over basal
Time (min)
1.2
1.1
1
0.9
0.8
0.7
0
10
20
30
40
50
60

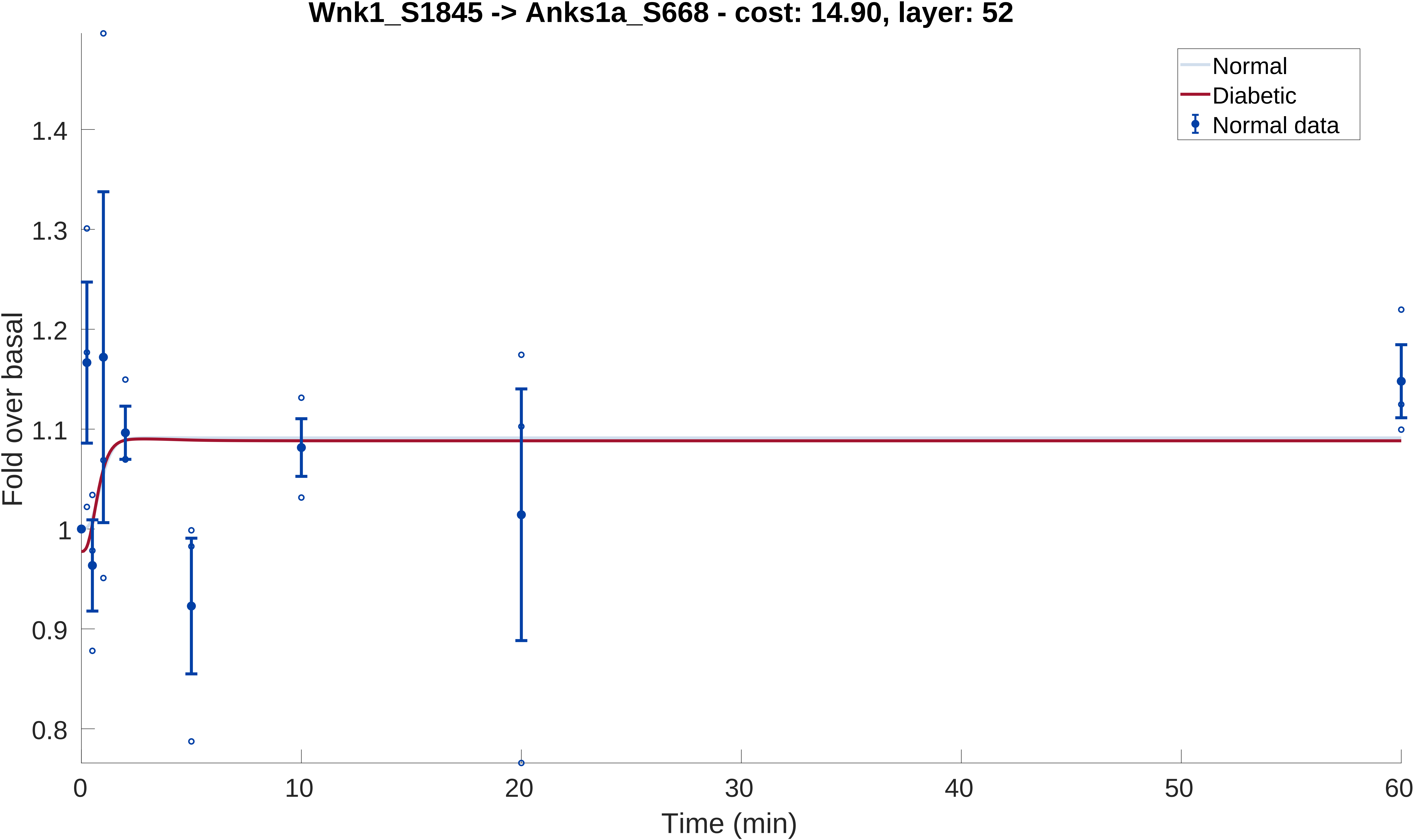
Wnk1_S1845 -> Anks1a_S668 - cost: 14.90, layer: 52
Normal
Diabetic
Normal data
Fold over basal
1.4
1.3
1.2
1.1
1
0.9
0.8
0
10
20
30
40
50
60
Time (min)

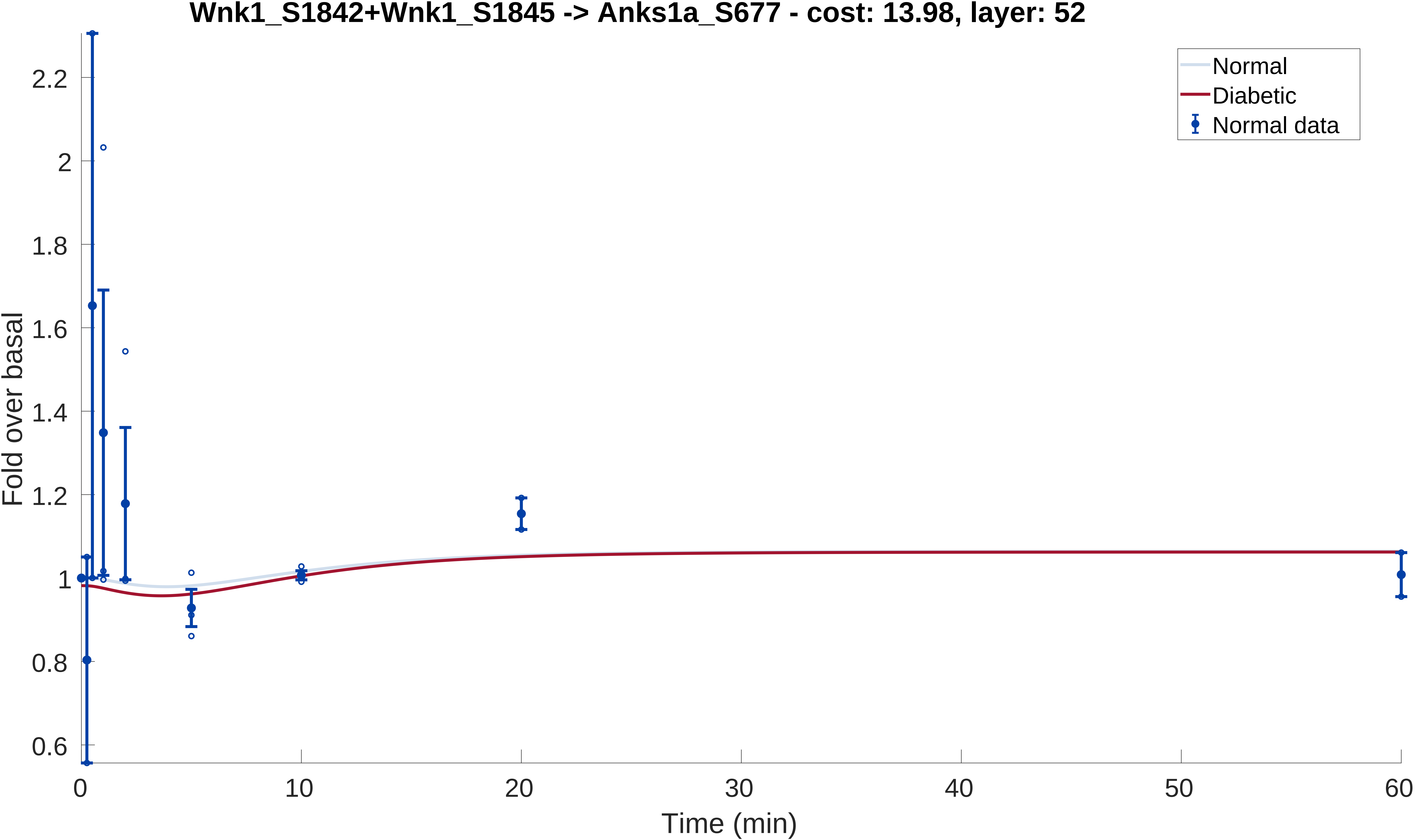

Wnk1_S1842+Wnk1_S1845 -> Anks1a_S677 - cost: 13.98, layer: 52
Normal
Diabetic
Normal data
Fold over basal
2.2
2
1.8
1.6
1.4
1.2
1
0.8
0.6
0
10
20
30
40
50
60
Time (min)

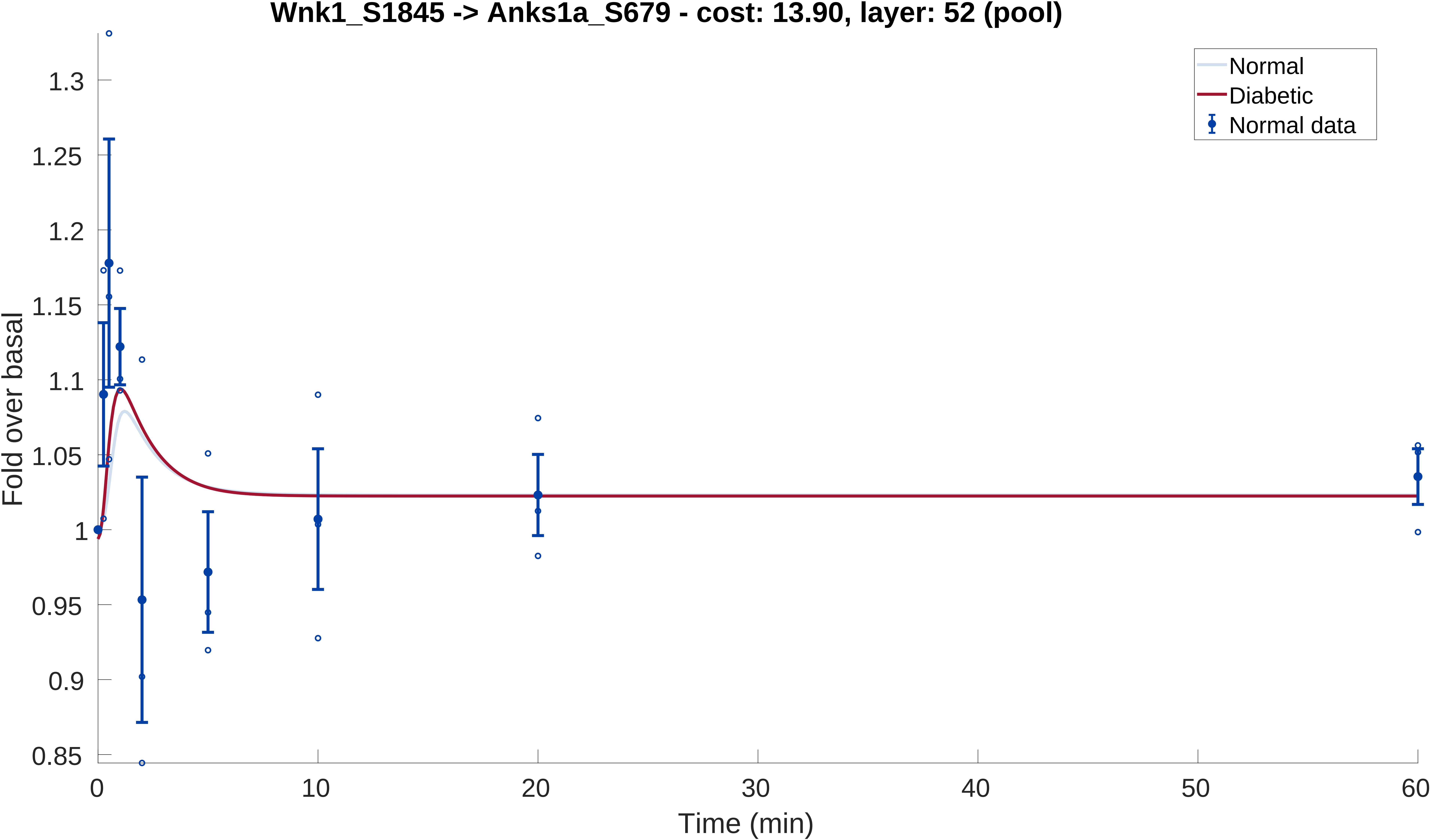

Wnk1_S1845 -> Anks1a_S679 - cost: 13.90, layer: 52 (pool)
Normal
Diabetic
Normal data
Fold over basal
1.3
1.25
1.2
1.15
1.1
1.05
1
0.95
0.9
0.85
0
10
20
30
40
50
60
Time (min)

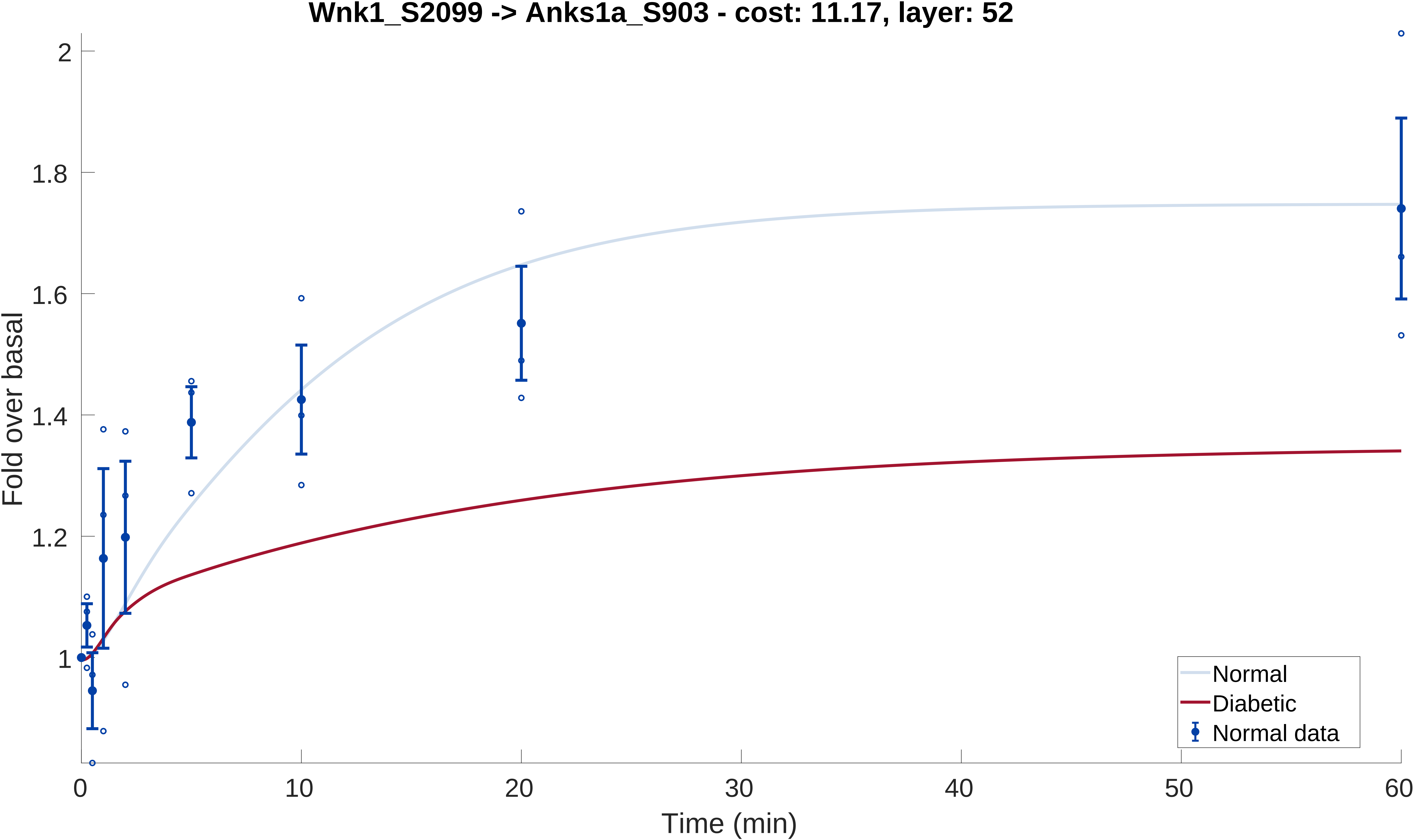
Wnk1_S2099 -> Anks1a_S903 - cost: 11.17, layer: 52
Fold over basal
2
1.8
1.6
1.4
1.2
1
0
10
20
30
40
50
60
Time (min)
Normal
Diabetic
Normal data

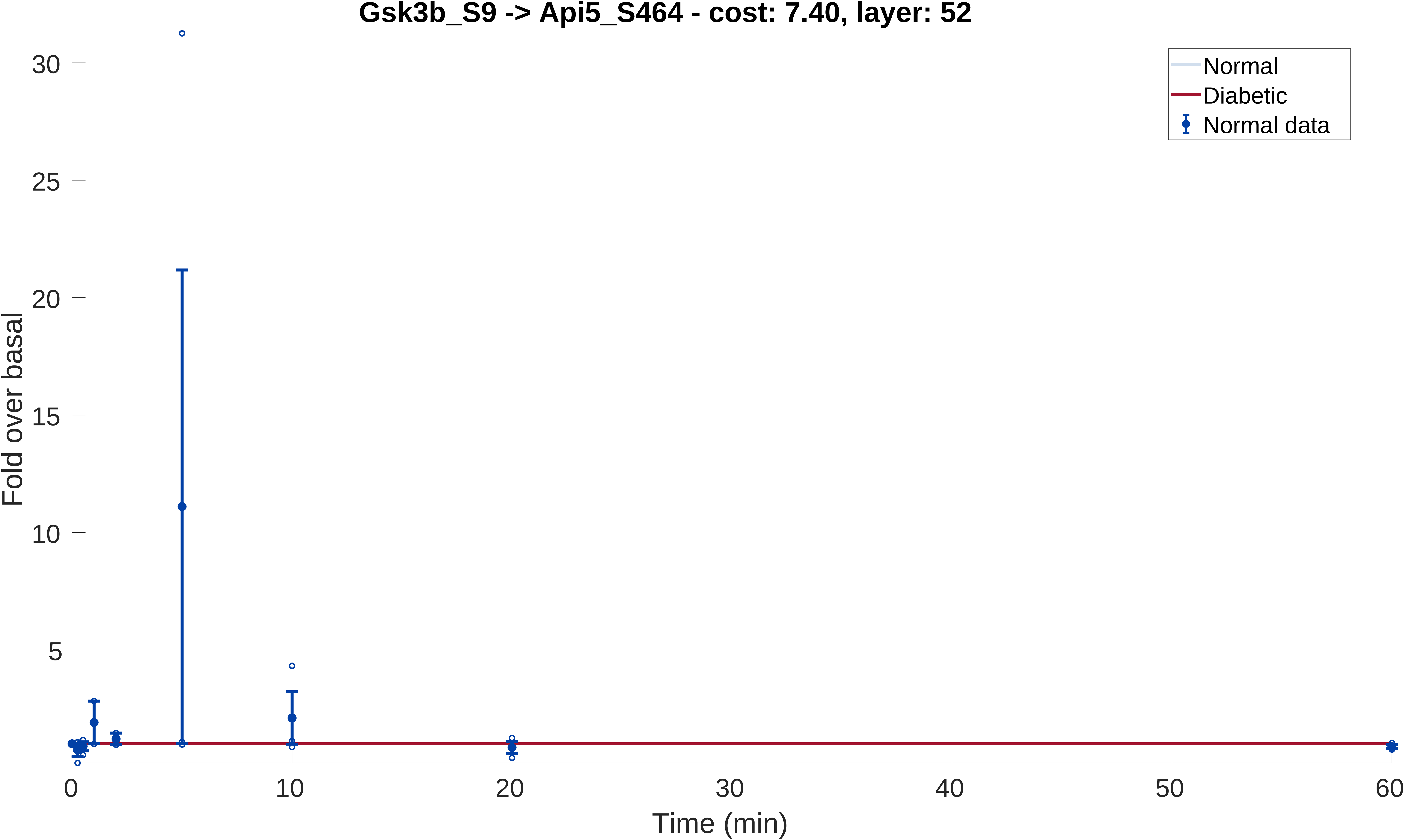

Gsk3b_S9 -> Api5_S464 - cost: 7.40, layer: 52
Fold over basal
Time (min)
Normal
Diabetic
Normal data
0
5
10
15
20
25
30
40
50
60

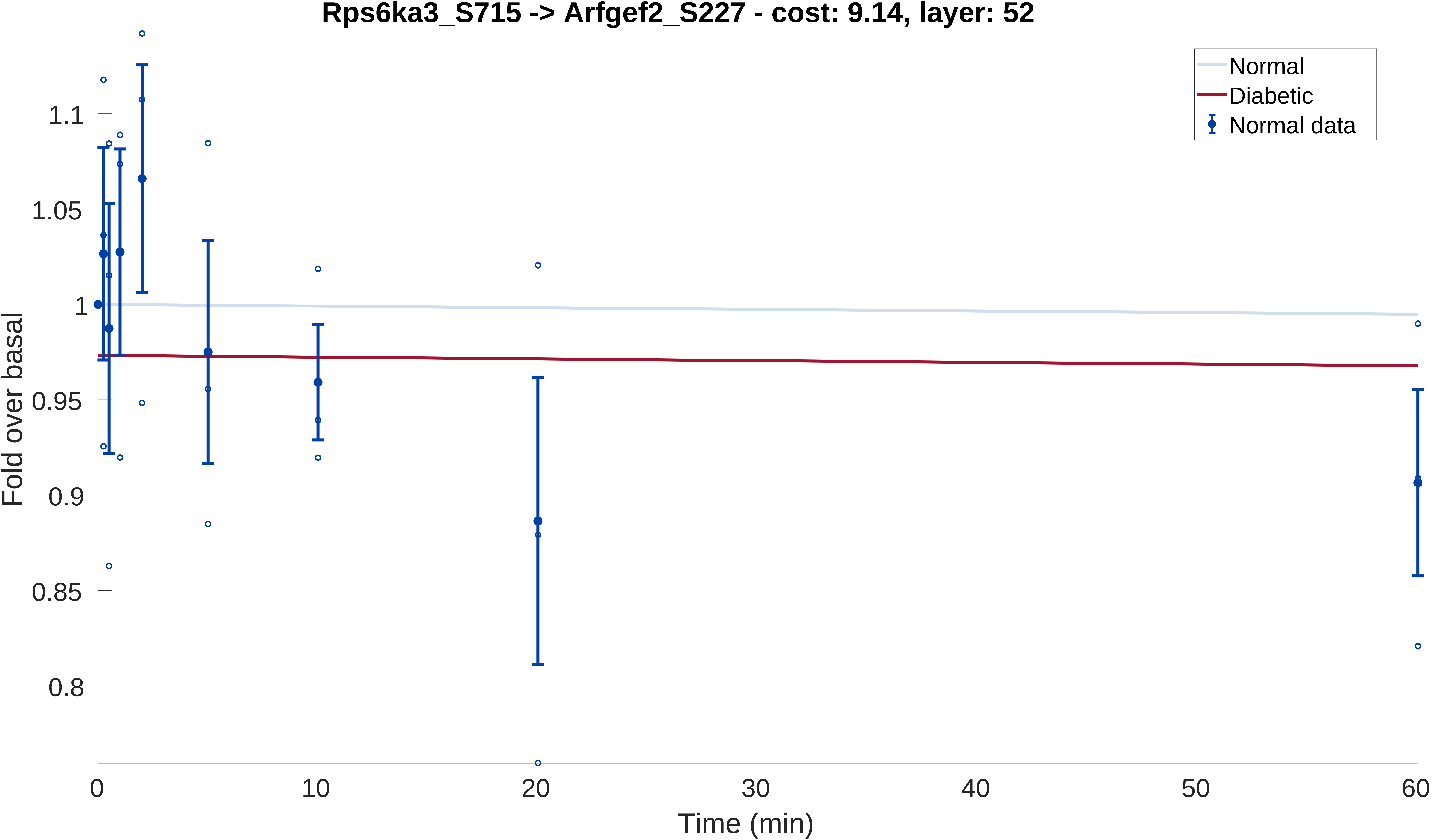

Rps6ka3_S715 -> Arfgef2_S227 - cost: 9.14, layer: 52
Normal
Diabetic
Normal data
Fold over basal
Time (min)
1.1
1.05
1
0.95
0.9
0.85
0.8
0
10
20
30
40
50
60

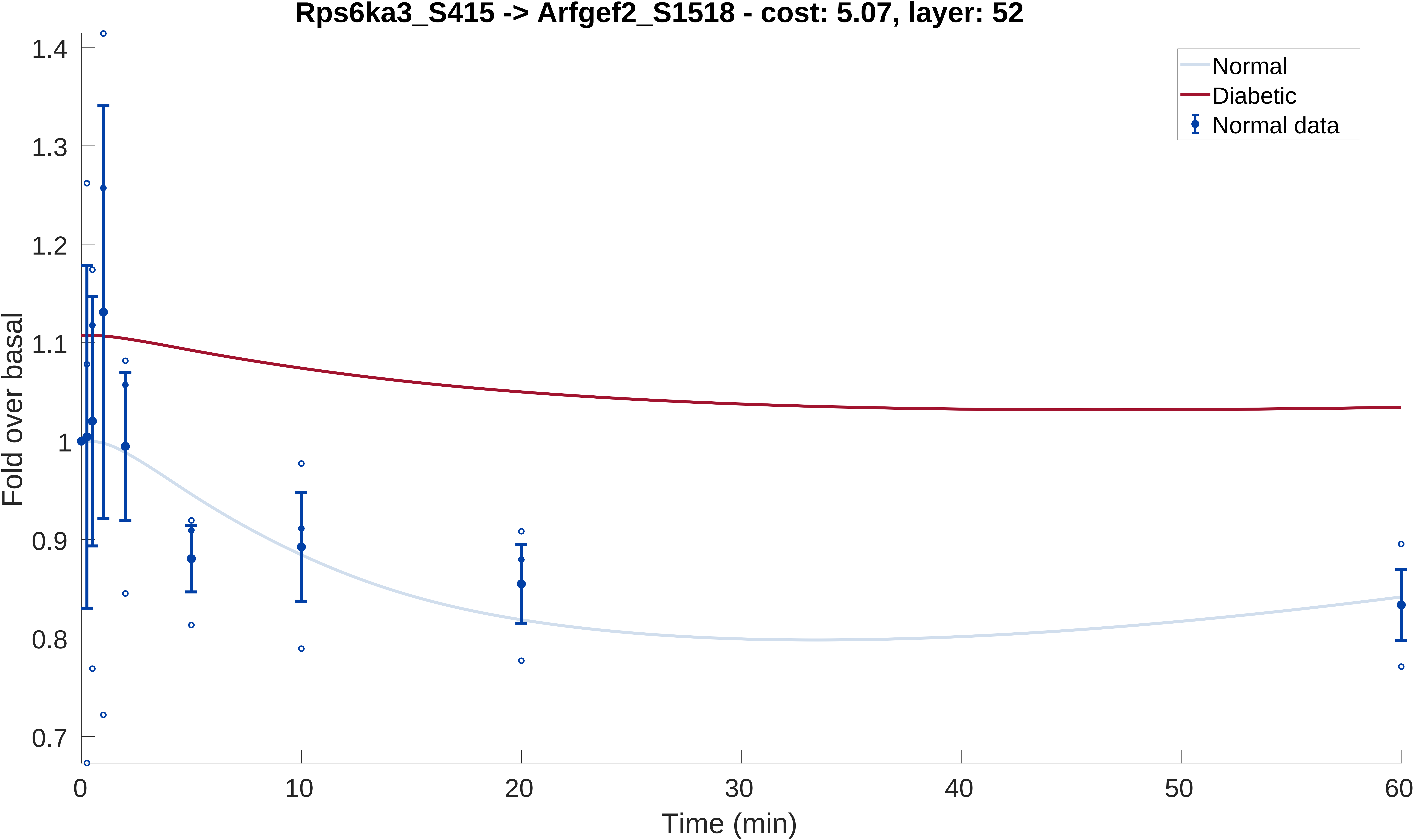
Rps6ka3_S415 -> Arfgef2_S1518 - cost: 5.07, layer: 52
Normal
Diabetic
Normal data
Fold over basal
Time (min)
1.4
1.3
1.2
1.1
1
0.9
0.8
0.7
0
10
20
30
40
50
60

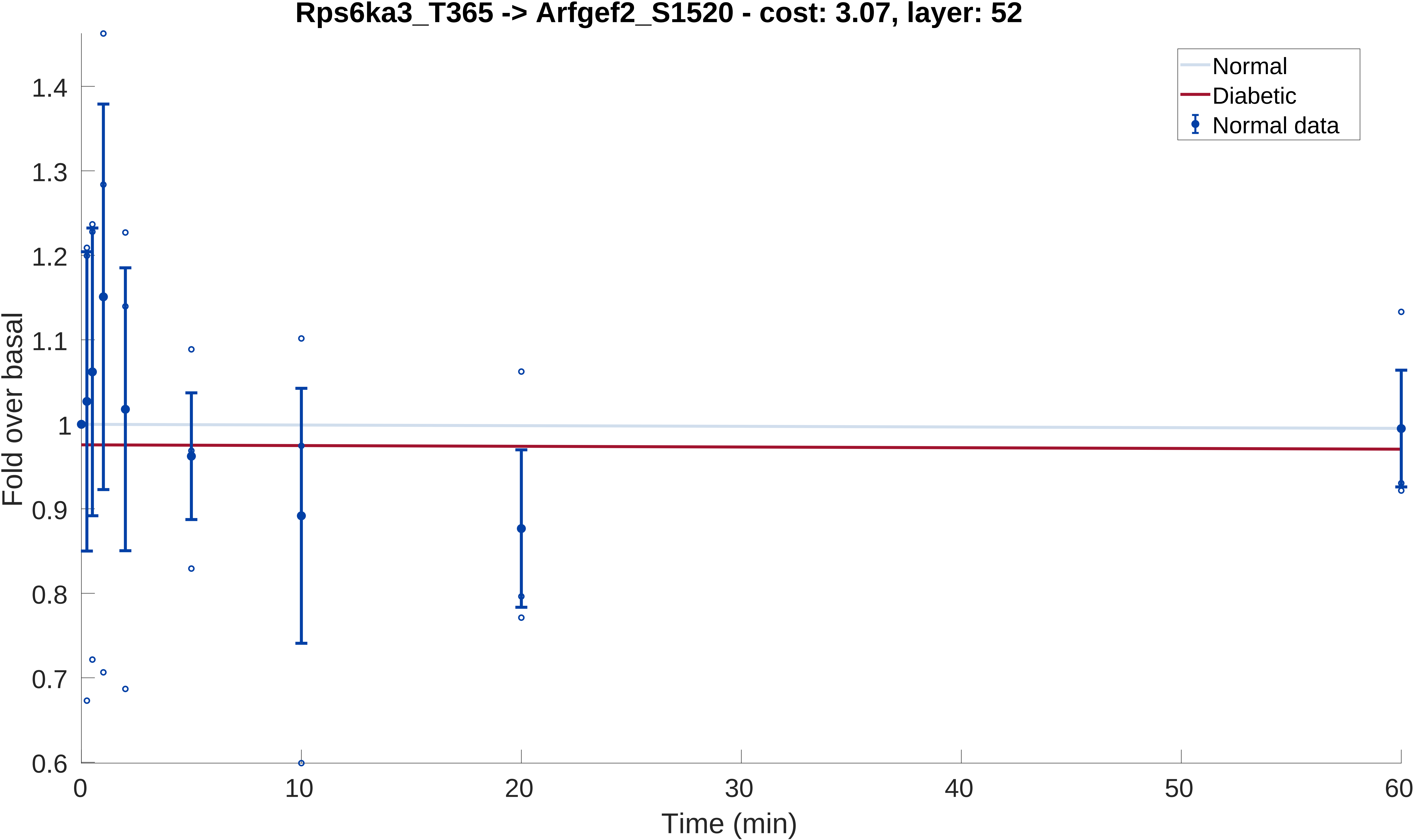

Rps6ka3_T365 -> Arfgef2_S1520 - cost: 3.07, layer: 52
Normal
Diabetic
Normal data
Fold over basal
1.4
1.3
1.2
1.1
1
0.9
0.8
0.7
0.6
0
10
20
30
40
50
60
Time (min)

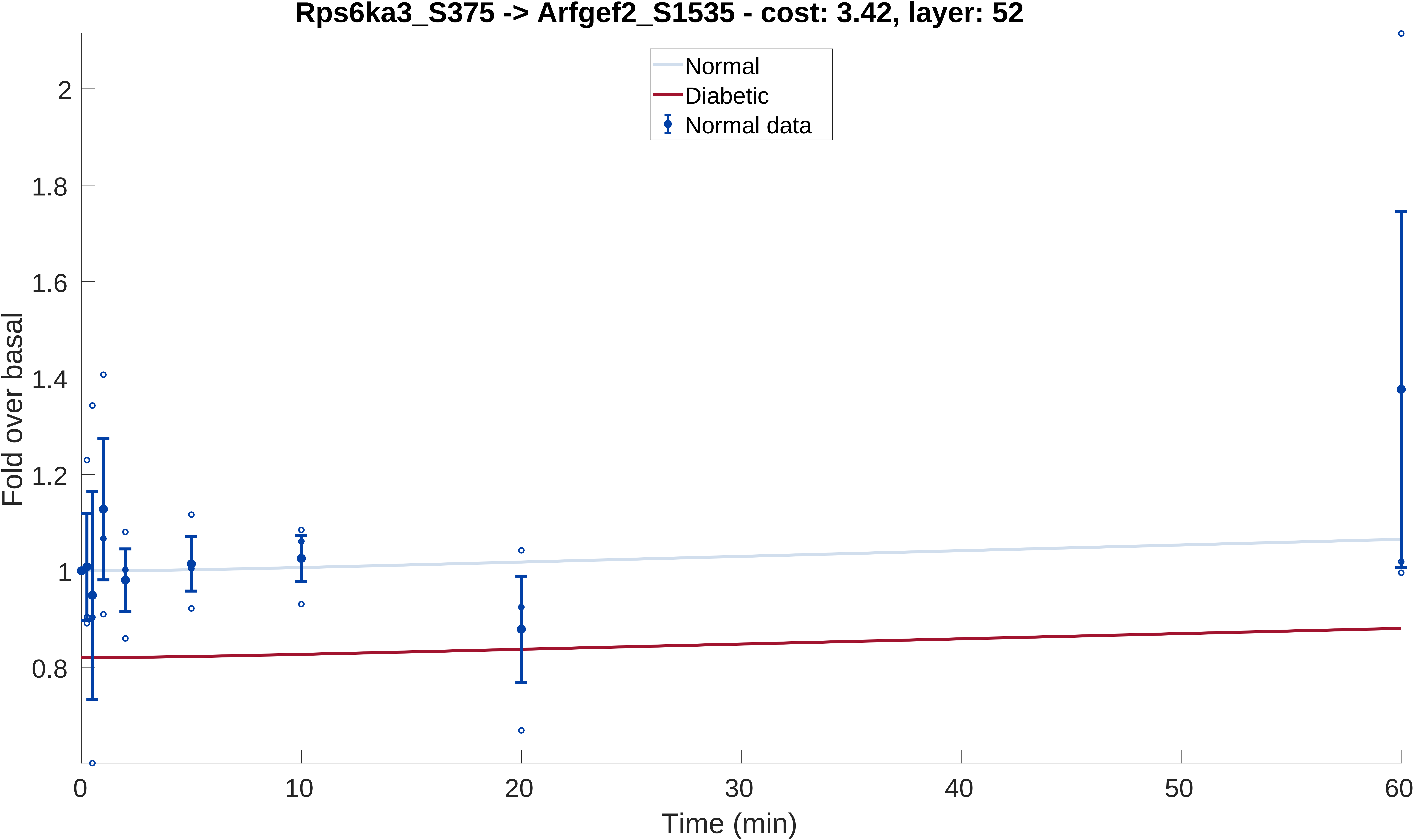

Rps6ka3_S375 -> Arfgef2_S1535 - cost: 3.42, layer: 52
Normal
Diabetic
Normal data
Fold over basal
Time (min)
0.8
1
1.2
1.4
1.6
1.8
2
0
10
20
30
40
50
60

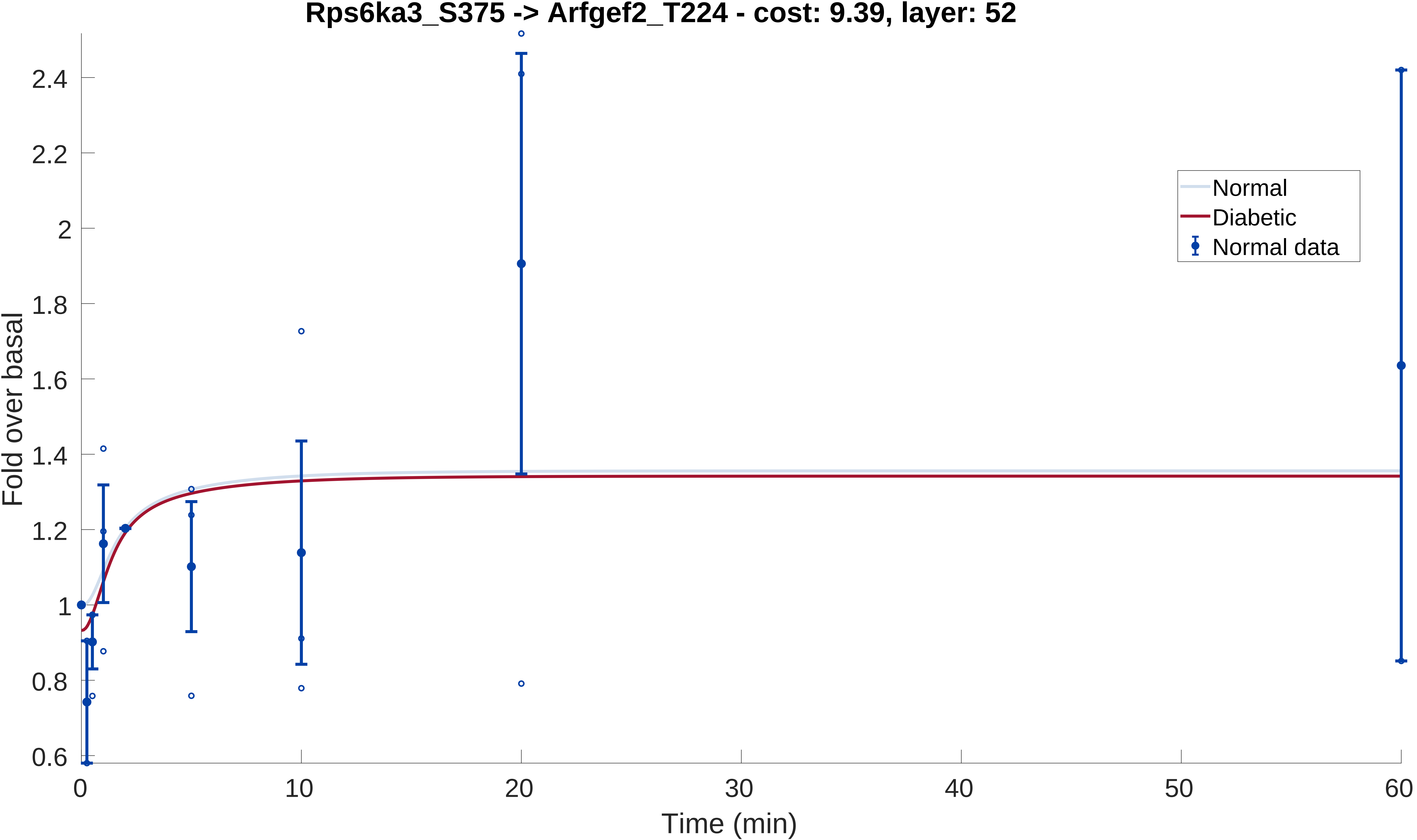

Rps6ka3_S375 -> Arfgef2_T224 - cost: 9.39, layer: 52
Fold over basal
Time (min)
0.6
0.8
1
1.2
1.4
1.6
1.8
2
2.2
2.4
0
10
20
30
40
50
60
Normal
Diabetic
Normal data

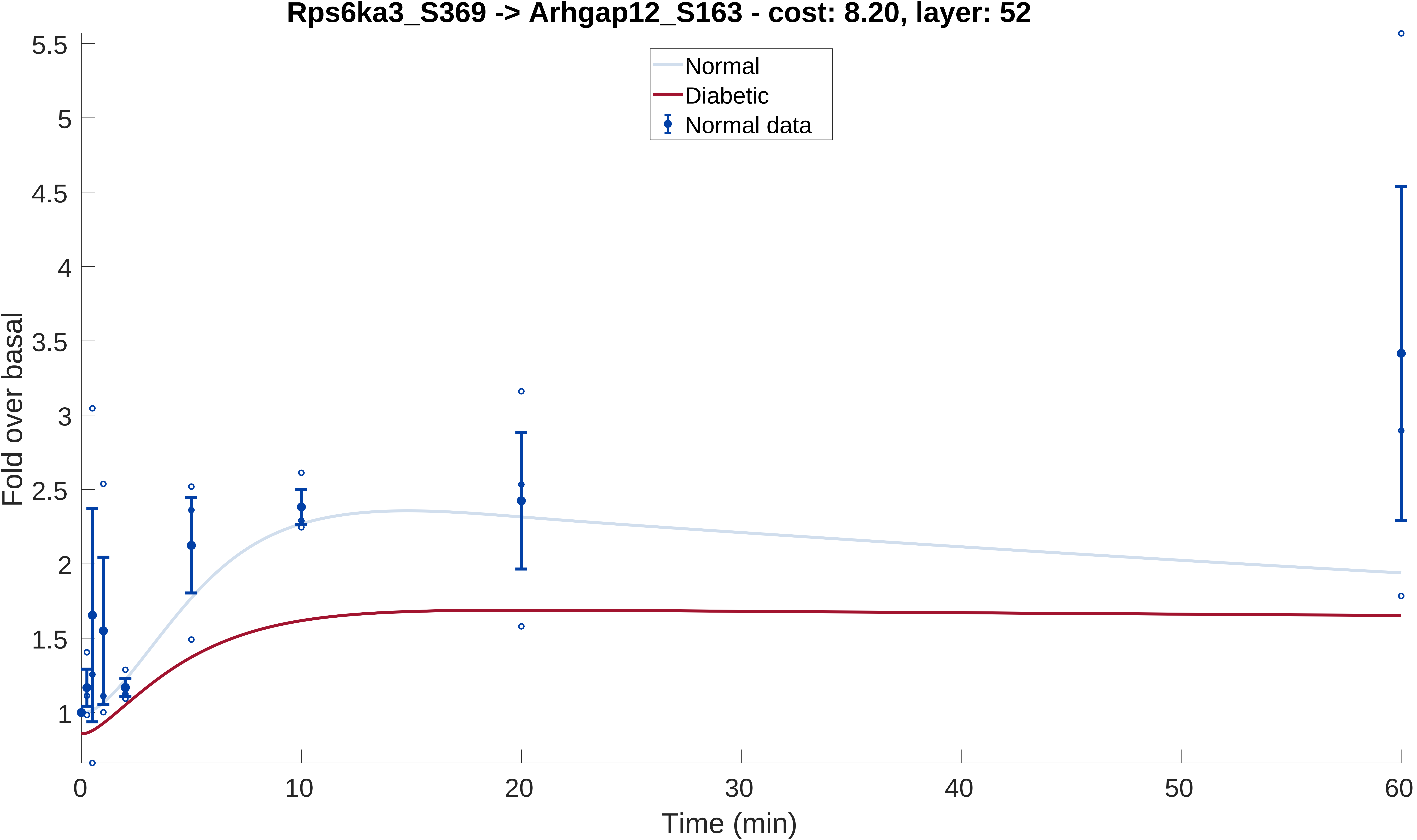

Rps6ka3_S369 -> Arhgap12_S163 - cost: 8.20, layer: 52
Normal
Diabetic
Normal data
Fold over basal
Time (min)
1
1.5
2
2.5
3
3.5
4
4.5
5
5.5
0
10
20
30
40
50
60

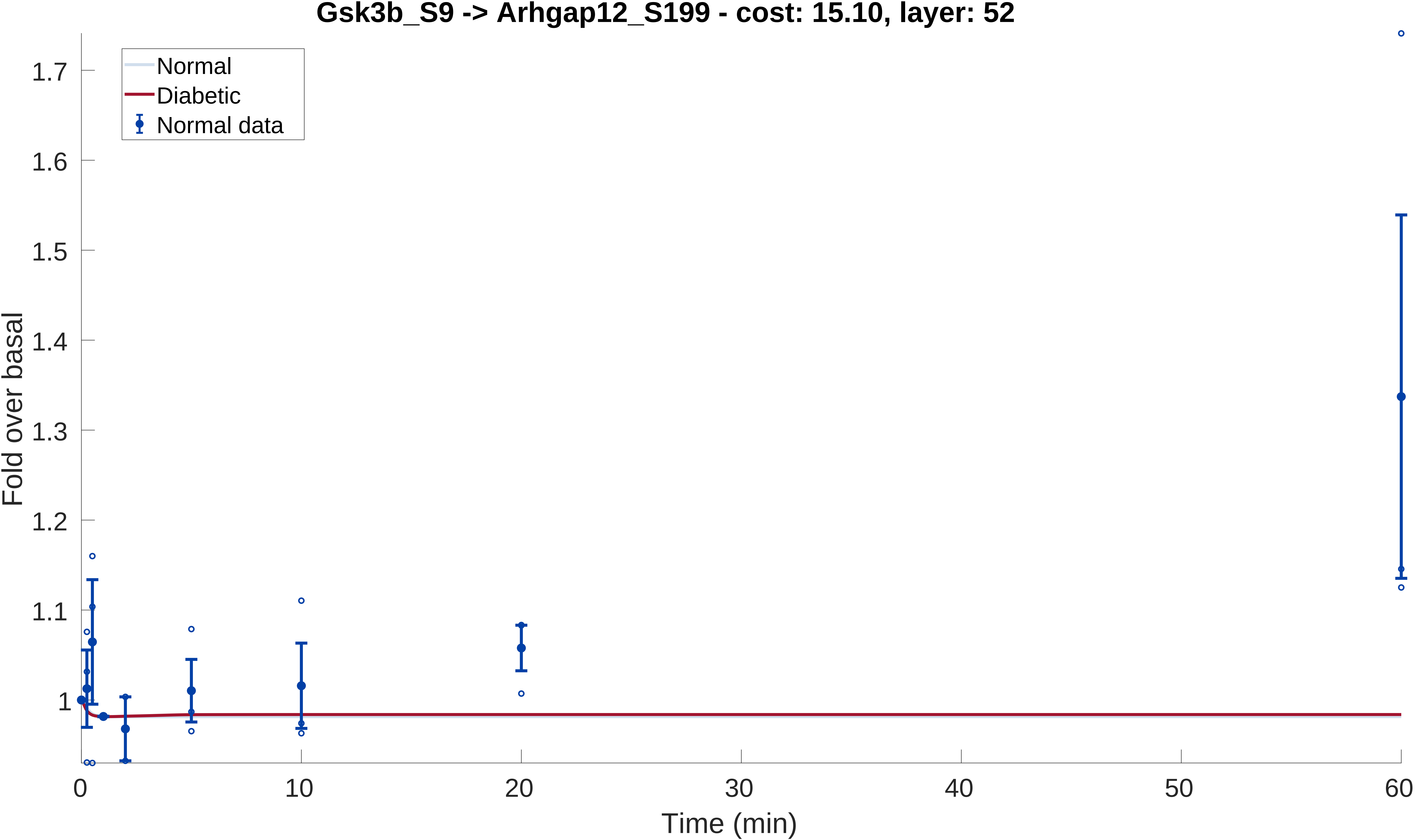

Gsk3b_S9 -> Arhgap12_S199 - cost: 15.10, layer: 52
Normal
Diabetic
Normal data
Fold over basal
1.7
1.6
1.5
1.4
1.3
1.2
1.1
1
0
10
20
30
40
50
60
Time (min)

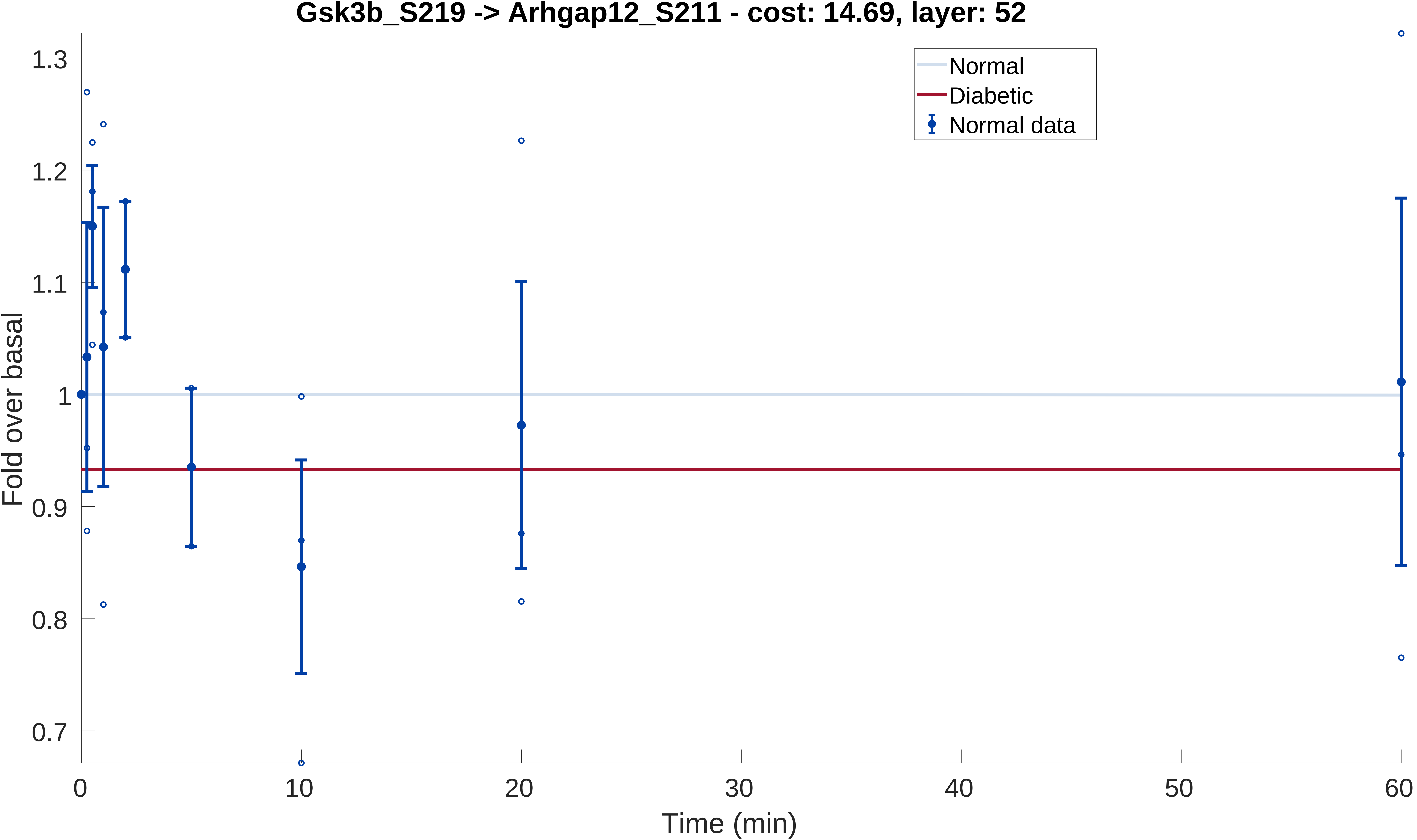

Gsk3b_S219 -> Arhgap12_S211 - cost: 14.69, layer: 52
Normal
Diabetic
Normal data
Fold over basal
1.3
1.2
1.1
1
0.9
0.8
0.7
0
10
20
30
40
50
60
Time (min)

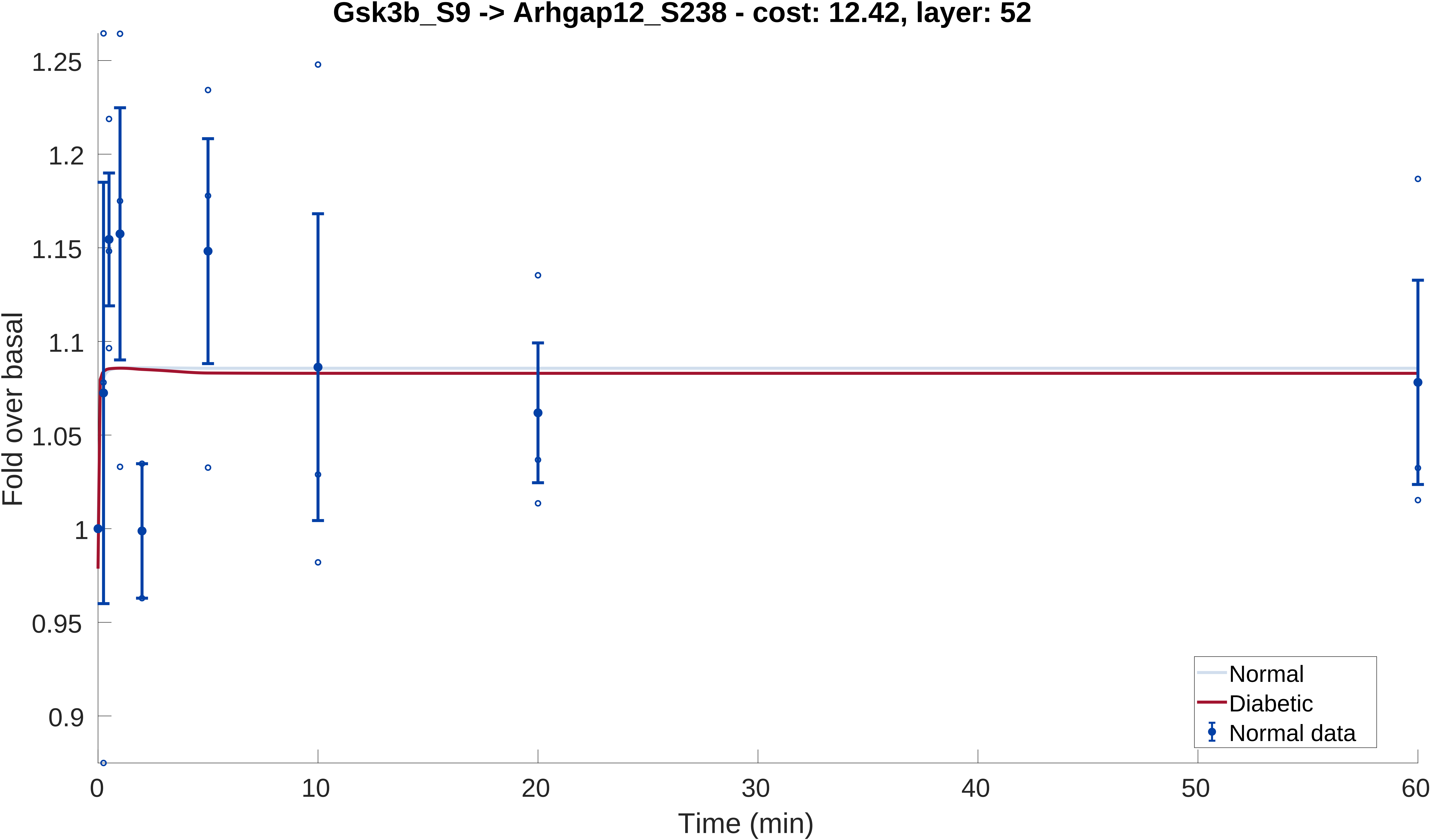
Gsk3b_S9 -> Arhgap12_S238 - cost: 12.42, layer: 52
Fold over basal
Time (min)
1.25
1.2
1.15
1.1
1.05
1
0.95
0.9
0
10
20
30
40
50
60
Normal
Diabetic
Normal data

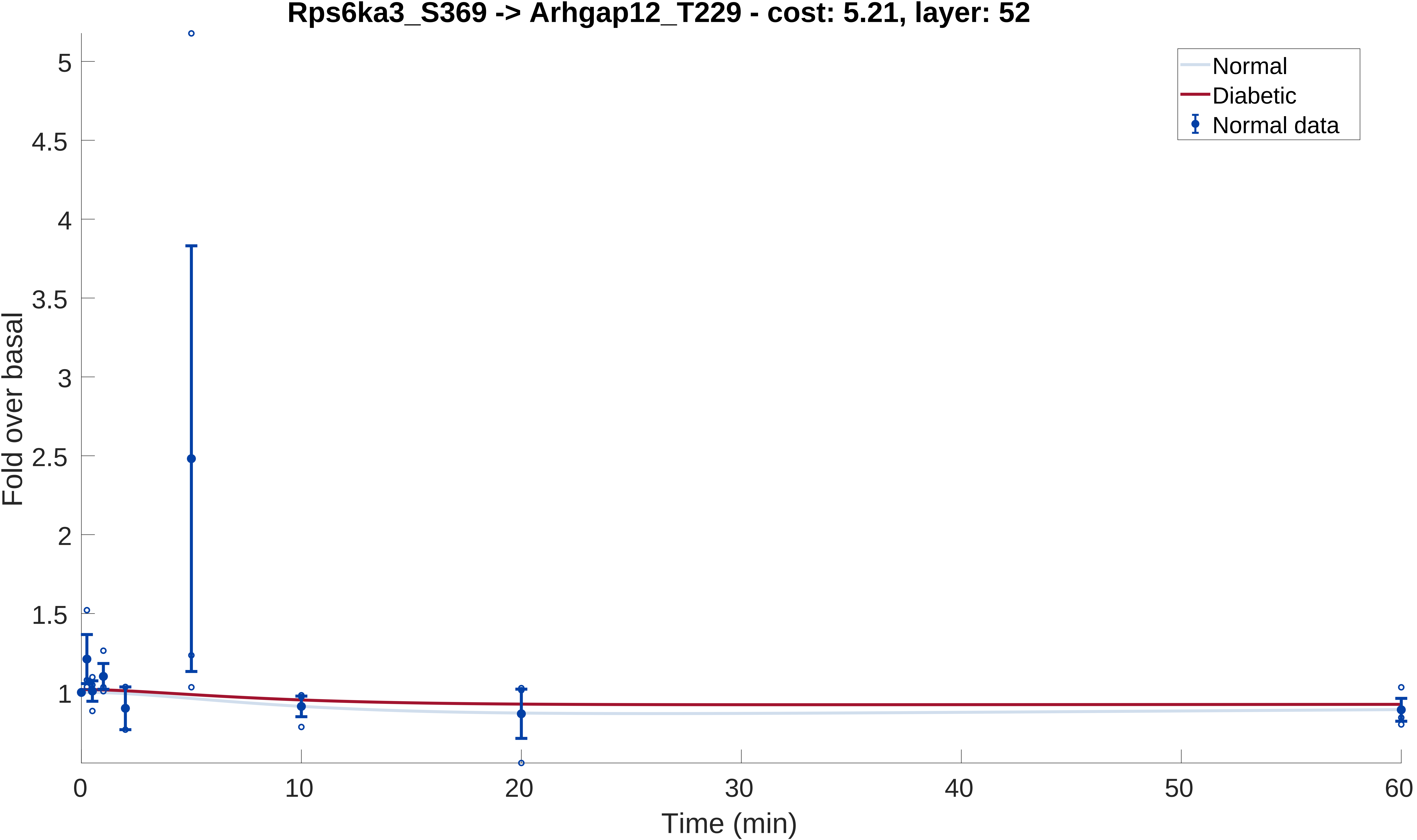

Rps6ka3_S369 -> Arhgap12_T229 - cost: 5.21, layer: 52
Normal
Diabetic
Normal data
Fold over basal
Time (min)
5
4.5
4
3.5
3
2.5
2
1.5
1
0
10
20
30
40
50
60

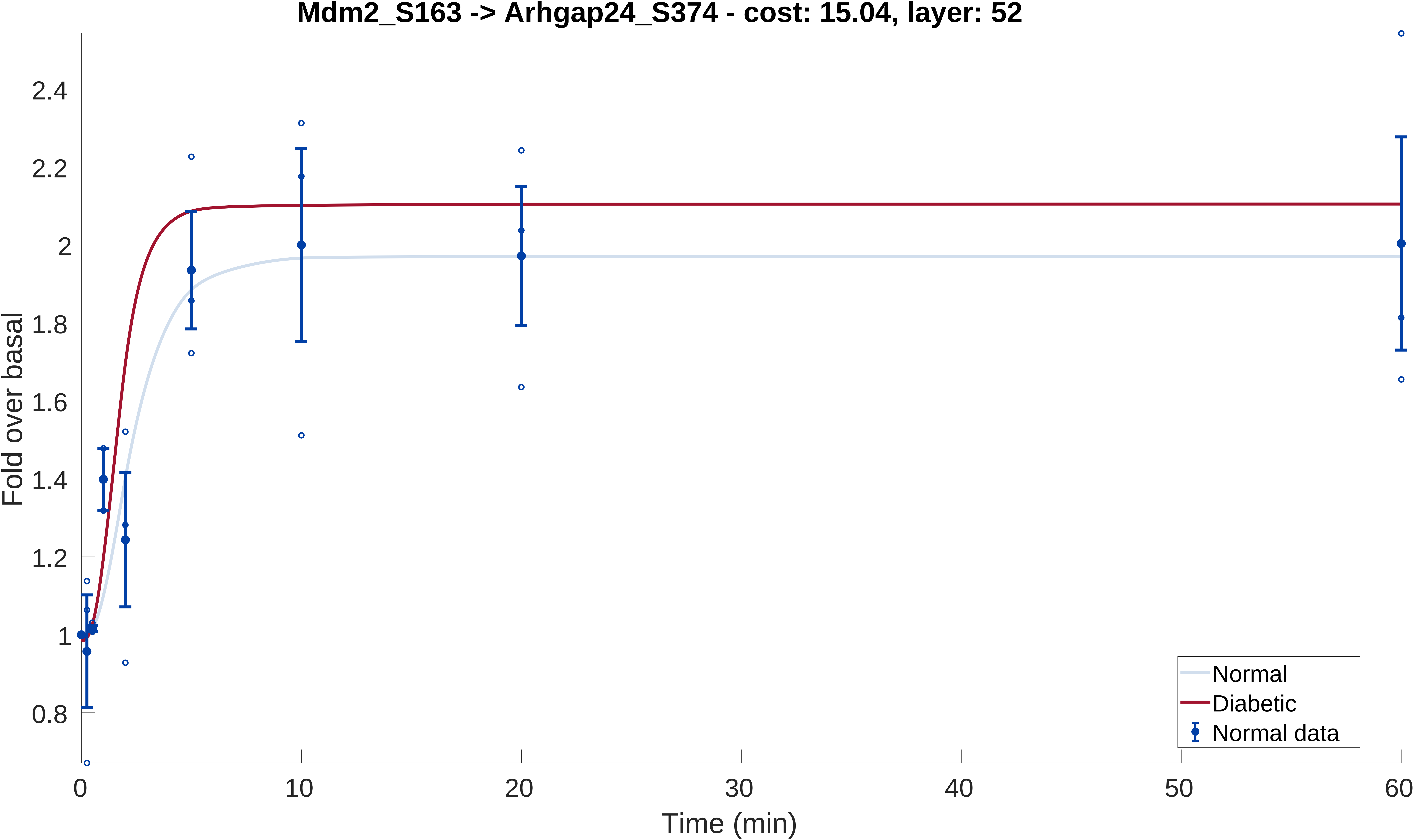
Mdm2_S163 -> Arhgap24_S374 - cost: 15.04, layer: 52
Fold over basal
2.4
2.2
2
1.8
1.6
1.4
1.2
1
0.8
0
10
20
30
40
50
60
Time (min)
Normal
Diabetic
Normal data

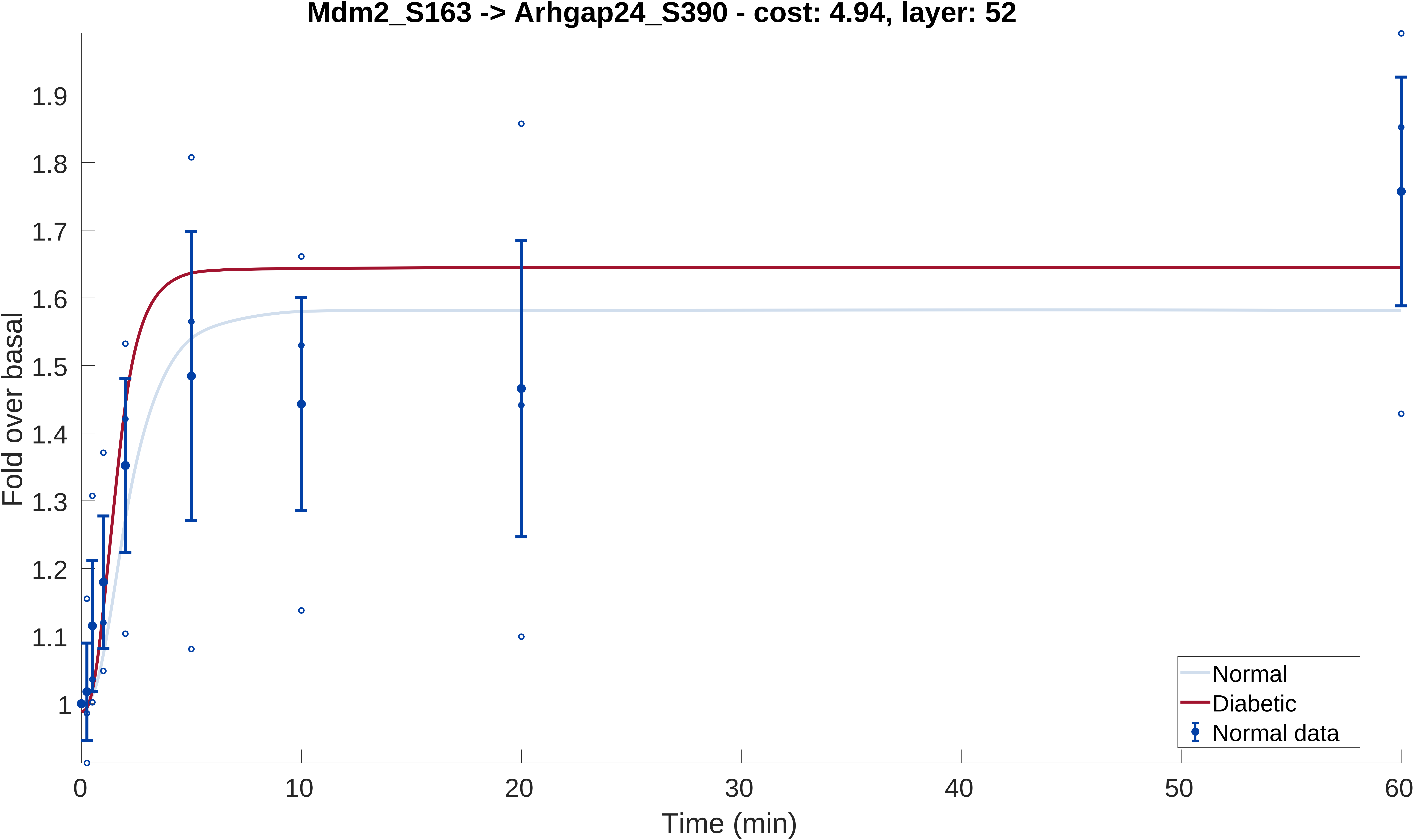

Mdm2_S163 -> Arhgap24_S390 - cost: 4.94, layer: 52
Fold over basal
1
1.1
1.2
1.3
1.4
1.5
1.6
1.7
1.8
1.9
0
10
20
30
40
50
60
Time (min)
Normal
Diabetic
Normal data

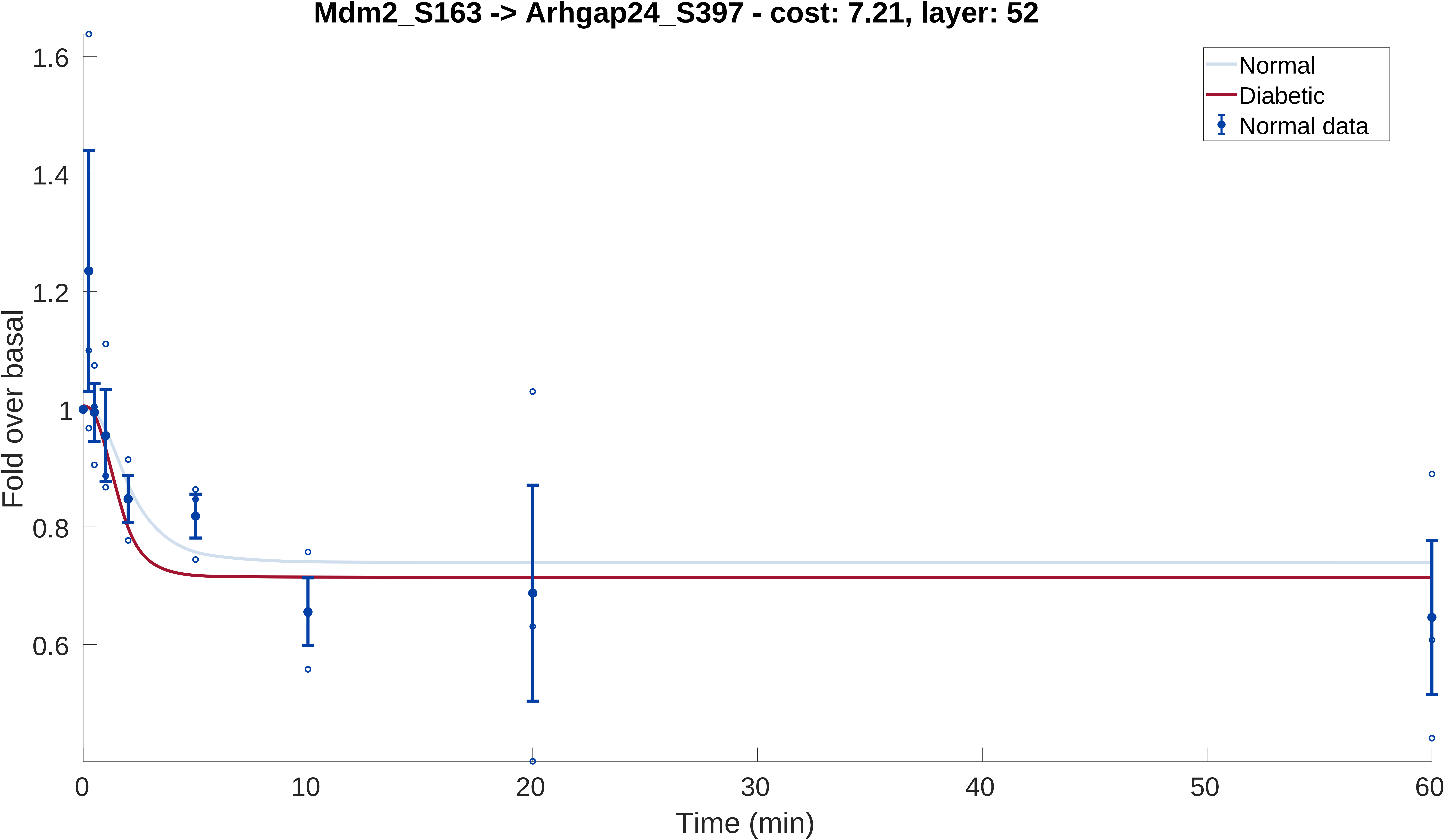
Mdm2_S163 -> Arhgap24_S397 - cost: 7.21, layer: 52
Fold over basal
1.6
1.4
1.2
1
0.8
0.6
0
10
20
30
40
50
60
Time (min)
Normal
Diabetic
Normal data

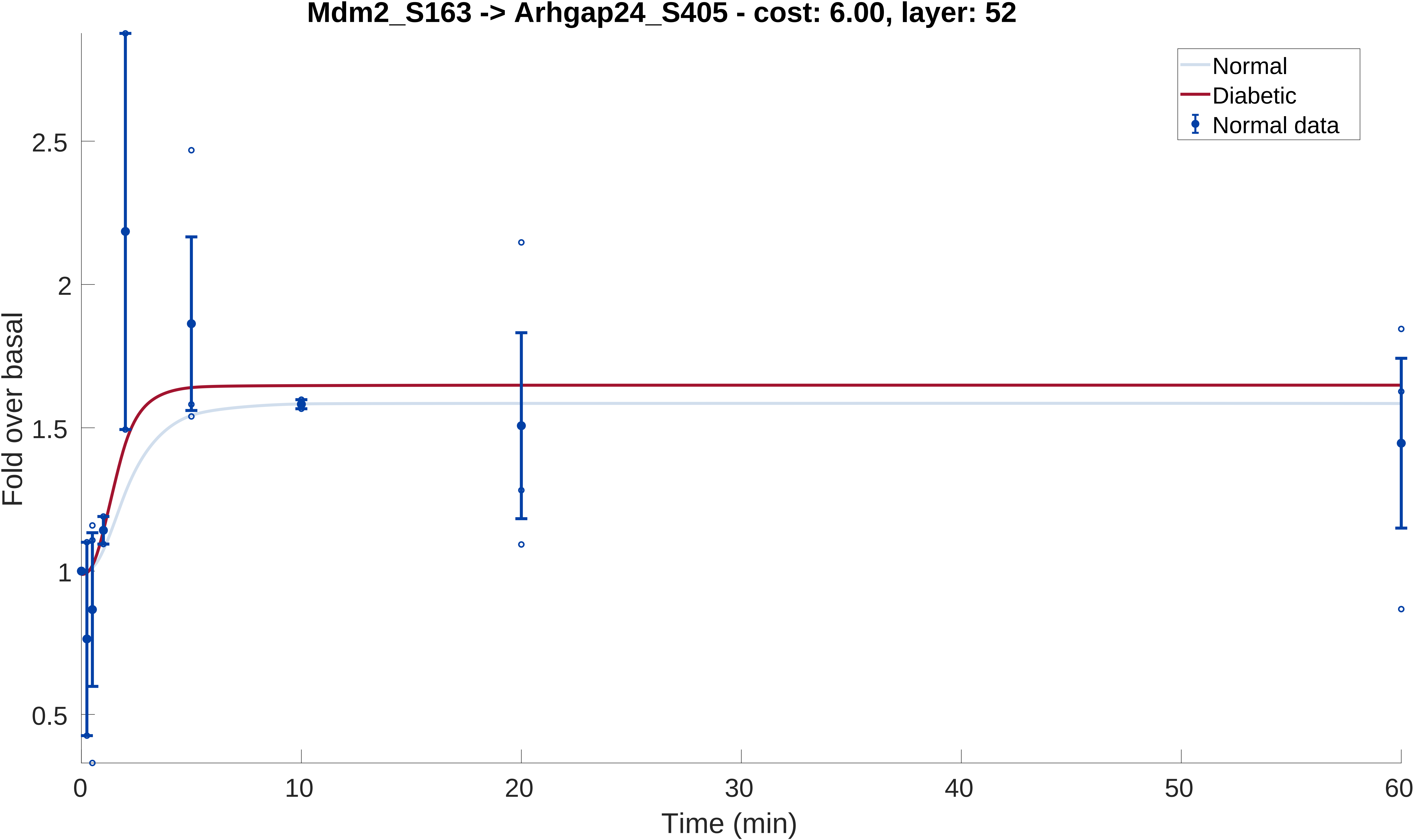

Mdm2_S163 -> Arhgap24_S405 - cost: 6.00, layer: 52
Normal
Diabetic
Normal data
Fold over basal
2.5
2
1.5
1
0.5
0
10
20
30
40
50
60
Time (min)

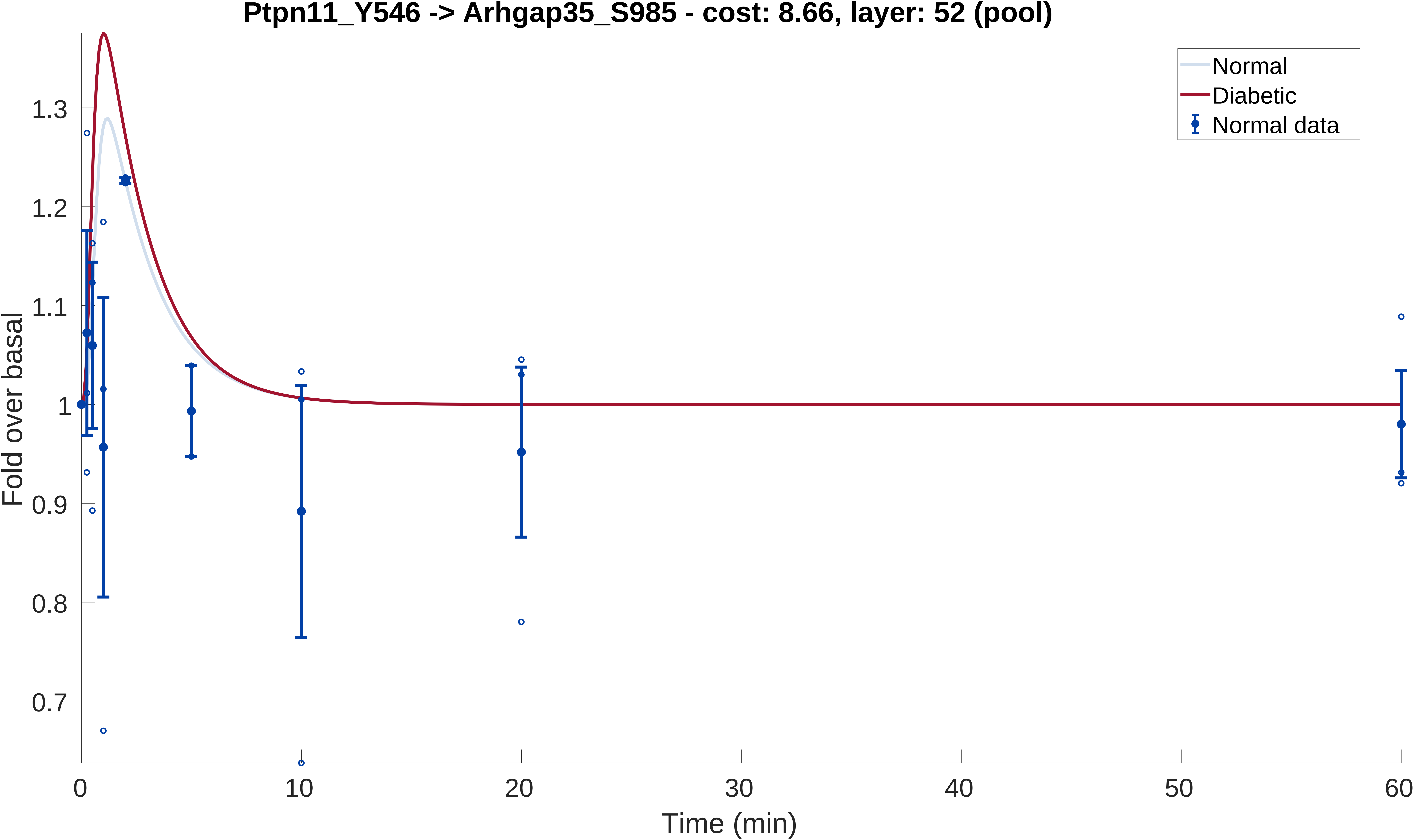
Ptpn11_Y546 -> Arhgap35_S985 - cost: 8.66, layer: 52 (pool)
Normal
Diabetic
Normal data
Fold over basal
Time (min)
1.3
1.2
1.1
1
0.9
0.8
0.7
0
10
20
30
40
50
60

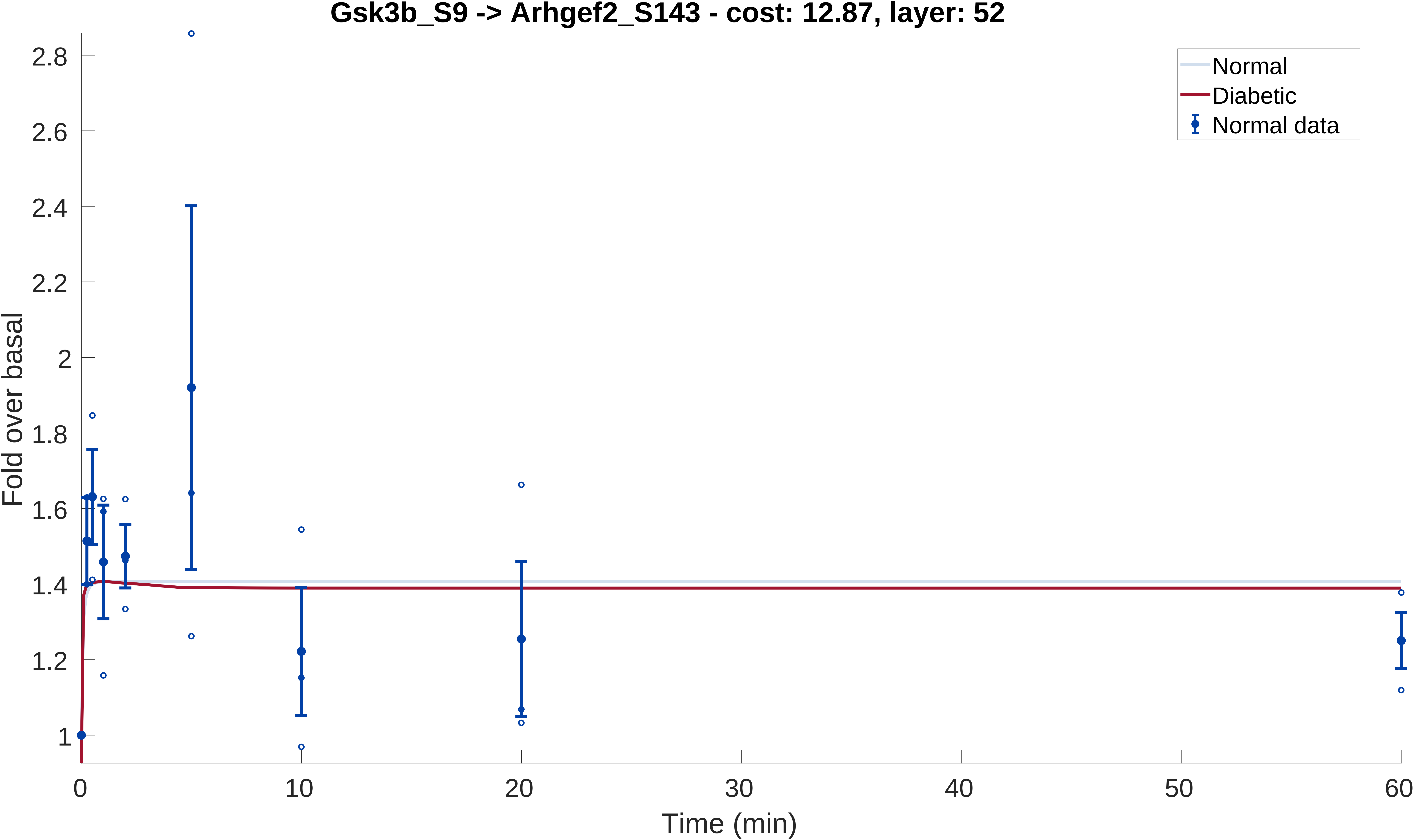

Gsk3b_S9 -> Arhgef2_S143 - cost: 12.87, layer: 52
Normal
Diabetic
Normal data
Fold over basal
Time (min)
1
1.2
1.4
1.6
1.8
2
2.2
2.4
2.6
2.8
0
10
20
30
40
50
60

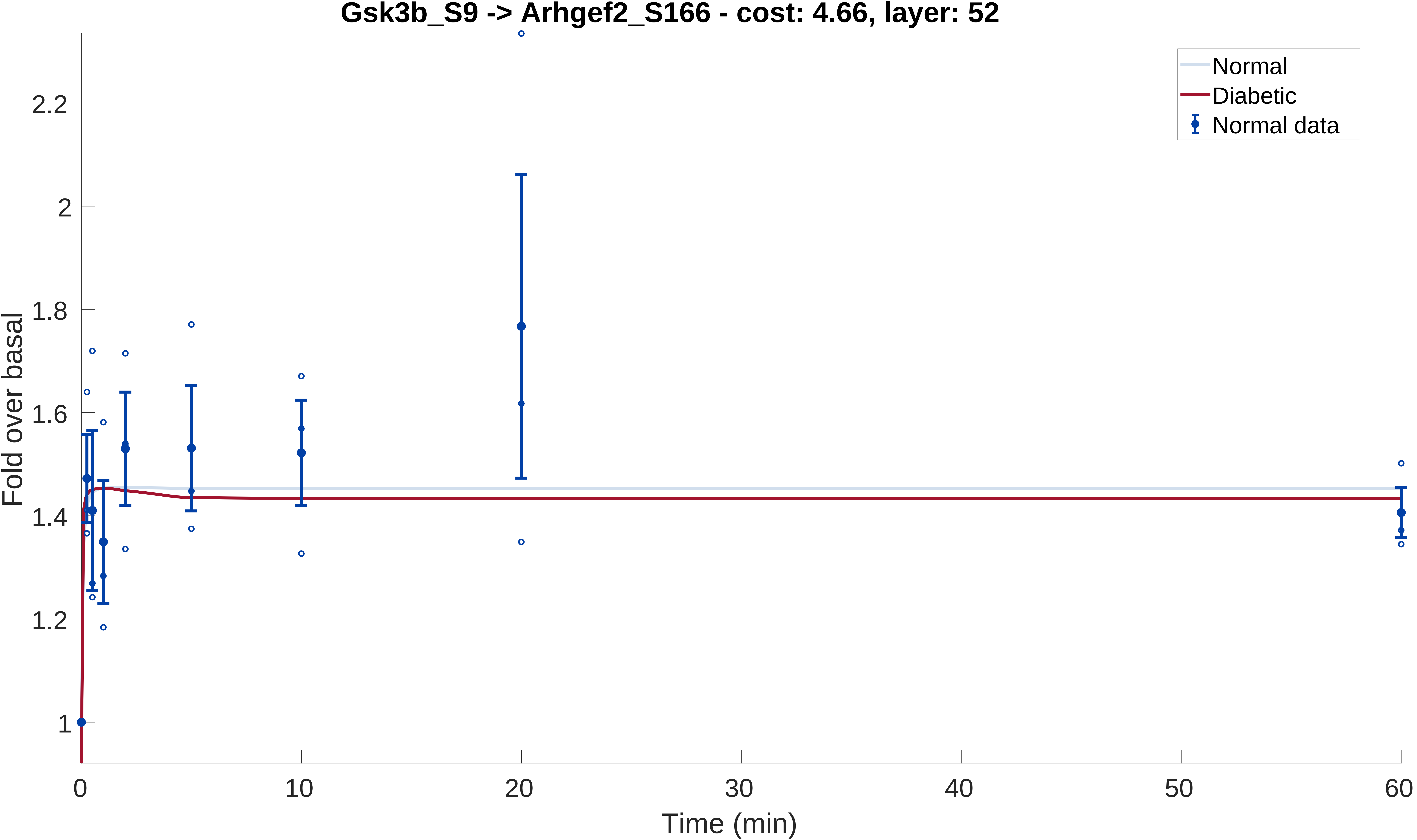

Gsk3b_S9 -> Arhgef2_S166 - cost: 4.66, layer: 52
Normal
Diabetic
Normal data
Fold over basal
Time (min)
2.2
2
1.8
1.6
1.4
1.2
1
0
10
20
30
40
50
60

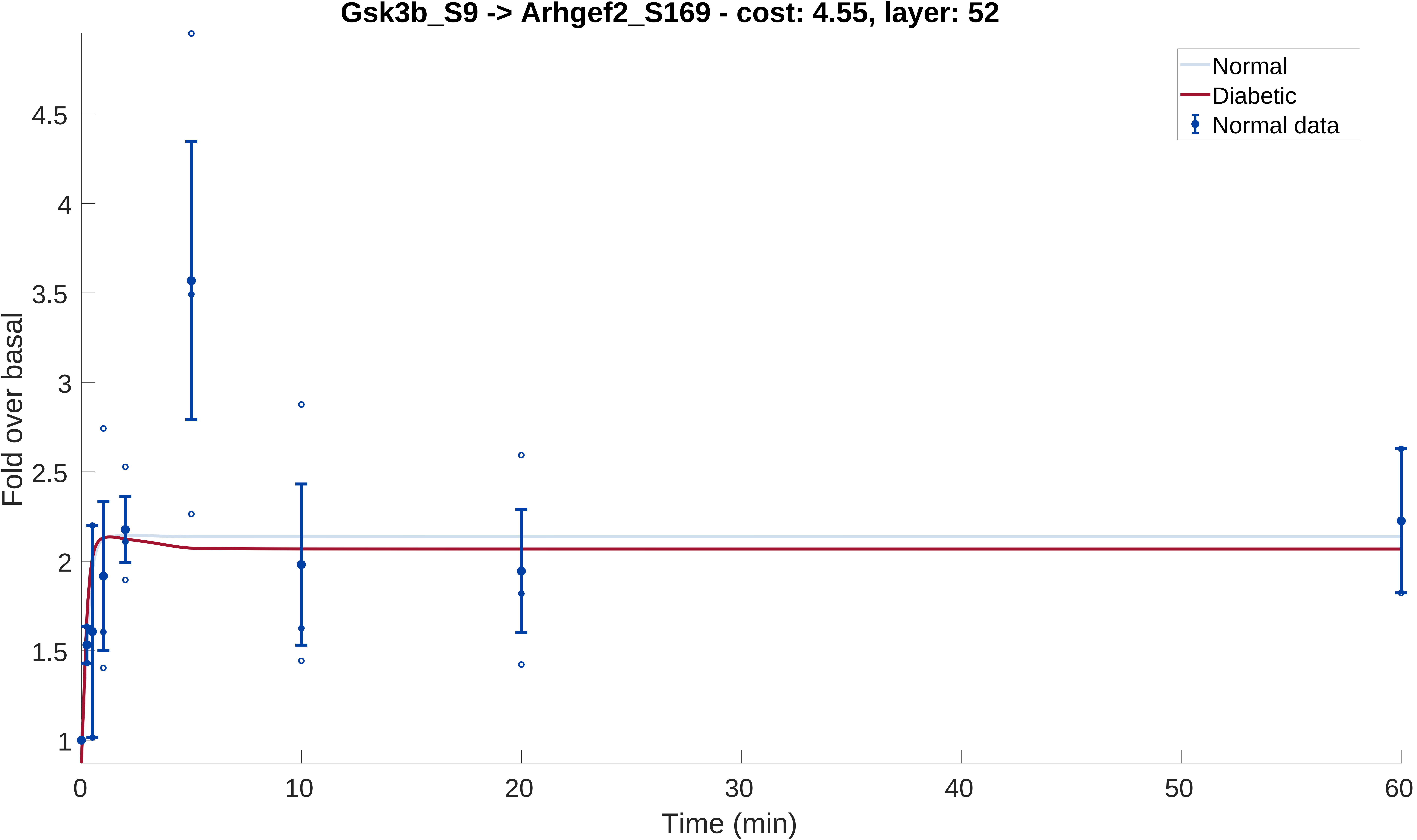
Gsk3b_S9 -> Arhgef2_S169 - cost: 4.55, layer: 52
Normal
Diabetic
Normal data
Fold over basal
4.5
4
3.5
3
2.5
2
1.5
1
0
10
20
30
40
50
60
Time (min)

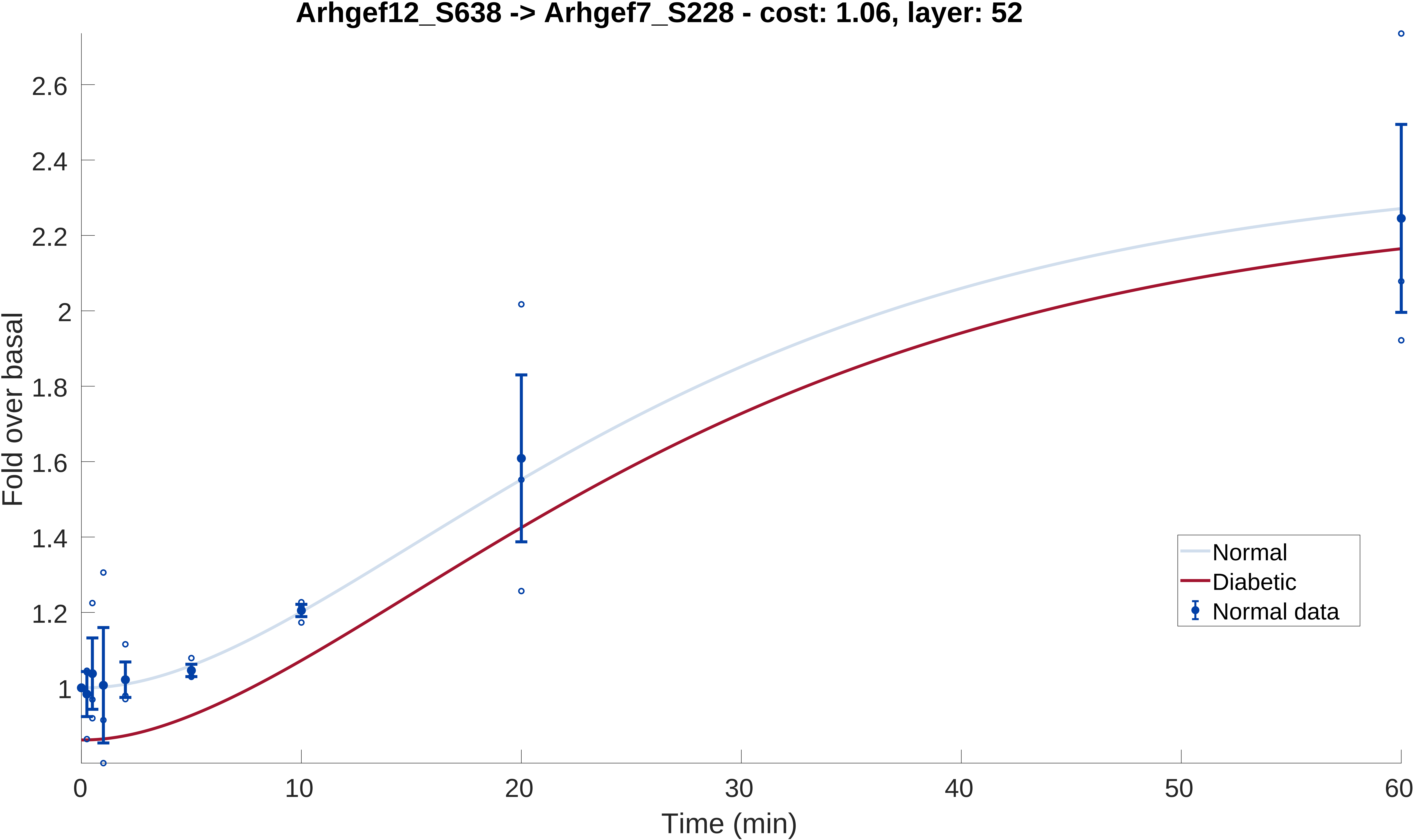
Arhgef12_S638 -> Arhgef7_S228 - cost: 1.06, layer: 52
Fold over basal
2.6
2.4
2.2
2
1.8
1.6
1.4
1.2
1
0
10
20
30
40
50
60
Time (min)
Normal
Diabetic
Normal data

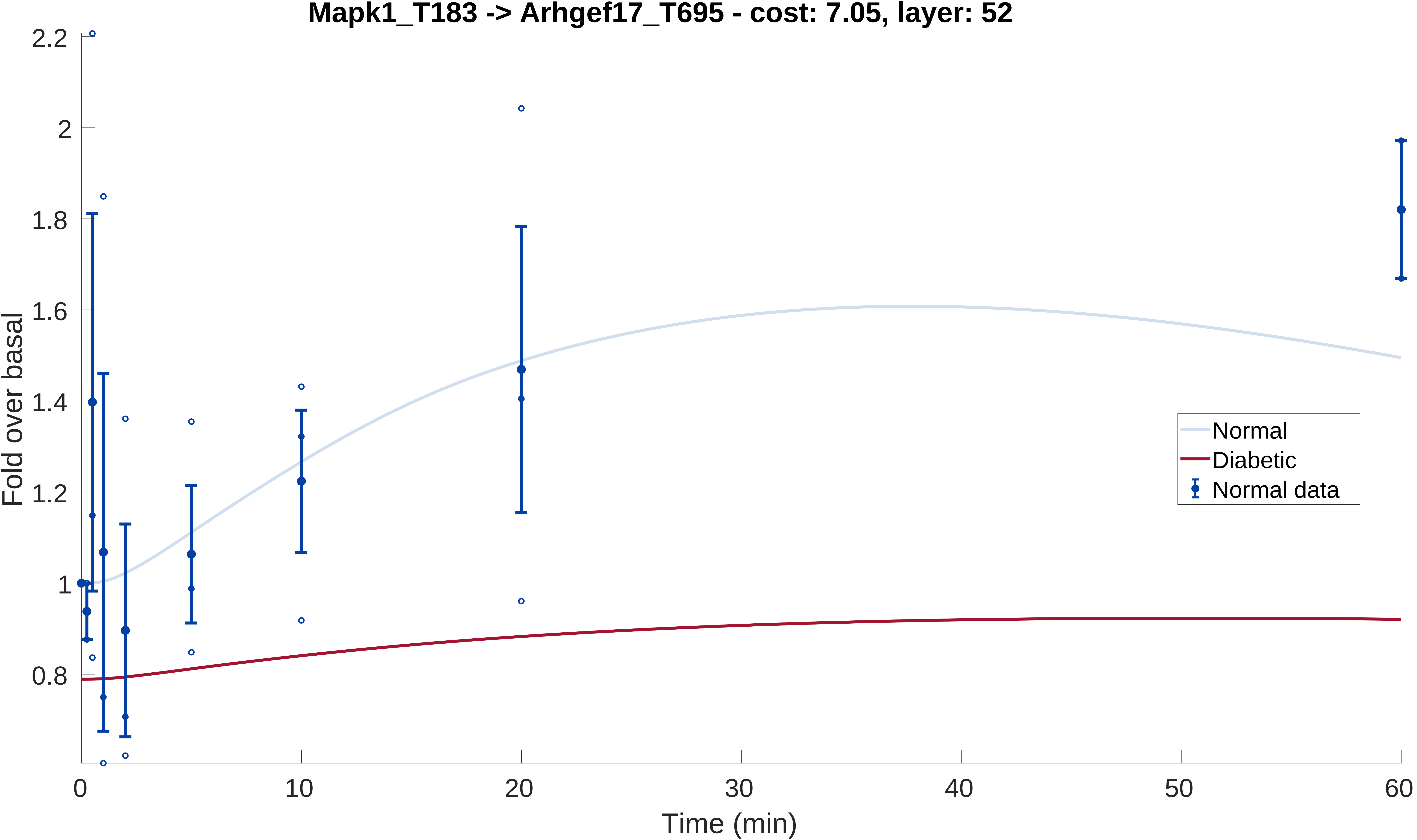
Mapk1_T183 -> Arhgef17_T695 - cost: 7.05, layer: 52
Fold over basal
Time (min)
0.8
1
1.2
1.4
1.6
1.8
2
2.2
0
10
20
30
40
50
60
Normal
Diabetic
Normal data

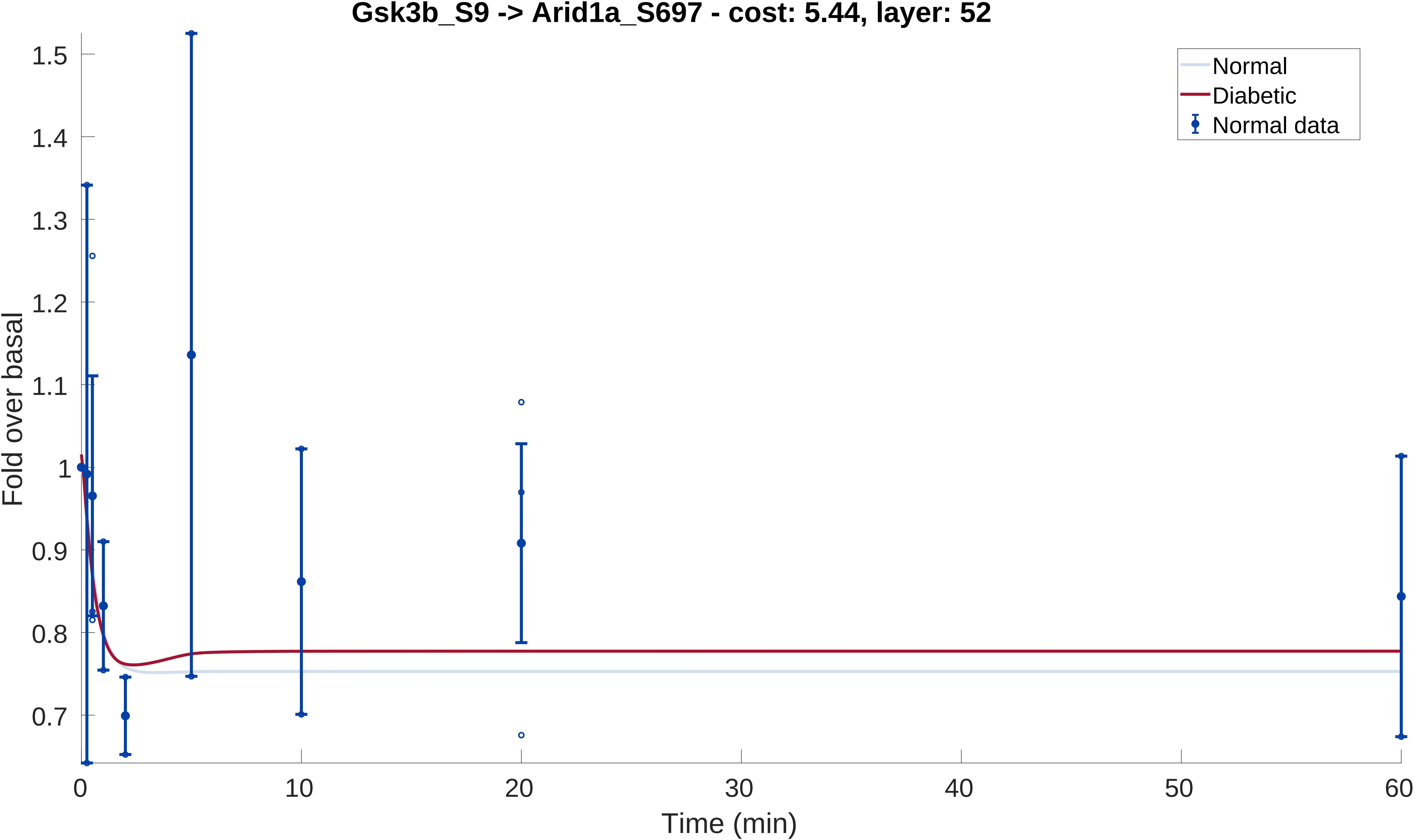

Gsk3b_S9 -> Arid1a_S697 - cost: 5.44, layer: 52
Normal
Diabetic
Normal data
Fold over basal
1.5
1.4
1.3
1.2
1.1
1
0.9
0.8
0.7
0
10
20
30
40
50
60
Time (min)

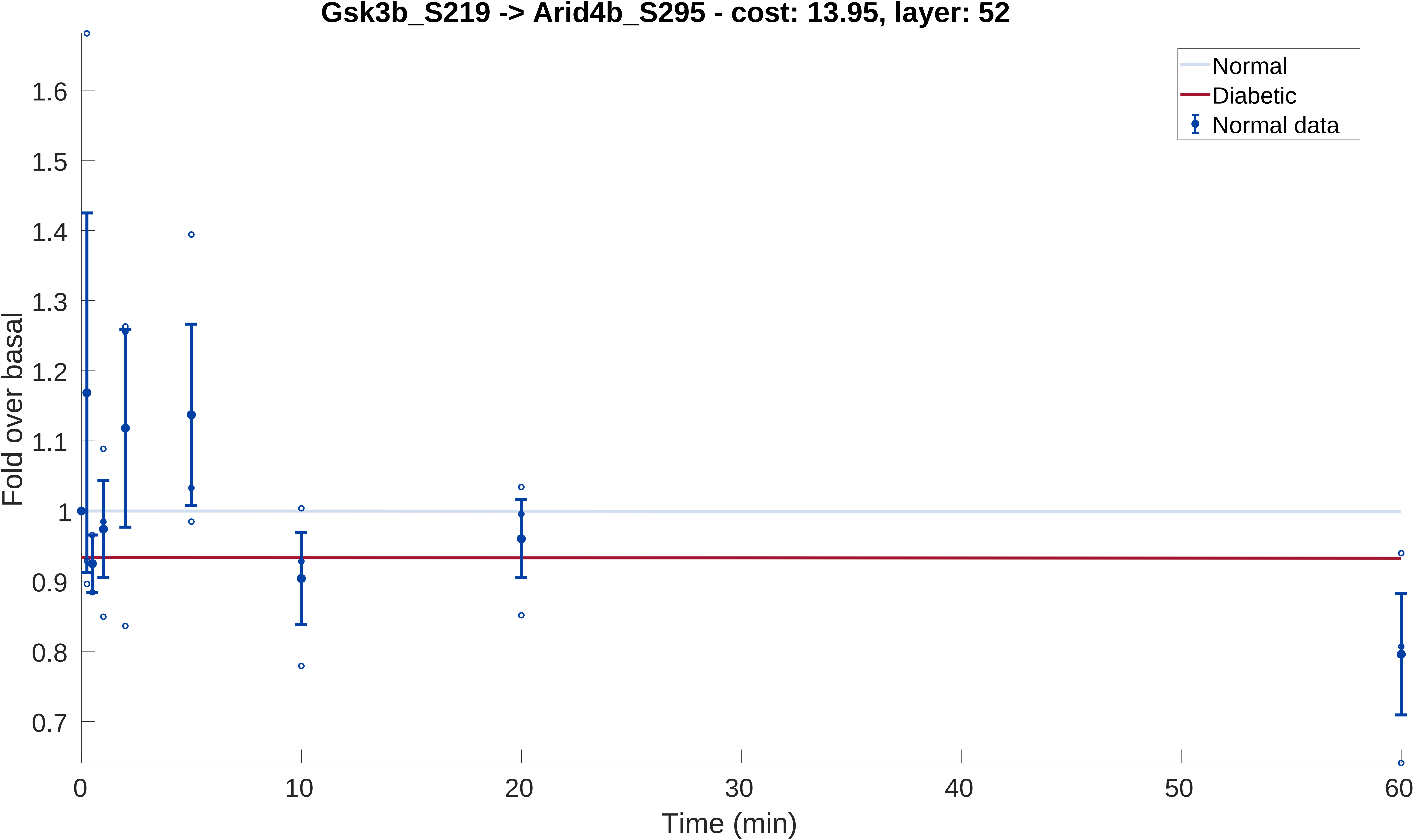

Gsk3b_S219 -> Arid4b_S295 - cost: 13.95, layer: 52
Normal
Diabetic
Normal data
Fold over basal
1.6
1.5
1.4
1.3
1.2
1.1
1
0.9
0.8
0.7
0
10
20
30
40
50
60
Time (min)

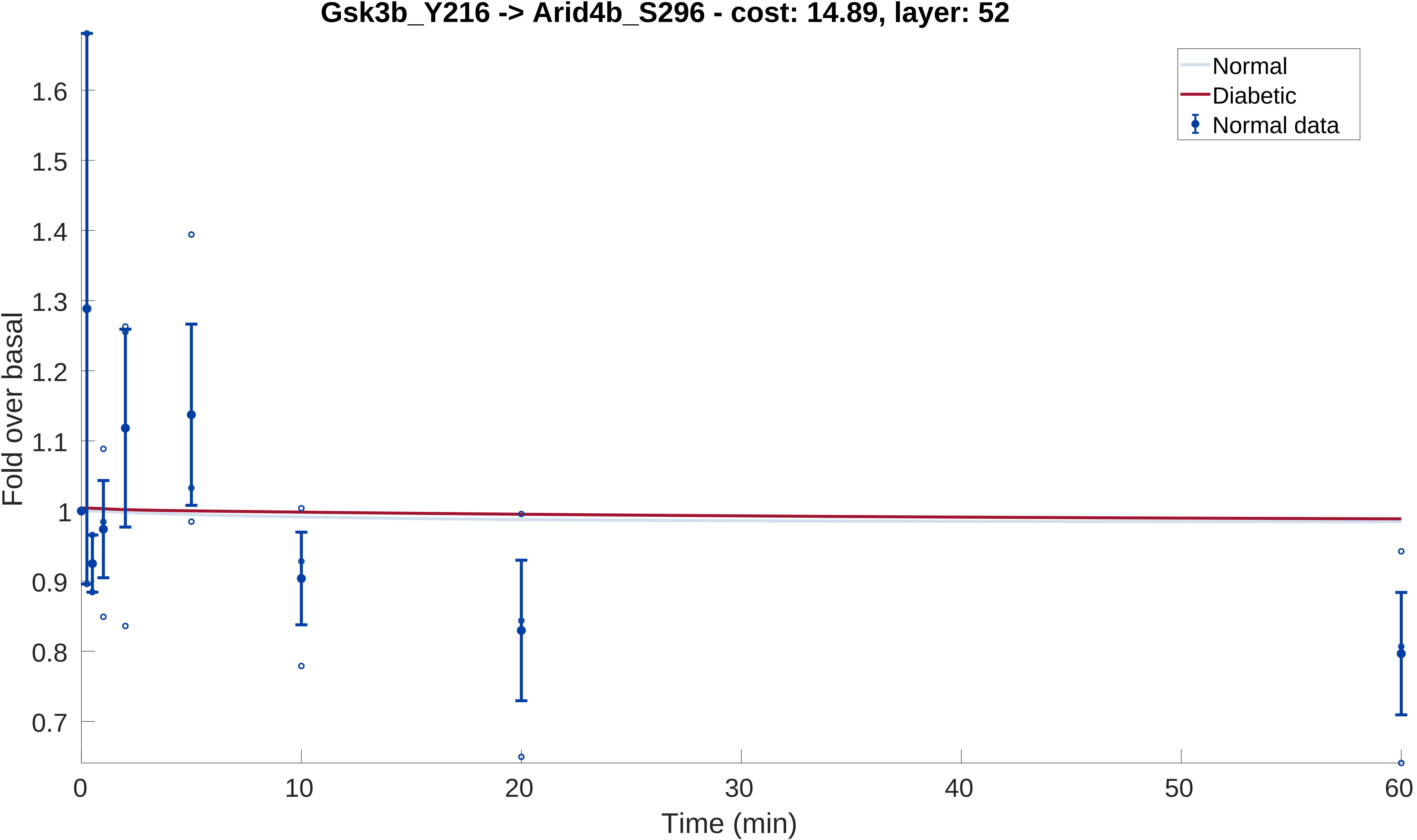
Gsk3b_Y216 -> Arid4b_S296 - cost: 14.89, layer: 52
Normal
Diabetic
Normal data
Fold over basal
0.7
0.8
0.9
1
1.1
1.2
1.3
1.4
1.5
1.6
0
10
20
30
40
50
60
Time (min)

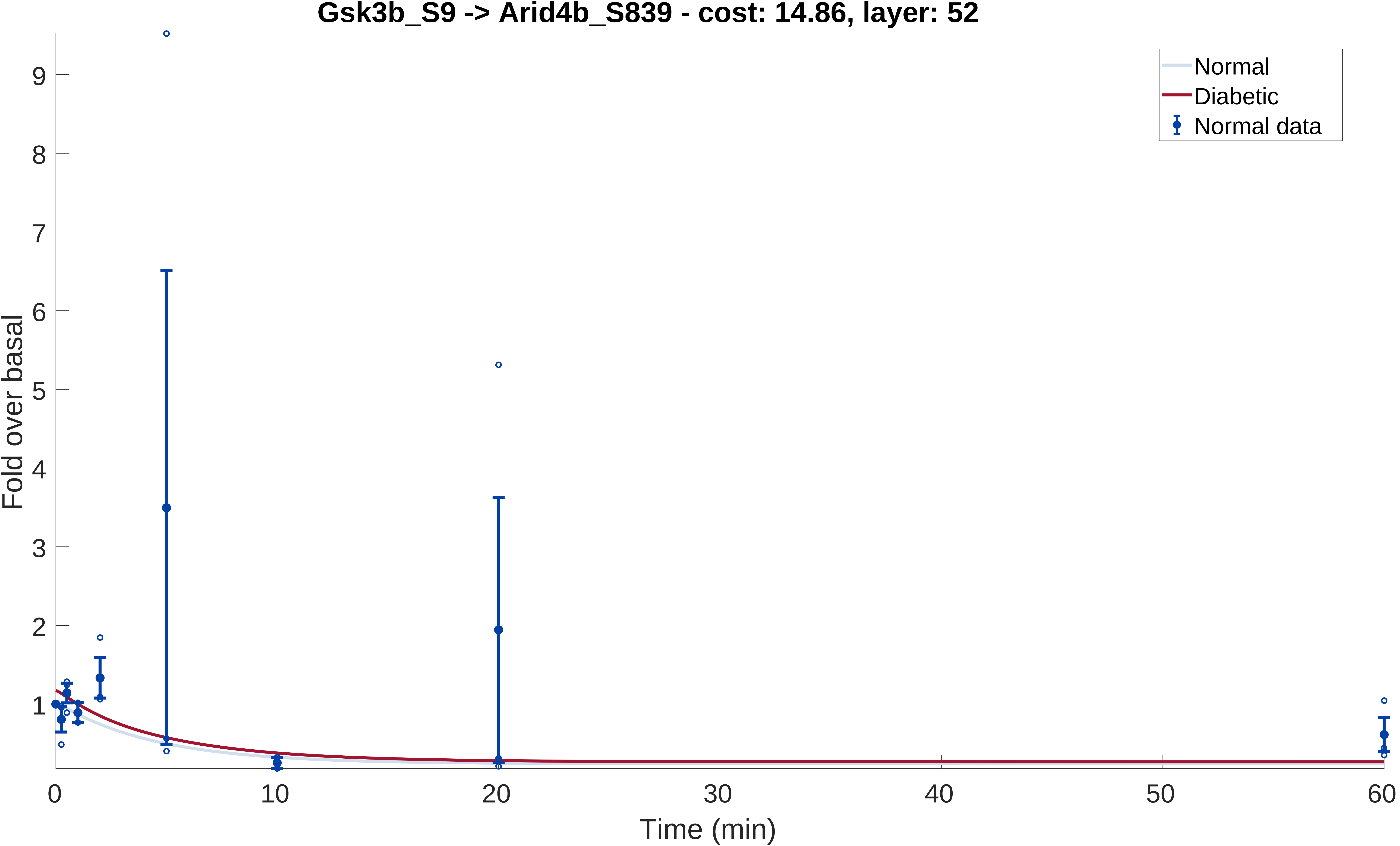

Gsk3b_S9 -> Arid4b_S839 - cost: 14.86, layer: 52
Normal
Diabetic
Normal data
Fold over basal
Time (min)
0
10
20
30
40
50
60
1
2
3
4
5
6
7
8
9

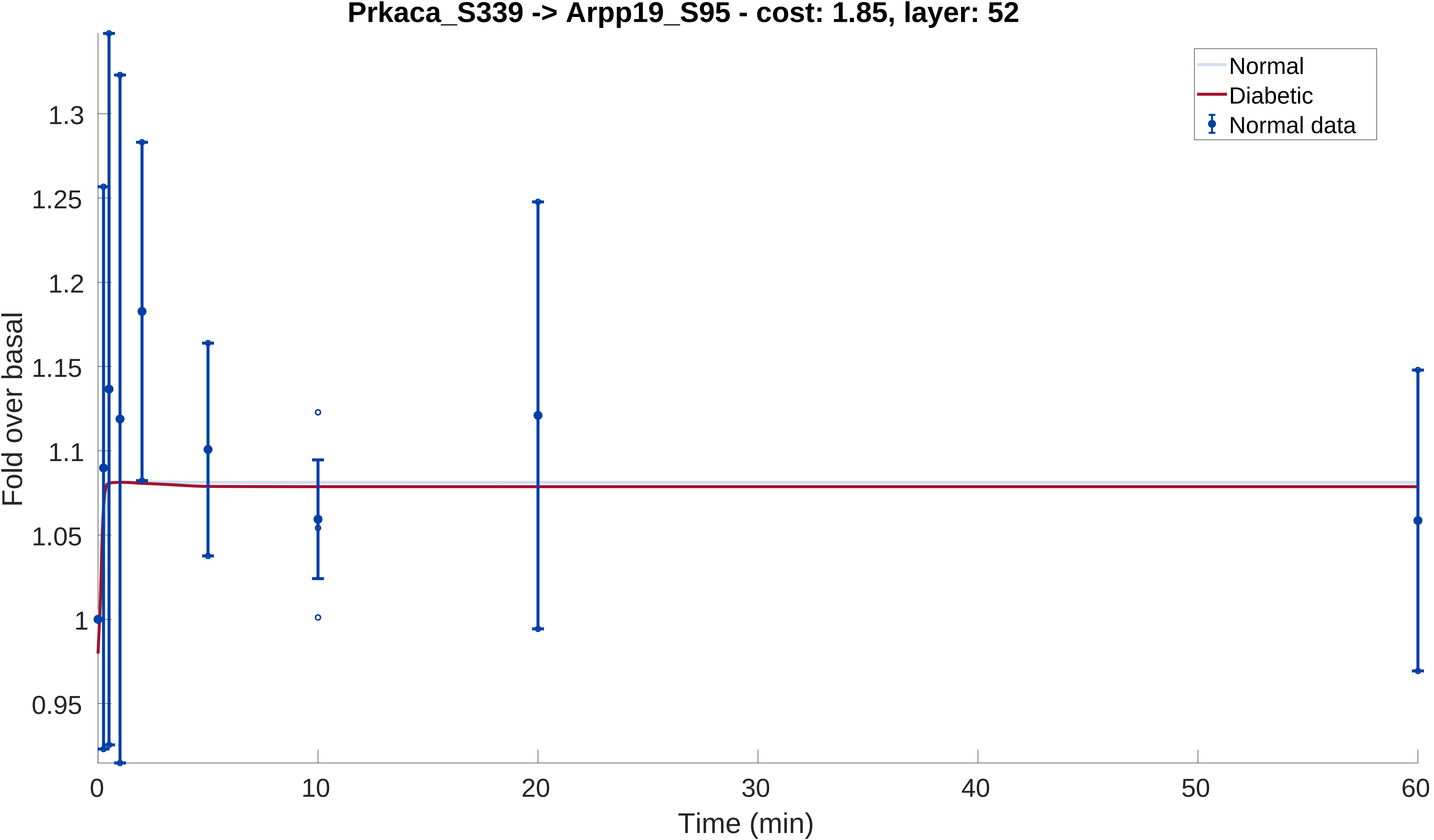
Prkaca_S339 -> Arpp19_S95 - cost: 1.85, layer: 52
Normal
Diabetic
Normal data
Fold over basal
1.3
1.25
1.2
1.15
1.1
1.05
1
0.95
0
10
20
30
40
50
60
Time (min)

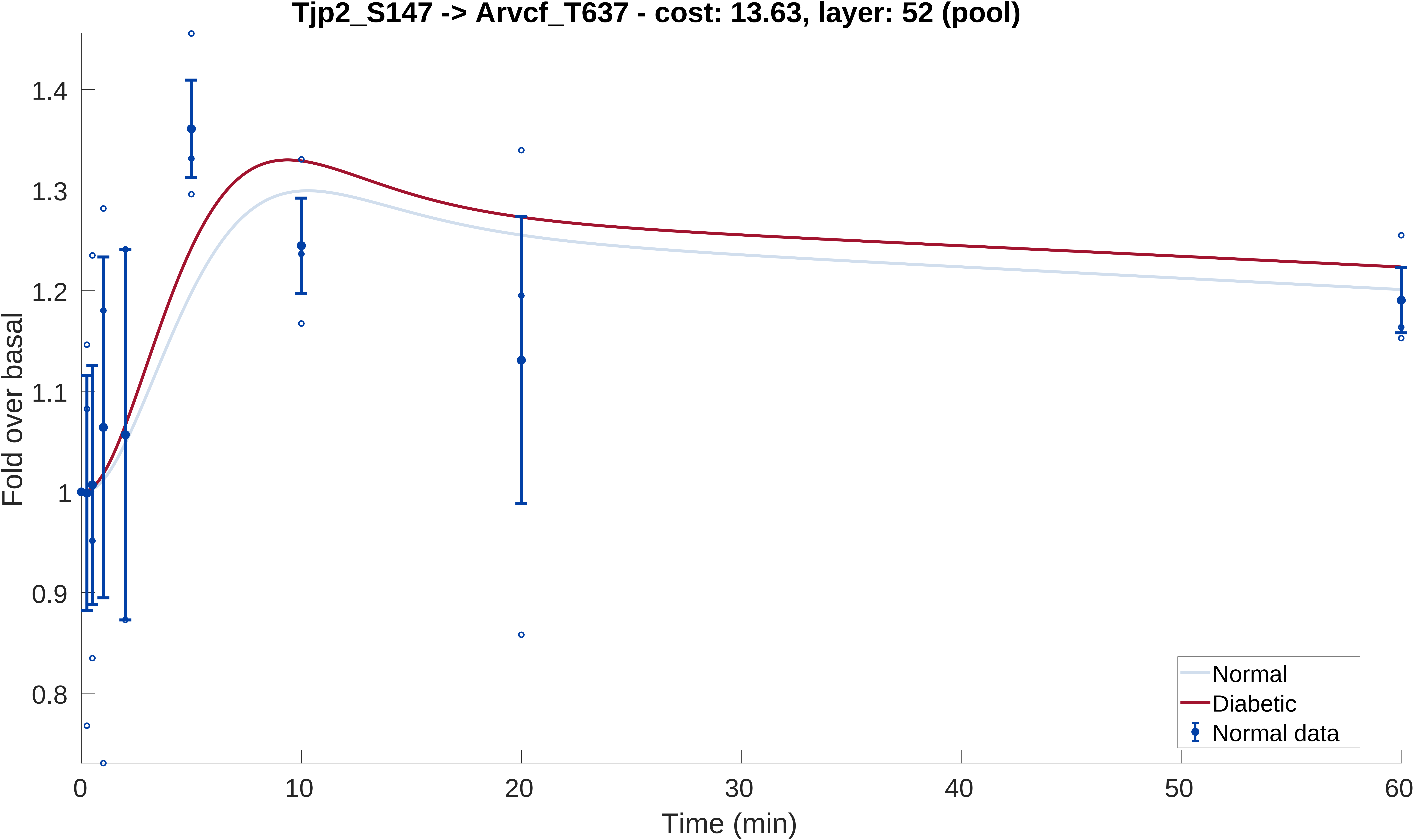

Tjp2_S147 -> Arvcf_T637 - cost: 13.63, layer: 52 (pool)
Fold over basal
1.4
1.3
1.2
1.1
1
0.9
0.8
0
10
20
30
40
50
60
Time (min)
Normal
Diabetic
Normal data

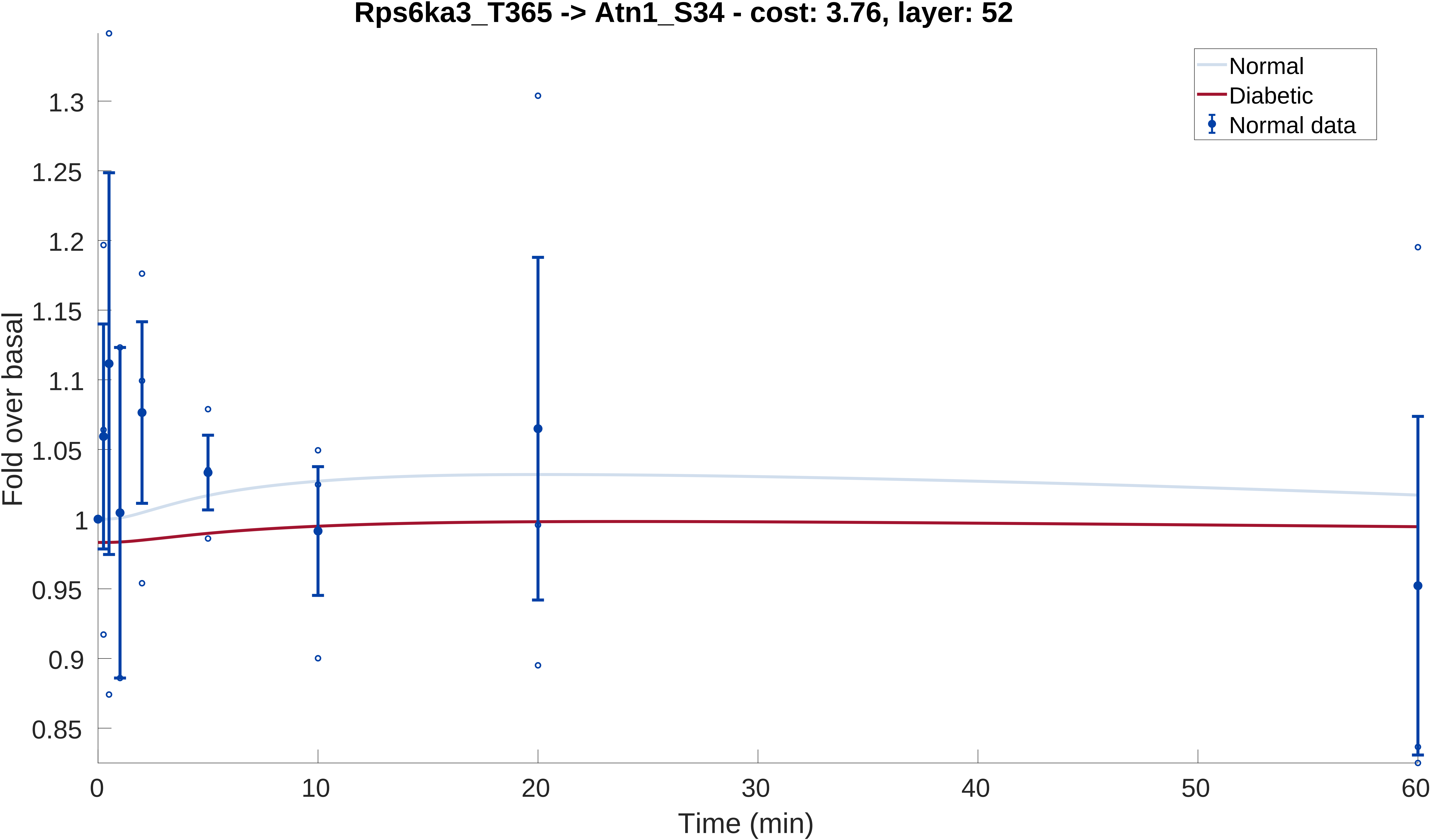

Rps6ka3_T365 -> Atn1_S34 - cost: 3.76, layer: 52
Normal
Diabetic
Normal data
Fold over basal
Time (min)
1.3
1.25
1.2
1.15
1.1
1.05
1
0.95
0.9
0.85
0
10
20
30
40
50
60

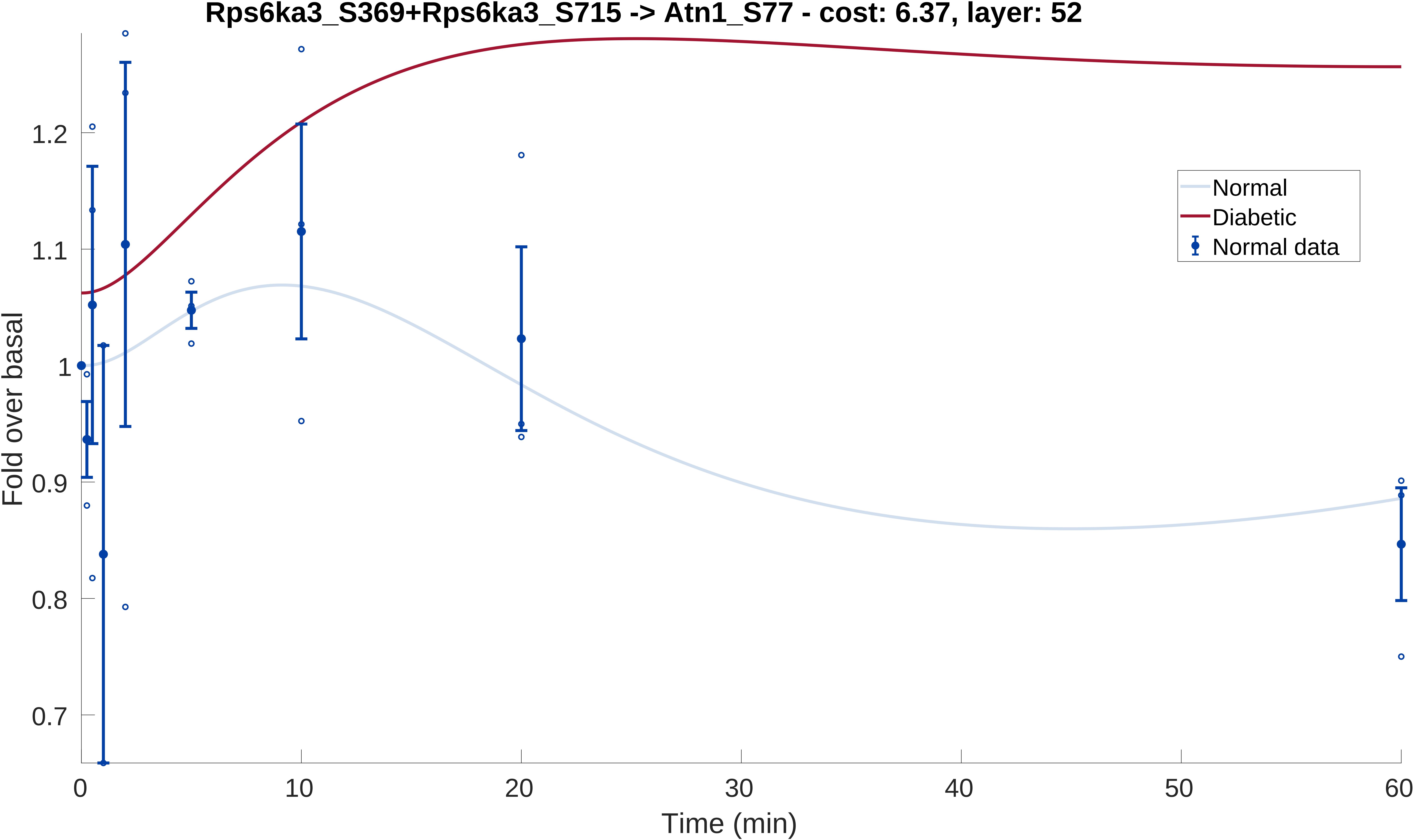

Rps6ka3_S369+Rps6ka3_S715 -> Atn1_S77 - cost: 6.37, layer: 52
Fold over basal
Time (min)
0.7
0.8
0.9
1
1.1
1.2
0
10
20
30
40
50
60
Normal
Diabetic
Normal data

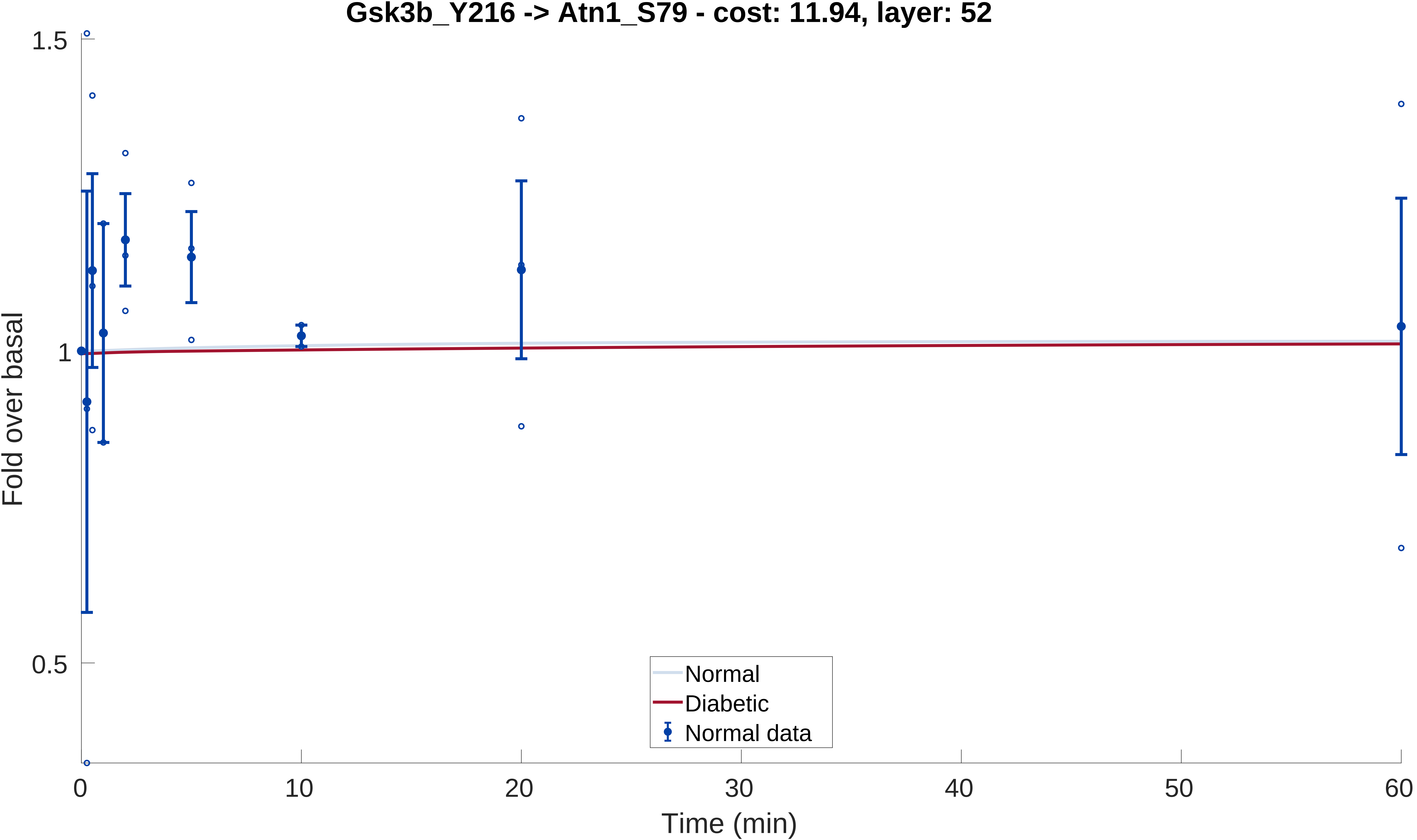

Gsk3b_Y216 -> Atn1_S79 - cost: 11.94, layer: 52
1.5
1
0.5
Fold over basal
0
10
20
30
40
50
60
Time (min)
Normal
Diabetic
Normal data

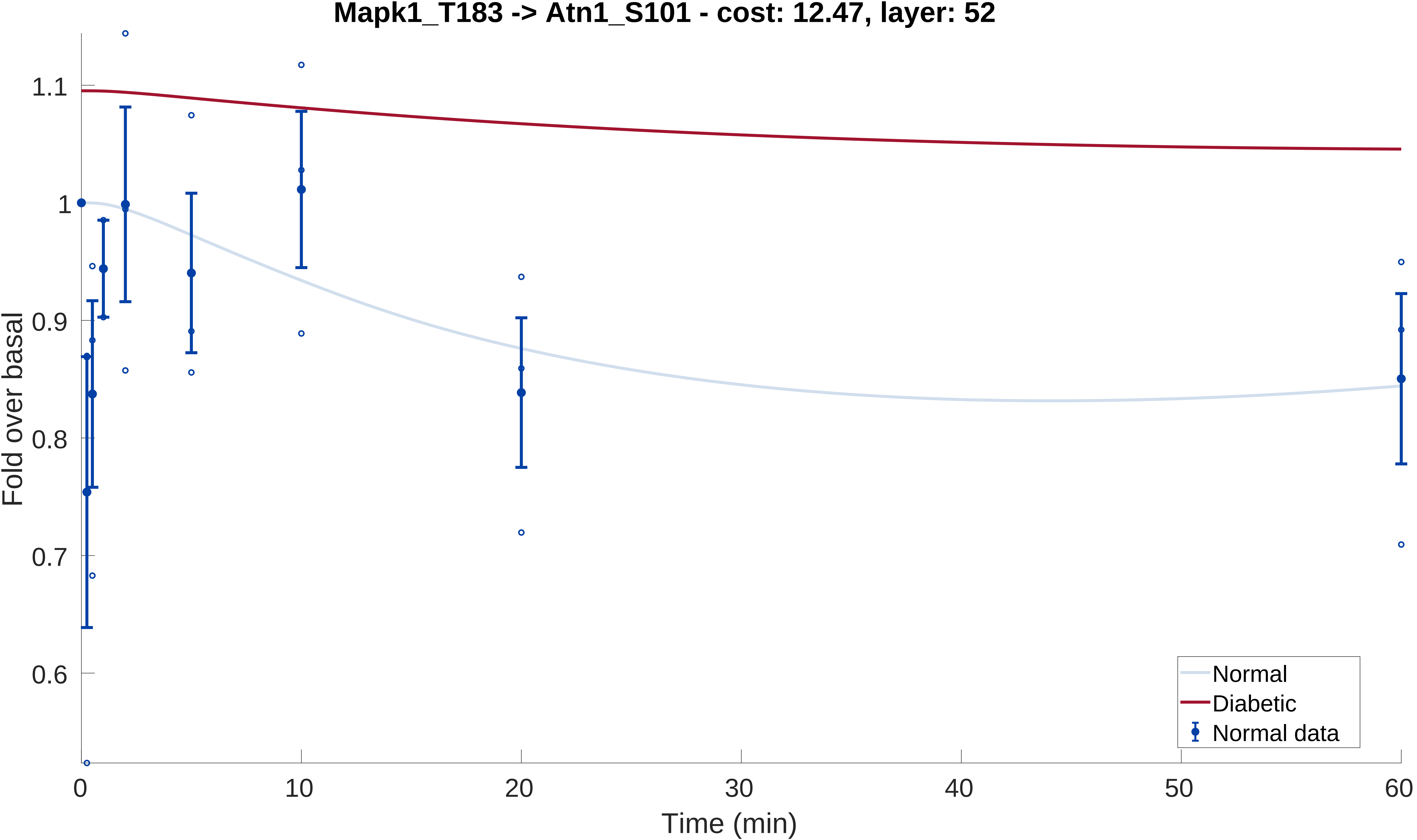
Mapk1_T183 -> Atn1_S101 - cost: 12.47, layer: 52
Fold over basal
1.1
1
0.9
0.8
0.7
0.6
0
10
20
30
40
50
60
Time (min)
Normal
Diabetic
Normal data

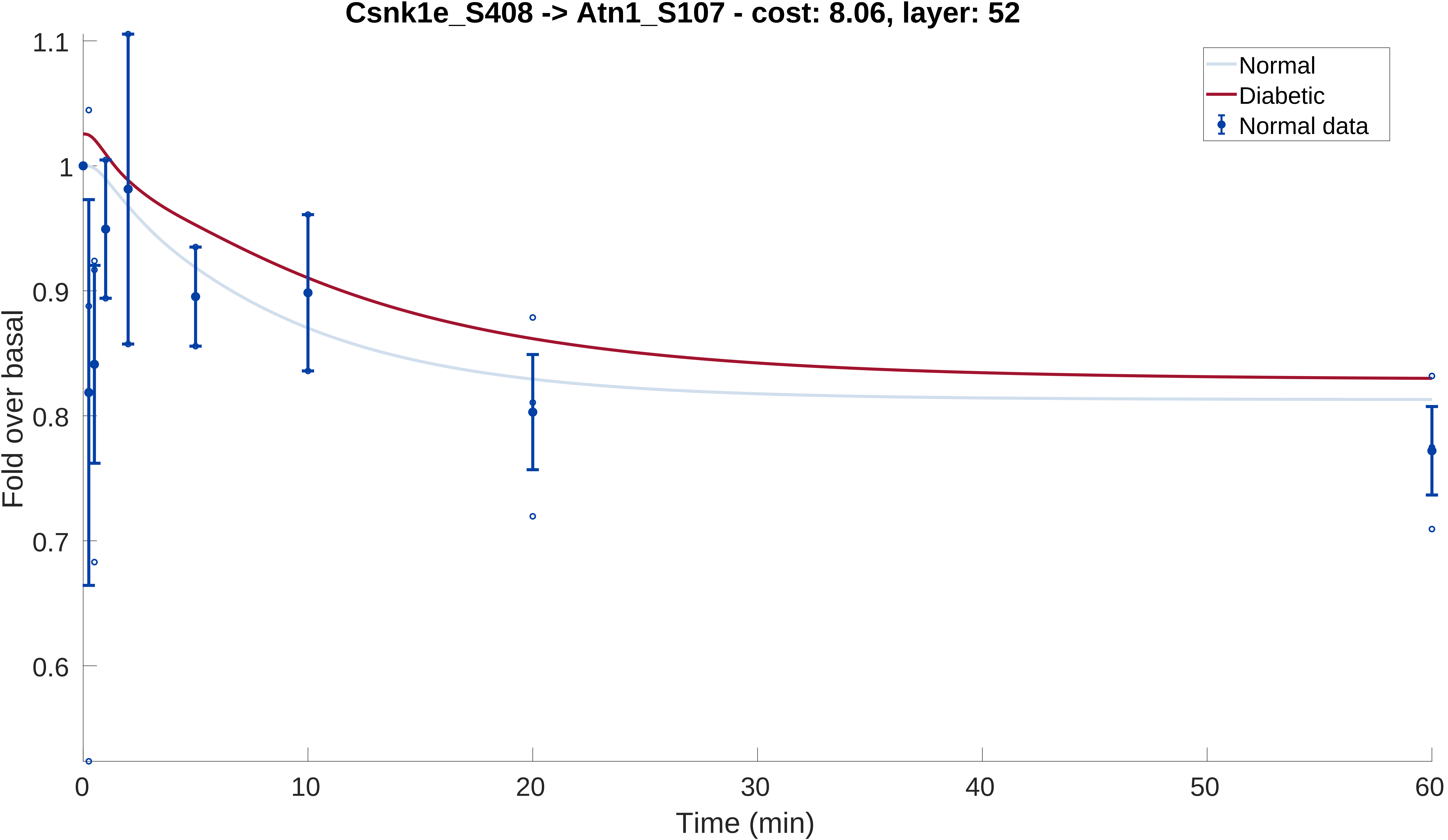

Csnk1e_S408 -> Atn1_S107 - cost: 8.06, layer: 52
Normal
Diabetic
Normal data
Fold over basal
1.1
1
0.9
0.8
0.7
0.6
0
10
20
30
40
50
60
Time (min)

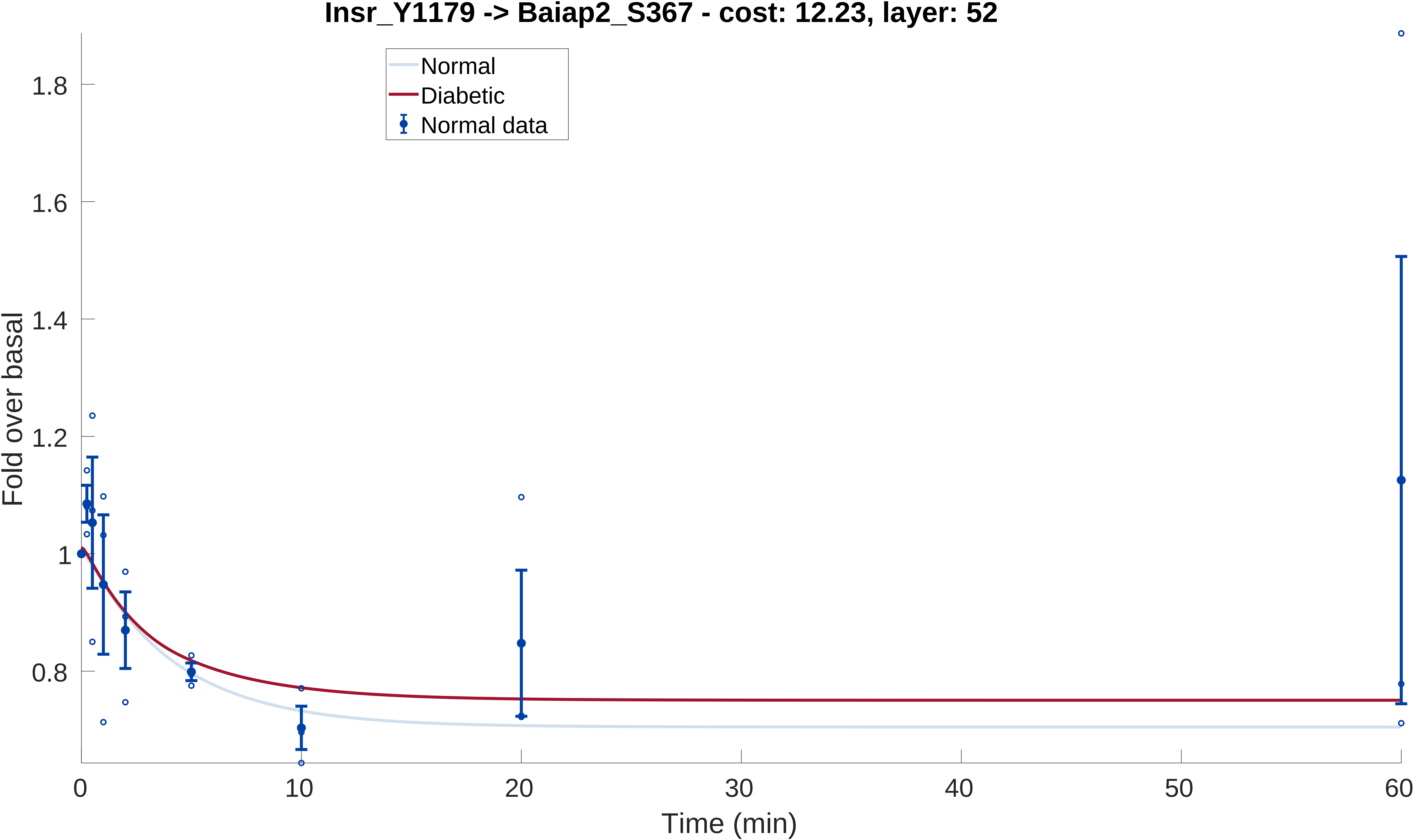

Insr_Y1179 -> Baiap2_S367 - cost: 12.23, layer: 52
Normal
Diabetic
Normal data
Fold over basal
1.8
1.6
1.4
1.2
1
0.8
0
10
20
30
40
50
60
Time (min)

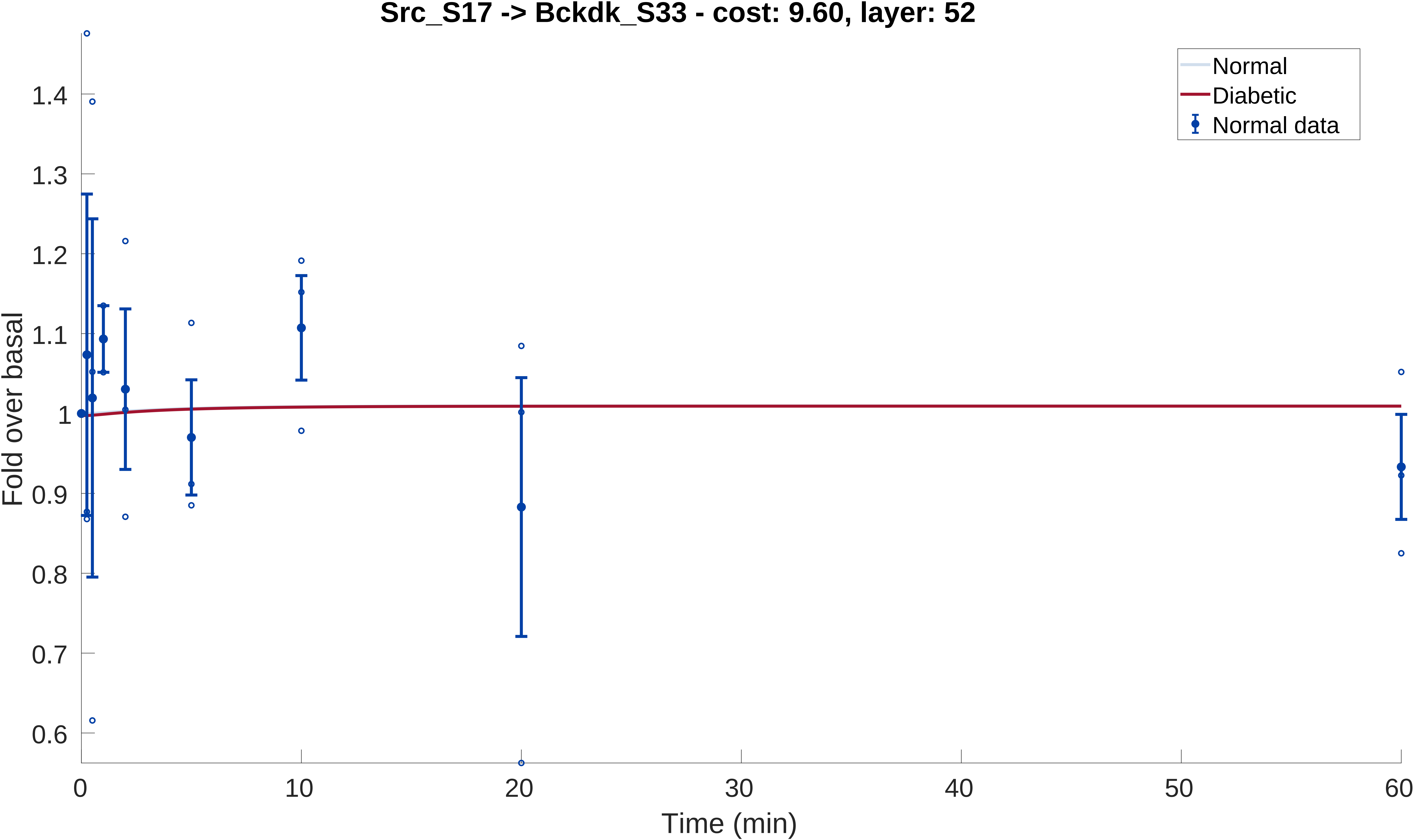

Src_S17 -> Bckdk_S33 - cost: 9.60, layer: 52
Normal
Diabetic
Normal data
Fold over basal
Time (min)
1.4
1.3
1.2
1.1
1
0.9
0.8
0.7
0.6
0
10
20
30
40
50
60

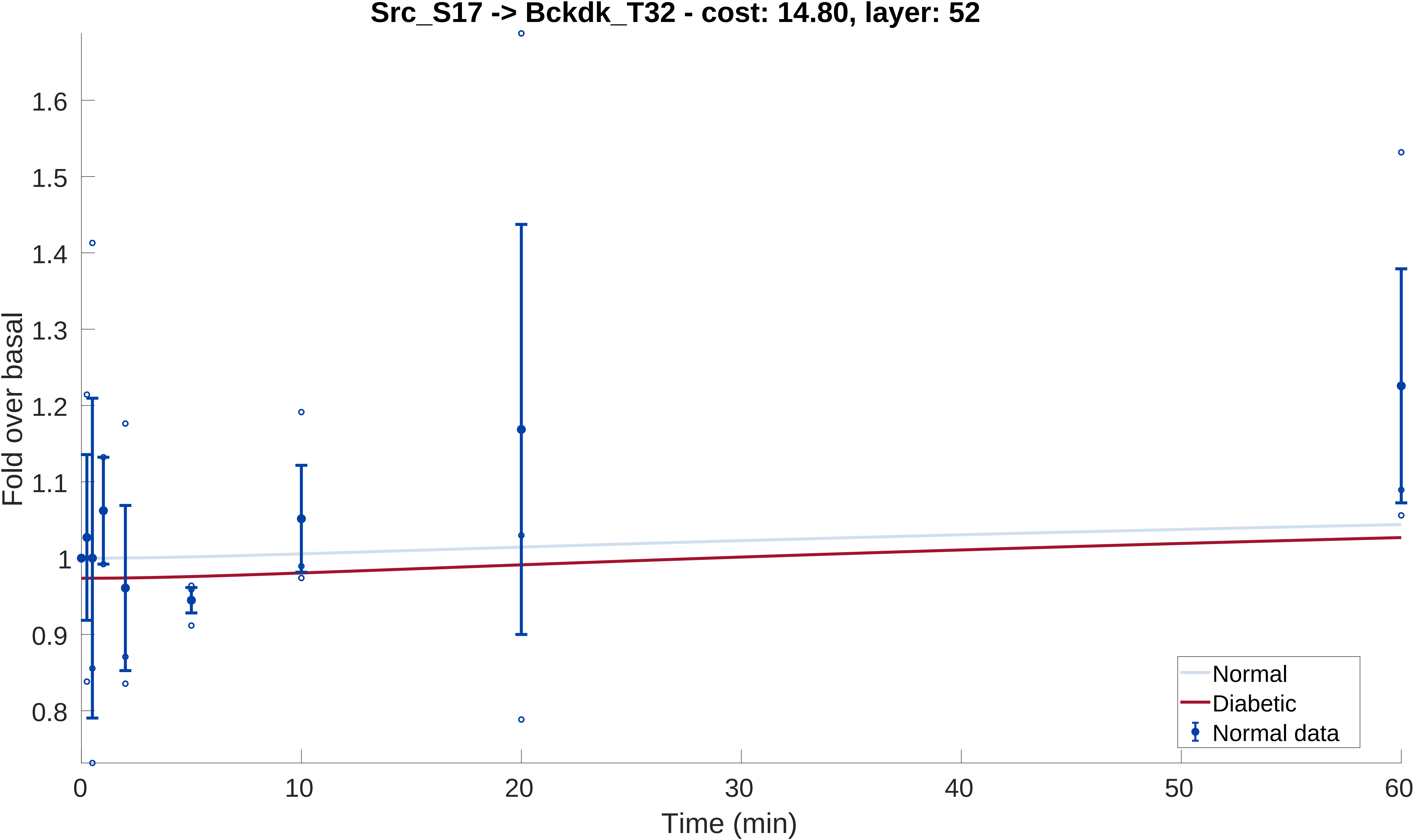

Src_S17 -> Bckdk_T32 - cost: 14.80, layer: 52
Fold over basal
1.6
1.5
1.4
1.3
1.2
1.1
1
0.9
0.8
0
10
20
30
40
50
60
Time (min)
Normal
Diabetic
Normal data

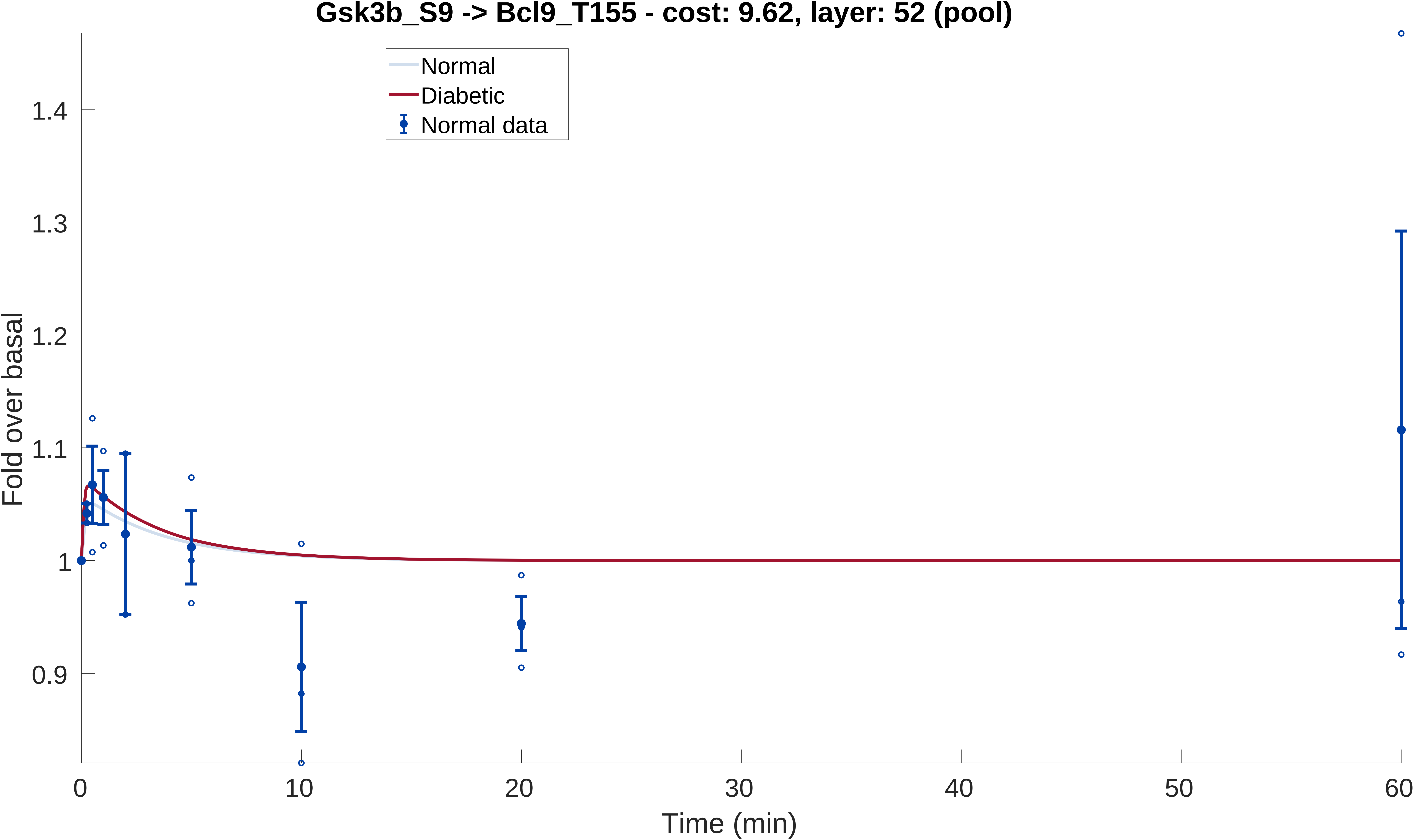
Gsk3b_S9 -> Bcl9_T155 - cost: 9.62, layer: 52 (pool)
Normal
Diabetic
Normal data
Fold over basal
1.4
1.3
1.2
1.1
1
0.9
0
10
20
30
40
50
60
Time (min)

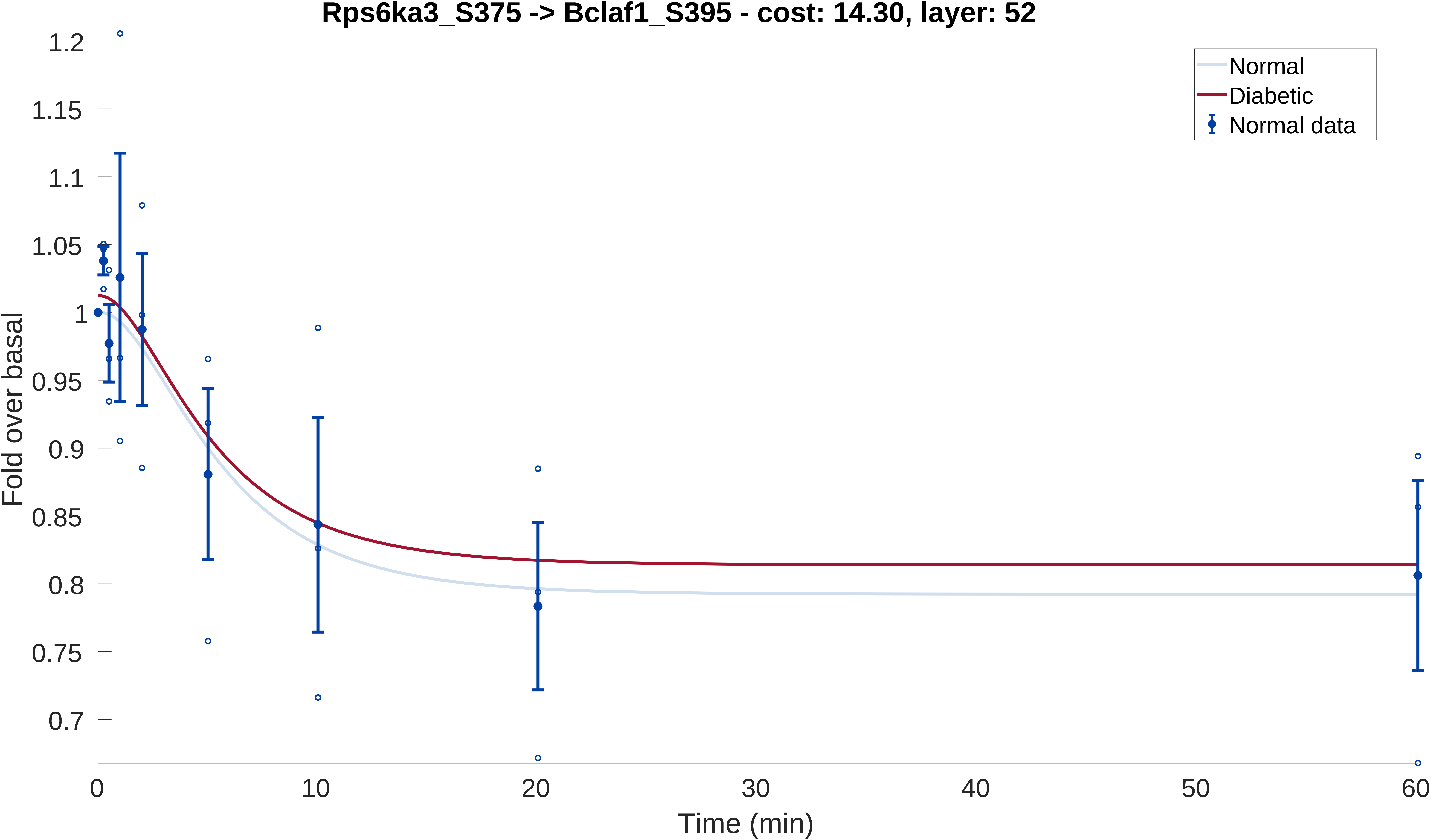

Rps6ka3_S375 -> Bclaf1_S395 - cost: 14.30, layer: 52
Normal
Diabetic
Normal data
Fold over basal
Time (min)
1.2
1.15
1.1
1.05
1
0.95
0.9
0.85
0.8
0.75
0.7
0
10
20
30
40
50
60

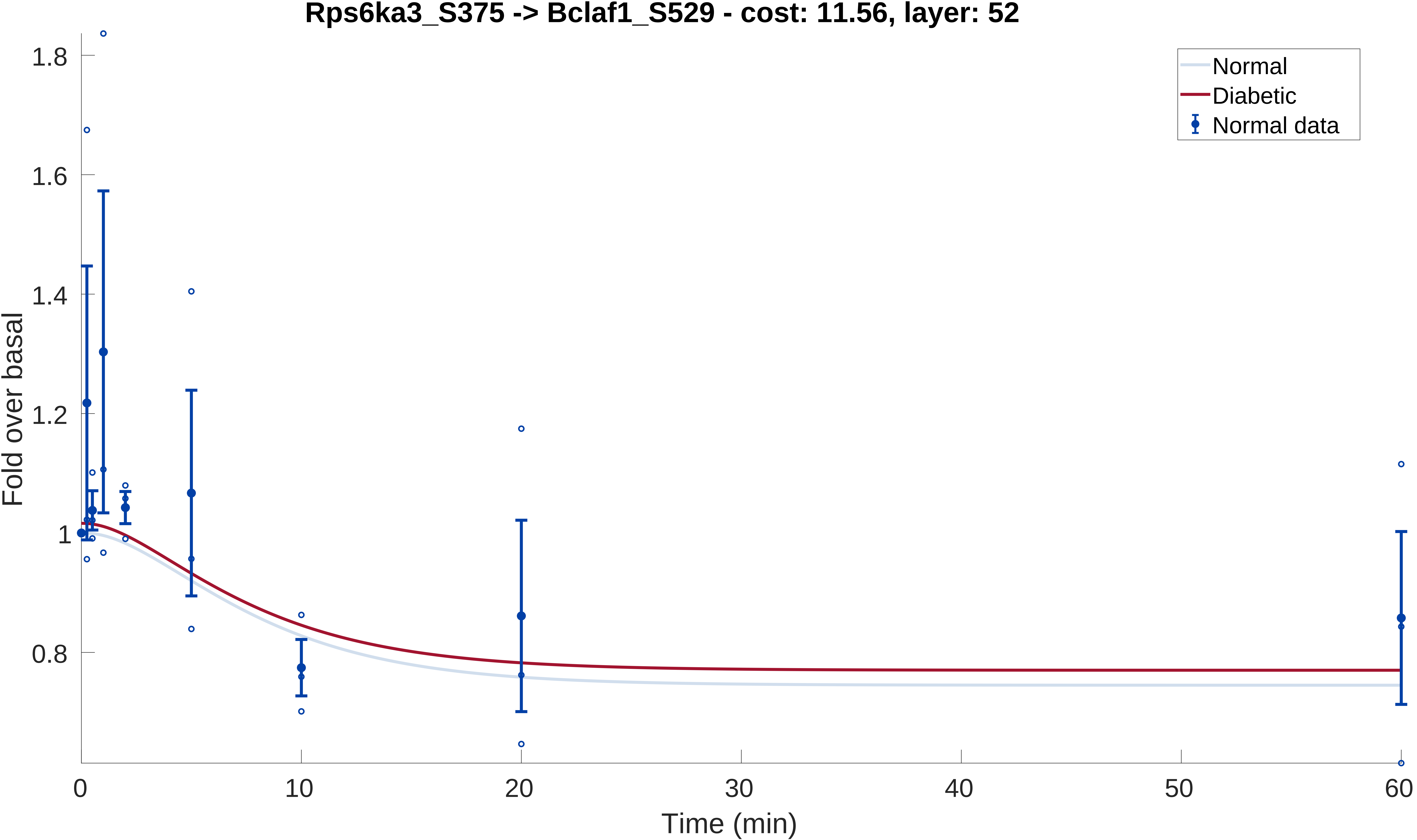

Rps6ka3_S375 -> Bclaf1_S529 - cost: 11.56, layer: 52
Normal
Diabetic
Normal data
Fold over basal
1.8
1.6
1.4
1.2
1
0.8
0
10
20
30
40
50
60
Time (min)

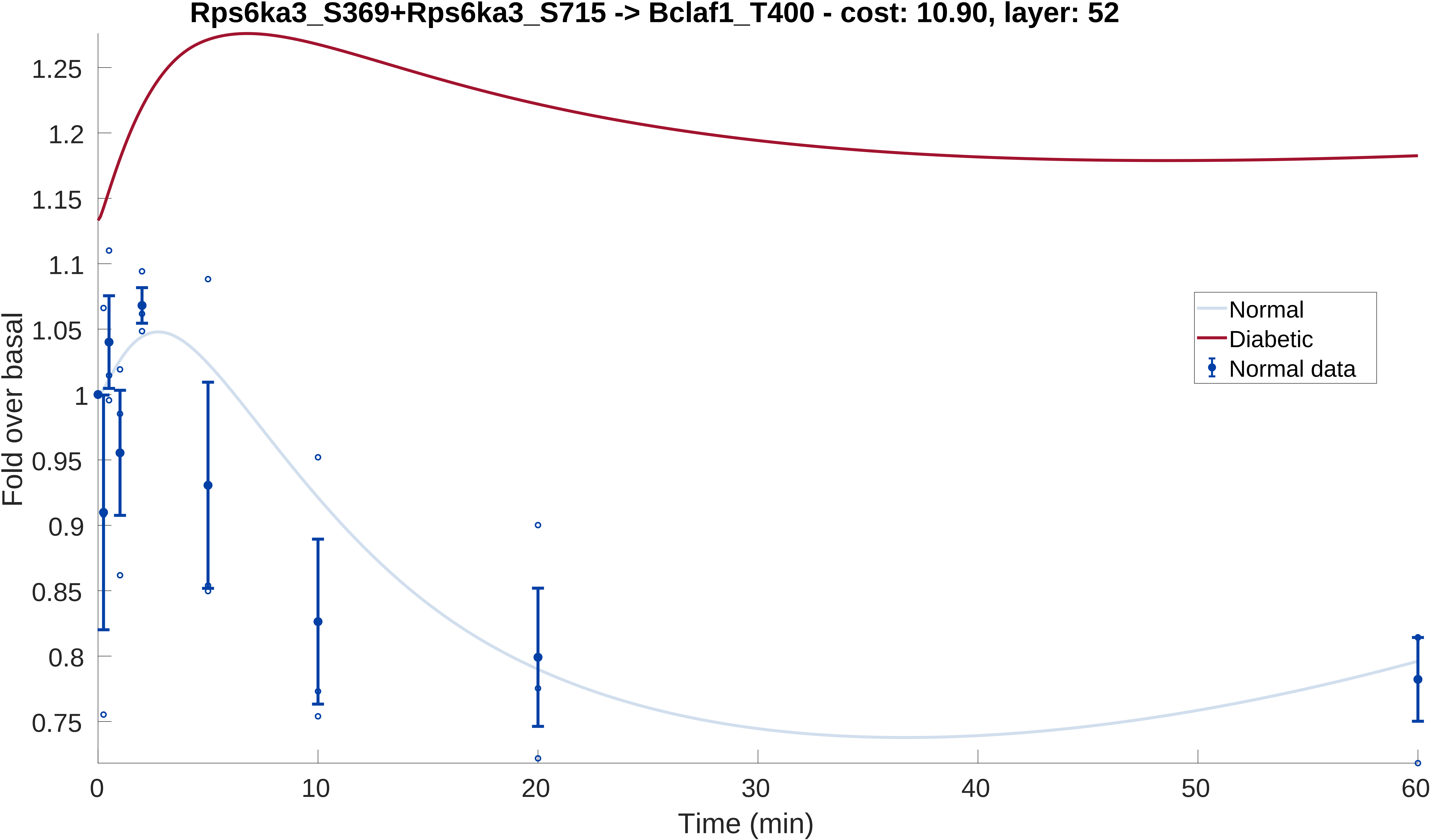
Rps6ka3_S369+Rps6ka3_S715 -> Bclaf1_T400 - cost: 10.90, layer: 52
Fold over basal
1.25
1.2
1.15
1.1
1.05
1
0.95
0.9
0.85
0.8
0.75
0
10
20
30
40
50
60
Time (min)
Normal
Diabetic
Normal data

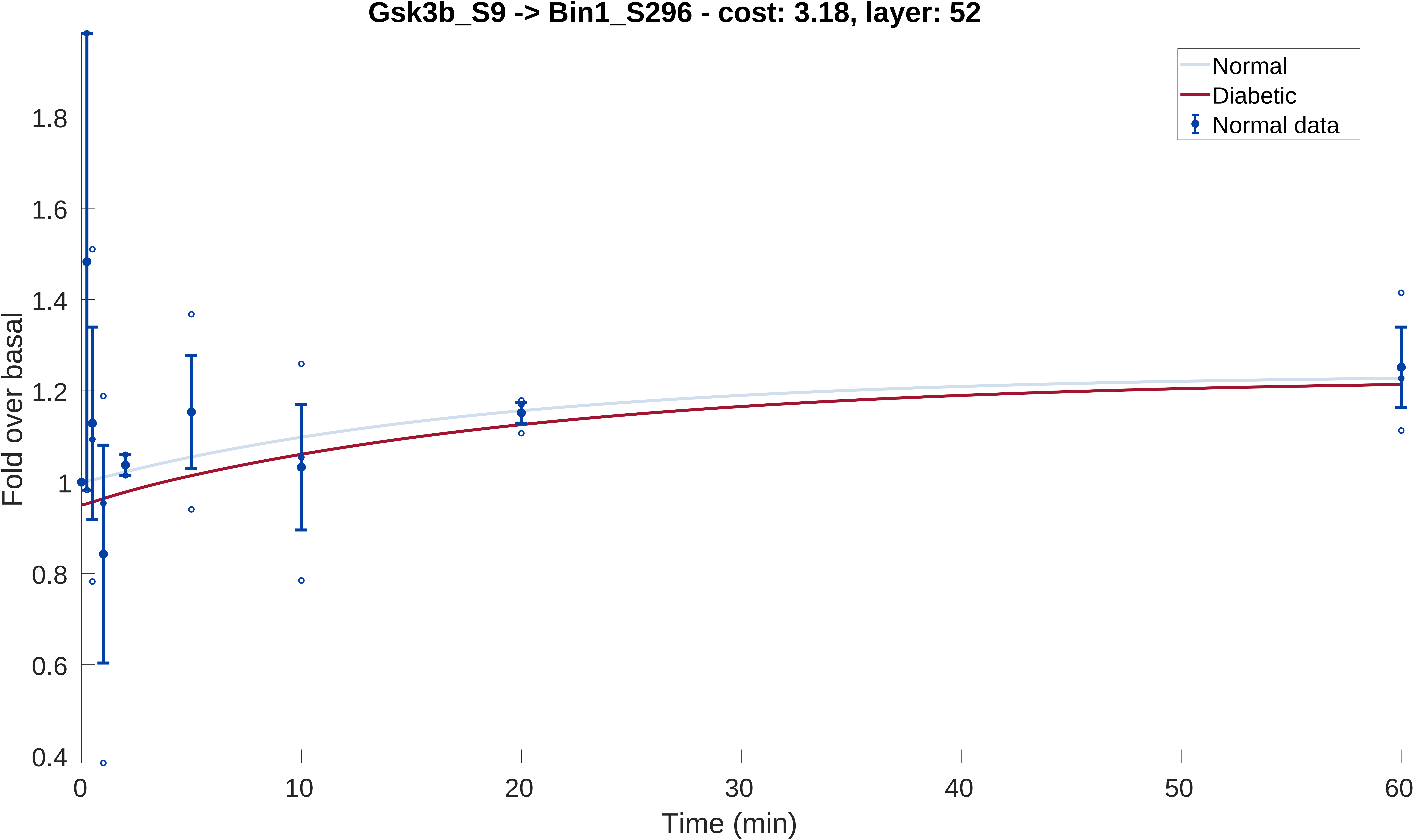
Gsk3b_S9 -> Bin1_S296 - cost: 3.18, layer: 52
Normal
Diabetic
Normal data
Fold over basal
1.8
1.6
1.4
1.2
1
0.8
0.6
0.4
0
10
20
30
40
50
60
Time (min)

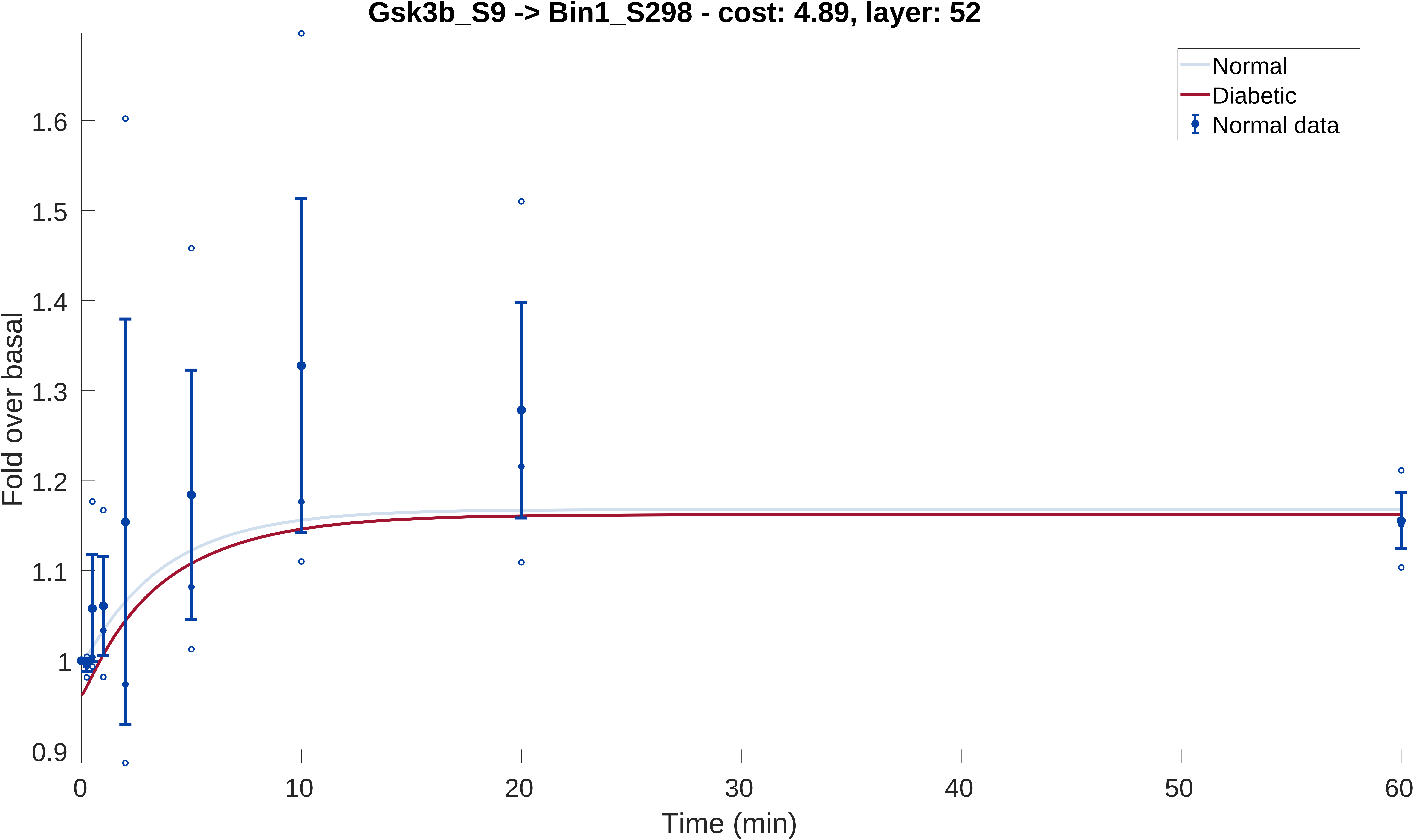

Gsk3b_S9 -> Bin1_S298 - cost: 4.89, layer: 52
Normal
Diabetic
Normal data
Fold over basal
1.6
1.5
1.4
1.3
1.2
1.1
1
0.9
0
10
20
30
40
50
60
Time (min)

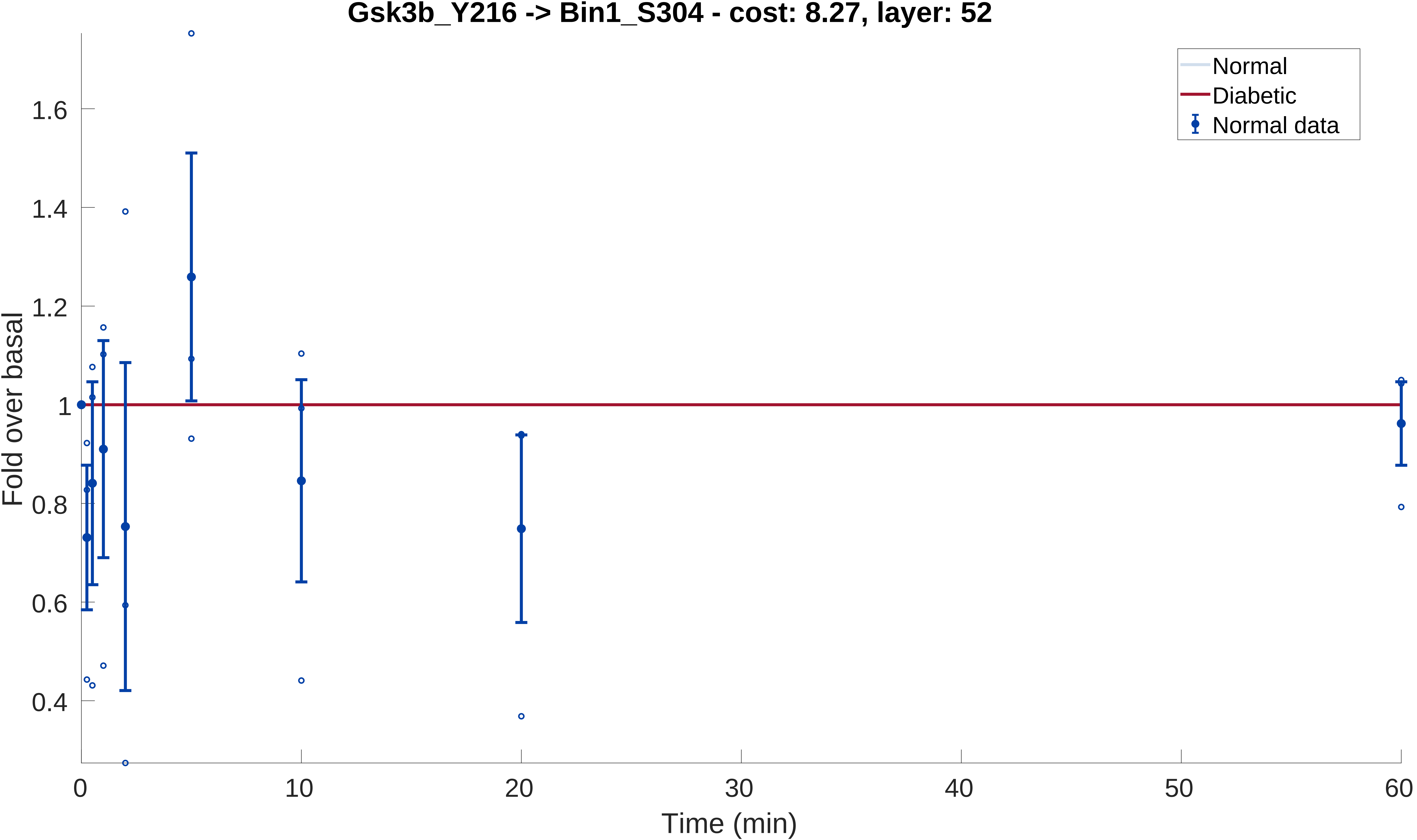

Gsk3b_Y216 -> Bin1_S304 - cost: 8.27, layer: 52
Normal
Diabetic
Normal data
Fold over basal
1.6
1.4
1.2
1
0.8
0.6
0.4
0
10
20
30
40
50
60
Time (min)

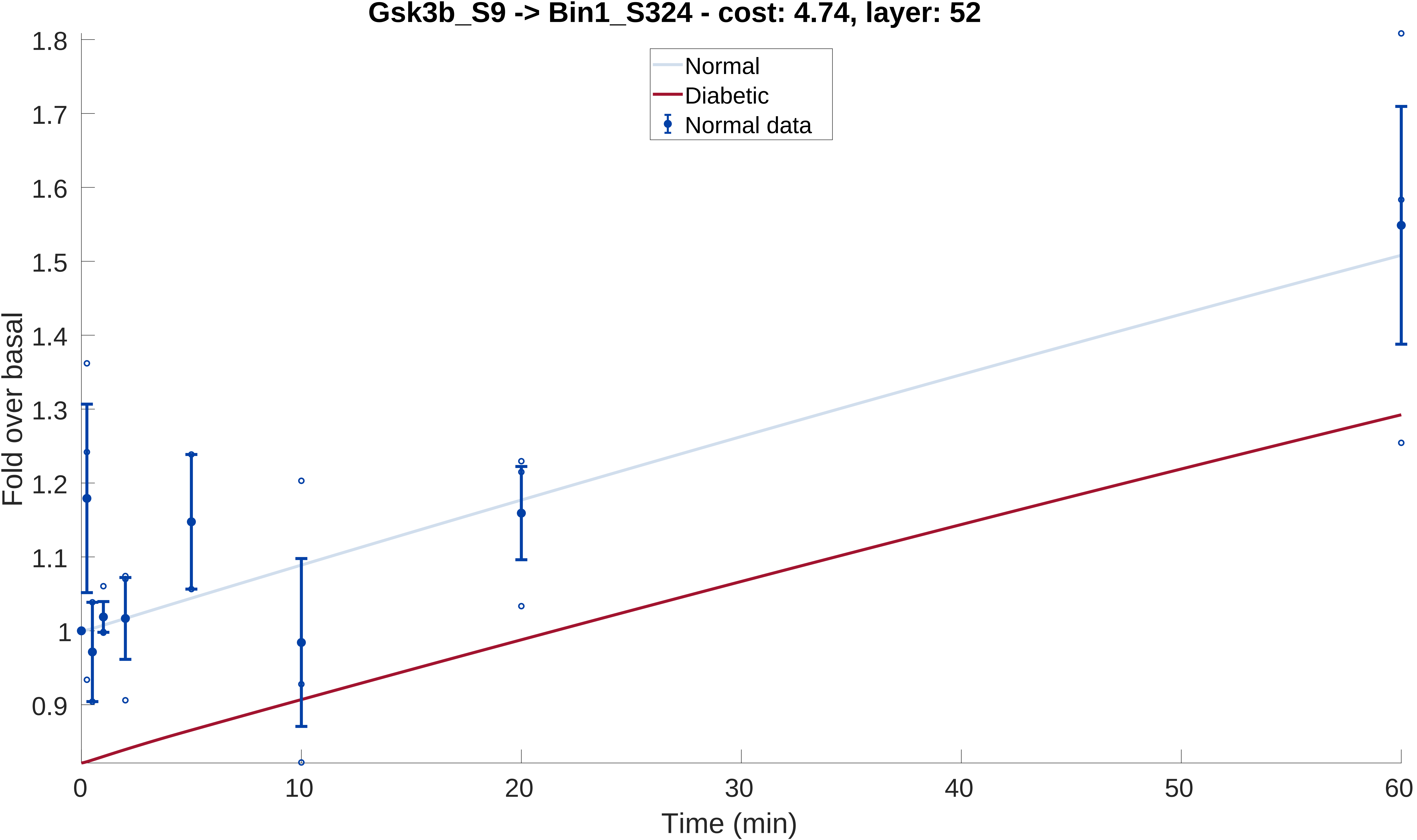

Gsk3b_S9 -> Bin1_S324 - cost: 4.74, layer: 52
Normal
Diabetic
Normal data
Fold over basal
Time (min)
1.8
1.7
1.6
1.5
1.4
1.3
1.2
1.1
1
0.9
0
10
20
30
40
50
60

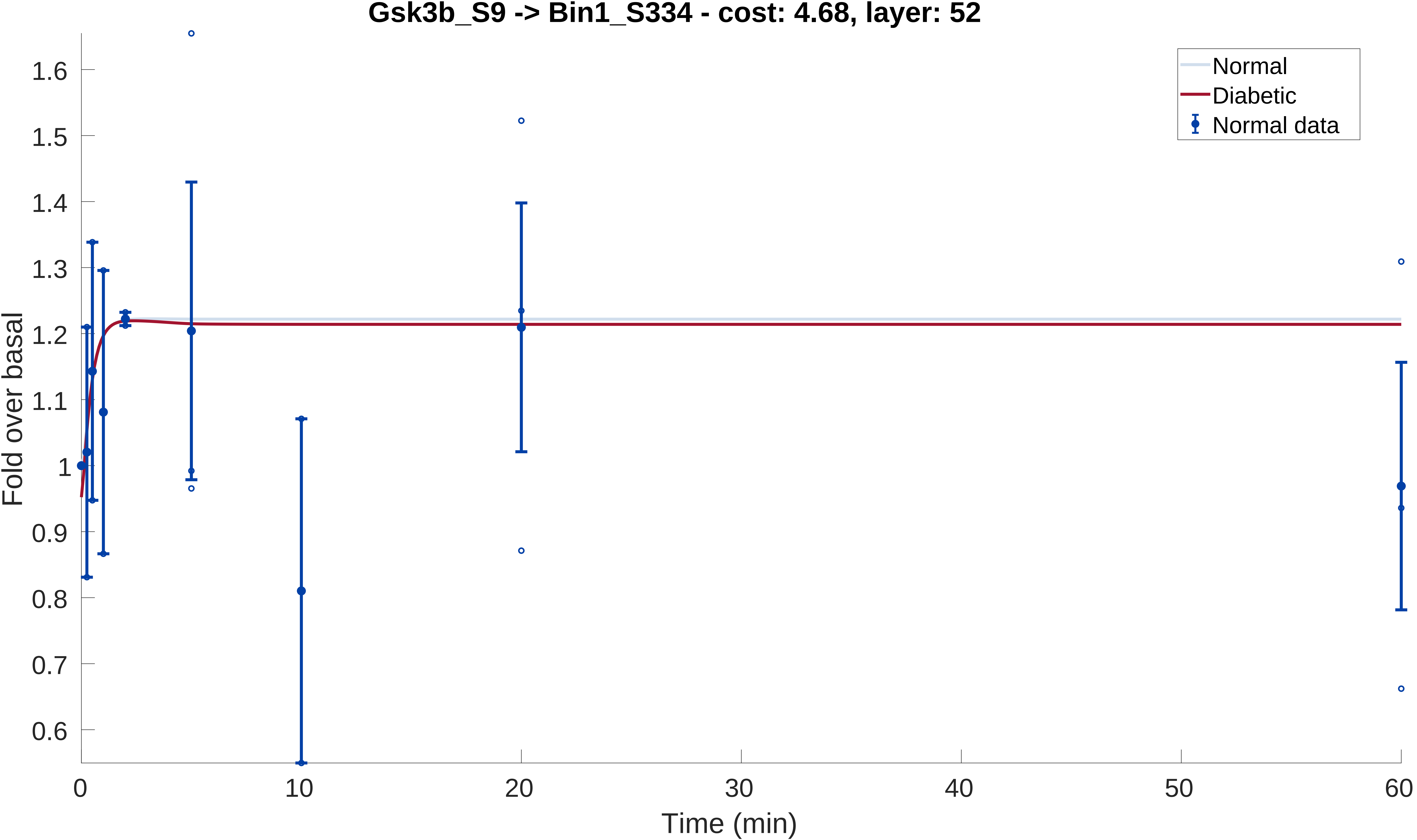
Gsk3b_S9 -> Bin1_S334 - cost: 4.68, layer: 52
Normal
Diabetic
Normal data
Fold over basal
Time (min)
0.6
0.7
0.8
0.9
1
1.1
1.2
1.3
1.4
1.5
1.6
0
10
20
30
40
50
60

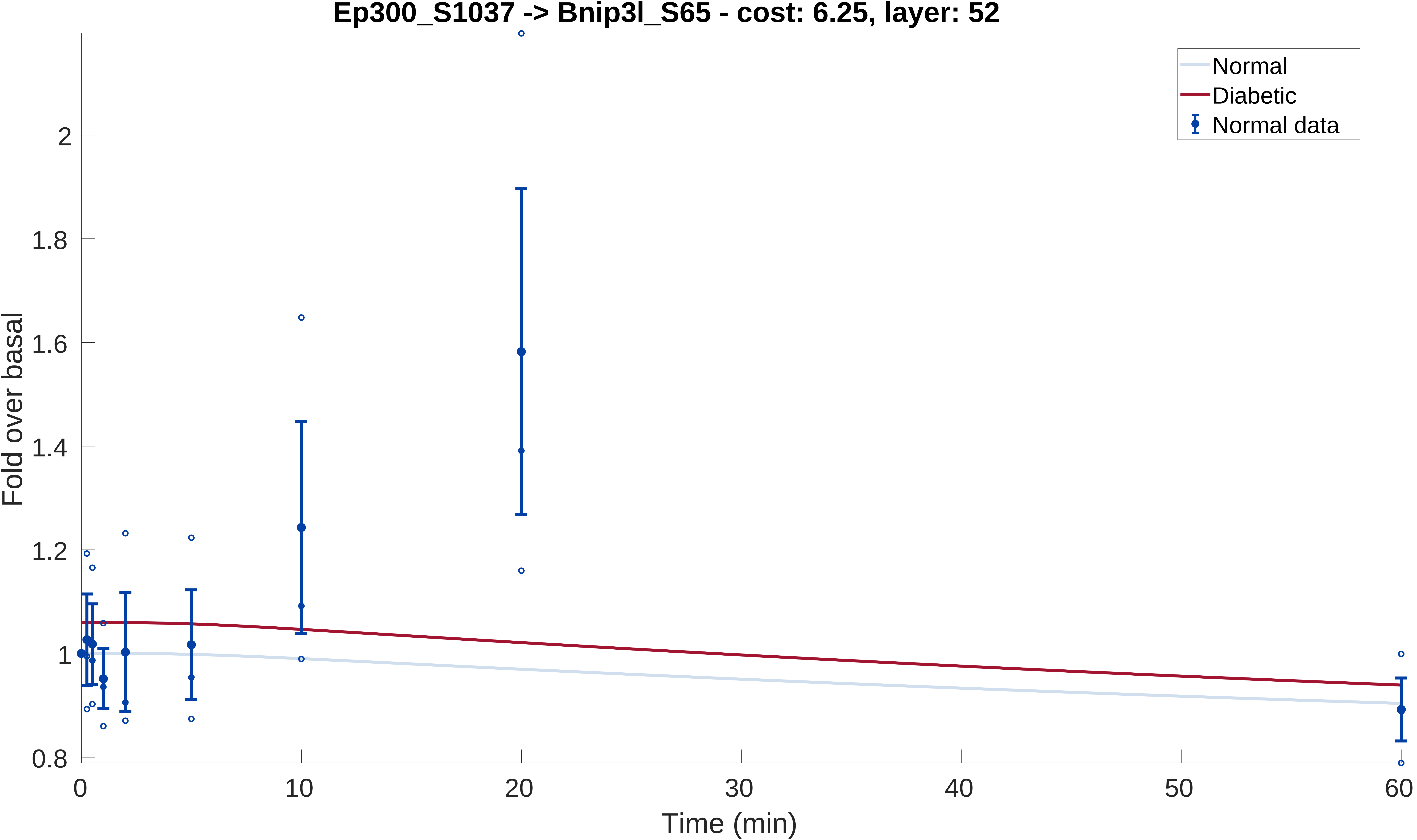
Ep300_S1037 -> Bnip3l_S65 - cost: 6.25, layer: 52
Normal
Diabetic
Normal data
Fold over basal
Time (min)
0.8
1
1.2
1.4
1.6
1.8
2
0
10
20
30
40
50
60

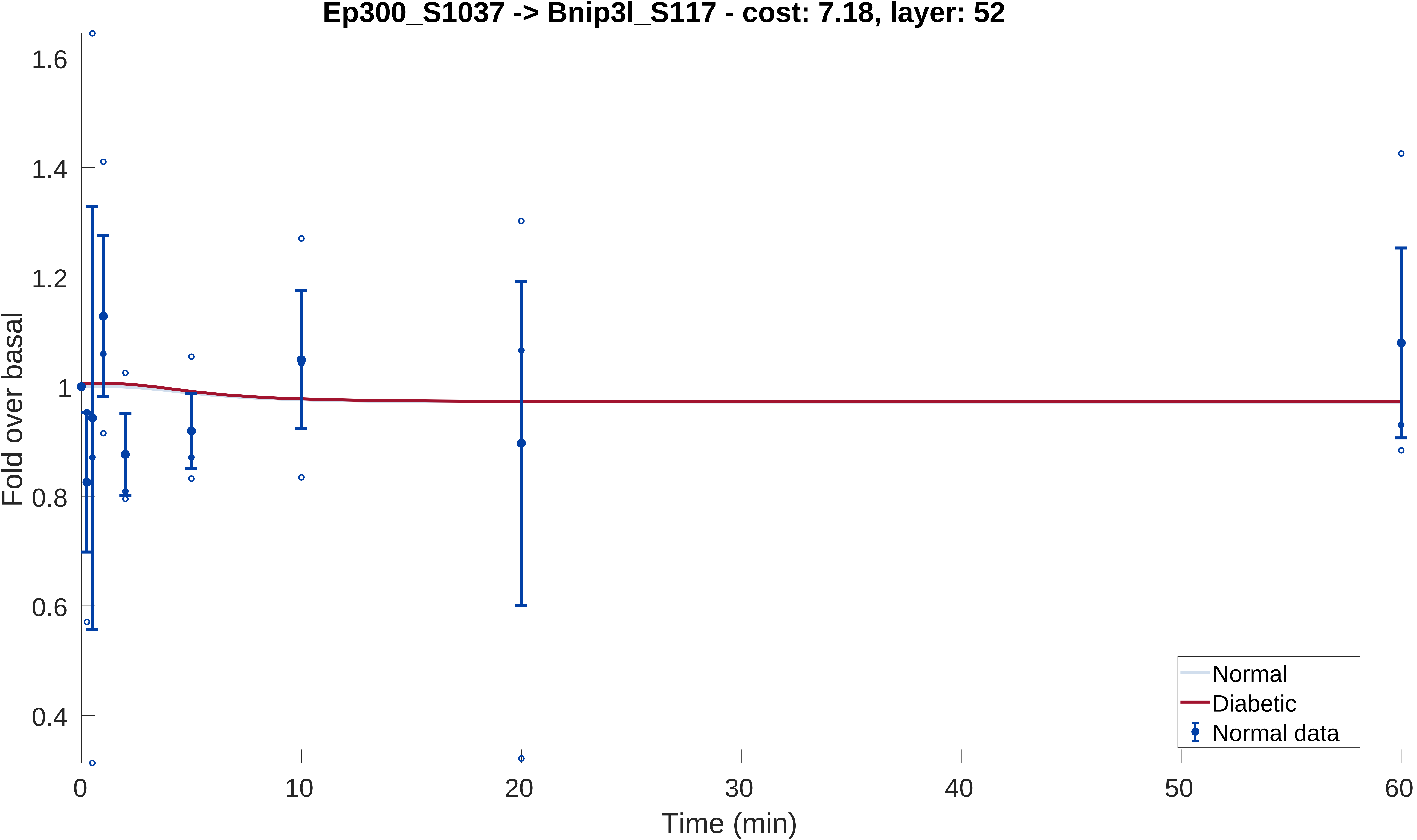

Ep300_S1037 -> Bnip3l_S117 - cost: 7.18, layer: 52
Fold over basal
1.6
1.4
1.2
1
0.8
0.6
0.4
0
10
20
30
40
50
60
Time (min)
Normal
Diabetic
Normal data

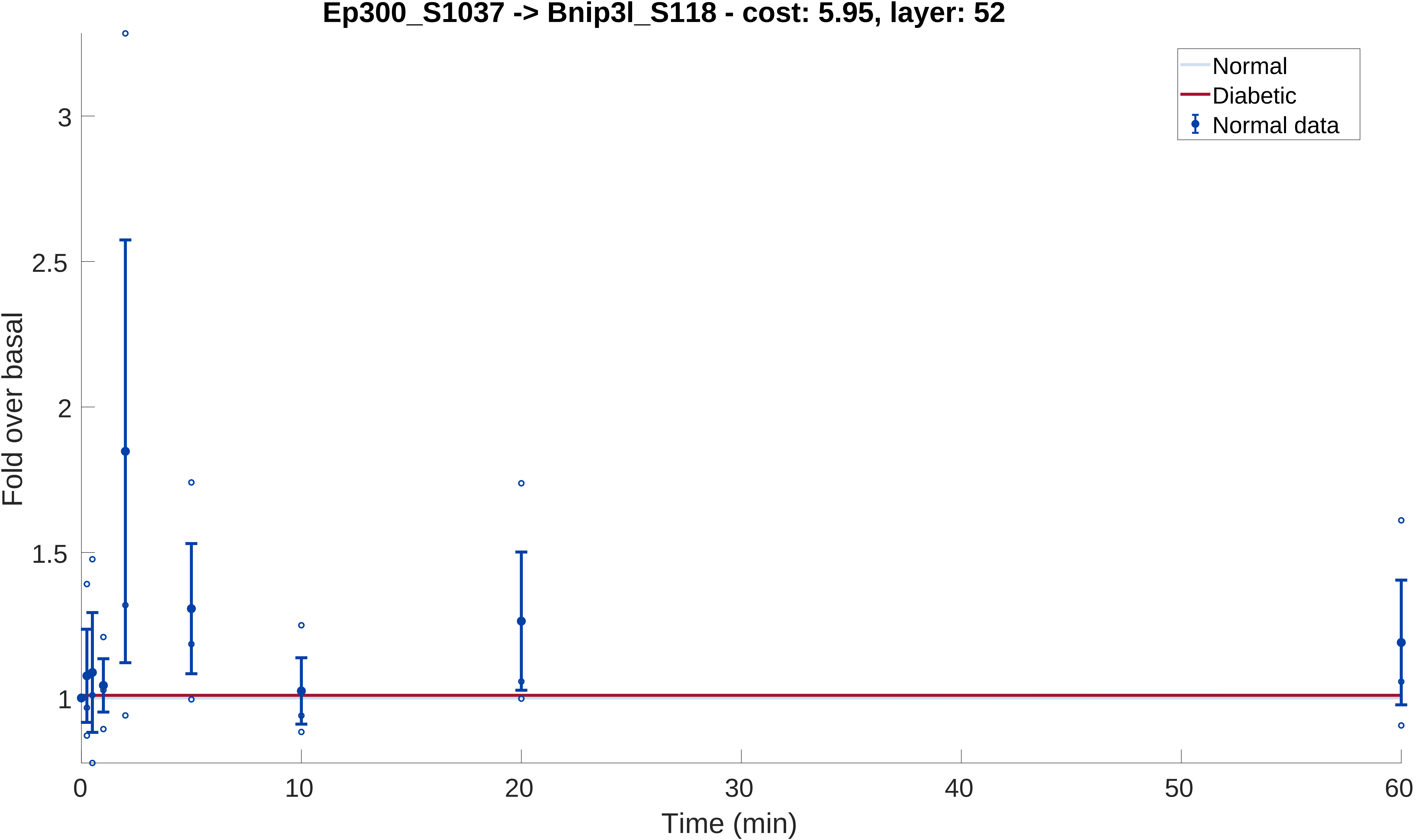

Ep300_S1037 -> Bnip3l_S118 - cost: 5.95, layer: 52
Normal
Diabetic
Normal data
Fold over basal
Time (min)
3
2.5
2
1.5
1
0
10
20
30
40
50
60

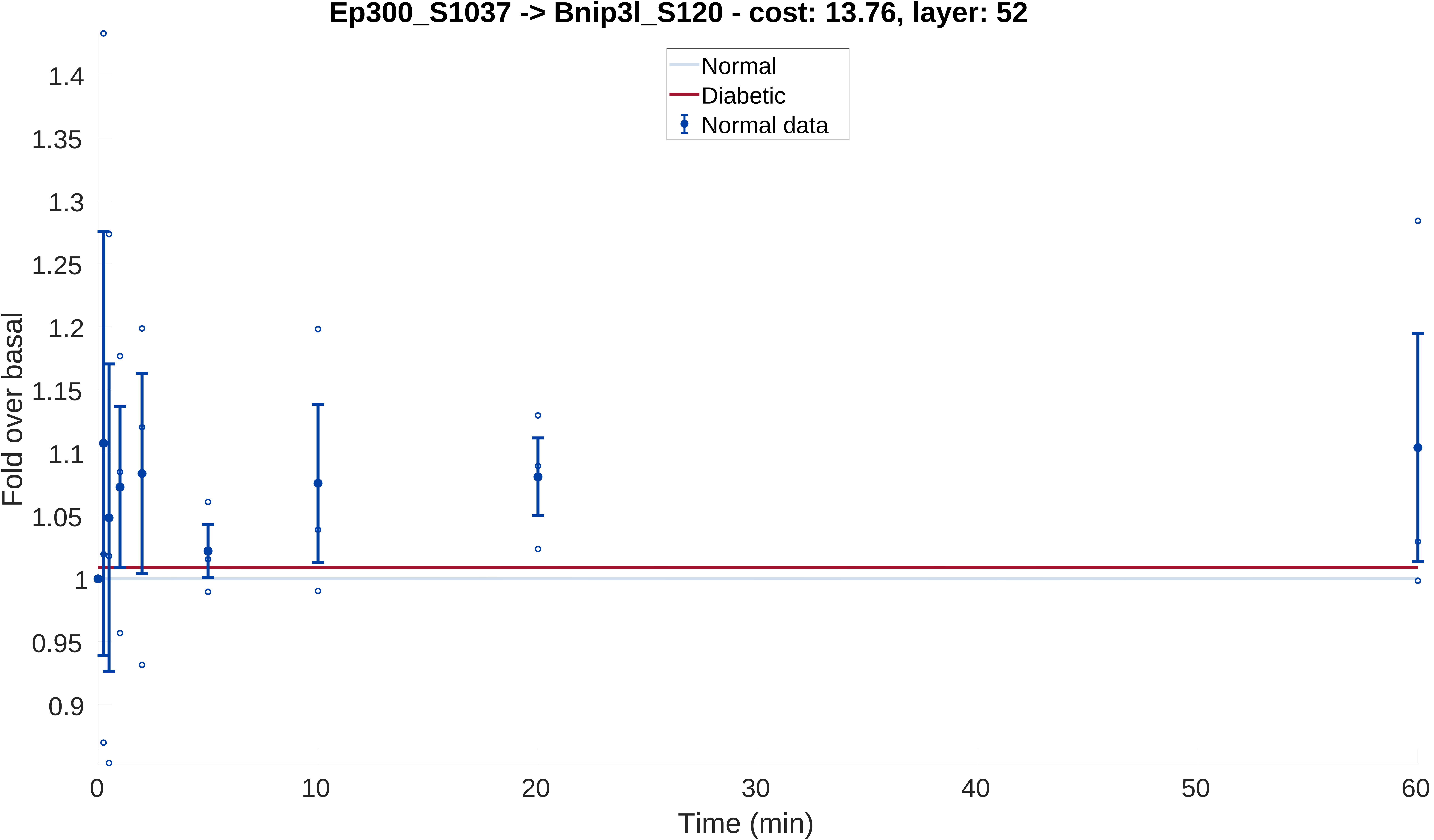

Ep300_S1037 -> Bnip3l_S120 - cost: 13.76, layer: 52
Normal
Diabetic
Normal data
Fold over basal
1.4
1.35
1.3
1.25
1.2
1.15
1.1
1.05
1
0.95
0.9
0
10
20
30
40
50
60
Time (min)

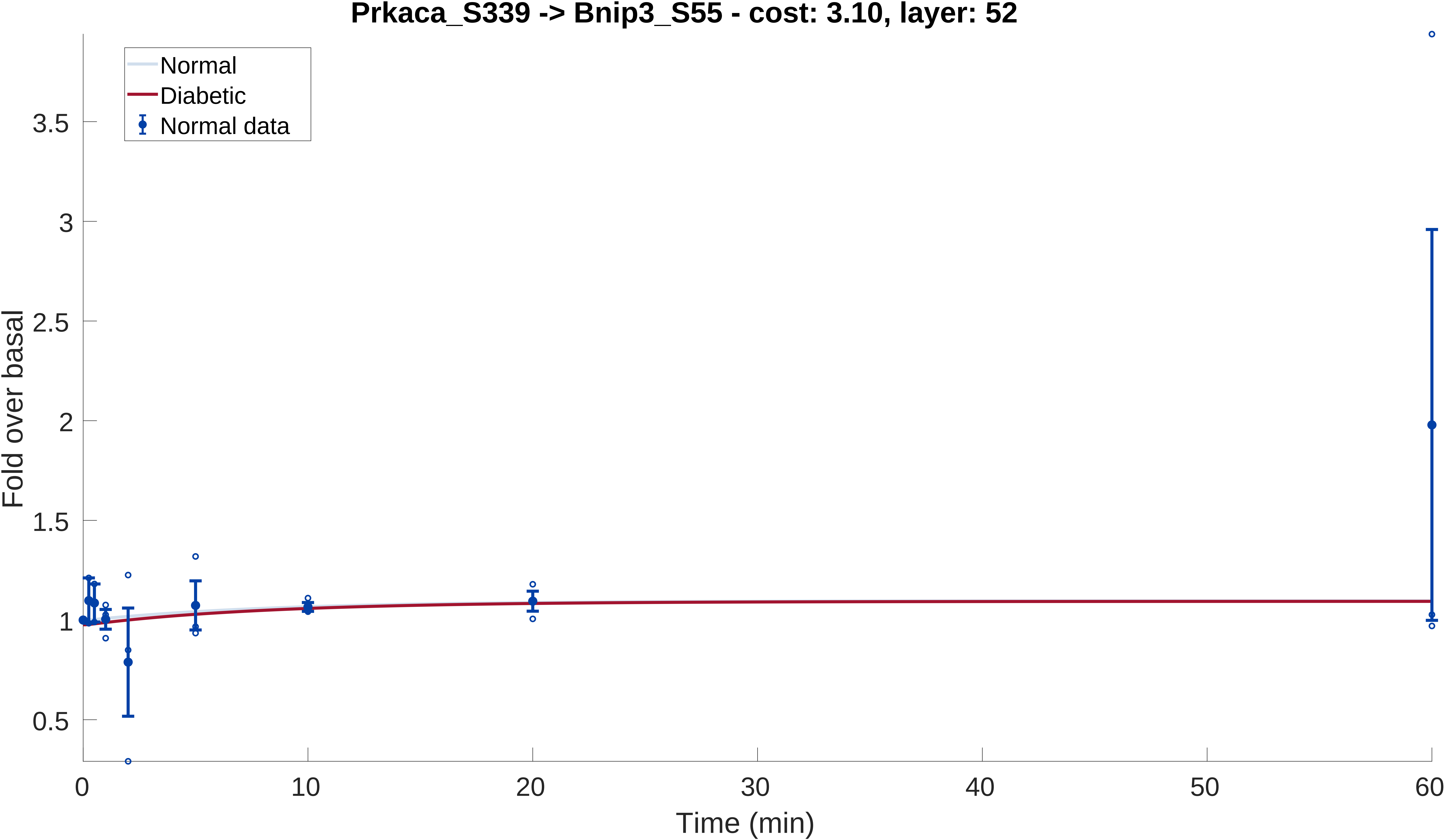

Prkaca_S339 -> Bnip3_S55 - cost: 3.10, layer: 52
Normal
Diabetic
Normal data
Fold over basal
3.5
3
2.5
2
1.5
1
0.5
0
10
20
30
40
50
60
Time (min)

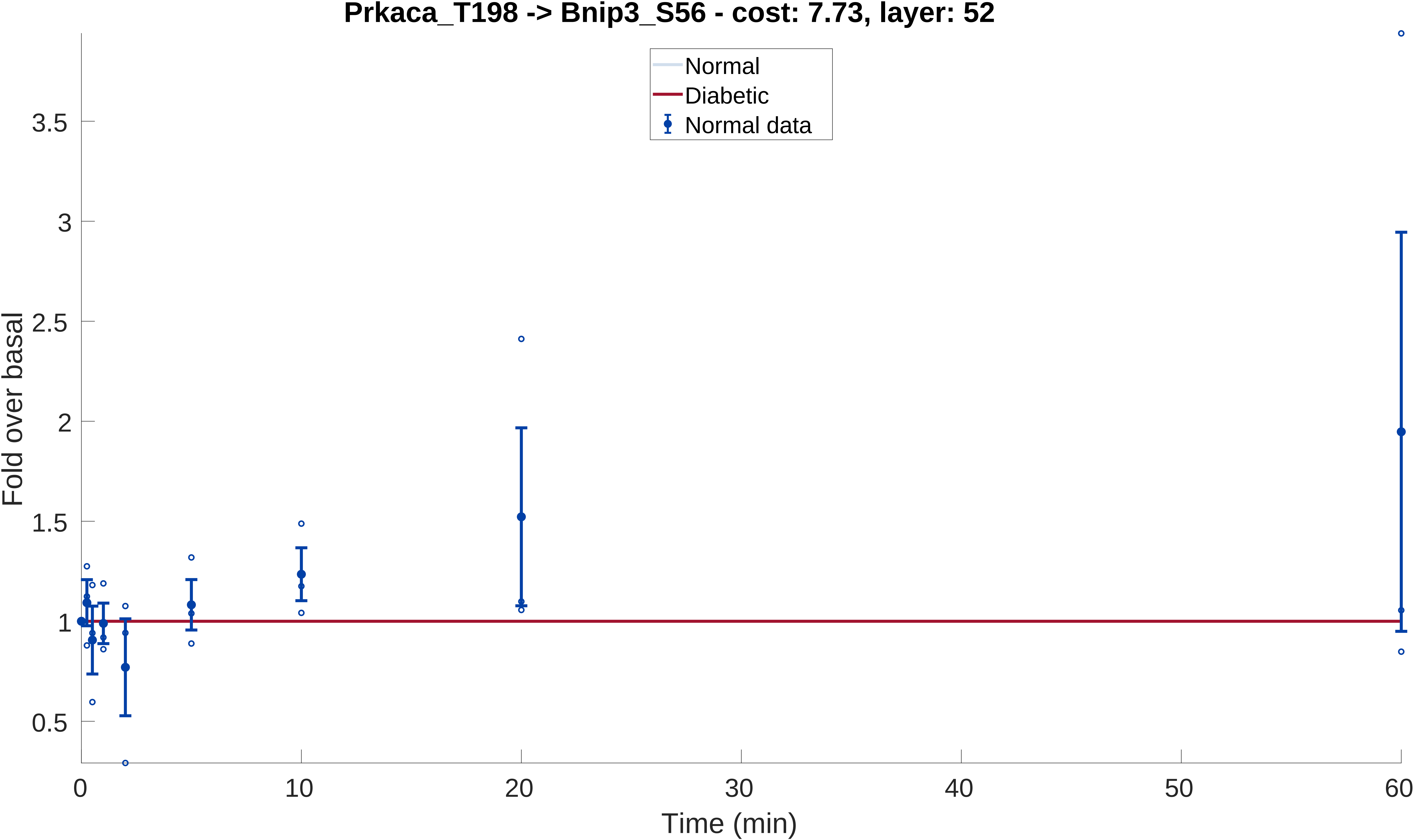

Prkaca_T198 -> Bnip3_S56 - cost: 7.73, layer: 52
Normal
Diabetic
Normal data
Fold over basal
3.5
3
2.5
2
1.5
1
0.5
0
10
20
30
40
50
60
Time (min)

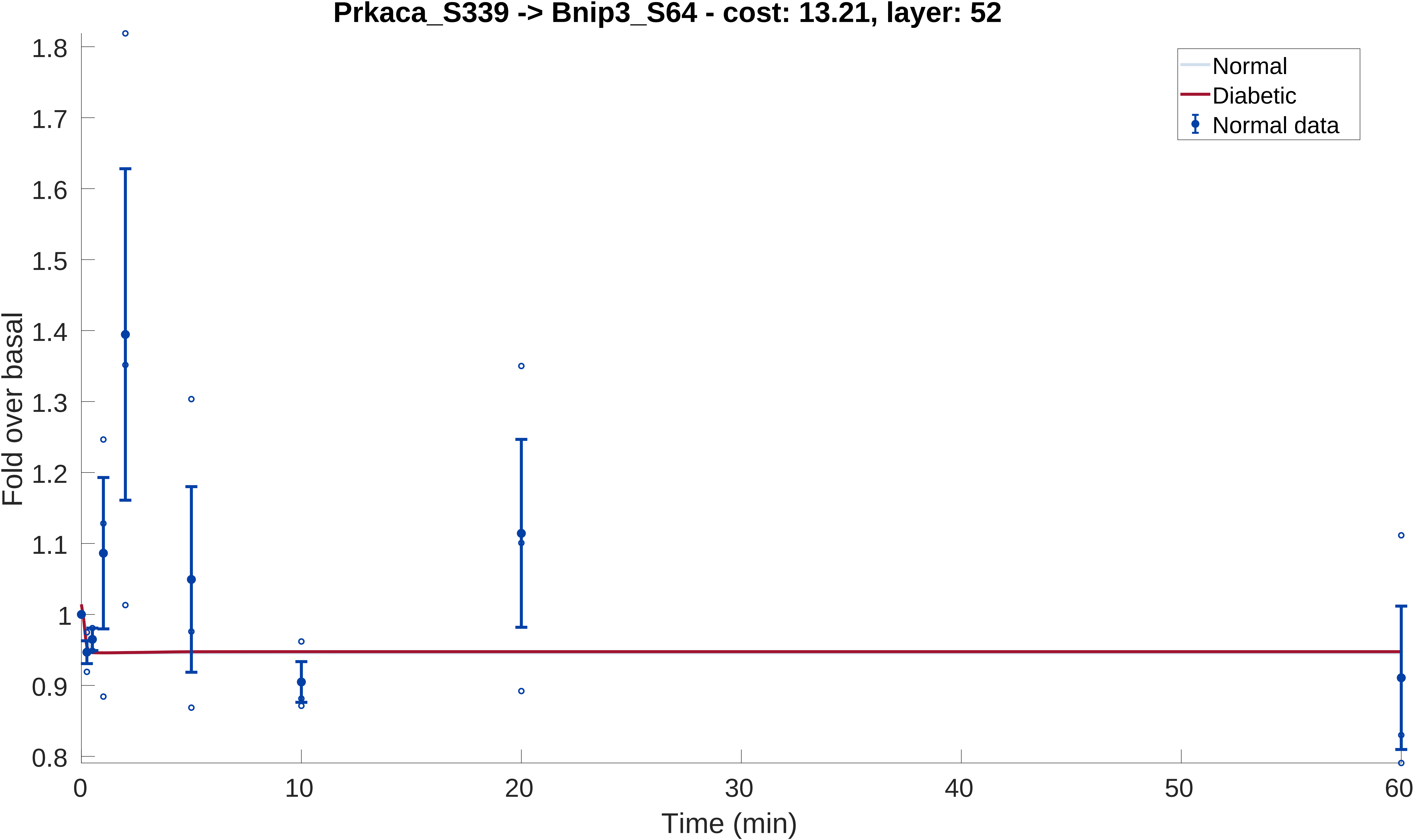

Prkaca_S339 -> Bnip3_S64 - cost: 13.21, layer: 52
Normal
Diabetic
Normal data
Fold over basal
1.8
1.7
1.6
1.5
1.4
1.3
1.2
1.1
1
0.9
0.8
0
10
20
30
40
50
60
Time (min)

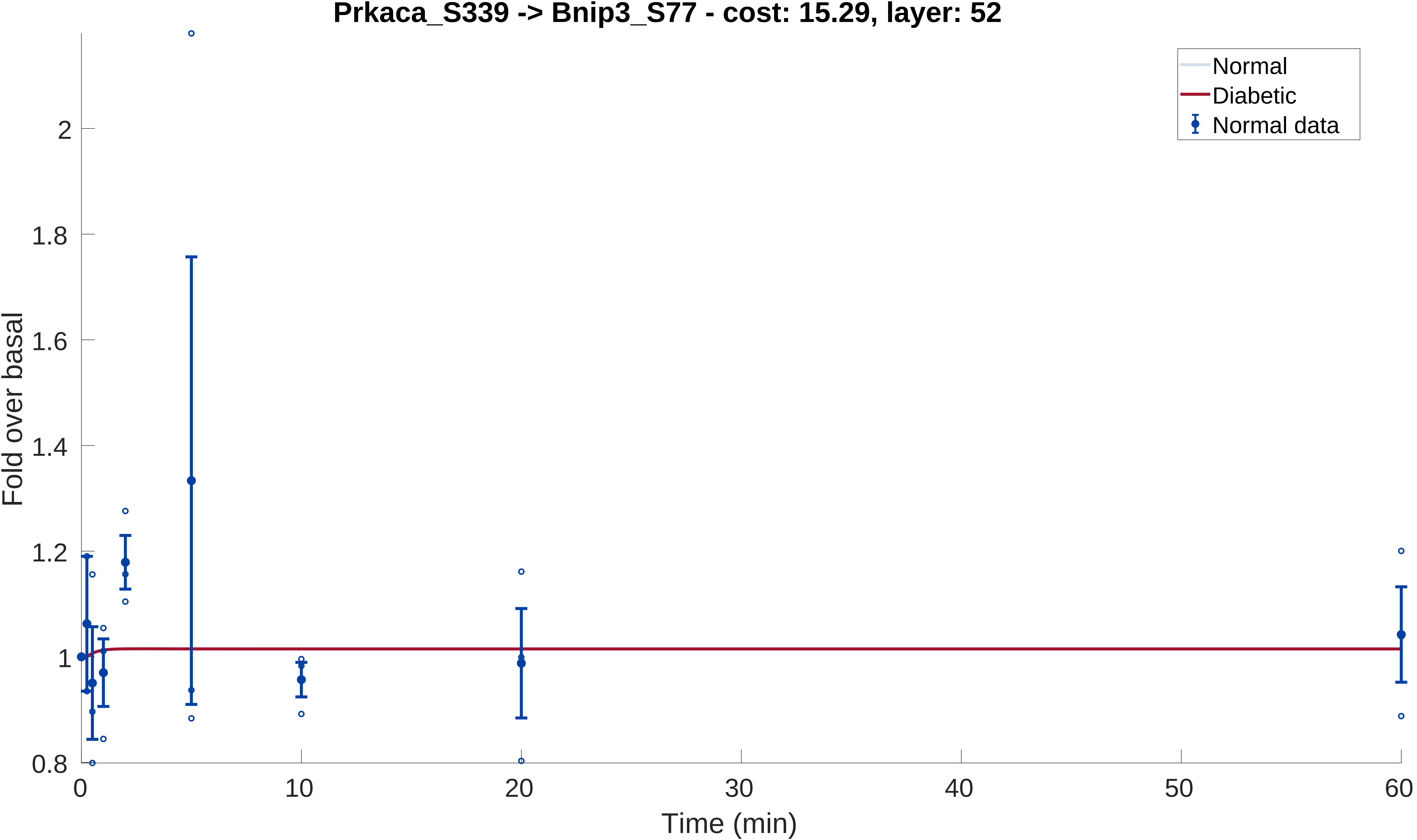

Prkaca_S339 -> Bnip3_S77 - cost: 15.29, layer: 52
Normal
Diabetic
Normal data
Fold over basal
Time (min)
0.8
1
1.2
1.4
1.6
1.8
2
0
10
20
30
40
50
60

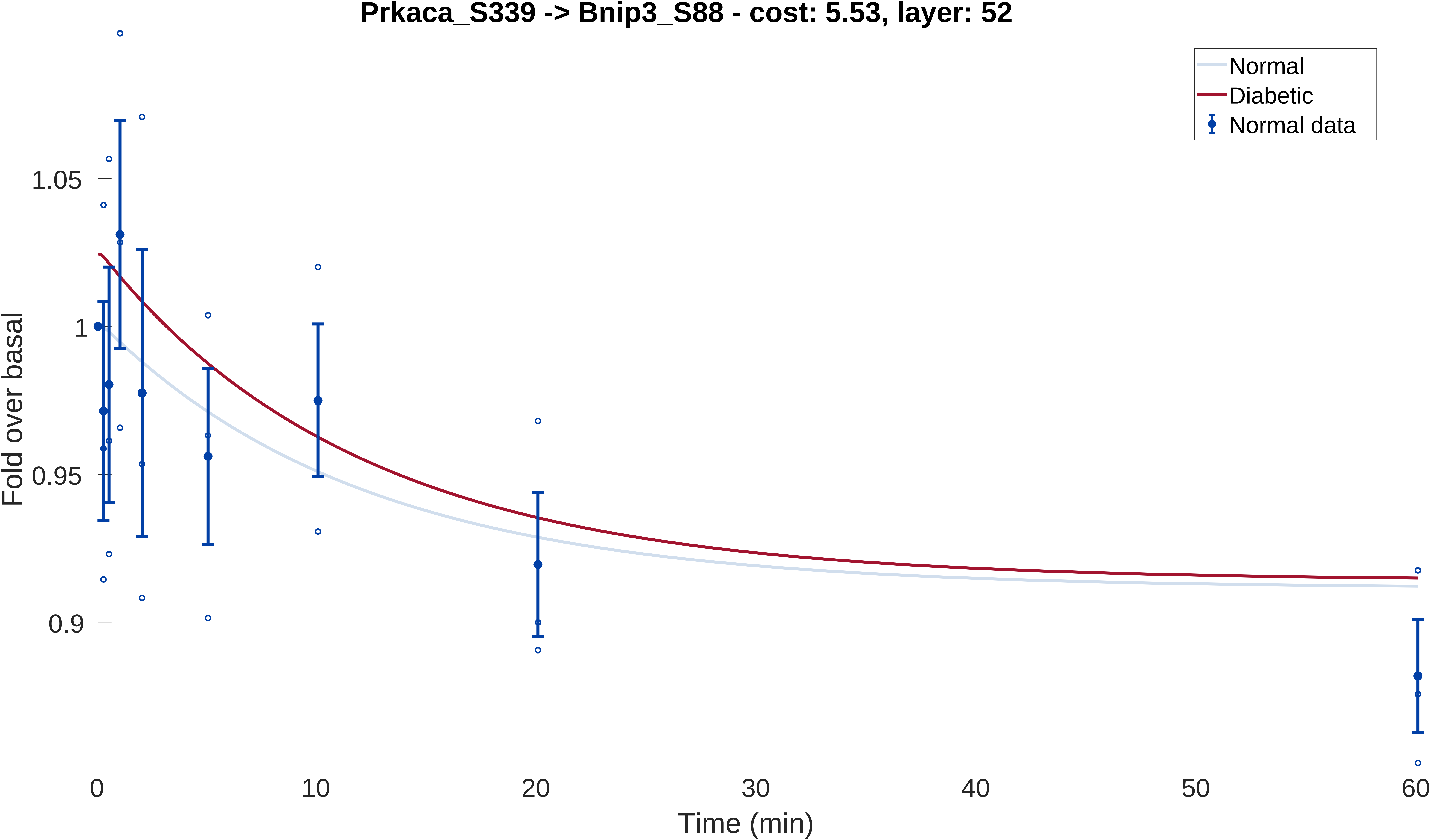

Prkaca_S339 -> Bnip3_S88 - cost: 5.53, layer: 52
Normal
Diabetic
Normal data
Fold over basal
1.05
1
0.95
0.9
0
10
20
30
40
50
60
Time (min)

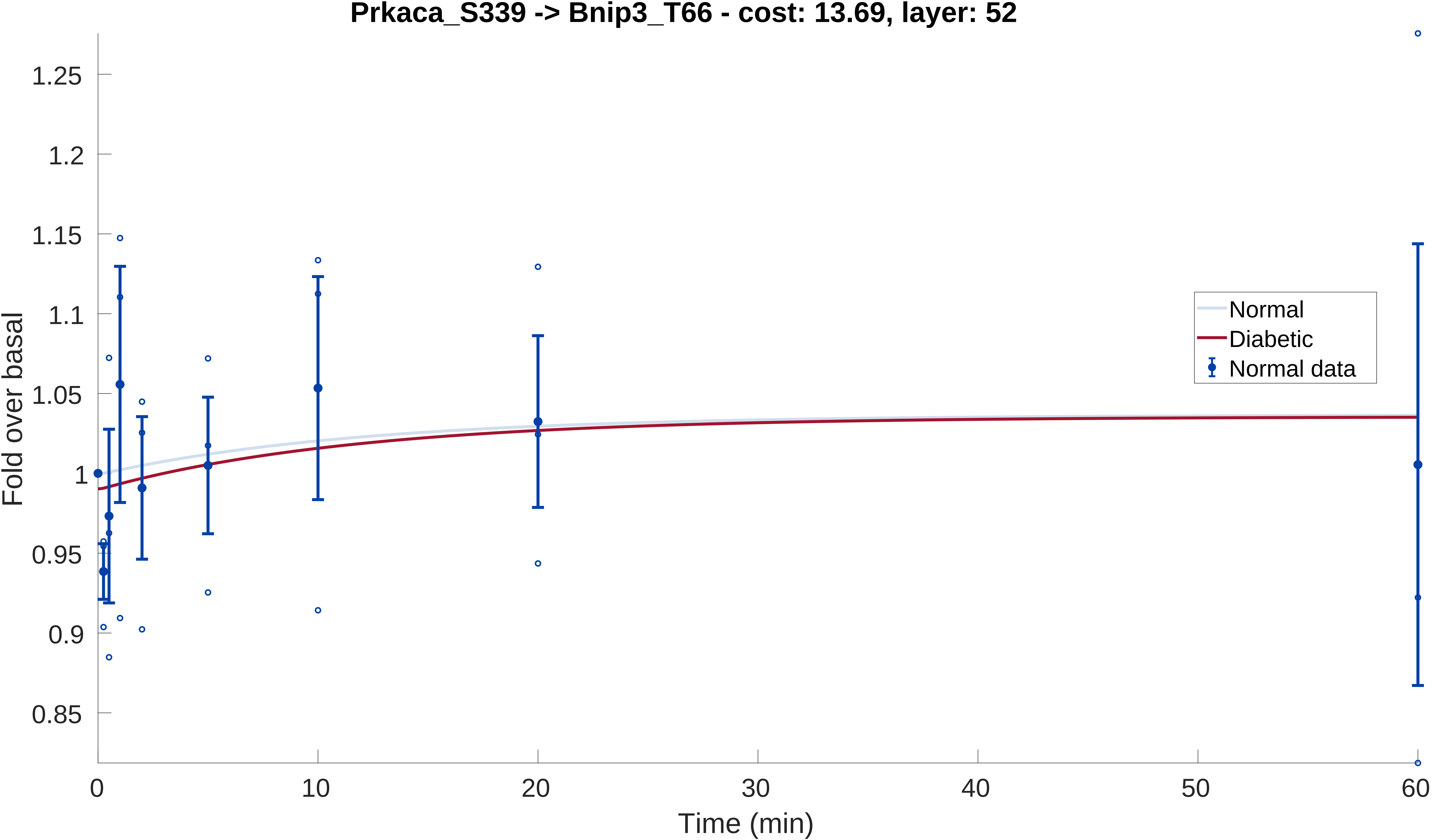
Prkaca_S339 -> Bnip3_T66 - cost: 13.69, layer: 52
Fold over basal
1.25
1.2
1.15
1.1
1.05
1
0.95
0.9
0.85
0
10
20
30
40
50
60
Time (min)
Normal
Diabetic
Normal data

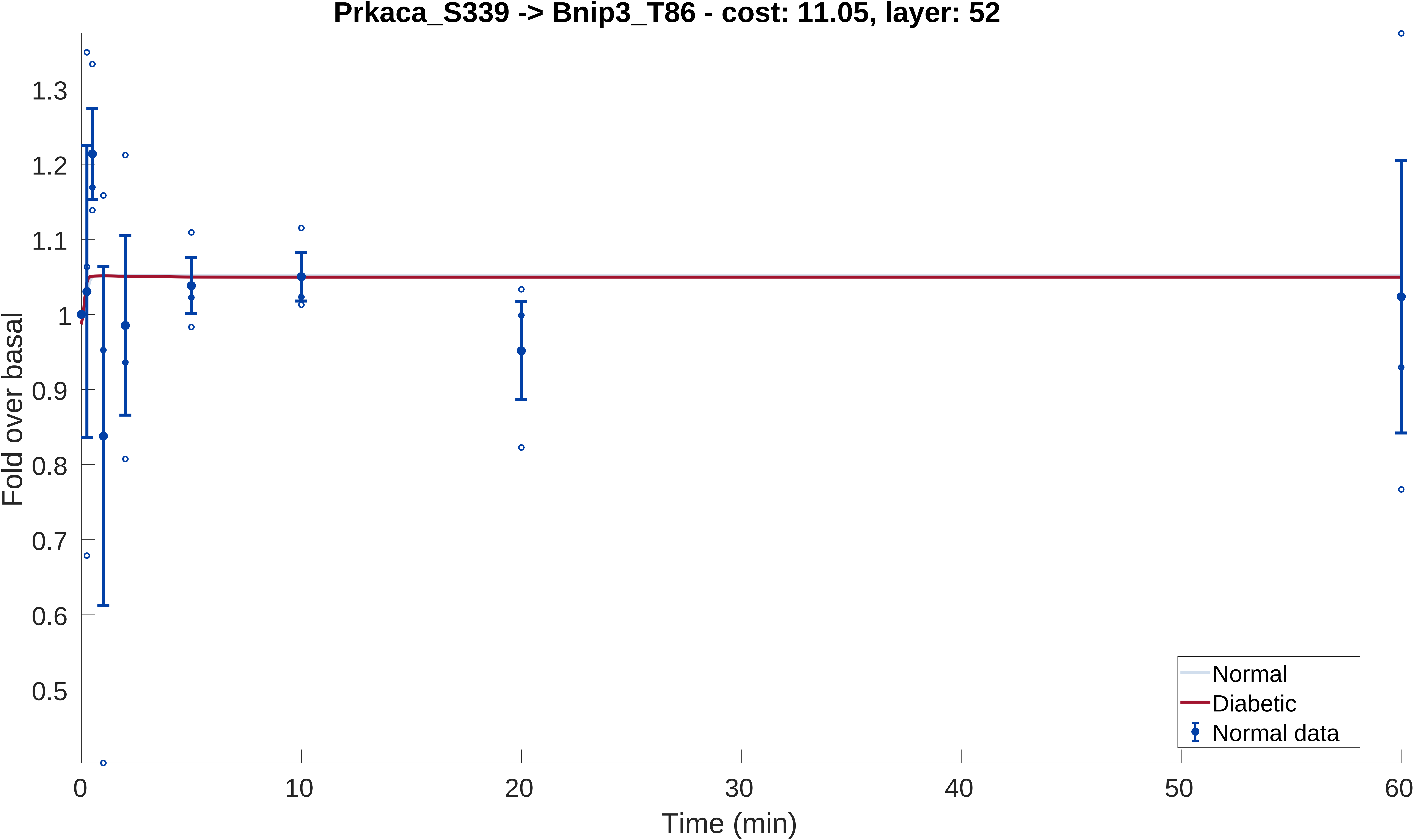

Prkaca_S339 -> Bnip3_T86 - cost: 11.05, layer: 52
Fold over basal
1.3
1.2
1.1
1
0.9
0.8
0.7
0.6
0.5
0
10
20
30
40
50
60
Time (min)
Normal
Diabetic
Normal data

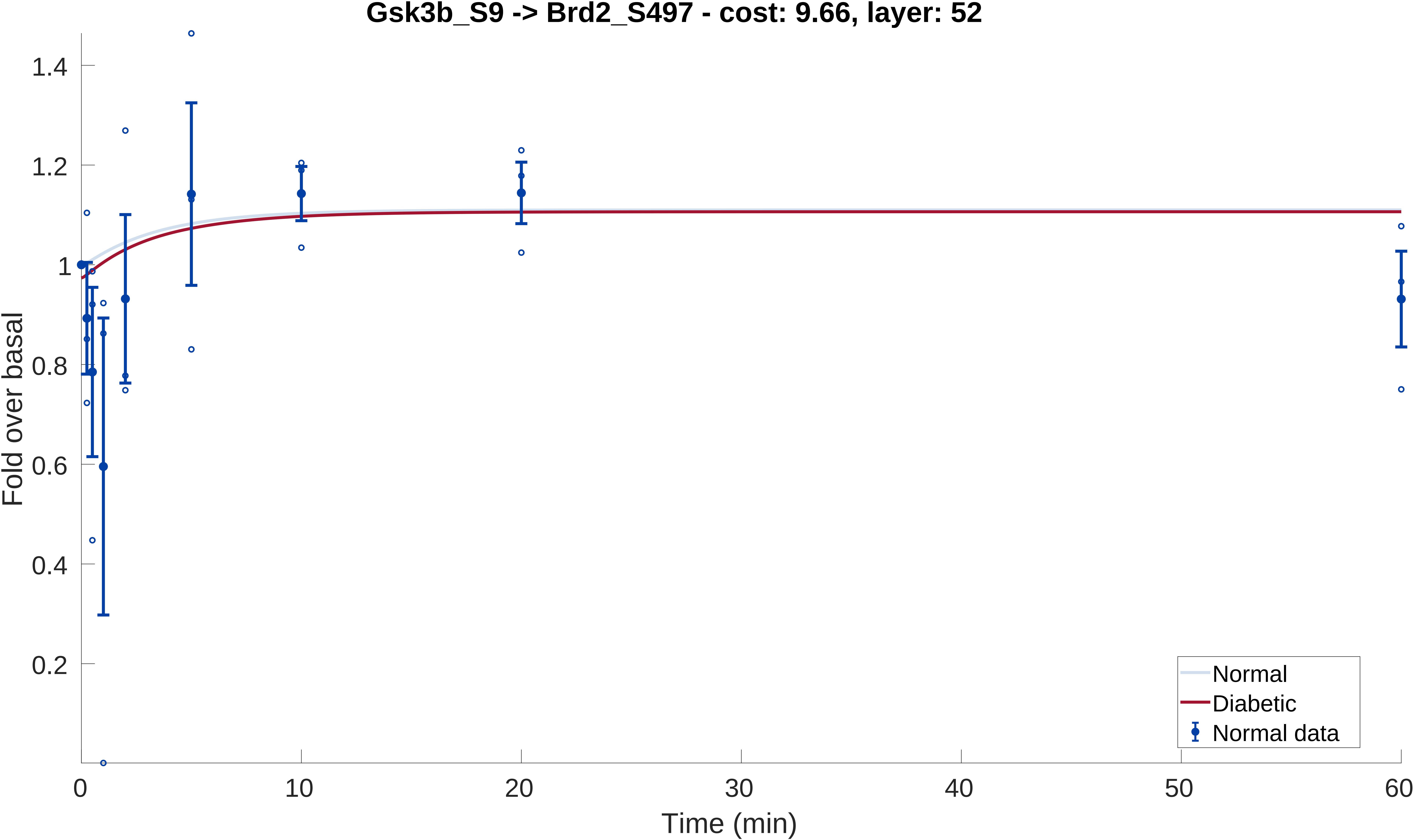

Gsk3b_S9 -> Brd2_S497 - cost: 9.66, layer: 52
Fold over basal
Time (min)
1.4
1.2
1
0.8
0.6
0.4
0.2
0
10
20
30
40
50
60
Normal
Diabetic
Normal data

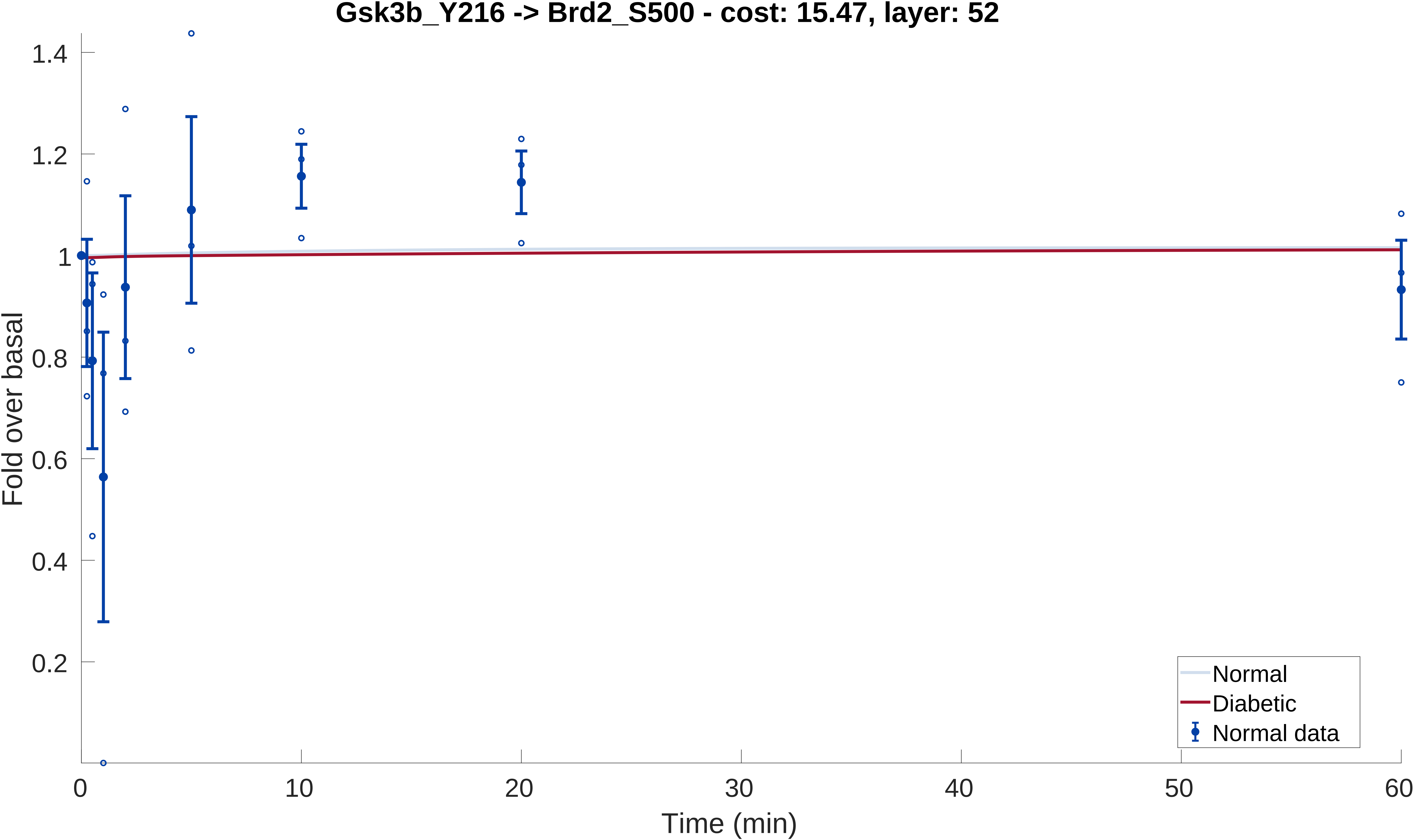

Gsk3b_Y216 -> Brd2_S500 - cost: 15.47, layer: 52
Fold over basal
1.4
1.2
1
0.8
0.6
0.4
0.2
0
10
20
30
40
50
60
Time (min)
Normal
Diabetic
Normal data

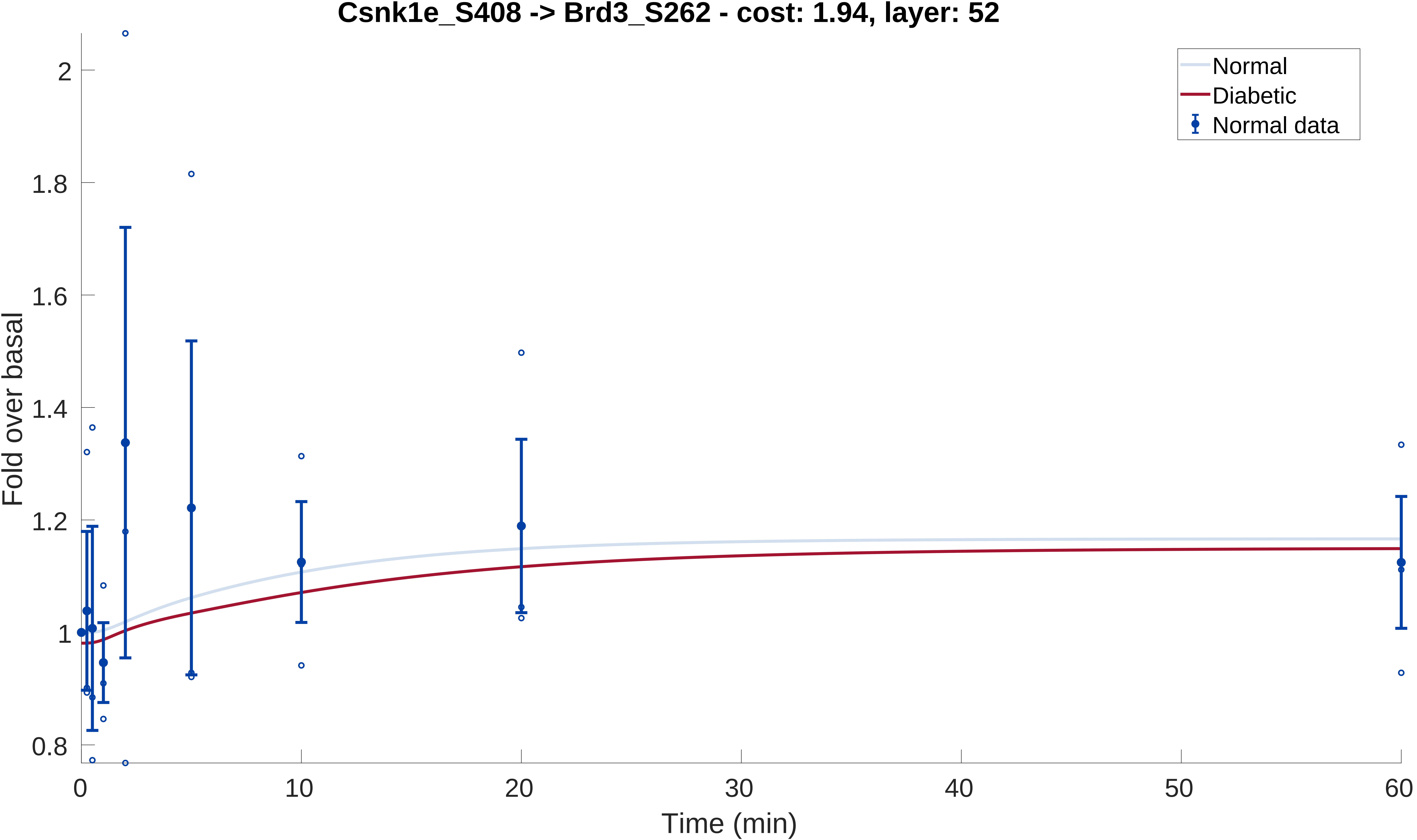

Csnk1e_S408 -> Brd3_S262 - cost: 1.94, layer: 52
Normal
Diabetic
Normal data
Fold over basal
Time (min)
0.8
1
1.2
1.4
1.6
1.8
2
0
10
20
30
40
50
60

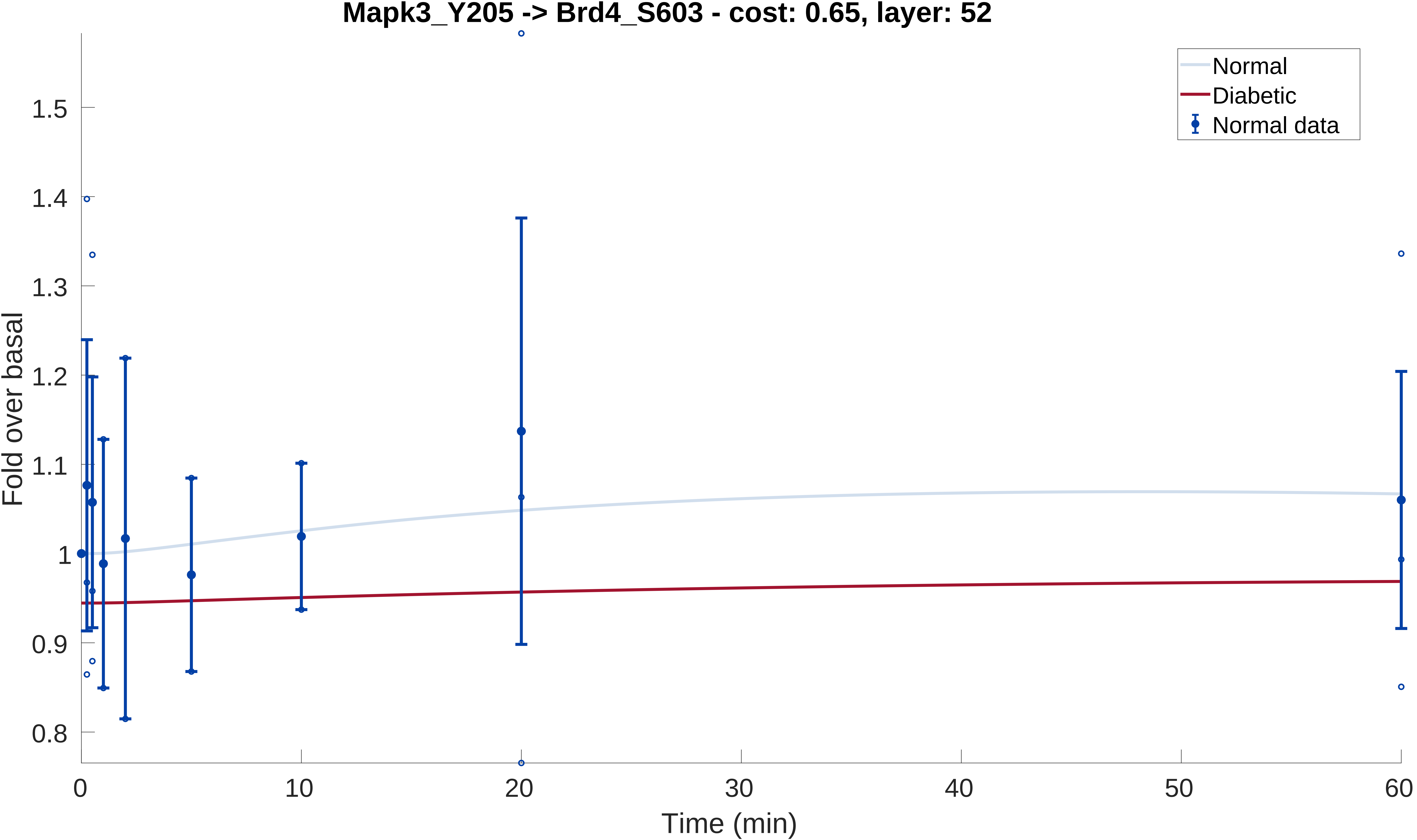

Mapk3_Y205 -> Brd4_S603 - cost: 0.65, layer: 52
Normal
Diabetic
Normal data
Fold over basal
Time (min)
0.8
0.9
1
1.1
1.2
1.3
1.4
1.5
0
10
20
30
40
50
60

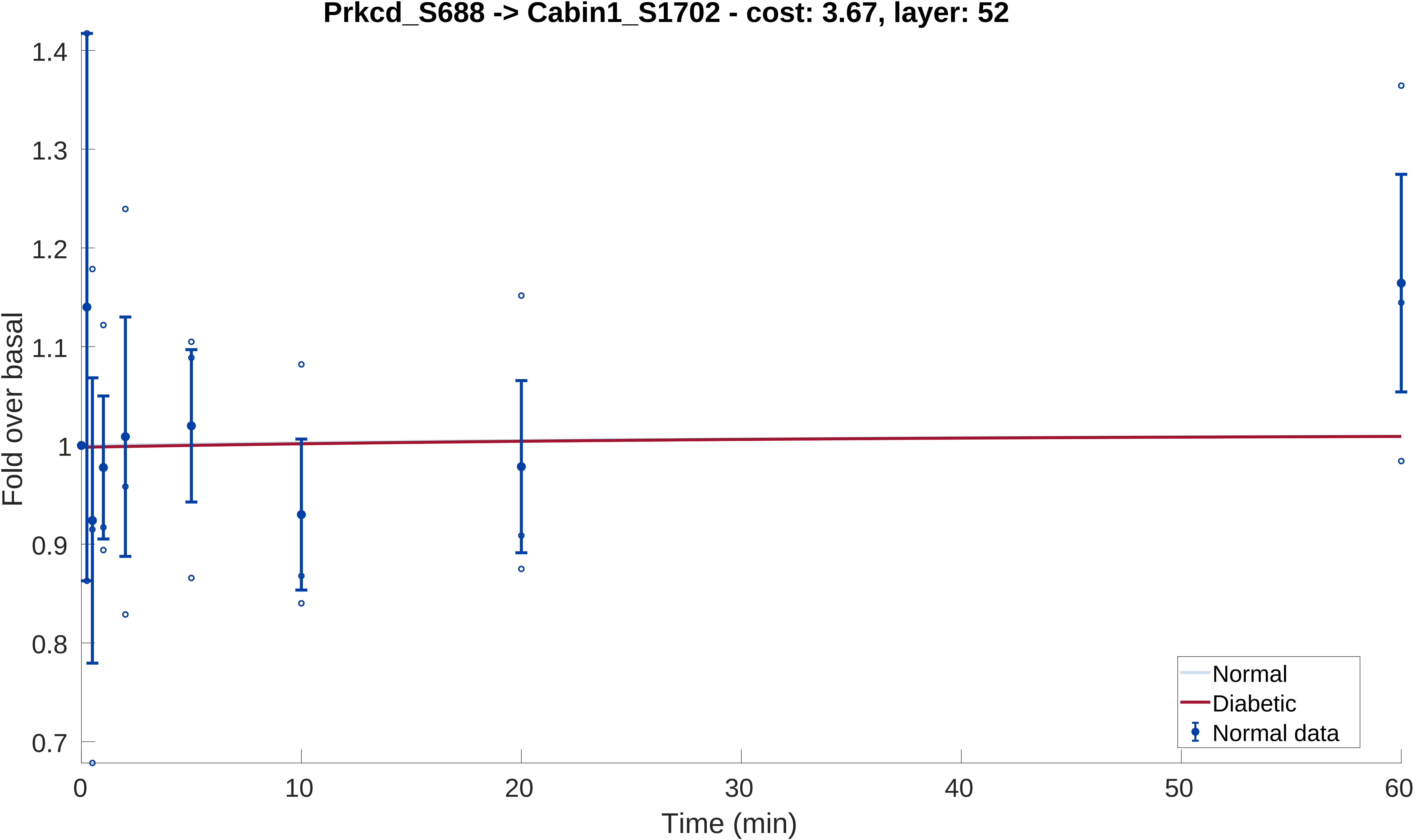

Prkcd_S688 -> Cabin1_S1702 - cost: 3.67, layer: 52
Fold over basal
1.4
1.3
1.2
1.1
1
0.9
0.8
0.7
0
10
20
30
40
50
60
Time (min)
Normal
Diabetic
Normal data

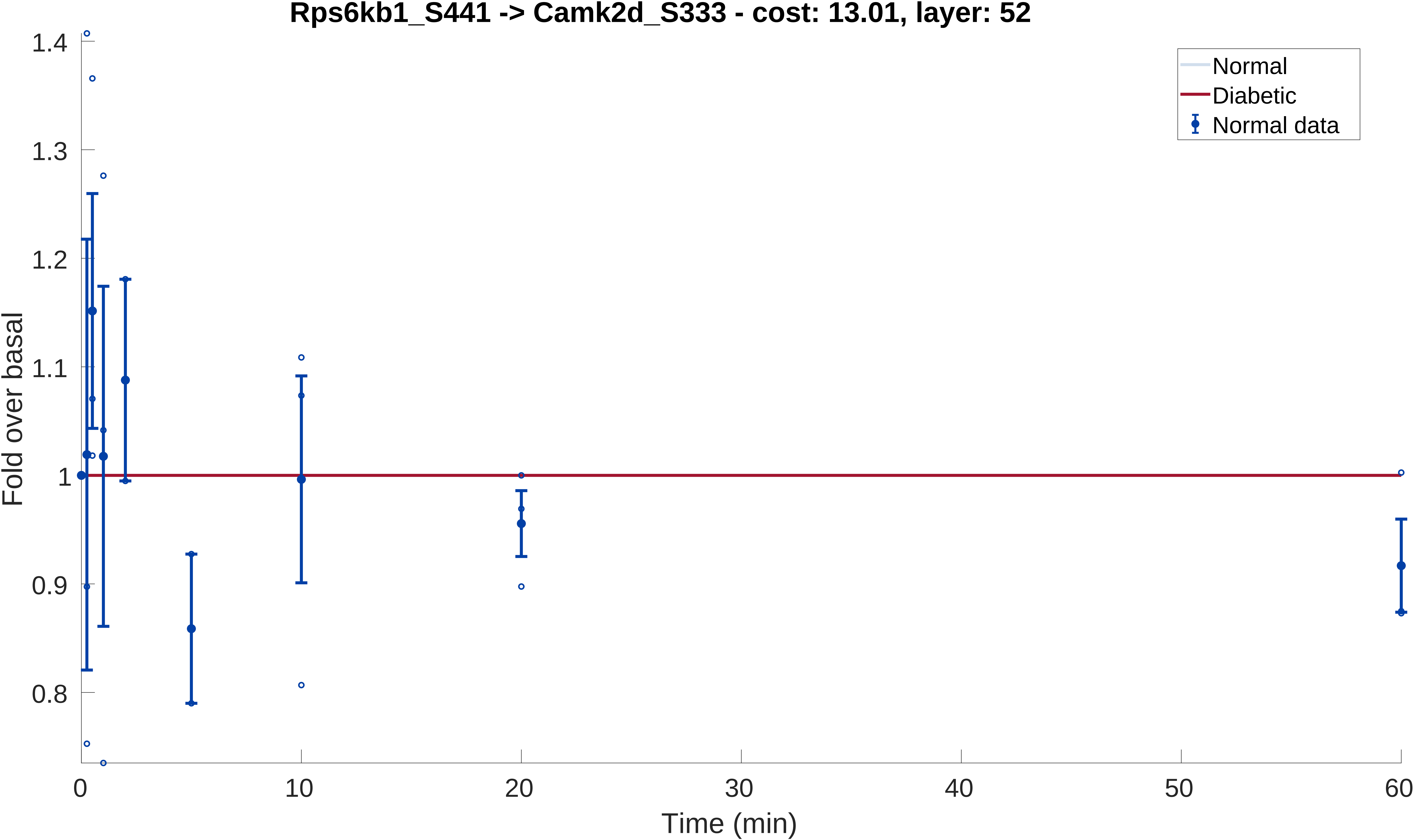

Rps6kb1_S441 -> Camk2d_S333 - cost: 13.01, layer: 52
Normal
Diabetic
Normal data
Fold over basal
Time (min)
1.4
1.3
1.2
1.1
1
0.9
0.8
0
10
20
30
40
50
60

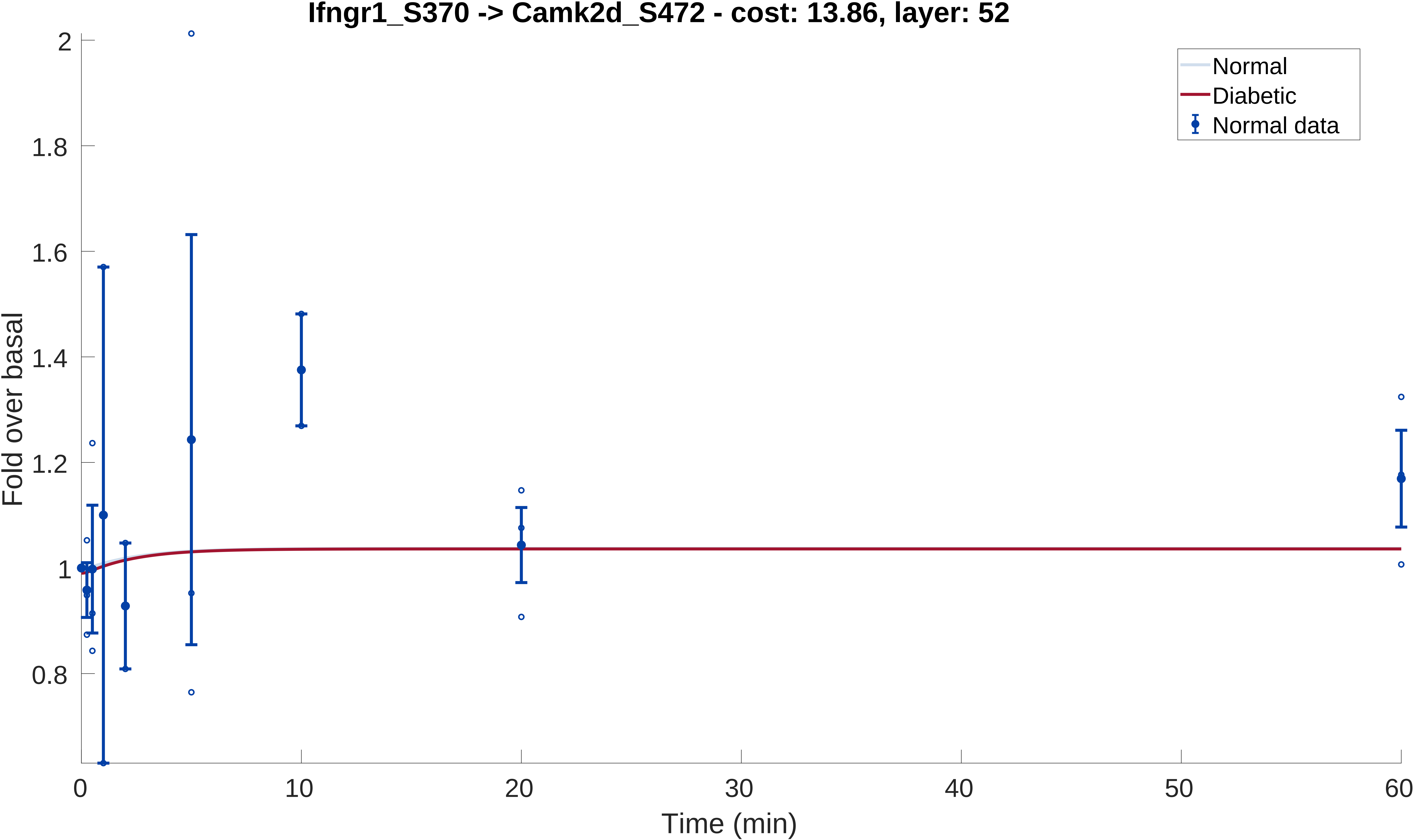

Ifngr1_S370 -> Camk2d_S472 - cost: 13.86, layer: 52
Normal
Diabetic
Normal data
Fold over basal
Time (min)
0.8
1
1.2
1.4
1.6
1.8
2
0
10
20
30
40
50
60

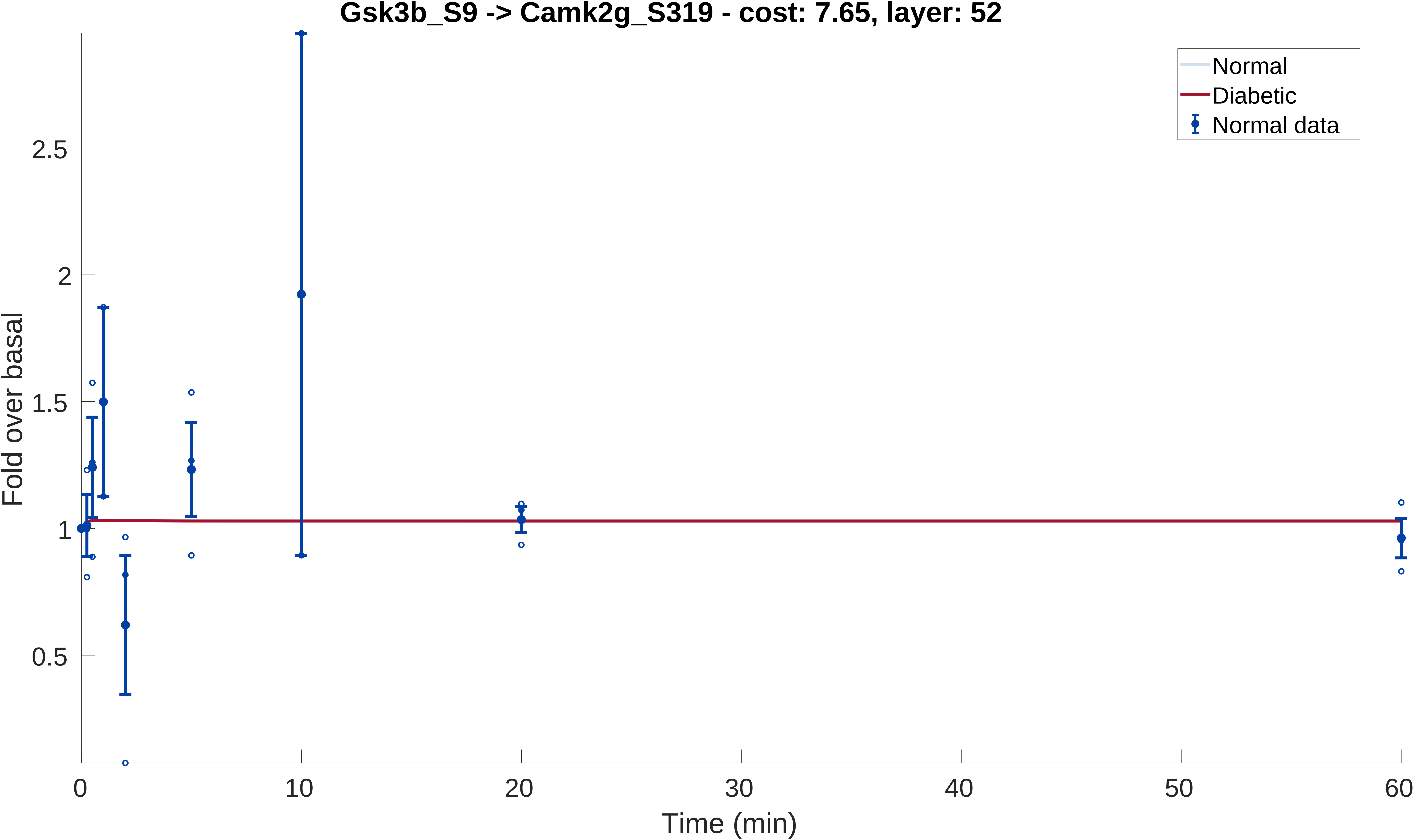
Gsk3b_S9 -> Camk2g_S319 - cost: 7.65, layer: 52
Normal
Diabetic
Normal data
Fold over basal
2.5
2
1.5
1
0.5
0
10
20
30
40
50
60
Time (min)

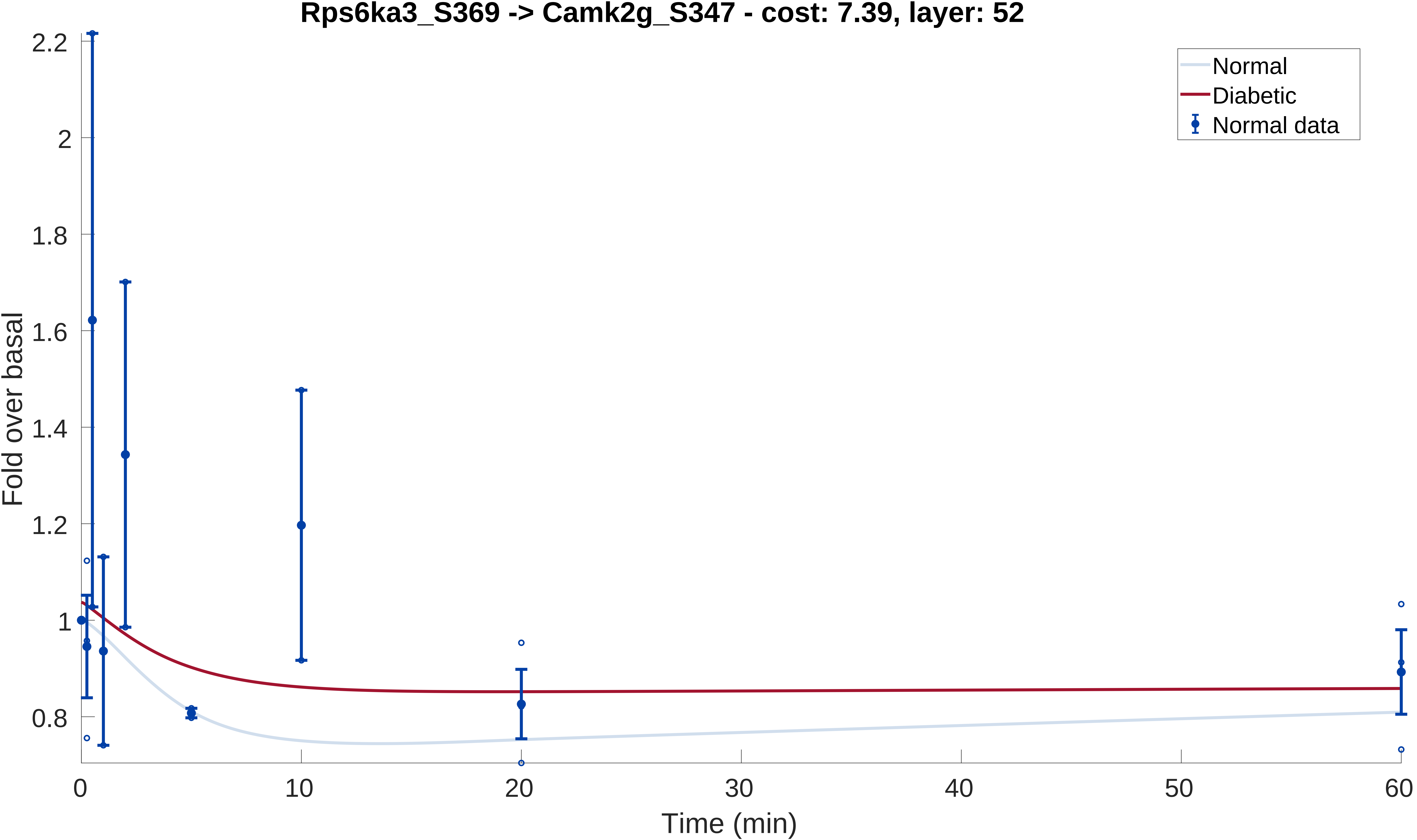

Rps6ka3_S369 -> Camk2g_S347 - cost: 7.39, layer: 52
Fold over basal
2.2
2
1.8
1.6
1.4
1.2
1
0.8
0
10
20
30
40
50
60
Time (min)
Normal
Diabetic
Normal data

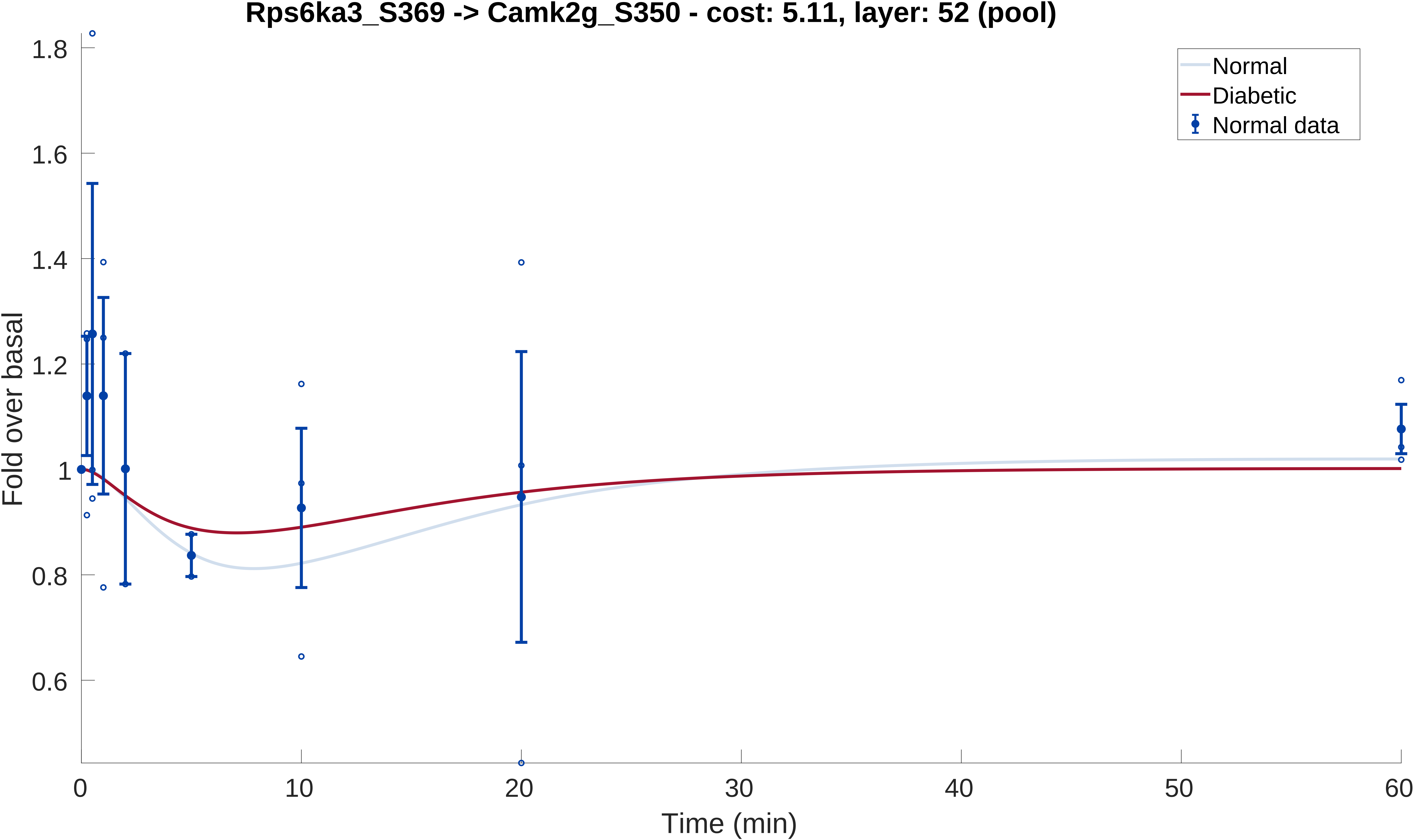
Rps6ka3_S369 -> Camk2g_S350 - cost: 5.11, layer: 52 (pool)
Normal
Diabetic
Normal data
Fold over basal
Time (min)
1.8
1.6
1.4
1.2
1
0.8
0.6
0
10
20
30
40
50
60

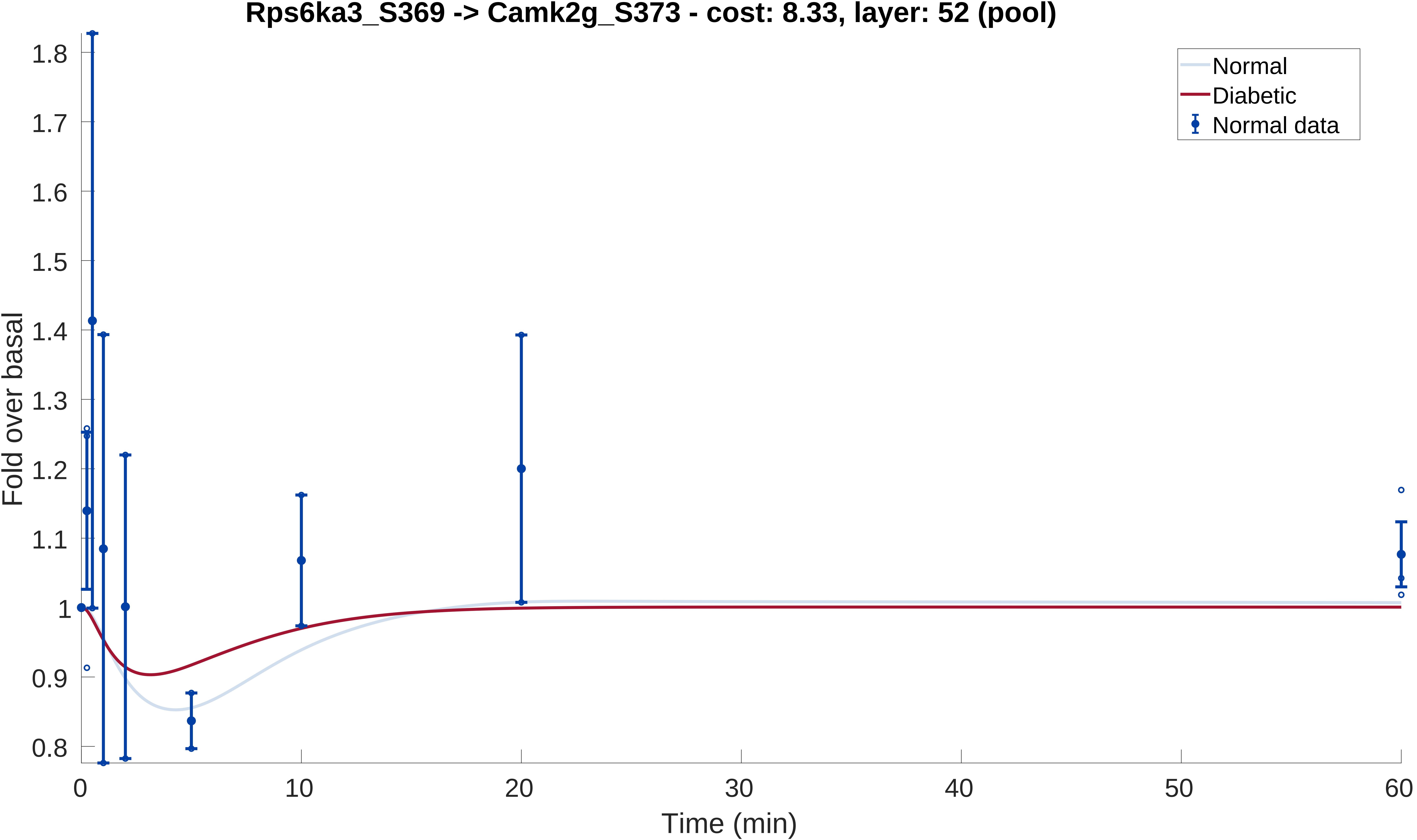

Rps6ka3_S369 -> Camk2g_S373 - cost: 8.33, layer: 52 (pool)
Fold over basal
Time (min)
Normal
Diabetic
Normal data
0.8
0.9
1
1.1
1.2
1.3
1.4
1.5
1.6
1.7
1.8
0
10
20
30
40
50
60

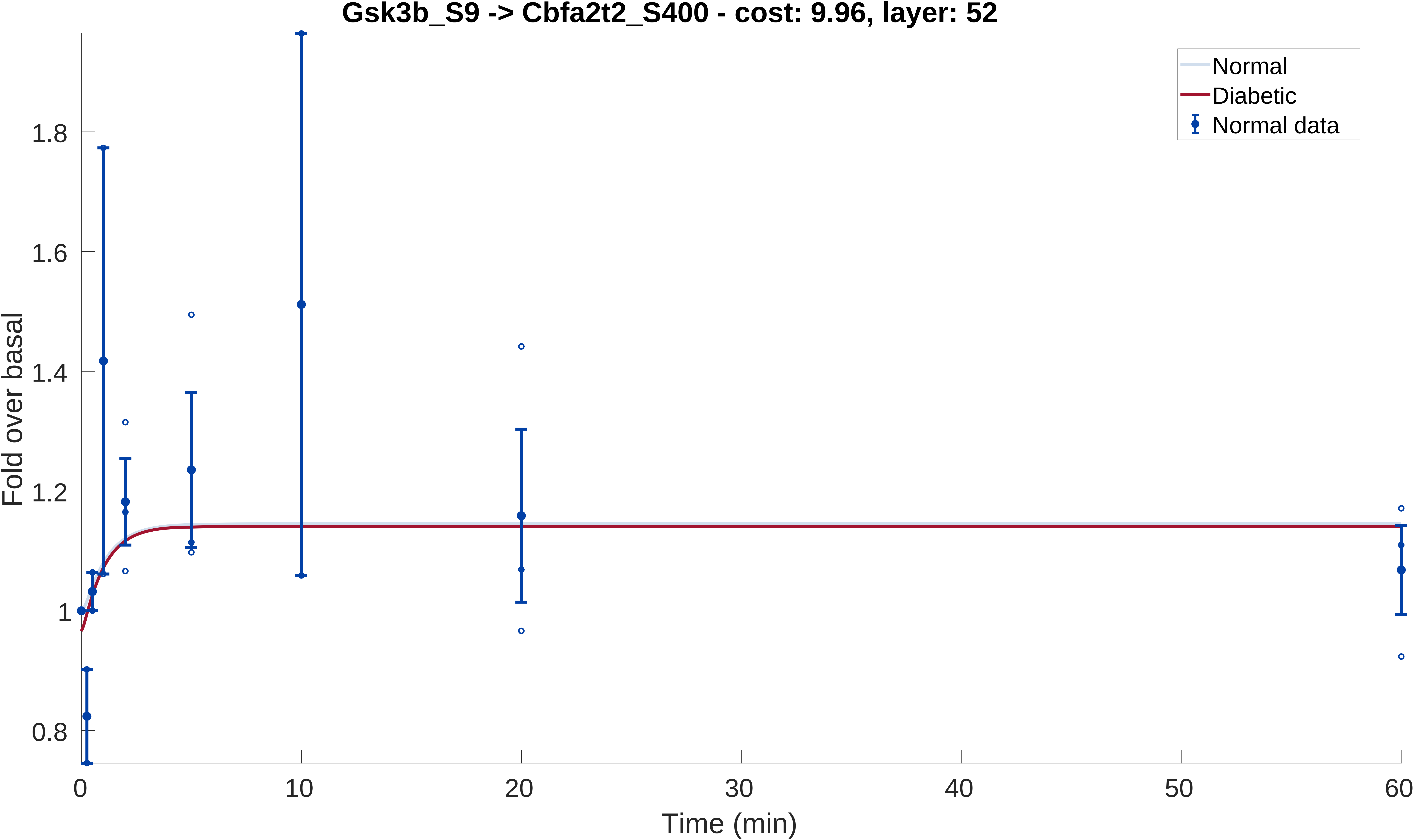

Gsk3b_S9 -> Cbfa2t2_S400 - cost: 9.96, layer: 52
Normal
Diabetic
Normal data
Fold over basal
1.8
1.6
1.4
1.2
1
0.8
0
10
20
30
40
50
60
Time (min)

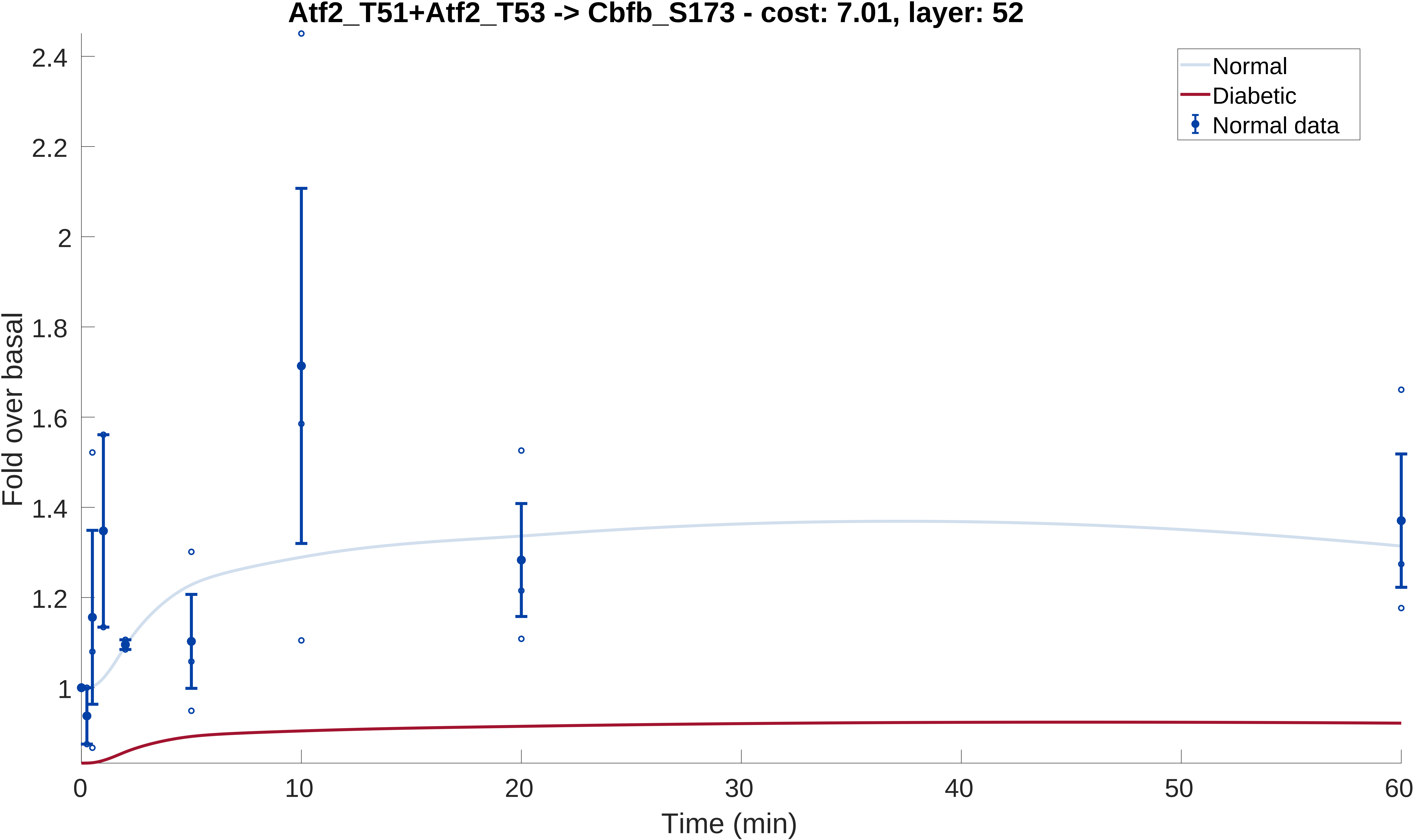

Atf2_T51+Atf2_T53 -> Cbfb_S173 - cost: 7.01, layer: 52
Normal
Diabetic
Normal data
Fold over basal
Time (min)
2.4
2.2
2
1.8
1.6
1.4
1.2
1
0
10
20
30
40
50
60

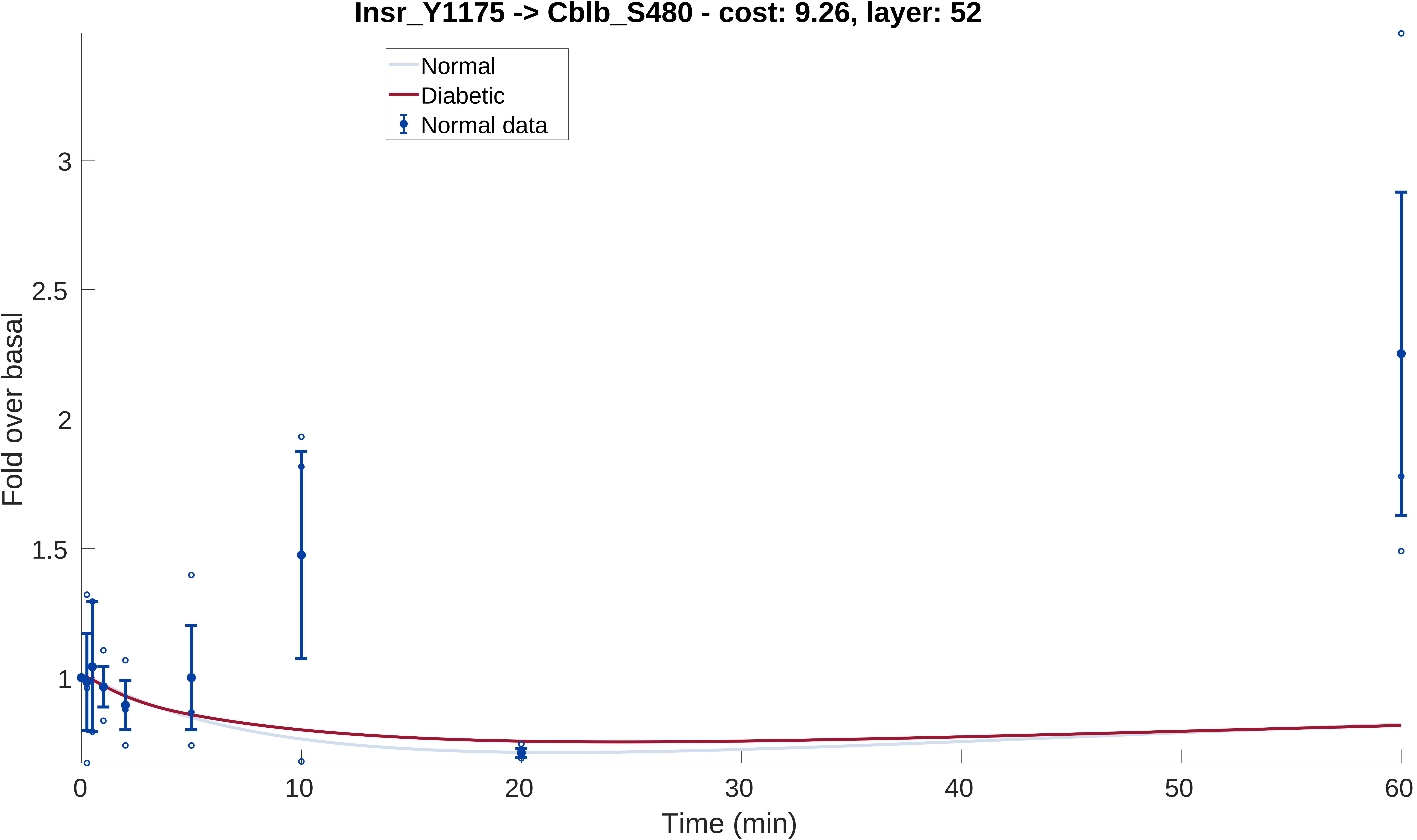

Insr_Y1175 -> Cblb_S480 - cost: 9.26, layer: 52
Normal
Diabetic
Normal data
Fold over basal
3
2.5
2
1.5
1
0
10
20
30
40
50
60
Time (min)

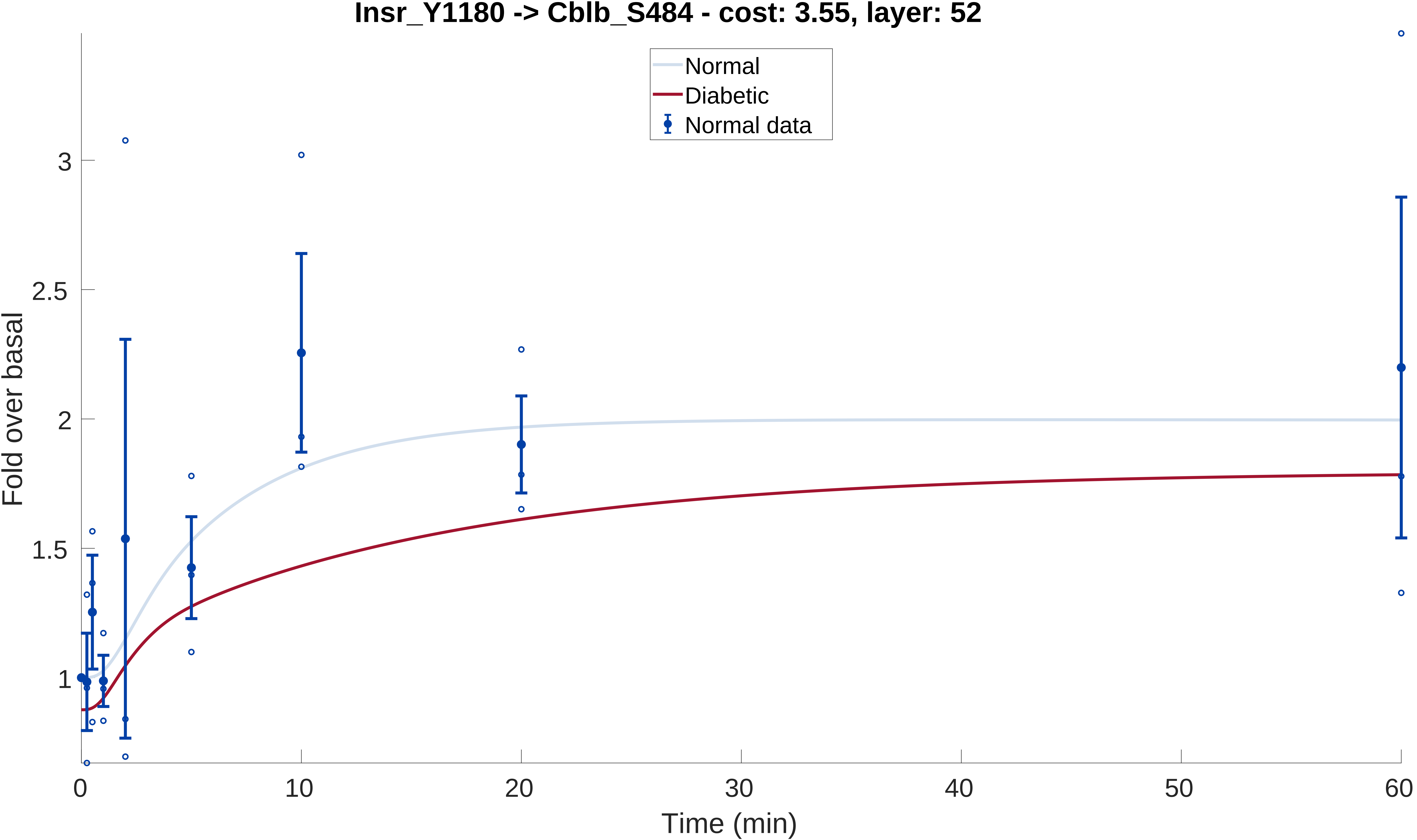
Insr_Y1180 -> Cblb_S484 - cost: 3.55, layer: 52
Normal
Diabetic
Normal data
Fold over basal
Time (min)
1
1.5
2
2.5
3
0
10
20
30
40
50
60

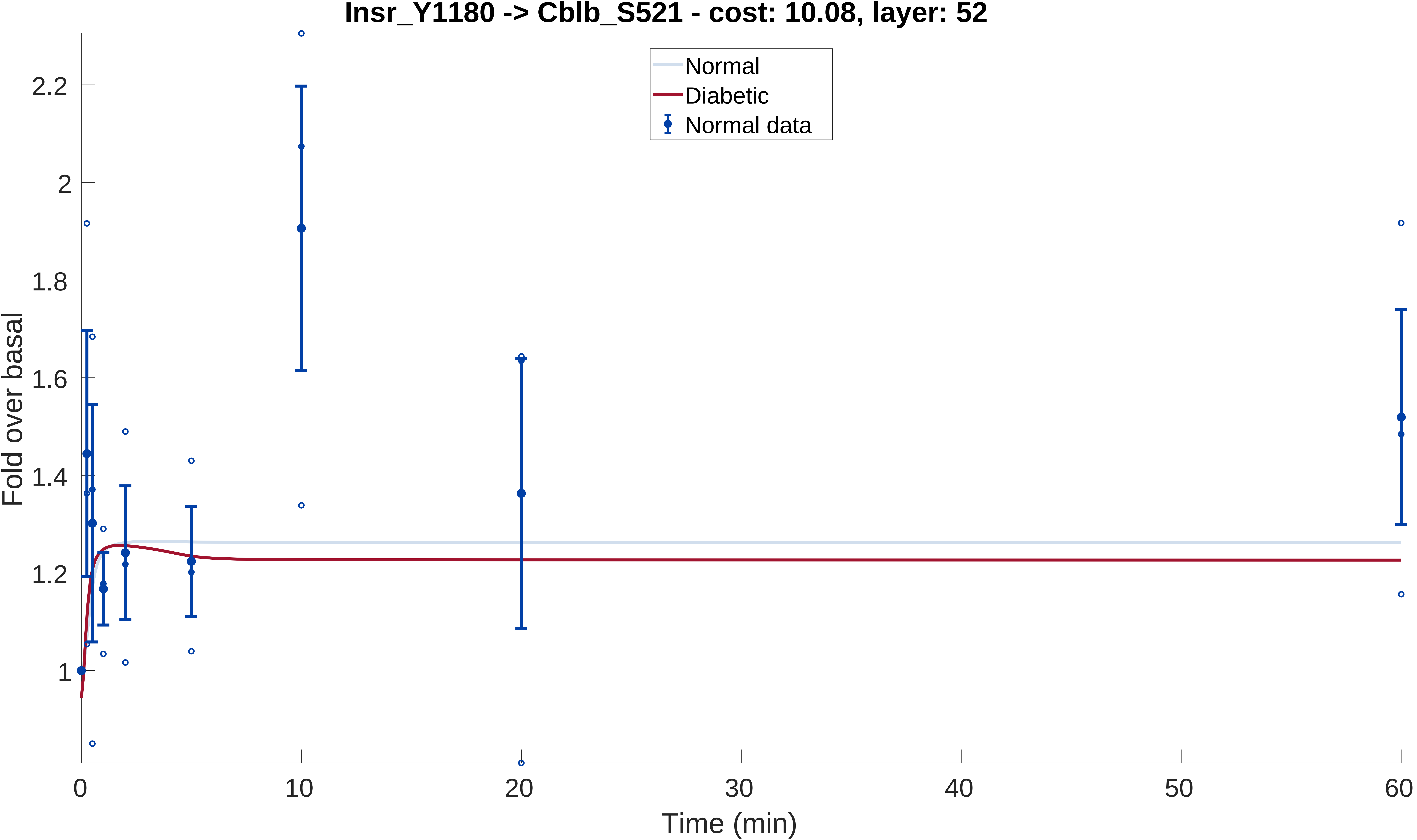
Insr_Y1180 -> Cblb_S521 - cost: 10.08, layer: 52
Normal
Diabetic
Normal data
Fold over basal
Time (min)
2.2
2
1.8
1.6
1.4
1.2
1
0
10
20
30
40
50
60

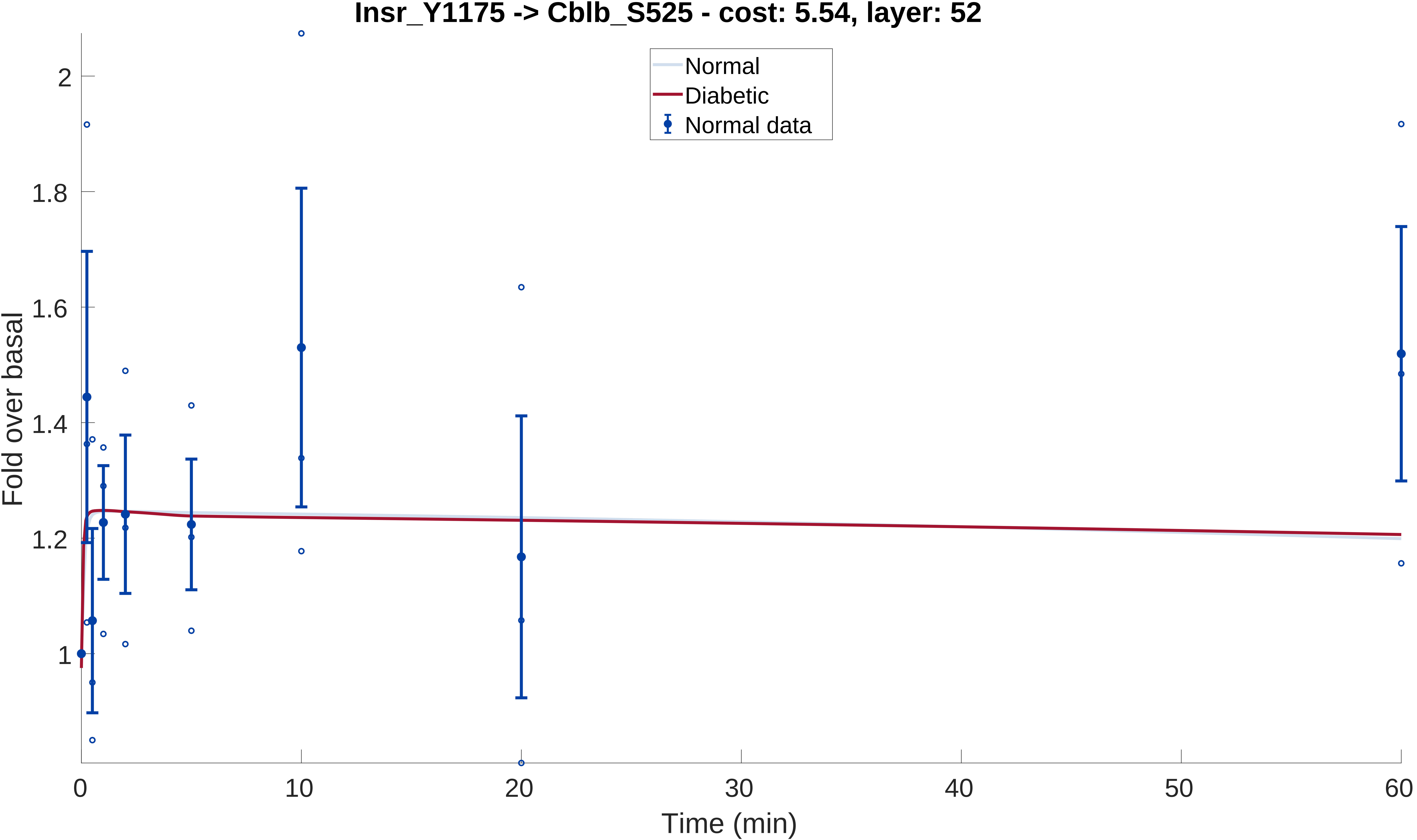
Insr_Y1175 -> Cblb_S525 - cost: 5.54, layer: 52
Normal
Diabetic
Normal data
Fold over basal
Time (min)
2
1.8
1.6
1.4
1.2
1
0
10
20
30
40
50
60

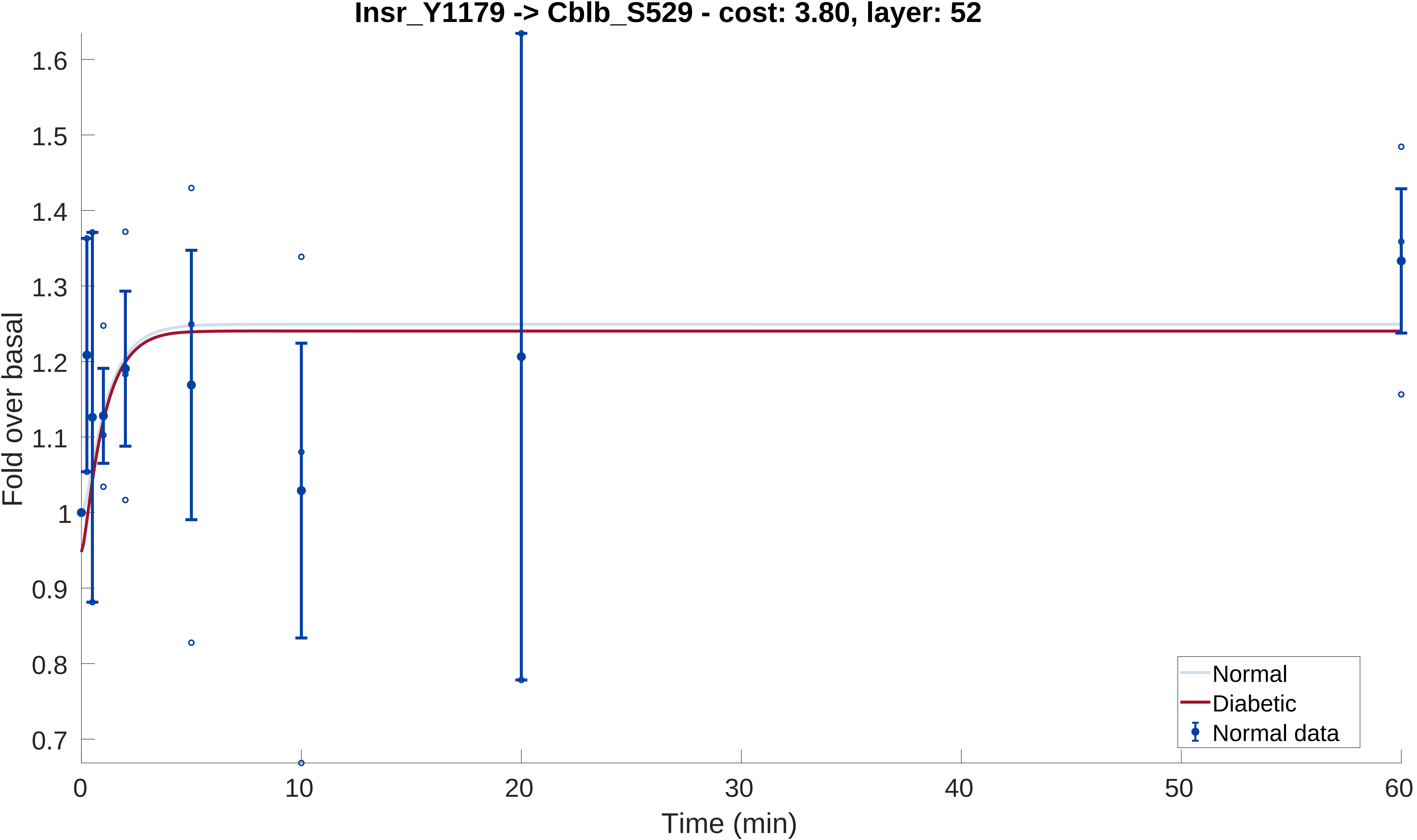
Insr_Y1179 -> Cblb_S529 - cost: 3.80, layer: 52
Fold over basal
Time (min)
1.6
1.5
1.4
1.3
1.2
1.1
1
0.9
0.8
0.7
0
10
20
30
40
50
60
Normal
Diabetic
Normal data

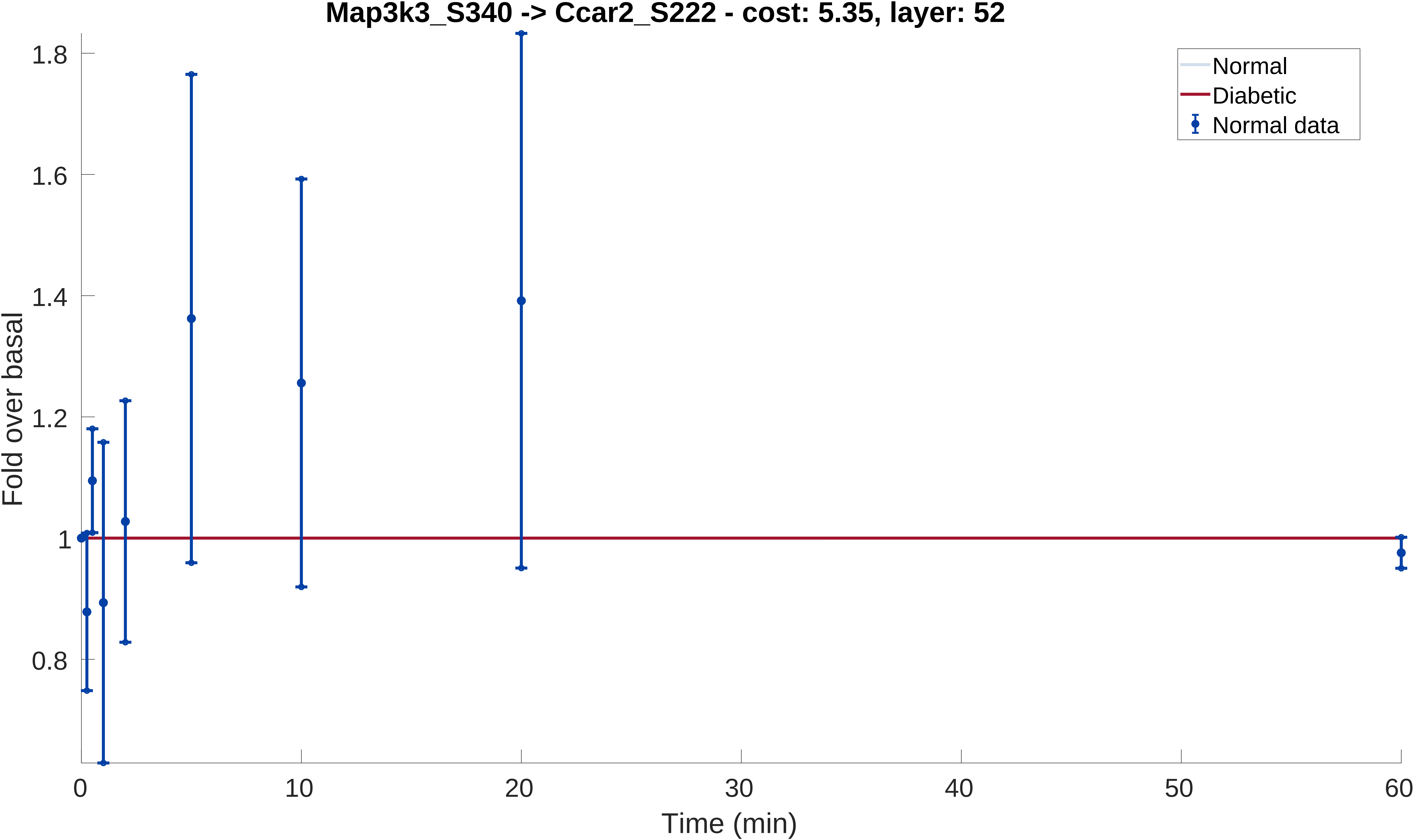

Map3k3_S340 -> Ccar2_S222 - cost: 5.35, layer: 52
Fold over basal
Time (min)
1.8
1.6
1.4
1.2
1
0.8
0
10
20
30
40
50
60
Normal
Diabetic
Normal data

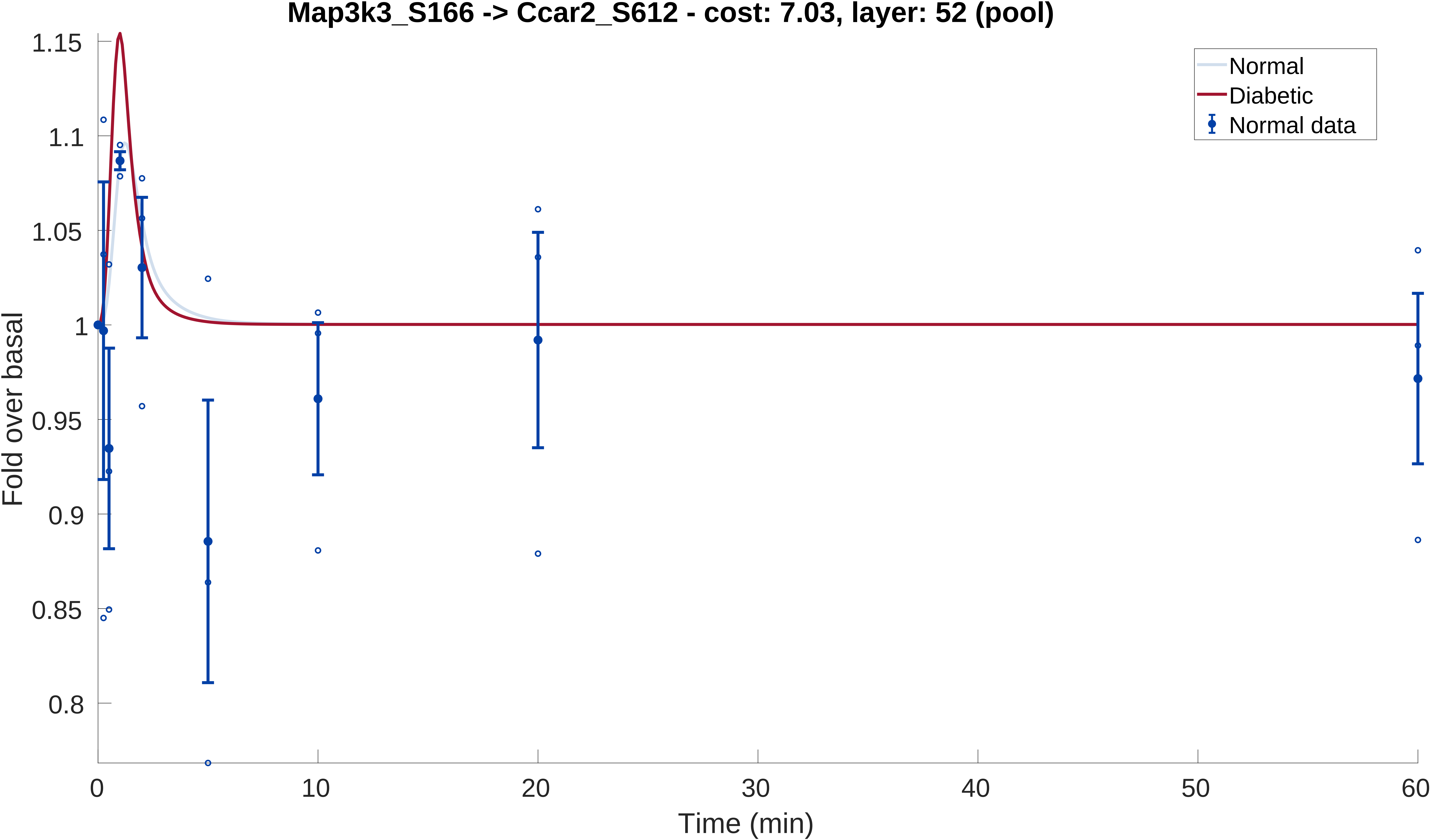
Map3k3_S166 -> Ccar2_S612 - cost: 7.03, layer: 52 (pool)
Normal
Diabetic
Normal data
Fold over basal
Time (min)
1.15
1.1
1.05
1
0.95
0.9
0.85
0.8
0
10
20
30
40
50
60

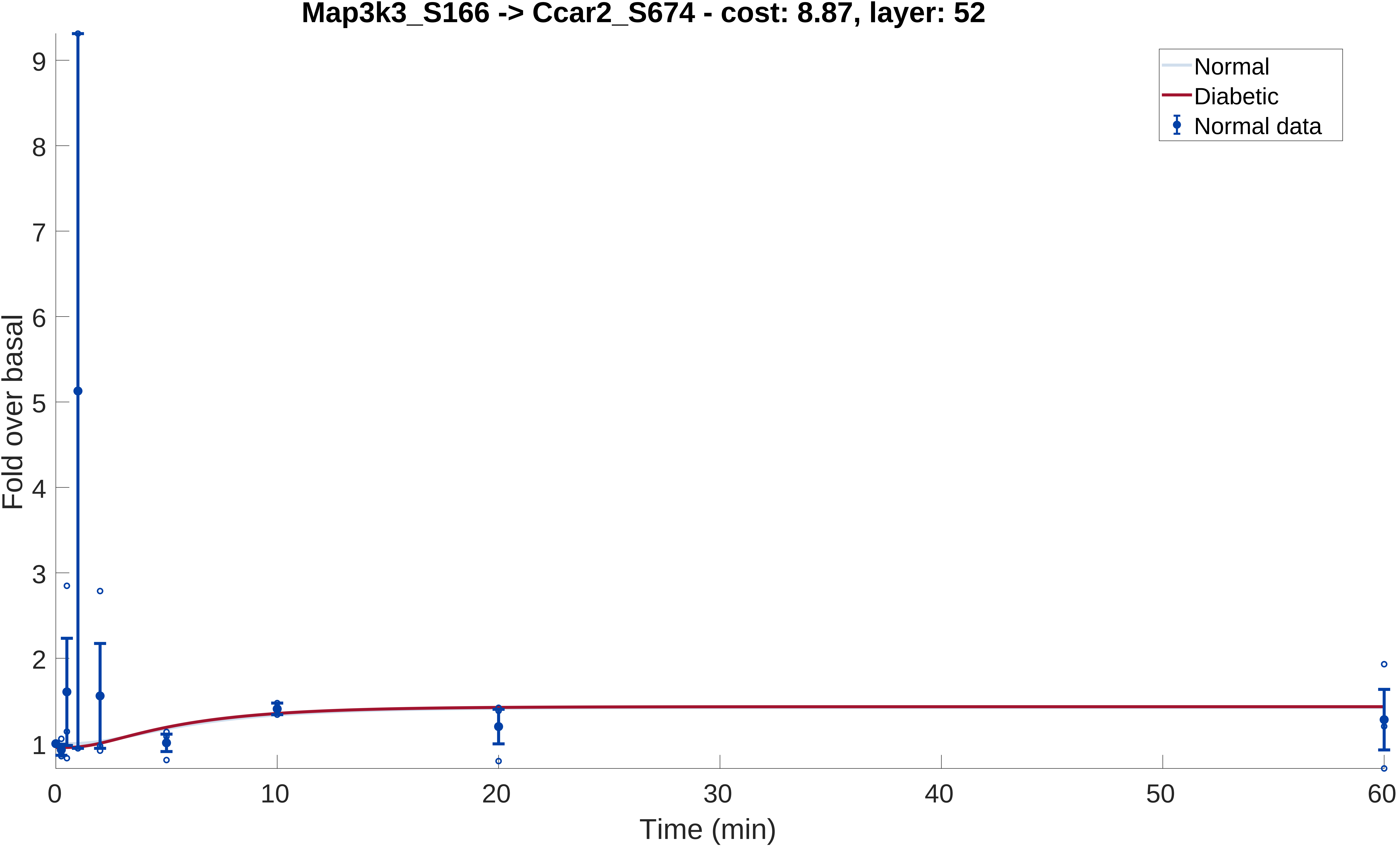
Map3k3_S166 -> Ccar2_S674 - cost: 8.87, layer: 52
Fold over basal
Time (min)
Normal
Diabetic
Normal data
1
2
3
4
5
6
7
8
9
0
10
20
30
40
50
60

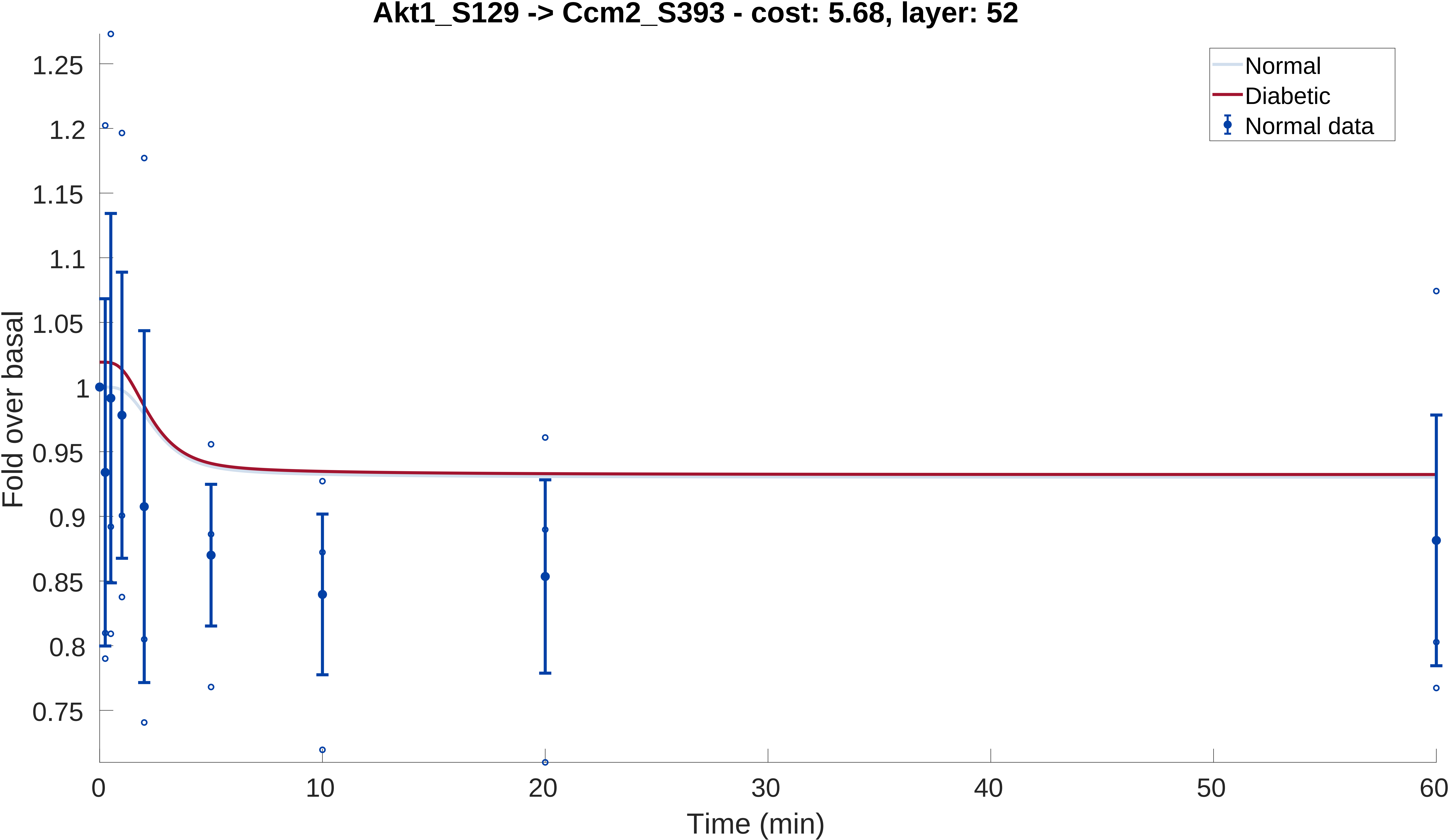

Akt1_S129 -> Ccm2_S393 - cost: 5.68, layer: 52
Normal
Diabetic
Normal data
Fold over basal
1.25
1.2
1.15
1.1
1.05
1
0.95
0.9
0.85
0.8
0.75
0
10
20
30
40
50
60
Time (min)

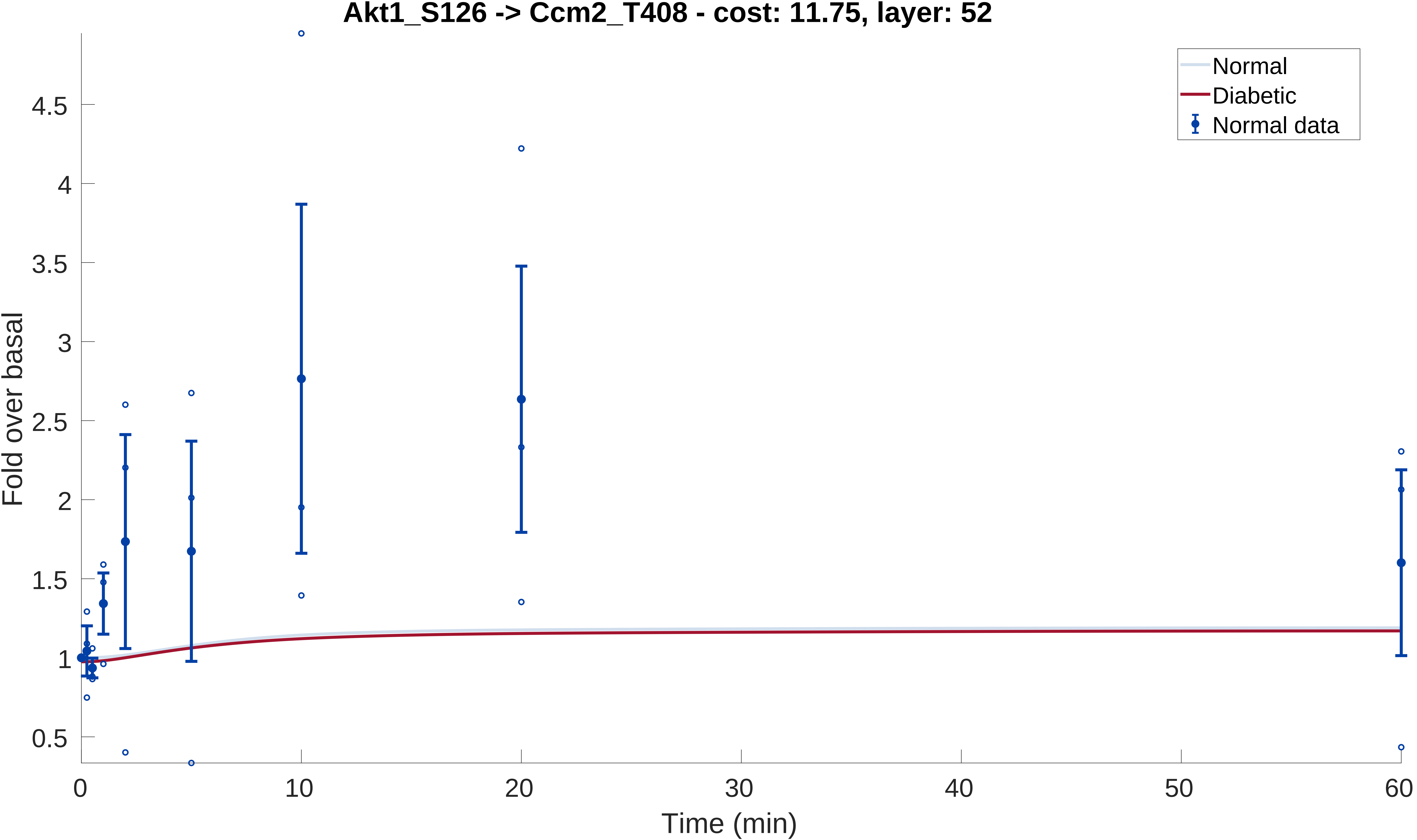
Akt1_S126 -> Ccm2_T408 - cost: 11.75, layer: 52
Normal
Diabetic
Normal data
Fold over basal
Time (min)
0.5
1
1.5
2
2.5
3
3.5
4
4.5
0
10
20
30
40
50
60

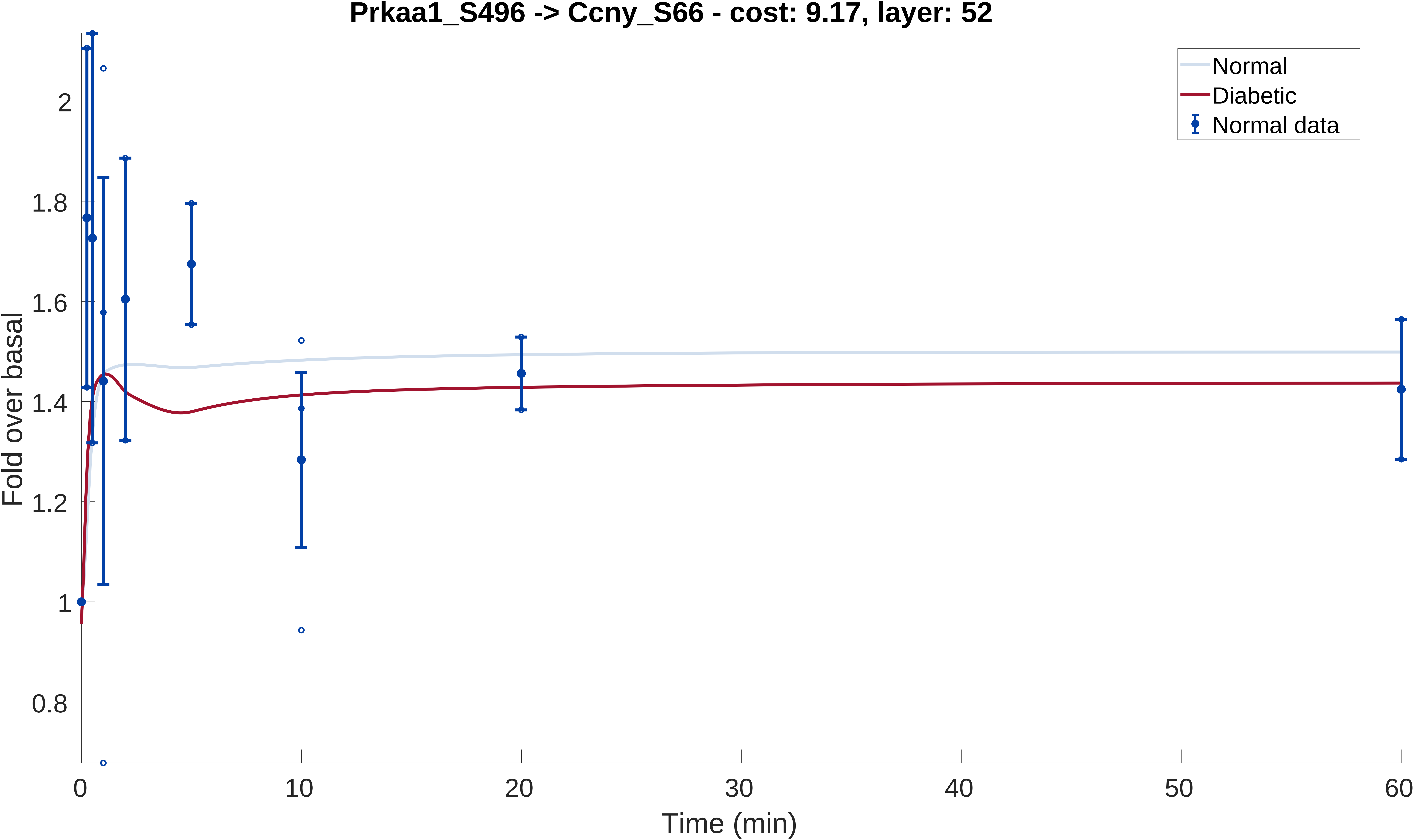

Prkaa1_S496 -> Ccny_S66 - cost: 9.17, layer: 52
Normal
Diabetic
Normal data
Fold over basal
Time (min)
0.8
1
1.2
1.4
1.6
1.8
2
0
10
20
30
40
50
60

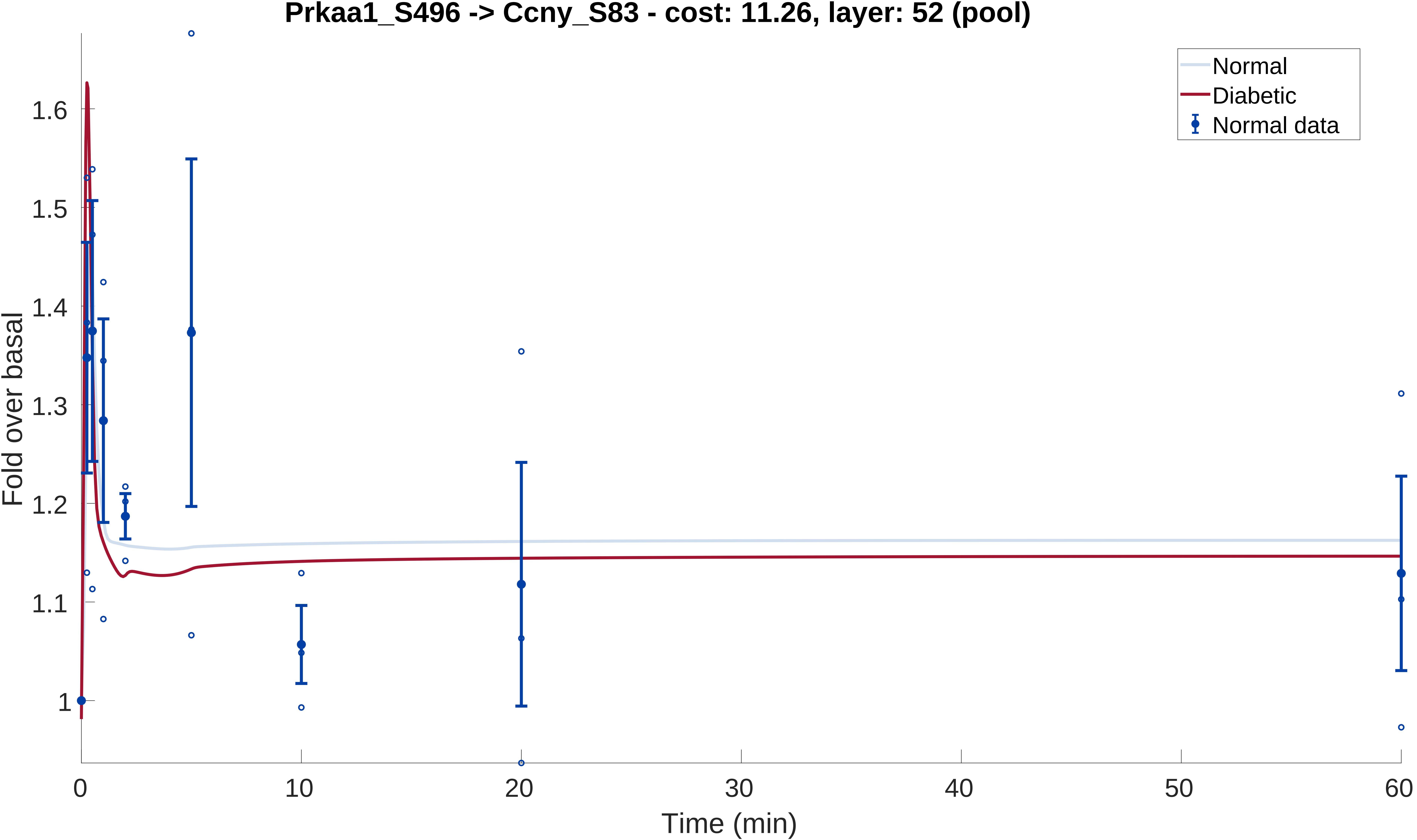

Prkaa1_S496 -> Ccny_S83 - cost: 11.26, layer: 52 (pool)
Normal
Diabetic
Normal data
Fold over basal
Time (min)
1.6
1.5
1.4
1.3
1.2
1.1
1
0
10
20
30
40
50
60

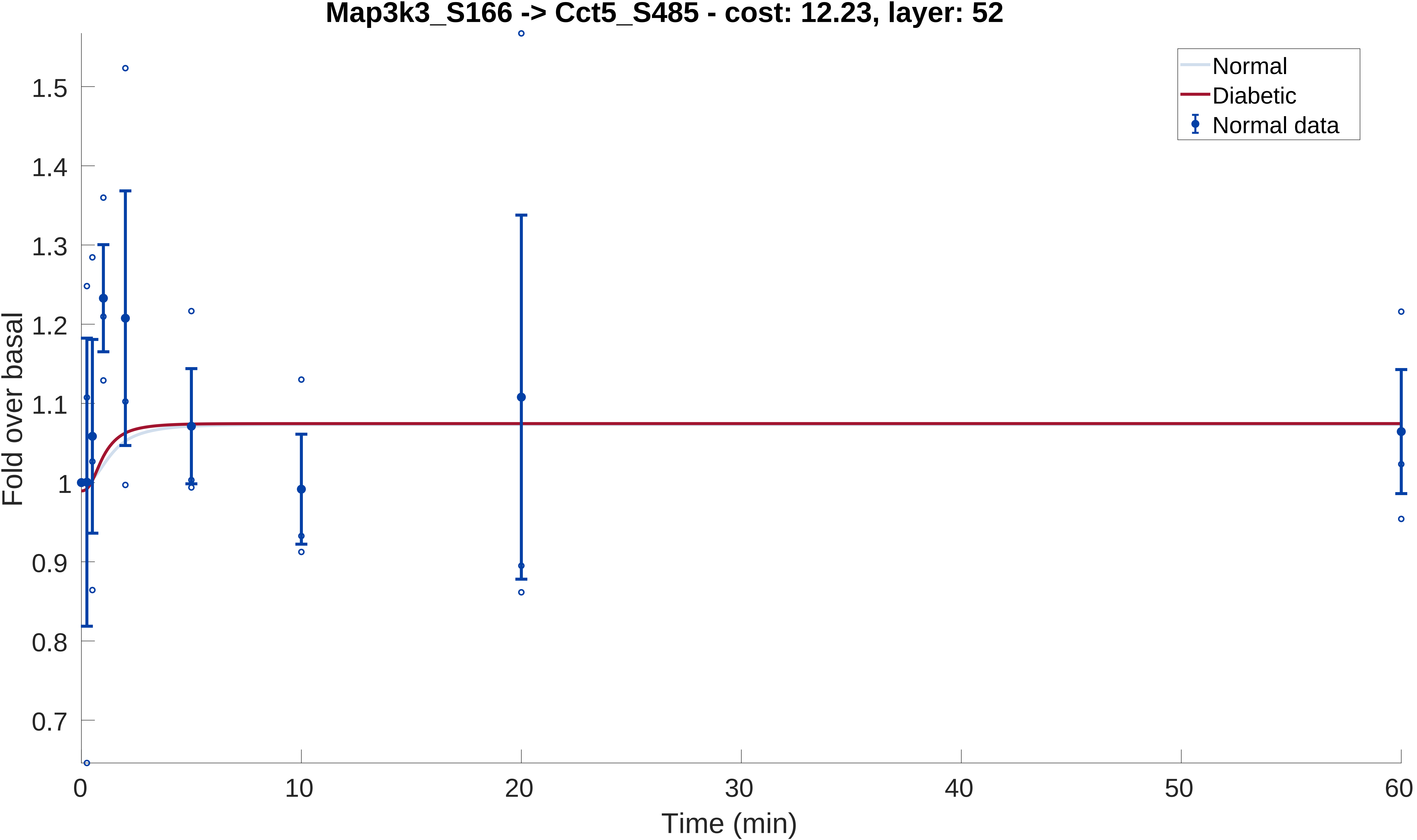

Map3k3_S166 -> Cct5_S485 - cost: 12.23, layer: 52
Normal
Diabetic
Normal data
Fold over basal
1.5
1.4
1.3
1.2
1.1
1
0.9
0.8
0.7
0
10
20
30
40
50
60
Time (min)

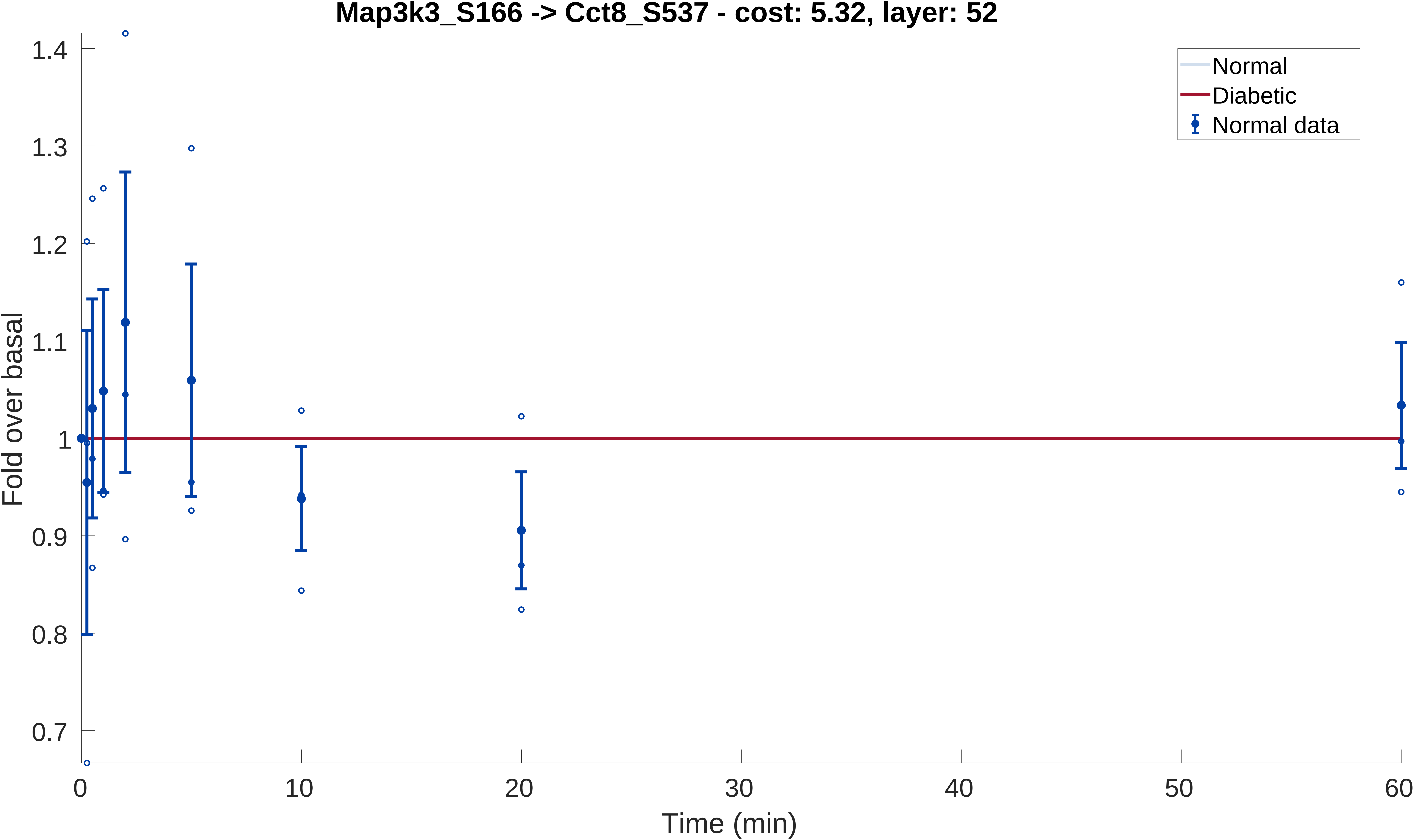

Map3k3_S166 -> Cct8_S537 - cost: 5.32, layer: 52
Normal
Diabetic
Normal data
Fold over basal
1.4
1.3
1.2
1.1
1
0.9
0.8
0.7
0
10
20
30
40
50
60
Time (min)

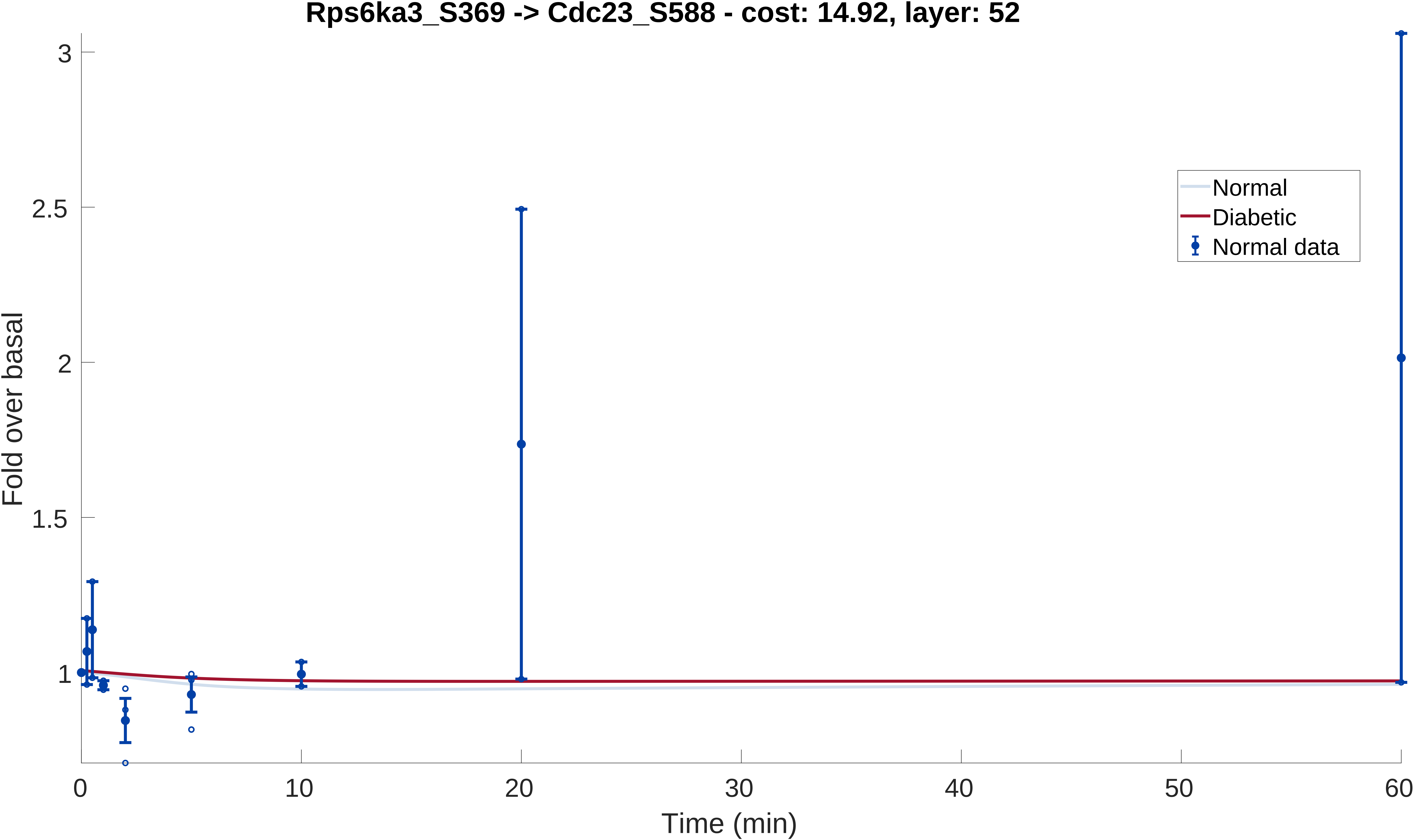

Rps6ka3_S369 -> Cdc23_S588 - cost: 14.92, layer: 52
Fold over basal
3
2.5
2
1.5
1
0
10
20
30
40
50
60
Time (min)
Normal
Diabetic
Normal data

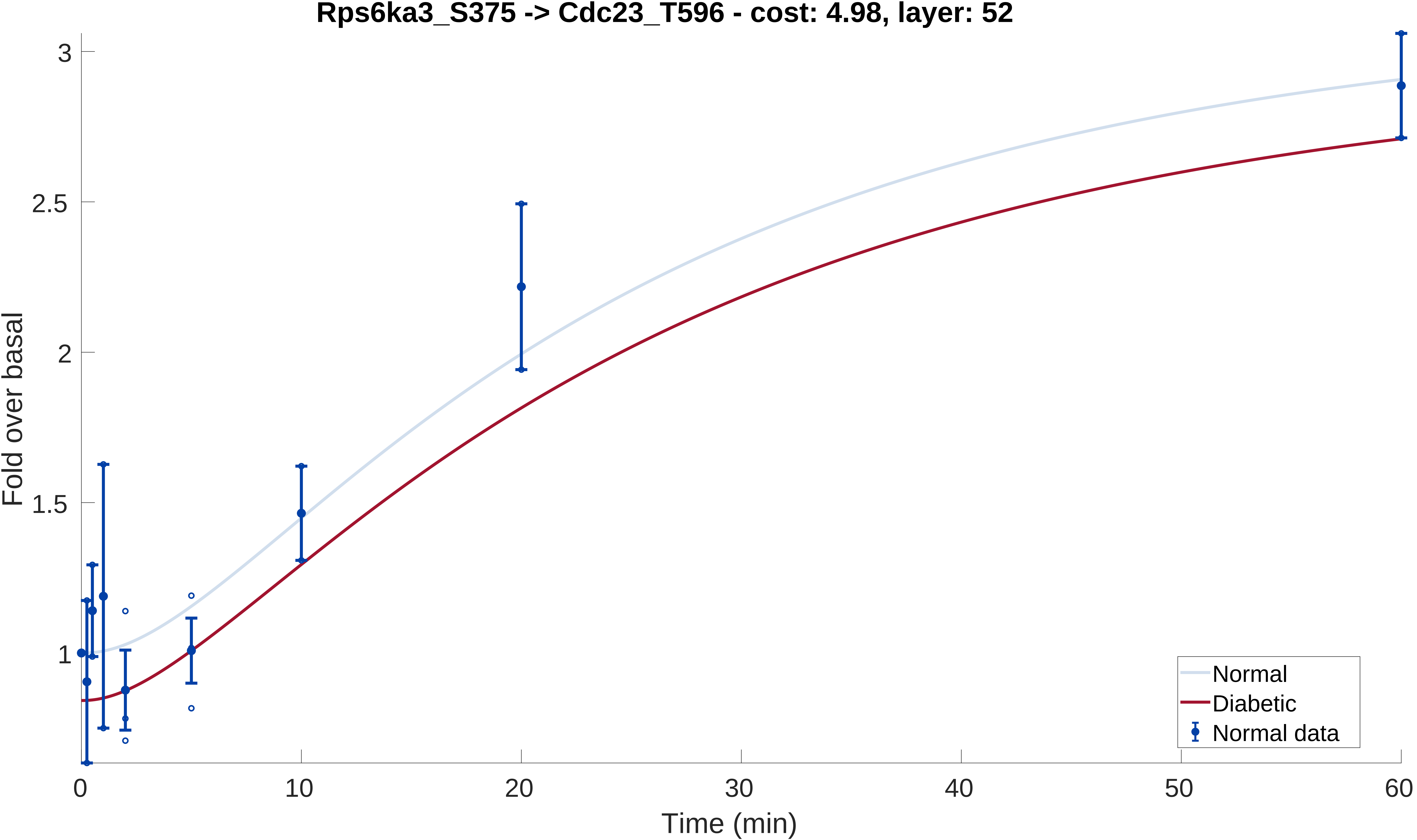
Rps6ka3_S375 -> Cdc23_T596 - cost: 4.98, layer: 52
Fold over basal
3
2.5
2
1.5
1
0
10
20
30
40
50
60
Time (min)
Normal
Diabetic
Normal data

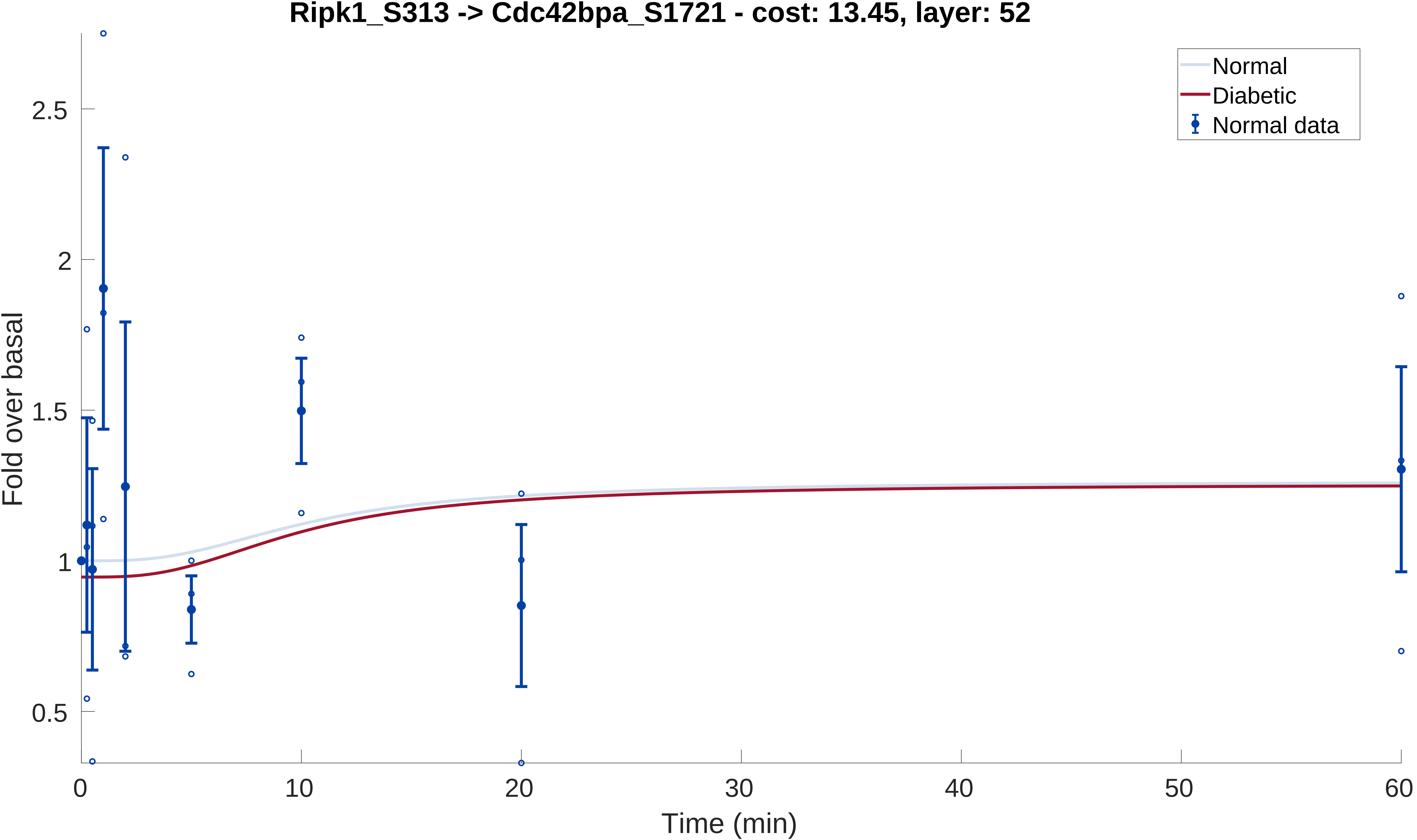

Ripk1_S313 -> Cdc42bpa_S1721 - cost: 13.45, layer: 52
Normal
Diabetic
Normal data
Fold over basal
2.5
2
1.5
1
0.5
0
10
20
30
40
50
60
Time (min)

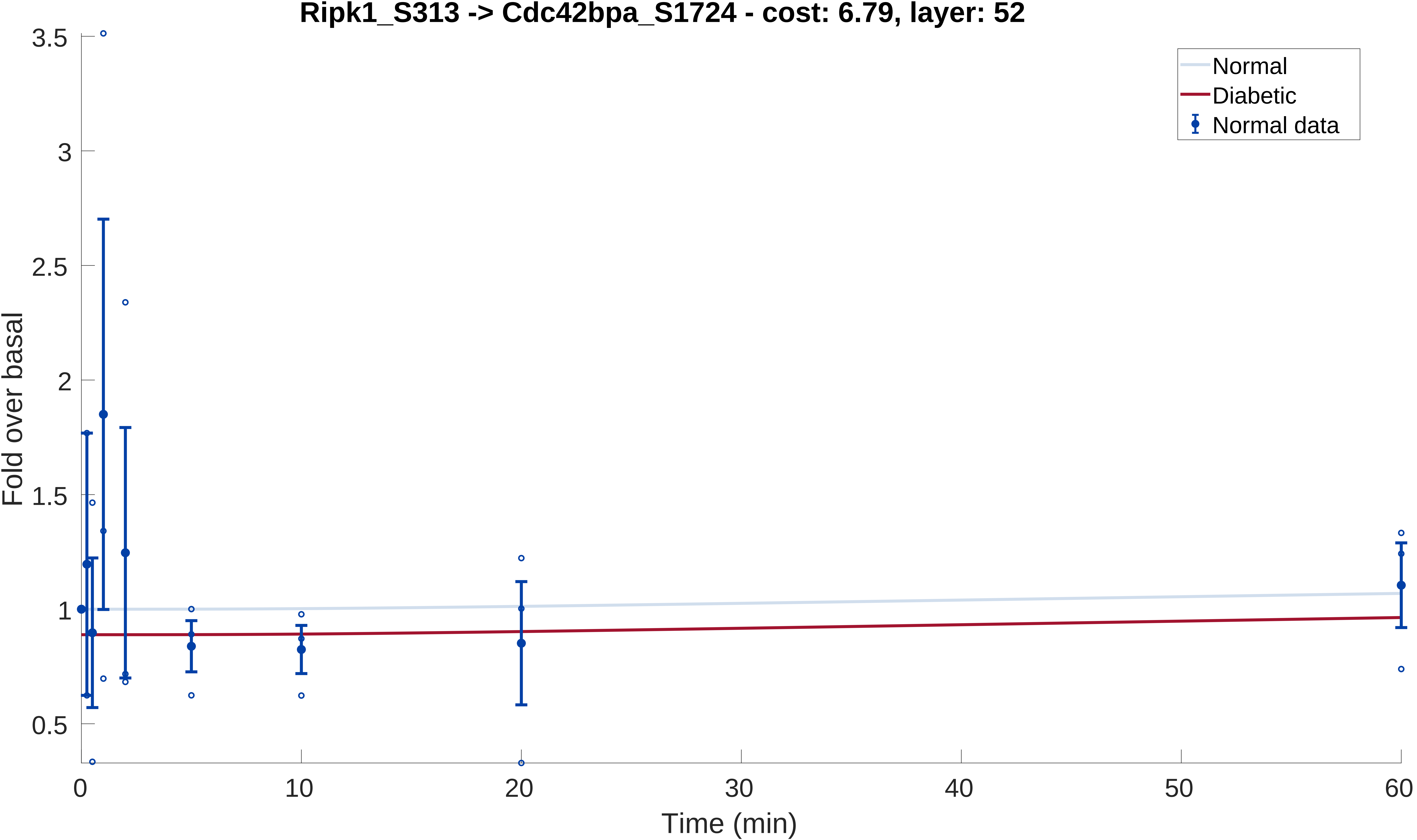

Ripk1_S313 -> Cdc42bpa_S1724 - cost: 6.79, layer: 52
Fold over basal
Time (min)
Normal
Diabetic
Normal data
3.5
3
2.5
2
1.5
1
0.5
0
10
20
30
40
50
60

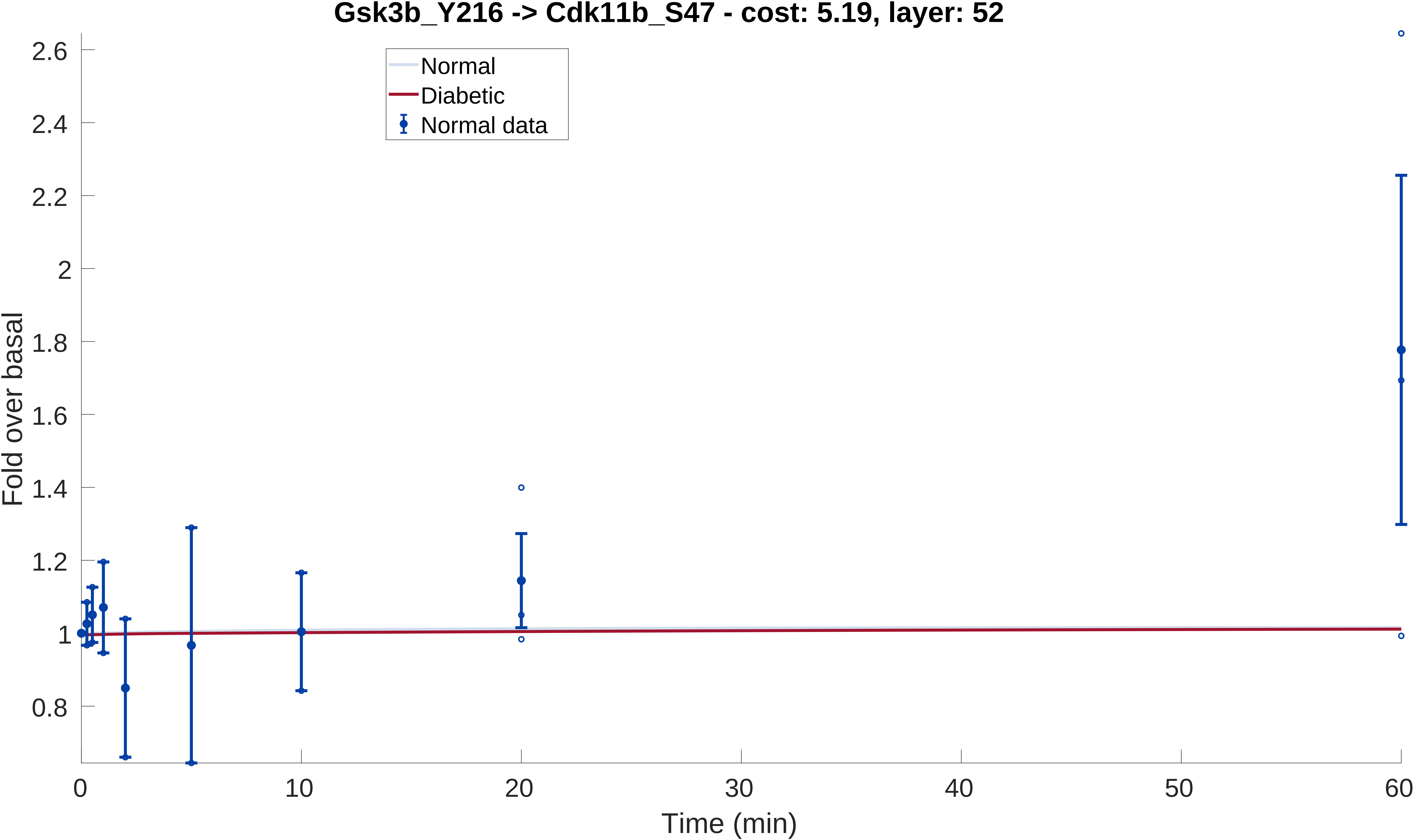

Gsk3b_Y216 -> Cdk11b_S47 - cost: 5.19, layer: 52
Normal
Diabetic
Normal data
Fold over basal
2.6
2.4
2.2
2
1.8
1.6
1.4
1.2
1
0.8
0
10
20
30
40
50
60
Time (min)

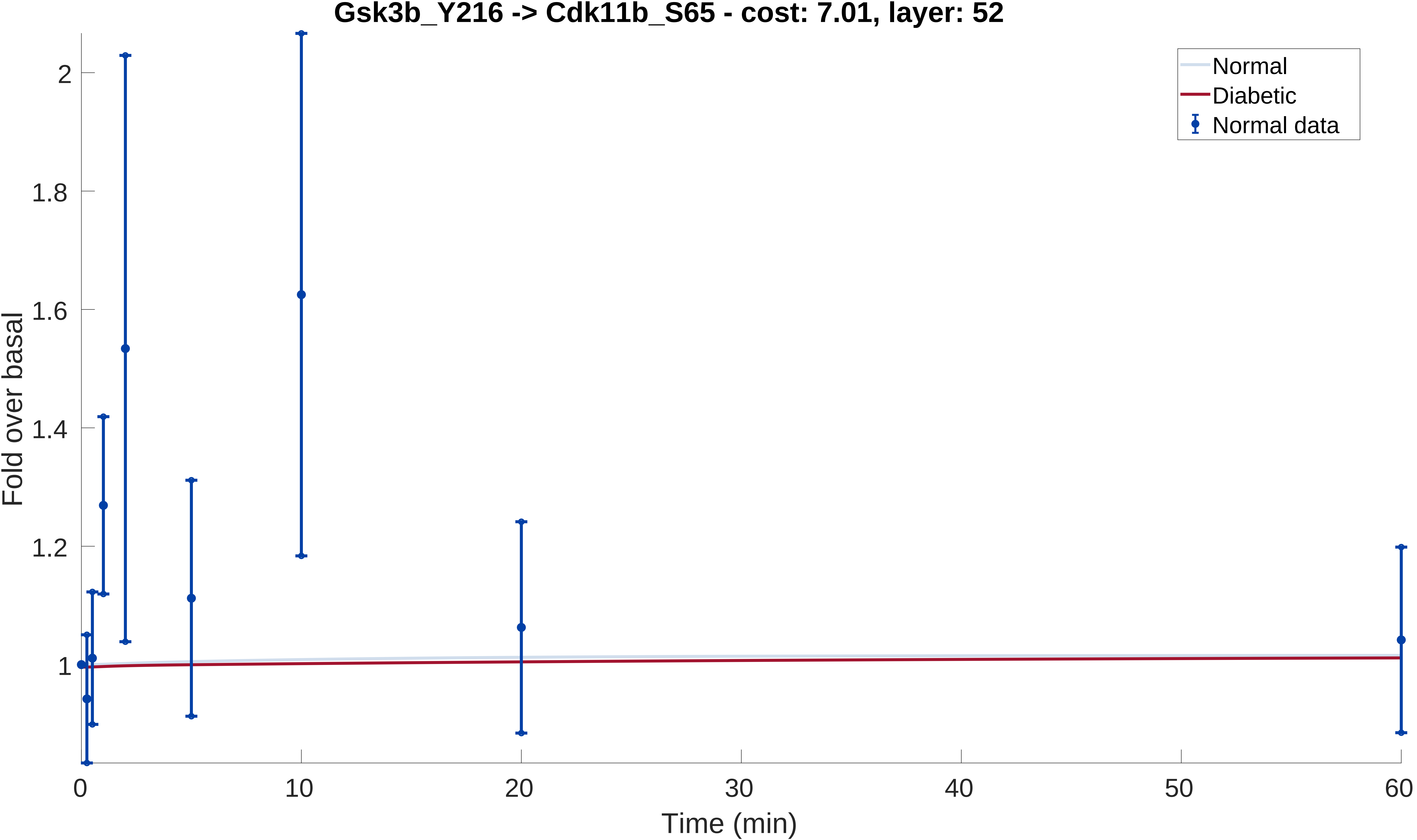
Gsk3b_Y216 -> Cdk11b_S65 - cost: 7.01, layer: 52
Normal
Diabetic
Normal data
Fold over basal
2
1.8
1.6
1.4
1.2
1
0
10
20
30
40
50
60
Time (min)

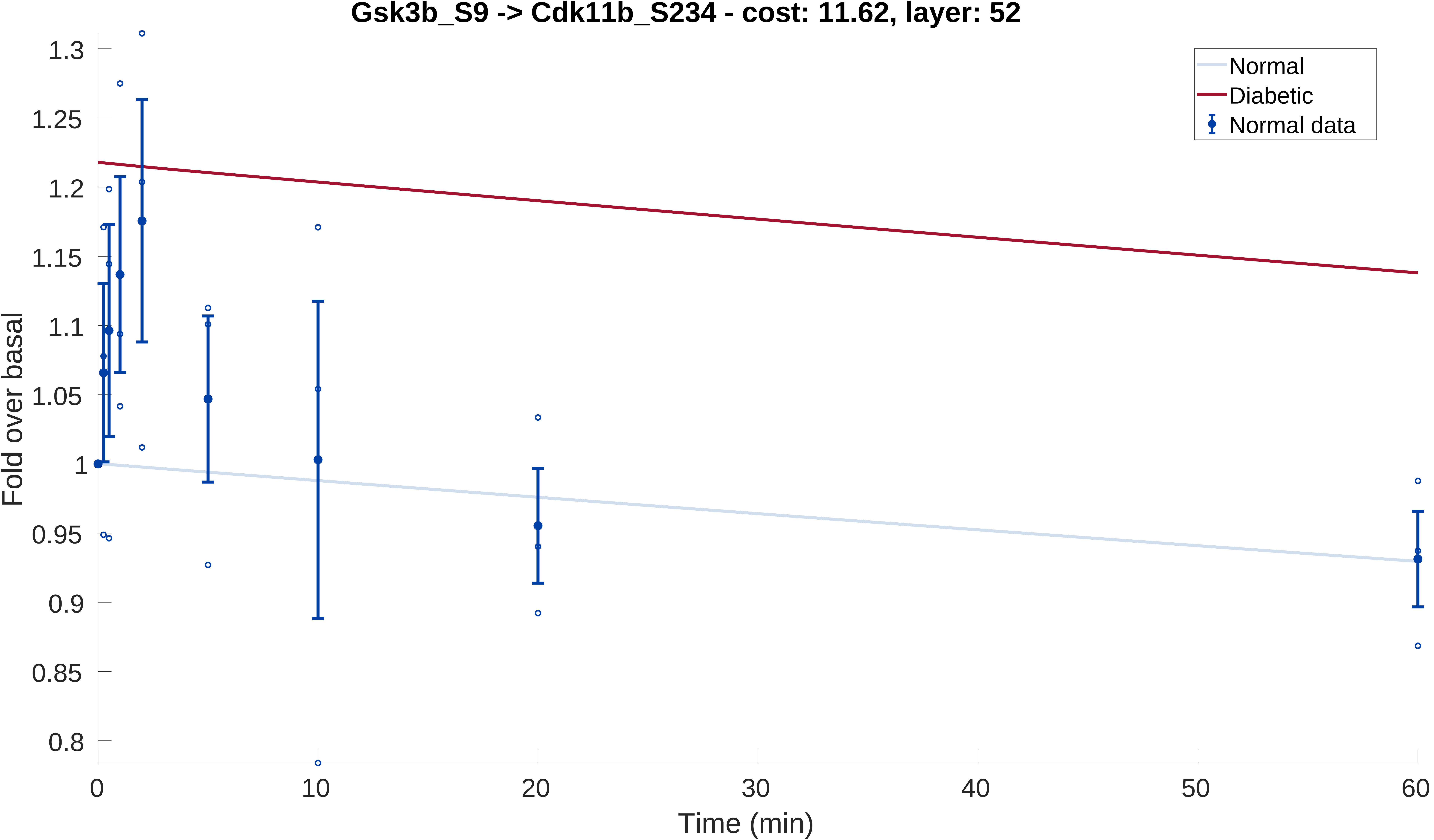

Gsk3b_S9 -> Cdk11b_S234 - cost: 11.62, layer: 52
Fold over basal
1.3
1.25
1.2
1.15
1.1
1.05
1
0.95
0.9
0.85
0.8
0
10
20
30
40
50
60
Time (min)
Normal
Diabetic
Normal data

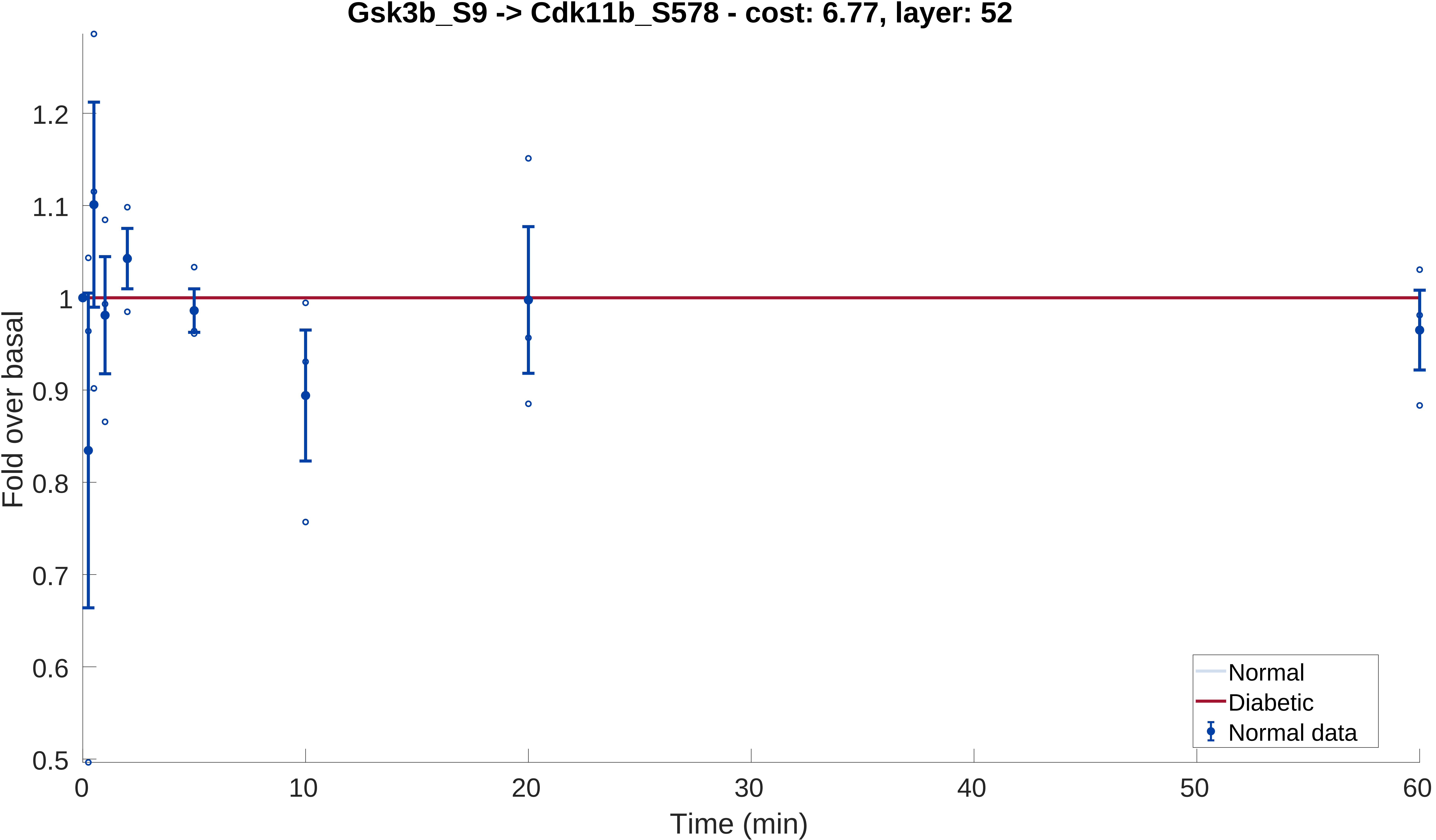

Gsk3b_S9 -> Cdk11b_S578 - cost: 6.77, layer: 52
Fold over basal
Time (min)
1.2
1.1
1
0.9
0.8
0.7
0.6
0.5
0
10
20
30
40
50
60
Normal
Diabetic
Normal data

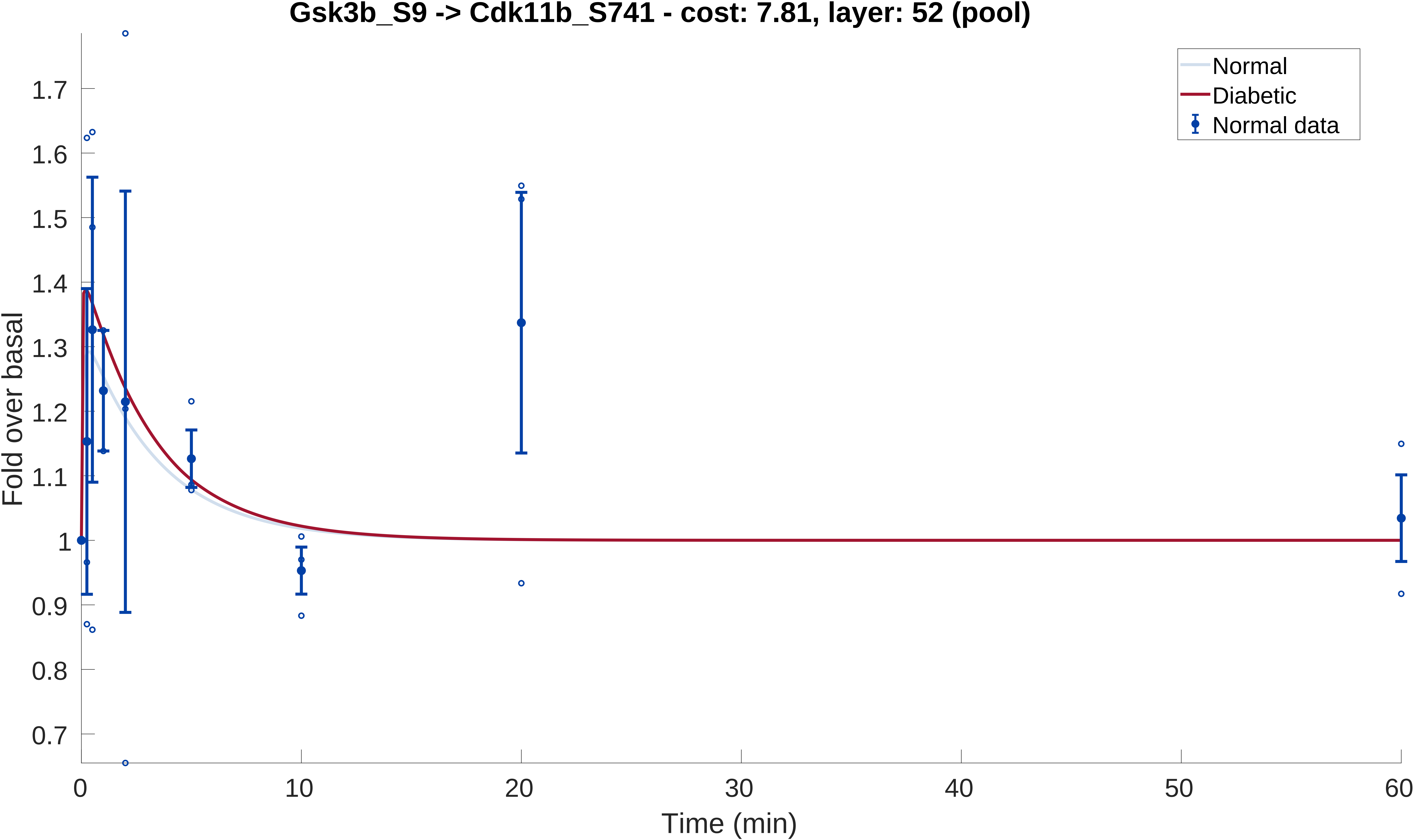

Gsk3b_S9 -> Cdk11b_S741 - cost: 7.81, layer: 52 (pool)
Normal
Diabetic
Normal data
1.7
1.6
1.5
1.4
1.3
1.2
1.1
1
0.9
0.8
0.7
Fold over basal
0
10
20
30
40
50
60
Time (min)

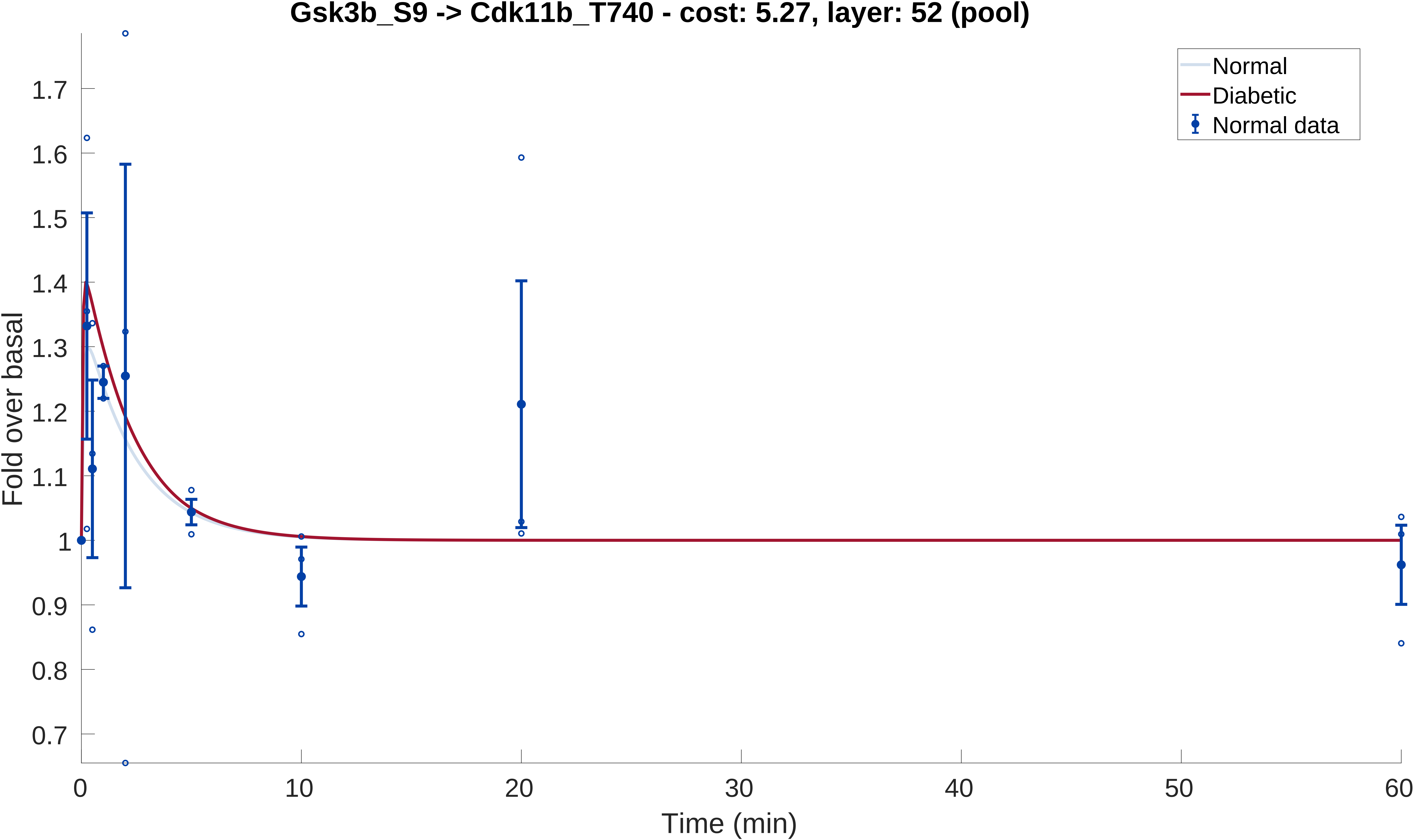

Gsk3b_S9 -> Cdk11b_T740 - cost: 5.27, layer: 52 (pool)
Normal
Diabetic
Normal data
Fold over basal
1.7
1.6
1.5
1.4
1.3
1.2
1.1
1
0.9
0.8
0.7
0
10
20
30
40
50
60
Time (min)

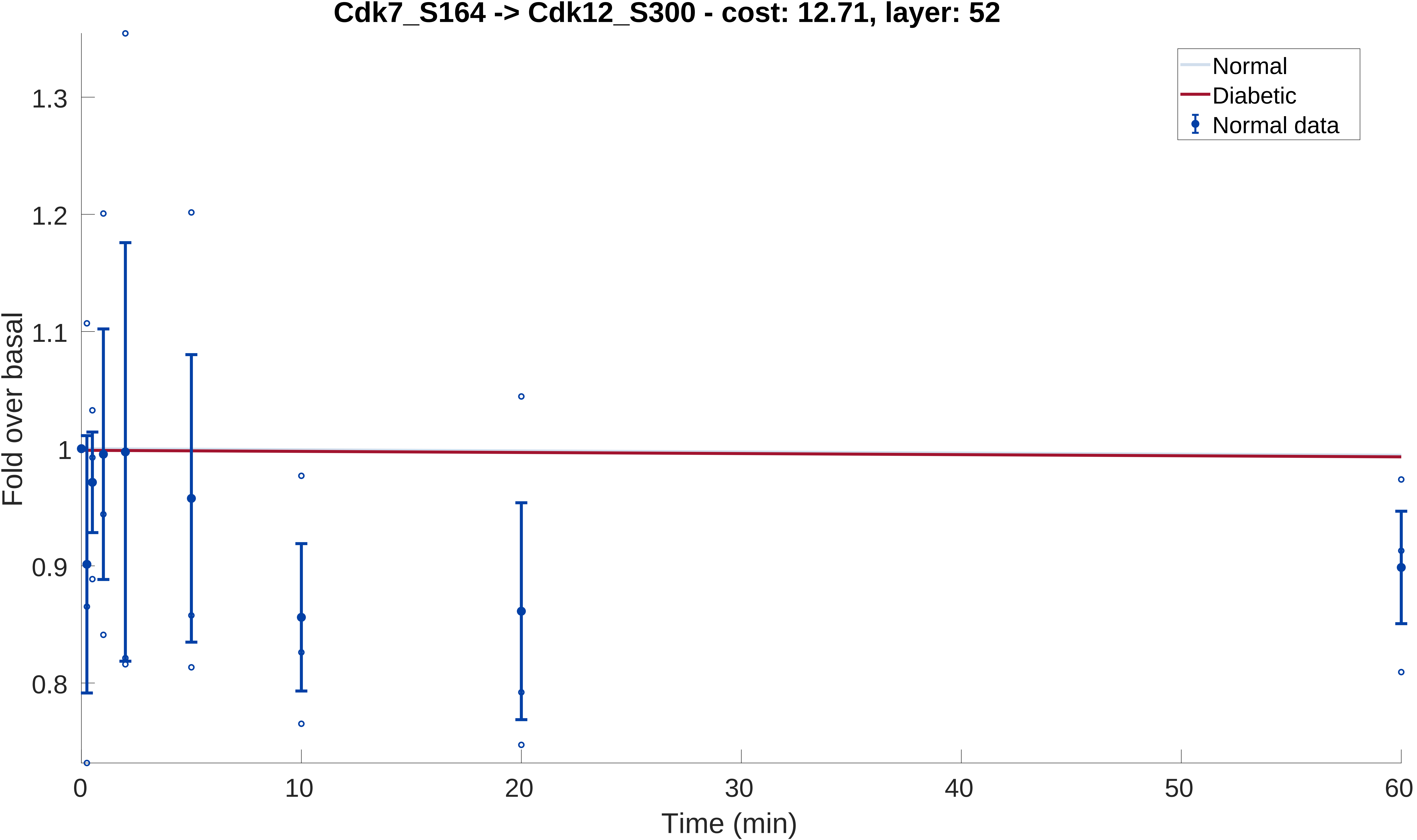

Cdk7_S164 -> Cdk12_S300 - cost: 12.71, layer: 52
Normal
Diabetic
Normal data
Fold over basal
Time (min)
1.3
1.2
1.1
1
0.9
0.8
0
10
20
30
40
50
60

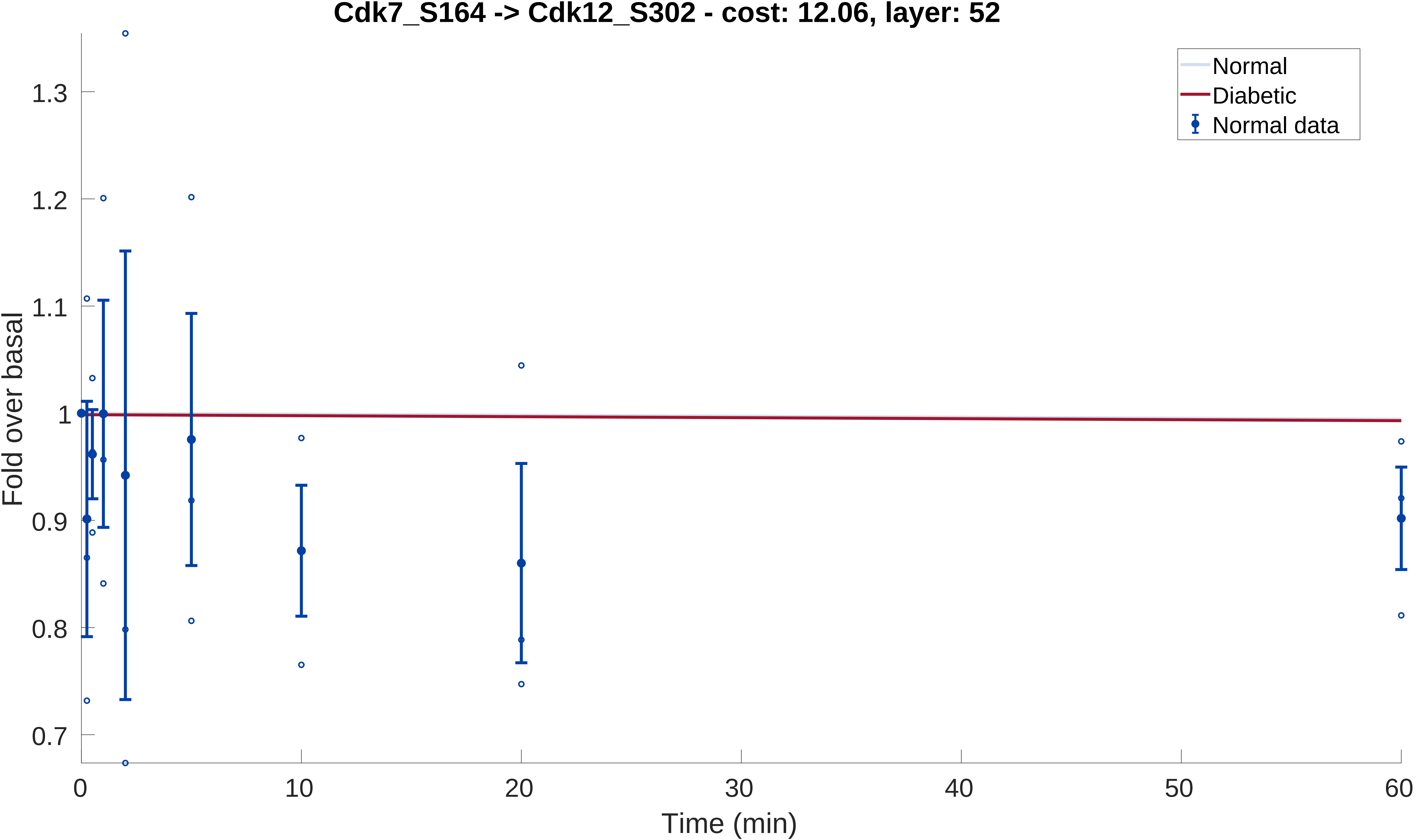
Cdk7_S164 -> Cdk12_S302 - cost: 12.06, layer: 52
Normal
Diabetic
Normal data
Fold over basal
Time (min)
0.7
0.8
0.9
1
1.1
1.2
1.3
0
10
20
30
40
50
60

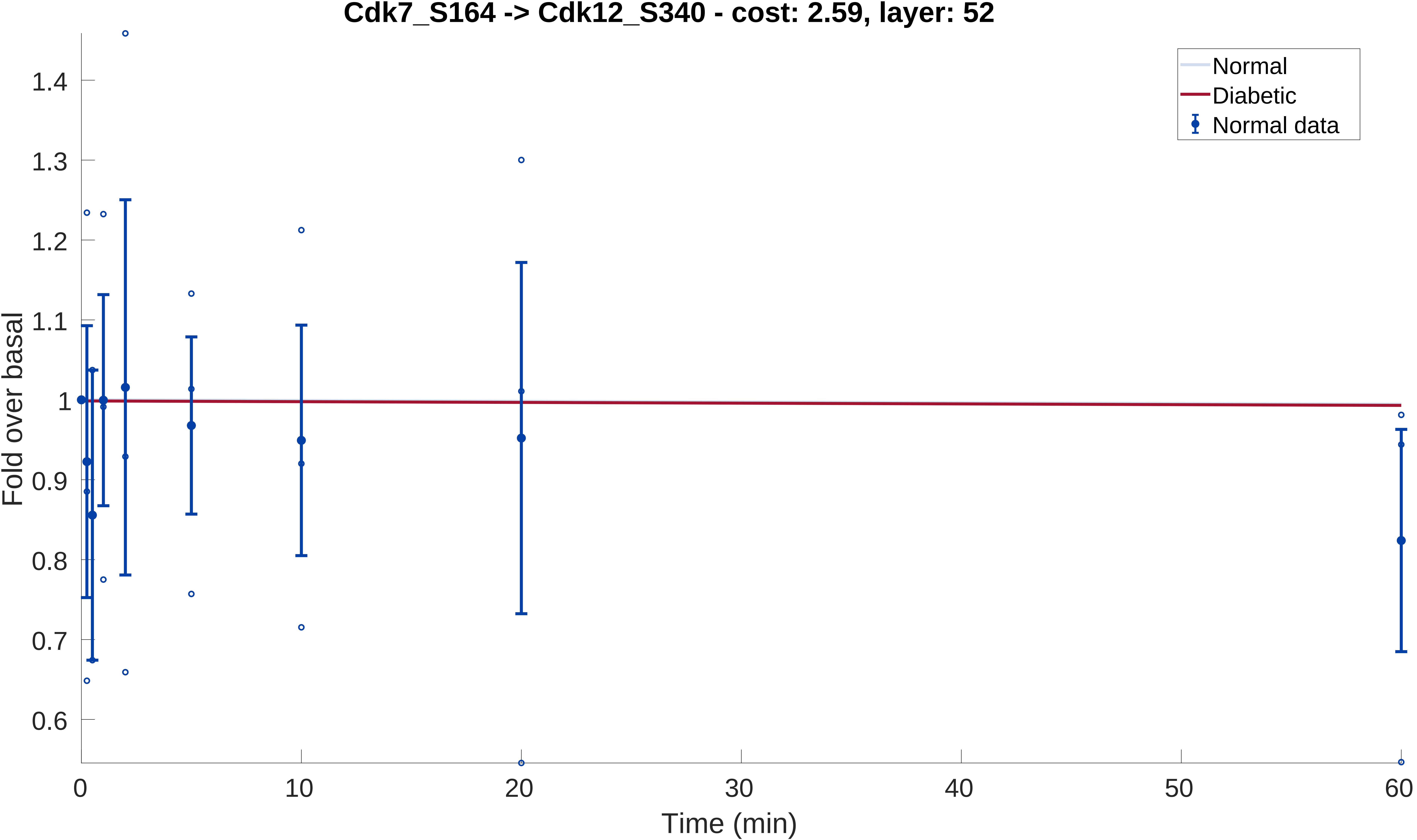

Cdk7_S164 -> Cdk12_S340 - cost: 2.59, layer: 52
Normal
Diabetic
Normal data
Fold over basal
Time (min)
1.4
1.3
1.2
1.1
1
0.9
0.8
0.7
0.6
0
10
20
30
40
50
60

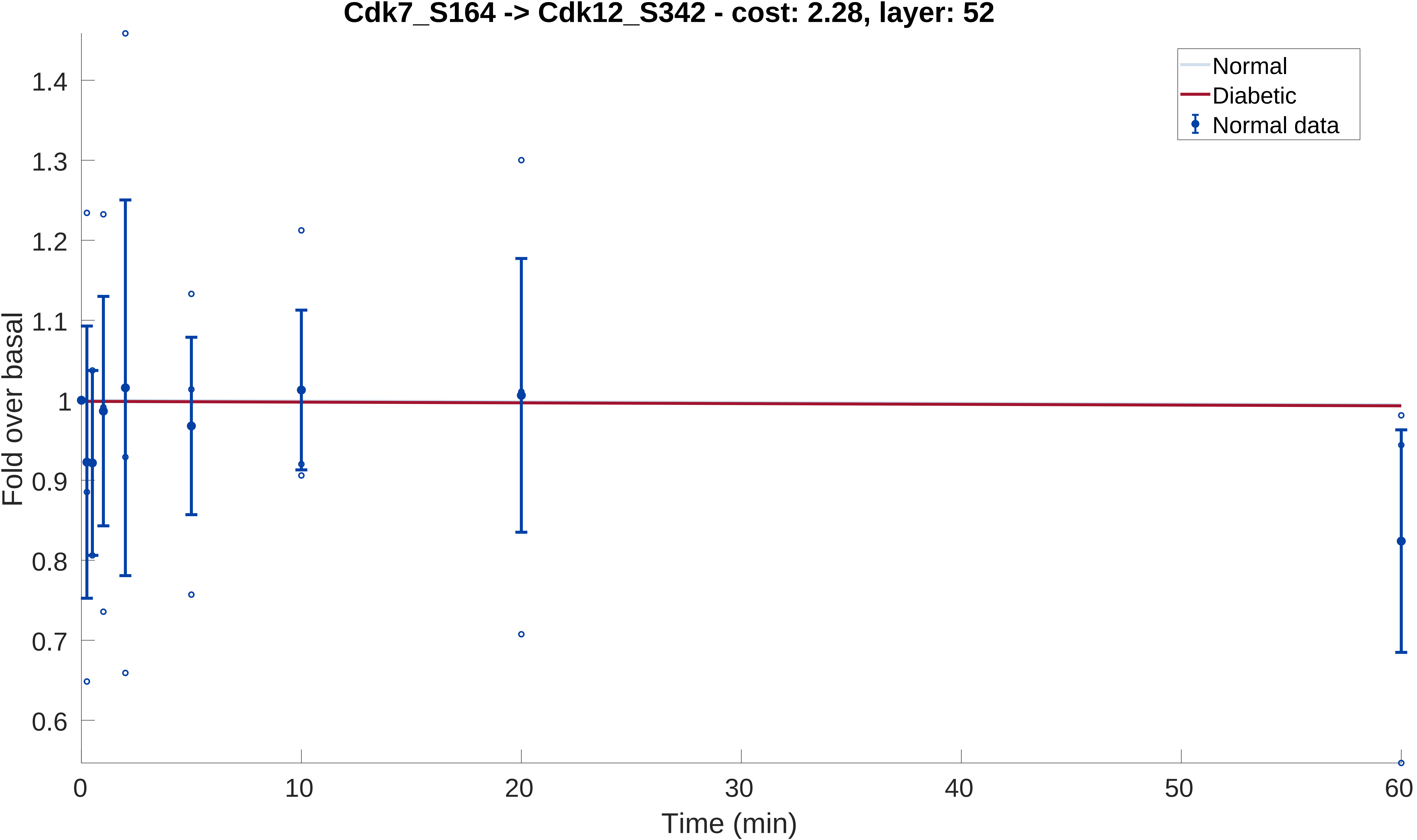

Cdk7_S164 -> Cdk12_S342 - cost: 2.28, layer: 52
Normal
Diabetic
Normal data
Fold over basal
Time (min)
0
10
20
30
40
50
60
0.6
0.7
0.8
0.9
1
1.1
1.2
1.3
1.4

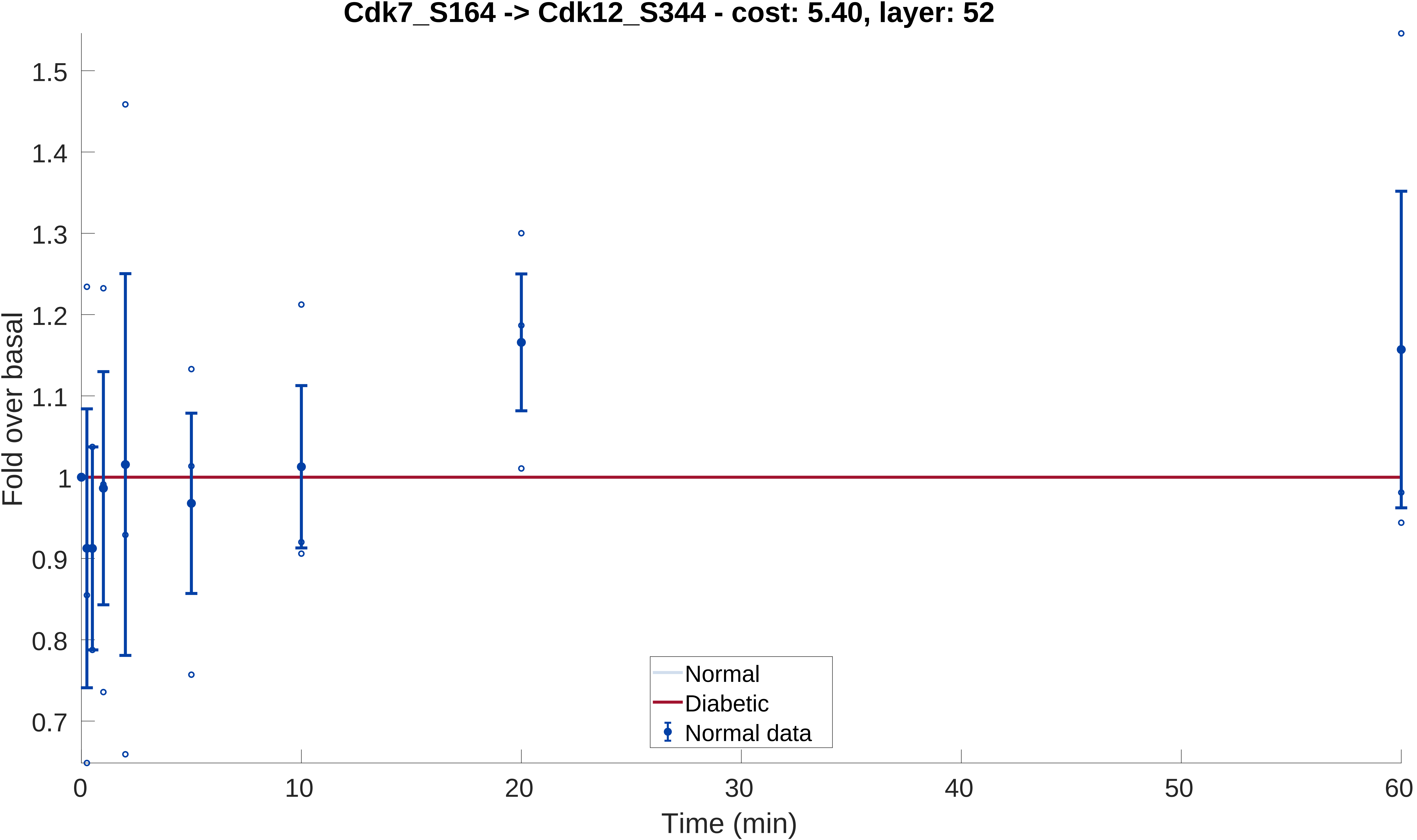
Cdk7_S164 -> Cdk12_S344 - cost: 5.40, layer: 52
Fold over basal
1.5
1.4
1.3
1.2
1.1
1
0.9
0.8
0.7
Time (min)
0
10
20
30
40
50
60
Normal
Diabetic
Normal data

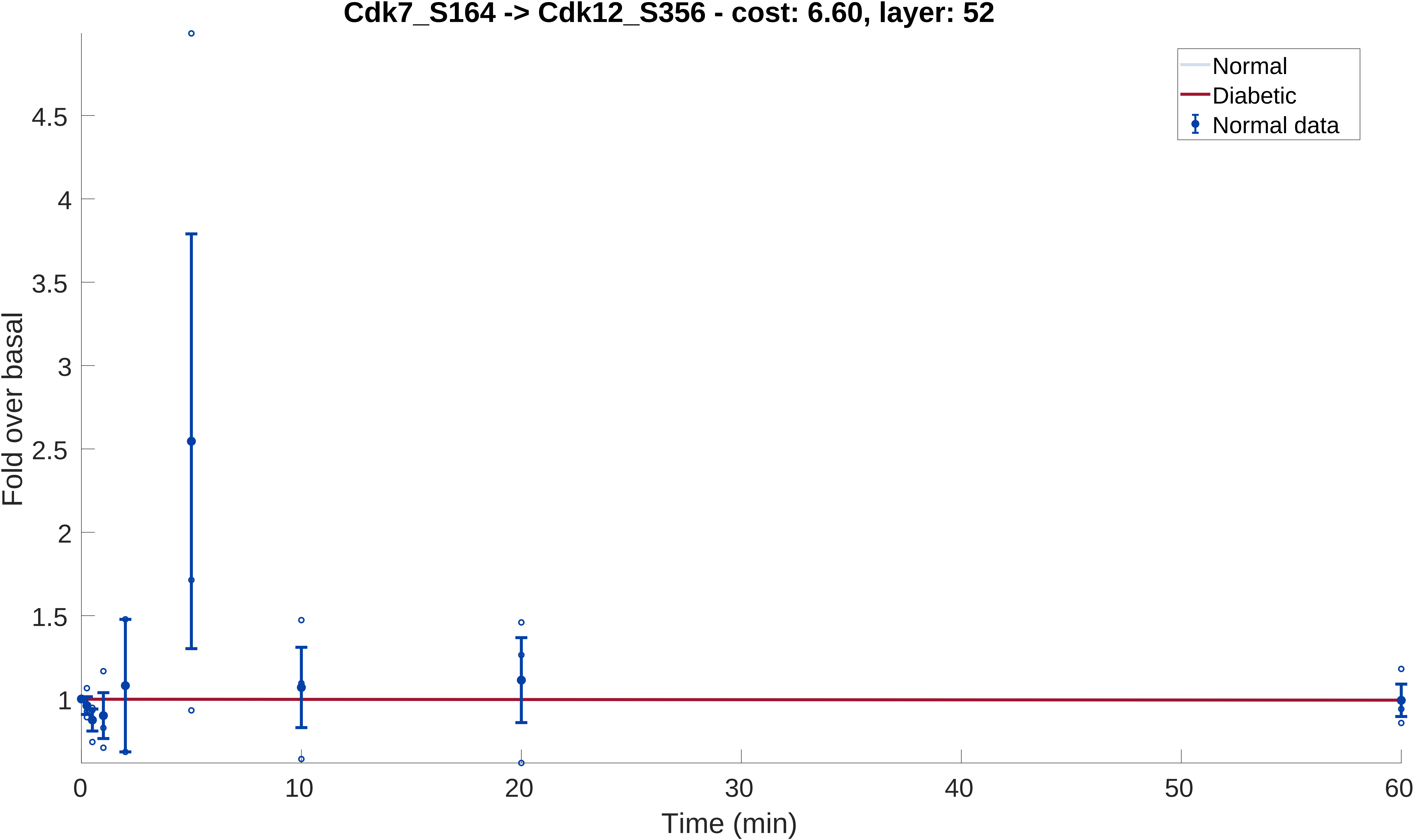

Cdk7_S164 -> Cdk12_S356 - cost: 6.60, layer: 52
Normal
Diabetic
Normal data
Fold over basal
Time (min)
4.5
4
3.5
3
2.5
2
1.5
1
0
10
20
30
40
50
60

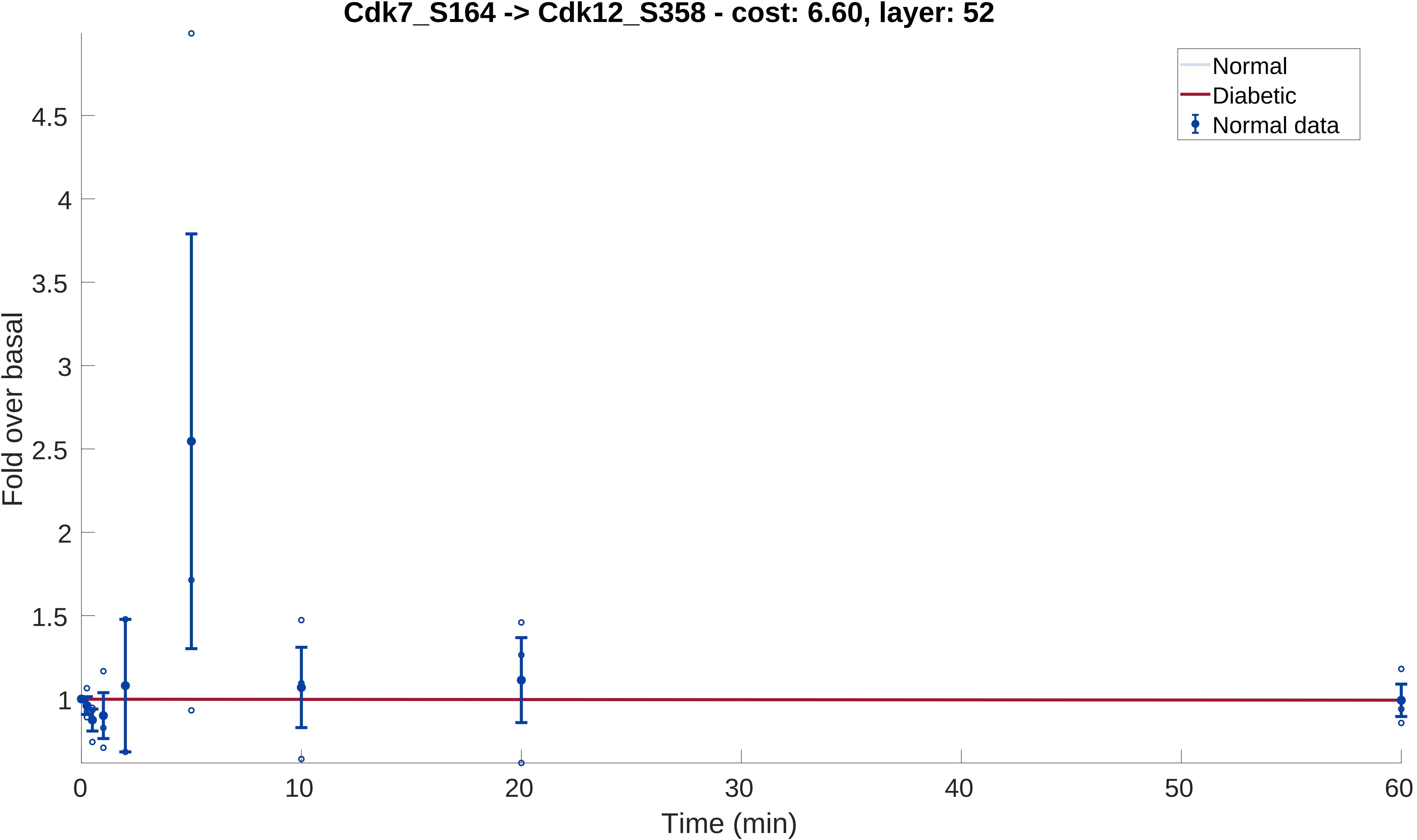
Cdk7_S164 -> Cdk12_S358 - cost: 6.60, layer: 52
Normal
Diabetic
Normal data
Fold over basal
4.5
4
3.5
3
2.5
2
1.5
1
0
10
20
30
40
50
60
Time (min)

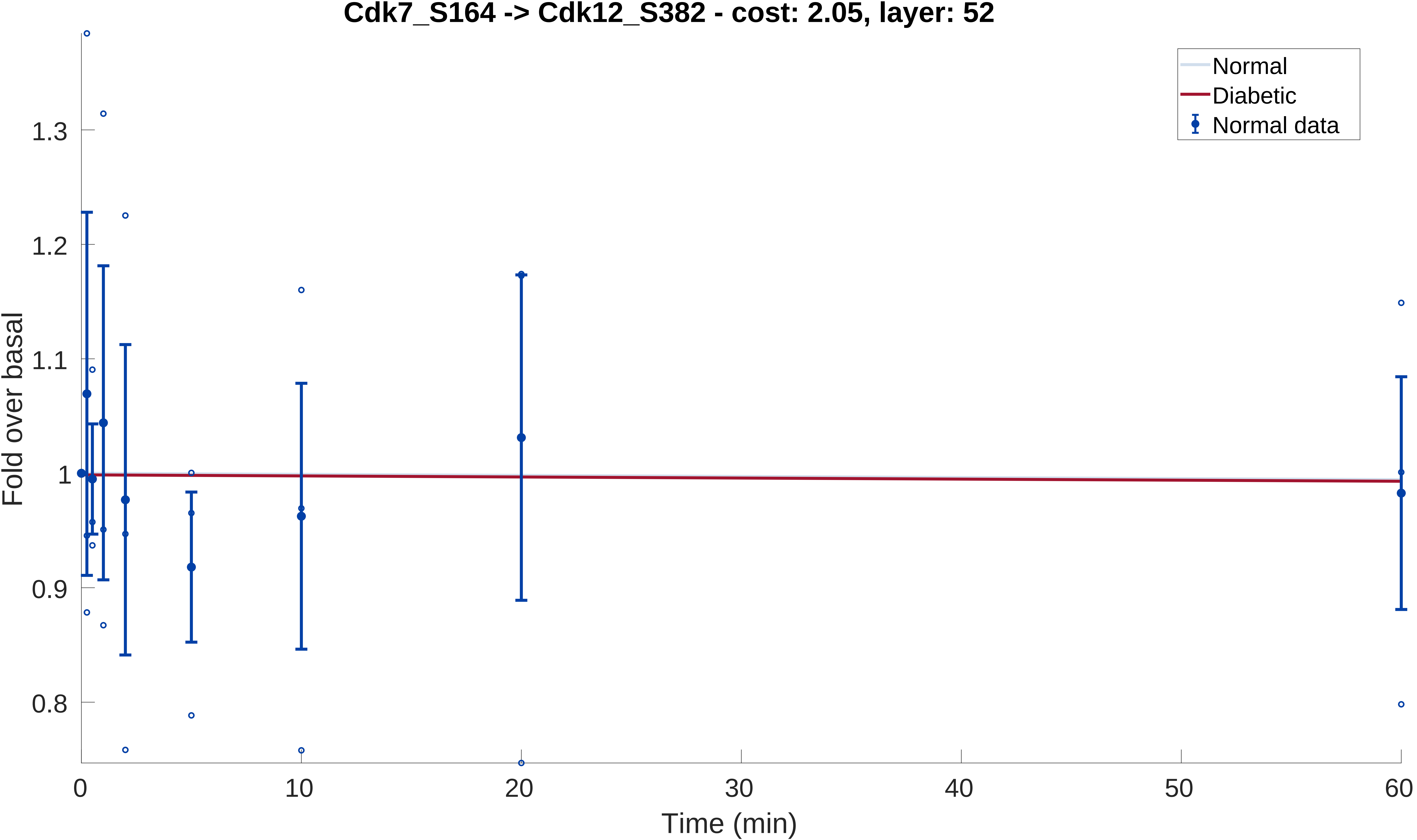

Cdk7_S164 -> Cdk12_S382 - cost: 2.05, layer: 52
Normal
Diabetic
Normal data
Fold over basal
1.3
1.2
1.1
1
0.9
0.8
0
10
20
30
40
50
60
Time (min)

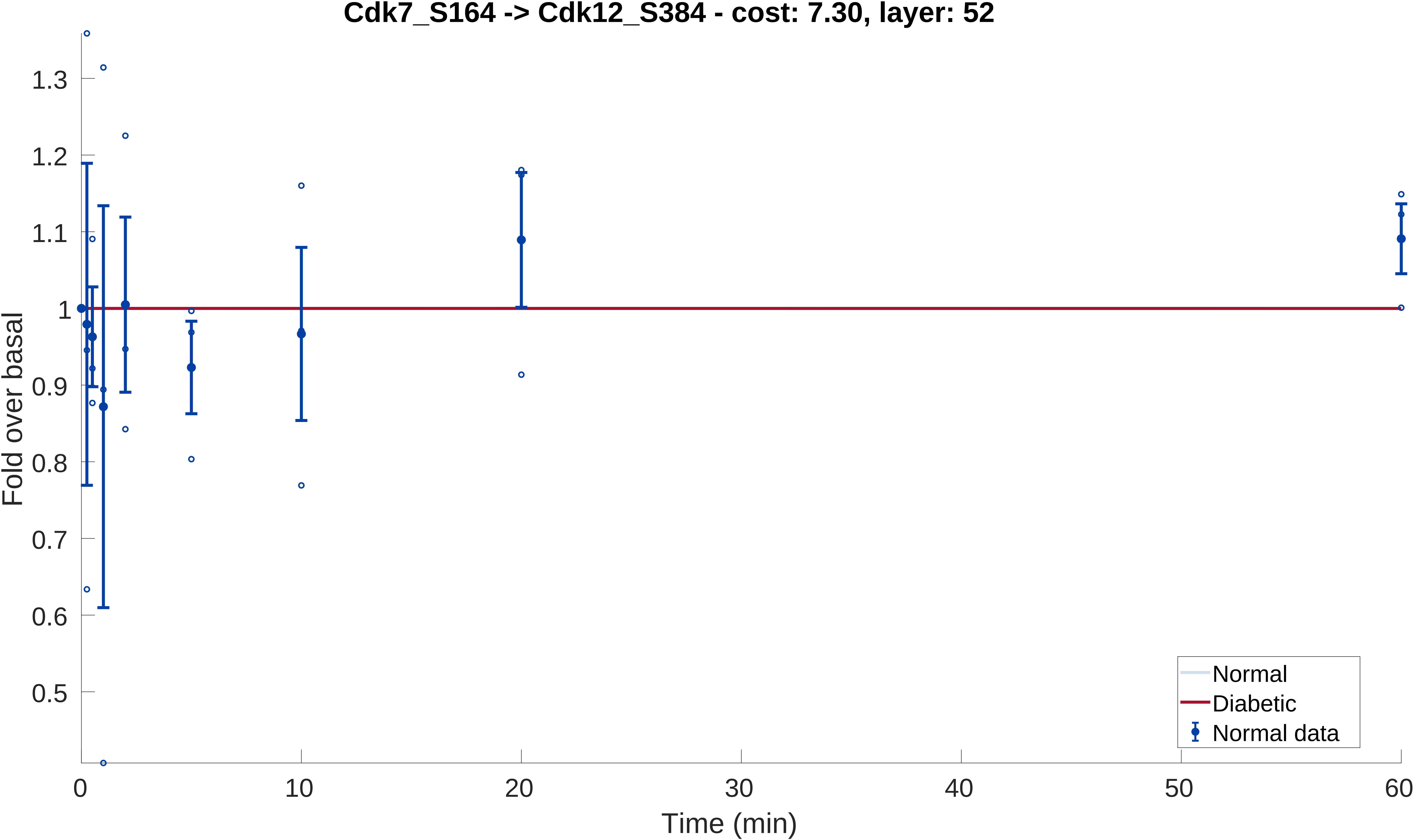
Cdk7_S164 -> Cdk12_S384 - cost: 7.30, layer: 52
Fold over basal
Time (min)
1.3
1.2
1.1
1
0.9
0.8
0.7
0.6
0.5
0
10
20
30
40
50
60
Normal
Diabetic
Normal data

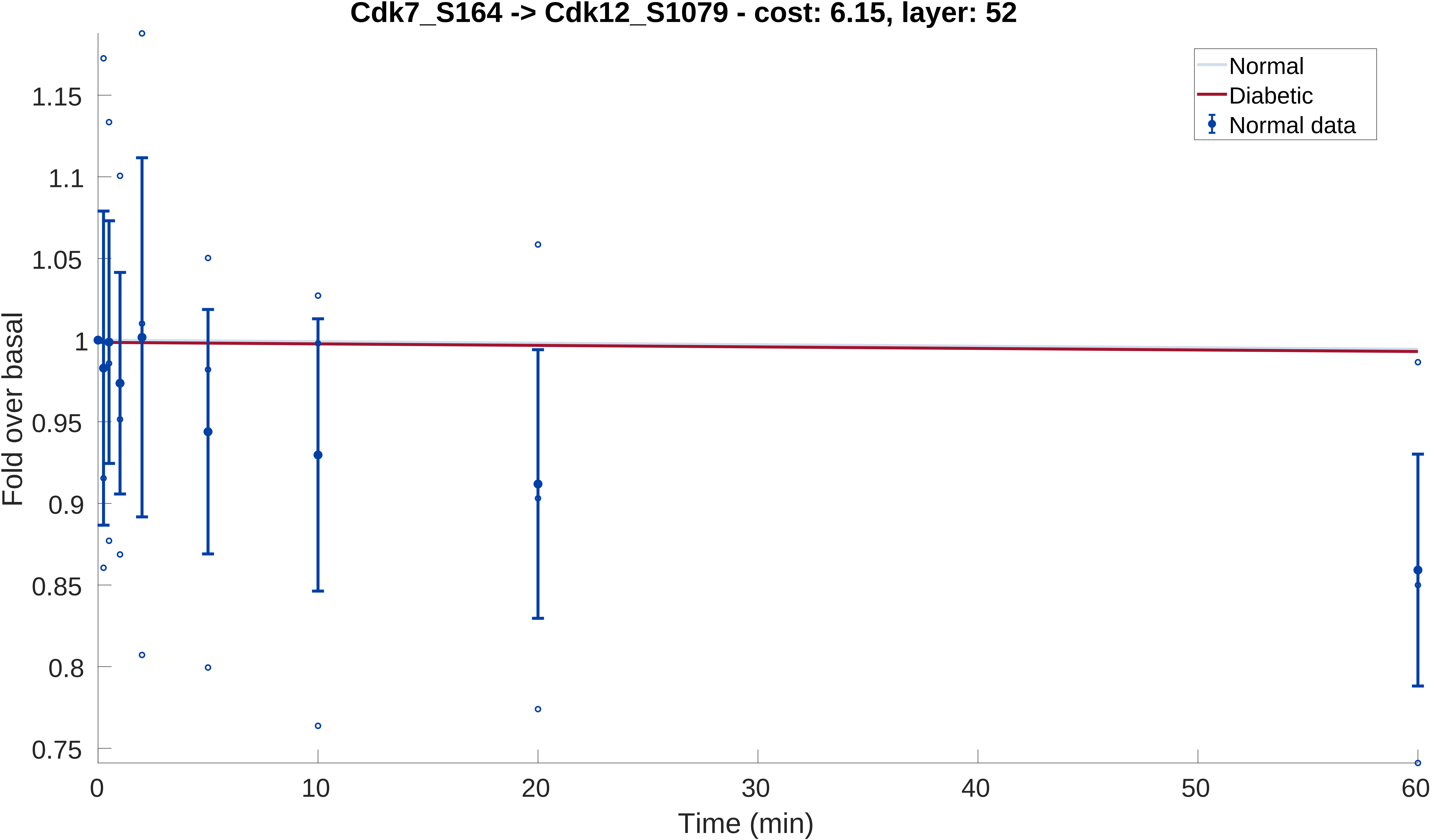

Cdk7_S164 -> Cdk12_S1079 - cost: 6.15, layer: 52
Normal
Diabetic
Normal data
Fold over basal
1.15
1.1
1.05
1
0.95
0.9
0.85
0.8
0.75
0
10
20
30
40
50
60
Time (min)

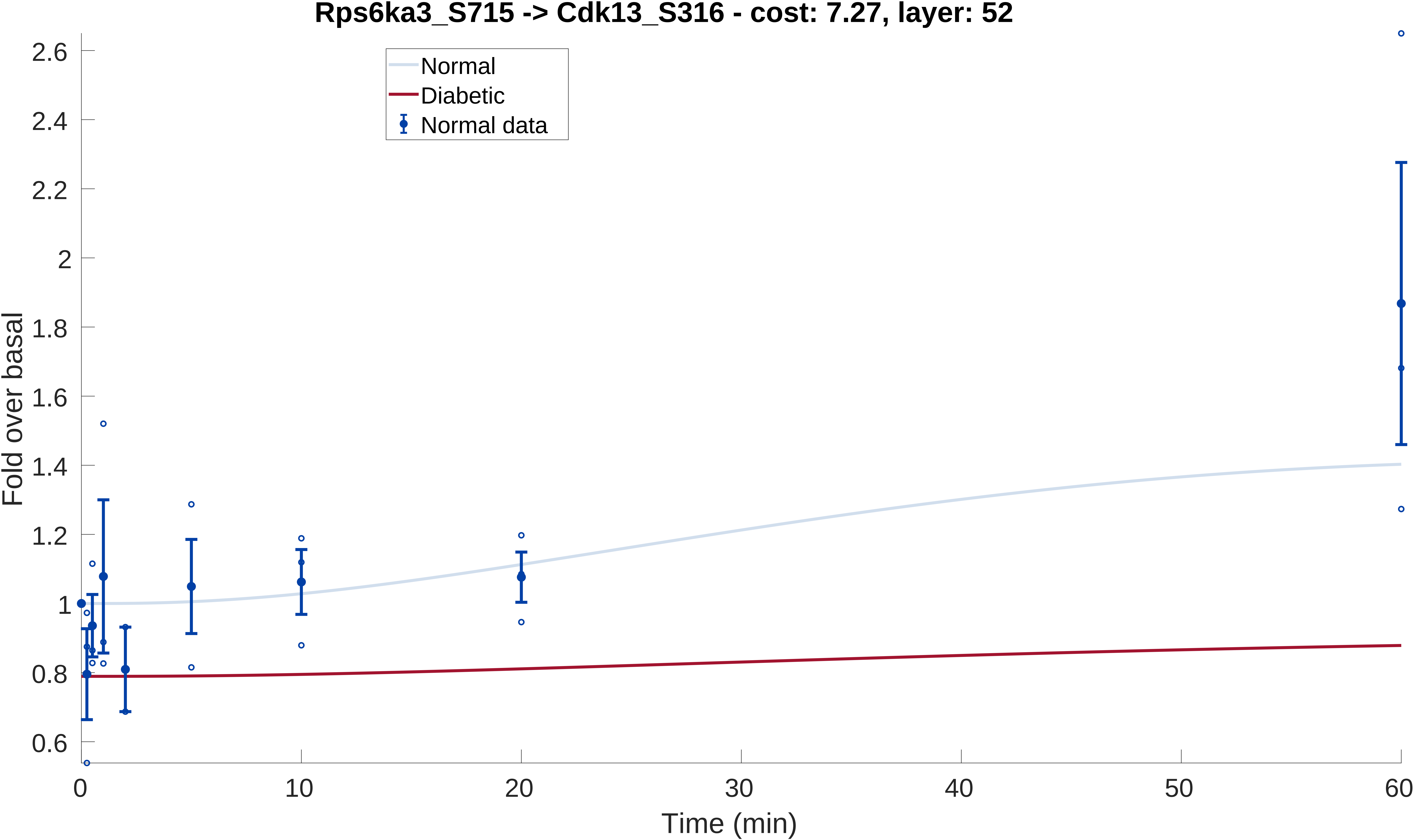
Rps6ka3_S715 -> Cdk13_S316 - cost: 7.27, layer: 52
Normal
Diabetic
Normal data
Fold over basal
Time (min)
0.6
0.8
1
1.2
1.4
1.6
1.8
2
2.2
2.4
2.6
0
10
20
30
40
50
60

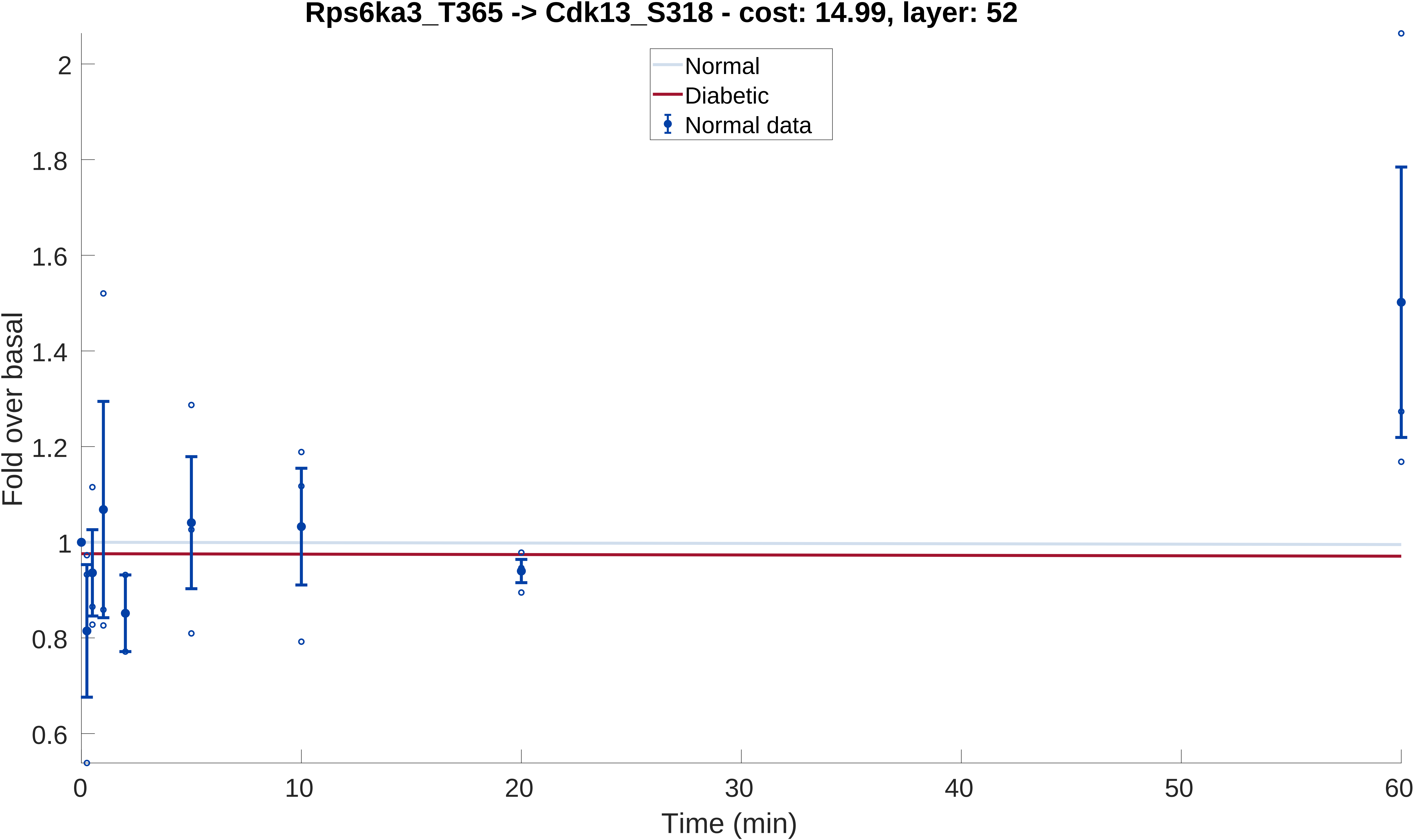
Rps6ka3_T365 -> Cdk13_S318 - cost: 14.99, layer: 52
Normal
Diabetic
Normal data
Fold over basal
Time (min)
2
1.8
1.6
1.4
1.2
1
0.8
0.6
0
10
20
30
40
50
60

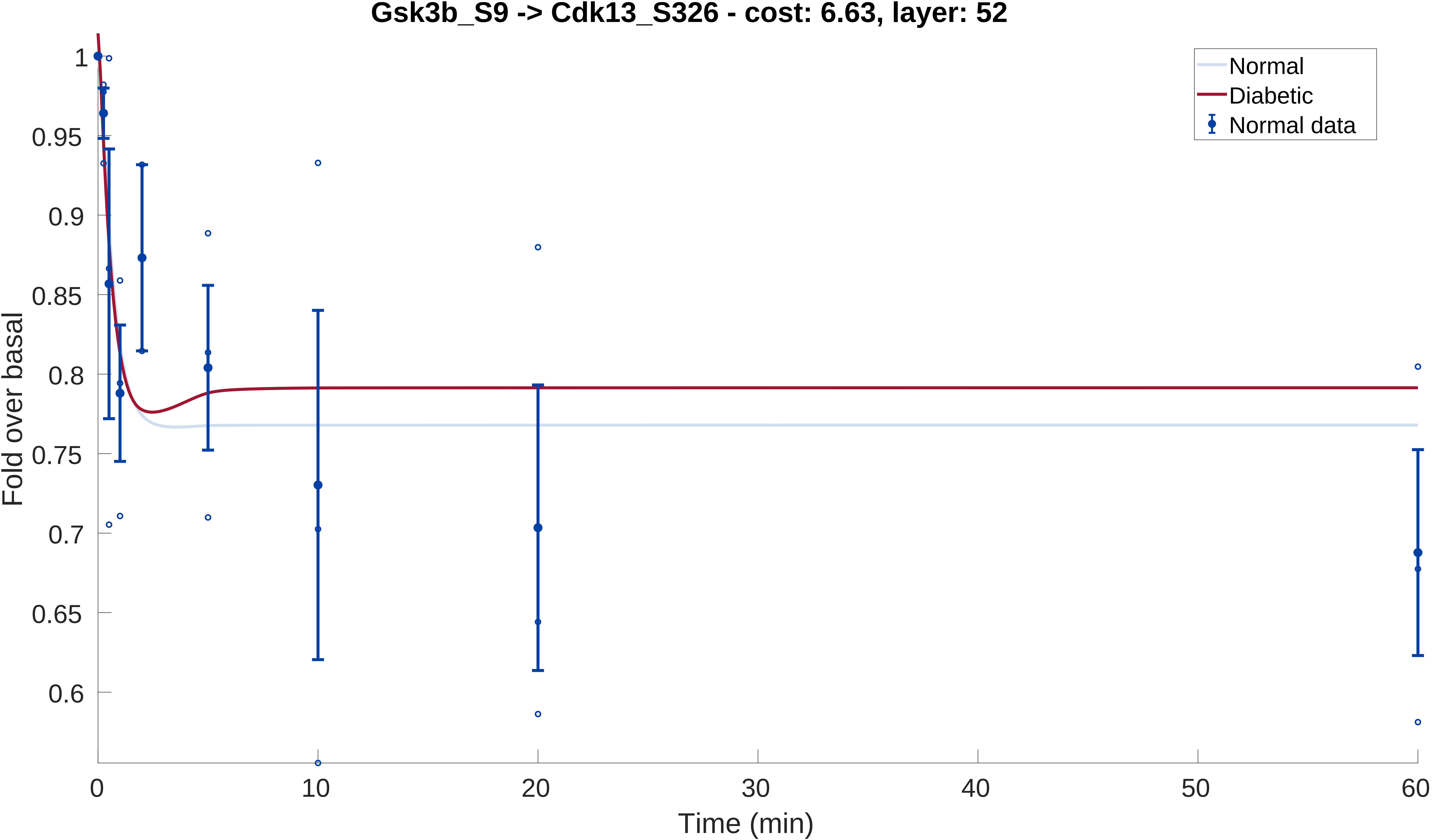

Gsk3b_S9 -> Cdk13_S326 - cost: 6.63, layer: 52
Normal
Diabetic
Normal data
Fold over basal
Time (min)
1
0.95
0.9
0.85
0.8
0.75
0.7
0.65
0.6
0
10
20
30
40
50
60

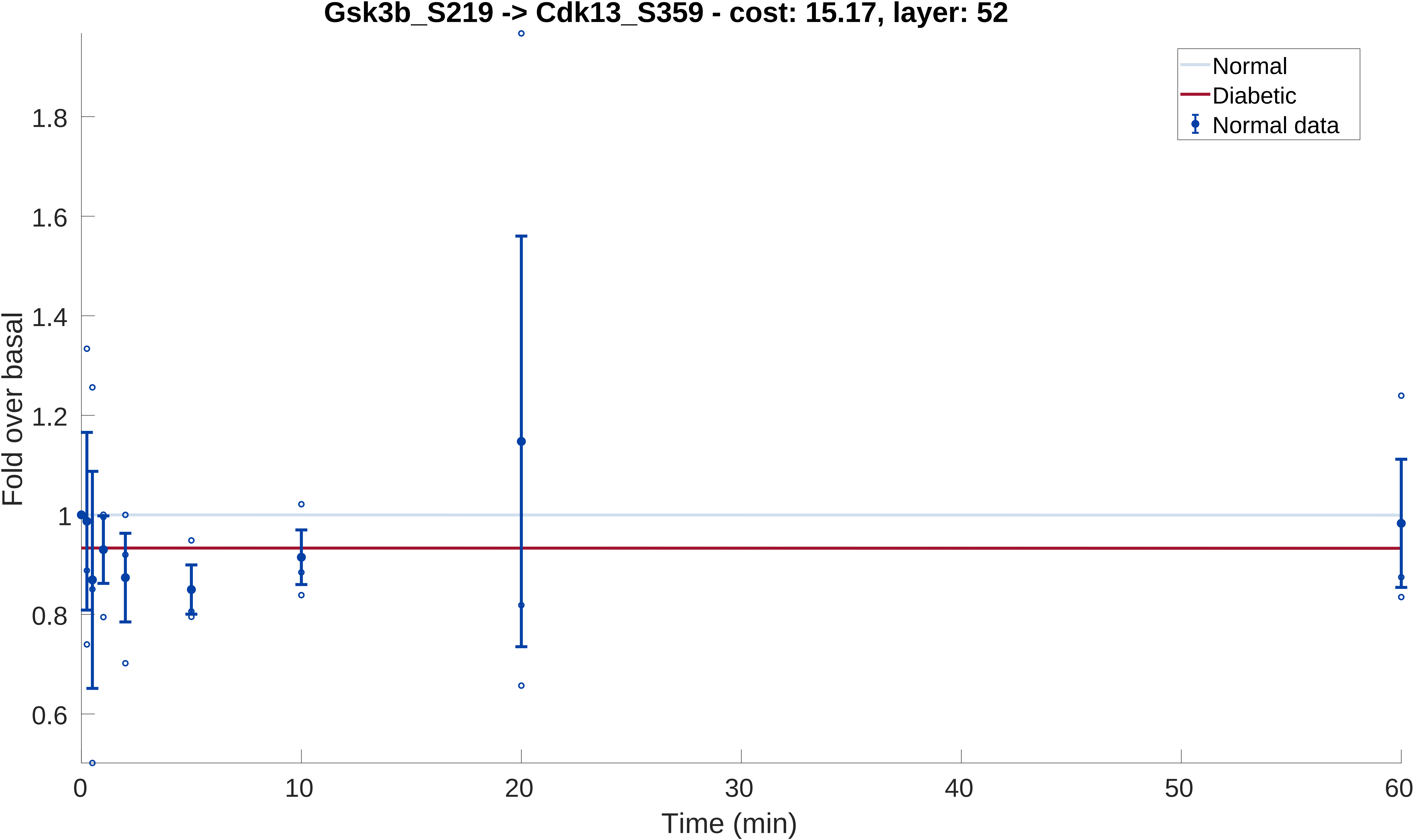

Gsk3b_S219 -> Cdk13_S359 - cost: 15.17, layer: 52
Normal
Diabetic
Normal data
1.8
1.6
1.4
1.2
1
0.8
0.6
Fold over basal
0
10
20
30
40
50
60
Time (min)

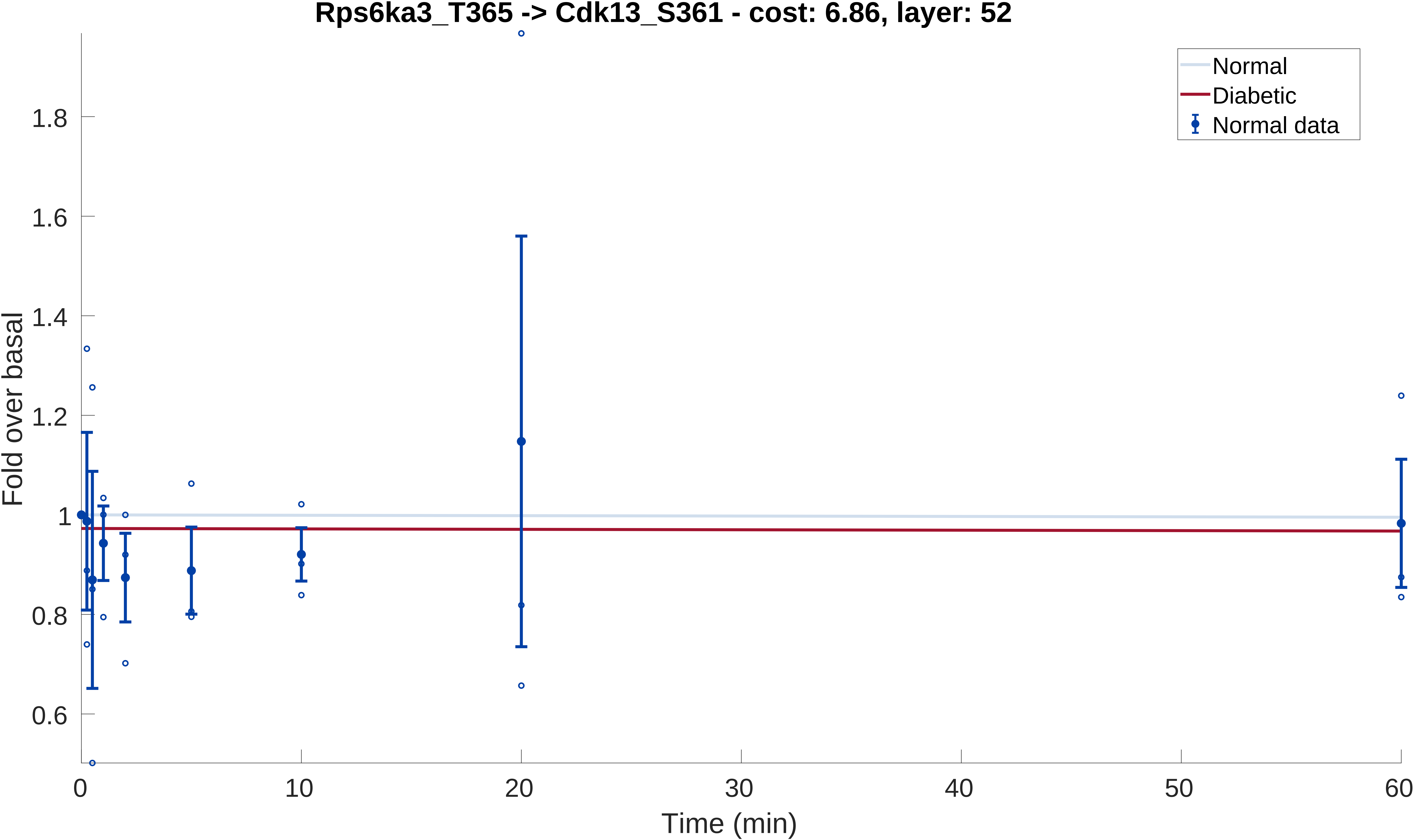

Rps6ka3_T365 -> Cdk13_S361 - cost: 6.86, layer: 52
Normal
Diabetic
Normal data
Fold over basal
Time (min)
1.8
1.6
1.4
1.2
1
0.8
0.6
0
10
20
30
40
50
60

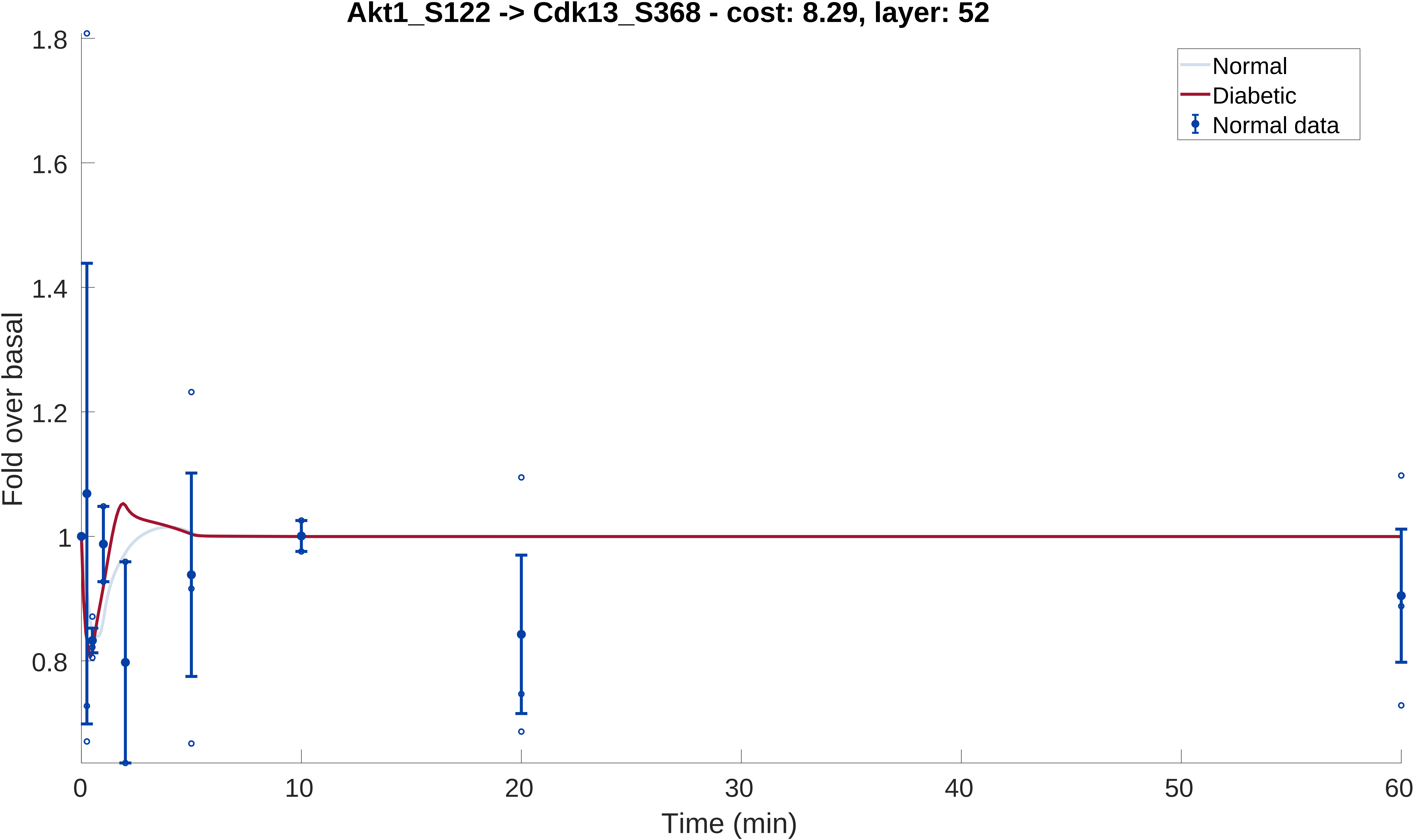

Akt1_S122 -> Cdk13_S368 - cost: 8.29, layer: 52
Normal
Diabetic
Normal data
Fold over basal
Time (min)
1.8
1.6
1.4
1.2
1
0.8
0
10
20
30
40
50
60

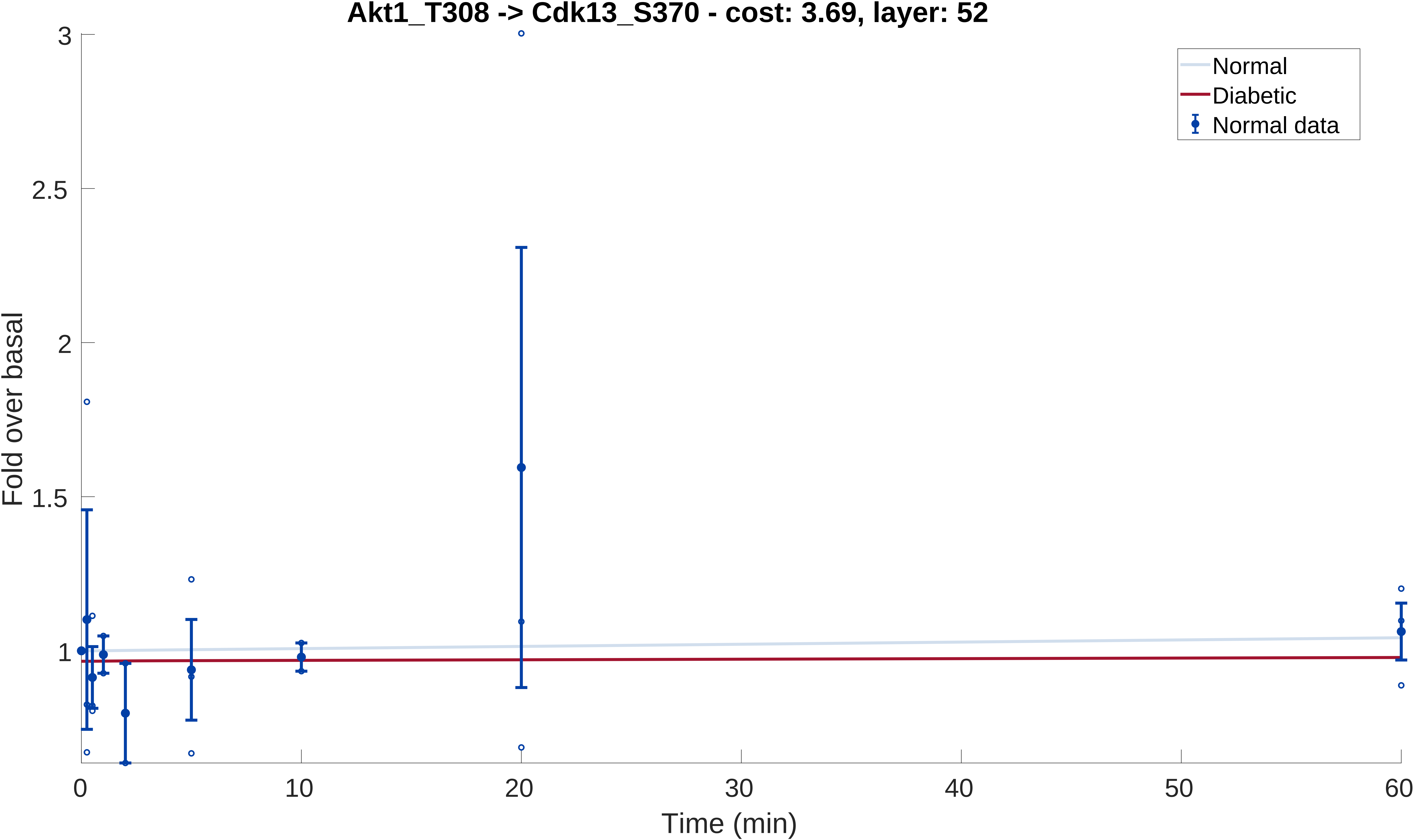

Akt1_T308 -> Cdk13_S370 - cost: 3.69, layer: 52
Fold over basal
3
2.5
2
1.5
1
Normal
Diabetic
Normal data
0
10
20
30
40
50
60
Time (min)

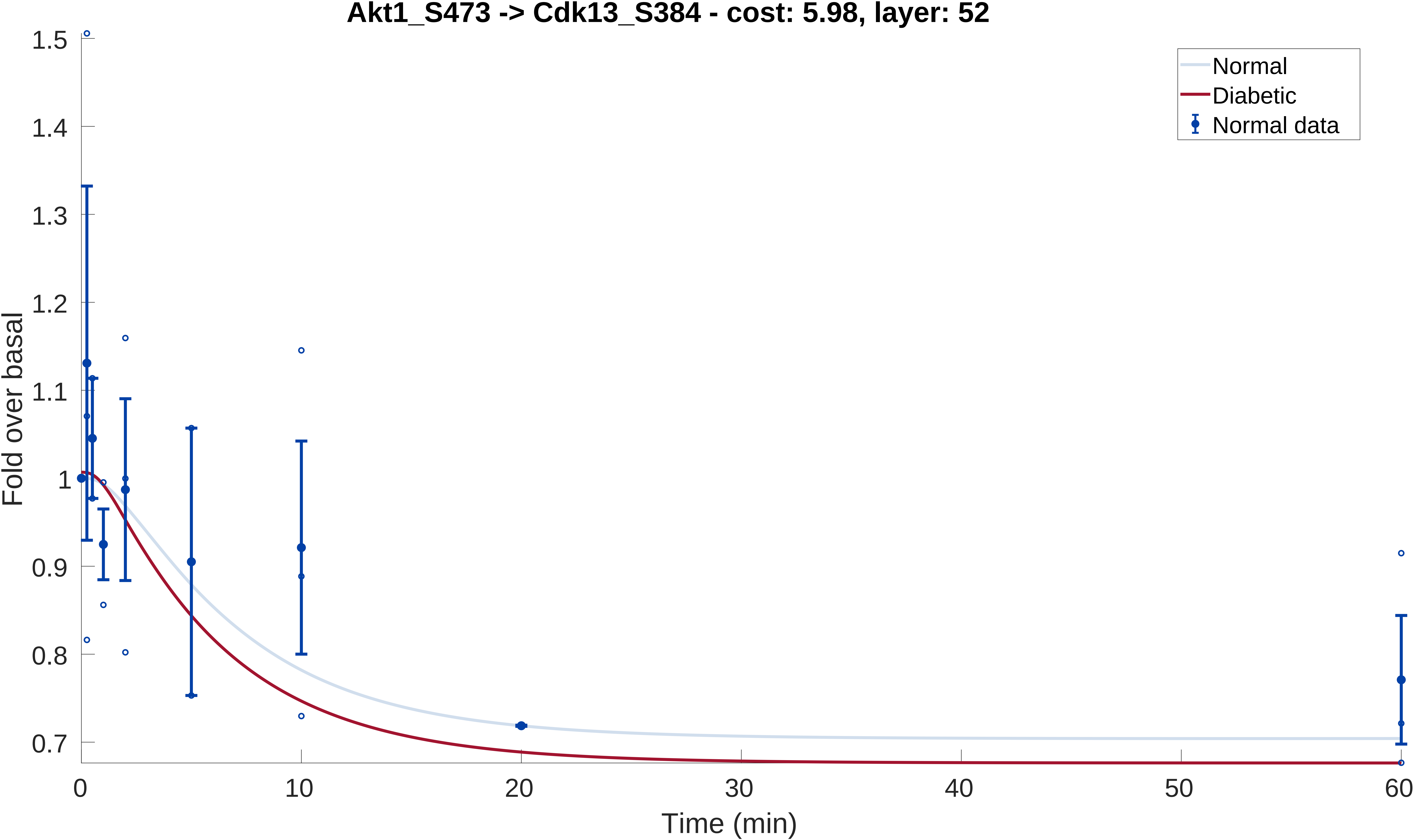

Akt1_S473 -> Cdk13_S384 - cost: 5.98, layer: 52
Normal
Diabetic
Normal data
Fold over basal
1.5
1.4
1.3
1.2
1.1
1
0.9
0.8
0.7
0
10
20
30
40
50
60
Time (min)

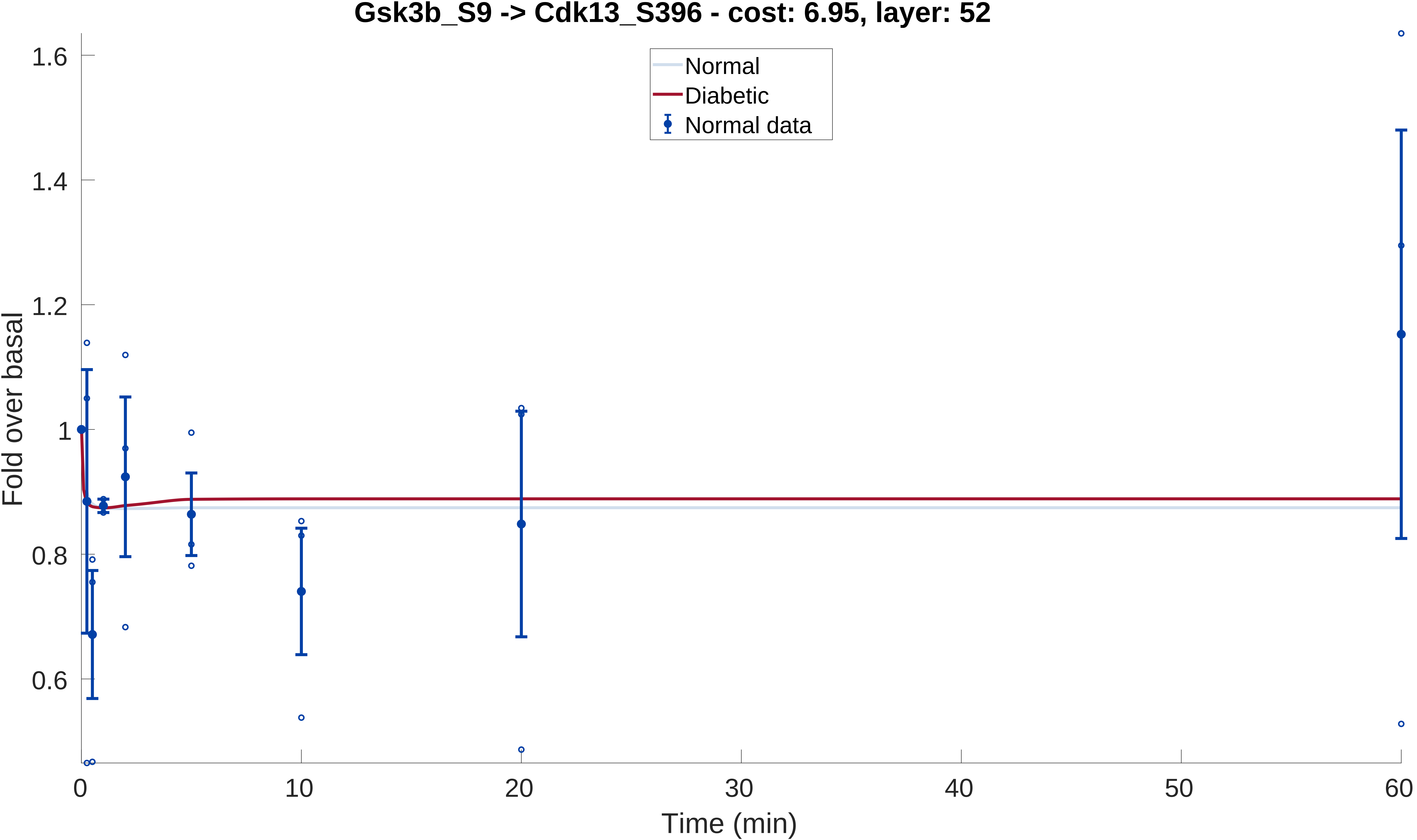

Gsk3b_S9 -> Cdk13_S396 - cost: 6.95, layer: 52
Normal
Diabetic
Normal data
Fold over basal
1.6
1.4
1.2
1
0.8
0.6
0
10
20
30
40
50
60
Time (min)

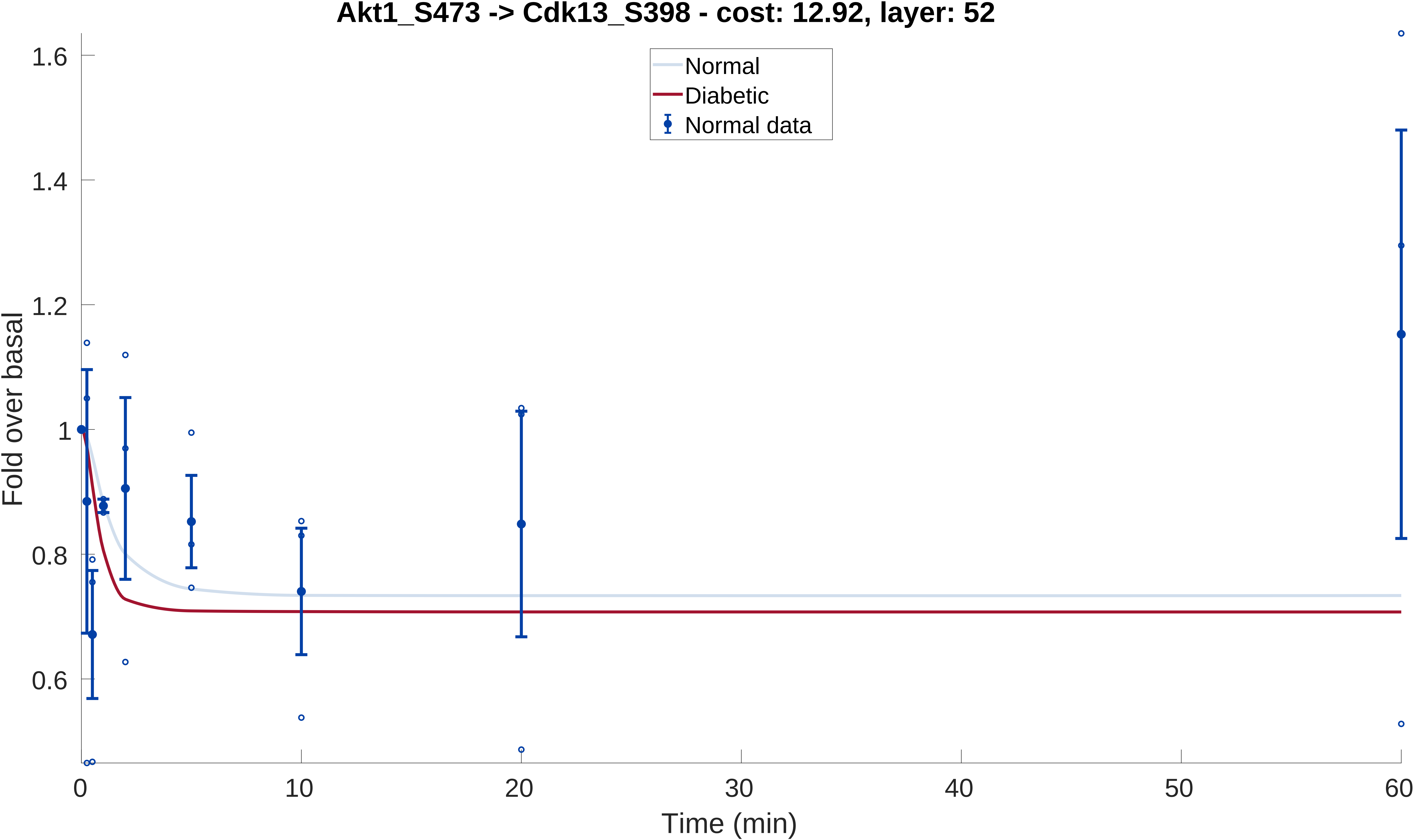

Akt1_S473 -> Cdk13_S398 - cost: 12.92, layer: 52
Normal
Diabetic
Normal data
Fold over basal
Time (min)
1.6
1.4
1.2
1
0.8
0.6
0
10
20
30
40
50
60

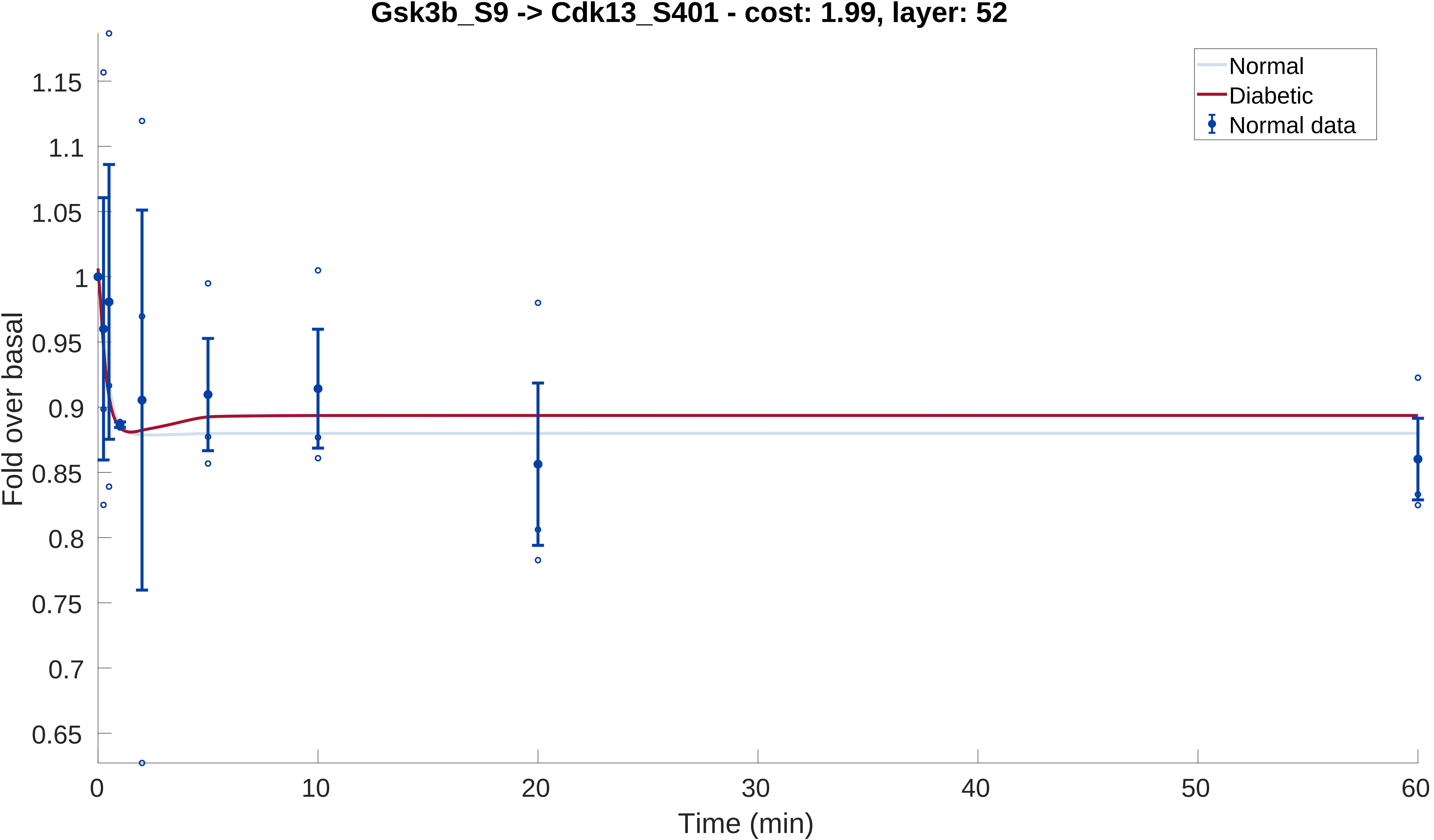
Gsk3b_S9 -> Cdk13_S401 - cost: 1.99, layer: 52
Normal
Diabetic
Normal data
Fold over basal
1.15
1.1
1.05
1
0.95
0.9
0.85
0.8
0.75
0.7
0.65
0
10
20
30
40
50
60
Time (min)

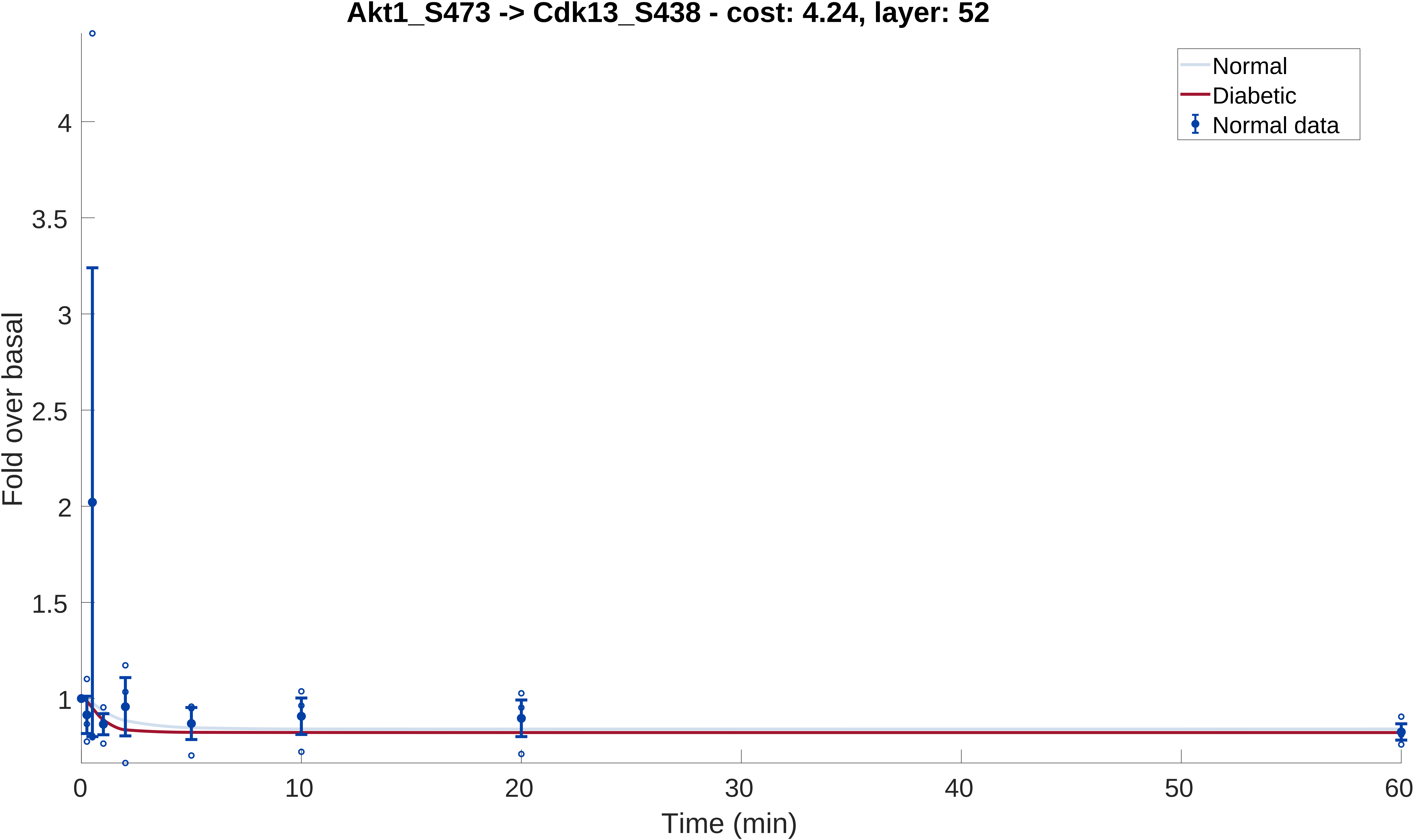
Akt1_S473 -> Cdk13_S438 - cost: 4.24, layer: 52
Normal
Diabetic
Normal data
Fold over basal
Time (min)
1
1.5
2
2.5
3
3.5
4
0
10
20
30
40
50
60

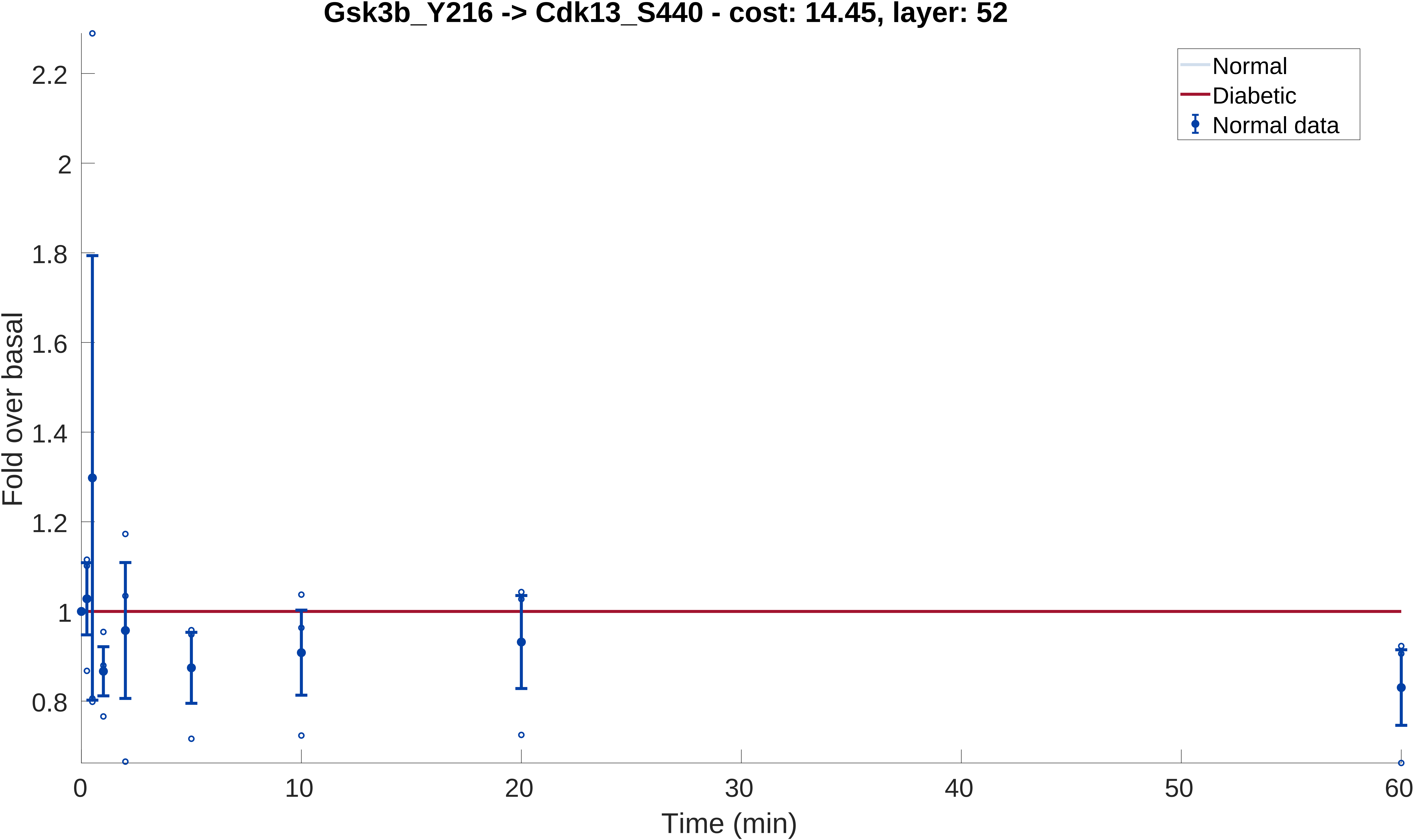

Gsk3b_Y216 -> Cdk13_S440 - cost: 14.45, layer: 52
Normal
Diabetic
Normal data
Fold over basal
2.2
2
1.8
1.6
1.4
1.2
1
0.8
0
10
20
30
40
50
60
Time (min)

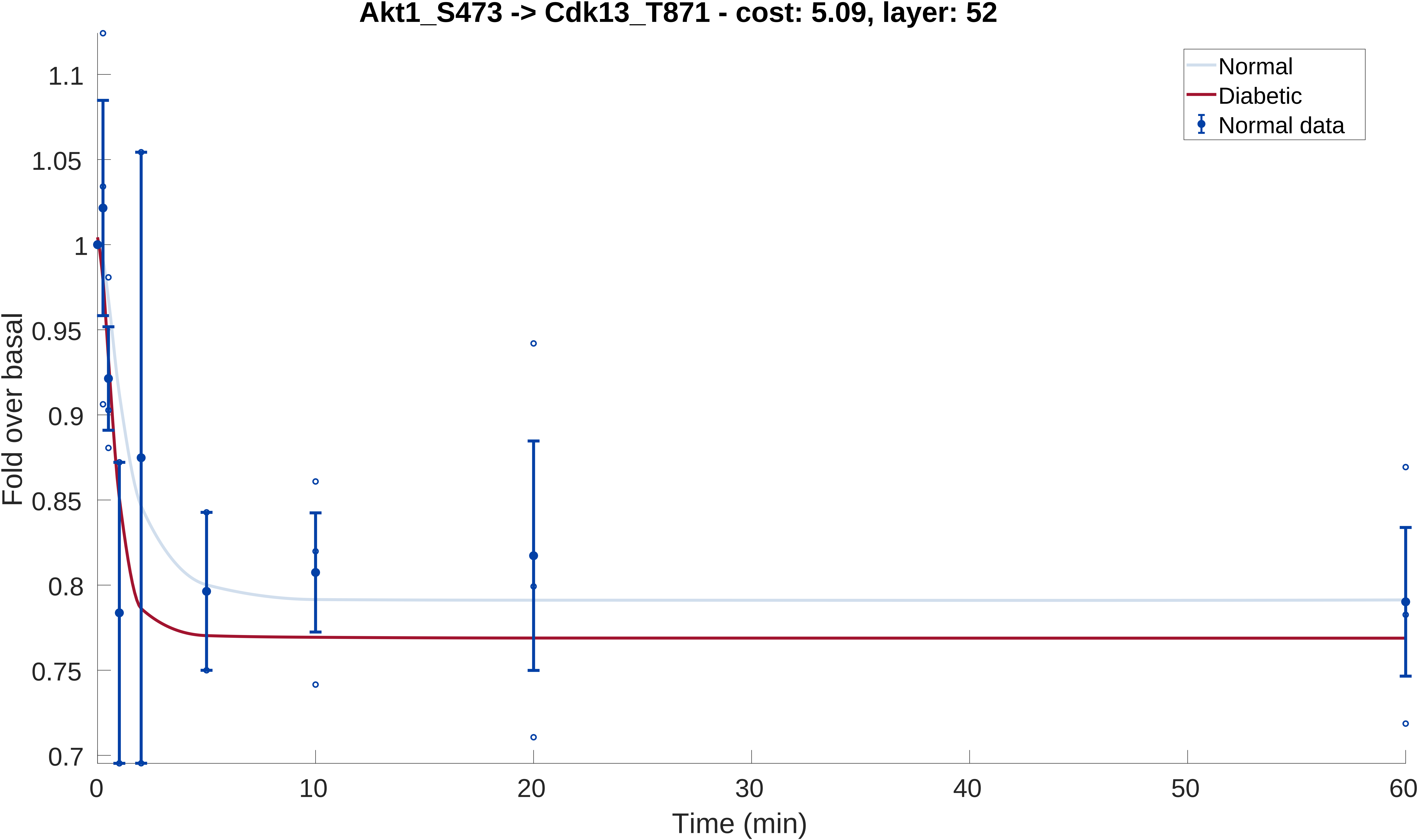
Akt1_S473 -> Cdk13_T871 - cost: 5.09, layer: 52
Normal
Diabetic
Normal data
Fold over basal
Time (min)
1.1
1.05
1
0.95
0.9
0.85
0.8
0.75
0.7
0
10
20
30
40
50
60

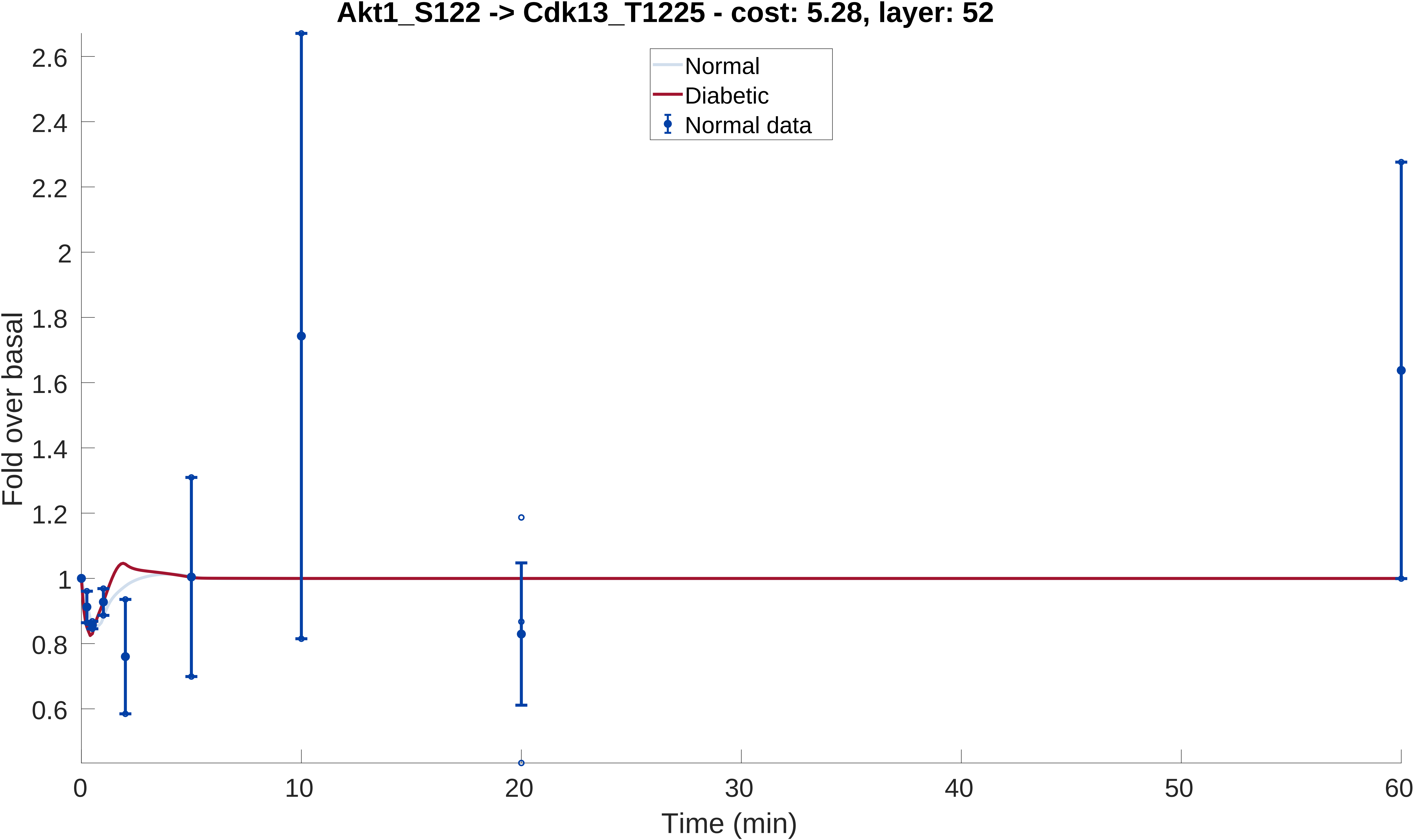
Akt1_S122 -> Cdk13_T1225 - cost: 5.28, layer: 52
Normal
Diabetic
Normal data
Fold over basal
2.6
2.4
2.2
2
1.8
1.6
1.4
1.2
1
0.8
0.6
0
10
20
30
40
50
60
Time (min)

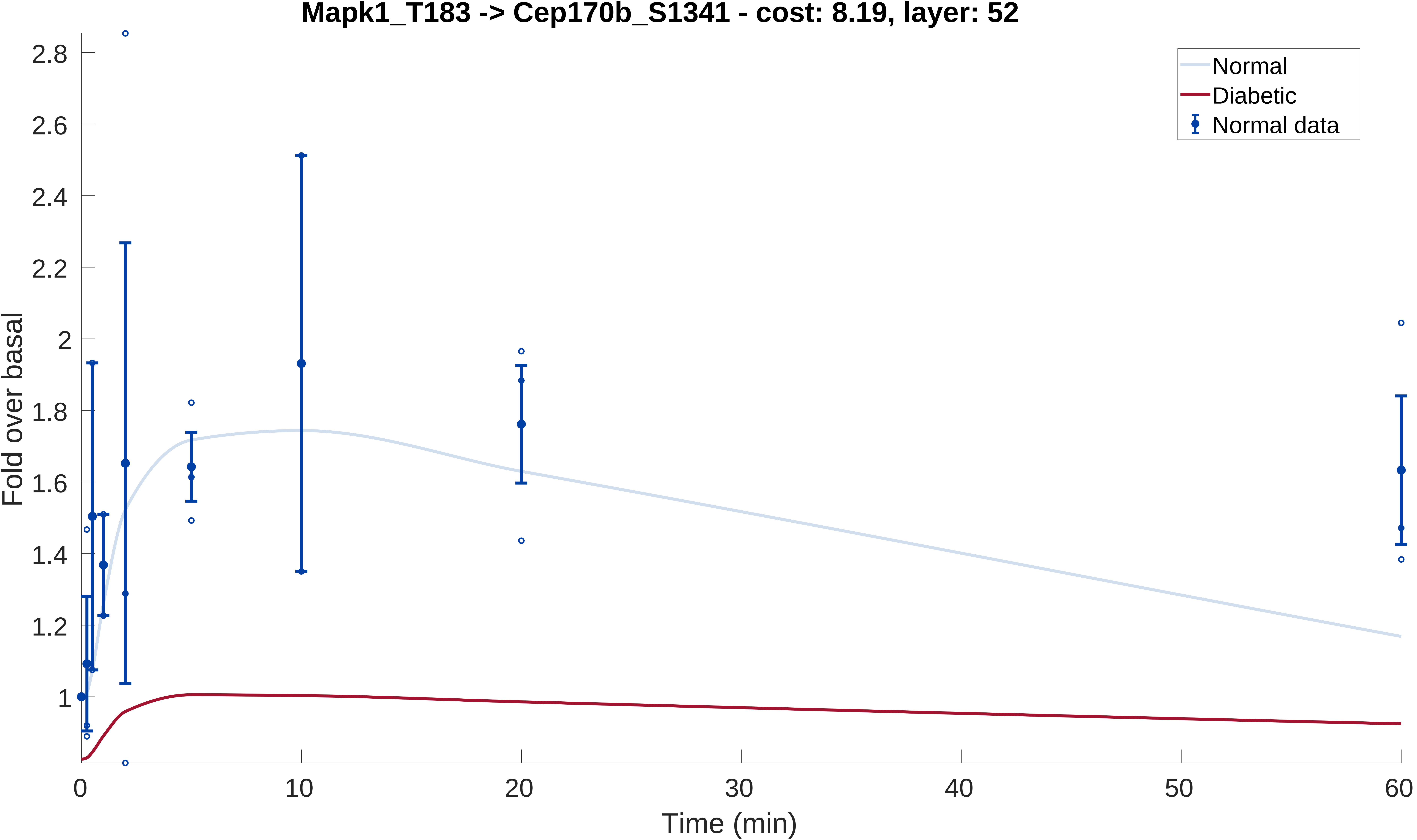

Mapk1_T183 -> Cep170b_S1341 - cost: 8.19, layer: 52
Fold over basal
Time (min)
Normal
Diabetic
Normal data
2.8
2.6
2.4
2.2
2
1.8
1.6
1.4
1.2
1
0
10
20
30
40
50
60

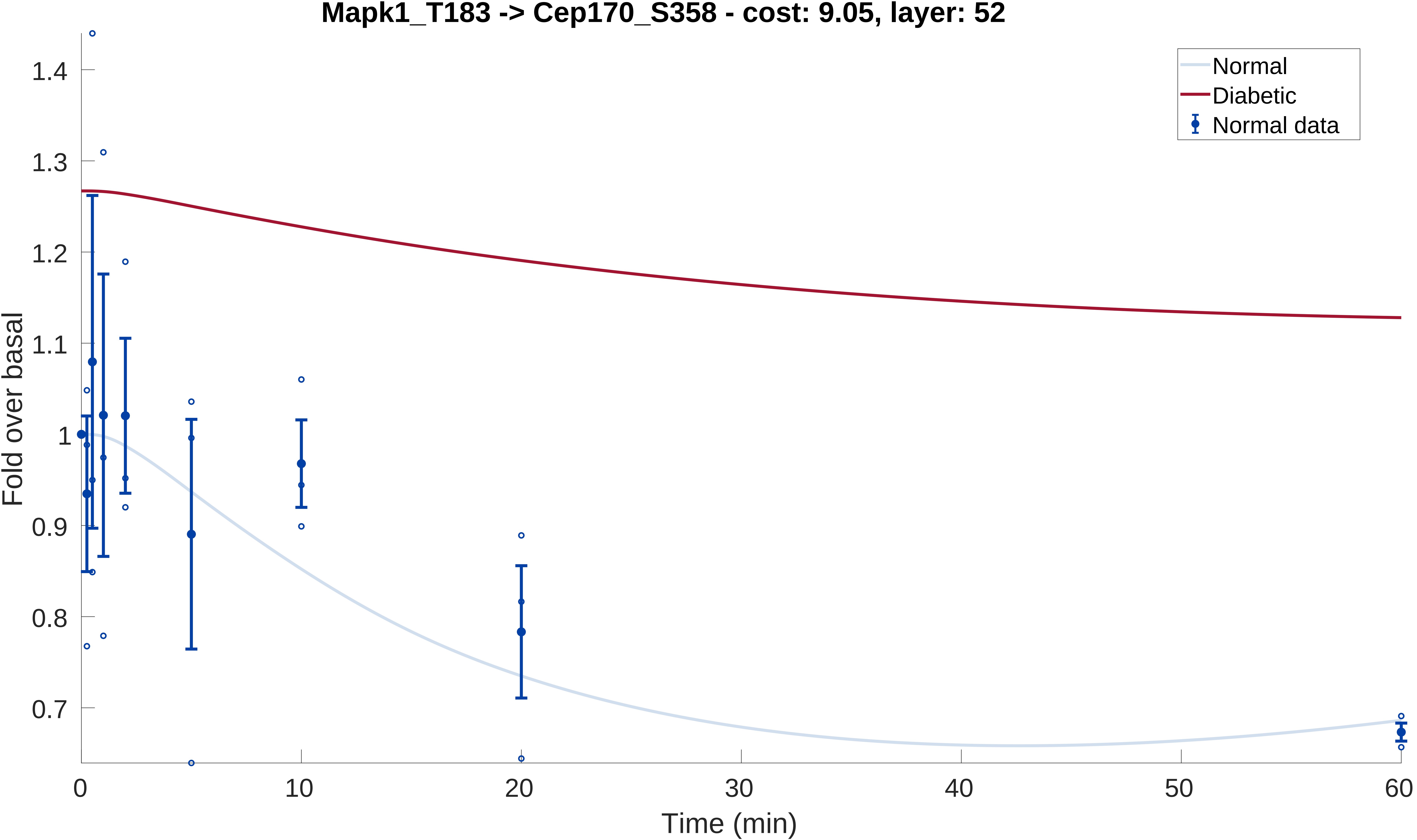

Mapk1_T183 -> Cep170_S358 - cost: 9.05, layer: 52
Fold over basal
1.4
1.3
1.2
1.1
1
0.9
0.8
0.7
0
10
20
30
40
50
60
Time (min)
Normal
Diabetic
Normal data

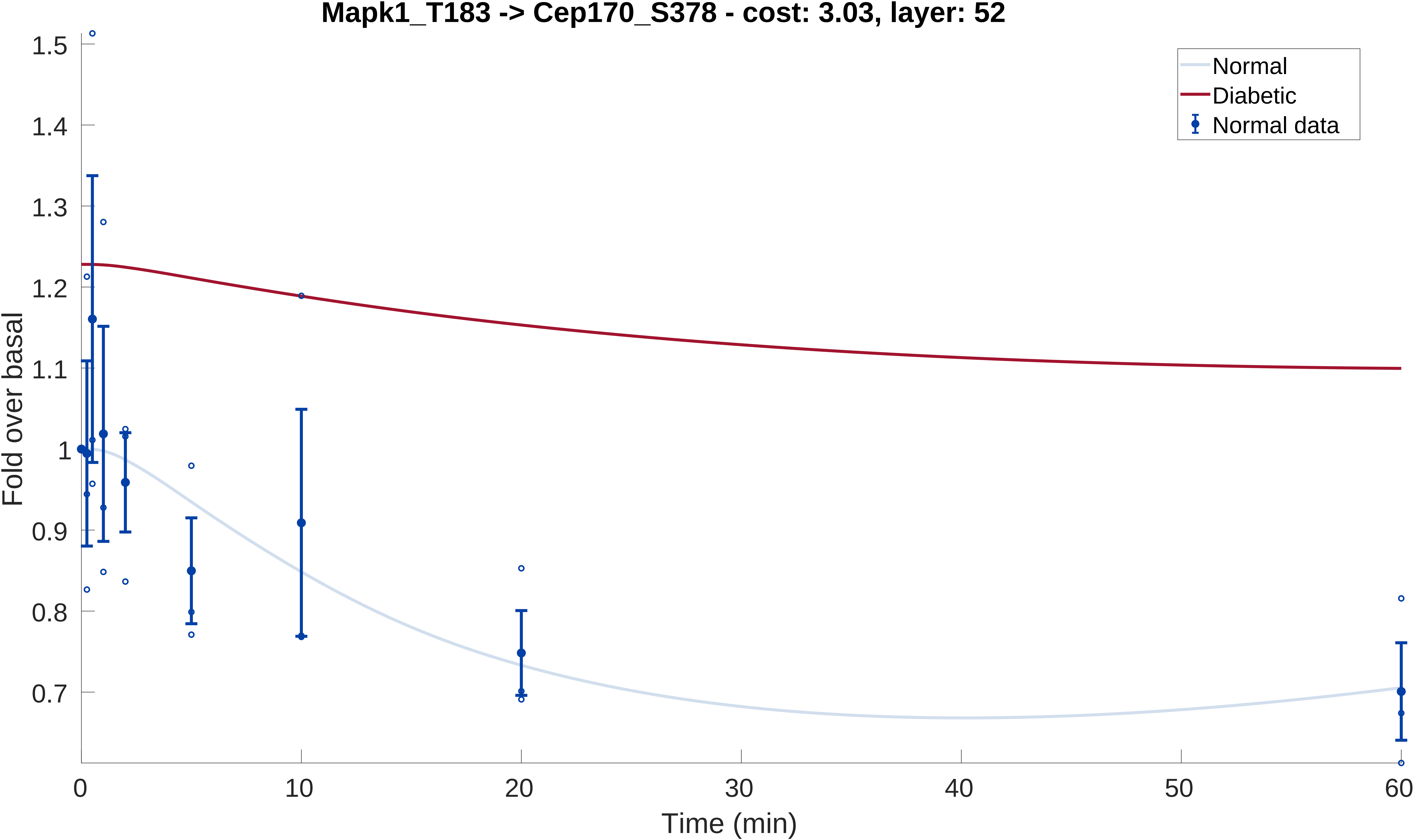

Mapk1_T183 -> Cep170_S378 - cost: 3.03, layer: 52
Normal
Diabetic
Normal data
Fold over basal
1.5
1.4
1.3
1.2
1.1
1
0.9
0.8
0.7
0
10
20
30
40
50
60
Time (min)

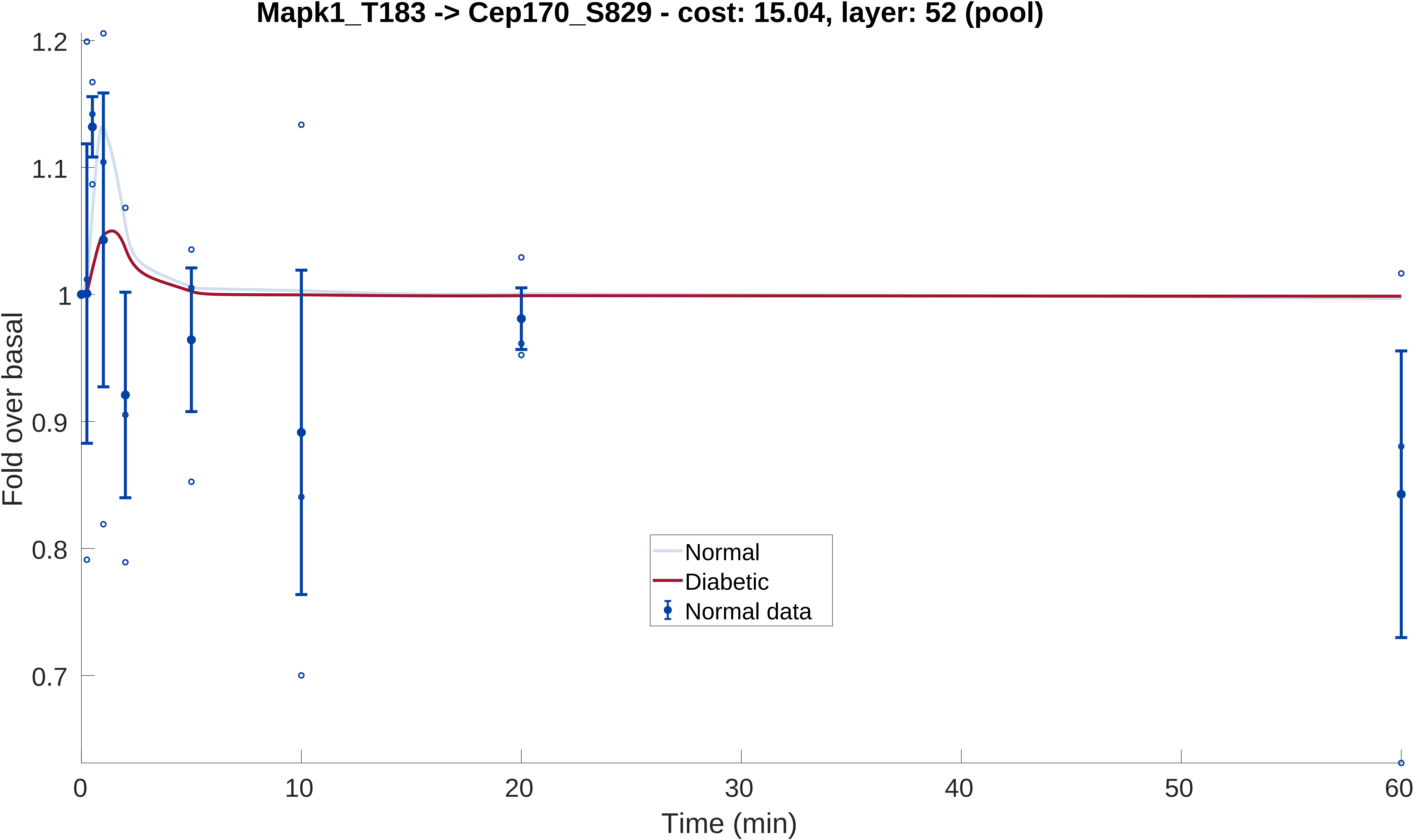

Mapk1_T183 -> Cep170_S829 - cost: 15.04, layer: 52 (pool)
Fold over basal
1.2
1.1
1
0.9
0.8
0.7
0
10
20
30
40
50
60
Time (min)
Normal
Diabetic
Normal data

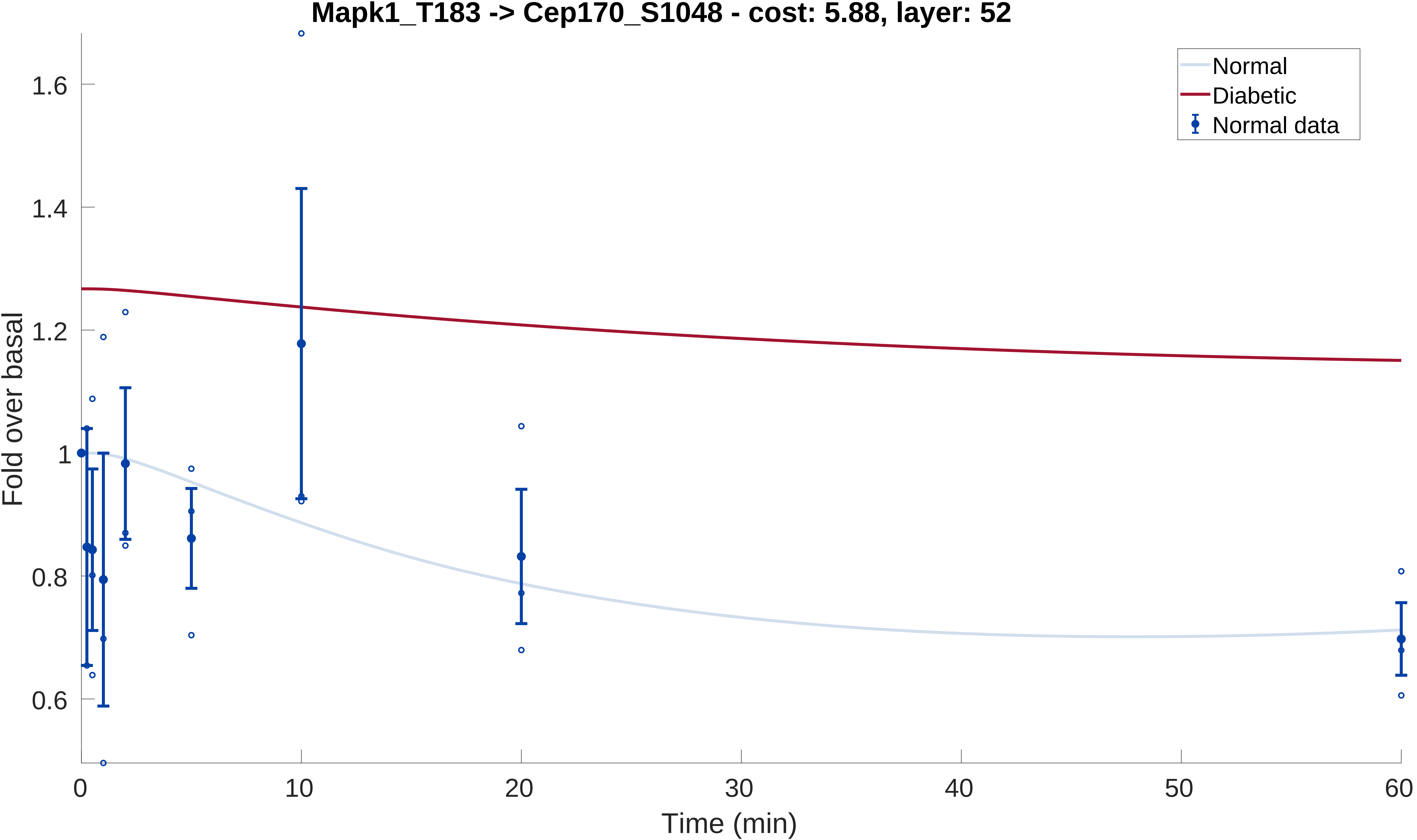

Mapk1_T183 -> Cep170_S1048 - cost: 5.88, layer: 52
Normal
Diabetic
Normal data
Fold over basal
Time (min)
1.6
1.4
1.2
1
0.8
0.6
0
10
20
30
40
50
60

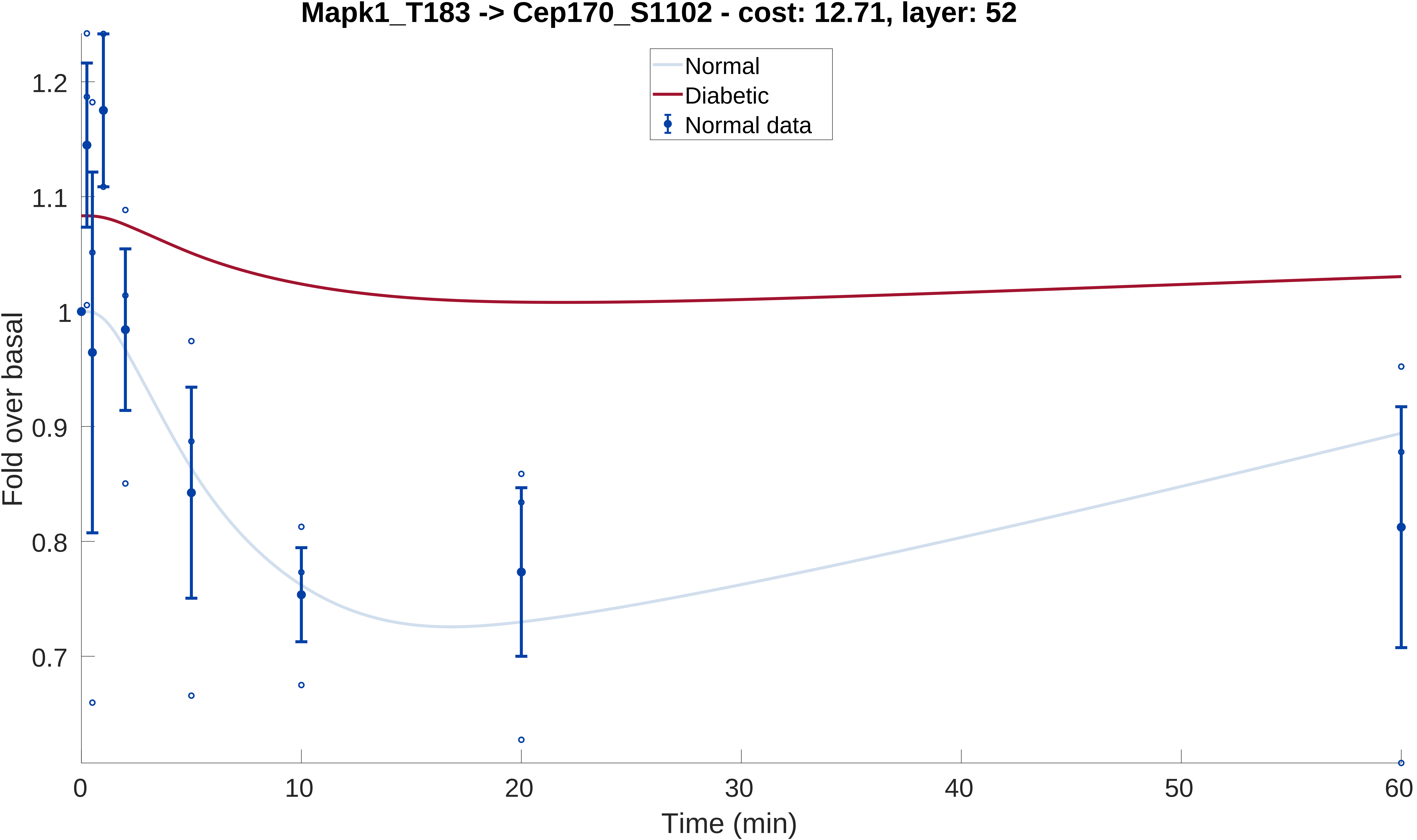

Mapk1_T183 -> Cep170_S1102 - cost: 12.71, layer: 52
Normal
Diabetic
Normal data
Fold over basal
1.2
1.1
1
0.9
0.8
0.7
0
10
20
30
40
50
60
Time (min)

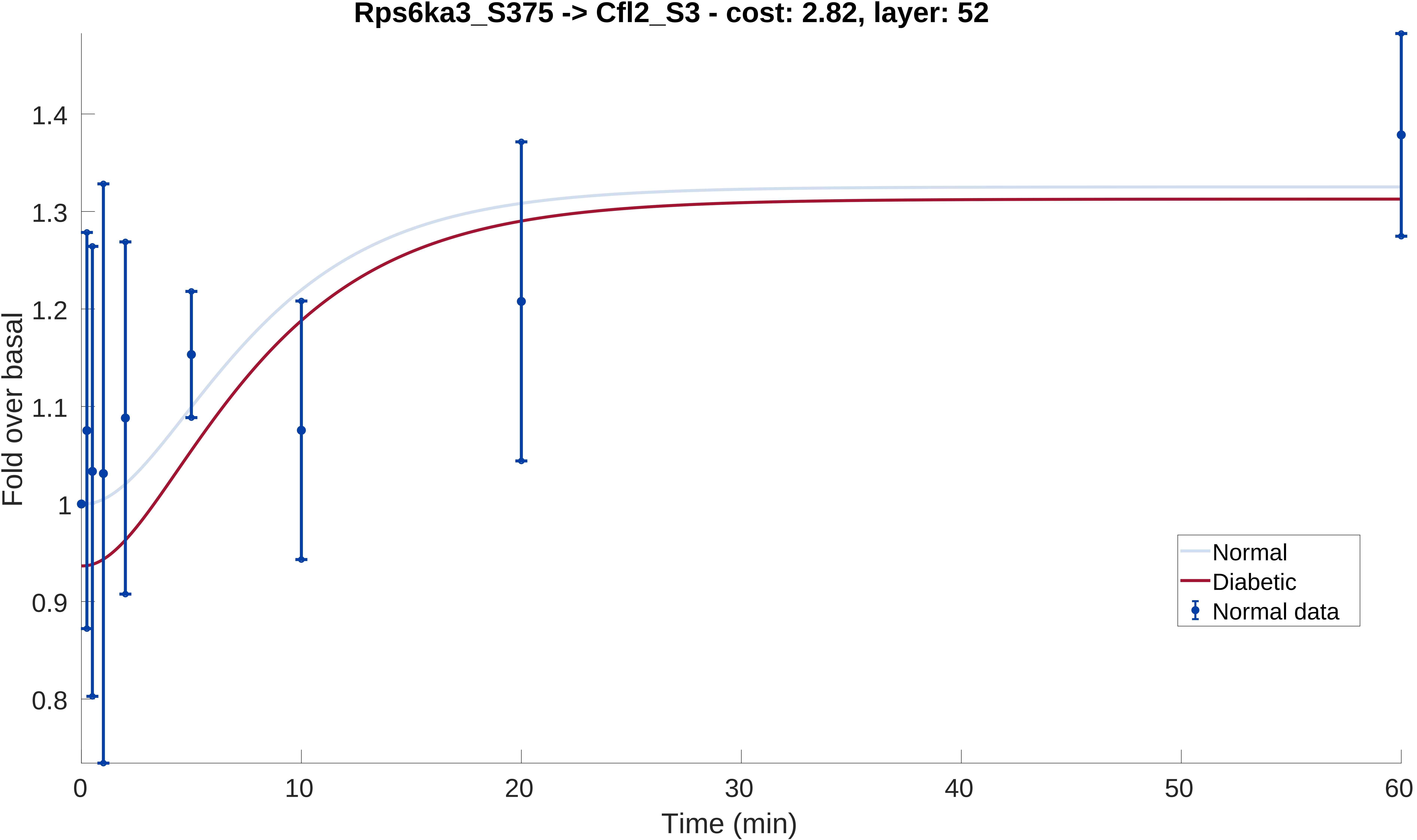

Rps6ka3_S375 -> Cfl2_S3 - cost: 2.82, layer: 52
Fold over basal
1.4
1.3
1.2
1.1
1
0.9
0.8
0
10
20
30
40
50
60
Time (min)
Normal
Diabetic
Normal data

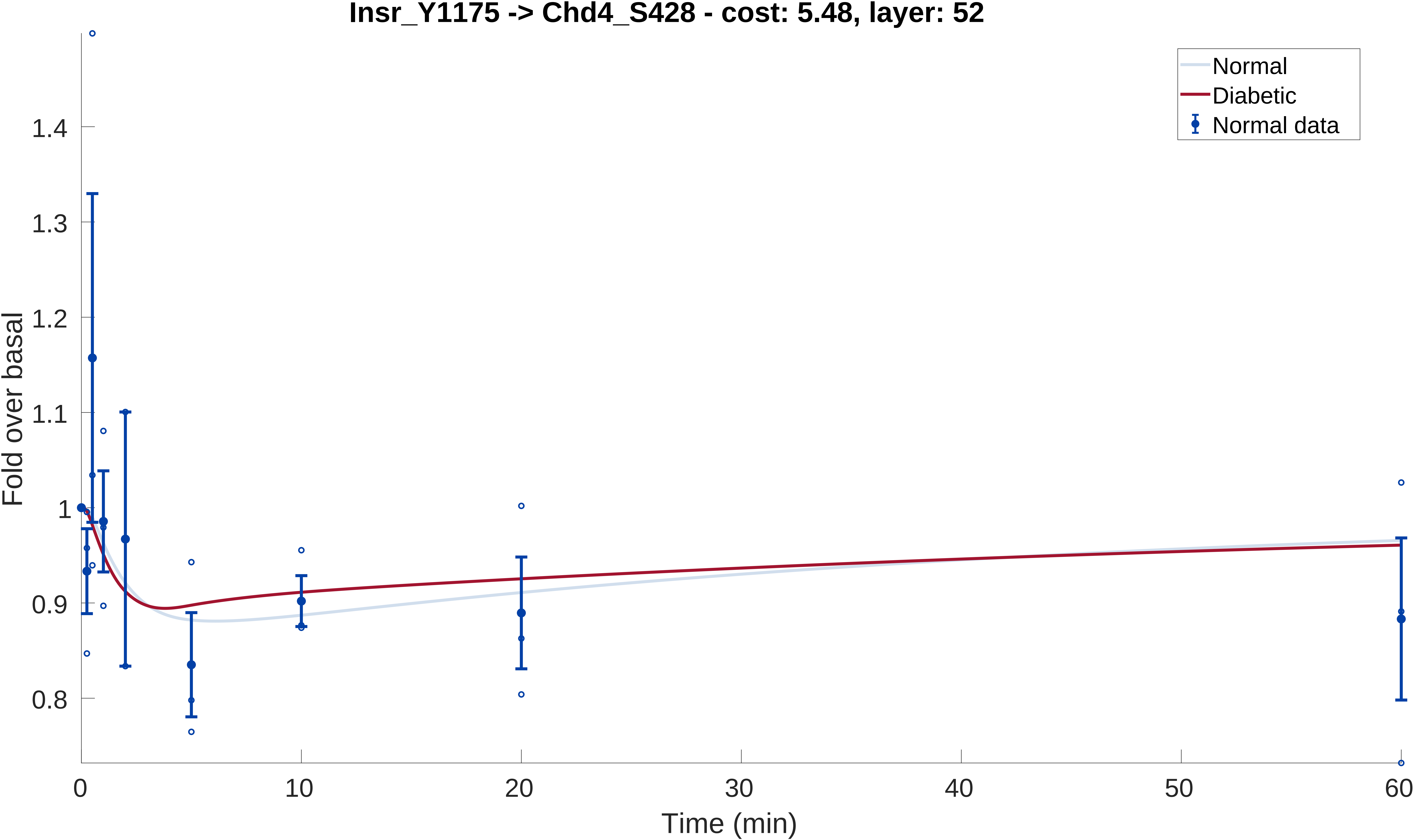
Insr_Y1175 -> Chd4_S428 - cost: 5.48, layer: 52
Normal
Diabetic
Normal data
Fold over basal
Time (min)
0.8
0.9
1
1.1
1.2
1.3
1.4
0
10
20
30
40
50
60

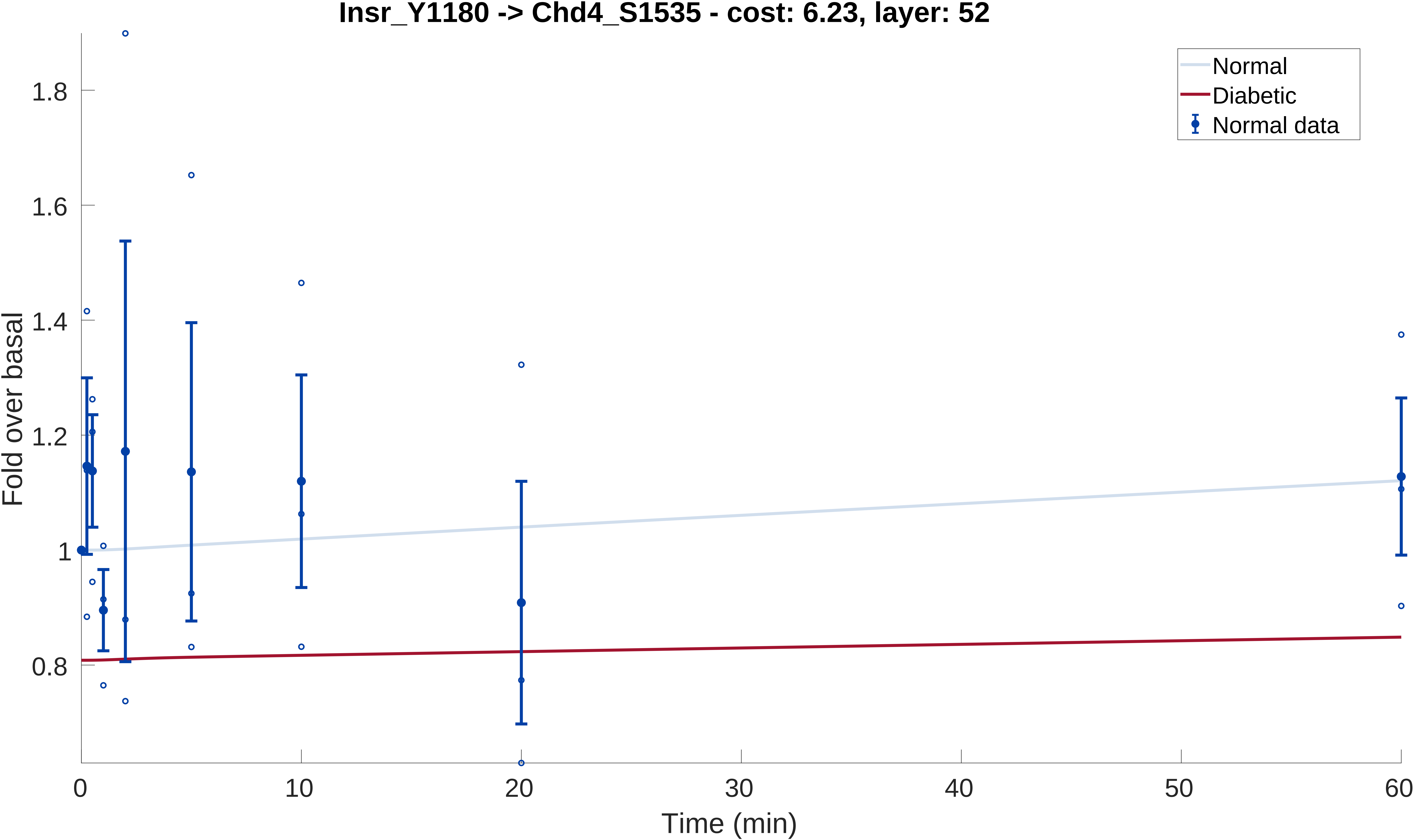

Insr_Y1180 -> Chd4_S1535 - cost: 6.23, layer: 52
Normal
Diabetic
Normal data
Fold over basal
1.8
1.6
1.4
1.2
1
0.8
0
10
20
30
40
50
60
Time (min)

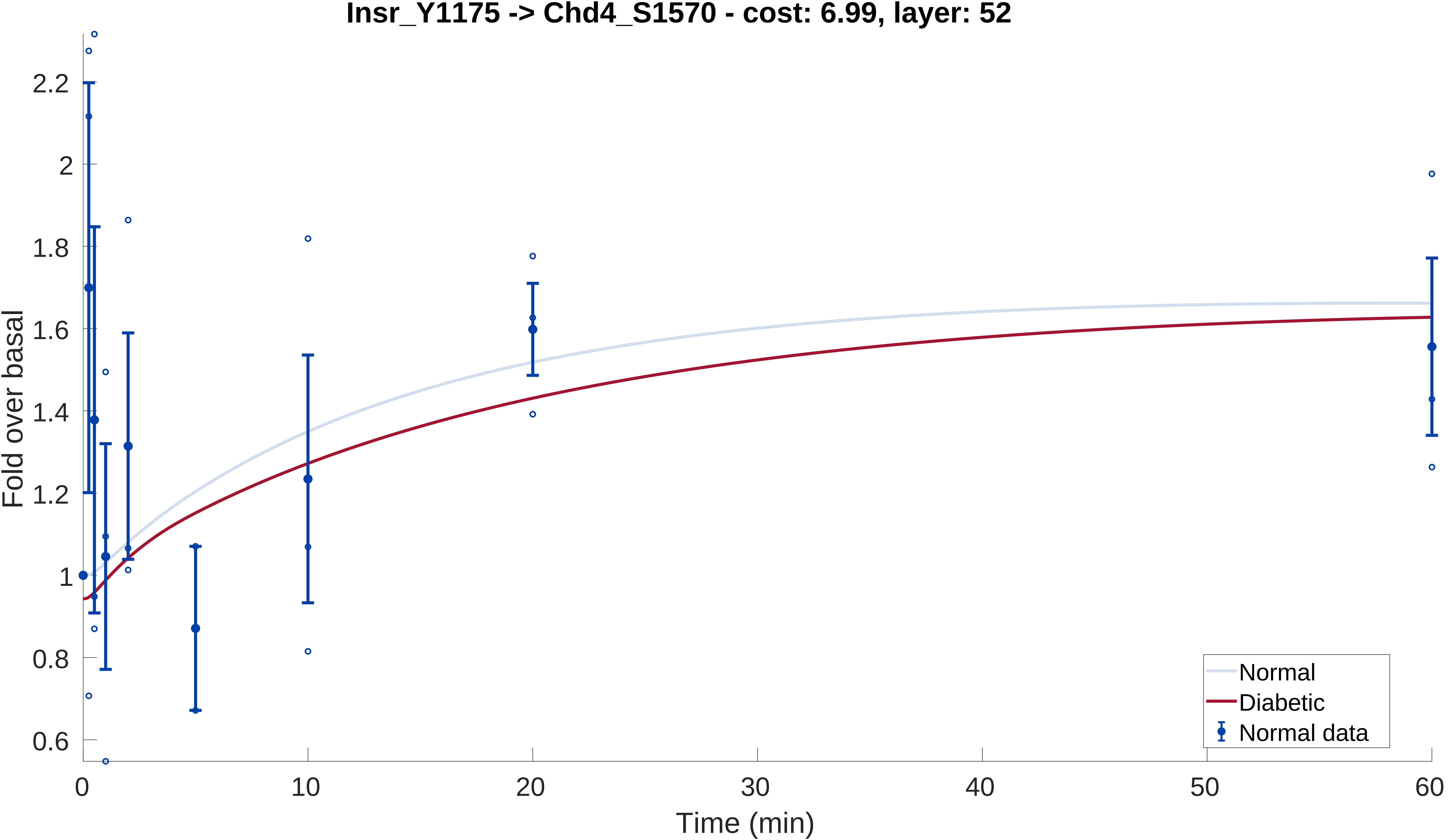

Insr_Y1175 -> Chd4_S1570 - cost: 6.99, layer: 52
Fold over basal
Time (min)
0.6
0.8
1
1.2
1.4
1.6
1.8
2
2.2
0
10
20
30
40
50
60
Normal
Diabetic
Normal data

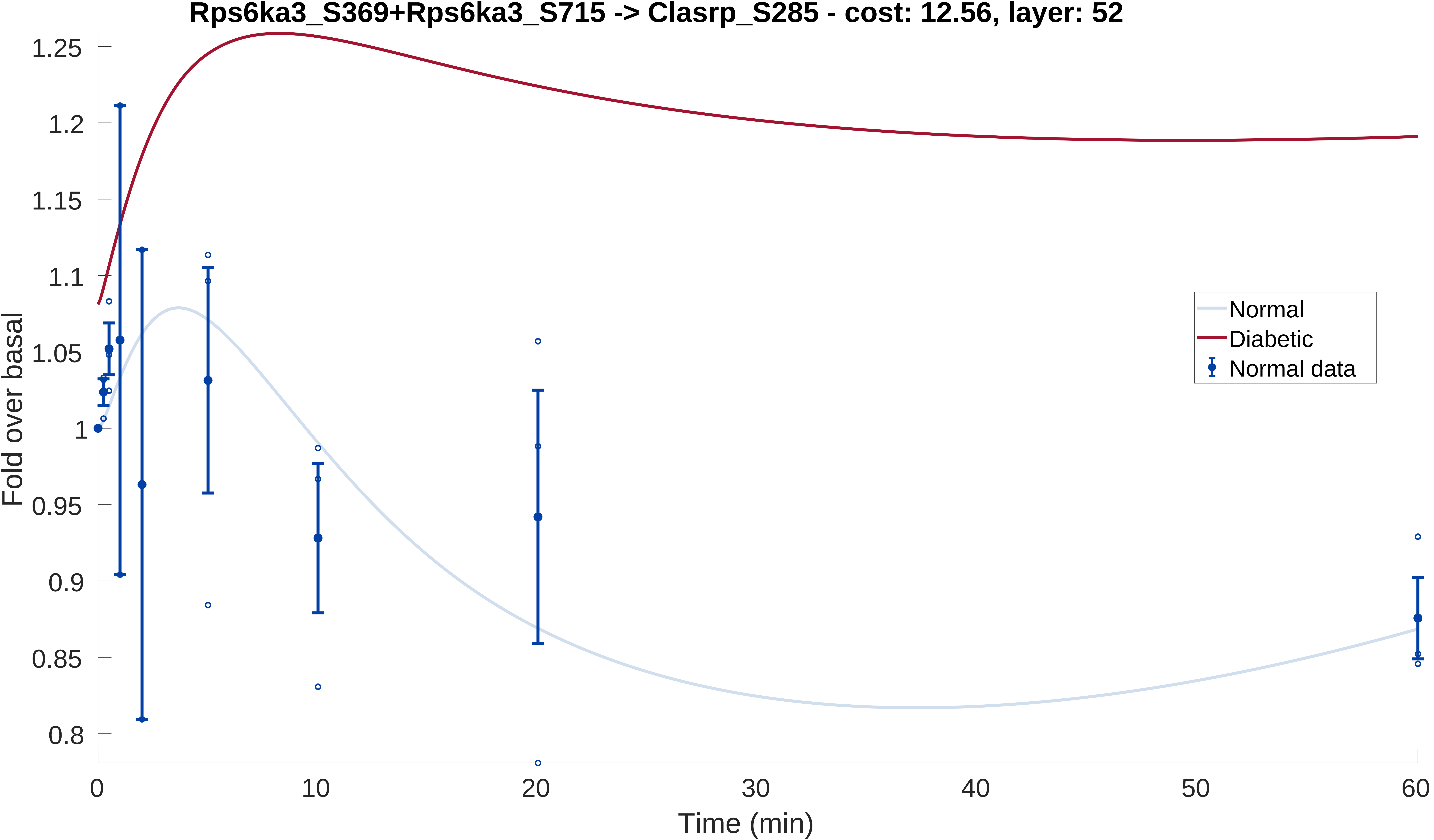
Rps6ka3_S369+Rps6ka3_S715 -> Clasrp_S285 - cost: 12.56, layer: 52
Fold over basal
1.25
1.2
1.15
1.1
1.05
1
0.95
0.9
0.85
0.8
0
10
20
30
40
50
60
Time (min)
Normal
Diabetic
Normal data

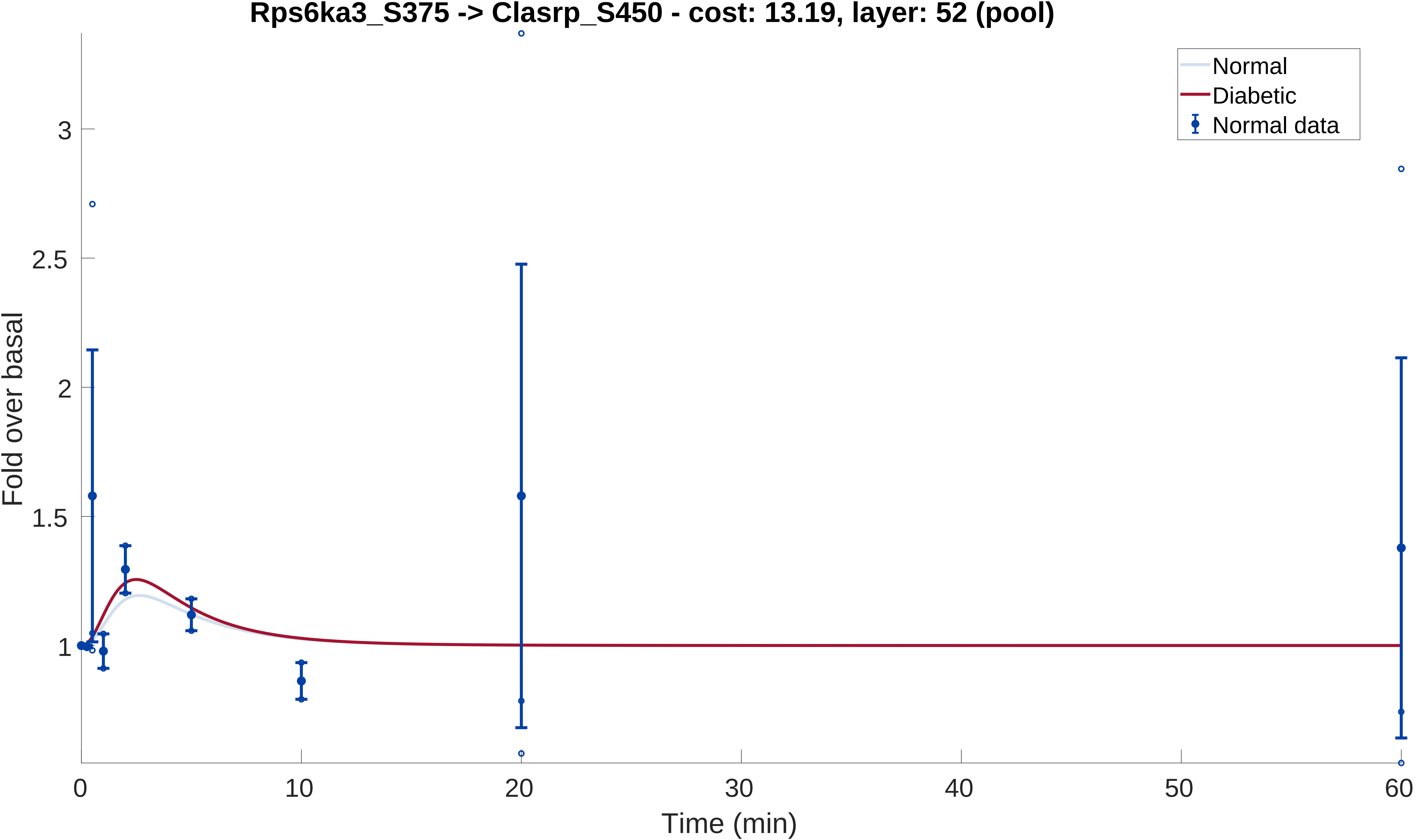
Rps6ka3_S375 -> Clasrp_S450 - cost: 13.19, layer: 52 (pool)
Normal
Diabetic
Normal data
Fold over basal
3
2.5
2
1.5
1
0
10
20
30
40
50
60
Time (min)

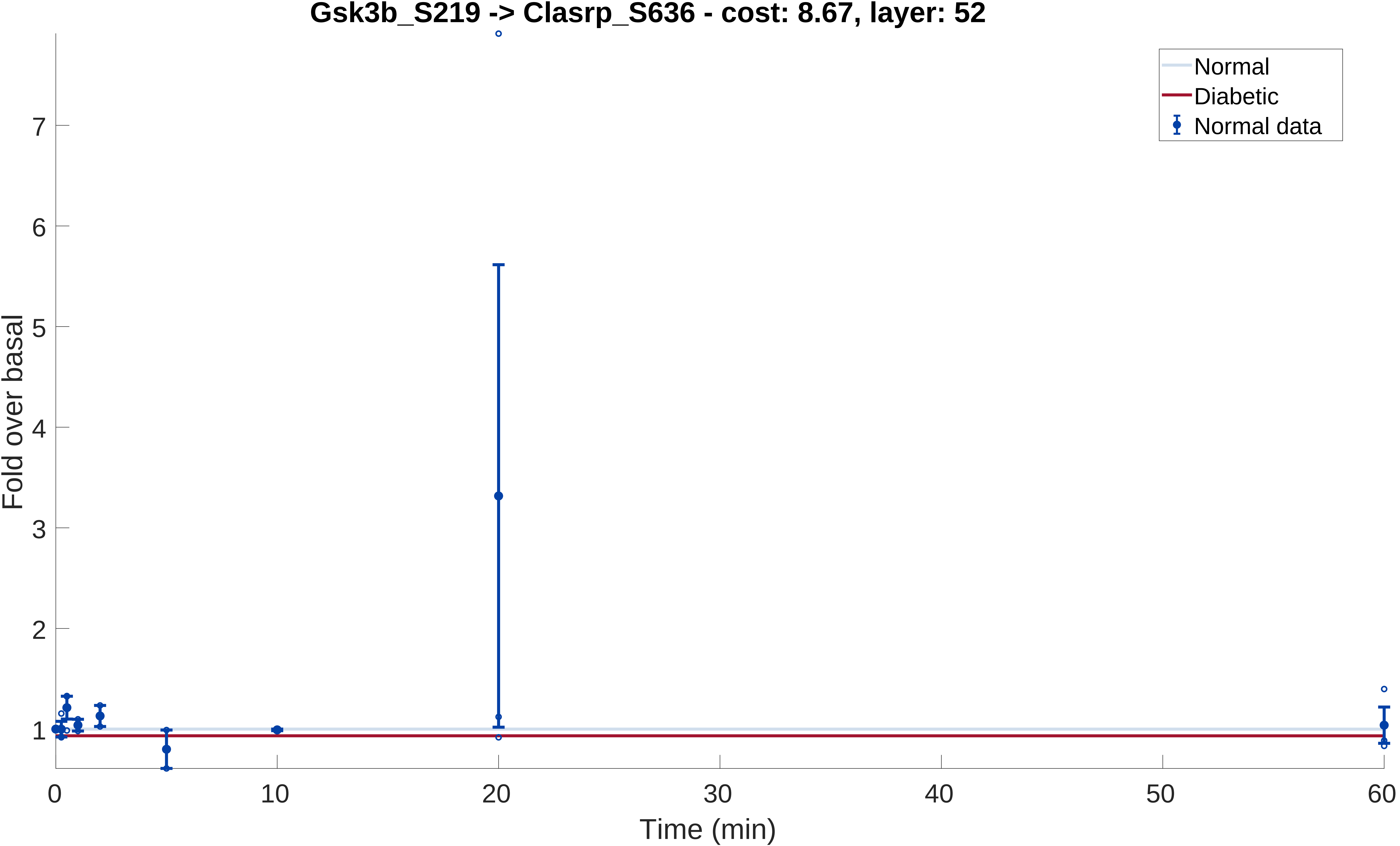

Gsk3b_S219 -> Clasrp_S636 - cost: 8.67, layer: 52
Normal
Diabetic
Normal data
Fold over basal
7
6
5
4
3
2
1
0
10
20
30
40
50
60
Time (min)

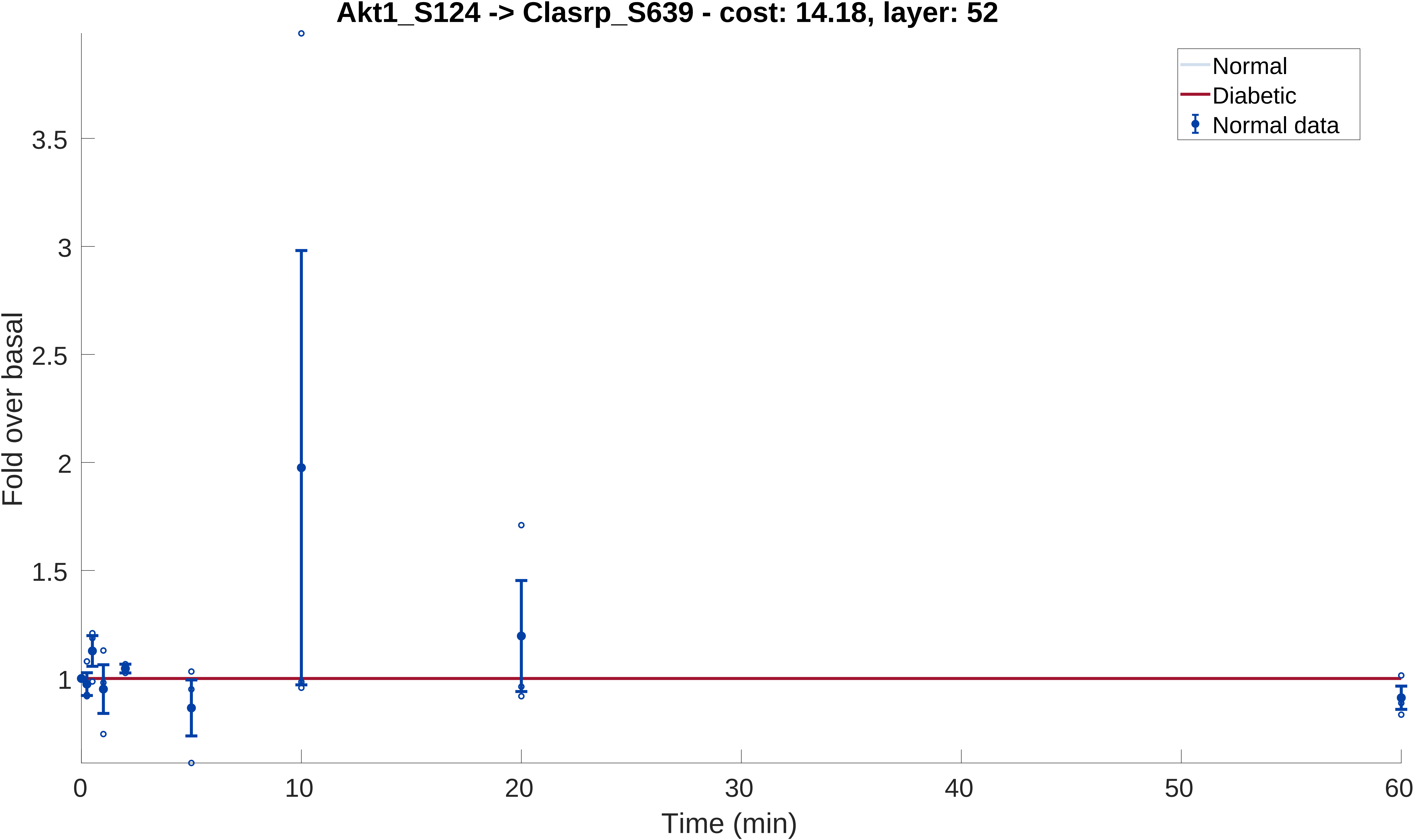
Akt1_S124 -> Clasrp_S639 - cost: 14.18, layer: 52
Normal
Diabetic
Normal data
Fold over basal
3.5
3
2.5
2
1.5
1
0
10
20
30
40
50
60
Time (min)

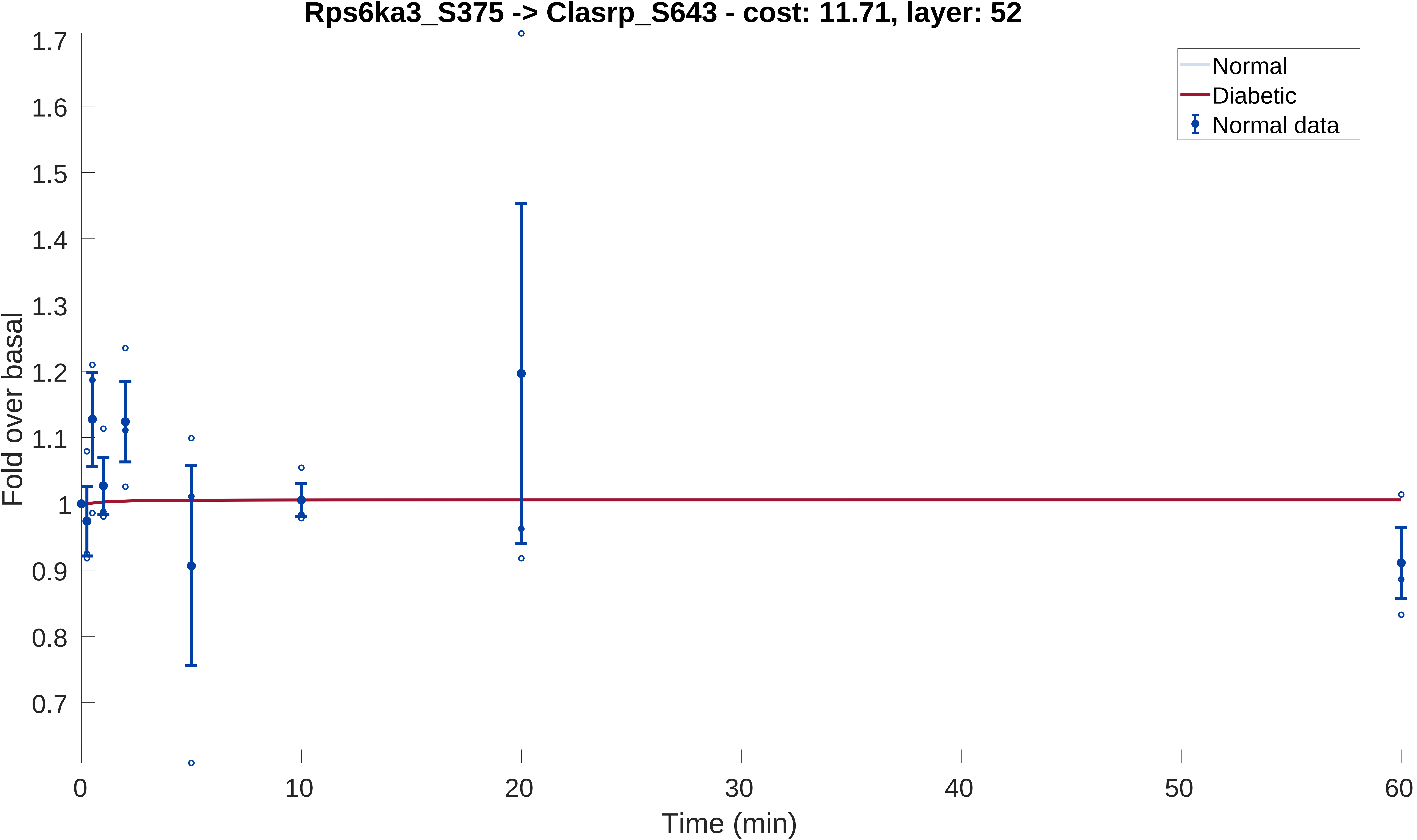
Rps6ka3_S375 -> Clasrp_S643 - cost: 11.71, layer: 52
Normal
Diabetic
Normal data
Fold over basal
Time (min)
1.7
1.6
1.5
1.4
1.3
1.2
1.1
1
0.9
0.8
0.7
0
10
20
30
40
50
60

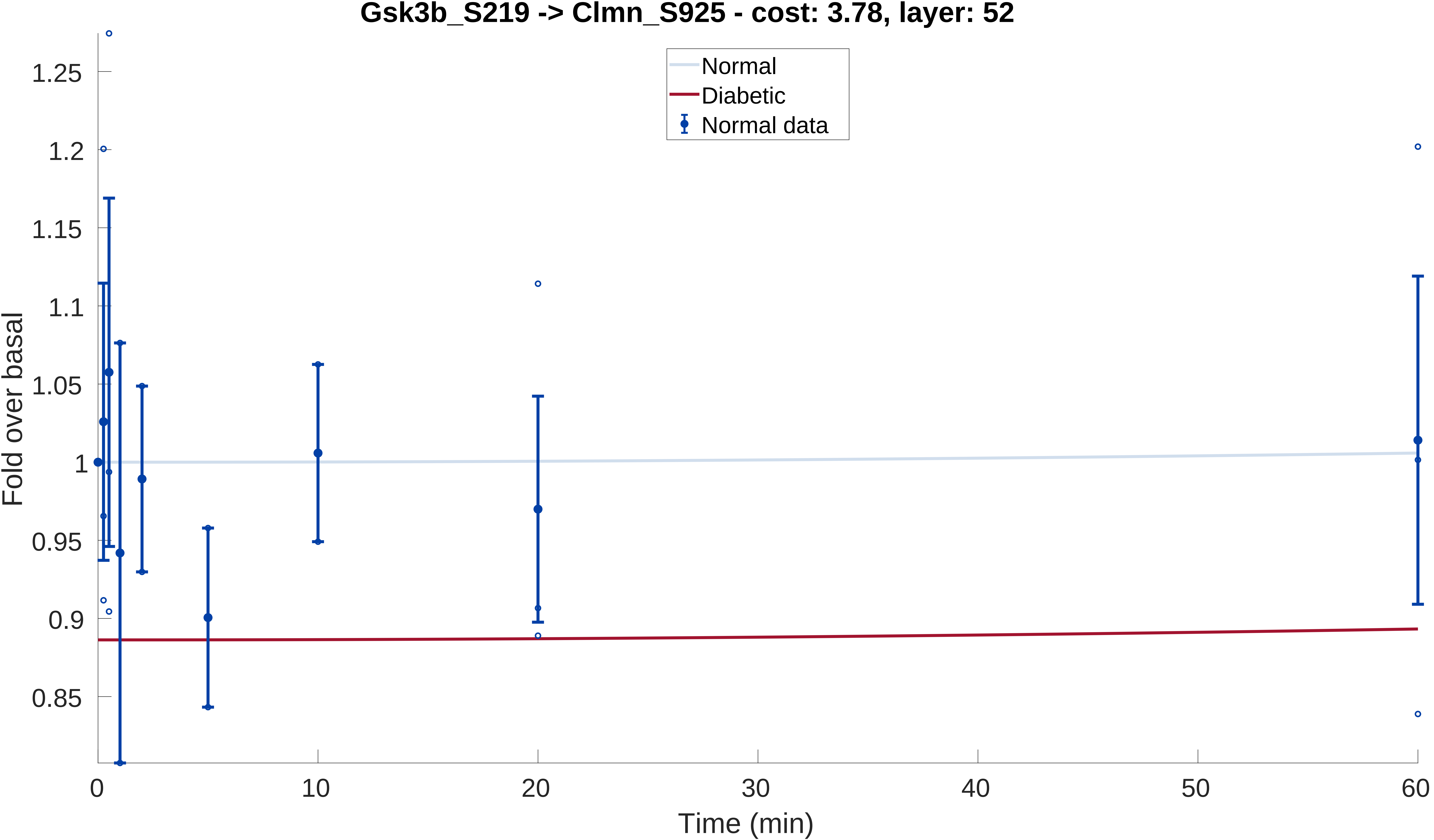

Gsk3b_S219 -> Clmn_S925 - cost: 3.78, layer: 52
Normal
Diabetic
Normal data
Fold over basal
Time (min)
1.25
1.2
1.15
1.1
1.05
1
0.95
0.9
0.85
0
10
20
30
40
50
60

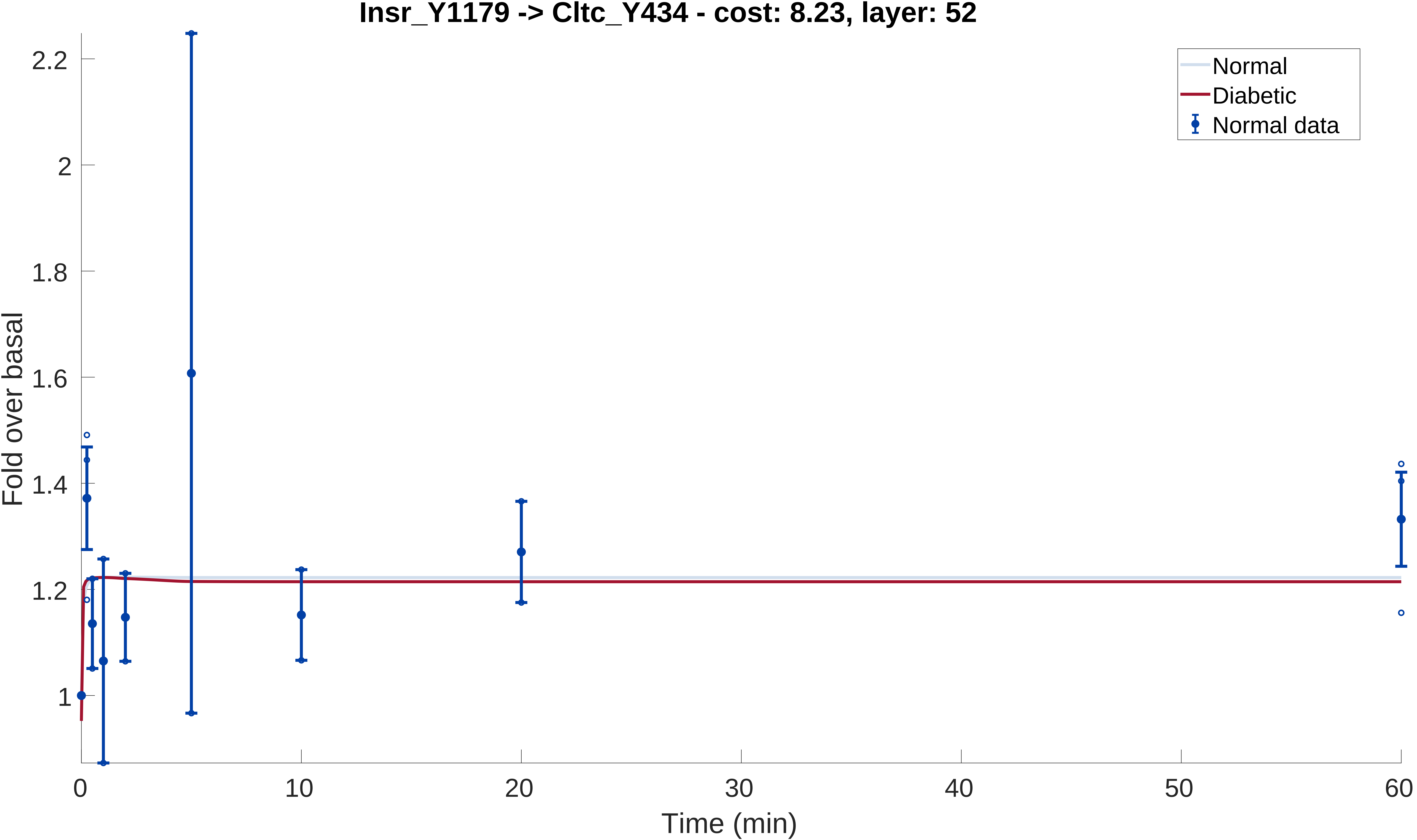

Insr_Y1179 -> Cltc_Y434 - cost: 8.23, layer: 52
Normal
Diabetic
Normal data
Fold over basal
2.2
2
1.8
1.6
1.4
1.2
1
0
10
20
30
40
50
60
Time (min)

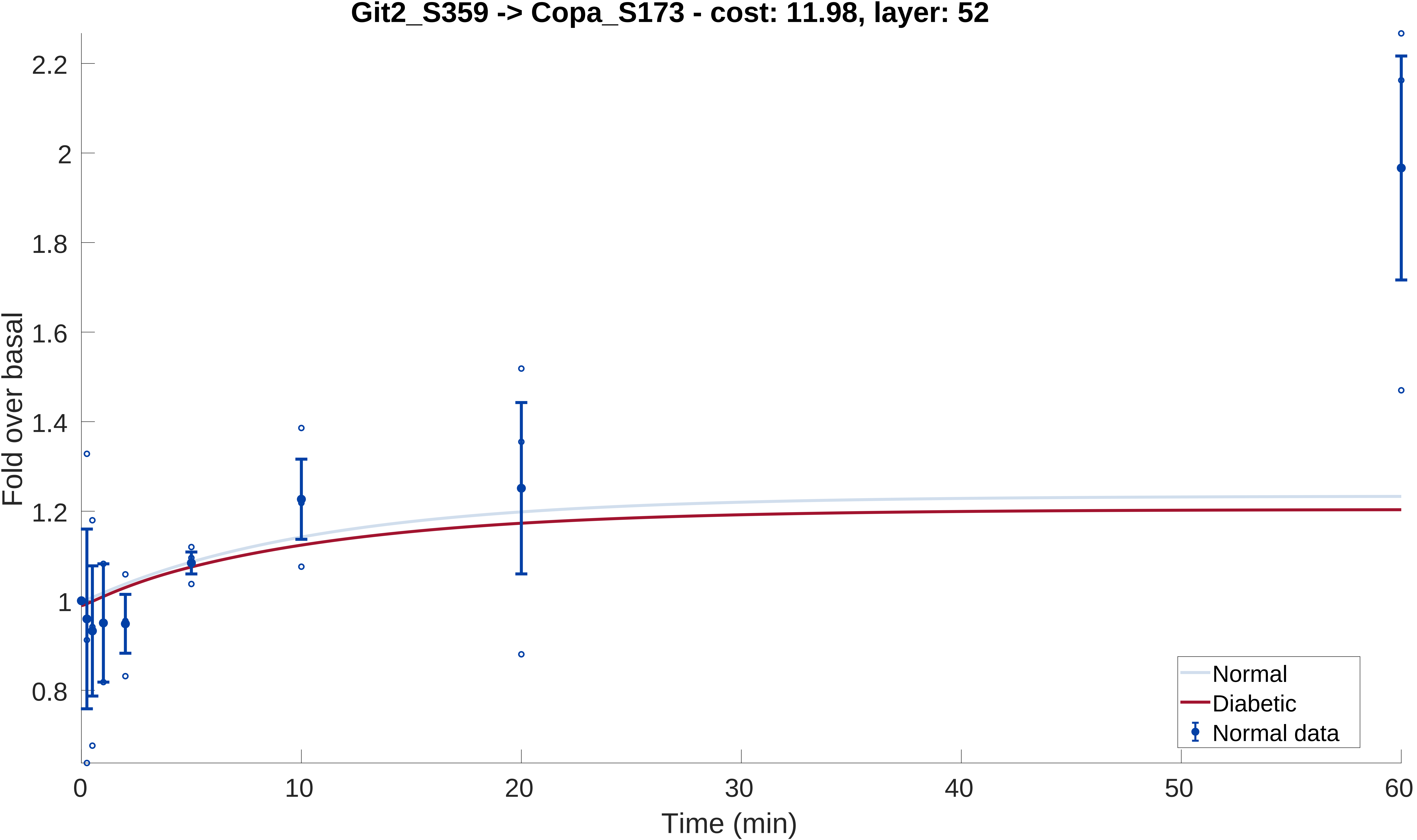

Git2_S359 -> Copa_S173 - cost: 11.98, layer: 52
Fold over basal
2.2
2
1.8
1.6
1.4
1.2
1
0.8
0
10
20
30
40
50
60
Time (min)
Normal
Diabetic
Normal data

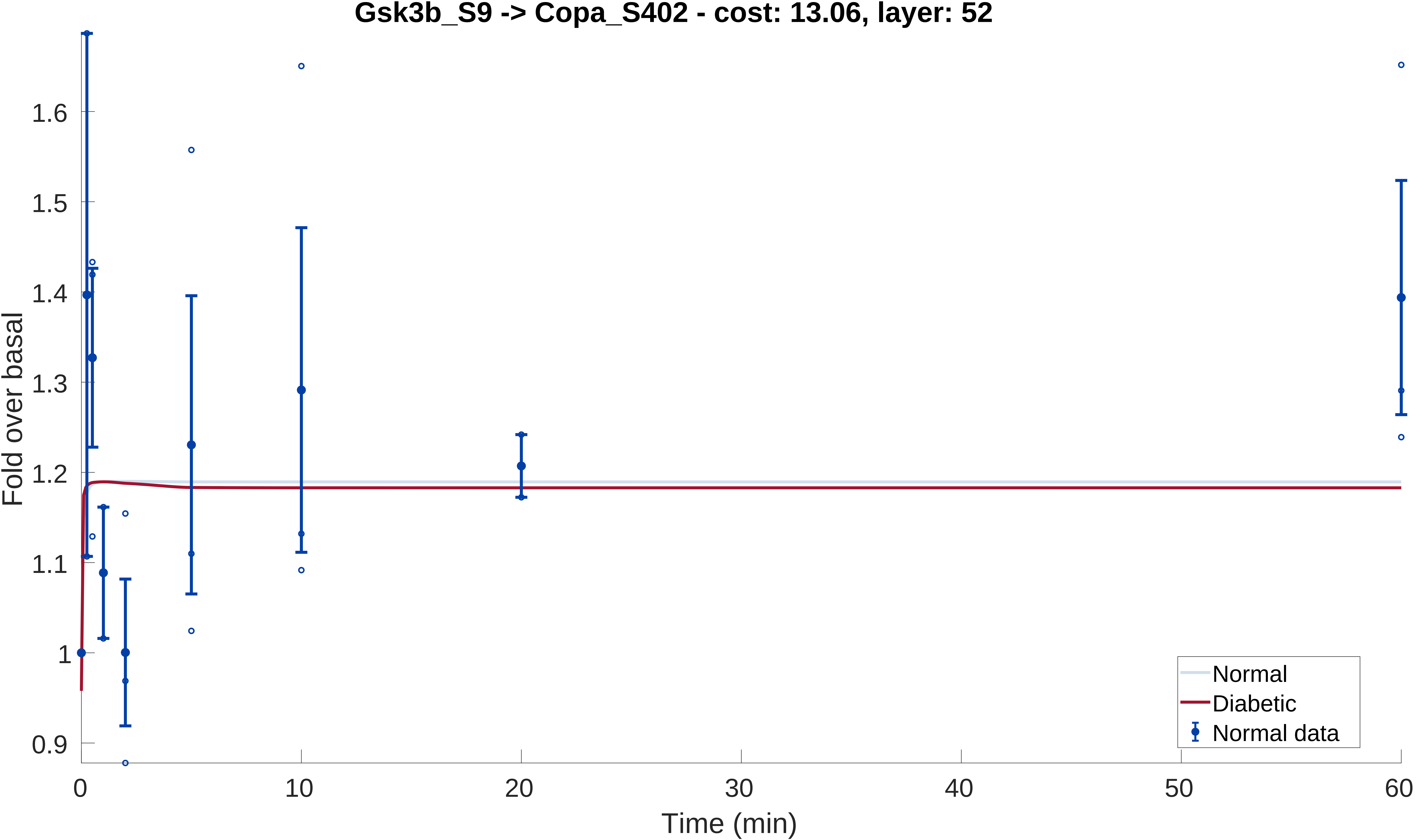
Gsk3b_S9 -> Copa_S402 - cost: 13.06, layer: 52
Fold over basal
0.9
1
1.1
1.2
1.3
1.4
1.5
1.6
0
10
20
30
40
50
60
Time (min)
Normal
Diabetic
Normal data

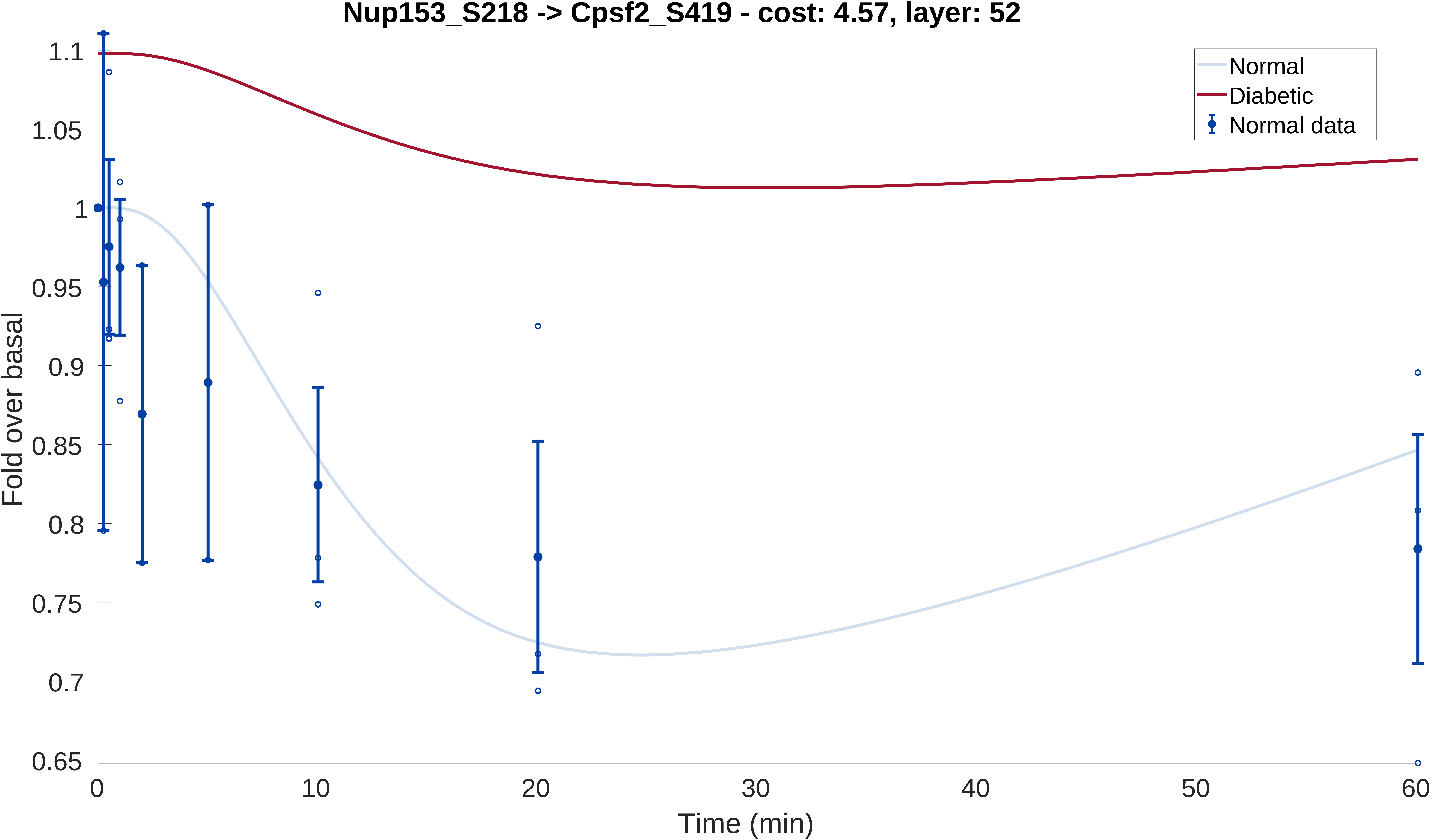

Nup153_S218 -> Cpsf2_S419 - cost: 4.57, layer: 52
Normal
Diabetic
Normal data
Fold over basal
Time (min)
1.1
1.05
1
0.95
0.9
0.85
0.8
0.75
0.7
0.65
0
10
20
30
40
50
60

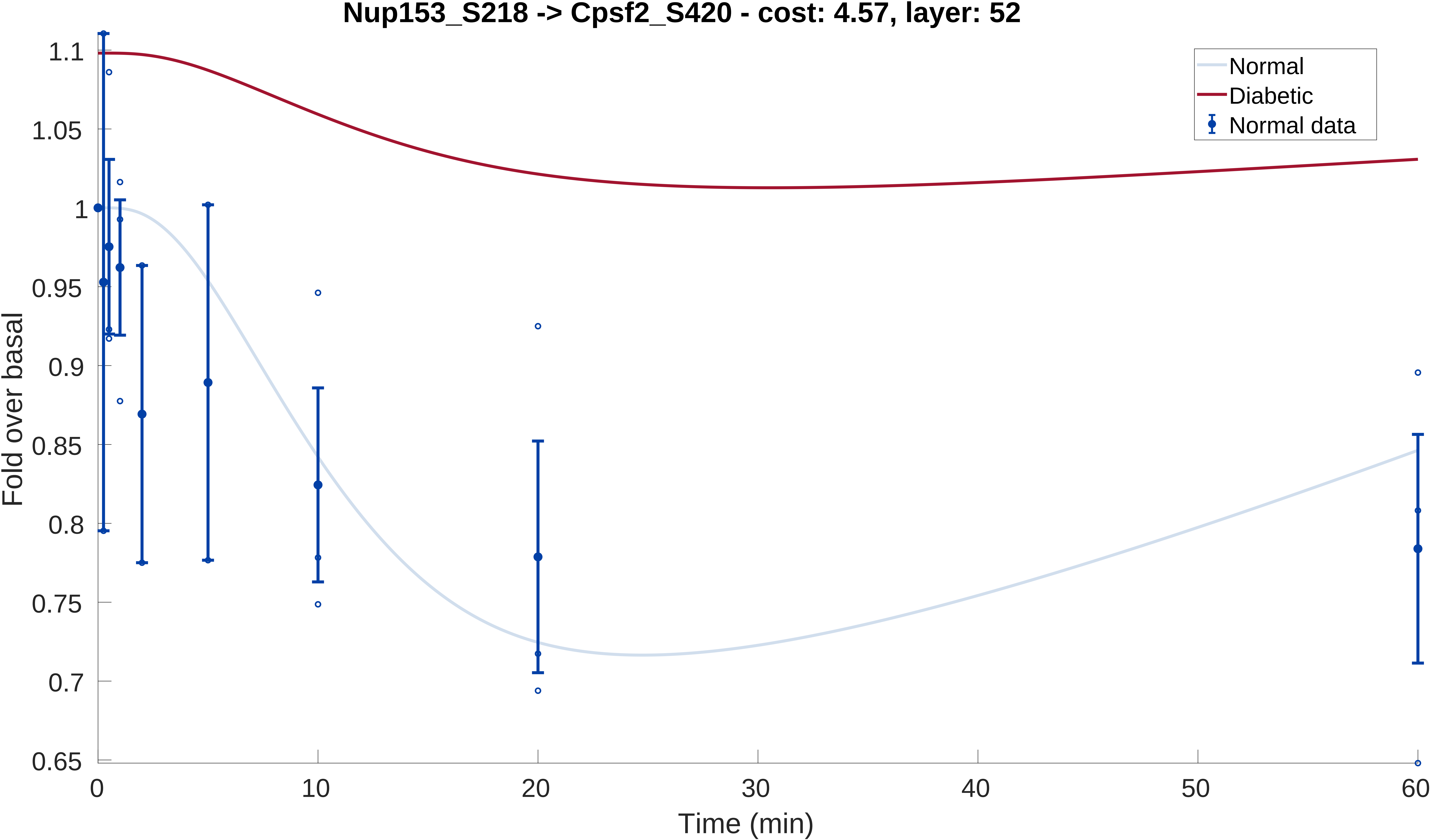

Nup153_S218 -> Cpsf2_S420 - cost: 4.57, layer: 52
Normal
Diabetic
Normal data
Fold over basal
1.1
1.05
1
0.95
0.9
0.85
0.8
0.75
0.7
0.65
0
10
20
30
40
50
60
Time (min)

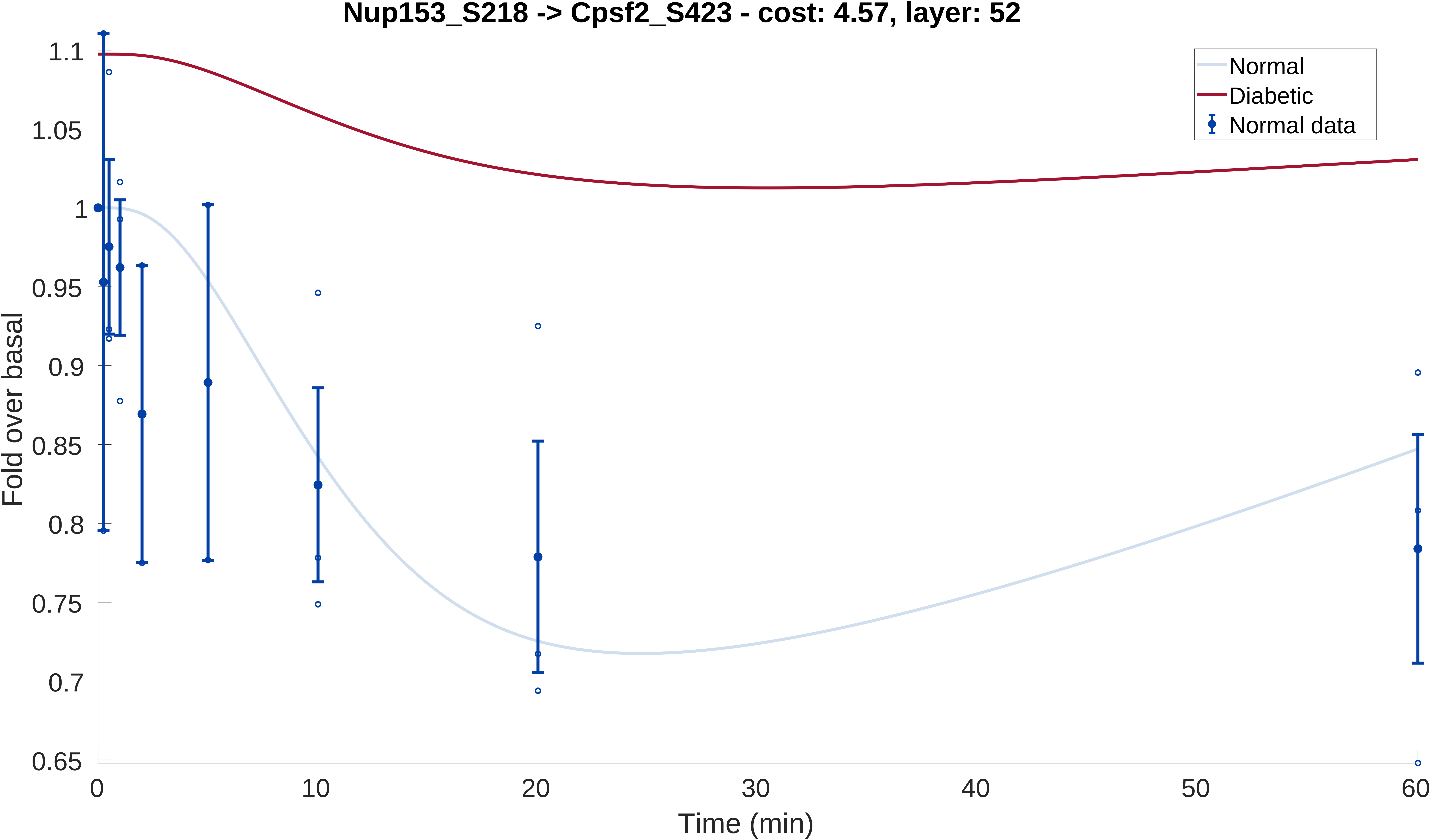

Nup153_S218 -> Cpsf2_S423 - cost: 4.57, layer: 52
Normal
Diabetic
Normal data
Fold over basal
Time (min)
1.1
1.05
1
0.95
0.9
0.85
0.8
0.75
0.7
0.65
0
10
20
30
40
50
60

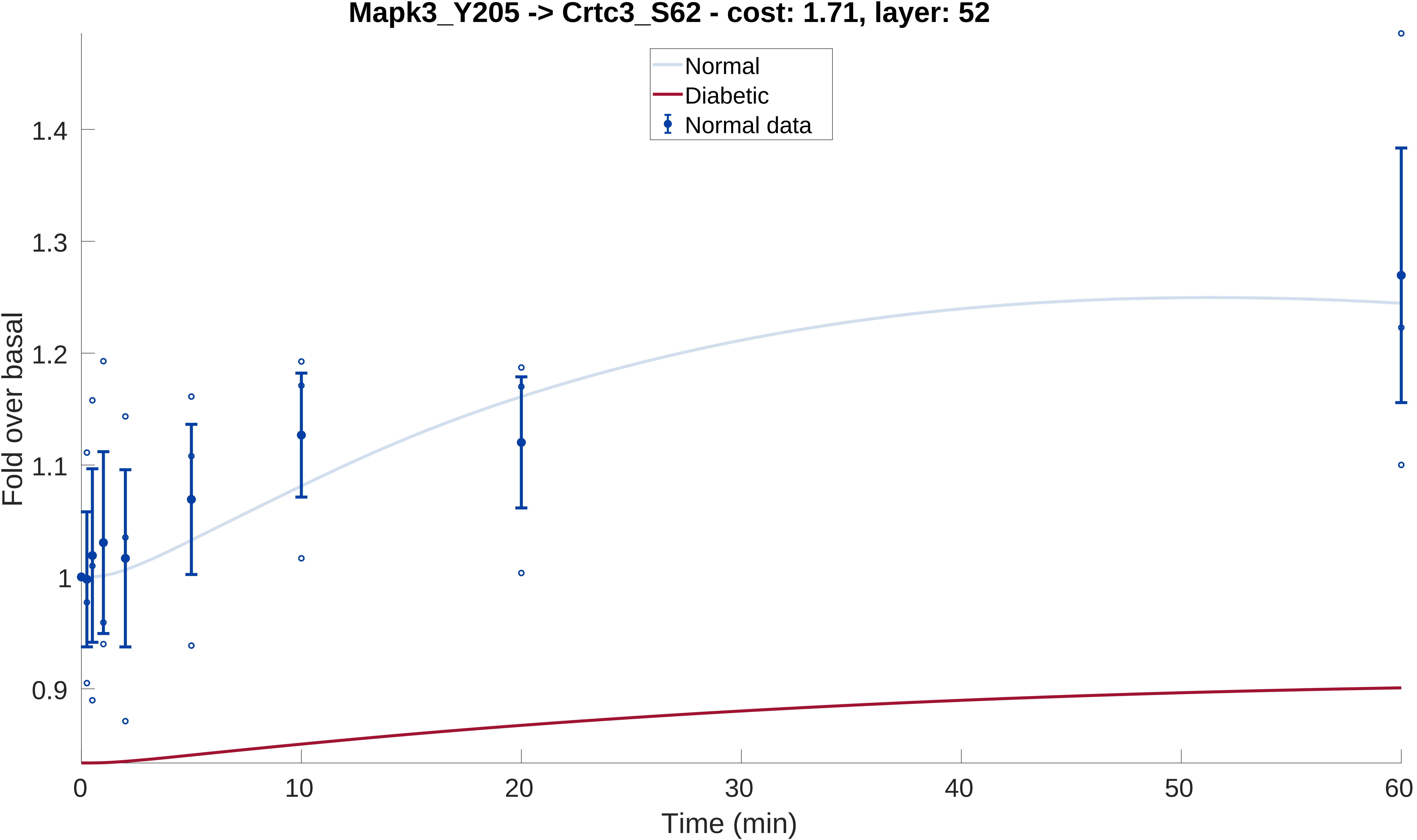

Mapk3_Y205 -> Crtc3_S62 - cost: 1.71, layer: 52
Normal
Diabetic
Normal data
Fold over basal
1.4
1.3
1.2
1.1
1
0.9
0
10
20
30
40
50
60
Time (min)

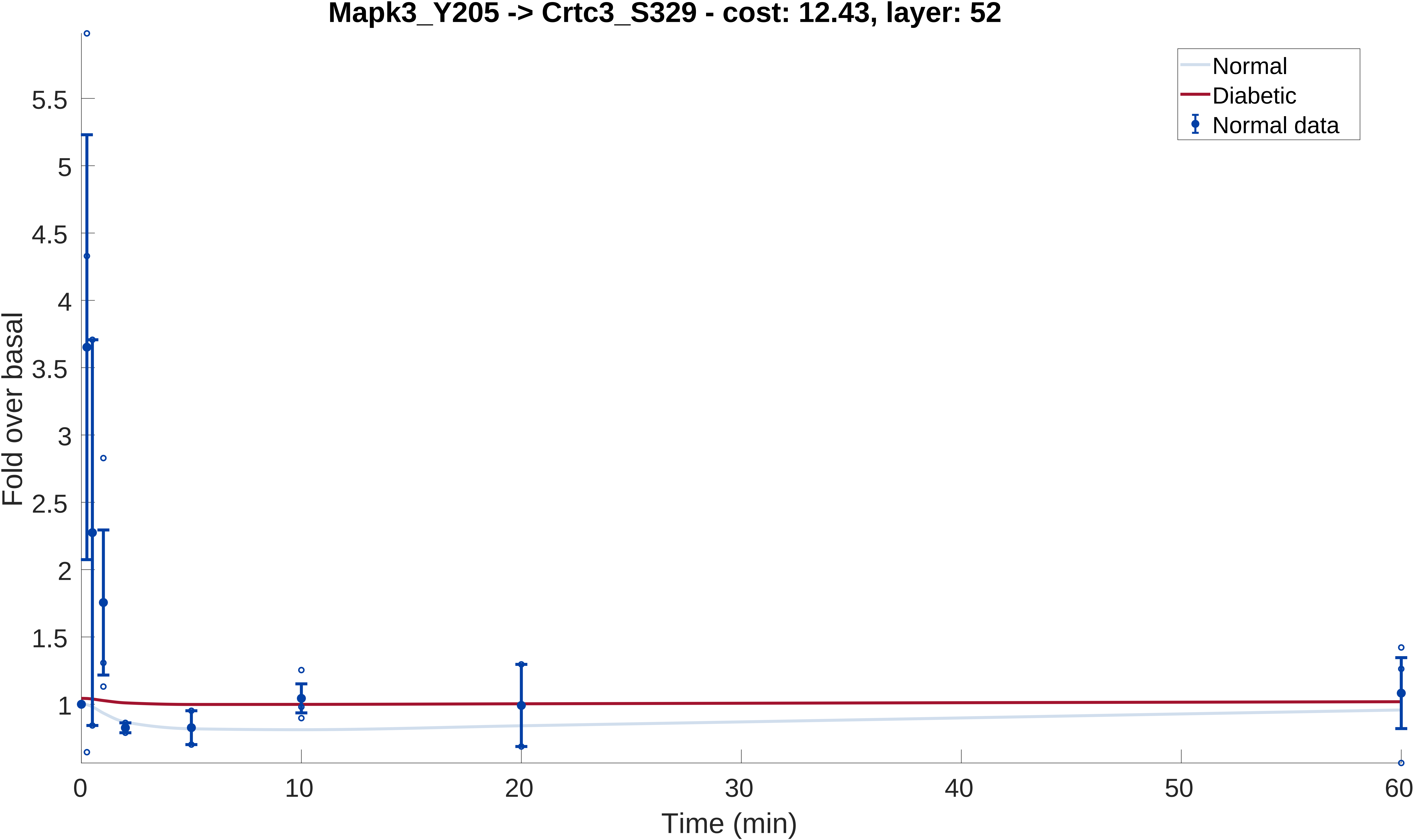

Mapk3_Y205 -> Crtc3_S329 - cost: 12.43, layer: 52
Normal
Diabetic
Normal data
Fold over basal
Time (min)
5.5
5
4.5
4
3.5
3
2.5
2
1.5
1
0
10
20
30
40
50
60

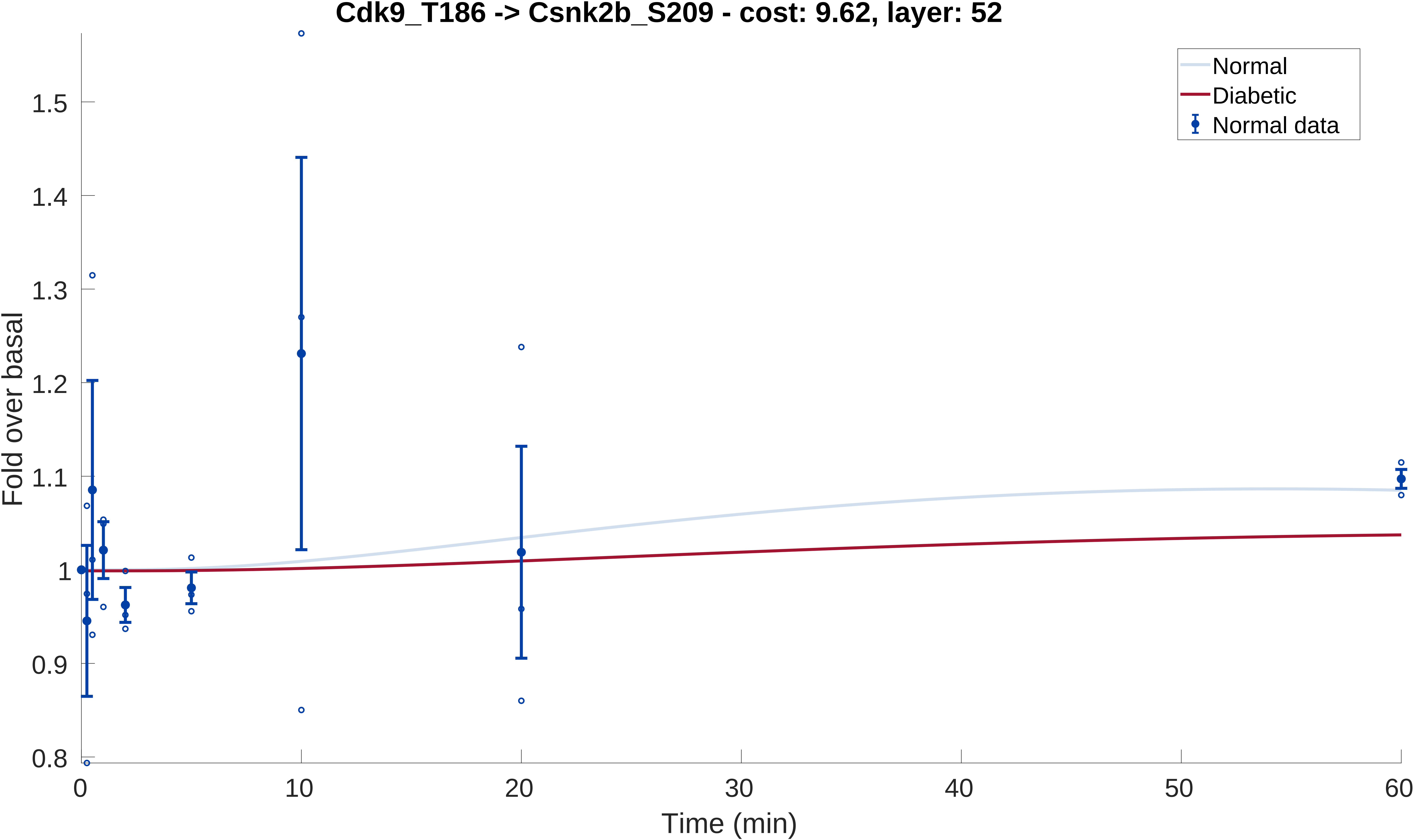
Cdk9_T186 -> Csnk2b_S209 - cost: 9.62, layer: 52
Normal
Diabetic
Normal data
Fold over basal
Time (min)
0.8
0.9
1
1.1
1.2
1.3
1.4
1.5
0
10
20
30
40
50
60

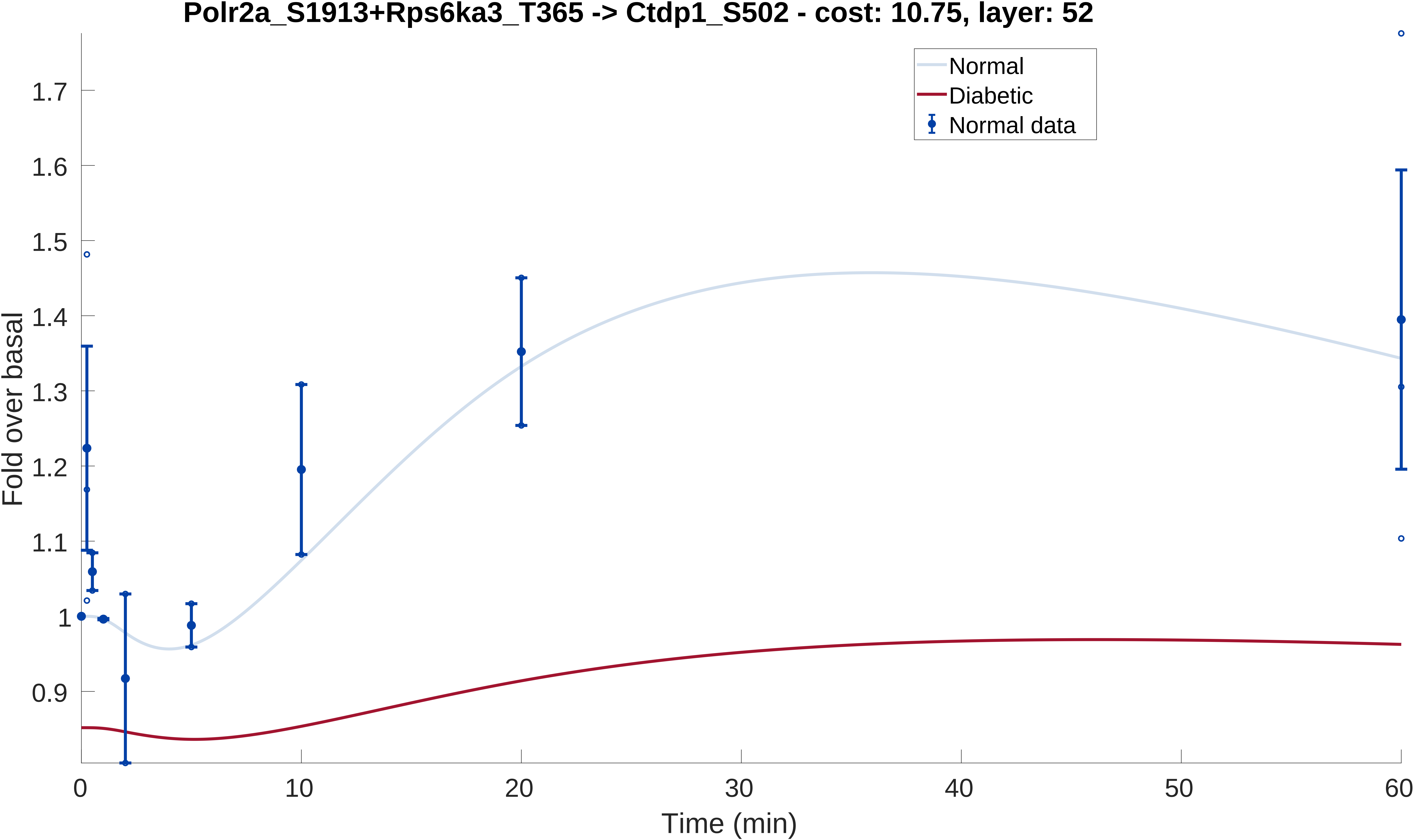

Polr2a_S1913+Rps6ka3_T365 -> Ctdp1_S502 - cost: 10.75, layer: 52
Normal
Diabetic
Normal data
Fold over basal
1.7
1.6
1.5
1.4
1.3
1.2
1.1
1
0.9
0
10
20
30
40
50
60
Time (min)

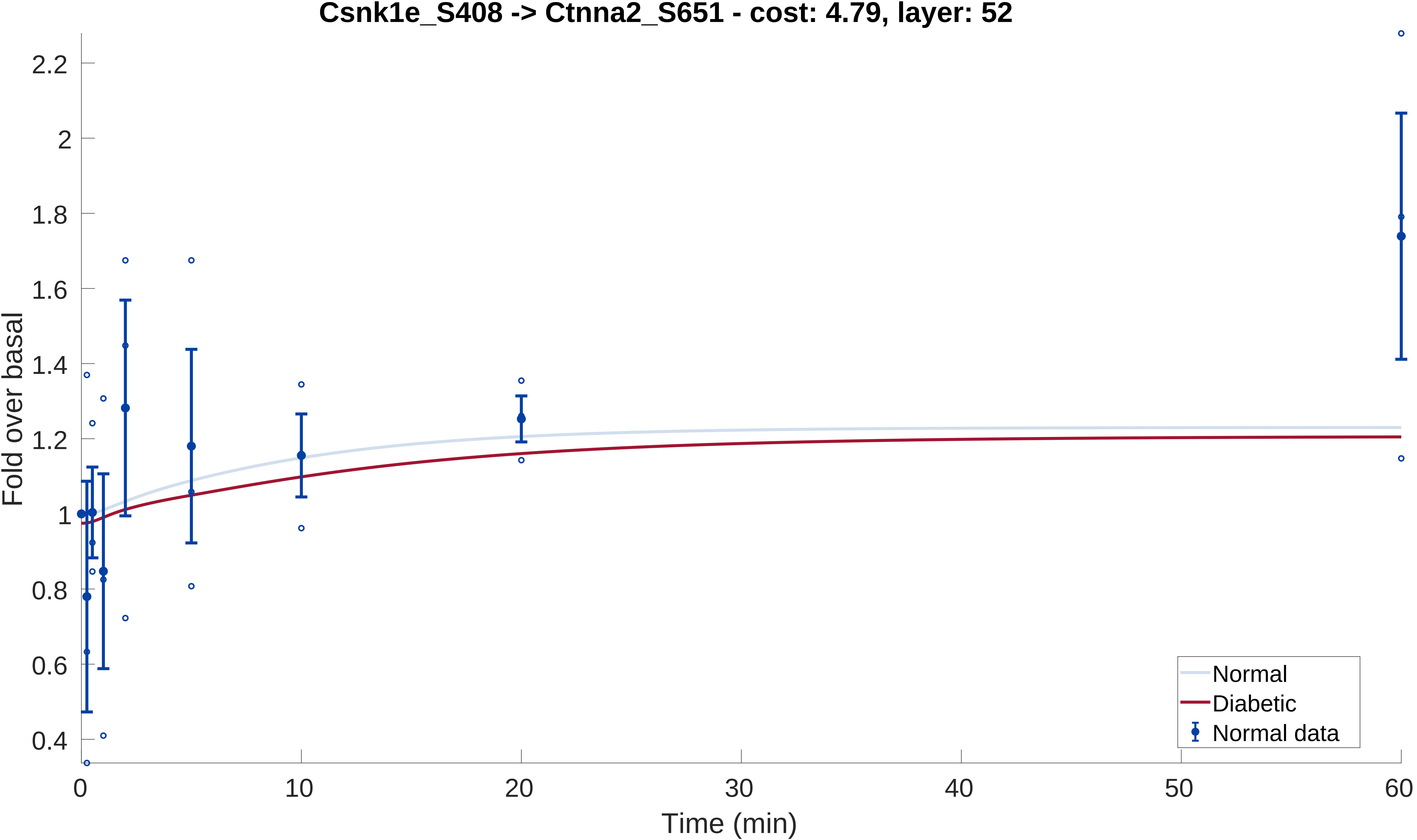

Csnk1e_S408 -> Ctnna2_S651 - cost: 4.79, layer: 52
Fold over basal
Time (min)
2.2
2
1.8
1.6
1.4
1.2
1
0.8
0.6
0.4
0
10
20
30
40
50
60
Normal
Diabetic
Normal data

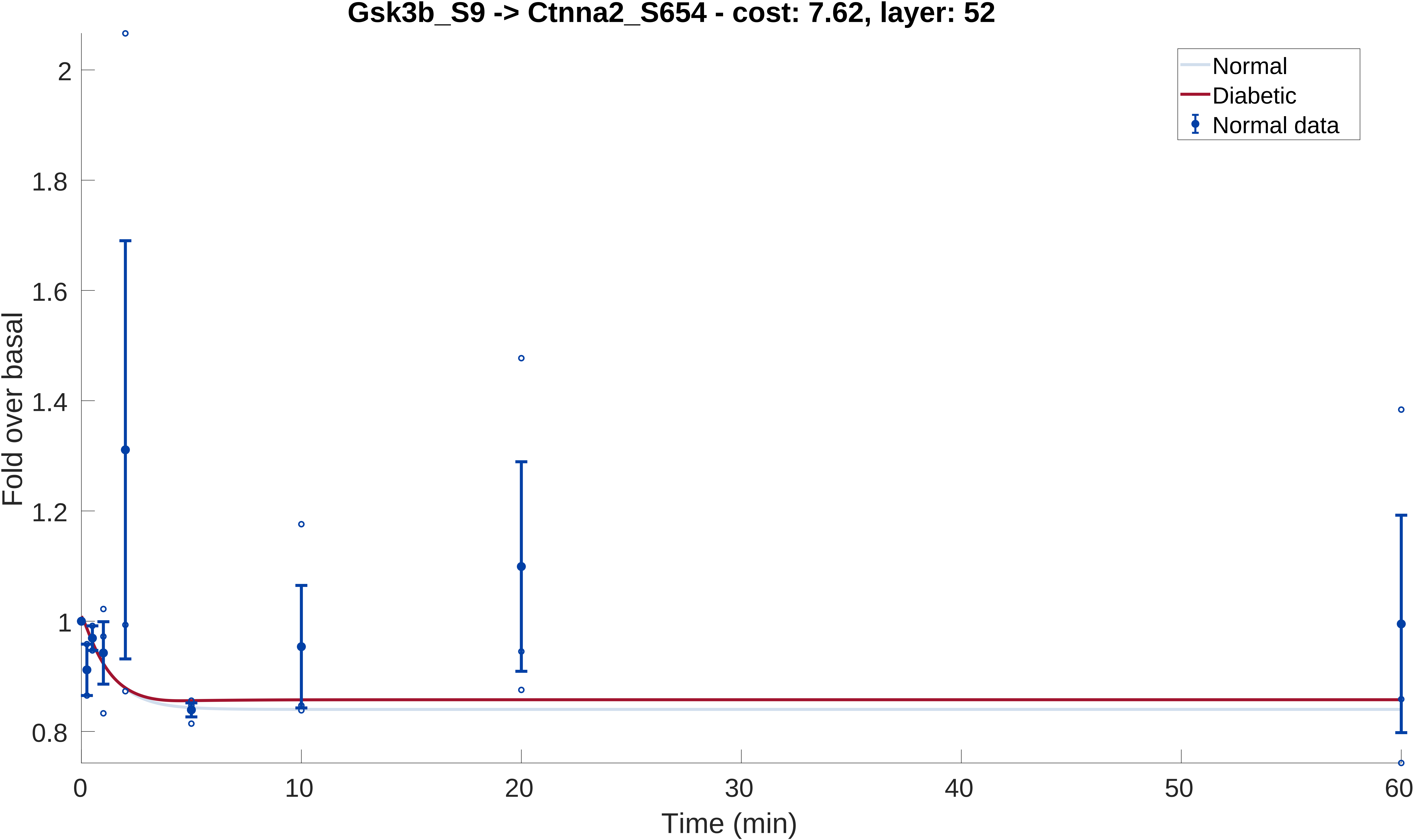
Gsk3b_S9 -> Ctnna2_S654 - cost: 7.62, layer: 52
Normal
Diabetic
Normal data
Fold over basal
0.8
1
1.2
1.4
1.6
1.8
2
Time (min)
0
10
20
30
40
50
60

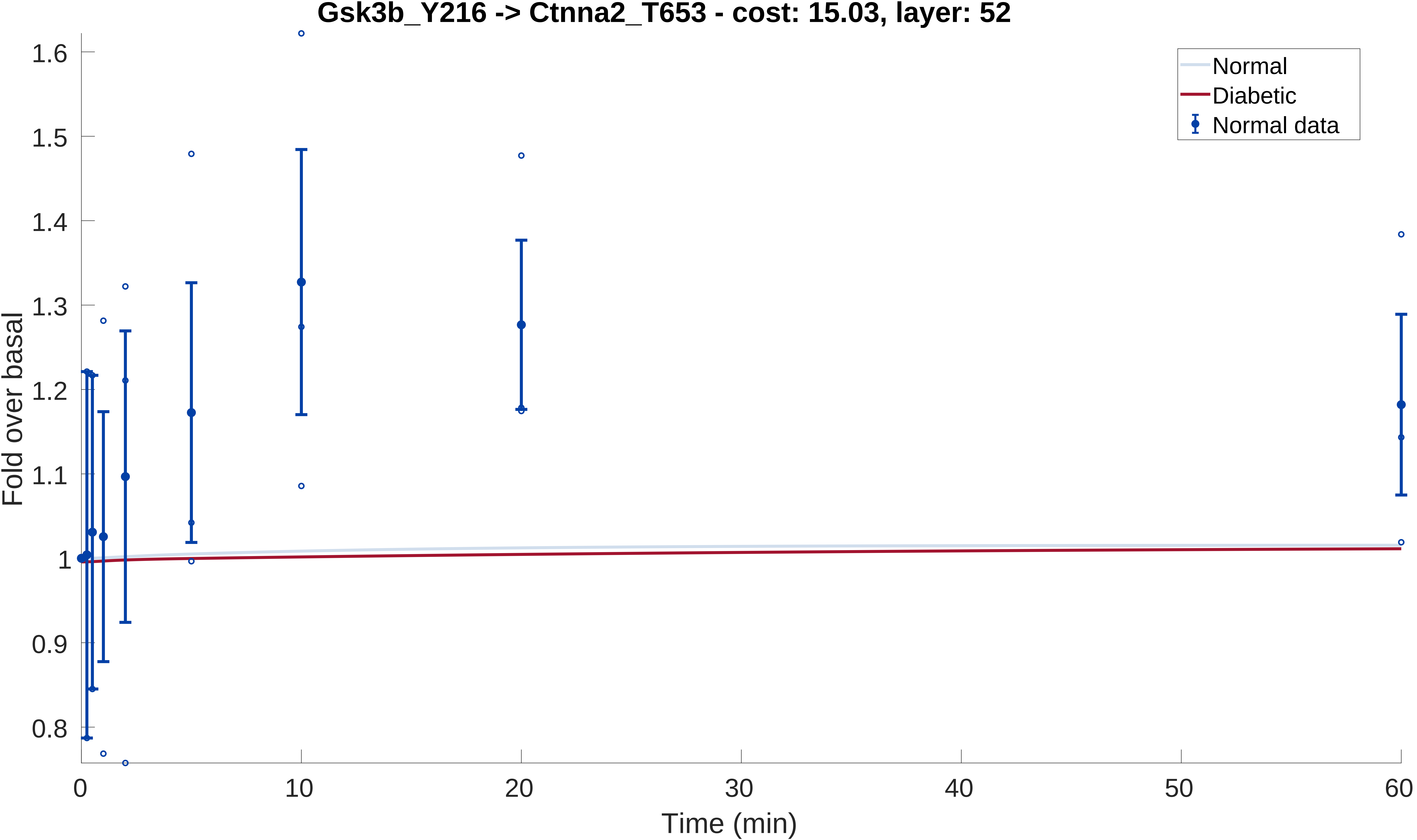
Gsk3b_Y216 -> Ctnna2_T653 - cost: 15.03, layer: 52
Fold over basal
1.6
1.5
1.4
1.3
1.2
1.1
1
0.9
0.8
0
10
20
30
40
50
60
Time (min)
Normal
Diabetic
Normal data

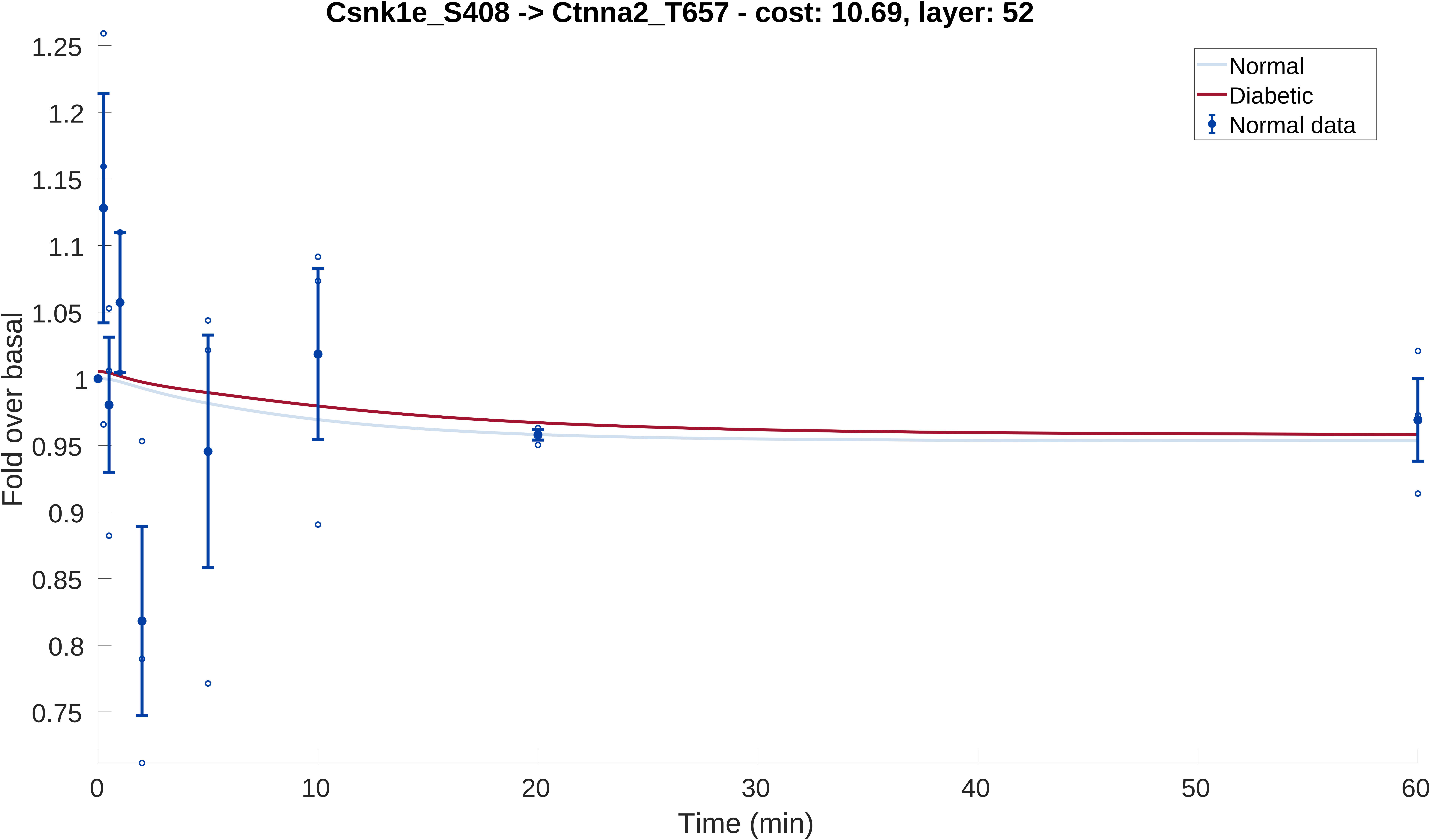

Csnk1e_S408 -> Ctnna2_T657 - cost: 10.69, layer: 52
Normal
Diabetic
Normal data
Fold over basal
Time (min)
1.25
1.2
1.15
1.1
1.05
1
0.95
0.9
0.85
0.8
0.75
0
10
20
30
40
50
60

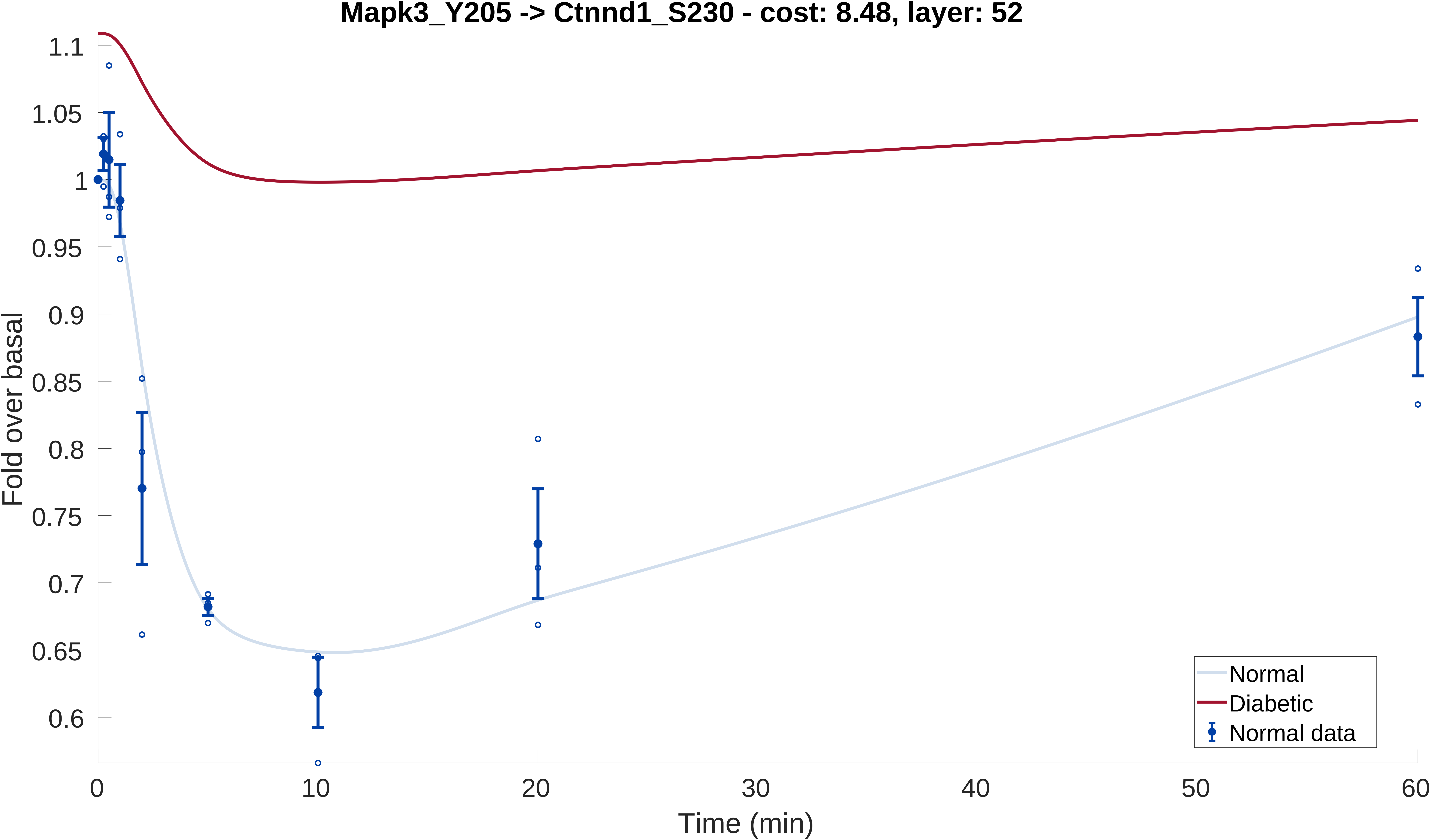
Mapk3_Y205 -> Ctnnd1_S230 - cost: 8.48, layer: 52
Fold over basal
Time (min)
1.1
1.05
1
0.95
0.9
0.85
0.8
0.75
0.7
0.65
0.6
0
10
20
30
40
50
60
Normal
Diabetic
Normal data

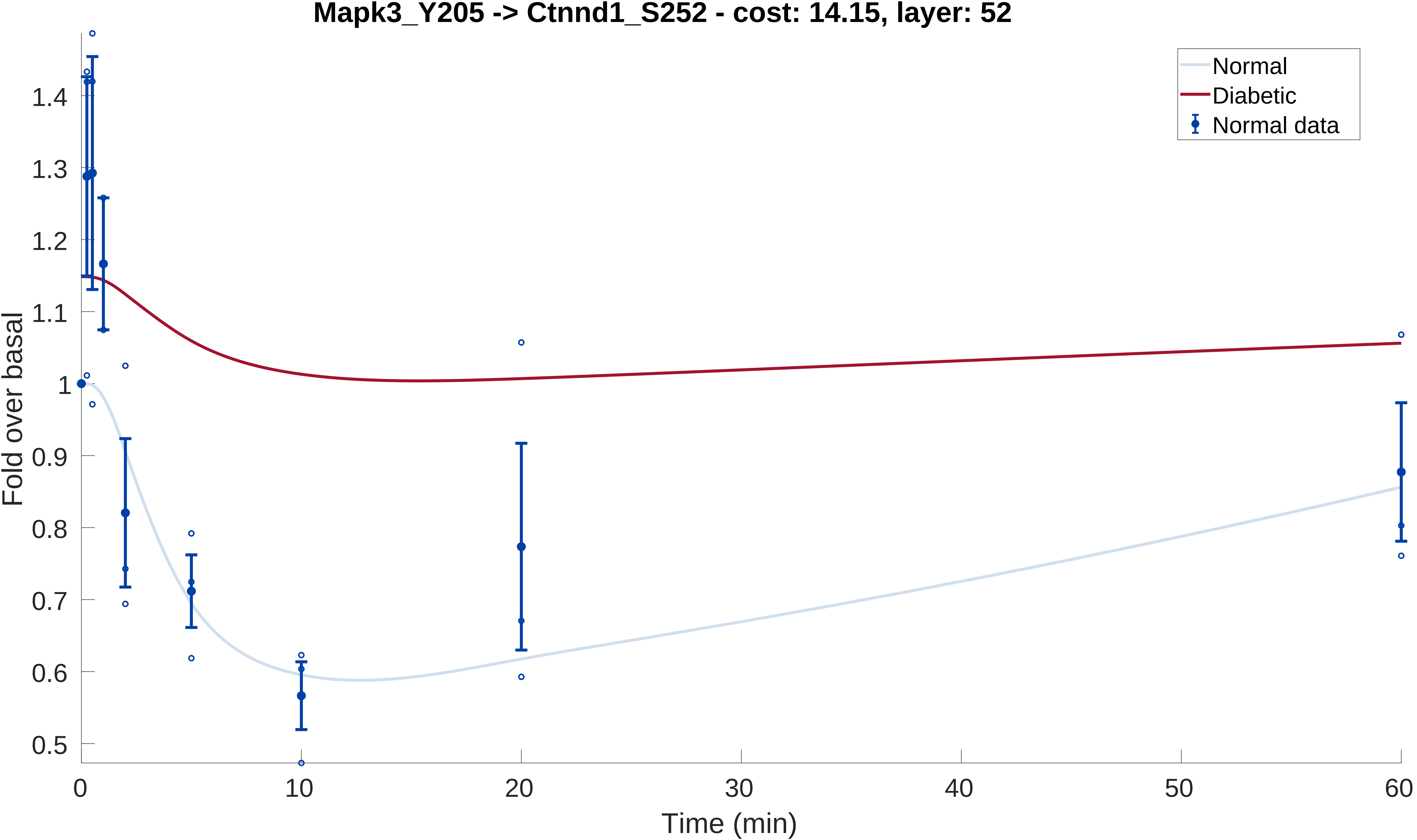

Mapk3_Y205 -> Ctnnd1_S252 - cost: 14.15, layer: 52
Normal
Diabetic
Normal data
Fold over basal
Time (min)
0
10
20
30
40
50
60
0.5
0.6
0.7
0.8
0.9
1
1.1
1.2
1.3
1.4

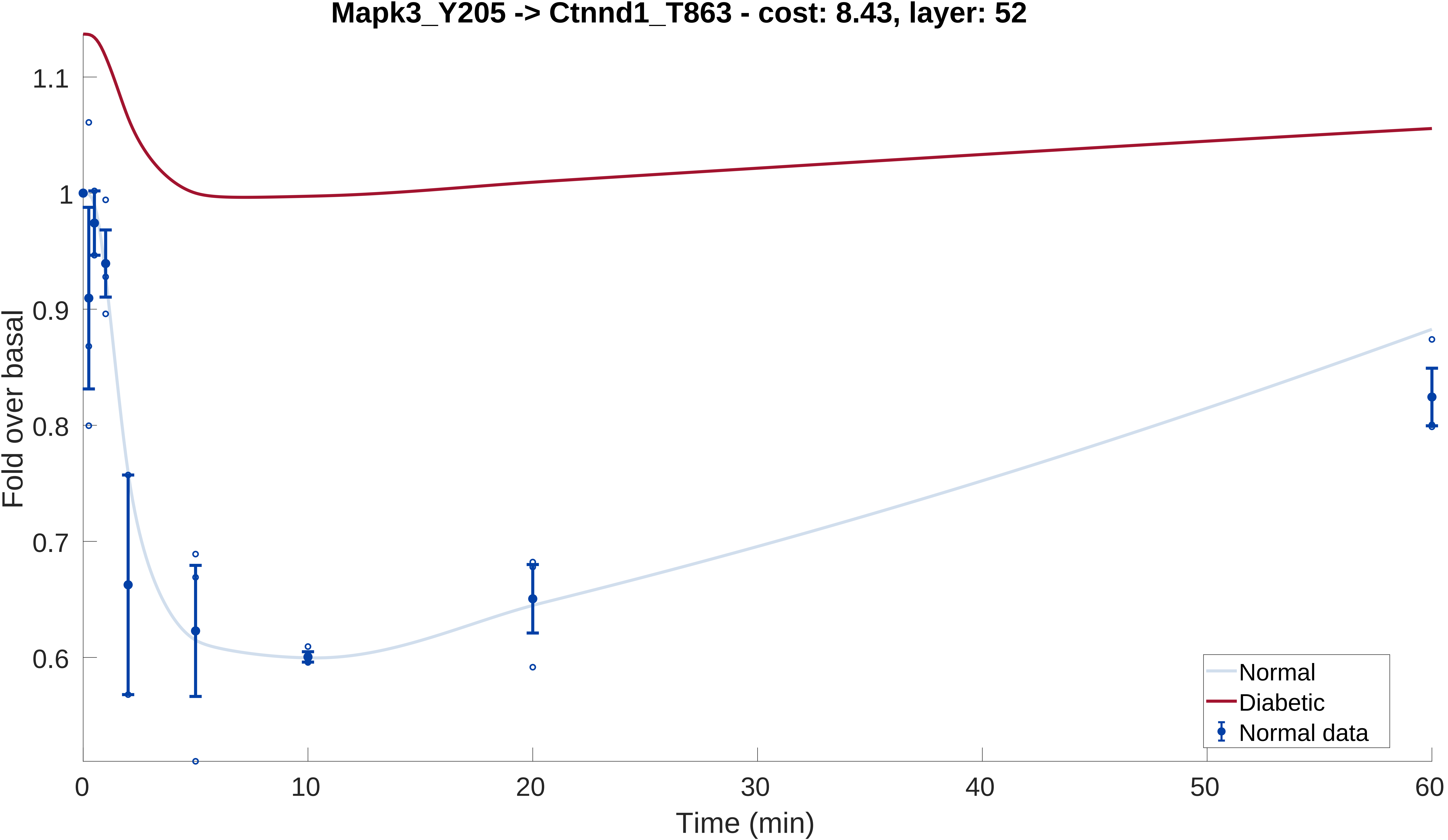

Mapk3_Y205 -> Ctnnd1_T863 - cost: 8.43, layer: 52
Fold over basal
1.1
1
0.9
0.8
0.7
0.6
0
10
20
30
40
50
60
Time (min)
Normal
Diabetic
Normal data

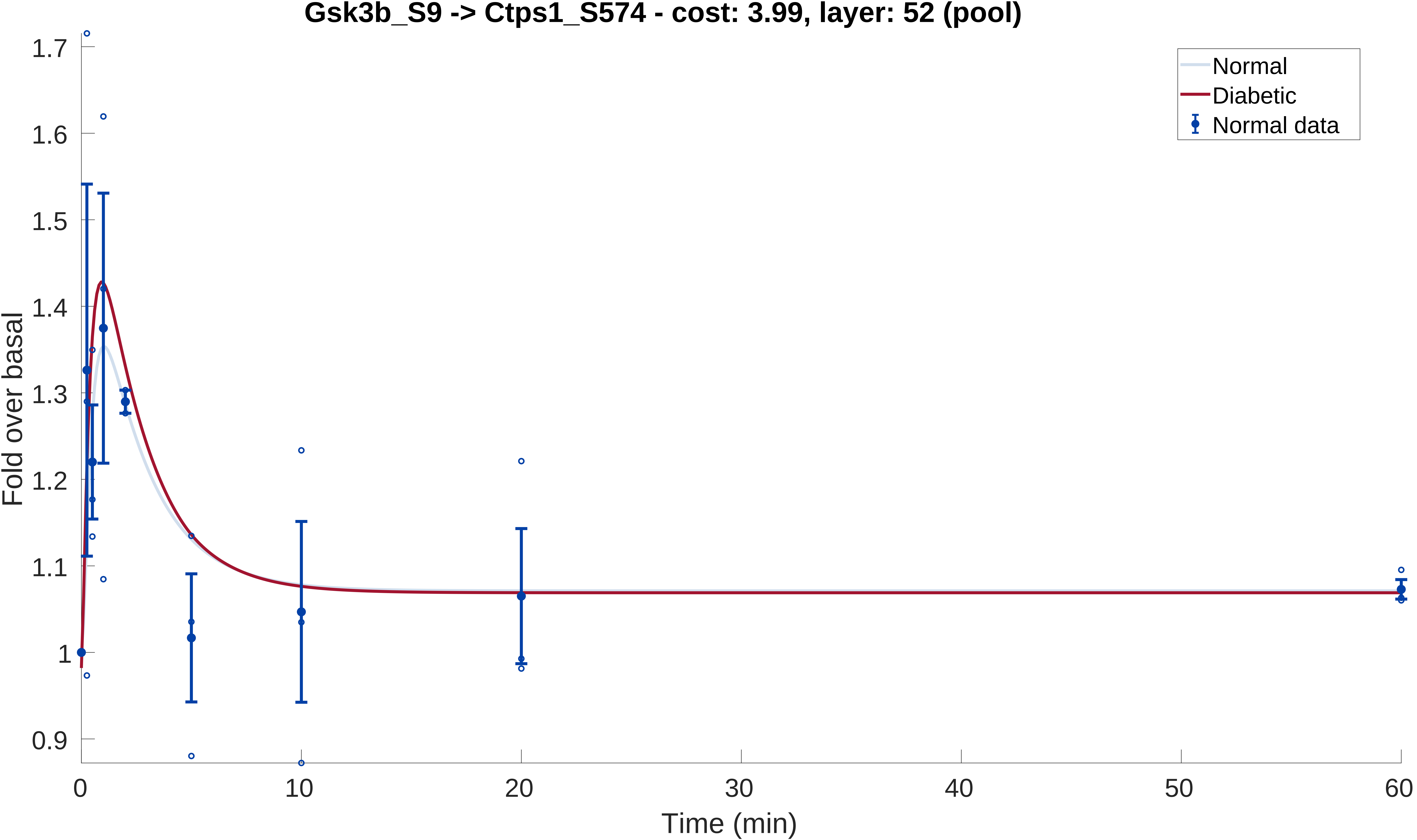

Gsk3b_S9 -> Ctps1_S574 - cost: 3.99, layer: 52 (pool)
Normal
Diabetic
Normal data
Fold over basal
Time (min)
1.7
1.6
1.5
1.4
1.3
1.2
1.1
1
0.9
0
10
20
30
40
50
60

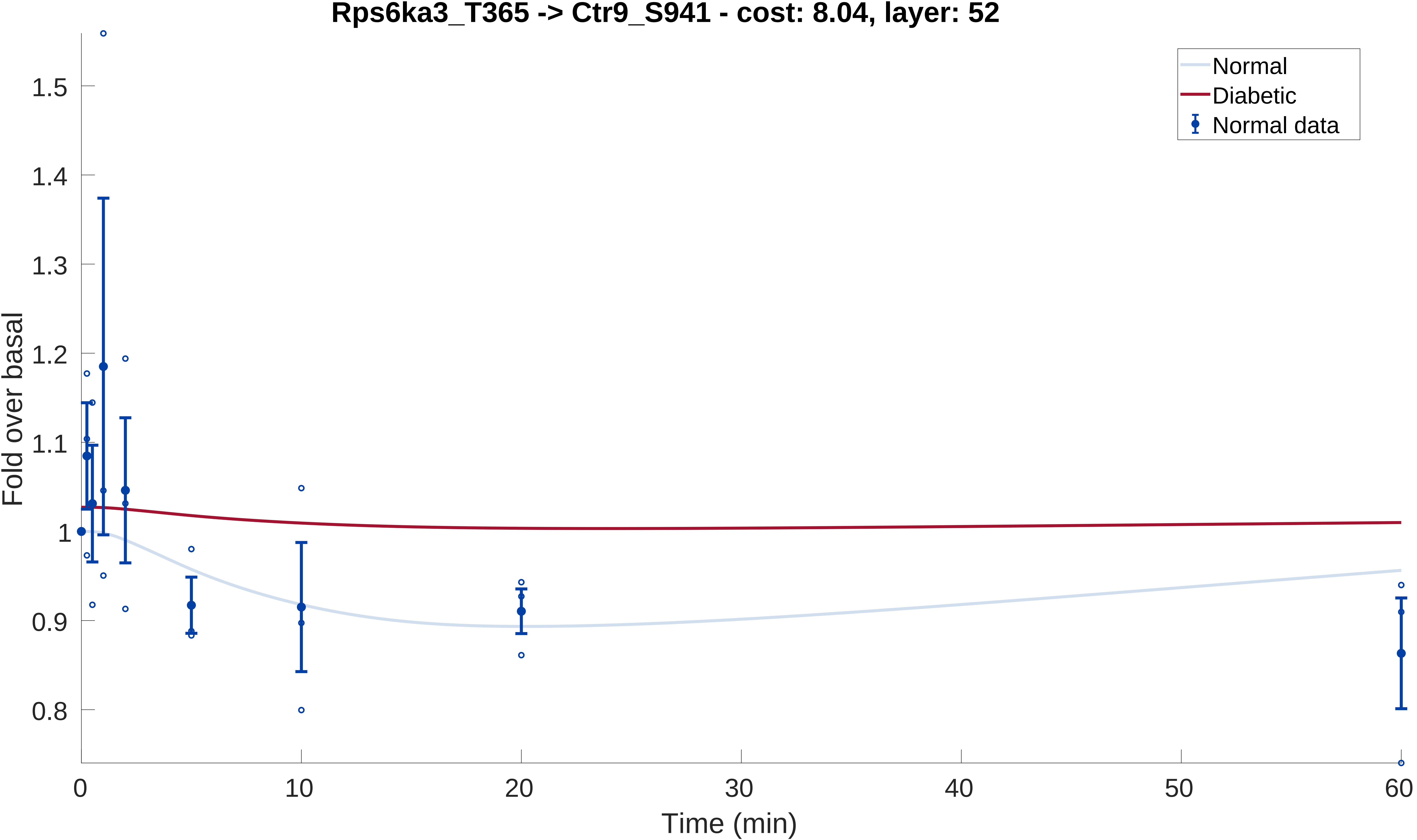

Rps6ka3_T365 -> Ctr9_S941 - cost: 8.04, layer: 52
Normal
Diabetic
Normal data
Fold over basal
1.5
1.4
1.3
1.2
1.1
1
0.9
0.8
0
10
20
30
40
50
60
Time (min)

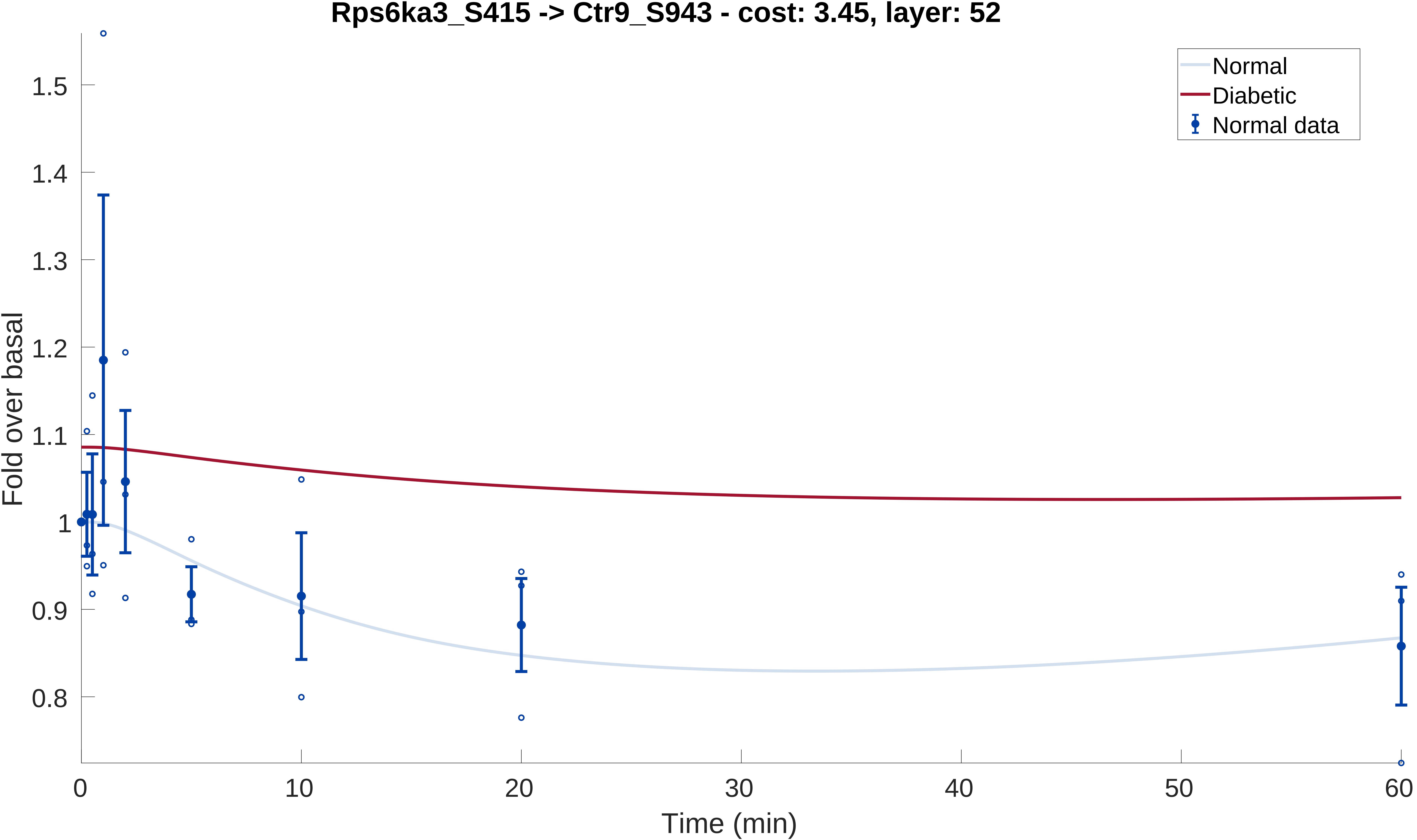
Rps6ka3_S415 -> Ctr9_S943 - cost: 3.45, layer: 52
Normal
Diabetic
Normal data
Fold over basal
Time (min)
1.5
1.4
1.3
1.2
1.1
1
0.9
0.8
0
10
20
30
40
50
60

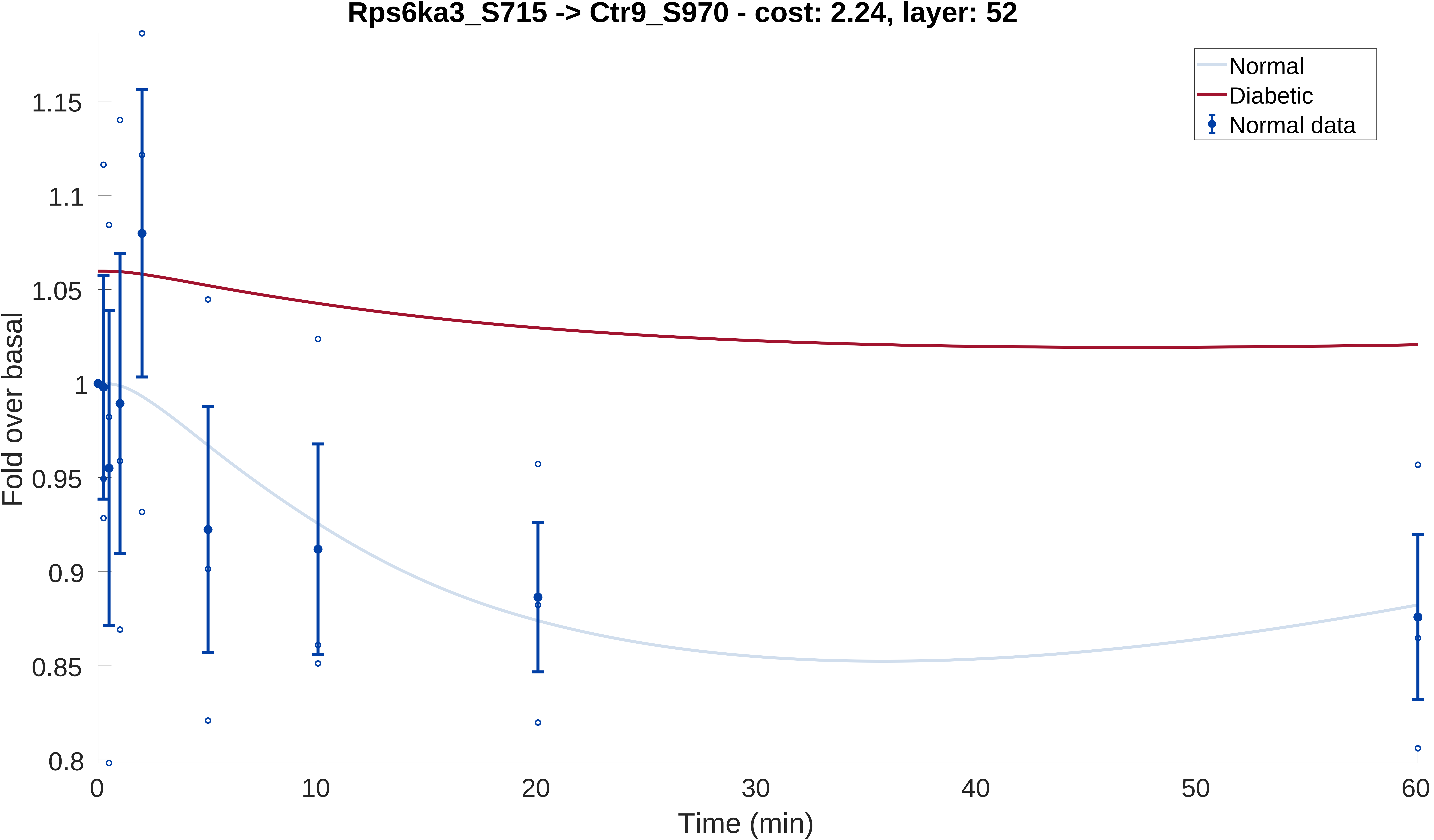

Rps6ka3_S715 -> Ctr9_S970 - cost: 2.24, layer: 52
Normal
Diabetic
Normal data
Fold over basal
1.15
1.1
1.05
1
0.95
0.9
0.85
0.8
0
10
20
30
40
50
60
Time (min)

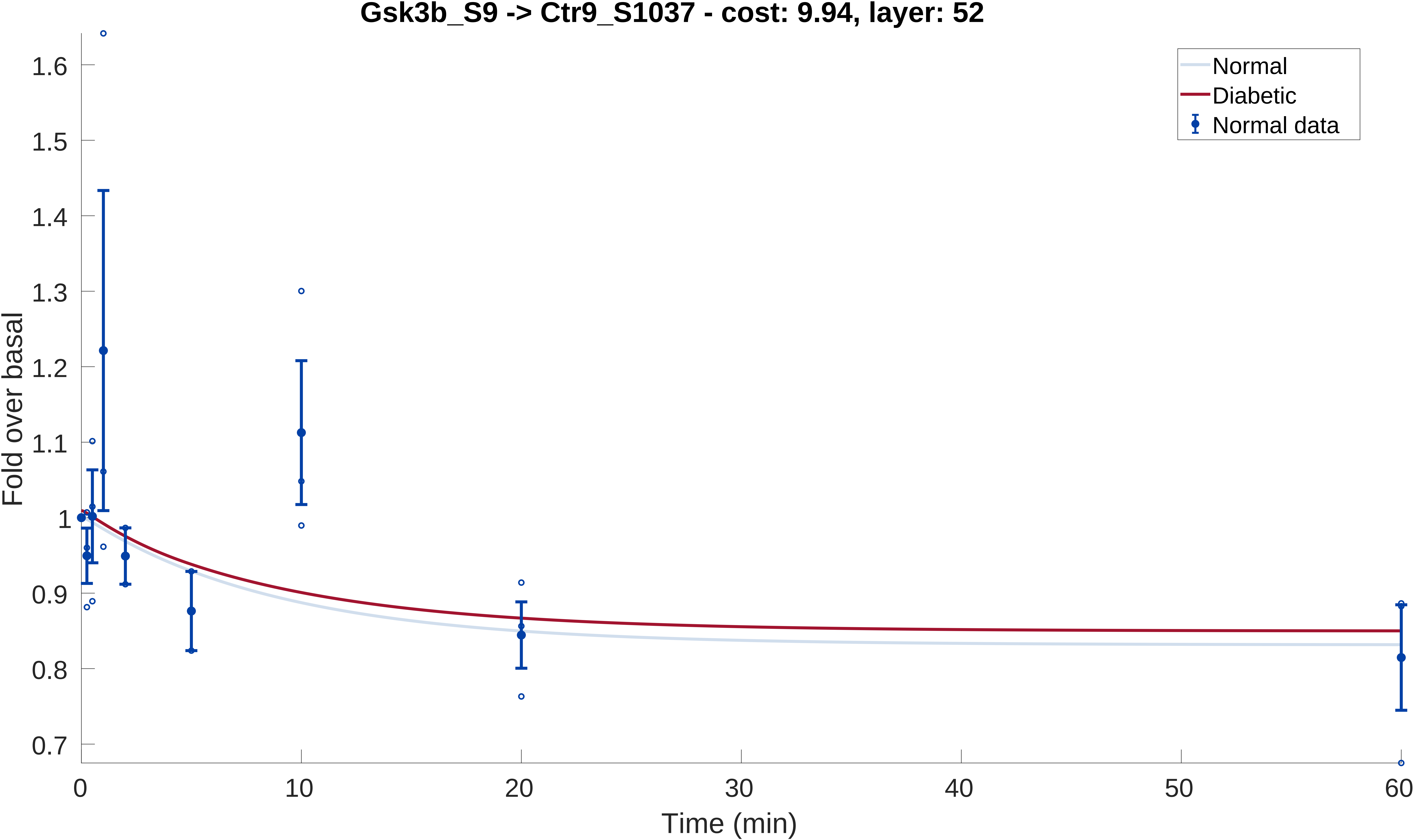

Gsk3b_S9 -> Ctr9_S1037 - cost: 9.94, layer: 52
Normal
Diabetic
Normal data
Fold over basal
Time (min)
1.6
1.5
1.4
1.3
1.2
1.1
1
0.9
0.8
0.7
0
10
20
30
40
50
60

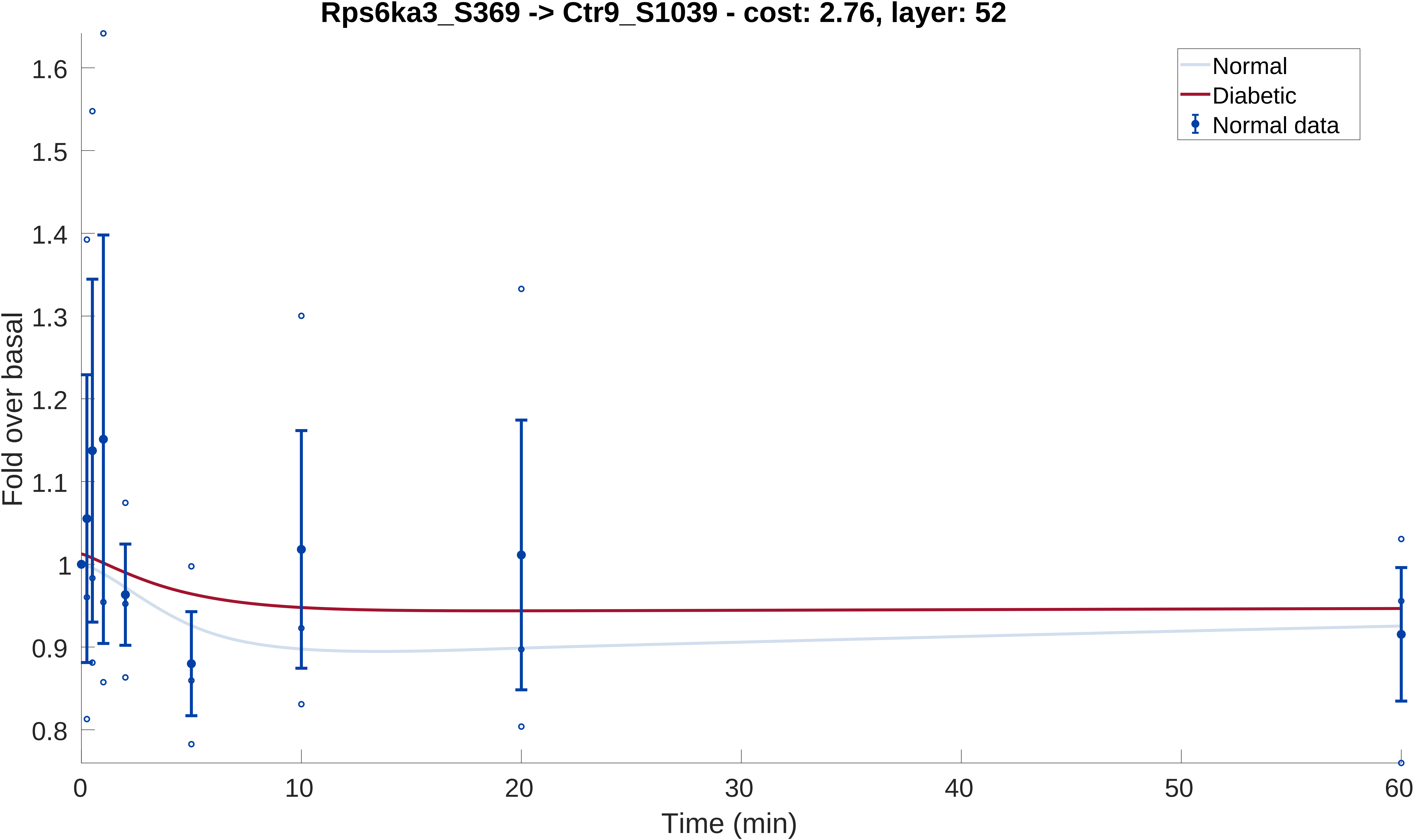

Rps6ka3_S369 -> Ctr9_S1039 - cost: 2.76, layer: 52
Fold over basal
1.6
1.5
1.4
1.3
1.2
1.1
1
0.9
0.8
0
10
20
30
40
50
60
Time (min)
Normal
Diabetic
Normal data

Rps6ka3_S375 -> Ctr9_S1041 - cost: 3.82, layer: 52

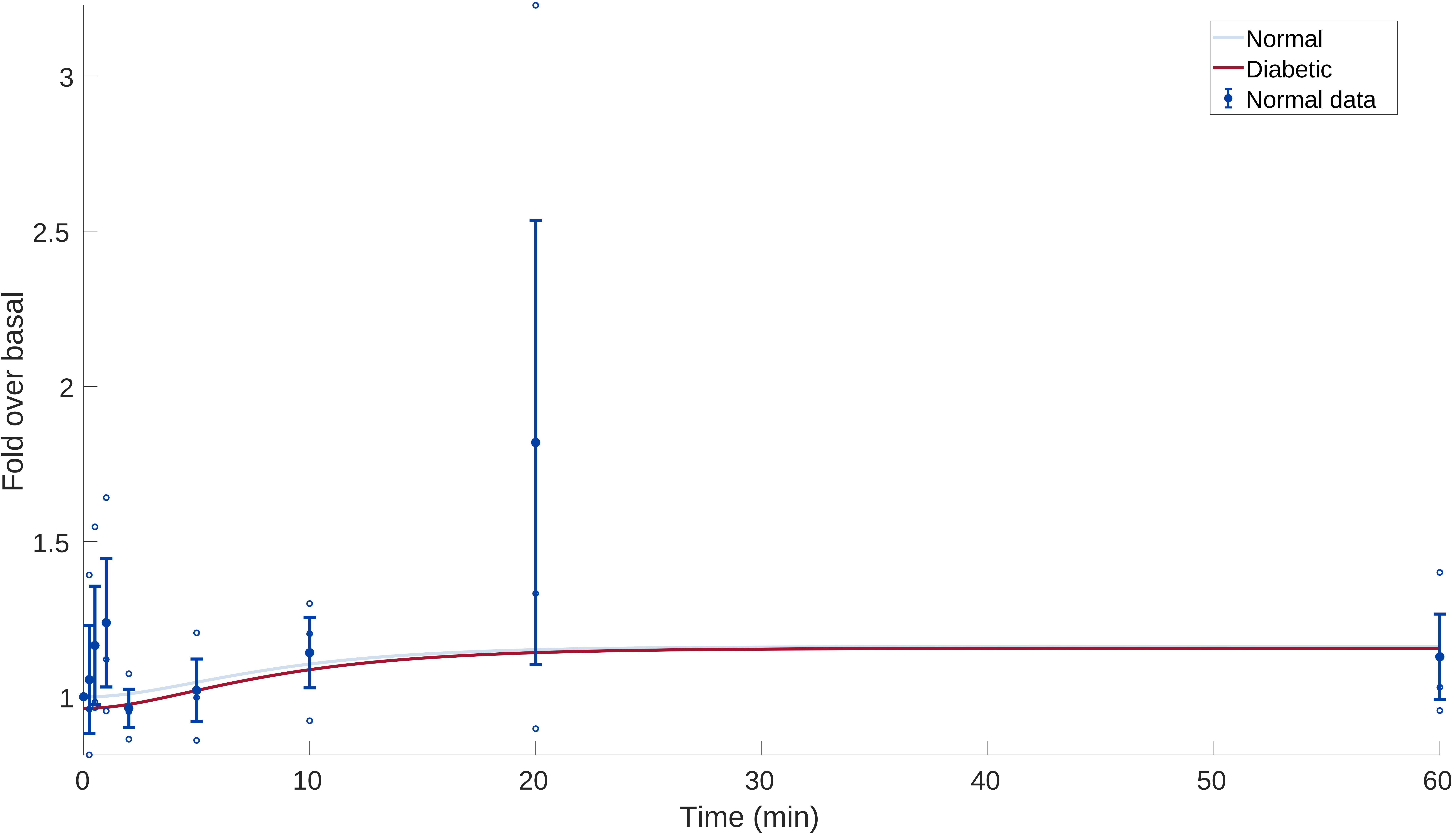

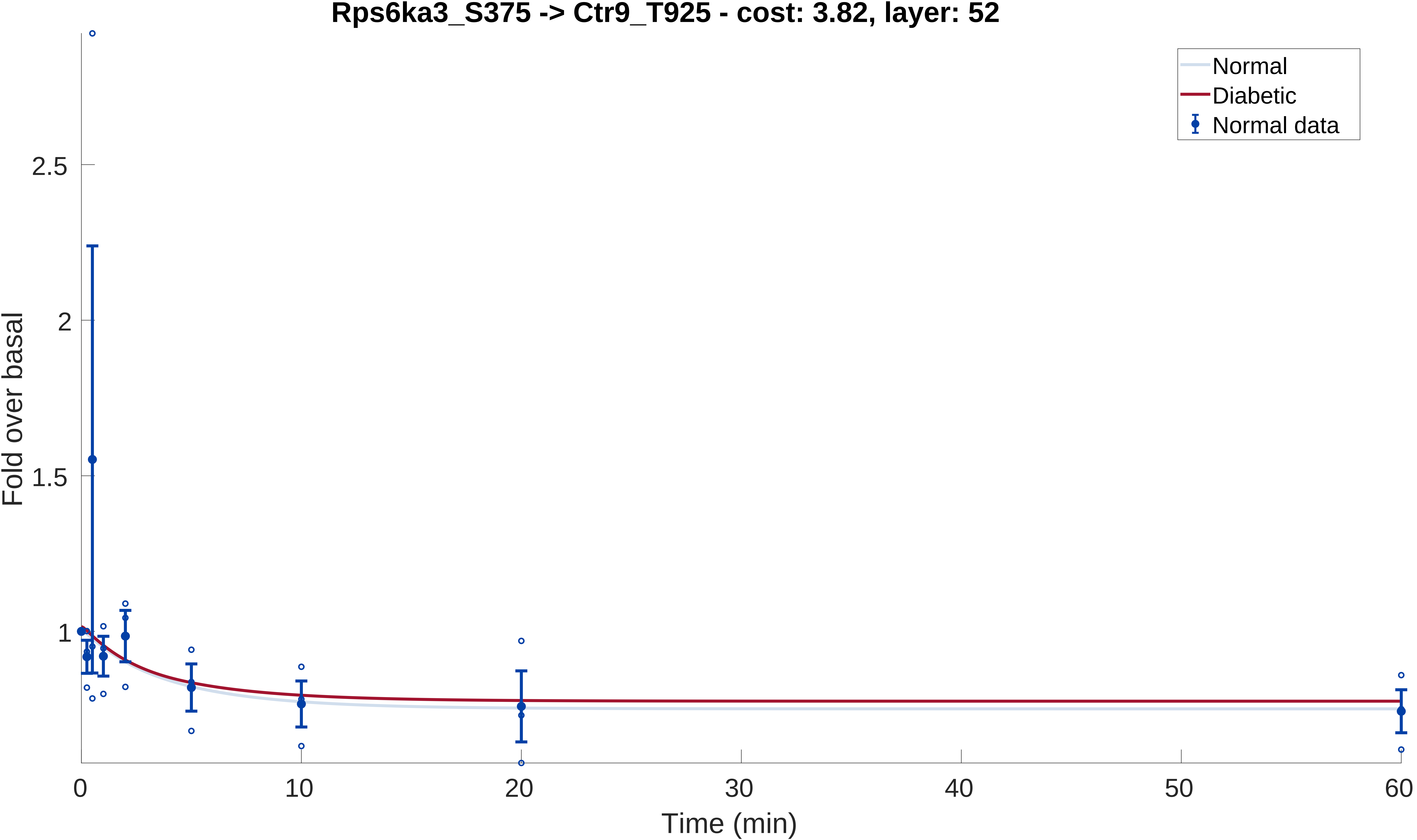

Rps6ka3_S375 -> Ctr9_T925 - cost: 3.82, layer: 52
Normal
Diabetic
Normal data
Fold over basal
2.5
2
1.5
1
0
10
20
30
40
50
60
Time (min)

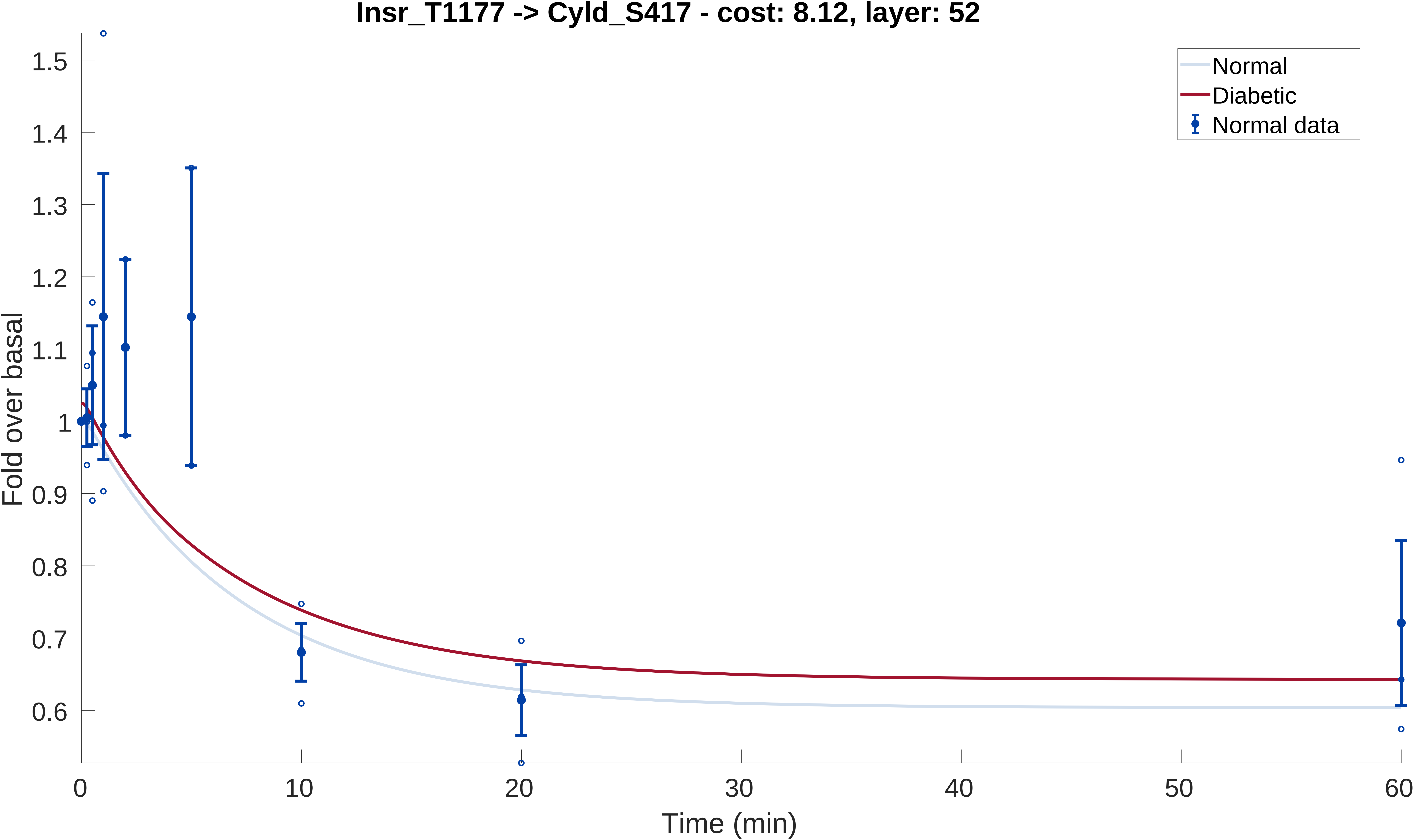

Insr_T1177 -> Cyld_S417 - cost: 8.12, layer: 52
Normal
Diabetic
Normal data
Fold over basal
1.5
1.4
1.3
1.2
1.1
1
0.9
0.8
0.7
0.6
0
10
20
30
40
50
60
Time (min)

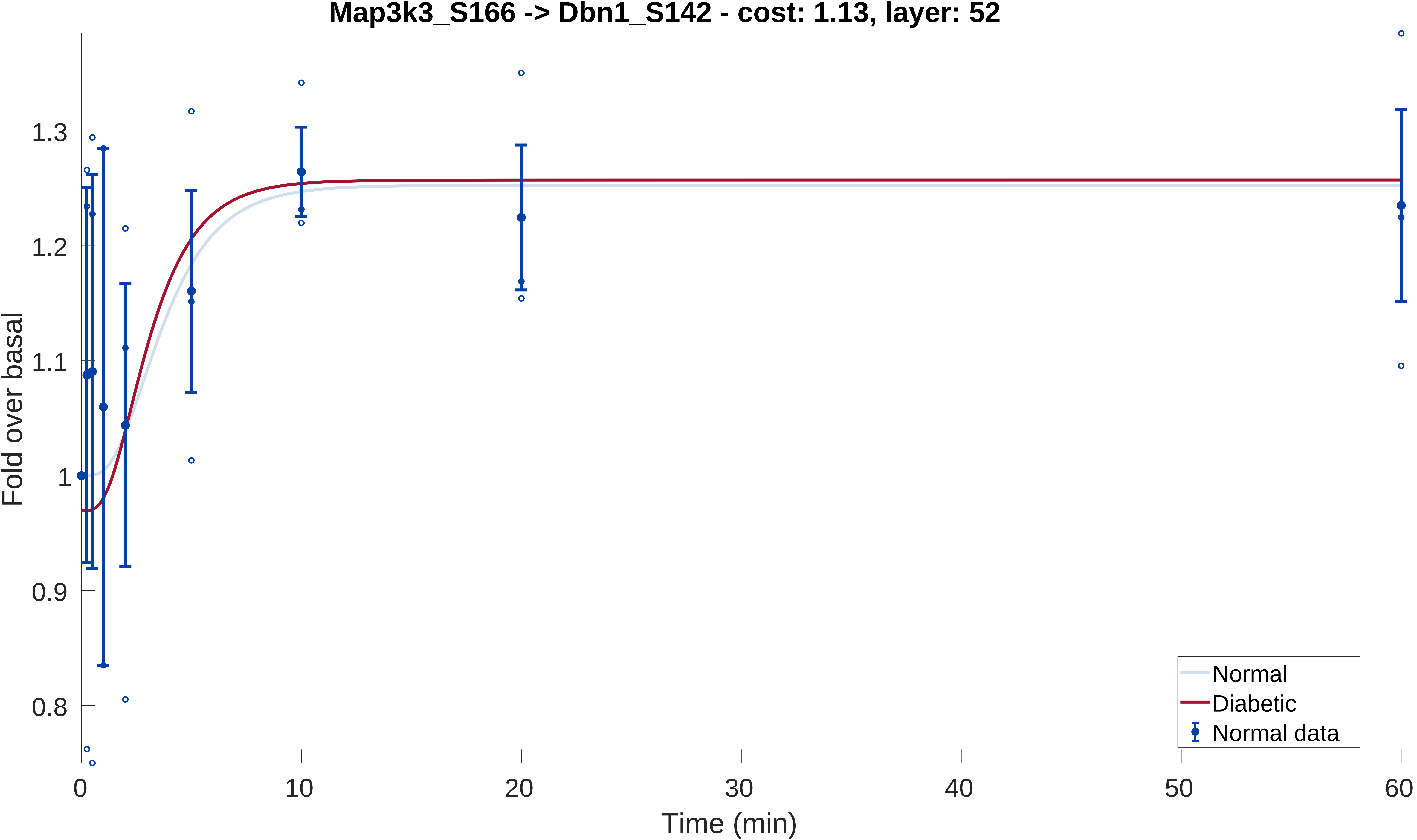

Map3k3_S166 -> Dbn1_S142 - cost: 1.13, layer: 52
Fold over basal
Time (min)
0.8
0.9
1
1.1
1.2
1.3
0
10
20
30
40
50
60
Normal
Diabetic
Normal data

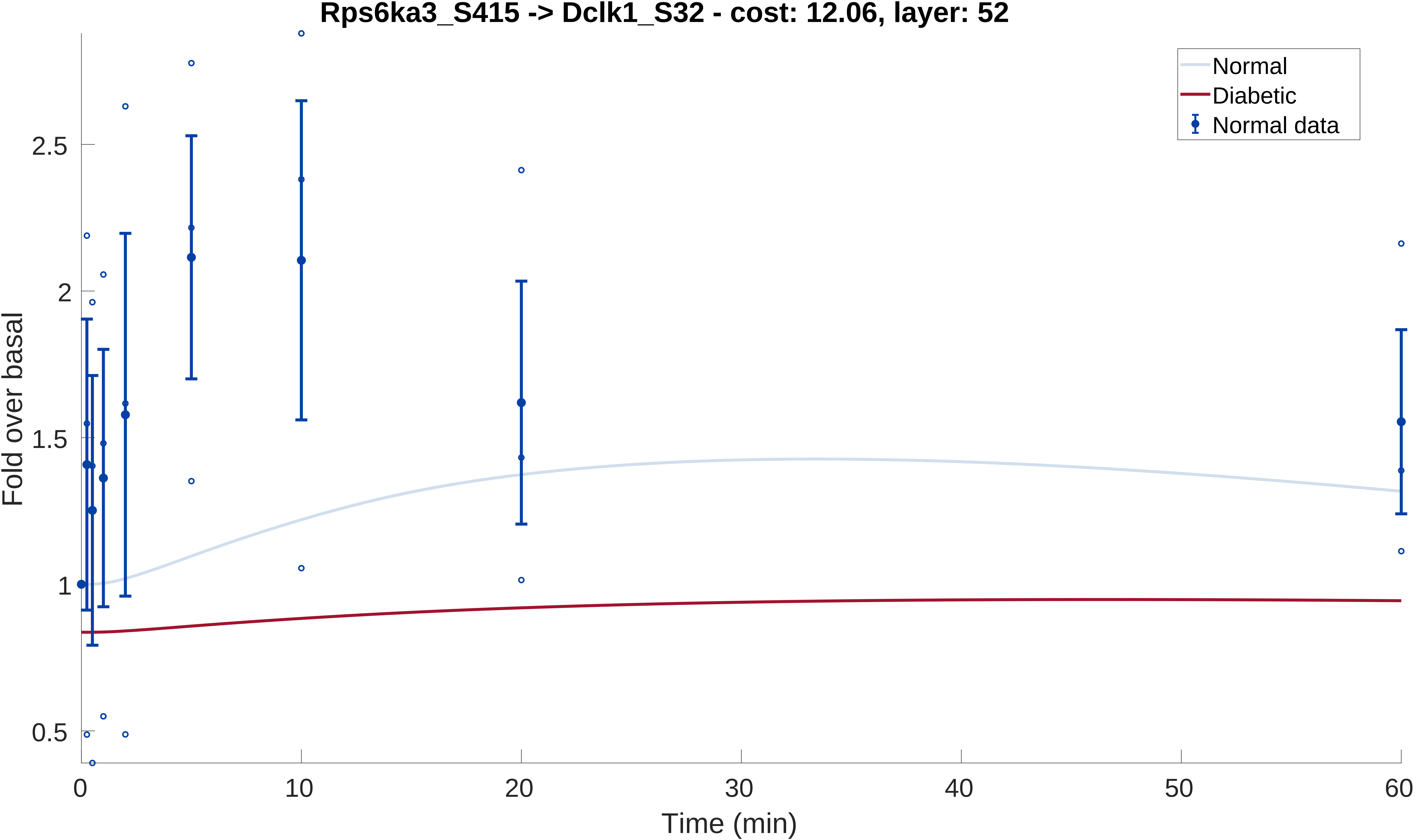
Rps6ka3_S415 -> Dclk1_S32 - cost: 12.06, layer: 52
Normal
Diabetic
Normal data
Fold over basal
2.5
2
1.5
1
0.5
0
10
20
30
40
50
60
Time (min)

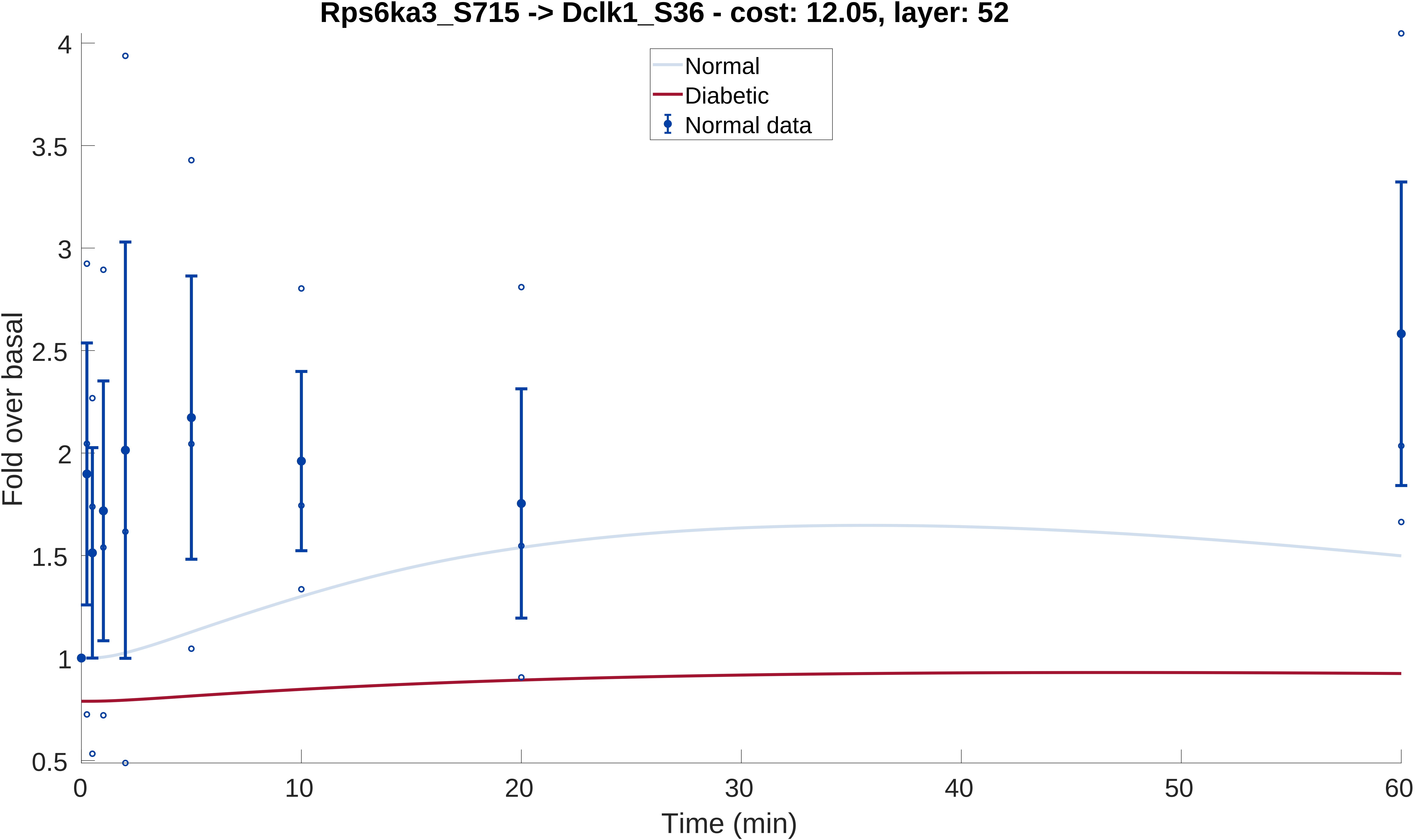

Rps6ka3_S715 -> Dclk1_S36 - cost: 12.05, layer: 52
Normal
Diabetic
Normal data
Fold over basal
Time (min)
0.5
1
1.5
2
2.5
3
3.5
4
0
10
20
30
40
50
60

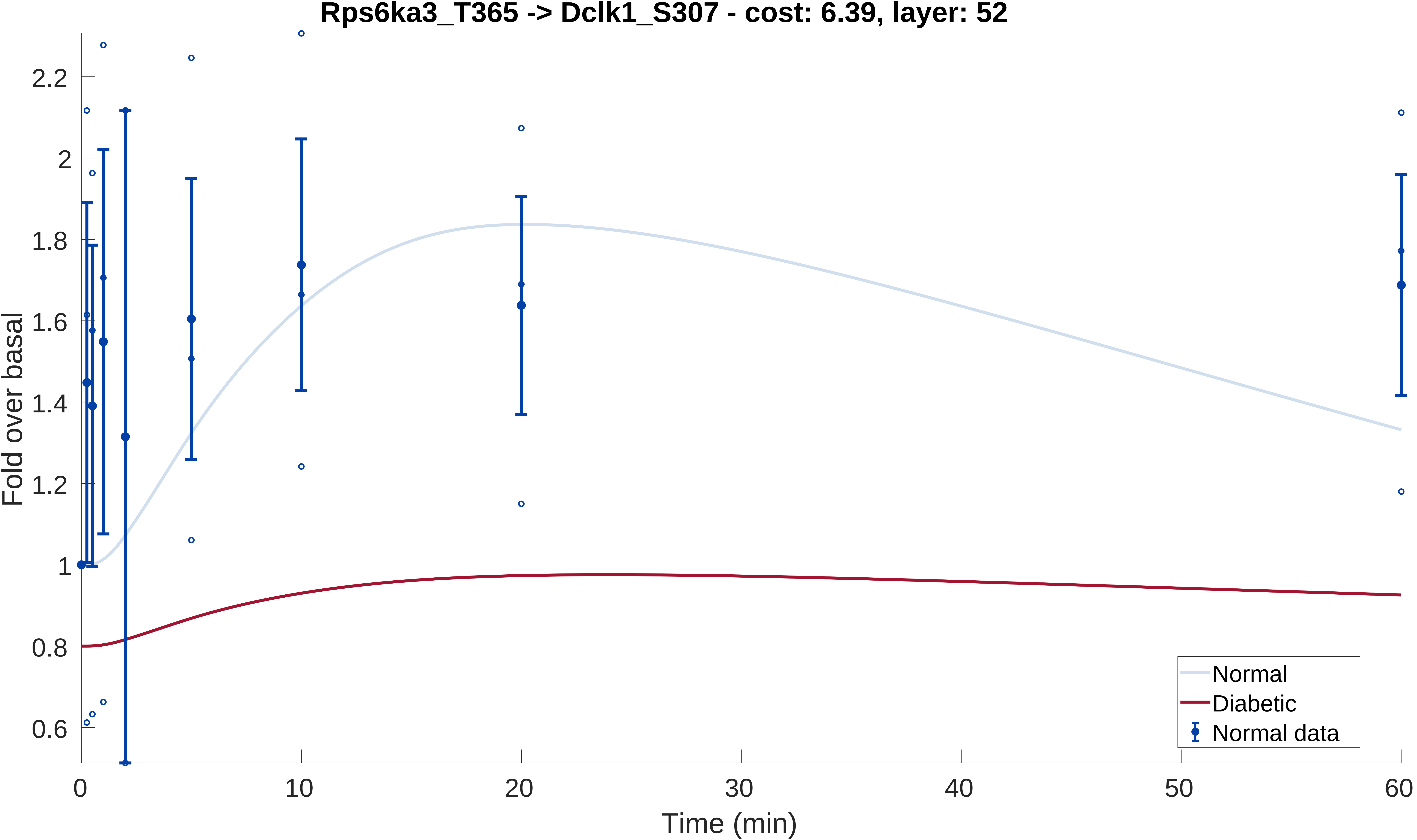
Rps6ka3_T365 -> Dclk1_S307 - cost: 6.39, layer: 52
Fold over basal
2.2
2
1.8
1.6
1.4
1.2
1
0.8
0.6
0
10
20
30
40
50
60
Time (min)
Normal
Diabetic
Normal data

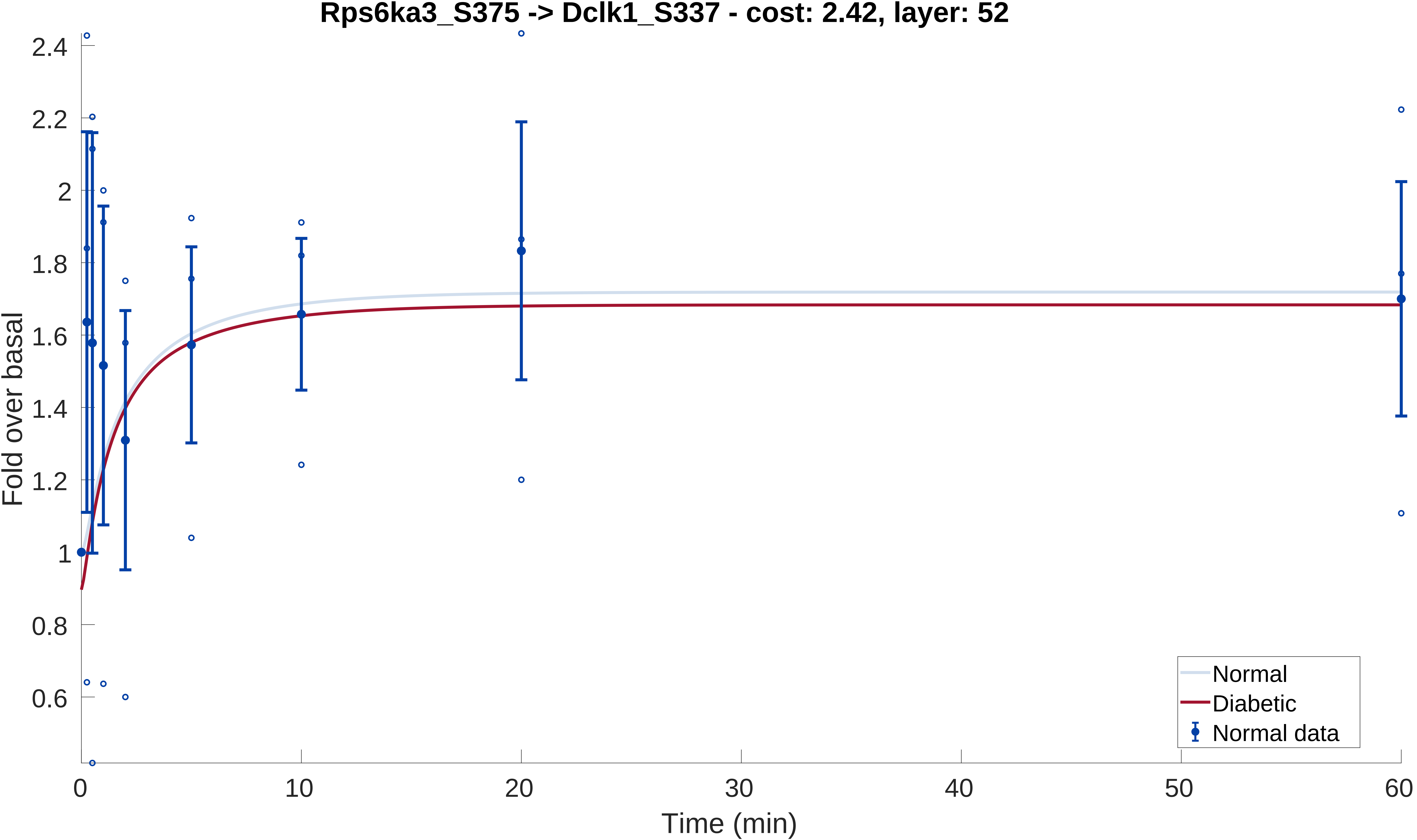

Rps6ka3_S375 -> Dclk1_S337 - cost: 2.42, layer: 52
Fold over basal
2.4
2.2
2
1.8
1.6
1.4
1.2
1
0.8
0.6
0
10
20
30
40
50
60
Time (min)
Normal
Diabetic
Normal data

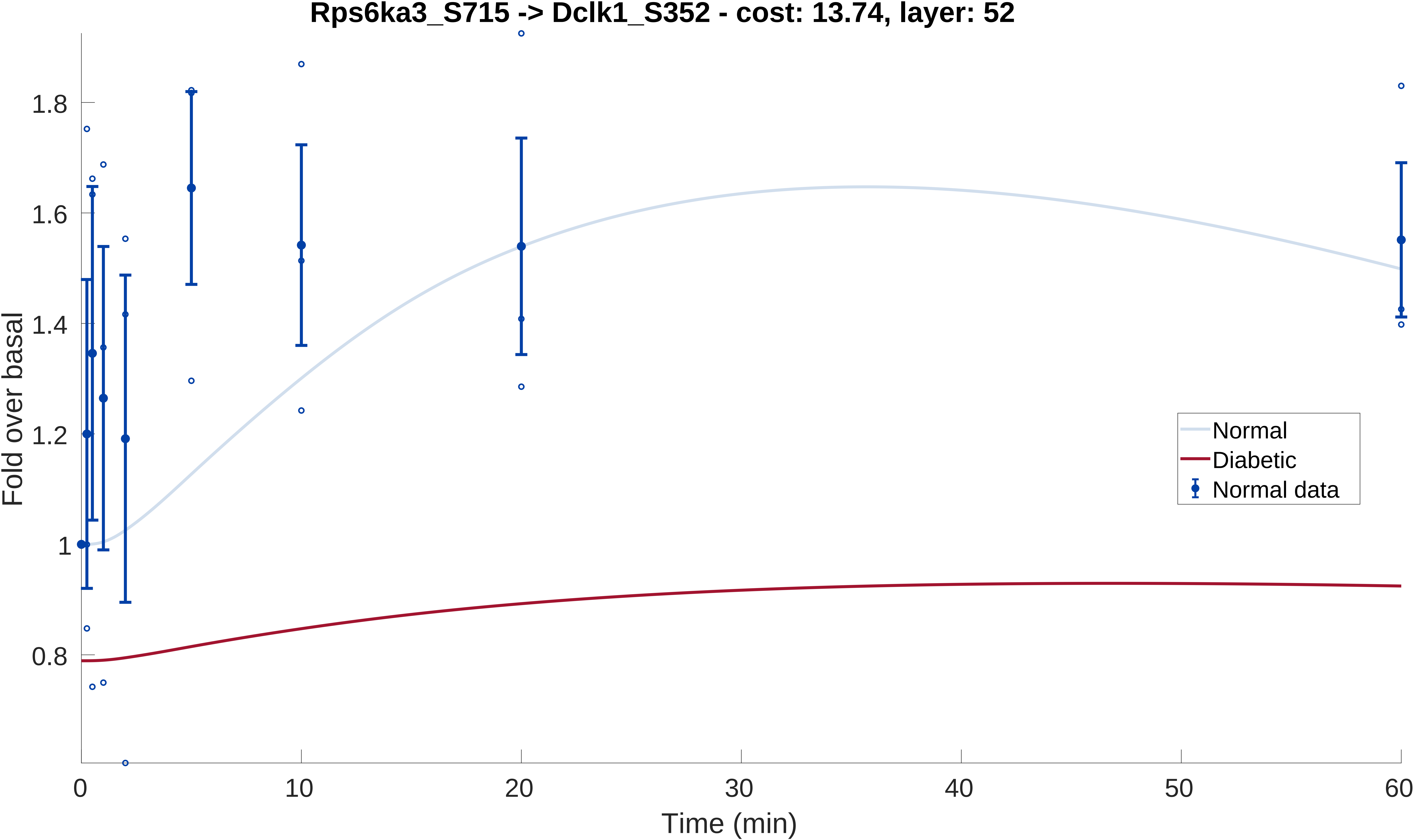

Rps6ka3_S715 -> Dclk1_S352 - cost: 13.74, layer: 52
Fold over basal
1.8
1.6
1.4
1.2
1
0.8
0
10
20
30
40
50
60
Time (min)
Normal
Diabetic
Normal data

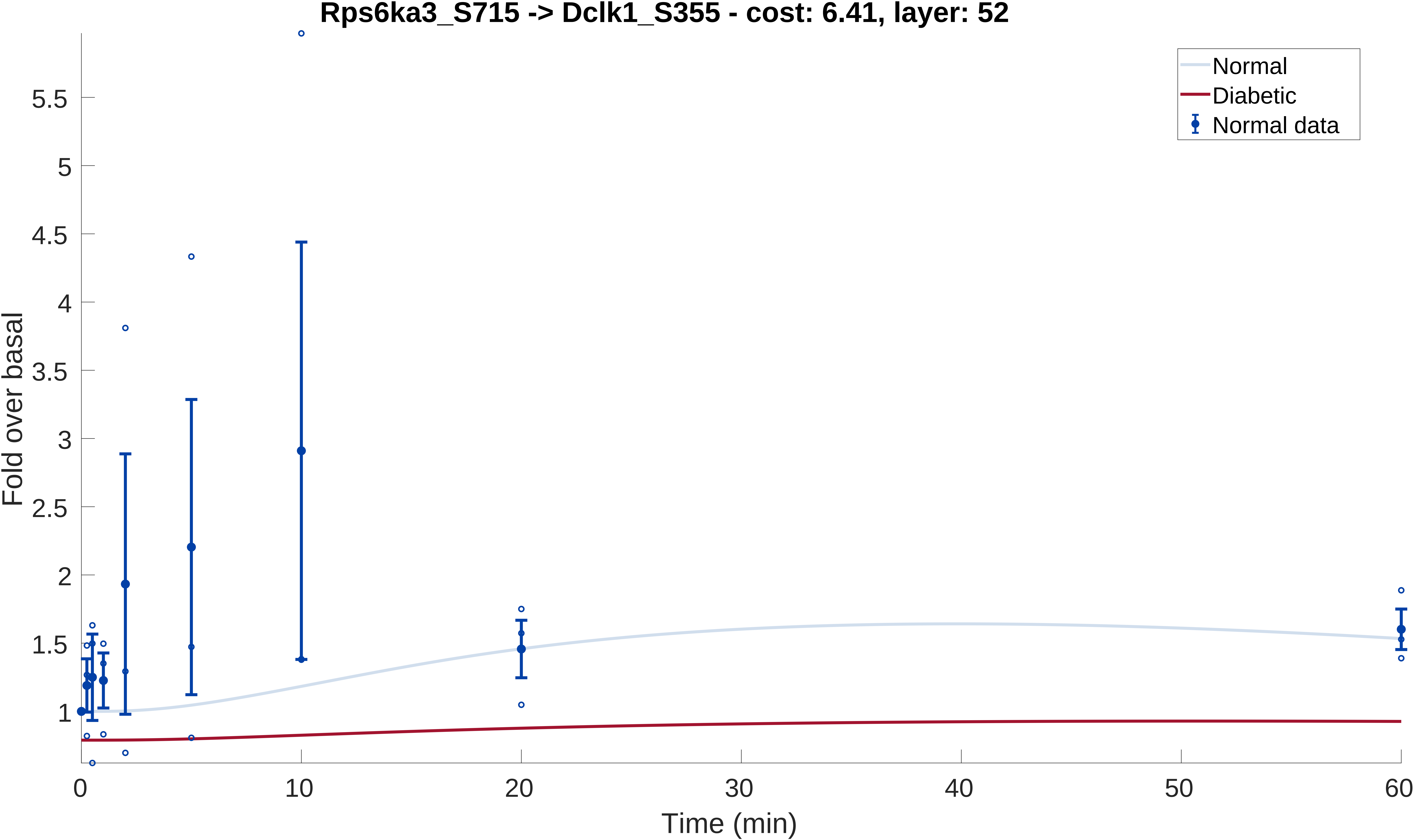

Rps6ka3_S715 -> Dclk1_S355 - cost: 6.41, layer: 52
Normal
Diabetic
Normal data
Fold over basal
5.5
5
4.5
4
3.5
3
2.5
2
1.5
1
0
10
20
30
40
50
60
Time (min)

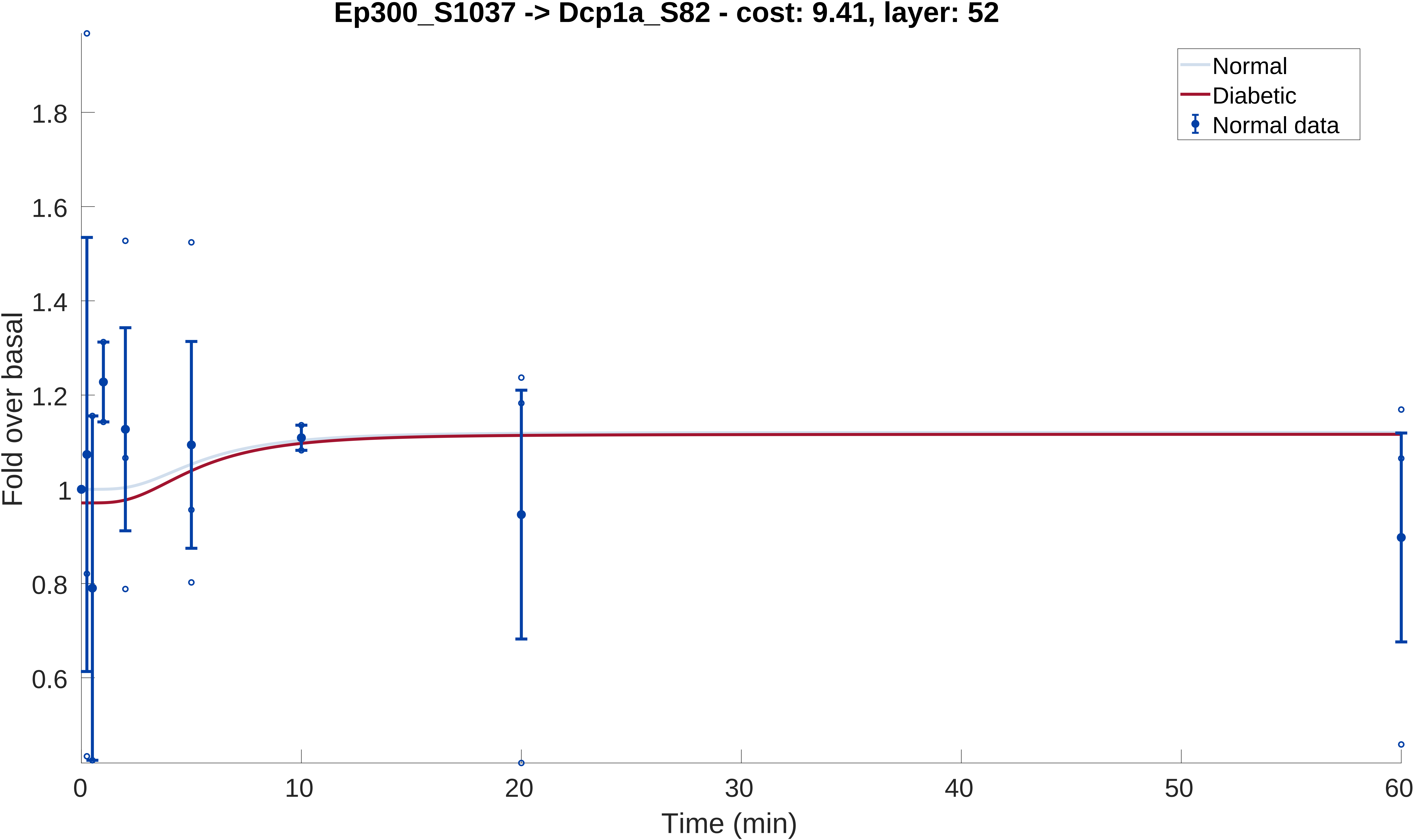
Ep300_S1037 -> Dcp1a_S82 - cost: 9.41, layer: 52
Normal
Diabetic
Normal data
Fold over basal
Time (min)
0.6
0.8
1
1.2
1.4
1.6
1.8
0
10
20
30
40
50
60

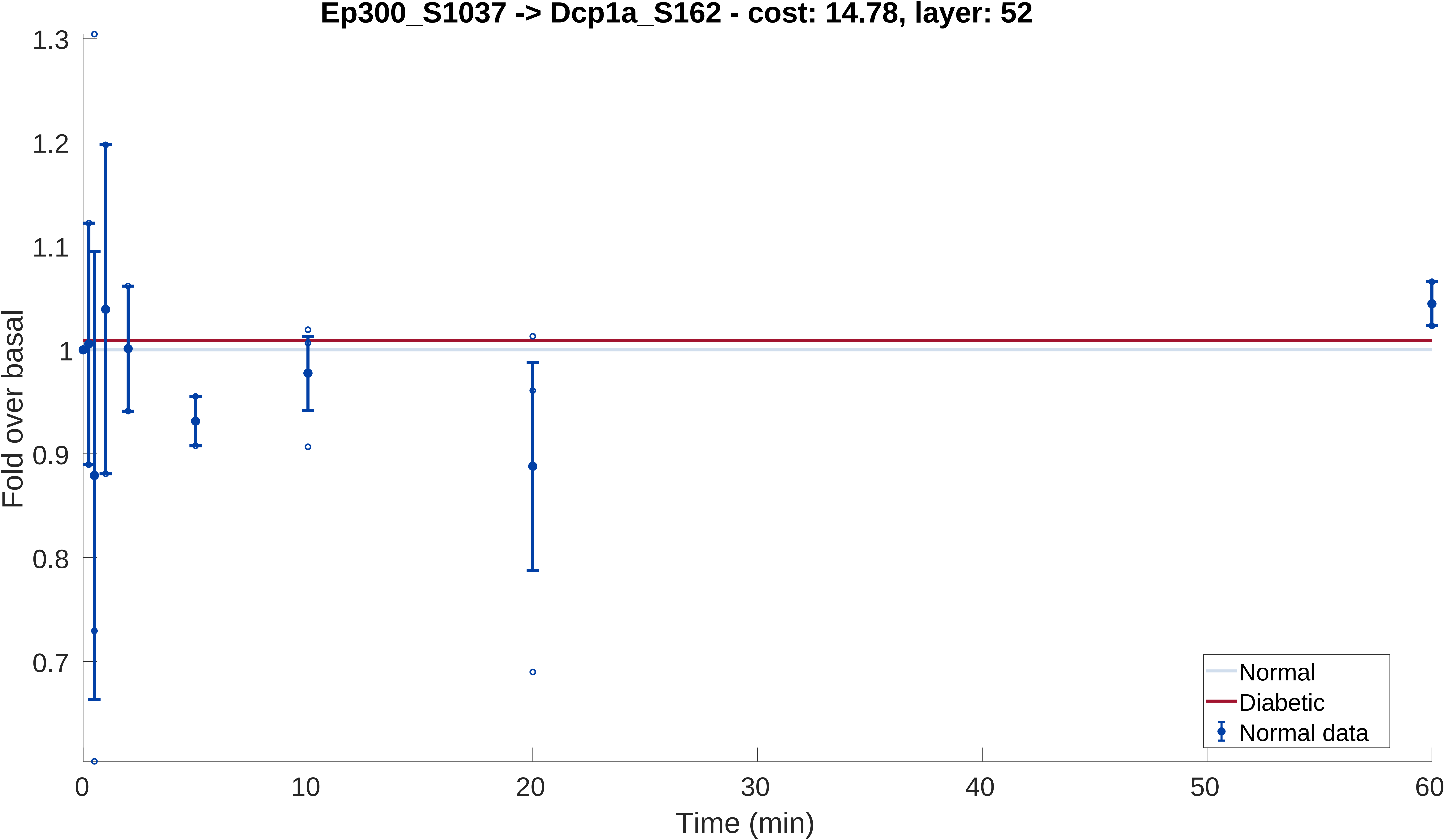

Ep300_S1037 -> Dcp1a_S162 - cost: 14.78, layer: 52
Fold over basal
1.3
1.2
1.1
1
0.9
0.8
0.7
0
10
20
30
40
50
60
Time (min)
Normal
Diabetic
Normal data

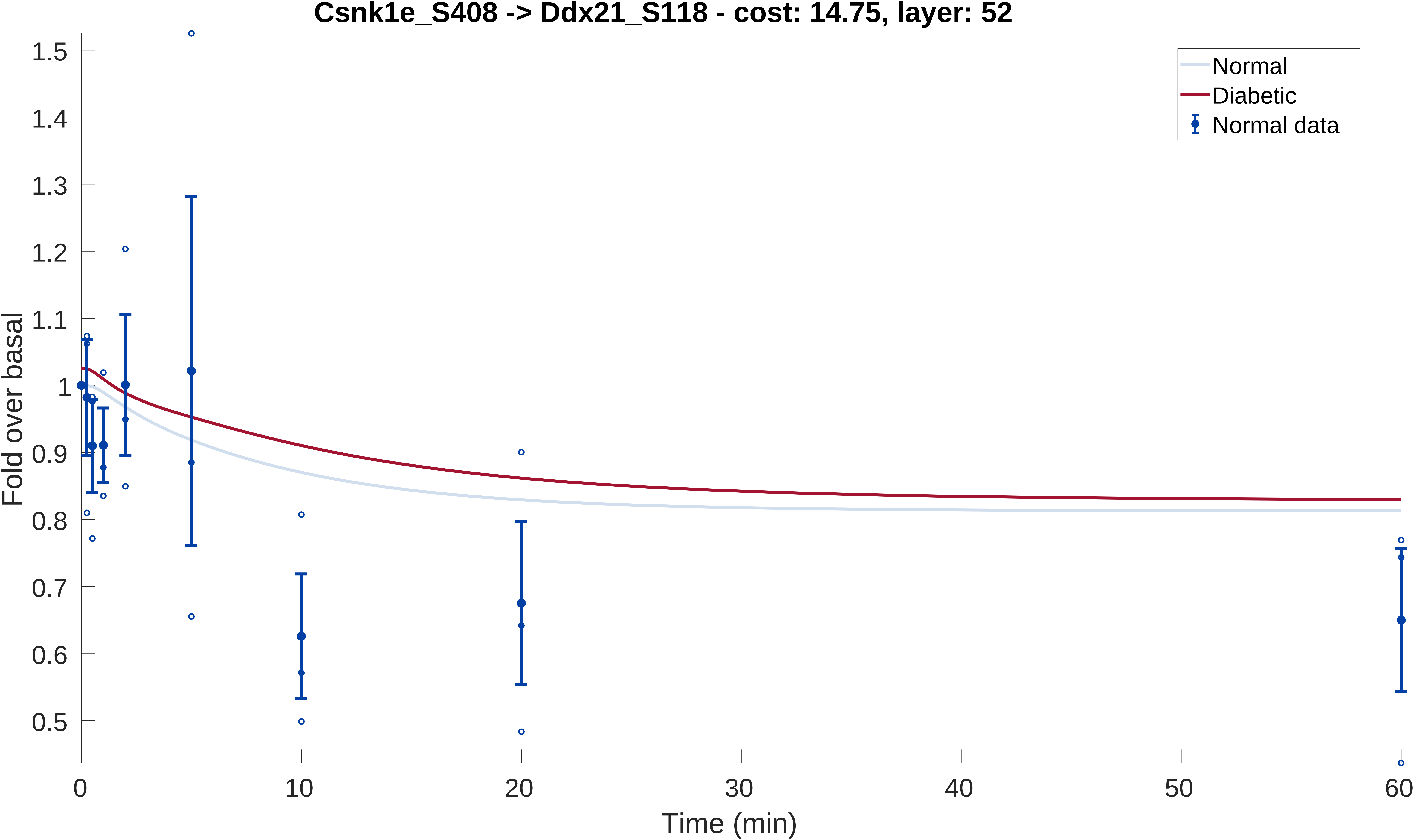
Csnk1e_S408 -> Ddx21_S118 - cost: 14.75, layer: 52
Normal
Diabetic
Normal data
Fold over basal
1.5
1.4
1.3
1.2
1.1
1
0.9
0.8
0.7
0.6
0.5
0
10
20
30
40
50
60
Time (min)

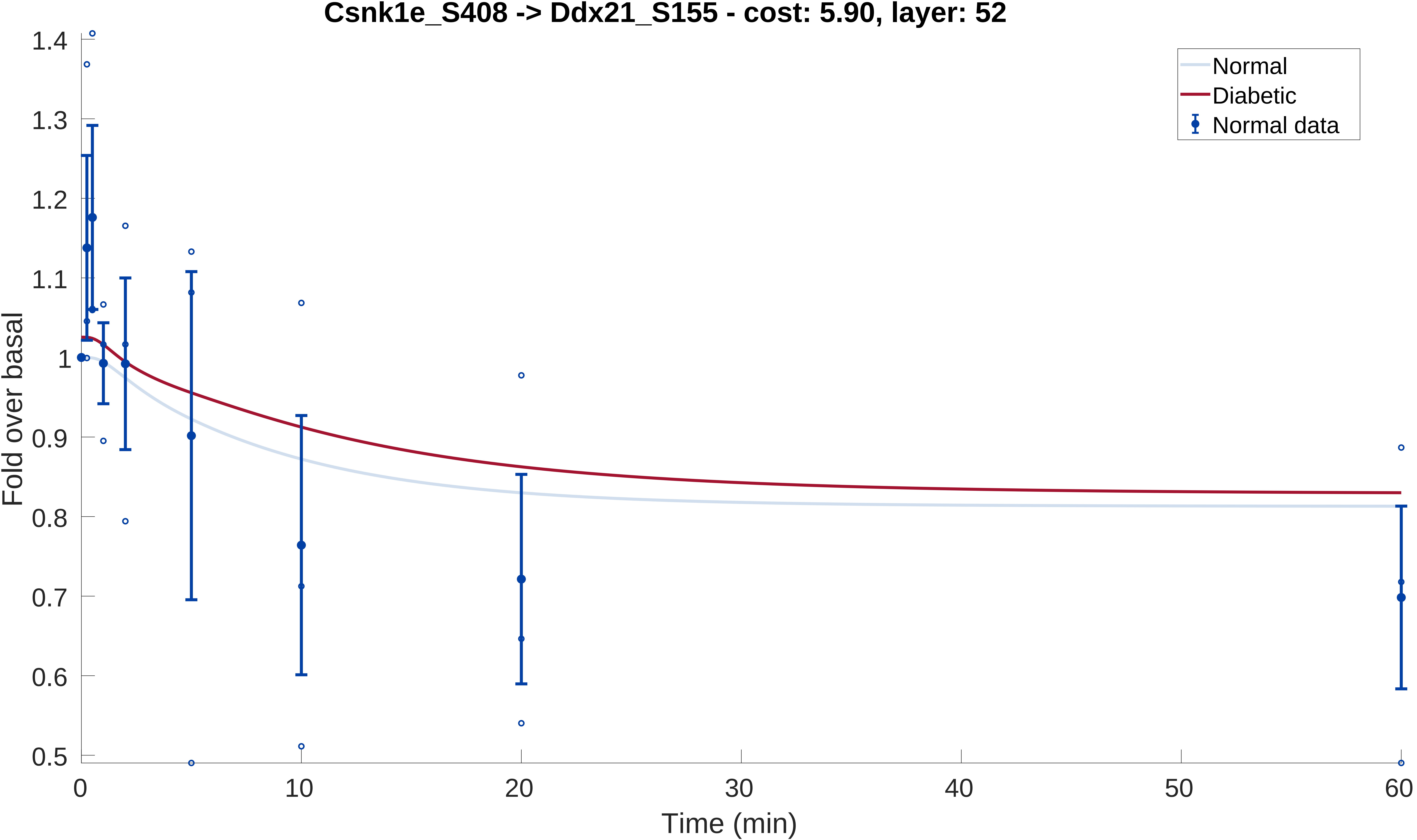

Csnk1e_S408 -> Ddx21_S155 - cost: 5.90, layer: 52
Normal
Diabetic
Normal data
Fold over basal
Time (min)
0.5
0.6
0.7
0.8
0.9
1
1.1
1.2
1.3
1.4
0
10
20
30
40
50
60

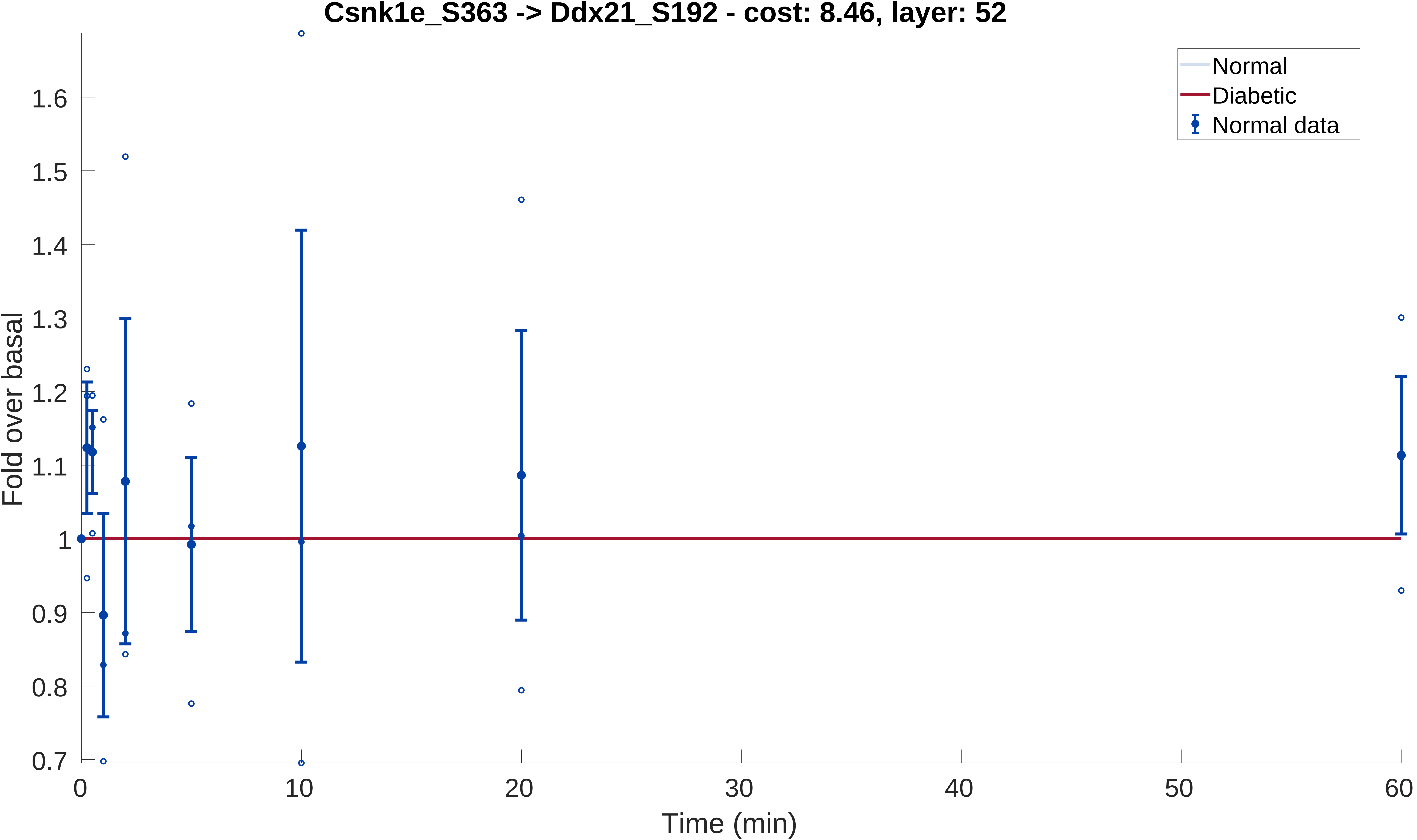

Csnk1e_S363 -> Ddx21_S192 - cost: 8.46, layer: 52
Normal
Diabetic
Normal data
Fold over basal
1.6
1.5
1.4
1.3
1.2
1.1
1
0.9
0.8
0.7
0
10
20
30
40
50
60
Time (min)

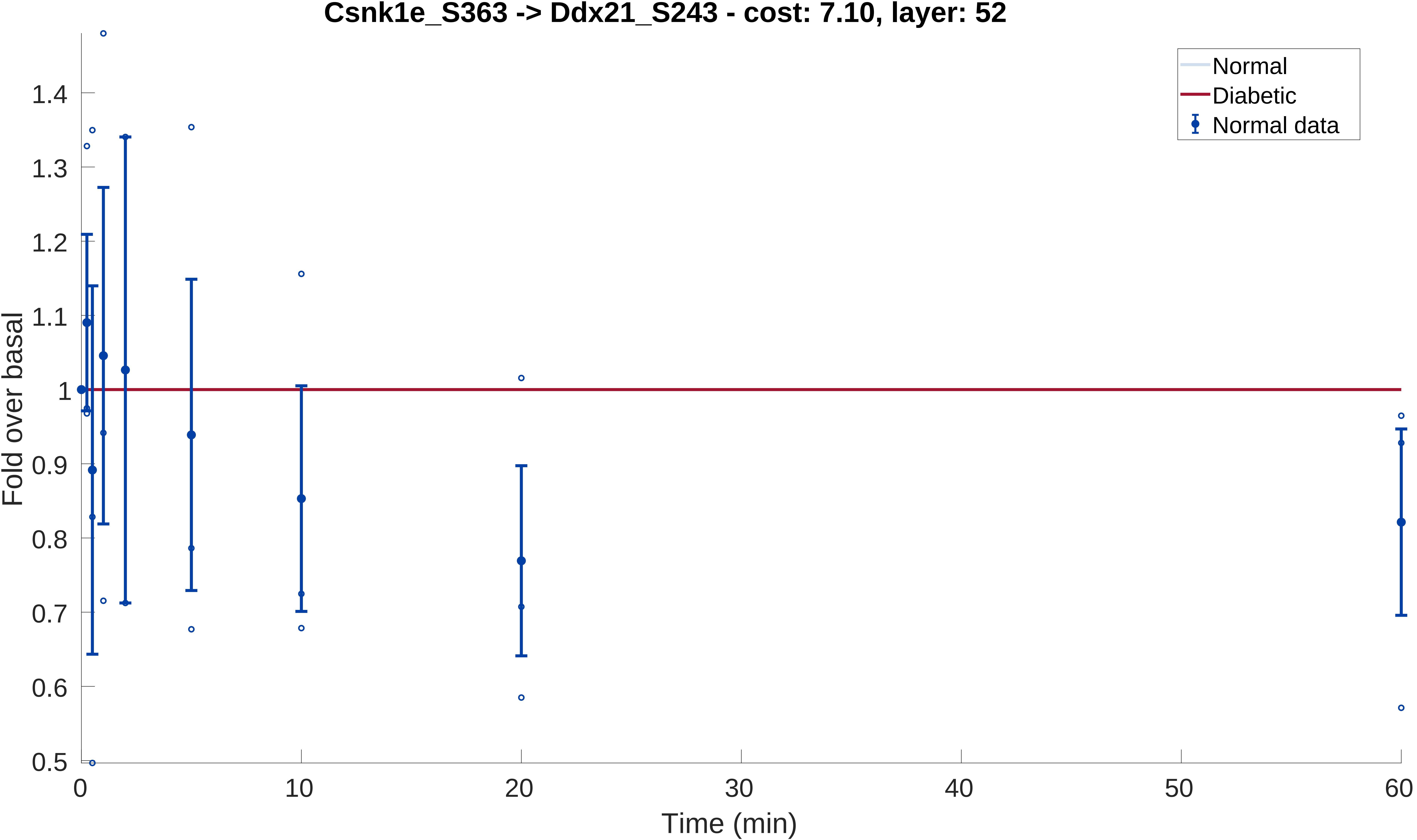

Csnk1e_S363 -> Ddx21_S243 - cost: 7.10, layer: 52
Normal
Diabetic
Normal data
Fold over basal
1.4
1.3
1.2
1.1
1
0.9
0.8
0.7
0.6
0.5
0
10
20
30
40
50
60
Time (min)

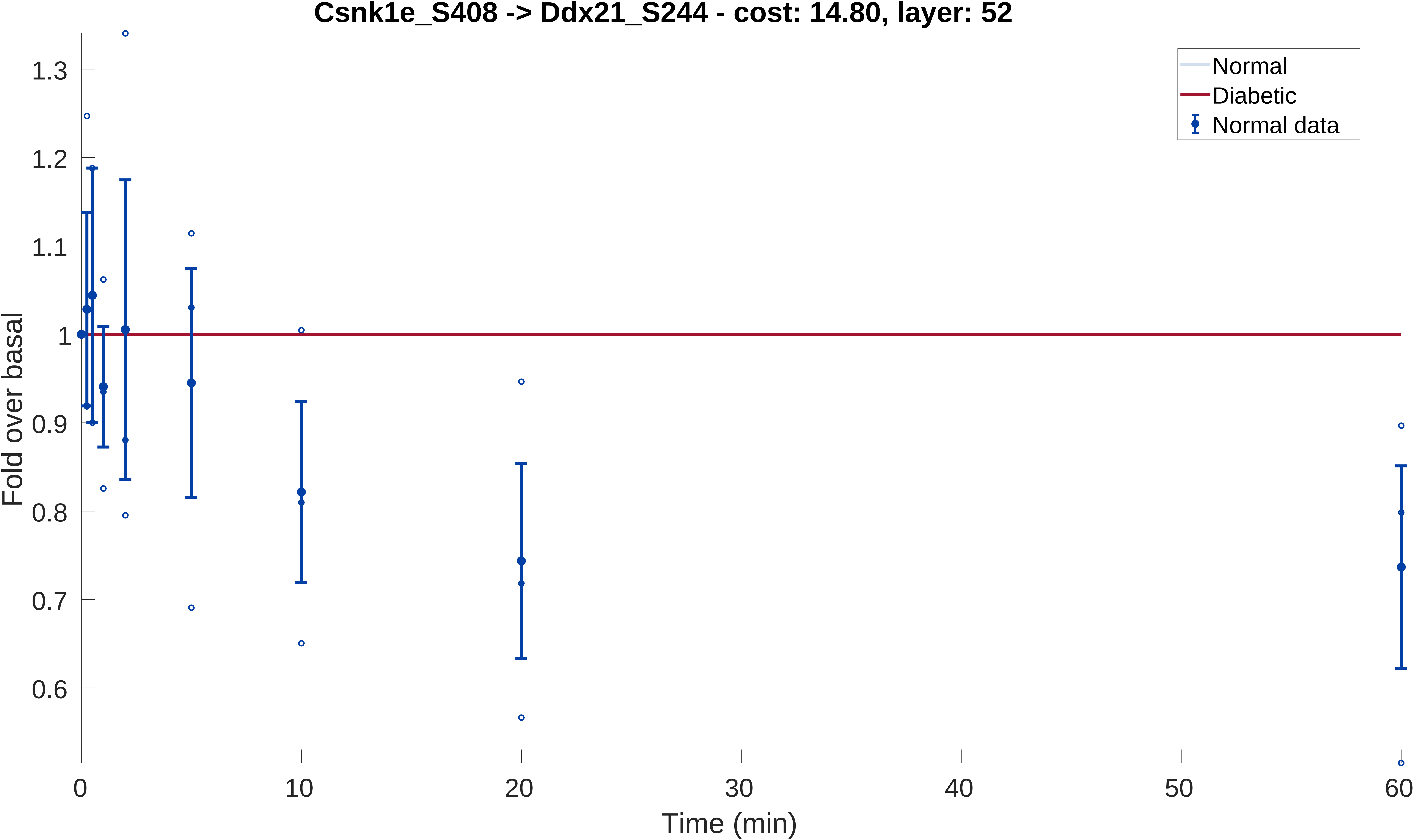

Csnk1e_S408 -> Ddx21_S244 - cost: 14.80, layer: 52
Normal
Diabetic
Normal data
Fold over basal
1.3
1.2
1.1
1
0.9
0.8
0.7
0.6
0
10
20
30
40
50
60
Time (min)

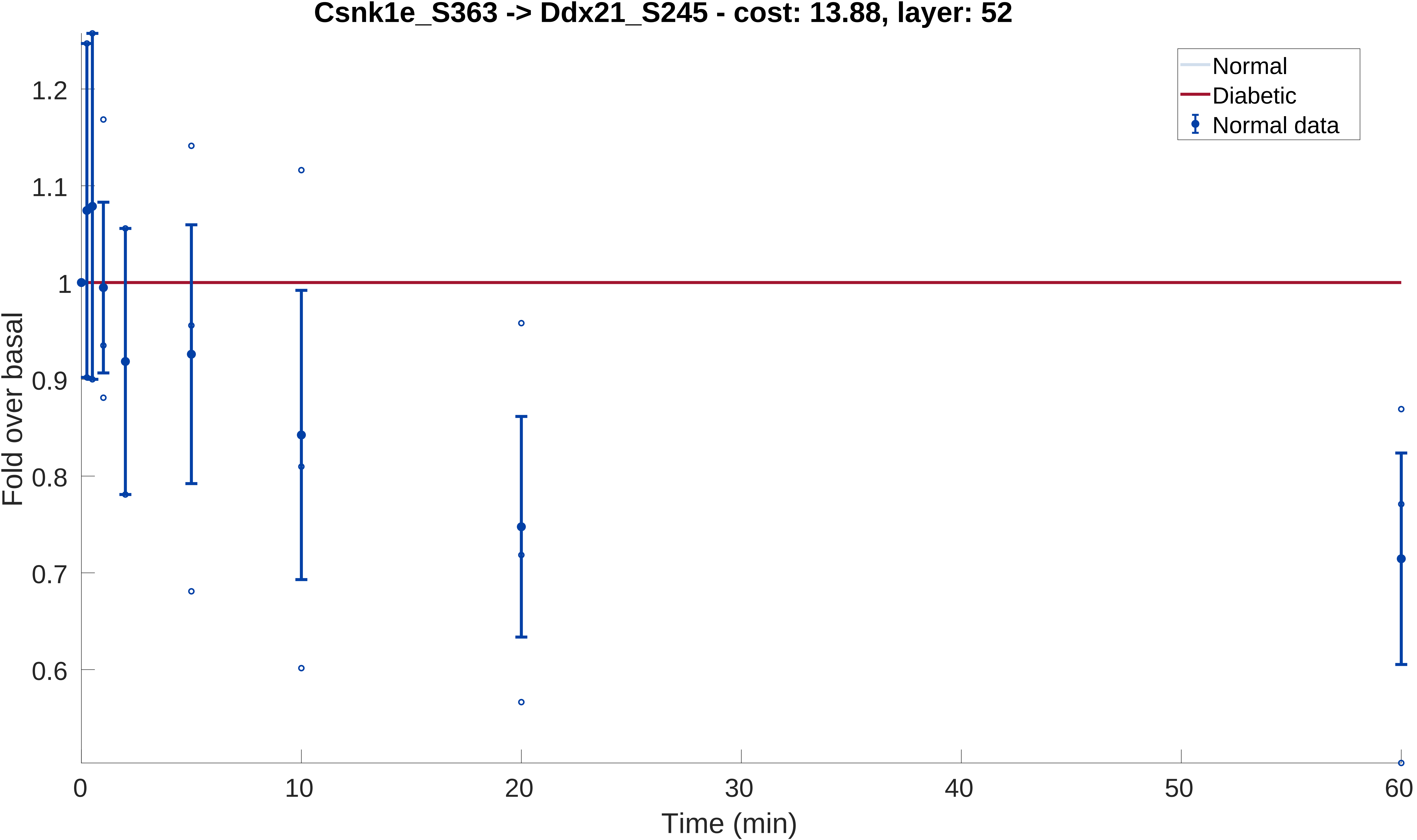

Csnk1e_S363 -> Ddx21_S245 - cost: 13.88, layer: 52
Normal
Diabetic
Normal data
Fold over basal
Time (min)
1.2
1.1
1
0.9
0.8
0.7
0.6
0
10
20
30
40
50
60

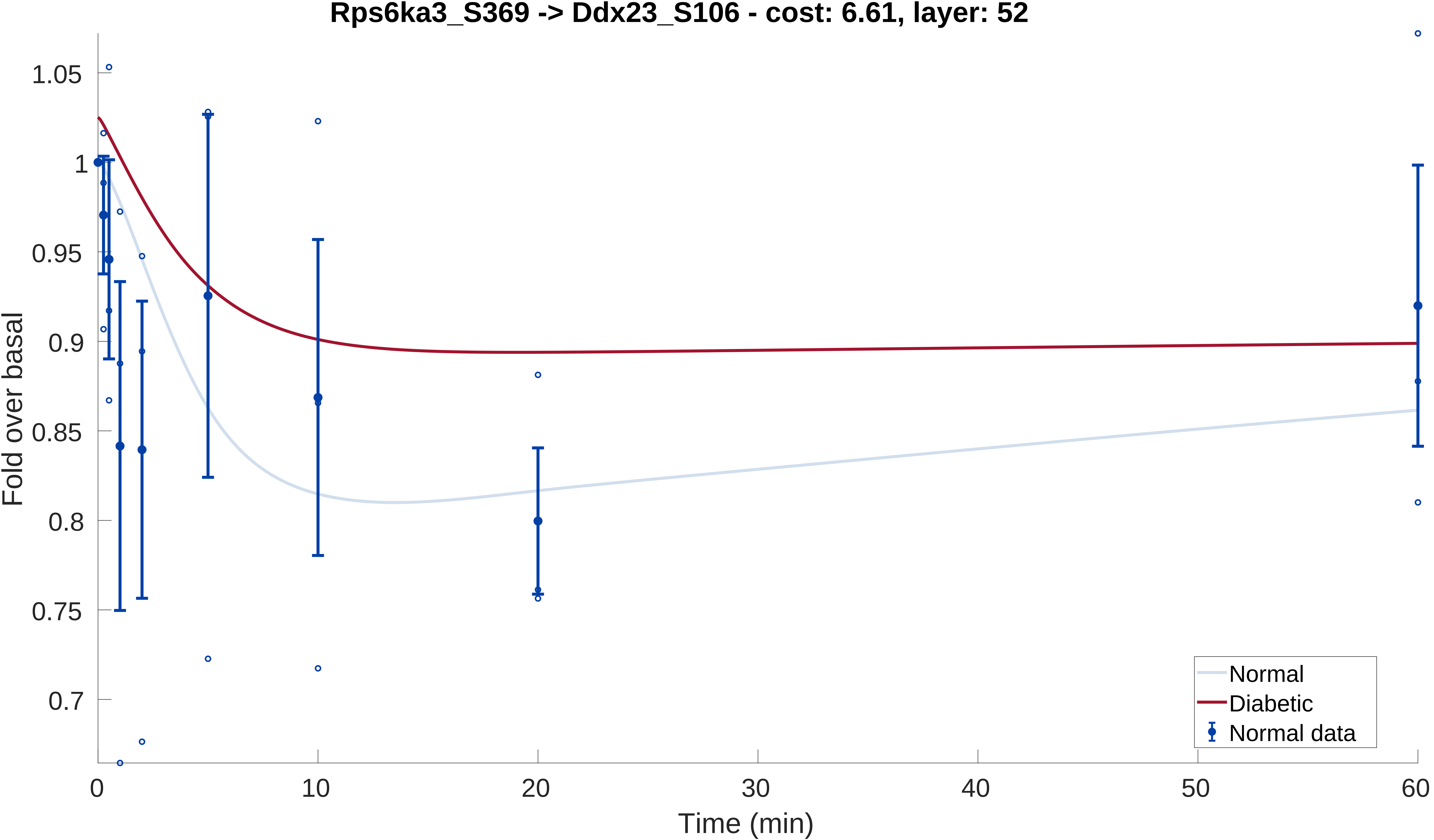
Rps6ka3_S369 -> Ddx23_S106 - cost: 6.61, layer: 52
Fold over basal
Time (min)
1.05
1
0.95
0.9
0.85
0.8
0.75
0.7
0
10
20
30
40
50
60
Normal
Diabetic
Normal data

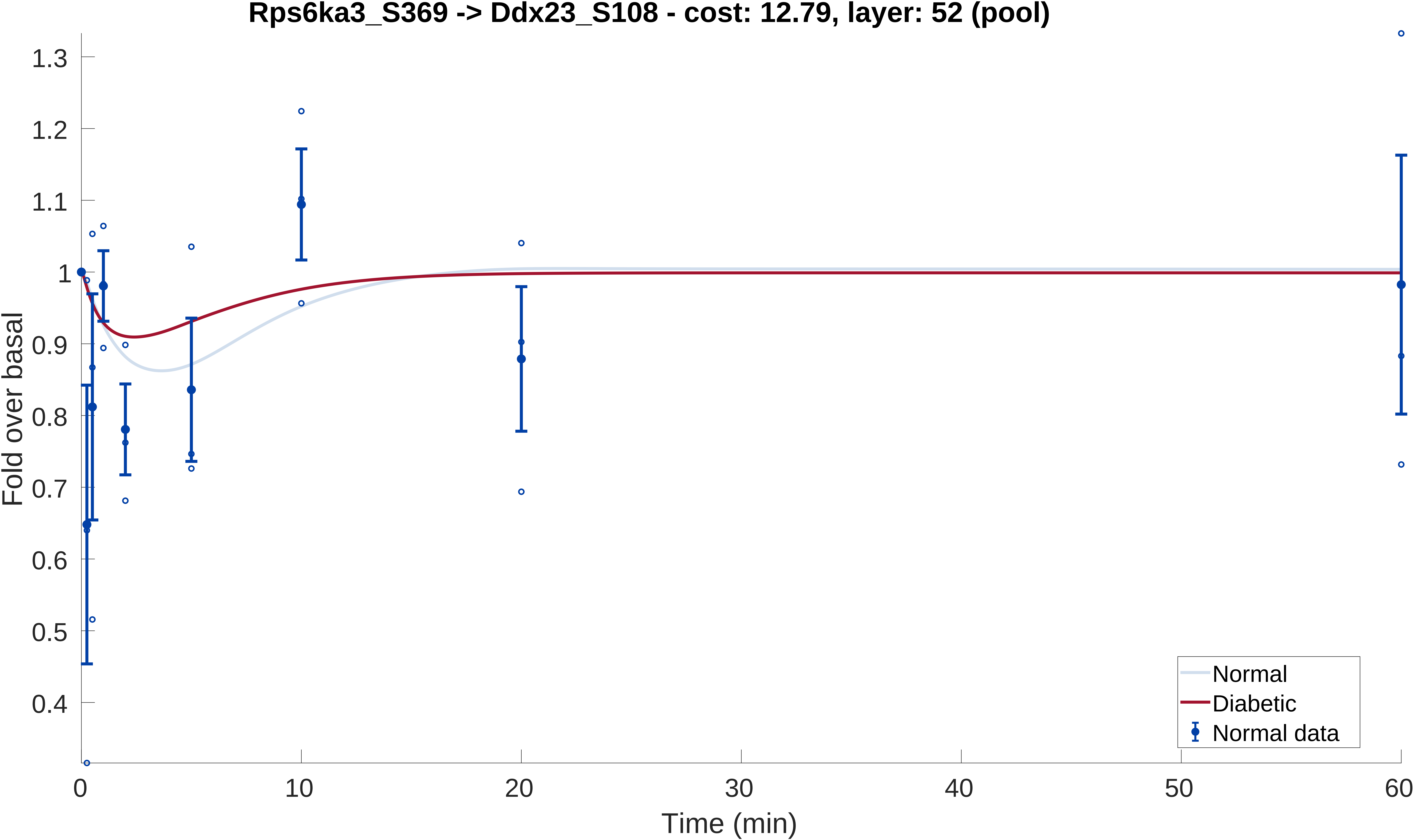
Rps6ka3_S369 -> Ddx23_S108 - cost: 12.79, layer: 52 (pool)
Fold over basal
Time (min)
1.3
1.2
1.1
1
0.9
0.8
0.7
0.6
0.5
0.4
0
10
20
30
40
50
60
Normal
Diabetic
Normal data

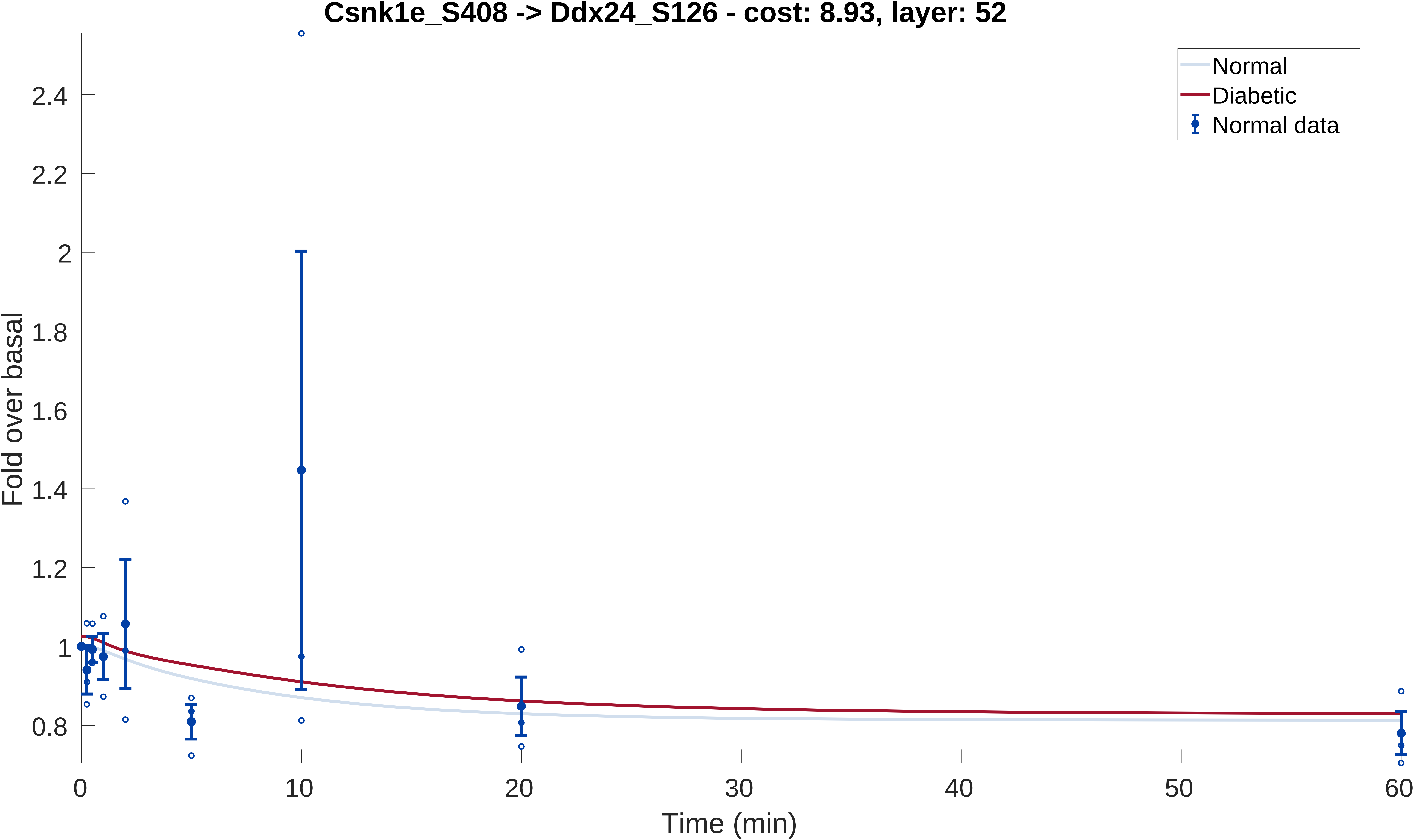
Csnk1e_S408 -> Ddx24_S126 - cost: 8.93, layer: 52
Normal
Diabetic
Normal data
Fold over basal
2.4
2.2
2
1.8
1.6
1.4
1.2
1
0.8
0
10
20
30
40
50
60
Time (min)

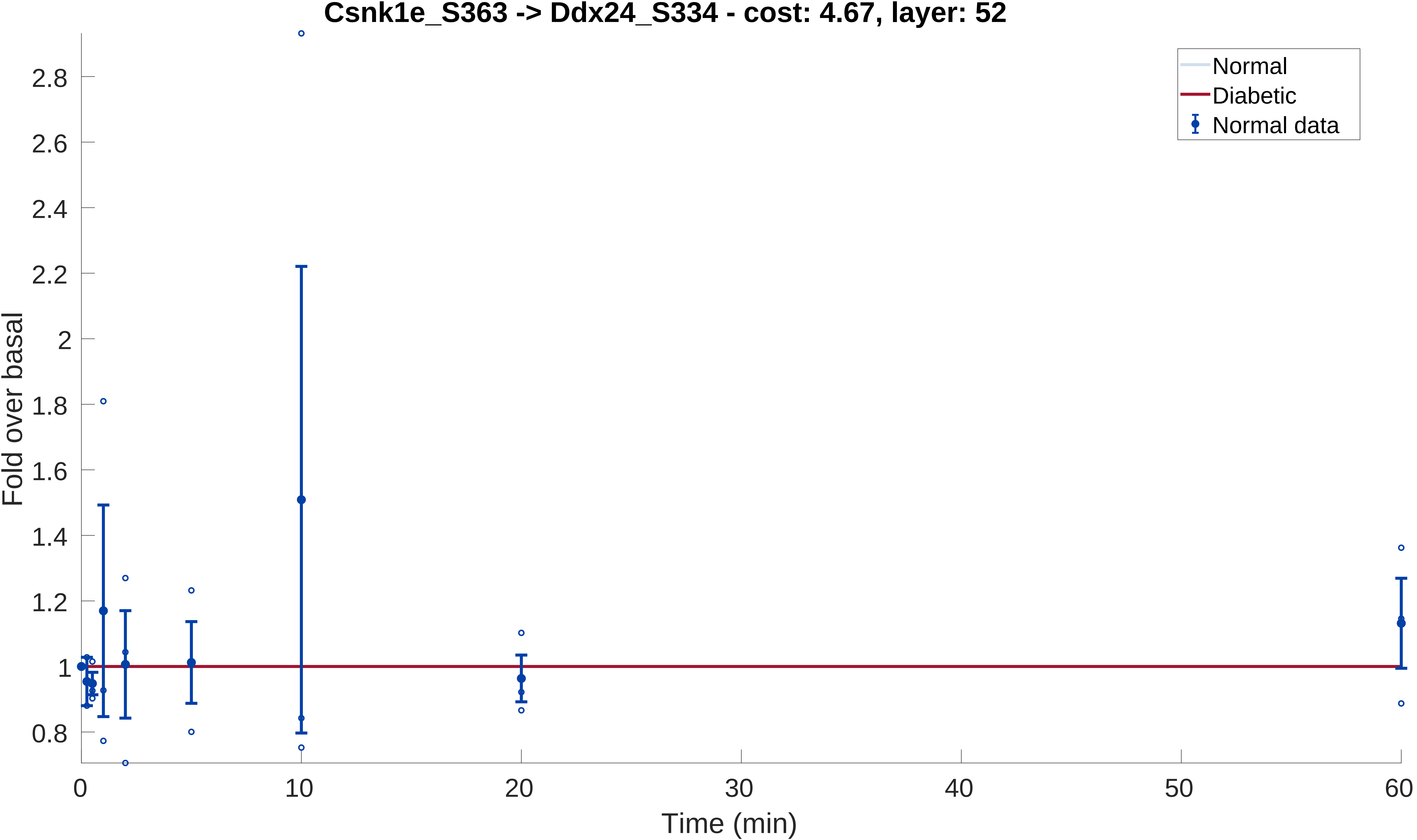

Csnk1e_S363 -> Ddx24_S334 - cost: 4.67, layer: 52
Normal
Diabetic
Normal data
Fold over basal
Time (min)
0.8
1
1.2
1.4
1.6
1.8
2
2.2
2.4
2.6
2.8
0
10
20
30
40
50
60

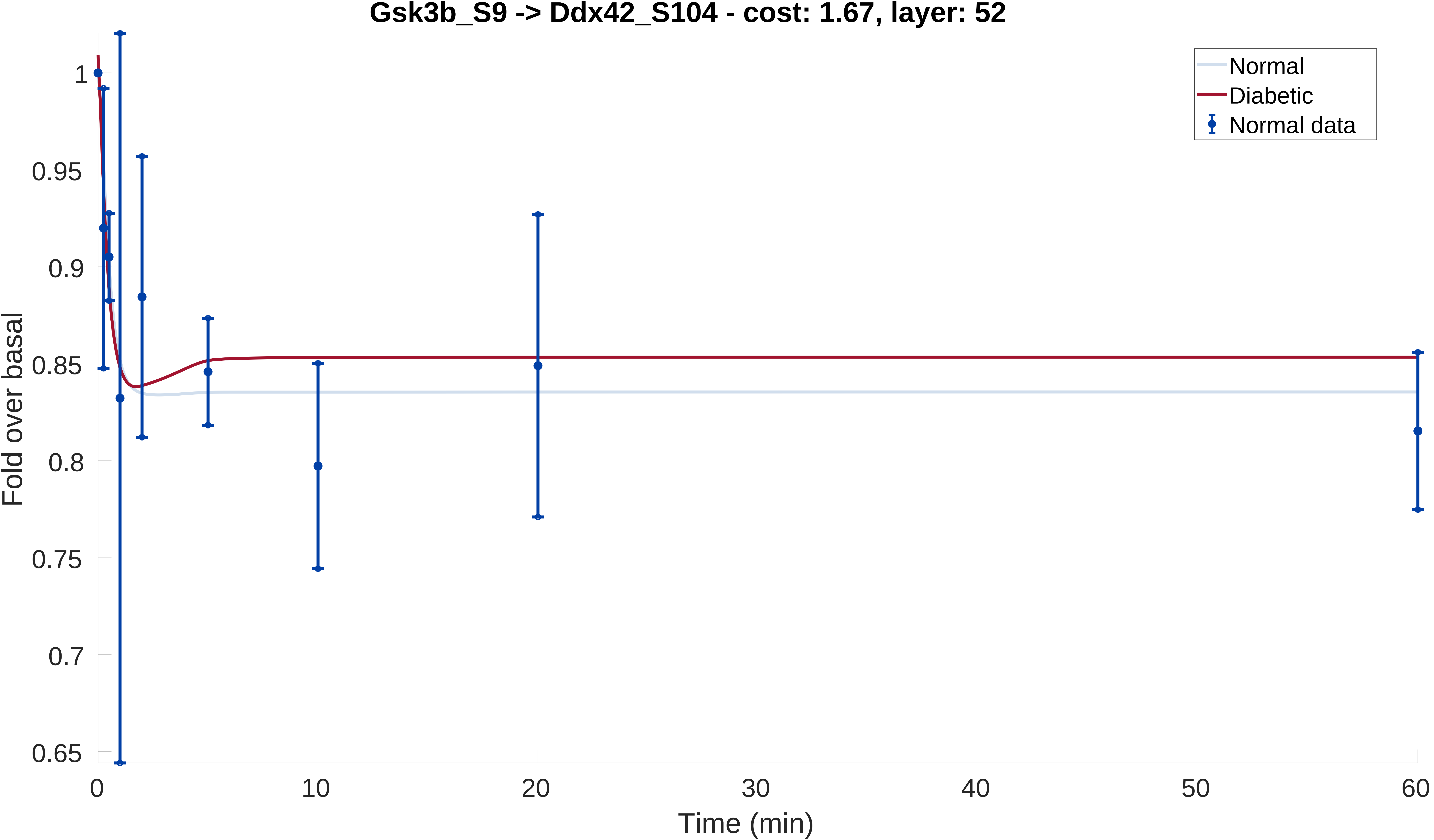
Gsk3b_S9 -> Ddx42_S104 - cost: 1.67, layer: 52
Normal
Diabetic
Normal data
Fold over basal
Time (min)
1
0.95
0.9
0.85
0.8
0.75
0.7
0.65
0
10
20
30
40
50
60

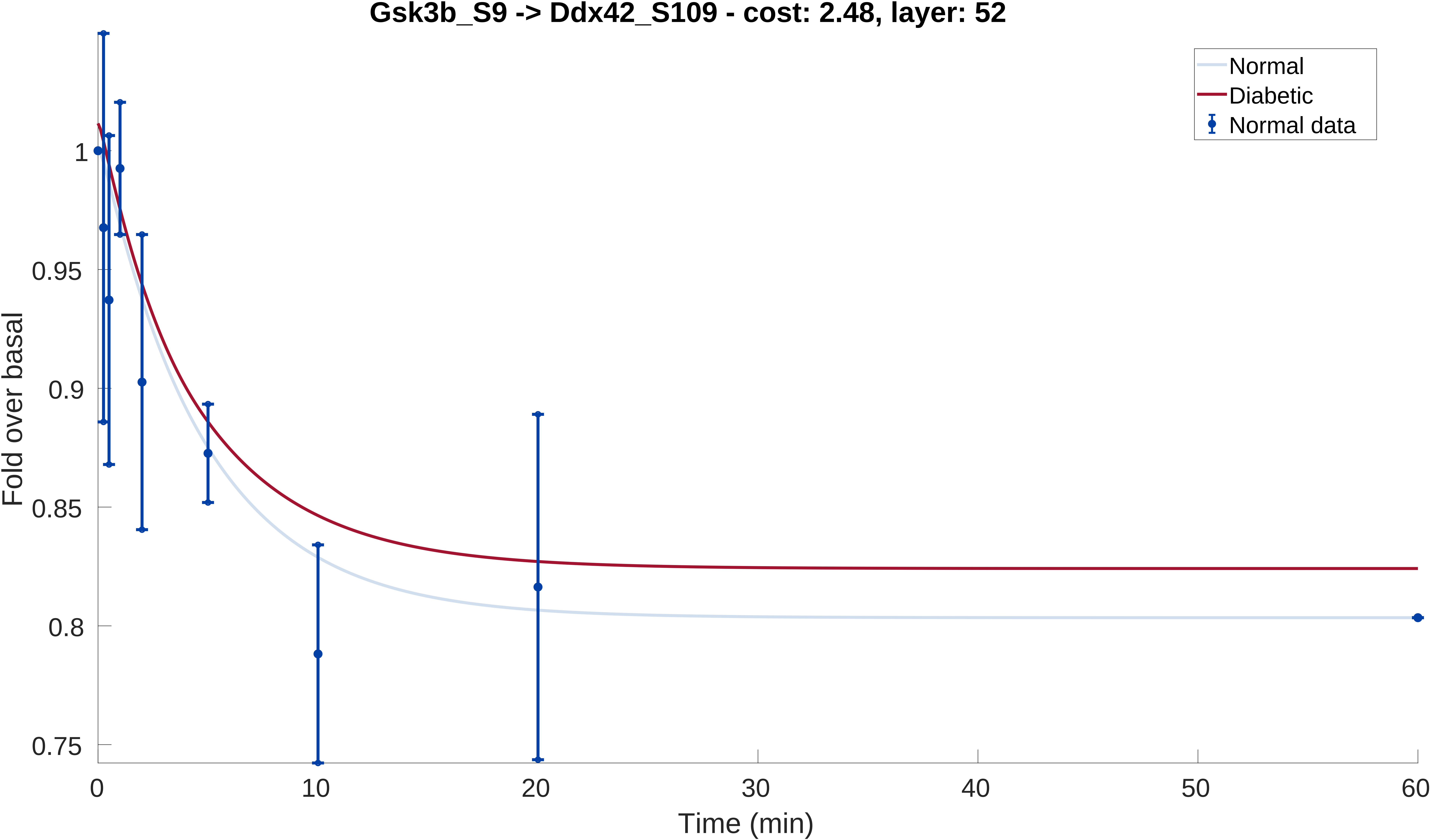

Gsk3b_S9 -> Ddx42_S109 - cost: 2.48, layer: 52
Normal
Diabetic
Normal data
Fold over basal
1
0.95
0.9
0.85
0.8
0.75
0
10
20
30
40
50
60
Time (min)

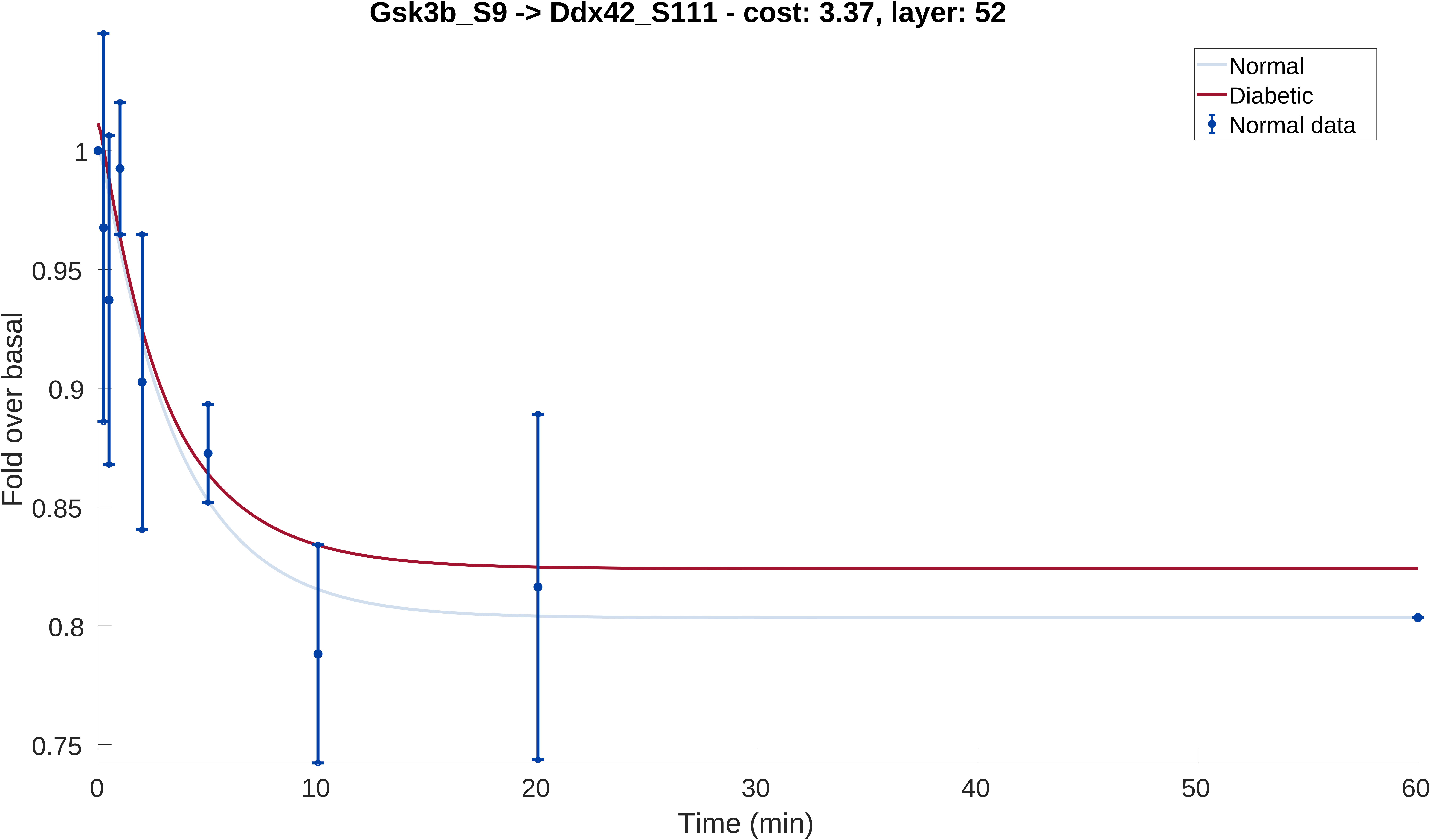

Gsk3b_S9 -> Ddx42_S111 - cost: 3.37, layer: 52
Normal
Diabetic
Normal data
Fold over basal
1
0.95
0.9
0.85
0.8
0.75
0
10
20
30
40
50
60
Time (min)

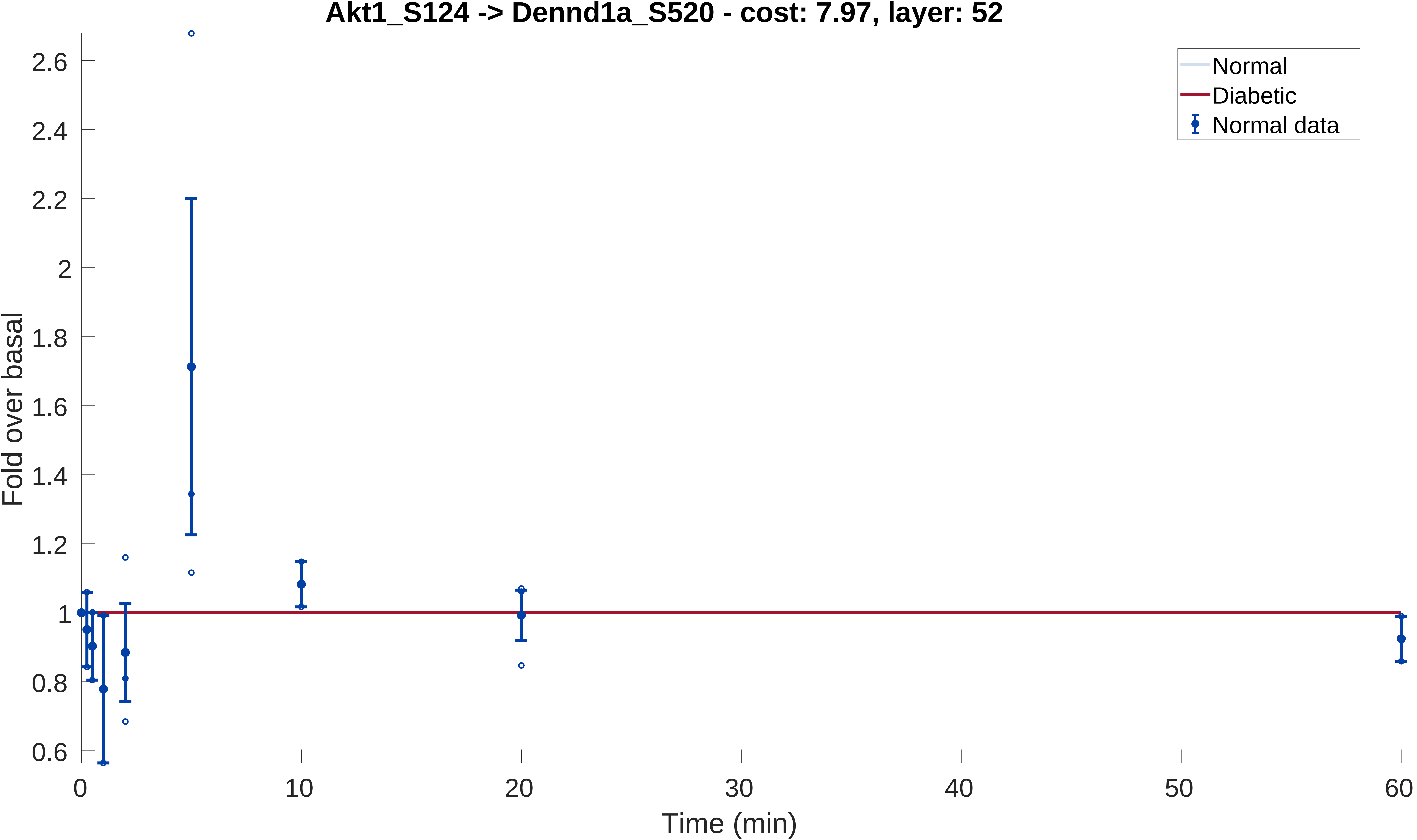

Akt1_S124 -> Dennd1a_S520 - cost: 7.97, layer: 52
Normal
Diabetic
Normal data
Fold over basal
2.6
2.4
2.2
2
1.8
1.6
1.4
1.2
1
0.8
0.6
0
10
20
30
40
50
60
Time (min)

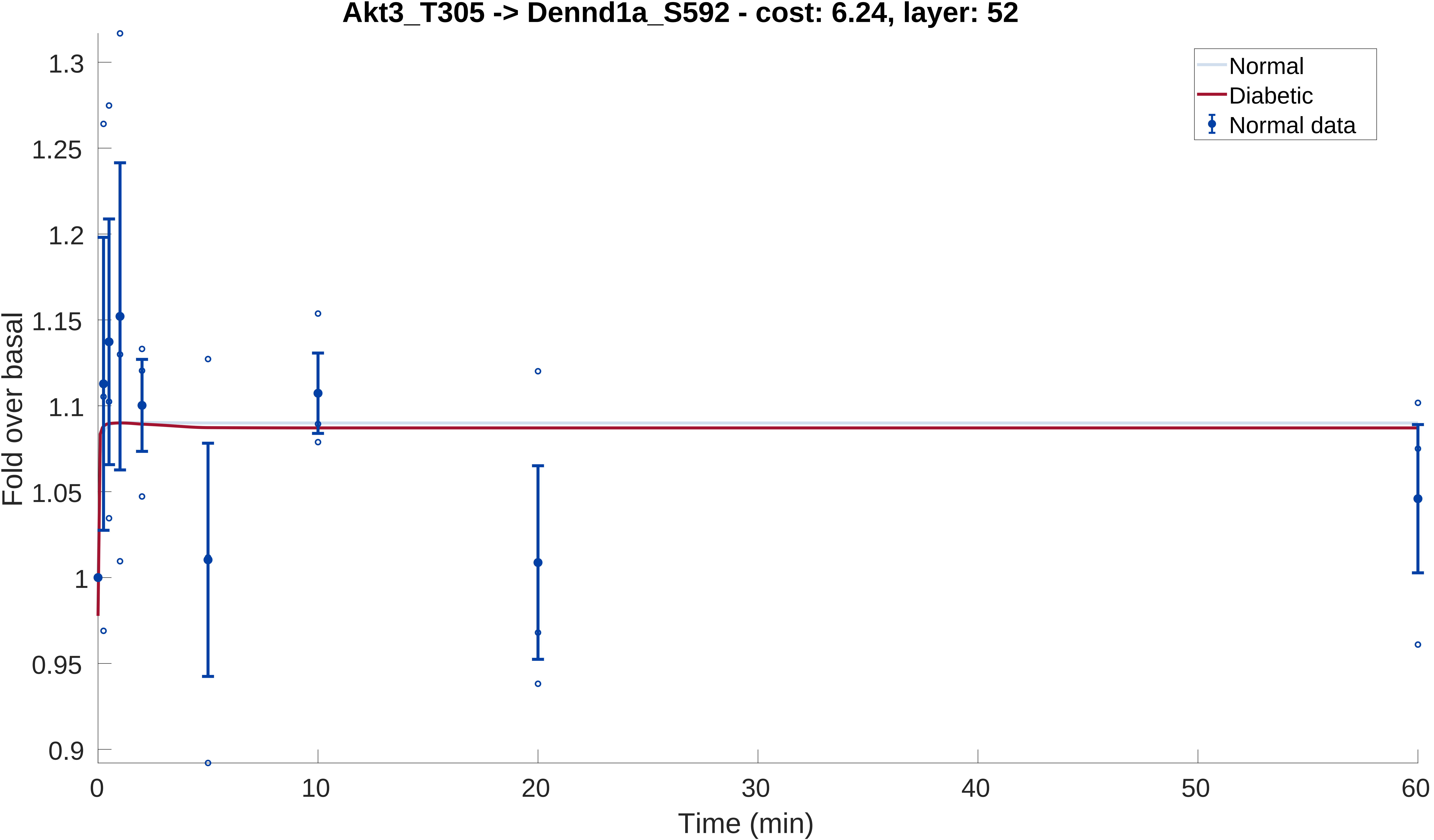
Akt3_T305 -> Dennd1a_S592 - cost: 6.24, layer: 52
Normal
Diabetic
Normal data
Fold over basal
1.3
1.25
1.2
1.15
1.1
1.05
1
0.95
0.9
0
10
20
30
40
50
60
Time (min)

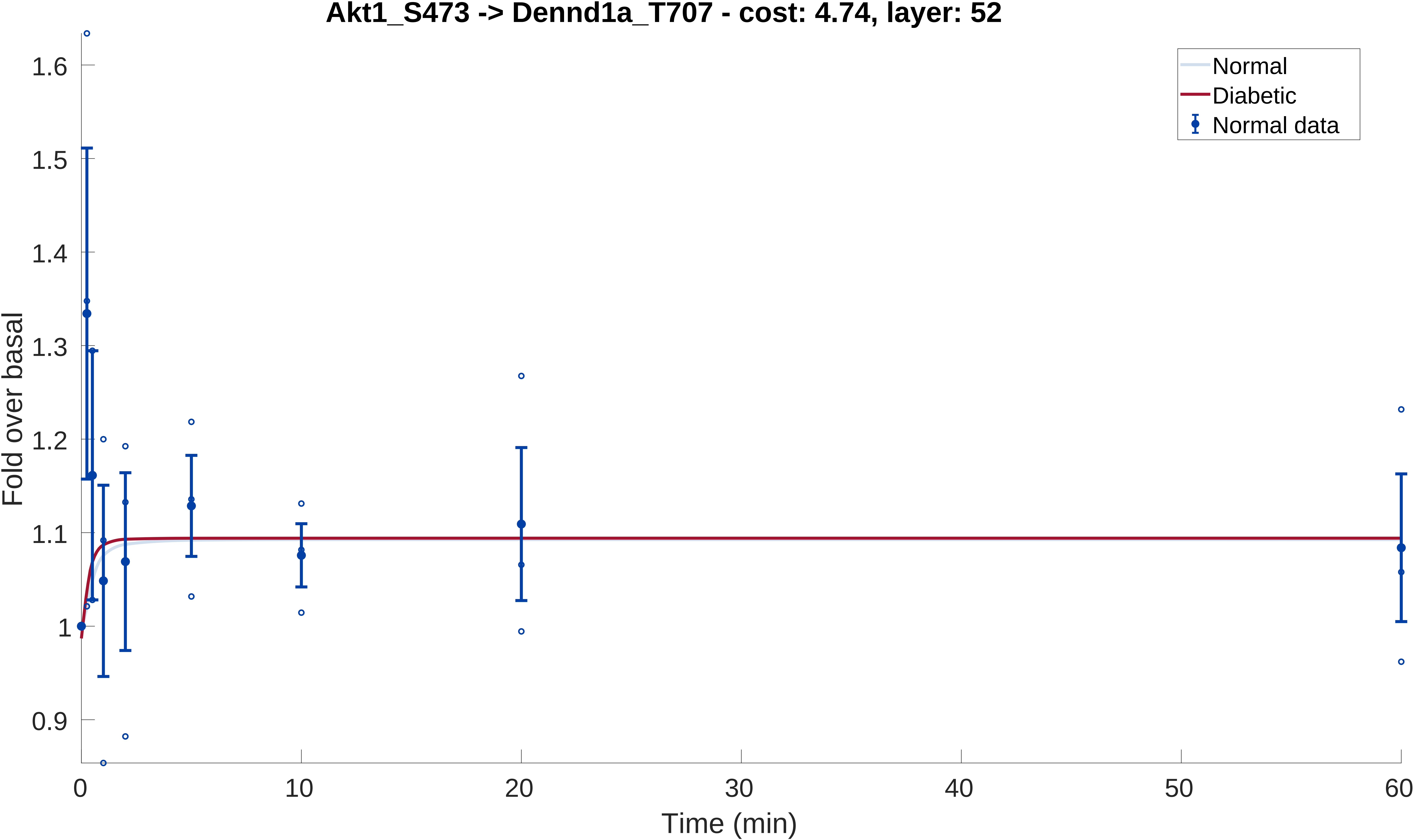
Akt1_S473 -> Dennd1a_T707 - cost: 4.74, layer: 52
Normal
Diabetic
Normal data
Fold over basal
Time (min)
1.6
1.5
1.4
1.3
1.2
1.1
1
0.9
0
10
20
30
40
50
60

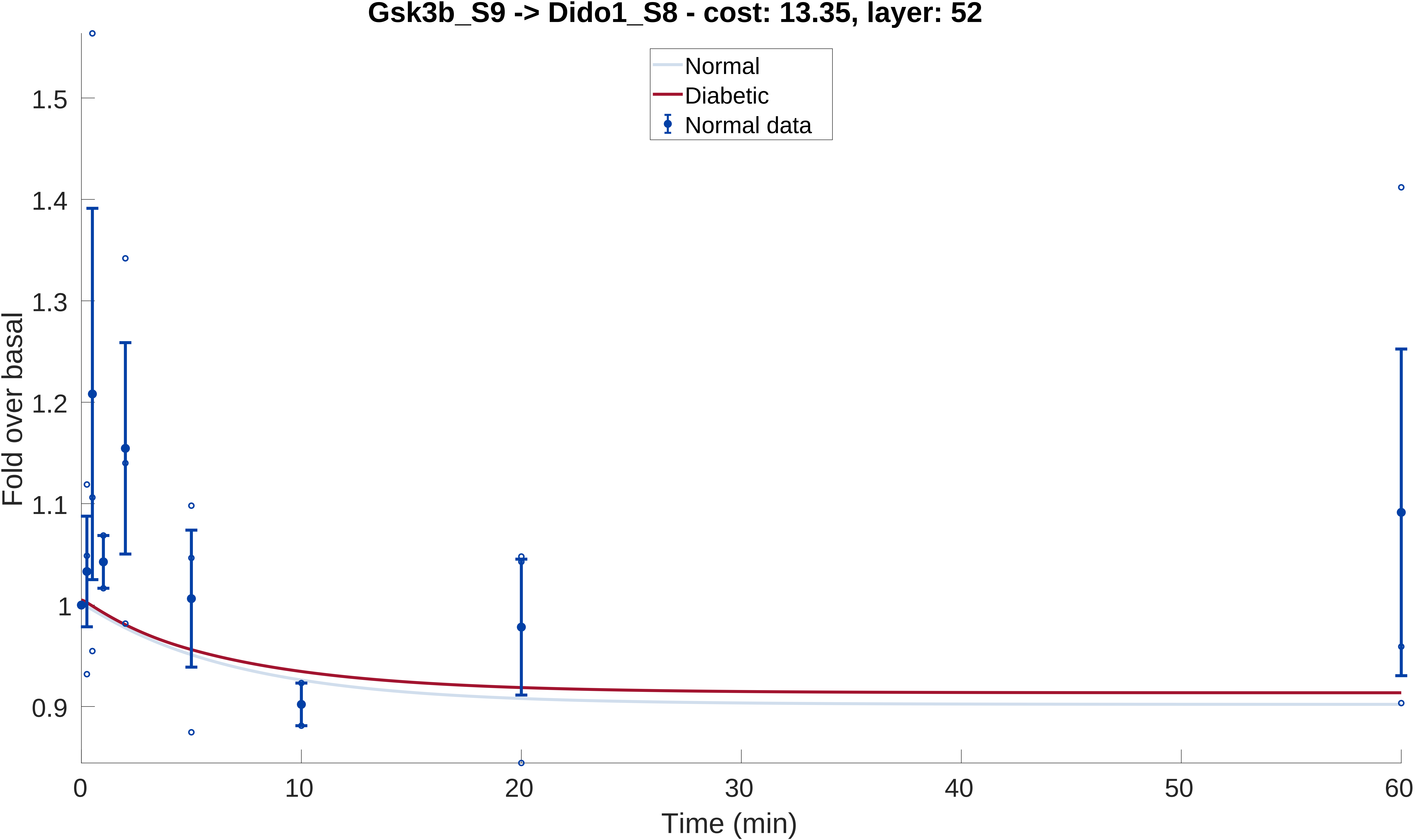

Gsk3b_S9 -> Dido1_S8 - cost: 13.35, layer: 52
Normal
Diabetic
Normal data
Fold over basal
1.5
1.4
1.3
1.2
1.1
1
0.9
0
10
20
30
40
50
60
Time (min)

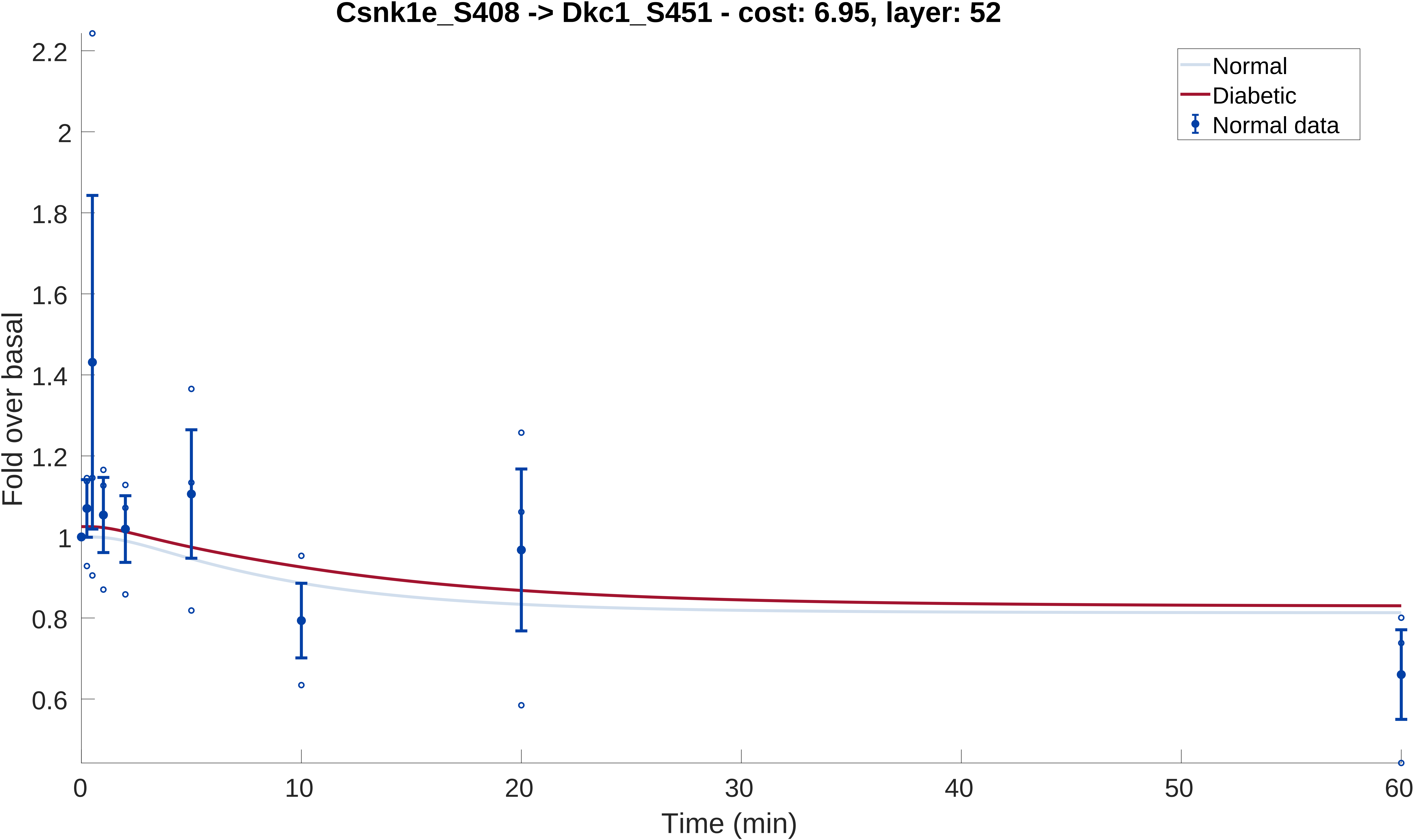
Csnk1e_S408 -> Dkc1_S451 - cost: 6.95, layer: 52
Fold over basal
Time (min)
Normal
Diabetic
Normal data
2.2
2
1.8
1.6
1.4
1.2
1
0.8
0.6
0
10
20
30
40
50
60

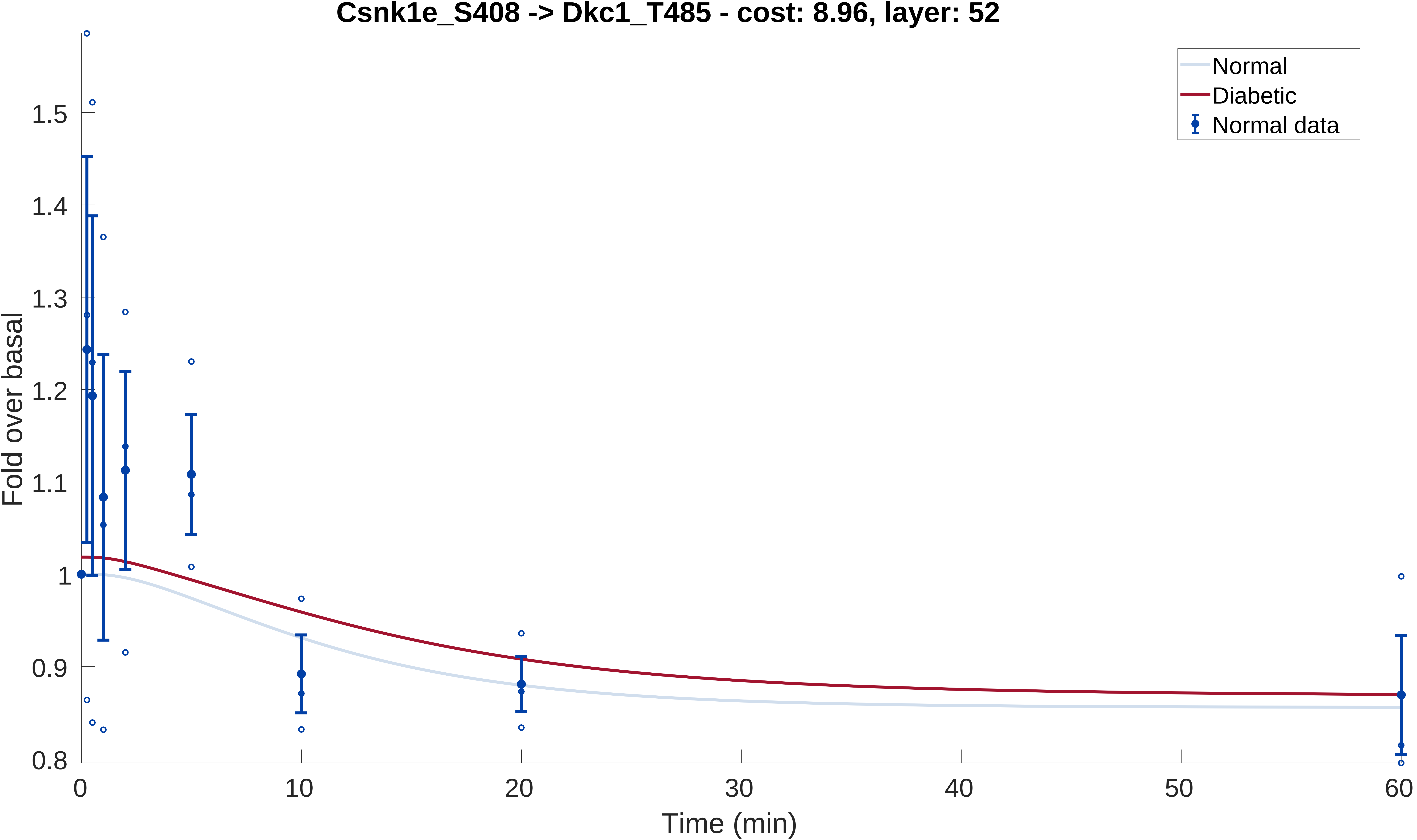

Csnk1e_S408 -> Dkc1_T485 - cost: 8.96, layer: 52
Normal
Diabetic
Normal data
Fold over basal
1.5
1.4
1.3
1.2
1.1
1
0.9
0.8
0
10
20
30
40
50
60
Time (min)

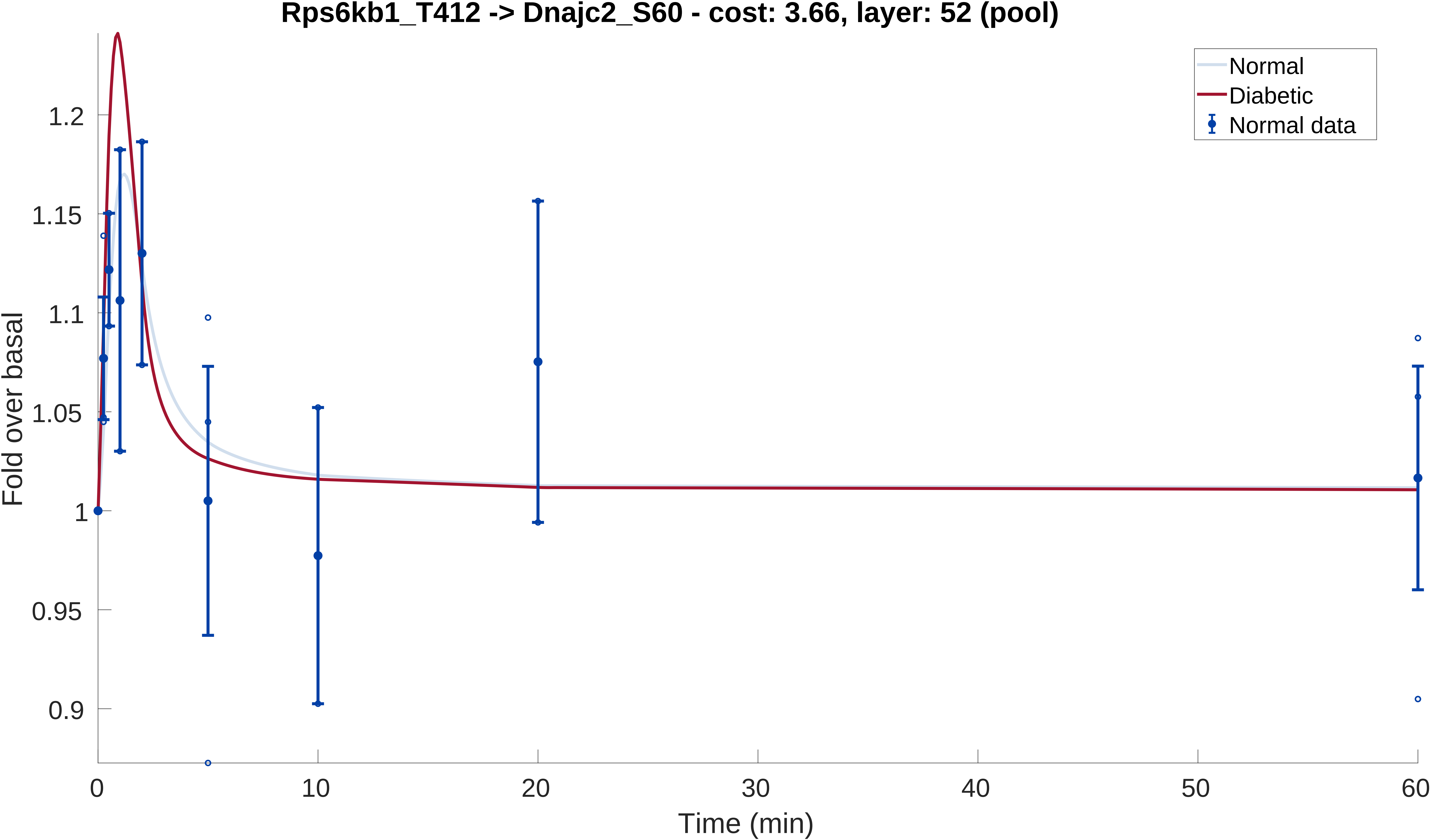

Rps6kb1_T412 -> Dnajc2_S60 - cost: 3.66, layer: 52 (pool)
Normal
Diabetic
Normal data
Fold over basal
1.2
1.15
1.1
1.05
1
0.95
0.9
0
10
20
30
40
50
60
Time (min)

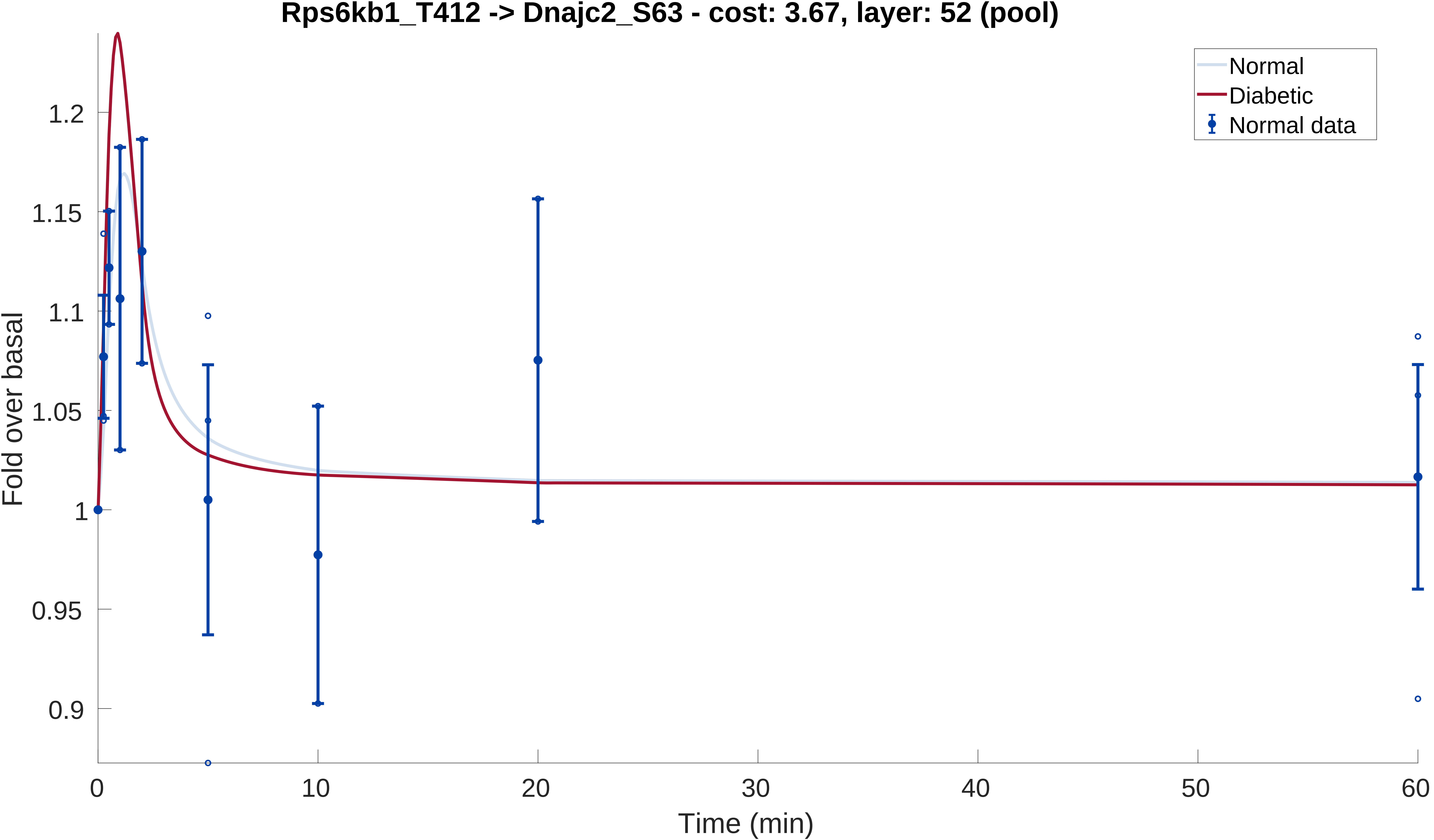
Rps6kb1_T412 -> Dnajc2_S63 - cost: 3.67, layer: 52 (pool)
Normal
Diabetic
Normal data
Fold over basal
1.2
1.15
1.1
1.05
1
0.95
0.9
0
10
20
30
40
50
60
Time (min)

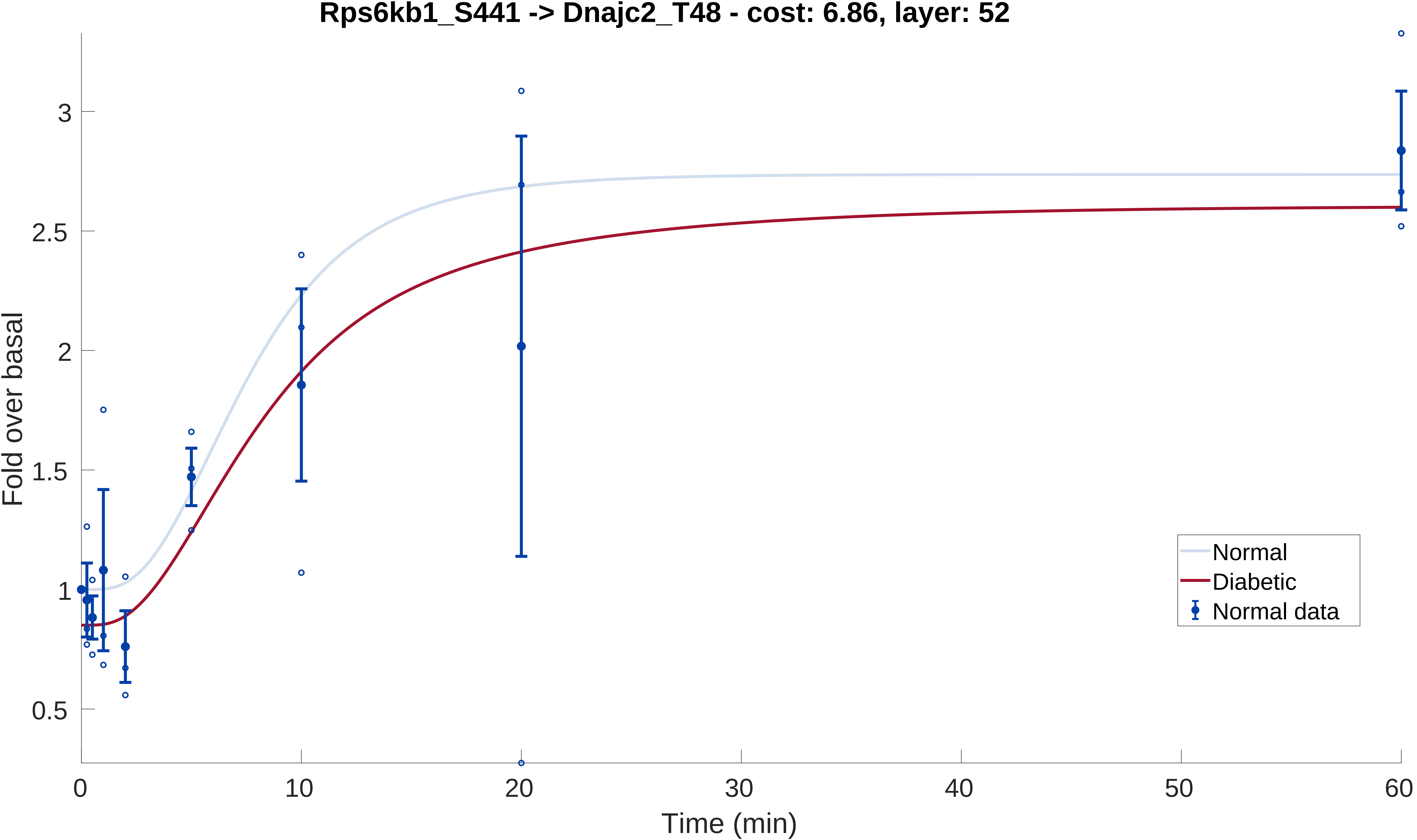
Rps6kb1_S441 -> Dnajc2_T48 - cost: 6.86, layer: 52
Fold over basal
3
2.5
2
1.5
1
0.5
0
10
20
30
40
50
60
Time (min)
Normal
Diabetic
Normal data

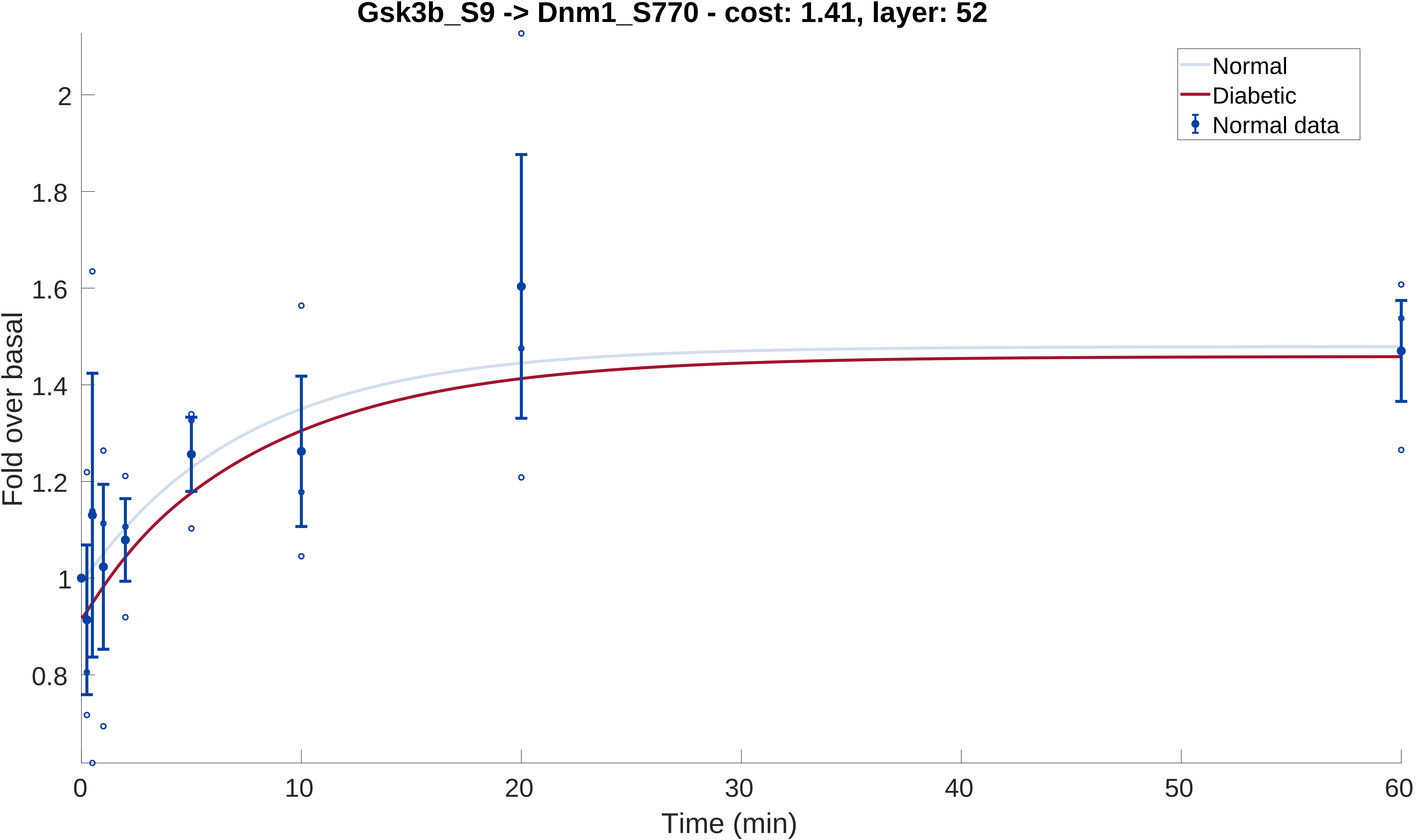
Gsk3b_S9 -> Dnm1_S770 - cost: 1.41, layer: 52
Normal
Diabetic
Normal data
Fold over basal
2
1.8
1.6
1.4
1.2
1
0.8
0
10
20
30
40
50
60
Time (min)

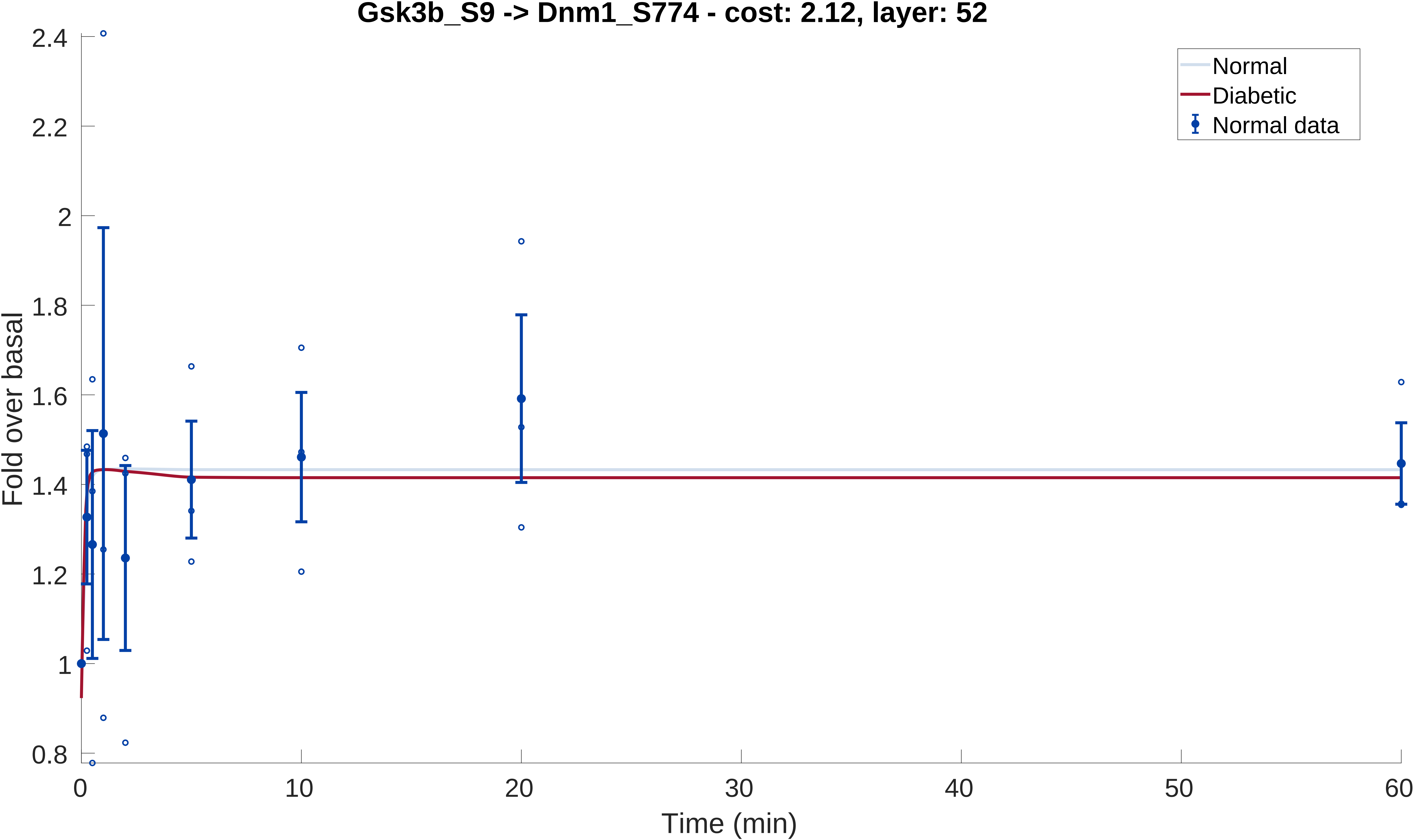

Gsk3b_S9 -> Dnm1_S774 - cost: 2.12, layer: 52
Normal
Diabetic
Normal data
Fold over basal
Time (min)
2.4
2.2
2
1.8
1.6
1.4
1.2
1
0.8
0
10
20
30
40
50
60

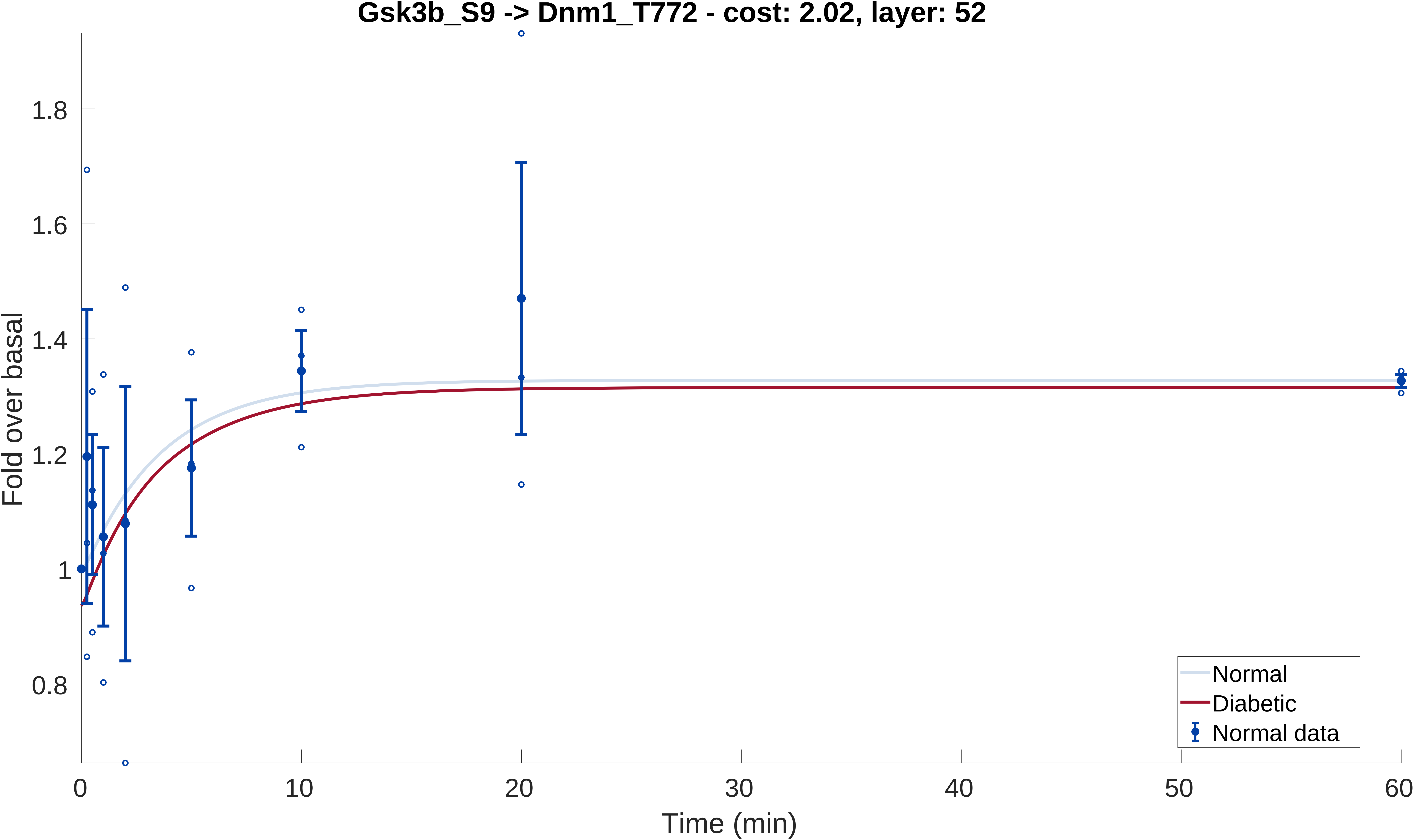
Gsk3b_S9 -> Dnm1_T772 - cost: 2.02, layer: 52
Fold over basal
Time (min)
0.8
1
1.2
1.4
1.6
1.8
0
10
20
30
40
50
60
Normal
Diabetic
Normal data

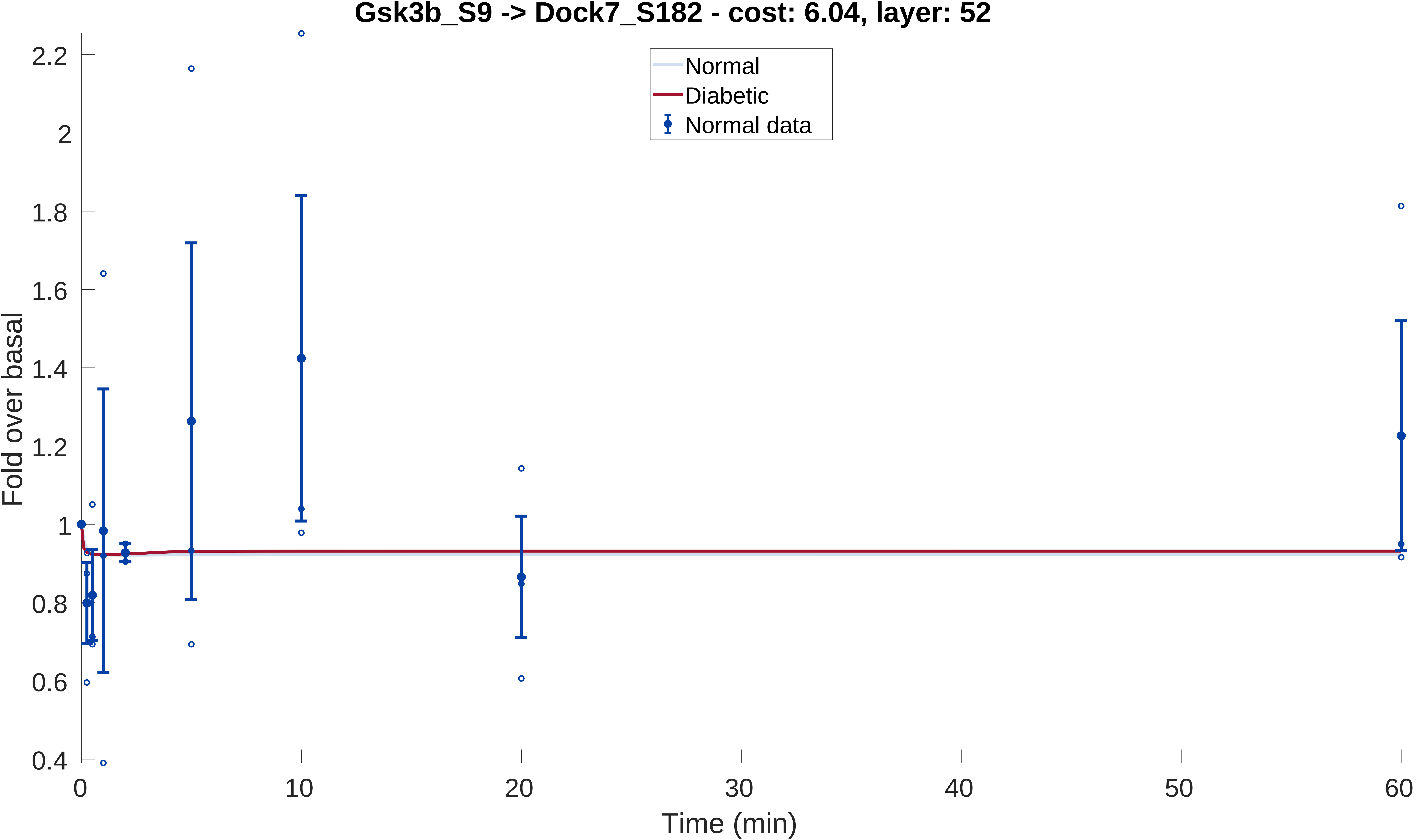
Gsk3b_S9 -> Dock7_S182 - cost: 6.04, layer: 52
Normal
Diabetic
Normal data
Fold over basal
Time (min)
0.4
0.6
0.8
1
1.2
1.4
1.6
1.8
2
2.2
0
10
20
30
40
50
60

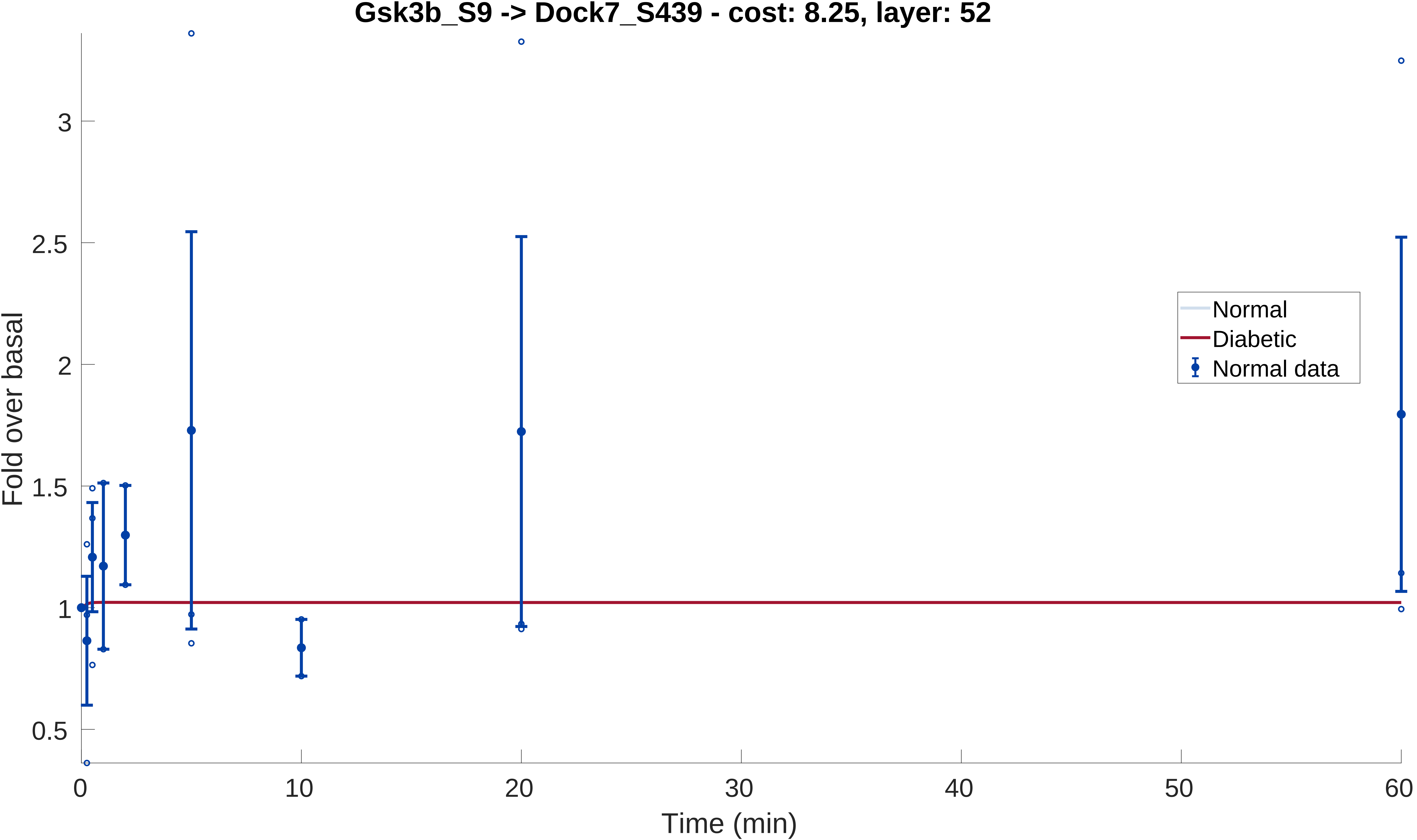
Gsk3b_S9 -> Dock7_S439 - cost: 8.25, layer: 52
Fold over basal
Time (min)
Normal
Diabetic
Normal data
0.5
1
1.5
2
2.5
3
0
10
20
30
40
50
60

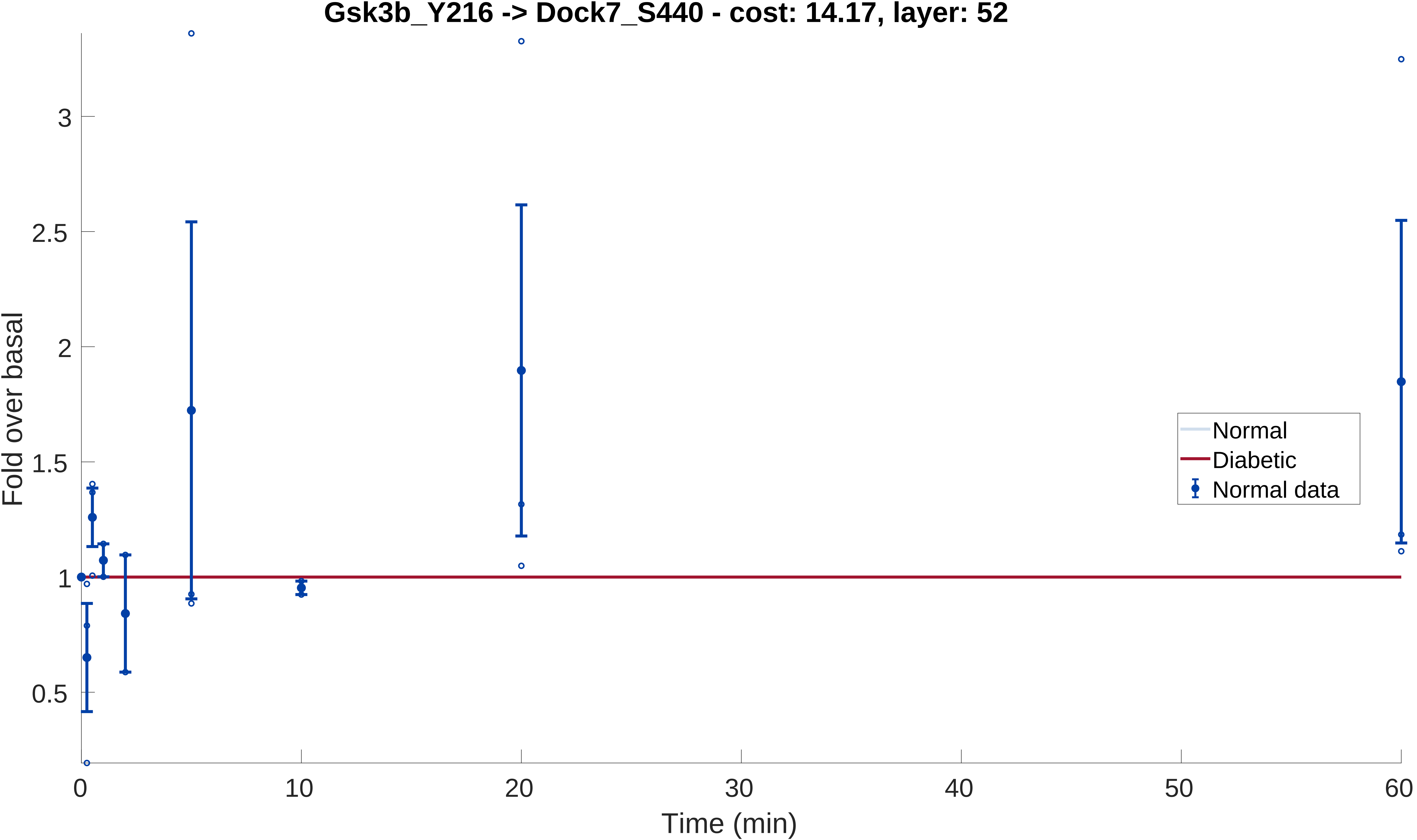
Gsk3b_Y216 -> Dock7_S440 - cost: 14.17, layer: 52
Fold over basal
3
2.5
2
1.5
1
0.5
0
10
20
30
40
50
60
Time (min)
Normal
Diabetic
Normal data

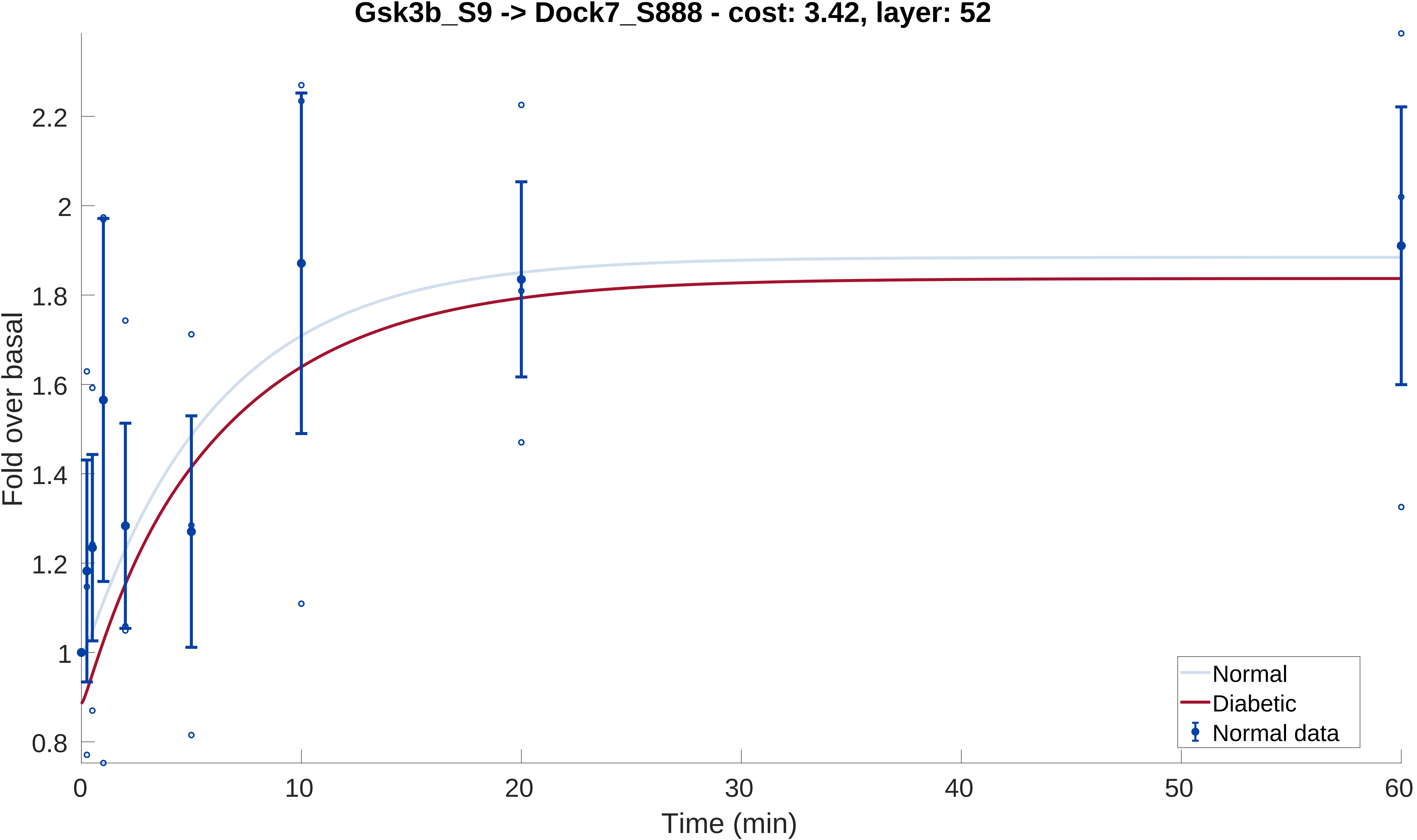
Gsk3b_S9 -> Dock7_S888 - cost: 3.42, layer: 52
Fold over basal
2.2
2
1.8
1.6
1.4
1.2
1
0.8
0
10
20
30
40
50
60
Time (min)
Normal
Diabetic
Normal data

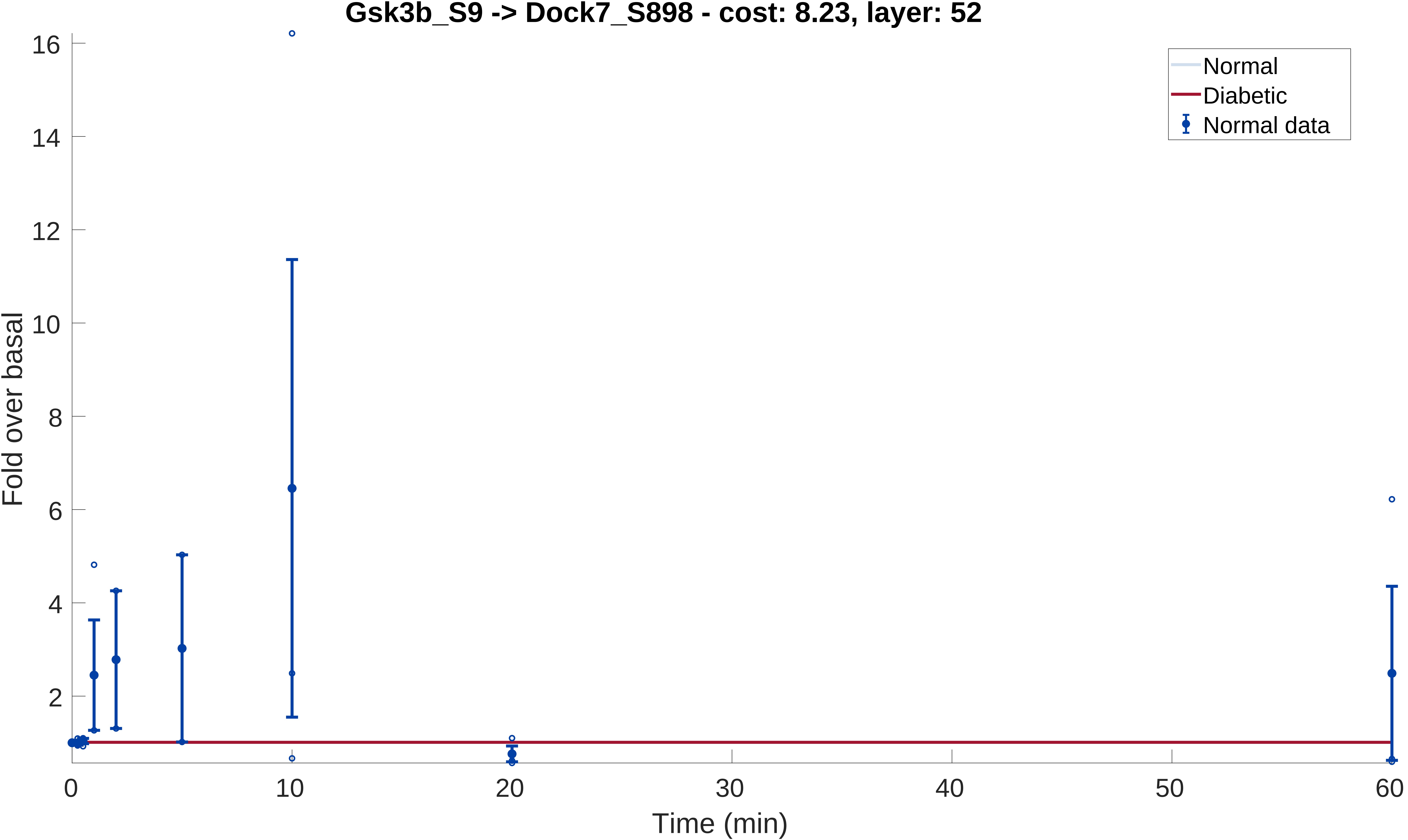
Gsk3b_S9 -> Dock7_S898 - cost: 8.23, layer: 52
Normal
Diabetic
Normal data
Fold over basal
Time (min)
0
10
20
30
40
50
60
2
4
6
8
10
12
14
16

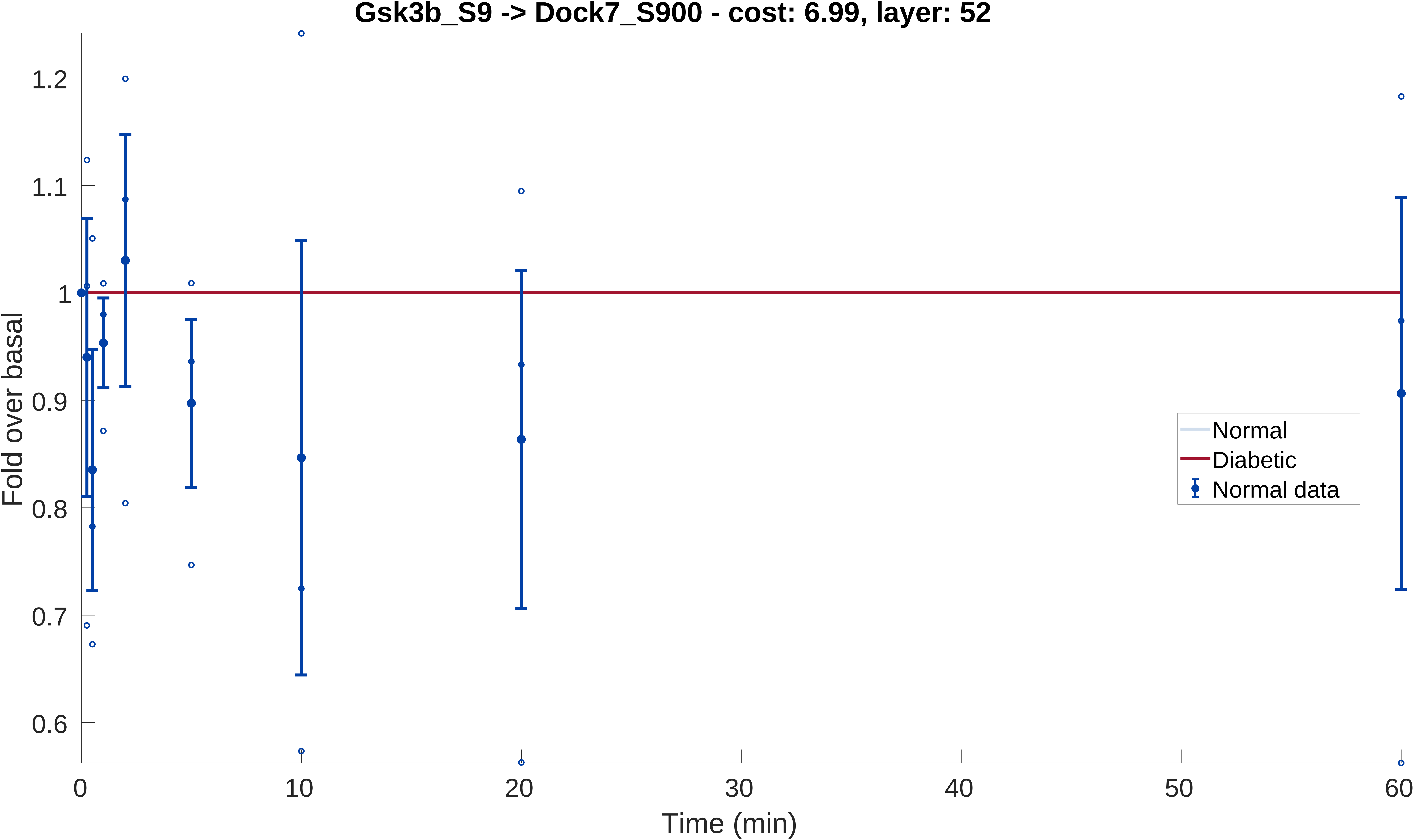
Gsk3b_S9 -> Dock7_S900 - cost: 6.99, layer: 52
Fold over basal
Time (min)
Normal
Diabetic
Normal data
1.2
1.1
1
0.9
0.8
0.7
0.6
0
10
20
30
40
50
60

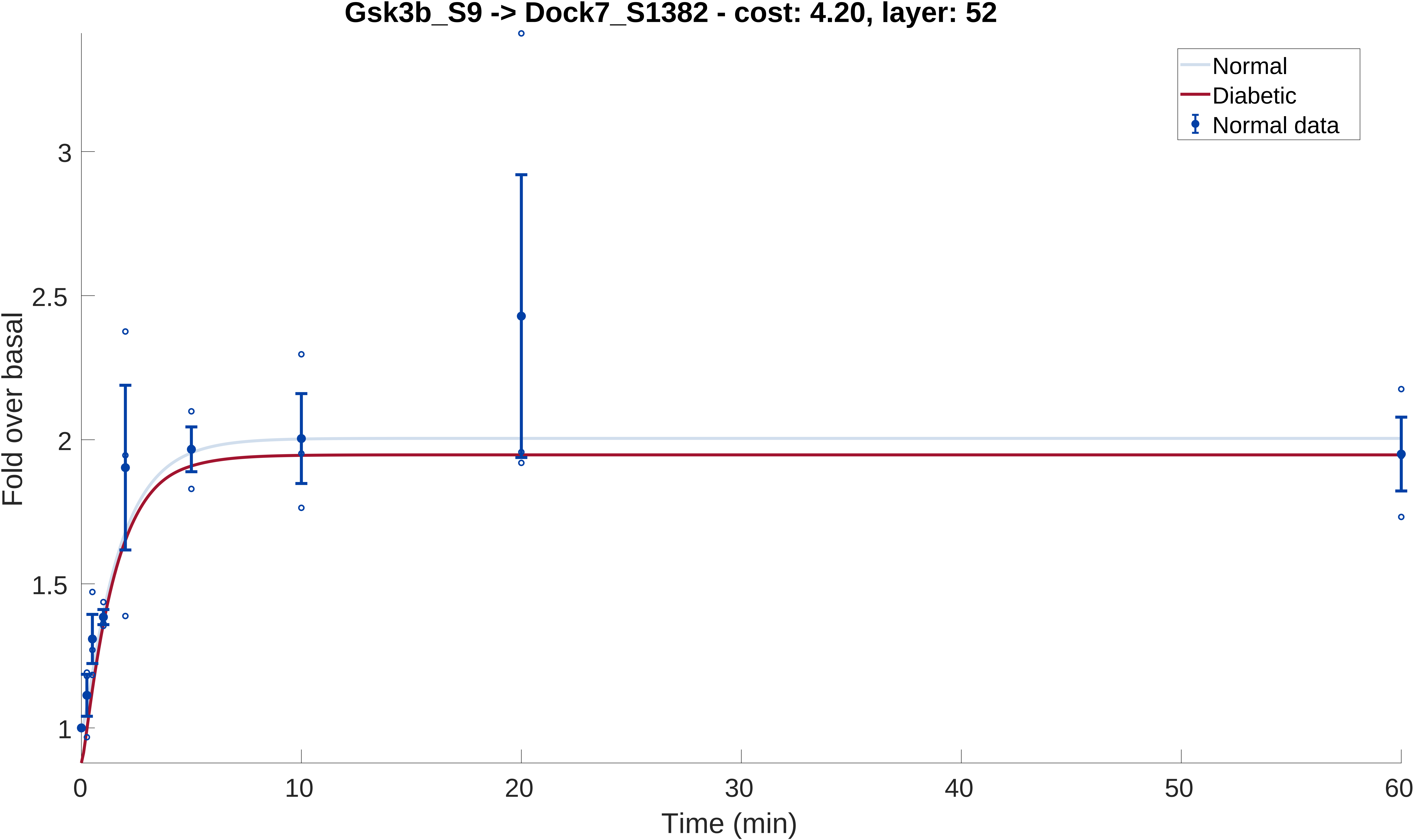

Gsk3b_S9 -> Dock7_S1382 - cost: 4.20, layer: 52
Normal
Diabetic
Normal data
Fold over basal
Time (min)
1
1.5
2
2.5
3
0
10
20
30
40
50
60

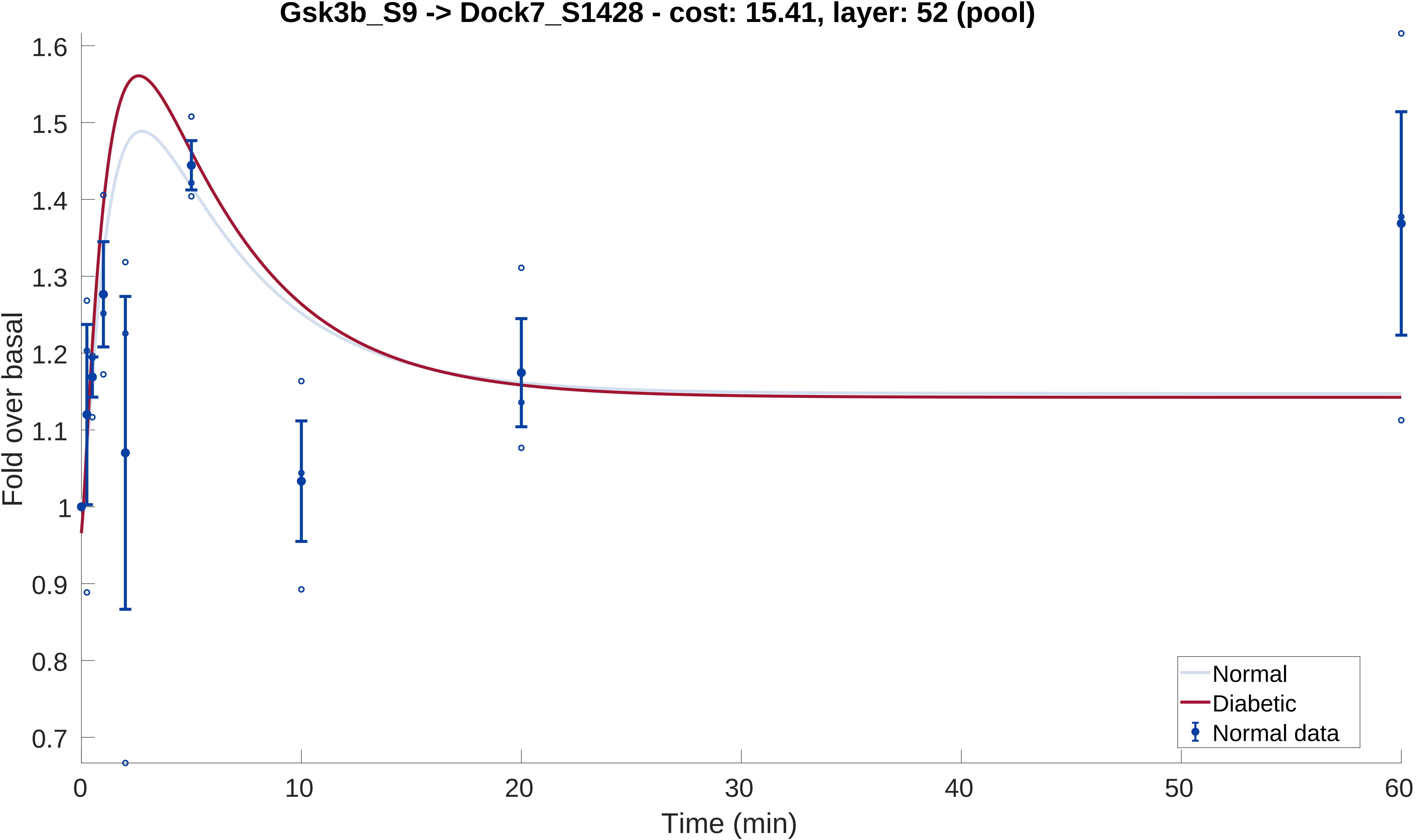
Gsk3b_S9 -> Dock7_S1428 - cost: 15.41, layer: 52 (pool)
Fold over basal
1.6
1.5
1.4
1.3
1.2
1.1
1
0.9
0.8
0.7
0
10
20
30
40
50
60
Time (min)
Normal
Diabetic
Normal data

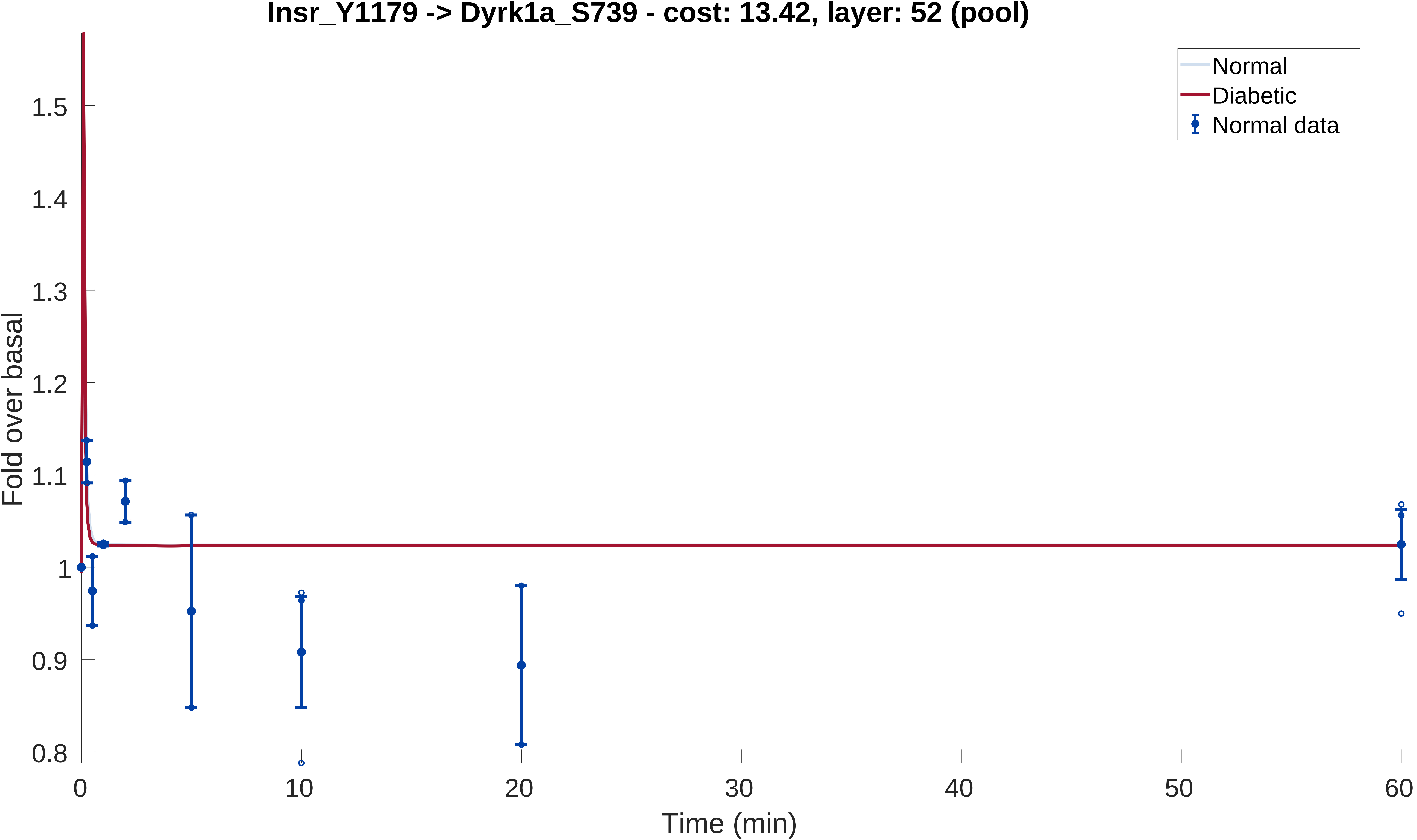

Insr_Y1179 -> Dyrk1a_S739 - cost: 13.42, layer: 52 (pool)
Normal
Diabetic
Normal data
Fold over basal
1.5
1.4
1.3
1.2
1.1
1
0.9
0.8
0
10
20
30
40
50
60
Time (min)

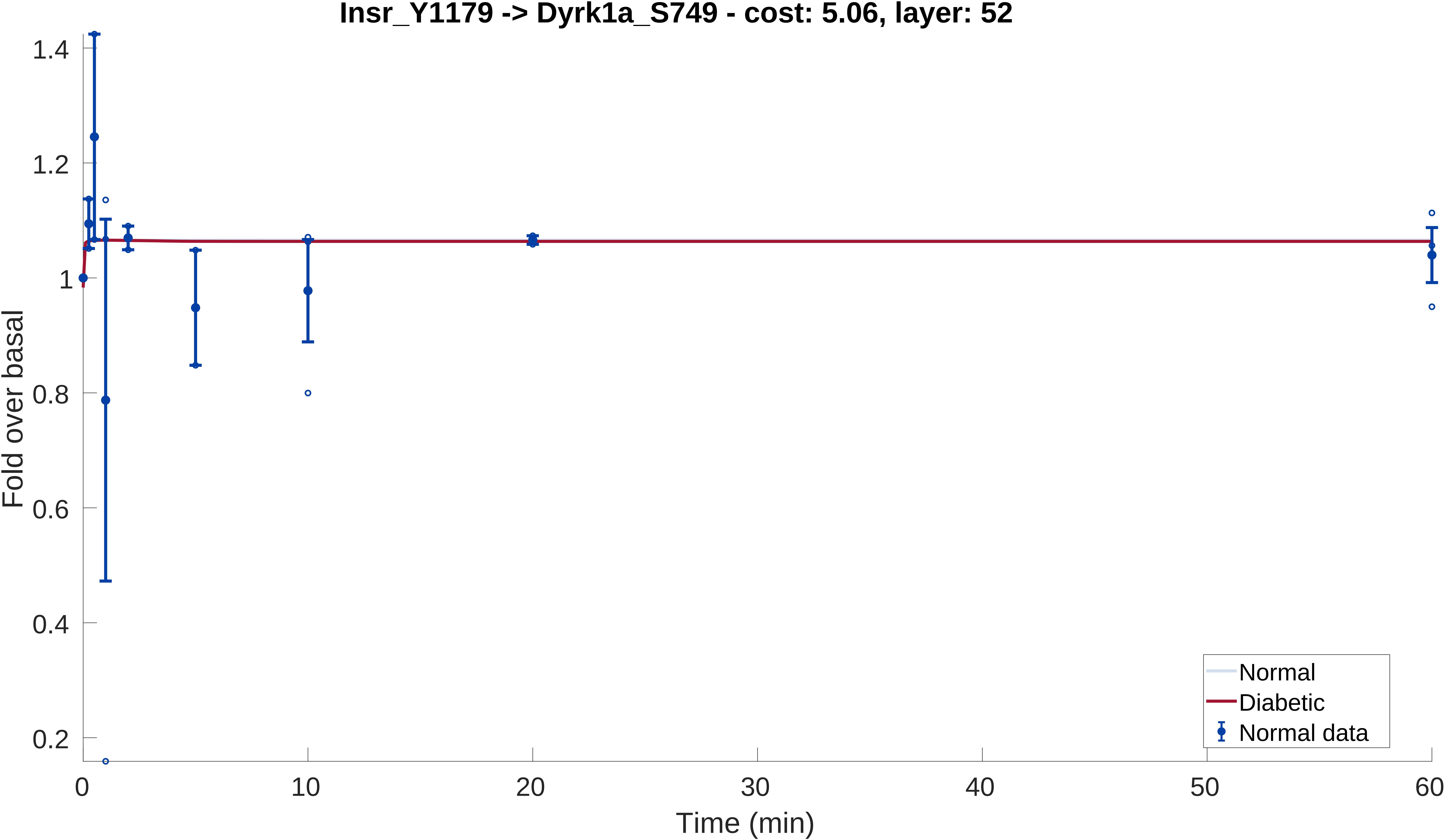

Insr_Y1179 -> Dyrk1a_S749 - cost: 5.06, layer: 52
Fold over basal
1.4
1.2
1
0.8
0.6
0.4
0.2
0
10
20
30
40
50
60
Time (min)
Normal
Diabetic
Normal data

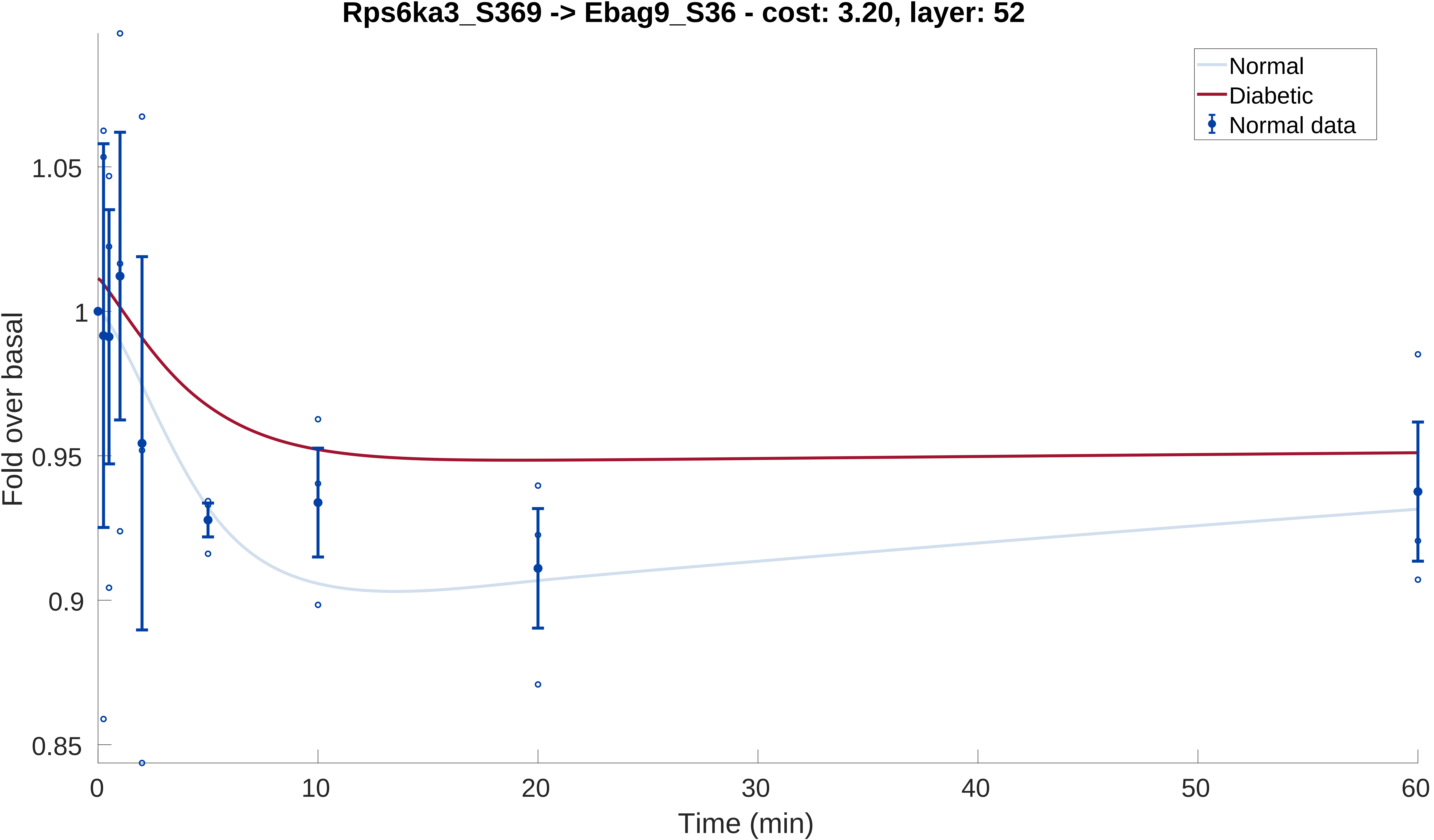

Rps6ka3_S369 -> Ebag9_S36 - cost: 3.20, layer: 52
Normal
Diabetic
Normal data
1.05
1
0.95
0.9
0.85
Fold over basal
0
10
20
30
40
50
60
Time (min)

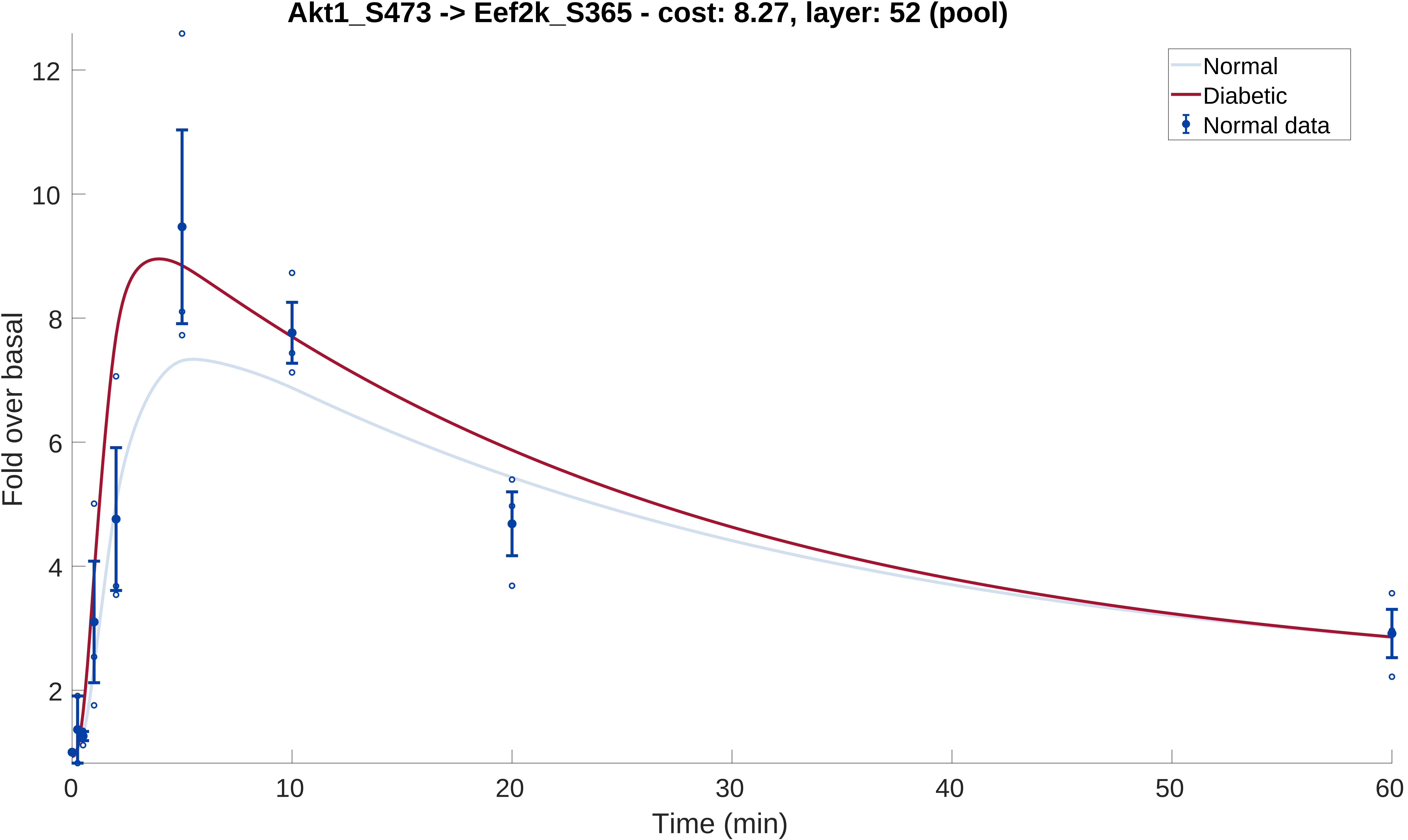

Akt1_S473 -> Eef2k_S365 - cost: 8.27, layer: 52 (pool)
Normal
Diabetic
Normal data
Fold over basal
Time (min)
0
2
4
6
8
10
12
0
10
20
30
40
50
60

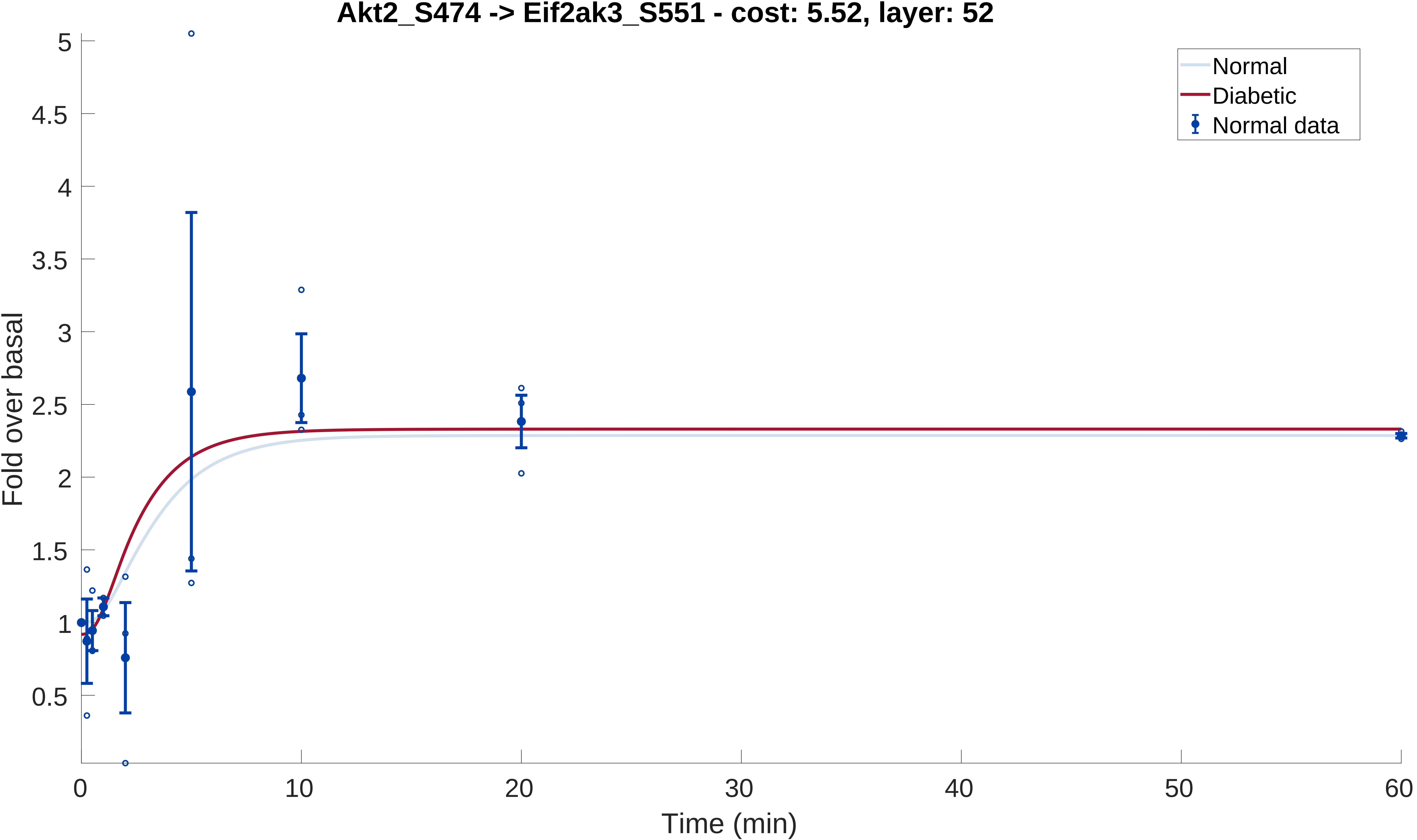

Akt2_S474 -> Eif2ak3_S551 - cost: 5.52, layer: 52
Normal
Diabetic
Normal data
Fold over basal
Time (min)
0
10
20
30
40
50
60
0.5
1
1.5
2
2.5
3
3.5
4
4.5
5

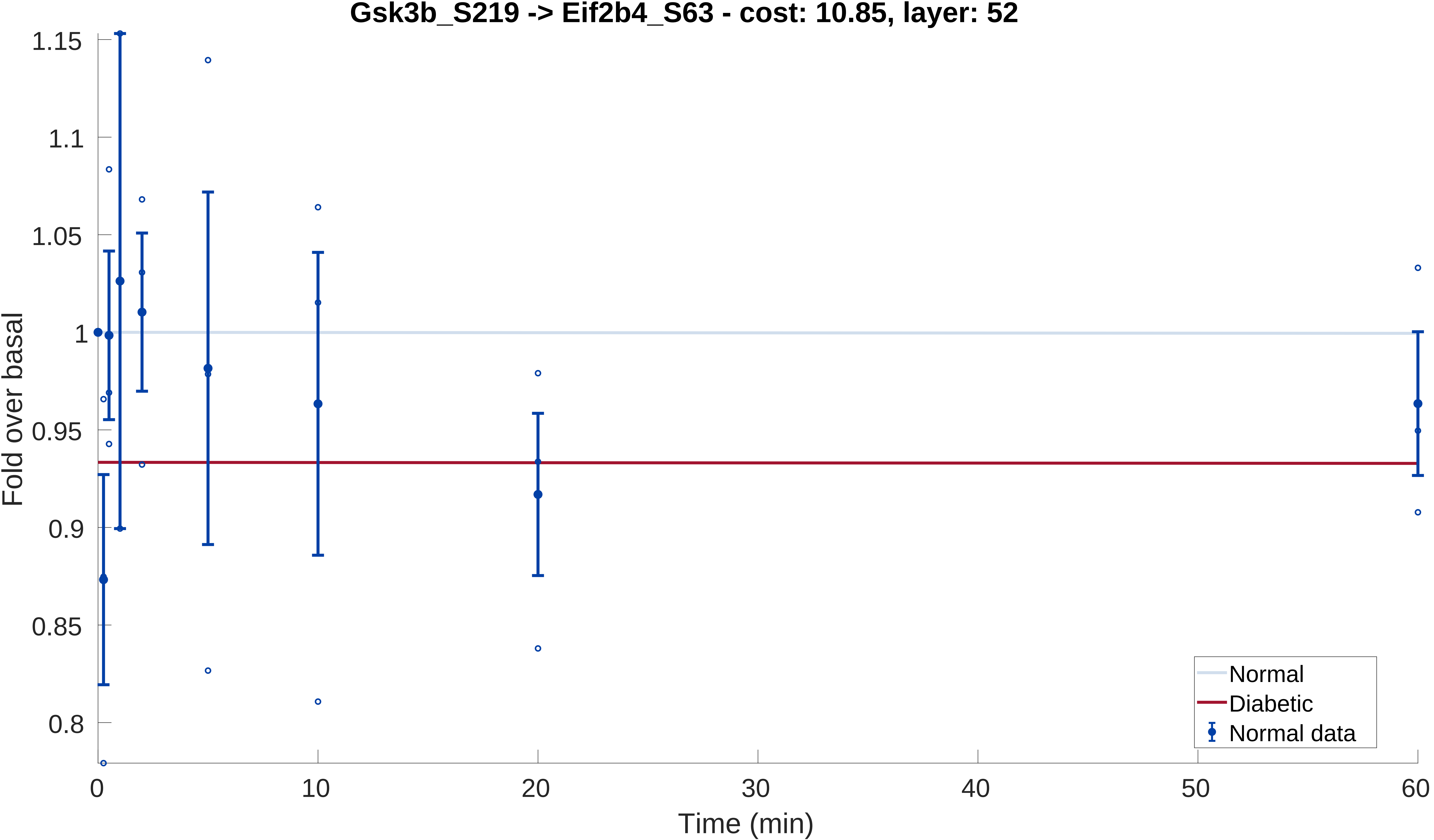

Gsk3b_S219 -> Eif2b4_S63 - cost: 10.85, layer: 52
1.15
1.1
1.05
1
0.95
0.9
0.85
0.8
Fold over basal
0
10
20
30
40
50
60
Time (min)
Normal
Diabetic
Normal data

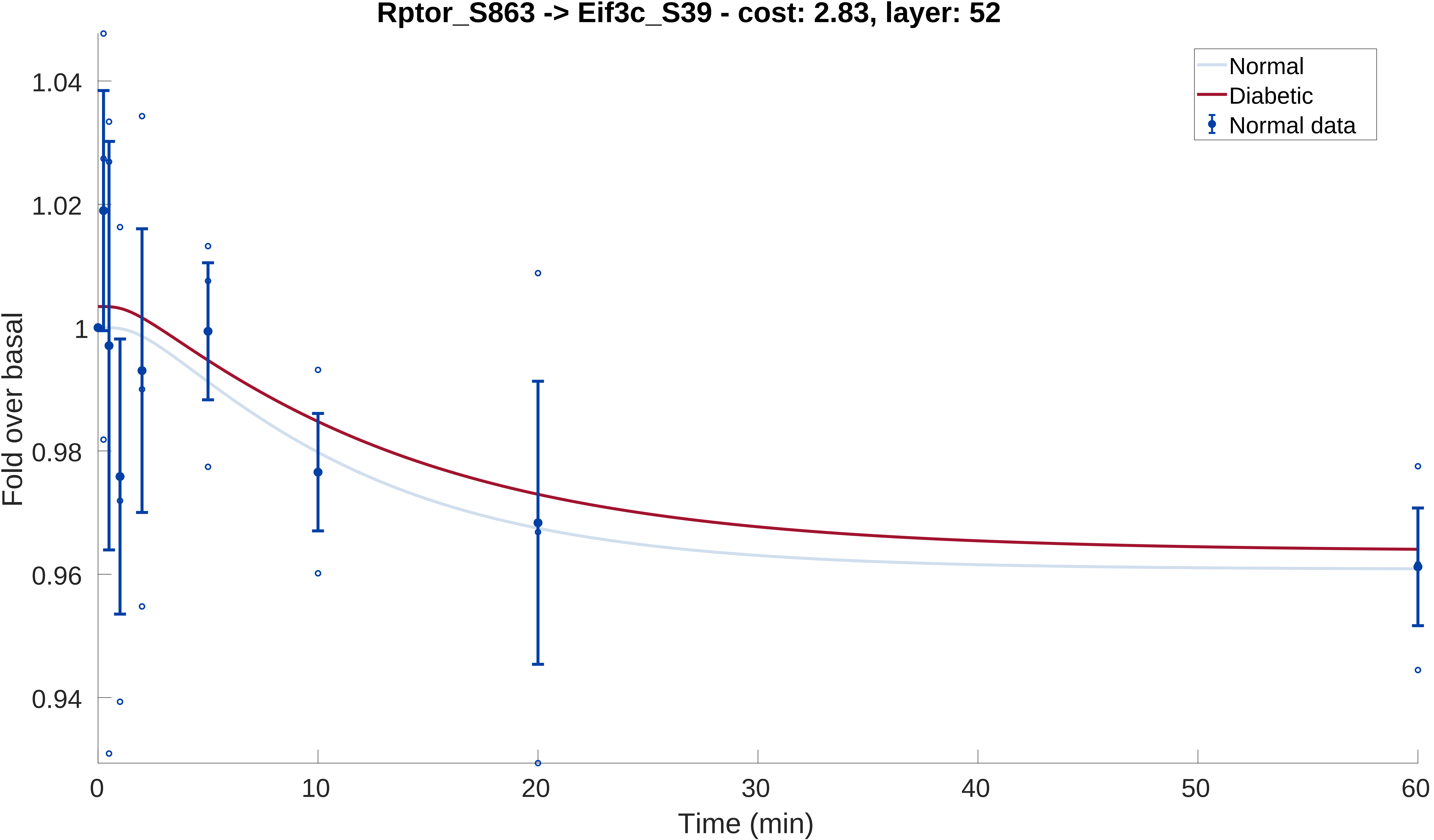

Rptor_S863 -> Eif3c_S39 - cost: 2.83, layer: 52
Normal
Diabetic
Normal data
Fold over basal
1.04
1.02
1
0.98
0.96
0.94
0
10
20
30
40
50
60
Time (min)

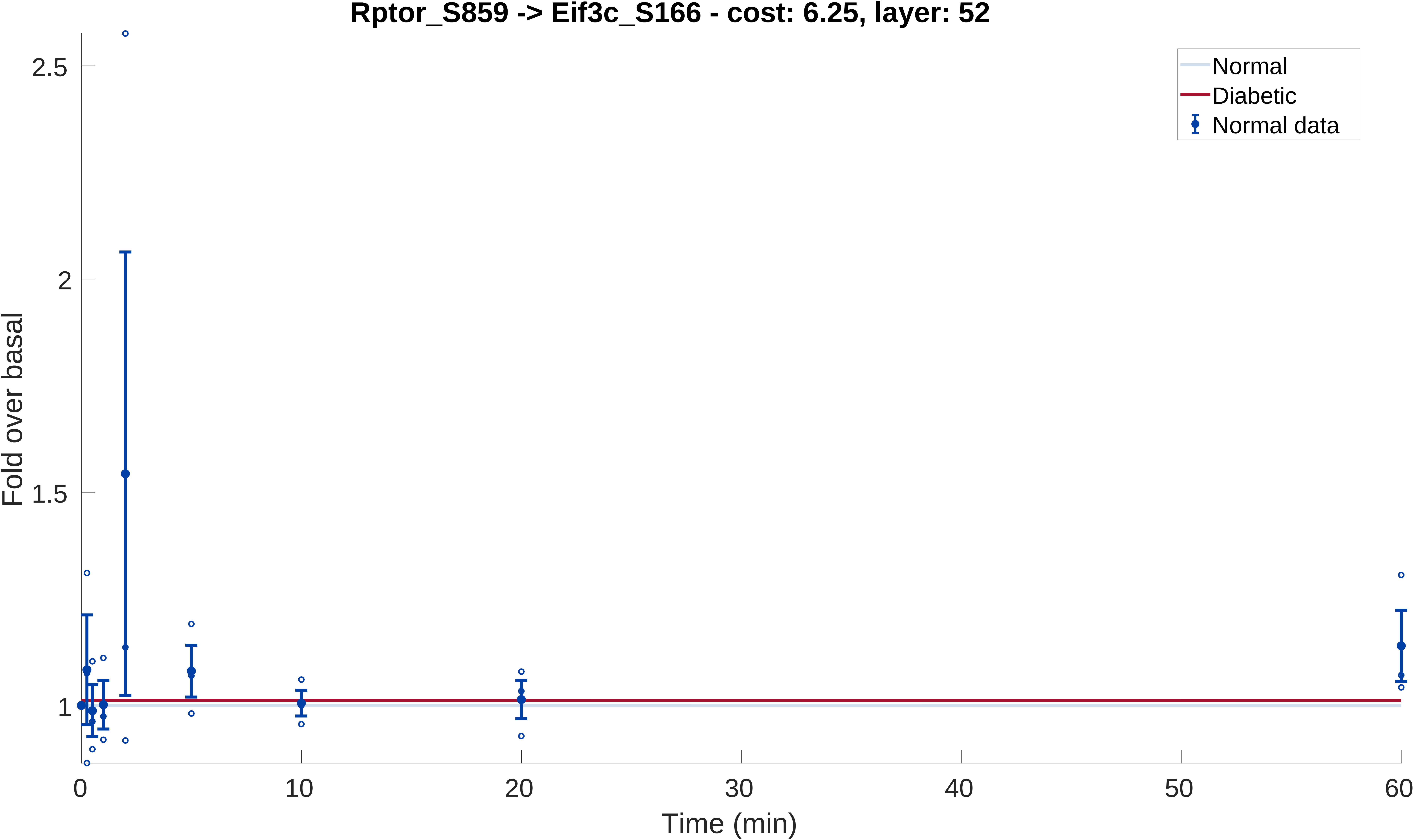

Rptor_S859 -> Eif3c_S166 - cost: 6.25, layer: 52
Normal
Diabetic
Normal data
Fold over basal
2.5
2
1.5
1
0
10
20
30
40
50
60
Time (min)

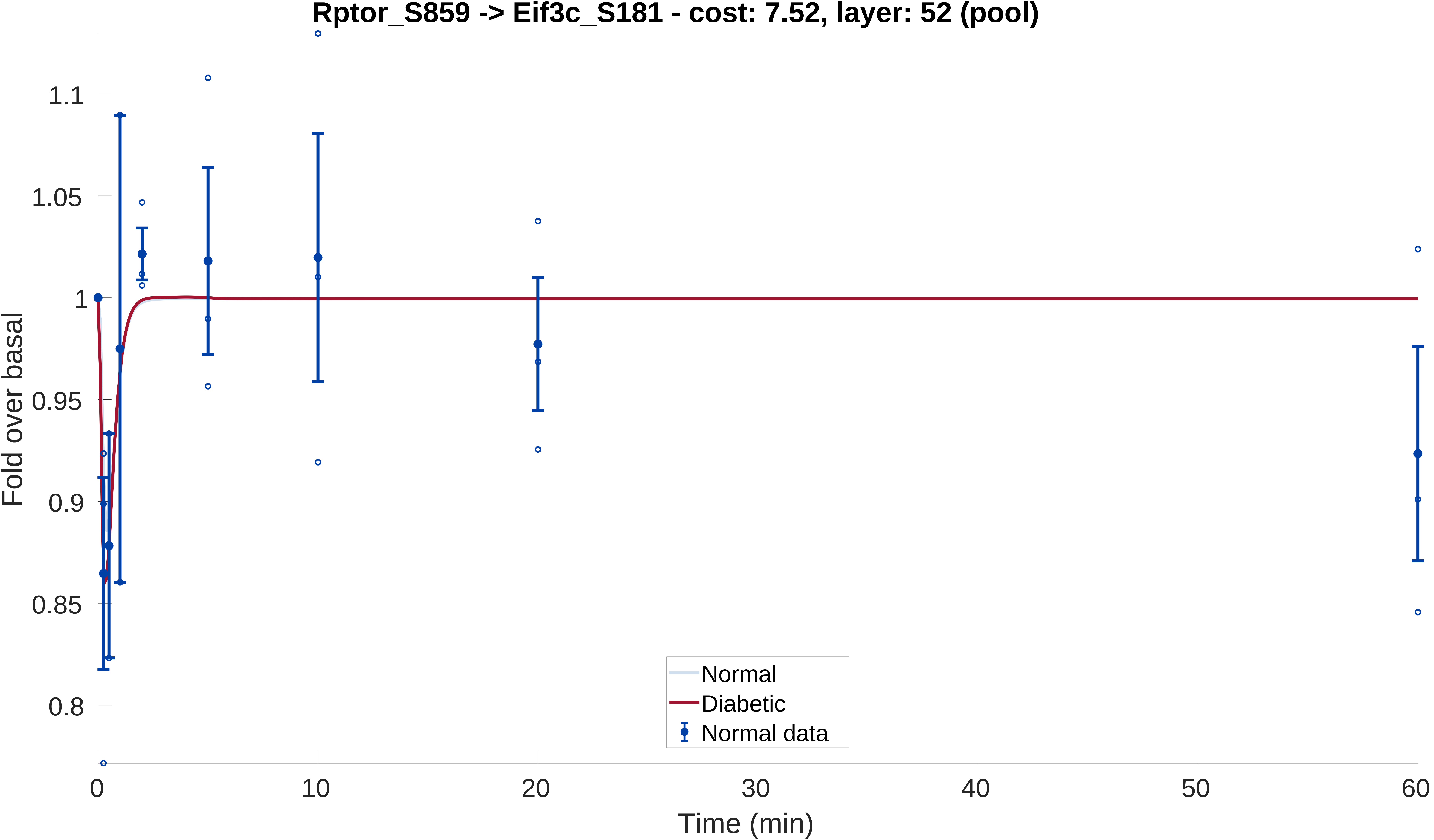

Rptor_S859 -> Eif3c_S181 - cost: 7.52, layer: 52 (pool)
Fold over basal
1.1
1.05
1
0.95
0.9
0.85
0.8
0
10
20
30
40
50
60
Time (min)
Normal
Diabetic
Normal data

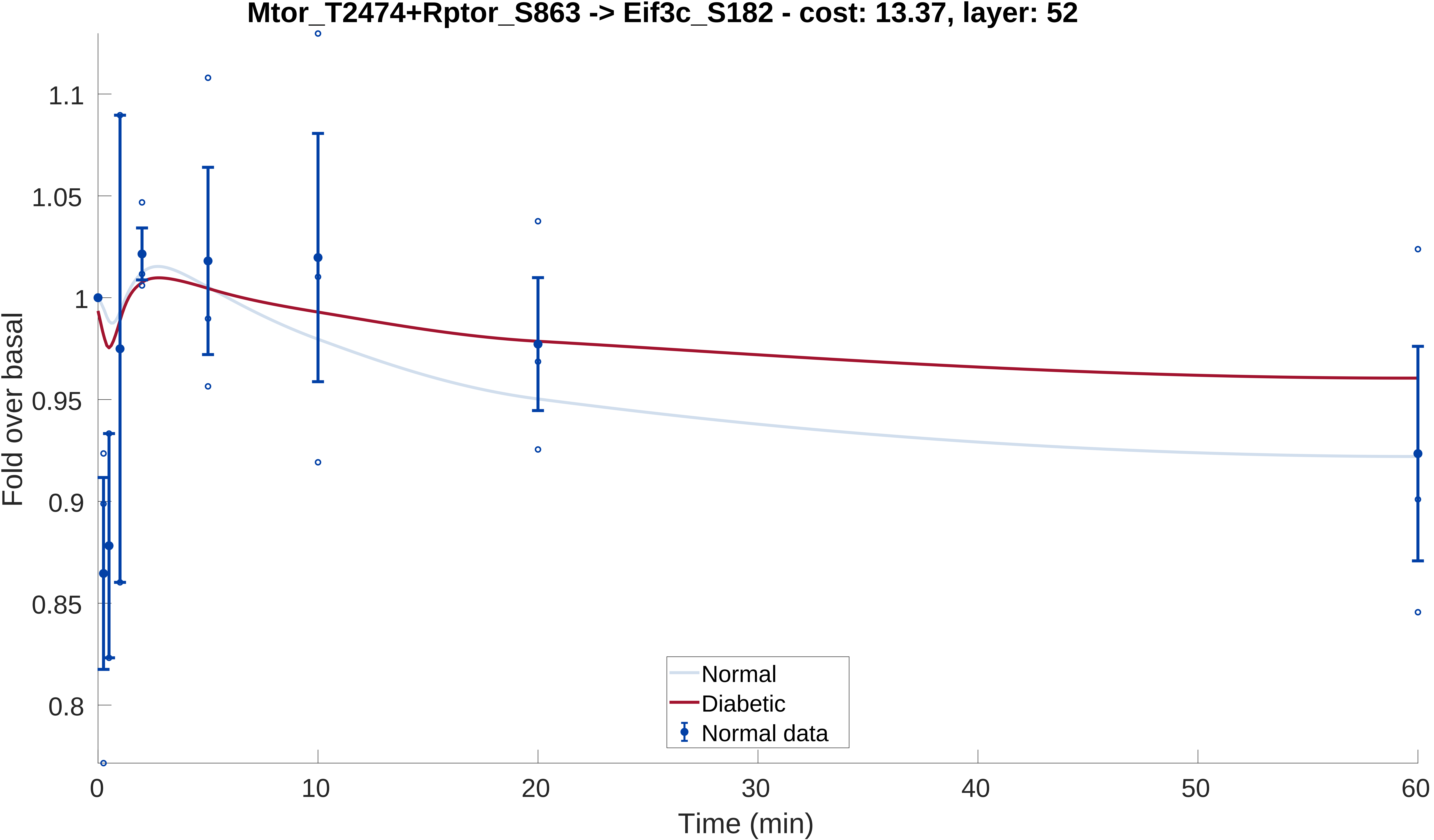

Mtor_T2474+Rptor_S863 -> Eif3c_S182 - cost: 13.37, layer: 52
Fold over basal
1.1
1.05
1
0.95
0.9
0.85
0.8
0
10
20
30
40
50
60
Time (min)
Normal
Diabetic
Normal data

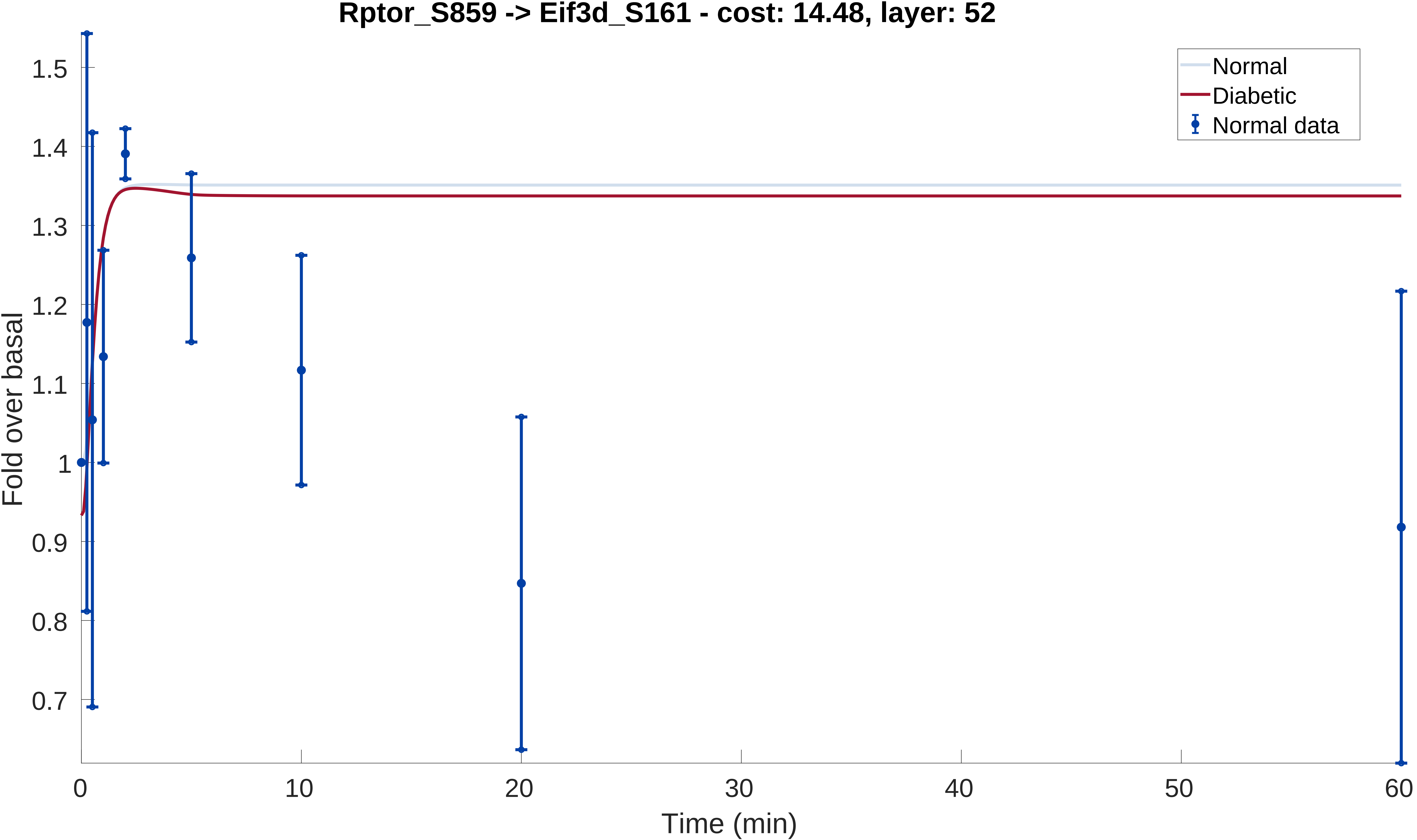

Rptor_S859 -> Eif3d_S161 - cost: 14.48, layer: 52
Normal
Diabetic
Normal data
Fold over basal
1.5
1.4
1.3
1.2
1.1
1
0.9
0.8
0.7
0
10
20
30
40
50
60
Time (min)

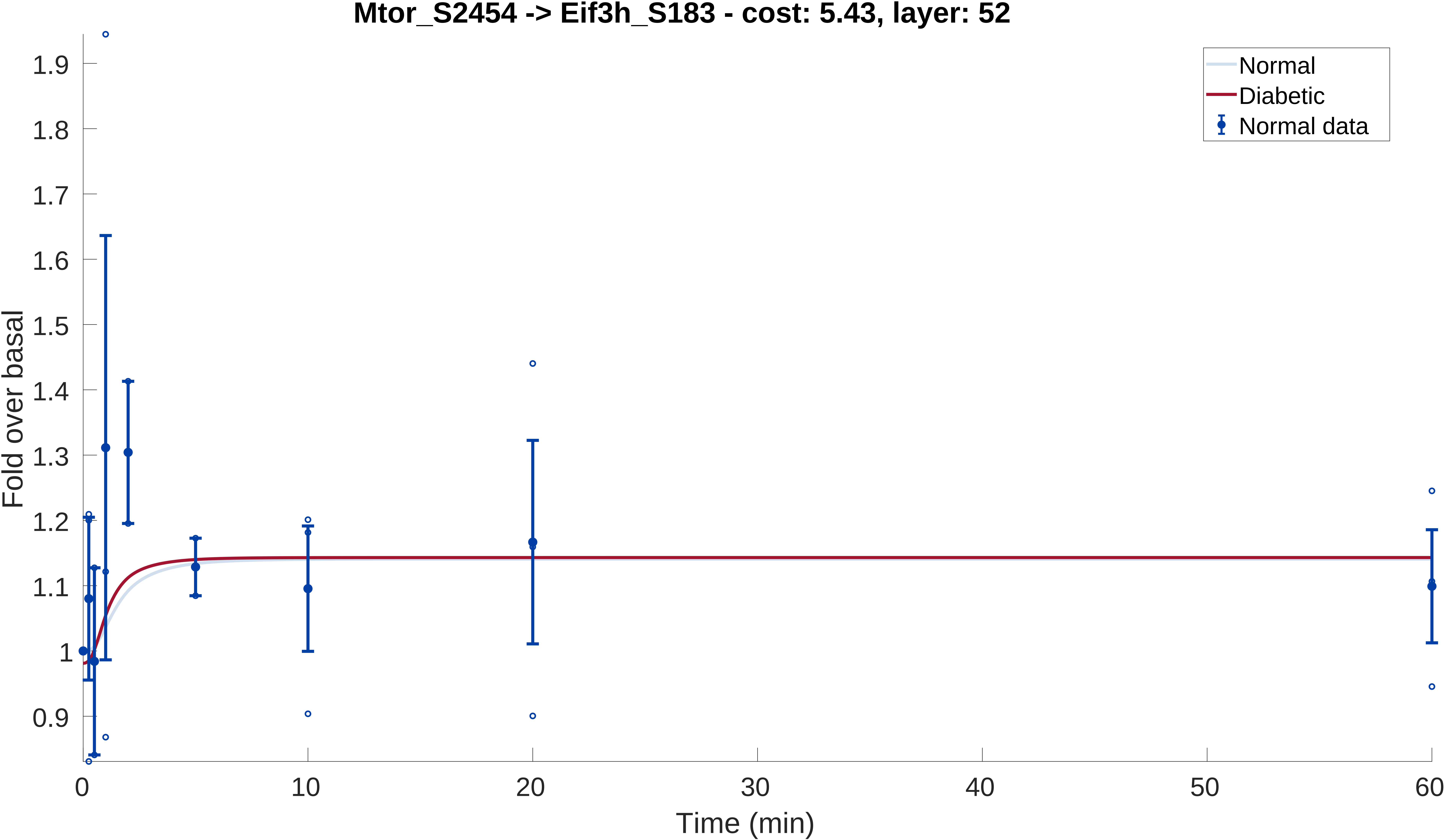

Mtor_S2454 -> Eif3h_S183 - cost: 5.43, layer: 52
Normal
Diabetic
Normal data
Fold over basal
1.9
1.8
1.7
1.6
1.5
1.4
1.3
1.2
1.1
1
0.9
0
10
20
30
40
50
60
Time (min)

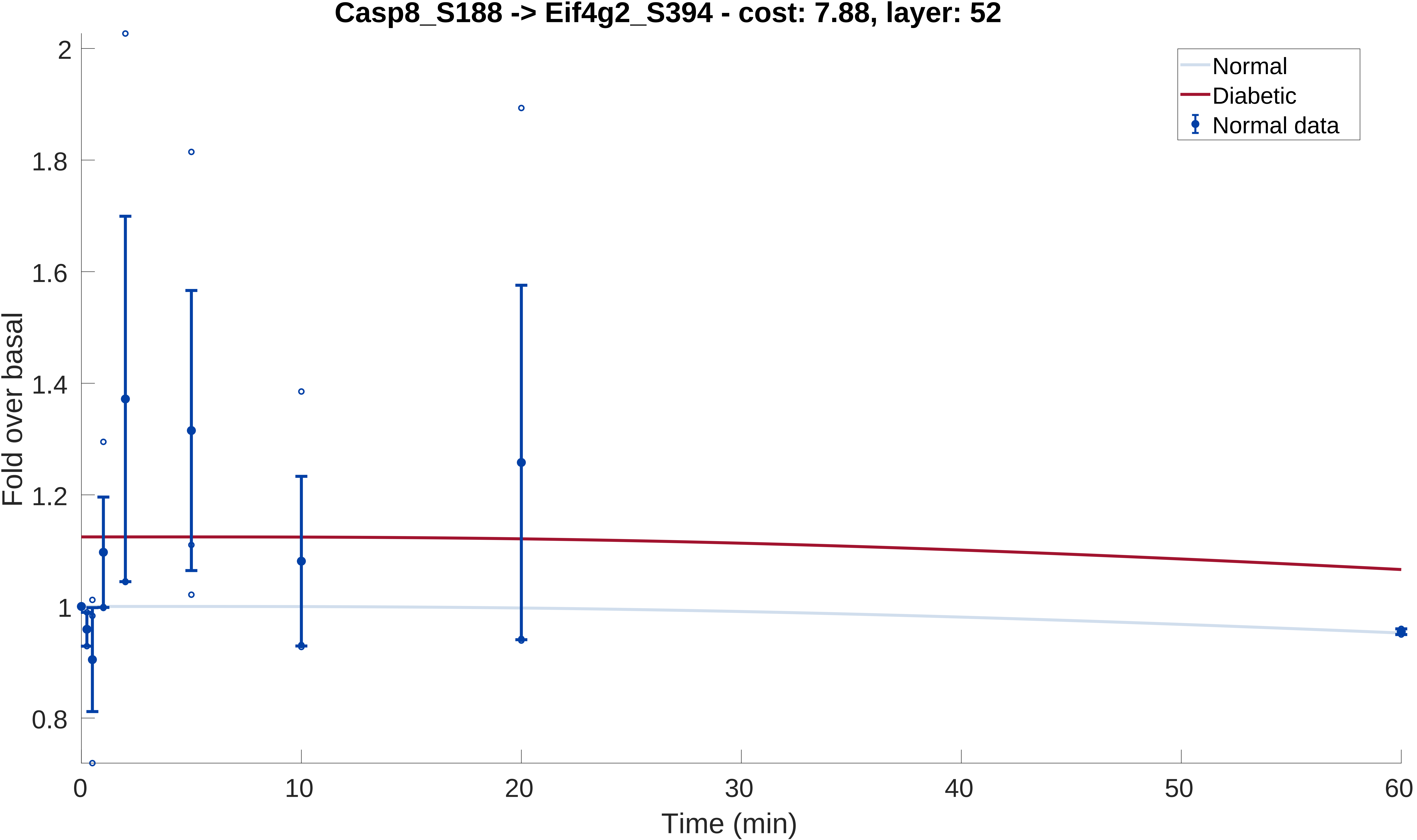
Casp8_S188 -> Eif4g2_S394 - cost: 7.88, layer: 52
Normal
Diabetic
Normal data
Fold over basal
Time (min)
2
1.8
1.6
1.4
1.2
1
0.8
0
10
20
30
40
50
60

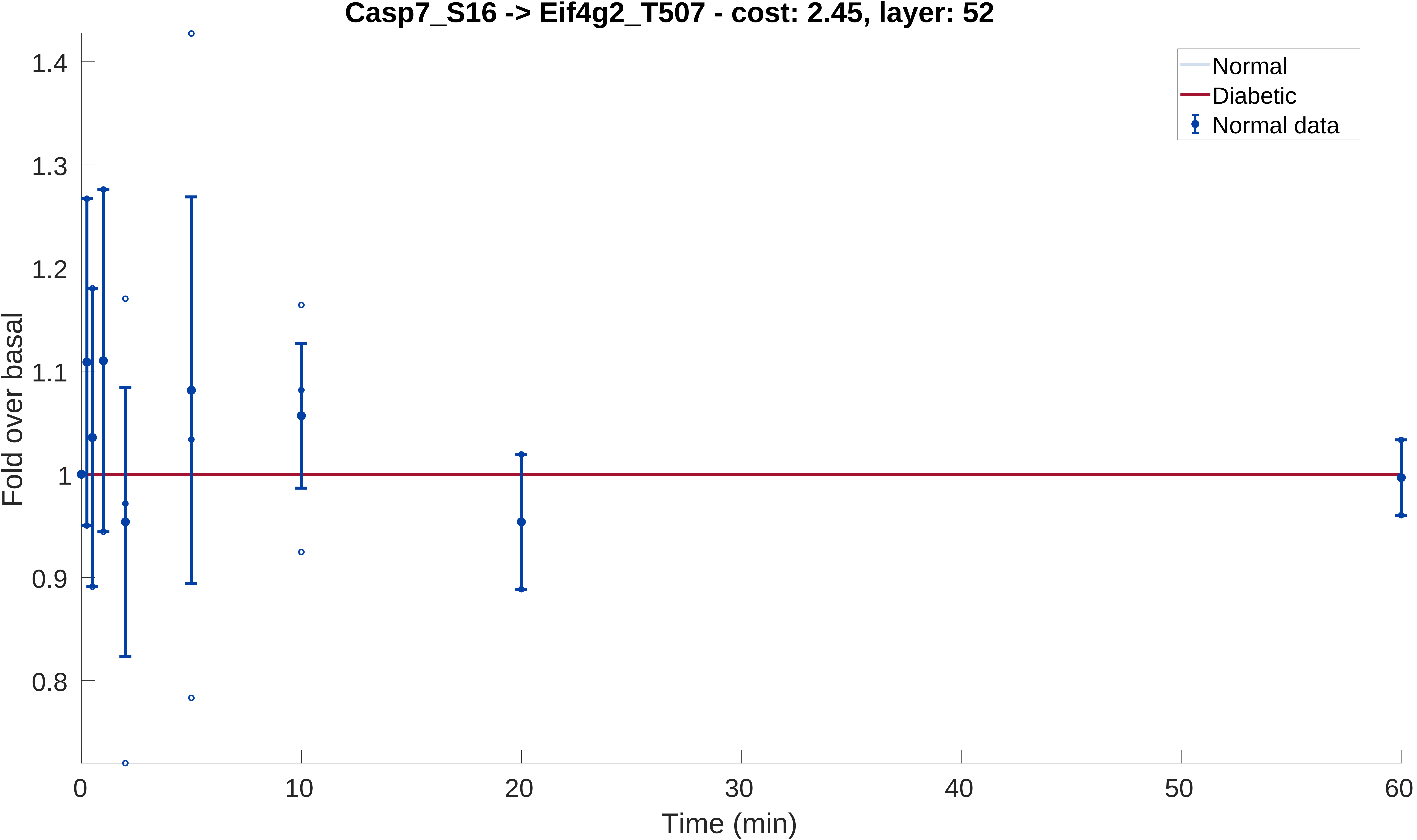

Casp7_S16 -> Eif4g2_T507 - cost: 2.45, layer: 52
Normal
Diabetic
Normal data
Fold over basal
1.4
1.3
1.2
1.1
1
0.9
0.8
Time (min)
0
10
20
30
40
50
60

Casp8_S188 -> Eif4g3_S480 - cost: 12.59, layer: 52

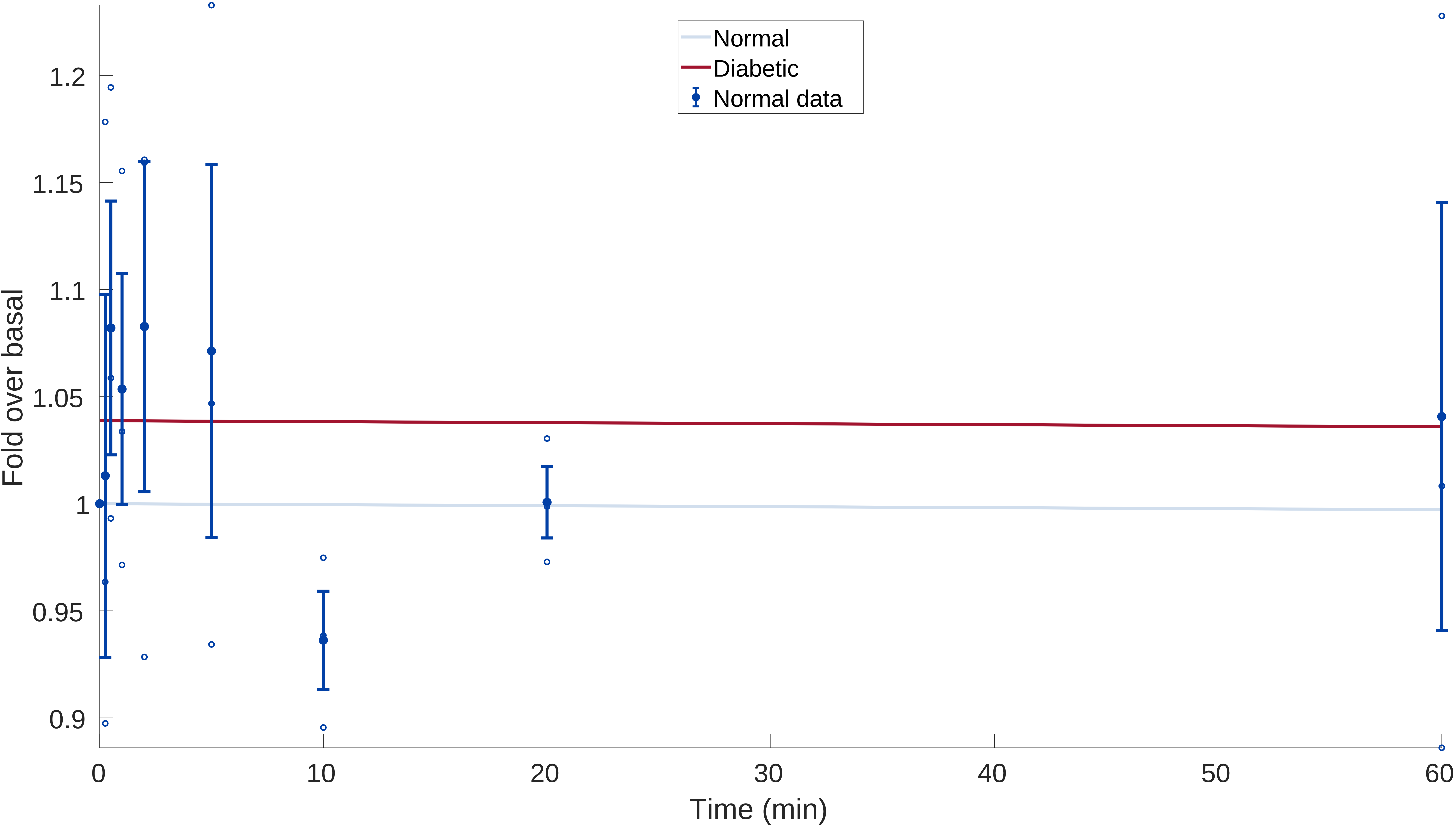

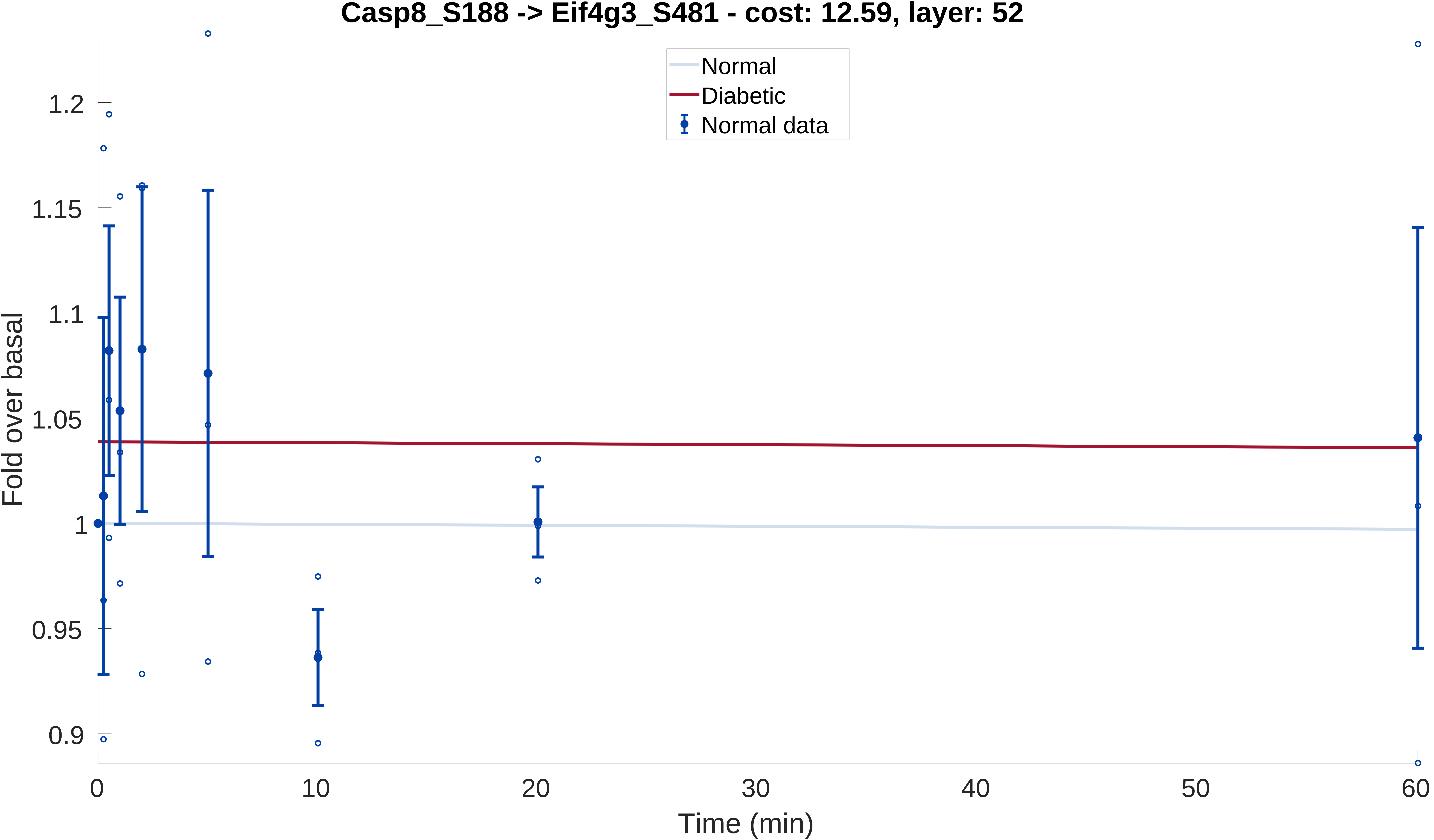

Casp8_S188 -> Eif4g3_S481 - cost: 12.59, layer: 52
Normal
Diabetic
Normal data
Fold over basal
1.2
1.15
1.1
1.05
1
0.95
0.9
0
10
20
30
40
50
60
Time (min)

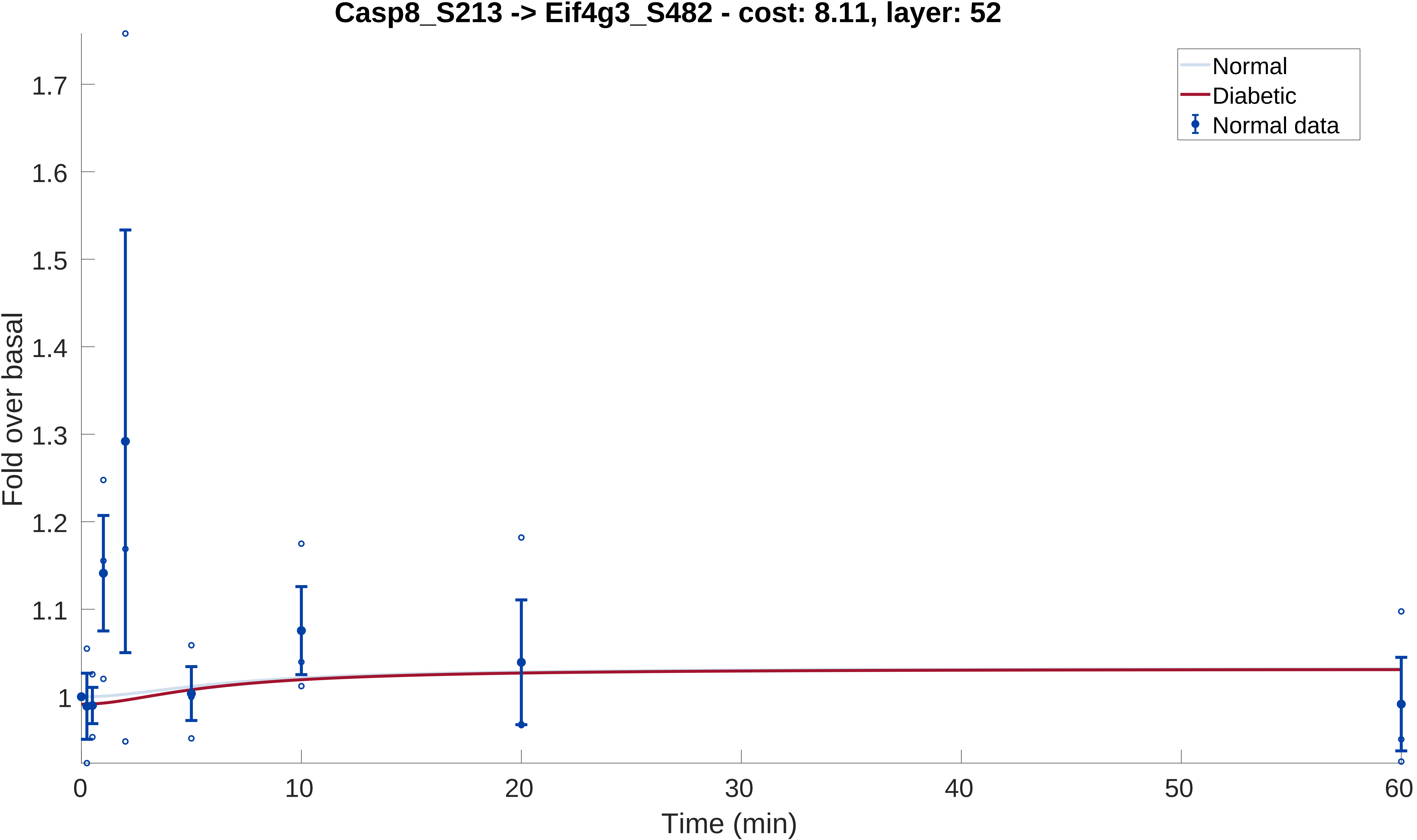
Casp8_S213 -> Eif4g3_S482 - cost: 8.11, layer: 52
Normal
Diabetic
Normal data
Fold over basal
1.7
1.6
1.5
1.4
1.3
1.2
1.1
1
0
10
20
30
40
50
60
Time (min)

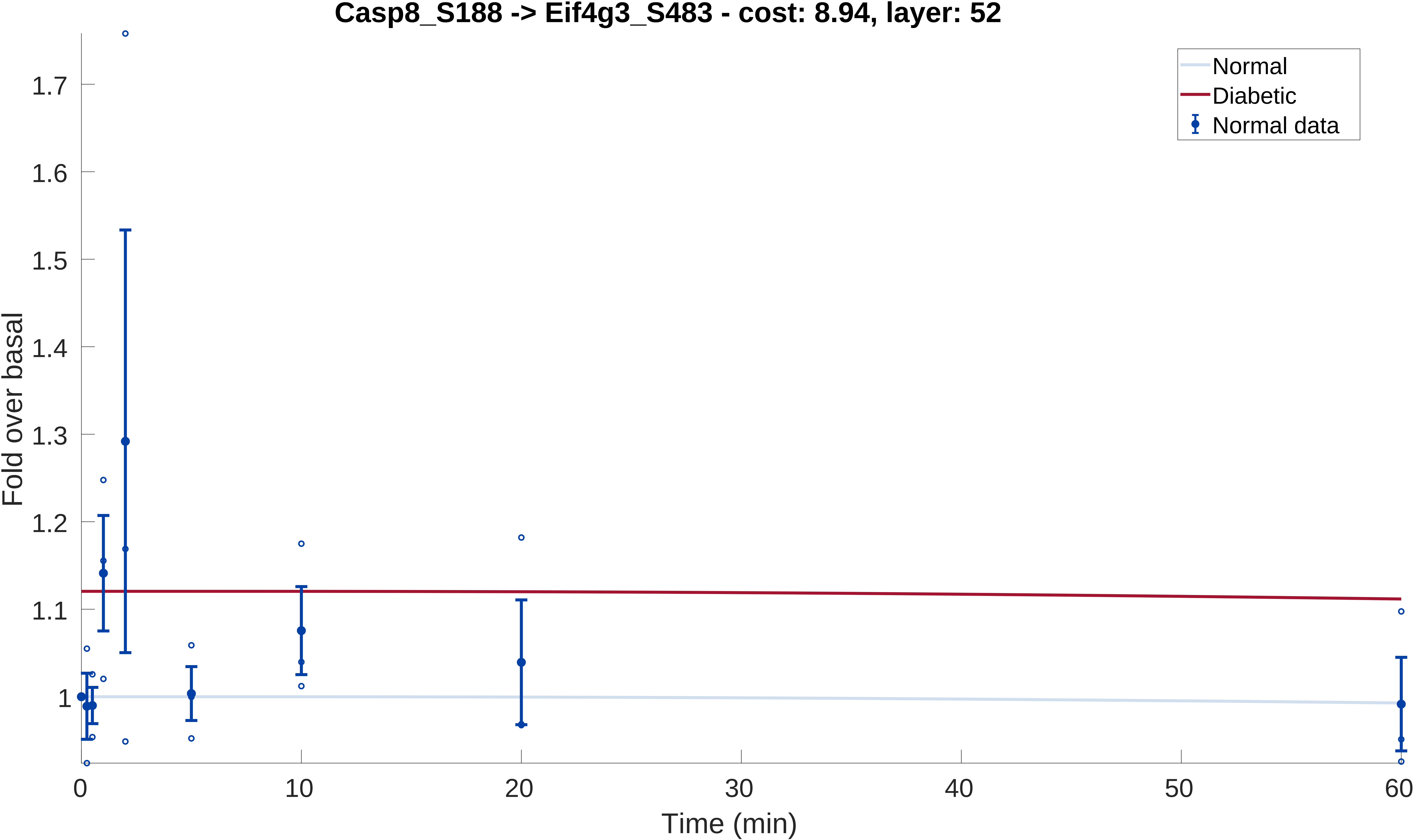

Casp8_S188 -> Eif4g3_S483 - cost: 8.94, layer: 52
Normal
Diabetic
Normal data
Fold over basal
1.7
1.6
1.5
1.4
1.3
1.2
1.1
1
Time (min)
0
10
20
30
40
50
60

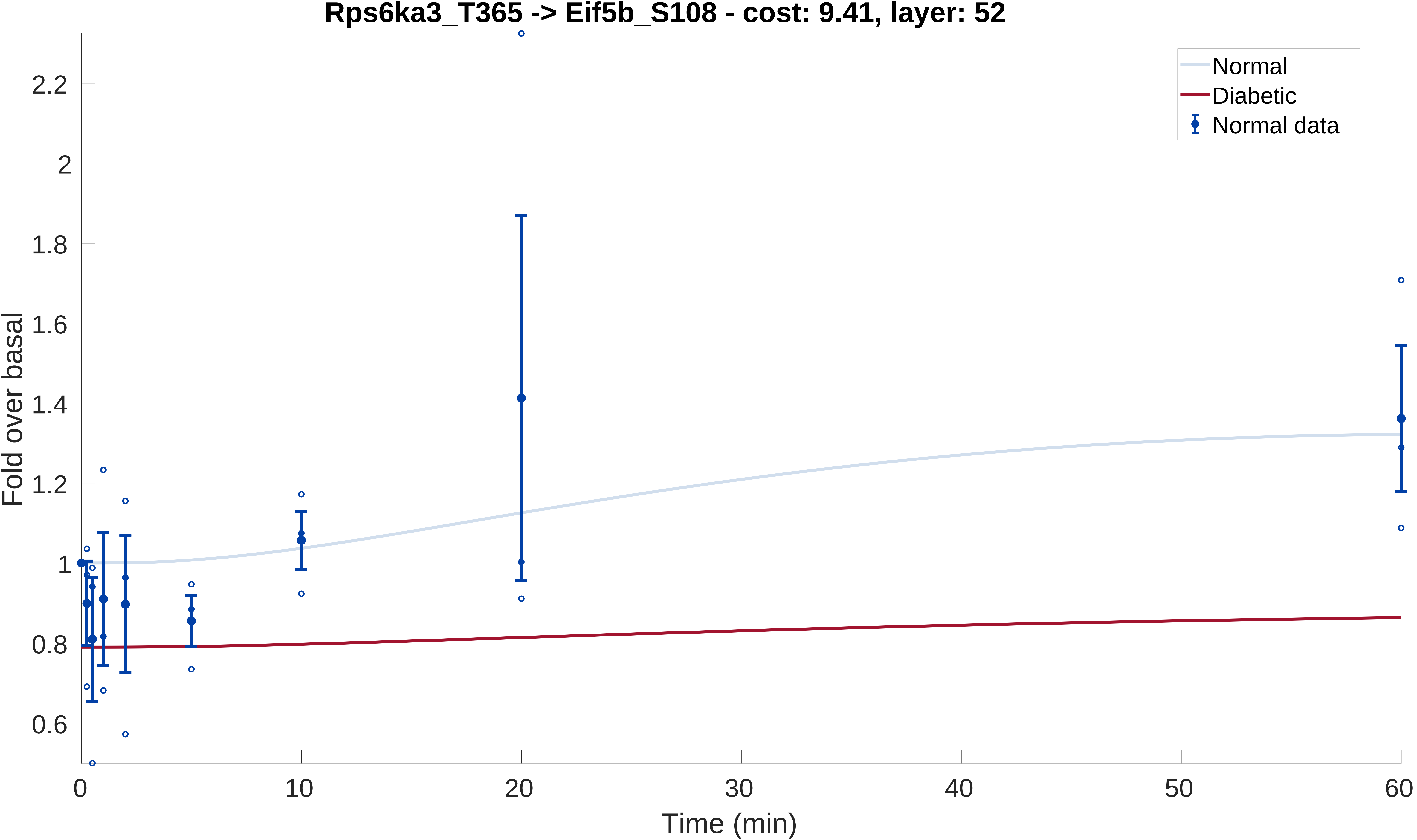

Rps6ka3_T365 -> Eif5b_S108 - cost: 9.41, layer: 52
Normal
Diabetic
Normal data
Fold over basal
Time (min)
2.2
2
1.8
1.6
1.4
1.2
1
0.8
0.6
0
10
20
30
40
50
60

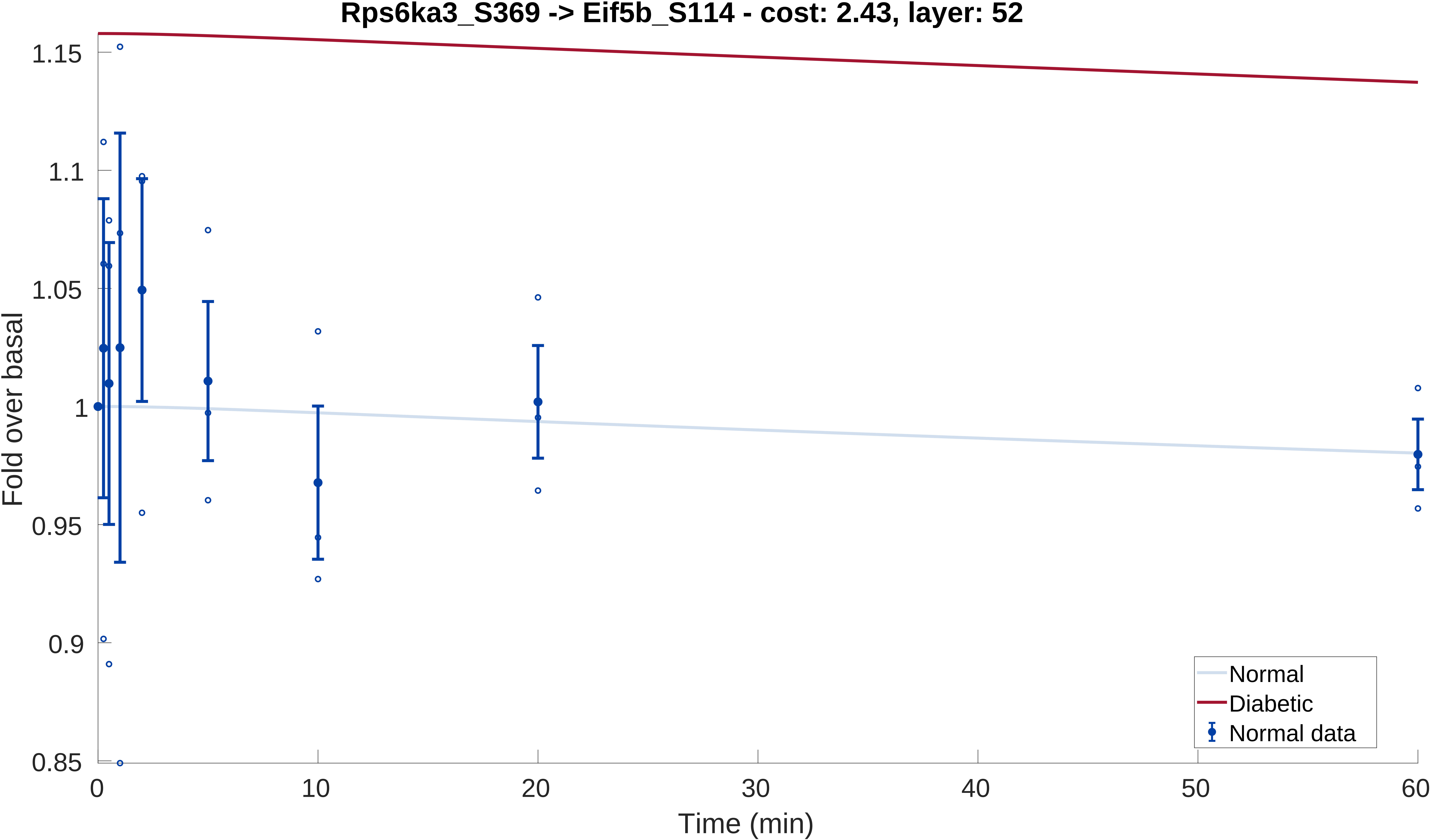

Rps6ka3_S369 -> Eif5b_S114 - cost: 2.43, layer: 52
Fold over basal
1.15
1.1
1.05
1
0.95
0.9
0.85
0
10
20
30
40
50
60
Time (min)
Normal
Diabetic
Normal data

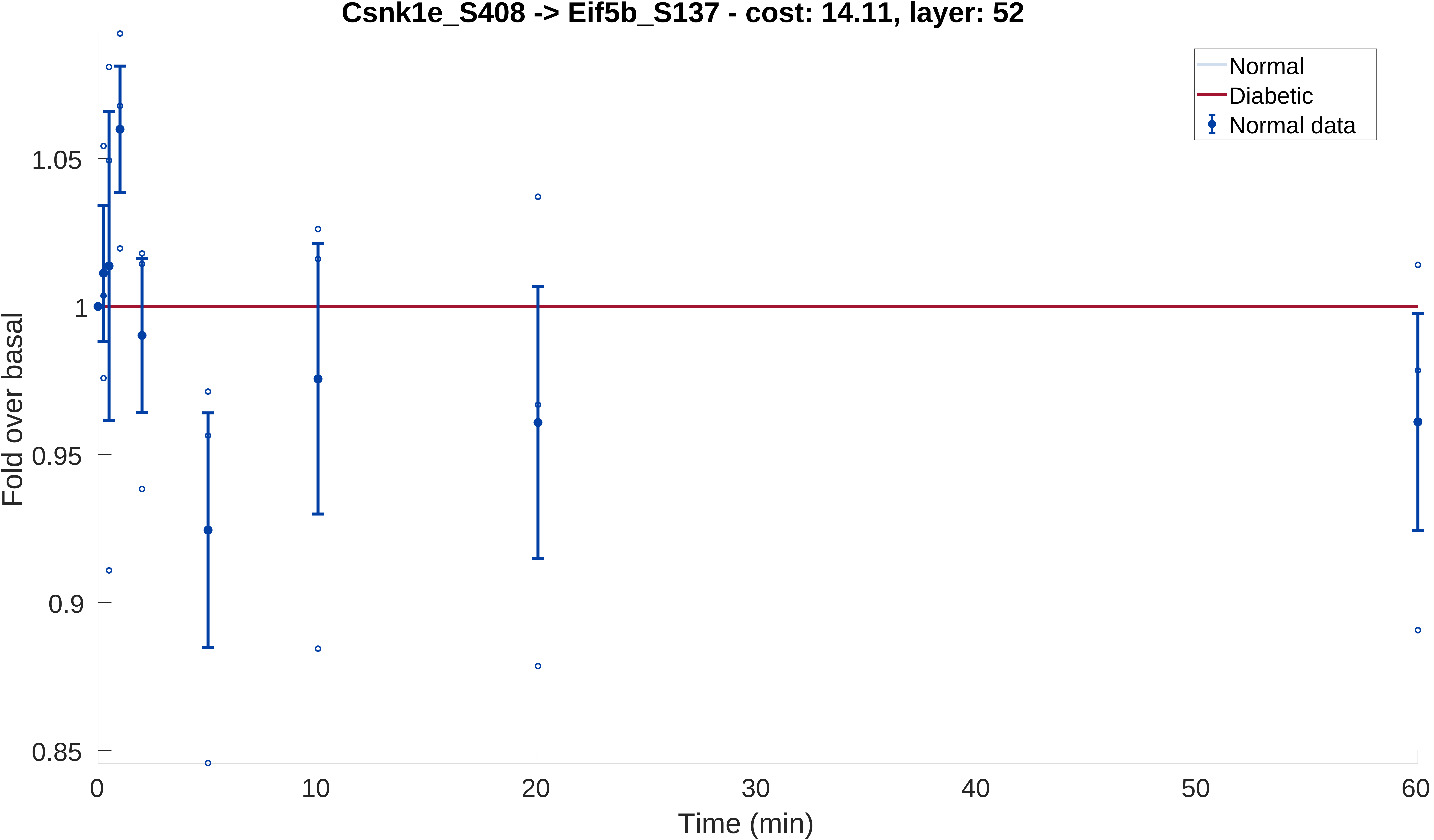
Csnk1e_S408 -> Eif5b_S137 - cost: 14.11, layer: 52
Normal
Diabetic
Normal data
Fold over basal
1.05
1
0.95
0.9
0.85
0
10
20
30
40
50
60
Time (min)

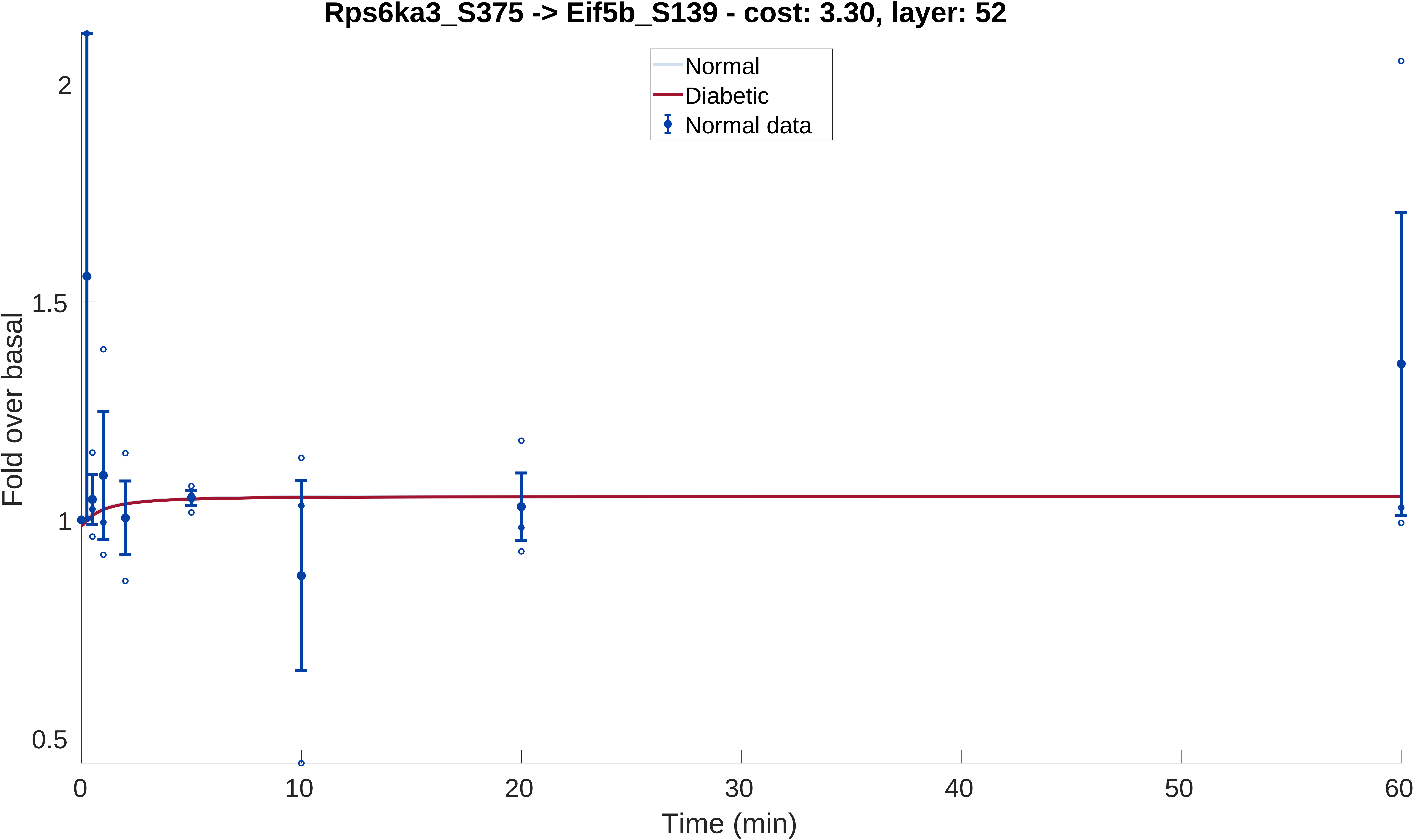

Rps6ka3_S375 -> Eif5b_S139 - cost: 3.30, layer: 52
Normal
Diabetic
Normal data
Fold over basal
2
1.5
1
0.5
0
10
20
30
40
50
60
Time (min)

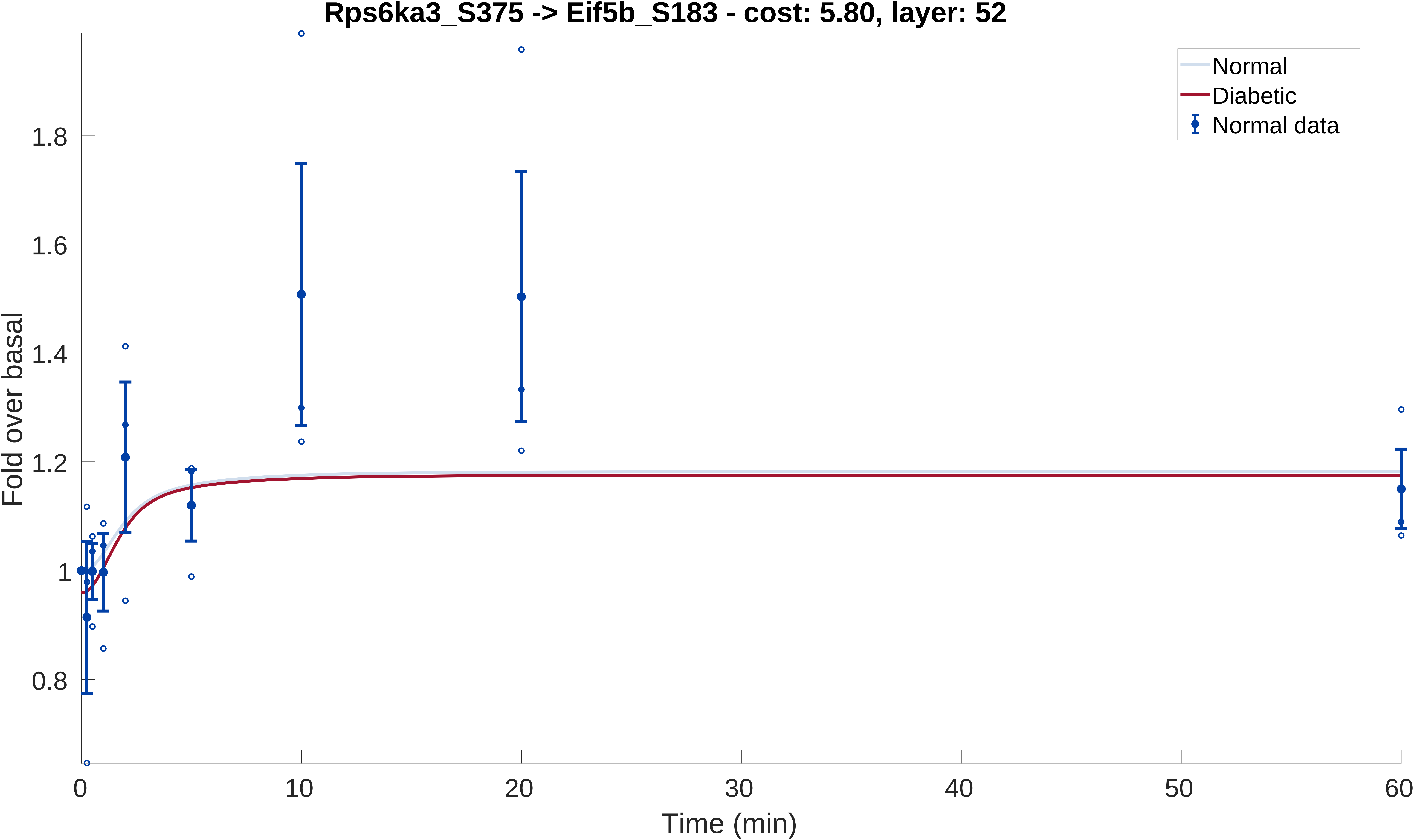

Rps6ka3_S375 -> Eif5b_S183 - cost: 5.80, layer: 52
Normal
Diabetic
Normal data
Fold over basal
1.8
1.6
1.4
1.2
1
0.8
0
10
20
30
40
50
60
Time (min)

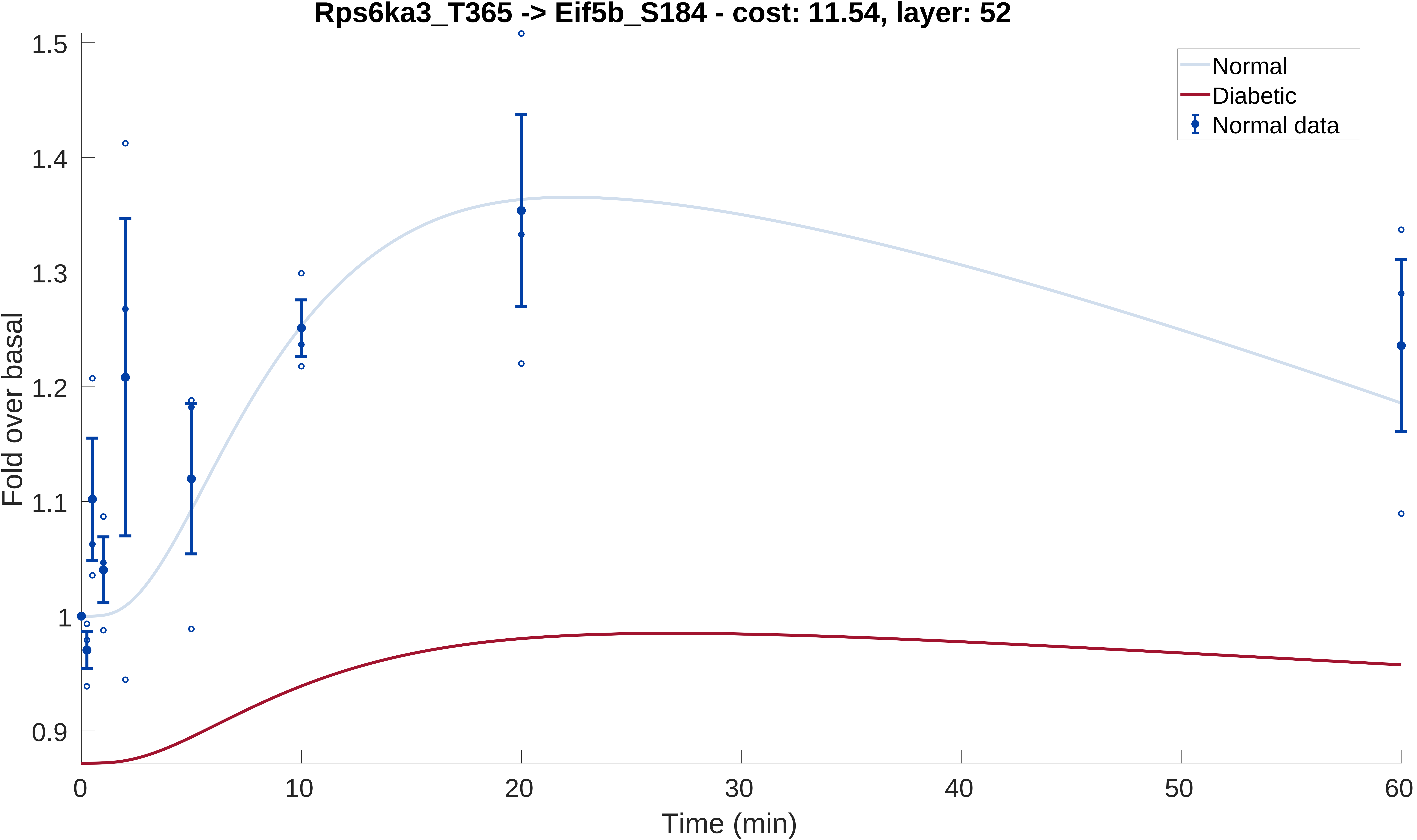

Rps6ka3_T365 -> Eif5b_S184 - cost: 11.54, layer: 52
Normal
Diabetic
Normal data
Fold over basal
Time (min)
1.5
1.4
1.3
1.2
1.1
1
0.9
0
10
20
30
40
50
60

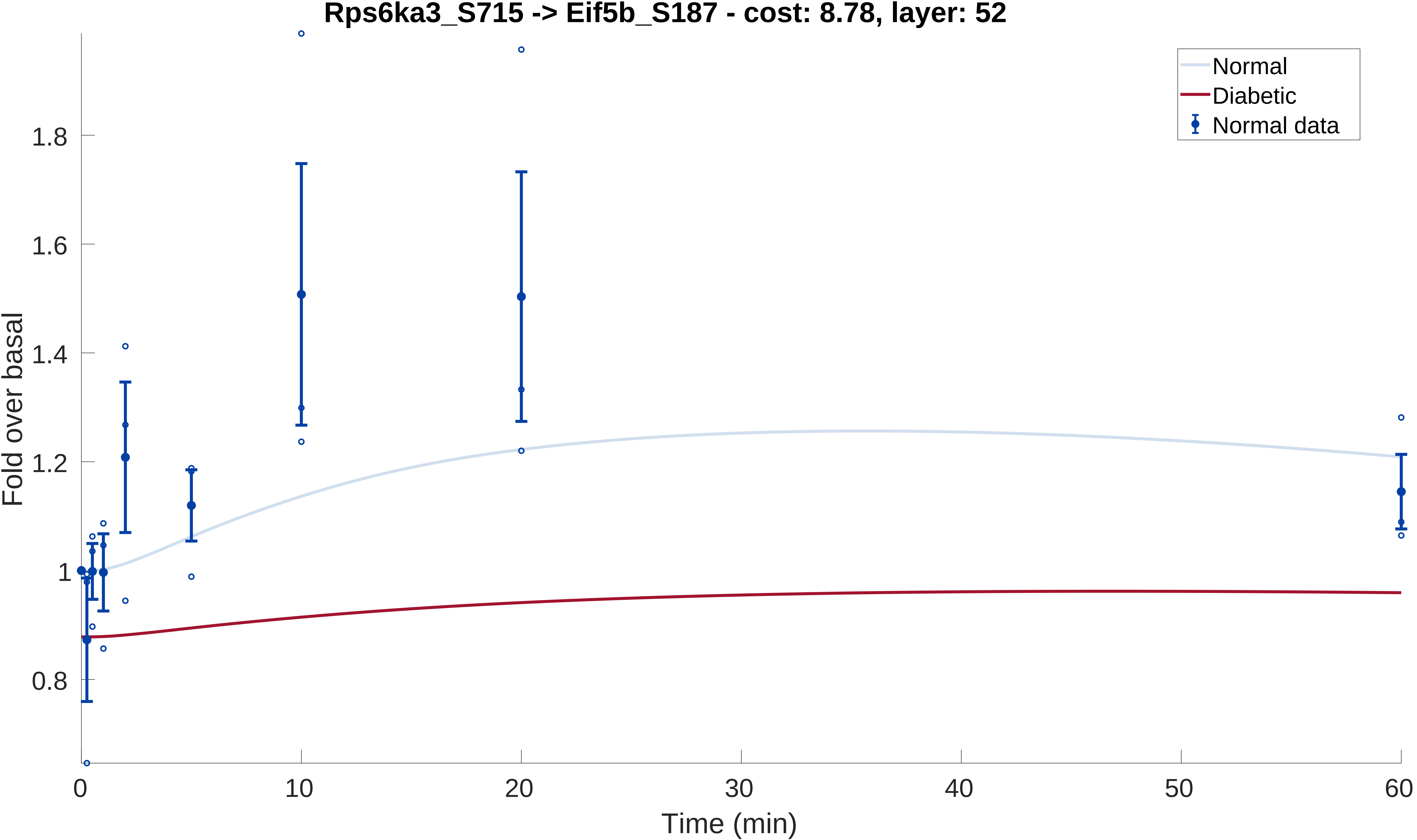

Rps6ka3_S715 -> Eif5b_S187 - cost: 8.78, layer: 52
Normal
Diabetic
Normal data
Fold over basal
1.8
1.6
1.4
1.2
1
0.8
0
10
20
30
40
50
60
Time (min)

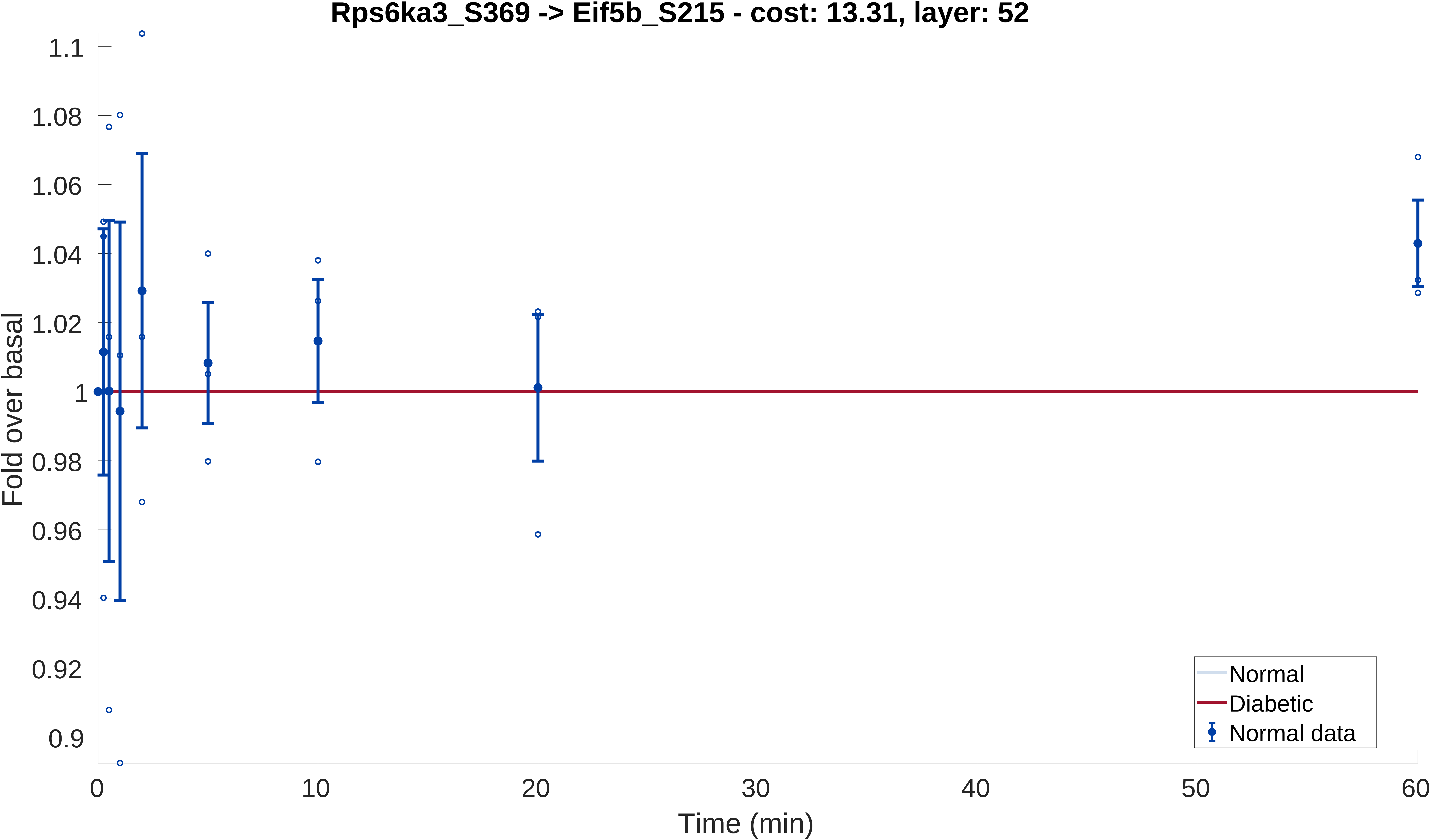

Rps6ka3_S369 -> Eif5b_S215 - cost: 13.31, layer: 52
Fold over basal
1.1
1.08
1.06
1.04
1.02
1
0.98
0.96
0.94
0.92
0.9
0
10
20
30
40
50
60
Time (min)
Normal
Diabetic
Normal data

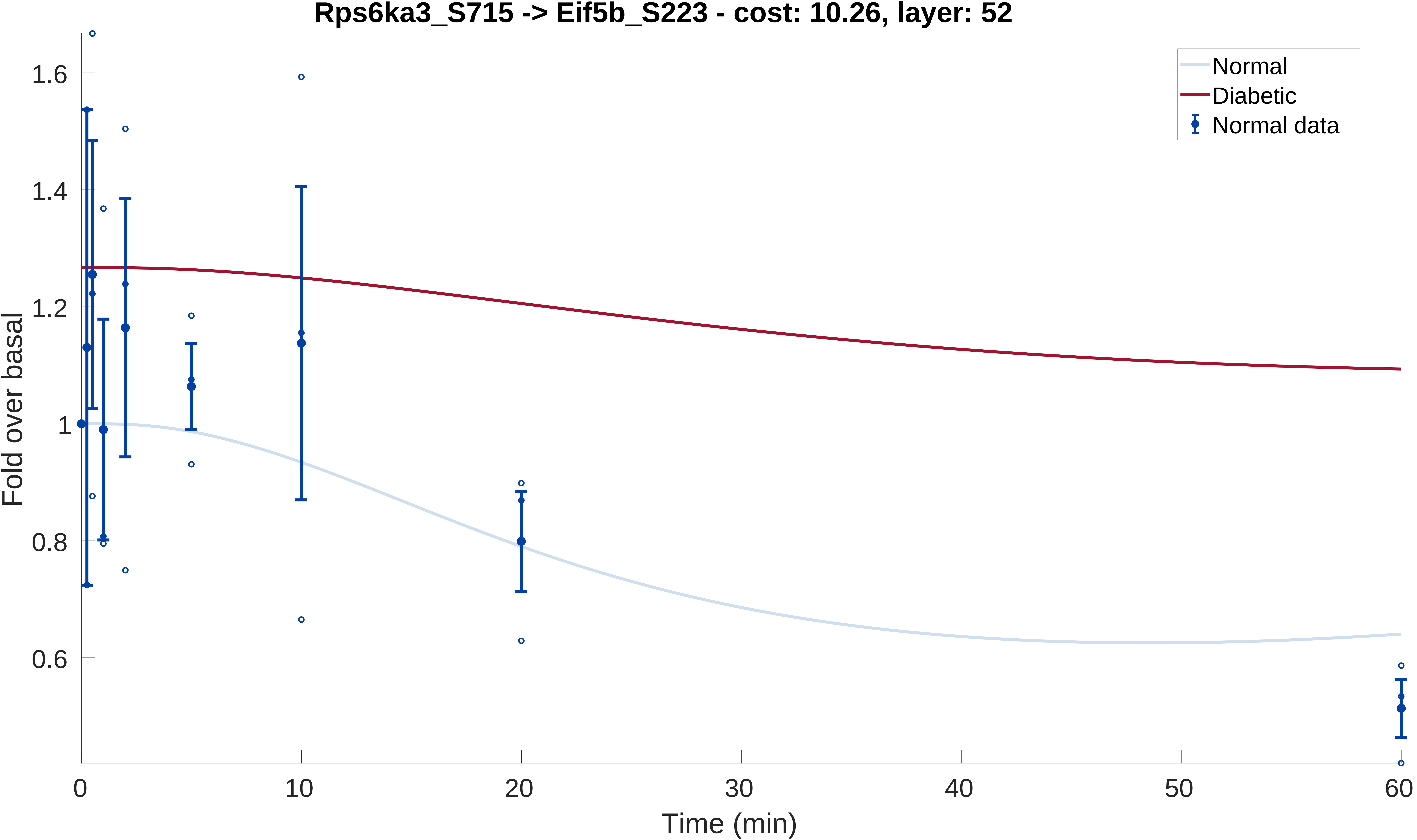

Rps6ka3_S715 -> Eif5b_S223 - cost: 10.26, layer: 52
Normal
Diabetic
Normal data
Fold over basal
1.6
1.4
1.2
1
0.8
0.6
0
10
20
30
40
50
60
Time (min)

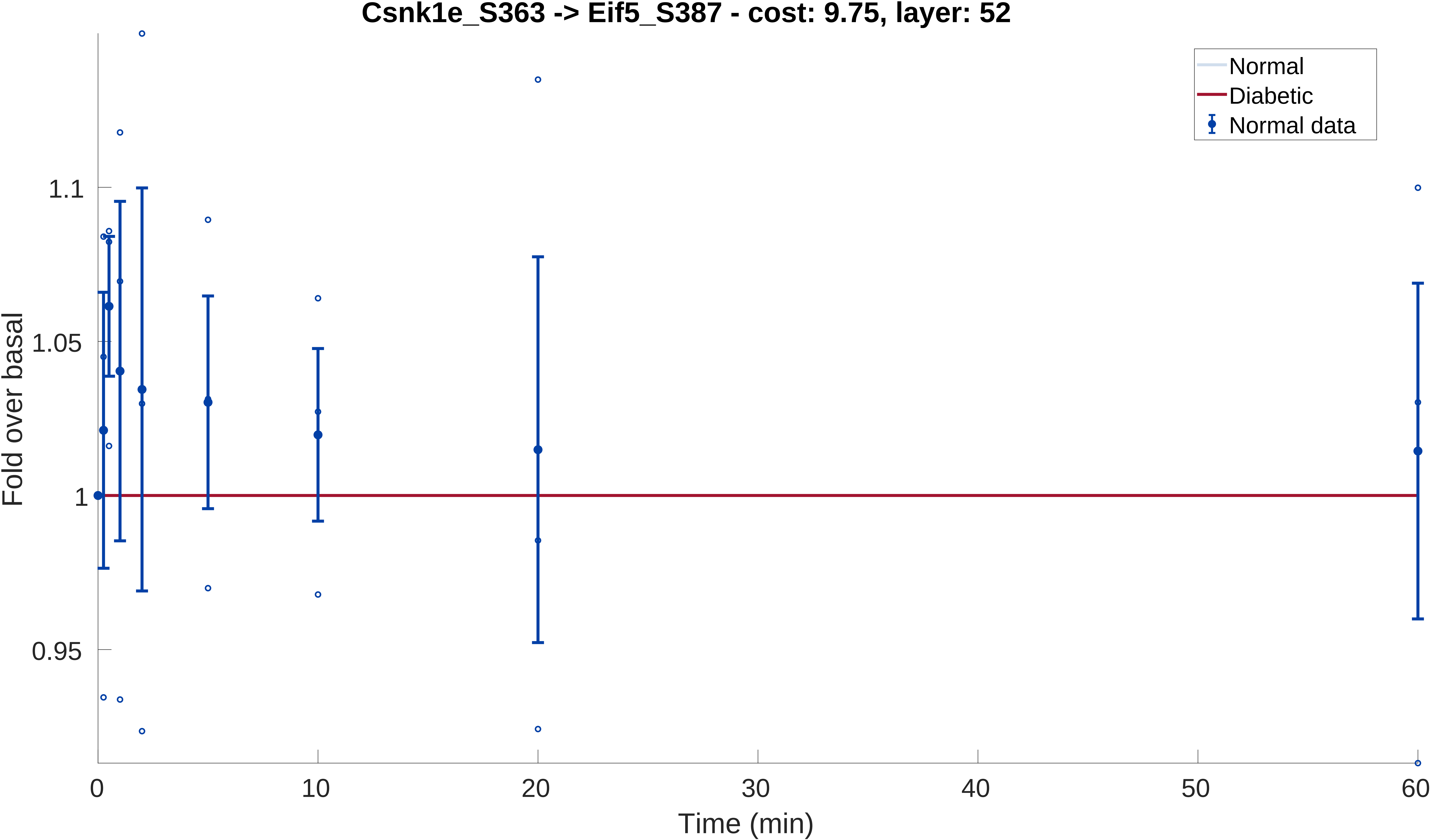

Csnk1e_S363 -> Eif5_S387 - cost: 9.75, layer: 52
Normal
Diabetic
Normal data
1.1
1.05
1
0.95
Fold over basal
0
10
20
30
40
50
60
Time (min)

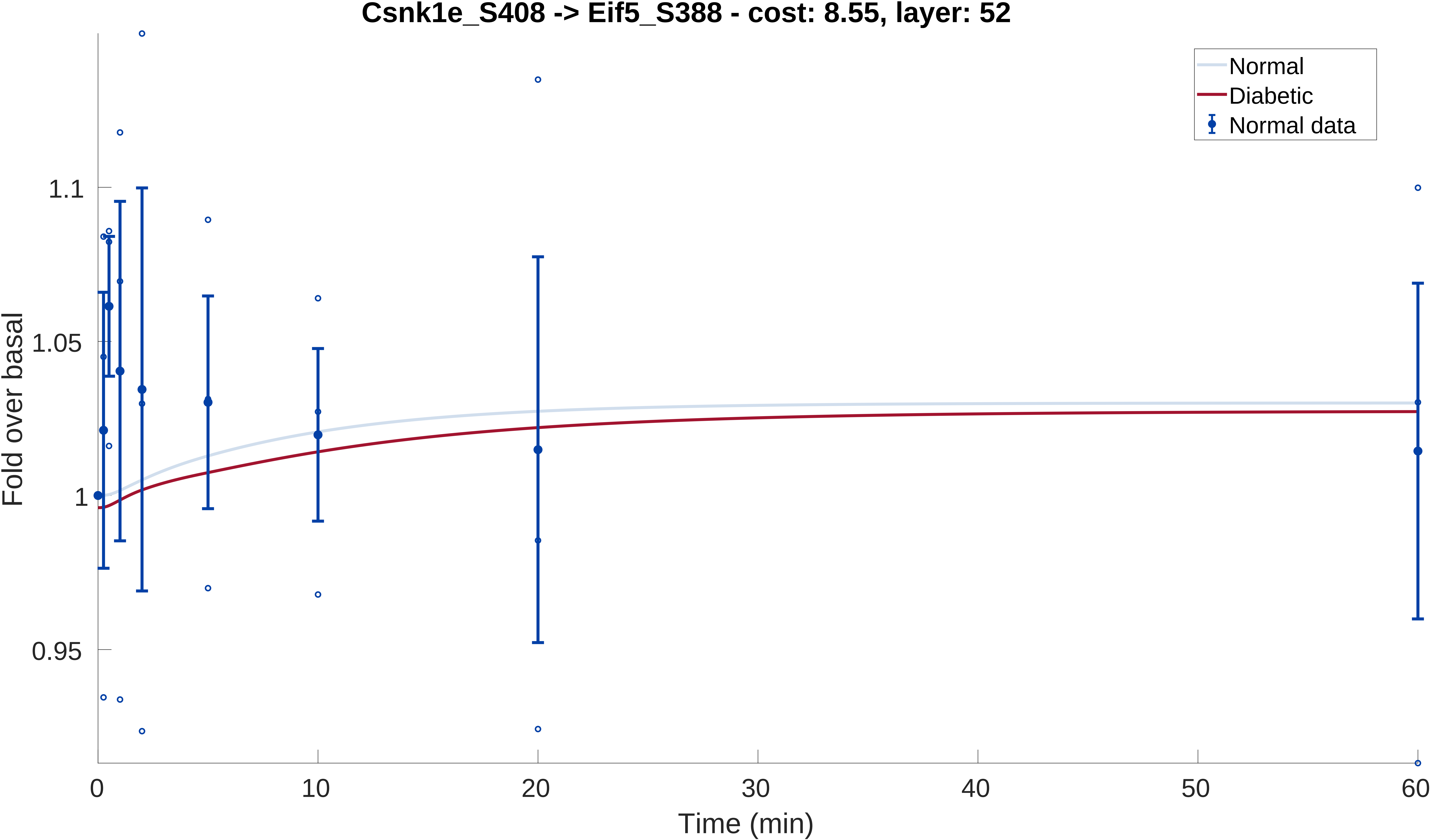

Csnk1e_S408 -> Eif5_S388 - cost: 8.55, layer: 52
Normal
Diabetic
Normal data
Fold over basal
1.1
1.05
1
0.95
0
10
20
30
40
50
60
Time (min)

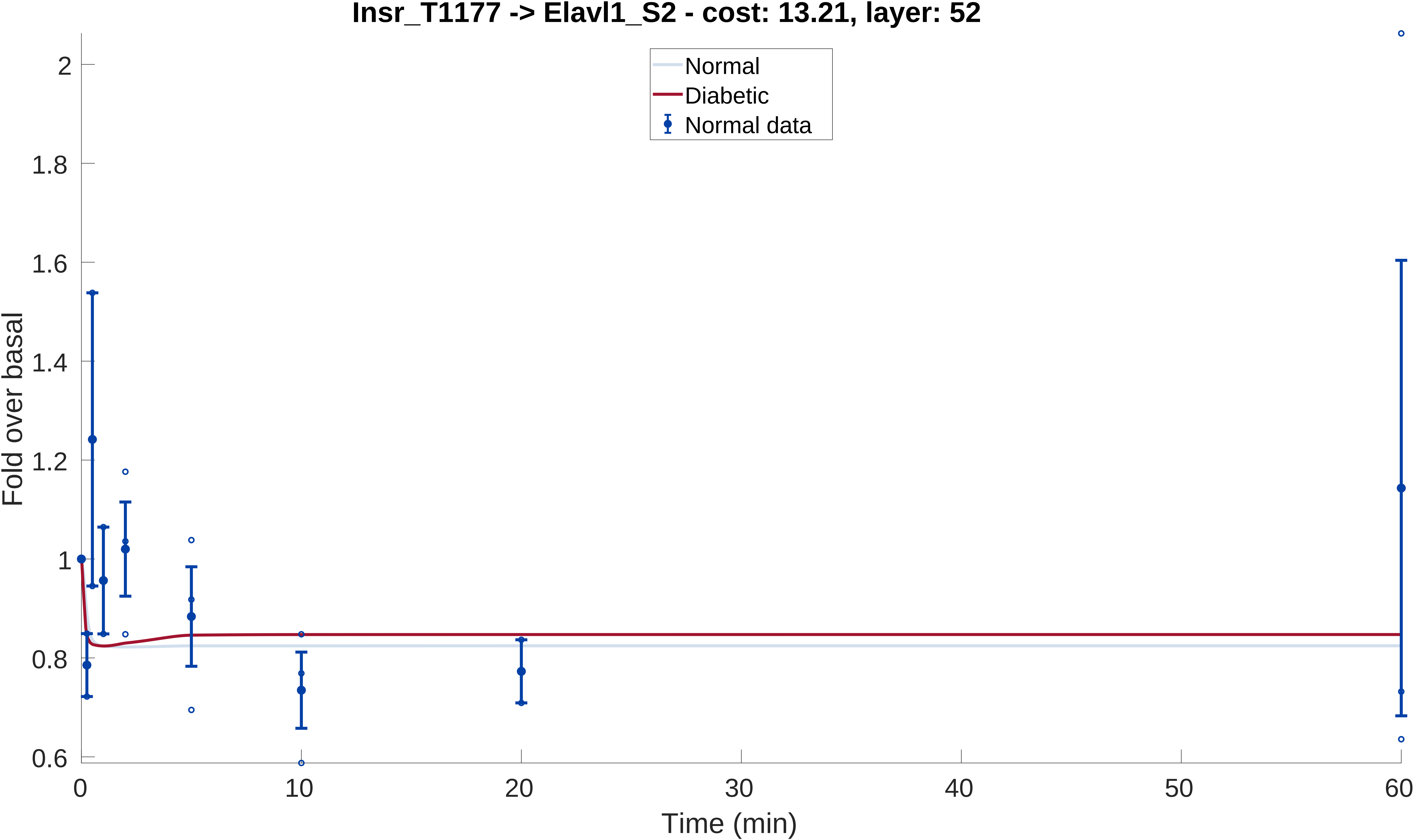

Insr_T1177 -> Elavl1_S2 - cost: 13.21, layer: 52
Normal
Diabetic
Normal data
Fold over basal
Time (min)
0.6
0.8
1
1.2
1.4
1.6
1.8
2
0
10
20
30
40
50
60

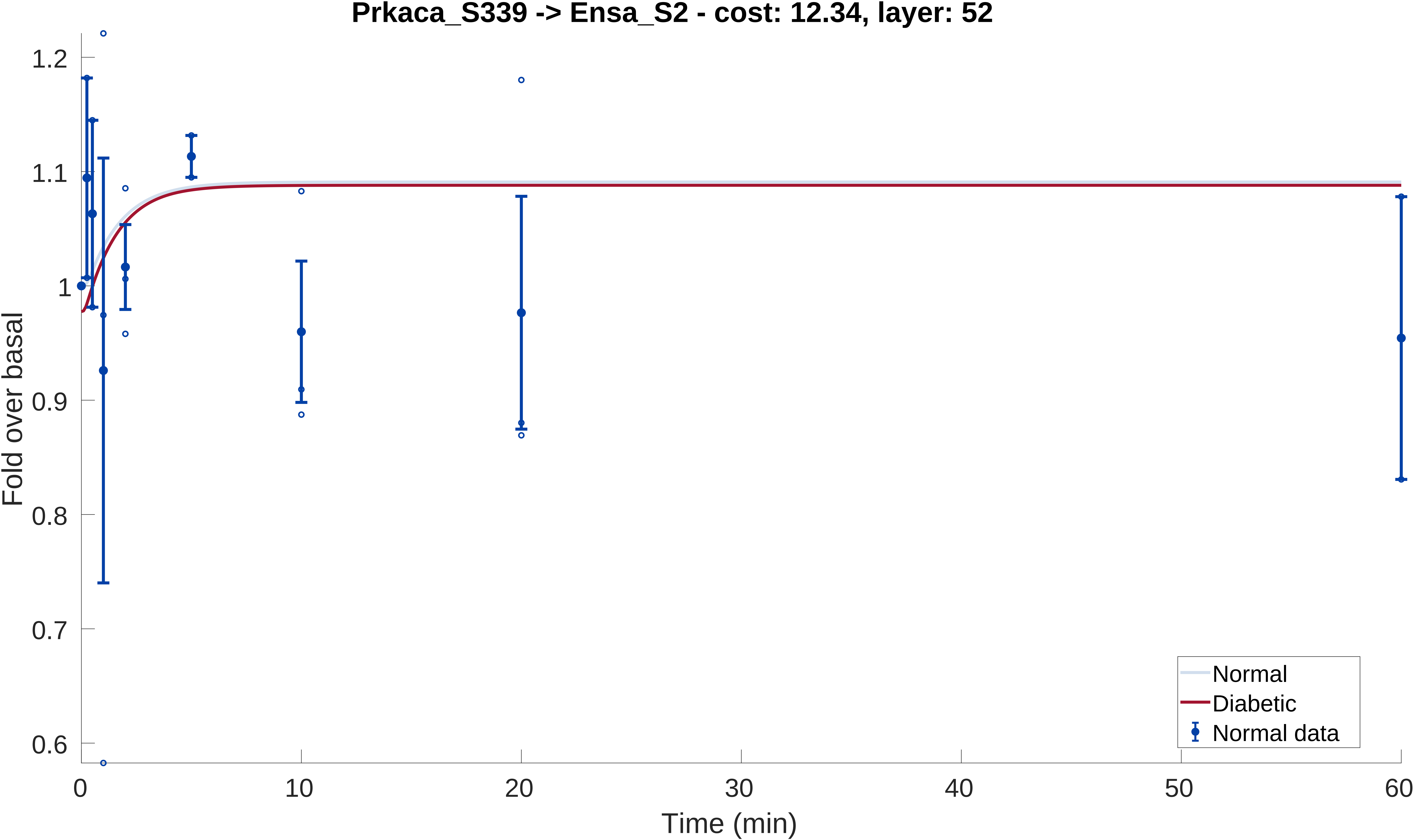
Prkaca_S339 -> Ensa_S2 - cost: 12.34, layer: 52
Fold over basal
Time (min)
1.2
1.1
1
0.9
0.8
0.7
0.6
0
10
20
30
40
50
60
Normal
Diabetic
Normal data

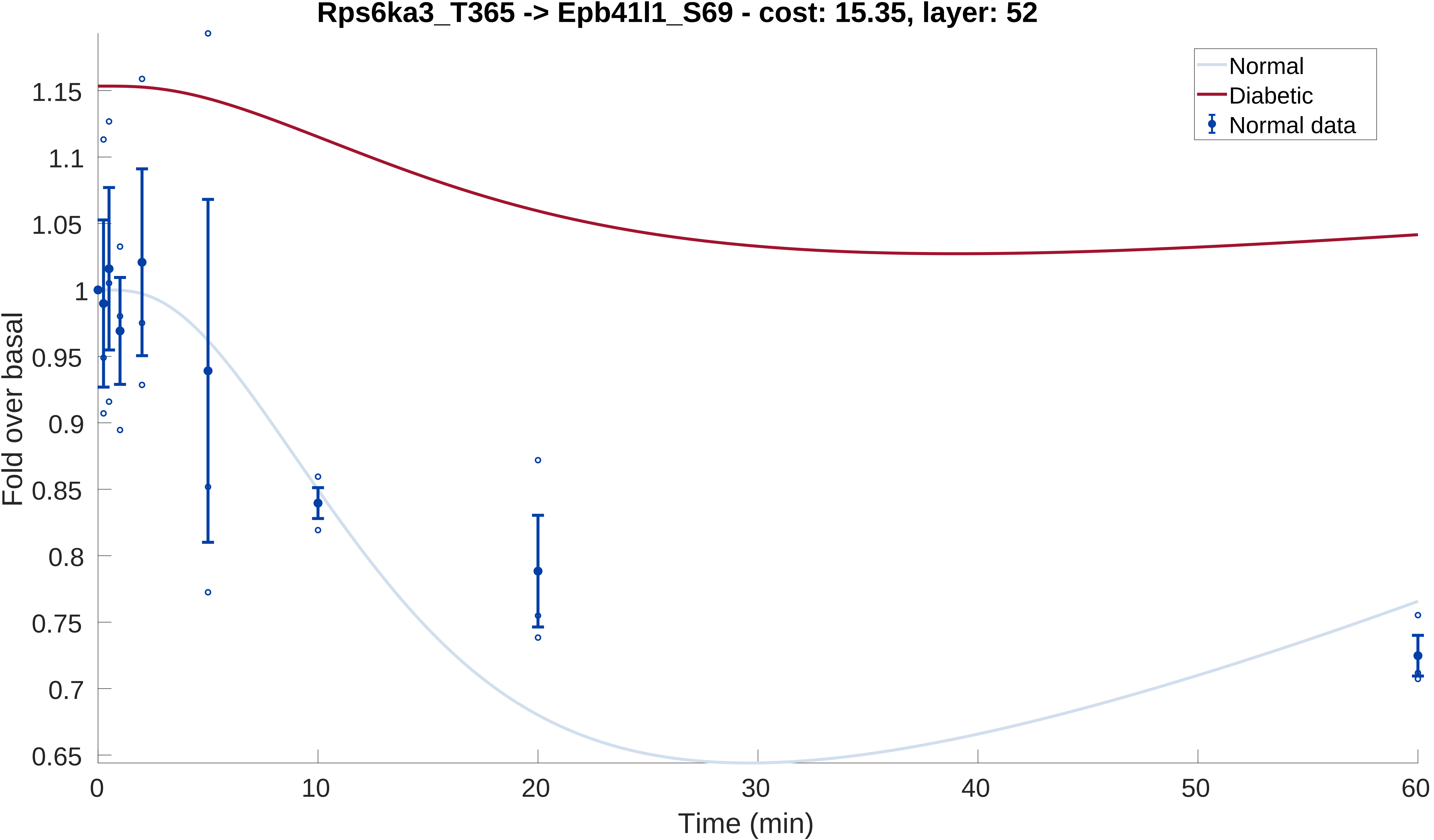

Rps6ka3_T365 -> Epb41l1_S69 - cost: 15.35, layer: 52
Normal
Diabetic
Normal data
Fold over basal
Time (min)
1.15
1.1
1.05
1
0.95
0.9
0.85
0.8
0.75
0.7
0.65
0
10
20
30
40
50
60

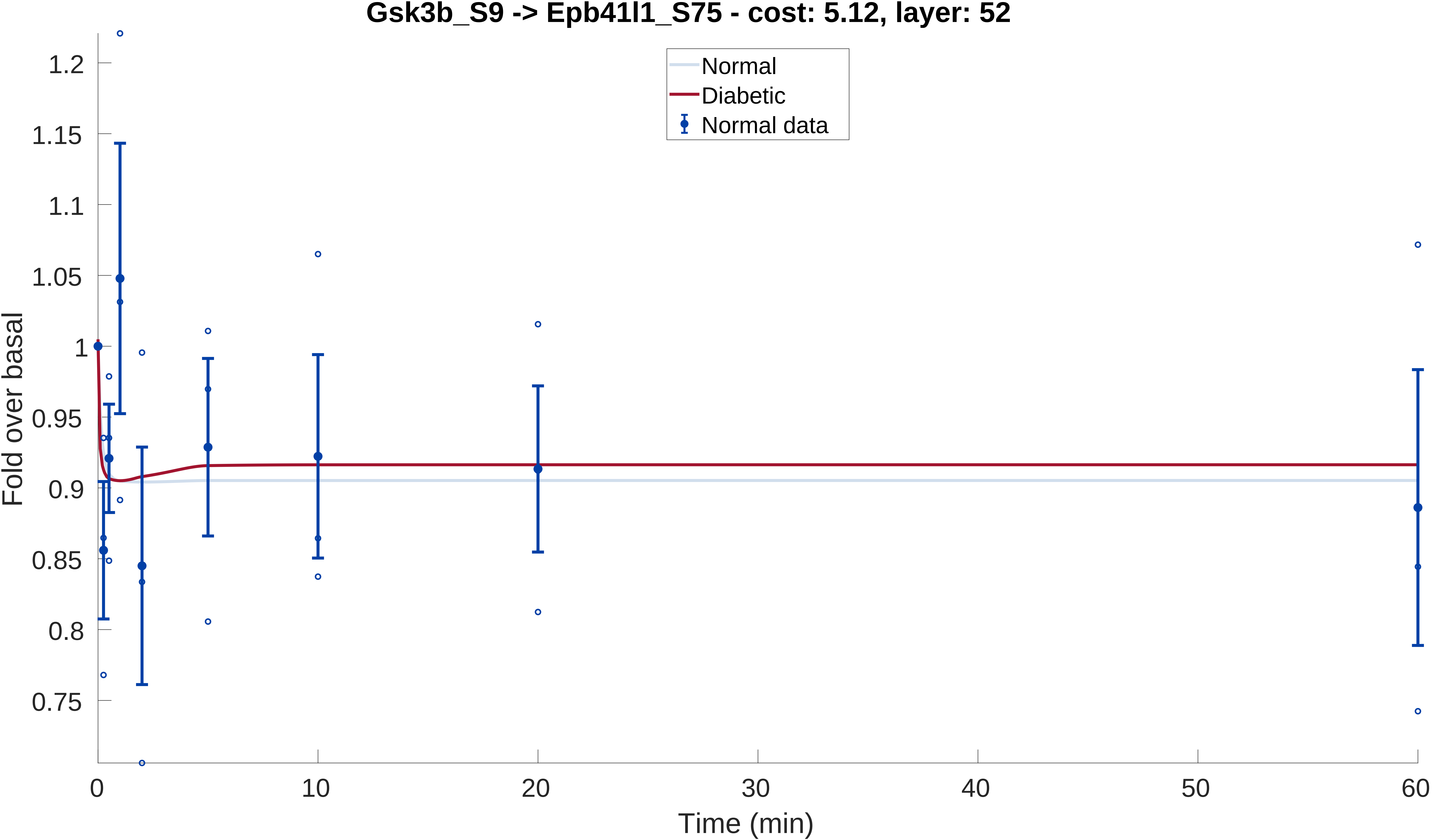

Gsk3b_S9 -> Epb41l1_S75 - cost: 5.12, layer: 52
Normal
Diabetic
Normal data
Fold over basal
1.2
1.15
1.1
1.05
1
0.95
0.9
0.85
0.8
0.75
0
10
20
30
40
50
60
Time (min)

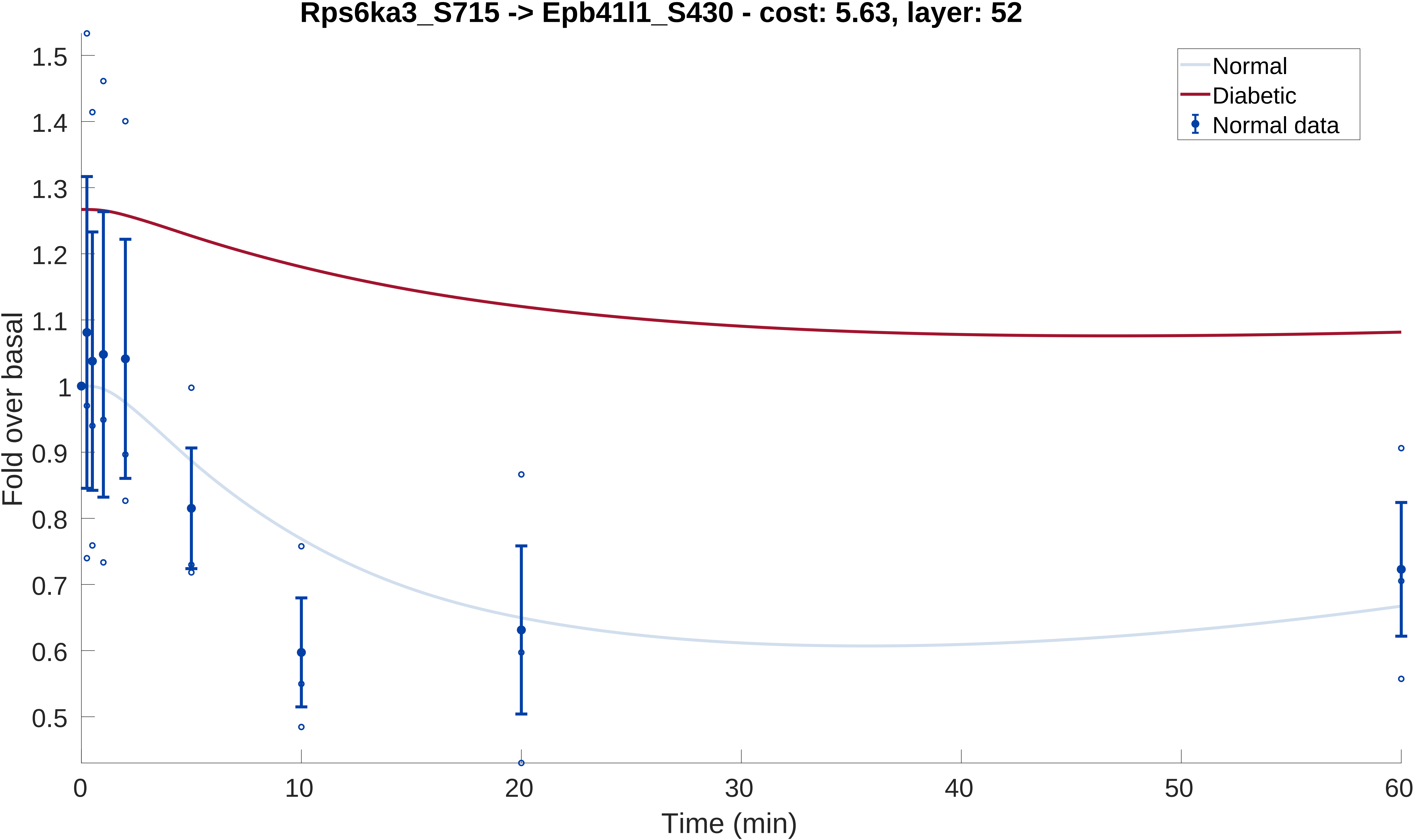
Rps6ka3_S715 -> Epb41l1_S430 - cost: 5.63, layer: 52
Normal
Diabetic
Normal data
Fold over basal
1.5
1.4
1.3
1.2
1.1
1
0.9
0.8
0.7
0.6
0.5
0
10
20
30
40
50
60
Time (min)

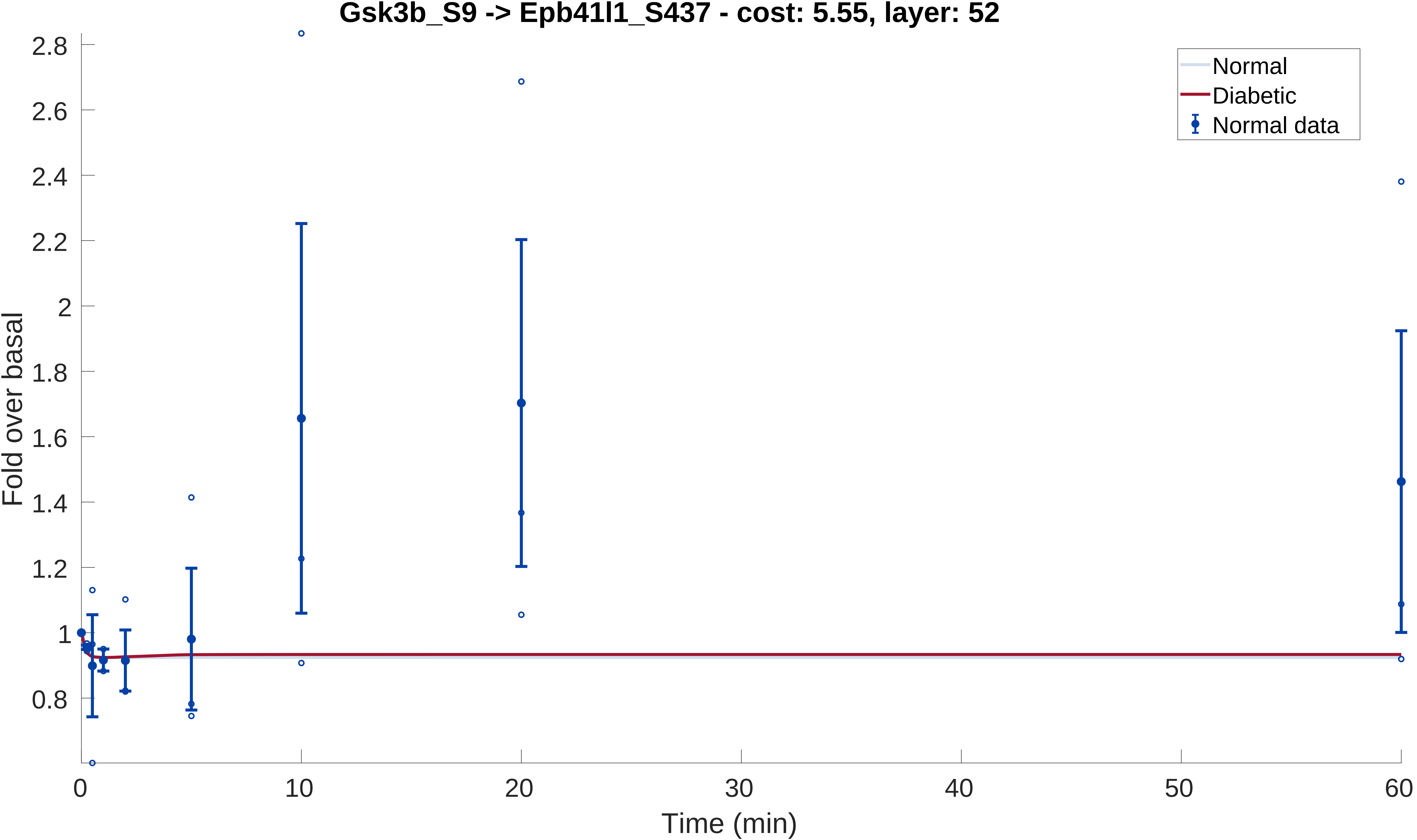

Gsk3b_S9 -> Epb41l1_S437 - cost: 5.55, layer: 52
Fold over basal
Time (min)
Normal
Diabetic
Normal data
0.8
1
1.2
1.4
1.6
1.8
2
2.2
2.4
2.6
2.8
0
10
20
30
40
50
60

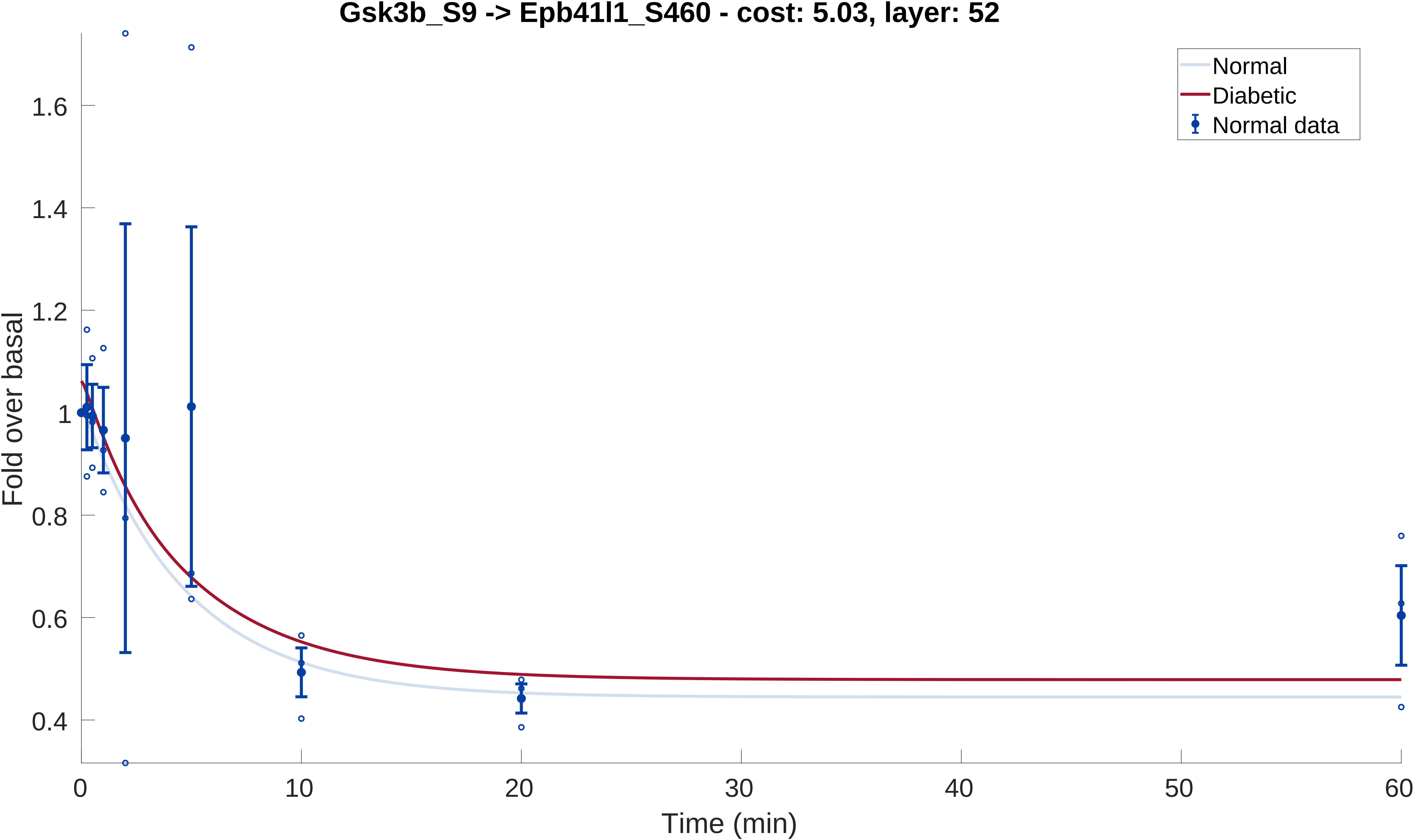
Gsk3b_S9 -> Epb41l1_S460 - cost: 5.03, layer: 52
Normal
Diabetic
Normal data
Fold over basal
Time (min)
0.4
0.6
0.8
1
1.2
1.4
1.6
0
10
20
30
40
50
60

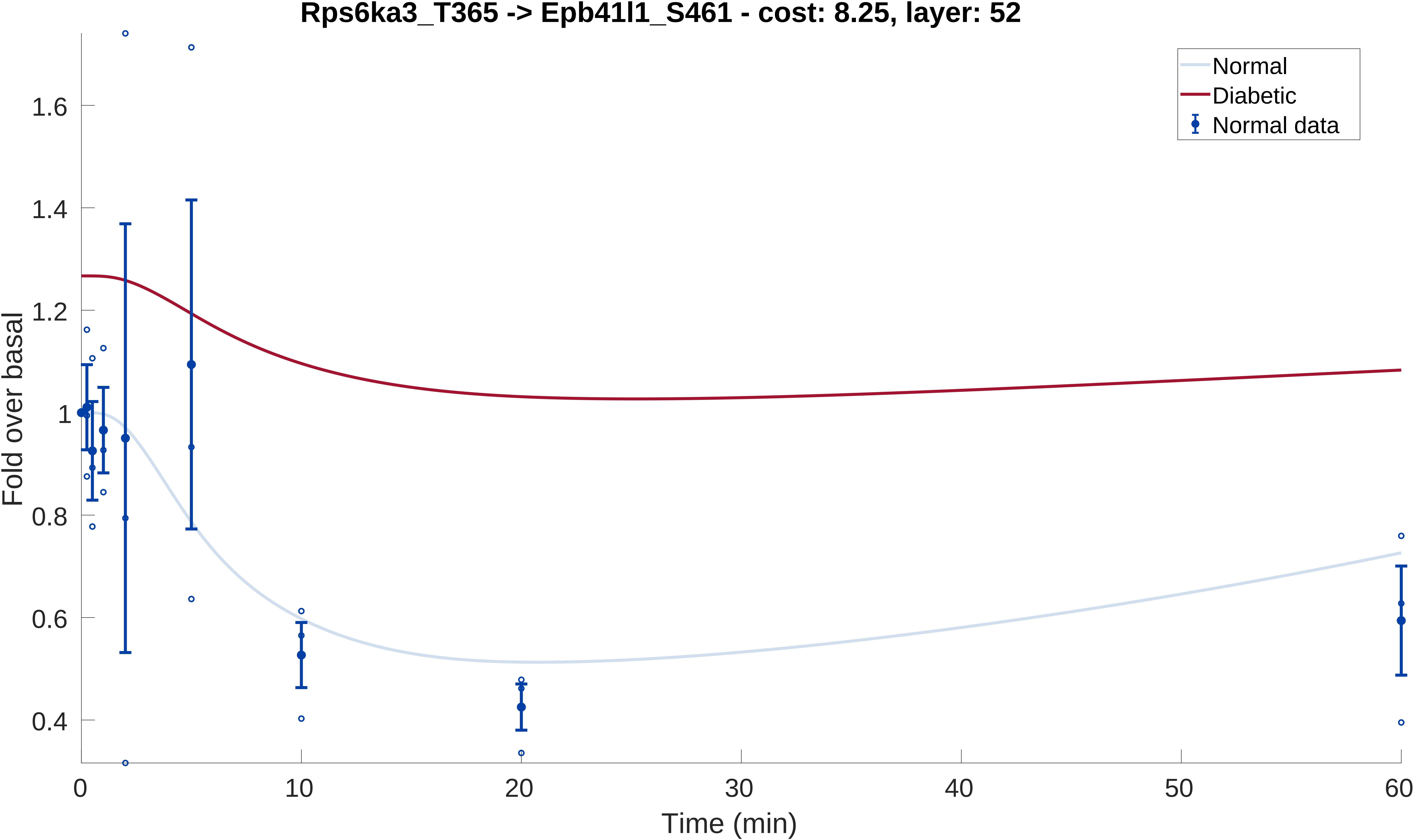

Rps6ka3_T365 -> Epb41l1_S461 - cost: 8.25, layer: 52
Normal
Diabetic
Normal data
Fold over basal
Time (min)
1.6
1.4
1.2
1
0.8
0.6
0.4
0
10
20
30
40
50
60

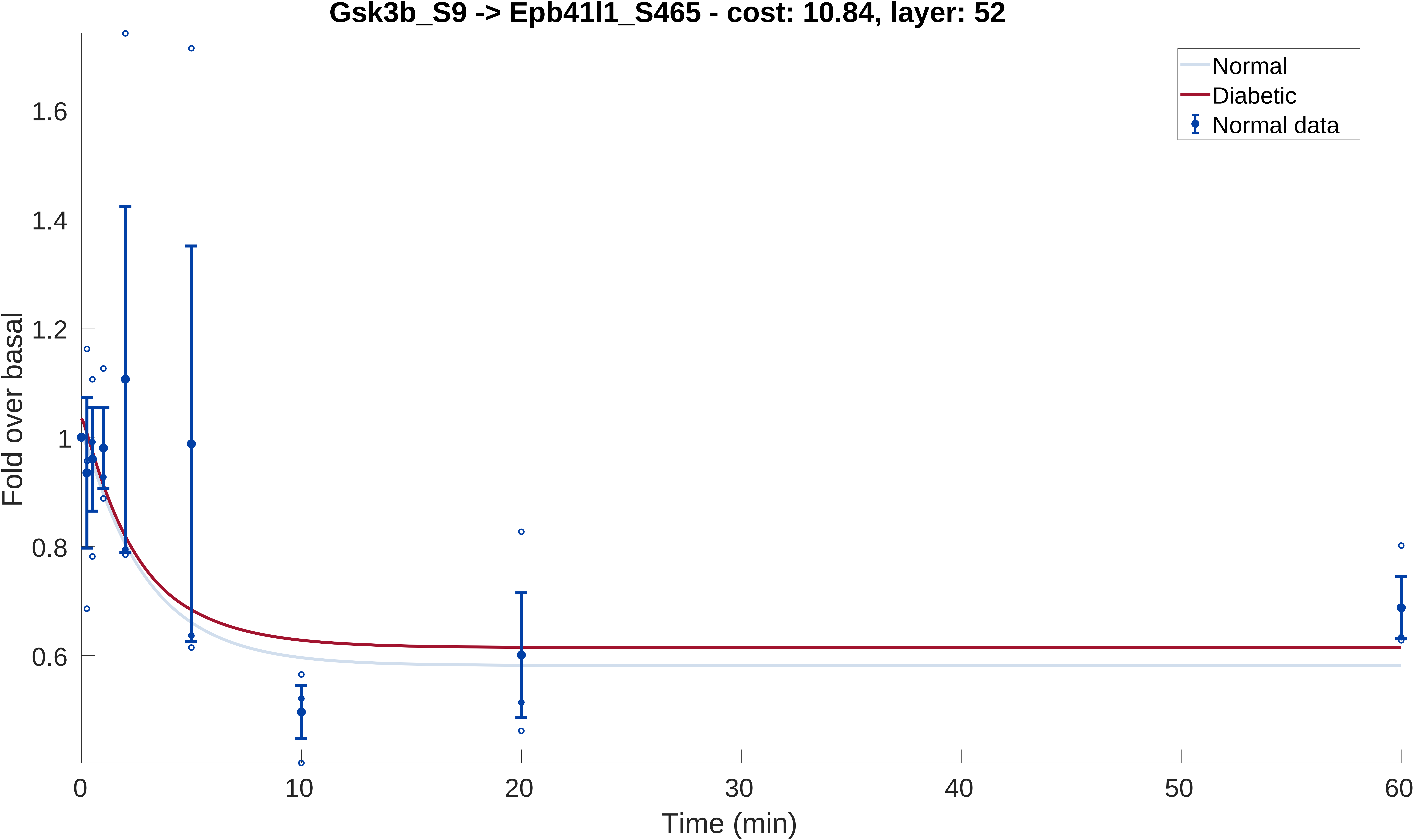

Gsk3b_S9 -> Epb41l1_S465 - cost: 10.84, layer: 52
Normal
Diabetic
Normal data
Fold over basal
1.6
1.4
1.2
1
0.8
0.6
0
10
20
30
40
50
60
Time (min)

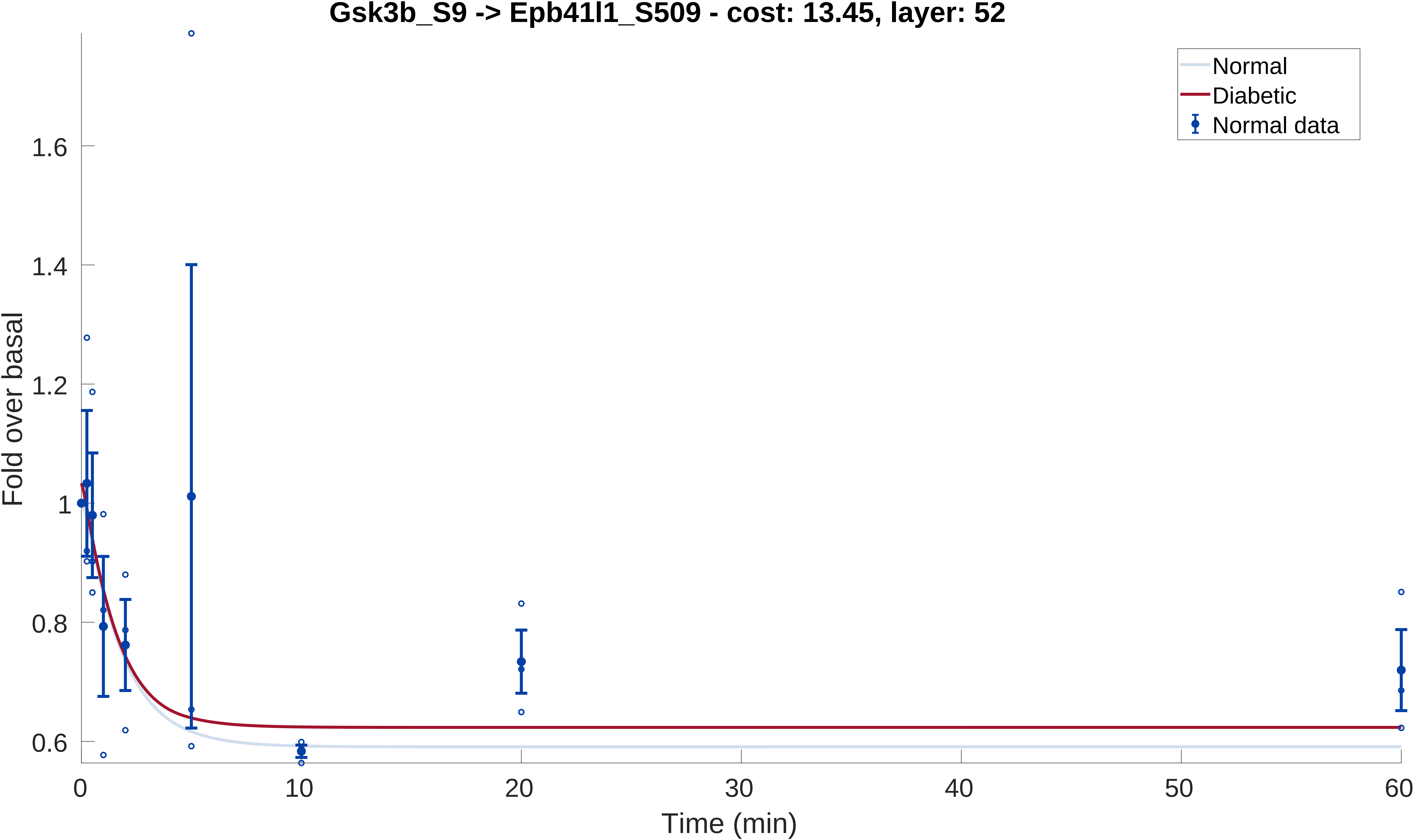

Gsk3b_S9 -> Epb41l1_S509 - cost: 13.45, layer: 52
Normal
Diabetic
Normal data
Fold over basal
1.6
1.4
1.2
1
0.8
0.6
0
10
20
30
40
50
60
Time (min)

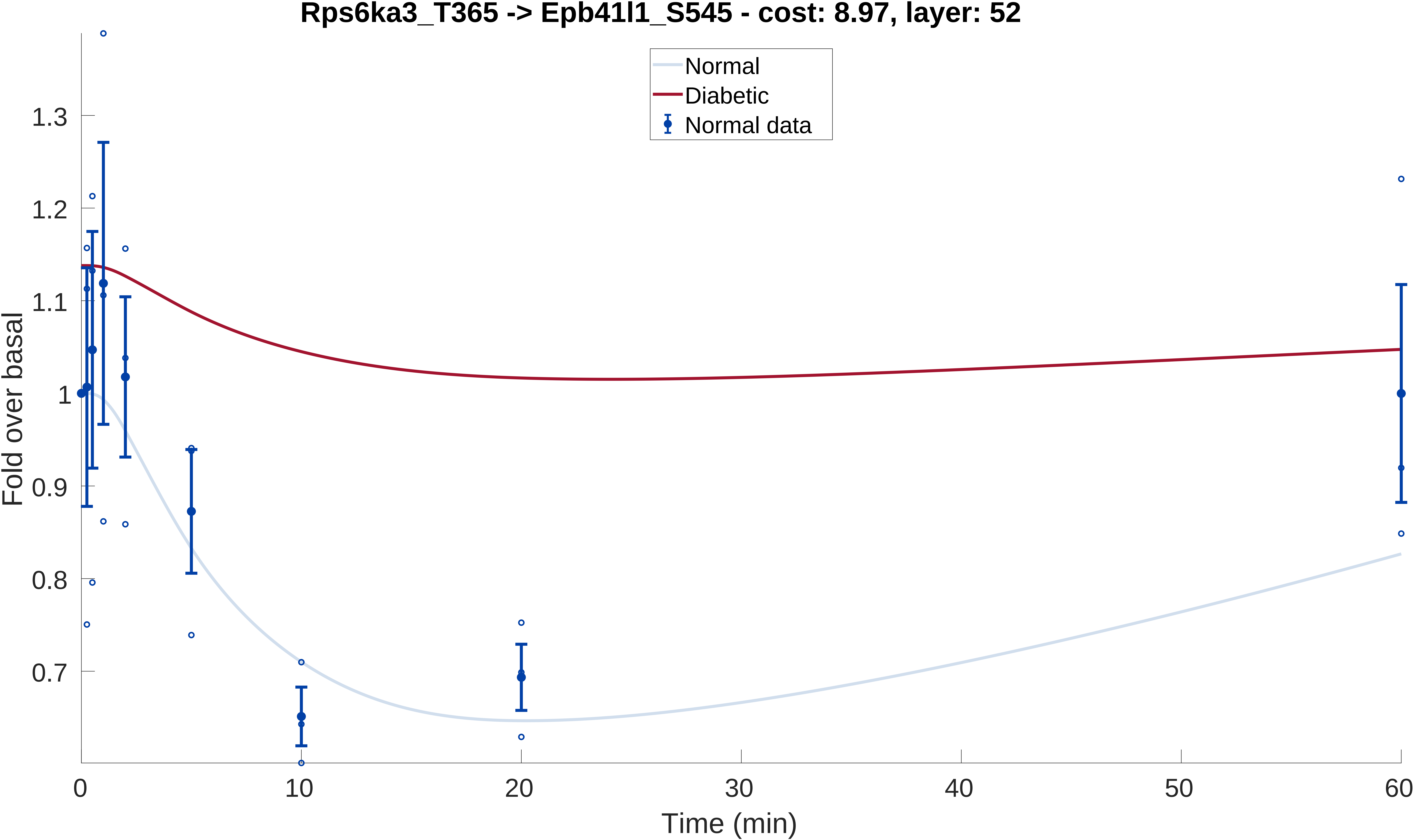
Rps6ka3_T365 -> Epb41l1_S545 - cost: 8.97, layer: 52
Normal
Diabetic
Normal data
Fold over basal
Time (min)
1.3
1.2
1.1
1
0.9
0.8
0.7
0
10
20
30
40
50
60

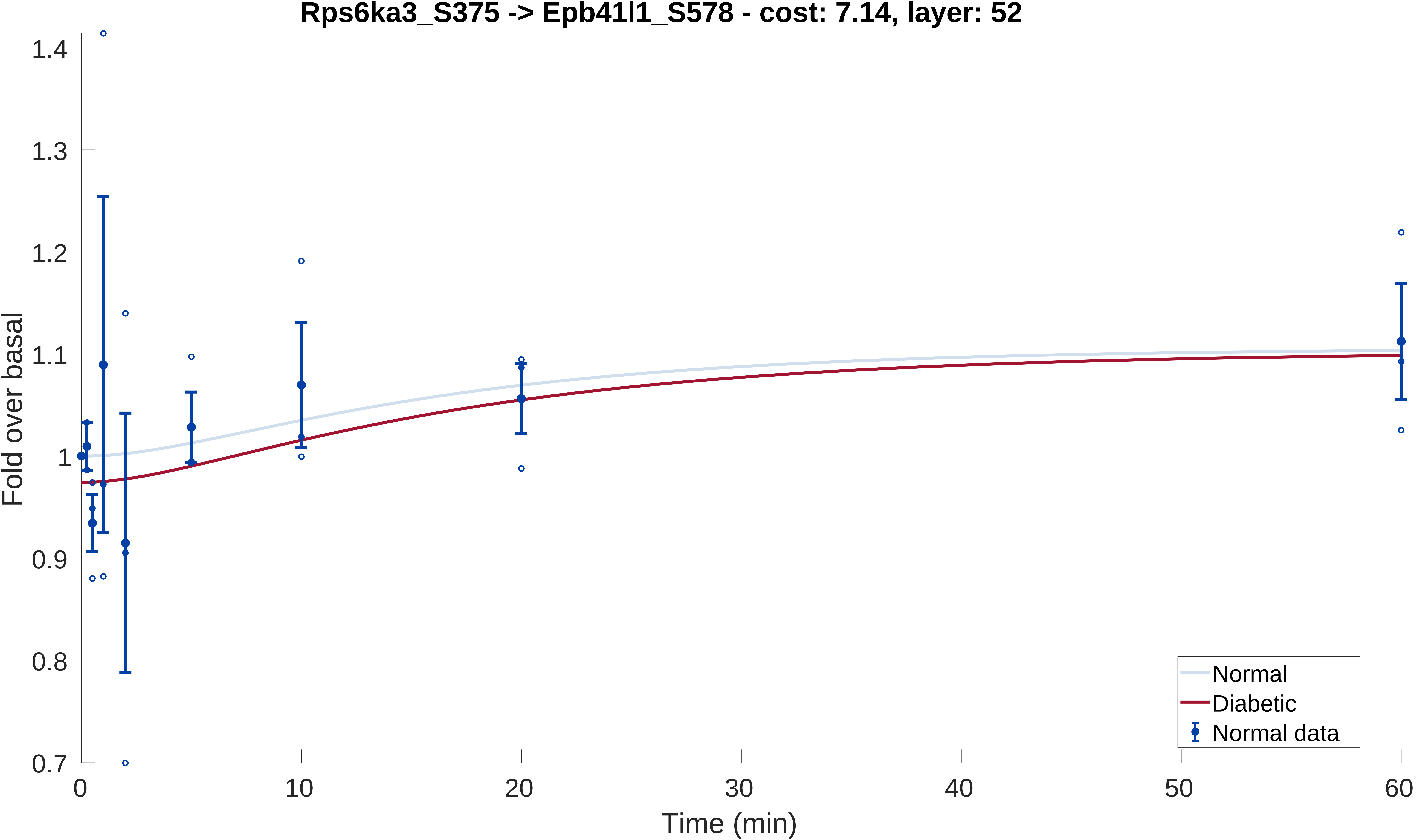

Rps6ka3_S375 -> Epb41l1_S578 - cost: 7.14, layer: 52
Fold over basal
Time (min)
0.7
0.8
0.9
1
1.1
1.2
1.3
1.4
0
10
20
30
40
50
60
Normal
Diabetic
Normal data

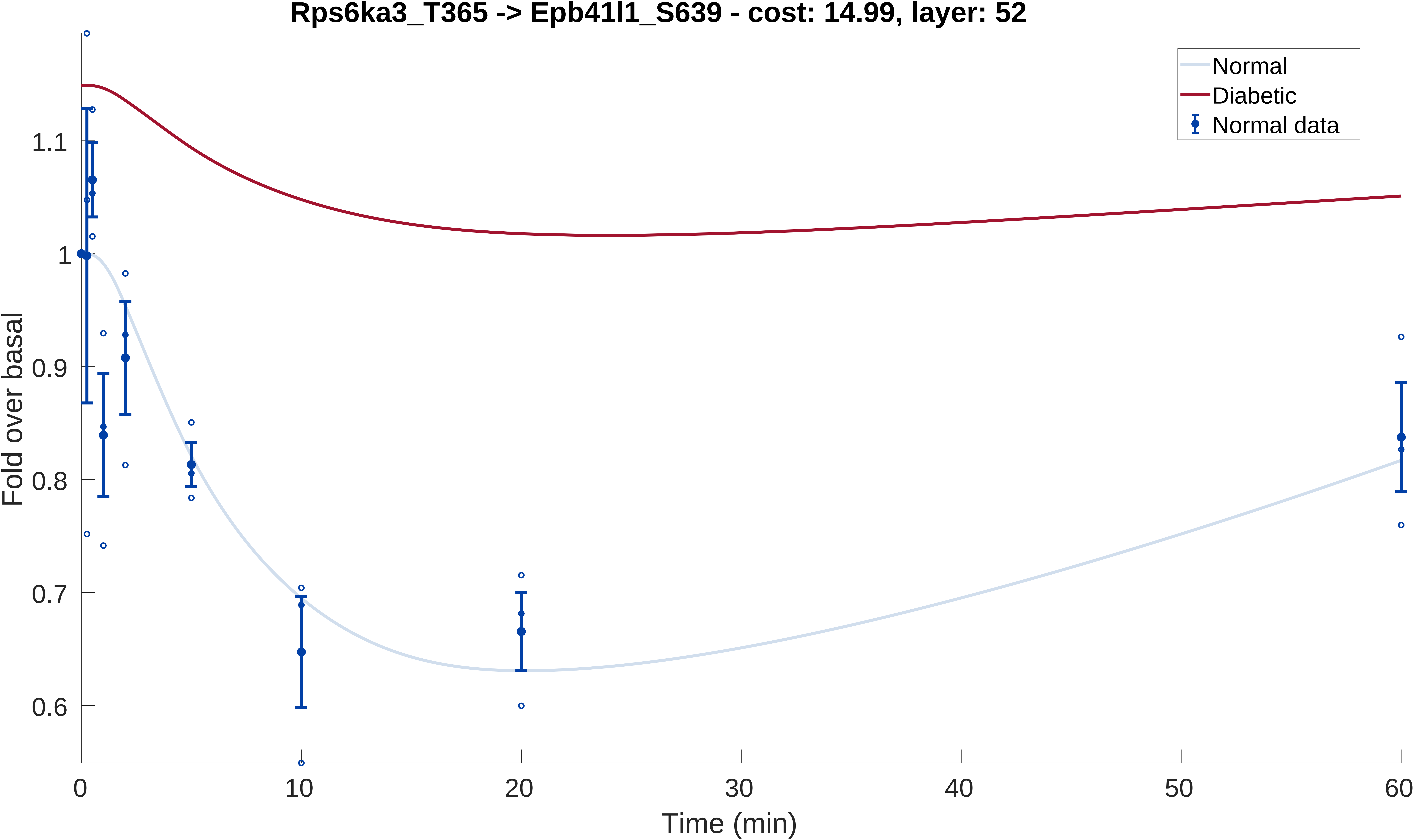

Rps6ka3_T365 -> Epb41l1_S639 - cost: 14.99, layer: 52
Normal
Diabetic
Normal data
Fold over basal
1.1
1
0.9
0.8
0.7
0.6
0
10
20
30
40
50
60
Time (min)

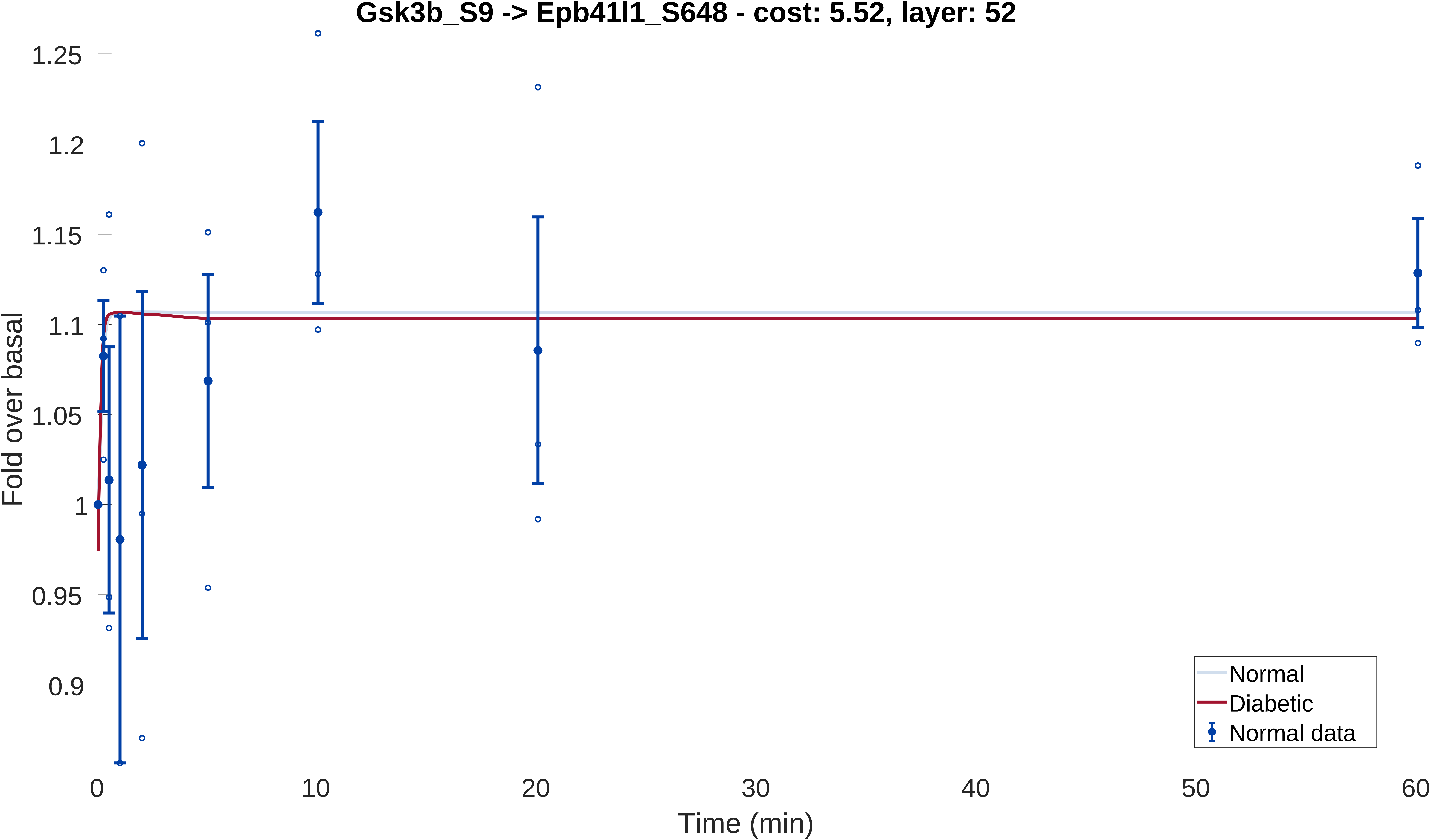

Gsk3b_S9 -> Epb41l1_S648 - cost: 5.52, layer: 52
Fold over basal
1.25
1.2
1.15
1.1
1.05
1
0.95
0.9
0
10
20
30
40
50
60
Time (min)
Normal
Diabetic
Normal data

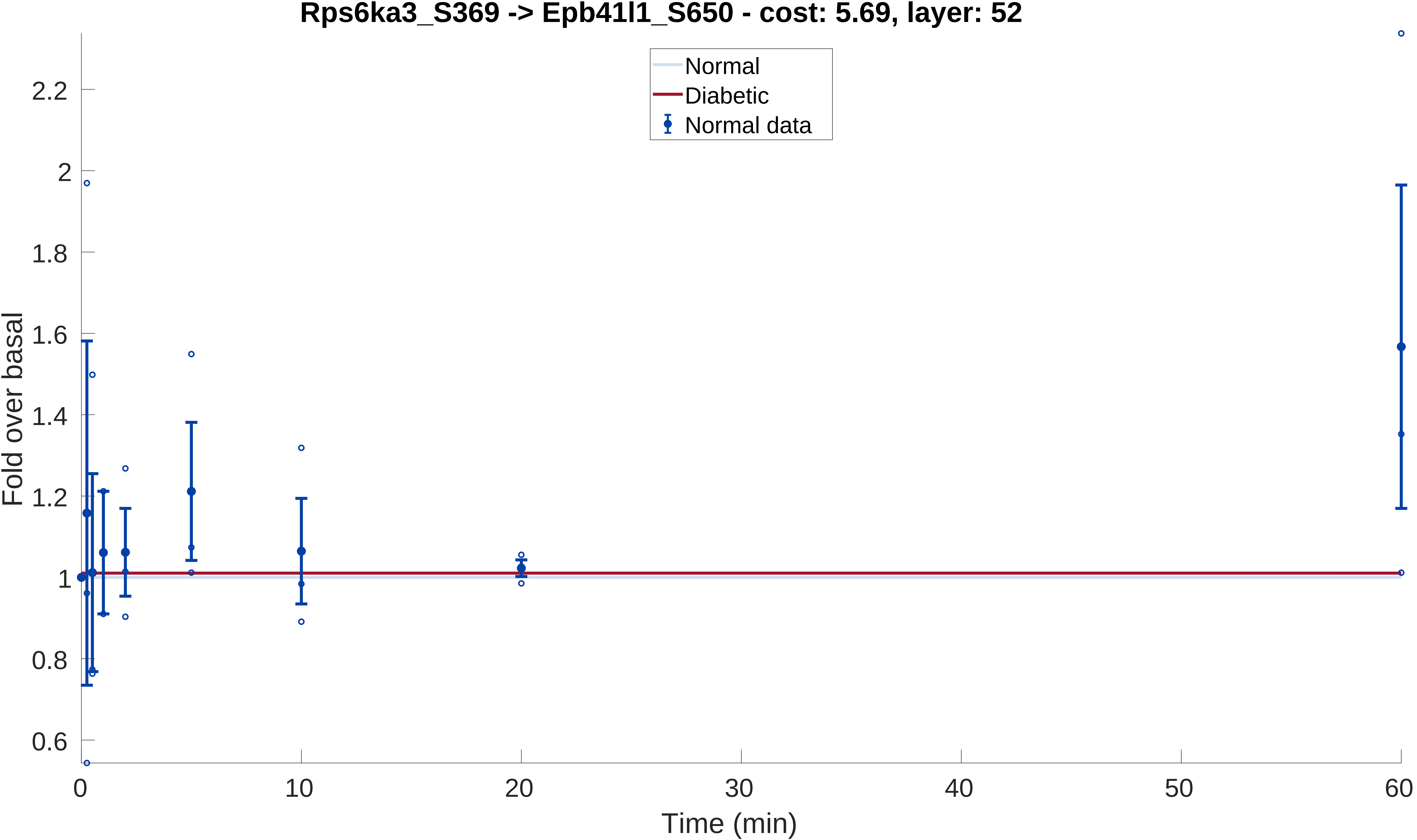

Rps6ka3_S369 -> Epb41l1_S650 - cost: 5.69, layer: 52
Normal
Diabetic
Normal data
Fold over basal
0.6
0.8
1
1.2
1.4
1.6
1.8
2
2.2
0
10
20
30
40
50
60
Time (min)

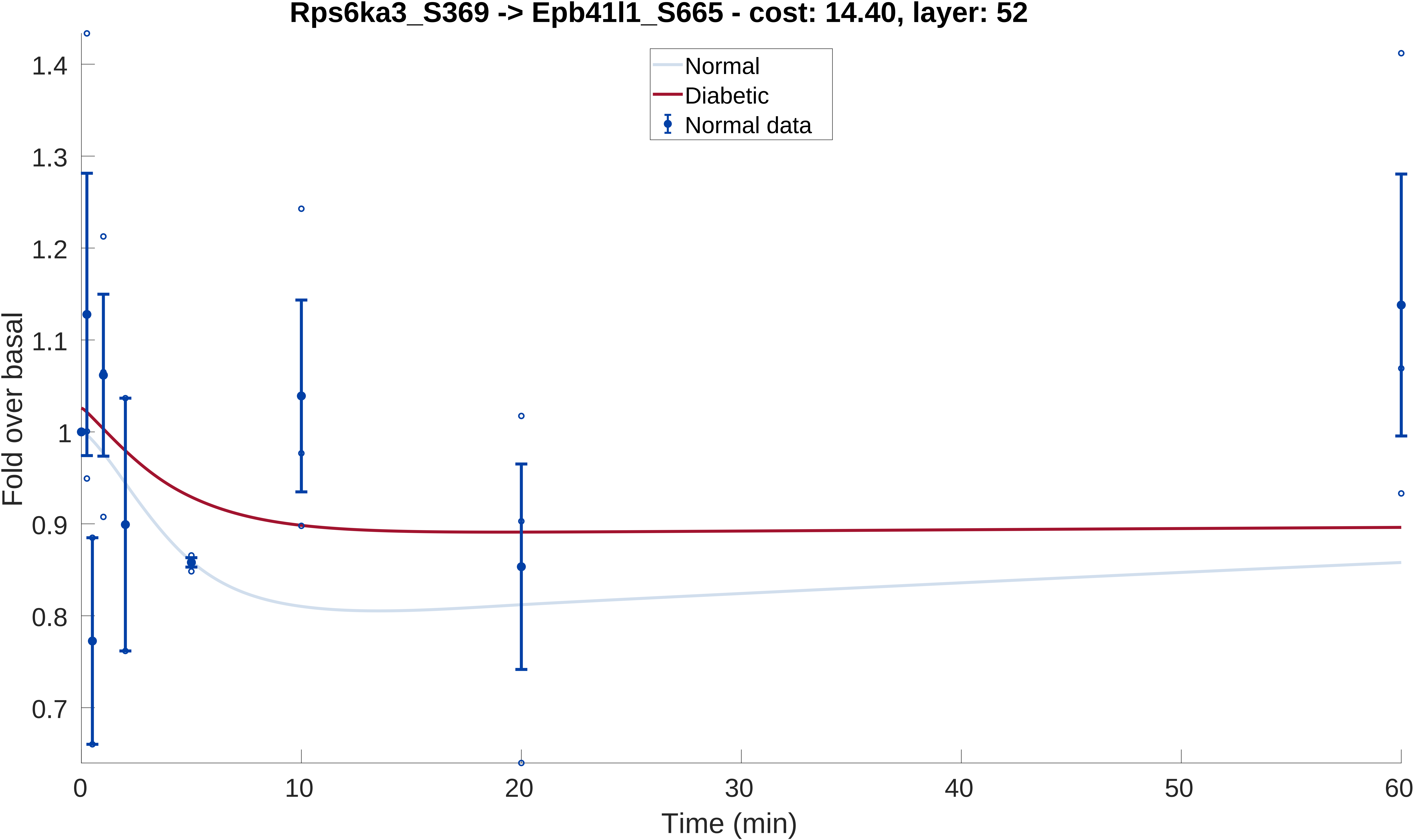

Rps6ka3_S369 -> Epb41l1_S665 - cost: 14.40, layer: 52
Normal
Diabetic
Normal data
Fold over basal
1.4
1.3
1.2
1.1
1
0.9
0.8
0.7
0
10
20
30
40
50
60
Time (min)

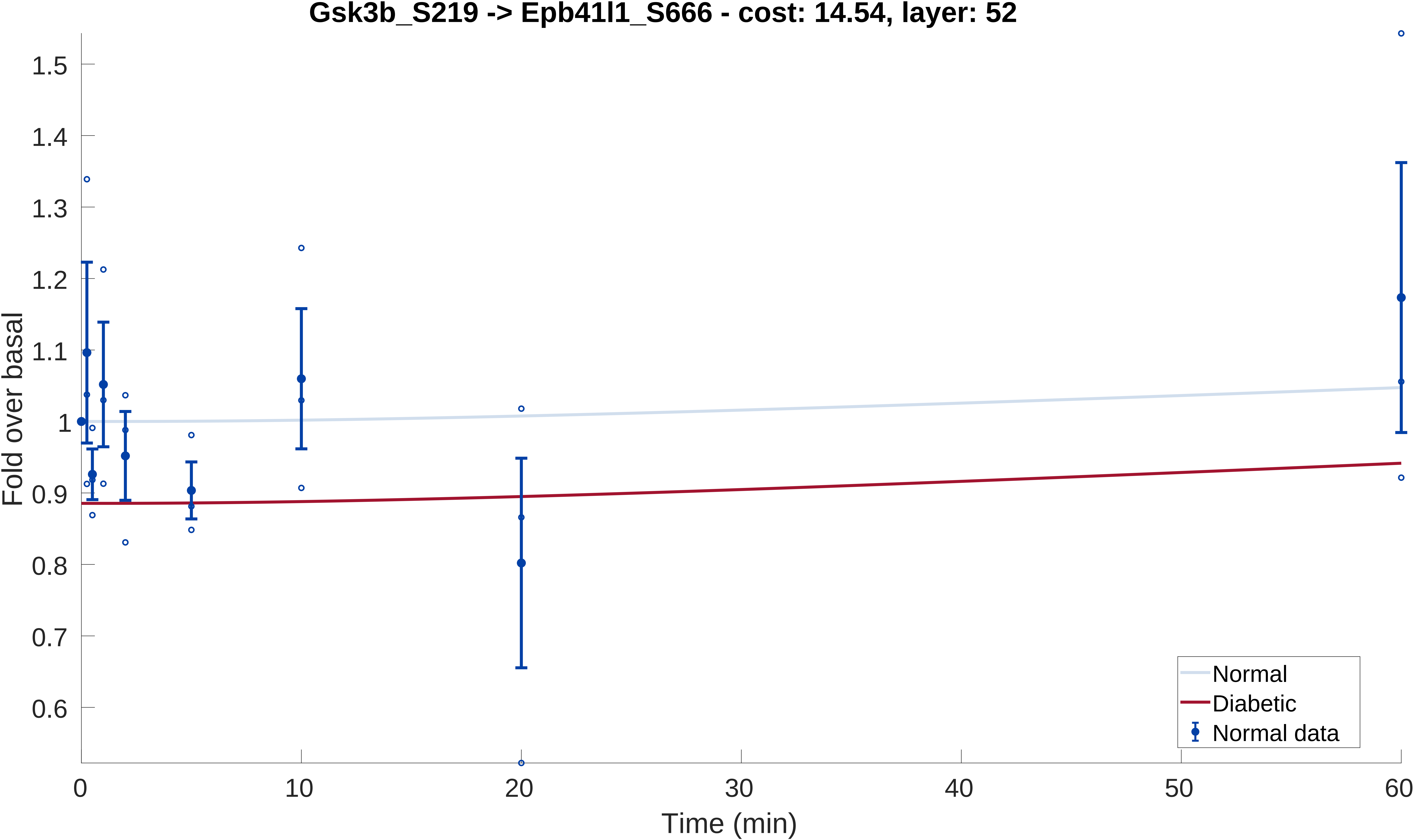
Gsk3b_S219 -> Epb41l1_S666 - cost: 14.54, layer: 52
Fold over basal
1.5
1.4
1.3
1.2
1.1
1
0.9
0.8
0.7
0.6
0
10
20
30
40
50
60
Time (min)
Normal
Diabetic
Normal data

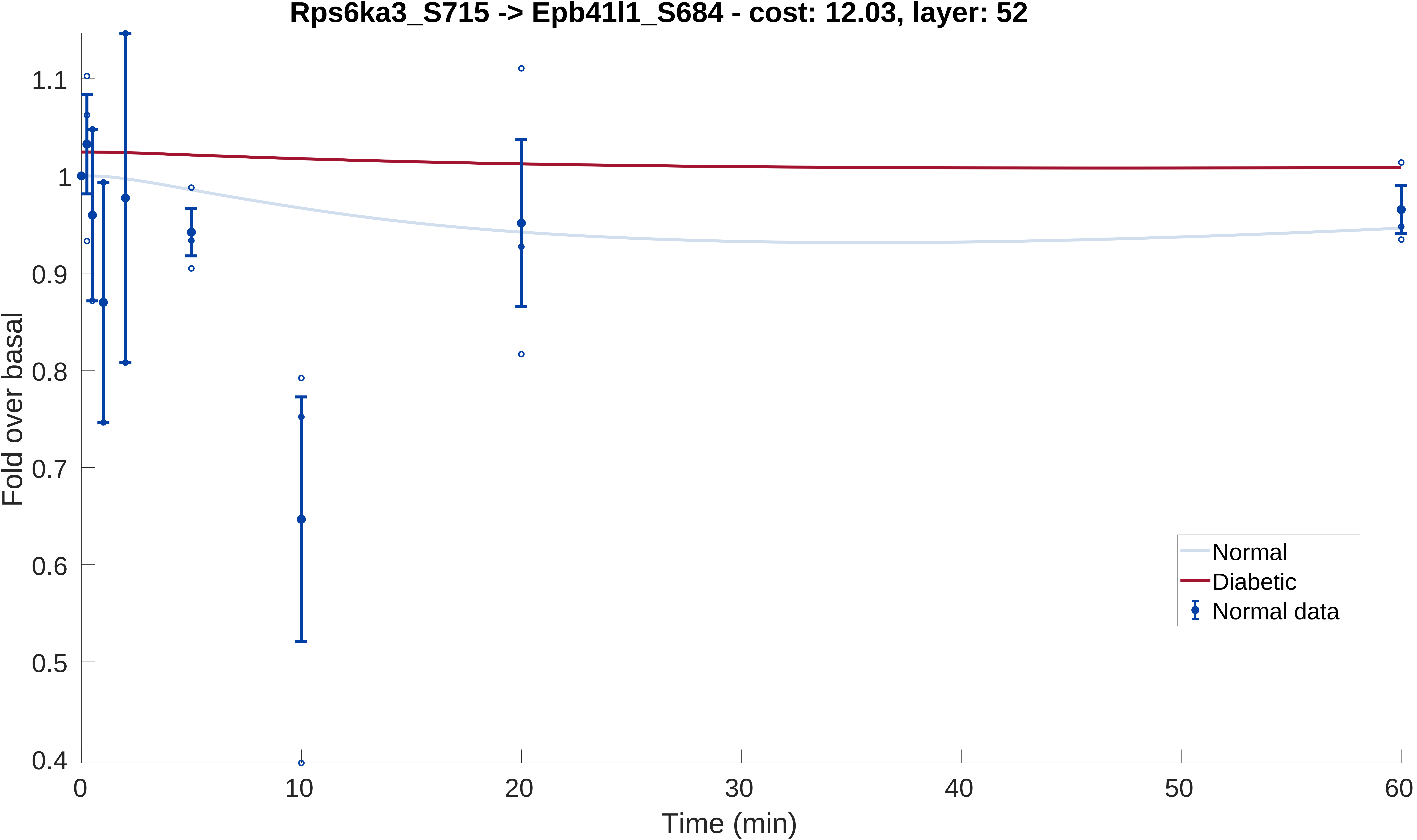

Rps6ka3_S715 -> Epb41l1_S684 - cost: 12.03, layer: 52
Fold over basal
1.1
1
0.9
0.8
0.7
0.6
0.5
0.4
0
10
20
30
40
50
60
Time (min)
Normal
Diabetic
Normal data

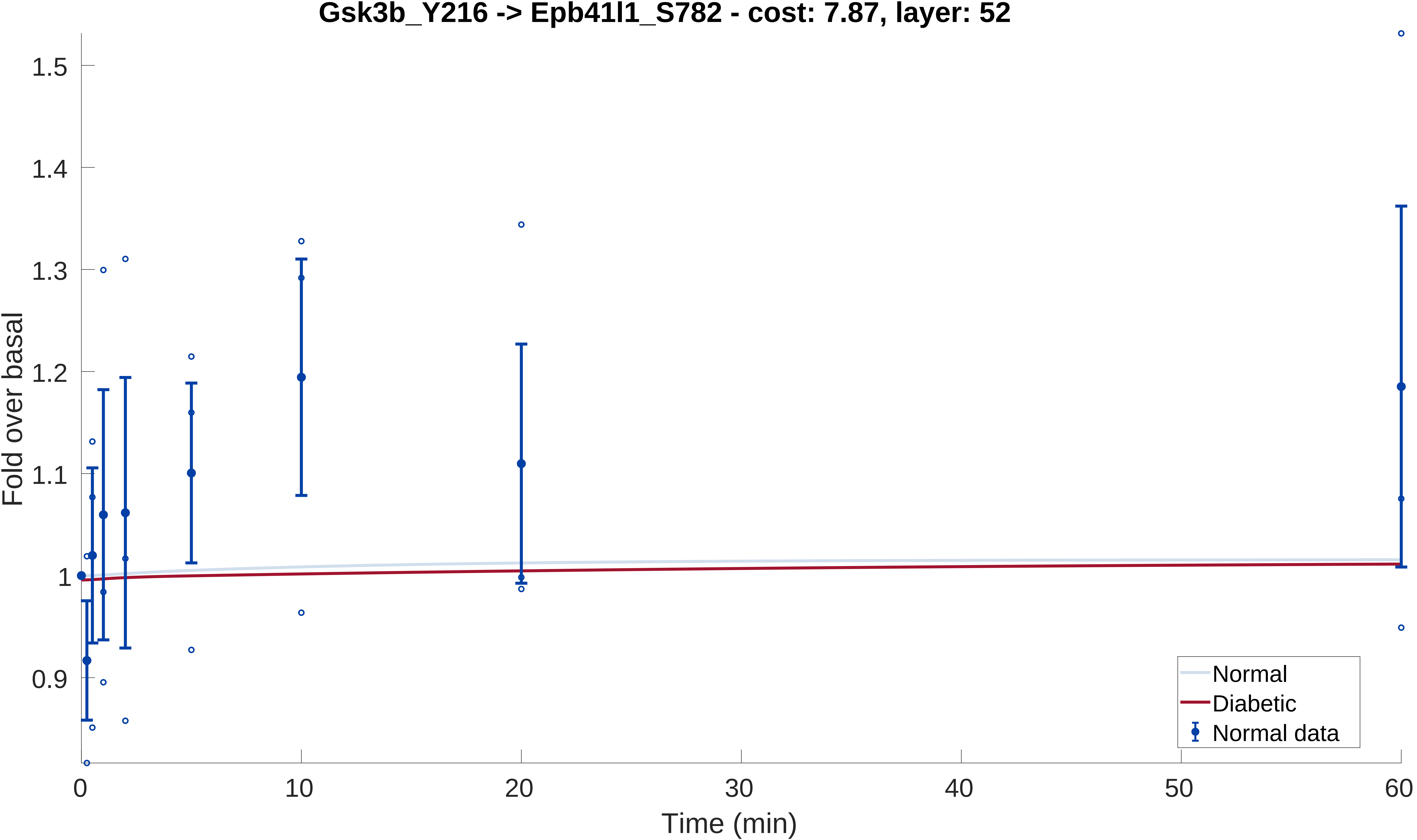

Gsk3b_Y216 -> Epb41l1_S782 - cost: 7.87, layer: 52
Fold over basal
1.5
1.4
1.3
1.2
1.1
1
0.9
0
10
20
30
40
50
60
Time (min)
Normal
Diabetic
Normal data

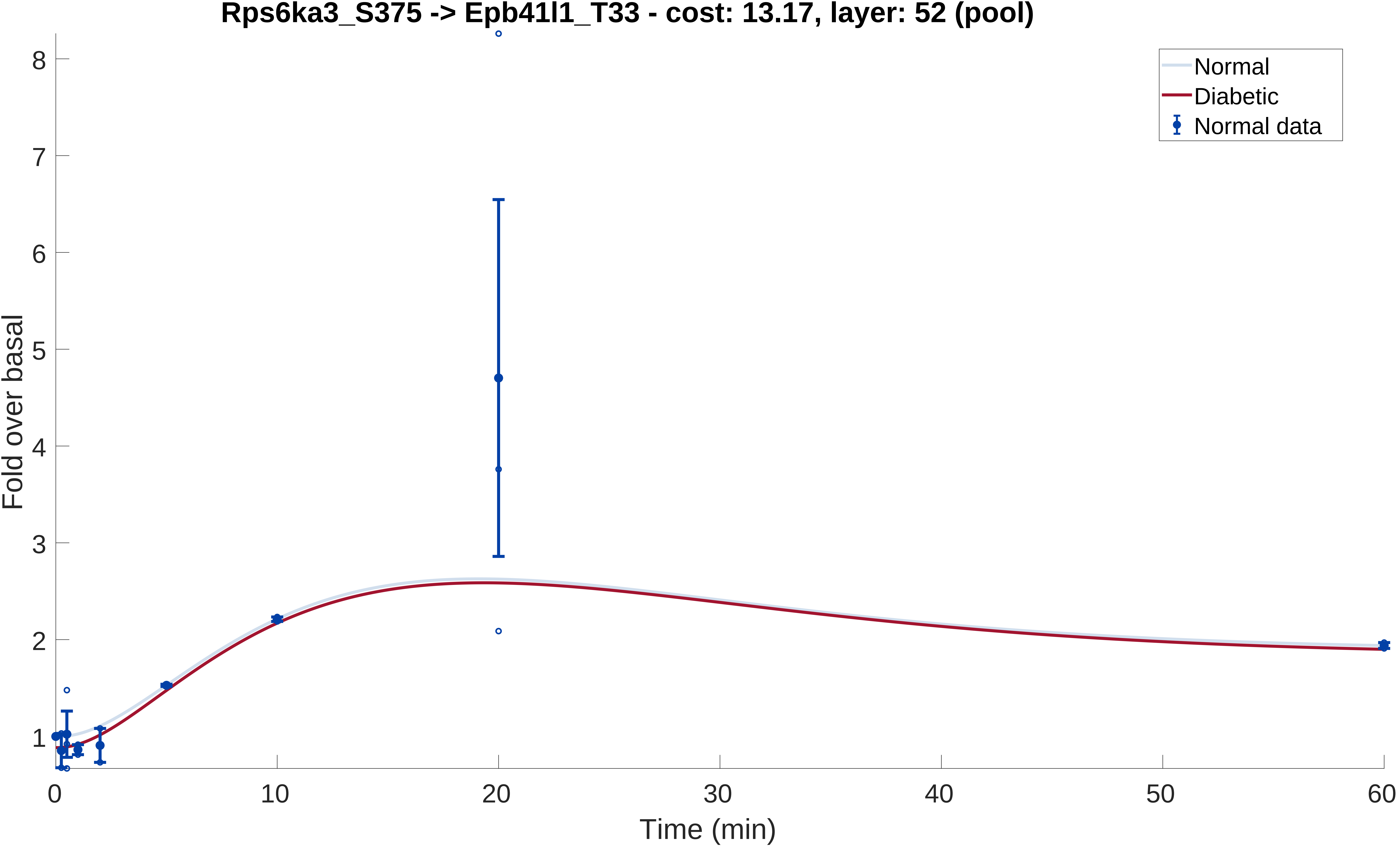

Rps6ka3_S375 -> Epb41l1_T33 - cost: 13.17, layer: 52 (pool)
Normal
Diabetic
Normal data
Fold over basal
Time (min)
0
10
20
30
40
50
60
1
2
3
4
5
6
7
8

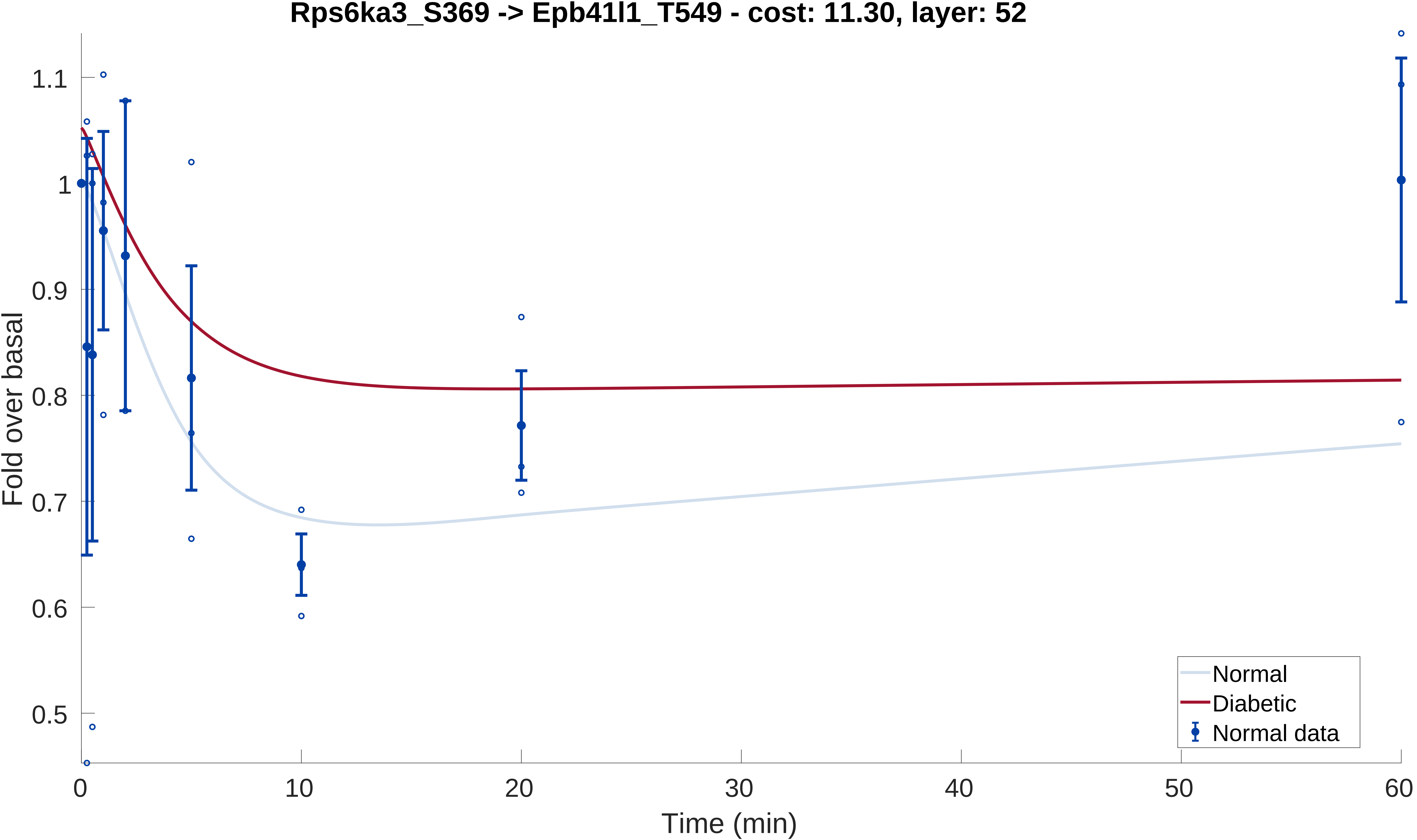
Rps6ka3_S369 -> Epb41l1_T549 - cost: 11.30, layer: 52
Fold over basal
Time (min)
1.1
1
0.9
0.8
0.7
0.6
0.5
0
10
20
30
40
50
60
Normal
Diabetic
Normal data

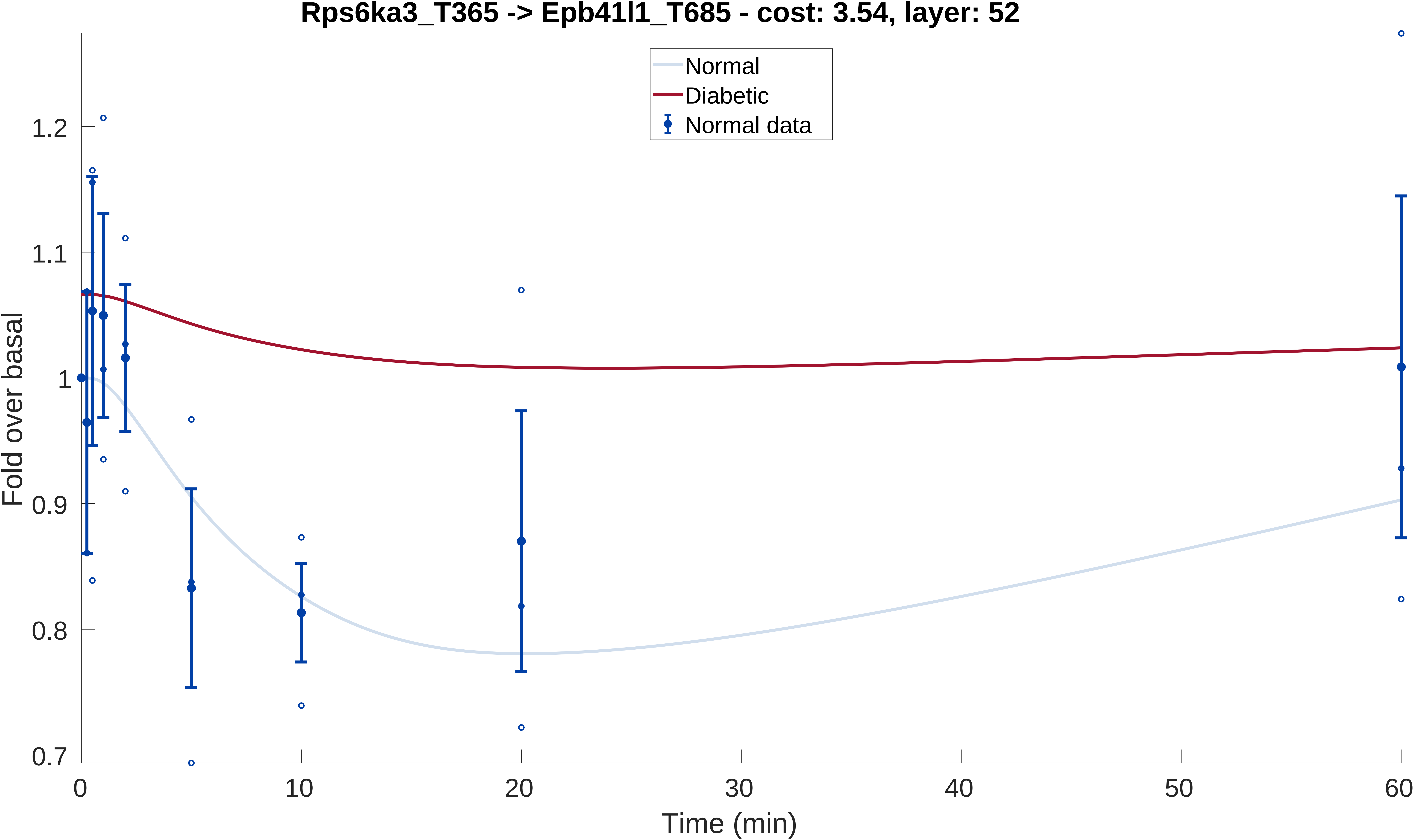

Rps6ka3_T365 -> Epb41l1_T685 - cost: 3.54, layer: 52
Normal
Diabetic
Normal data
Fold over basal
Time (min)
0.7
0.8
0.9
1
1.1
1.2
0
10
20
30
40
50
60

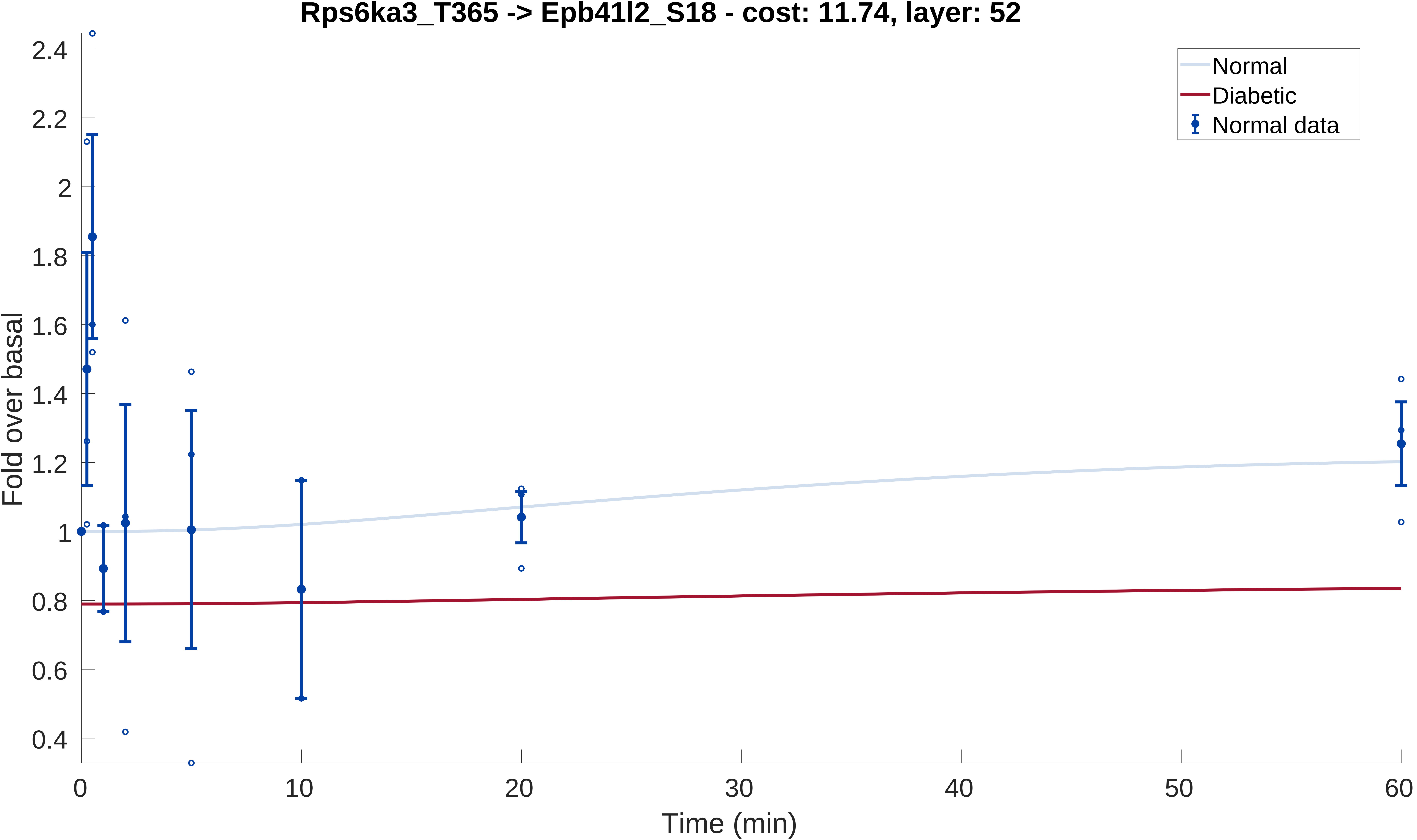

Rps6ka3_T365 -> Epb41l2_S18 - cost: 11.74, layer: 52
Normal
Diabetic
Normal data
Fold over basal
Time (min)
2.4
2.2
2
1.8
1.6
1.4
1.2
1
0.8
0.6
0.4
0
10
20
30
40
50
60

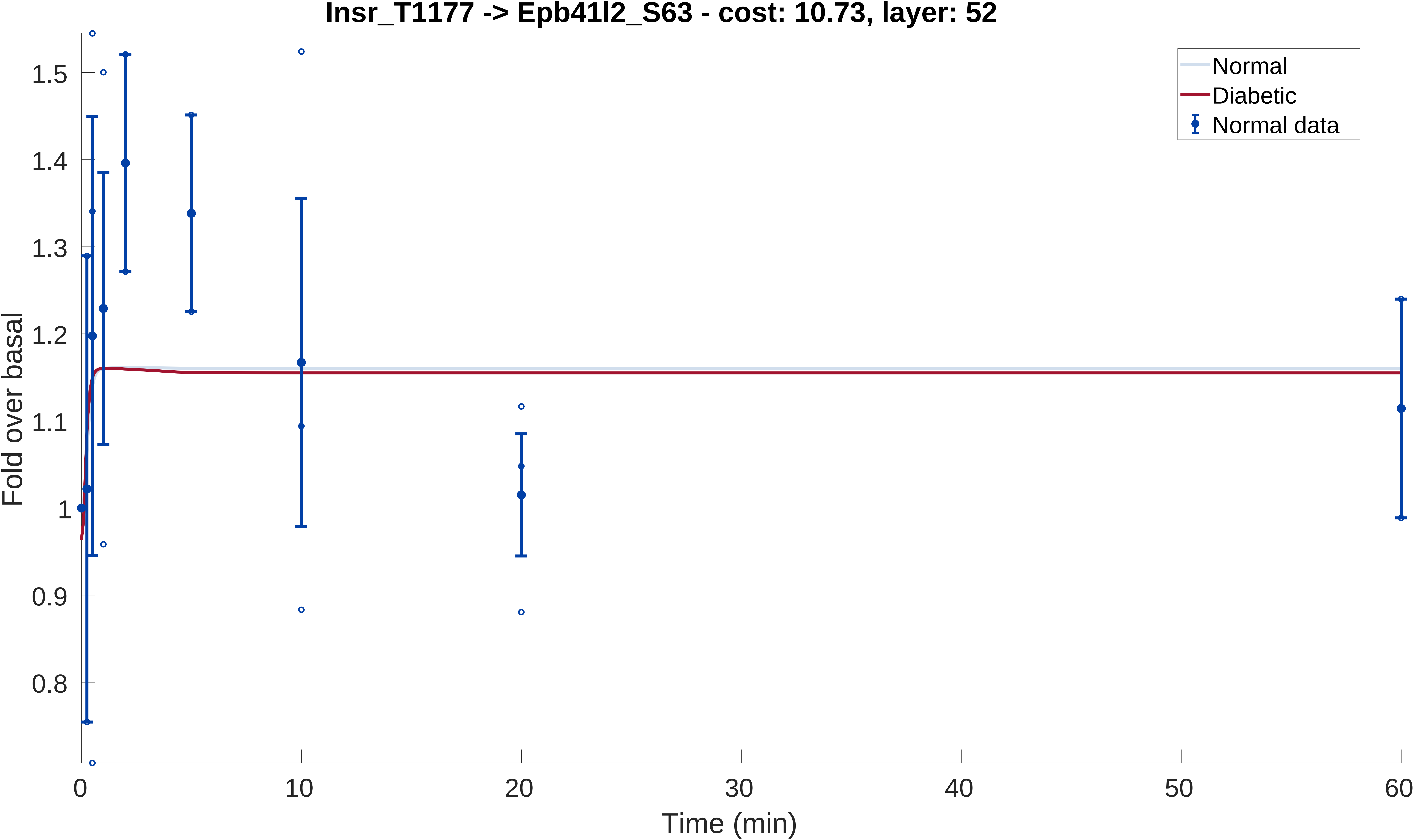

Insr_T1177 -> Epb41l2_S63 - cost: 10.73, layer: 52
Normal
Diabetic
Normal data
Fold over basal
1.5
1.4
1.3
1.2
1.1
1
0.9
0.8
0
10
20
30
40
50
60
Time (min)

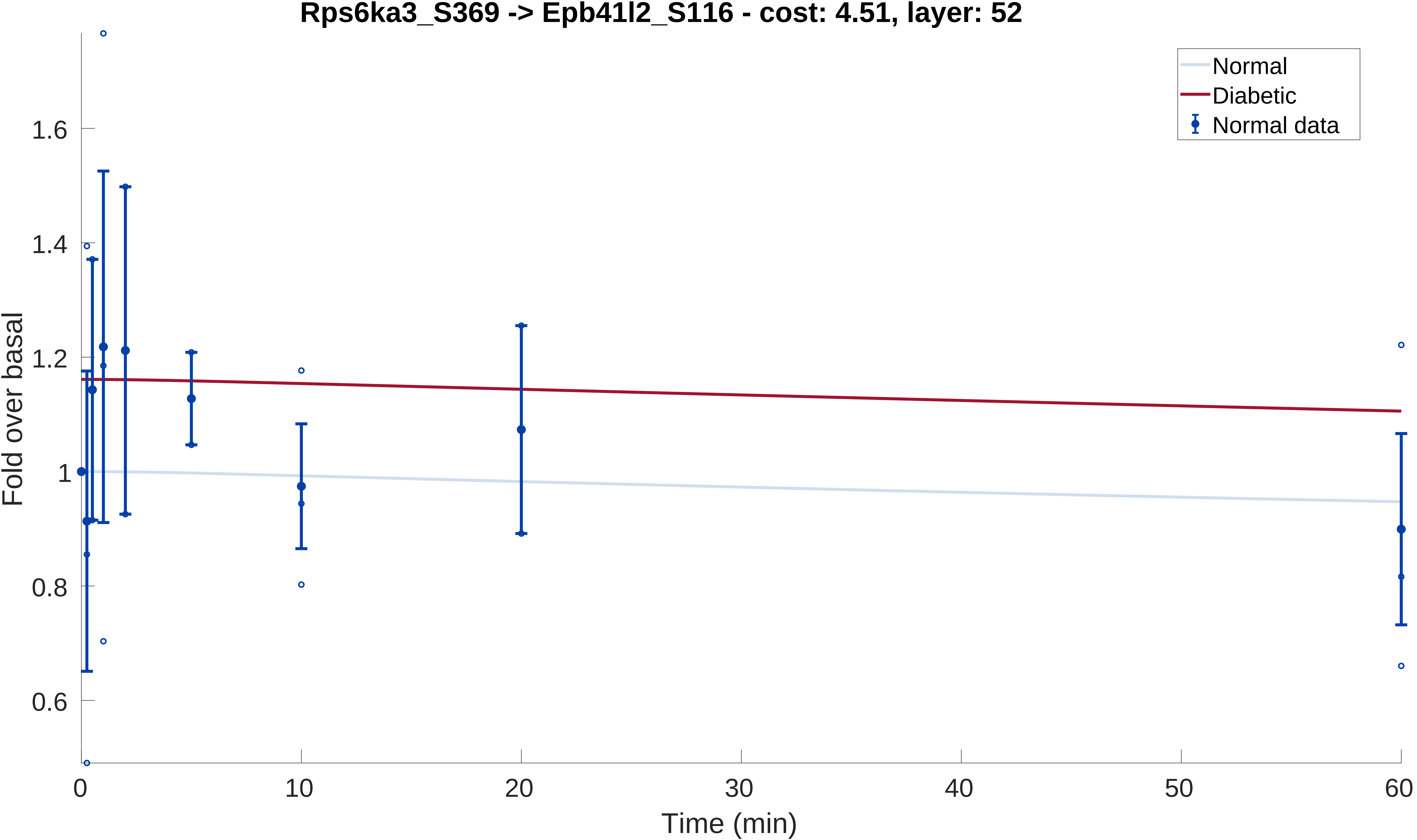

Rps6ka3_S369 -> Epb41l2_S116 - cost: 4.51, layer: 52
Normal
Diabetic
Normal data
Fold over basal
1.6
1.4
1.2
1
0.8
0.6
0
10
20
30
40
50
60
Time (min)

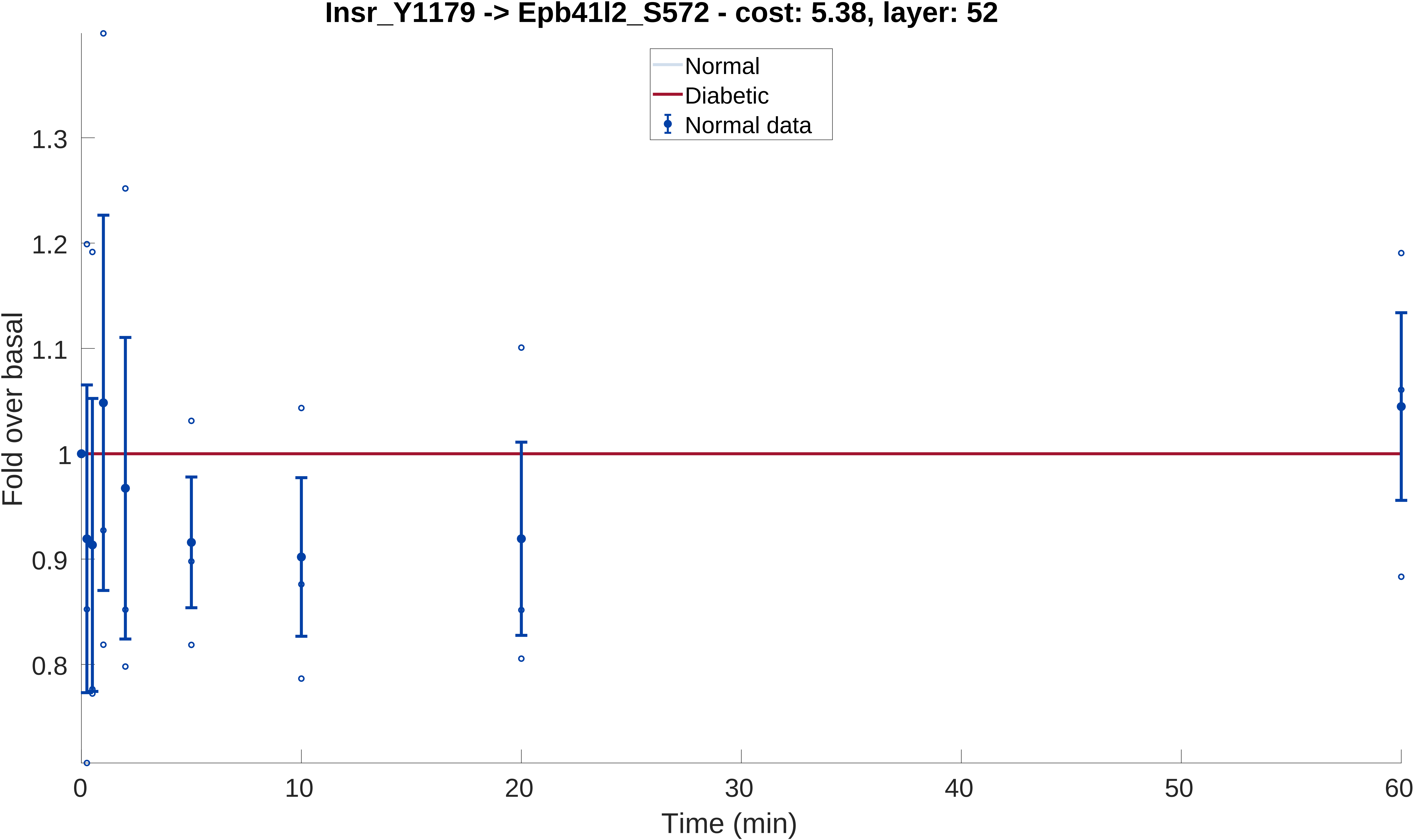

Insr_Y1179 -> Epb41l2_S572 - cost: 5.38, layer: 52
Normal
Diabetic
Normal data
Fold over basal
1.3
1.2
1.1
1
0.9
0.8
0
10
20
30
40
50
60
Time (min)

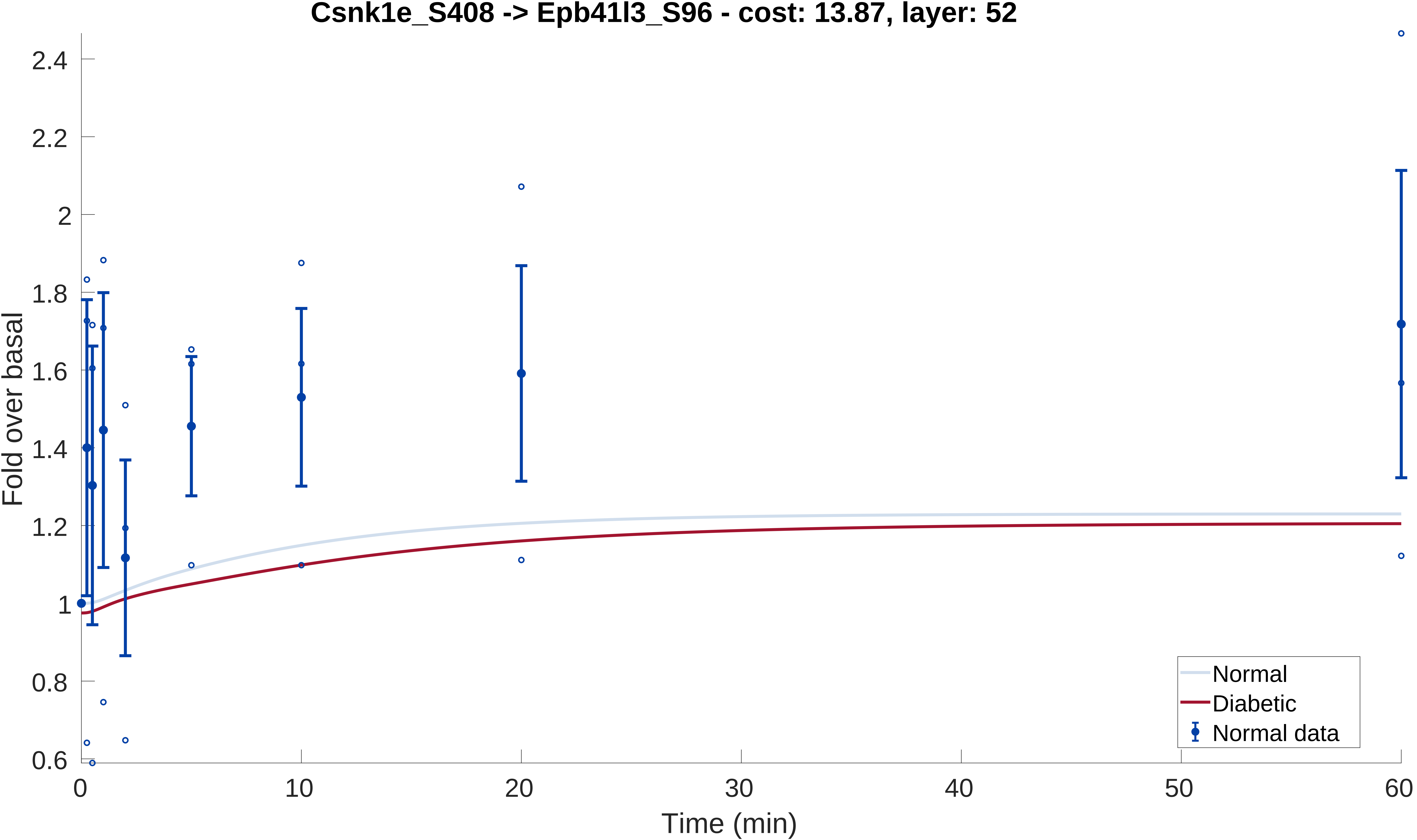

Csnk1e_S408 -> Epb41l3_S96 - cost: 13.87, layer: 52
Fold over basal
Time (min)
2.4
2.2
2
1.8
1.6
1.4
1.2
1
0.8
0.6
0
10
20
30
40
50
60
Normal
Diabetic
Normal data

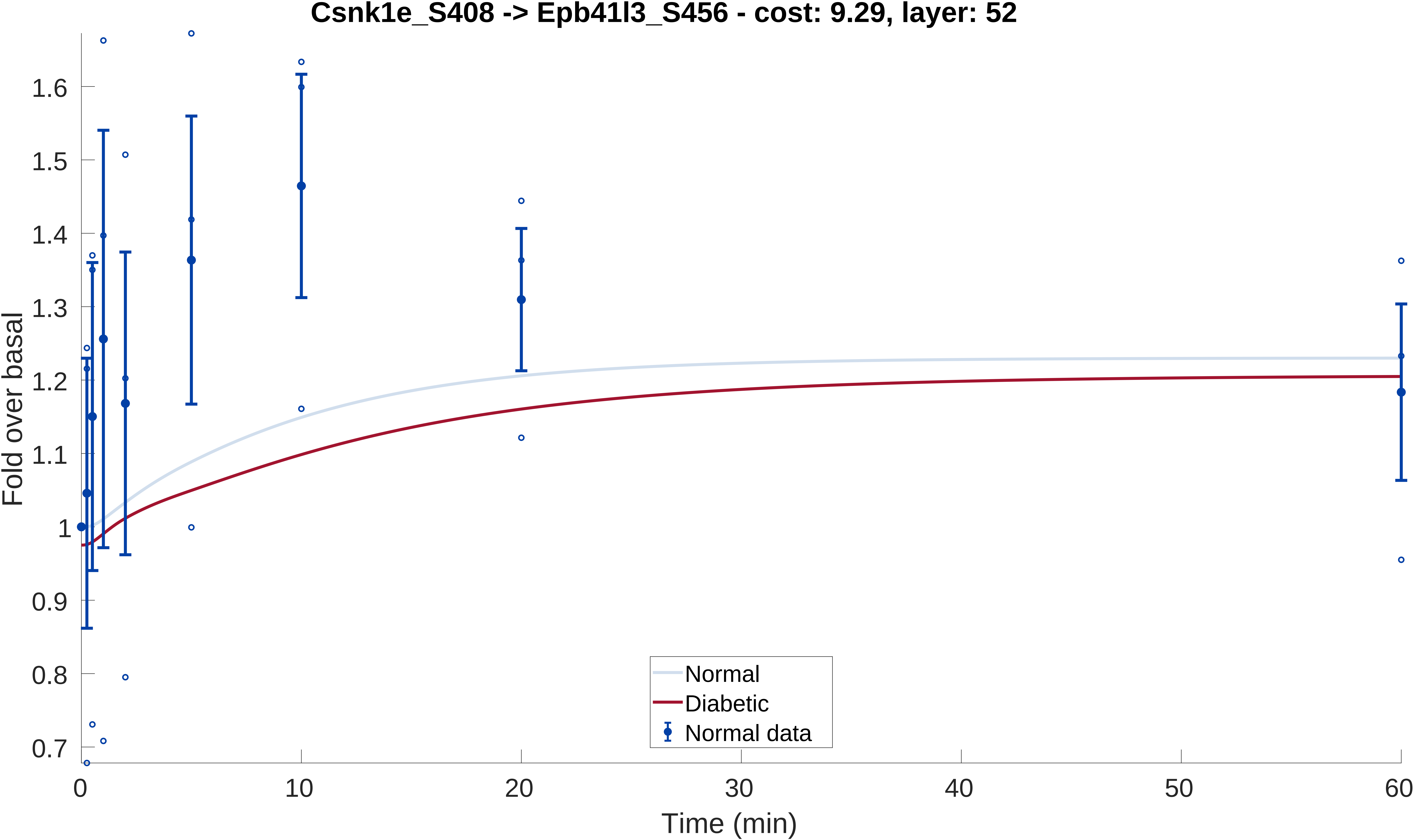

Csnk1e_S408 -> Epb41l3_S456 - cost: 9.29, layer: 52
Fold over basal
Time (min)
Normal
Diabetic
Normal data
0.7
0.8
0.9
1
1.1
1.2
1.3
1.4
1.5
1.6
0
10
20
30
40
50
60

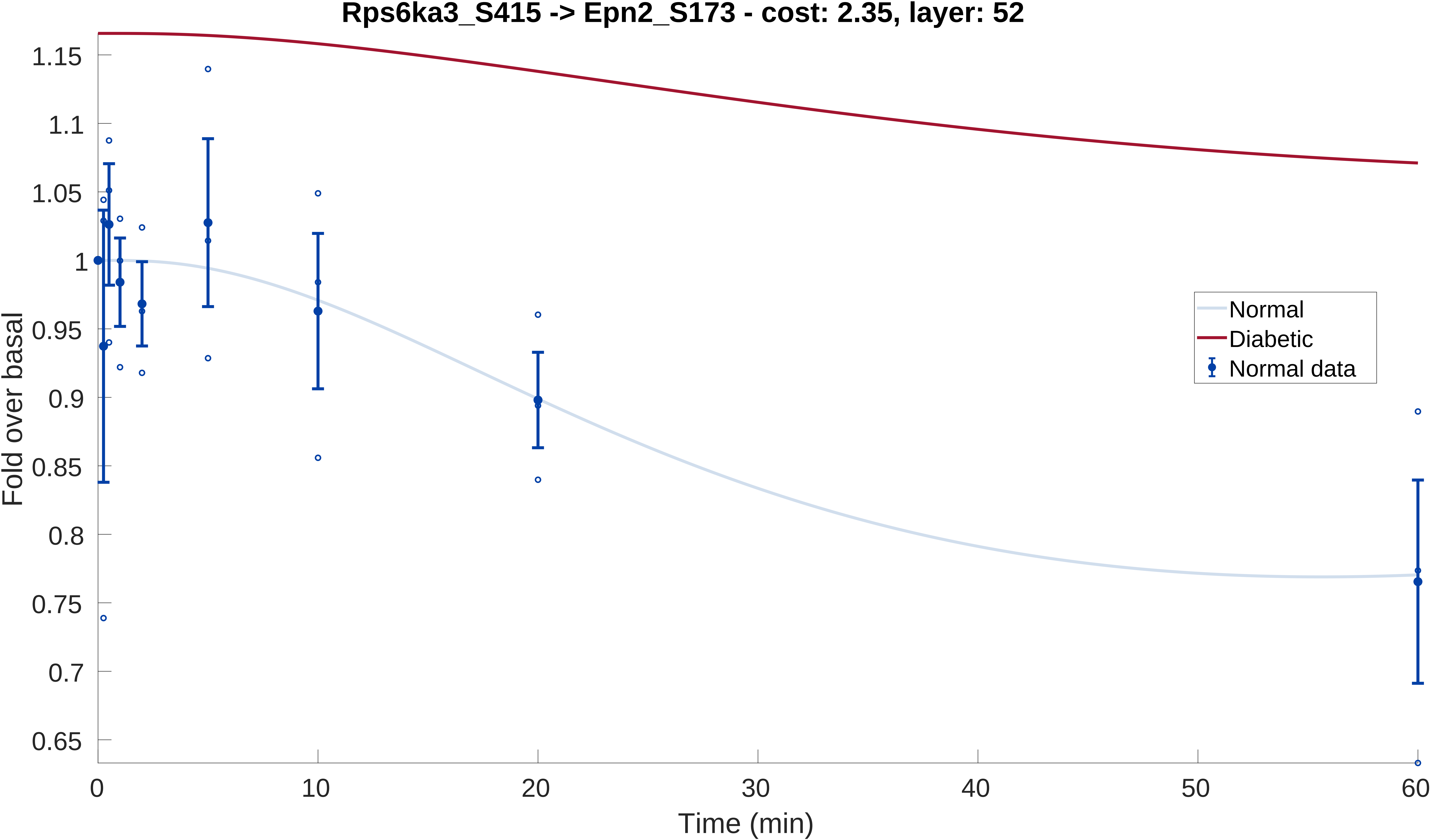

Rps6ka3_S415 -> Epn2_S173 - cost: 2.35, layer: 52
1.15
1.1
1.05
1
0.95
0.9
0.85
0.8
0.75
0.7
0.65
Fold over basal
0
10
20
30
40
50
60
Time (min)
Normal
Diabetic
Normal data

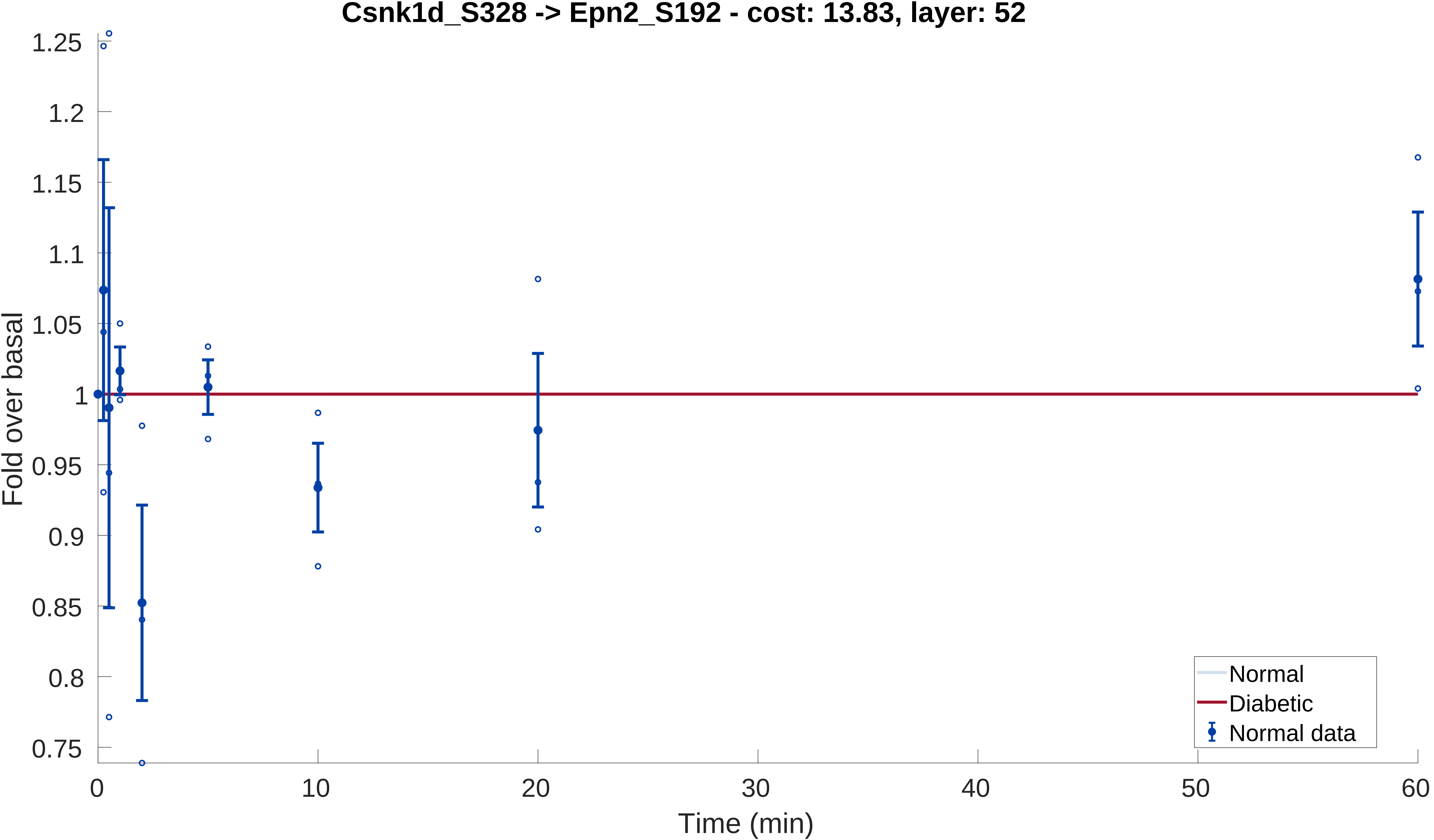

Csnk1d_S328 -> Epn2_S192 - cost: 13.83, layer: 52
Fold over basal
Time (min)
1.25
1.2
1.15
1.1
1.05
1
0.95
0.9
0.85
0.8
0.75
0
10
20
30
40
50
60
Normal
Diabetic
Normal data

Rps6ka3_S375 -> Epn2_S195 - cost: 4.88, layer: 52

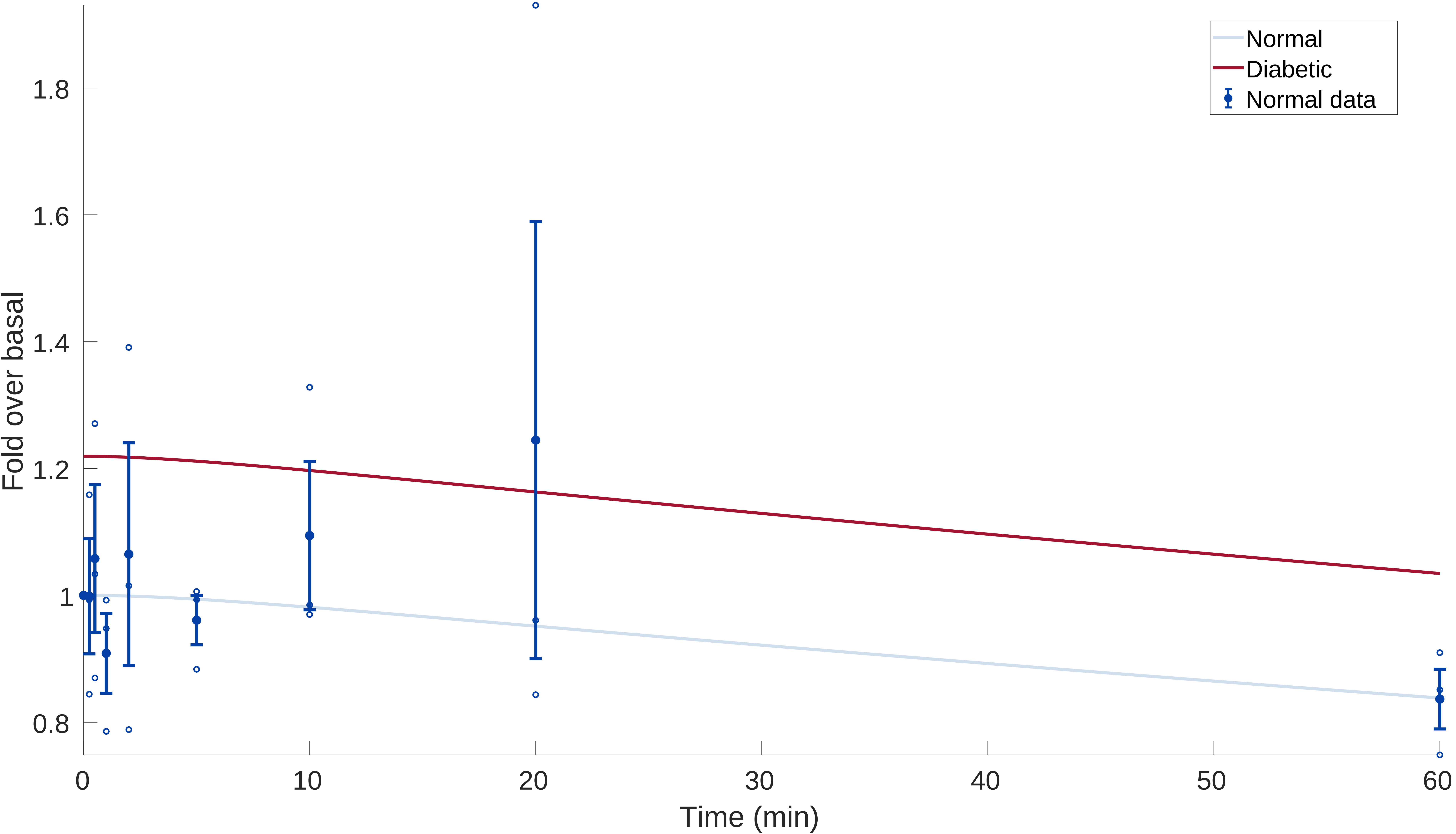

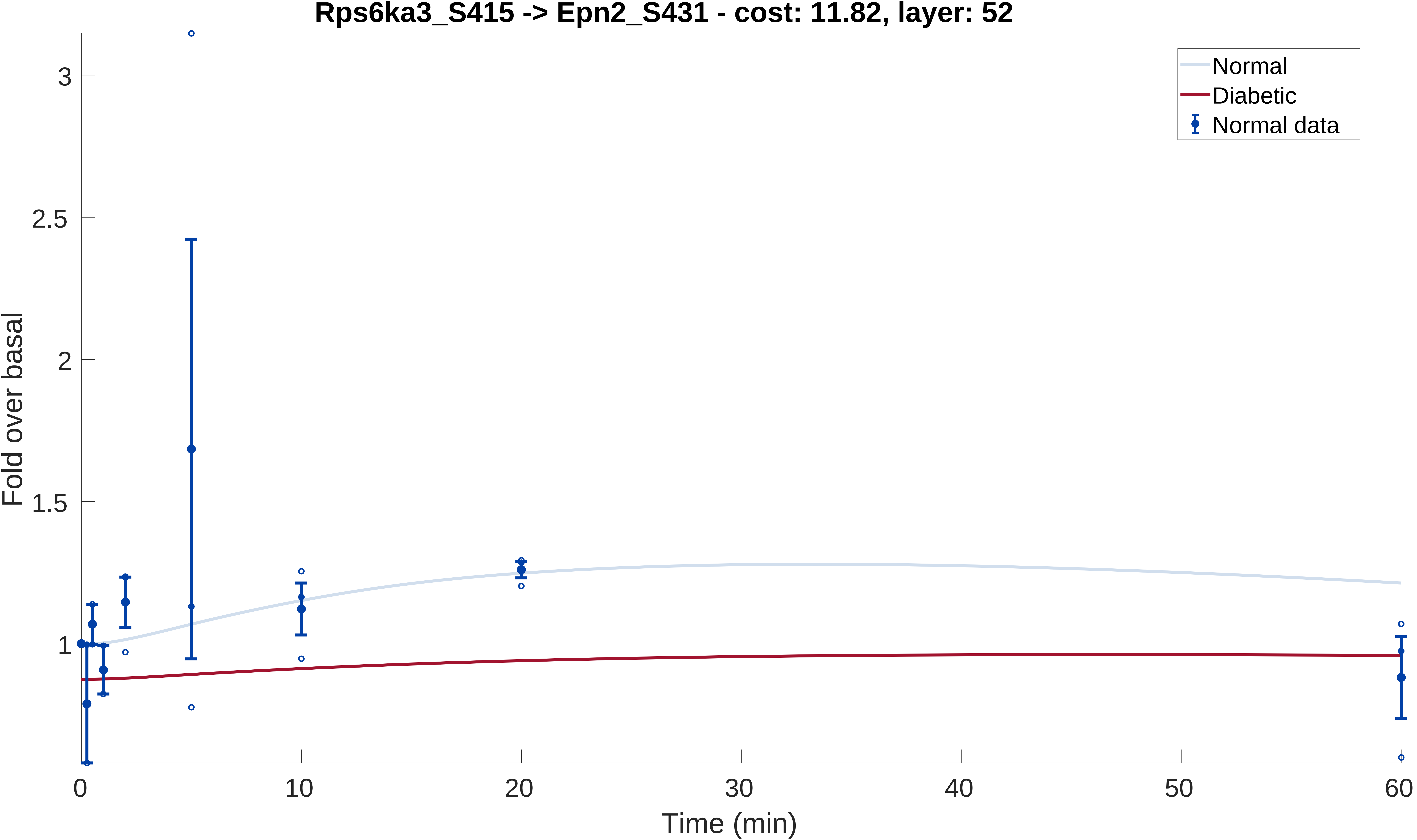
Rps6ka3_S415 -> Epn2_S431 - cost: 11.82, layer: 52
Fold over basal
Time (min)
Normal
Diabetic
Normal data
3
2.5
2
1.5
1
0
10
20
30
40
50
60

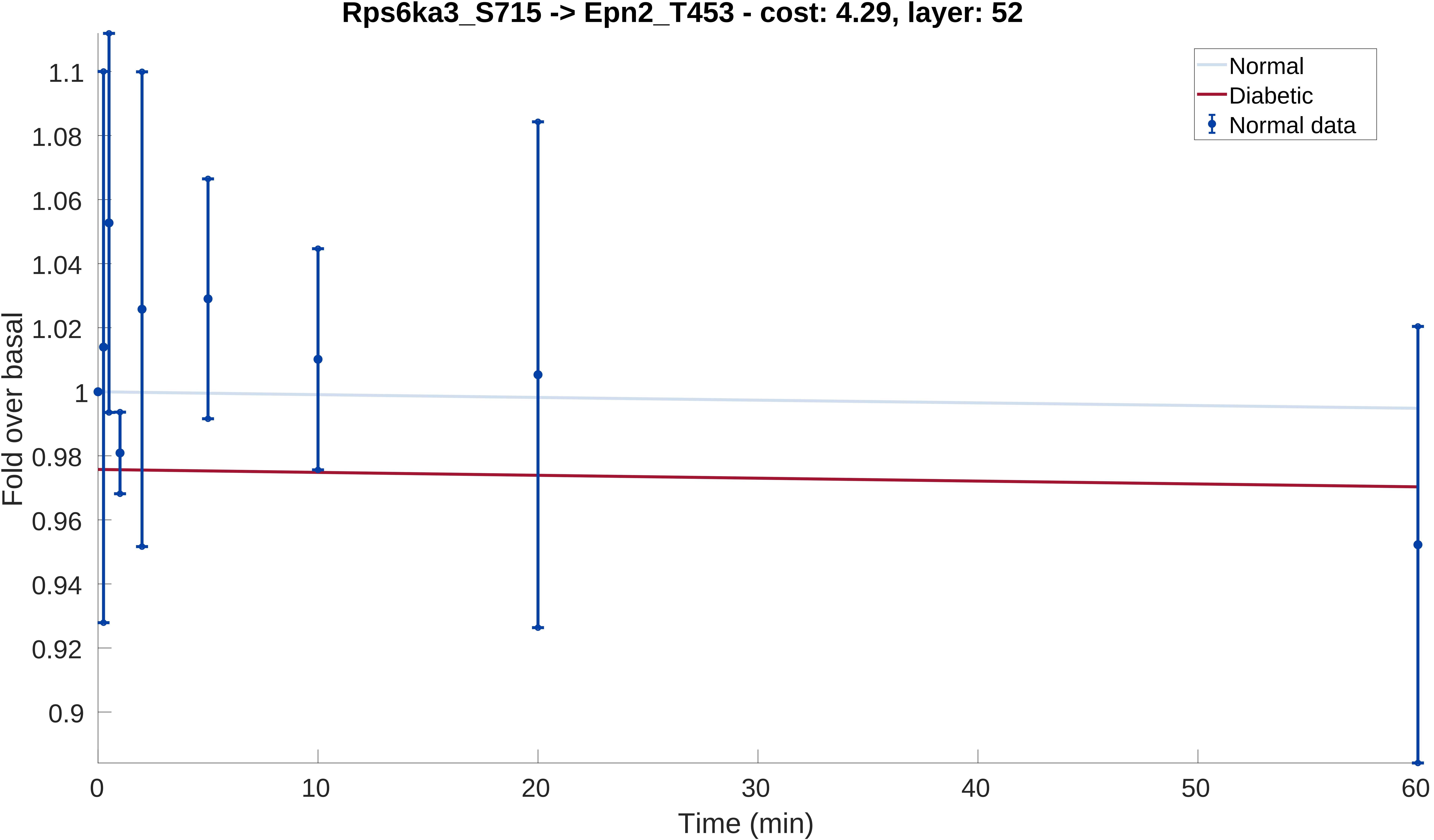

Rps6ka3_S715 -> Epn2_T453 - cost: 4.29, layer: 52
Normal
Diabetic
Normal data
Fold over basal
1.1
1.08
1.06
1.04
1.02
1
0.98
0.96
0.94
0.92
0.9
0
10
20
30
40
50
60
Time (min)

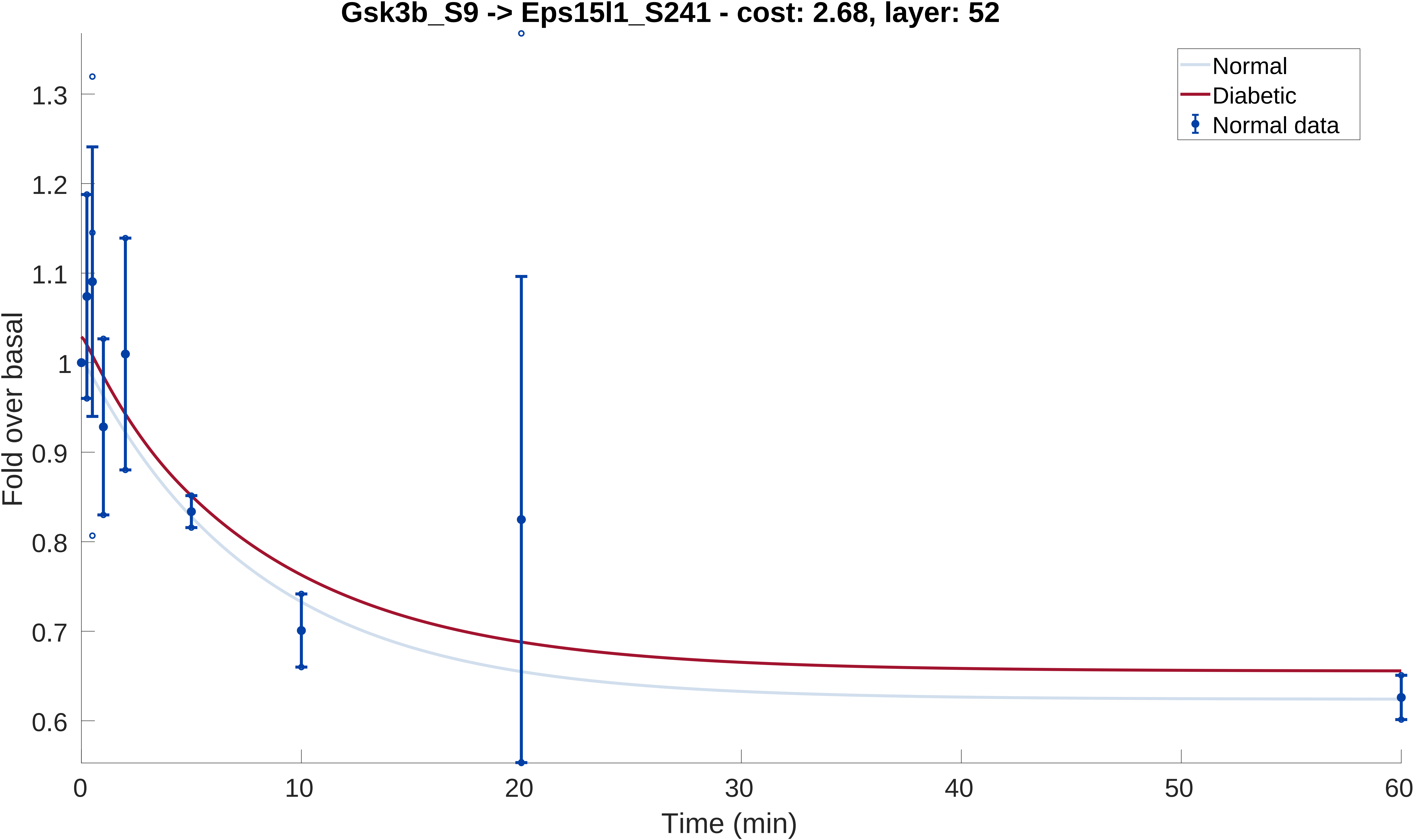

Gsk3b_S9 -> Eps15l1_S241 - cost: 2.68, layer: 52
Normal
Diabetic
Normal data
Fold over basal
1.3
1.2
1.1
1
0.9
0.8
0.7
0.6
0
10
20
30
40
50
60
Time (min)

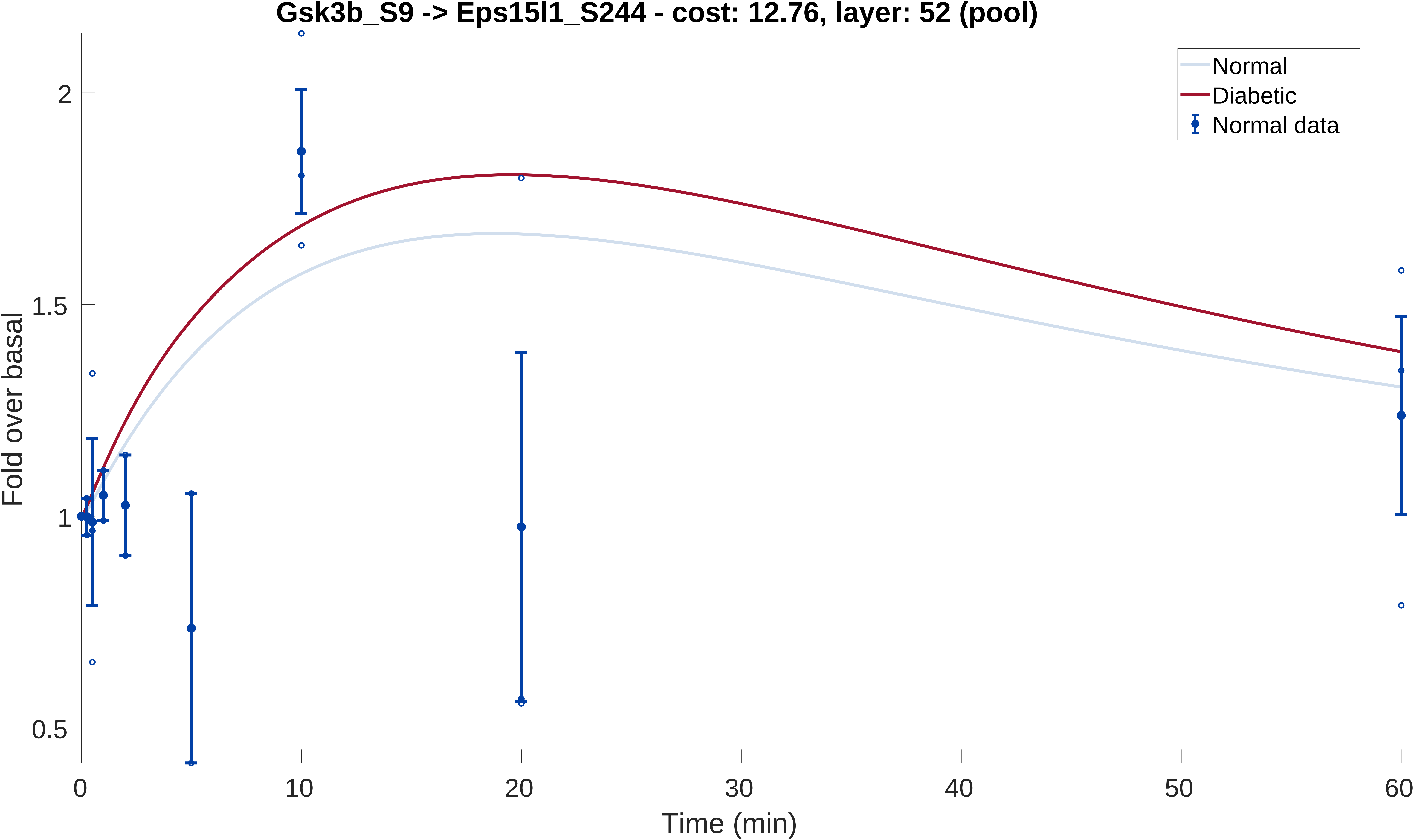

Gsk3b_S9 -> Eps15l1_S244 - cost: 12.76, layer: 52 (pool)
Normal
Diabetic
Normal data
Fold over basal
Time (min)
0.5
1
1.5
2
0
10
20
30
40
50
60

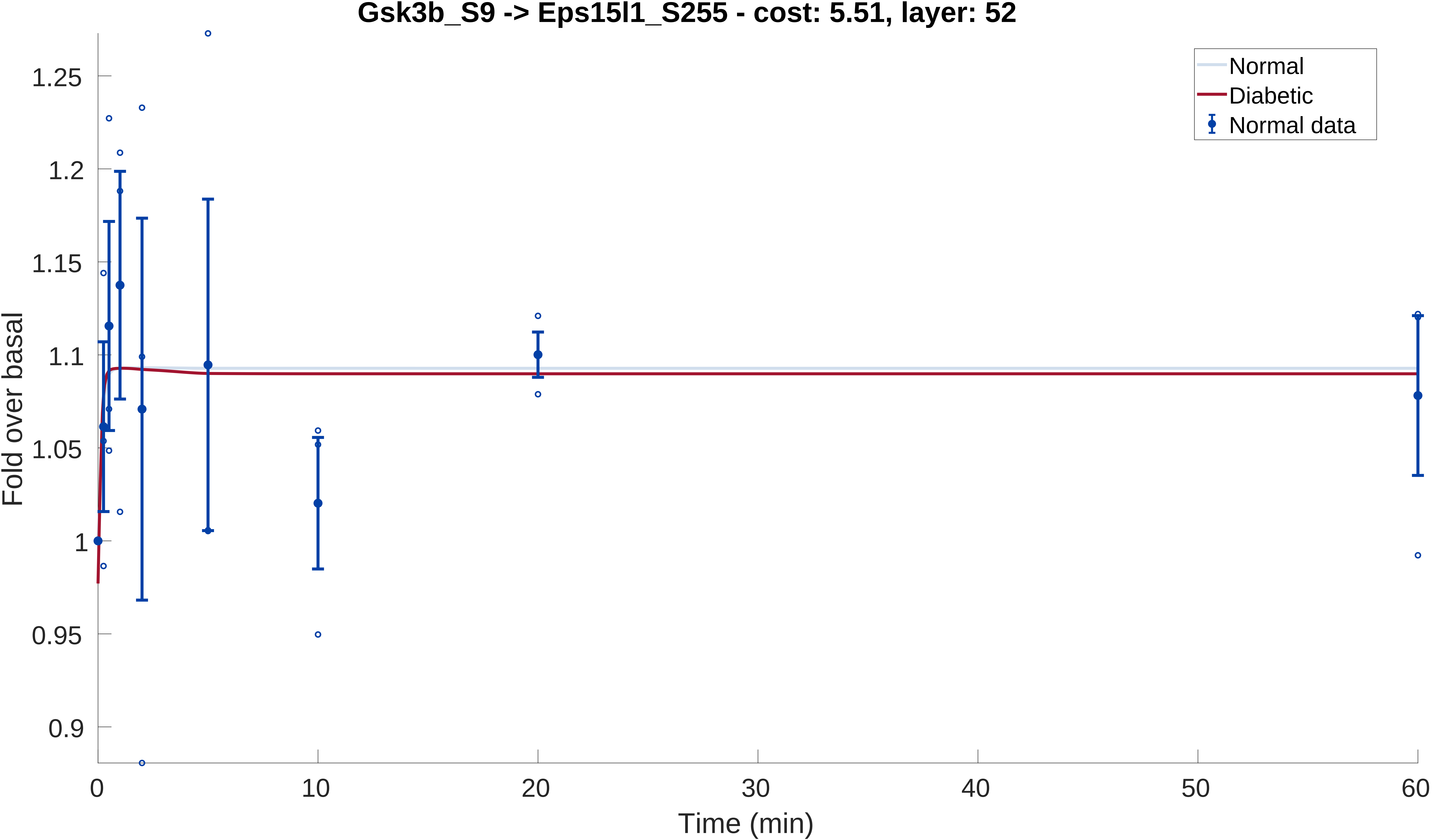

Gsk3b_S9 -> Eps15l1_S255 - cost: 5.51, layer: 52
Normal
Diabetic
Normal data
Fold over basal
1.25
1.2
1.15
1.1
1.05
1
0.95
0.9
0
10
20
30
40
50
60
Time (min)

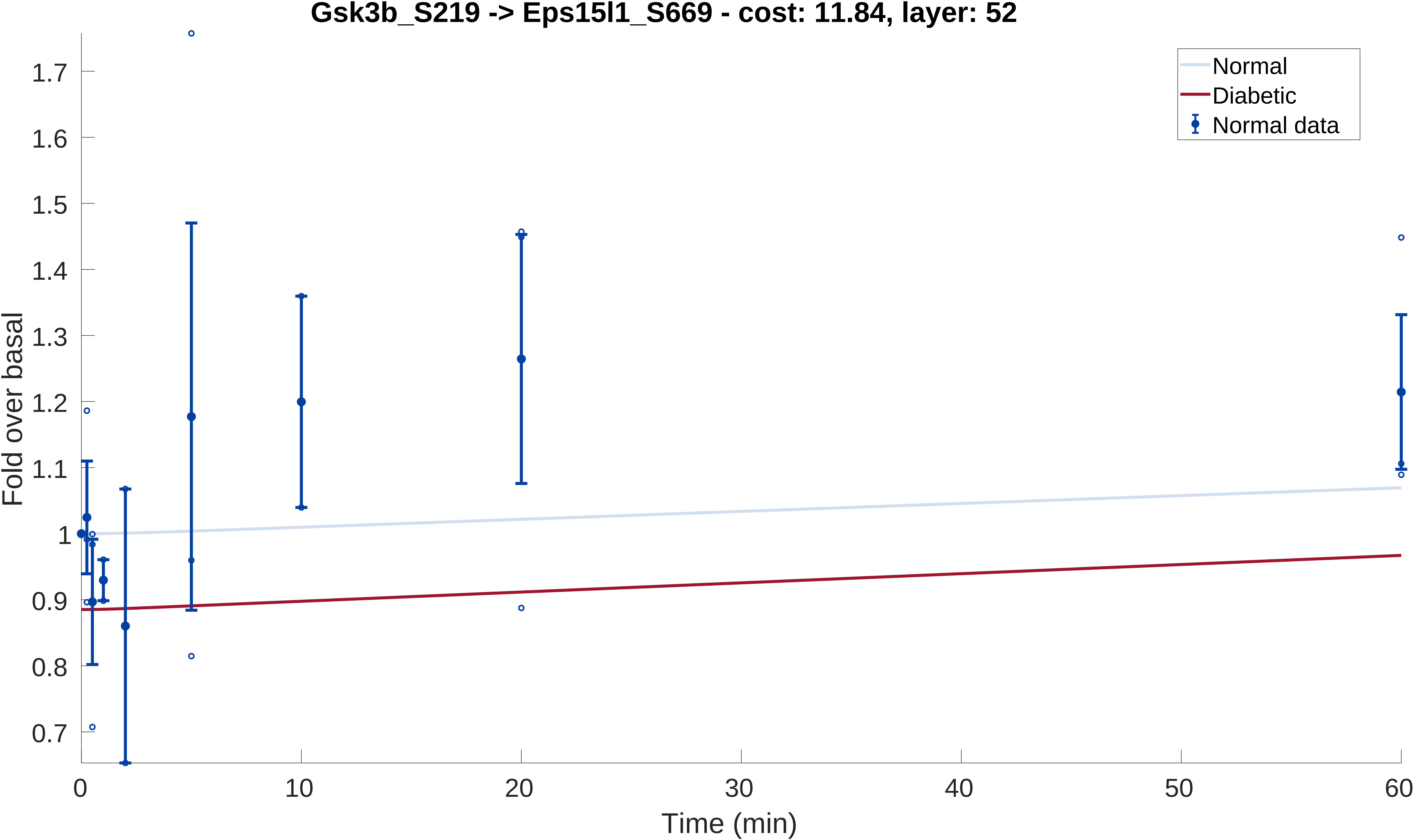

Gsk3b_S219 -> Eps15l1_S669 - cost: 11.84, layer: 52
Normal
Diabetic
Normal data
Fold over basal
1.7
1.6
1.5
1.4
1.3
1.2
1.1
1
0.9
0.8
0.7
0
10
20
30
40
50
60
Time (min)

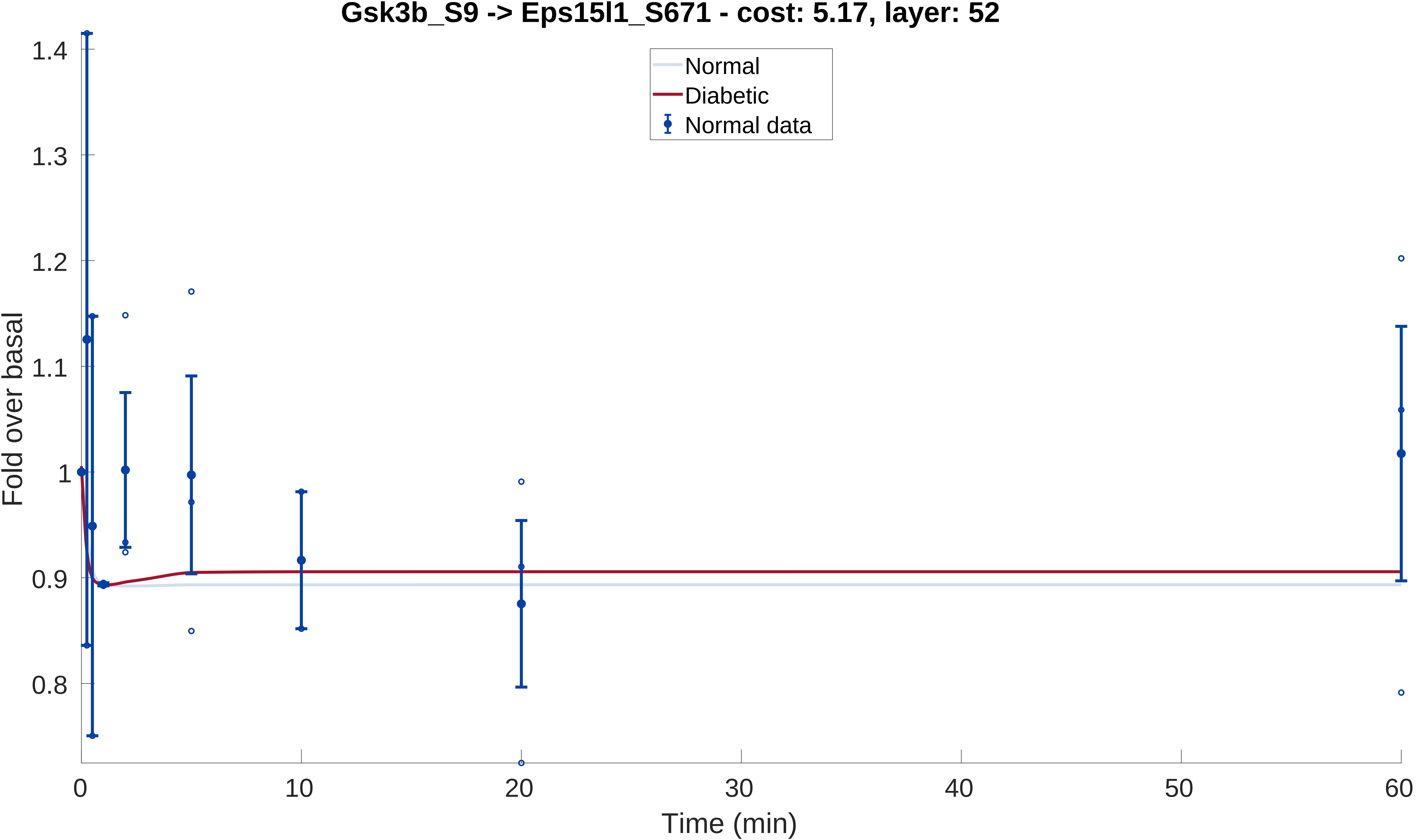

Gsk3b_S9 -> Eps15l1_S671 - cost: 5.17, layer: 52
Normal
Diabetic
Normal data
Fold over basal
1.4
1.3
1.2
1.1
1
0.9
0.8
0
10
20
30
40
50
60
Time (min)

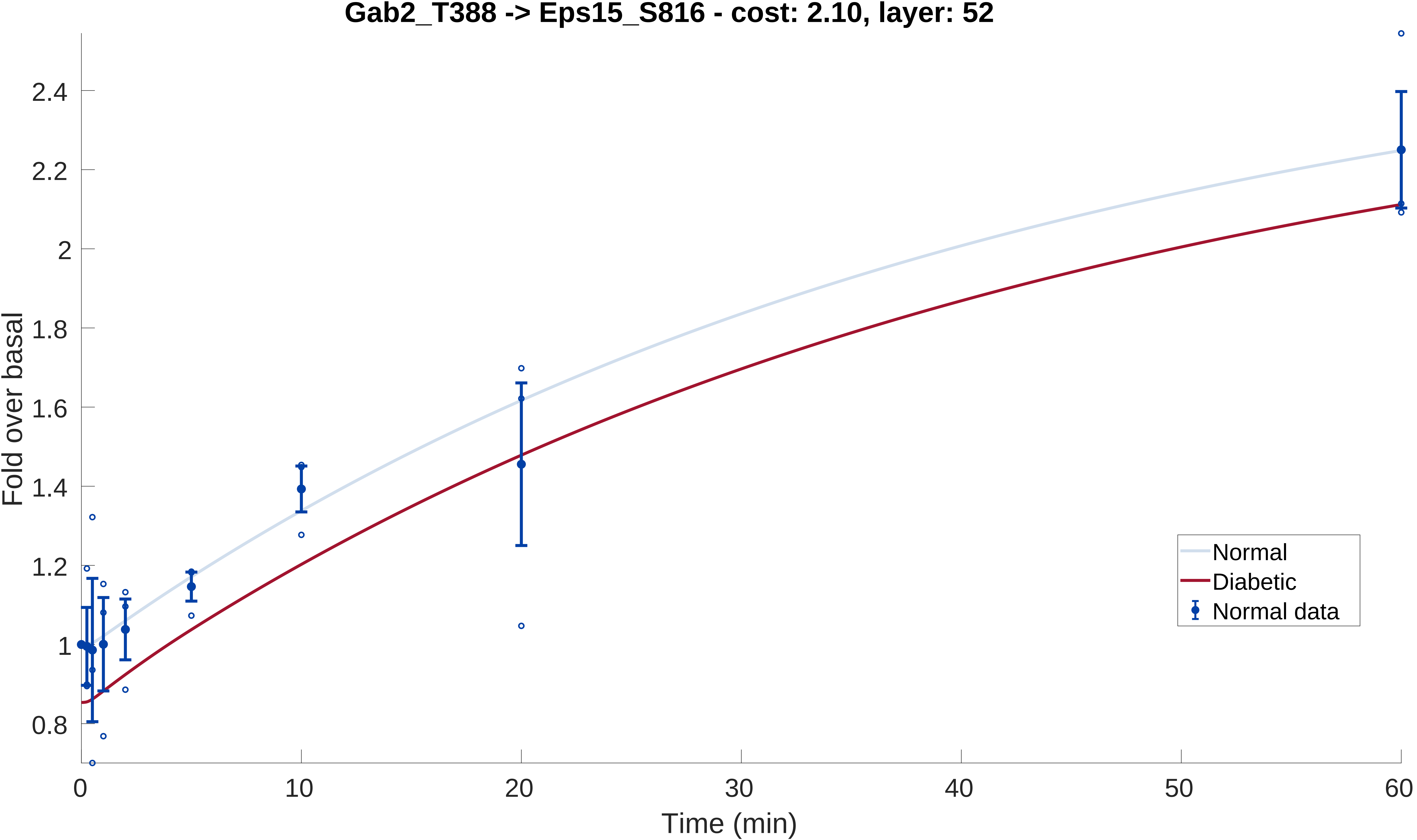
Gab2_T388 -> Eps15_S816 - cost: 2.10, layer: 52
Fold over basal
2.4
2.2
2
1.8
1.6
1.4
1.2
1
0.8
0
10
20
30
40
50
60
Time (min)
Normal
Diabetic
Normal data

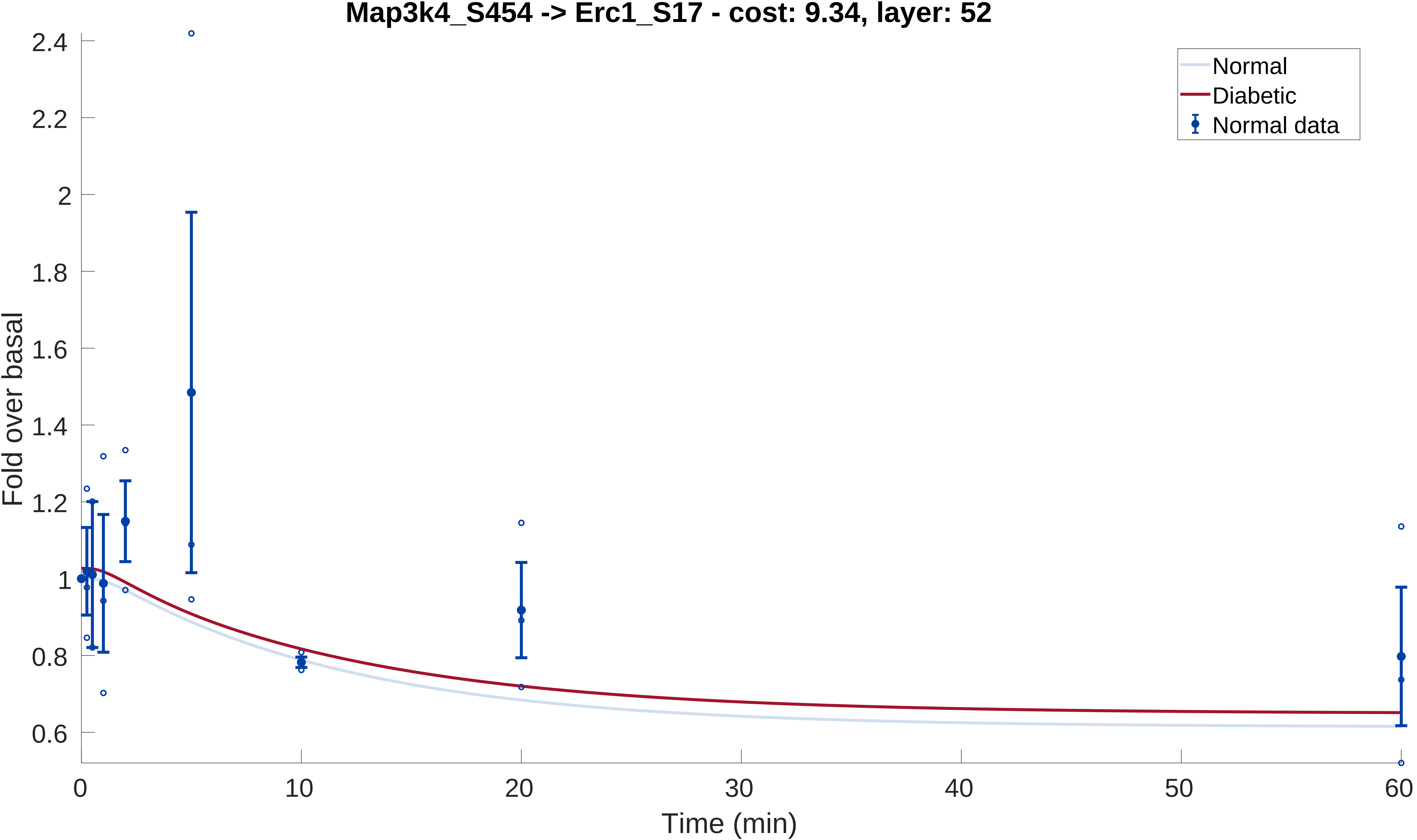
Map3k4_S454 -> Erc1_S17 - cost: 9.34, layer: 52
Normal
Diabetic
Normal data
Fold over basal
Time (min)
2.4
2.2
2
1.8
1.6
1.4
1.2
1
0.8
0.6
0
10
20
30
40
50
60

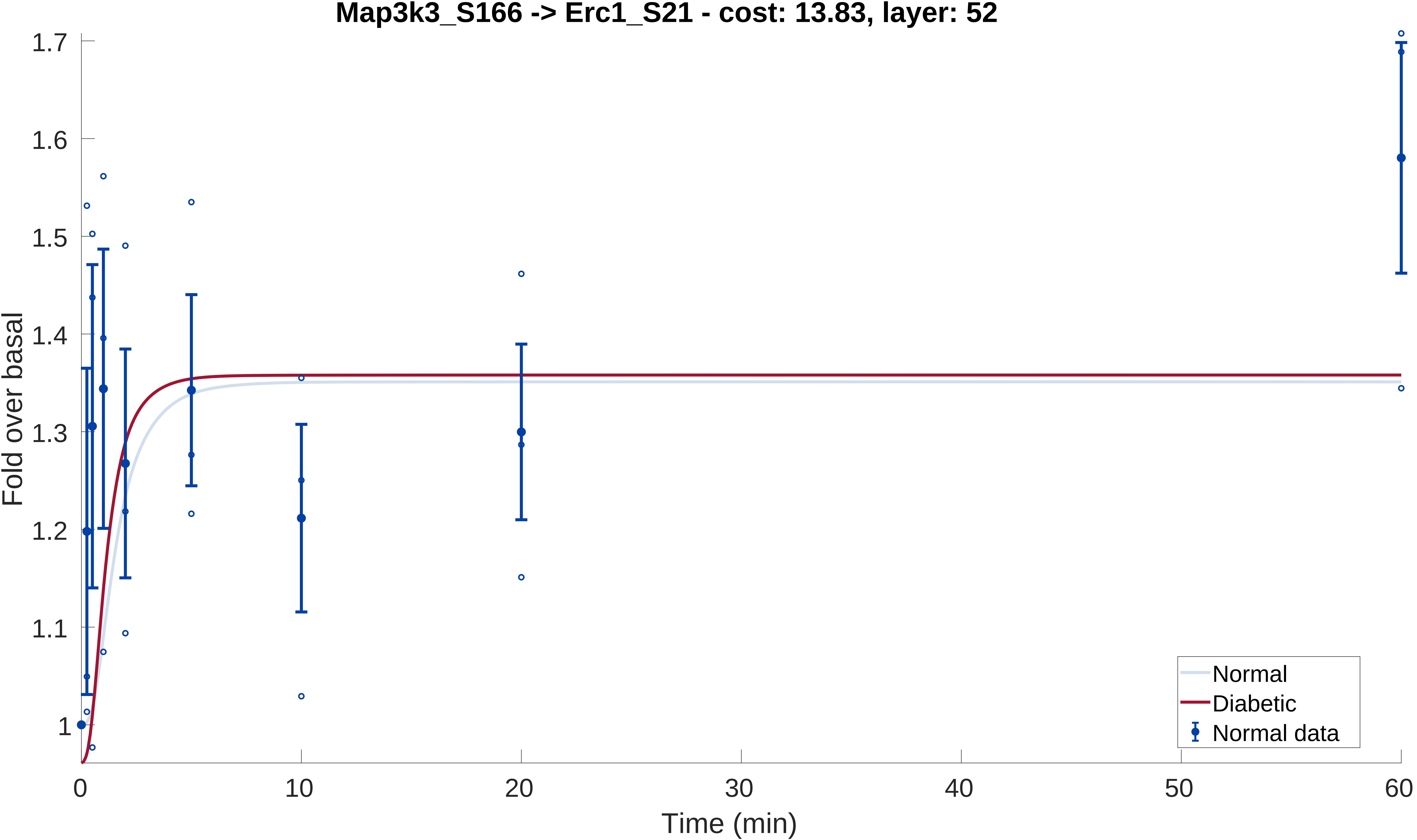

Map3k3_S166 -> Erc1_S21 - cost: 13.83, layer: 52
Fold over basal
1.7
1.6
1.5
1.4
1.3
1.2
1.1
1
0
10
20
30
40
50
60
Time (min)
Normal
Diabetic
Normal data

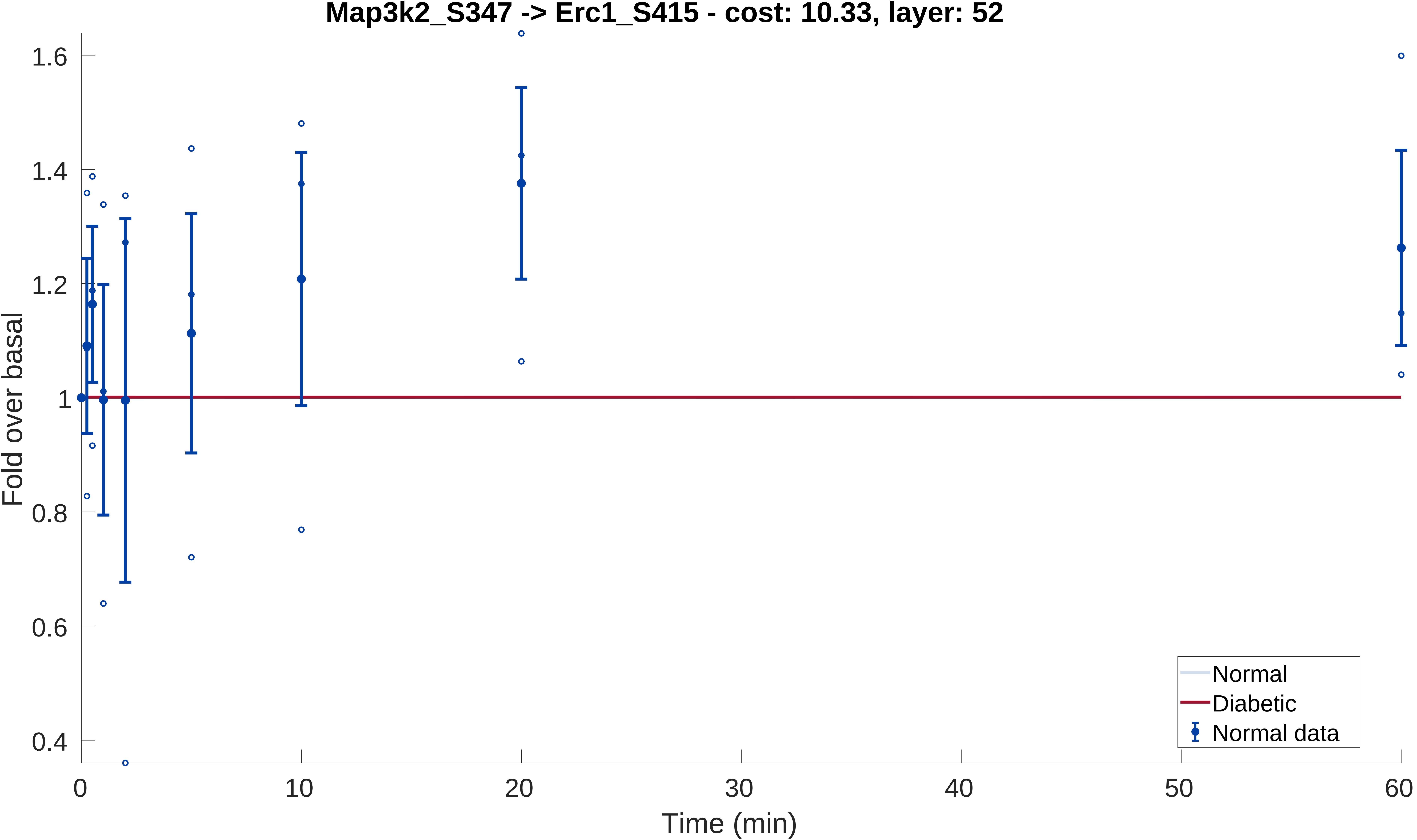

Map3k2_S347 -> Erc1_S415 - cost: 10.33, layer: 52
Fold over basal
1.6
1.4
1.2
1
0.8
0.6
0.4
0
10
20
30
40
50
60
Time (min)
Normal
Diabetic
Normal data

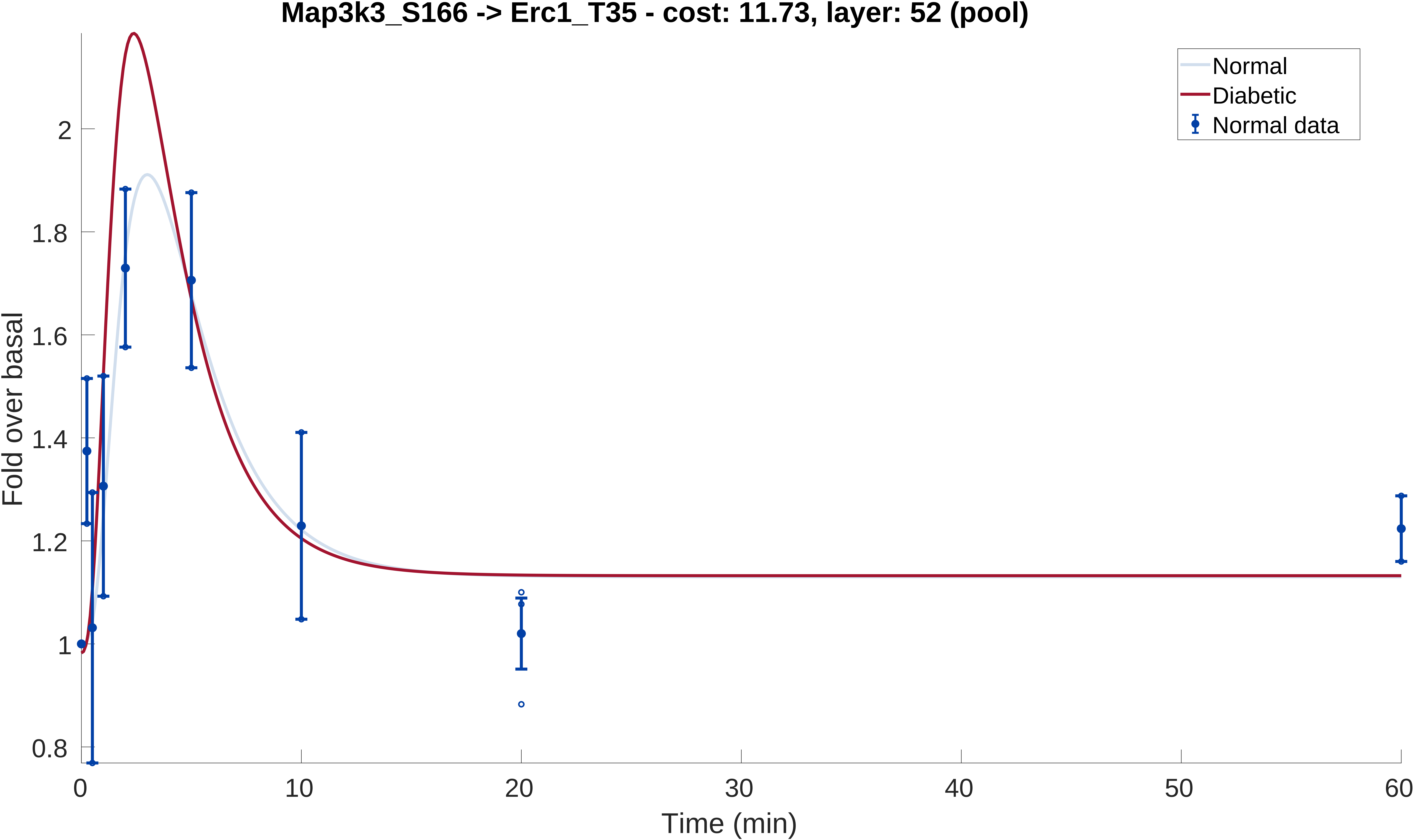

Map3k3_S166 -> Erc1_T35 - cost: 11.73, layer: 52 (pool)
Normal
Diabetic
Normal data
Fold over basal
Time (min)
0.8
1
1.2
1.4
1.6
1.8
2
0
10
20
30
40
50
60

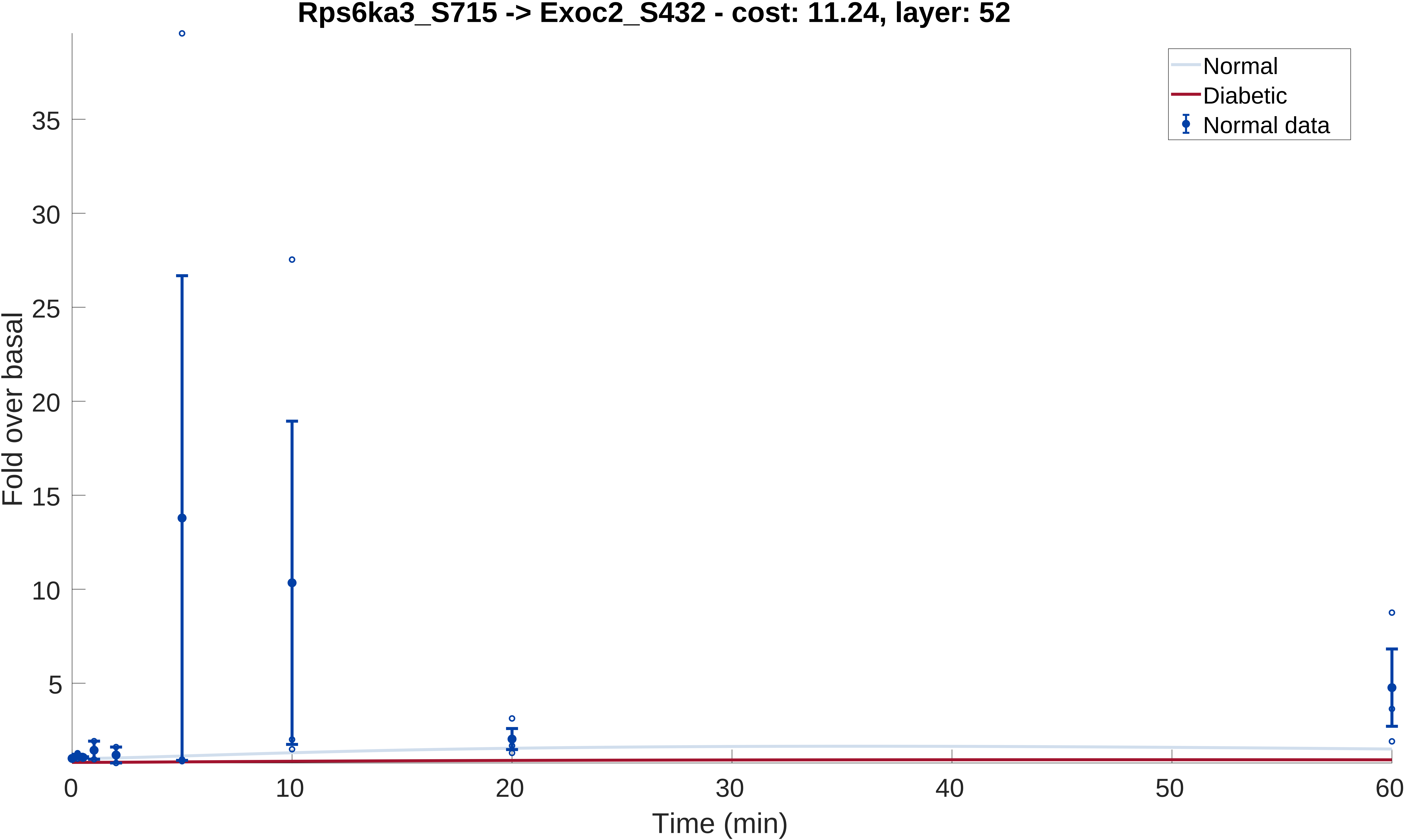

Rps6ka3_S715 -> Exoc2_S432 - cost: 11.24, layer: 52
Normal
Diabetic
Normal data
Fold over basal
Time (min)
0
10
20
30
40
50
60
5
10
15
20
25
30
35

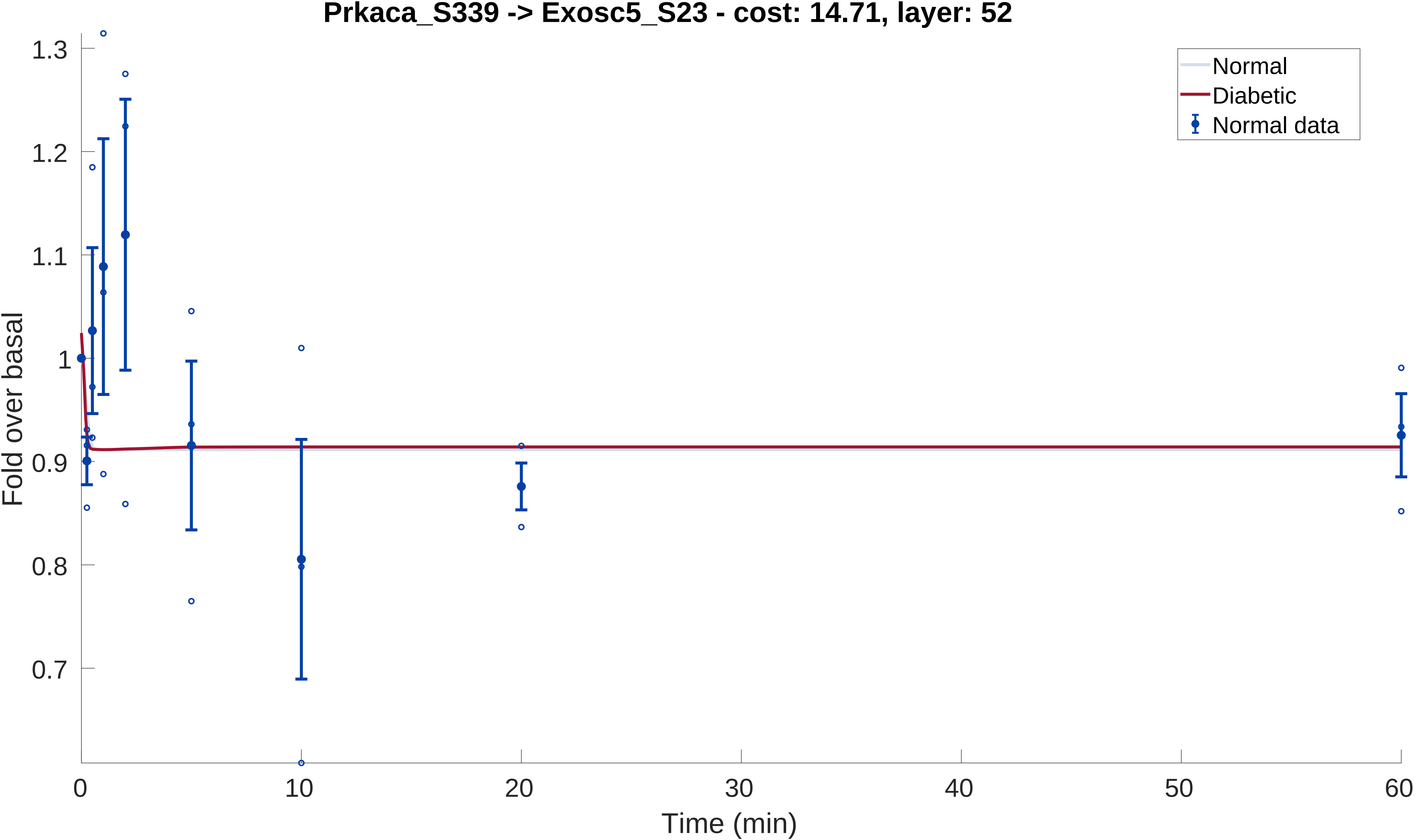

Prkaca_S339 -> Exosc5_S23 - cost: 14.71, layer: 52
Normal
Diabetic
Normal data
Fold over basal
Time (min)
1.3
1.2
1.1
1
0.9
0.8
0.7
0
10
20
30
40
50
60

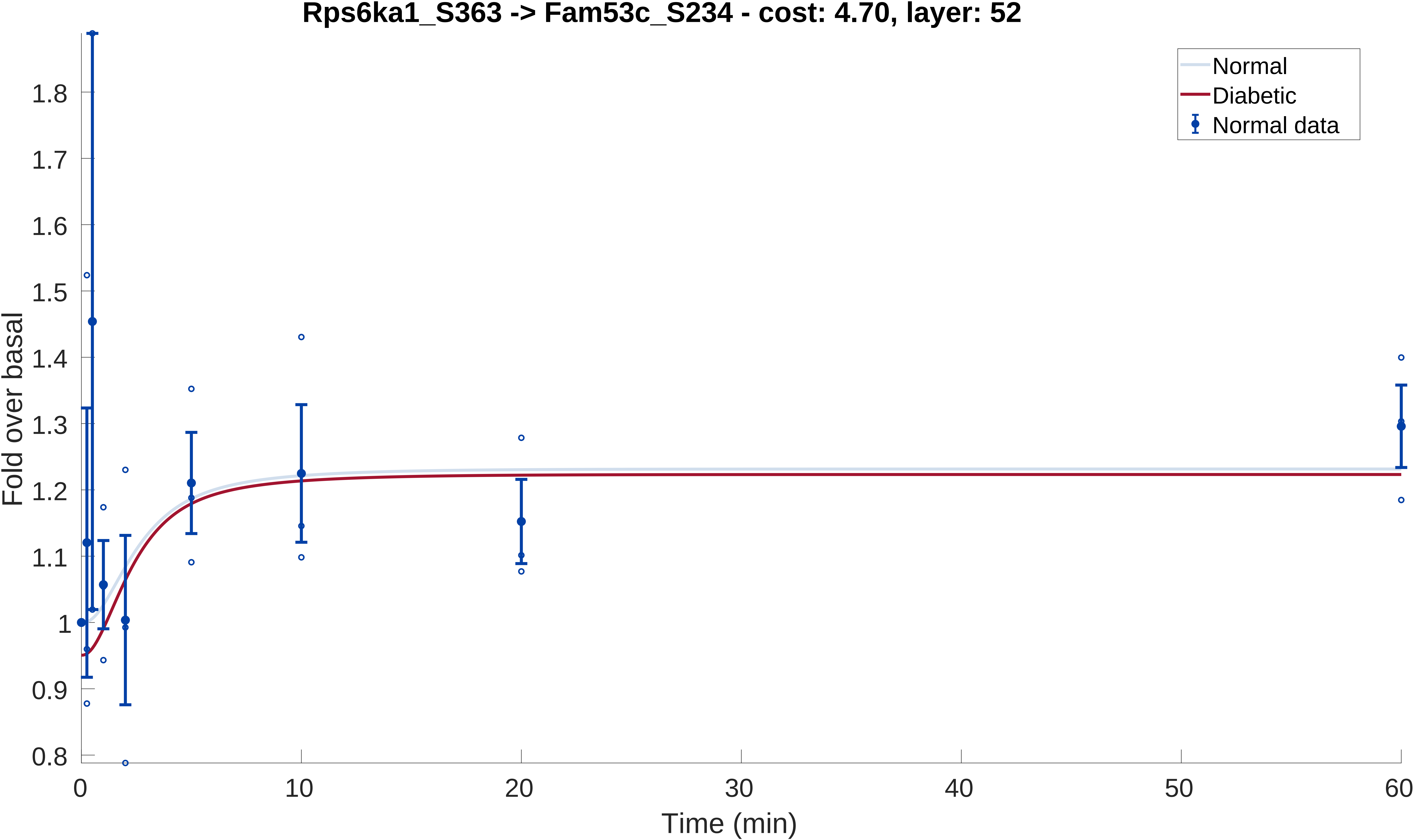

Rps6ka1_S363 -> Fam53c_S234 - cost: 4.70, layer: 52
Normal
Diabetic
Normal data
Fold over basal
Time (min)
0
10
20
30
40
50
60
0.8
0.9
1
1.1
1.2
1.3
1.4
1.5
1.6
1.7
1.8

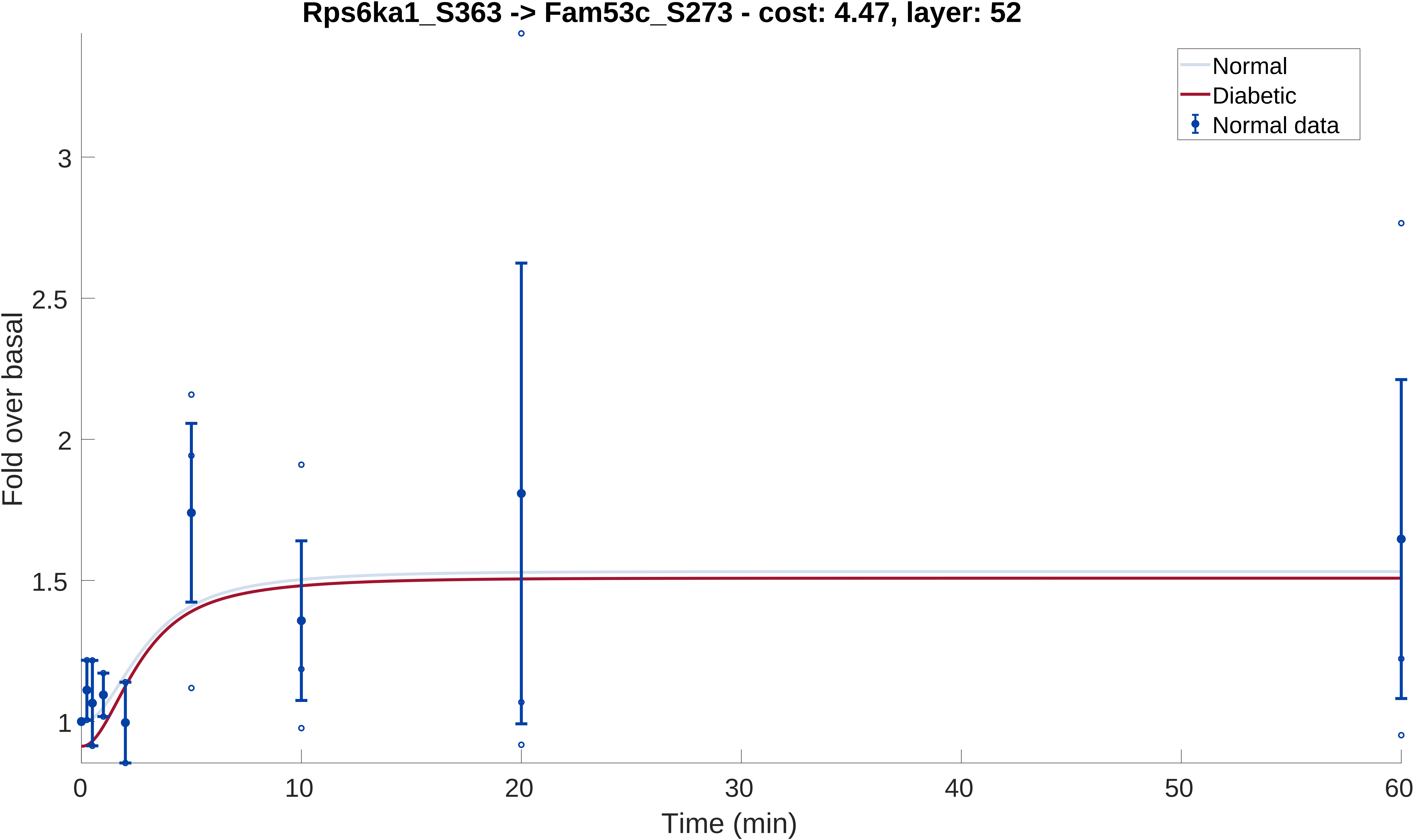

Rps6ka1_S363 -> Fam53c_S273 - cost: 4.47, layer: 52
Normal
Diabetic
Normal data
Fold over basal
Time (min)
1
1.5
2
2.5
3
0
10
20
30
40
50
60

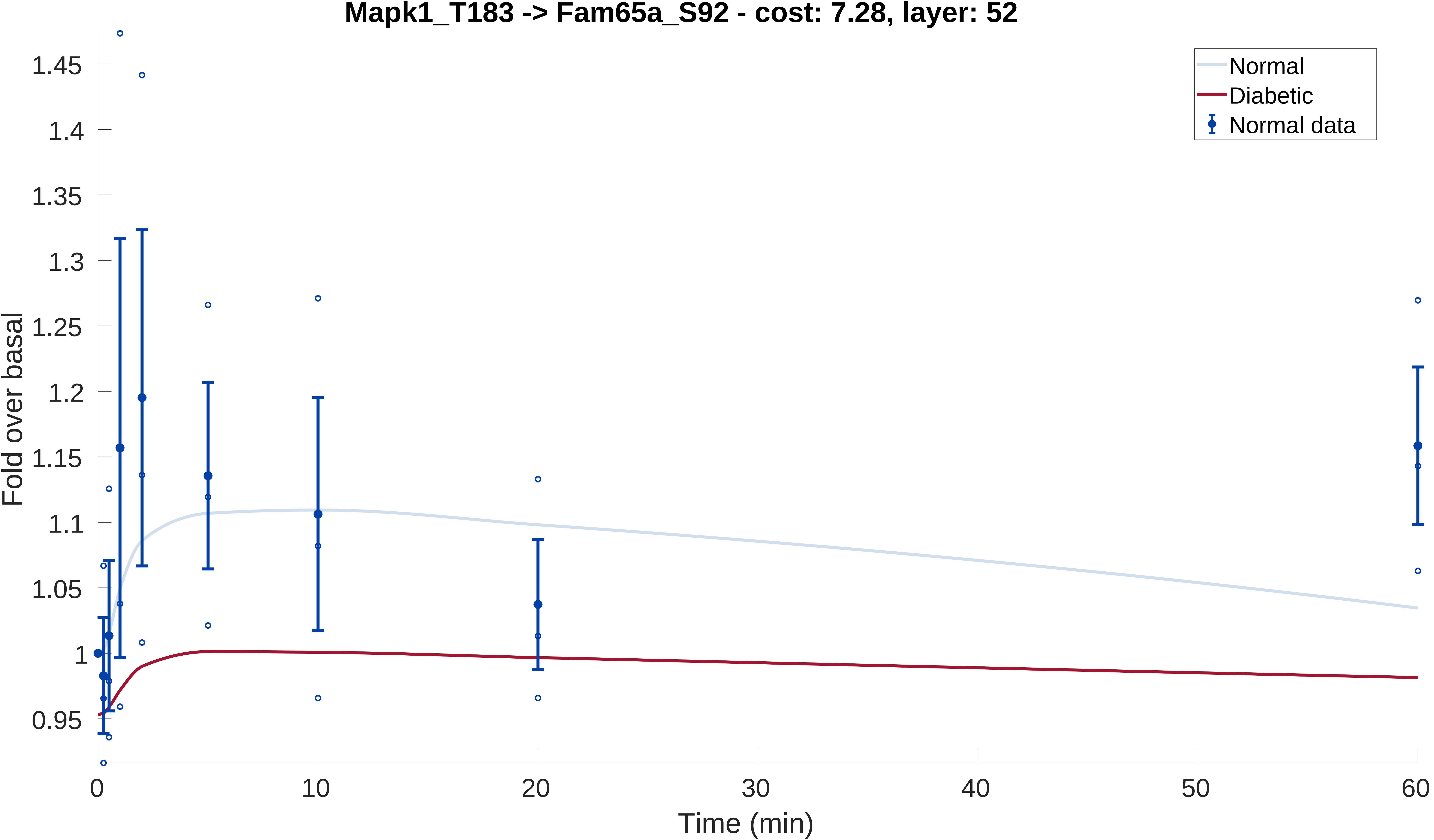

Mapk1_T183 -> Fam65a_S92 - cost: 7.28, layer: 52
Normal
Diabetic
Normal data
Fold over basal
1.45
1.4
1.35
1.3
1.25
1.2
1.15
1.1
1.05
1
0.95
0
10
20
30
40
50
60
Time (min)

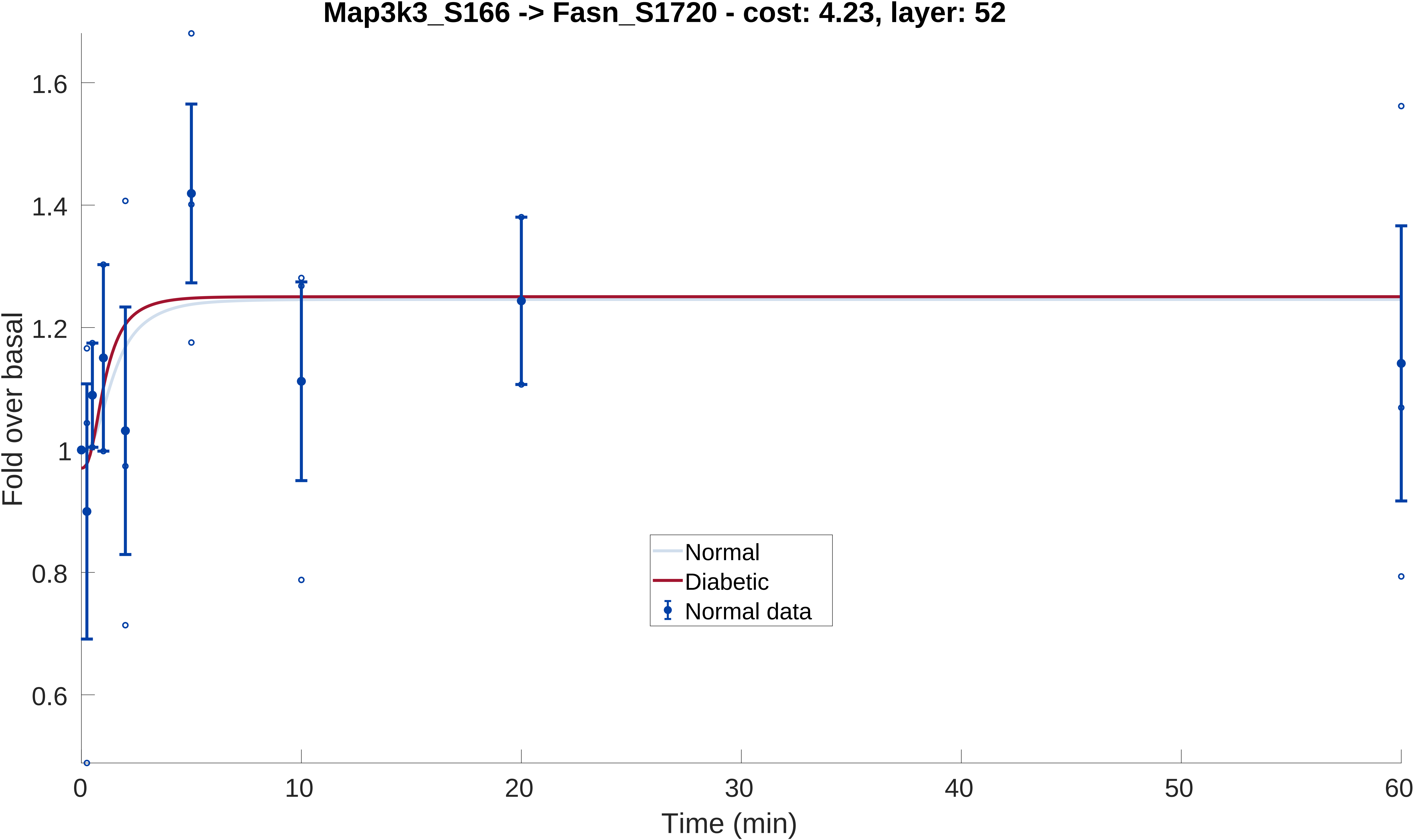
Map3k3_S166 -> Fasn_S1720 - cost: 4.23, layer: 52
Fold over basal
1.6
1.4
1.2
1
0.8
0.6
0
10
20
30
40
50
60
Time (min)
Normal
Diabetic
Normal data

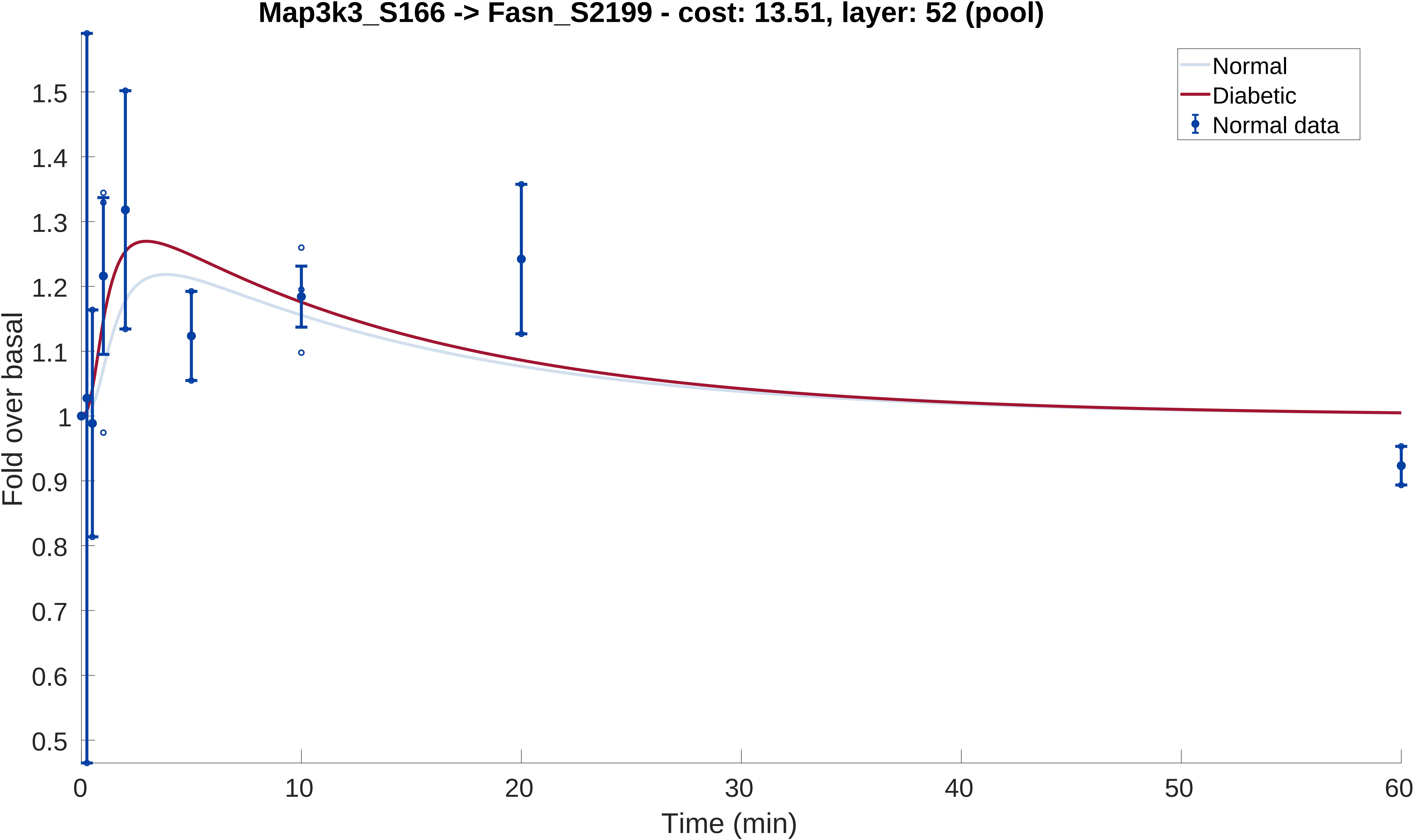

Map3k3_S166 -> Fasn_S2199 - cost: 13.51, layer: 52 (pool)
Normal
Diabetic
Normal data
Fold over basal
Time (min)
0.5
0.6
0.7
0.8
0.9
1
1.1
1.2
1.3
1.4
1.5
0
10
20
30
40
50
60

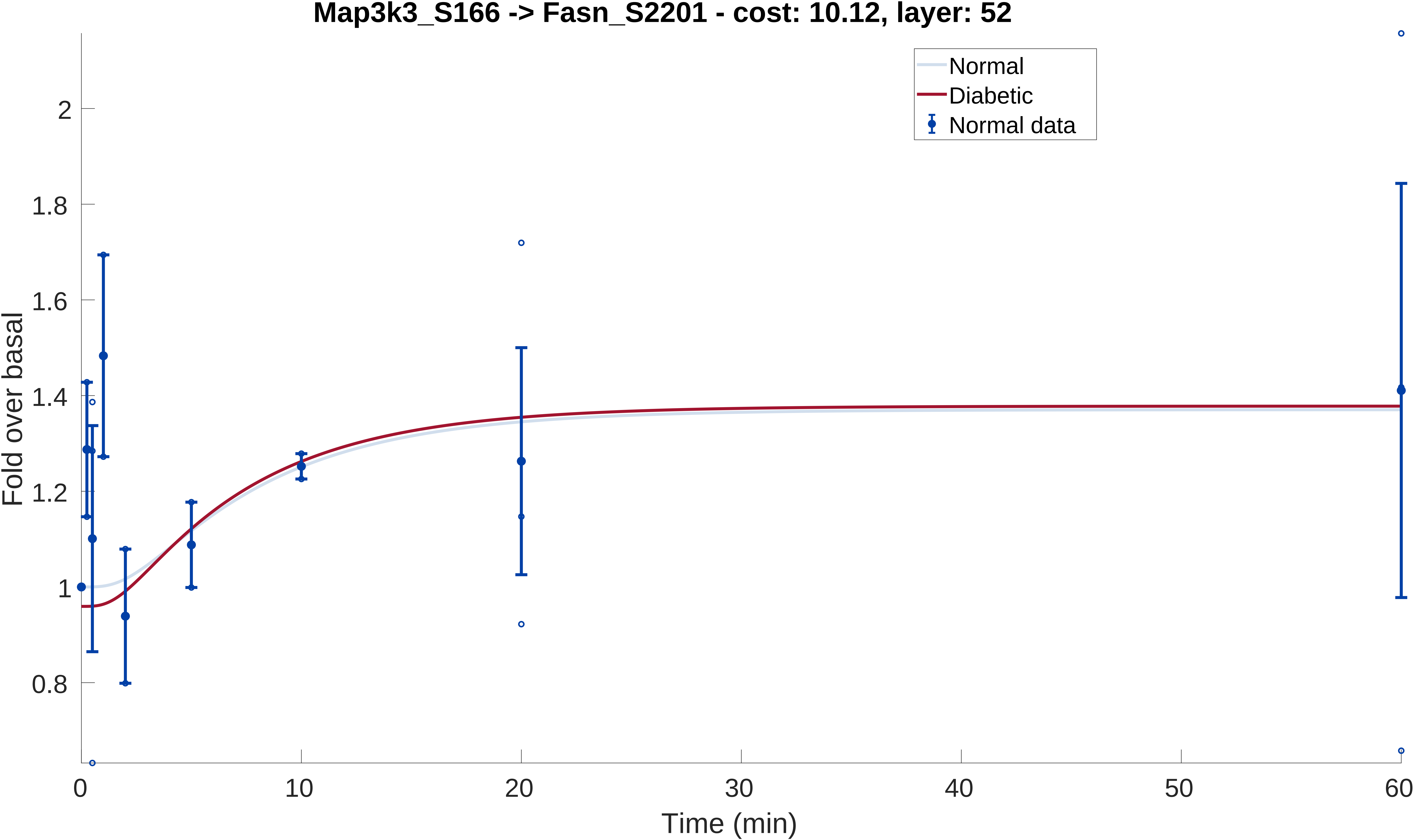

Map3k3_S166 -> Fasn_S2201 - cost: 10.12, layer: 52
Normal
Diabetic
Normal data
Fold over basal
Time (min)
0
10
20
30
40
50
60
0.8
1
1.2
1.4
1.6
1.8
2

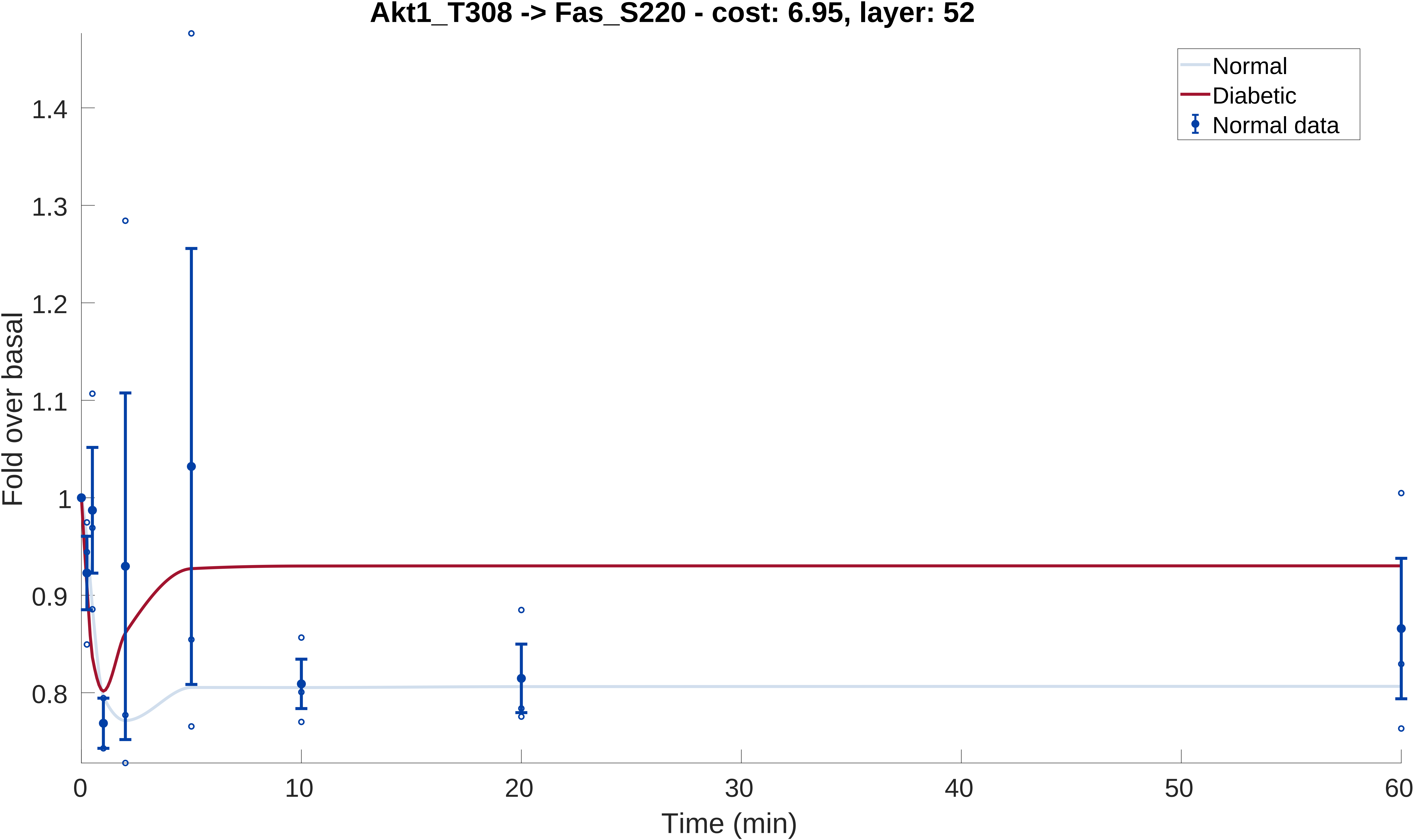
Akt1_T308 -> Fas_S220 - cost: 6.95, layer: 52
Normal
Diabetic
Normal data
Fold over basal
1.4
1.3
1.2
1.1
1
0.9
0.8
0
10
20
30
40
50
60
Time (min)

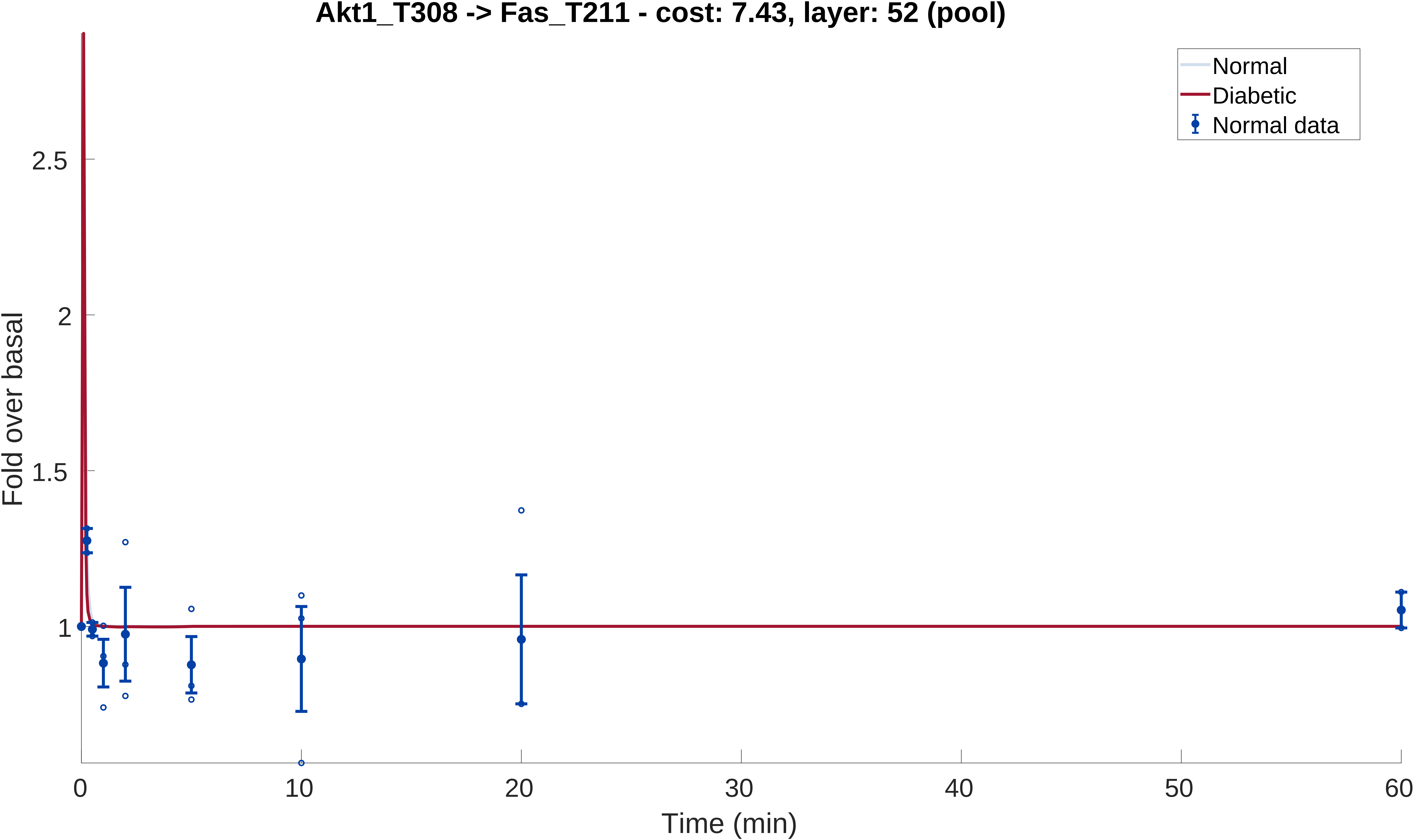

Akt1_T308 -> Fas_T211 - cost: 7.43, layer: 52 (pool)
Normal
Diabetic
Normal data
Fold over basal
2.5
2
1.5
1
0
10
20
30
40
50
60
Time (min)

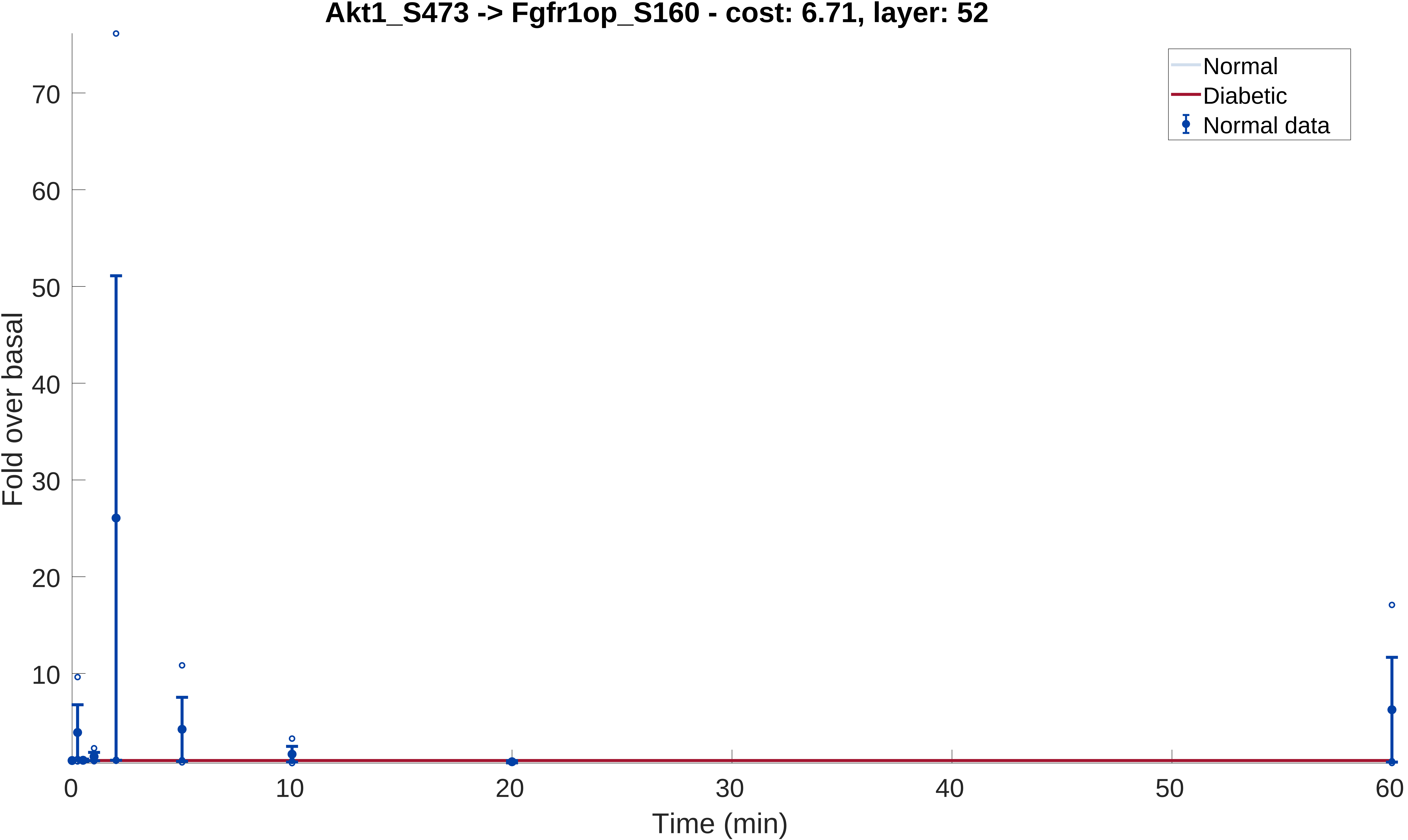

Akt1_S473 -> Fgfr1op_S160 - cost: 6.71, layer: 52
Normal
Diabetic
Normal data
Fold over basal
Time (min)
0
10
20
30
40
50
60
70

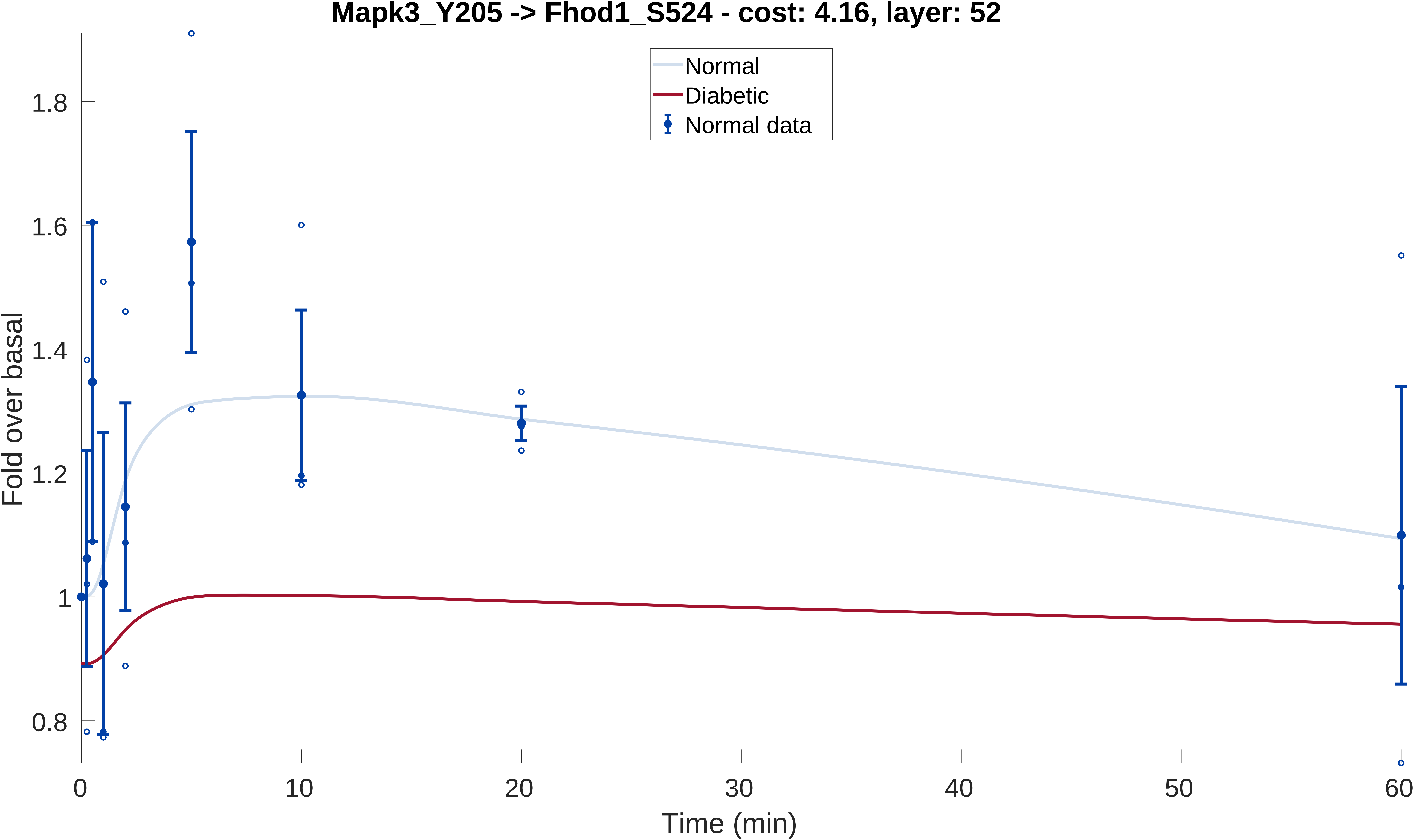

Mapk3_Y205 -> Fhod1_S524 - cost: 4.16, layer: 52
Normal
Diabetic
Normal data
Fold over basal
1.8
1.6
1.4
1.2
1
0.8
0
10
20
30
40
50
60
Time (min)

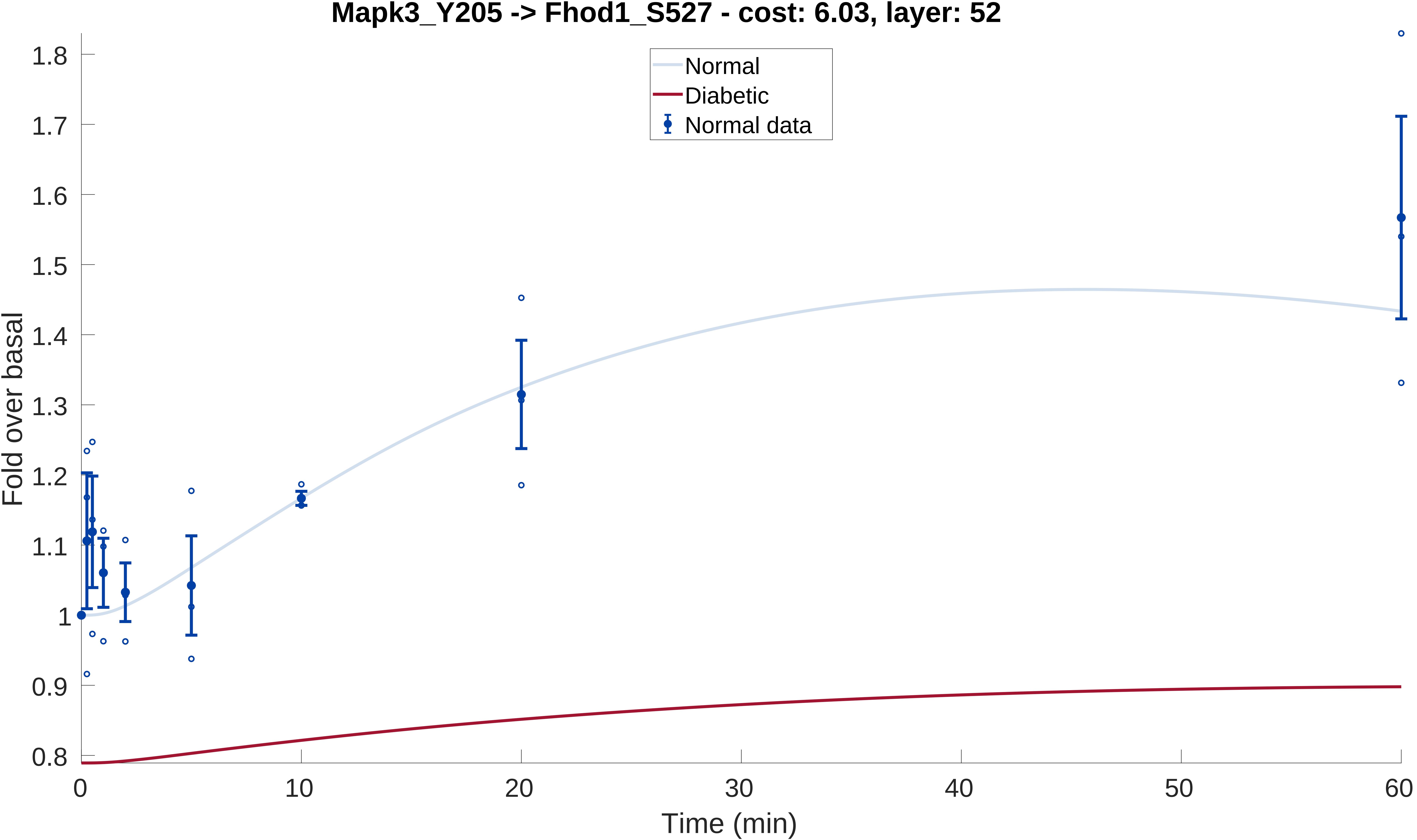

Mapk3_Y205 -> Fhod1_S527 - cost: 6.03, layer: 52
Normal
Diabetic
Normal data
Fold over basal
1.8
1.7
1.6
1.5
1.4
1.3
1.2
1.1
1
0.9
0.8
0
10
20
30
40
50
60
Time (min)

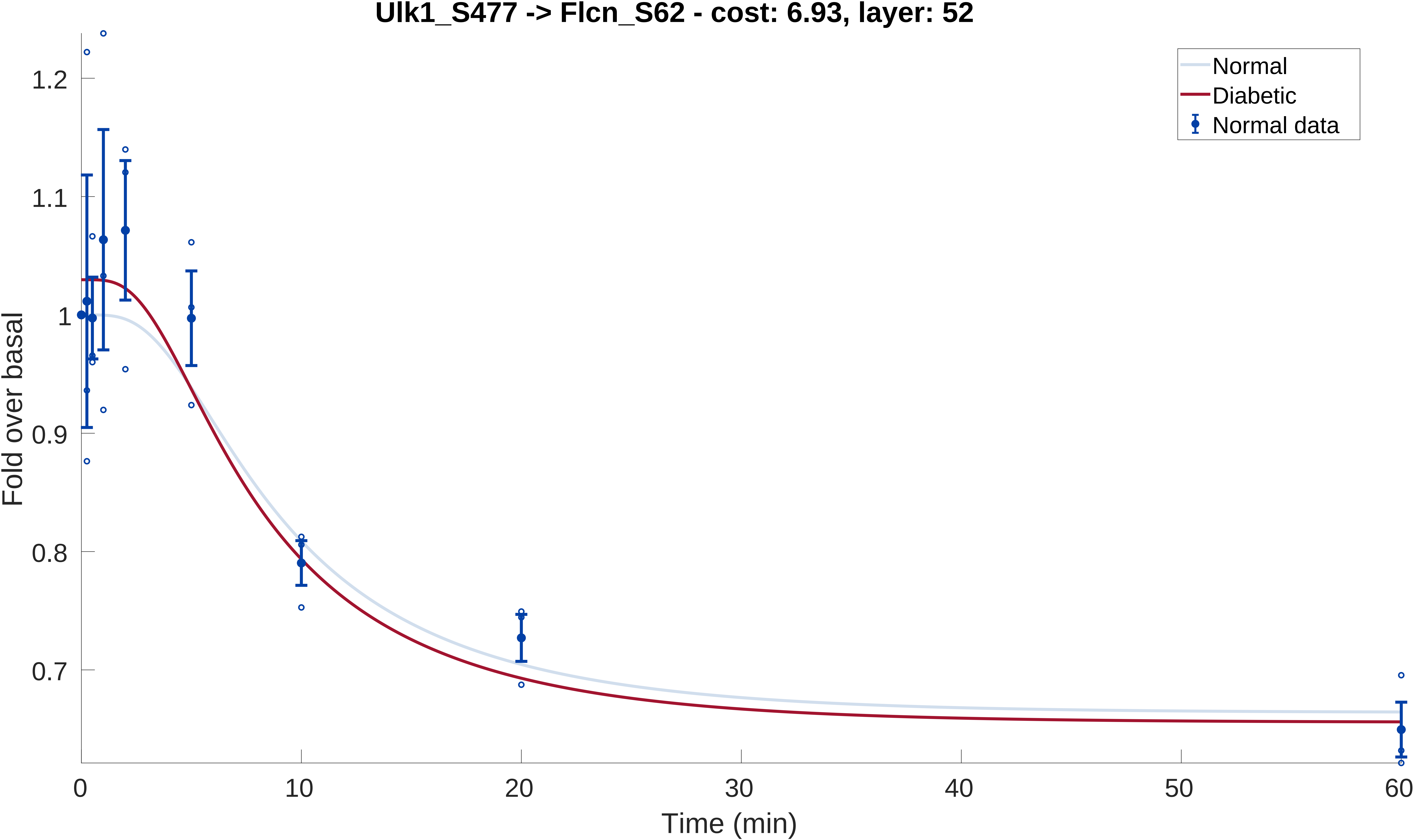

Ulk1_S477 -> Flcn_S62 - cost: 6.93, layer: 52
Normal
Diabetic
Normal data
Fold over basal
Time (min)
1.2
1.1
1
0.9
0.8
0.7
0
10
20
30
40
50
60

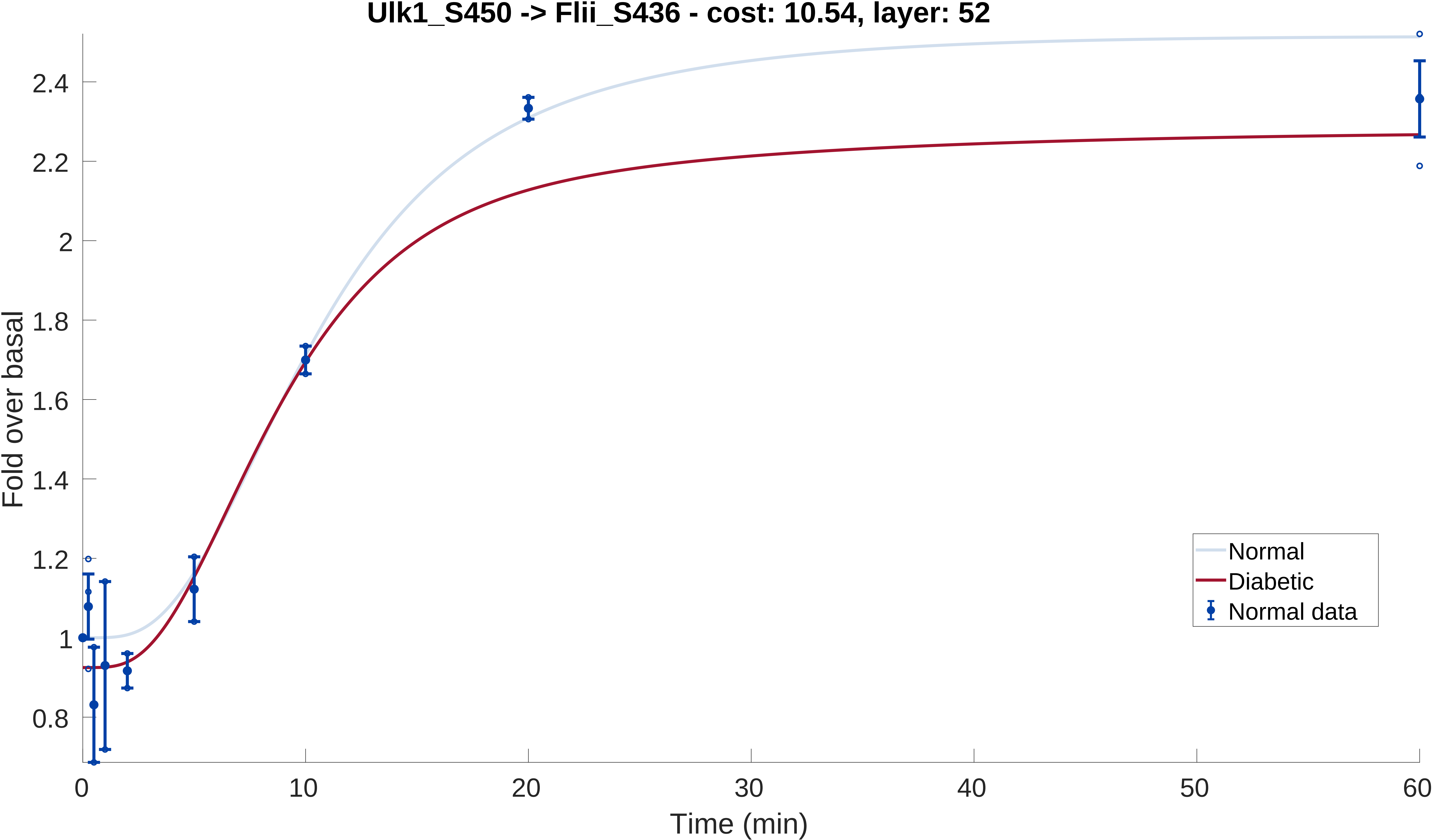
Ulk1_S450 -> Flii_S436 - cost: 10.54, layer: 52
Fold over basal
2.4
2.2
2
1.8
1.6
1.4
1.2
1
0.8
0
10
20
30
40
50
60
Time (min)
Normal
Diabetic
Normal data

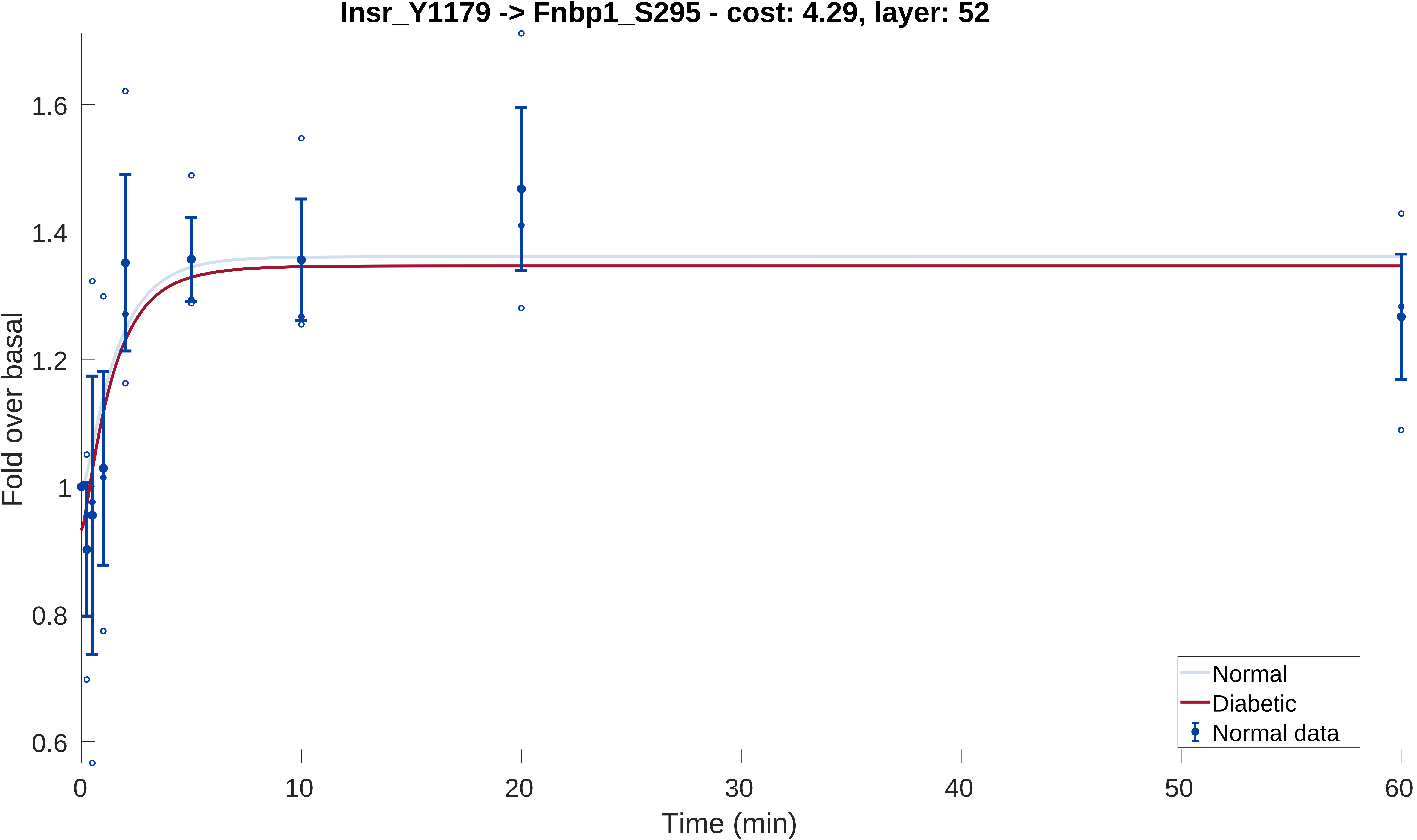

Insr_Y1179 -> Fnbp1_S295 - cost: 4.29, layer: 52
Fold over basal
1.6
1.4
1.2
1
0.8
0.6
0
10
20
30
40
50
60
Time (min)
Normal
Diabetic
Normal data

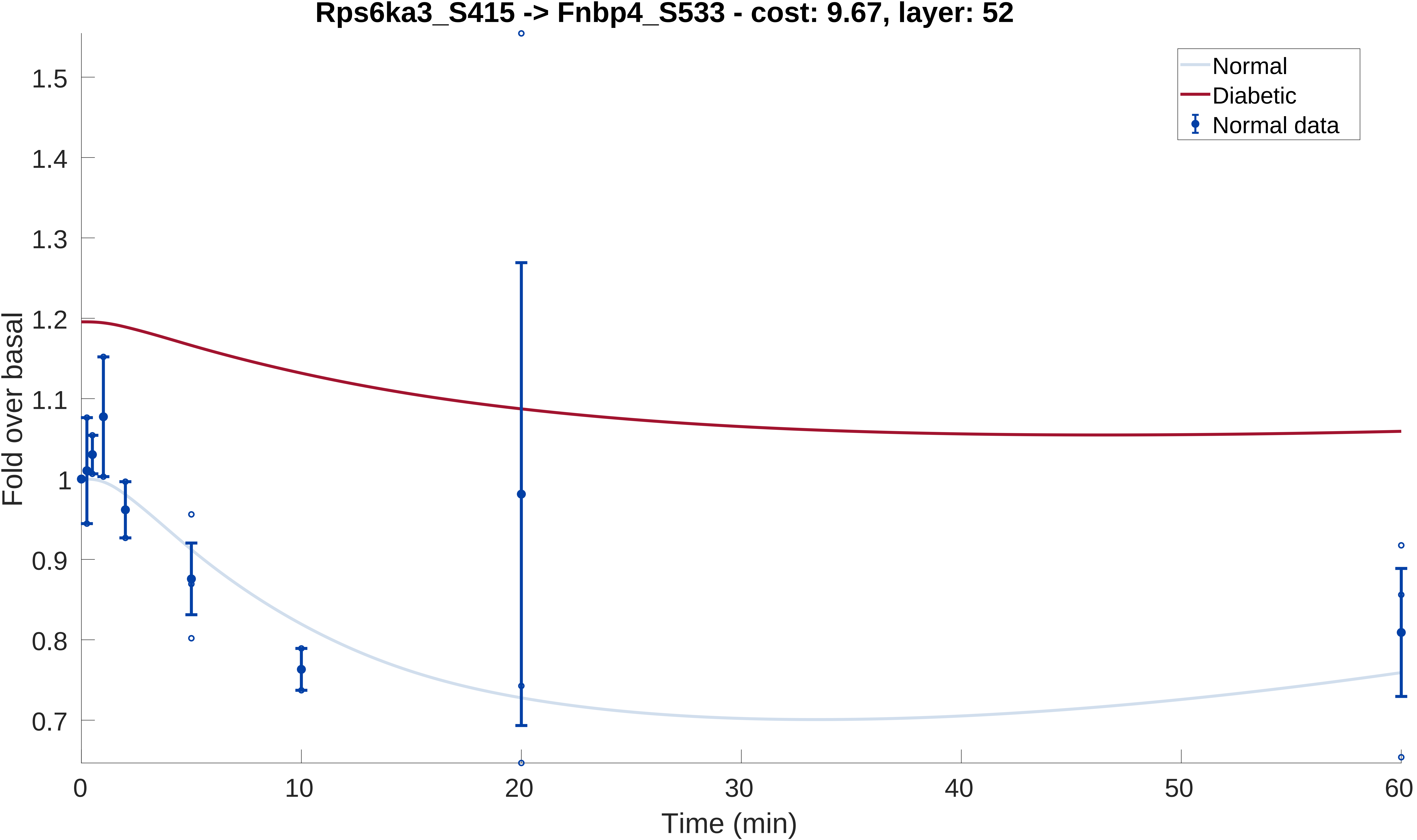
Rps6ka3_S415 -> Fnbp4_S533 - cost: 9.67, layer: 52
Normal
Diabetic
Normal data
Fold over basal
Time (min)
1.5
1.4
1.3
1.2
1.1
1
0.9
0.8
0.7
0
10
20
30
40
50
60

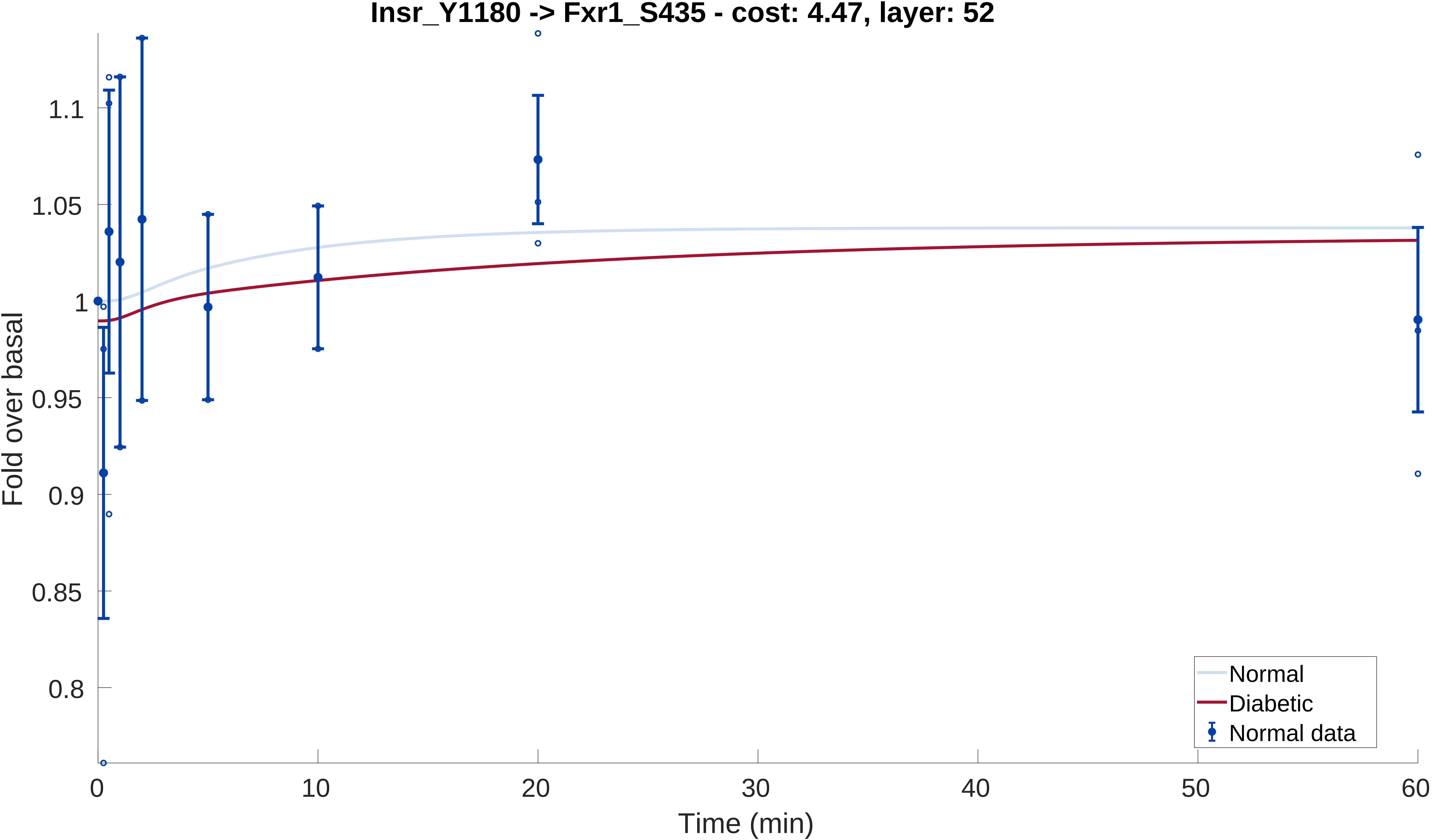

Insr_Y1180 -> Fxr1_S435 - cost: 4.47, layer: 52
Fold over basal
1.1
1.05
1
0.95
0.9
0.85
0.8
0
10
20
30
40
50
60
Time (min)
Normal
Diabetic
Normal data

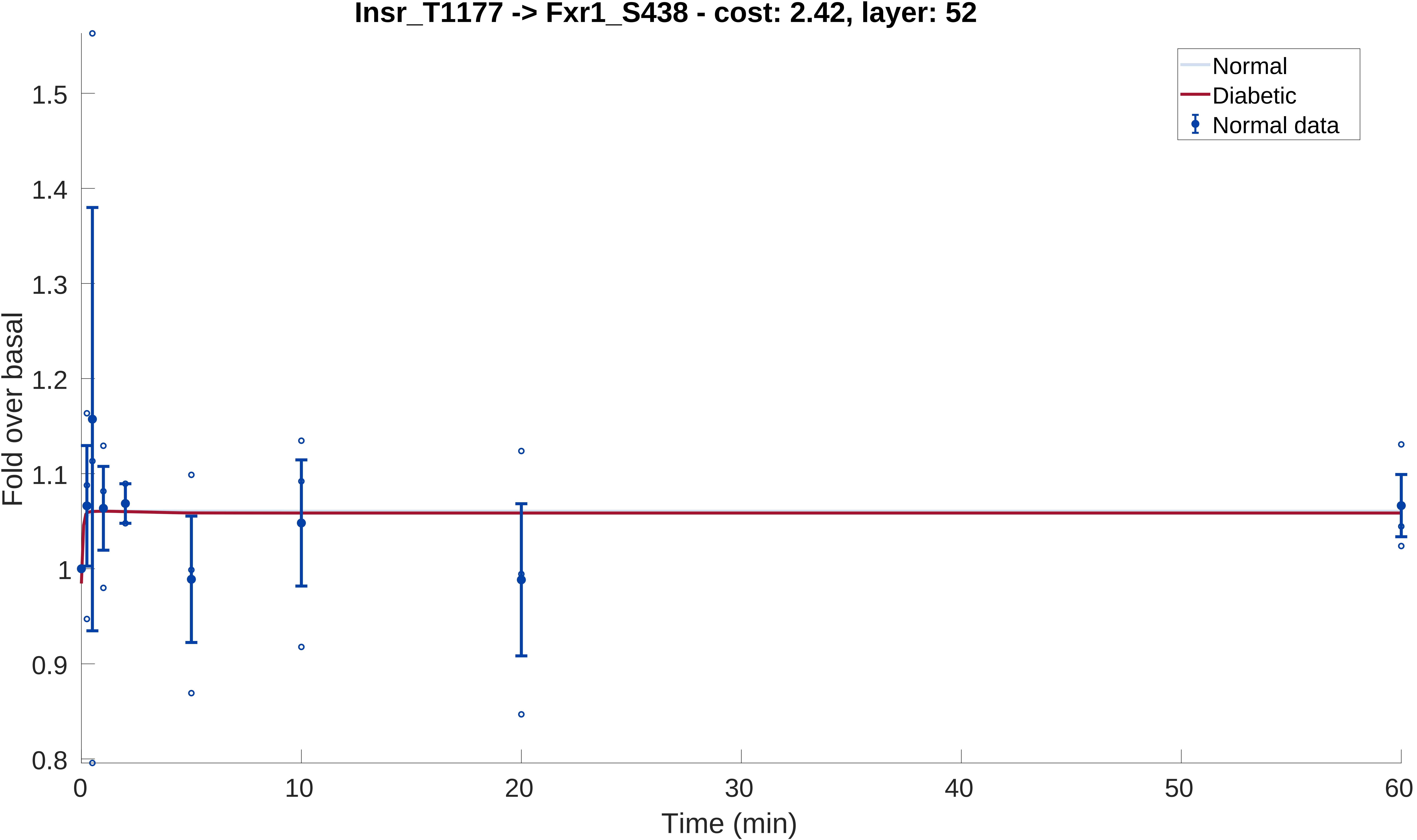

Insr_T1177 -> Fxr1_S438 - cost: 2.42, layer: 52
Normal
Diabetic
Normal data
Fold over basal
1.5
1.4
1.3
1.2
1.1
1
0.9
0.8
0
10
20
30
40
50
60
Time (min)

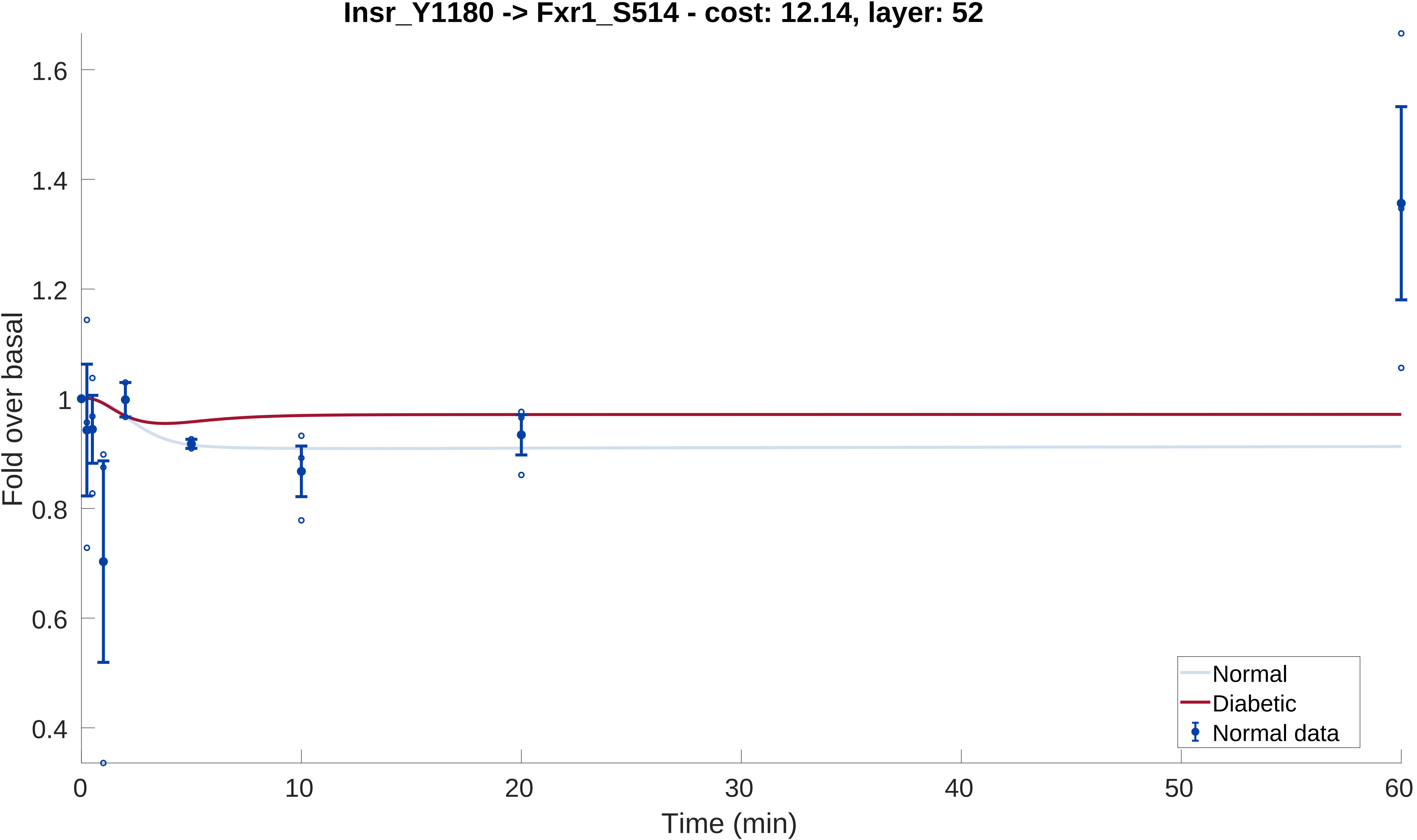

Insr_Y1180 -> Fxr1_S514 - cost: 12.14, layer: 52
Fold over basal
1.6
1.4
1.2
1
0.8
0.6
0.4
0
10
20
30
40
50
60
Time (min)
Normal
Diabetic
Normal data

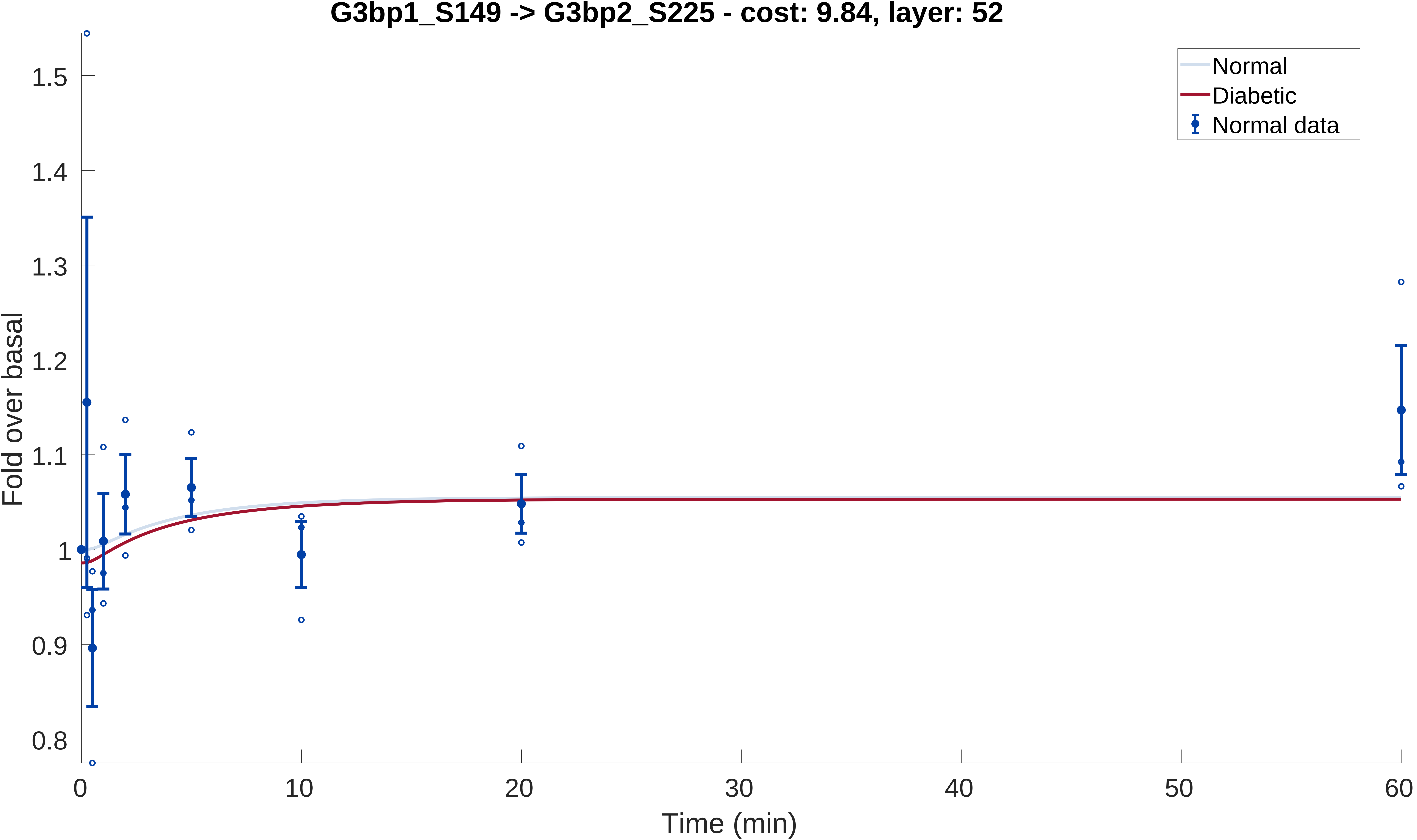
G3bp1_S149 -> G3bp2_S225 - cost: 9.84, layer: 52
Normal
Diabetic
Normal data
Fold over basal
1.5
1.4
1.3
1.2
1.1
1
0.9
0.8
0
10
20
30
40
50
60
Time (min)

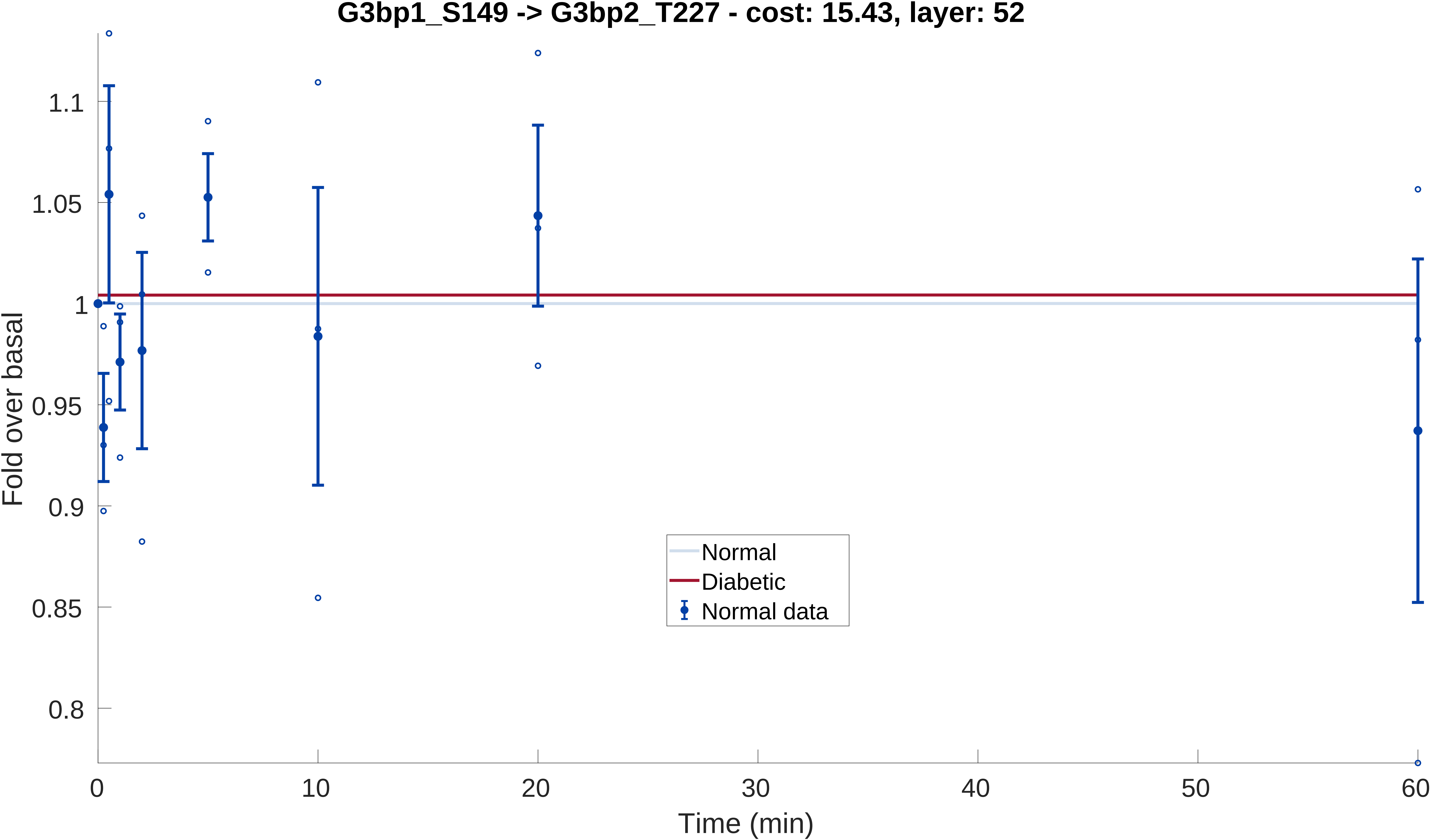

G3bp1_S149 -> G3bp2_T227 - cost: 15.43, layer: 52
Fold over basal
1.1
1.05
1
0.95
0.9
0.85
0.8
0
10
20
30
40
50
60
Time (min)
Normal
Diabetic
Normal data

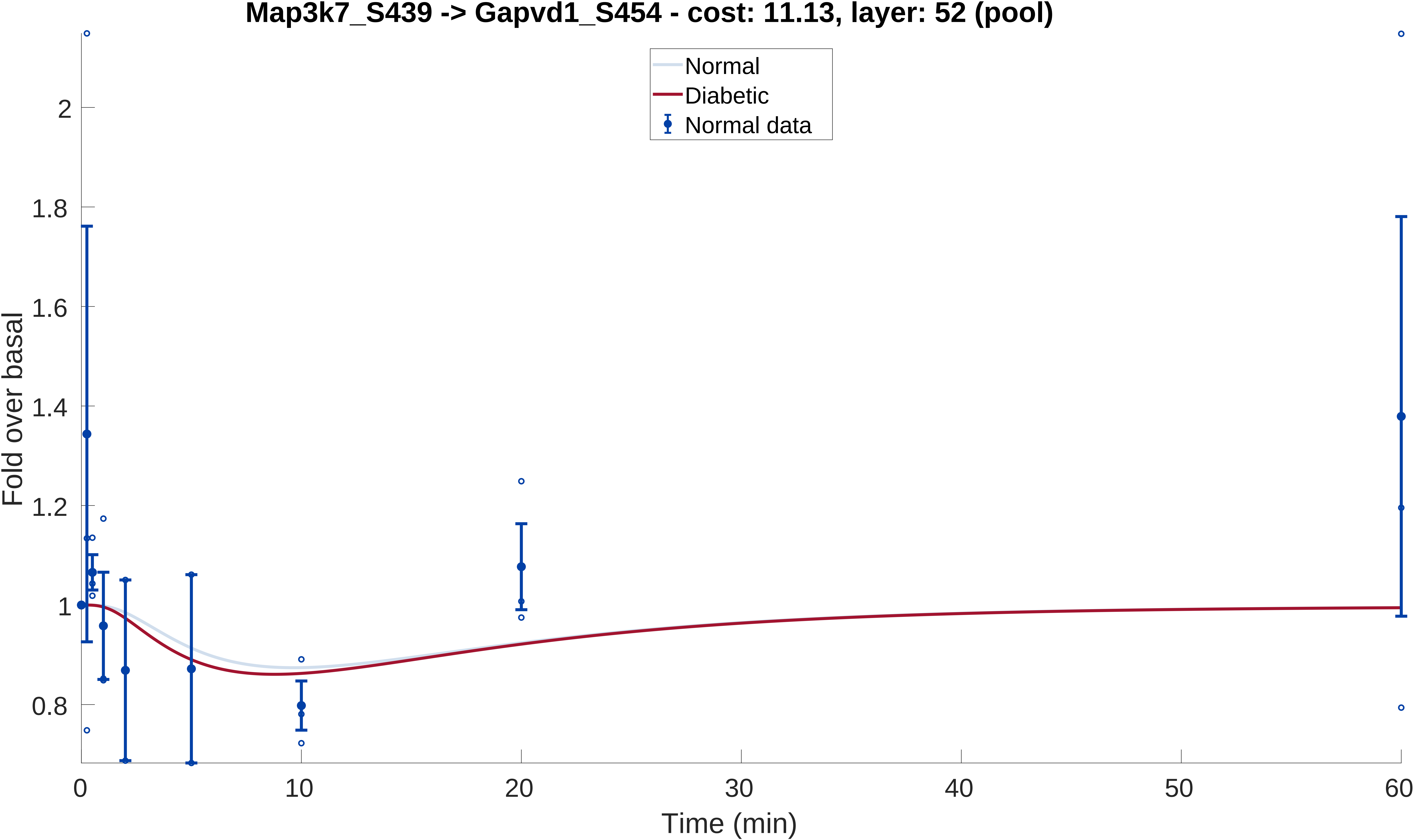
Map3k7_S439 -> Gapvd1_S454 - cost: 11.13, layer: 52 (pool)
Normal
Diabetic
Normal data
Fold over basal
Time (min)
0.8
1
1.2
1.4
1.6
1.8
2
0
10
20
30
40
50
60

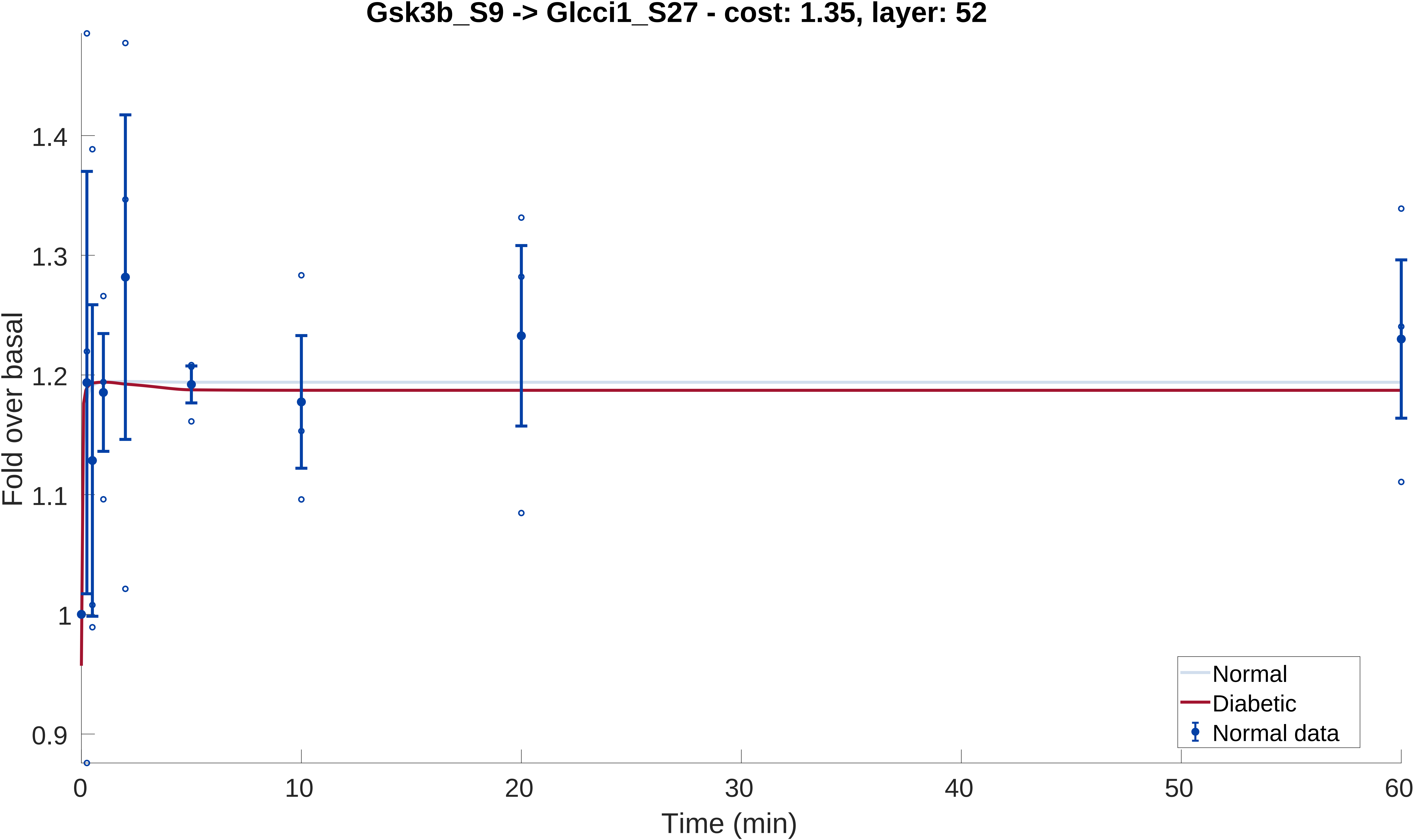

Gsk3b_S9 -> Glcci1_S27 - cost: 1.35, layer: 52
Fold over basal
1.4
1.3
1.2
1.1
1
0.9
0
10
20
30
40
50
60
Time (min)
Normal
Diabetic
Normal data

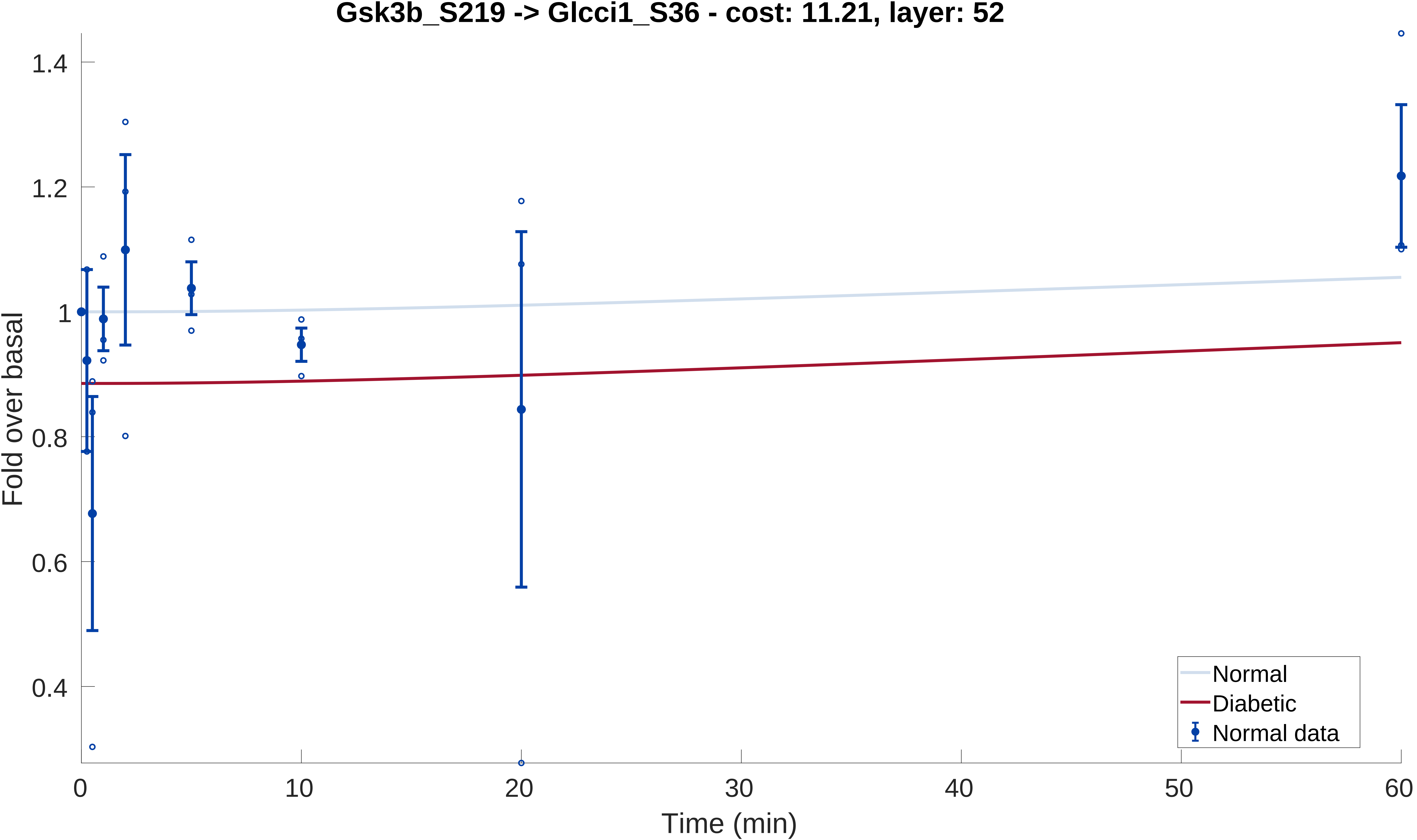

Gsk3b_S219 -> Glcci1_S36 - cost: 11.21, layer: 52
1.4
1.2
1
0.8
0.6
0.4
Fold over basal
0
10
20
30
40
50
60
Time (min)
Normal
Diabetic
Normal data

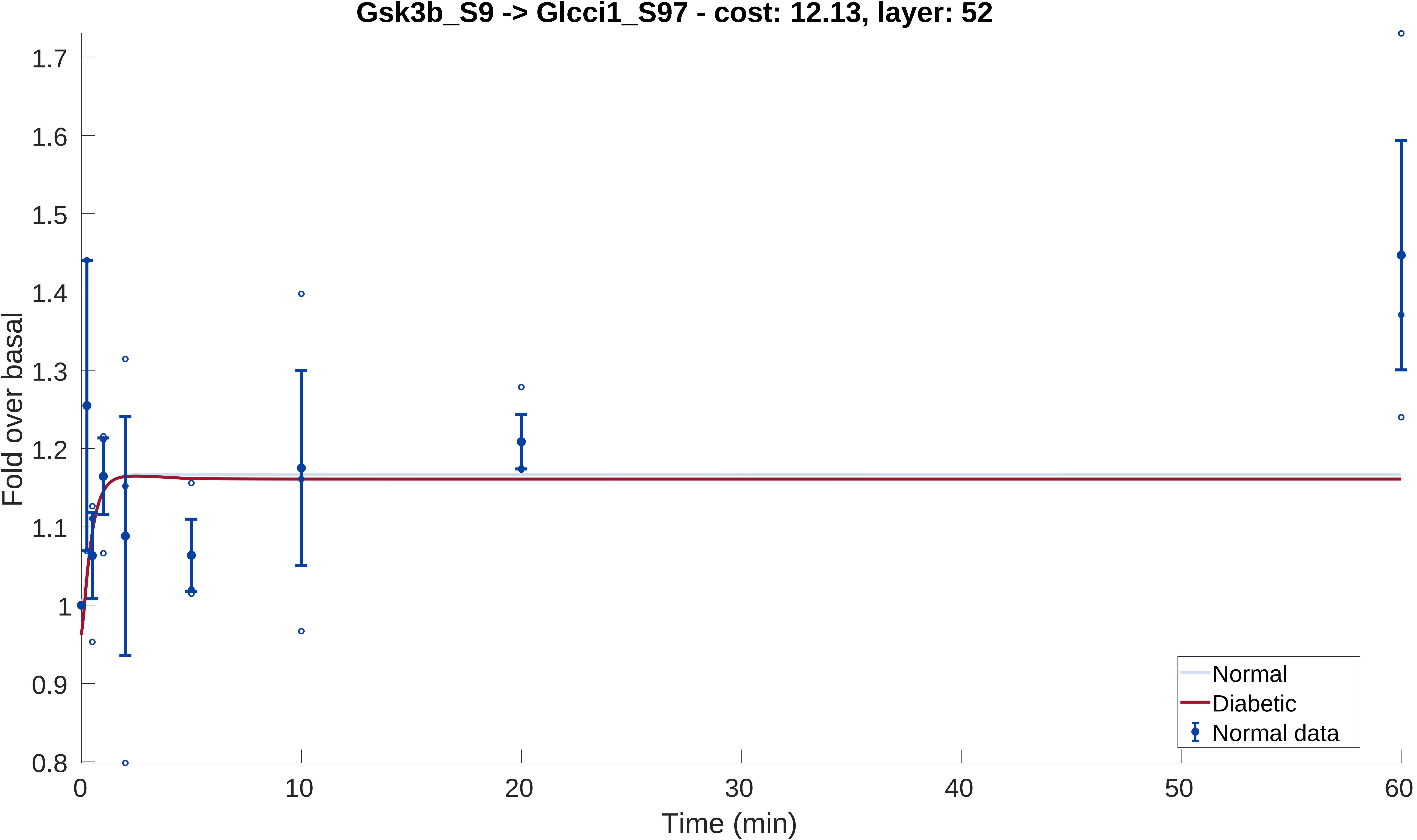
Gsk3b_S9 -> Glcci1_S97 - cost: 12.13, layer: 52
Fold over basal
Time (min)
0.8
0.9
1
1.1
1.2
1.3
1.4
1.5
1.6
1.7
0
10
20
30
40
50
60
Normal
Diabetic
Normal data

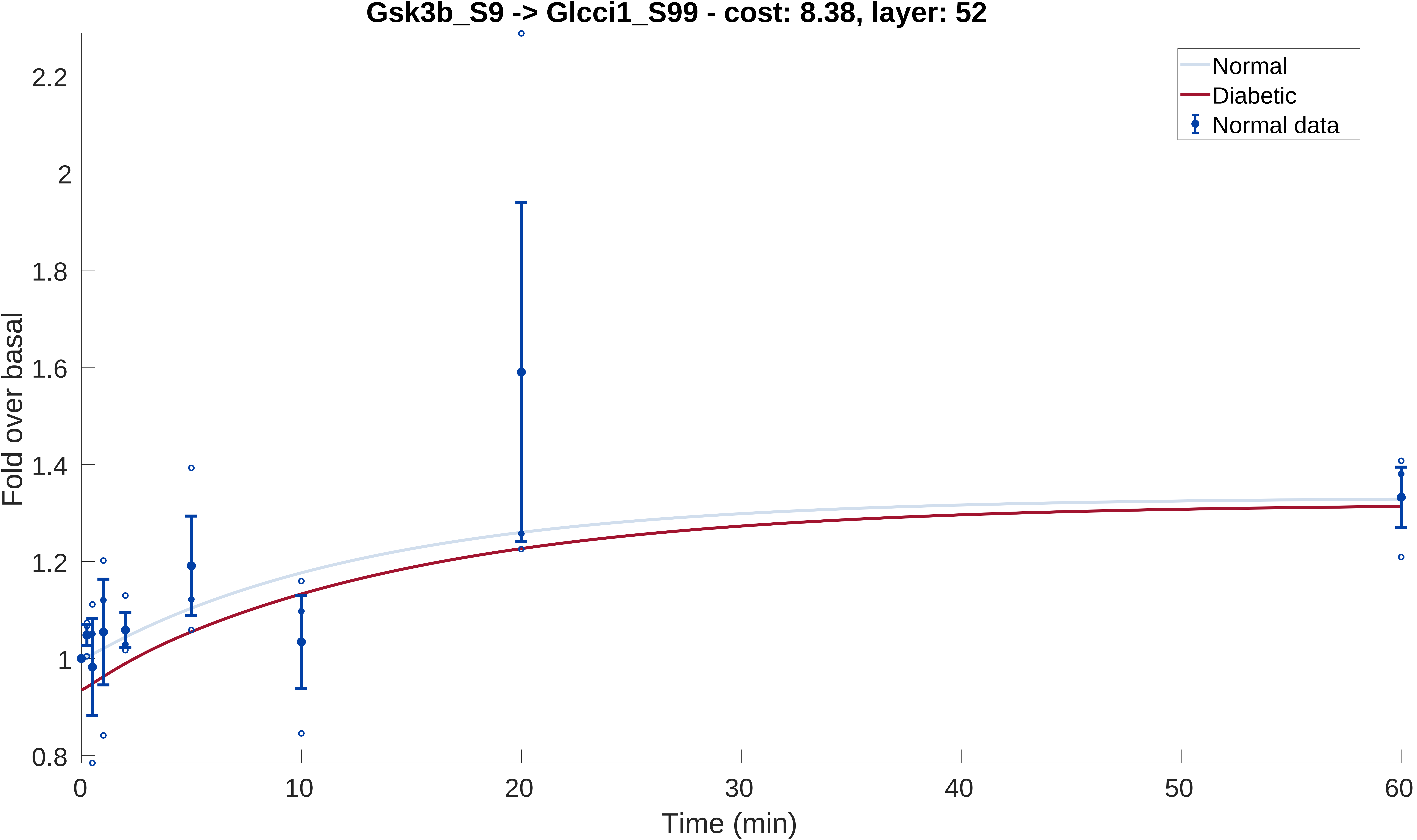

Gsk3b_S9 -> Glcci1_S99 - cost: 8.38, layer: 52
Normal
Diabetic
Normal data
Fold over basal
2.2
2
1.8
1.6
1.4
1.2
1
0.8
0
10
20
30
40
50
60
Time (min)

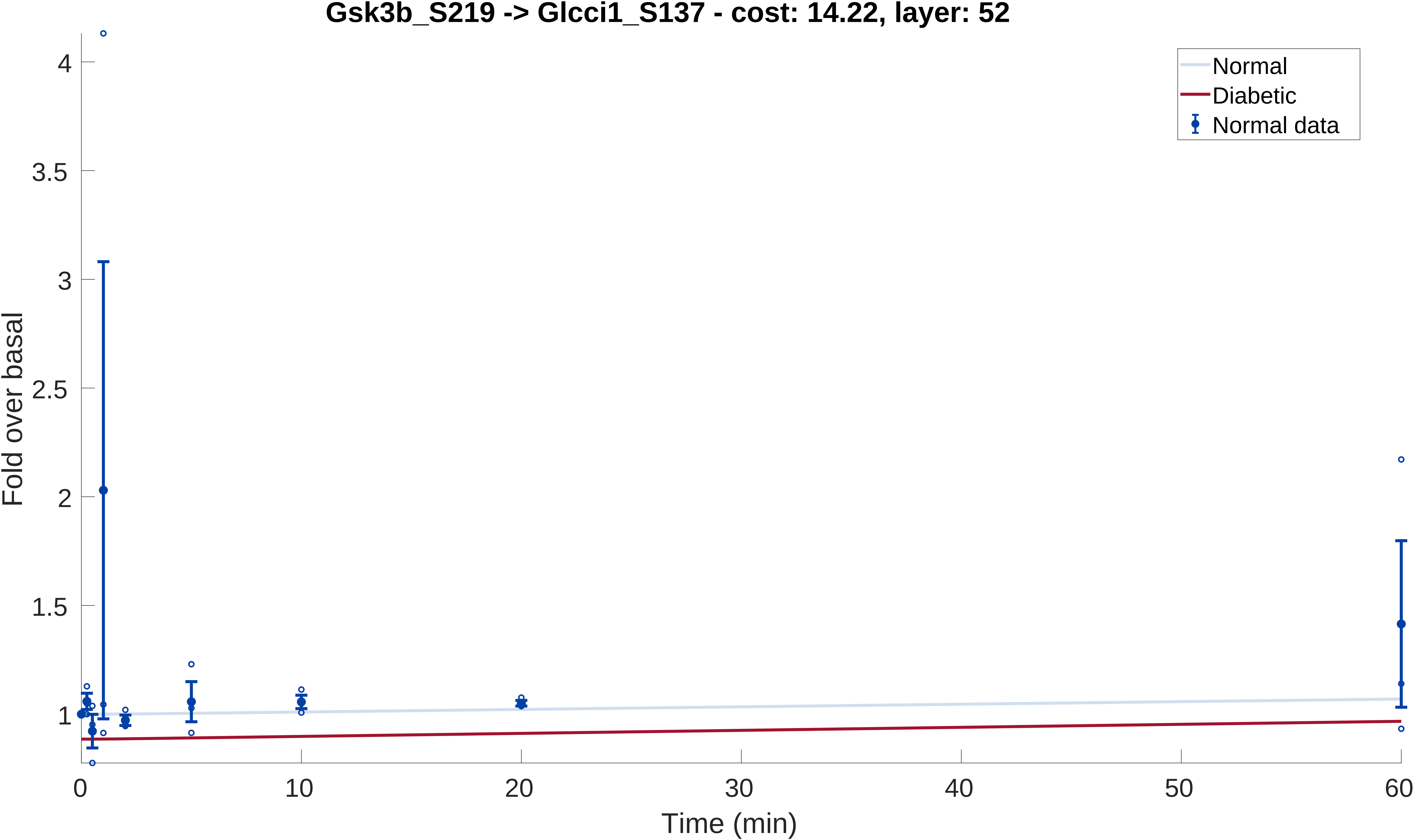

Gsk3b_S219 -> Glcci1_S137 - cost: 14.22, layer: 52
Normal
Diabetic
Normal data
Fold over basal
Time (min)
4
3.5
3
2.5
2
1.5
1
0
10
20
30
40
50
60

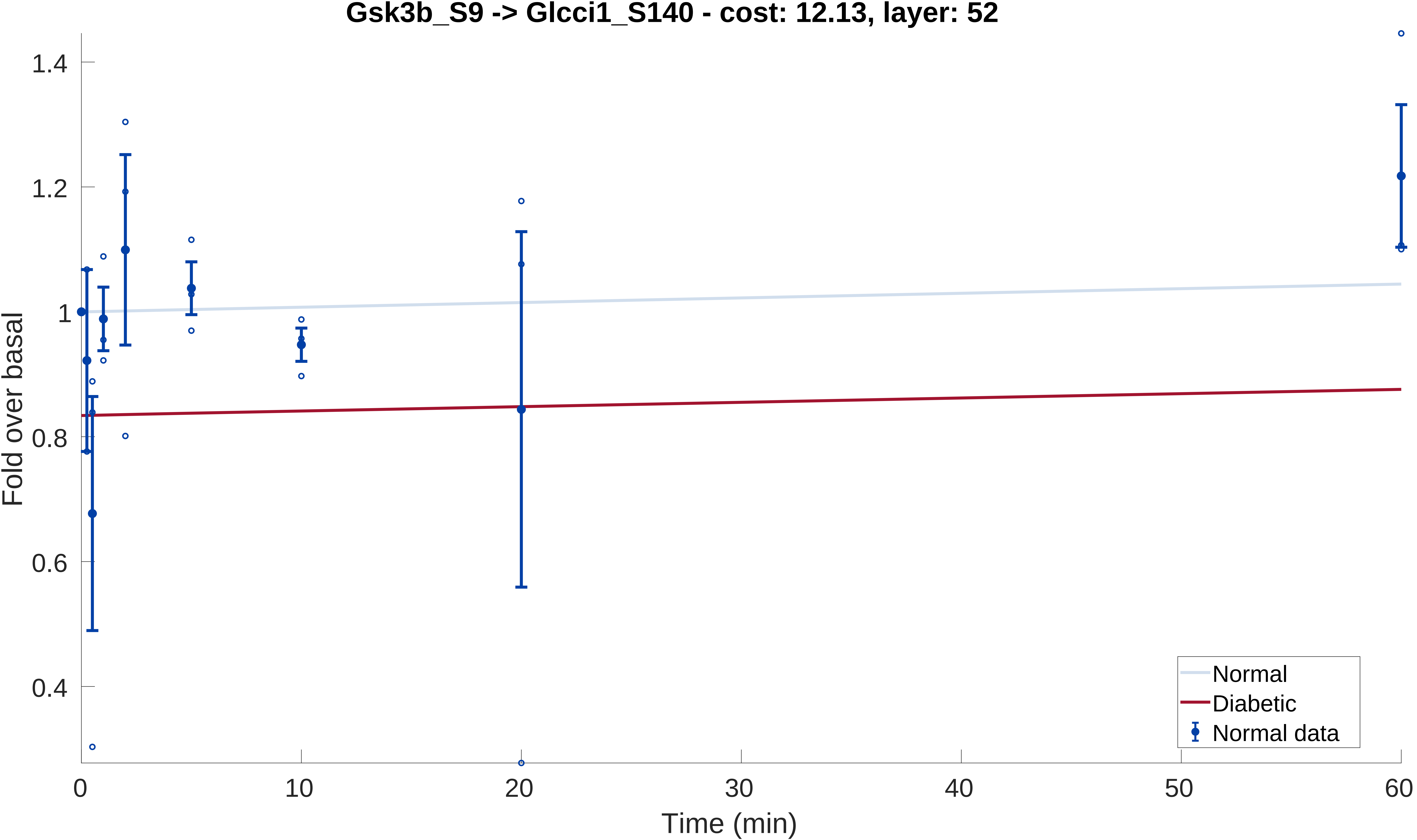
Gsk3b_S9 -> Glcci1_S140 - cost: 12.13, layer: 52
Fold over basal
1.4
1.2
1
0.8
0.6
0.4
0
10
20
30
40
50
60
Time (min)
Normal
Diabetic
Normal data

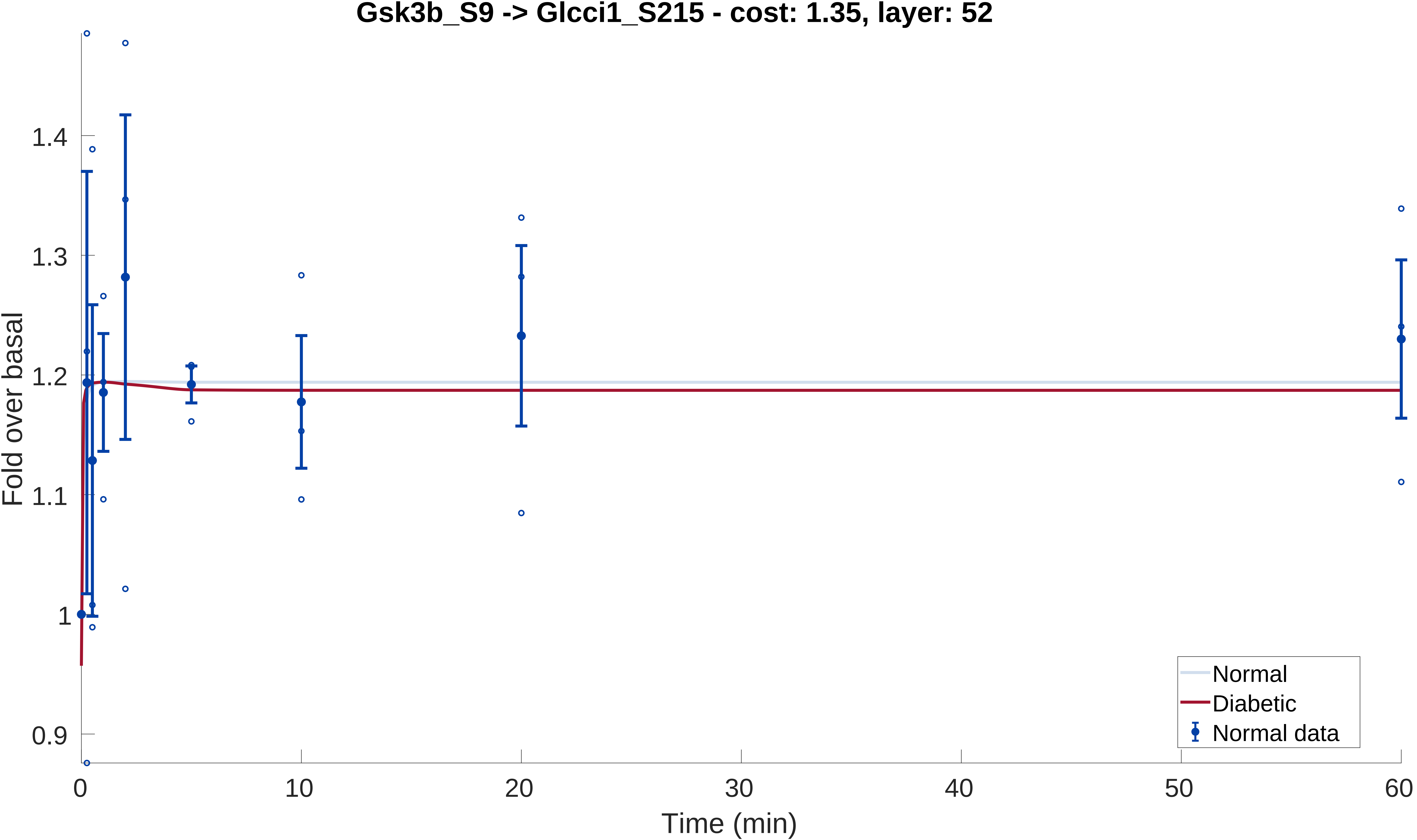

Gsk3b_S9 -> Glcci1_S215 - cost: 1.35, layer: 52
1.4
1.3
1.2
1.1
1
0.9
Fold over basal
0
10
20
30
40
50
60
Time (min)
Normal
Diabetic
Normal data

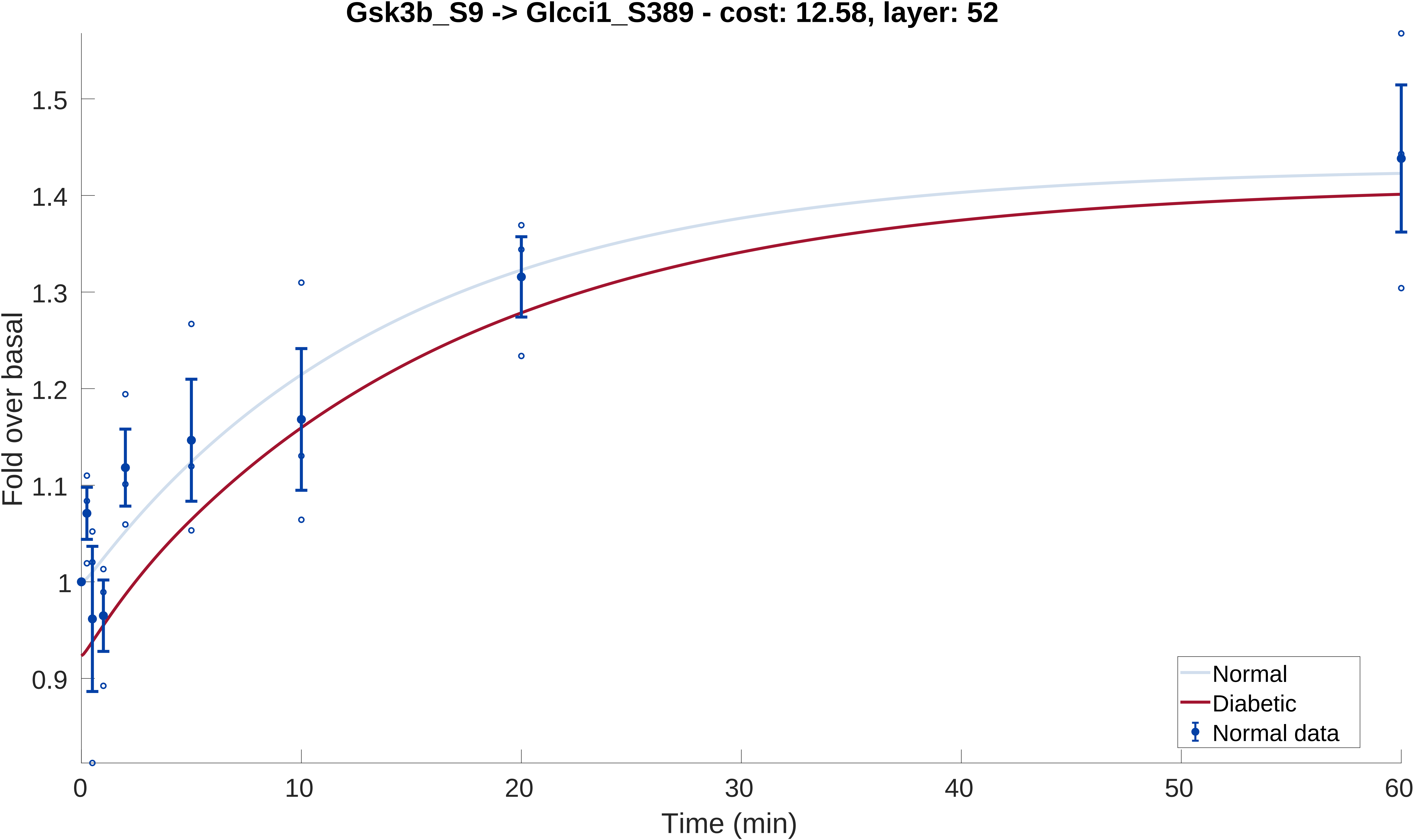

Gsk3b_S9 -> Glcci1_S389 - cost: 12.58, layer: 52
Fold over basal
Time (min)
0.9
1
1.1
1.2
1.3
1.4
1.5
0
10
20
30
40
50
60
Normal
Diabetic
Normal data

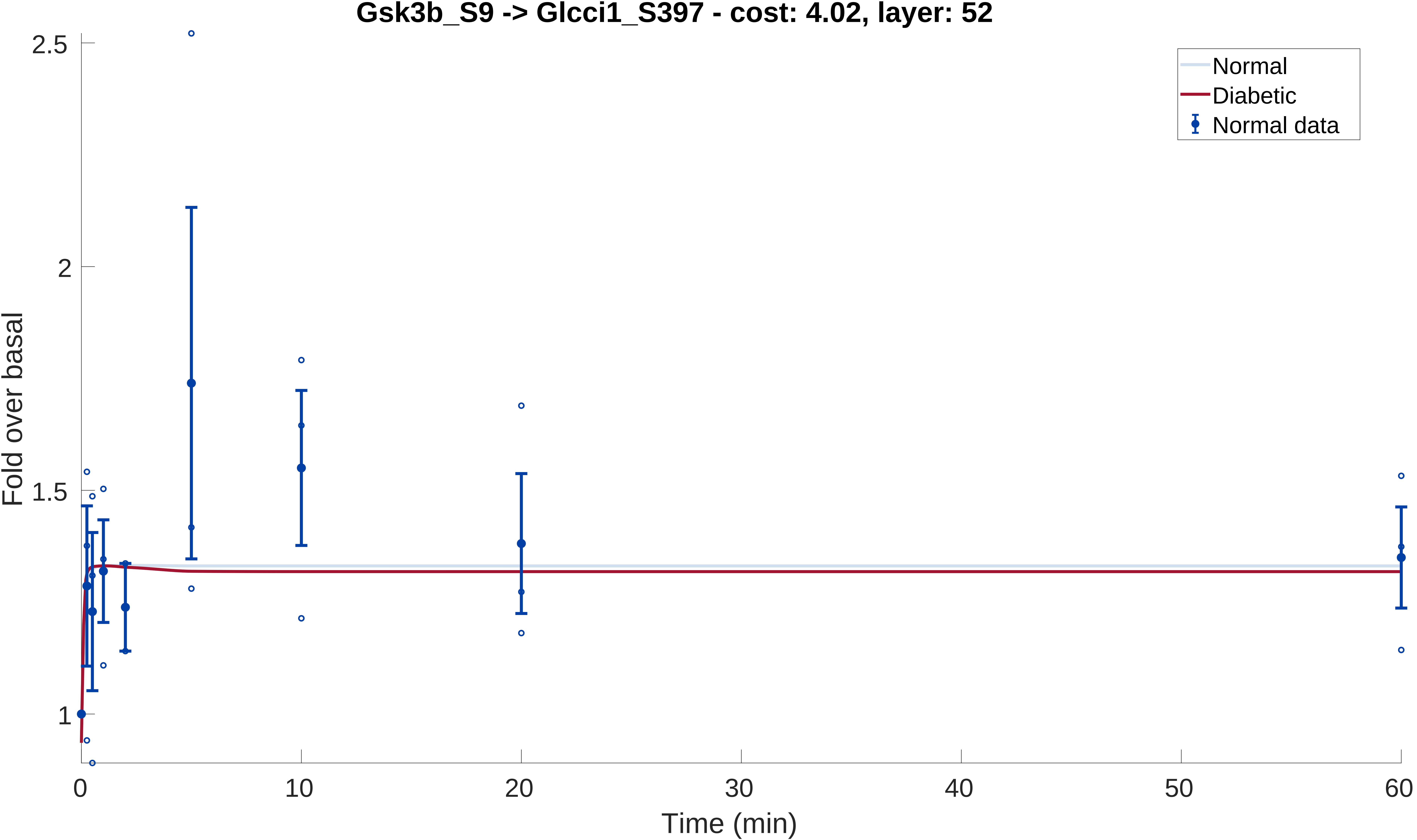

Gsk3b_S9 -> Glcci1_S397 - cost: 4.02, layer: 52
Normal
Diabetic
Normal data
Fold over basal
Time (min)
2.5
2
1.5
1
0
10
20
30
40
50
60

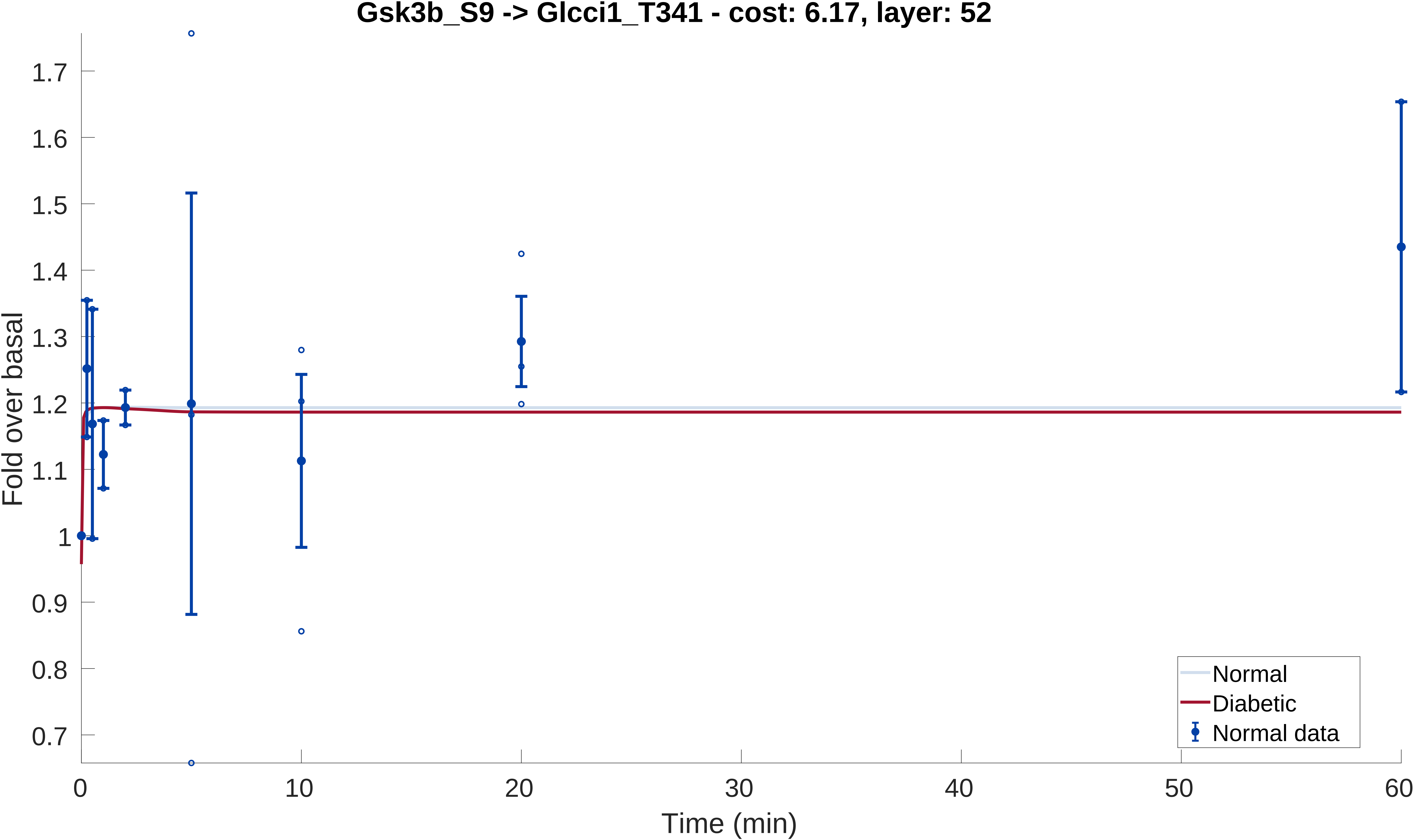

Gsk3b_S9 -> Glcci1_T341 - cost: 6.17, layer: 52
Fold over basal
1.7
1.6
1.5
1.4
1.3
1.2
1.1
1
0.9
0.8
0.7
0
10
20
30
40
50
60
Time (min)
Normal
Diabetic
Normal data

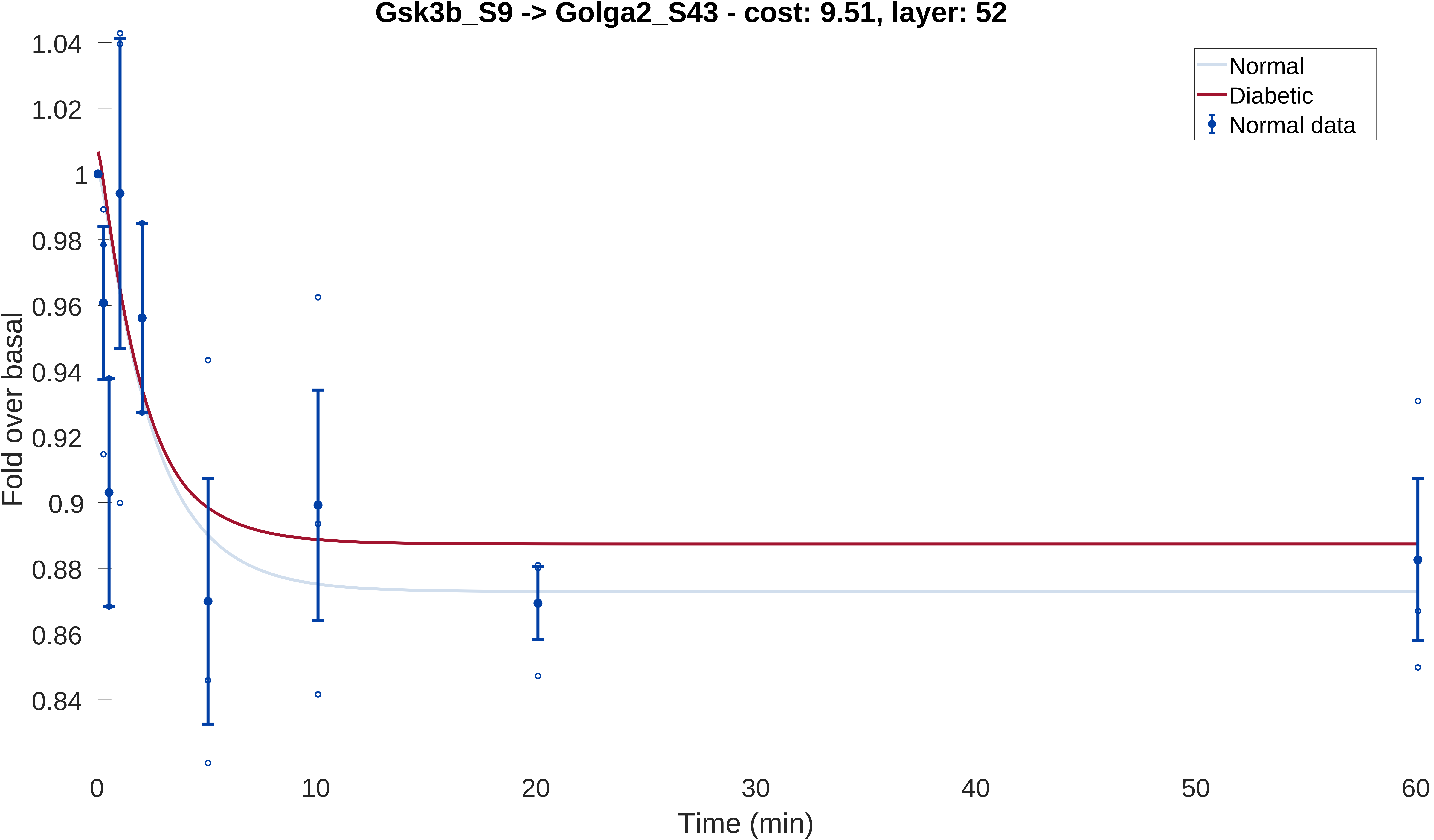

Gsk3b_S9 -> Golga2_S43 - cost: 9.51, layer: 52
Normal
Diabetic
Normal data
Fold over basal
Time (min)
1.04
1.02
1
0.98
0.96
0.94
0.92
0.9
0.88
0.86
0.84
0
10
20
30
40
50
60

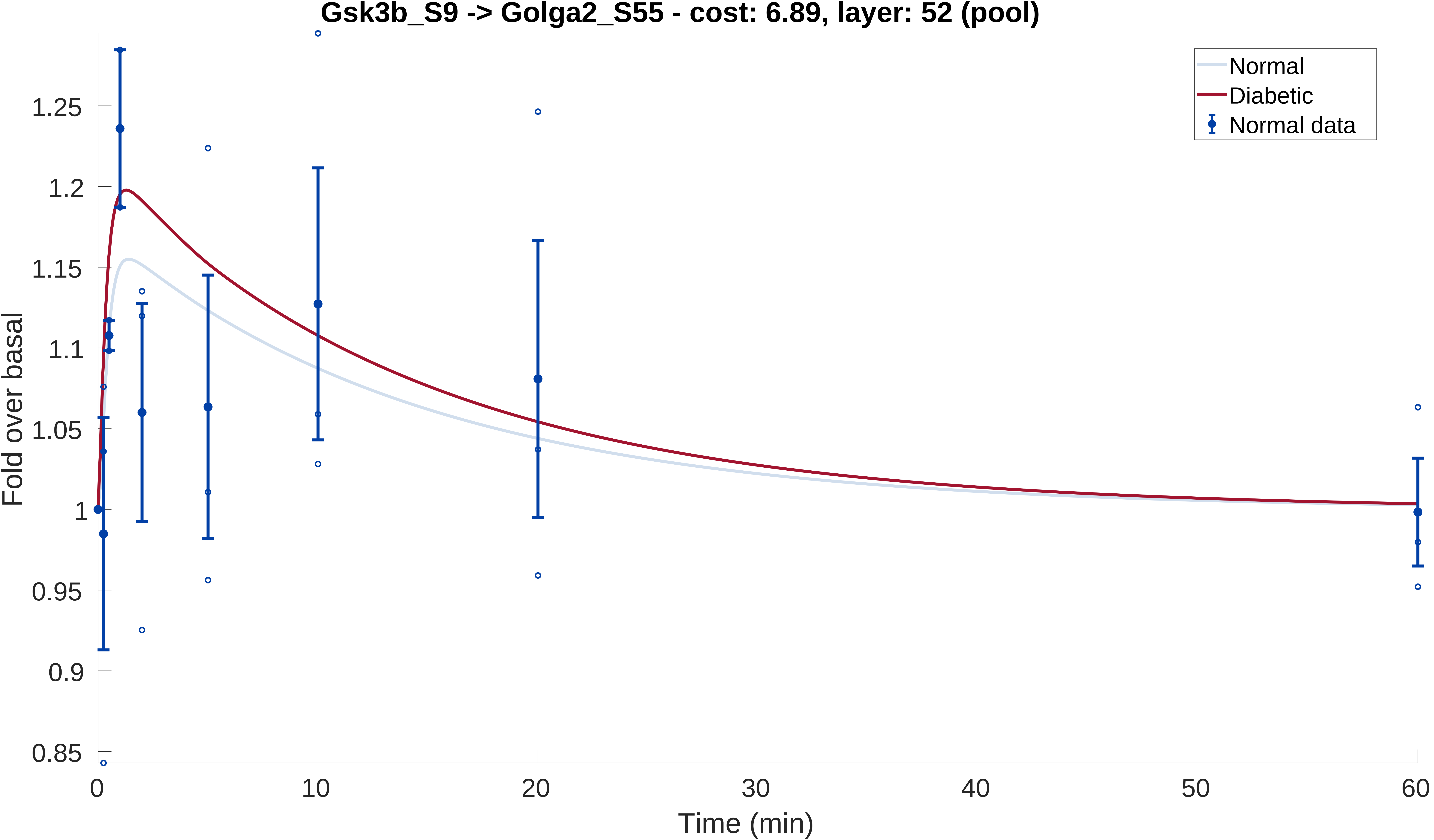

Gsk3b_S9 -> Golga2_S55 - cost: 6.89, layer: 52 (pool)
Normal
Diabetic
Normal data
Fold over basal
1.25
1.2
1.15
1.1
1.05
1
0.95
0.9
0.85
0
10
20
30
40
50
60
Time (min)

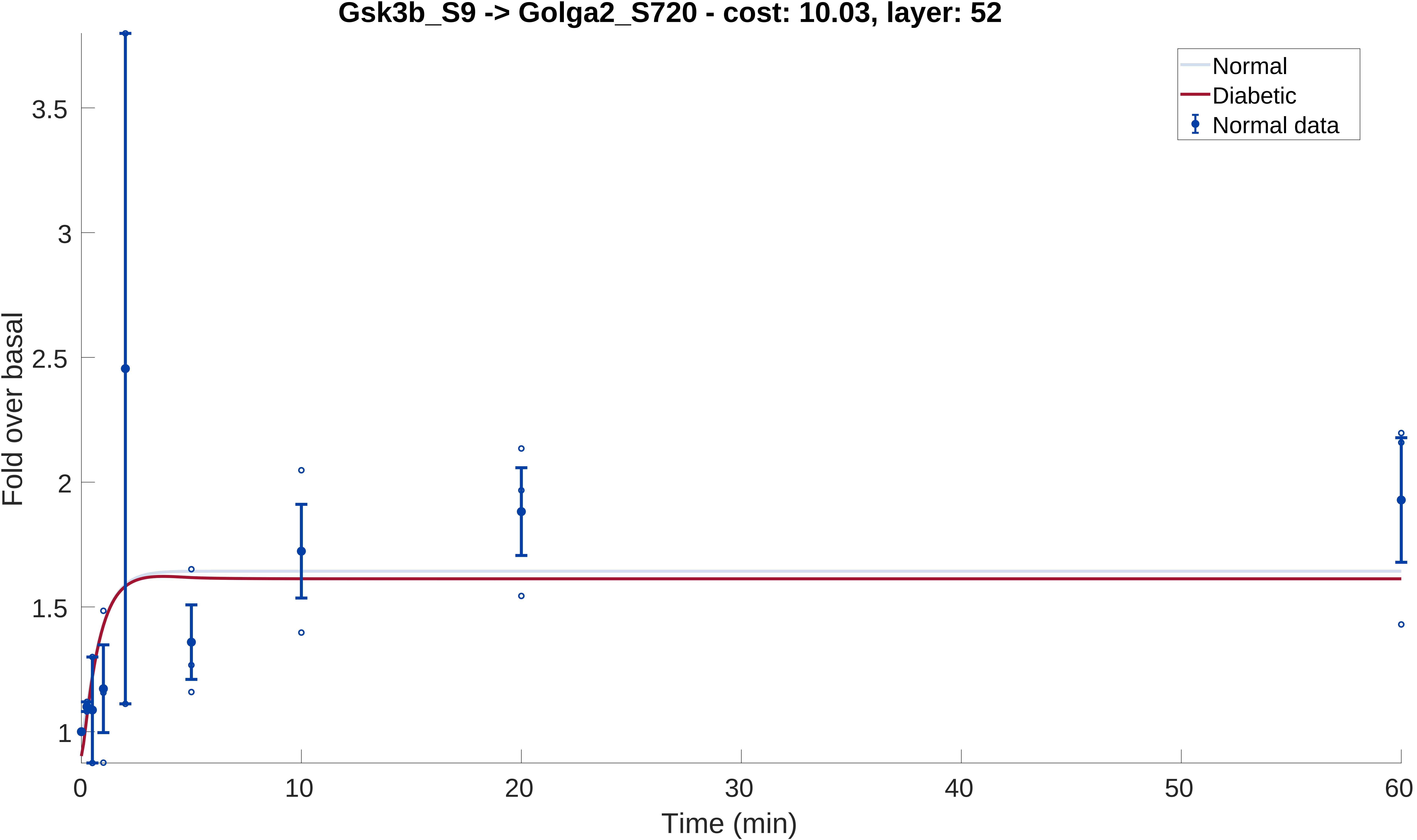

Gsk3b_S9 -> Golga2_S720 - cost: 10.03, layer: 52
Normal
Diabetic
Normal data
Fold over basal
3.5
3
2.5
2
1.5
1
Time (min)
0
10
20
30
40
50
60

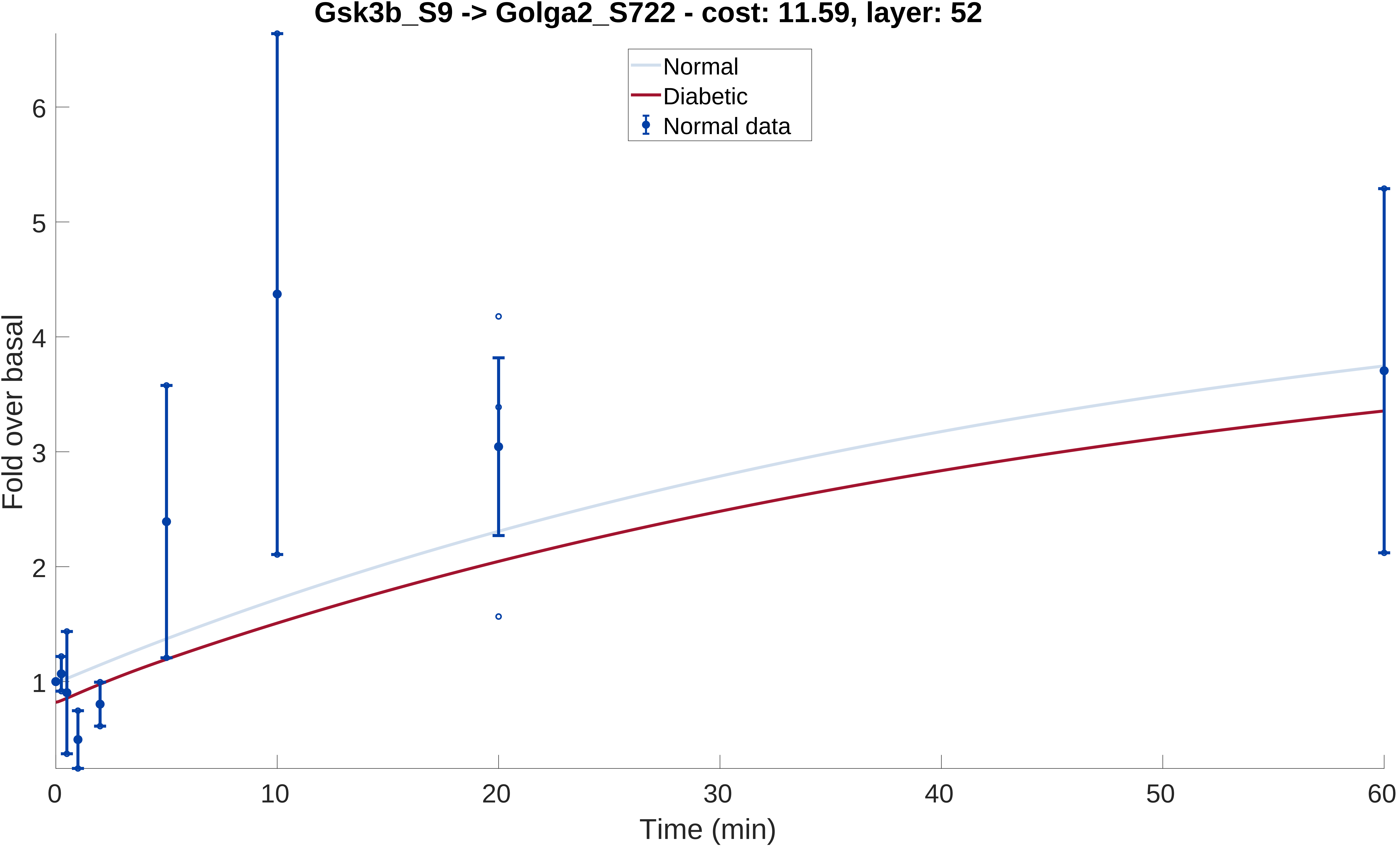

Gsk3b_S9 -> Golga2_S722 - cost: 11.59, layer: 52
Normal
Diabetic
Normal data
Fold over basal
Time (min)
0
10
20
30
40
50
60
1
2
3
4
5
6

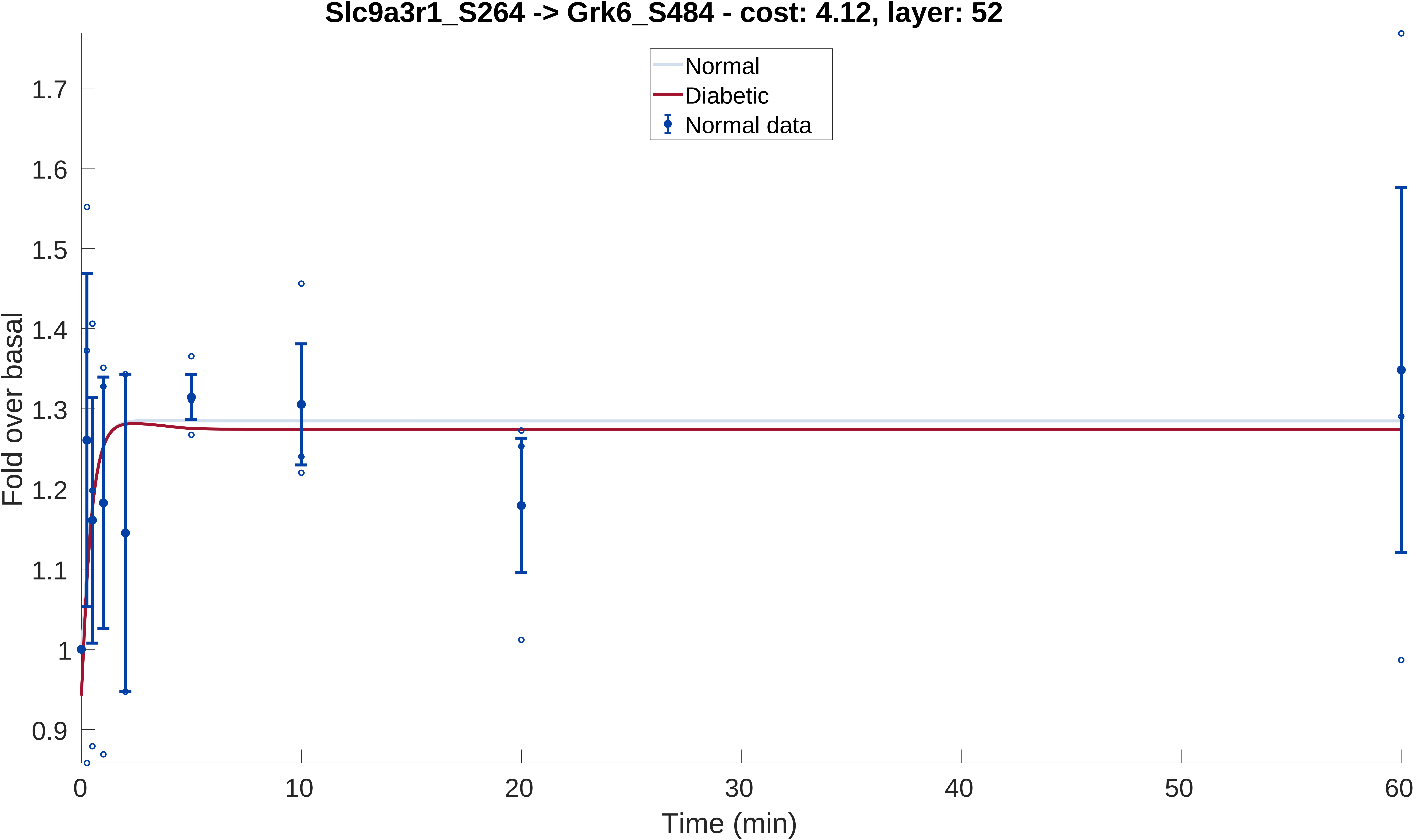
Slc9a3r1_S264 -> Grk6_S484 - cost: 4.12, layer: 52
Normal
Diabetic
Normal data
Fold over basal
Time (min)
0.9
1
1.1
1.2
1.3
1.4
1.5
1.6
1.7
0
10
20
30
40
50
60

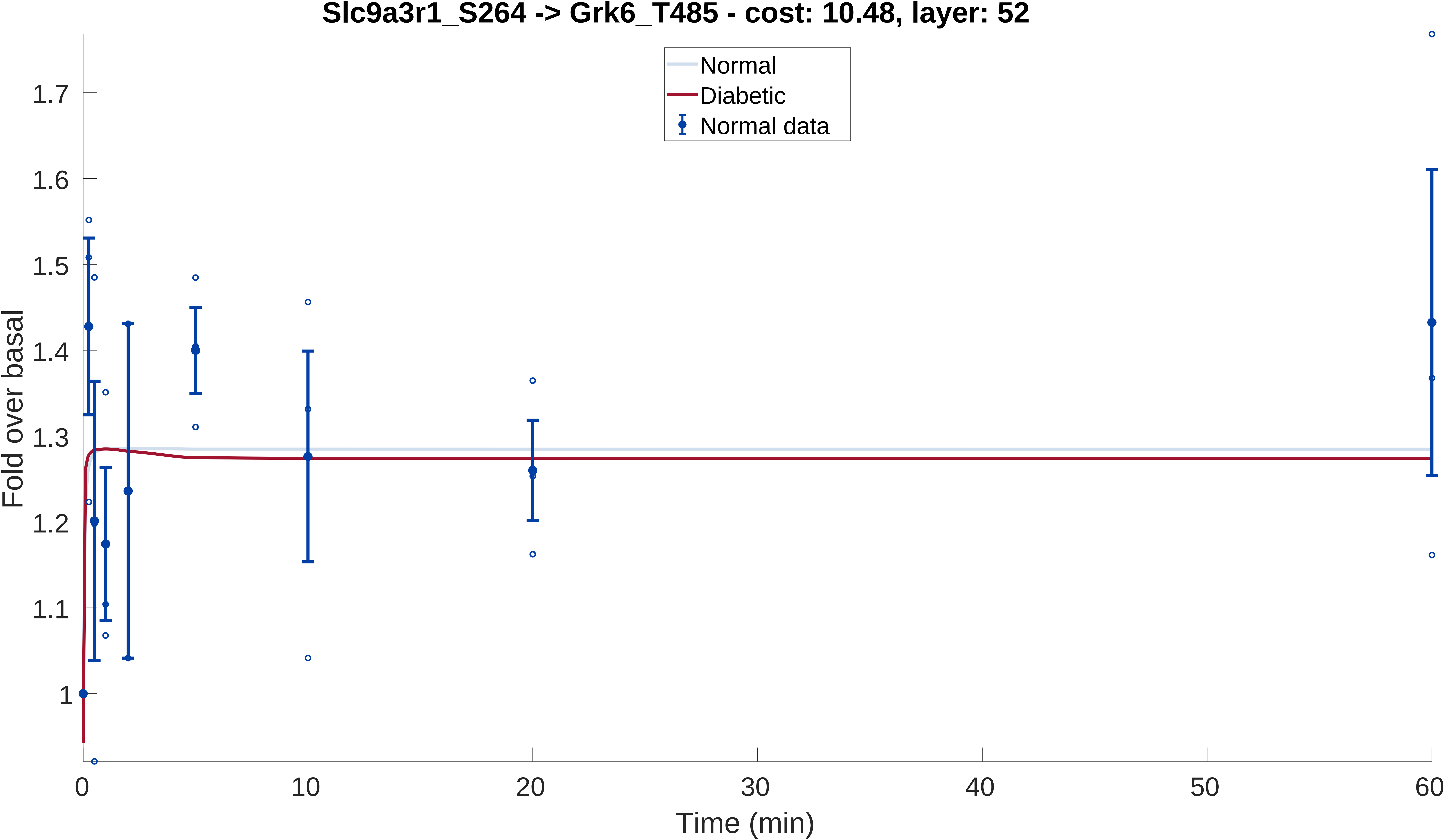

Slc9a3r1_S264 -> Grk6_T485 - cost: 10.48, layer: 52
Normal
Diabetic
Normal data
Fold over basal
1.7
1.6
1.5
1.4
1.3
1.2
1.1
1
0
10
20
30
40
50
60
Time (min)

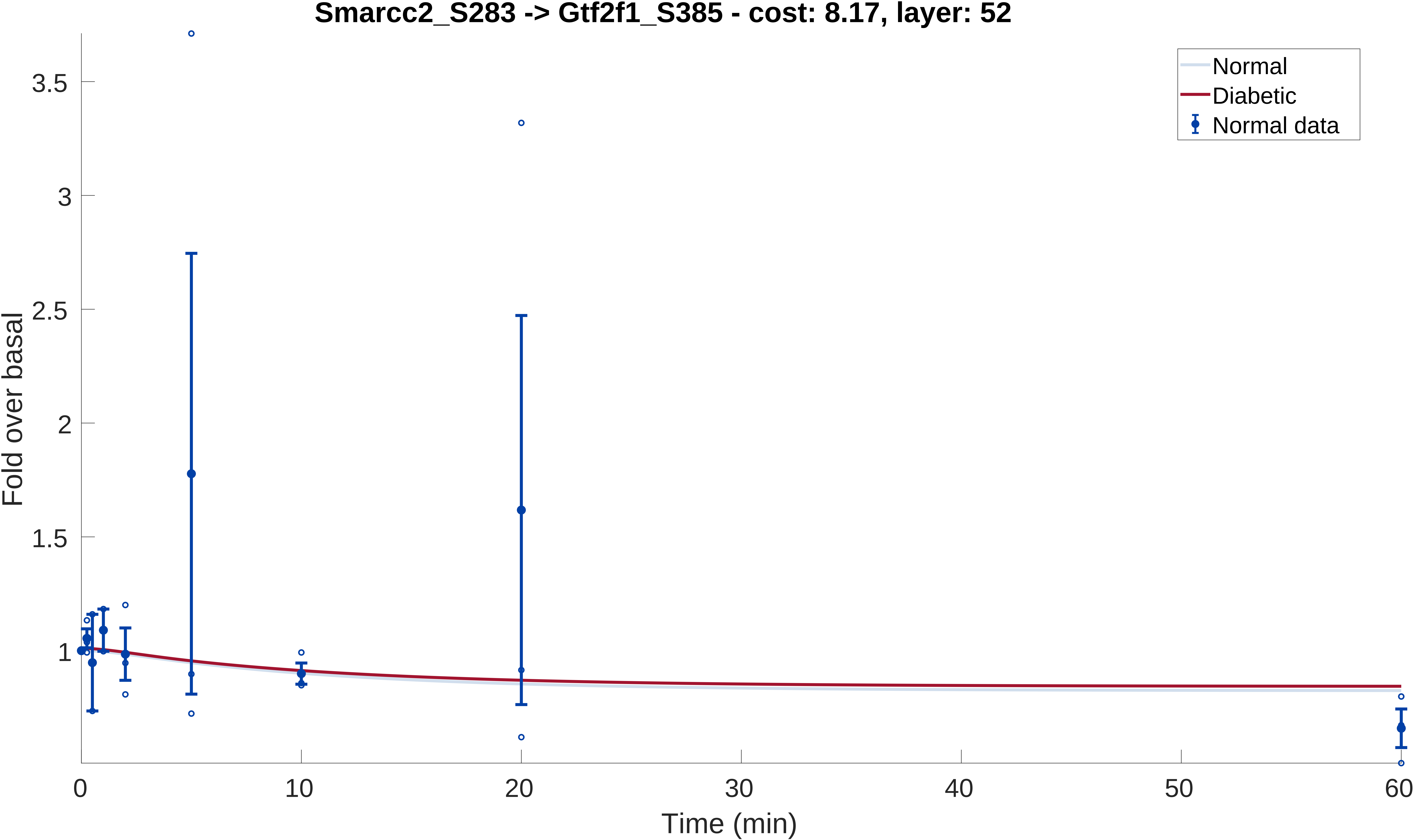

Smarcc2_S283 -> Gtf2f1_S385 - cost: 8.17, layer: 52
Normal
Diabetic
Normal data
Fold over basal
3.5
3
2.5
2
1.5
1
0
10
20
30
40
50
60
Time (min)

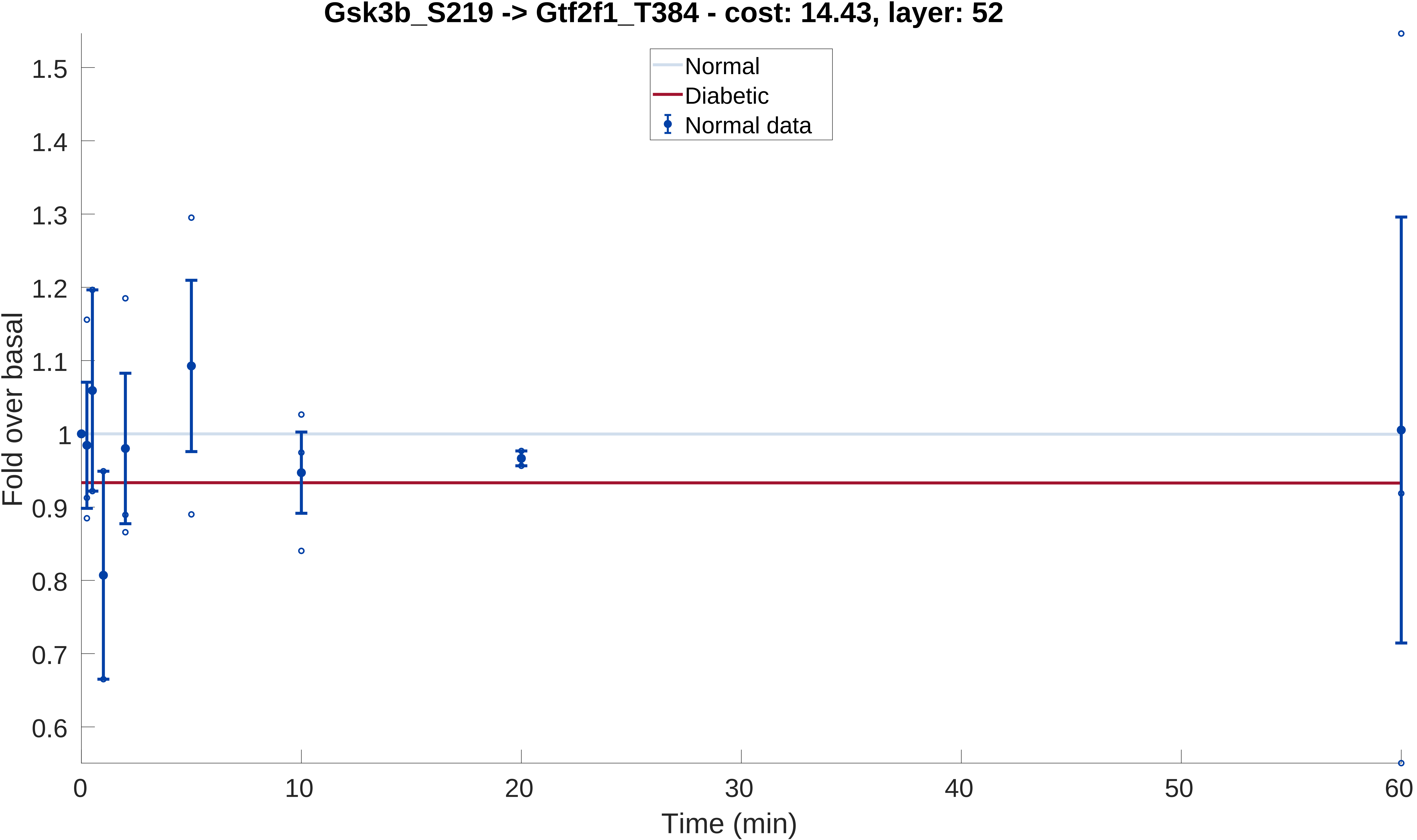

Gsk3b_S219 -> Gtf2f1_T384 - cost: 14.43, layer: 52
Normal
Diabetic
Normal data
Fold over basal
Time (min)
1.5
1.4
1.3
1.2
1.1
1
0.9
0.8
0.7
0.6
0
10
20
30
40
50
60

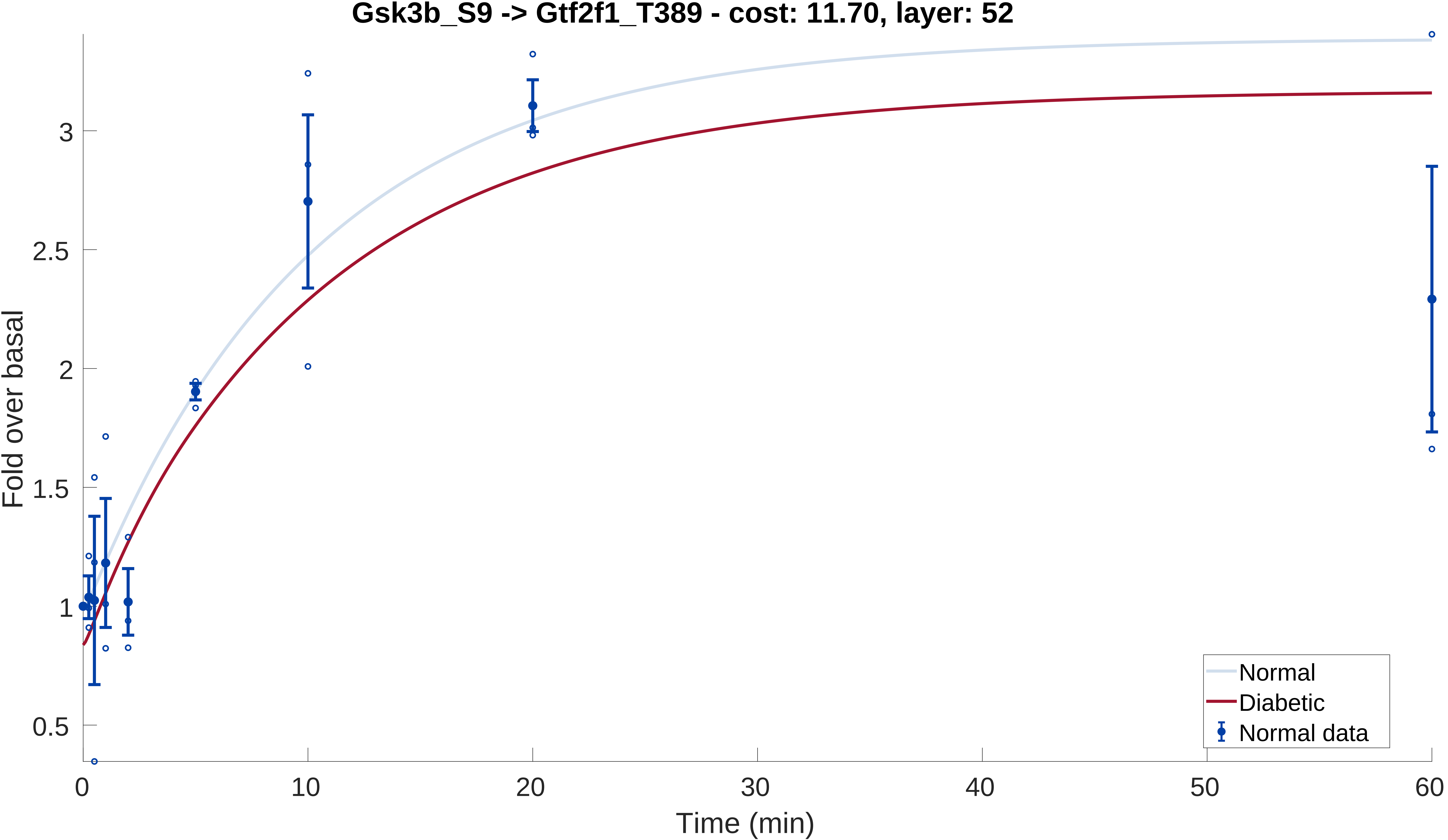

Gsk3b_S9 -> Gtf2f1_T389 - cost: 11.70, layer: 52
Fold over basal
0.5
1
1.5
2
2.5
3
0
10
20
30
40
50
60
Time (min)
Normal
Diabetic
Normal data

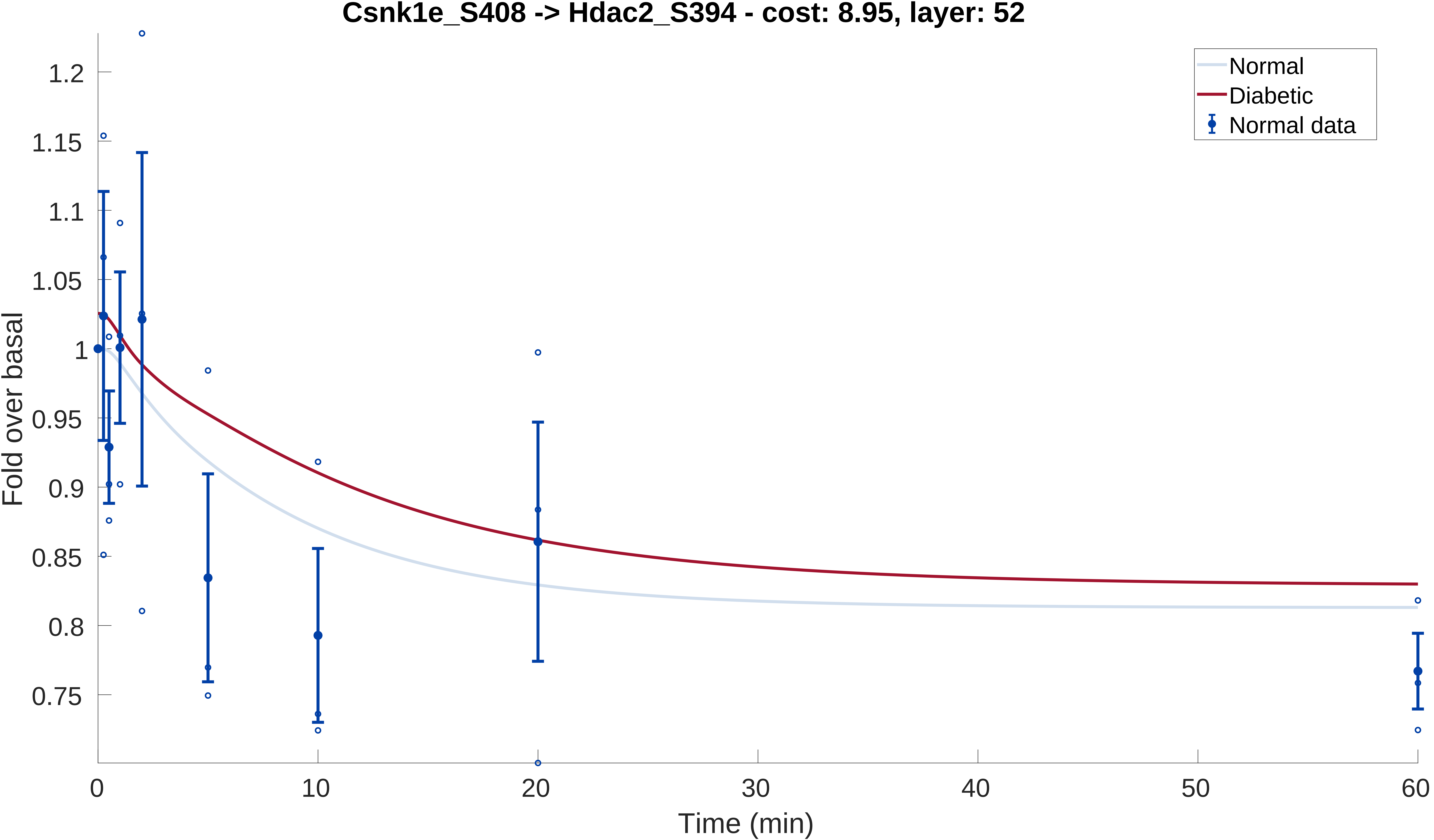

Csnk1e_S408 -> Hdac2_S394 - cost: 8.95, layer: 52
Normal
Diabetic
Normal data
Fold over basal
1.2
1.15
1.1
1.05
1
0.95
0.9
0.85
0.8
0.75
0
10
20
30
40
50
60
Time (min)

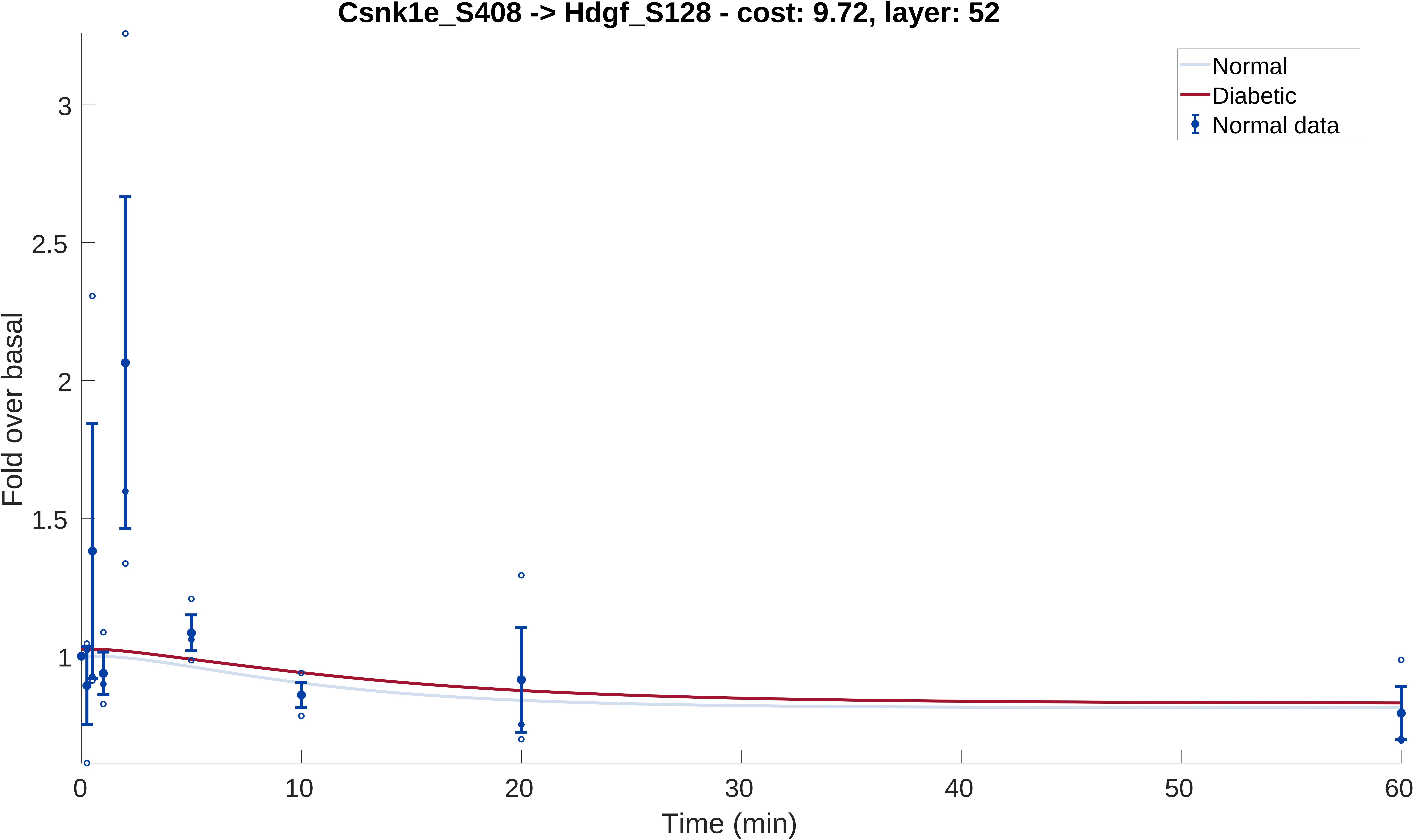
Csnk1e_S408 -> Hdgf_S128 - cost: 9.72, layer: 52
Normal
Diabetic
Normal data
Fold over basal
3
2.5
2
1.5
1
0
10
20
30
40
50
60
Time (min)

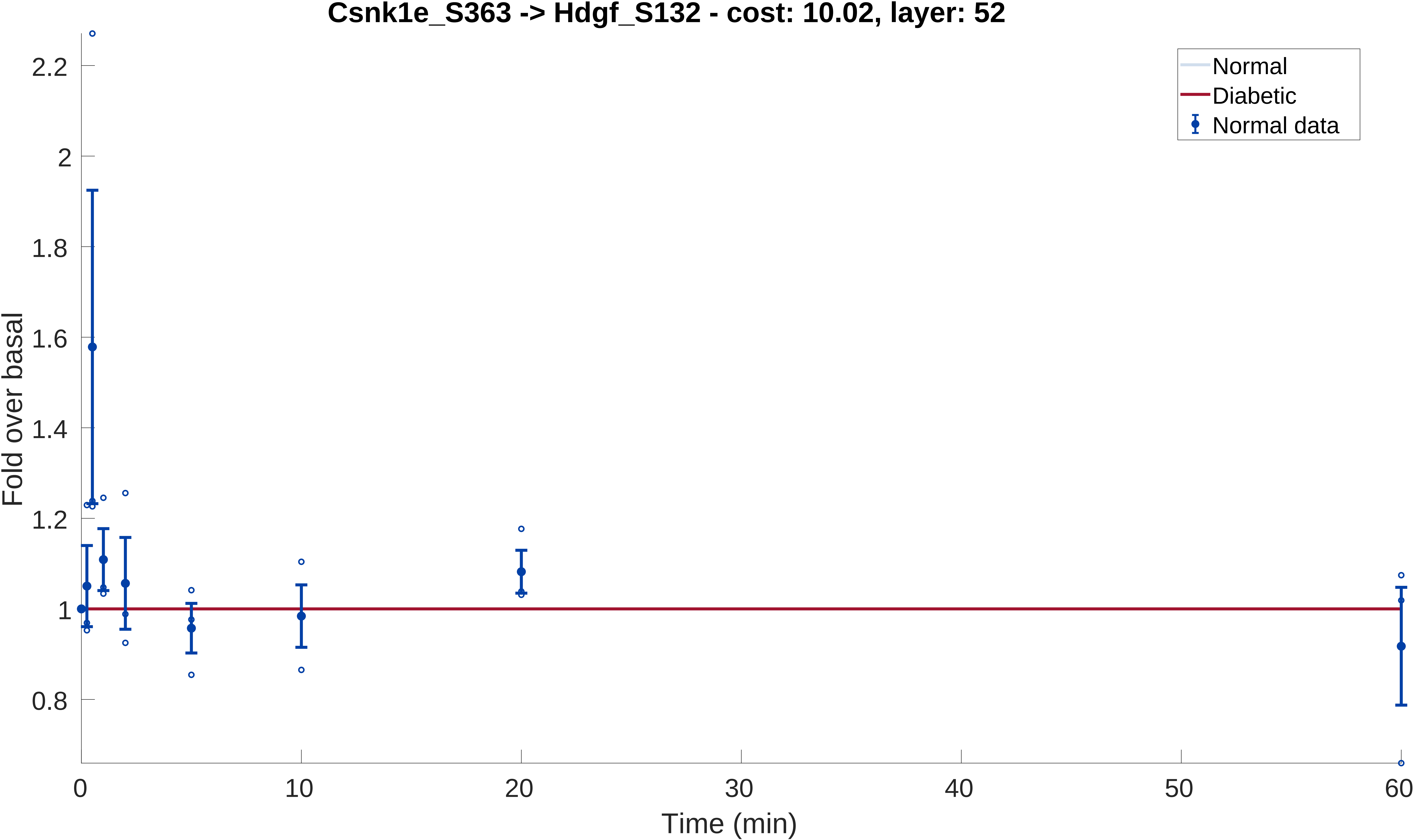

Csnk1e_S363 -> Hdgf_S132 - cost: 10.02, layer: 52
Normal
Diabetic
Normal data
Fold over basal
2.2
2
1.8
1.6
1.4
1.2
1
0.8
0
10
20
30
40
50
60
Time (min)

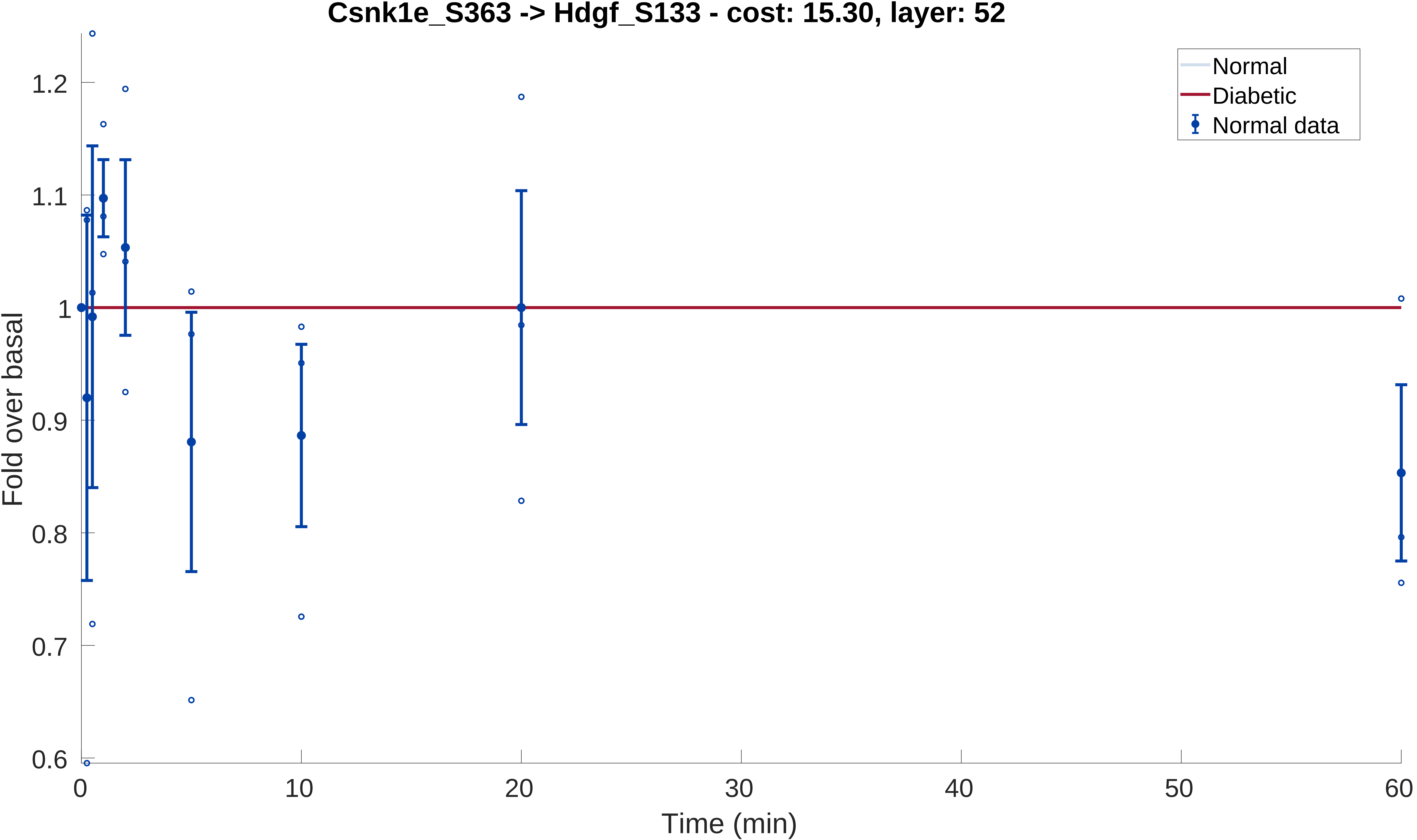

Csnk1e_S363 -> Hdgf_S133 - cost: 15.30, layer: 52
Normal
Diabetic
Normal data
Fold over basal
Time (min)
0.6
0.7
0.8
0.9
1
1.1
1.2
0
10
20
30
40
50
60

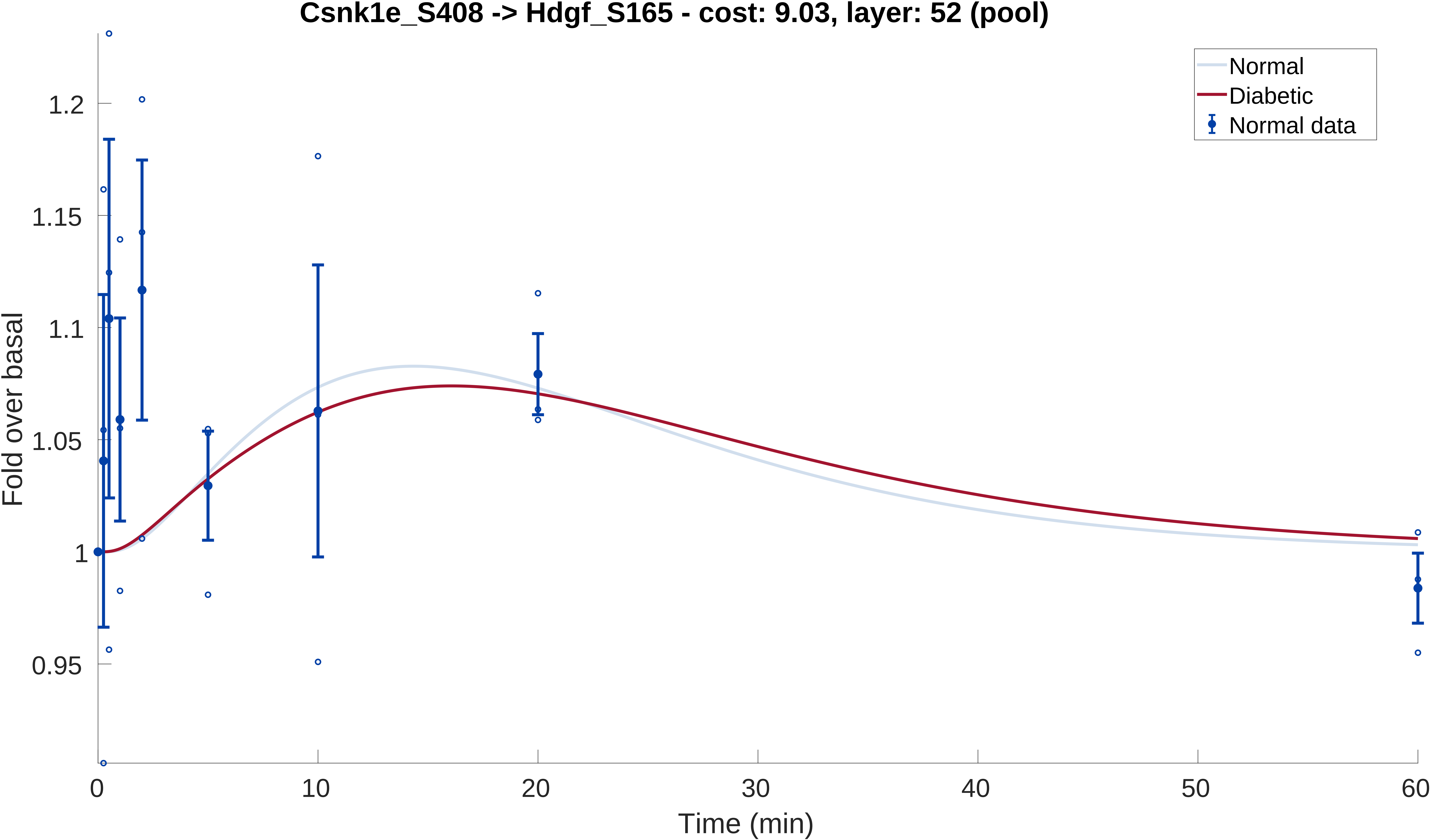

Csnk1e_S408 -> Hdgf_S165 - cost: 9.03, layer: 52 (pool)
Normal
Diabetic
Normal data
Fold over basal
1.2
1.15
1.1
1.05
1
0.95
0
10
20
30
40
50
60
Time (min)

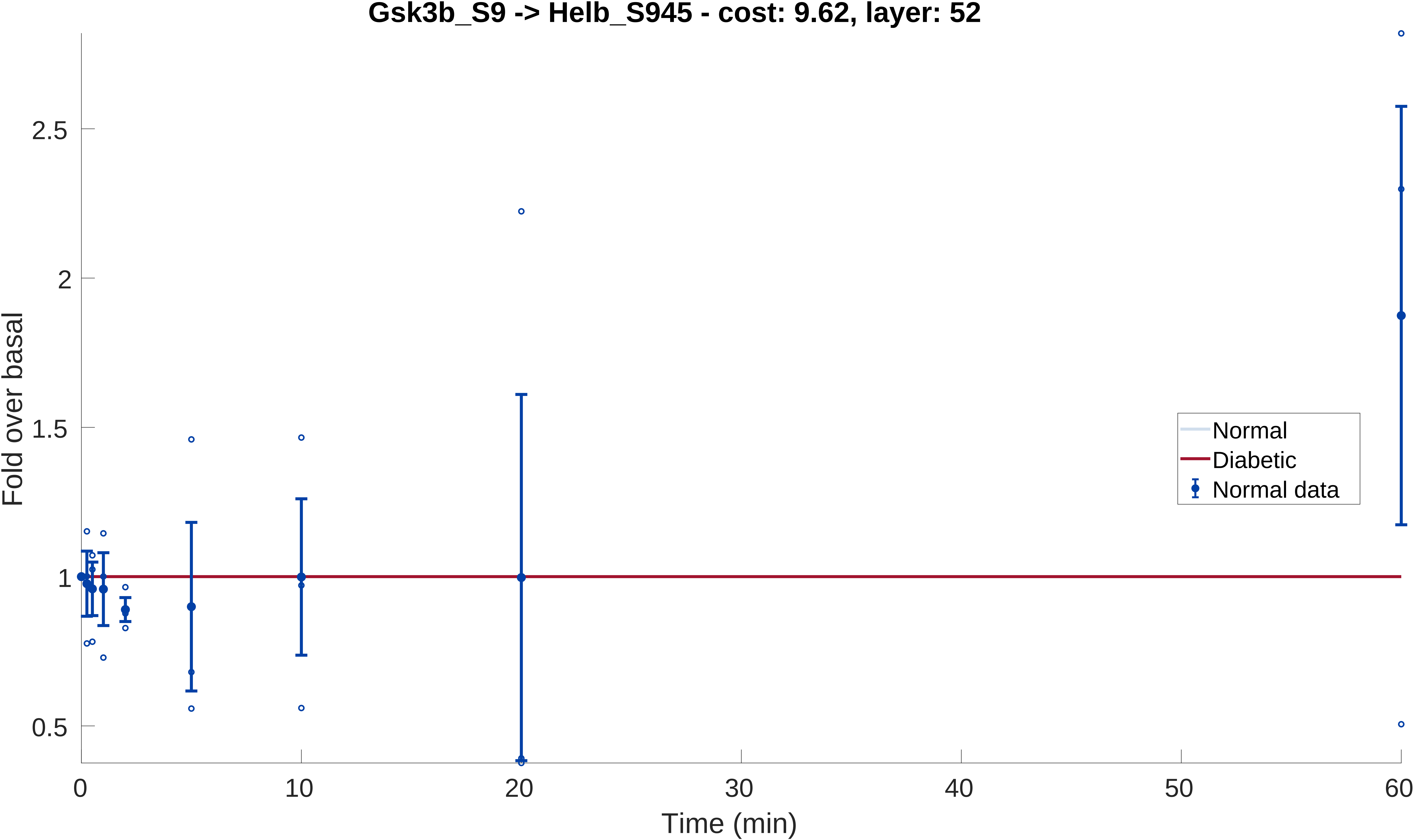
Gsk3b_S9 -> Helb_S945 - cost: 9.62, layer: 52
Fold over basal
2.5
2
1.5
1
0.5
0
10
20
30
40
50
60
Time (min)
Normal
Diabetic
Normal data

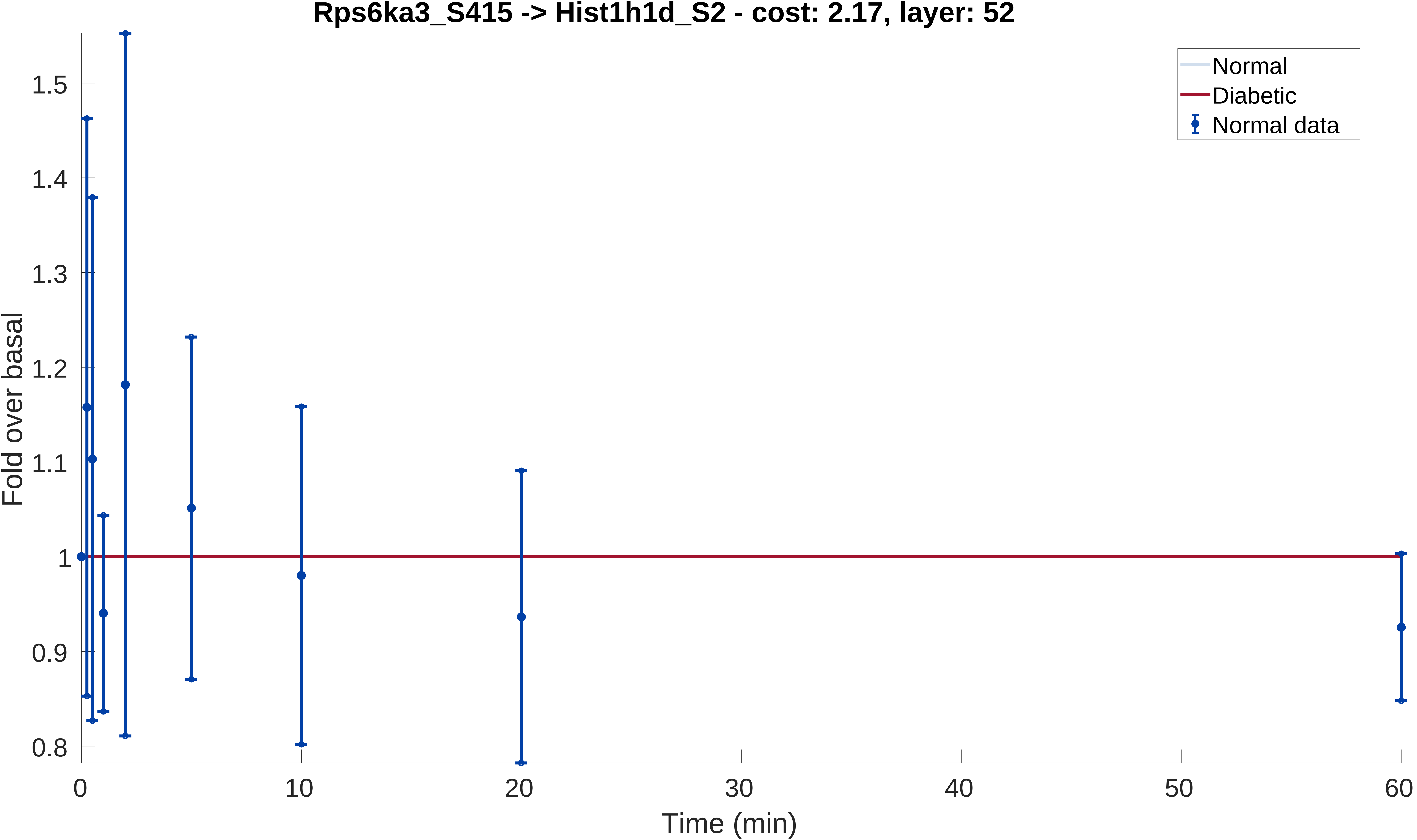

Rps6ka3_S415 -> Hist1h1d_S2 - cost: 2.17, layer: 52
Normal
Diabetic
Normal data
Fold over basal
1.5
1.4
1.3
1.2
1.1
1
0.9
0.8
0
10
20
30
40
50
60
Time (min)

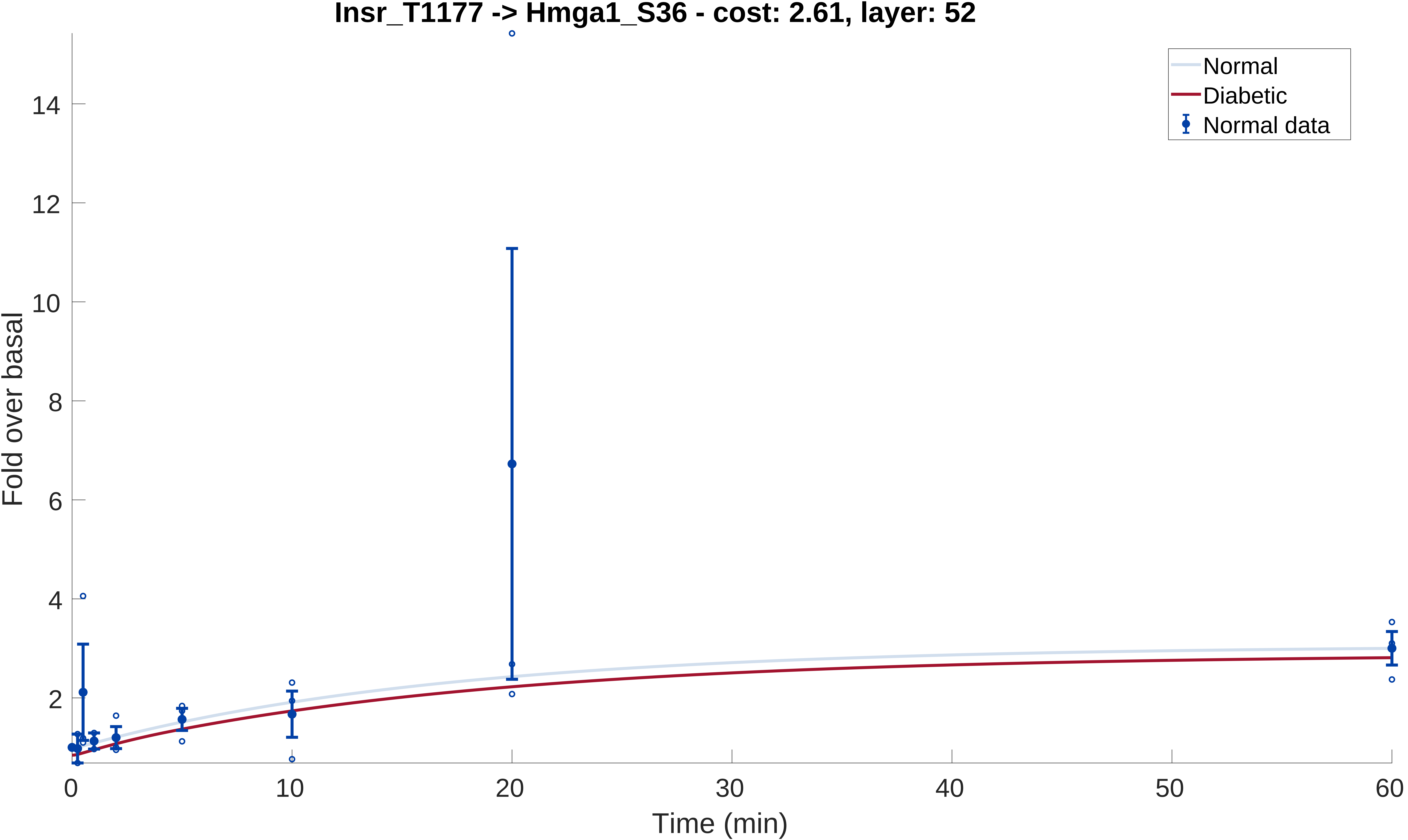
Insr_T1177 -> Hmga1_S36 - cost: 2.61, layer: 52
Normal
Diabetic
Normal data
Fold over basal
14
12
10
8
6
4
2
0
10
20
30
40
50
60
Time (min)

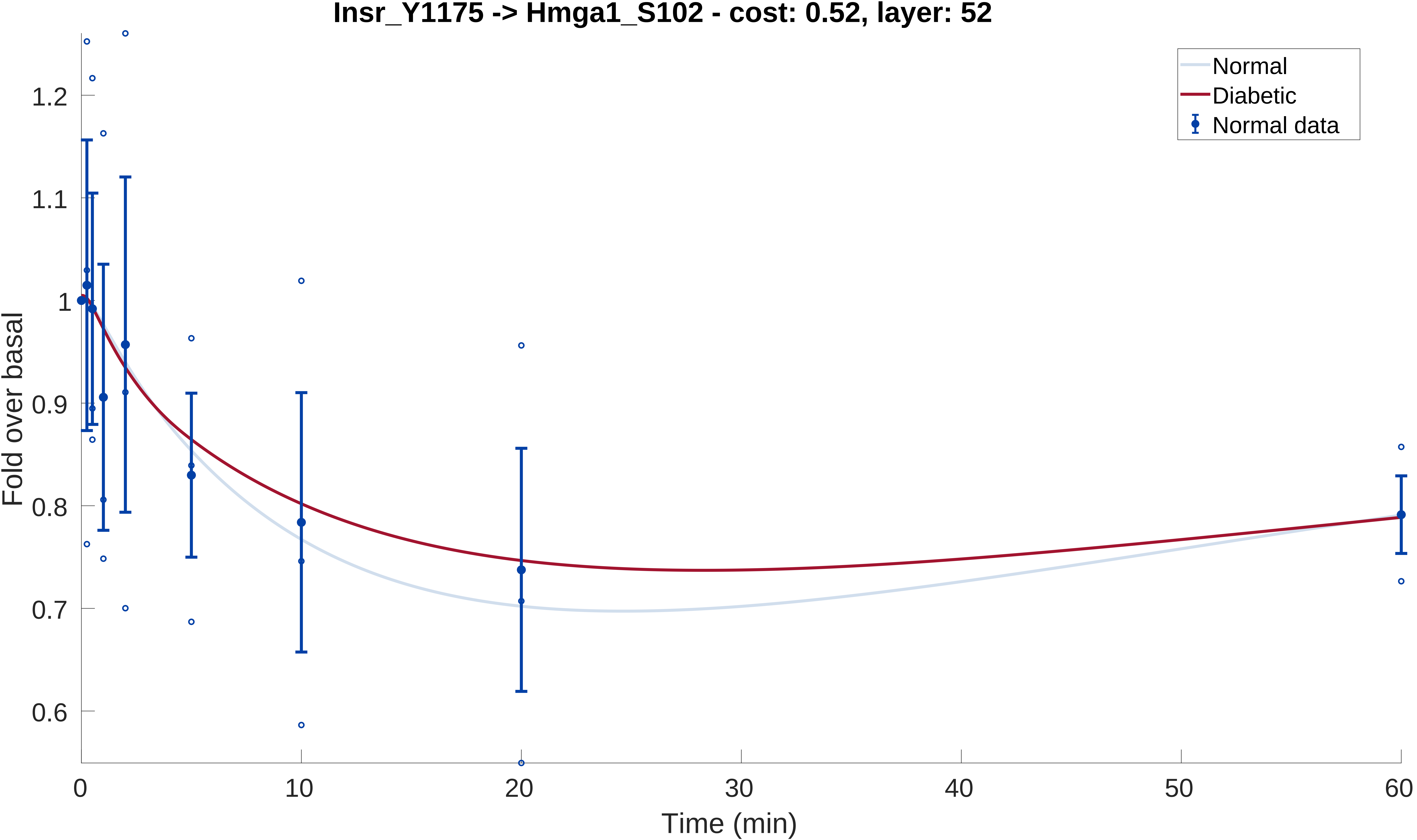

Insr_Y1175 -> Hmga1_S102 - cost: 0.52, layer: 52
Normal
Diabetic
Normal data
Fold over basal
1.2
1.1
1
0.9
0.8
0.7
0.6
0
10
20
30
40
50
60
Time (min)

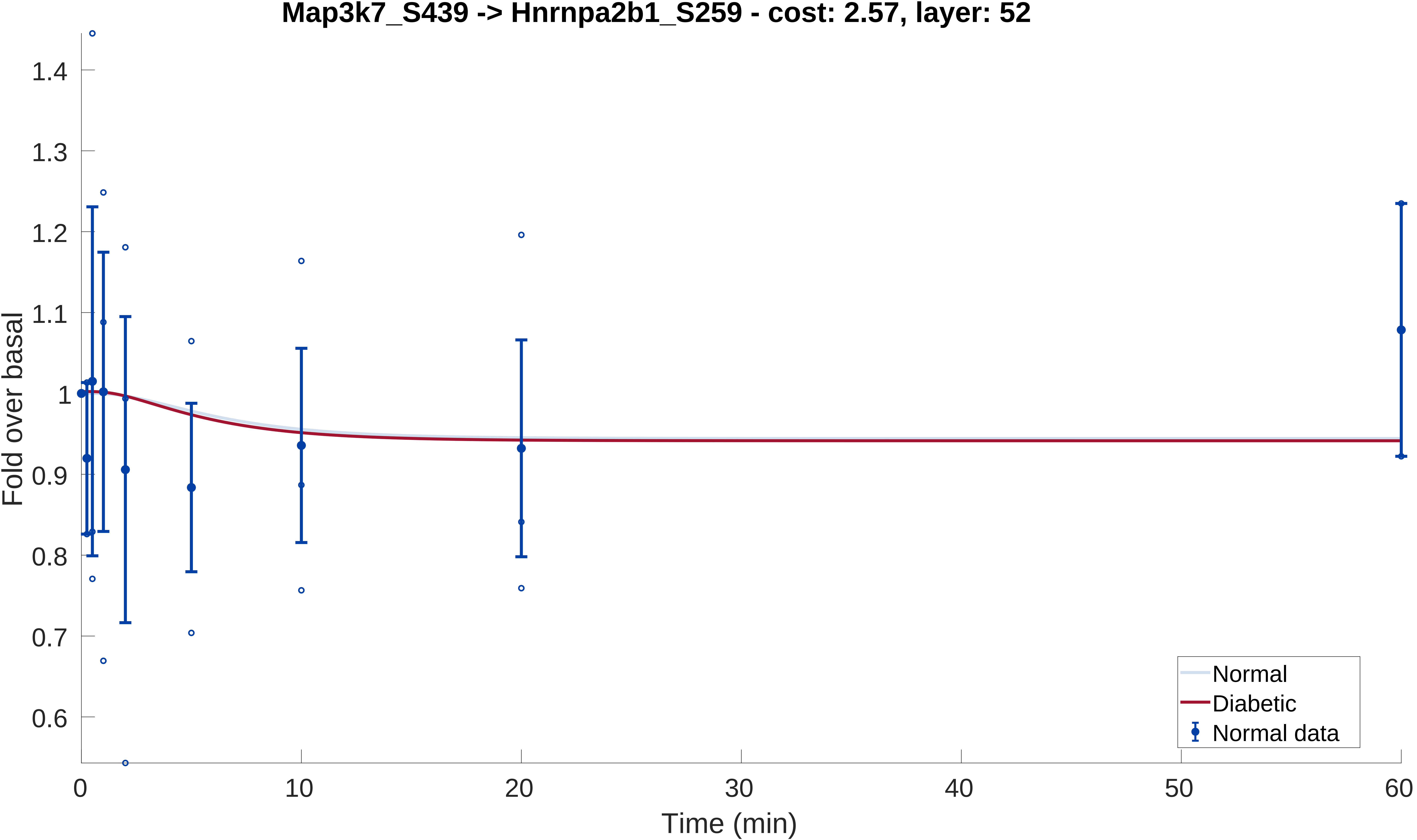

Map3k7_S439 -> Hnrnpa2b1_S259 - cost: 2.57, layer: 52
Fold over basal
Time (min)
Normal
Diabetic
Normal data

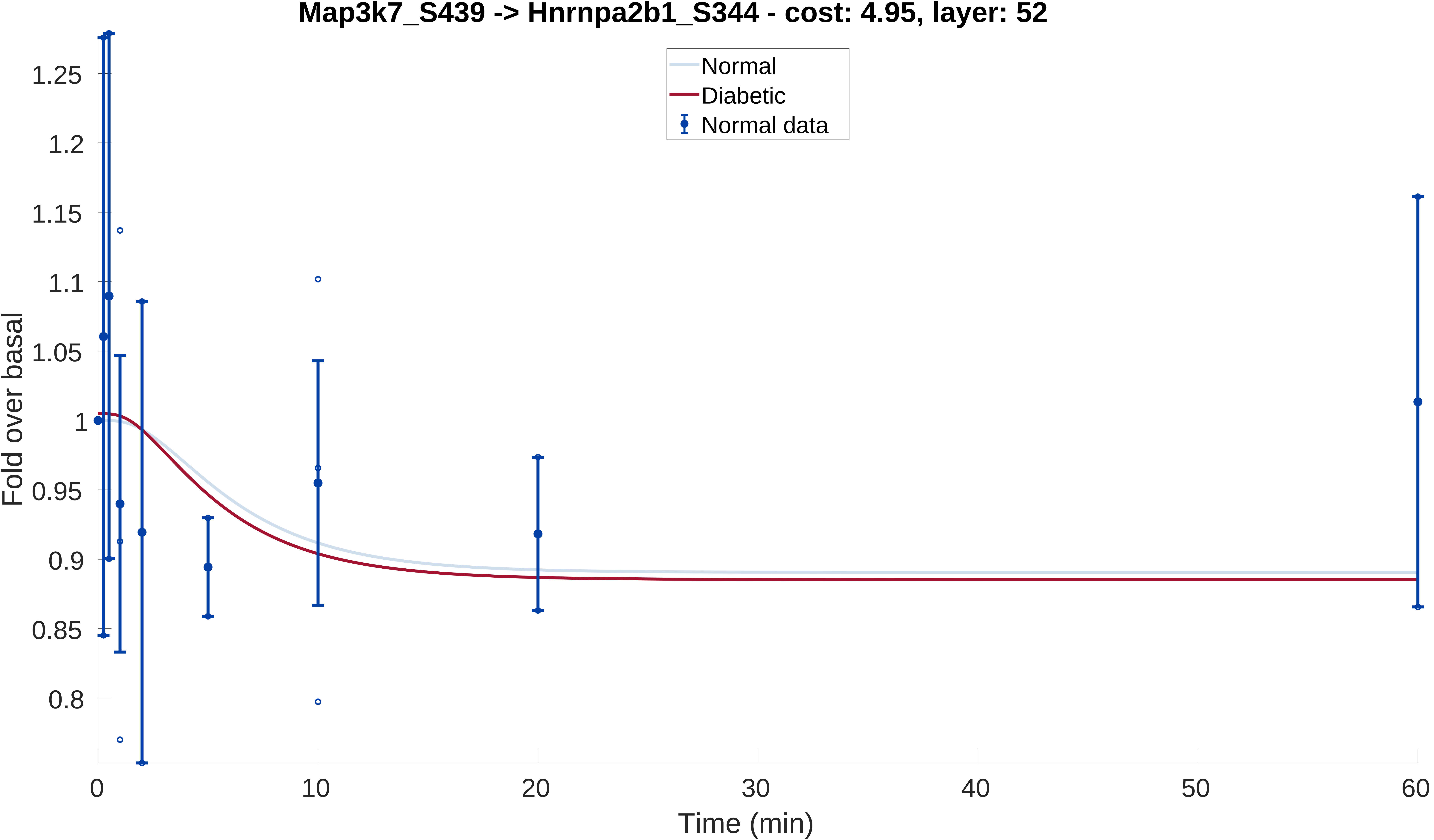

Map3k7_S439 -> Hnrnpa2b1_S344 - cost: 4.95, layer: 52
Normal
Diabetic
Normal data
Fold over basal
1.25
1.2
1.15
1.1
1.05
1
0.95
0.9
0.85
0.8
0
10
20
30
40
50
60
Time (min)

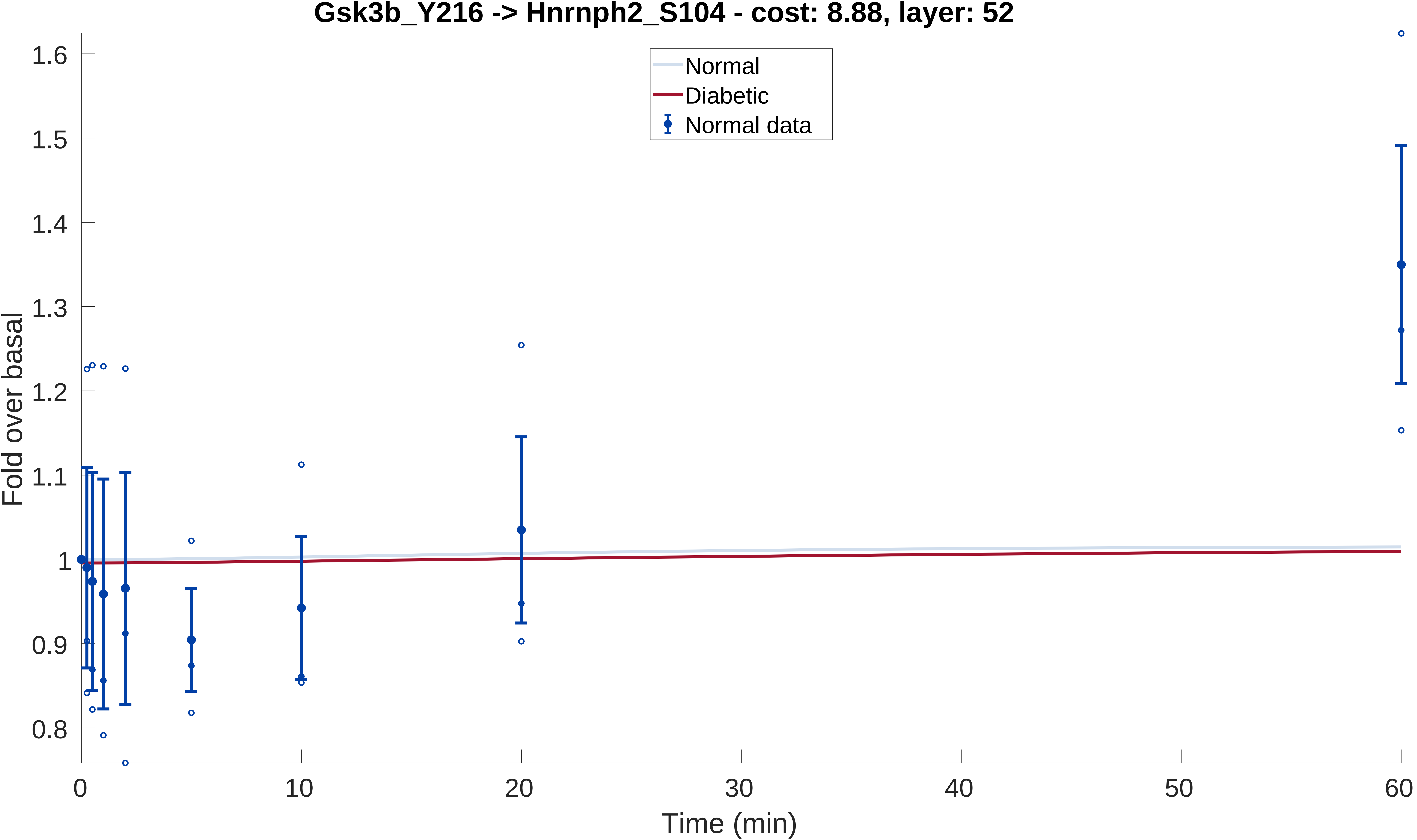
Gsk3b_Y216 -> Hnrnph2_S104 - cost: 8.88, layer: 52
Normal
Diabetic
Normal data
Fold over basal
1.6
1.5
1.4
1.3
1.2
1.1
1
0.9
0.8
0
10
20
30
40
50
60
Time (min)

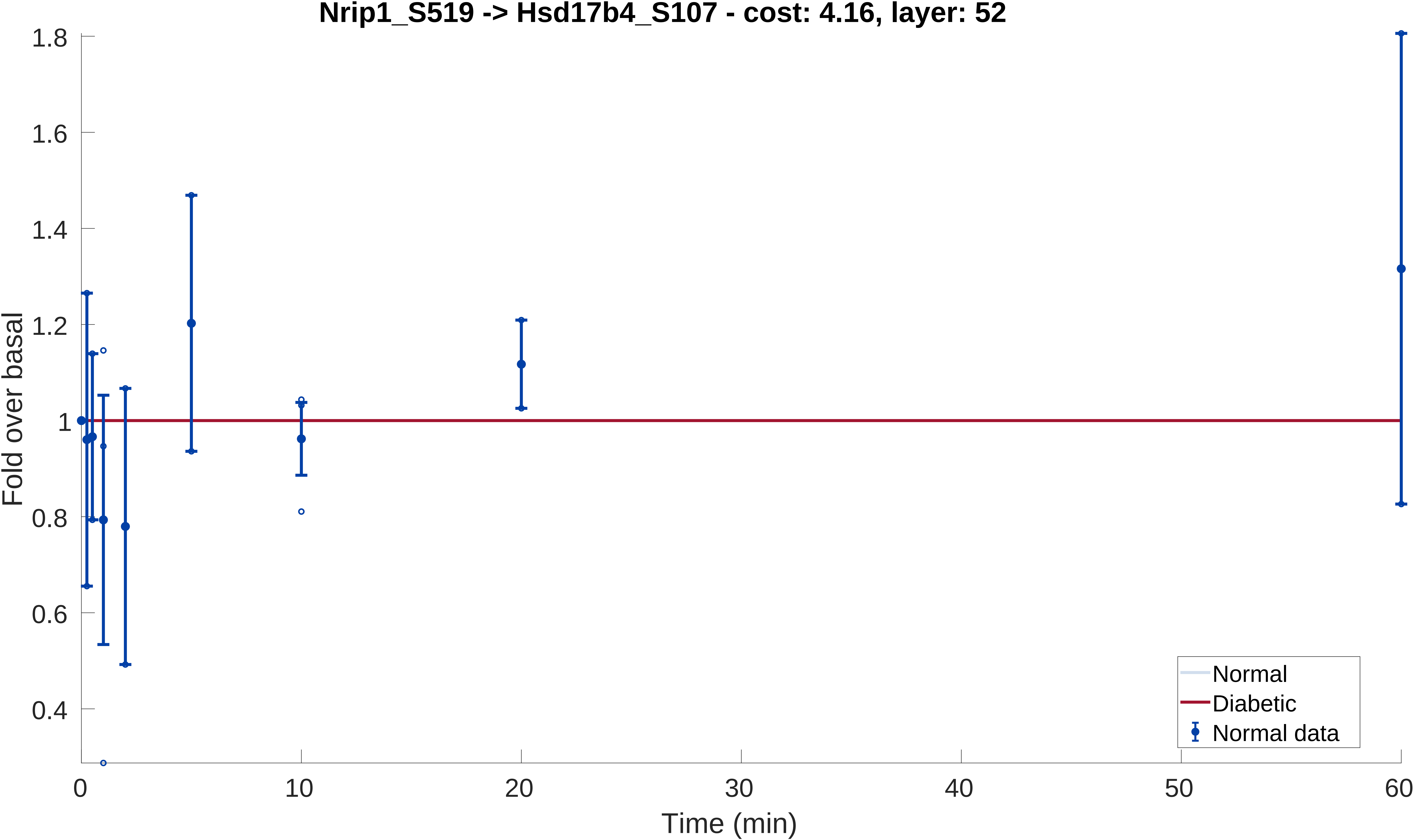

Nrip1_S519 -> Hsd17b4_S107 - cost: 4.16, layer: 52
Fold over basal
1.8
1.6
1.4
1.2
1
0.8
0.6
0.4
0
10
20
30
40
50
60
Time (min)
Normal
Diabetic
Normal data

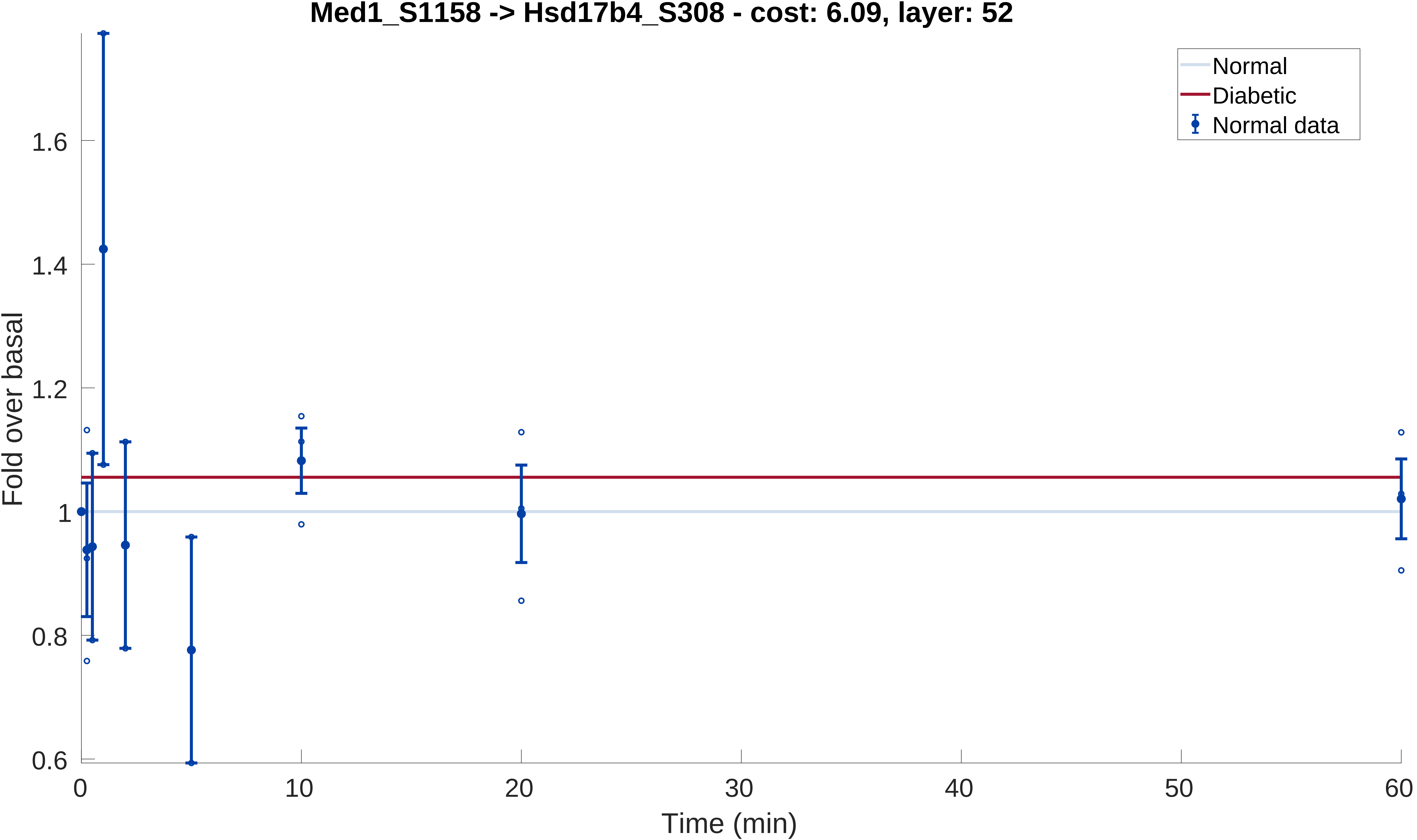

Med1_S1158 -> Hsd17b4_S308 - cost: 6.09, layer: 52
Normal
Diabetic
Normal data
Fold over basal
0.6
0.8
1
1.2
1.4
1.6
0
10
20
30
40
50
60
Time (min)

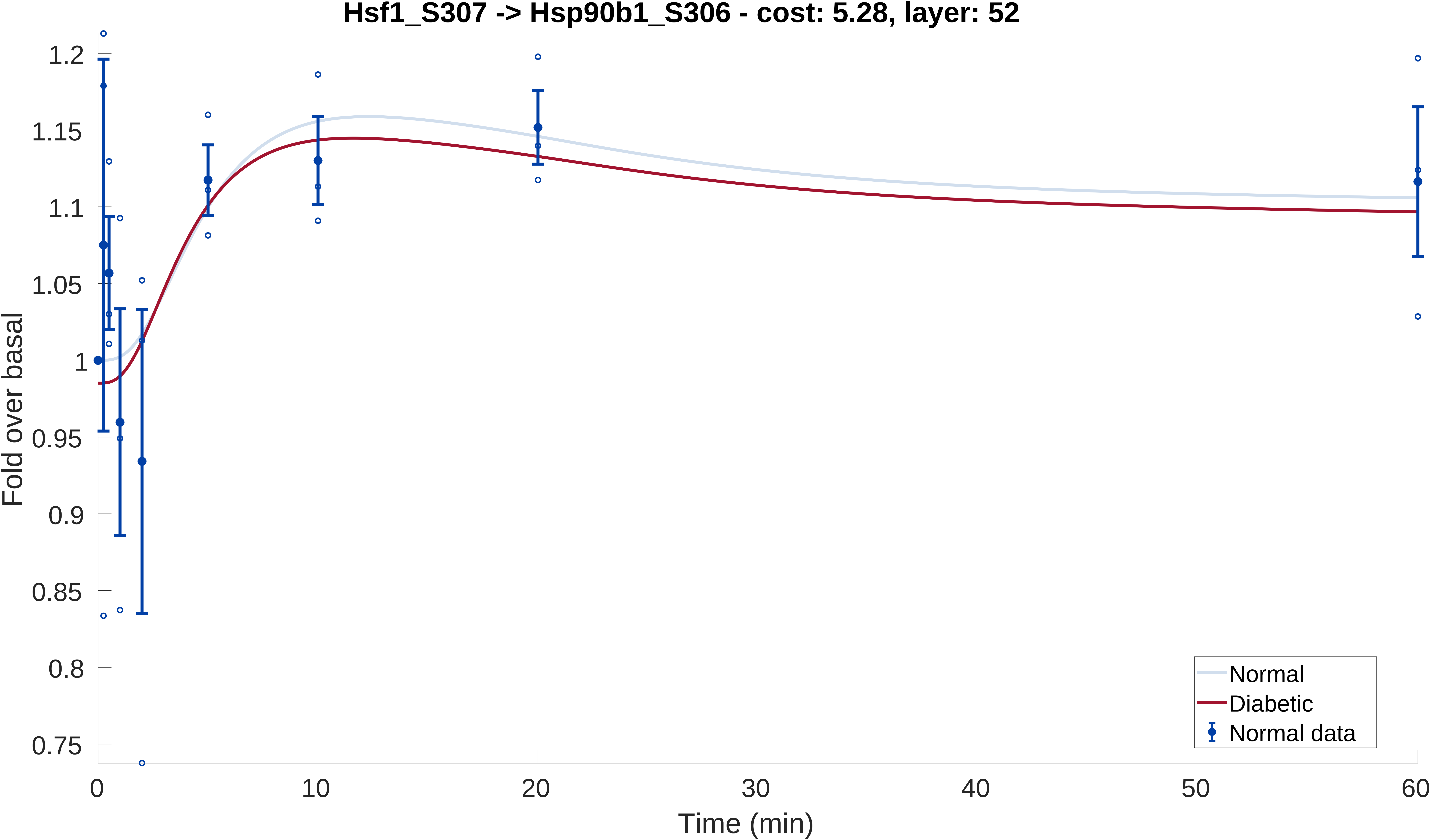

Hsf1_S307 -> Hsp90b1_S306 - cost: 5.28, layer: 52
Fold over basal
Time (min)
1.2
1.15
1.1
1.05
1
0.95
0.9
0.85
0.8
0.75
0
10
20
30
40
50
60
Normal
Diabetic
Normal data

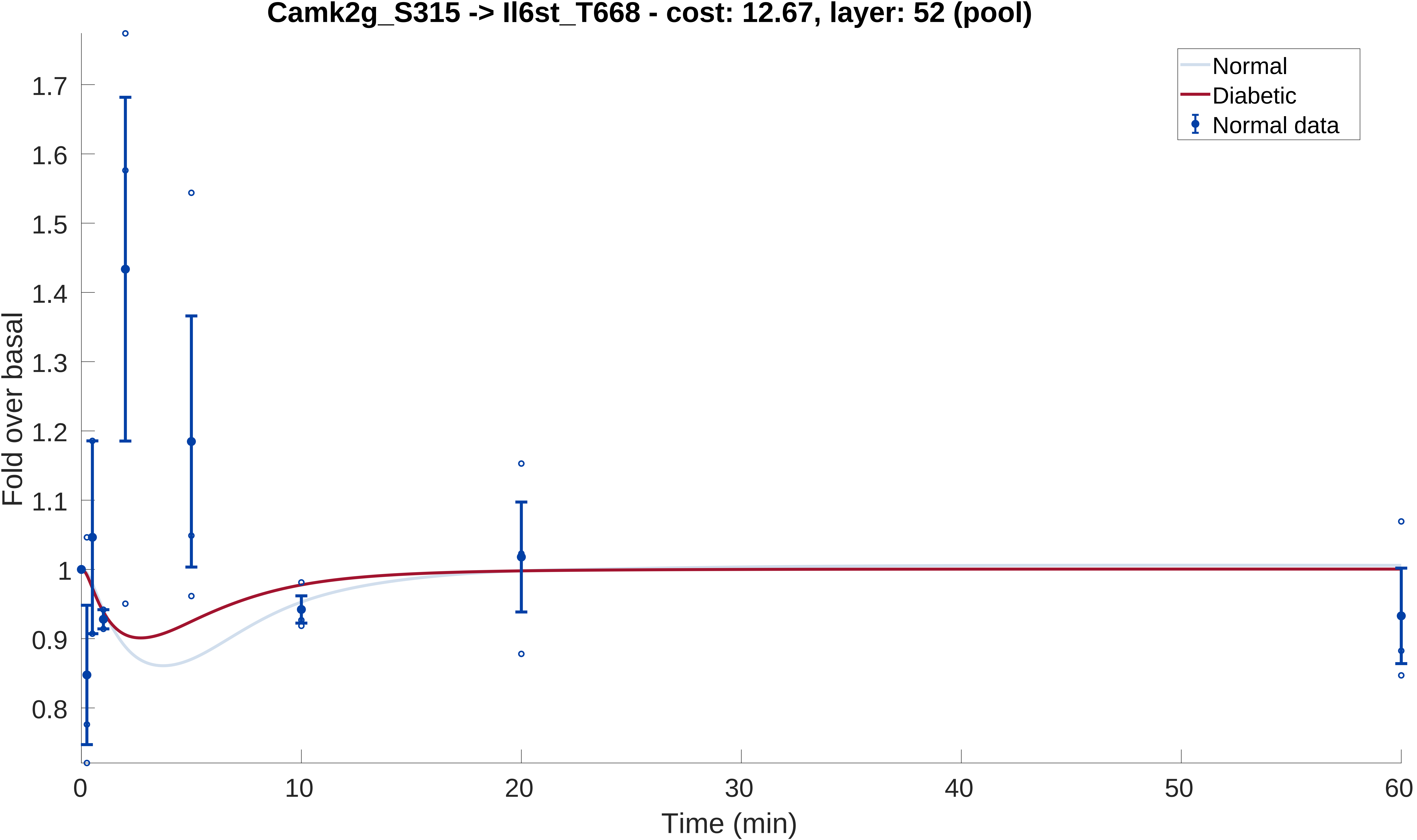
Camk2g_S315 -> Il6st_T668 - cost: 12.67, layer: 52 (pool)
Normal
Diabetic
Normal data
Fold over basal
Time (min)
0
10
20
30
40
50
60
0.8
0.9
1
1.1
1.2
1.3
1.4
1.5
1.6
1.7

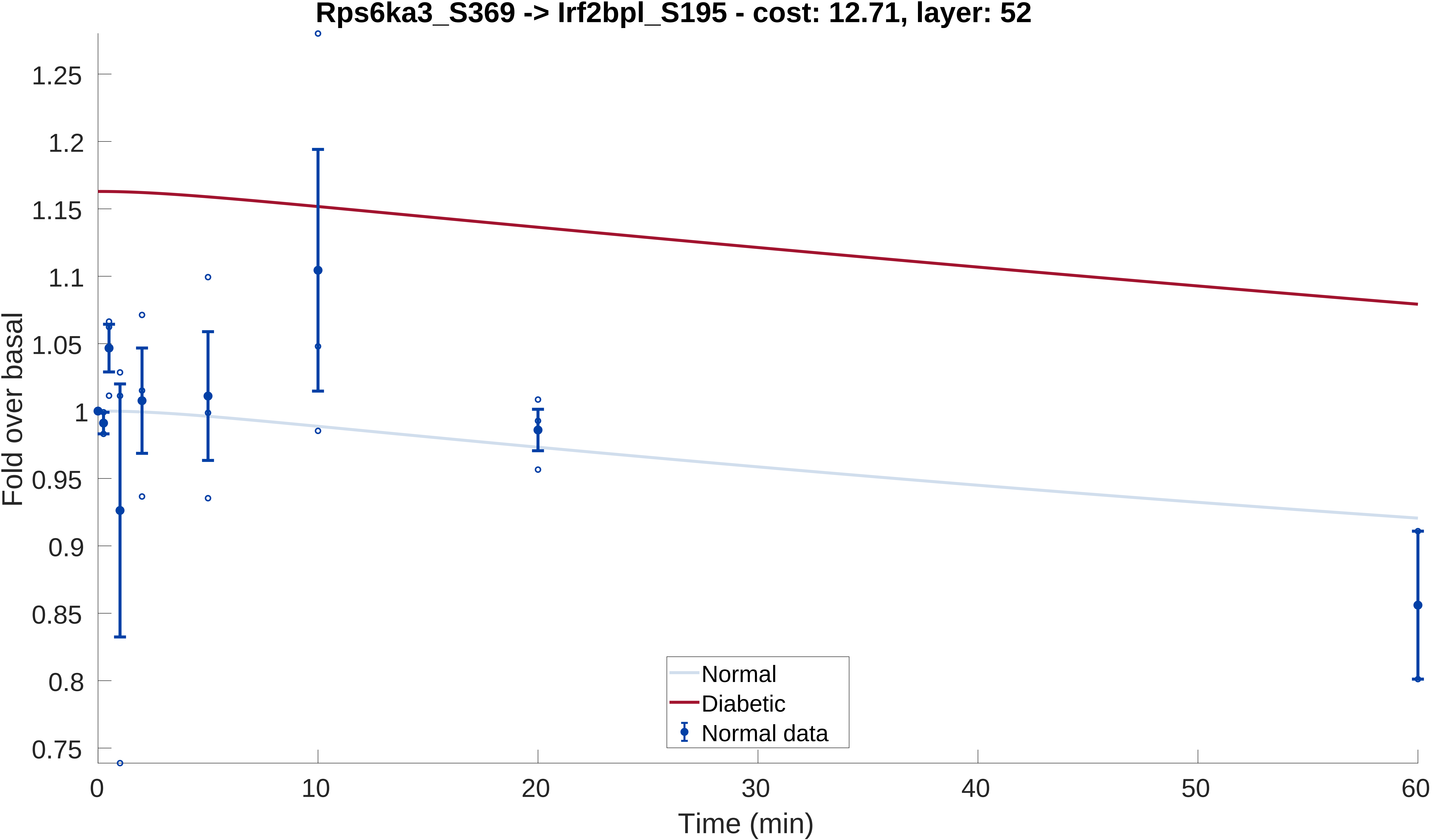

Rps6ka3_S369 -> Irf2bpl_S195 - cost: 12.71, layer: 52
Fold over basal
Time (min)
1.25
1.2
1.15
1.1
1.05
1
0.95
0.9
0.85
0.8
0.75
0
10
20
30
40
50
60
Normal
Diabetic
Normal data

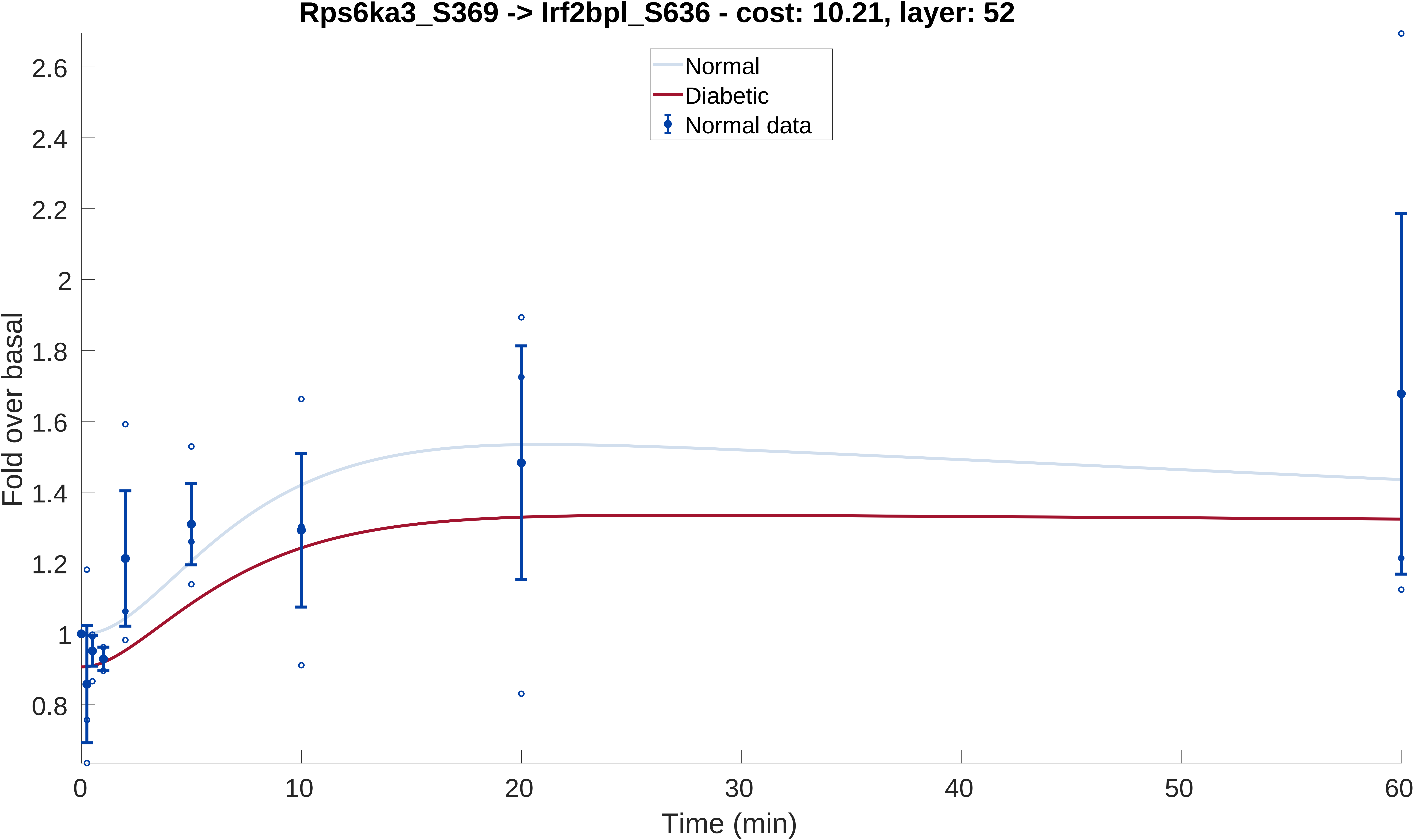

Rps6ka3_S369 -> Irf2bpl_S636 - cost: 10.21, layer: 52
Normal
Diabetic
Normal data
Fold over basal
2.6
2.4
2.2
2
1.8
1.6
1.4
1.2
1
0.8
0
10
20
30
40
50
60
Time (min)

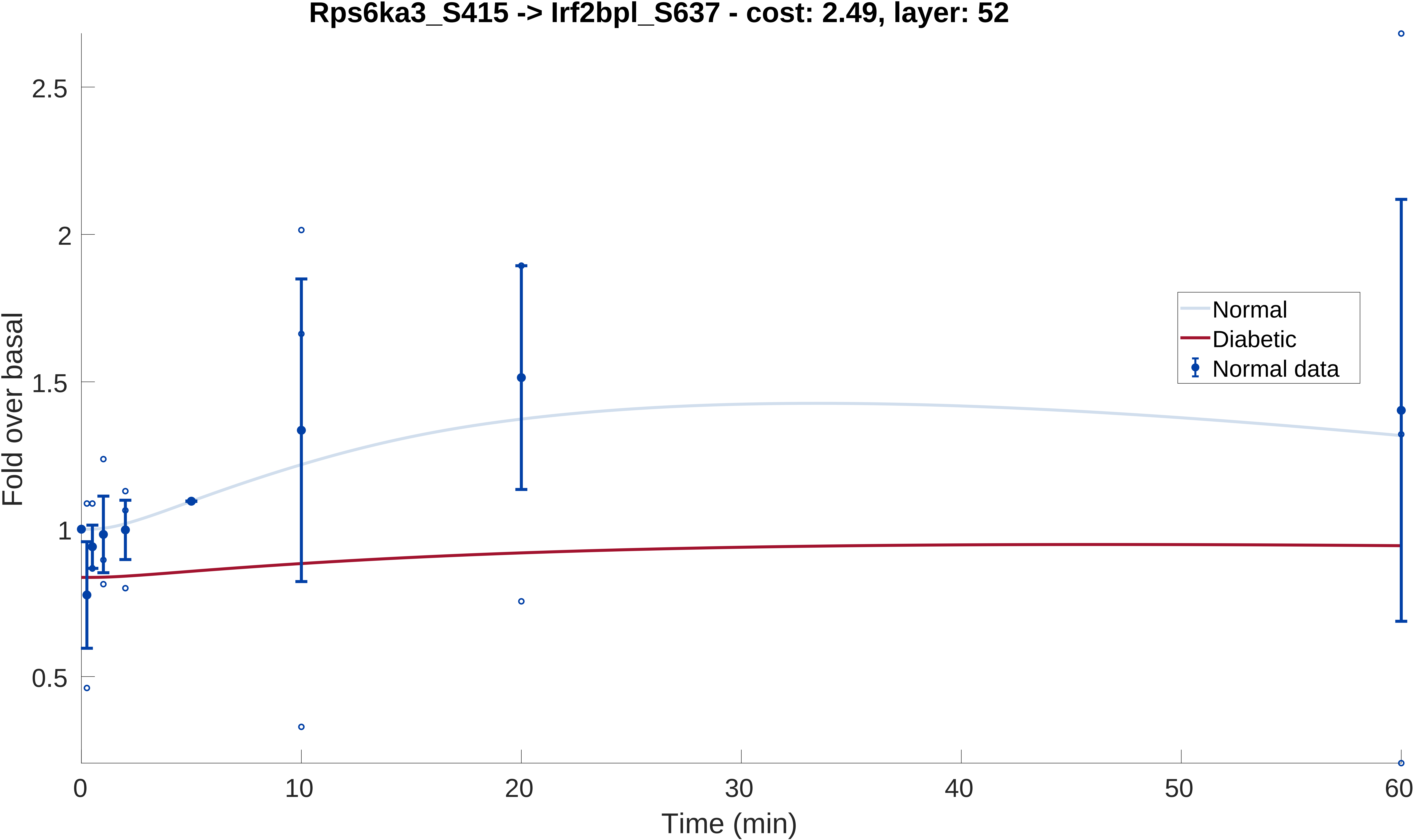

Rps6ka3_S415 -> Irf2bpl_S637 - cost: 2.49, layer: 52
Fold over basal
Time (min)
2.5
2
1.5
1
0.5
0
10
20
30
40
50
60
Normal
Diabetic
Normal data

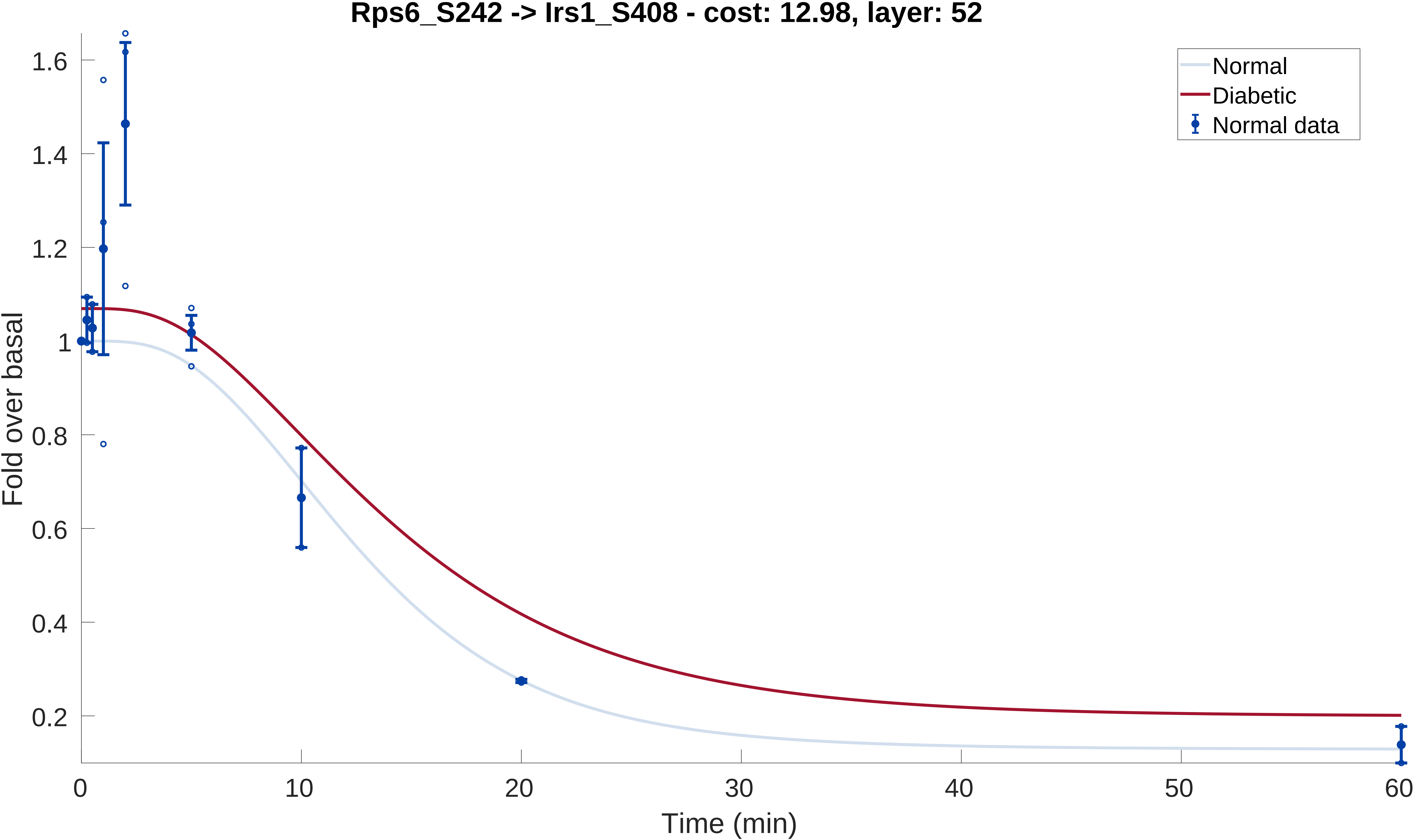
Rps6_S242 -> Irs1_S408 - cost: 12.98, layer: 52
Fold over basal
Time (min)
Normal
Diabetic
Normal data
0
10
20
30
40
50
60
0.2
0.4
0.6
0.8
1
1.2
1.4
1.6

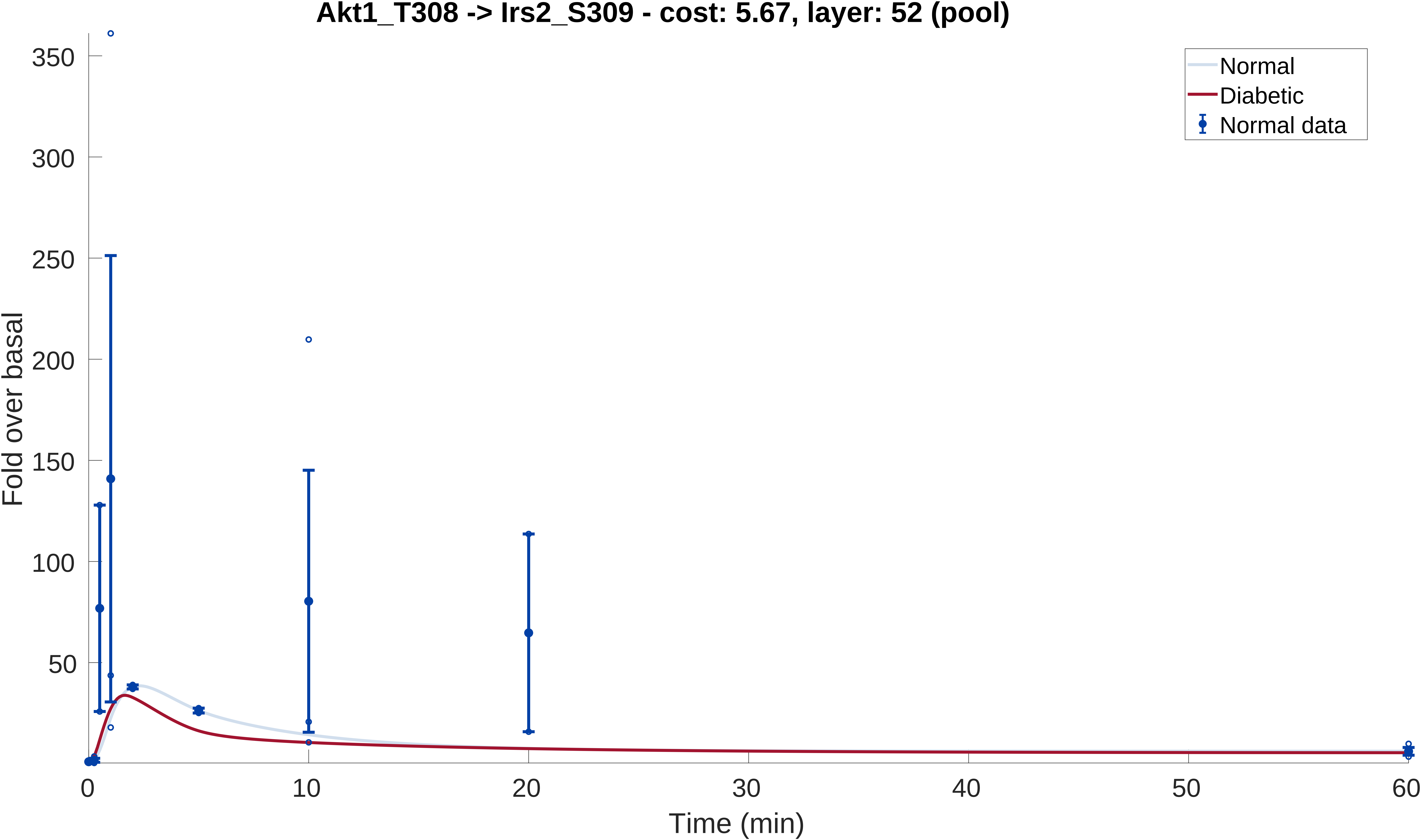

Akt1_T308 -> Irs2_S309 - cost: 5.67, layer: 52 (pool)
Normal
Diabetic
Normal data
Fold over basal
350
300
250
200
150
100
50
0
10
20
30
40
50
60
Time (min)

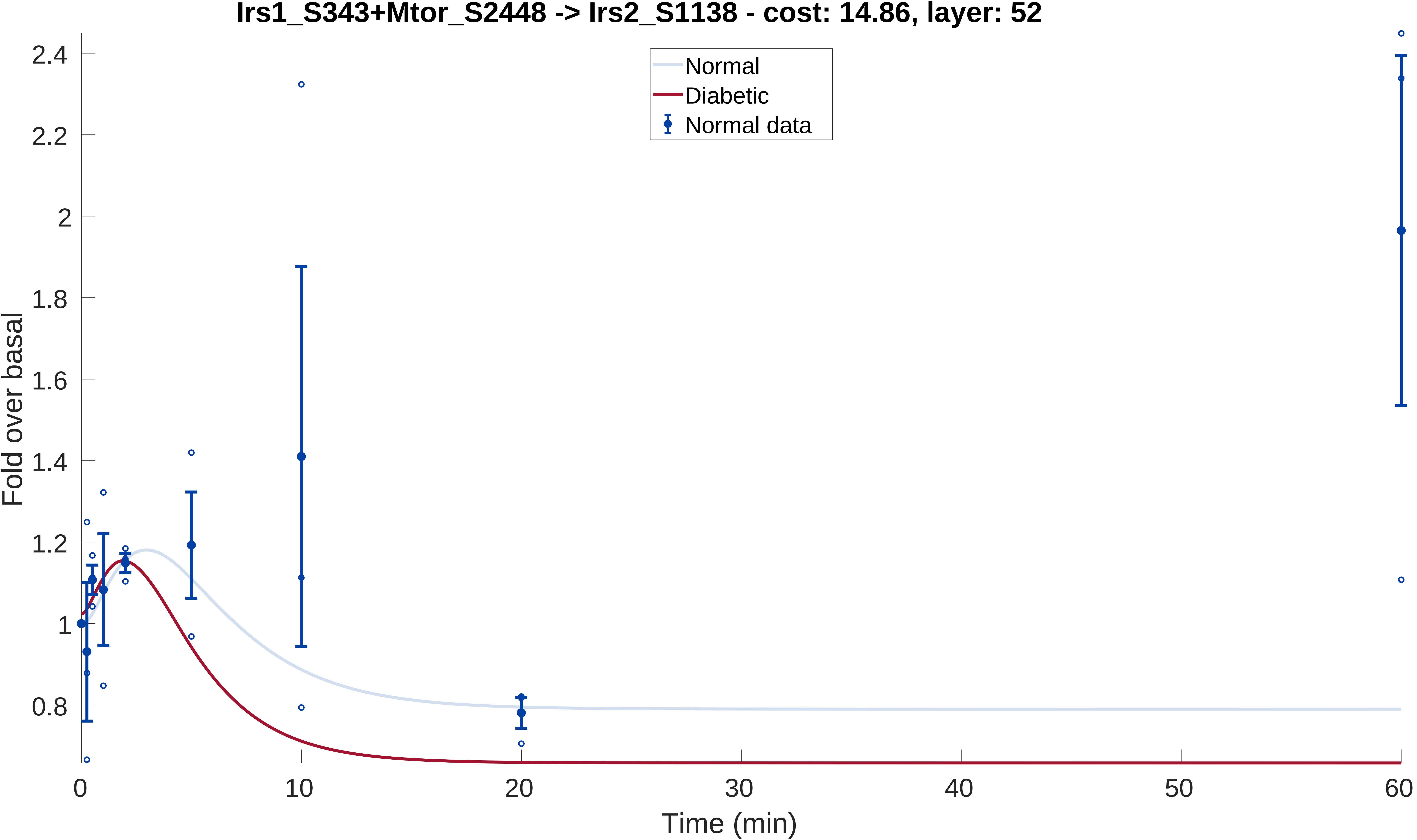

Irs1_S343+Mtor_S2448 -> Irs2_S1138 - cost: 14.86, layer: 52
Normal
Diabetic
Normal data
Fold over basal
2.4
2.2
2
1.8
1.6
1.4
1.2
1
0.8
0
10
20
30
40
50
60
Time (min)

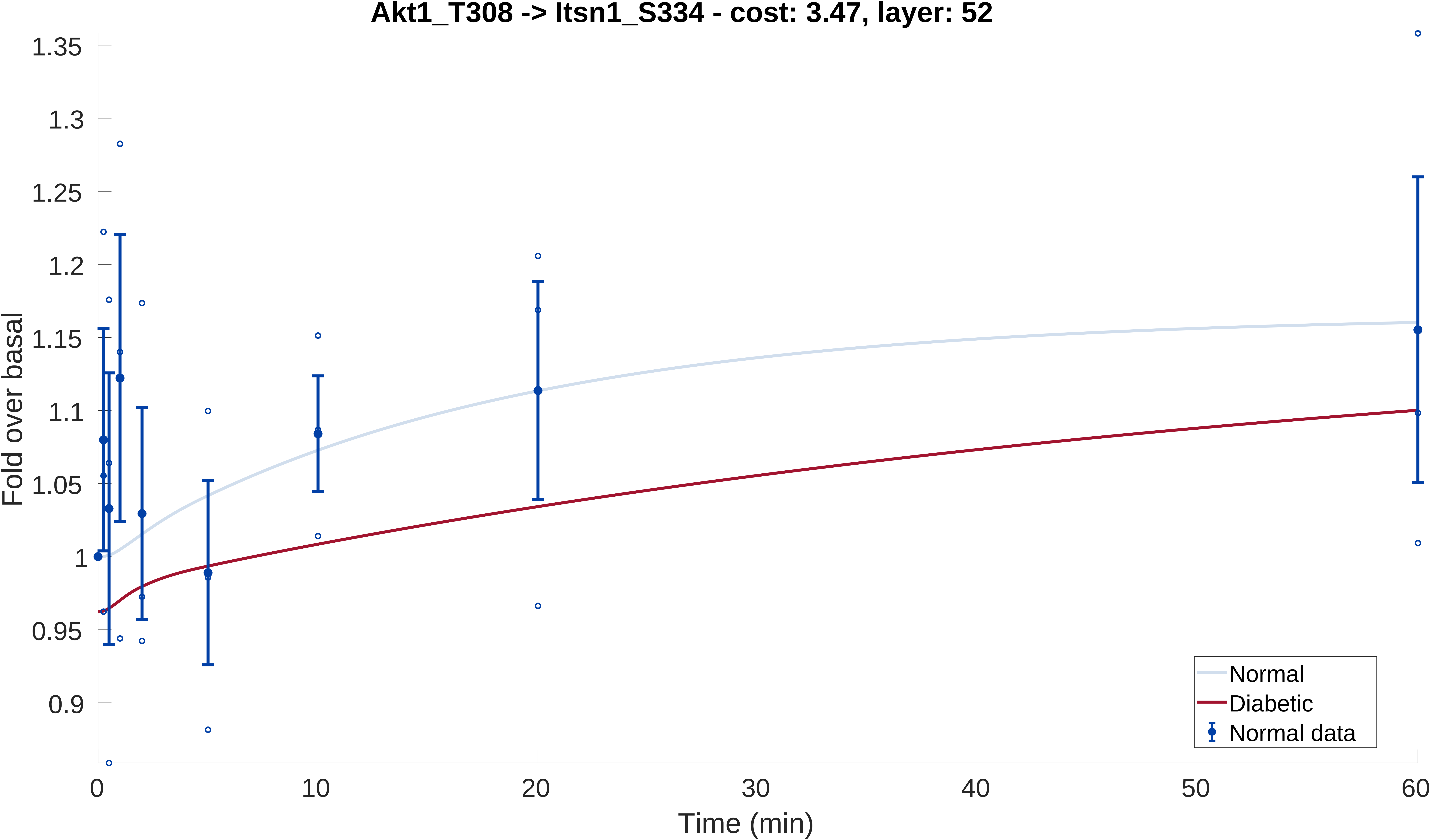

Akt1_T308 -> Itsn1_S334 - cost: 3.47, layer: 52
1.35
1.3
1.25
1.2
1.15
1.1
1.05
1
0.95
0.9
Fold over basal
0
10
20
30
40
50
60
Time (min)
Normal
Diabetic
Normal data

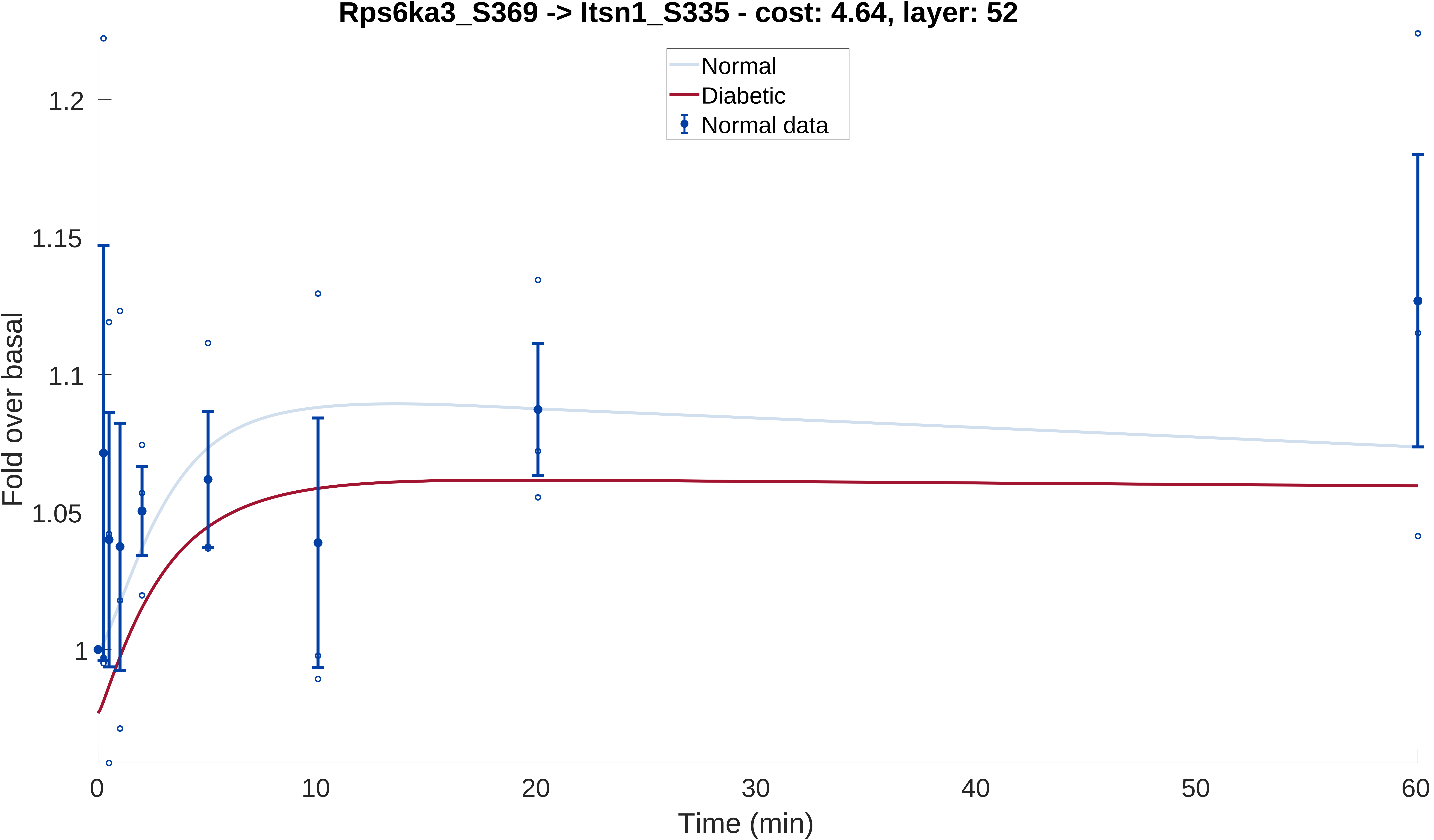

Rps6ka3_S369 -> Itsn1_S335 - cost: 4.64, layer: 52
Normal
Diabetic
Normal data
Fold over basal
1.2
1.15
1.1
1.05
1
0
10
20
30
40
50
60
Time (min)

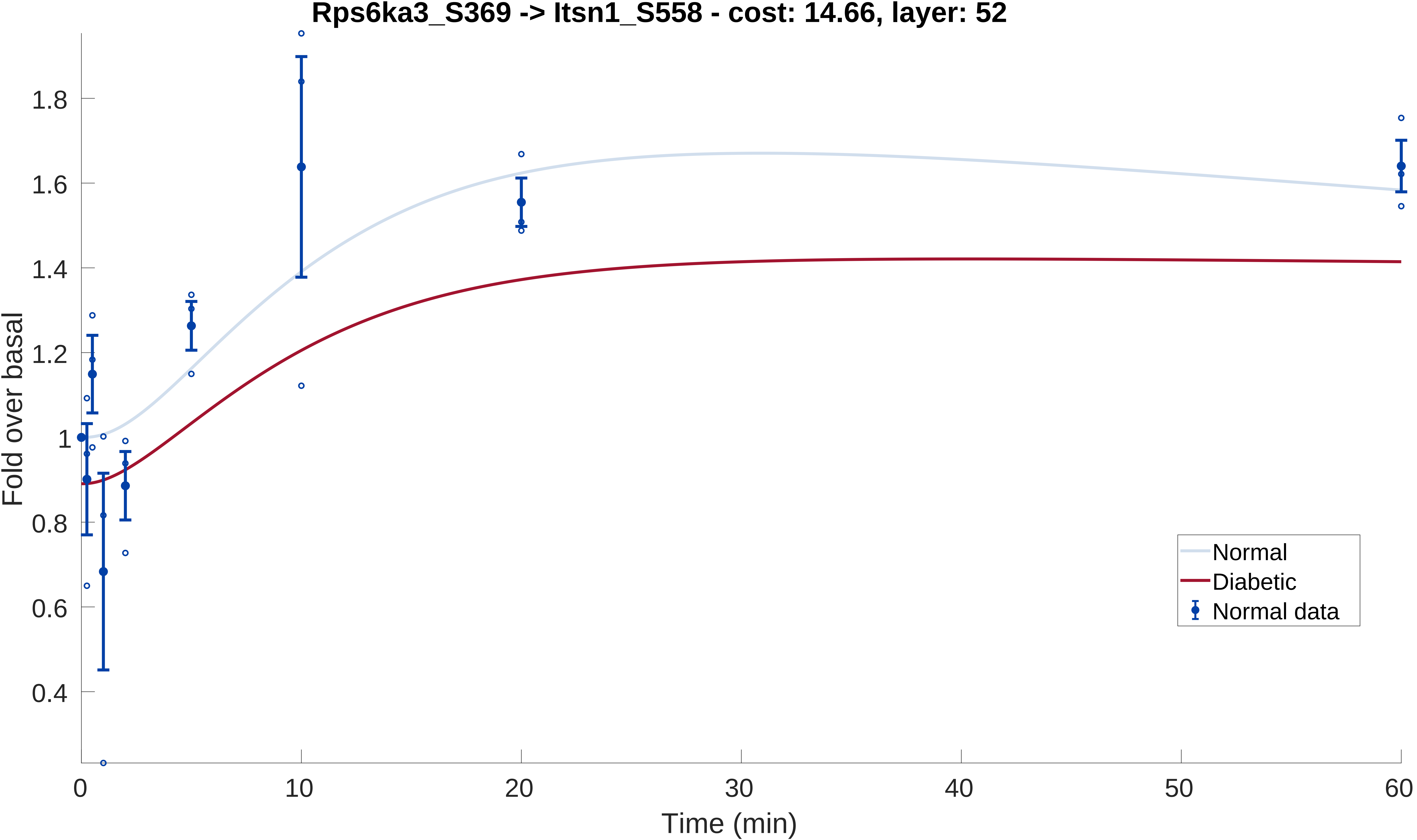

Rps6ka3_S369 -> Itsn1_S558 - cost: 14.66, layer: 52
Fold over basal
Time (min)
0.4
0.6
0.8
1
1.2
1.4
1.6
1.8
0
10
20
30
40
50
60
Normal
Diabetic
Normal data

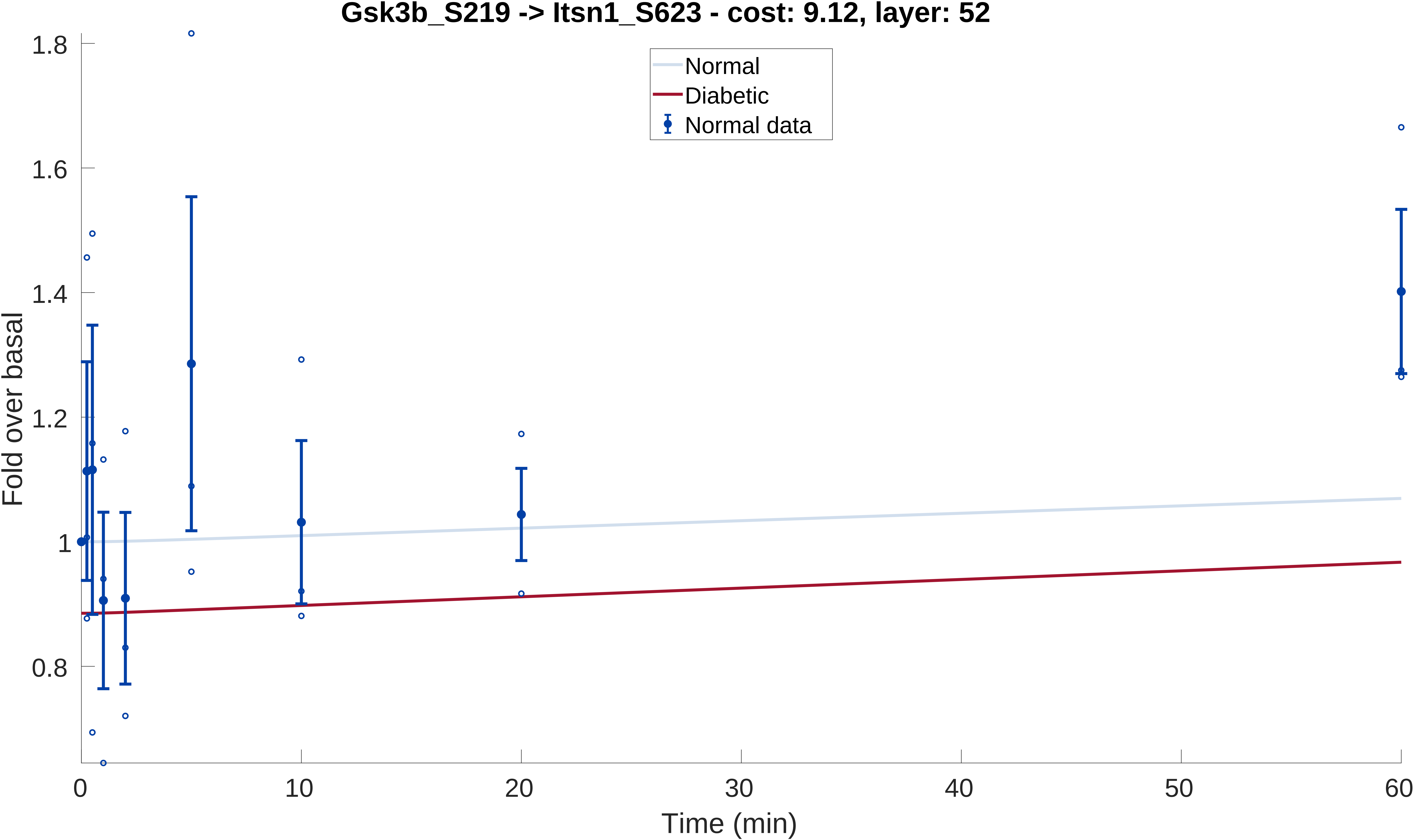

Gsk3b_S219 -> Itsn1_S623 - cost: 9.12, layer: 52
Normal
Diabetic
Normal data
Fold over basal
Time (min)
1.8
1.6
1.4
1.2
1
0.8
0
10
20
30
40
50
60

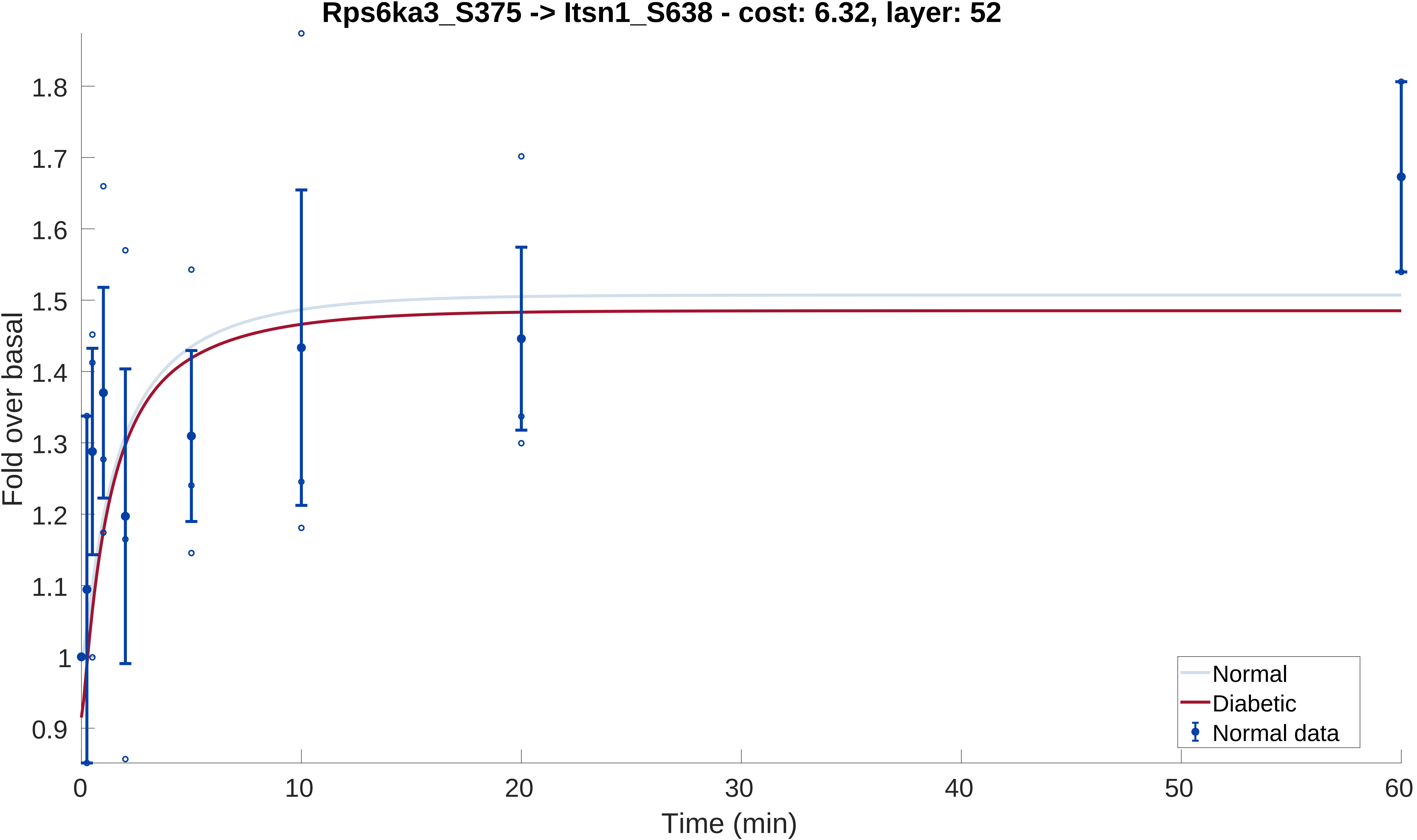
Rps6ka3_S375 -> Itsn1_S638 - cost: 6.32, layer: 52
Fold over basal
Time (min)
Normal
Diabetic
Normal data
0.9
1
1.1
1.2
1.3
1.4
1.5
1.6
1.7
1.8
0
10
20
30
40
50
60

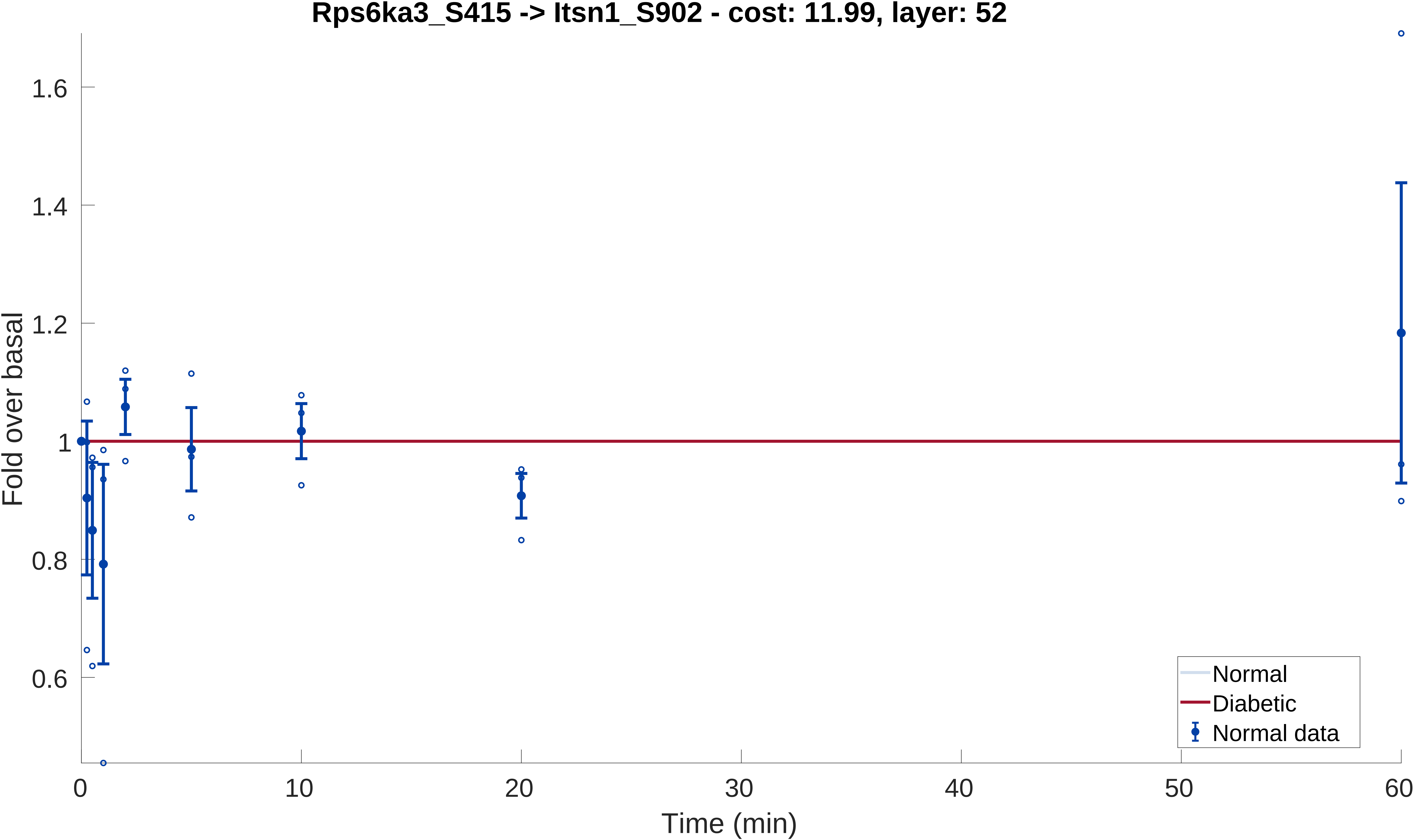

Rps6ka3_S415 -> Itsn1_S902 - cost: 11.99, layer: 52
Fold over basal
Time (min)
0.6
0.8
1
1.2
1.4
1.6
0
10
20
30
40
50
60
Normal
Diabetic
Normal data

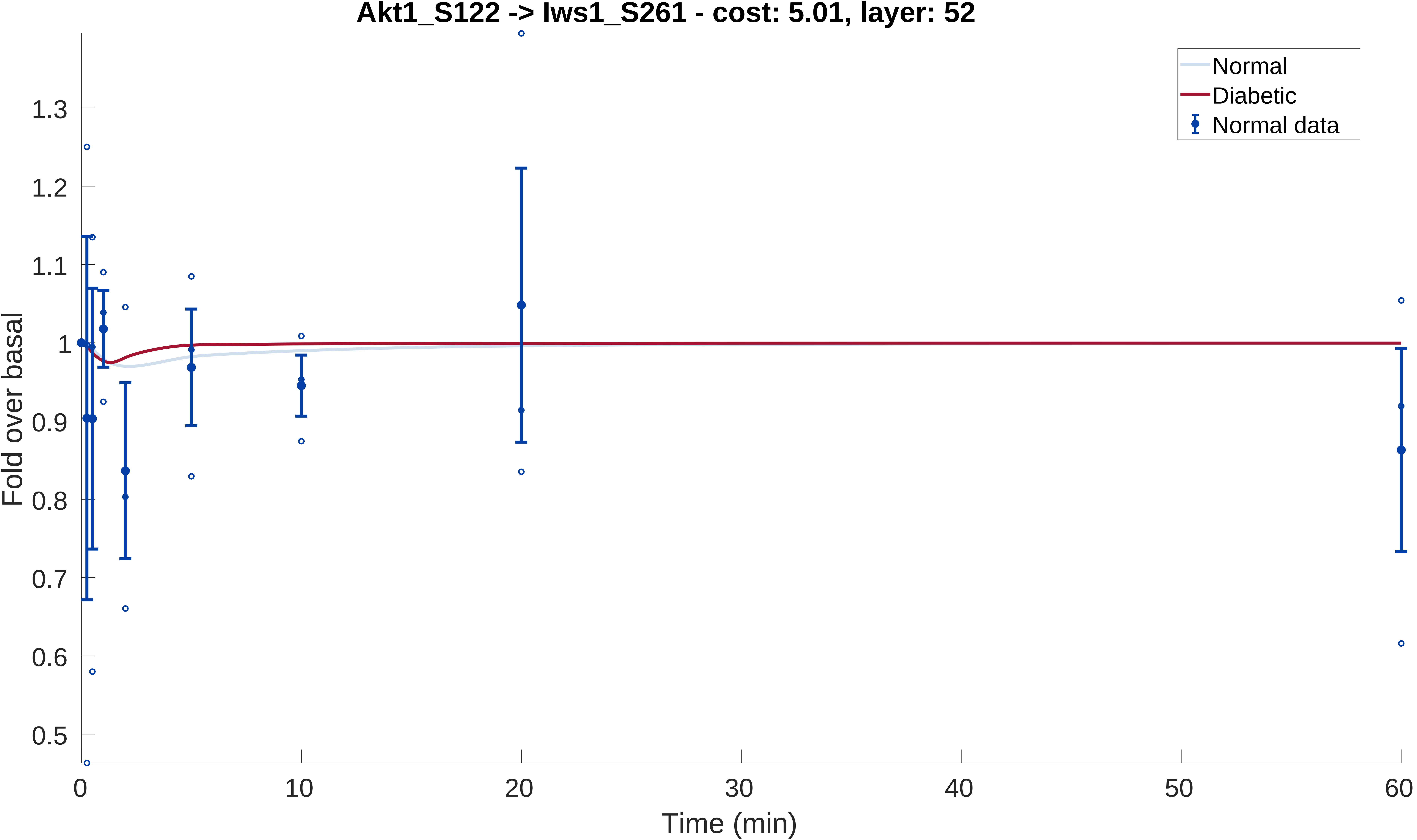
Akt1_S122 -> Iws1_S261 - cost: 5.01, layer: 52
Normal
Diabetic
Normal data
Fold over basal
Time (min)
1.3
1.2
1.1
1
0.9
0.8
0.7
0.6
0.5
0
10
20
30
40
50
60

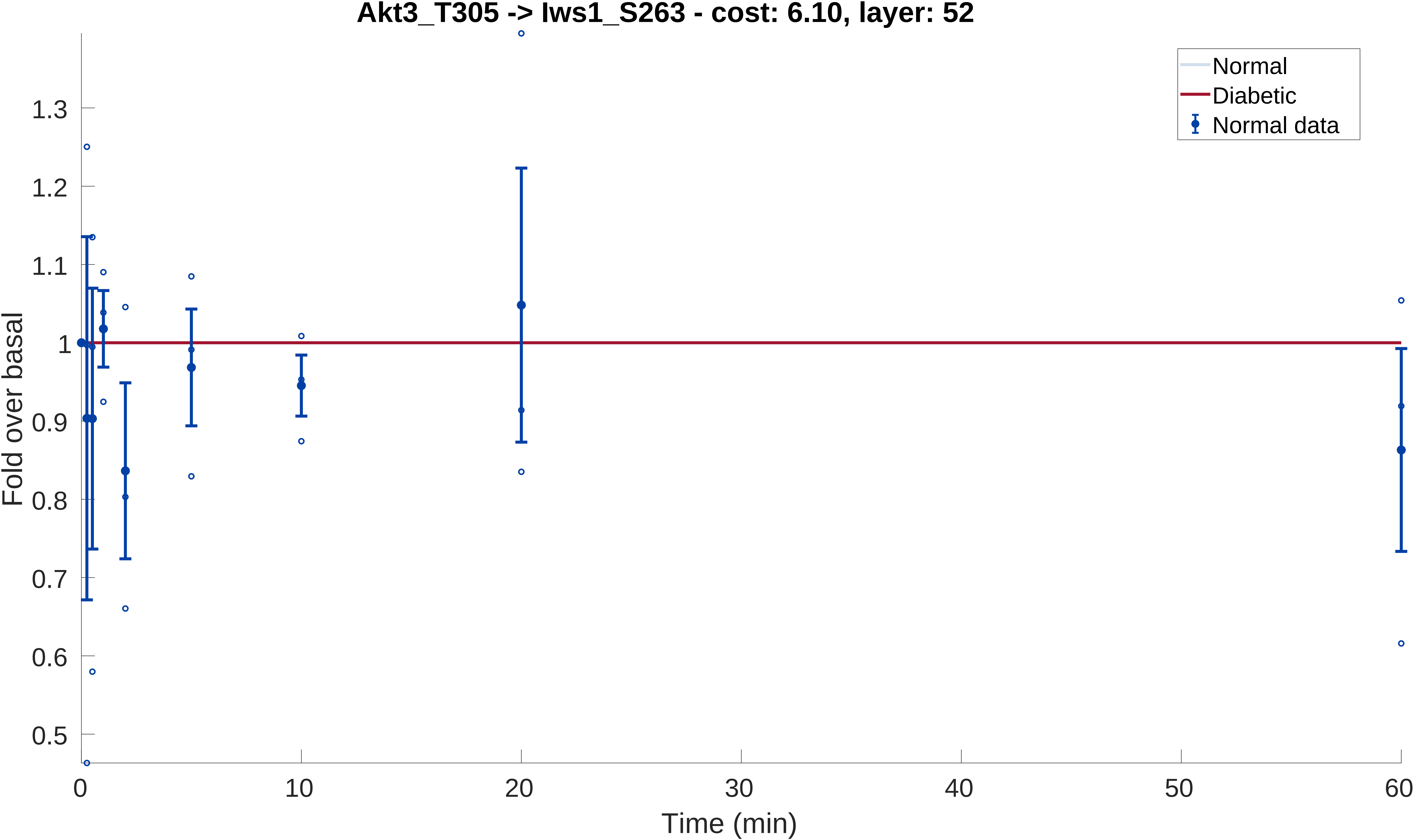

Akt3_T305 -> Iws1_S263 - cost: 6.10, layer: 52
Normal
Diabetic
Normal data
Fold over basal
Time (min)
1.3
1.2
1.1
1
0.9
0.8
0.7
0.6
0.5
0
10
20
30
40
50
60

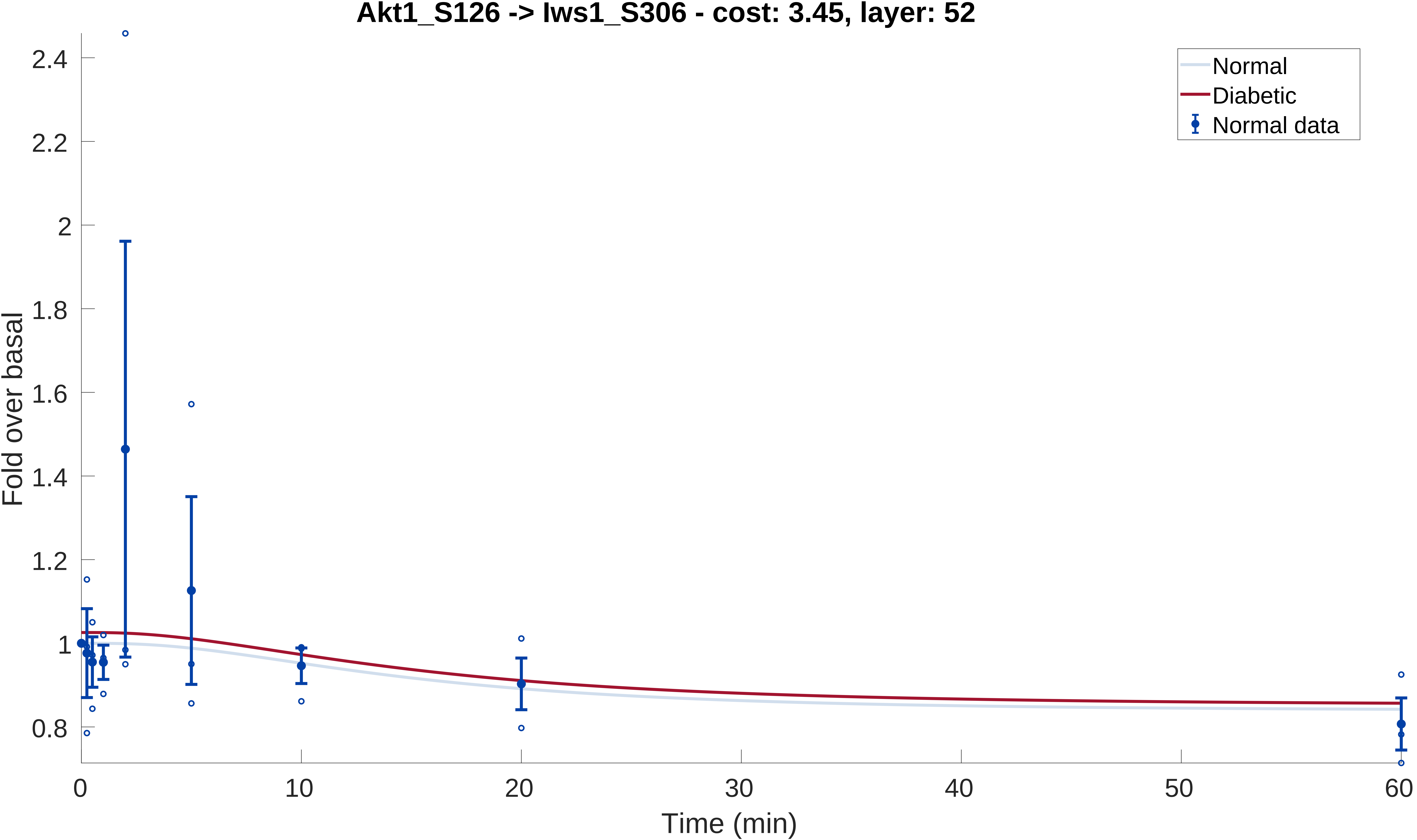
Akt1_S126 -> Iws1_S306 - cost: 3.45, layer: 52
Fold over basal
2.4
2.2
2
1.8
1.6
1.4
1.2
1
0.8
0
10
20
30
40
50
60
Time (min)
Normal
Diabetic
Normal data

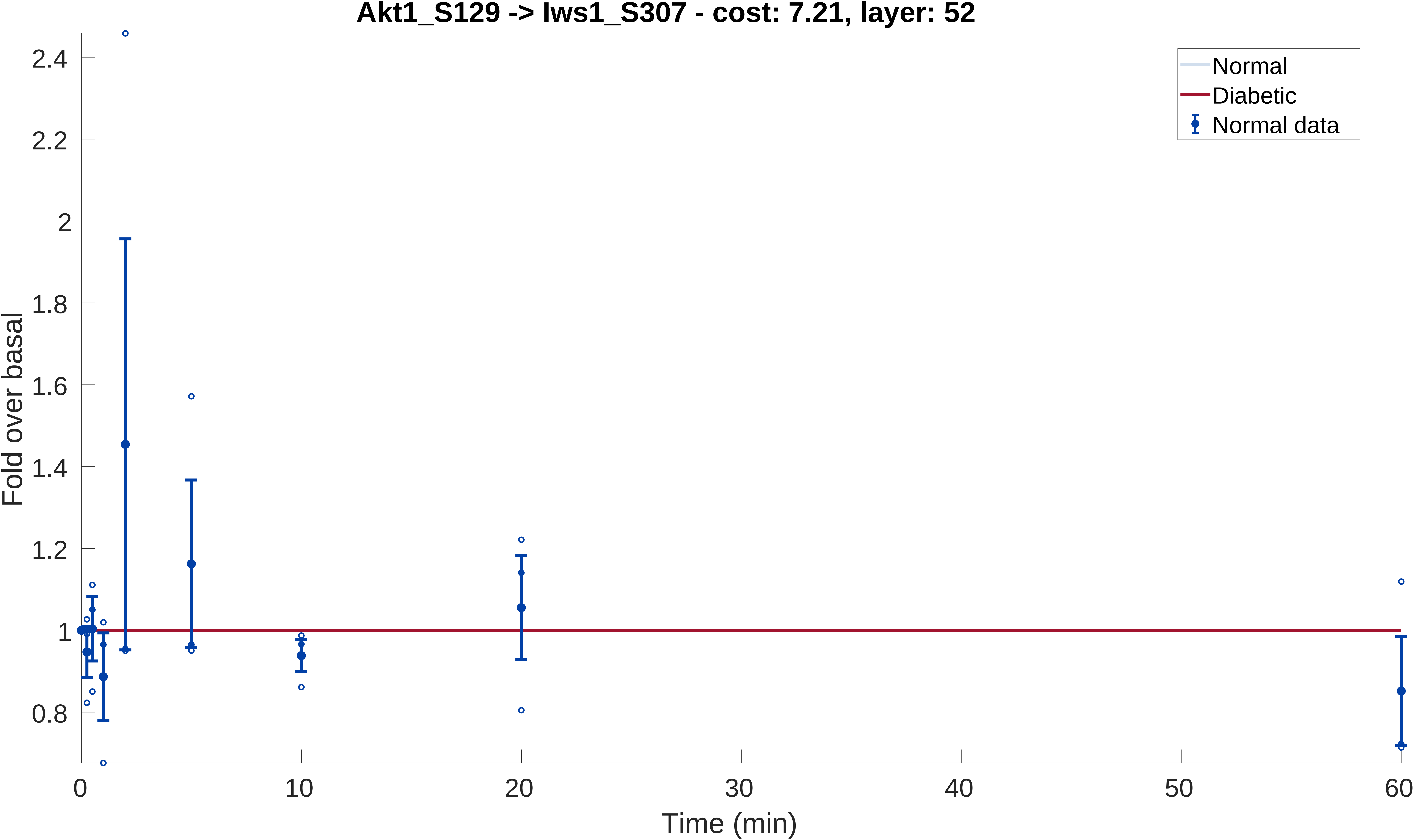
Akt1_S129 -> Iws1_S307 - cost: 7.21, layer: 52
Fold over basal
Time (min)
Normal
Diabetic
Normal data
2.4
2.2
2
1.8
1.6
1.4
1.2
1
0.8
0
10
20
30
40
50
60

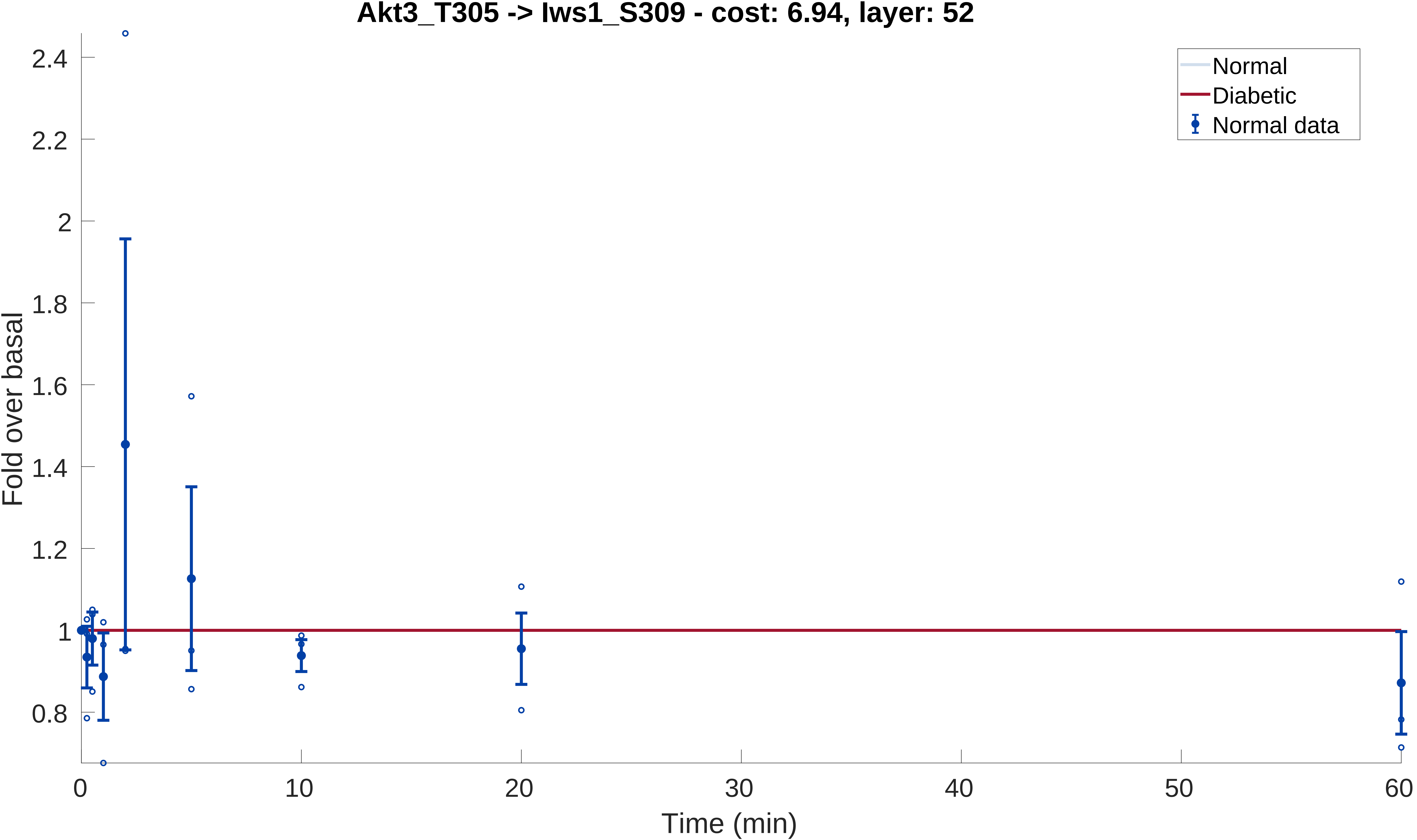

Akt3_T305 -> Iws1_S309 - cost: 6.94, layer: 52
Normal
Diabetic
Normal data
Fold over basal
Time (min)
2.4
2.2
2
1.8
1.6
1.4
1.2
1
0.8
0
10
20
30
40
50
60

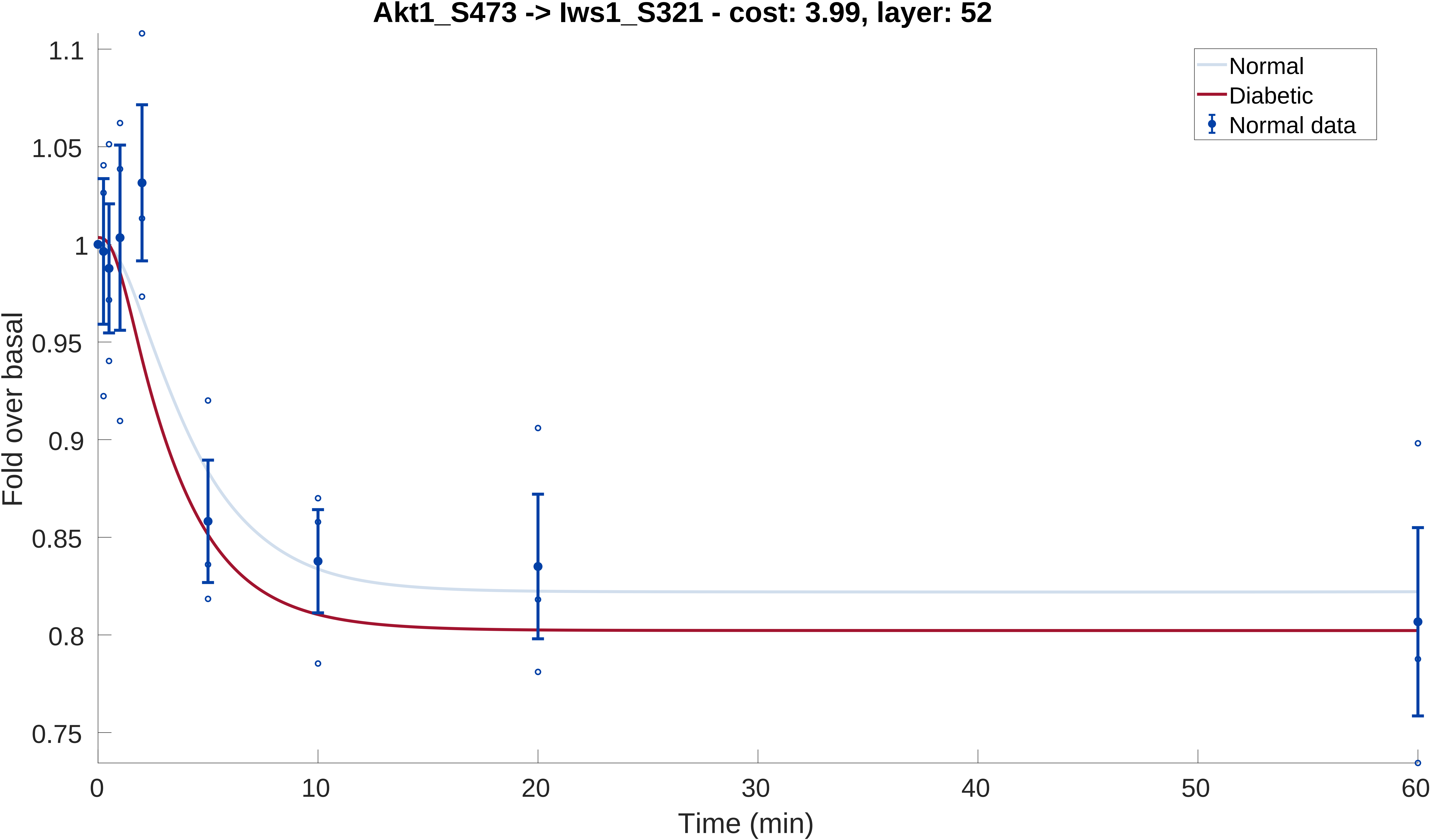

Akt1_S473 -> Iws1_S321 - cost: 3.99, layer: 52
Normal
Diabetic
Normal data
Fold over basal
1.1
1.05
1
0.95
0.9
0.85
0.8
0.75
0
10
20
30
40
50
60
Time (min)

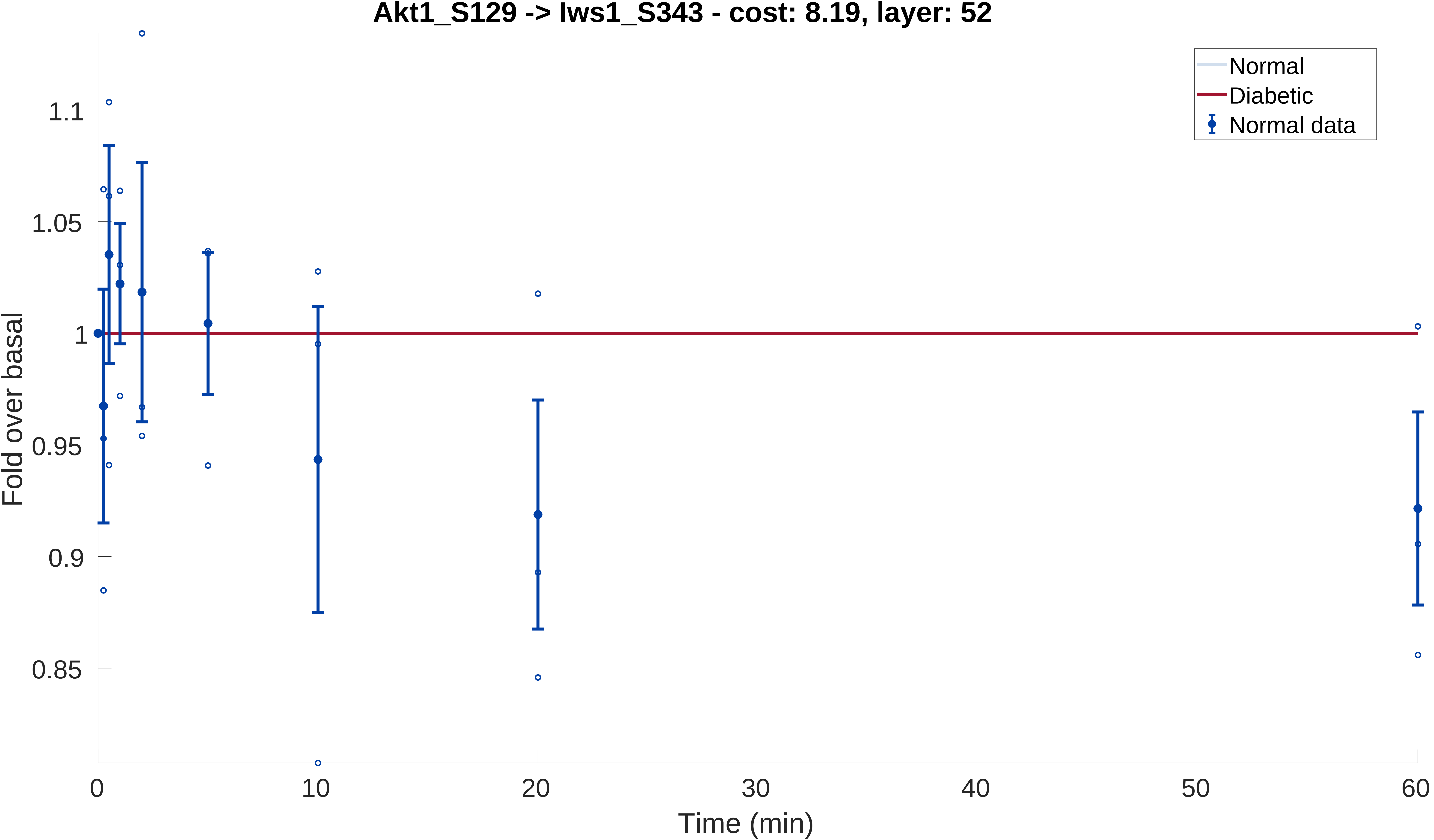

Akt1_S129 -> Iws1_S343 - cost: 8.19, layer: 52
Normal
Diabetic
Normal data
Fold over basal
1.1
1.05
1
0.95
0.9
0.85
0
10
20
30
40
50
60
Time (min)

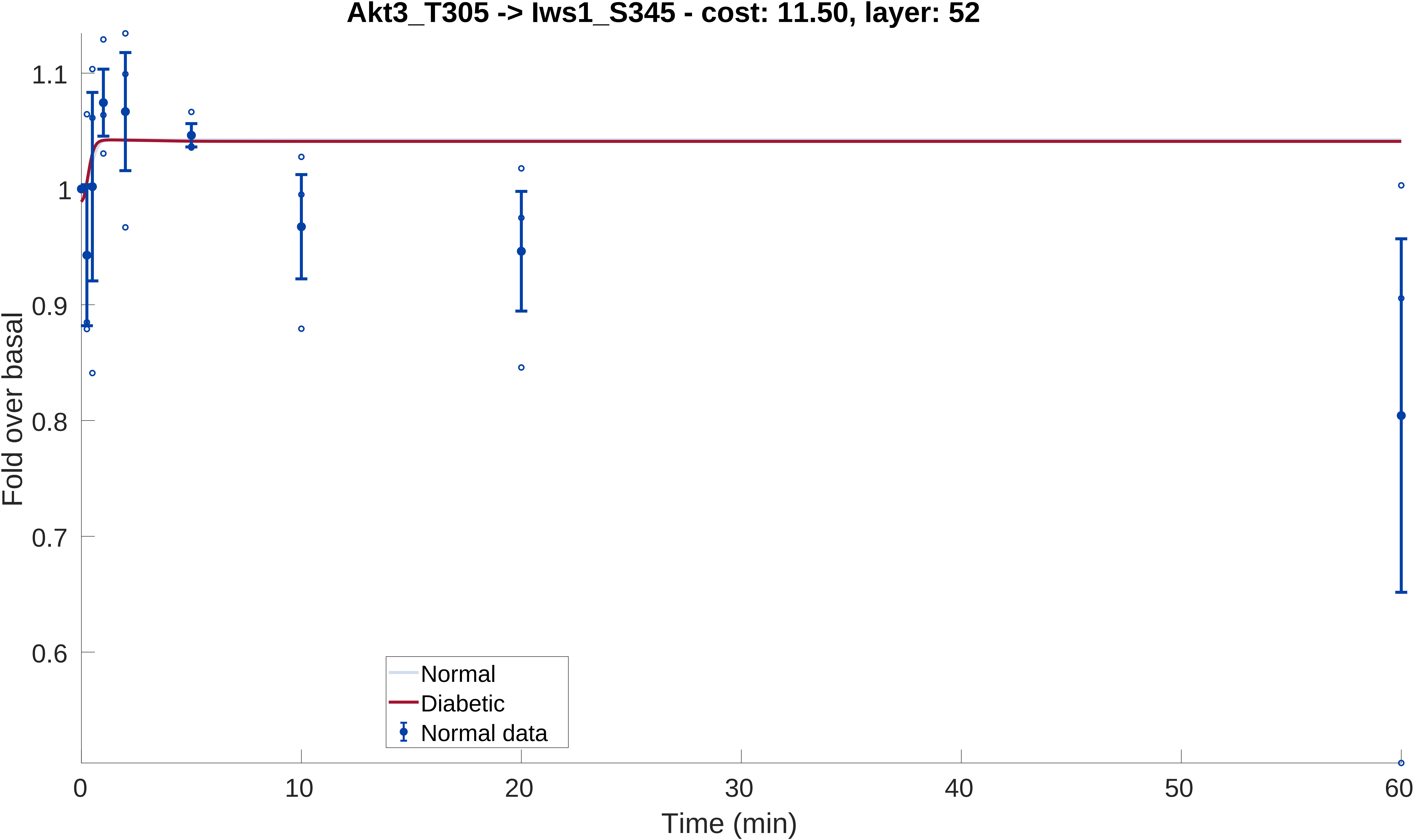

Akt3_T305 -> Iws1_S345 - cost: 11.50, layer: 52
1.1
1
0.9
0.8
0.7
0.6
Fold over basal
0
10
20
30
40
50
60
Time (min)
Normal
Diabetic
Normal data

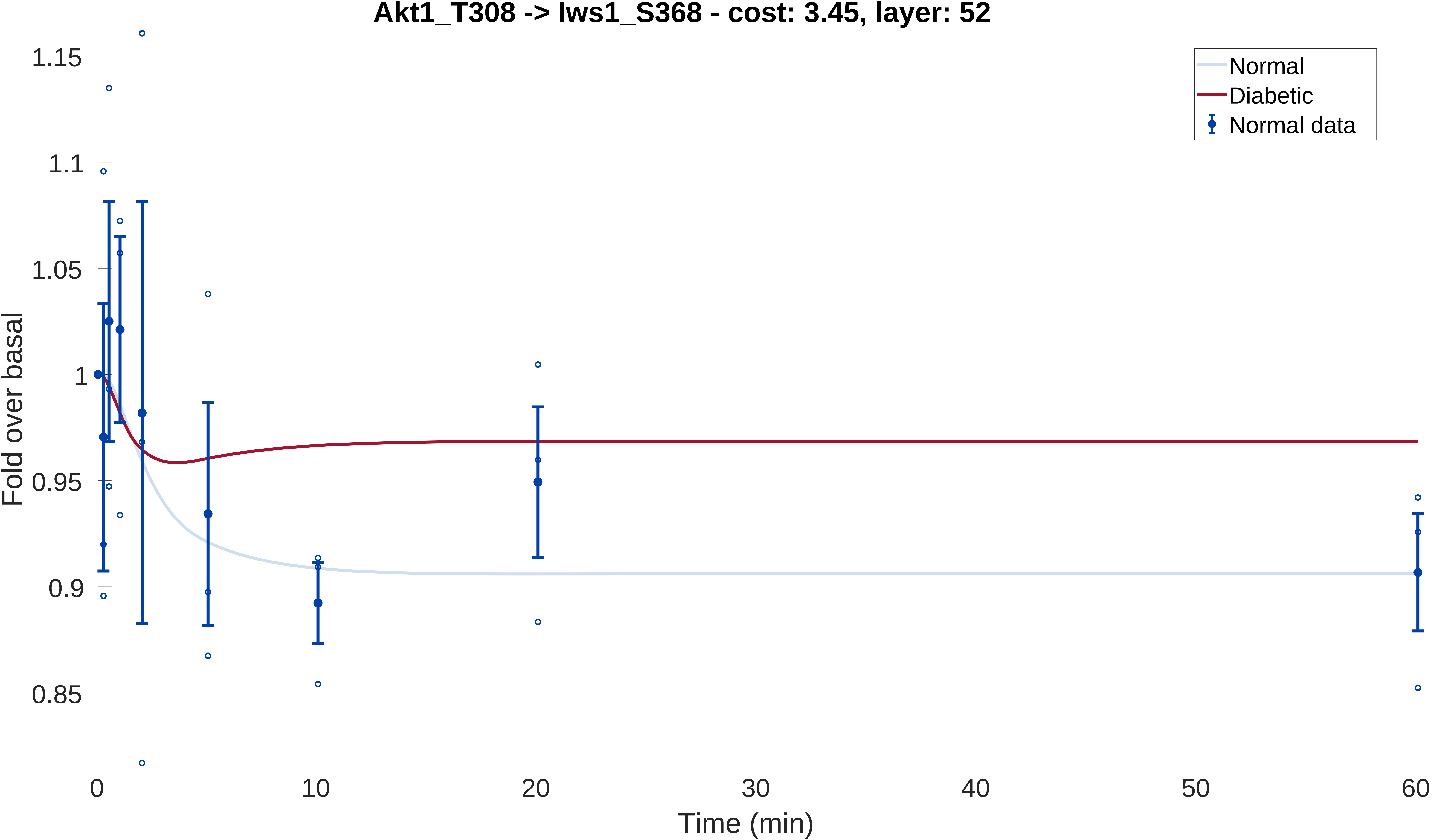
Akt1_T308 -> Iws1_S368 - cost: 3.45, layer: 52
Fold over basal
Time (min)
Normal
Diabetic
Normal data
1.15
1.1
1.05
1
0.95
0.9
0.85
0
10
20
30
40
50
60

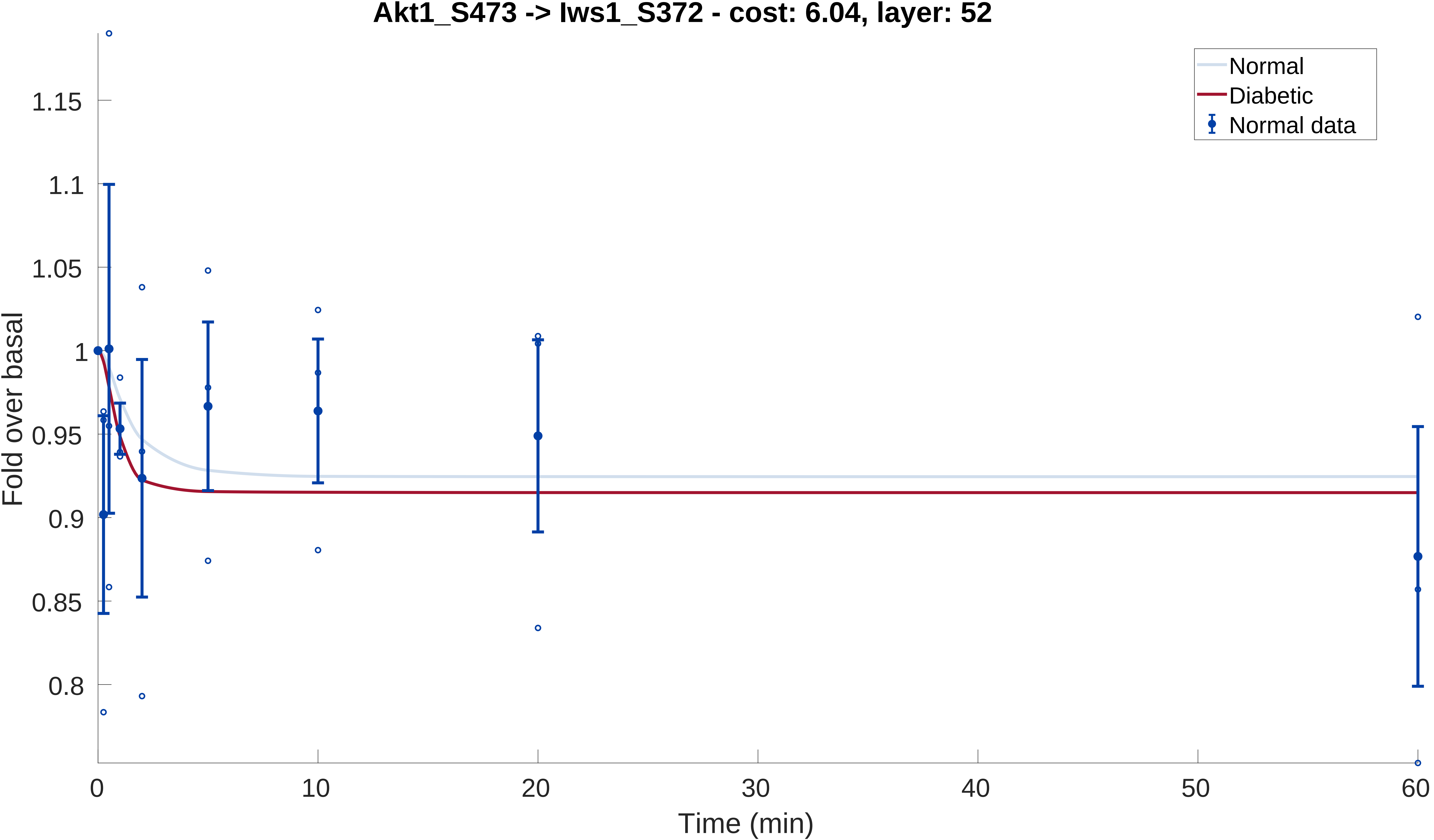
Akt1_S473 -> Iws1_S372 - cost: 6.04, layer: 52
Normal
Diabetic
Normal data
Fold over basal
1.15
1.1
1.05
1
0.95
0.9
0.85
0.8
0
10
20
30
40
50
60
Time (min)

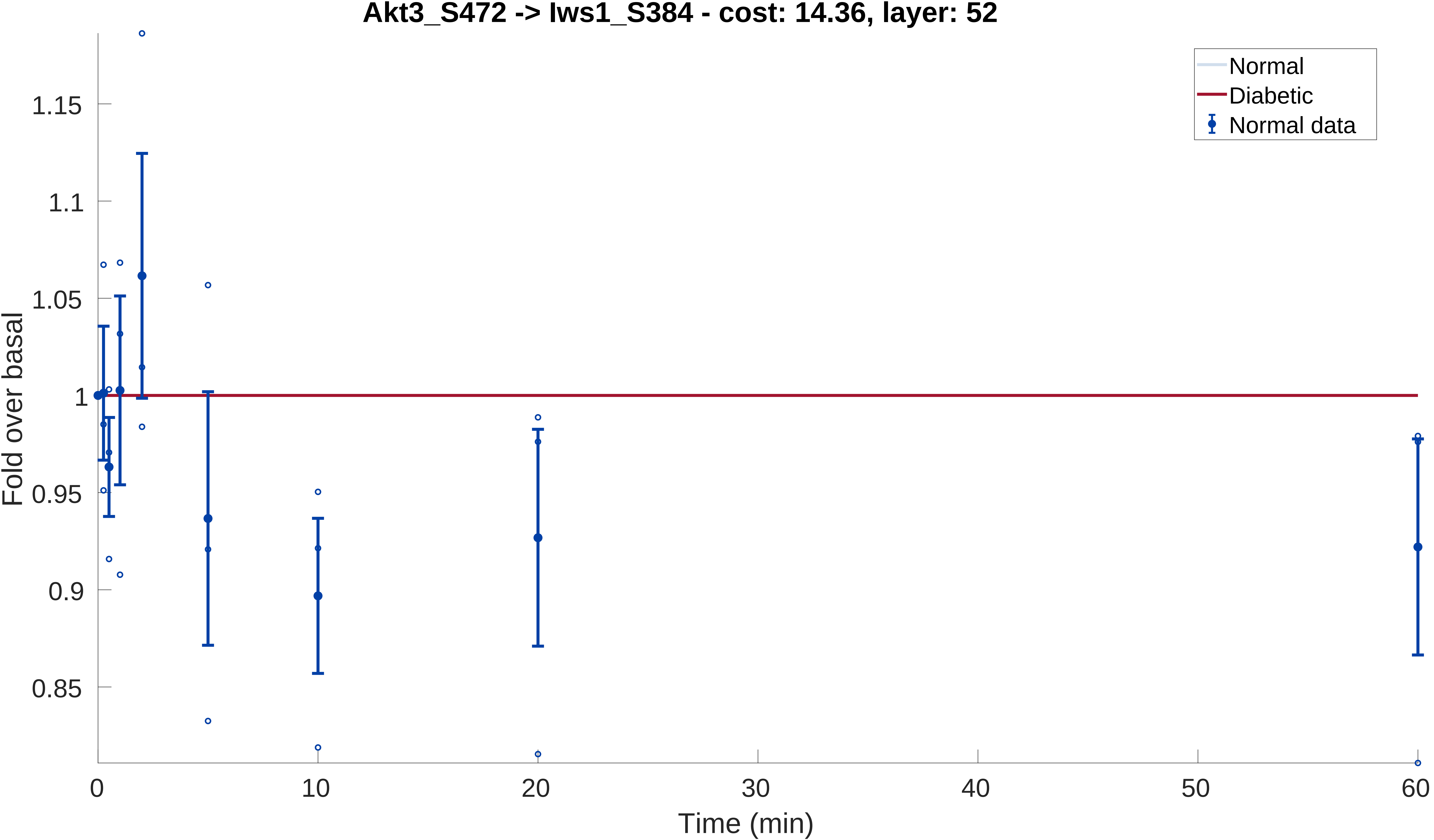

Akt3_S472 -> Iws1_S384 - cost: 14.36, layer: 52
Normal
Diabetic
Normal data
Fold over basal
1.15
1.1
1.05
1
0.95
0.9
0.85
0
10
20
30
40
50
60
Time (min)

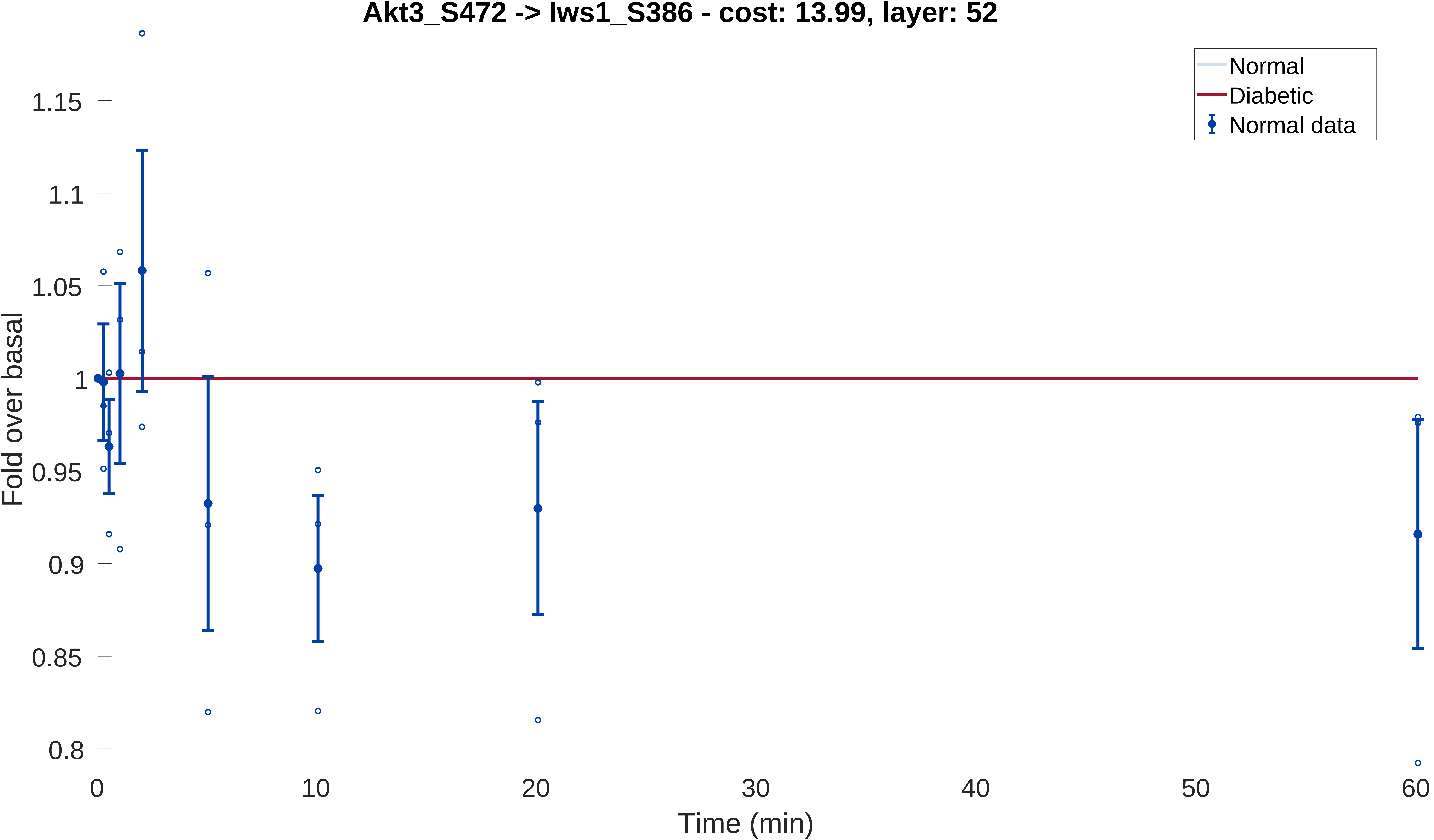

Akt3_S472 -> Iws1_S386 - cost: 13.99, layer: 52
Normal
Diabetic
Normal data
Fold over basal
1.15
1.1
1.05
1
0.95
0.9
0.85
0.8
0
10
20
30
40
50
60
Time (min)

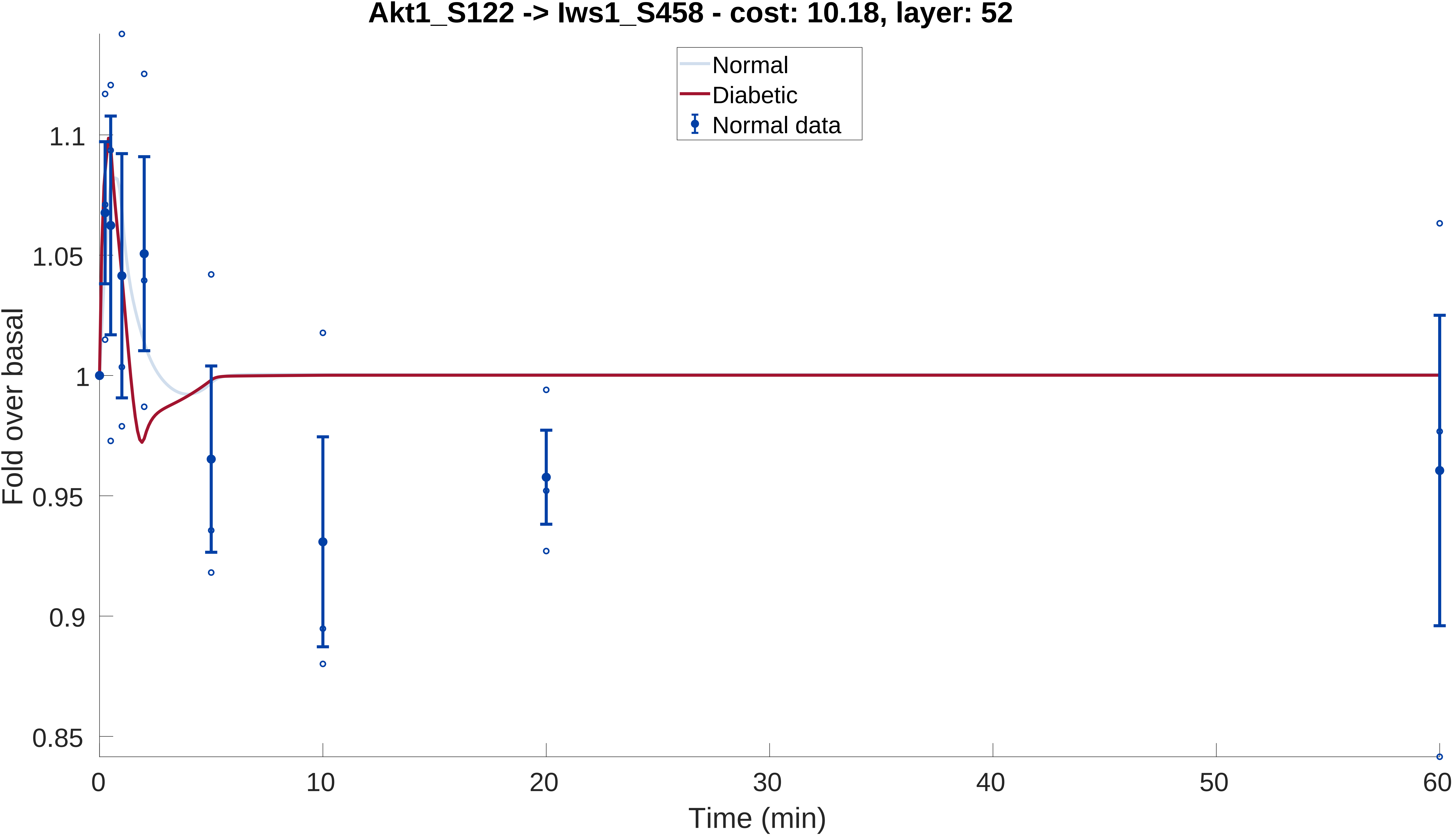
Akt1_S122 -> Iws1_S458 - cost: 10.18, layer: 52
Normal
Diabetic
Normal data
1.1
1.05
1
0.95
0.9
0.85
Fold over basal
0
10
20
30
40
50
60
Time (min)

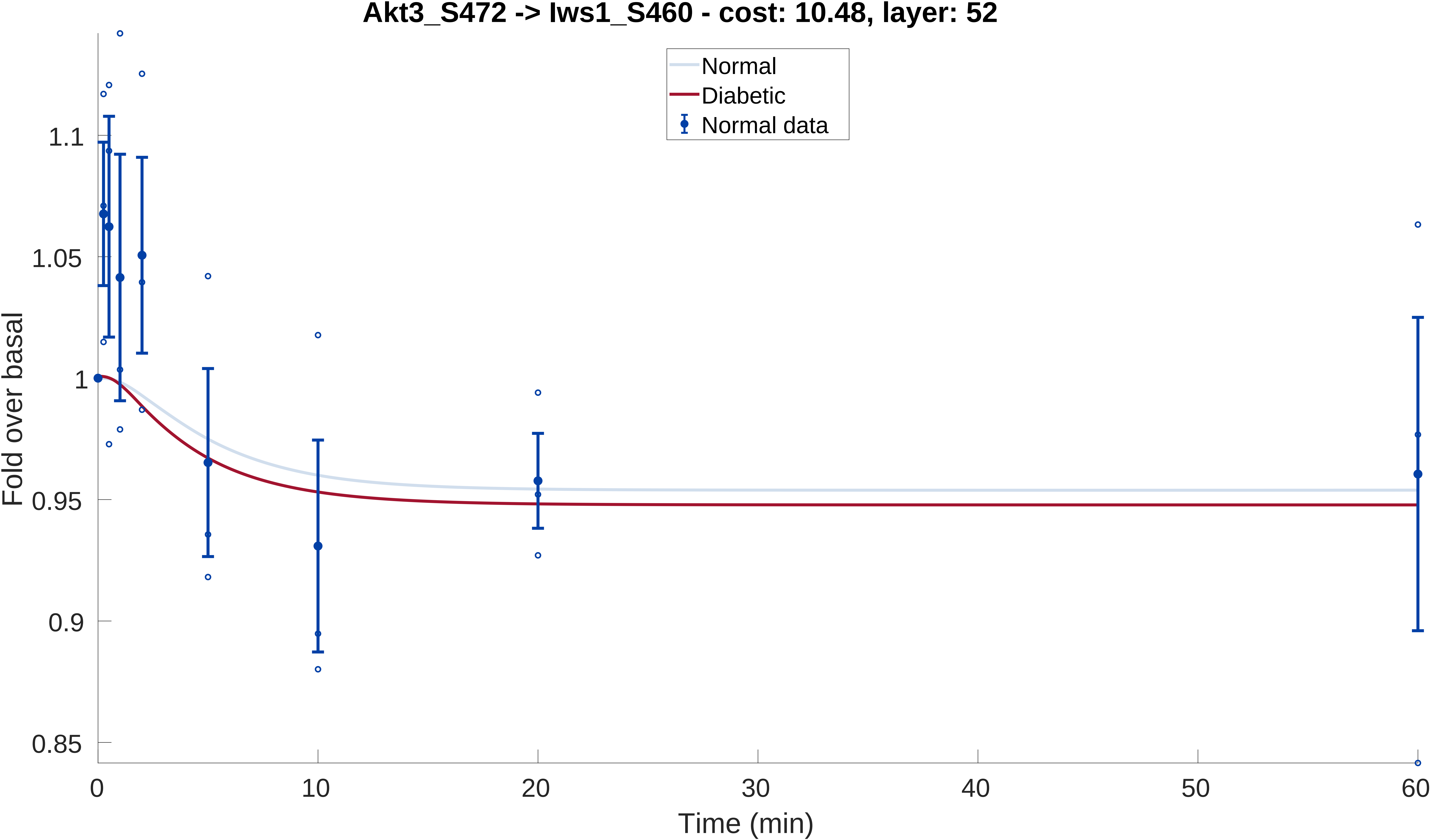

Akt3_S472 -> Iws1_S460 - cost: 10.48, layer: 52
Normal
Diabetic
Normal data
1.1
1.05
1
0.95
0.9
0.85
Fold over basal
0
10
20
30
40
50
60
Time (min)

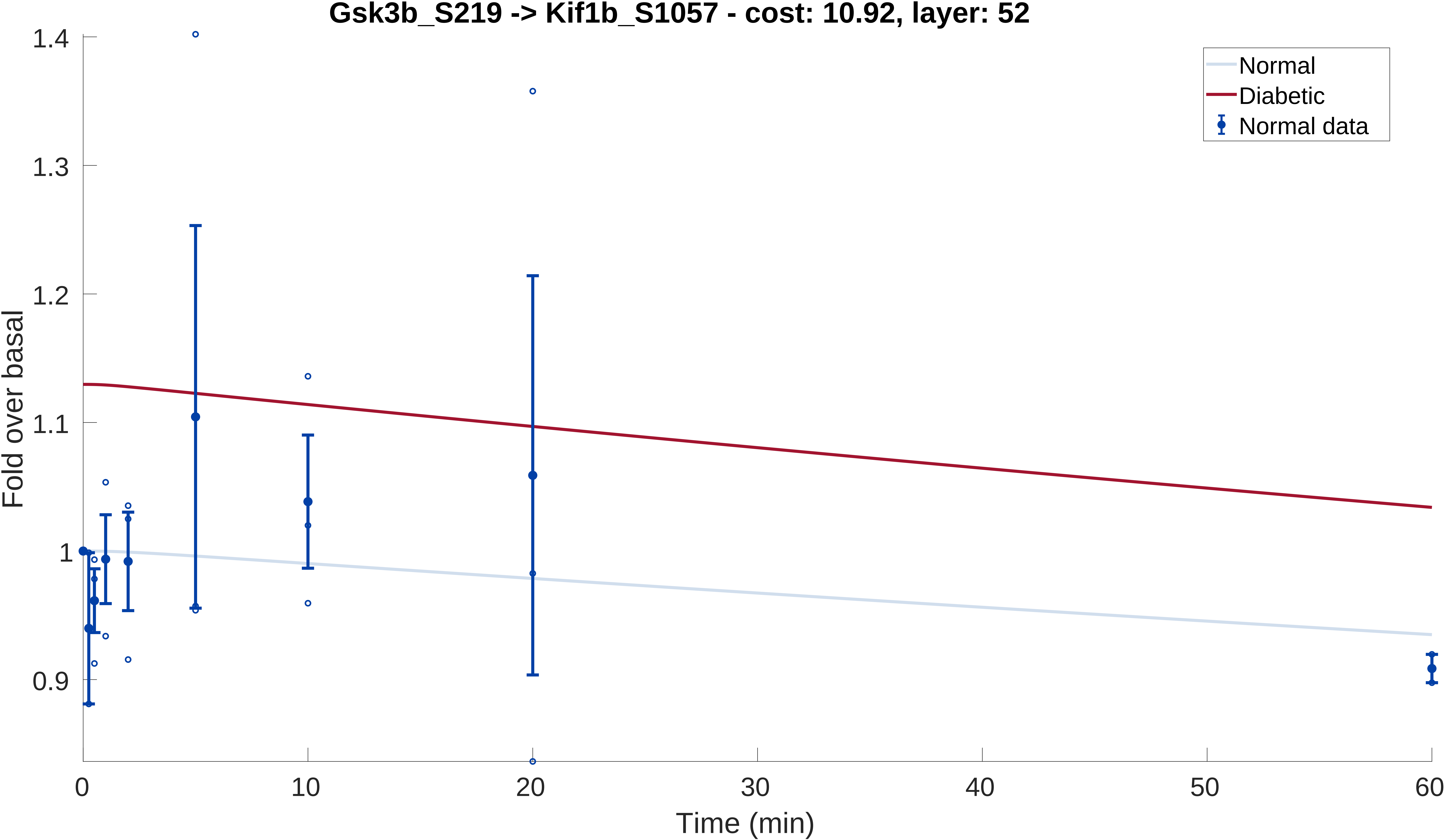

Gsk3b_S219 -> Kif1b_S1057 - cost: 10.92, layer: 52
Fold over basal
Time (min)
Normal
Diabetic
Normal data
1.4
1.3
1.2
1.1
1
0.9
0
10
20
30
40
50
60

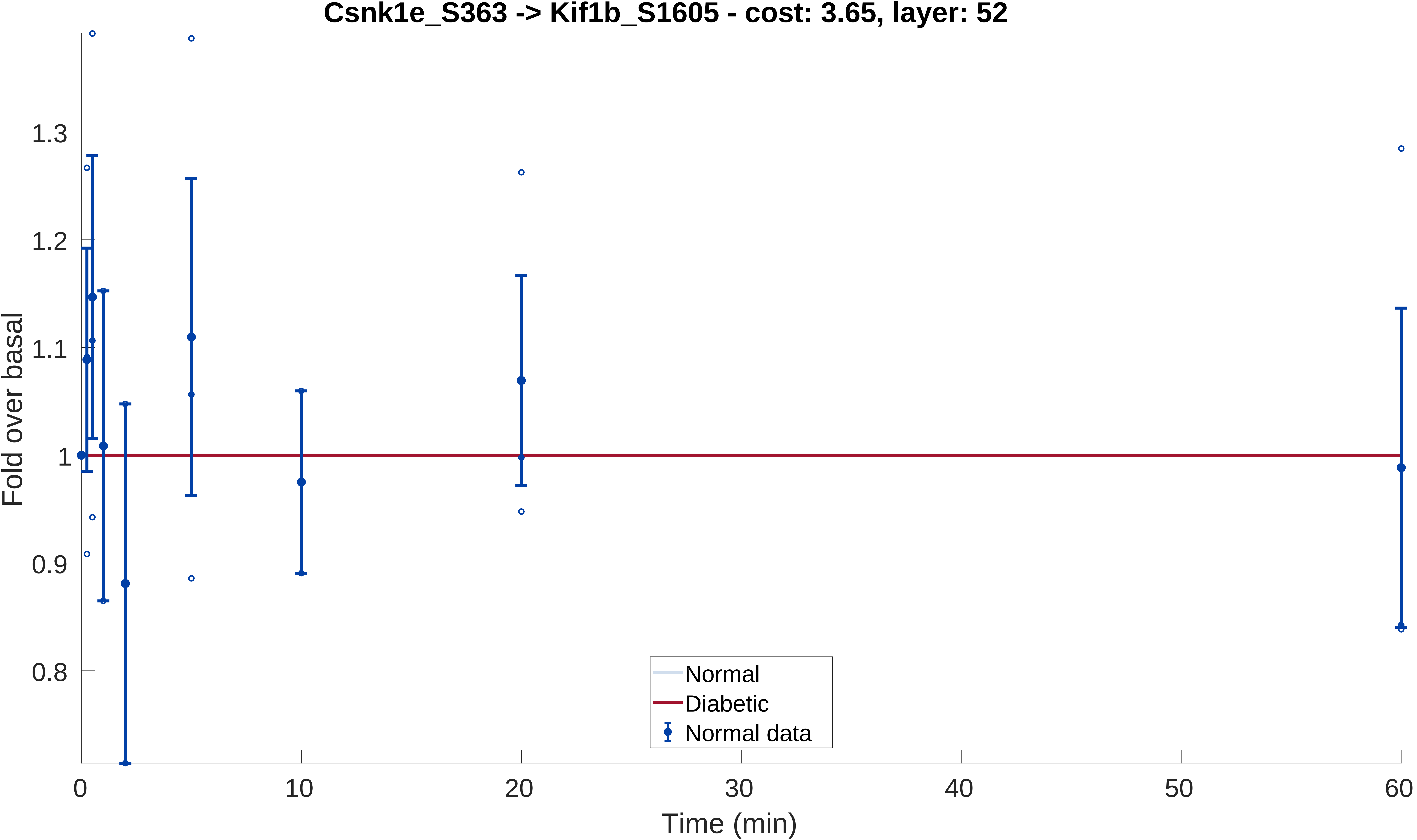
Csnk1e_S363 -> Kif1b_S1605 - cost: 3.65, layer: 52
Fold over basal
1.3
1.2
1.1
1
0.9
0.8
0
10
20
30
40
50
60
Time (min)
Normal
Diabetic
Normal data

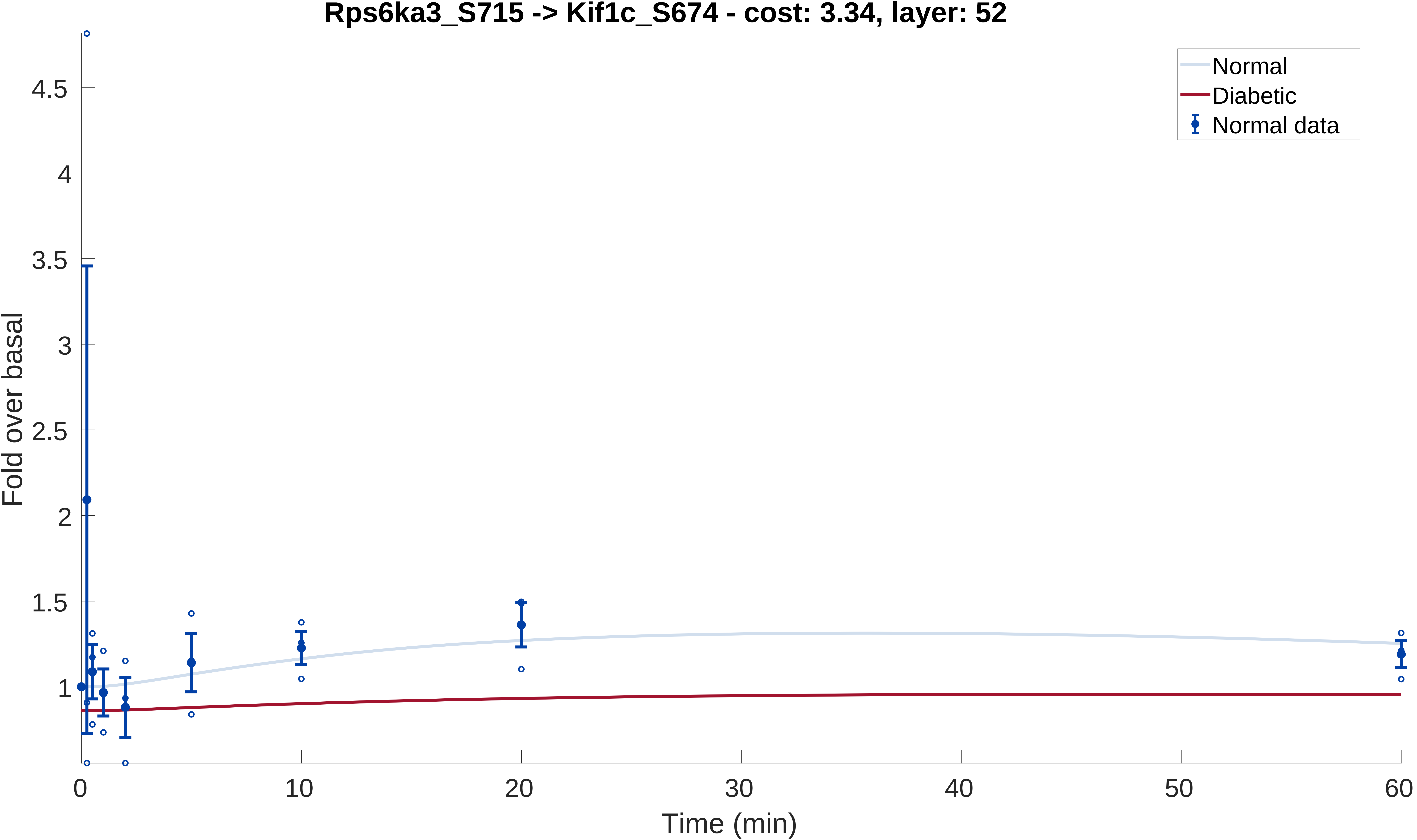
Rps6ka3_S715 -> Kif1c_S674 - cost: 3.34, layer: 52
Normal
Diabetic
Normal data
Fold over basal
4.5
4
3.5
3
2.5
2
1.5
1
0
10
20
30
40
50
60
Time (min)

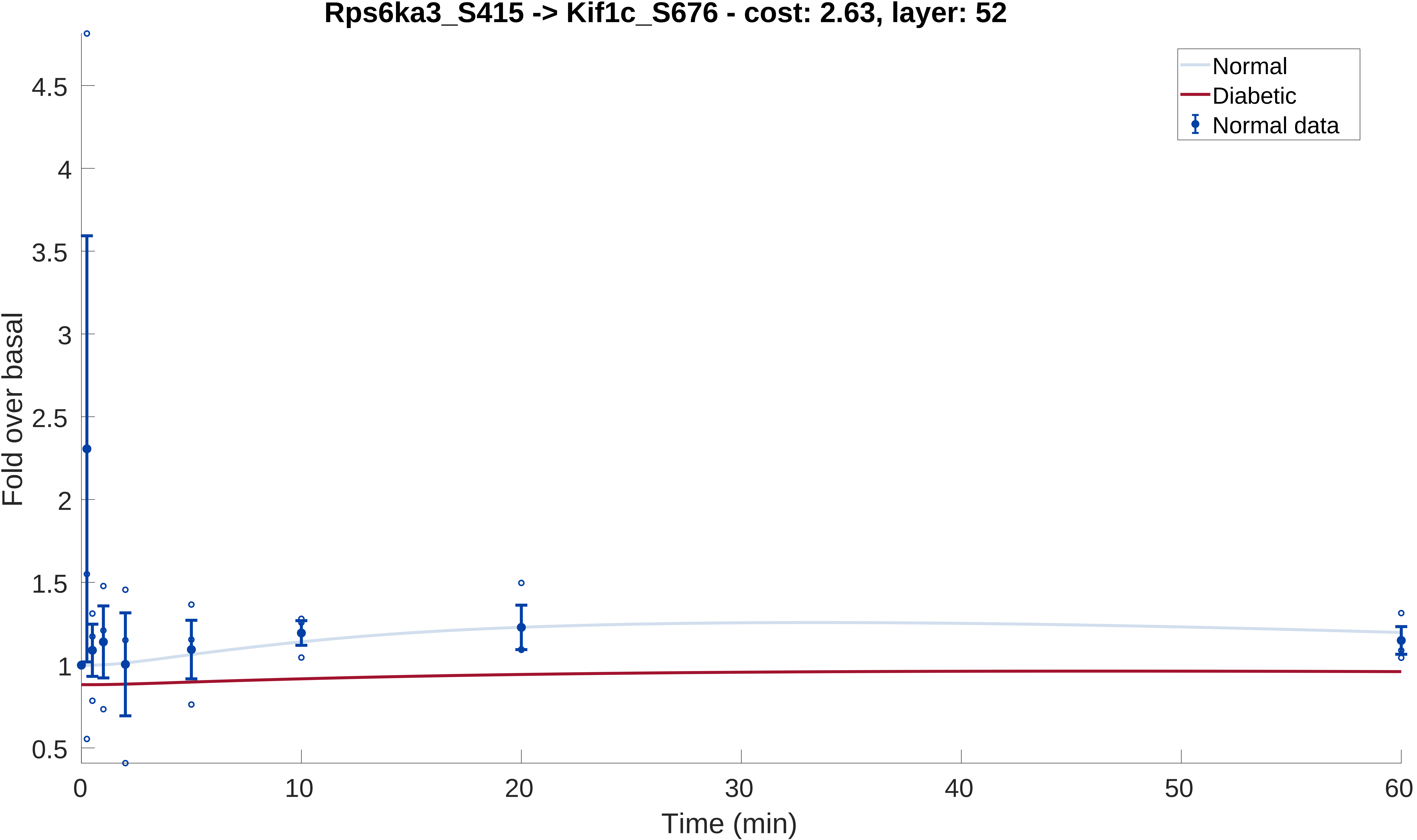

Rps6ka3_S415 -> Kif1c_S676 - cost: 2.63, layer: 52
Normal
Diabetic
Normal data
Fold over basal
Time (min)
0.5
1
1.5
2
2.5
3
3.5
4
4.5
0
10
20
30
40
50
60

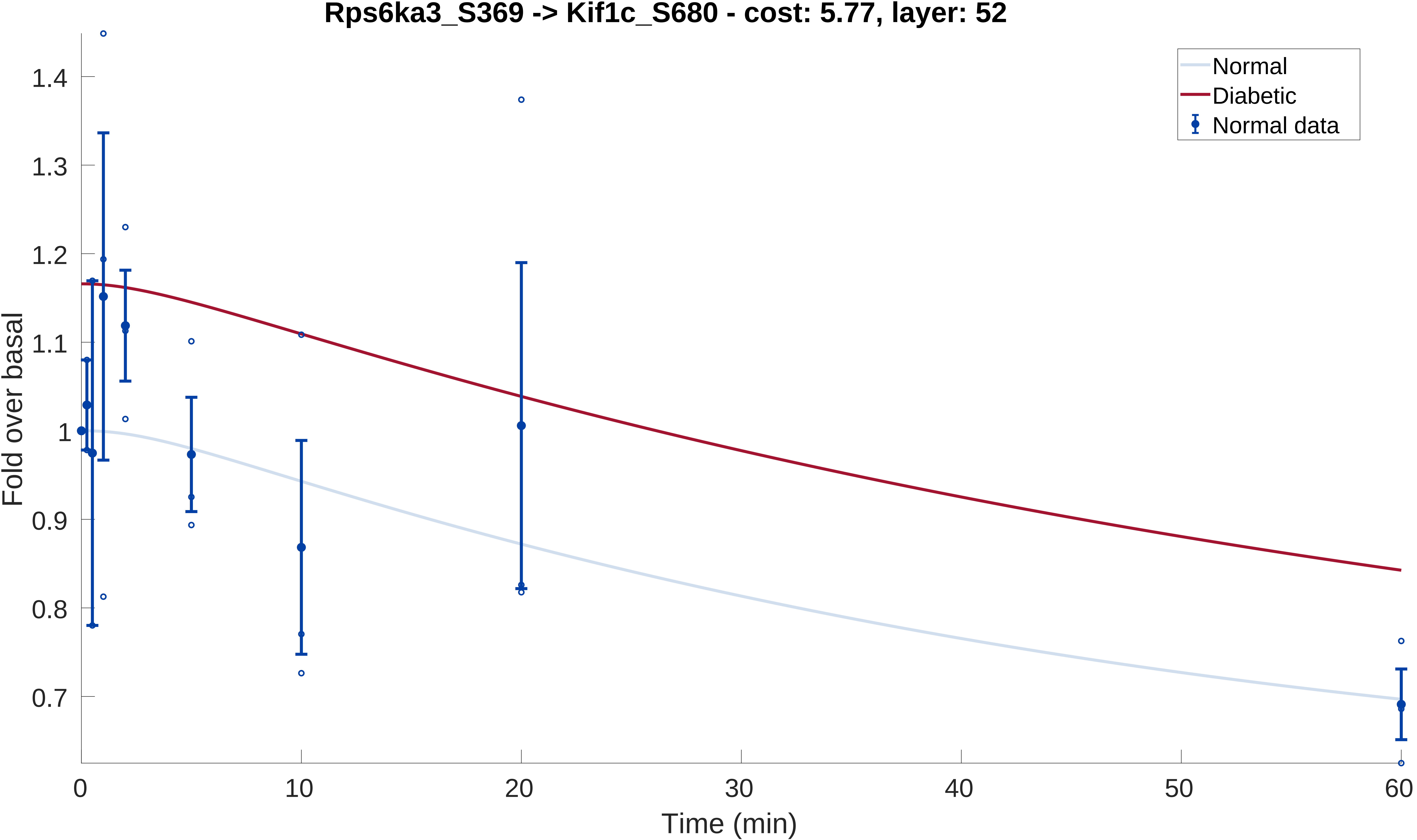

Rps6ka3_S369 -> Kif1c_S680 - cost: 5.77, layer: 52
Normal
Diabetic
Normal data
Fold over basal
1.4
1.3
1.2
1.1
1
0.9
0.8
0.7
0
10
20
30
40
50
60
Time (min)

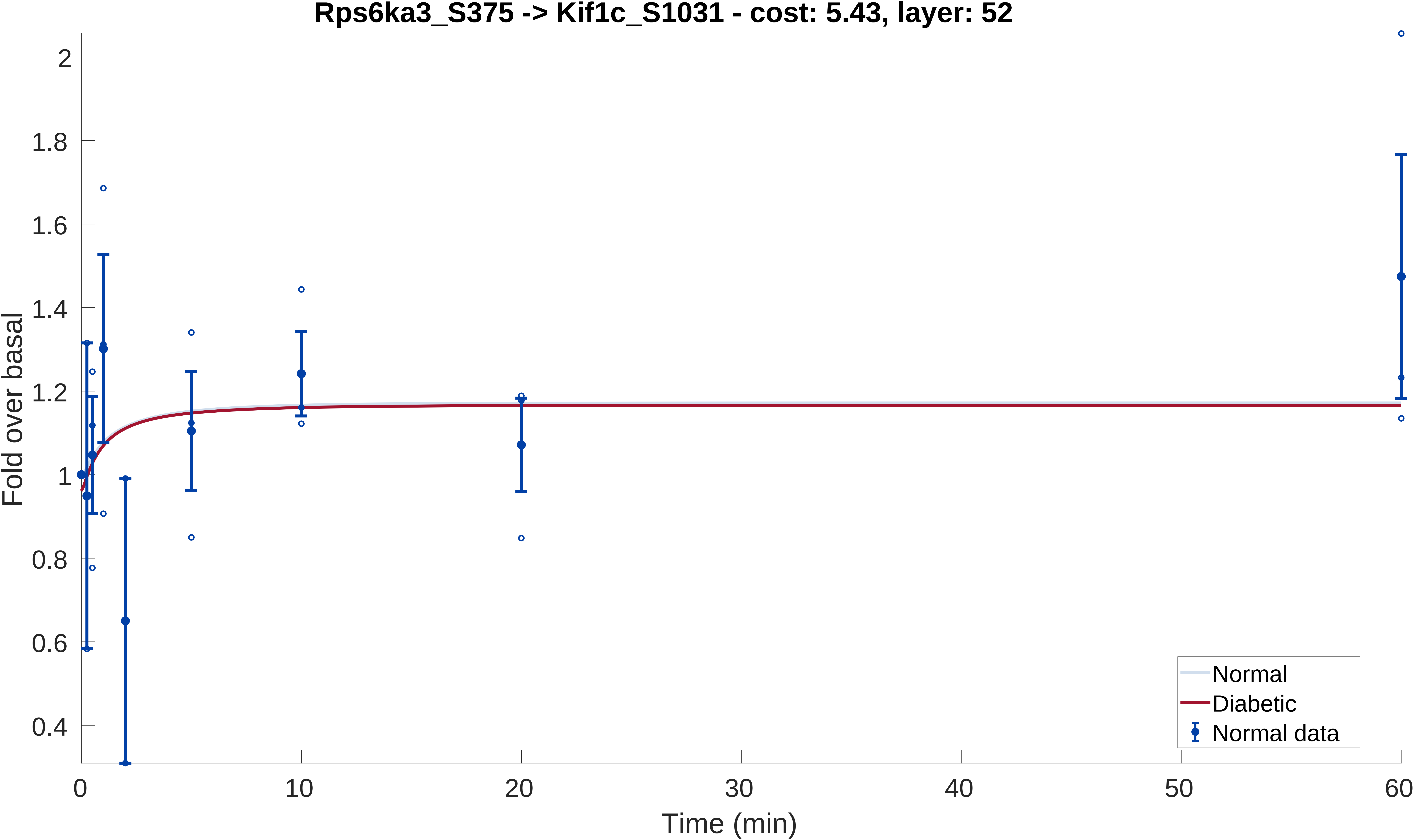

Rps6ka3_S375 -> Kif1c_S1031 - cost: 5.43, layer: 52
Fold over basal
Time (min)
0
10
20
30
40
50
60
0.4
0.6
0.8
1
1.2
1.4
1.6
1.8
2
Normal
Diabetic
Normal data

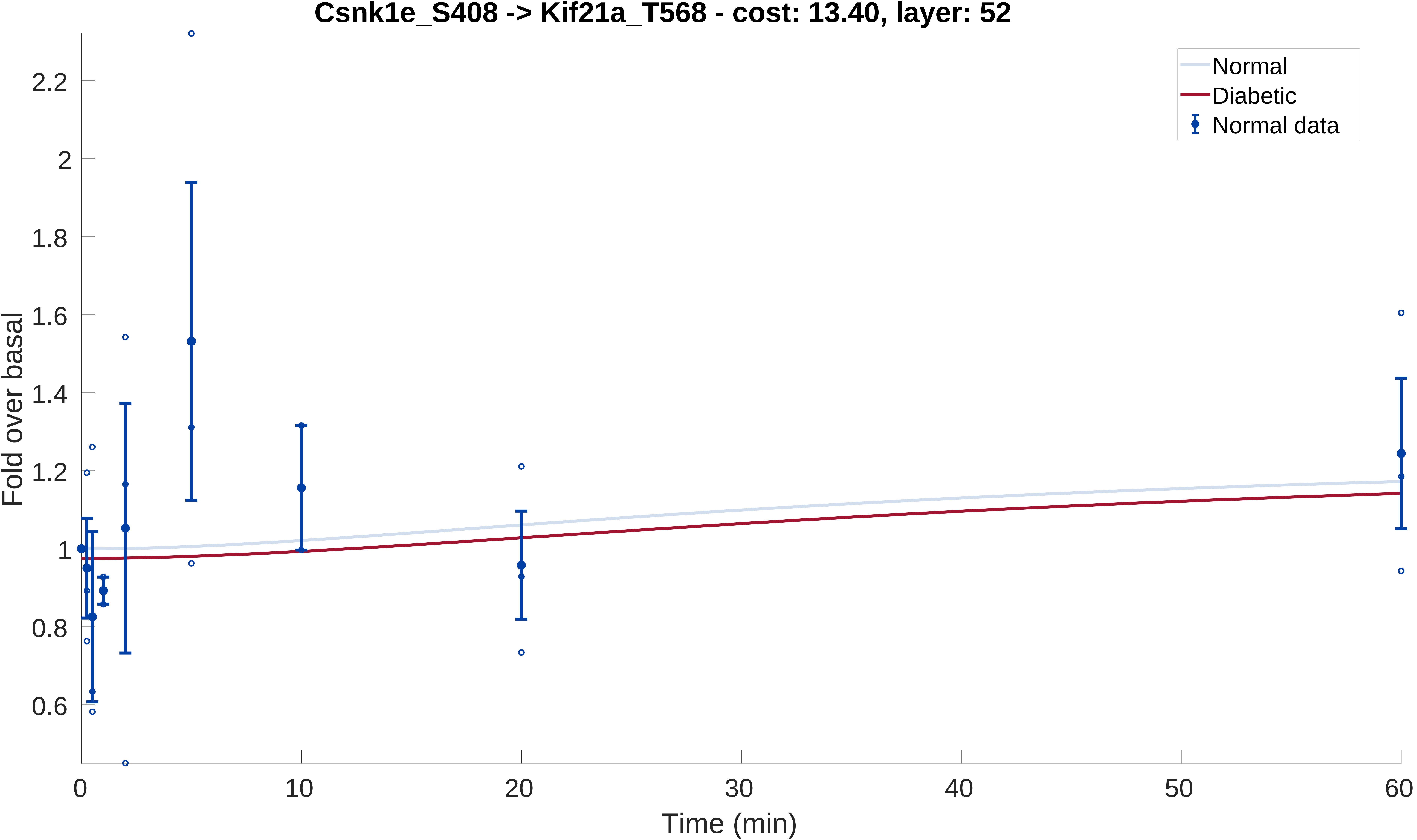

Csnk1e_S408 -> Kif21a_T568 - cost: 13.40, layer: 52
Normal
Diabetic
Normal data
Fold over basal
Time (min)
2.2
2
1.8
1.6
1.4
1.2
1
0.8
0.6
0
10
20
30
40
50
60

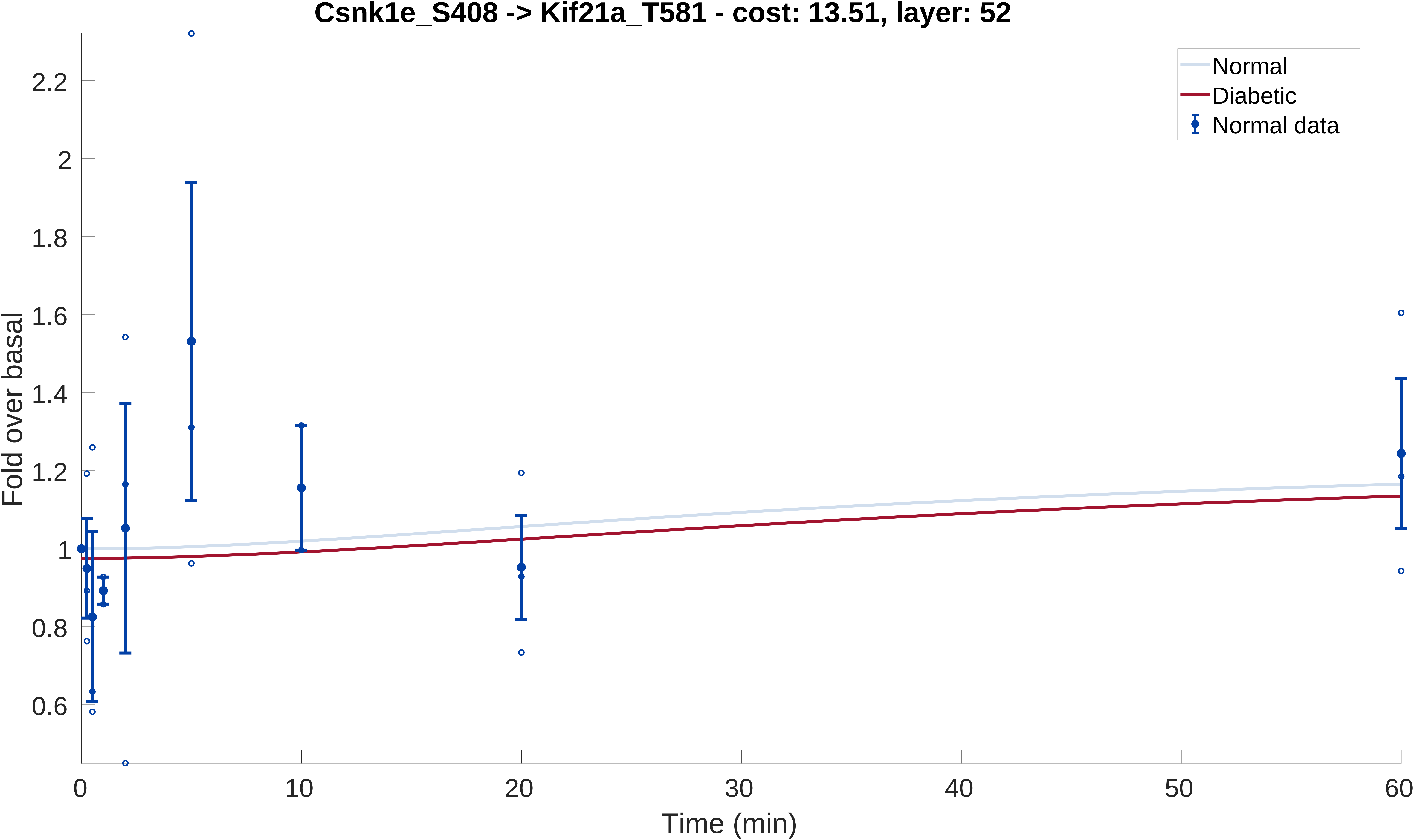
Csnk1e_S408 -> Kif21a_T581 - cost: 13.51, layer: 52
Normal
Diabetic
Normal data
Fold over basal
Time (min)
2.2
2
1.8
1.6
1.4
1.2
1
0.8
0.6
0
10
20
30
40
50
60

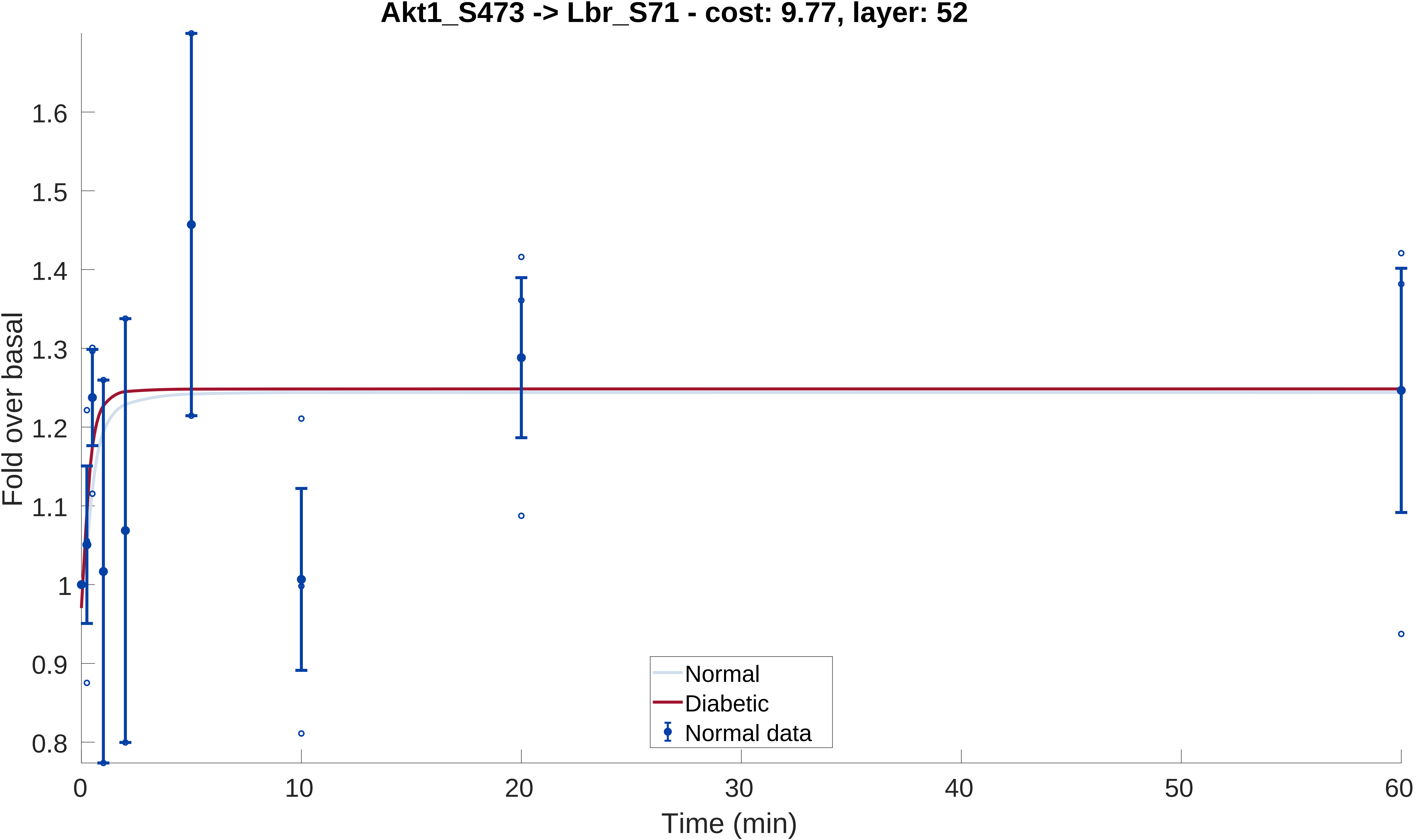

Akt1_S473 -> Lbr_S71 - cost: 9.77, layer: 52
Fold over basal
Time (min)
0
10
20
30
40
50
60
0.8
0.9
1
1.1
1.2
1.3
1.4
1.5
1.6
Normal
Diabetic
Normal data

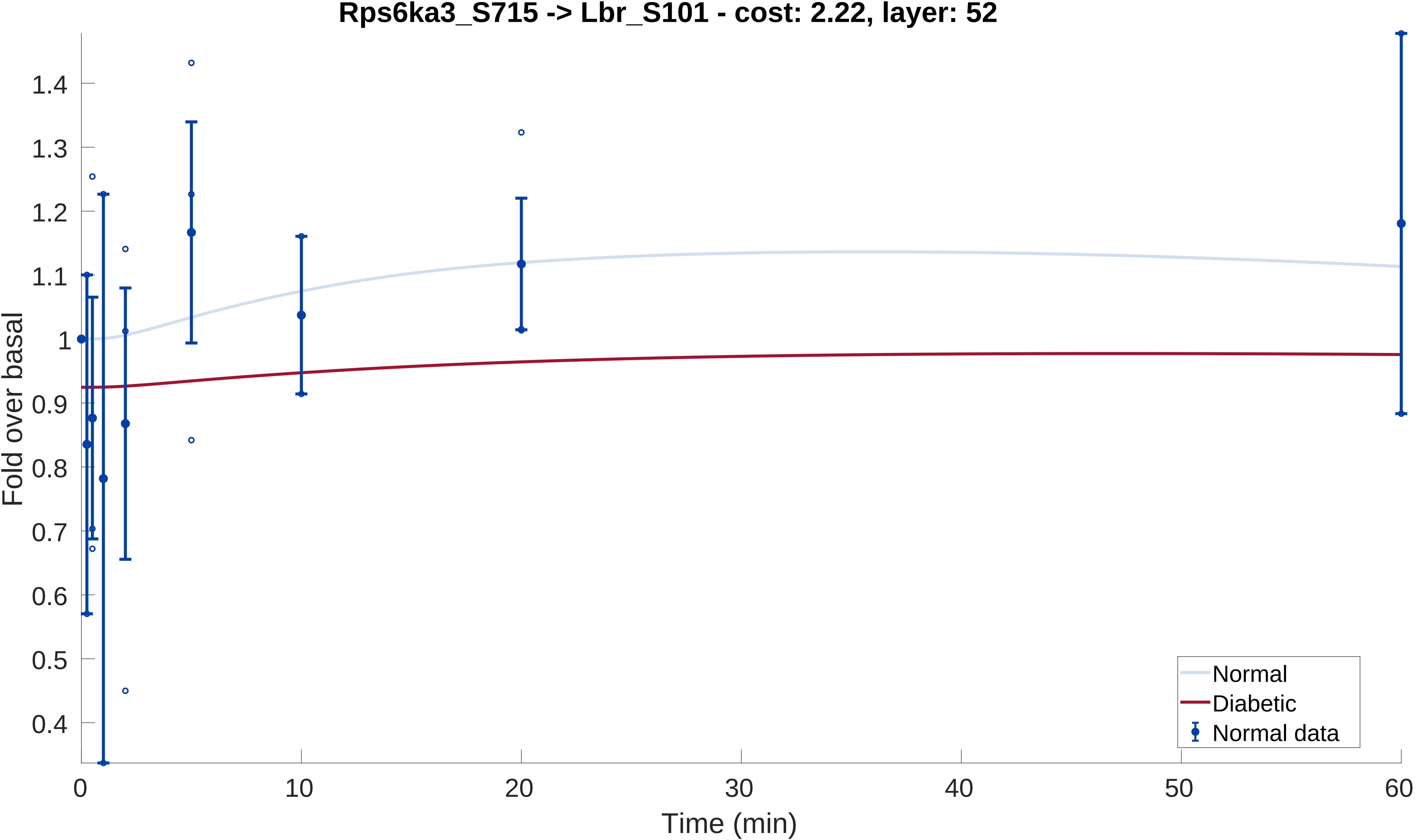

Rps6ka3_S715 -> Lbr_S101 - cost: 2.22, layer: 52
Fold over basal
Time (min)
0
10
20
30
40
50
60
0.4
0.5
0.6
0.7
0.8
0.9
1
1.1
1.2
1.3
1.4
Normal
Diabetic
Normal data

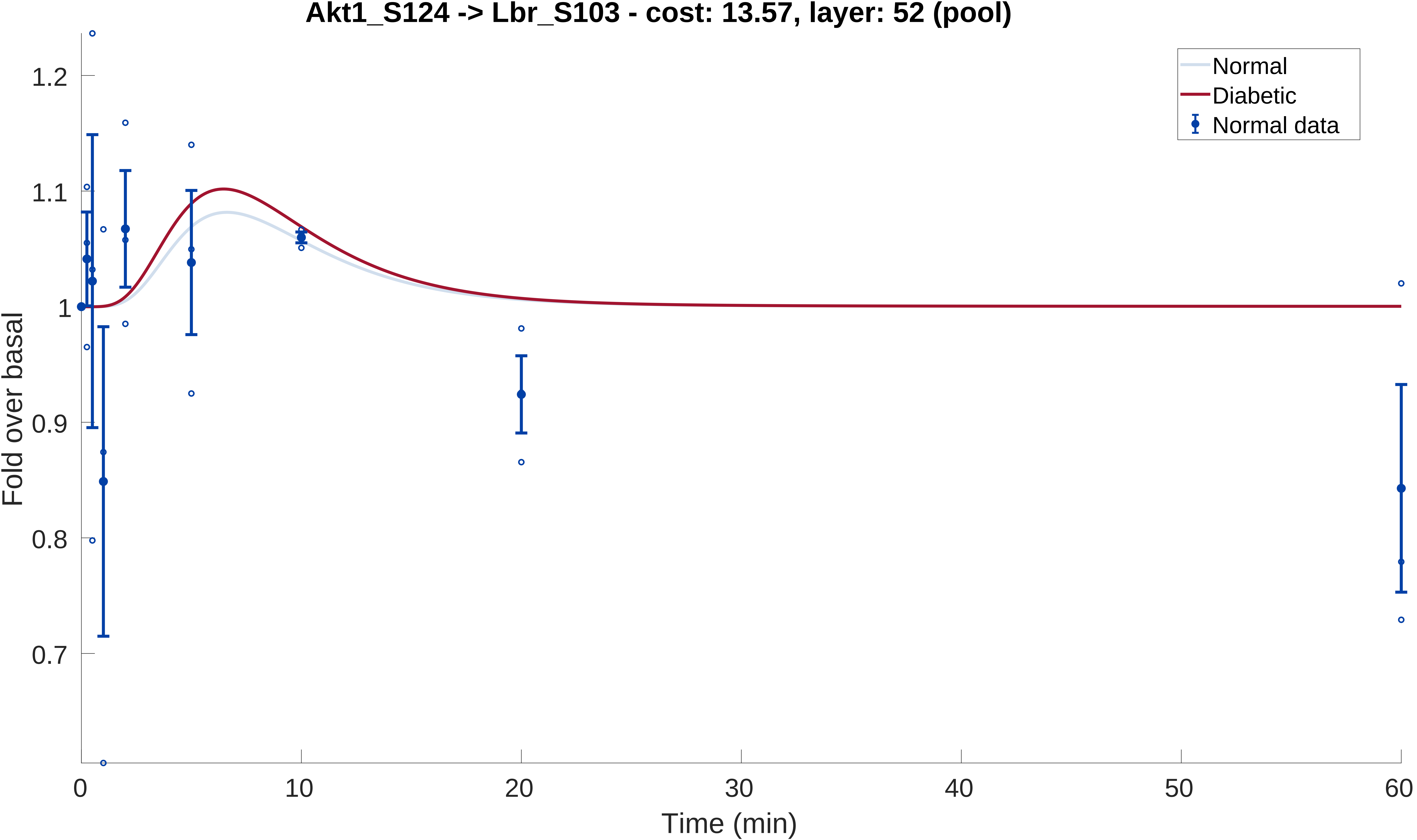

Akt1_S124 -> Lbr_S103 - cost: 13.57, layer: 52 (pool)
Normal
Diabetic
Normal data
Fold over basal
Time (min)
1.2
1.1
1
0.9
0.8
0.7
0
10
20
30
40
50
60

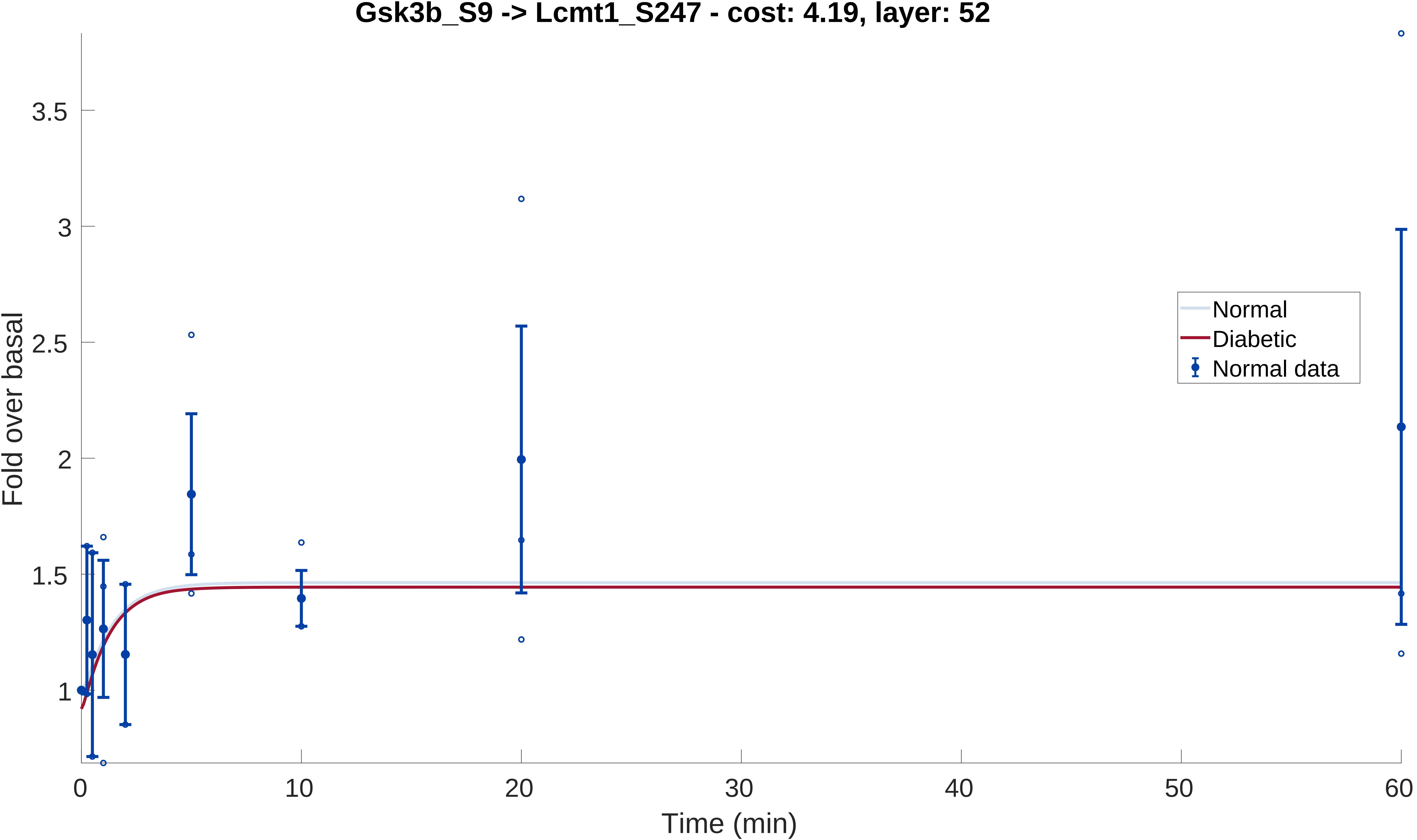

Gsk3b_S9 -> Lcmt1_S247 - cost: 4.19, layer: 52
Fold over basal
Time (min)
Normal
Diabetic
Normal data
0
10
20
30
40
50
60
1
1.5
2
2.5
3
3.5

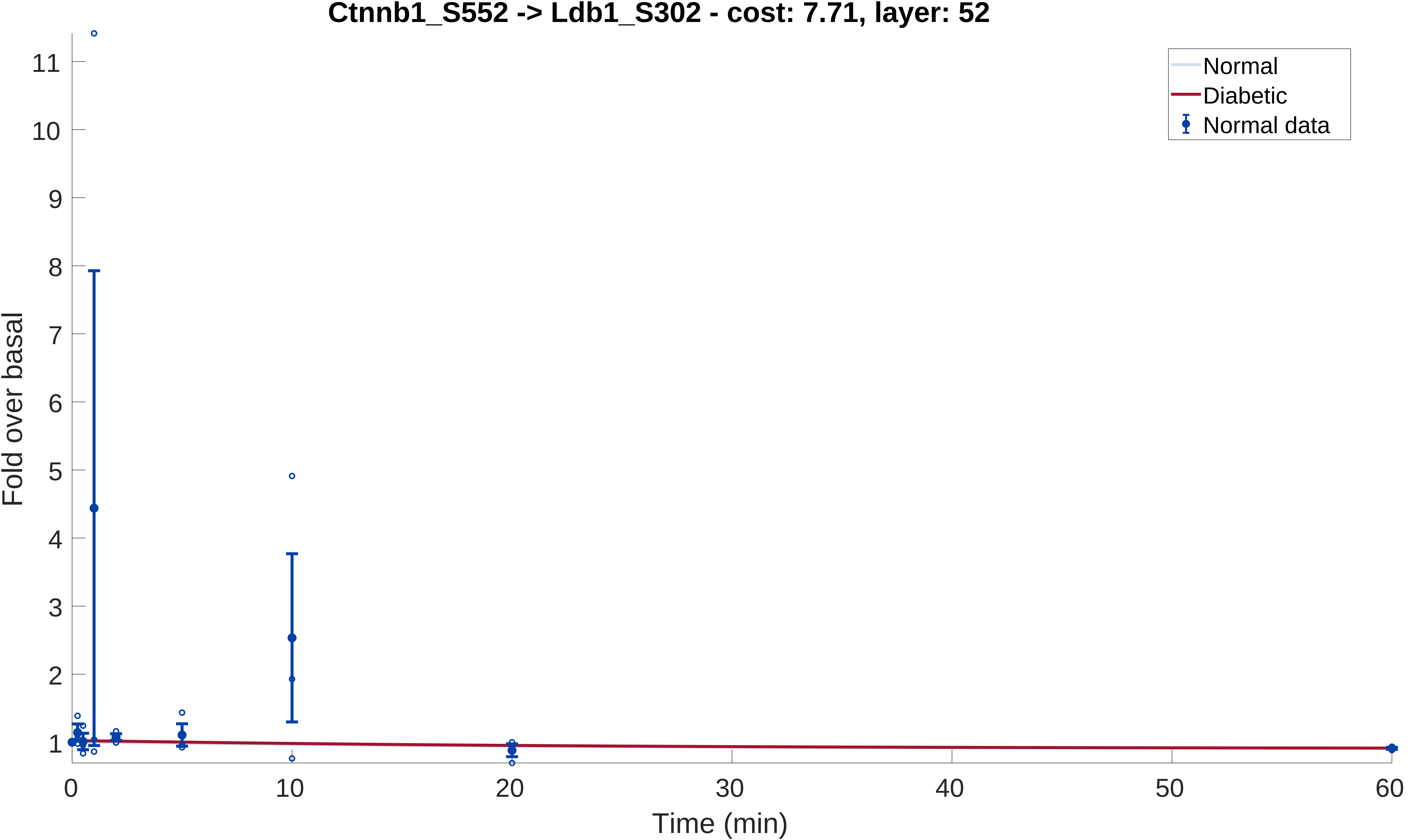
Ctnnb1_S552 -> Ldb1_S302 - cost: 7.71, layer: 52
Fold over basal
Time (min)
Normal
Diabetic
Normal data
0
10
20
30
40
50
60
1
2
3
4
5
6
7
8
9
10
11

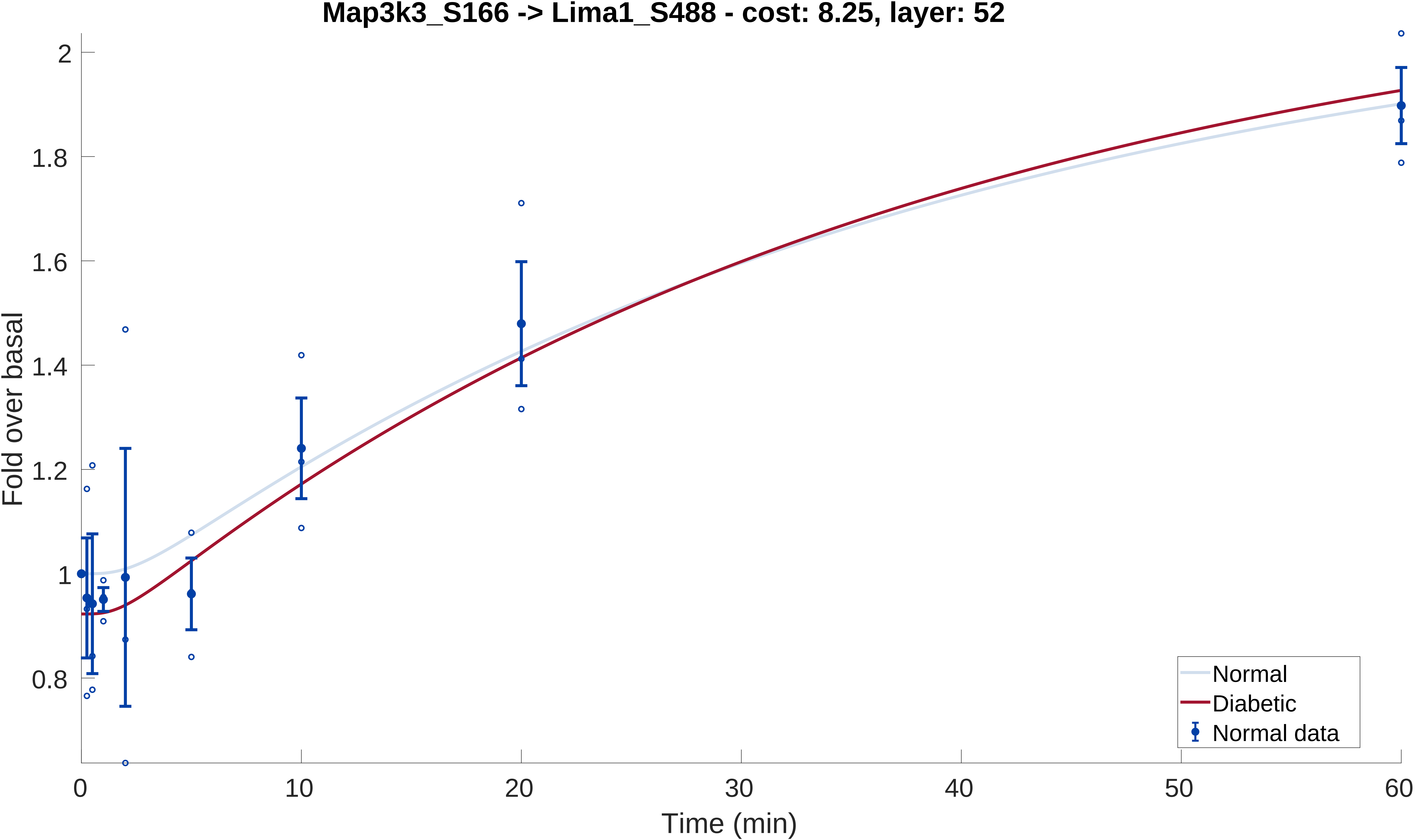

Map3k3_S166 -> Lima1_S488 - cost: 8.25, layer: 52
Fold over basal
2
1.8
1.6
1.4
1.2
1
0.8
0
10
20
30
40
50
60
Time (min)
Normal
Diabetic
Normal data

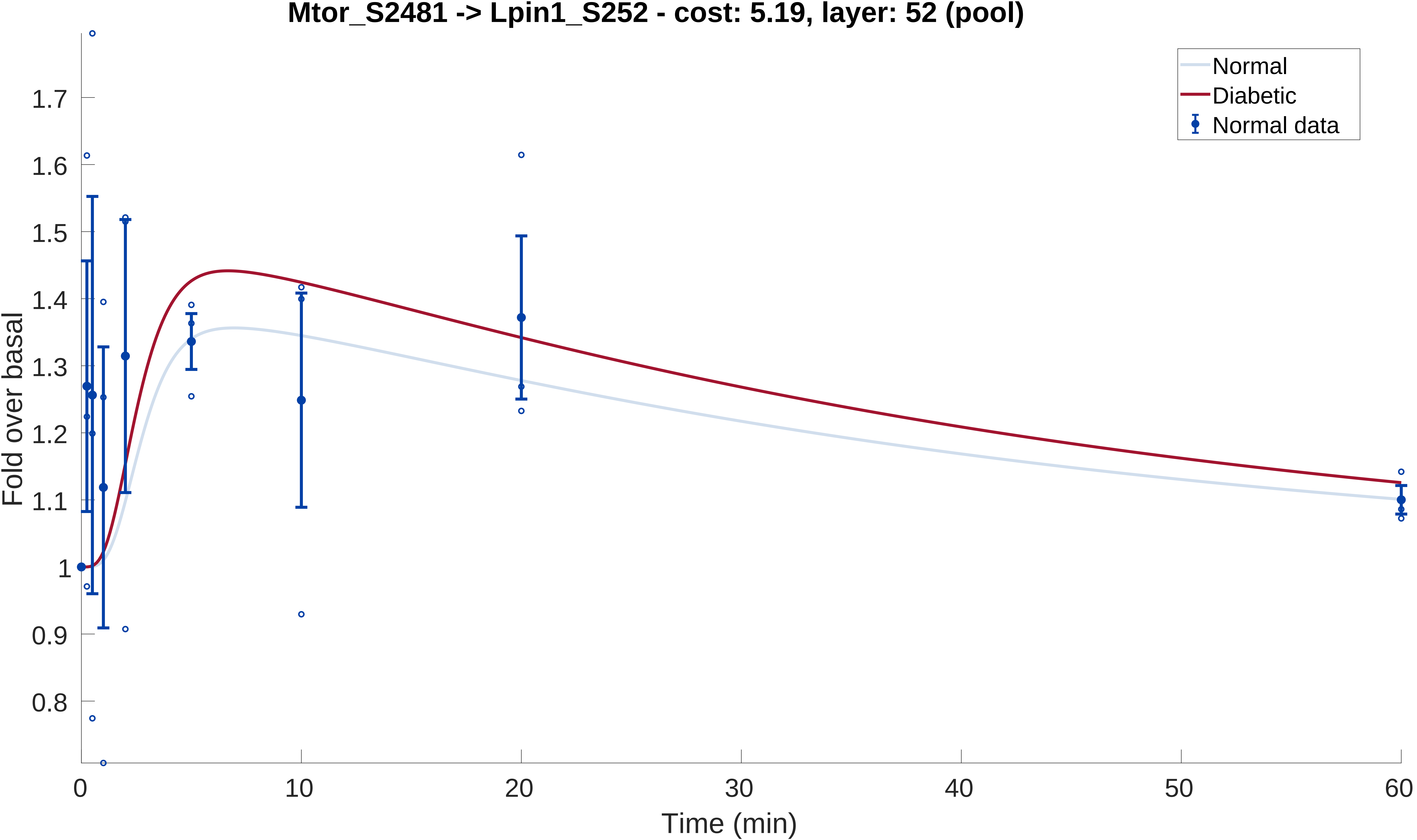
Mtor_S2481 -> Lpin1_S252 - cost: 5.19, layer: 52 (pool)
Normal
Diabetic
Normal data
Fold over basal
Time (min)
0.8
0.9
1
1.1
1.2
1.3
1.4
1.5
1.6
1.7
0
10
20
30
40
50
60

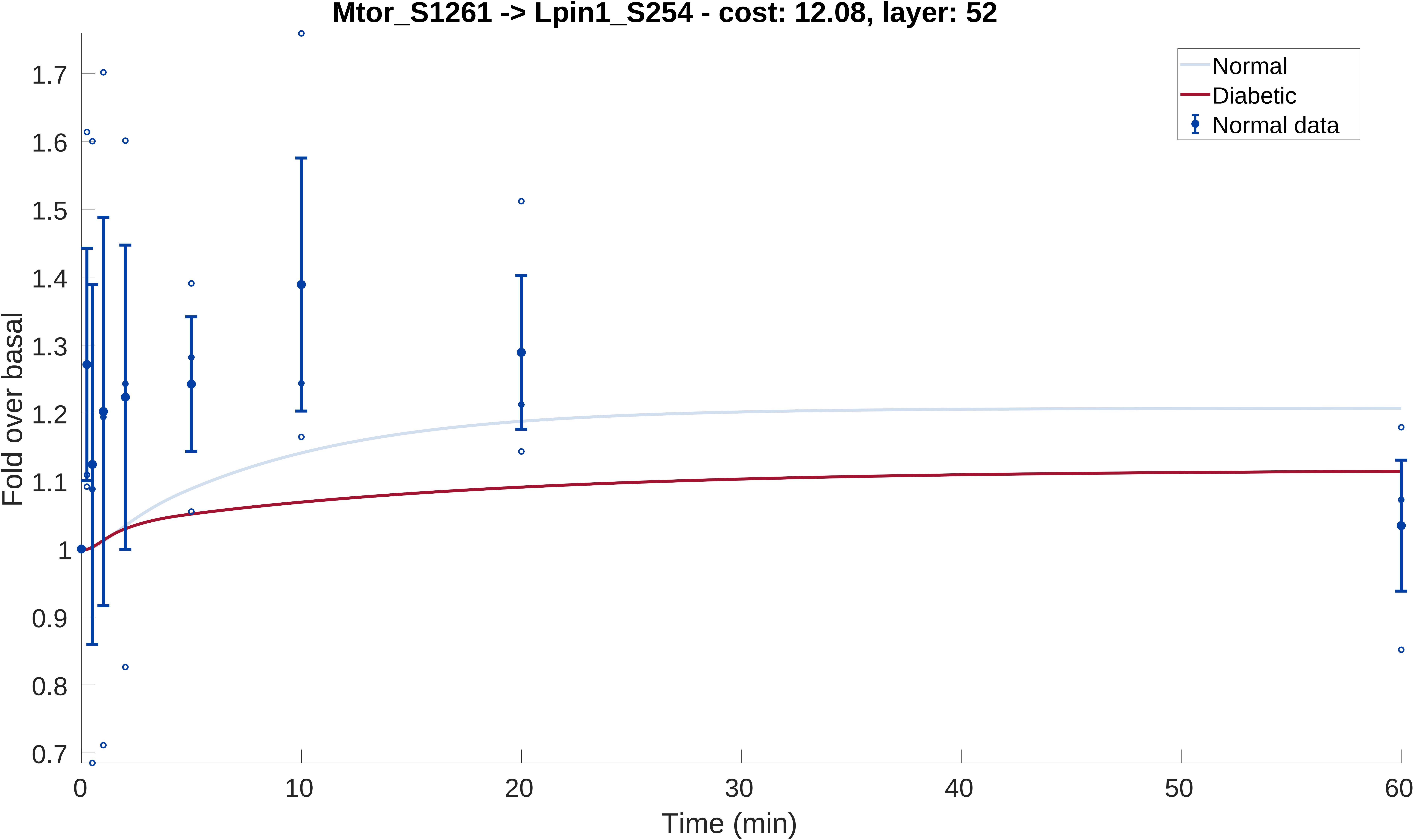
Mtor_S1261 -> Lpin1_S254 - cost: 12.08, layer: 52
Normal
Diabetic
Normal data
Fold over basal
Time (min)
0.7
0.8
0.9
1
1.1
1.2
1.3
1.4
1.5
1.6
1.7
0
10
20
30
40
50
60

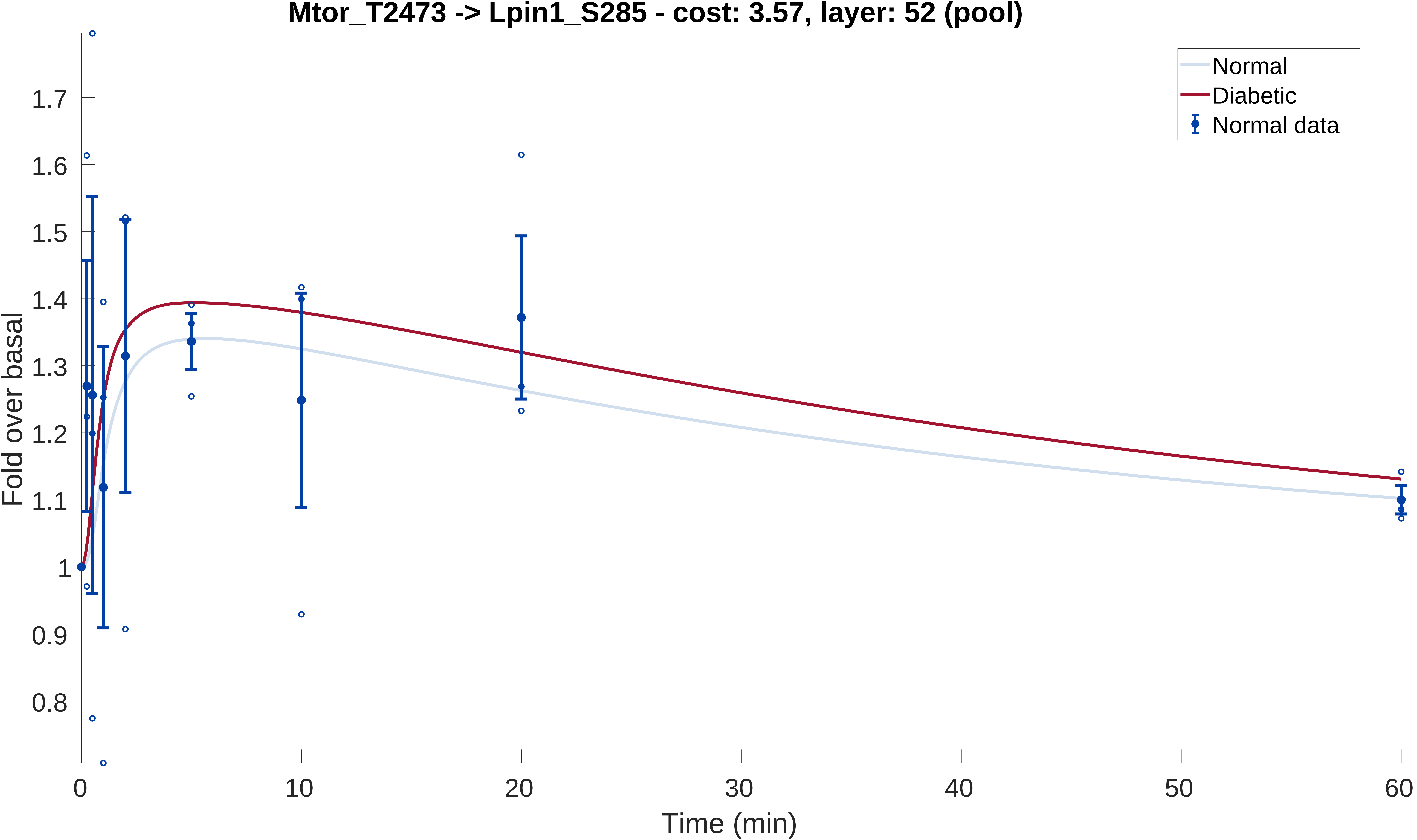
Mtor_T2473 -> Lpin1_S285 - cost: 3.57, layer: 52 (pool)
Normal
Diabetic
Normal data
Fold over basal
1.7
1.6
1.5
1.4
1.3
1.2
1.1
1
0.9
0.8
0
10
20
30
40
50
60
Time (min)

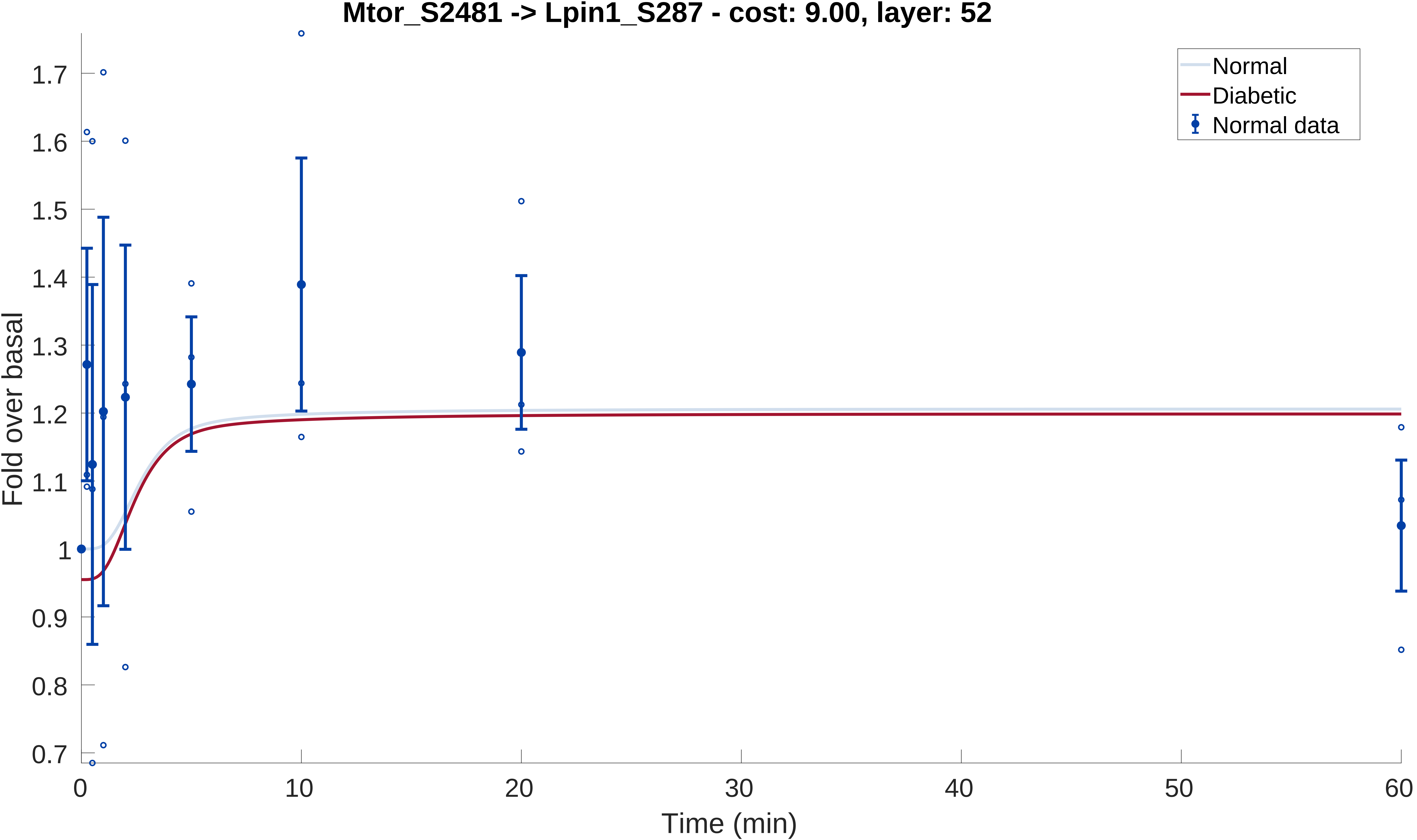

Mtor_S2481 -> Lpin1_S287 - cost: 9.00, layer: 52
Normal
Diabetic
Normal data
Fold over basal
Time (min)
0
10
20
30
40
50
60
0.7
0.8
0.9
1
1.1
1.2
1.3
1.4
1.5
1.6
1.7

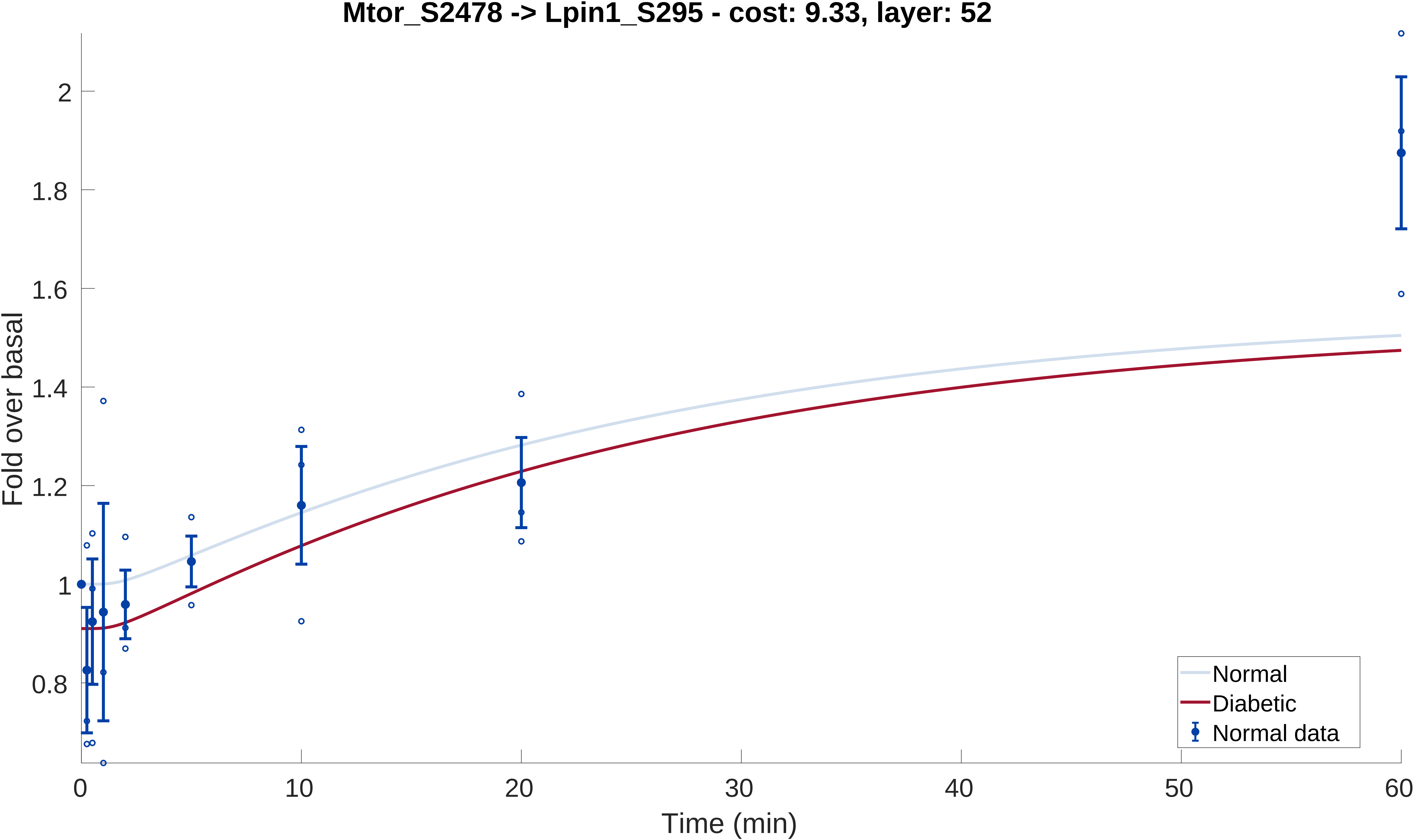

Mtor_S2478 -> Lpin1_S295 - cost: 9.33, layer: 52
Fold over basal
Time (min)
0
10
20
30
40
50
60
0.8
1
1.2
1.4
1.6
1.8
2
Normal
Diabetic
Normal data

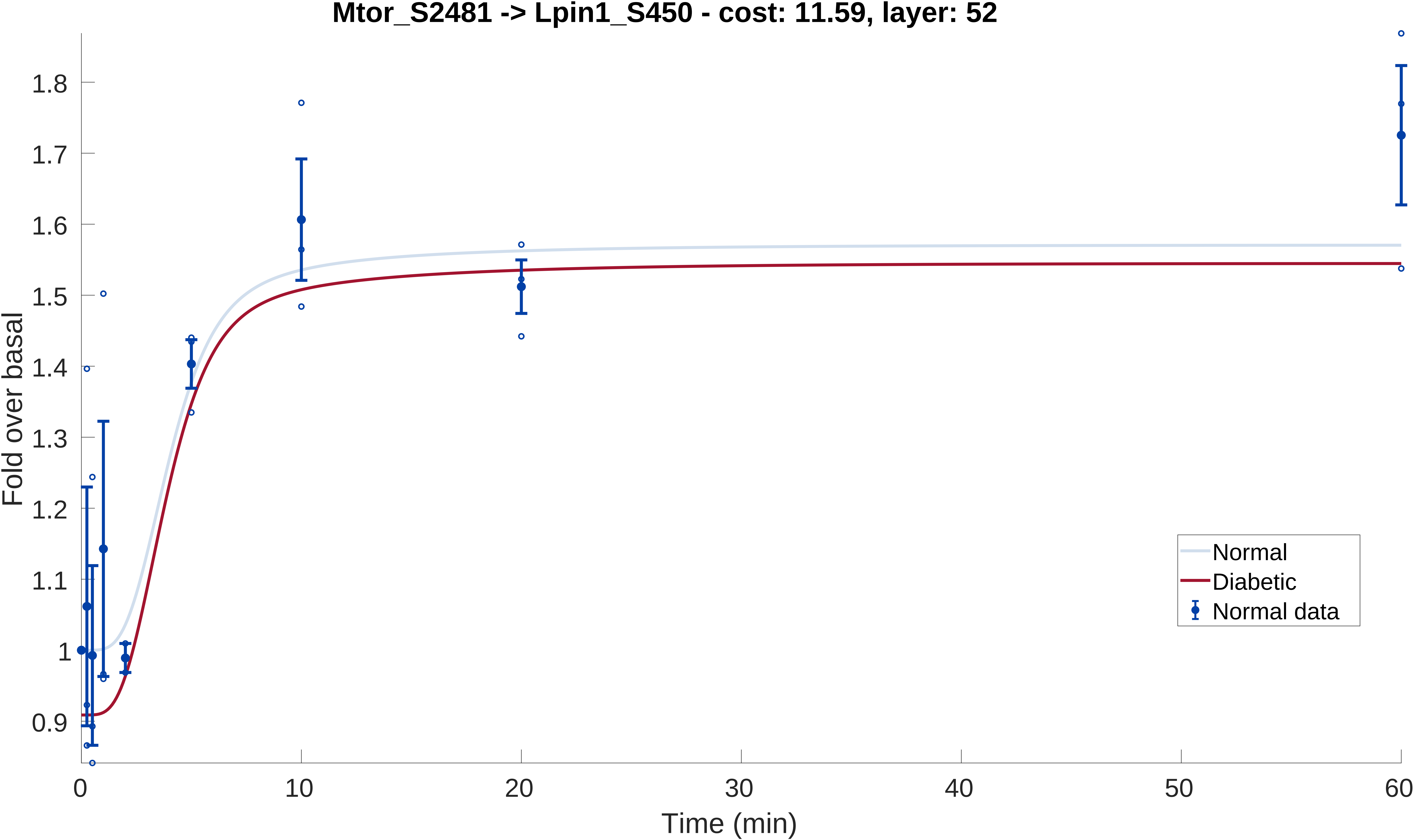

Mtor_S2481 -> Lpin1_S450 - cost: 11.59, layer: 52
Fold over basal
0.9
1
1.1
1.2
1.3
1.4
1.5
1.6
1.7
1.8
0
10
20
30
40
50
60
Time (min)
Normal
Diabetic
Normal data

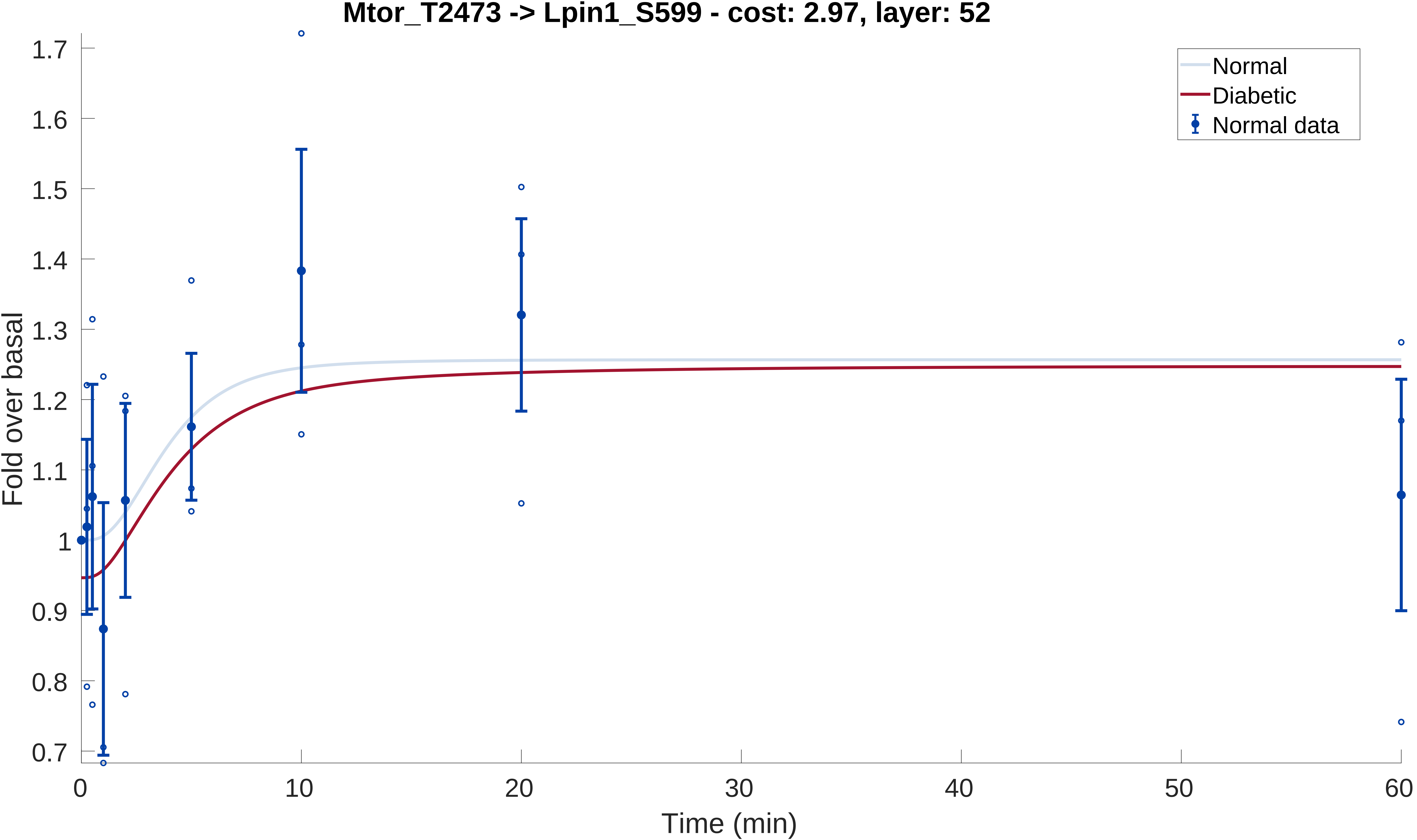
Mtor_T2473 -> Lpin1_S599 - cost: 2.97, layer: 52
Fold over basal
1.7
1.6
1.5
1.4
1.3
1.2
1.1
1
0.9
0.8
0.7
0
10
20
30
40
50
60
Time (min)
Normal
Diabetic
Normal data

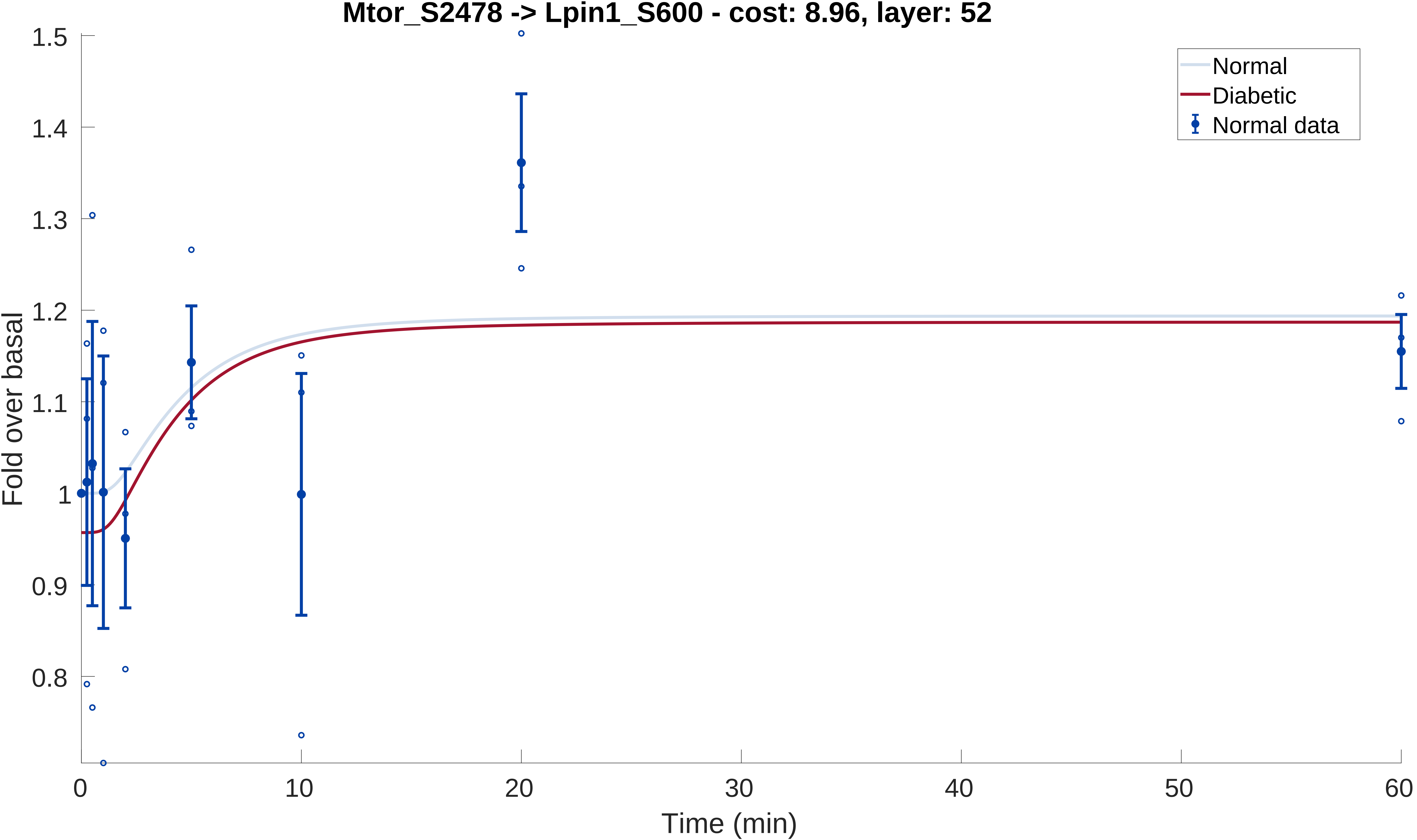
Mtor_S2478 -> Lpin1_S600 - cost: 8.96, layer: 52
Normal
Diabetic
Normal data
Fold over basal
1.5
1.4
1.3
1.2
1.1
1
0.9
0.8
0
10
20
30
40
50
60
Time (min)

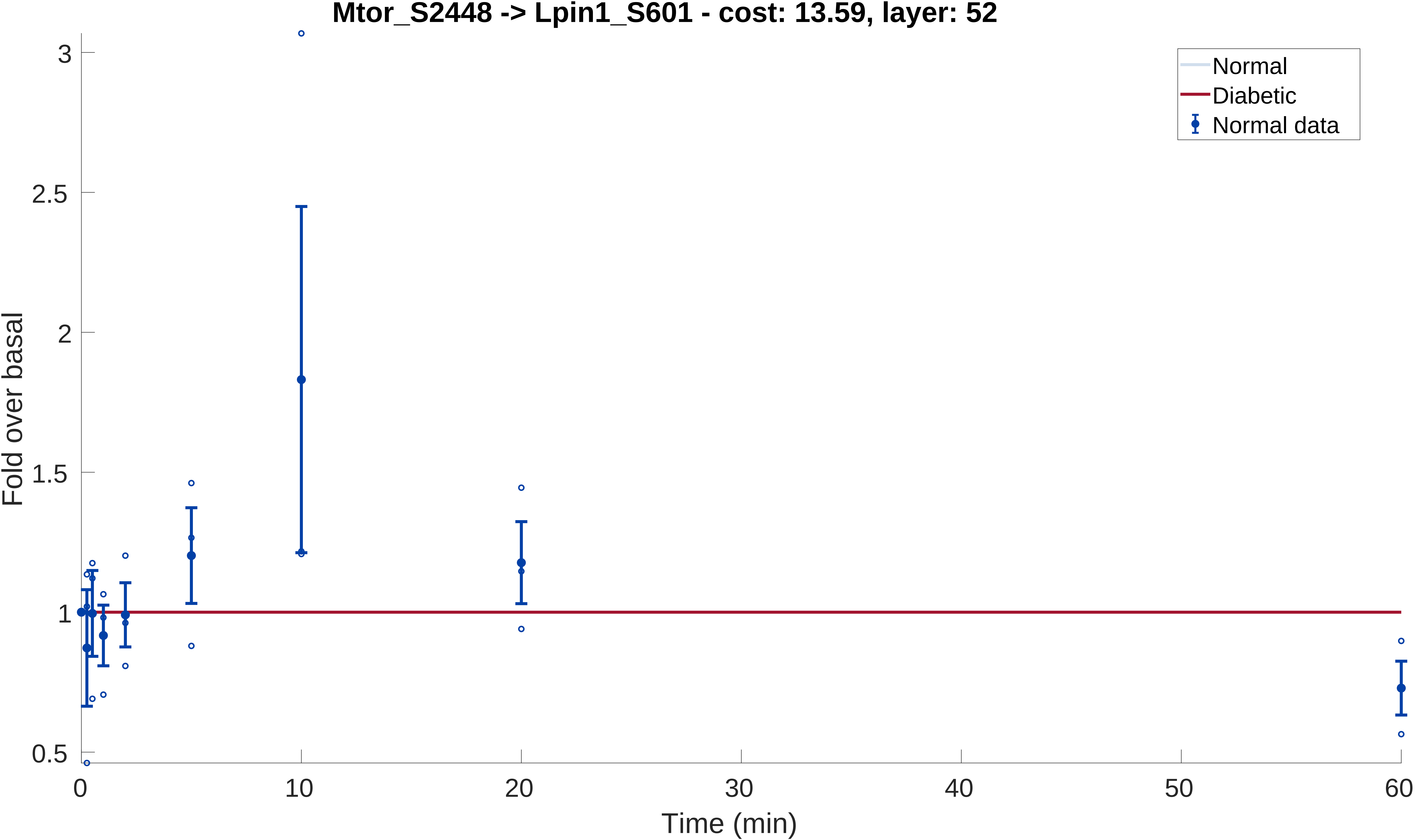

Mtor_S2448 -> Lpin1_S601 - cost: 13.59, layer: 52
Normal
Diabetic
Normal data
Fold over basal
0.5
1
1.5
2
2.5
3
0
10
20
30
40
50
60
Time (min)

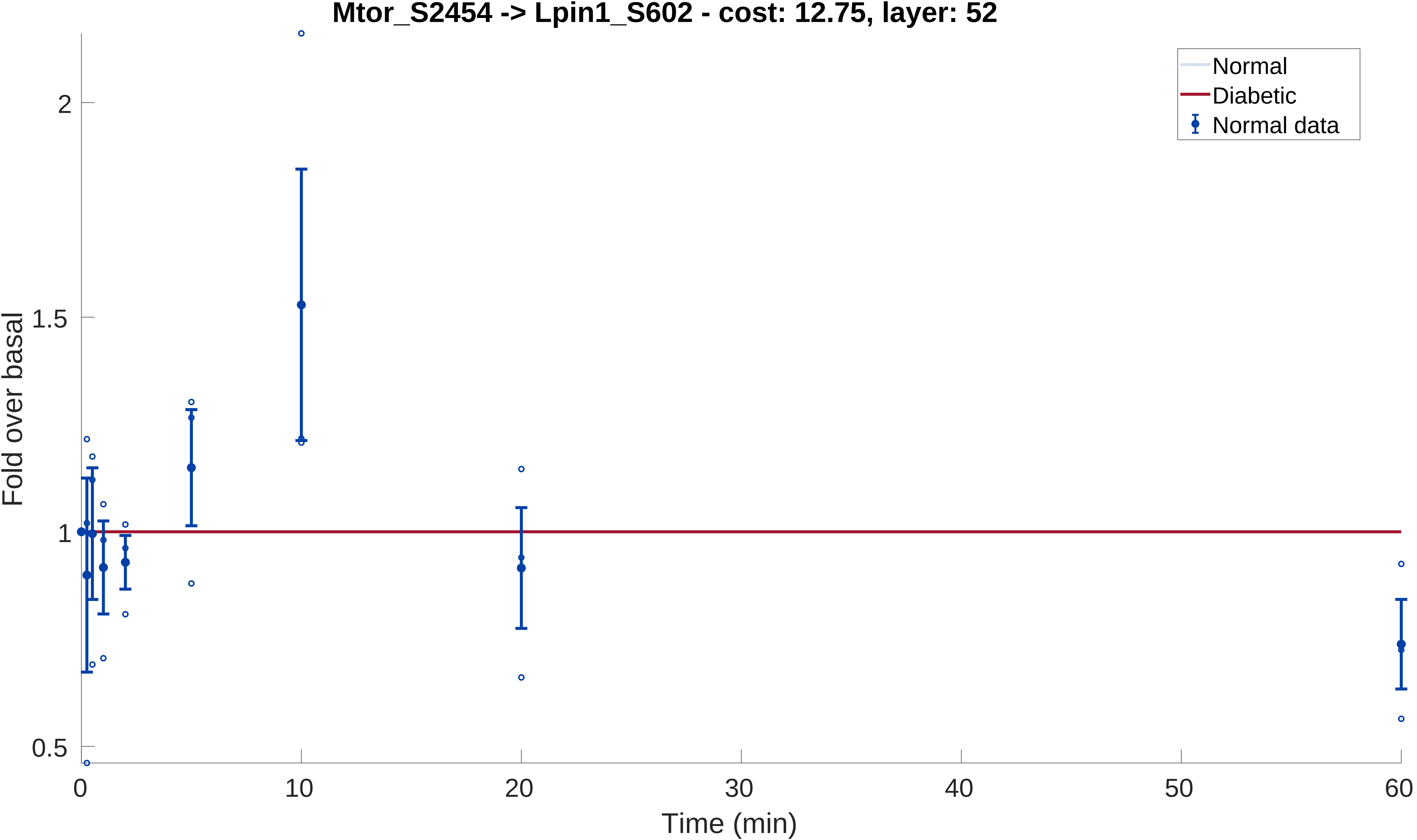

Mtor_S2454 -> Lpin1_S602 - cost: 12.75, layer: 52
Normal
Diabetic
Normal data
Fold over basal
Time (min)
0.5
1
1.5
2
0
10
20
30
40
50
60

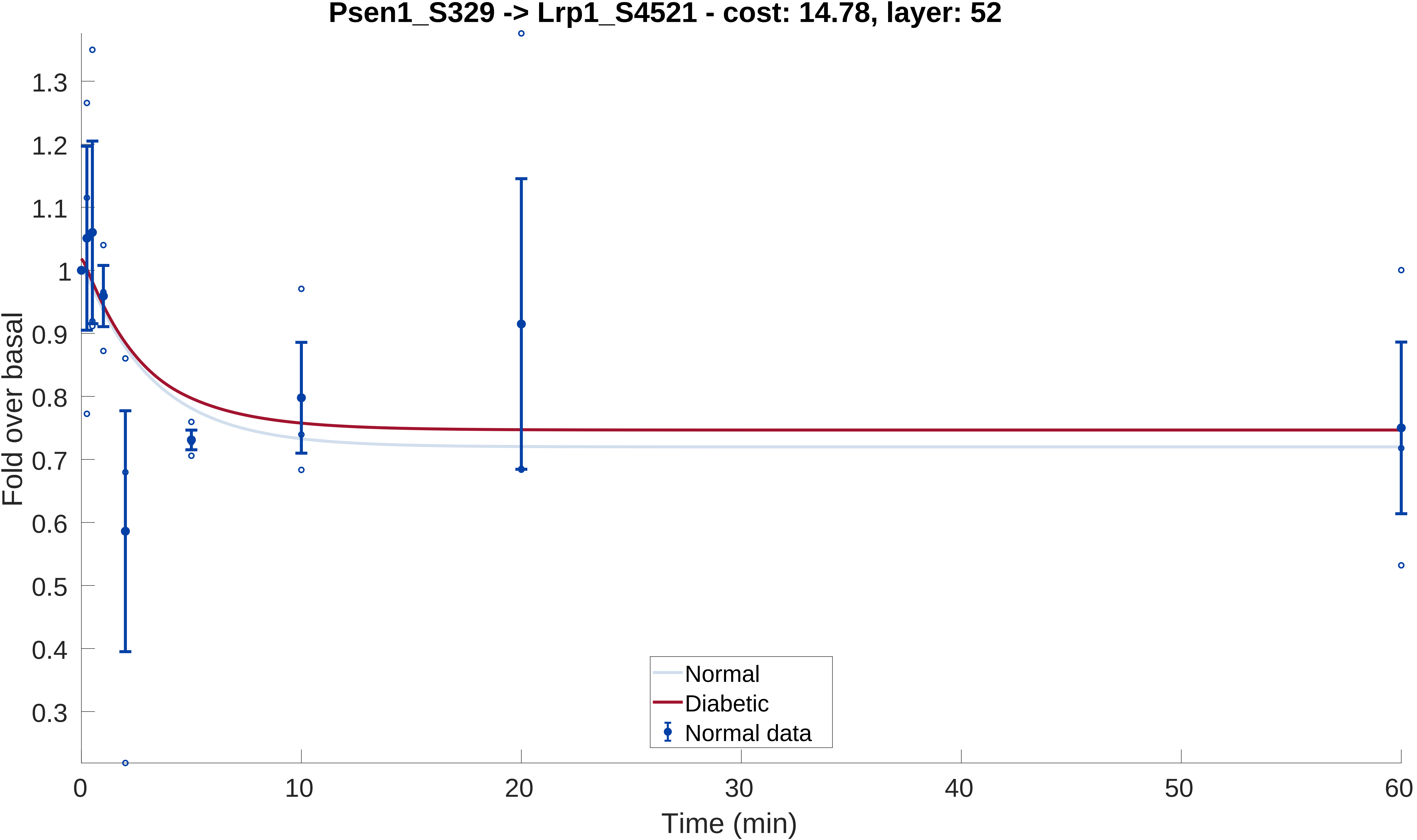

Psen1_S329 -> Lrp1_S4521 - cost: 14.78, layer: 52
Fold over basal
1.3
1.2
1.1
1
0.9
0.8
0.7
0.6
0.5
0.4
0.3
0
10
20
30
40
50
60
Time (min)
Normal
Diabetic
Normal data

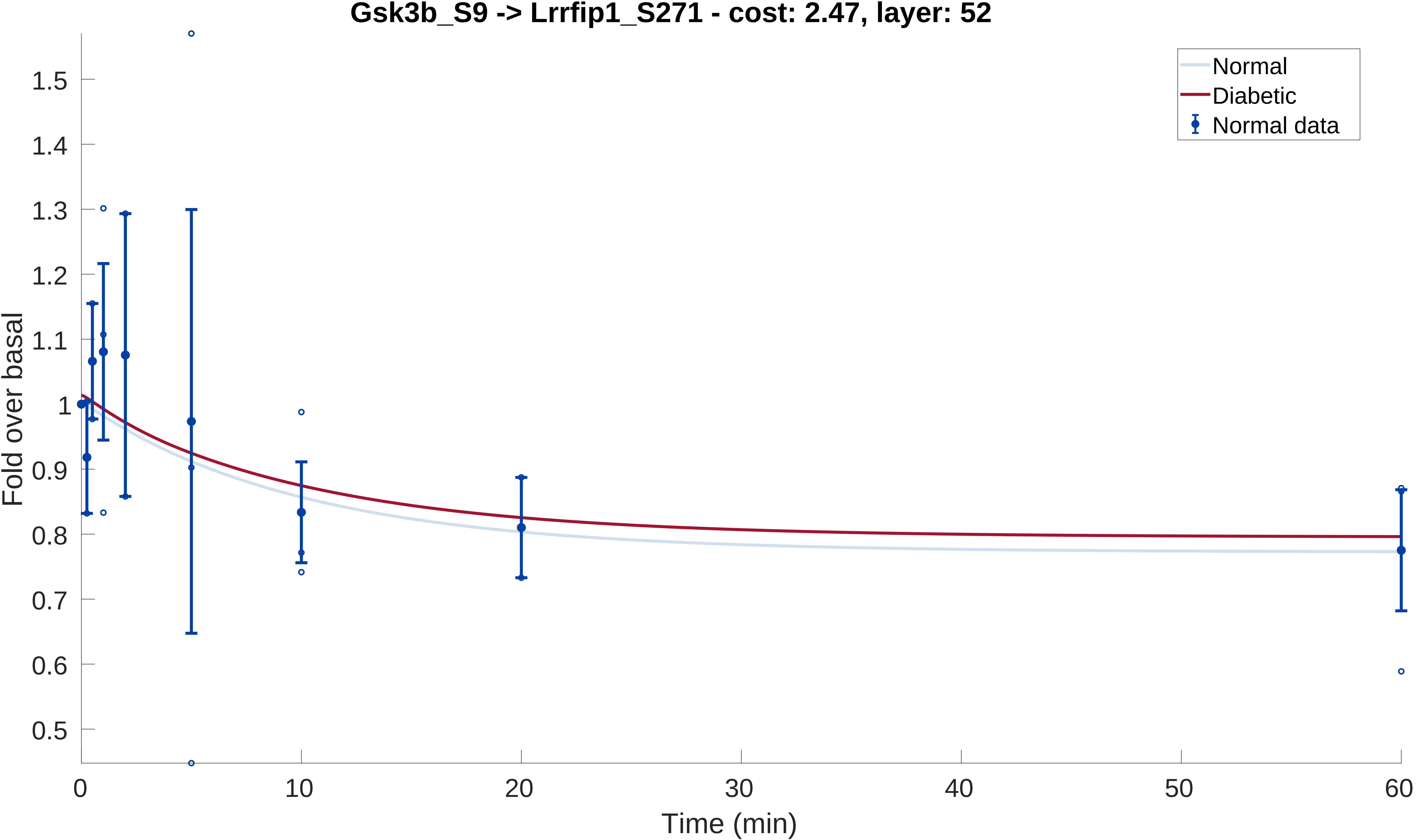

Gsk3b_S9 -> Lrrfip1_S271 - cost: 2.47, layer: 52
Normal
Diabetic
Normal data
Fold over basal
1.5
1.4
1.3
1.2
1.1
1
0.9
0.8
0.7
0.6
0.5
0
10
20
30
40
50
60
Time (min)

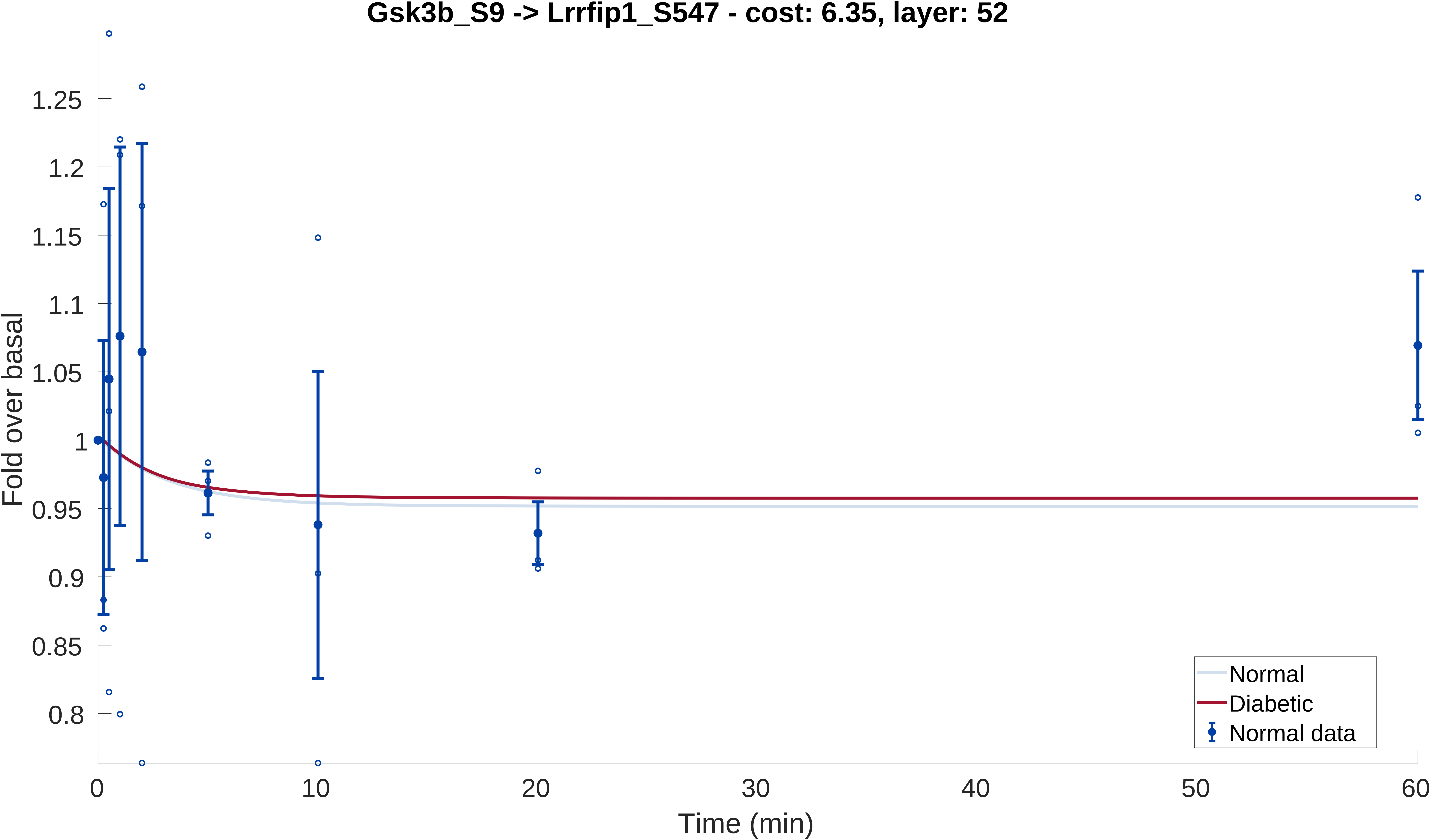
Gsk3b_S9 -> Lrrfip1_S547 - cost: 6.35, layer: 52
Fold over basal
1.25
1.2
1.15
1.1
1.05
1
0.95
0.9
0.85
0.8
0
10
20
30
40
50
60
Time (min)
Normal
Diabetic
Normal data

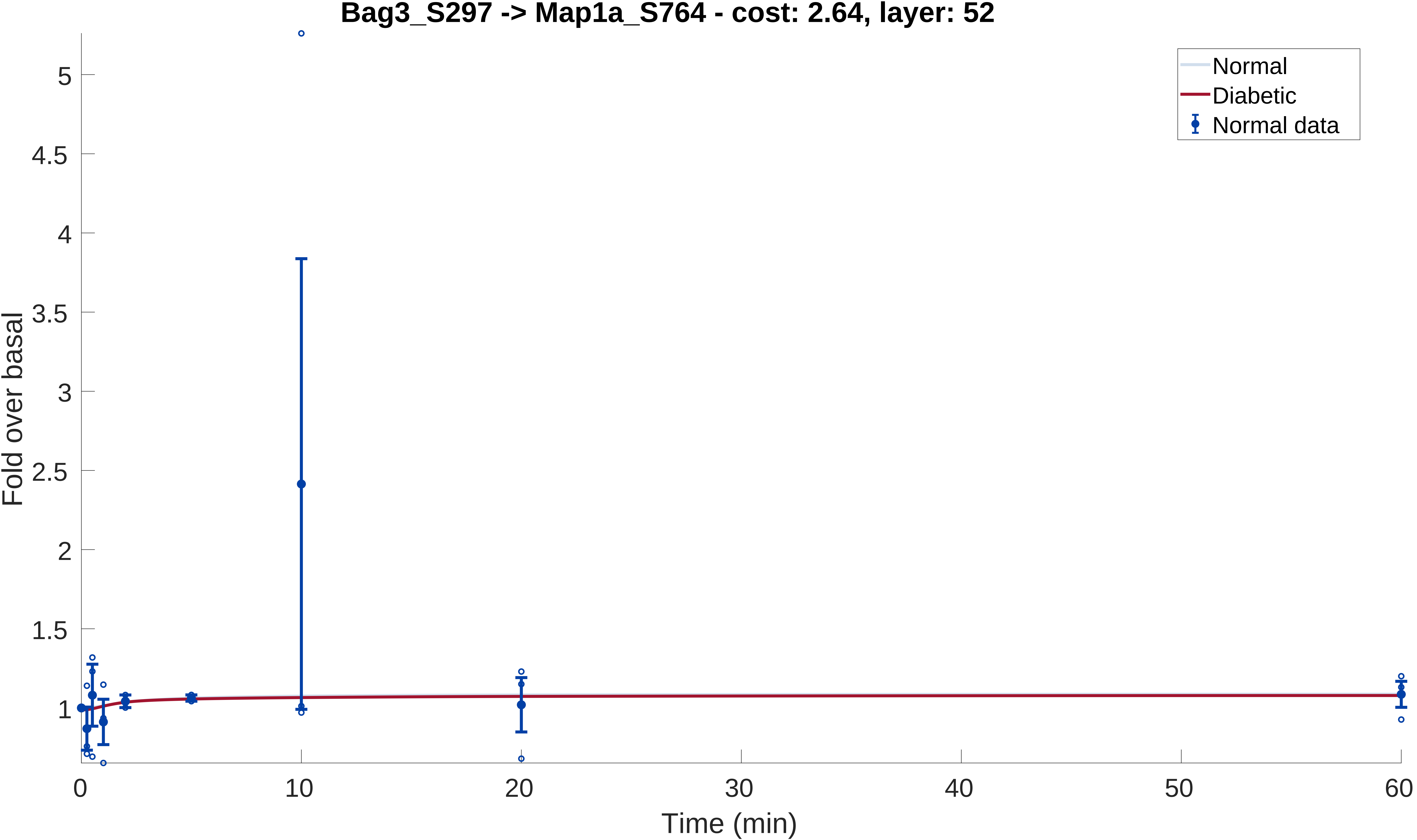

Bag3_S297 -> Map1a_S764 - cost: 2.64, layer: 52
Fold over basal
Time (min)
Normal
Diabetic
Normal data
5
4.5
4
3.5
3
2.5
2
1.5
1
0
10
20
30
40
50
60

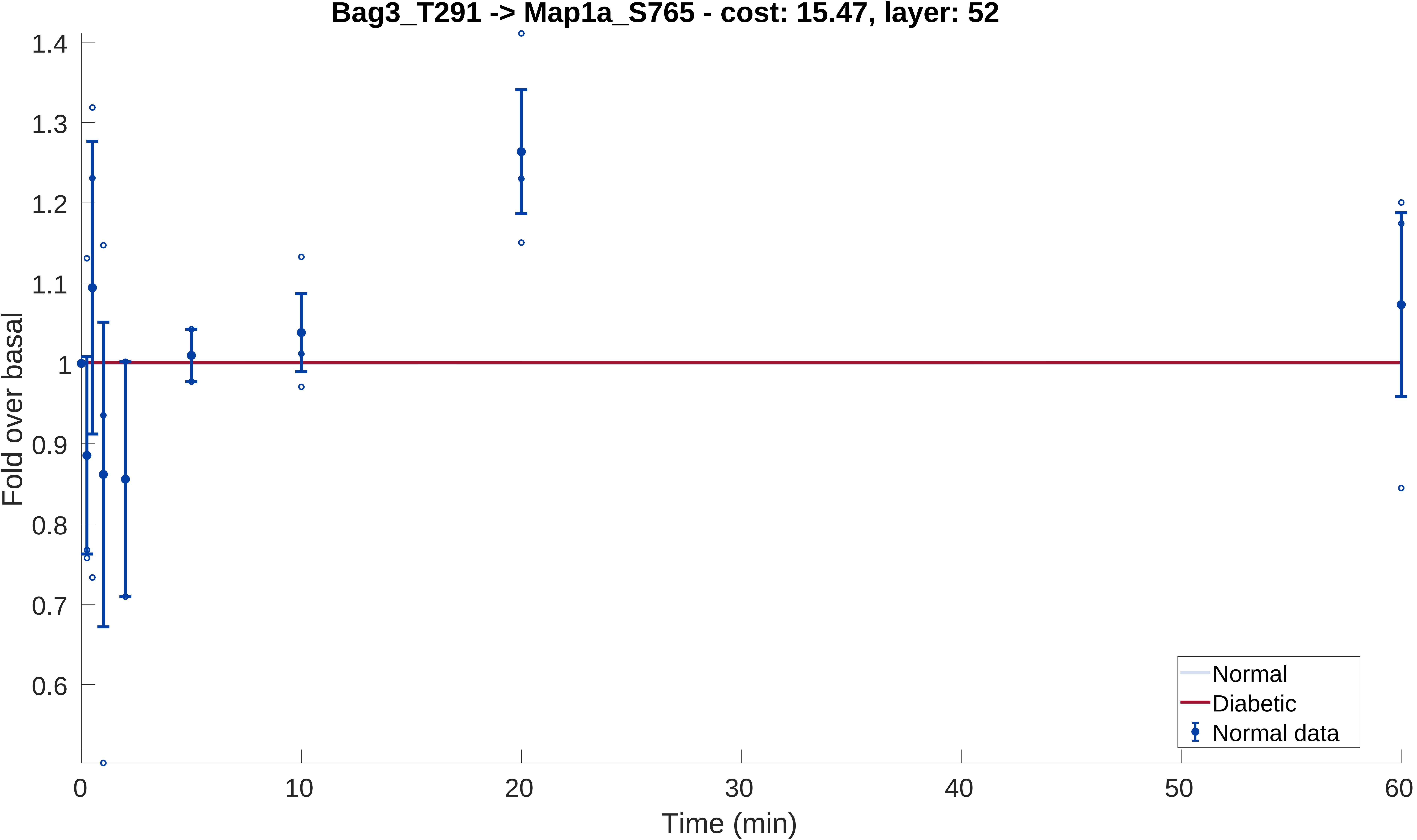
Bag3_T291 -> Map1a_S765 - cost: 15.47, layer: 52
Fold over basal
Time (min)
1.4
1.3
1.2
1.1
1
0.9
0.8
0.7
0.6
0
10
20
30
40
50
60
Normal
Diabetic
Normal data

Bag3_S270+Bag3_S390 -> Map1a_S882 - cost: 16.64, layer: 52

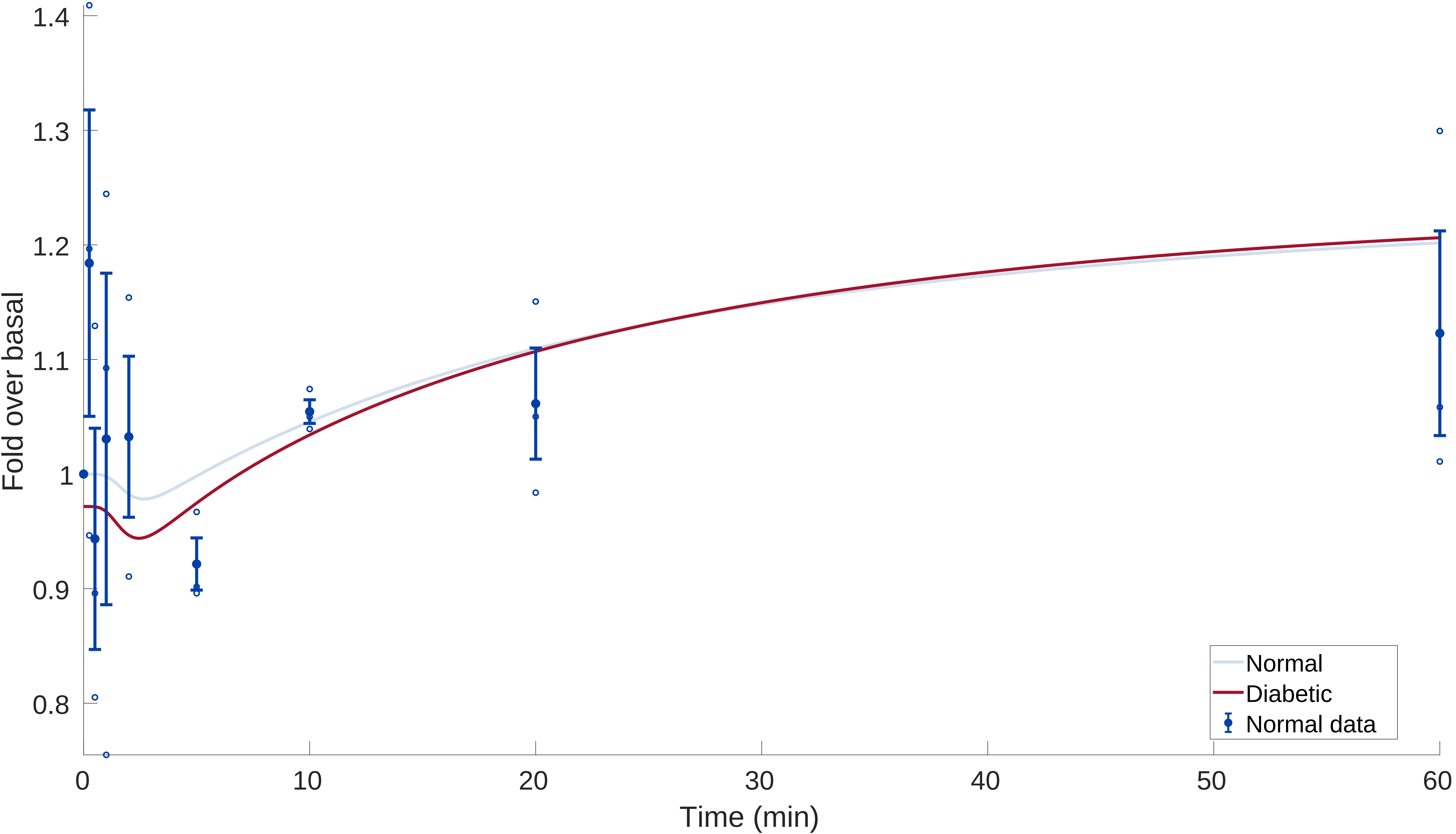

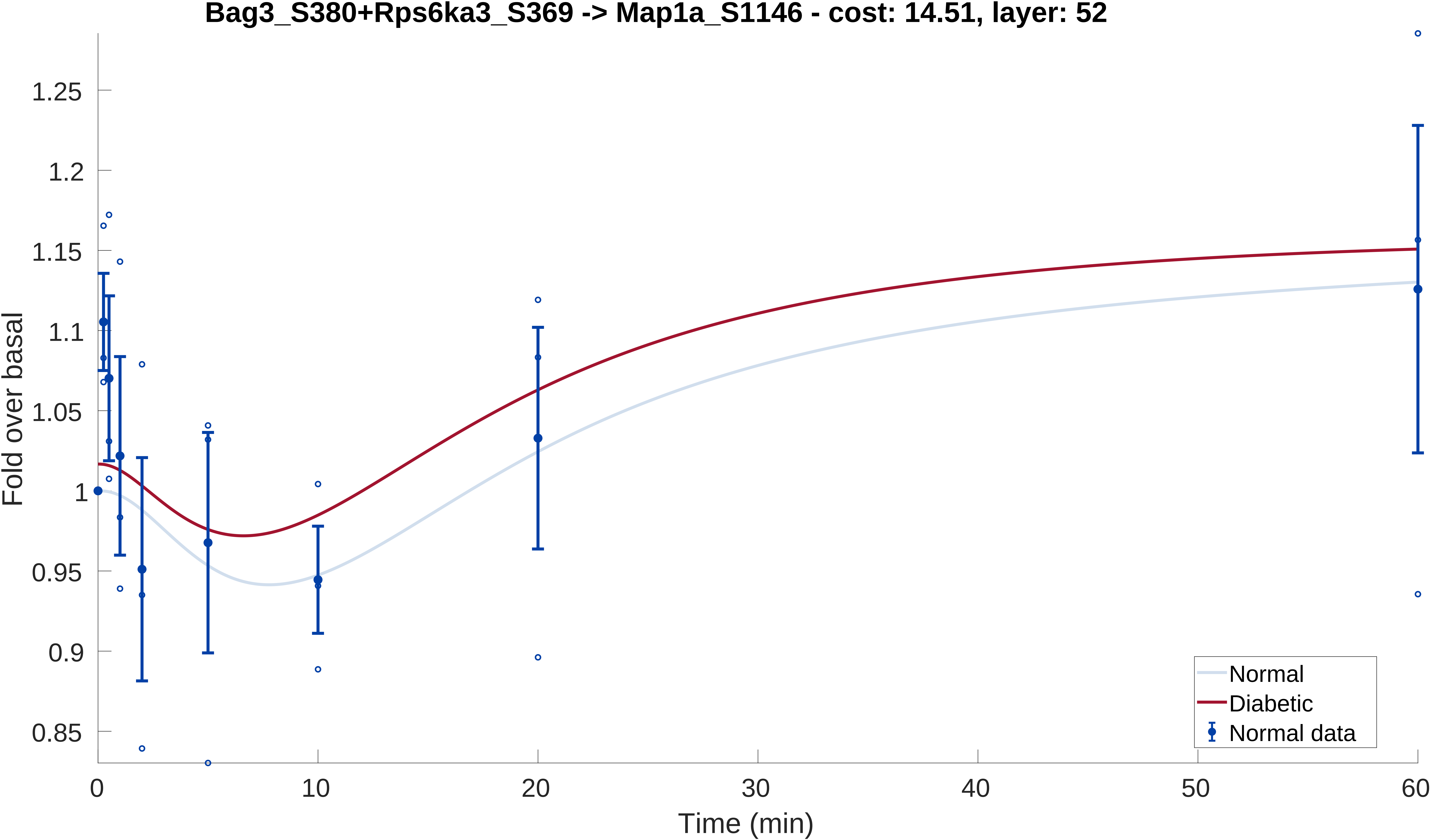

Bag3_S380+Rps6ka3_S369 -> Map1a_S1146 - cost: 14.51, layer: 52
Fold over basal
1.25
1.2
1.15
1.1
1.05
1
0.95
0.9
0.85
0
10
20
30
40
50
60
Time (min)
Normal
Diabetic
Normal data

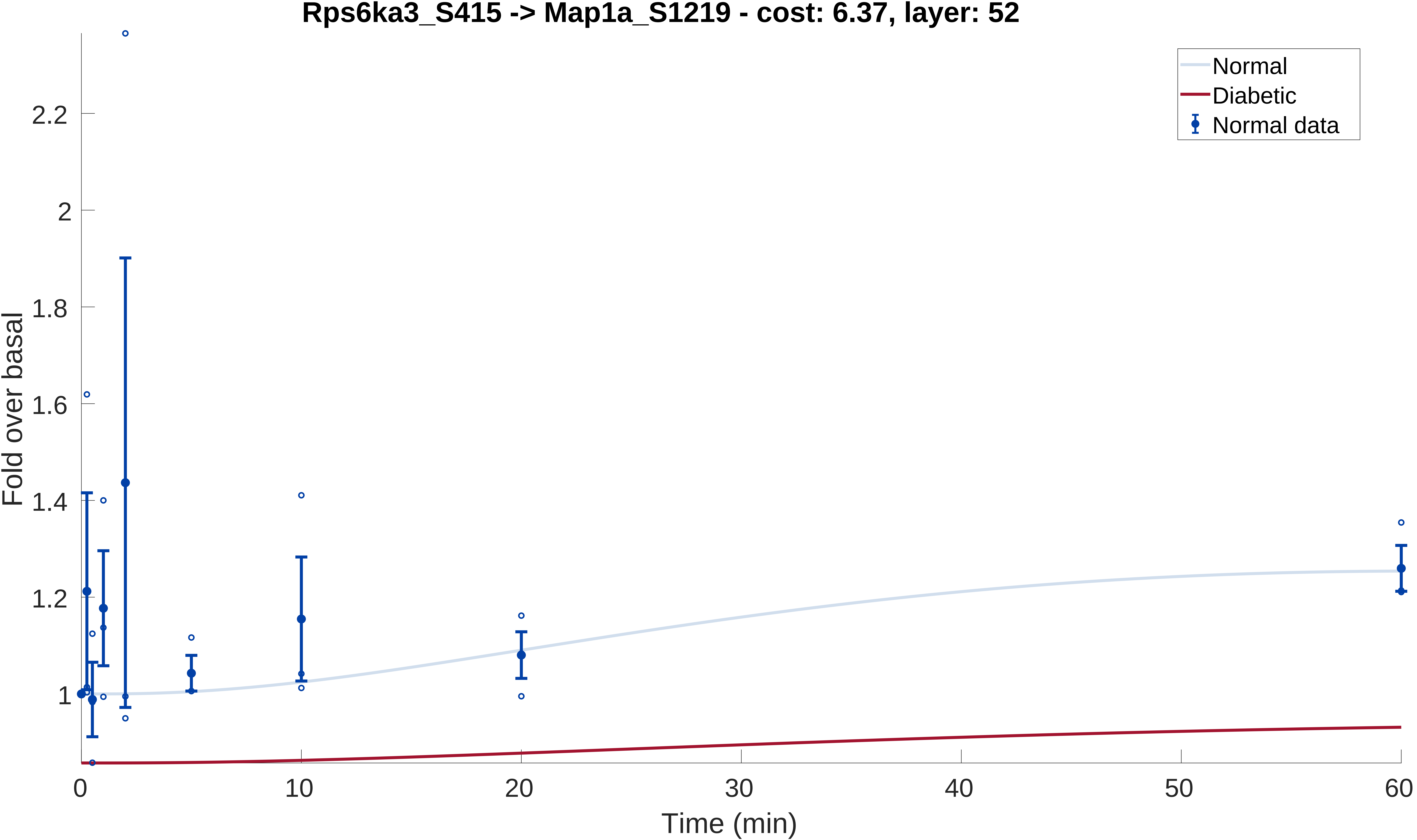

Rps6ka3_S415 -> Map1a_S1219 - cost: 6.37, layer: 52
Normal
Diabetic
Normal data
Fold over basal
Time (min)
2.2
2
1.8
1.6
1.4
1.2
1
0
10
20
30
40
50
60

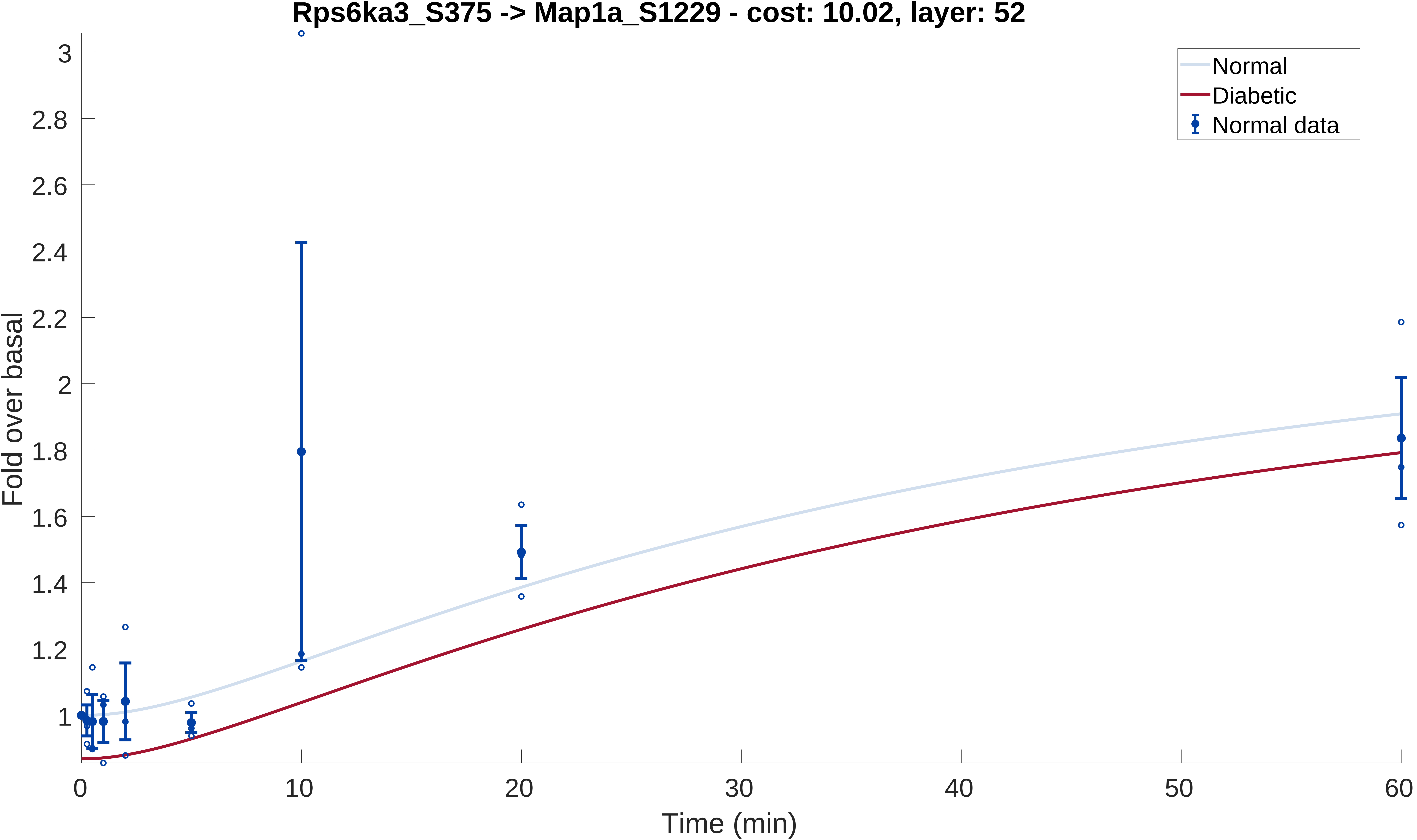
Rps6ka3_S375 -> Map1a_S1229 - cost: 10.02, layer: 52
Normal
Diabetic
Normal data
Fold over basal
Time (min)
3
2.8
2.6
2.4
2.2
2
1.8
1.6
1.4
1.2
1
0
10
20
30
40
50
60

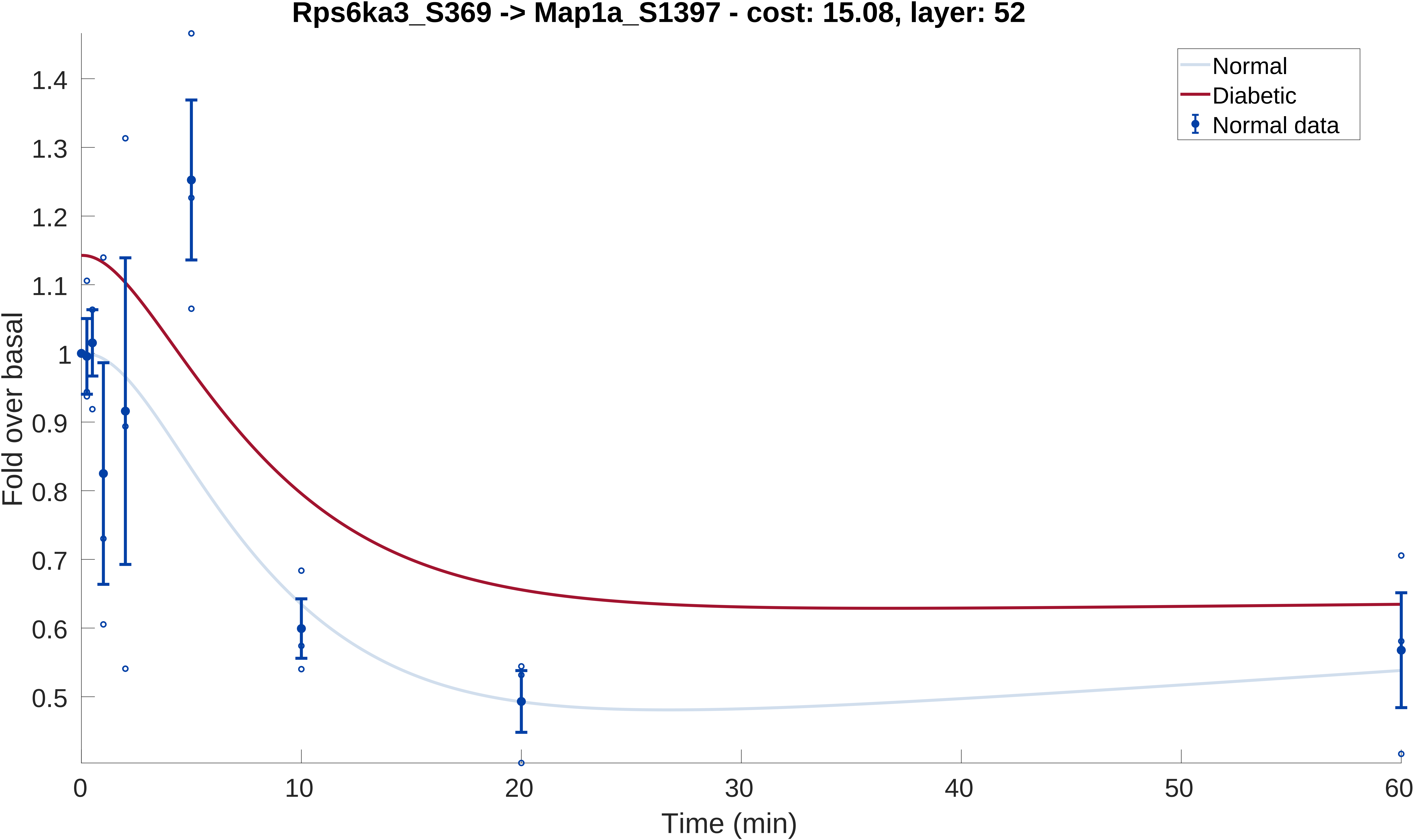

Rps6ka3_S369 -> Map1a_S1397 - cost: 15.08, layer: 52
Normal
Diabetic
Normal data
Fold over basal
Time (min)
1.4
1.3
1.2
1.1
1
0.9
0.8
0.7
0.6
0.5
0
10
20
30
40
50
60

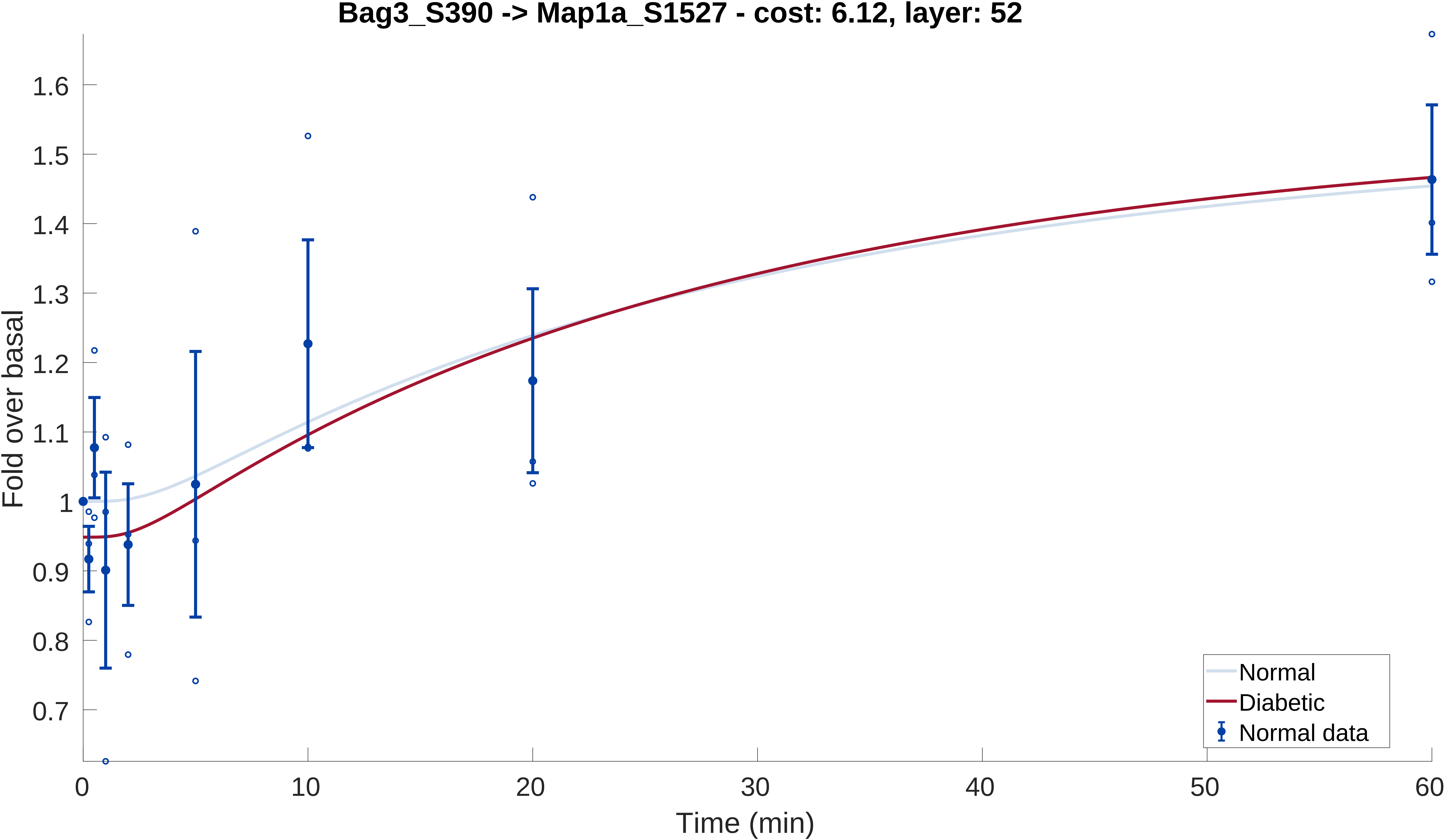

Bag3_S390 -> Map1a_S1527 - cost: 6.12, layer: 52
Fold over basal
1.6
1.5
1.4
1.3
1.2
1.1
1
0.9
0.8
0.7
0
10
20
30
40
50
60
Time (min)
Normal
Diabetic
Normal data

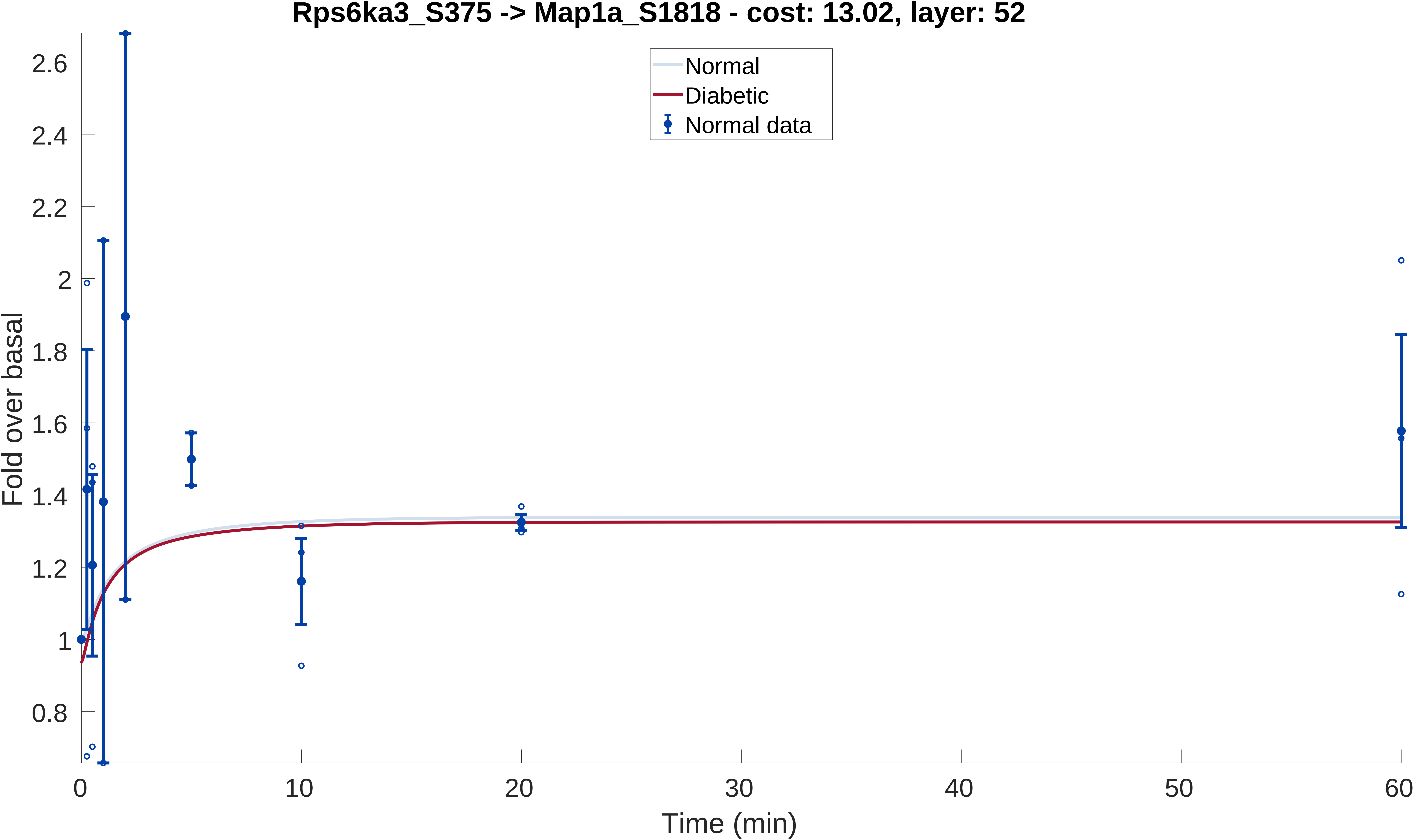

Rps6ka3_S375 -> Map1a_S1818 - cost: 13.02, layer: 52
Normal
Diabetic
Normal data
Fold over basal
Time (min)
0.8
1
1.2
1.4
1.6
1.8
2
2.2
2.4
2.6
0
10
20
30
40
50
60

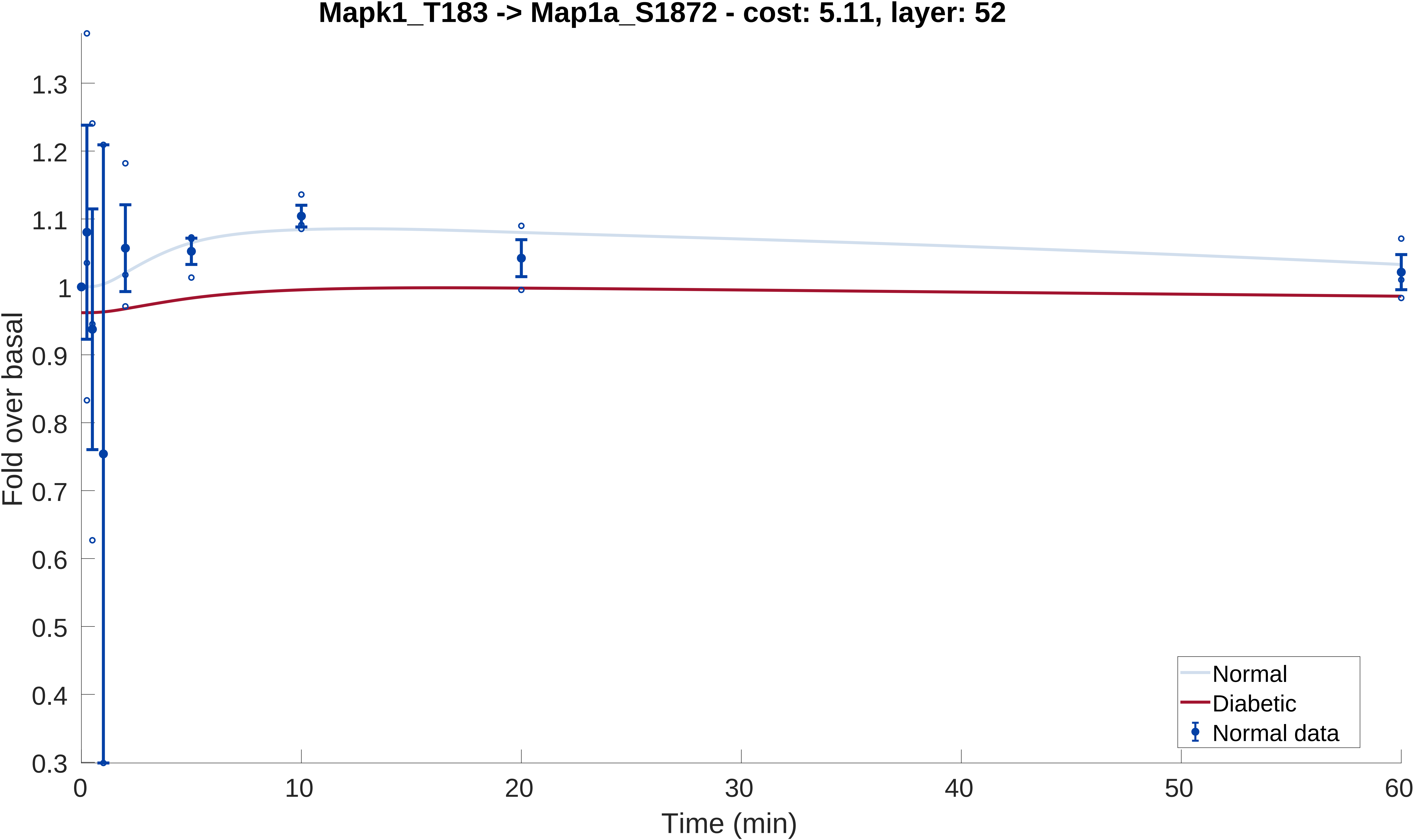

Mapk1_T183 -> Map1a_S1872 - cost: 5.11, layer: 52
1.3
1.2
1.1
1
0.9
0.8
0.7
0.6
0.5
0.4
0.3
Fold over basal
0
10
20
30
40
50
60
Time (min)
Normal
Diabetic
Normal data

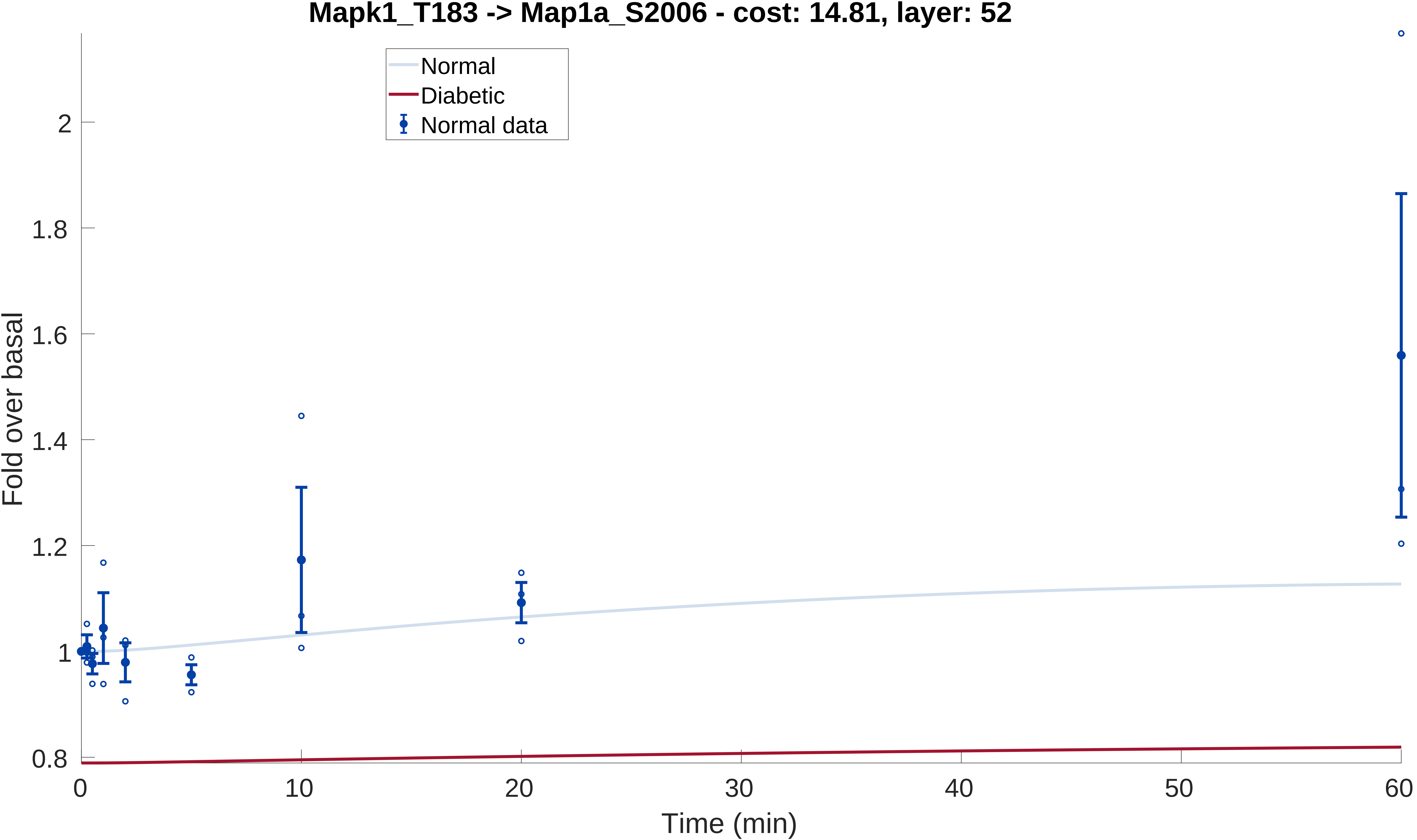
Mapk1_T183 -> Map1a_S2006 - cost: 14.81, layer: 52
Normal
Diabetic
Normal data
Fold over basal
2
1.8
1.6
1.4
1.2
1
0.8
0
10
20
30
40
50
60
Time (min)

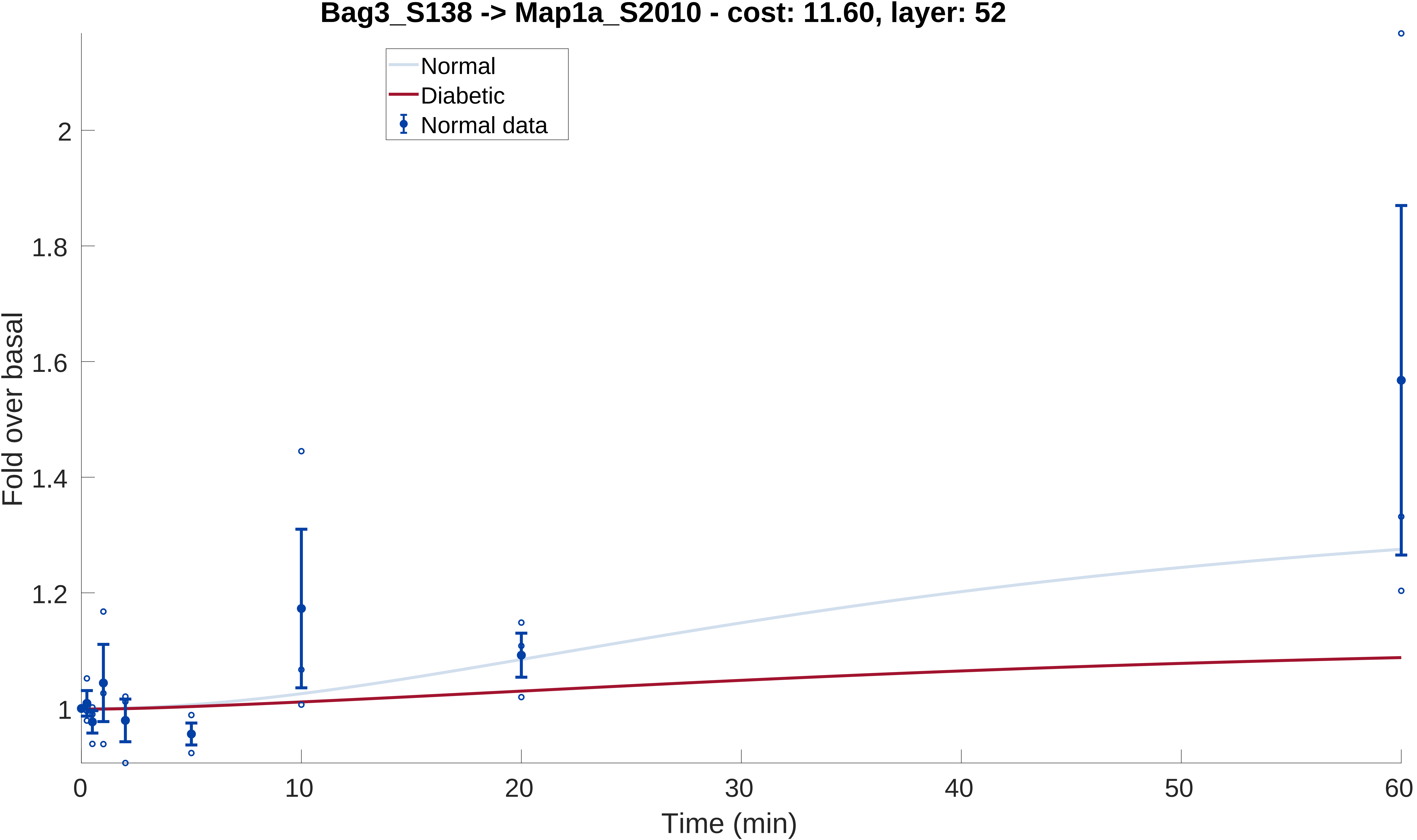
Bag3_S138 -> Map1a_S2010 - cost: 11.60, layer: 52
Normal
Diabetic
Normal data
Fold over basal
2
1.8
1.6
1.4
1.2
1
Time (min)
0
10
20
30
40
50
60

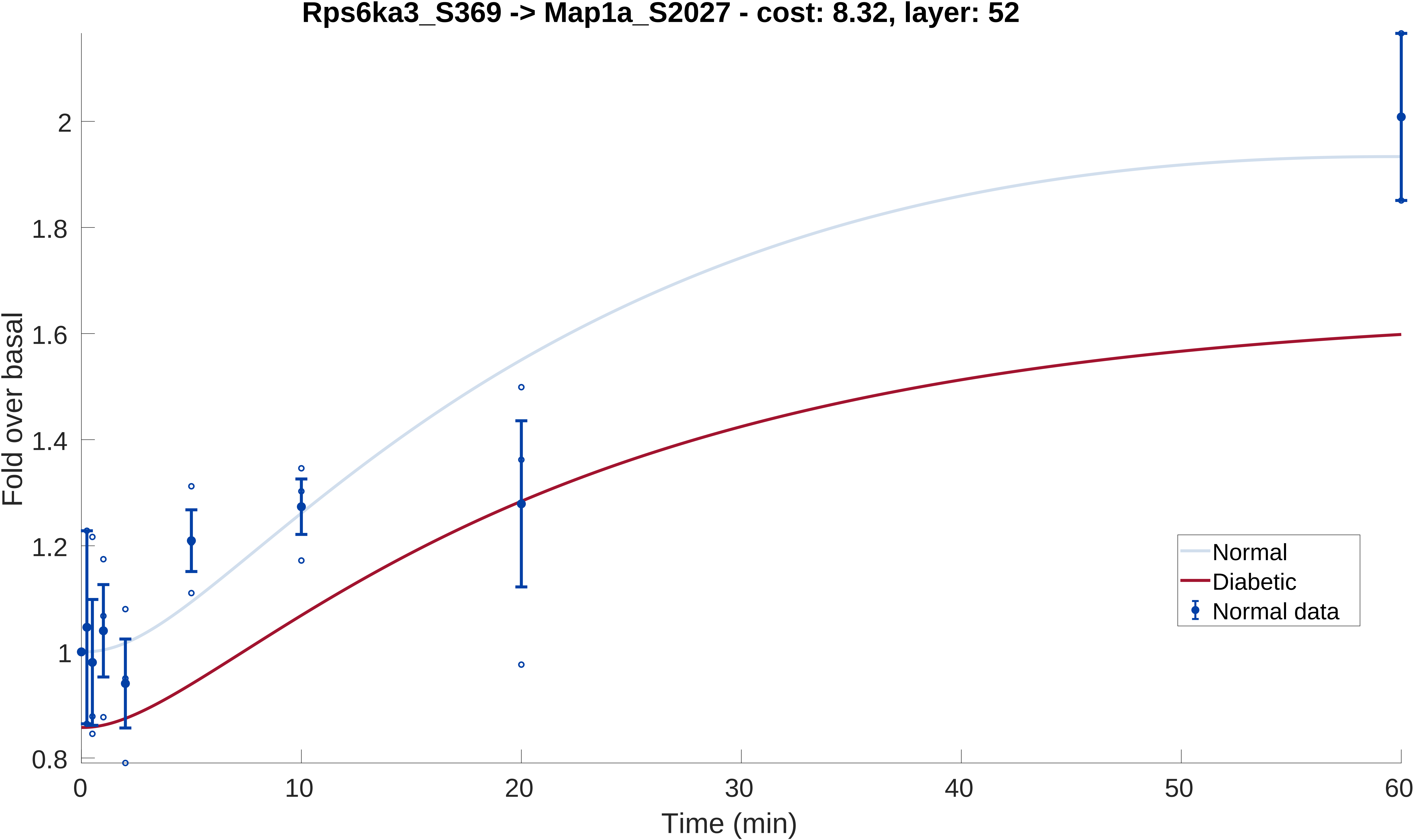

Rps6ka3_S369 -> Map1a_S2027 - cost: 8.32, layer: 52
Fold over basal
0.8
1
1.2
1.4
1.6
1.8
2
0
10
20
30
40
50
60
Time (min)
Normal
Diabetic
Normal data

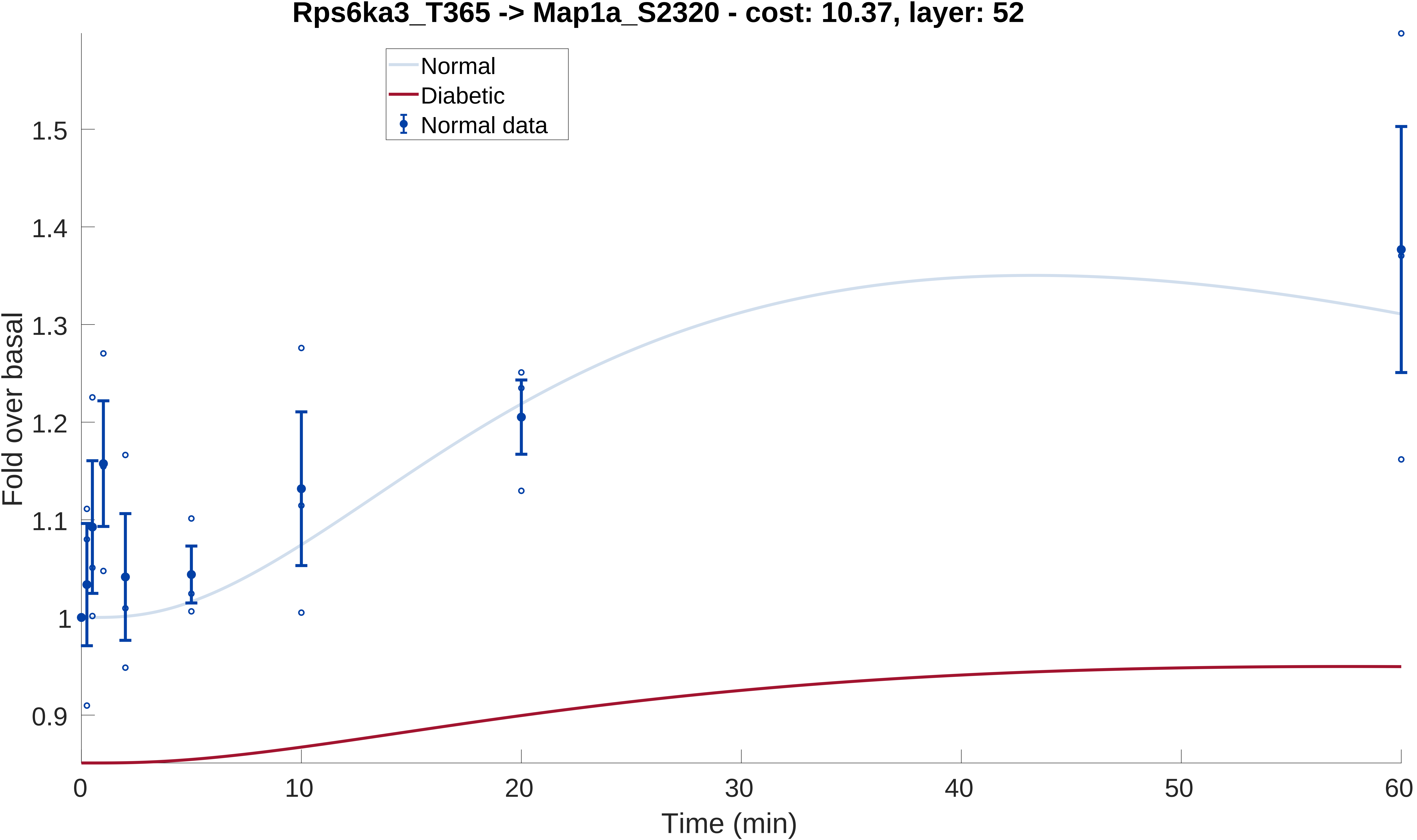

Rps6ka3_T365 -> Map1a_S2320 - cost: 10.37, layer: 52
Normal
Diabetic
Normal data
Fold over basal
1.5
1.4
1.3
1.2
1.1
1
0.9
0
10
20
30
40
50
60
Time (min)

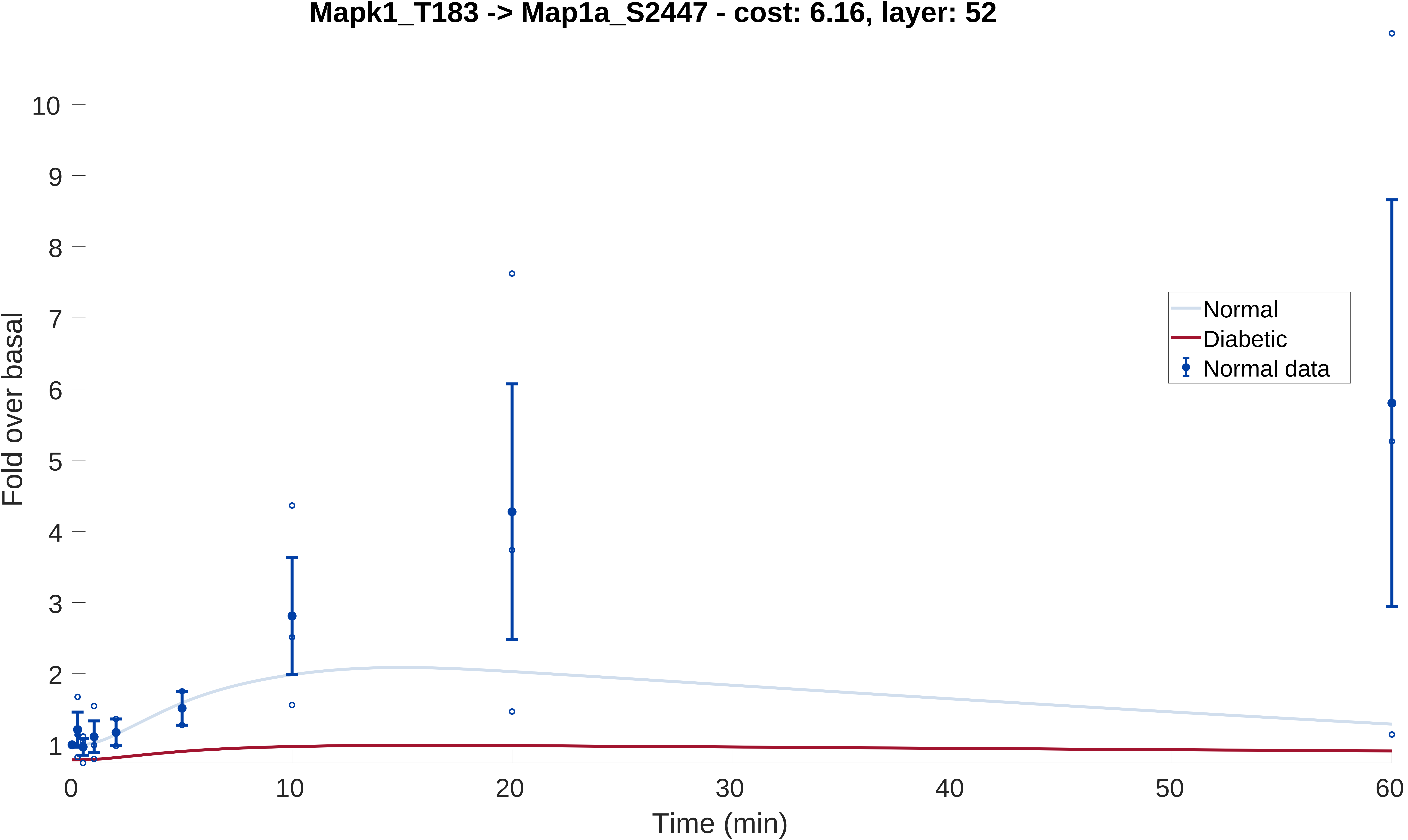

Mapk1_T183 -> Map1a_S2447 - cost: 6.16, layer: 52
Fold over basal
Time (min)
Normal
Diabetic
Normal data
0
10
20
30
40
50
60
1
2
3
4
5
6
7
8
9
10

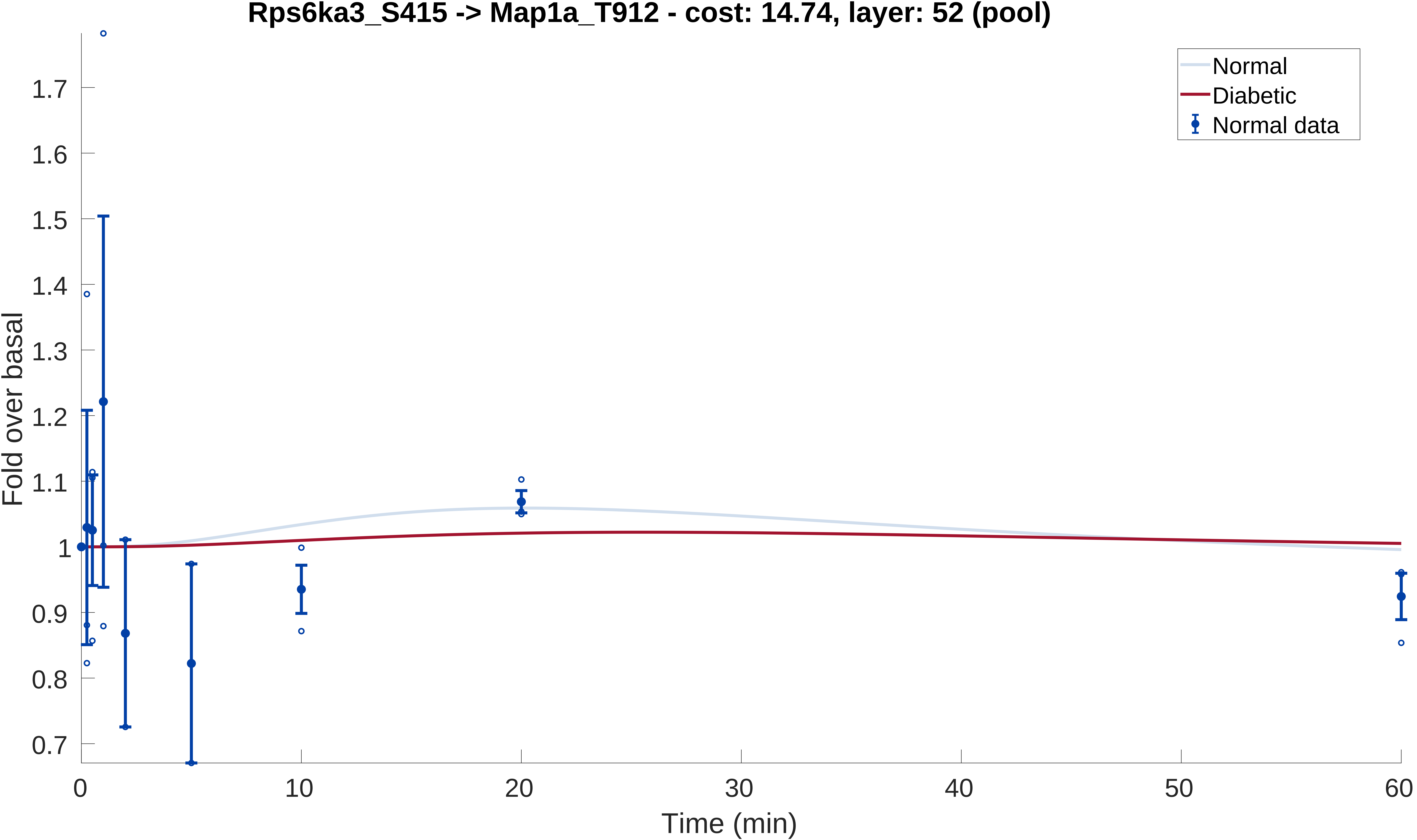

Rps6ka3_S415 -> Map1a_T912 - cost: 14.74, layer: 52 (pool)
Normal
Diabetic
Normal data
Fold over basal
1.7
1.6
1.5
1.4
1.3
1.2
1.1
1
0.9
0.8
0.7
0
10
20
30
40
50
60
Time (min)

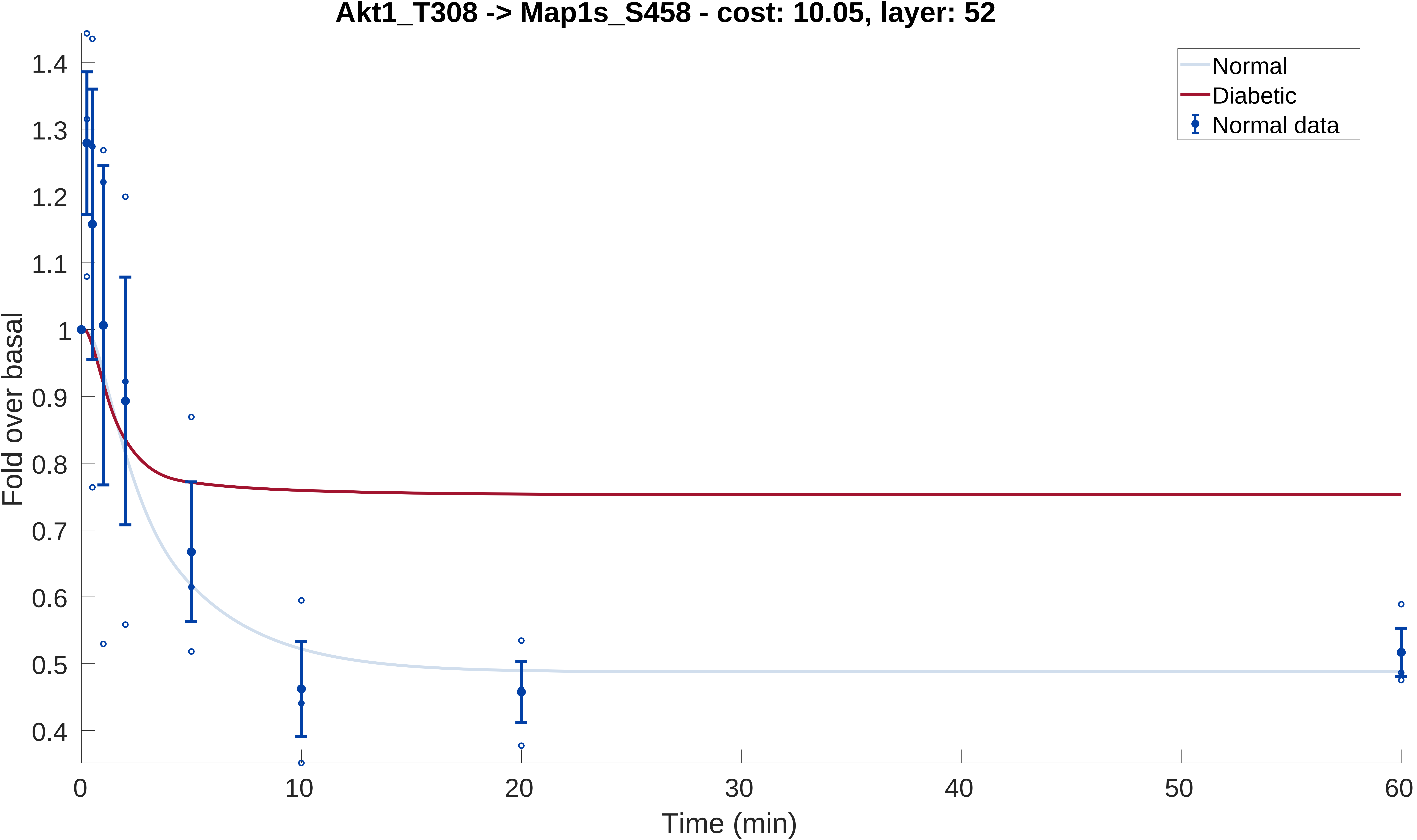

Akt1_T308 -> Map1s_S458 - cost: 10.05, layer: 52
Normal
Diabetic
Normal data
Fold over basal
Time (min)
1.4
1.3
1.2
1.1
1
0.9
0.8
0.7
0.6
0.5
0.4
0
10
20
30
40
50
60

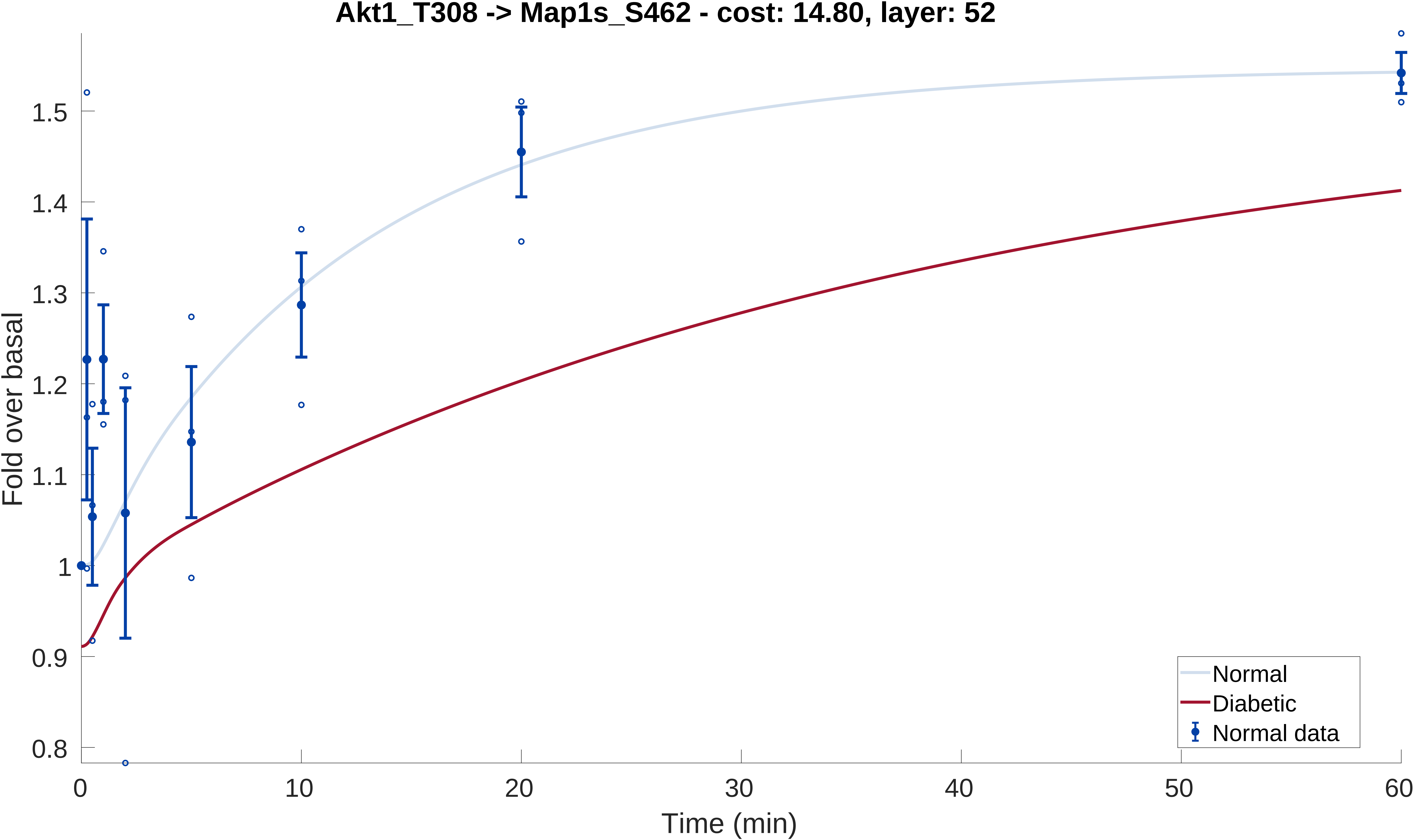
Akt1_T308 -> Map1s_S462 - cost: 14.80, layer: 52
Fold over basal
Time (min)
0.8
0.9
1
1.1
1.2
1.3
1.4
1.5
0
10
20
30
40
50
60
Normal
Diabetic
Normal data

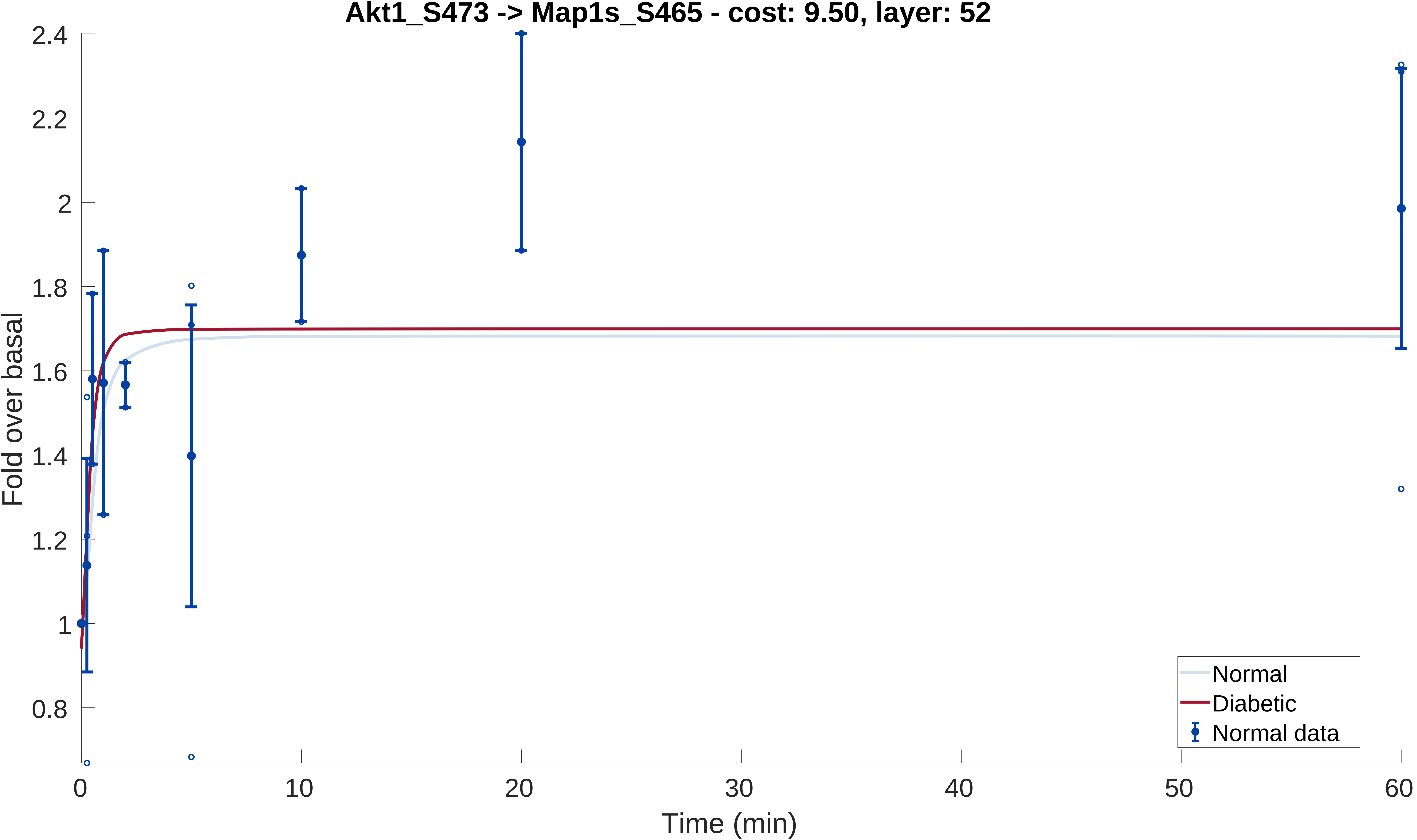

Akt1_S473 -> Map1s_S465 - cost: 9.50, layer: 52
Fold over basal
Time (min)
2.4
2.2
2
1.8
1.6
1.4
1.2
1
0.8
0
10
20
30
40
50
60
Normal
Diabetic
Normal data

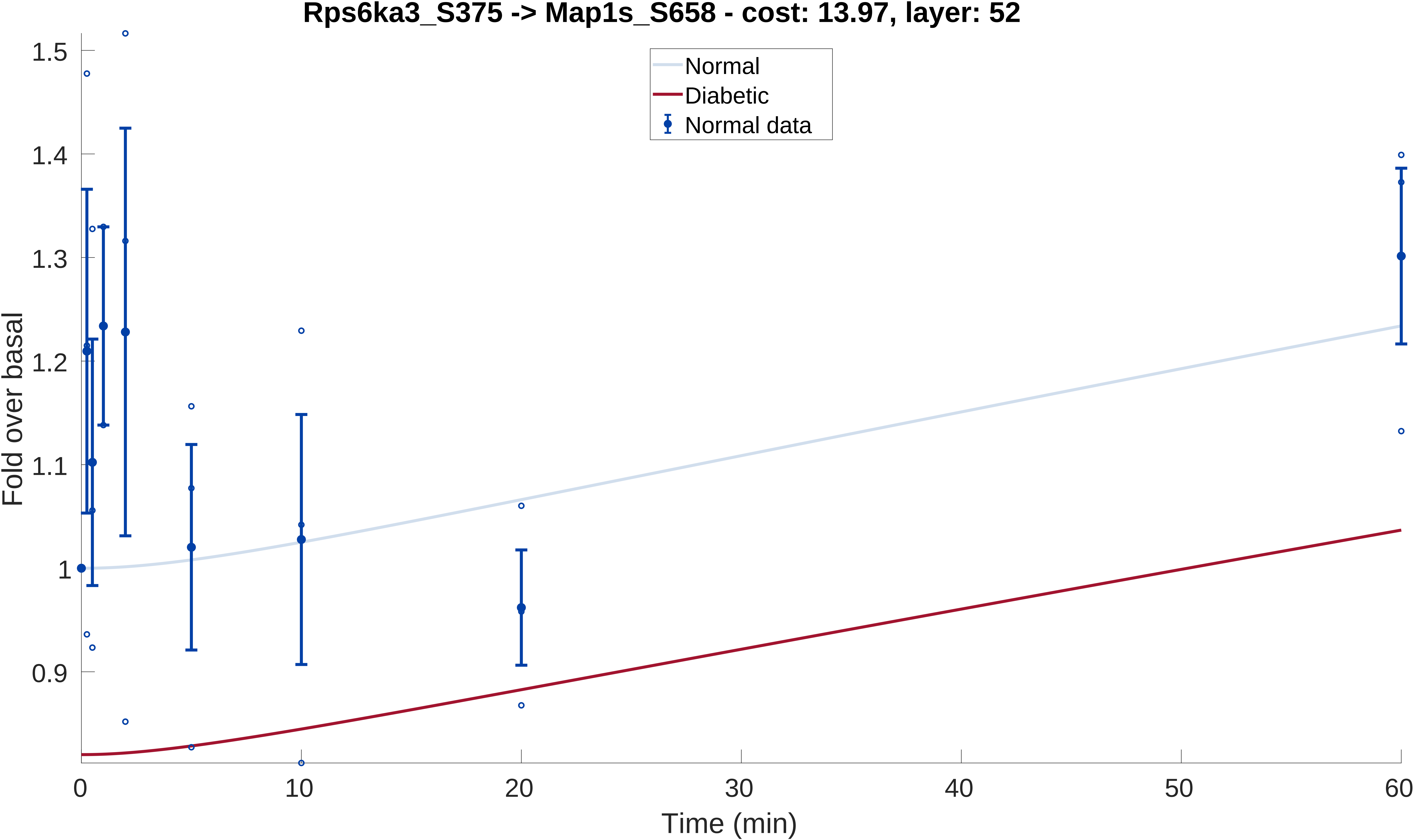

Rps6ka3_S375 -> Map1s_S658 - cost: 13.97, layer: 52
Normal
Diabetic
Normal data
Fold over basal
1.5
1.4
1.3
1.2
1.1
1
0.9
0
10
20
30
40
50
60
Time (min)

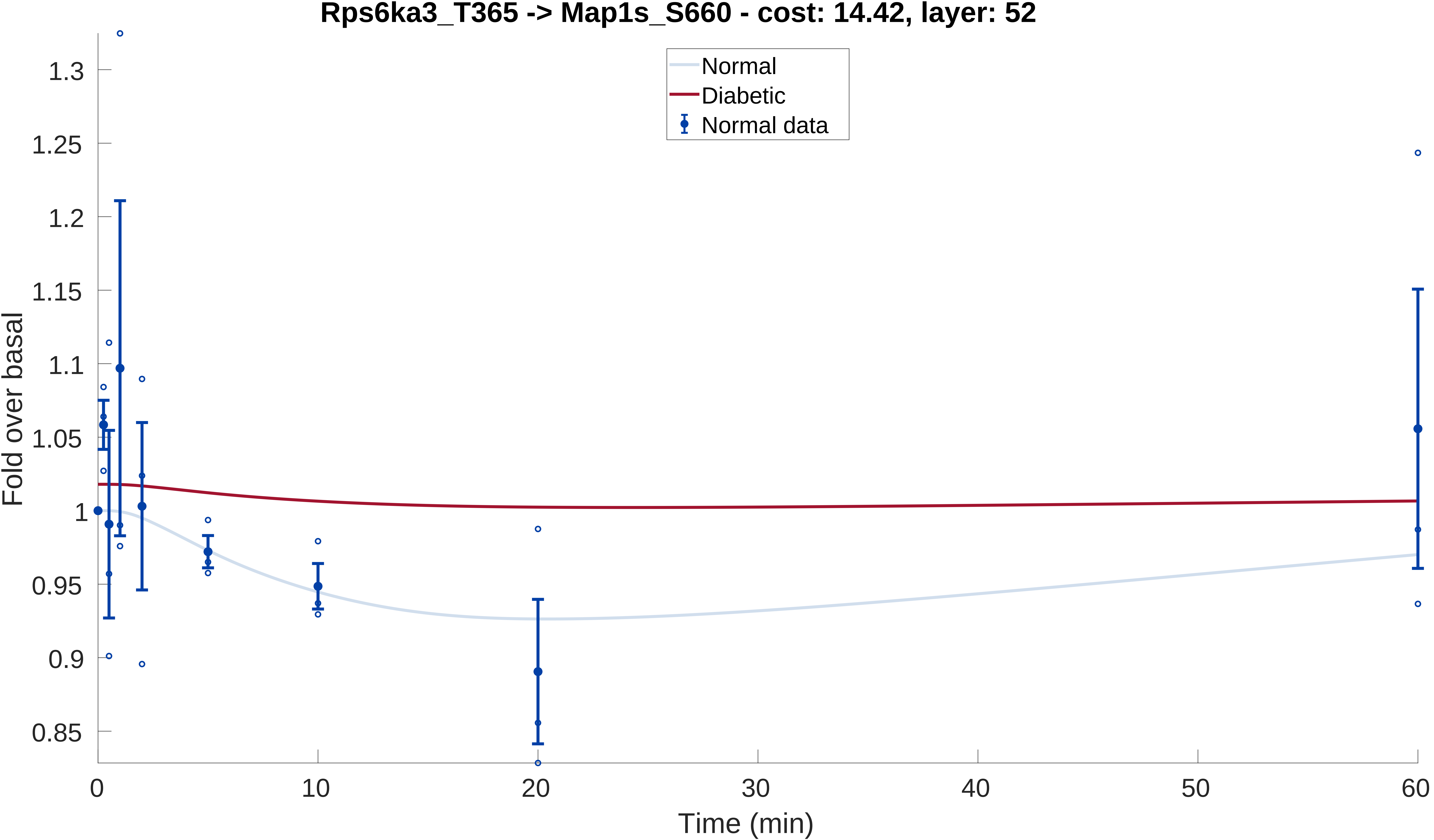

Rps6ka3_T365 -> Map1s_S660 - cost: 14.42, layer: 52
Normal
Diabetic
Normal data
Fold over basal
Time (min)
1.3
1.25
1.2
1.15
1.1
1.05
1
0.95
0.9
0.85
0
10
20
30
40
50
60

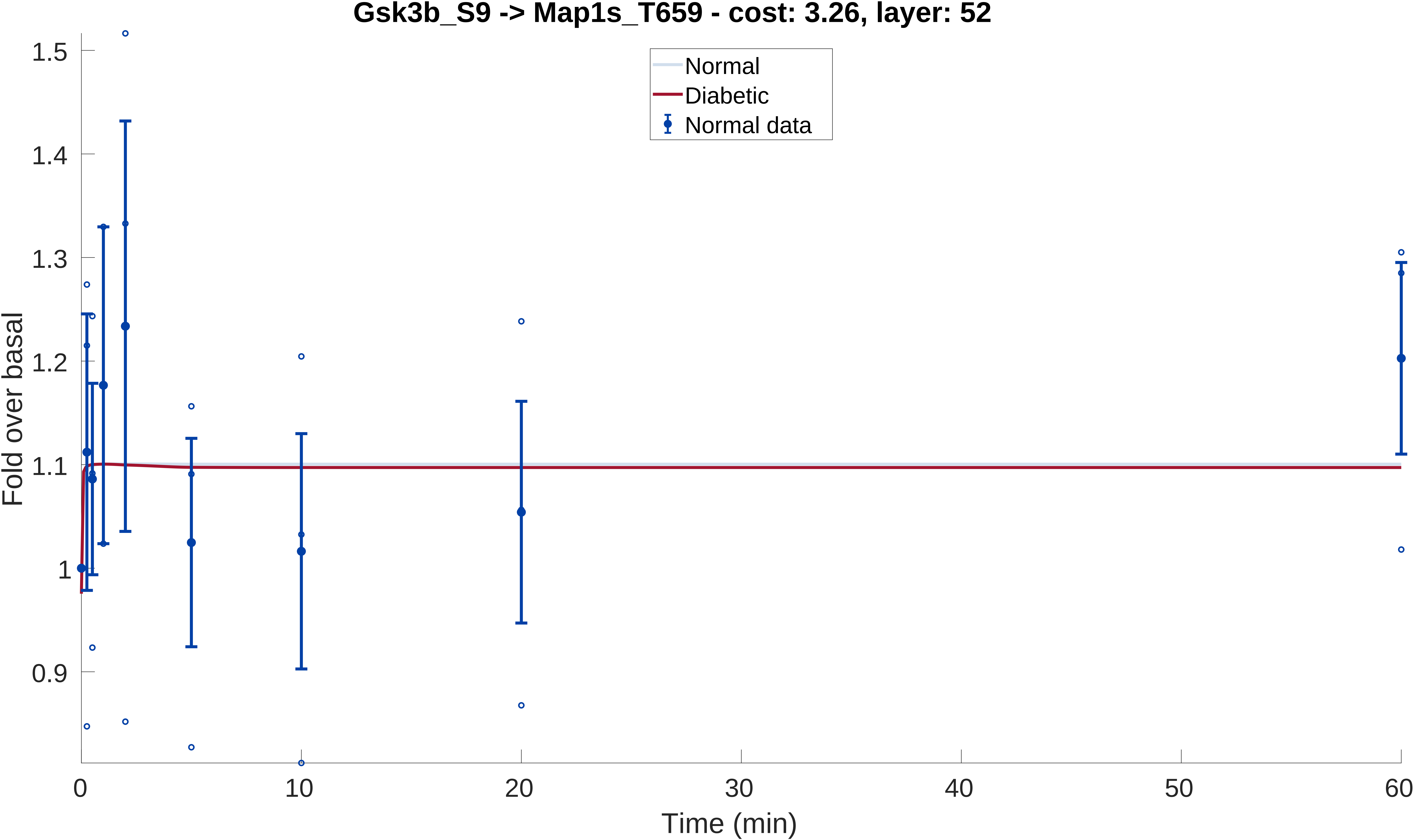
Gsk3b_S9 -> Map1s_T659 - cost: 3.26, layer: 52
Normal
Diabetic
Normal data
Fold over basal
Time (min)
1.5
1.4
1.3
1.2
1.1
1
0.9
0
10
20
30
40
50
60

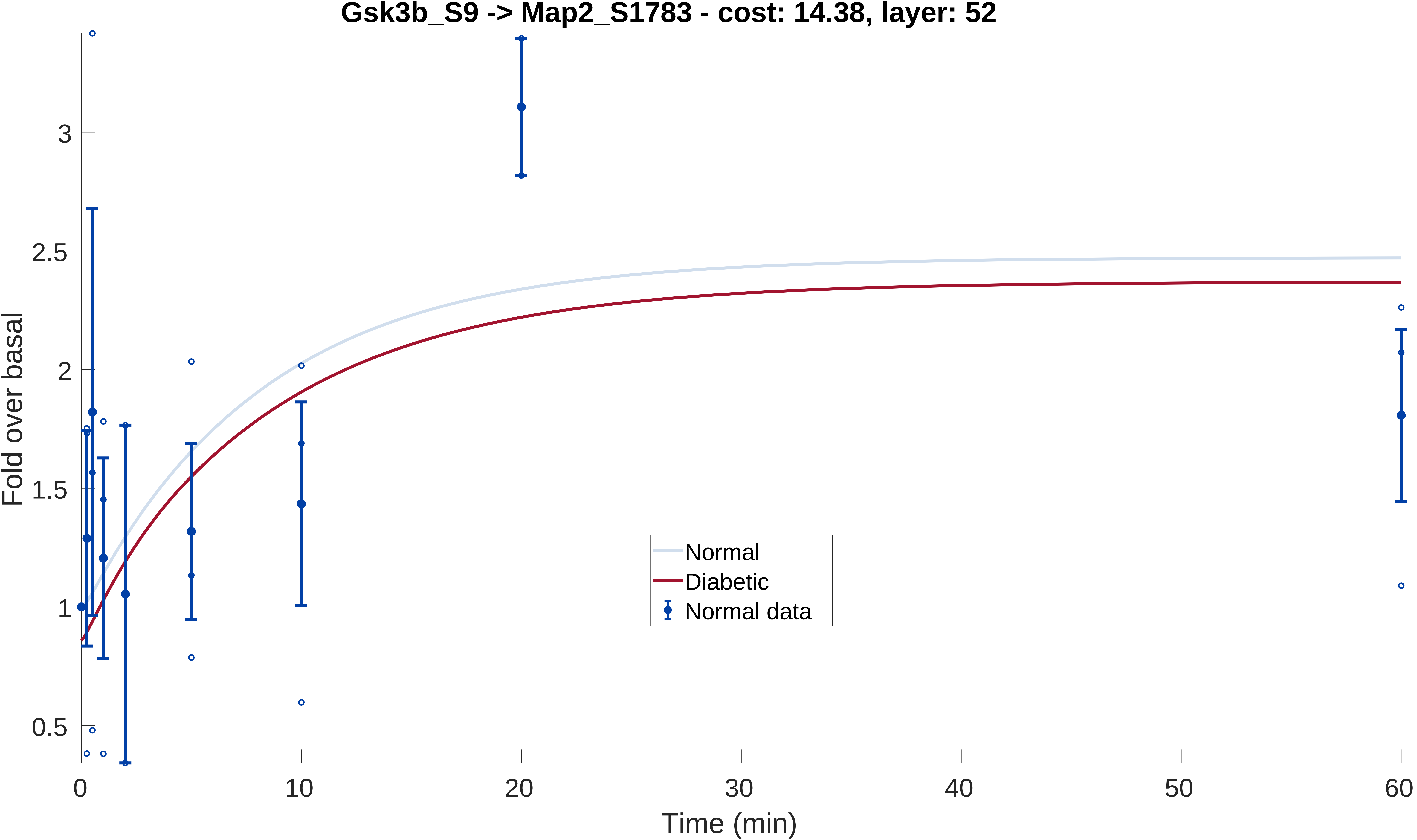

Gsk3b_S9 -> Map2_S1783 - cost: 14.38, layer: 52
Fold over basal
Time (min)
Normal
Diabetic
Normal data
0.5
1
1.5
2
2.5
3
0
10
20
30
40
50
60

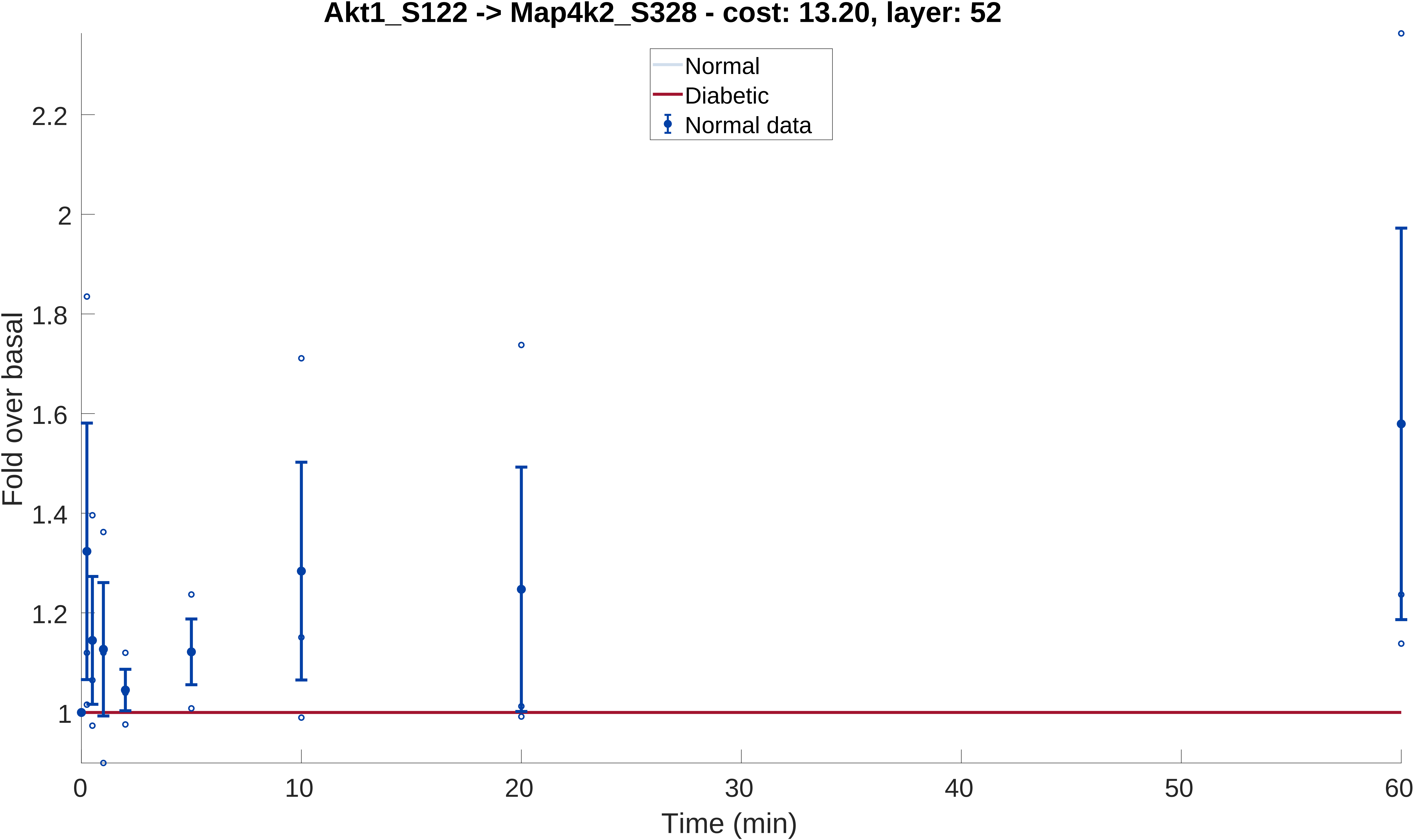

Akt1_S122 -> Map4k2_S328 - cost: 13.20, layer: 52
Normal
Diabetic
Normal data
Fold over basal
2.2
2
1.8
1.6
1.4
1.2
1
0
10
20
30
40
50
60
Time (min)

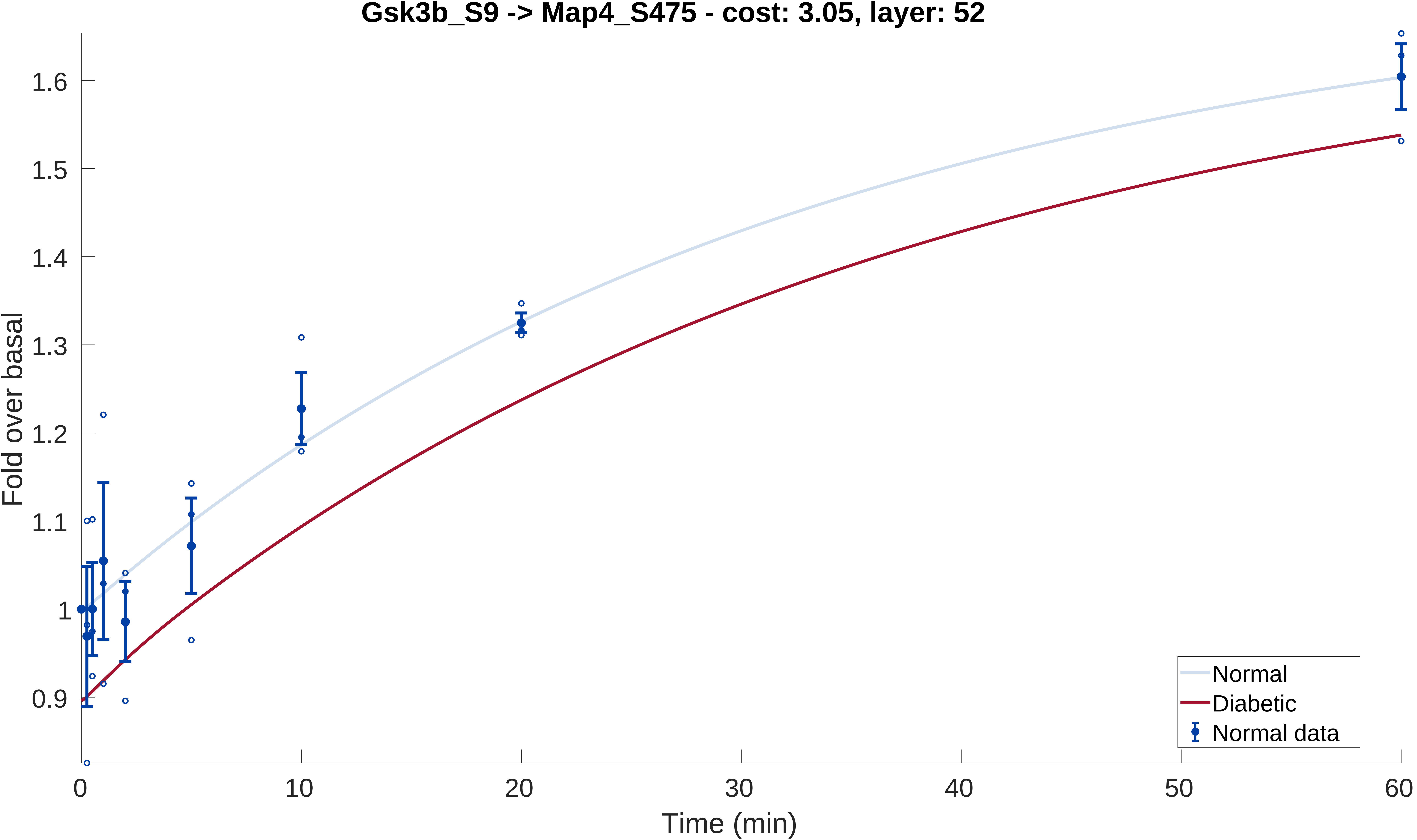

Gsk3b_S9 -> Map4_S475 - cost: 3.05, layer: 52
Fold over basal
1.6
1.5
1.4
1.3
1.2
1.1
1
0.9
0
10
20
30
40
50
60
Time (min)
Normal
Diabetic
Normal data

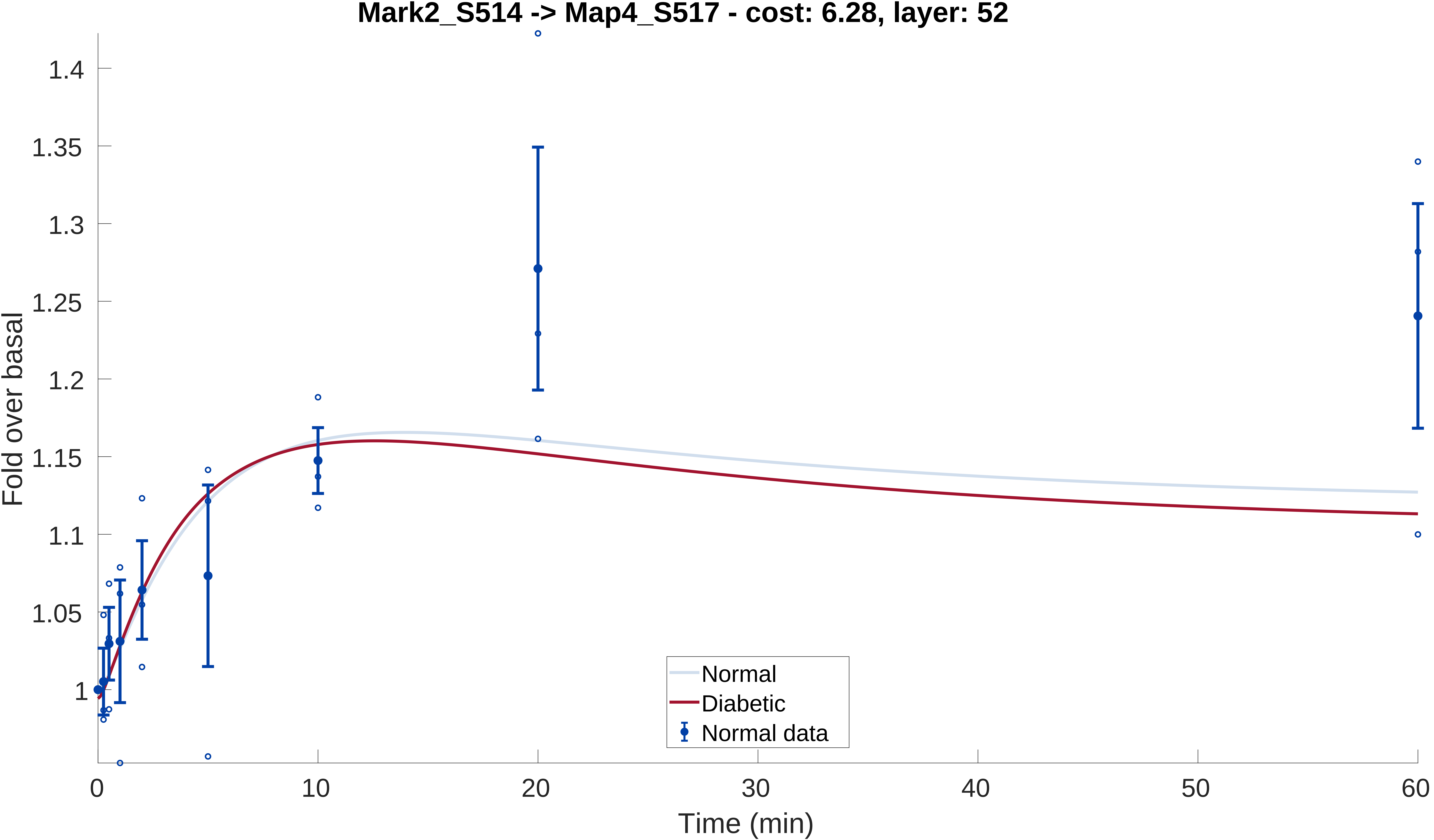
Mark2_S514 -> Map4_S517 - cost: 6.28, layer: 52
Fold over basal
1.4
1.35
1.3
1.25
1.2
1.15
1.1
1.05
1
0
10
20
30
40
50
60
Time (min)
Normal
Diabetic
Normal data

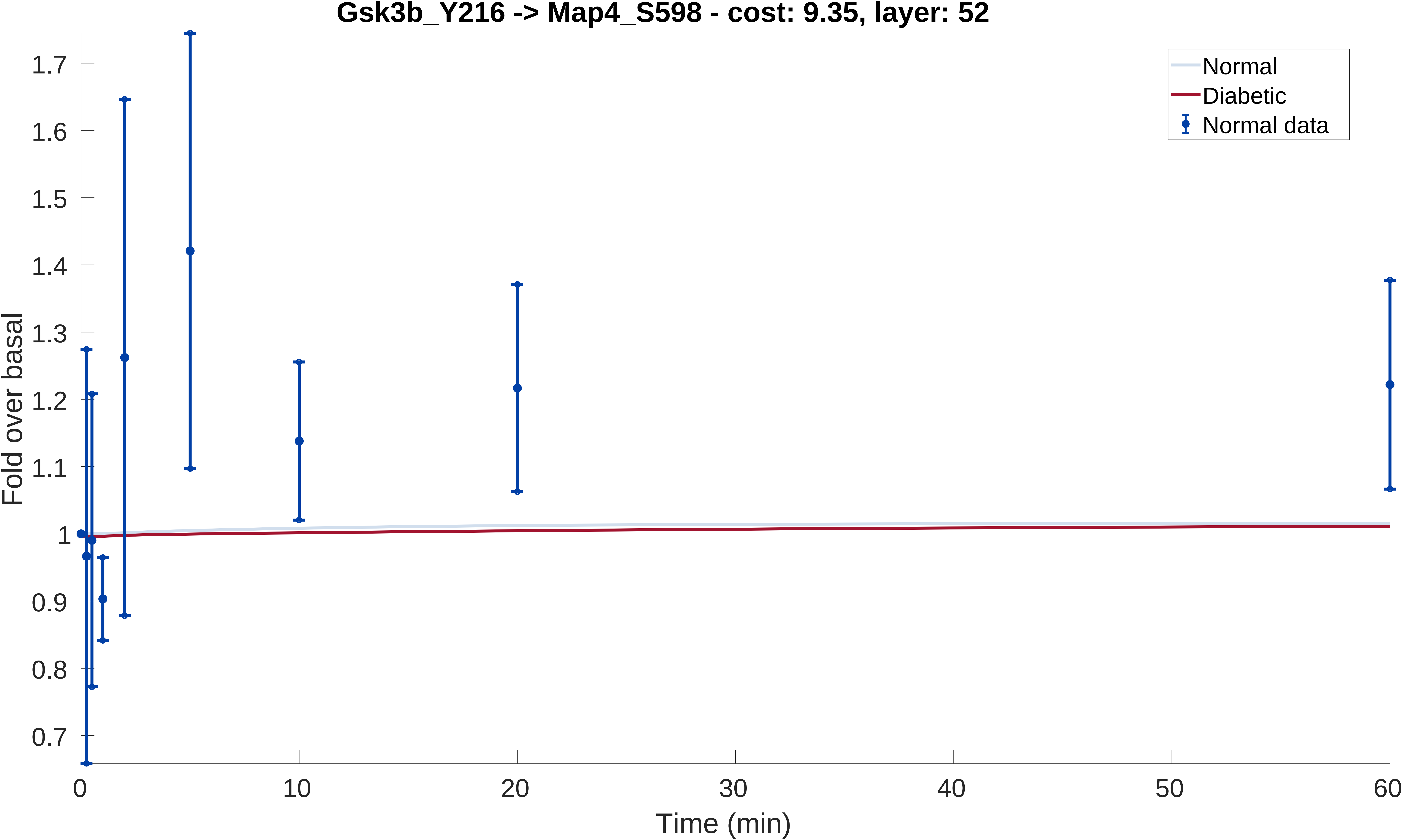

Gsk3b_Y216 -> Map4_S598 - cost: 9.35, layer: 52
Normal
Diabetic
Normal data
Fold over basal
Time (min)
0.7
0.8
0.9
1
1.1
1.2
1.3
1.4
1.5
1.6
1.7
0
10
20
30
40
50
60

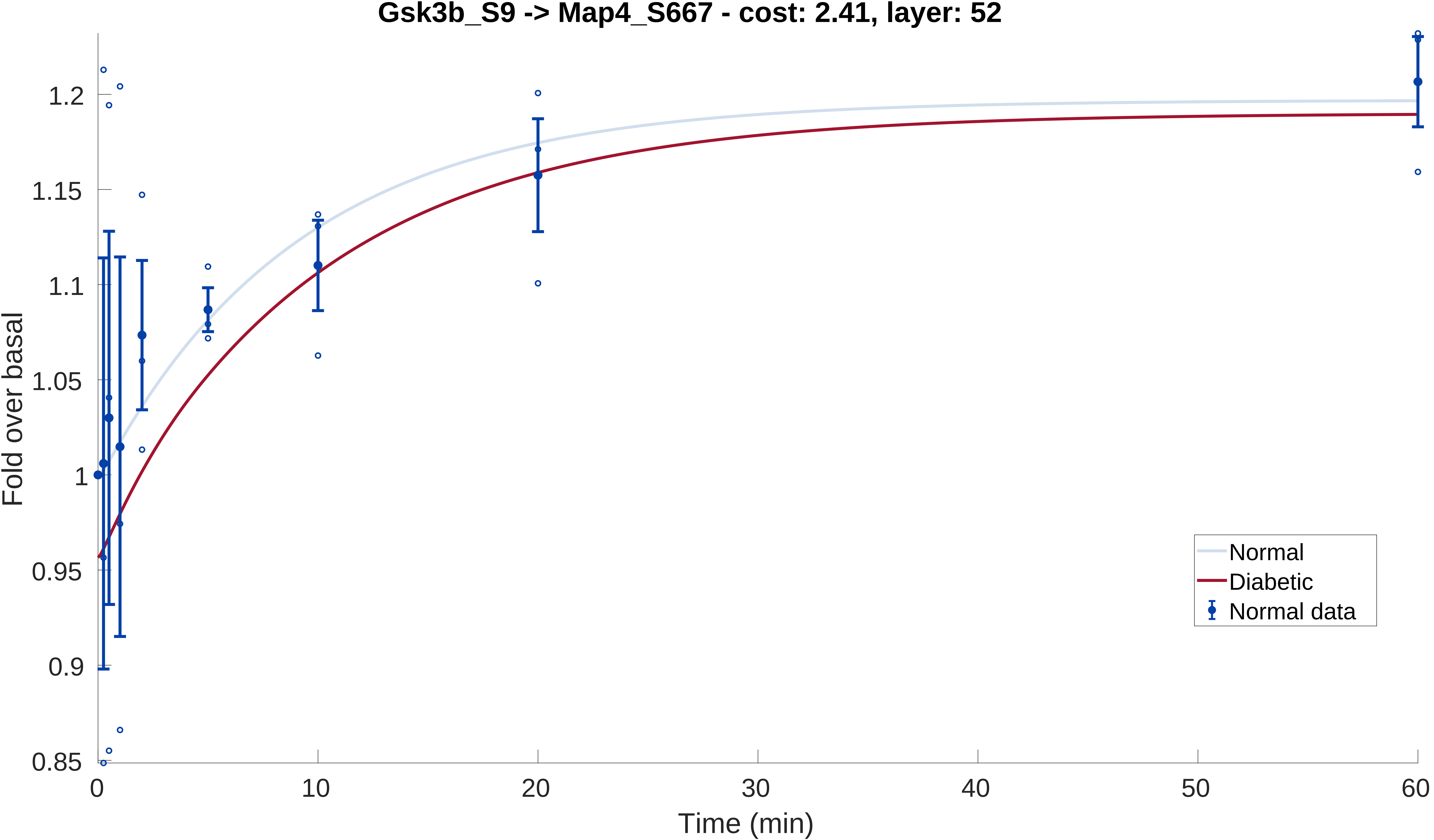
Gsk3b_S9 -> Map4_S667 - cost: 2.41, layer: 52
Fold over basal
Time (min)
1.2
1.15
1.1
1.05
1
0.95
0.9
0.85
0
10
20
30
40
50
60
Normal
Diabetic
Normal data

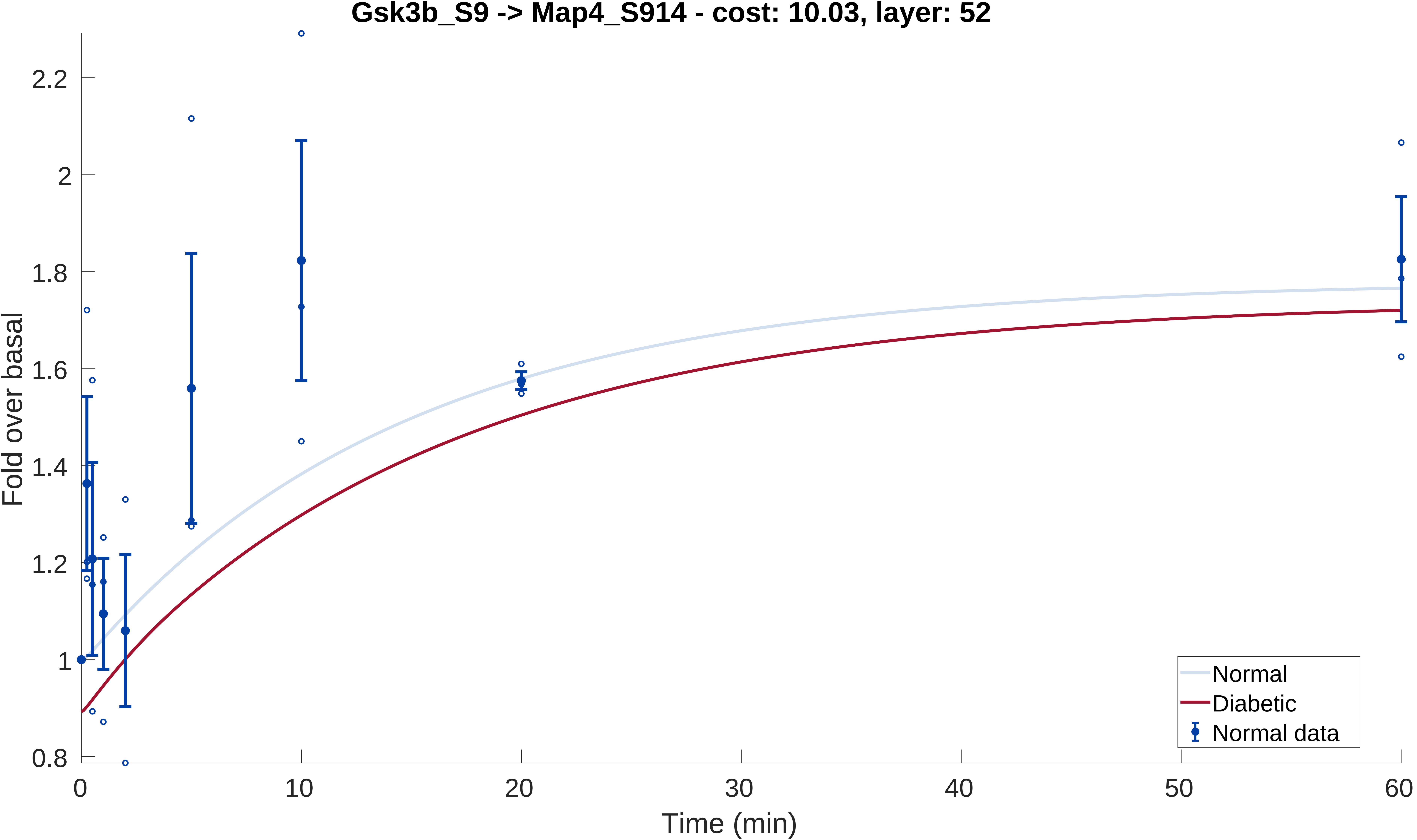
Gsk3b_S9 -> Map4_S914 - cost: 10.03, layer: 52
Fold over basal
Time (min)
2.2
2
1.8
1.6
1.4
1.2
1
0.8
0
10
20
30
40
50
60
Normal
Diabetic
Normal data

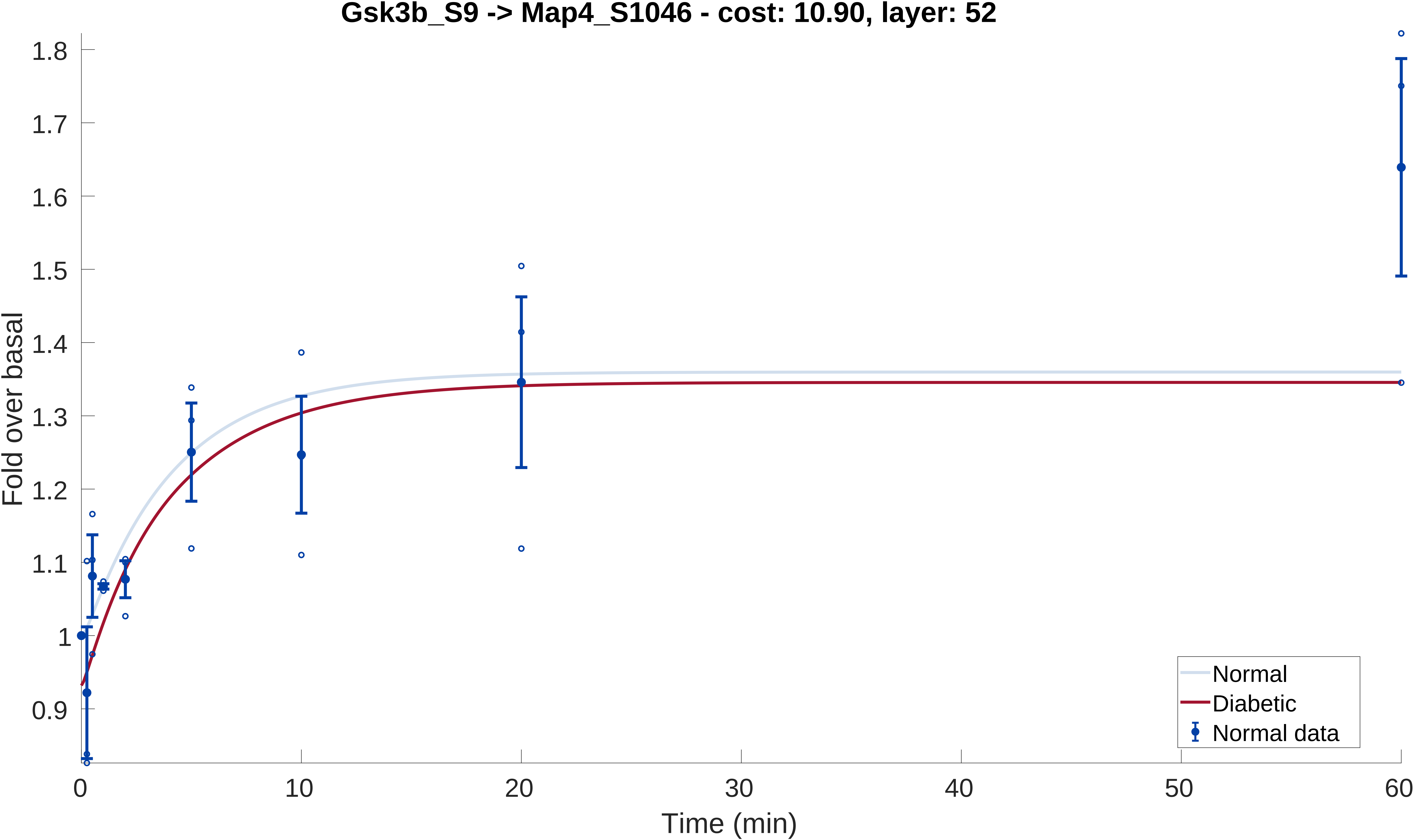

Gsk3b_S9 -> Map4_S1046 - cost: 10.90, layer: 52
Fold over basal
1.8
1.7
1.6
1.5
1.4
1.3
1.2
1.1
1
0.9
0
10
20
30
40
50
60
Time (min)
Normal
Diabetic
Normal data

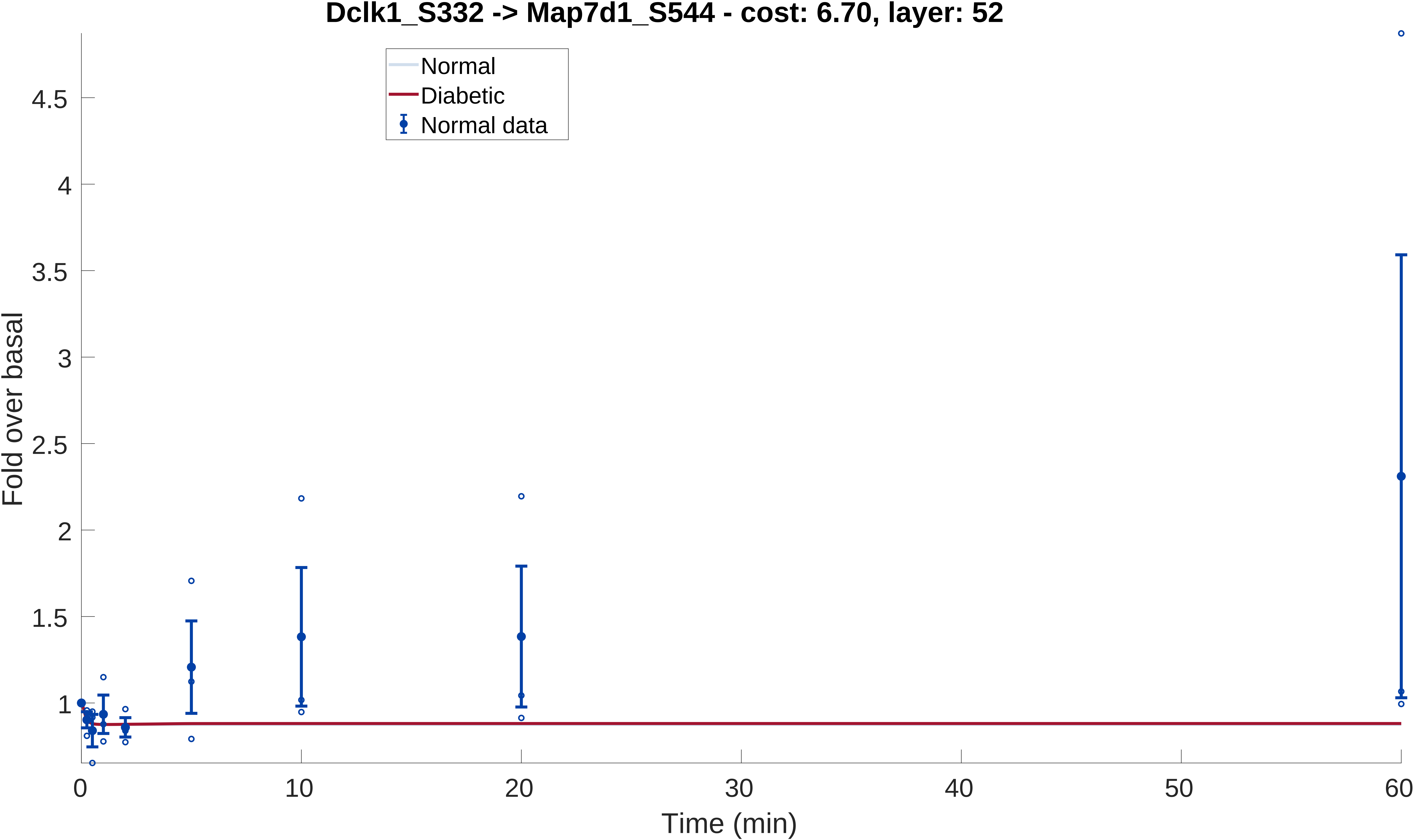

Dclk1_S332 -> Map7d1_S544 - cost: 6.70, layer: 52
Normal
Diabetic
Normal data
Fold over basal
4.5
4
3.5
3
2.5
2
1.5
1
0
10
20
30
40
50
60
Time (min)

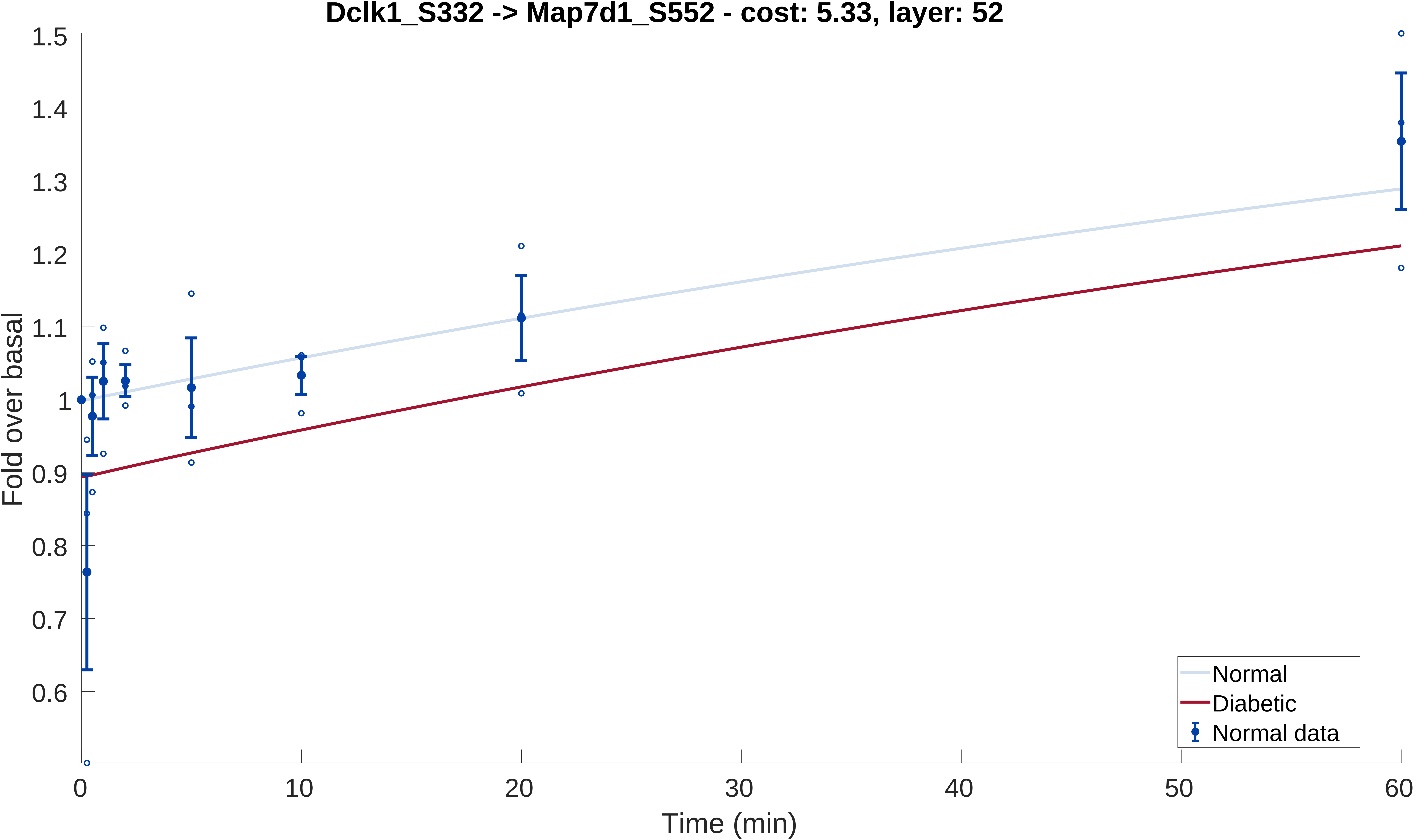
Dclk1_S332 -> Map7d1_S552 - cost: 5.33, layer: 52
1.5
1.4
1.3
1.2
1.1
1
0.9
0.8
0.7
0.6
Fold over basal
0
10
20
30
40
50
60
Time (min)
Normal
Diabetic
Normal data

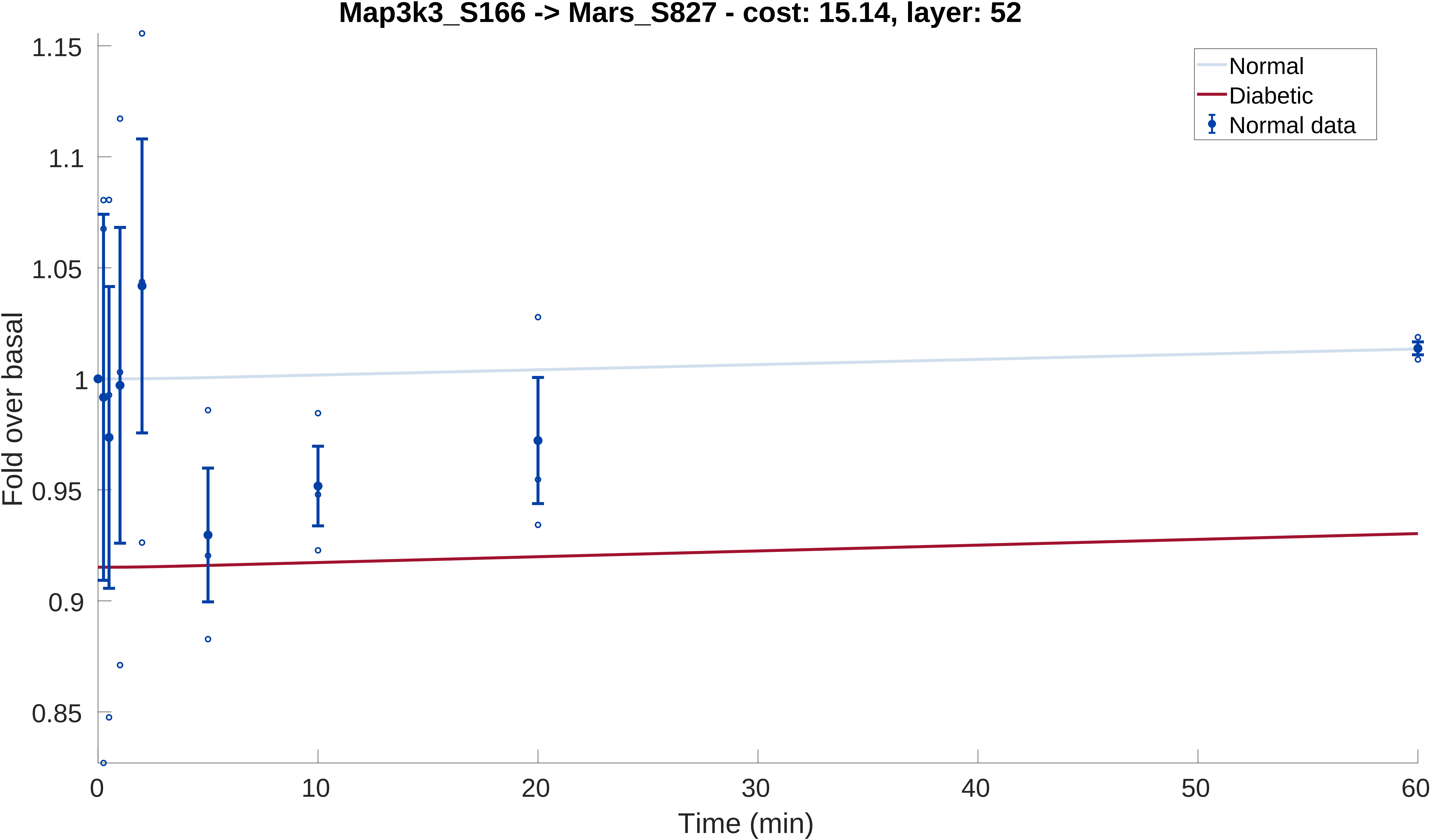

Map3k3_S166 -> Mars_S827 - cost: 15.14, layer: 52
Normal
Diabetic
Normal data
Fold over basal
Time (min)
1.15
1.1
1.05
1
0.95
0.9
0.85
0
10
20
30
40
50
60

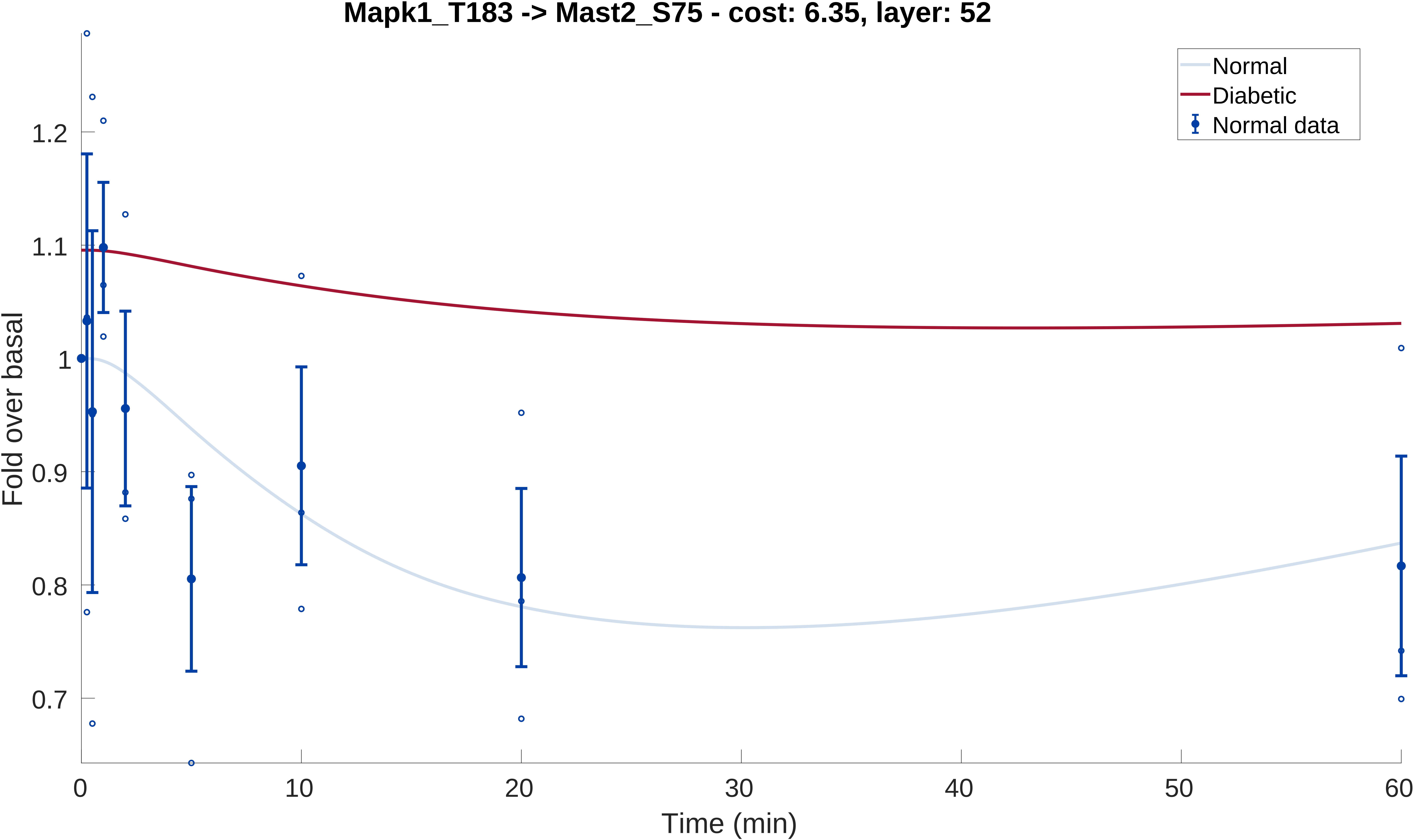

Mapk1_T183 -> Mast2_S75 - cost: 6.35, layer: 52
Normal
Diabetic
Normal data
Fold over basal
1.2
1.1
1
0.9
0.8
0.7
0
10
20
30
40
50
60
Time (min)

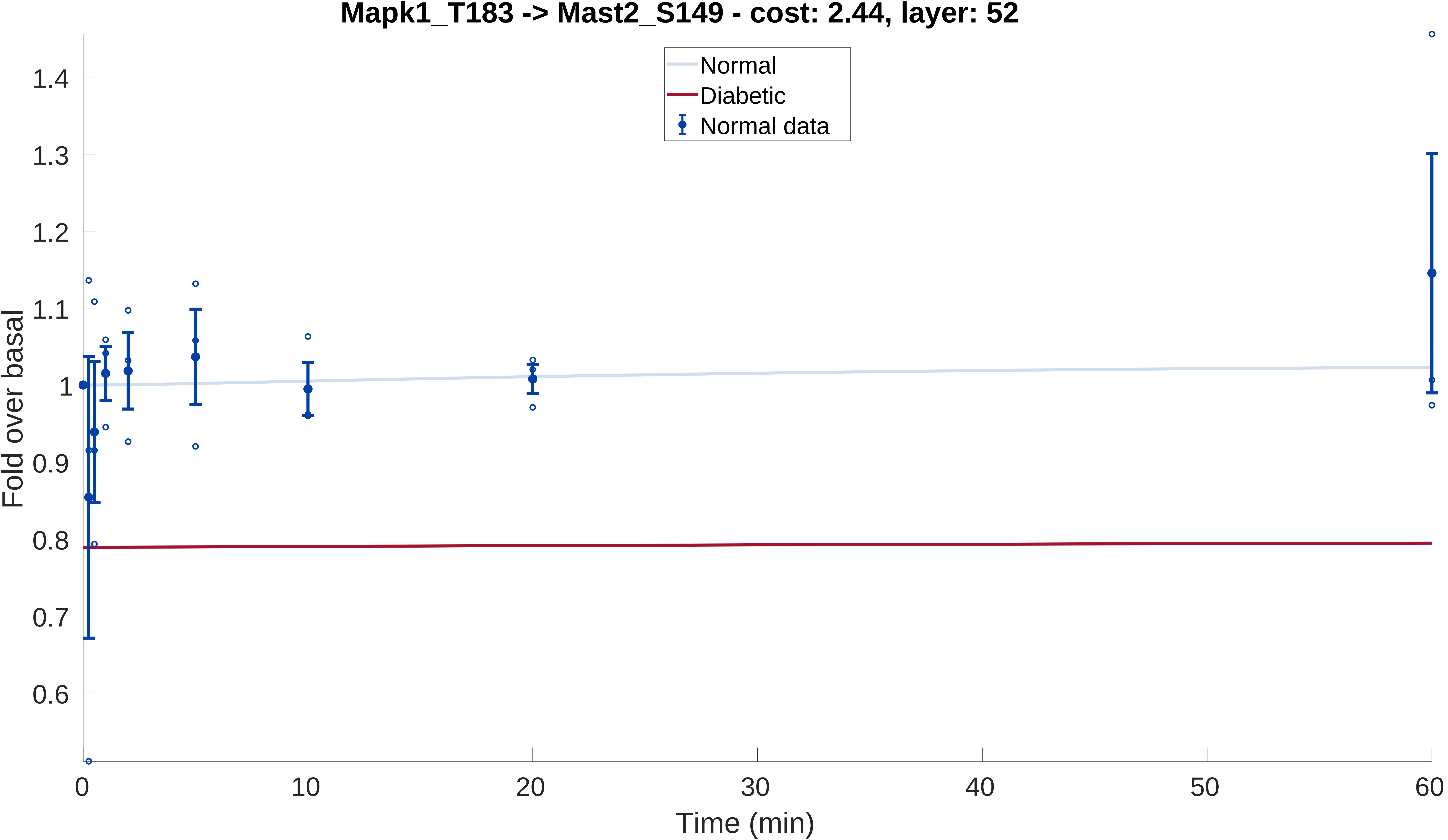

Mapk1_T183 -> Mast2_S149 - cost: 2.44, layer: 52
Normal
Diabetic
Normal data
Fold over basal
1.4
1.3
1.2
1.1
1
0.9
0.8
0.7
0.6
0
10
20
30
40
50
60
Time (min)

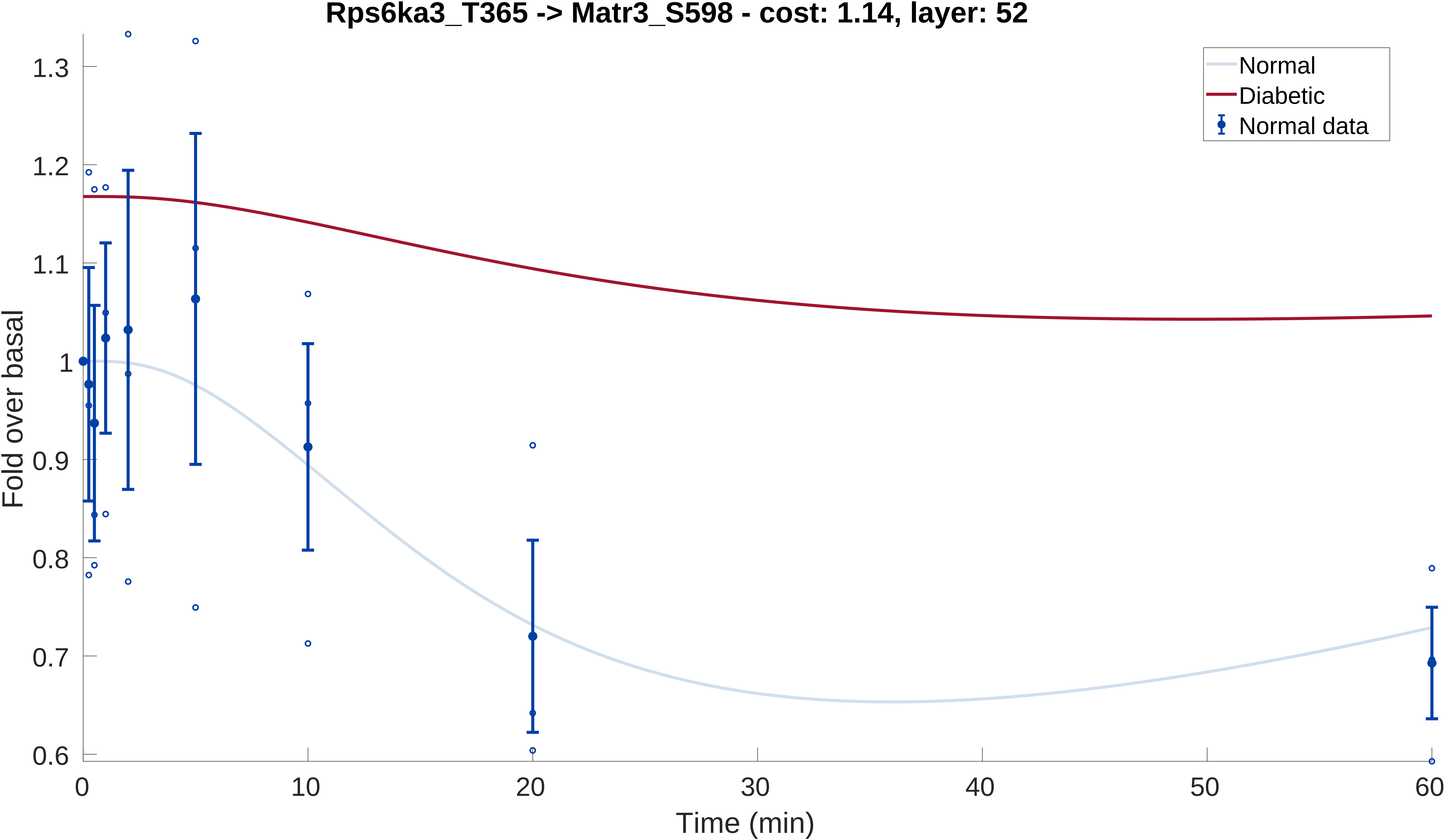

Rps6ka3_T365 -> Matr3_S598 - cost: 1.14, layer: 52
Normal
Diabetic
Normal data
Fold over basal
Time (min)
1.3
1.2
1.1
1
0.9
0.8
0.7
0.6
0
10
20
30
40
50
60

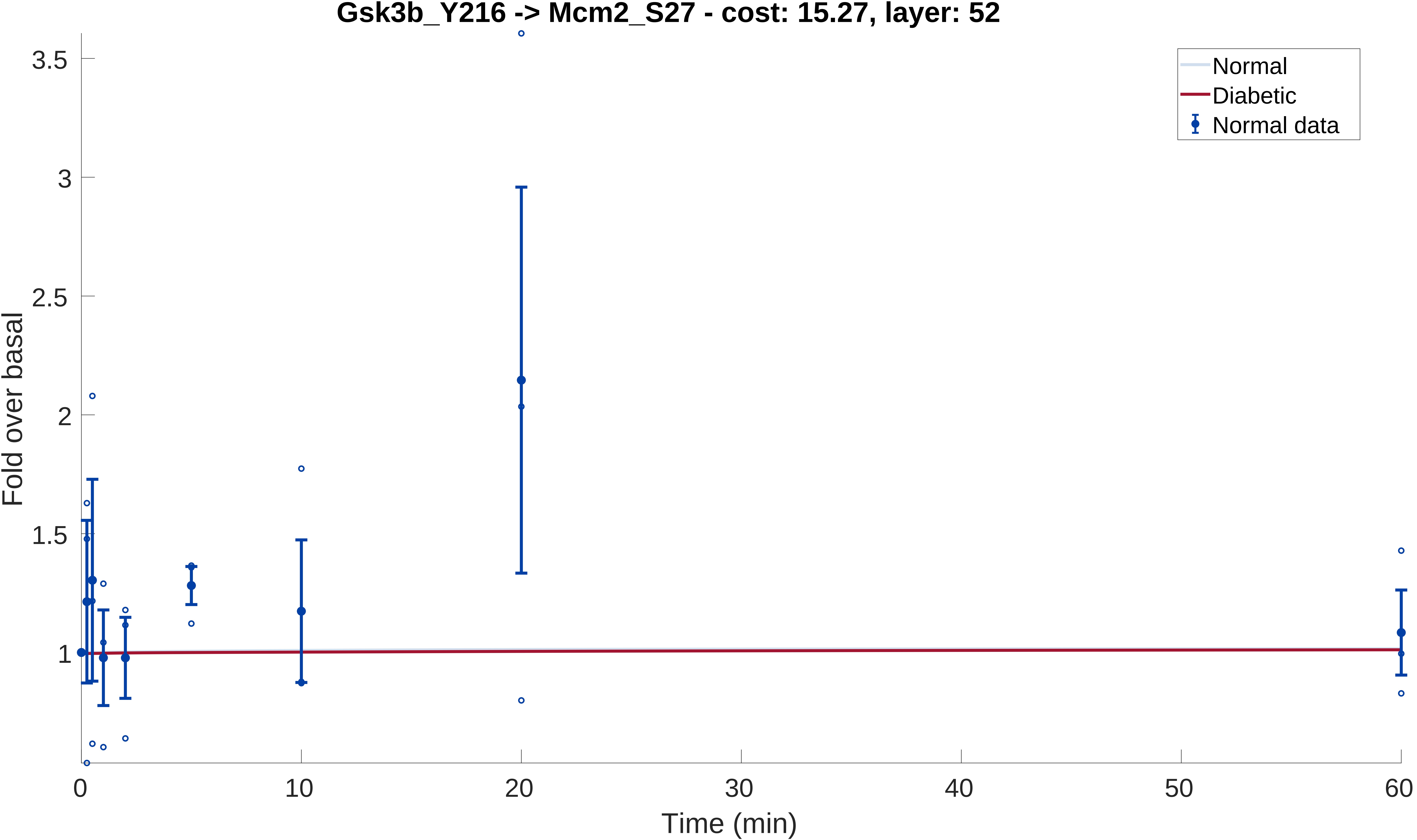

Gsk3b_Y216 -> Mcm2_S27 - cost: 15.27, layer: 52
Normal
Diabetic
Normal data
Fold over basal
Time (min)
3.5
3
2.5
2
1.5
1
0
10
20
30
40
50
60

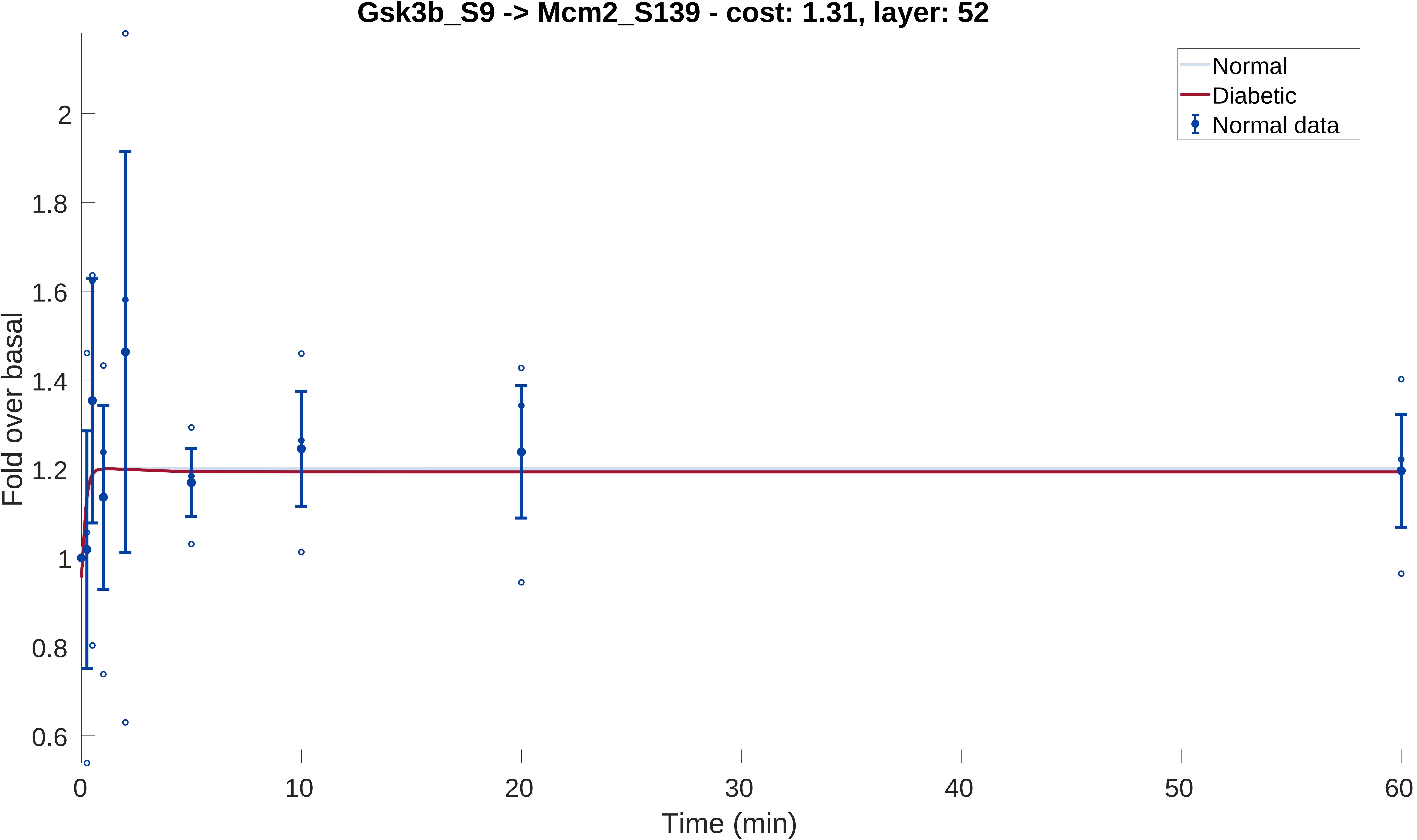

Gsk3b_S9 -> Mcm2_S139 - cost: 1.31, layer: 52
Normal
Diabetic
Normal data
Fold over basal
Time (min)
0.6
0.8
1
1.2
1.4
1.6
1.8
2
0
10
20
30
40
50
60

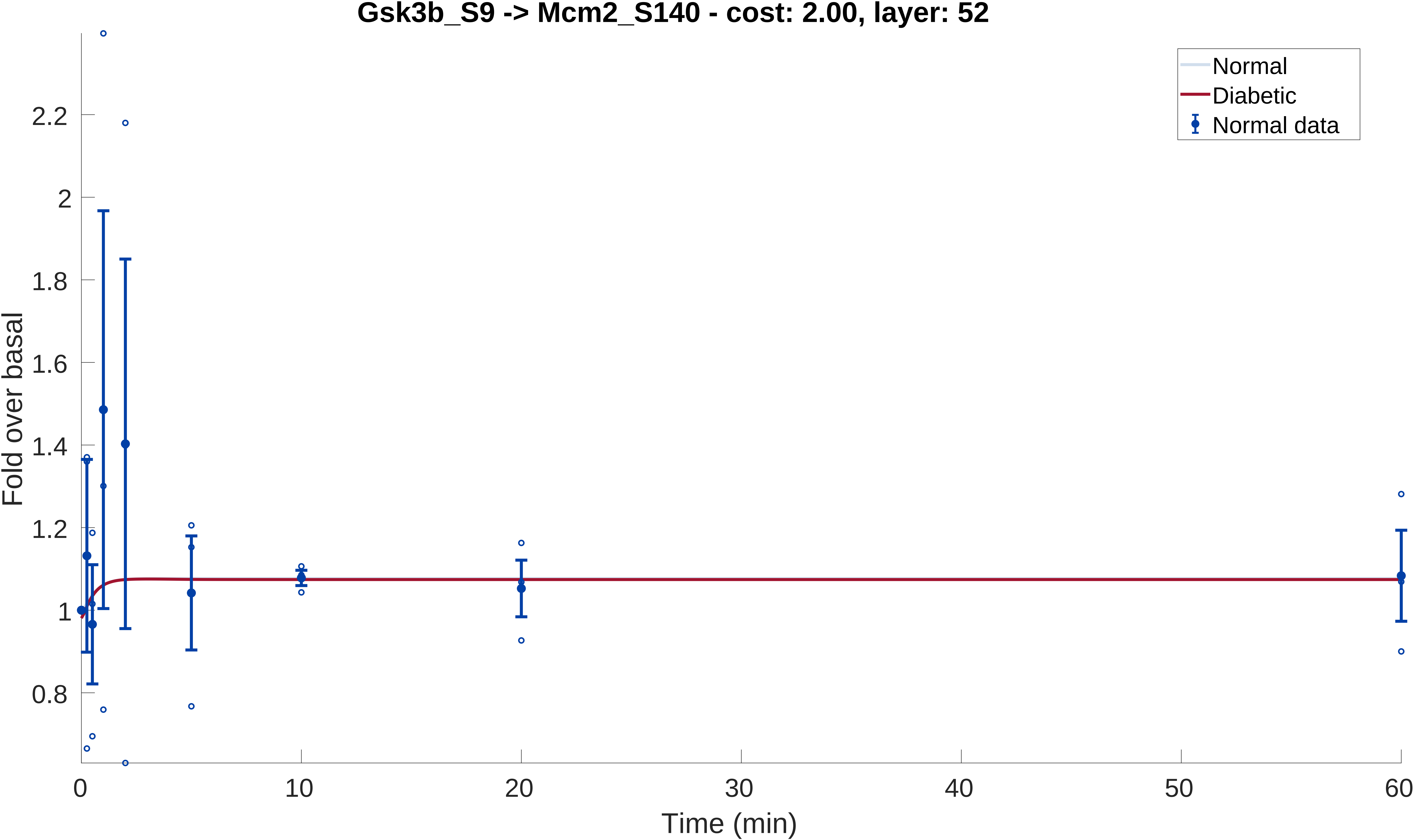
Gsk3b_S9 -> Mcm2_S140 - cost: 2.00, layer: 52
Normal
Diabetic
Normal data
Fold over basal
0.8
1
1.2
1.4
1.6
1.8
2
2.2
0
10
20
30
40
50
60
Time (min)

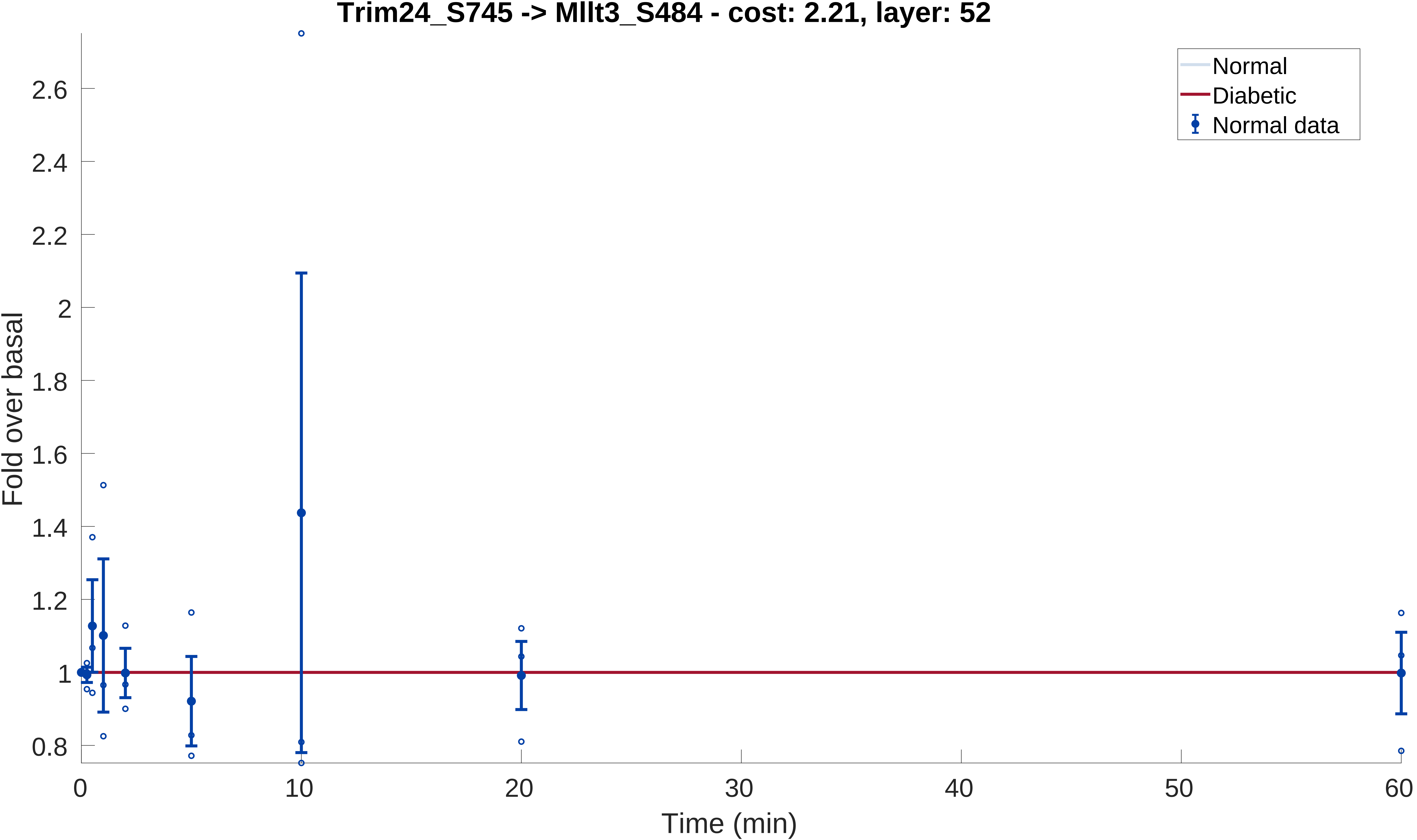
Trim24_S745 -> Mllt3_S484 - cost: 2.21, layer: 52
Normal
Diabetic
Normal data
Fold over basal
Time (min)
2.6
2.4
2.2
2
1.8
1.6
1.4
1.2
1
0.8
0
10
20
30
40
50
60

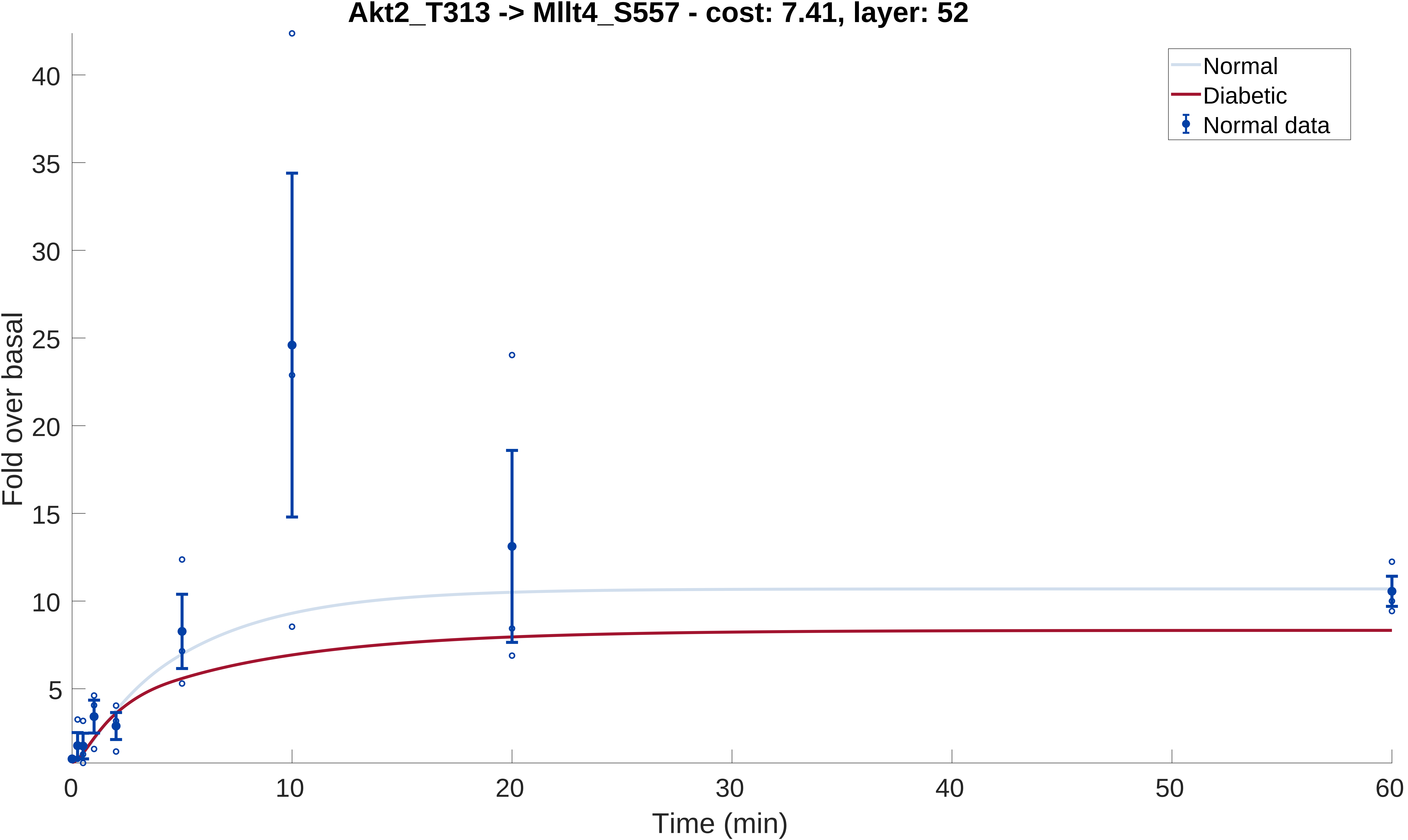
Akt2_T313 -> Mllt4_S557 - cost: 7.41, layer: 52
Normal
Diabetic
Normal data
Fold over basal
Time (min)
0
5
10
15
20
25
30
35
40
50
60

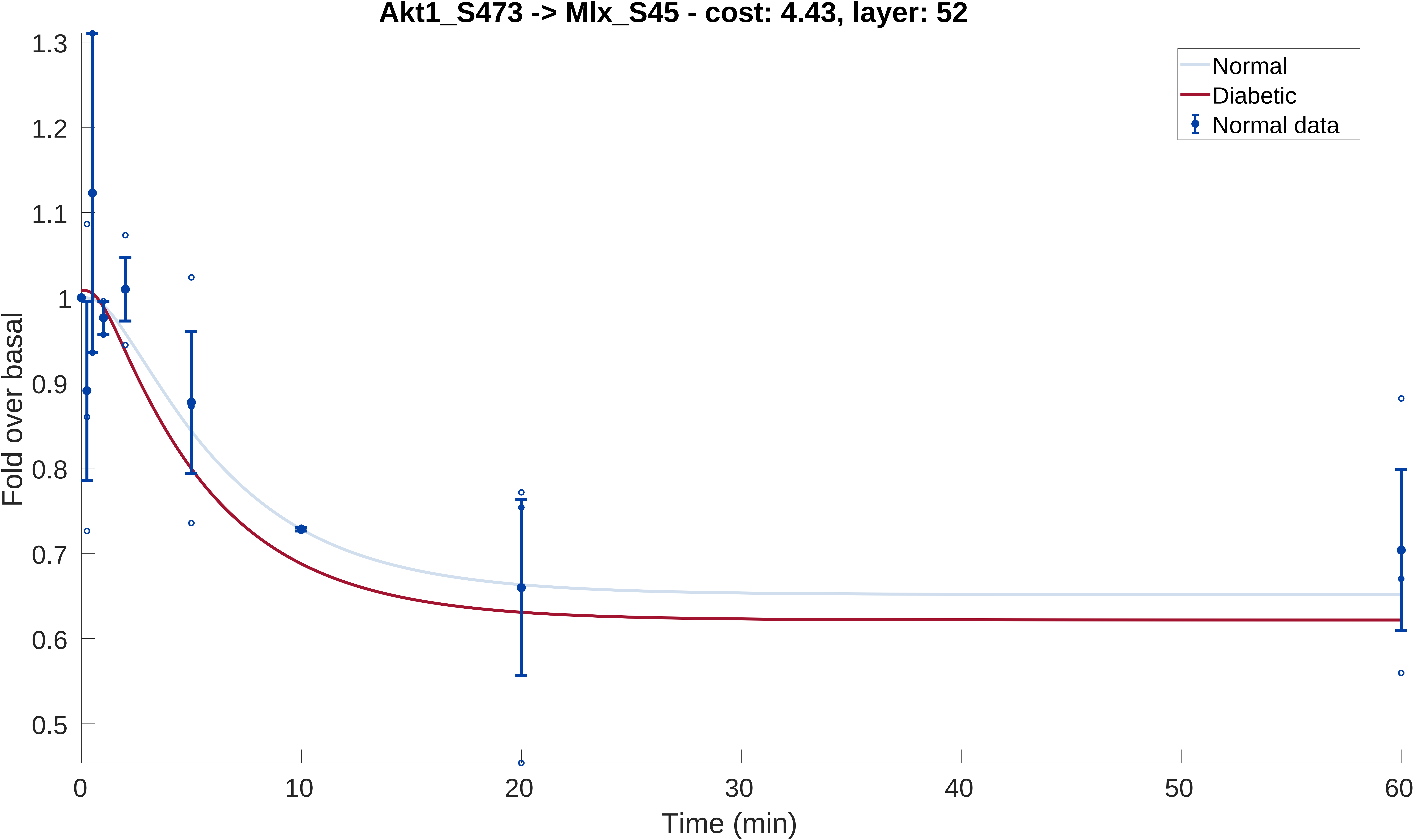

Akt1_S473 -> Mlx_S45 - cost: 4.43, layer: 52
Normal
Diabetic
Normal data
Fold over basal
Time (min)
1.3
1.2
1.1
1
0.9
0.8
0.7
0.6
0.5
0
10
20
30
40
50
60

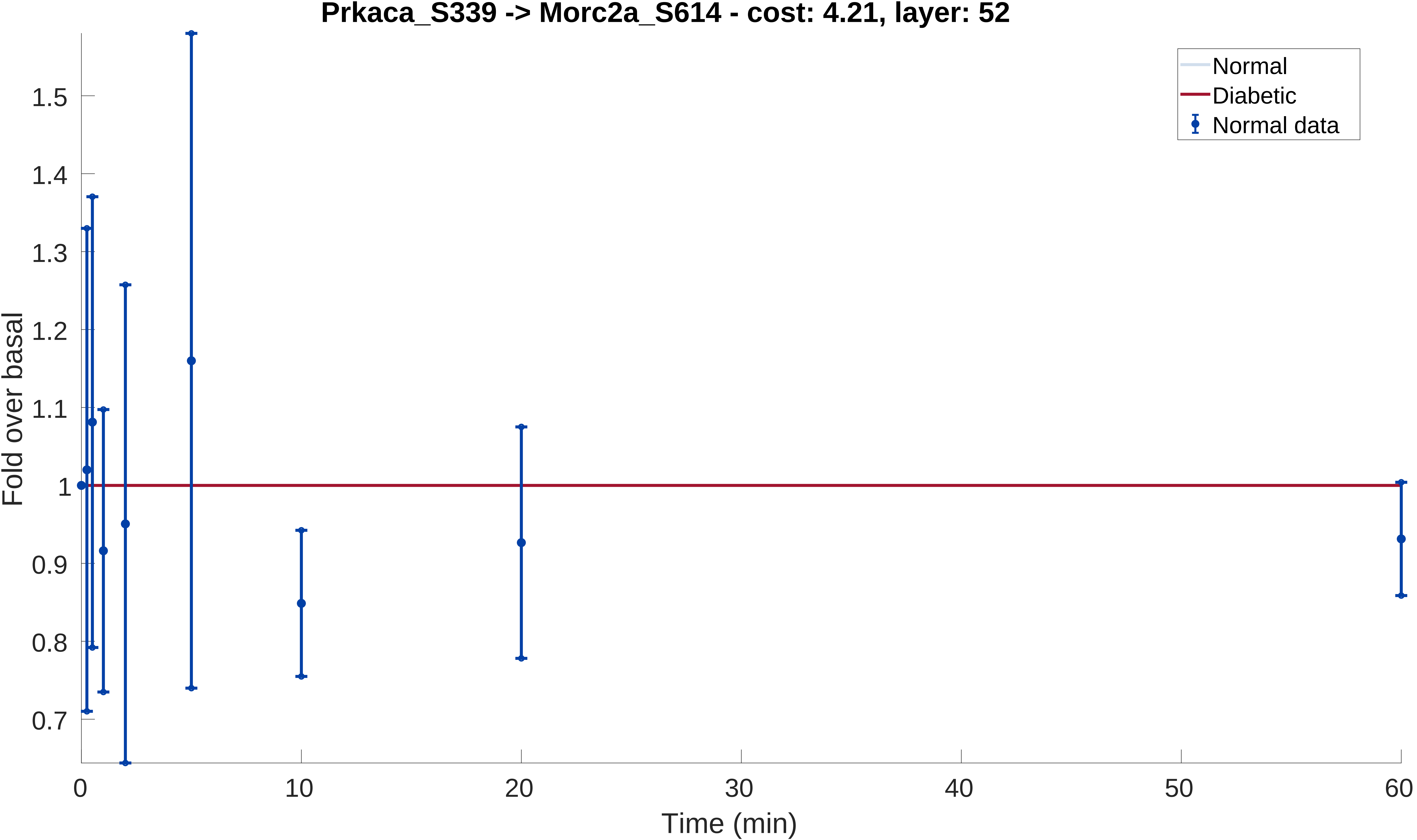

Prkaca_S339 -> Morc2a_S614 - cost: 4.21, layer: 52
Normal
Diabetic
Normal data
Fold over basal
1.5
1.4
1.3
1.2
1.1
1
0.9
0.8
0.7
0
10
20
30
40
50
60
Time (min)

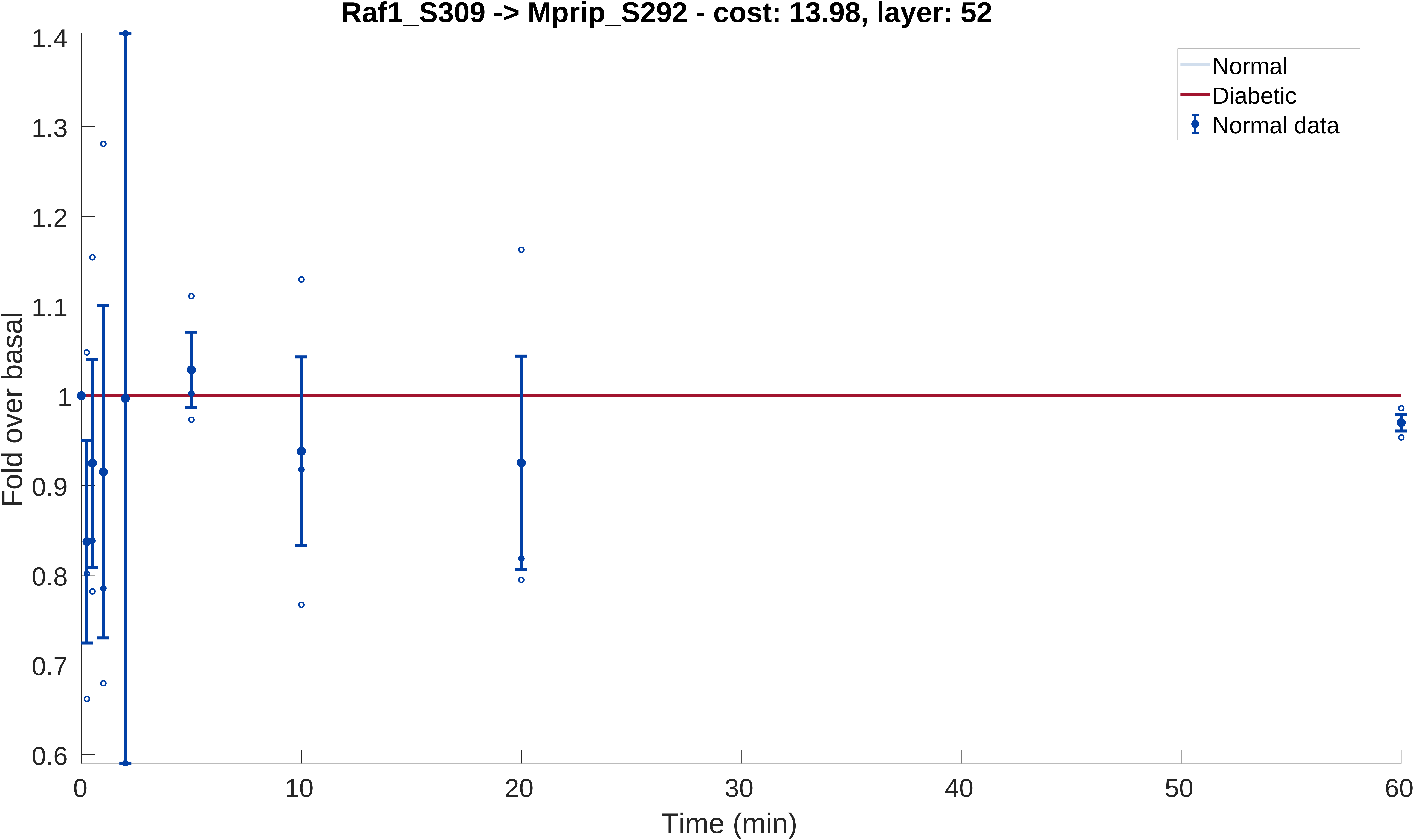

Raf1_S309 -> Mprip_S292 - cost: 13.98, layer: 52
Normal
Diabetic
Normal data
Fold over basal
Time (min)
1.4
1.3
1.2
1.1
1
0.9
0.8
0.7
0.6
0
10
20
30
40
50
60

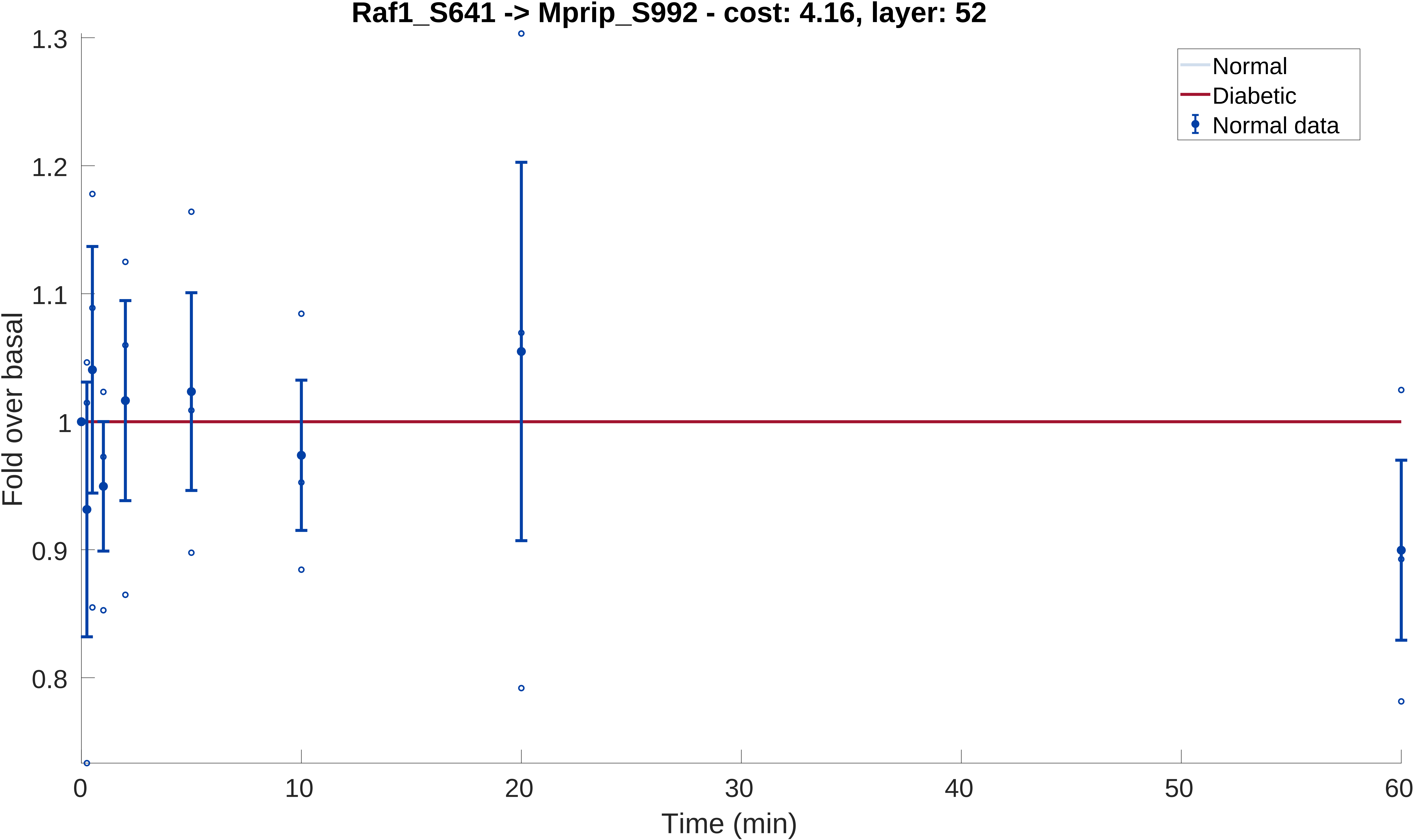

Raf1_S641 -> Mprip_S992 - cost: 4.16, layer: 52
Normal
Diabetic
Normal data
Fold over basal
1.3
1.2
1.1
1
0.9
0.8
0
10
20
30
40
50
60
Time (min)

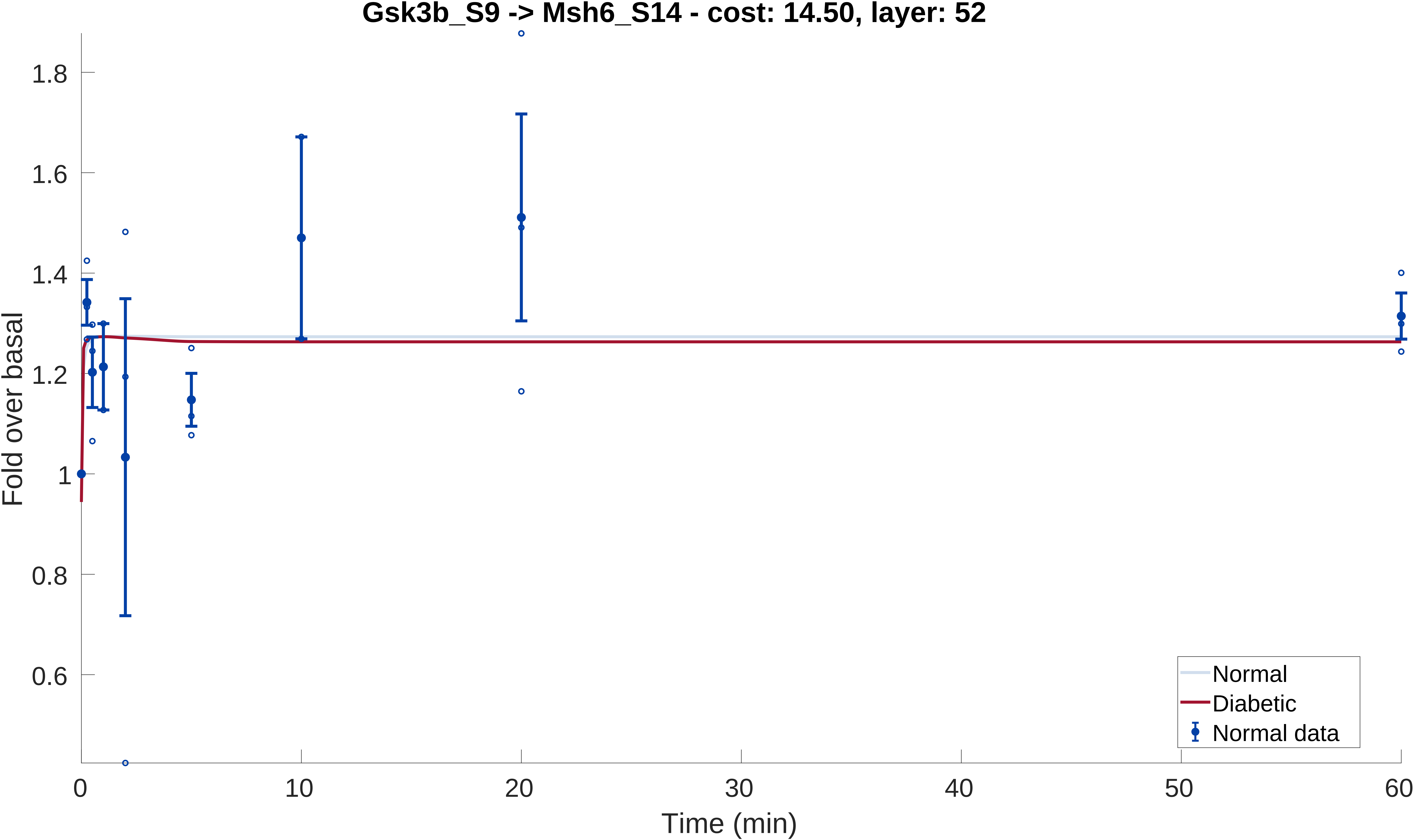

Gsk3b_S9 -> Msh6_S14 - cost: 14.50, layer: 52
Fold over basal
1.8
1.6
1.4
1.2
1
0.8
0.6
0
10
20
30
40
50
60
Time (min)
Normal
Diabetic
Normal data

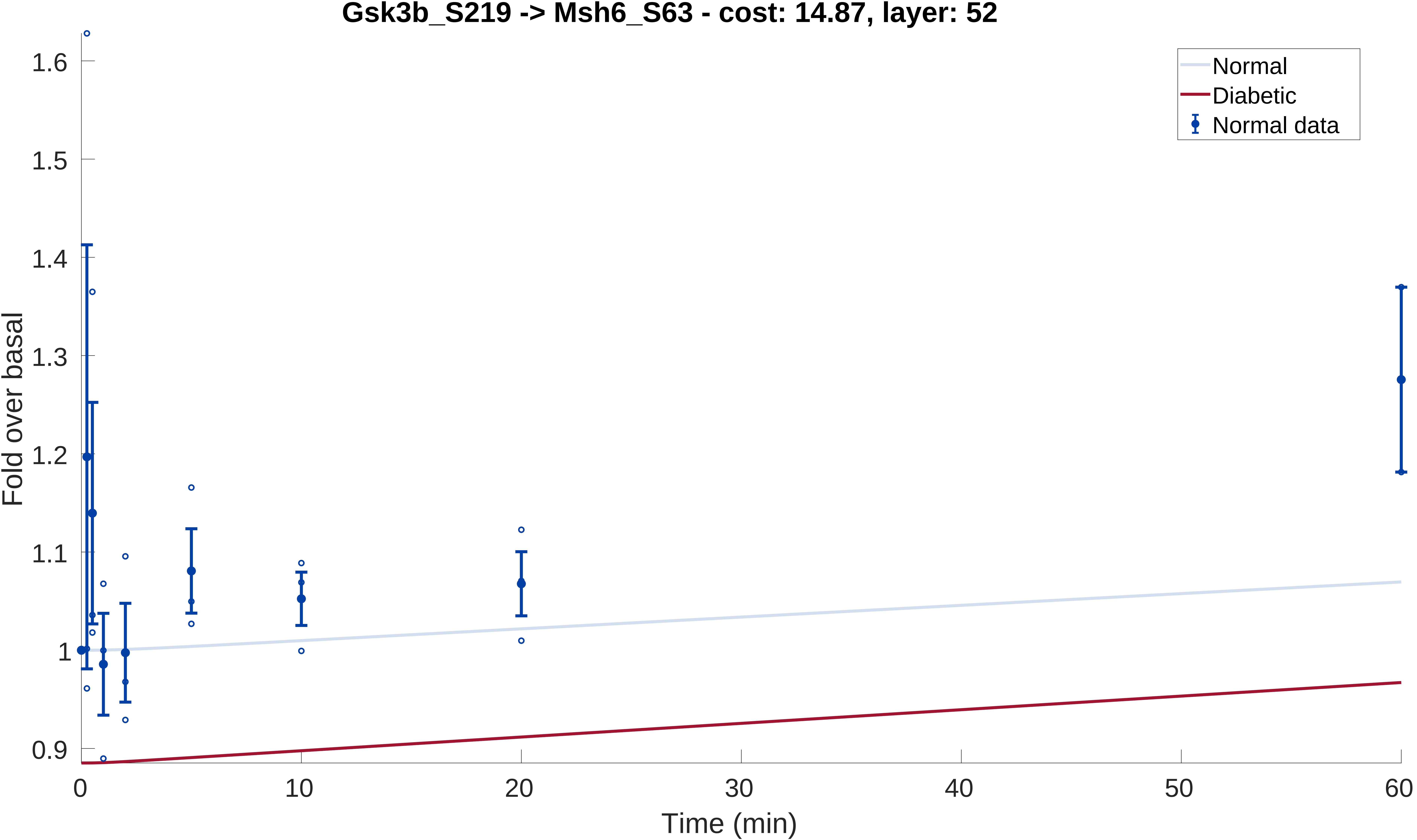

Gsk3b_S219 -> Msh6_S63 - cost: 14.87, layer: 52
Normal
Diabetic
Normal data
Fold over basal
Time (min)
1.6
1.5
1.4
1.3
1.2
1.1
1
0.9
0
10
20
30
40
50
60

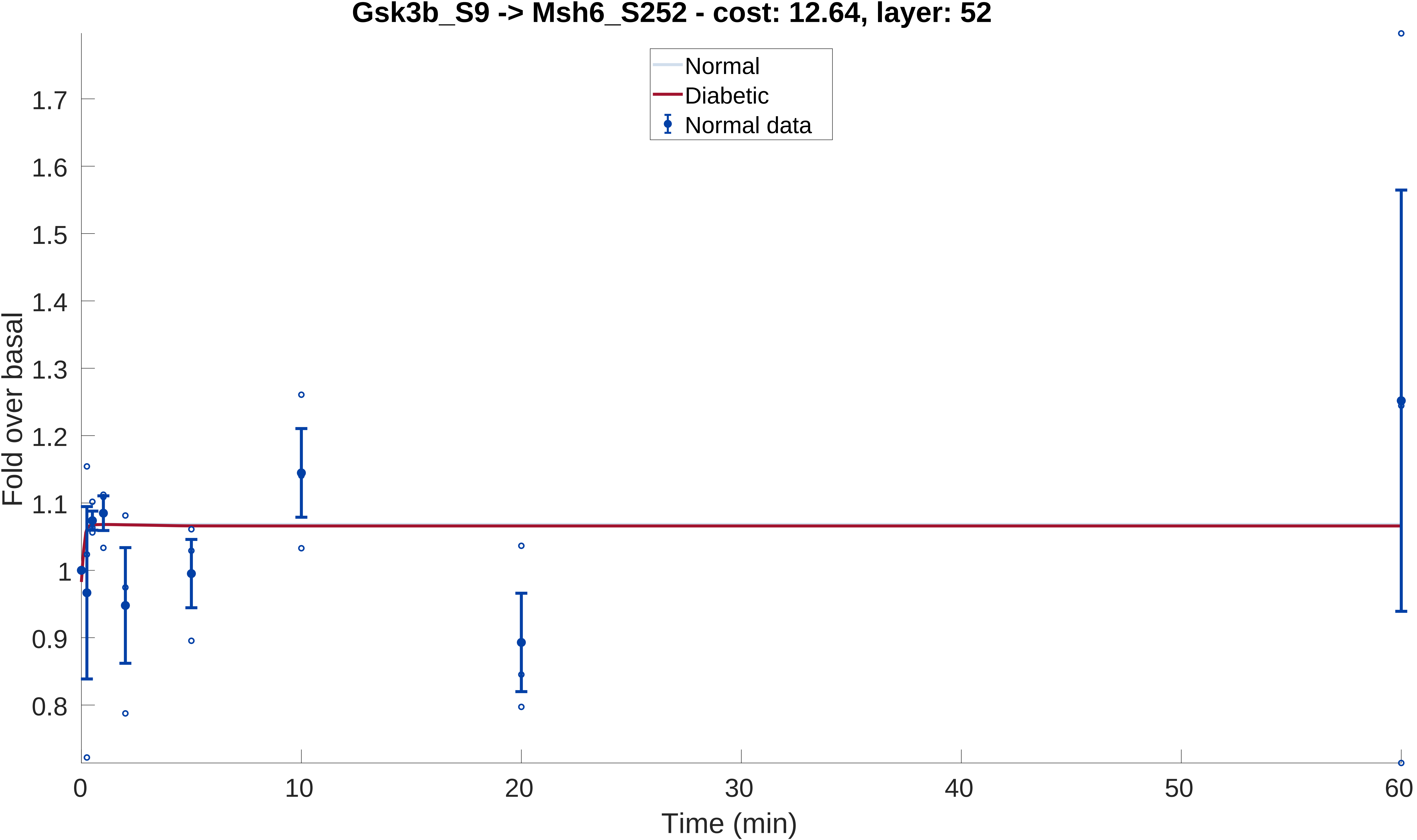
Gsk3b_S9 -> Msh6_S252 - cost: 12.64, layer: 52
Normal
Diabetic
Normal data
Fold over basal
1.7
1.6
1.5
1.4
1.3
1.2
1.1
1
0.9
0.8
0
10
20
30
40
50
60
Time (min)

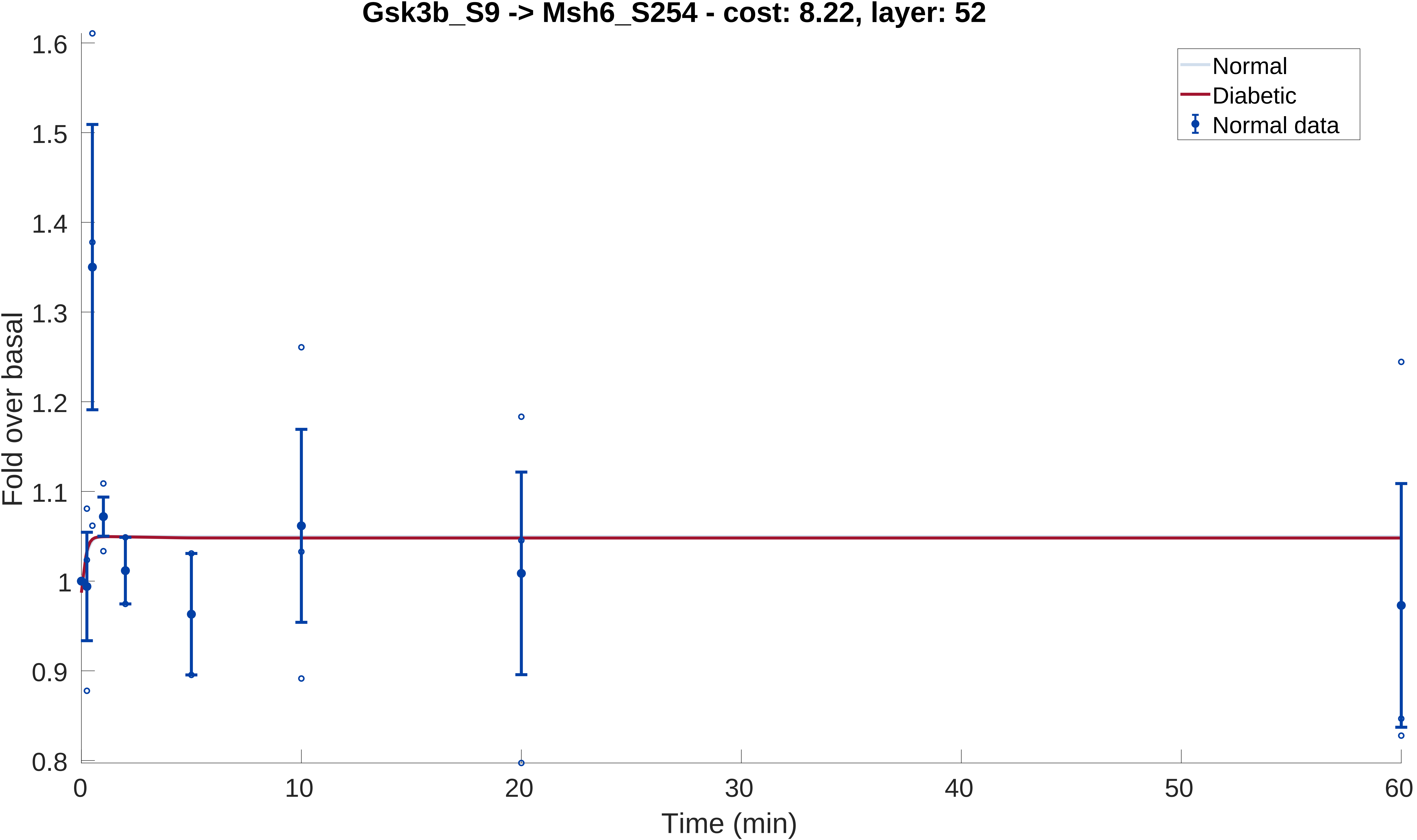
Gsk3b_S9 -> Msh6_S254 - cost: 8.22, layer: 52
Normal
Diabetic
Normal data
Fold over basal
Time (min)
0.8
0.9
1
1.1
1.2
1.3
1.4
1.5
1.6
0
10
20
30
40
50
60

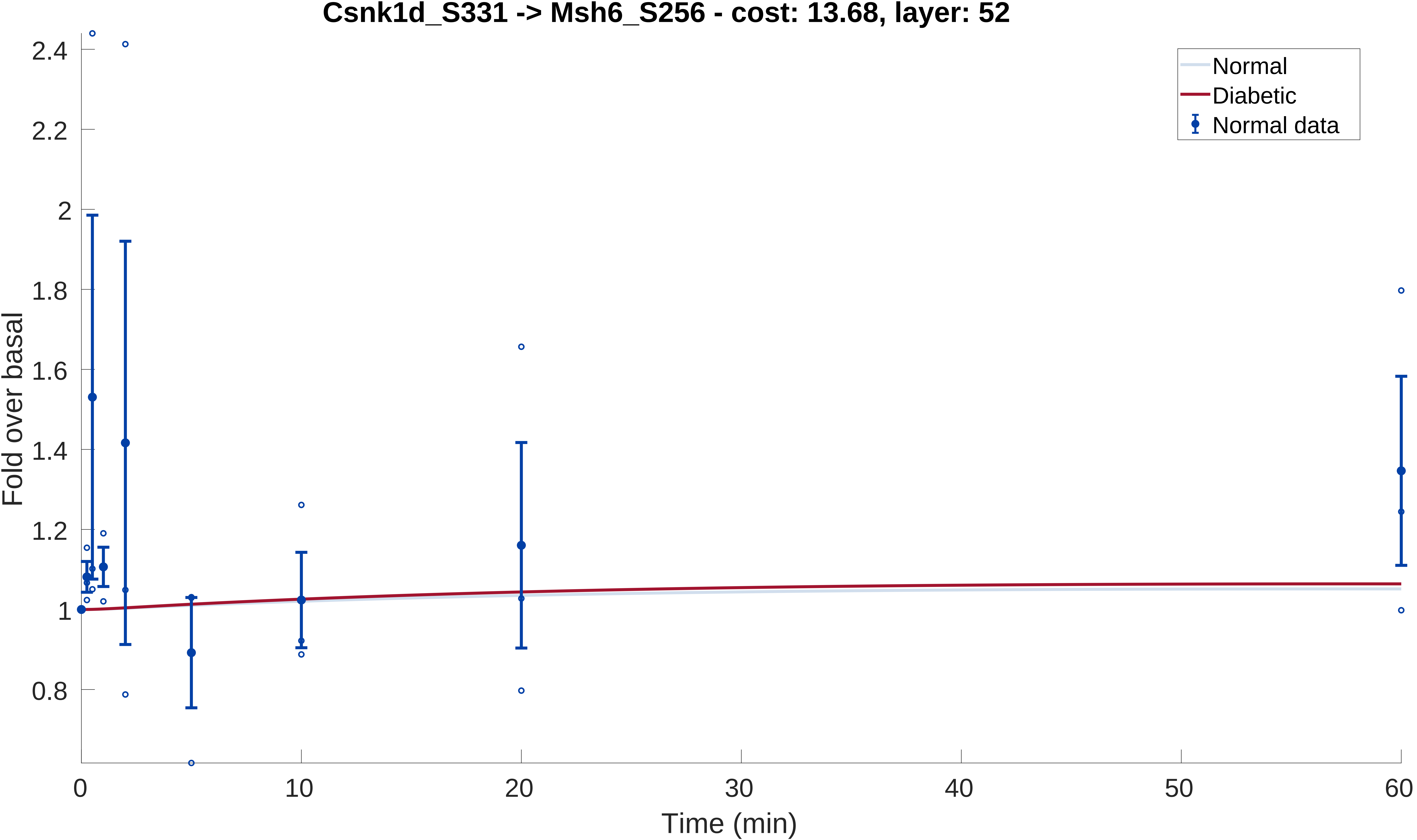

Csnk1d_S331 -> Msh6_S256 - cost: 13.68, layer: 52
Normal
Diabetic
Normal data
Fold over basal
2.4
2.2
2
1.8
1.6
1.4
1.2
1
0.8
0
10
20
30
40
50
60
Time (min)

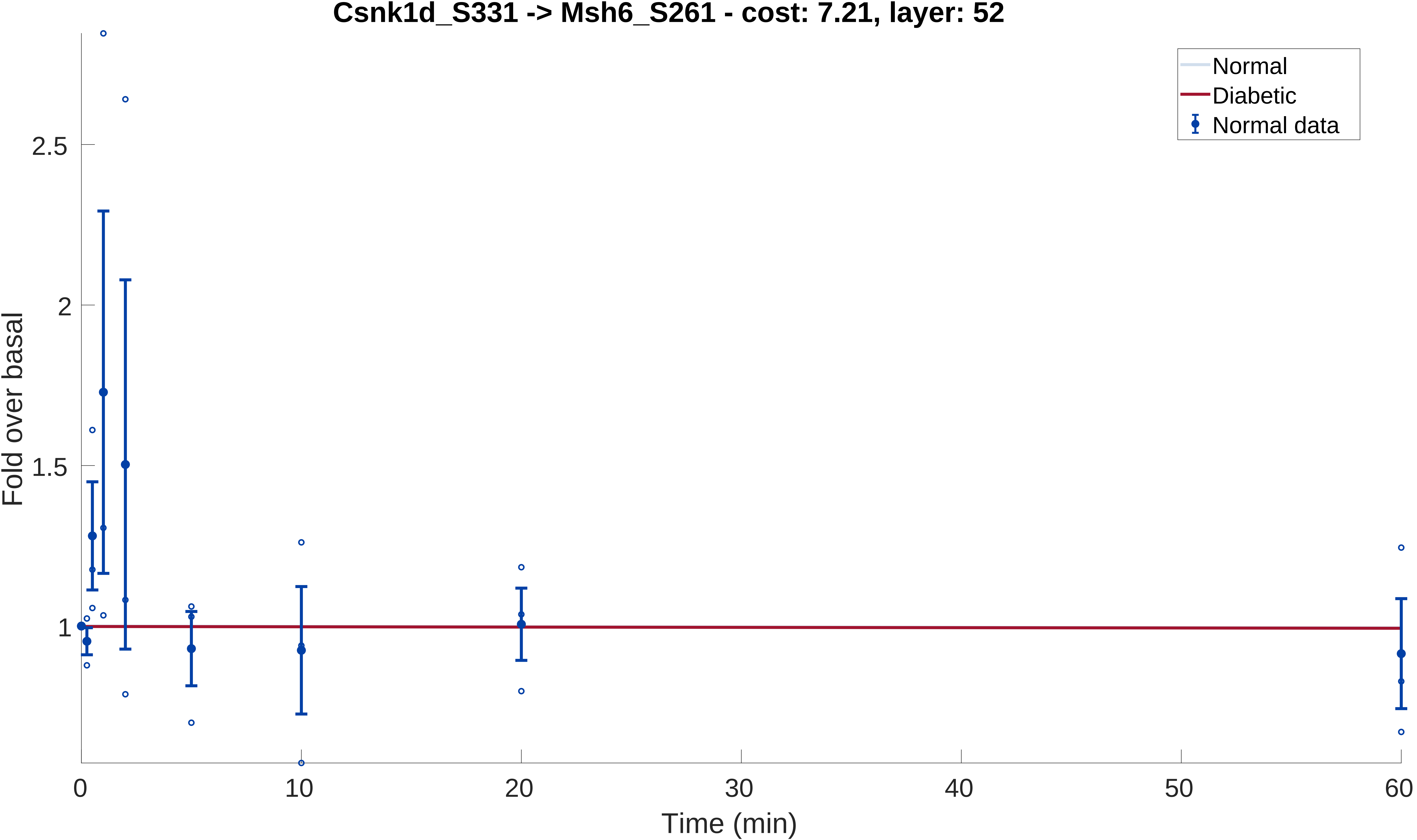

Csnk1d_S331 -> Msh6_S261 - cost: 7.21, layer: 52
Normal
Diabetic
Normal data
Fold over basal
2.5
2
1.5
1
0
10
20
30
40
50
60
Time (min)

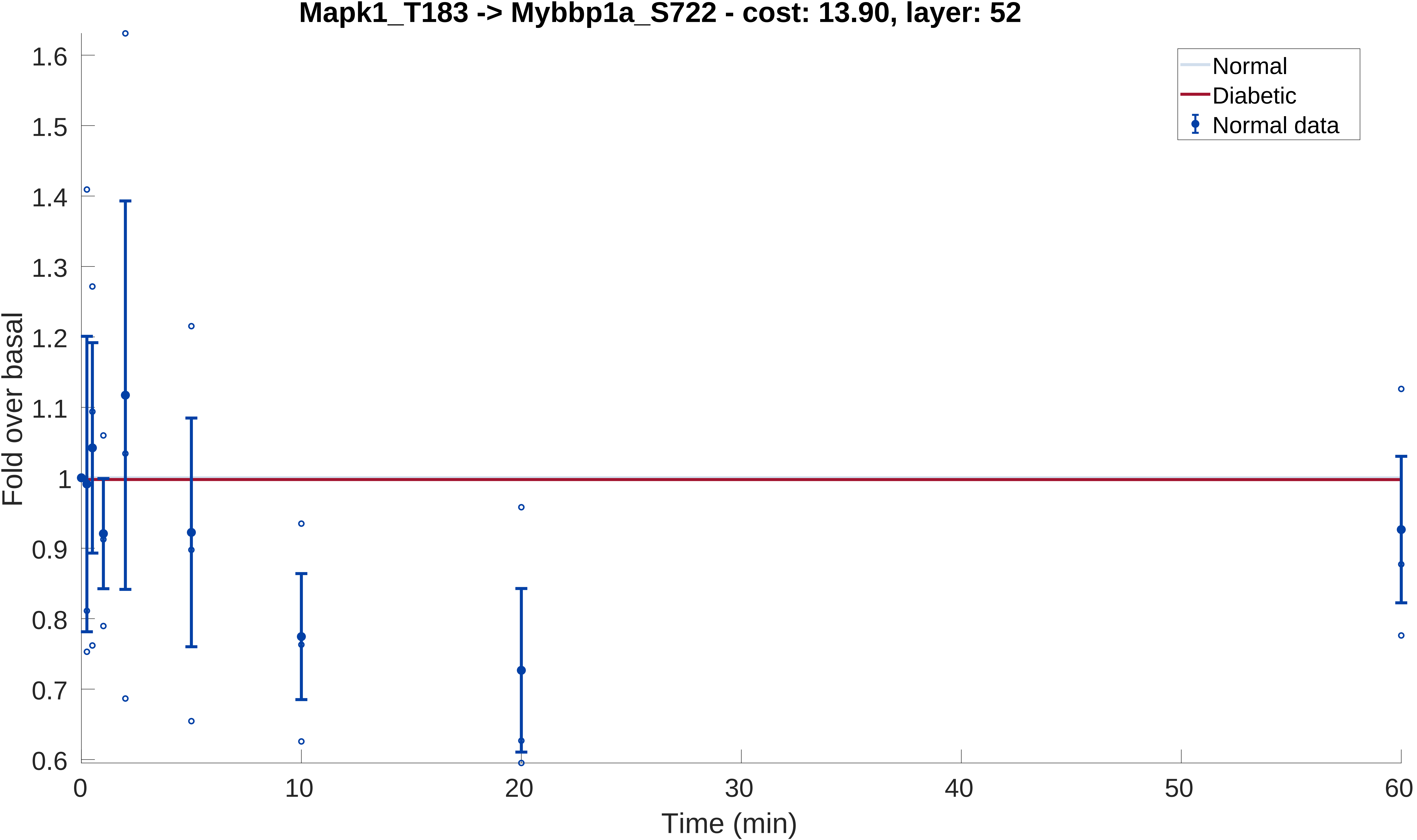

Mapk1_T183 -> Mybbp1a_S722 - cost: 13.90, layer: 52
Normal
Diabetic
Normal data
Fold over basal
Time (min)
0.6
0.7
0.8
0.9
1
1.1
1.2
1.3
1.4
1.5
1.6
0
10
20
30
40
50
60

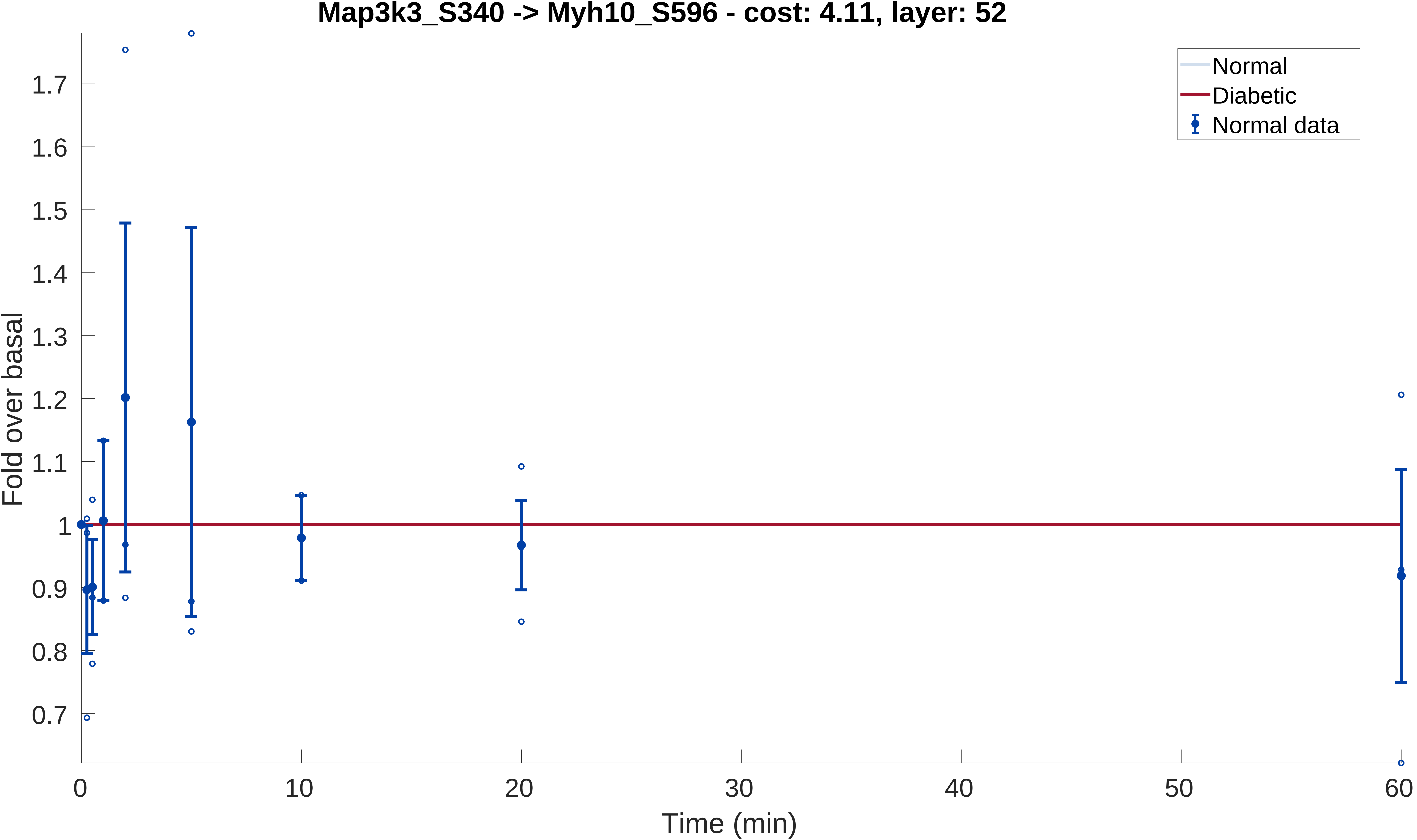

Map3k3_S340 -> Myh10_S596 - cost: 4.11, layer: 52
Normal
Diabetic
Normal data
Fold over basal
Time (min)
1.7
1.6
1.5
1.4
1.3
1.2
1.1
1
0.9
0.8
0.7
0
10
20
30
40
50
60

Map3k3_S340 -> Myh10_S1993 - cost: 5.60, layer: 52

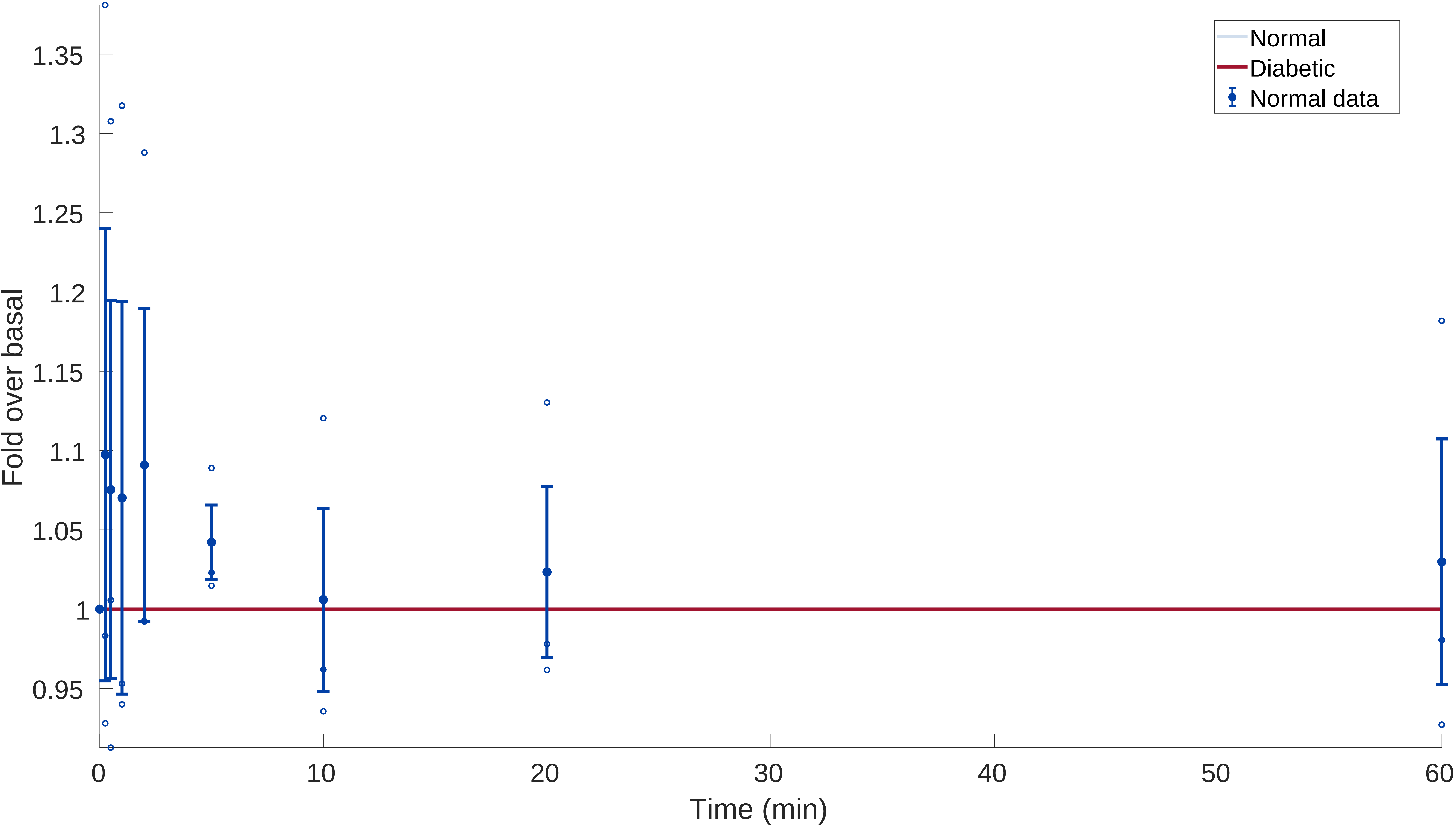

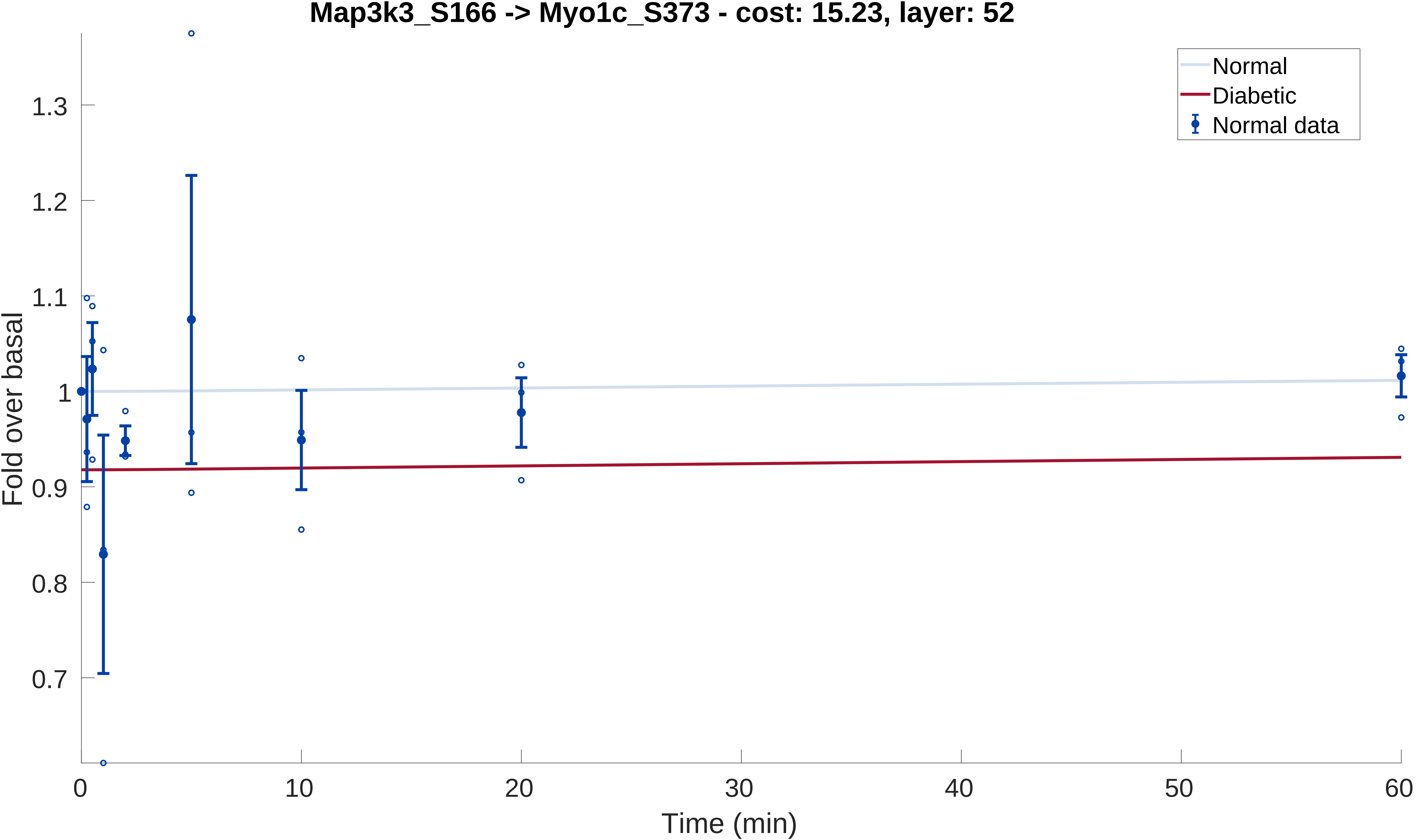
Map3k3_S166 -> Myo1c_S373 - cost: 15.23, layer: 52
Normal
Diabetic
Normal data
Fold over basal
Time (min)
0.7
0.8
0.9
1
1.1
1.2
1.3
0
10
20
30
40
50
60

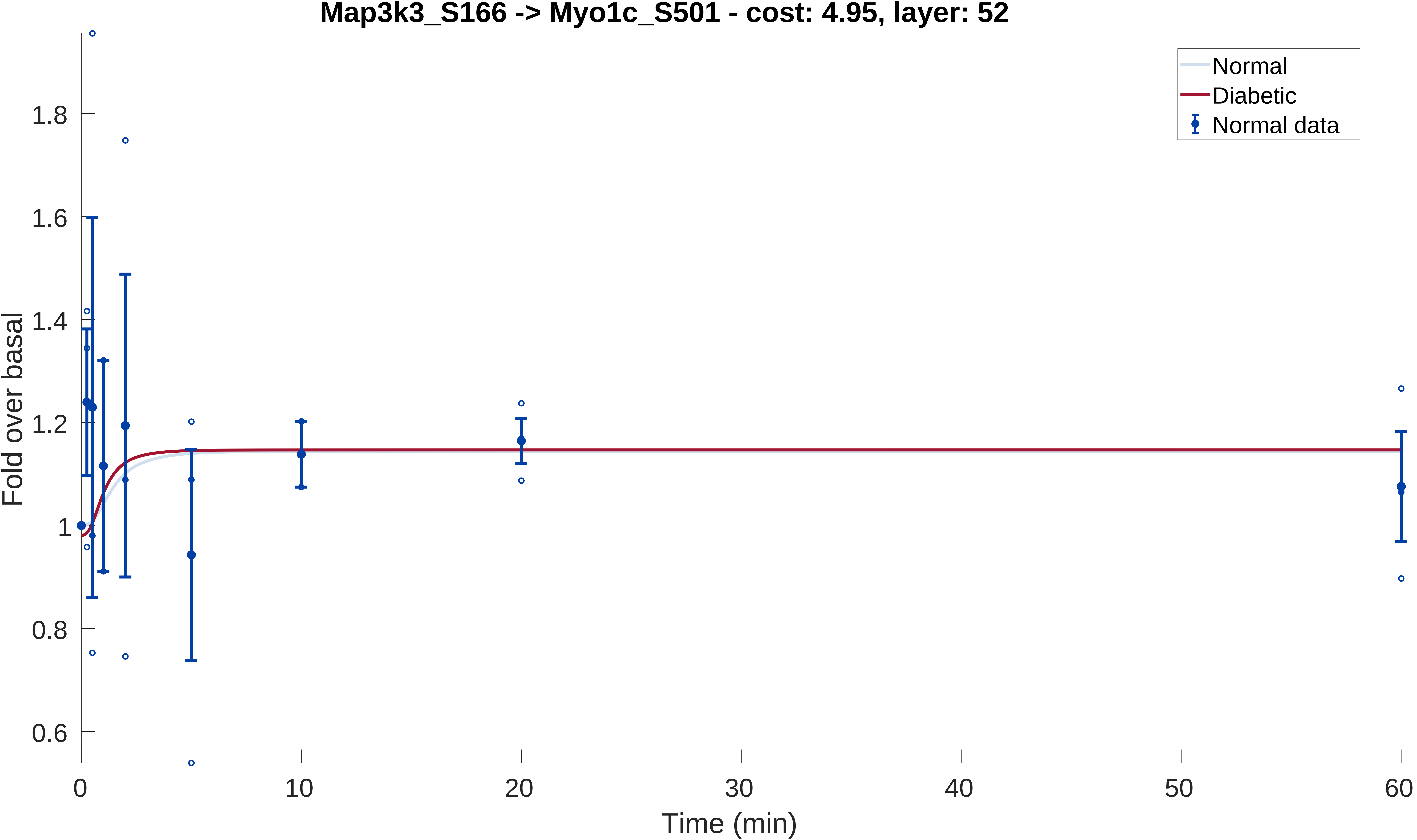

Map3k3_S166 -> Myo1c_S501 - cost: 4.95, layer: 52
Normal
Diabetic
Normal data
Fold over basal
1.8
1.6
1.4
1.2
1
0.8
0.6
0
10
20
30
40
50
60
Time (min)

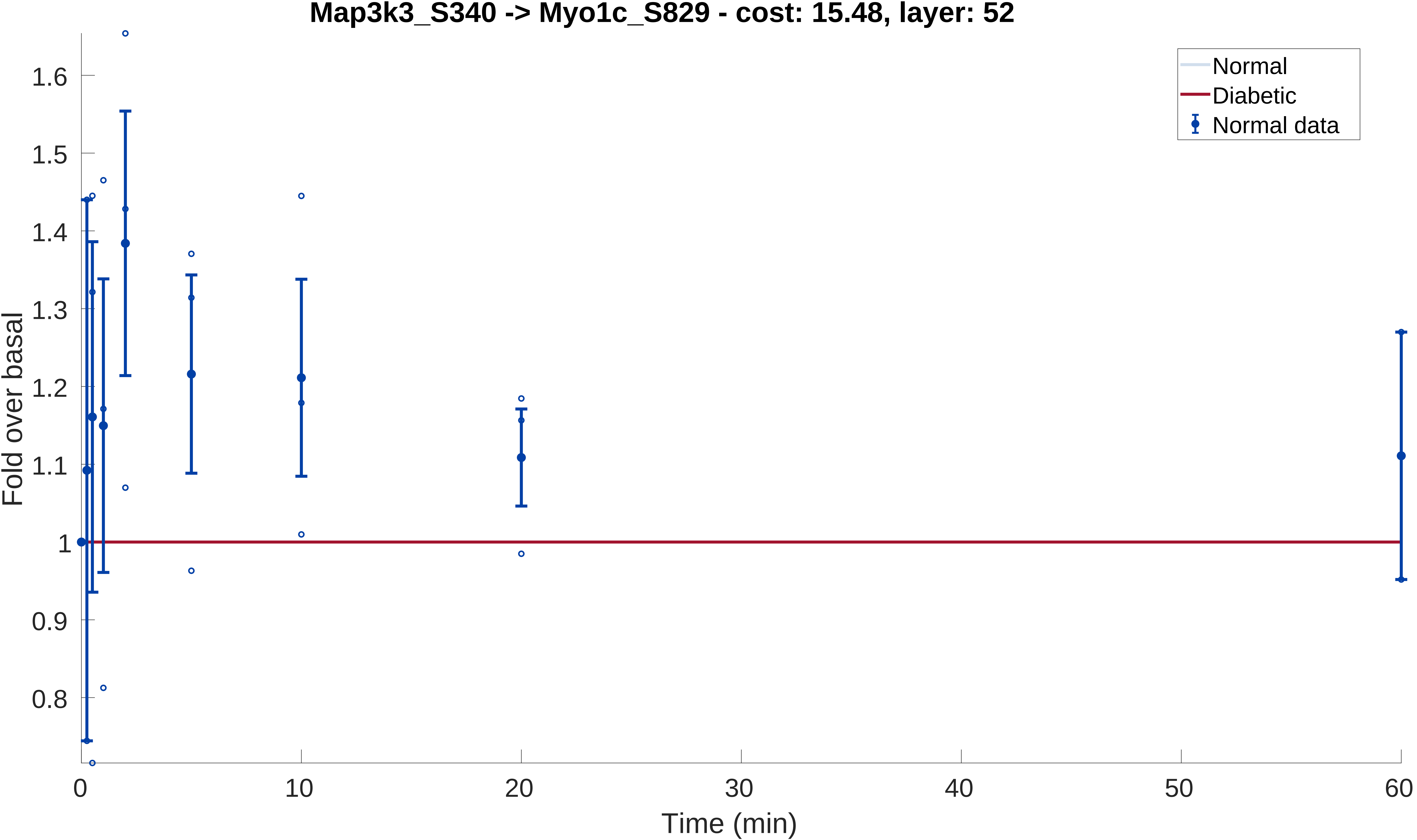

Map3k3_S340 -> Myo1c_S829 - cost: 15.48, layer: 52
Normal
Diabetic
Normal data
Fold over basal
Time (min)
1.6
1.5
1.4
1.3
1.2
1.1
1
0.9
0.8
0
10
20
30
40
50
60

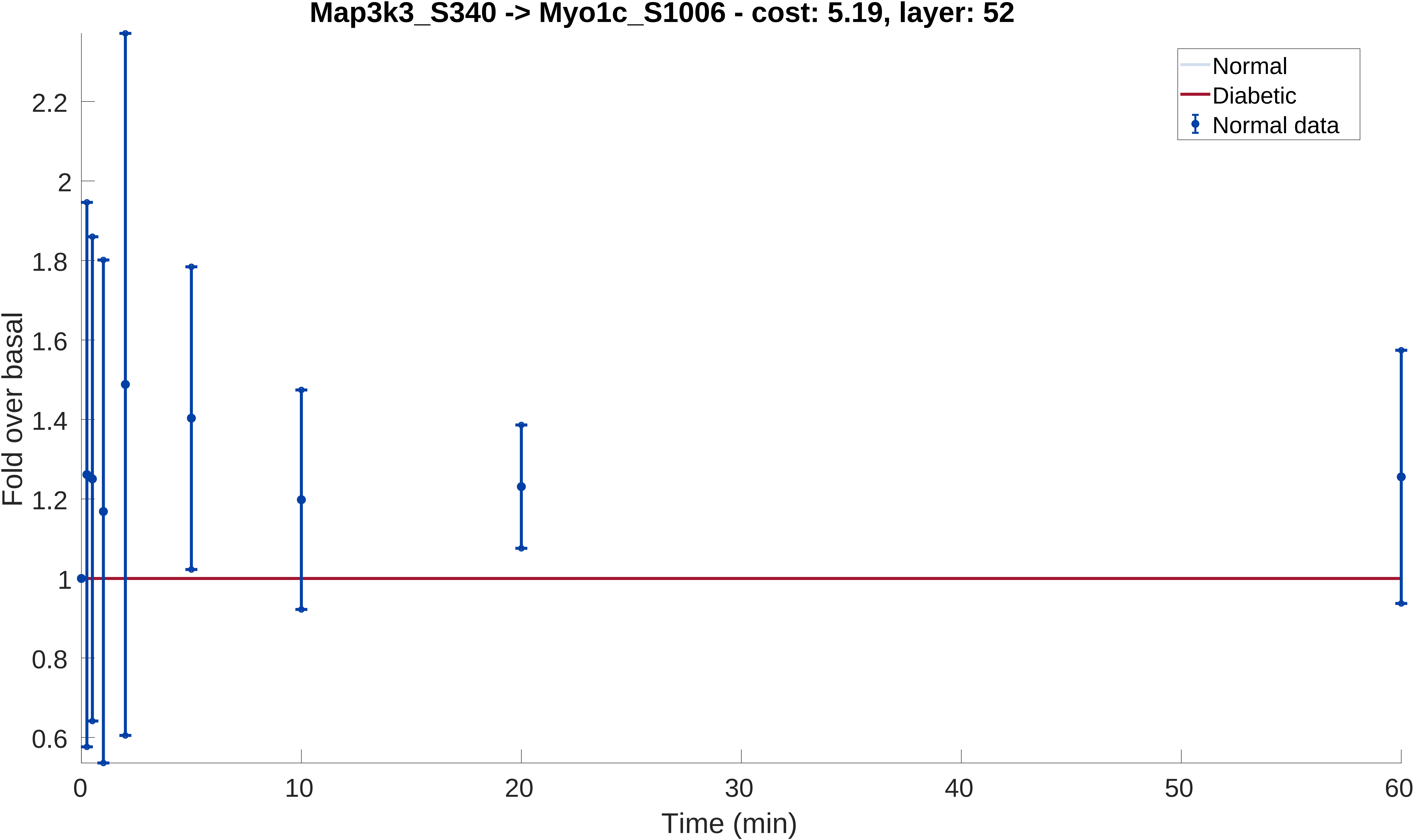

Map3k3_S340 -> Myo1c_S1006 - cost: 5.19, layer: 52
Normal
Diabetic
Normal data
Fold over basal
Time (min)
0.6
0.8
1
1.2
1.4
1.6
1.8
2
2.2
0
10
20
30
40
50
60

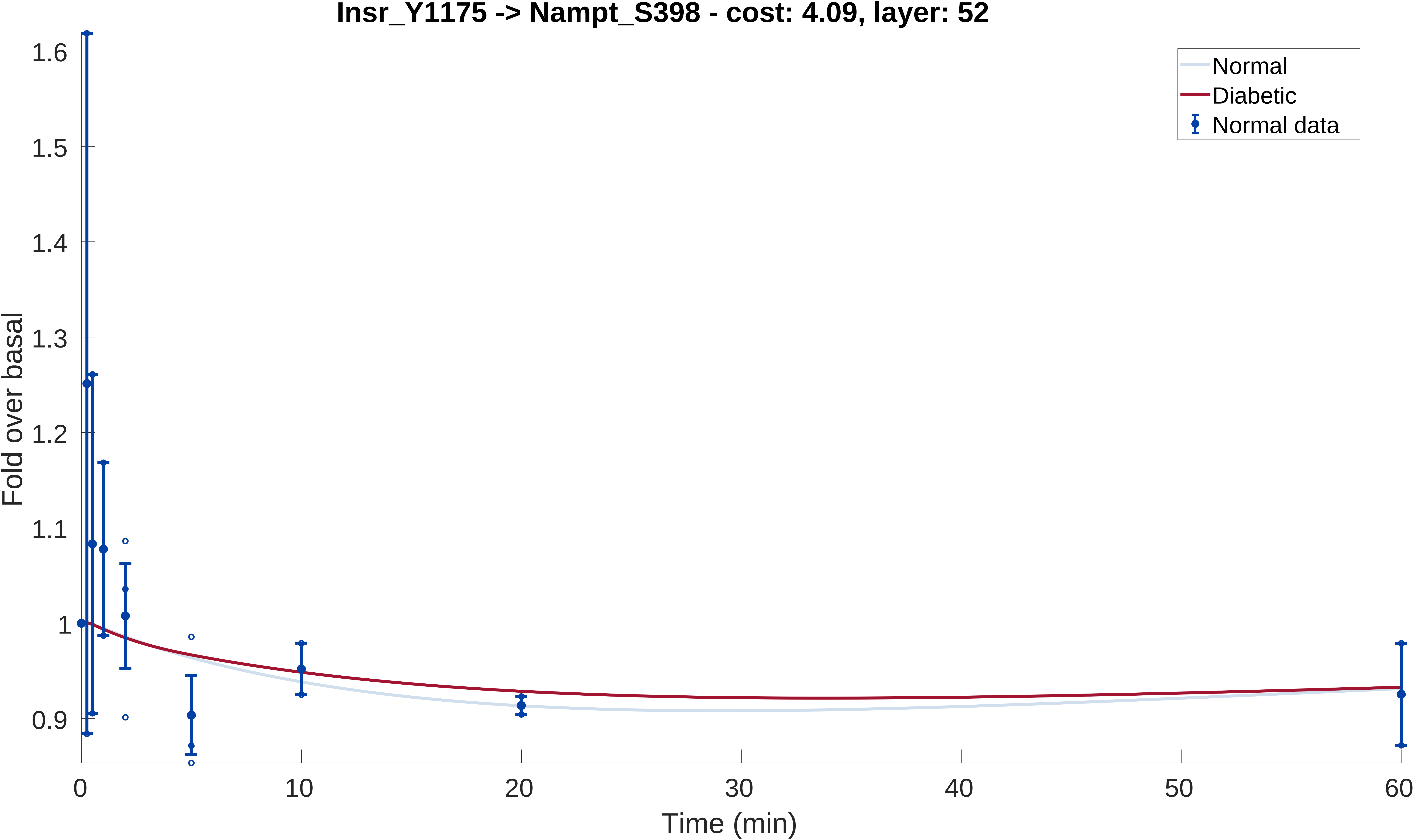

Insr_Y1175 -> Nampt_S398 - cost: 4.09, layer: 52
Normal
Diabetic
Normal data
Fold over basal
1.6
1.5
1.4
1.3
1.2
1.1
1
0.9
0
10
20
30
40
50
60
Time (min)

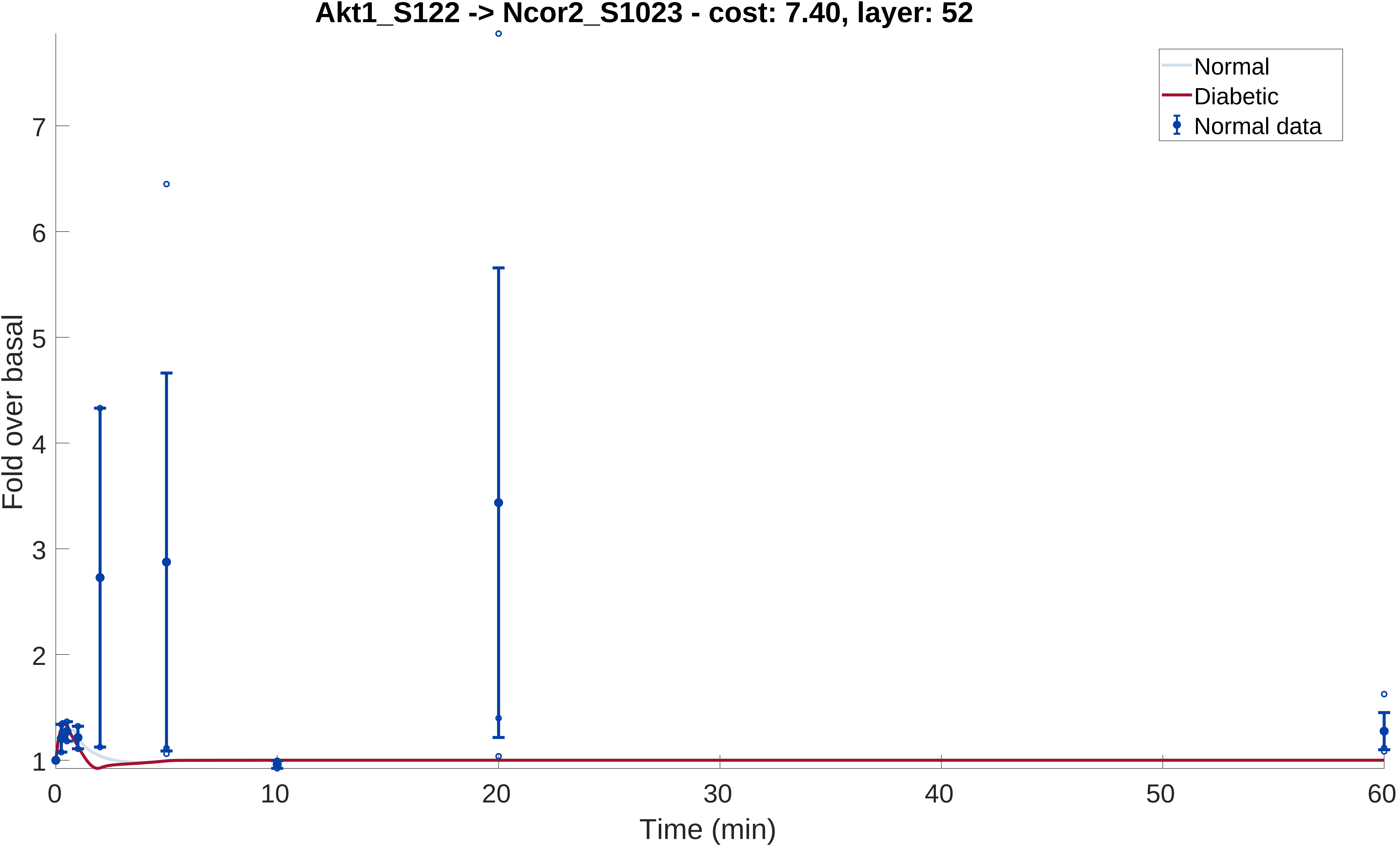
Akt1_S122 -> Ncor2_S1023 - cost: 7.40, layer: 52
Normal
Diabetic
Normal data
Fold over basal
Time (min)
1
2
3
4
5
6
7
0
10
20
30
40
50
60

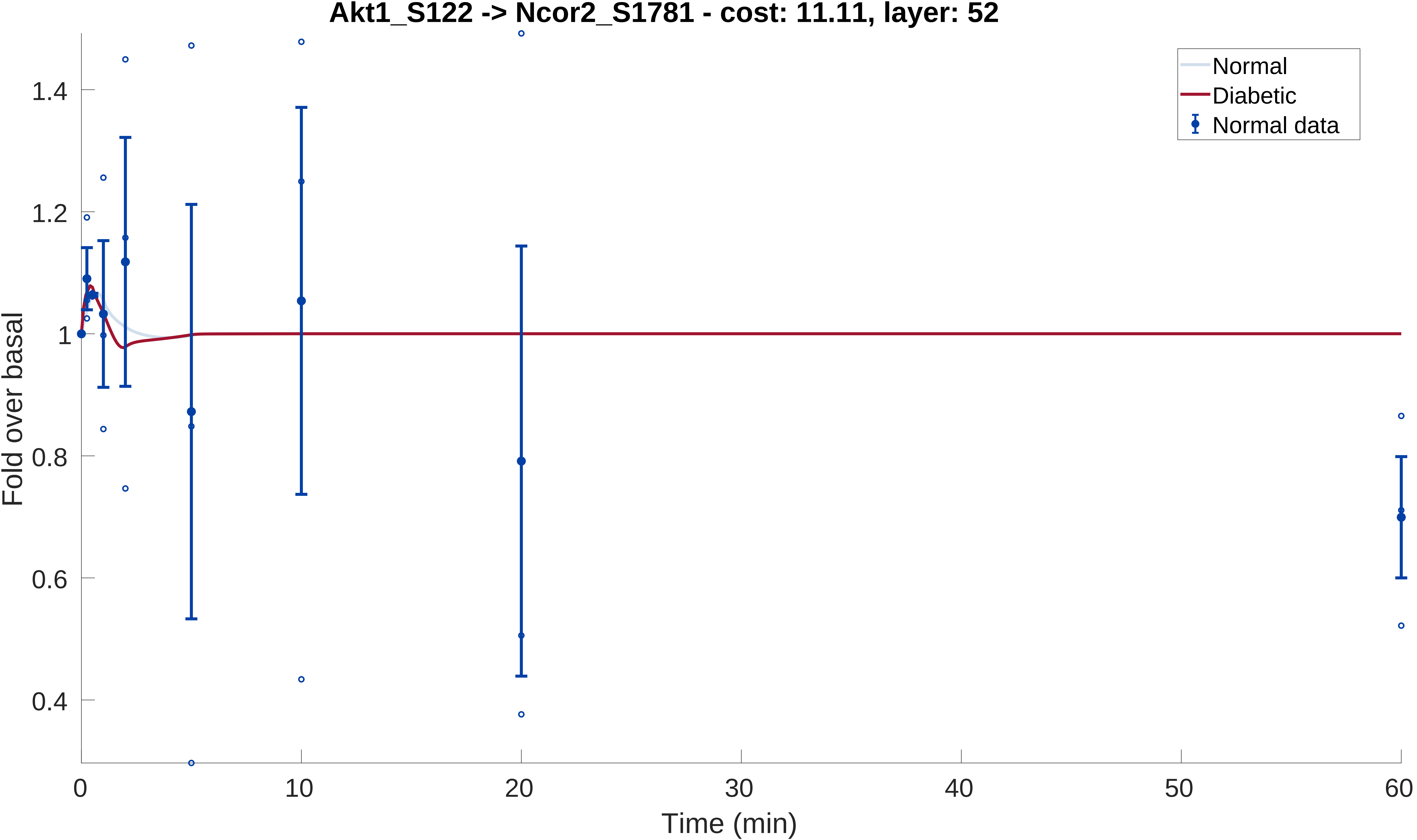

Akt1_S122 -> Ncor2_S1781 - cost: 11.11, layer: 52
Normal
Diabetic
Normal data
Fold over basal
1.4
1.2
1
0.8
0.6
0.4
0
10
20
30
40
50
60
Time (min)

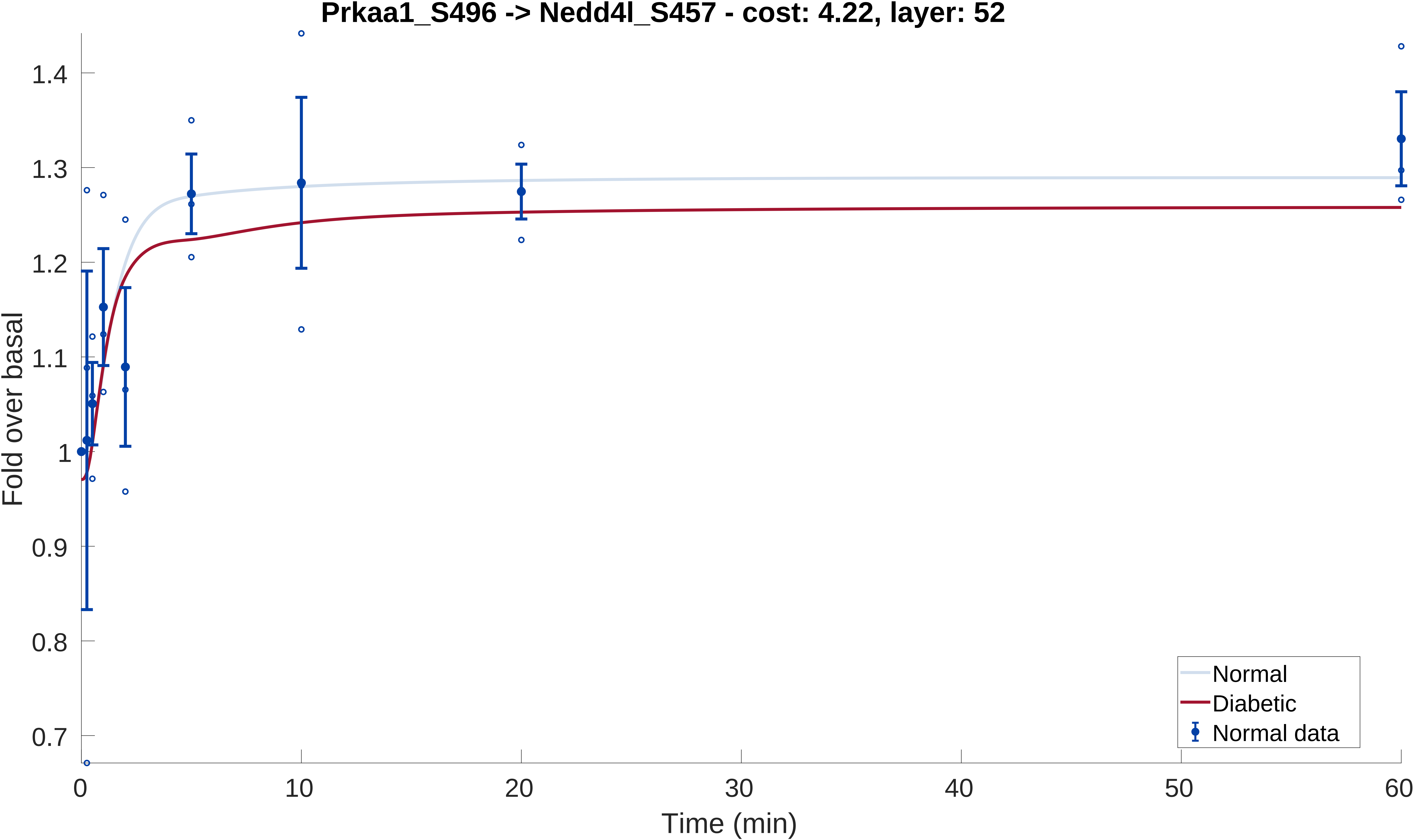

Prkaa1_S496 -> Nedd4l_S457 - cost: 4.22, layer: 52
Fold over basal
1.4
1.3
1.2
1.1
1
0.9
0.8
0.7
0
10
20
30
40
50
60
Time (min)
Normal
Diabetic
Normal data

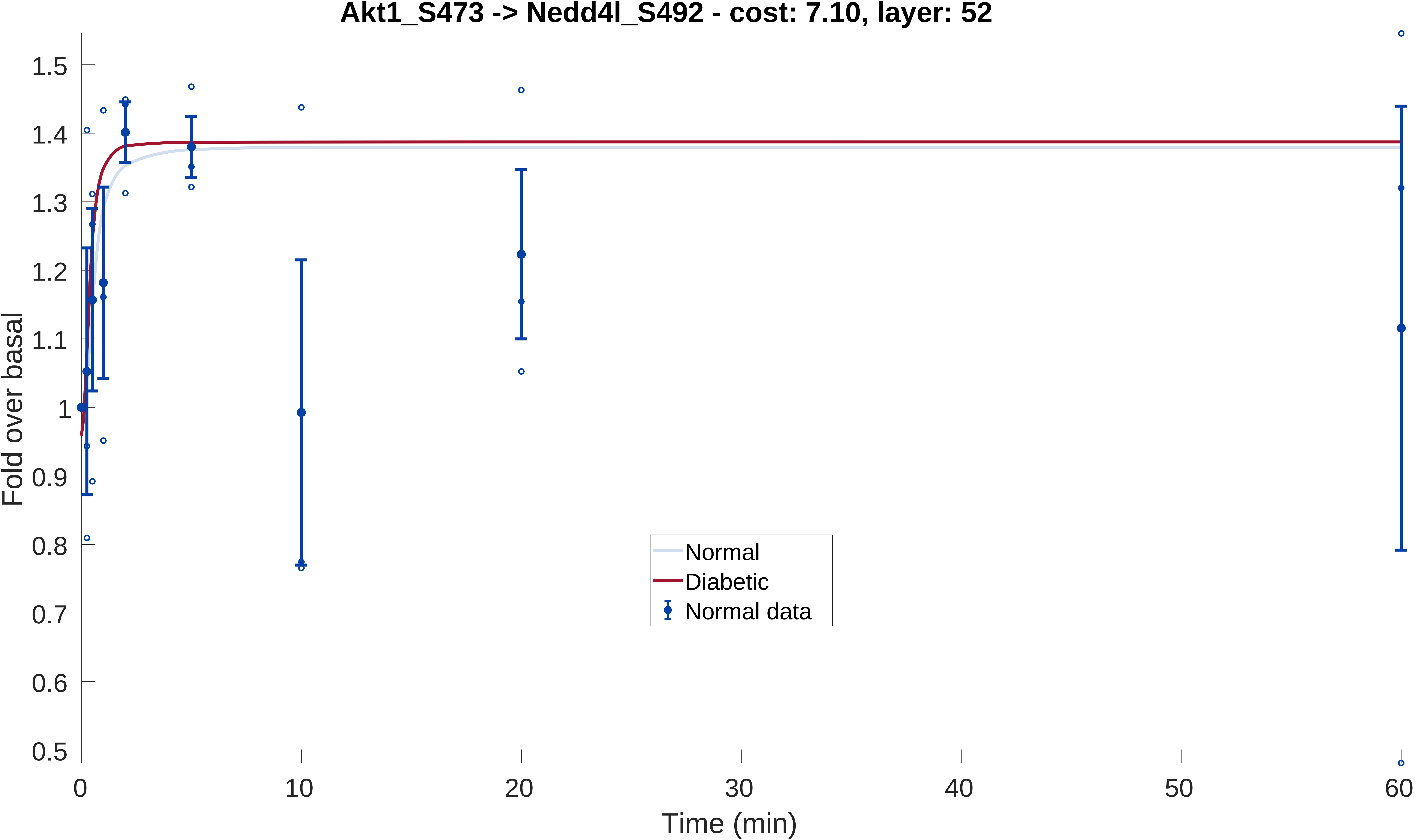

Akt1_S473 -> Nedd4l_S492 - cost: 7.10, layer: 52
Fold over basal
1.5
1.4
1.3
1.2
1.1
1
0.9
0.8
0.7
0.6
0.5
0
10
20
30
40
50
60
Time (min)
Normal
Diabetic
Normal data

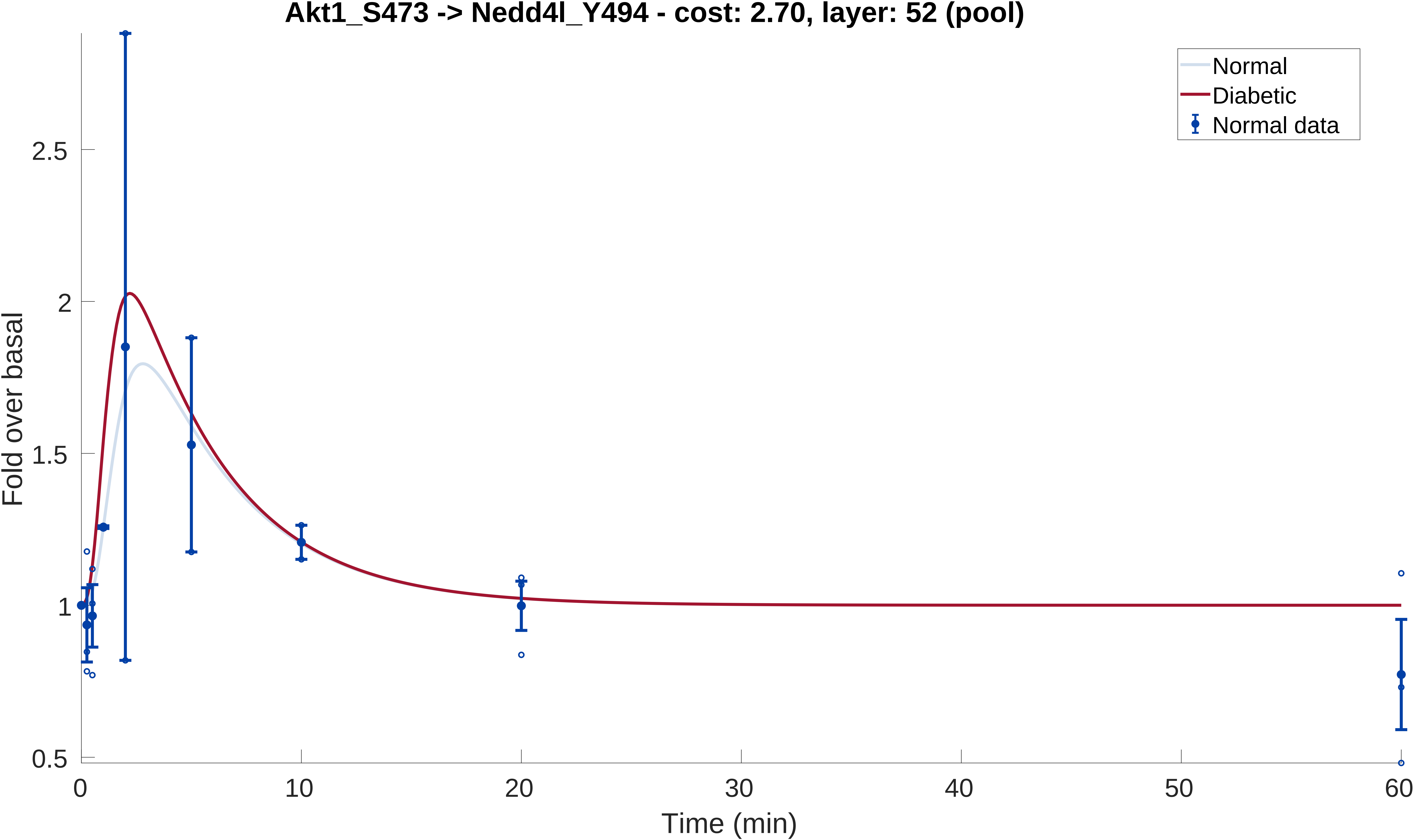

Akt1_S473 -> Nedd4l_Y494 - cost: 2.70, layer: 52 (pool)
Normal
Diabetic
Normal data
Fold over basal
0.5
1
1.5
2
2.5
0
10
20
30
40
50
60
Time (min)

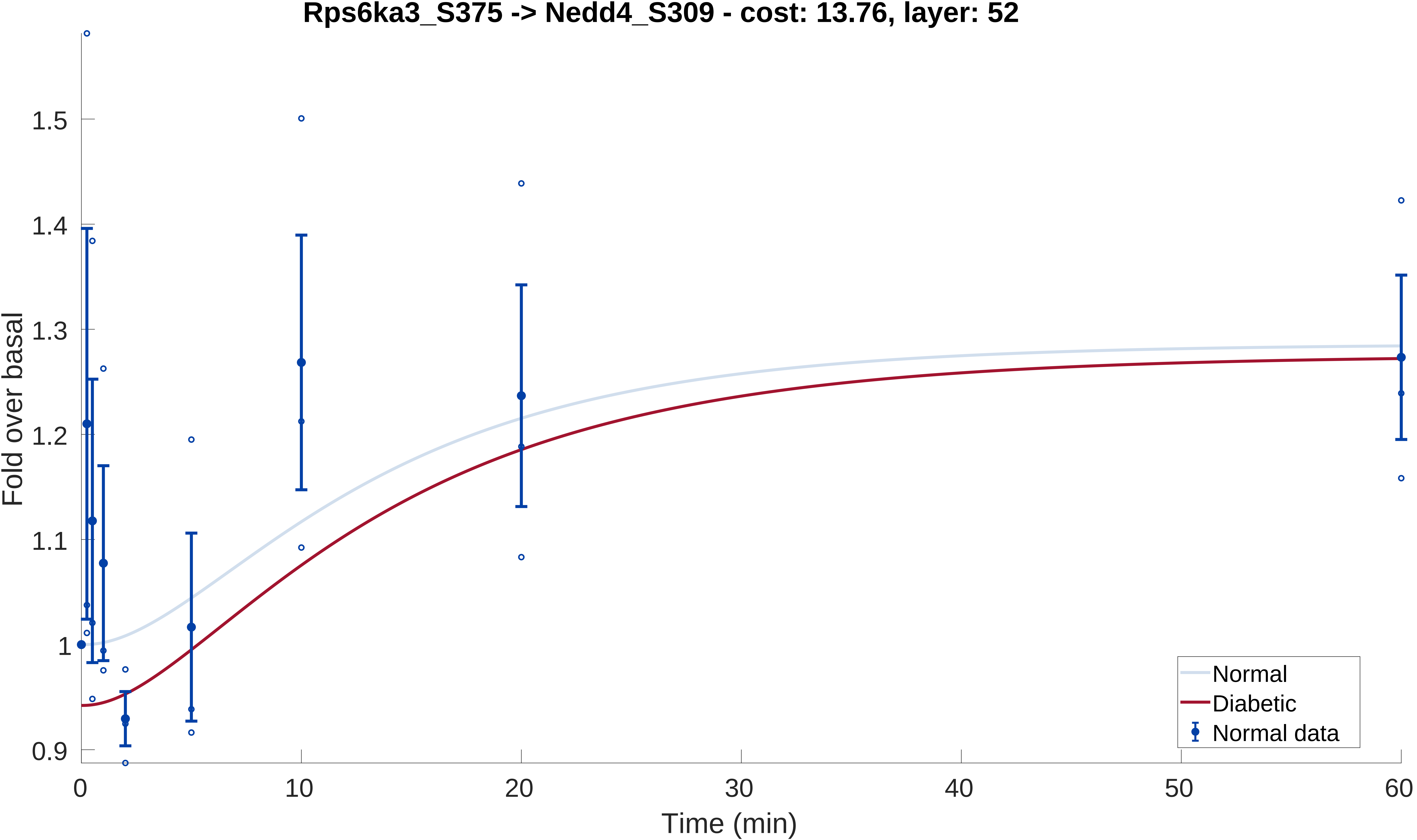

Rps6ka3_S375 -> Nedd4_S309 - cost: 13.76, layer: 52
Fold over basal
Time (min)
1.5
1.4
1.3
1.2
1.1
1
0.9
0
10
20
30
40
50
60
Normal
Diabetic
Normal data

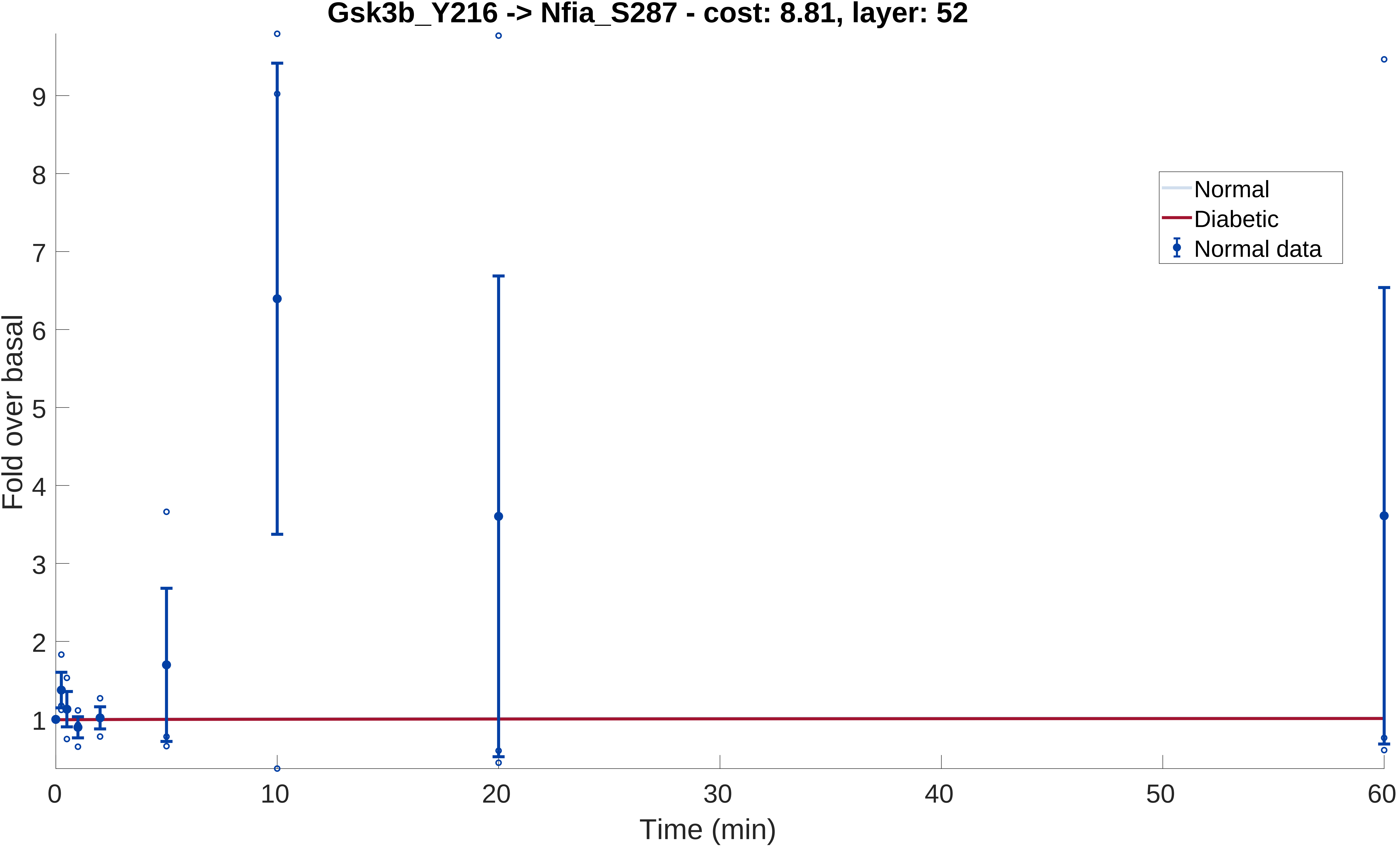

Gsk3b_Y216 -> Nfia_S287 - cost: 8.81, layer: 52
Fold over basal
Time (min)
Normal
Diabetic
Normal data
0
10
20
30
40
50
60
1
2
3
4
5
6
7
8
9

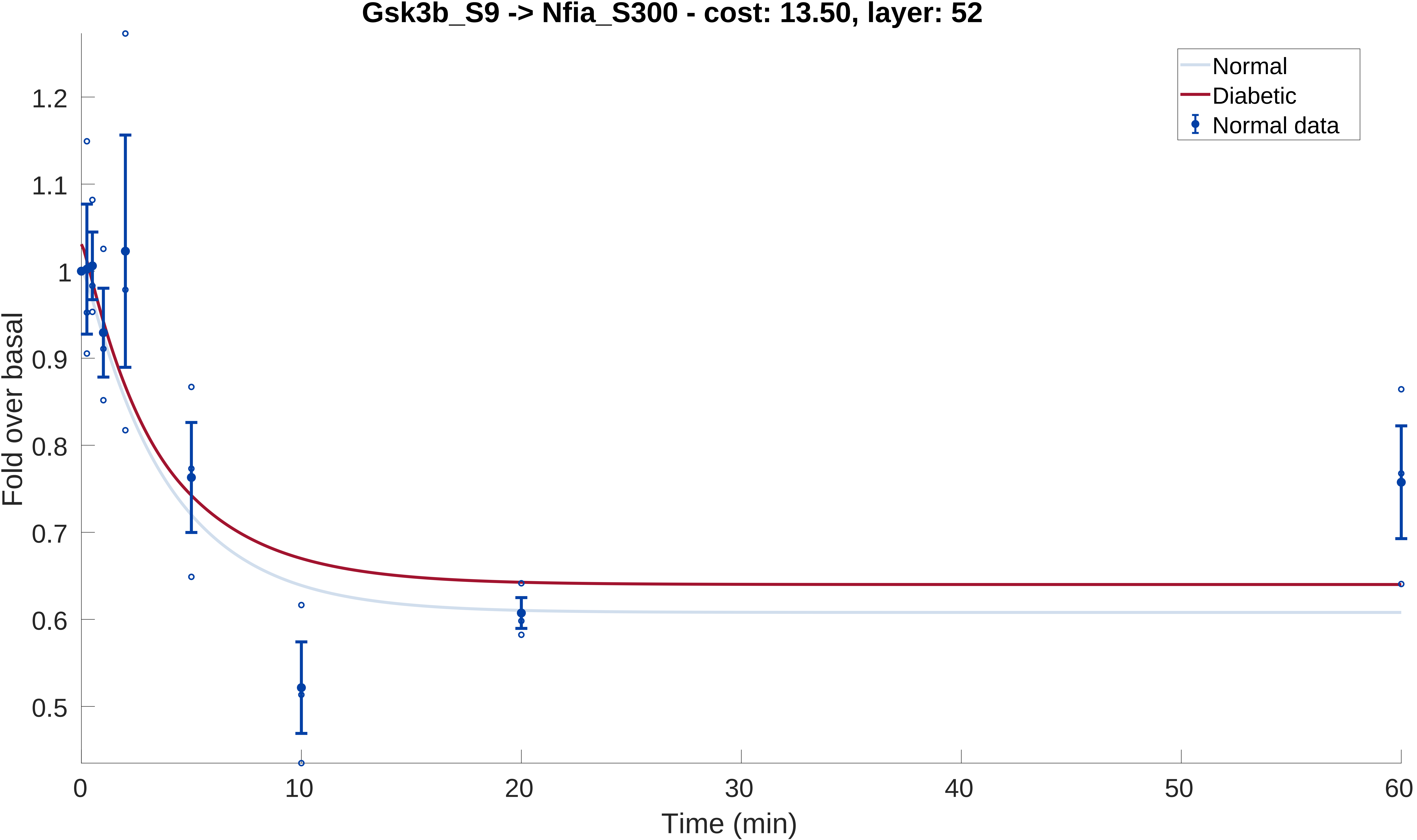

Gsk3b_S9 -> Nfia_S300 - cost: 13.50, layer: 52
Normal
Diabetic
Normal data
Fold over basal
Time (min)
1.2
1.1
1
0.9
0.8
0.7
0.6
0.5
0
10
20
30
40
50
60

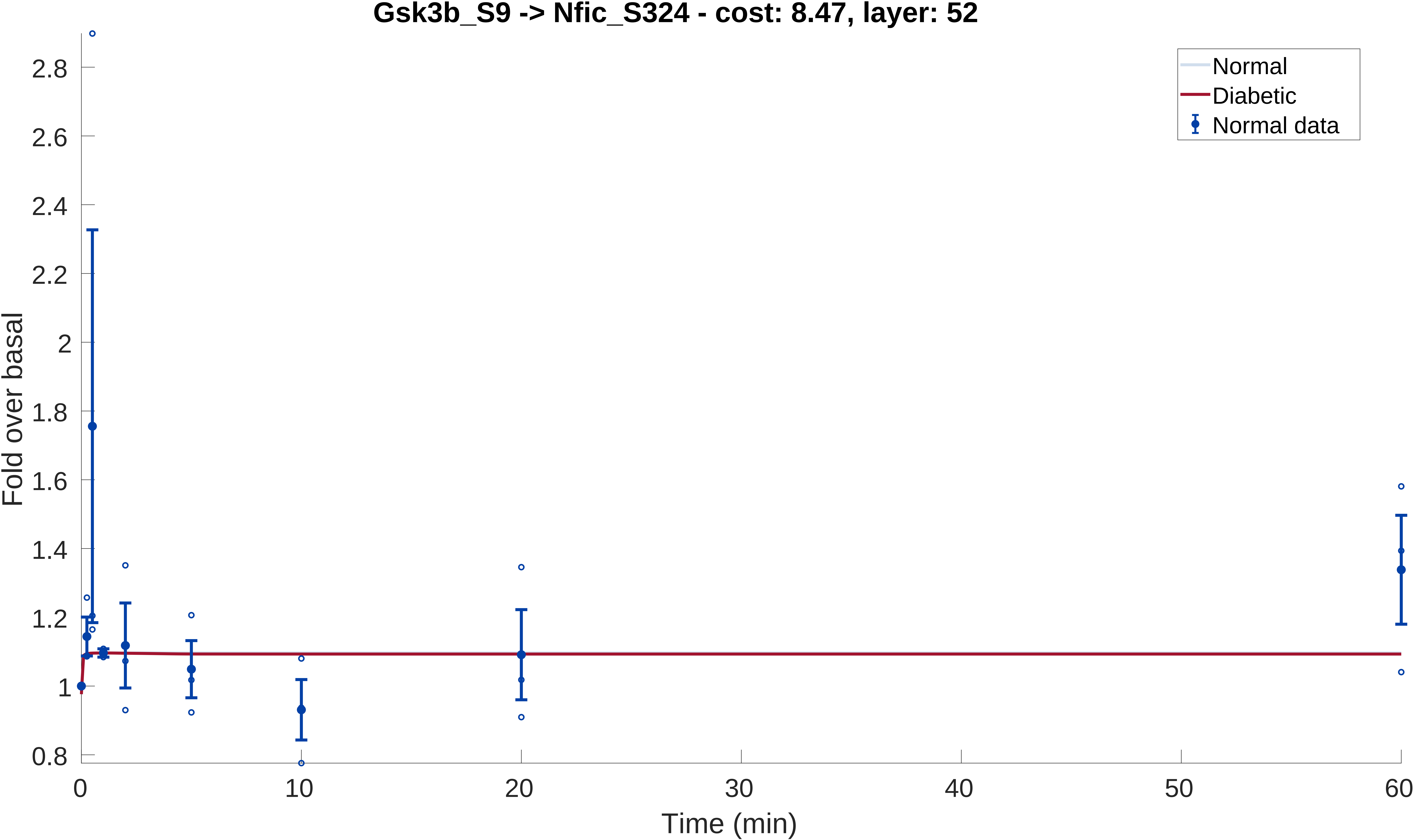

Gsk3b_S9 -> Nfic_S324 - cost: 8.47, layer: 52
Normal
Diabetic
Normal data
Fold over basal
Time (min)
0.8
1
1.2
1.4
1.6
1.8
2
2.2
2.4
2.6
2.8
0
10
20
30
40
50
60

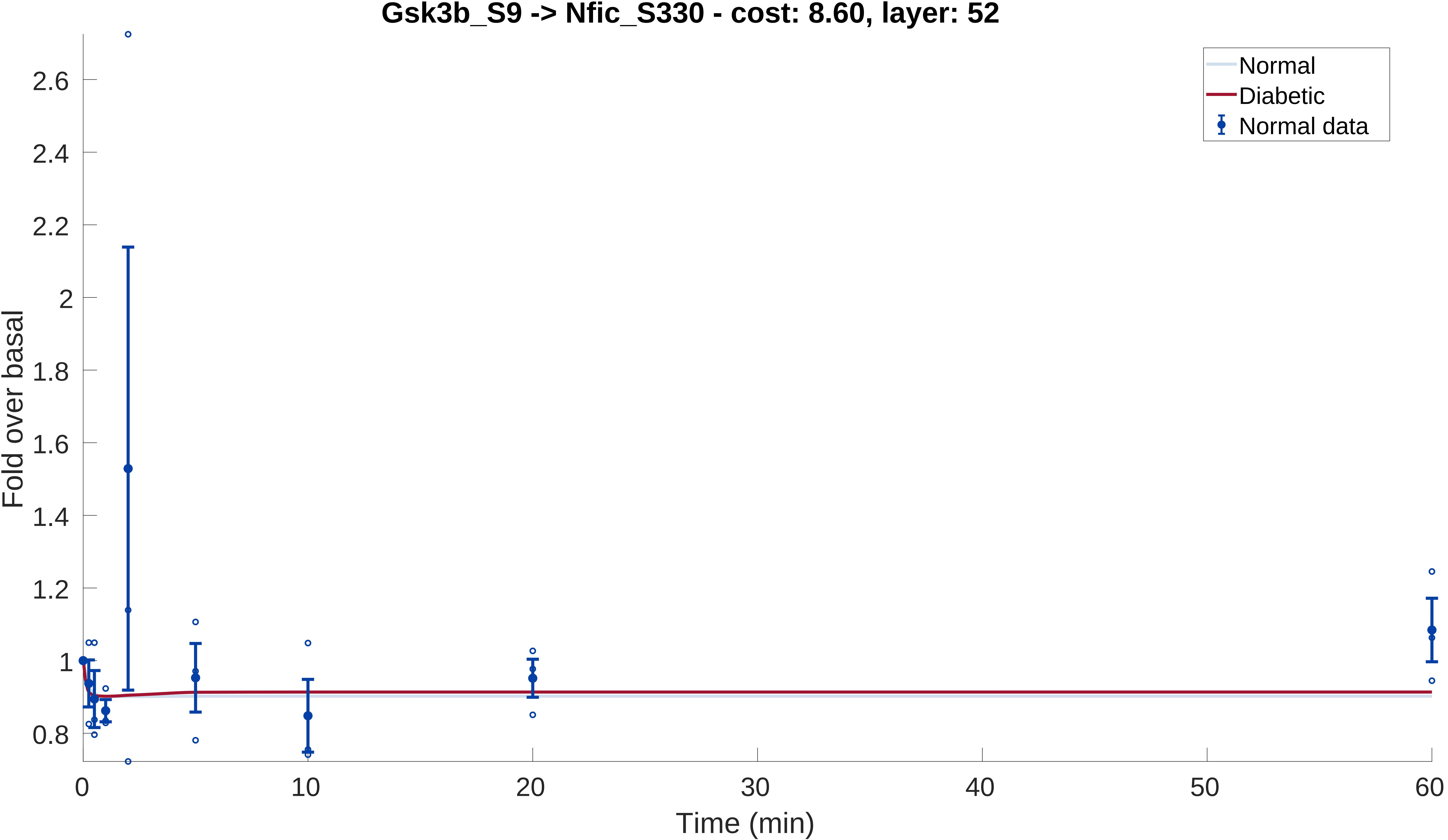
Gsk3b_S9 -> Nfic_S330 - cost: 8.60, layer: 52
Normal
Diabetic
Normal data
Fold over basal
2.6
2.4
2.2
2
1.8
1.6
1.4
1.2
1
0.8
0
10
20
30
40
50
60
Time (min)

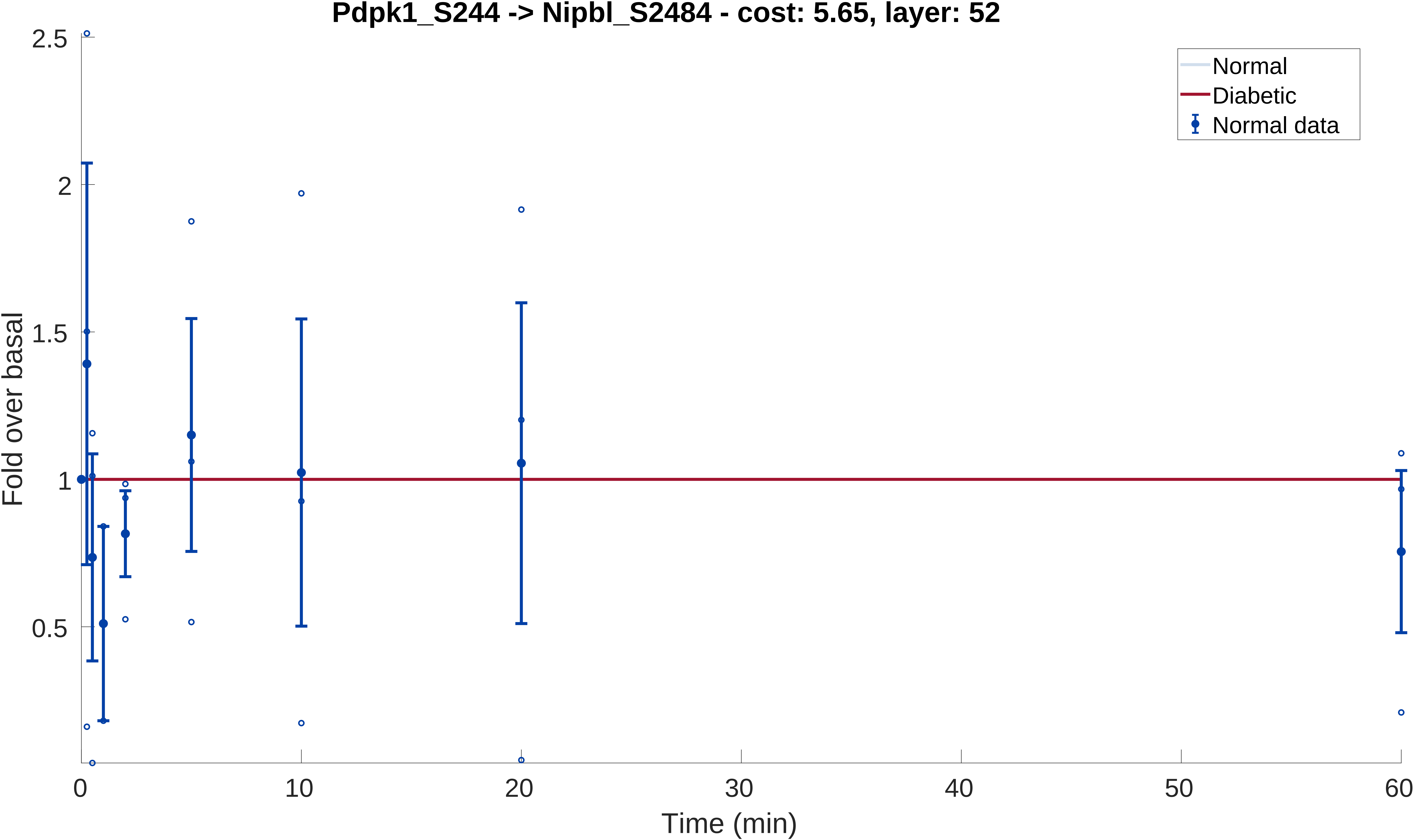

Pdpk1_S244 -> Nipbl_S2484 - cost: 5.65, layer: 52
Normal
Diabetic
Normal data
Fold over basal
Time (min)
0
10
20
30
40
50
60
0.5
1
1.5
2
2.5

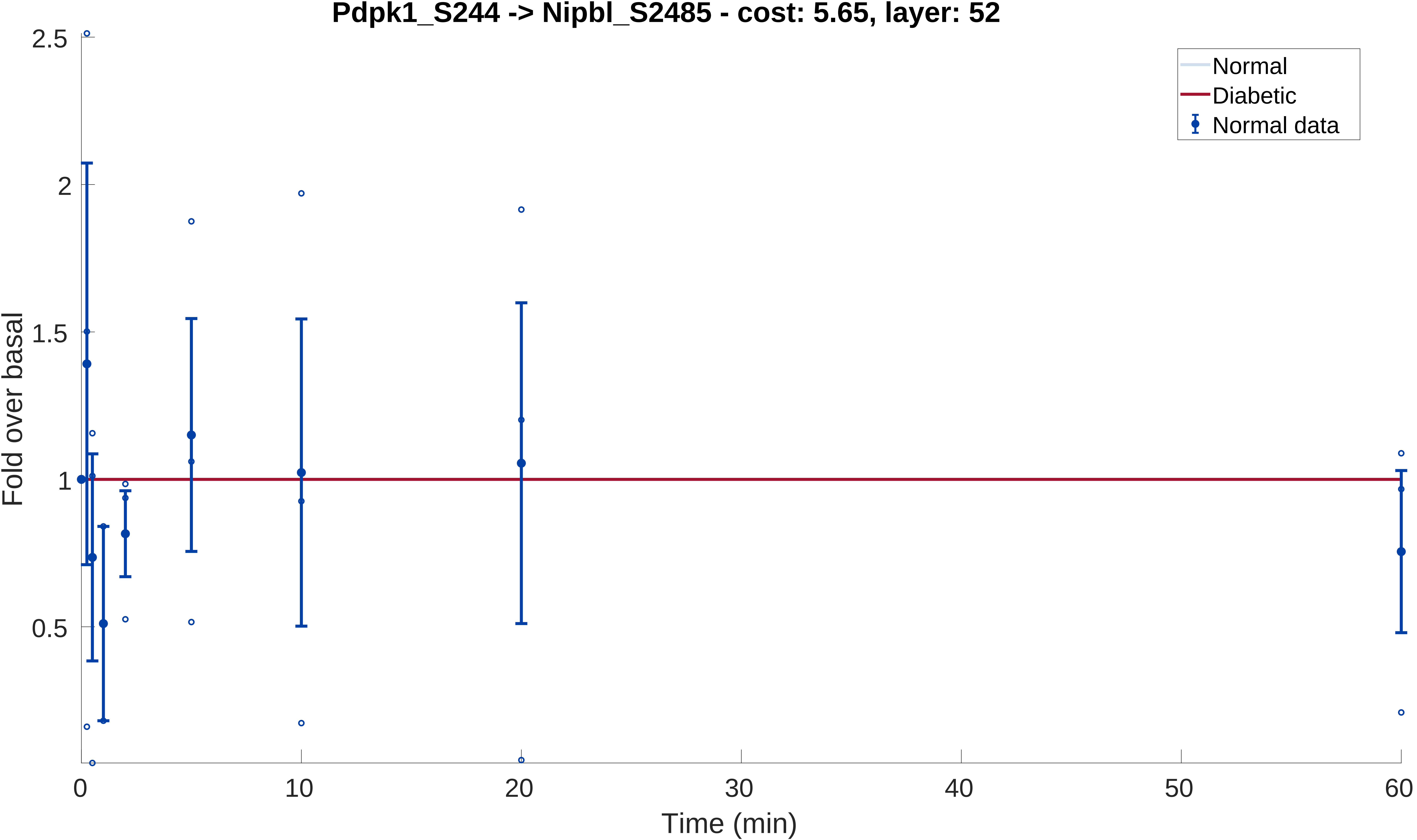

Pdpk1_S244 -> Nipbl_S2485 - cost: 5.65, layer: 52
Normal
Diabetic
Normal data
Fold over basal
Time (min)
2.5
2
1.5
1
0.5
0
10
20
30
40
50
60

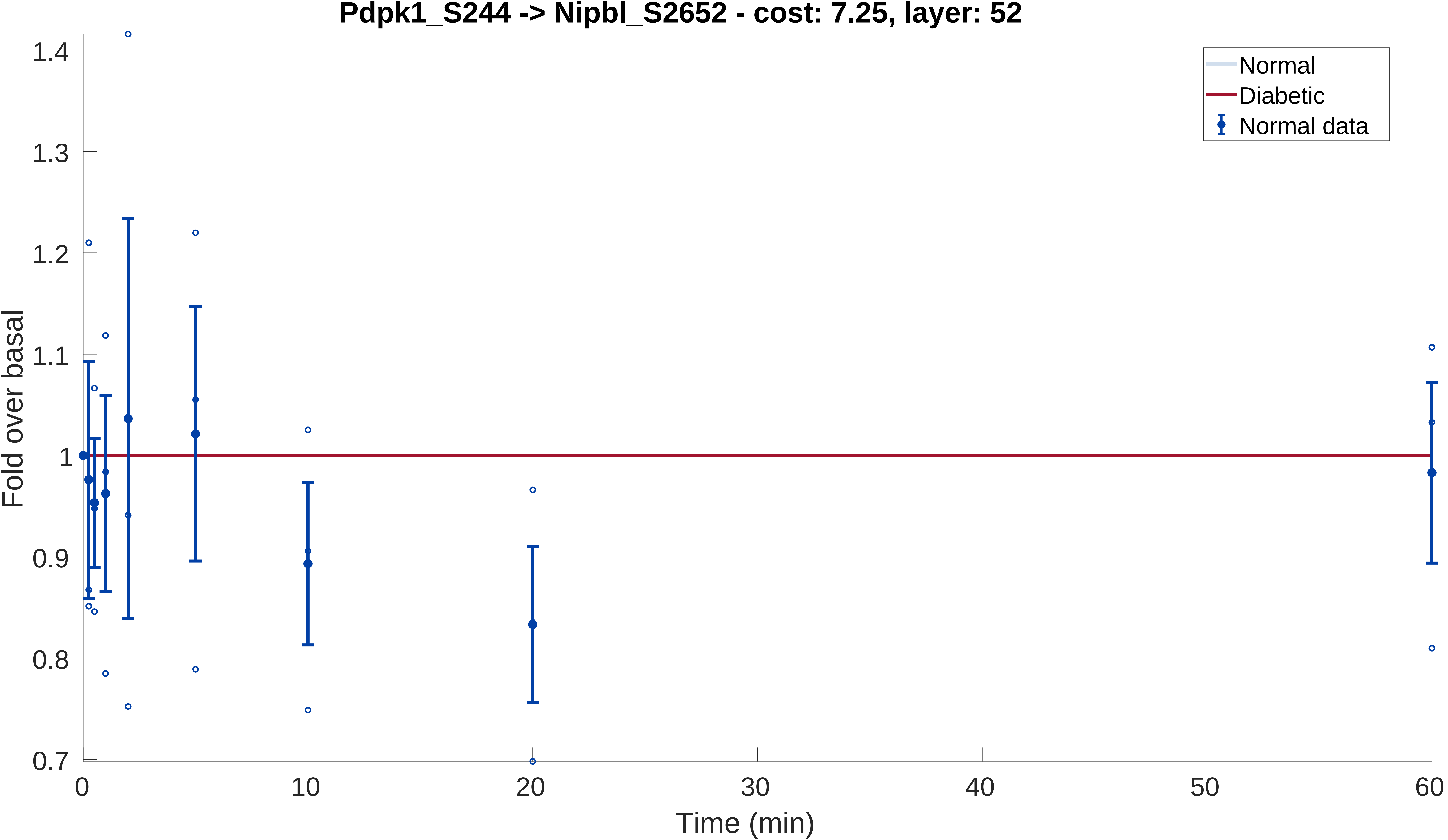

Pdpk1_S244 -> Nipbl_S2652 - cost: 7.25, layer: 52
Normal
Diabetic
Normal data
Fold over basal
Time (min)
1.4
1.3
1.2
1.1
1
0.9
0.8
0.7
0
10
20
30
40
50
60

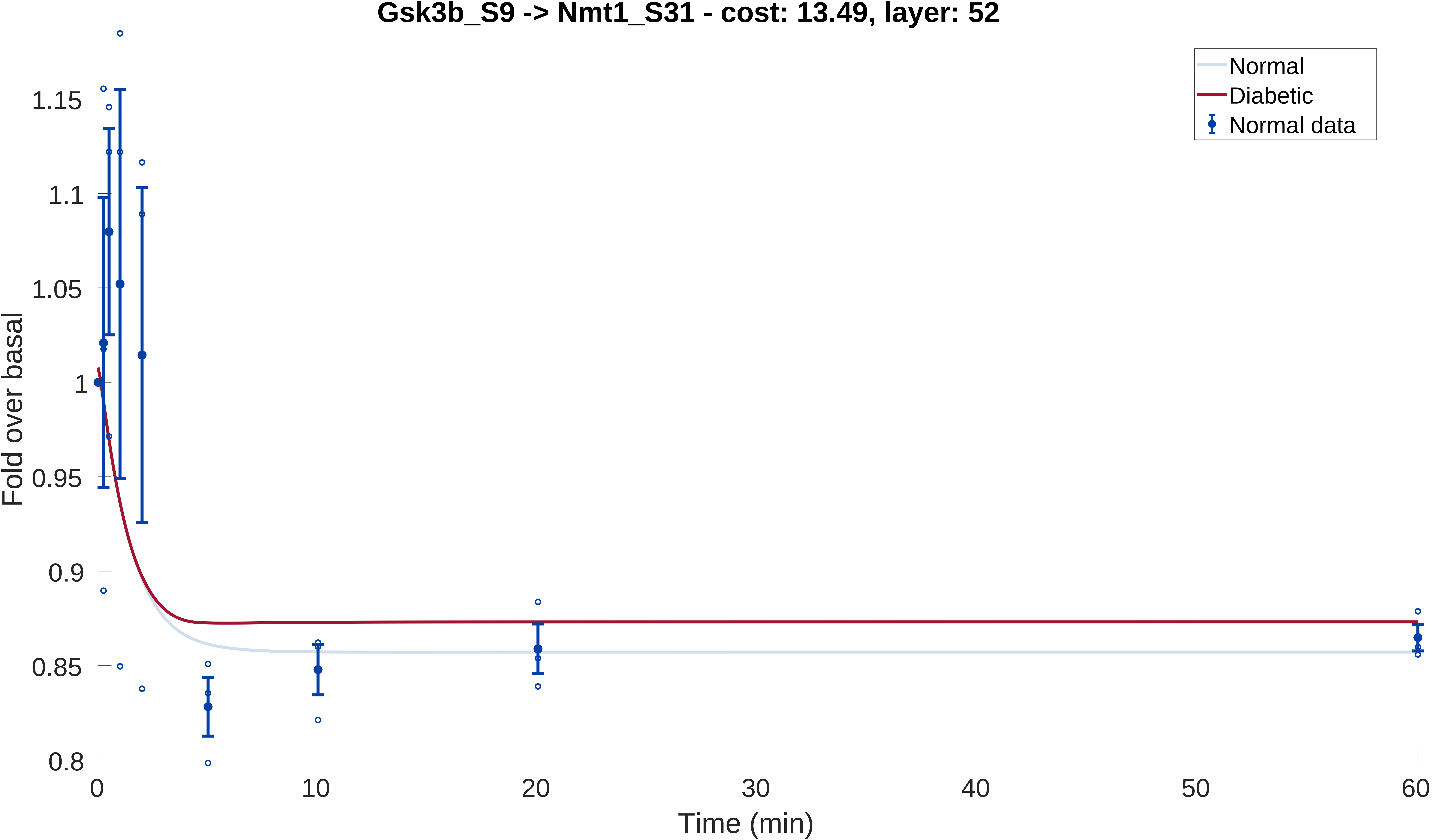

Gsk3b_S9 -> Nmt1_S31 - cost: 13.49, layer: 52
Normal
Diabetic
Normal data
Fold over basal
1.15
1.1
1.05
1
0.95
0.9
0.85
0.8
0
10
20
30
40
50
60
Time (min)

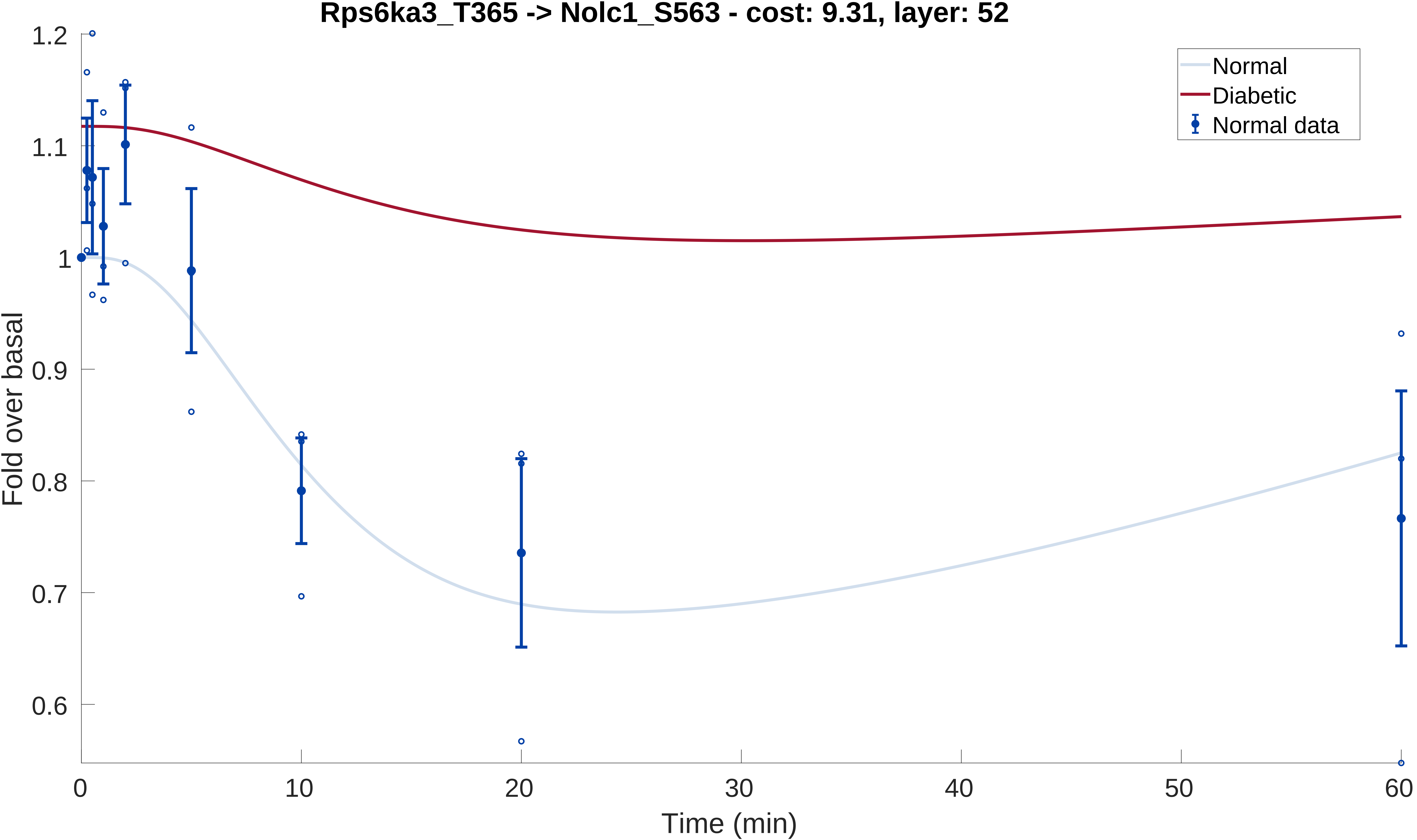

Rps6ka3_T365 -> Nolc1_S563 - cost: 9.31, layer: 52
Fold over basal
Time (min)
Normal
Diabetic
Normal data
1.2
1.1
1
0.9
0.8
0.7
0.6
0
10
20
30
40
50
60

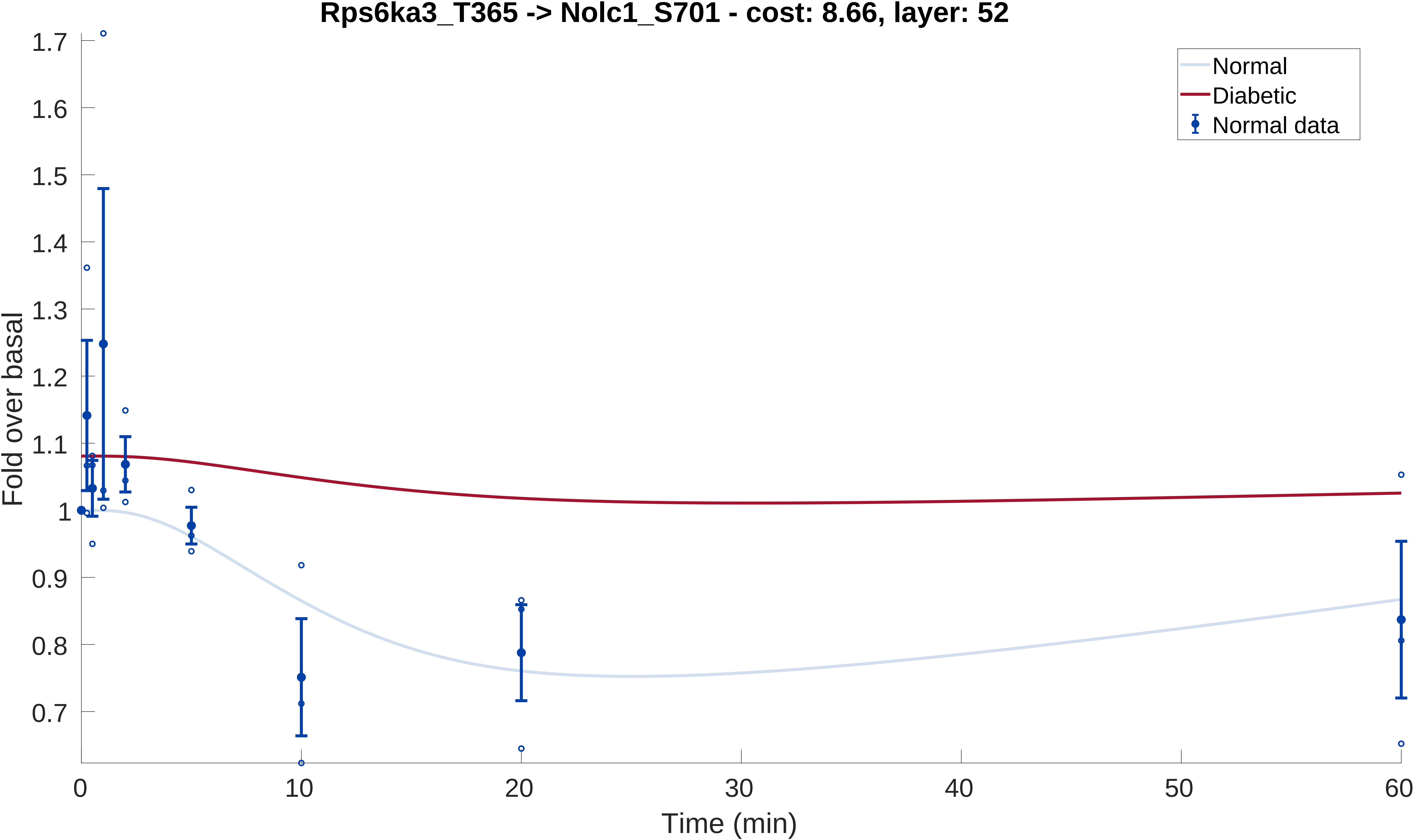

Rps6ka3_T365 -> Nolc1_S701 - cost: 8.66, layer: 52
Normal
Diabetic
Normal data
Fold over basal
Time (min)
1.7
1.6
1.5
1.4
1.3
1.2
1.1
1
0.9
0.8
0.7
0
10
20
30
40
50
60

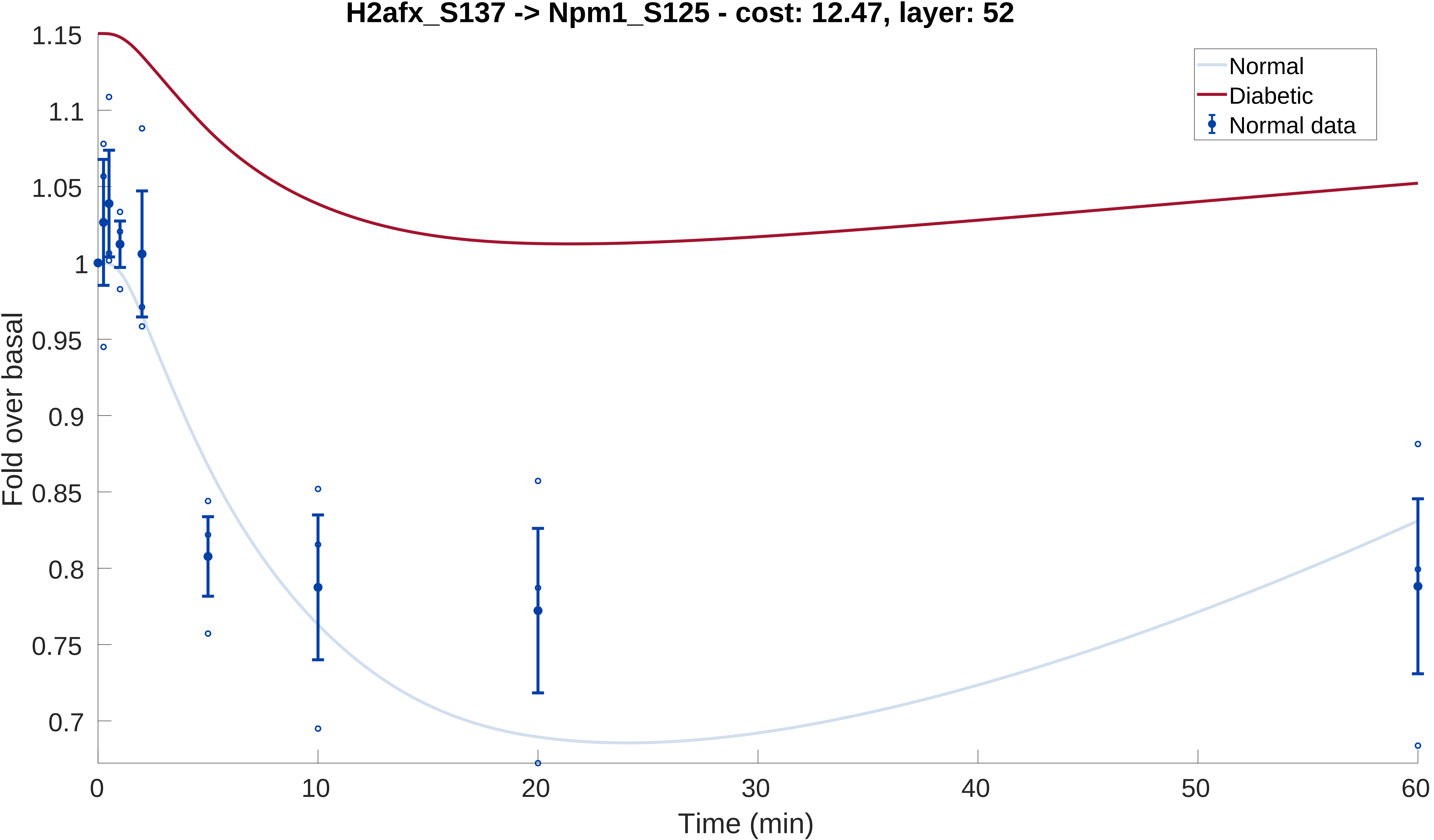

H2afx_S137 -> Npm1_S125 - cost: 12.47, layer: 52
Normal
Diabetic
Normal data
Fold over basal
1.15
1.1
1.05
1
0.95
0.9
0.85
0.8
0.75
0.7
0
10
20
30
40
50
60
Time (min)

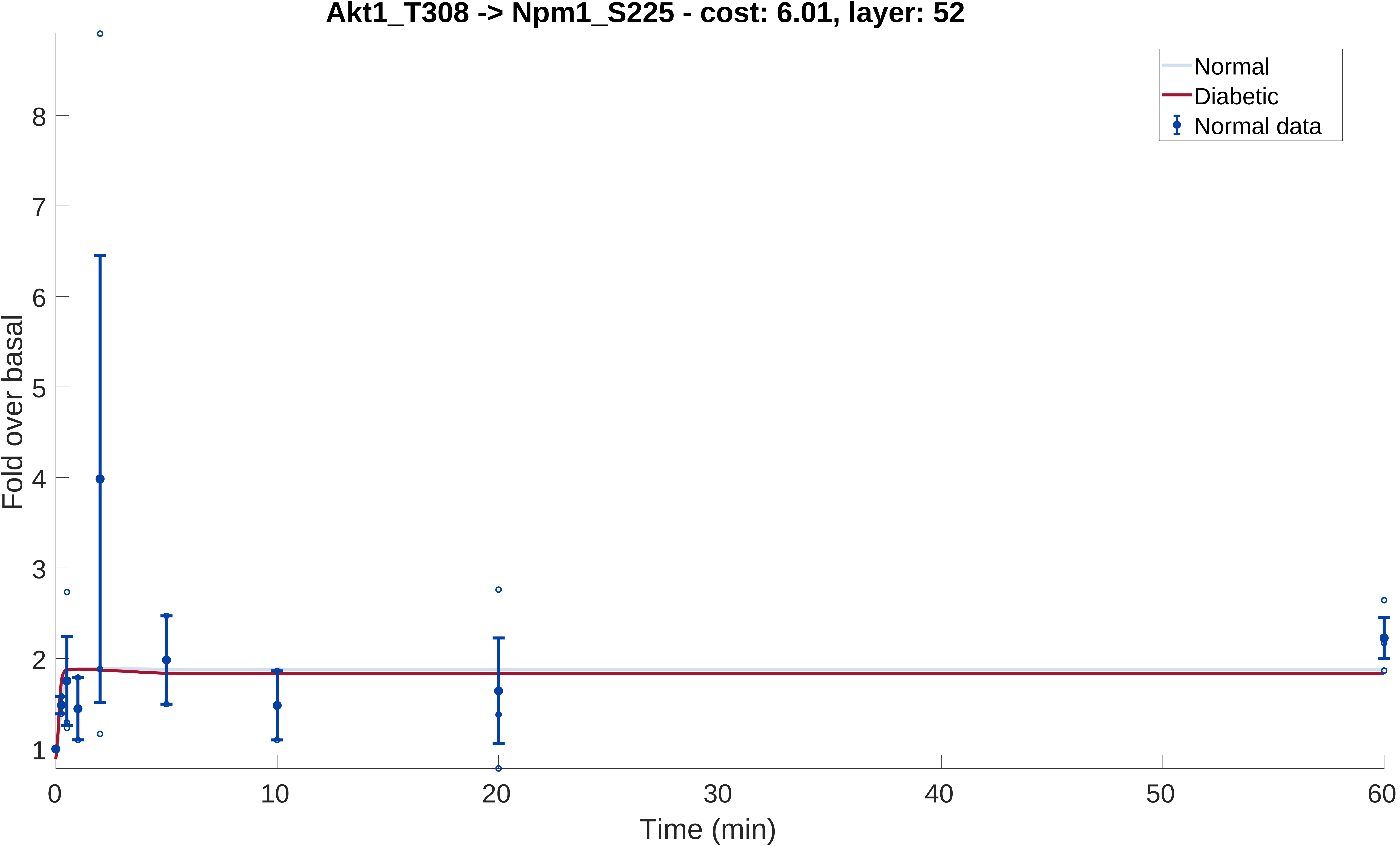

Akt1_T308 -> Npm1_S225 - cost: 6.01, layer: 52
Normal
Diabetic
Normal data
Fold over basal
1
2
3
4
5
6
7
8
0
10
20
30
40
50
60
Time (min)

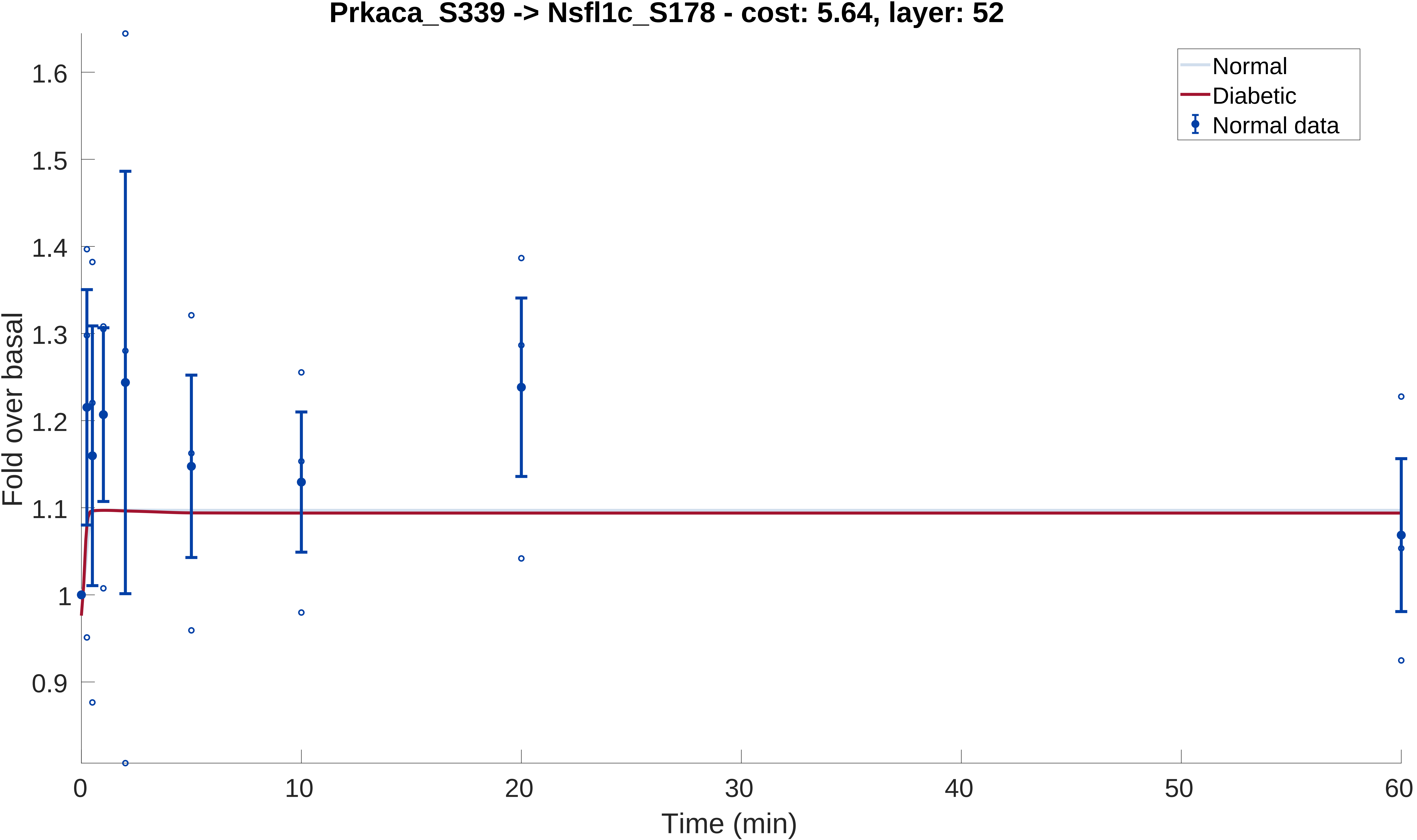

Prkaca_S339 -> Nsfl1c_S178 - cost: 5.64, layer: 52
Normal
Diabetic
Normal data
Fold over basal
1.6
1.5
1.4
1.3
1.2
1.1
1
0.9
0
10
20
30
40
50
60
Time (min)

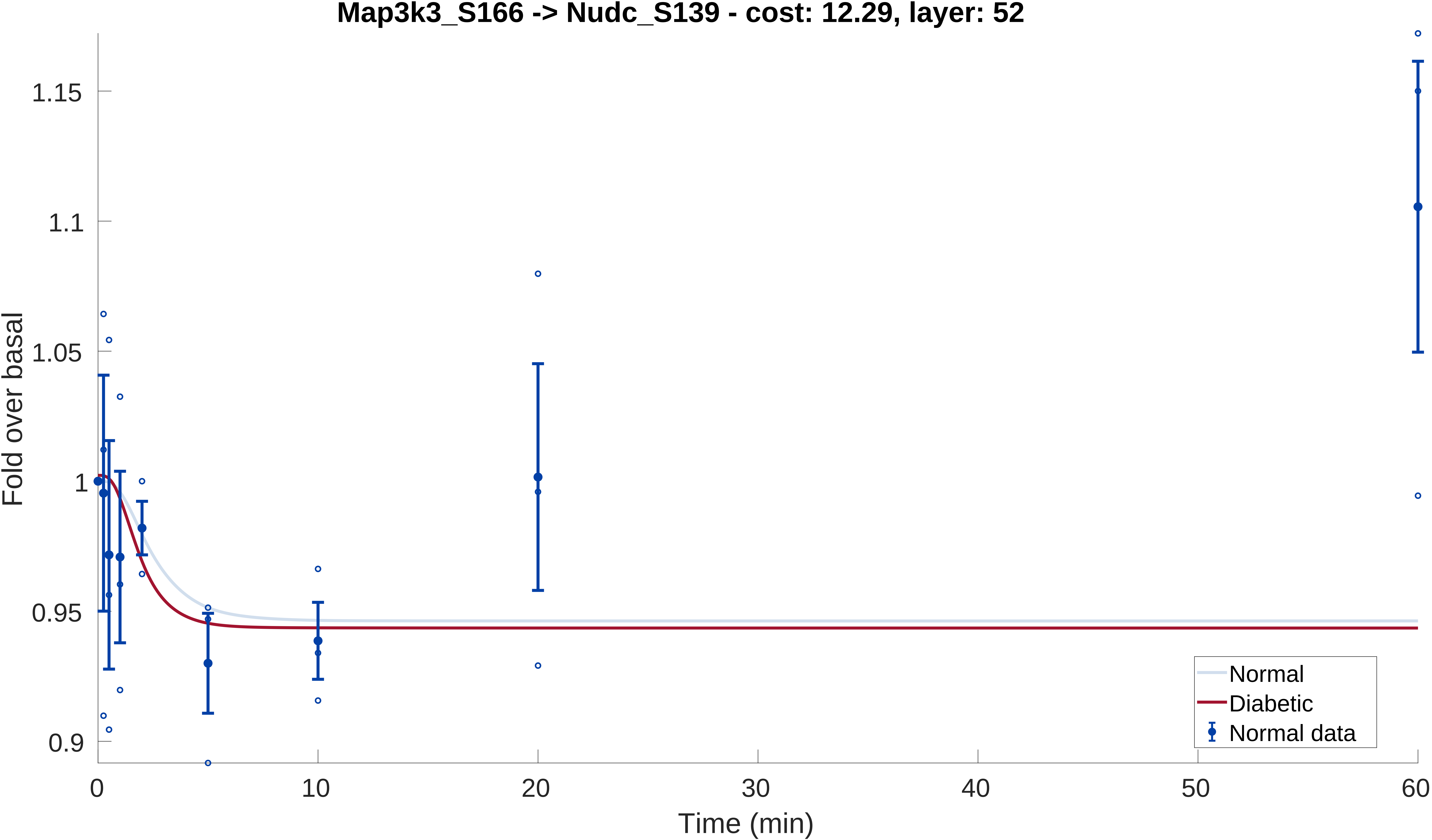

Map3k3_S166 -> Nudc_S139 - cost: 12.29, layer: 52
Fold over basal
1.15
1.1
1.05
1
0.95
0.9
0
10
20
30
40
50
60
Time (min)
Normal
Diabetic
Normal data

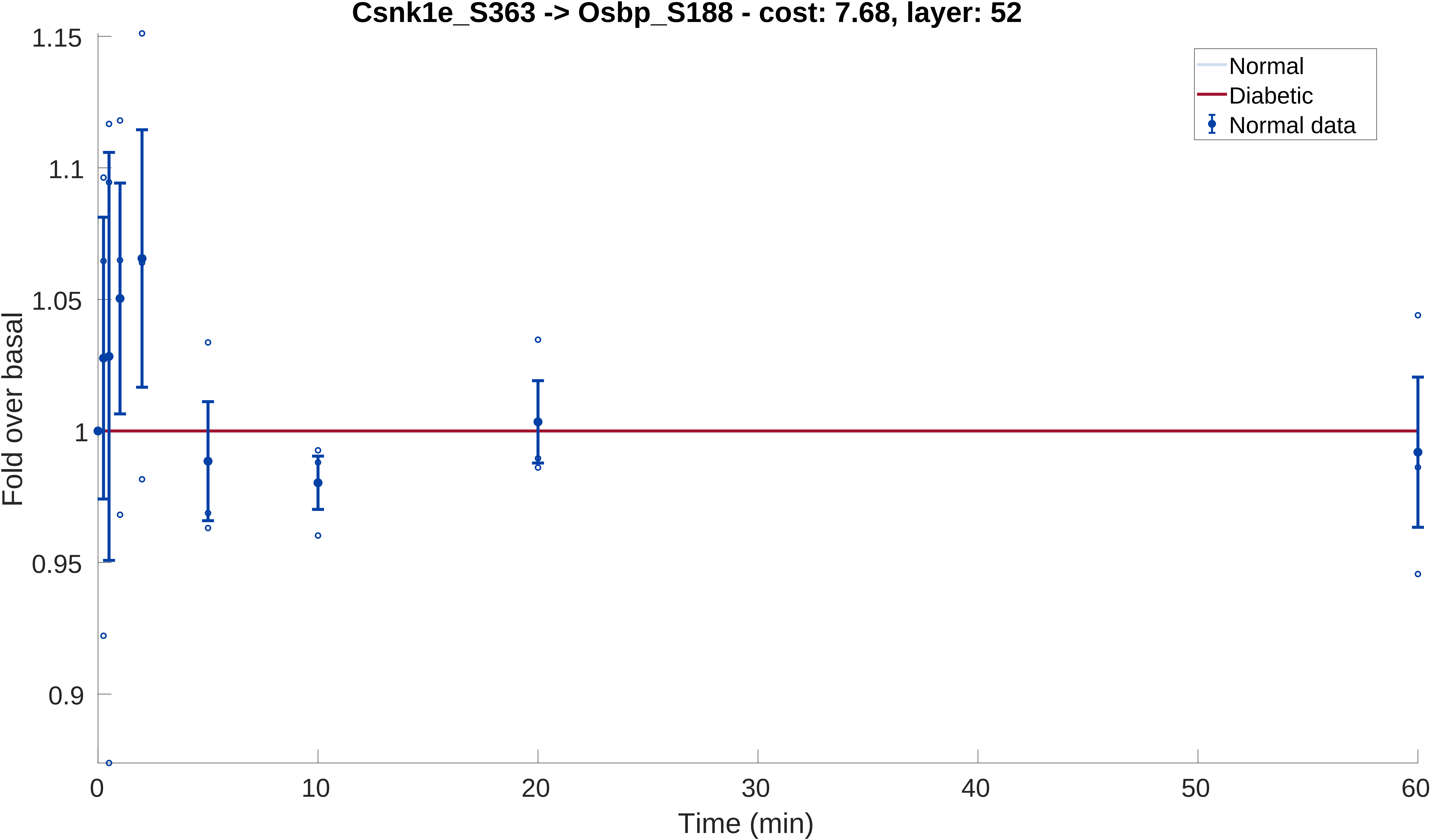

Csnk1e_S363 -> Osbp_S188 - cost: 7.68, layer: 52
Normal
Diabetic
Normal data
Fold over basal
1.15
1.1
1.05
1
0.95
0.9
0
10
20
30
40
50
60
Time (min)

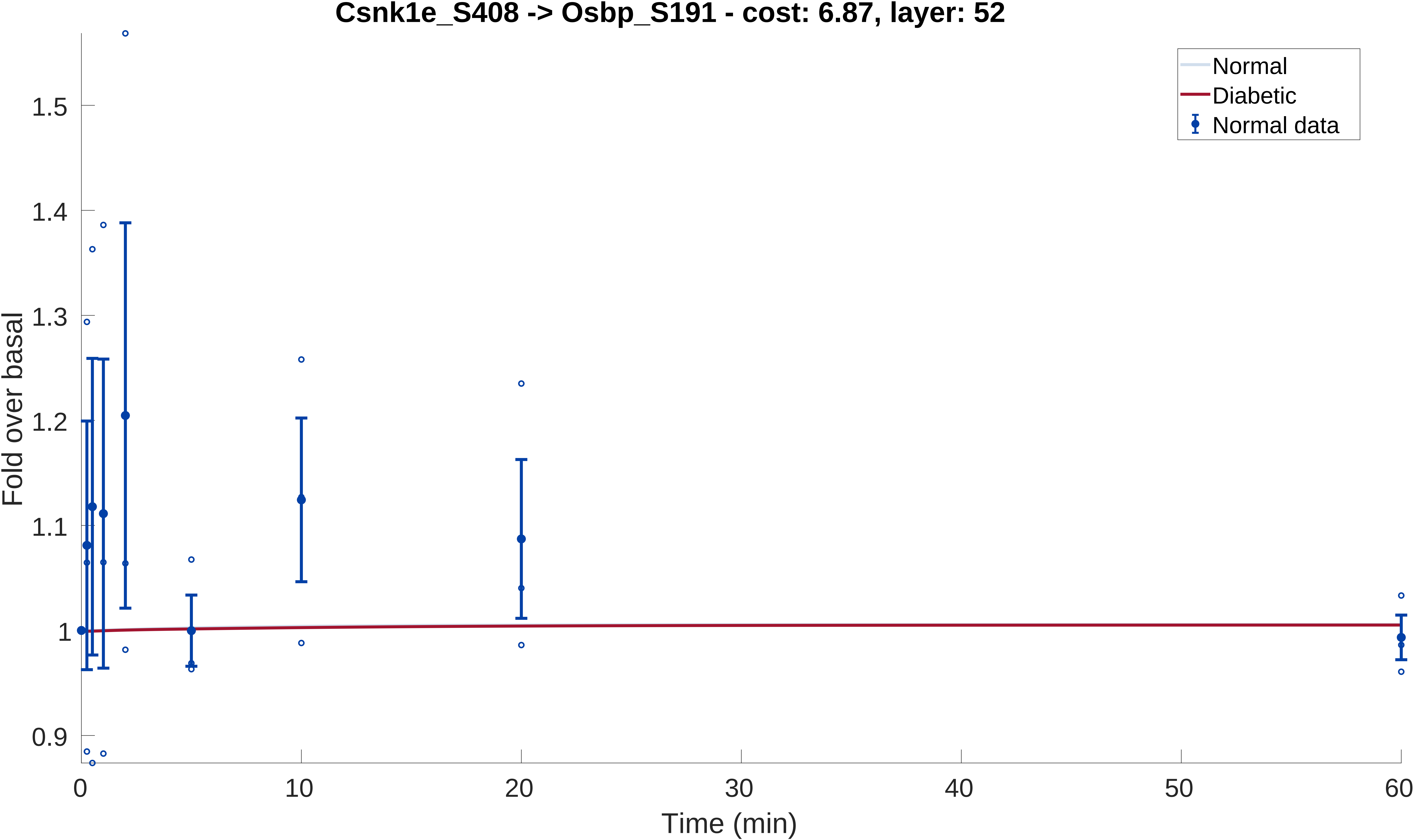
Csnk1e_S408 -> Osbp_S191 - cost: 6.87, layer: 52
Fold over basal
Time (min)
Normal
Diabetic
Normal data
0.9
1
1.1
1.2
1.3
1.4
1.5
0
10
20
30
40
50
60

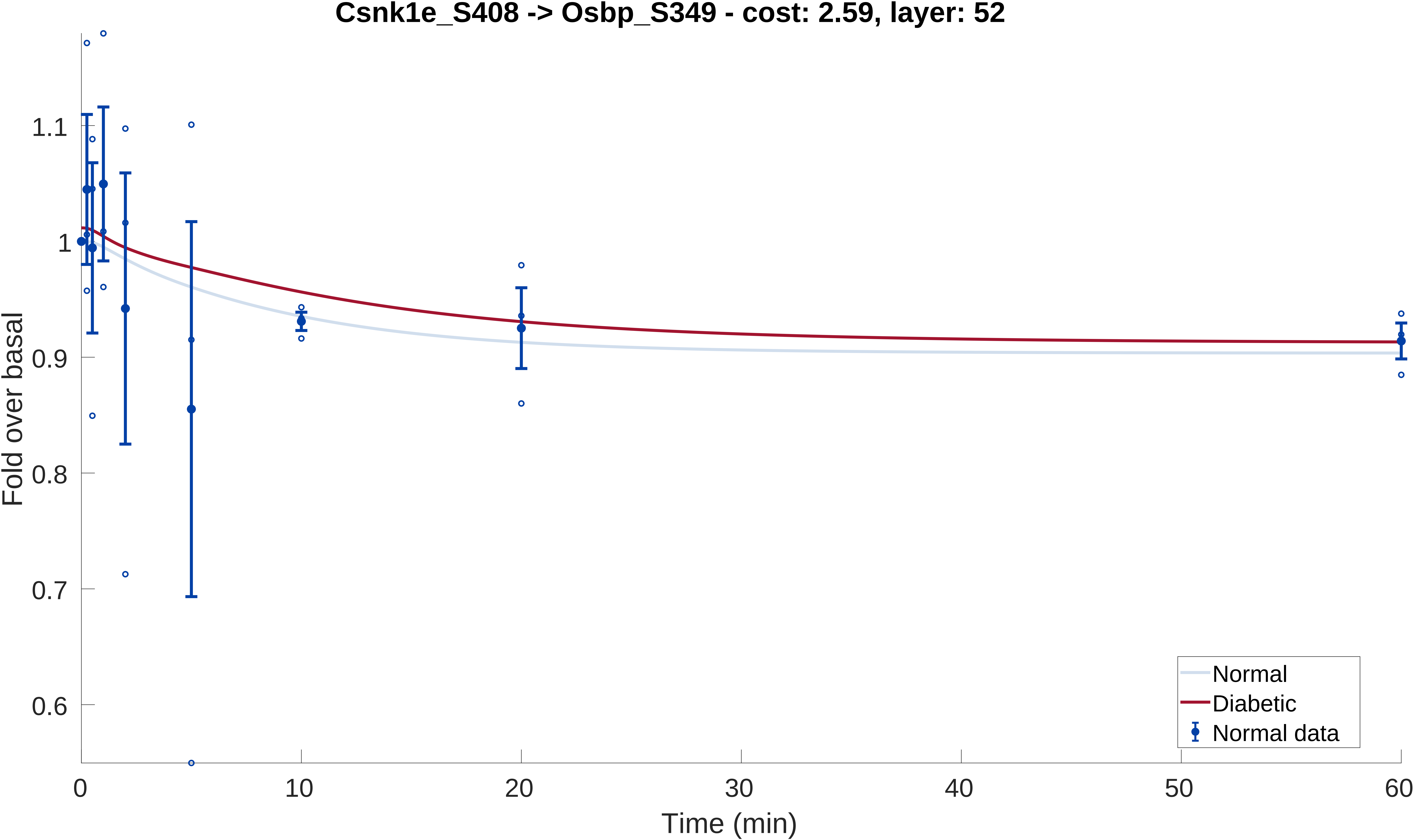

Csnk1e_S408 -> Osbp_S349 - cost: 2.59, layer: 52
Fold over basal
1.1
1
0.9
0.8
0.7
0.6
0
10
20
30
40
50
60
Time (min)
Normal
Diabetic
Normal data

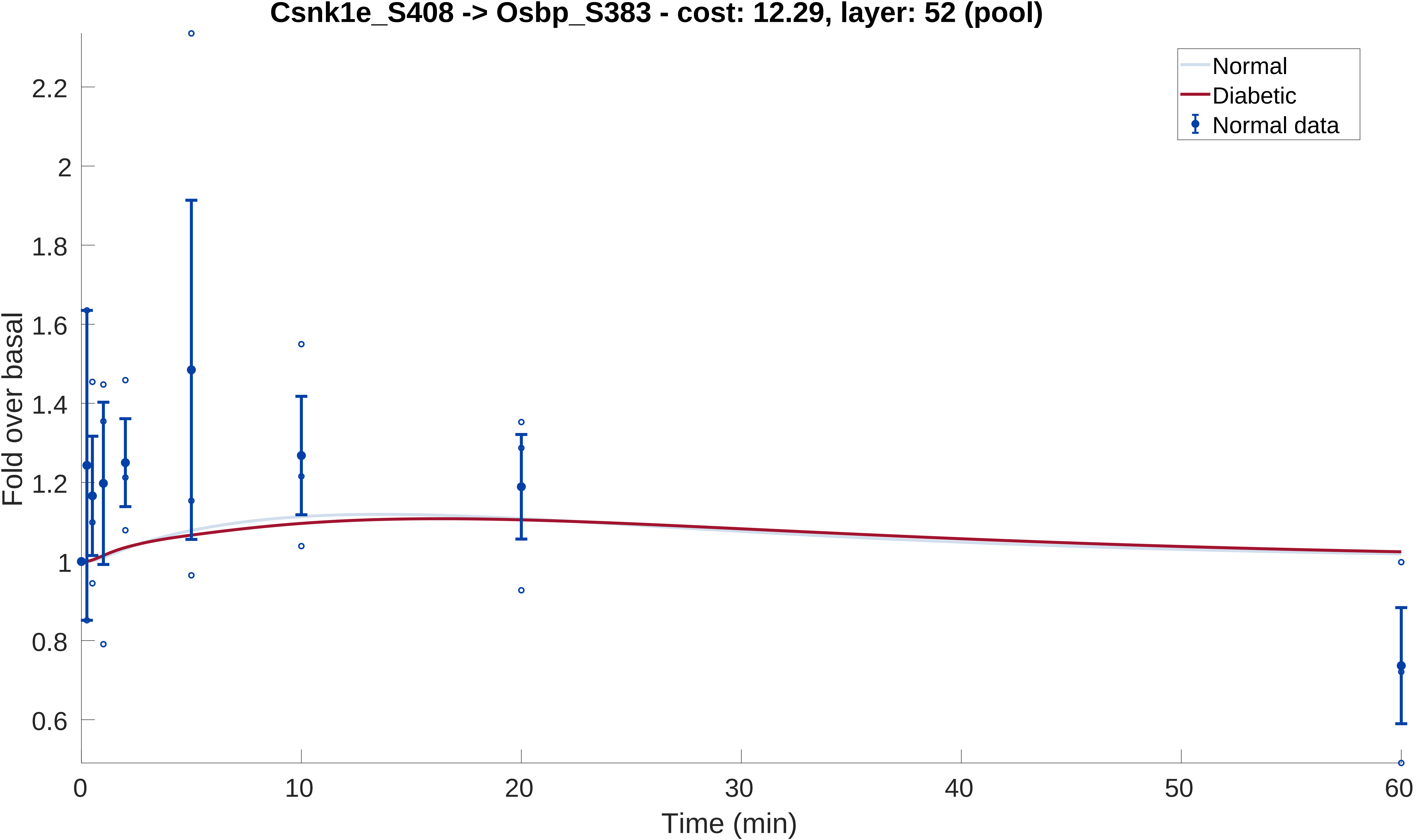
Csnk1e_S408 -> Osbp_S383 - cost: 12.29, layer: 52 (pool)
Normal
Diabetic
Normal data
Fold over basal
Time (min)
0.6
0.8
1
1.2
1.4
1.6
1.8
2
2.2
0
10
20
30
40
50
60

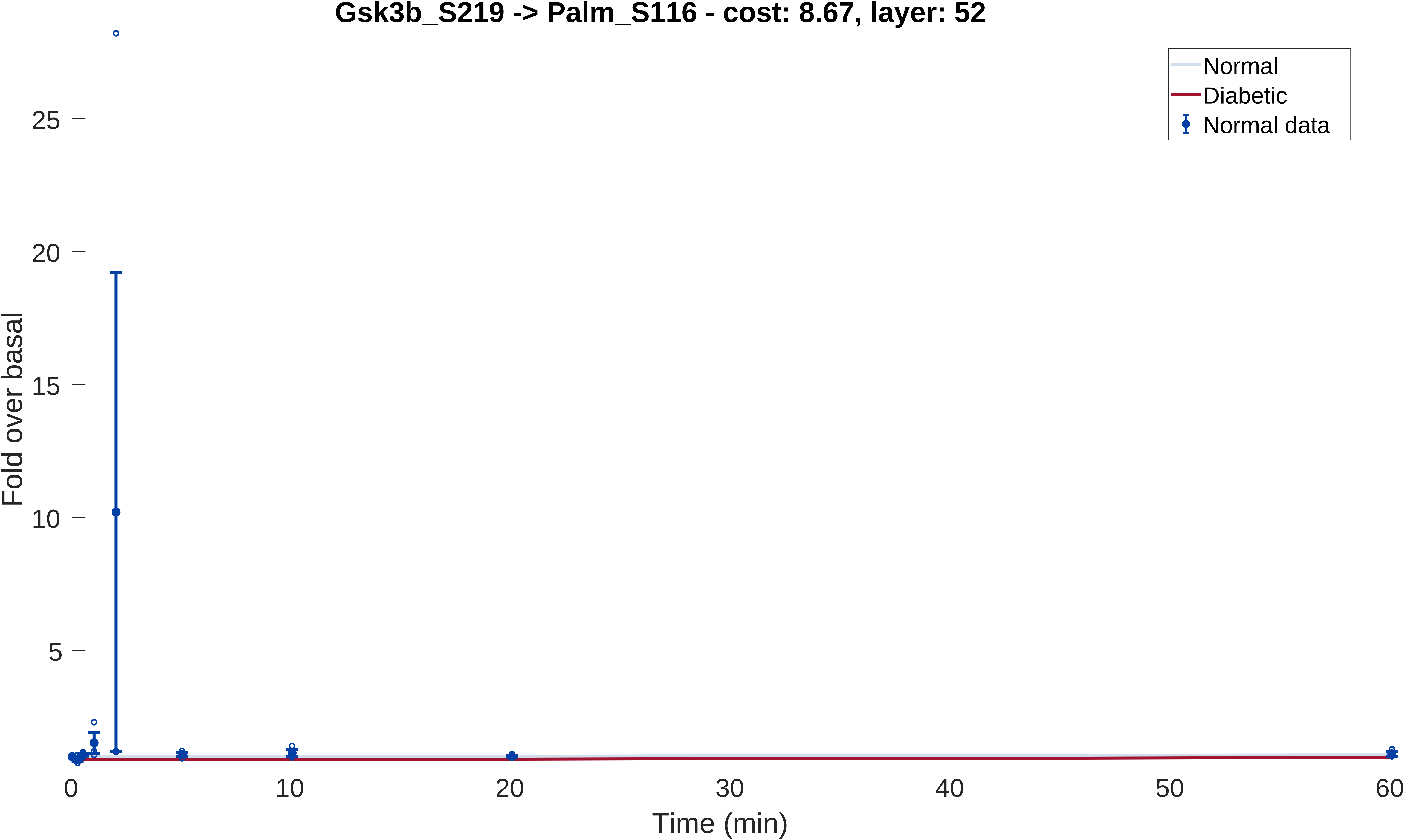
Gsk3b_S219 -> Palm_S116 - cost: 8.67, layer: 52
Normal
Diabetic
Normal data
Fold over basal
Time (min)
0
10
20
30
40
50
60
5
10
15
20
25

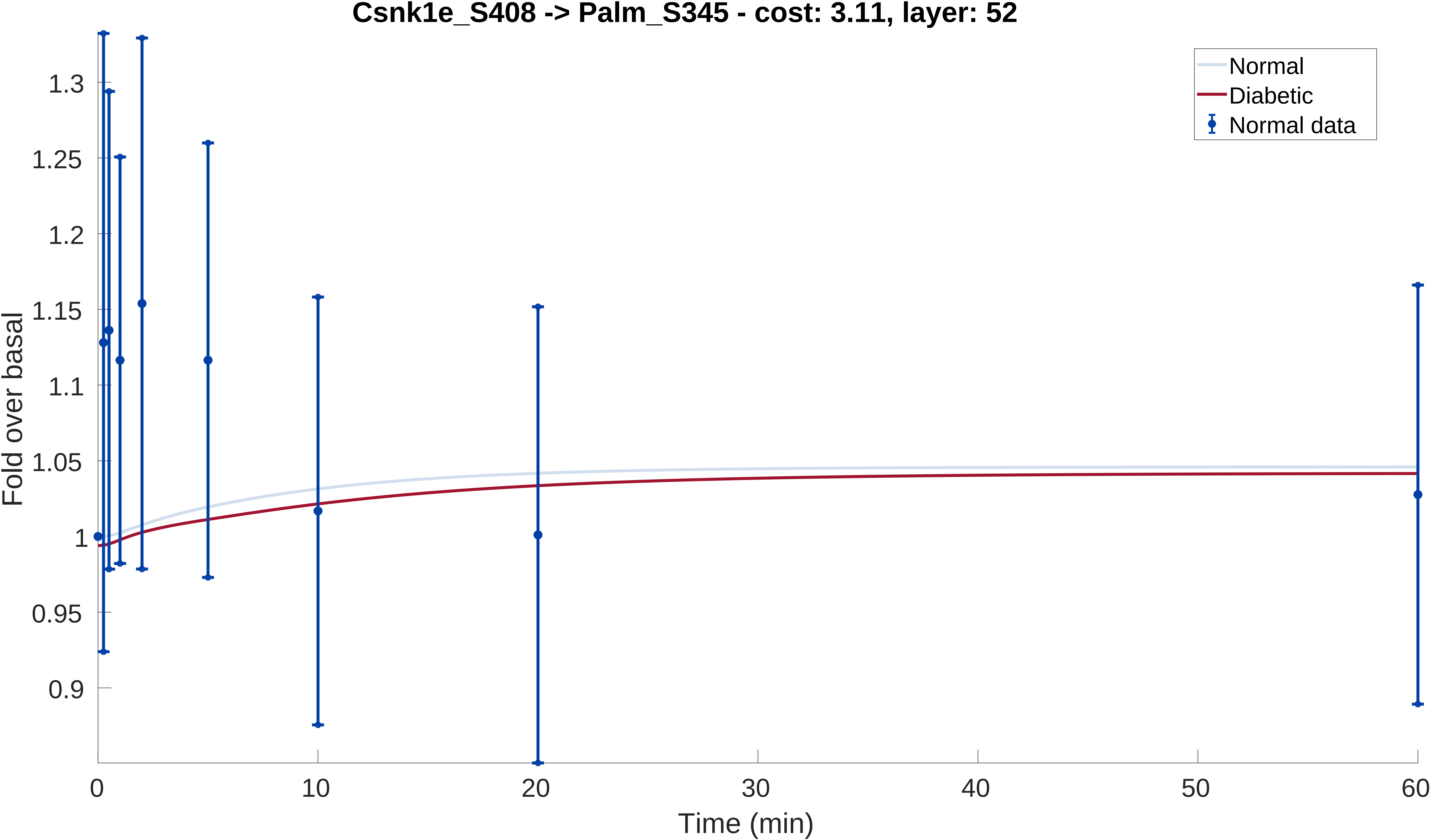

Csnk1e_S408 -> Palm_S345 - cost: 3.11, layer: 52
Normal
Diabetic
Normal data
Fold over basal
1.3
1.25
1.2
1.15
1.1
1.05
1
0.95
0.9
0
10
20
30
40
50
60
Time (min)

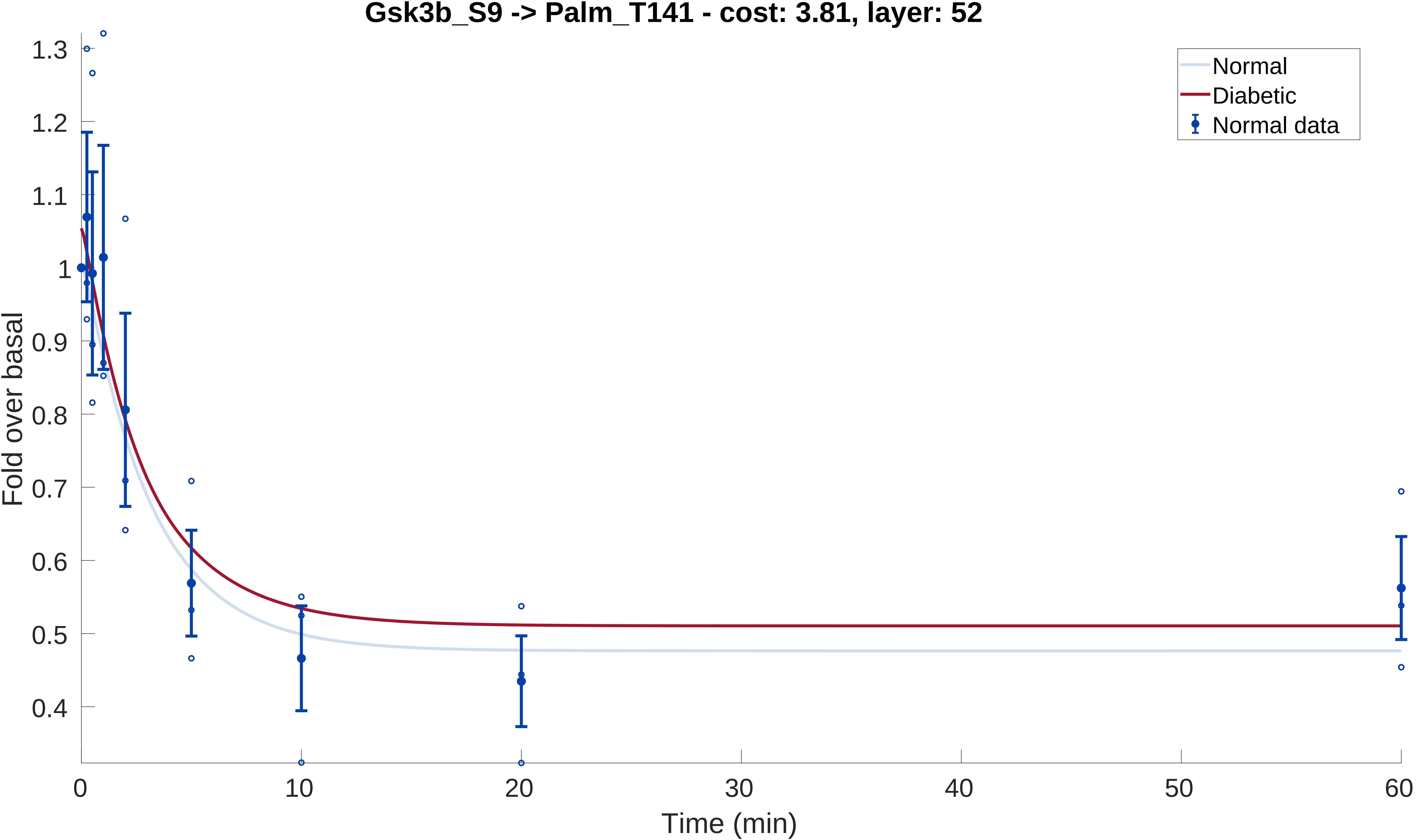

Gsk3b_S9 -> Palm_T141 - cost: 3.81, layer: 52
Normal
Diabetic
Normal data
Fold over basal
1.3
1.2
1.1
1
0.9
0.8
0.7
0.6
0.5
0.4
0
10
20
30
40
50
60
Time (min)

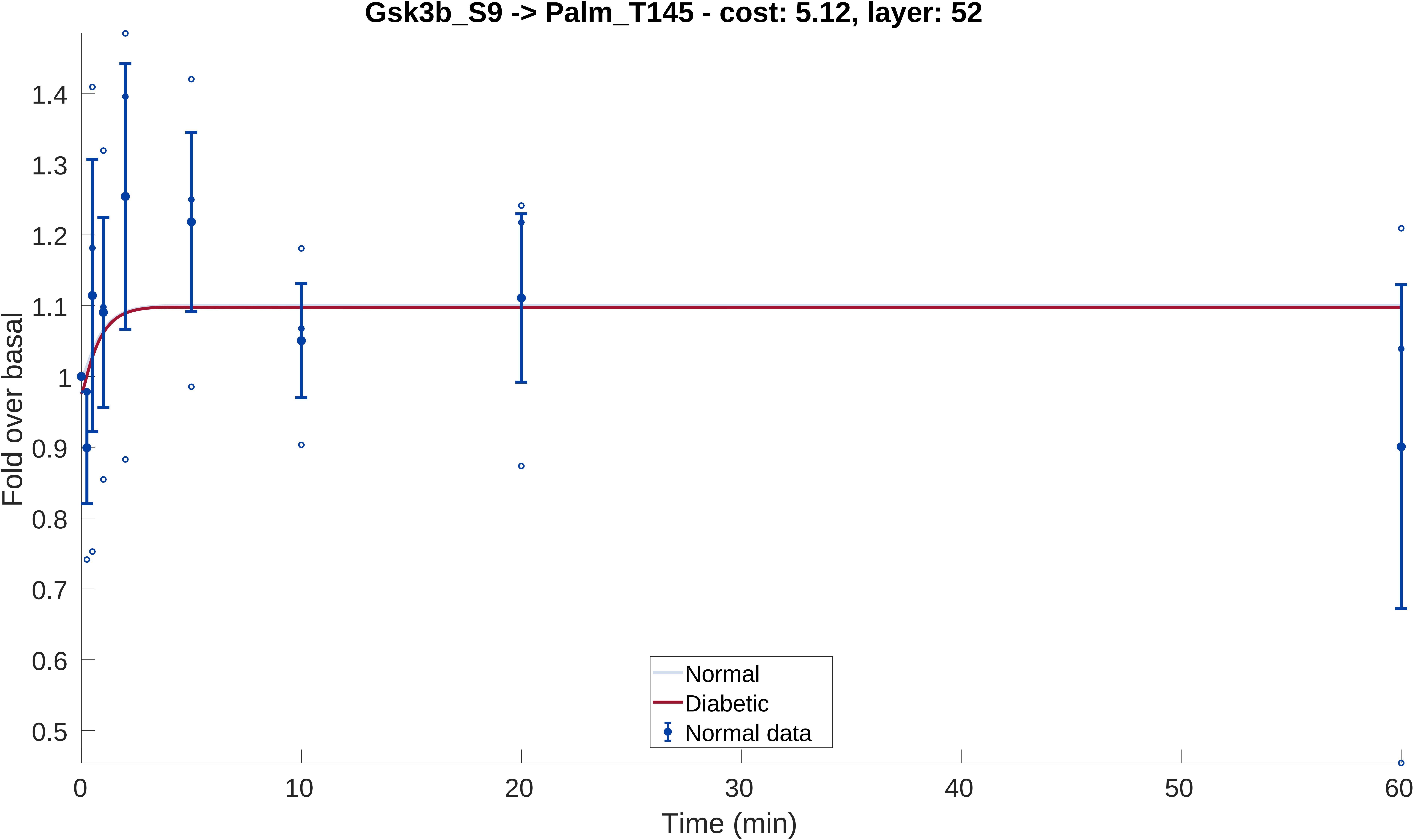

Gsk3b_S9 -> Palm_T145 - cost: 5.12, layer: 52
Fold over basal
Time (min)
0.5
0.6
0.7
0.8
0.9
1
1.1
1.2
1.3
1.4
0
10
20
30
40
50
60
Normal
Diabetic
Normal data

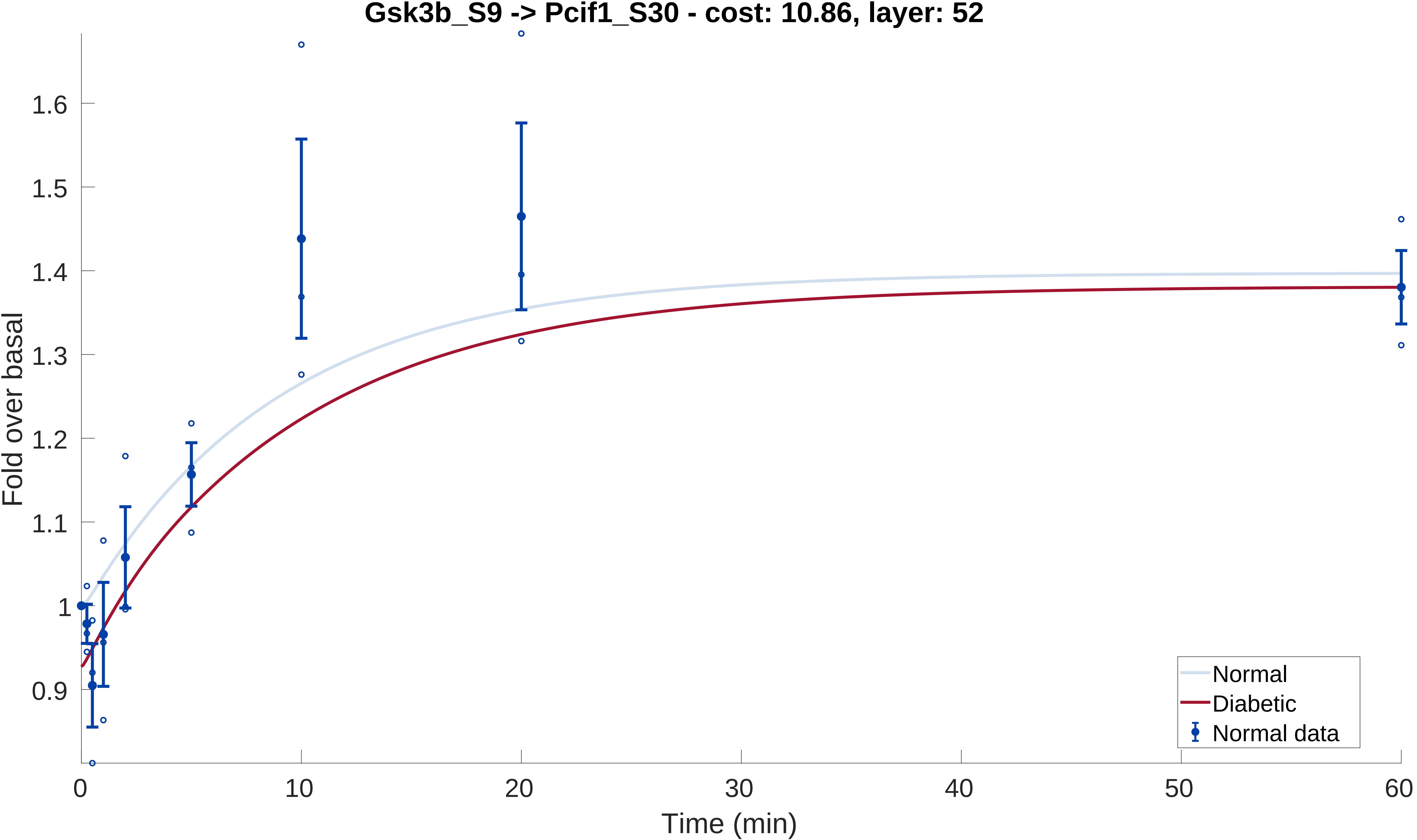
Gsk3b_S9 -> Pcif1_S30 - cost: 10.86, layer: 52
Fold over basal
Time (min)
0.9
1
1.1
1.2
1.3
1.4
1.5
1.6
0
10
20
30
40
50
60
Normal
Diabetic
Normal data

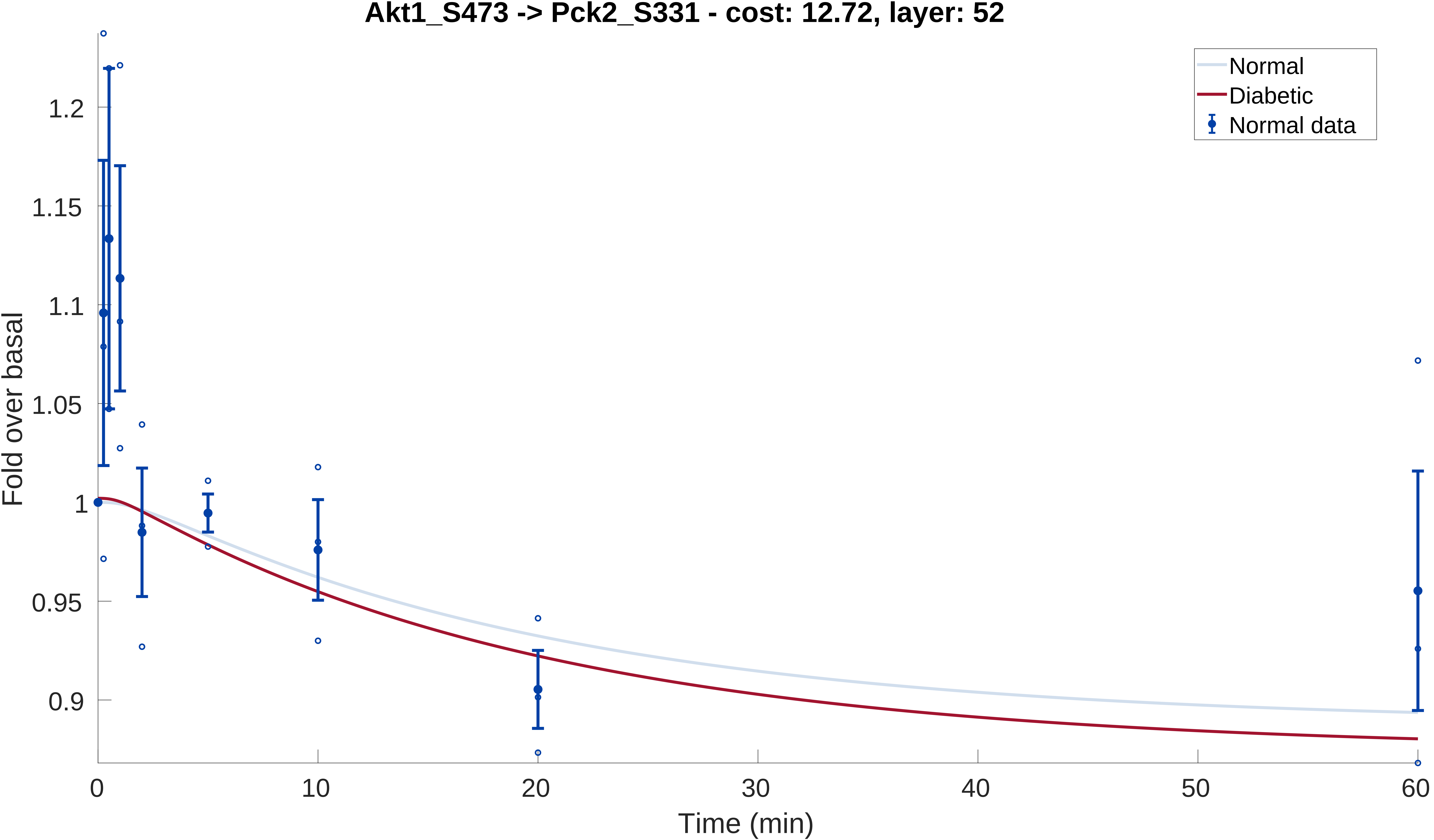

Akt1_S473 -> Pck2_S331 - cost: 12.72, layer: 52
Normal
Diabetic
Normal data
Fold over basal
1.2
1.15
1.1
1.05
1
0.95
0.9
0
10
20
30
40
50
60
Time (min)

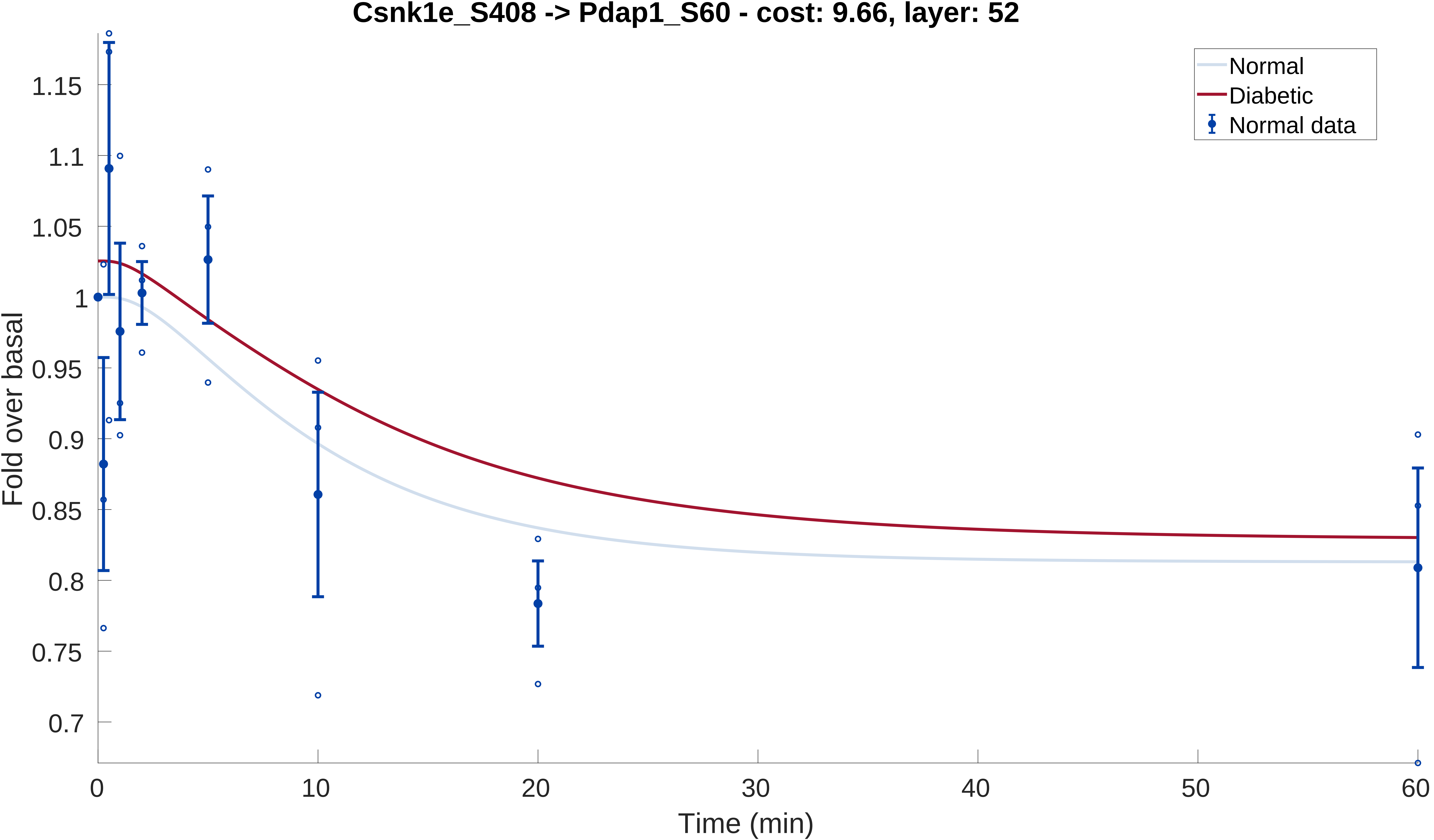

Csnk1e_S408 -> Pdap1_S60 - cost: 9.66, layer: 52
Normal
Diabetic
Normal data
Fold over basal
Time (min)
0.7
0.75
0.8
0.85
0.9
0.95
1
1.05
1.1
1.15
0
10
20
30
40
50
60

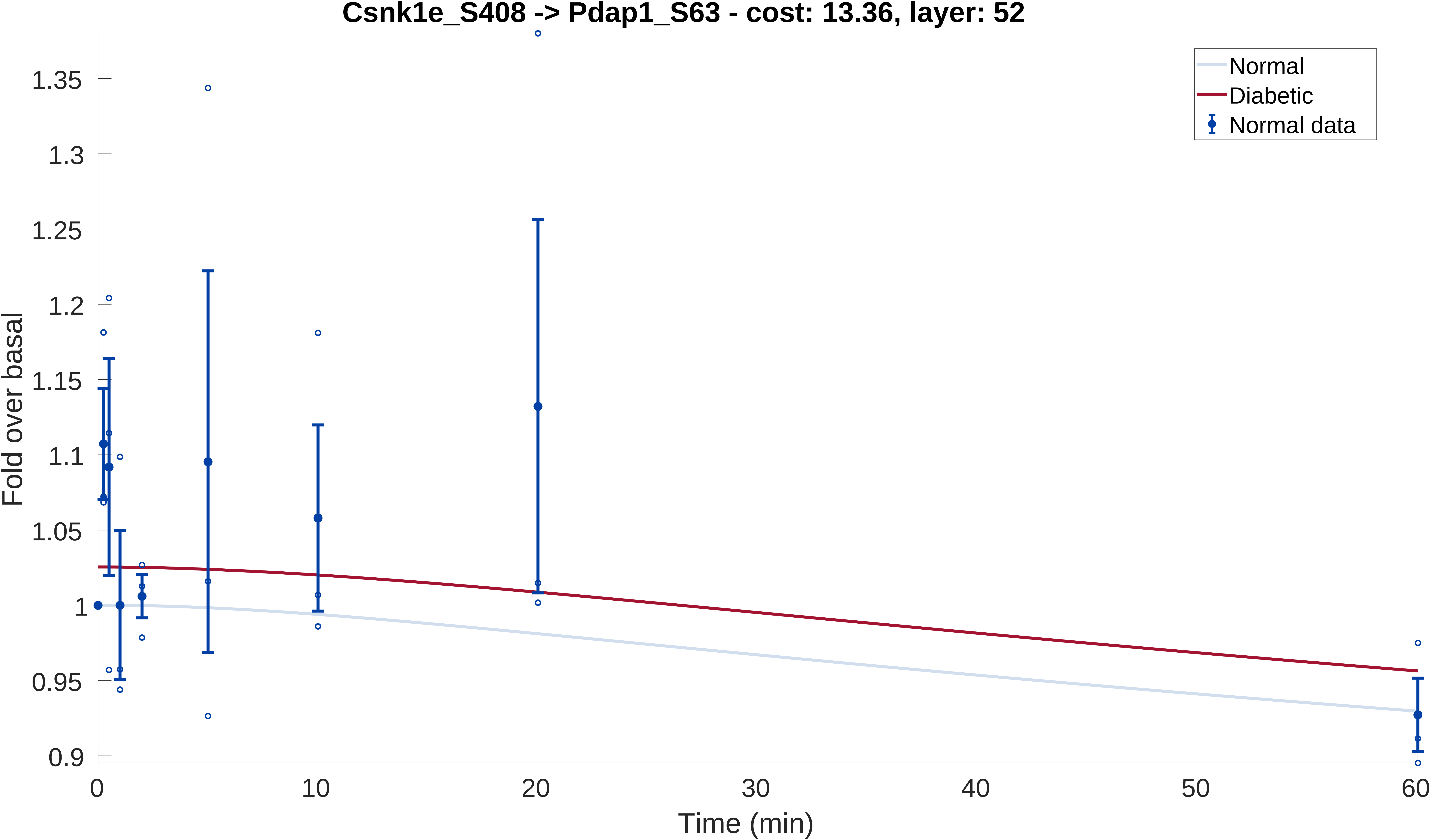

Csnk1e_S408 -> Pdap1_S63 - cost: 13.36, layer: 52
Normal
Diabetic
Normal data
Fold over basal
1.35
1.3
1.25
1.2
1.15
1.1
1.05
1
0.95
0.9
0
10
20
30
40
50
60
Time (min)

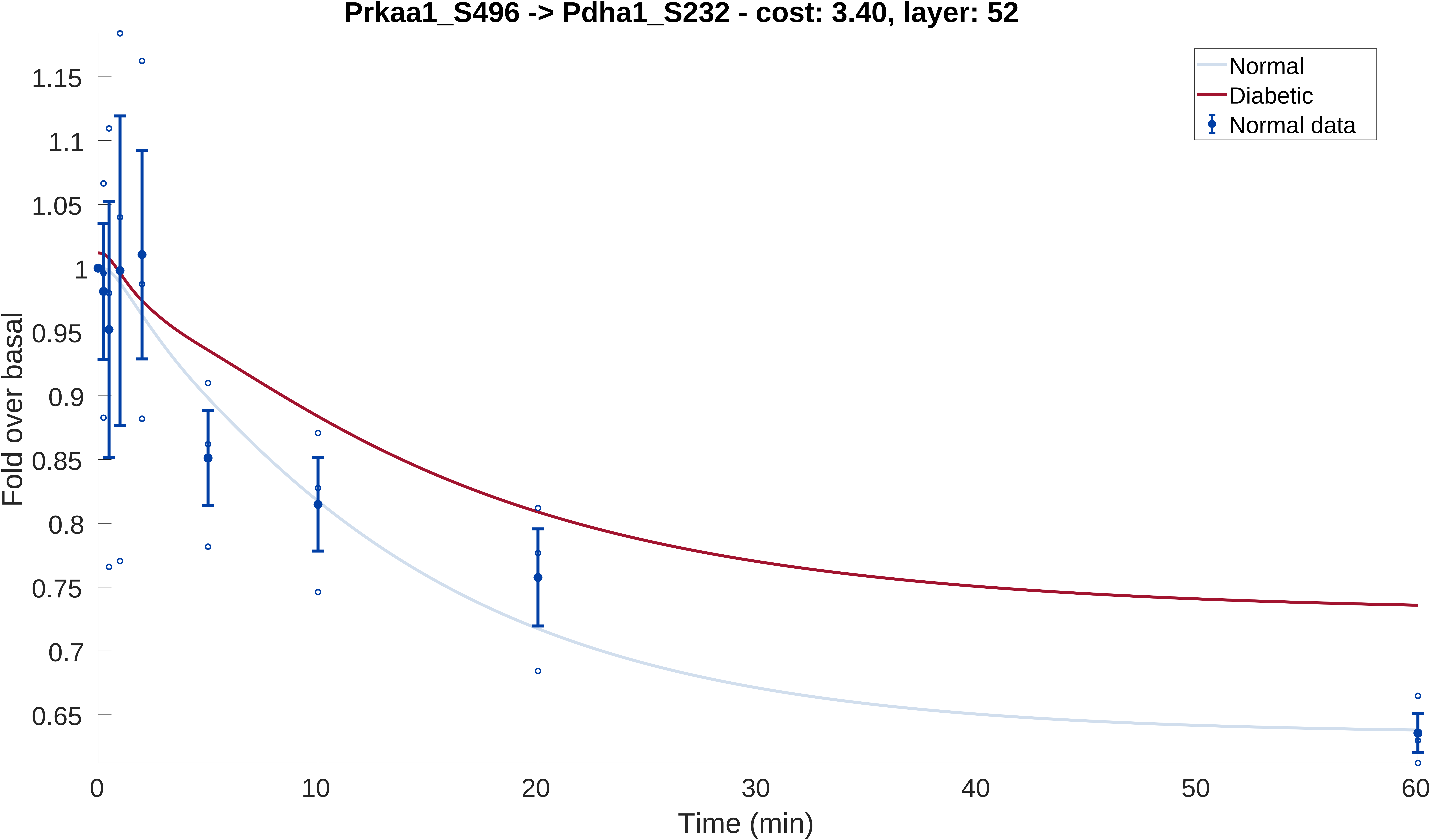

Prkaa1_S496 -> Pdha1_S232 - cost: 3.40, layer: 52
Normal
Diabetic
Normal data
Fold over basal
1.15
1.1
1.05
1
0.95
0.9
0.85
0.8
0.75
0.7
0.65
0
10
20
30
40
50
60
Time (min)

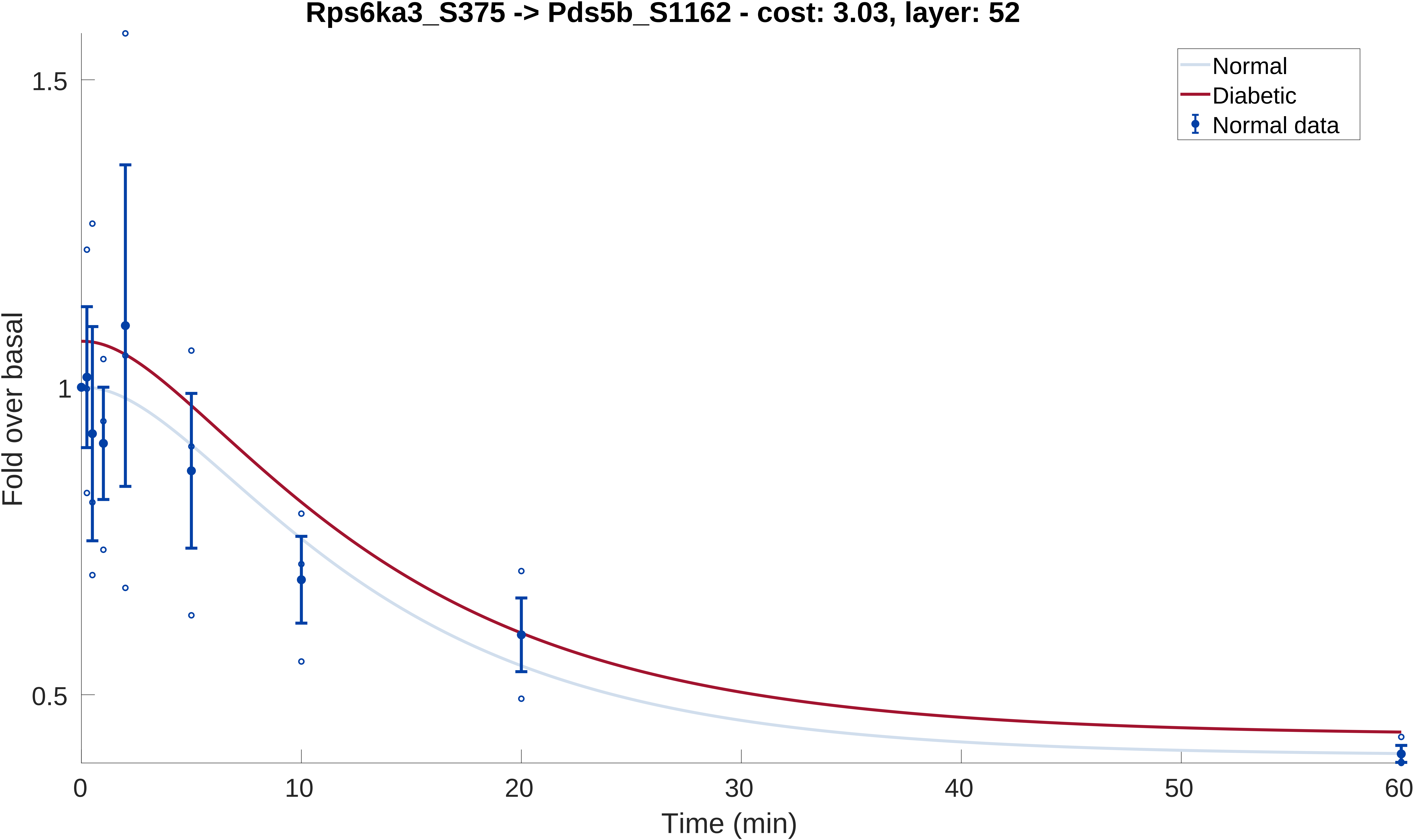

Rps6ka3_S375 -> Pds5b_S1162 - cost: 3.03, layer: 52
Normal
Diabetic
Normal data
Fold over basal
1.5
1
0.5
0
10
20
30
40
50
60
Time (min)

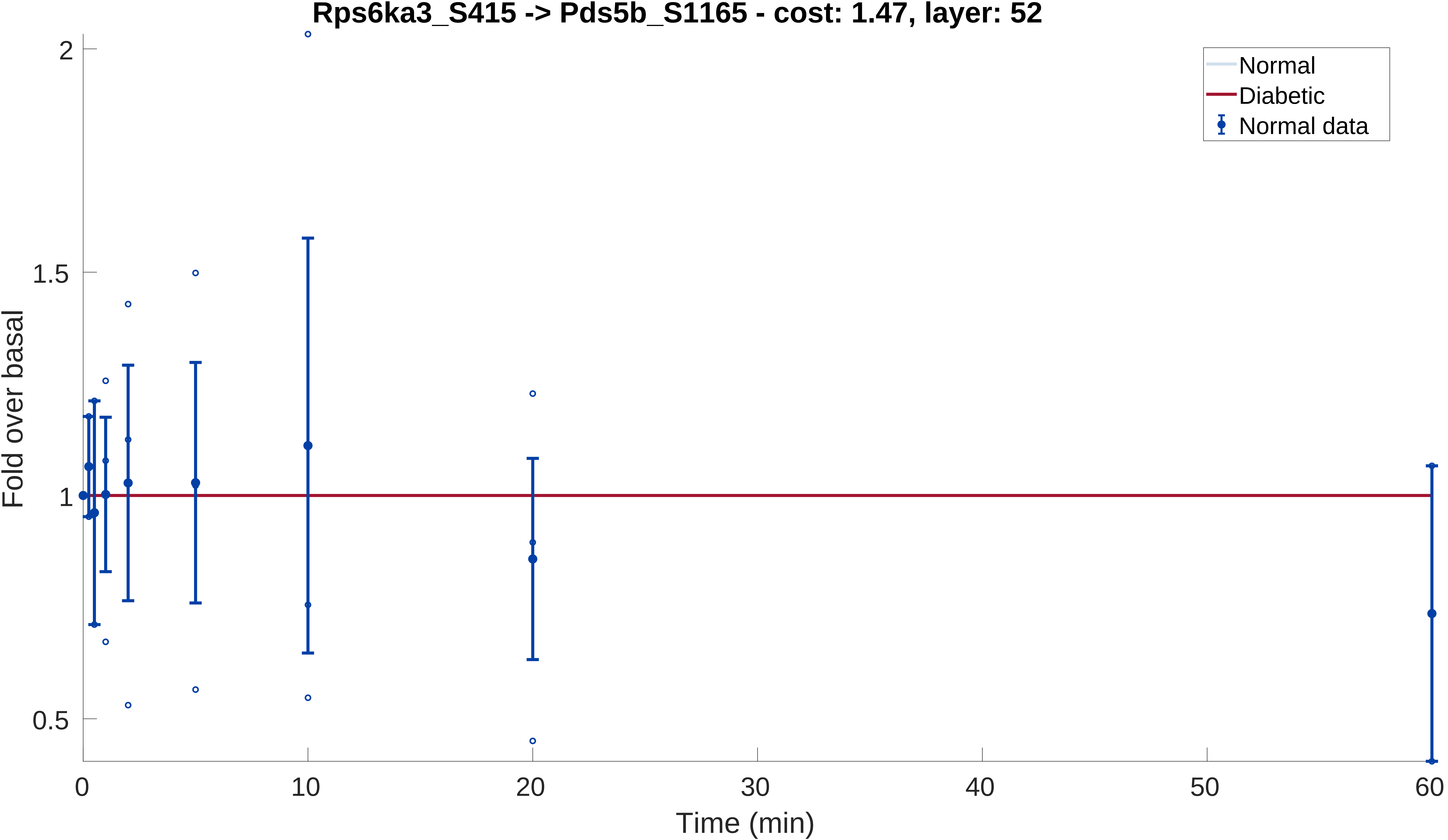

Rps6ka3_S415 -> Pds5b_S1165 - cost: 1.47, layer: 52
Fold over basal
Time (min)
0
10
20
30
40
50
60
0.5
1
1.5
2
Normal
Diabetic
Normal data

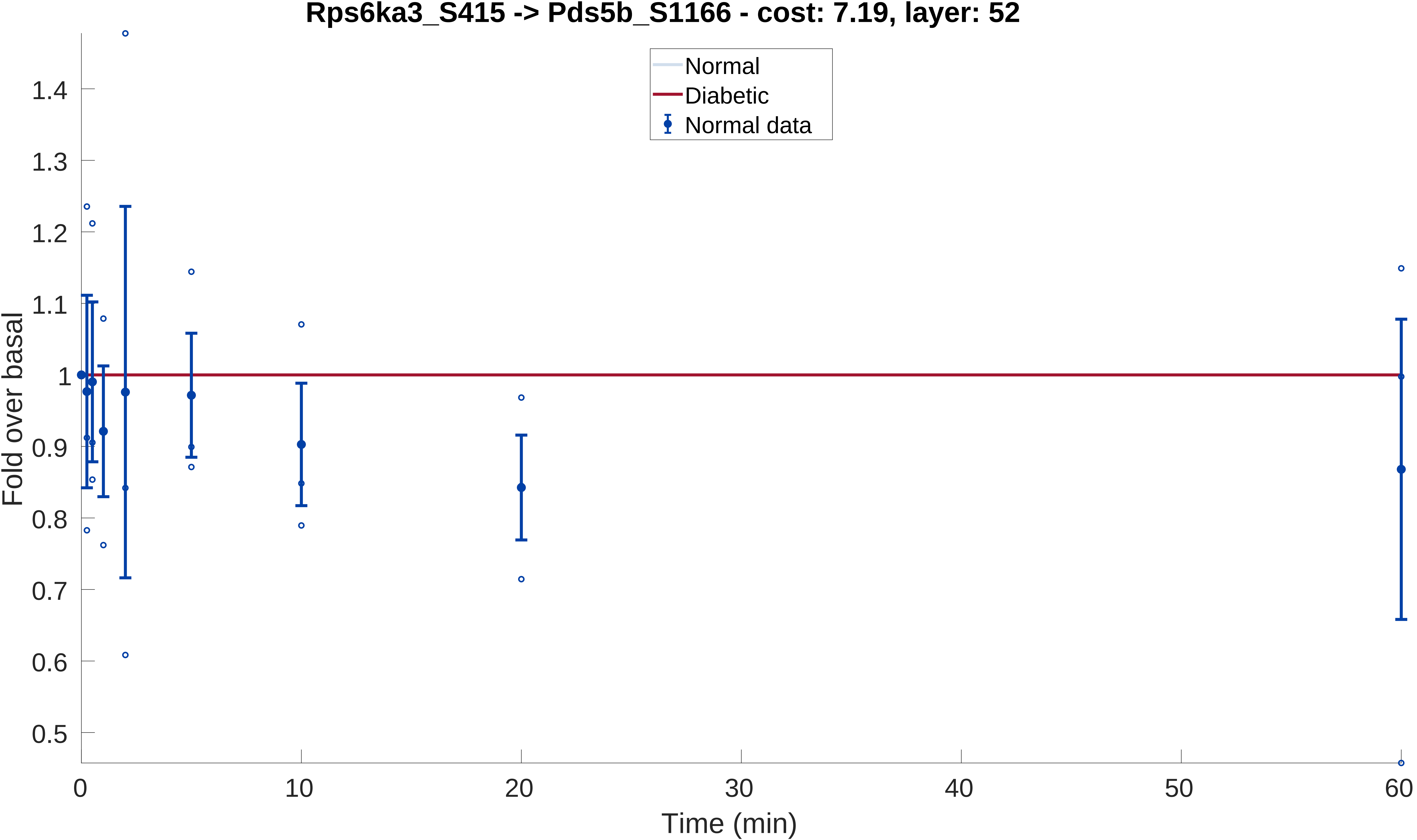

Rps6ka3_S415 -> Pds5b_S1166 - cost: 7.19, layer: 52
Normal
Diabetic
Normal data
Fold over basal
1.4
1.3
1.2
1.1
1
0.9
0.8
0.7
0.6
0.5
0
10
20
30
40
50
60
Time (min)

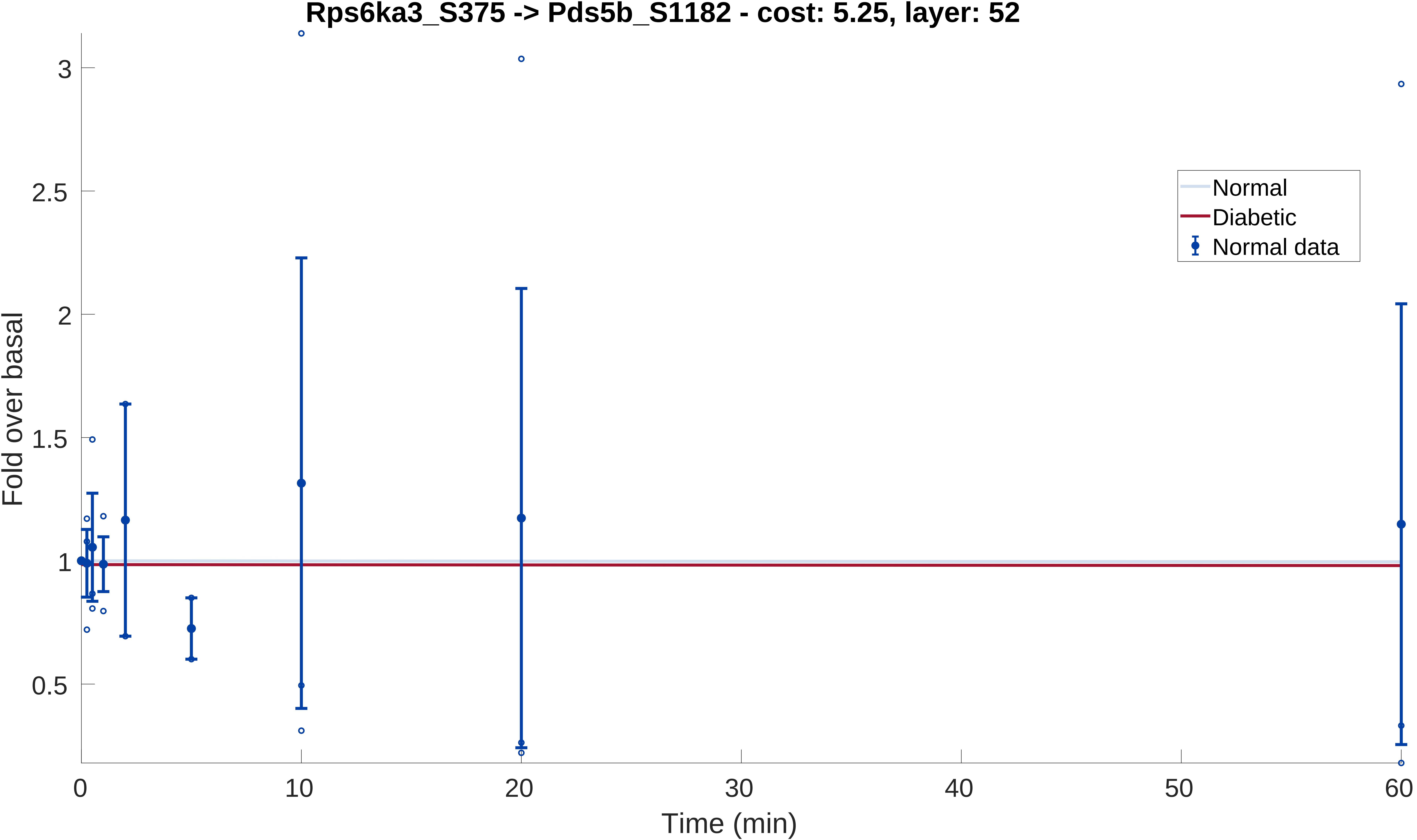

Rps6ka3_S375 -> Pds5b_S1182 - cost: 5.25, layer: 52
Fold over basal
Time (min)
Normal
Diabetic
Normal data
0
0.5
1
1.5
2
2.5
3
10
20
30
40
50
60

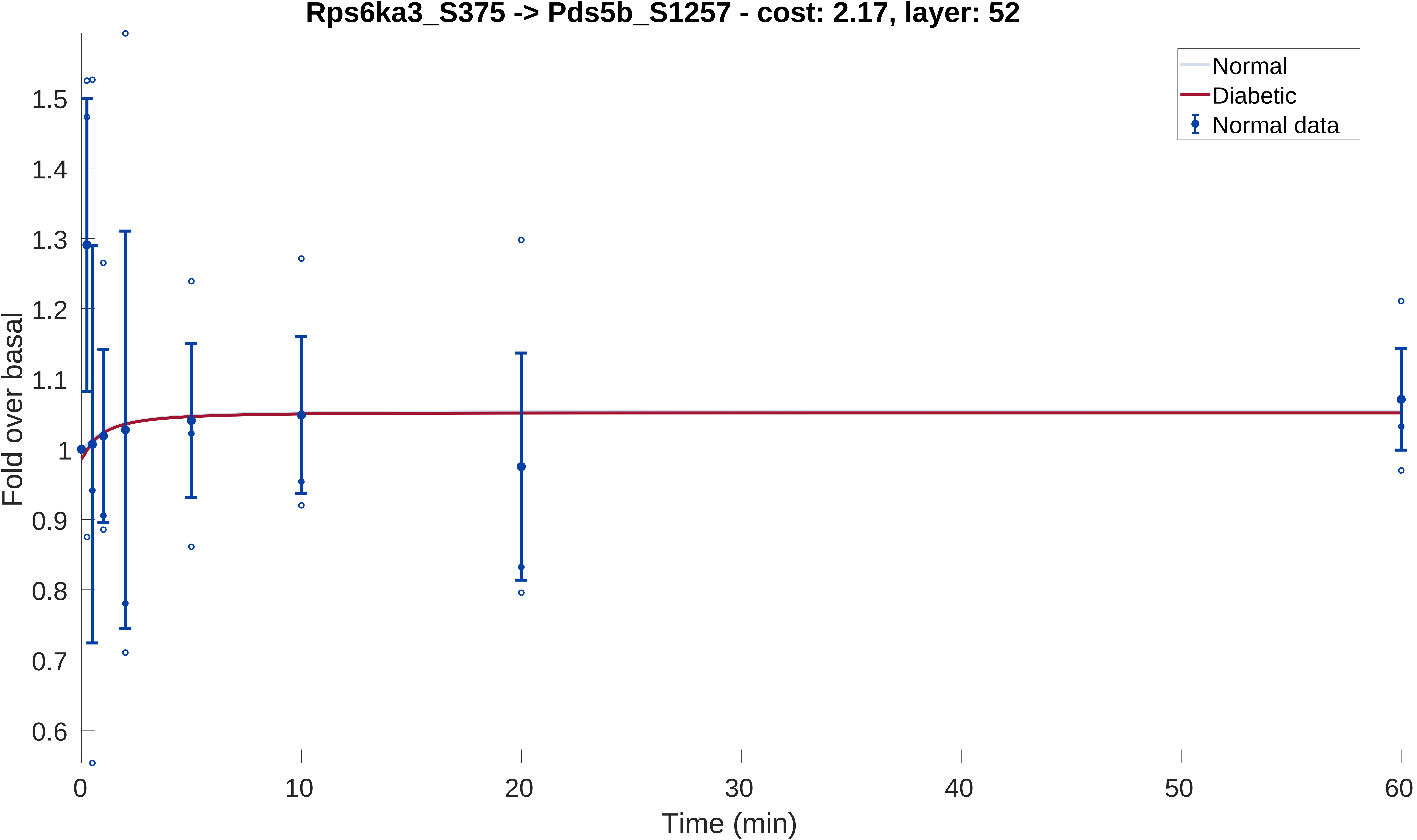

Rps6ka3_S375 -> Pds5b_S1257 - cost: 2.17, layer: 52
Normal
Diabetic
Normal data
Fold over basal
Time (min)
0.6
0.7
0.8
0.9
1
1.1
1.2
1.3
1.4
1.5
0
10
20
30
40
50
60

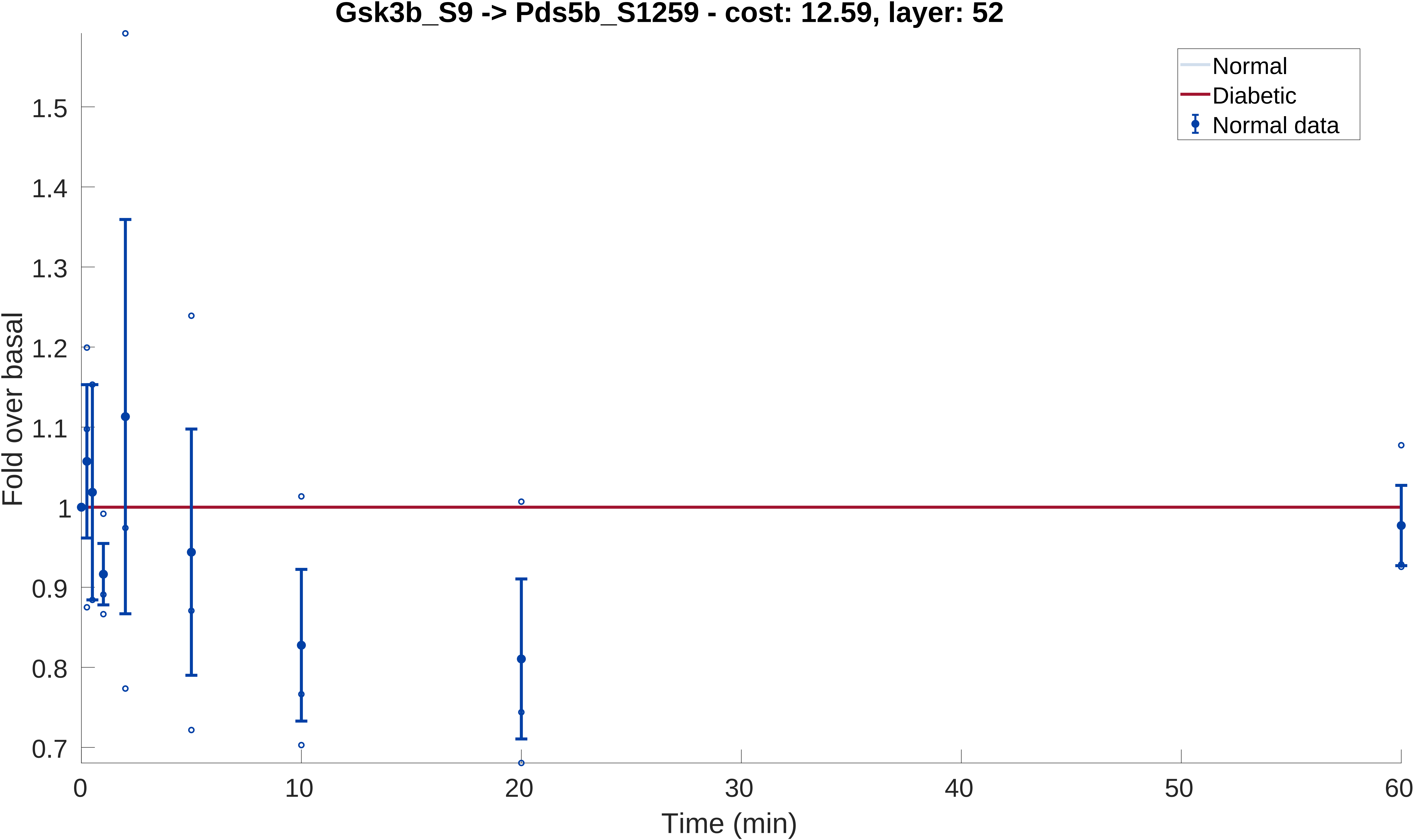

Gsk3b_S9 -> Pds5b_S1259 - cost: 12.59, layer: 52
Normal
Diabetic
Normal data
Fold over basal
Time (min)
1.5
1.4
1.3
1.2
1.1
1
0.9
0.8
0.7
0
10
20
30
40
50
60

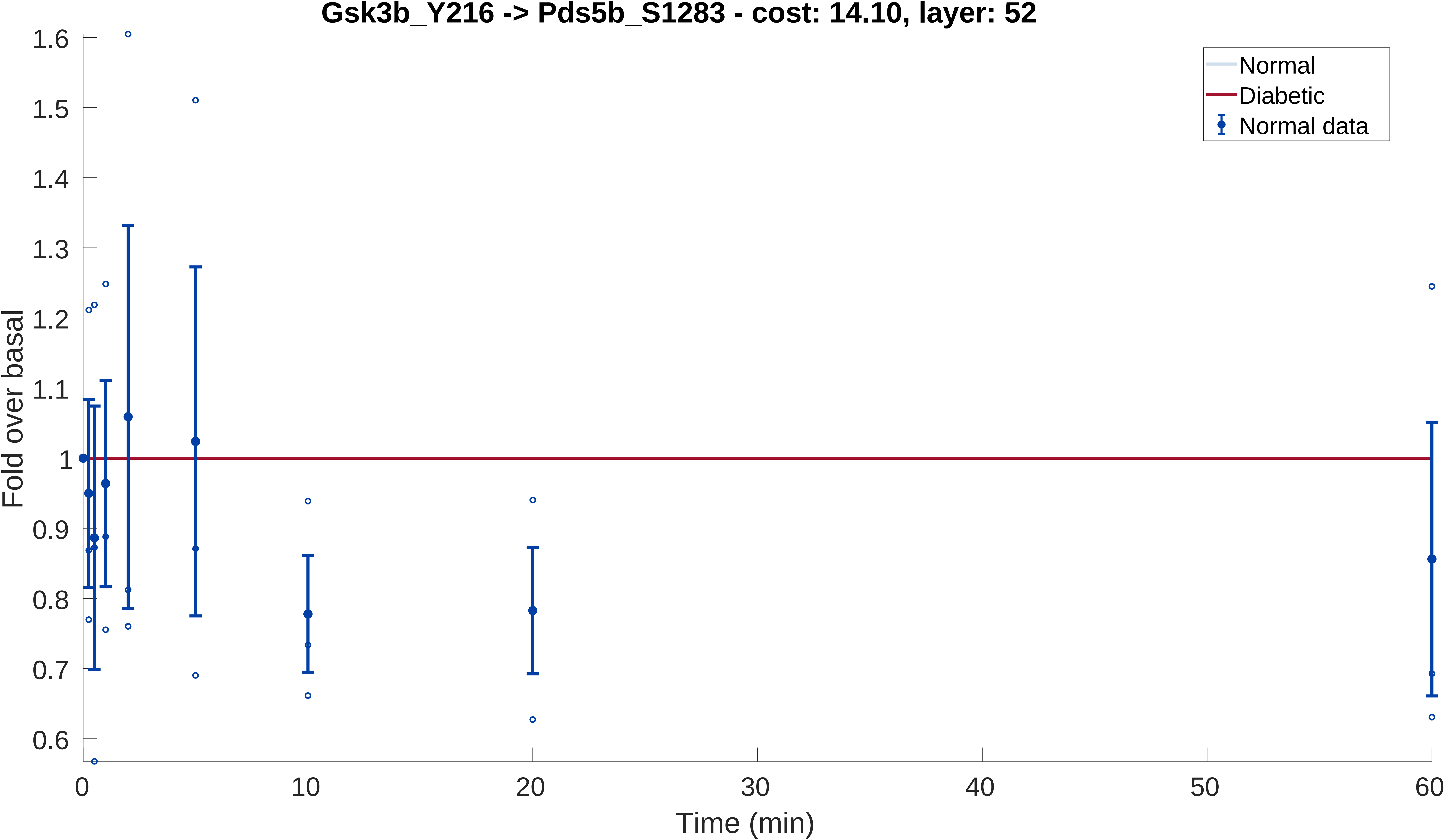

Gsk3b_Y216 -> Pds5b_S1283 - cost: 14.10, layer: 52
Fold over basal
Time (min)
Normal
Diabetic
Normal data
1.6
1.5
1.4
1.3
1.2
1.1
1
0.9
0.8
0.7
0.6
0
10
20
30
40
50
60

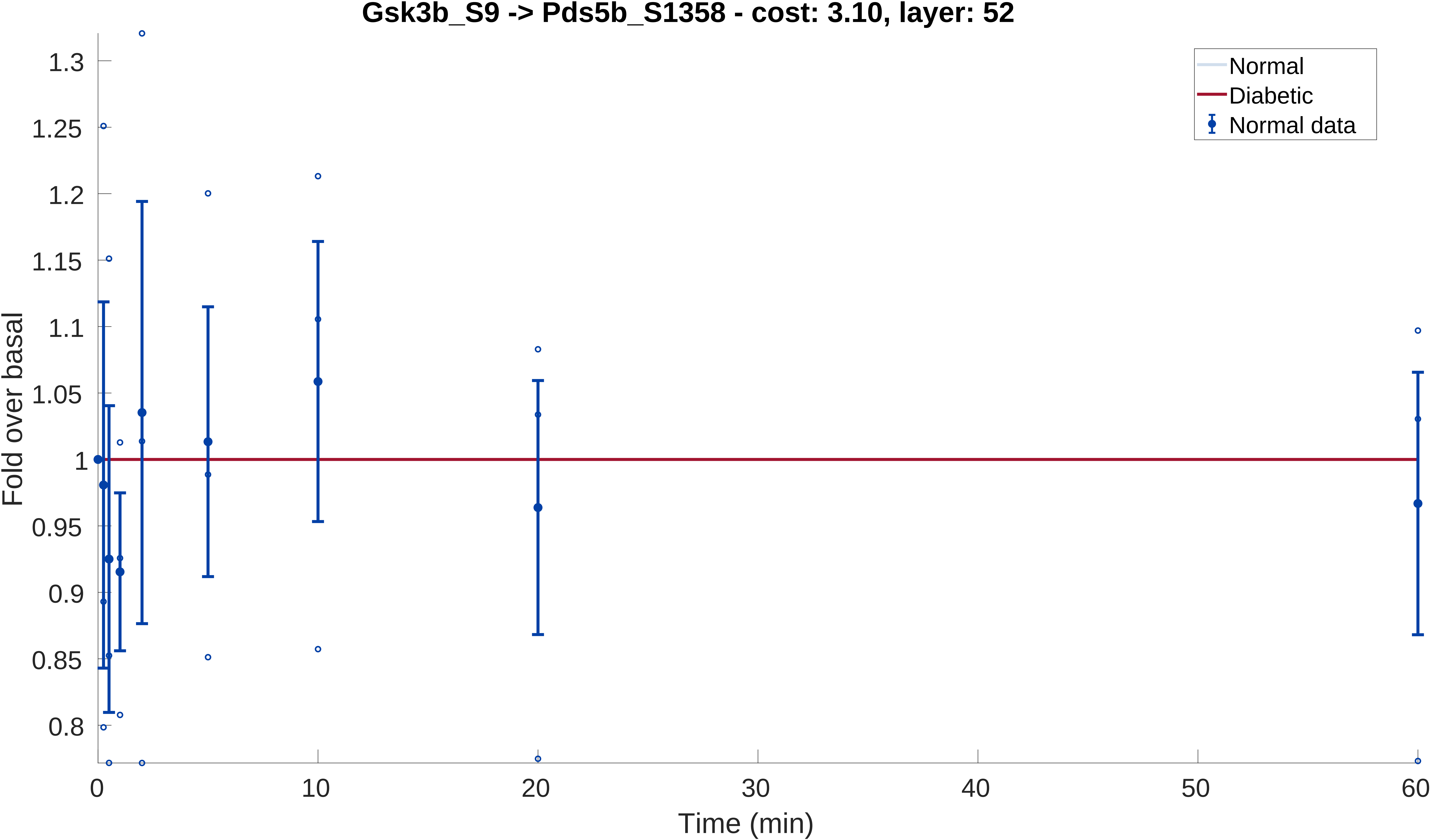

Gsk3b_S9 -> Pds5b_S1358 - cost: 3.10, layer: 52
Normal
Diabetic
Normal data
Fold over basal
1.3
1.25
1.2
1.15
1.1
1.05
1
0.95
0.9
0.85
0.8
0
10
20
30
40
50
60
Time (min)

Gsk3b_S9 -> Pds5b_S1383 - cost: 4.71, layer: 52

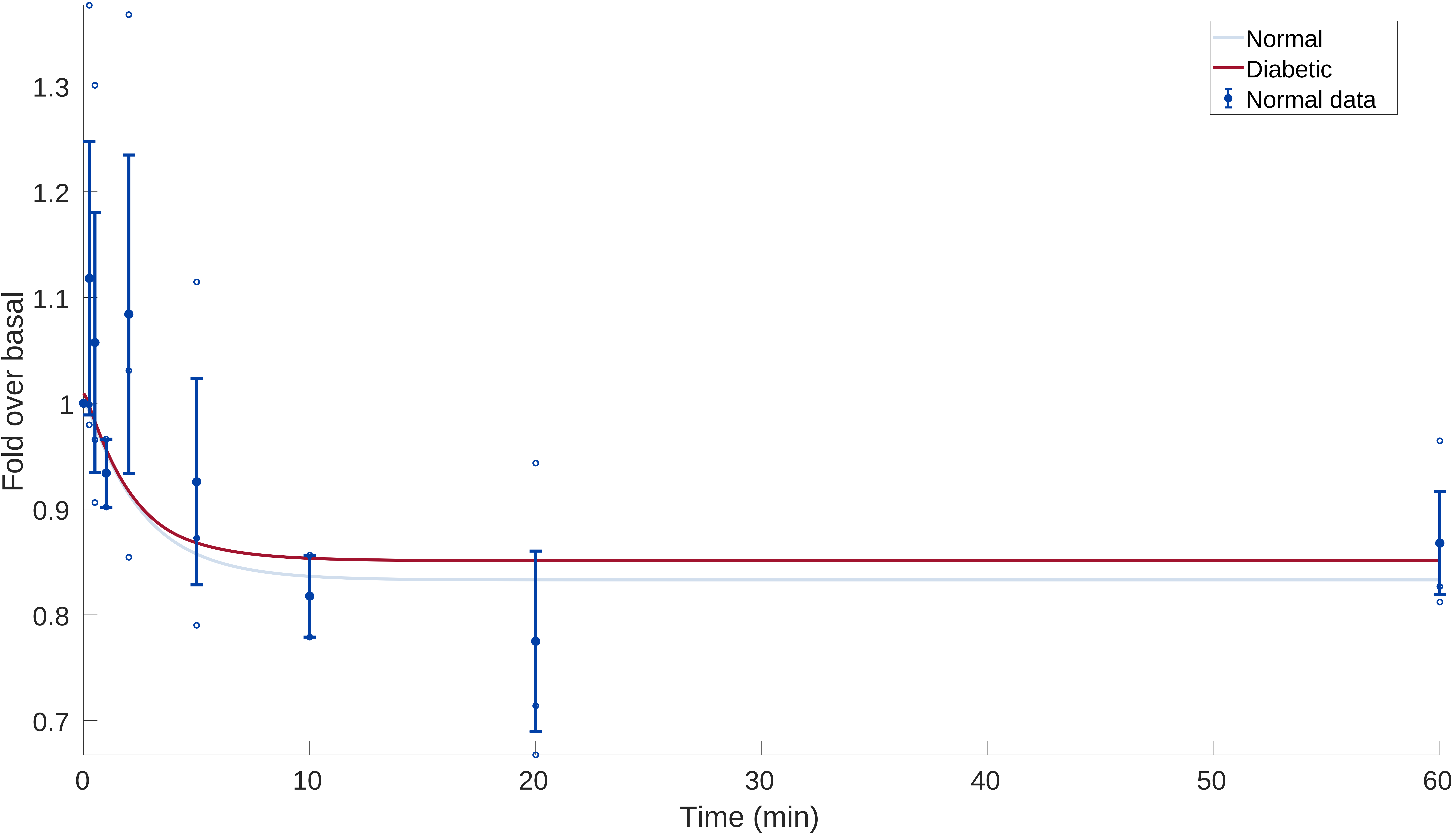

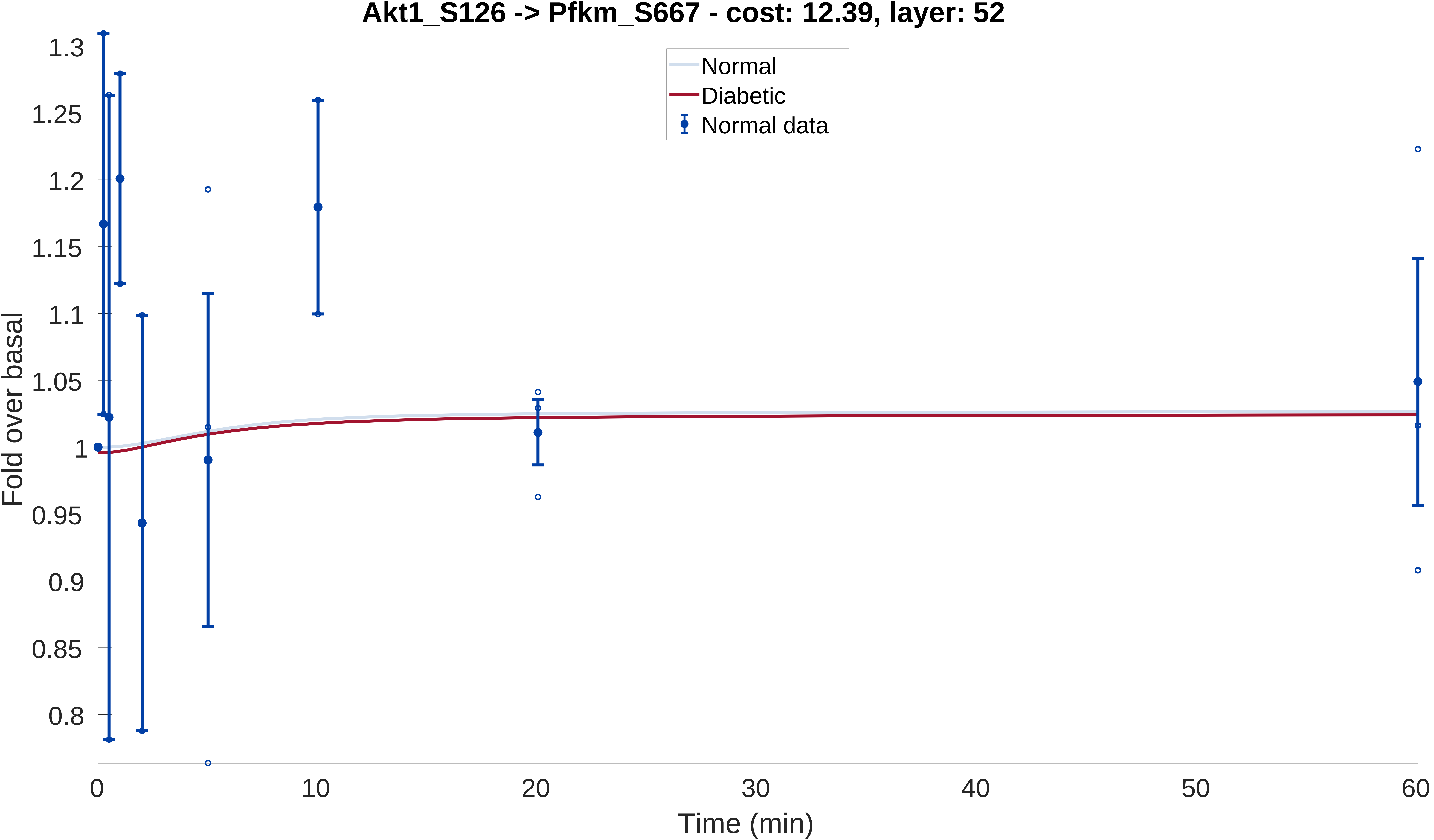

Akt1_S126 -> Pfkm_S667 - cost: 12.39, layer: 52
Normal
Diabetic
Normal data
Fold over basal
Time (min)
1.3
1.25
1.2
1.15
1.1
1.05
1
0.95
0.9
0.85
0.8
0
10
20
30
40
50
60

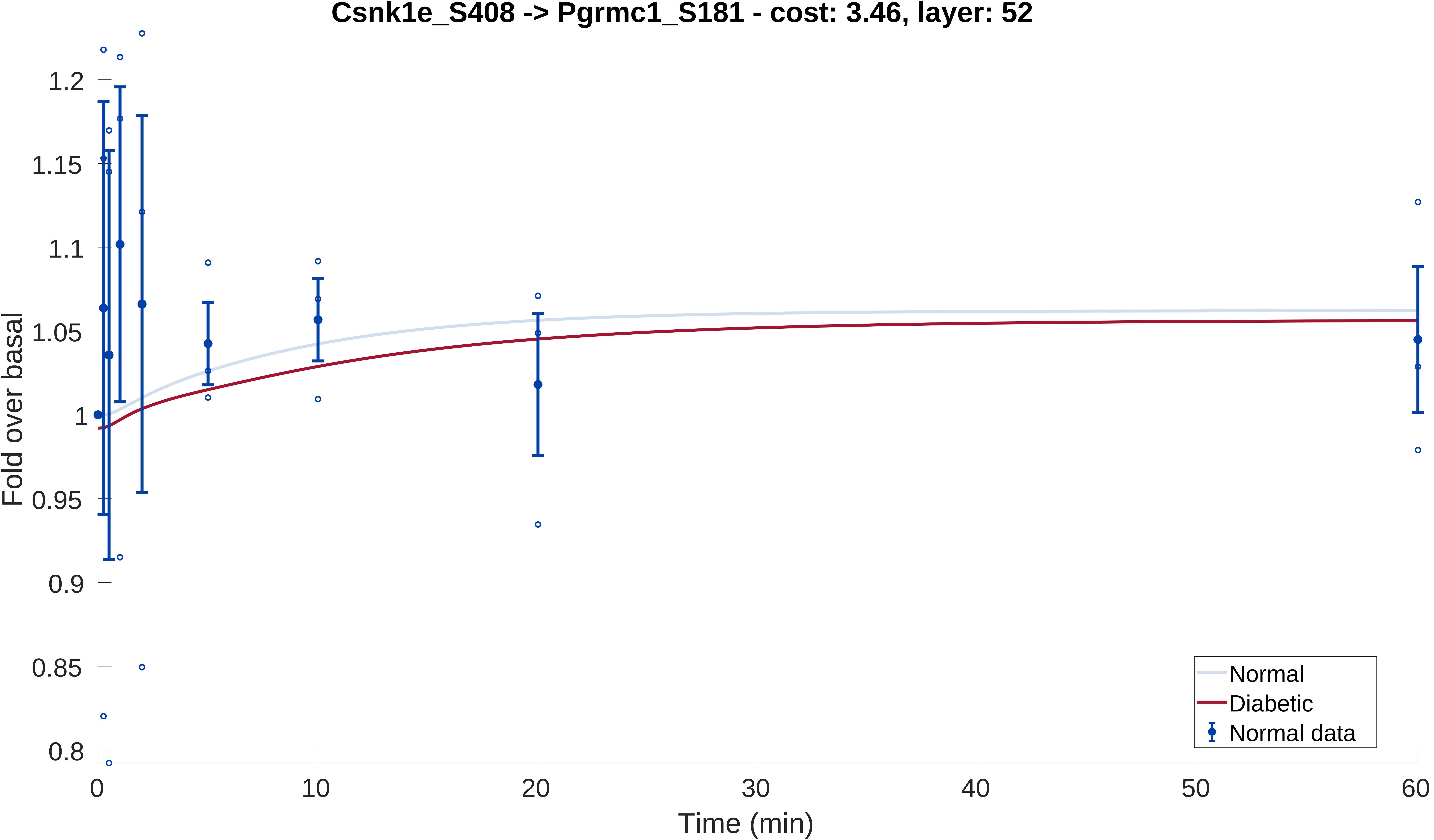

Csnk1e_S408 -> Pgrmc1_S181 - cost: 3.46, layer: 52
Fold over basal
1.2
1.15
1.1
1.05
1
0.95
0.9
0.85
0.8
0
10
20
30
40
50
60
Time (min)
Normal
Diabetic
Normal data

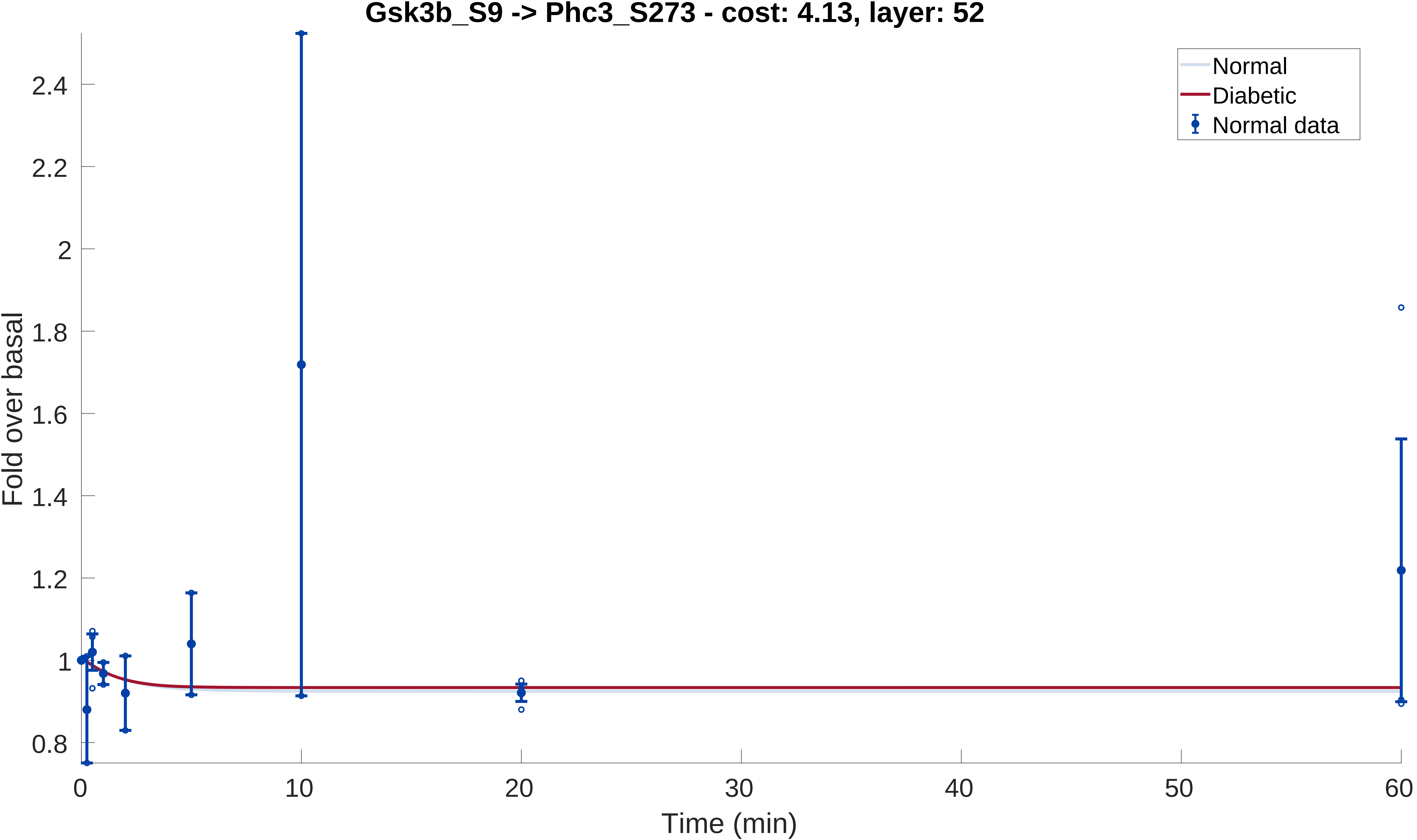

Gsk3b_S9 -> Phc3_S273 - cost: 4.13, layer: 52
Normal
Diabetic
Normal data
Fold over basal
2.4
2.2
2
1.8
1.6
1.4
1.2
1
0.8
0
10
20
30
40
50
60
Time (min)

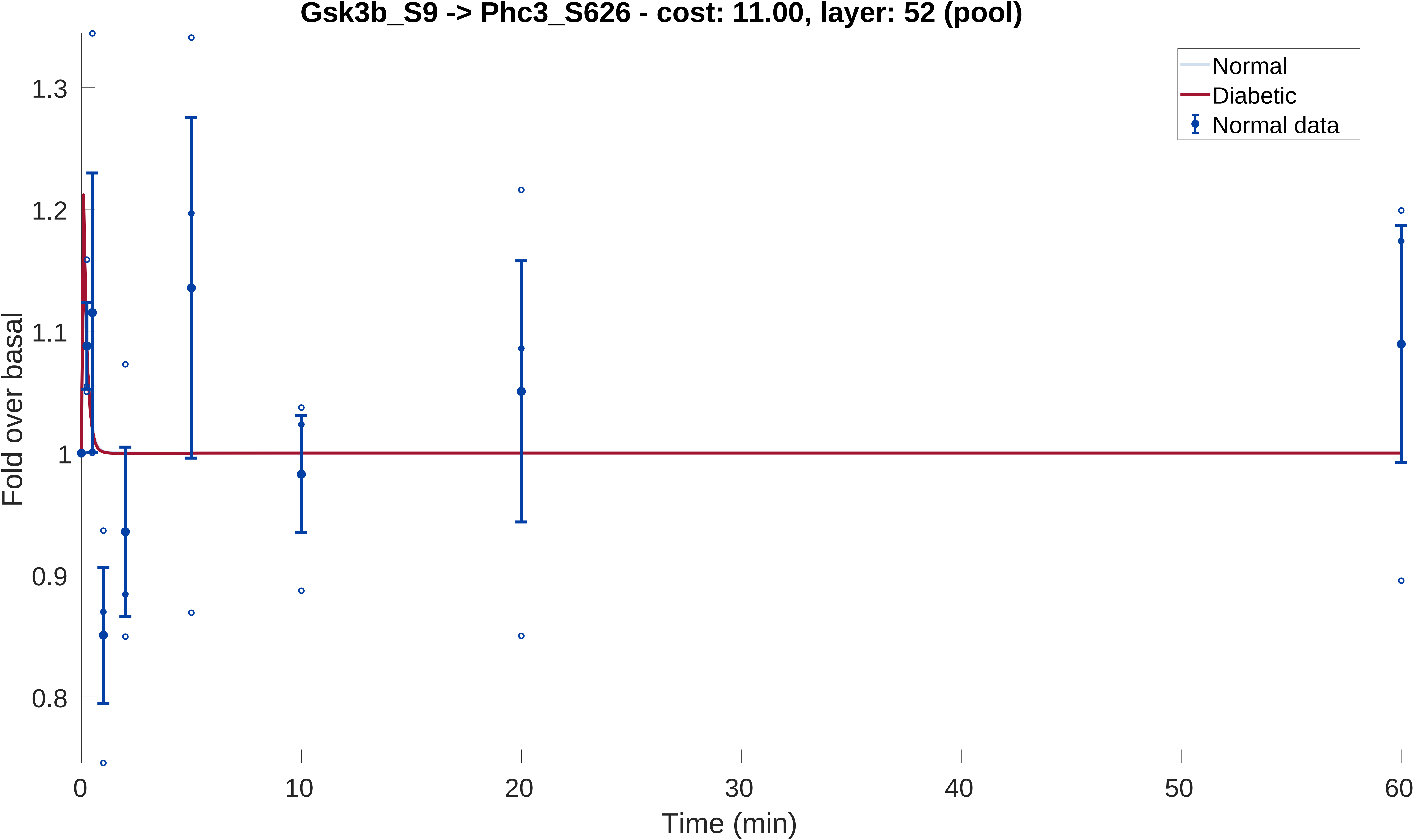

Gsk3b_S9 -> Phc3_S626 - cost: 11.00, layer: 52 (pool)
Normal
Diabetic
Normal data
Fold over basal
Time (min)
1.3
1.2
1.1
1
0.9
0.8
0
10
20
30
40
50
60

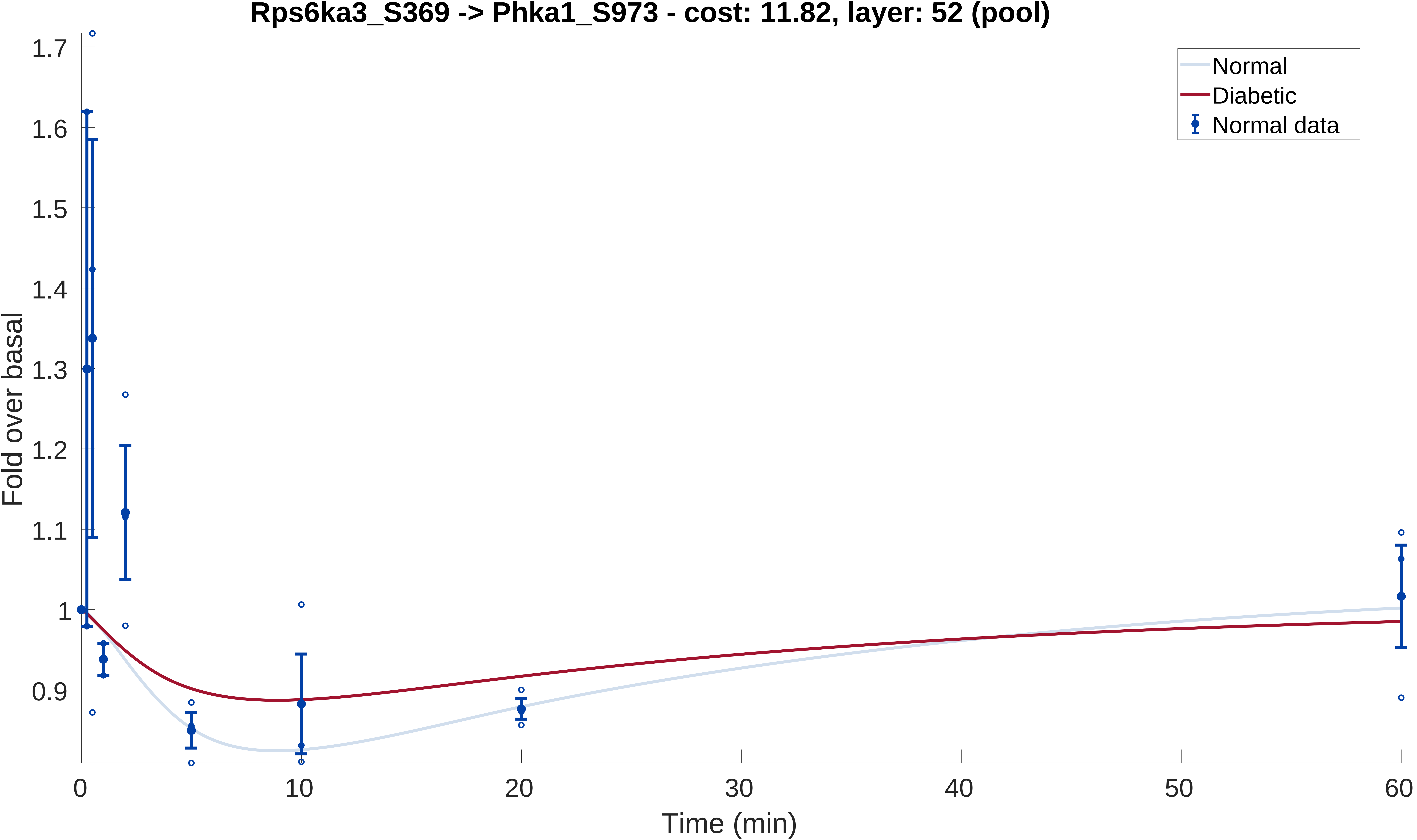
Rps6ka3_S369 -> Phka1_S973 - cost: 11.82, layer: 52 (pool)
Normal
Diabetic
Normal data
Fold over basal
Time (min)
1.7
1.6
1.5
1.4
1.3
1.2
1.1
1
0.9
0
10
20
30
40
50
60

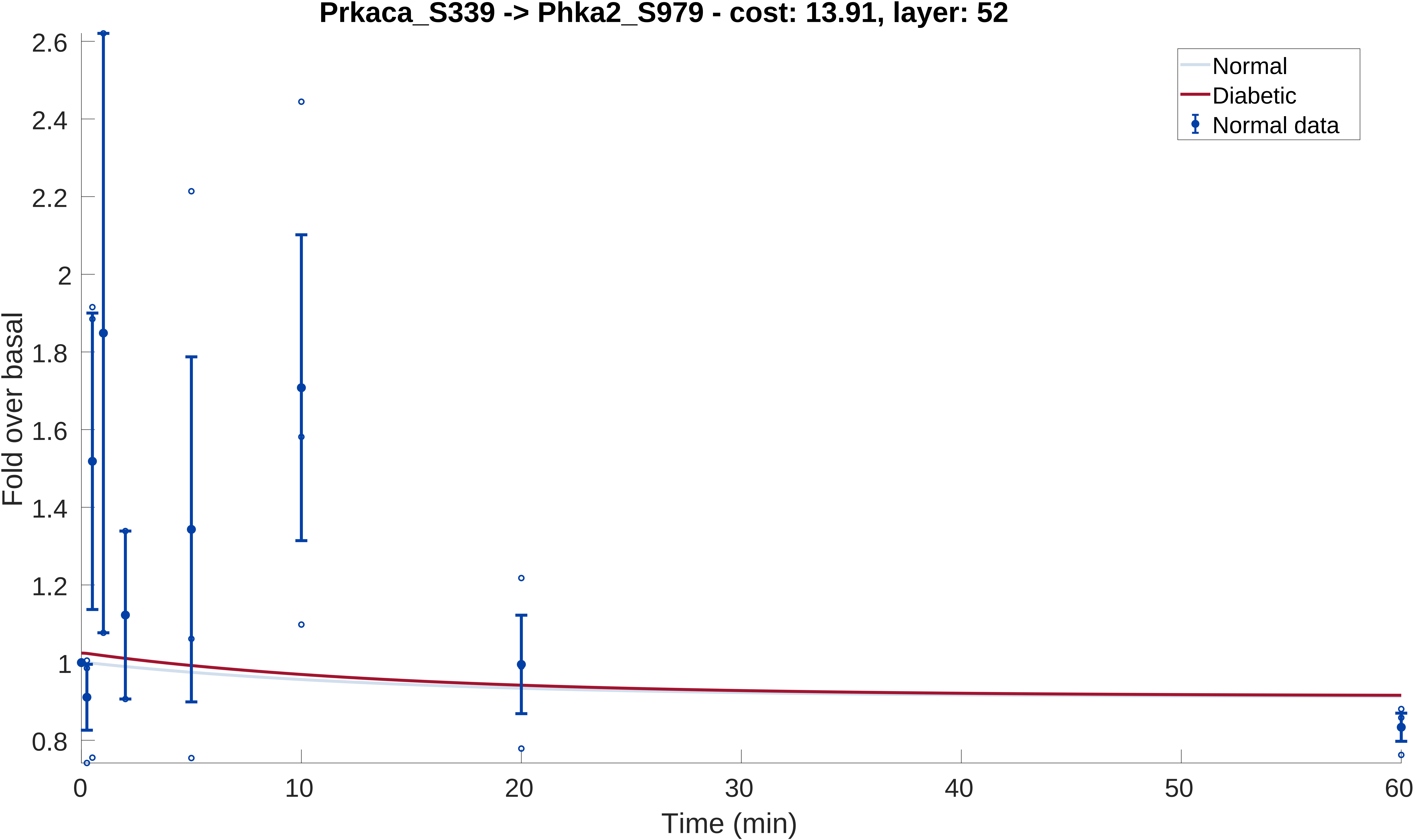
Prkaca_S339 -> Phka2_S979 - cost: 13.91, layer: 52
Normal
Diabetic
Normal data
Fold over basal
Time (min)
2.6
2.4
2.2
2
1.8
1.6
1.4
1.2
1
0.8
0
10
20
30
40
50
60

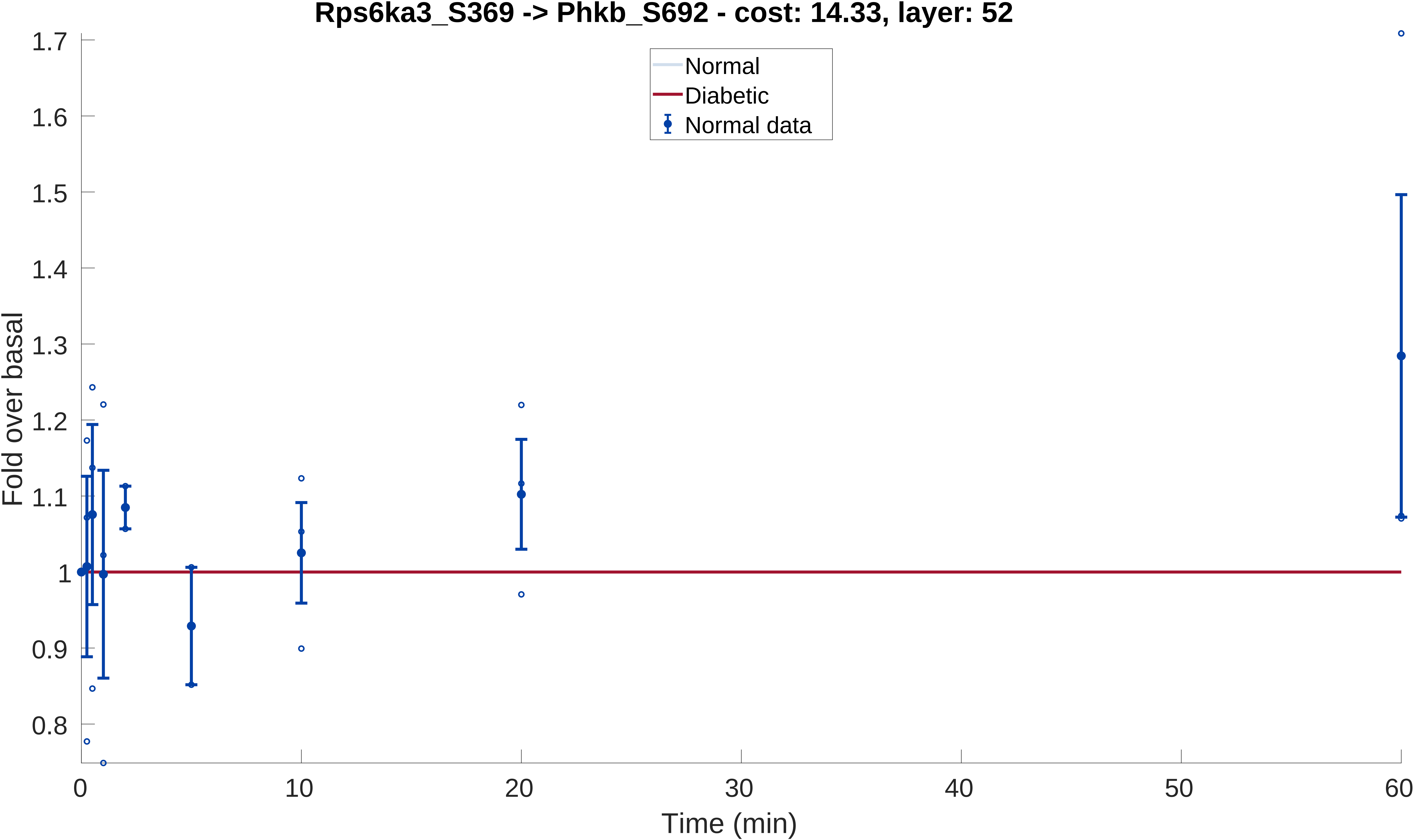

Rps6ka3_S369 -> Phkb_S692 - cost: 14.33, layer: 52
Normal
Diabetic
Normal data
Fold over basal
Time (min)
1.7
1.6
1.5
1.4
1.3
1.2
1.1
1
0.9
0.8
0
10
20
30
40
50
60

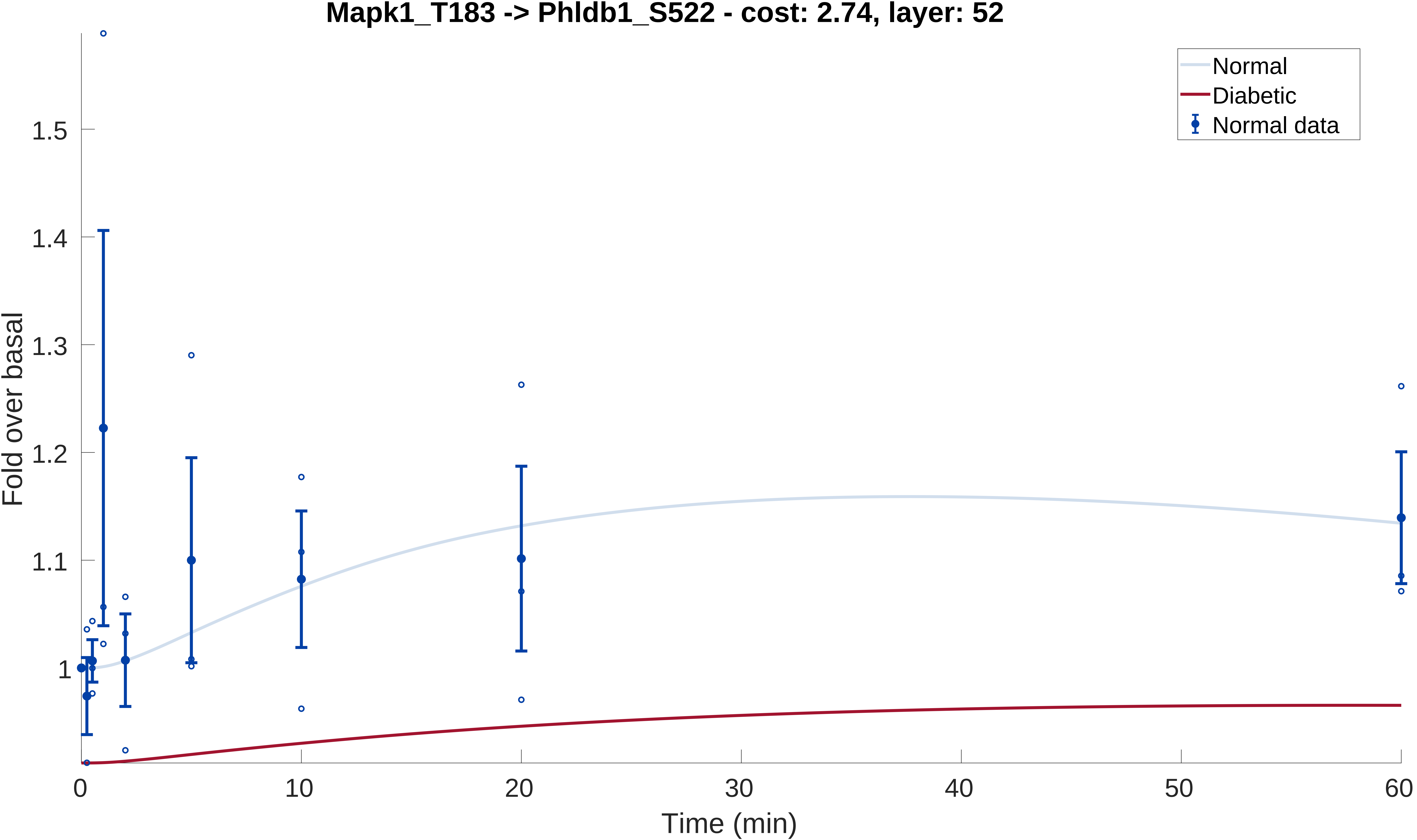

Mapk1_T183 -> Phldb1_S522 - cost: 2.74, layer: 52
Normal
Diabetic
Normal data
Fold over basal
1.5
1.4
1.3
1.2
1.1
1
0
10
20
30
40
50
60
Time (min)

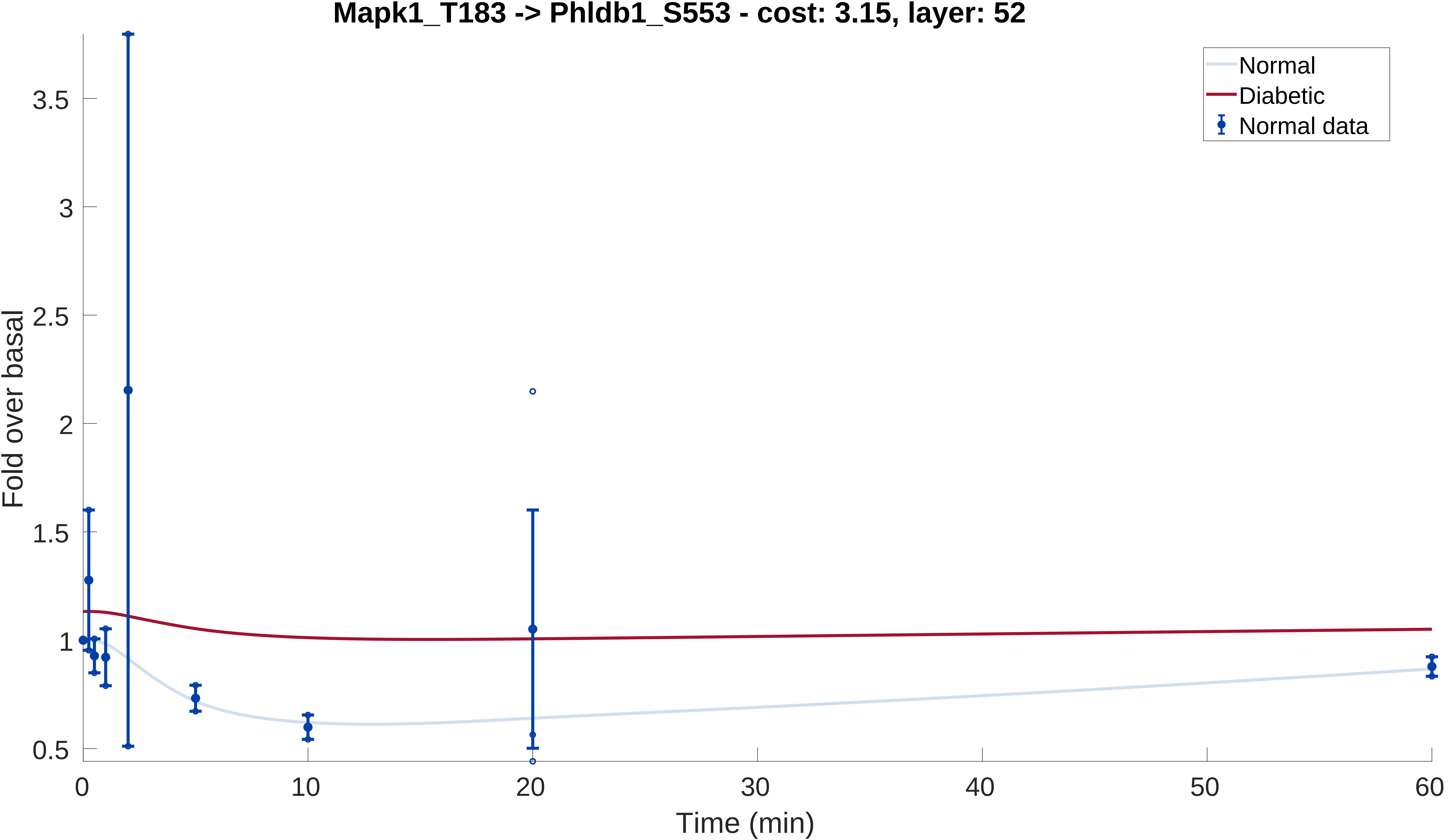

Mapk1_T183 -> Phldb1_S553 - cost: 3.15, layer: 52
Normal
Diabetic
Normal data
Fold over basal
3.5
3
2.5
2
1.5
1
0.5
0
10
20
30
40
50
60
Time (min)

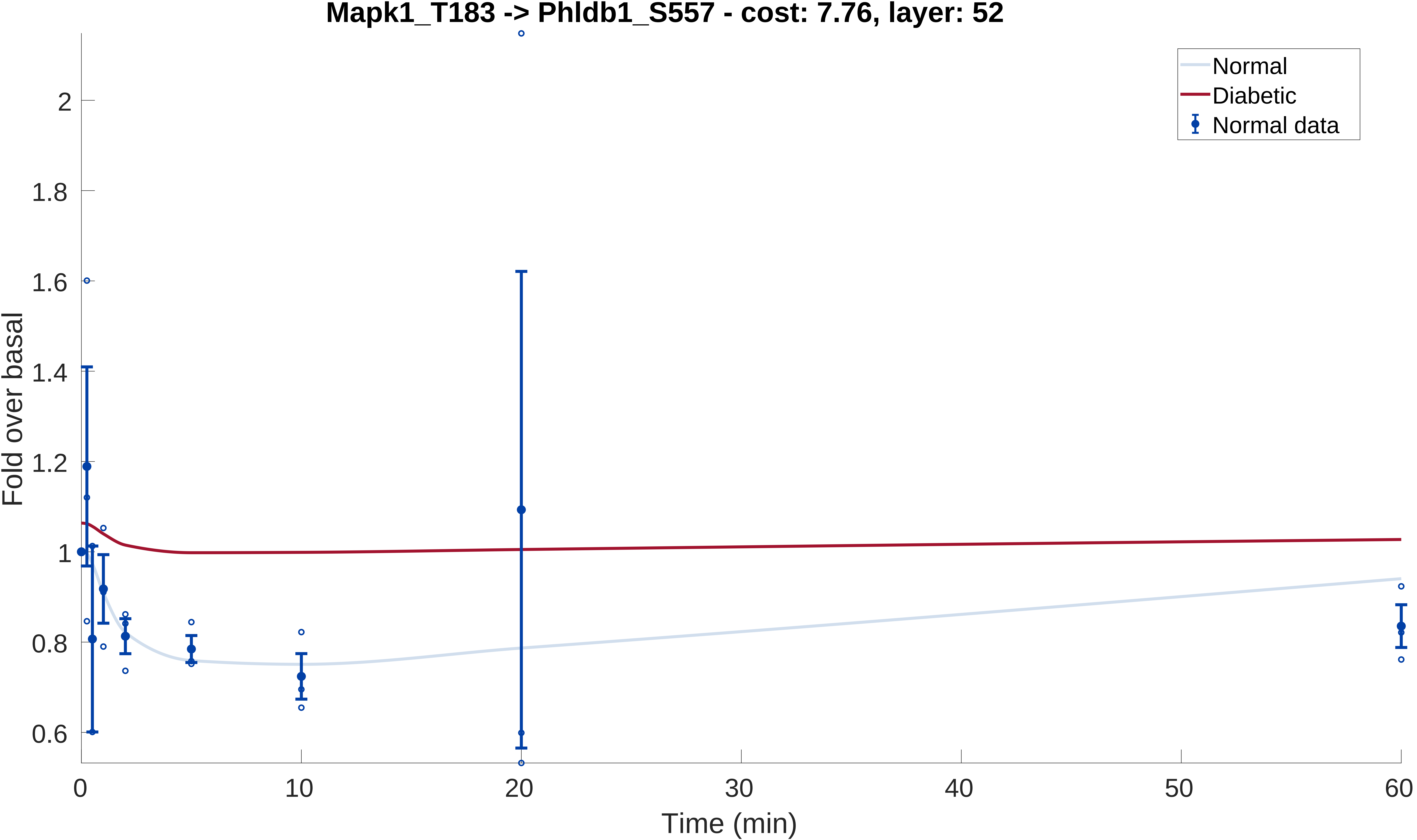

Mapk1_T183 -> Phldb1_S557 - cost: 7.76, layer: 52
Normal
Diabetic
Normal data
Fold over basal
Time (min)
0.6
0.8
1
1.2
1.4
1.6
1.8
2
0
10
20
30
40
50
60

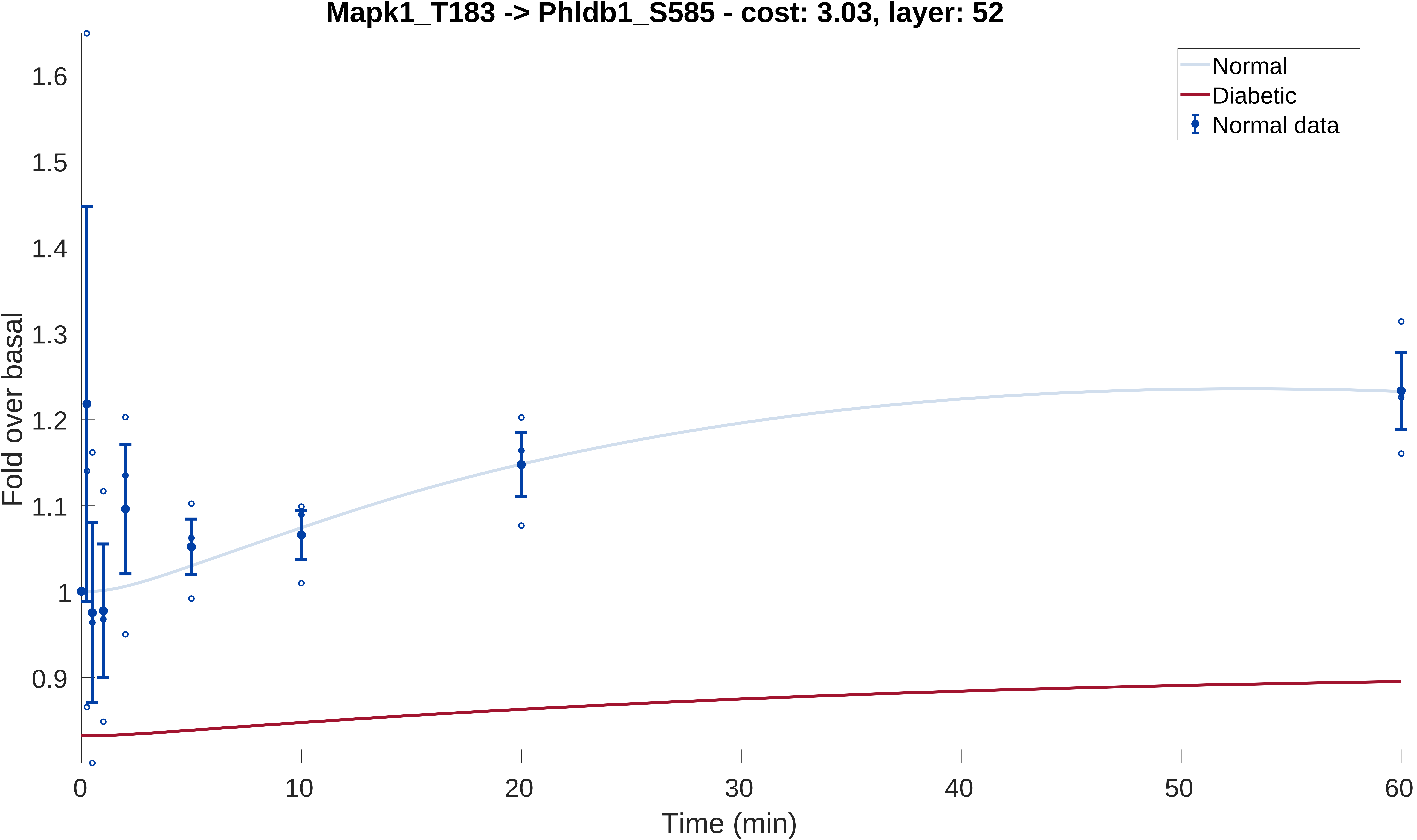
Mapk1_T183 -> Phldb1_S585 - cost: 3.03, layer: 52
Normal
Diabetic
Normal data
Fold over basal
1.6
1.5
1.4
1.3
1.2
1.1
1
0.9
0
10
20
30
40
50
60
Time (min)

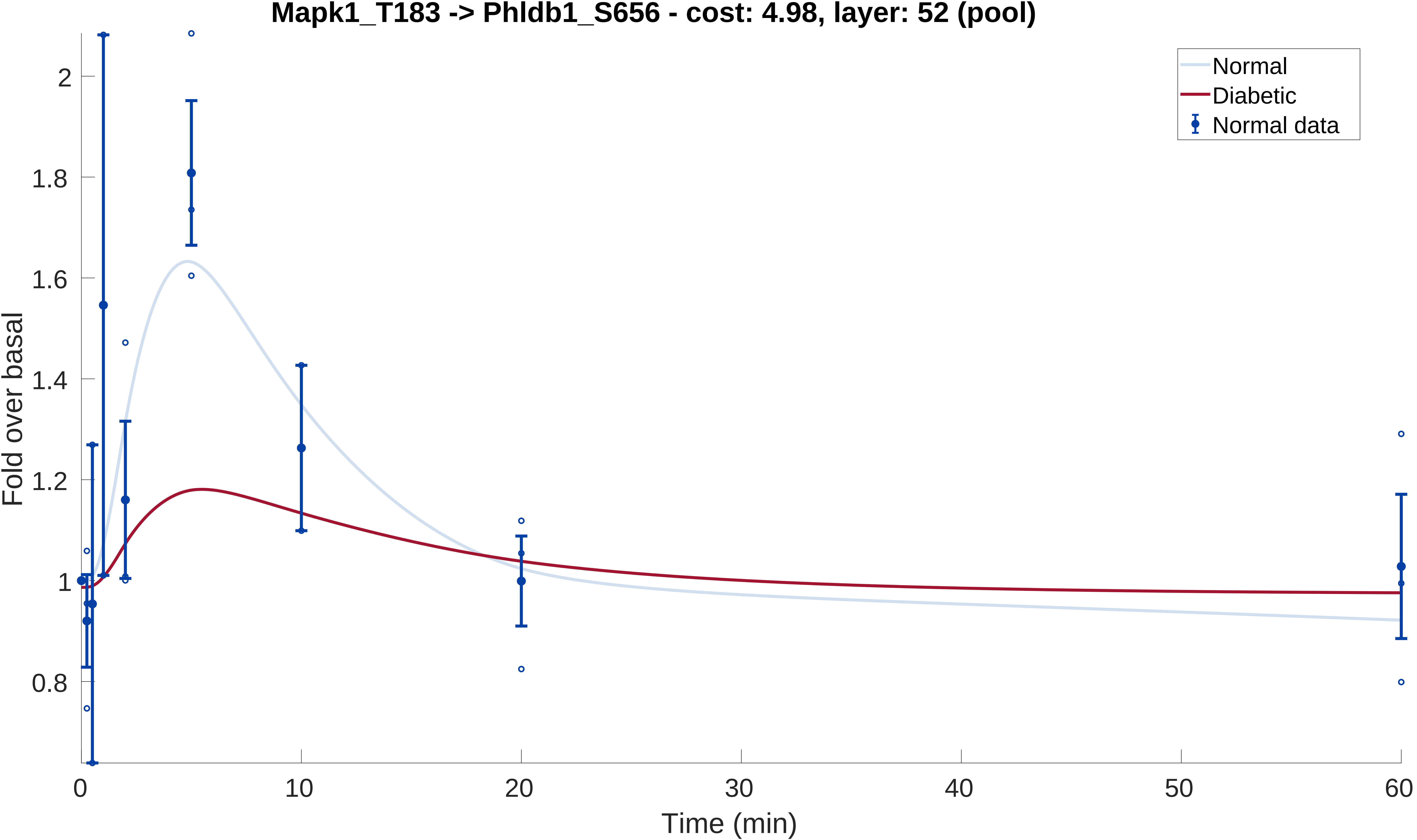
Mapk1_T183 -> Phldb1_S656 - cost: 4.98, layer: 52 (pool)
Fold over basal
Time (min)
0.8
1
1.2
1.4
1.6
1.8
2
0
10
20
30
40
50
60
Normal
Diabetic
Normal data

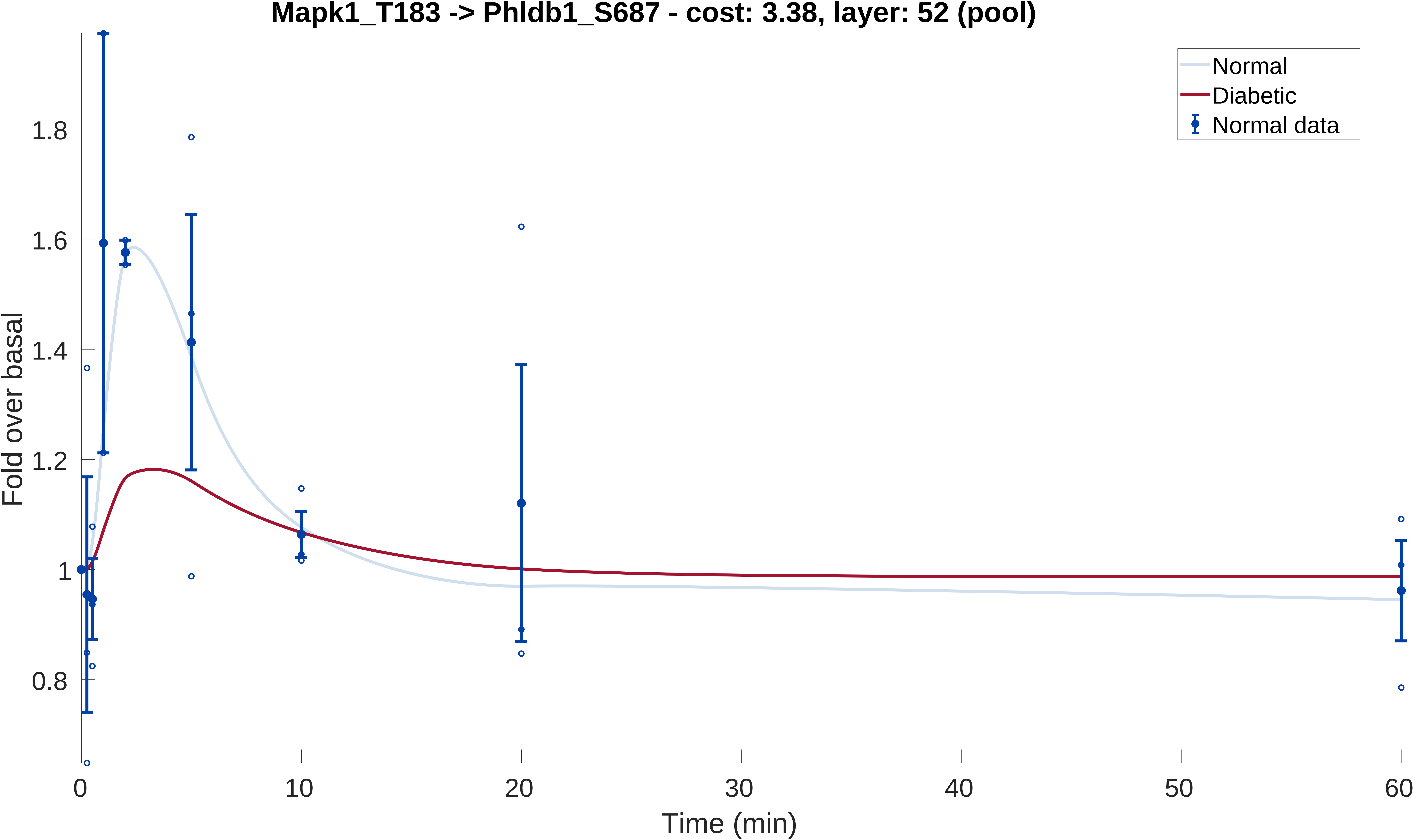

Mapk1_T183 -> Phldb1_S687 - cost: 3.38, layer: 52 (pool)
Normal
Diabetic
Normal data
Fold over basal
1.8
1.6
1.4
1.2
1
0.8
0
10
20
30
40
50
60
Time (min)

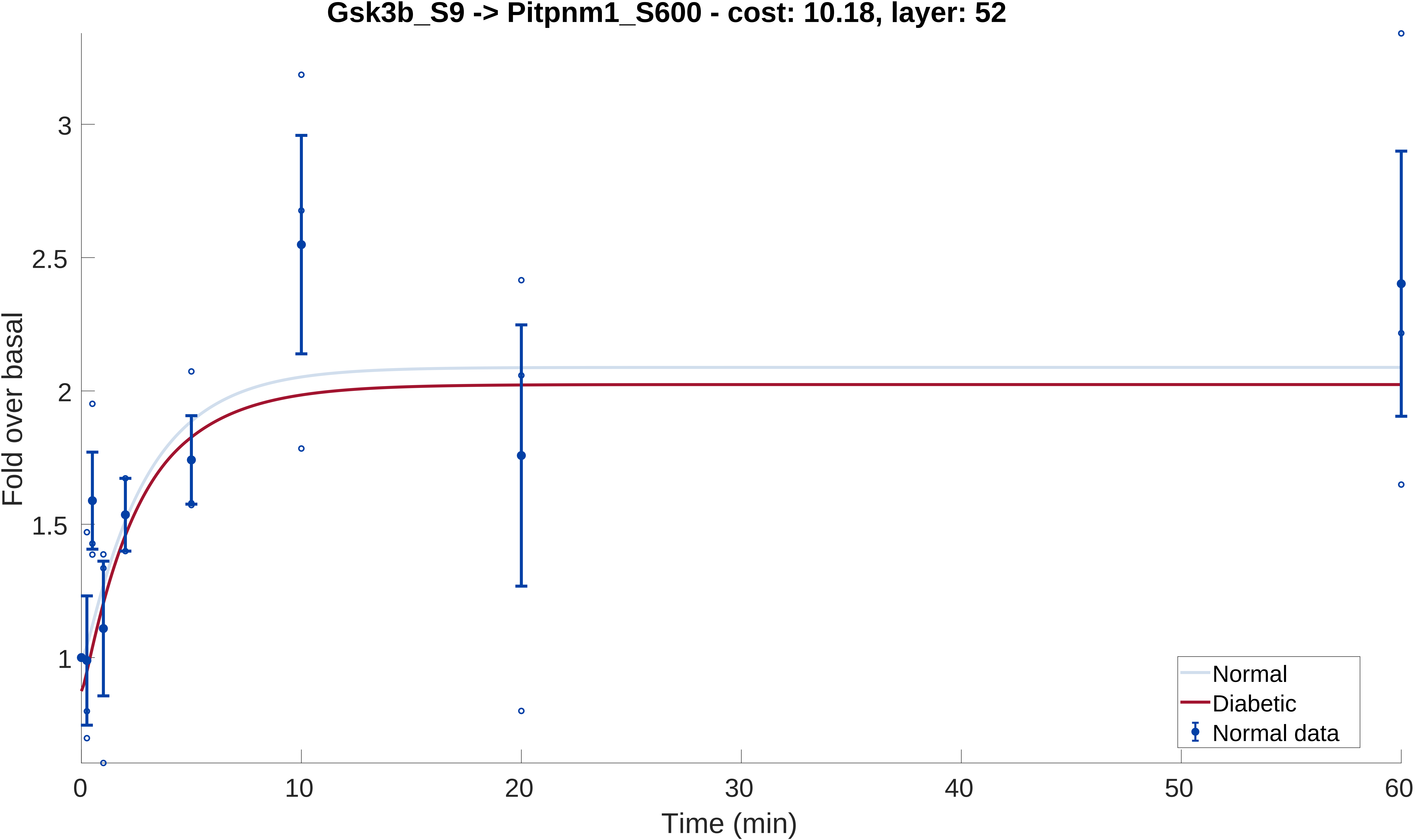
Gsk3b_S9 -> Pitpnm1_S600 - cost: 10.18, layer: 52
Fold over basal
Time (min)
Normal
Diabetic
Normal data
0
10
20
30
40
50
60
1
1.5
2
2.5
3

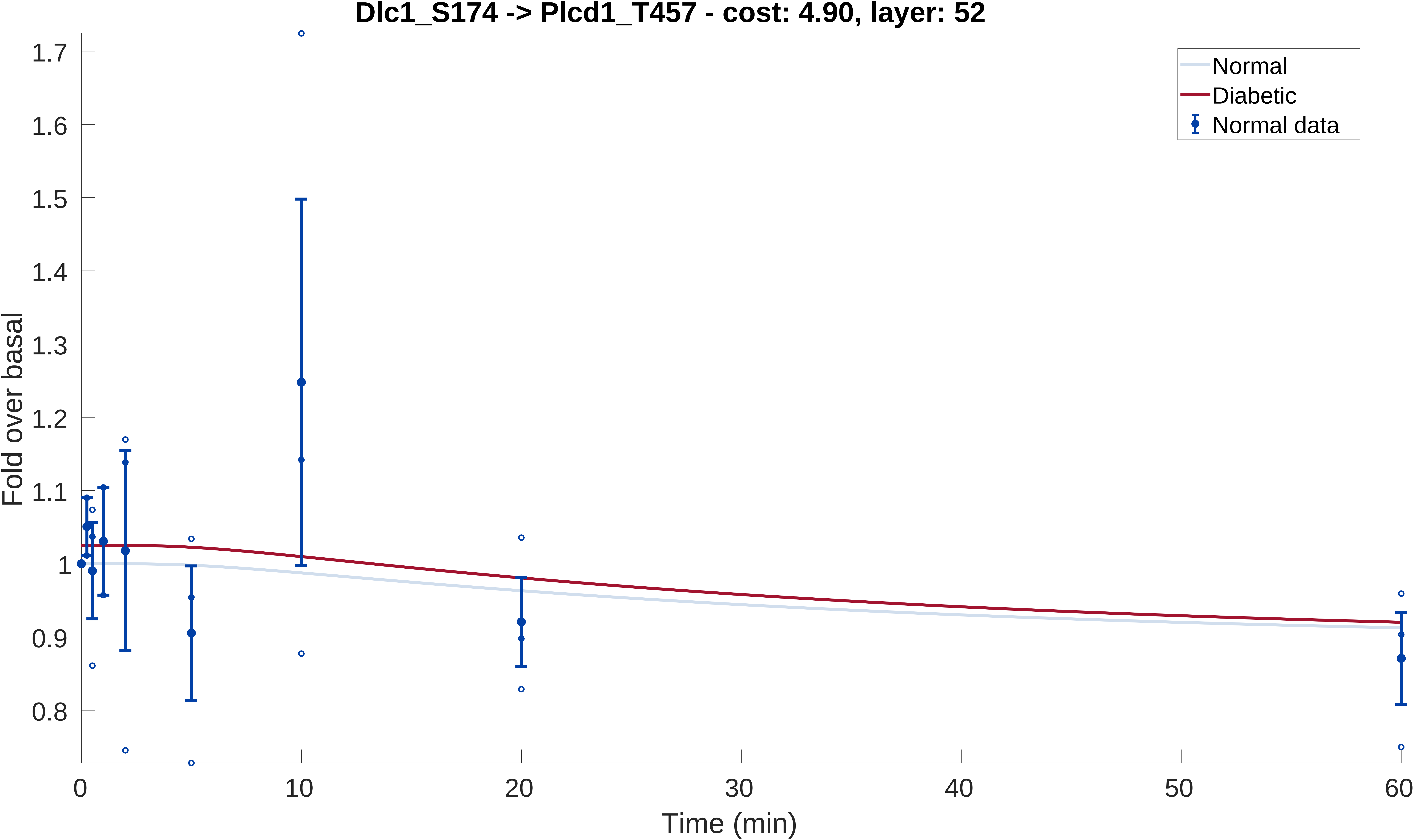
Dlc1_S174 -> Plcd1_T457 - cost: 4.90, layer: 52
Fold over basal
1.7
1.6
1.5
1.4
1.3
1.2
1.1
1
0.9
0.8
0
10
20
30
40
50
60
Time (min)
Normal
Diabetic
Normal data

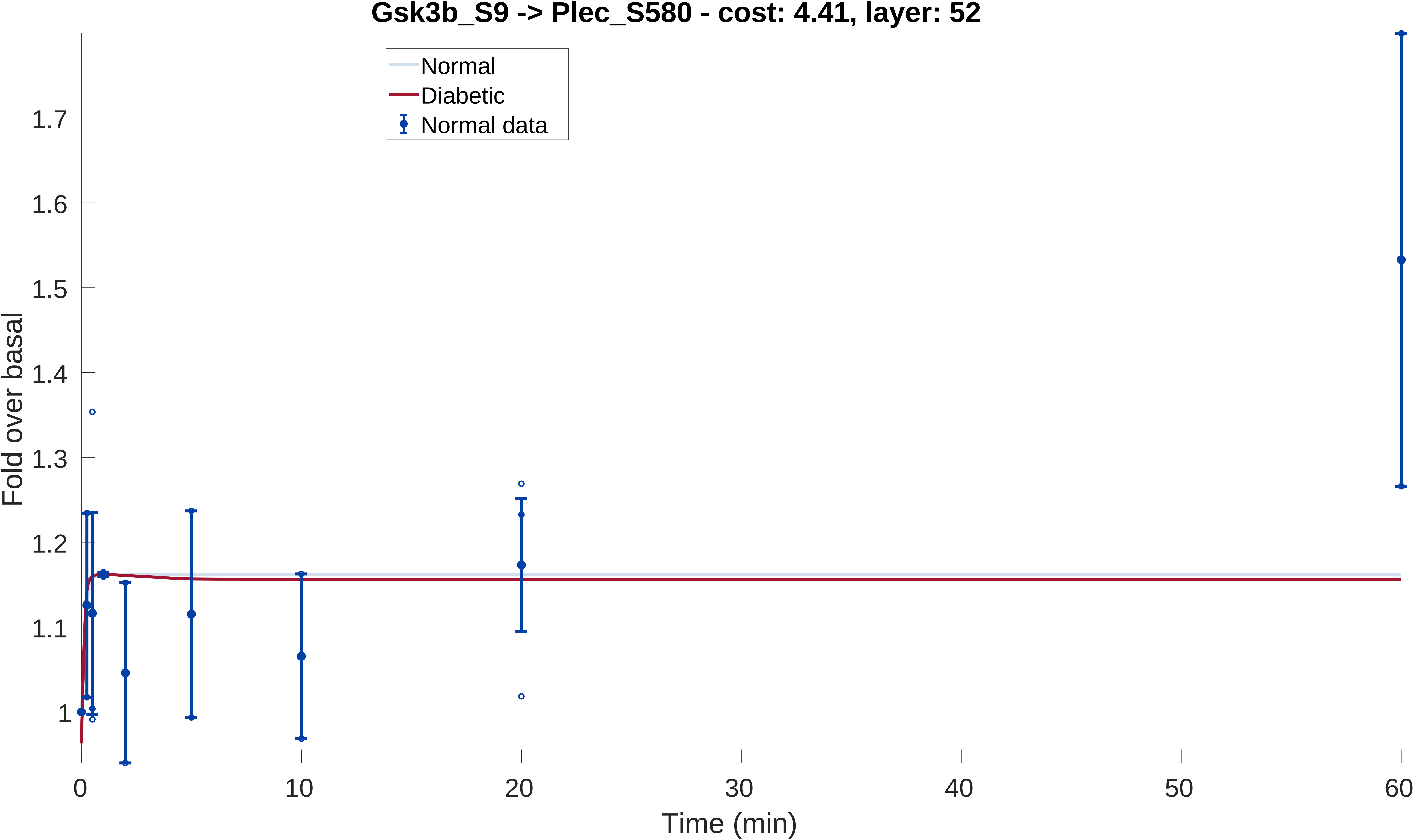
Gsk3b_S9 -> Plec_S580 - cost: 4.41, layer: 52
Normal
Diabetic
Normal data
Fold over basal
1.7
1.6
1.5
1.4
1.3
1.2
1.1
1
0
10
20
30
40
50
60
Time (min)

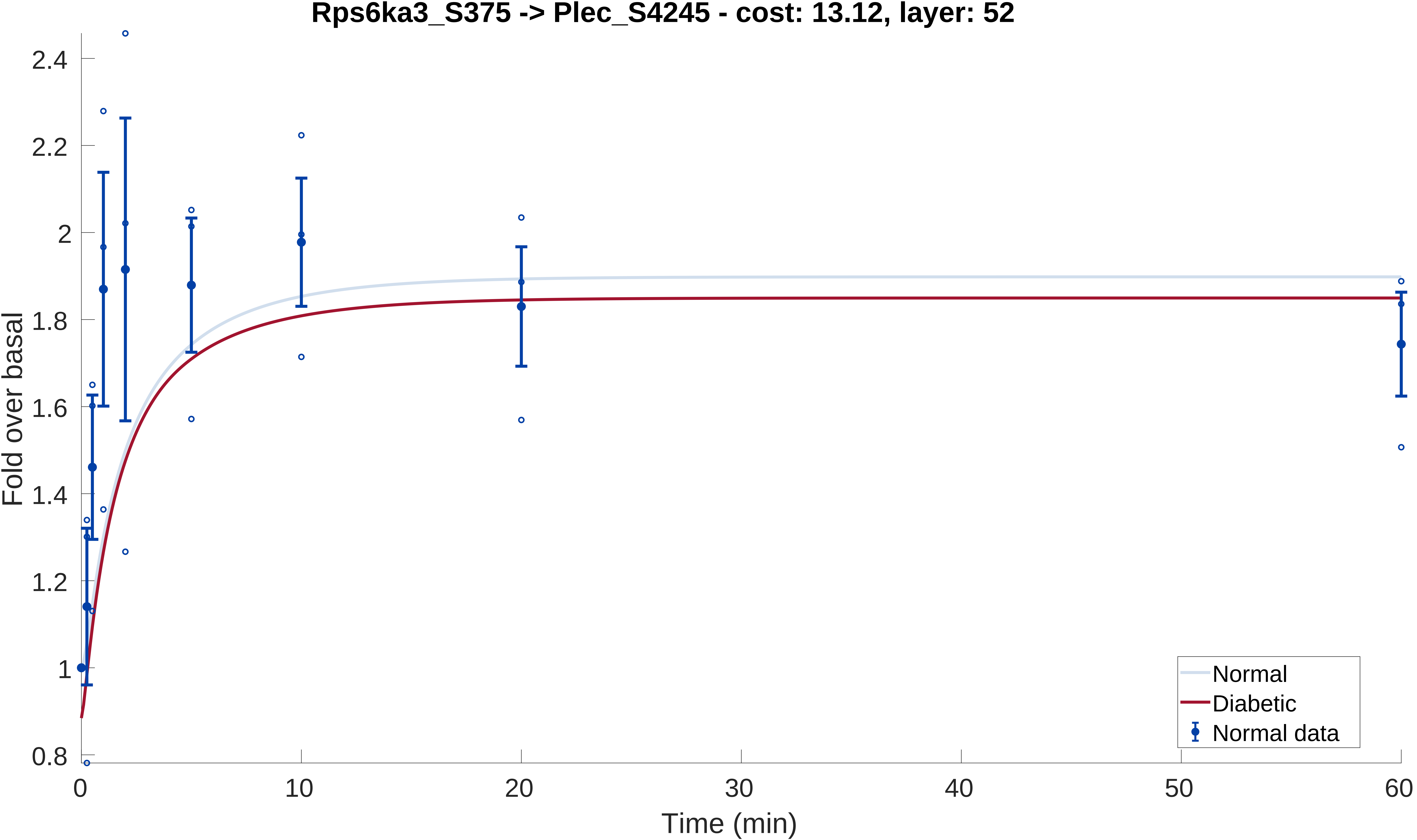

Rps6ka3_S375 -> Plec_S4245 - cost: 13.12, layer: 52
Fold over basal
2.4
2.2
2
1.8
1.6
1.4
1.2
1
0.8
0
10
20
30
40
50
60
Time (min)
Normal
Diabetic
Normal data

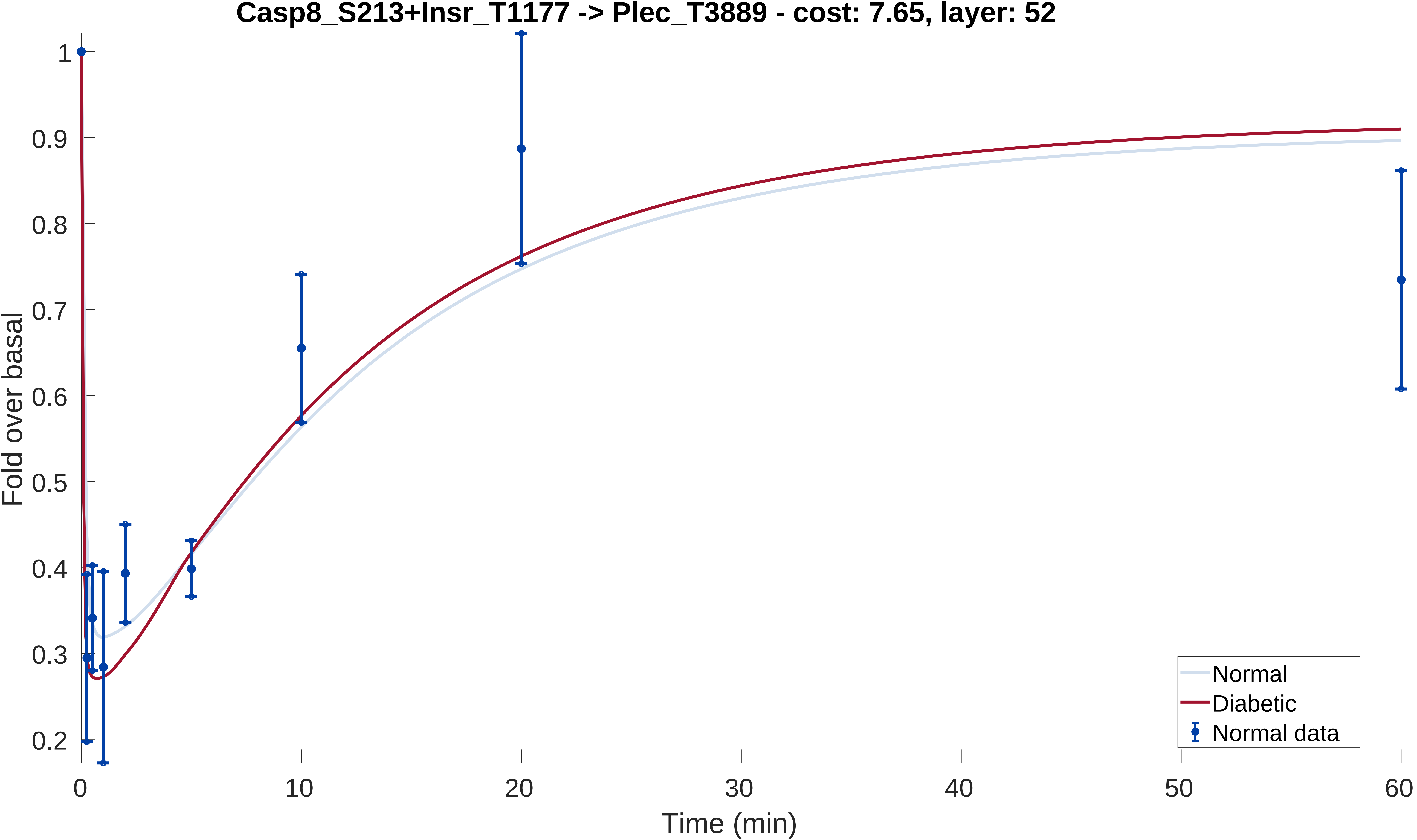
Casp8_S213+Insr_T1177 -> Plec_T3889 - cost: 7.65, layer: 52
Fold over basal
1
0.9
0.8
0.7
0.6
0.5
0.4
0.3
0.2
0
10
20
30
40
50
60
Time (min)
Normal
Diabetic
Normal data

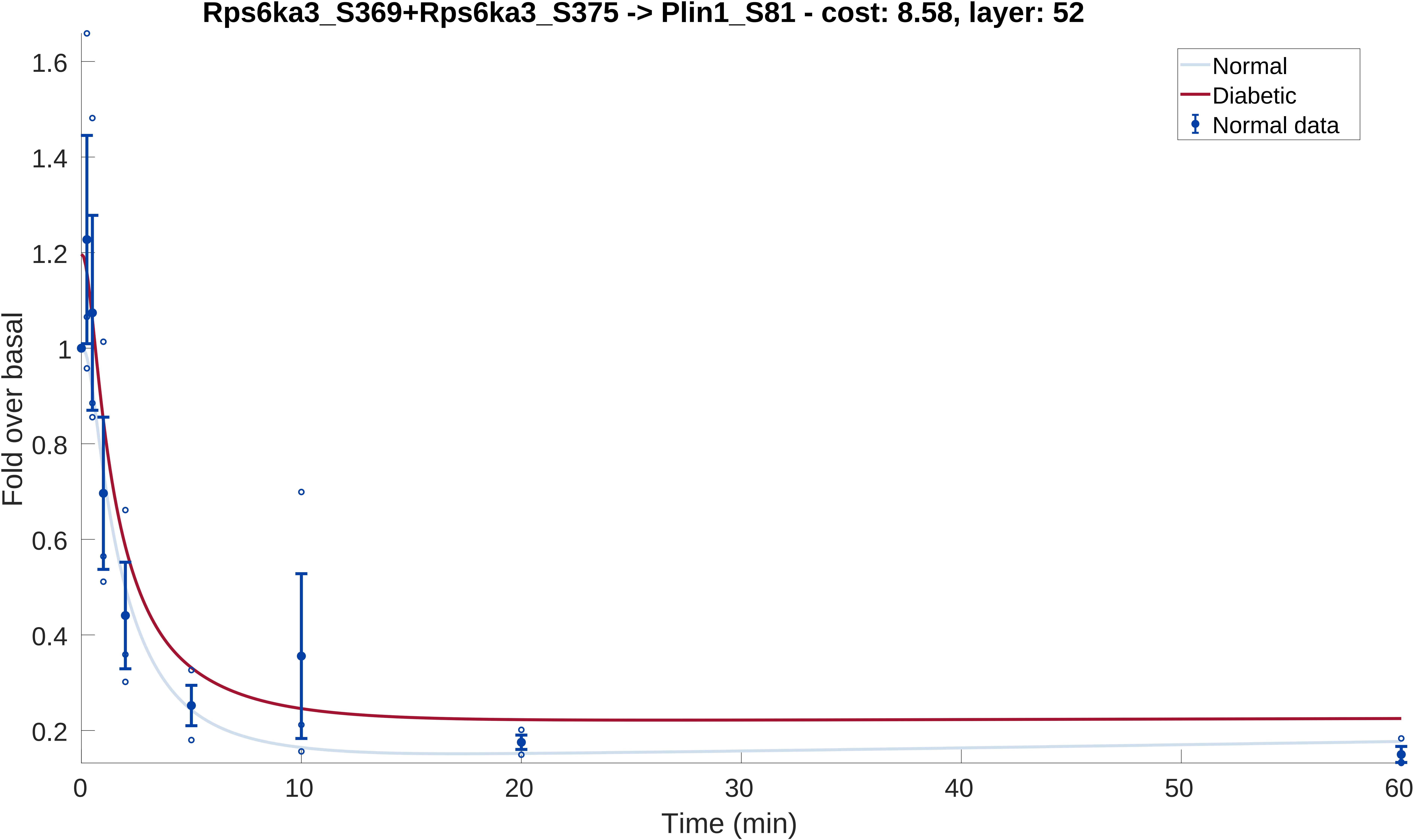

Rps6ka3_S369+Rps6ka3_S375 -> Plin1_S81 - cost: 8.58, layer: 52
Normal
Diabetic
Normal data
Fold over basal
Time (min)
1.6
1.4
1.2
1
0.8
0.6
0.4
0.2
0
10
20
30
40
50
60

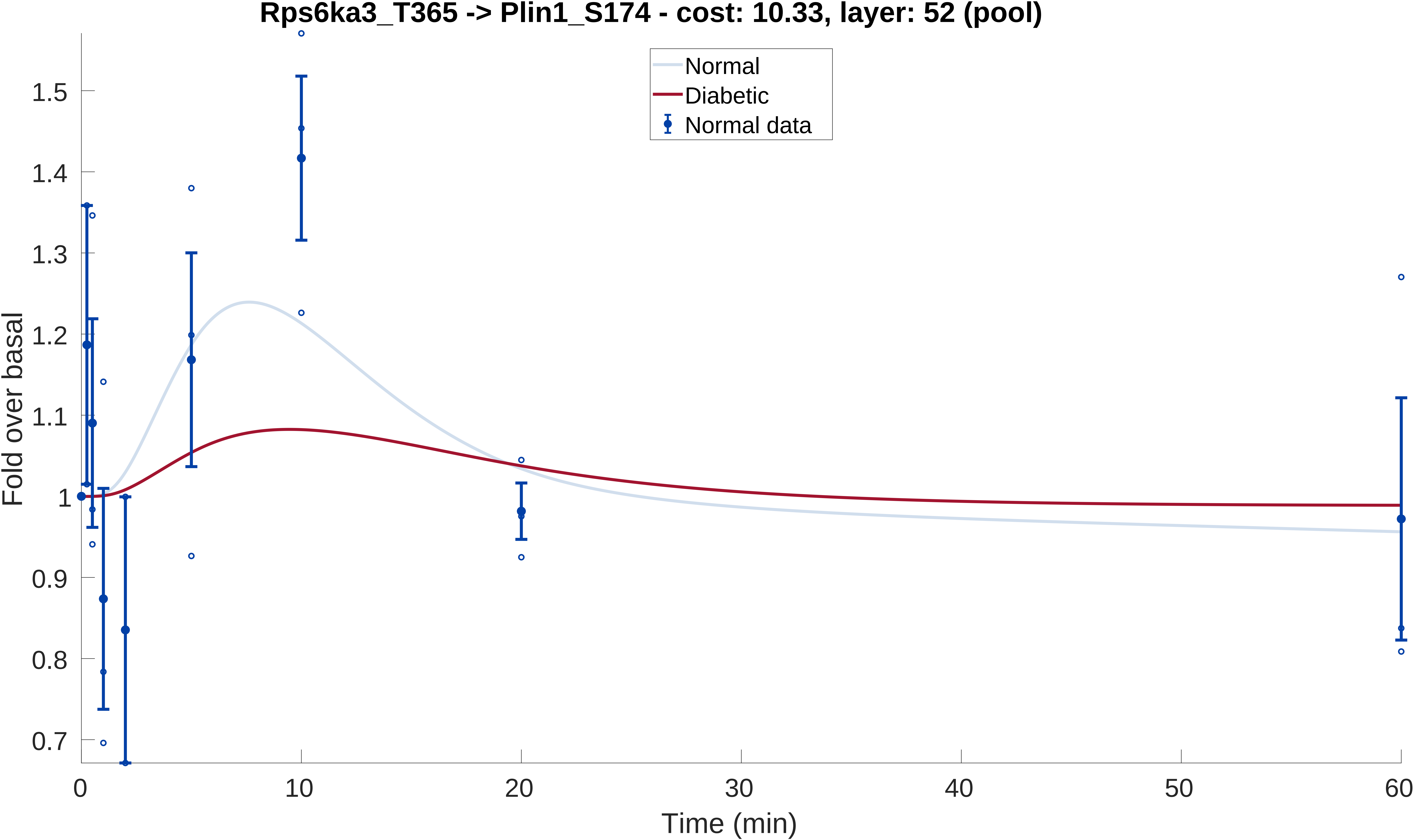

Rps6ka3_T365 -> Plin1_S174 - cost: 10.33, layer: 52 (pool)
Normal
Diabetic
Normal data
Fold over basal
Time (min)
1.5
1.4
1.3
1.2
1.1
1
0.9
0.8
0.7
0
10
20
30
40
50
60

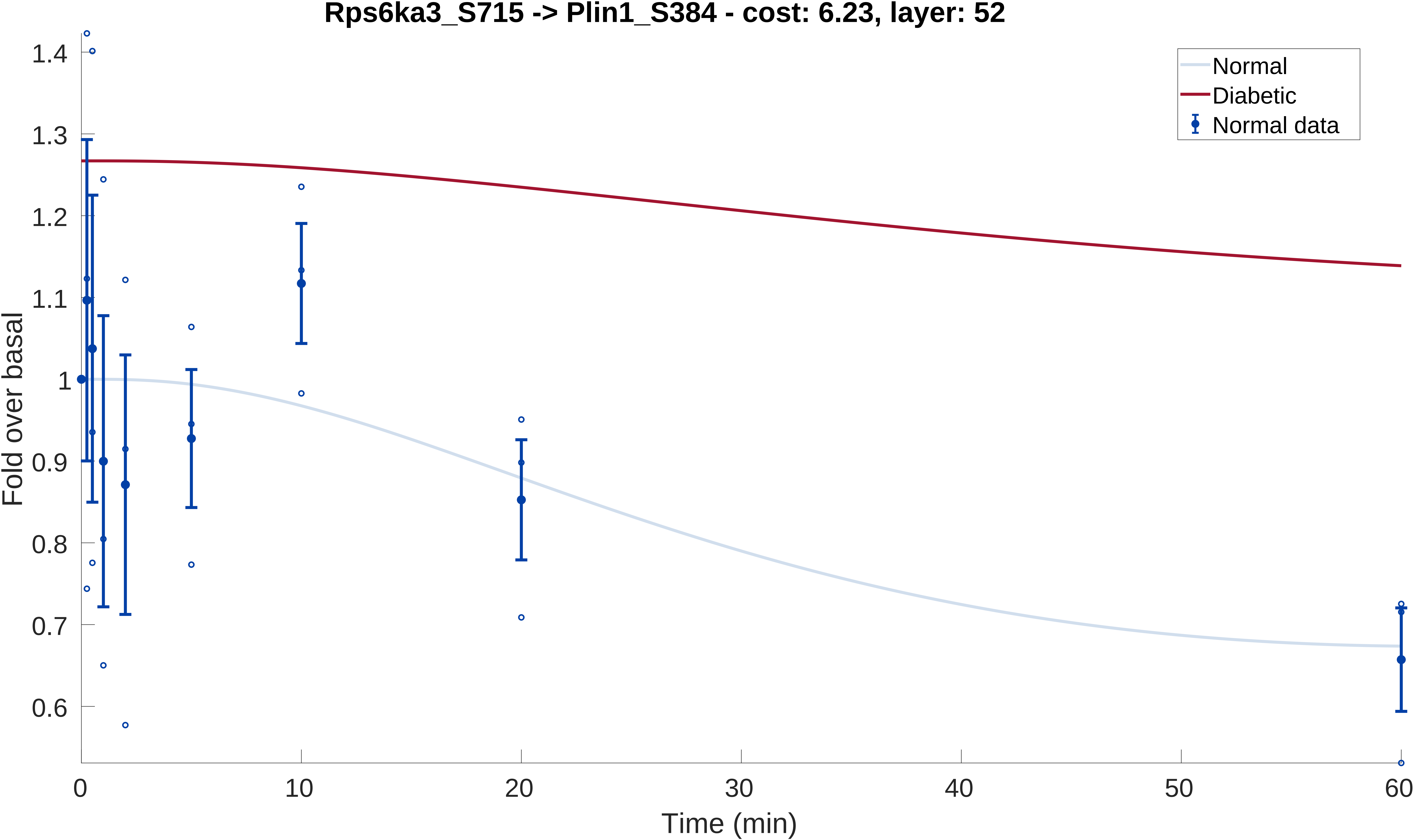

Rps6ka3_S715 -> Plin1_S384 - cost: 6.23, layer: 52
Normal
Diabetic
Normal data
Fold over basal
Time (min)
1.4
1.3
1.2
1.1
1
0.9
0.8
0.7
0.6
0
10
20
30
40
50
60

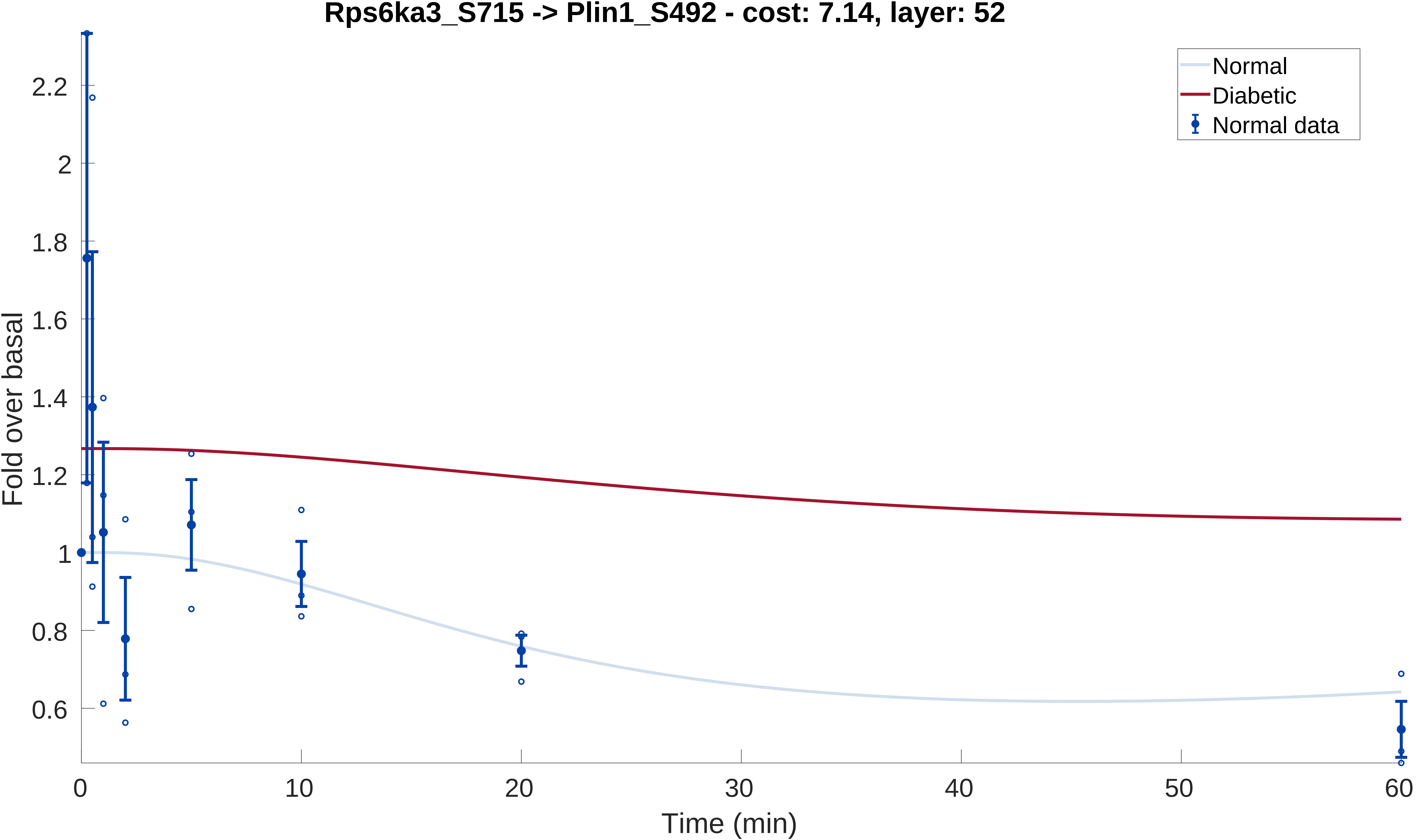
Rps6ka3_S715 -> Plin1_S492 - cost: 7.14, layer: 52
Normal
Diabetic
Normal data
Fold over basal
2.2
2
1.8
1.6
1.4
1.2
1
0.8
0.6
0
10
20
30
40
50
60
Time (min)

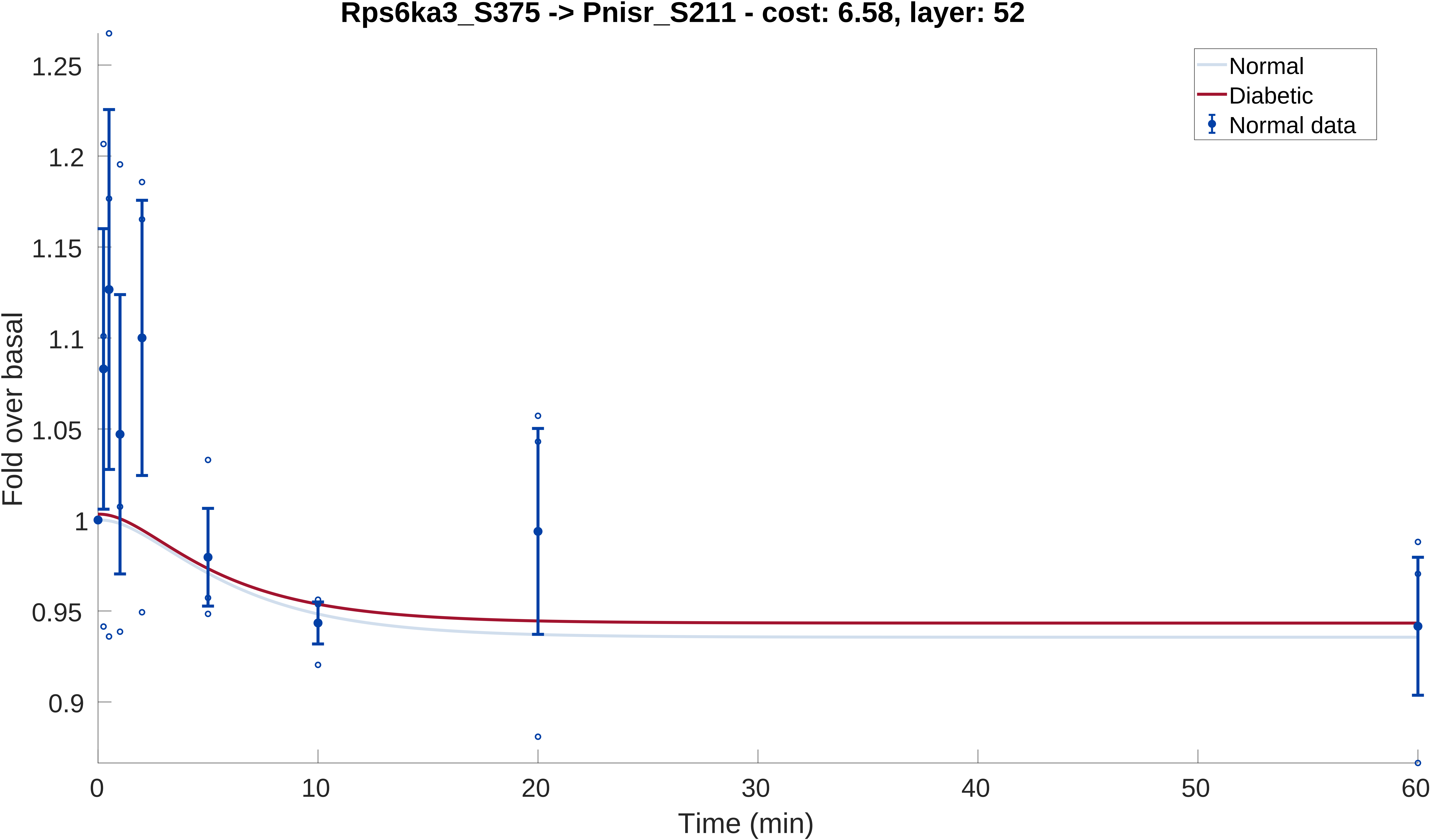

Rps6ka3_S375 -> Pnisr_S211 - cost: 6.58, layer: 52
Normal
Diabetic
Normal data
Fold over basal
1.25
1.2
1.15
1.1
1.05
1
0.95
0.9
0
10
20
30
40
50
60
Time (min)

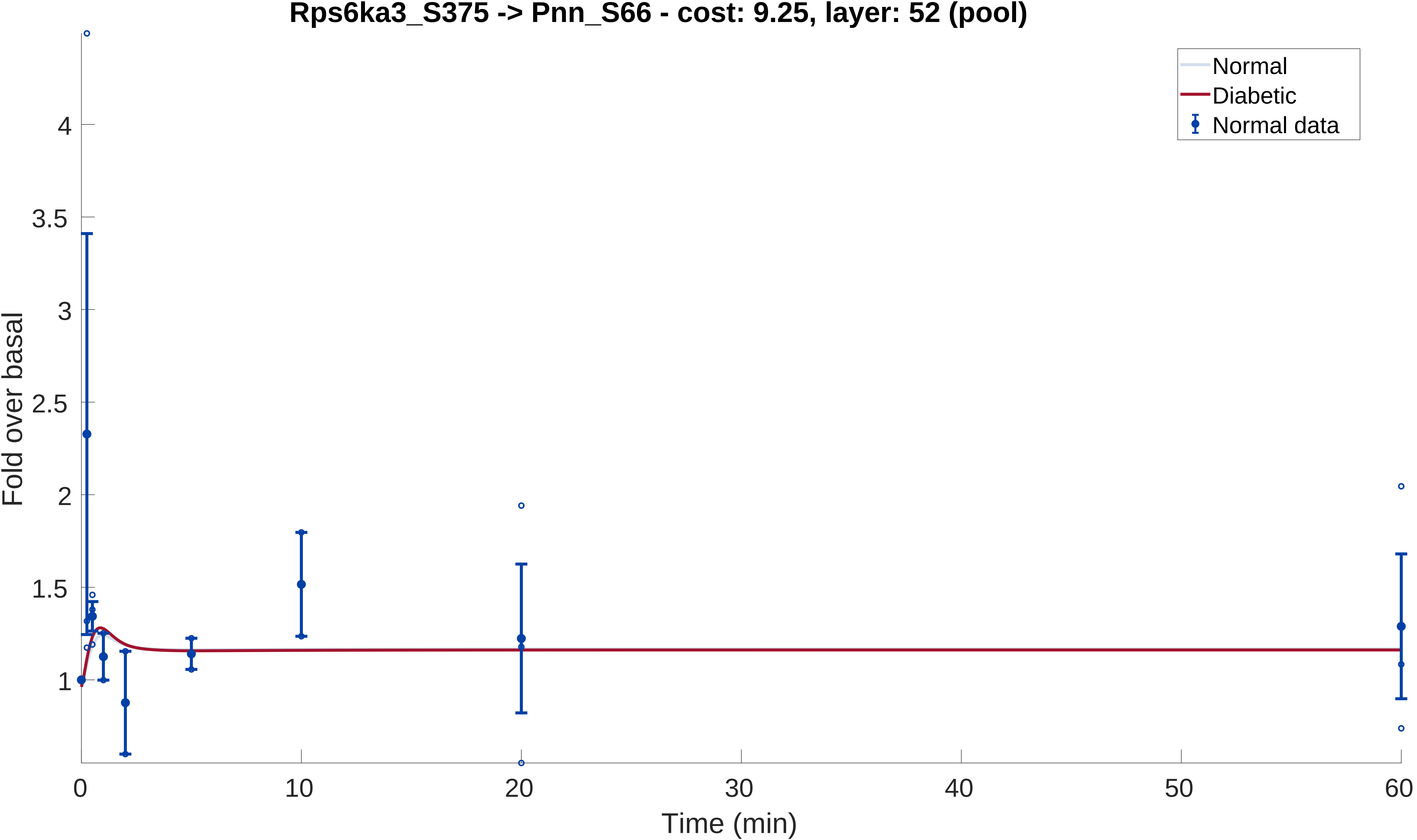

Rps6ka3_S375 -> Pnn_S66 - cost: 9.25, layer: 52 (pool)
Normal
Diabetic
Normal data
Fold over basal
Time (min)
4
3.5
3
2.5
2
1.5
1
0
10
20
30
40
50
60

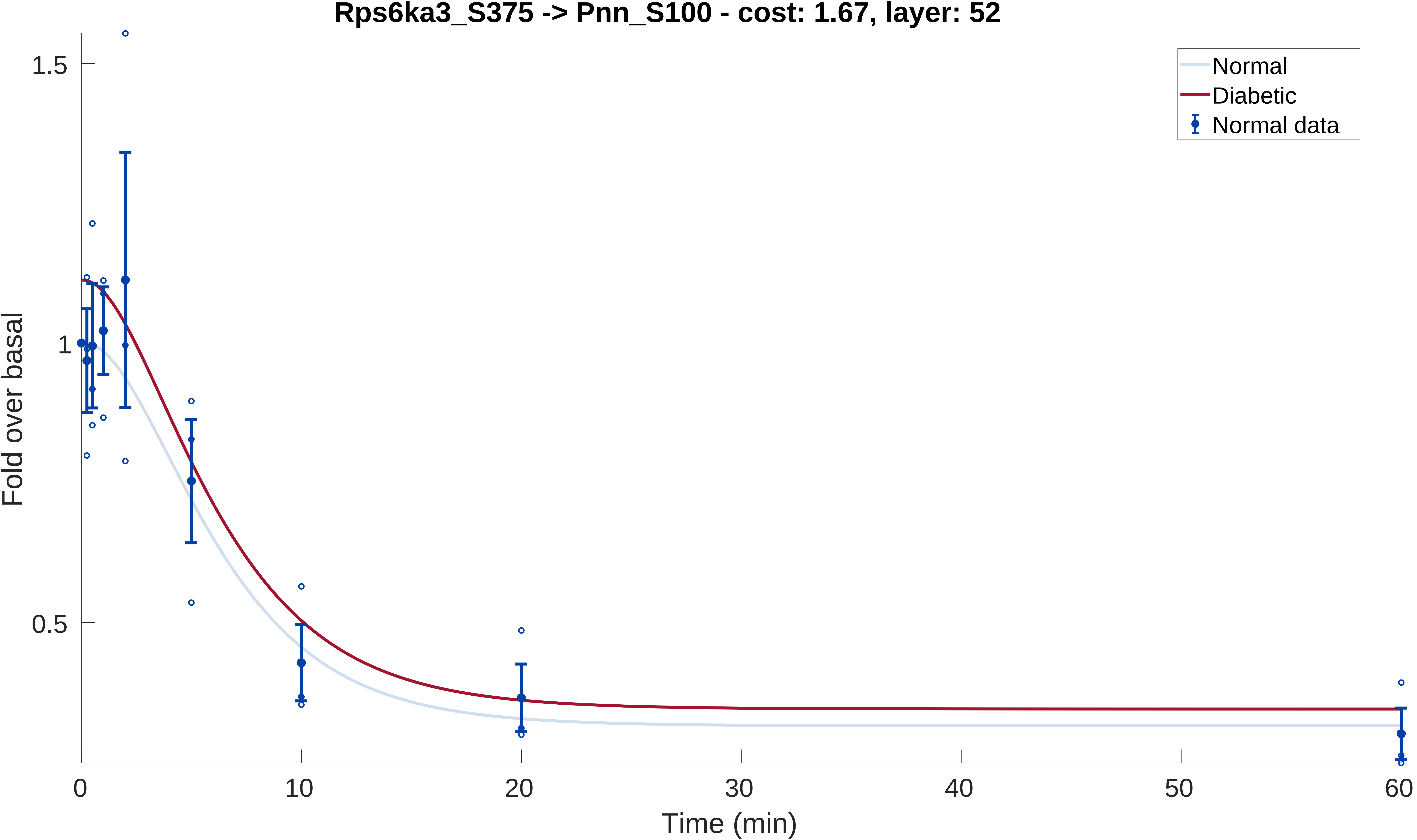

Rps6ka3_S375 -> Pnn_S100 - cost: 1.67, layer: 52
Normal
Diabetic
Normal data
1.5
1
0.5
Fold over basal
0
10
20
30
40
50
60
Time (min)

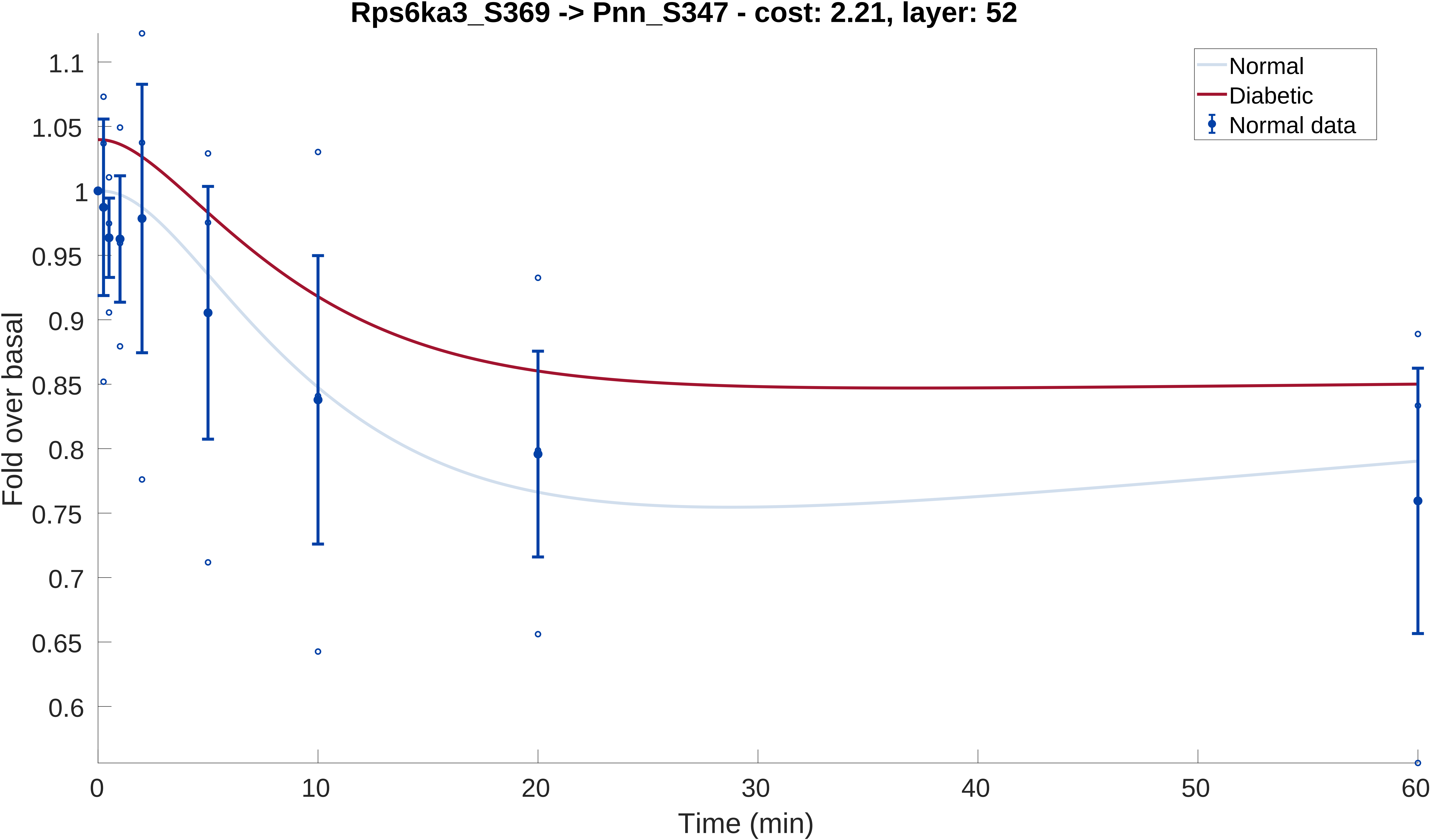

Rps6ka3_S369 -> Pnn_S347 - cost: 2.21, layer: 52
Normal
Diabetic
Normal data
Fold over basal
Time (min)
1.1
1.05
1
0.95
0.9
0.85
0.8
0.75
0.7
0.65
0.6
0
10
20
30
40
50
60

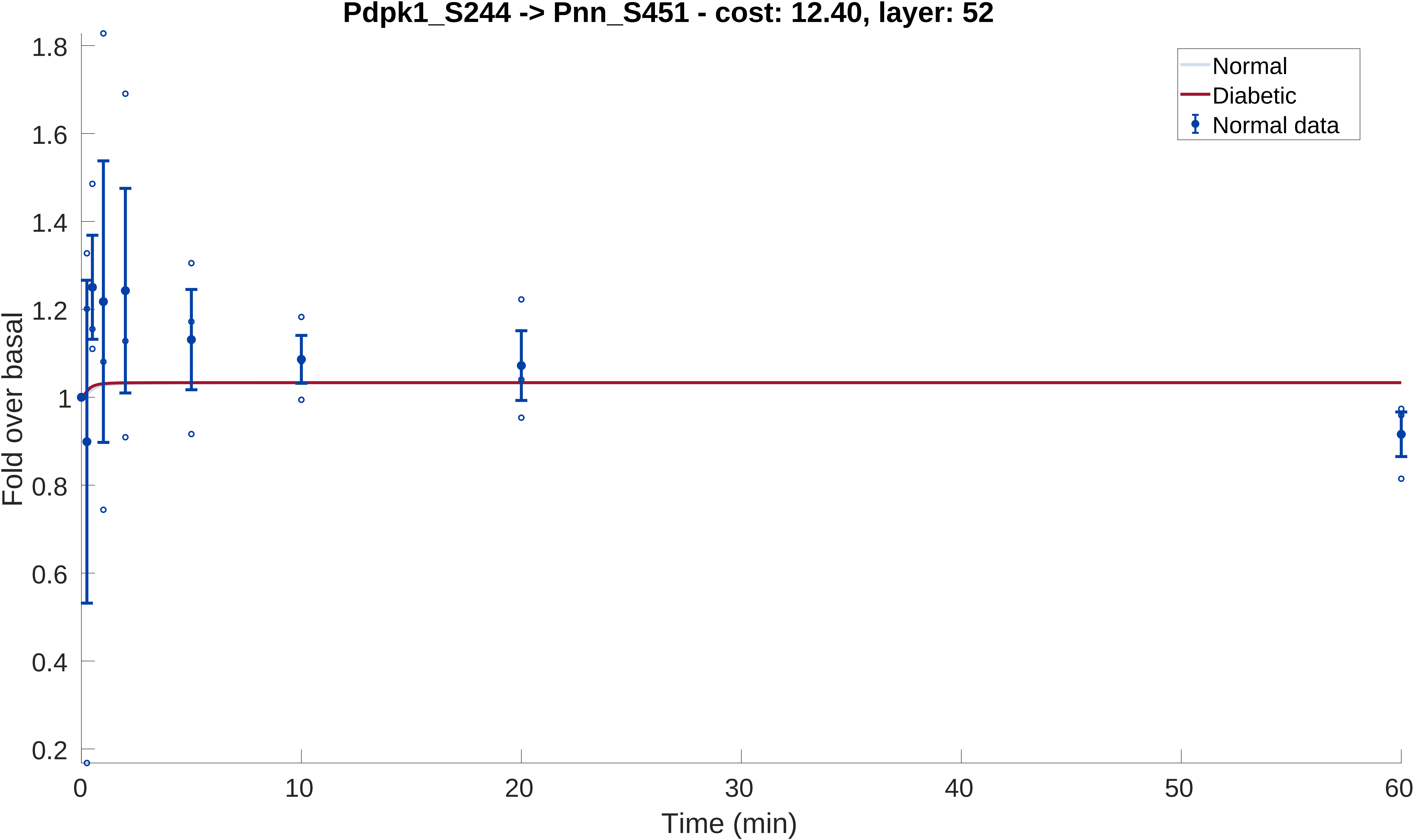

Pdpk1_S244 -> Pnn_S451 - cost: 12.40, layer: 52
Fold over basal
Time (min)
Normal
Diabetic
Normal data
1.8
1.6
1.4
1.2
1
0.8
0.6
0.4
0.2
0
10
20
30
40
50
60

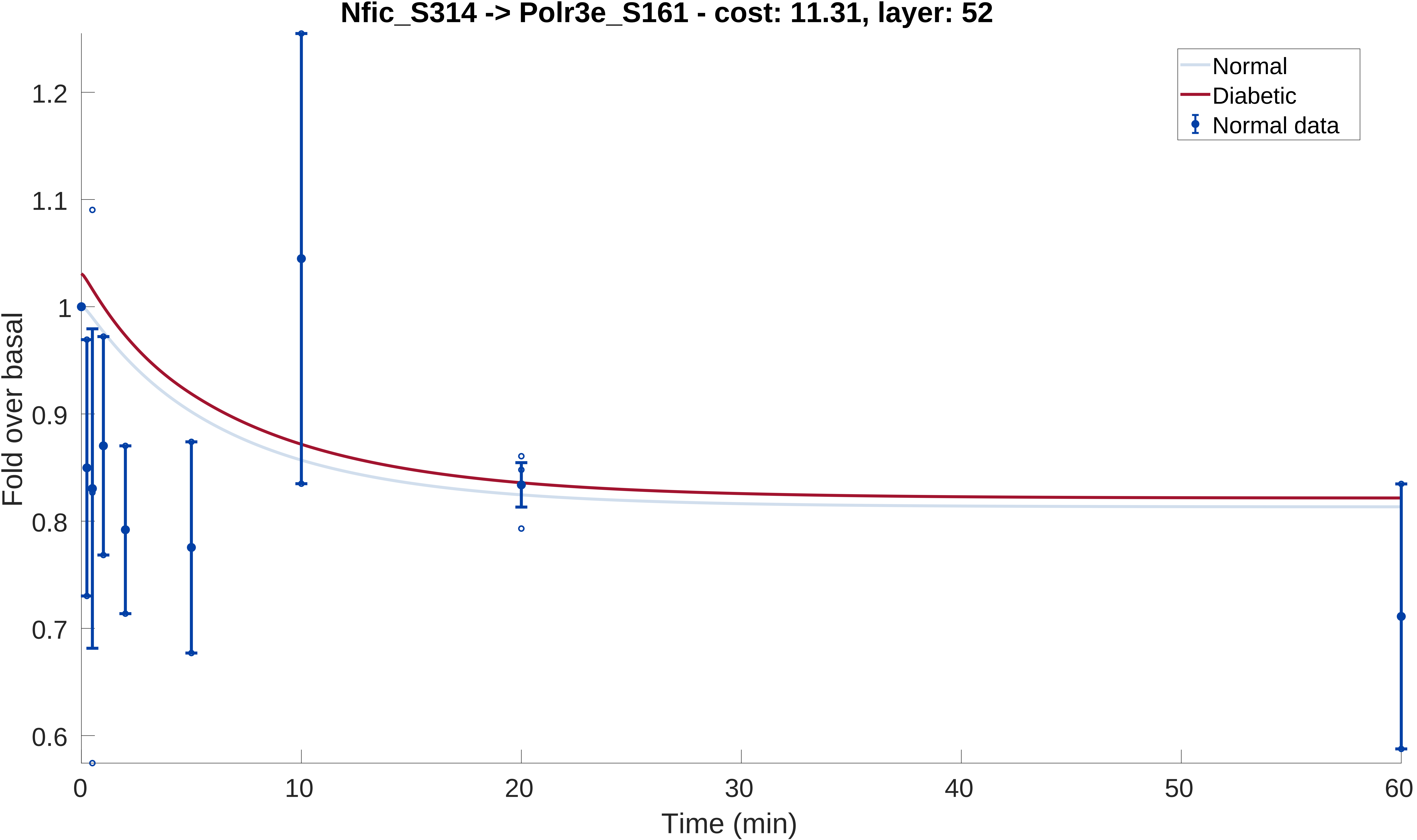

Nfic_S314 -> Polr3e_S161 - cost: 11.31, layer: 52
Normal
Diabetic
Normal data
Fold over basal
Time (min)
0.6
0.7
0.8
0.9
1
1.1
1.2
0
10
20
30
40
50
60

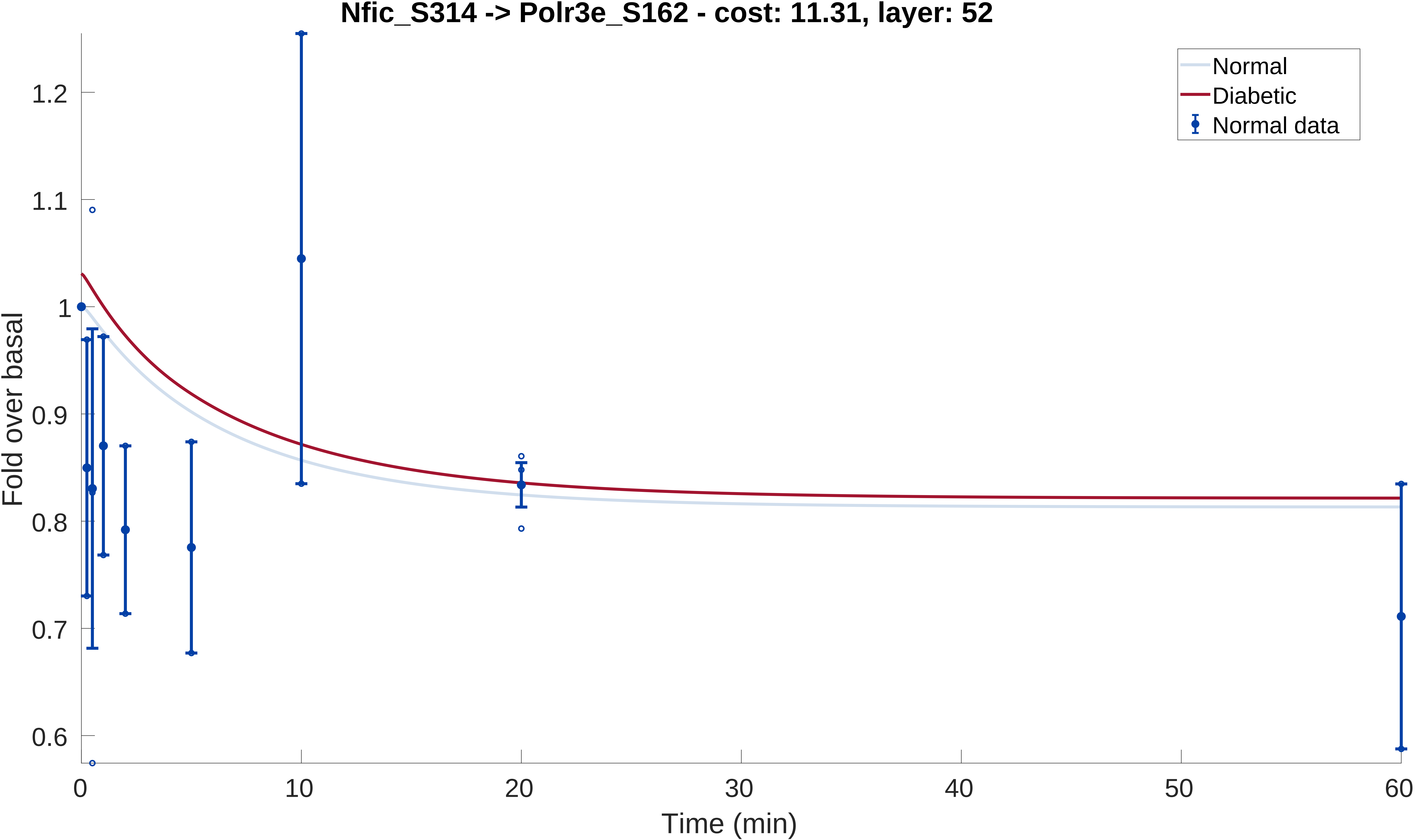
Nfic_S314 -> Polr3e_S162 - cost: 11.31, layer: 52
Normal
Diabetic
Normal data
Fold over basal
1.2
1.1
1
0.9
0.8
0.7
0.6
0
10
20
30
40
50
60
Time (min)

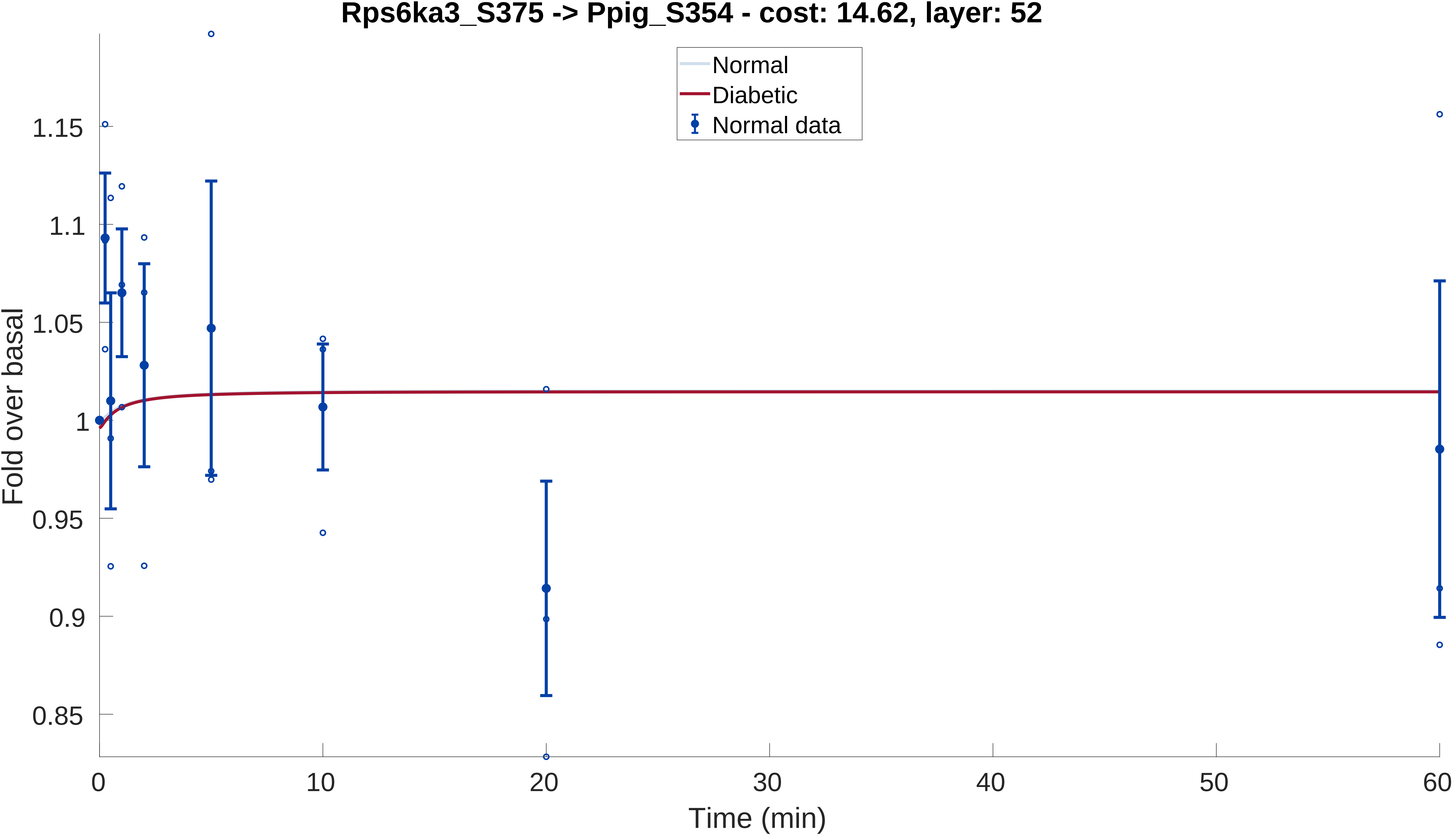

Rps6ka3_S375 -> Ppig_S354 - cost: 14.62, layer: 52
Normal
Diabetic
Normal data
Fold over basal
1.15
1.1
1.05
1
0.95
0.9
0.85
0
10
20
30
40
50
60
Time (min)

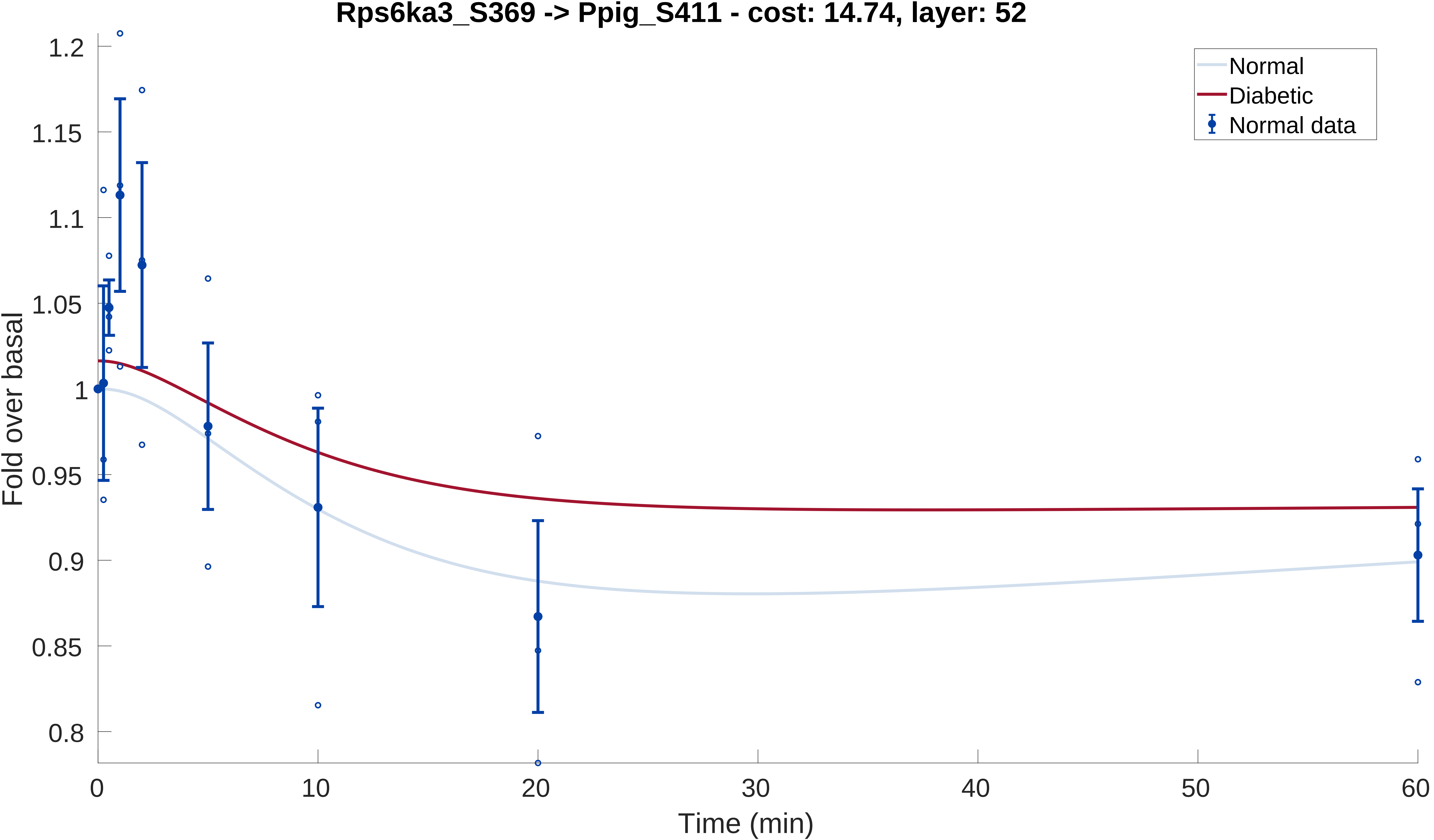

Rps6ka3_S369 -> Ppig_S411 - cost: 14.74, layer: 52
Fold over basal
Time (min)
Normal
Diabetic
Normal data
1.2
1.15
1.1
1.05
1
0.95
0.9
0.85
0.8
0
10
20
30
40
50
60

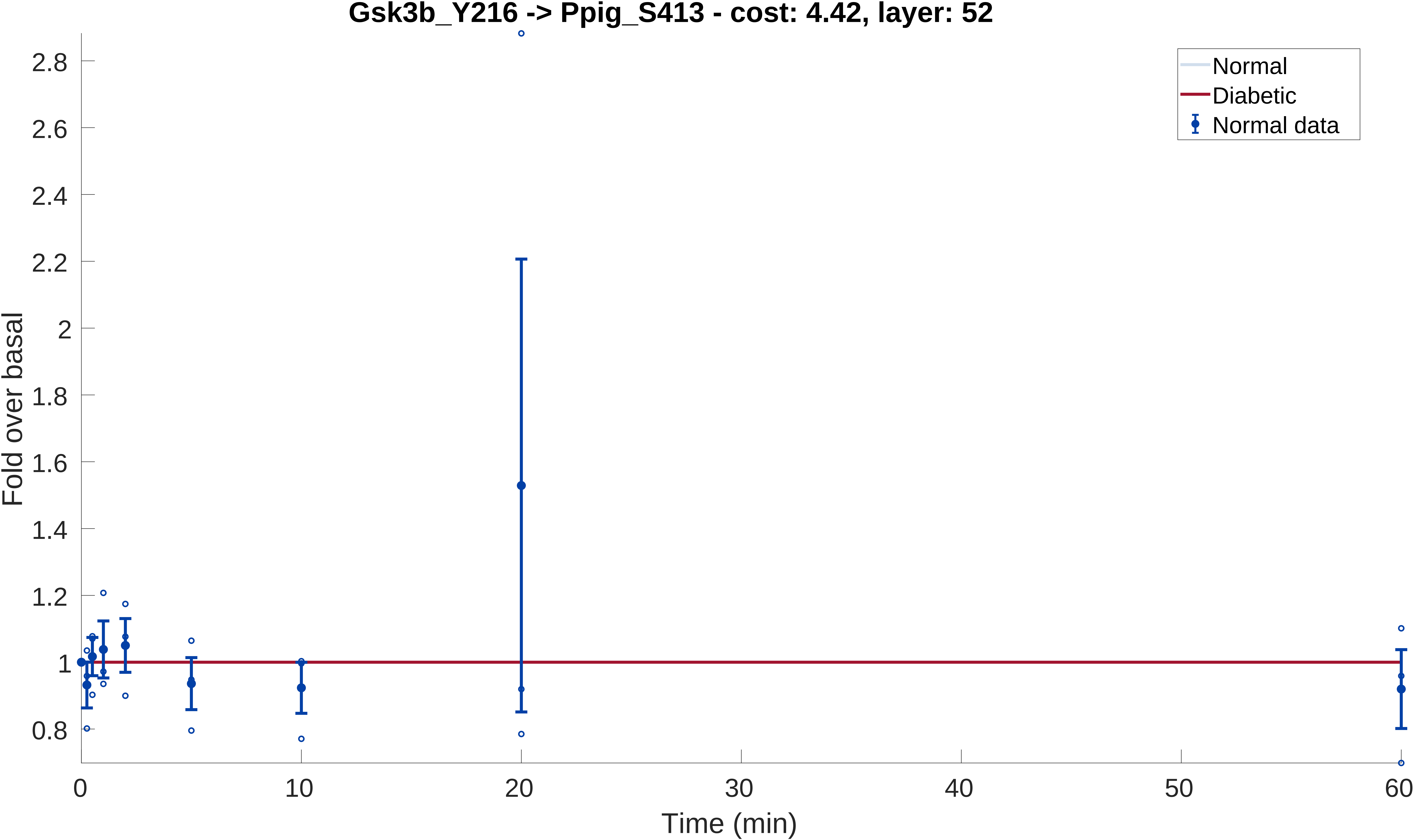
Gsk3b_Y216 -> Ppig_S413 - cost: 4.42, layer: 52
Fold over basal
2.8
2.6
2.4
2.2
2
1.8
1.6
1.4
1.2
1
0.8
0
10
20
30
40
50
60
Time (min)
Normal
Diabetic
Normal data

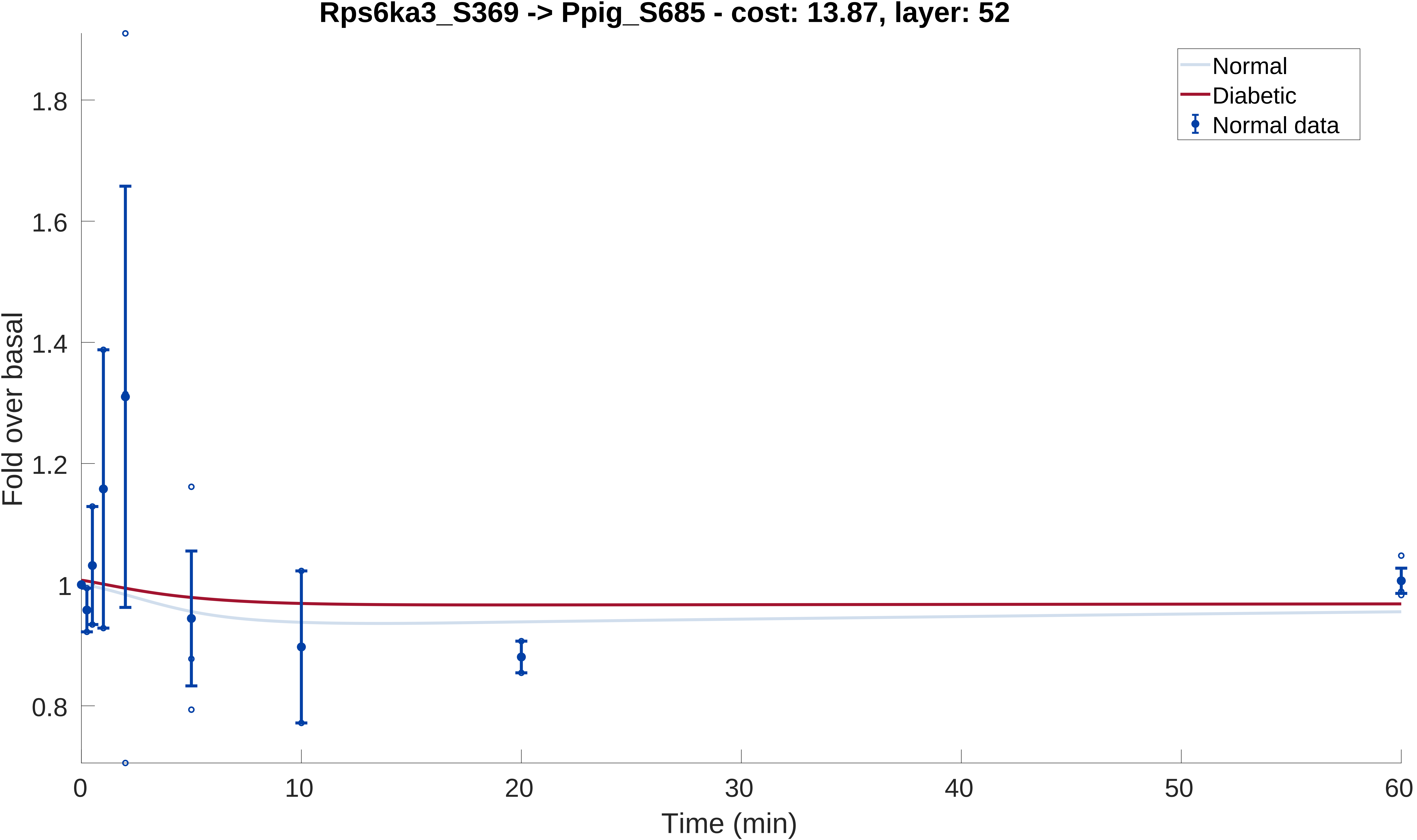

Rps6ka3_S369 -> Ppig_S685 - cost: 13.87, layer: 52
Normal
Diabetic
Normal data
Fold over basal
1.8
1.6
1.4
1.2
1
0.8
0
10
20
30
40
50
60
Time (min)

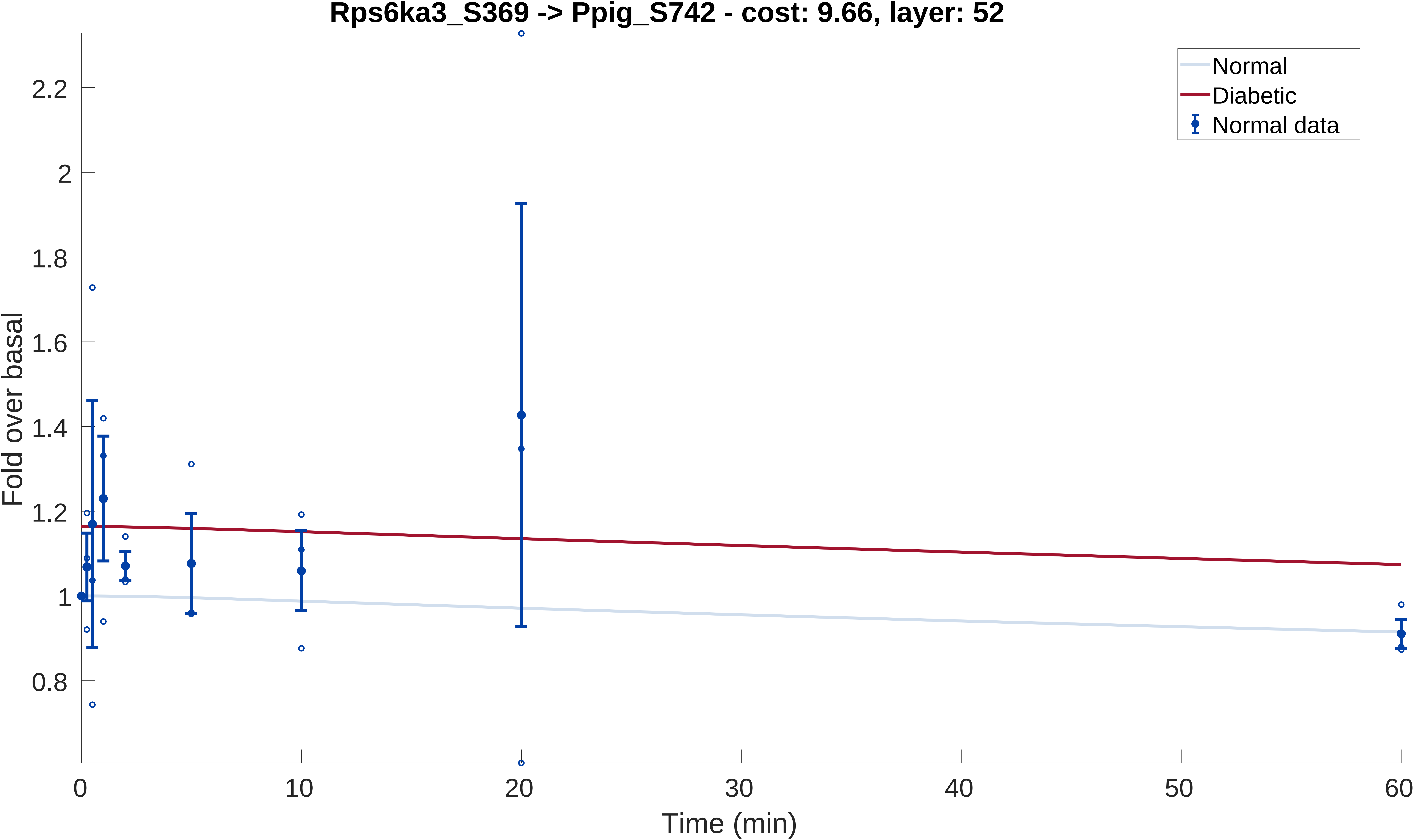
Rps6ka3_S369 -> Ppig_S742 - cost: 9.66, layer: 52
Normal
Diabetic
Normal data
2.2
2
1.8
1.6
1.4
1.2
1
0.8
Fold over basal
0
10
20
30
40
50
60
Time (min)

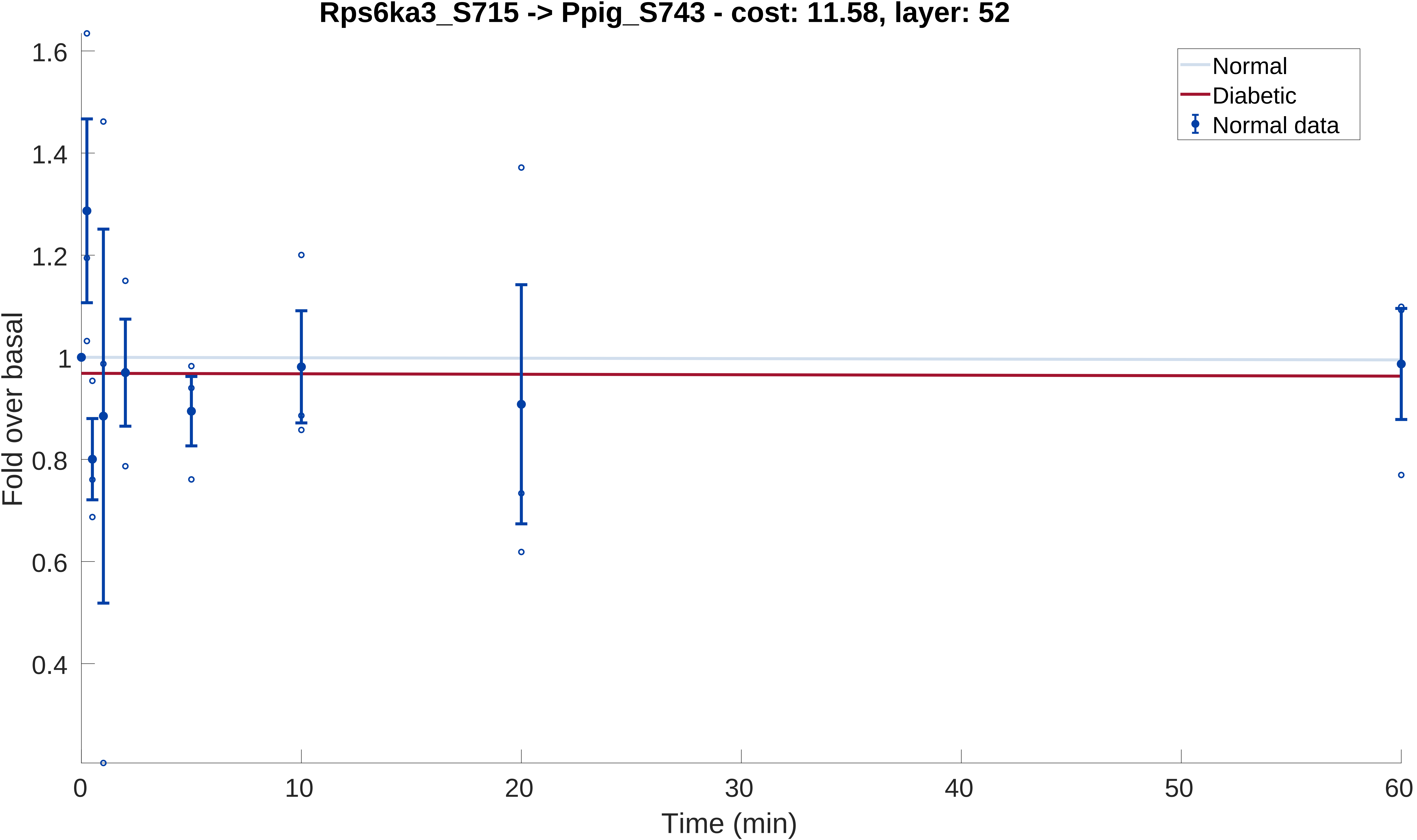

Rps6ka3_S715 -> Ppig_S743 - cost: 11.58, layer: 52
Normal
Diabetic
Normal data
Fold over basal
Time (min)
1.6
1.4
1.2
1
0.8
0.6
0.4
0
10
20
30
40
50
60

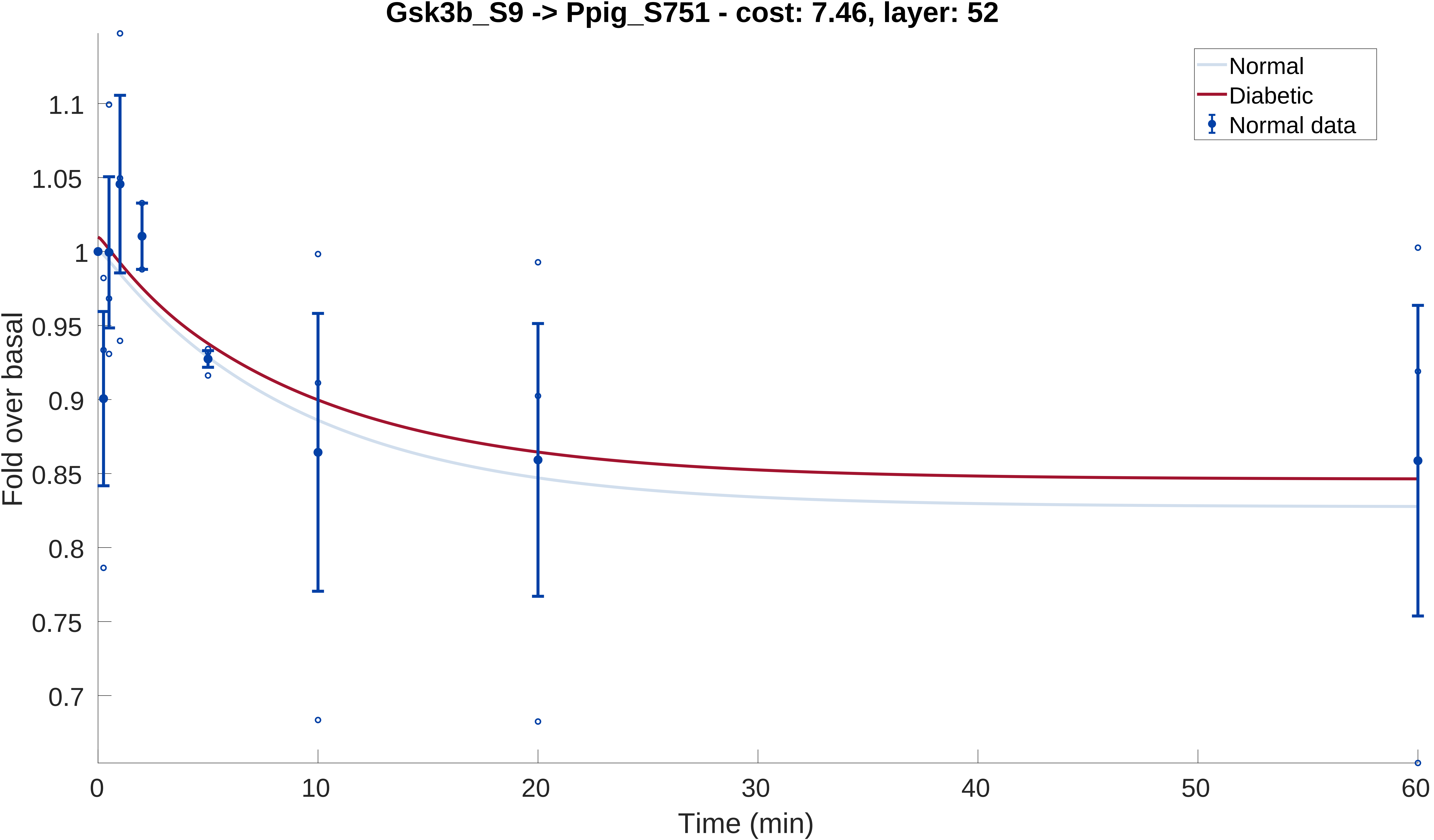

Gsk3b_S9 -> Ppig_S751 - cost: 7.46, layer: 52
Normal
Diabetic
Normal data
Fold over basal
1.1
1.05
1
0.95
0.9
0.85
0.8
0.75
0.7
0
10
20
30
40
50
60
Time (min)

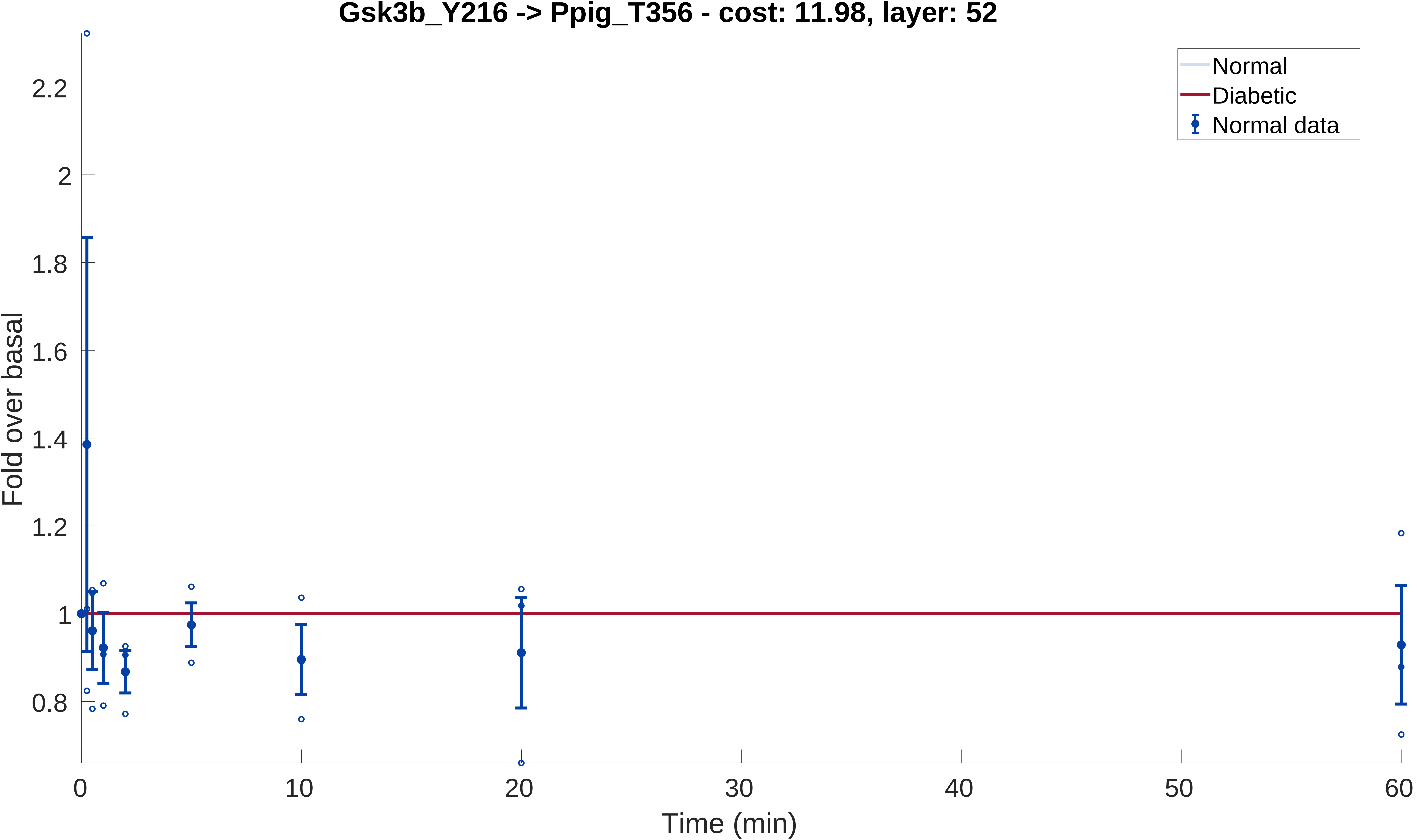
Gsk3b_Y216 -> Ppig_T356 - cost: 11.98, layer: 52
Normal
Diabetic
Normal data
Fold over basal
2.2
2
1.8
1.6
1.4
1.2
1
0.8
0
10
20
30
40
50
60
Time (min)

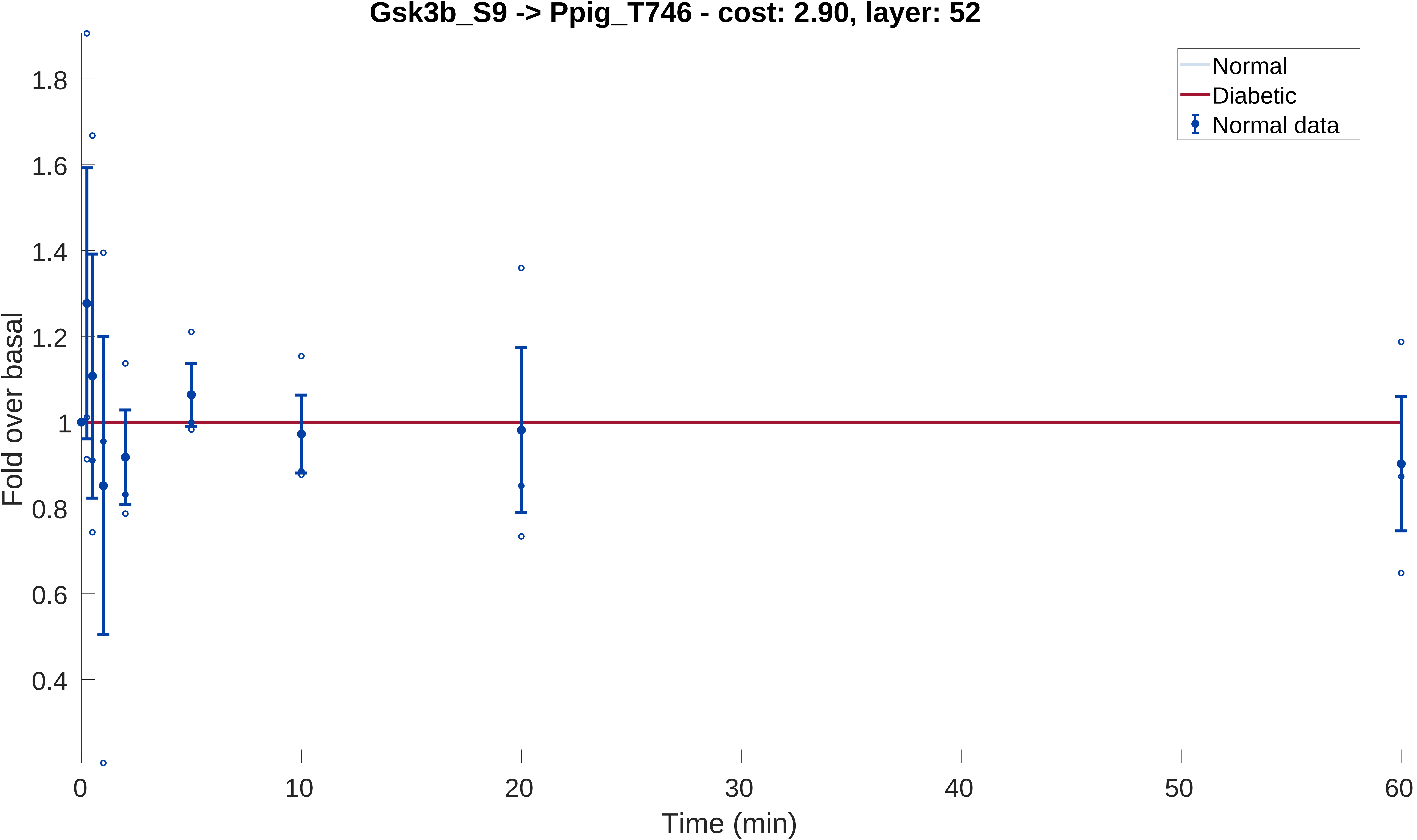

Gsk3b_S9 -> Ppig_T746 - cost: 2.90, layer: 52
Normal
Diabetic
Normal data
1.8
1.6
1.4
1.2
1
0.8
0.6
0.4
Fold over basal
0
10
20
30
40
50
60
Time (min)

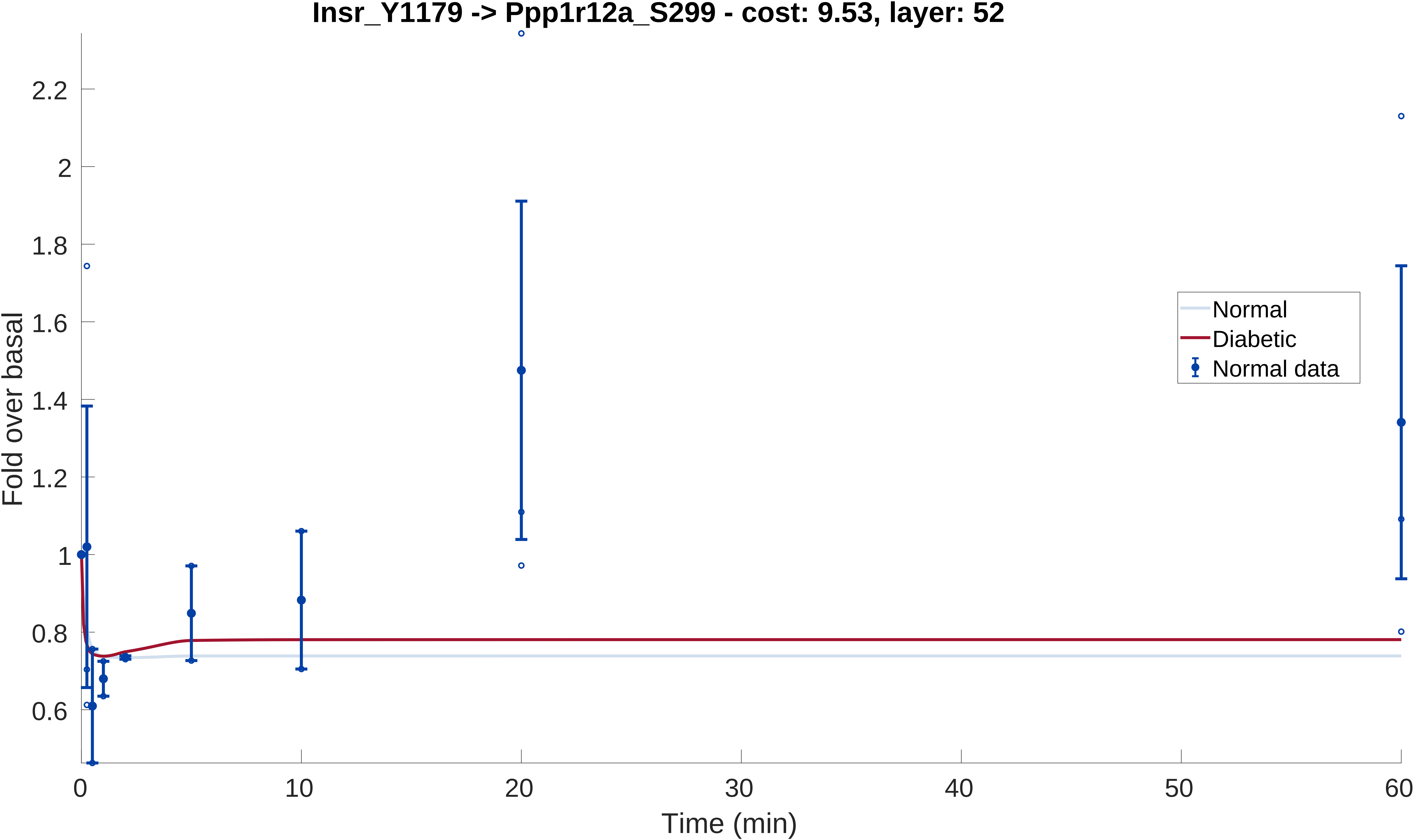

Insr_Y1179 -> Ppp1r12a_S299 - cost: 9.53, layer: 52
Fold over basal
Time (min)
Normal
Diabetic
Normal data
2.2
2
1.8
1.6
1.4
1.2
1
0.8
0.6
0
10
20
30
40
50
60

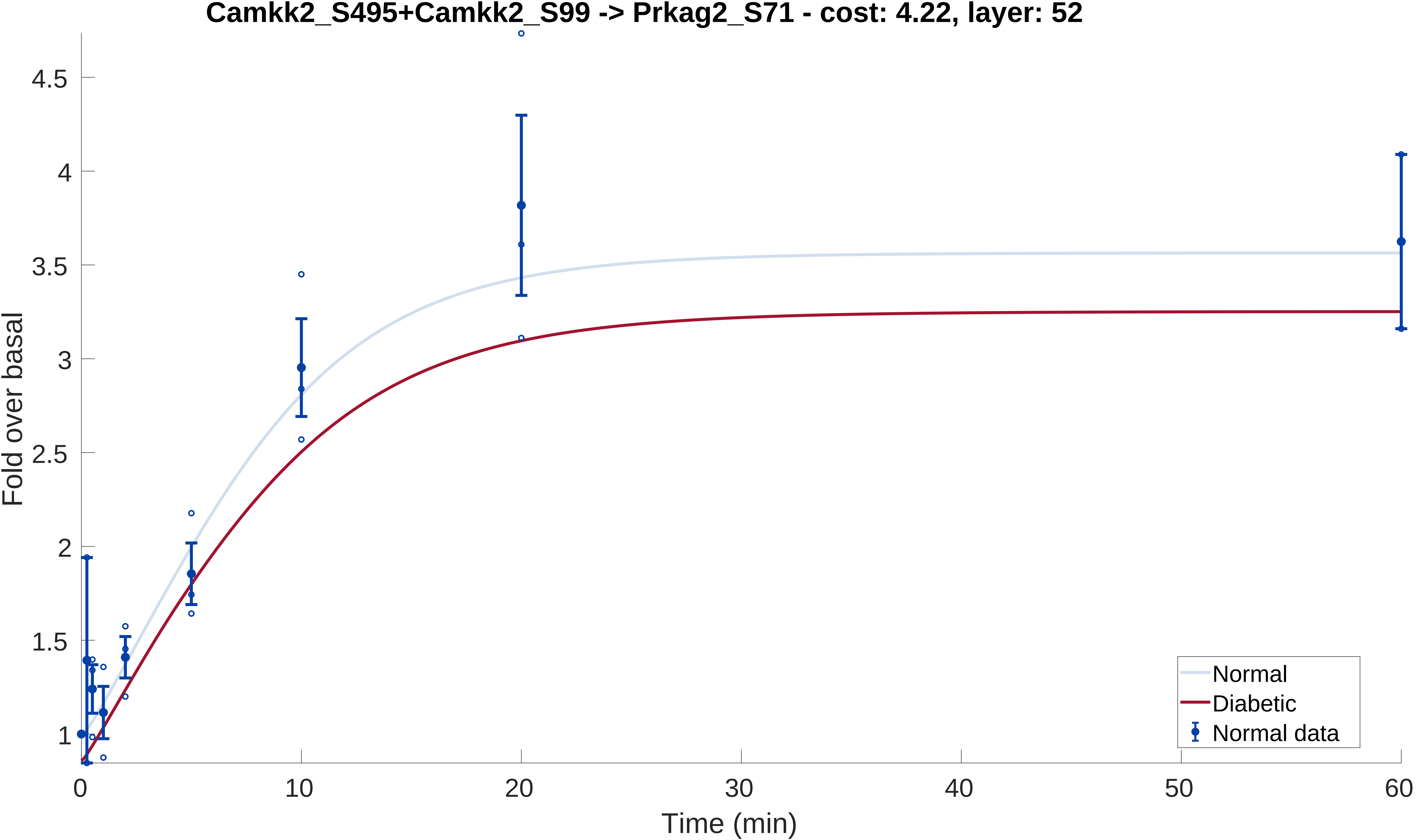

Camkk2_S495+Camkk2_S99 -> Prkag2_S71 - cost: 4.22, layer: 52
Fold over basal
4.5
4
3.5
3
2.5
2
1.5
1
0
10
20
30
40
50
60
Time (min)
Normal
Diabetic
Normal data

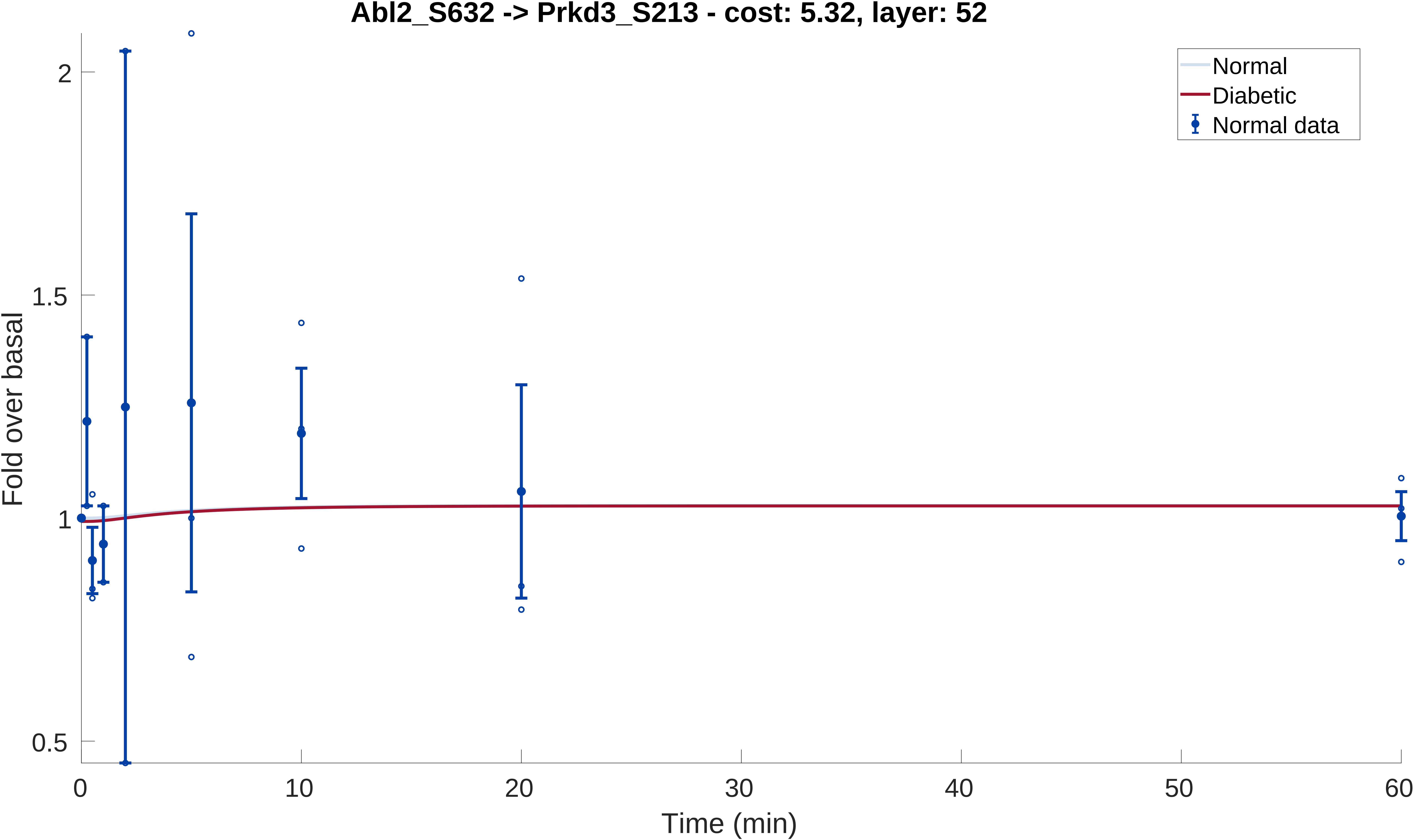

Abl2_S632 -> Prkd3_S213 - cost: 5.32, layer: 52
Normal
Diabetic
Normal data
Fold over basal
Time (min)
0.5
1
1.5
2
0
10
20
30
40
50
60

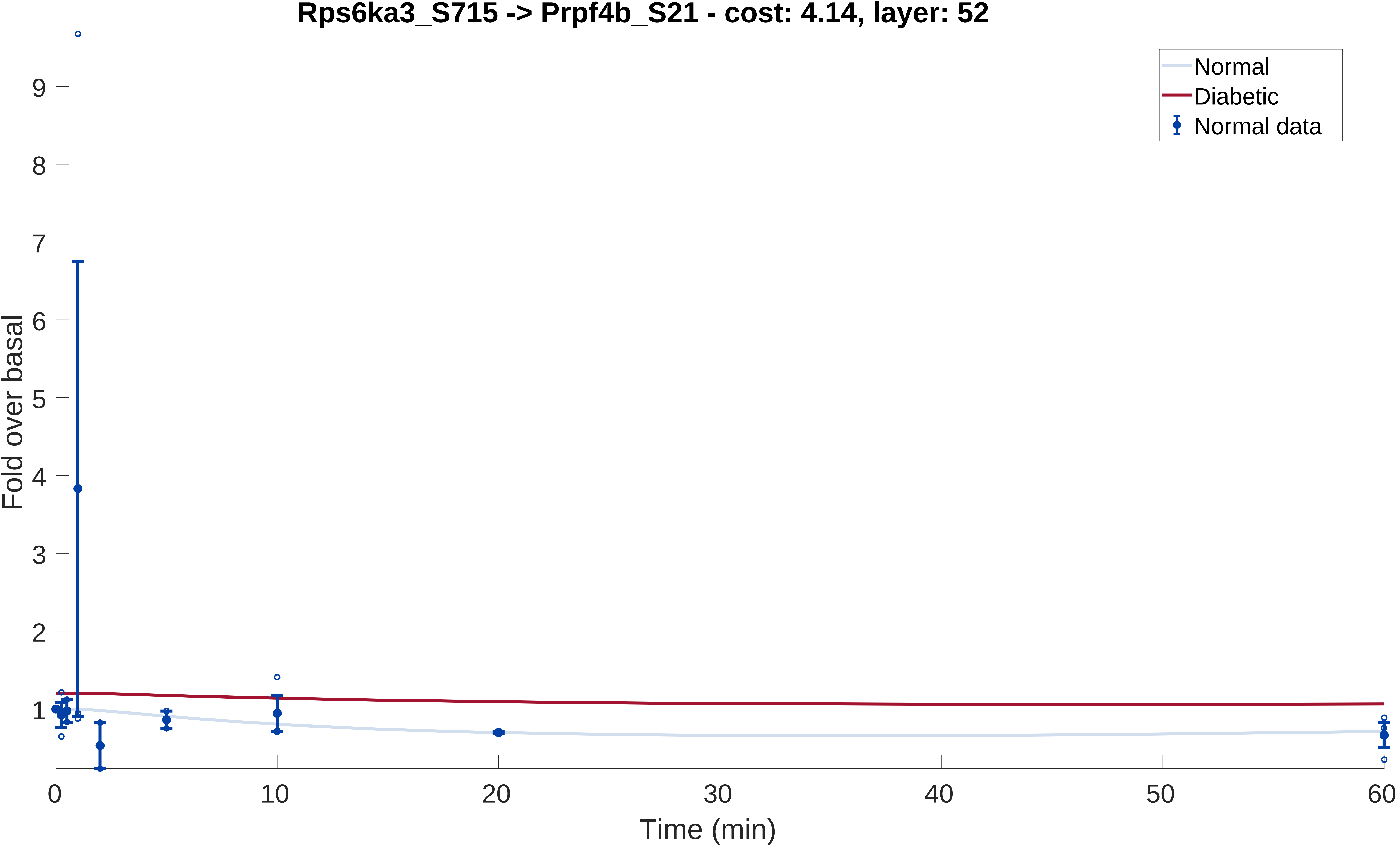

Rps6ka3_S715 -> Prpf4b_S21 - cost: 4.14, layer: 52
Normal
Diabetic
Normal data
Fold over basal
Time (min)
9
8
7
6
5
4
3
2
1
0
10
20
30
40
50
60

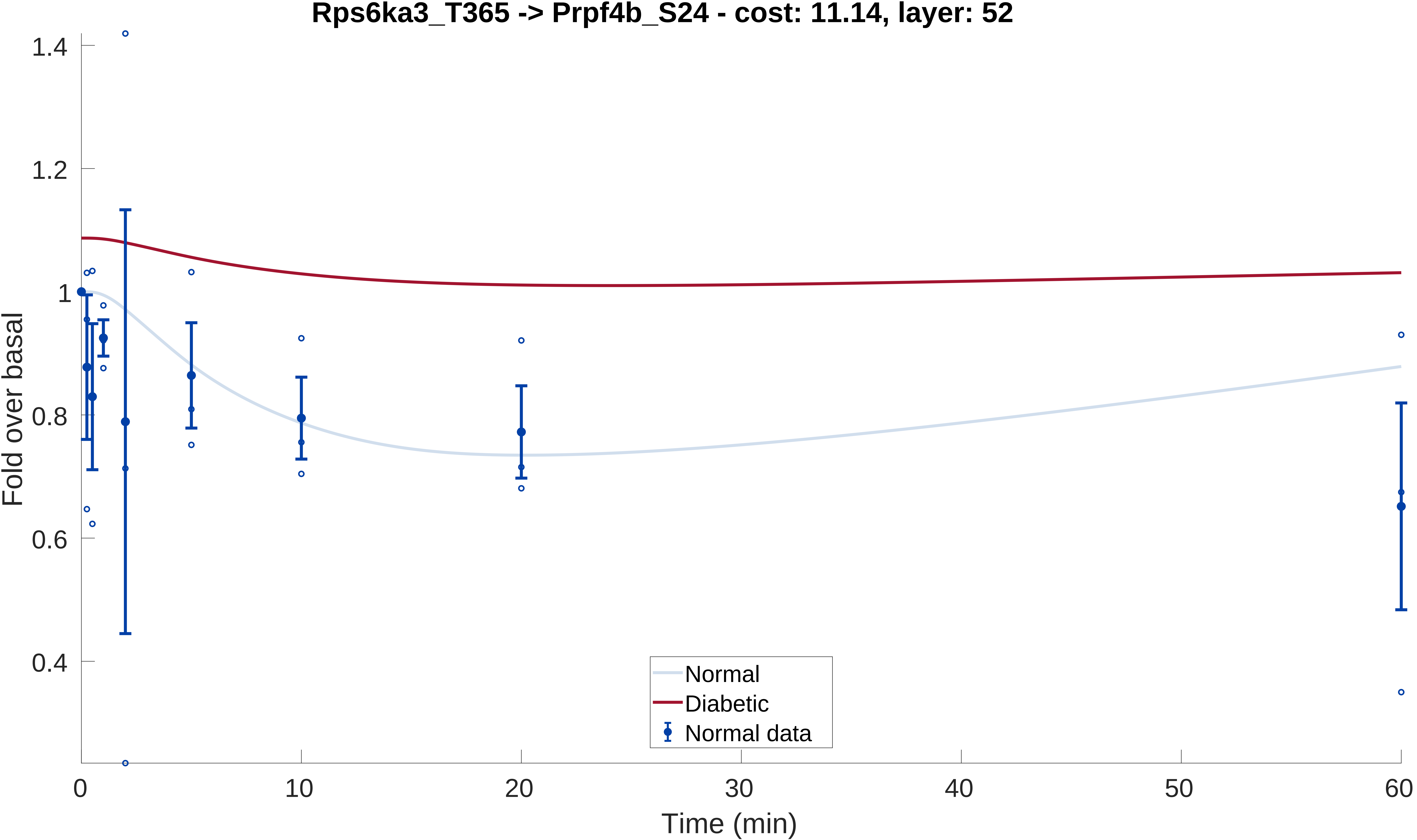
Rps6ka3_T365 -> Prpf4b_S24 - cost: 11.14, layer: 52
Fold over basal
1.4
1.2
1
0.8
0.6
0.4
0
10
20
30
40
50
60
Time (min)
Normal
Diabetic
Normal data

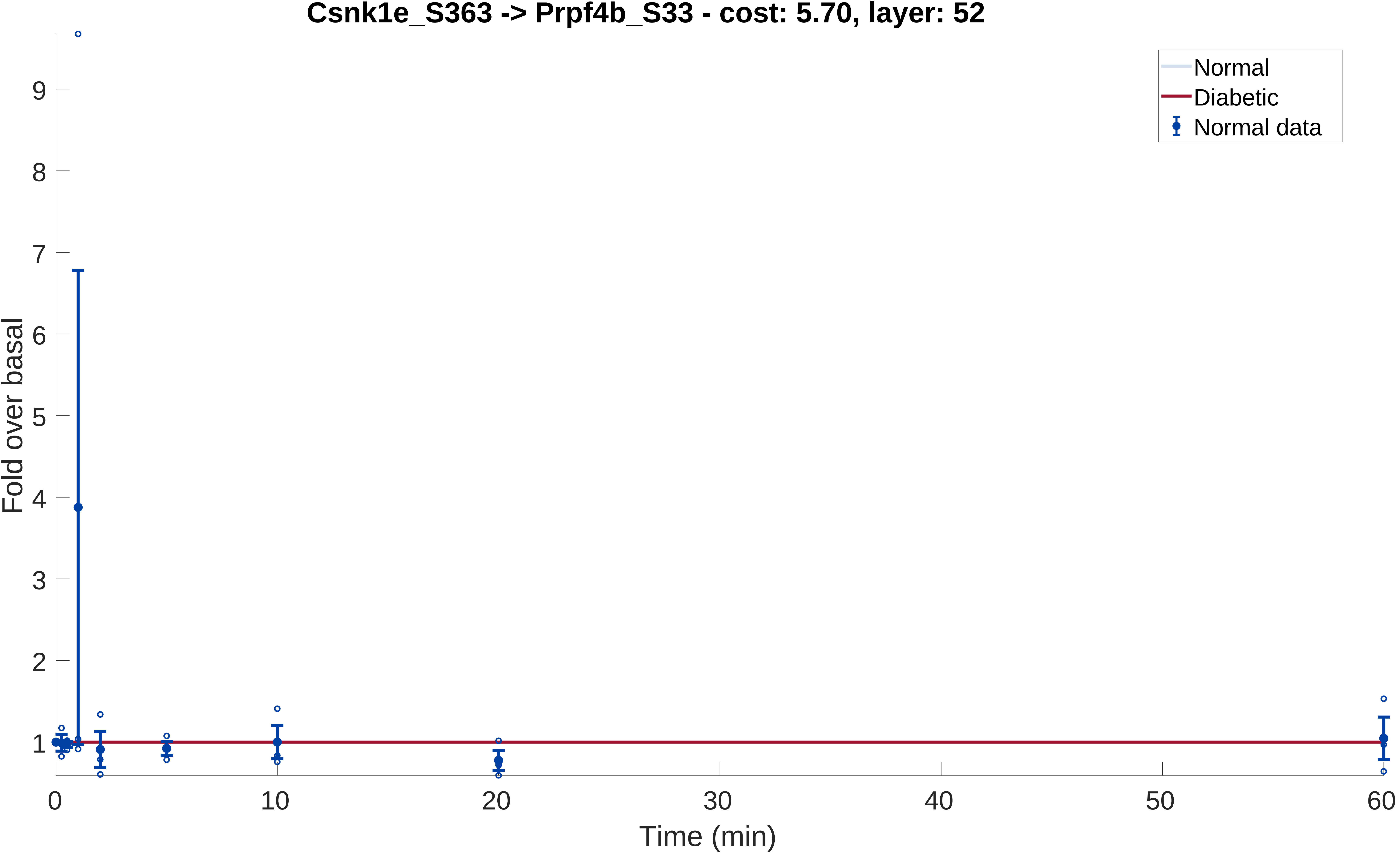

Csnk1e_S363 -> Prpf4b_S33 - cost: 5.70, layer: 52
Normal
Diabetic
Normal data
Fold over basal
Time (min)
0
10
20
30
40
50
60
1
2
3
4
5
6
7
8
9

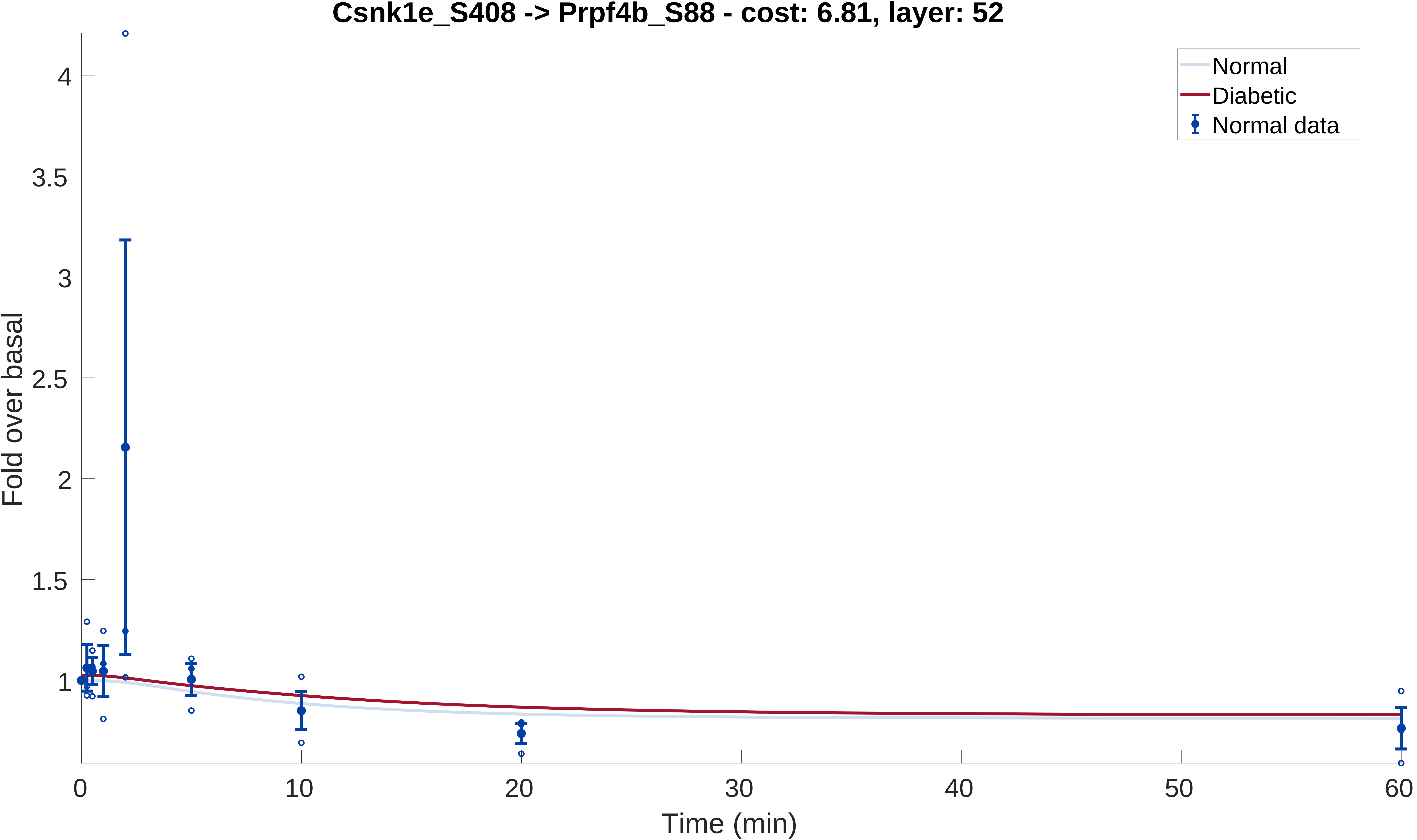

Csnk1e_S408 -> Prpf4b_S88 - cost: 6.81, layer: 52
Normal
Diabetic
Normal data
Fold over basal
4
3.5
3
2.5
2
1.5
1
0
10
20
30
40
50
60
Time (min)

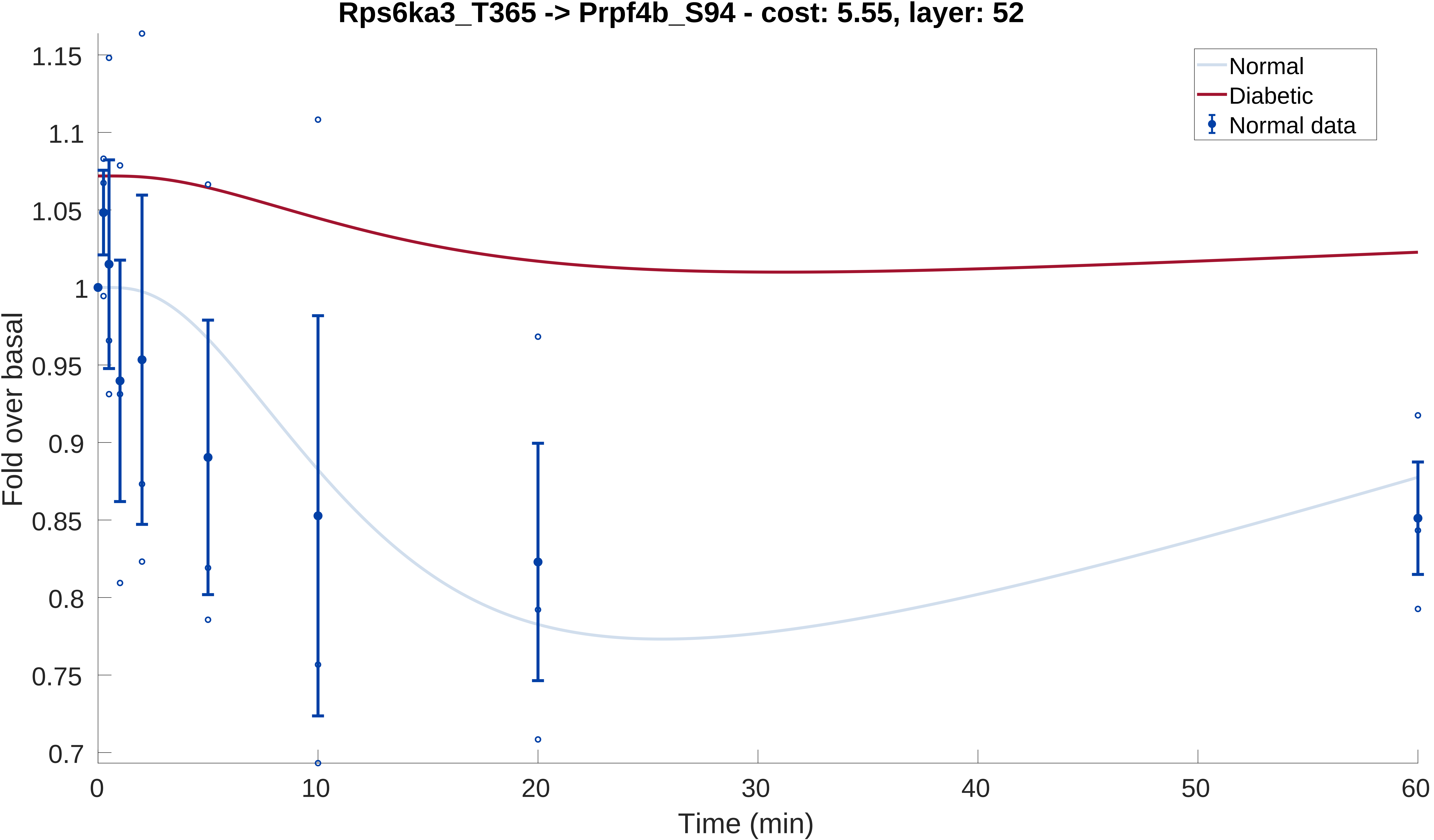

Rps6ka3_T365 -> Prpf4b_S94 - cost: 5.55, layer: 52
Normal
Diabetic
Normal data
Fold over basal
Time (min)
1.15
1.1
1.05
1
0.95
0.9
0.85
0.8
0.75
0.7
0
10
20
30
40
50
60

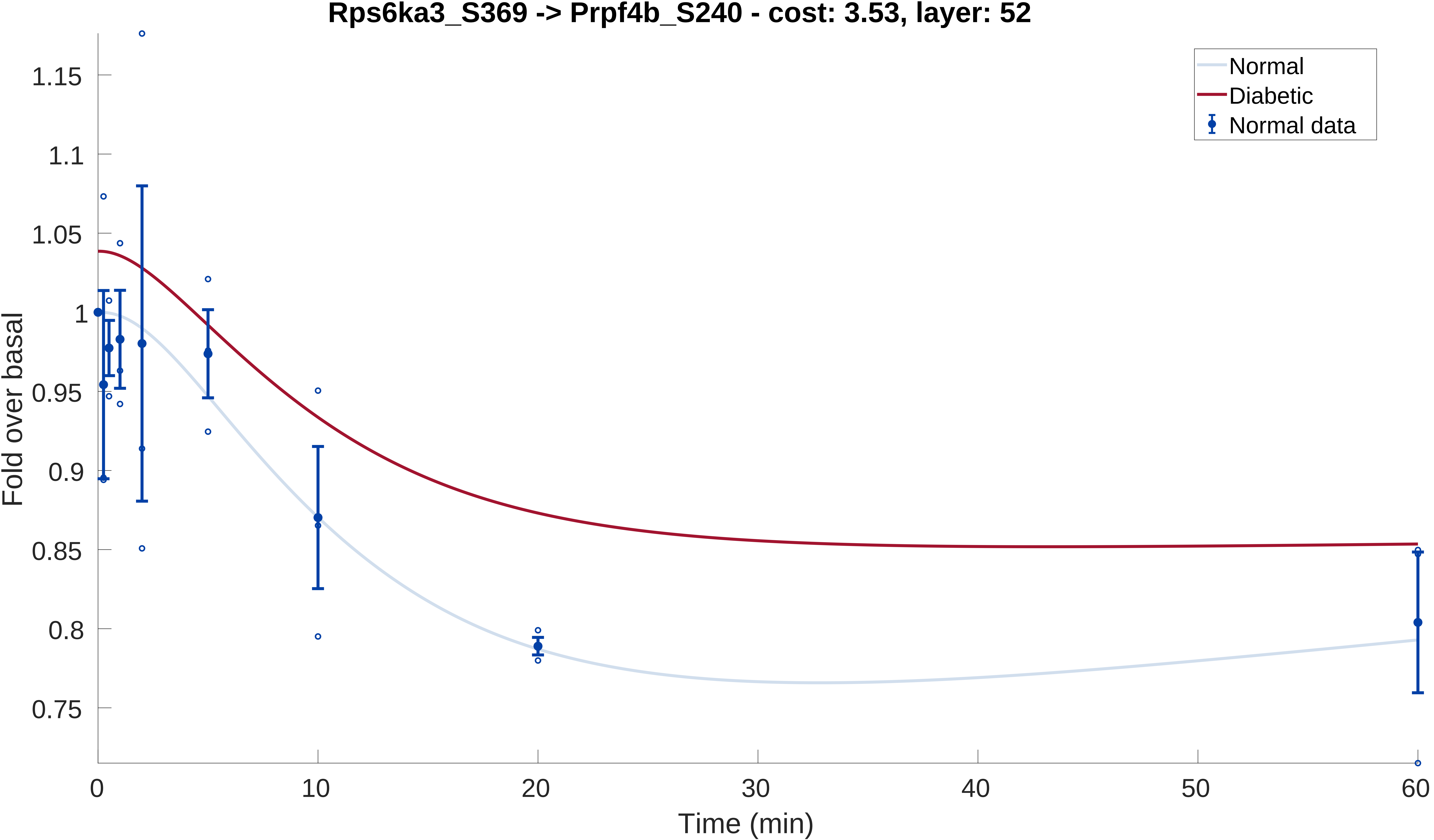

Rps6ka3_S369 -> Prpf4b_S240 - cost: 3.53, layer: 52
Normal
Diabetic
Normal data
Fold over basal
1.15
1.1
1.05
1
0.95
0.9
0.85
0.8
0.75
0
10
20
30
40
50
60
Time (min)

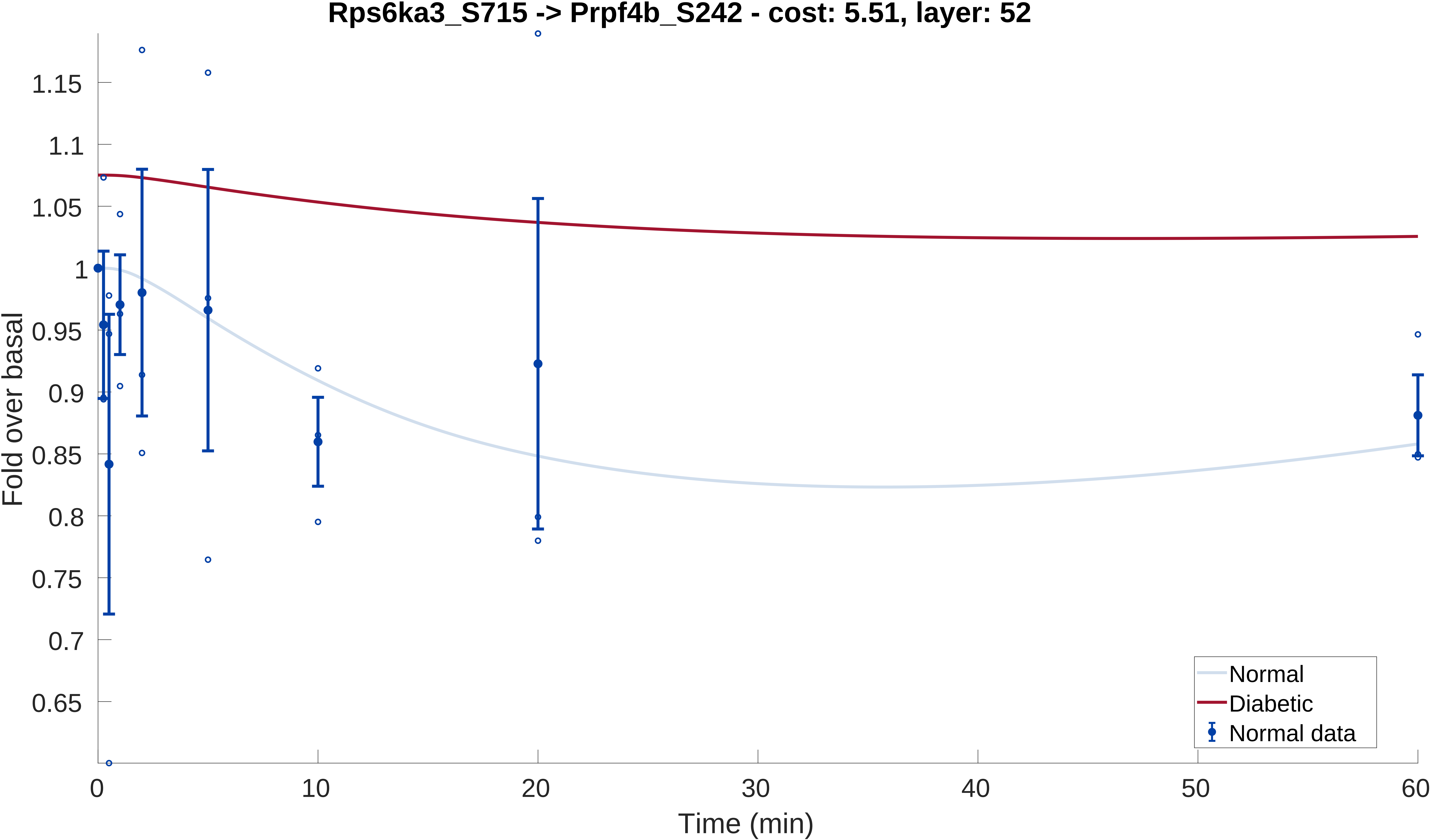

Rps6ka3_S715 -> Prpf4b_S242 - cost: 5.51, layer: 52
1.15
1.1
1.05
1
0.95
0.9
0.85
0.8
0.75
0.7
0.65
Fold over basal
0
10
20
30
40
50
60
Time (min)
Normal
Diabetic
Normal data

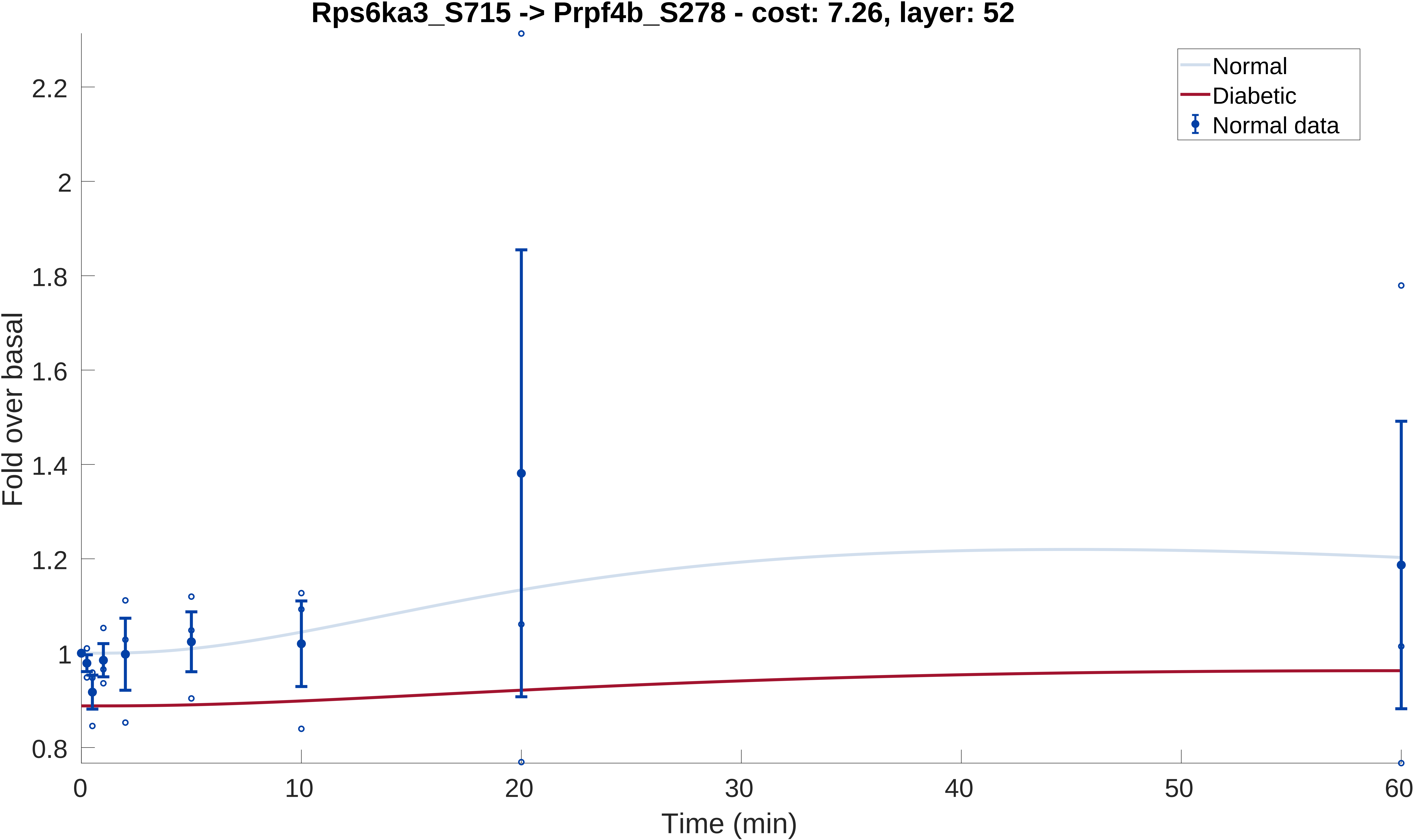

Rps6ka3_S715 -> Prpf4b_S278 - cost: 7.26, layer: 52
Normal
Diabetic
Normal data
Fold over basal
Time (min)
0.8
1
1.2
1.4
1.6
1.8
2
2.2
0
10
20
30
40
50
60

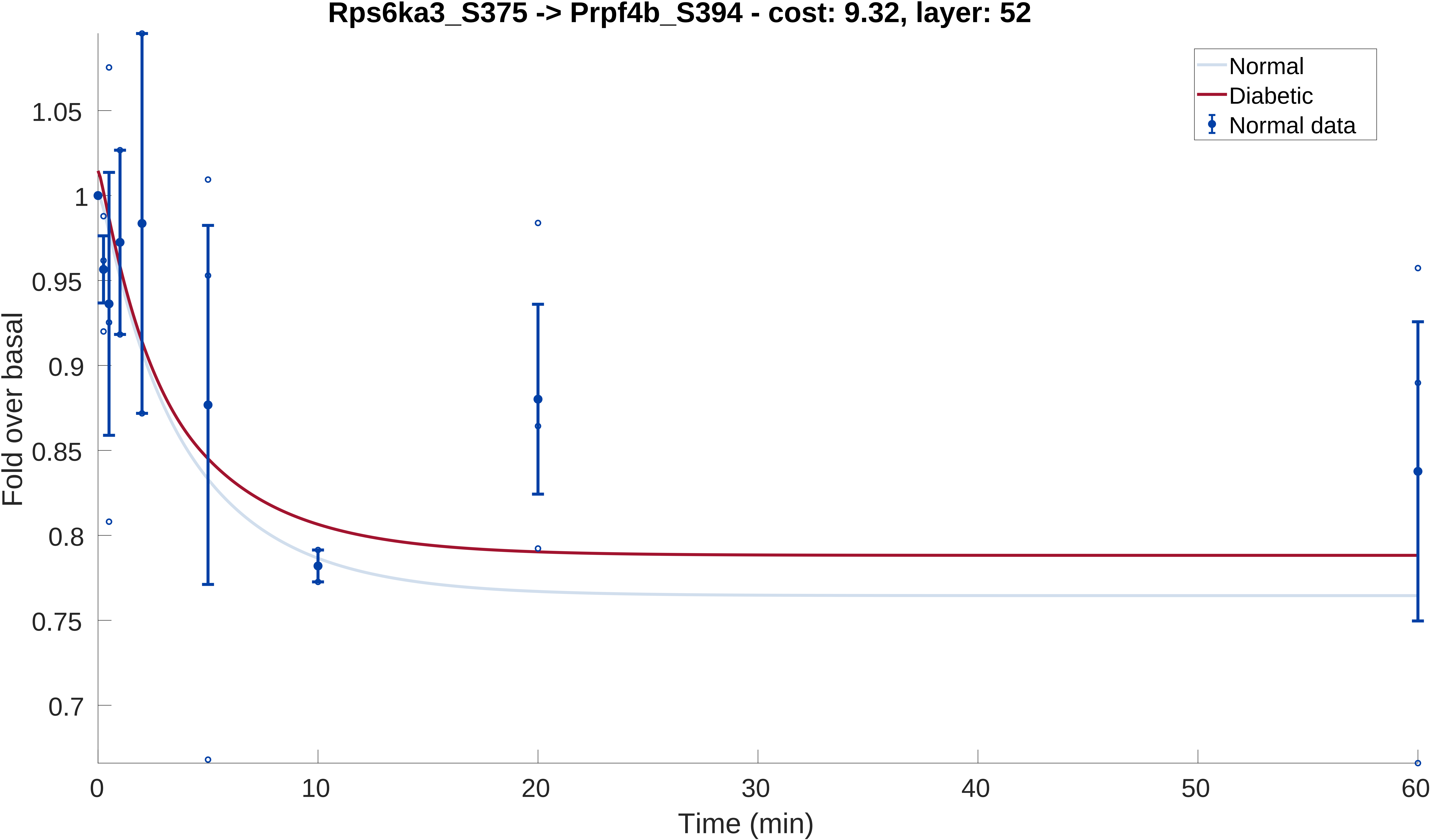

Rps6ka3_S375 -> Prpf4b_S394 - cost: 9.32, layer: 52
Normal
Diabetic
Normal data
Fold over basal
1.05
1
0.95
0.9
0.85
0.8
0.75
0.7
0
10
20
30
40
50
60
Time (min)

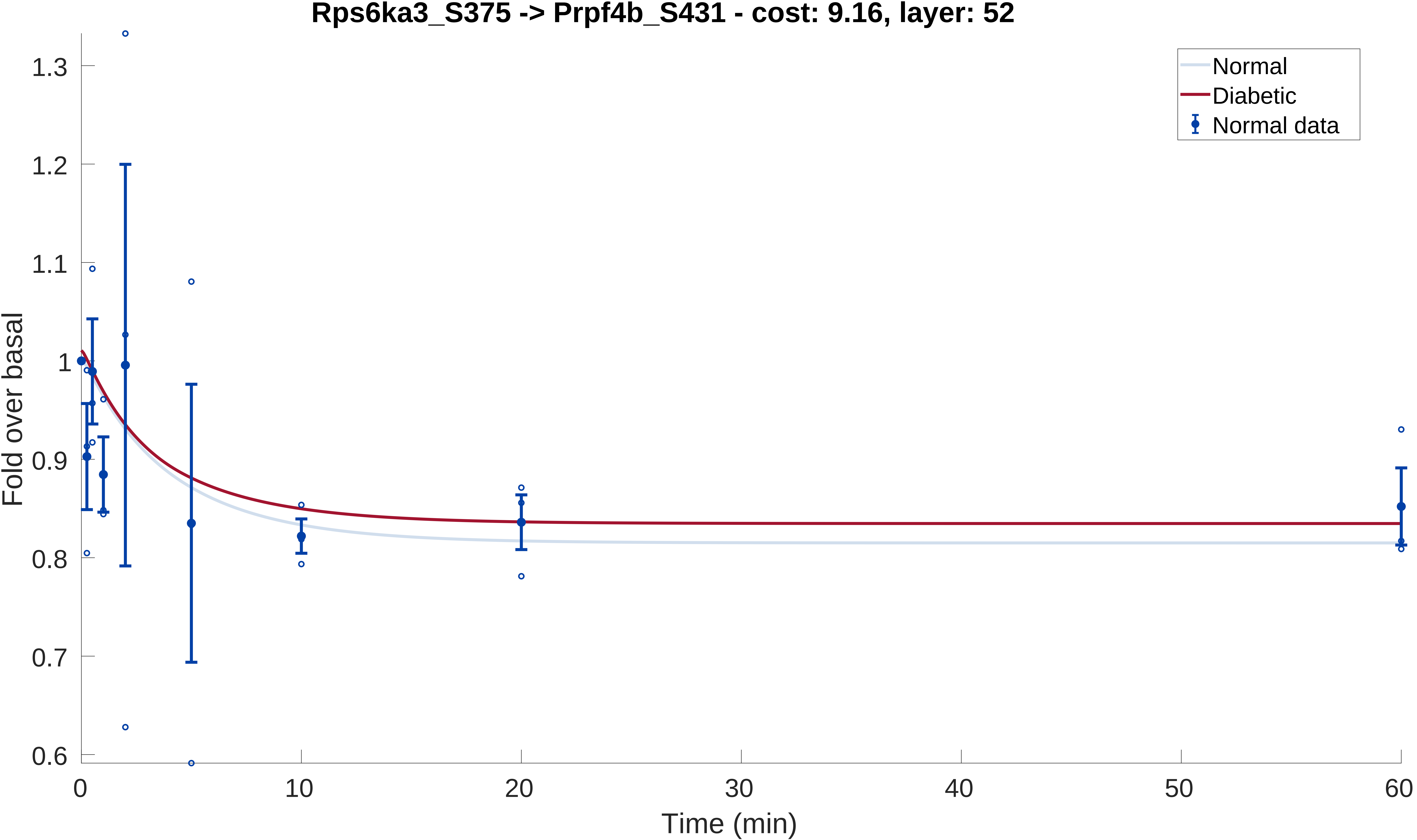

Rps6ka3_S375 -> Prpf4b_S431 - cost: 9.16, layer: 52
Normal
Diabetic
Normal data
Fold over basal
Time (min)
1.3
1.2
1.1
1
0.9
0.8
0.7
0.6
0
10
20
30
40
50
60

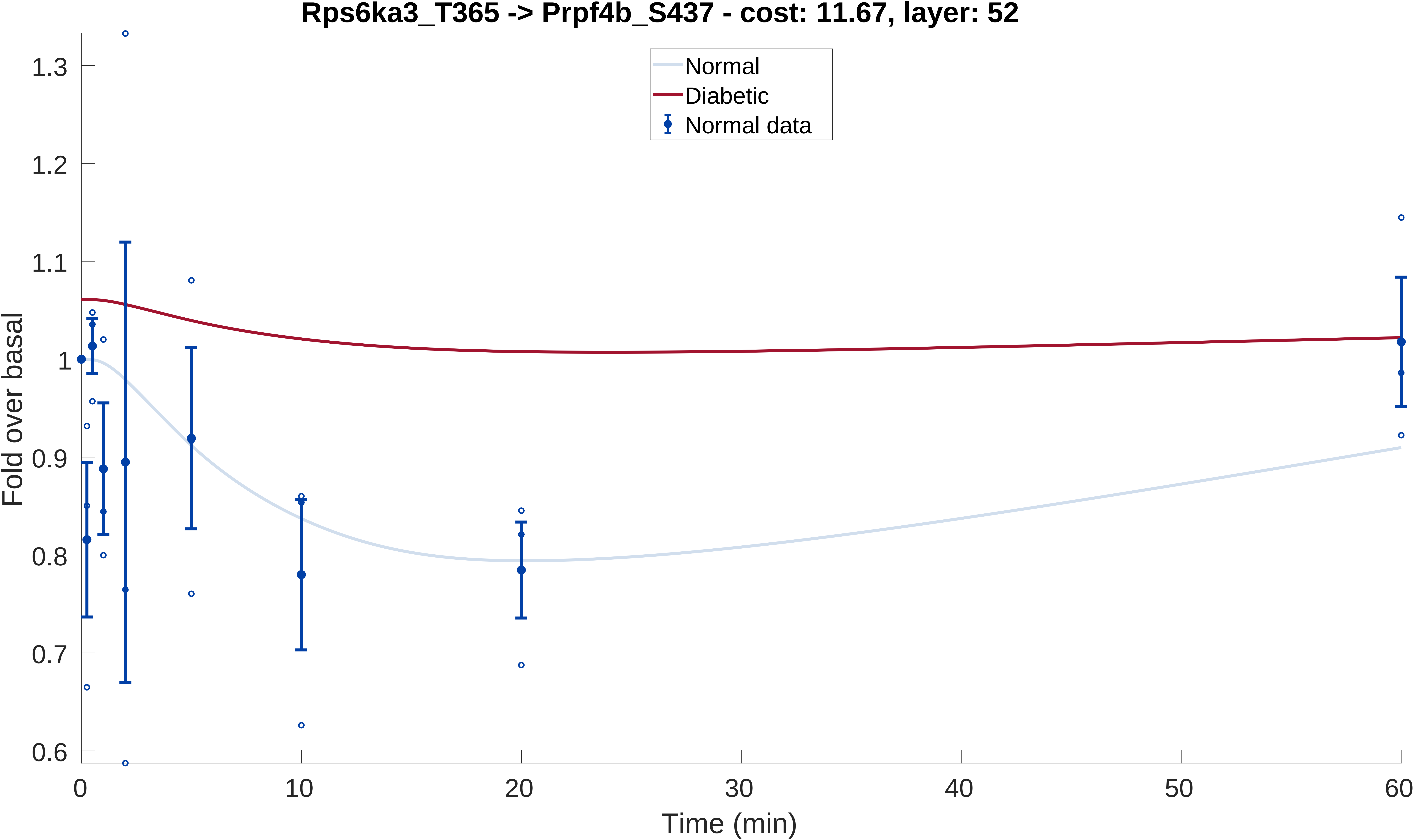

Rps6ka3_T365 -> Prpf4b_S437 - cost: 11.67, layer: 52
Normal
Diabetic
Normal data
Fold over basal
Time (min)
0.6
0.7
0.8
0.9
1
1.1
1.2
1.3
0
10
20
30
40
50
60

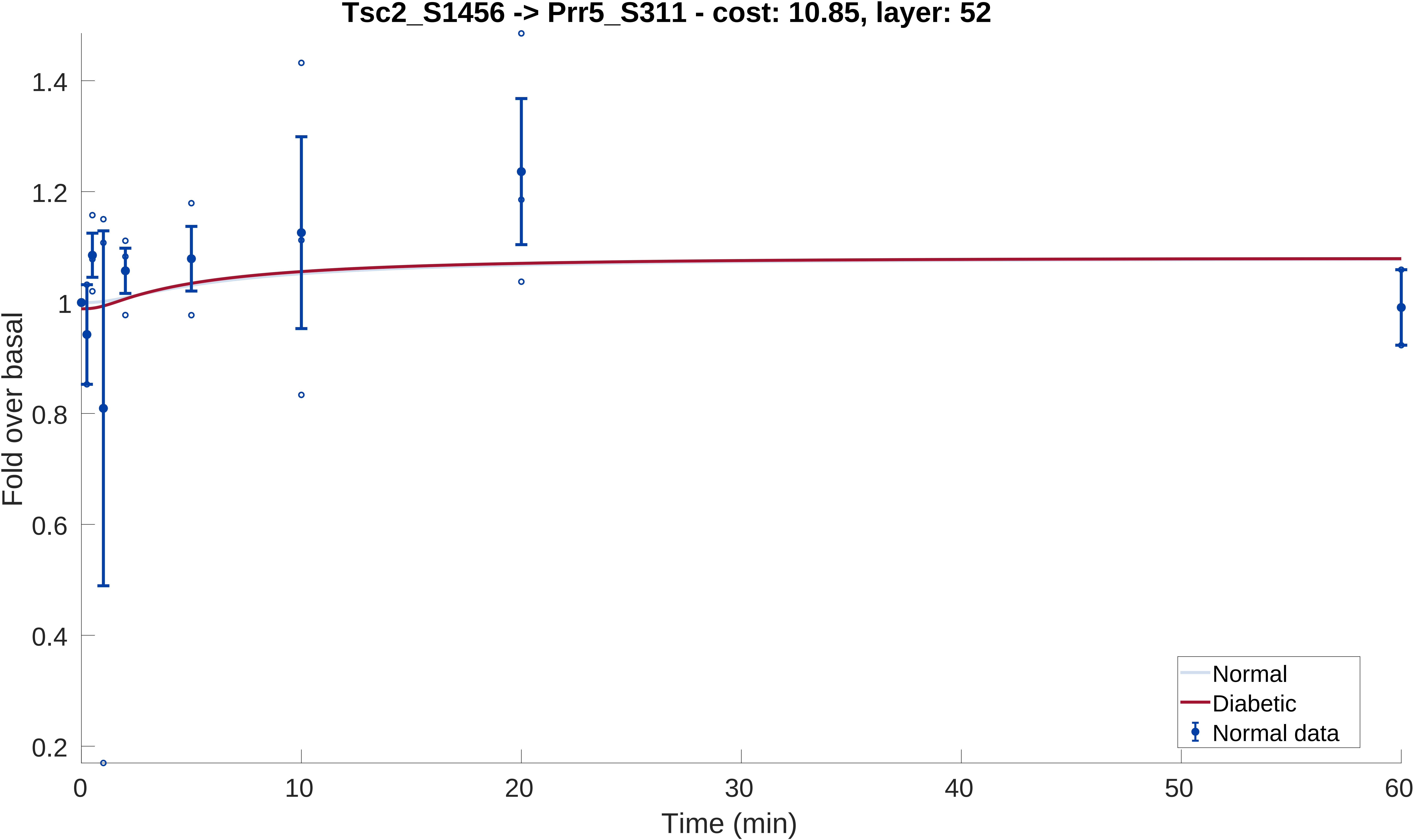

Tsc2_S1456 -> Prr5_S311 - cost: 10.85, layer: 52
Fold over basal
Time (min)
1.4
1.2
1
0.8
0.6
0.4
0.2
0
10
20
30
40
50
60
Normal
Diabetic
Normal data

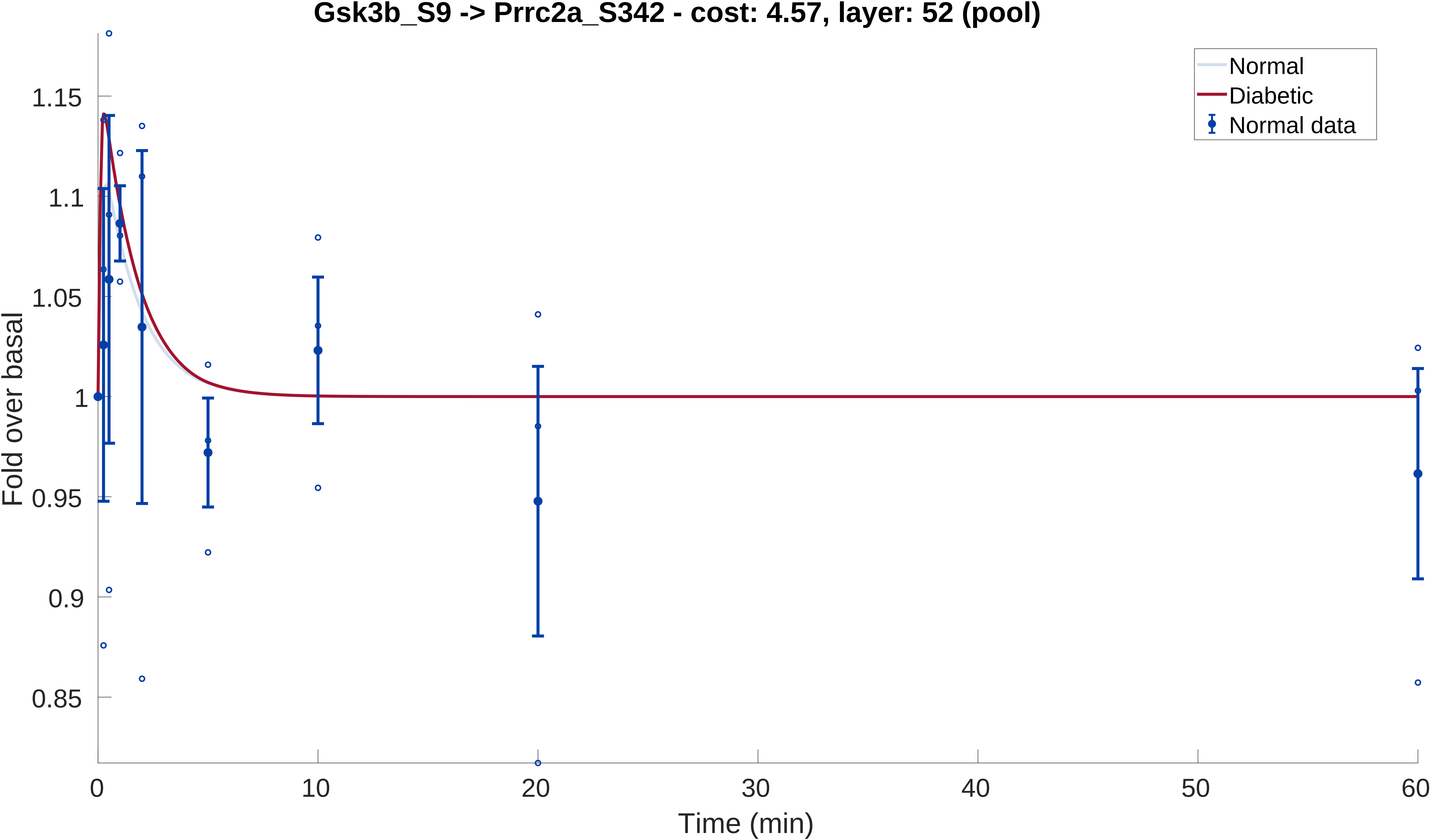

Gsk3b_S9 -> Prrc2a_S342 - cost: 4.57, layer: 52 (pool)
Fold over basal
Time (min)
Normal
Diabetic
Normal data
1.15
1.1
1.05
1
0.95
0.9
0.85
0
10
20
30
40
50
60

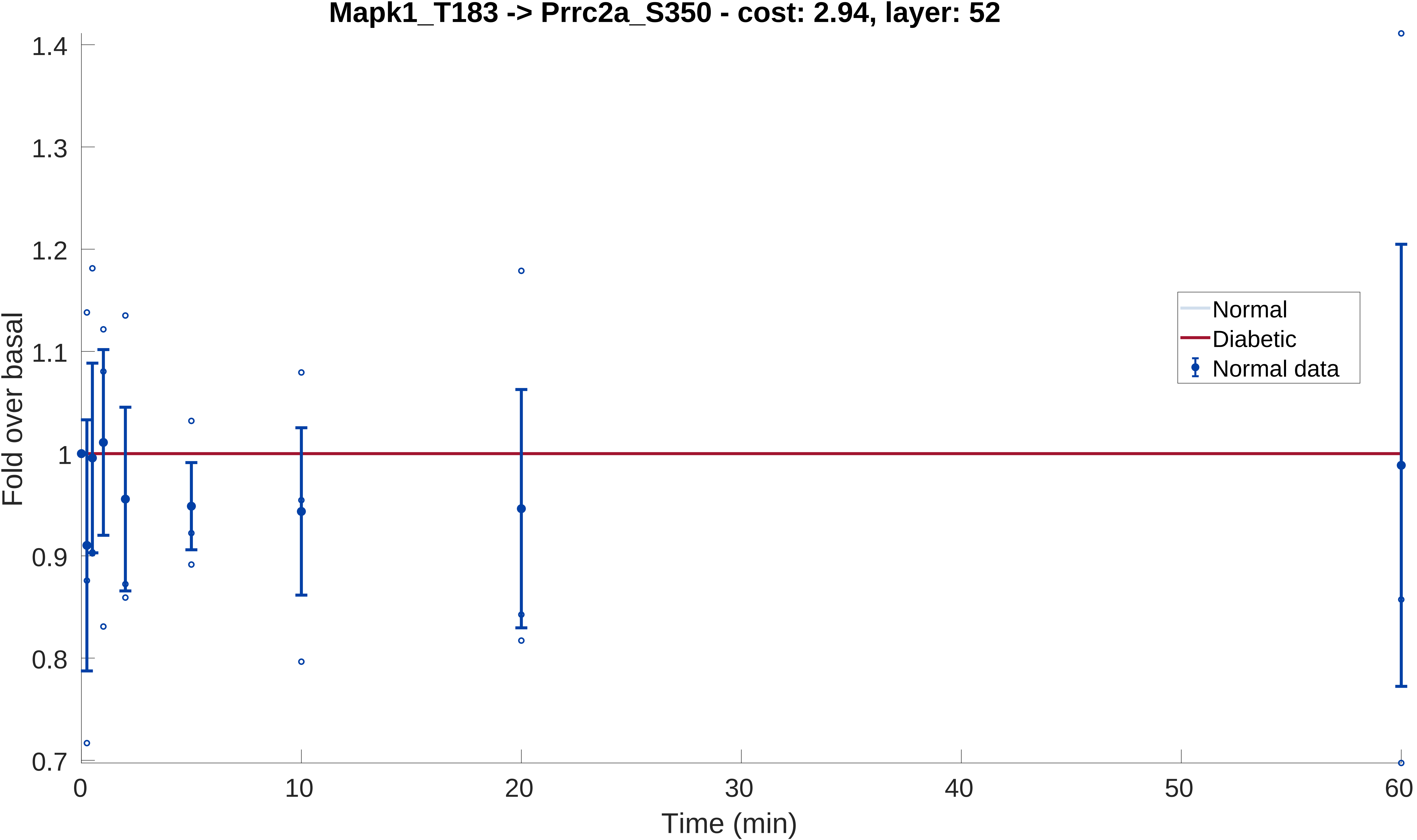

Mapk1_T183 -> Prrc2a_S350 - cost: 2.94, layer: 52
Fold over basal
Time (min)
1.4
1.3
1.2
1.1
1
0.9
0.8
0.7
0
10
20
30
40
50
60
Normal
Diabetic
Normal data

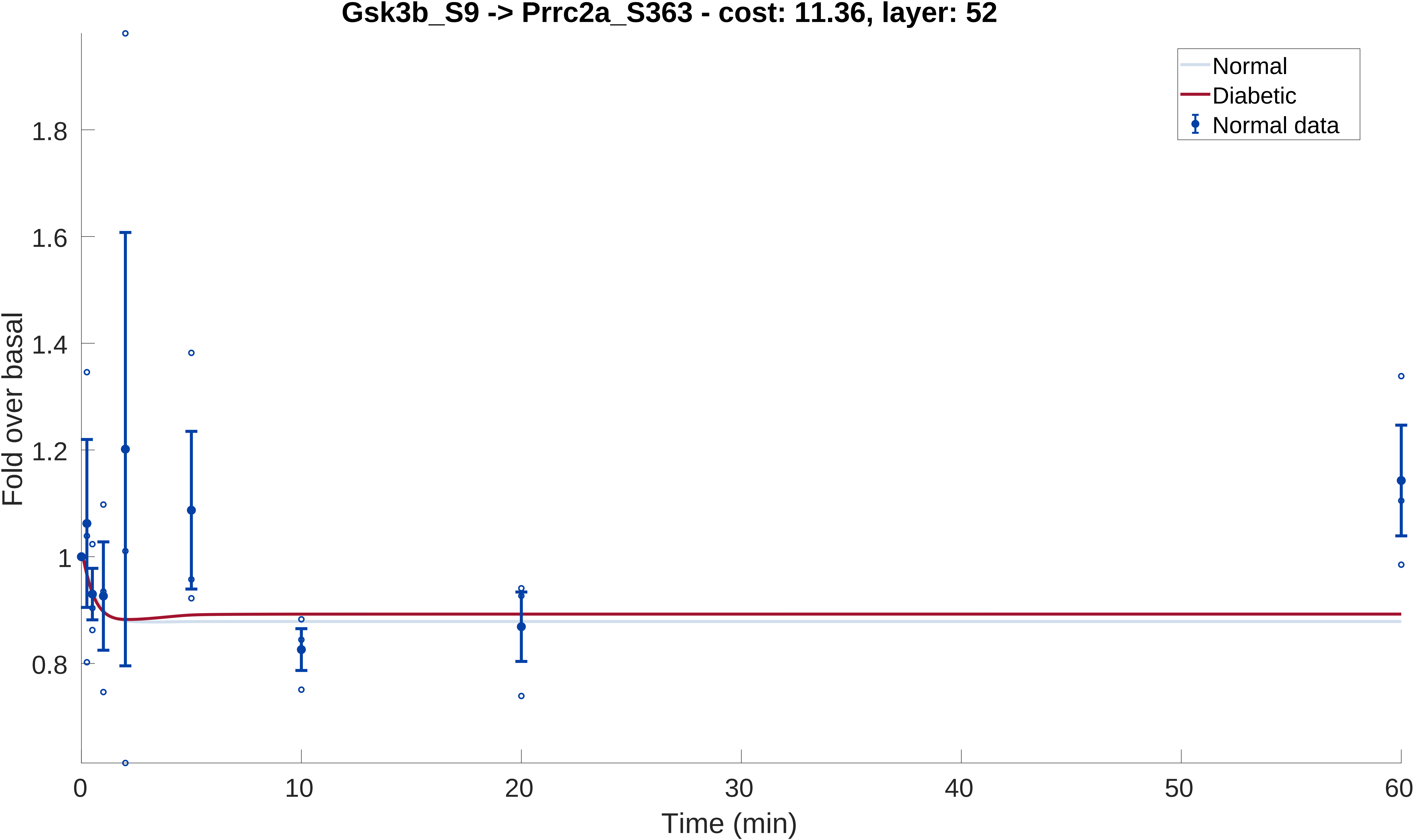

Gsk3b_S9 -> Prrc2a_S363 - cost: 11.36, layer: 52
Normal
Diabetic
Normal data
Fold over basal
1.8
1.6
1.4
1.2
1
0.8
0
10
20
30
40
50
60
Time (min)

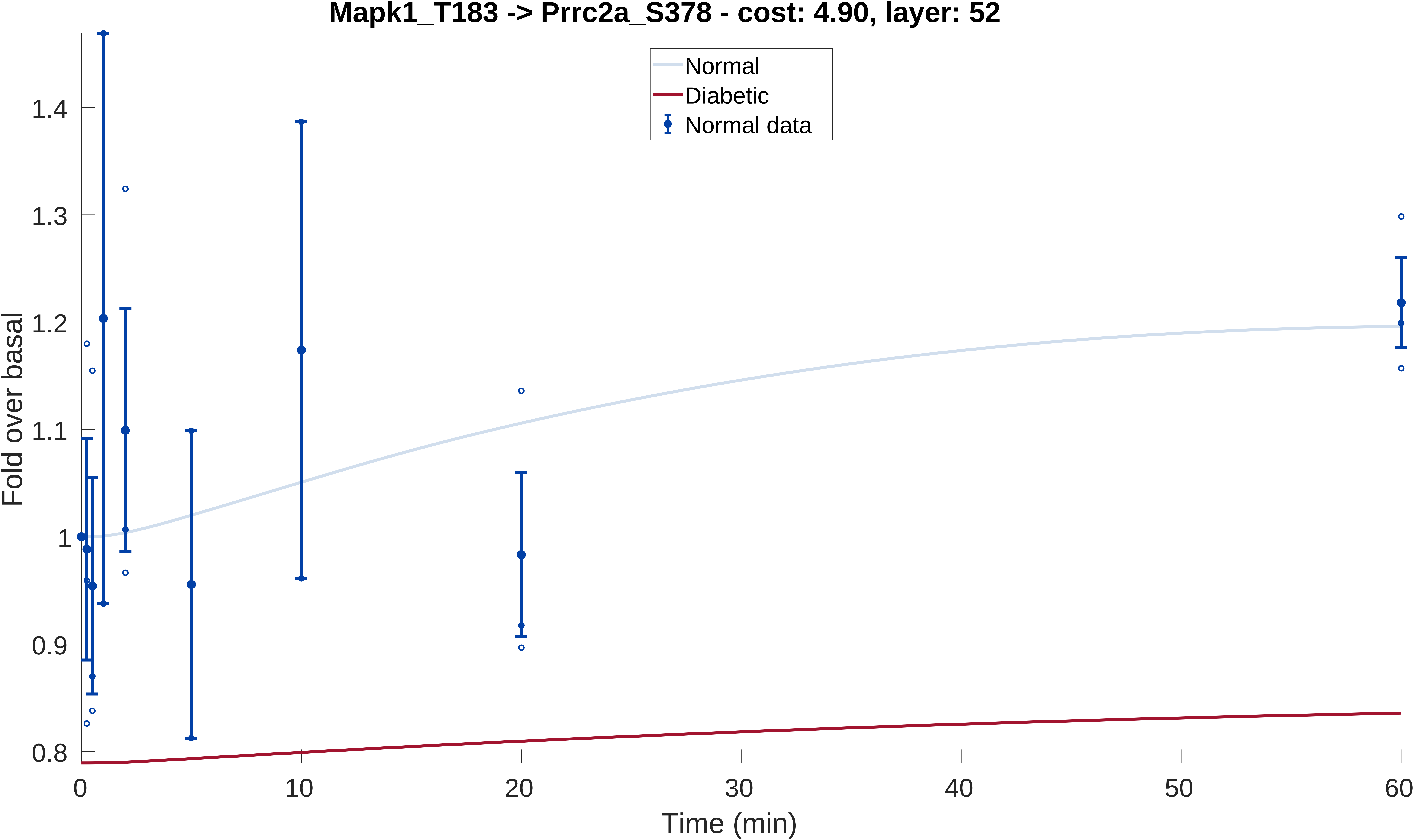

Mapk1_T183 -> Prrc2a_S378 - cost: 4.90, layer: 52
Normal
Diabetic
Normal data
Fold over basal
Time (min)
0.8
0.9
1
1.1
1.2
1.3
1.4
0
10
20
30
40
50
60

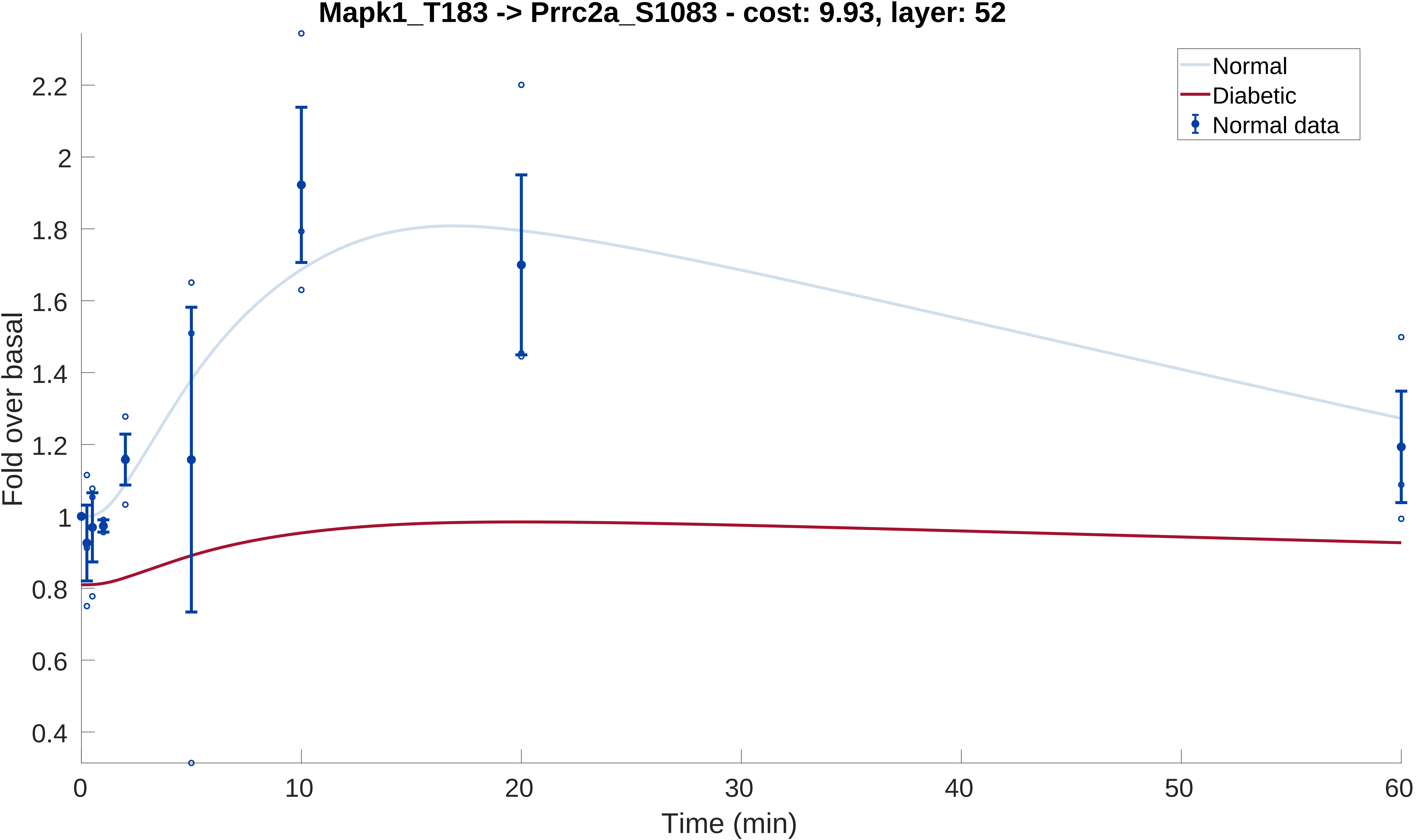
Mapk1_T183 -> Prrc2a_S1083 - cost: 9.93, layer: 52
Fold over basal
Time (min)
Normal
Diabetic
Normal data
0.4
0.6
0.8
1
1.2
1.4
1.6
1.8
2
2.2
0
10
20
30
40
50
60

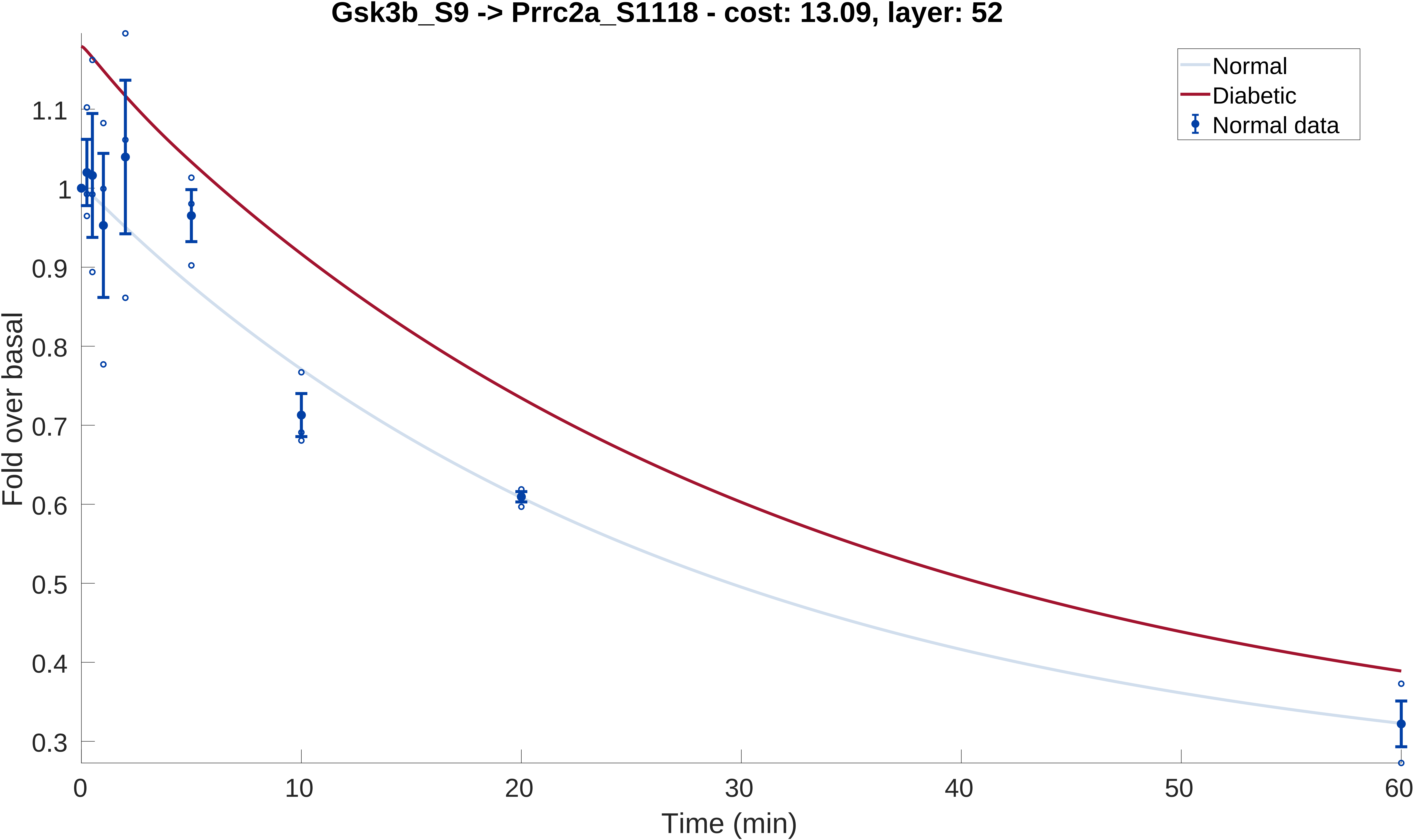

Gsk3b_S9 -> Prrc2a_S1118 - cost: 13.09, layer: 52
Normal
Diabetic
Normal data
Fold over basal
Time (min)
1.1
1
0.9
0.8
0.7
0.6
0.5
0.4
0.3
0
10
20
30
40
50
60

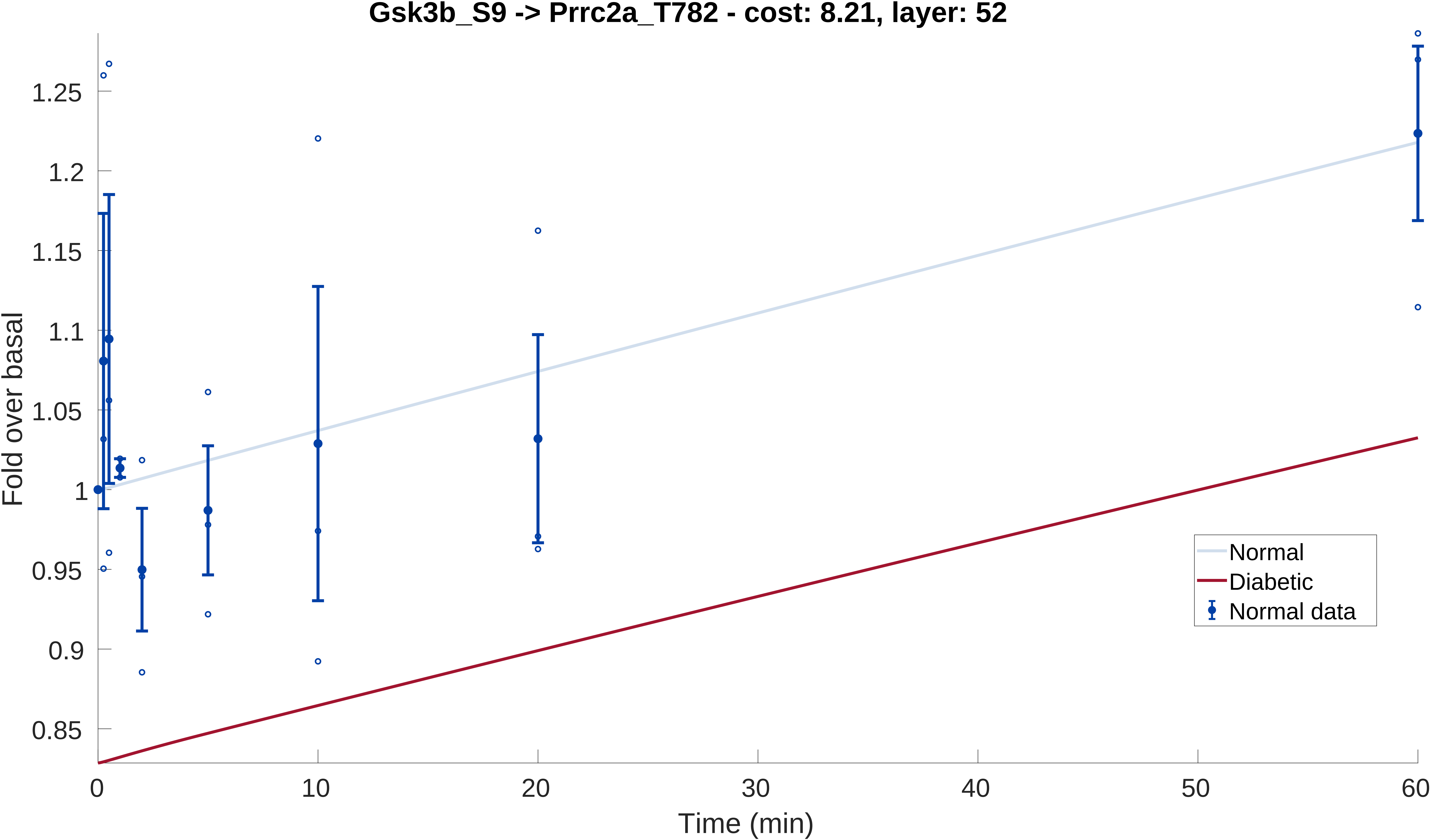

Gsk3b_S9 -> Prrc2a_T782 - cost: 8.21, layer: 52
Fold over basal
1.25
1.2
1.15
1.1
1.05
1
0.95
0.9
0.85
0
10
20
30
40
50
60
Time (min)
Normal
Diabetic
Normal data

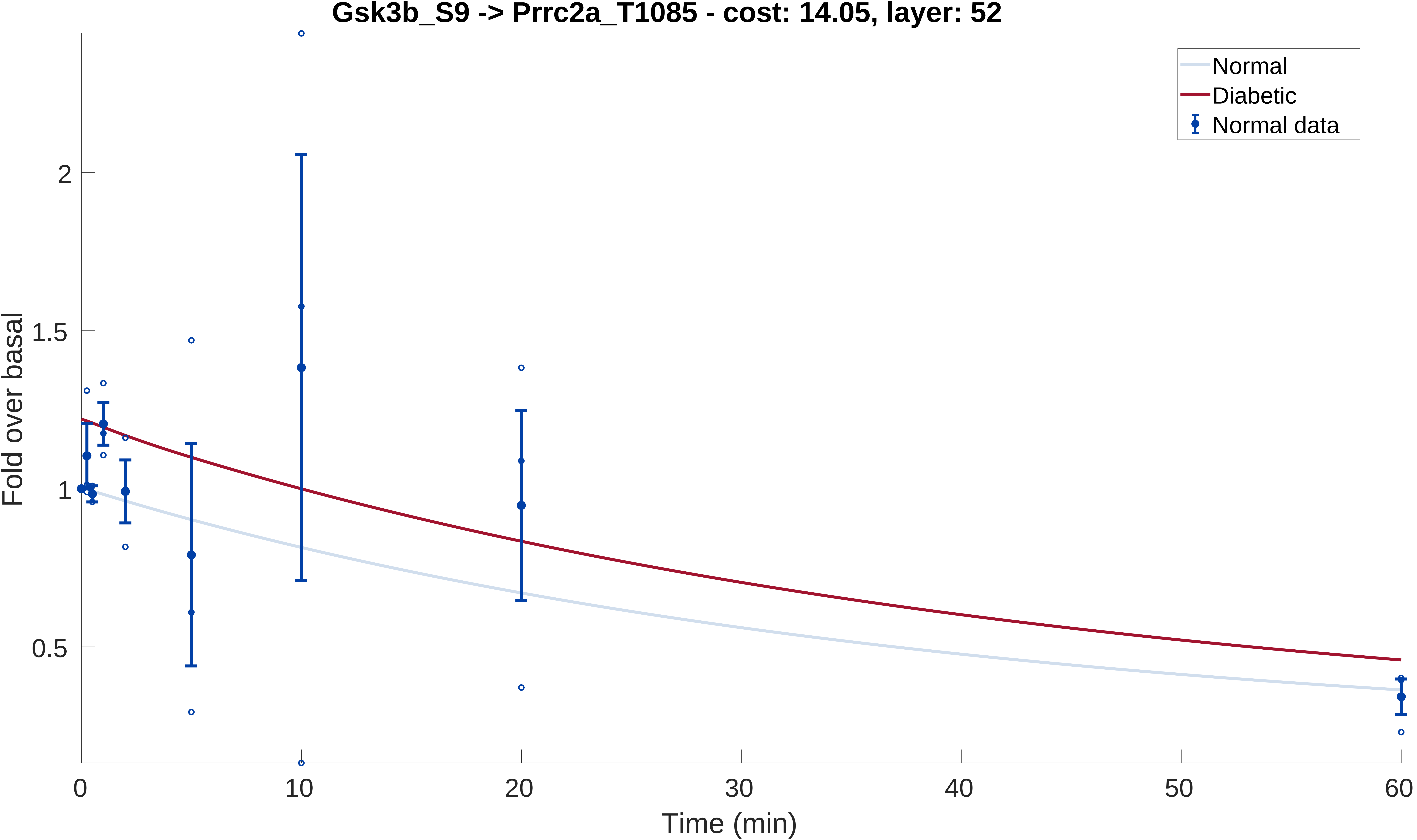

Gsk3b_S9 -> Prrc2a_T1085 - cost: 14.05, layer: 52
Normal
Diabetic
Normal data
Fold over basal
2
1.5
1
0.5
0
10
20
30
40
50
60
Time (min)

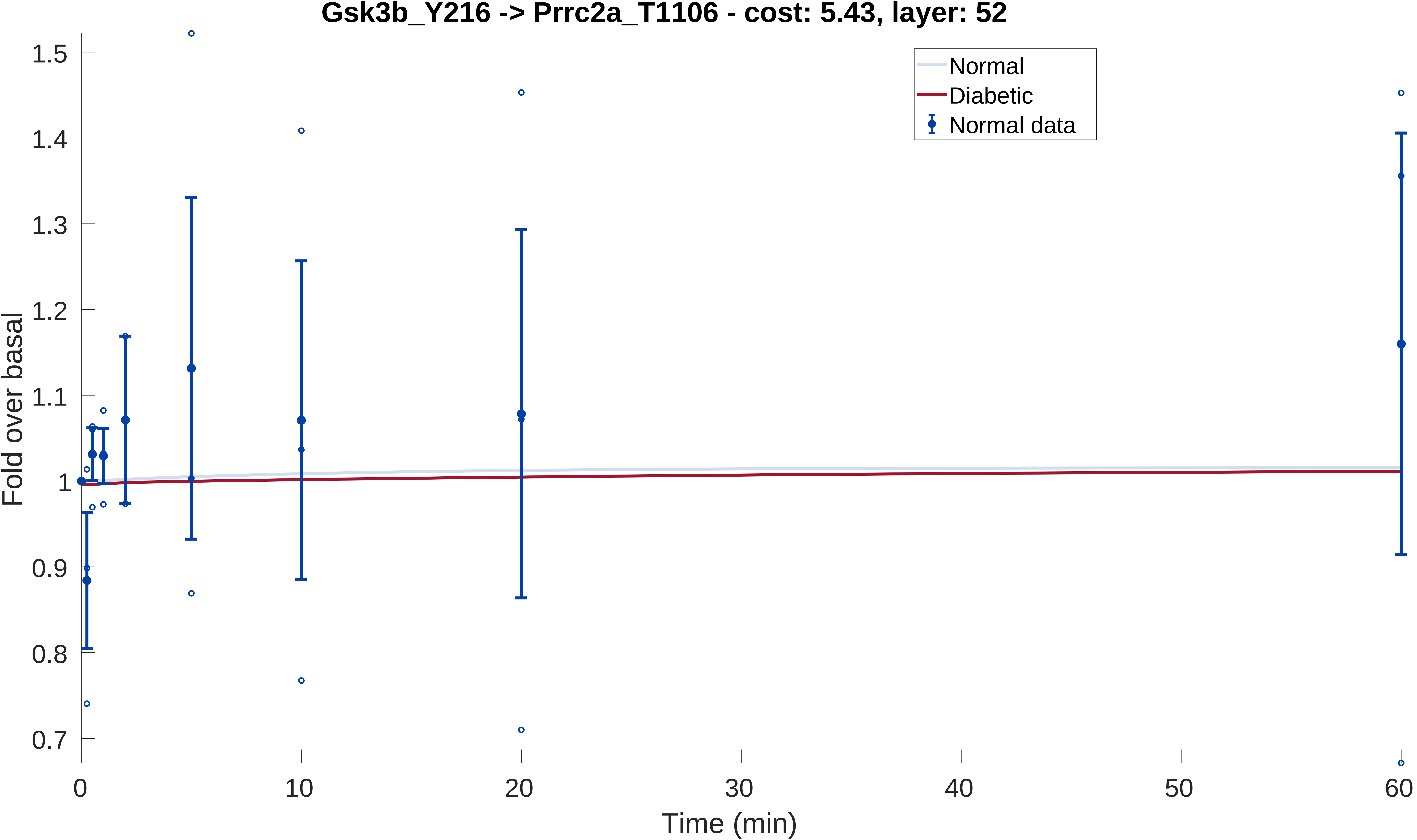
Gsk3b_Y216 -> Prrc2a_T1106 - cost: 5.43, layer: 52
Normal
Diabetic
Normal data
Fold over basal
Time (min)
0.7
0.8
0.9
1
1.1
1.2
1.3
1.4
1.5
0
10
20
30
40
50
60

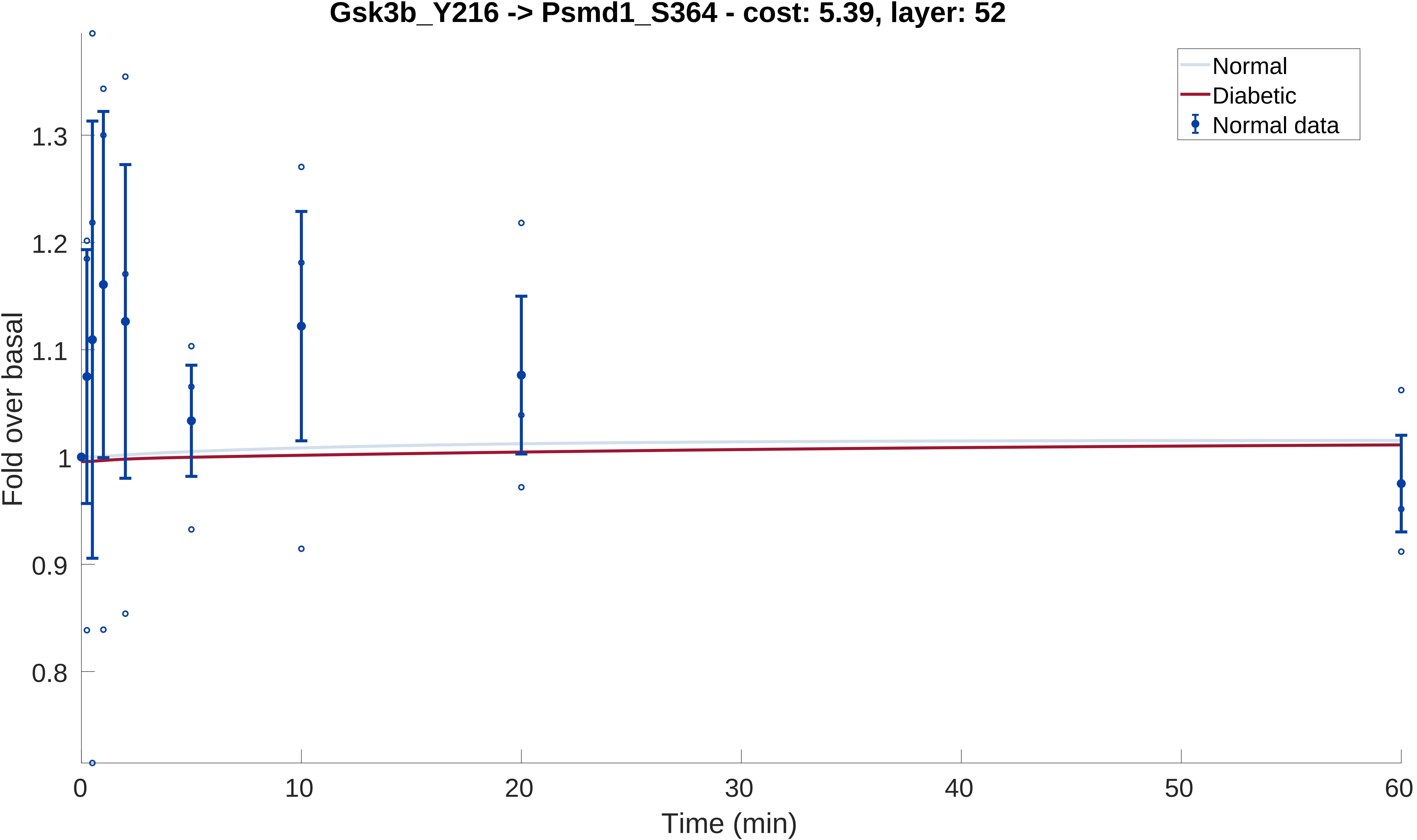
Gsk3b_Y216 -> Psmd1_S364 - cost: 5.39, layer: 52
Normal
Diabetic
Normal data
Fold over basal
1.3
1.2
1.1
1
0.9
0.8
0
10
20
30
40
50
60
Time (min)

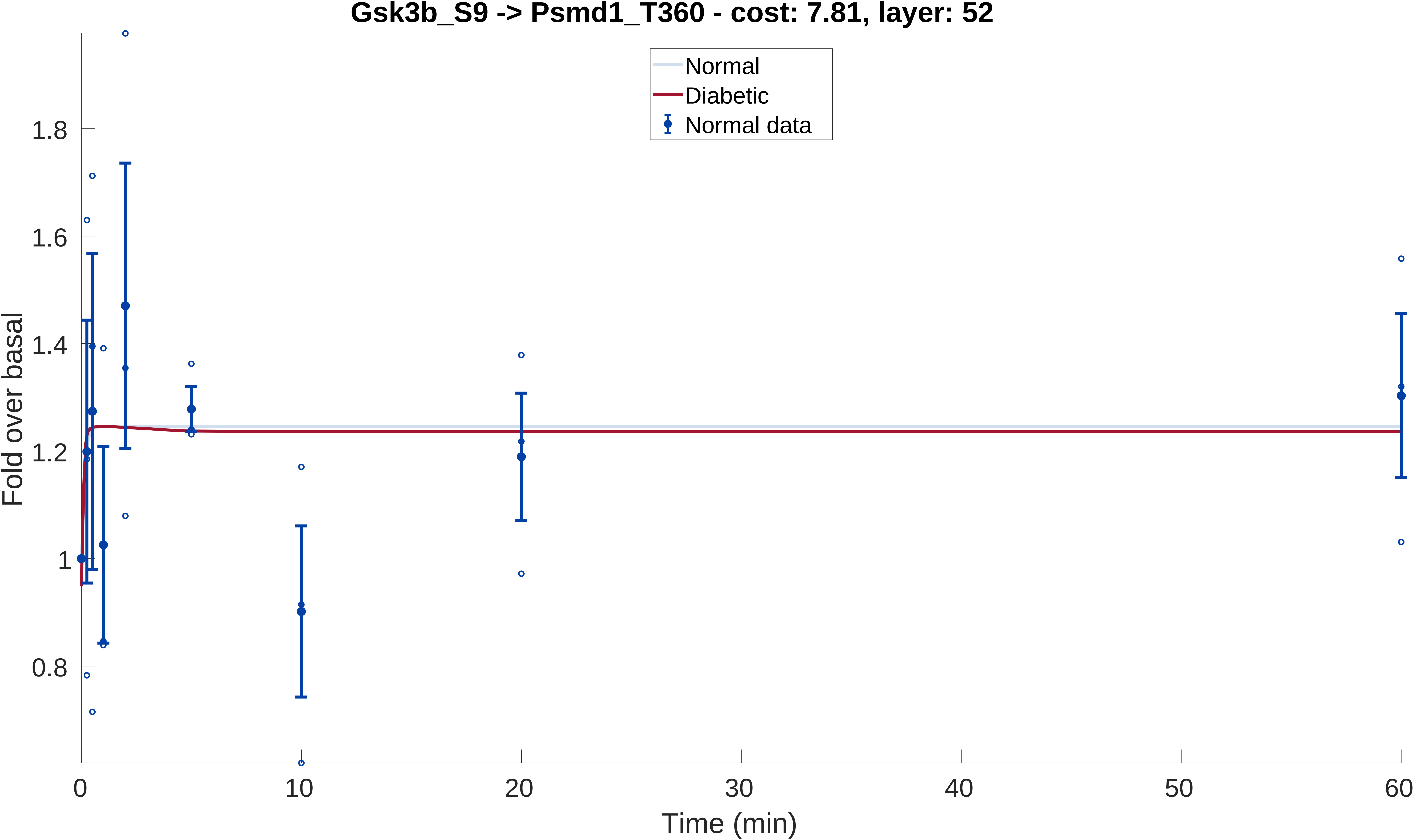

Gsk3b_S9 -> Psmd1_T360 - cost: 7.81, layer: 52
Normal
Diabetic
Normal data
Fold over basal
1.8
1.6
1.4
1.2
1
0.8
0
10
20
30
40
50
60
Time (min)

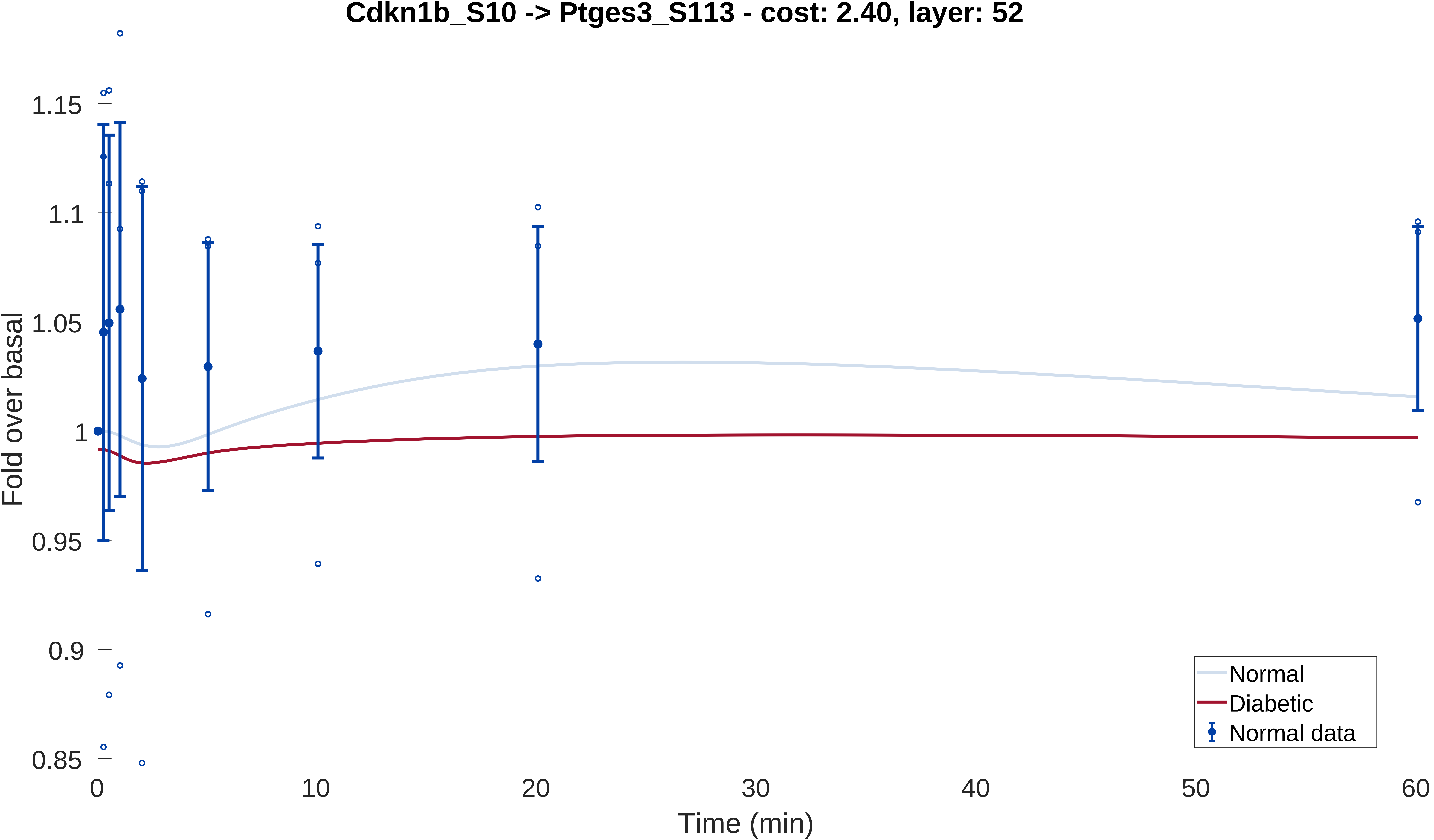
Cdkn1b_S10 -> Ptges3_S113 - cost: 2.40, layer: 52
Fold over basal
1.15
1.1
1.05
1
0.95
0.9
0.85
0
10
20
30
40
50
60
Time (min)
Normal
Diabetic
Normal data

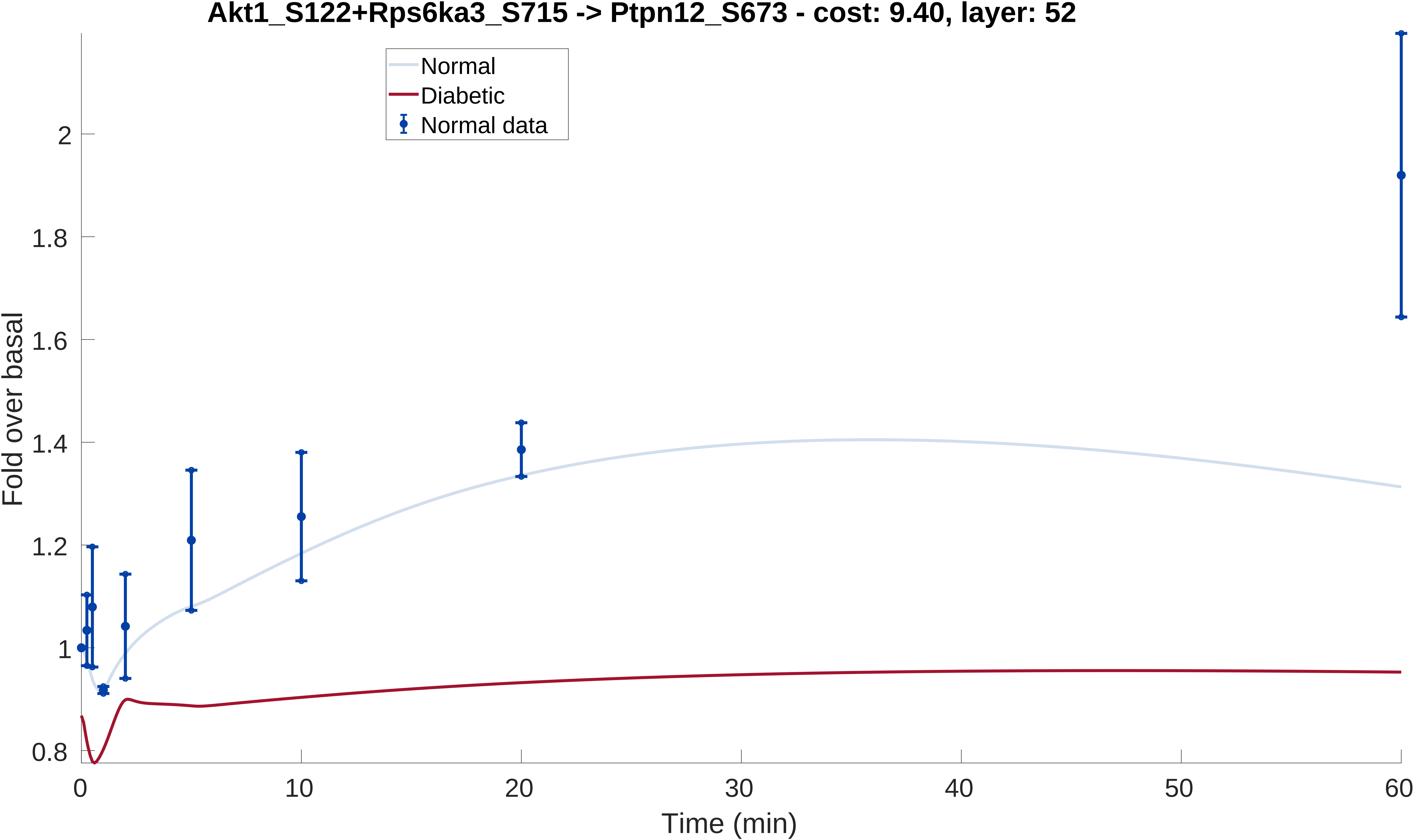

Akt1_S122+Rps6ka3_S715 -> Ptpn12_S673 - cost: 9.40, layer: 52
Normal
Diabetic
Normal data
Fold over basal
2
1.8
1.6
1.4
1.2
1
0.8
0
10
20
30
40
50
60
Time (min)

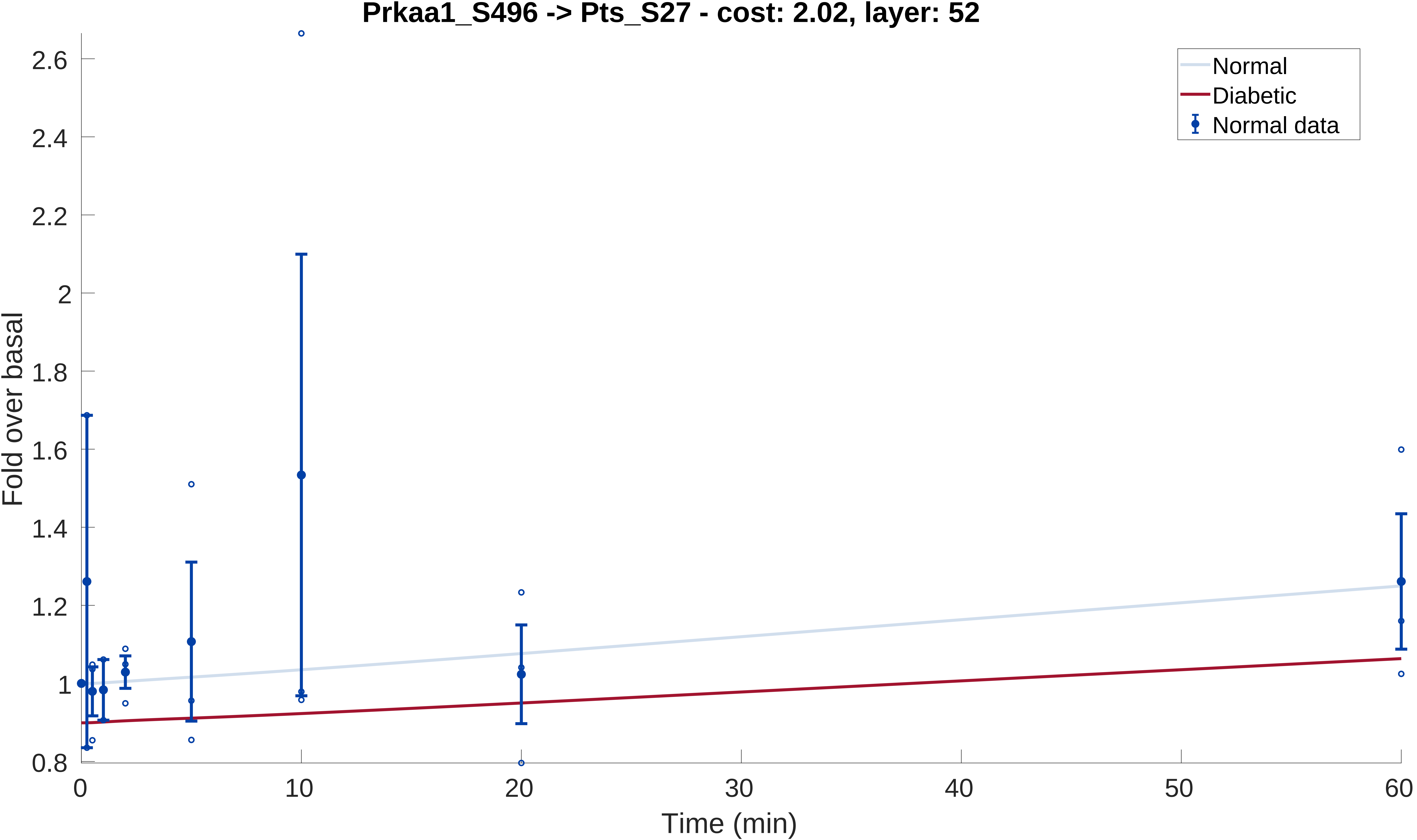

Prrkaa1_S496 -> Pts_S27 - cost: 2.02, layer: 52
Normal
Diabetic
Normal data
Fold over basal
Time (min)
0.8
1
1.2
1.4
1.6
1.8
2
2.2
2.4
2.6
0
10
20
30
40
50
60

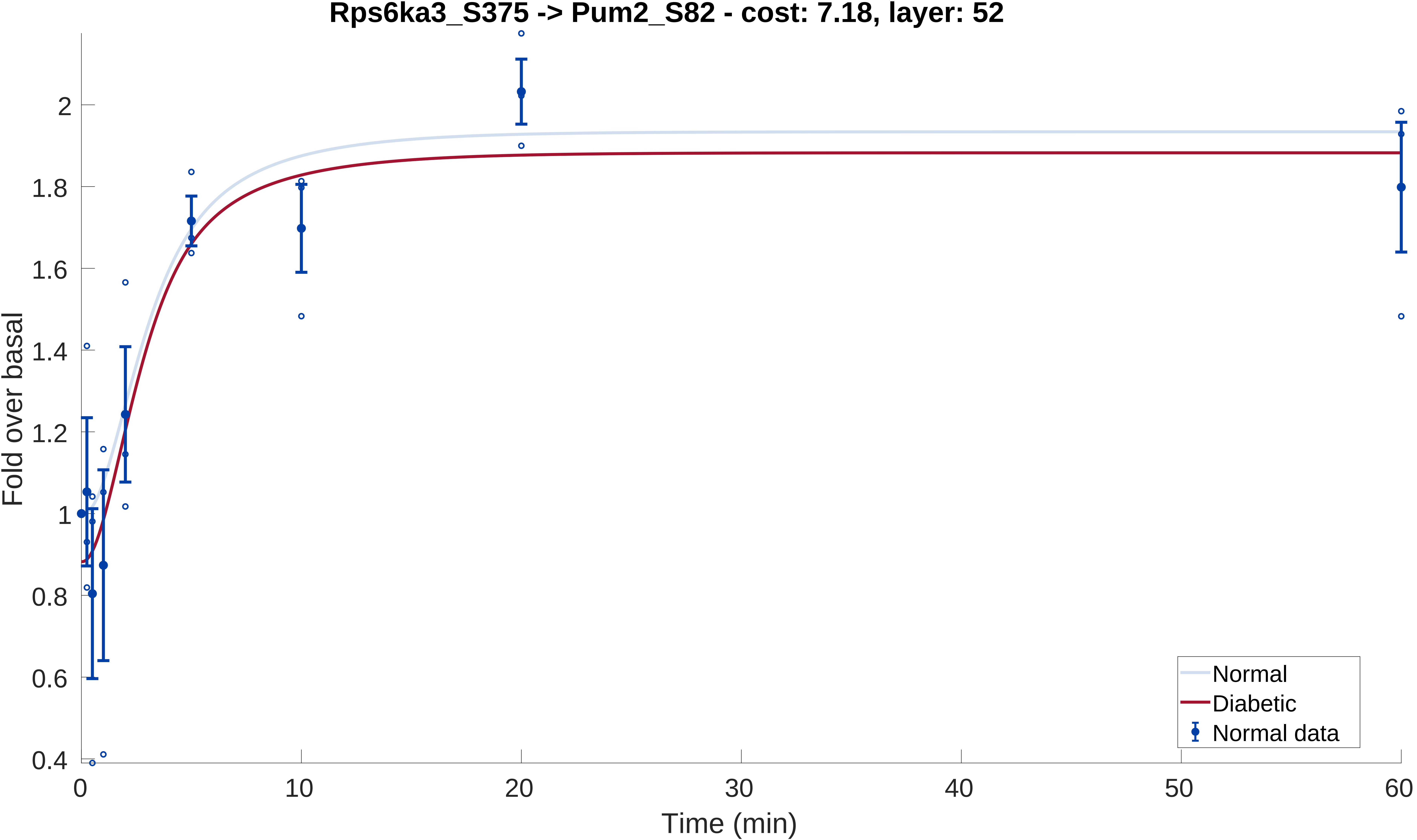

Rps6ka3_S375 -> Pum2_S82 - cost: 7.18, layer: 52
Fold over basal
Time (min)
0
10
20
30
40
50
60
0.4
0.6
0.8
1
1.2
1.4
1.6
1.8
2
Normal
Diabetic
Normal data

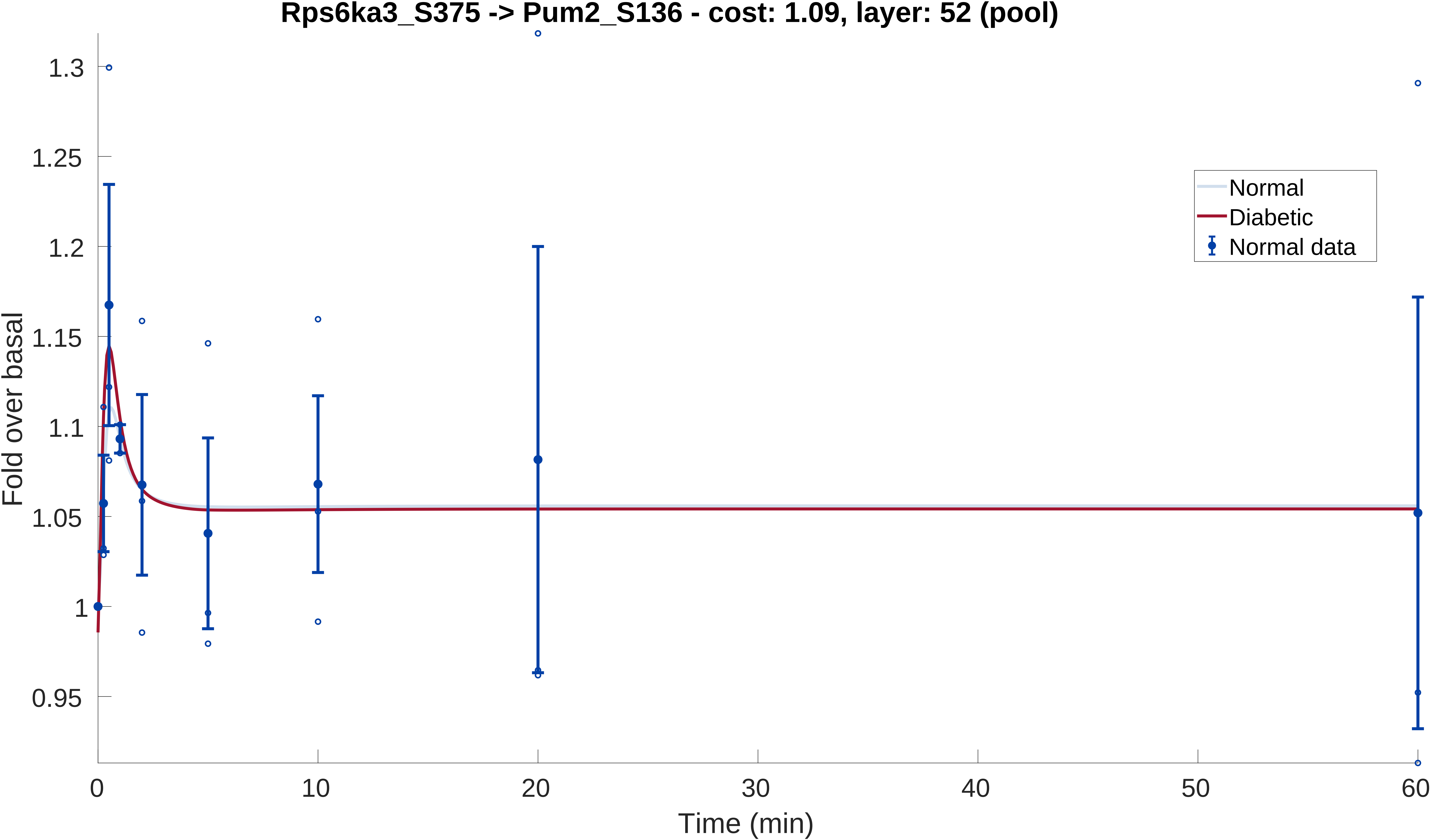

Rps6ka3_S375 -> Pum2_S136 - cost: 1.09, layer: 52 (pool)
Fold over basal
1.3
1.25
1.2
1.15
1.1
1.05
1
0.95
0
10
20
30
40
50
60
Time (min)
Normal
Diabetic
Normal data

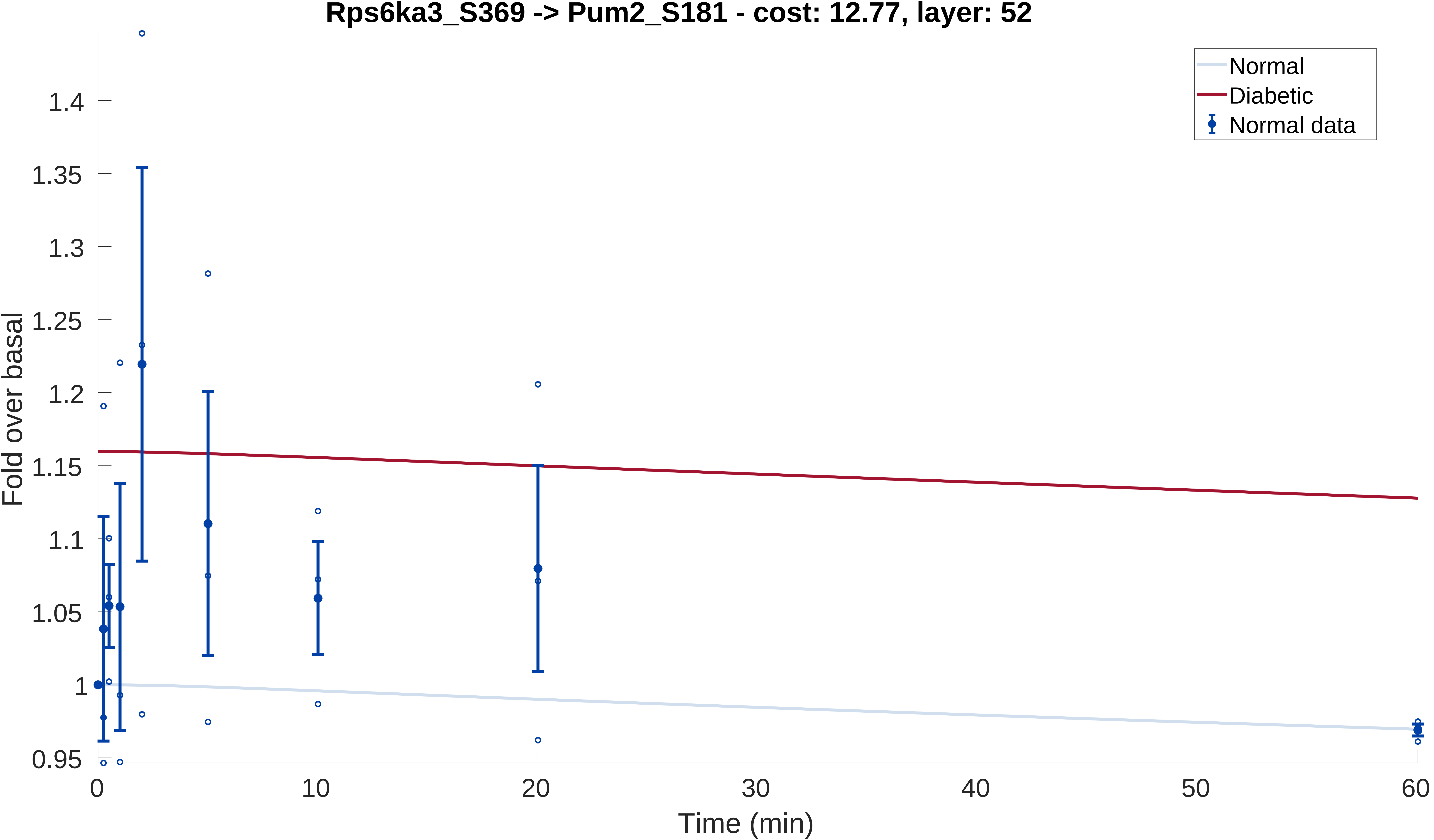

Rps6ka3_S369 -> Pum2_S181 - cost: 12.77, layer: 52
Normal
Diabetic
Normal data
Fold over basal
1.4
1.35
1.3
1.25
1.2
1.15
1.1
1.05
1
0.95
0
10
20
30
40
50
60
Time (min)

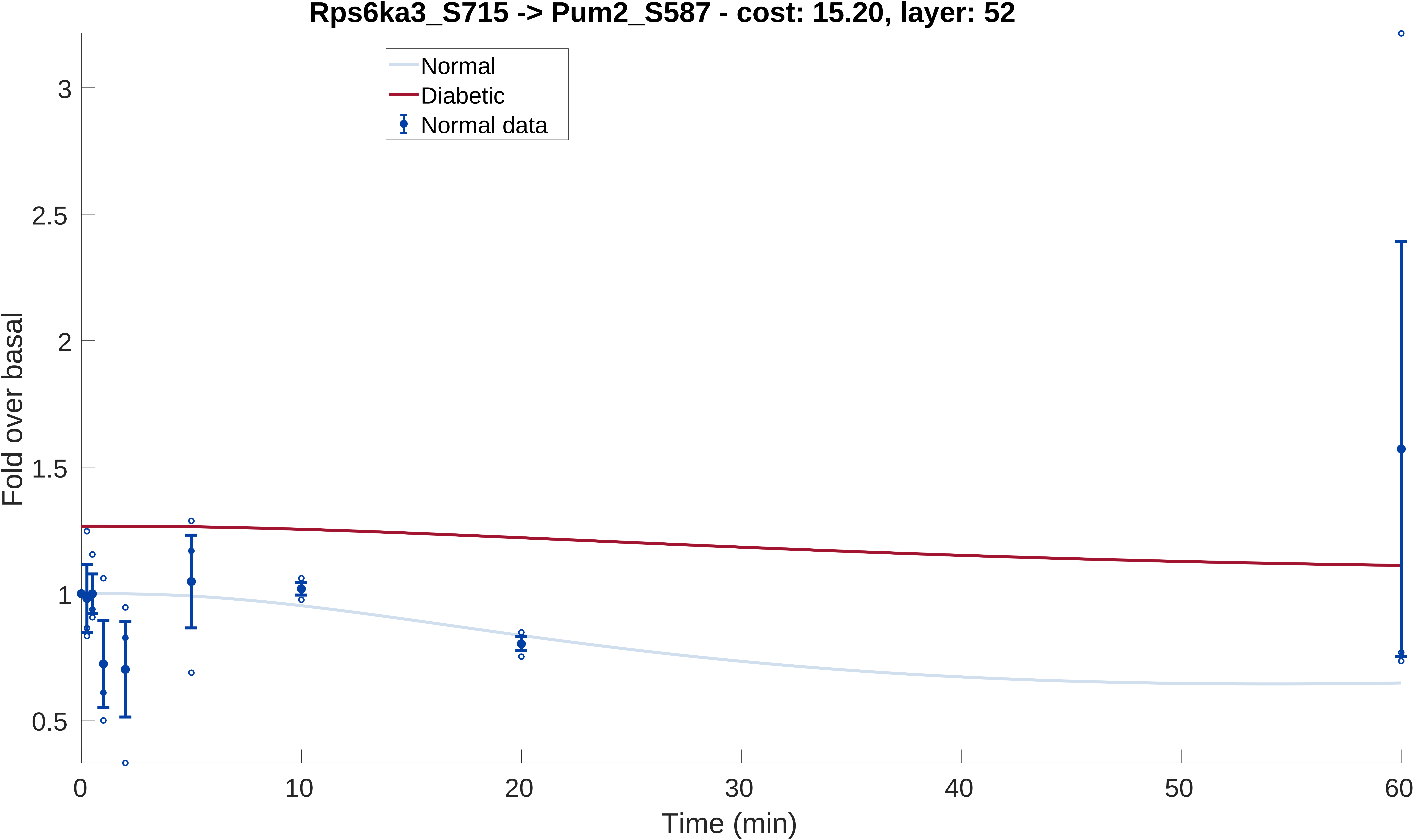

Rps6ka3_S715 -> Pum2_S587 - cost: 15.20, layer: 52
Normal
Diabetic
Normal data
Fold over basal
3
2.5
2
1.5
1
0.5
0
10
20
30
40
50
60
Time (min)

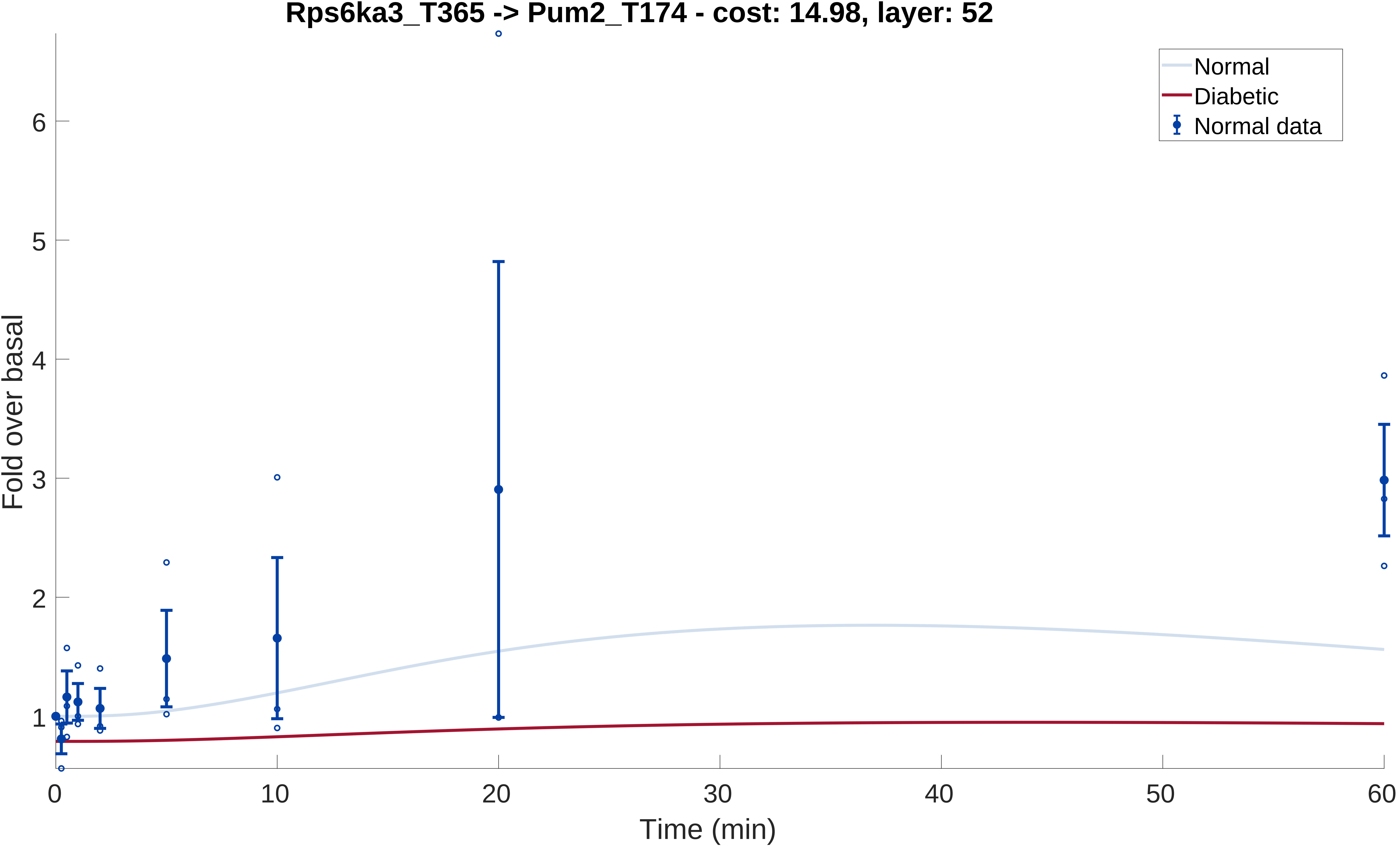

Rps6ka3_T365 -> Pum2_T174 - cost: 14.98, layer: 52
Normal
Diabetic
Normal data
Fold over basal
Time (min)
1
2
3
4
5
6
0
10
20
30
40
50
60

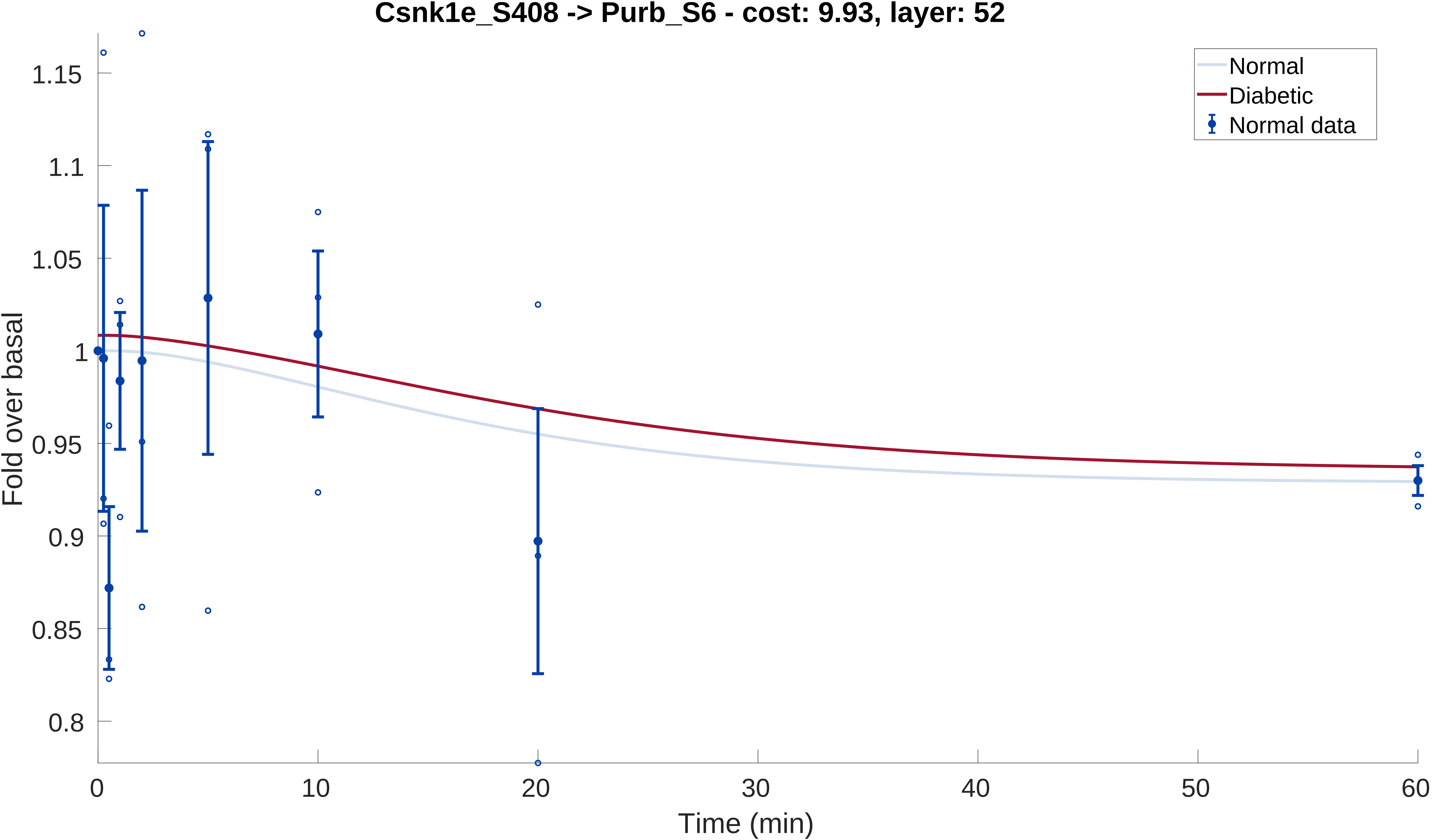

Csnk1e_S408 -> Purb_S6 - cost: 9.93, layer: 52
Normal
Diabetic
Normal data
Fold over basal
1.15
1.1
1.05
1
0.95
0.9
0.85
0.8
0
10
20
30
40
50
60
Time (min)

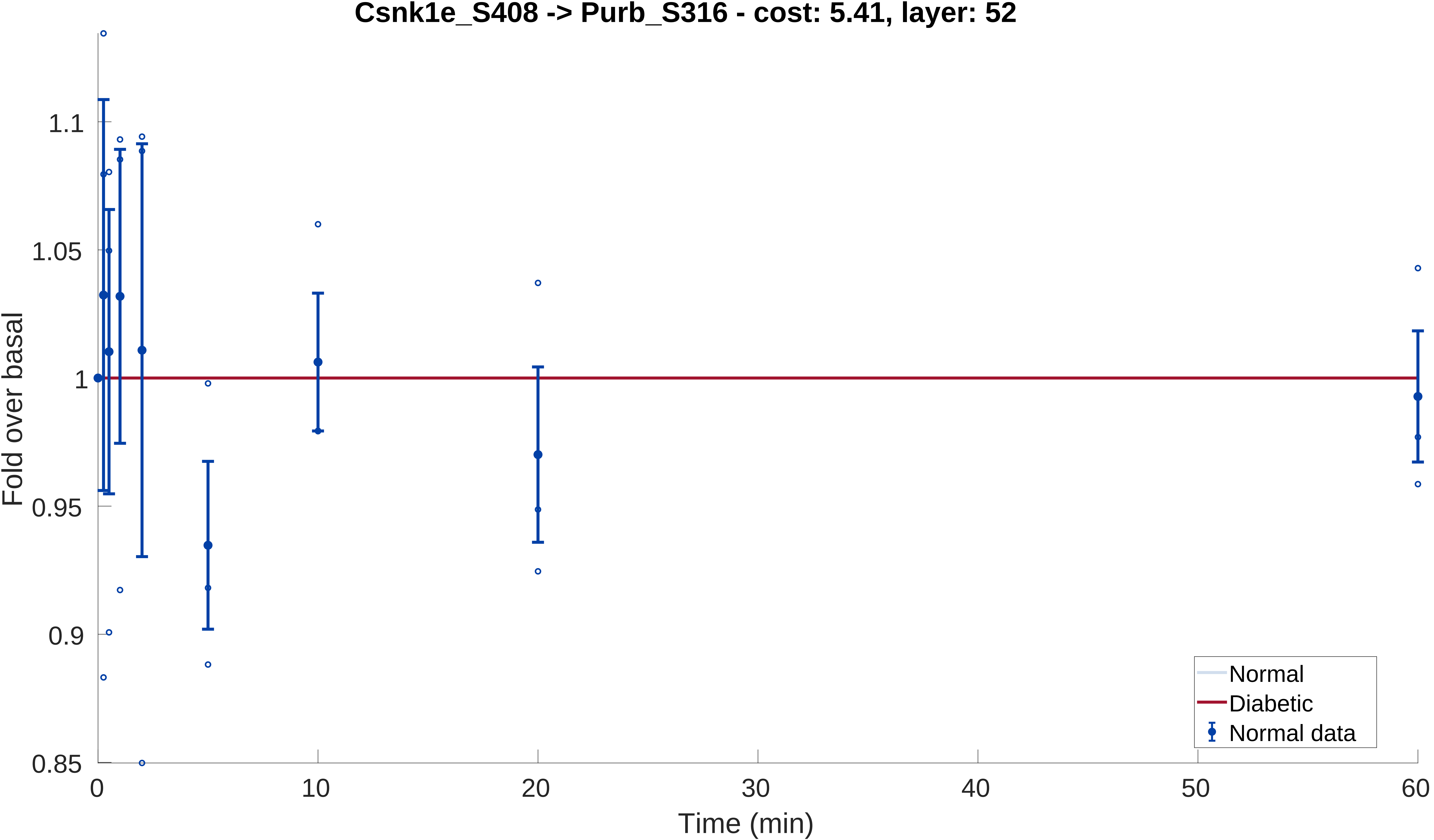

Csnk1e_S408 -> Purb_S316 - cost: 5.41, layer: 52
1.1
1.05
1
0.95
0.9
0.85
Fold over basal
0
10
20
30
40
50
60
Time (min)
Normal
Diabetic
Normal data

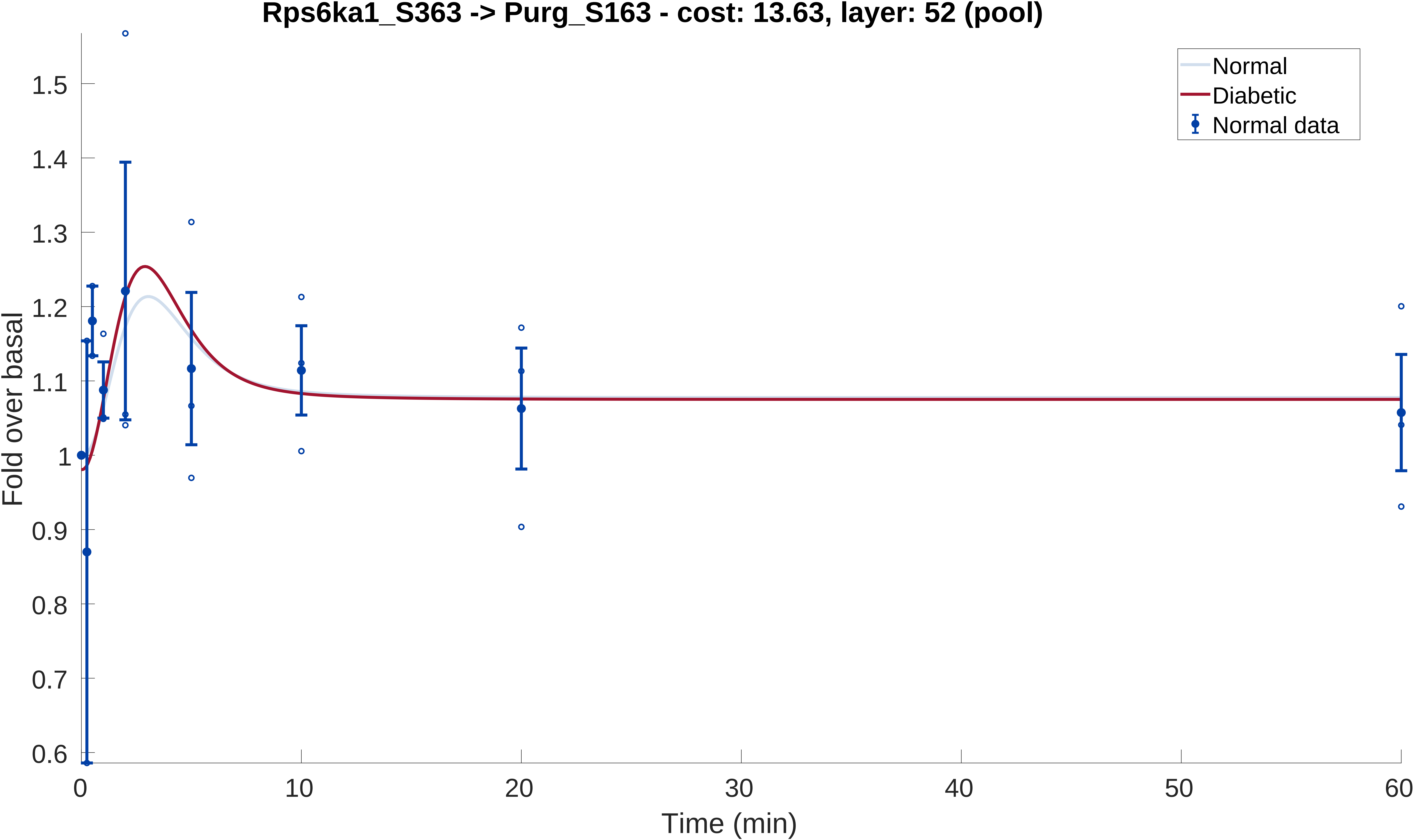

Rps6ka1_S363 -> Purg_S163 - cost: 13.63, layer: 52 (pool)
Normal
Diabetic
Normal data
Fold over basal
Time (min)
0.6
0.7
0.8
0.9
1
1.1
1.2
1.3
1.4
1.5
0
10
20
30
40
50
60

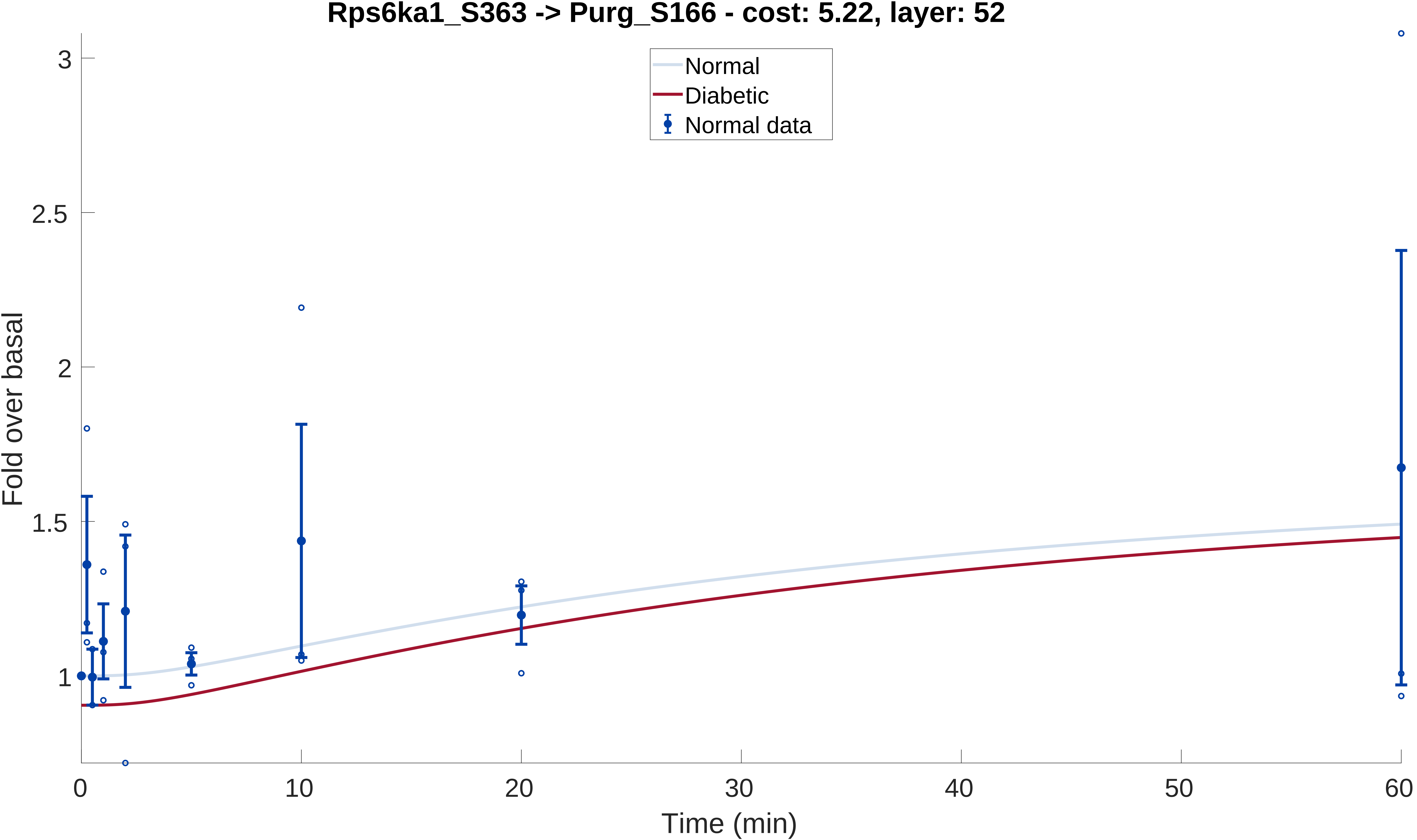

Rps6ka1_S363 -> Purg_S166 - cost: 5.22, layer: 52
Normal
Diabetic
Normal data
Fold over basal
3
2.5
2
1.5
1
0
10
20
30
40
50
60
Time (min)

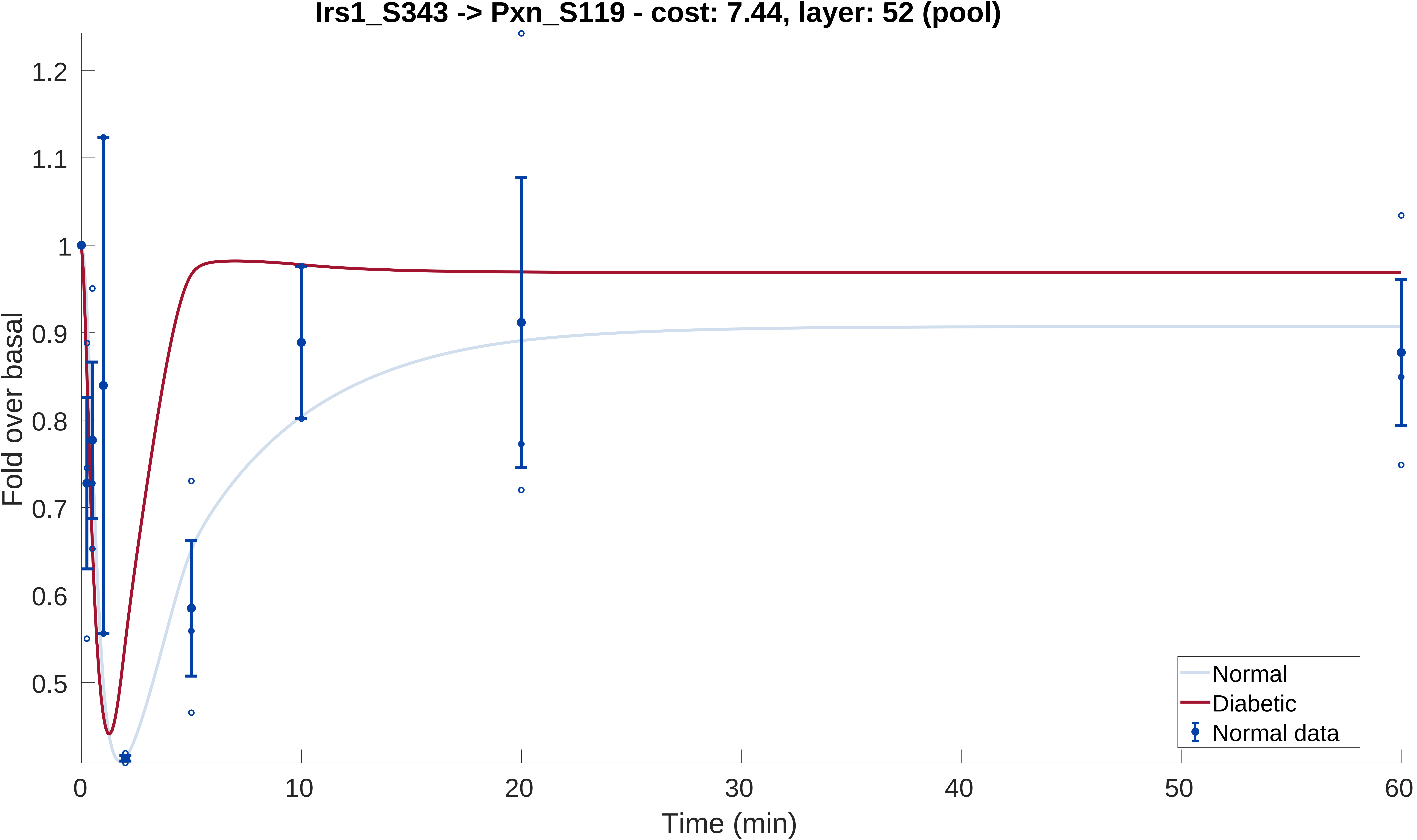
Irs1_S343 -> Pxn_S119 - cost: 7.44, layer: 52 (pool)
Fold over basal
1.2
1.1
1
0.9
0.8
0.7
0.6
0.5
0
10
20
30
40
50
60
Time (min)
Normal
Diabetic
Normal data

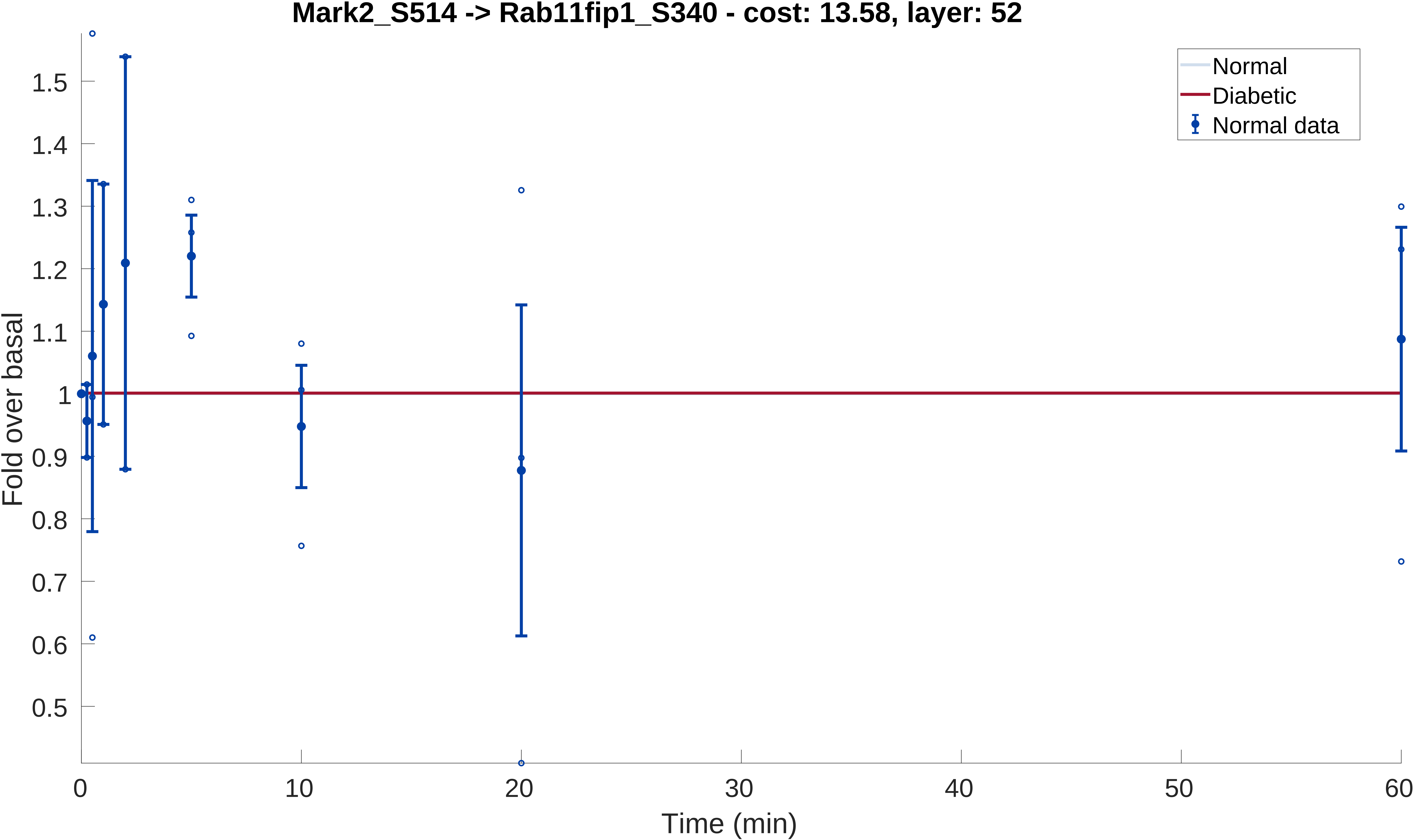

Mark2_S514 -> Rab11fip1_S340 - cost: 13.58, layer: 52
Fold over basal
Time (min)
Normal
Diabetic
Normal data
1.5
1.4
1.3
1.2
1.1
1
0.9
0.8
0.7
0.6
0.5
0
10
20
30
40
50
60

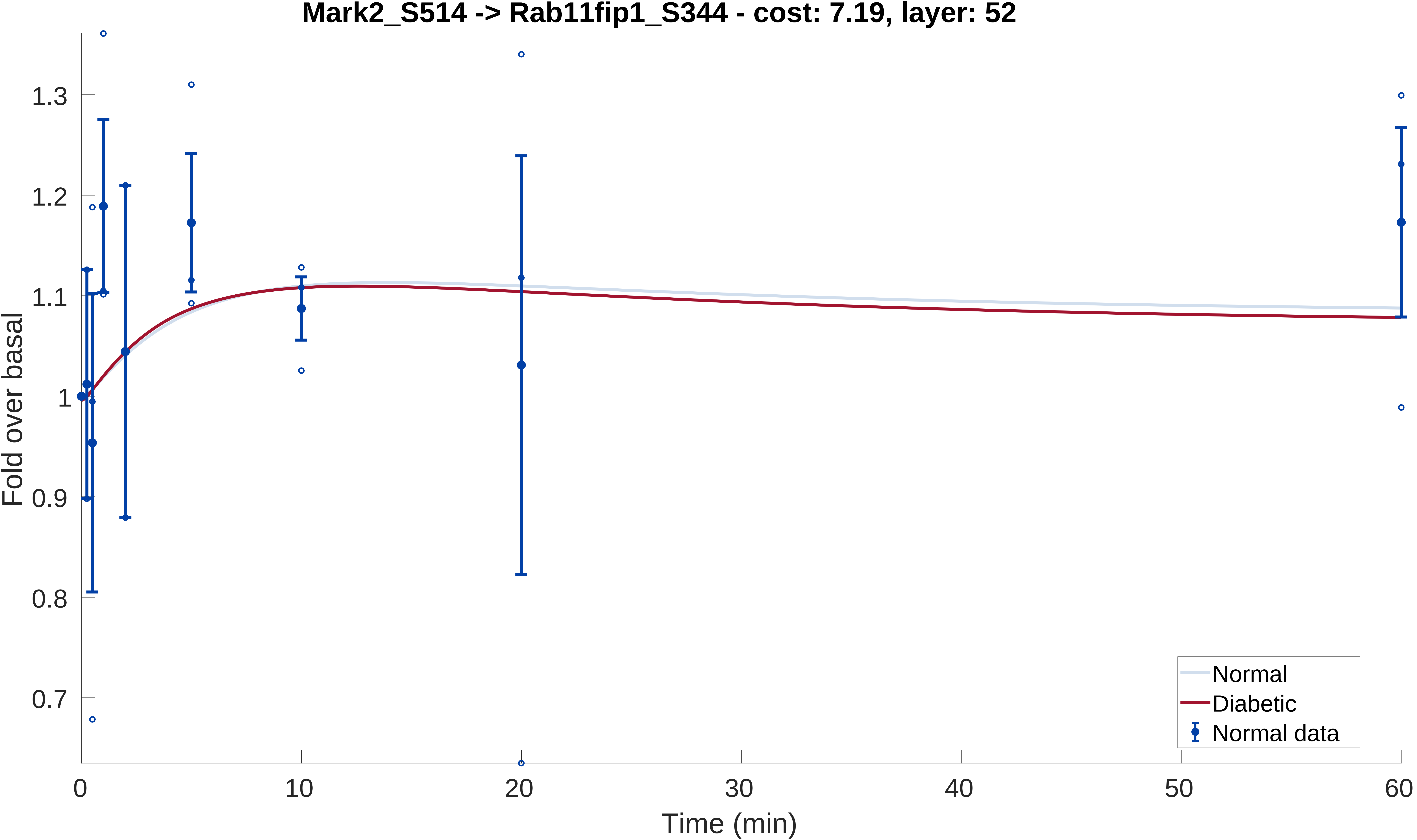

Mark2_S514 -> Rab11fip1_S344 - cost: 7.19, layer: 52
Fold over basal
1.3
1.2
1.1
1
0.9
0.8
0.7
0
10
20
30
40
50
60
Time (min)
Normal
Diabetic
Normal data

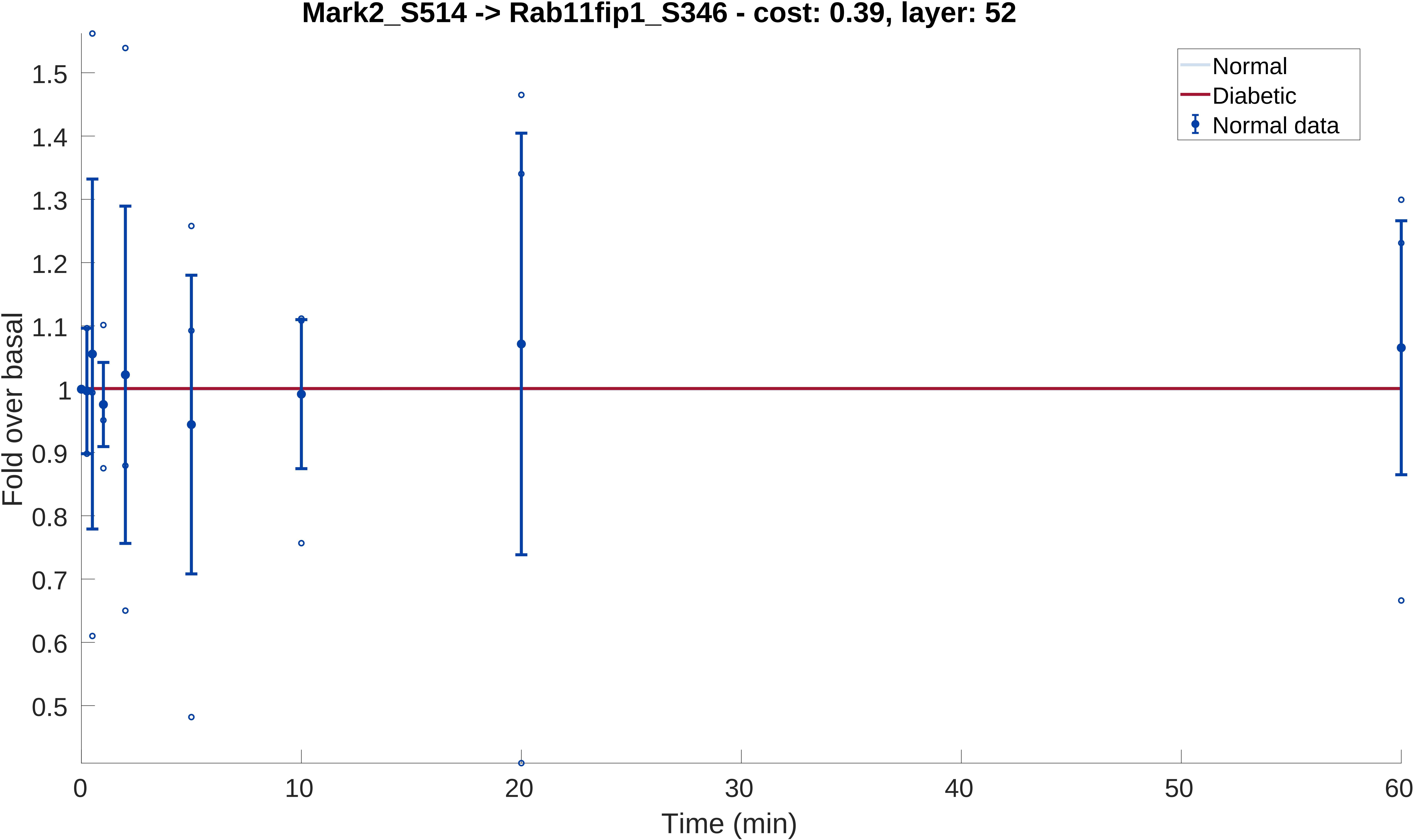

Mark2_S514 -> Rab11fip1_S346 - cost: 0.39, layer: 52
Normal
Diabetic
Normal data
Fold over basal
Time (min)
0.5
0.6
0.7
0.8
0.9
1
1.1
1.2
1.3
1.4
1.5
0
10
20
30
40
50
60

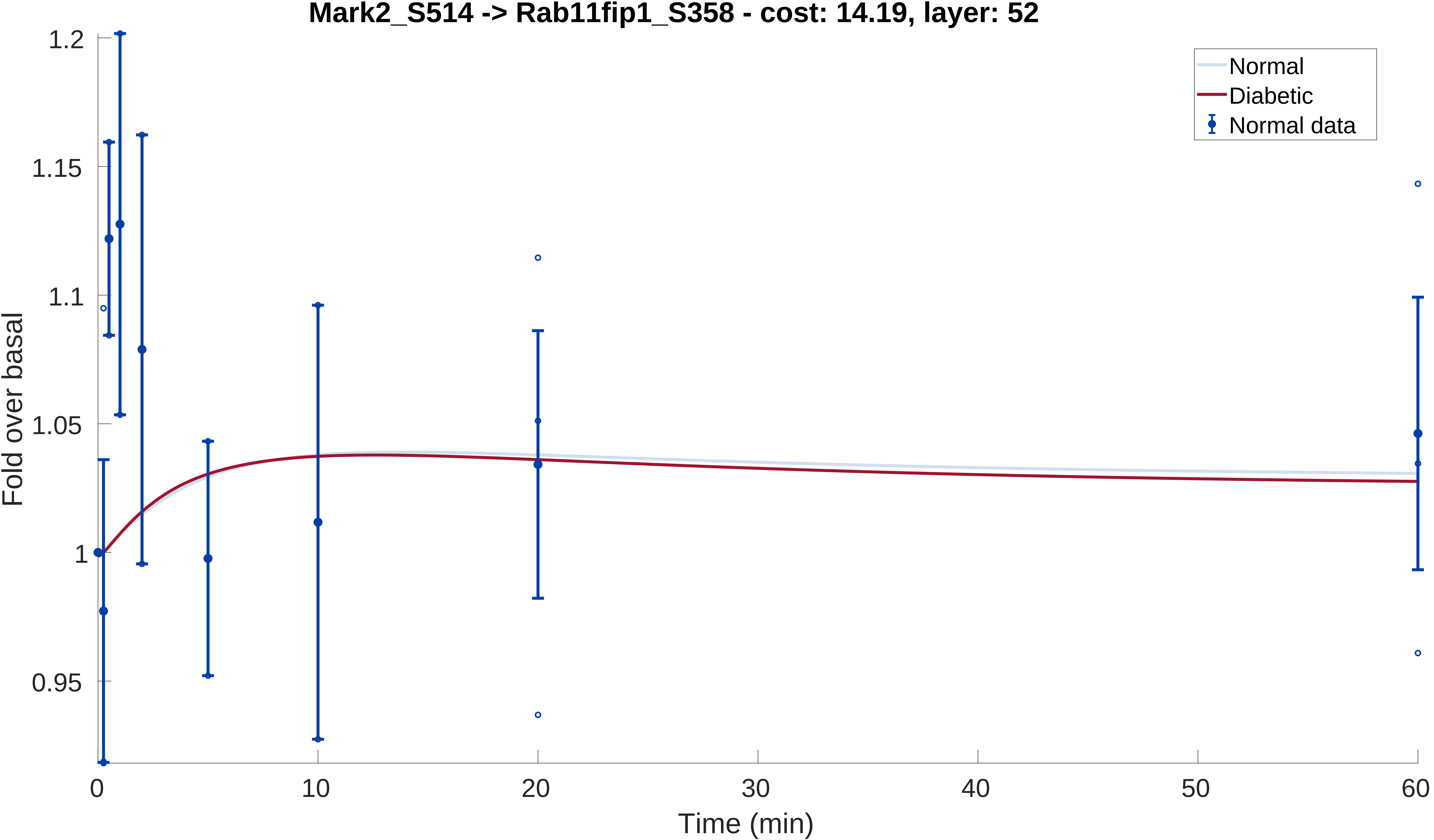

Mark2_S514 -> Rab11fip1_S358 - cost: 14.19, layer: 52
1.2
1.15
1.1
1.05
1
0.95
Fold over basal
0
10
20
30
40
50
60
Time (min)
Normal
Diabetic
Normal data

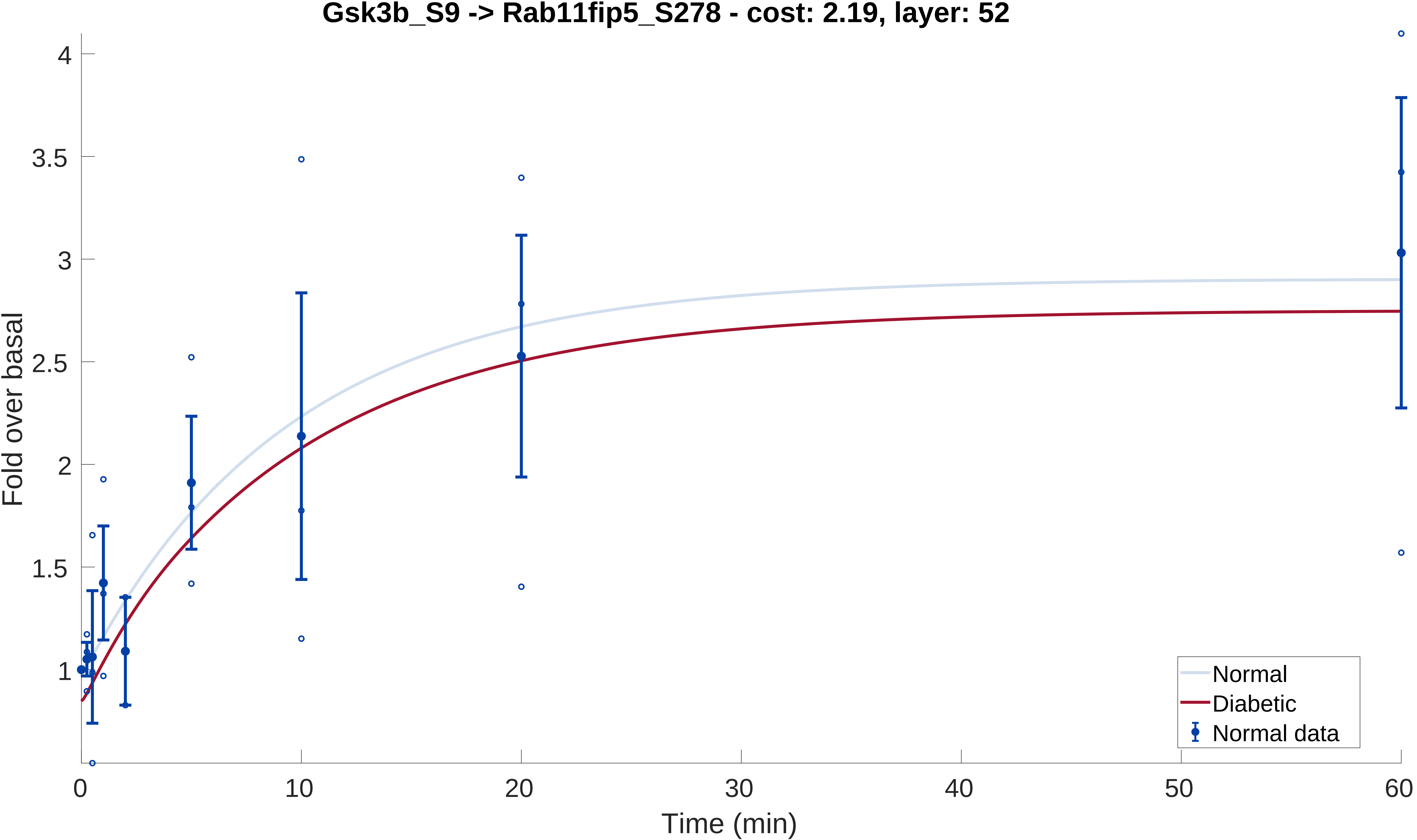
Gsk3b_S9 -> Rab11fip5_S278 - cost: 2.19, layer: 52
Fold over basal
4
3.5
3
2.5
2
1.5
1
0
10
20
30
40
50
60
Time (min)
Normal
Diabetic
Normal data

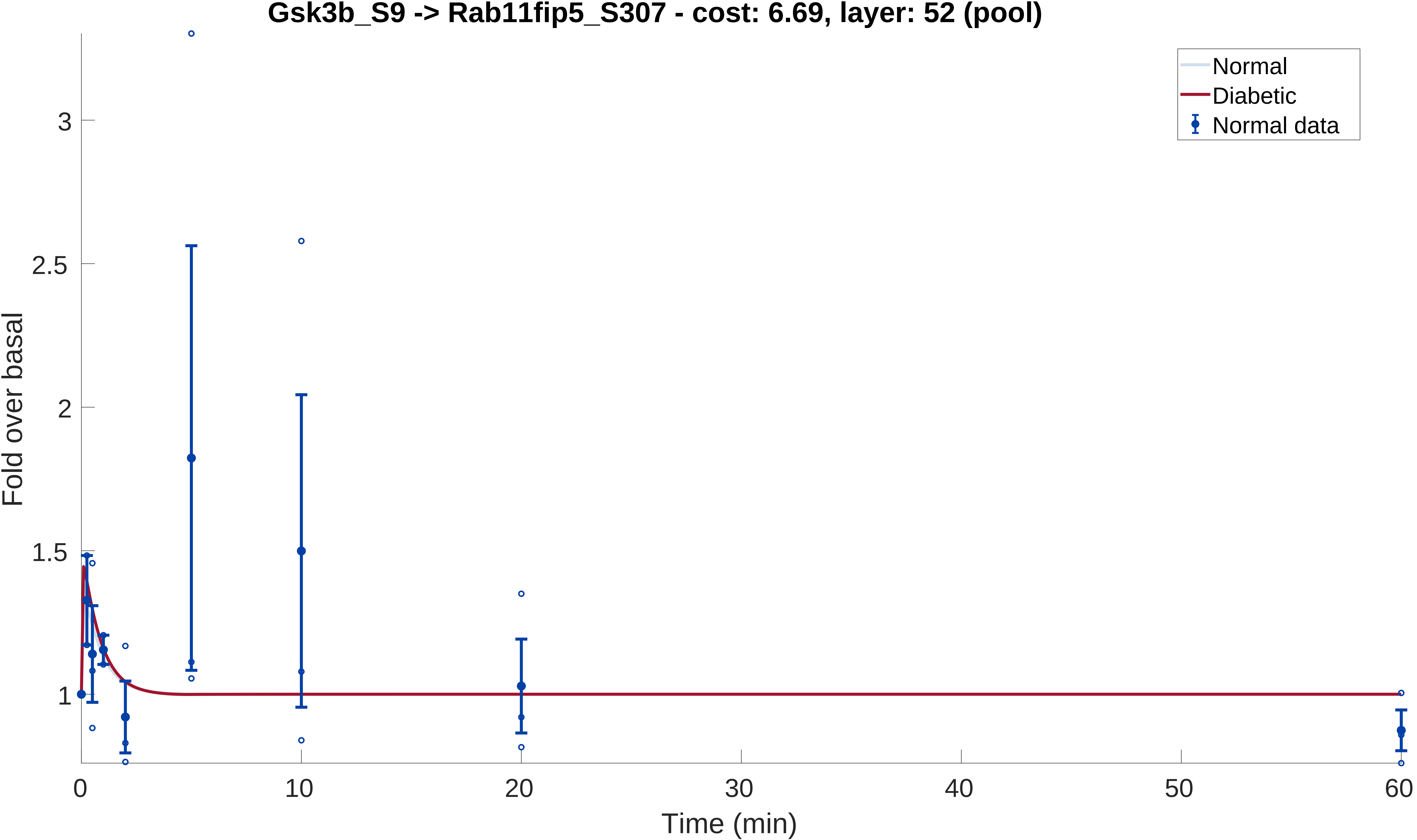
Gsk3b_S9 -> Rab11fip5_S307 - cost: 6.69, layer: 52 (pool)
Normal
Diabetic
Normal data
Fold over basal
Time (min)
0
10
20
30
40
50
60
1
1.5
2
2.5
3

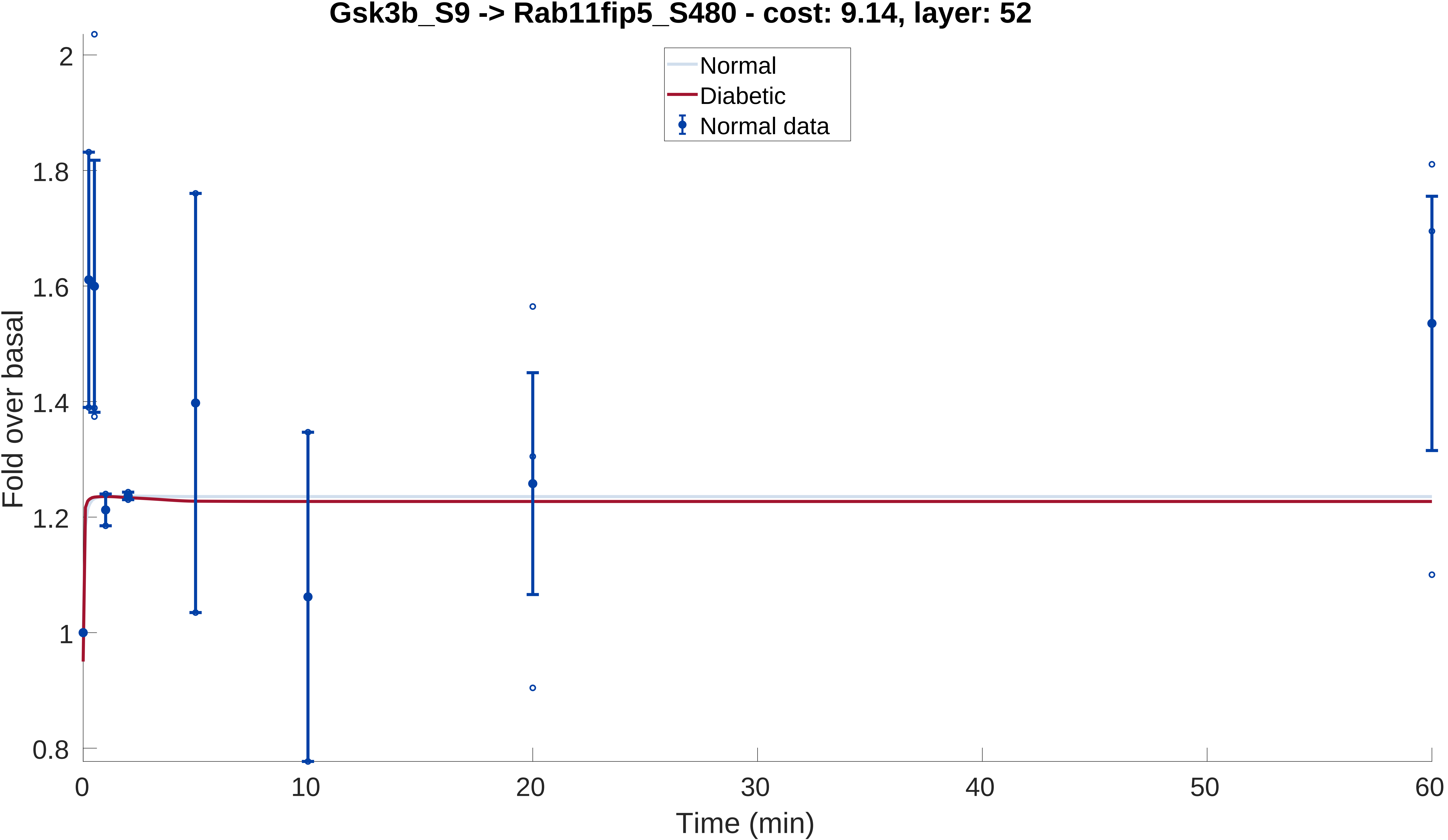

Gsk3b_S9 -> Rab11fip5_S480 - cost: 9.14, layer: 52
Normal
Diabetic
Normal data
Fold over basal
2
1.8
1.6
1.4
1.2
1
0.8
0
10
20
30
40
50
60
Time (min)

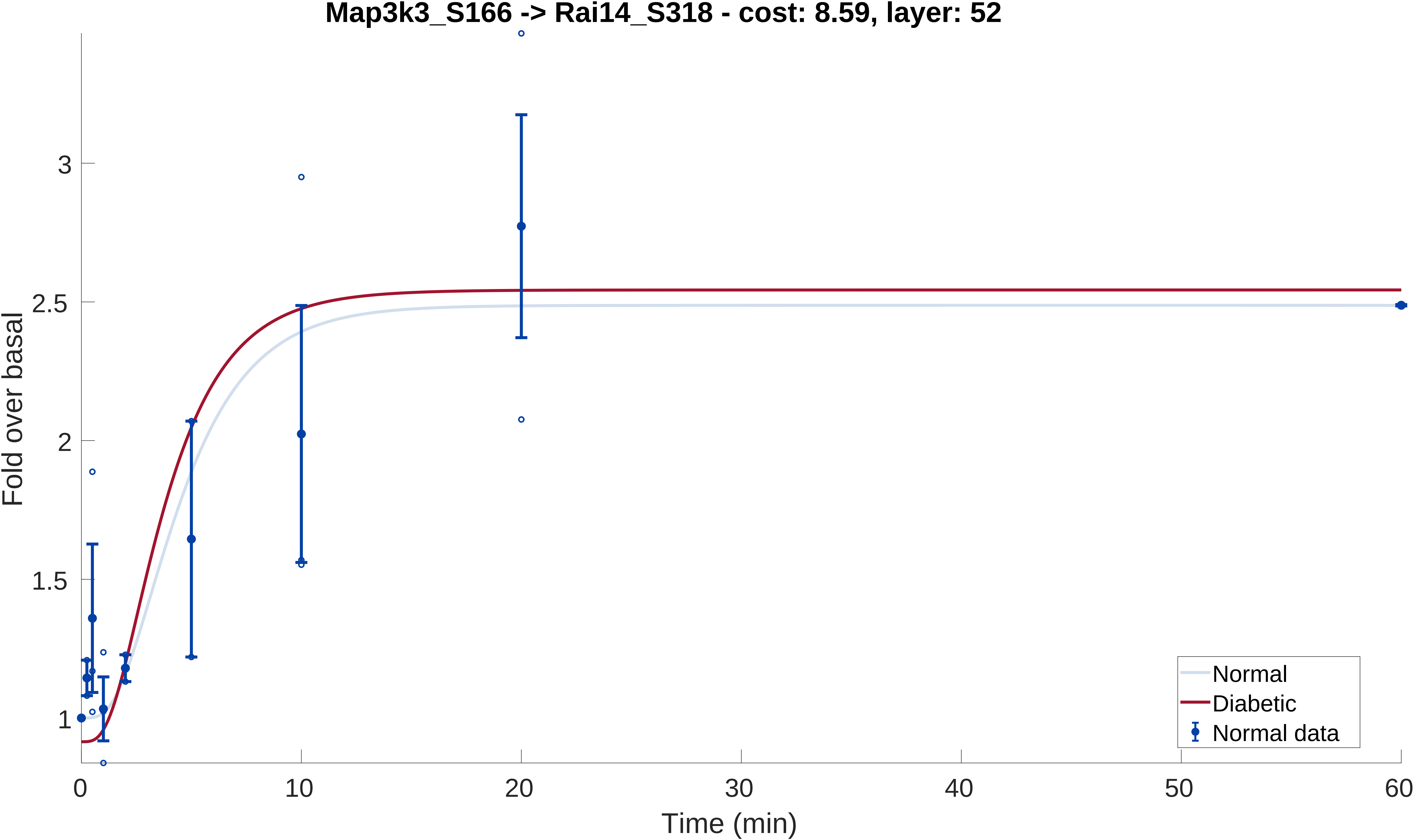

Map3k3_S166 -> Rai14_S318 - cost: 8.59, layer: 52
Fold over basal
3
2.5
2
1.5
1
0
10
20
30
40
50
60
Time (min)
Normal
Diabetic
Normal data

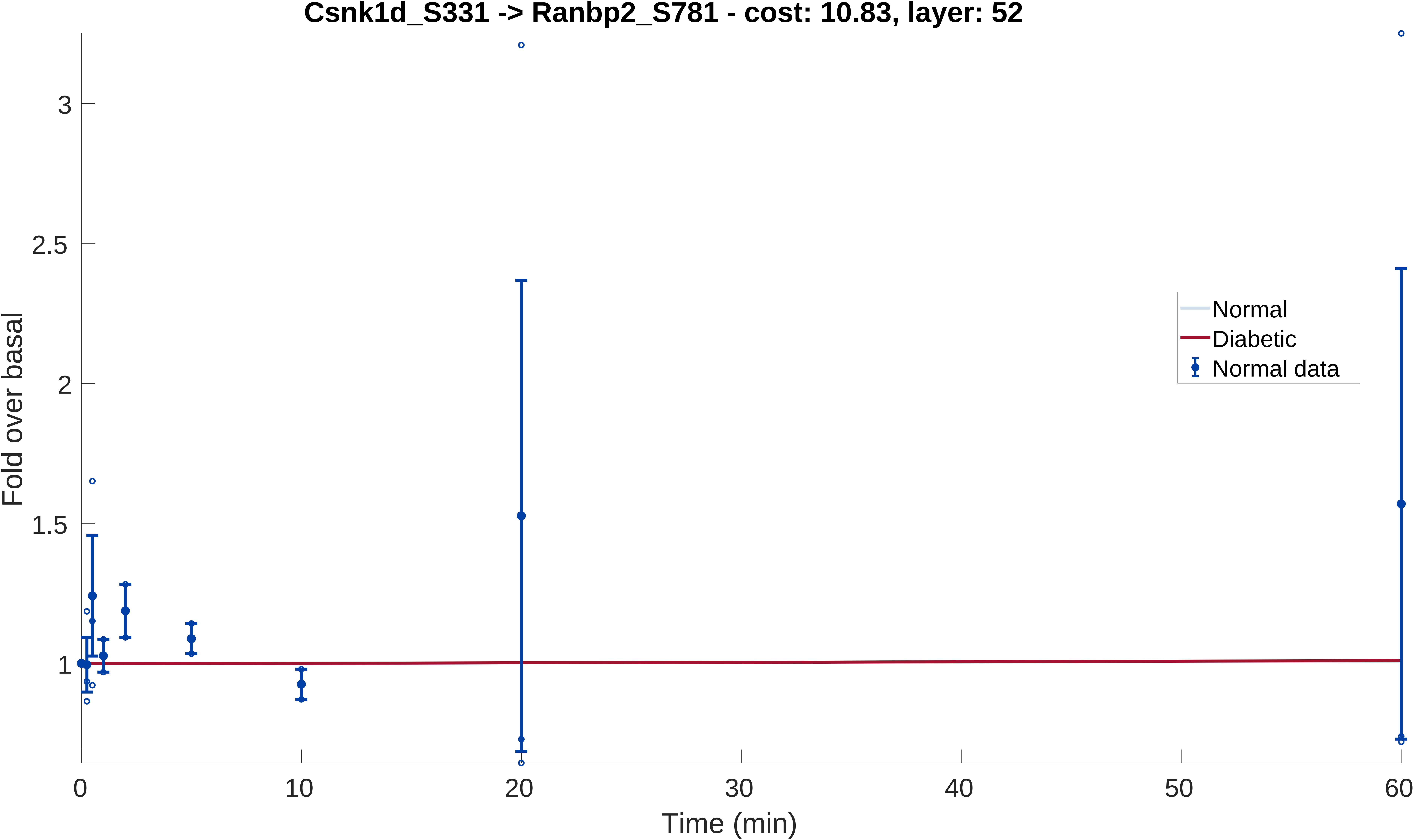

Csnk1d_S331 -> Ranbp2_S781 - cost: 10.83, layer: 52
Fold over basal
Time (min)
3
2.5
2
1.5
1
0
10
20
30
40
50
60
Normal
Diabetic
Normal data

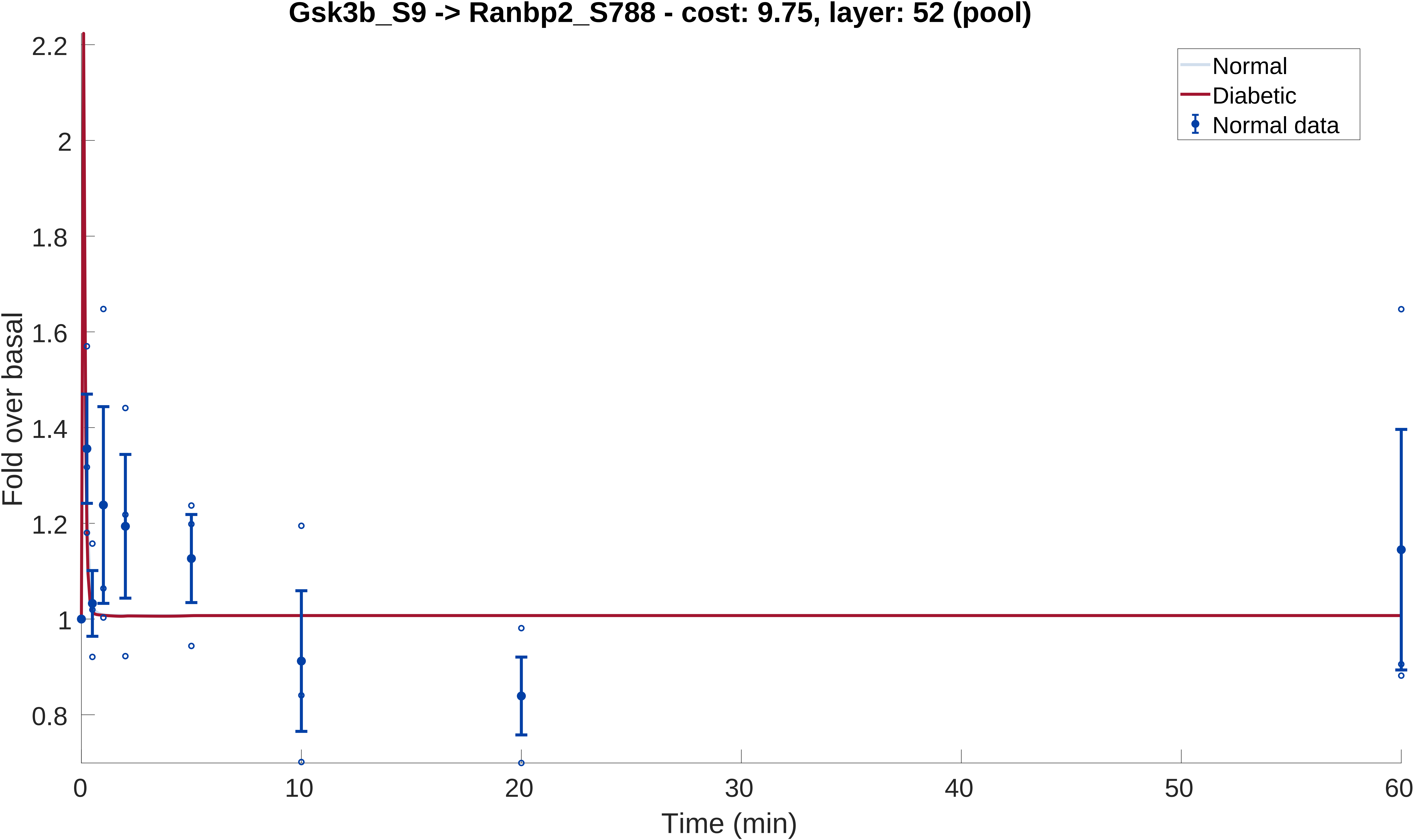
Gsk3b_S9 -> Ranbp2_S788 - cost: 9.75, layer: 52 (pool)
Normal
Diabetic
Normal data
Fold over basal
Time (min)
2.2
2
1.8
1.6
1.4
1.2
1
0.8
0
10
20
30
40
50
60

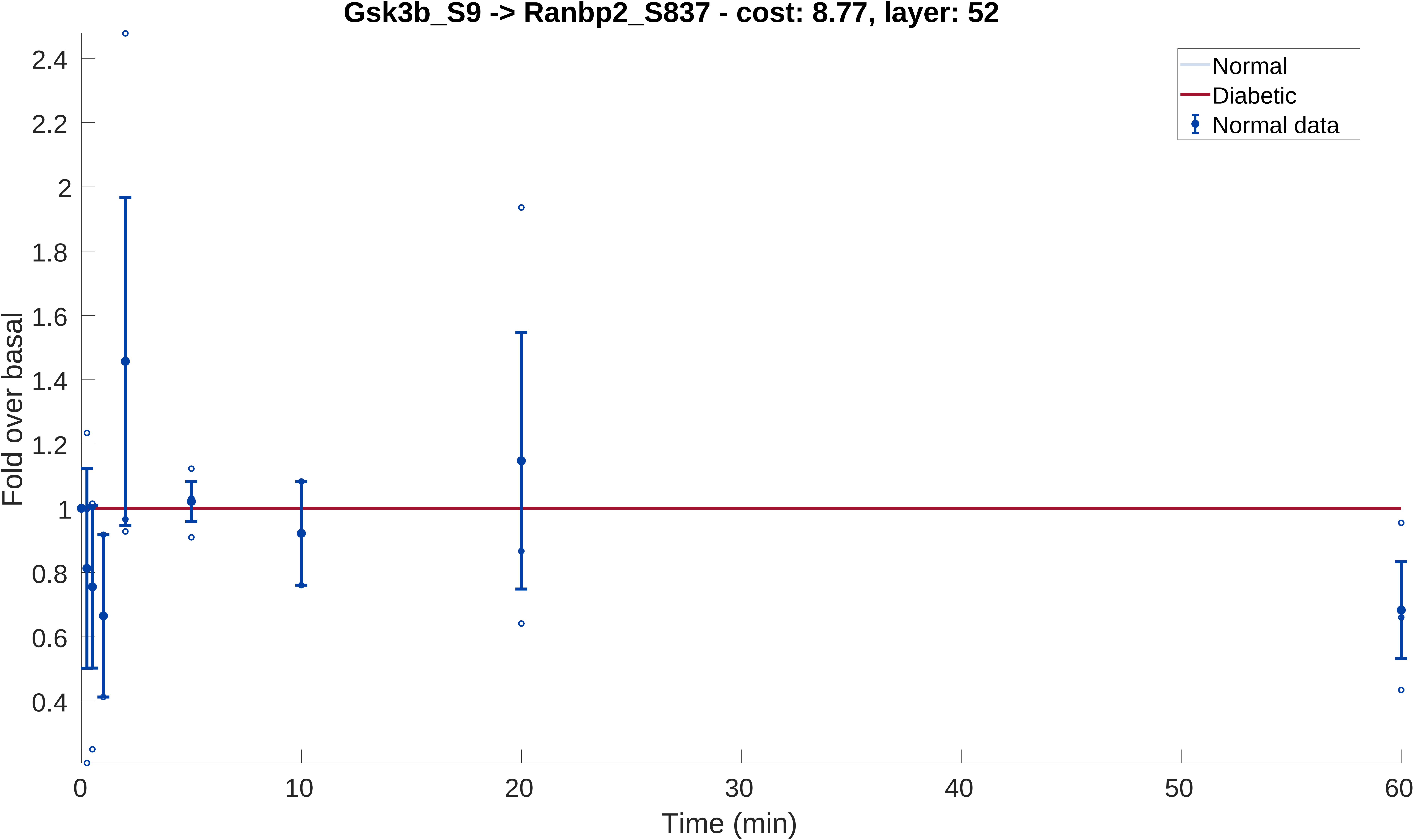
Gsk3b_S9 -> Ranbp2_S837 - cost: 8.77, layer: 52
Normal
Diabetic
Normal data
Fold over basal
Time (min)
0.4
0.6
0.8
1
1.2
1.4
1.6
1.8
2
2.2
2.4
0
10
20
30
40
50
60

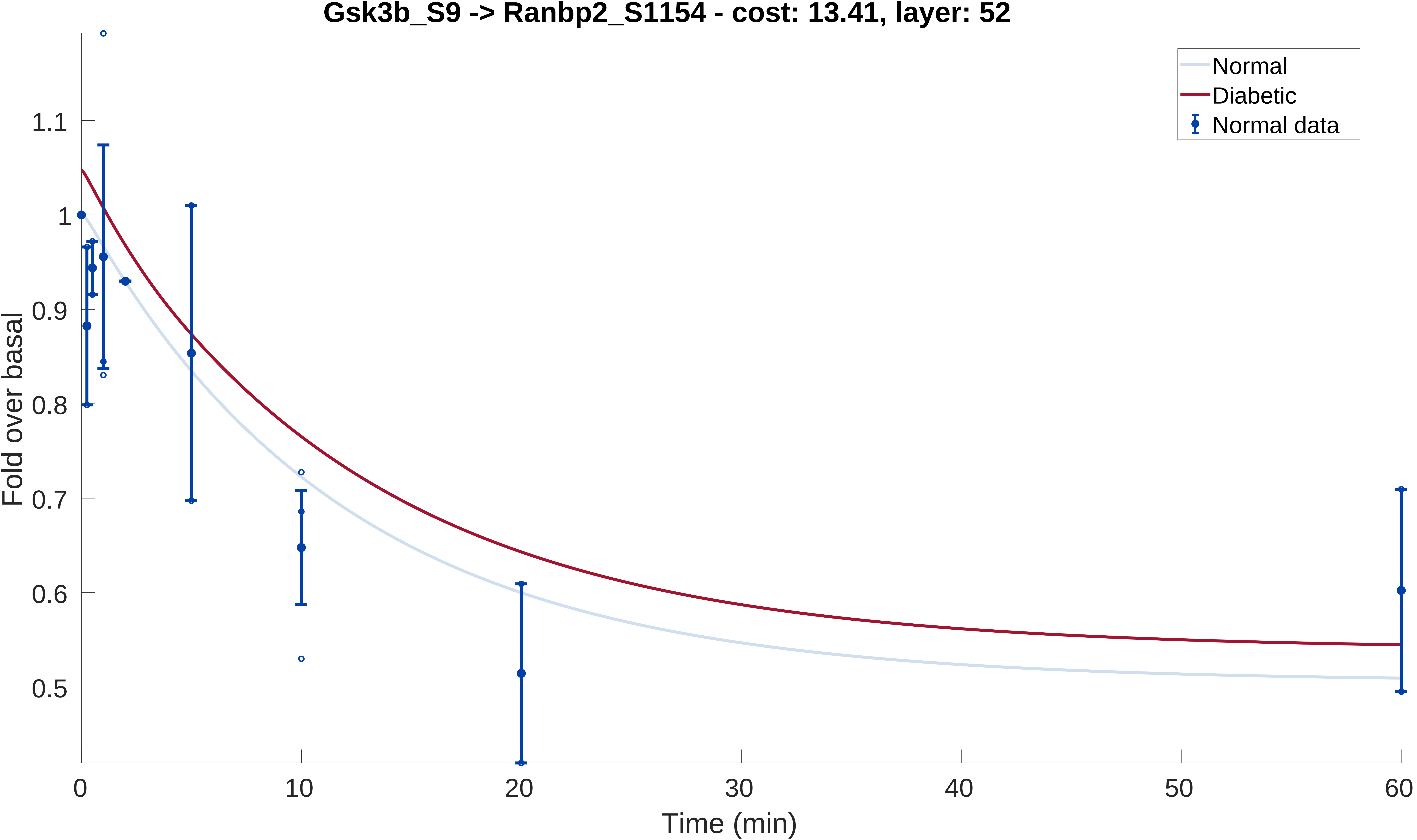

Gsk3b_S9 -> Ranbp2_S1154 - cost: 13.41, layer: 52
Normal
Diabetic
Normal data
Fold over basal
Time (min)
1.1
1
0.9
0.8
0.7
0.6
0.5
0
10
20
30
40
50
60

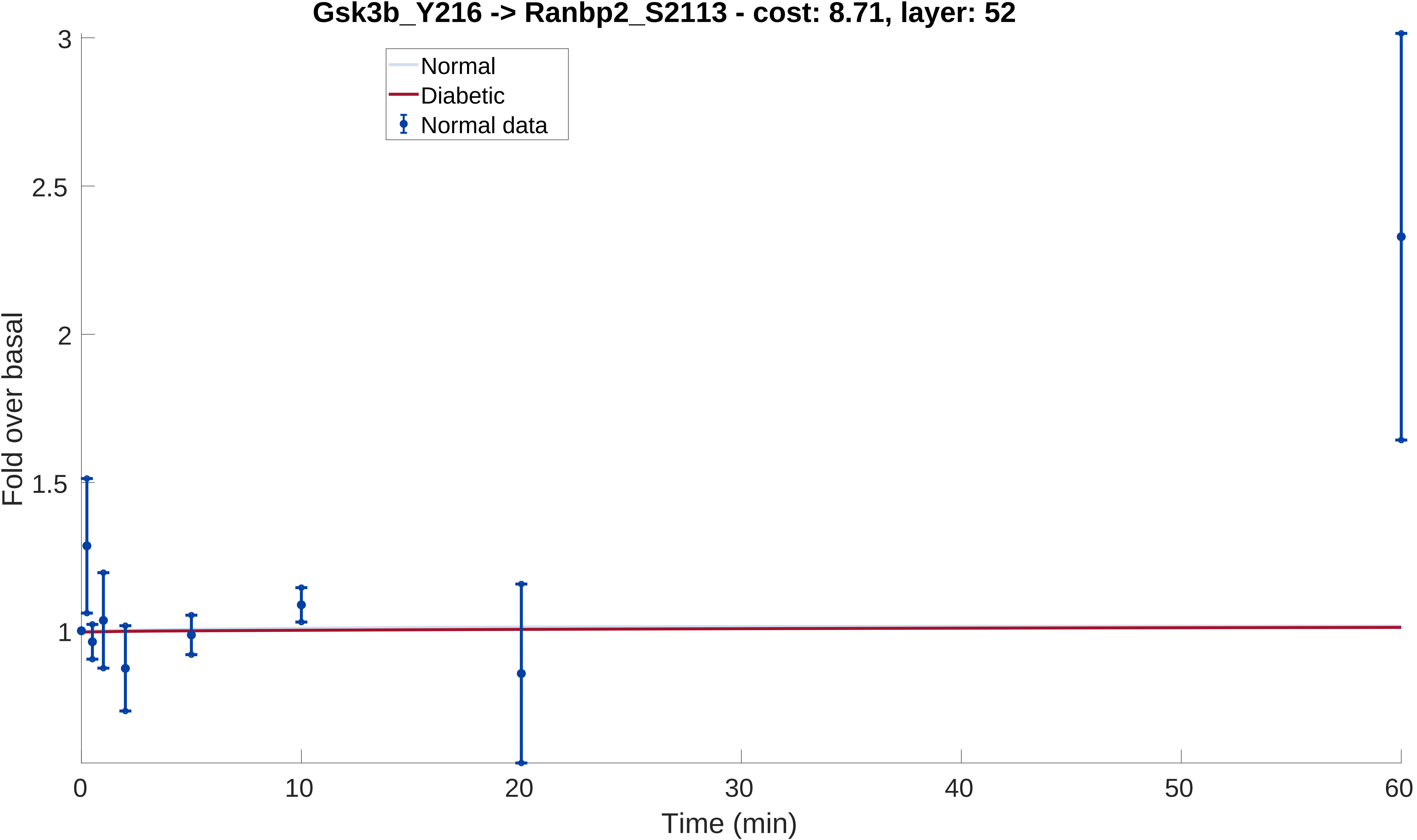

Gsk3b_Y216 -> Ranbp2_S2113 - cost: 8.71, layer: 52
Normal
Diabetic
Normal data
Fold over basal
Time (min)
3
2.5
2
1.5
1
0
10
20
30
40
50
60

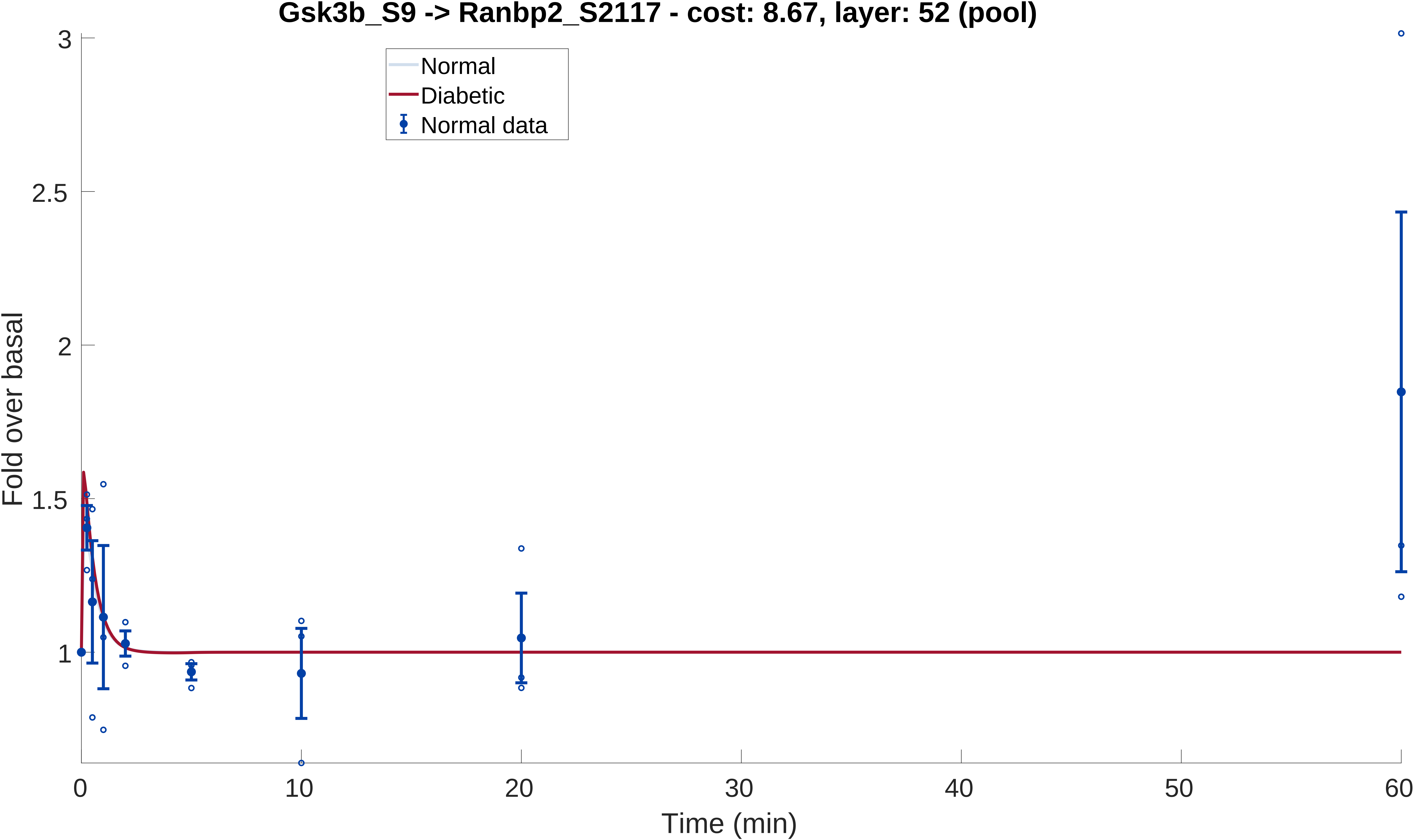

Gsk3b_S9 -> Ranbp2_S2117 - cost: 8.67, layer: 52 (pool)
Normal
Diabetic
Normal data
Fold over basal
Time (min)
3
2.5
2
1.5
1
0
10
20
30
40
50
60

Gsk3b_S9 -> Ranbp2_S2641 - cost: 13.46, layer: 52

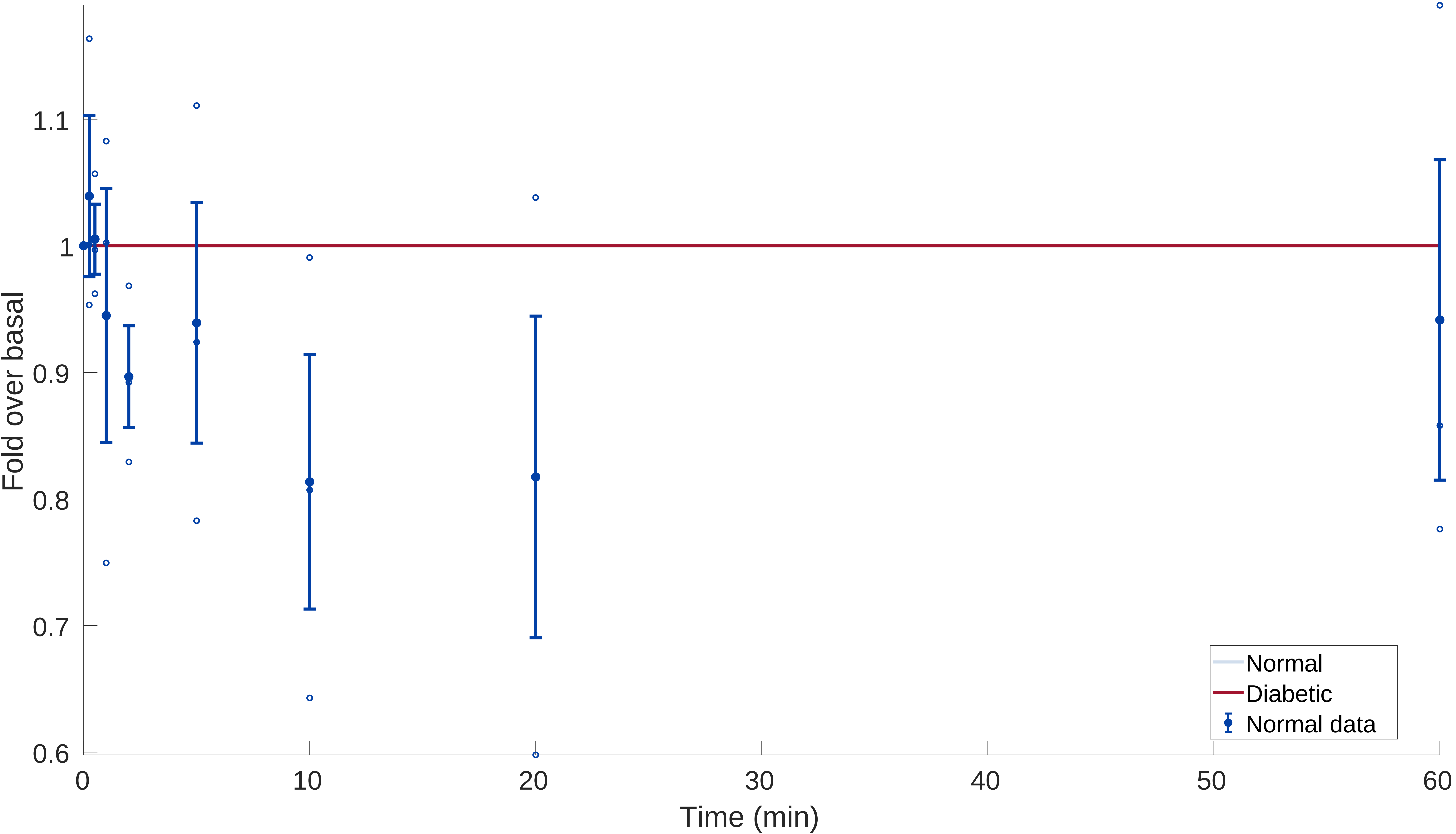

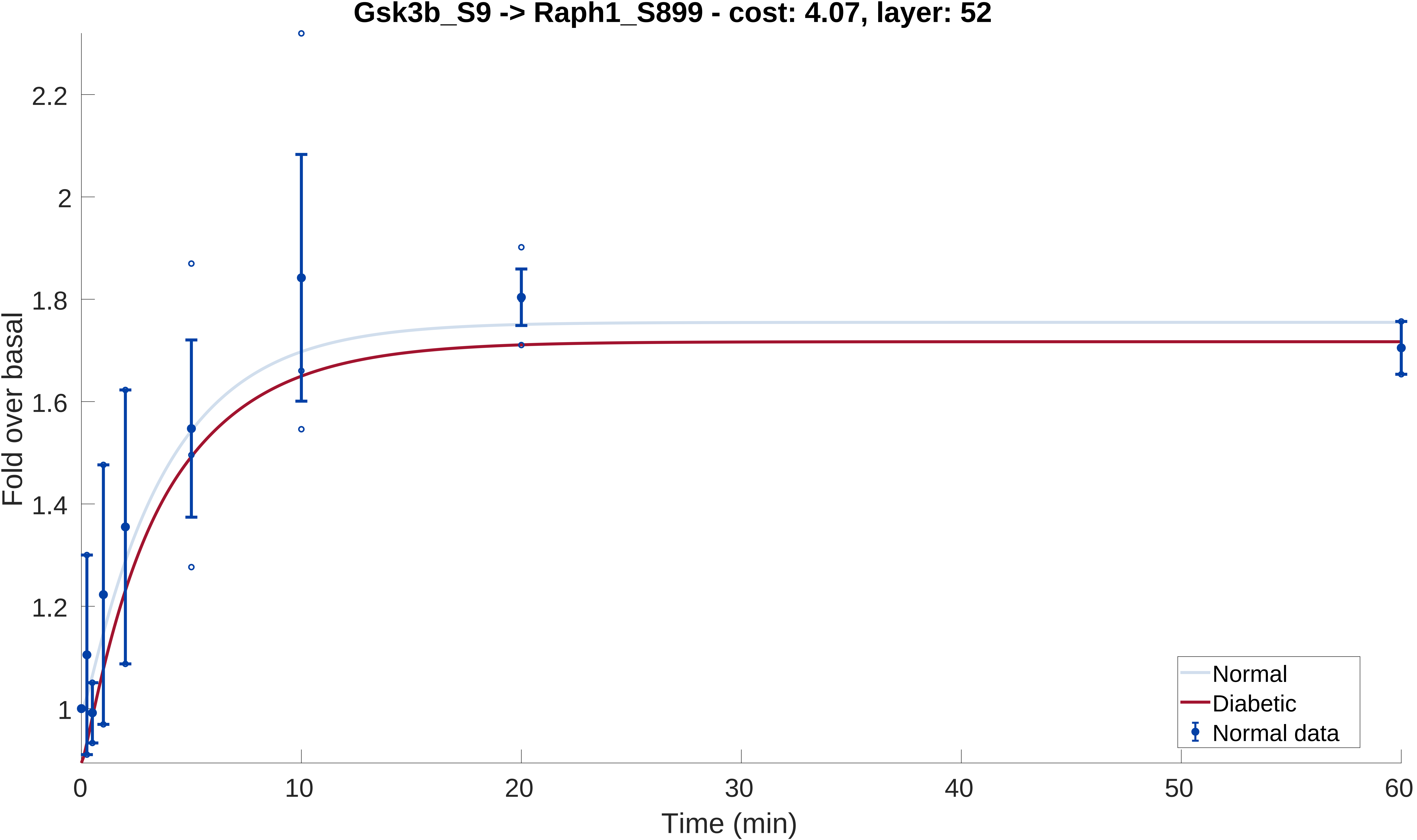

Gsk3b_S9 -> Raph1_S899 - cost: 4.07, layer: 52
Fold over basal
2.2
2
1.8
1.6
1.4
1.2
1
0
10
20
30
40
50
60
Time (min)
Normal
Diabetic
Normal data

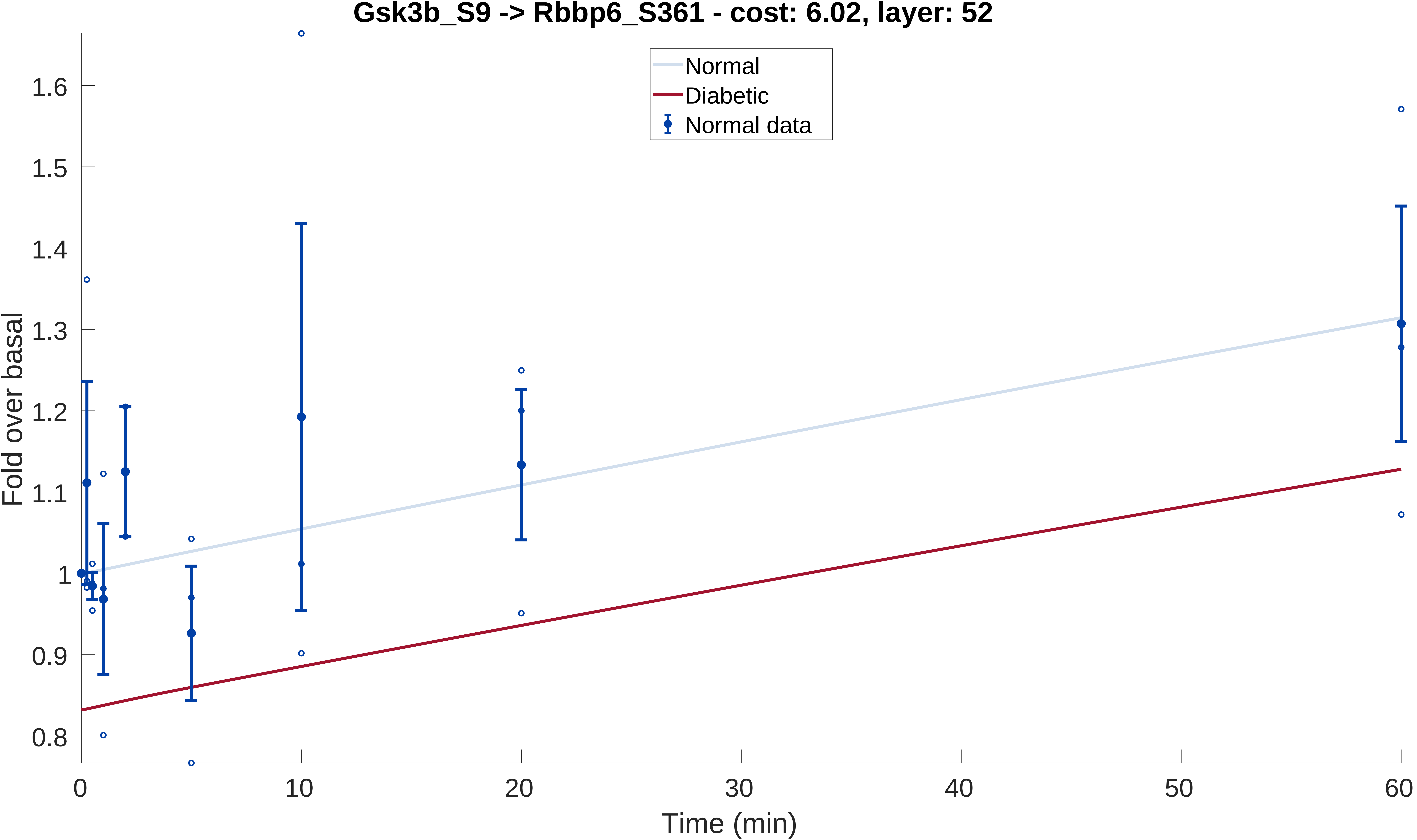

Gsk3b_S9 -> Rbbp6_S361 - cost: 6.02, layer: 52
Normal
Diabetic
Normal data
Fold over basal
Time (min)
0.8
0.9
1
1.1
1.2
1.3
1.4
1.5
1.6
0
10
20
30
40
50
60

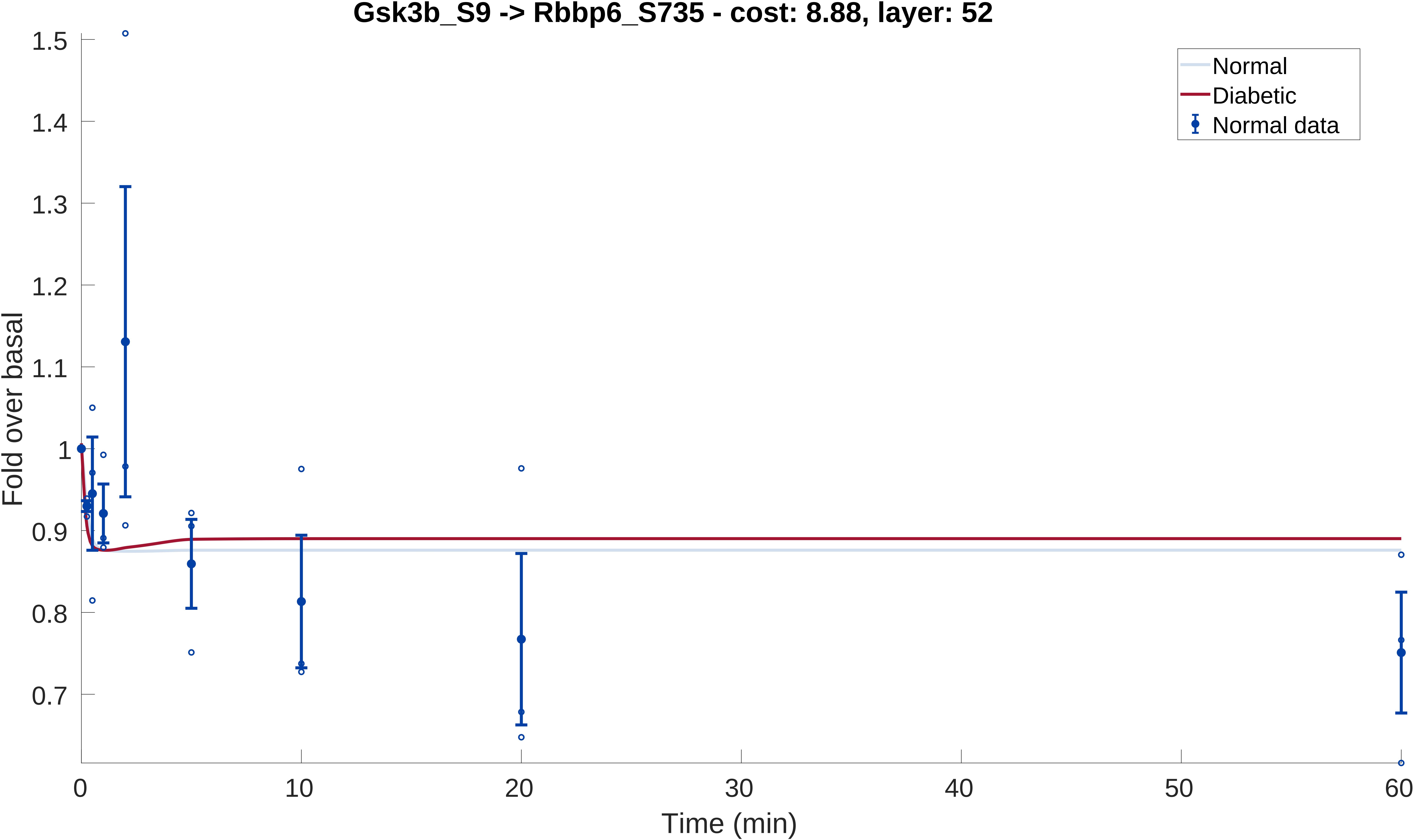
Gsk3b_S9 -> Rbbp6_S735 - cost: 8.88, layer: 52
Normal
Diabetic
Normal data
Fold over basal
Time (min)
1.5
1.4
1.3
1.2
1.1
1
0.9
0.8
0.7
0
10
20
30
40
50
60

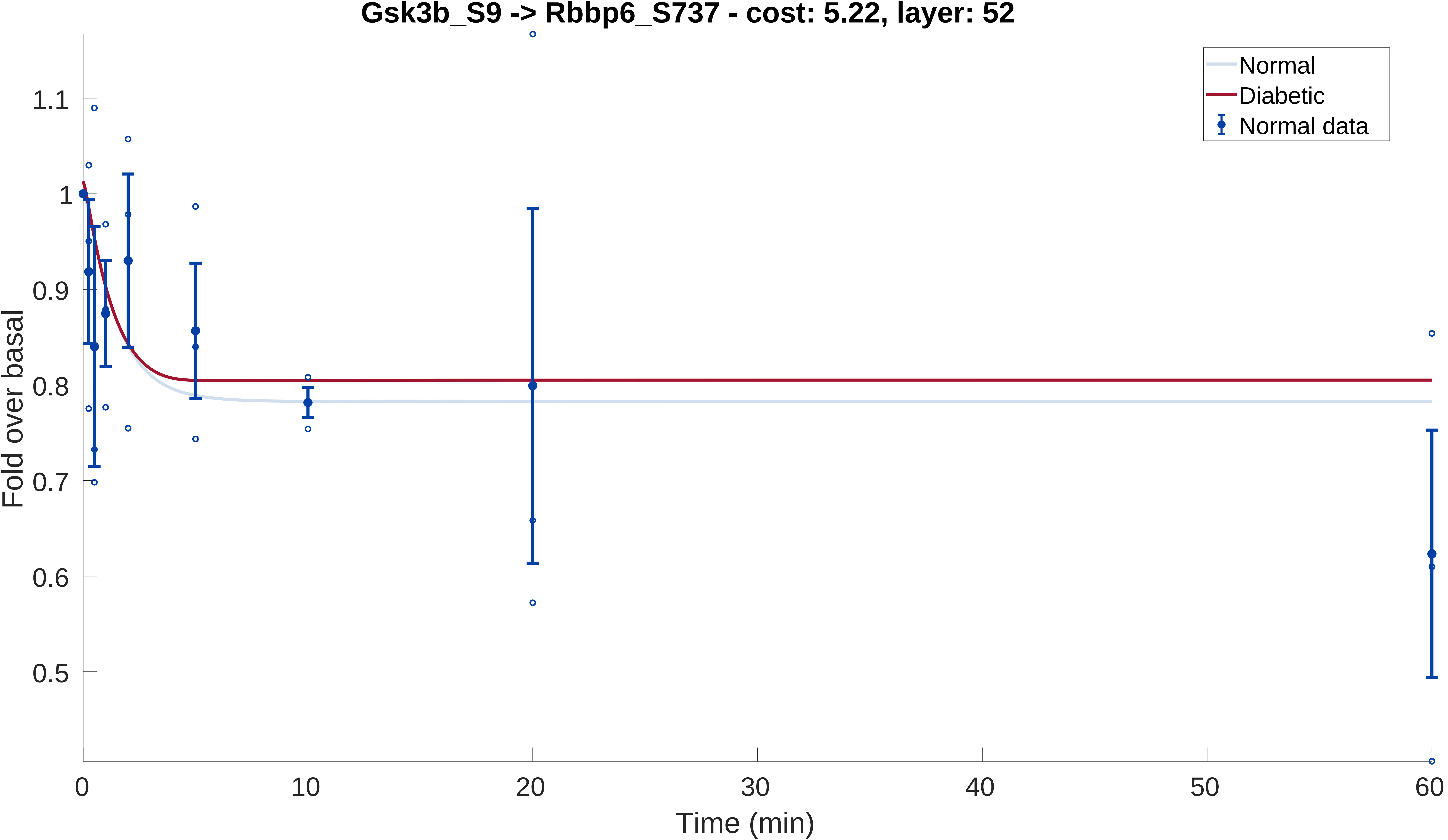
Gsk3b_S9 -> Rbbp6_S737 - cost: 5.22, layer: 52
Normal
Diabetic
Normal data
Fold over basal
Time (min)
1.1
1
0.9
0.8
0.7
0.6
0.5
0
10
20
30
40
50
60

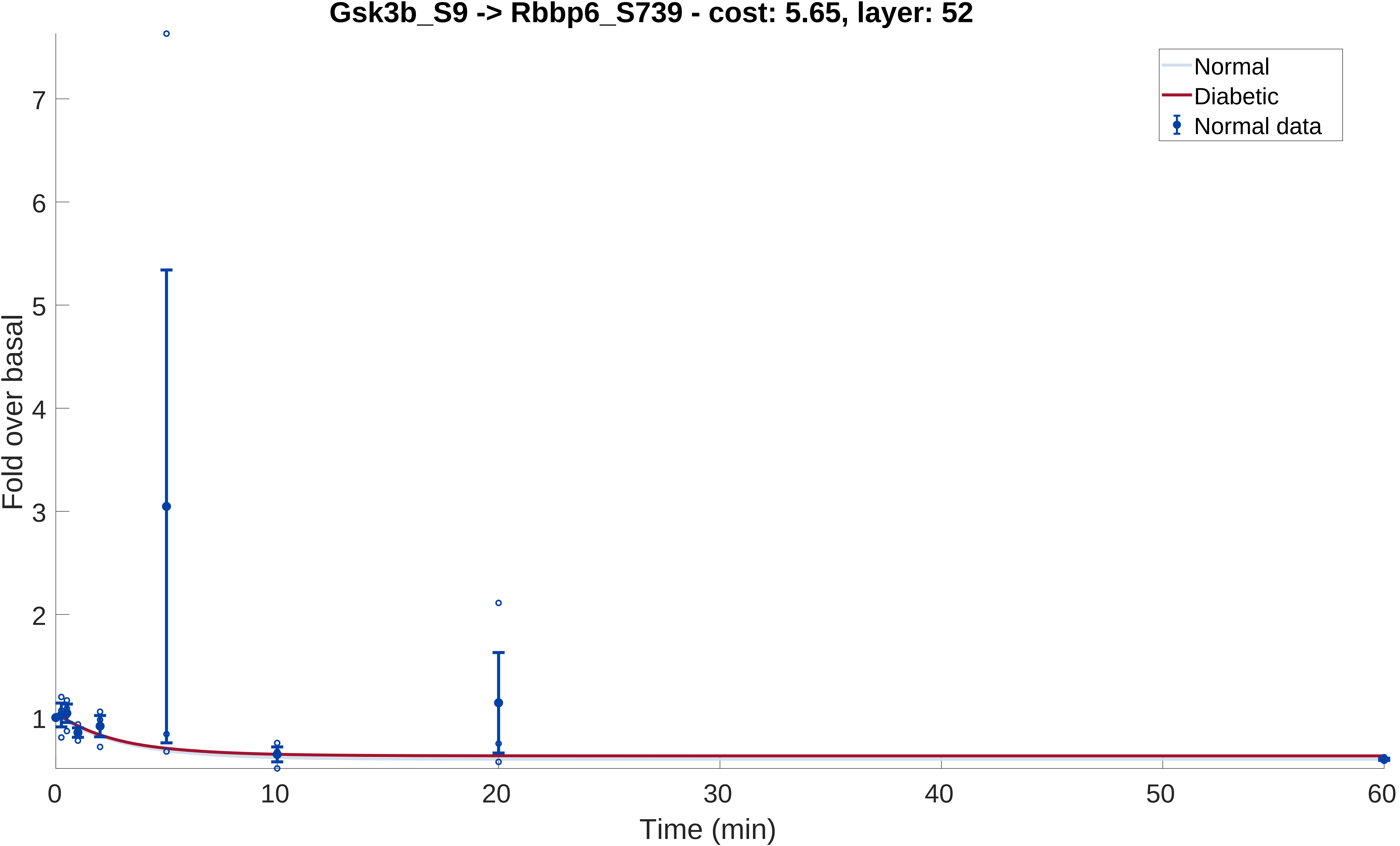

Gsk3b_S9 -> Rbbp6_S739 - cost: 5.65, layer: 52
Normal
Diabetic
Normal data
Fold over basal
Time (min)
0
10
20
30
40
50
60
1
2
3
4
5
6
7

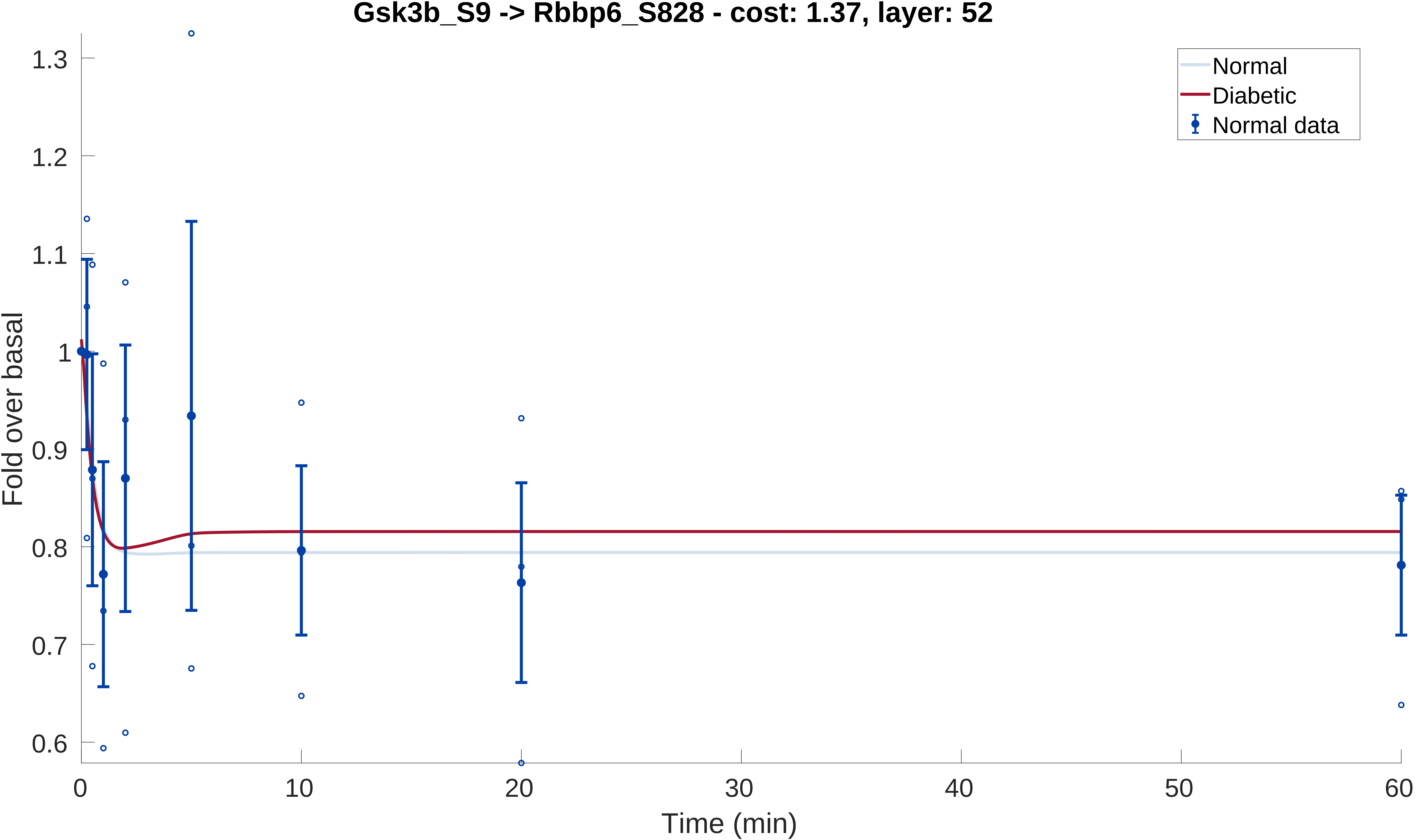

Gsk3b_S9 -> Rbbp6_S828 - cost: 1.37, layer: 52
Normal
Diabetic
Normal data
Fold over basal
Time (min)
1.3
1.2
1.1
1
0.9
0.8
0.7
0.6
0
10
20
30
40
50
60

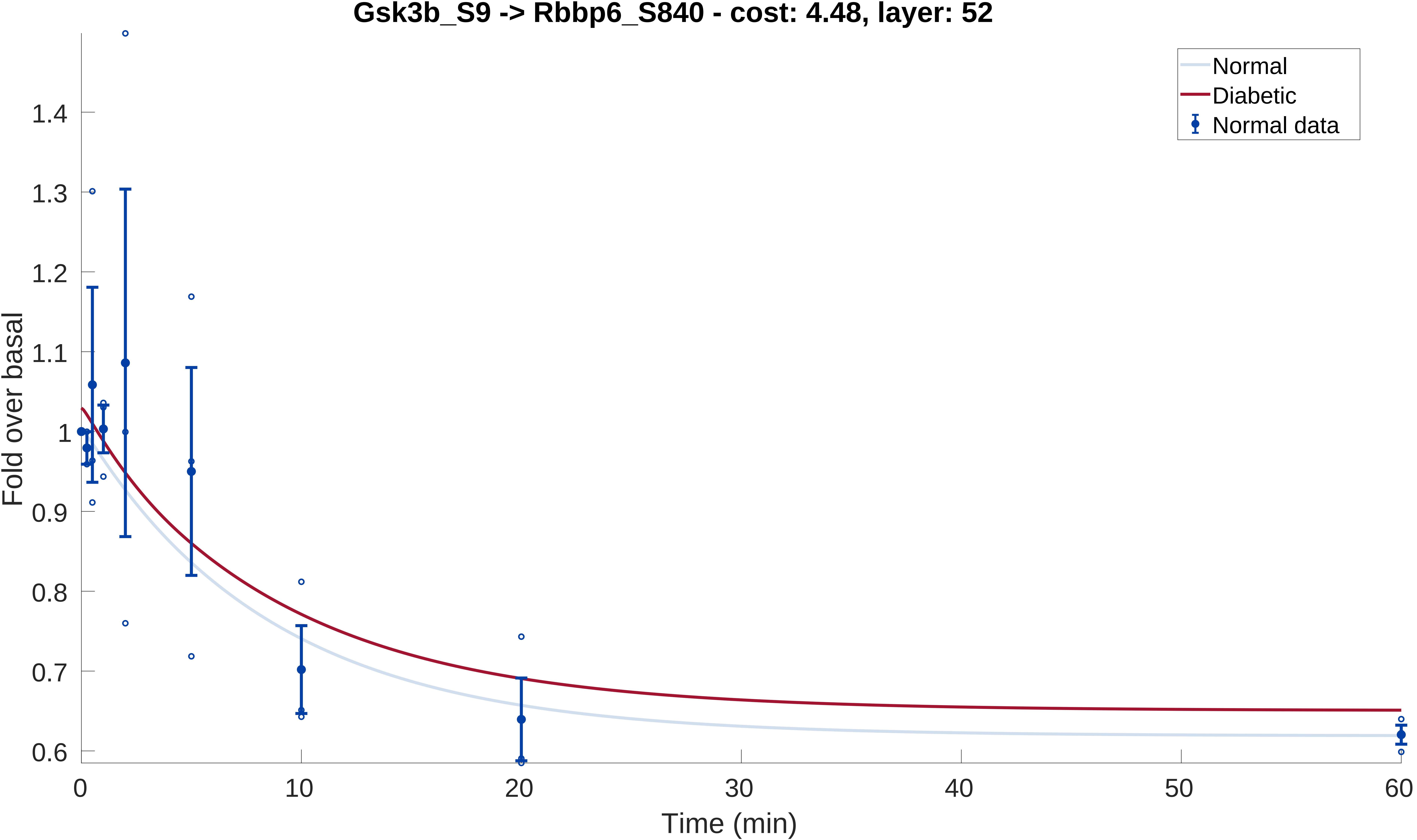
Gsk3b_S9 -> Rbbp6_S840 - cost: 4.48, layer: 52
Normal
Diabetic
Normal data
Fold over basal
Time (min)
1.4
1.3
1.2
1.1
1
0.9
0.8
0.7
0.6
0
10
20
30
40
50
60

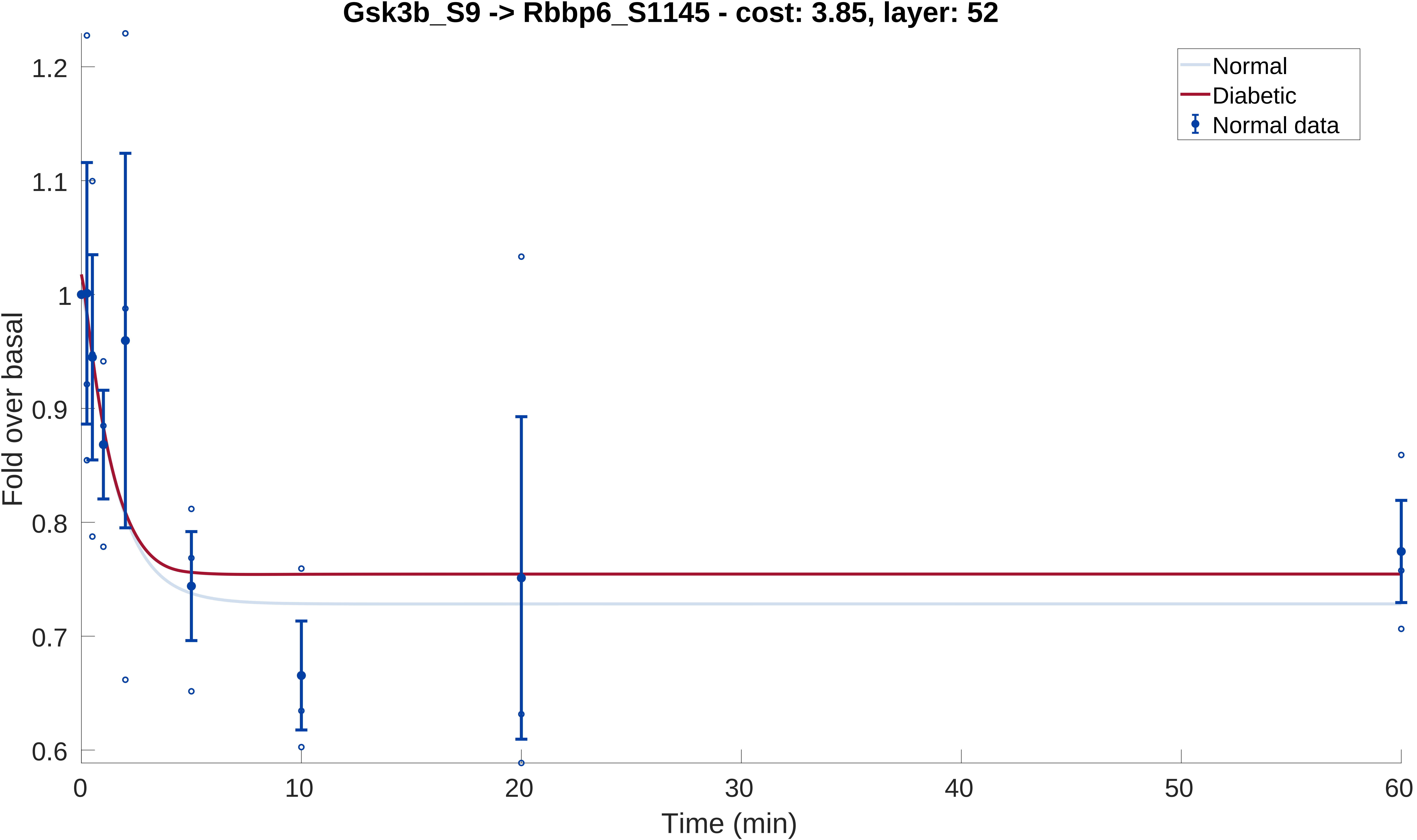
Gsk3b_S9 -> Rbbp6_S1145 - cost: 3.85, layer: 52
Normal
Diabetic
Normal data
Fold over basal
1.2
1.1
1
0.9
0.8
0.7
0.6
0
10
20
30
40
50
60
Time (min)

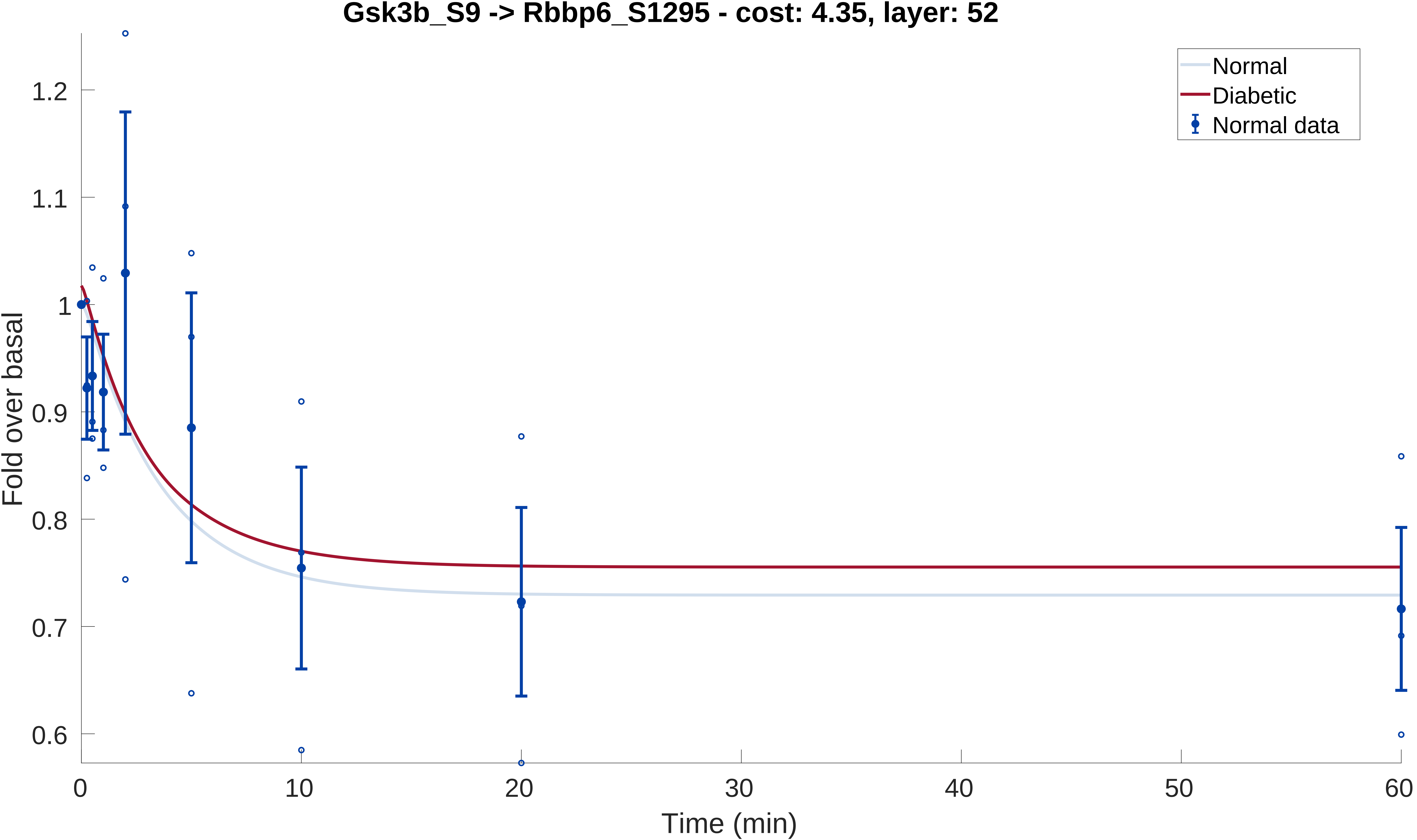

Gsk3b_S9 -> Rbbp6_S1295 - cost: 4.35, layer: 52
Normal
Diabetic
Normal data
Fold over basal
Time (min)
1.2
1.1
1
0.9
0.8
0.7
0.6
0
10
20
30
40
50
60

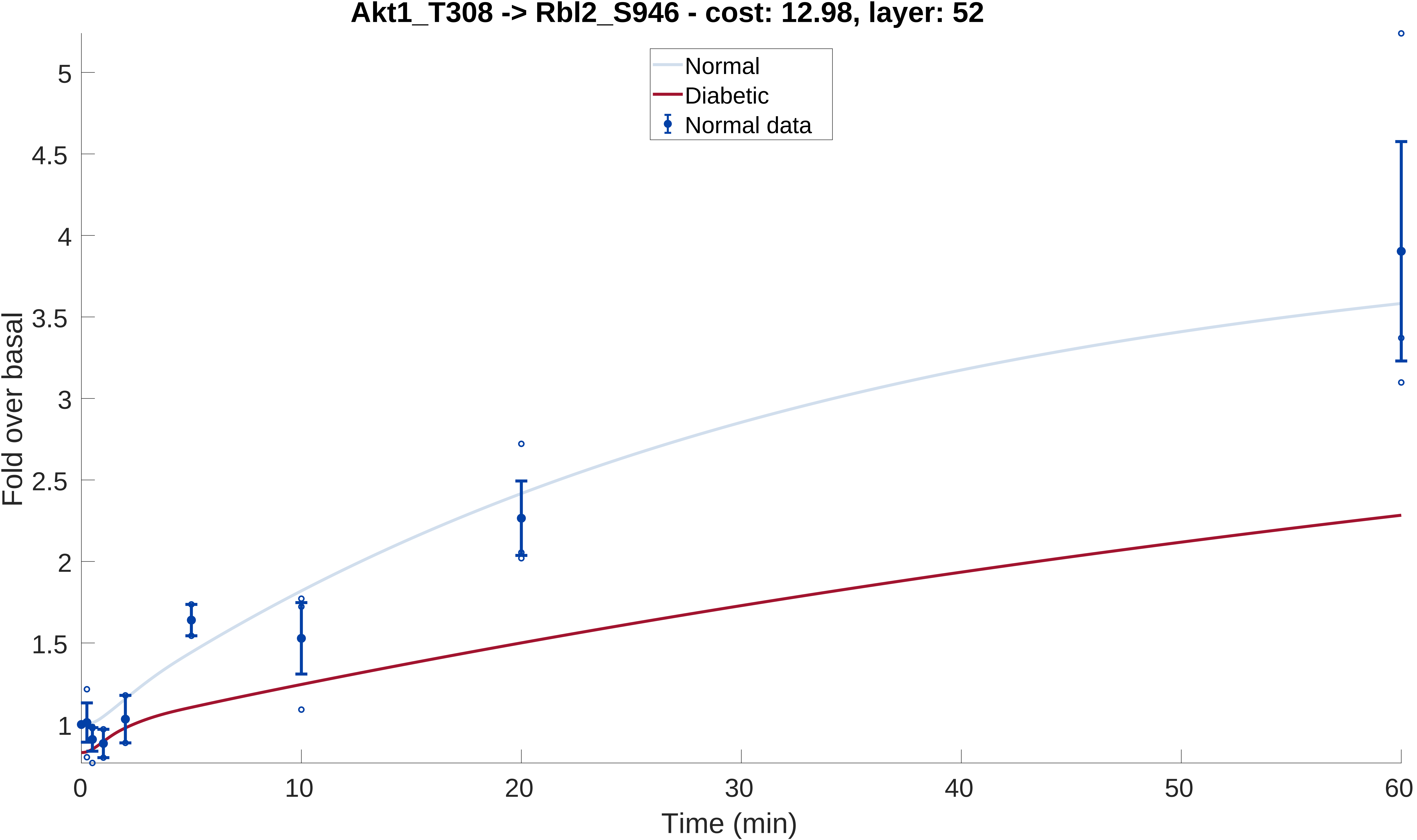

Akt1_T308 -> Rbl2_S946 - cost: 12.98, layer: 52
Normal
Diabetic
Normal data
Fold over basal
Time (min)
5
4.5
4
3.5
3
2.5
2
1.5
1
0
10
20
30
40
50
60

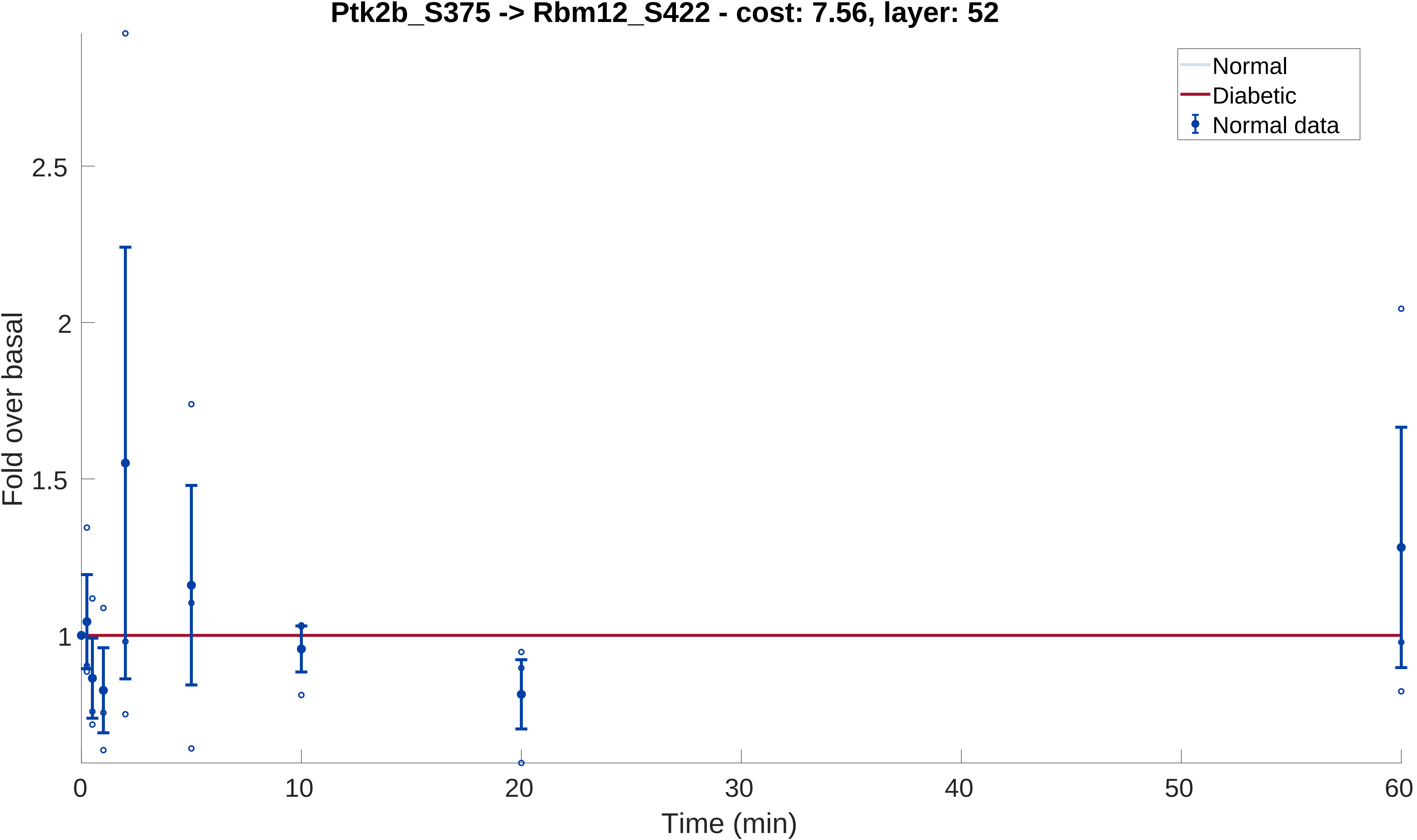

Ptk2b_S375 -> Rbm12_S422 - cost: 7.56, layer: 52
Normal
Diabetic
Normal data
Fold over basal
2.5
2
1.5
1
0
10
20
30
40
50
60
Time (min)

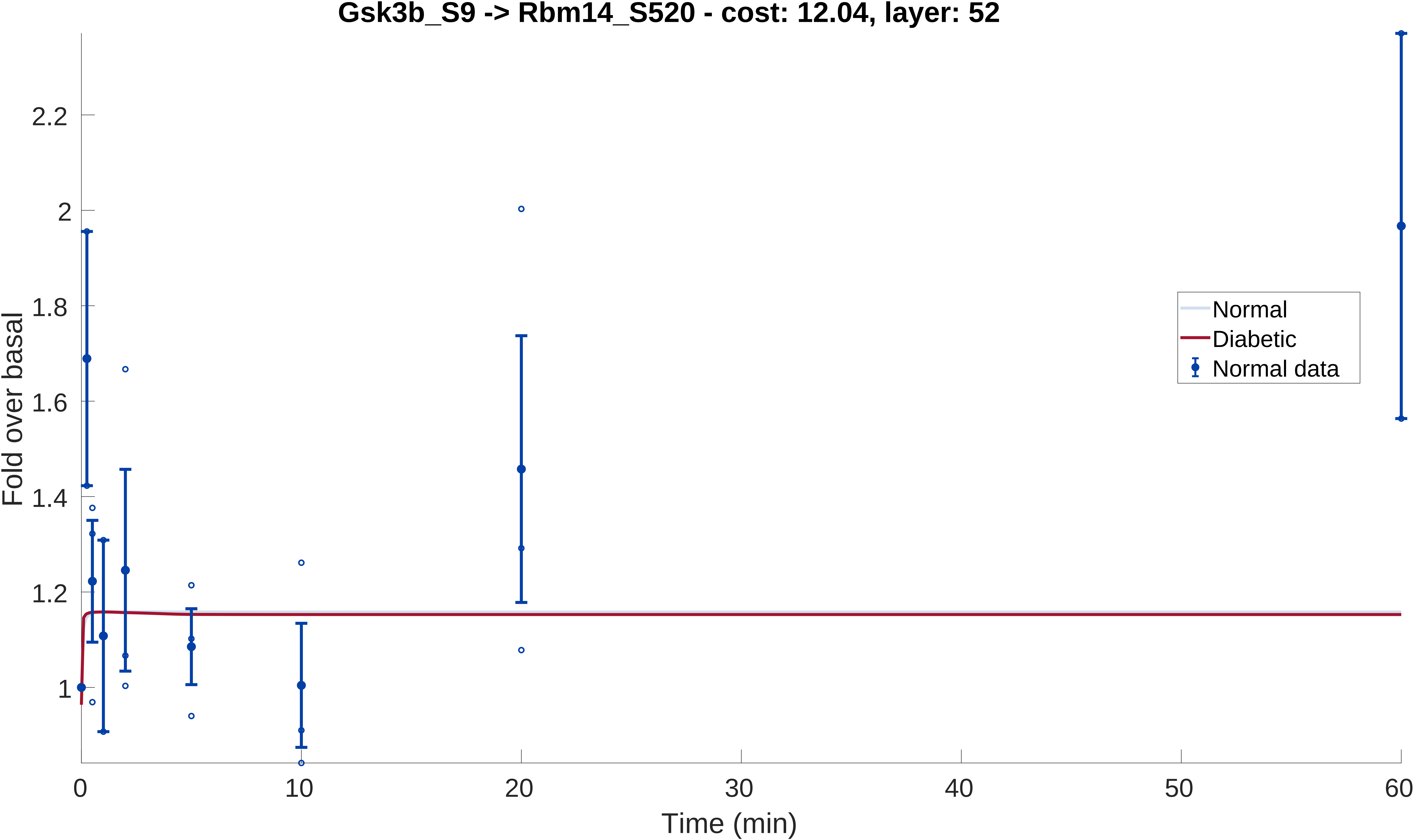
Gsk3b_S9 -> Rbm14_S520 - cost: 12.04, layer: 52
Fold over basal
Time (min)
Normal
Diabetic
Normal data
2.2
2
1.8
1.6
1.4
1.2
1
0
10
20
30
40
50
60

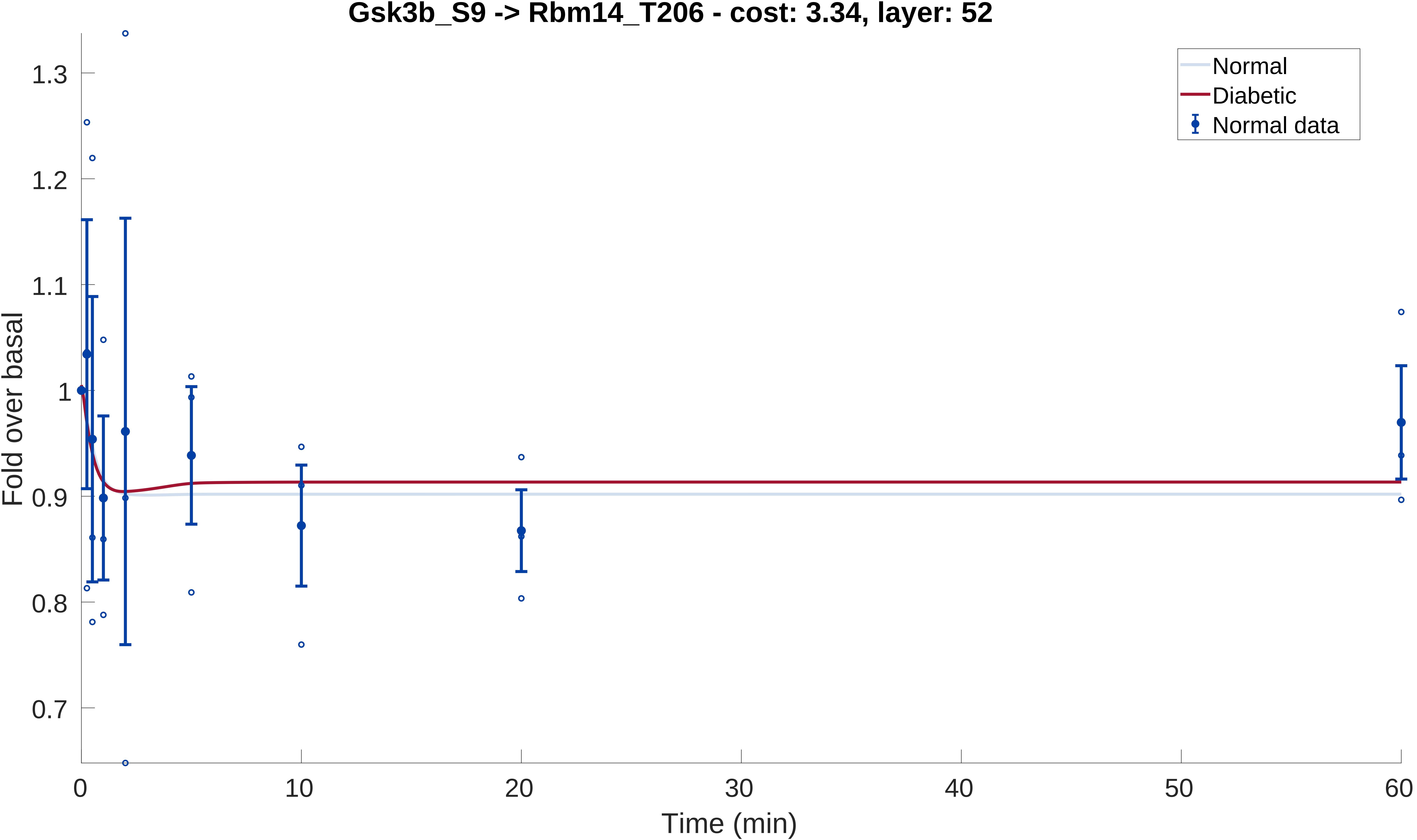

Gsk3b_S9 -> Rbm14_T206 - cost: 3.34, layer: 52
Normal
Diabetic
Normal data
Fold over basal
Time (min)
1.3
1.2
1.1
1
0.9
0.8
0.7
0
10
20
30
40
50
60

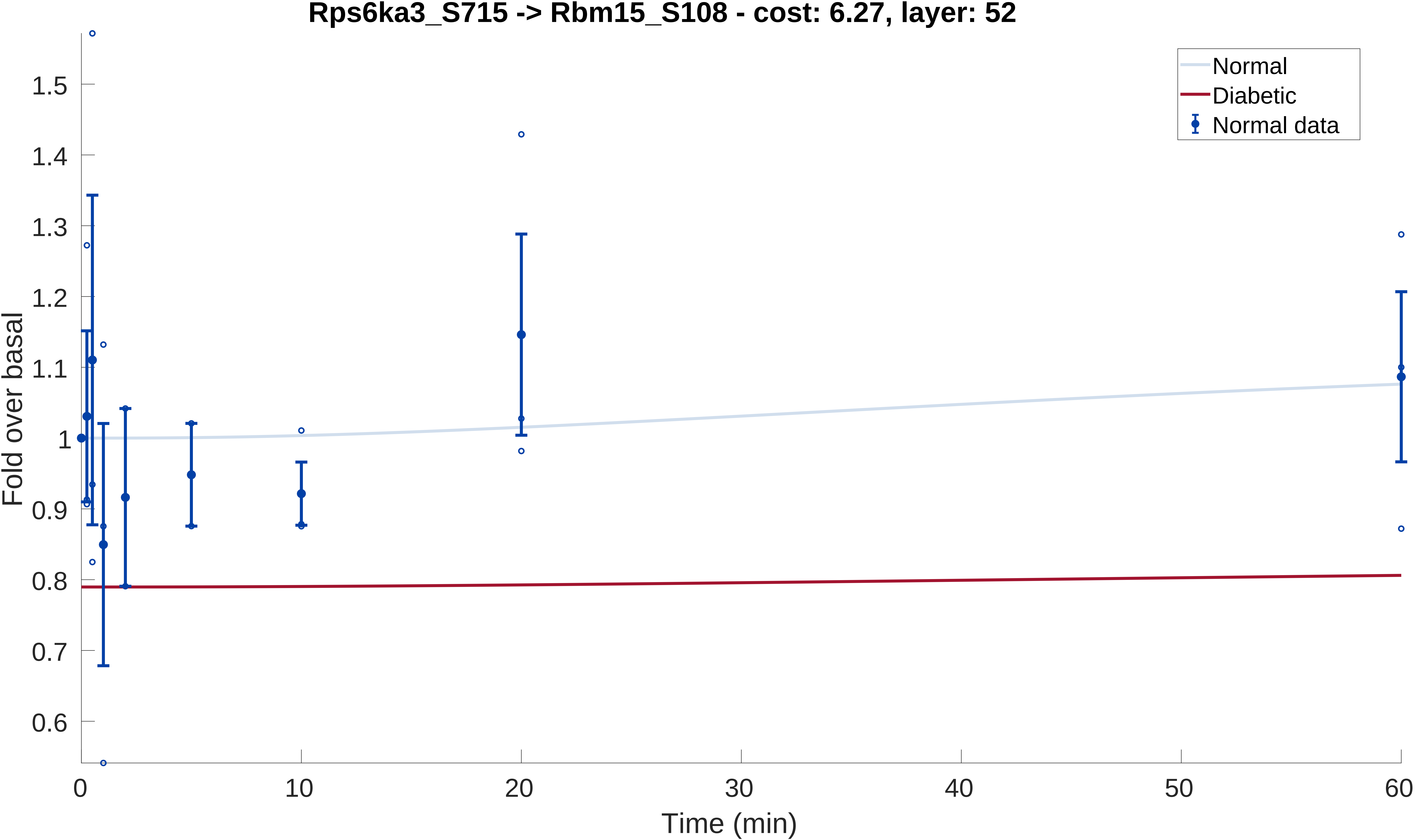

Rps6ka3_S715 -> Rbm15_S108 - cost: 6.27, layer: 52
Normal
Diabetic
Normal data
Fold over basal
1.5
1.4
1.3
1.2
1.1
1
0.9
0.8
0.7
0.6
0
10
20
30
40
50
60
Time (min)

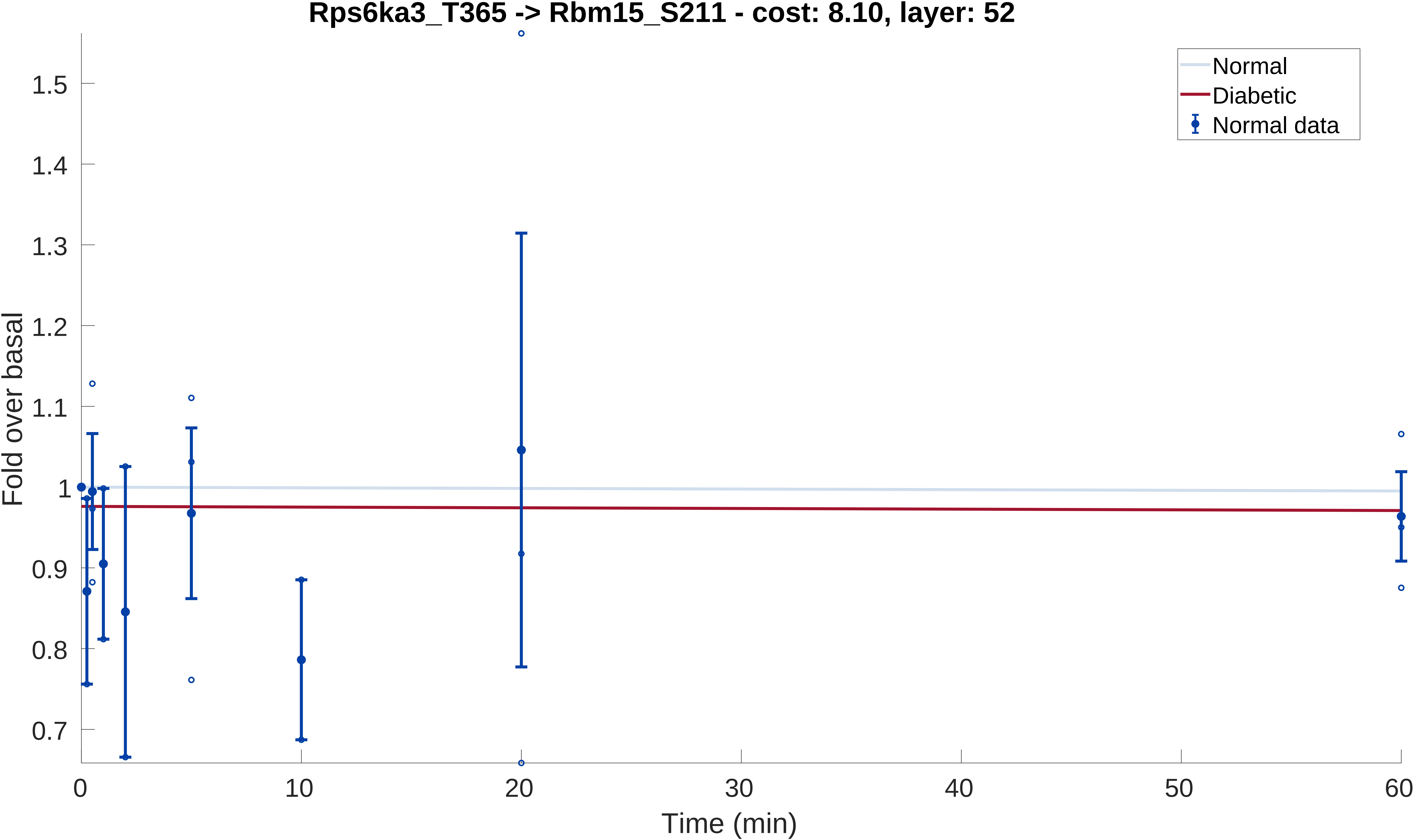
Rps6ka3_T365 -> Rbm15_S211 - cost: 8.10, layer: 52
Normal
Diabetic
Normal data
Fold over basal
1.5
1.4
1.3
1.2
1.1
1
0.9
0.8
0.7
0
10
20
30
40
50
60
Time (min)

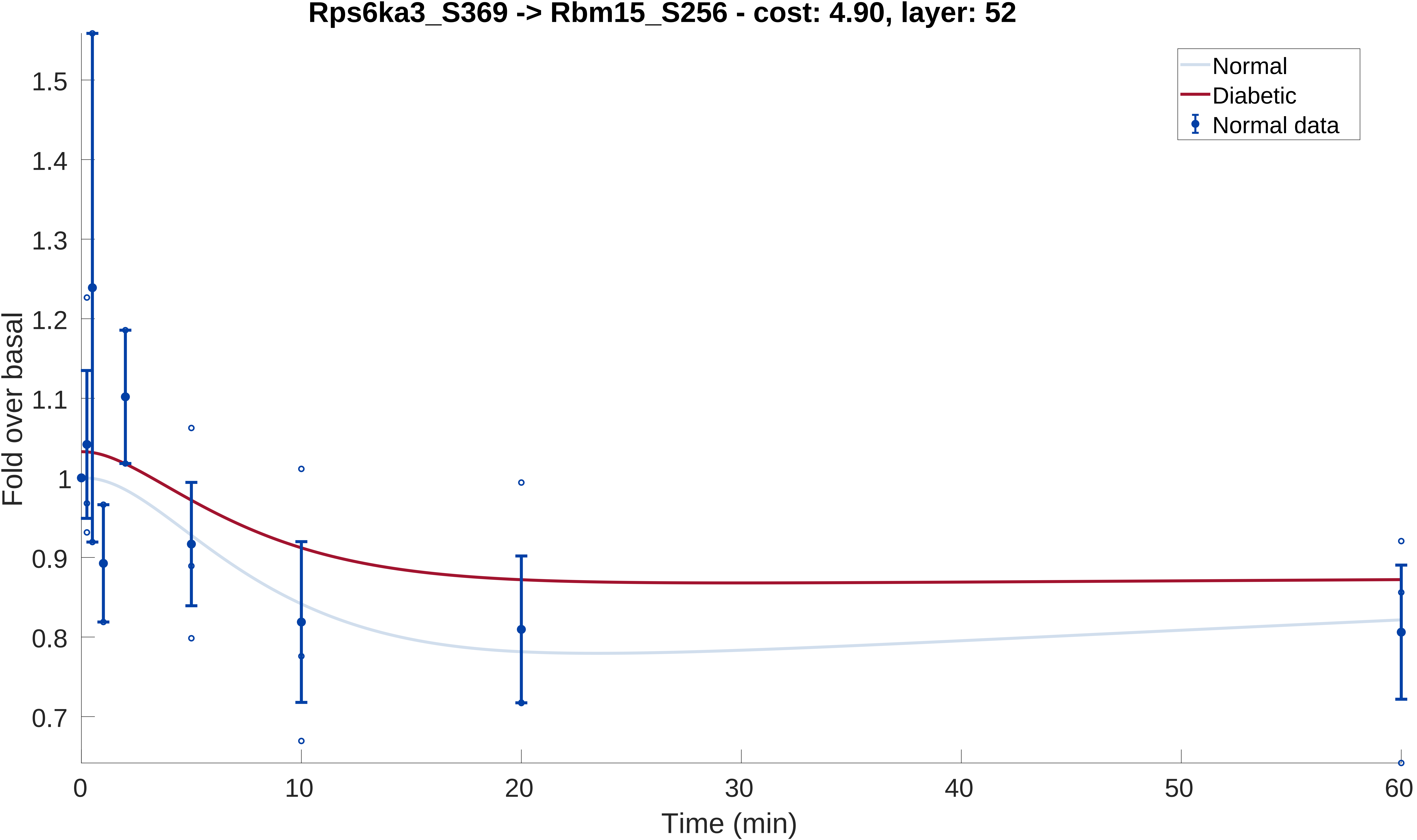

Rps6ka3_S369 -> Rbm15_S256 - cost: 4.90, layer: 52
Normal
Diabetic
Normal data
Fold over basal
Time (min)
0.7
0.8
0.9
1
1.1
1.2
1.3
1.4
1.5
0
10
20
30
40
50
60

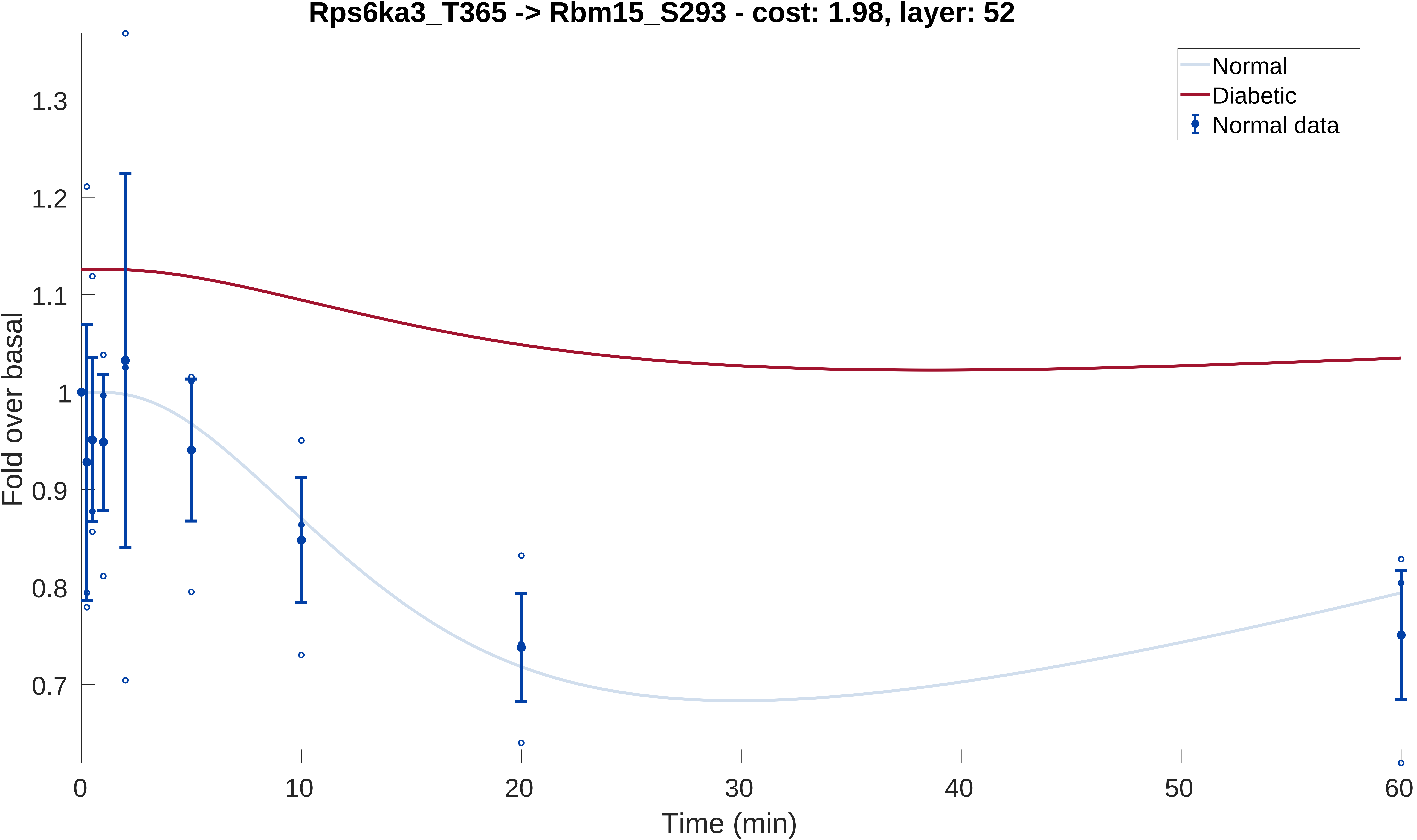
Rps6ka3_T365 -> Rbm15_S293 - cost: 1.98, layer: 52
Normal
Diabetic
Normal data
Fold over basal
Time (min)
0
10
20
30
40
50
60
0.7
0.8
0.9
1
1.1
1.2
1.3

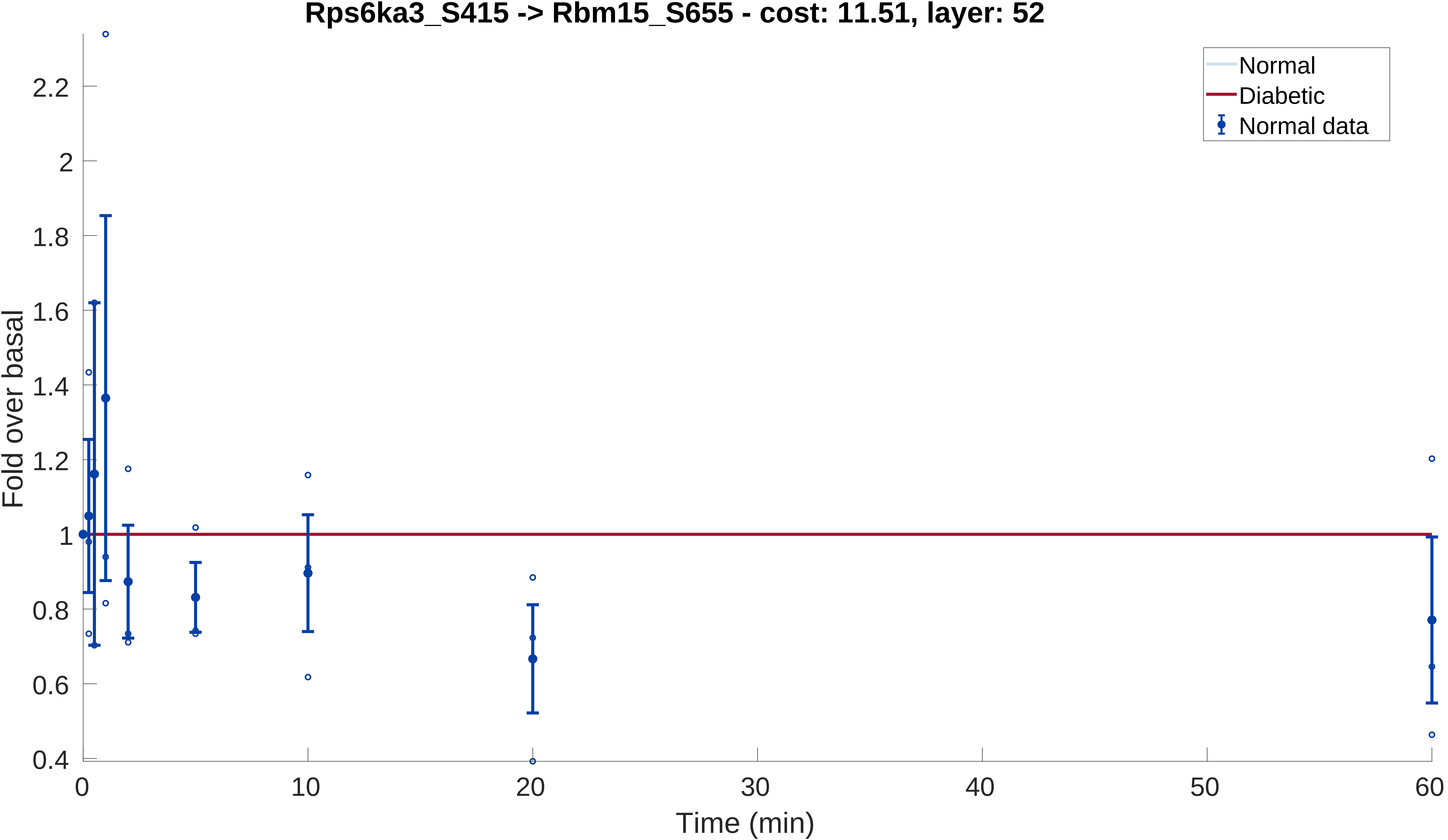

Rps6ka3_S415 -> Rbm15_S655 - cost: 11.51, layer: 52
Normal
Diabetic
Normal data
Fold over basal
Time (min)
0.4
0.6
0.8
1
1.2
1.4
1.6
1.8
2
2.2
0
10
20
30
40
50
60

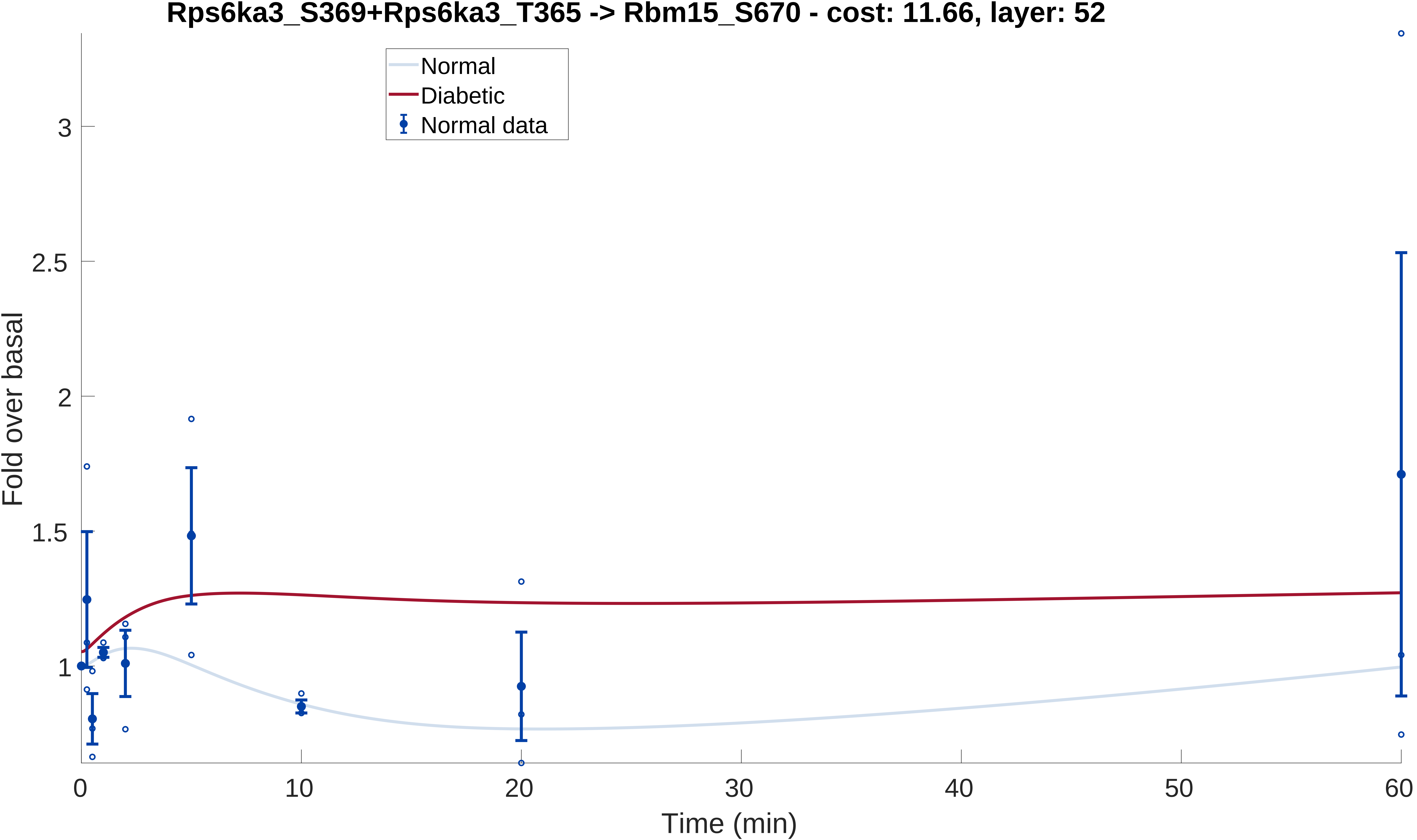

Rps6ka3_S369+Rps6ka3_T365 -> Rbm15_S670 - cost: 11.66, layer: 52
Normal
Diabetic
Normal data
Fold over basal
Time (min)
3
2.5
2
1.5
1
0
10
20
30
40
50
60

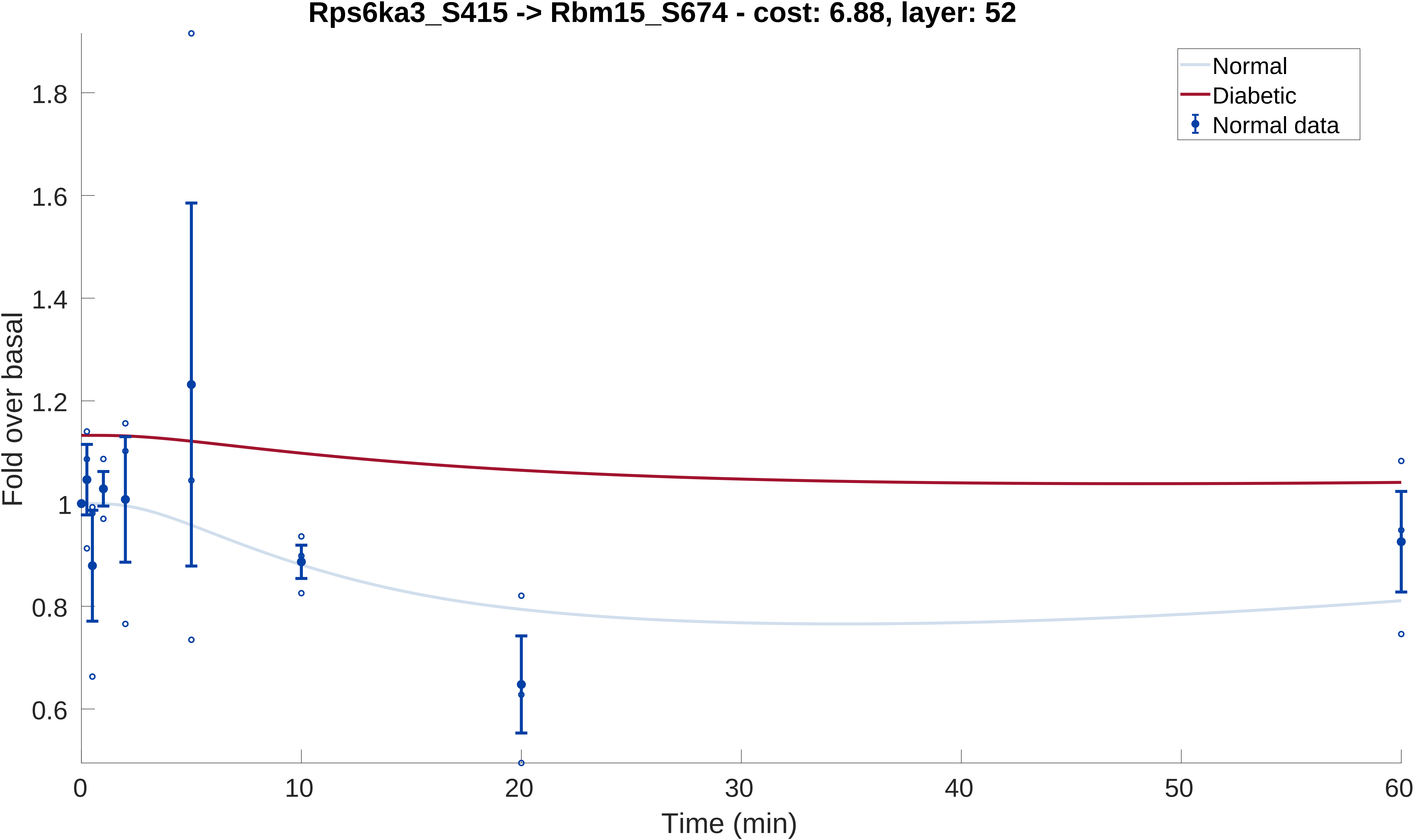

Rps6ka3_S415 -> Rbm15_S674 - cost: 6.88, layer: 52
Normal
Diabetic
Normal data
Fold over basal
1.8
1.6
1.4
1.2
1
0.8
0.6
0
10
20
30
40
50
60
Time (min)

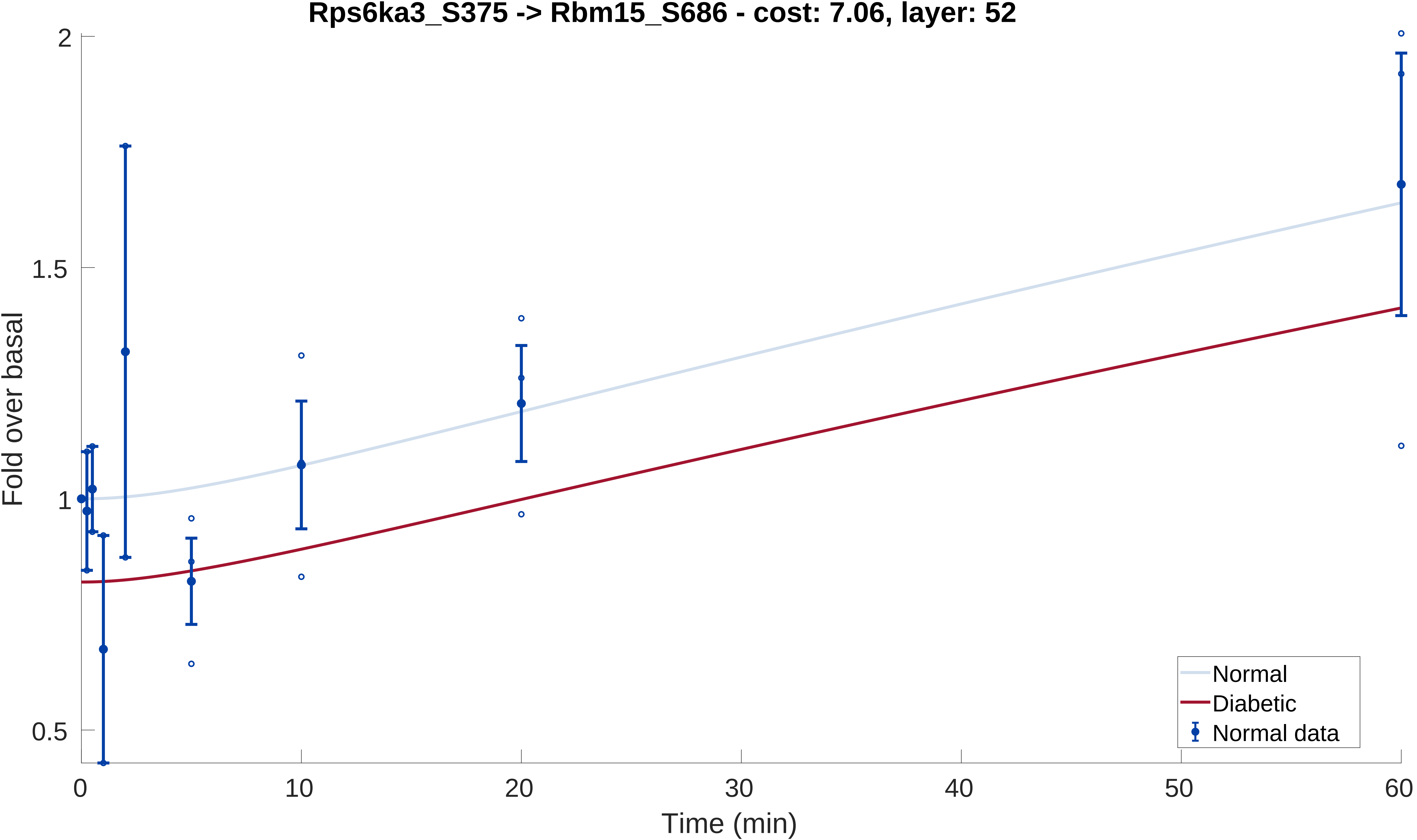

Rps6ka3_S375 -> Rbm15_S686 - cost: 7.06, layer: 52
Fold over basal
2
1.5
1
0.5
0
10
20
30
40
50
60
Time (min)
Normal
Diabetic
Normal data

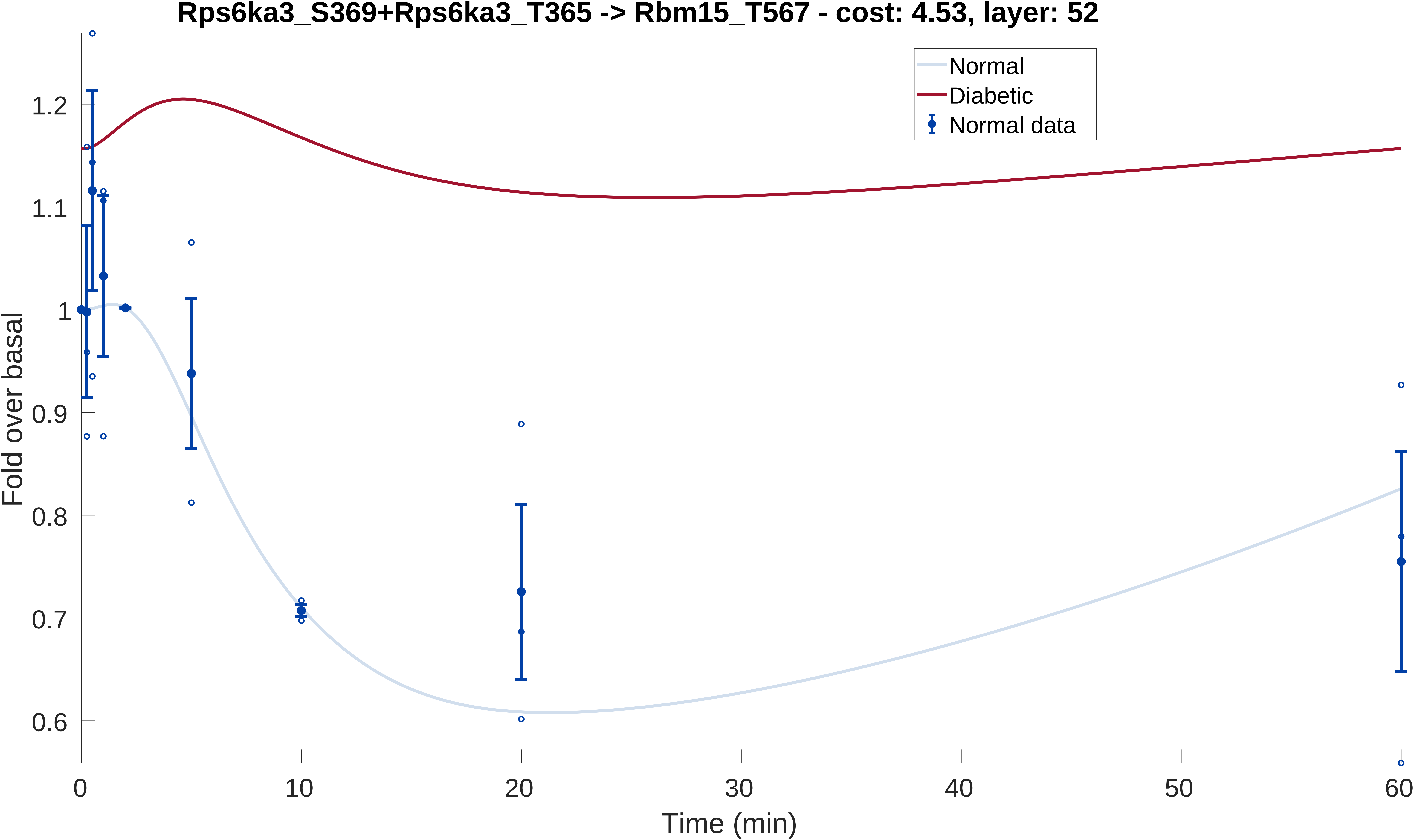
Rps6ka3_S369+Rps6ka3_T365 -> Rbm15_T567 - cost: 4.53, layer: 52
Normal
Diabetic
Normal data
Fold over basal
Time (min)
0.6
0.7
0.8
0.9
1
1.1
1.2
0
10
20
30
40
50
60

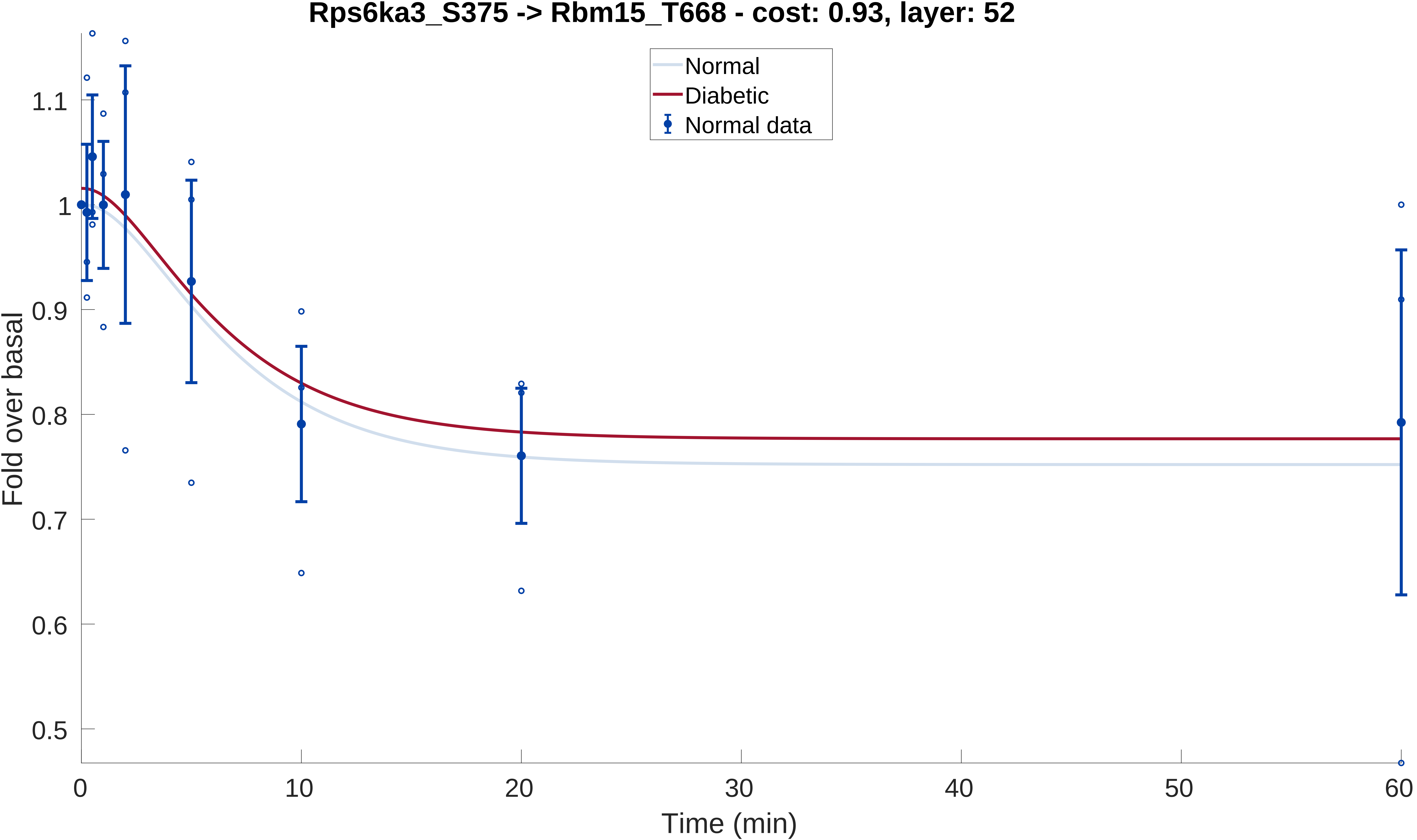

Rps6ka3_S375 -> Rbm15_T668 - cost: 0.93, layer: 52
Normal
Diabetic
Normal data
Fold over basal
Time (min)
0.5
0.6
0.7
0.8
0.9
1
1.1
0
10
20
30
40
50
60

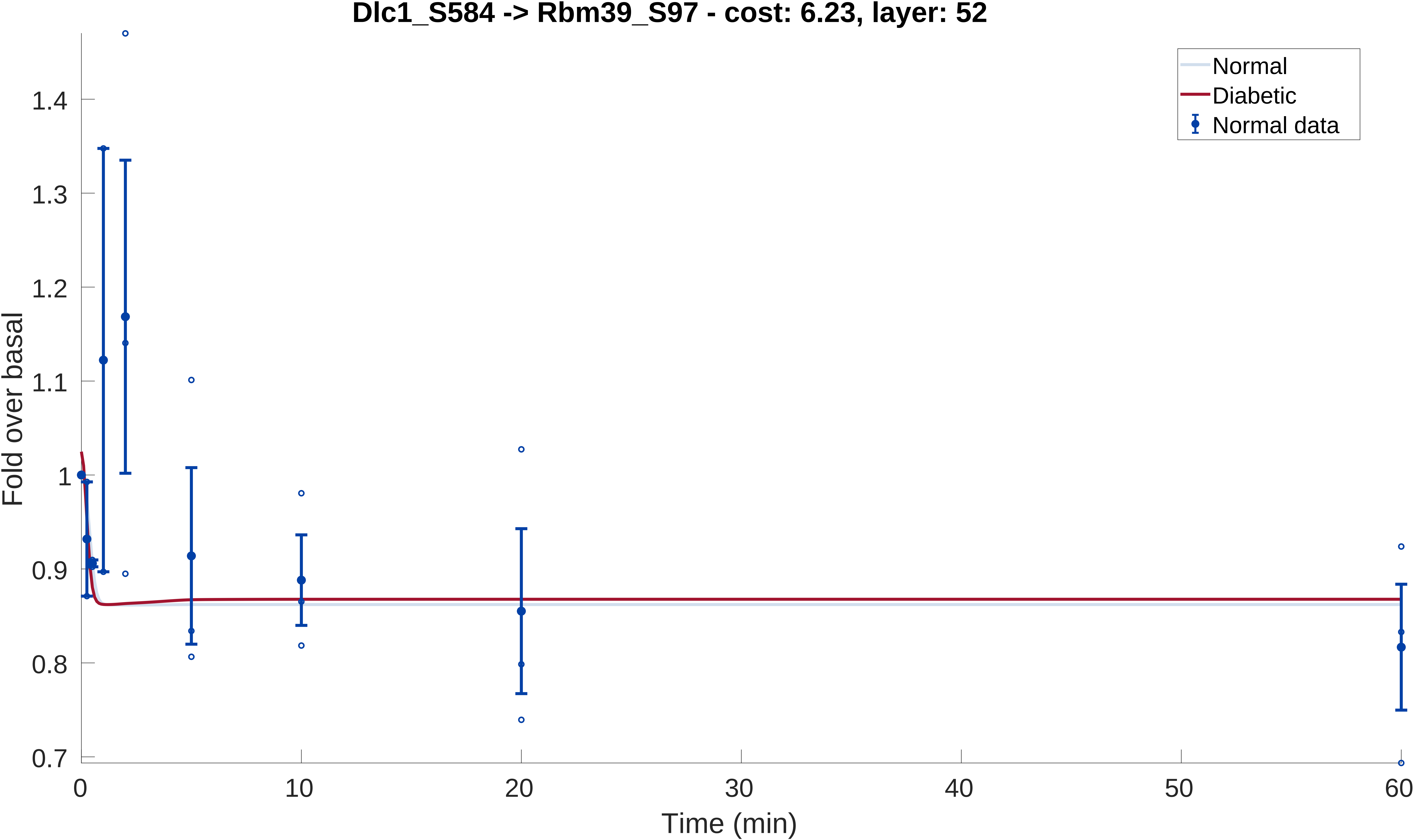

Dlc1_S584 -> Rbm39_S97 - cost: 6.23, layer: 52
Normal
Diabetic
Normal data
Fold over basal
Time (min)
1.4
1.3
1.2
1.1
1
0.9
0.8
0.7
0
10
20
30
40
50
60

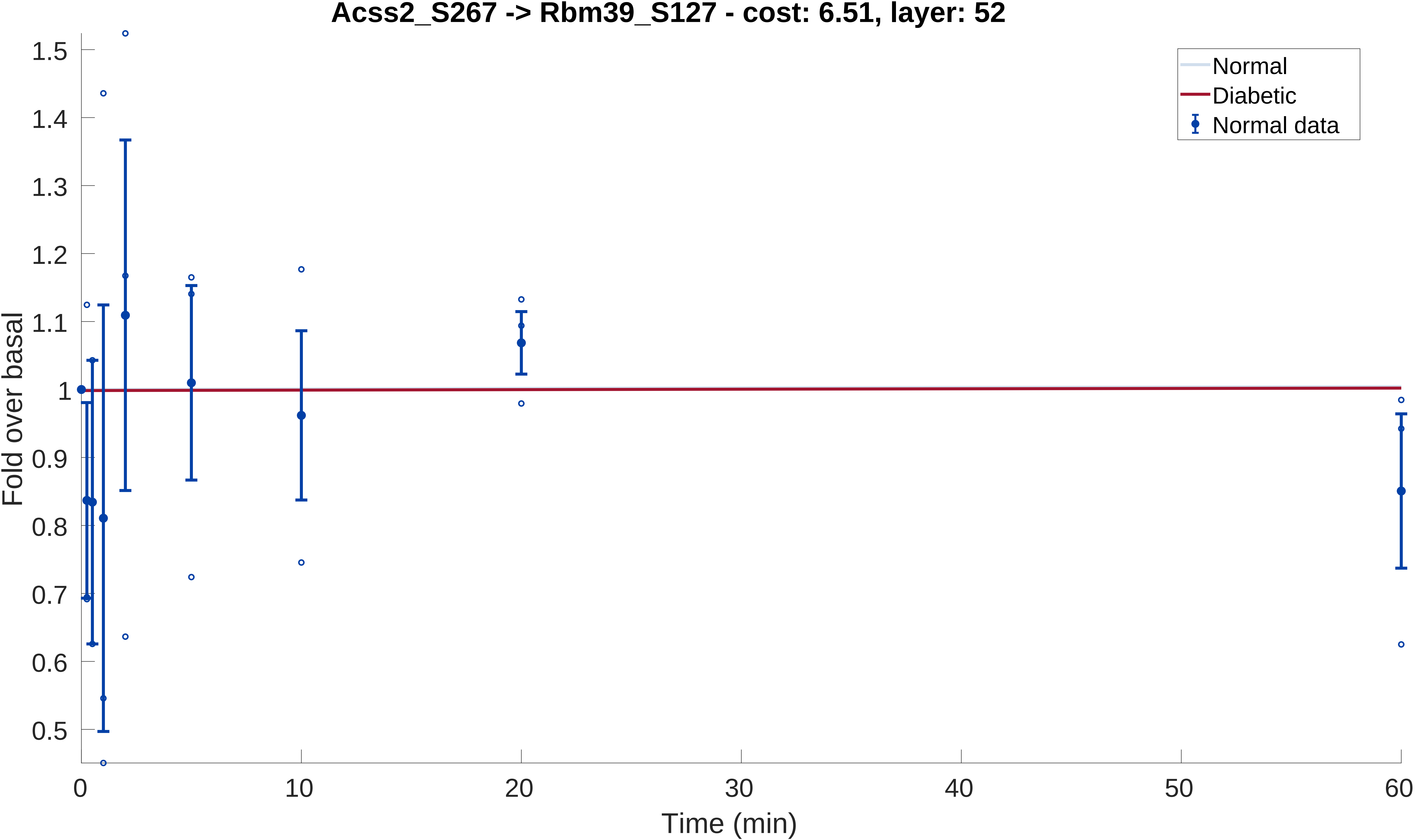

Acss2_S267 -> Rbm39_S127 - cost: 6.51, layer: 52
Normal
Diabetic
Normal data
Fold over basal
1.5
1.4
1.3
1.2
1.1
1
0.9
0.8
0.7
0.6
0.5
0
10
20
30
40
50
60
Time (min)

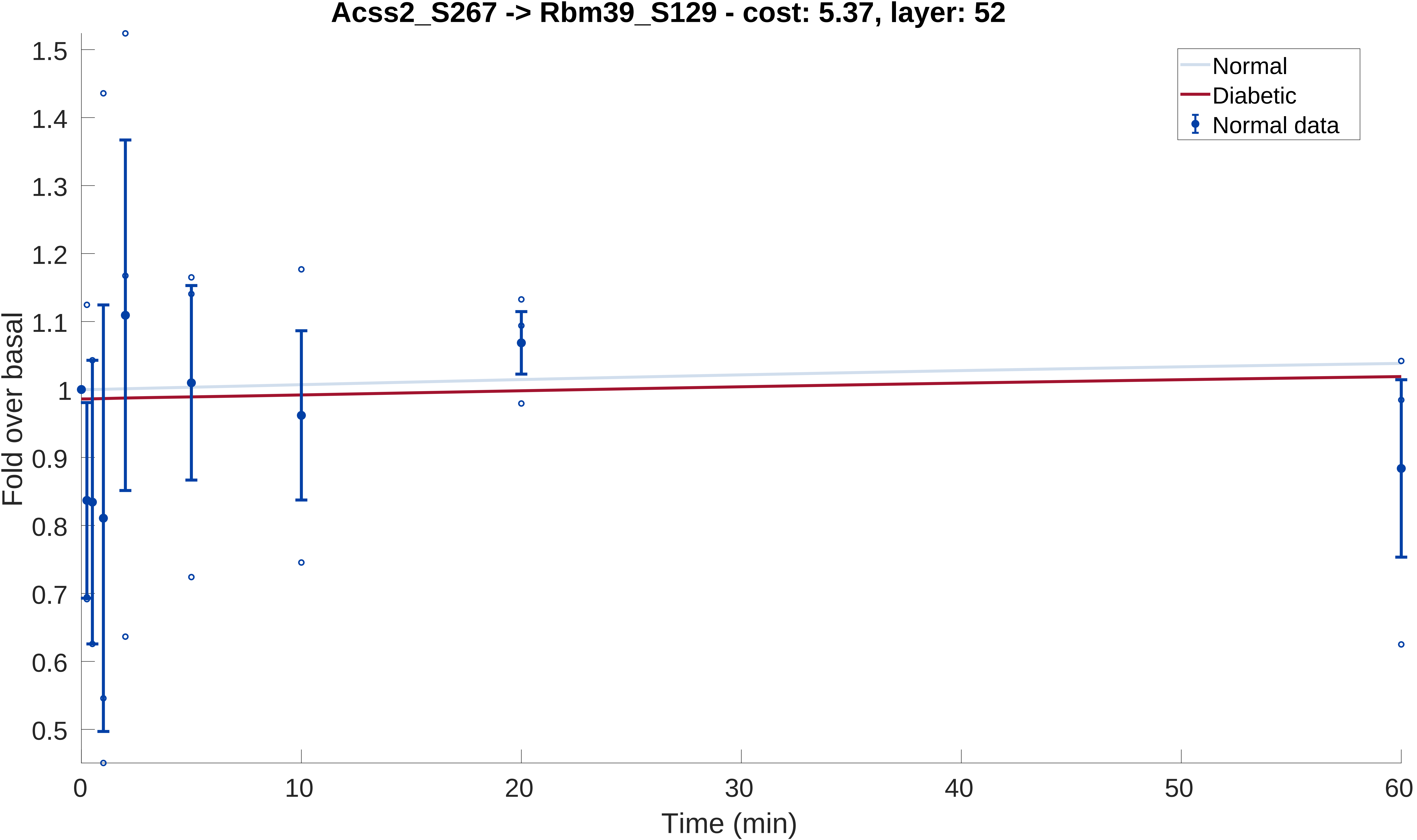

Acss2_S267 -> Rbm39_S129 - cost: 5.37, layer: 52
Fold over basal
Time (min)
Normal
Diabetic
Normal data
0.5
0.6
0.7
0.8
0.9
1
1.1
1.2
1.3
1.4
1.5
0
10
20
30
40
50
60

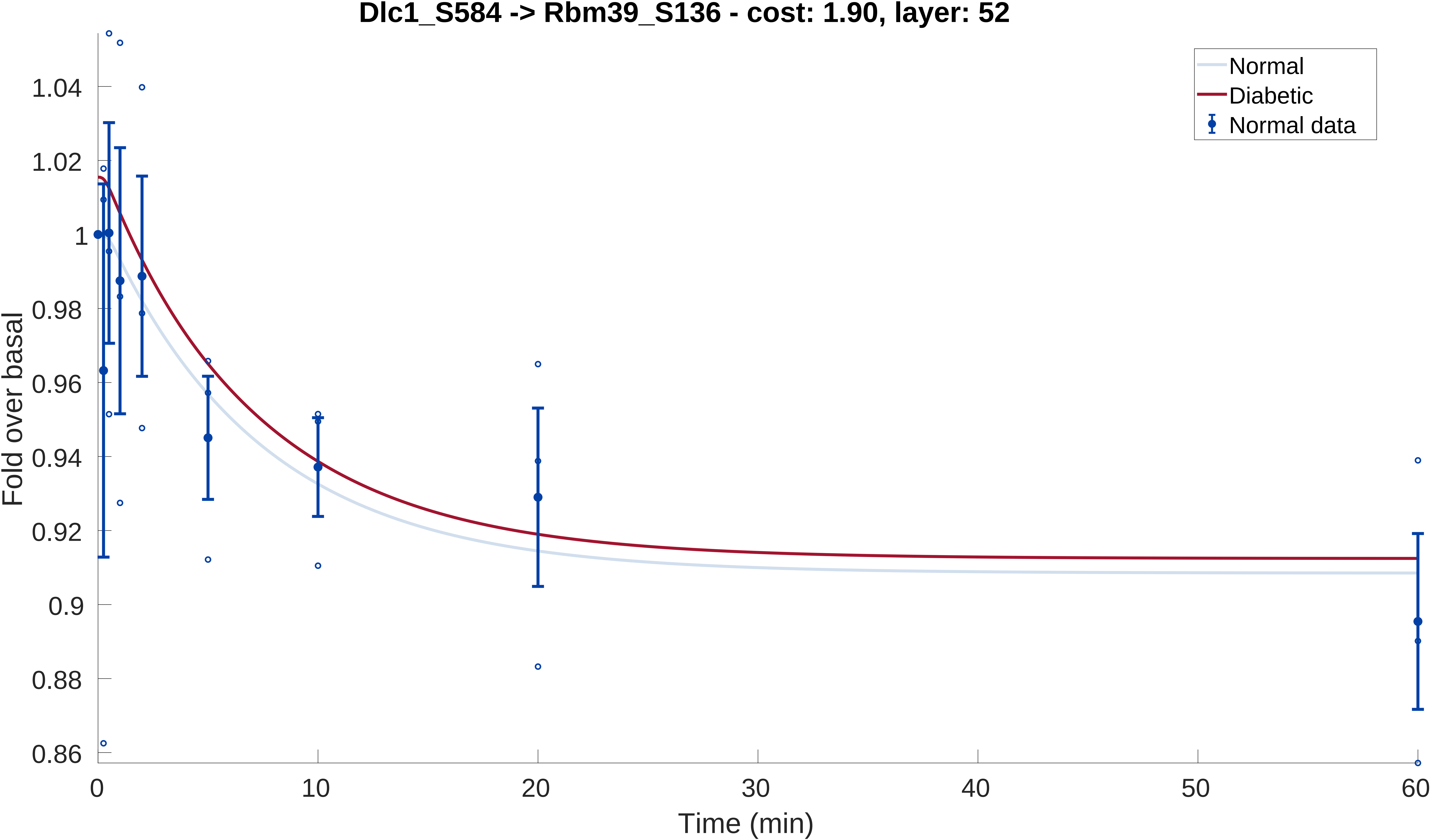

Dlc1_S584 -> Rbm39_S136 - cost: 1.90, layer: 52
Normal
Diabetic
Normal data
Fold over basal
Time (min)
1.04
1.02
1
0.98
0.96
0.94
0.92
0.9
0.88
0.86
0
10
20
30
40
50
60

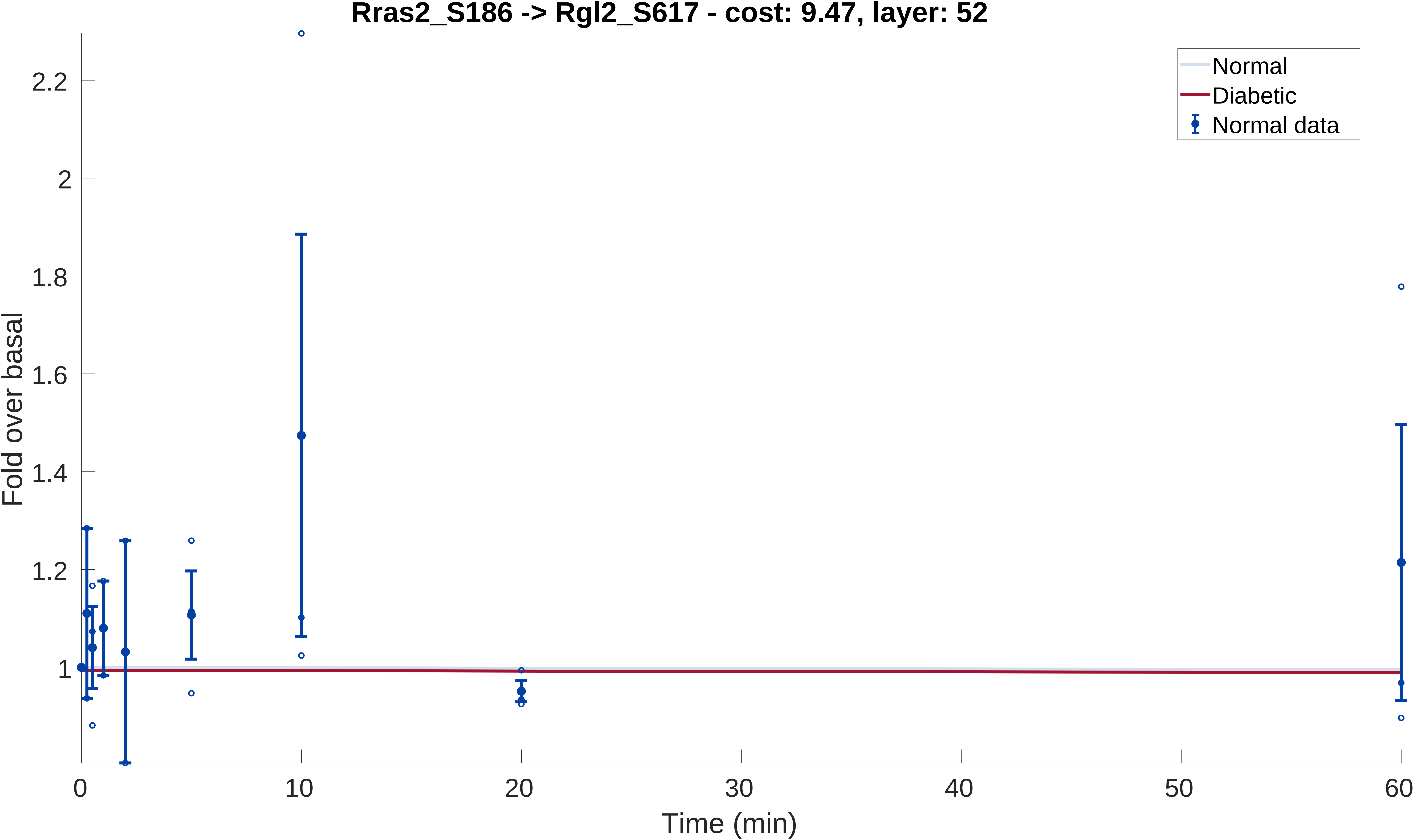
Rras2_S186 -> Rgl2_S617 - cost: 9.47, layer: 52
Normal
Diabetic
Normal data
Fold over basal
Time (min)
2.2
2
1.8
1.6
1.4
1.2
1
0
10
20
30
40
50
60

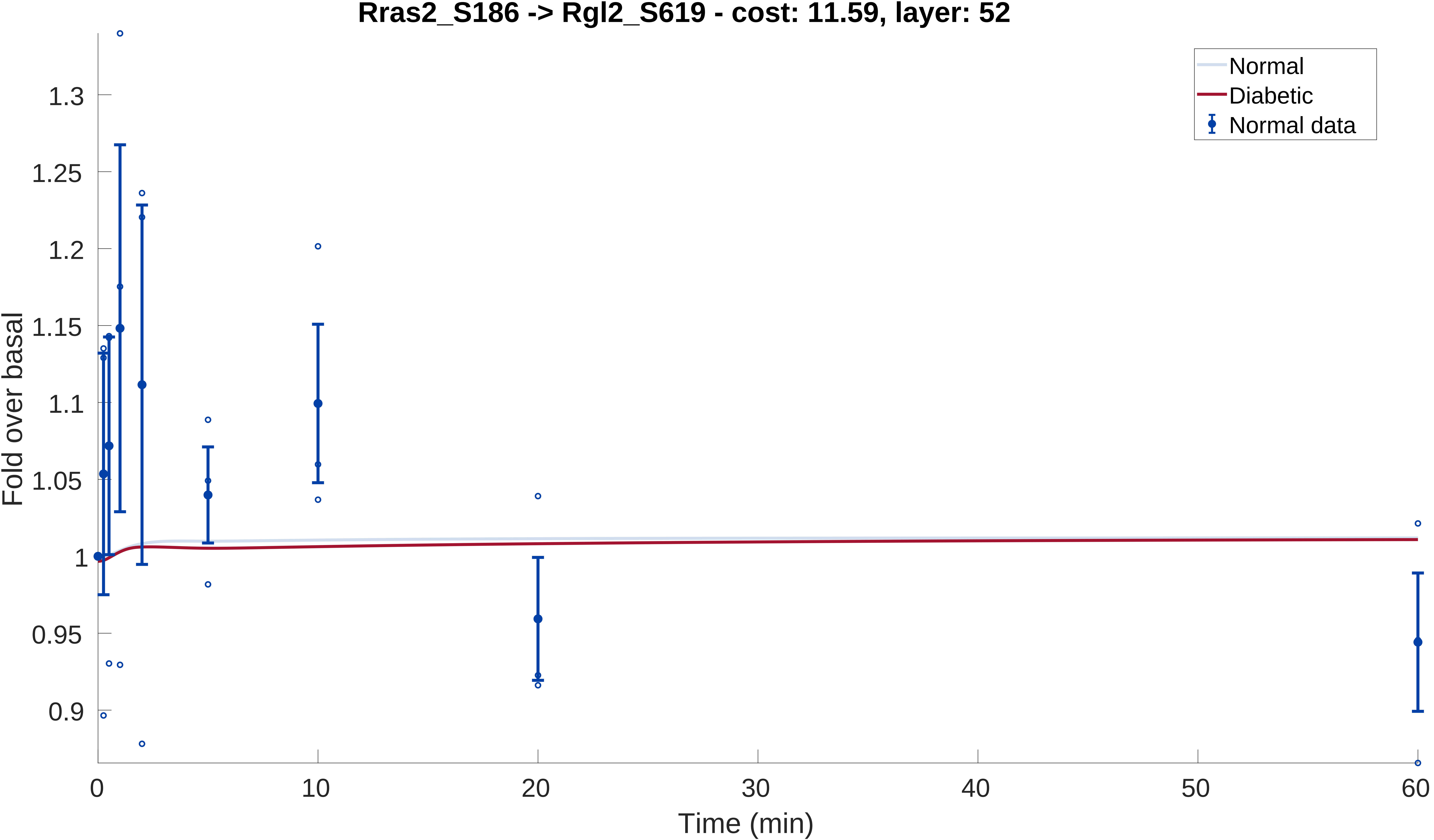

Rras2_S186 -> Rgl2_S619 - cost: 11.59, layer: 52
Normal
Diabetic
Normal data
Fold over basal
Time (min)
1.3
1.25
1.2
1.15
1.1
1.05
1
0.95
0.9
0
10
20
30
40
50
60

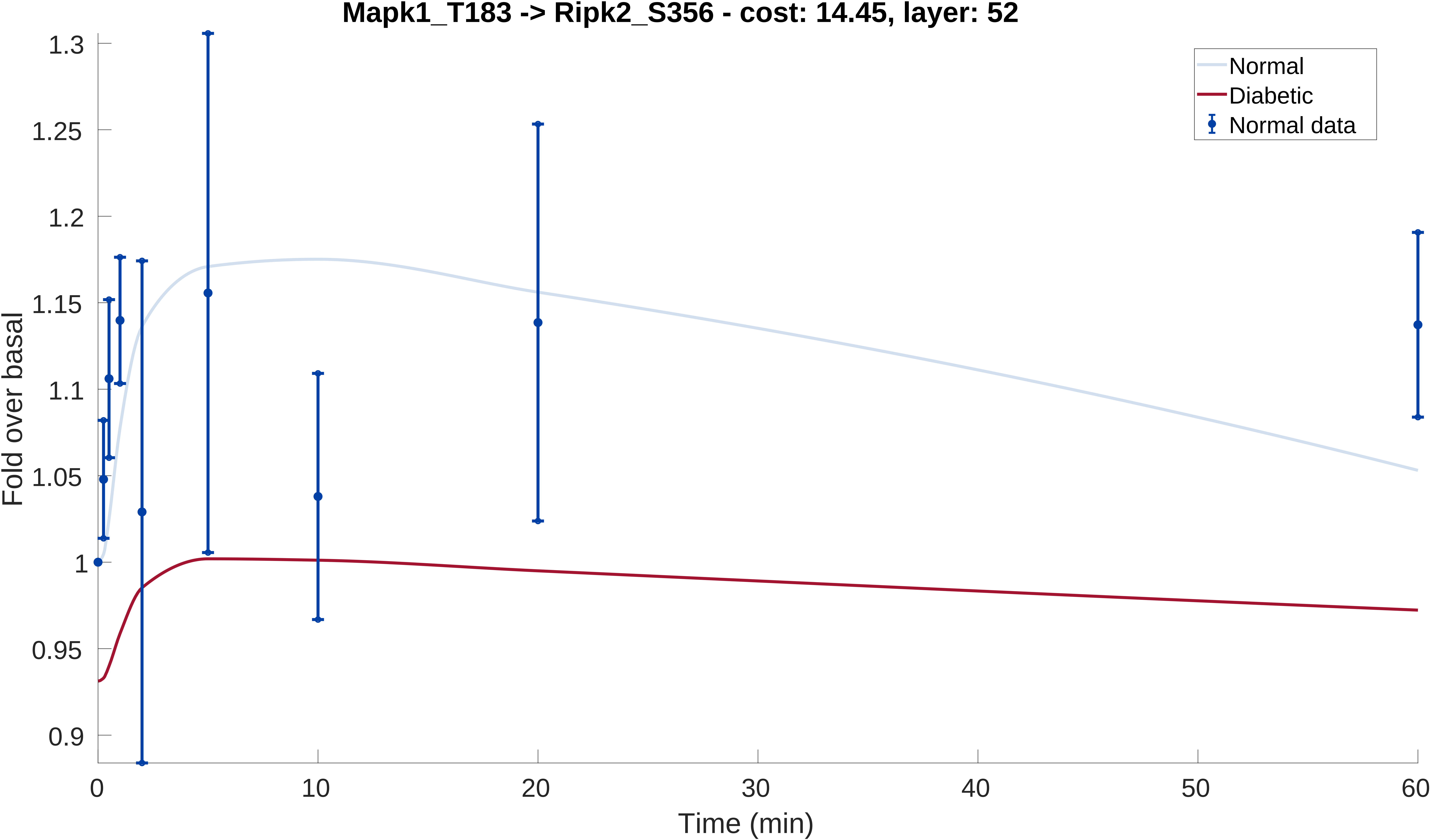
Mapk1_T183 -> Ripk2_S356 - cost: 14.45, layer: 52
Normal
Diabetic
Normal data
Fold over basal
Time (min)
1.3
1.25
1.2
1.15
1.1
1.05
1
0.95
0.9
0
10
20
30
40
50
60

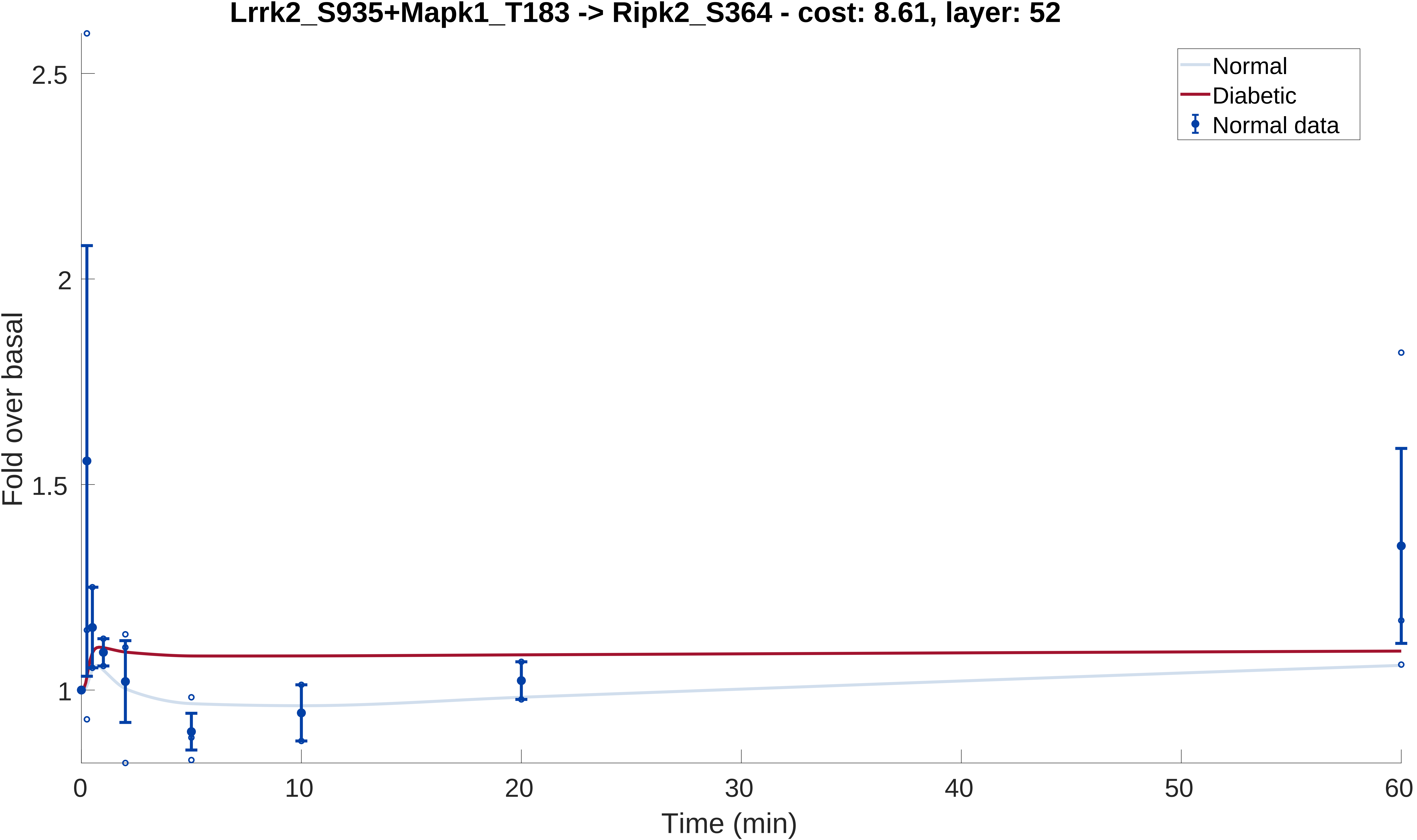

Lrrk2_S935+Mapk1_T183 -> Ripk2_S364 - cost: 8.61, layer: 52
Fold over basal
2.5
2
1.5
1
0
10
20
30
40
50
60
Time (min)
Normal
Diabetic
Normal data

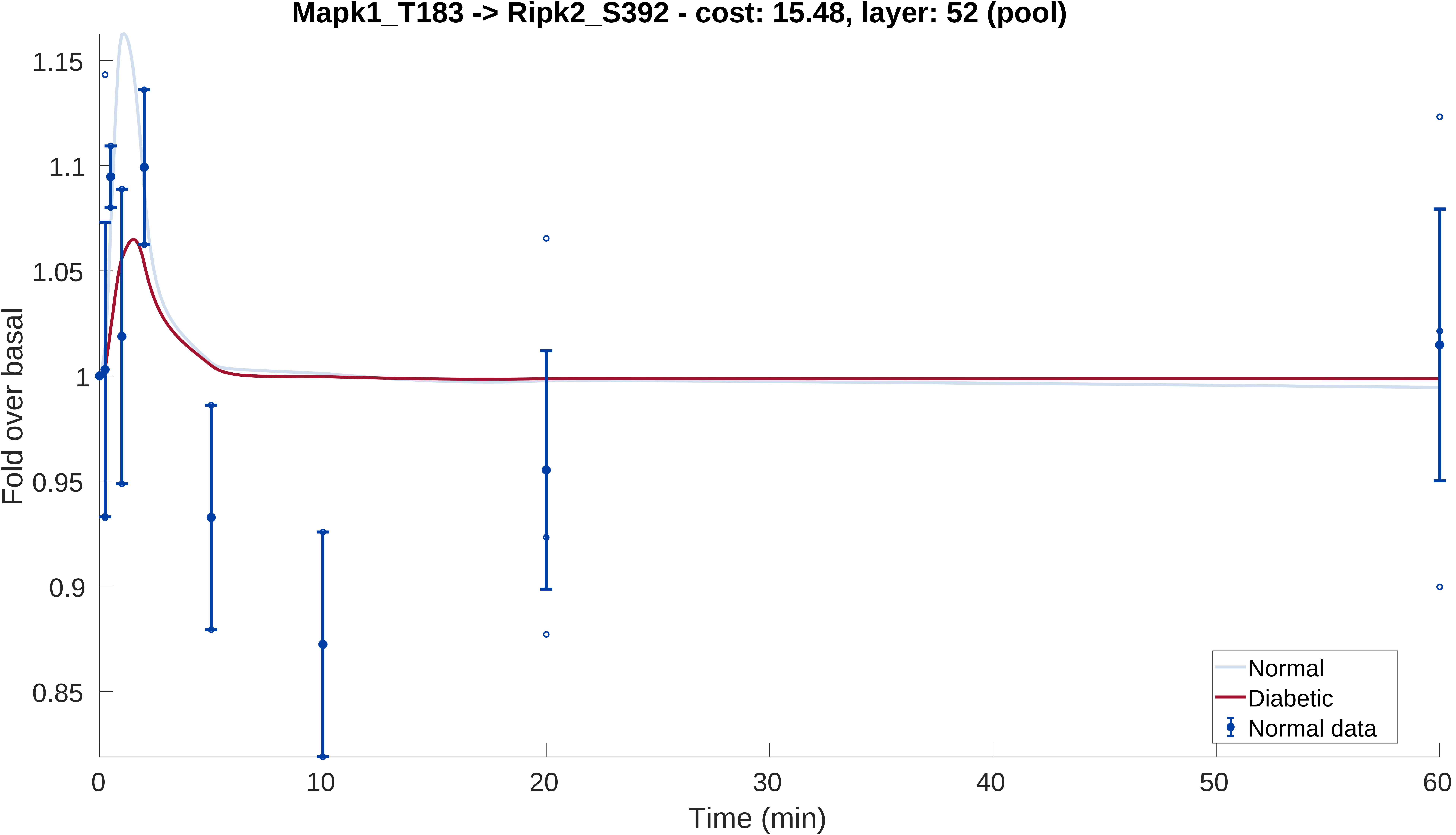

Mapk1_T183 -> Ripk2_S392 - cost: 15.48, layer: 52 (pool)
Fold over basal
1.15
1.1
1.05
1
0.95
0.9
0.85
Time (min)
0
10
20
30
40
50
60
Normal
Diabetic
Normal data

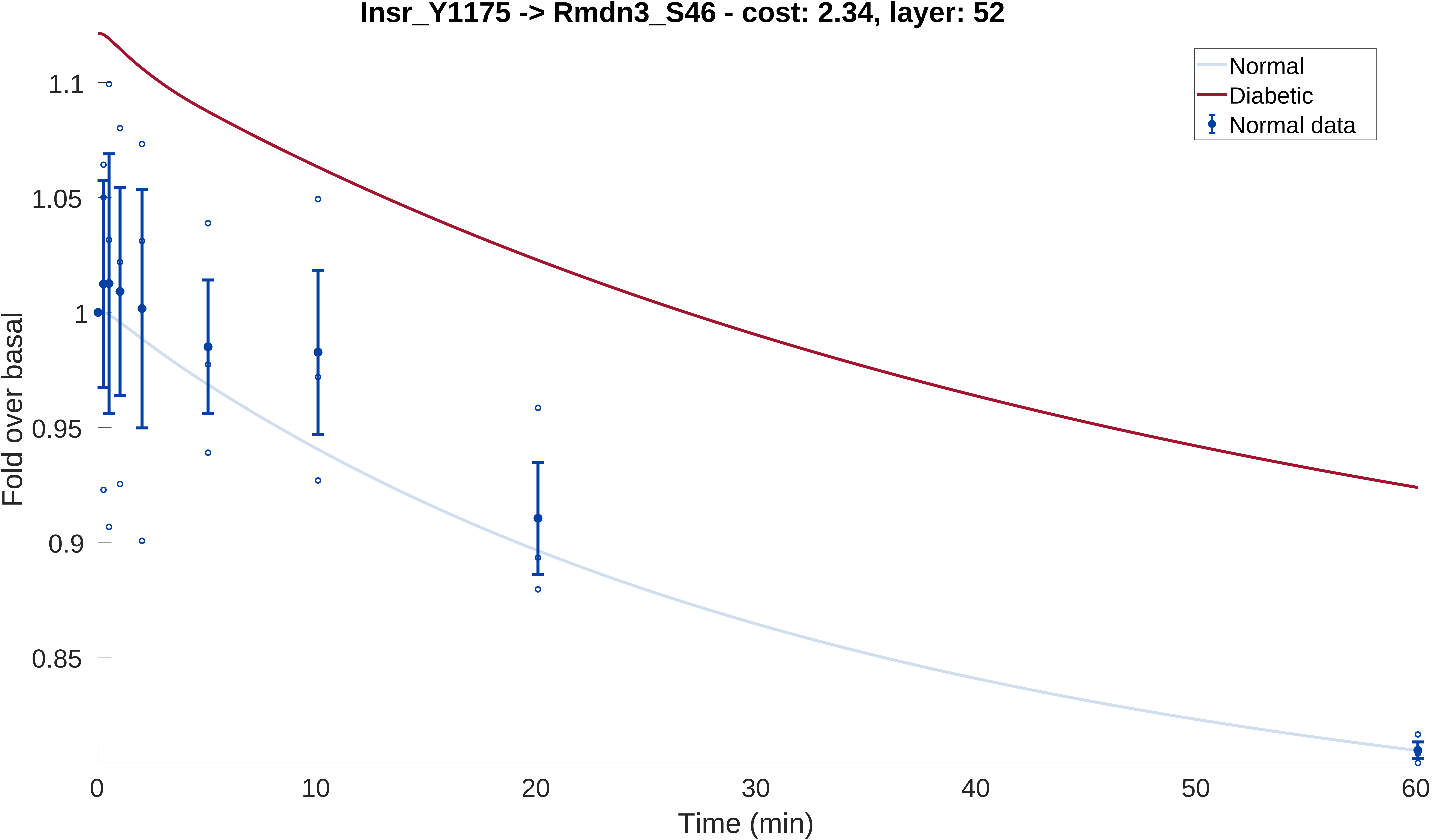

Insr_Y1175 -> Rmdn3_S46 - cost: 2.34, layer: 52
Normal
Diabetic
Normal data
Fold over basal
1.1
1.05
1
0.95
0.9
0.85
0
10
20
30
40
50
60
Time (min)

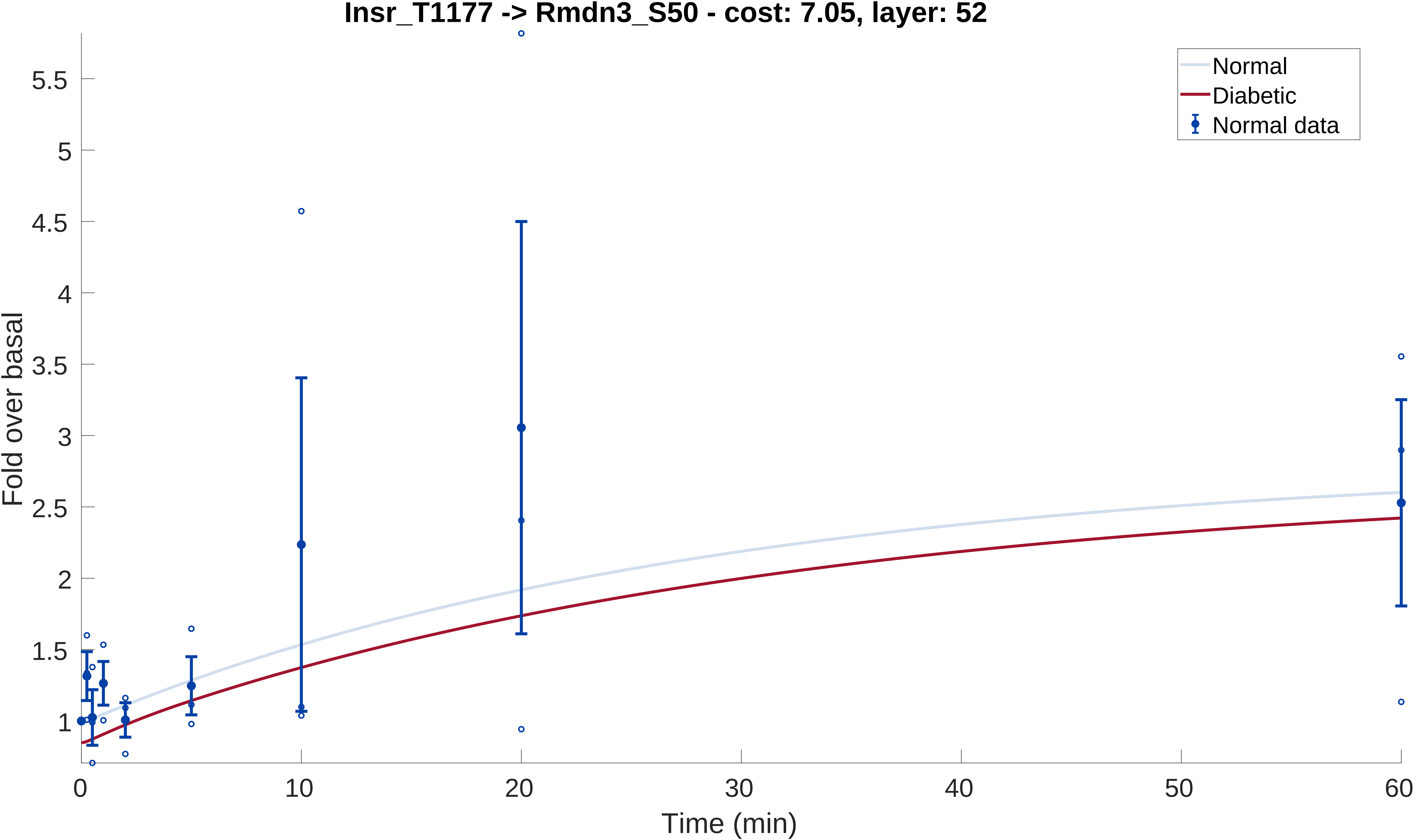

Insr_T1177 -> Rmdn3_S50 - cost: 7.05, layer: 52
Normal
Diabetic
Normal data
Fold over basal
Time (min)
5.5
5
4.5
4
3.5
3
2.5
2
1.5
1
0
10
20
30
40
50
60

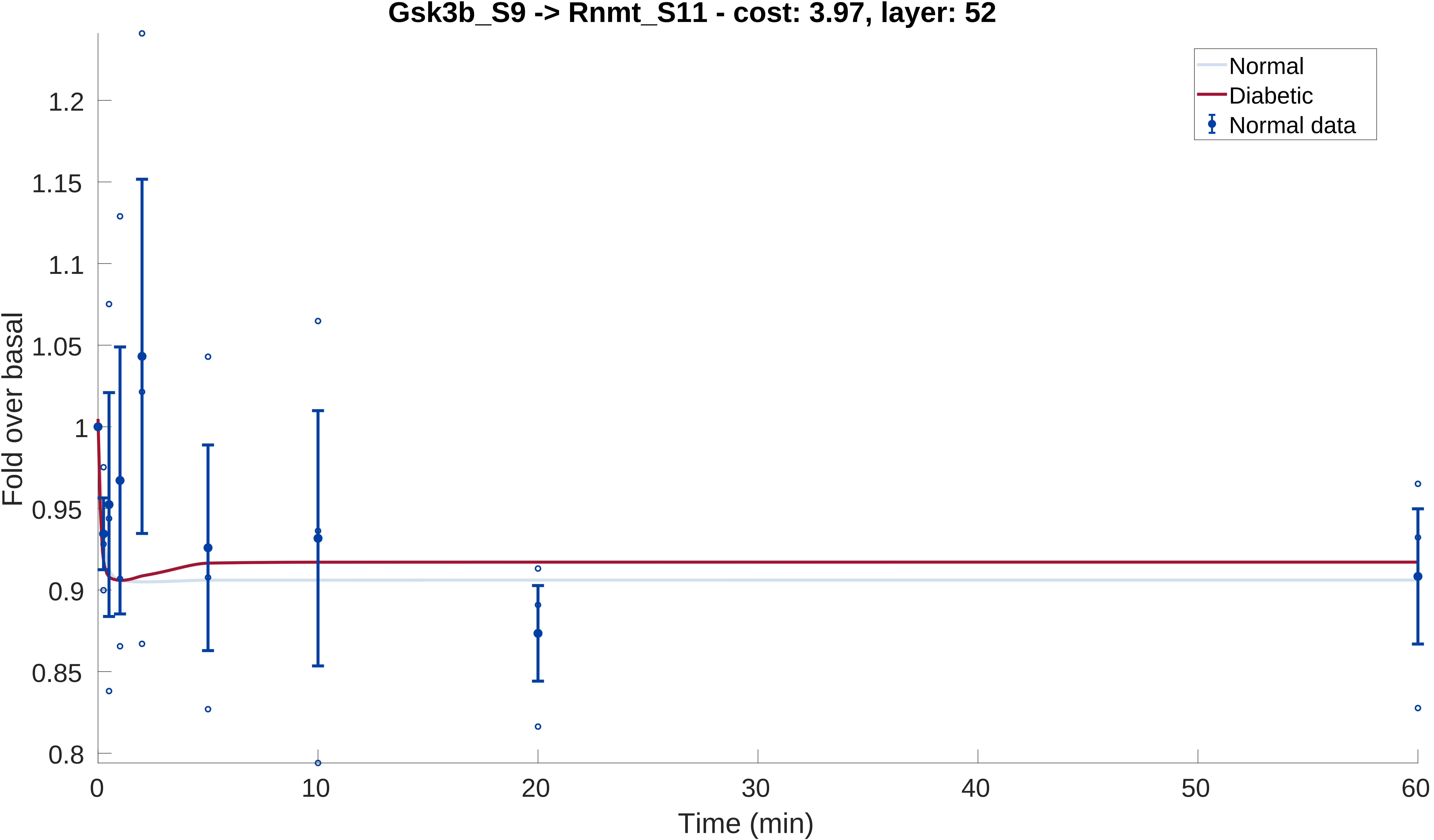
Gsk3b_S9 -> Rnmt_S11 - cost: 3.97, layer: 52
Normal
Diabetic
Normal data
Fold over basal
Time (min)
1.2
1.15
1.1
1.05
1
0.95
0.9
0.85
0.8
0
10
20
30
40
50
60

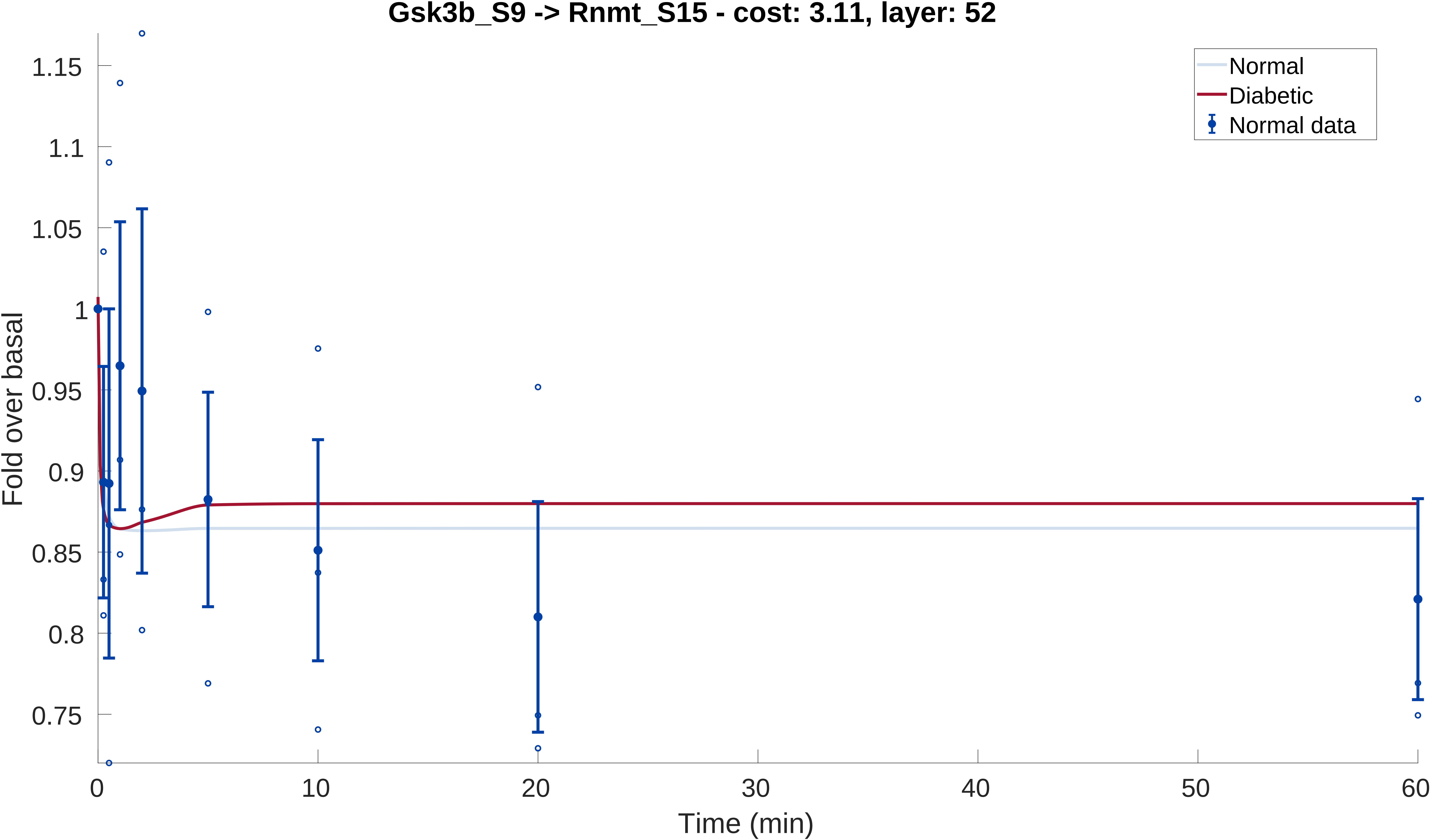

Gsk3b_S9 -> Rnmt_S15 - cost: 3.11, layer: 52
Normal
Diabetic
Normal data
Fold over basal
Time (min)
1.15
1.1
1.05
1
0.95
0.9
0.85
0.8
0.75
0
10
20
30
40
50
60

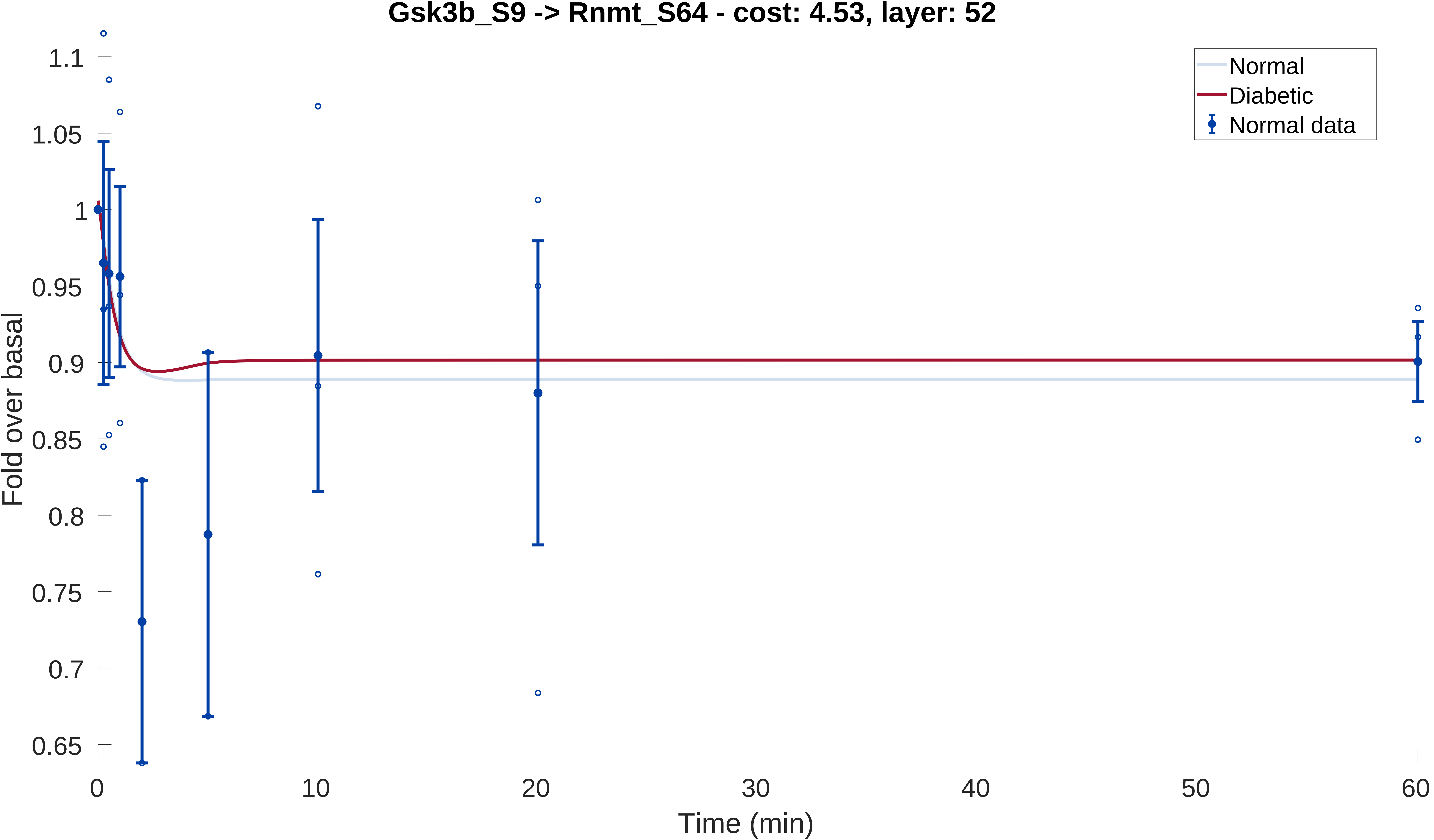

Gsk3b_S9 -> Rnmt_S64 - cost: 4.53, layer: 52
Normal
Diabetic
Normal data
1.1
1.05
1
0.95
0.9
0.85
0.8
0.75
0.7
0.65
Fold over basal
0
10
20
30
40
50
60
Time (min)

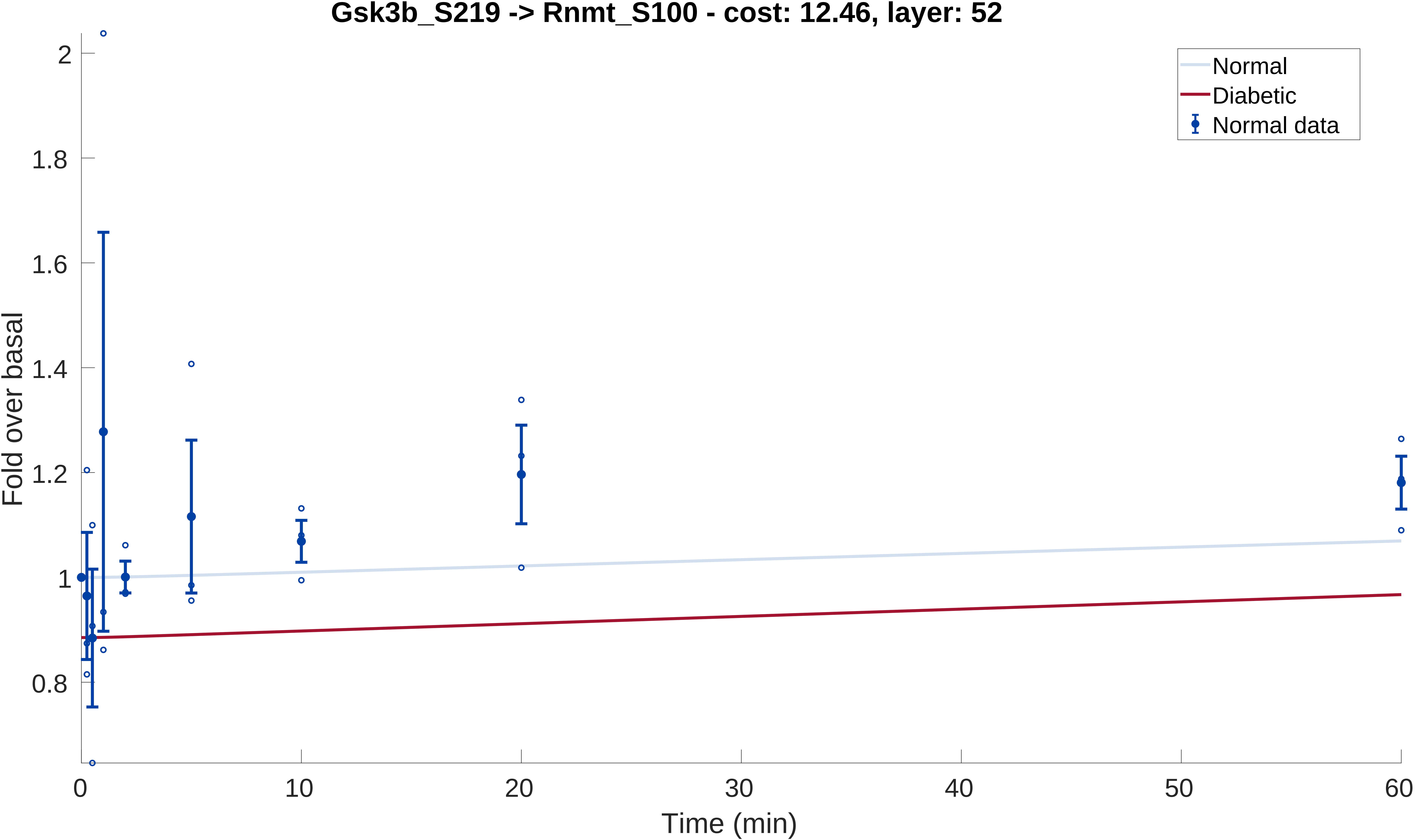

Gsk3b_S219 -> Rnmt_S100 - cost: 12.46, layer: 52
Normal
Diabetic
Normal data
Fold over basal
0.8
1
1.2
1.4
1.6
1.8
2
Time (min)
0
10
20
30
40
50
60

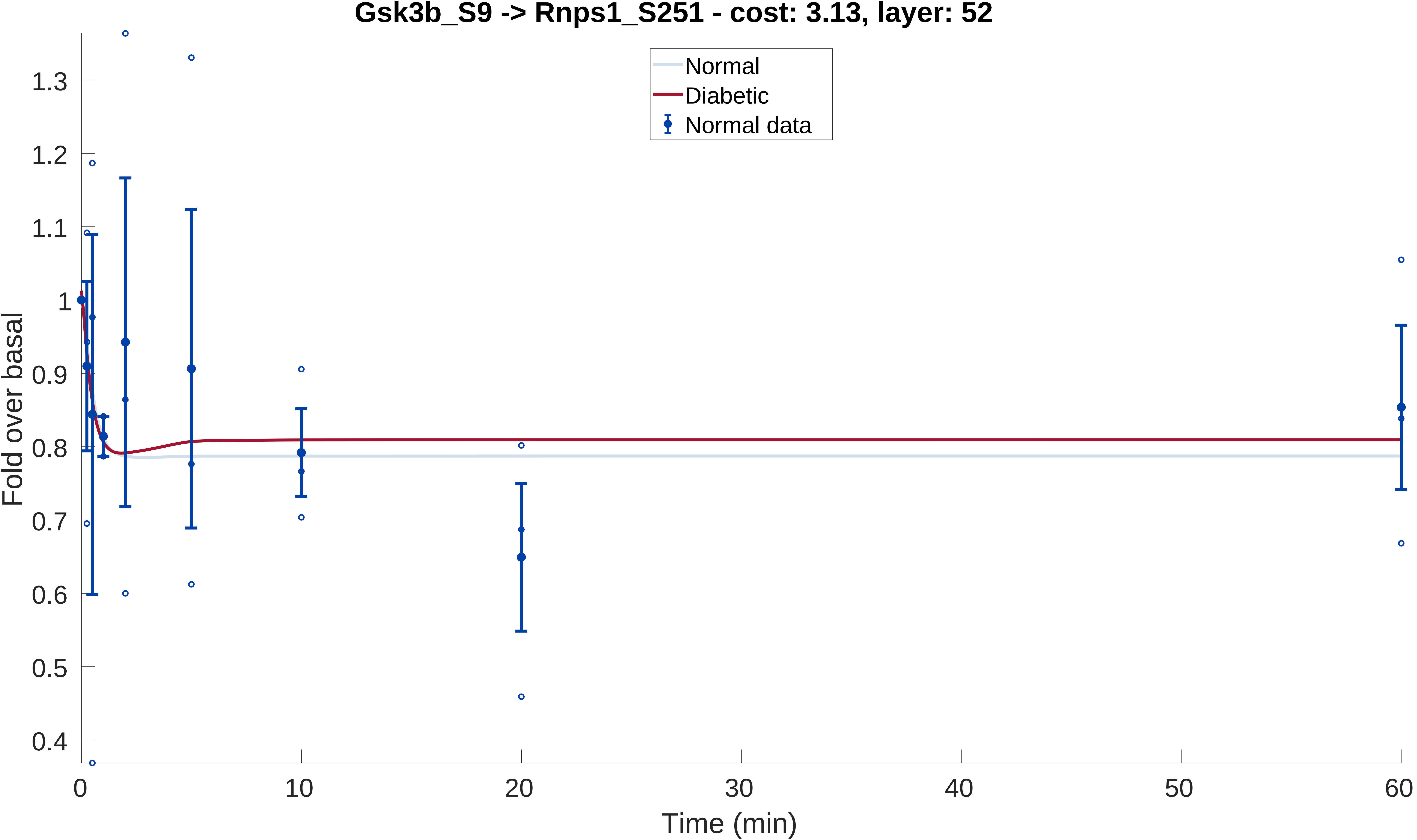

Gsk3b_S9 -> Rnps1_S251 - cost: 3.13, layer: 52
Normal
Diabetic
Normal data
Fold over basal
Time (min)
1.3
1.2
1.1
1
0.9
0.8
0.7
0.6
0.5
0.4
0
10
20
30
40
50
60

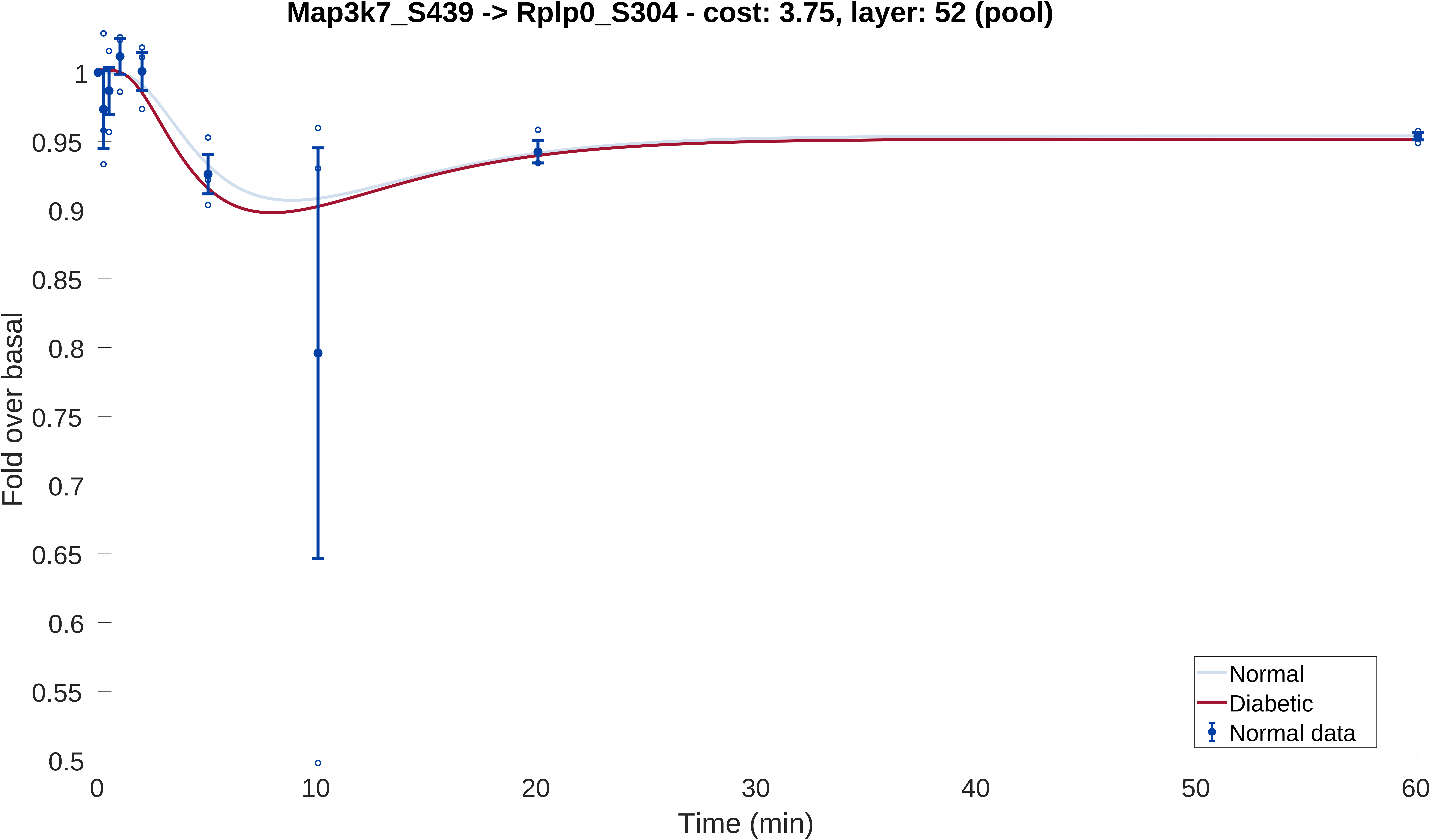

Map3k7_S439 -> Rplp0_S304 - cost: 3.75, layer: 52 (pool)
Fold over basal
1
0.95
0.9
0.85
0.8
0.75
0.7
0.65
0.6
0.55
0.5
0
10
20
30
40
50
60
Time (min)
Normal
Diabetic
Normal data

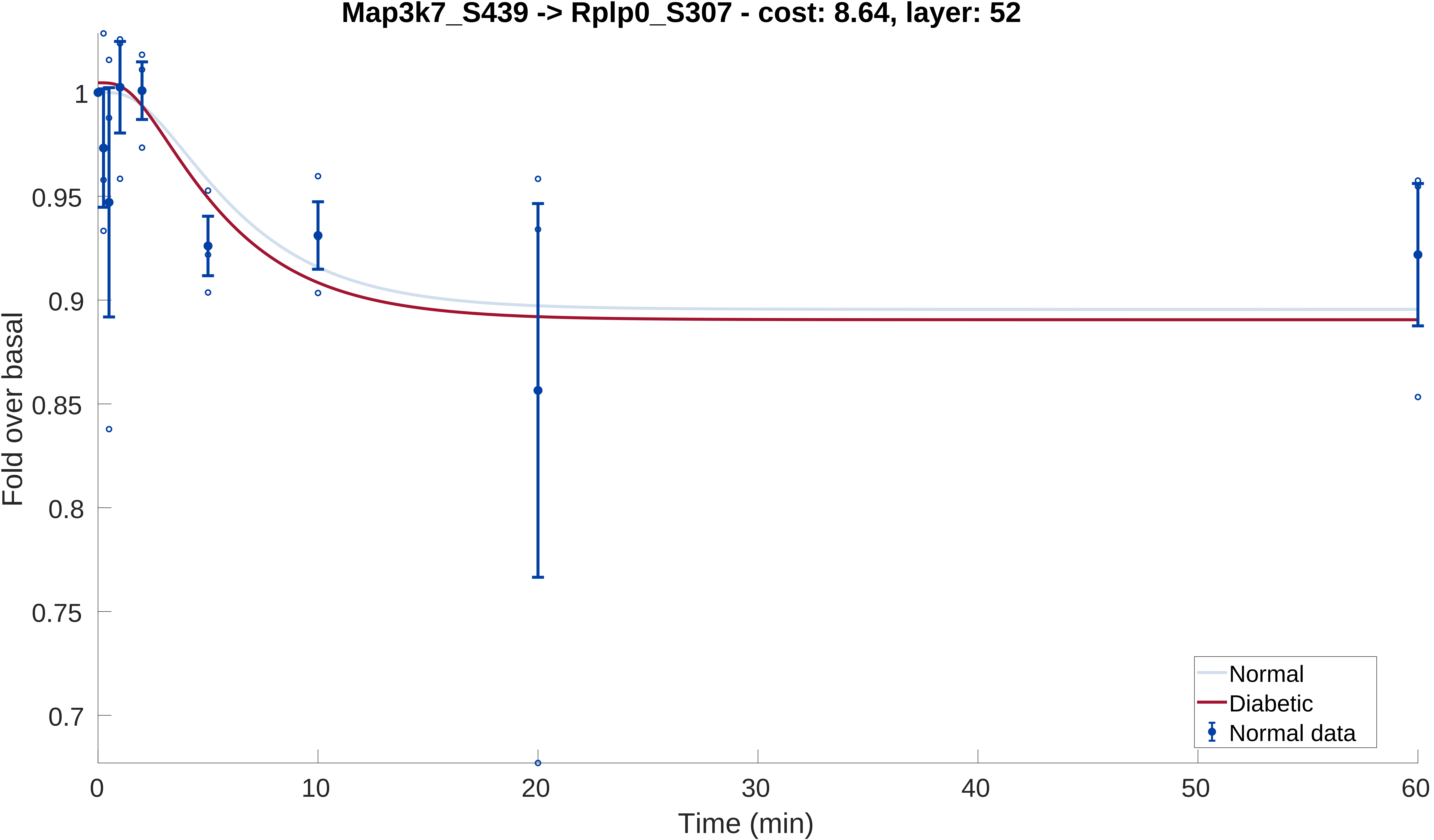

Map3k7_S439 -> Rplp0_S307 - cost: 8.64, layer: 52
Fold over basal
1
0.95
0.9
0.85
0.8
0.75
0.7
0
10
20
30
40
50
60
Time (min)
Normal
Diabetic
Normal data

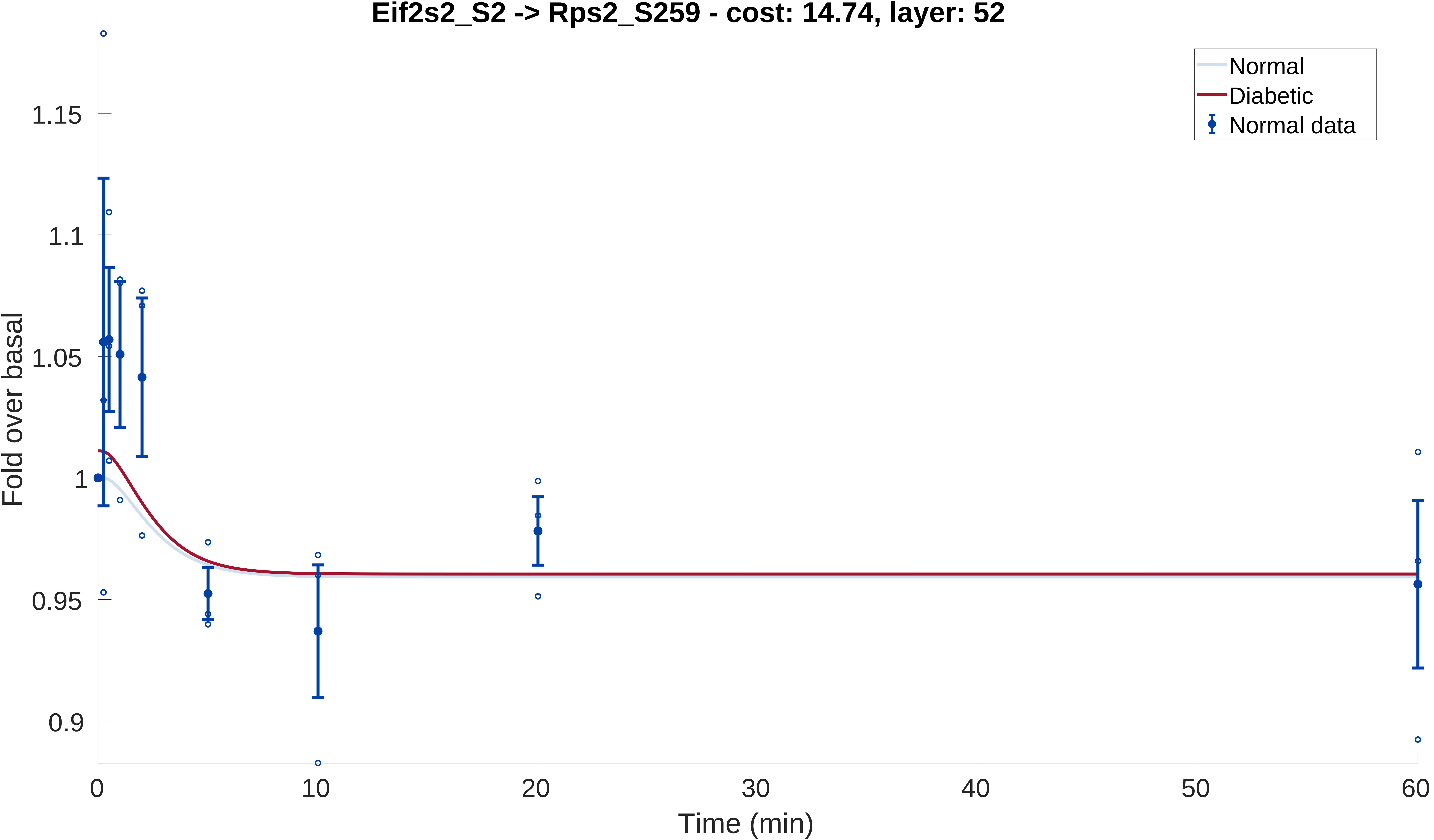

Eif2s2_S2 -> Rps2_S259 - cost: 14.74, layer: 52
Normal
Diabetic
Normal data
1.15
1.1
1.05
1
0.95
0.9
Fold over basal
0
10
20
30
40
50
60
Time (min)

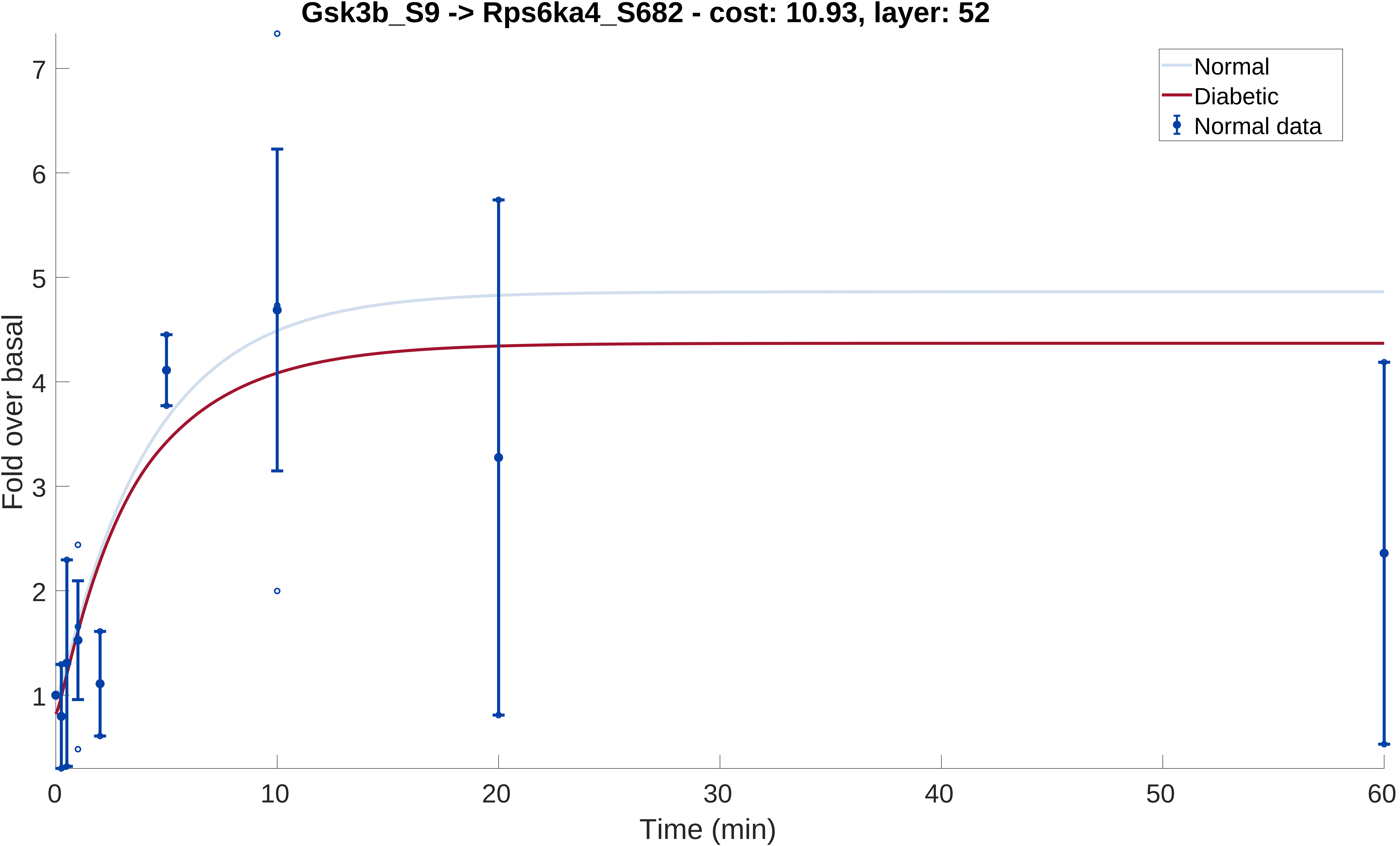

Gsk3b_S9 -> Rps6ka4_S682 - cost: 10.93, layer: 52
Normal
Diabetic
Normal data
Fold over basal
Time (min)
0
10
20
30
40
50
60
1
2
3
4
5
6
7

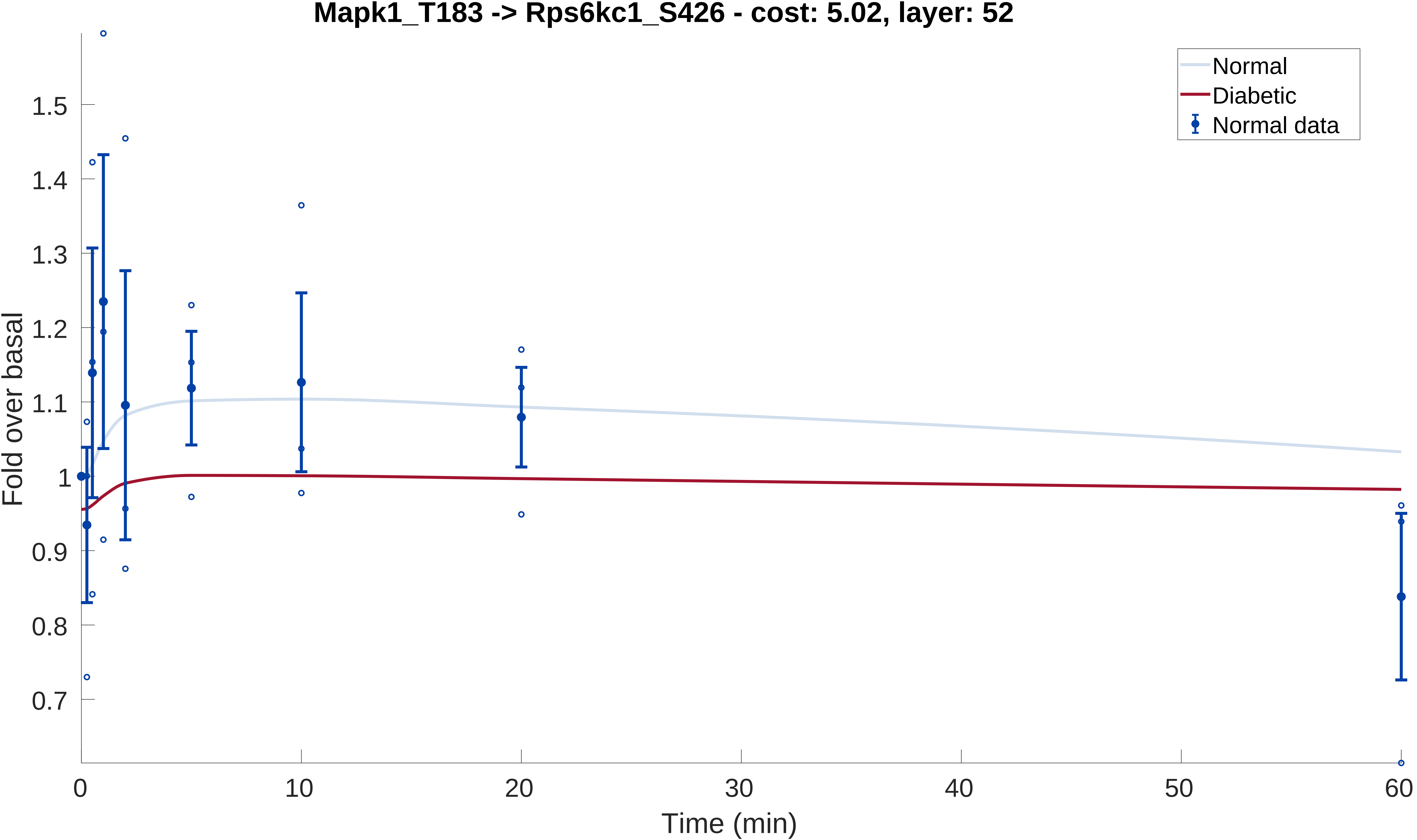
Mapk1_T183 -> Rps6kc1_S426 - cost: 5.02, layer: 52
Normal
Diabetic
Normal data
Fold over basal
1.5
1.4
1.3
1.2
1.1
1
0.9
0.8
0.7
0
10
20
30
40
50
60
Time (min)

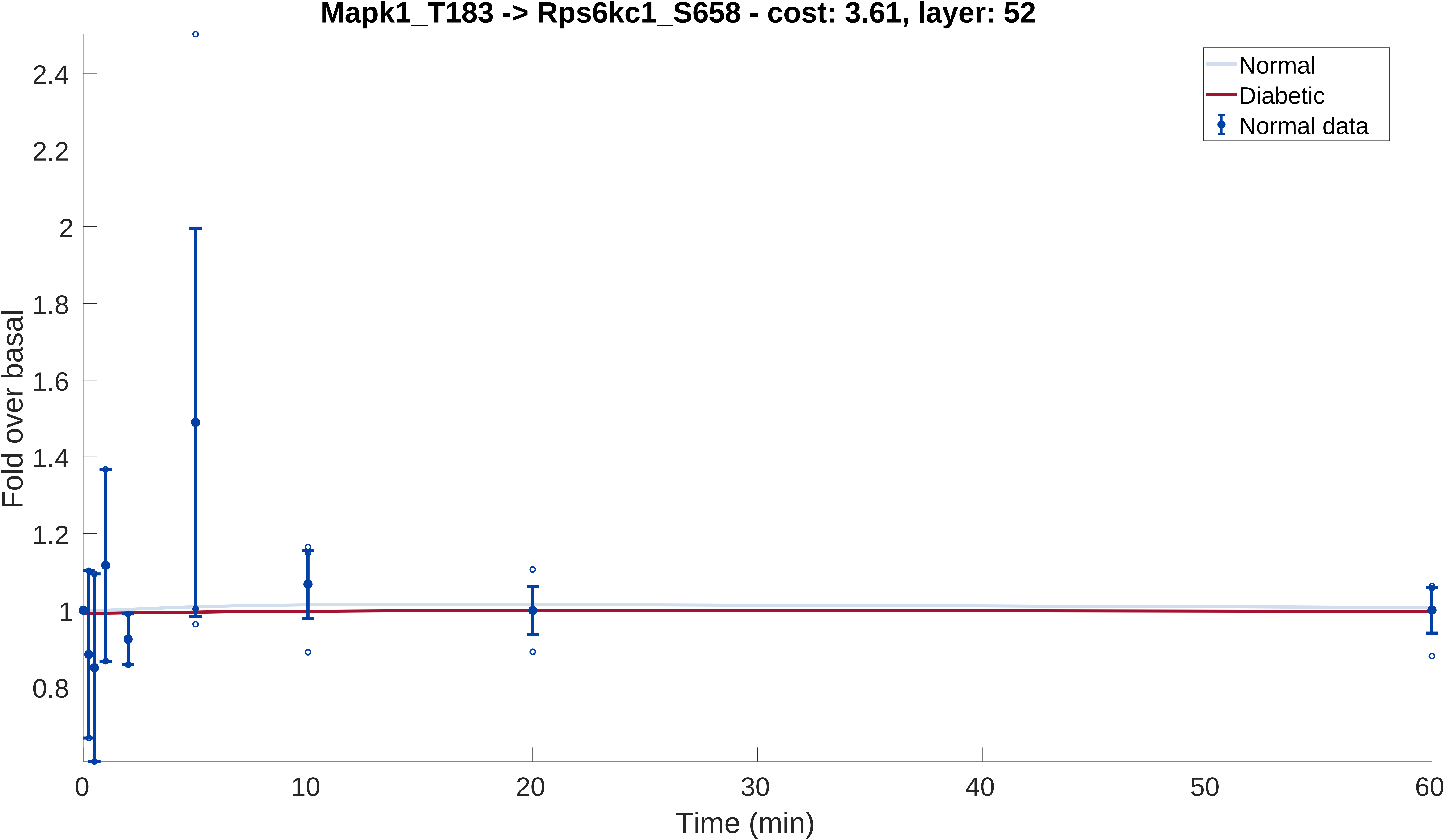

Mapk1_T183 -> Rps6kc1_S658 - cost: 3.61, layer: 52
Normal
Diabetic
Normal data
Fold over basal
2.4
2.2
2
1.8
1.6
1.4
1.2
1
0.8
0
10
20
30
40
50
60
Time (min)

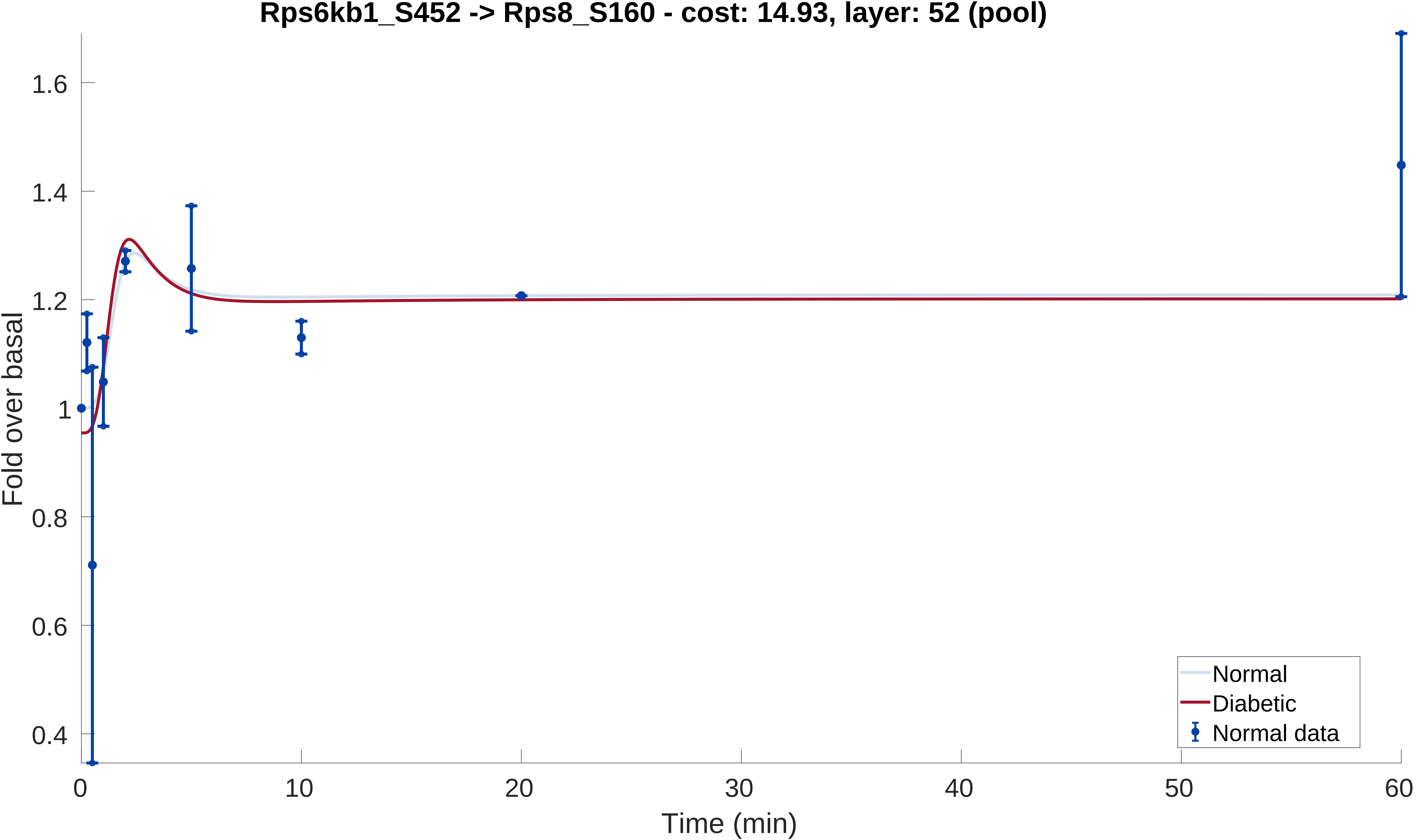
Rps6kb1_S452 -> Rps8_S160 - cost: 14.93, layer: 52 (pool)
Fold over basal
Time (min)
1.6
1.4
1.2
1
0.8
0.6
0.4
0
10
20
30
40
50
60
Normal
Diabetic
Normal data

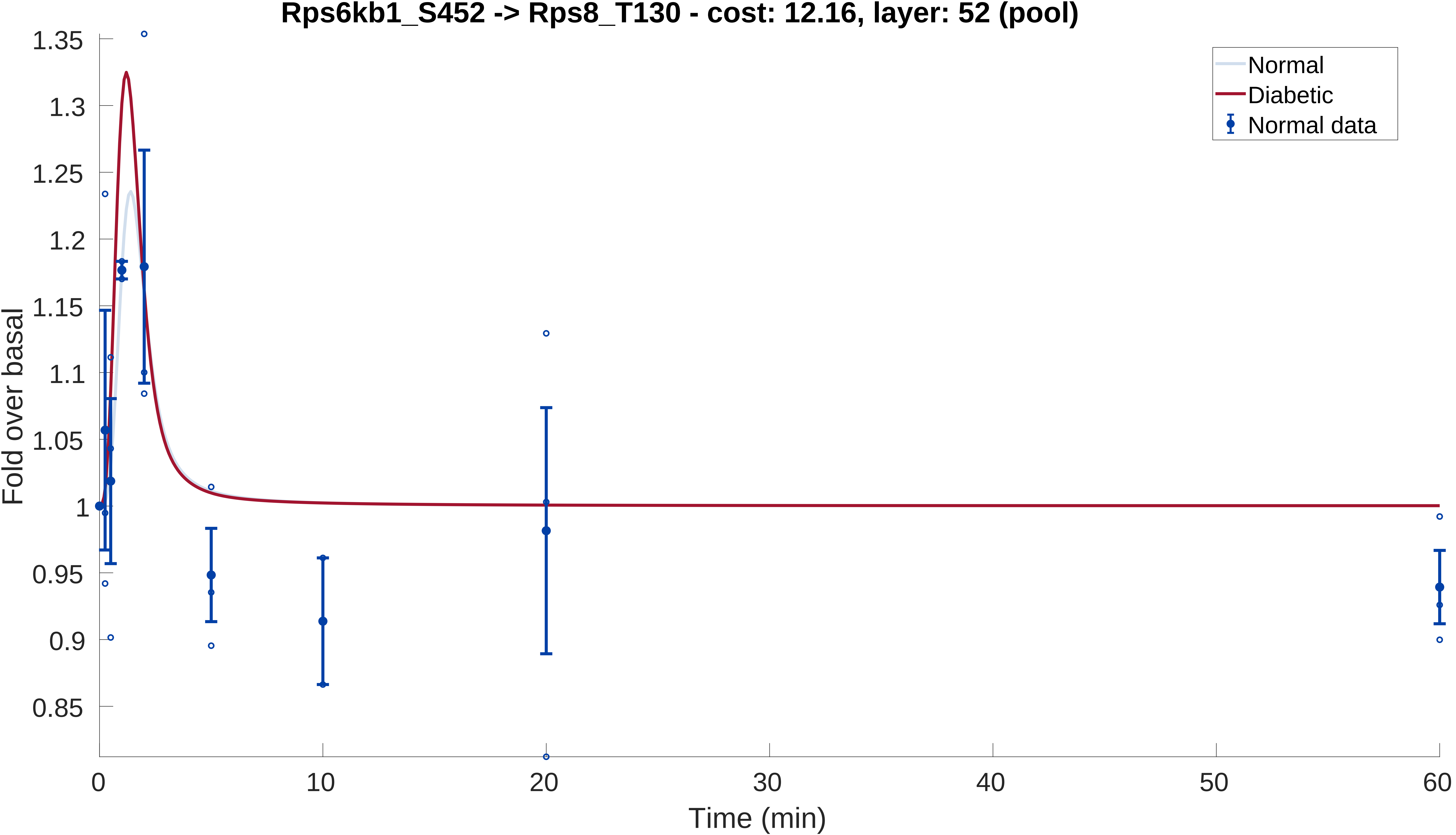
Rps6kb1_S452 -> Rps8_T130 - cost: 12.16, layer: 52 (pool)
Normal
Diabetic
Normal data
Fold over basal
Time (min)
1.35
1.3
1.25
1.2
1.15
1.1
1.05
1
0.95
0.9
0.85
0
10
20
30
40
50
60

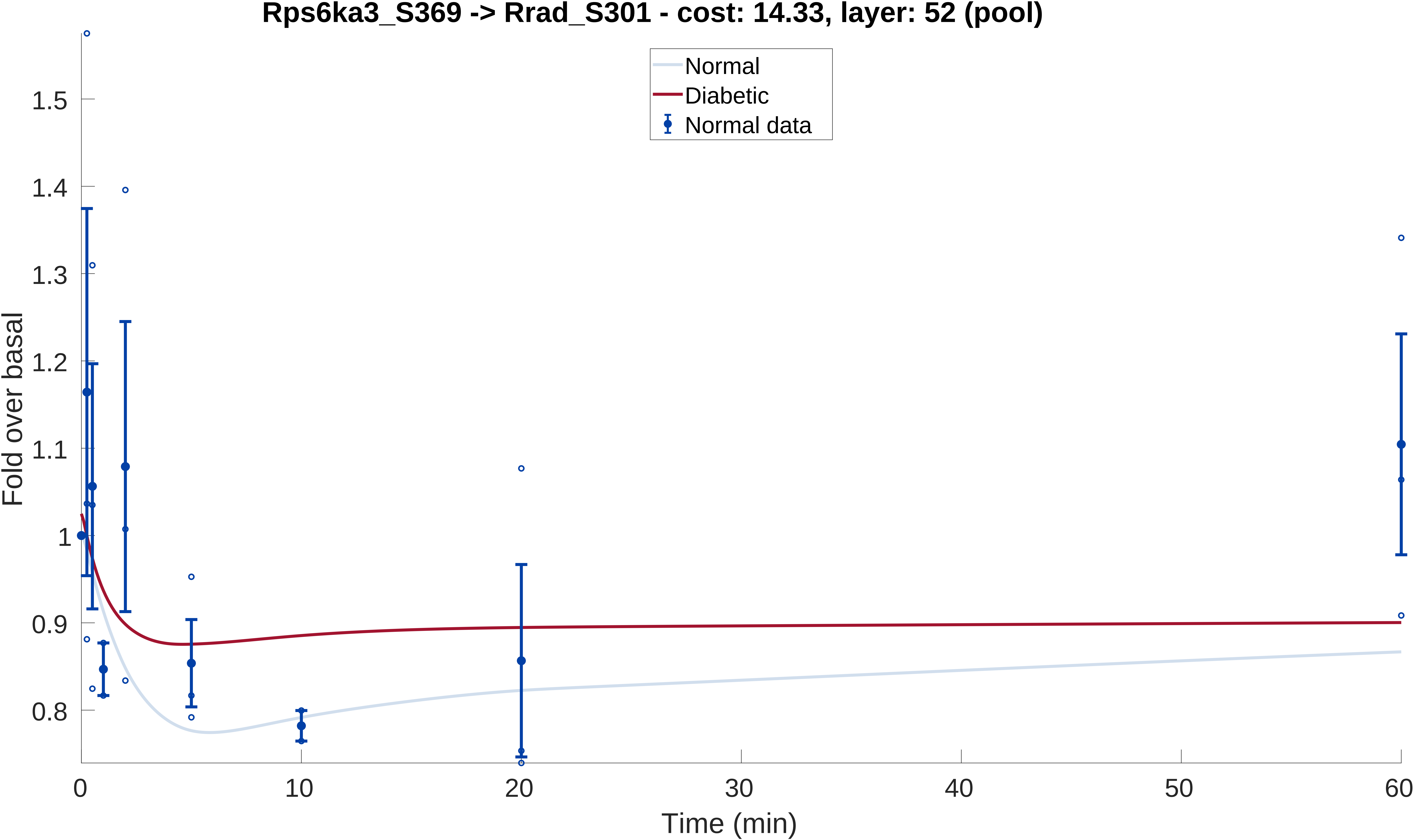

Rps6ka3_S369 -> Rrad_S301 - cost: 14.33, layer: 52 (pool)
Normal
Diabetic
Normal data
Fold over basal
1.5
1.4
1.3
1.2
1.1
1
0.9
0.8
0
10
20
30
40
50
60
Time (min)

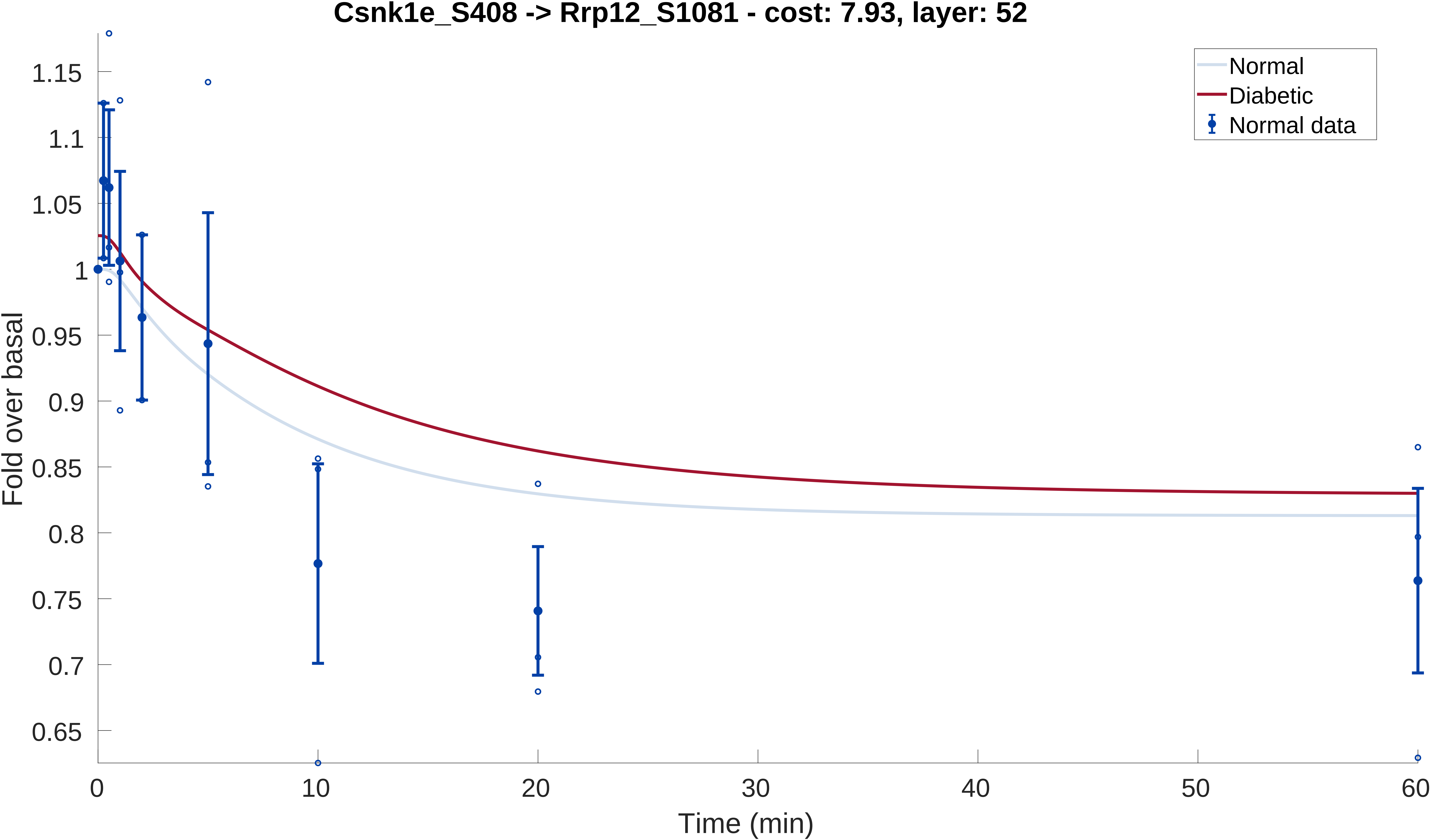

Csnk1e_S408 -> Rrp12_S1081 - cost: 7.93, layer: 52
Normal
Diabetic
Normal data
Fold over basal
Time (min)
0.65
0.7
0.75
0.8
0.85
0.9
0.95
1
1.05
1.1
1.15
0
10
20
30
40
50
60

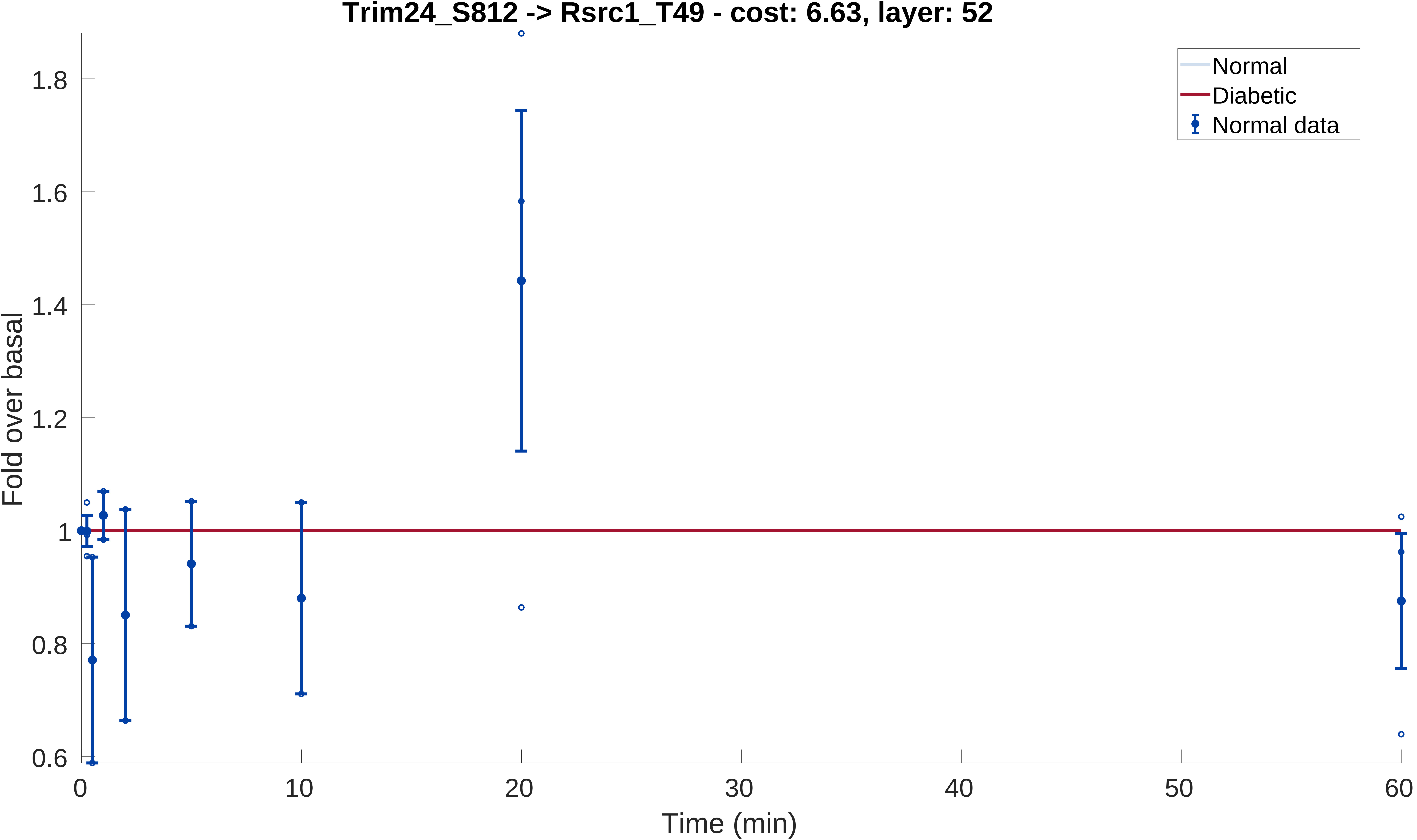

Trim24_S812 -> Rsrc1_T49 - cost: 6.63, layer: 52
Normal
Diabetic
Normal data
Fold over basal
1.8
1.6
1.4
1.2
1
0.8
0.6
0
10
20
30
40
50
60
Time (min)

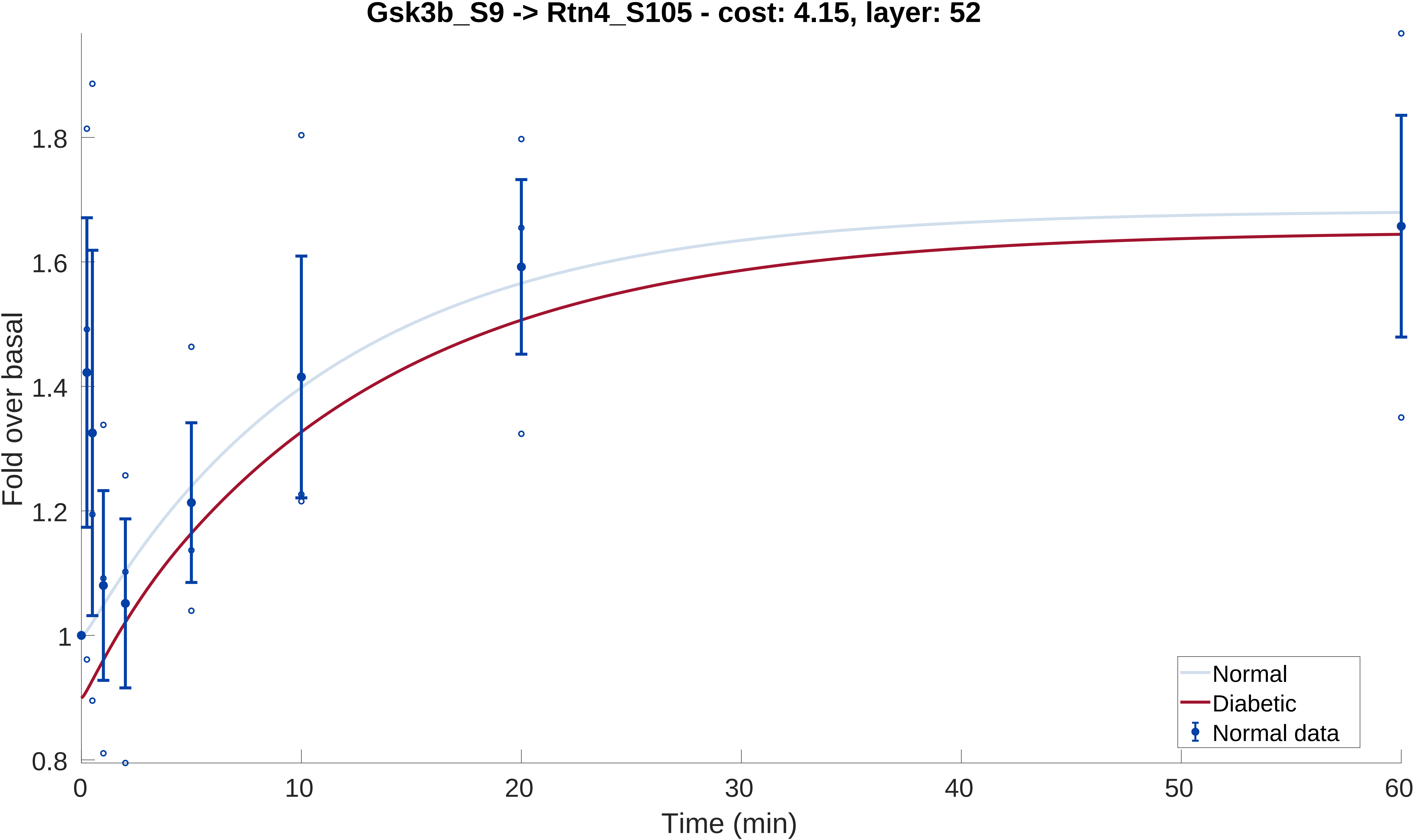

Gsk3b_S9 -> Rtn4_S105 - cost: 4.15, layer: 52
Fold over basal
1.8
1.6
1.4
1.2
1
0.8
0
10
20
30
40
50
60
Time (min)
Normal
Diabetic
Normal data

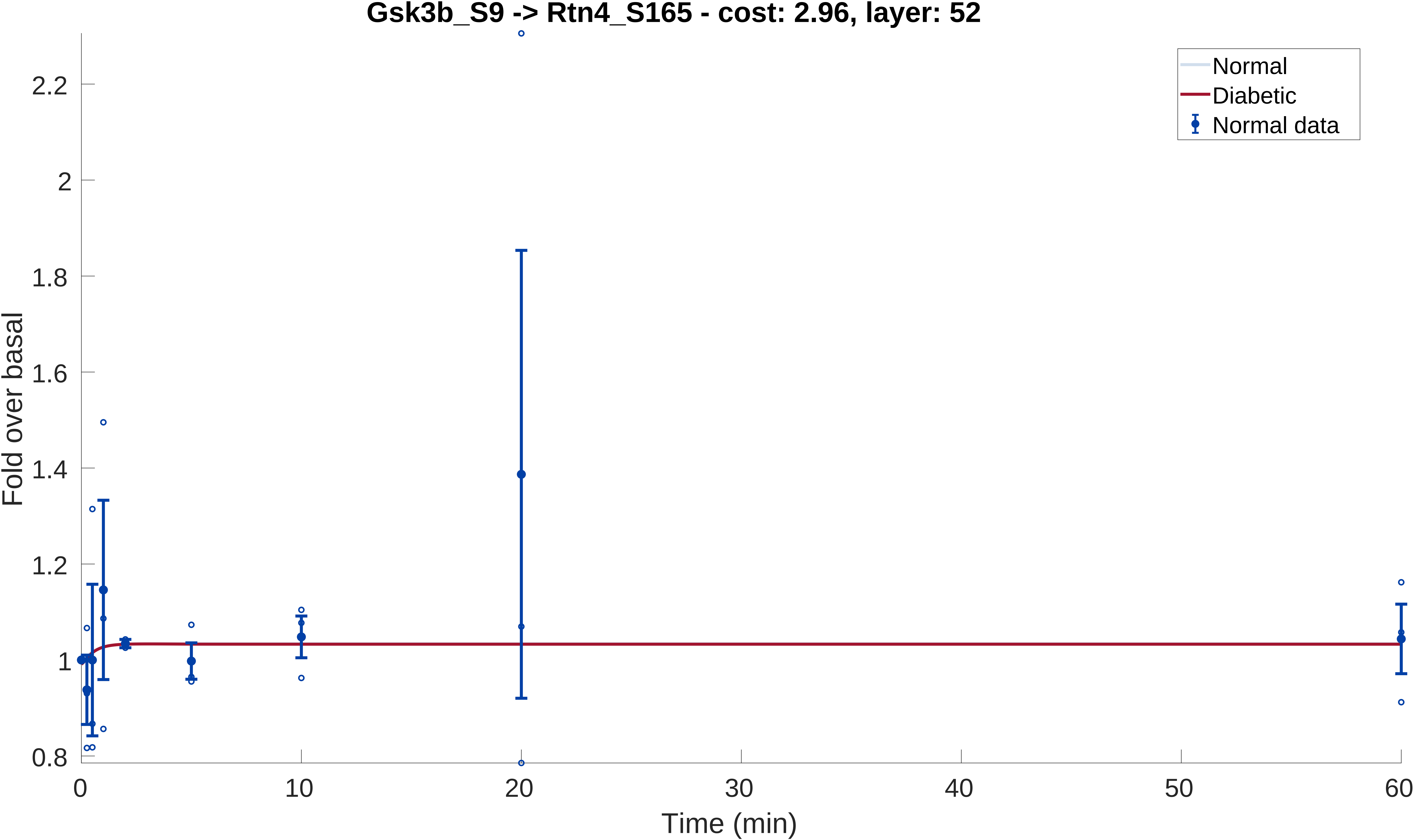

Gsk3b_S9 -> Rtn4_S165 - cost: 2.96, layer: 52
Normal
Diabetic
Normal data
Fold over basal
0.8
1
1.2
1.4
1.6
1.8
2
2.2
0
10
20
30
40
50
60
Time (min)

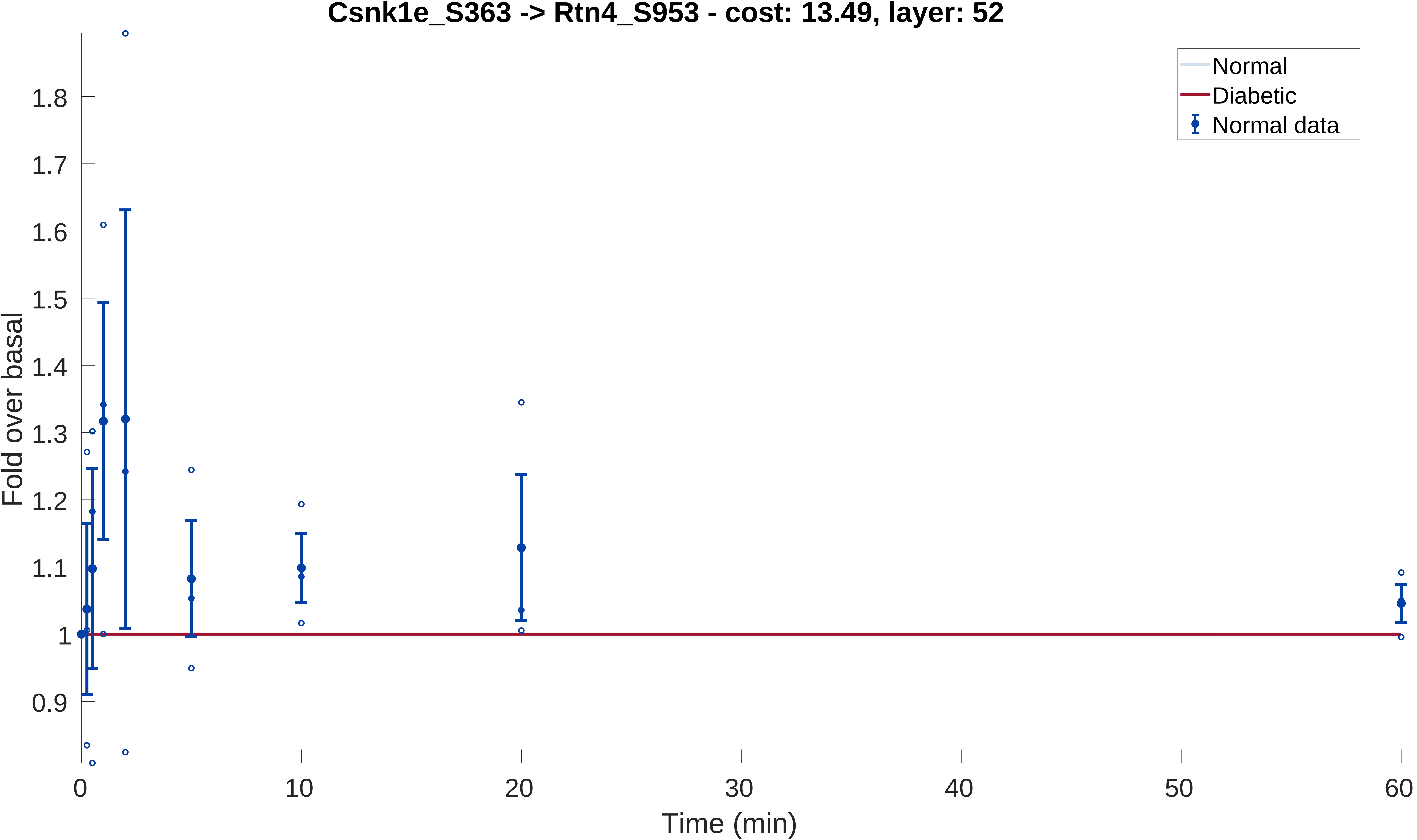

Csnk1e_S363 -> Rtn4_S953 - cost: 13.49, layer: 52
Normal
Diabetic
Normal data
Fold over basal
1.8
1.7
1.6
1.5
1.4
1.3
1.2
1.1
1
0.9
0
10
20
30
40
50
60
Time (min)

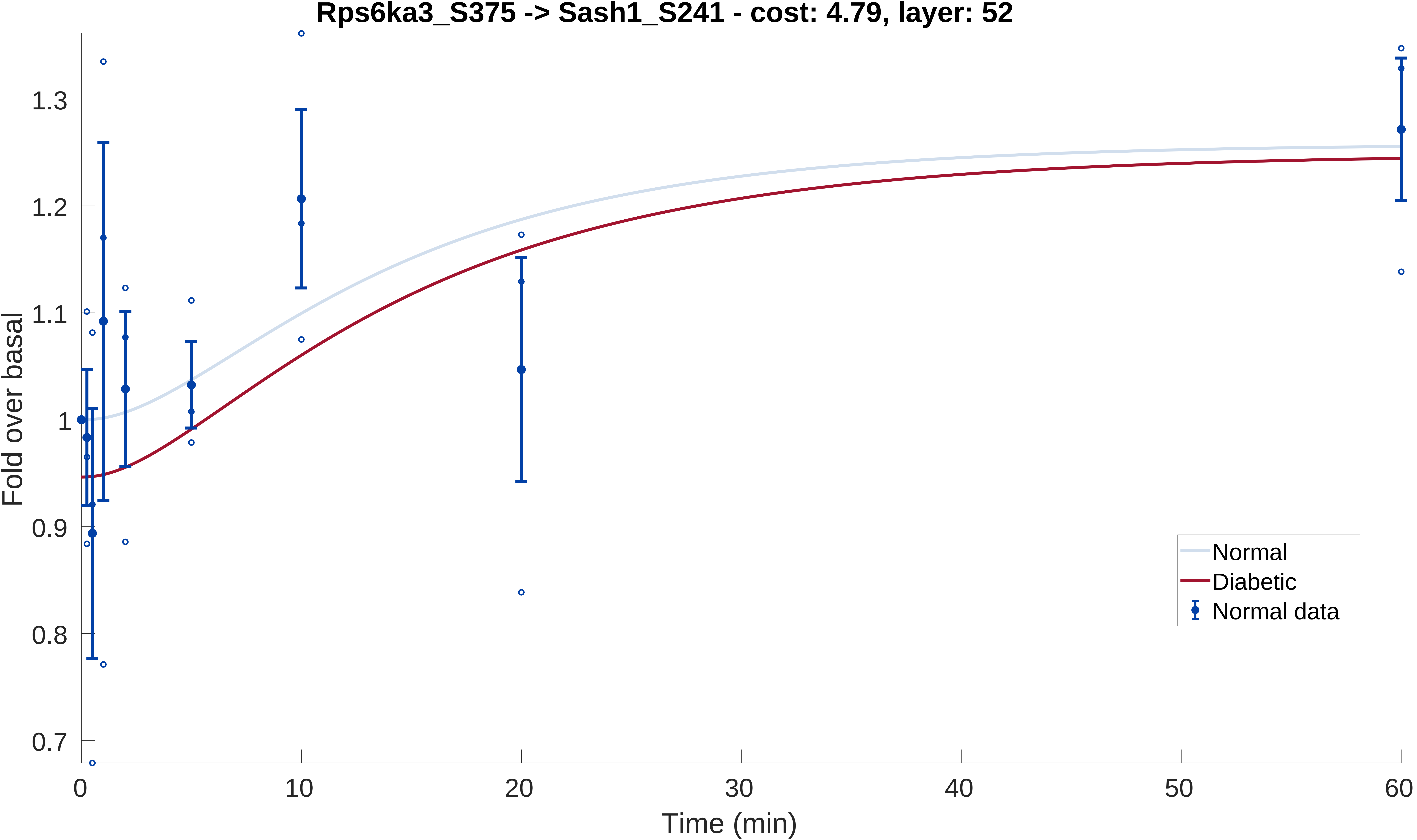

Rps6ka3_S375 -> Sash1_S241 - cost: 4.79, layer: 52
Fold over basal
Time (min)
0.7
0.8
0.9
1
1.1
1.2
1.3
0
10
20
30
40
50
60
Normal
Diabetic
Normal data

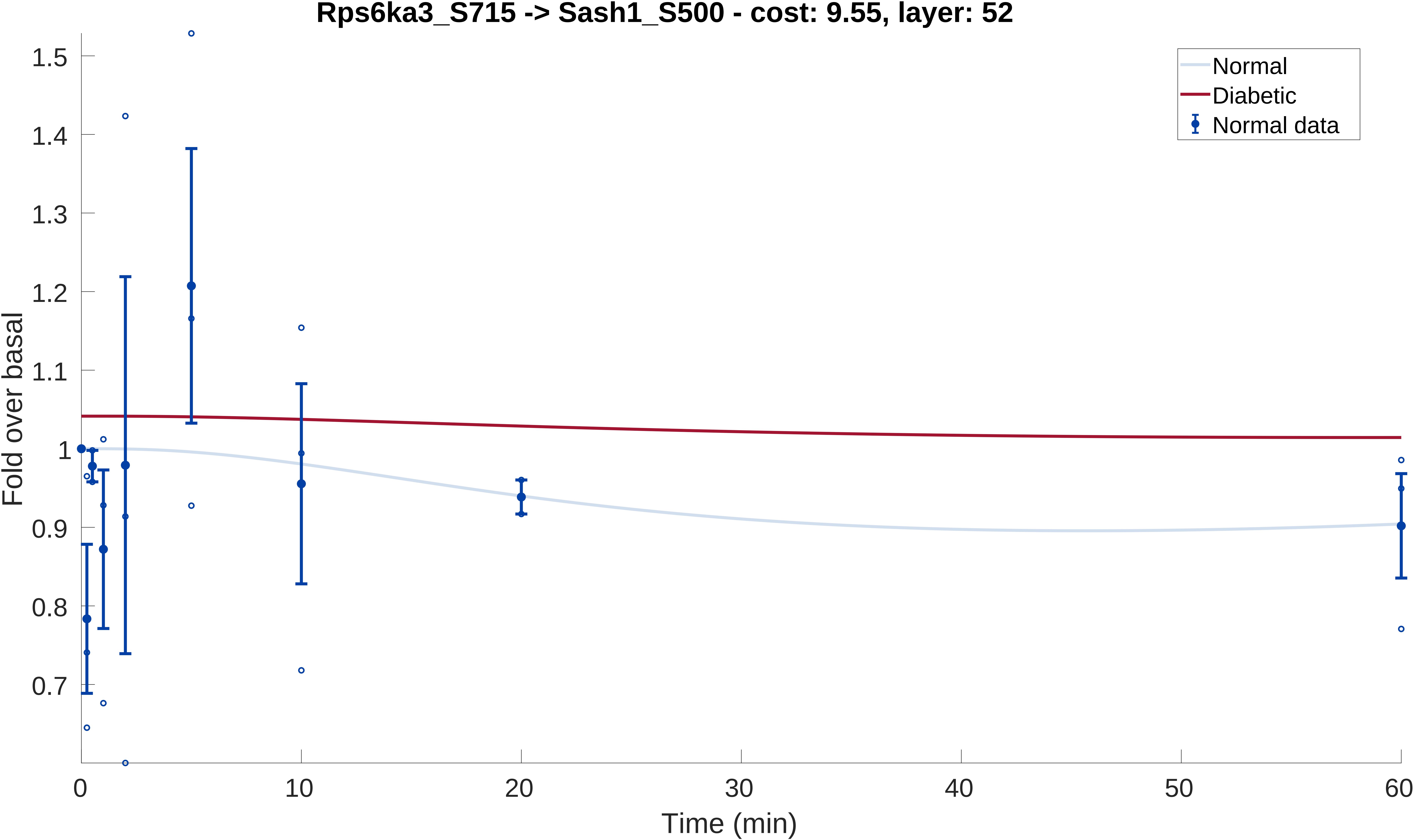

Rps6ka3_S715 -> Sash1_S500 - cost: 9.55, layer: 52
Normal
Diabetic
Normal data
Fold over basal
Time (min)
1.5
1.4
1.3
1.2
1.1
1
0.9
0.8
0.7
0
10
20
30
40
50
60

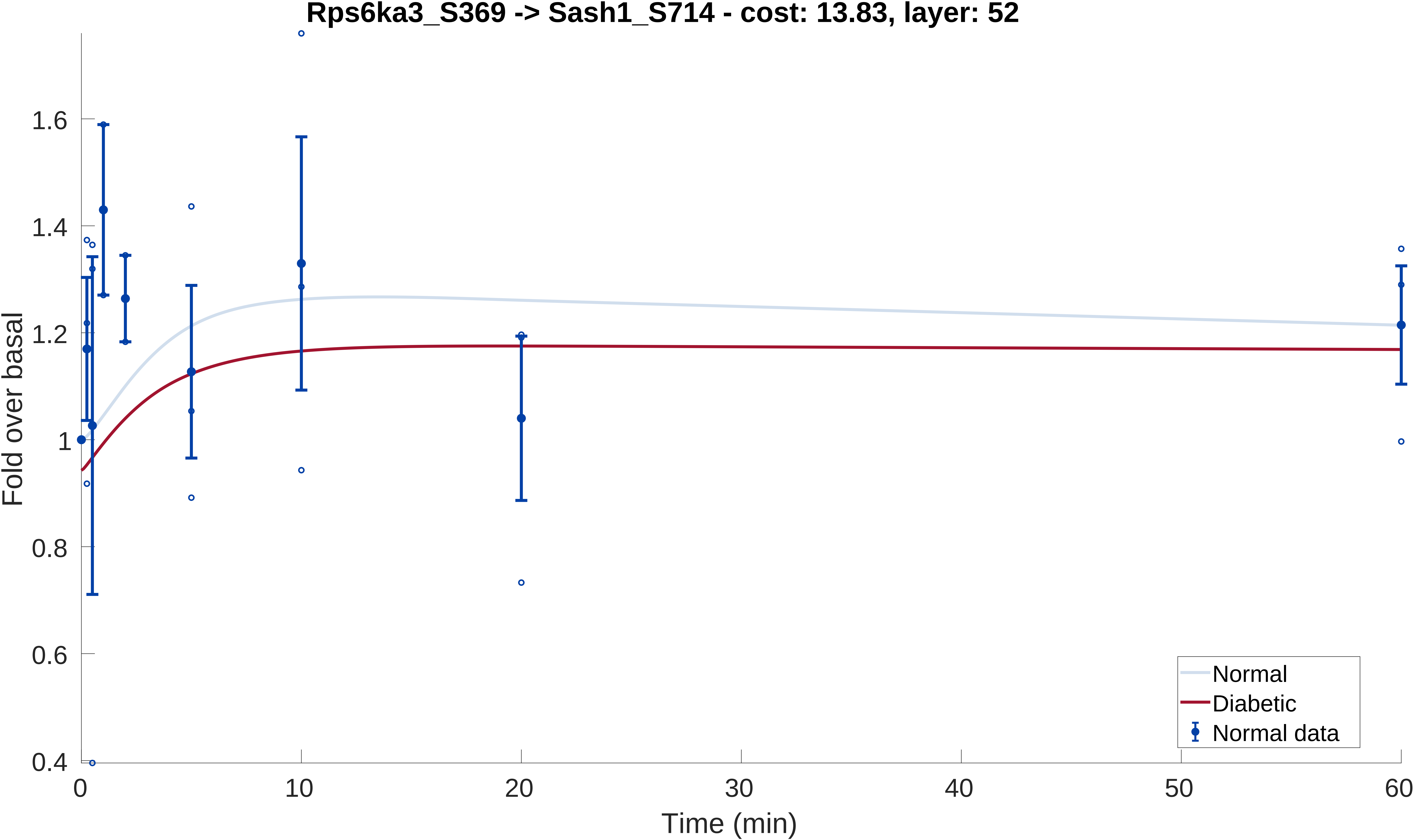
Rps6ka3_S369 -> Sash1_S714 - cost: 13.83, layer: 52
Fold over basal
Time (min)
1.6
1.4
1.2
1
0.8
0.6
0.4
0
10
20
30
40
50
60
Normal
Diabetic
Normal data

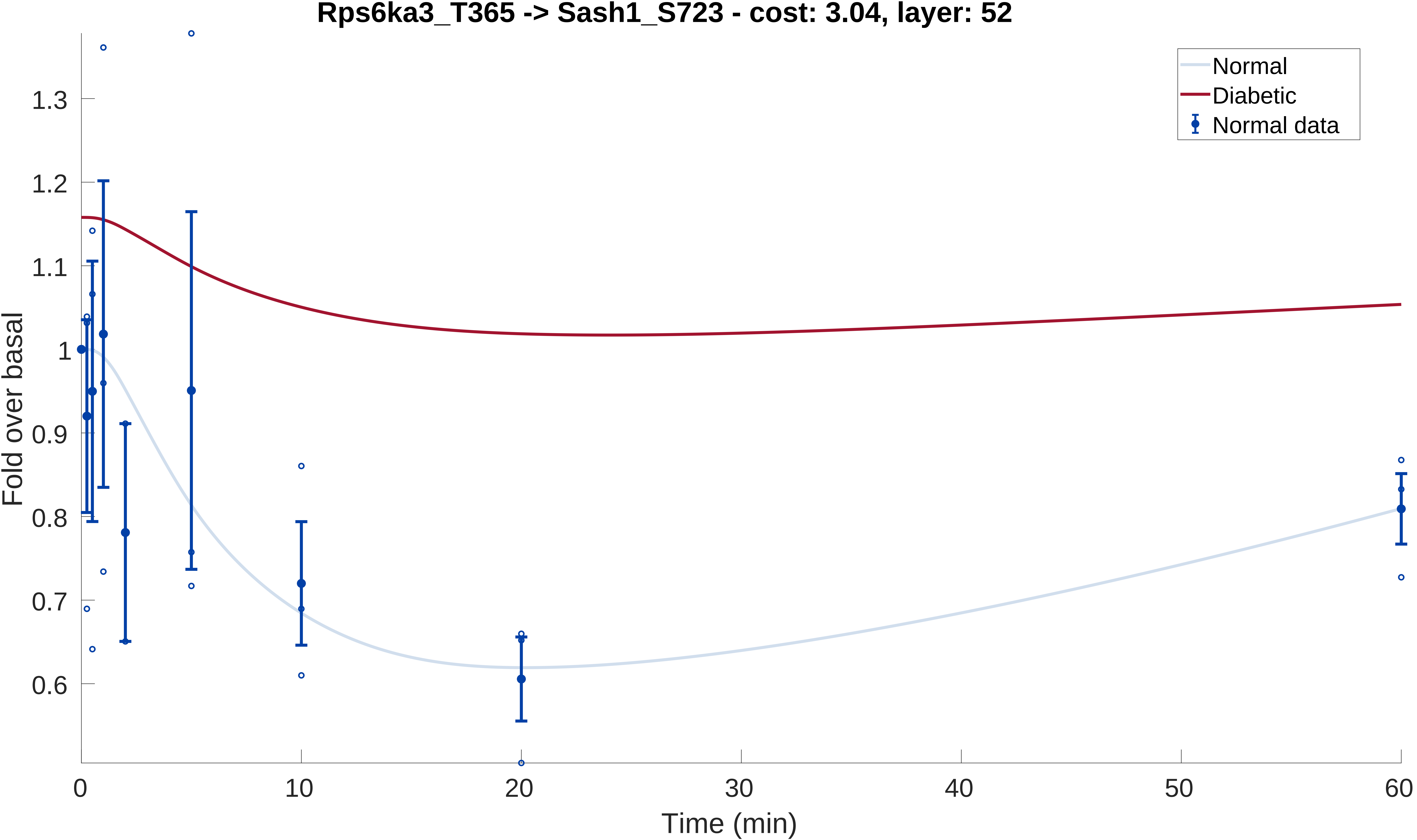
Rps6ka3_T365 -> Sash1_S723 - cost: 3.04, layer: 52
Normal
Diabetic
Normal data
Fold over basal
Time (min)
1.3
1.2
1.1
1
0.9
0.8
0.7
0.6
0
10
20
30
40
50
60

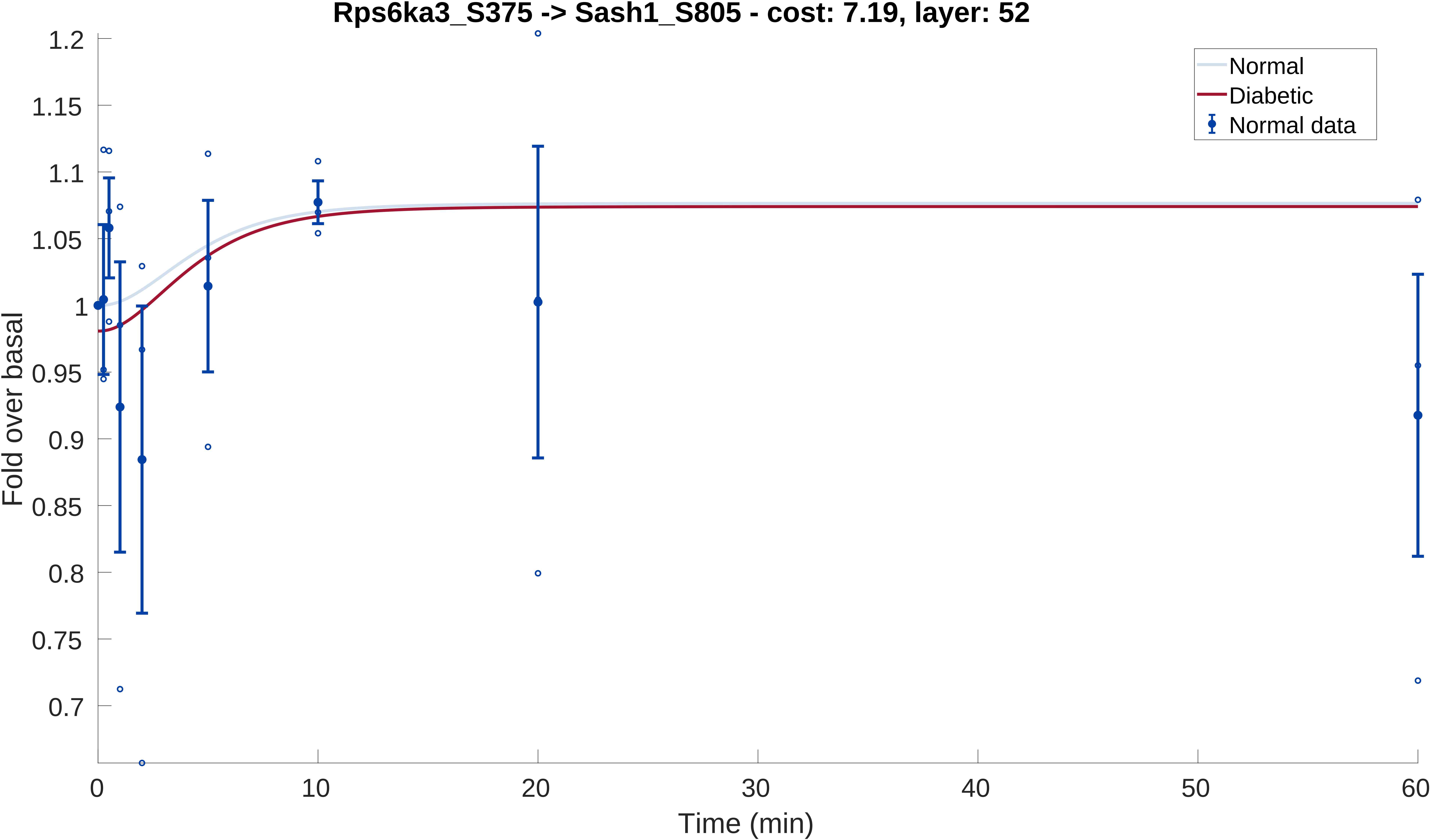

Rps6ka3_S375 -> Sash1_S805 - cost: 7.19, layer: 52
Fold over basal
Time (min)
Normal
Diabetic
Normal data
1.2
1.15
1.1
1.05
1
0.95
0.9
0.85
0.8
0.75
0.7
0
10
20
30
40
50
60

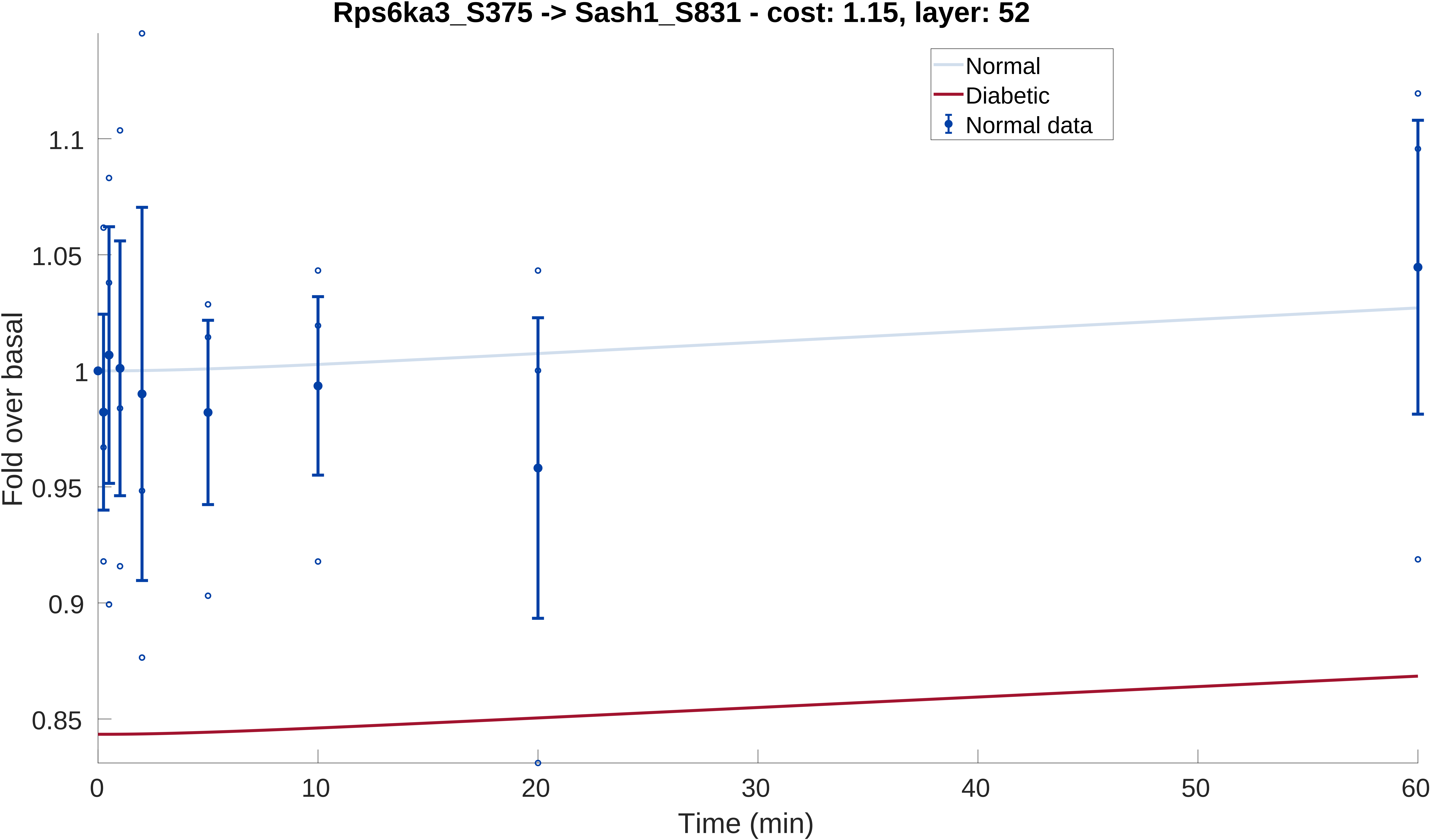

Rps6ka3_S375 -> Sash1_S831 - cost: 1.15, layer: 52
Normal
Diabetic
Normal data
Fold over basal
1.1
1.05
1
0.95
0.9
0.85
0
10
20
30
40
50
60
Time (min)

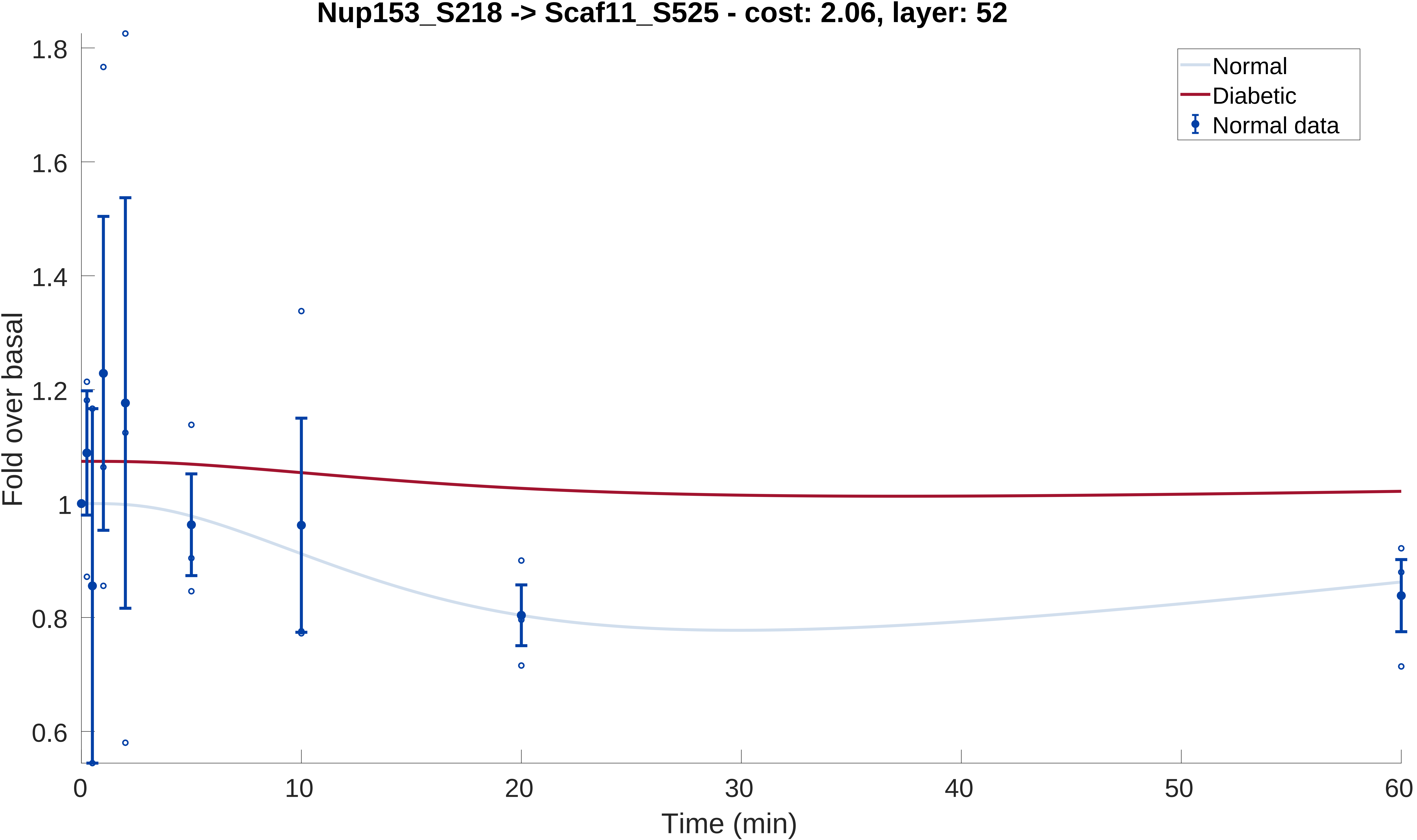

Nup153_S218 -> Scaf11_S525 - cost: 2.06, layer: 52
Normal
Diabetic
Normal data
Fold over basal
Time (min)
0.6
0.8
1
1.2
1.4
1.6
1.8
0
10
20
30
40
50
60

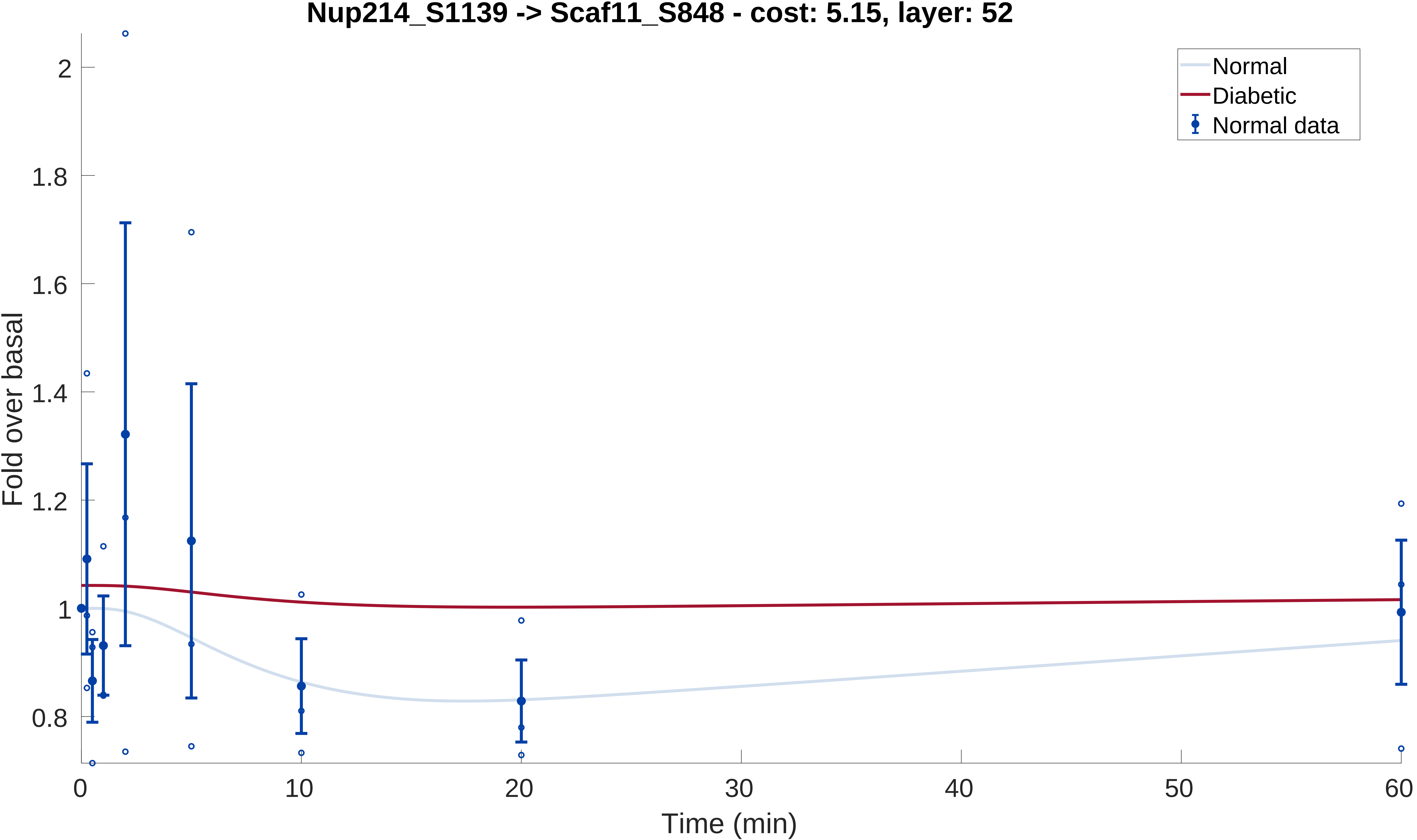

Nup214_S1139 -> Scaf11_S848 - cost: 5.15, layer: 52
Normal
Diabetic
Normal data
Fold over basal
Time (min)
0.8
1
1.2
1.4
1.6
1.8
2
0
10
20
30
40
50
60

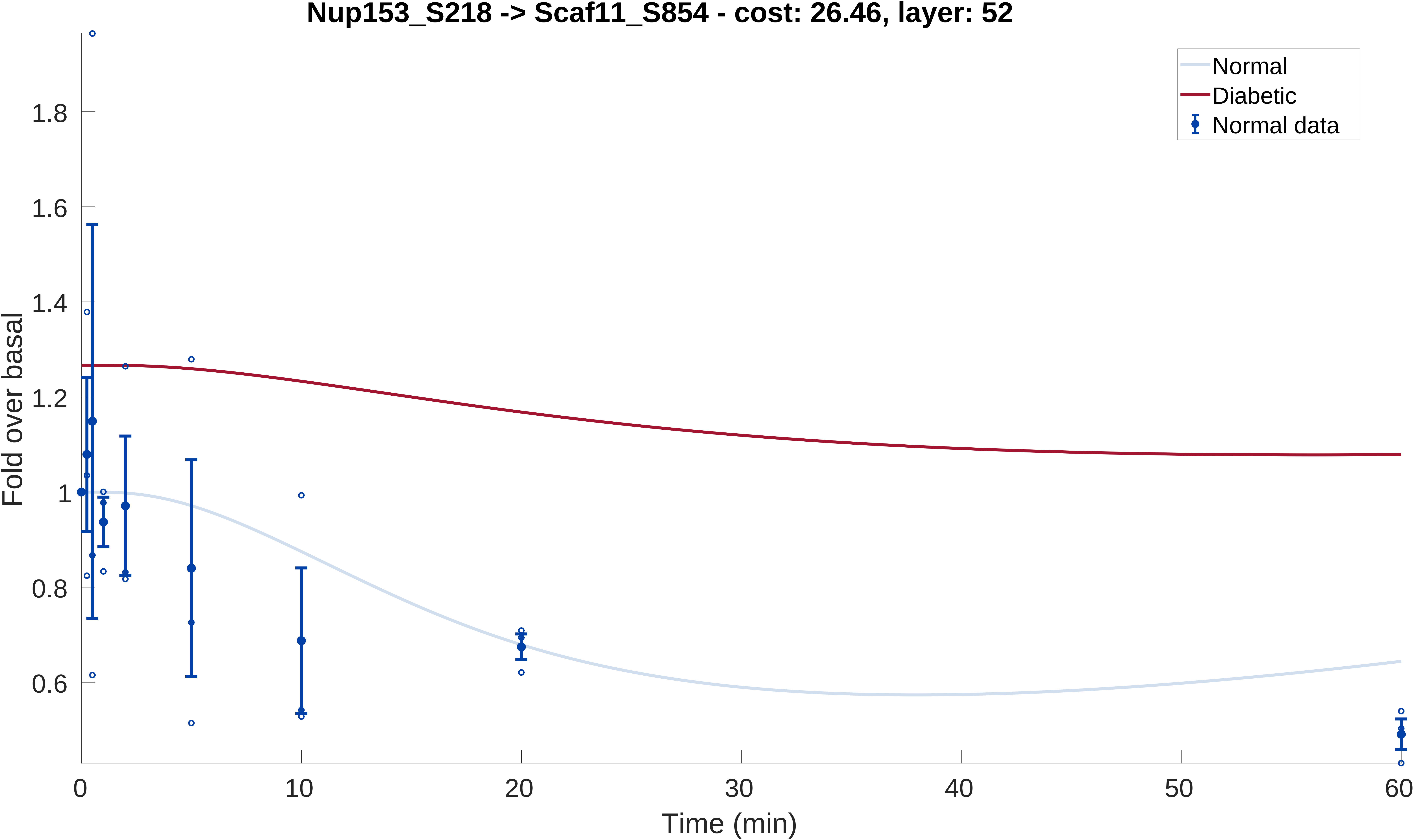

Nup153_S218 -> Scaf11_S854 - cost: 26.46, layer: 52
Normal
Diabetic
Normal data
Fold over basal
Time (min)
0.6
0.8
1
1.2
1.4
1.6
1.8
0
10
20
30
40
50
60

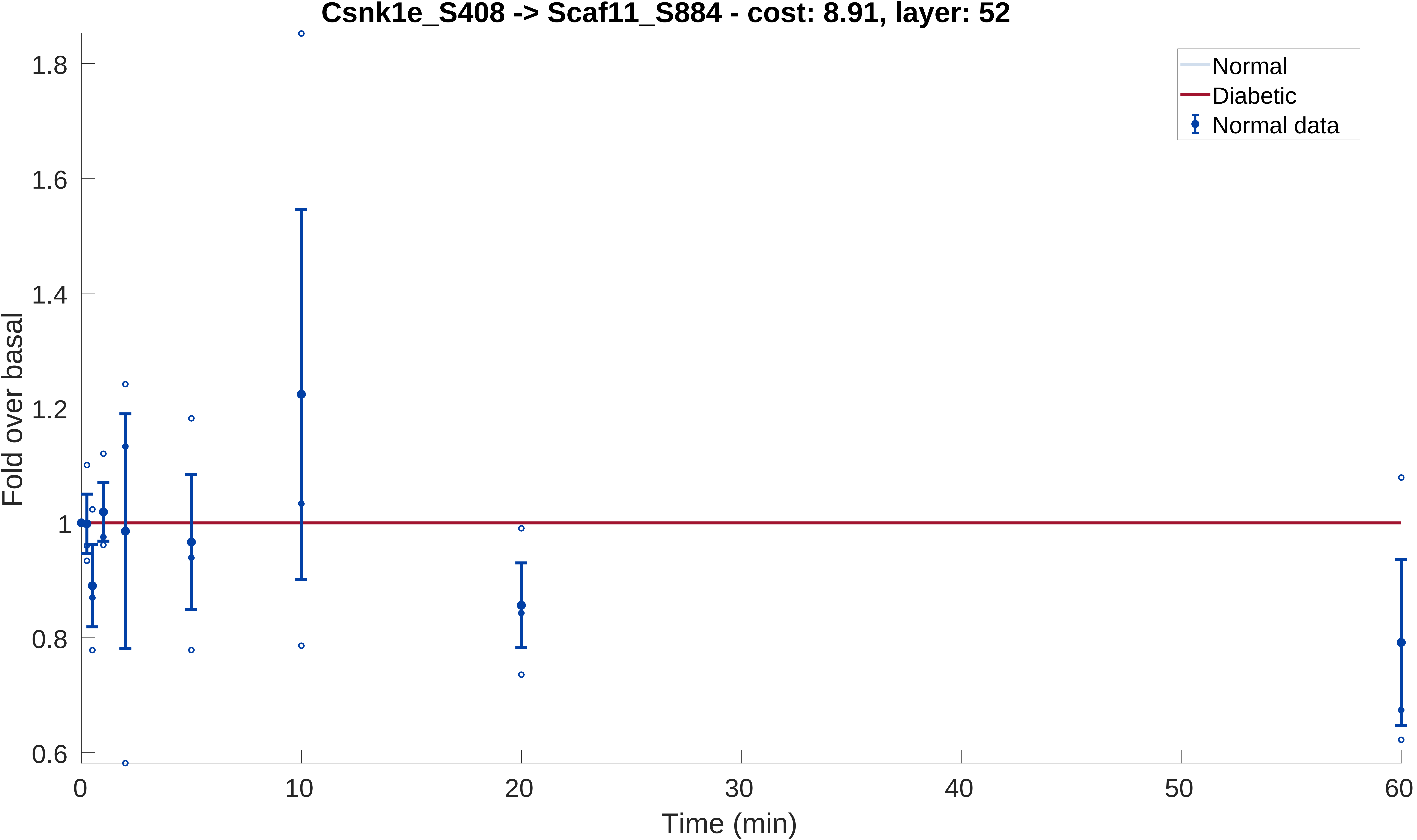

Csnk1e_S408 -> Scaf11_S884 - cost: 8.91, layer: 52
Normal
Diabetic
Normal data
Fold over basal
1.8
1.6
1.4
1.2
1
0.8
0.6
0
10
20
30
40
50
60
Time (min)

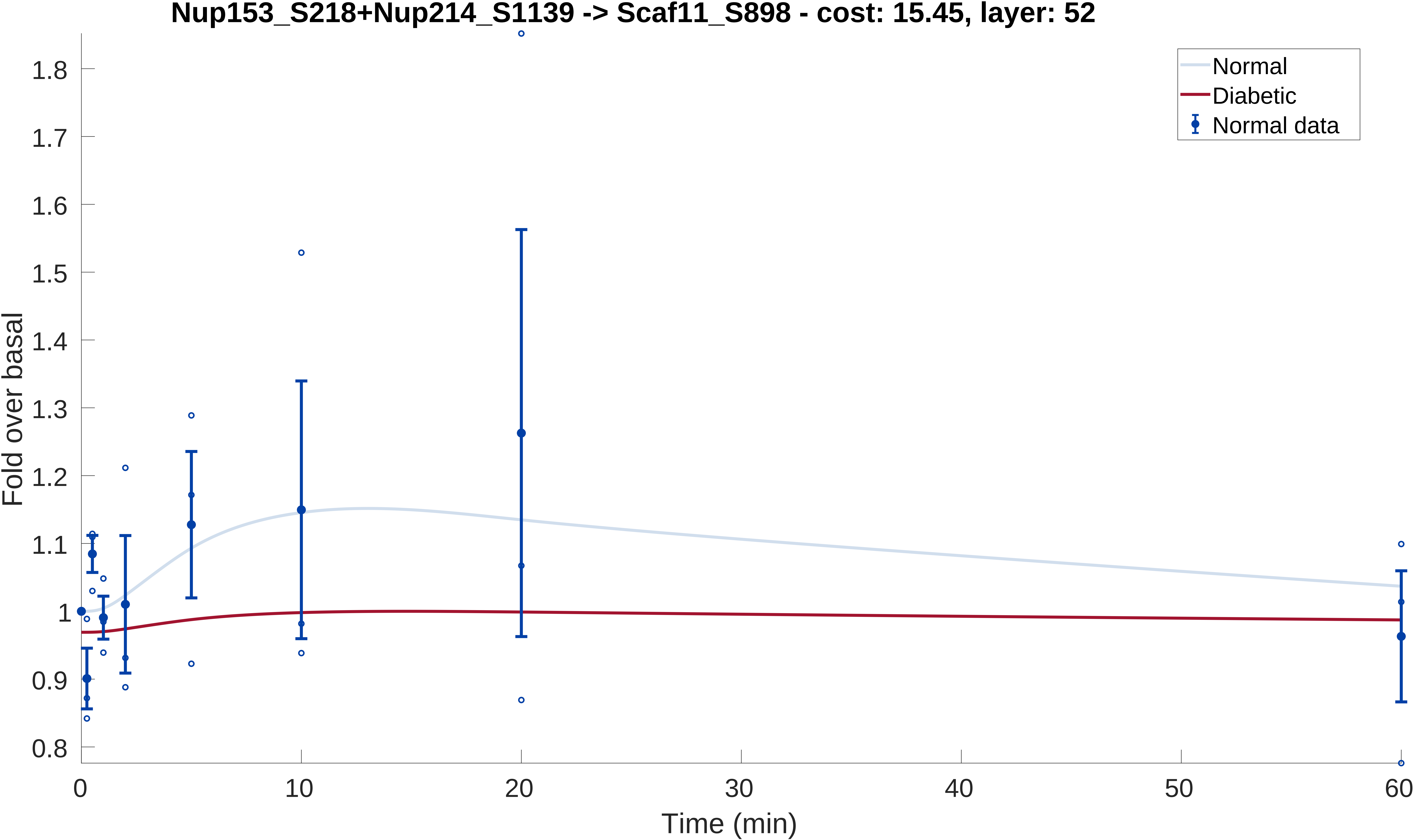

Nup153_S218+Nup214_S1139 -> Scaf11_S898 - cost: 15.45, layer: 52
Normal
Diabetic
Normal data
Fold over basal
Time (min)
1.8
1.7
1.6
1.5
1.4
1.3
1.2
1.1
1
0.9
0.8
0
10
20
30
40
50
60

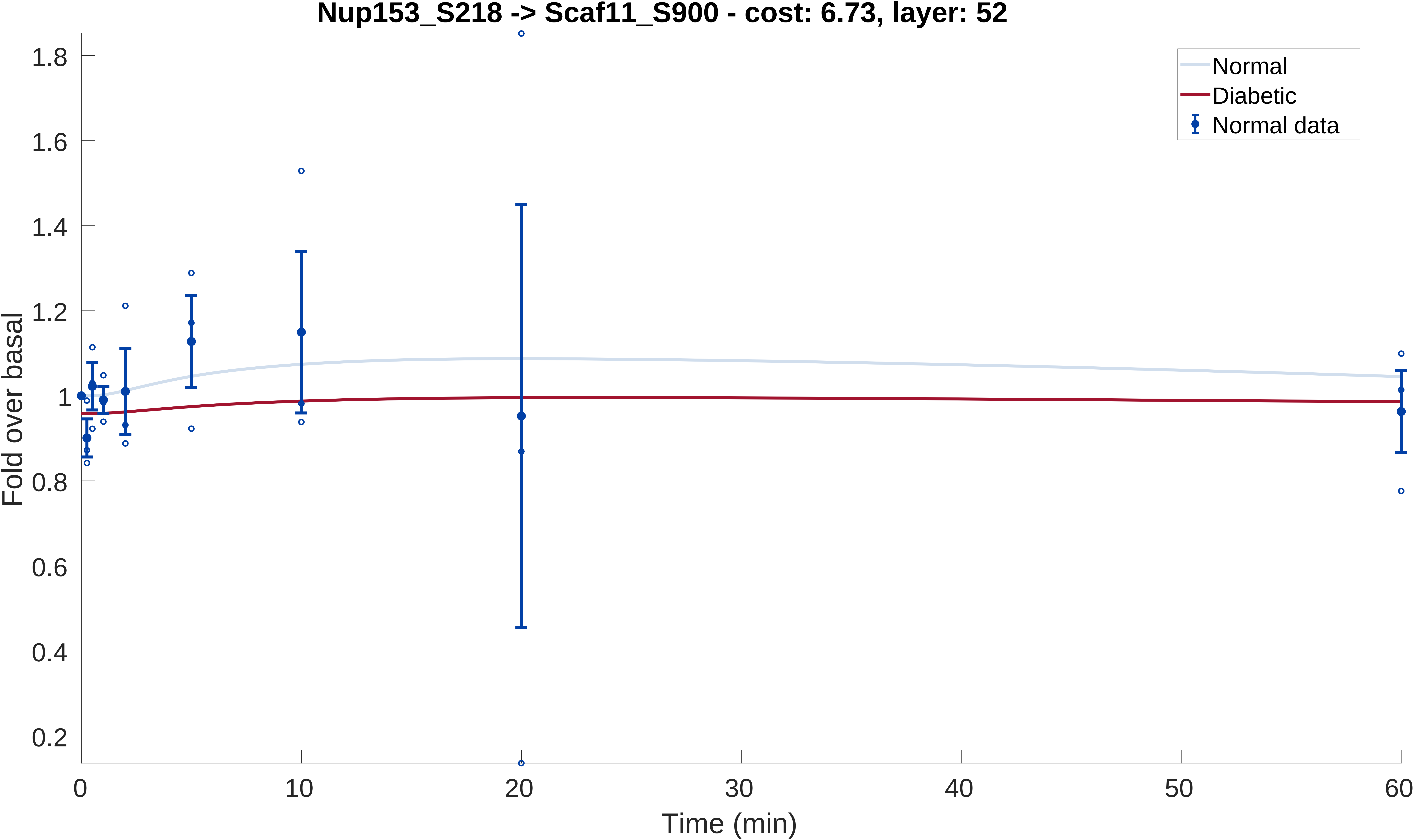

Nup153_S218 -> Scaf11_S900 - cost: 6.73, layer: 52
Normal
Diabetic
Normal data
Fold over basal
Time (min)
0.2
0.4
0.6
0.8
1
1.2
1.4
1.6
1.8
0
10
20
30
40
50
60

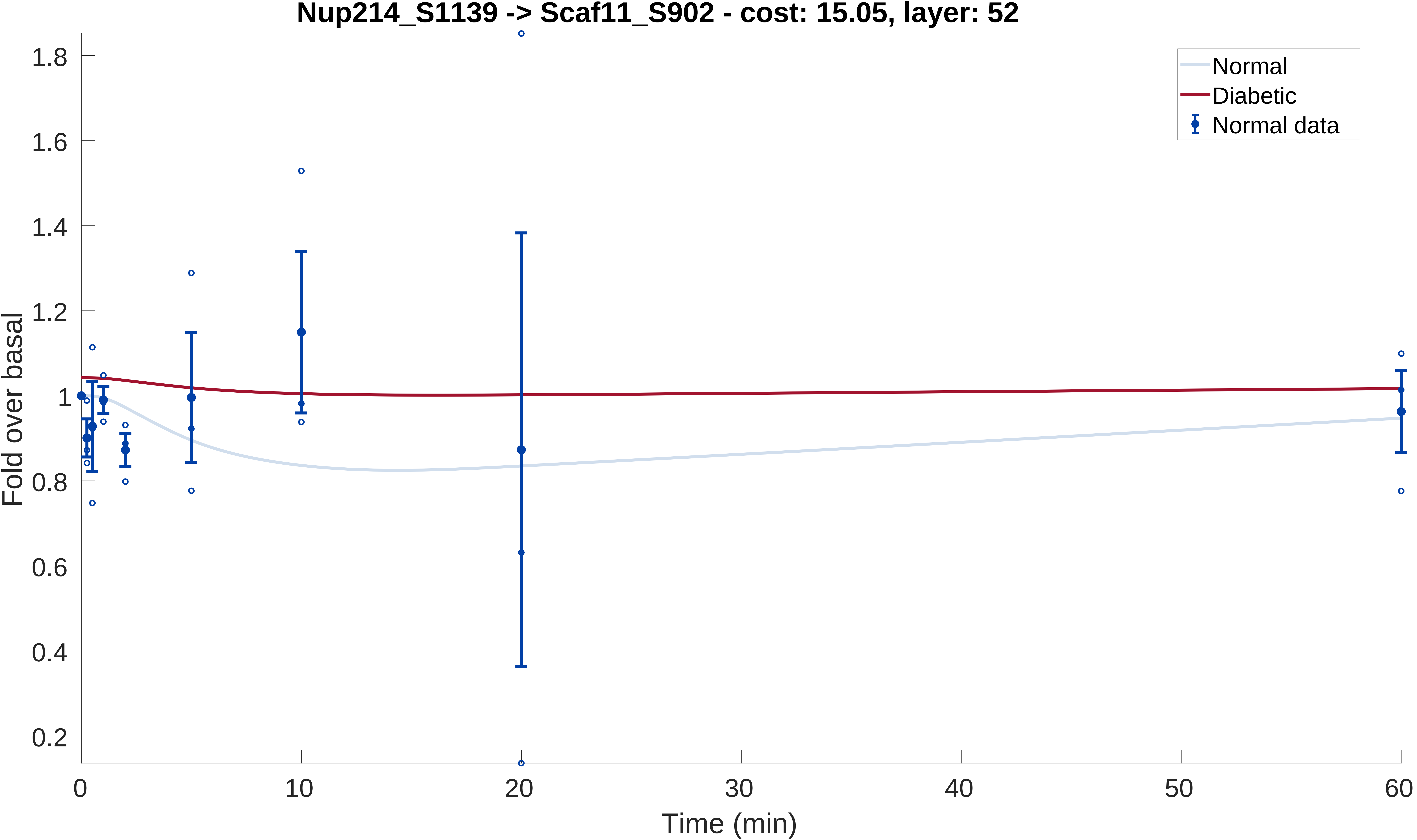

Nup214_S1139 -> Scaf11_S902 - cost: 15.05, layer: 52
Normal
Diabetic
Normal data
Fold over basal
1.8
1.6
1.4
1.2
1
0.8
0.6
0.4
0.2
0
10
20
30
40
50
60
Time (min)

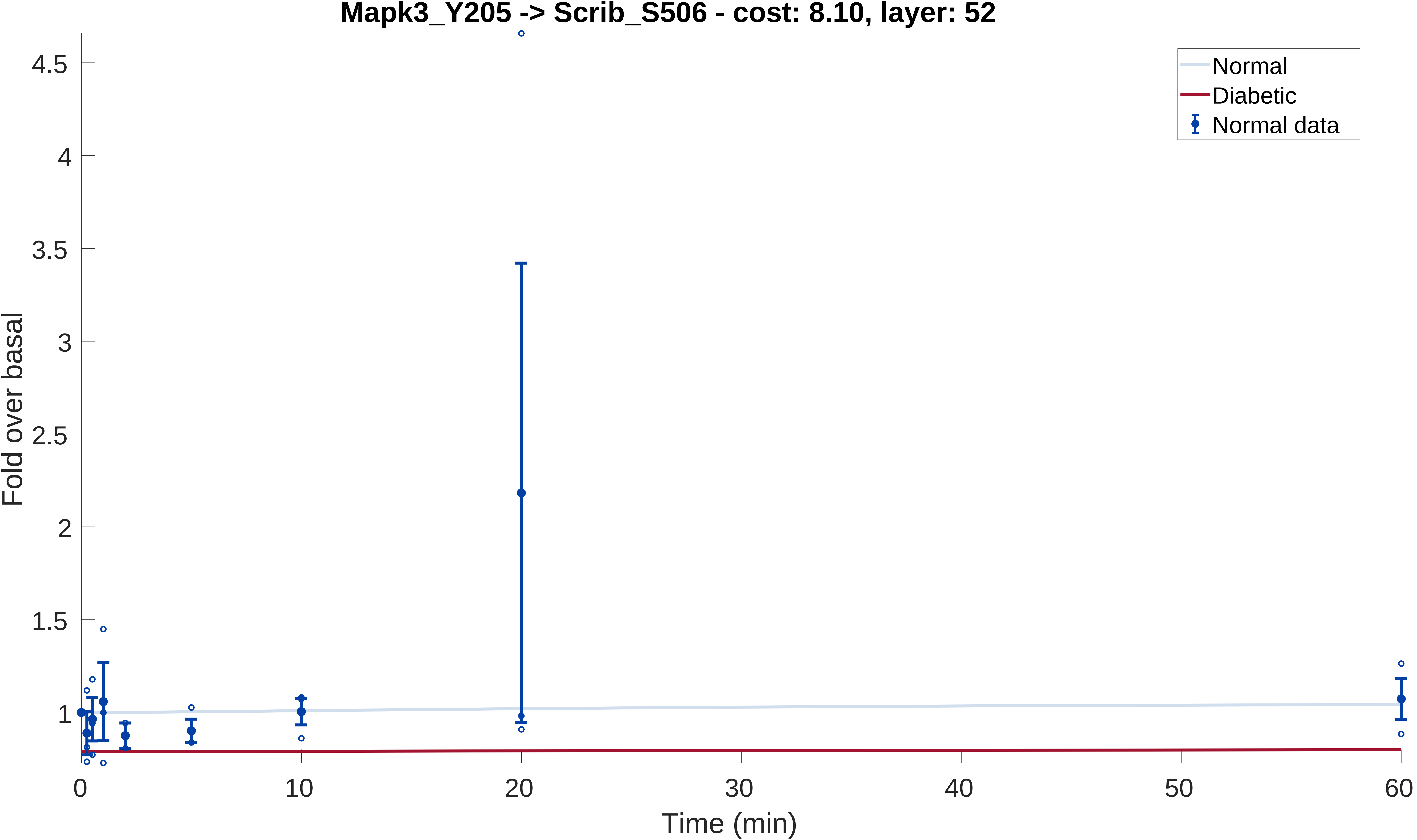

Mapk3_Y205 -> Scrib_S506 - cost: 8.10, layer: 52
Normal
Diabetic
Normal data
Fold over basal
Time (min)
4.5
4
3.5
3
2.5
2
1.5
1
0
10
20
30
40
50
60

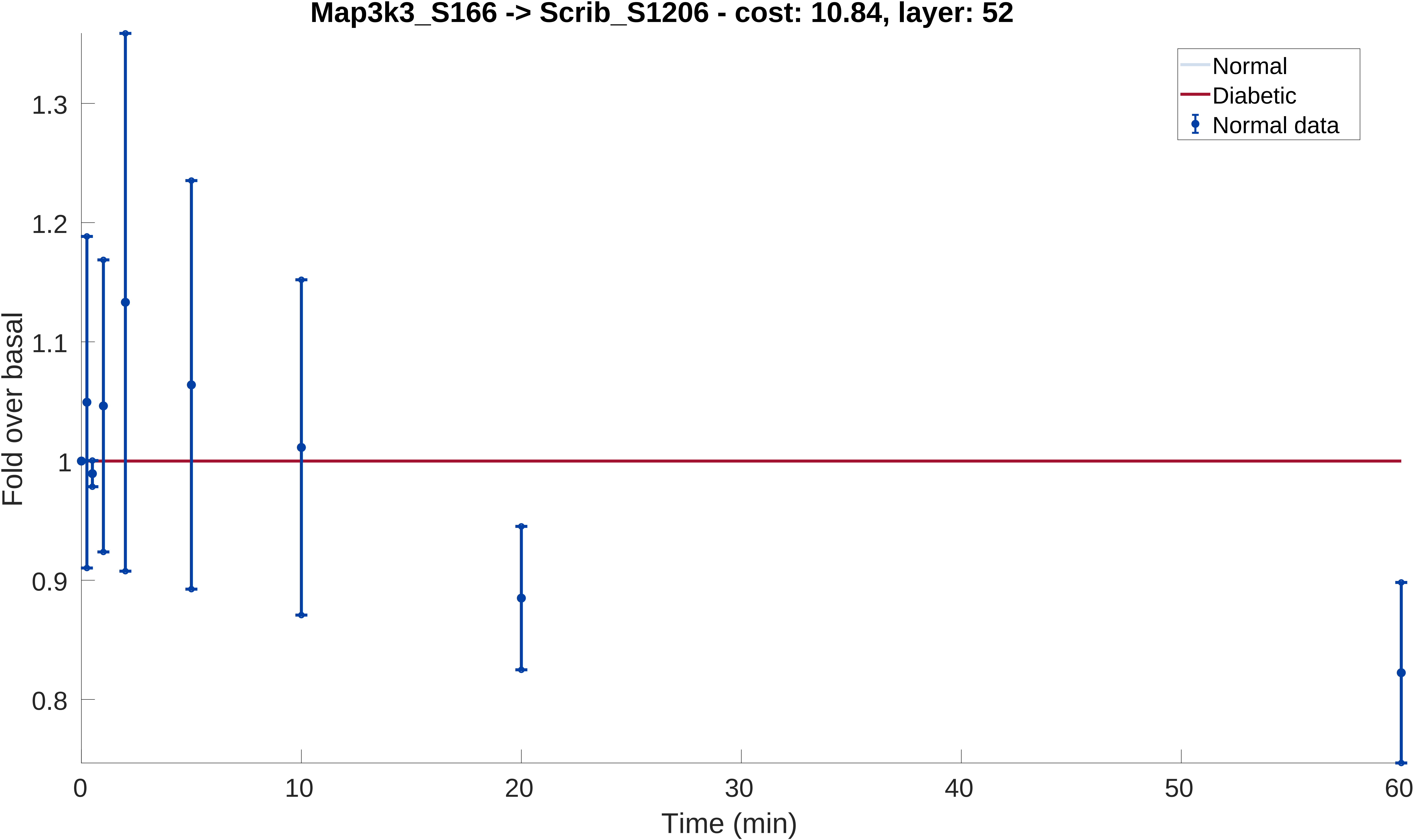

Map3k3_S166 -> Scrib_S1206 - cost: 10.84, layer: 52
Normal
Diabetic
Normal data
Fold over basal
1.3
1.2
1.1
1
0.9
0.8
0
10
20
30
40
50
60
Time (min)

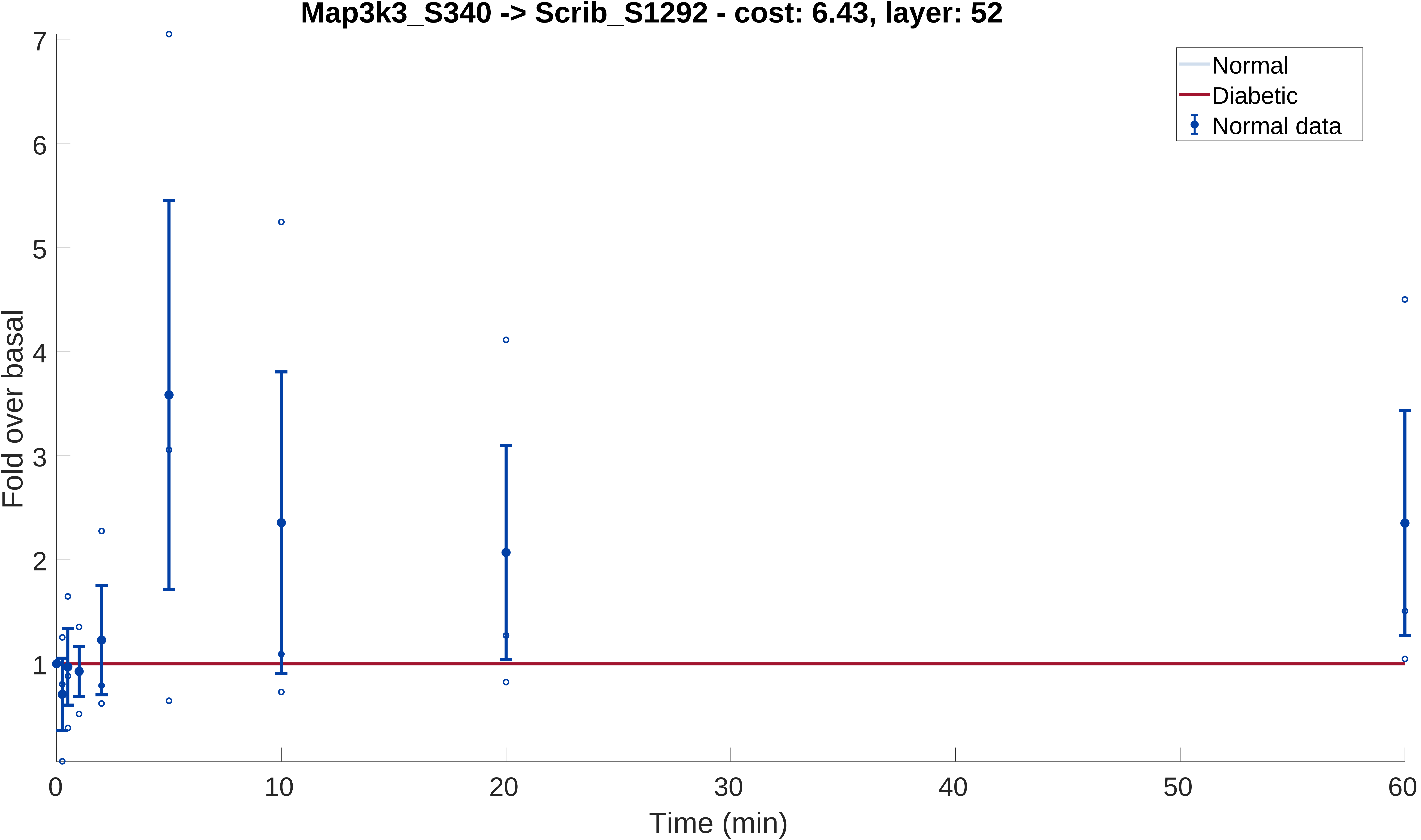
Map3k3_S340 -> Scrib_S1292 - cost: 6.43, layer: 52
Normal
Diabetic
Normal data
Fold over basal
Time (min)
0
10
20
30
40
50
60
1
2
3
4
5
6
7

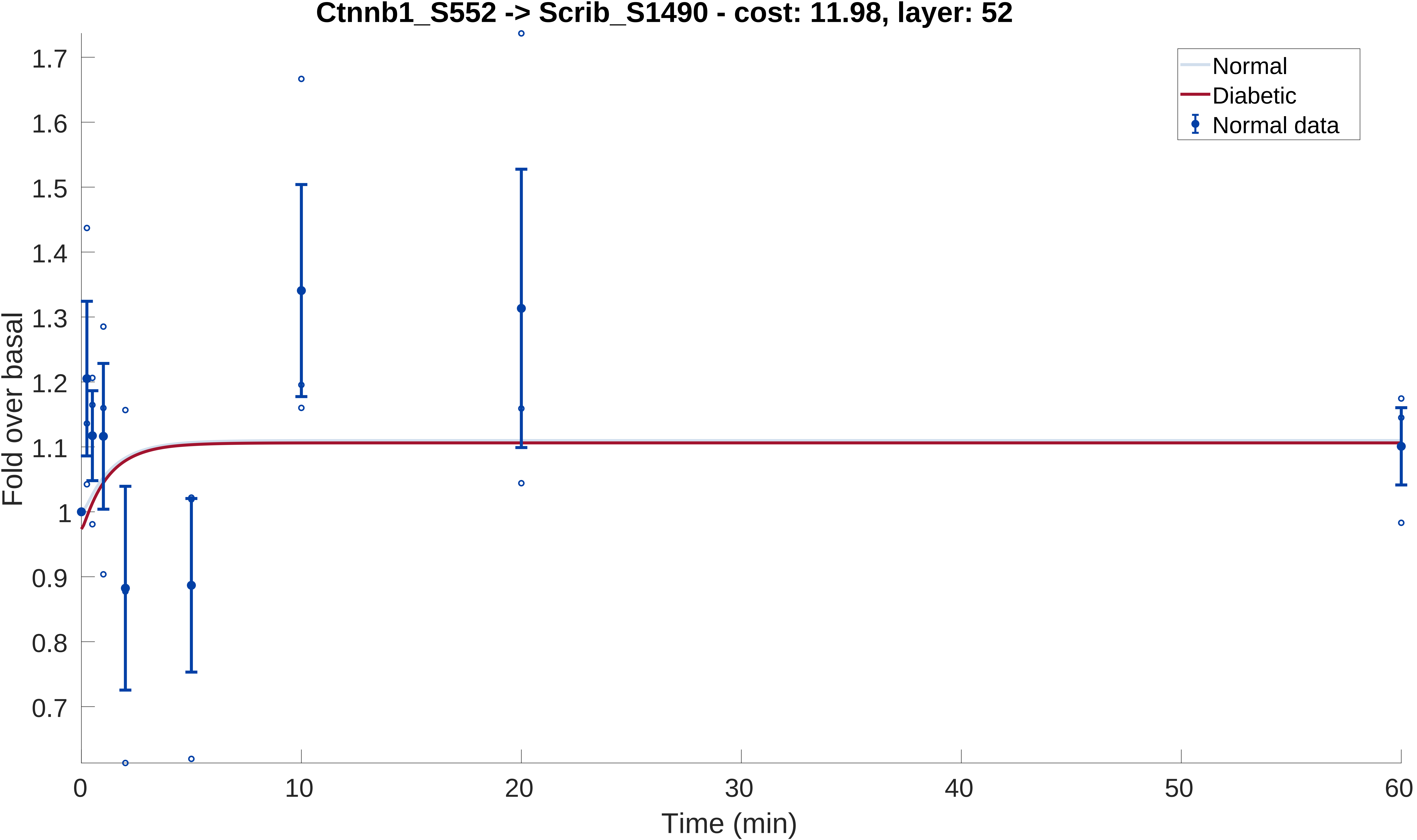
Ctnnb1_S552 -> Scrib_S1490 - cost: 11.98, layer: 52
Fold over basal
Time (min)
Normal
Diabetic
Normal data
0.7
0.8
0.9
1
1.1
1.2
1.3
1.4
1.5
1.6
1.7
0
10
20
30
40
50
60

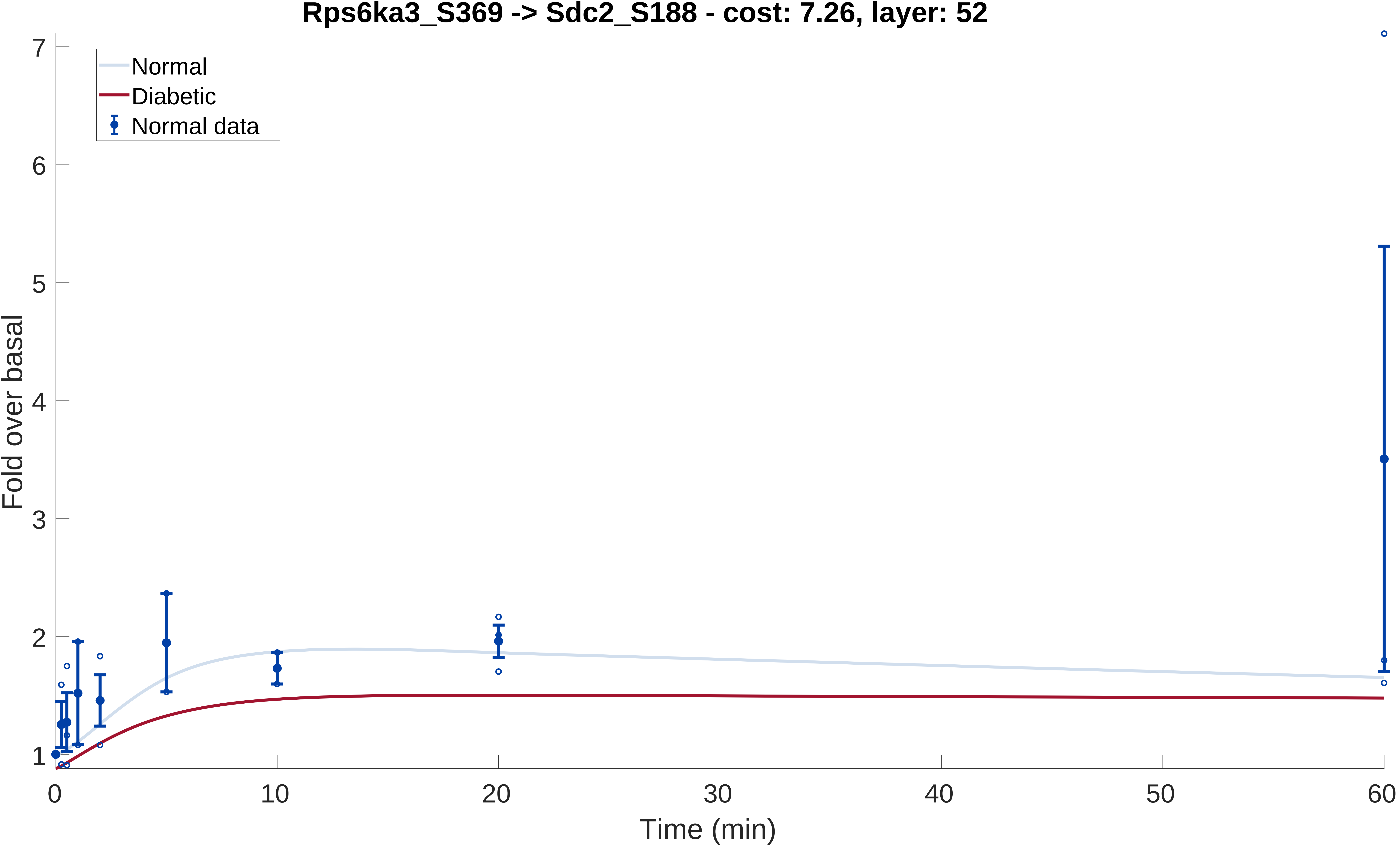
Rps6ka3_S369 -> Sdc2_S188 - cost: 7.26, layer: 52
Normal
Diabetic
Normal data
Fold over basal
Time (min)
1
2
3
4
5
6
7
0
10
20
30
40
50
60

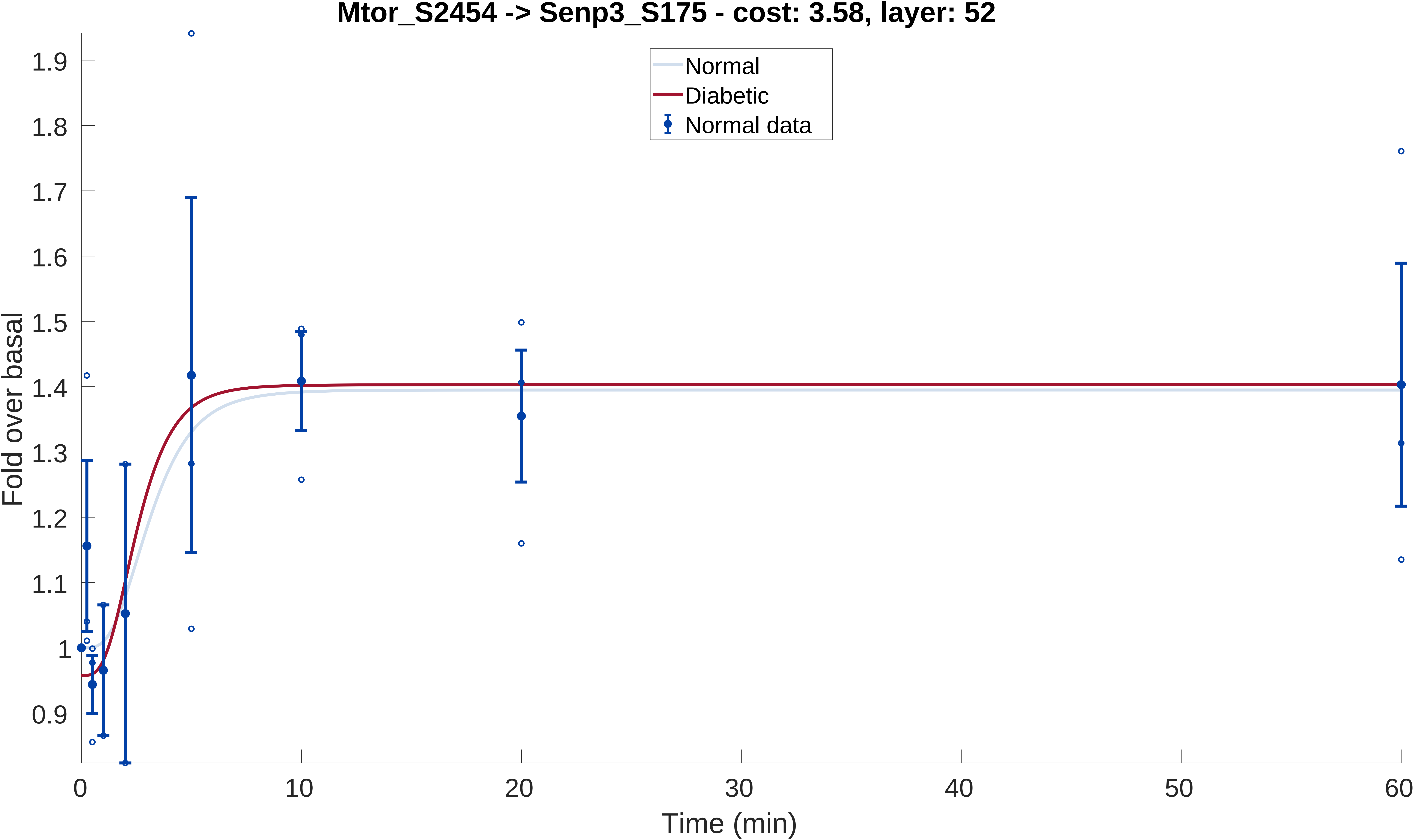

Mtor_S2454 -> Senp3_S175 - cost: 3.58, layer: 52
Normal
Diabetic
Normal data
Fold over basal
1.9
1.8
1.7
1.6
1.5
1.4
1.3
1.2
1.1
1
0.9
0
10
20
30
40
50
60
Time (min)

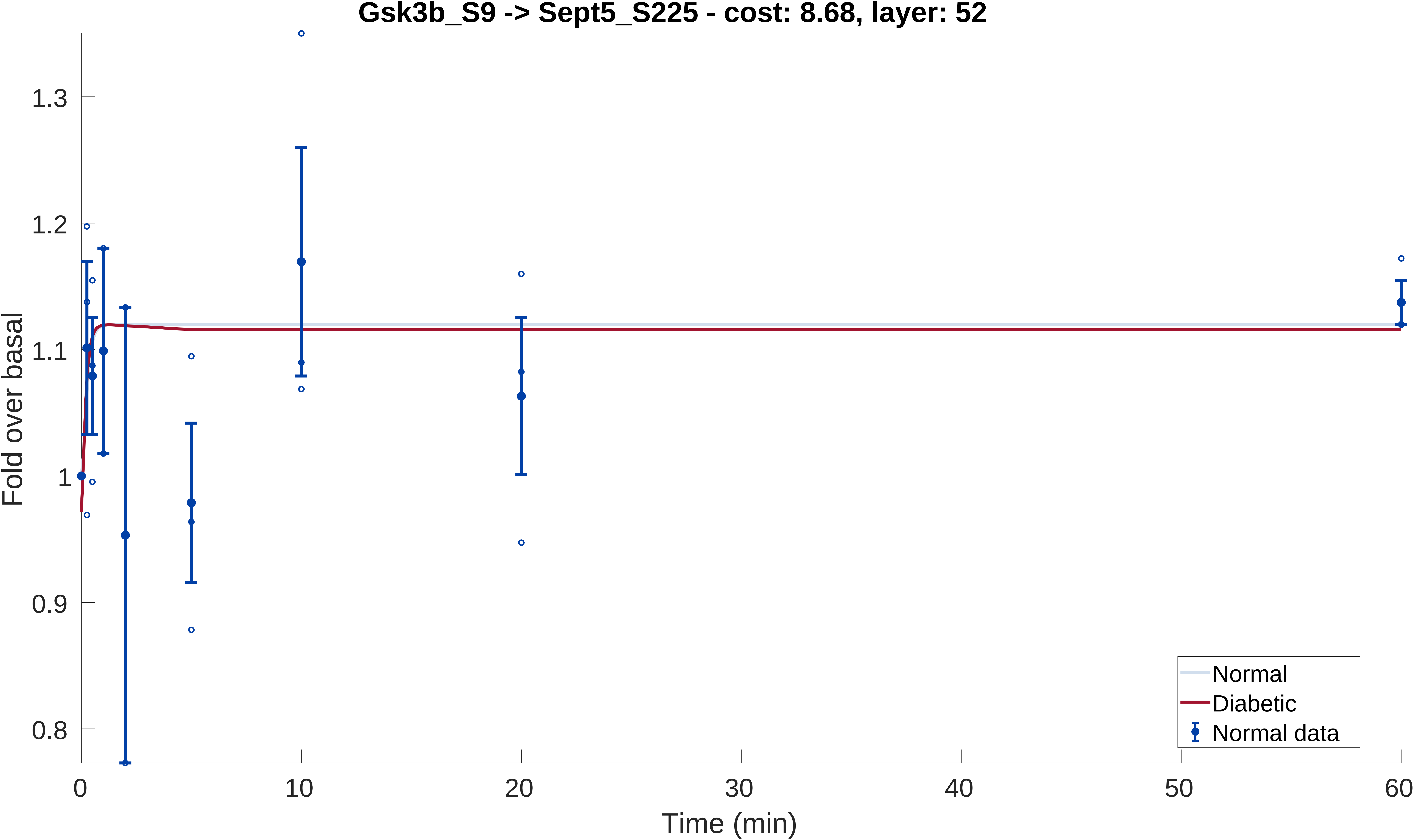
Gsk3b_S9 -> Sept5_S225 - cost: 8.68, layer: 52
Fold over basal
1.3
1.2
1.1
1
0.9
0.8
0
10
20
30
40
50
60
Time (min)
Normal
Diabetic
Normal data

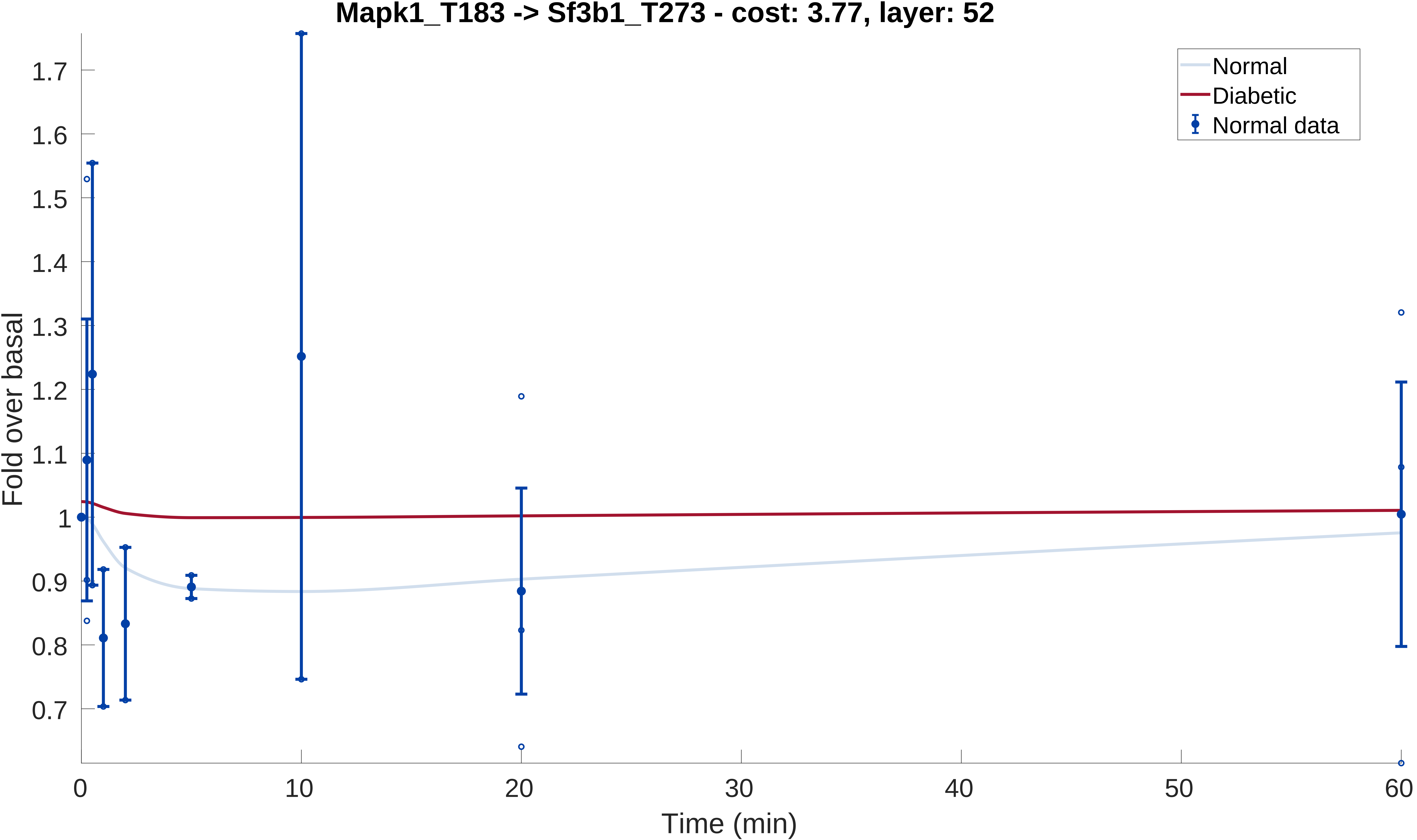

Mapk1_T183 -> Sf3b1_T273 - cost: 3.77, layer: 52
Normal
Diabetic
Normal data
Fold over basal
1.7
1.6
1.5
1.4
1.3
1.2
1.1
1
0.9
0.8
0.7
0
10
20
30
40
50
60
Time (min)

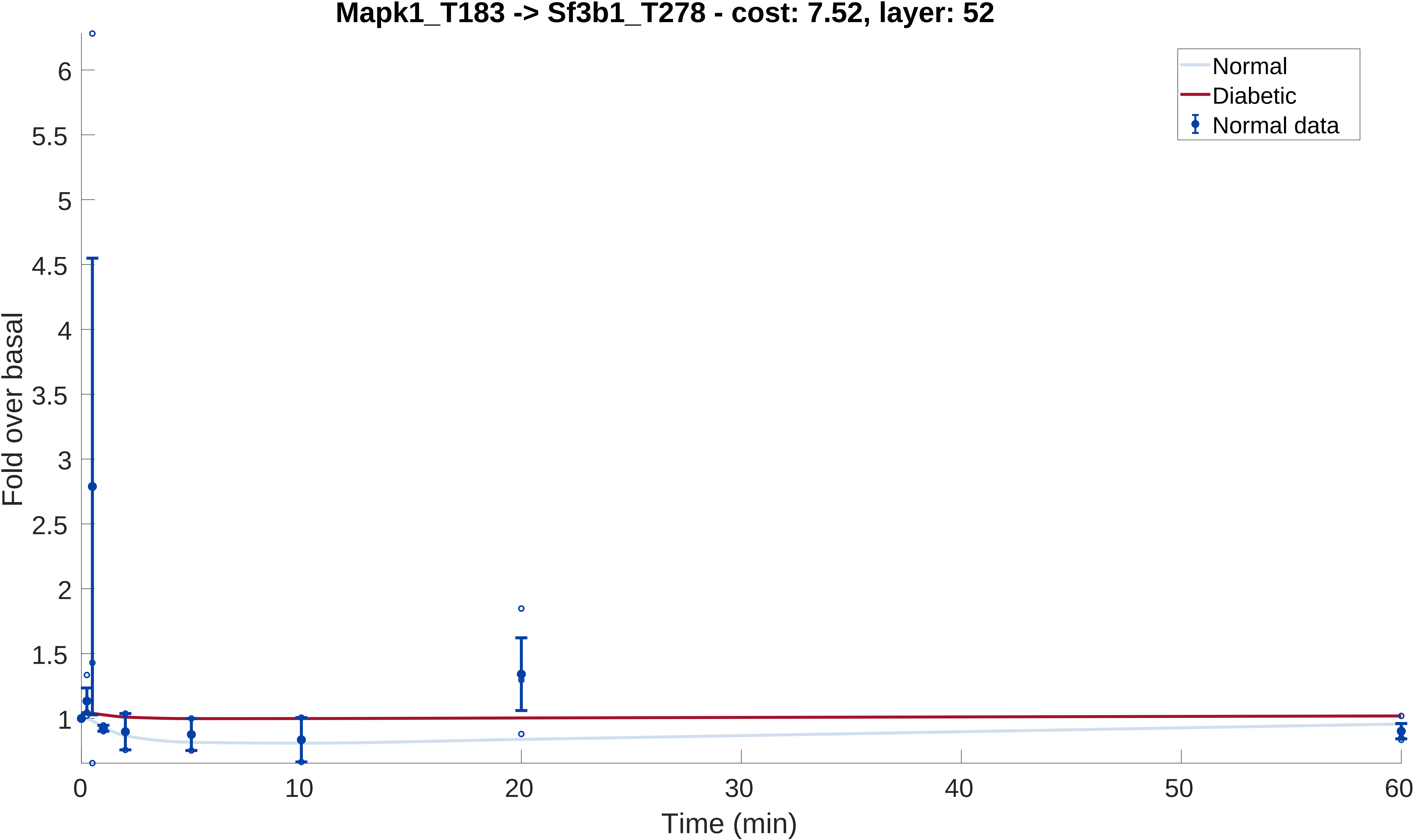
Mapk1_T183 -> Sf3b1_T278 - cost: 7.52, layer: 52
Normal
Diabetic
Normal data
Fold over basal
Time (min)
6
5.5
5
4.5
4
3.5
3
2.5
2
1.5
1
0
10
20
30
40
50
60

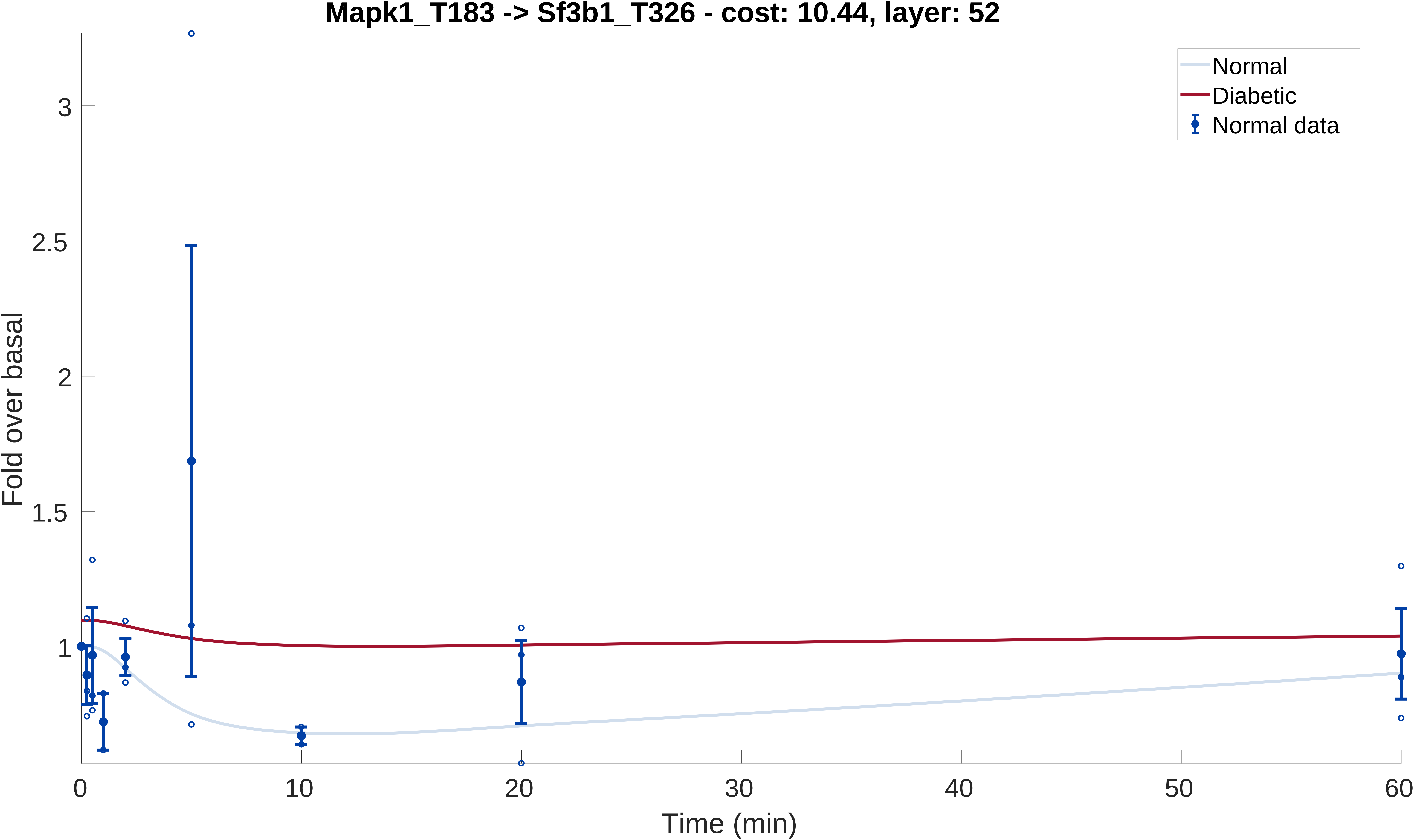

Mapk1_T183 -> Sf3b1_T326 - cost: 10.44, layer: 52
Normal
Diabetic
Normal data
Fold over basal
Time (min)
3
2.5
2
1.5
1
0
10
20
30
40
50
60

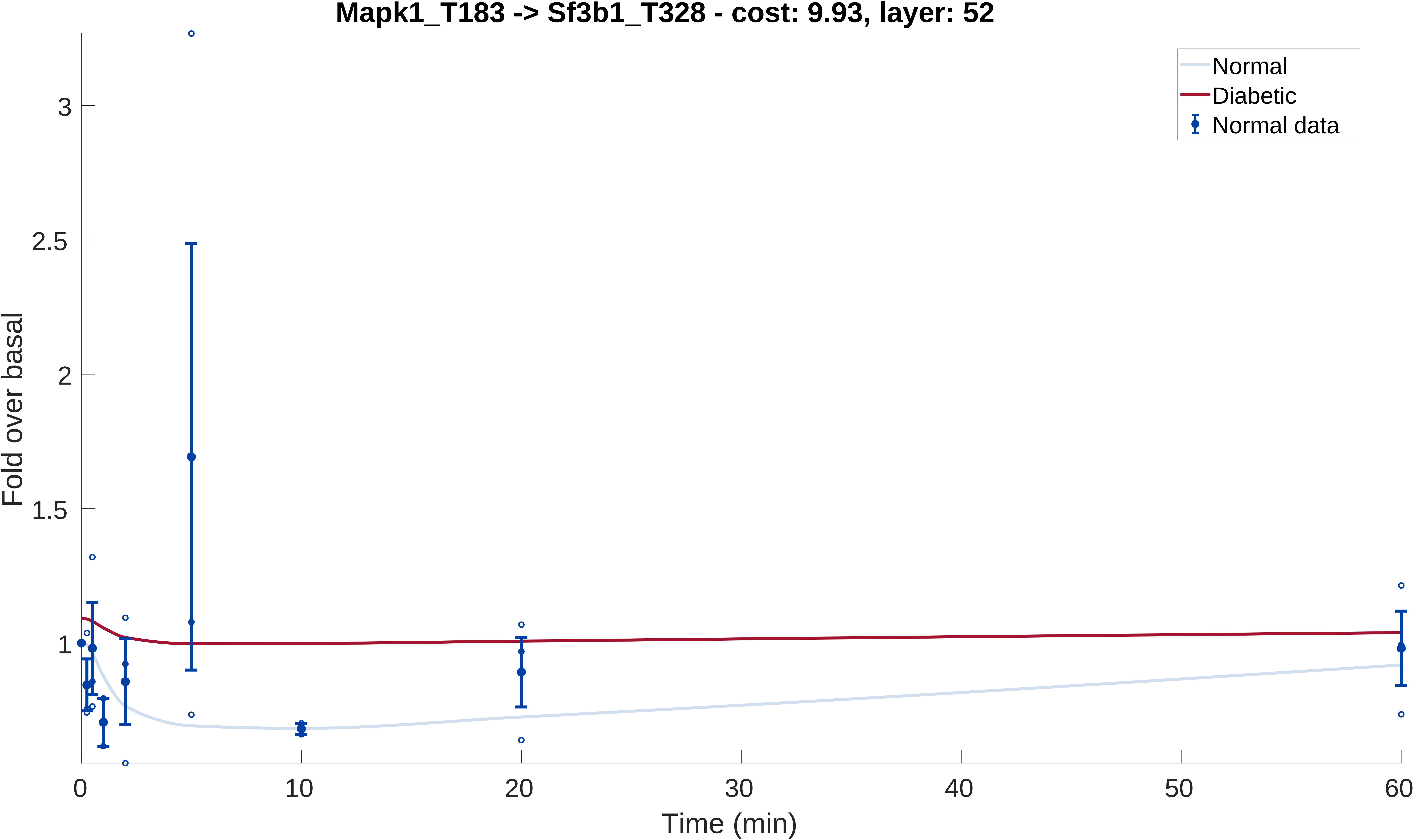
Mapk1_T183 -> Sf3b1_T328 - cost: 9.93, layer: 52
Normal
Diabetic
Normal data
Fold over basal
3
2.5
2
1.5
1
0
10
20
30
40
50
60
Time (min)

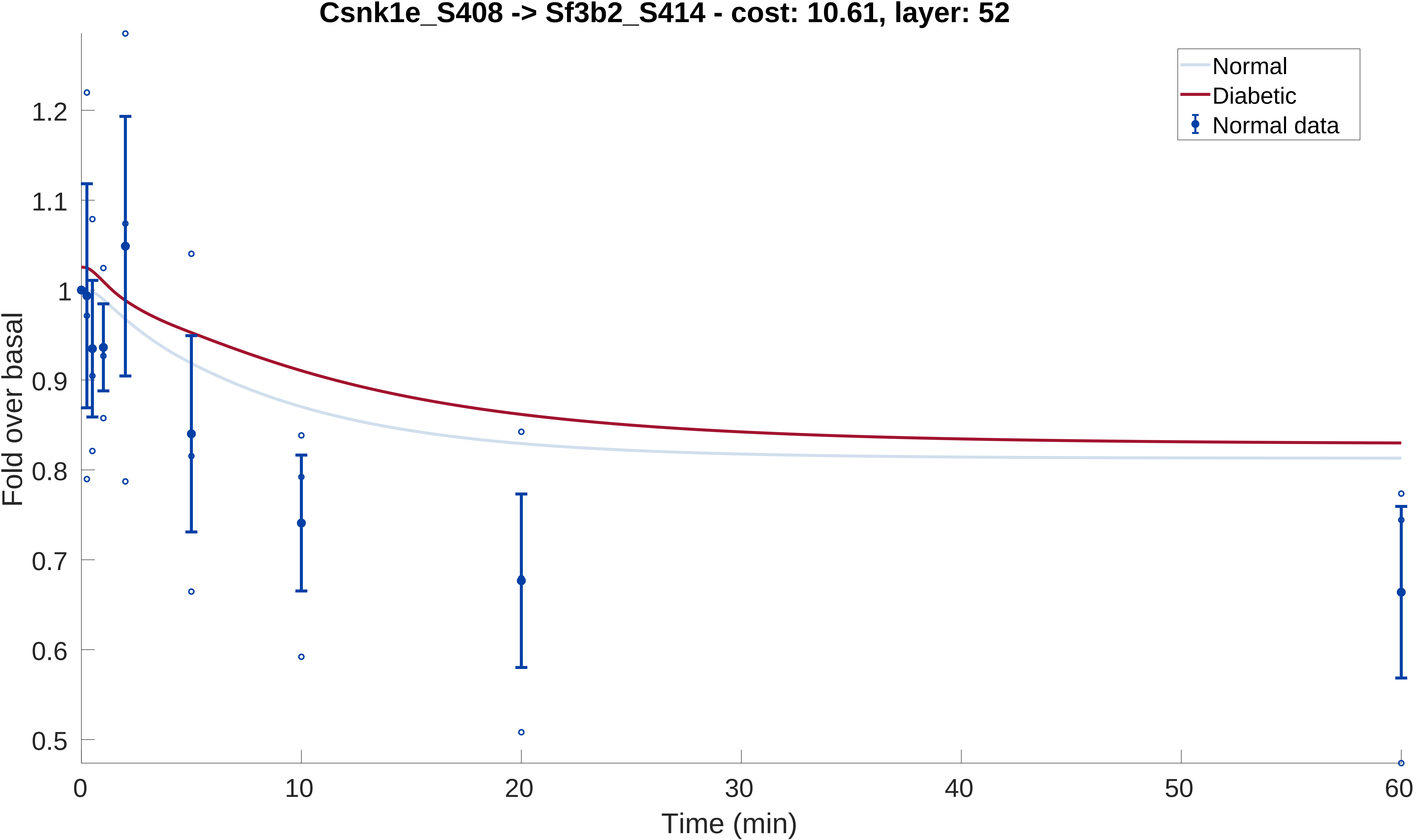

Csnk1e_S408 -> Sf3b2_S414 - cost: 10.61, layer: 52
Normal
Diabetic
Normal data
Fold over basal
Time (min)
0.5
0.6
0.7
0.8
0.9
1
1.1
1.2
0
10
20
30
40
50
60

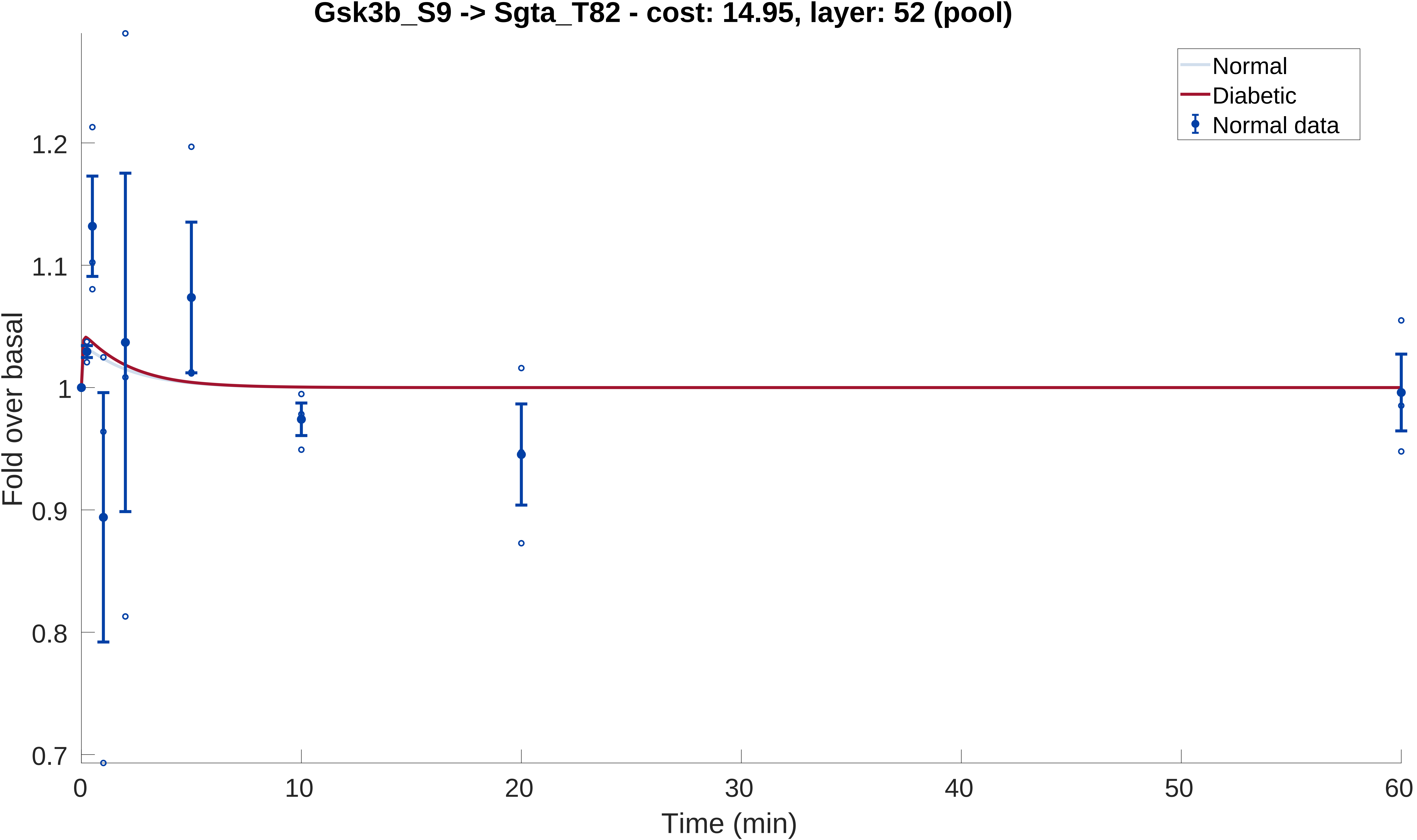
Gsk3b_S9 -> Sgta_T82 - cost: 14.95, layer: 52 (pool)
Normal
Diabetic
Normal data
Fold over basal
Time (min)
0.7
0.8
0.9
1
1.1
1.2
0
10
20
30
40
50
60

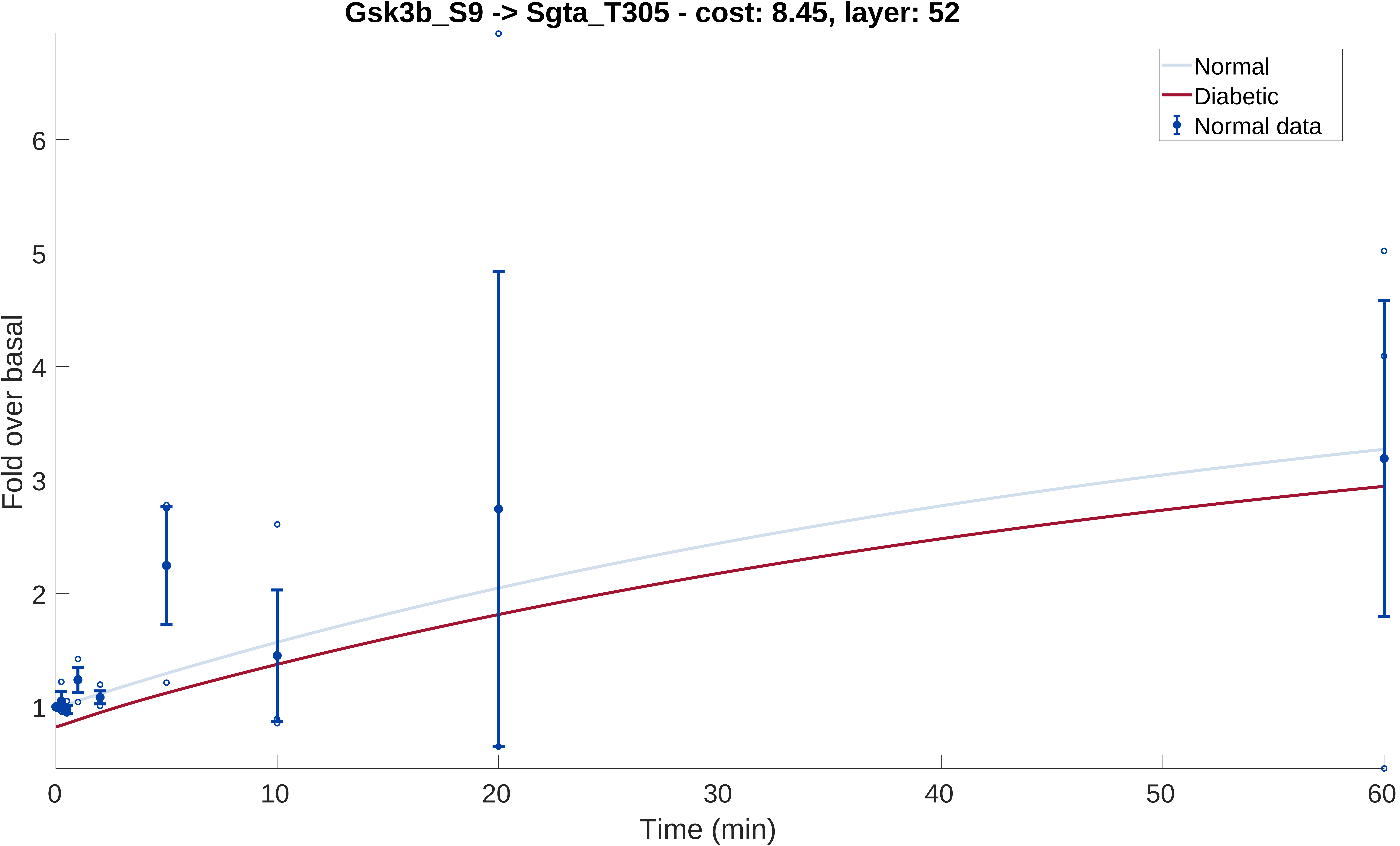
Gsk3b_S9 -> Sgta_T305 - cost: 8.45, layer: 52
Normal
Diabetic
Normal data
Fold over basal
1
2
3
4
5
6
0
10
20
30
40
50
60
Time (min)

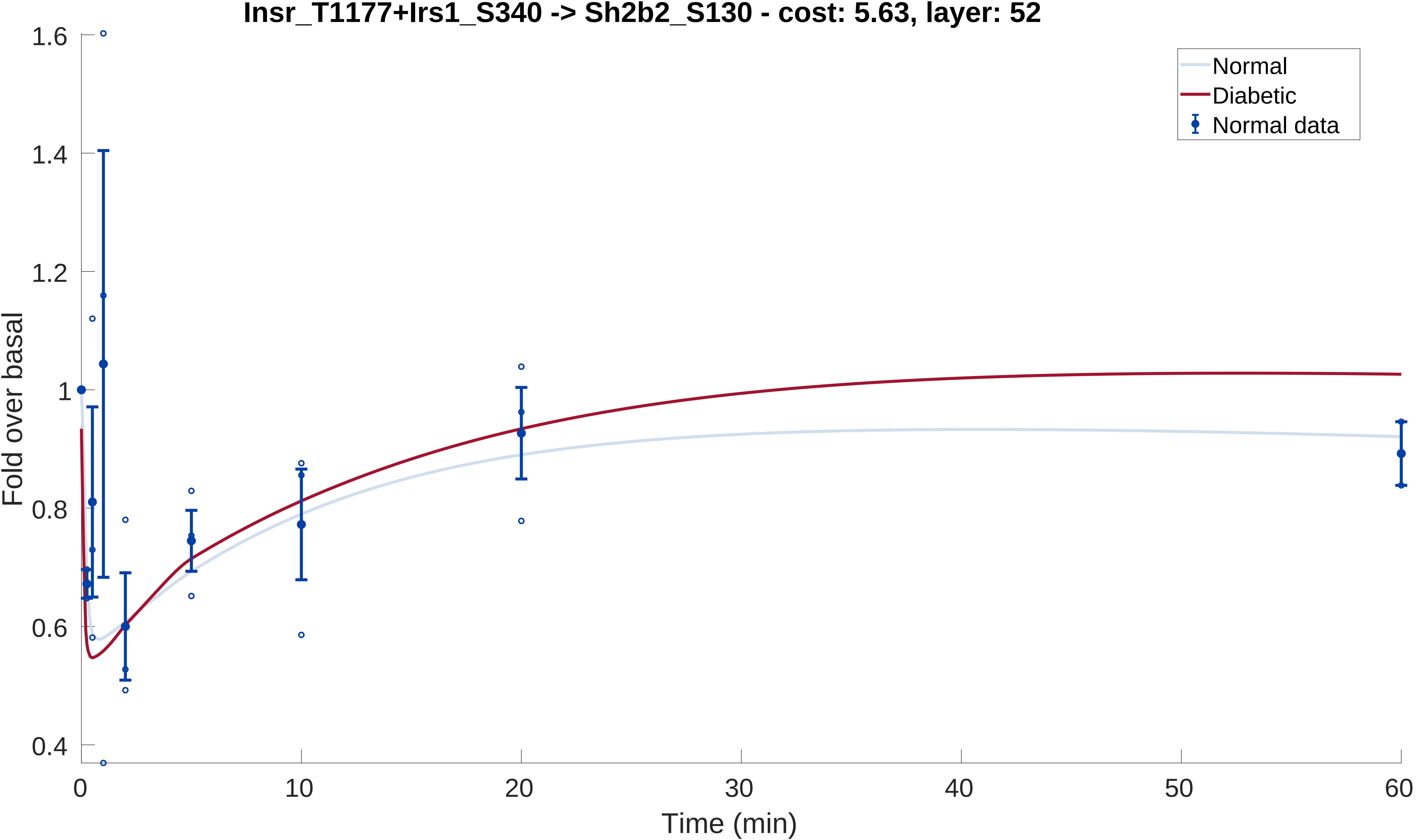
Insr_T1177+Irs1_S340 -> Sh2b2_S130 - cost: 5.63, layer: 52
Normal
Diabetic
Normal data
Fold over basal
Time (min)
0.4
0.6
0.8
1
1.2
1.4
1.6
0
10
20
30
40
50
60

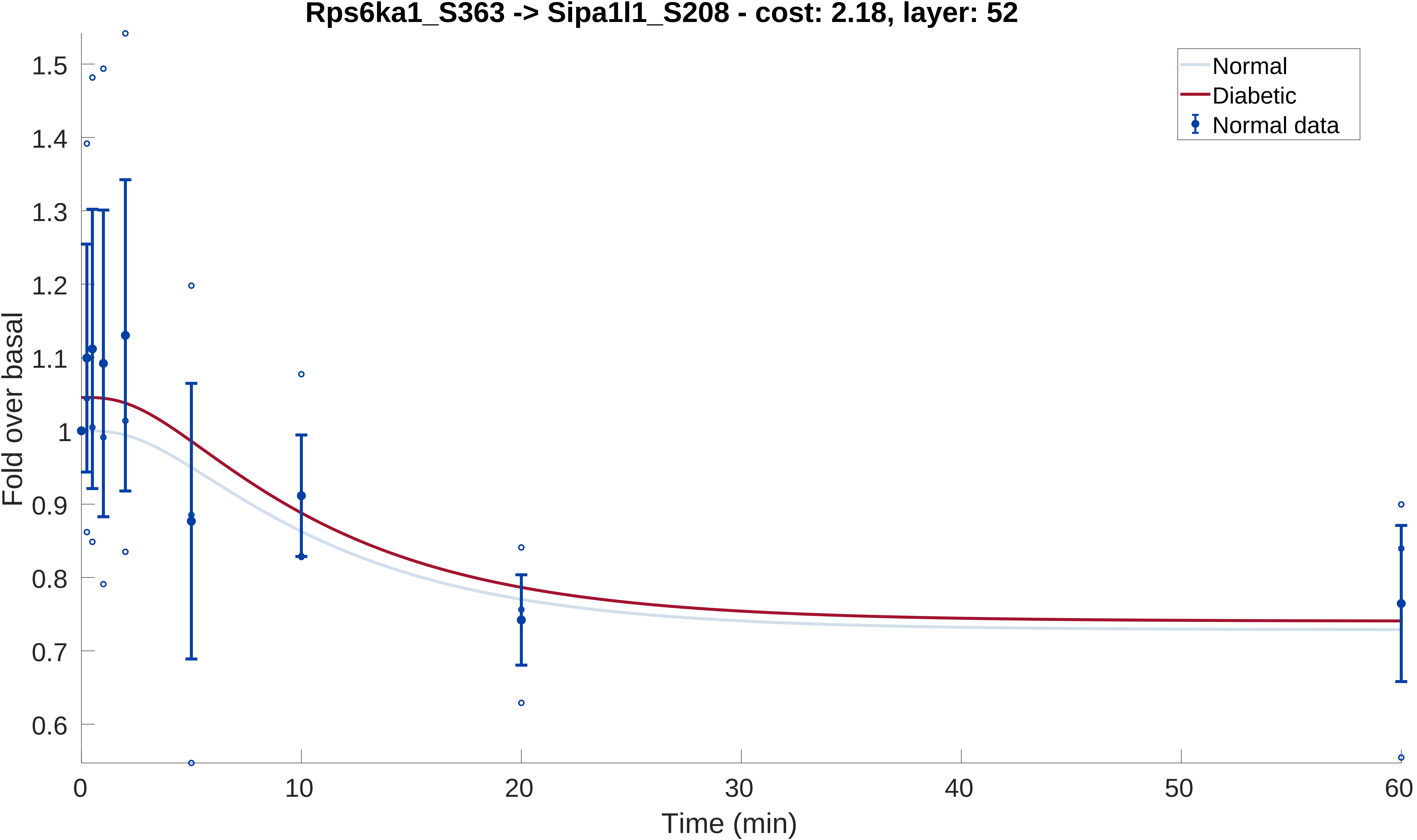

Rps6ka1_S363 -> Sipa1l1_S208 - cost: 2.18, layer: 52
Normal
Diabetic
Normal data
Fold over basal
Time (min)
0.6
0.7
0.8
0.9
1
1.1
1.2
1.3
1.4
1.5
0
10
20
30
40
50
60

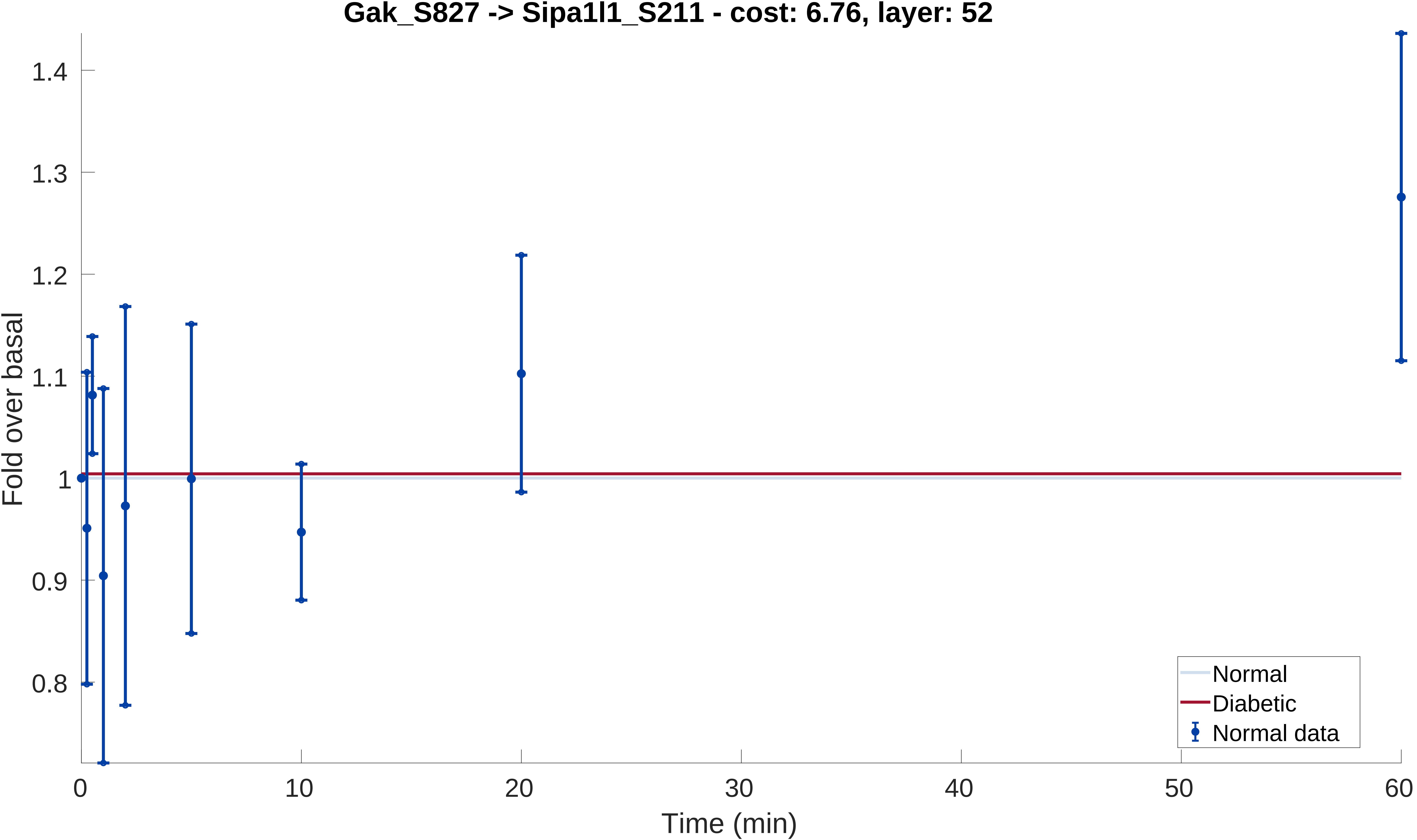
Gak_S827 -> Sipa1l1_S211 - cost: 6.76, layer: 52
Fold over basal
1.4
1.3
1.2
1.1
1
0.9
0.8
0
10
20
30
40
50
60
Time (min)
Normal
Diabetic
Normal data

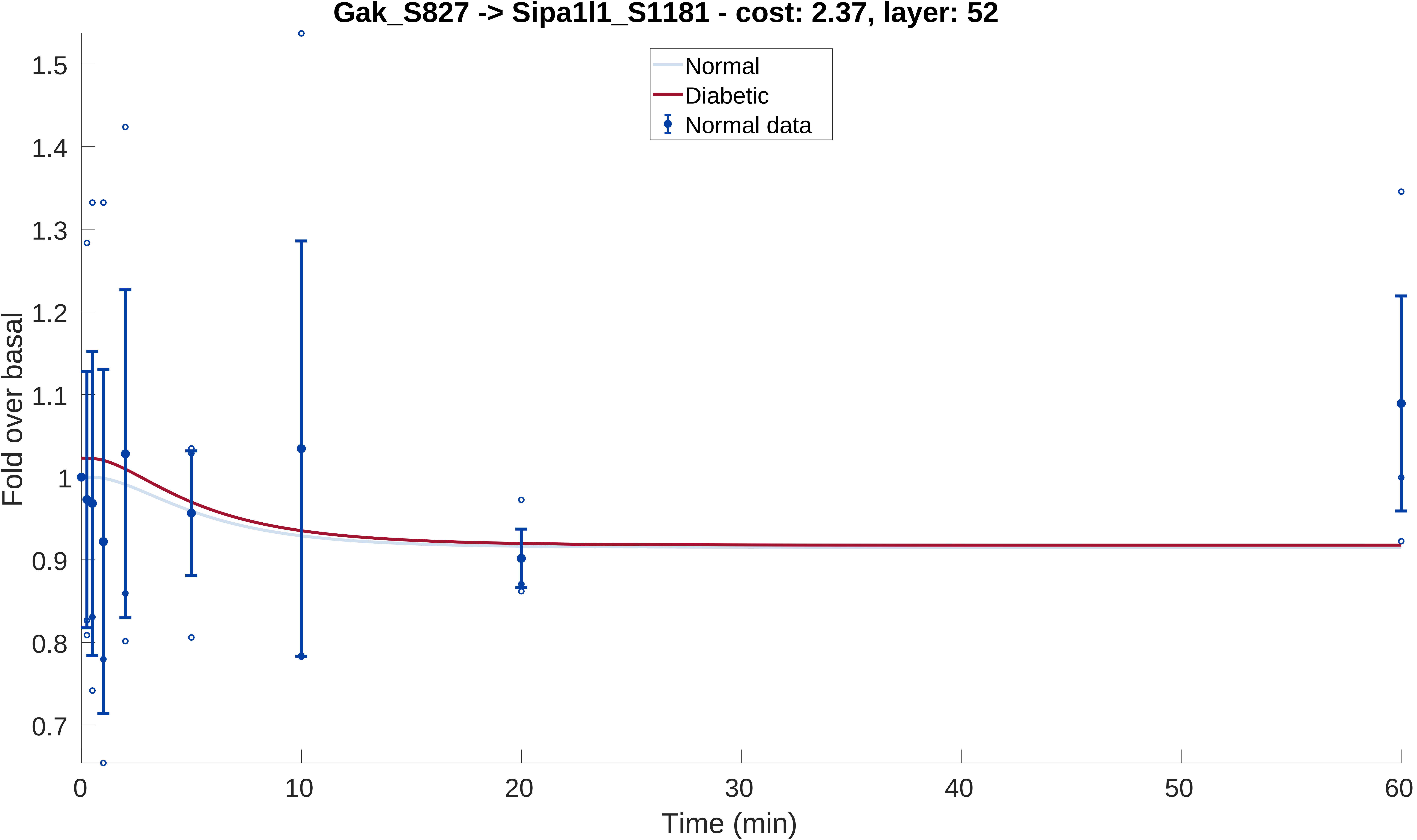

Gak_S827 -> Sipa1l1_S1181 - cost: 2.37, layer: 52
Normal
Diabetic
Normal data
Fold over basal
Time (min)
0.7
0.8
0.9
1
1.1
1.2
1.3
1.4
1.5
0
10
20
30
40
50
60

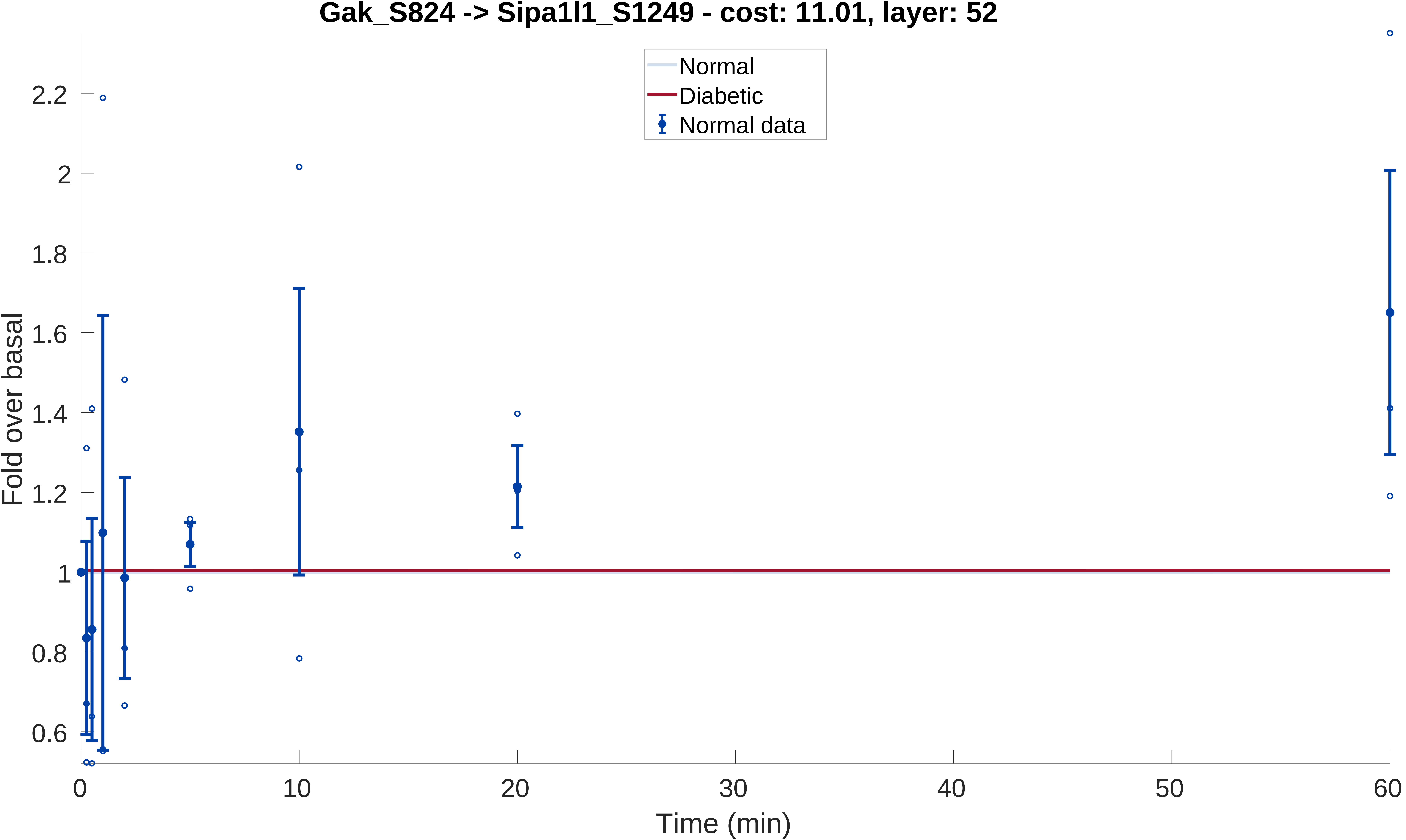
Gak_S824 -> Sipa1l1_S1249 - cost: 11.01, layer: 52
Normal
Diabetic
Normal data
Fold over basal
Time (min)
2.2
2
1.8
1.6
1.4
1.2
1
0.8
0.6
0
10
20
30
40
50
60

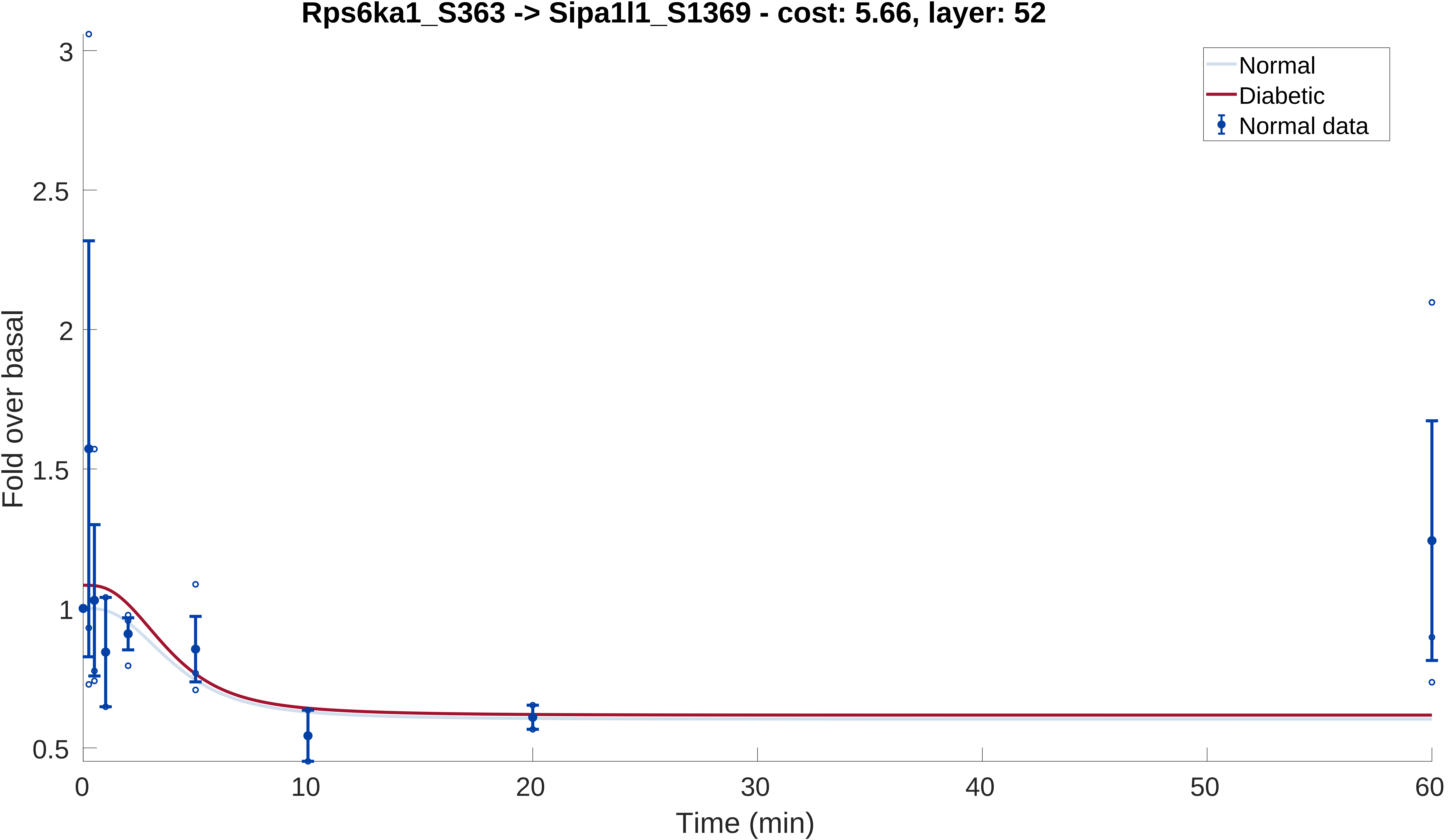

Rps6ka1_S363 -> Sipa1l1_S1369 - cost: 5.66, layer: 52
Normal
Diabetic
Normal data
Fold over basal
Time (min)
0.5
1
1.5
2
2.5
3
0
10
20
30
40
50
60

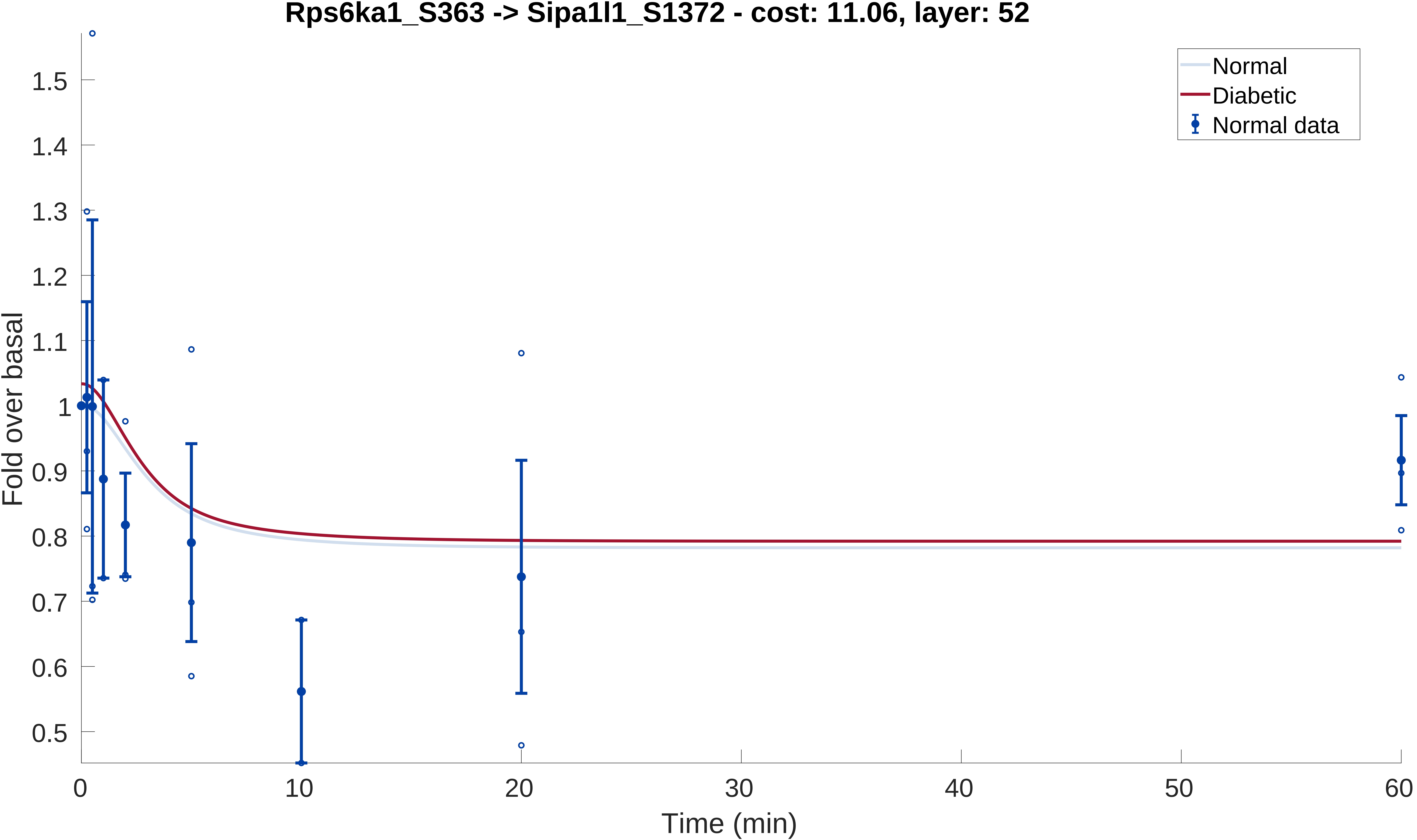

Rps6ka1_S363 -> Sipa1l1_S1372 - cost: 11.06, layer: 52
Normal
Diabetic
Normal data
Fold over basal
Time (min)
1.5
1.4
1.3
1.2
1.1
1
0.9
0.8
0.7
0.6
0.5
0
10
20
30
40
50
60

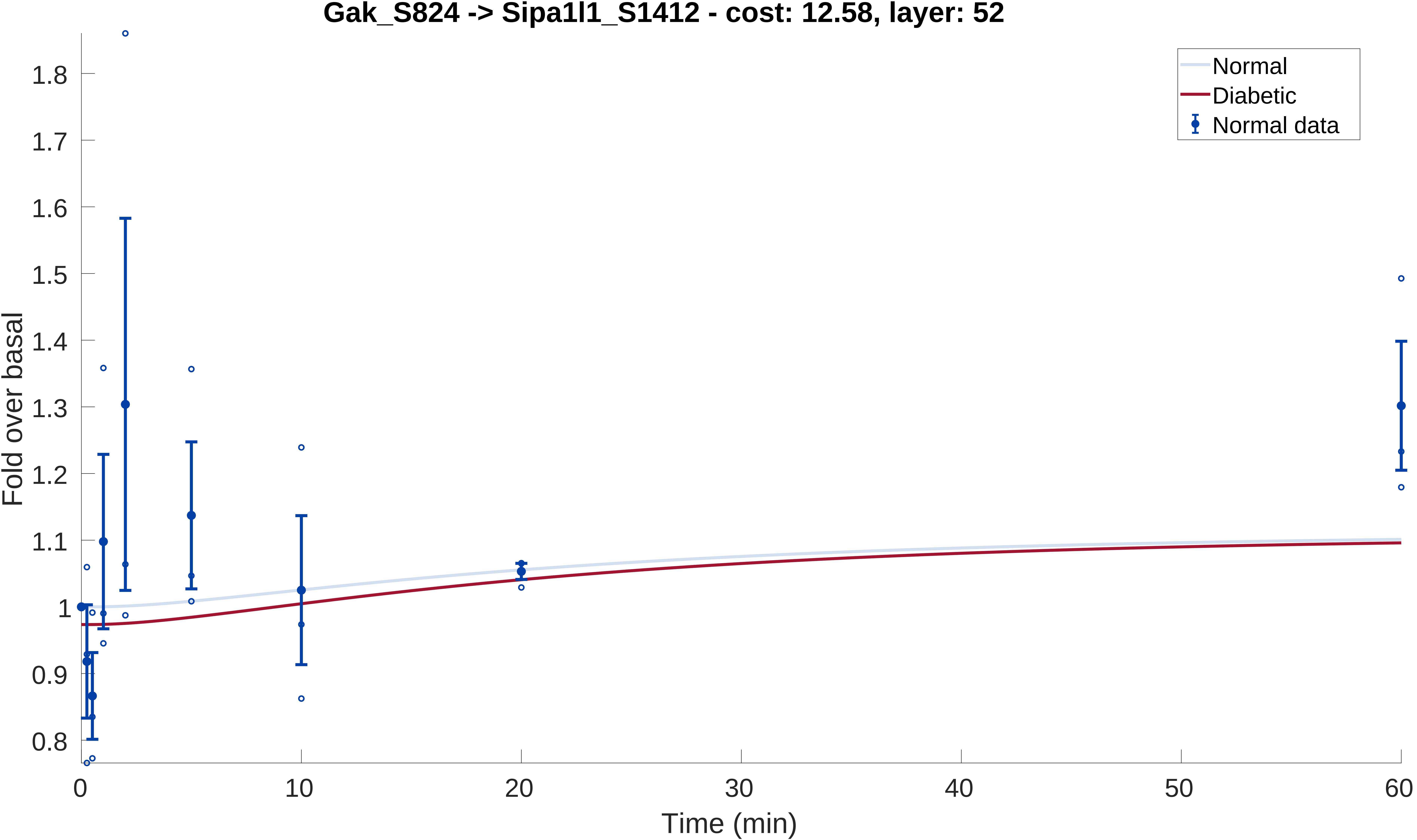

Gak_S824 -> Sipa1l1_S1412 - cost: 12.58, layer: 52
Normal
Diabetic
Normal data
Fold over basal
0.8
0.9
1
1.1
1.2
1.3
1.4
1.5
1.6
1.7
1.8
0
10
20
30
40
50
60
Time (min)

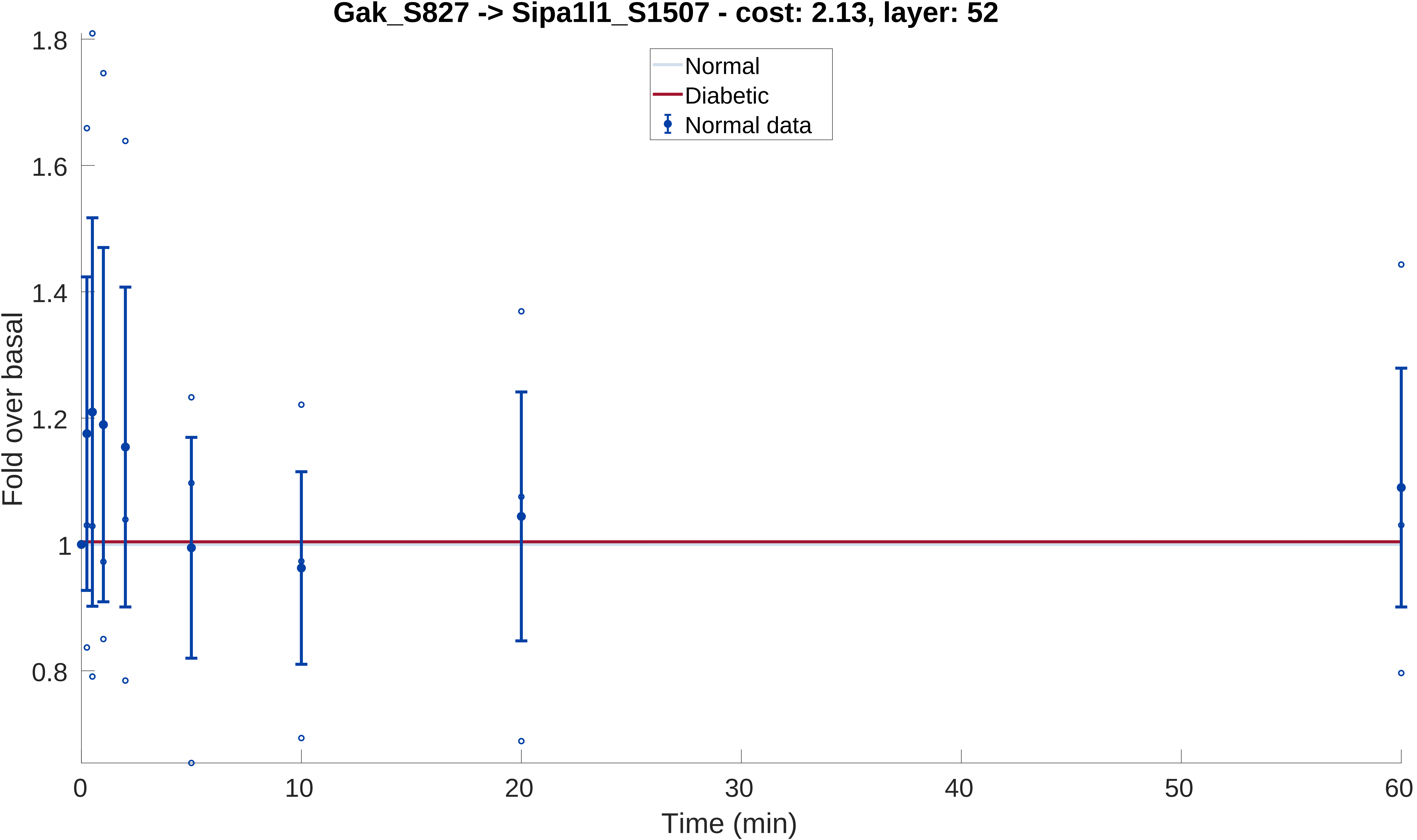

Gak_S827 -> Sipa1l1_S1507 - cost: 2.13, layer: 52
Normal
Diabetic
Normal data
Fold over basal
1.8
1.6
1.4
1.2
1
0.8
0
10
20
30
40
50
60
Time (min)

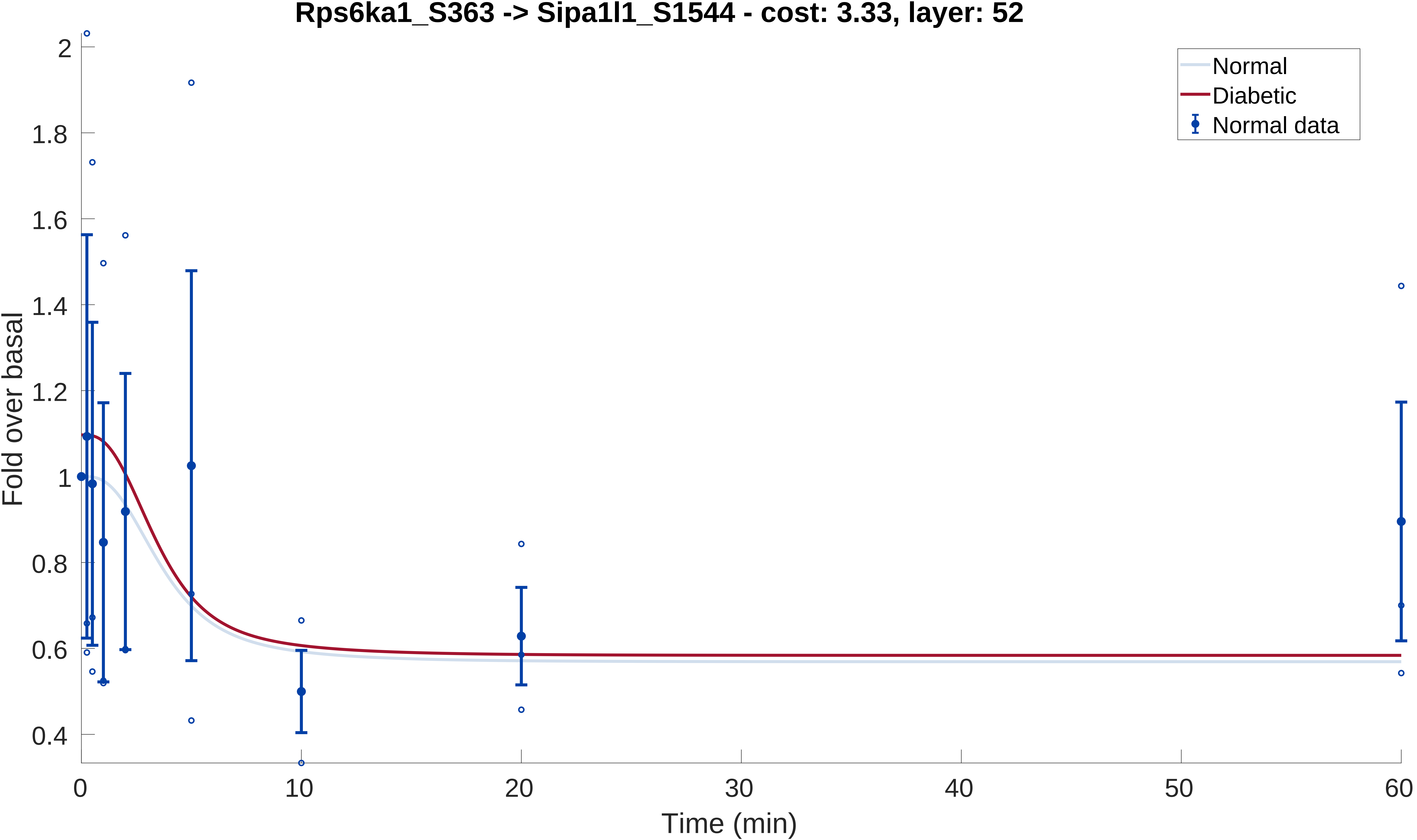
Rps6ka1_S363 -> Sipa1l1_S1544 - cost: 3.33, layer: 52
Fold over basal
Time (min)
Normal
Diabetic
Normal data
0.4
0.6
0.8
1
1.2
1.4
1.6
1.8
2
0
10
20
30
40
50
60

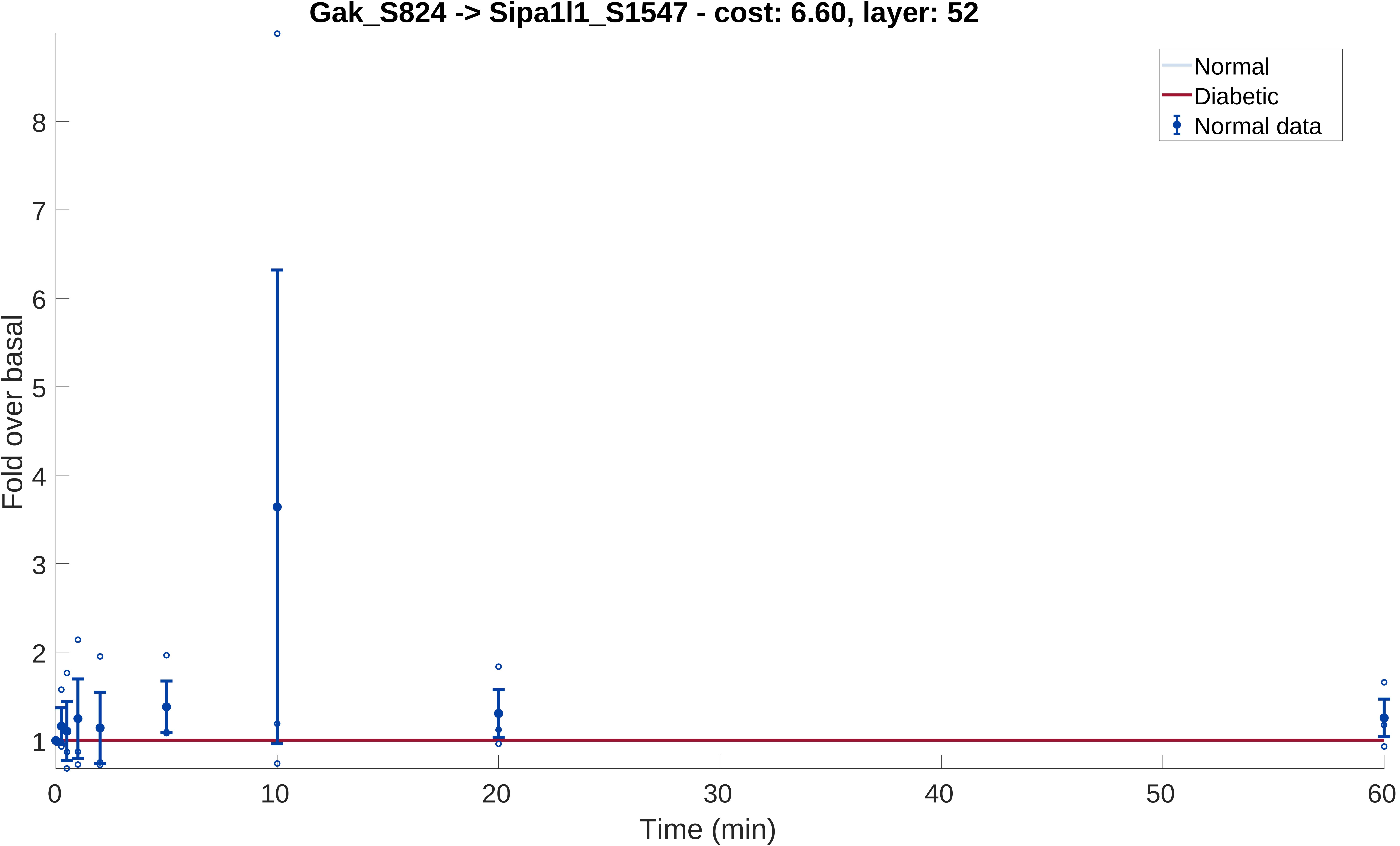

Gak_S824 -> Sipa1l1_S1547 - cost: 6.60, layer: 52
Normal
Diabetic
Normal data
Fold over basal
1
2
3
4
5
6
7
8
0
10
20
30
40
50
60
Time (min)

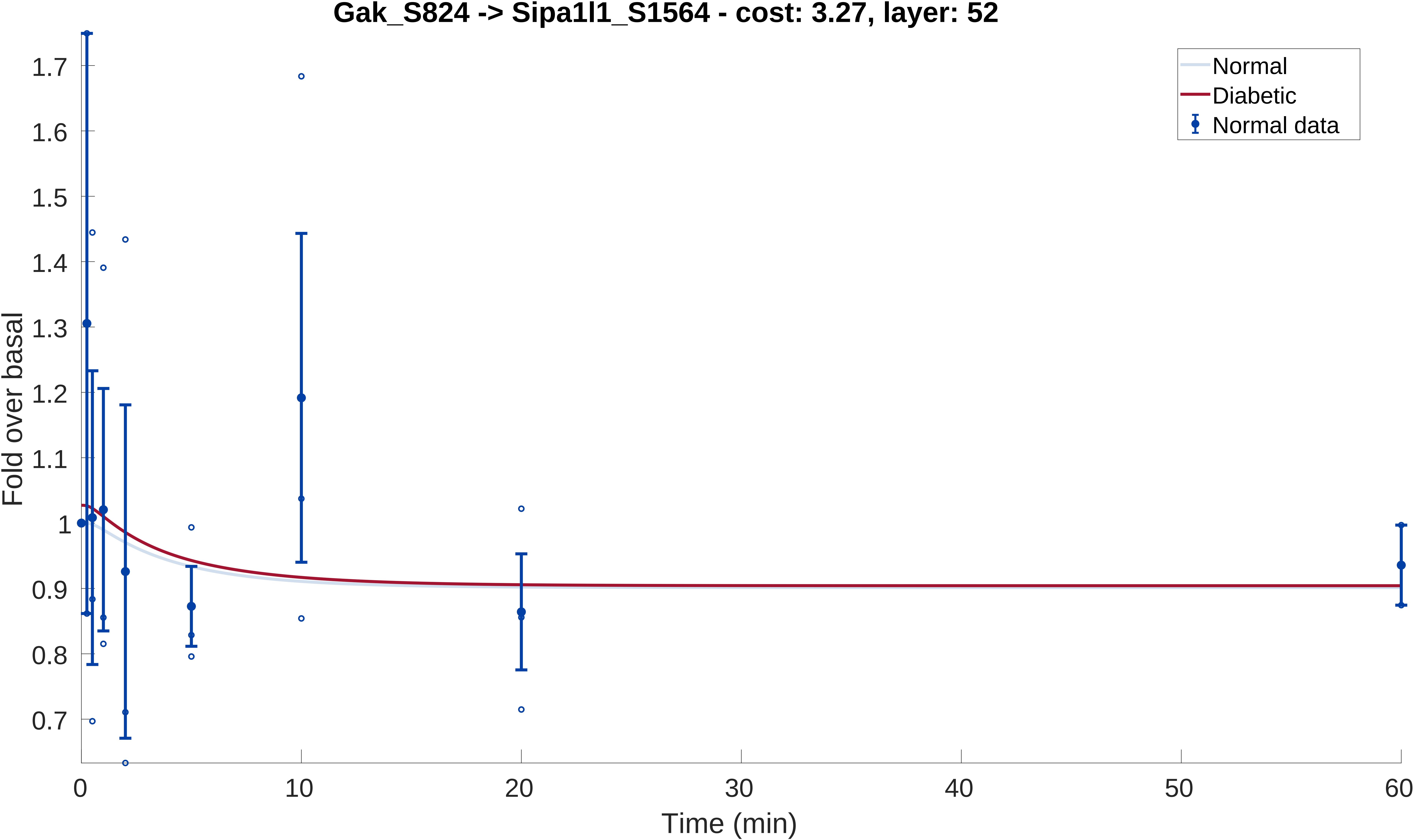
Gak_S824 -> Sipa1l1_S1564 - cost: 3.27, layer: 52
Normal
Diabetic
Normal data
Fold over basal
1.7
1.6
1.5
1.4
1.3
1.2
1.1
1
0.9
0.8
0.7
0
10
20
30
40
50
60
Time (min)

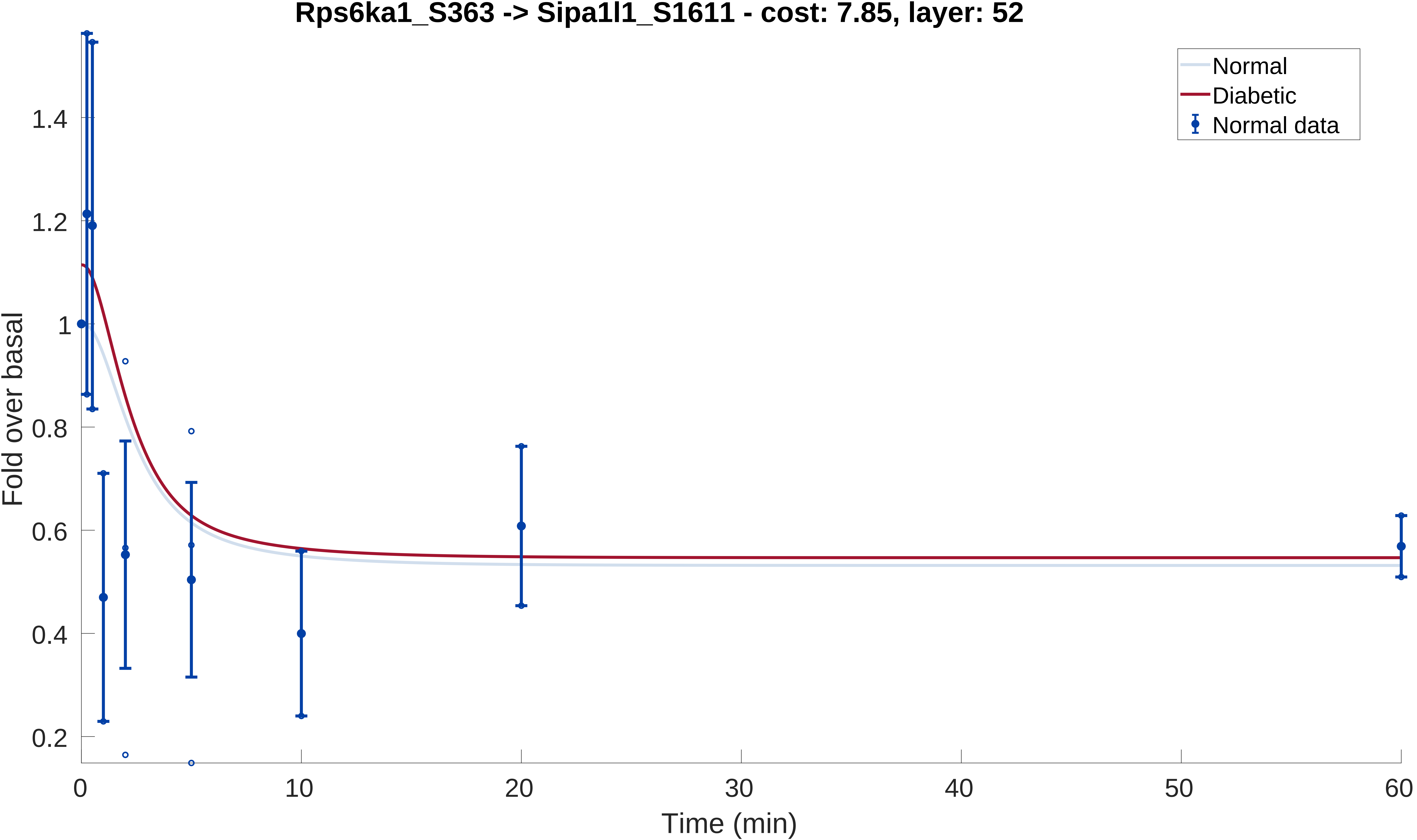
Rps6ka1_S363 -> Sipa1l1_S1611 - cost: 7.85, layer: 52
Normal
Diabetic
Normal data
Fold over basal
1.4
1.2
1
0.8
0.6
0.4
0.2
0
10
20
30
40
50
60
Time (min)

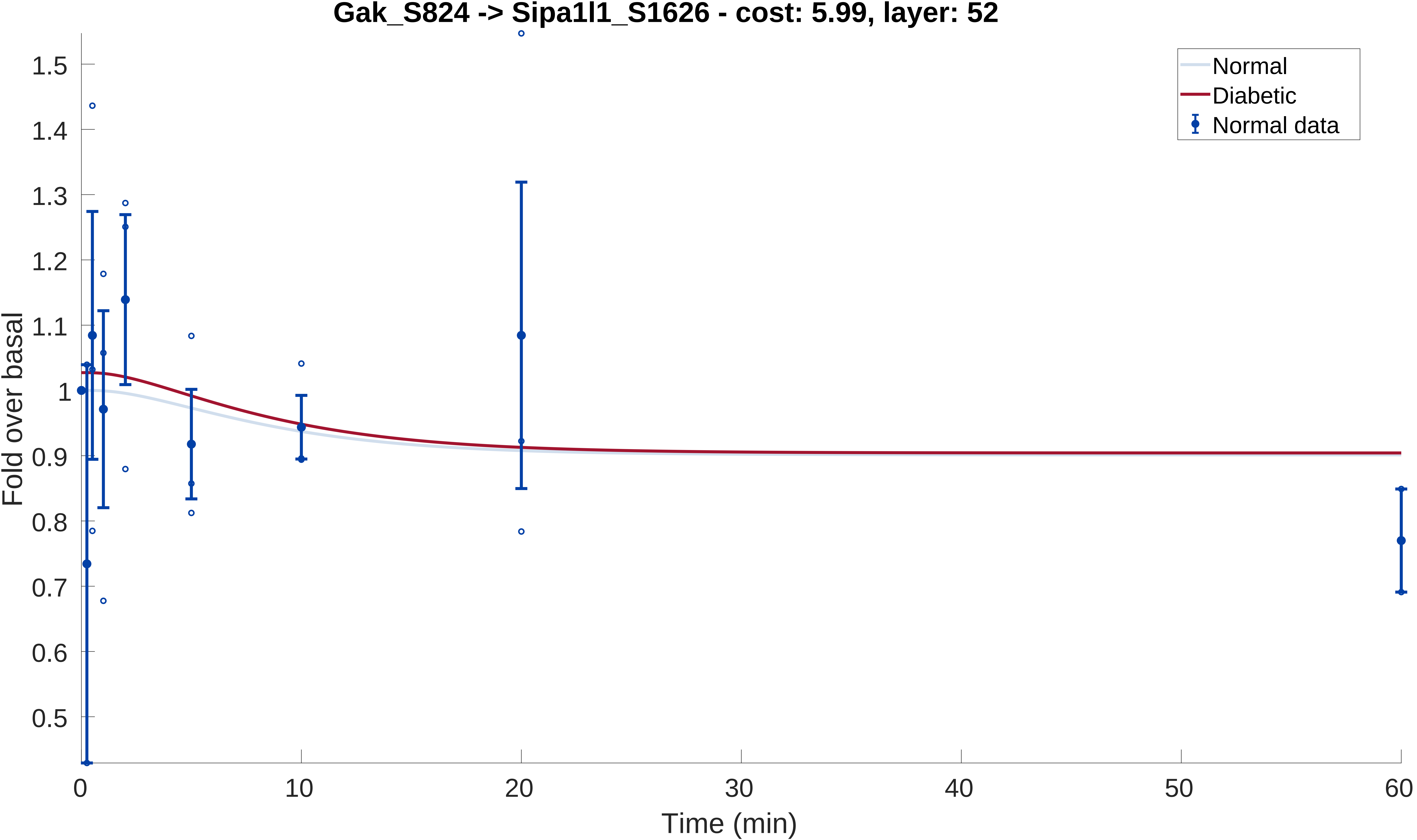
Gak_S824 -> Sipa1l1_S1626 - cost: 5.99, layer: 52
Normal
Diabetic
Normal data
Fold over basal
Time (min)
1.5
1.4
1.3
1.2
1.1
1
0.9
0.8
0.7
0.6
0.5
0
10
20
30
40
50
60

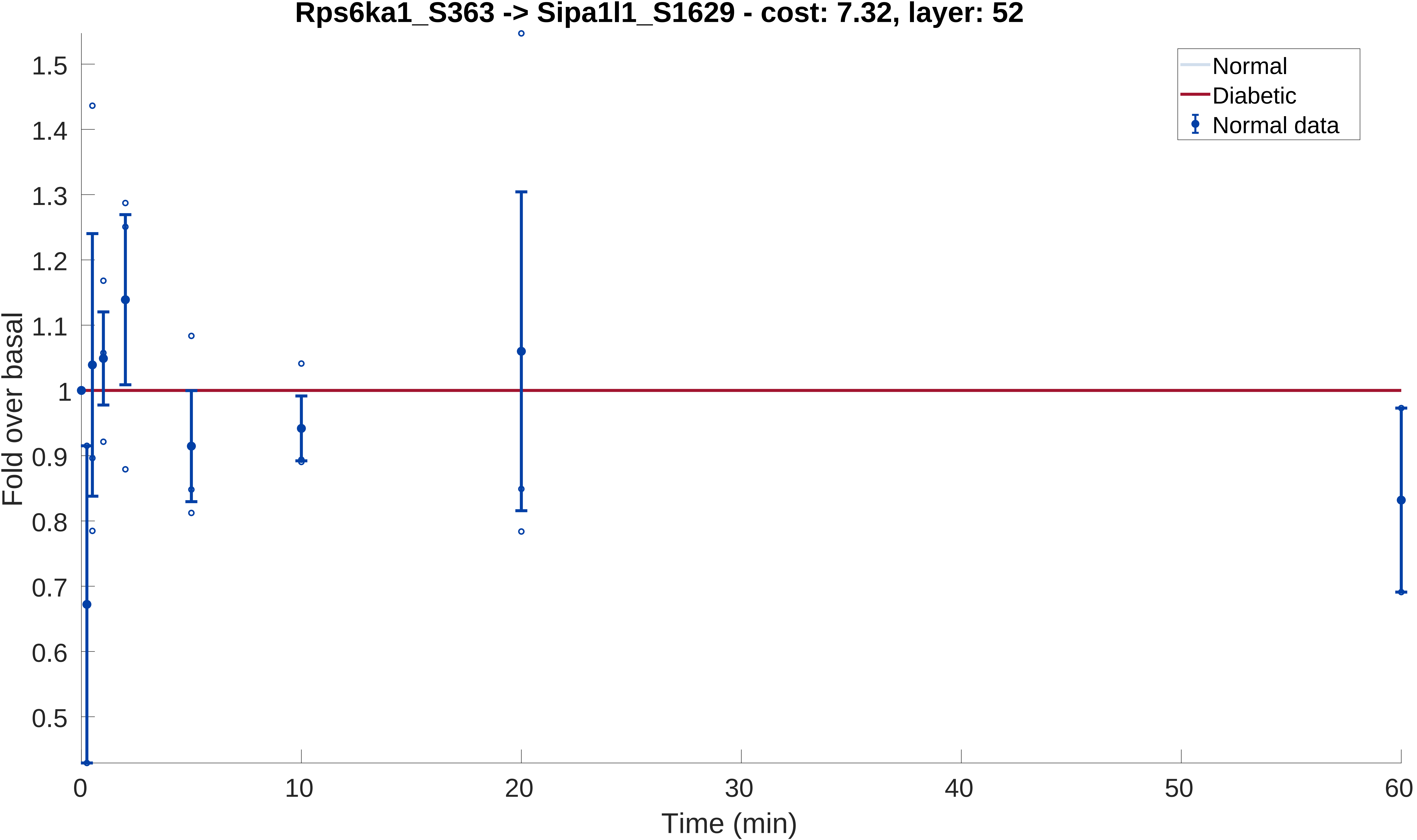

Rps6ka1_S363 -> Sipa1l1_S1629 - cost: 7.32, layer: 52
Normal
Diabetic
Normal data
Fold over basal
Time (min)
1.5
1.4
1.3
1.2
1.1
1
0.9
0.8
0.7
0.6
0.5
0
10
20
30
40
50
60

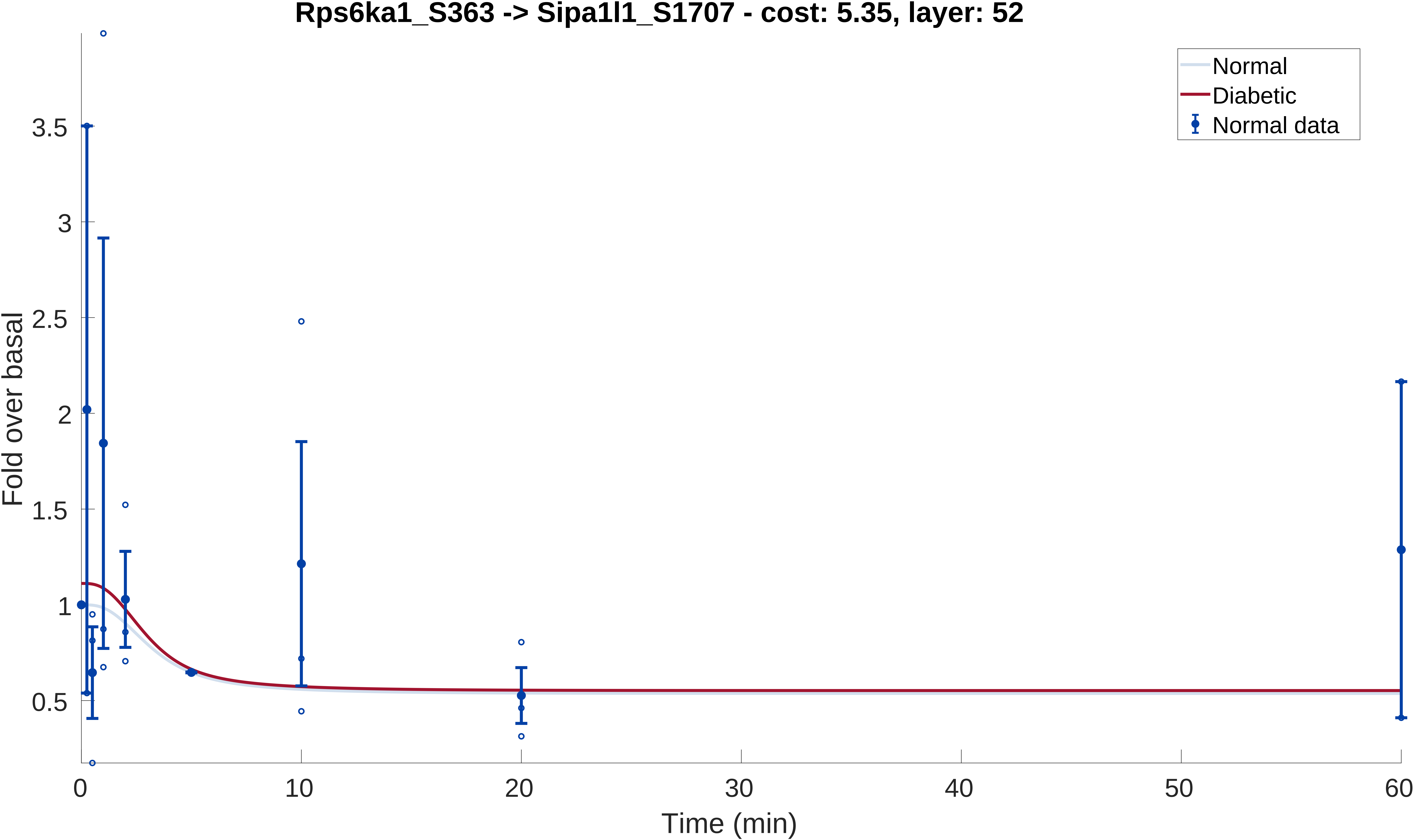
Rps6ka1_S363 -> Sipa1l1_S1707 - cost: 5.35, layer: 52
Normal
Diabetic
Normal data
Fold over basal
3.5
3
2.5
2
1.5
1
0.5
0
10
20
30
40
50
60
Time (min)

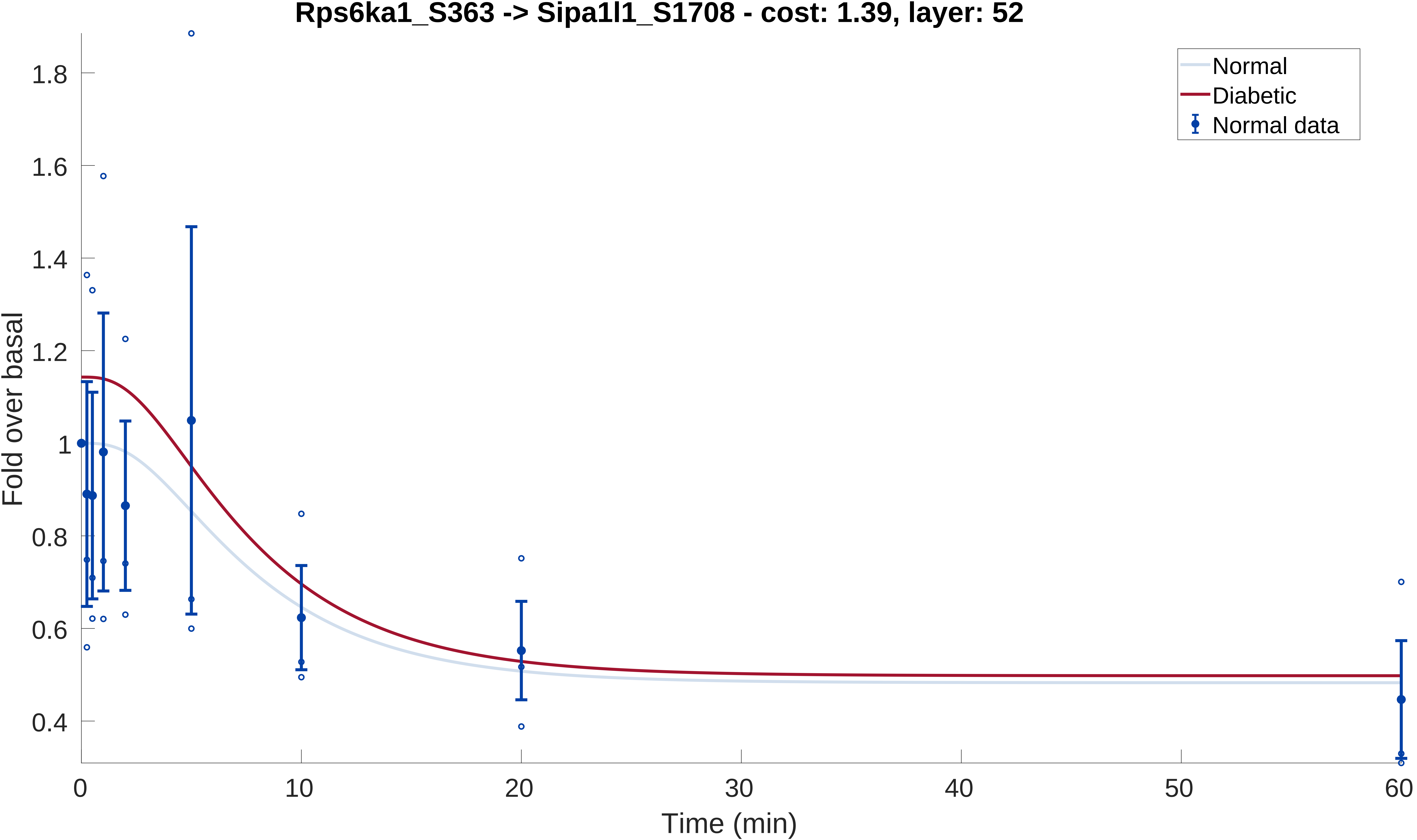
Rps6ka1_S363 -> Sipa1l1_S1708 - cost: 1.39, layer: 52
Normal
Diabetic
Normal data
Fold over basal
Time (min)
1.8
1.6
1.4
1.2
1
0.8
0.6
0.4
0
10
20
30
40
50
60

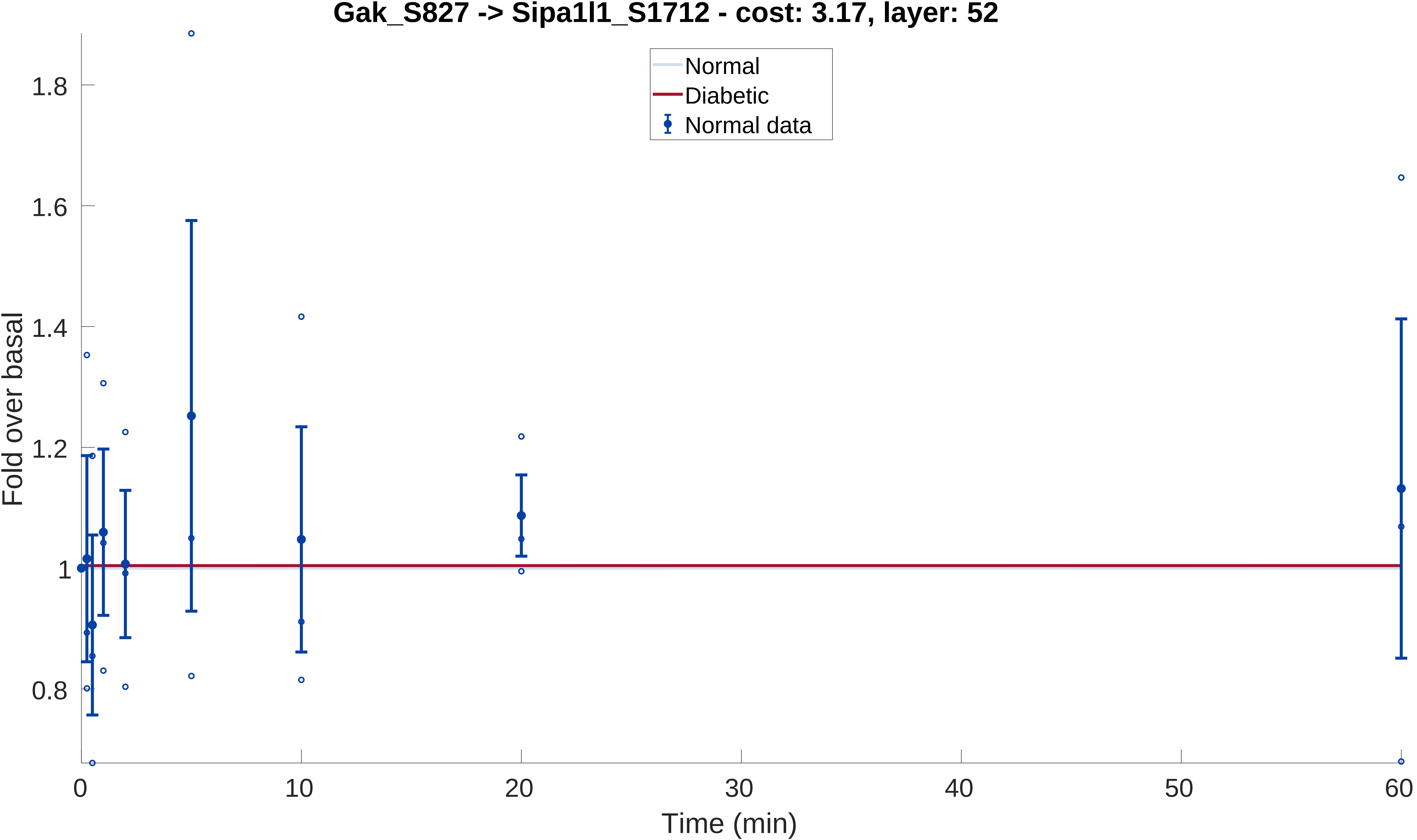

Gak_S827 -> Sipa1l1_S1712 - cost: 3.17, layer: 52
Normal
Diabetic
Normal data
Fold over basal
Time (min)
0.8
1
1.2
1.4
1.6
1.8
0
10
20
30
40
50
60

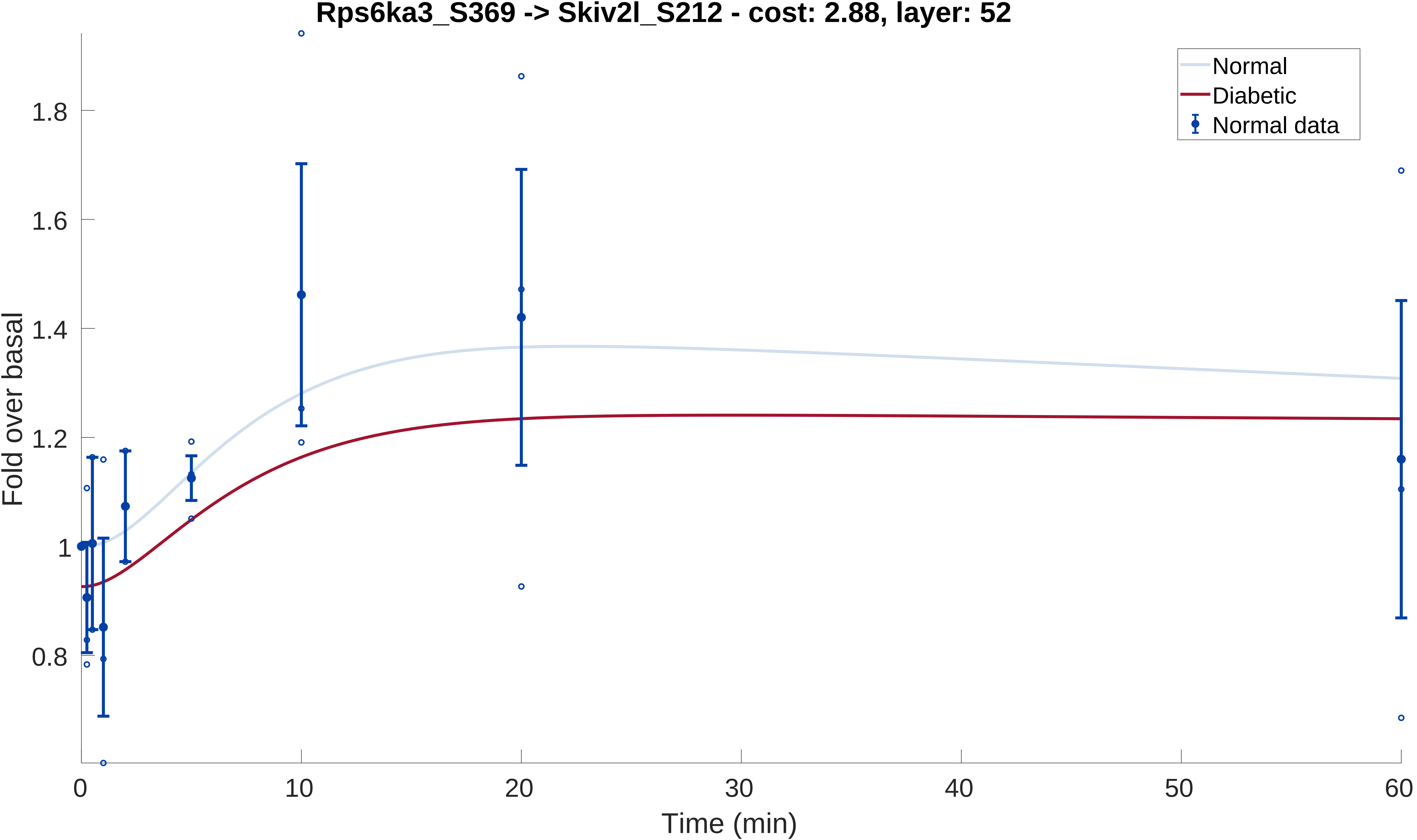

Rps6ka3_S369 -> Skiv2l_S212 - cost: 2.88, layer: 52
Normal
Diabetic
Normal data
Fold over basal
0.8
1
1.2
1.4
1.6
1.8
0
10
20
30
40
50
60
Time (min)

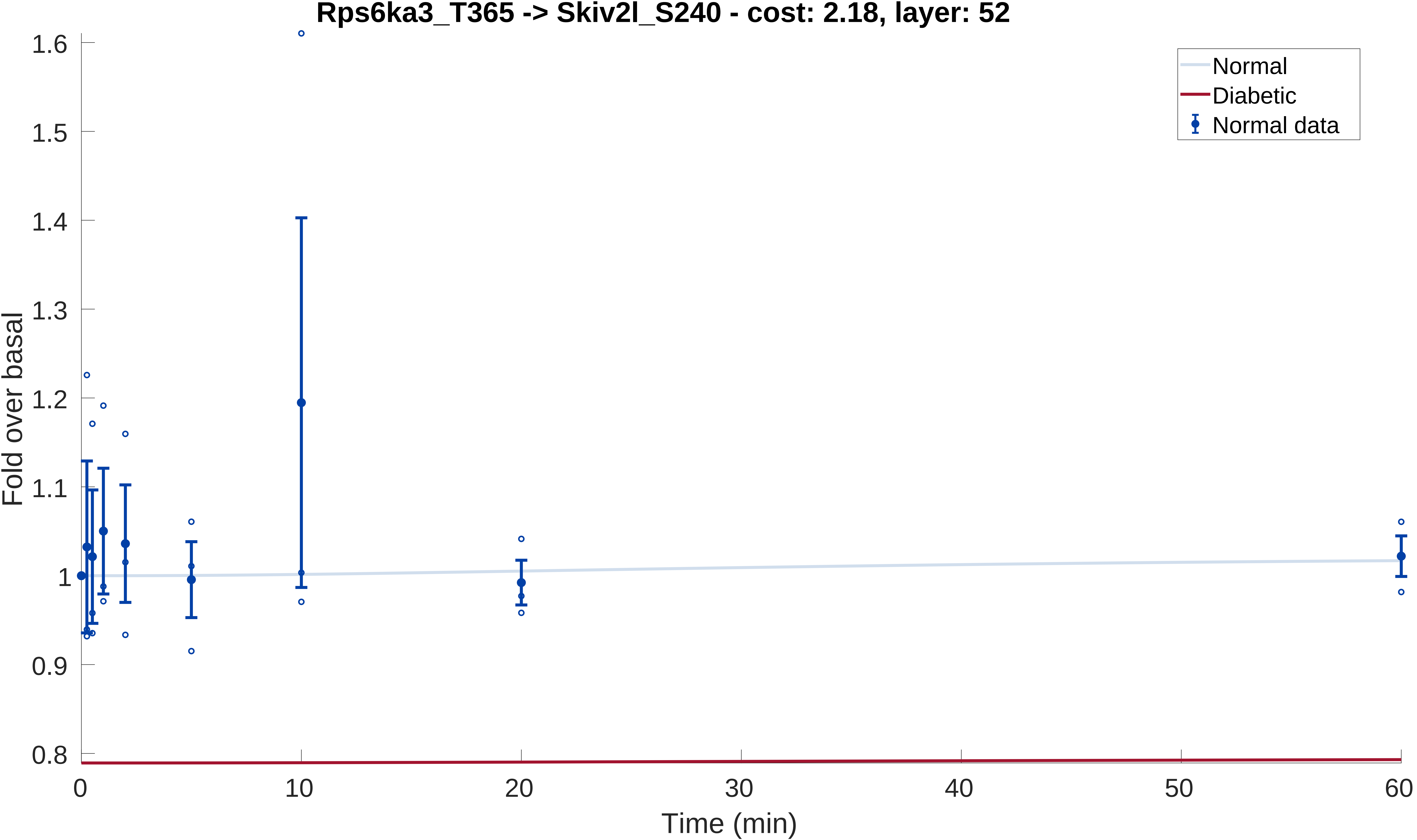

Rps6ka3_T365 -> Skiv2l_S240 - cost: 2.18, layer: 52
Normal
Diabetic
Normal data
Fold over basal
1.6
1.5
1.4
1.3
1.2
1.1
1
0.9
0.8
0
10
20
30
40
50
60
Time (min)

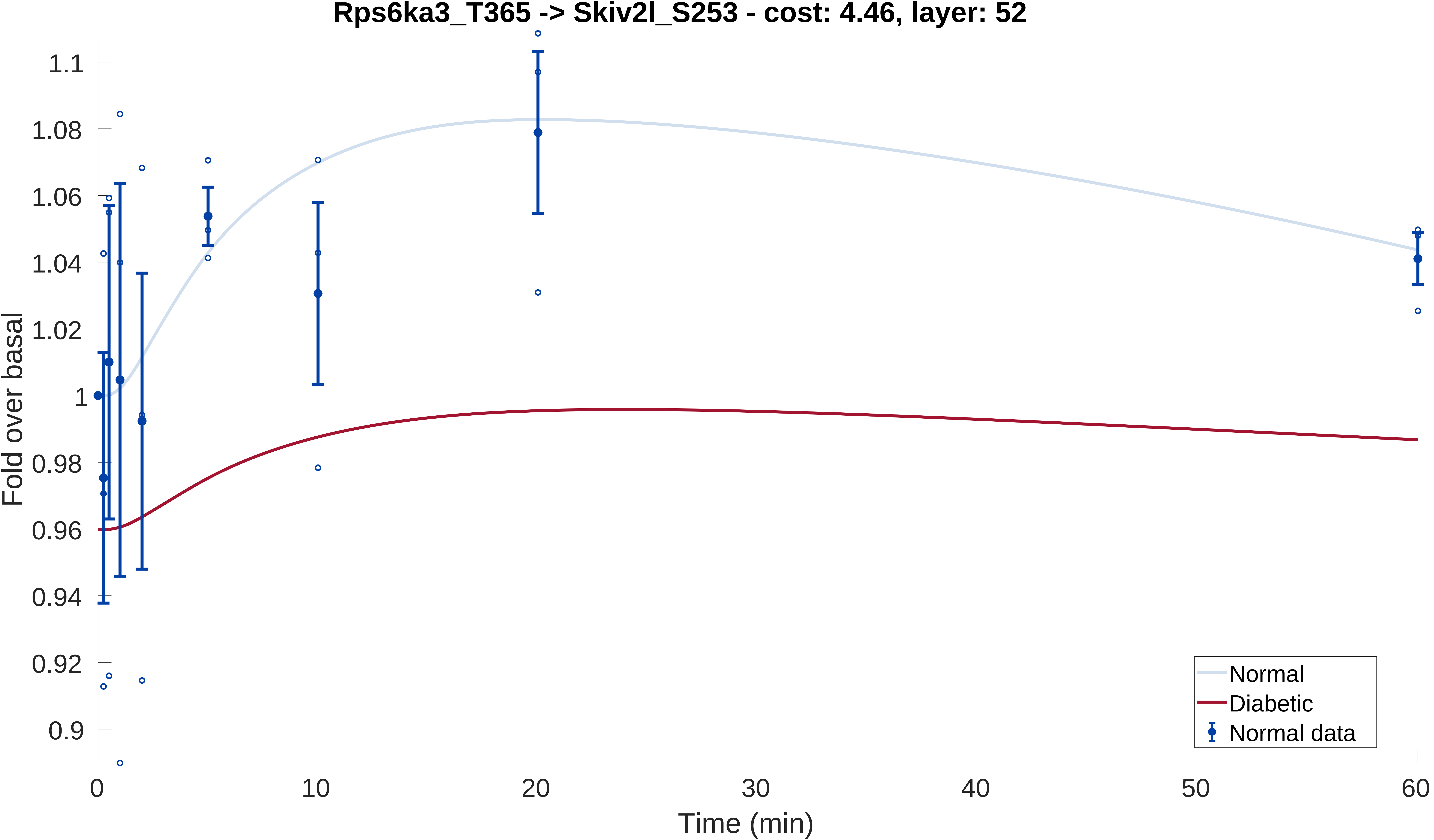

Rps6ka3_T365 -> Skiv2l_S253 - cost: 4.46, layer: 52
1.1
1.08
1.06
1.04
1.02
1
0.98
0.96
0.94
0.92
0.9
Fold over basal
0
10
20
30
40
50
60
Time (min)
Normal
Diabetic
Normal data

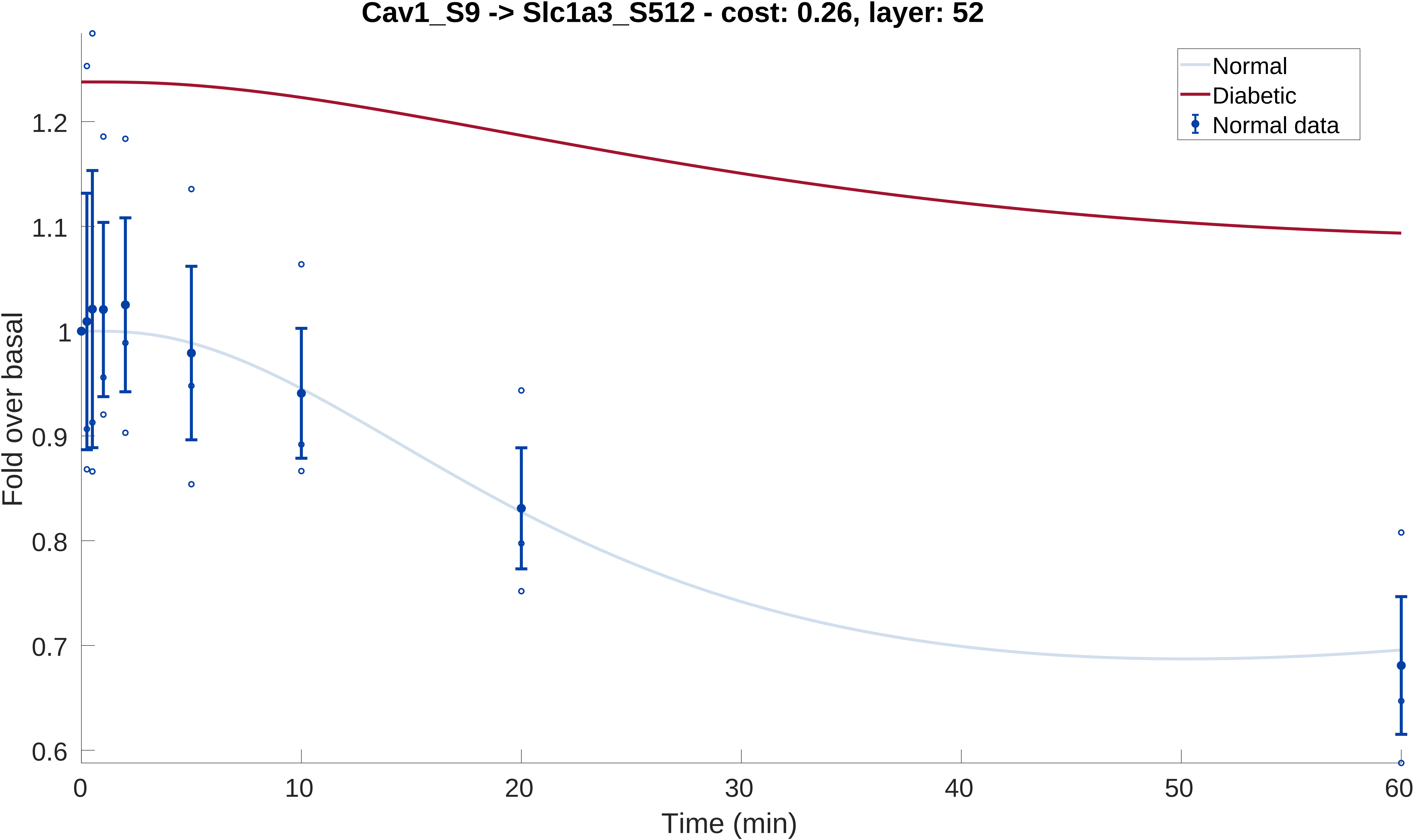

Cav1_S9 -> Slc1a3_S512 - cost: 0.26, layer: 52
Normal
Diabetic
Normal data
Fold over basal
1.2
1.1
1
0.9
0.8
0.7
0.6
0
10
20
30
40
50
60
Time (min)

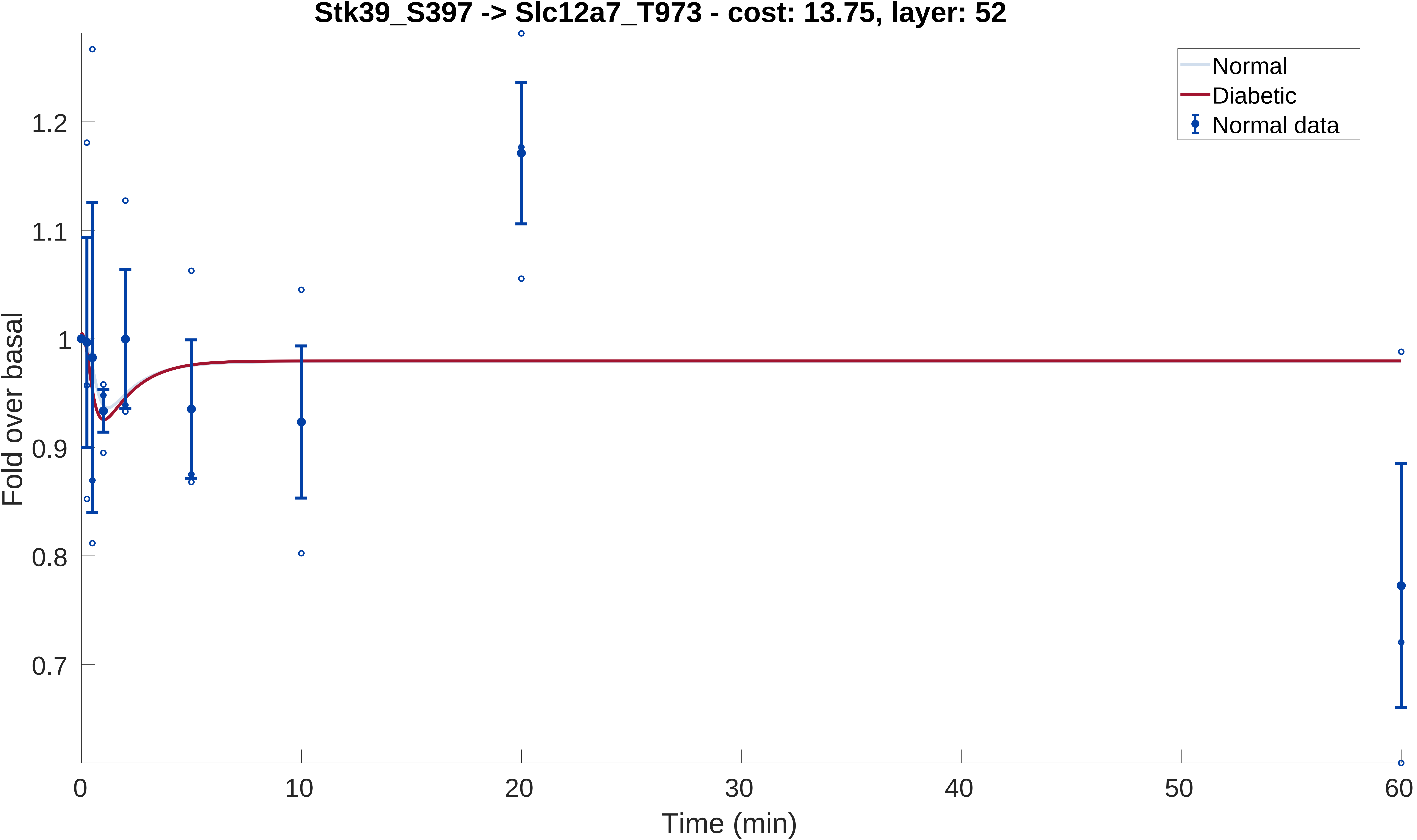
Stk39_S397 -> Slc12a7_T973 - cost: 13.75, layer: 52
Normal
Diabetic
Normal data
Fold over basal
Time (min)
1.2
1.1
1
0.9
0.8
0.7
0
10
20
30
40
50
60

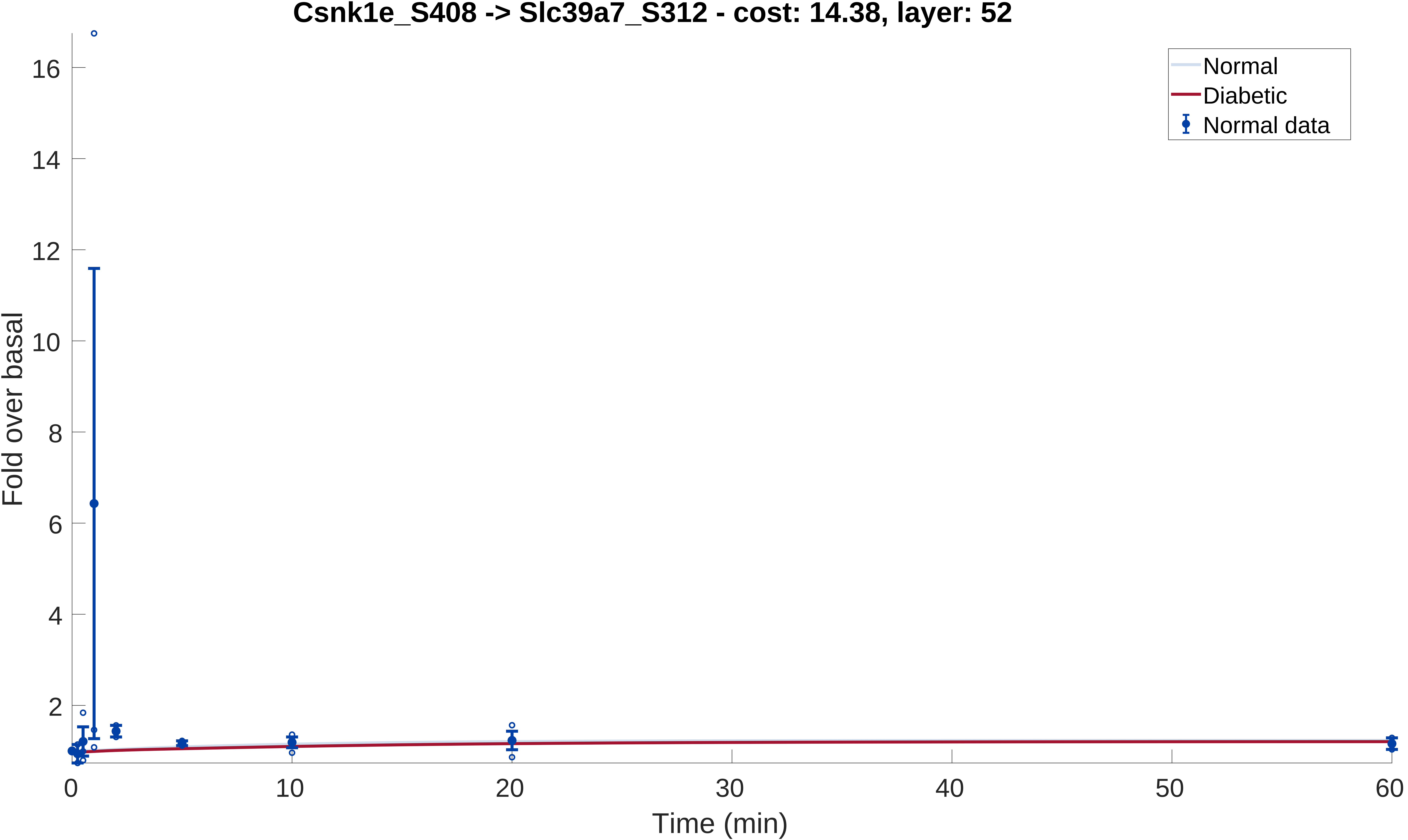

Csnk1e_S408 -> Slc39a7_S312 - cost: 14.38, layer: 52
Normal
Diabetic
Normal data
Fold over basal
Time (min)
0
10
20
30
40
50
60
2
4
6
8
10
12
14
16

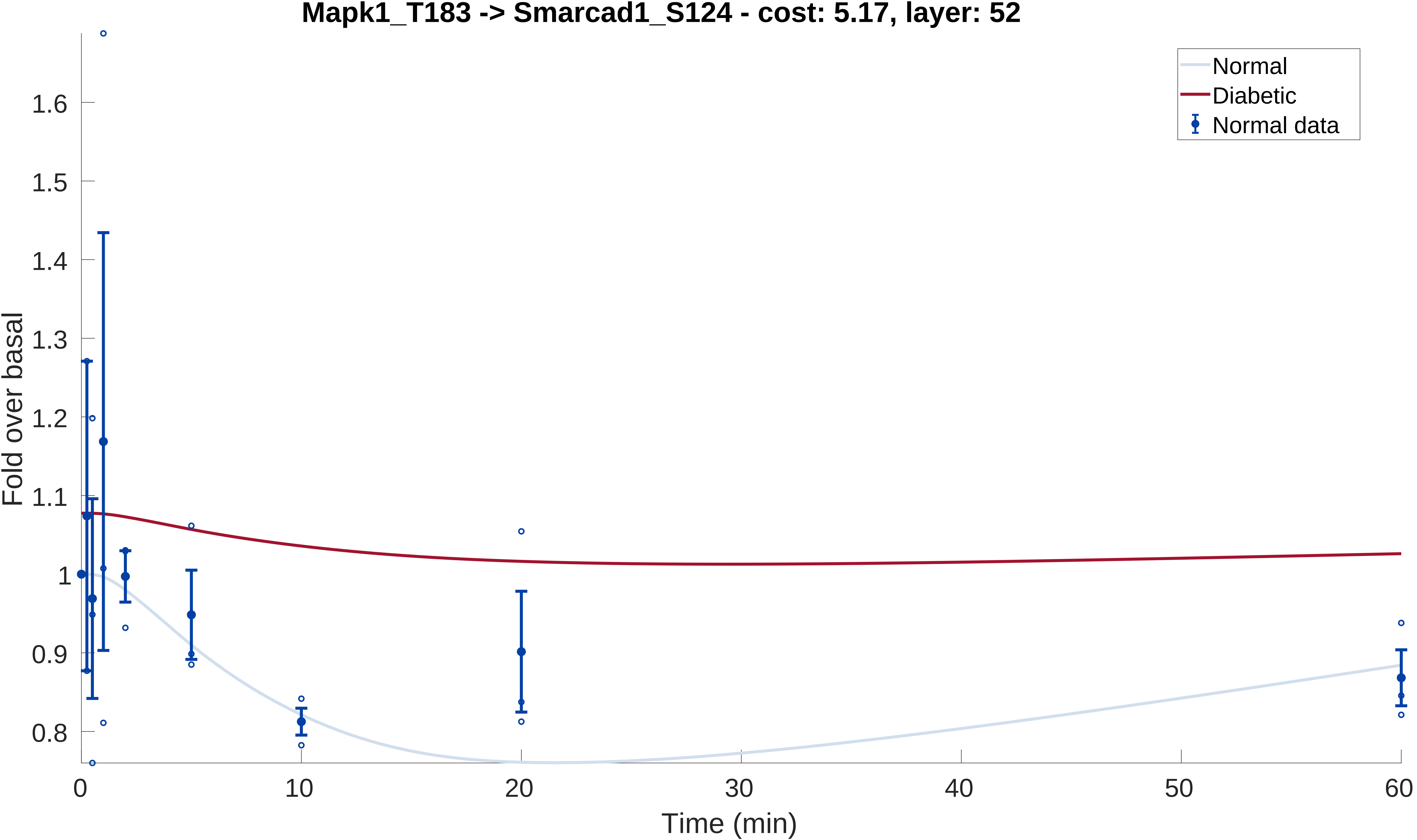
Mapk1_T183 -> Smarcad1_S124 - cost: 5.17, layer: 52
Normal
Diabetic
Normal data
Fold over basal
1.6
1.5
1.4
1.3
1.2
1.1
1
0.9
0.8
0
10
20
30
40
50
60
Time (min)

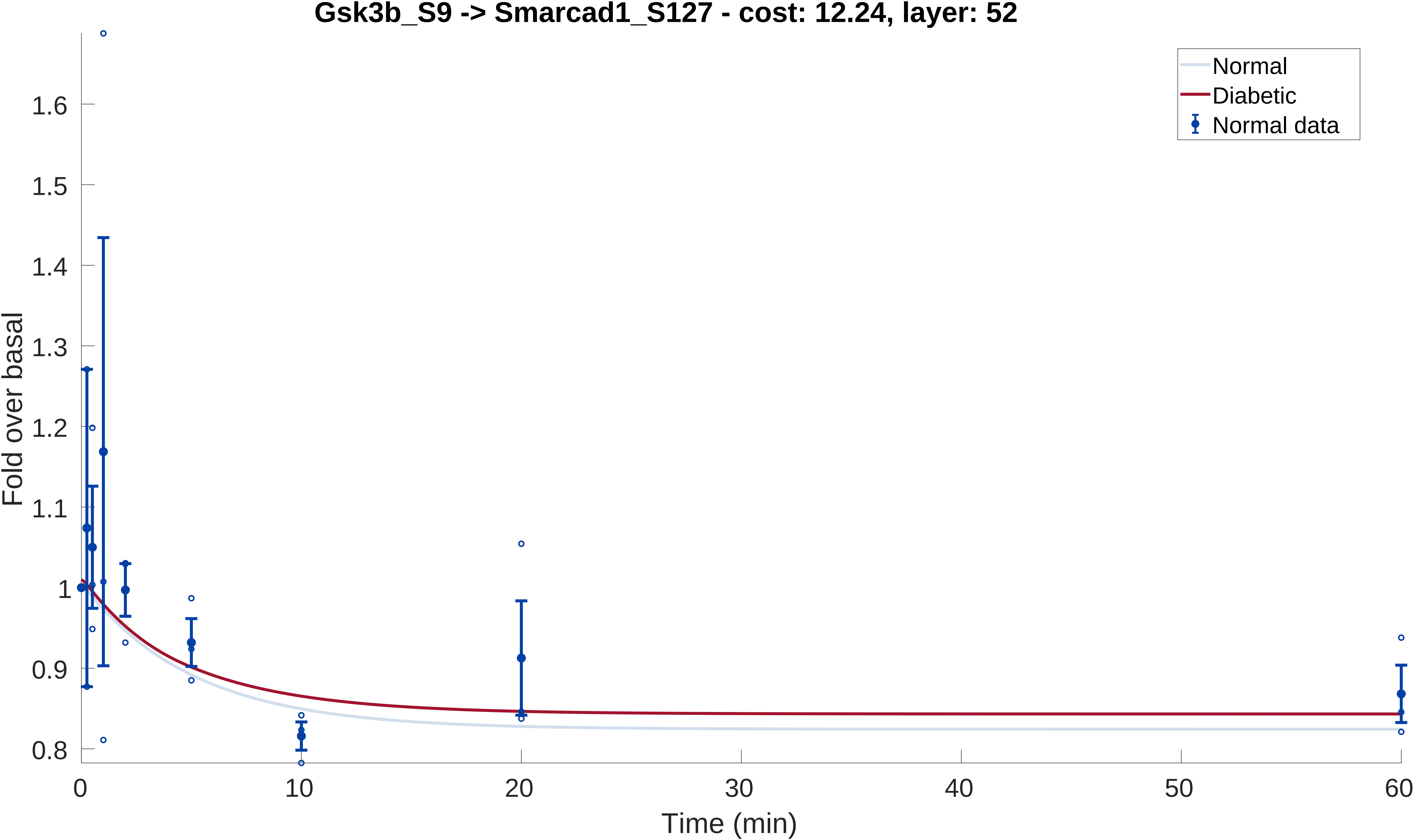

Gsk3b_S9 -> Smarcad1_S127 - cost: 12.24, layer: 52
Normal
Diabetic
Normal data
Fold over basal
1.6
1.5
1.4
1.3
1.2
1.1
1
0.9
0.8
0
10
20
30
40
50
60
Time (min)

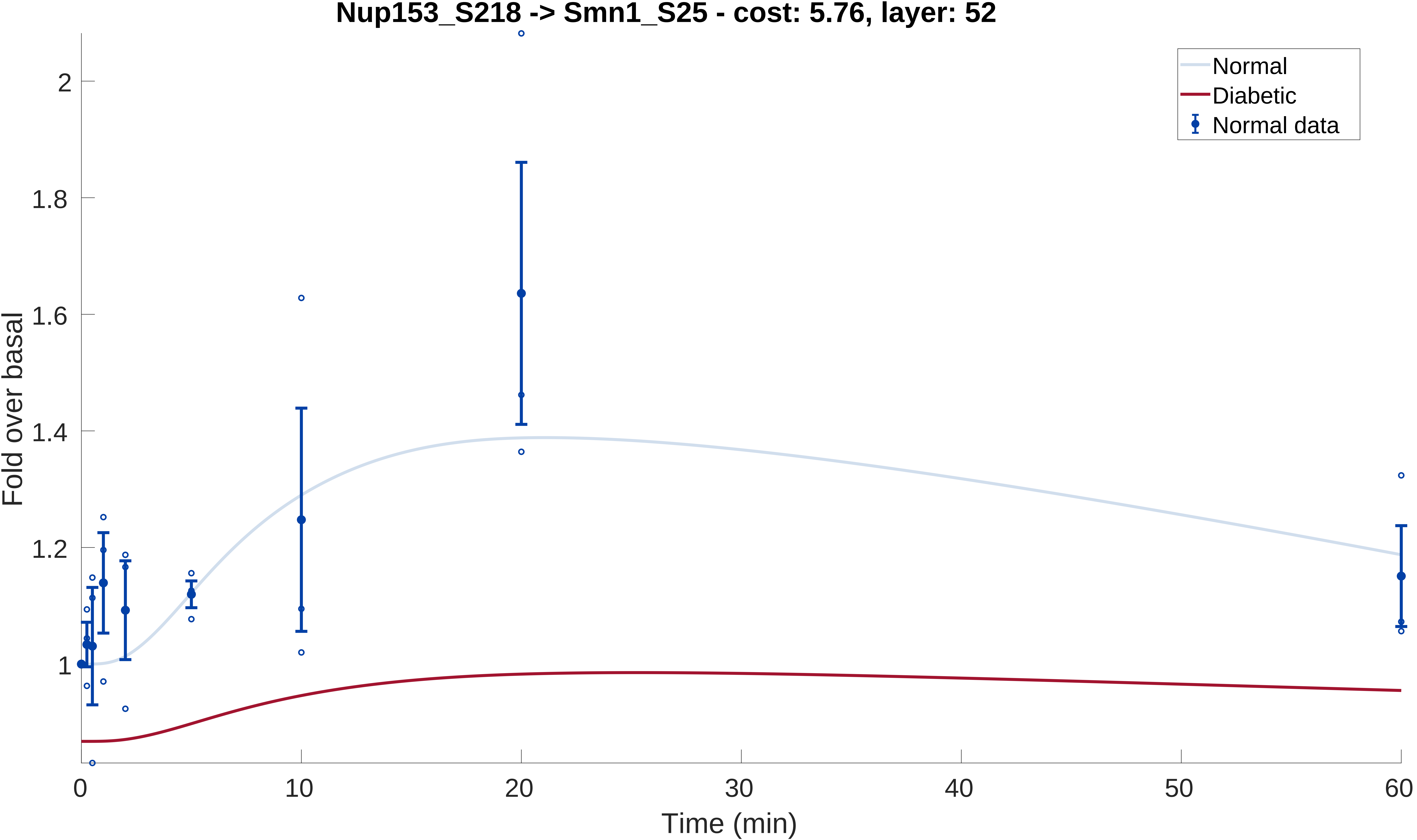

Nup153_S218 -> Smn1_S25 - cost: 5.76, layer: 52
Normal
Diabetic
Normal data
Fold over basal
2
1.8
1.6
1.4
1.2
1
0
10
20
30
40
50
60
Time (min)

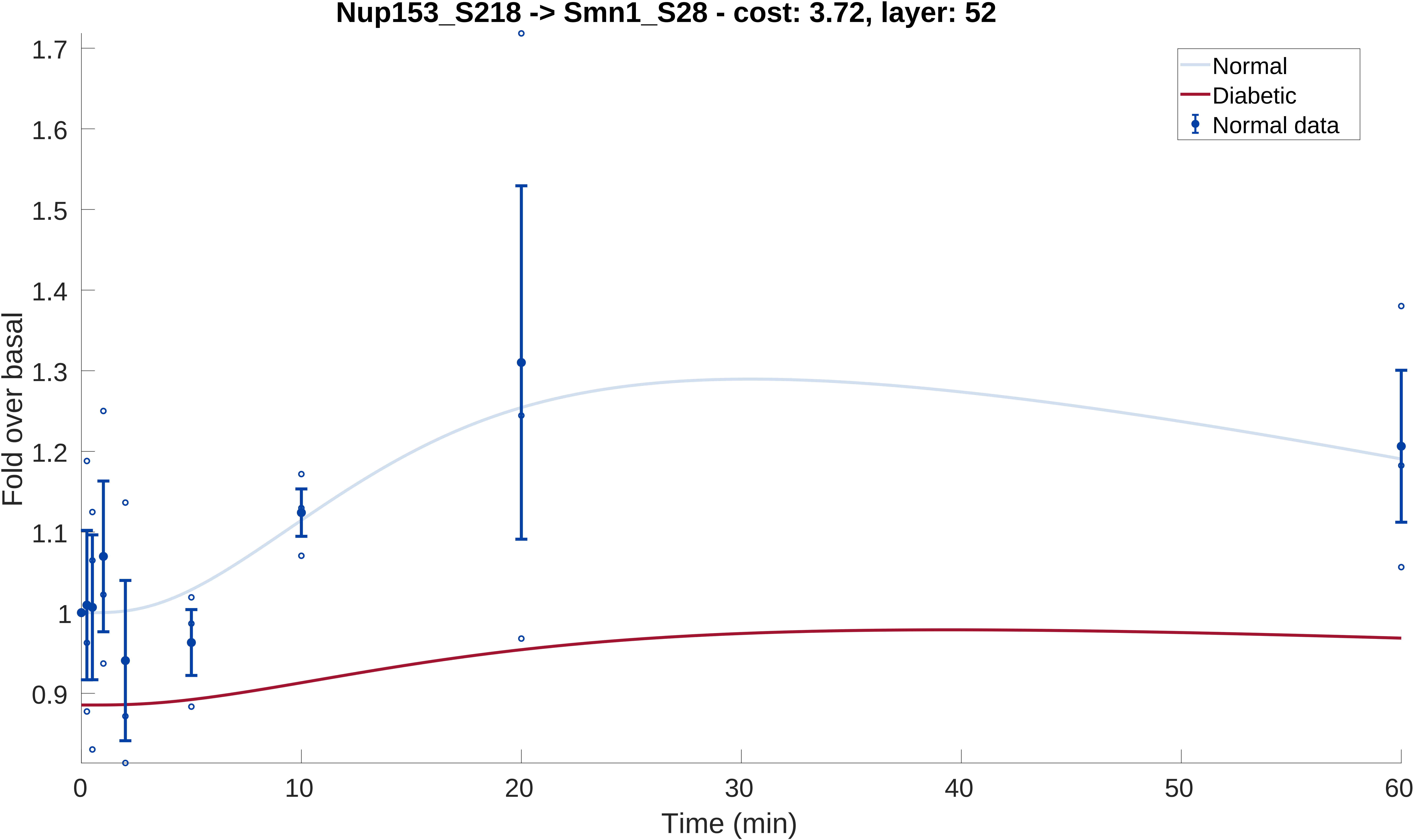

Nup153_S218 -> Smn1_S28 - cost: 3.72, layer: 52
Normal
Diabetic
Normal data
Fold over basal
Time (min)
0.9
1
1.1
1.2
1.3
1.4
1.5
1.6
1.7
0
10
20
30
40
50
60

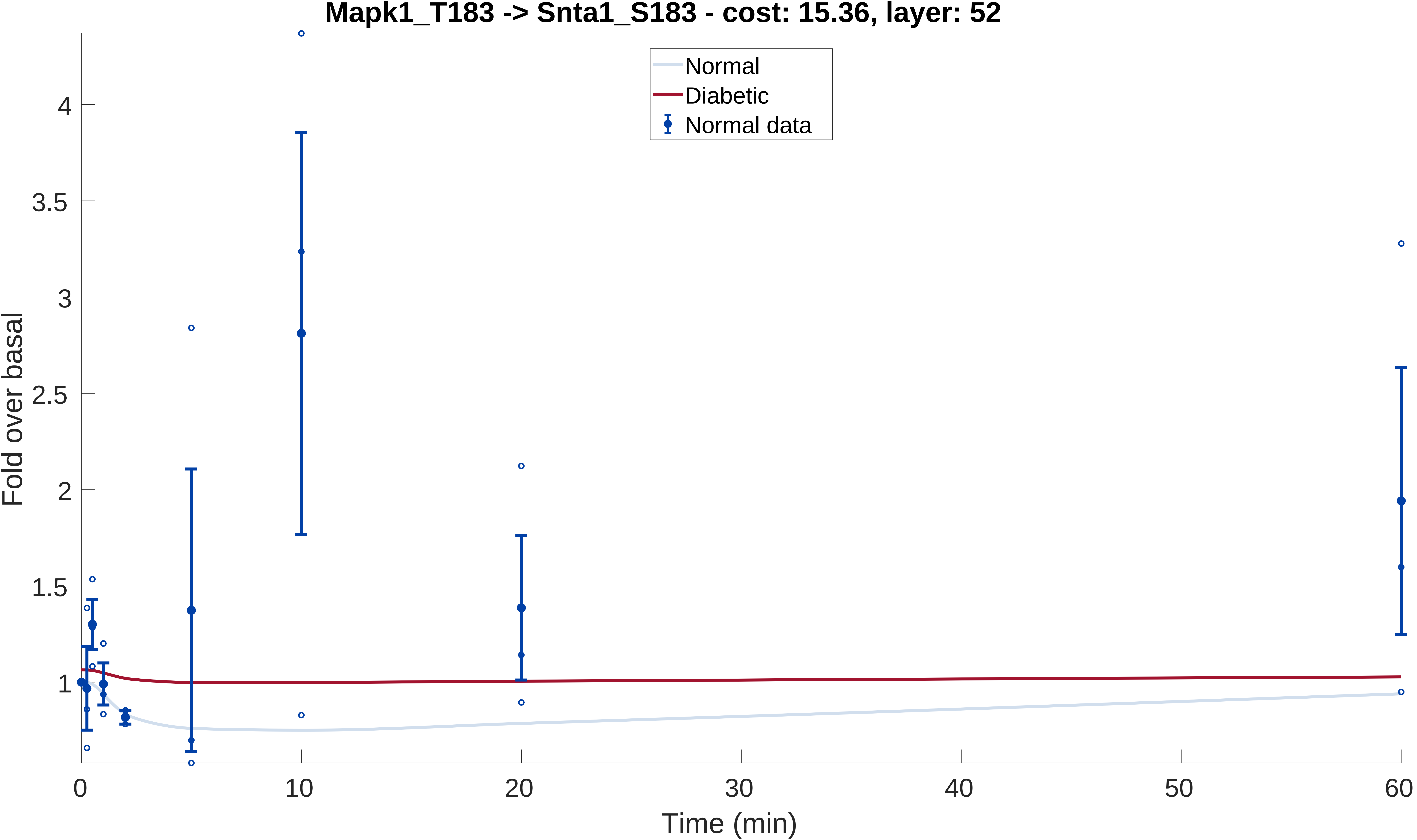

Mapk1_T183 -> Snta1_S183 - cost: 15.36, layer: 52
Normal
Diabetic
Normal data
Fold over basal
Time (min)
0
10
20
30
40
50
60
1
1.5
2
2.5
3
3.5
4

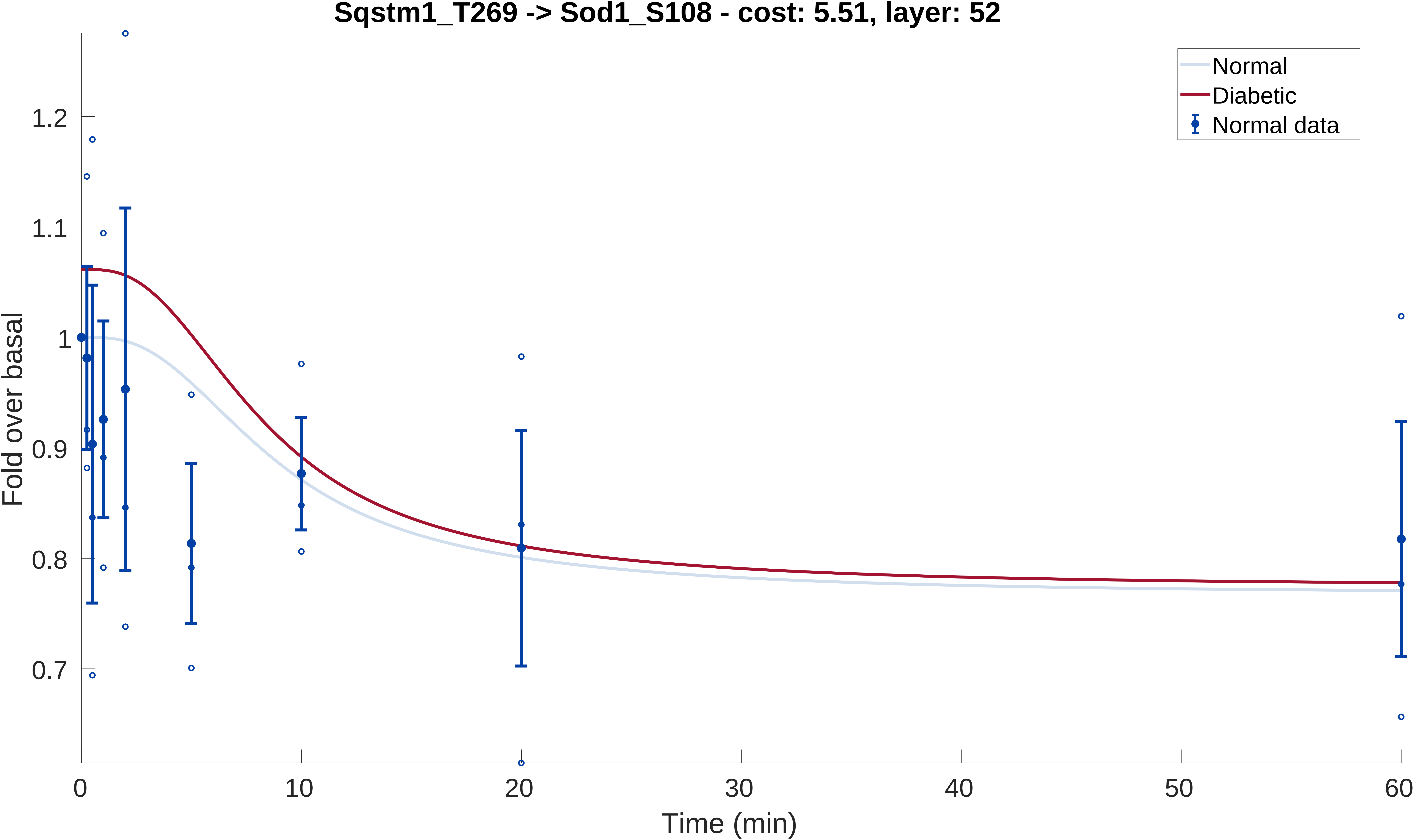

Sqstm1_T269 -> Sod1_S108 - cost: 5.51, layer: 52
Normal
Diabetic
Normal data
Fold over basal
1.2
1.1
1
0.9
0.8
0.7
0
10
20
30
40
50
60
Time (min)

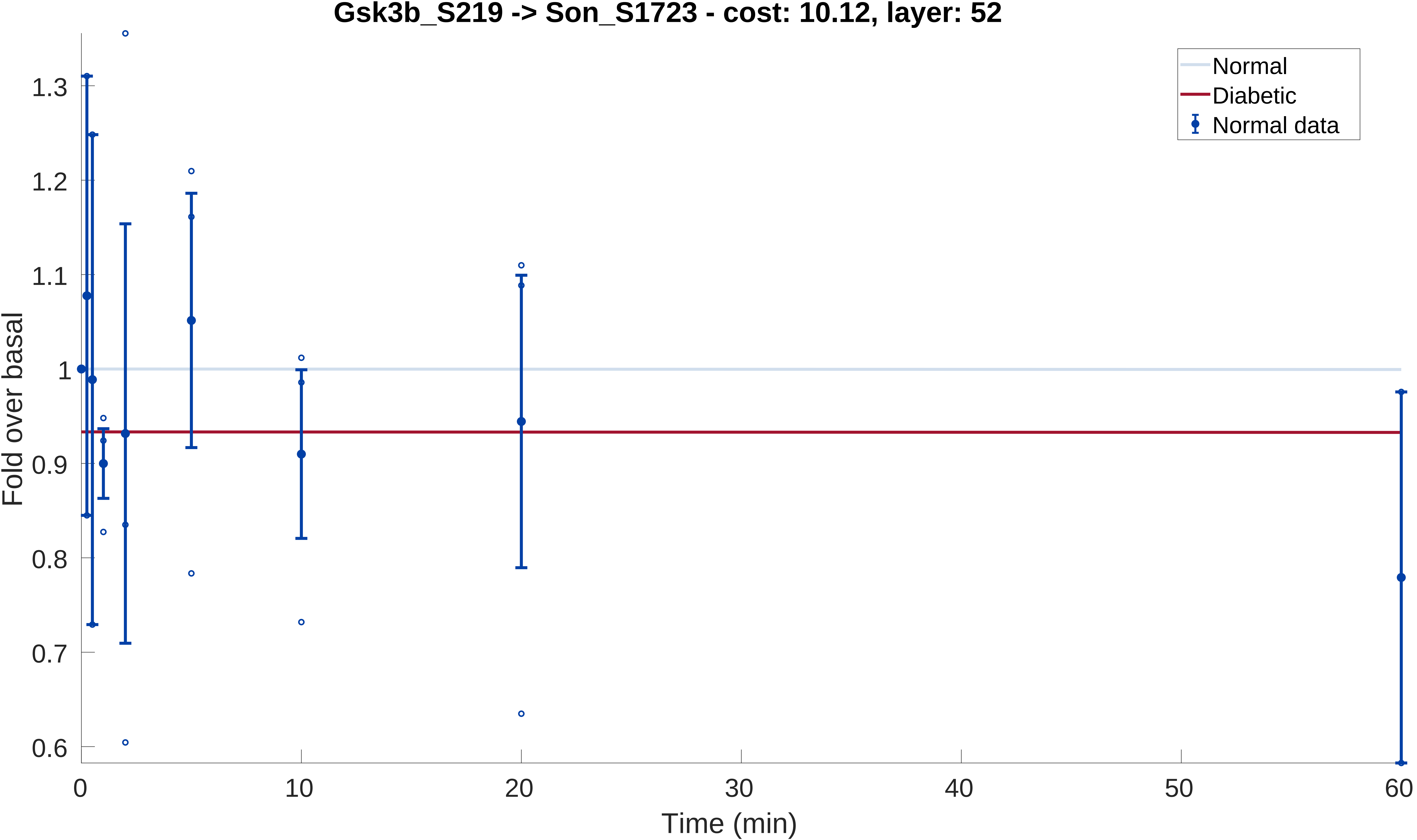

Gsk3b_S219 -> Son_S1723 - cost: 10.12, layer: 52
Normal
Diabetic
Normal data
Fold over basal
1.3
1.2
1.1
1
0.9
0.8
0.7
0.6
0
10
20
30
40
50
60
Time (min)

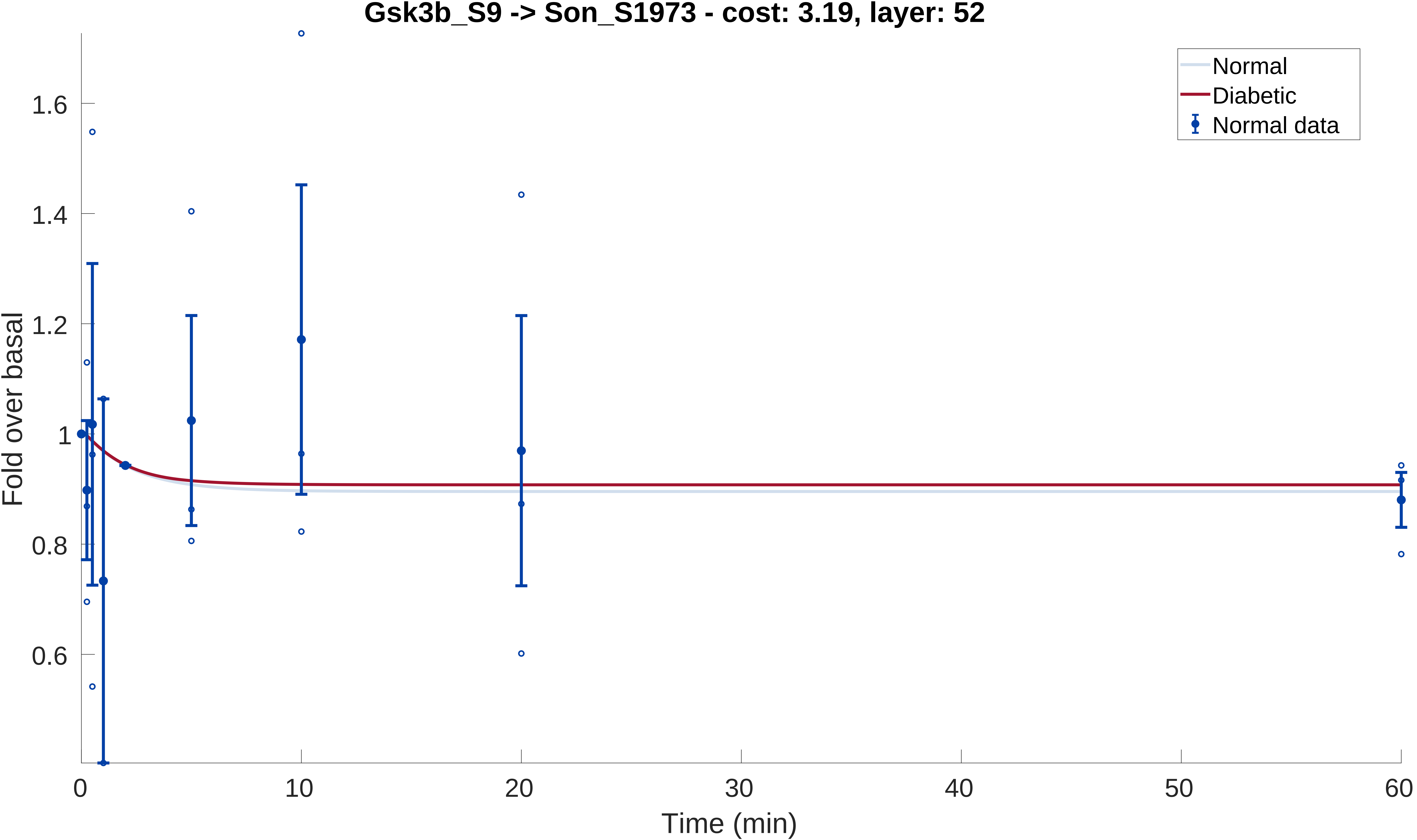

Gsk3b_S9 -> Son_S1973 - cost: 3.19, layer: 52
Normal
Diabetic
Normal data
Fold over basal
1.6
1.4
1.2
1
0.8
0.6
0
10
20
30
40
50
60
Time (min)

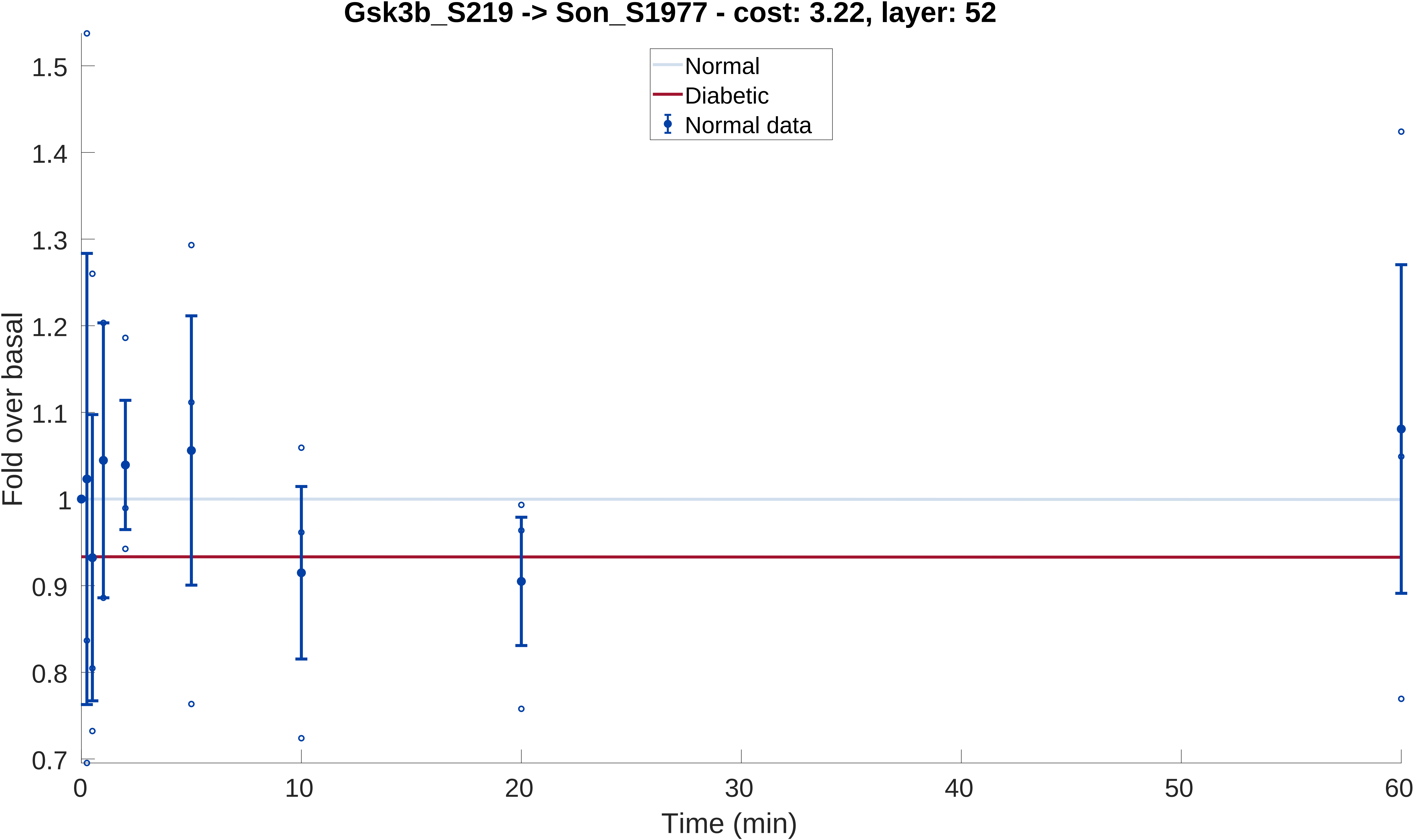

Gsk3b_S219 -> Son_S1977 - cost: 3.22, layer: 52
Normal
Diabetic
Normal data
Fold over basal
Time (min)
1.5
1.4
1.3
1.2
1.1
1
0.9
0.8
0.7
0
10
20
30
40
50
60

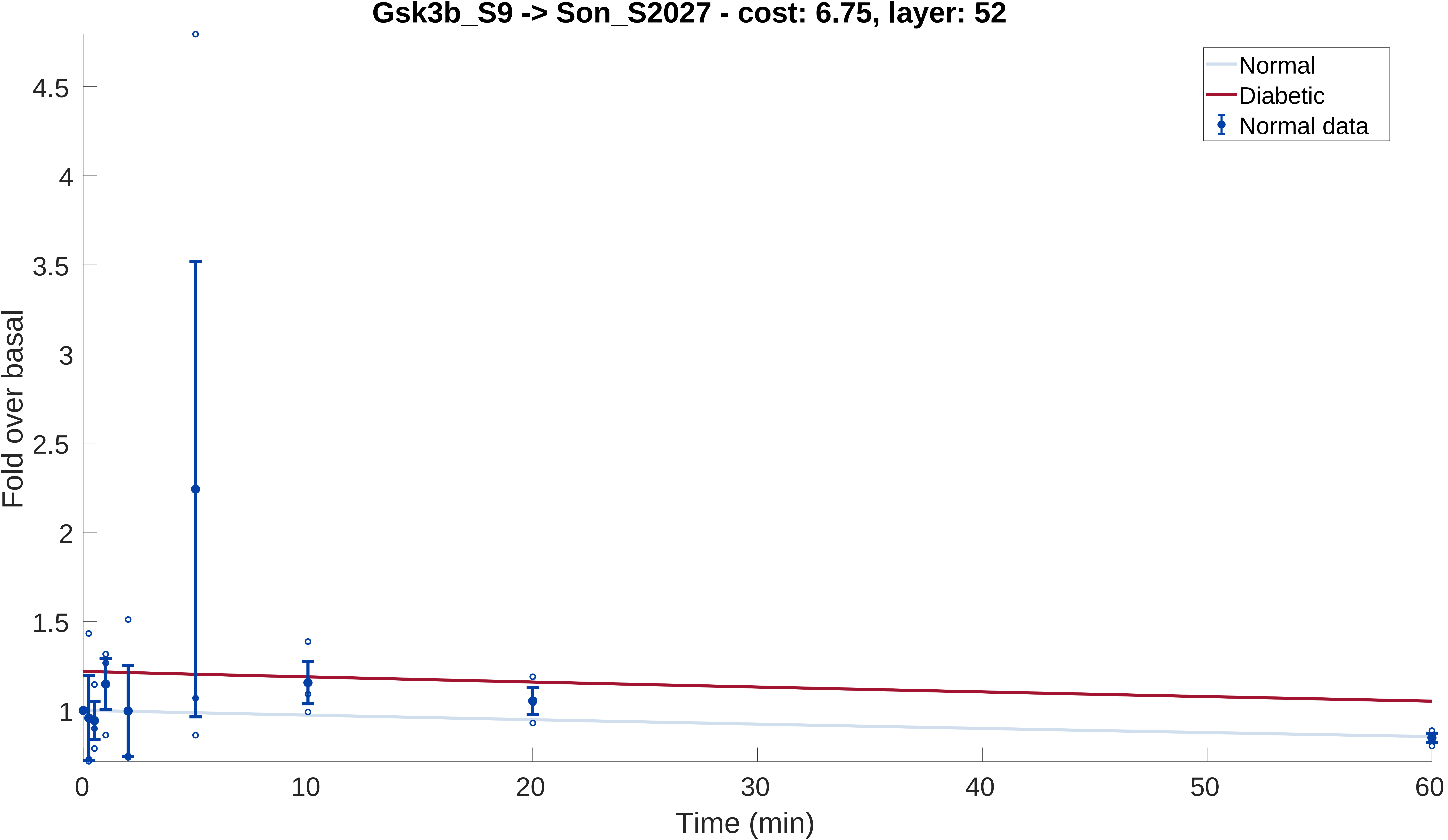

Gsk3b_S9 -> Son_S2027 - cost: 6.75, layer: 52
Normal
Diabetic
Normal data
Fold over basal
4.5
4
3.5
3
2.5
2
1.5
1
0
10
20
30
40
50
60
Time (min)

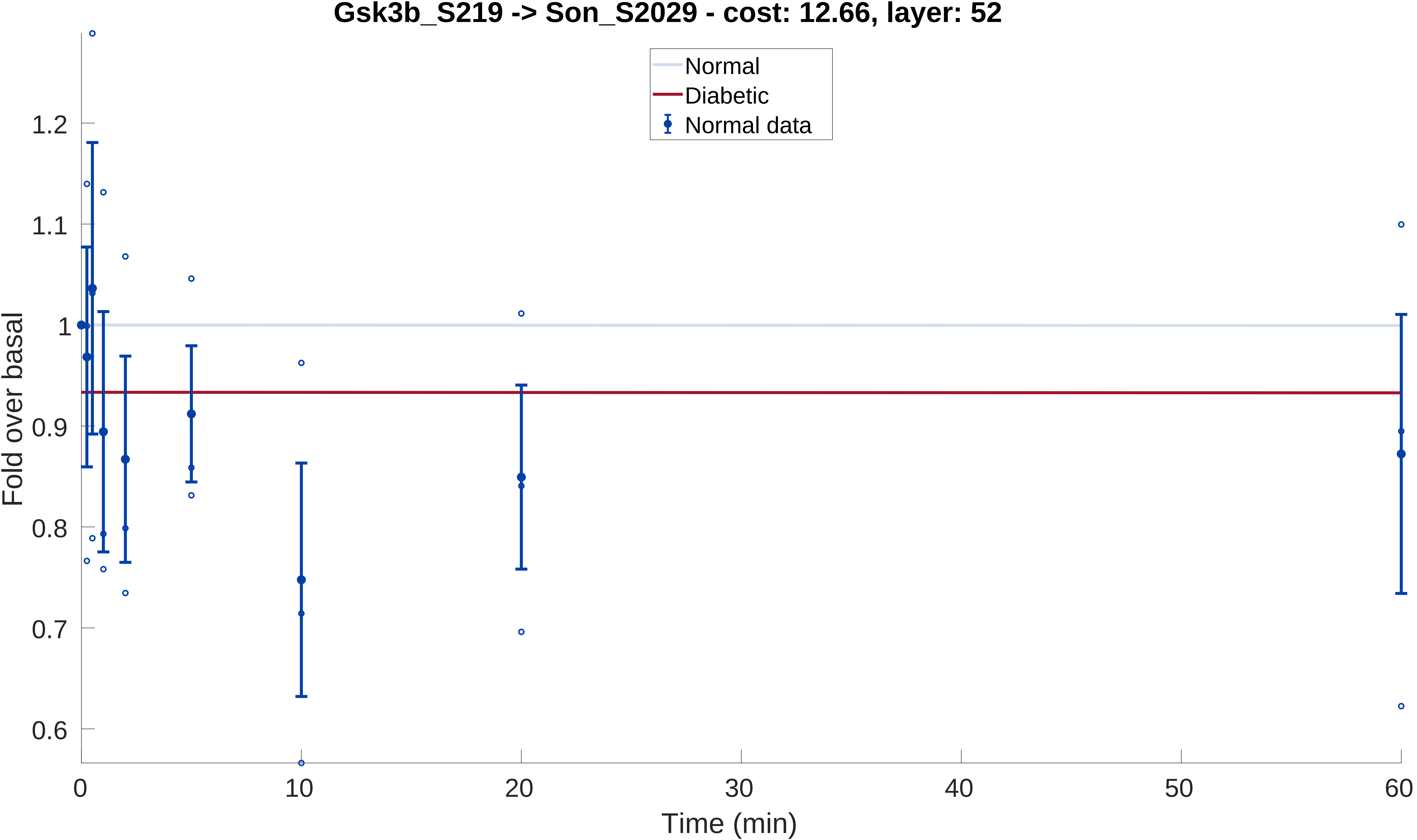

Gsk3b_S219 -> Son_S2029 - cost: 12.66, layer: 52
Normal
Diabetic
Normal data
Fold over basal
1.2
1.1
1
0.9
0.8
0.7
0.6
0
10
20
30
40
50
60
Time (min)

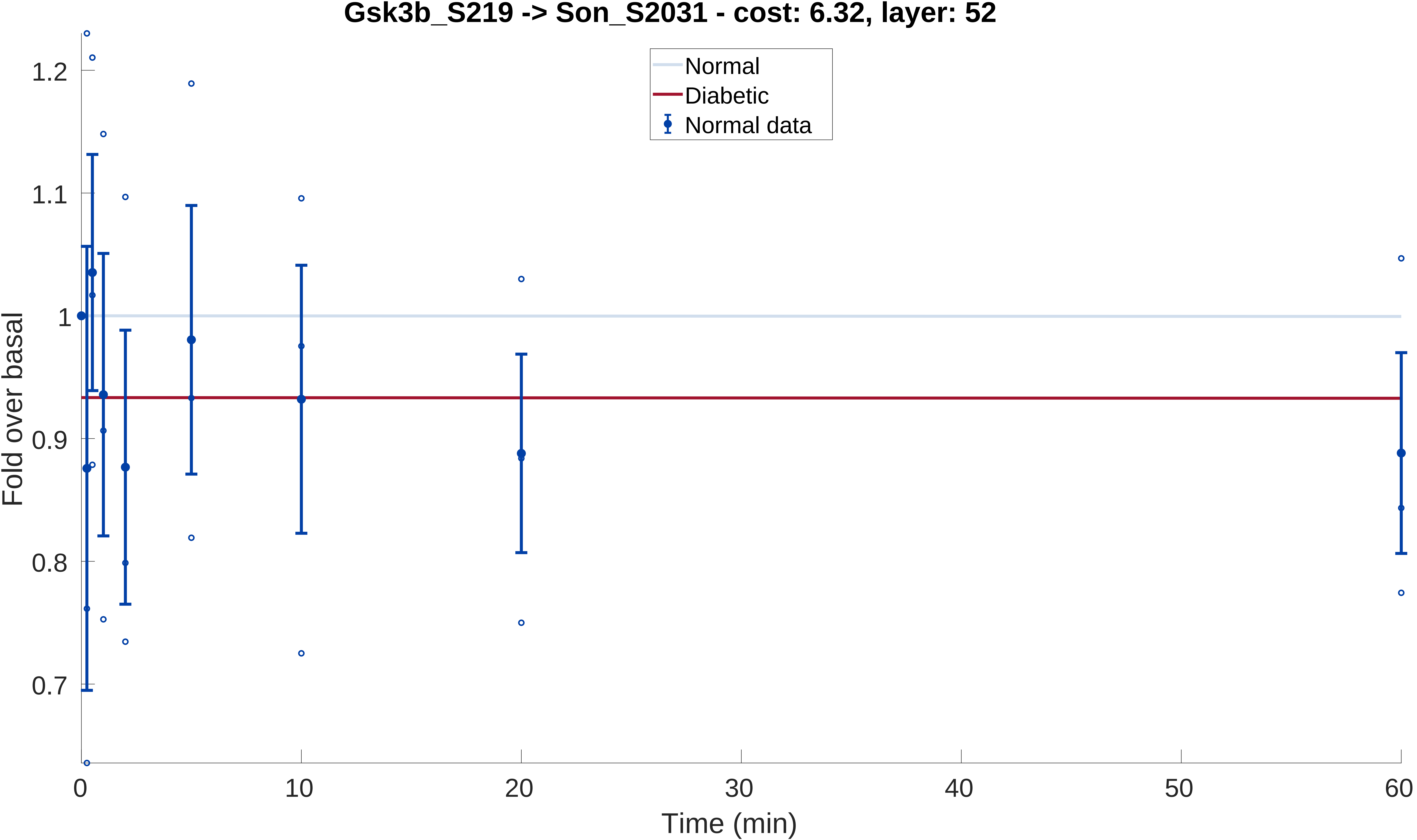

Gsk3b_S219 -> Son_S2031 - cost: 6.32, layer: 52
Normal
Diabetic
Normal data
1.2
1.1
1
0.9
0.8
0.7
Fold over basal
0
10
20
30
40
50
60
Time (min)

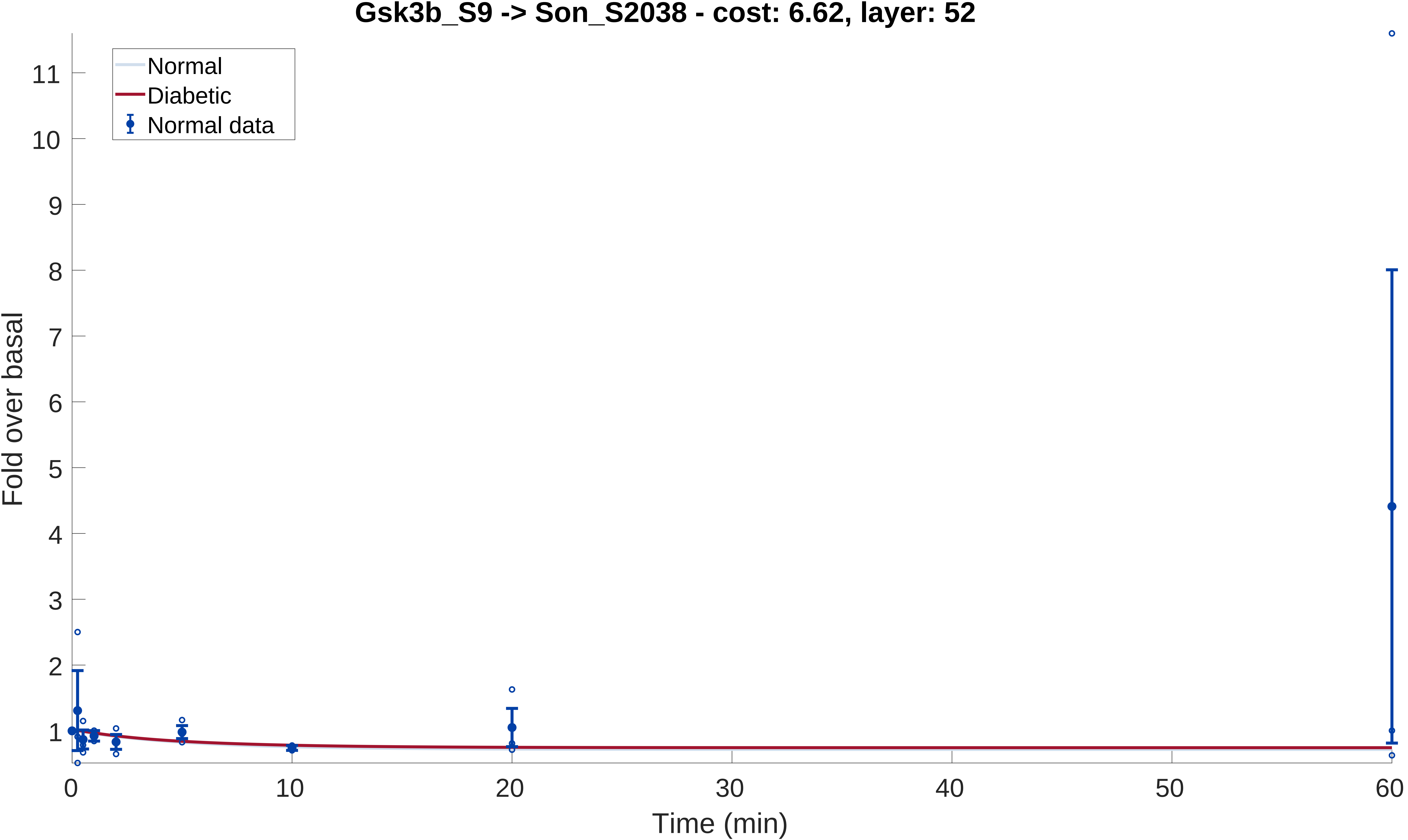

Gsk3b_S9 -> Son_S2038 - cost: 6.62, layer: 52
Normal
Diabetic
Normal data
Fold over basal
Time (min)
11
10
9
8
7
6
5
4
3
2
1
0
10
20
30
40
50
60

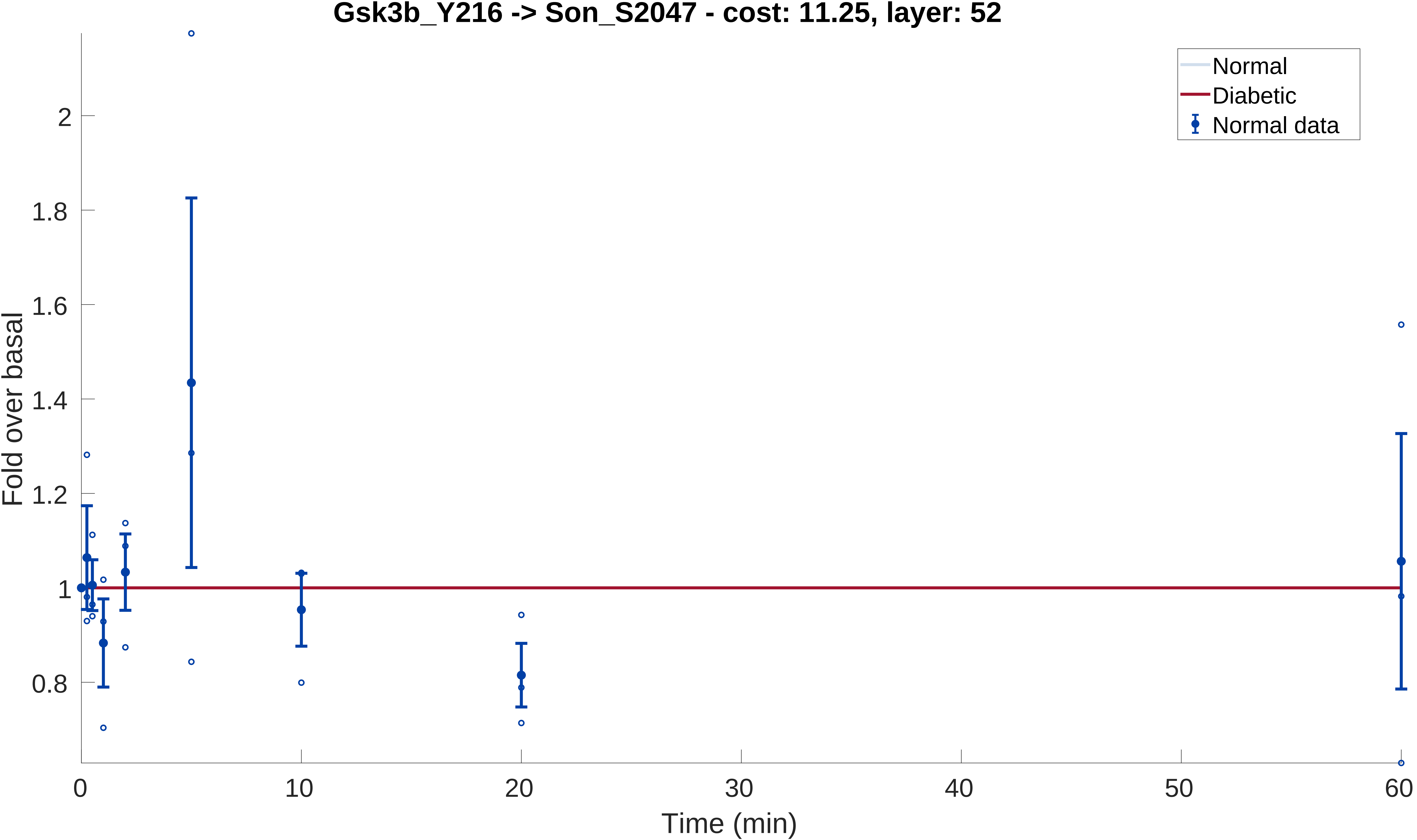
Gsk3b_Y216 -> Son_S2047 - cost: 11.25, layer: 52
Normal
Diabetic
Normal data
Fold over basal
2
1.8
1.6
1.4
1.2
1
0.8
0
10
20
30
40
50
60
Time (min)

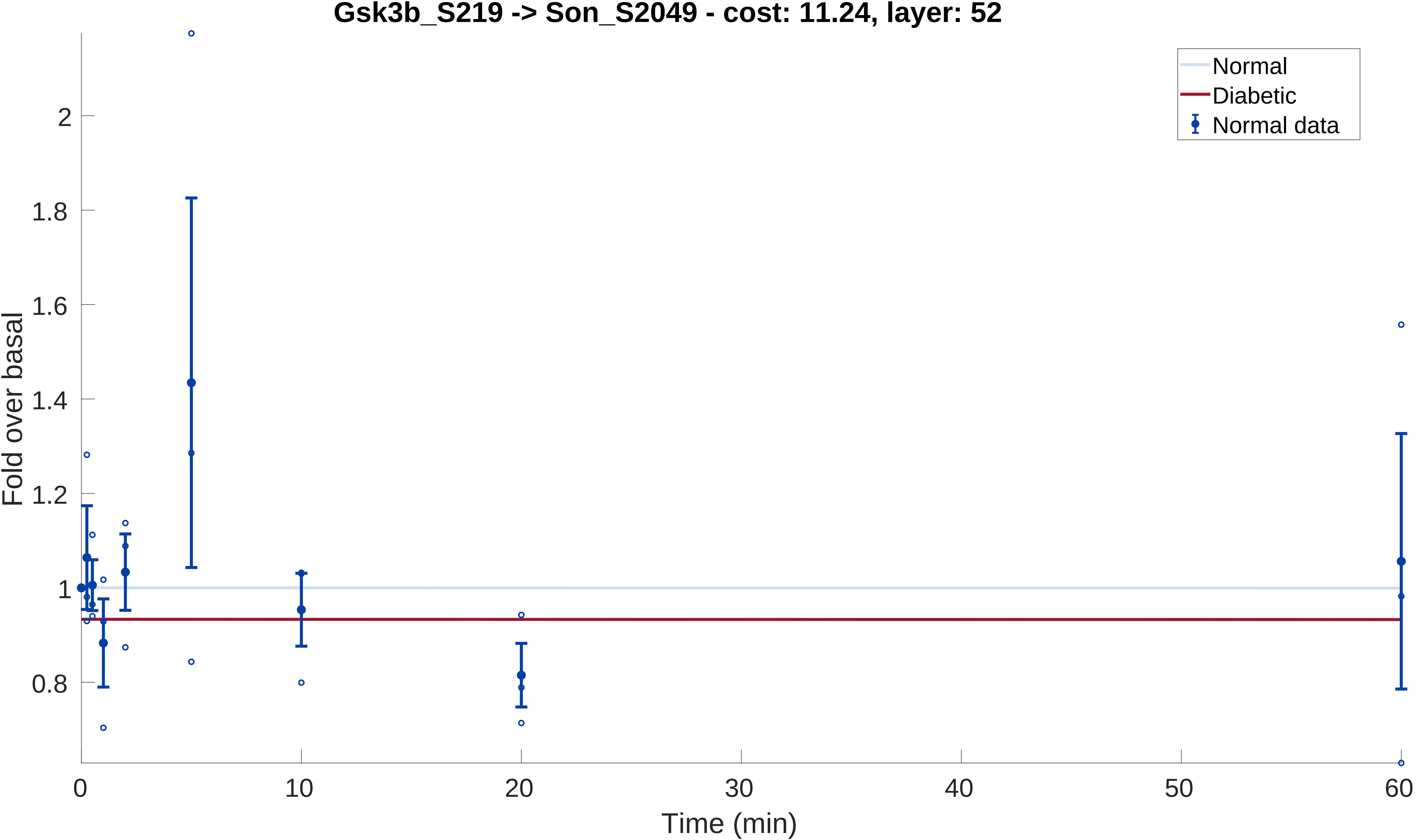

Gsk3b_S219 -> Son_S2049 - cost: 11.24, layer: 52
Normal
Diabetic
Normal data
Fold over basal
Time (min)
2
1.8
1.6
1.4
1.2
1
0.8
0
10
20
30
40
50
60

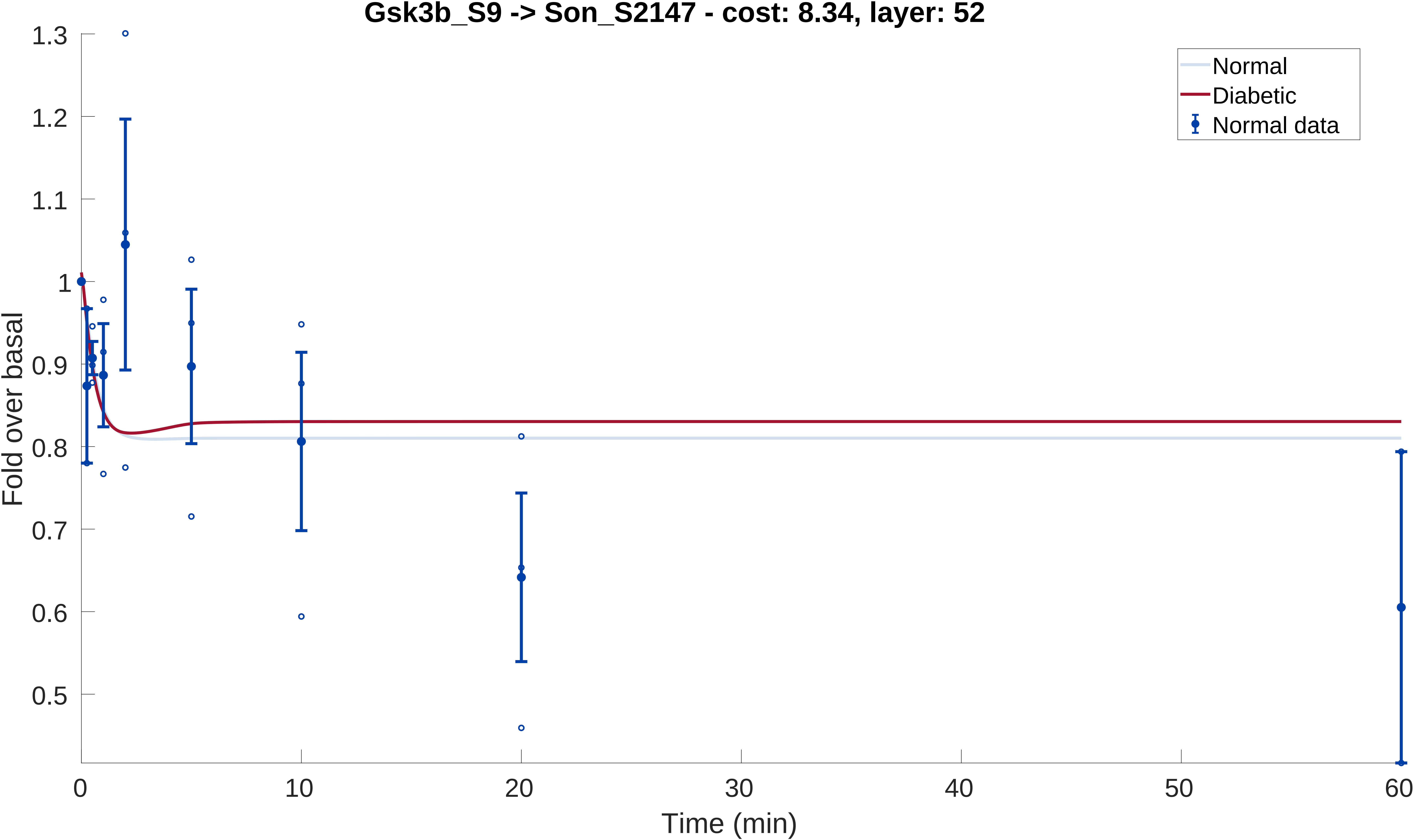
Gsk3b_S9 -> Son_S2147 - cost: 8.34, layer: 52
Fold over basal
Time (min)
Normal
Diabetic
Normal data
1.3
1.2
1.1
1
0.9
0.8
0.7
0.6
0.5
0
10
20
30
40
50
60

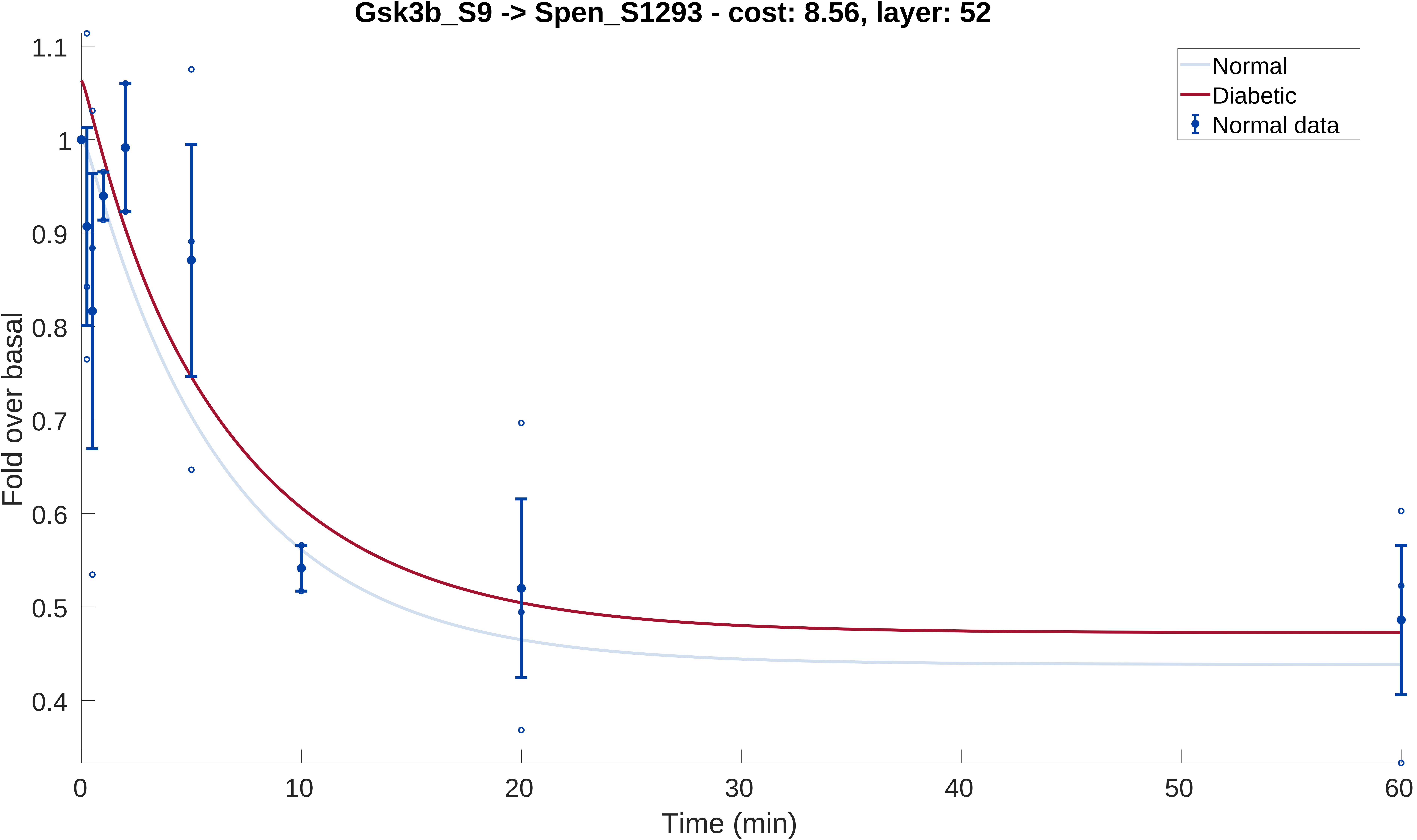

Gsk3b_S9 -> Spen_S1293 - cost: 8.56, layer: 52
Normal
Diabetic
Normal data
Fold over basal
Time (min)
1.1
1
0.9
0.8
0.7
0.6
0.5
0.4
0
10
20
30
40
50
60

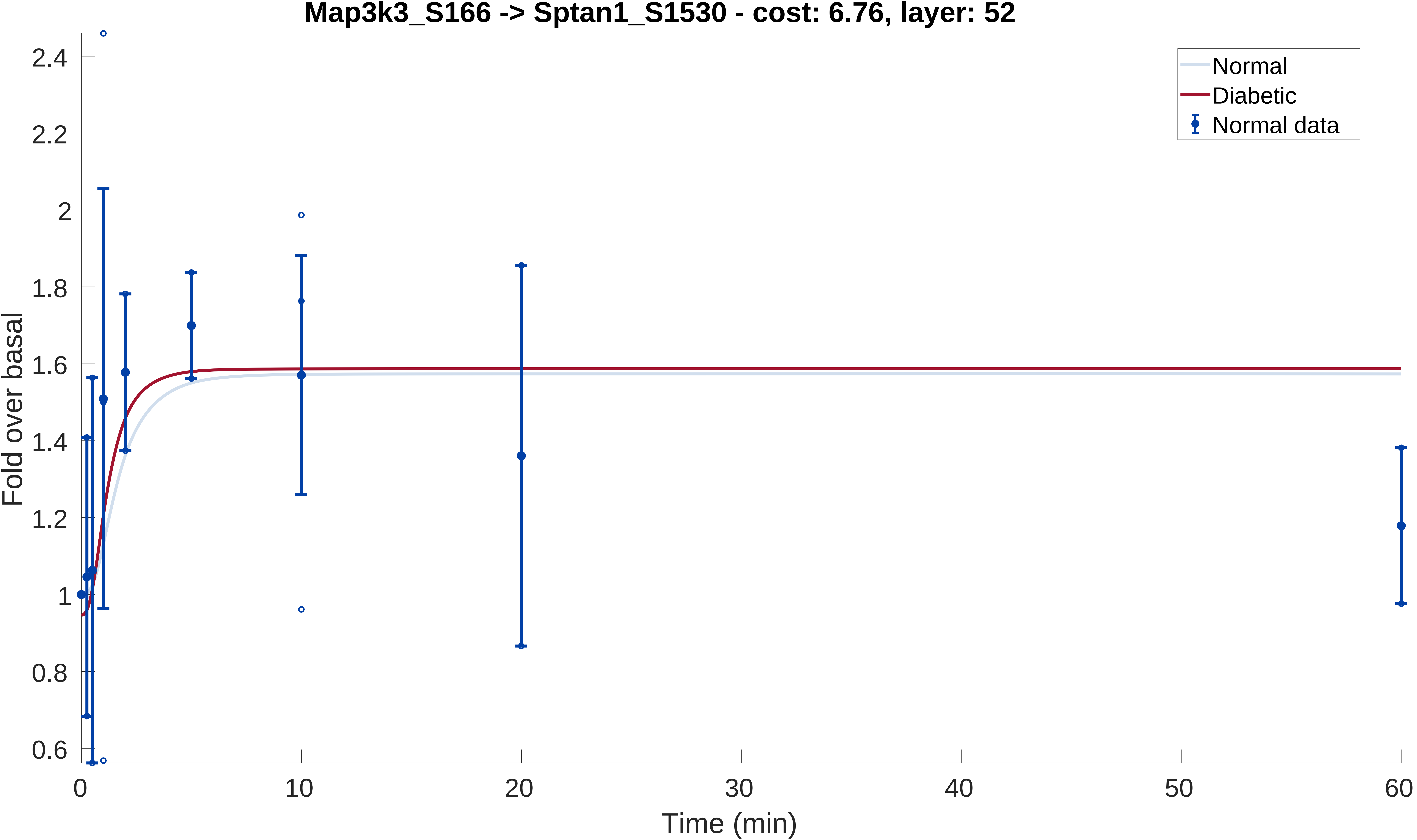

Map3k3_S166 -> Sptan1_S1530 - cost: 6.76, layer: 52
Normal
Diabetic
Normal data
Fold over basal
Time (min)
2.4
2.2
2
1.8
1.6
1.4
1.2
1
0.8
0.6
0
10
20
30
40
50
60

Map3k3_S166 -> Sptbn1_S2318 - cost: 5.56, layer: 52

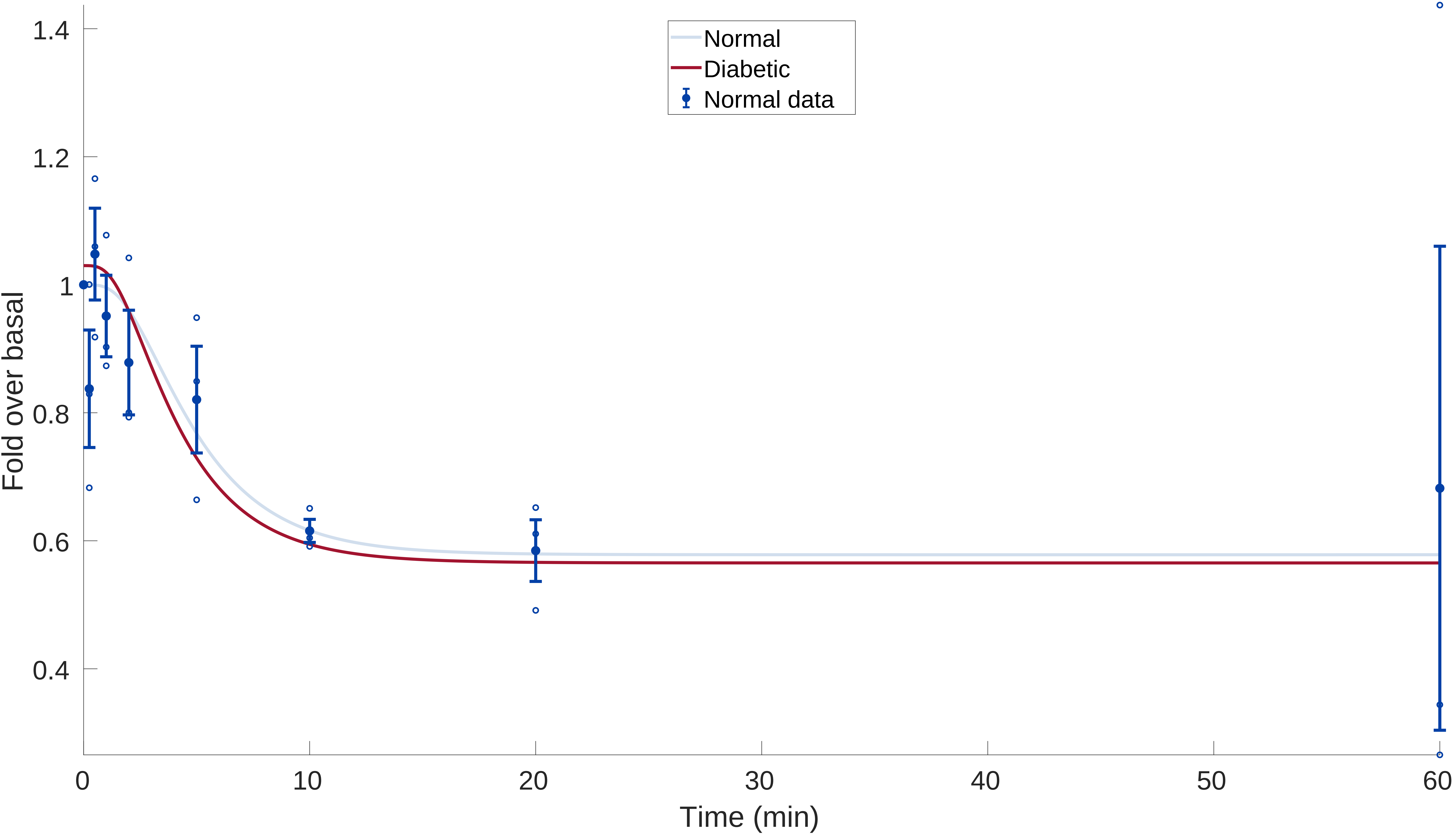

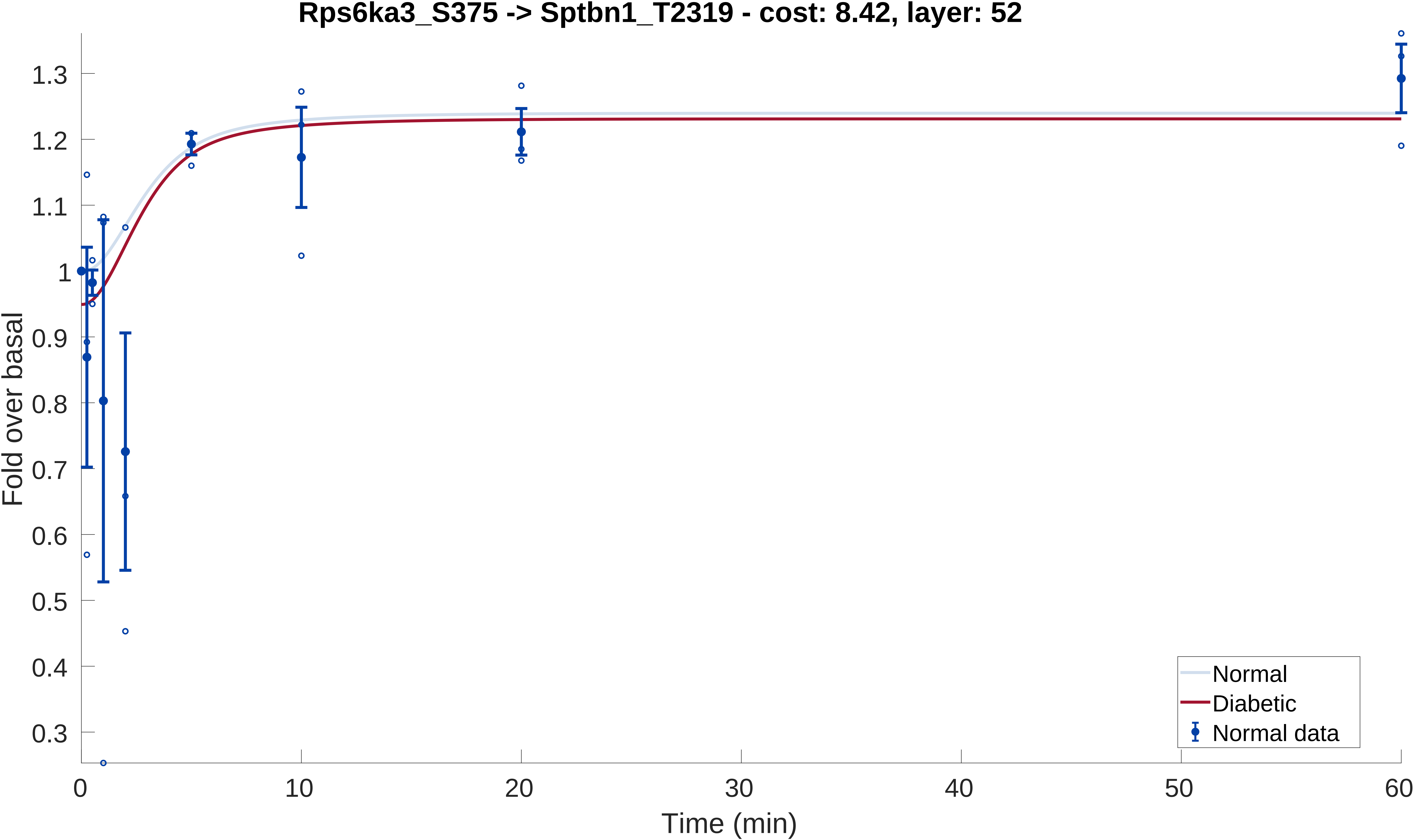
Rps6ka3_S375 -> Sptbn1_T2319 - cost: 8.42, layer: 52
Fold over basal
1.3
1.2
1.1
1
0.9
0.8
0.7
0.6
0.5
0.4
0.3
0
10
20
30
40
50
60
Time (min)
Normal
Diabetic
Normal data

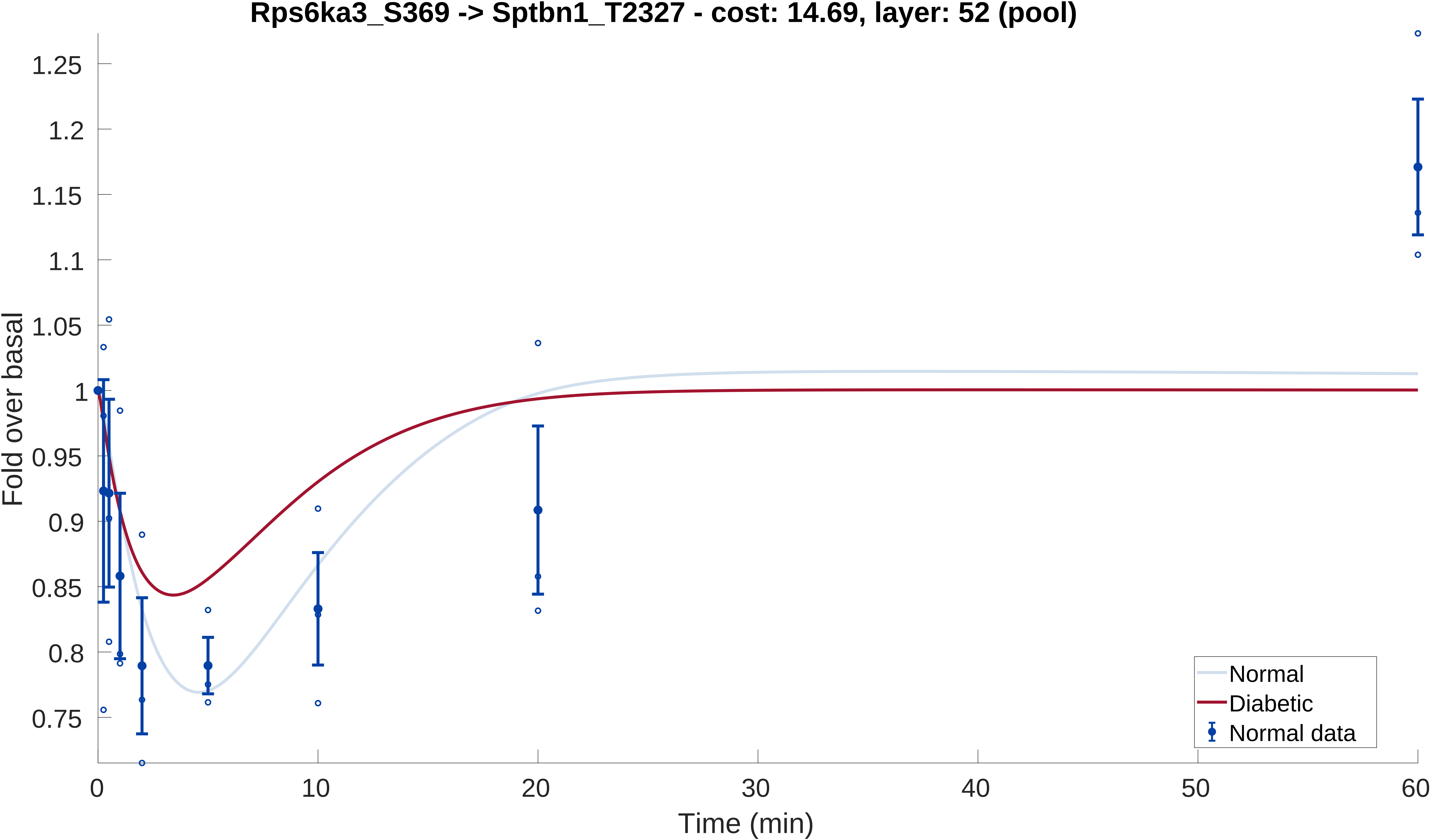

Rps6ka3_S369 -> Sptbn1_T2327 - cost: 14.69, layer: 52 (pool)
Fold over basal
Time (min)
1.25
1.2
1.15
1.1
1.05
1
0.95
0.9
0.85
0.8
0.75
0
10
20
30
40
50
60
Normal
Diabetic
Normal data

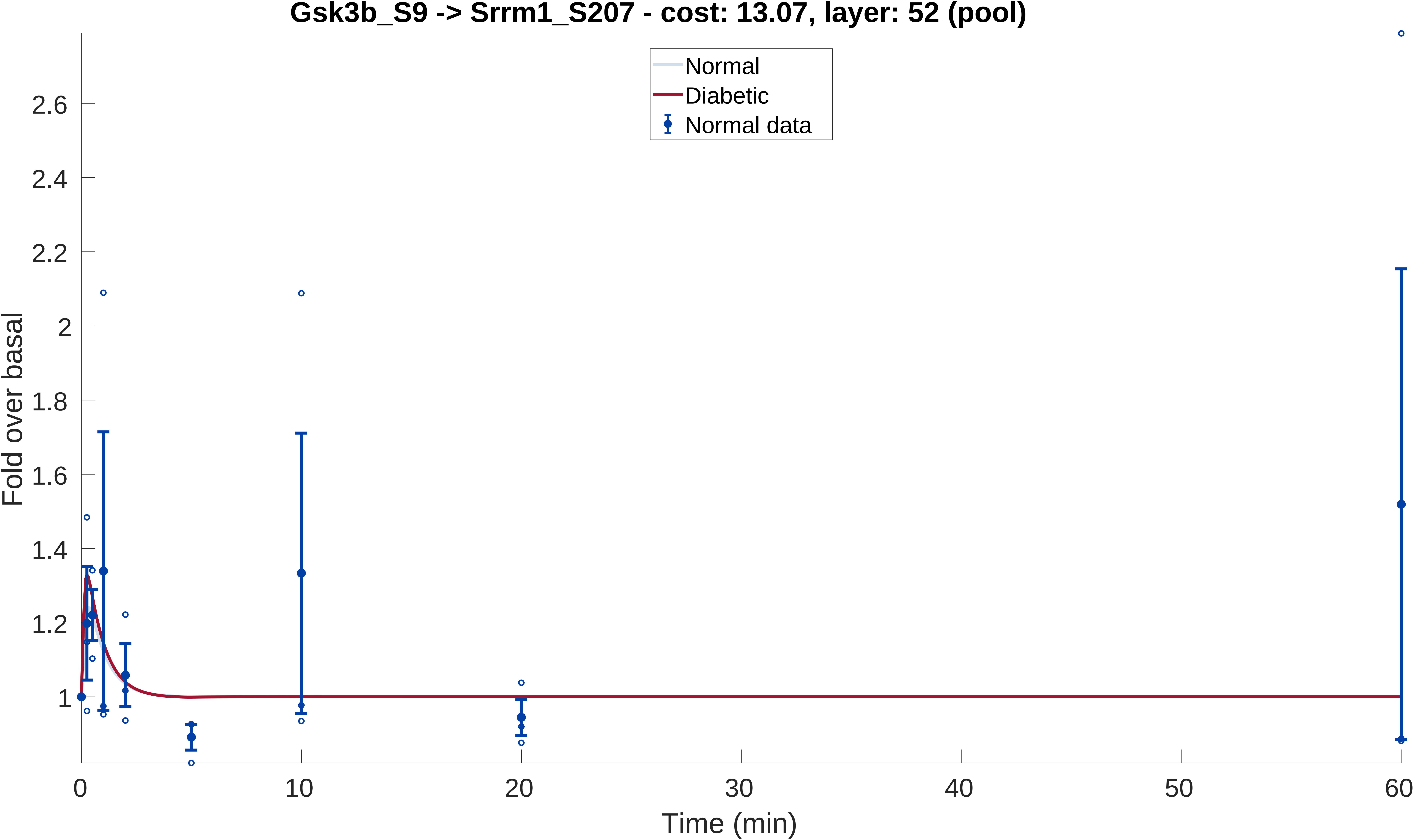

Gsk3b_S9 -> Srrm1_S207 - cost: 13.07, layer: 52 (pool)
Normal
Diabetic
Normal data
Fold over basal
2.6
2.4
2.2
2
1.8
1.6
1.4
1.2
1
0
10
20
30
40
50
60
Time (min)

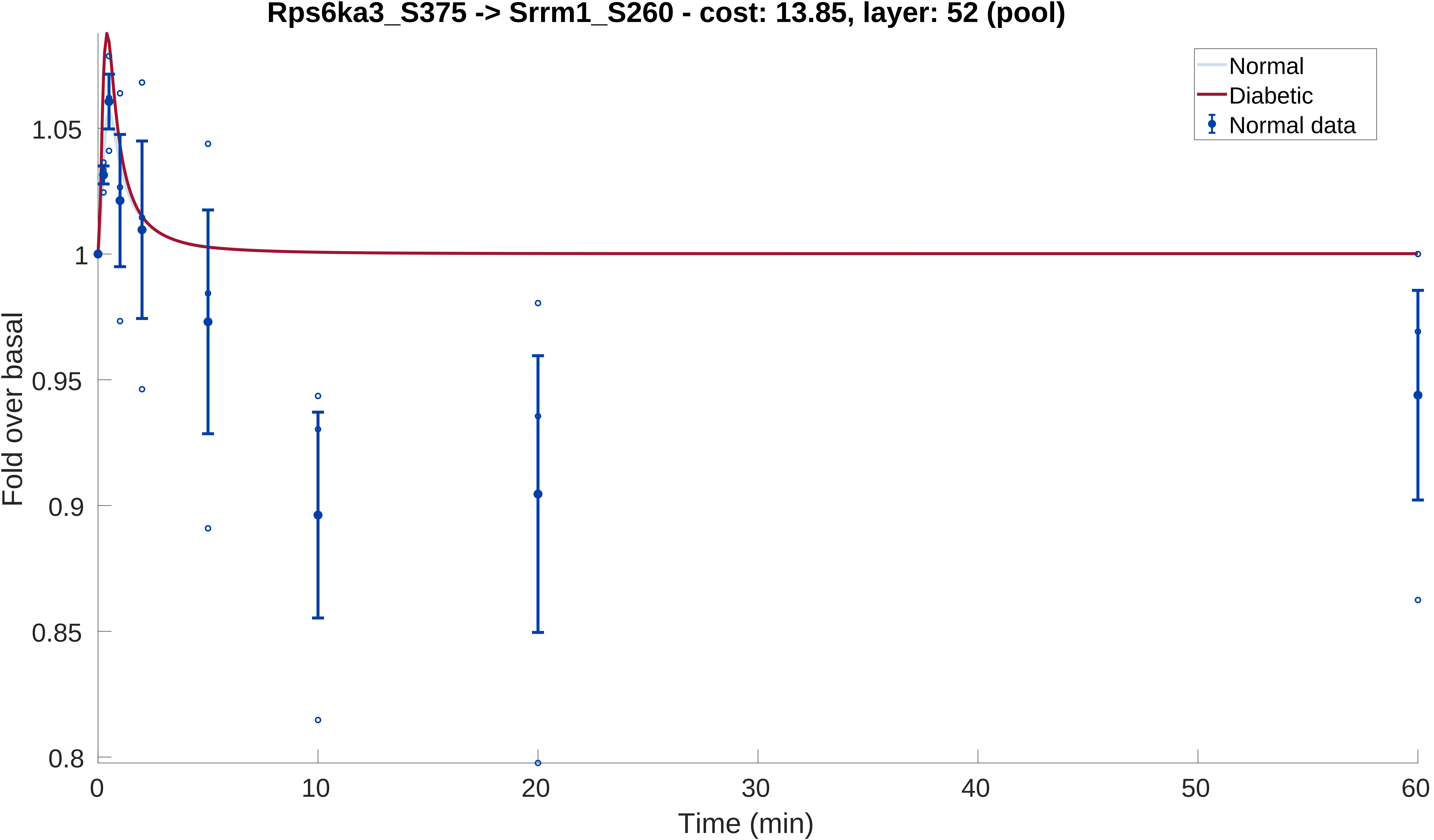

Rps6ka3_S375 -> Srrm1_S260 - cost: 13.85, layer: 52 (pool)
Normal
Diabetic
Normal data
Fold over basal
1.05
1
0.95
0.9
0.85
0.8
0
10
20
30
40
50
60
Time (min)

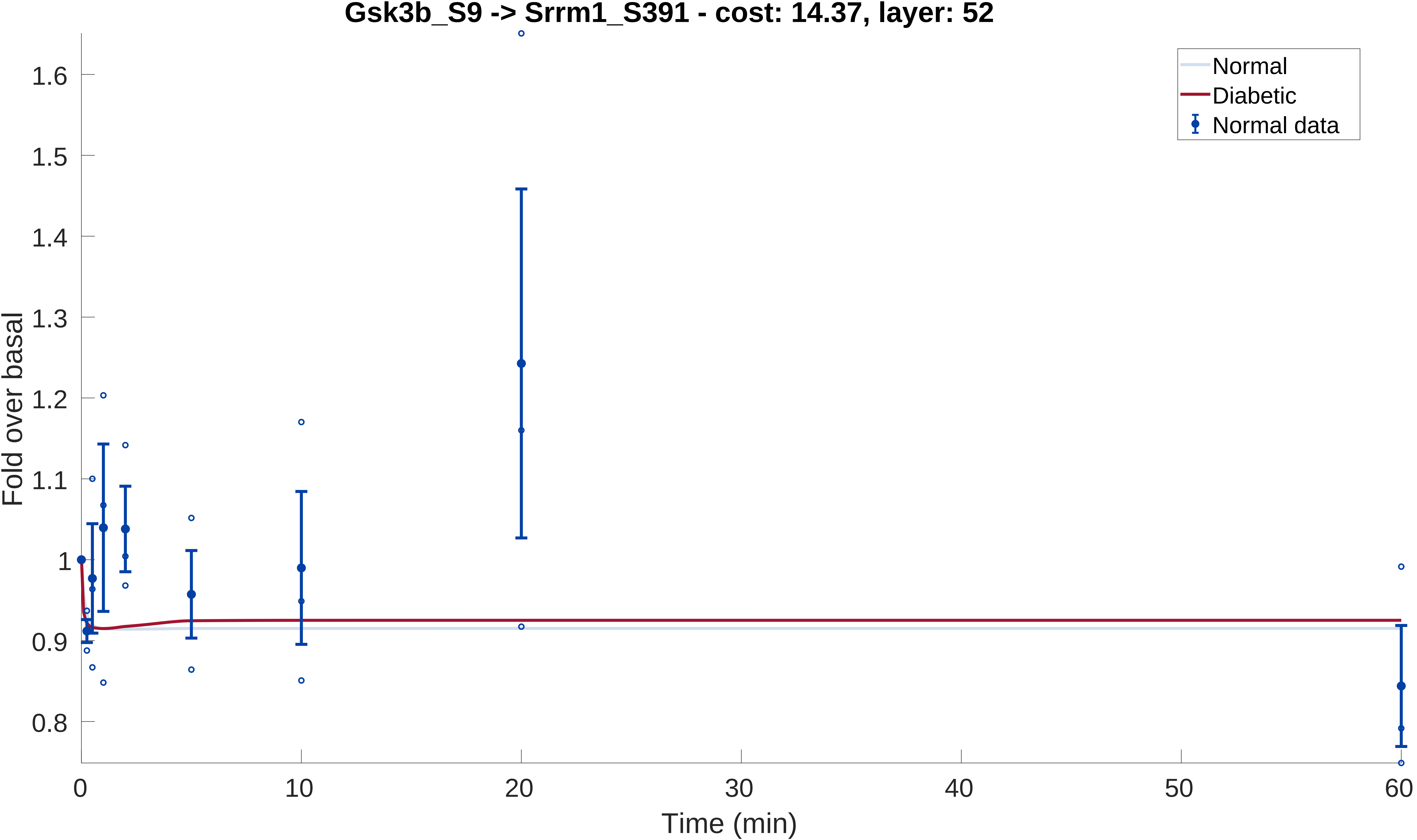
Gsk3b_S9 -> Srrm1_S391 - cost: 14.37, layer: 52
Normal
Diabetic
Normal data
Fold over basal
1.6
1.5
1.4
1.3
1.2
1.1
1
0.9
0.8
0
10
20
30
40
50
60
Time (min)

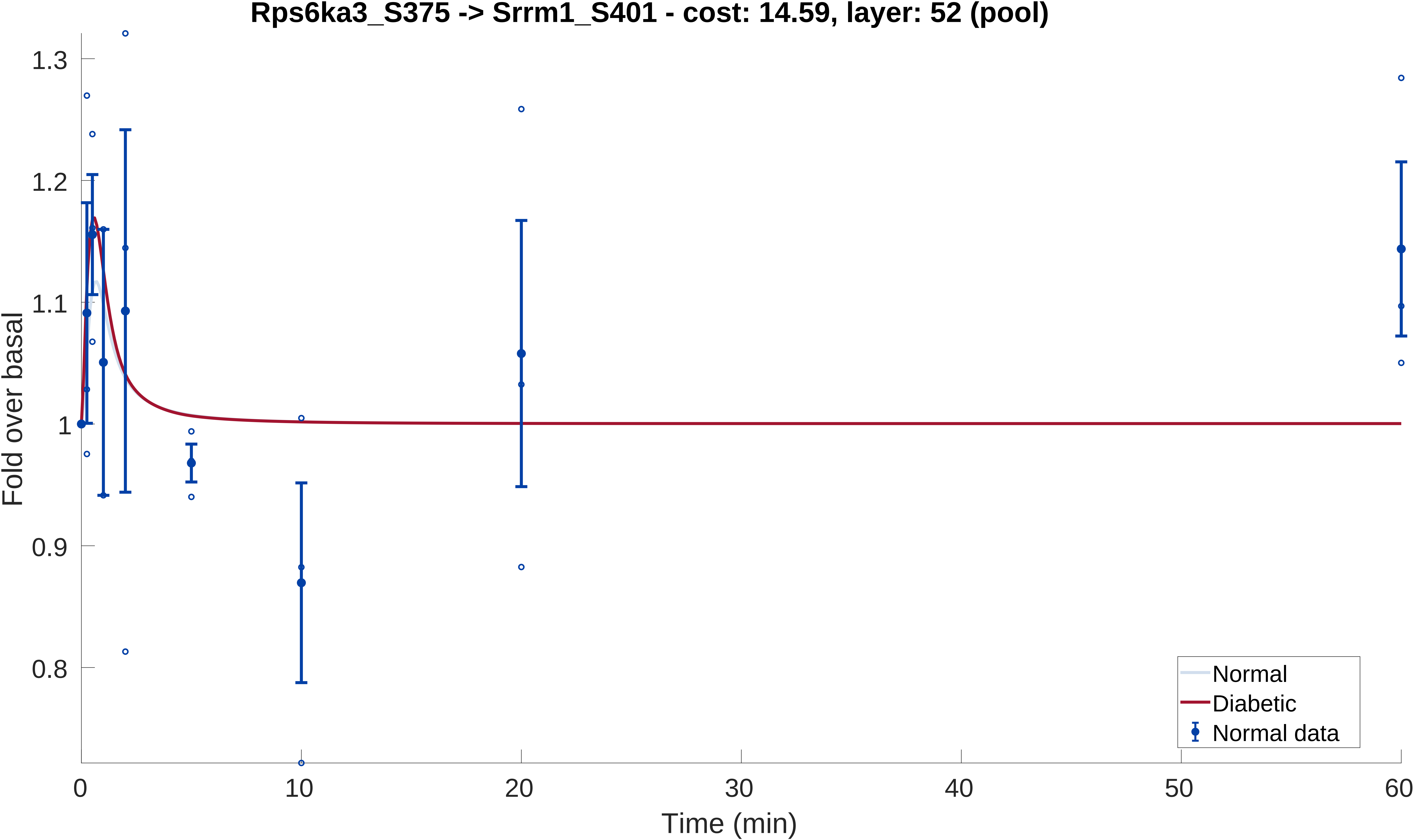

Rps6ka3_S375 -> Srrm1_S401 - cost: 14.59, layer: 52 (pool)
Fold over basal
1.3
1.2
1.1
1
0.9
0.8
0
10
20
30
40
50
60
Time (min)
Normal
Diabetic
Normal data

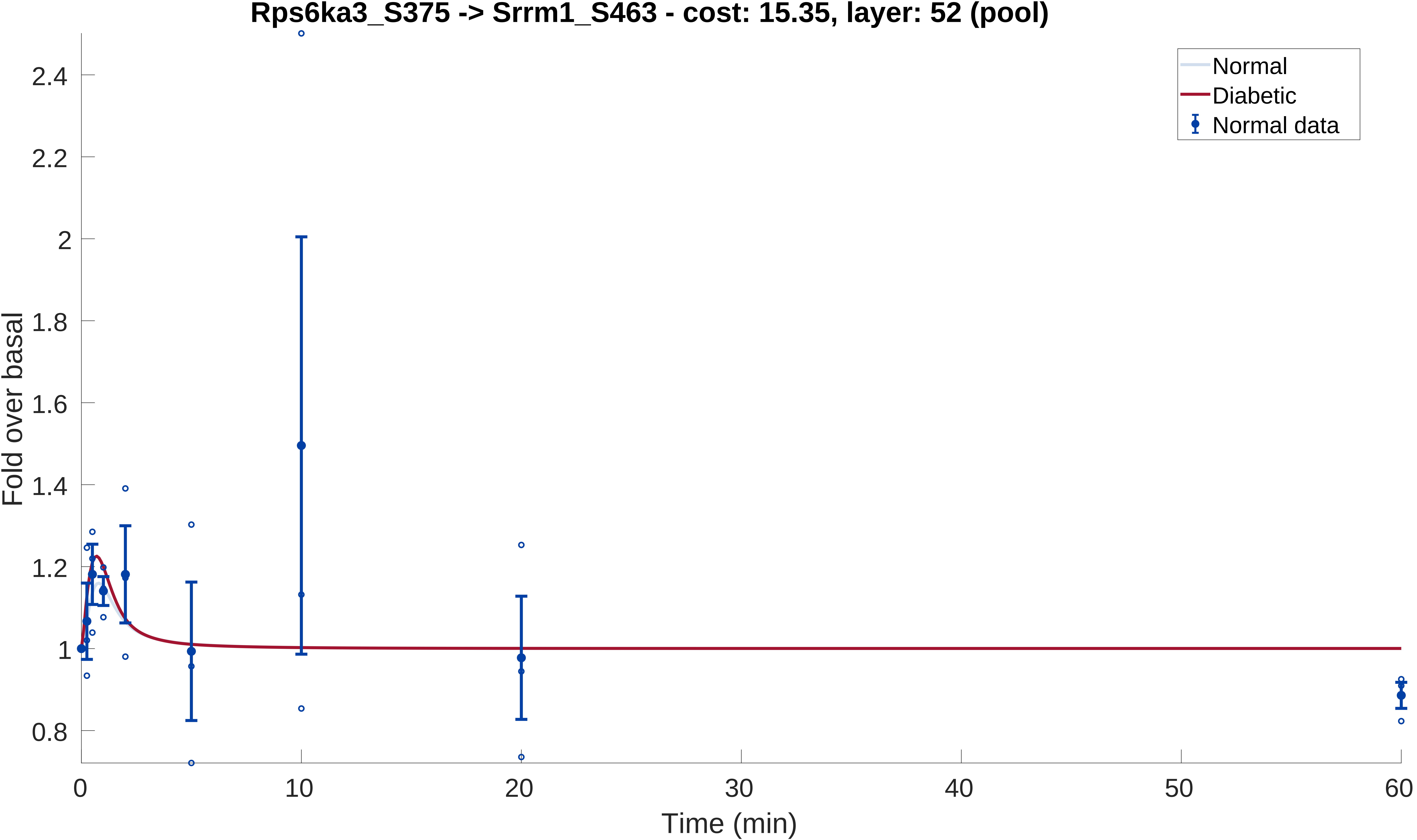
Rps6ka3_S375 -> Srrm1_S463 - cost: 15.35, layer: 52 (pool)
Normal
Diabetic
Normal data
Fold over basal
Time (min)
0.8
1
1.2
1.4
1.6
1.8
2
2.2
2.4
0
10
20
30
40
50
60

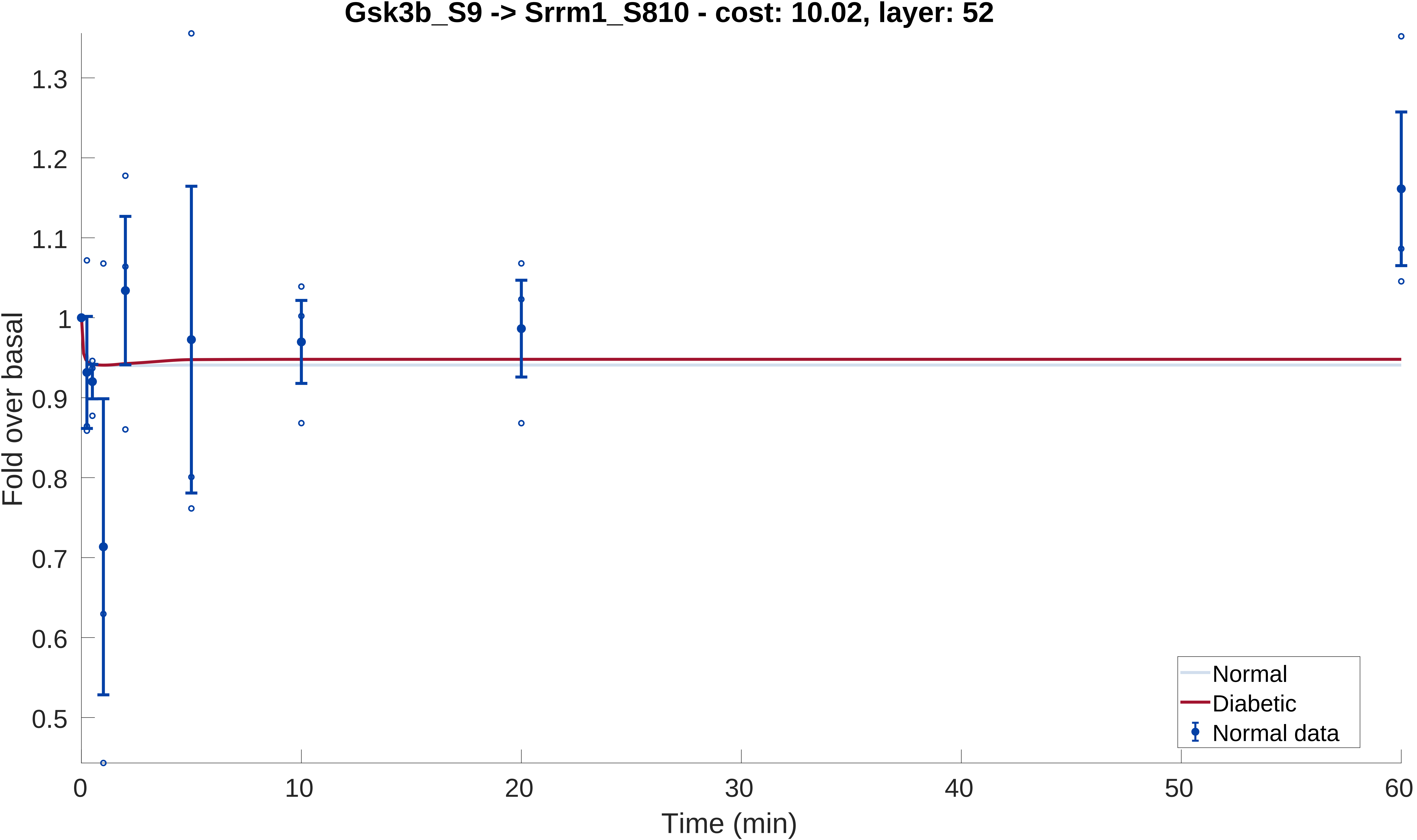

Gsk3b_S9 -> Srrm1_S810 - cost: 10.02, layer: 52
Fold over basal
Time (min)
1.3
1.2
1.1
1
0.9
0.8
0.7
0.6
0.5
0
10
20
30
40
50
60
Normal
Diabetic
Normal data

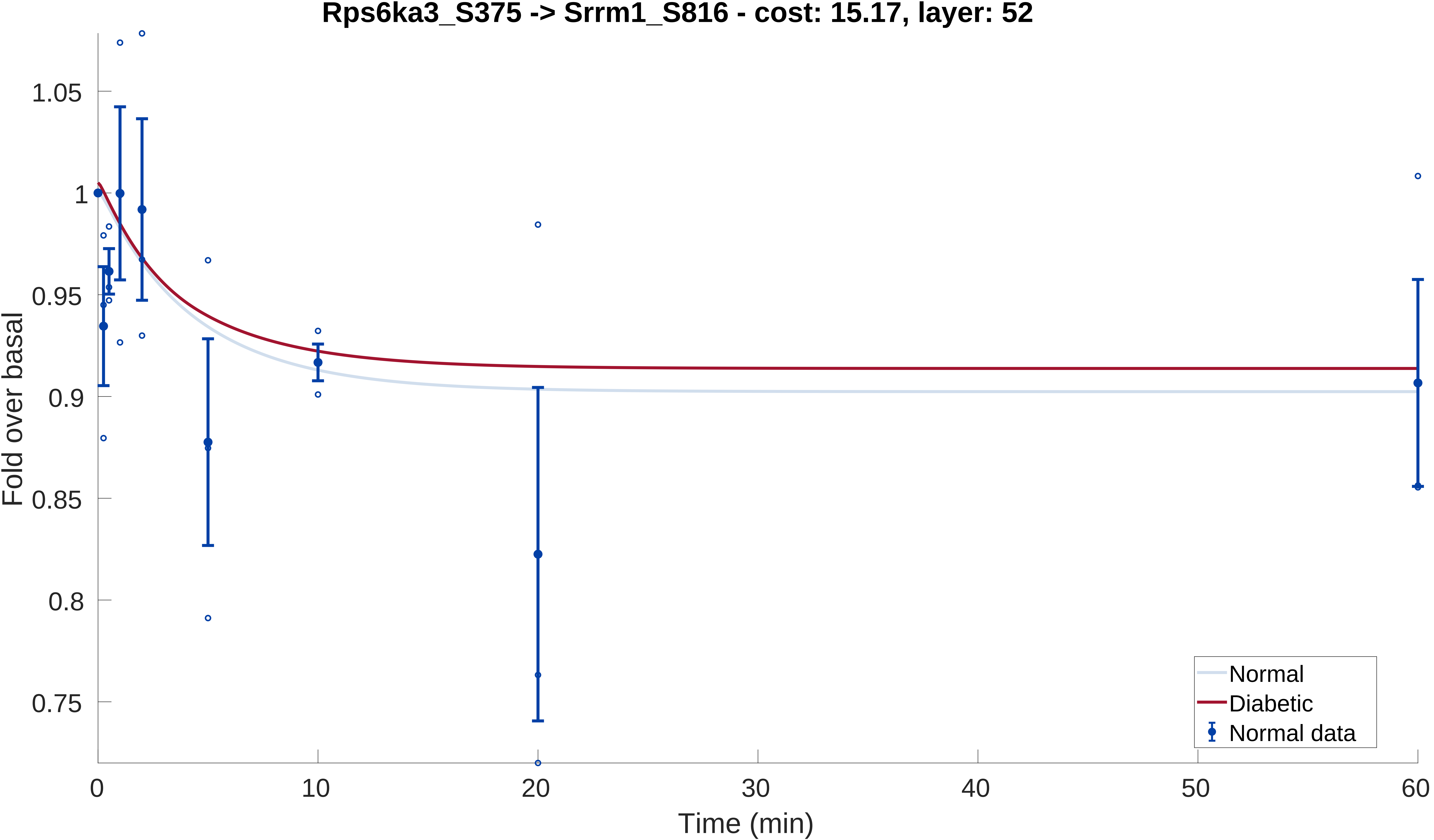

Rps6ka3_S375 -> Srrm1_S816 - cost: 15.17, layer: 52
Fold over basal
1.05
1
0.95
0.9
0.85
0.8
0.75
0
10
20
30
40
50
60
Time (min)
Normal
Diabetic
Normal data

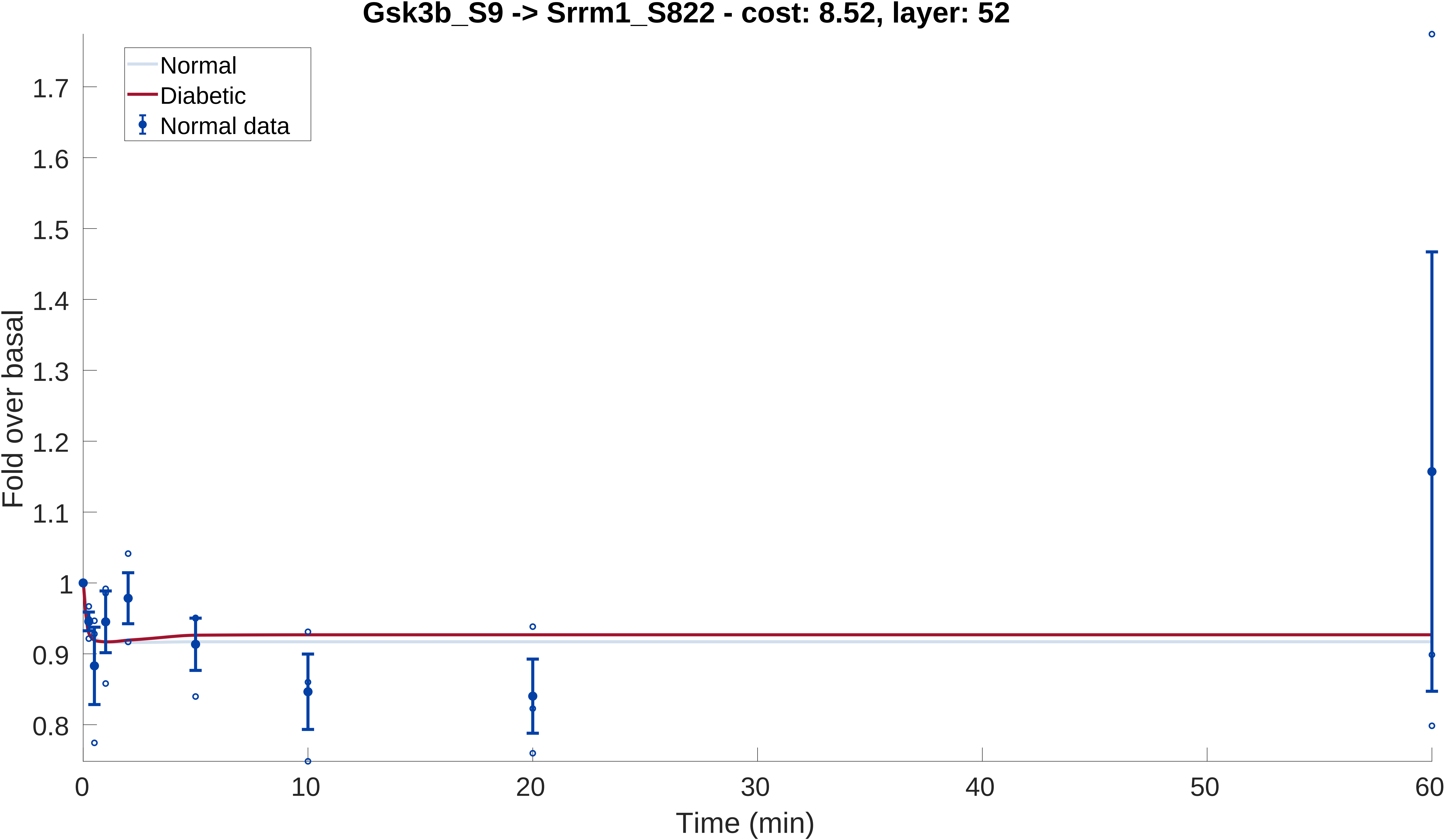

Gsk3b_S9 -> Srrm1_S822 - cost: 8.52, layer: 52
Normal
Diabetic
Normal data
Fold over basal
Time (min)
1.7
1.6
1.5
1.4
1.3
1.2
1.1
1
0.9
0.8
0
10
20
30
40
50
60

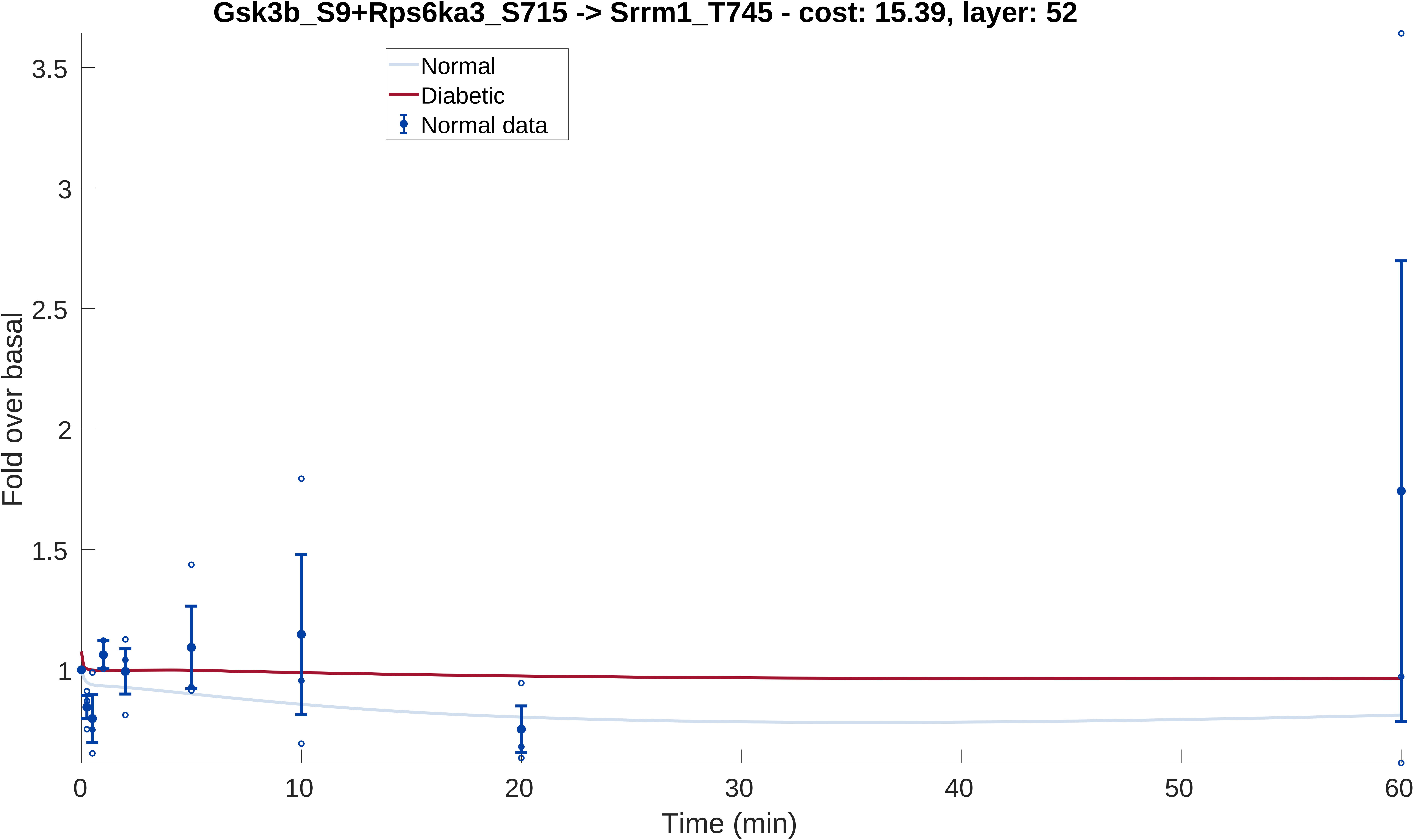
Gsk3b_S9+Rps6ka3_S715 -> Srrm1_T745 - cost: 15.39, layer: 52
Normal
Diabetic
Normal data
Fold over basal
Time (min)
3.5
3
2.5
2
1.5
1
0
10
20
30
40
50
60

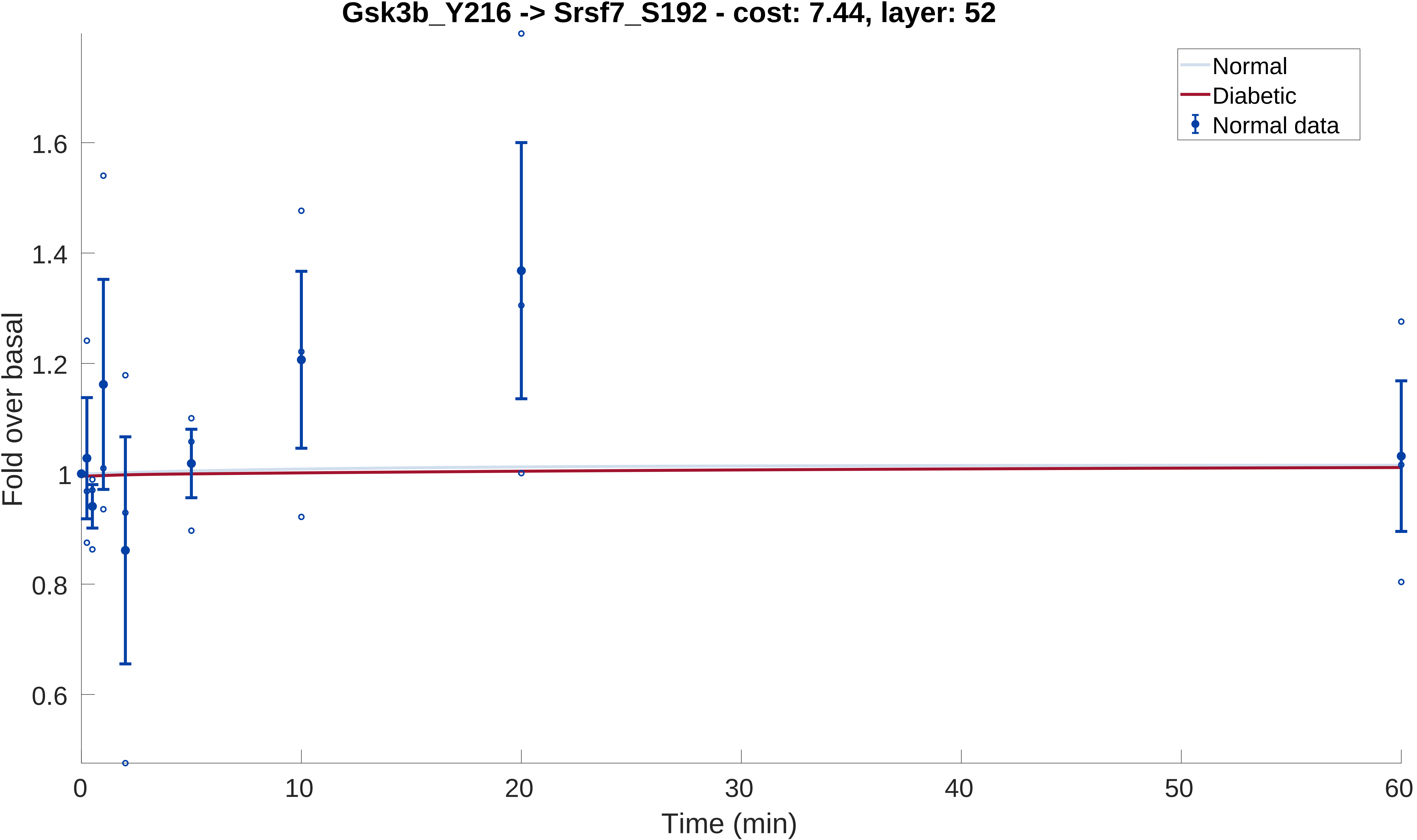

Gsk3b_Y216 -> Srsf7_S192 - cost: 7.44, layer: 52
Normal
Diabetic
Normal data
Fold over basal
1.6
1.4
1.2
1
0.8
0.6
0
10
20
30
40
50
60
Time (min)

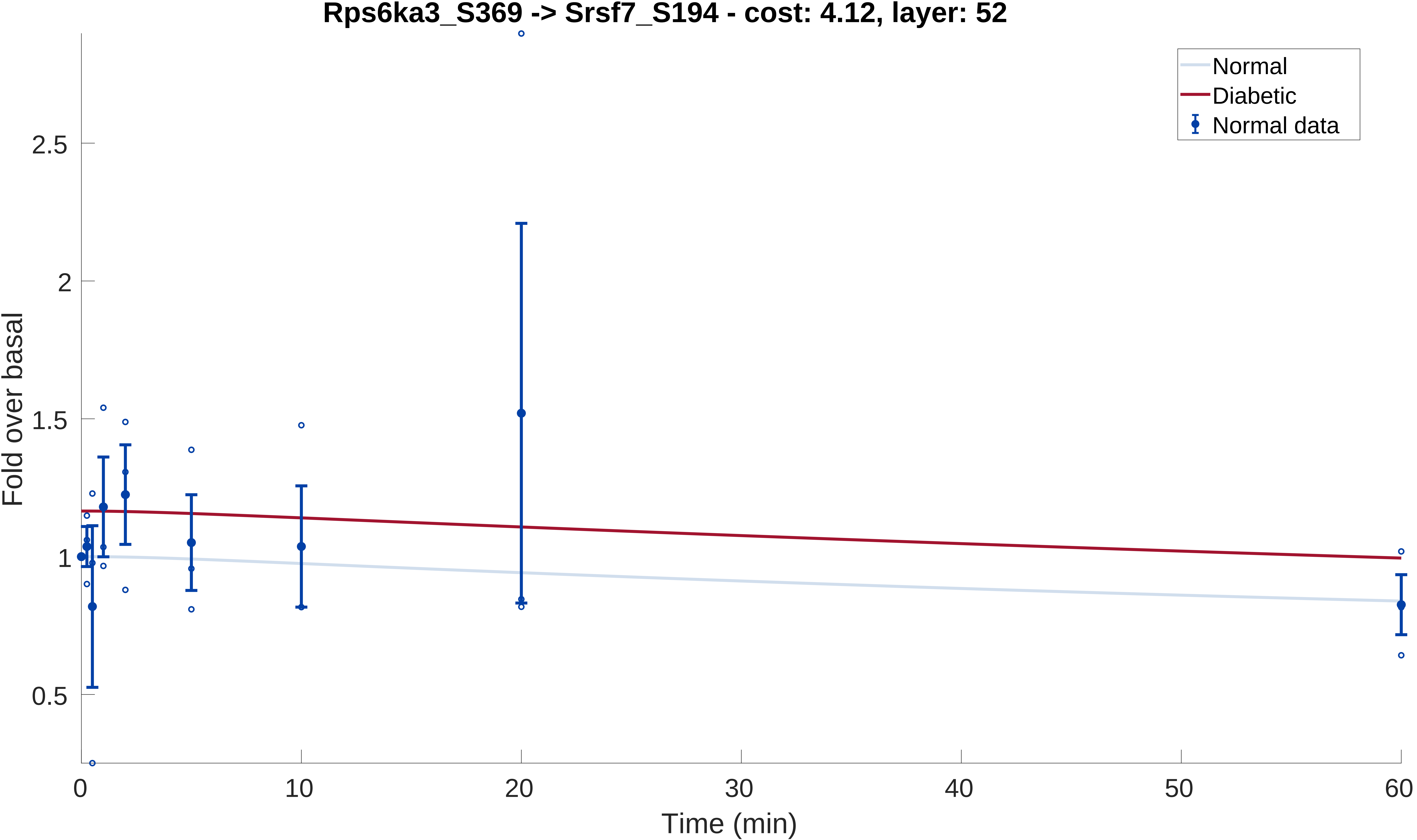

Rps6ka3_S369 -> Srsf7_S194 - cost: 4.12, layer: 52
Normal
Diabetic
Normal data
Fold over basal
2.5
2
1.5
1
0.5
0
10
20
30
40
50
60
Time (min)

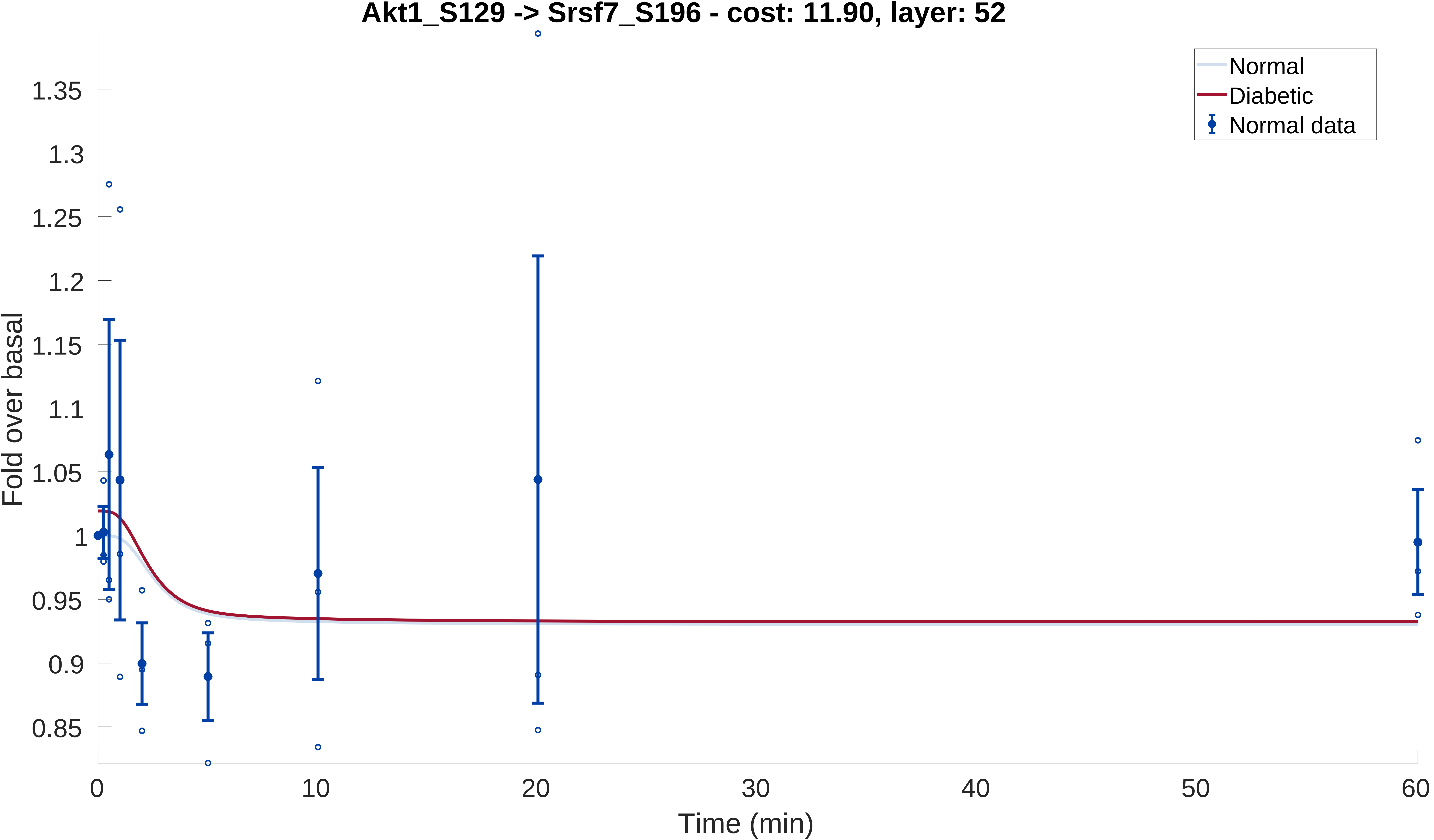

Akt1_S129 -> Srsf7_S196 - cost: 11.90, layer: 52
Normal
Diabetic
Normal data
Fold over basal
1.35
1.3
1.25
1.2
1.15
1.1
1.05
1
0.95
0.9
0.85
0
10
20
30
40
50
60
Time (min)

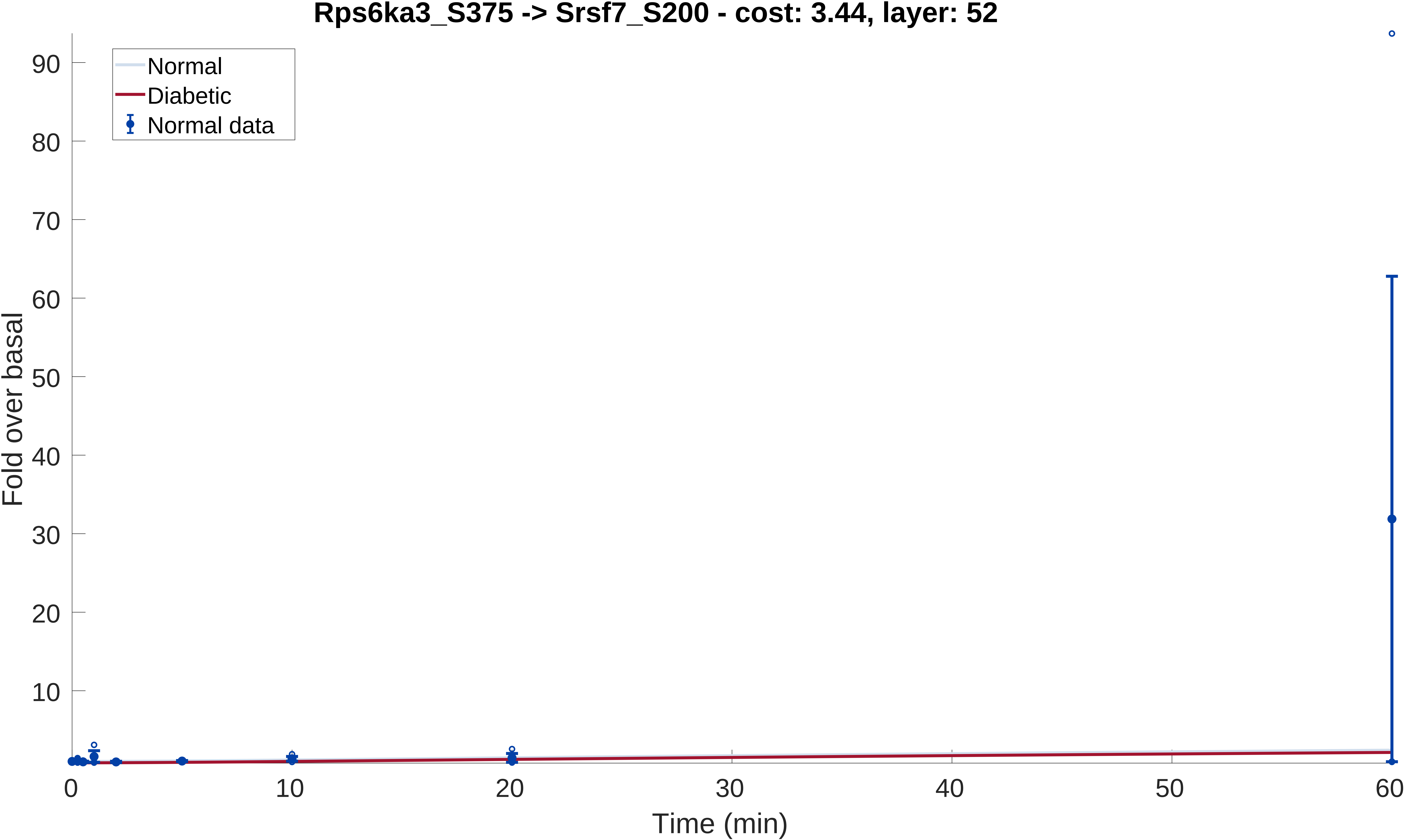

Rps6ka3_S375 -> Srsf7_S200 - cost: 3.44, layer: 52
Normal
Diabetic
Normal data
Fold over basal
Time (min)
90
80
70
60
50
40
30
20
10
0
10
20
30
40
50
60

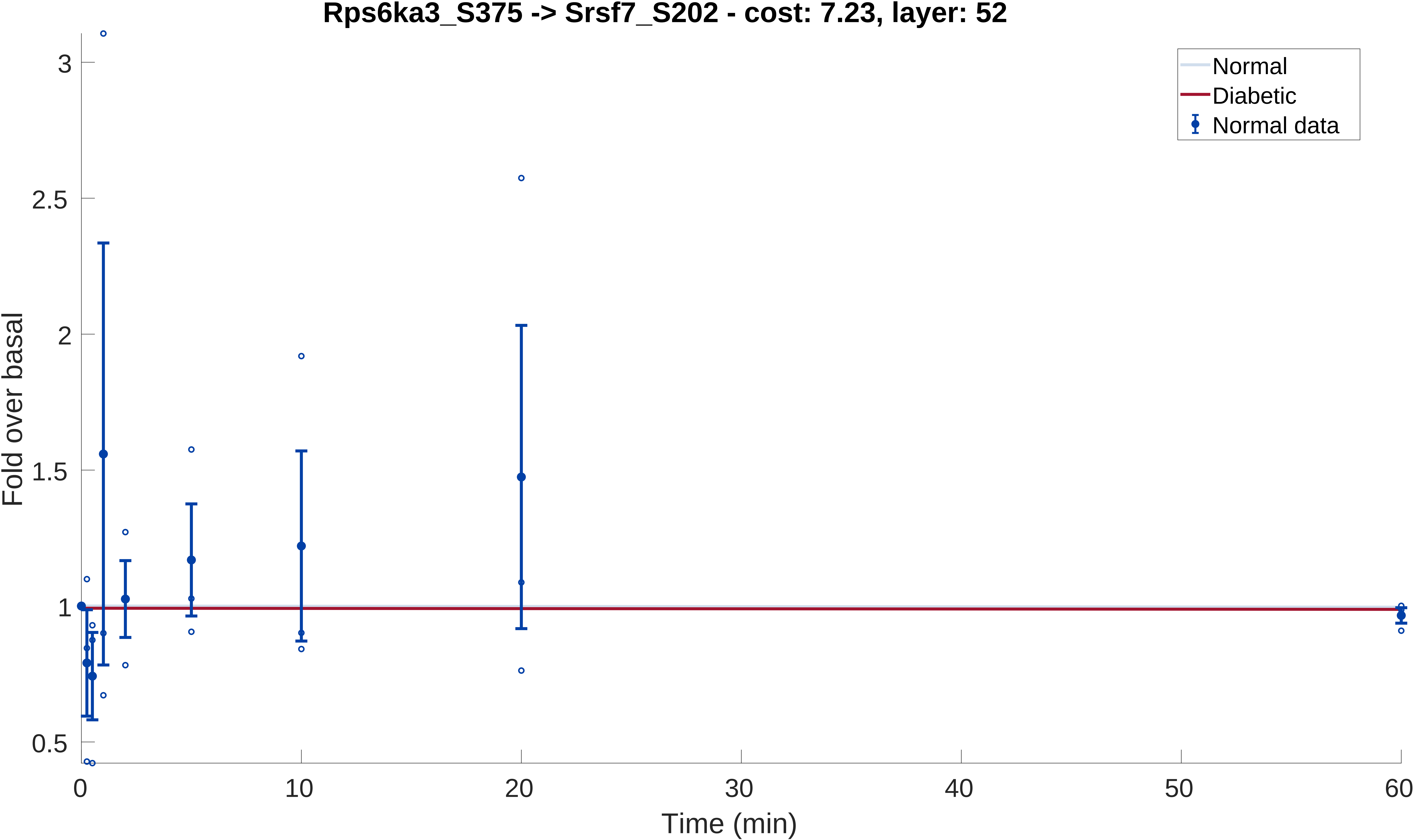
Rps6ka3_S375 -> Srsf7_S202 - cost: 7.23, layer: 52
Normal
Diabetic
Normal data
Fold over basal
3
2.5
2
1.5
1
0.5
0
10
20
30
40
50
60
Time (min)

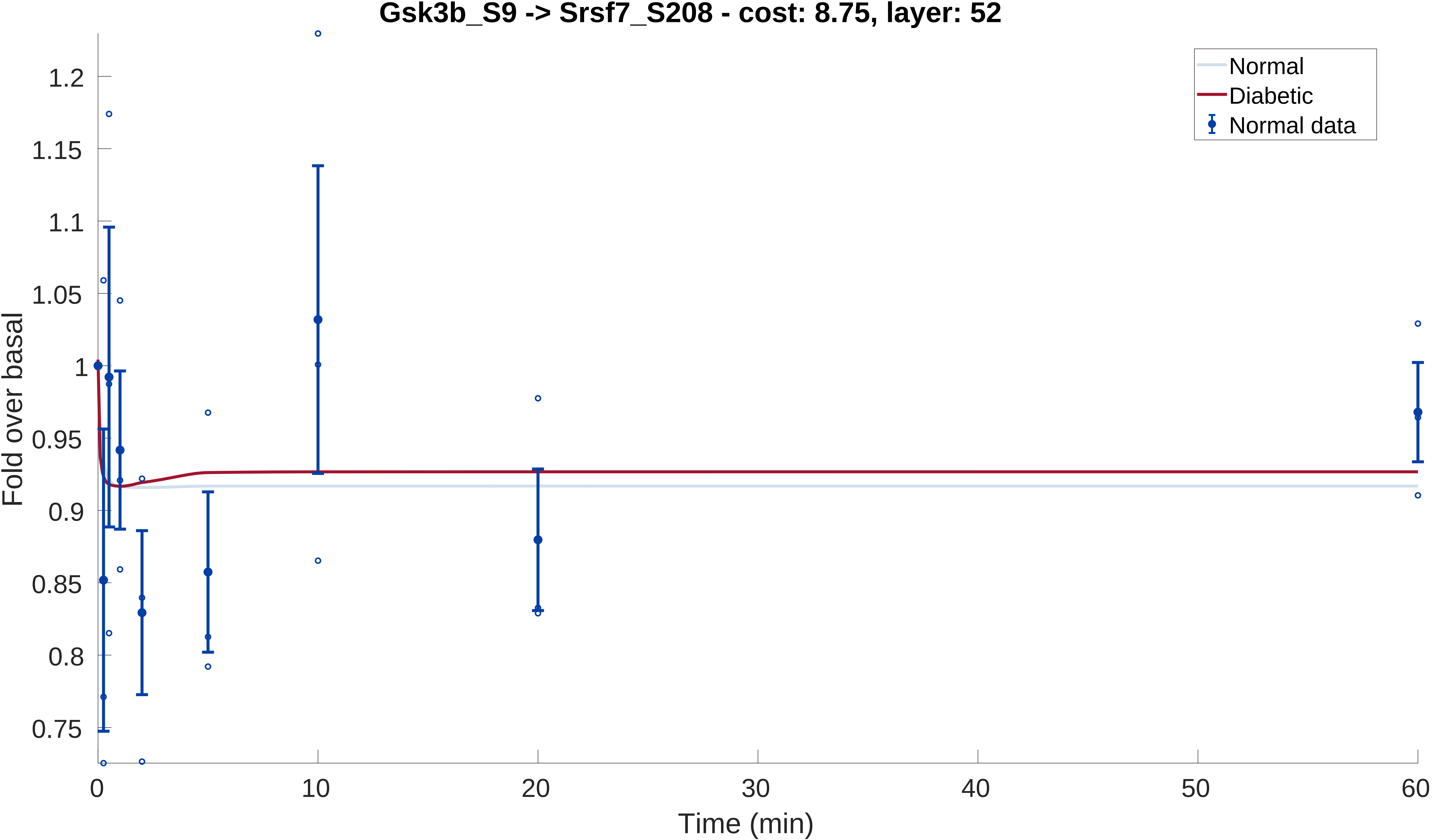

Gsk3b_S9 -> Srsf7_S208 - cost: 8.75, layer: 52
Normal
Diabetic
Normal data
Fold over basal
1.2
1.15
1.1
1.05
1
0.95
0.9
0.85
0.8
0.75
0
10
20
30
40
50
60
Time (min)

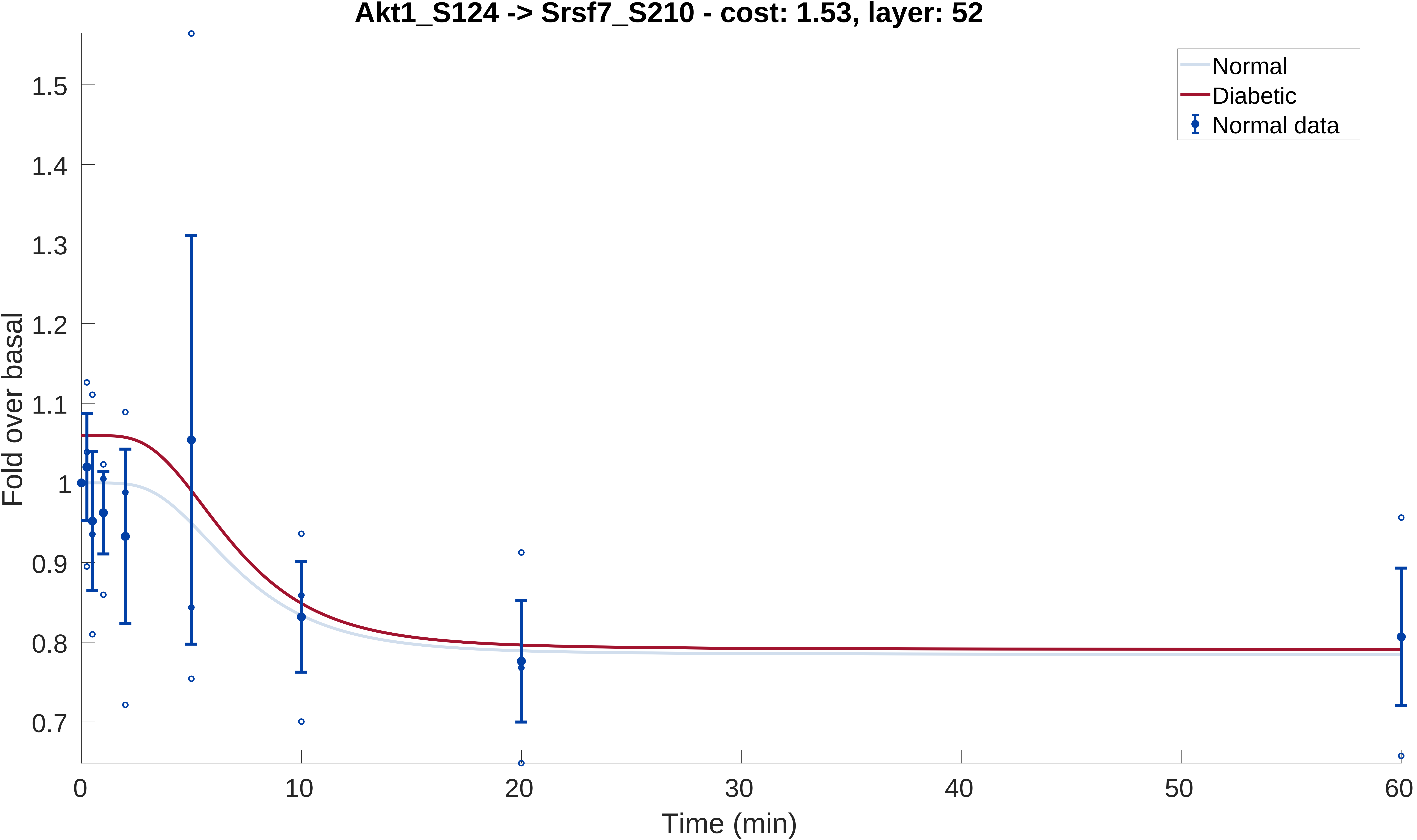
Akt1_S124 -> Srsf7_S210 - cost: 1.53, layer: 52
Normal
Diabetic
Normal data
Fold over basal
1.5
1.4
1.3
1.2
1.1
1
0.9
0.8
0.7
0
10
20
30
40
50
60
Time (min)

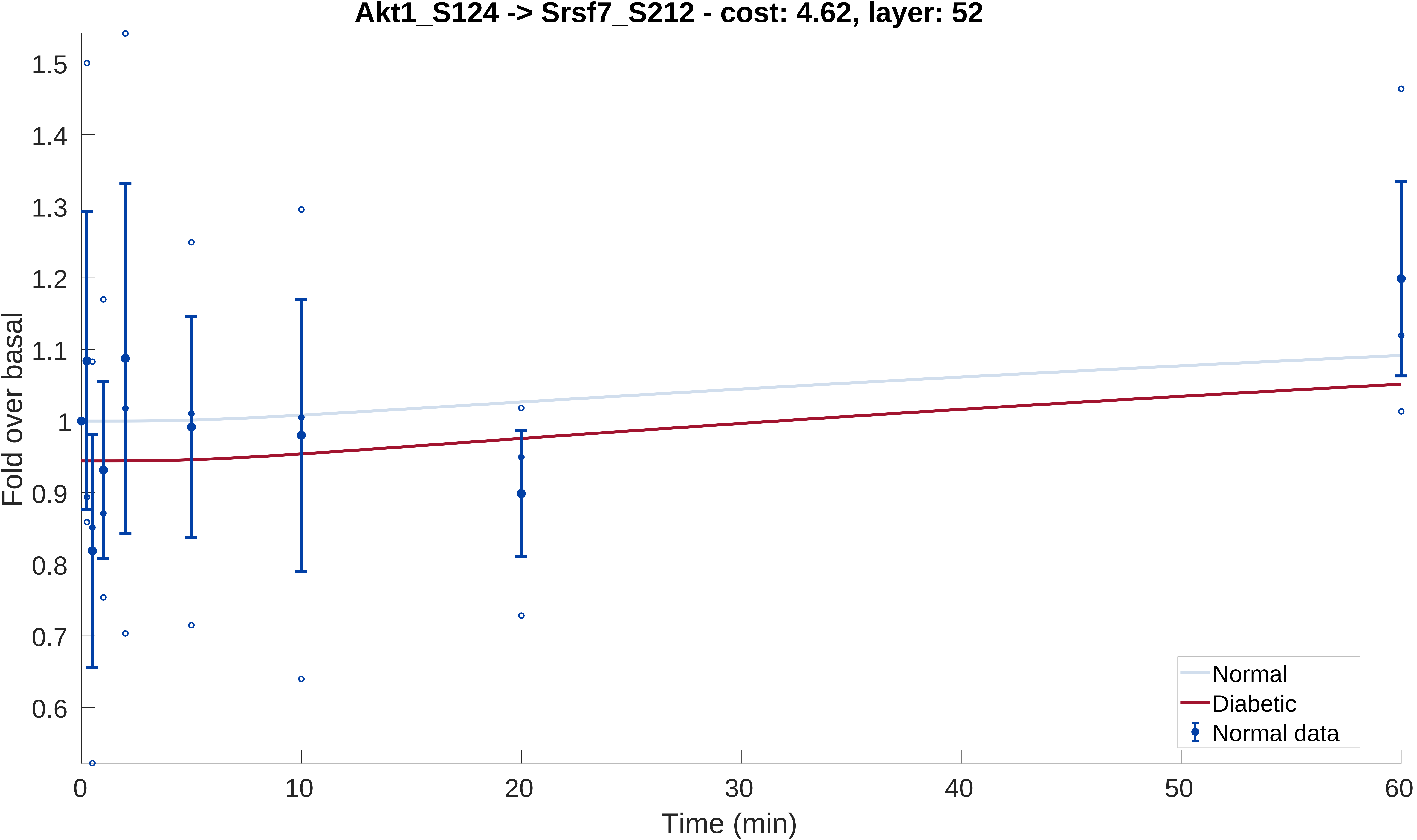

Akt1_S124 -> Srsf7_S212 - cost: 4.62, layer: 52
Fold over basal
1.5
1.4
1.3
1.2
1.1
1
0.9
0.8
0.7
0.6
0
10
20
30
40
50
60
Time (min)
Normal
Diabetic
Normal data

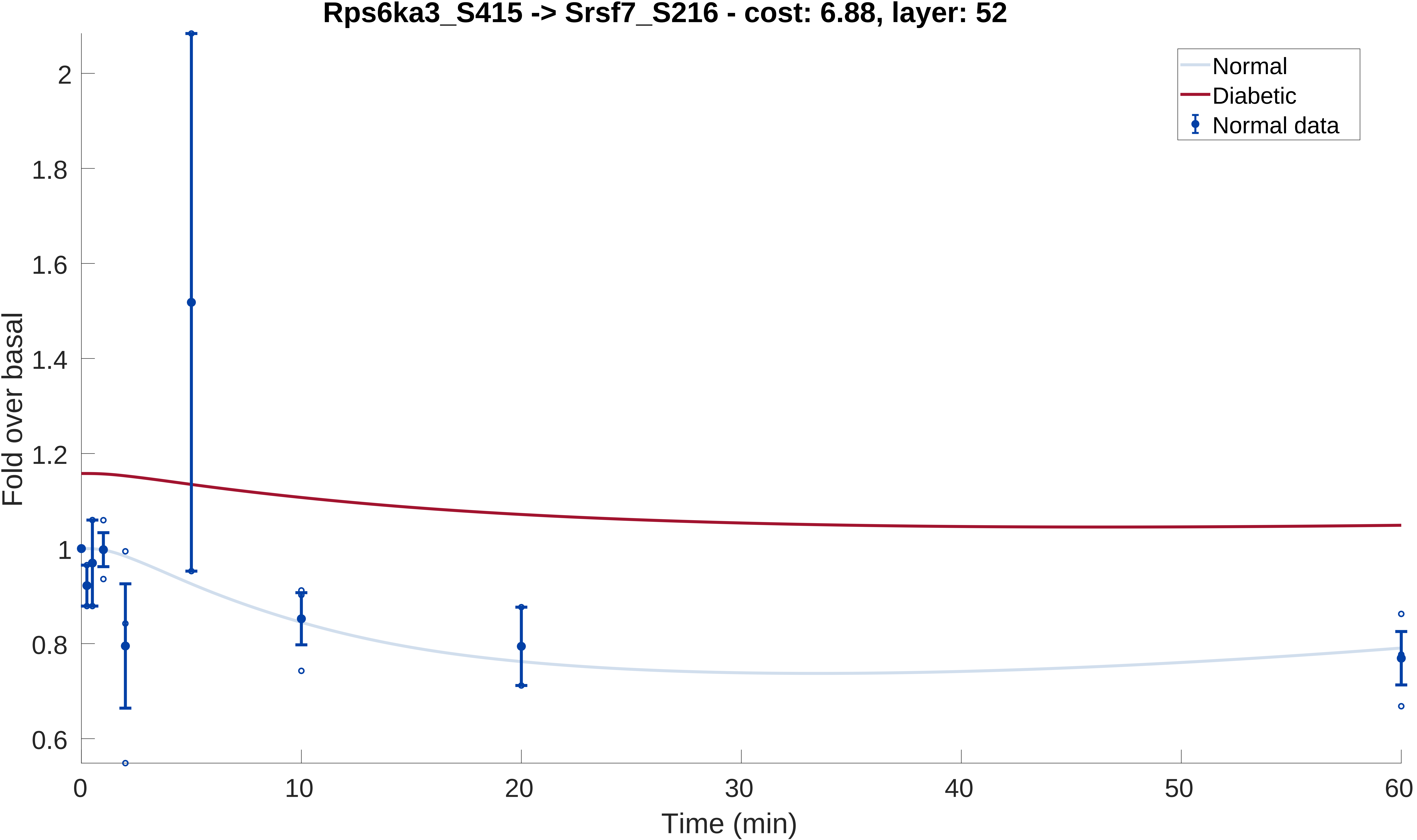
Rps6ka3_S415 -> Srsf7_S216 - cost: 6.88, layer: 52
Fold over basal
Time (min)
Normal
Diabetic
Normal data
0.6
0.8
1
1.2
1.4
1.6
1.8
2
0
10
20
30
40
50
60

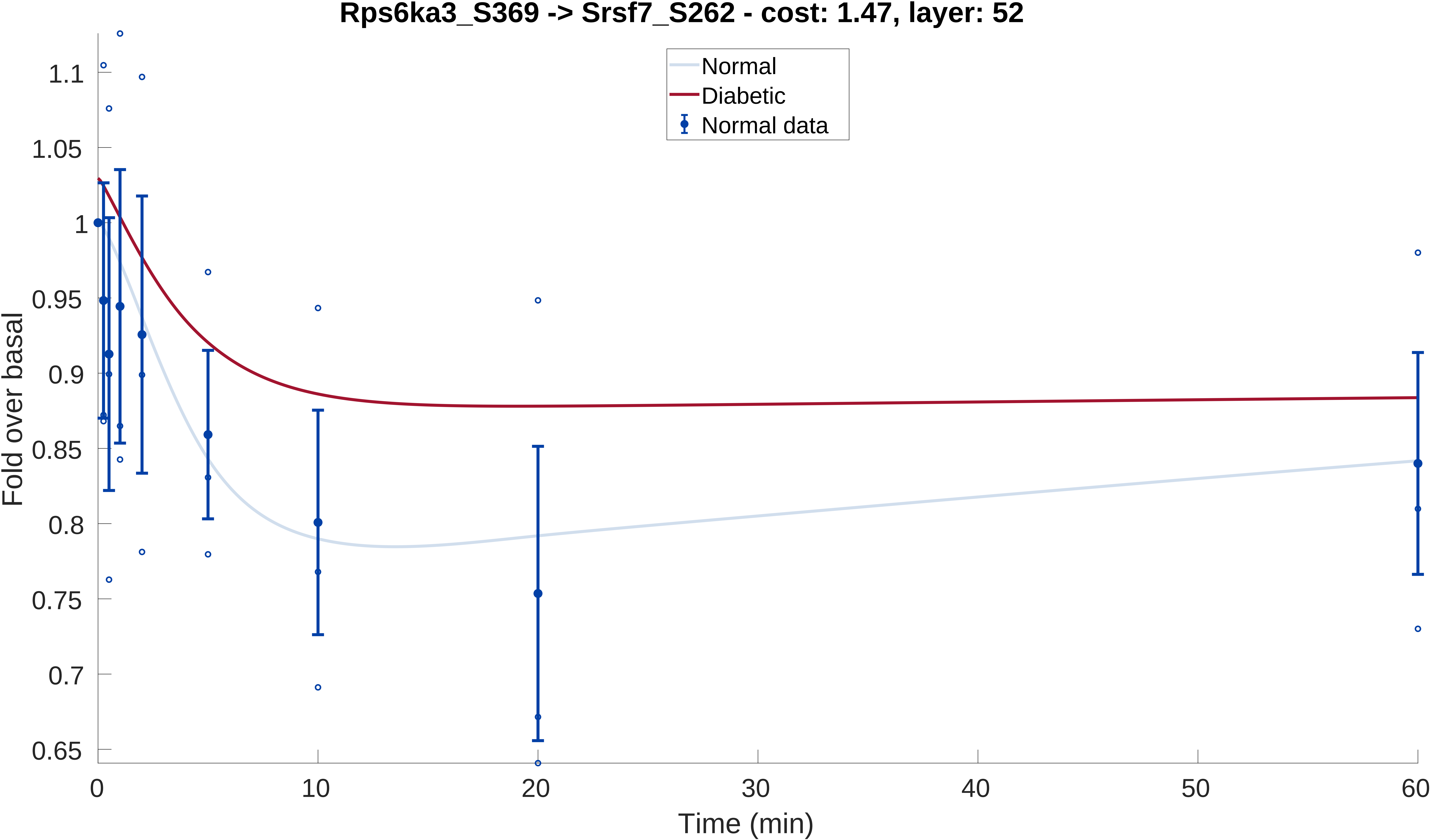

Rps6ka3_S369 -> Srsf7_S262 - cost: 1.47, layer: 52
Normal
Diabetic
Normal data
Fold over basal
Time (min)
1.1
1.05
1
0.95
0.9
0.85
0.8
0.75
0.7
0.65
0
10
20
30
40
50
60

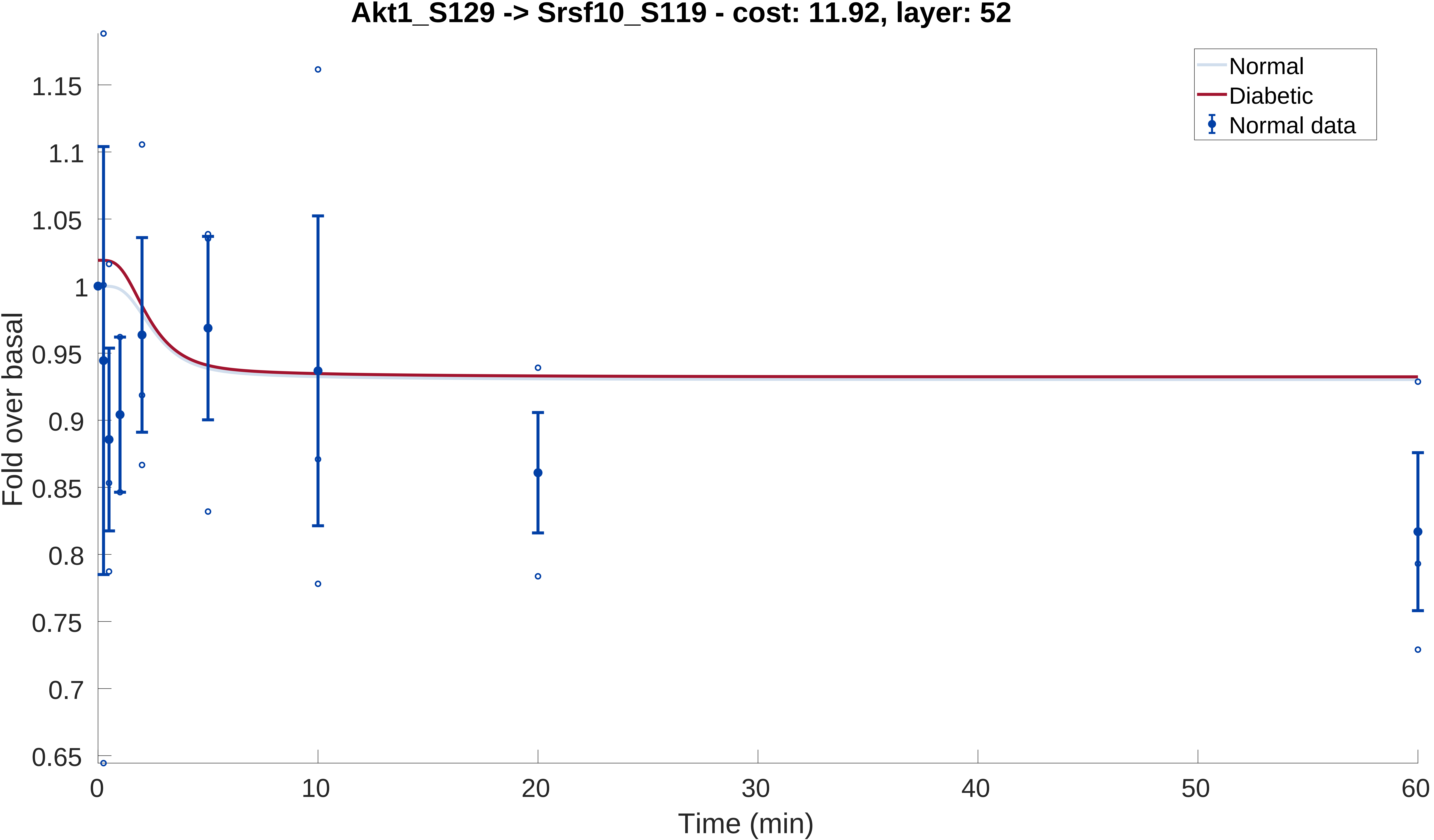
Akt1_S129 -> Srsf10_S119 - cost: 11.92, layer: 52
Normal
Diabetic
Normal data
Fold over basal
Time (min)
0.65
0.7
0.75
0.8
0.85
0.9
0.95
1
1.05
1.1
1.15
0
10
20
30
40
50
60

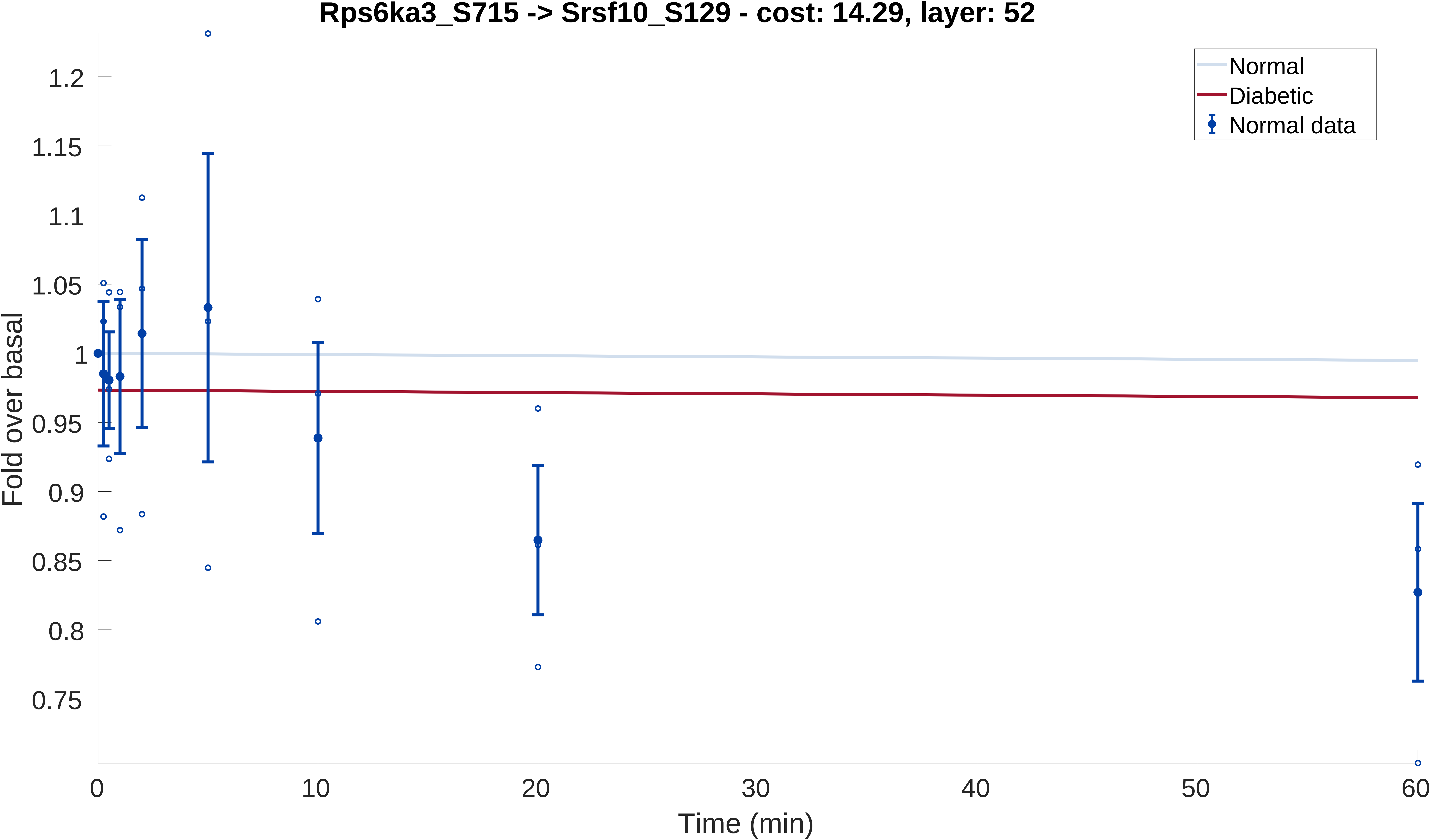

Rps6ka3_S715 -> Srsf10_S129 - cost: 14.29, layer: 52
Normal
Diabetic
Normal data
Fold over basal
Time (min)
1.2
1.15
1.1
1.05
1
0.95
0.9
0.85
0.8
0.75
0
10
20
30
40
50
60

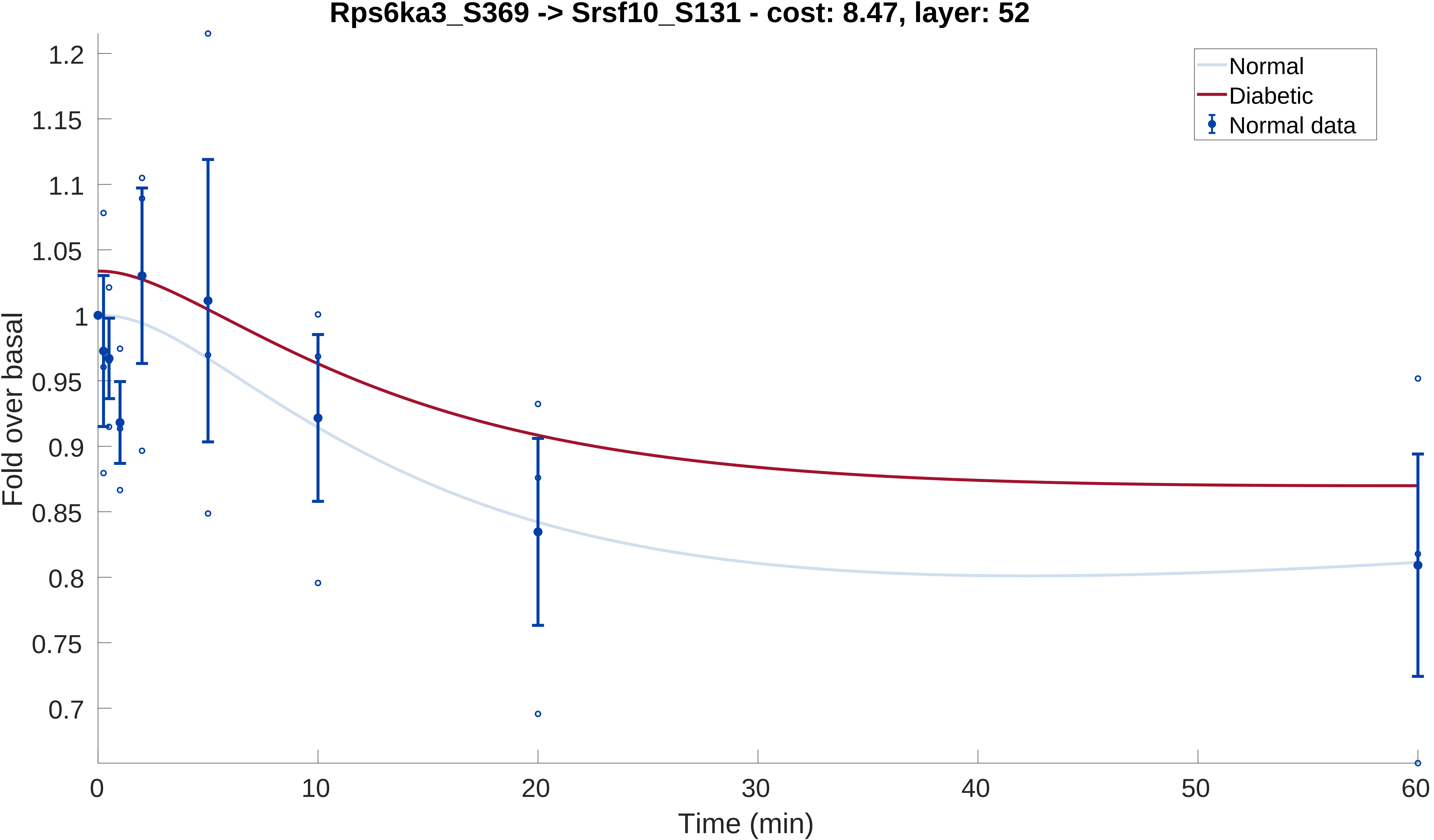
Rps6ka3_S369 -> Srsf10_S131 - cost: 8.47, layer: 52
Fold over basal
1.2
1.15
1.1
1.05
1
0.95
0.9
0.85
0.8
0.75
0.7
0
10
20
30
40
50
60
Time (min)
Normal
Diabetic
Normal data

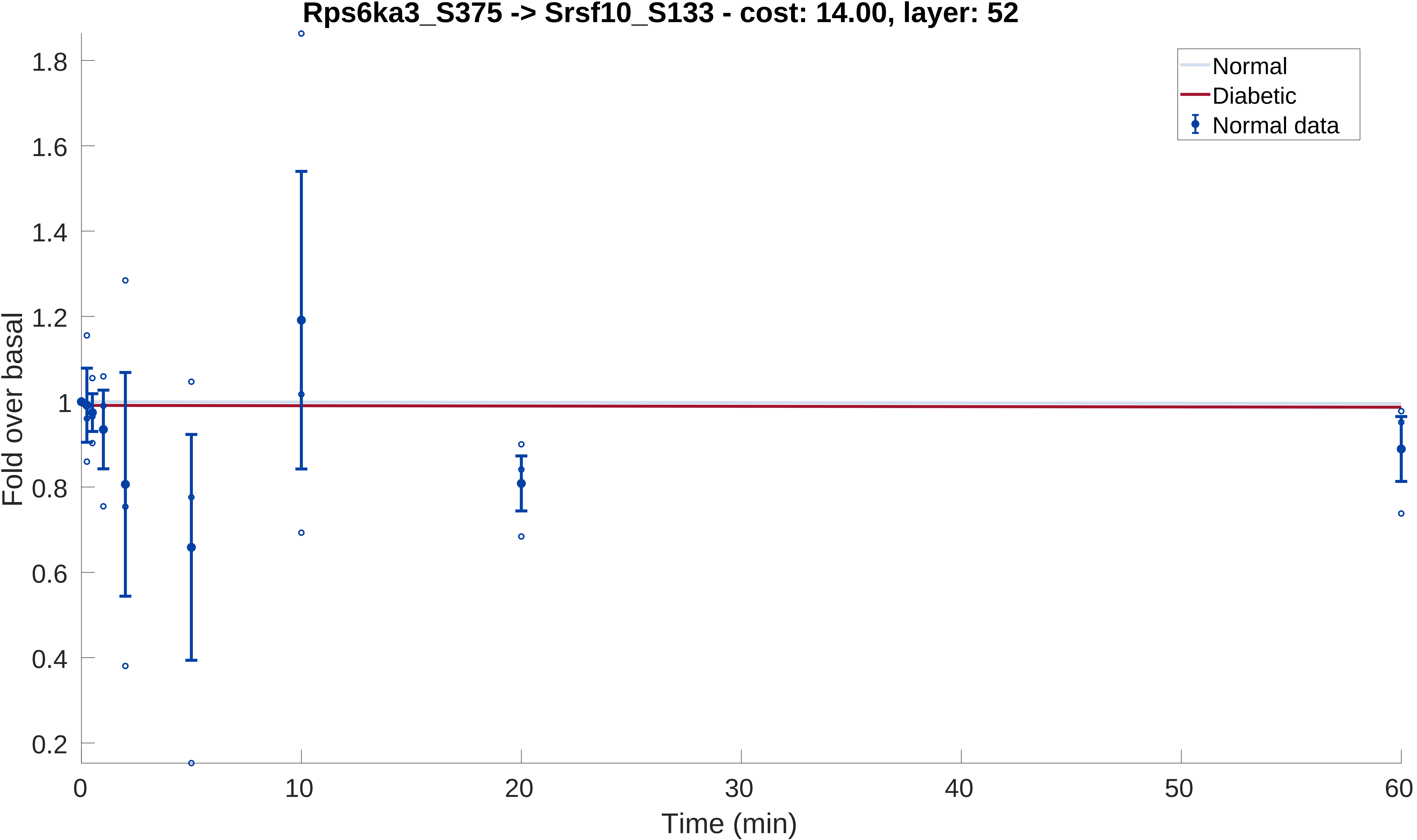
Rps6ka3_S375 -> Srsf10_S133 - cost: 14.00, layer: 52
Normal
Diabetic
Normal data
Fold over basal
Time (min)
0.2
0.4
0.6
0.8
1
1.2
1.4
1.6
1.8
0
10
20
30
40
50
60

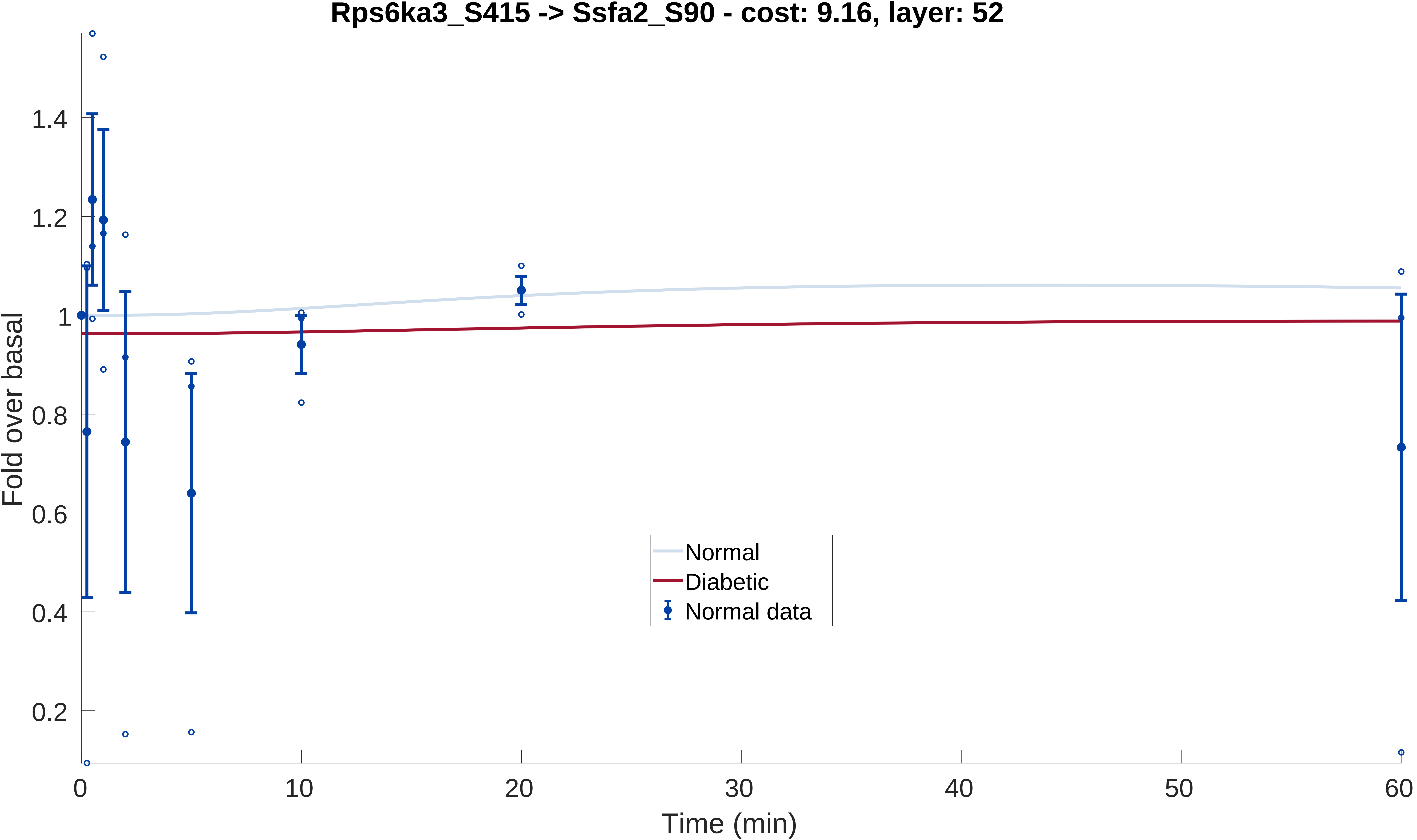

Rps6ka3_S415 -> Ssfa2_S90 - cost: 9.16, layer: 52
Fold over basal
Time (min)
Normal
Diabetic
Normal data
0.2
0.4
0.6
0.8
1
1.2
1.4
0
10
20
30
40
50
60

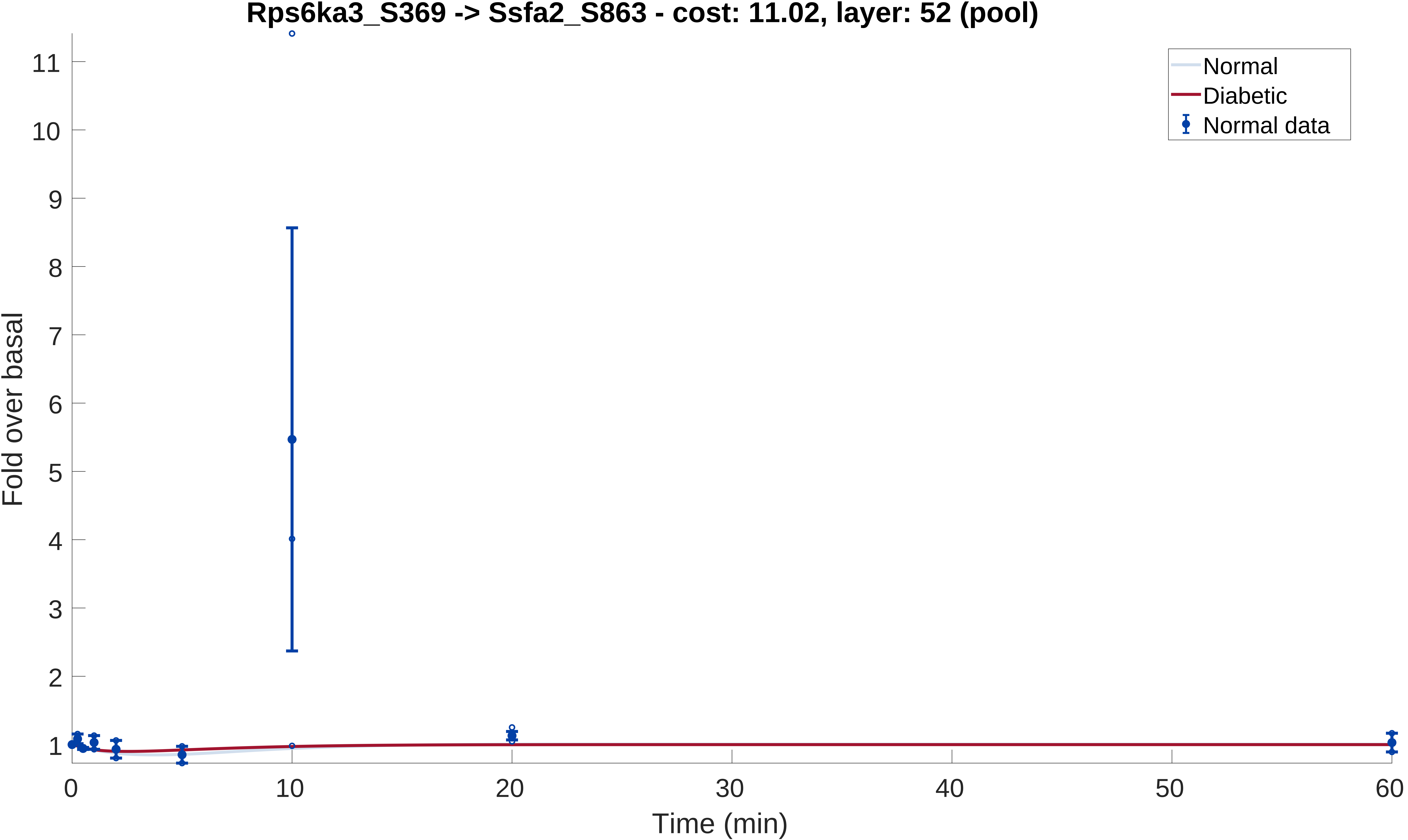
Rps6ka3_S369 -> Ssfa2_S863 - cost: 11.02, layer: 52 (pool)
Normal
Diabetic
Normal data
Fold over basal
Time (min)
0
10
20
30
40
50
60
1
2
3
4
5
6
7
8
9
10
11

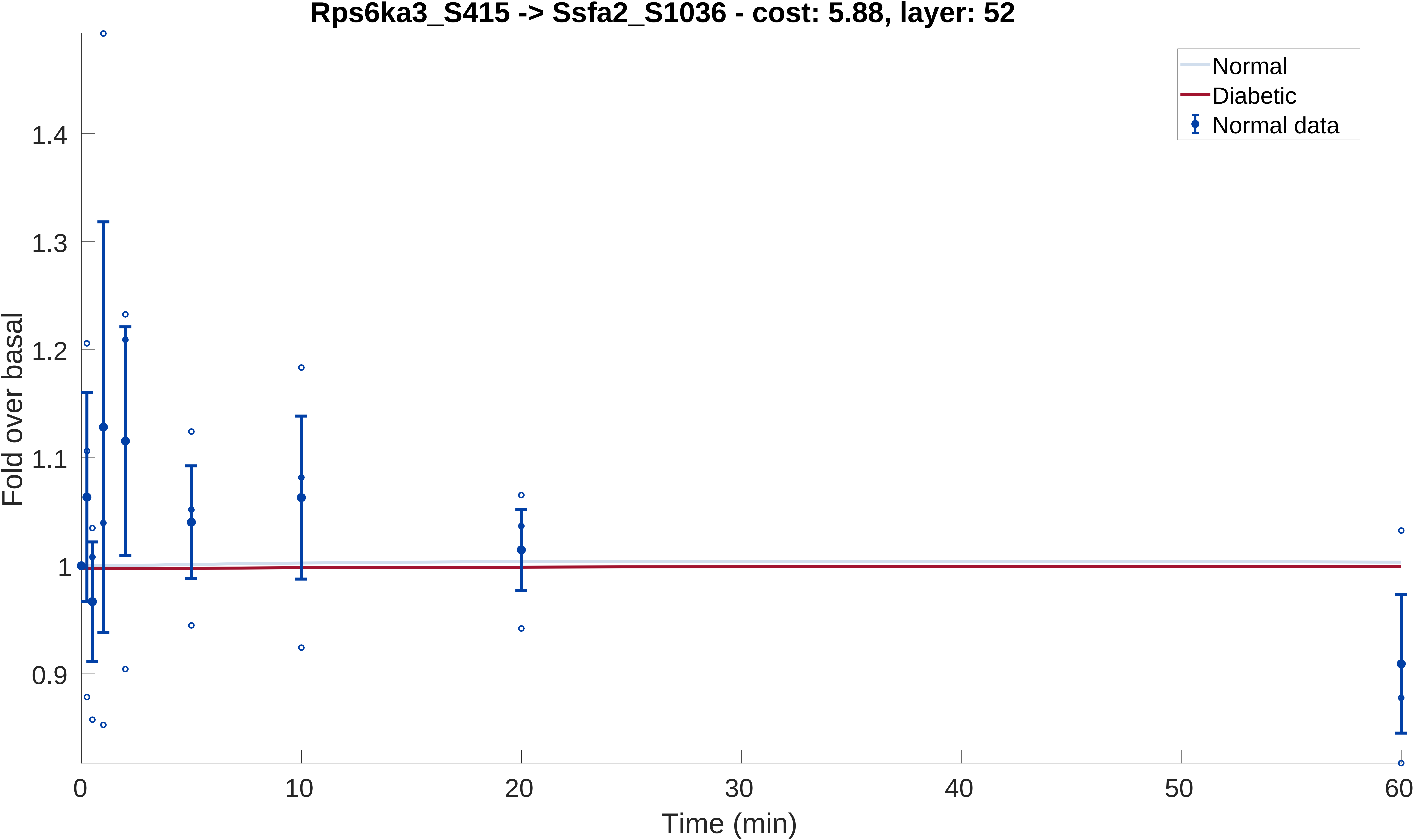

Rps6ka3_S415 -> Ssfa2_S1036 - cost: 5.88, layer: 52
Normal
Diabetic
Normal data
Fold over basal
1.4
1.3
1.2
1.1
1
0.9
0
10
20
30
40
50
60
Time (min)

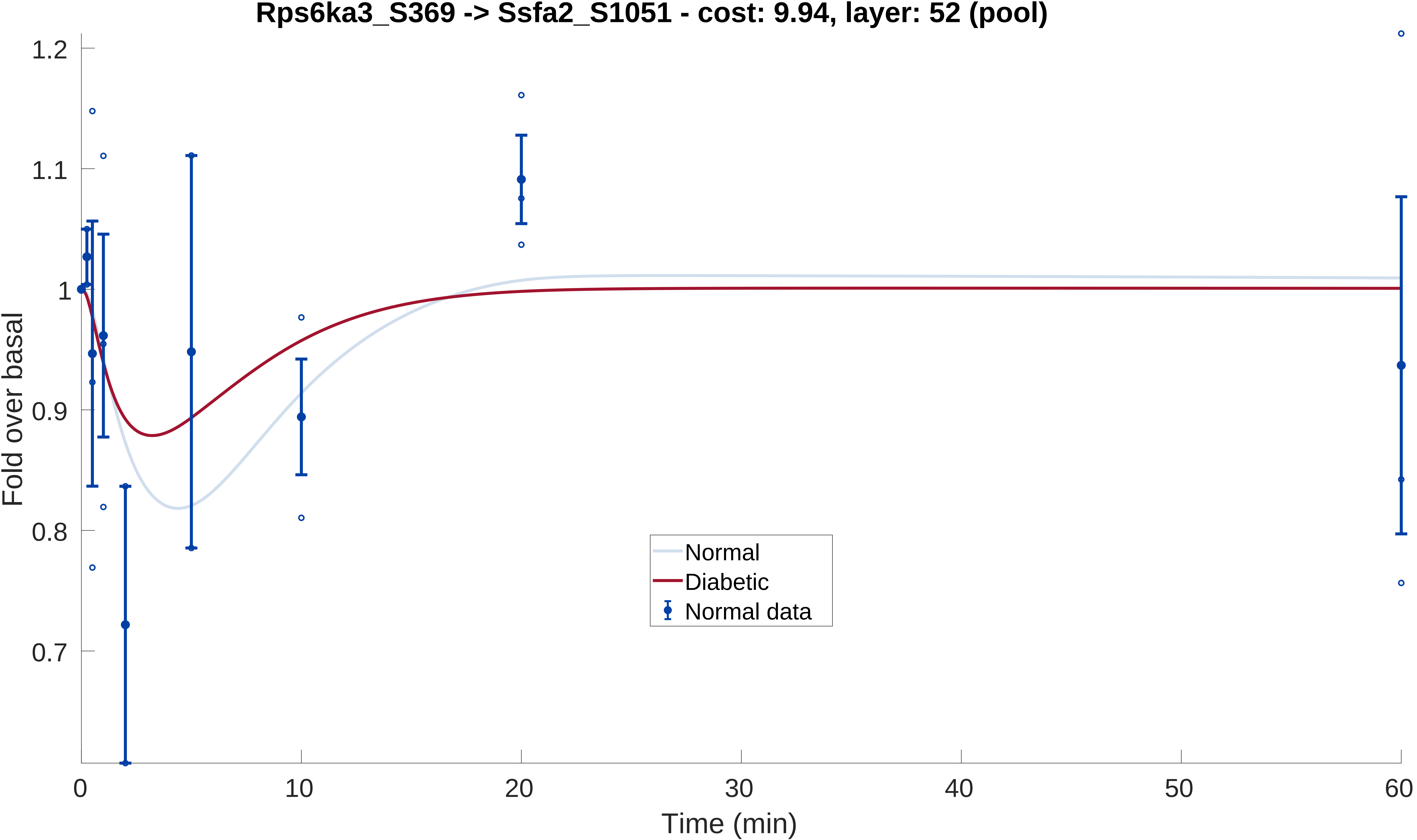
Rps6ka3_S369 -> Ssfa2_S1051 - cost: 9.94, layer: 52 (pool)
Fold over basal
Time (min)
1.2
1.1
1
0.9
0.8
0.7
0
10
20
30
40
50
60
Normal
Diabetic
Normal data

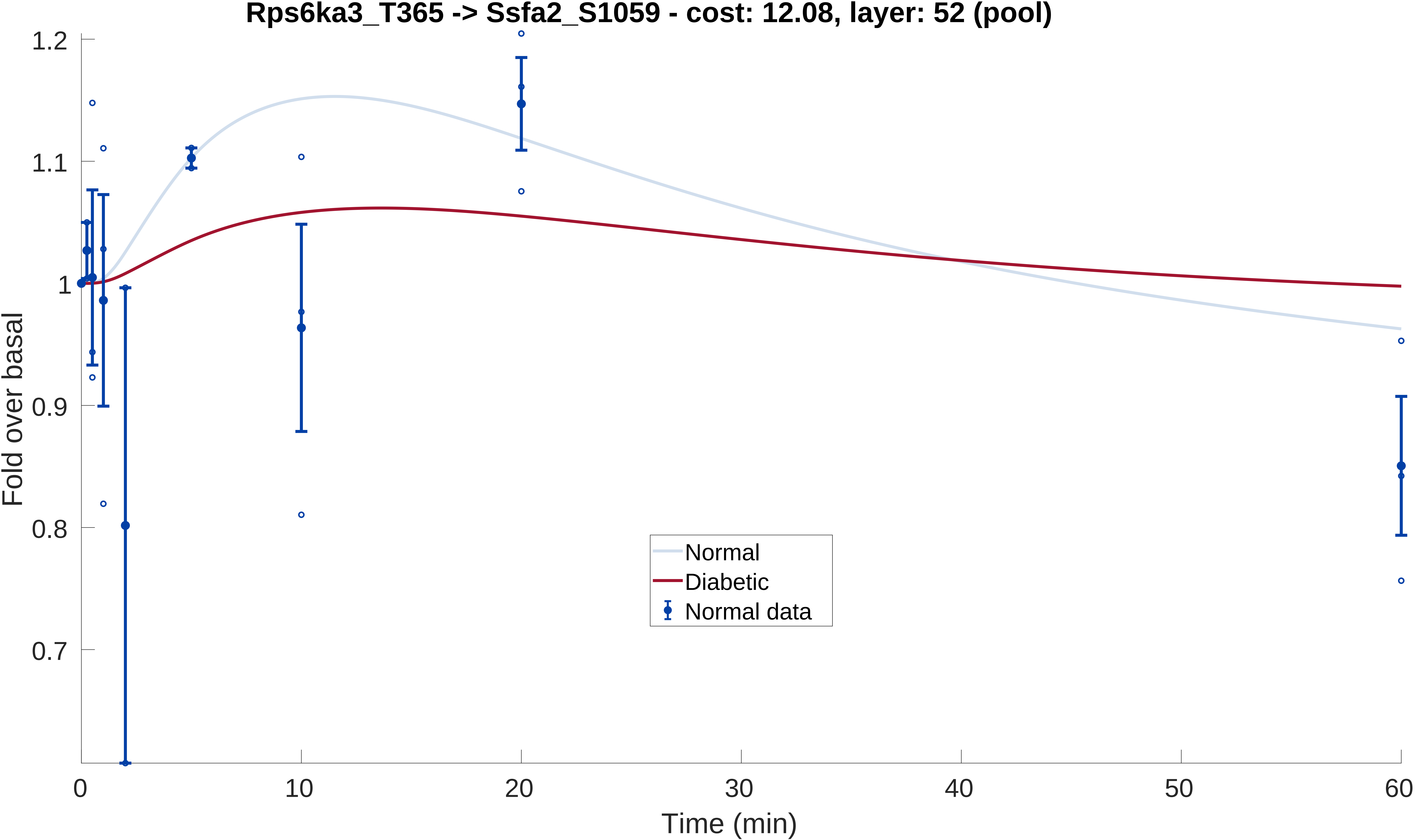

Rps6ka3_T365 -> Ssfa2_S1059 - cost: 12.08, layer: 52 (pool)
Fold over basal
1.2
1.1
1
0.9
0.8
0.7
0
10
20
30
40
50
60
Time (min)
Normal
Diabetic
Normal data

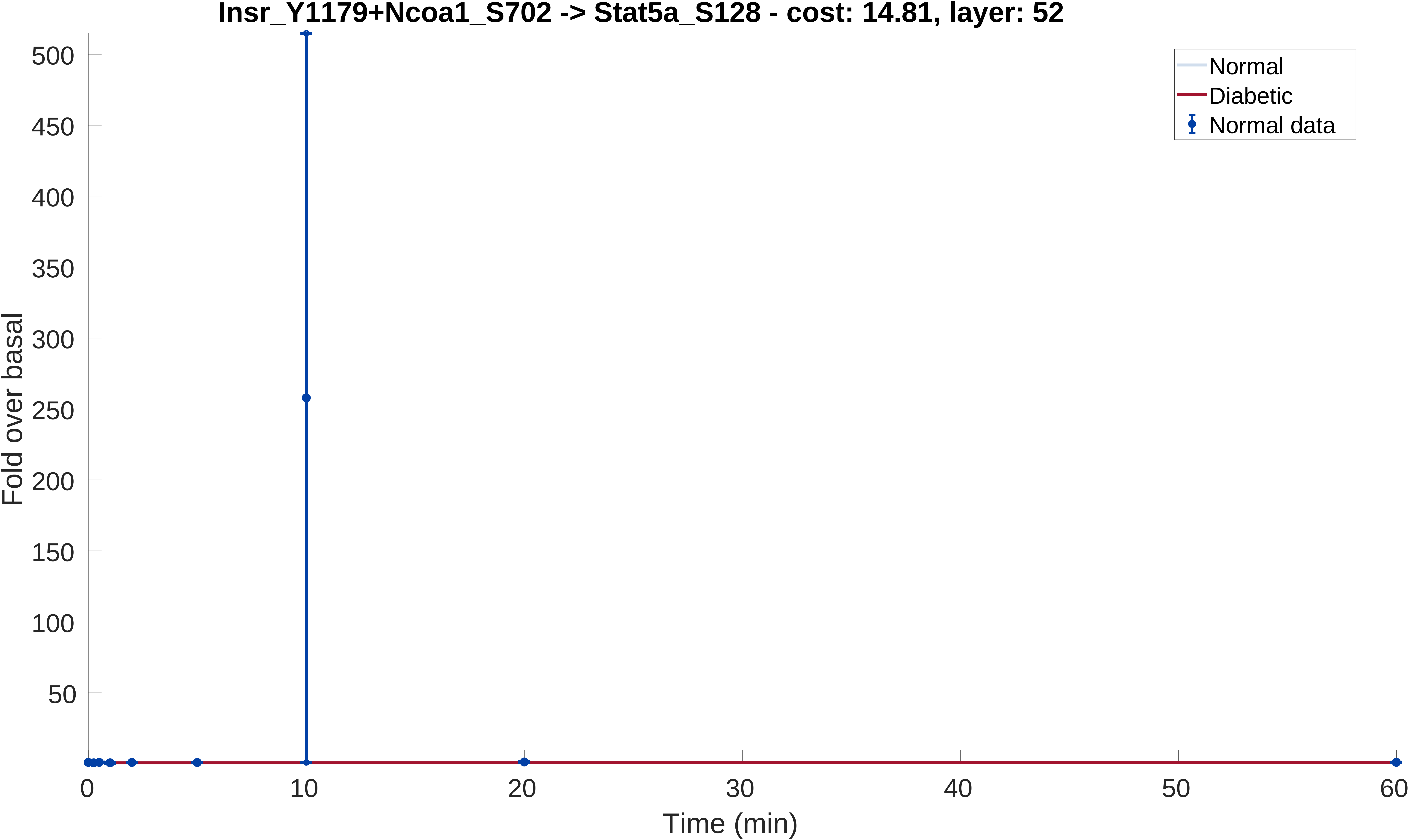
Insr_Y1179+Ncoa1_S702 -> Stat5a_S128 - cost: 14.81, layer: 52
Normal
Diabetic
Normal data
Fold over basal
Time (min)
0
10
20
30
40
50
60
50
100
150
200
250
300
350
400
450
500

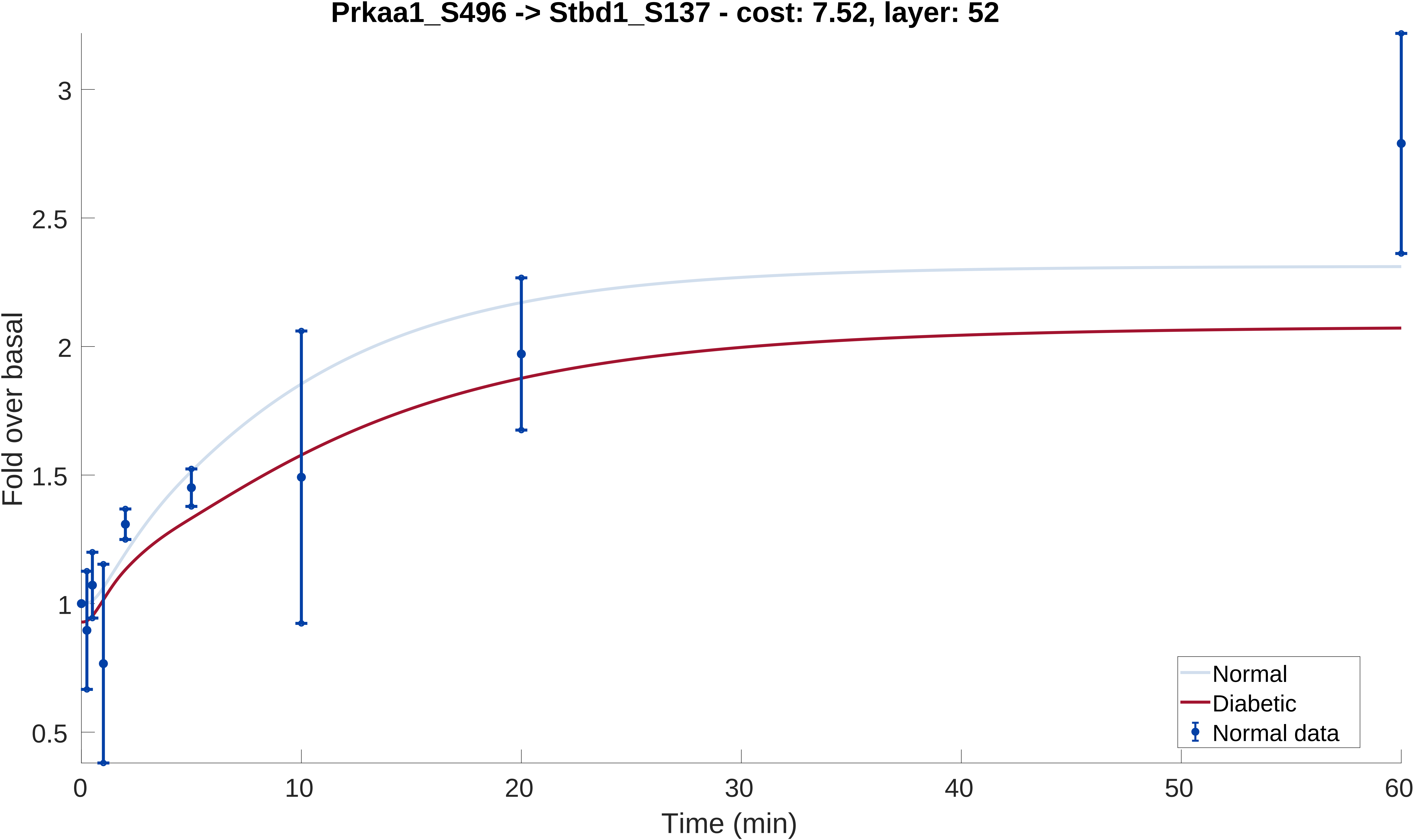
Prkaa1_S496 -> Stbd1_S137 - cost: 7.52, layer: 52
Fold over basal
Time (min)
0
10
20
30
40
50
60
0.5
1
1.5
2
2.5
3
Normal
Diabetic
Normal data

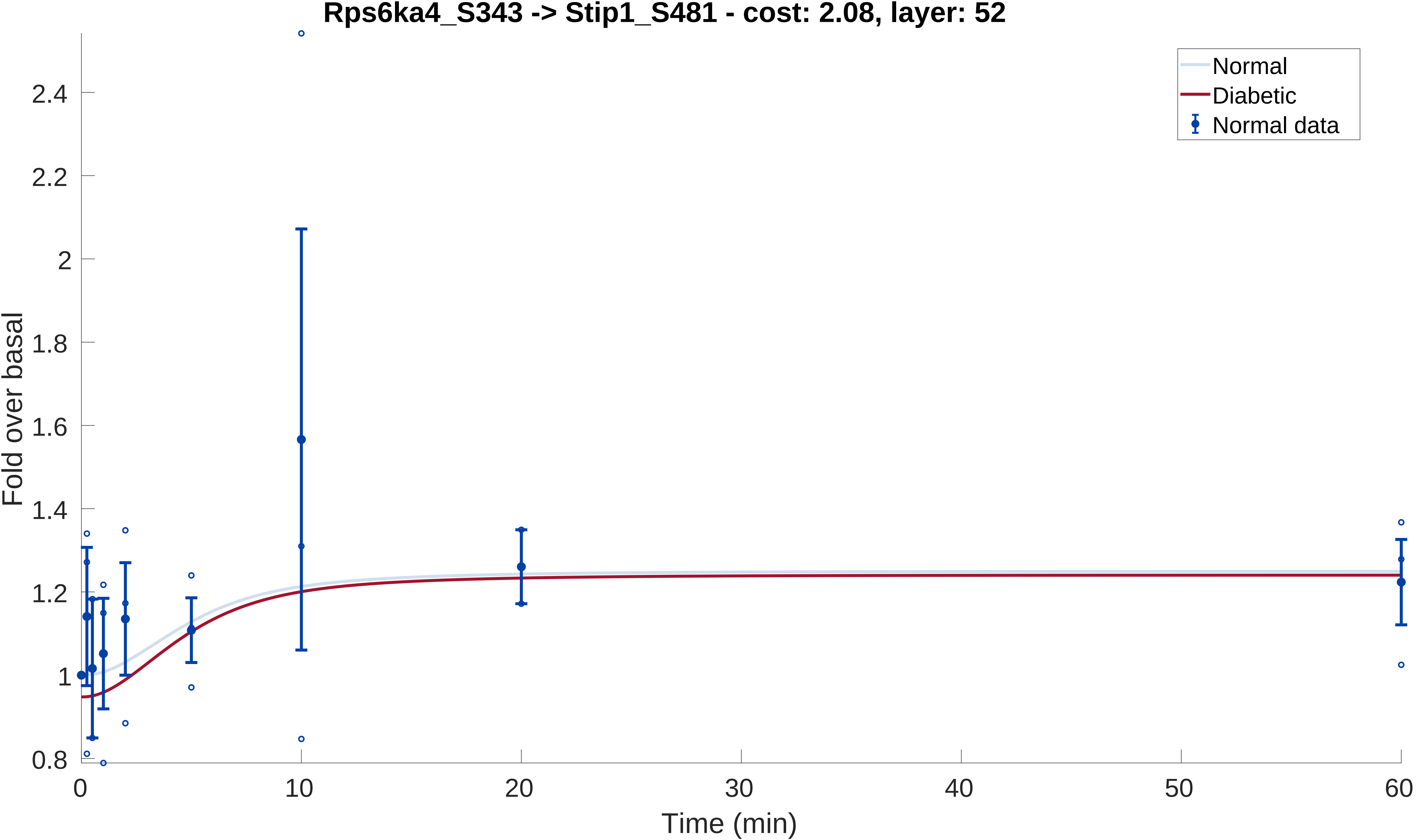

Rps6ka4_S343 -> Stip1_S481 - cost: 2.08, layer: 52
Normal
Diabetic
Normal data
Fold over basal
Time (min)
0.8
1
1.2
1.4
1.6
1.8
2
2.2
2.4
0
10
20
30
40
50
60

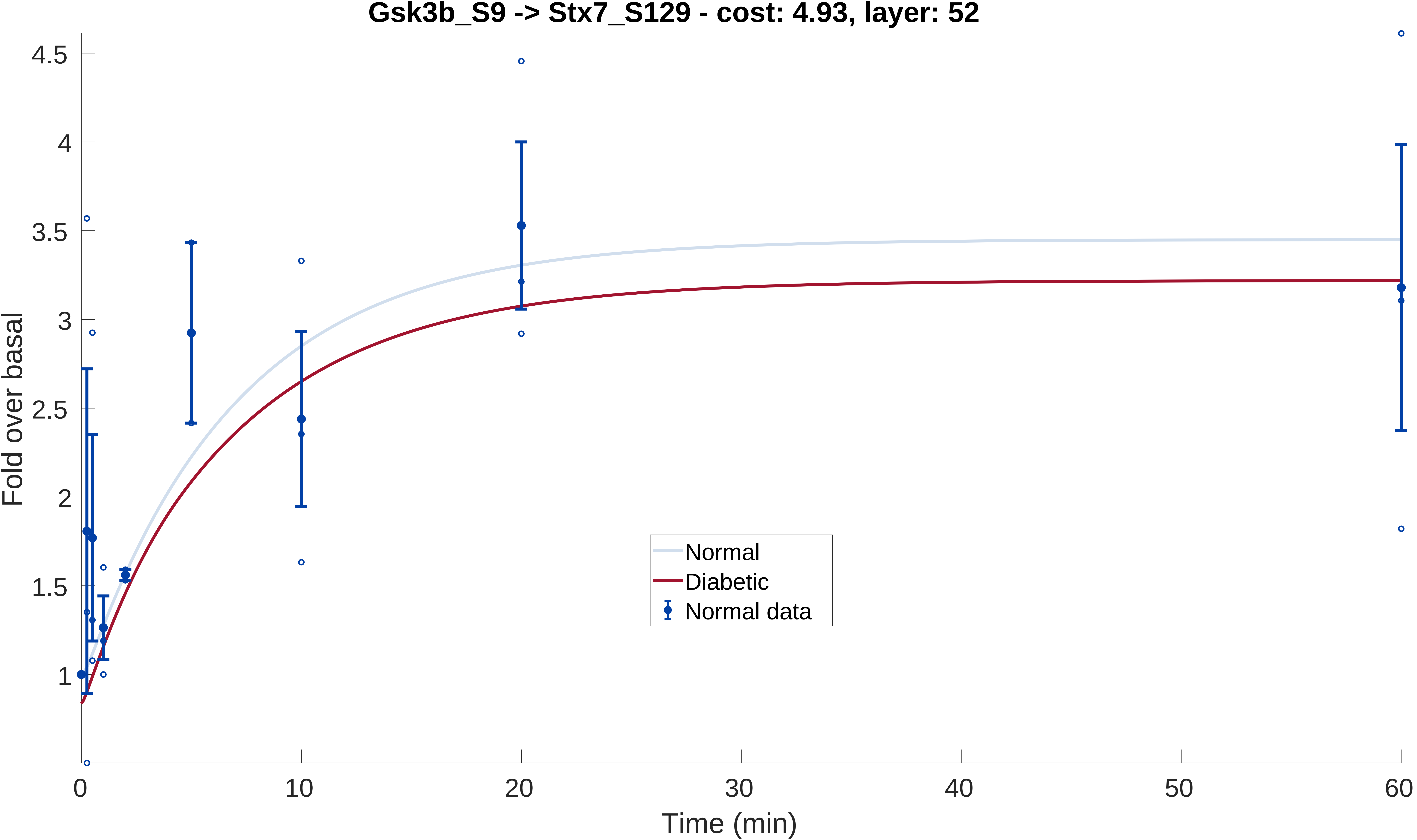

Gsk3b_S9 -> Stx7_S129 - cost: 4.93, layer: 52
Fold over basal
Time (min)
4.5
4
3.5
3
2.5
2
1.5
1
0
10
20
30
40
50
60
Normal
Diabetic
Normal data

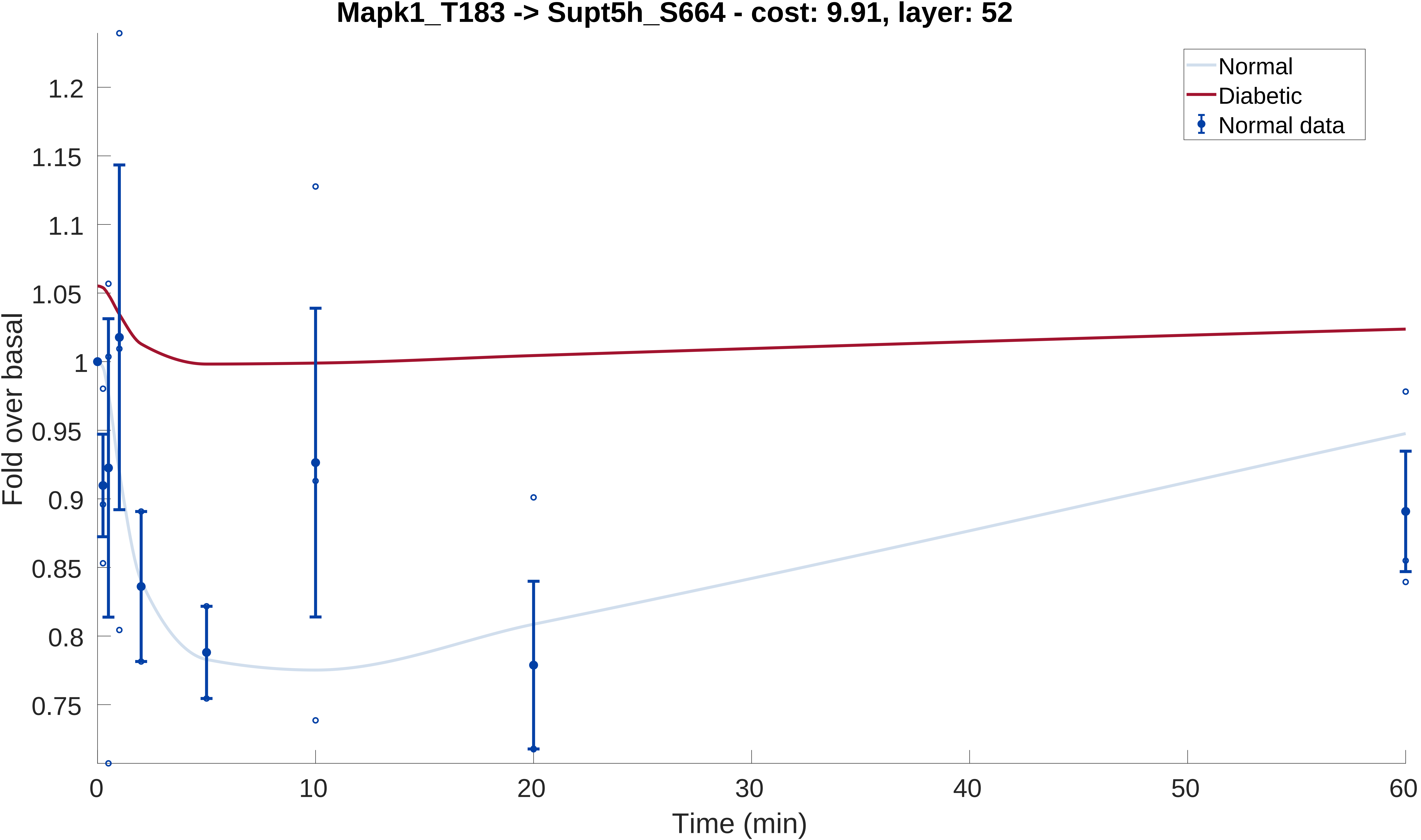

Mapk1_T183 -> Supt5h_S664 - cost: 9.91, layer: 52
Normal
Diabetic
Normal data
Fold over basal
1.2
1.15
1.1
1.05
1
0.95
0.9
0.85
0.8
0.75
0
10
20
30
40
50
60
Time (min)

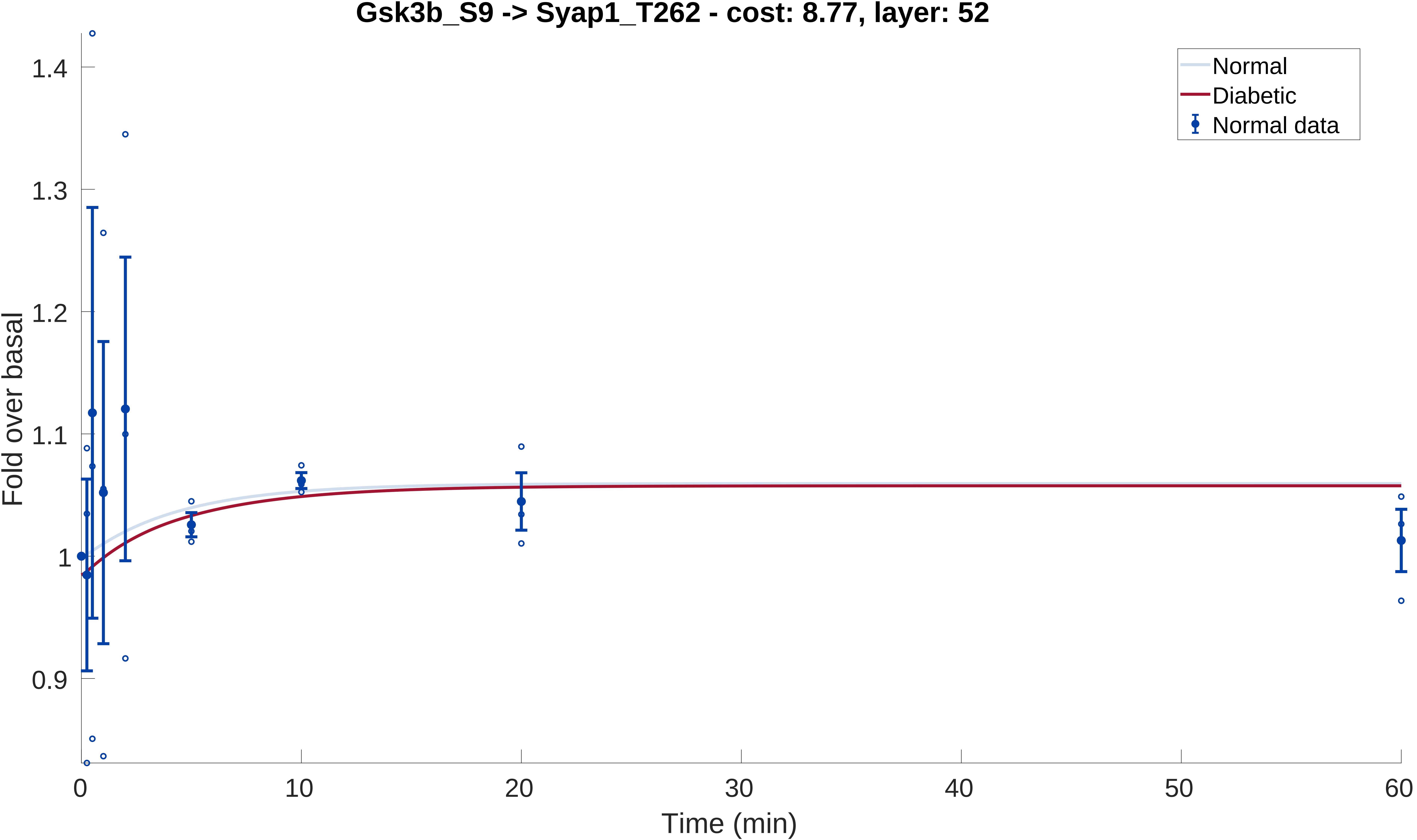

Gsk3b_S9 -> Syap1_T262 - cost: 8.77, layer: 52
Normal
Diabetic
Normal data
Fold over basal
1.4
1.3
1.2
1.1
1
0.9
0
10
20
30
40
50
60
Time (min)

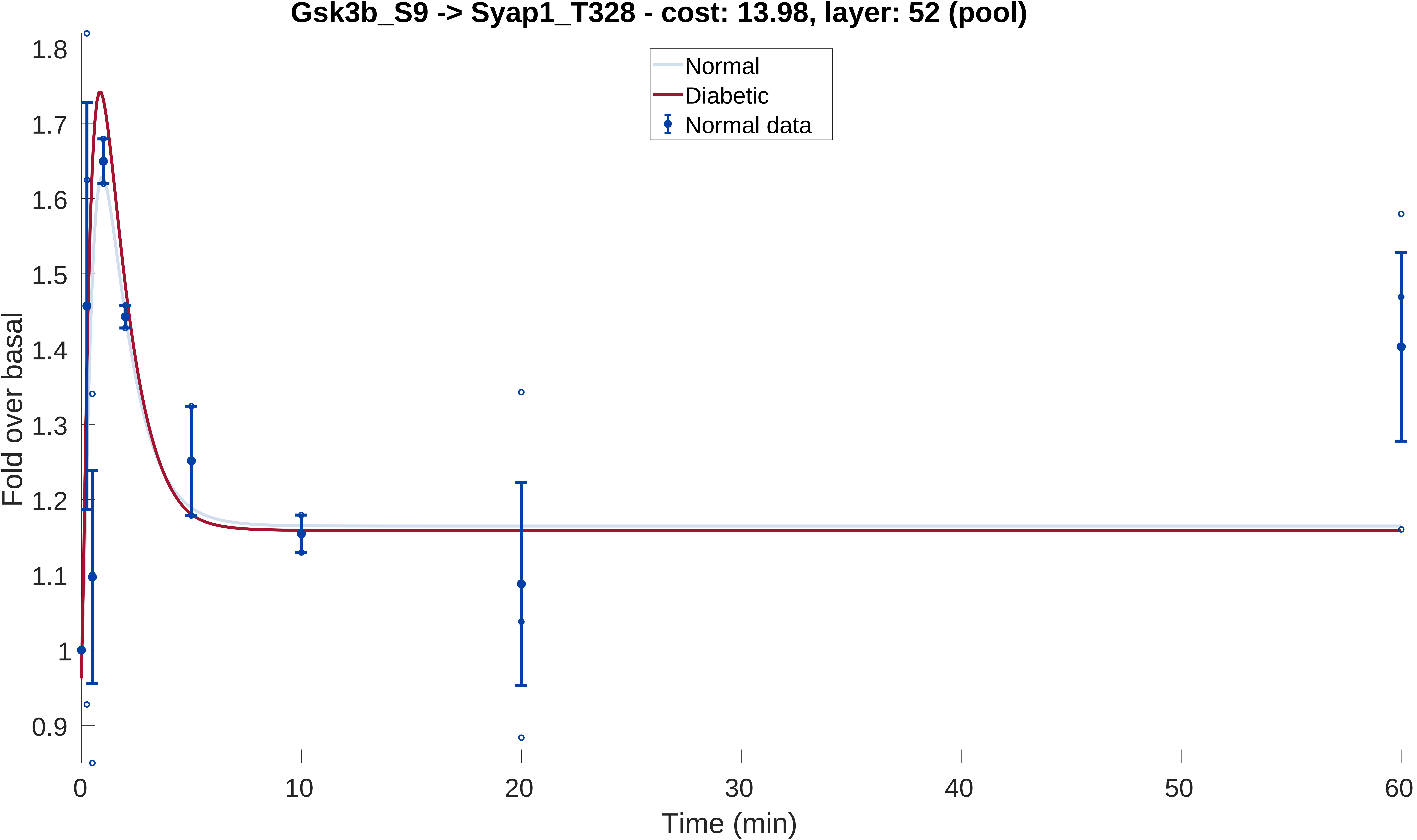

Gsk3b_S9 -> Syap1_T328 - cost: 13.98, layer: 52 (pool)
Normal
Diabetic
Normal data
Fold over basal
Time (min)
1.8
1.7
1.6
1.5
1.4
1.3
1.2
1.1
1
0.9
0
10
20
30
40
50
60

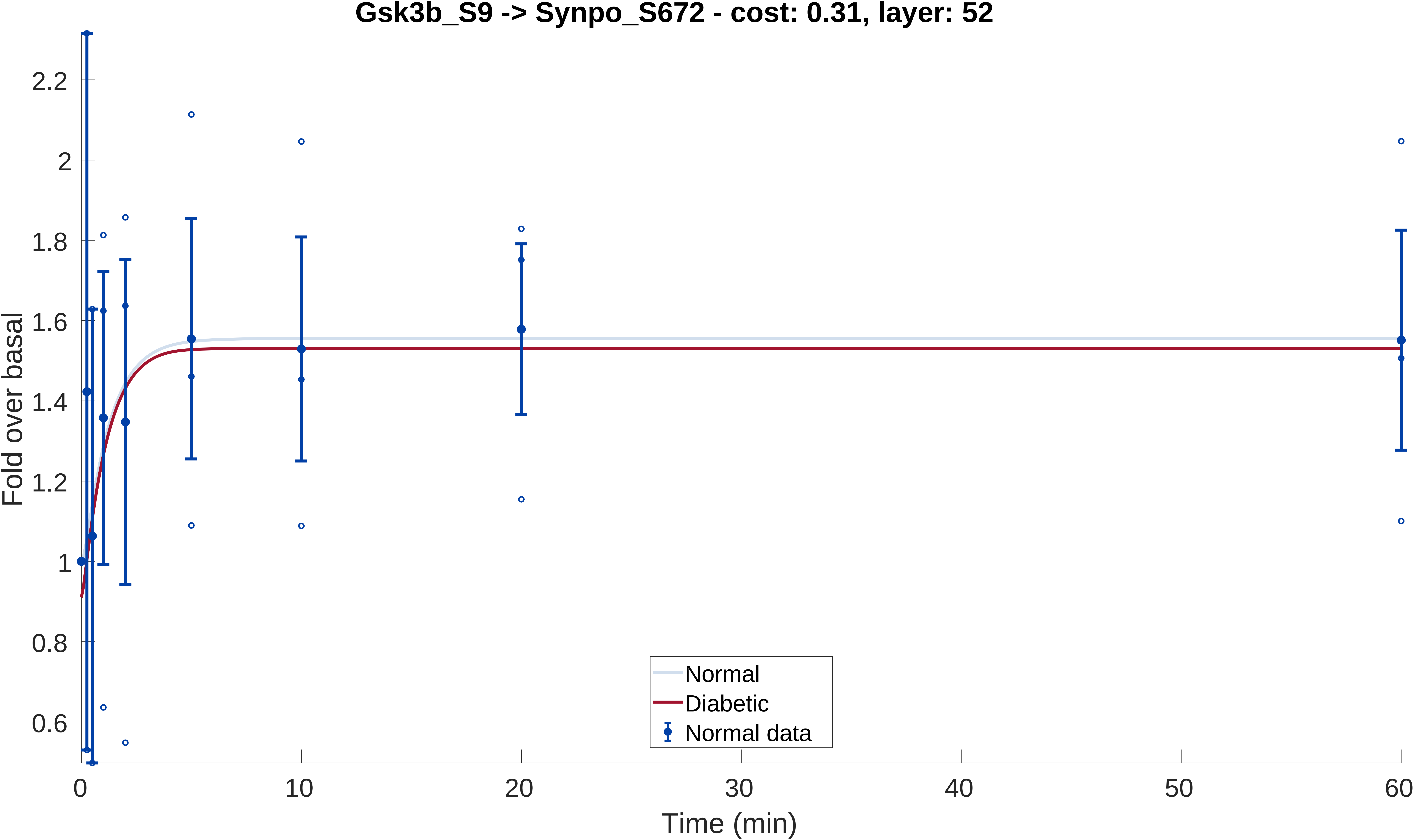
Gsk3b_S9 -> Synpo_S672 - cost: 0.31, layer: 52
Fold over basal
2.2
2
1.8
1.6
1.4
1.2
1
0.8
0.6
0
10
20
30
40
50
60
Time (min)
Normal
Diabetic
Normal data

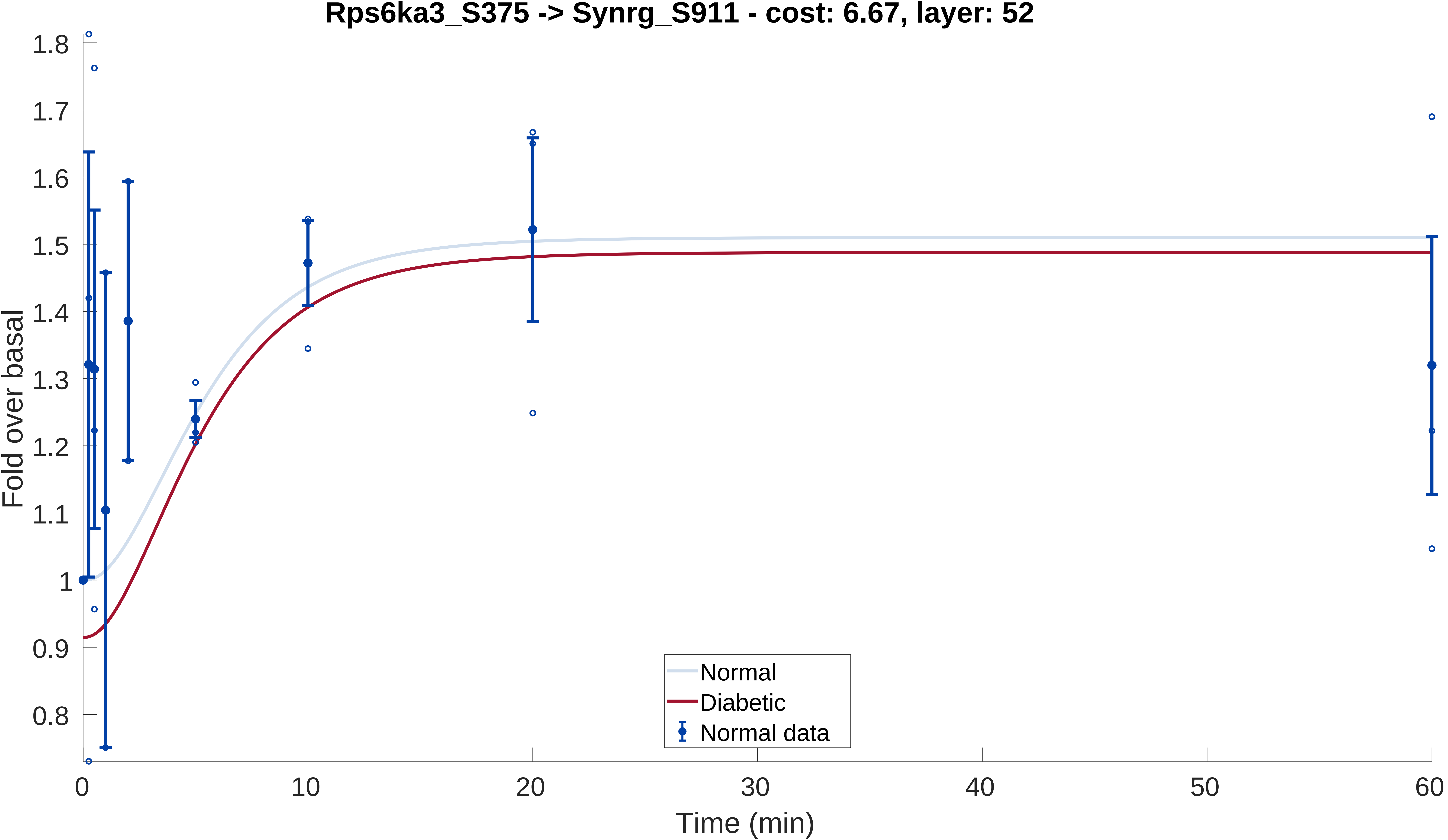
Rps6ka3_S375 -> Synrg_S911 - cost: 6.67, layer: 52
Fold over basal
Time (min)
0
10
20
30
40
50
60
0.8
0.9
1
1.1
1.2
1.3
1.4
1.5
1.6
1.7
1.8
Normal
Diabetic
Normal data

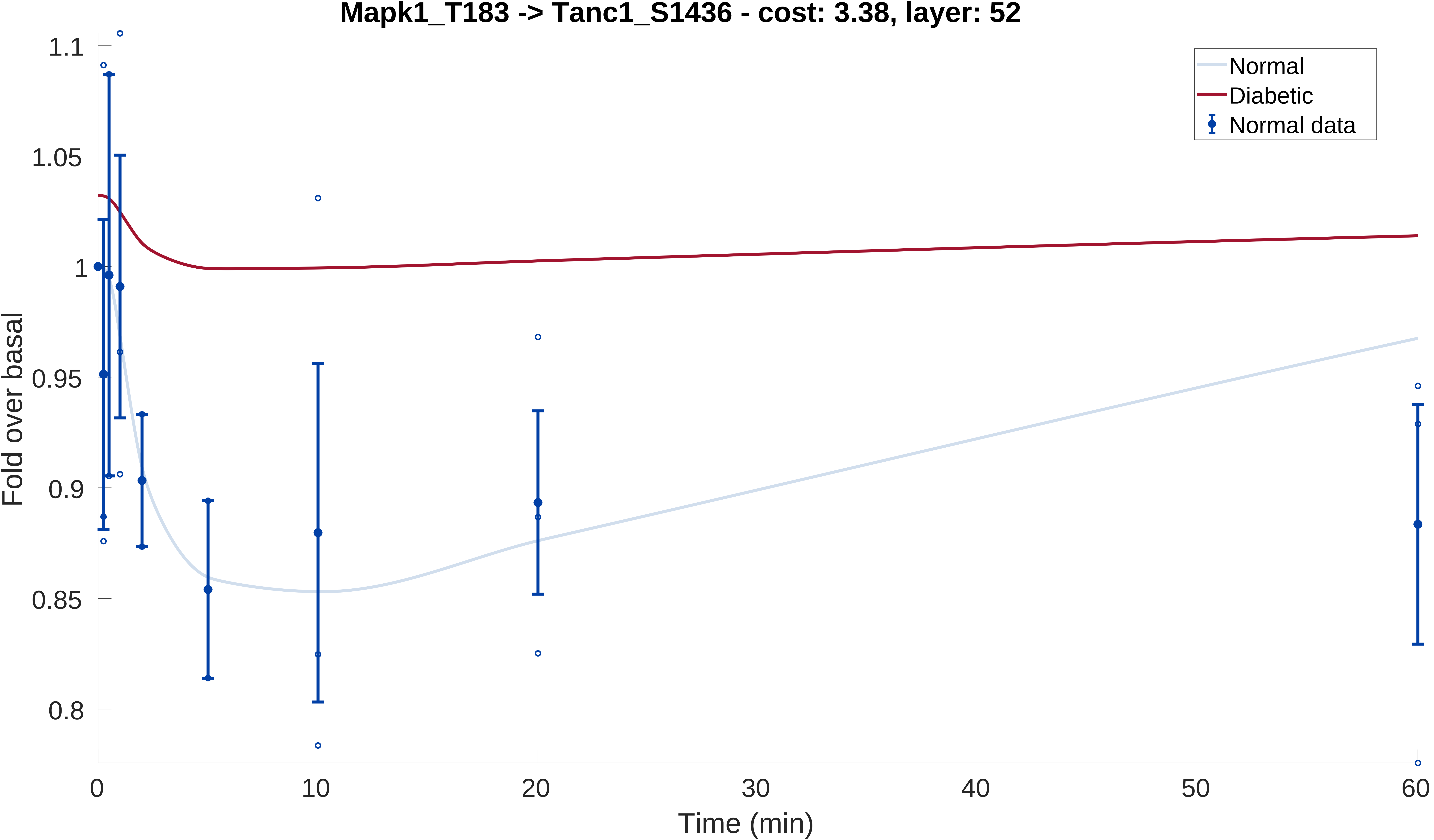

Mapk1_T183 -> Tanc1_S1436 - cost: 3.38, layer: 52
Normal
Diabetic
Normal data
Fold over basal
Time (min)
1.1
1.05
1
0.95
0.9
0.85
0.8
0
10
20
30
40
50
60

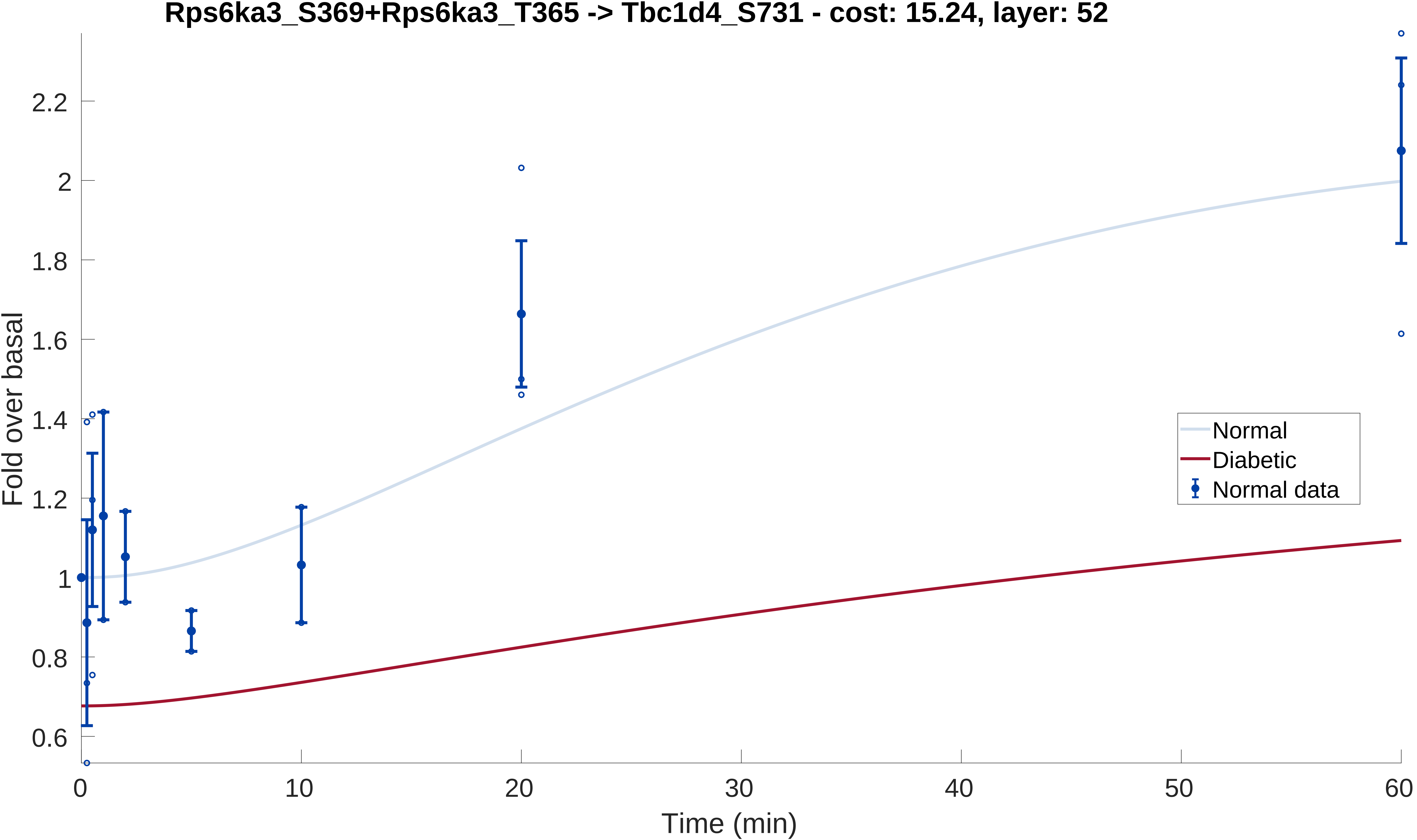
Rps6ka3_S369+Rps6ka3_T365 -> Tbc1d4_S731 - cost: 15.24, layer: 52
Fold over basal
0.6
0.8
1
1.2
1.4
1.6
1.8
2
2.2
Time (min)
0
10
20
30
40
50
60
Normal
Diabetic
Normal data

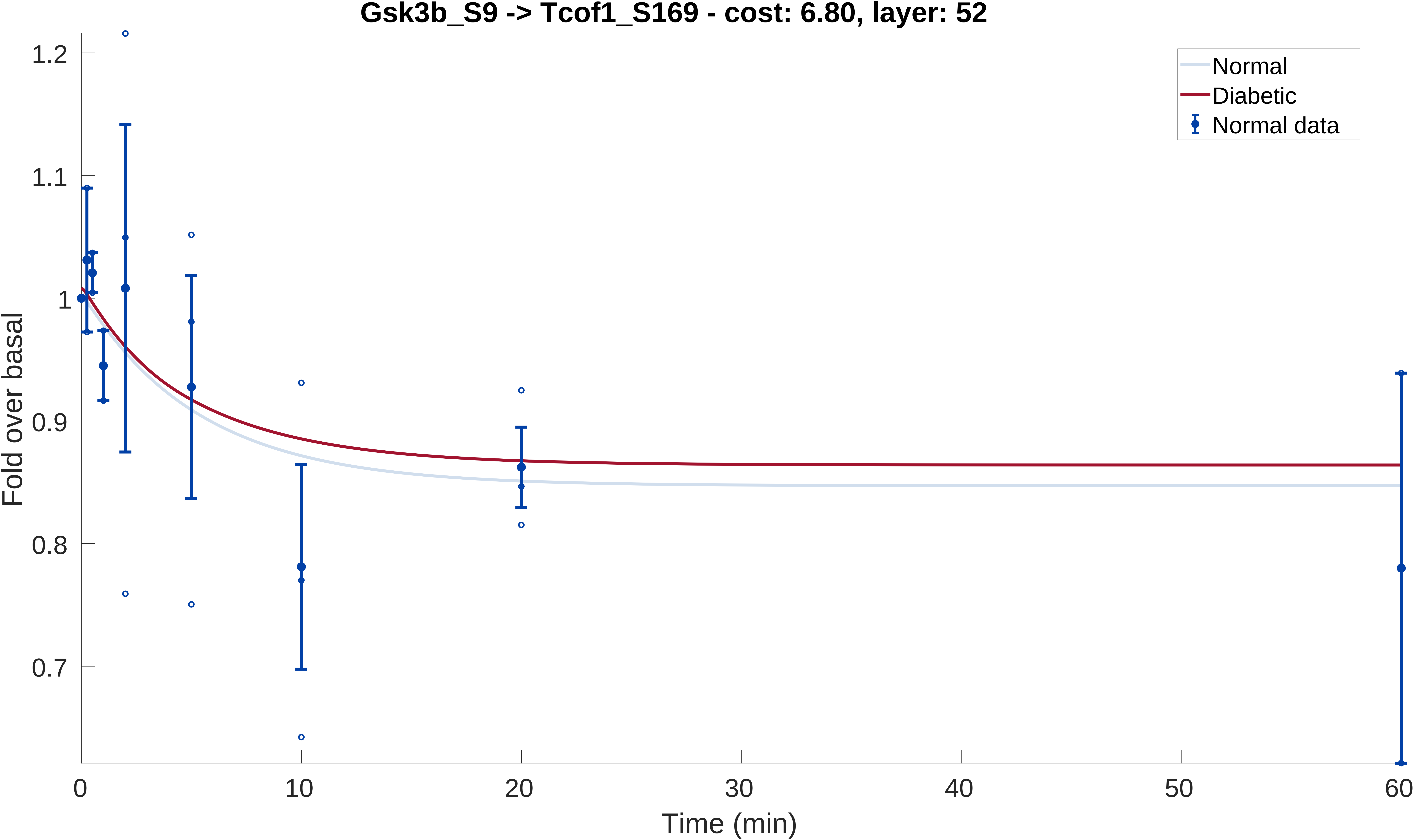
Gsk3b_S9 -> Tcof1_S169 - cost: 6.80, layer: 52
Normal
Diabetic
Normal data
Fold over basal
Time (min)
1.2
1.1
1
0.9
0.8
0.7
0
10
20
30
40
50
60

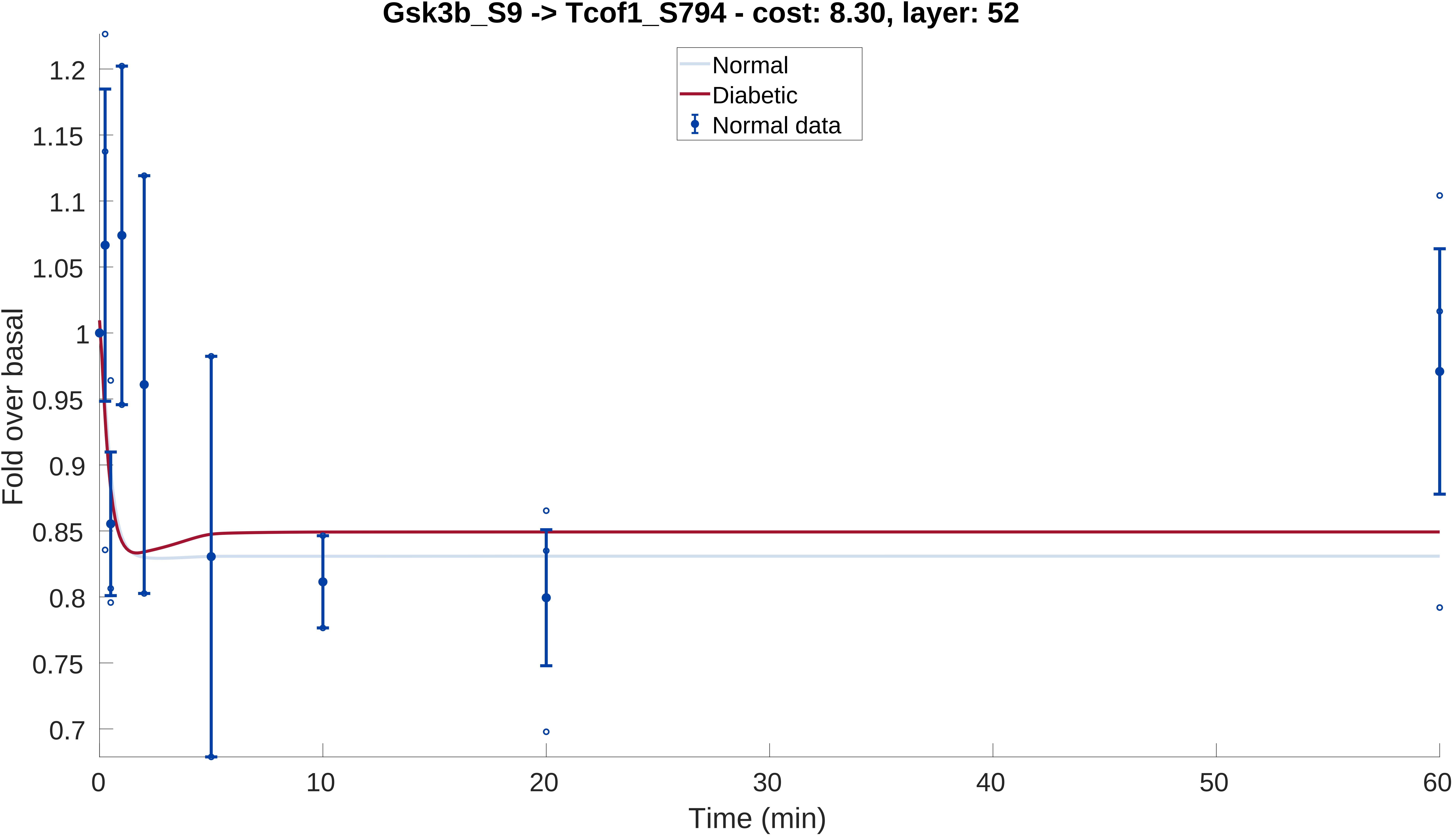
Gsk3b_S9 -> Tcof1_S794 - cost: 8.30, layer: 52
Normal
Diabetic
Normal data
Fold over basal
Time (min)
0.7
0.75
0.8
0.85
0.9
0.95
1
1.05
1.1
1.15
1.2
0
10
20
30
40
50
60

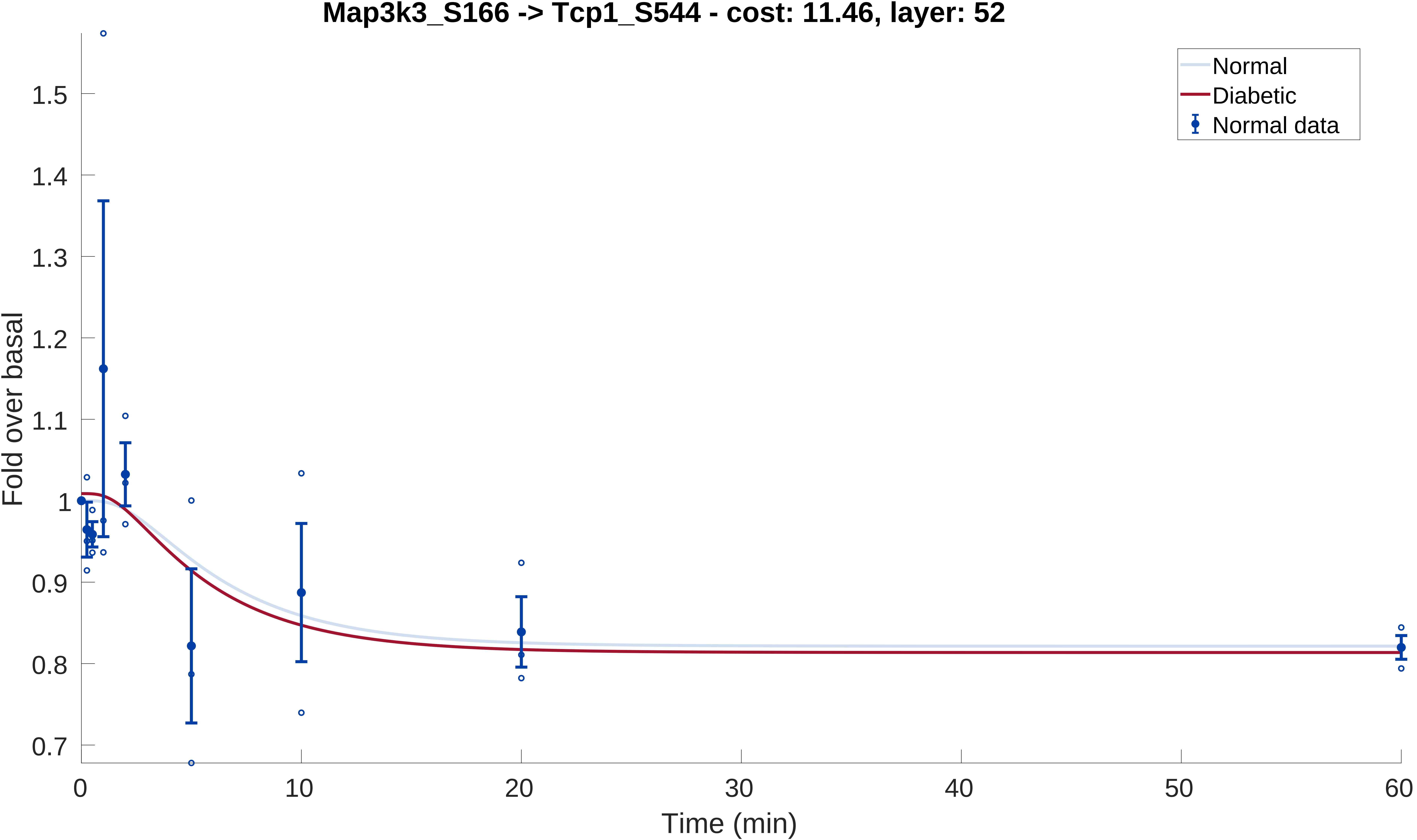

Map3k3_S166 -> Tcp1_S544 - cost: 11.46, layer: 52
Normal
Diabetic
Normal data
Fold over basal
Time (min)
0.7
0.8
0.9
1
1.1
1.2
1.3
1.4
1.5
0
10
20
30
40
50
60

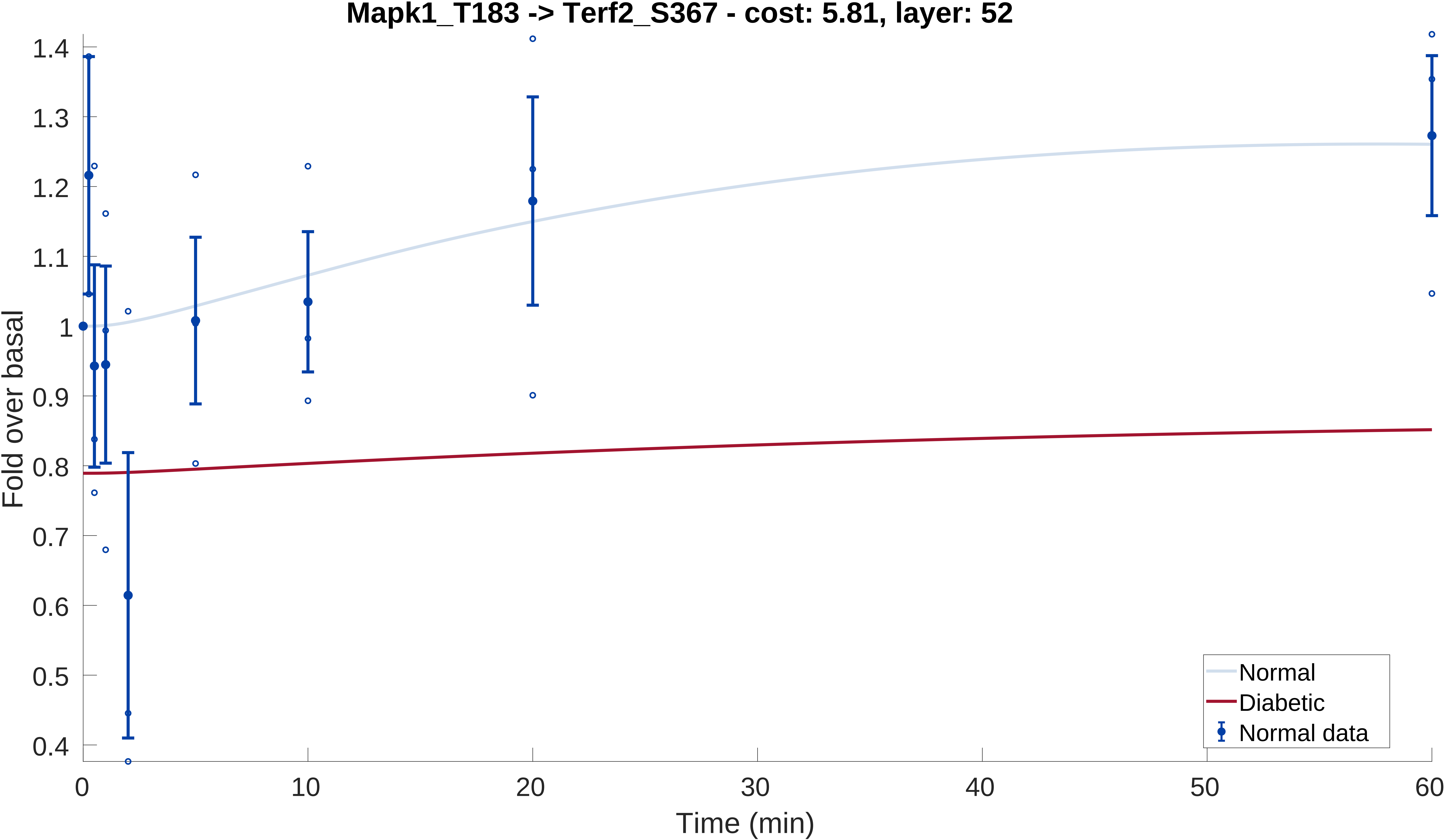

Mapk1_T183 -> Terf2_S367 - cost: 5.81, layer: 52
Fold over basal
1.4
1.3
1.2
1.1
1
0.9
0.8
0.7
0.6
0.5
0.4
0
10
20
30
40
50
60
Time (min)
Normal
Diabetic
Normal data

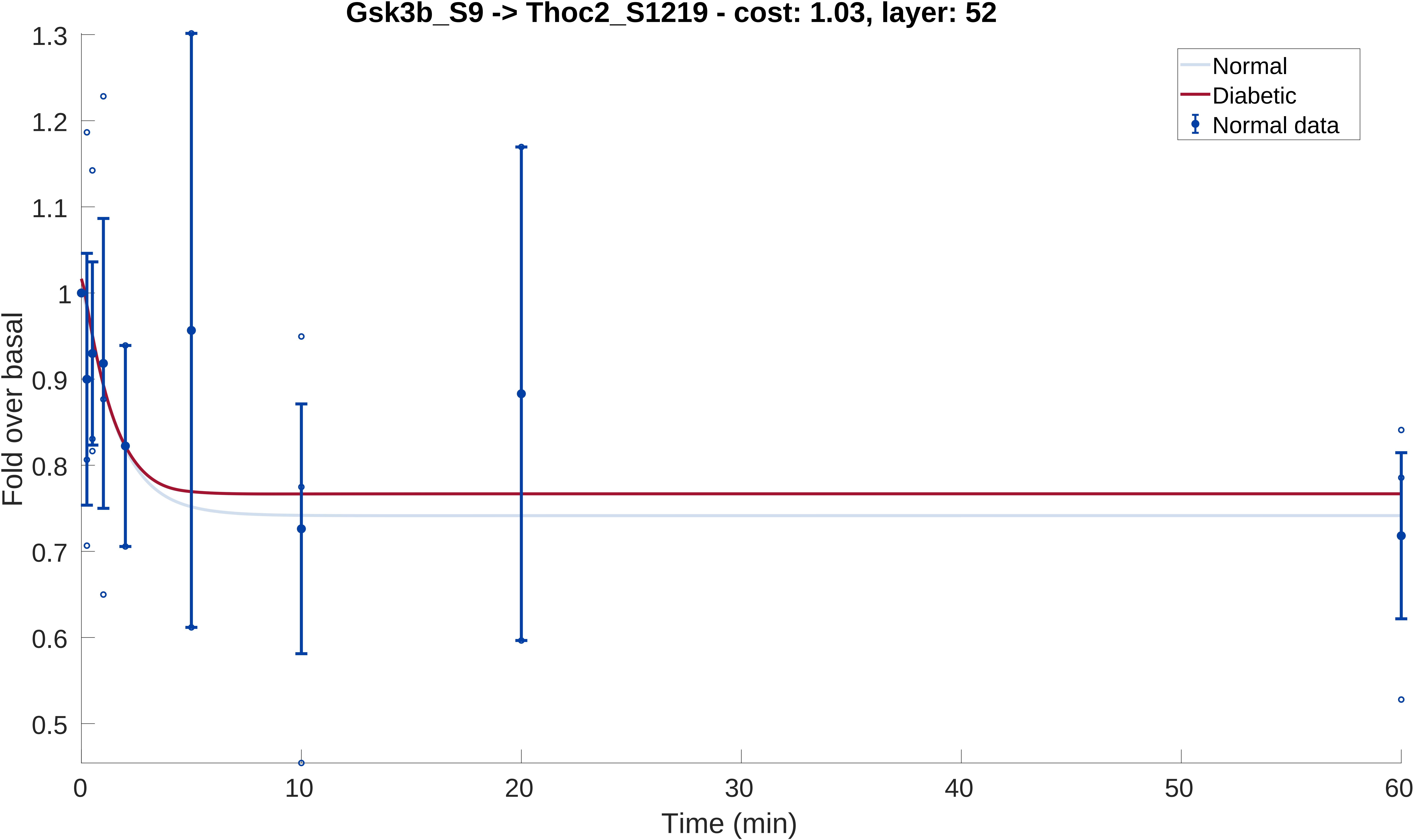

Gsk3b_S9 -> Thoc2_S1219 - cost: 1.03, layer: 52
Normal
Diabetic
Normal data
Fold over basal
Time (min)
1.3
1.2
1.1
1
0.9
0.8
0.7
0.6
0.5
0
10
20
30
40
50
60

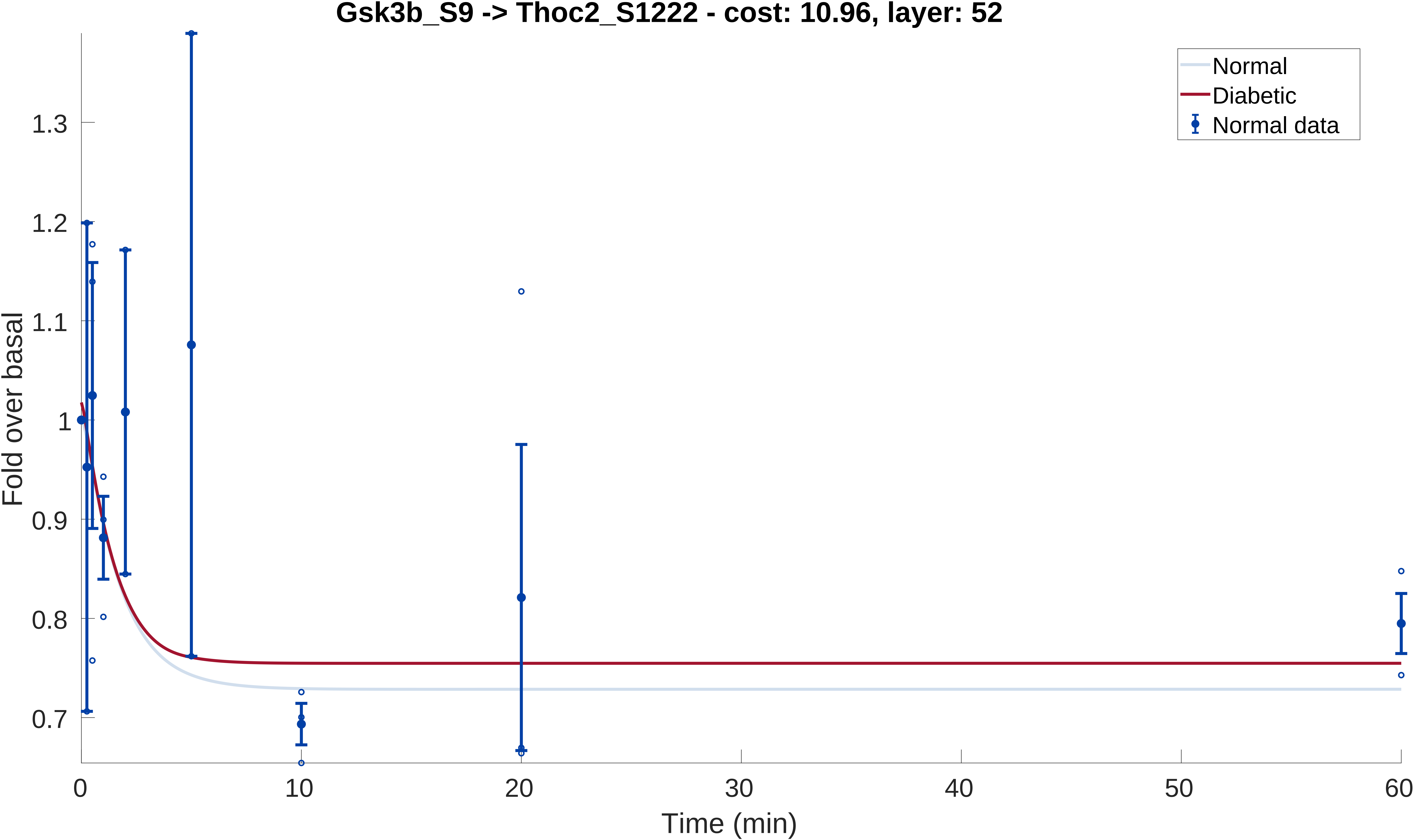
Gsk3b_S9 -> Thoc2_S1222 - cost: 10.96, layer: 52
Normal
Diabetic
Normal data
Fold over basal
Time (min)
1.3
1.2
1.1
1
0.9
0.8
0.7
0
10
20
30
40
50
60

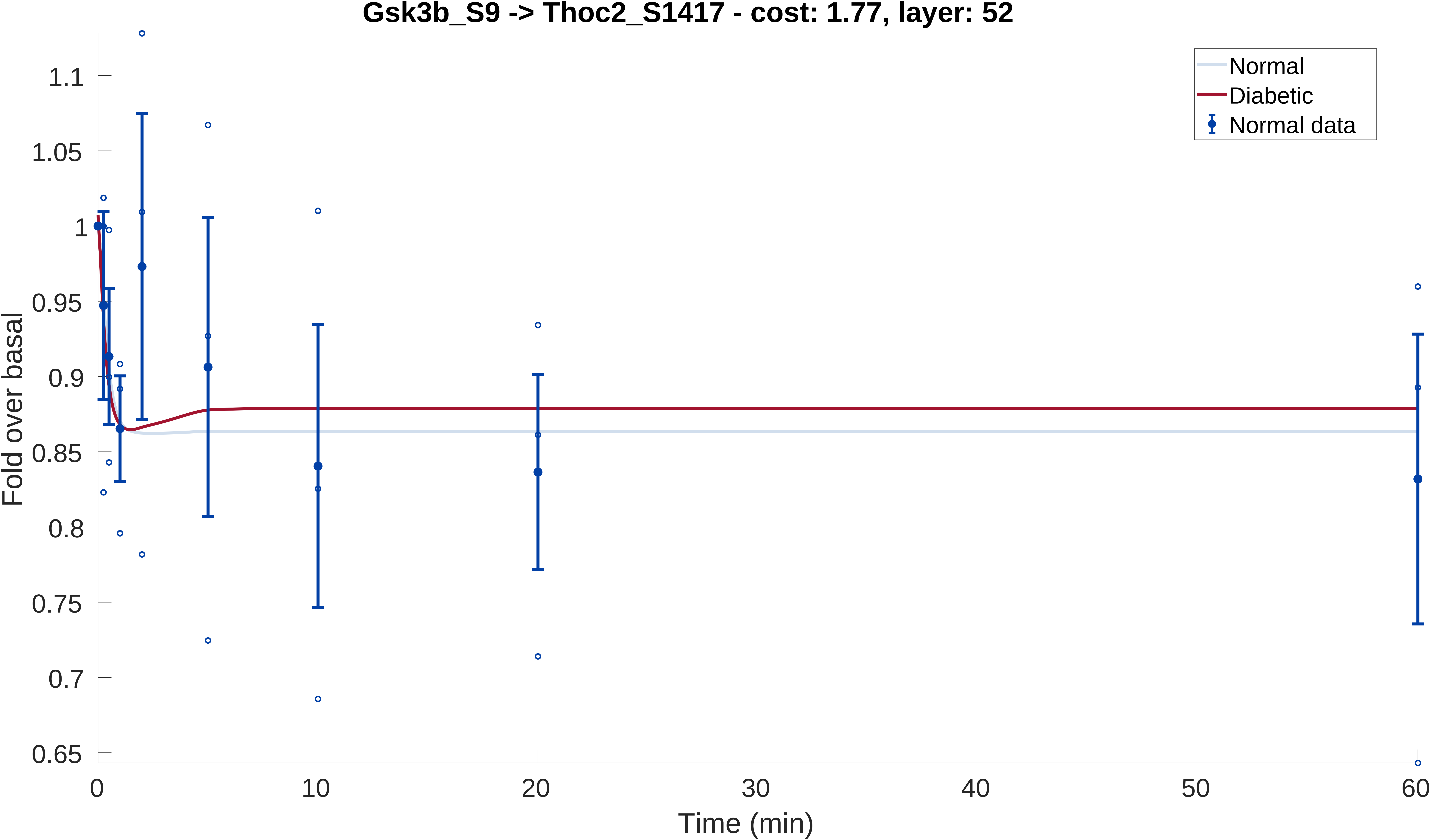

Gsk3b_S9 -> Thoc2_S1417 - cost: 1.77, layer: 52
Normal
Diabetic
Normal data
Fold over basal
Time (min)
1.1
1.05
1
0.95
0.9
0.85
0.8
0.75
0.7
0.65
0
10
20
30
40
50
60

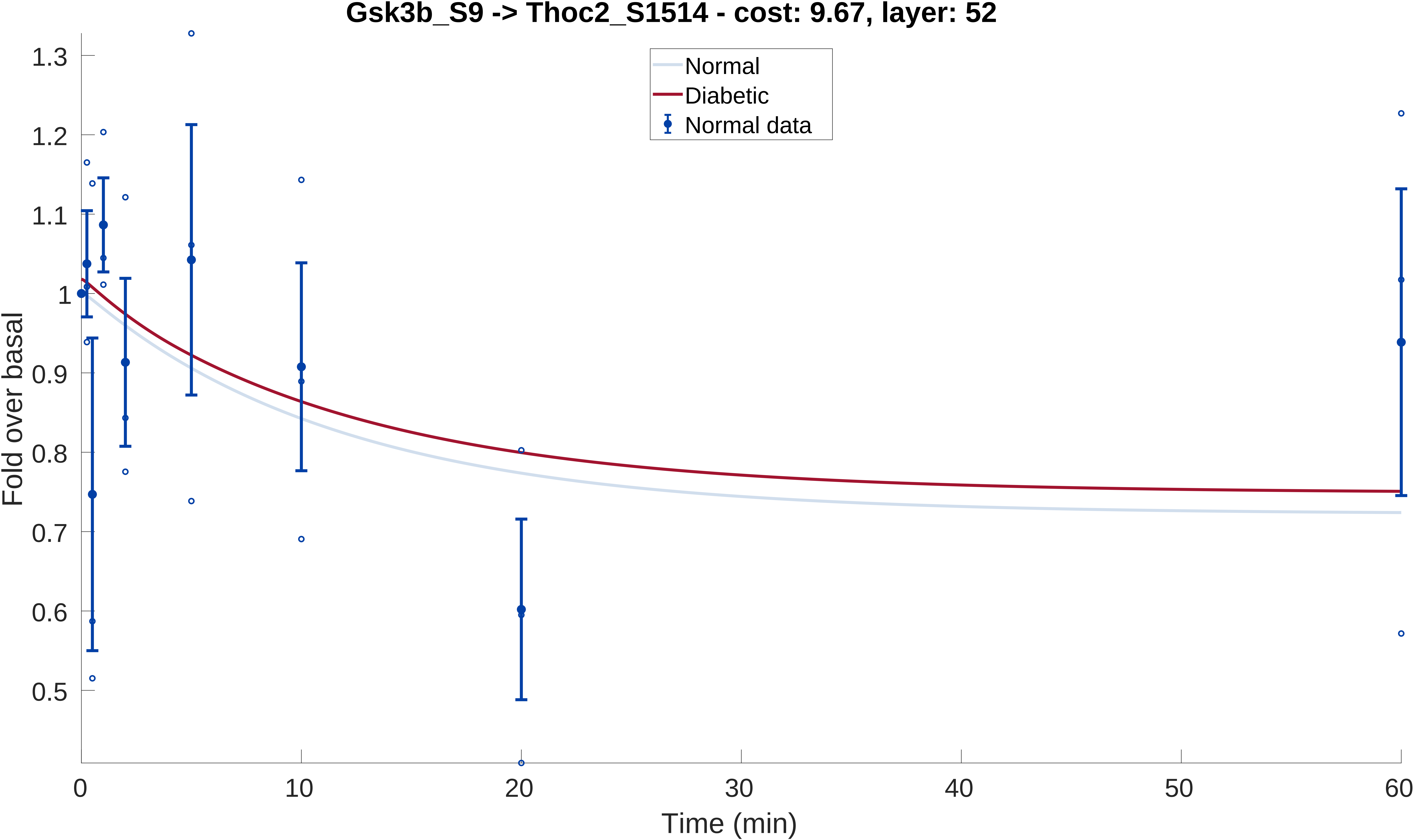

Gsk3b_S9 -> Thoc2_S1514 - cost: 9.67, layer: 52
Normal
Diabetic
Normal data
Fold over basal
1.3
1.2
1.1
1
0.9
0.8
0.7
0.6
0.5
0
10
20
30
40
50
60
Time (min)

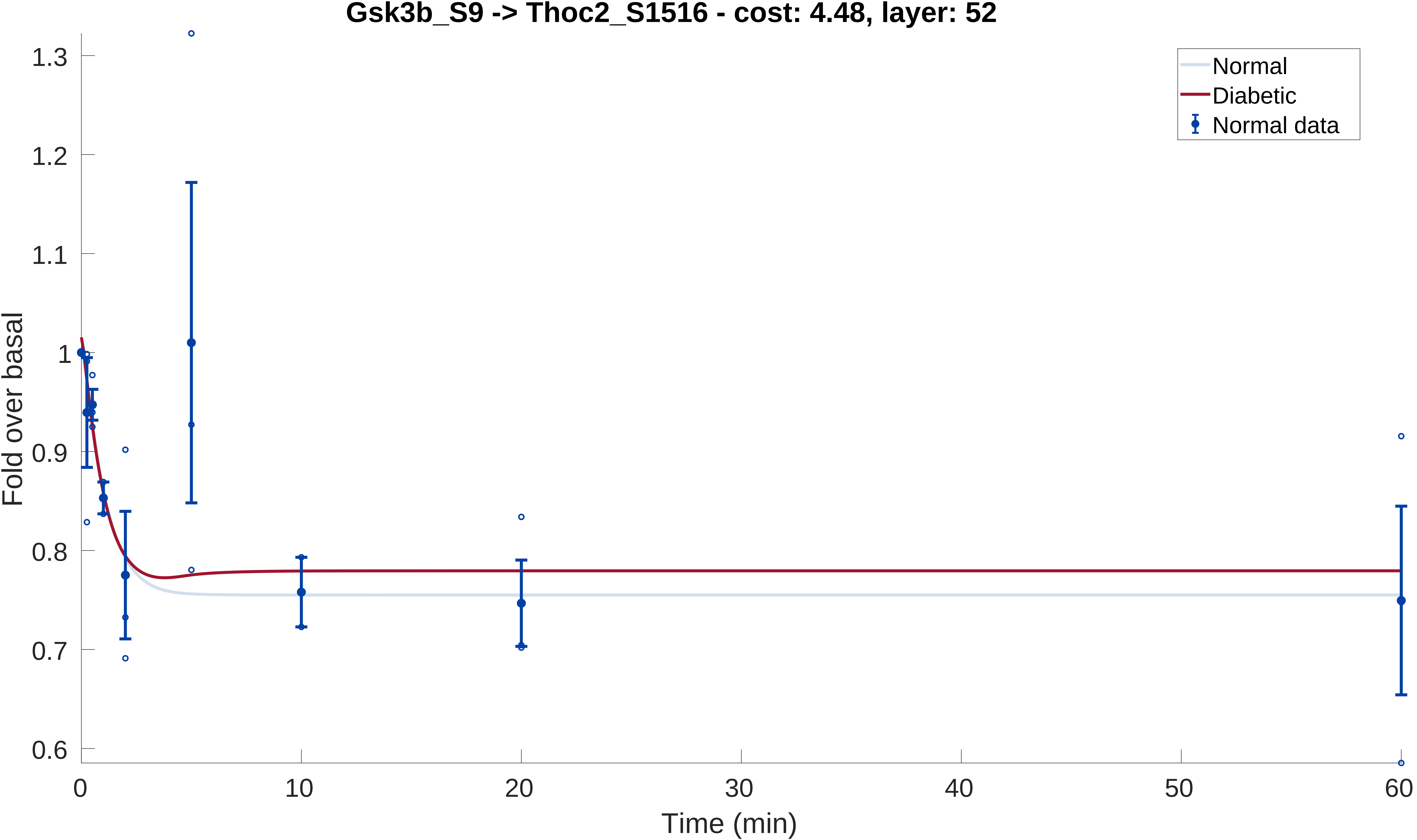
Gsk3b_S9 -> Thoc2_S1516 - cost: 4.48, layer: 52
Fold over basal
1.3
1.2
1.1
1
0.9
0.8
0.7
0.6
0
10
20
30
40
50
60
Time (min)
Normal
Diabetic
Normal data

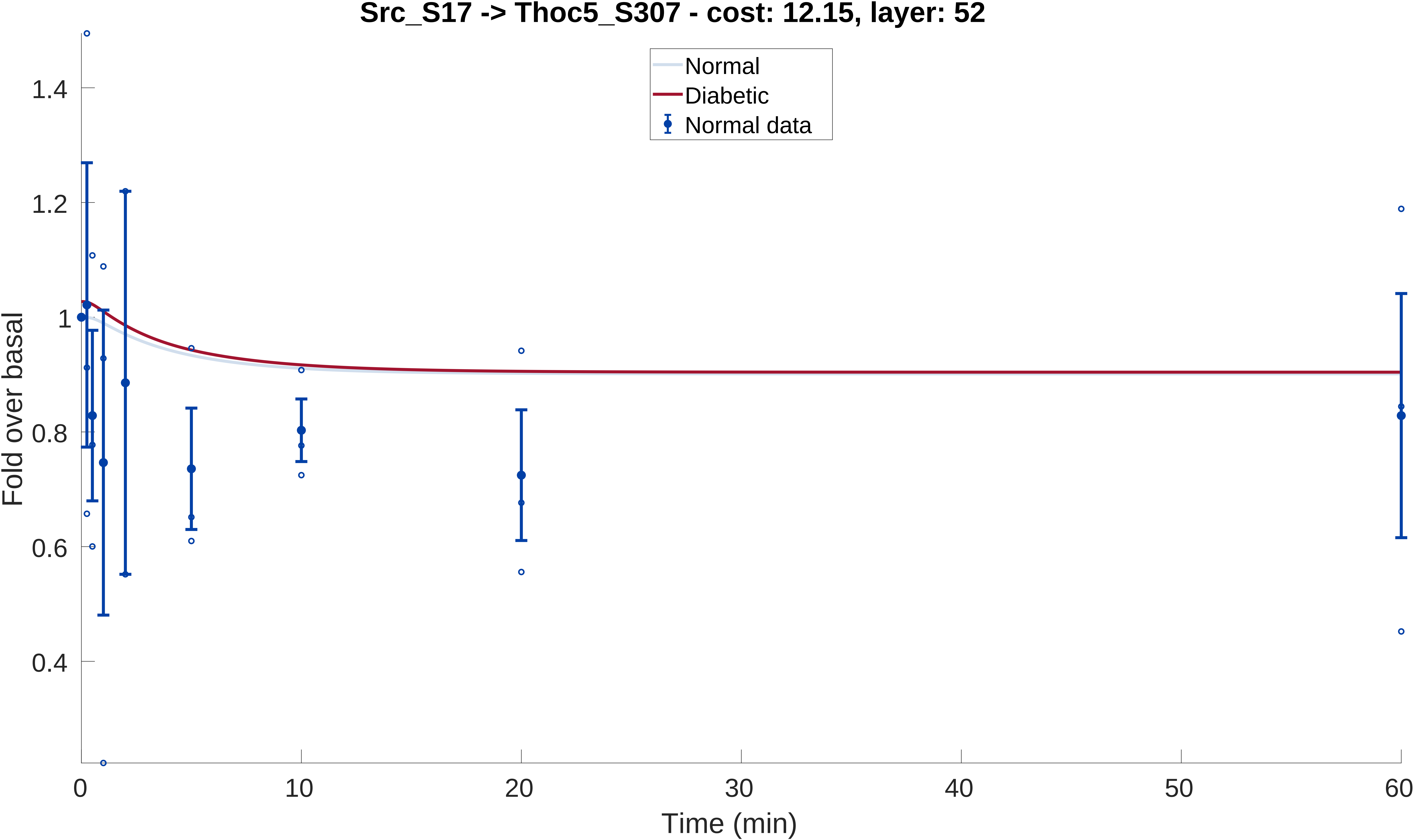
Src_S17 -> Thoc5_S307 - cost: 12.15, layer: 52
Normal
Diabetic
Normal data
1.4
1.2
1
0.8
0.6
0.4
Fold over basal
0
10
20
30
40
50
60
Time (min)

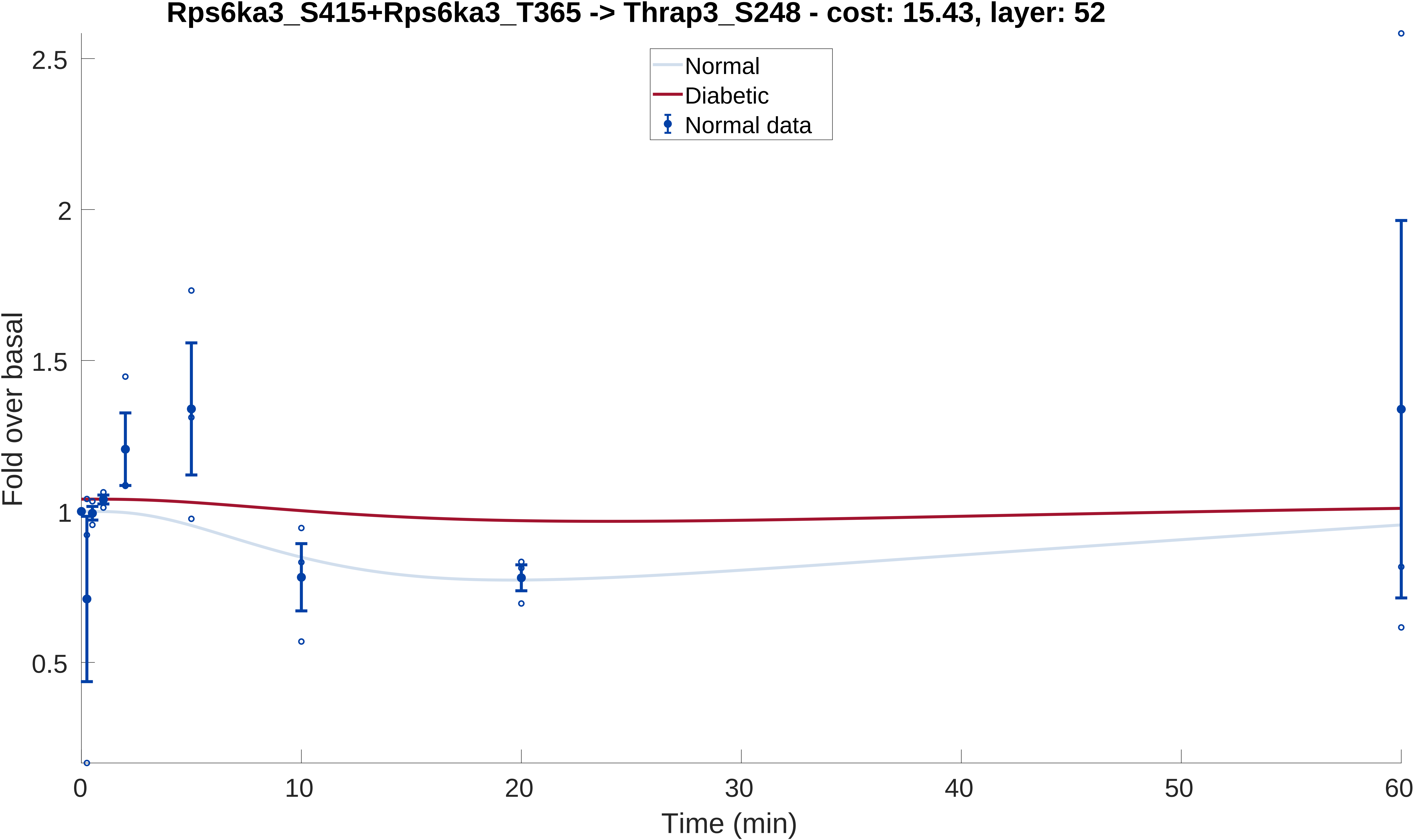

Rps6ka3_S415+Rps6ka3_T365 -> Thrap3_S248 - cost: 15.43, layer: 52
Normal
Diabetic
Normal data
Fold over basal
Time (min)
0.5
1
1.5
2
2.5
0
10
20
30
40
50
60

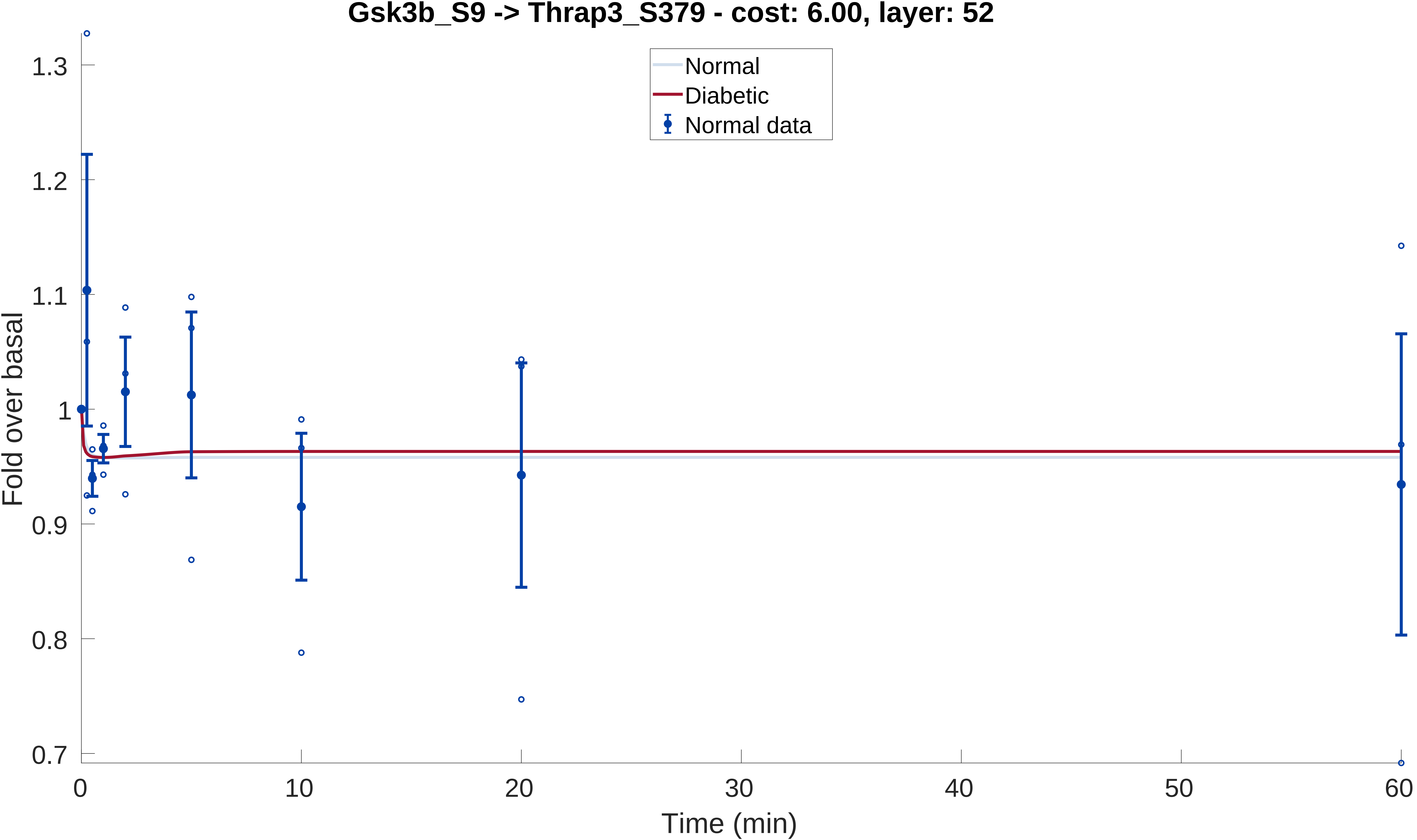

Gsk3b_S9 -> Thrap3_S379 - cost: 6.00, layer: 52
Normal
Diabetic
Normal data
Fold over basal
1.3
1.2
1.1
1
0.9
0.8
0.7
0
10
20
30
40
50
60
Time (min)

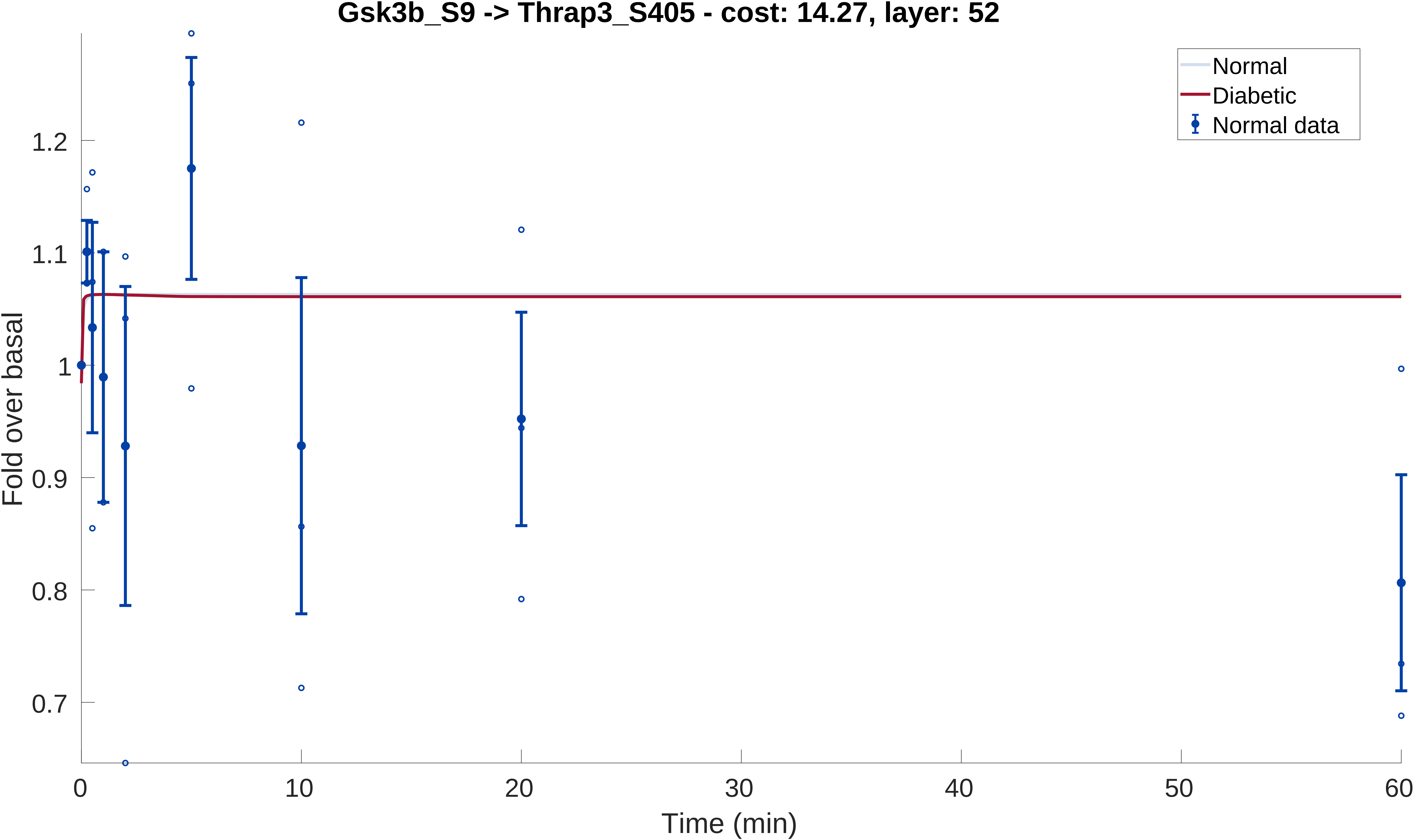

Gsk3b_S9 -> Thrap3_S405 - cost: 14.27, layer: 52
Normal
Diabetic
Normal data
Fold over basal
1.2
1.1
1
0.9
0.8
0.7
0
10
20
30
40
50
60
Time (min)

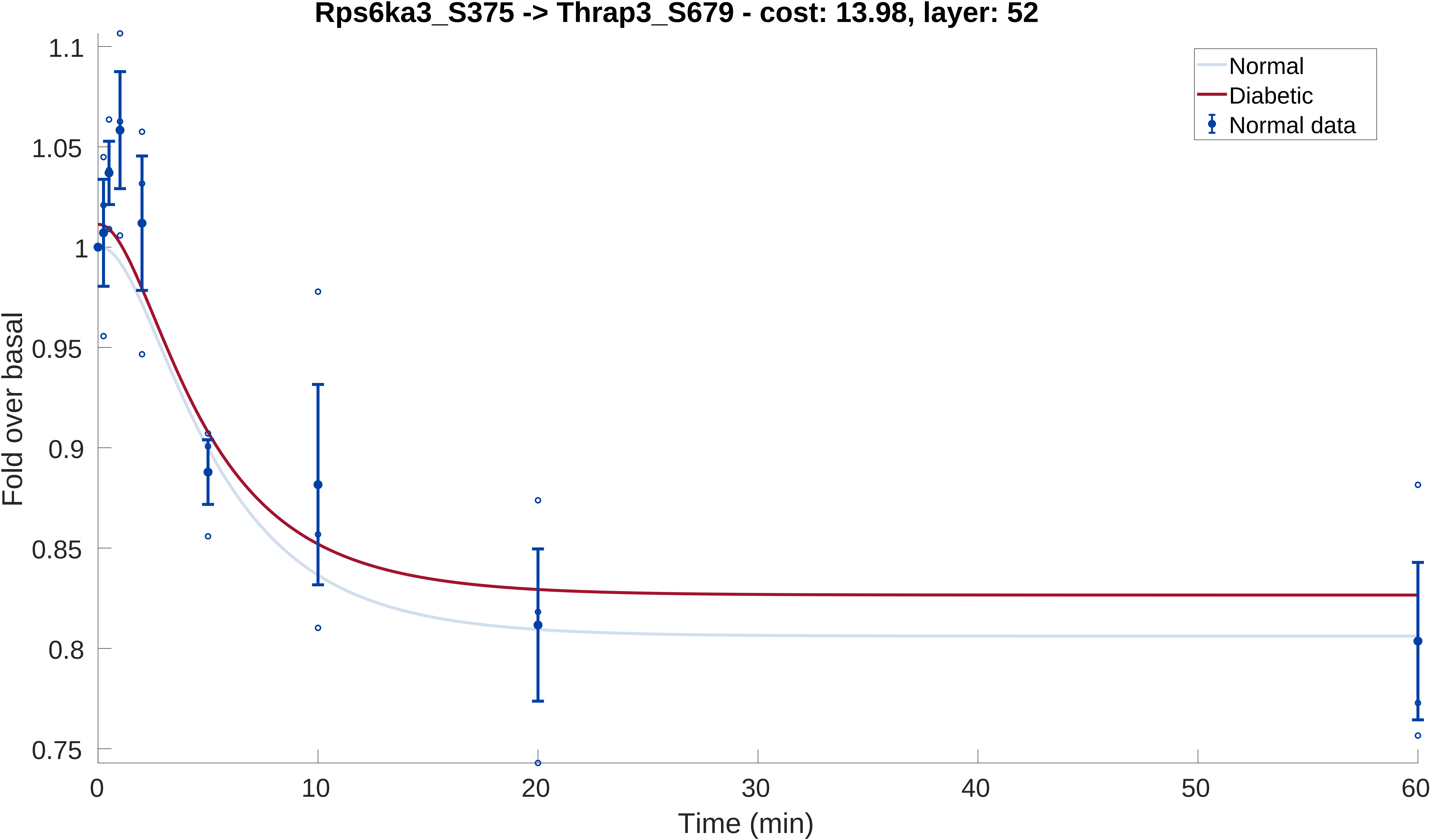

Rps6ka3_S375 -> Thrap3_S679 - cost: 13.98, layer: 52
Normal
Diabetic
Normal data
Fold over basal
1.1
1.05
1
0.95
0.9
0.85
0.8
0.75
0
10
20
30
40
50
60
Time (min)

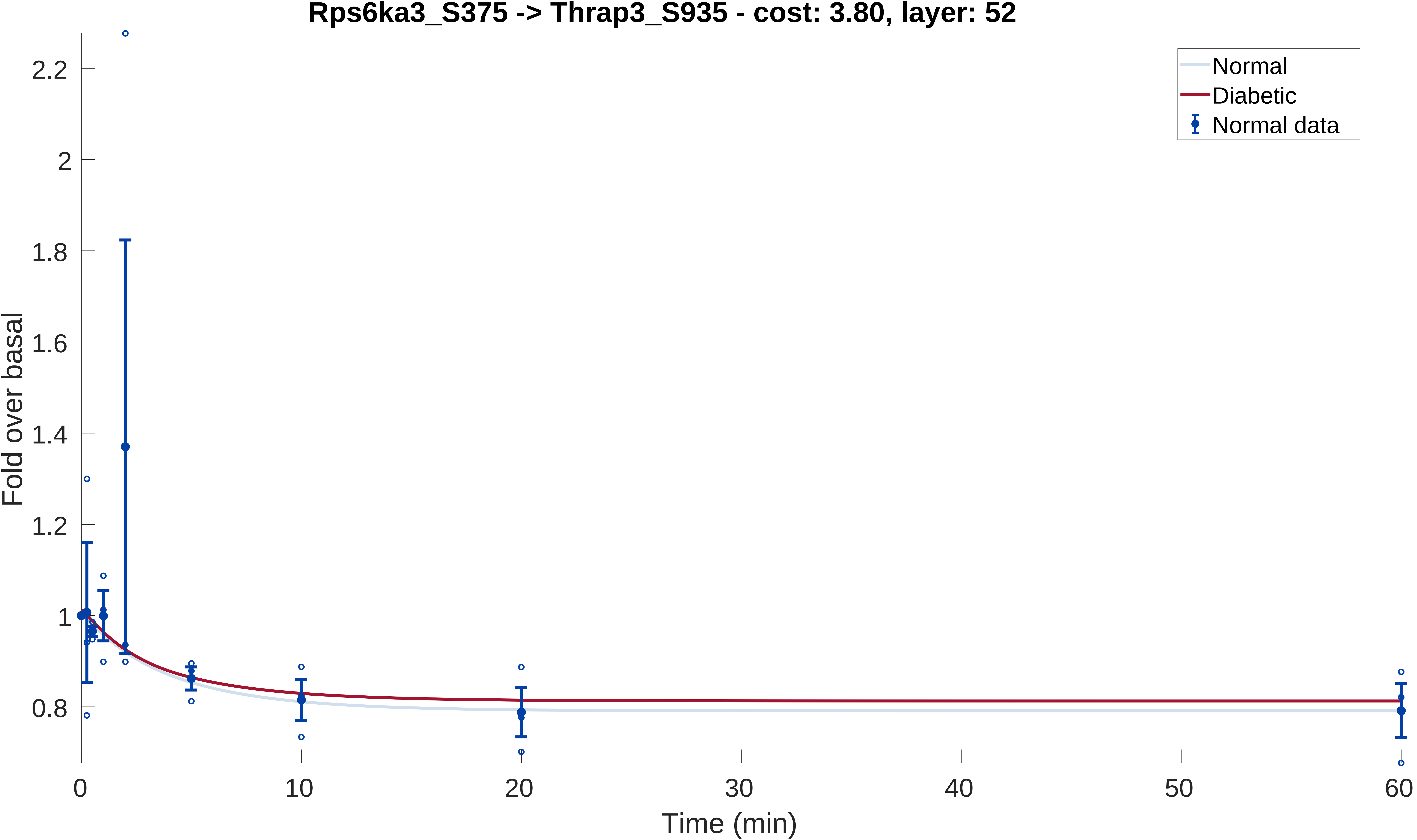
Rps6ka3_S375 -> Thrap3_S935 - cost: 3.80, layer: 52
Fold over basal
2.2
2
1.8
1.6
1.4
1.2
1
0.8
0
10
20
30
40
50
60
Time (min)
Normal
Diabetic
Normal data

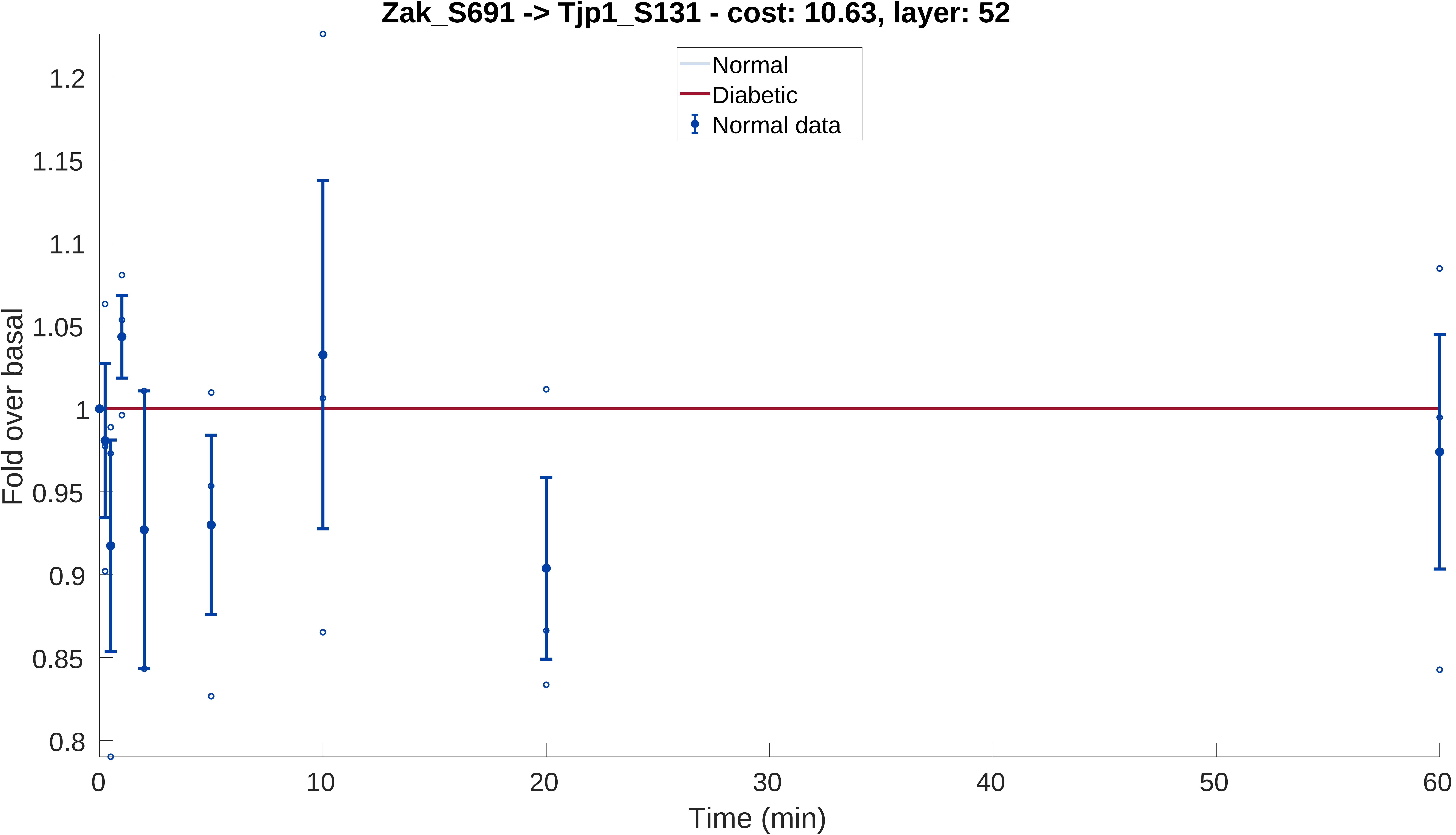

Zak_S691 -> Tjp1_S131 - cost: 10.63, layer: 52
Normal
Diabetic
Normal data
Fold over basal
1.2
1.15
1.1
1.05
1
0.95
0.9
0.85
0.8
0
10
20
30
40
50
60
Time (min)

Prkcd_S669 -> Tjp1_S175 - cost: 10.24, layer: 52

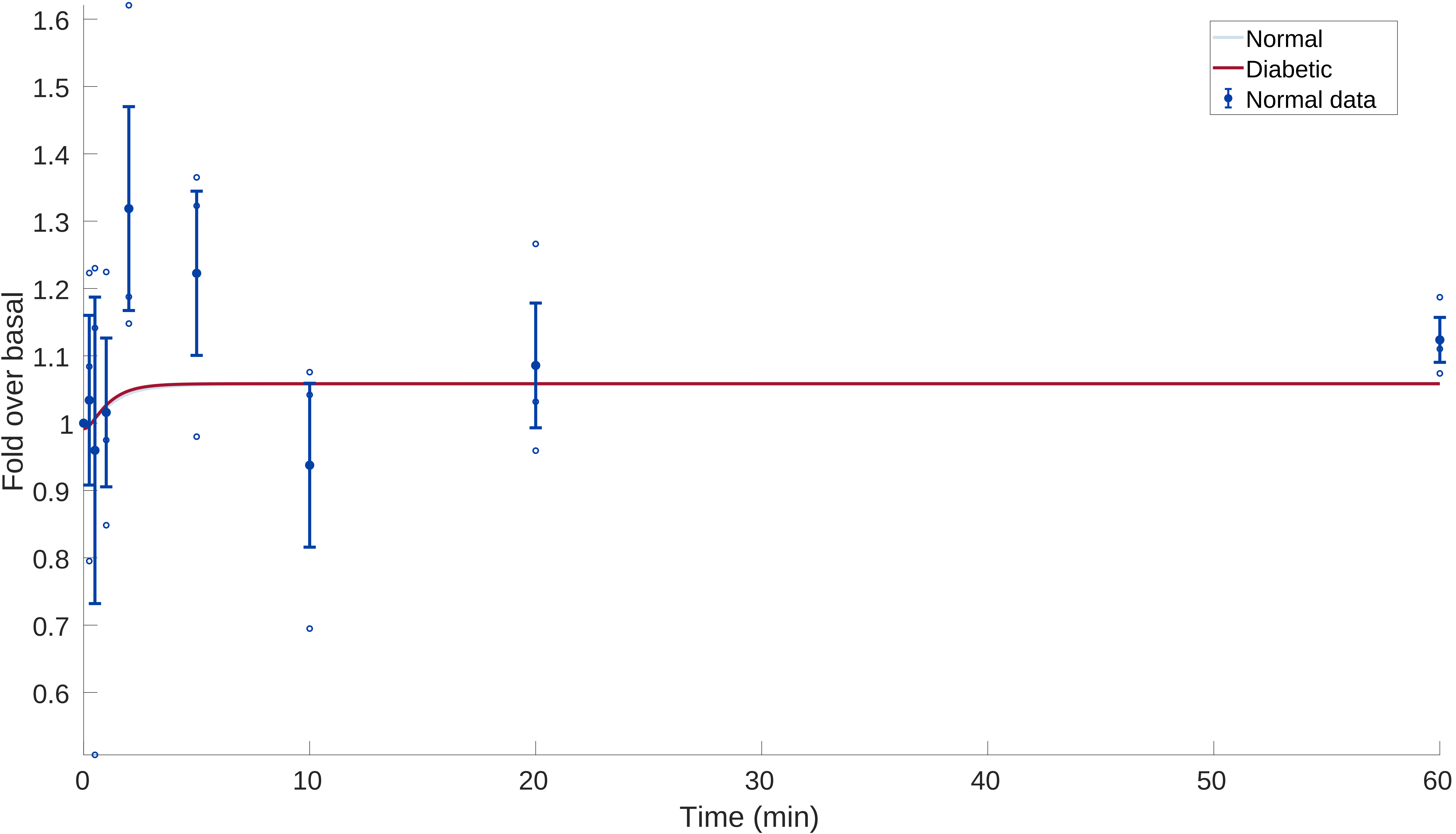

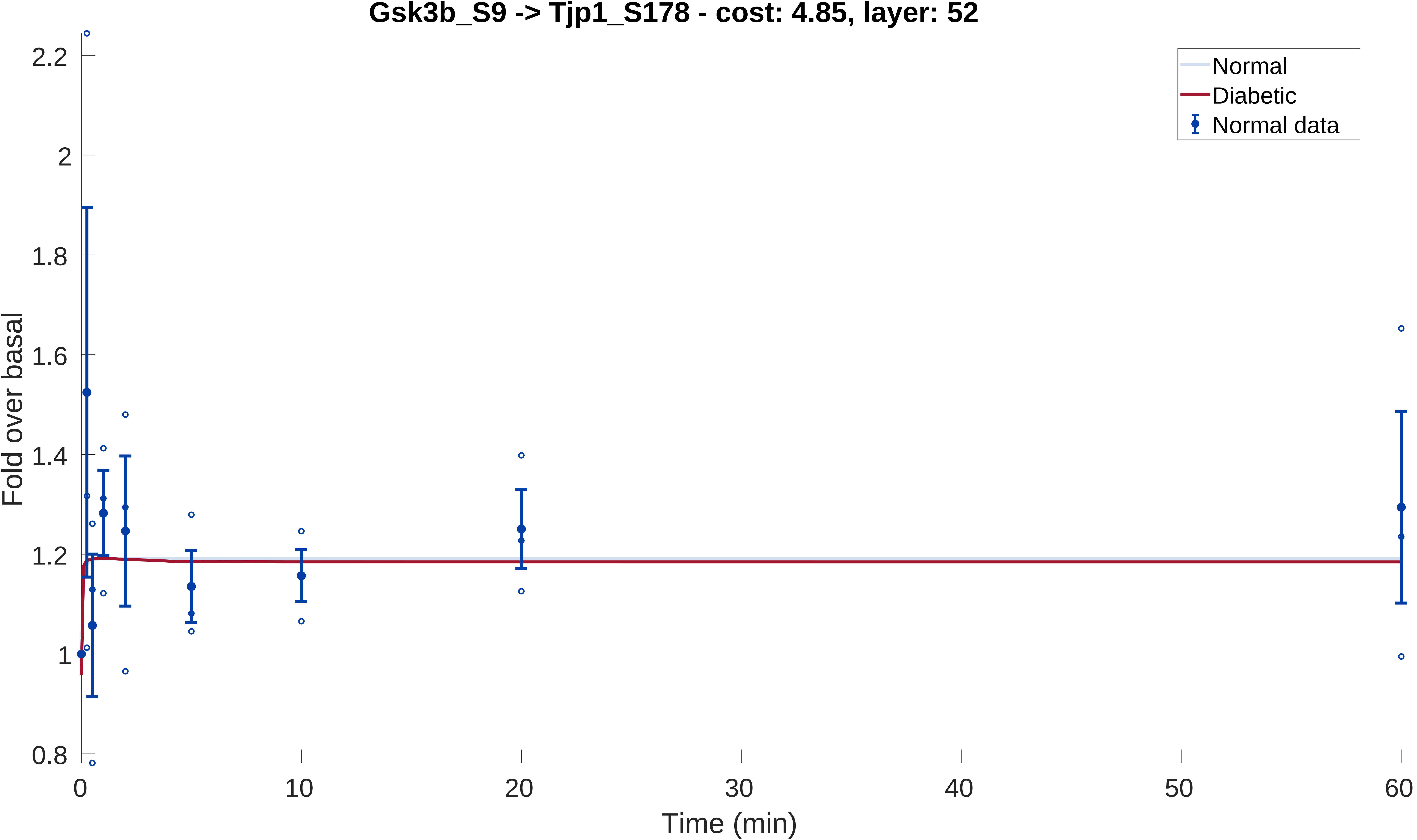

Gsk3b_S9 -> Tjp1_S178 - cost: 4.85, layer: 52
Normal
Diabetic
Normal data
Fold over basal
Time (min)
0.8
1
1.2
1.4
1.6
1.8
2
2.2
0
10
20
30
40
50
60

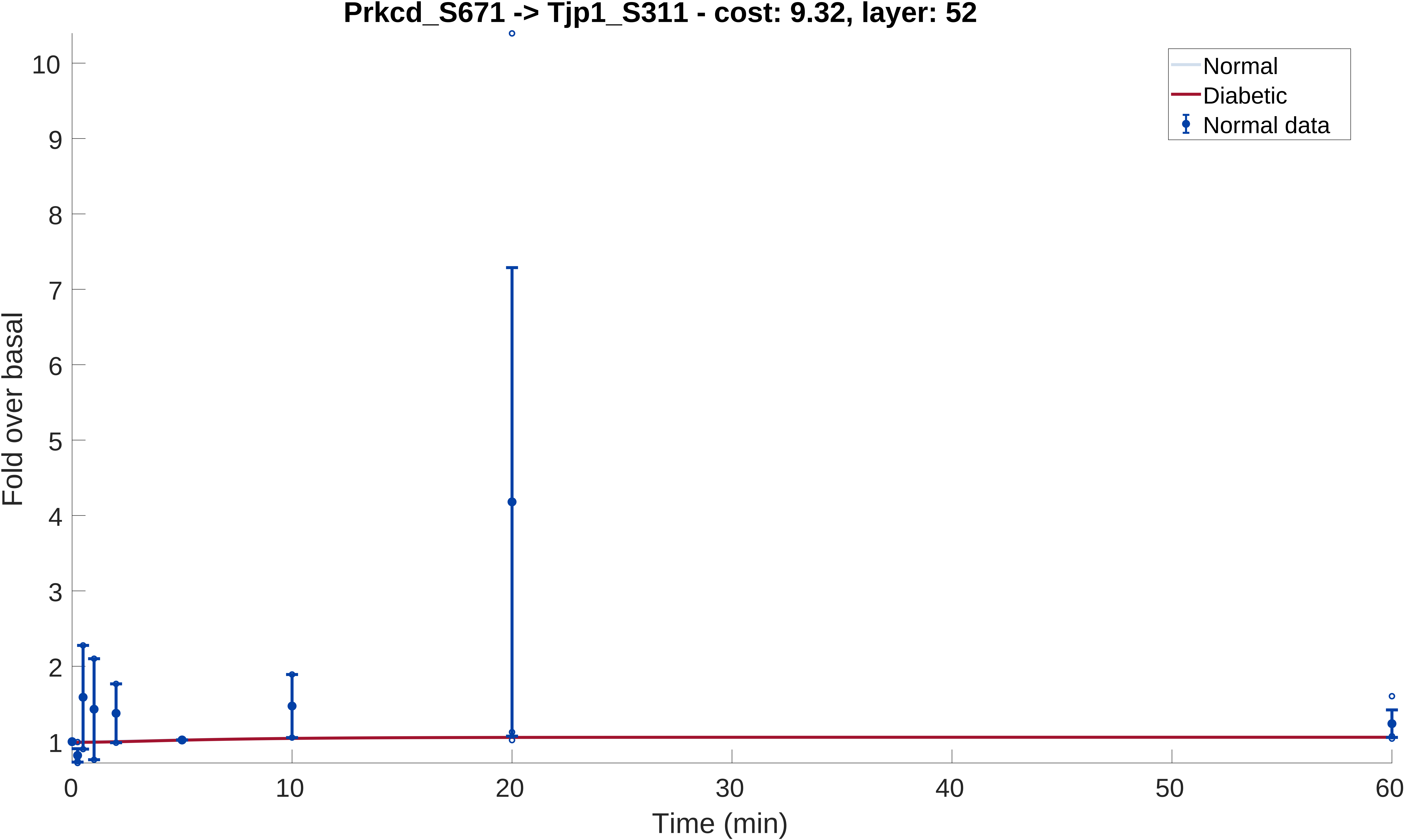

Prkcd_S671 -> Tjp1_S311 - cost: 9.32, layer: 52
Normal
Diabetic
Normal data
Fold over basal
Time (min)
0
10
20
30
40
50
60
1
2
3
4
5
6
7
8
9
10

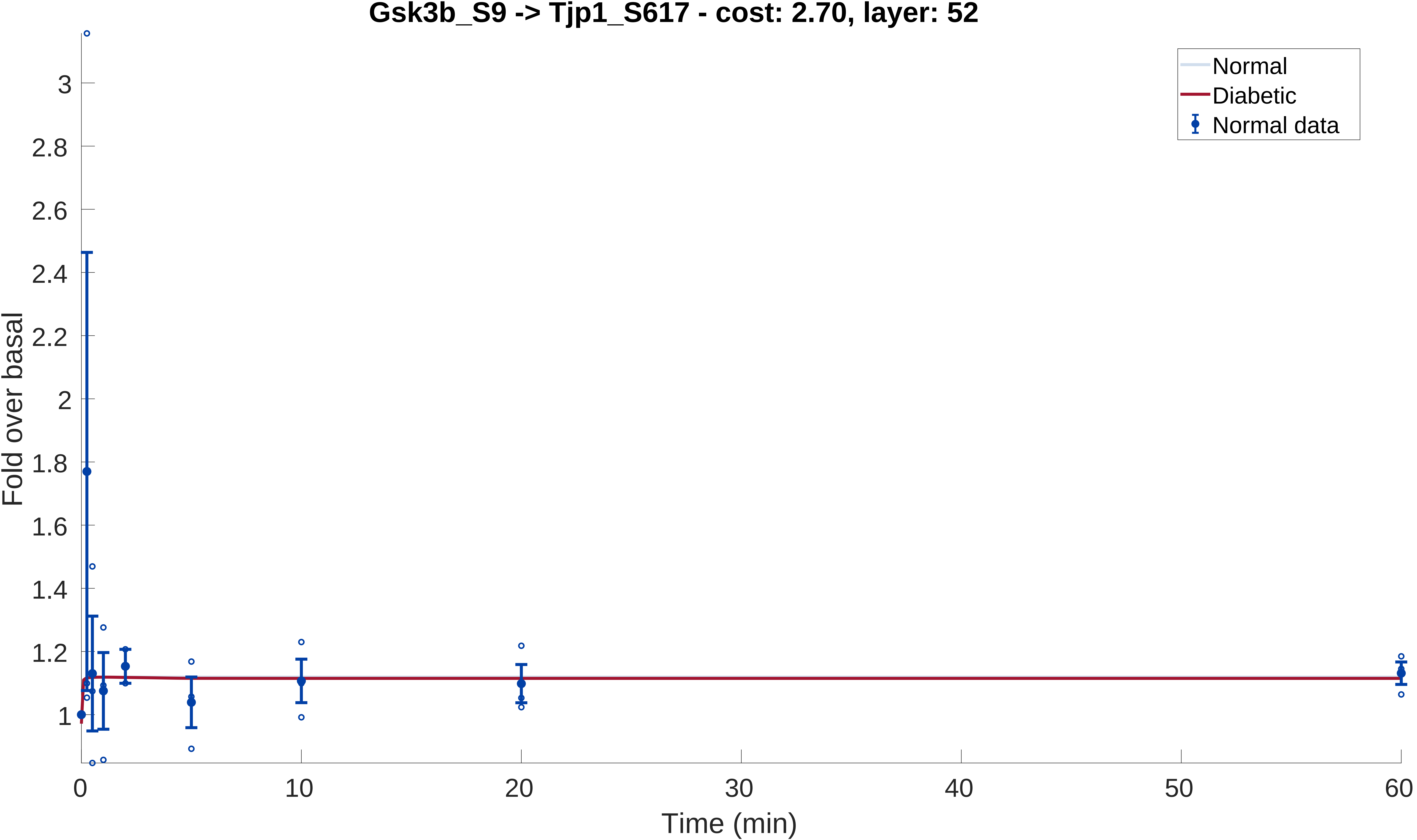

Gsk3b_S9 -> Tjp1_S617 - cost: 2.70, layer: 52
Normal
Diabetic
Normal data
Fold over basal
3
2.8
2.6
2.4
2.2
2
1.8
1.6
1.4
1.2
1
0
10
20
30
40
50
60
Time (min)

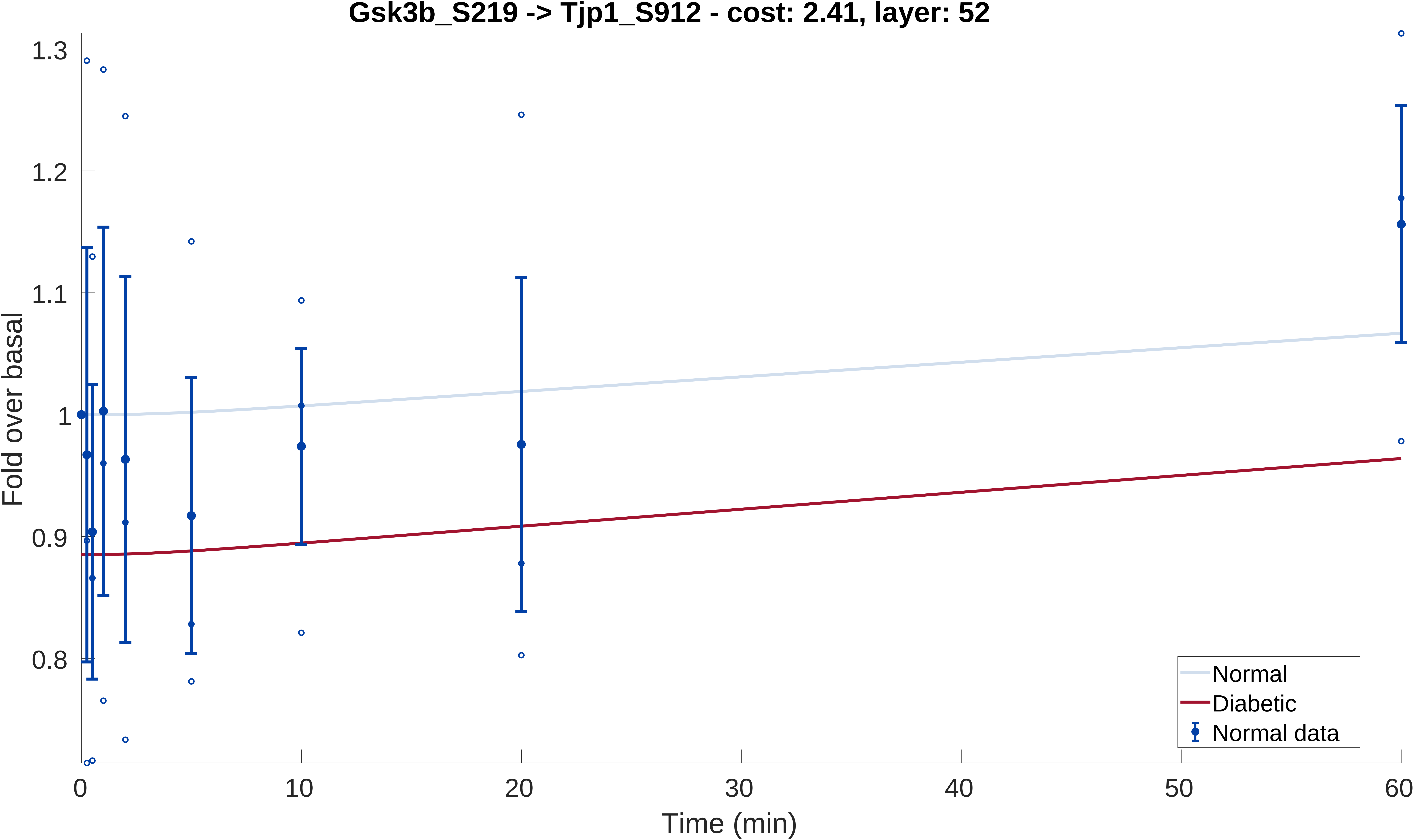
Gsk3b_S219 -> Tjp1_S912 - cost: 2.41, layer: 52
Fold over basal
1.3
1.2
1.1
1
0.9
0.8
0
10
20
30
40
50
60
Time (min)
Normal
Diabetic
Normal data

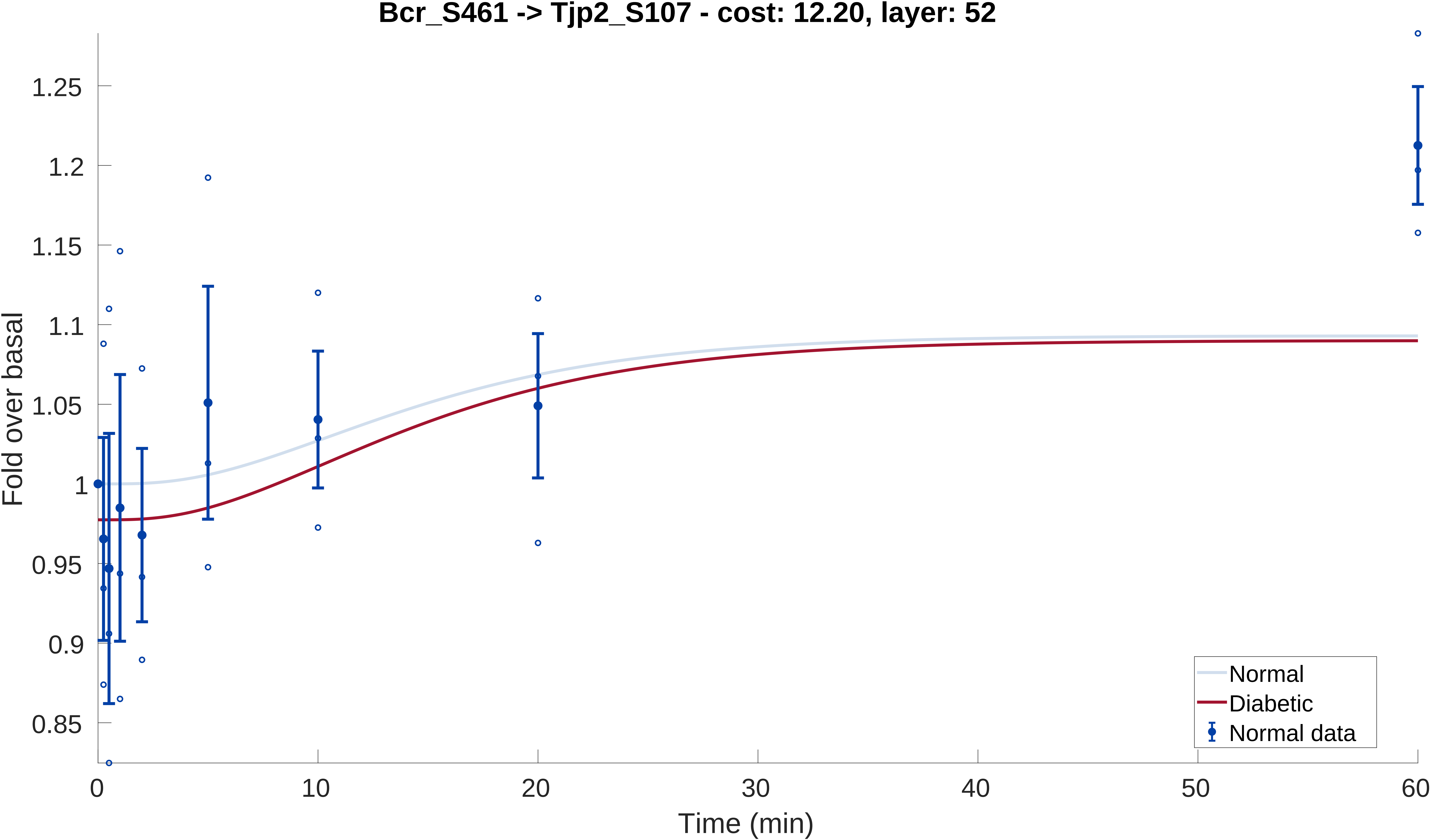

Bcr_S461 -> Tjp2_S107 - cost: 12.20, layer: 52
Fold over basal
1.25
1.2
1.15
1.1
1.05
1
0.95
0.9
0.85
0
10
20
30
40
50
60
Time (min)
Normal
Diabetic
Normal data

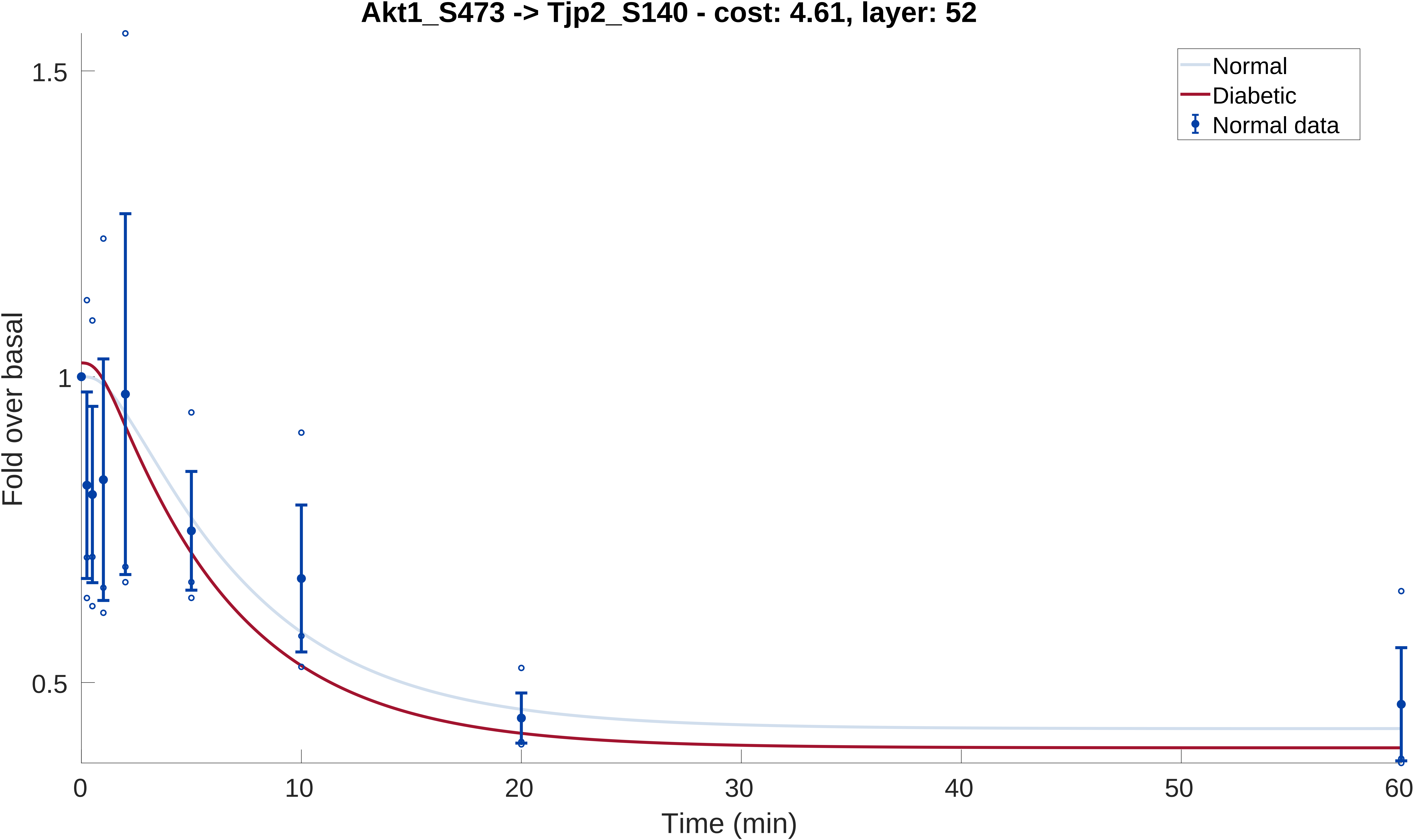

Akt1_S473 -> Tjp2_S140 - cost: 4.61, layer: 52
Normal
Diabetic
Normal data
Fold over basal
1.5
1
0.5
0
10
20
30
40
50
60
Time (min)

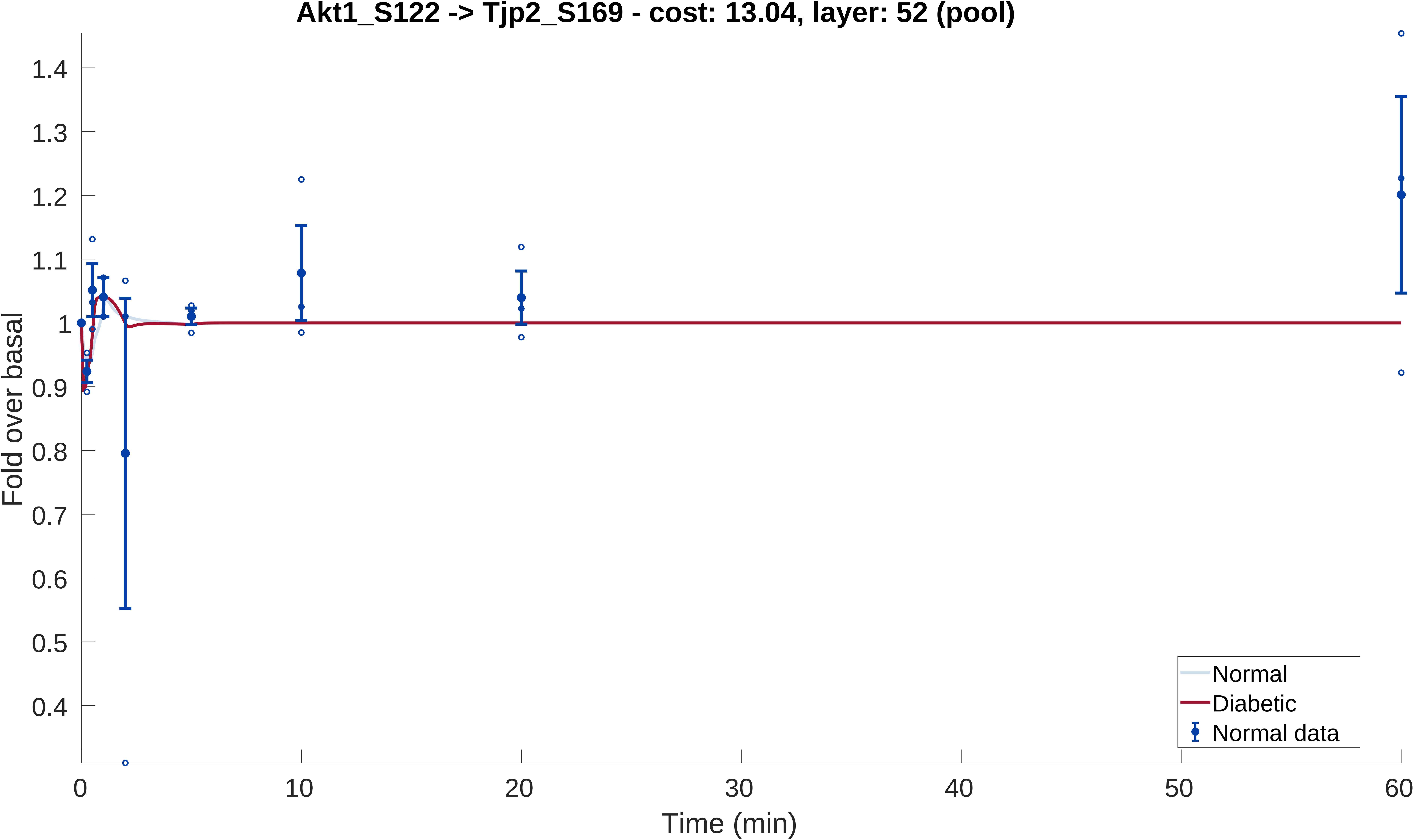
Akt1_S122 -> Tjp2_S169 - cost: 13.04, layer: 52 (pool)
Fold over basal
Time (min)
1.4
1.3
1.2
1.1
1
0.9
0.8
0.7
0.6
0.5
0.4
0
10
20
30
40
50
60
Normal
Diabetic
Normal data

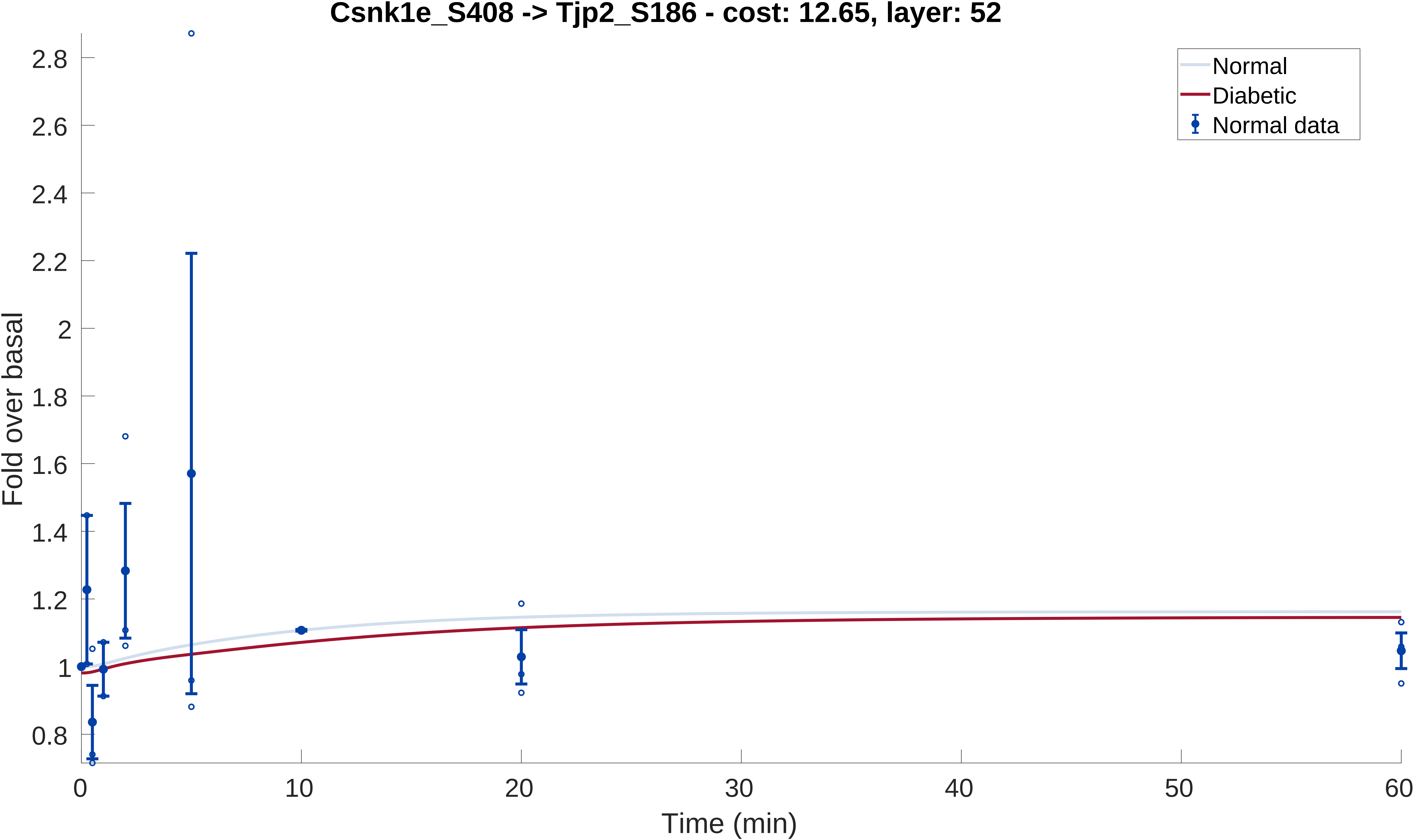

Csnk1e_S408 -> Tjp2_S186 - cost: 12.65, layer: 52
Normal
Diabetic
Normal data
Fold over basal
Time (min)
0.8
1
1.2
1.4
1.6
1.8
2
2.2
2.4
2.6
2.8
0
10
20
30
40
50
60

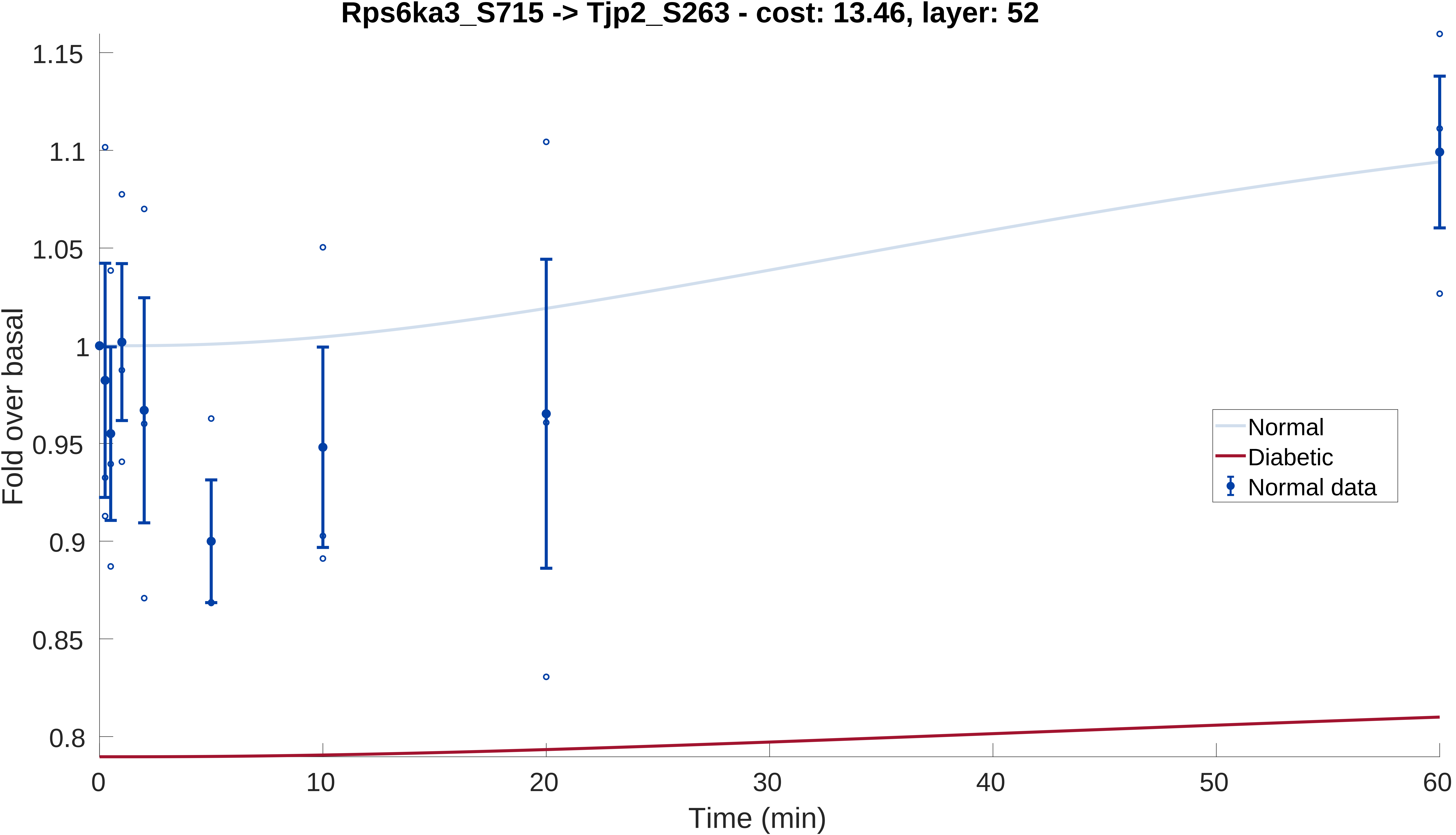

Rps6ka3_S715 -> Tjp2_S263 - cost: 13.46, layer: 52
Fold over basal
Time (min)
1.15
1.1
1.05
1
0.95
0.9
0.85
0.8
0
10
20
30
40
50
60
Normal
Diabetic
Normal data

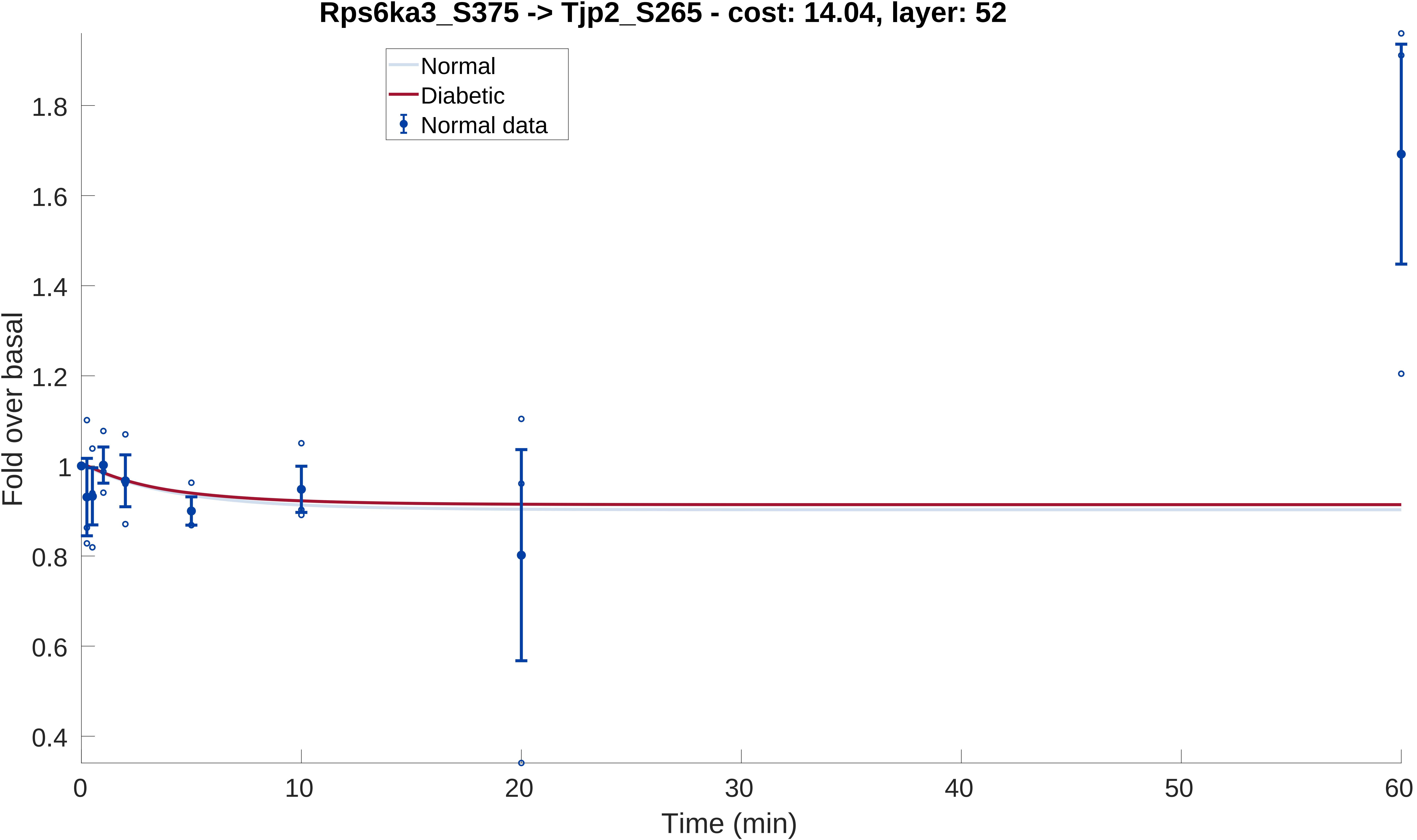

Rps6ka3_S375 -> Tjp2_S265 - cost: 14.04, layer: 52
Normal
Diabetic
Normal data
Fold over basal
1.8
1.6
1.4
1.2
1
0.8
0.6
0.4
0
10
20
30
40
50
60
Time (min)

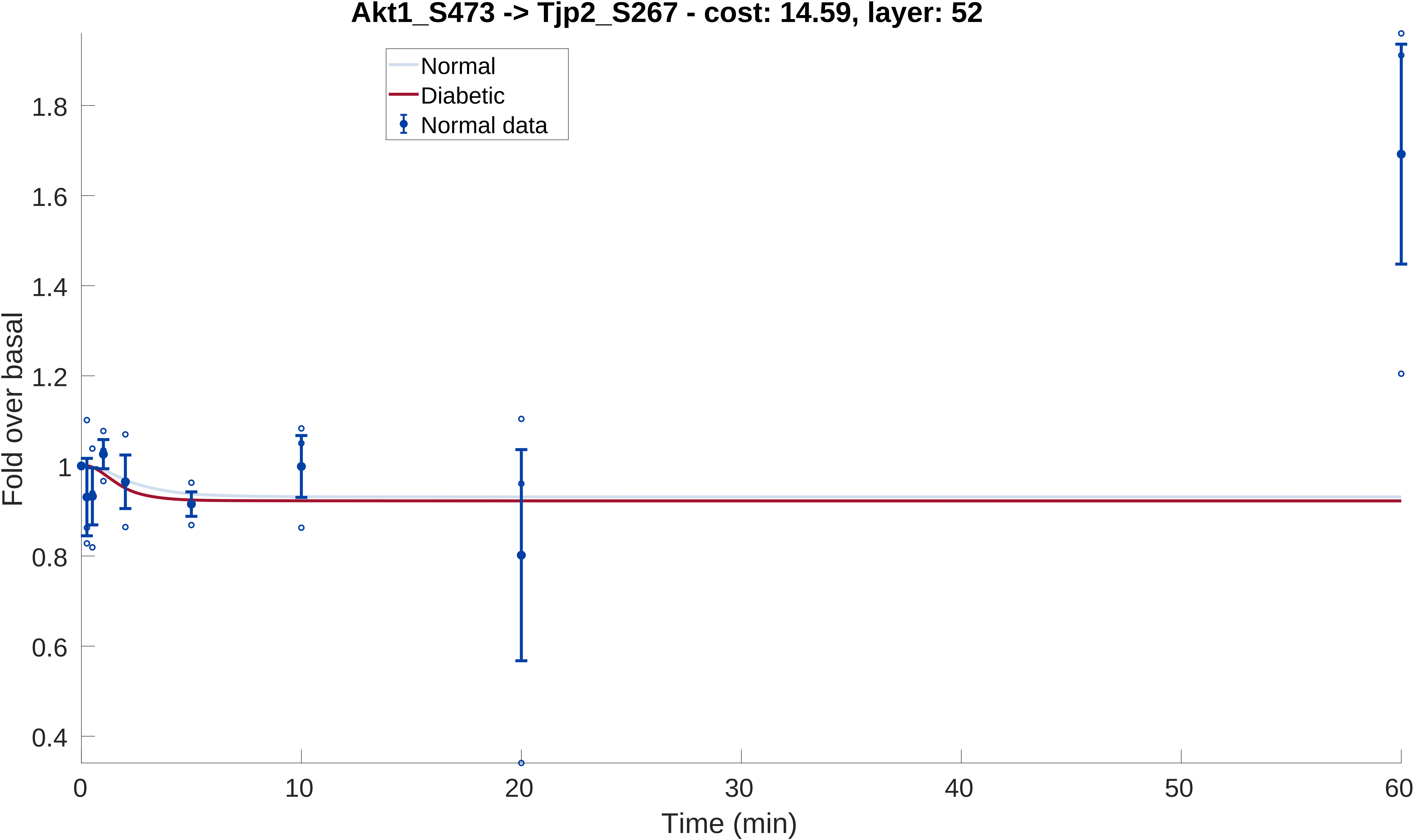
Akt1_S473 -> Tjp2_S267 - cost: 14.59, layer: 52
Normal
Diabetic
Normal data
Fold over basal
Time (min)
0.4
0.6
0.8
1
1.2
1.4
1.6
1.8
0
10
20
30
40
50
60

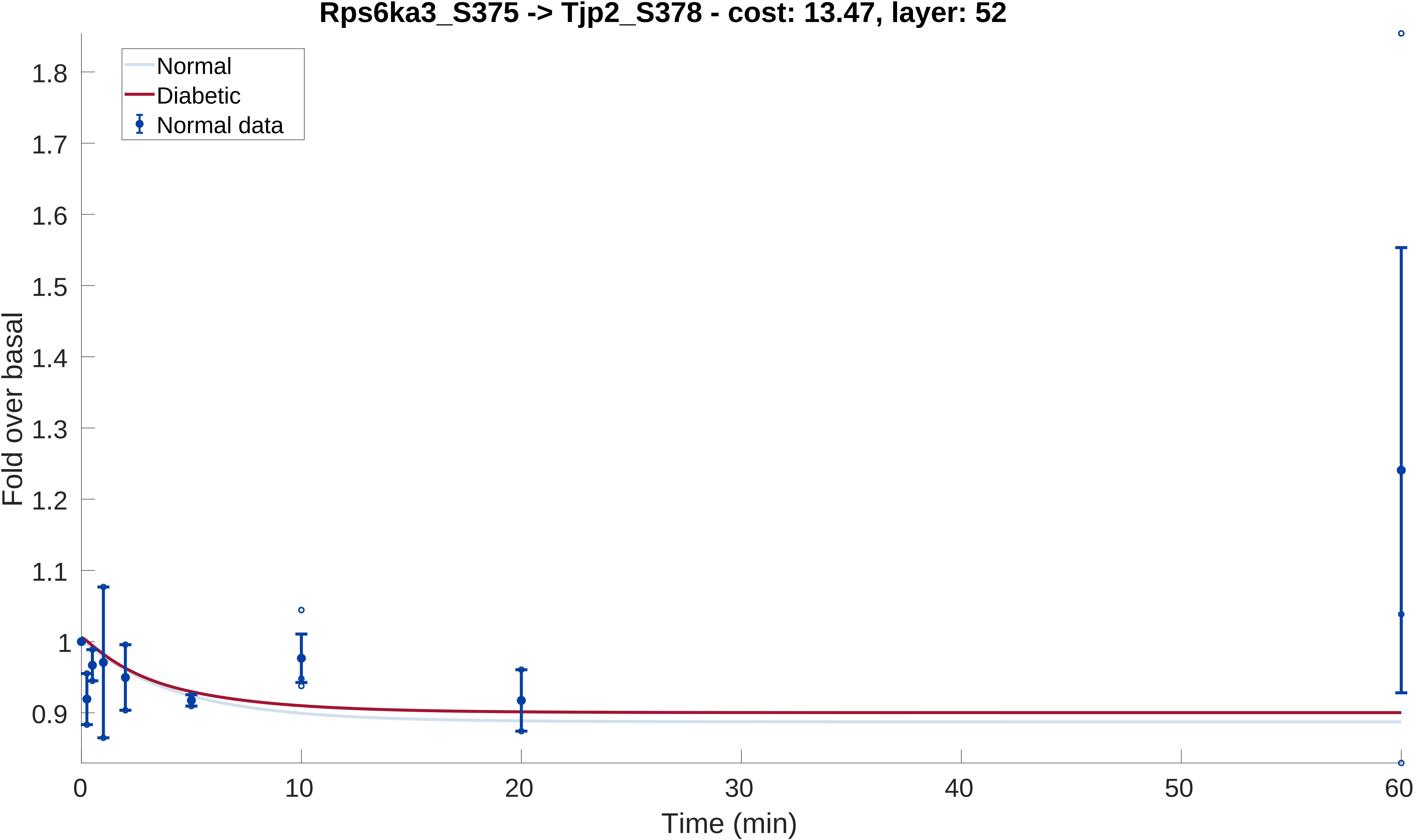

Rps6ka3_S375 -> Tjp2_S378 - cost: 13.47, layer: 52
Normal
Diabetic
Normal data
Fold over basal
Time (min)
1.8
1.7
1.6
1.5
1.4
1.3
1.2
1.1
1
0.9
0
10
20
30
40
50
60

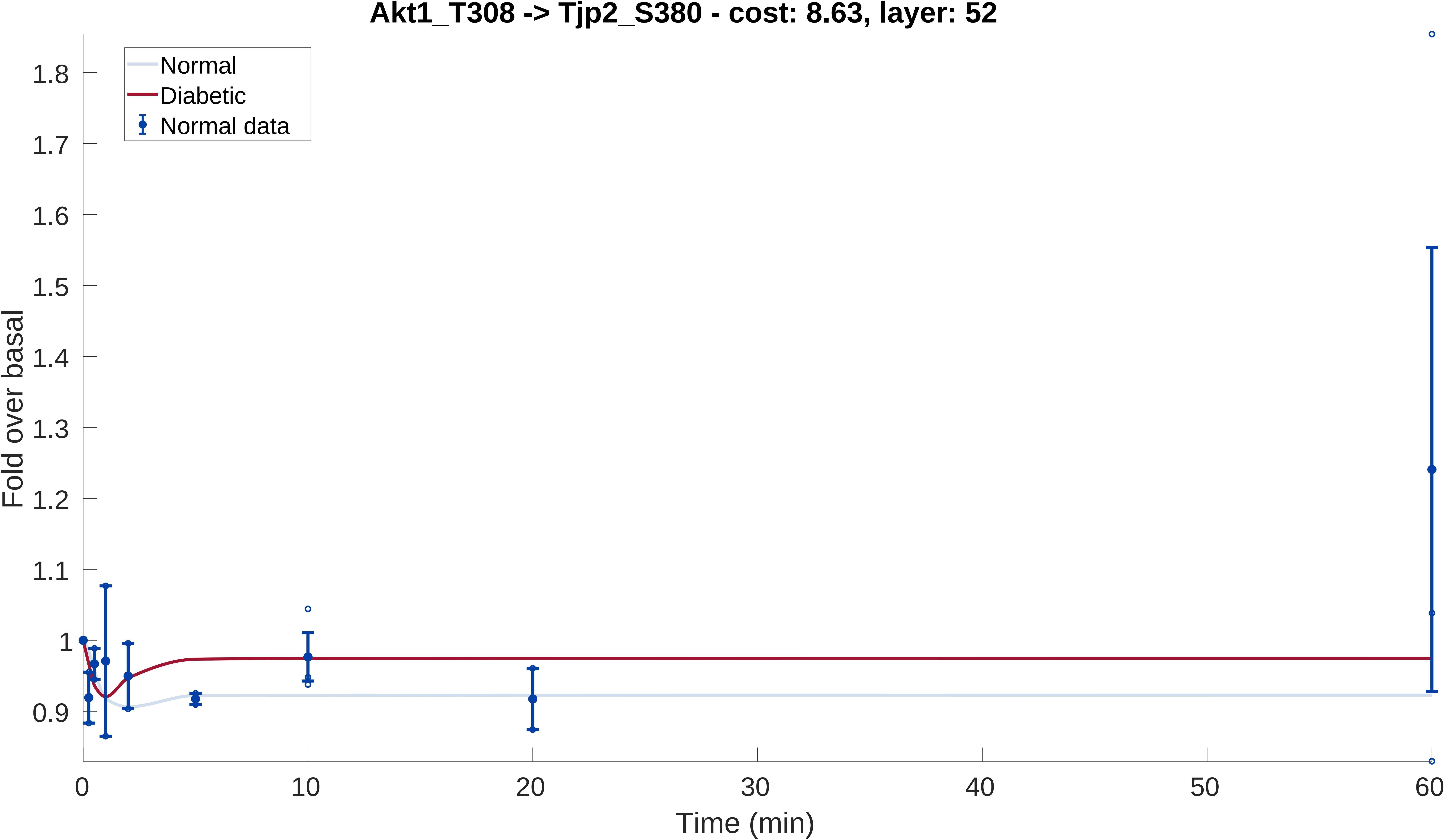

Akt1_T308 -> Tjp2_S380 - cost: 8.63, layer: 52
Normal
Diabetic
Normal data
Fold over basal
1.8
1.7
1.6
1.5
1.4
1.3
1.2
1.1
1
0.9
0
10
20
30
40
50
60
Time (min)

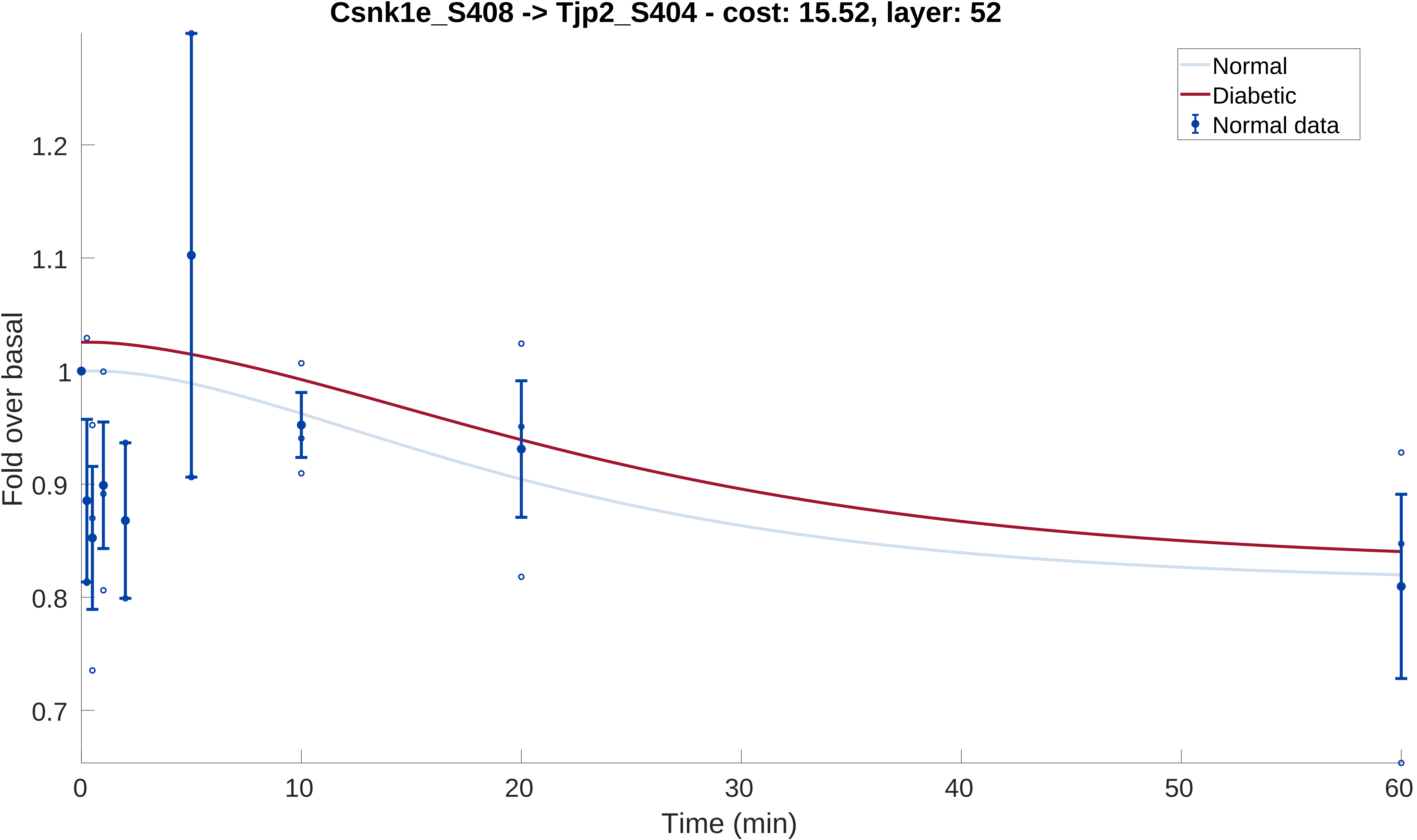

Csnk1e_S408 -> Tjp2_S404 - cost: 15.52, layer: 52
Normal
Diabetic
Normal data
Fold over basal
1.2
1.1
1
0.9
0.8
0.7
0
10
20
30
40
50
60
Time (min)

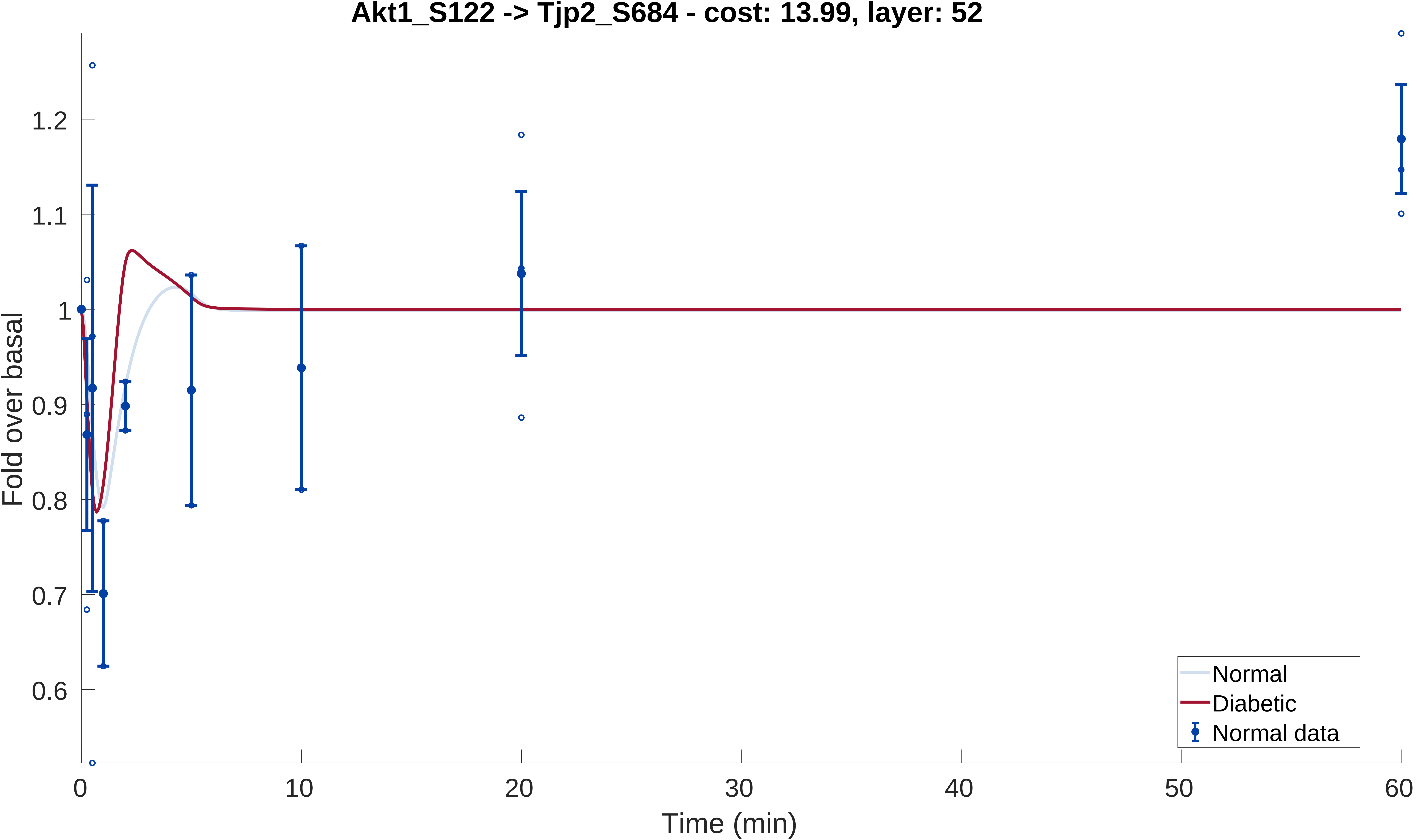

Akt1_S122 -> Tjp2_S684 - cost: 13.99, layer: 52
Fold over basal
Time (min)
0.6
0.7
0.8
0.9
1
1.1
1.2
0
10
20
30
40
50
60
Normal
Diabetic
Normal data

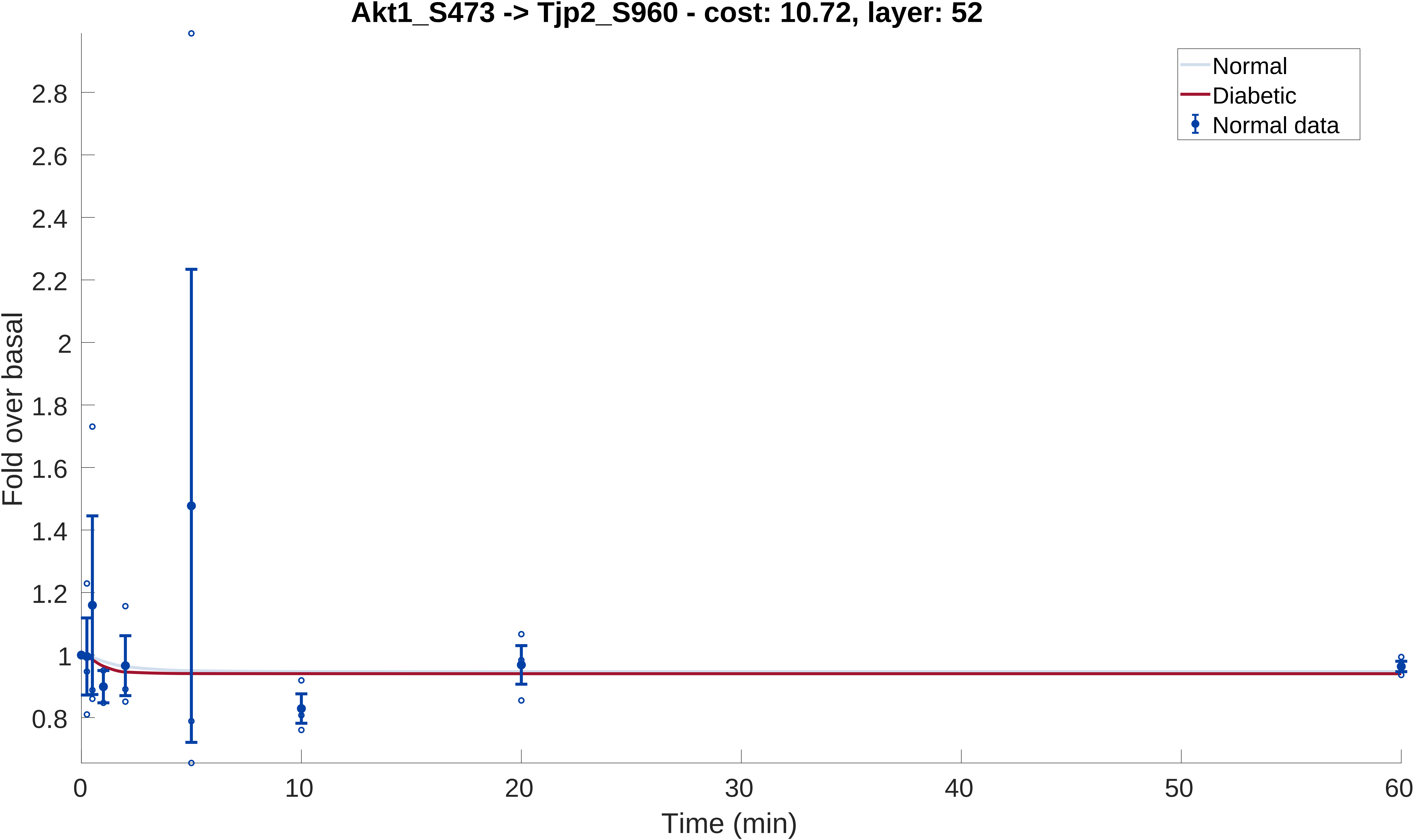

Akt1_S473 -> Tjp2_S960 - cost: 10.72, layer: 52
Normal
Diabetic
Normal data
Fold over basal
2.8
2.6
2.4
2.2
2
1.8
1.6
1.4
1.2
1
0.8
0
10
20
30
40
50
60
Time (min)

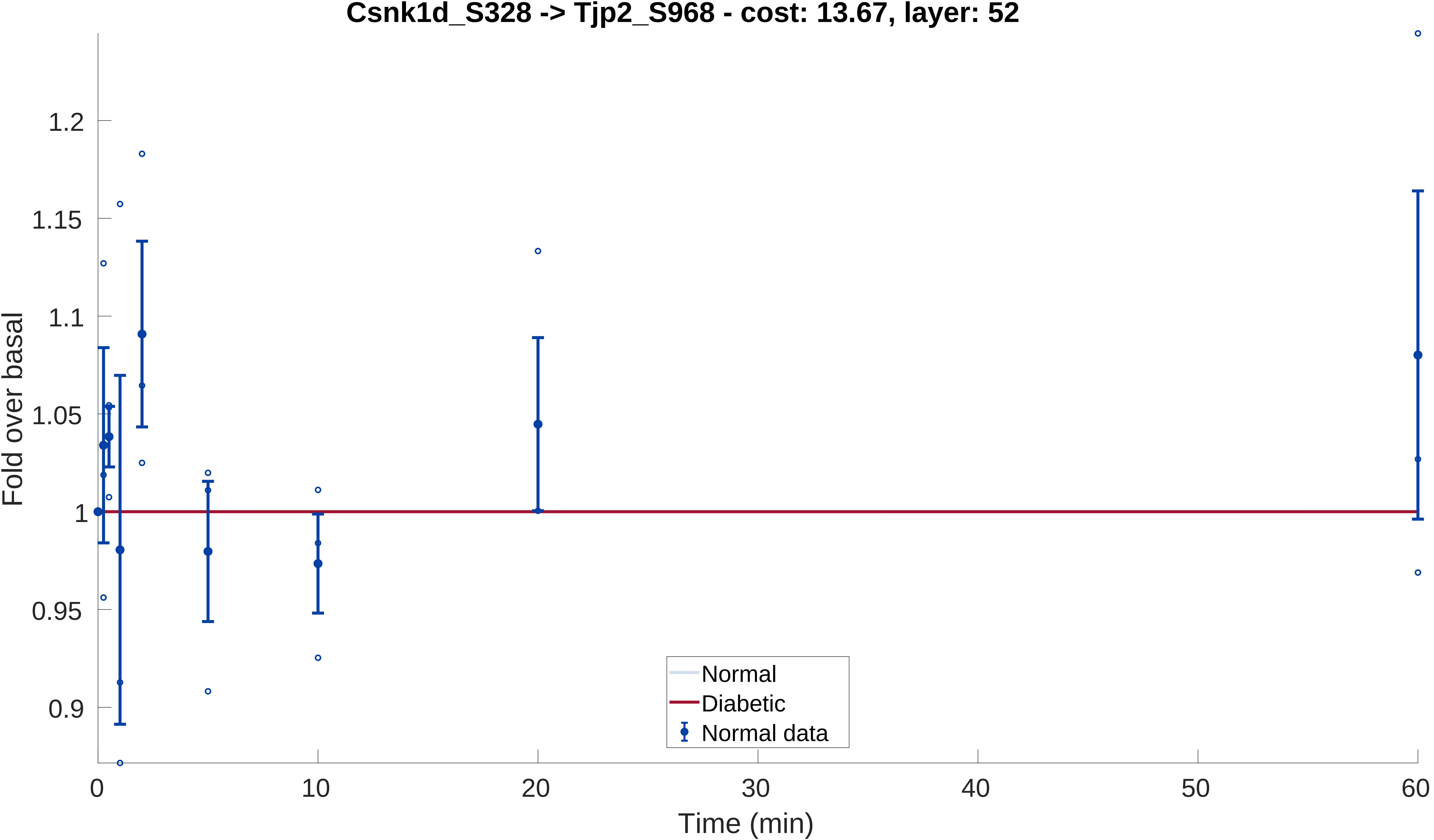

Csnk1d_S328 -> Tjp2_S968 - cost: 13.67, layer: 52
Fold over basal
1.2
1.15
1.1
1.05
1
0.95
0.9
0
10
20
30
40
50
60
Time (min)
Normal
Diabetic
Normal data

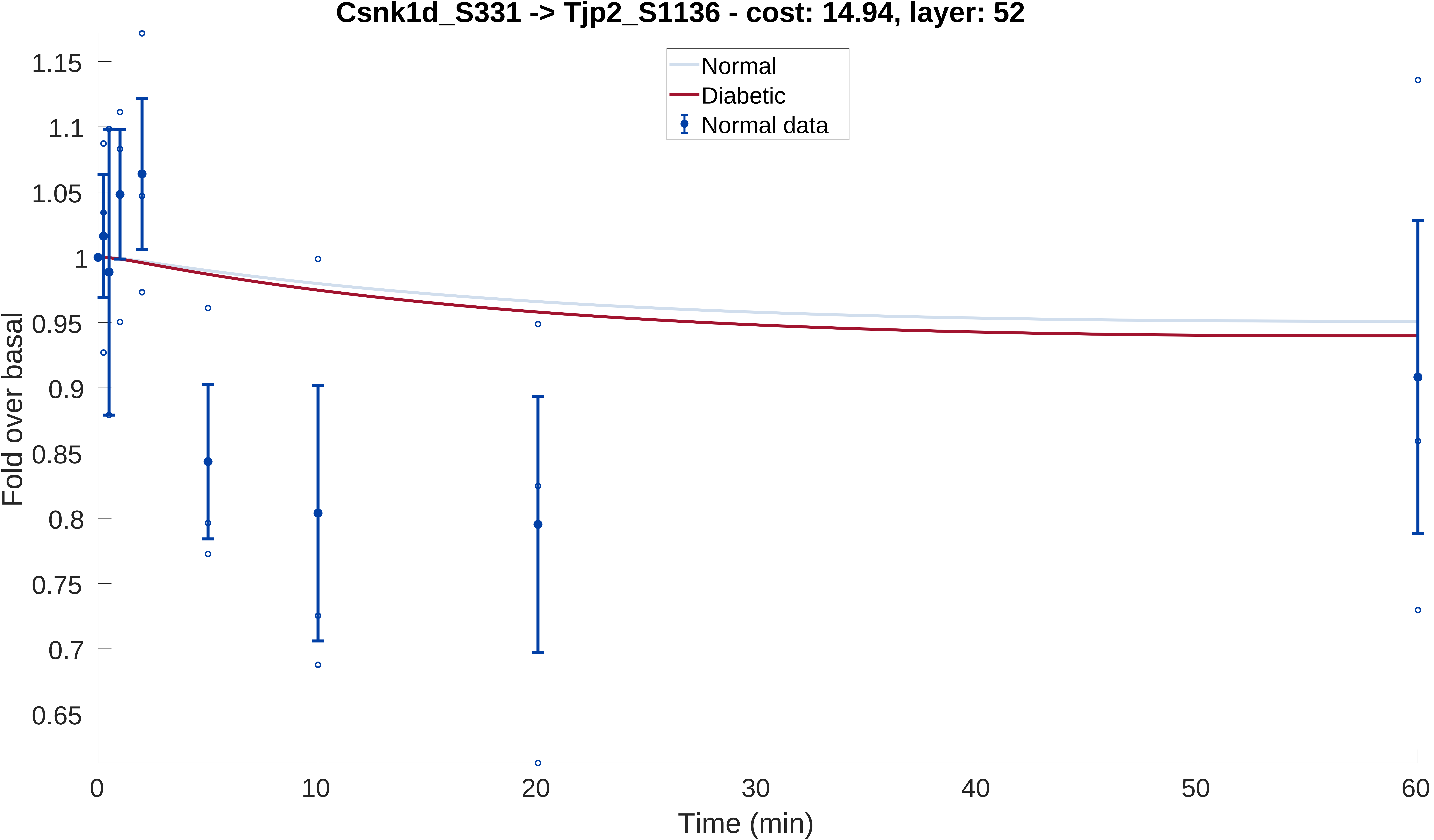

Csnk1d_S331 -> Tjp2_S1136 - cost: 14.94, layer: 52
Normal
Diabetic
Normal data
Fold over basal
1.15
1.1
1.05
1
0.95
0.9
0.85
0.8
0.75
0.7
0.65
0
10
20
30
40
50
60
Time (min)

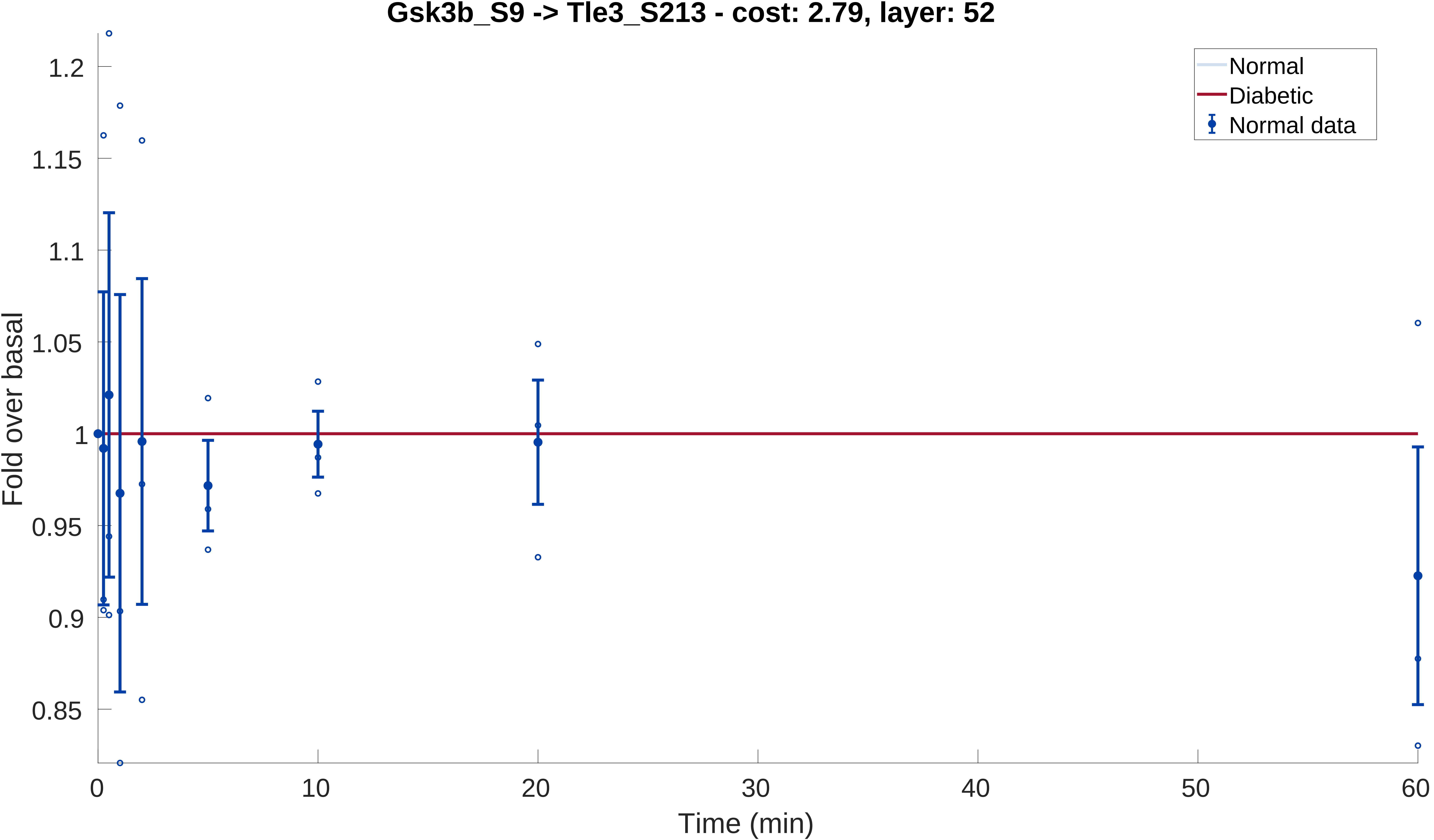

Gsk3b_S9 -> Tle3_S213 - cost: 2.79, layer: 52
Normal
Diabetic
Normal data
Fold over basal
1.2
1.15
1.1
1.05
1
0.95
0.9
0.85
0
10
20
30
40
50
60
Time (min)

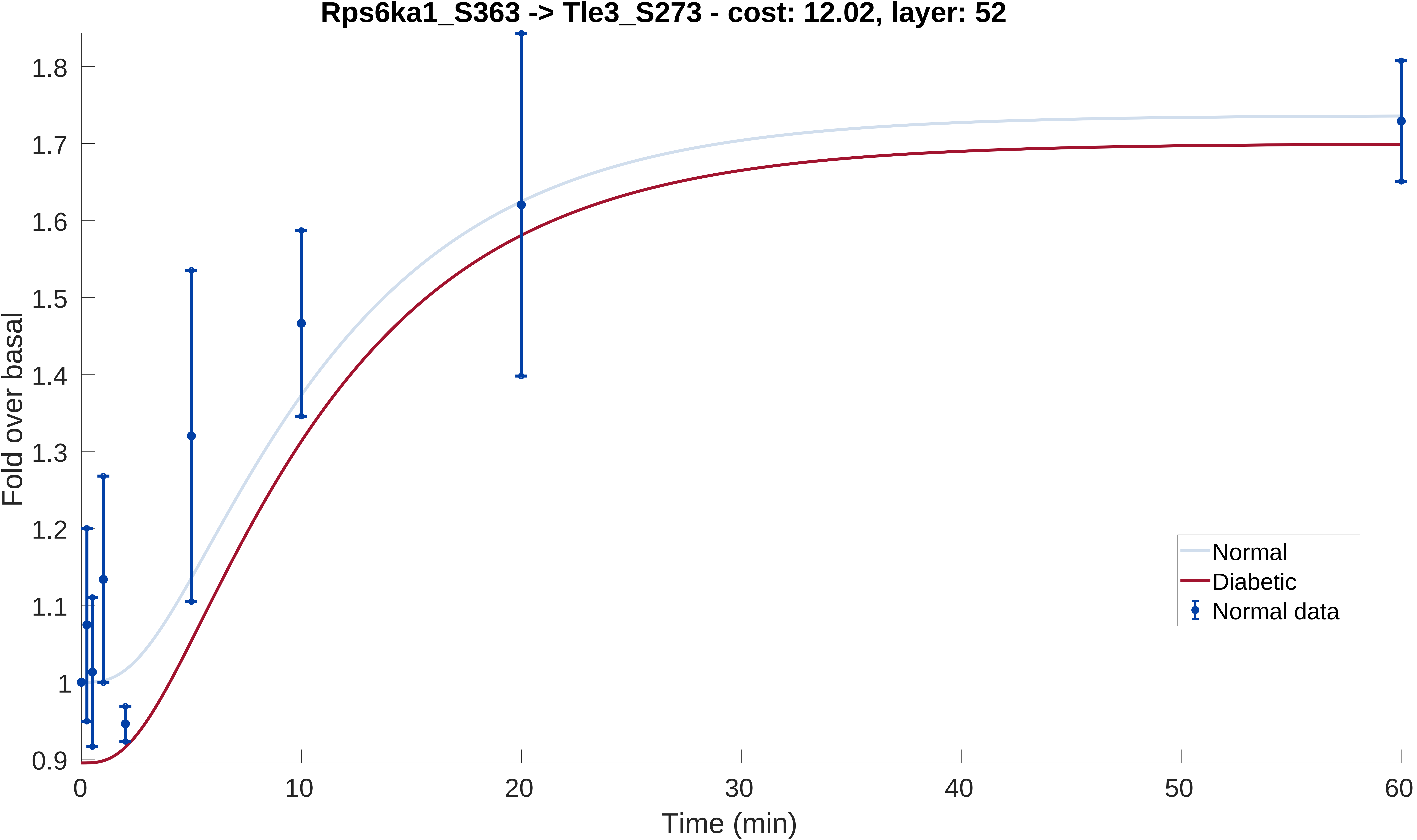

Rps6ka1_S363 -> Tle3_S273 - cost: 12.02, layer: 52
Fold over basal
1.8
1.7
1.6
1.5
1.4
1.3
1.2
1.1
1
0.9
0
10
20
30
40
50
60
Time (min)
Normal
Diabetic
Normal data

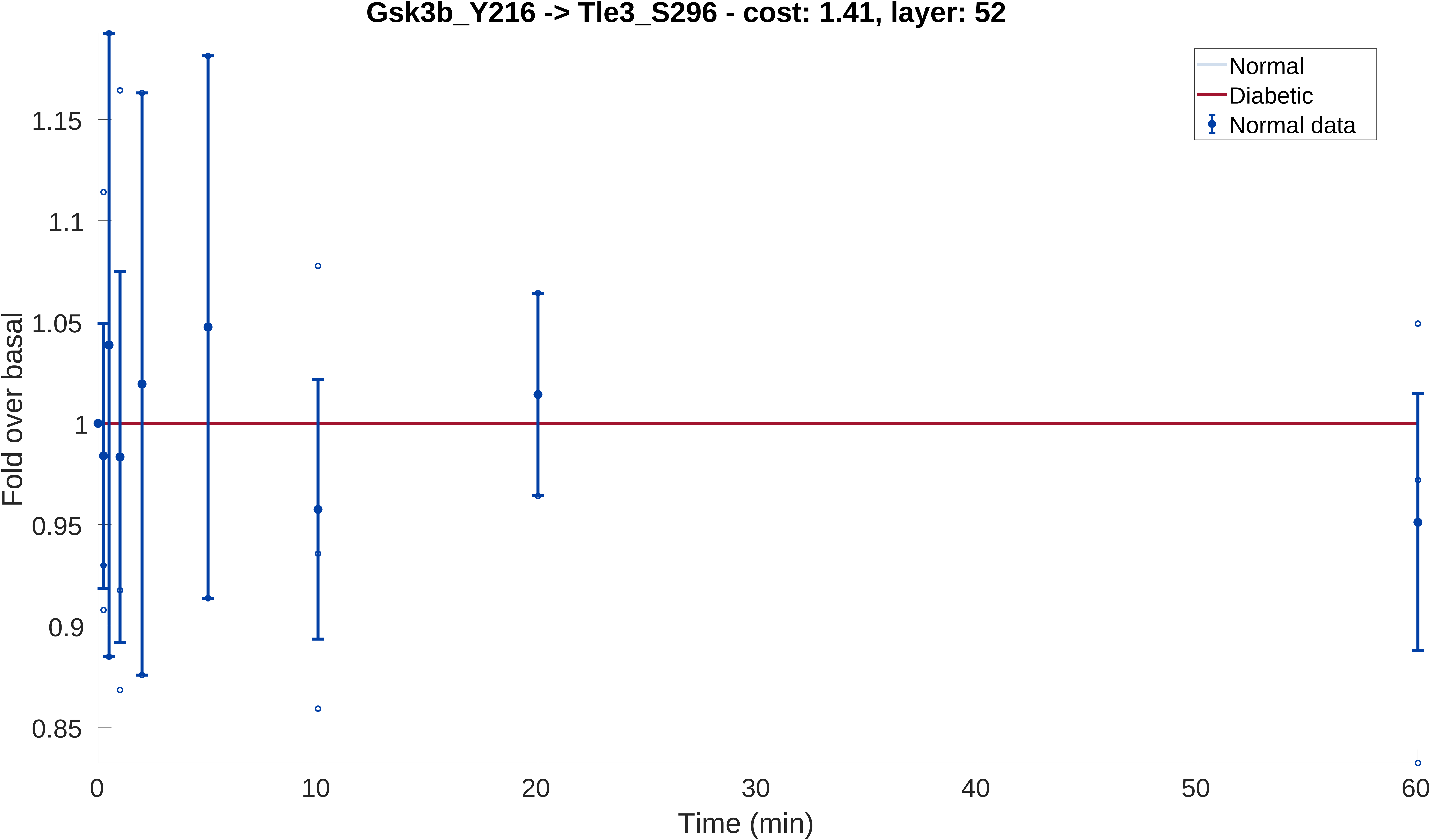

Gsk3b_Y216 -> Tle3_S296 - cost: 1.41, layer: 52
Normal
Diabetic
Normal data
Fold over basal
1.15
1.1
1.05
1
0.95
0.9
0.85
0
10
20
30
40
50
60
Time (min)

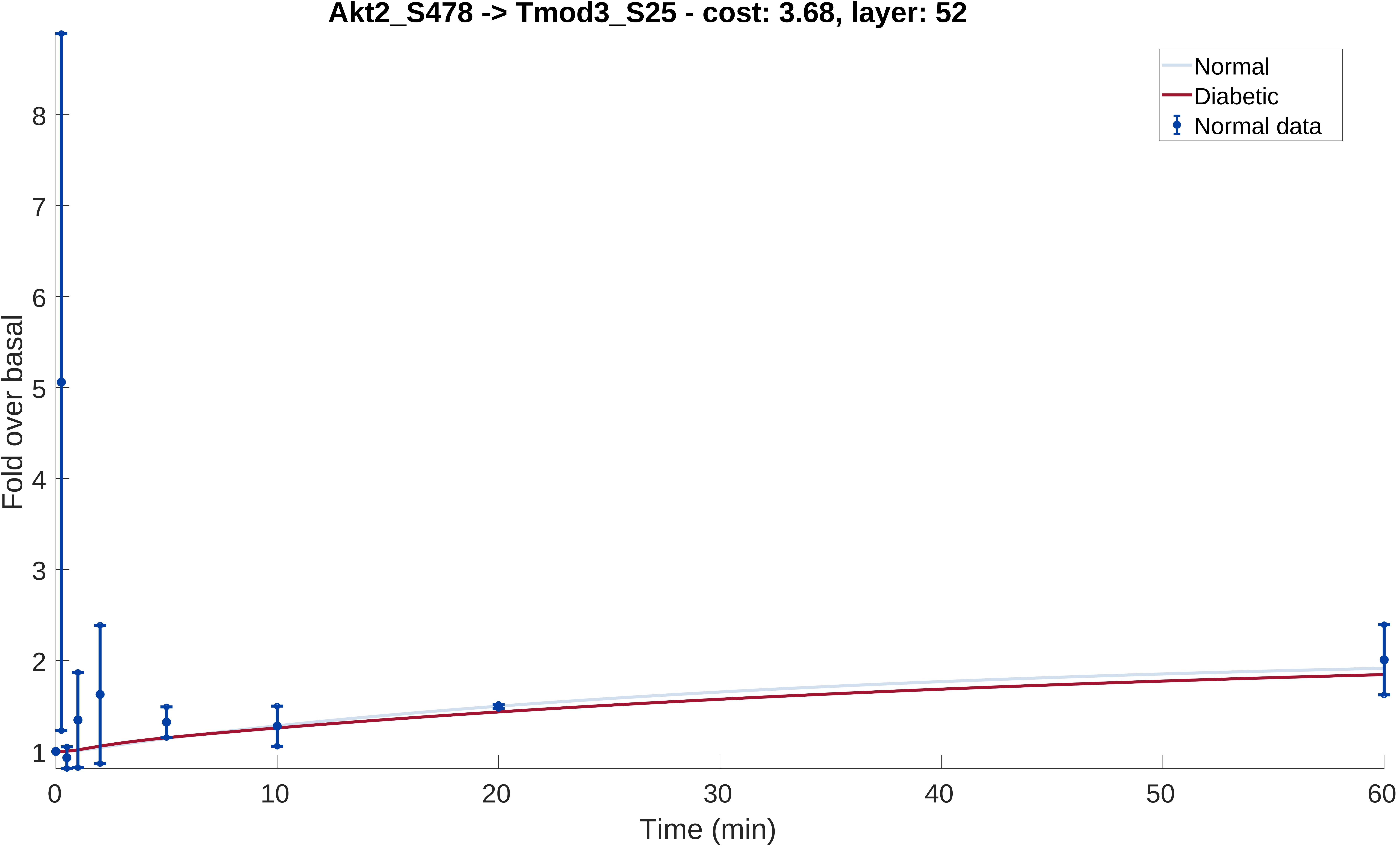

Akt2_S478 -> Tmod3_S25 - cost: 3.68, layer: 52
Normal
Diabetic
Normal data
Fold over basal
Time (min)
1
2
3
4
5
6
7
8
0
10
20
30
40
50
60

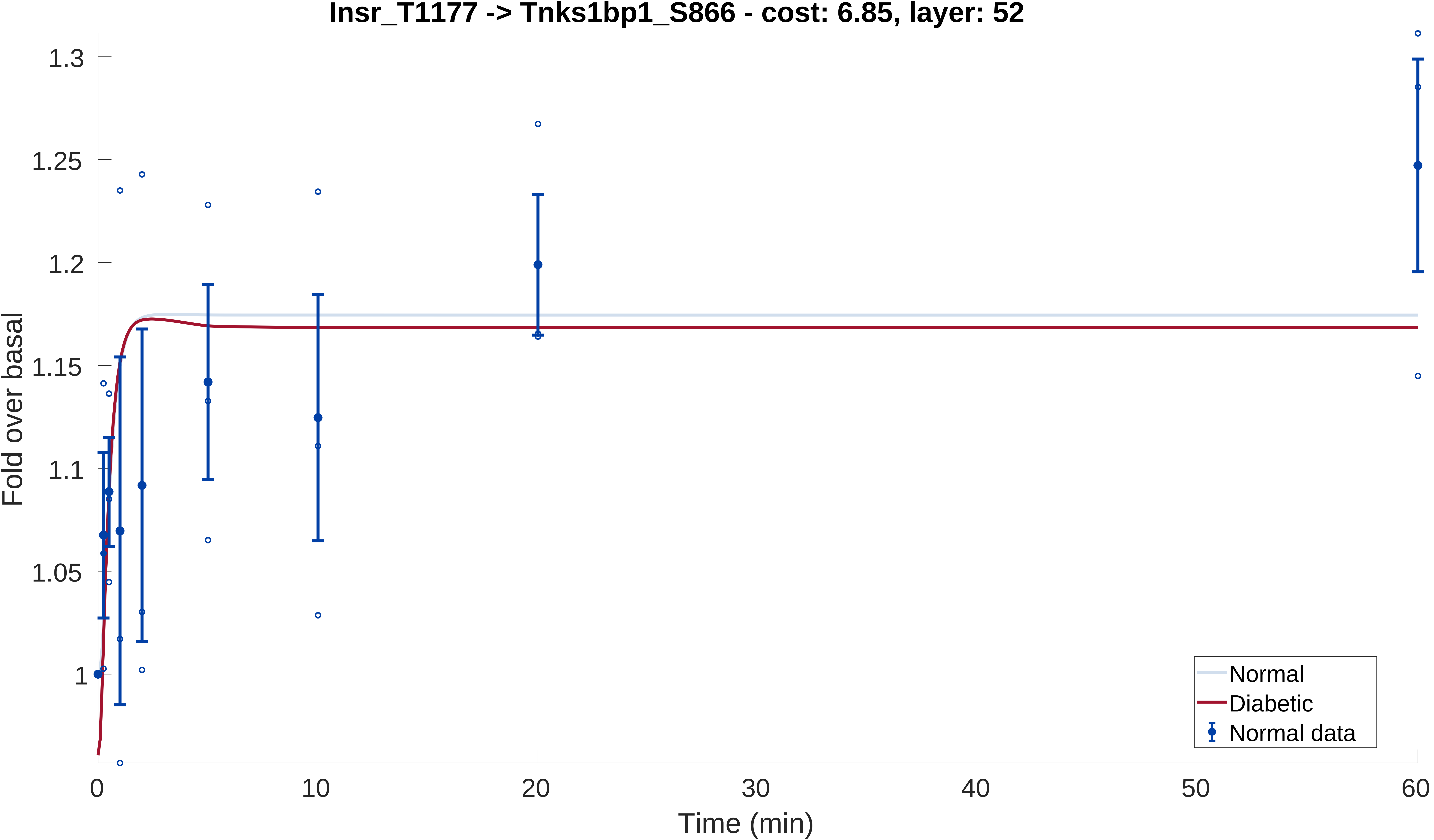

Insr_T1177 -> Tnks1bp1_S866 - cost: 6.85, layer: 52
Fold over basal
1.3
1.25
1.2
1.15
1.1
1.05
1
0
10
20
30
40
50
60
Time (min)
Normal
Diabetic
Normal data

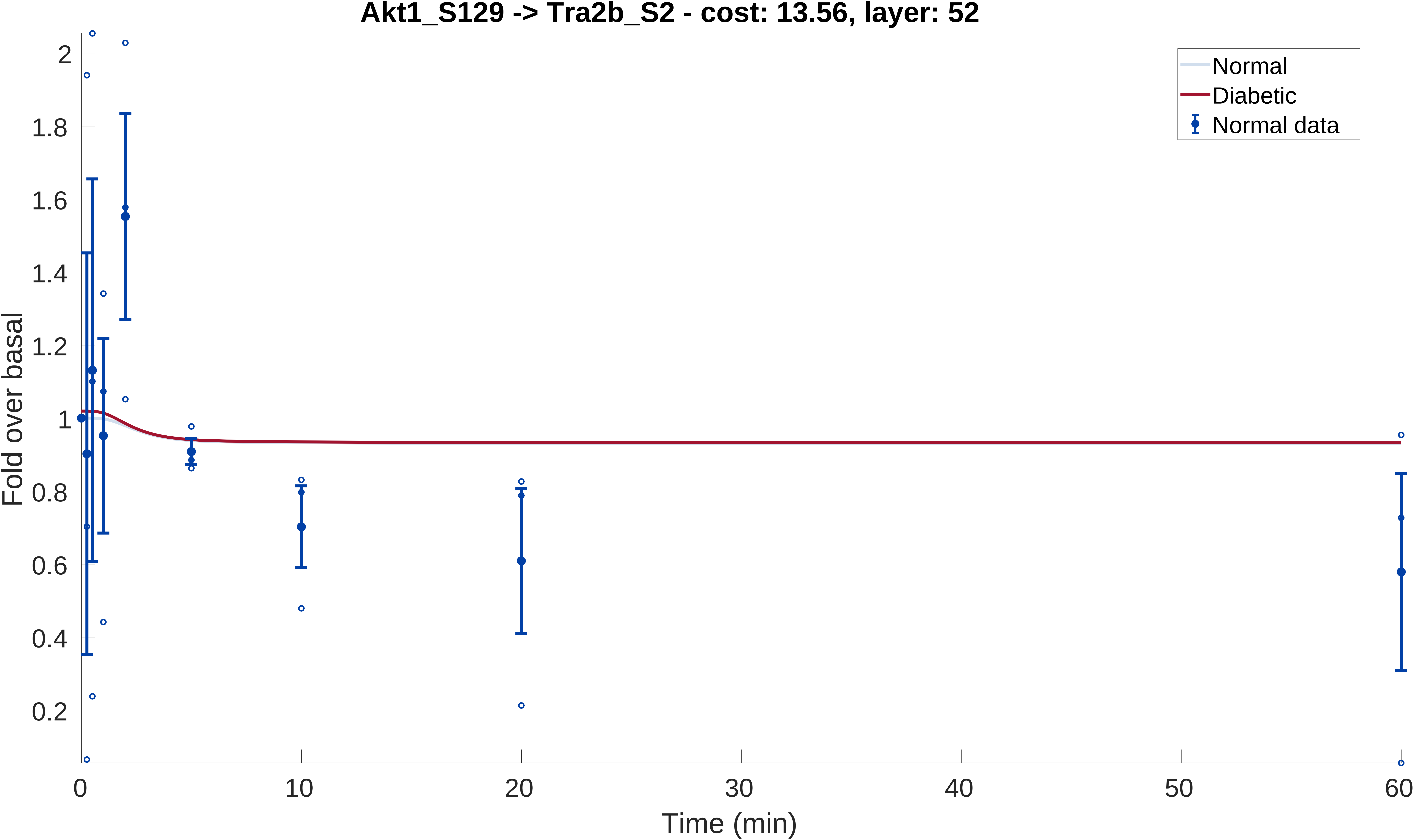
Akt1_S129 -> Tra2b_S2 - cost: 13.56, layer: 52
Normal
Diabetic
Normal data
Fold over basal
Time (min)
2
1.8
1.6
1.4
1.2
1
0.8
0.6
0.4
0.2
0
10
20
30
40
50
60

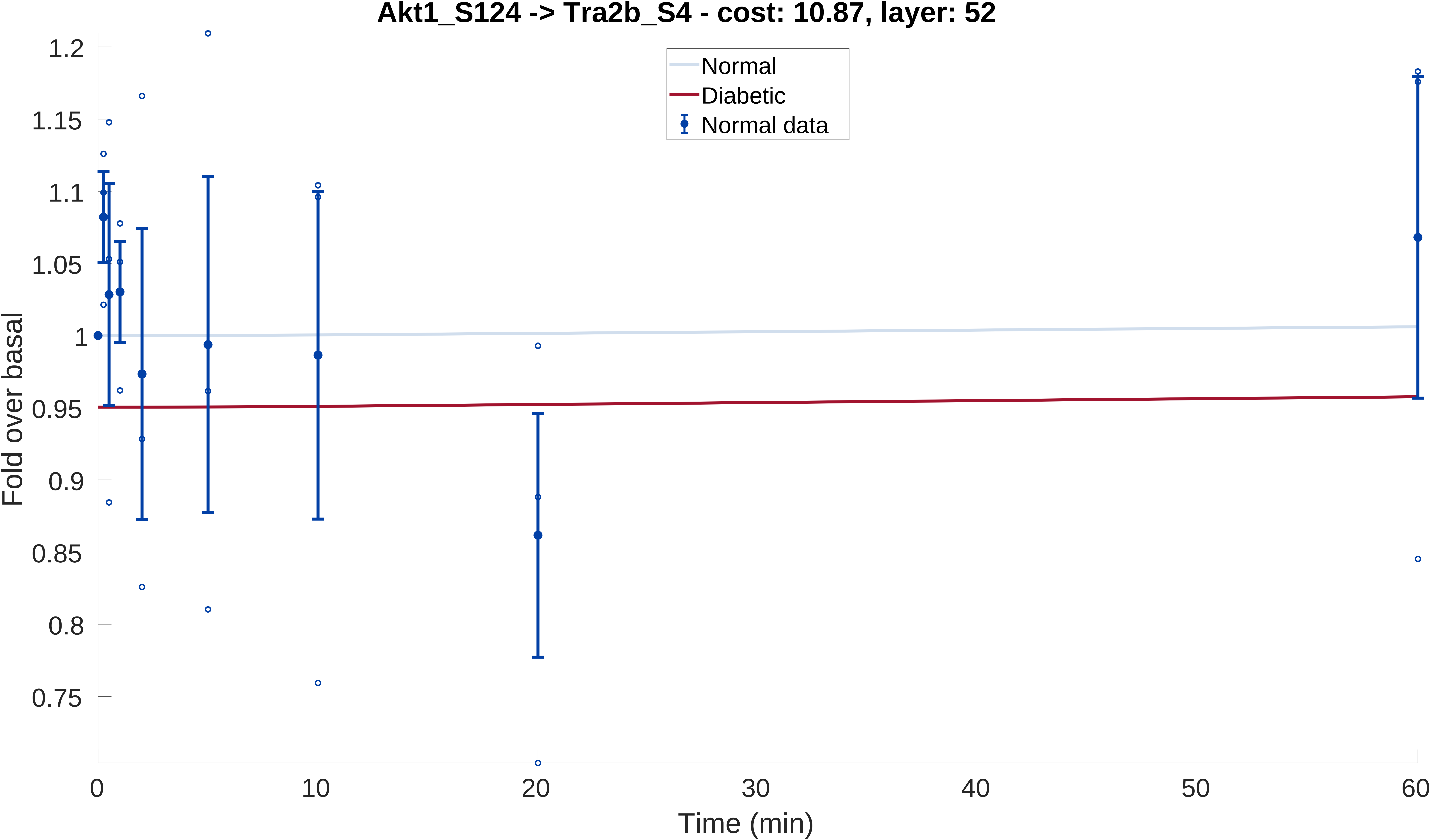
Akt1_S124 -> Tra2b_S4 - cost: 10.87, layer: 52
Normal
Diabetic
Normal data
Fold over basal
1.2
1.15
1.1
1.05
1
0.95
0.9
0.85
0.8
0.75
0
10
20
30
40
50
60
Time (min)

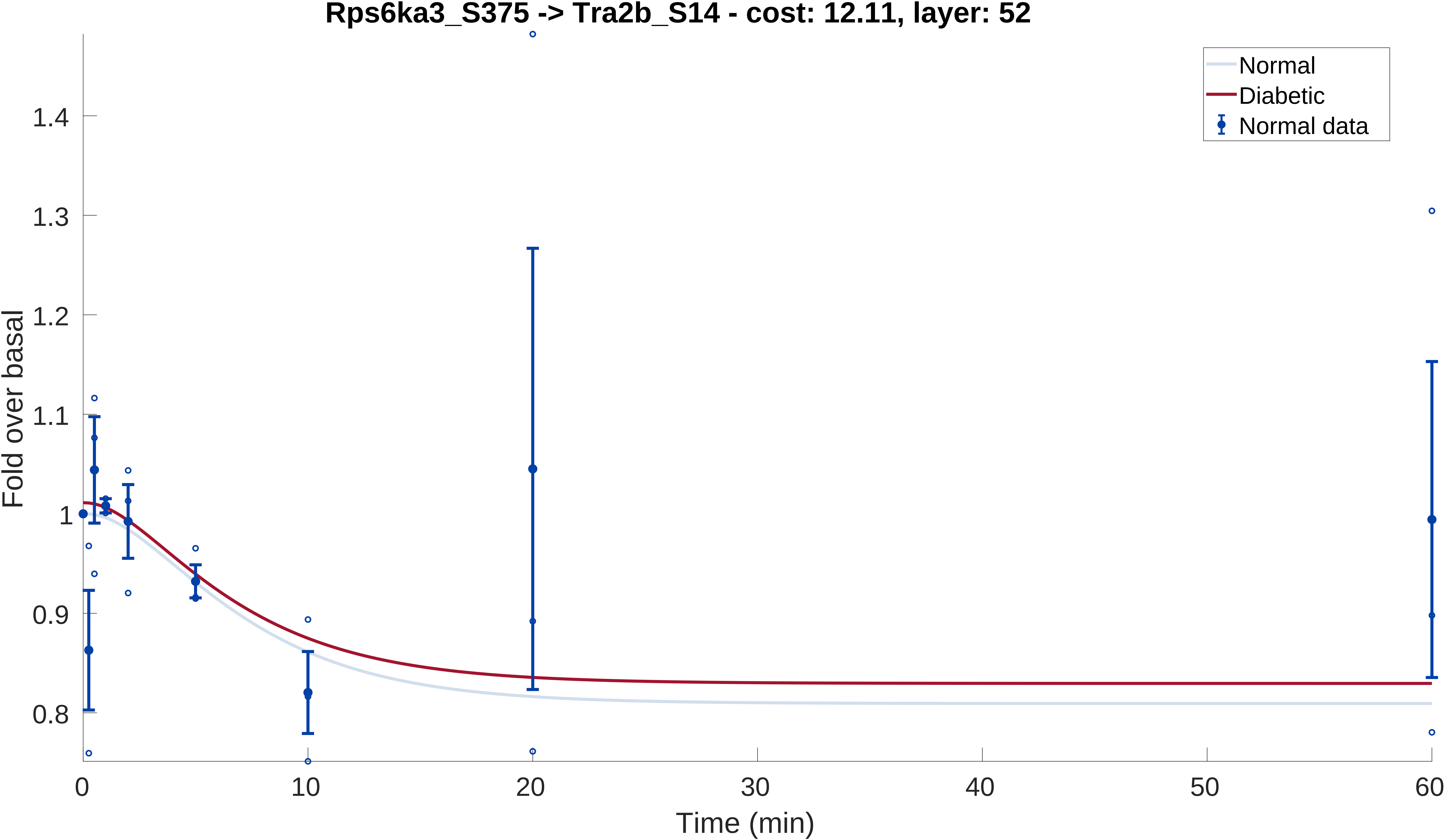

Rps6ka3_S375 -> Tra2b_S14 - cost: 12.11, layer: 52
Normal
Diabetic
Normal data
Fold over basal
Time (min)
1.4
1.3
1.2
1.1
1
0.9
0.8
0
10
20
30
40
50
60

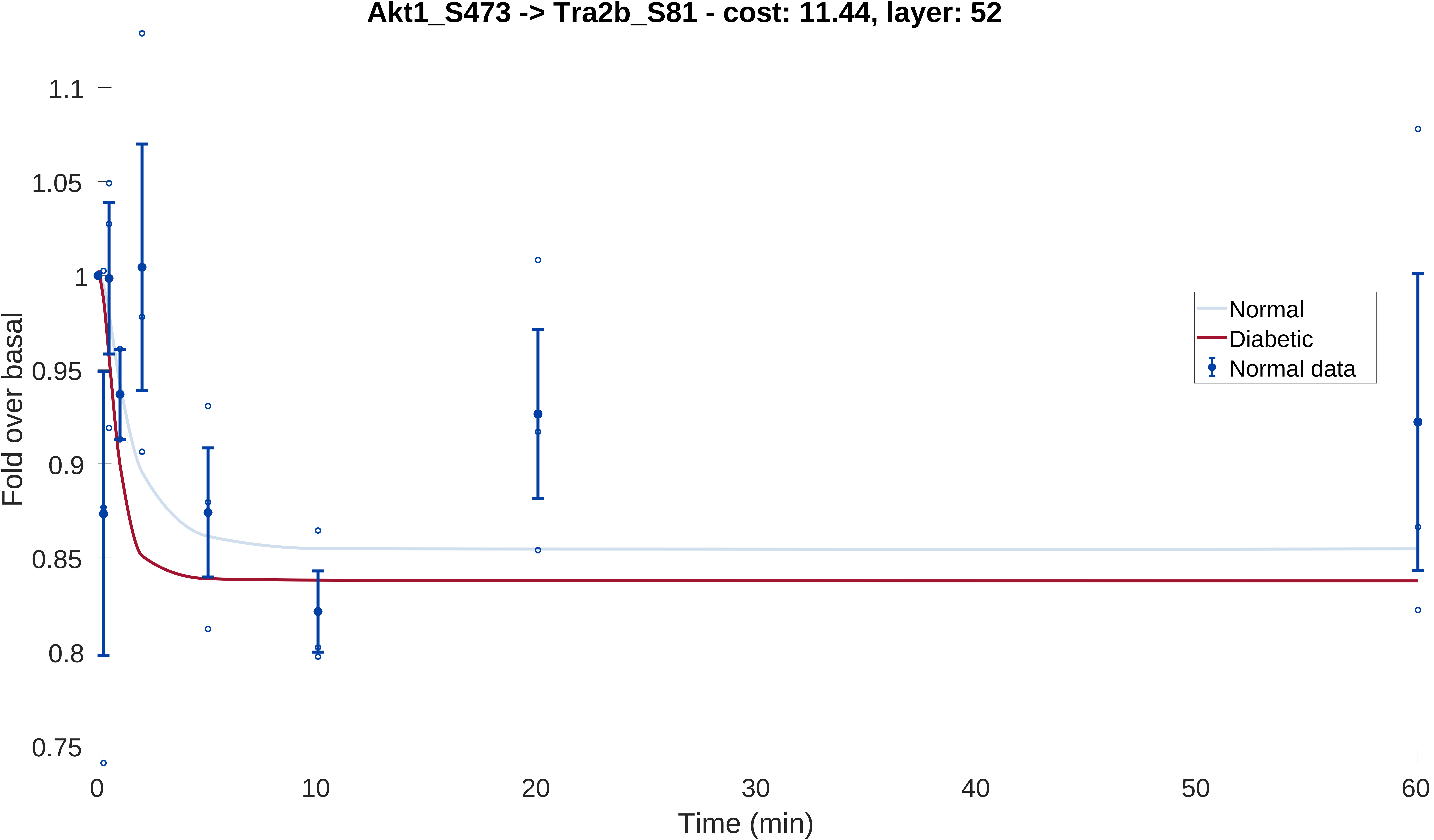

Akt1_S473 -> Tra2b_S81 - cost: 11.44, layer: 52
Fold over basal
1.1
1.05
1
0.95
0.9
0.85
0.8
0.75
0
10
20
30
40
50
60
Time (min)
Normal
Diabetic
Normal data

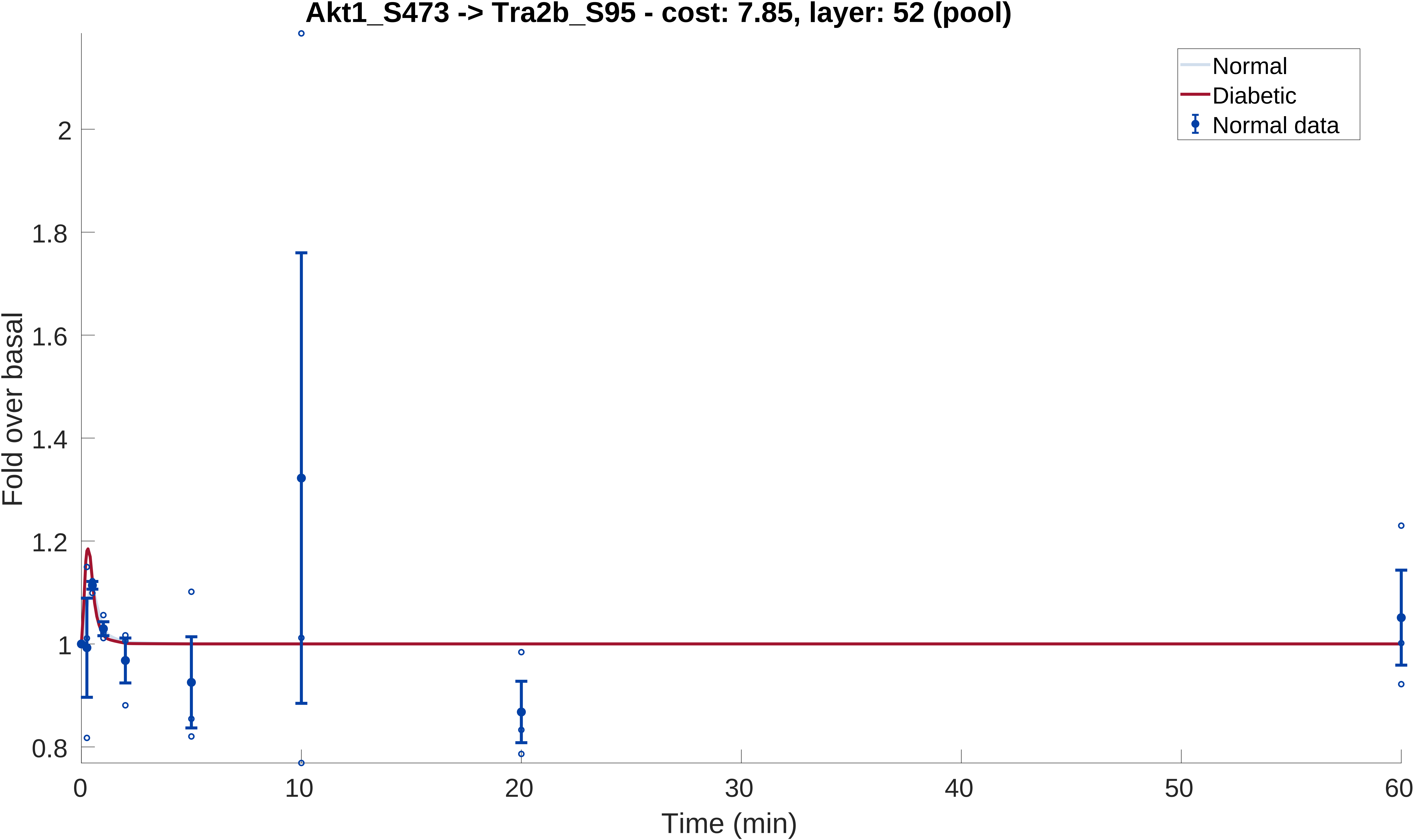

Akt1_S473 -> Tra2b_S95 - cost: 7.85, layer: 52 (pool)
Normal
Diabetic
Normal data
Fold over basal
2
1.8
1.6
1.4
1.2
1
0.8
0
10
20
30
40
50
60
Time (min)

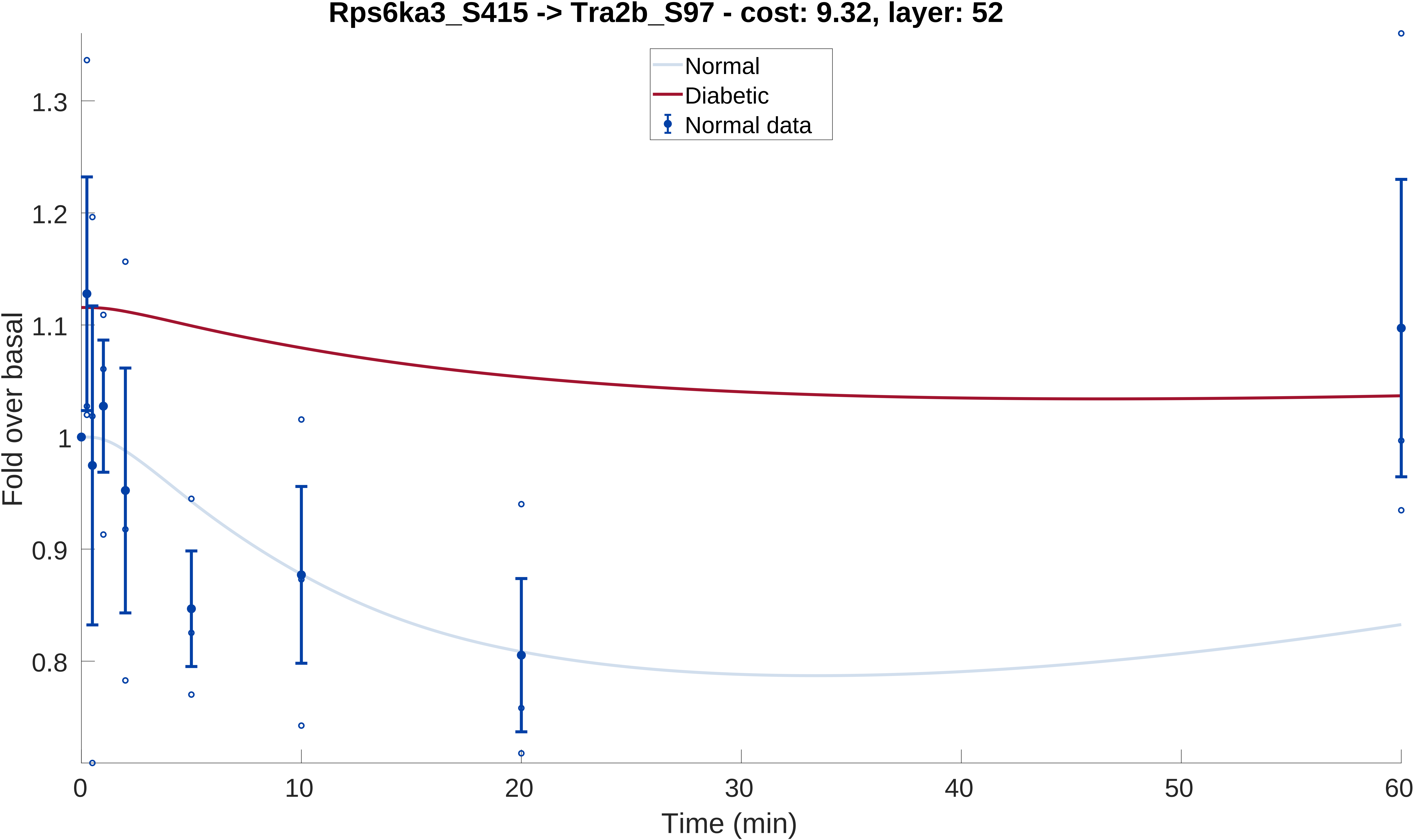
Rps6ka3_S415 -> Tra2b_S97 - cost: 9.32, layer: 52
Normal
Diabetic
Normal data
Fold over basal
Time (min)
1.3
1.2
1.1
1
0.9
0.8
0
10
20
30
40
50
60

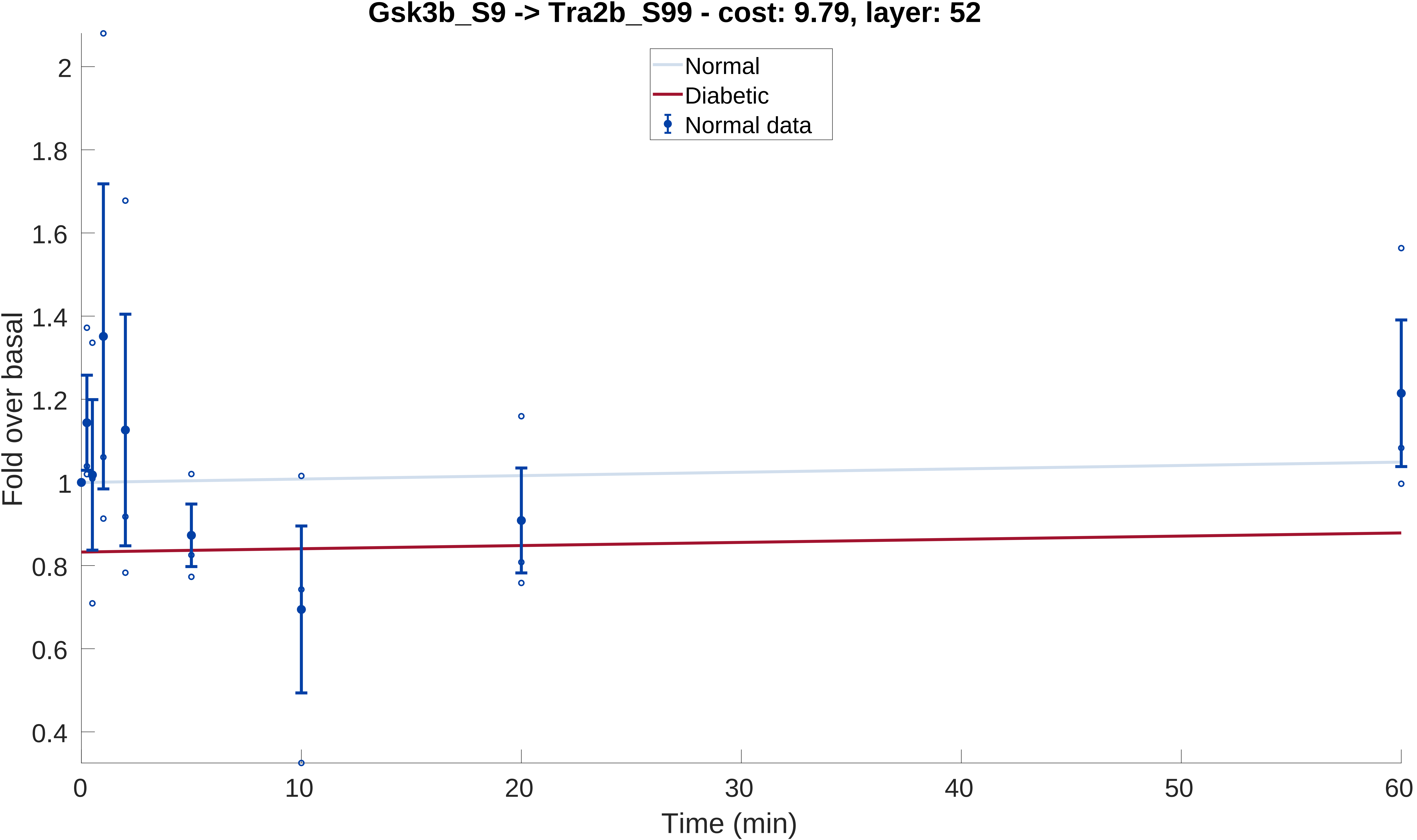

Gsk3b_S9 -> Tra2b_S99 - cost: 9.79, layer: 52
Normal
Diabetic
Normal data
Fold over basal
Time (min)
0.4
0.6
0.8
1
1.2
1.4
1.6
1.8
2
0
10
20
30
40
50
60

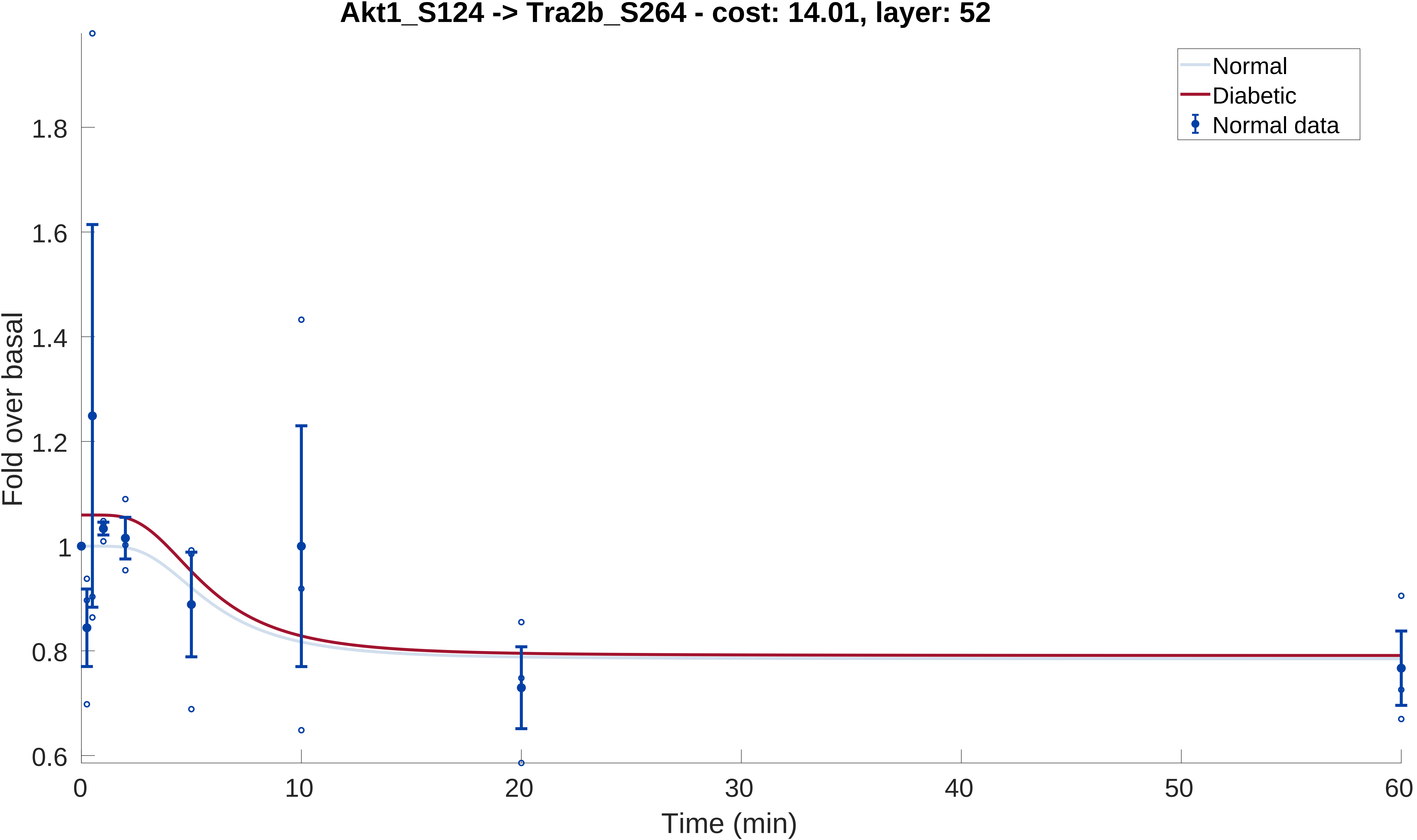
Akt1_S124 -> Tra2b_S264 - cost: 14.01, layer: 52
Normal
Diabetic
Normal data
Fold over basal
Time (min)
0.6
0.8
1
1.2
1.4
1.6
1.8
0
10
20
30
40
50
60

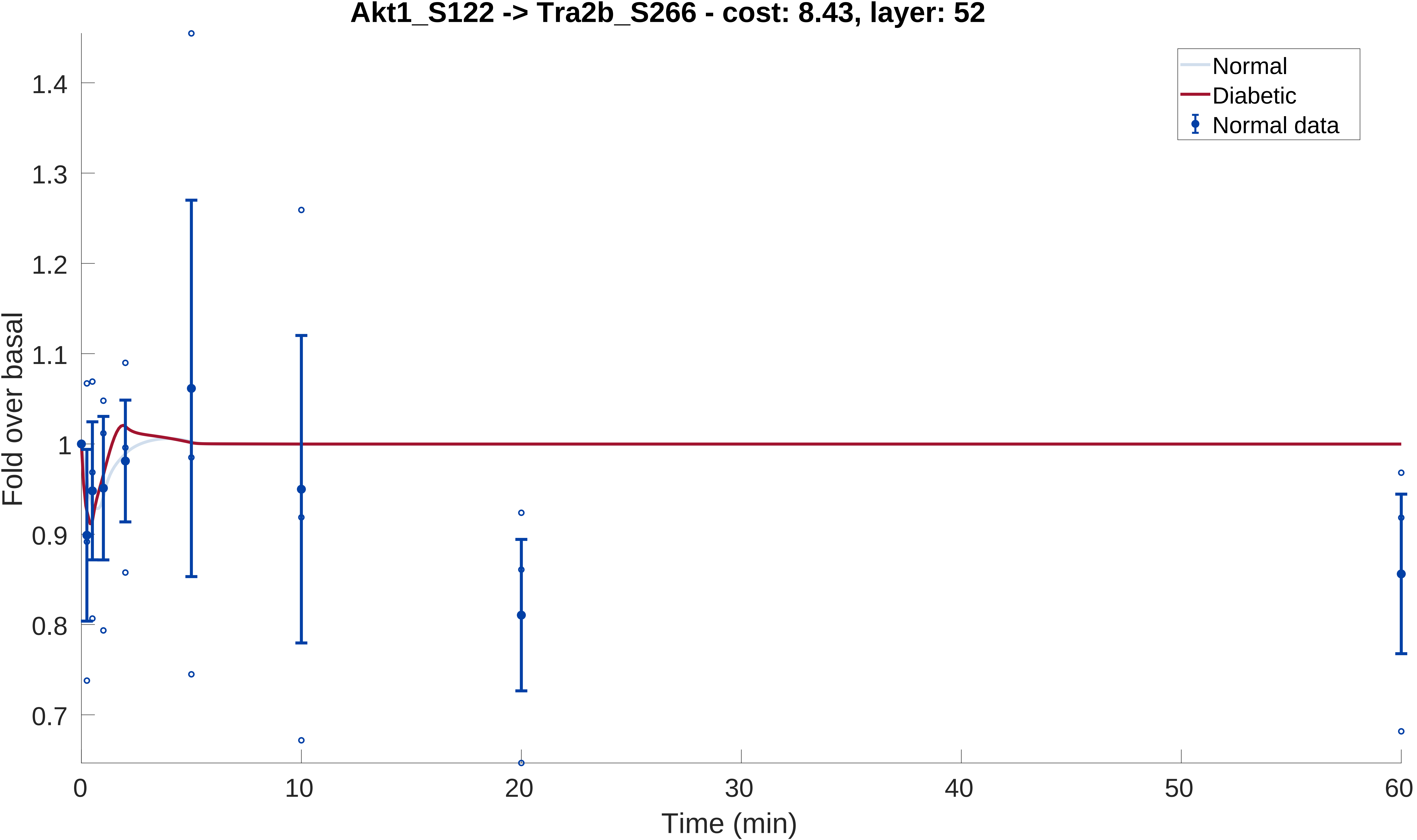

Akt1_S122 -> Tra2b_S266 - cost: 8.43, layer: 52
Normal
Diabetic
Normal data
Fold over basal
1.4
1.3
1.2
1.1
1
0.9
0.8
0.7
0
10
20
30
40
50
60
Time (min)

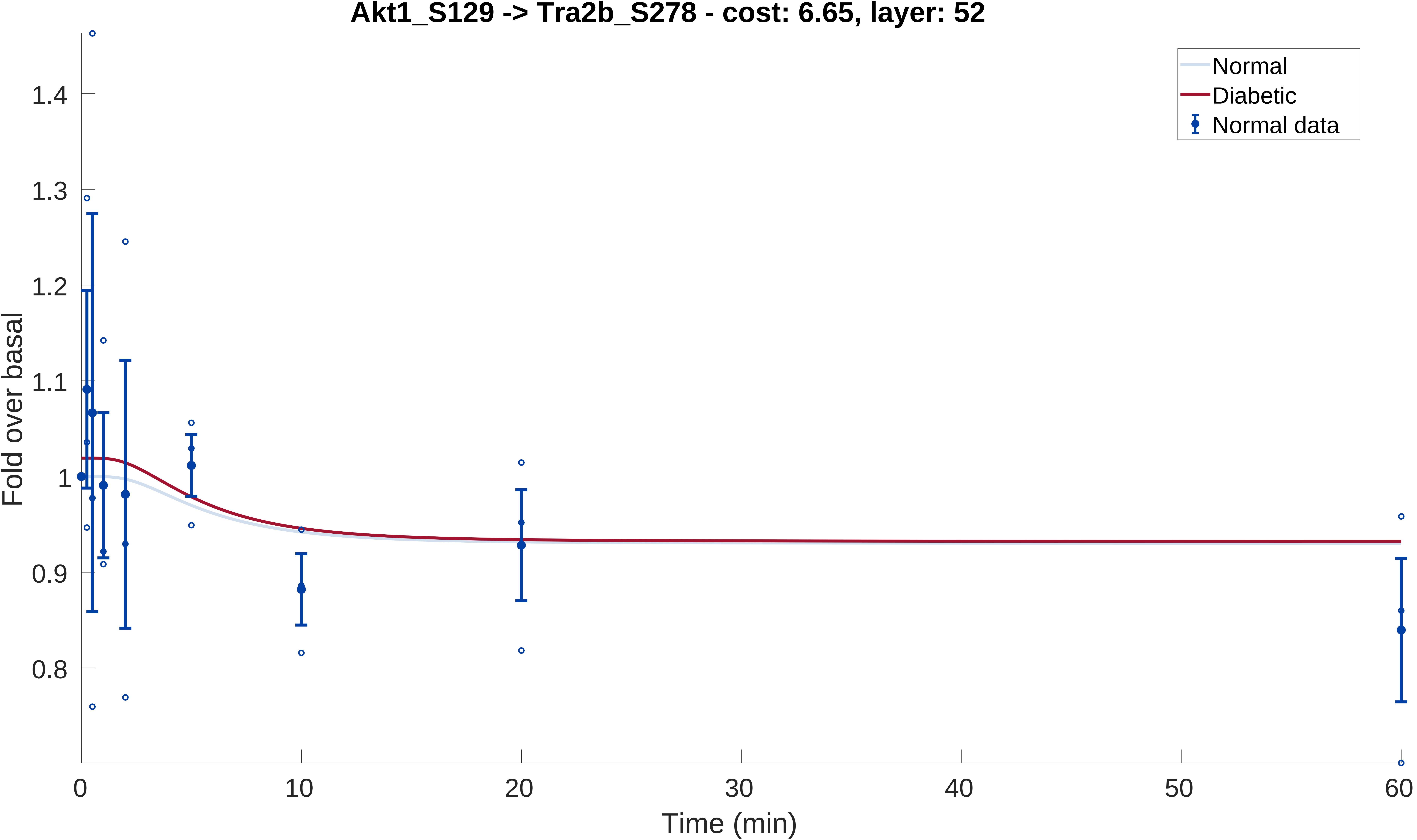
Akt1_S129 -> Tra2b_S278 - cost: 6.65, layer: 52
Normal
Diabetic
Normal data
Fold over basal
Time (min)
1.4
1.3
1.2
1.1
1
0.9
0.8
0
10
20
30
40
50
60

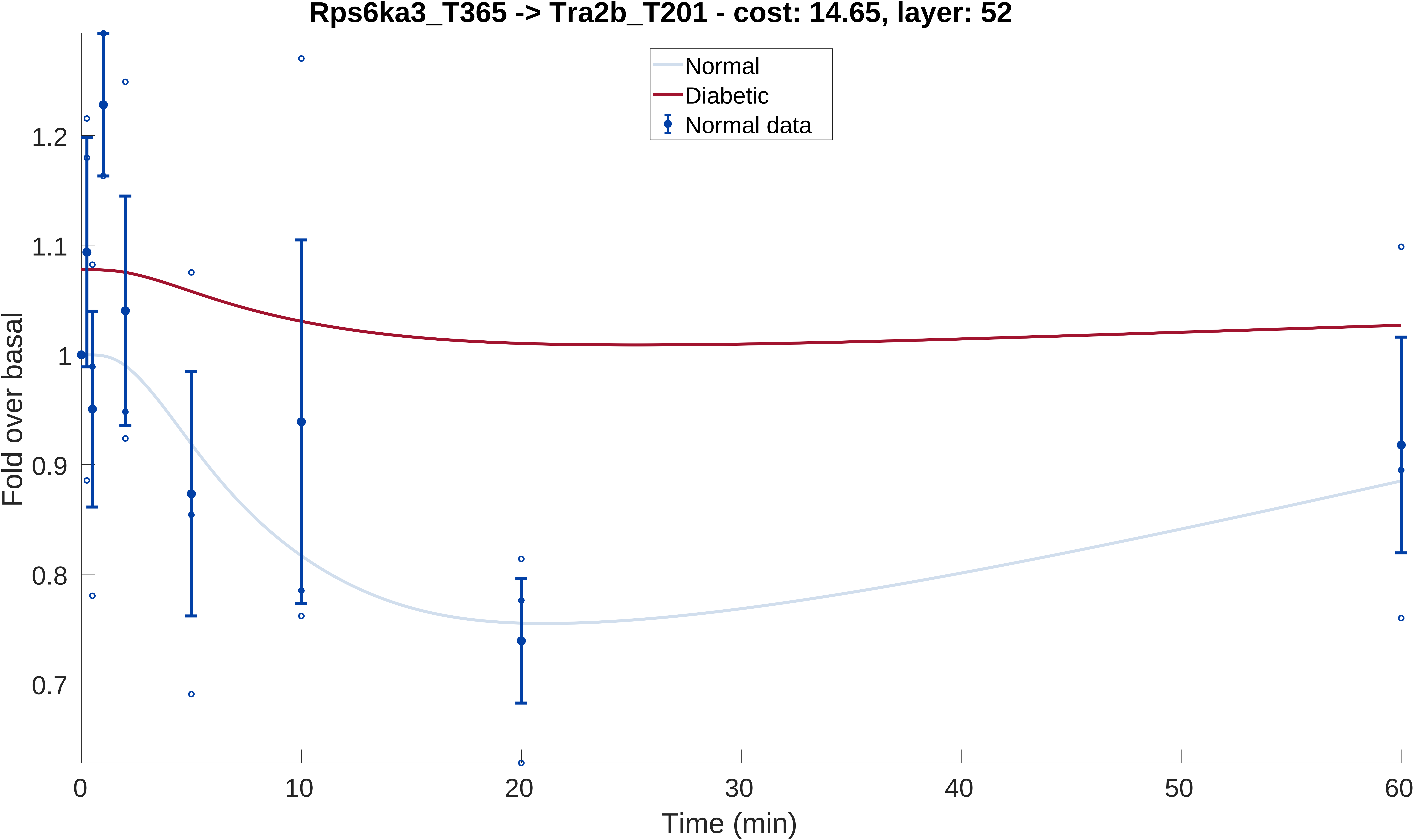
Rps6ka3_T365 -> Tra2b_T201 - cost: 14.65, layer: 52
Normal
Diabetic
Normal data
Fold over basal
1.2
1.1
1
0.9
0.8
0.7
0
10
20
30
40
50
60
Time (min)

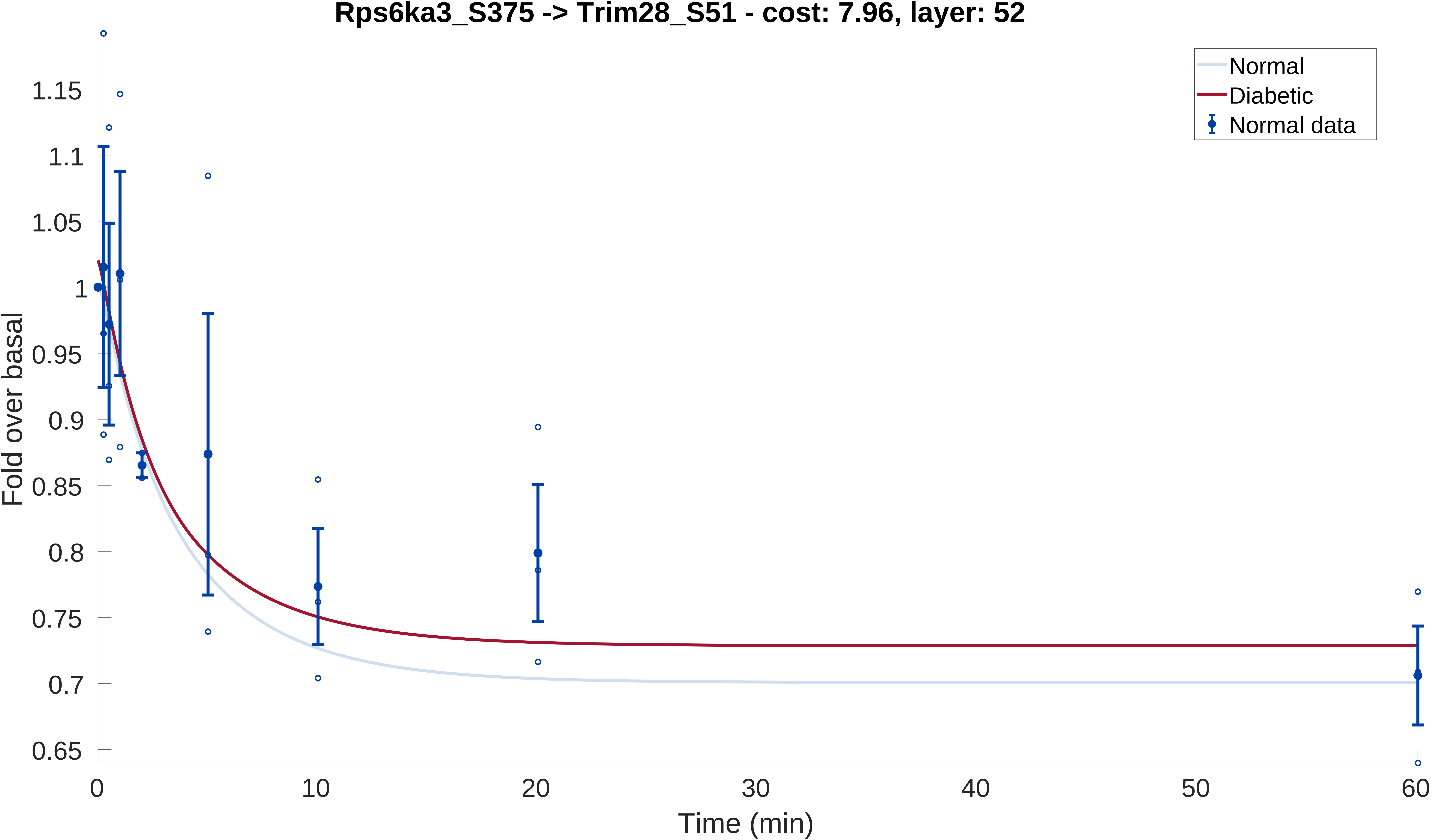
Rps6ka3_S375 -> Trim28_S51 - cost: 7.96, layer: 52
Normal
Diabetic
Normal data
Fold over basal
1.15
1.1
1.05
1
0.95
0.9
0.85
0.8
0.75
0.7
0.65
0
10
20
30
40
50
60
Time (min)

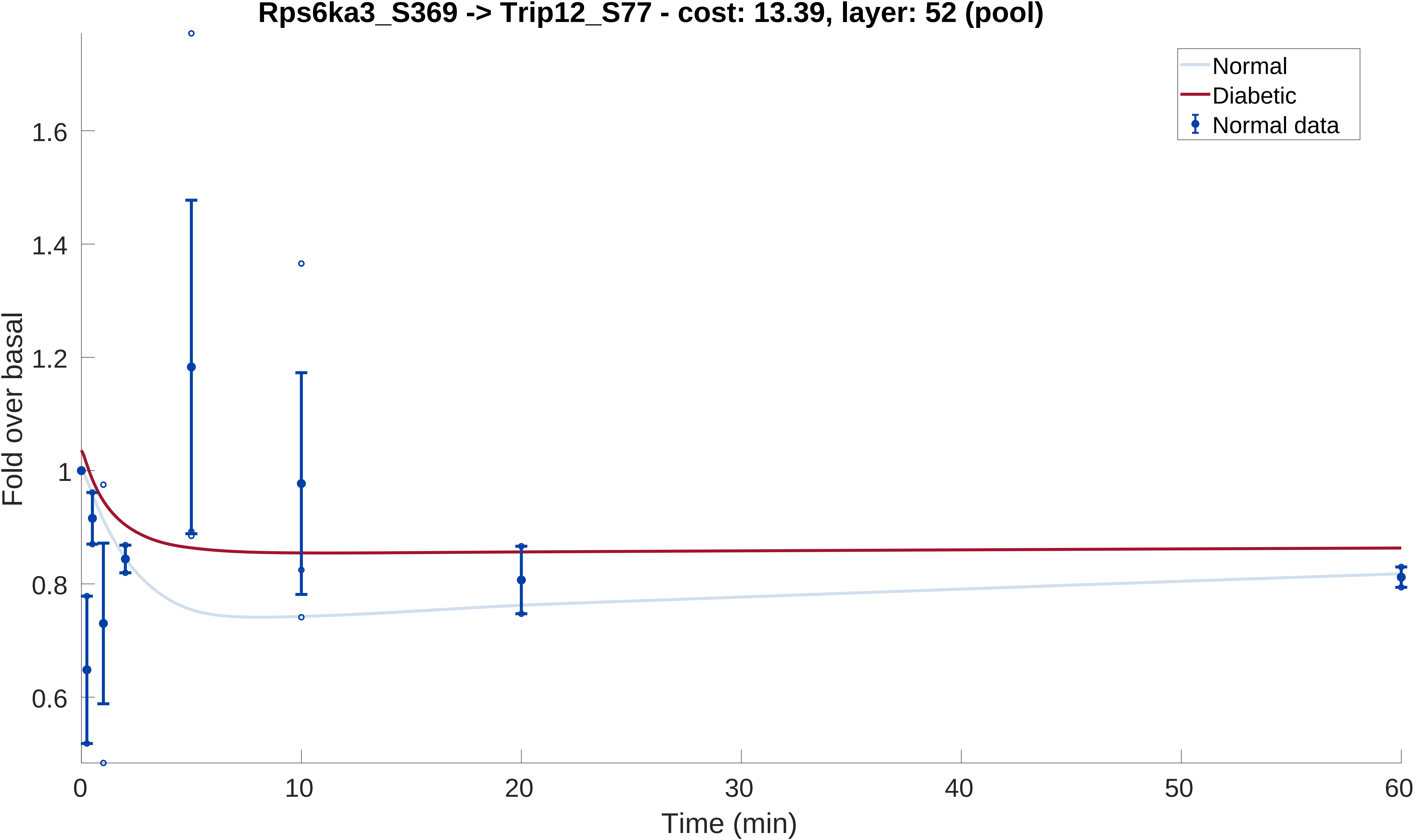
Rps6ka3_S369 -> Trip12_S77 - cost: 13.39, layer: 52 (pool)
Normal
Diabetic
Normal data
Fold over basal
Time (min)
0.6
0.8
1
1.2
1.4
1.6
0
10
20
30
40
50
60

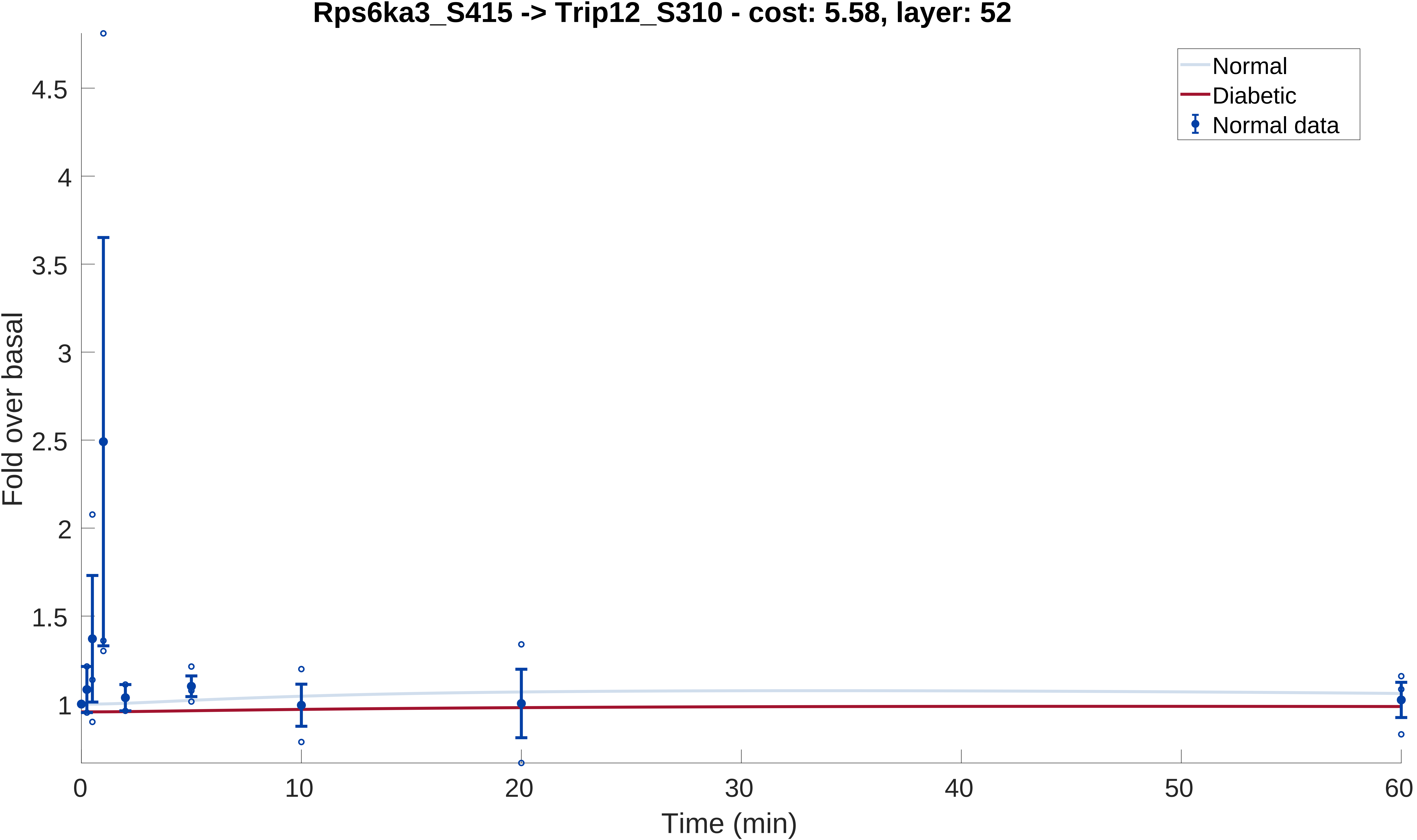
Rps6ka3_S415 -> Trip12_S310 - cost: 5.58, layer: 52
Normal
Diabetic
Normal data
Fold over basal
4.5
4
3.5
3
2.5
2
1.5
1
0
10
20
30
40
50
60
Time (min)

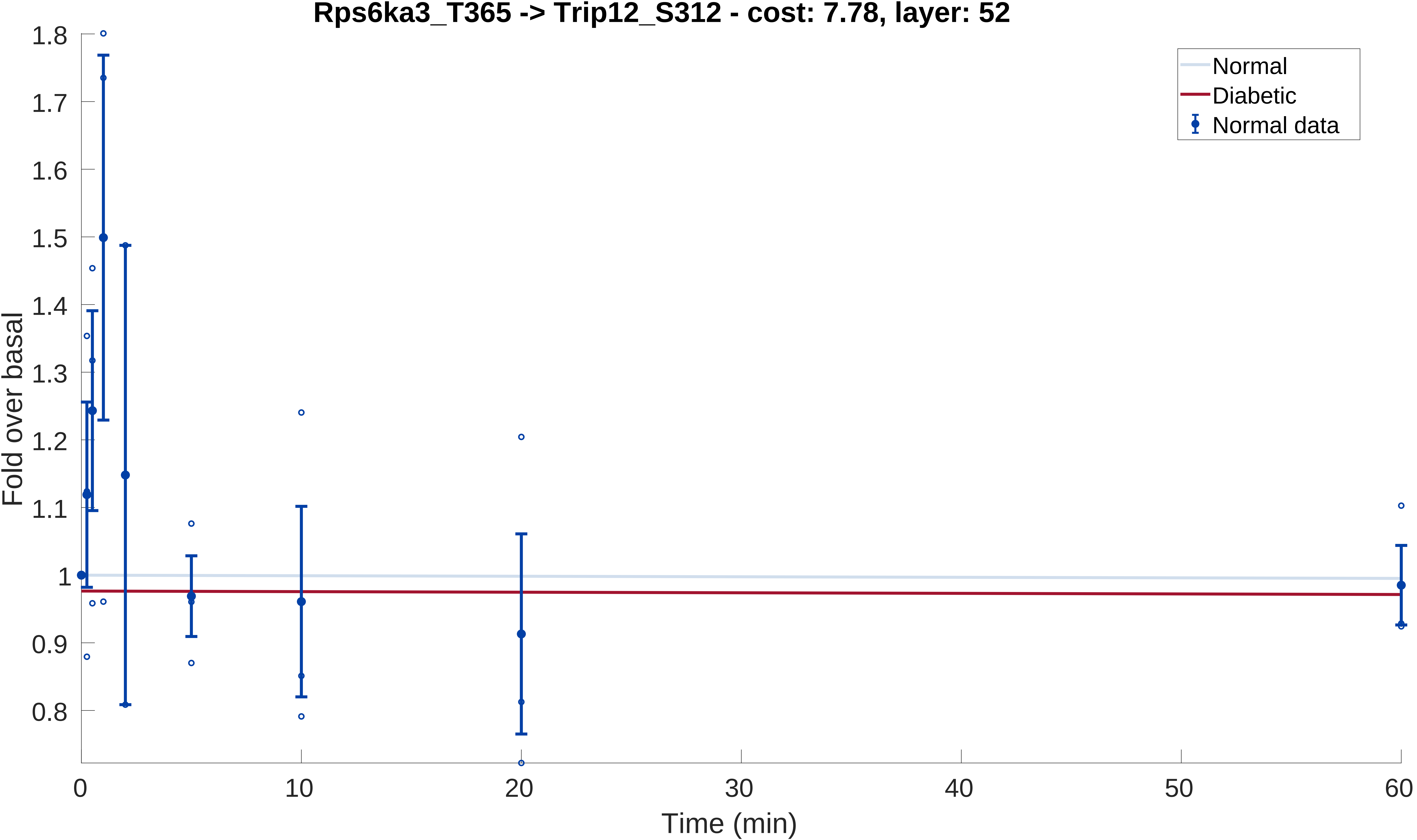

Rps6ka3_T365 -> Trip12_S312 - cost: 7.78, layer: 52
Fold over basal
Time (min)
Normal
Diabetic
Normal data
1.8
1.7
1.6
1.5
1.4
1.3
1.2
1.1
1
0.9
0.8
0
10
20
30
40
50
60

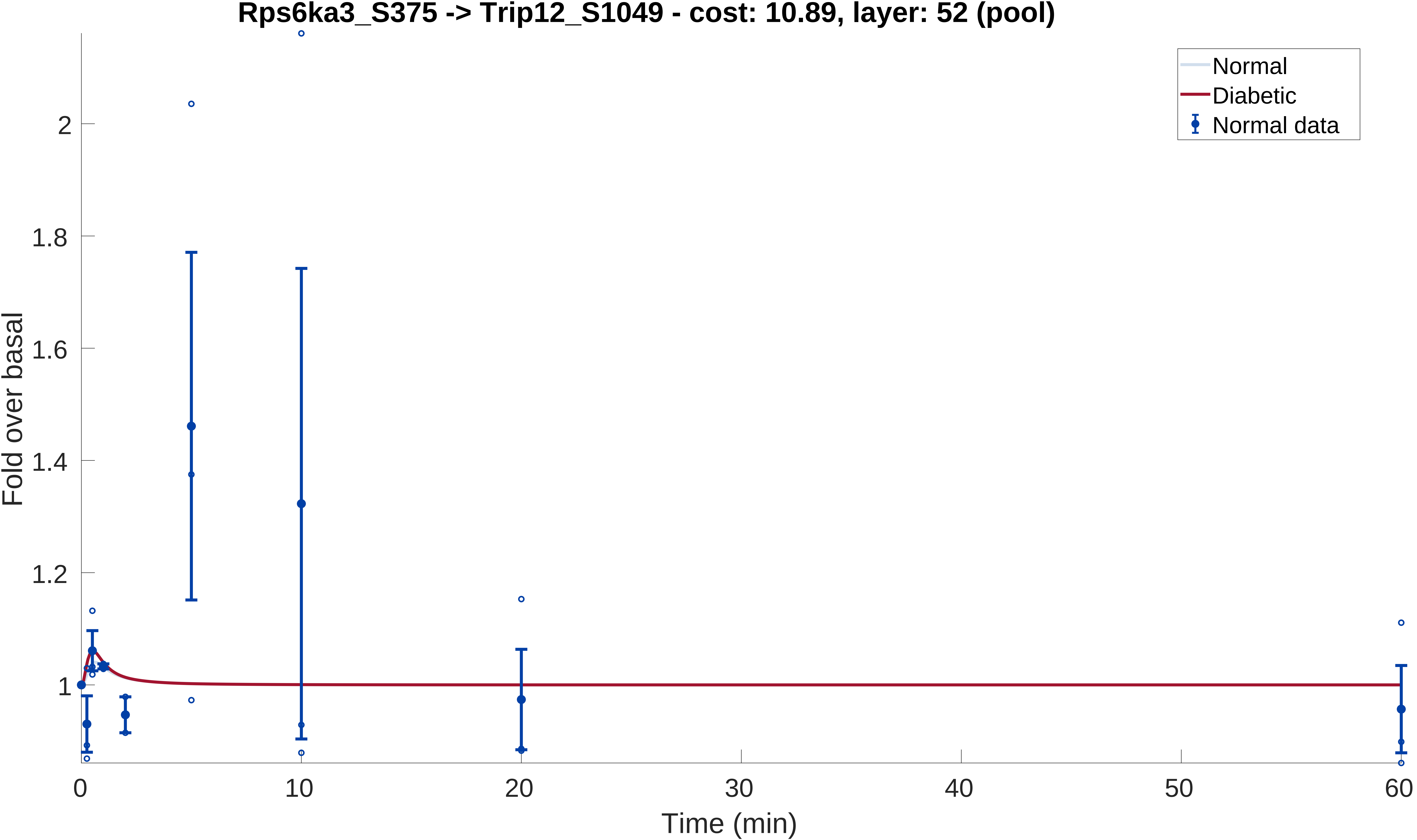
Rps6ka3_S375 -> Trip12_S1049 - cost: 10.89, layer: 52 (pool)
Normal
Diabetic
Normal data
Fold over basal
Time (min)
2
1.8
1.6
1.4
1.2
1
0
10
20
30
40
50
60

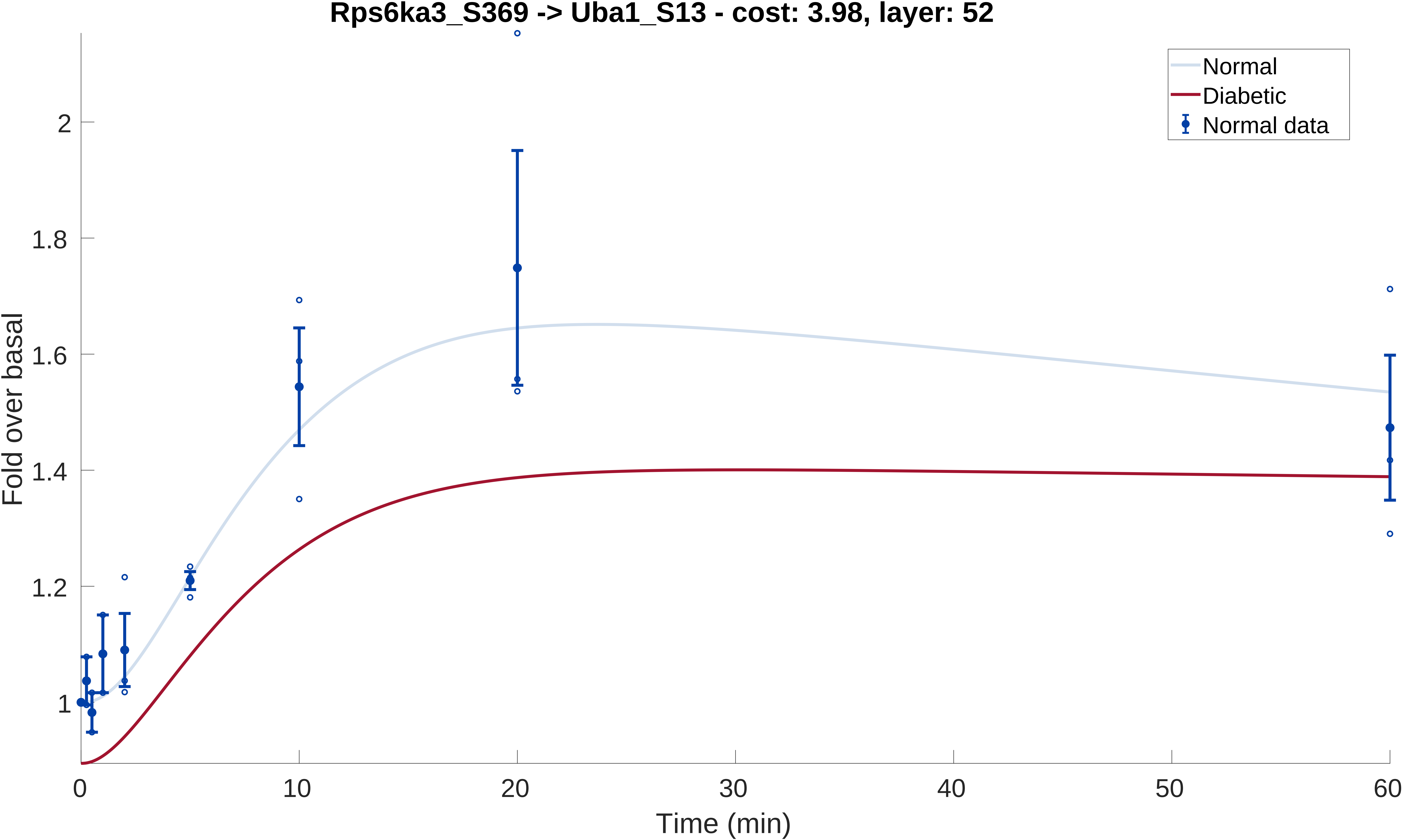

Rps6ka3_S369 -> Uba1_S13 - cost: 3.98, layer: 52
Normal
Diabetic
Normal data
Fold over basal
2
1.8
1.6
1.4
1.2
1
0
10
20
30
40
50
60
Time (min)

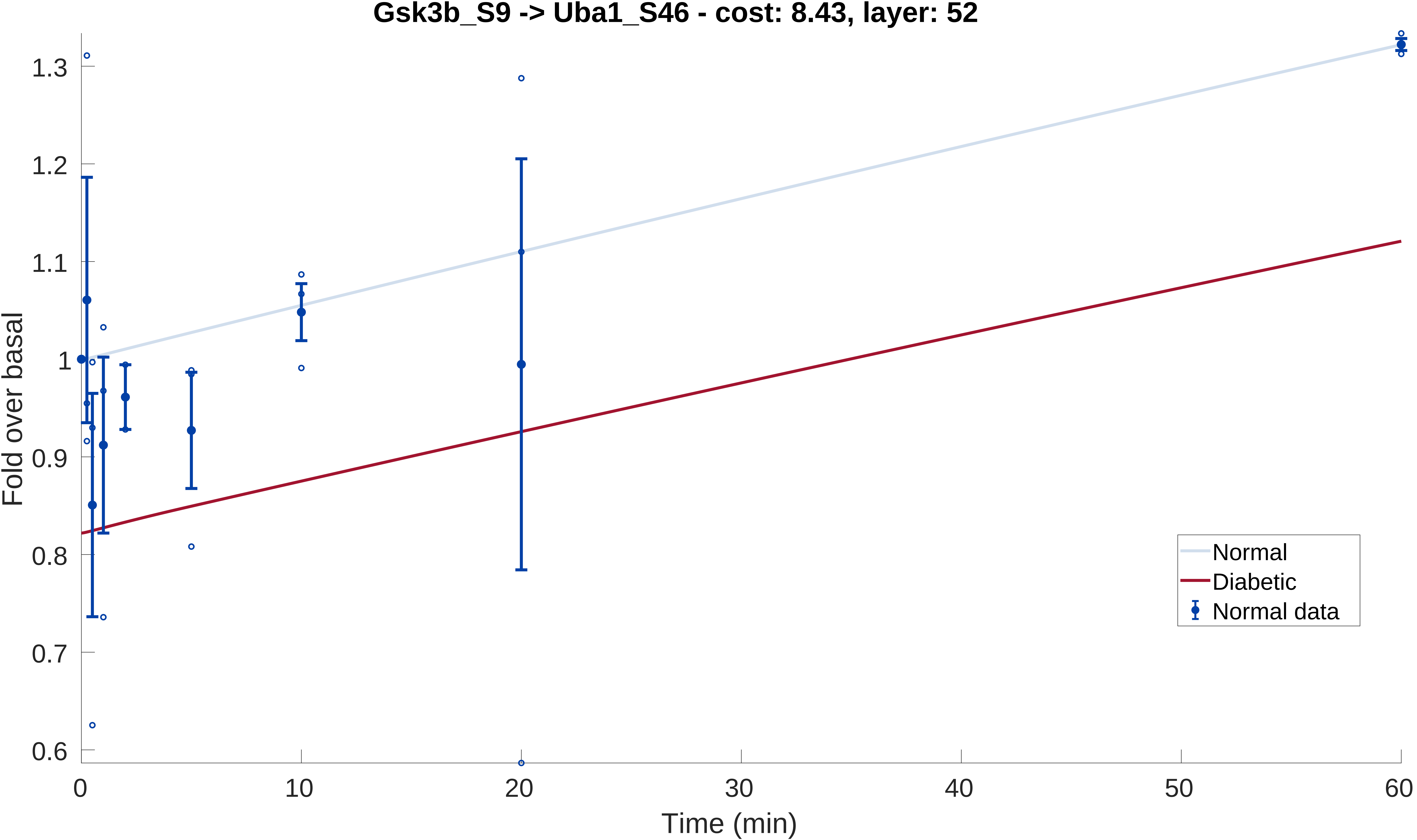

Gsk3b_S9 -> Uba1_S46 - cost: 8.43, layer: 52
Fold over basal
1.3
1.2
1.1
1
0.9
0.8
0.7
0.6
0
10
20
30
40
50
60
Time (min)
Normal
Diabetic
Normal data

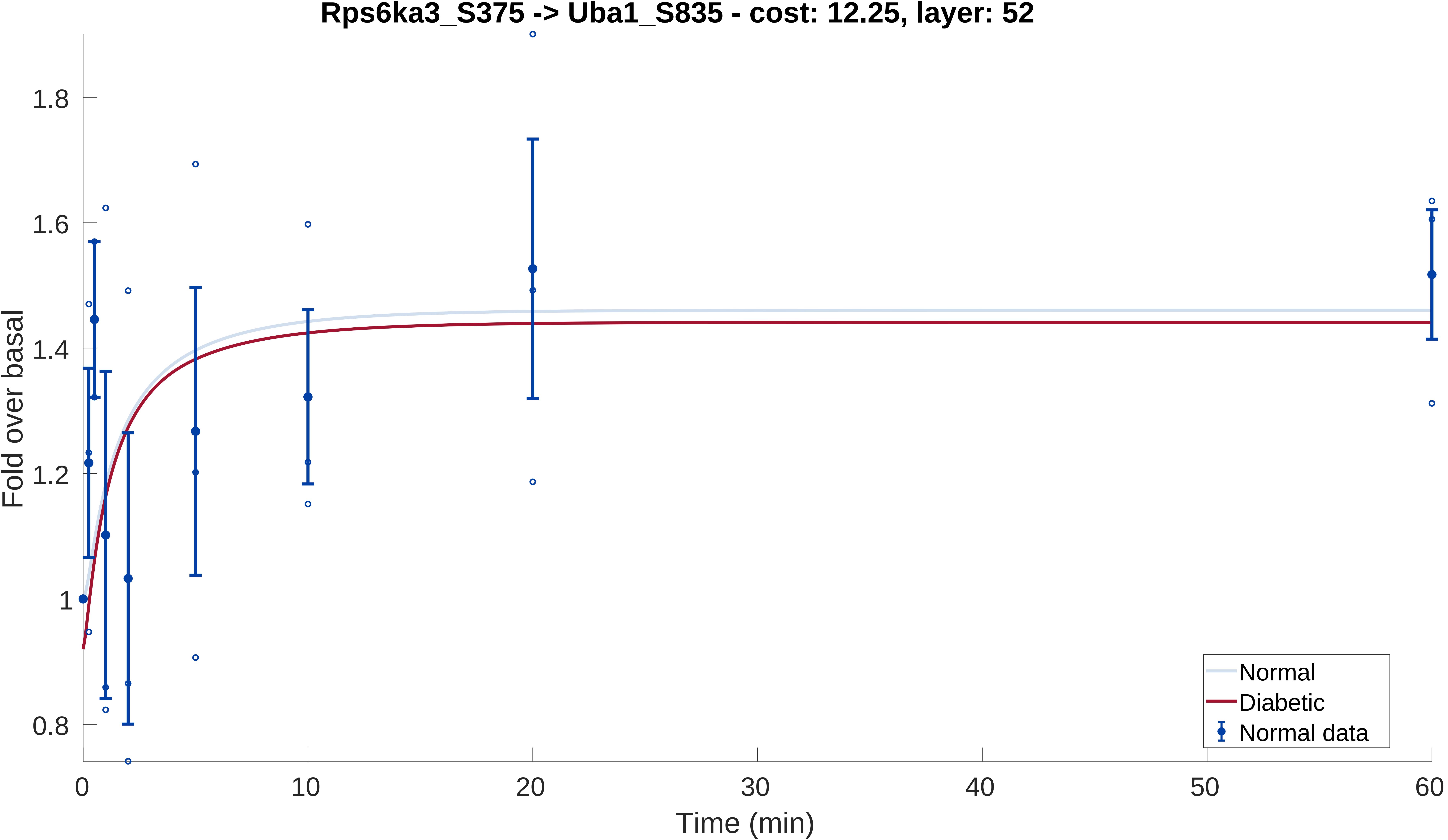

Rps6ka3_S375 -> Uba1_S835 - cost: 12.25, layer: 52
Fold over basal
1.8
1.6
1.4
1.2
1
0.8
0
10
20
30
40
50
60
Time (min)
Normal
Diabetic
Normal data

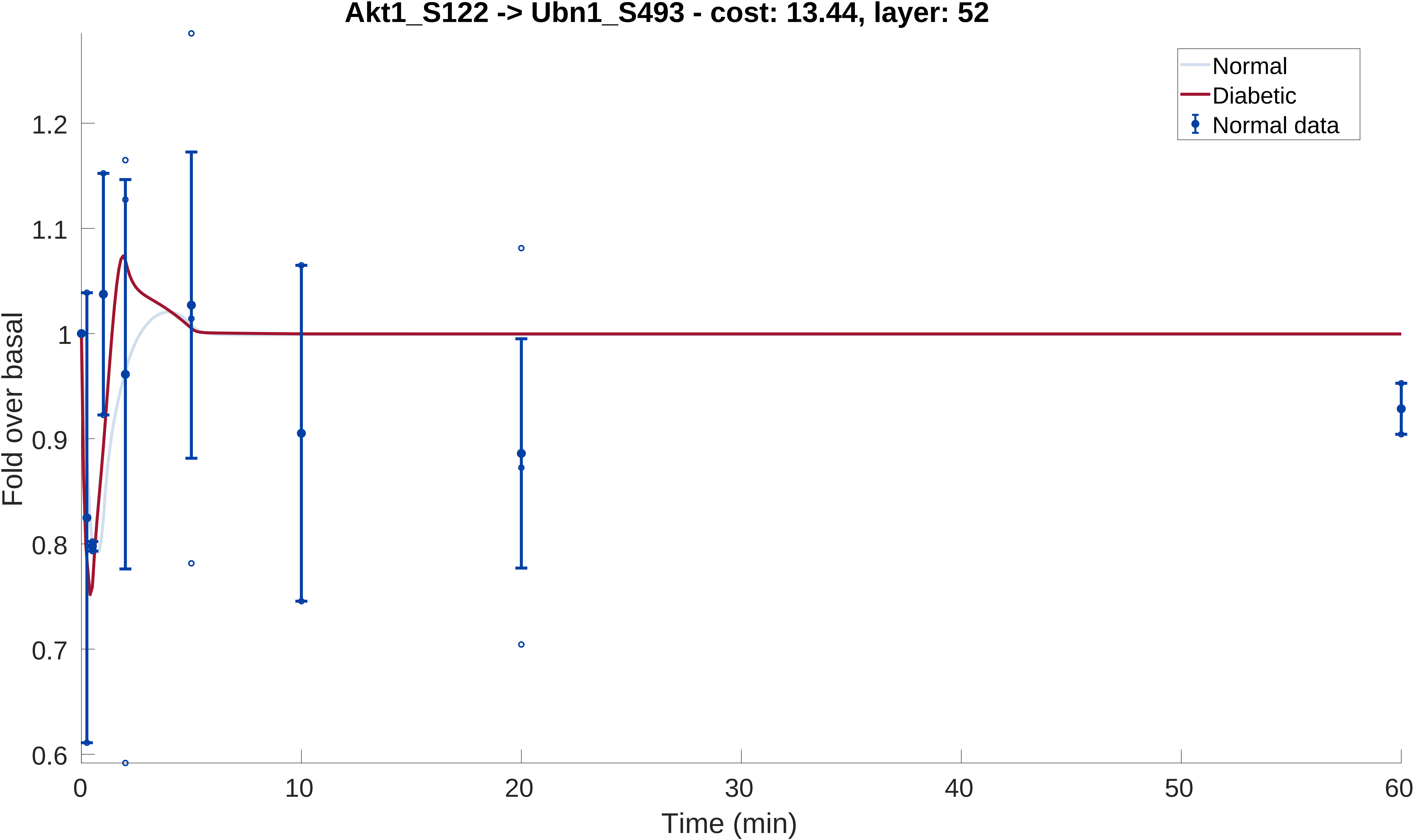

Akt1_S122 -> Ubn1_S493 - cost: 13.44, layer: 52
Normal
Diabetic
Normal data
Fold over basal
0.6
0.7
0.8
0.9
1
1.1
1.2
0
10
20
30
40
50
60
Time (min)

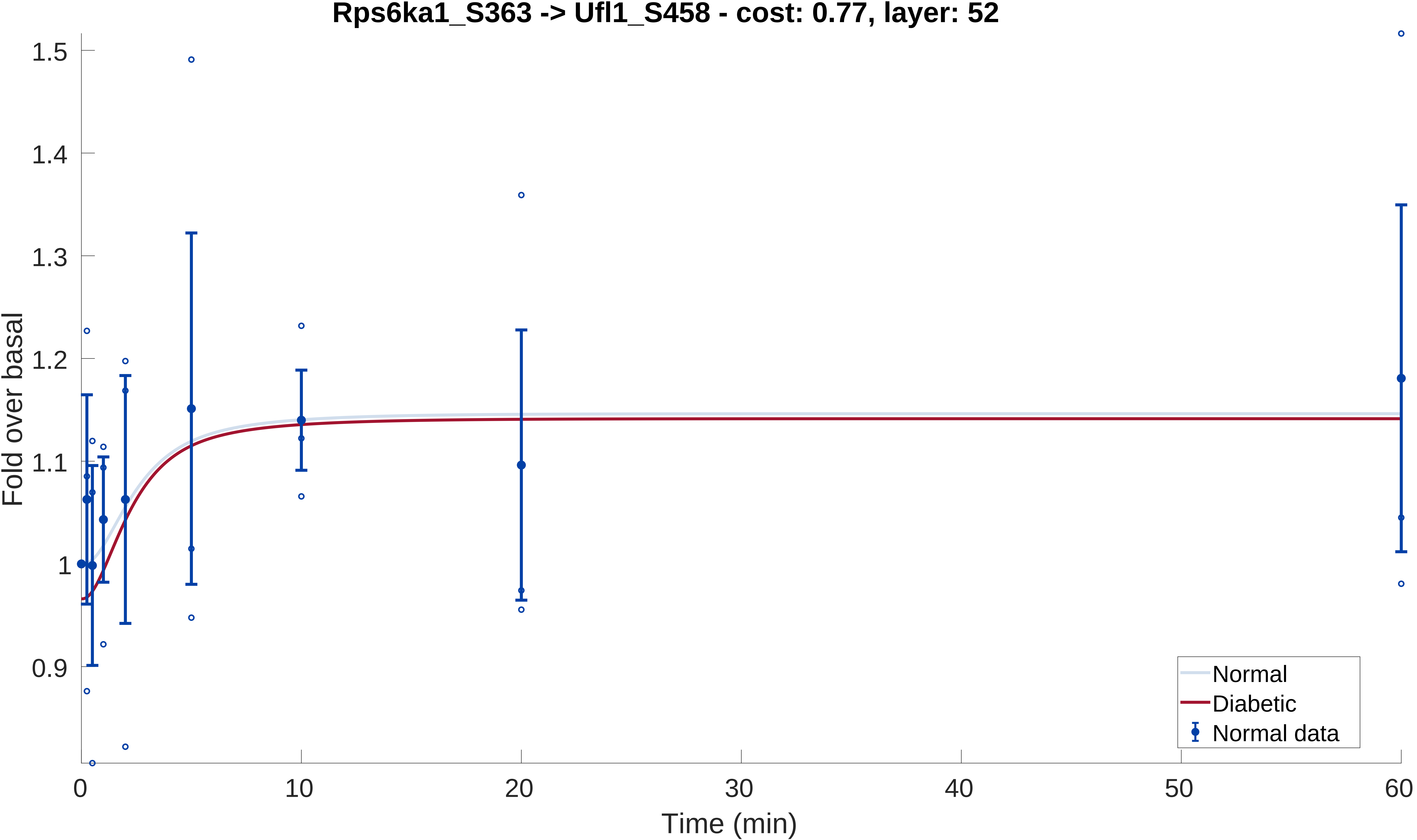

Rps6ka1_S363 -> Ufl1_S458 - cost: 0.77, layer: 52
Fold over basal
1.5
1.4
1.3
1.2
1.1
1
0.9
0
10
20
30
40
50
60
Time (min)
Normal
Diabetic
Normal data

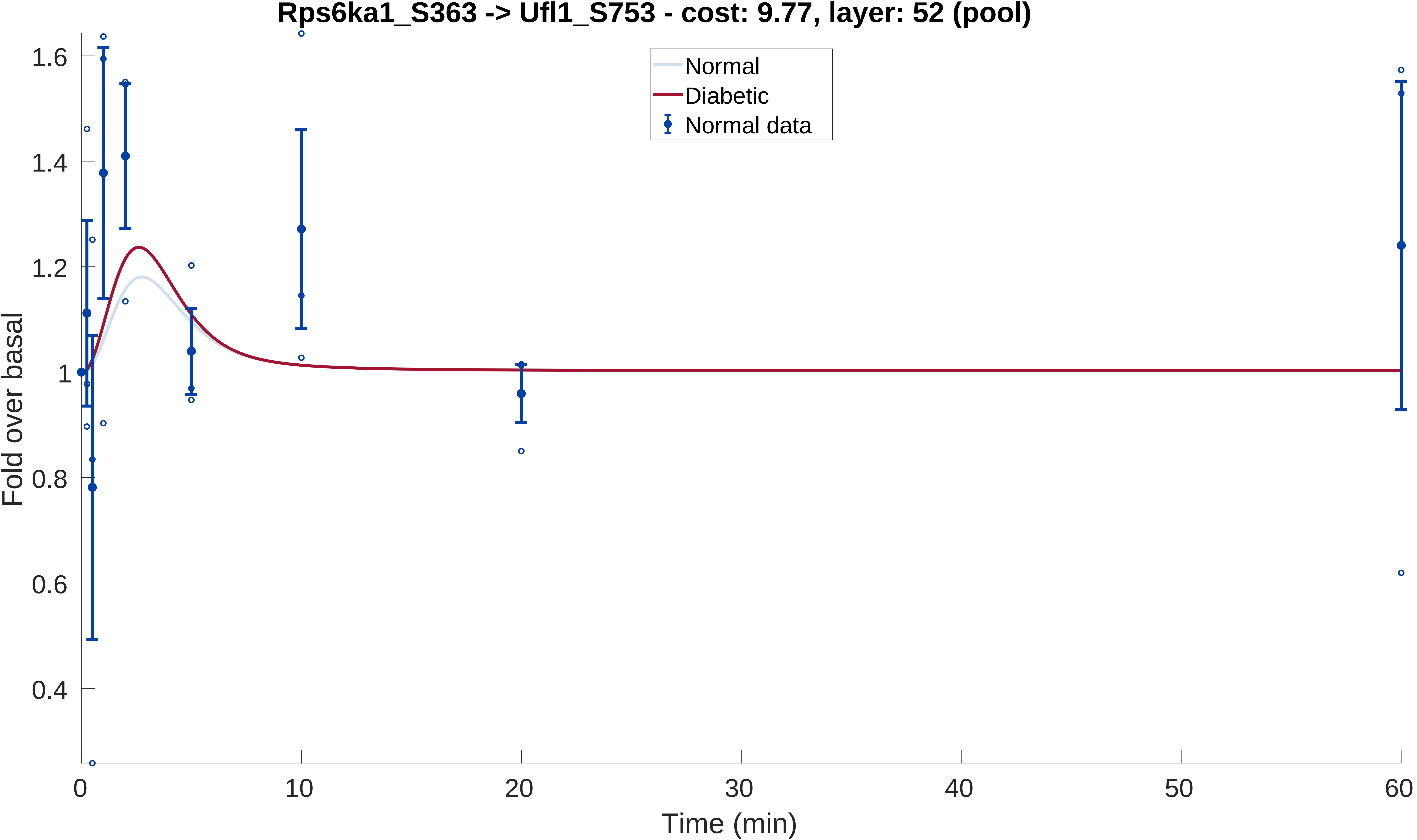

Rps6ka1_S363 -> Ufl1_S753 - cost: 9.77, layer: 52 (pool)
Normal
Diabetic
Normal data
Fold over basal
1.6
1.4
1.2
1
0.8
0.6
0.4
0
10
20
30
40
50
60
Time (min)

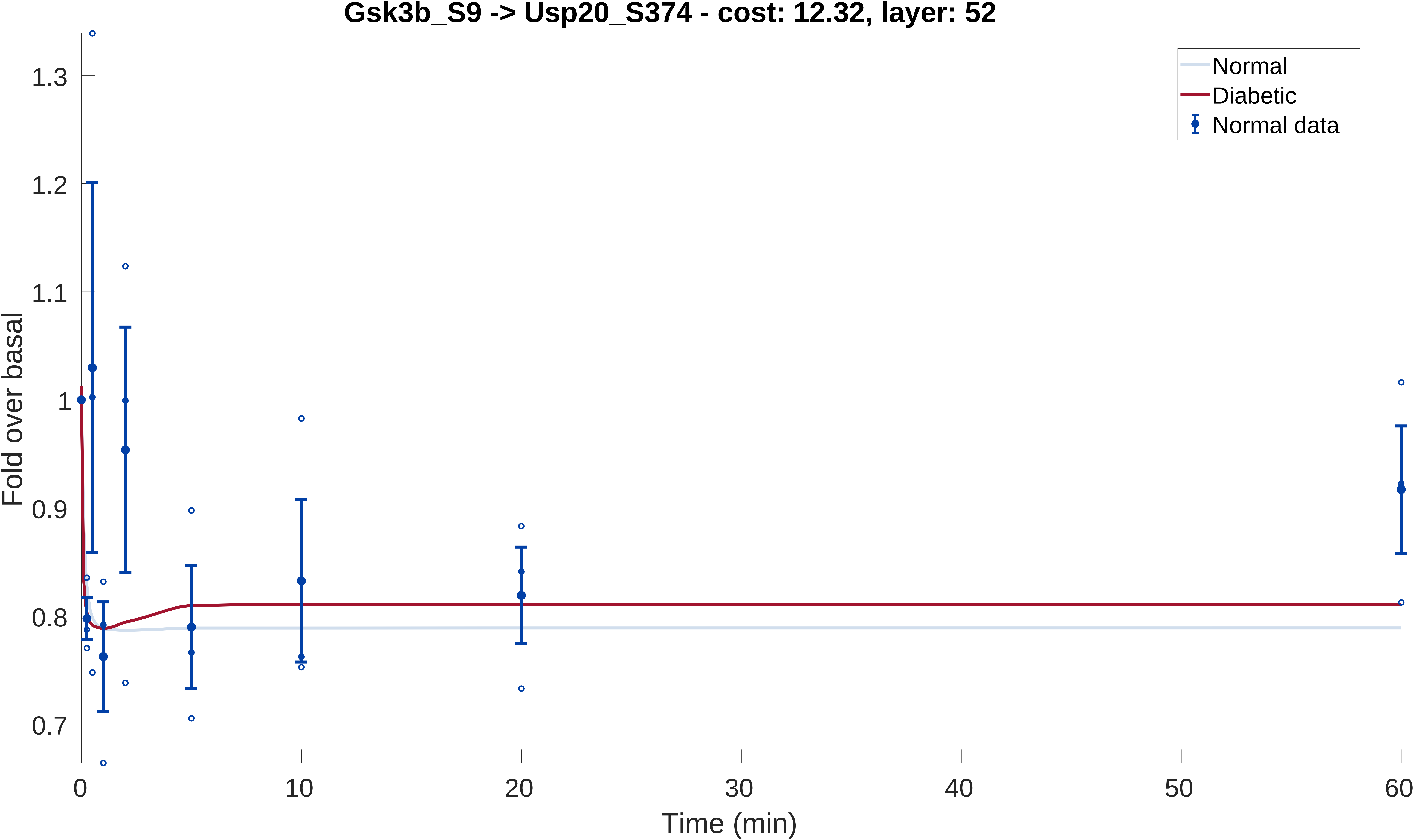

Gsk3b_S9 -> Usp20_S374 - cost: 12.32, layer: 52
Fold over basal
1.3
1.2
1.1
1
0.9
0.8
0.7
0
10
20
30
40
50
60
Time (min)
Normal
Diabetic
Normal data

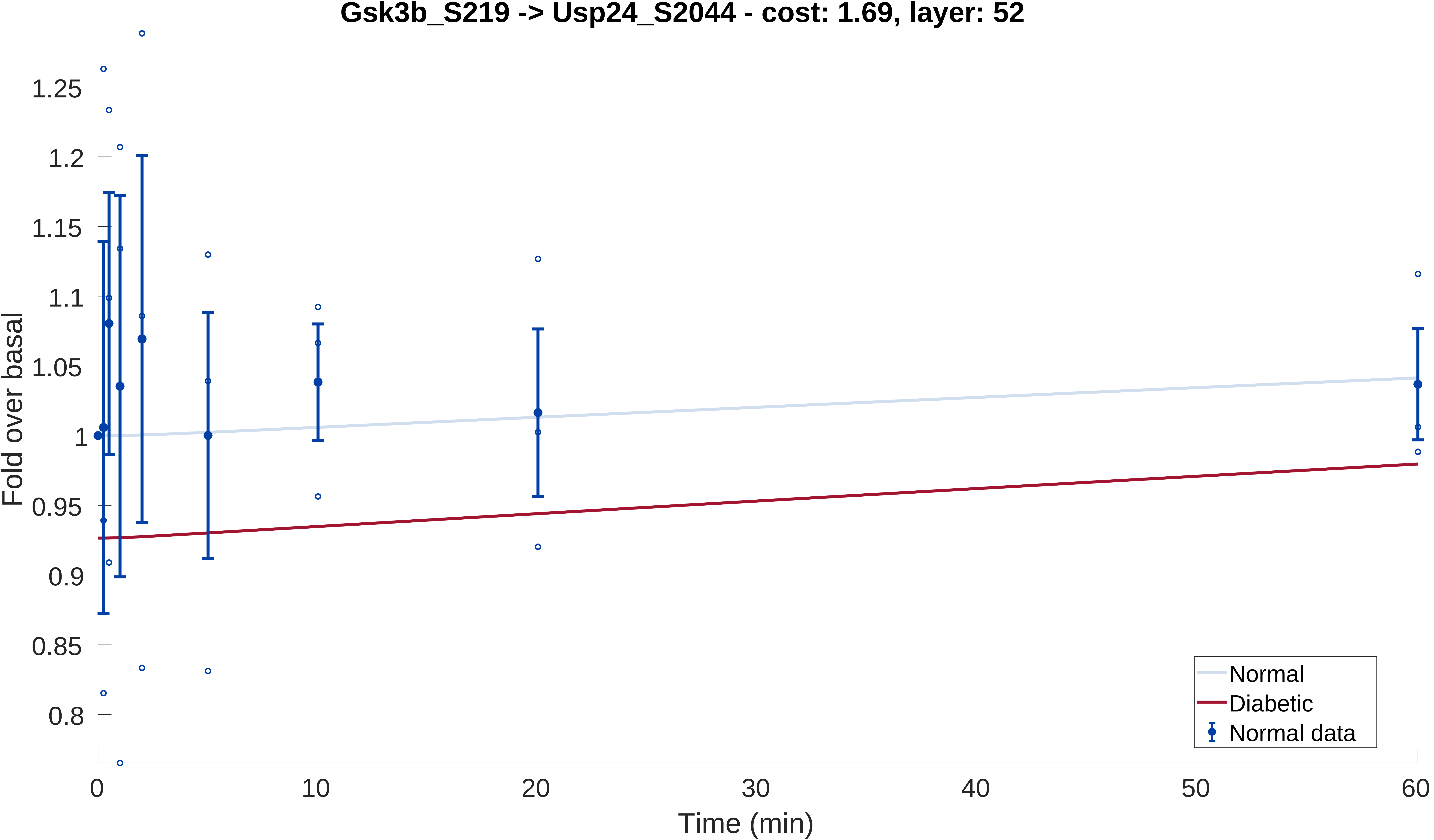
Gsk3b_S219 -> Usp24_S2044 - cost: 1.69, layer: 52
Fold over basal
1.25
1.2
1.15
1.1
1.05
1
0.95
0.9
0.85
0.8
0
10
20
30
40
50
60
Time (min)
Normal
Diabetic
Normal data

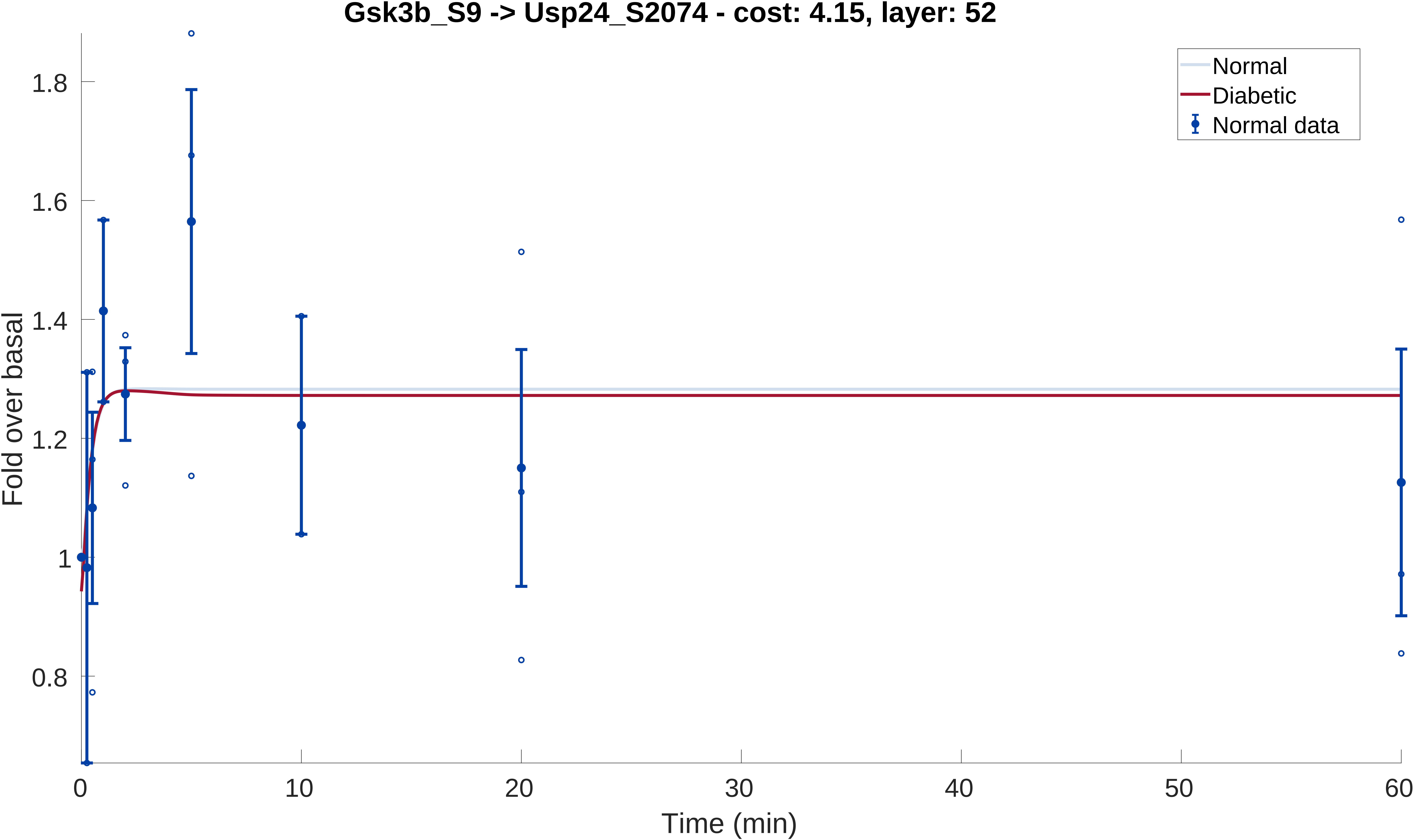

Gsk3b_S9 -> Usp24_S2074 - cost: 4.15, layer: 52
Normal
Diabetic
Normal data
Fold over basal
1.8
1.6
1.4
1.2
1
0.8
0
10
20
30
40
50
60
Time (min)

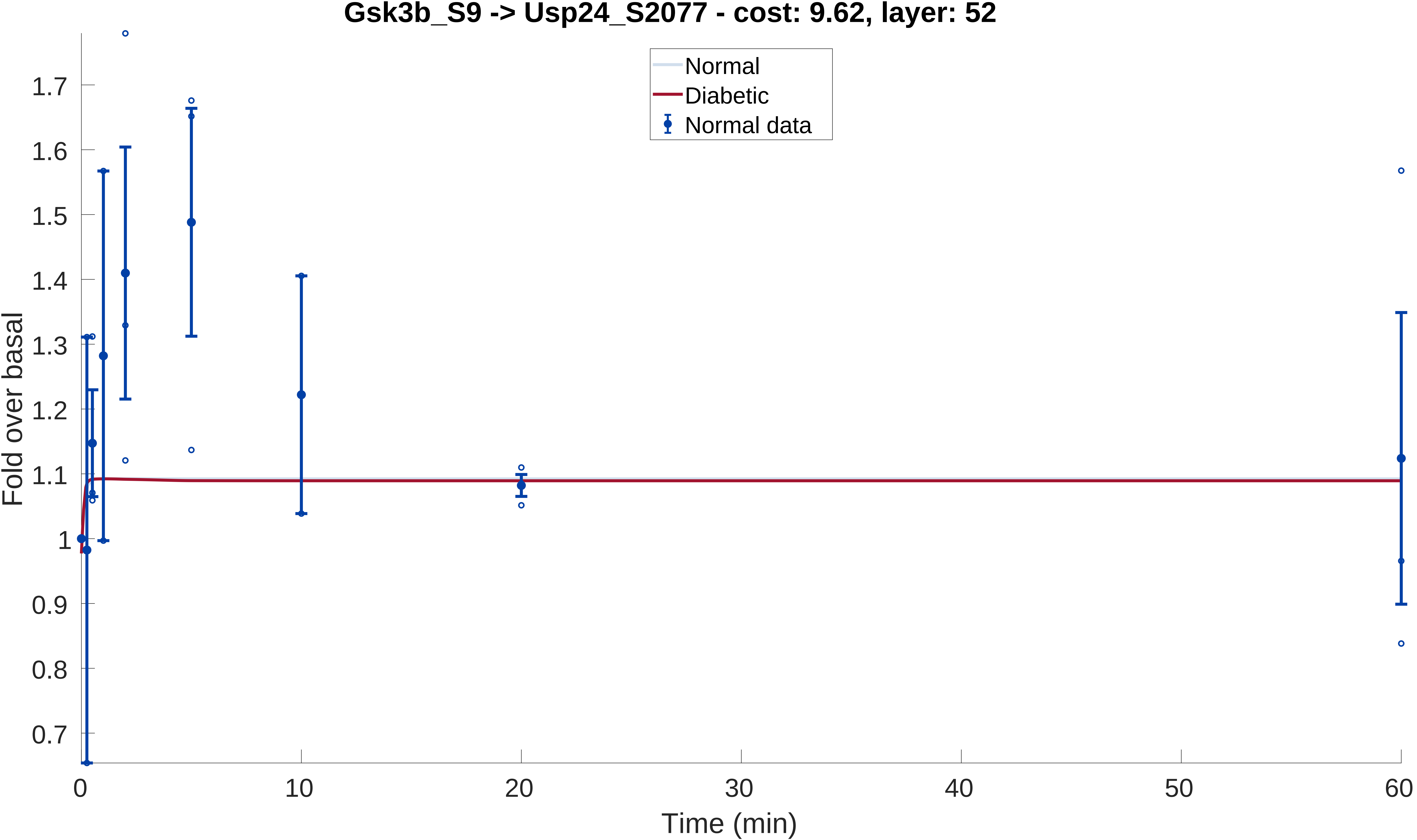

Gsk3b_S9 -> Usp24_S2077 - cost: 9.62, layer: 52
Normal
Diabetic
Normal data
Fold over basal
1.7
1.6
1.5
1.4
1.3
1.2
1.1
1
0.9
0.8
0.7
0
10
20
30
40
50
60
Time (min)

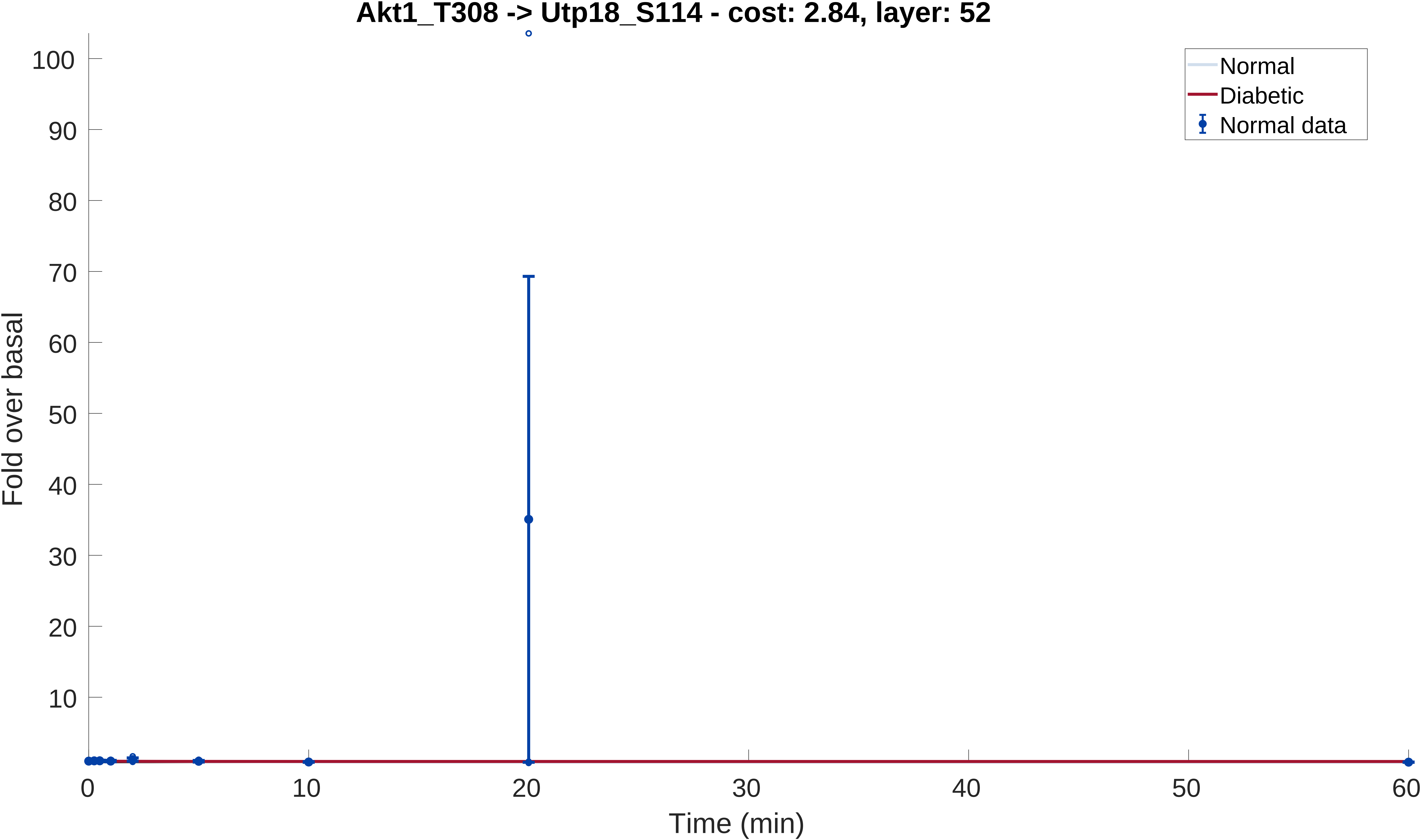
Akt1_T308 -> Utp18_S114 - cost: 2.84, layer: 52
Normal
Diabetic
Normal data
Fold over basal
Time (min)
100
90
80
70
60
50
40
30
20
10
0
10
20
30
40
50
60

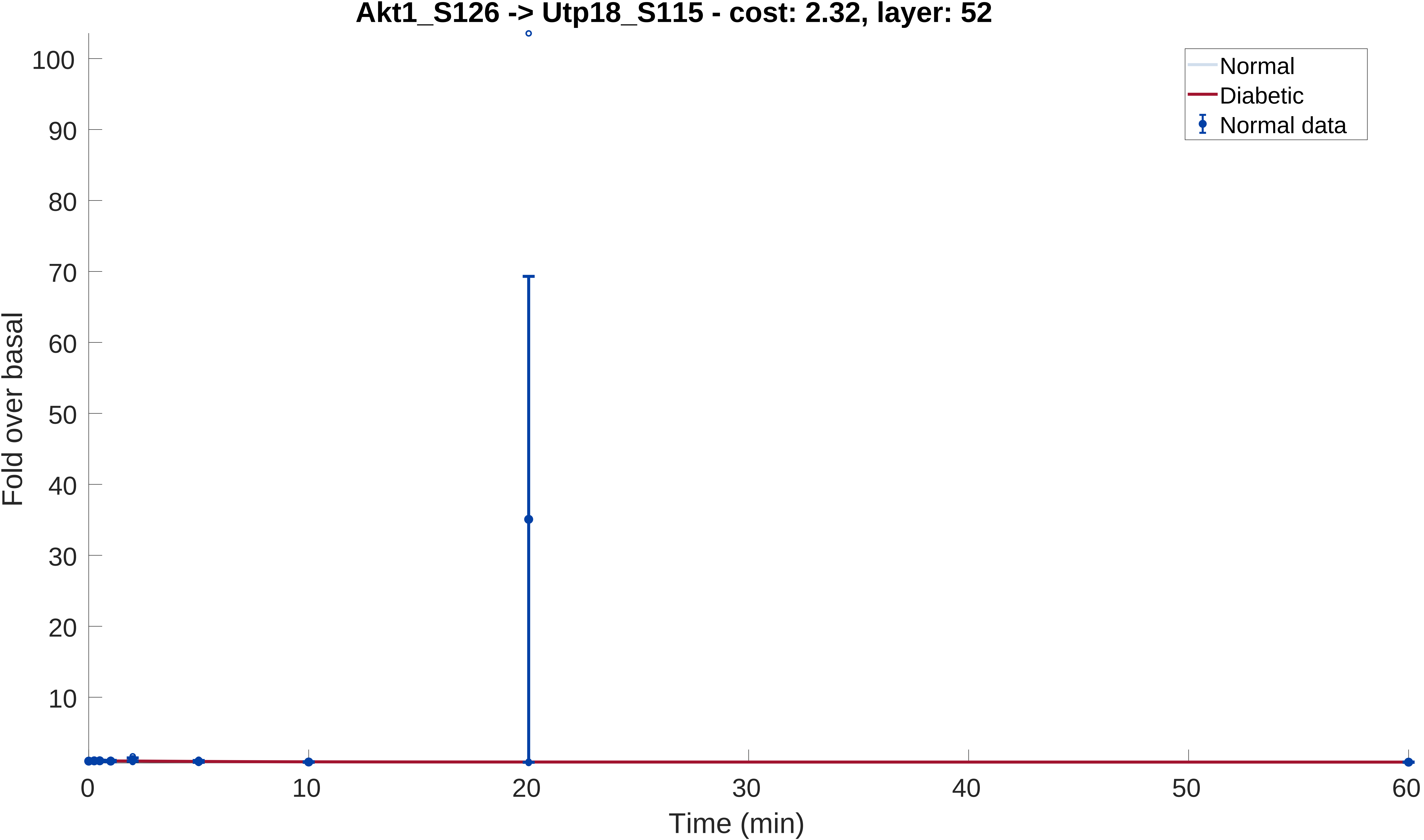
Akt1_S126 -> Utp18_S115 - cost: 2.32, layer: 52
Normal
Diabetic
Normal data
Fold over basal
100
90
80
70
60
50
40
30
20
10
0
10
20
30
40
50
60
Time (min)

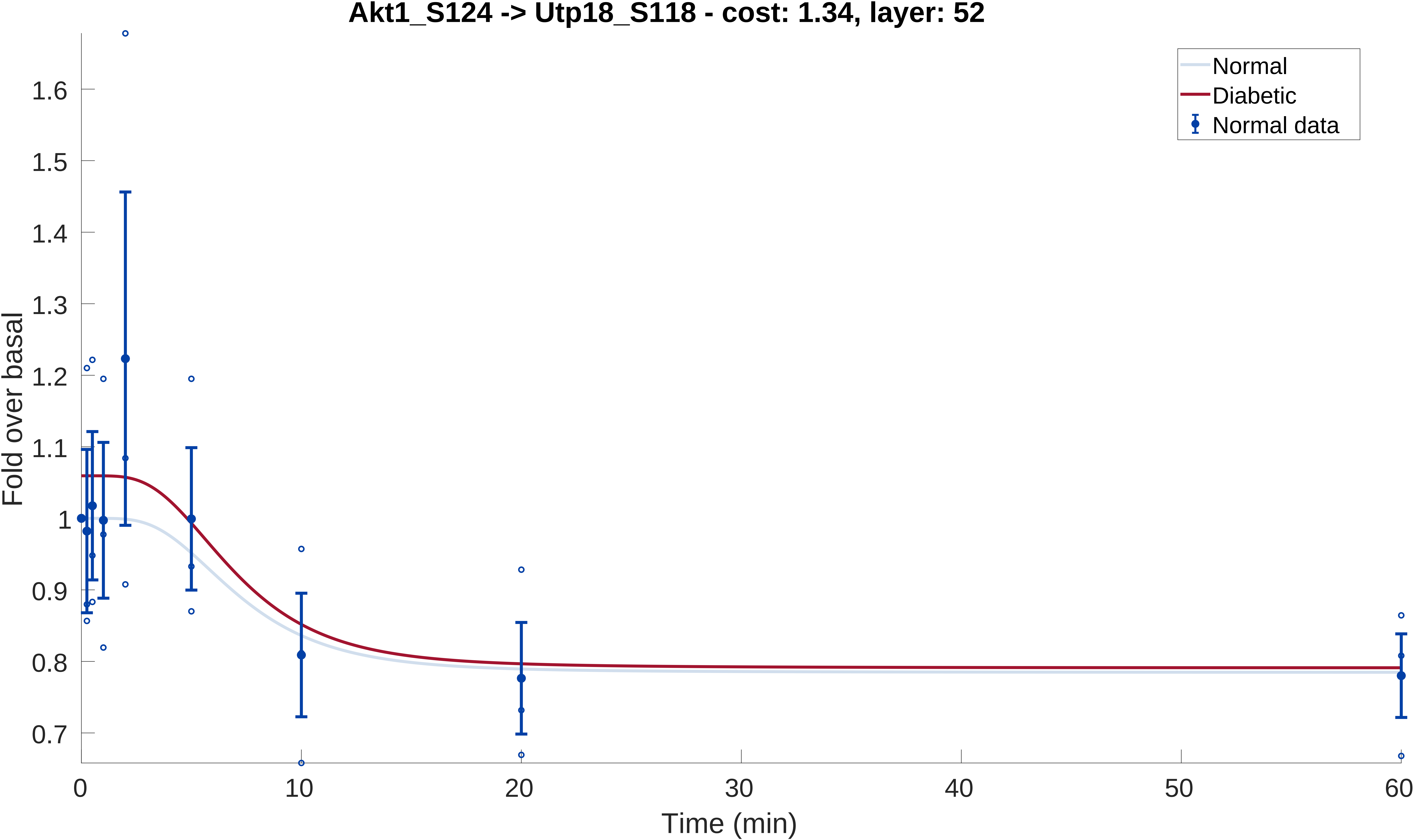
Akt1_S124 -> Utp18_S118 - cost: 1.34, layer: 52
Normal
Diabetic
Normal data
Fold over basal
1.6
1.5
1.4
1.3
1.2
1.1
1
0.9
0.8
0.7
0
10
20
30
40
50
60
Time (min)

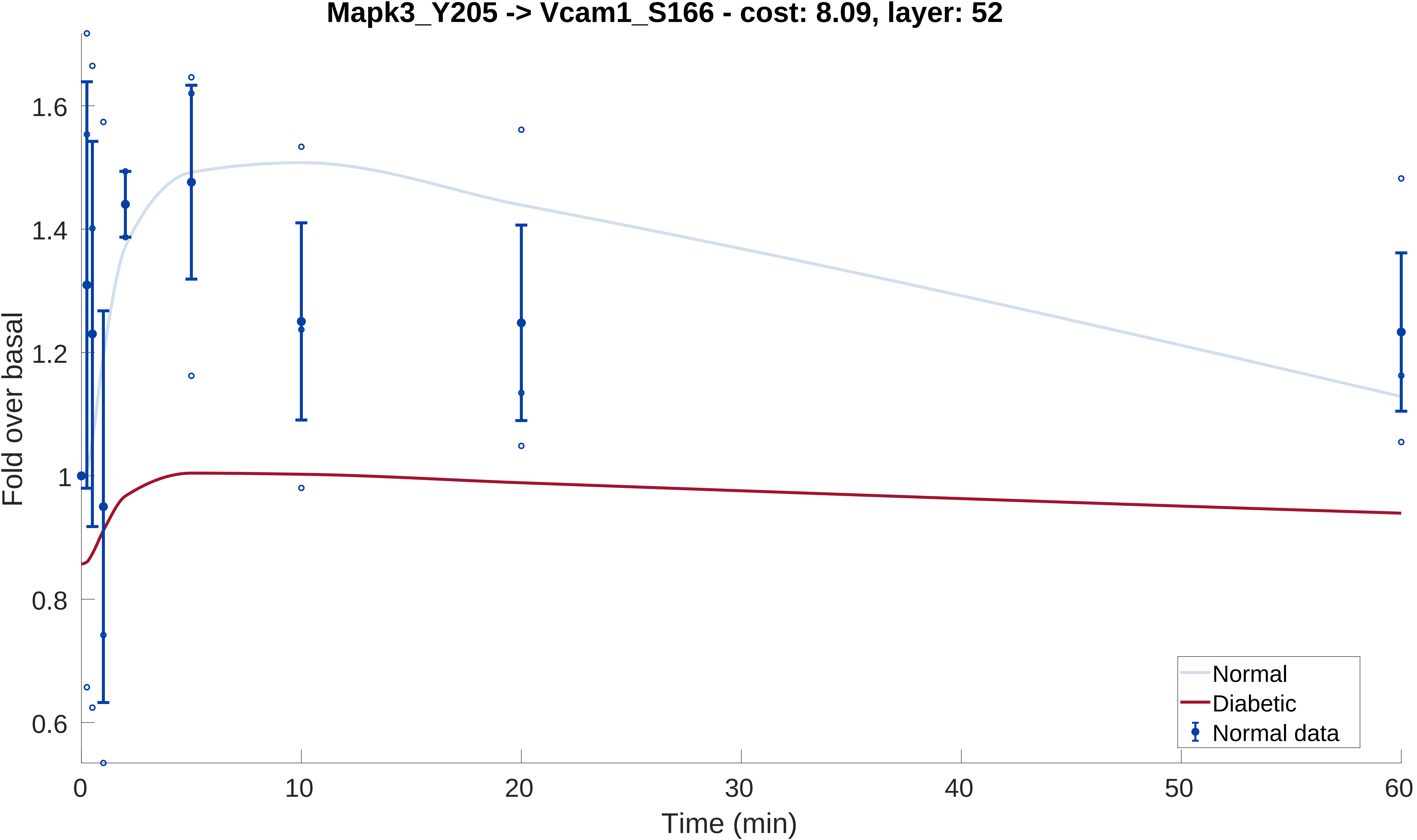

Mapk3_Y205 -> Vcam1_S166 - cost: 8.09, layer: 52
Fold over basal
1.6
1.4
1.2
1
0.8
0.6
0
10
20
30
40
50
60
Time (min)
Normal
Diabetic
Normal data

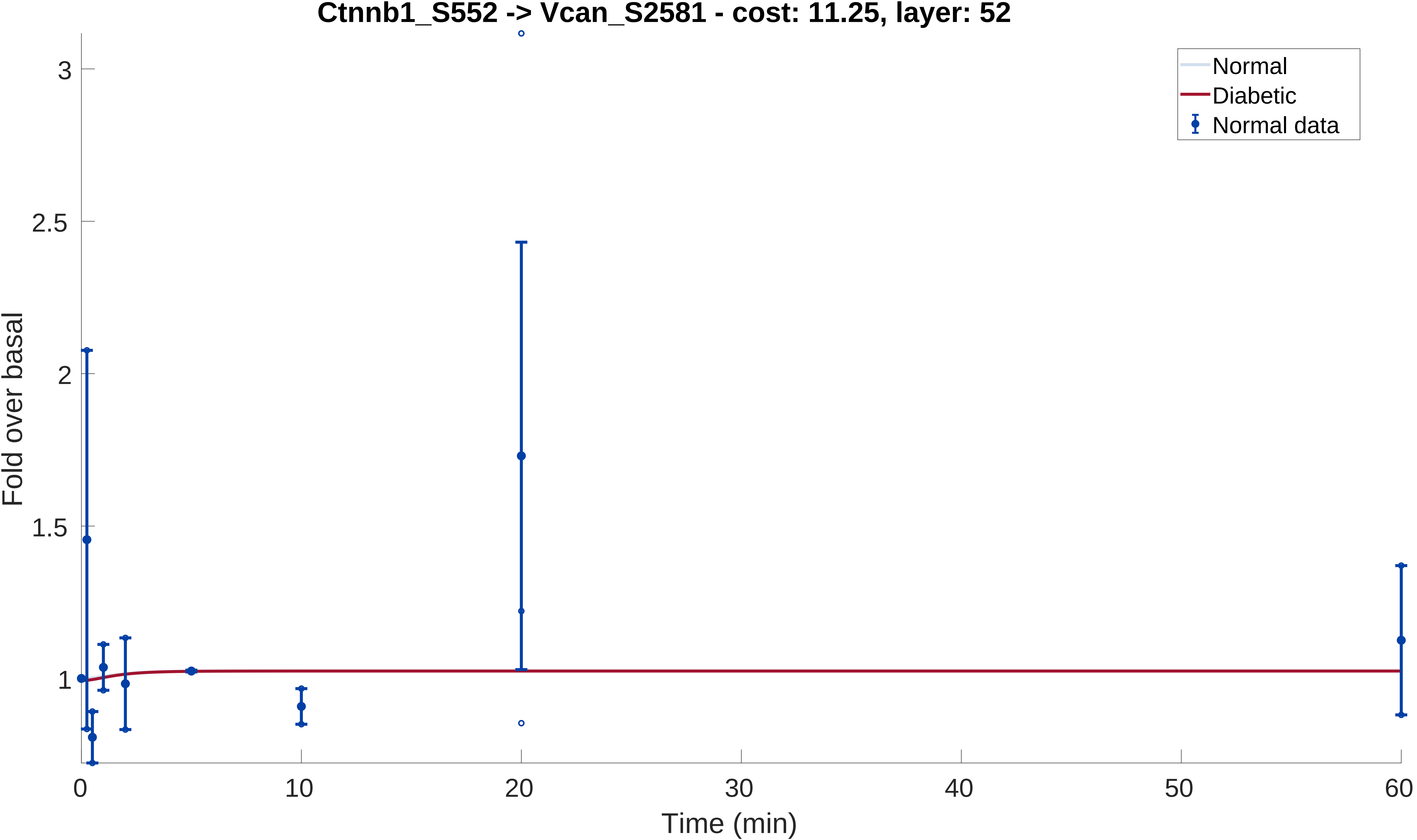

Ctnnb1_S552 -> Vcan_S2581 - cost: 11.25, layer: 52
Normal
Diabetic
Normal data
Fold over basal
Time (min)
3
2.5
2
1.5
1
0
10
20
30
40
50
60

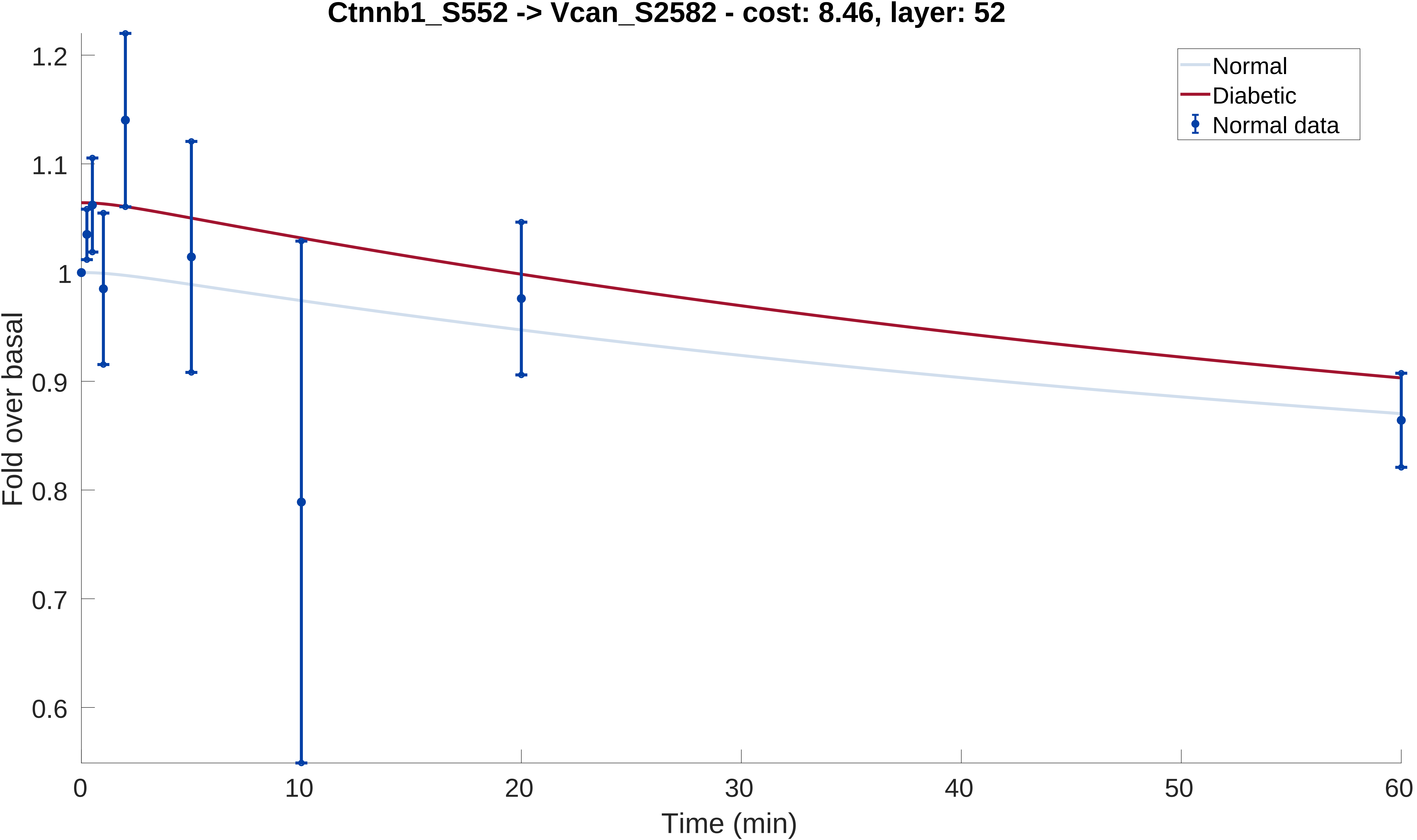

Ctnnb1_S552 -> Vcan_S2582 - cost: 8.46, layer: 52
Normal
Diabetic
Normal data
Fold over basal
Time (min)
1.2
1.1
1
0.9
0.8
0.7
0.6
0
10
20
30
40
50
60

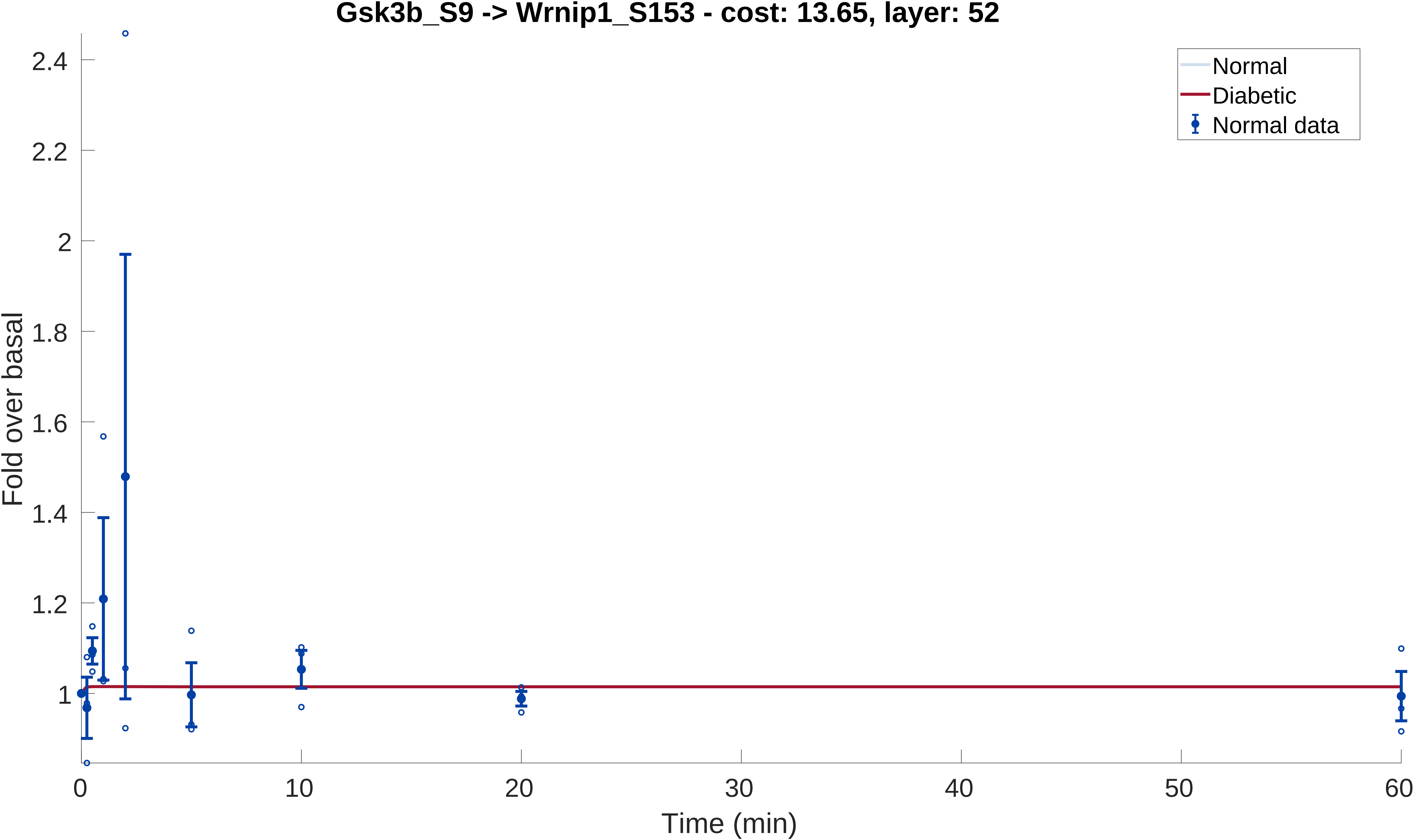
Gsk3b_S9 -> Wrnip1_S153 - cost: 13.65, layer: 52
Normal
Diabetic
Normal data
Fold over basal
Time (min)
2.4
2.2
2
1.8
1.6
1.4
1.2
1
0
10
20
30
40
50
60

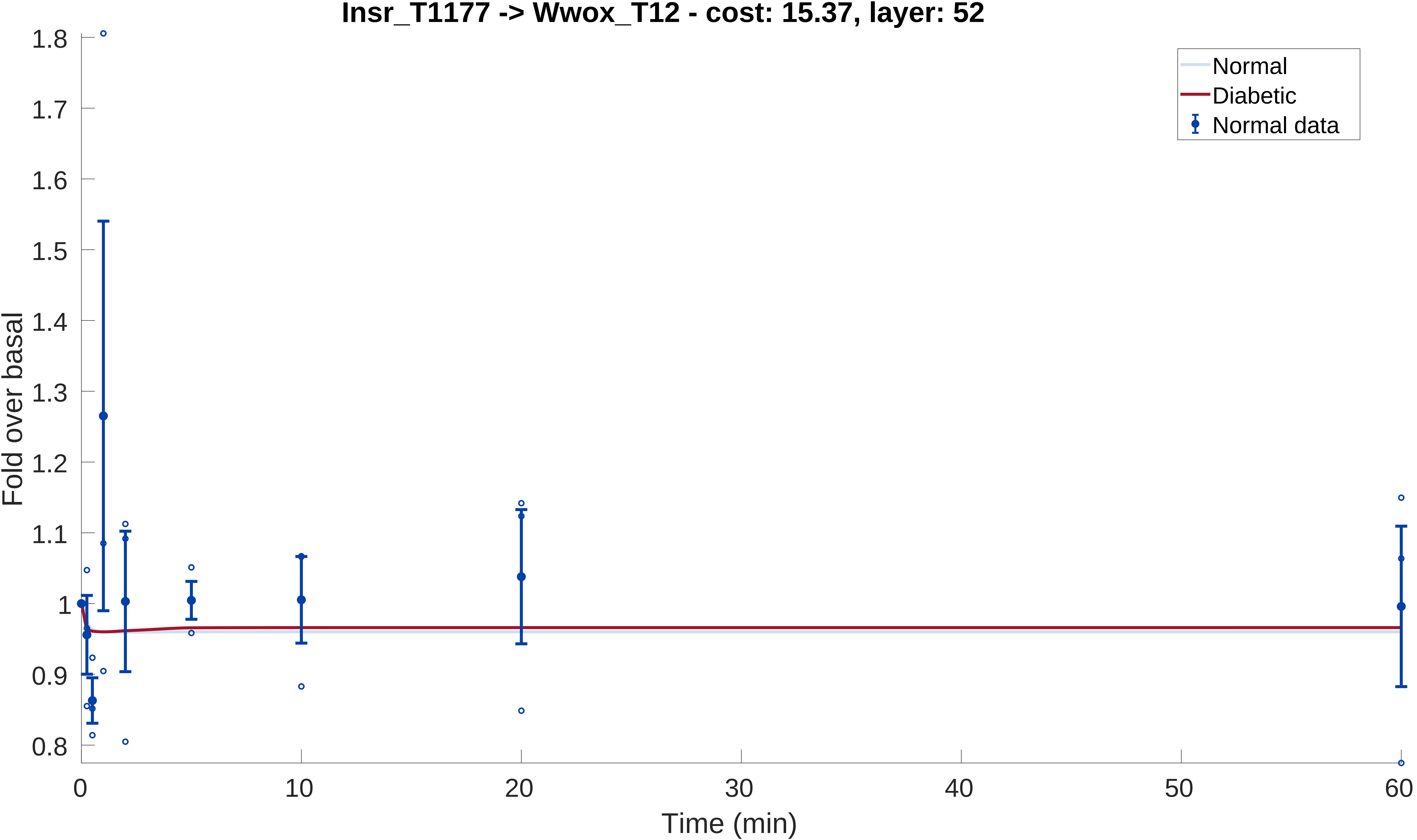

Insr_T1177 -> Wwox_T12 - cost: 15.37, layer: 52
Normal
Diabetic
Normal data
Fold over basal
1.8
1.7
1.6
1.5
1.4
1.3
1.2
1.1
1
0.9
0.8
0
10
20
30
40
50
60
Time (min)

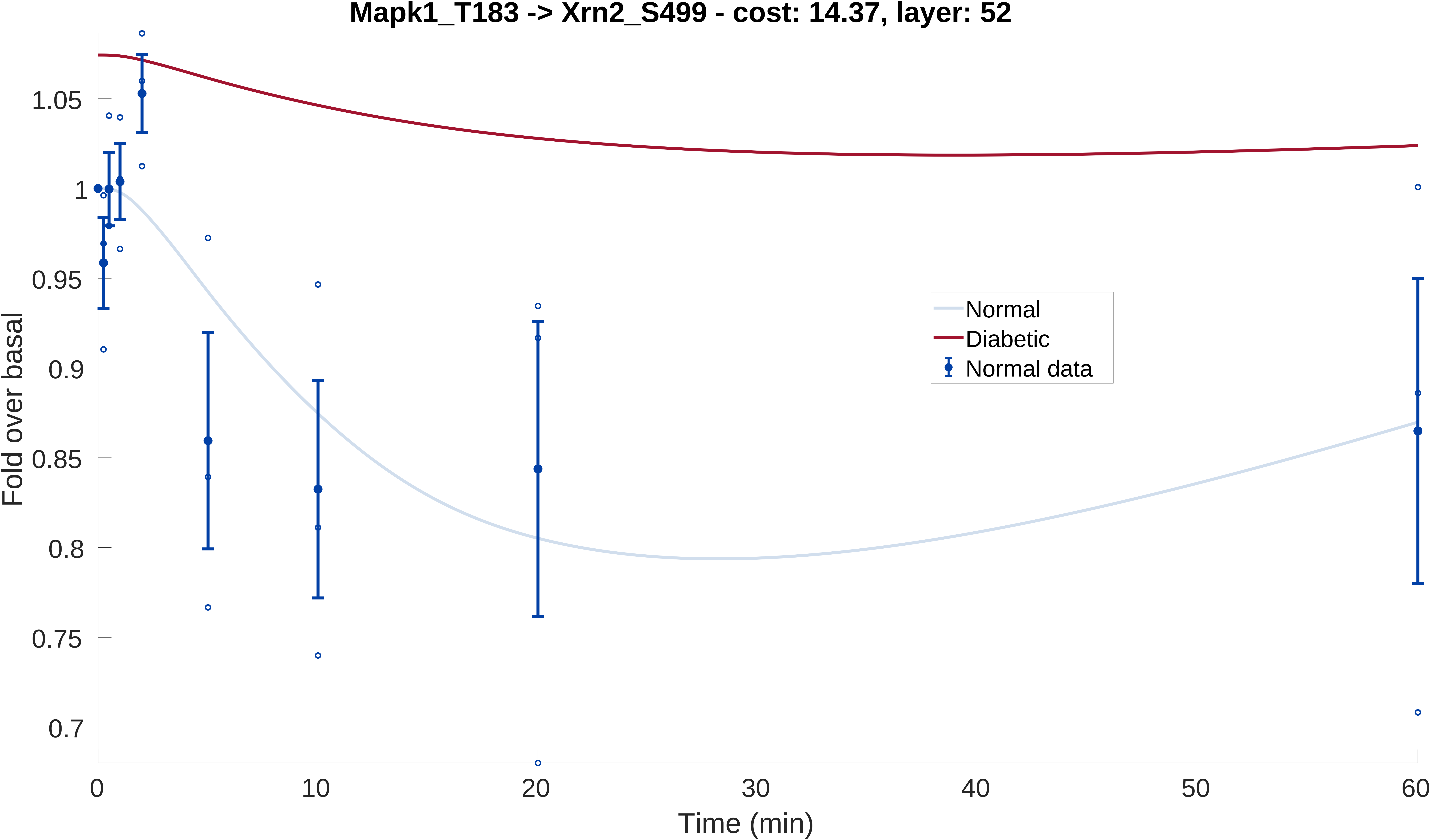

Mapk1_T183 -> Xrn2_S499 - cost: 14.37, layer: 52
1.05
1
0.95
0.9
0.85
0.8
0.75
0.7
Fold over basal
0
10
20
30
40
50
60
Time (min)
Normal
Diabetic
Normal data

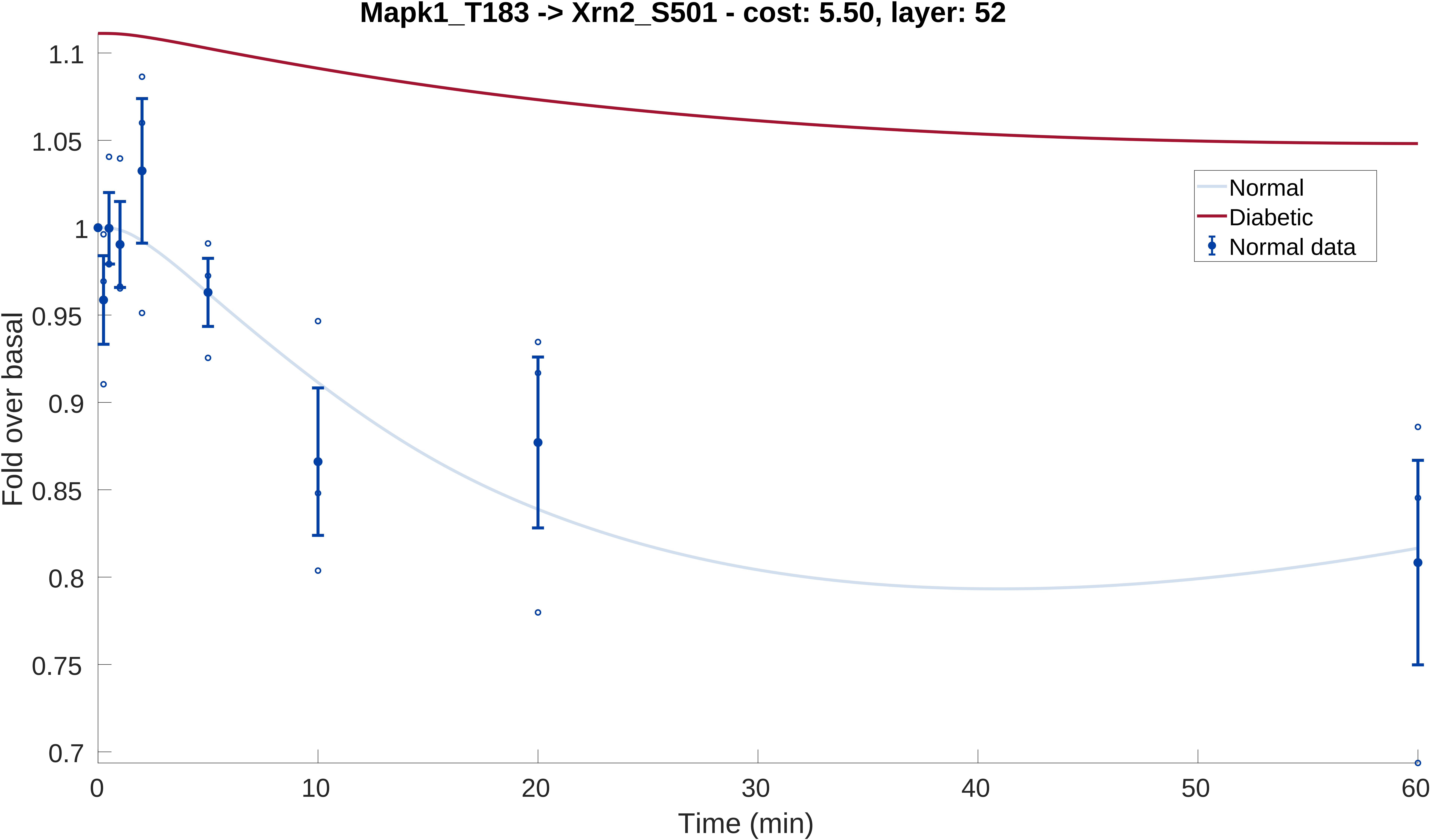

Mapk1_T183 -> Xrn2_S501 - cost: 5.50, layer: 52
Fold over basal
1.1
1.05
1
0.95
0.9
0.85
0.8
0.75
0.7
0
10
20
30
40
50
60
Time (min)
Normal
Diabetic
Normal data

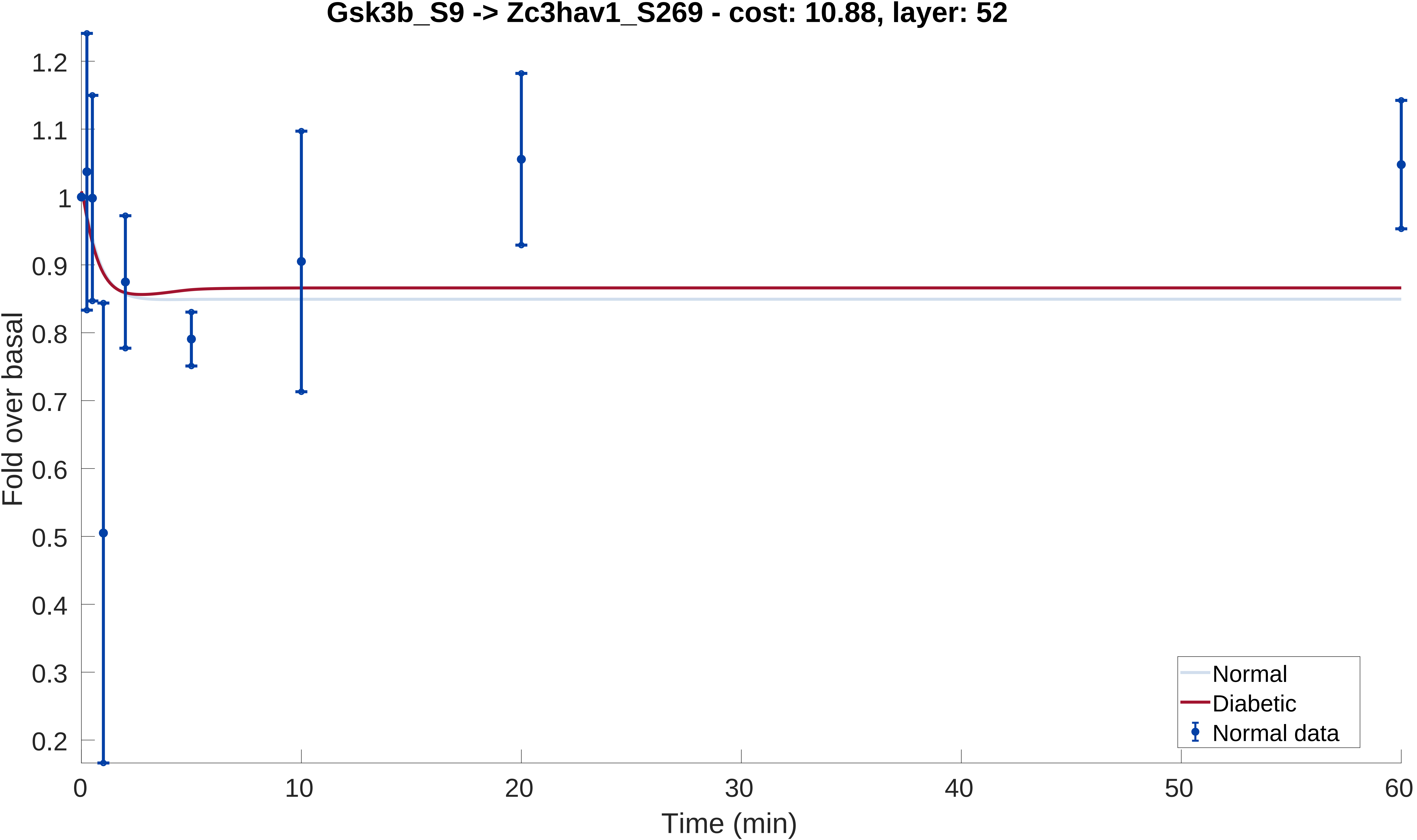
Gsk3b_S9 -> Zc3hav1_S269 - cost: 10.88, layer: 52
Fold over basal
1.2
1.1
1
0.9
0.8
0.7
0.6
0.5
0.4
0.3
0.2
0
10
20
30
40
50
60
Time (min)
Normal
Diabetic
Normal data

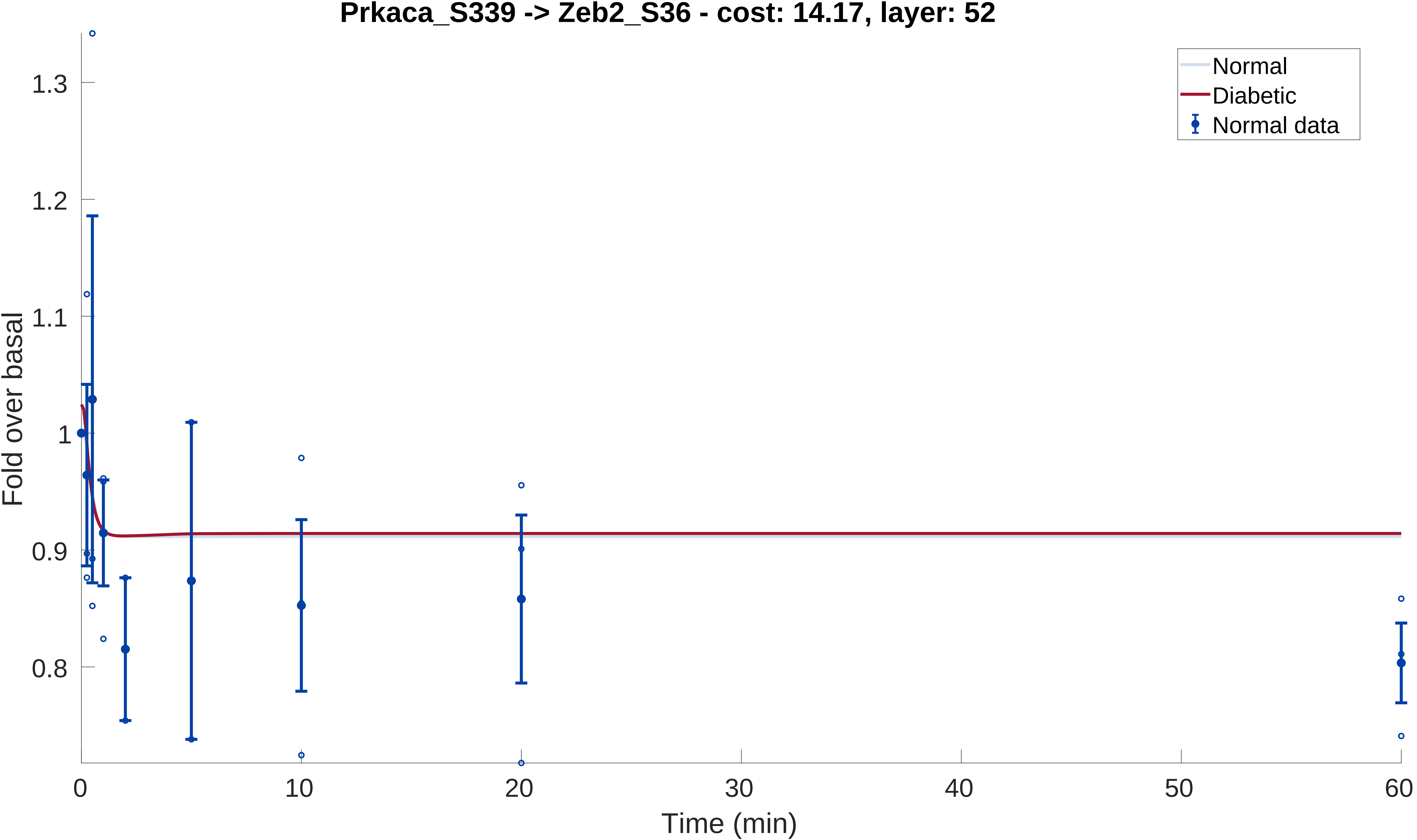

Prkaca_S339 -> Zeb2_S36 - cost: 14.17, layer: 52
Normal
Diabetic
Normal data
Fold over basal
1.3
1.2
1.1
1
0.9
0.8
0
10
20
30
40
50
60
Time (min)

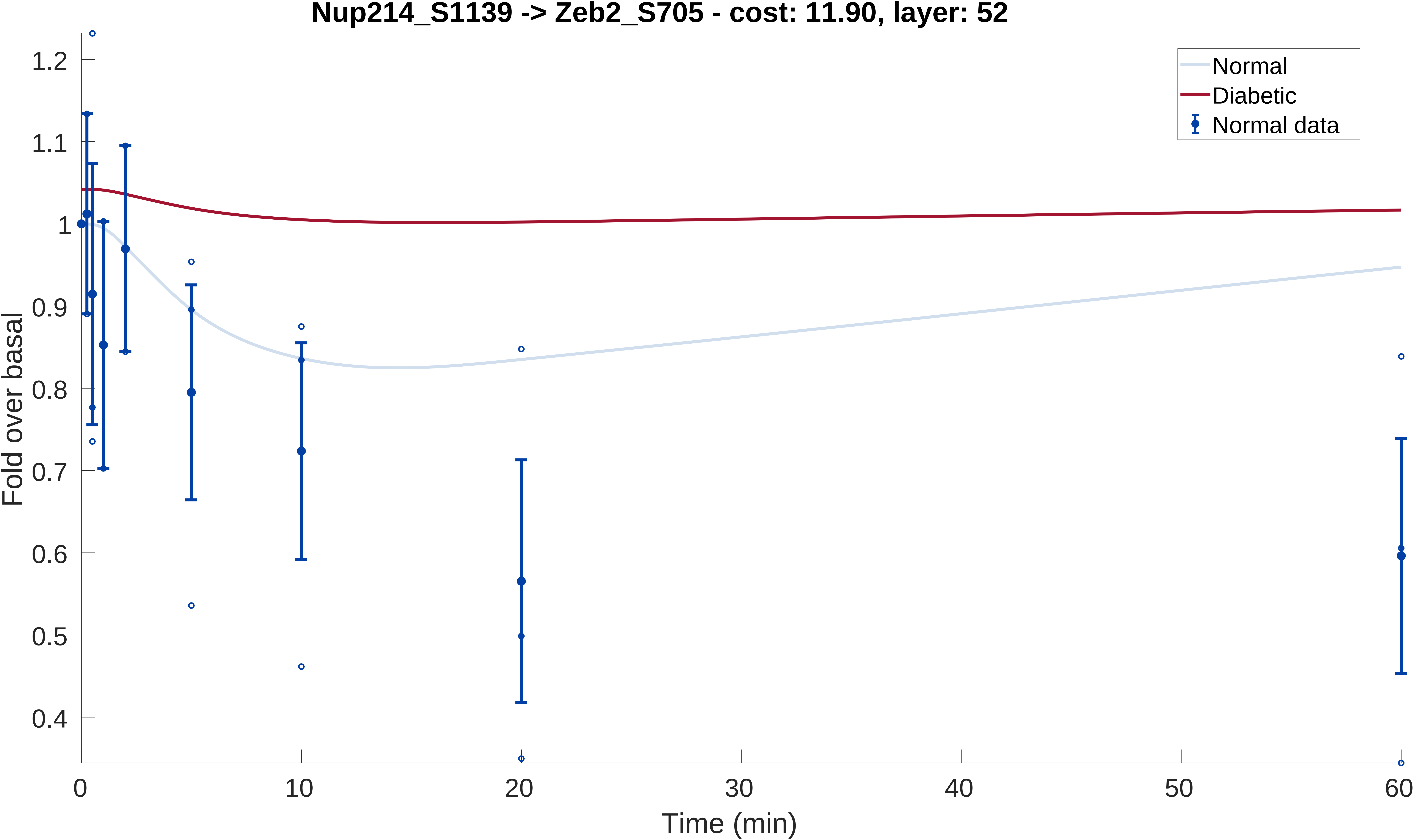
Nup214_S1139 -> Zeb2_S705 - cost: 11.90, layer: 52
Normal
Diabetic
Normal data
Fold over basal
Time (min)
1.2
1.1
1
0.9
0.8
0.7
0.6
0.5
0.4
0
10
20
30
40
50
60

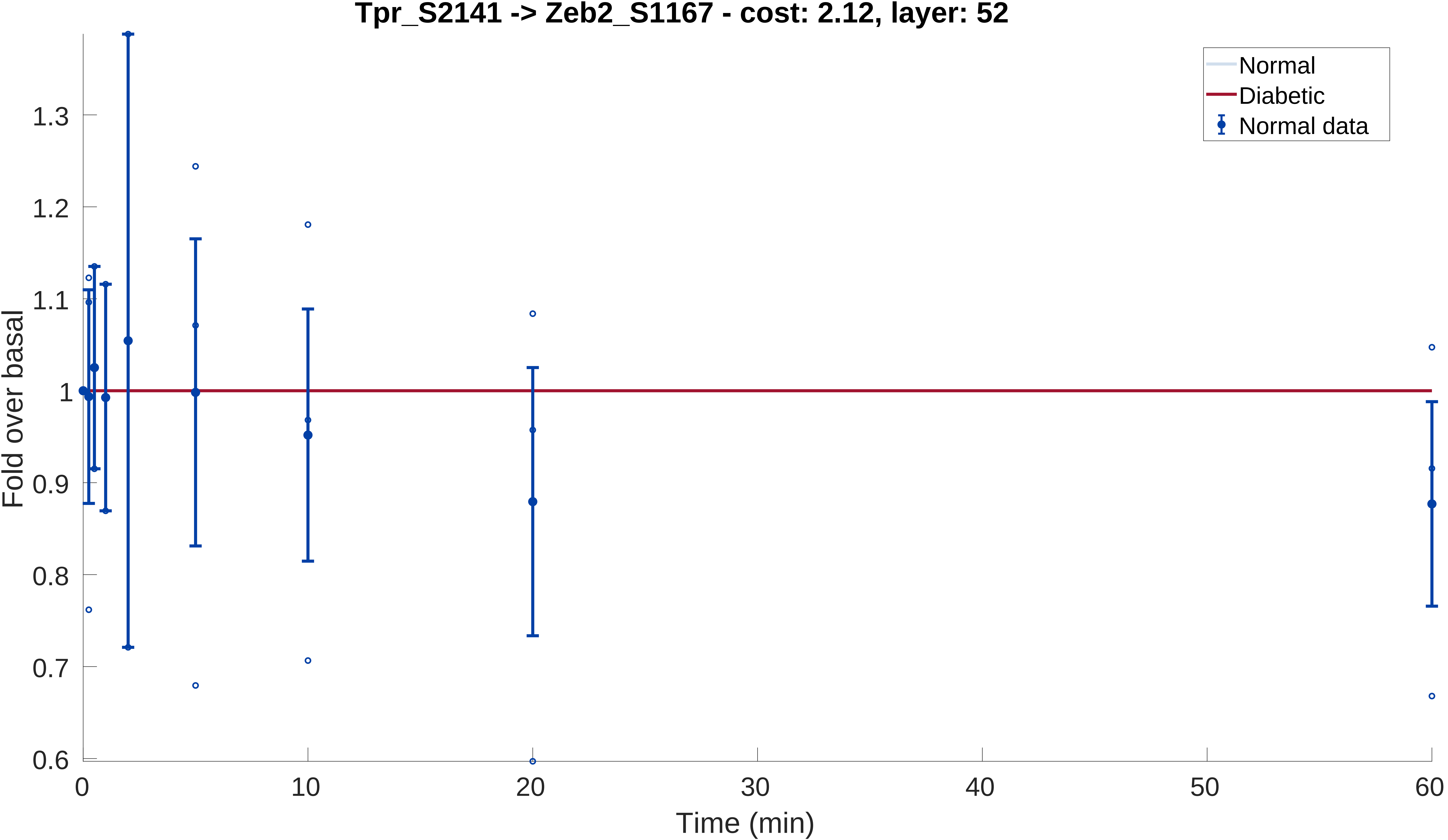

Tpr_S2141 -> Zeb2_S1167 - cost: 2.12, layer: 52
Normal
Diabetic
Normal data
Fold over basal
1.3
1.2
1.1
1
0.9
0.8
0.7
0.6
0
10
20
30
40
50
60
Time (min)

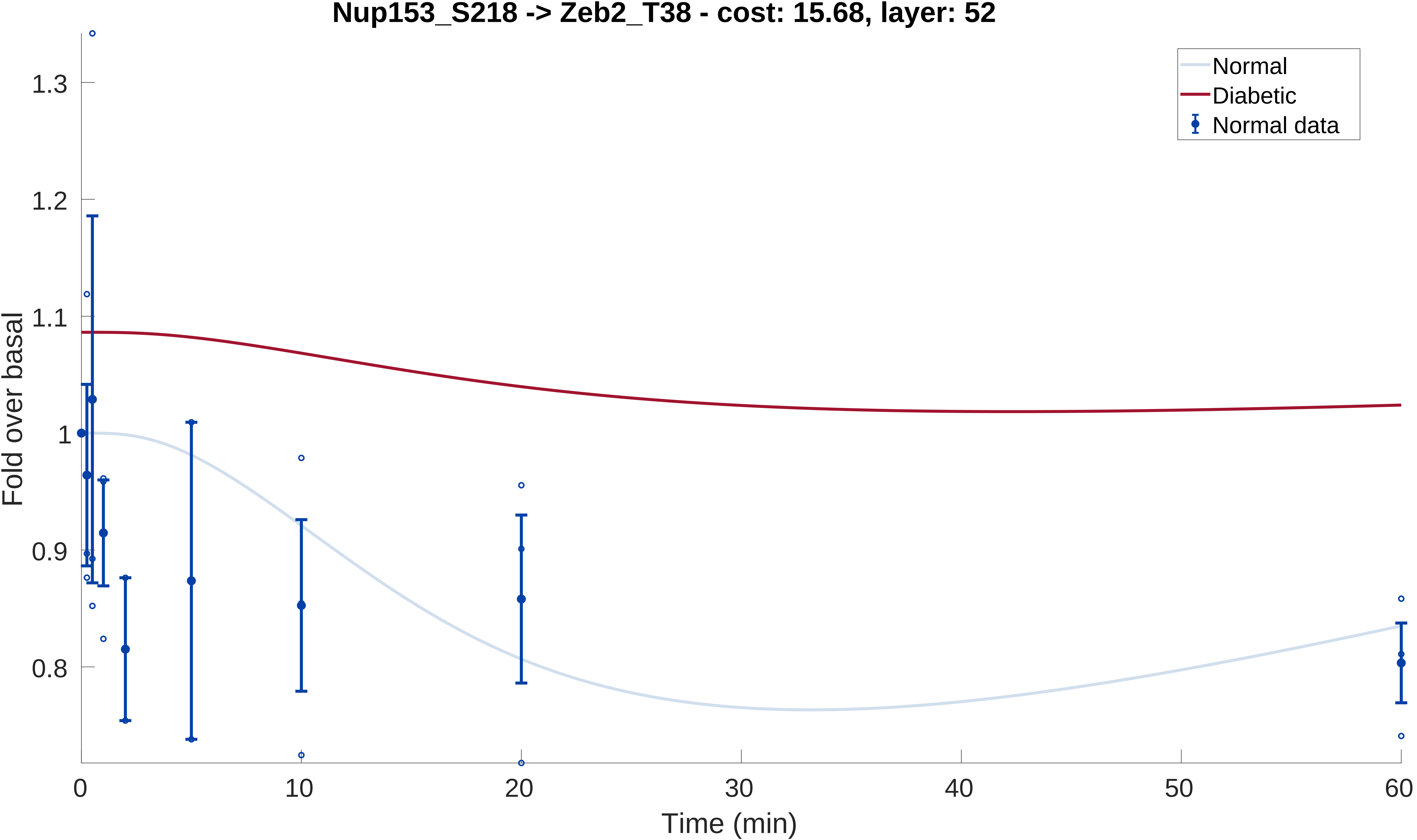

Nup153_S218 -> Zeb2_T38 - cost: 15.68, layer: 52
Normal
Diabetic
Normal data
1.3
1.2
1.1
1
0.9
0.8
Fold over basal
0
10
20
30
40
50
60
Time (min)

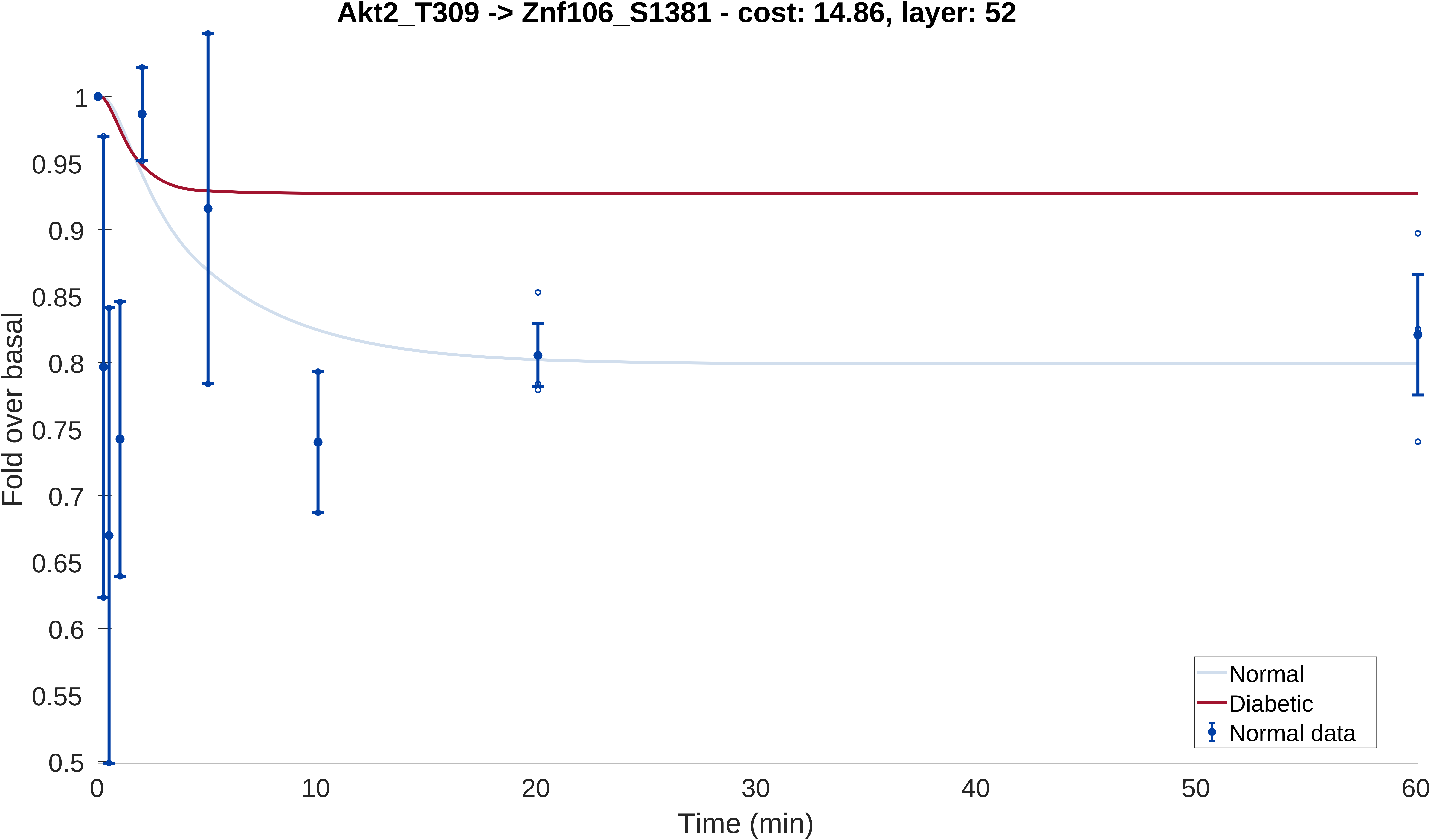

Akt2_T309 -> Znf106_S1381 - cost: 14.86, layer: 52
Fold over basal
1
0.95
0.9
0.85
0.8
0.75
0.7
0.65
0.6
0.55
0.5
0
10
20
30
40
50
60
Time (min)
Normal
Diabetic
Normal data

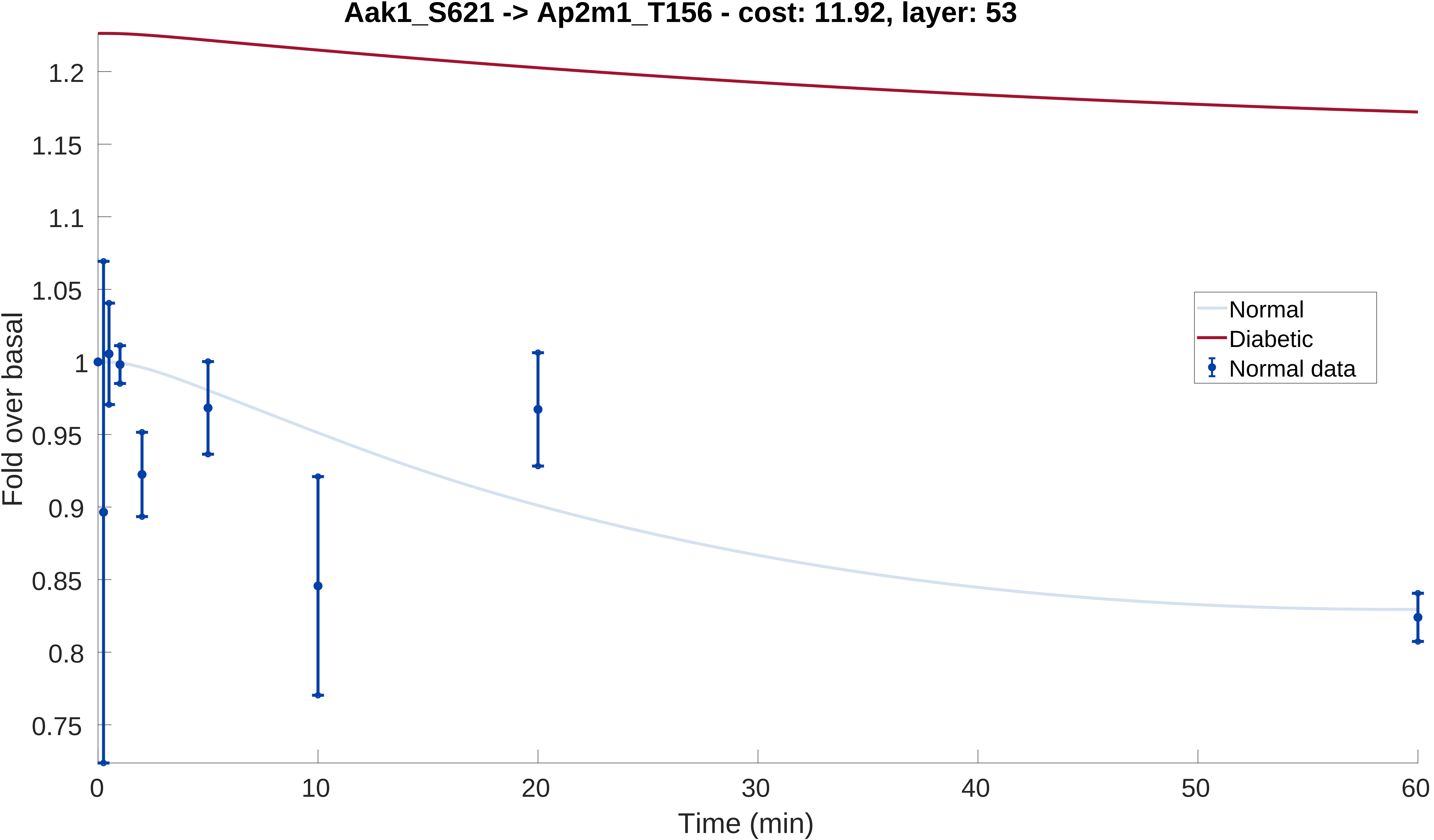

Aak1_S621 -> Ap2m1_T156 - cost: 11.92, layer: 53
Fold over basal
1.2
1.15
1.1
1.05
1
0.95
0.9
0.85
0.8
0.75
0
10
20
30
40
50
60
Time (min)
Normal
Diabetic
Normal data

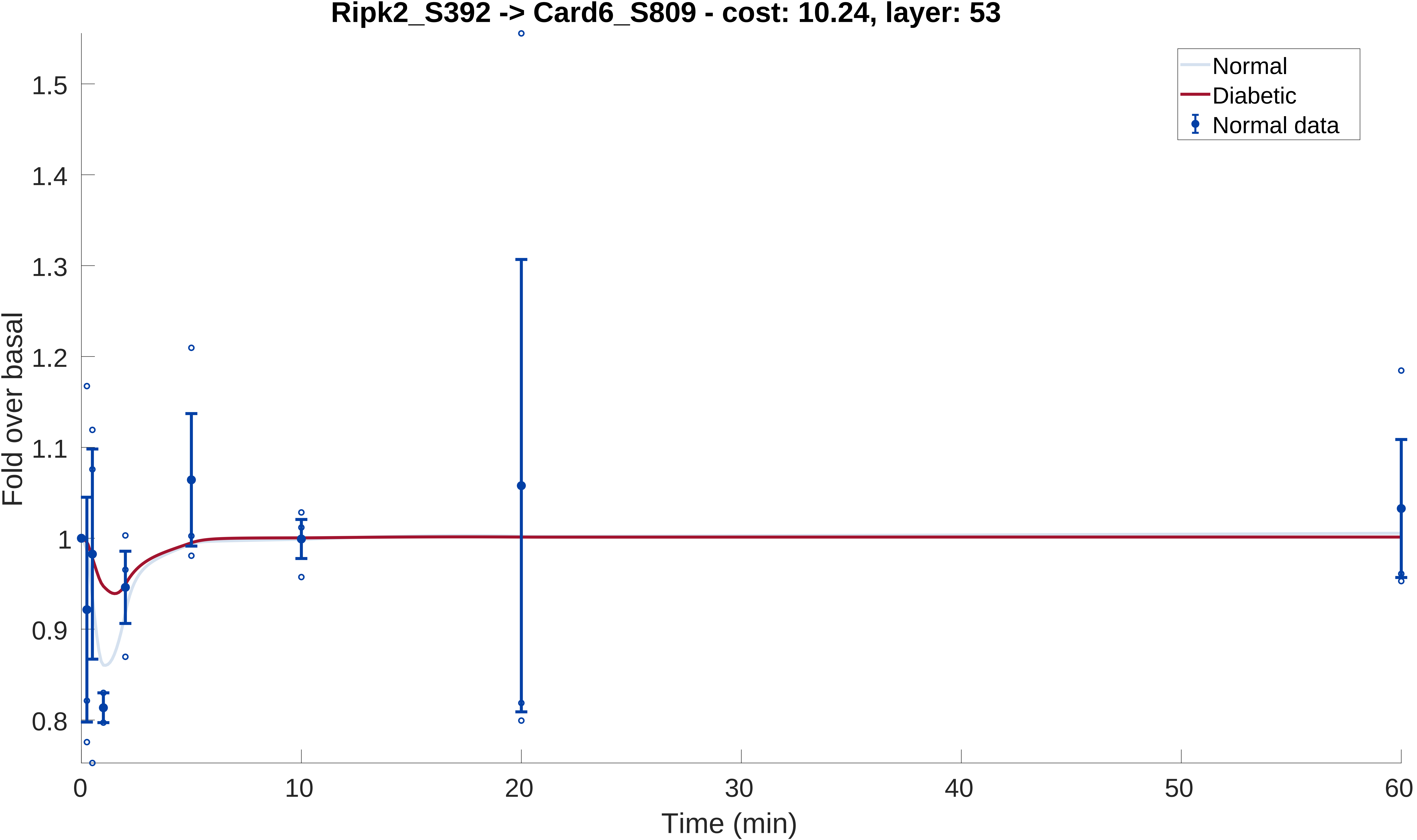

Ripk2_S392 -> Card6_S809 - cost: 10.24, layer: 53
Normal
Diabetic
Normal data
Fold over basal
1.5
1.4
1.3
1.2
1.1
1
0.9
0.8
0
10
20
30
40
50
60
Time (min)

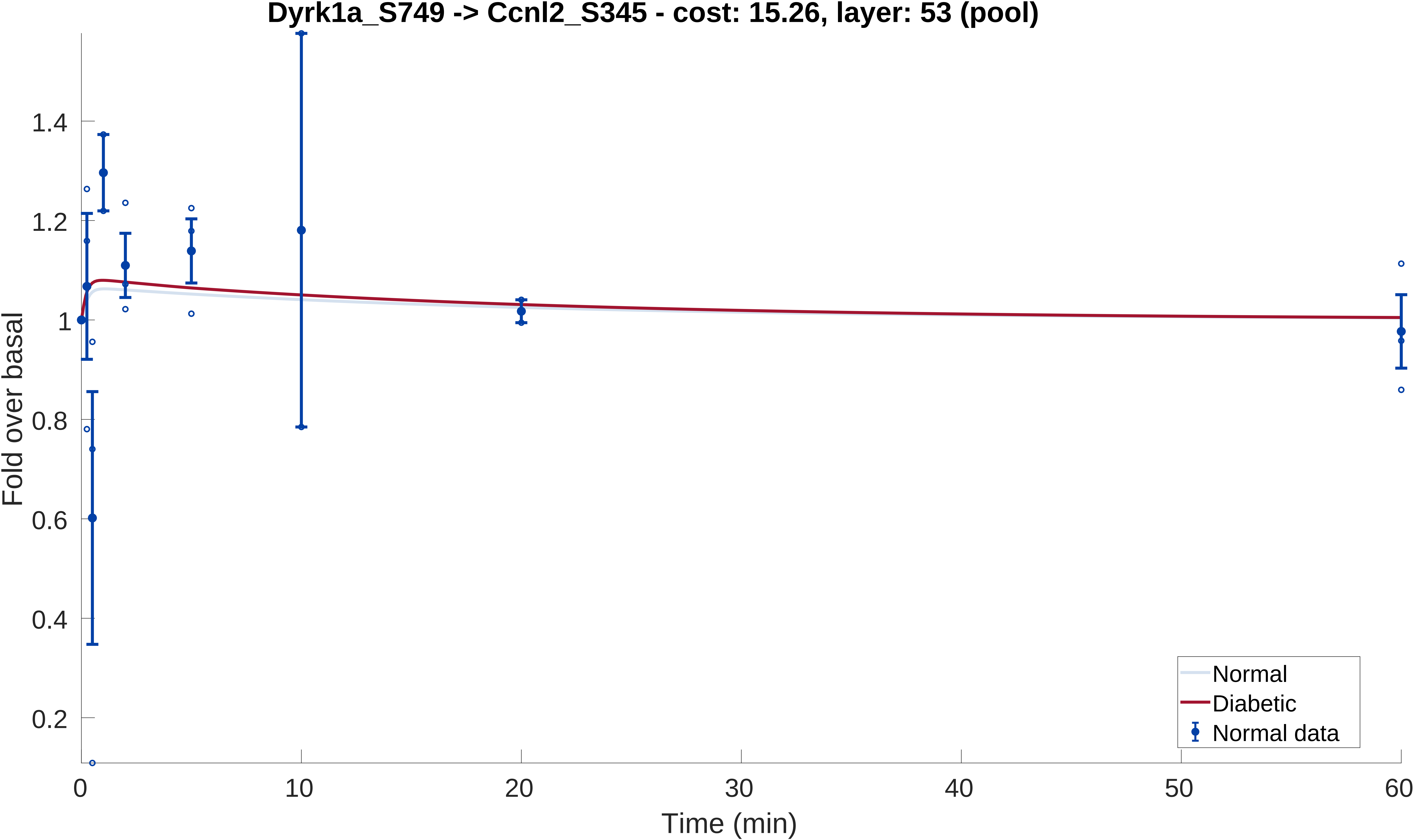

Dyrk1a_S749 -> Ccnl2_S345 - cost: 15.26, layer: 53 (pool)
Fold over basal
1.4
1.2
1
0.8
0.6
0.4
0.2
0
10
20
30
40
50
60
Time (min)
Normal
Diabetic
Normal data

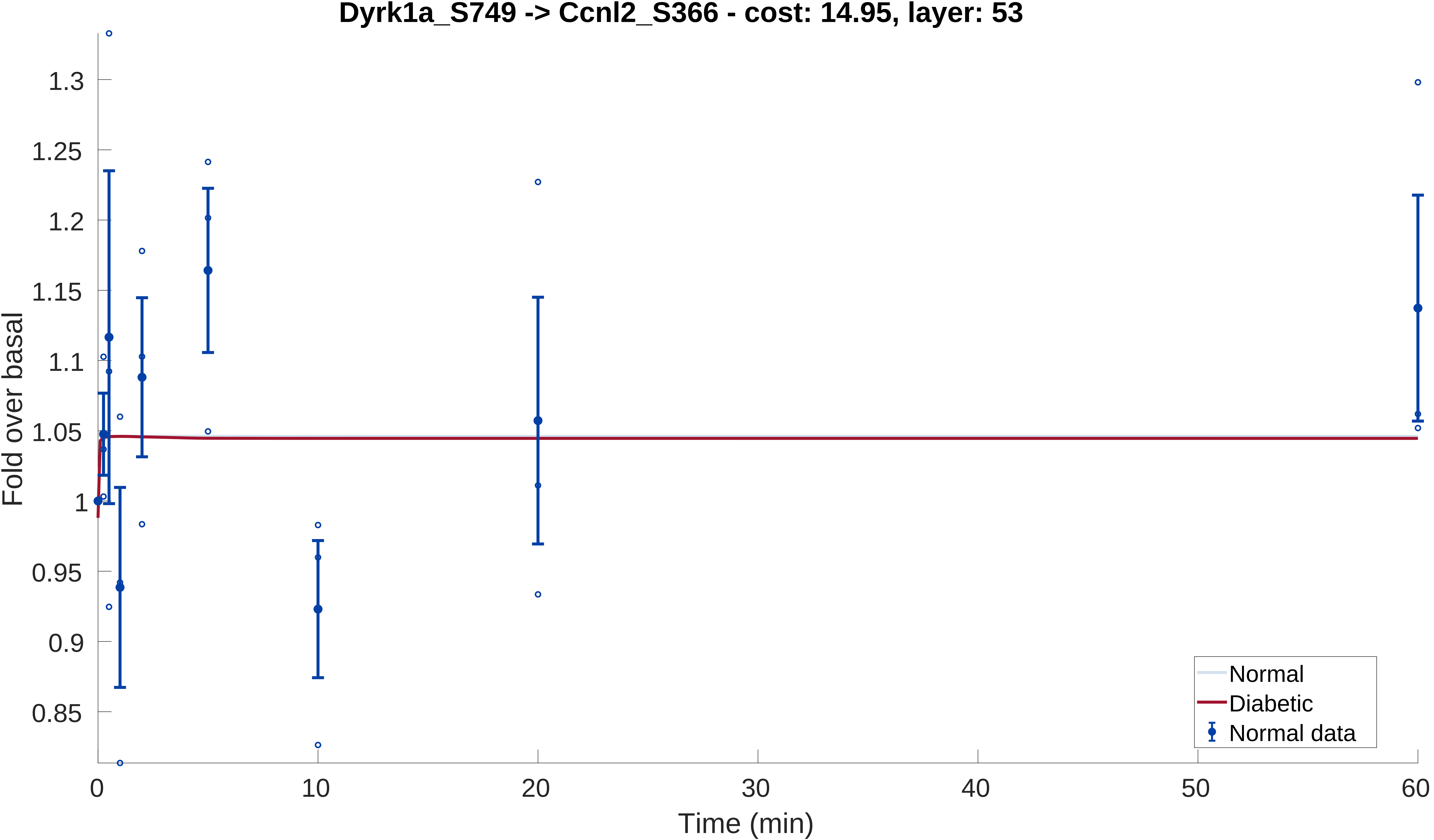

Dyrk1a_S749 -> Ccnl2_S366 - cost: 14.95, layer: 53
Fold over basal
1.3
1.25
1.2
1.15
1.1
1.05
1
0.95
0.9
0.85
0
10
20
30
40
50
60
Time (min)
Normal
Diabetic
Normal data

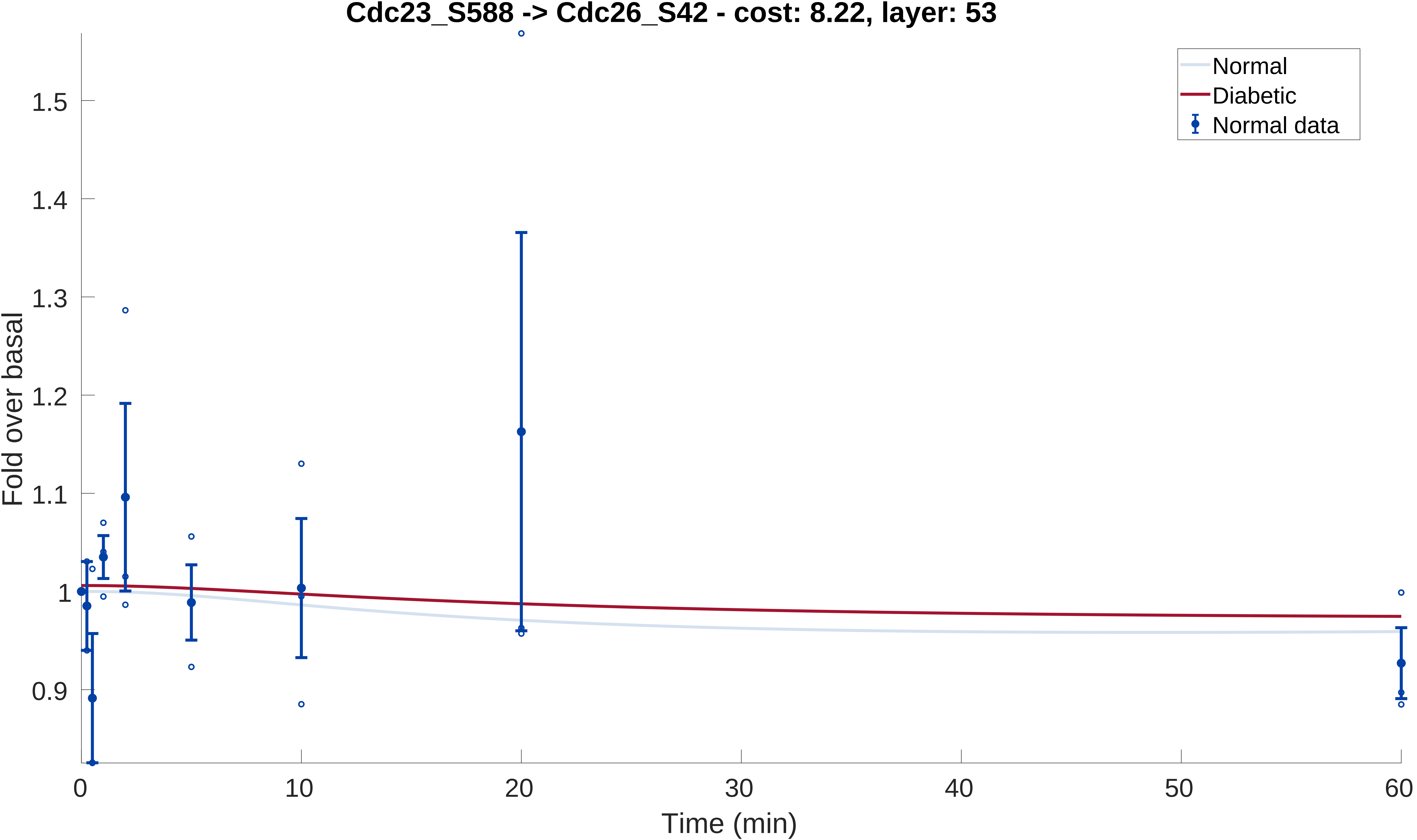

Cdc23_S588 -> Cdc26_S42 - cost: 8.22, layer: 53
Normal
Diabetic
Normal data
Fold over basal
1.5
1.4
1.3
1.2
1.1
1
0.9
0
10
20
30
40
50
60
Time (min)

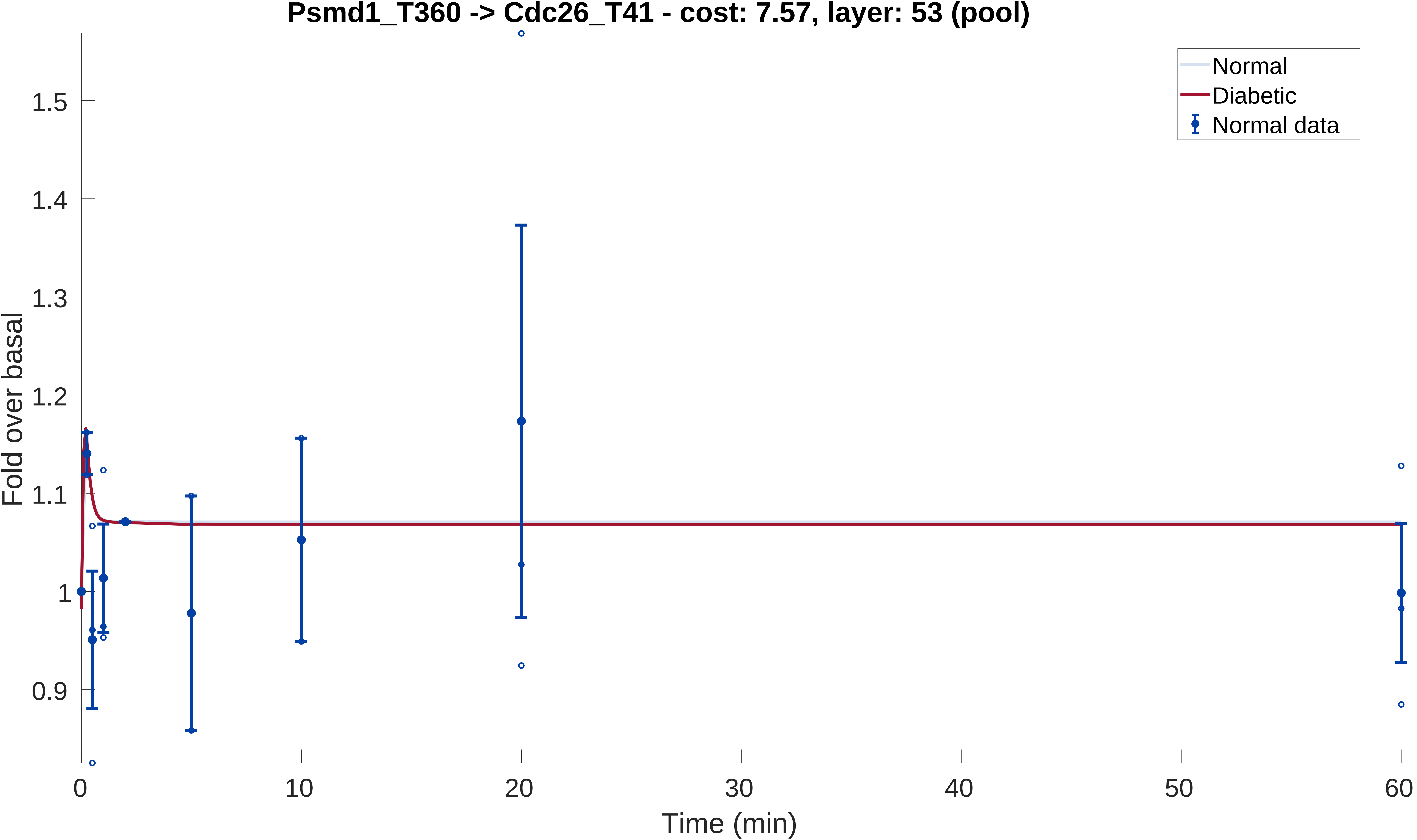

Psmd1_T360 -> Cdc26_T41 - cost: 7.57, layer: 53 (pool)
Normal
Diabetic
Normal data
Fold over basal
1.5
1.4
1.3
1.2
1.1
1
0.9
0
10
20
30
40
50
60
Time (min)

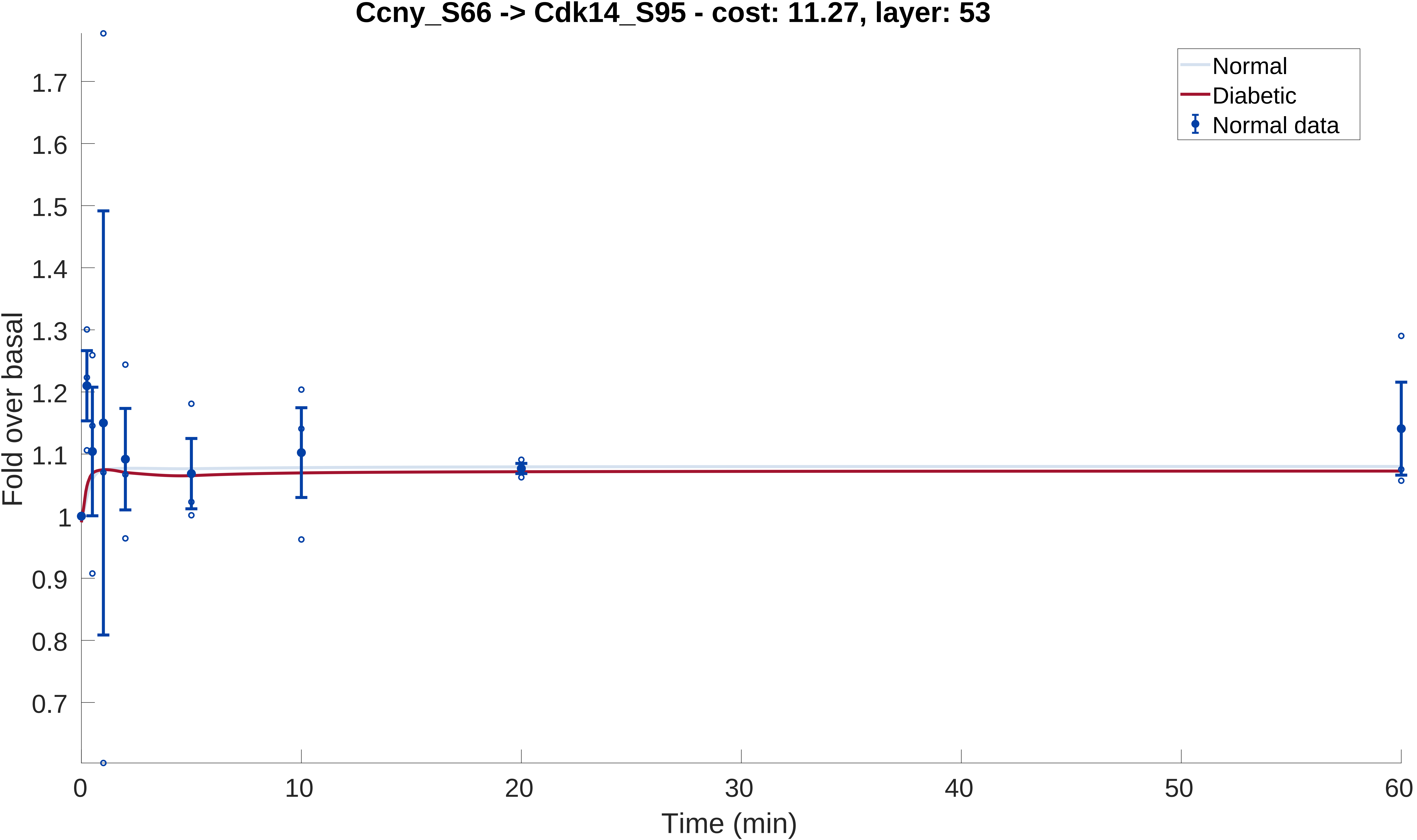

Ccny_S66 -> Cdk14_S95 - cost: 11.27, layer: 53
Normal
Diabetic
Normal data
Fold over basal
Time (min)
1.7
1.6
1.5
1.4
1.3
1.2
1.1
1
0.9
0.8
0.7
0
10
20
30
40
50
60

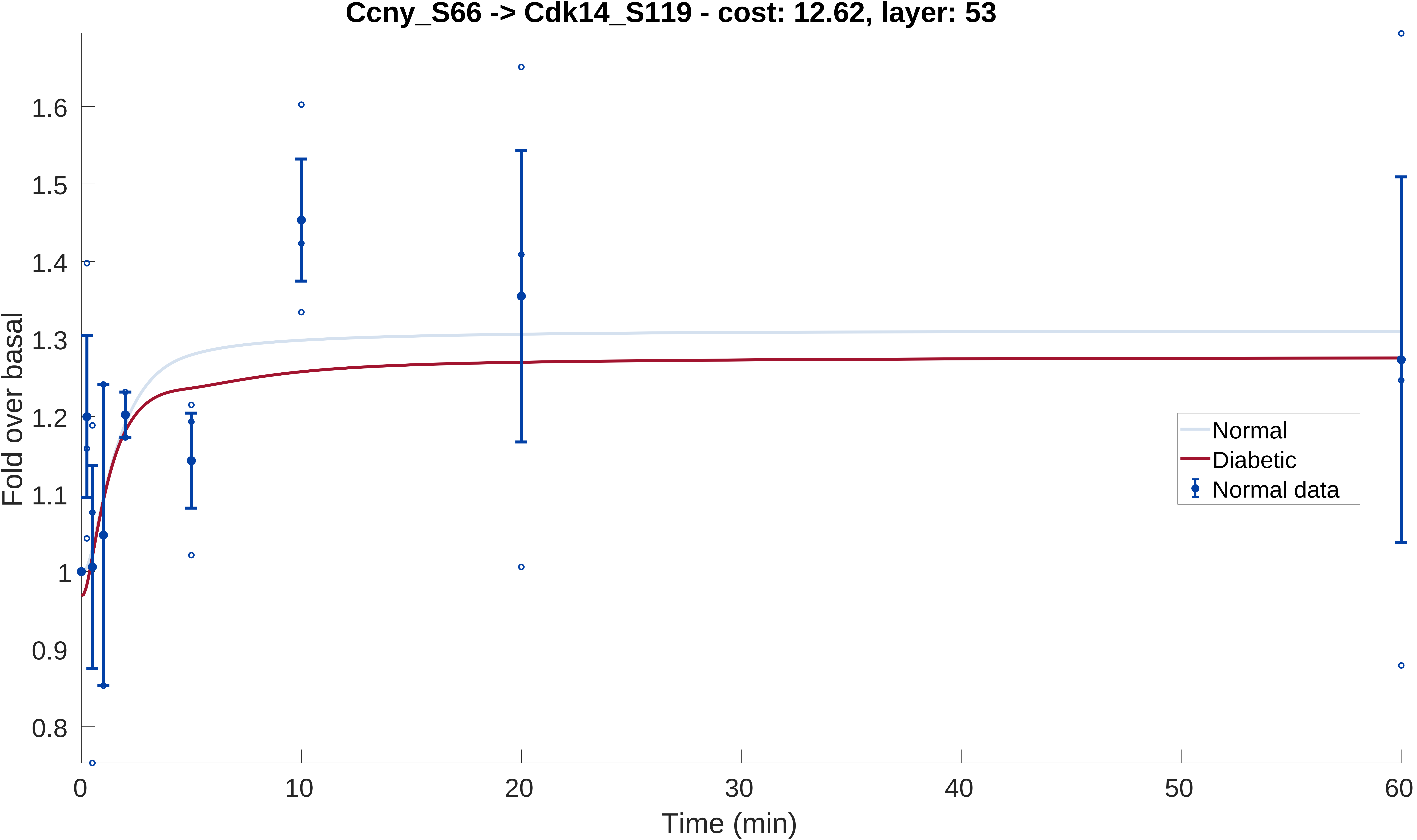

Ccny_S66 -> Cdk14_S119 - cost: 12.62, layer: 53
Fold over basal
Time (min)
0.8
0.9
1
1.1
1.2
1.3
1.4
1.5
1.6
0
10
20
30
40
50
60
Normal
Diabetic
Normal data

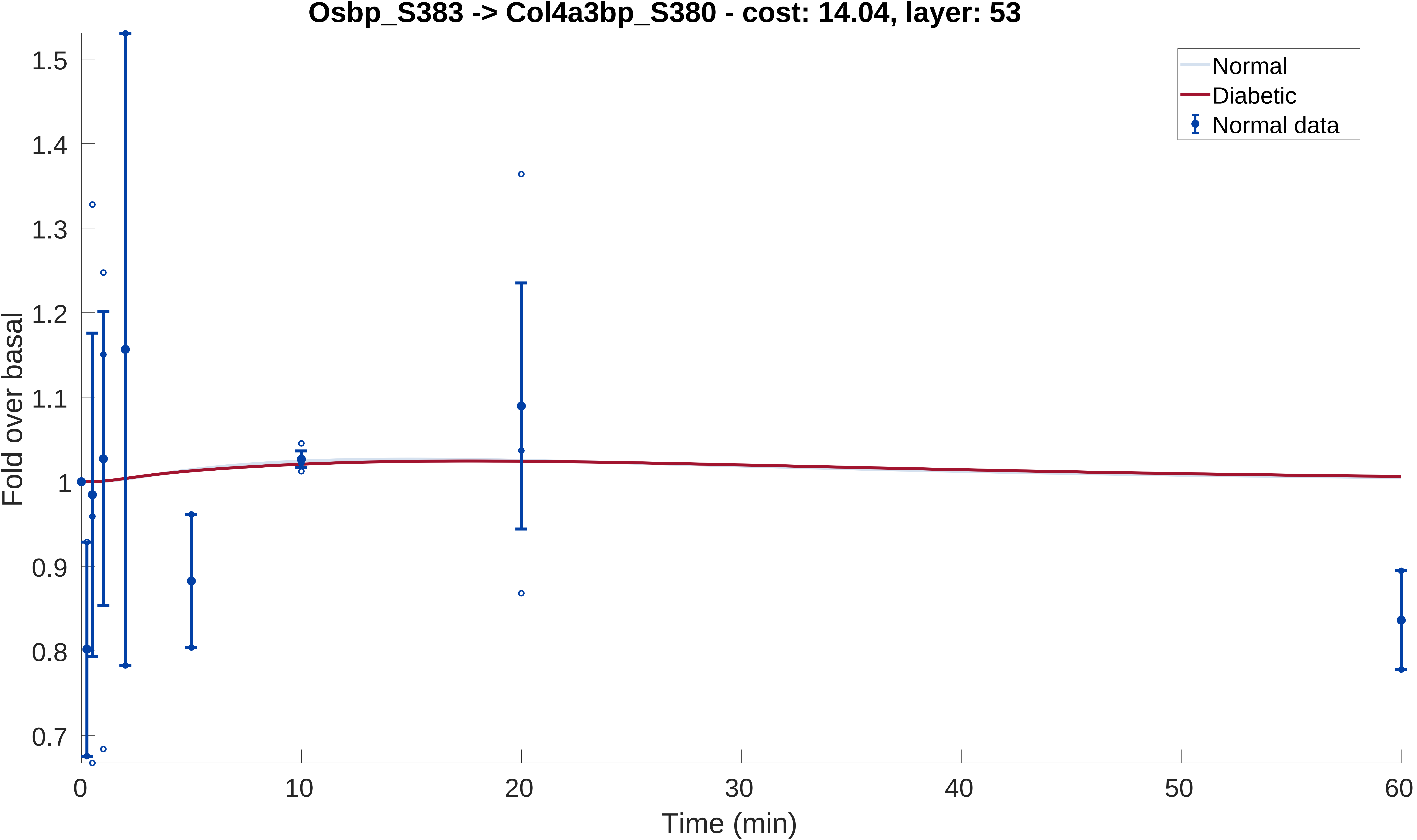

Osbp_S383 -> Col4a3bp_S380 - cost: 14.04, layer: 53
Normal
Diabetic
Normal data
Fold over basal
Time (min)
1.5
1.4
1.3
1.2
1.1
1
0.9
0.8
0.7
0
10
20
30
40
50
60

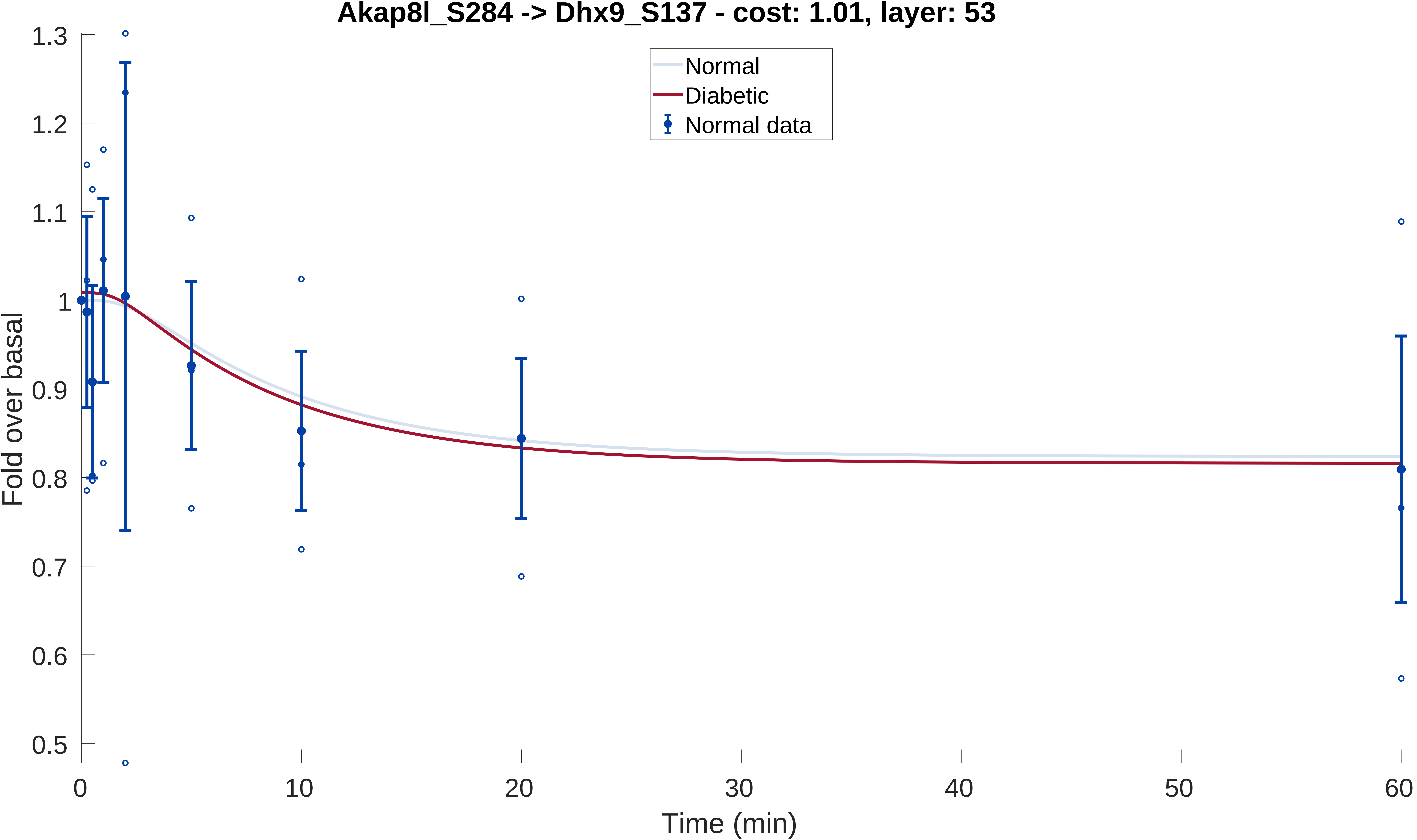

Akap8l_S284 -> Dhx9_S137 - cost: 1.01, layer: 53
Normal
Diabetic
Normal data
Fold over basal
Time (min)
1.3
1.2
1.1
1
0.9
0.8
0.7
0.6
0.5
0
10
20
30
40
50
60

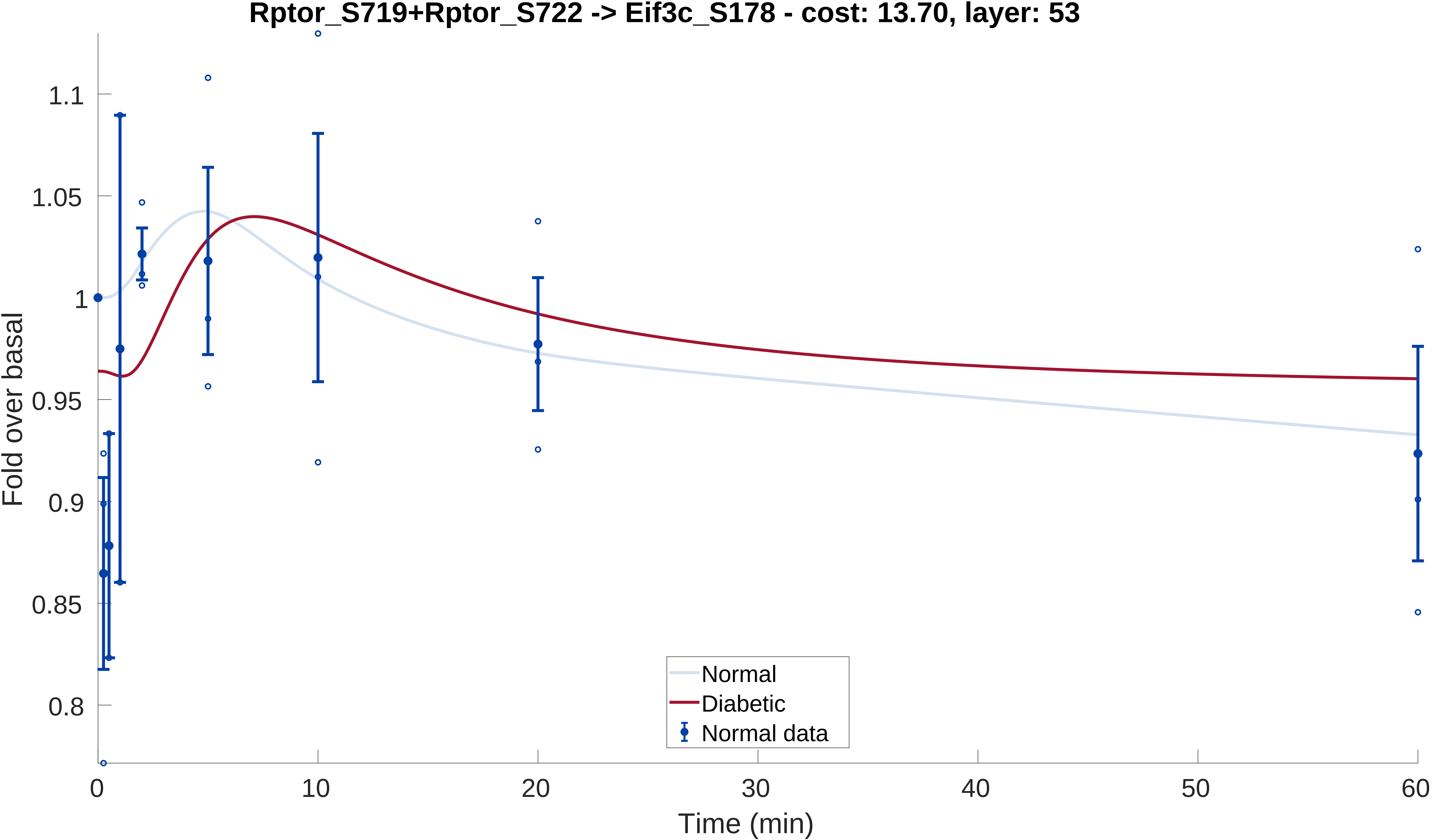

Rptor_S719+Rptor_S722 -> Eif3c_S178 - cost: 13.70, layer: 53
Fold over basal
1.1
1.05
1
0.95
0.9
0.85
0.8
0
10
20
30
40
50
60
Time (min)
Normal
Diabetic
Normal data

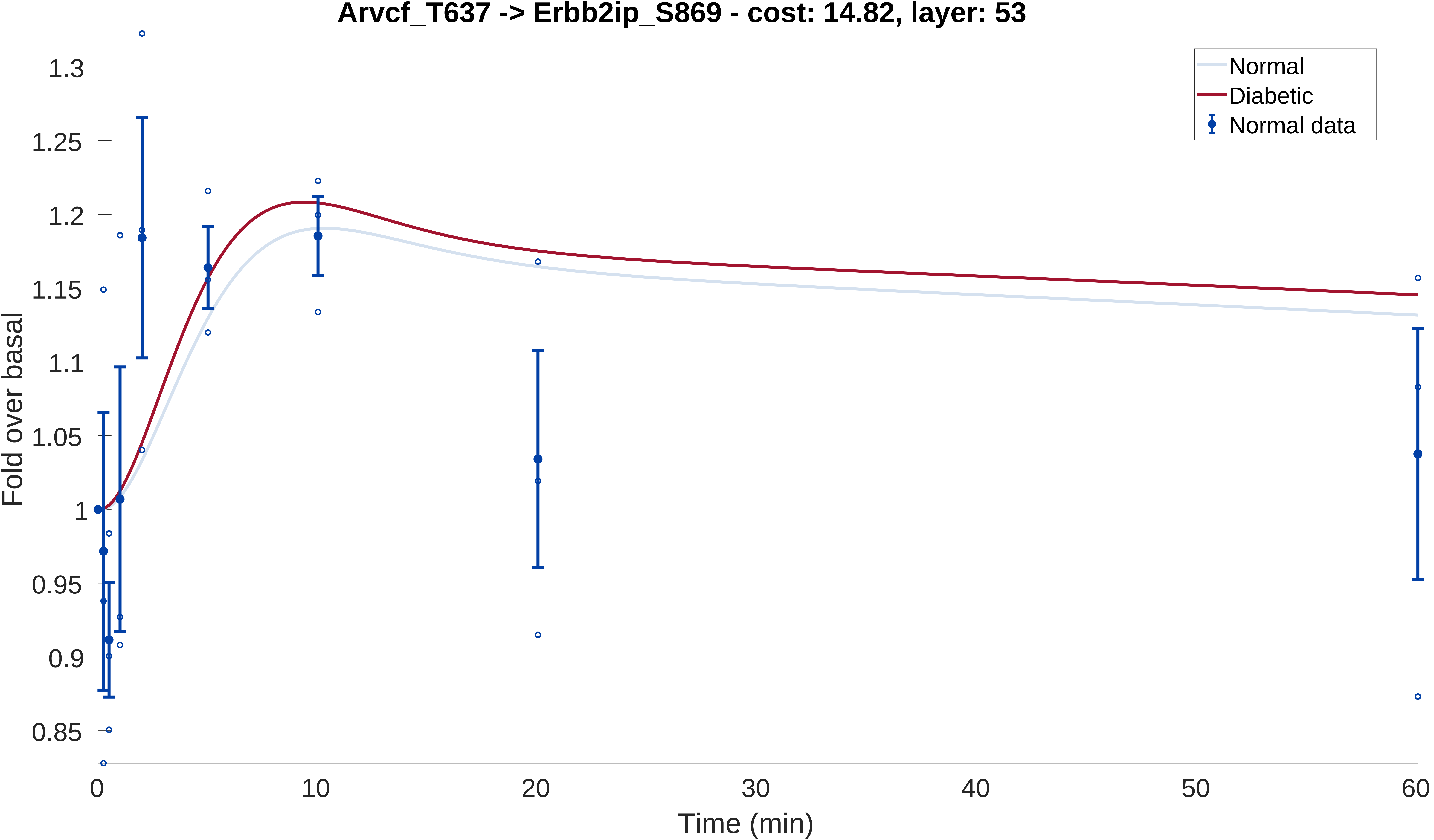

Arvcf_T637 -> Erbb2ip_S869 - cost: 14.82, layer: 53
Normal
Diabetic
Normal data
Fold over basal
Time (min)
1.3
1.25
1.2
1.15
1.1
1.05
1
0.95
0.9
0.85
0
10
20
30
40
50
60

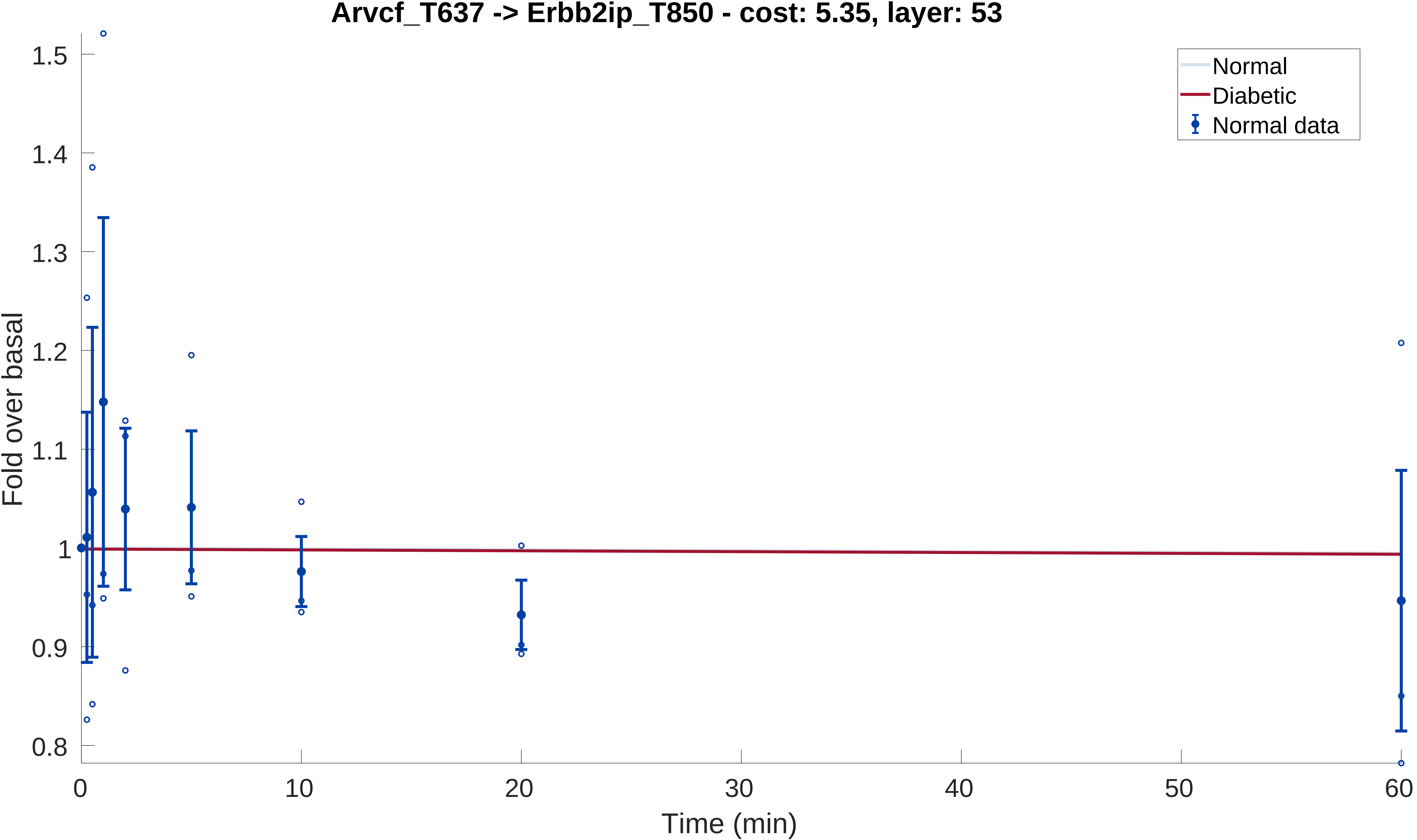
Arvcf_T637 -> Erbb2ip_T850 - cost: 5.35, layer: 53
Normal
Diabetic
Normal data
Fold over basal
Time (min)
1.5
1.4
1.3
1.2
1.1
1
0.9
0.8
0
10
20
30
40
50
60

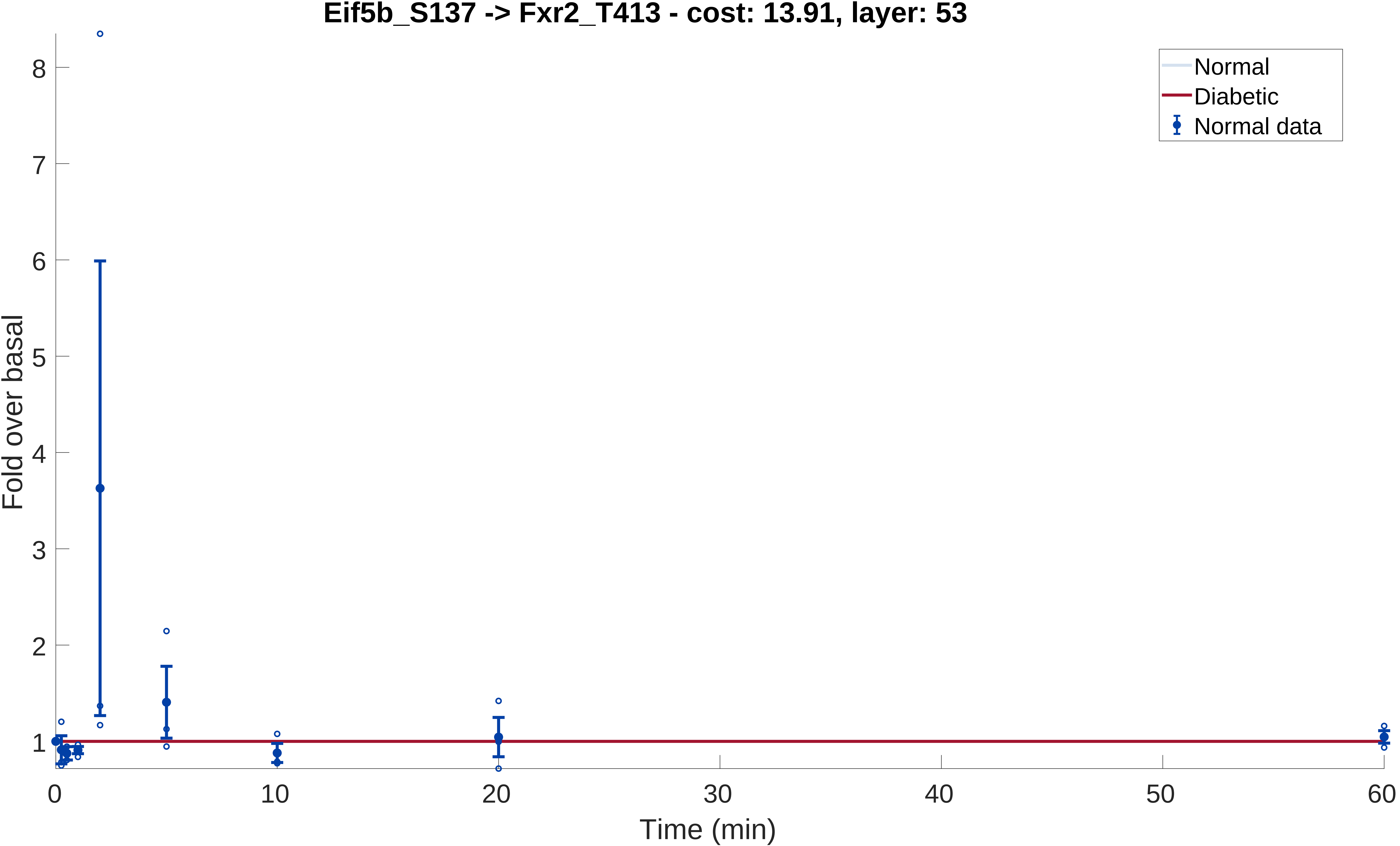

Eif5b_S137 -> Fxr2_T413 - cost: 13.91, layer: 53
Normal
Diabetic
Normal data
Fold over basal
Time (min)
1
2
3
4
5
6
7
8
0
10
20
30
40
50
60

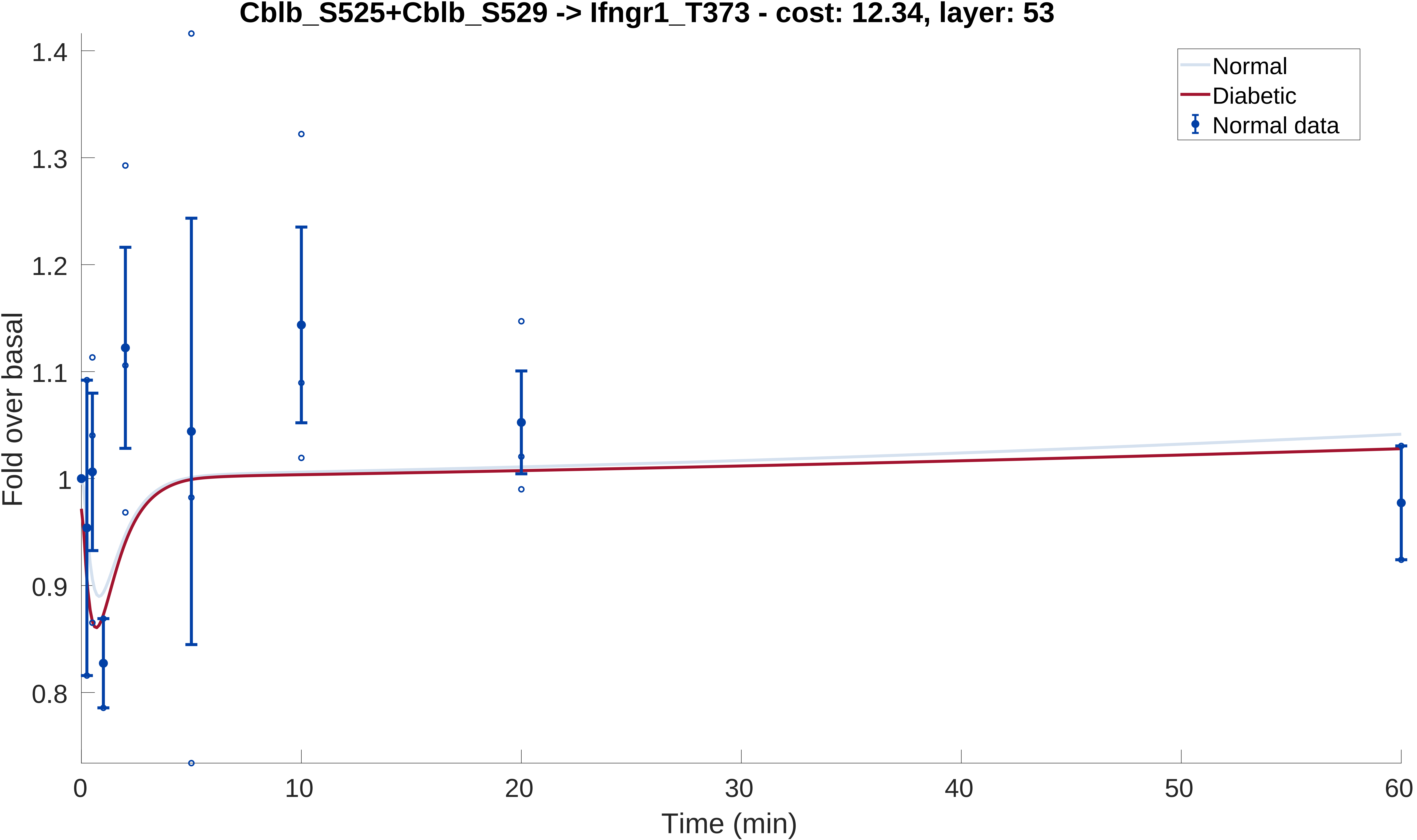

Cblb_S525+Cblb_S529 -> Ifngr1_T373 - cost: 12.34, layer: 53
Normal
Diabetic
Normal data
Fold over basal
Time (min)
1.4
1.3
1.2
1.1
1
0.9
0.8
0
10
20
30
40
50
60

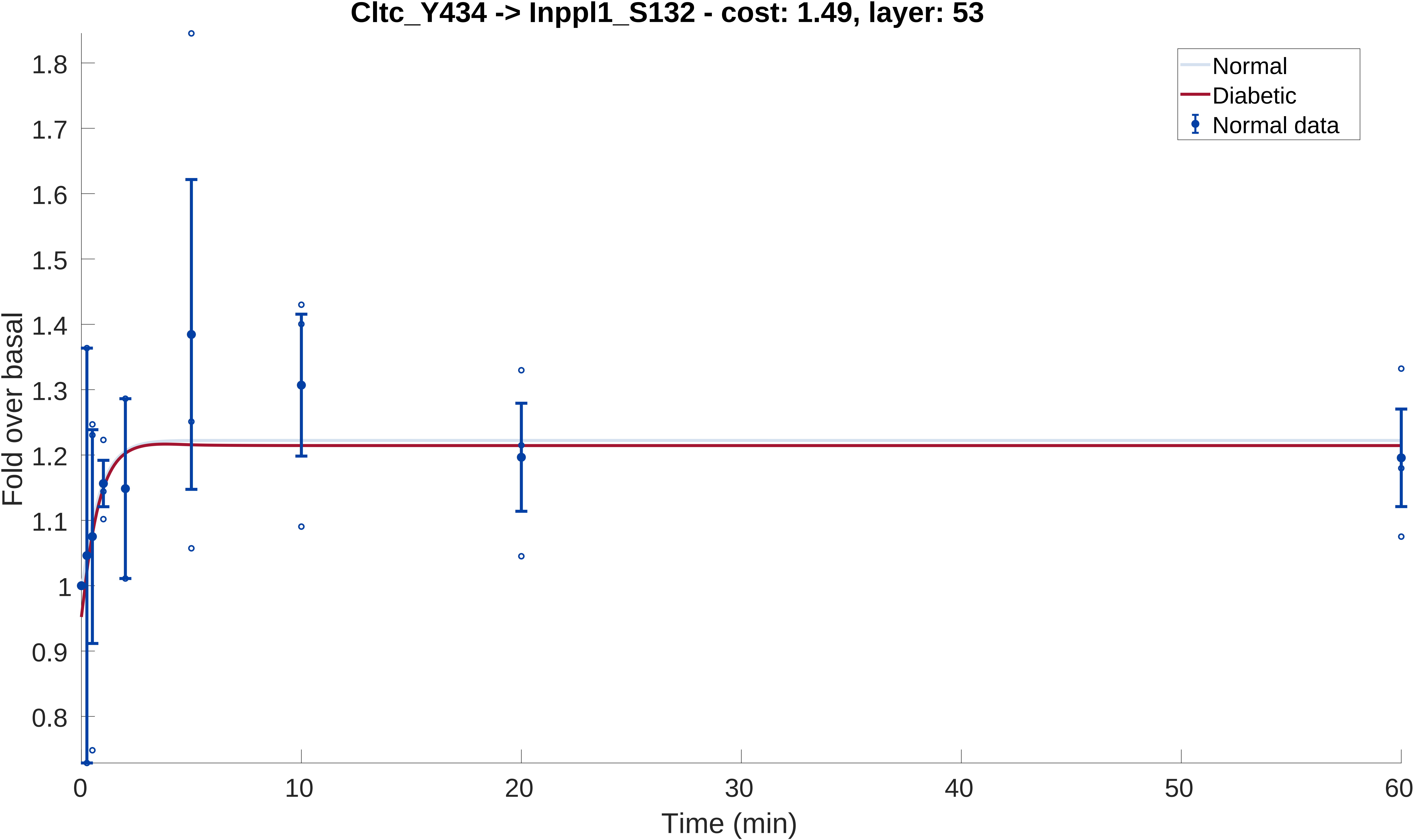
Cltc_Y434 -> Inppl1_S132 - cost: 1.49, layer: 53
Normal
Diabetic
Normal data
Fold over basal
Time (min)
1.8
1.7
1.6
1.5
1.4
1.3
1.2
1.1
1
0.9
0.8
0
10
20
30
40
50
60

Gsk3b_S9+Mtor_T2474 -> Irs2_S573 - cost: 11.20, layer: 53

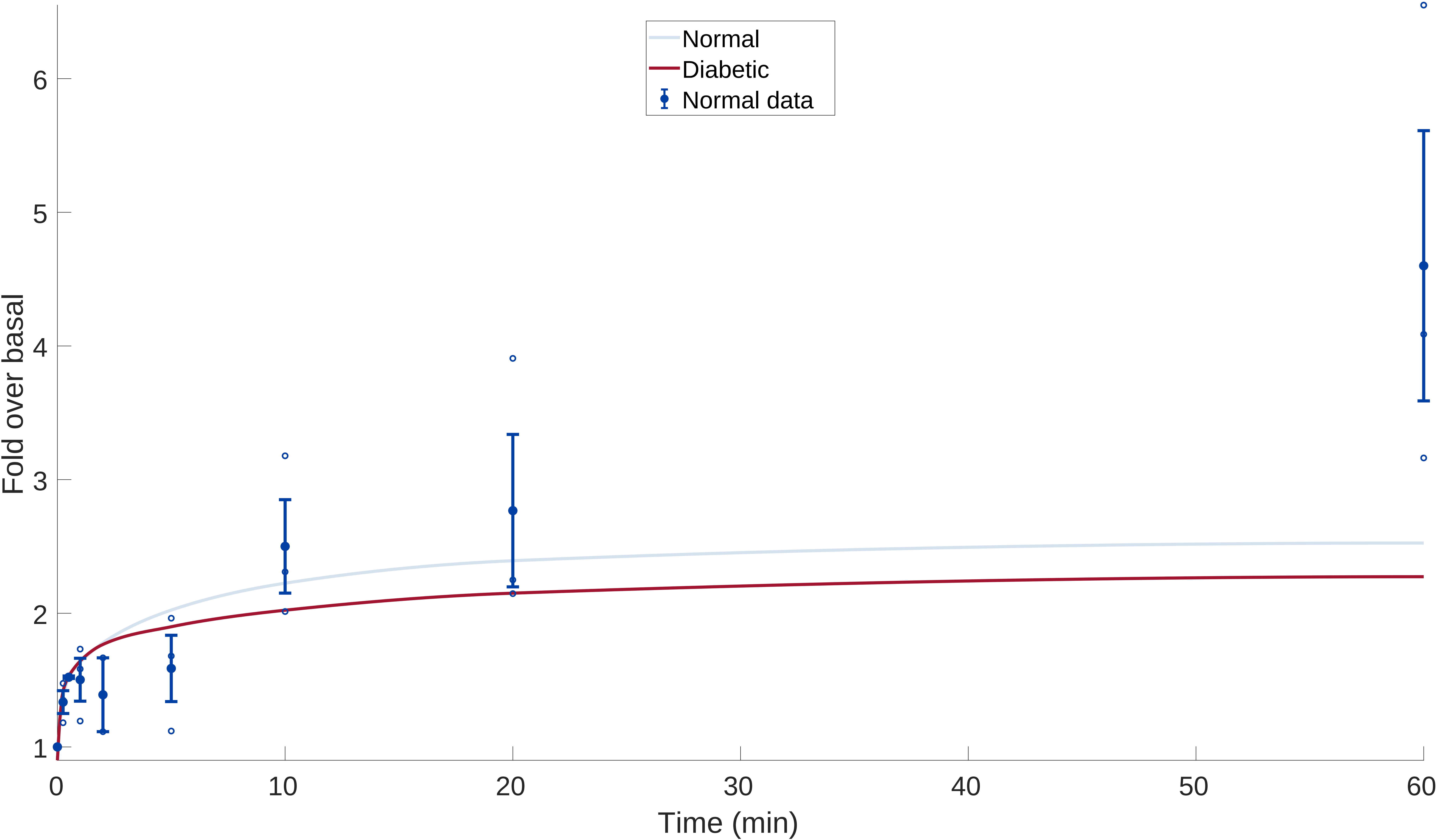

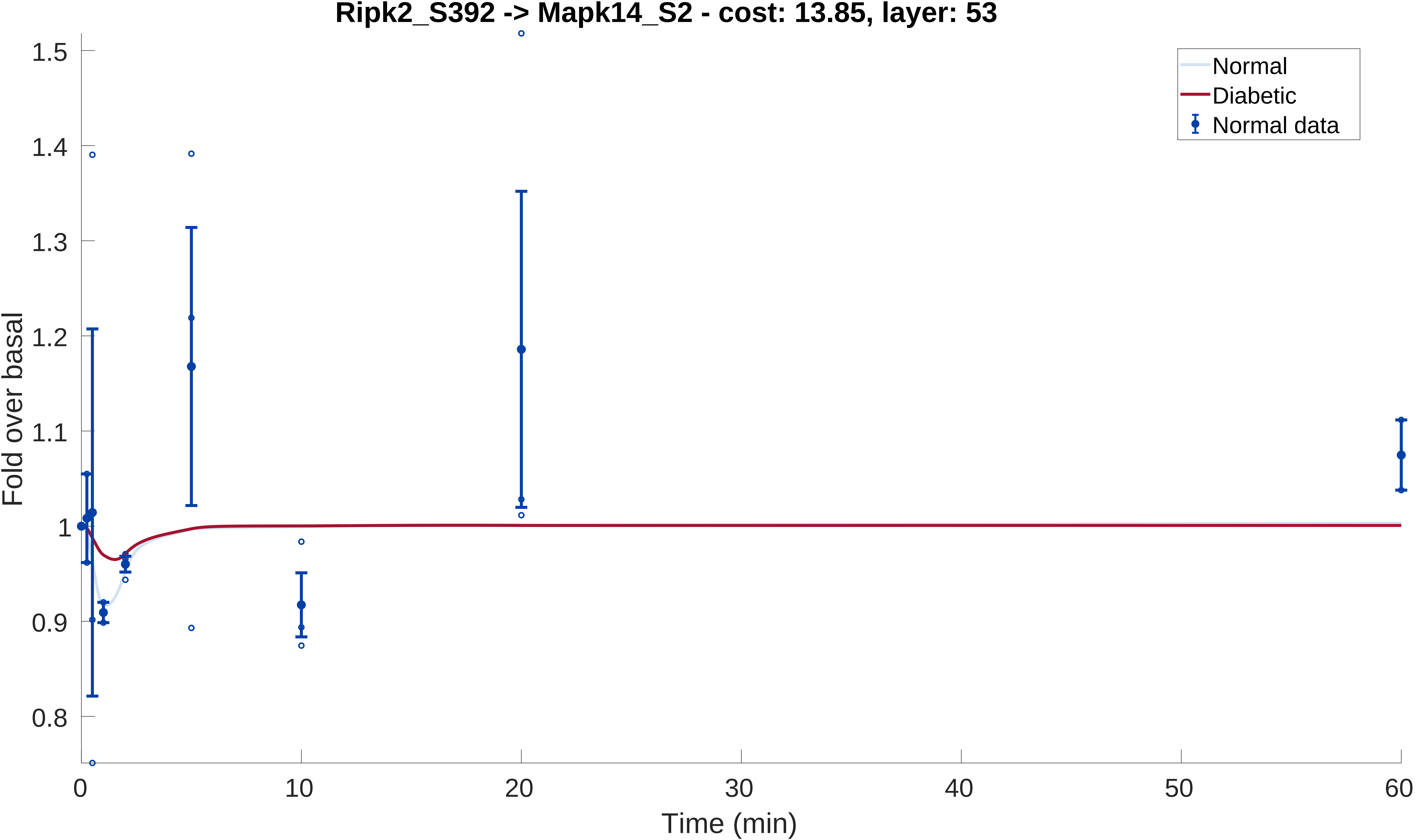

Ripk2_S392 -> Mapk14_S2 - cost: 13.85, layer: 53
Normal
Diabetic
Normal data
Fold over basal
Time (min)
1.5
1.4
1.3
1.2
1.1
1
0.9
0.8
0
10
20
30
40
50
60

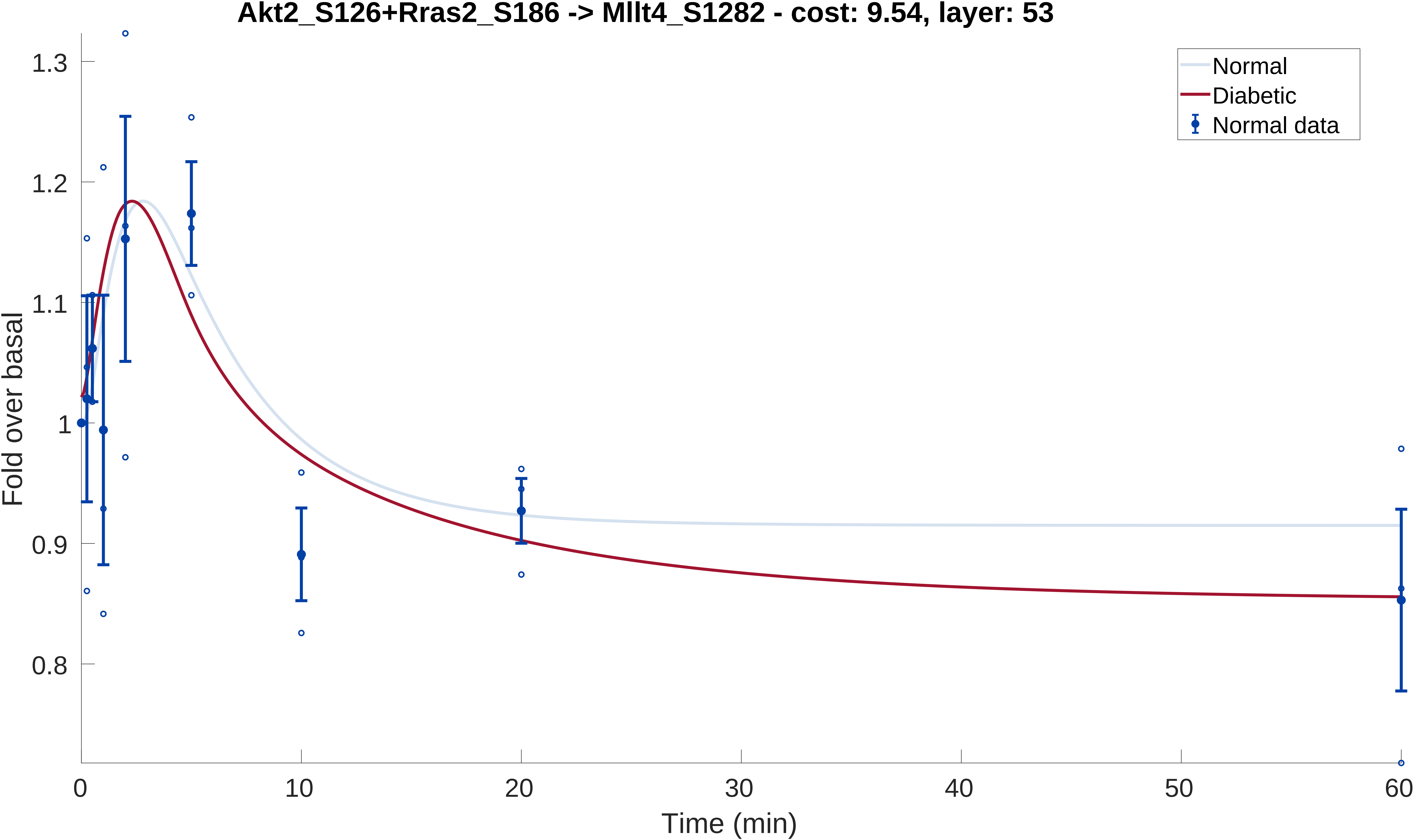

Akt2_S126+Rras2_S186 -> Mllt4_S1282 - cost: 9.54, layer: 53
Normal
Diabetic
Normal data
Fold over basal
1.3
1.2
1.1
1
0.9
0.8
0
10
20
30
40
50
60
Time (min)

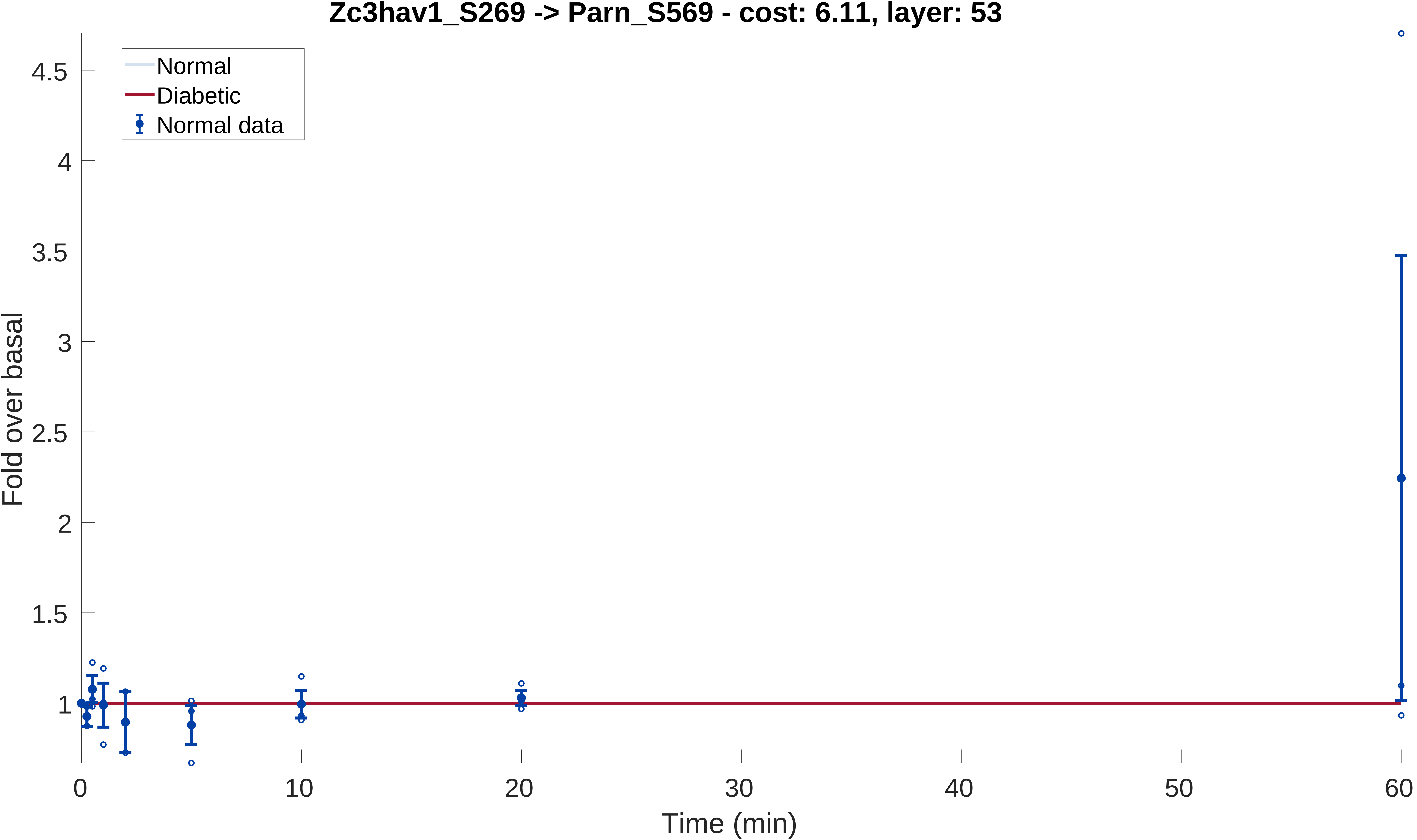

Zc3hav1_S269 -> Parn_S569 - cost: 6.11, layer: 53
Normal
Diabetic
Normal data
Fold over basal
4.5
4
3.5
3
2.5
2
1.5
1
0
10
20
30
40
50
60
Time (min)

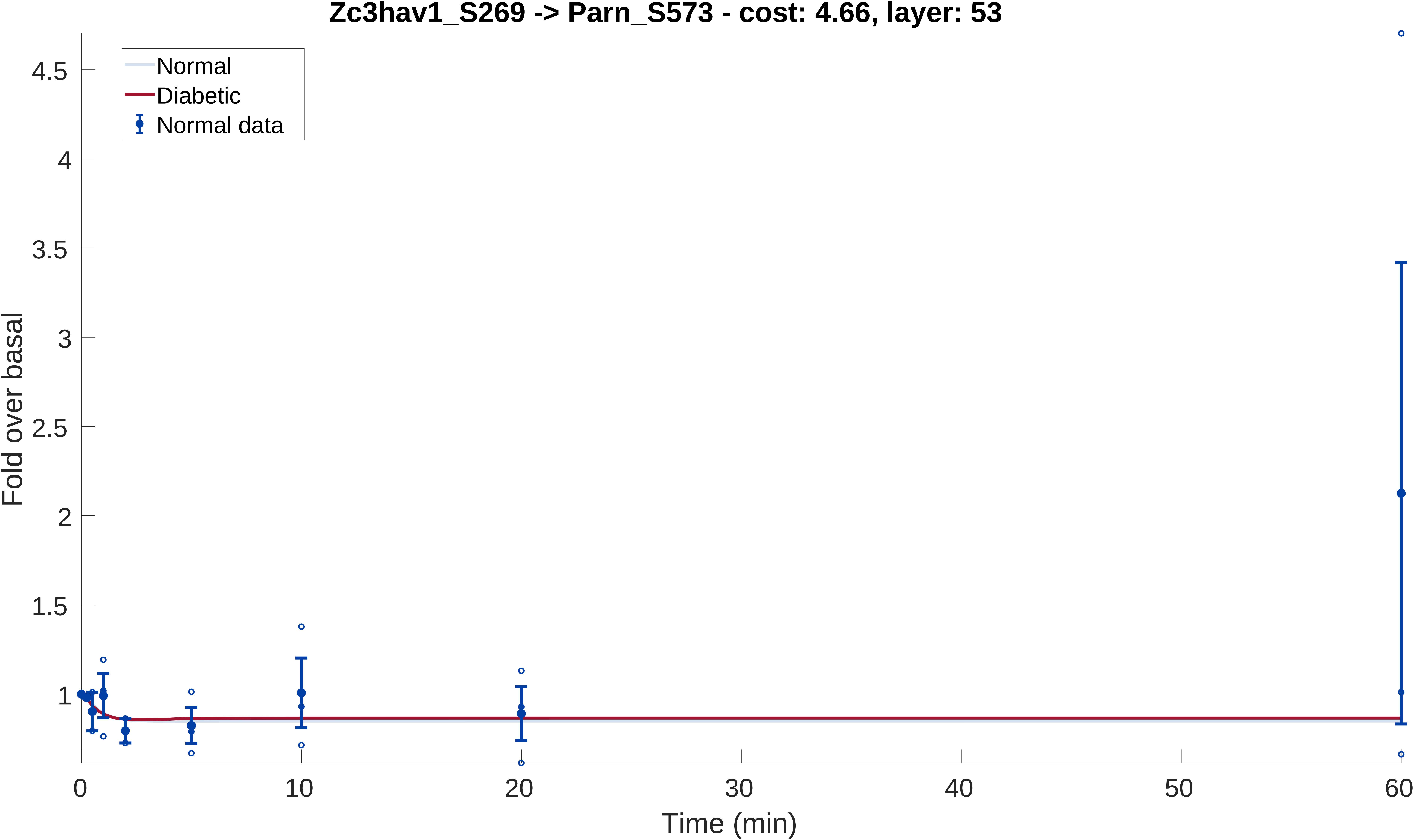

Zc3hav1_S269 -> Parn_S573 - cost: 4.66, layer: 53
Normal
Diabetic
Normal data
Fold over basal
Time (min)
4.5
4
3.5
3
2.5
2
1.5
1
0
10
20
30
40
50
60

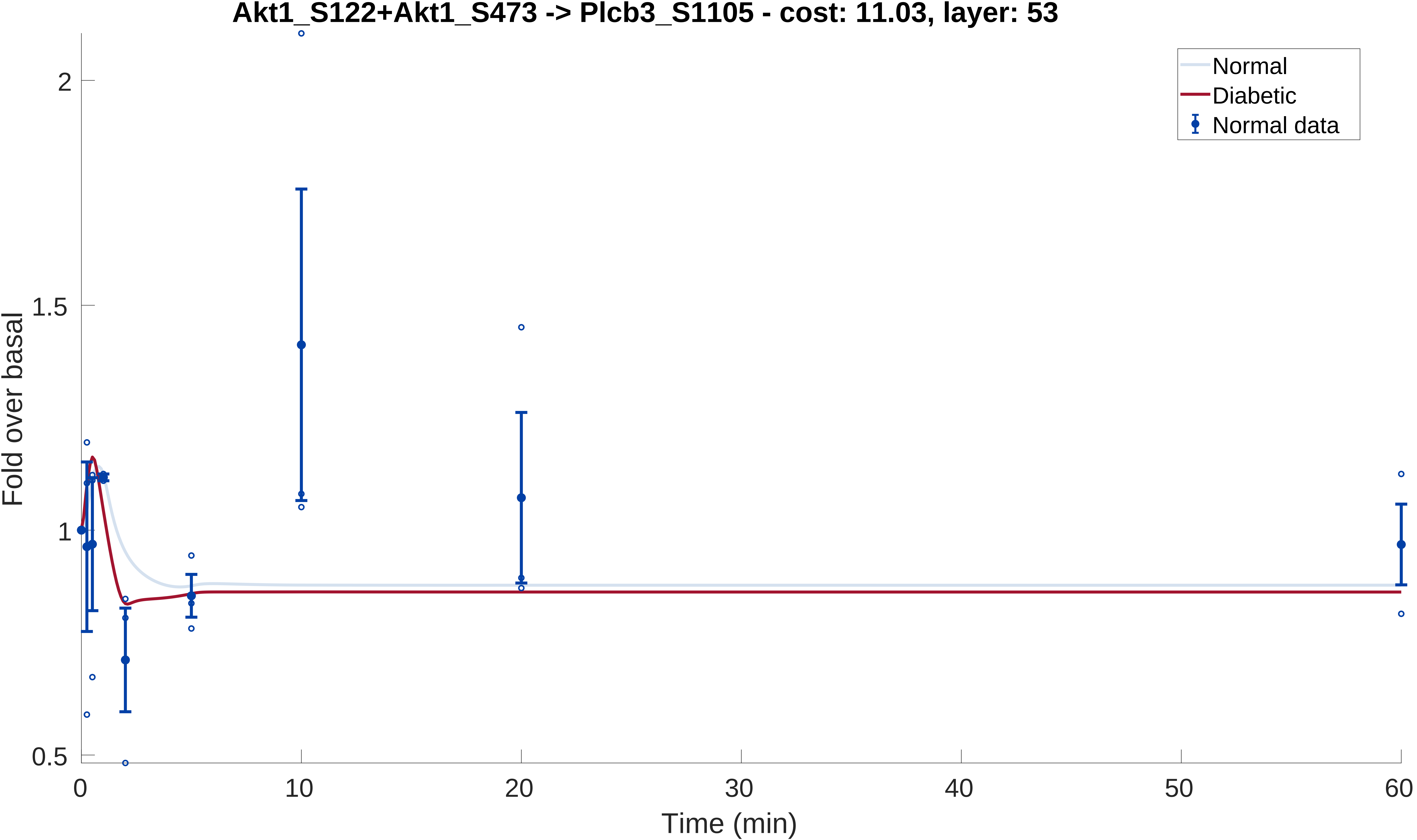
Akt1_S122+Akt1_S473 -> Plcb3_S1105 - cost: 11.03, layer: 53
Normal
Diabetic
Normal data
Fold over basal
Time (min)
0.5
1
1.5
2
0
10
20
30
40
50
60

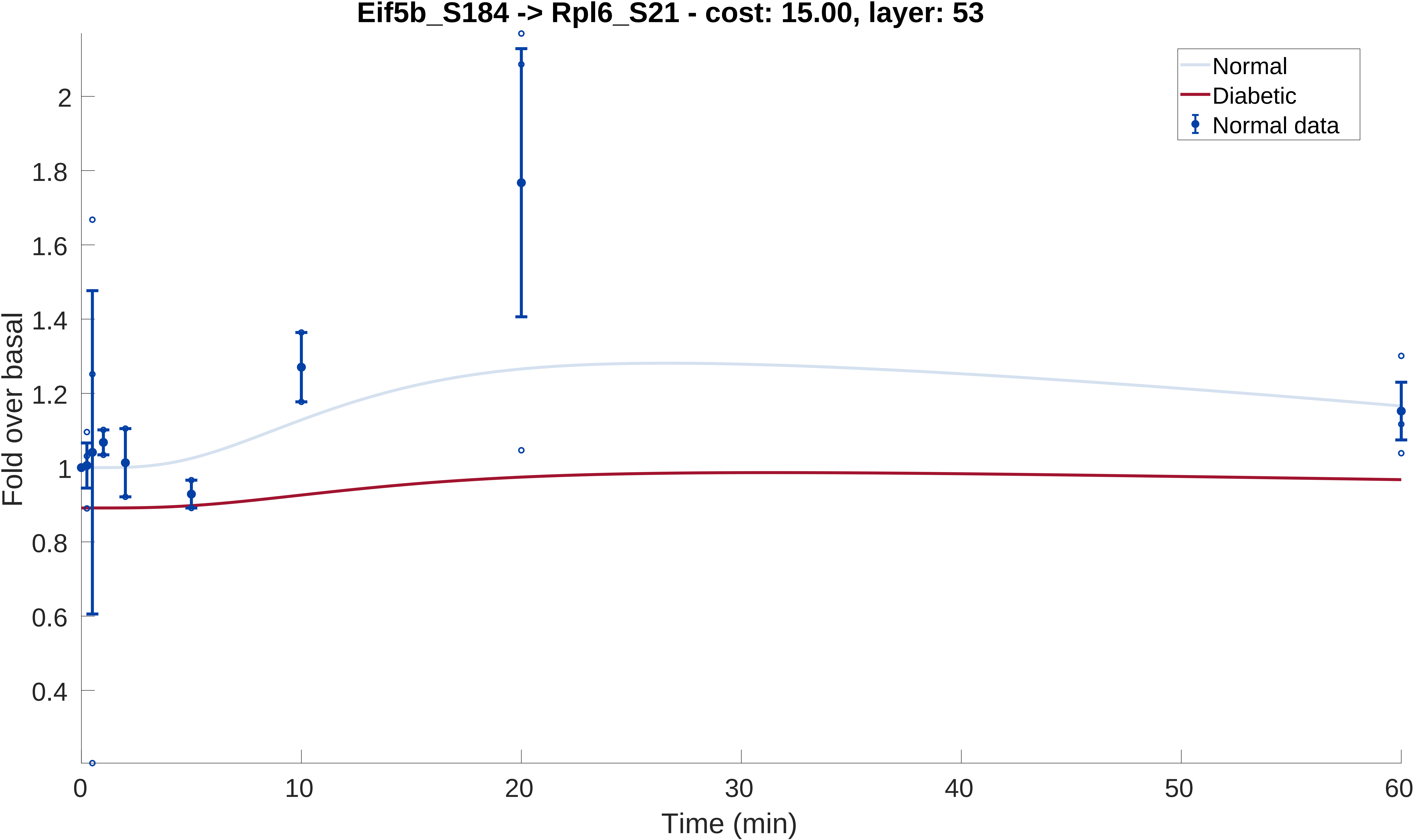

Eif5b_S184 -> Rpl6_S21 - cost: 15.00, layer: 53
Normal
Diabetic
Normal data
Fold over basal
0.4
0.6
0.8
1
1.2
1.4
1.6
1.8
2
0
10
20
30
40
50
60
Time (min)

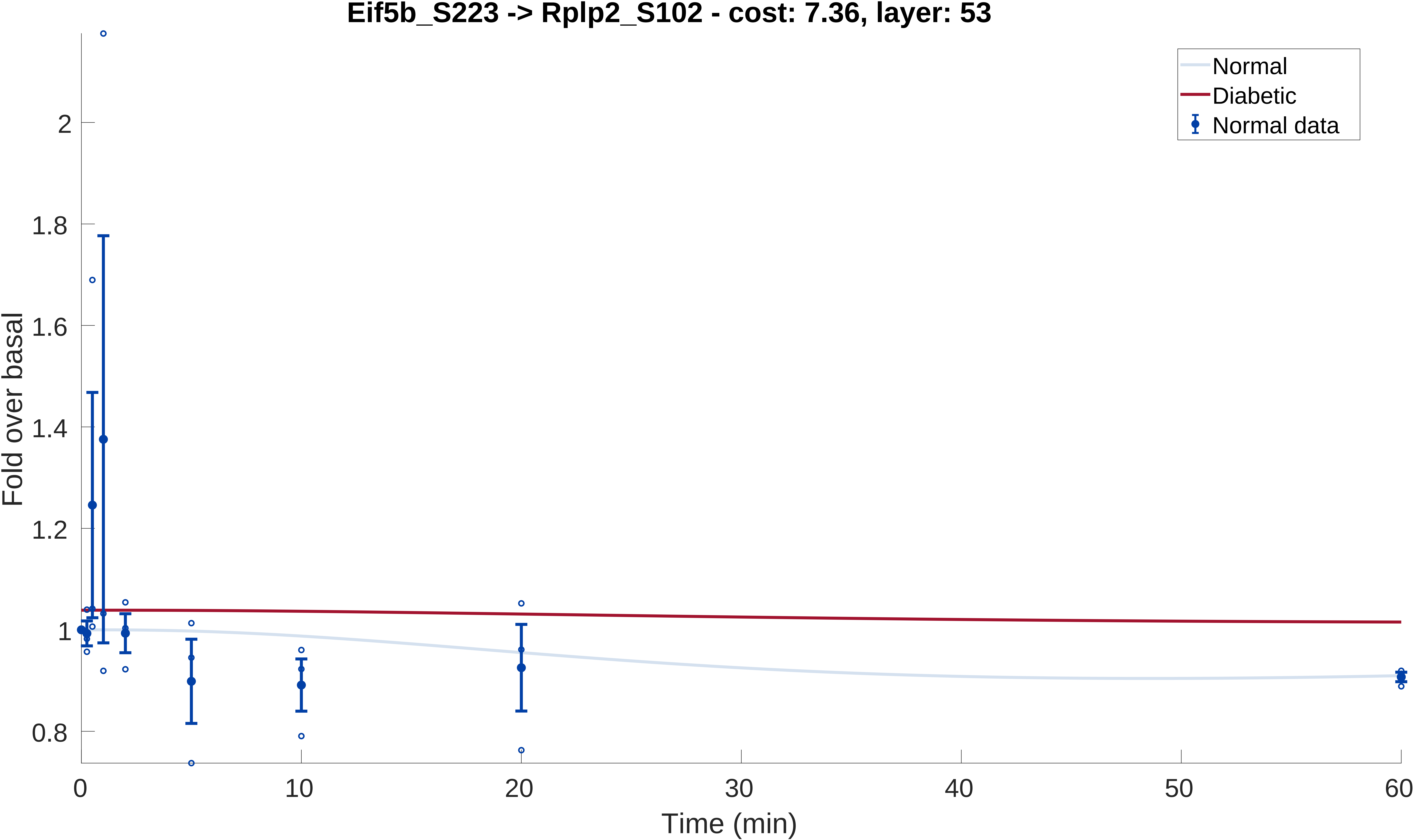

Eif5b_S223 -> Rplp2_S102 - cost: 7.36, layer: 53
Normal
Diabetic
Normal data
Fold over basal
2
1.8
1.6
1.4
1.2
1
0.8
0
10
20
30
40
50
60
Time (min)

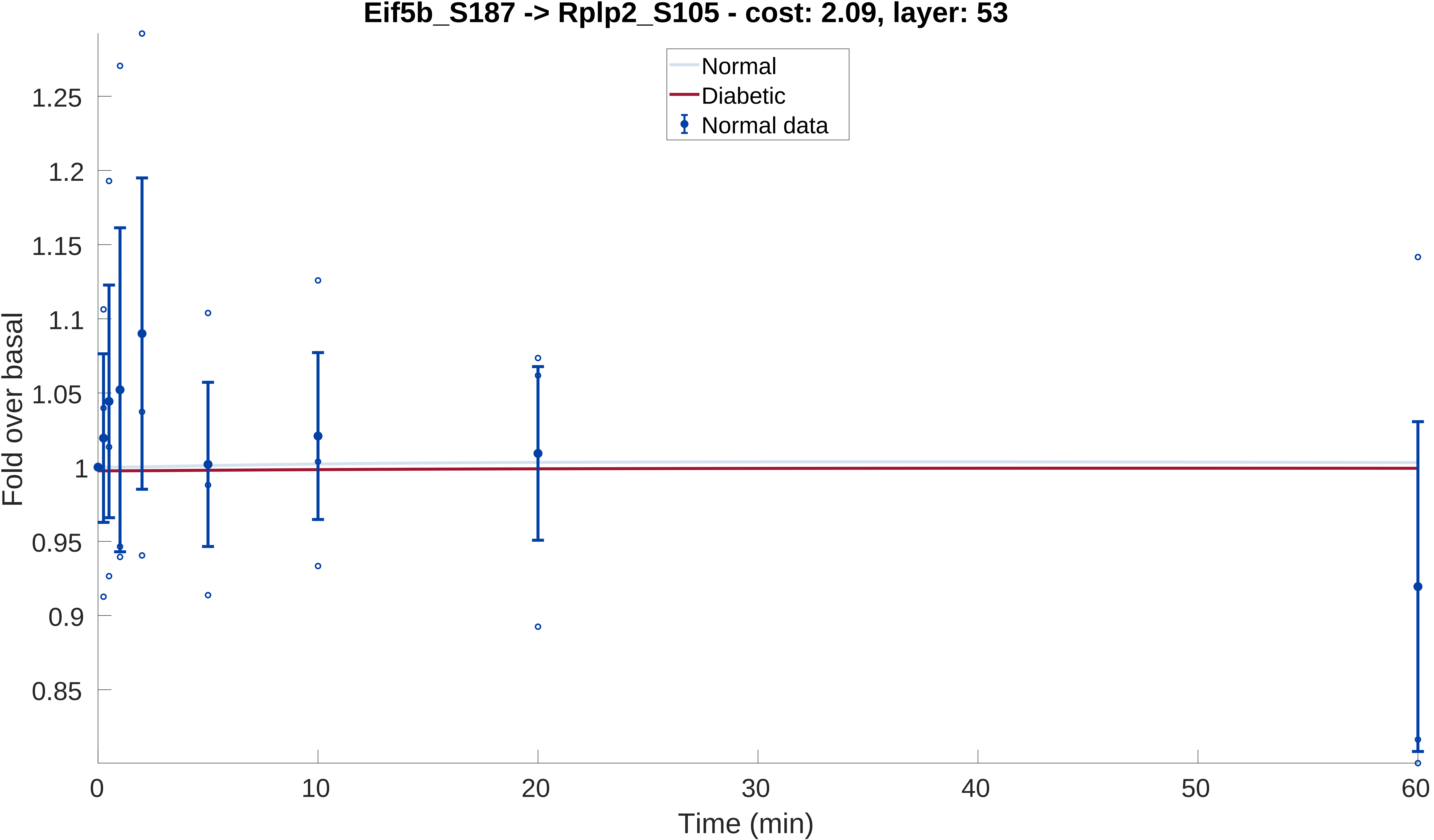
Eif5b_S187 -> Rplp2_S105 - cost: 2.09, layer: 53
Normal
Diabetic
Normal data
Fold over basal
1.25
1.2
1.15
1.1
1.05
1
0.95
0.9
0.85
0
10
20
30
40
50
60
Time (min)

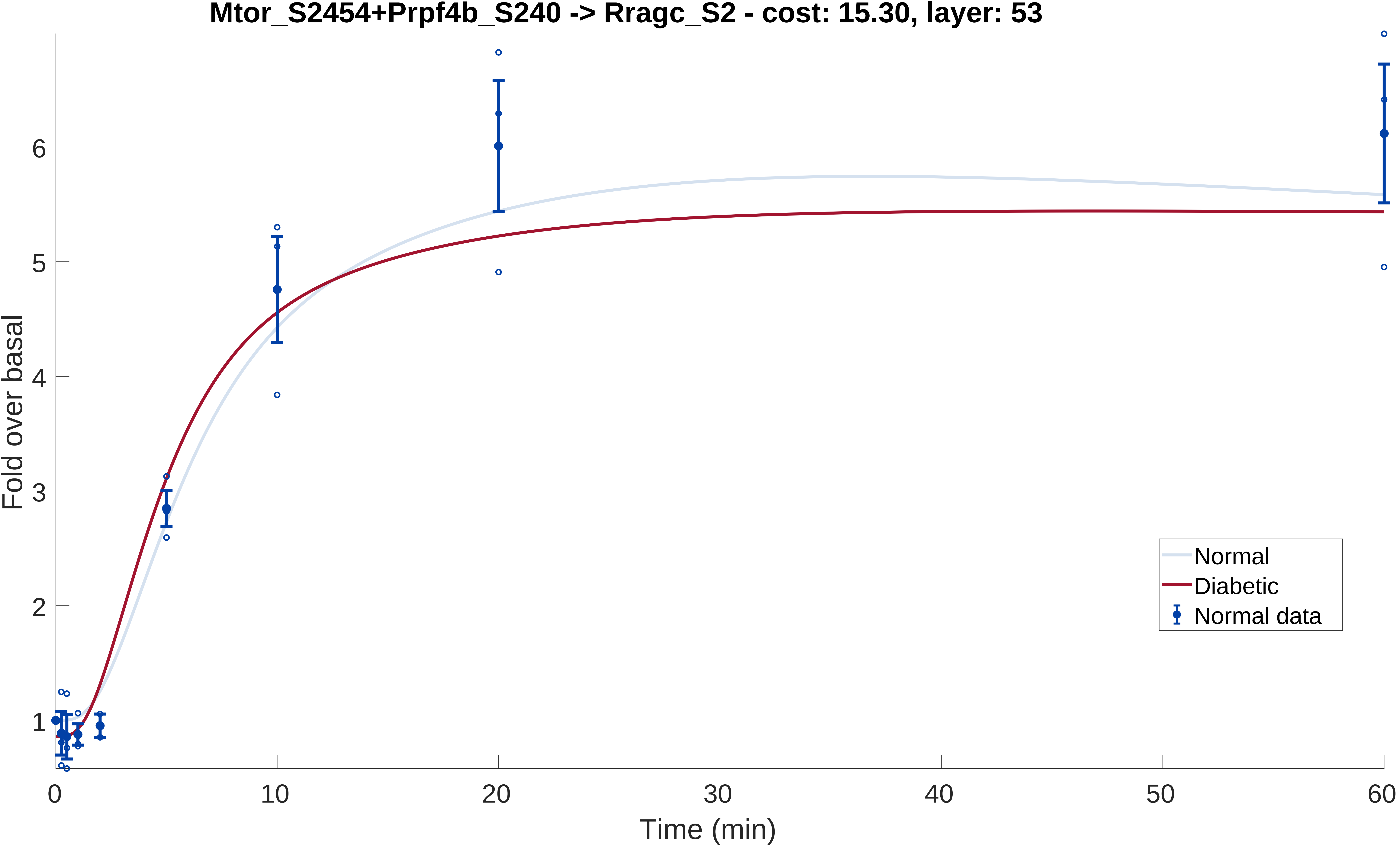

Mtor_S2454+Prpf4b_S240 -> Rragc_S2 - cost: 15.30, layer: 53
Fold over basal
Time (min)
Normal
Diabetic
Normal data
0
10
20
30
40
50
60
1
2
3
4
5
6

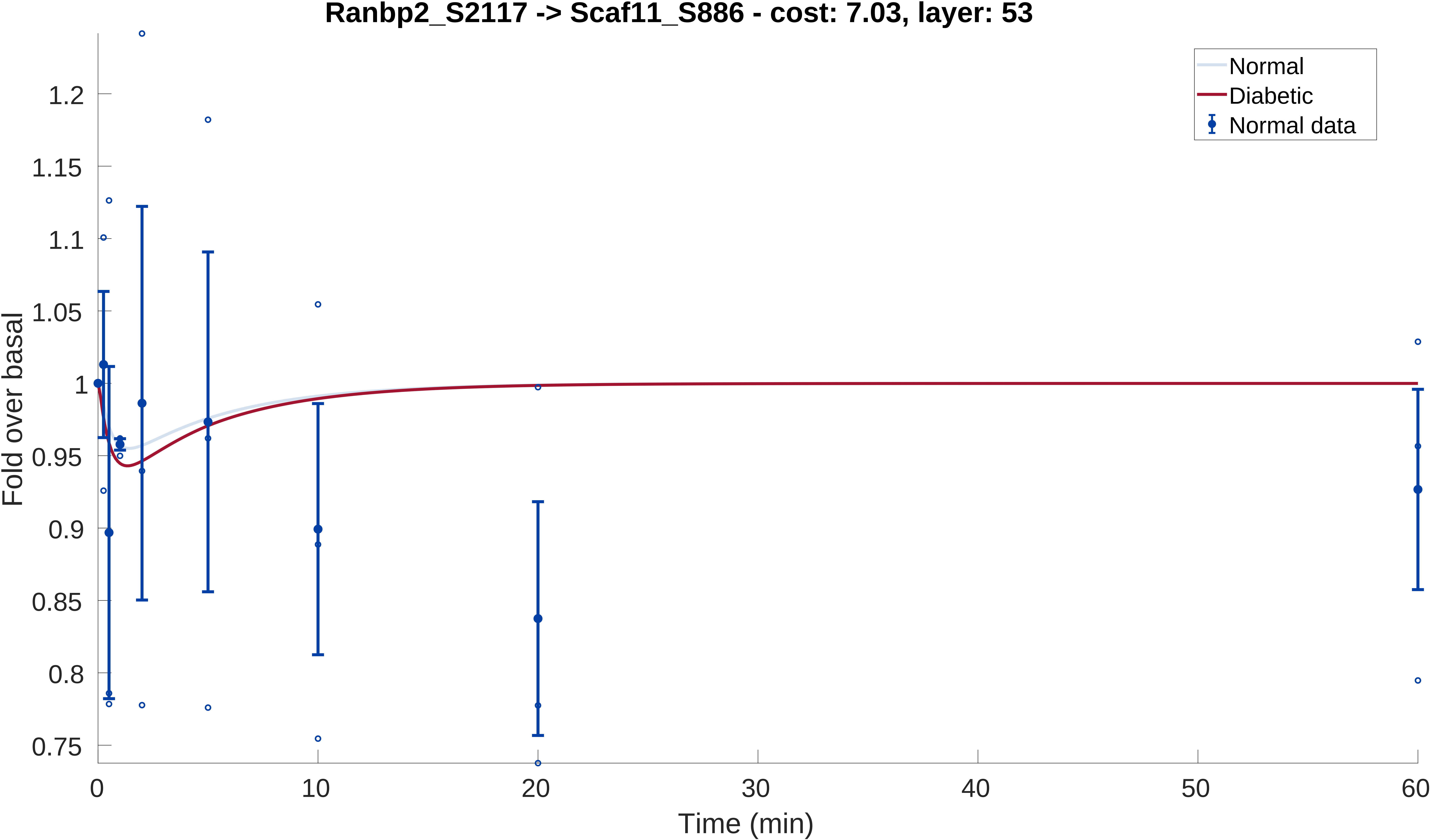

Ranbp2_S2117 -> Scaf11_S886 - cost: 7.03, layer: 53
Normal
Diabetic
Normal data
Fold over basal
1.2
1.15
1.1
1.05
1
0.95
0.9
0.85
0.8
0.75
0
10
20
30
40
50
60
Time (min)

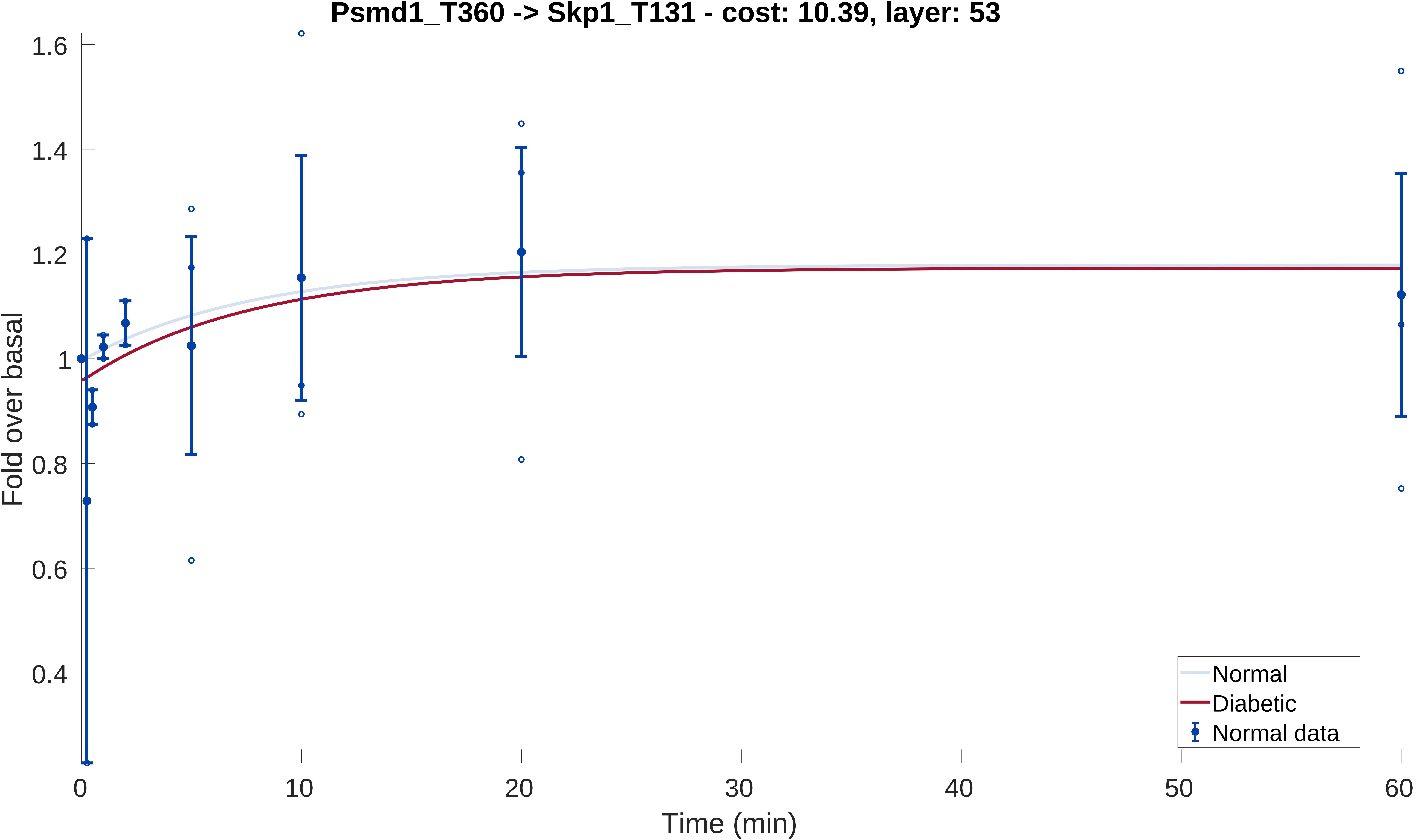

Psmd1_T360 -> Skp1_T131 - cost: 10.39, layer: 53
Fold over basal
Time (min)
Normal
Diabetic
Normal data
1.6
1.4
1.2
1
0.8
0.6
0.4
0
10
20
30
40
50
60

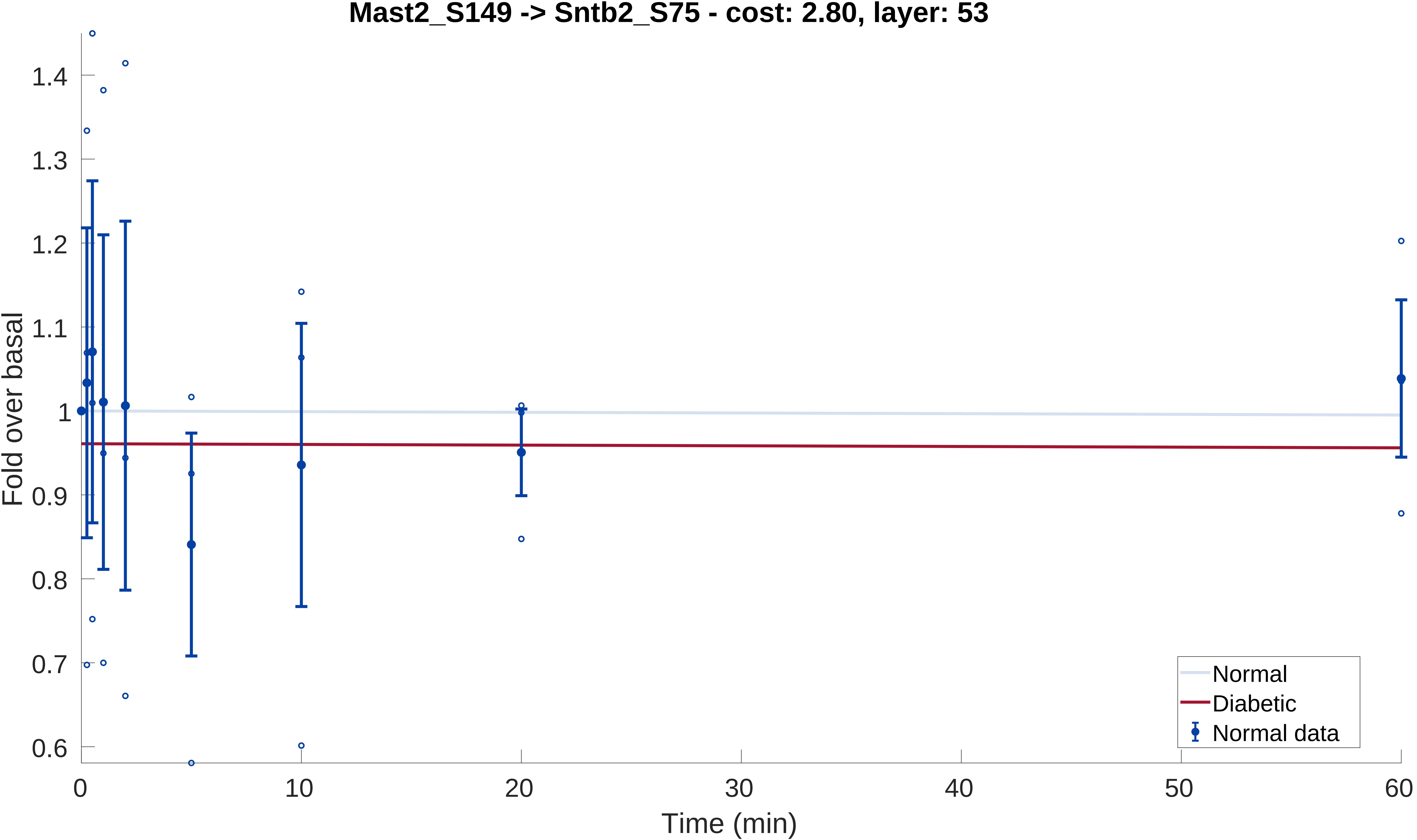

Mast2_S149 -> Sntb2_S75 - cost: 2.80, layer: 53
Fold over basal
Time (min)
0.6
0.7
0.8
0.9
1
1.1
1.2
1.3
1.4
0
10
20
30
40
50
60
Normal
Diabetic
Normal data

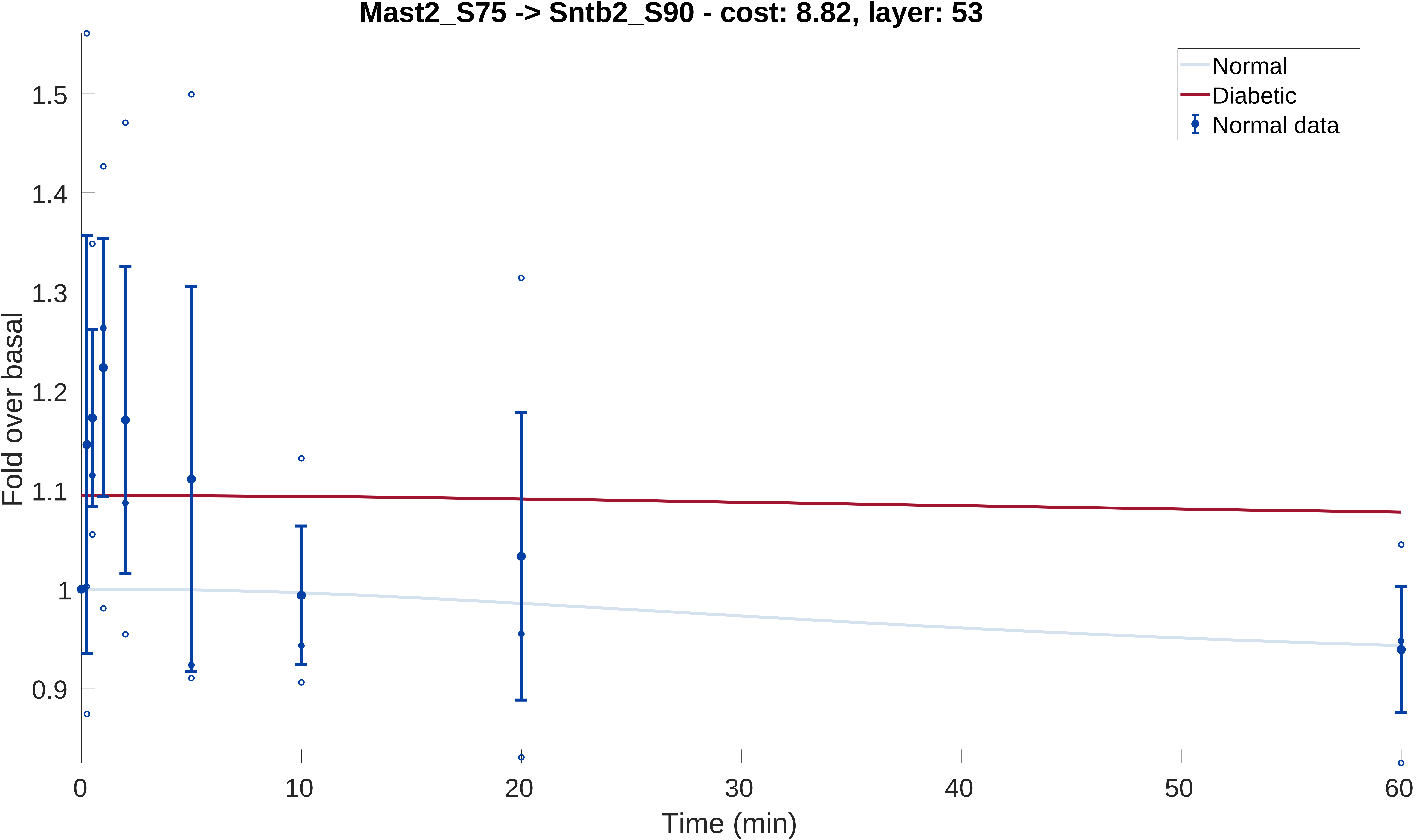

Mast2_S75 -> Sntb2_S90 - cost: 8.82, layer: 53
Normal
Diabetic
Normal data
Fold over basal
Time (min)
0.9
1
1.1
1.2
1.3
1.4
1.5
0
10
20
30
40
50
60

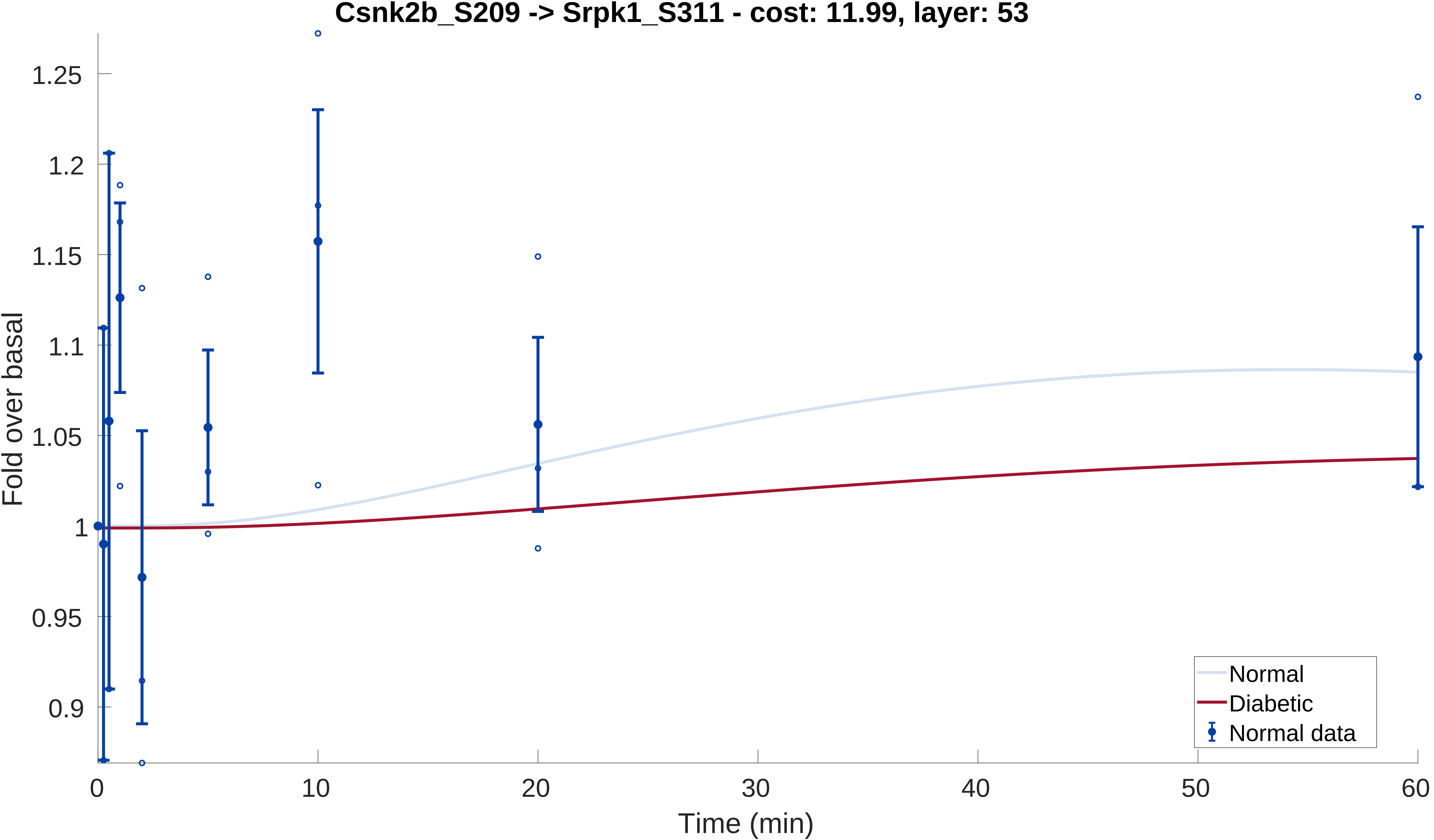

Csnk2b_S209 -> Srpk1_S311 - cost: 11.99, layer: 53
Fold over basal
1.25
1.2
1.15
1.1
1.05
1
0.95
0.9
0
10
20
30
40
50
60
Time (min)
Normal
Diabetic
Normal data

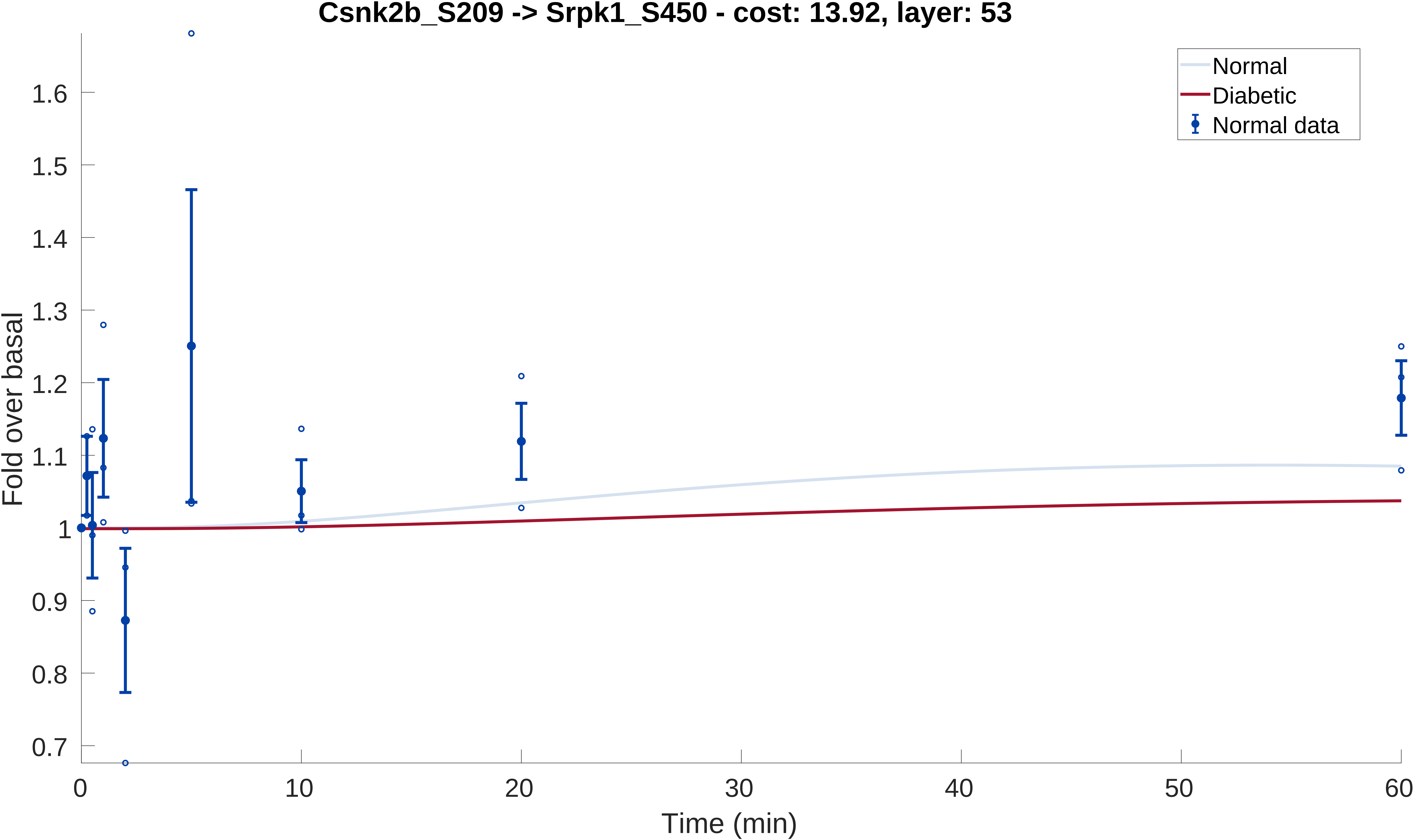

Csnk2b_S209 -> Srpk1_S450 - cost: 13.92, layer: 53
Normal
Diabetic
Normal data
Fold over basal
Time (min)
0.7
0.8
0.9
1
1.1
1.2
1.3
1.4
1.5
1.6
0
10
20
30
40
50
60

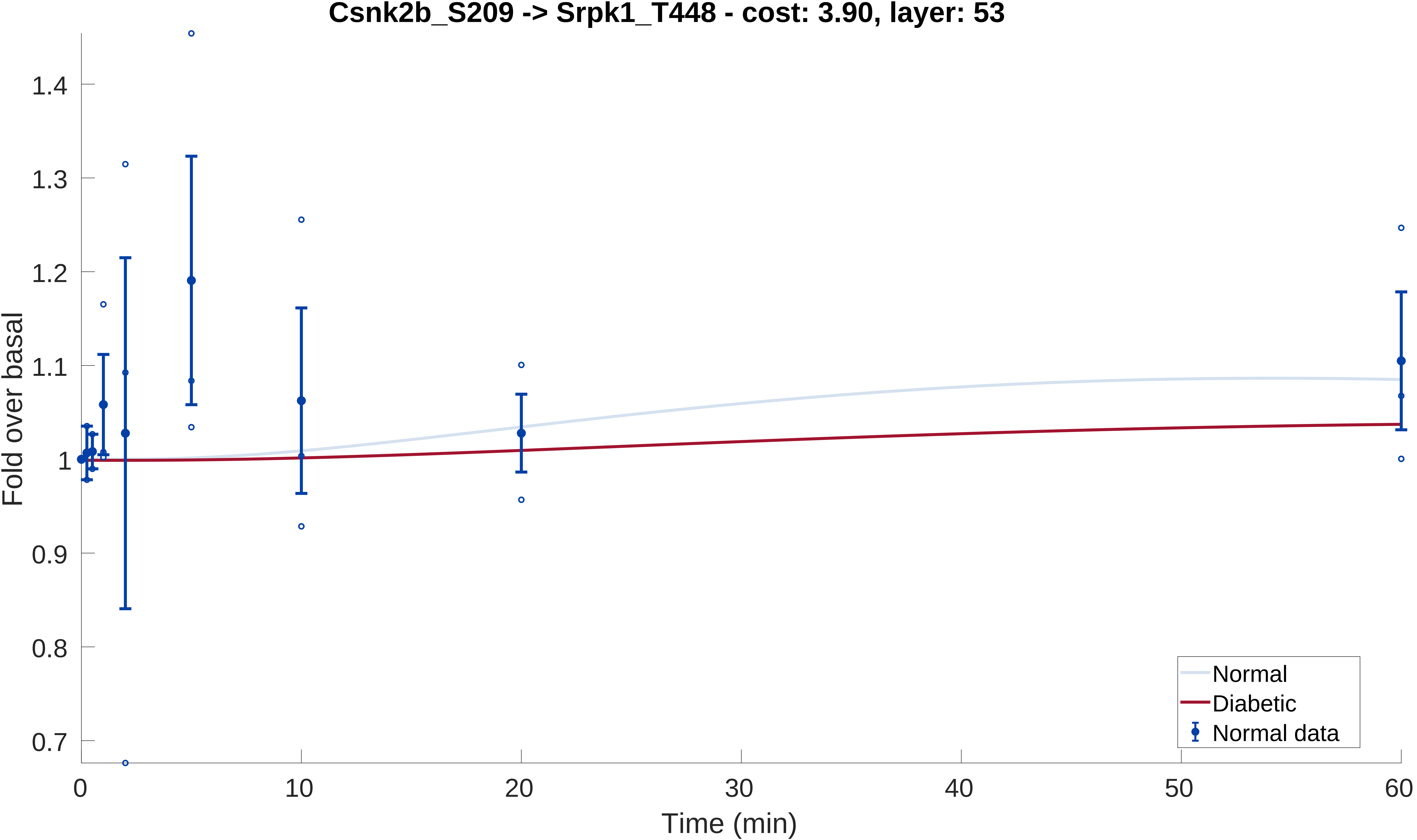

Csnk2b_S209 -> Srpk1_T448 - cost: 3.90, layer: 53
Fold over basal
1.4
1.3
1.2
1.1
1
0.9
0.8
0.7
0
10
20
30
40
50
60
Time (min)
Normal
Diabetic
Normal data

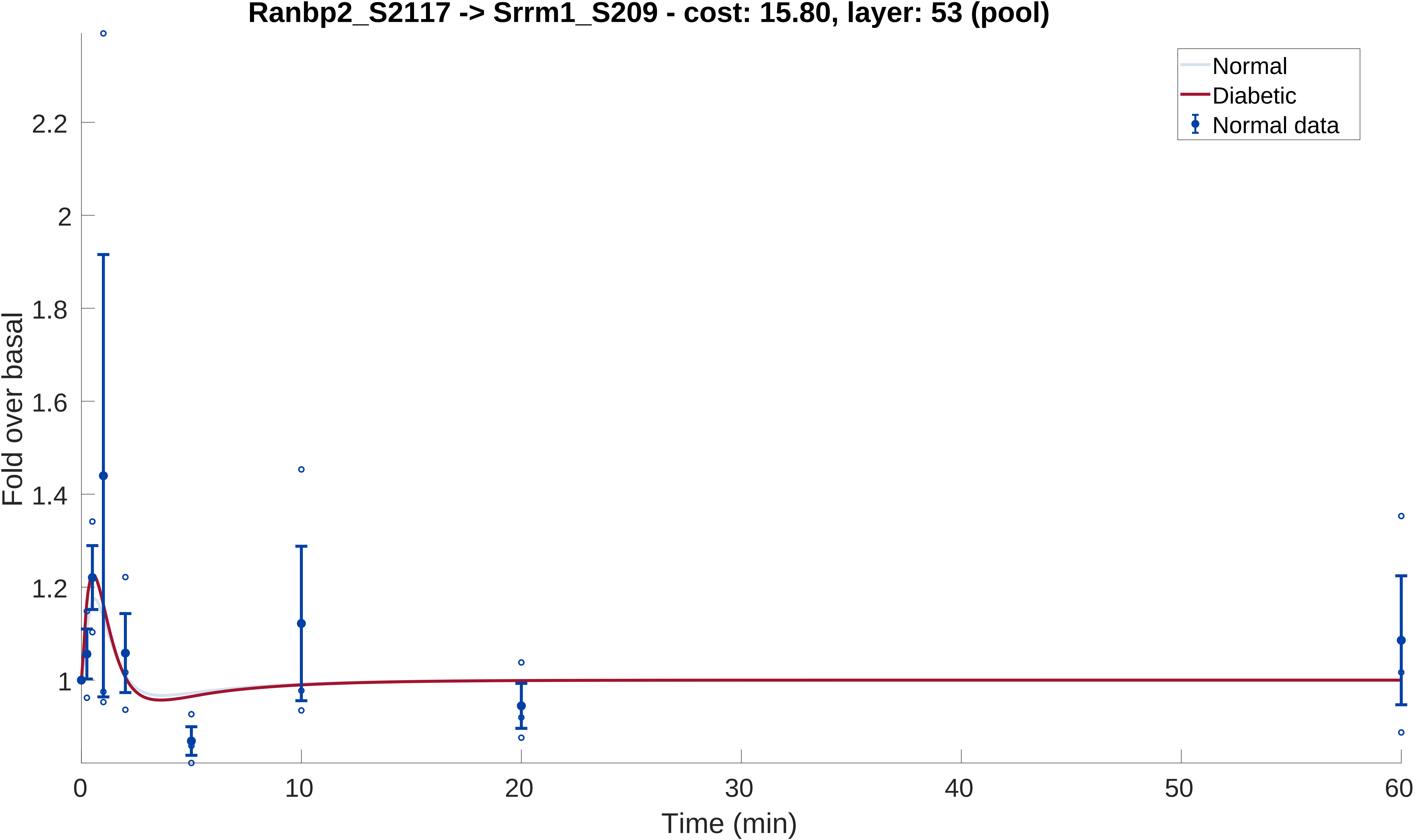

Ranbp2_S2117 -> Srrm1_S209 - cost: 15.80, layer: 53 (pool)
Normal
Diabetic
Normal data
Fold over basal
2.2
2
1.8
1.6
1.4
1.2
1
0
10
20
30
40
50
60
Time (min)

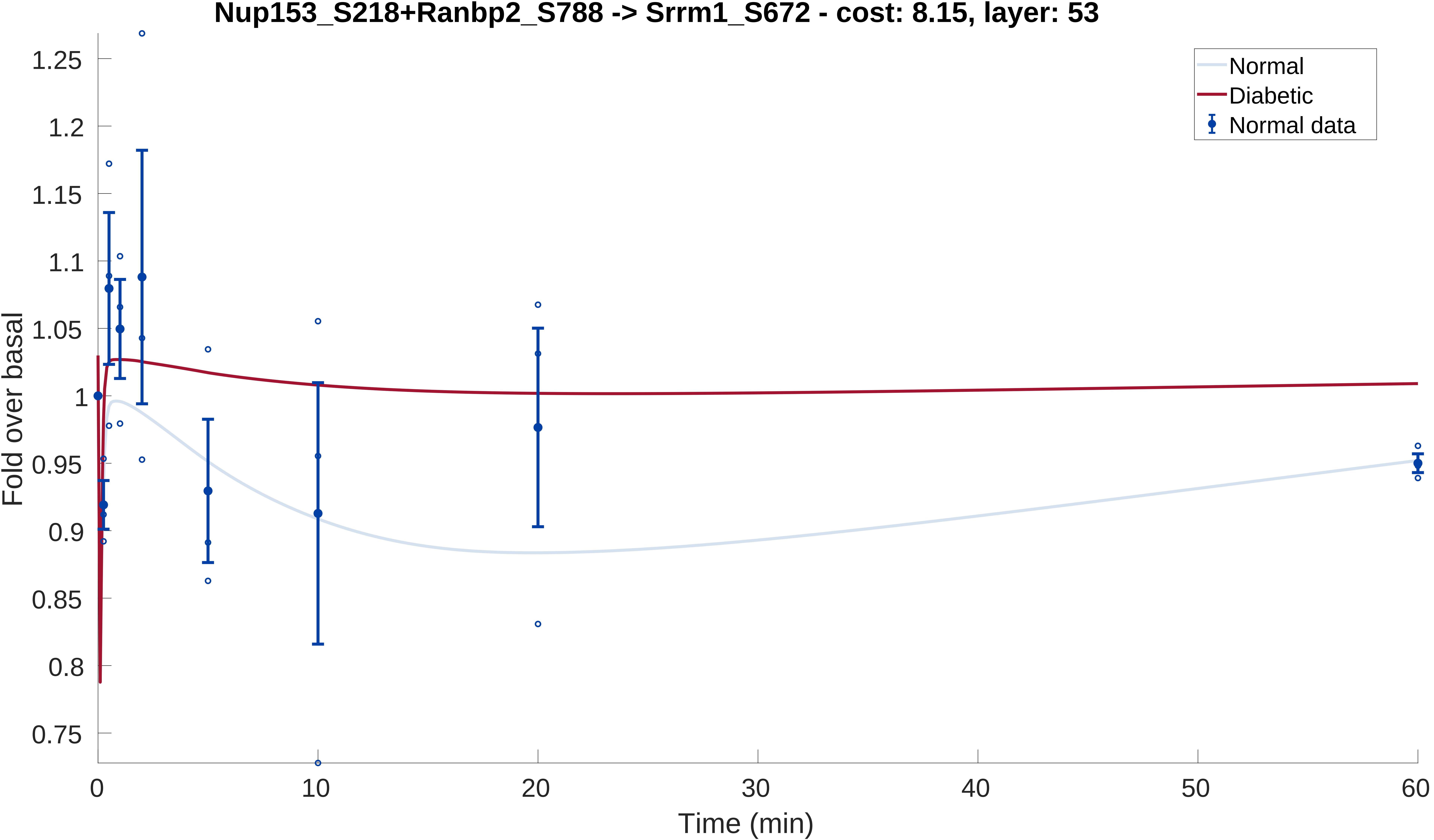

Nup153_S218+Ranbp2_S788 -> Srrm1_S672 - cost: 8.15, layer: 53
Normal
Diabetic
Normal data
Fold over basal
Time (min)
1.25
1.2
1.15
1.1
1.05
1
0.95
0.9
0.85
0.8
0.75
0
10
20
30
40
50
60

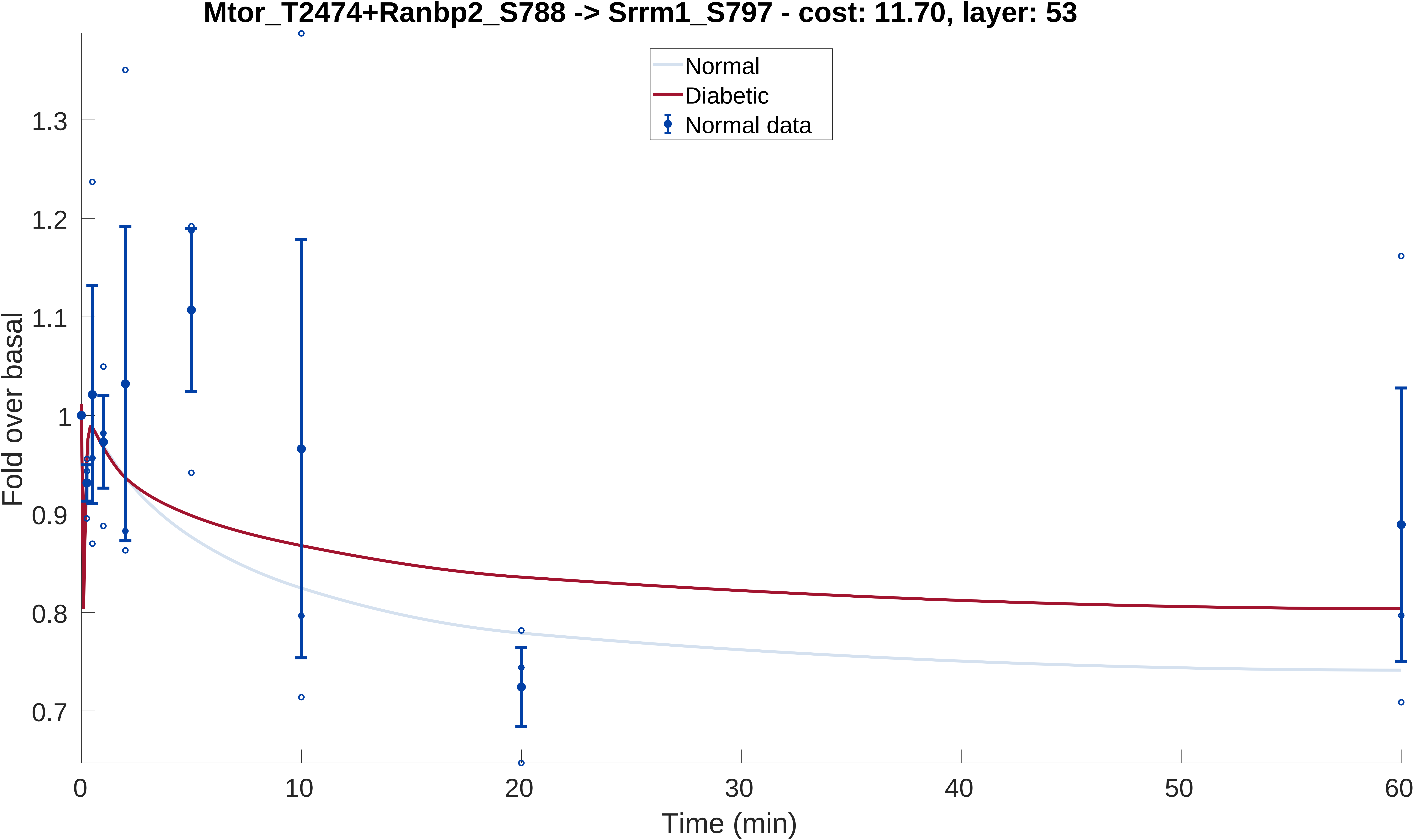

Mtor_T2474+Ranbp2_S788 -> Srrm1_S797 - cost: 11.70, layer: 53
Normal
Diabetic
Normal data
Fold over basal
Time (min)
1.3
1.2
1.1
1
0.9
0.8
0.7
0
10
20
30
40
50
60

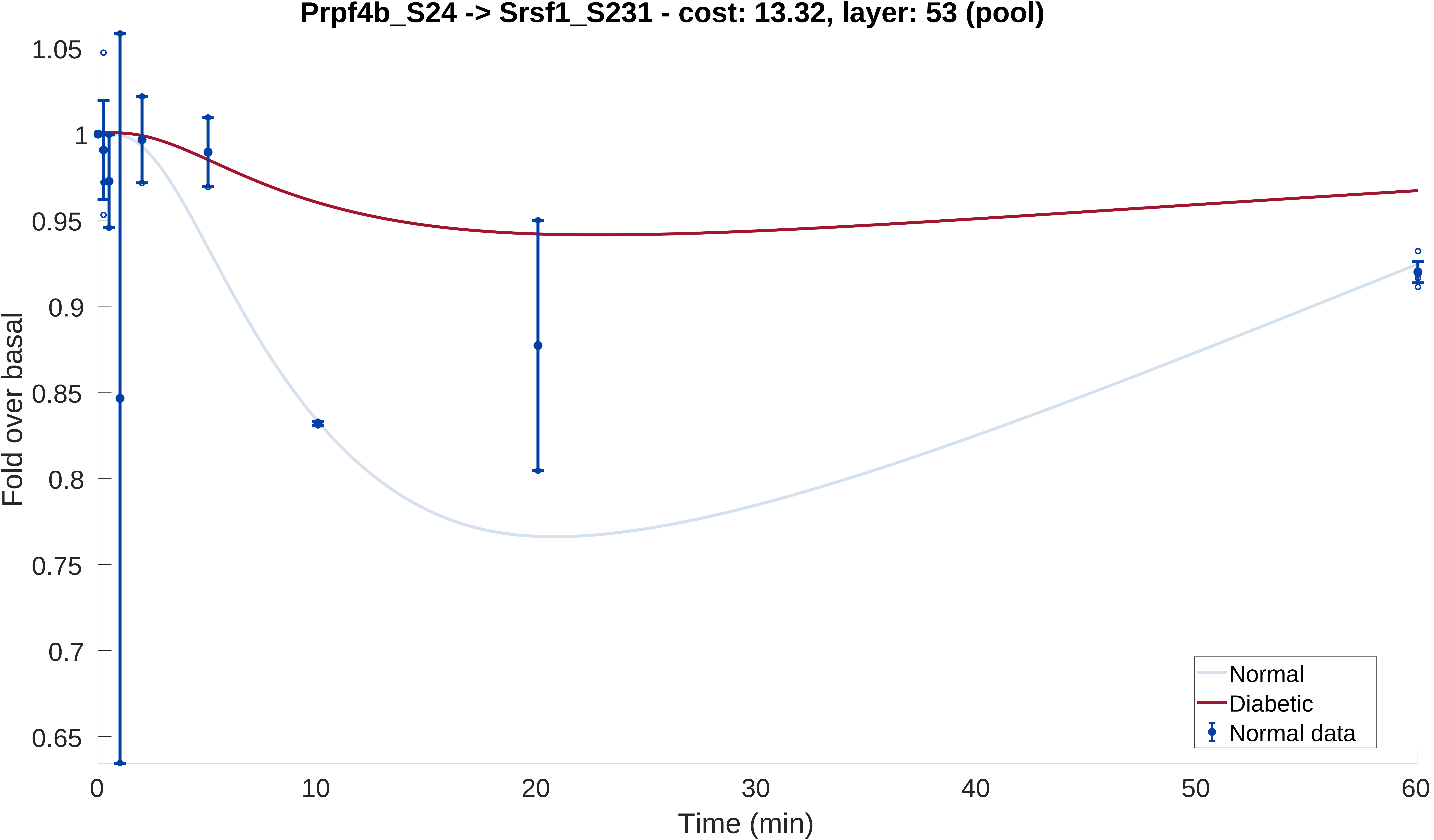
Prpf4b_S24 -> Srsf1_S231 - cost: 13.32, layer: 53 (pool)
Fold over basal
1.05
1
0.95
0.9
0.85
0.8
0.75
0.7
0.65
0
10
20
30
40
50
60
Time (min)
Normal
Diabetic
Normal data

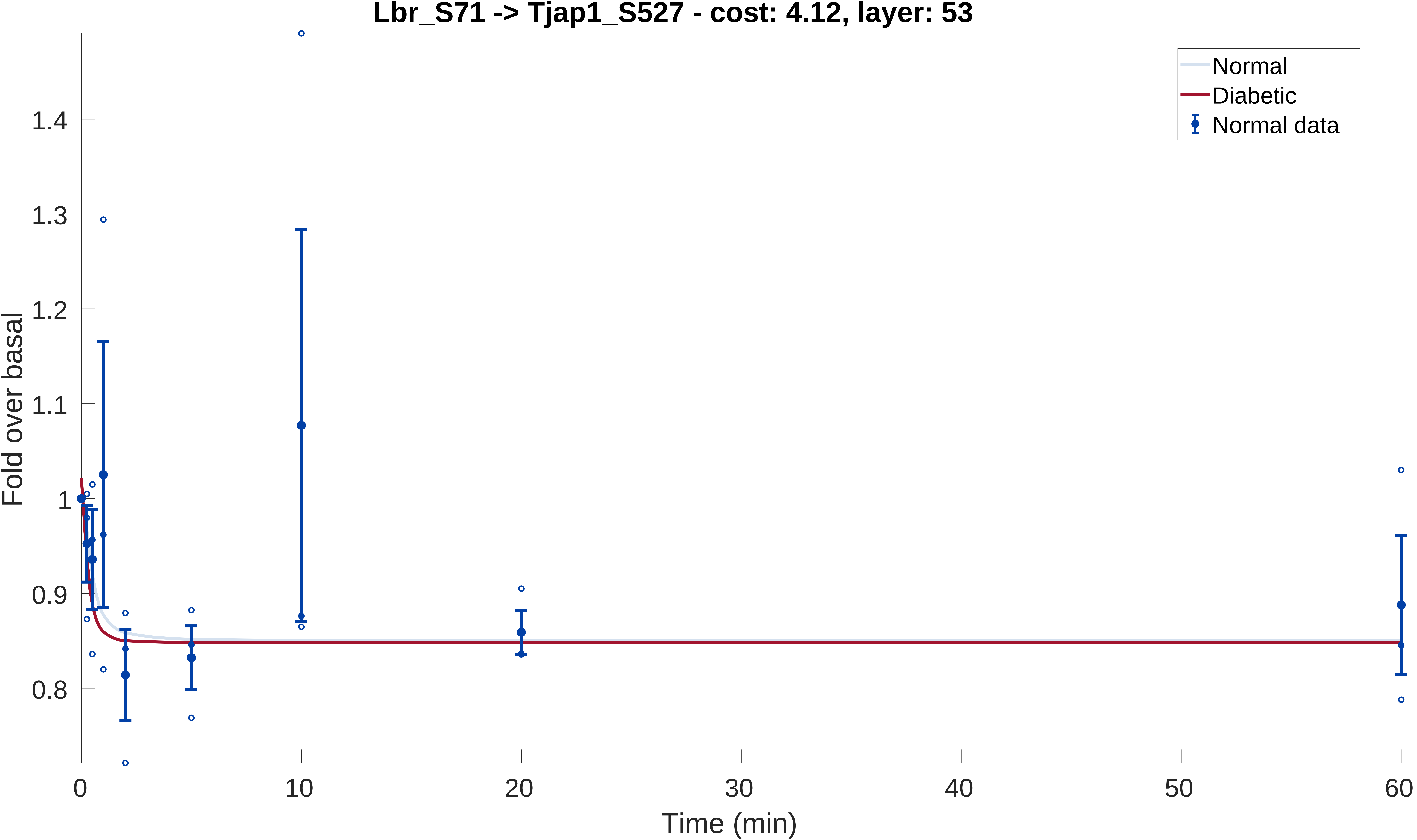

Lbr_S71 -> Tjap1_S527 - cost: 4.12, layer: 53
Normal
Diabetic
Normal data
Fold over basal
1.4
1.3
1.2
1.1
1
0.9
0.8
0
10
20
30
40
50
60
Time (min)

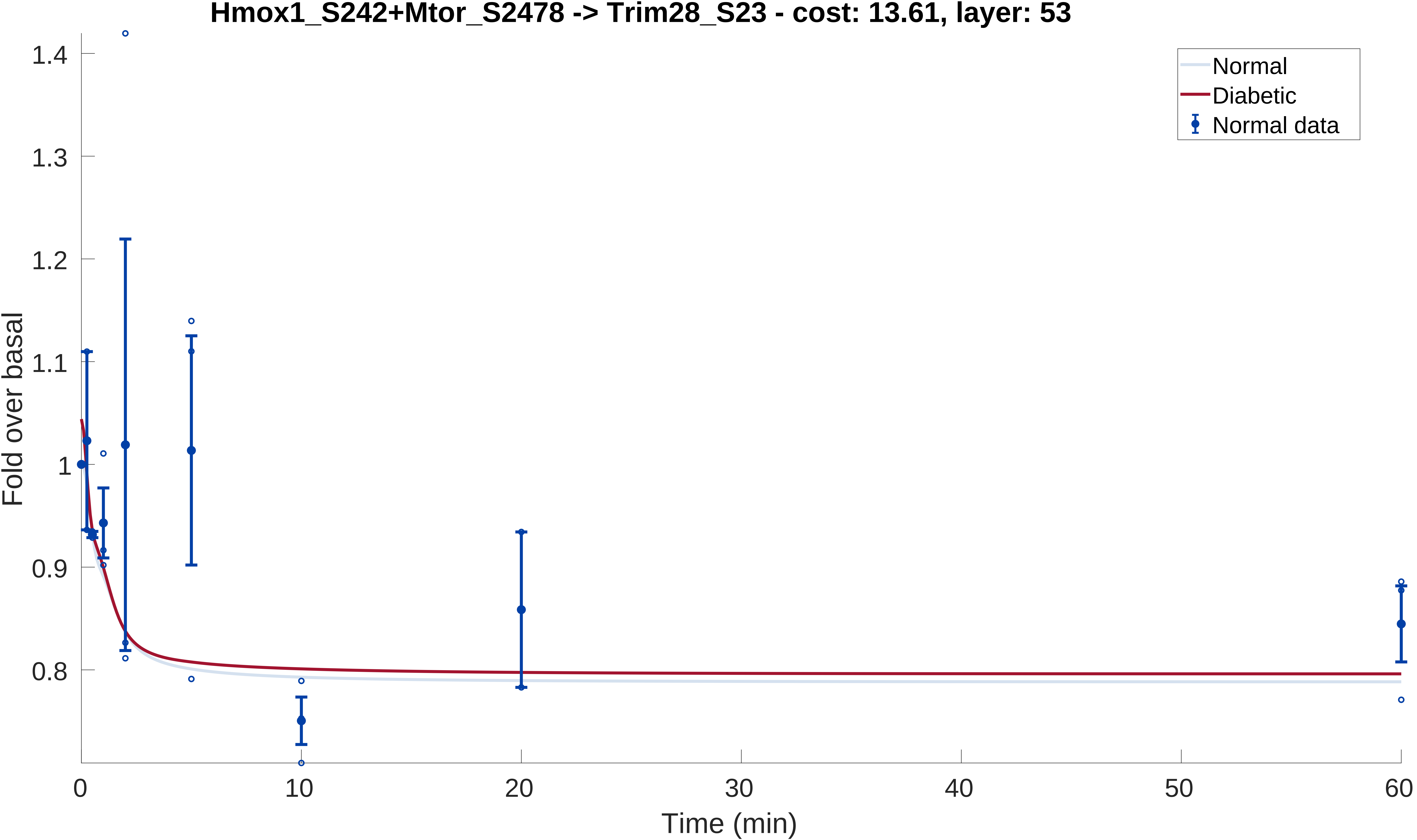
Hmox1_S242+Mtor_S2478 -> Trim28_S23 - cost: 13.61, layer: 53
Fold over basal
1.4
1.3
1.2
1.1
1
0.9
0.8
0
10
20
30
40
50
60
Time (min)
Normal
Diabetic
Normal data

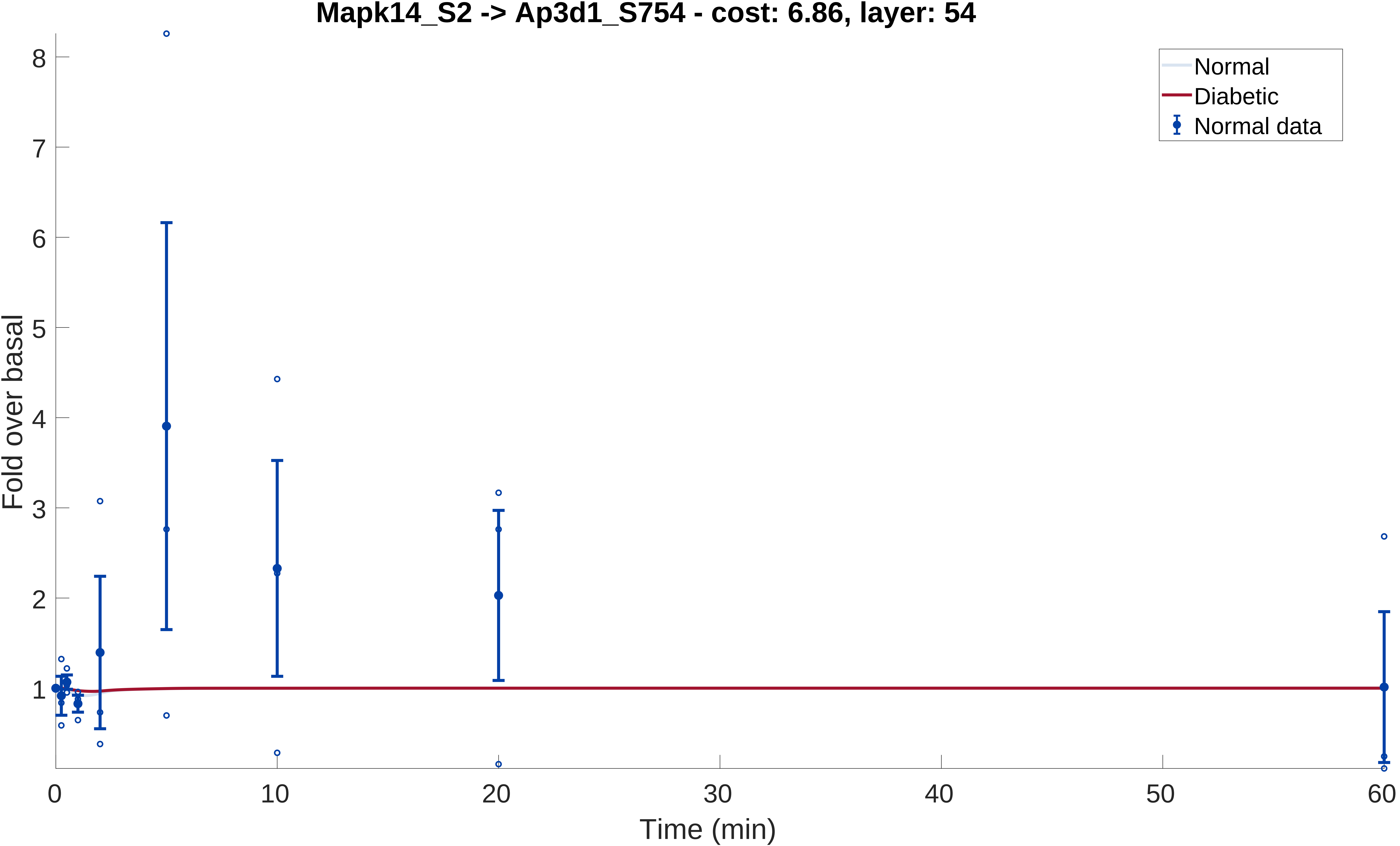

Mapk14_S2 -> Ap3d1_S754 - cost: 6.86, layer: 54
Normal
Diabetic
Normal data
Fold over basal
Time (min)
0
10
20
30
40
50
60
1
2
3
4
5
6
7
8

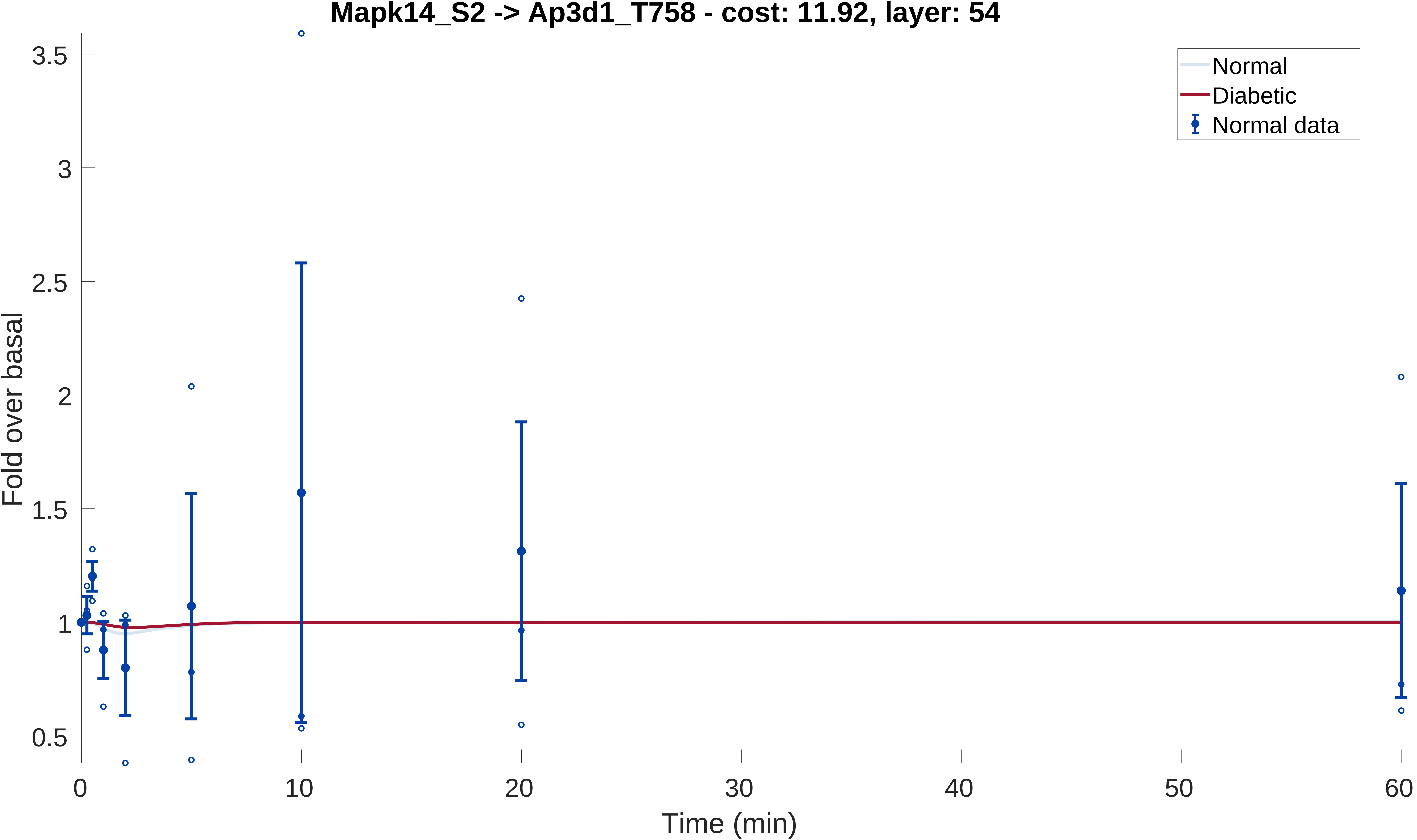

Mapk14_S2 -> Ap3d1_T758 - cost: 11.92, layer: 54
Normal
Diabetic
Normal data
Fold over basal
Time (min)
0.5
1
1.5
2
2.5
3
3.5
0
10
20
30
40
50
60

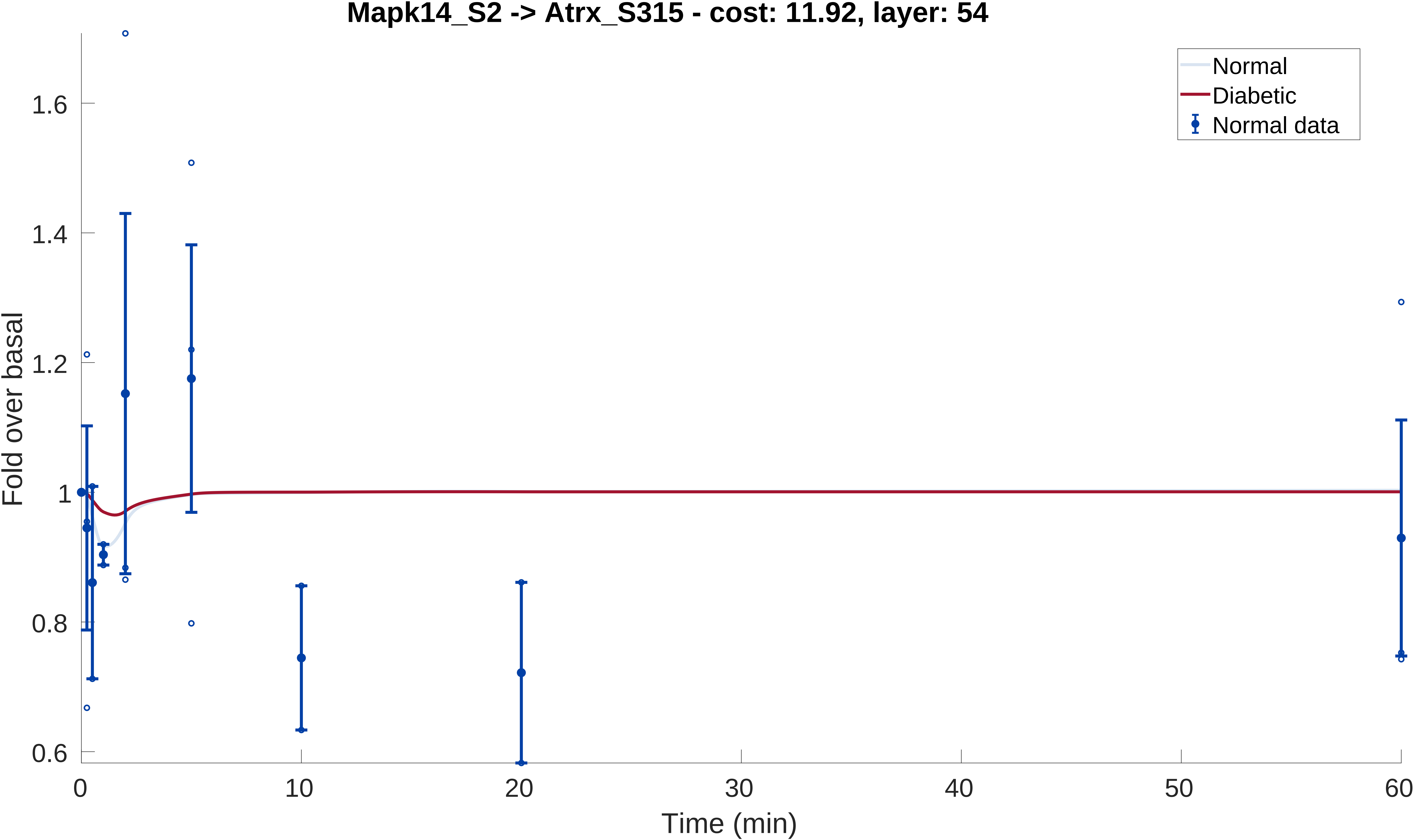
Mapk14_S2 -> Atrx_S315 - cost: 11.92, layer: 54
Normal
Diabetic
Normal data
Fold over basal
Time (min)
1.6
1.4
1.2
1
0.8
0.6
0
10
20
30
40
50
60

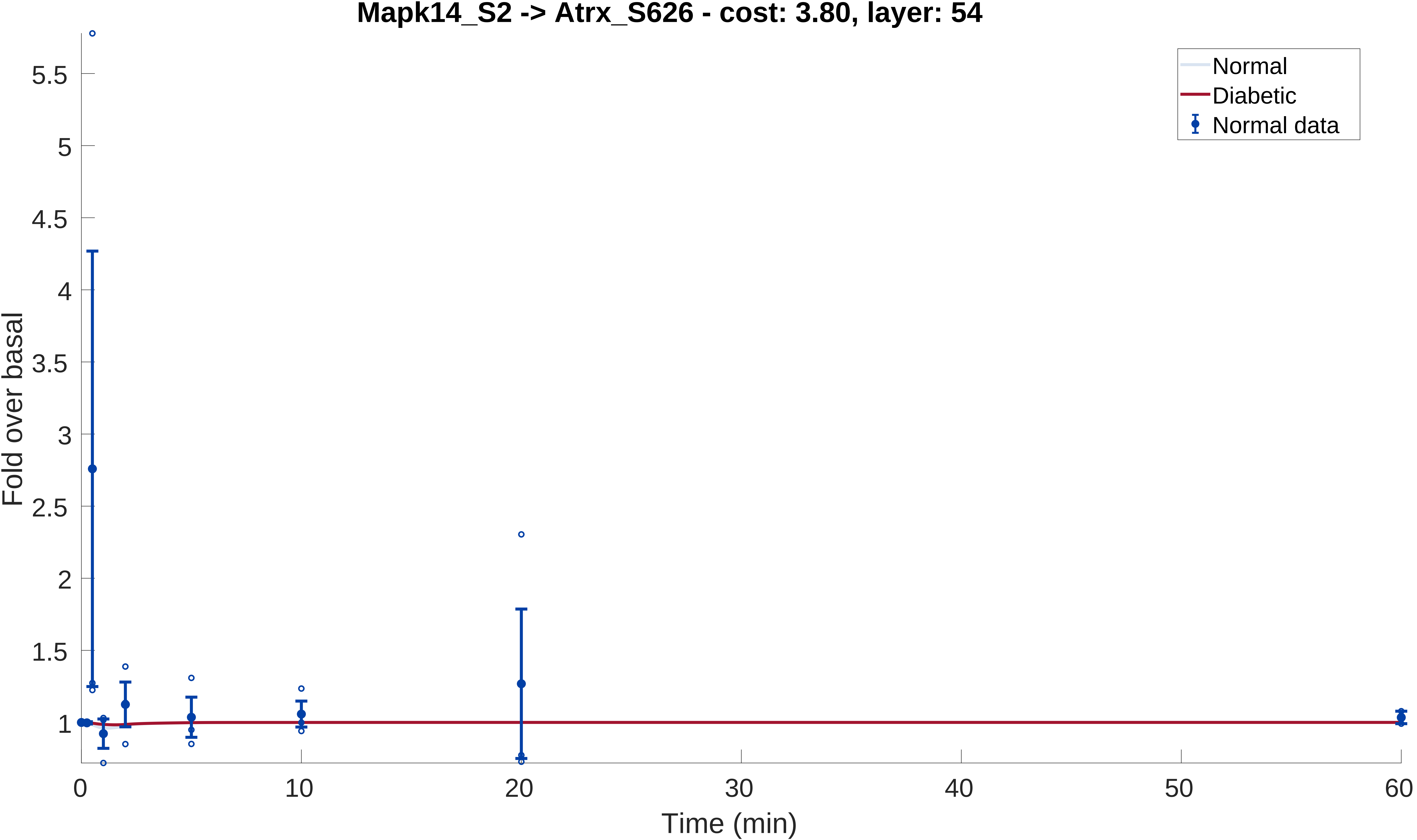

Mapk14_S2 -> Atrx_S626 - cost: 3.80, layer: 54
Normal
Diabetic
Normal data
Fold over basal
Time (min)
5.5
5
4.5
4
3.5
3
2.5
2
1.5
1
0
10
20
30
40
50
60

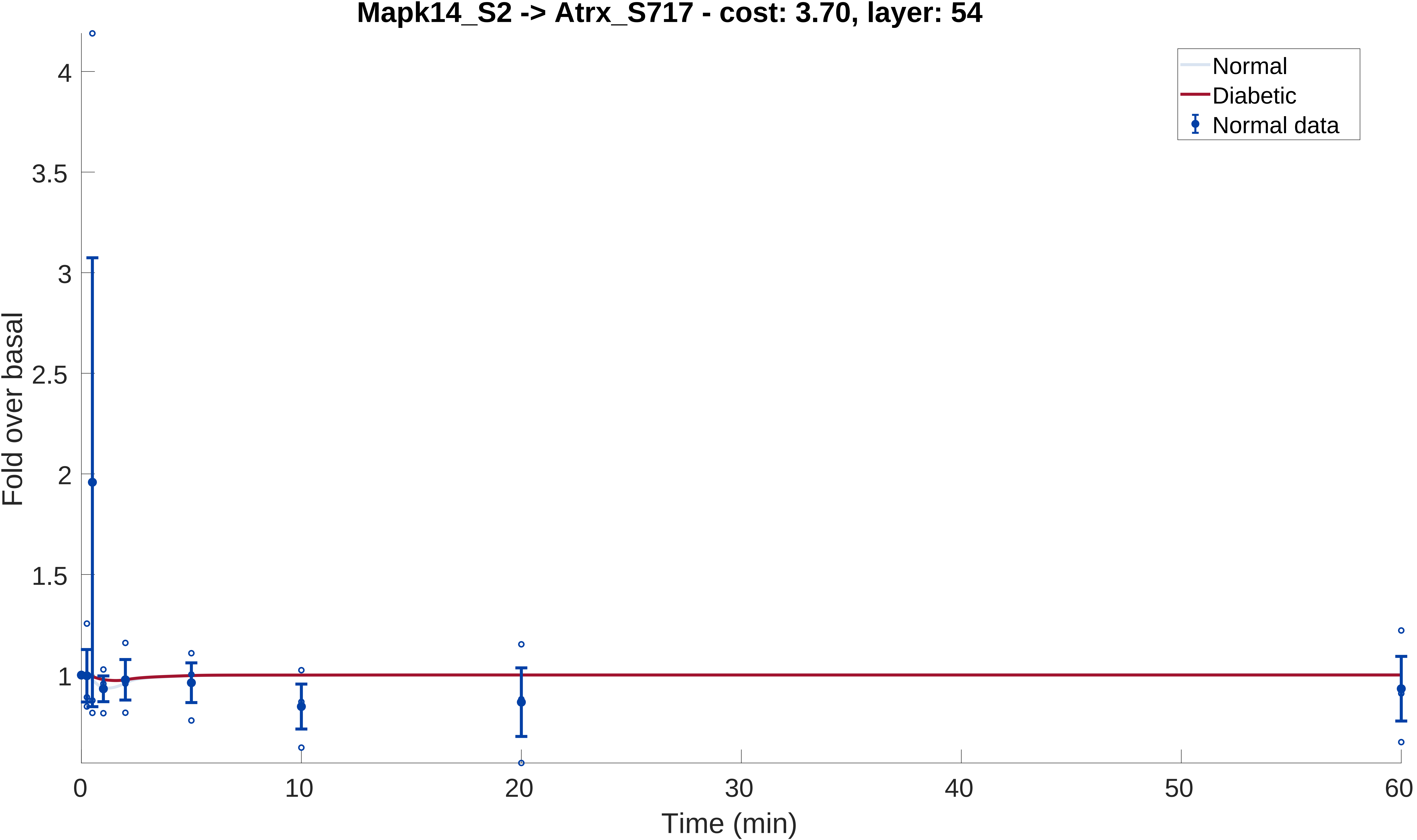

Mapk14_S2 -> Atrx_S717 - cost: 3.70, layer: 54
Normal
Diabetic
Normal data
Fold over basal
Time (min)
4
3.5
3
2.5
2
1.5
1
0
10
20
30
40
50
60

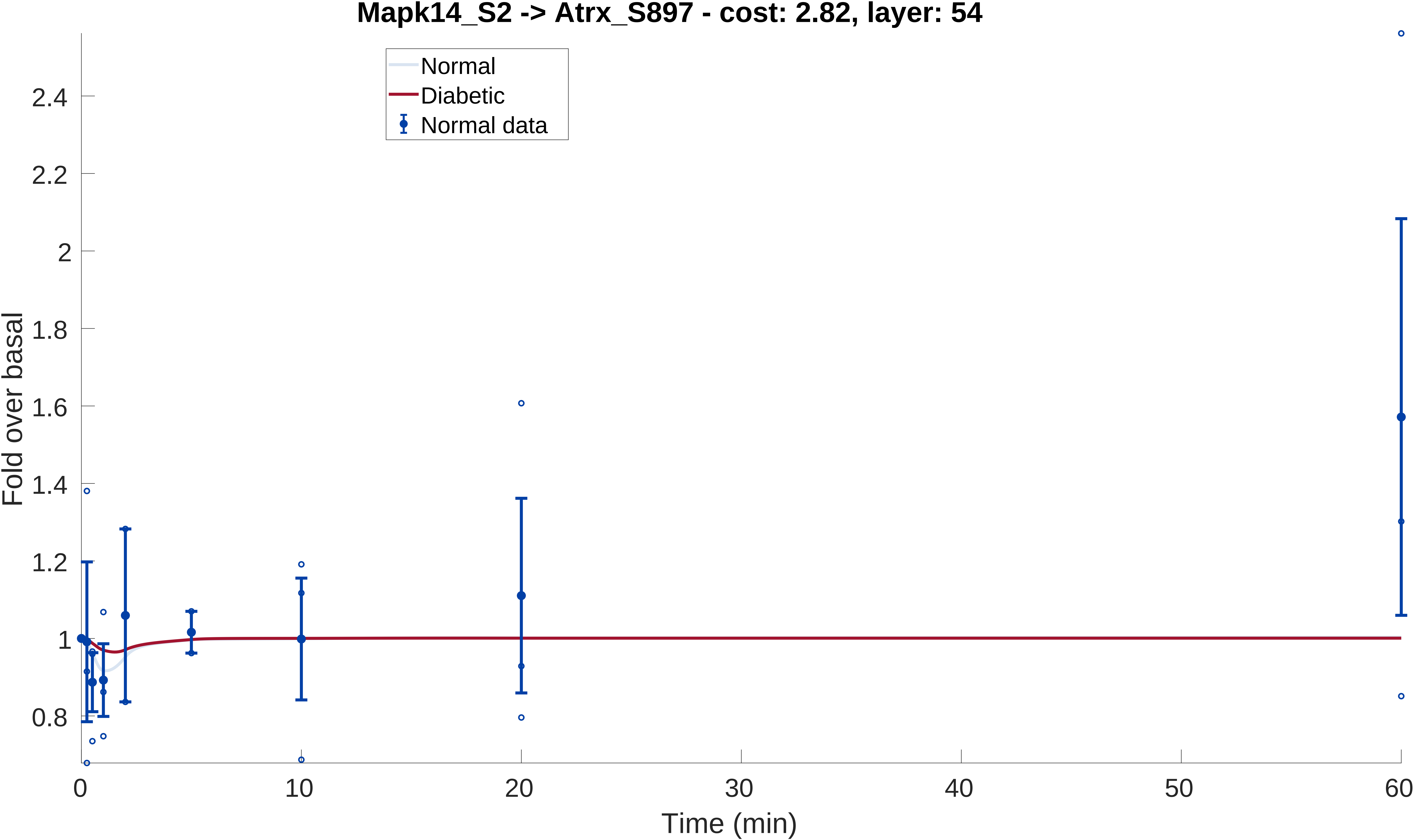

Mapk14_S2 -> Atrx_S897 - cost: 2.82, layer: 54
Normal
Diabetic
Normal data
2.4
2.2
2
1.8
1.6
1.4
1.2
1
0.8
Fold over basal
0
10
20
30
40
50
60
Time (min)

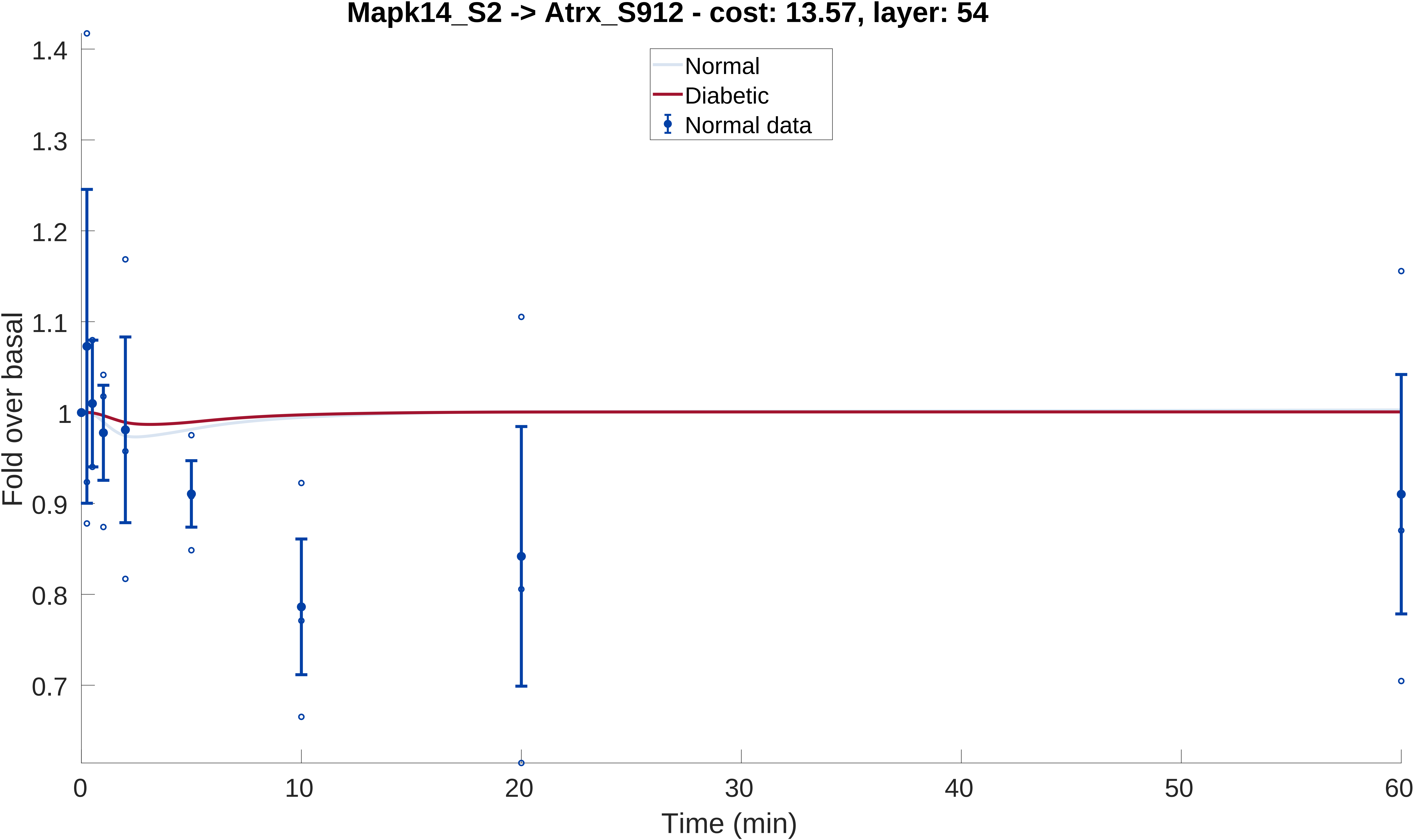

Mapk14_S2 -> Atrx_S912 - cost: 13.57, layer: 54
Normal
Diabetic
Normal data
1.4
1.3
1.2
1.1
1
0.9
0.8
0.7
Fold over basal
0
10
20
30
40
50
60
Time (min)

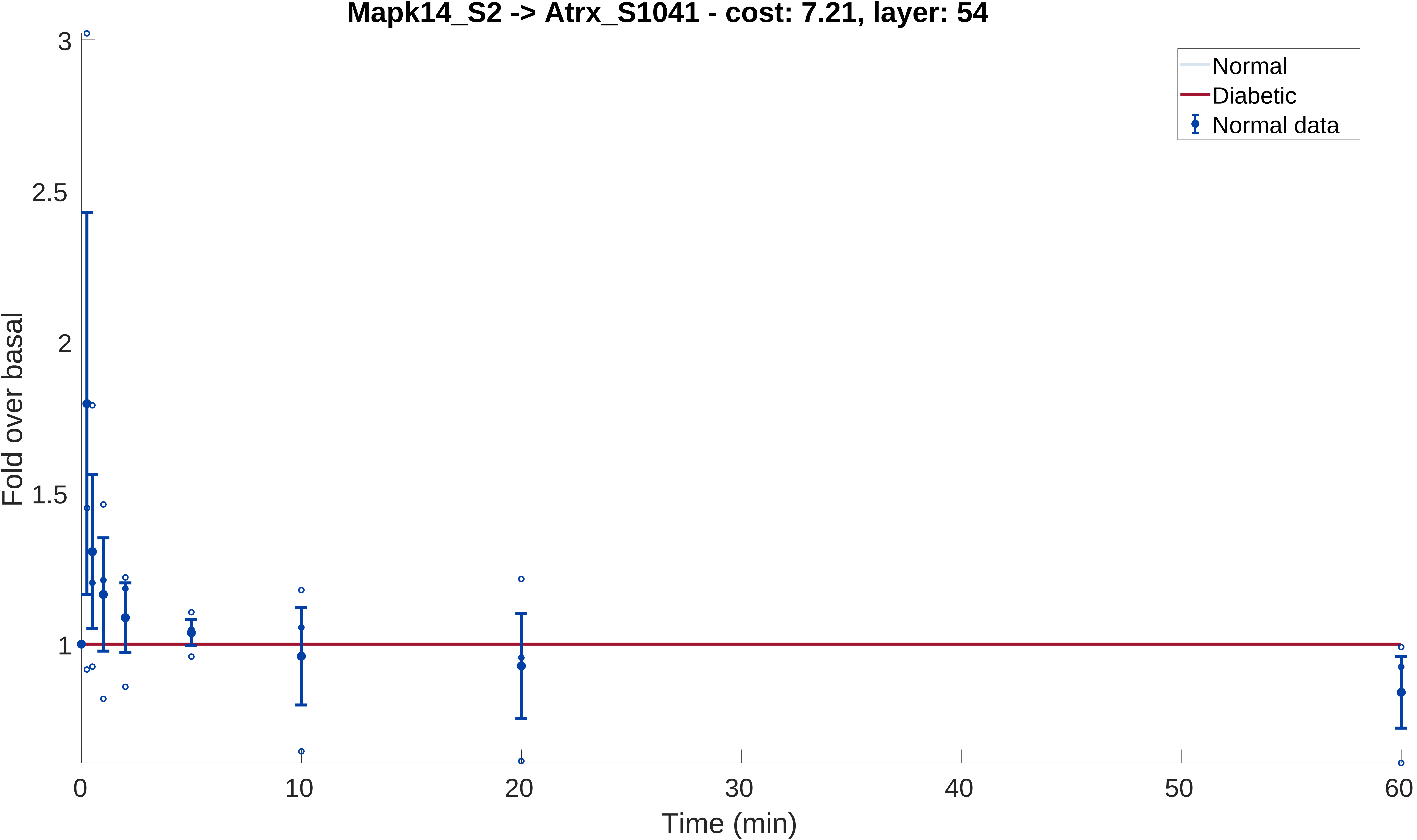

Mapk14_S2 -> Atrx_S1041 - cost: 7.21, layer: 54
Normal
Diabetic
Normal data
Fold over basal
Time (min)
3
2.5
2
1.5
1
0
10
20
30
40
50
60

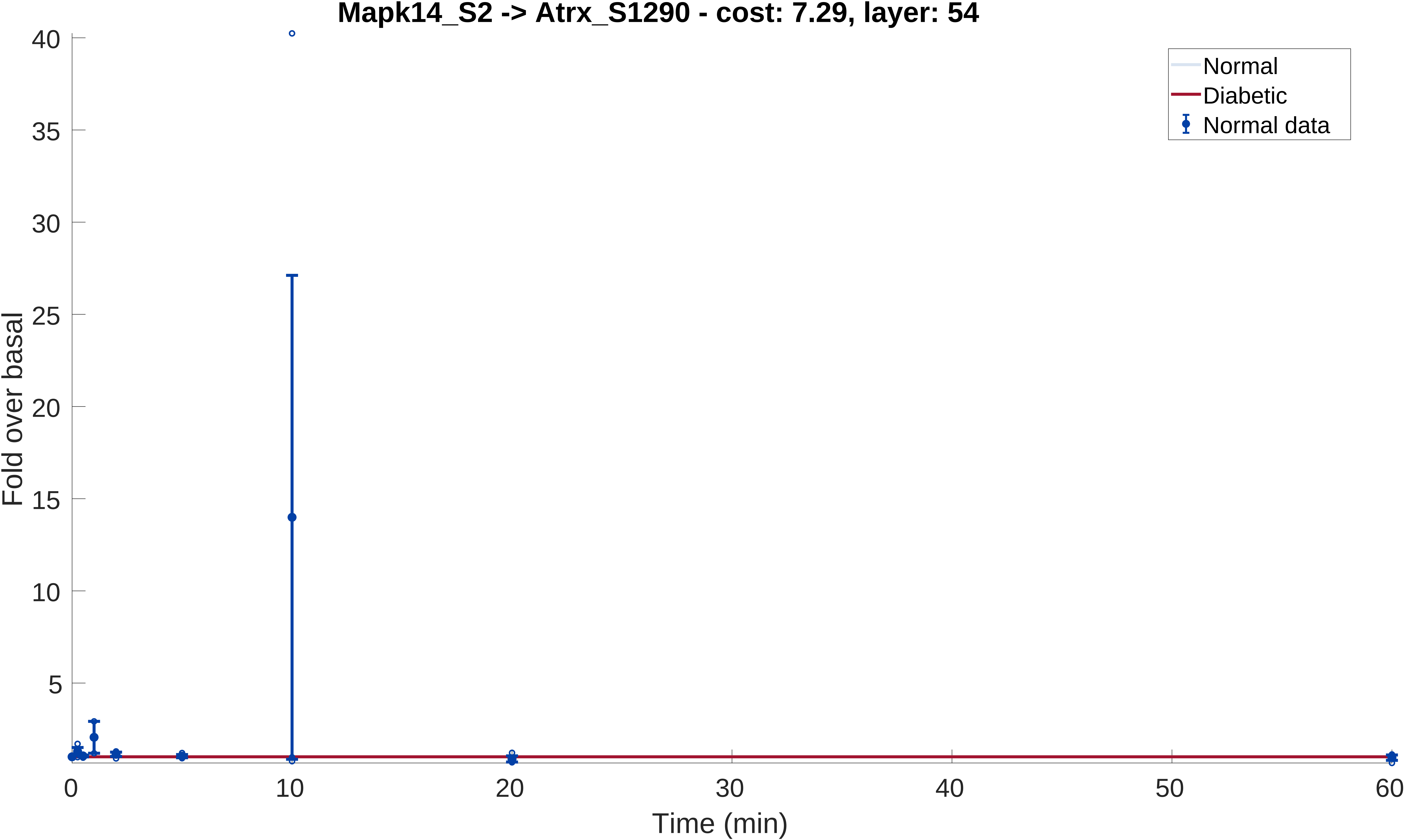

Mapk14_S2 -> Atrx_S1290 - cost: 7.29, layer: 54
Normal
Diabetic
Normal data
Fold over basal
40
35
30
25
20
15
10
5
0
10
20
30
40
50
60
Time (min)

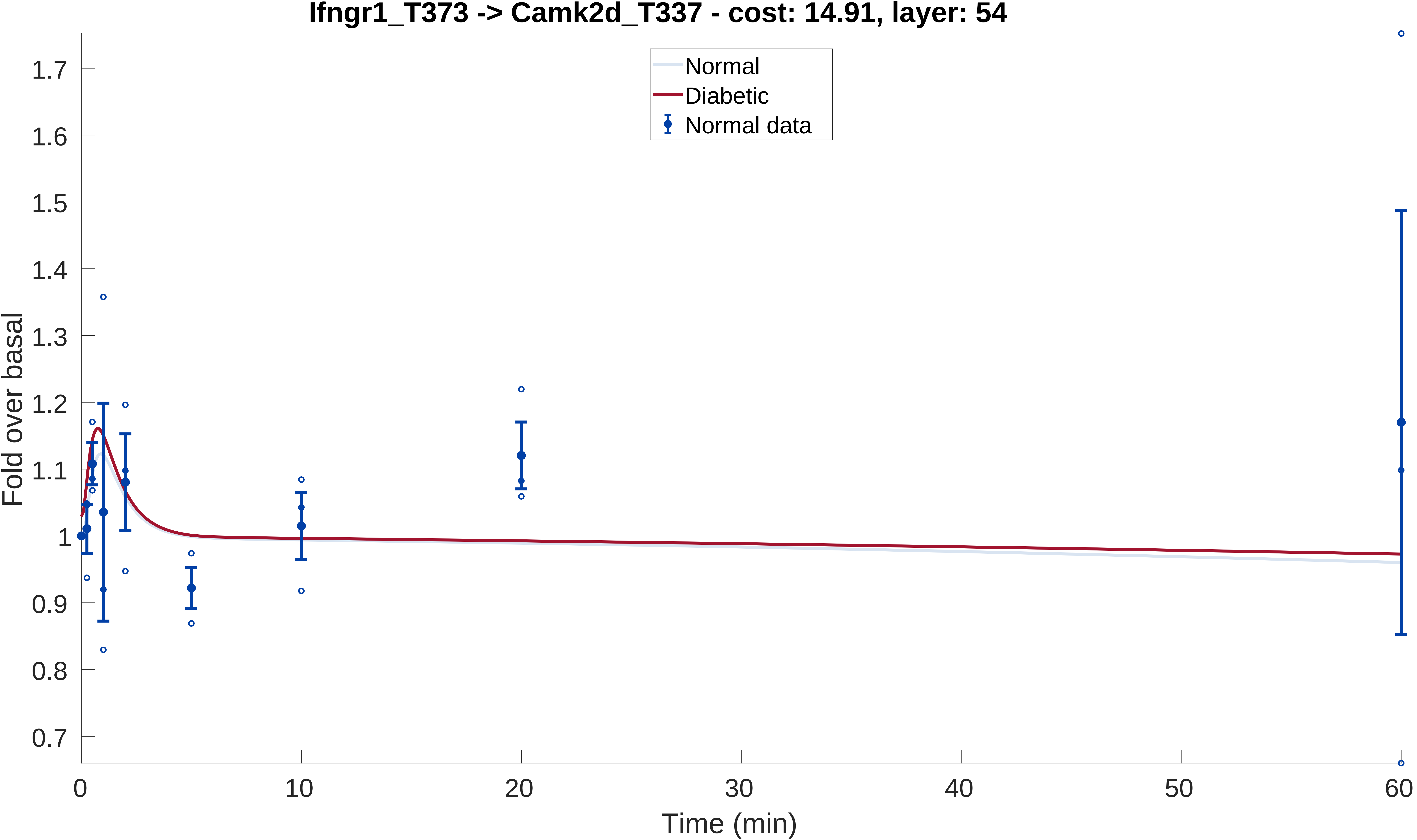

Ifngr1_T373 -> Camk2d_T337 - cost: 14.91, layer: 54
Normal
Diabetic
Normal data
Fold over basal
Time (min)
0.7
0.8
0.9
1
1.1
1.2
1.3
1.4
1.5
1.6
1.7
0
10
20
30
40
50
60

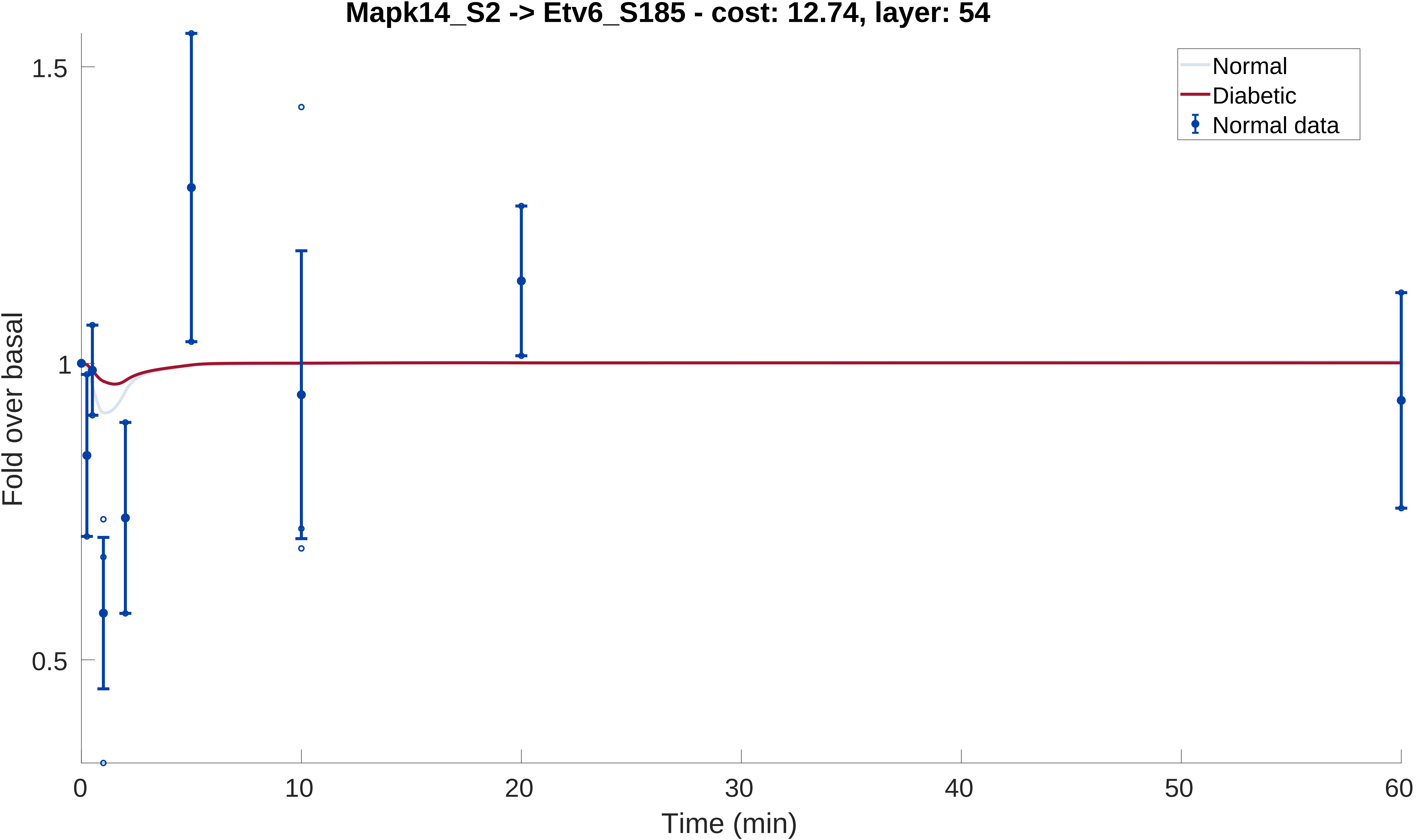
Mapk14_S2 -> Etv6_S185 - cost: 12.74, layer: 54
Normal
Diabetic
Normal data
Fold over basal
1.5
1
0.5
0
10
20
30
40
50
60
Time (min)

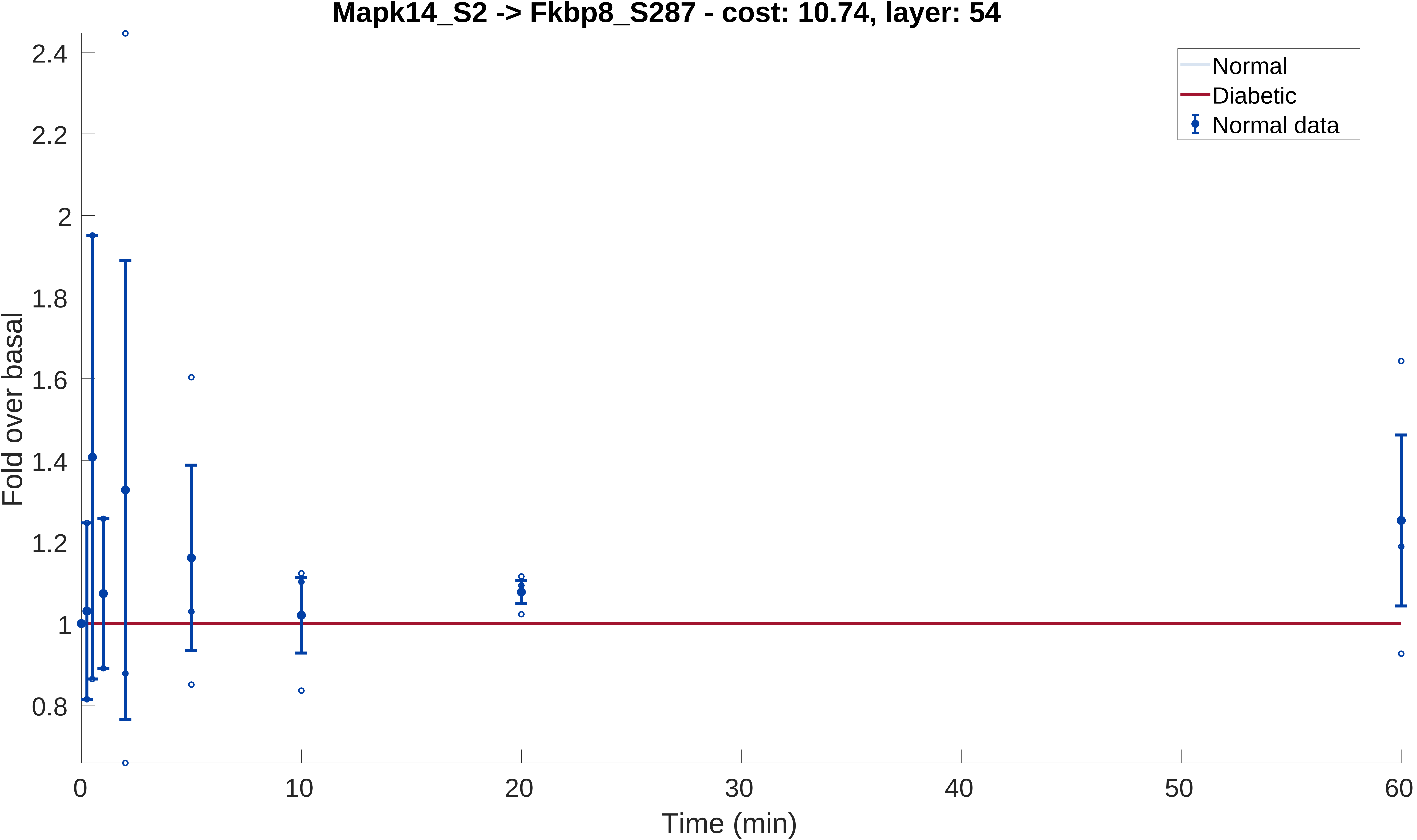

Mapk14_S2 -> Fkbp8_S287 - cost: 10.74, layer: 54
Normal
Diabetic
Normal data
Fold over basal
Time (min)
0.8
1
1.2
1.4
1.6
1.8
2
2.2
2.4
0
10
20
30
40
50
60

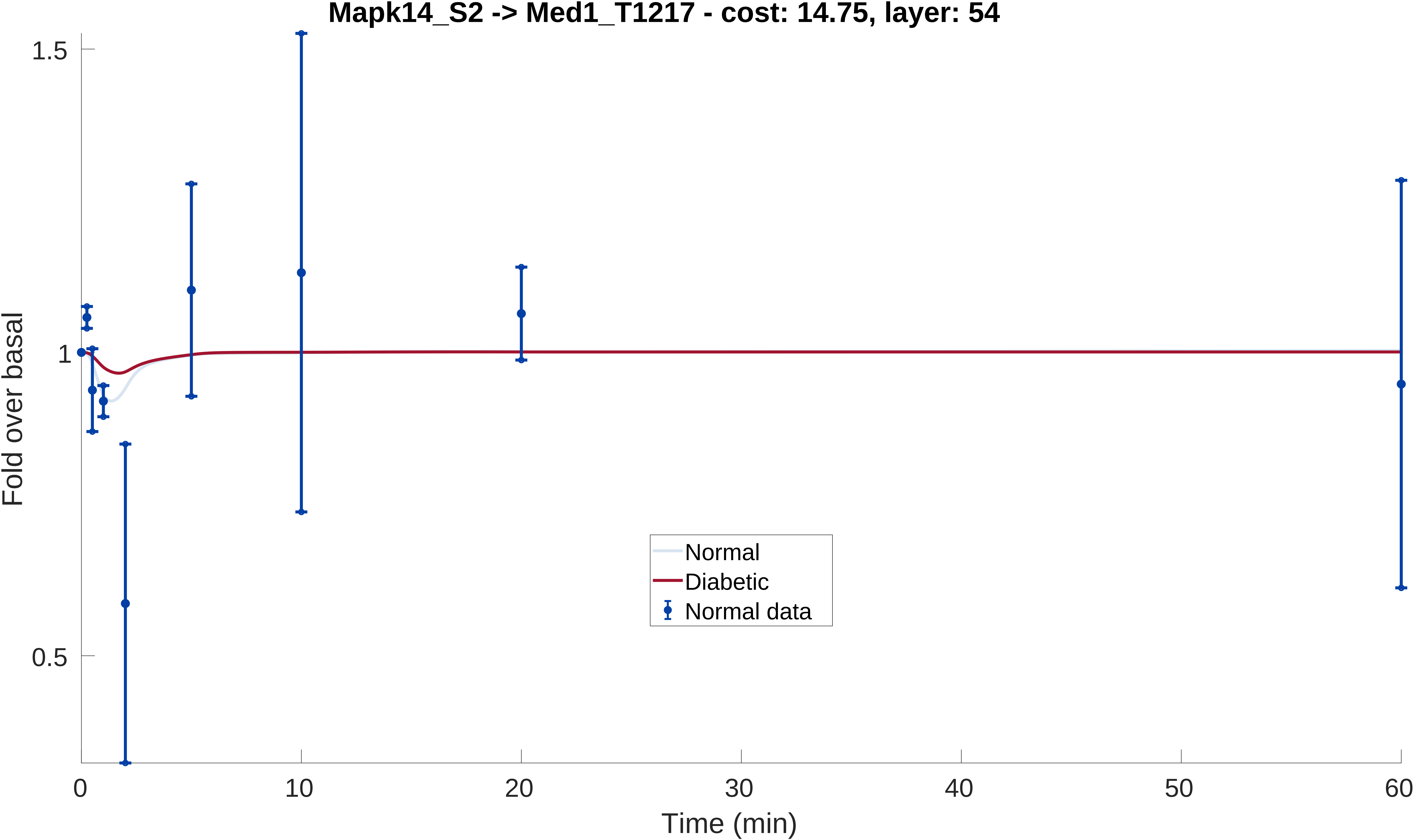

Mapk14_S2 -> Med1_T1217 - cost: 14.75, layer: 54
Fold over basal
1.5
1
0.5
0
10
20
30
40
50
60
Time (min)
Normal
Diabetic
Normal data

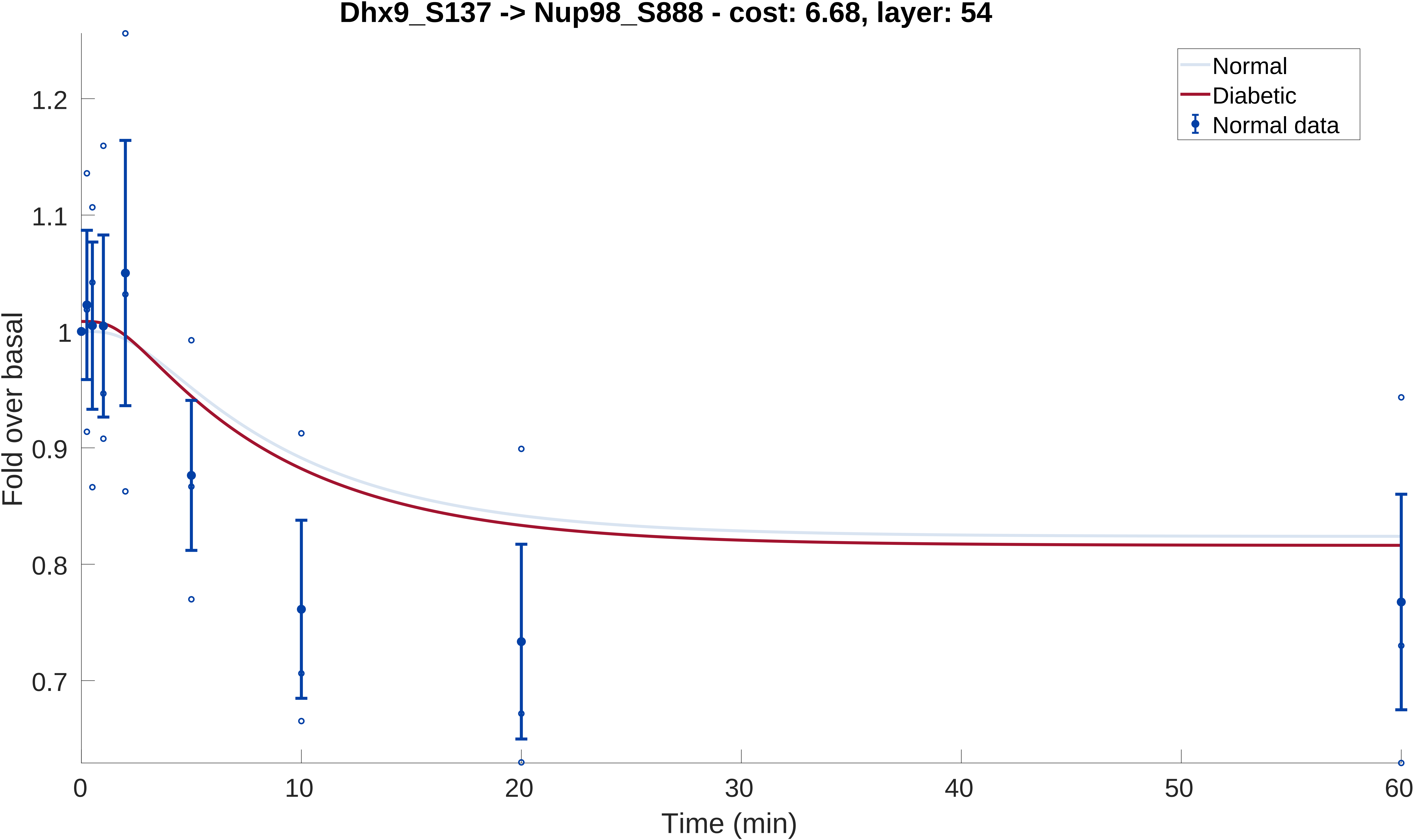
Dhx9_S137 -> Nup98_S888 - cost: 6.68, layer: 54
Normal
Diabetic
Normal data
1.2
1.1
1
0.9
0.8
0.7
Fold over basal
0
10
20
30
40
50
60
Time (min)

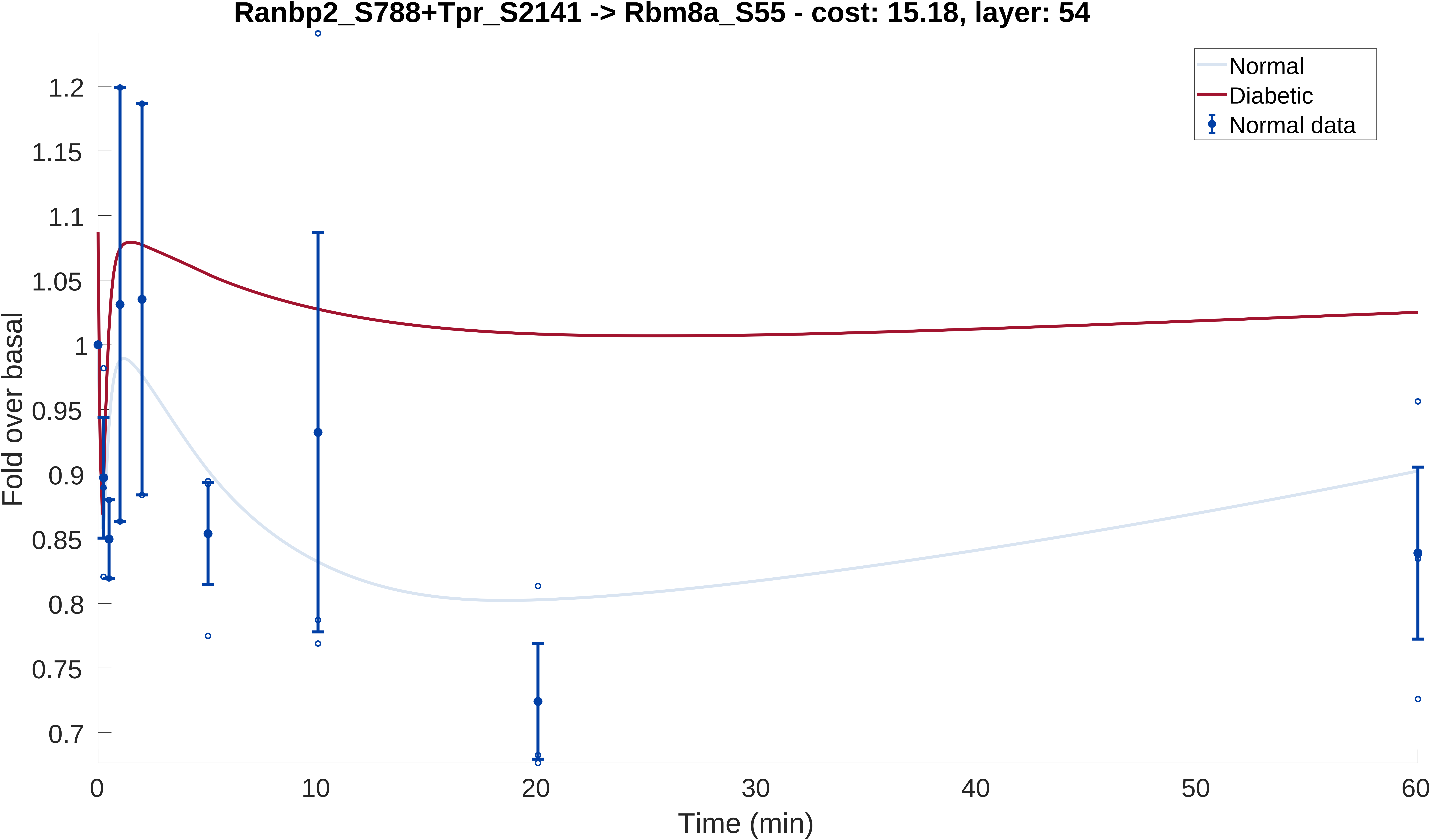

Ranbp2_S788+Tpr_S2141 -> Rbm8a_S55 - cost: 15.18, layer: 54
Normal
Diabetic
Normal data
Fold over basal
Time (min)
0.7
0.75
0.8
0.85
0.9
0.95
1
1.05
1.1
1.15
1.2
0
10
20
30
40
50
60

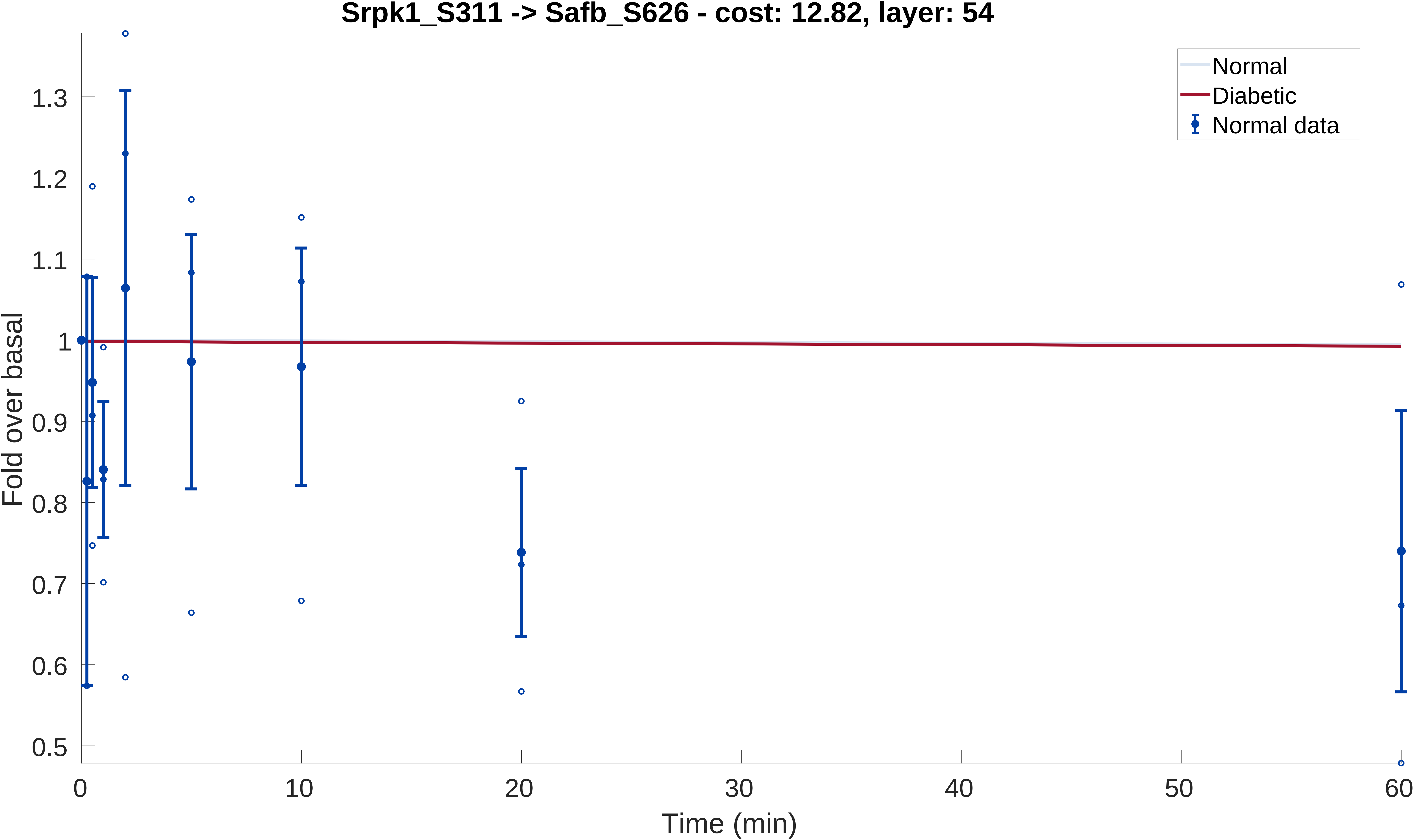

Srpk1_S311 -> Safb_S626 - cost: 12.82, layer: 54
Normal
Diabetic
Normal data
Fold over basal
Time (min)
0.5
0.6
0.7
0.8
0.9
1
1.1
1.2
1.3
0
10
20
30
40
50
60

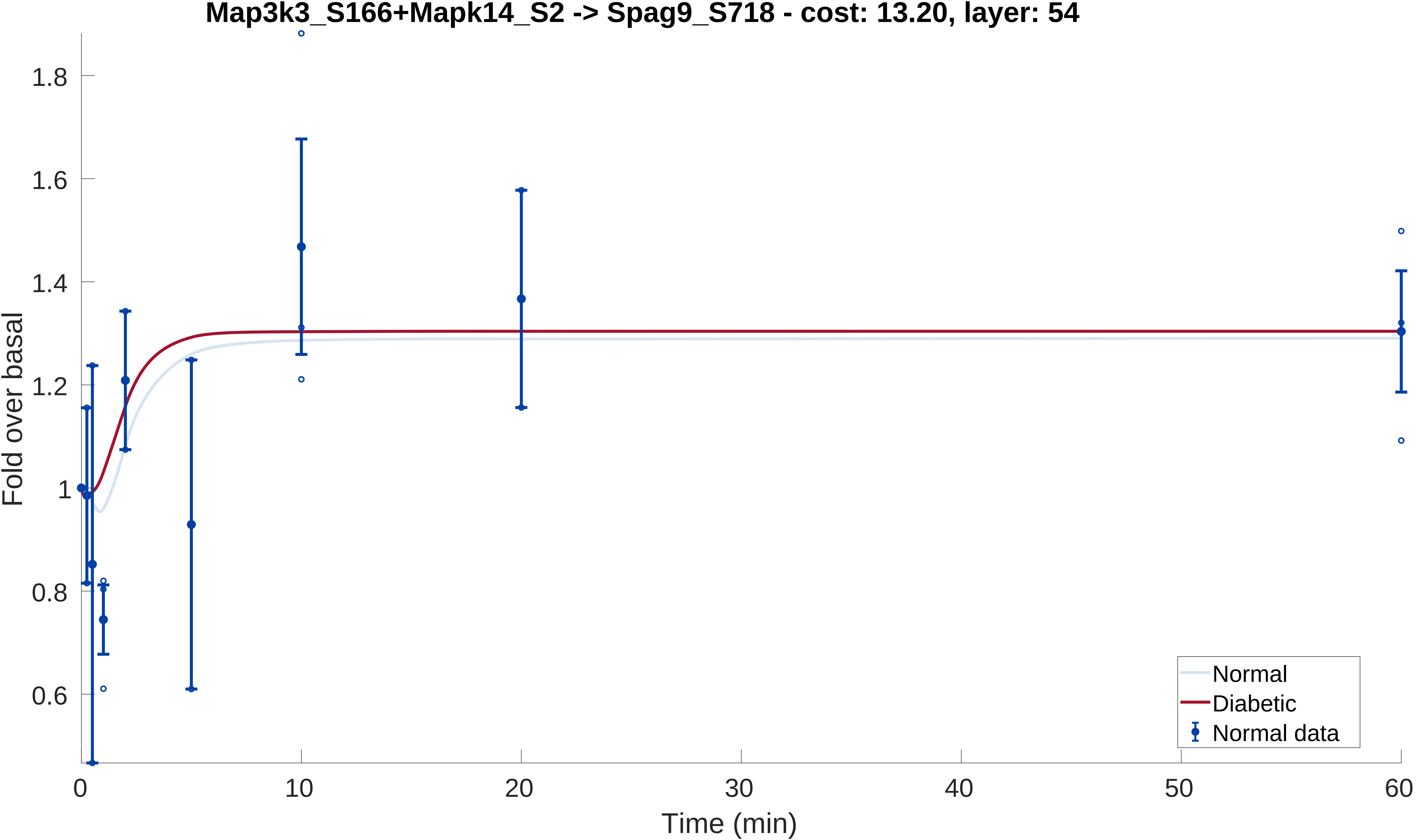

Map3k3_S166+Mapk14_S2 -> Spag9_S718 - cost: 13.20, layer: 54
Fold over basal
1.8
1.6
1.4
1.2
1
0.8
0.6
0
10
20
30
40
50
60
Time (min)
Normal
Diabetic
Normal data

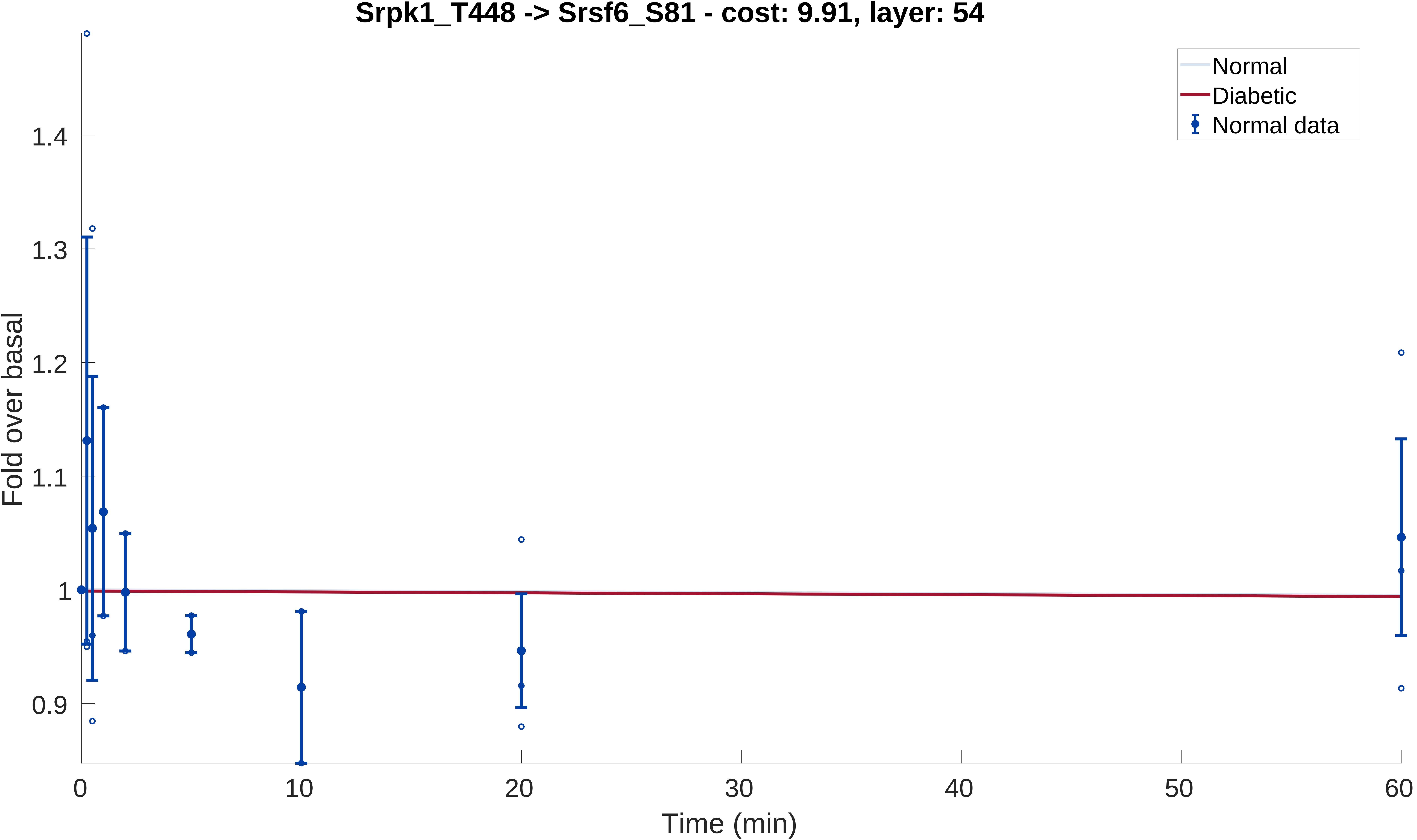

Srpk1_T448 -> Srsf6_S81 - cost: 9.91, layer: 54
Normal
Diabetic
Normal data
Fold over basal
1.4
1.3
1.2
1.1
1
0.9
0
10
20
30
40
50
60
Time (min)

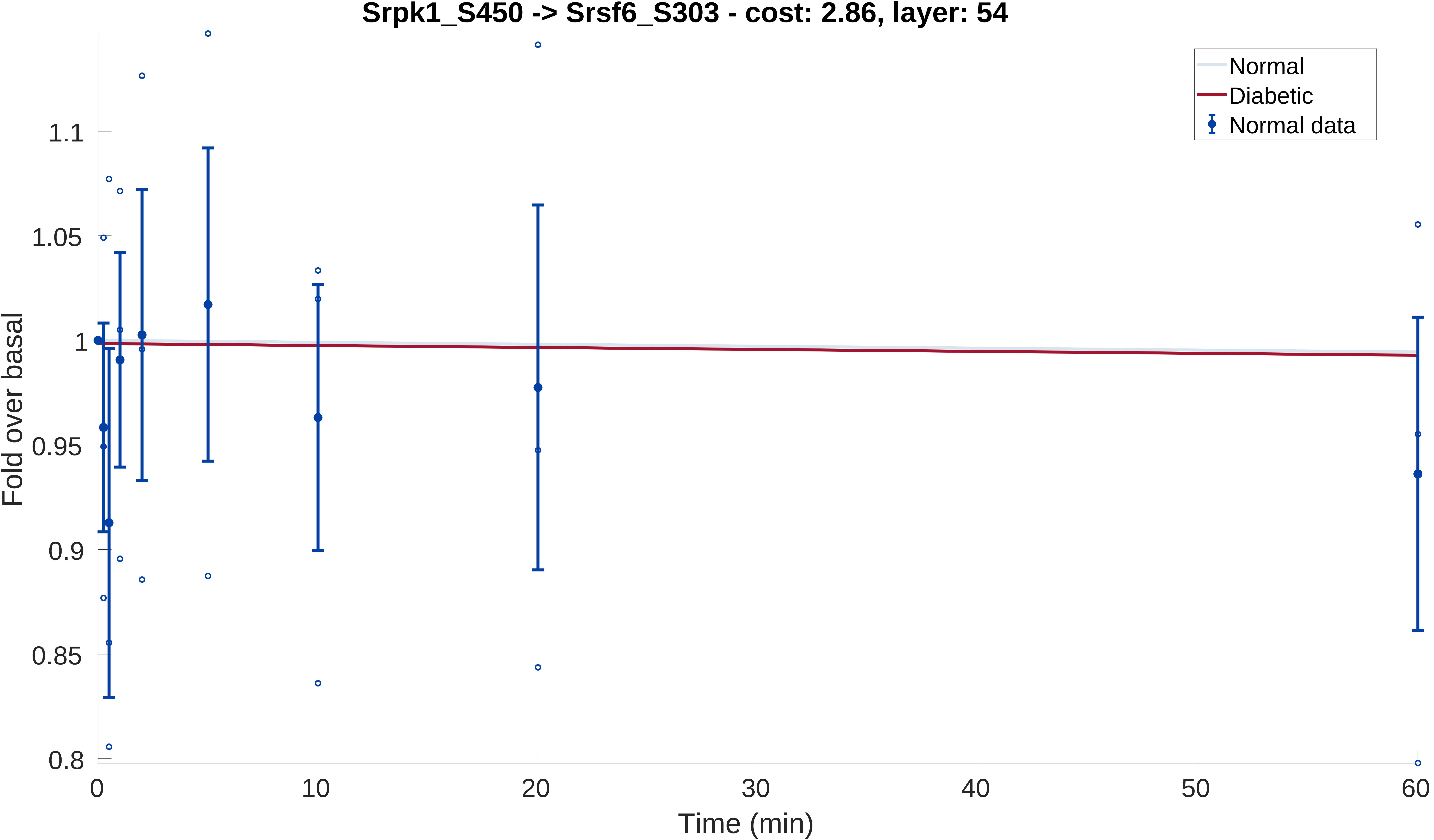

Srpk1_S450 -> Srsf6_S303 - cost: 2.86, layer: 54
Normal
Diabetic
Normal data
Fold over basal
Time (min)
1.1
1.05
1
0.95
0.9
0.85
0.8
0
10
20
30
40
50
60

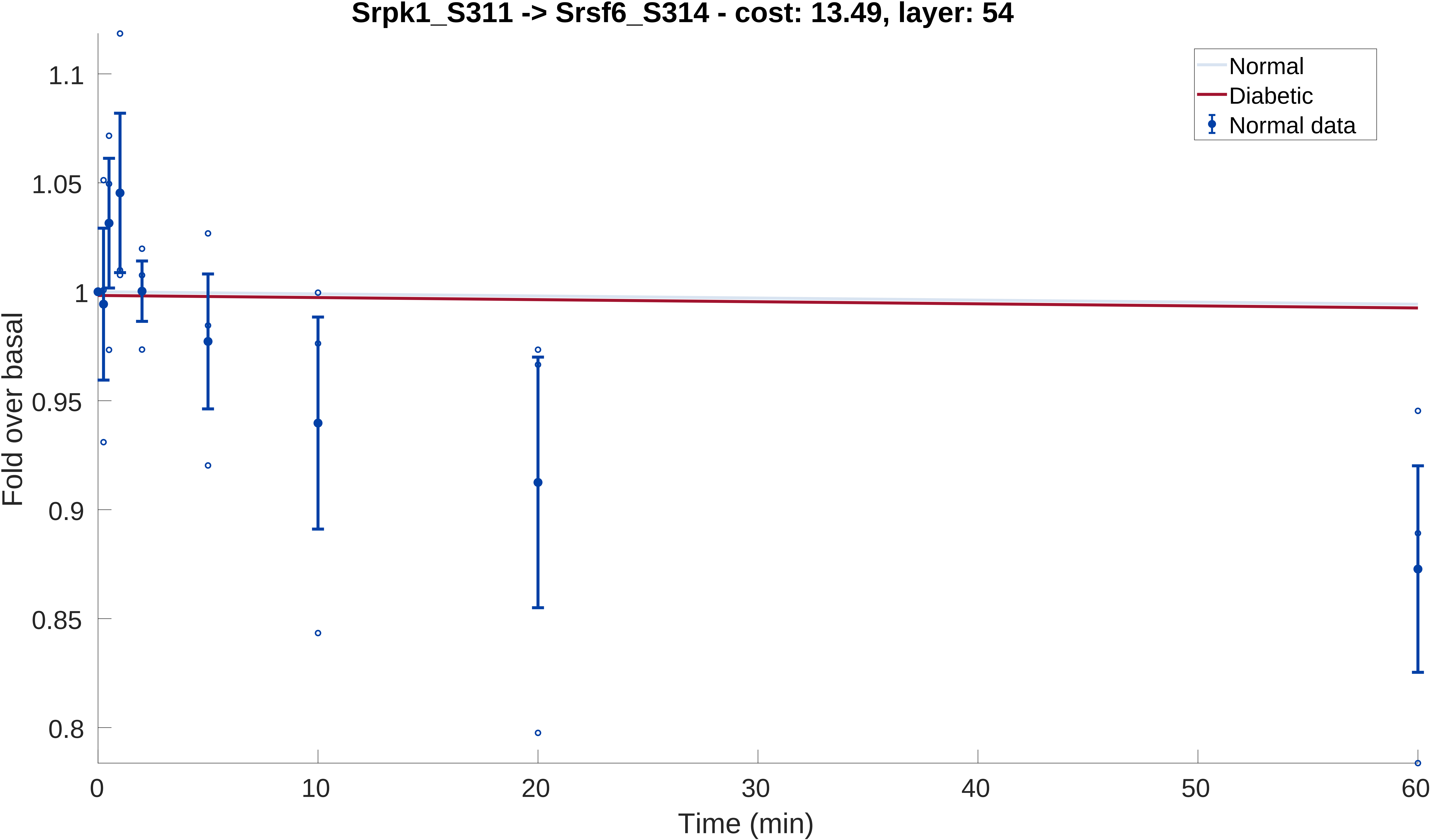

Srpk1_S311 -> Srsf6_S314 - cost: 13.49, layer: 54
Normal
Diabetic
Normal data
Fold over basal
Time (min)
1.1
1.05
1
0.95
0.9
0.85
0.8
0
10
20
30
40
50
60

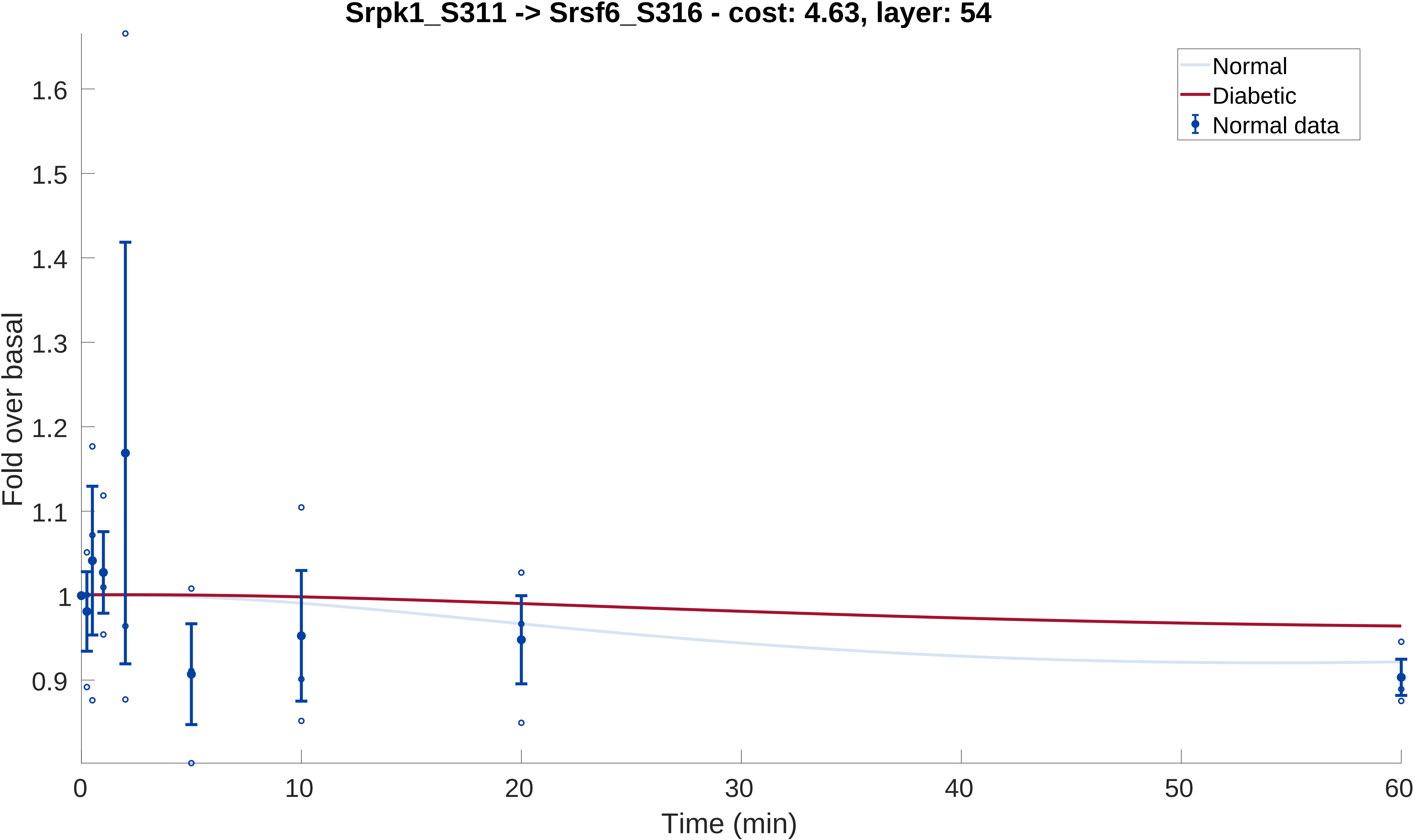

Srpk1_S311 -> Srsf6_S316 - cost: 4.63, layer: 54
Normal
Diabetic
Normal data
Fold over basal
1.6
1.5
1.4
1.3
1.2
1.1
1
0.9
0
10
20
30
40
50
60
Time (min)

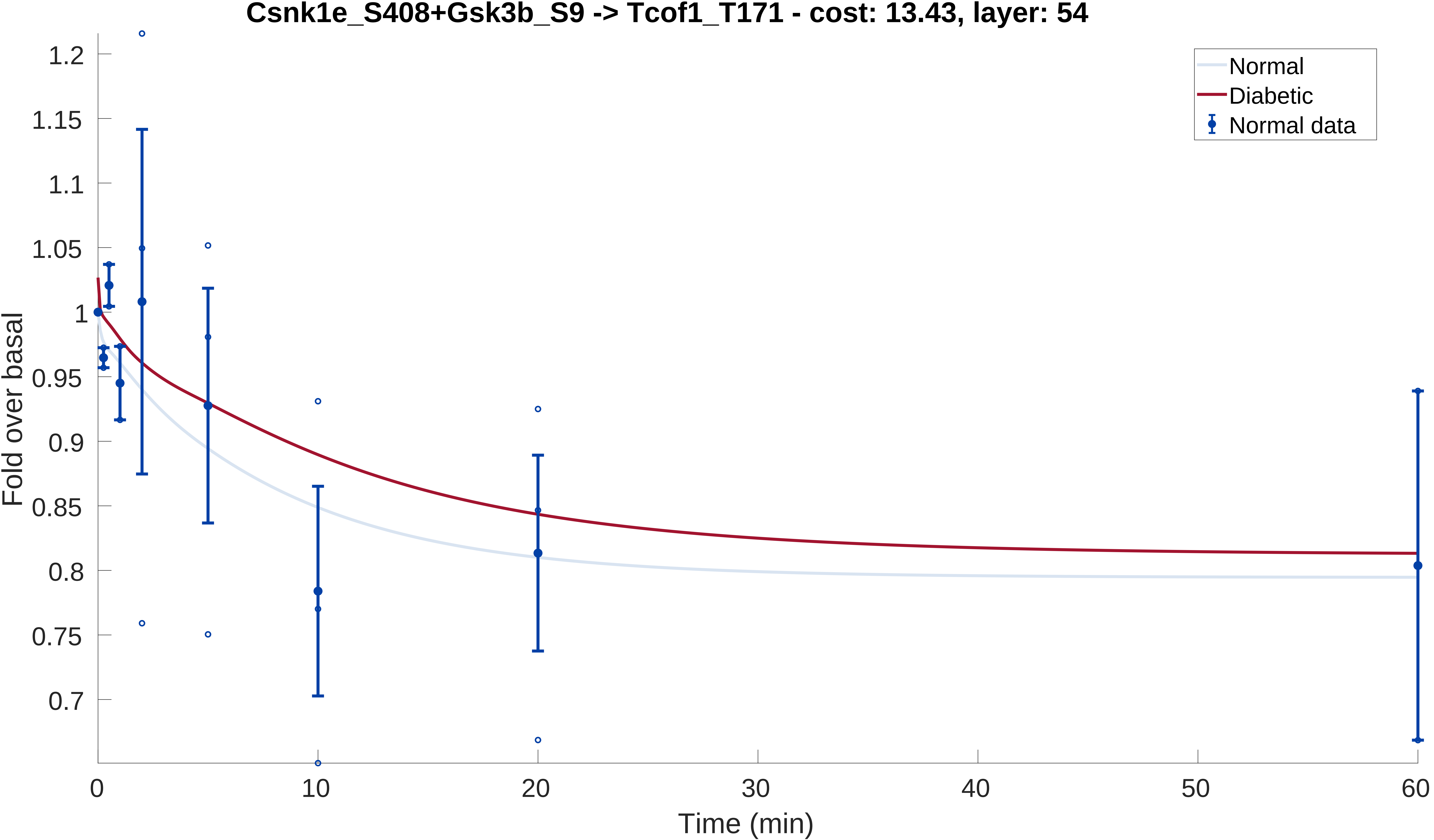
Csnk1e_S408+Gsk3b_S9 -> Tcof1_T171 - cost: 13.43, layer: 54
Normal
Diabetic
Normal data
Fold over basal
Time (min)
1.2
1.15
1.1
1.05
1
0.95
0.9
0.85
0.8
0.75
0.7
0
10
20
30
40
50
60

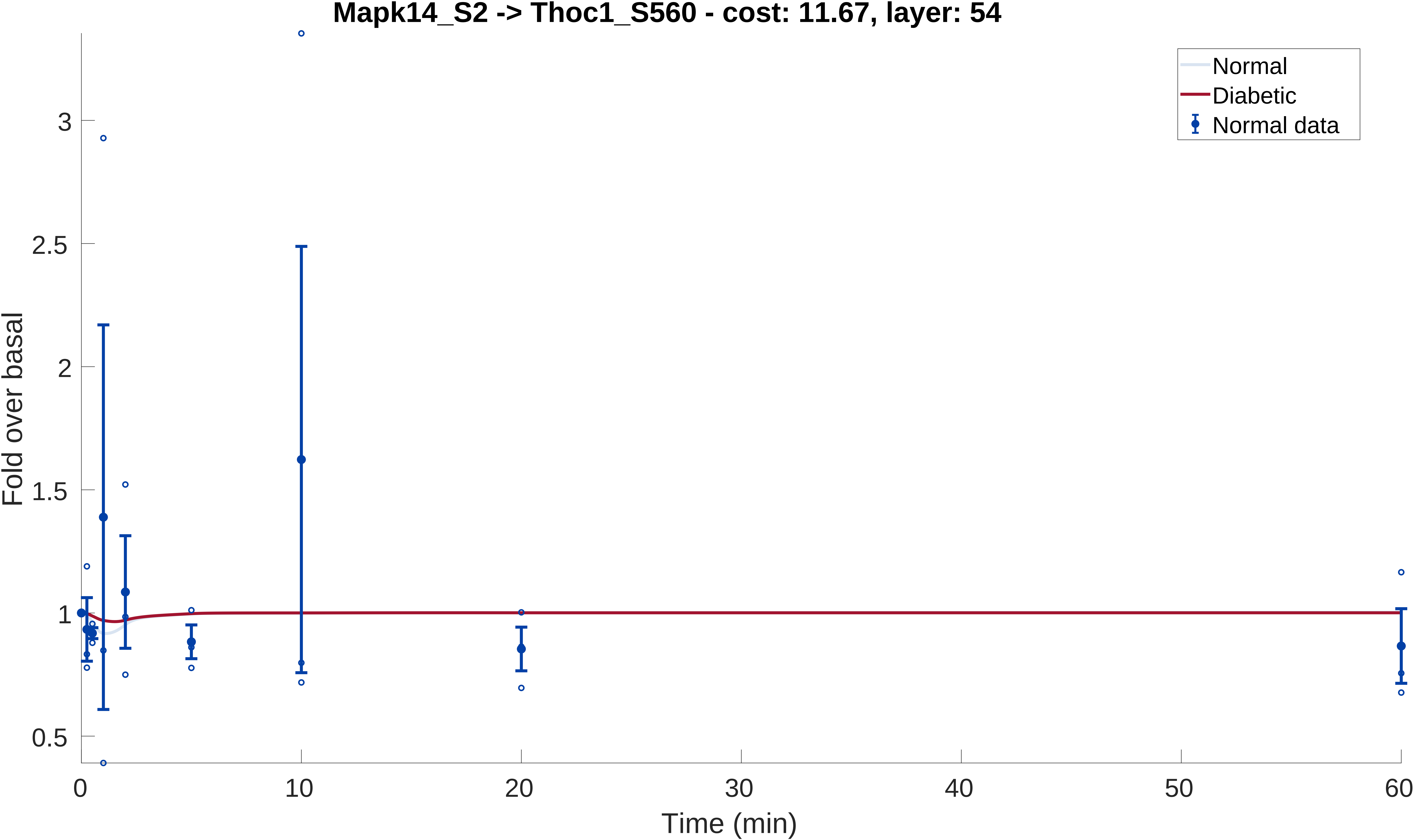

Mapk14_S2 -> Thoc1_S560 - cost: 11.67, layer: 54
Normal
Diabetic
Normal data
Fold over basal
Time (min)
3
2.5
2
1.5
1
0.5
0
10
20
30
40
50
60

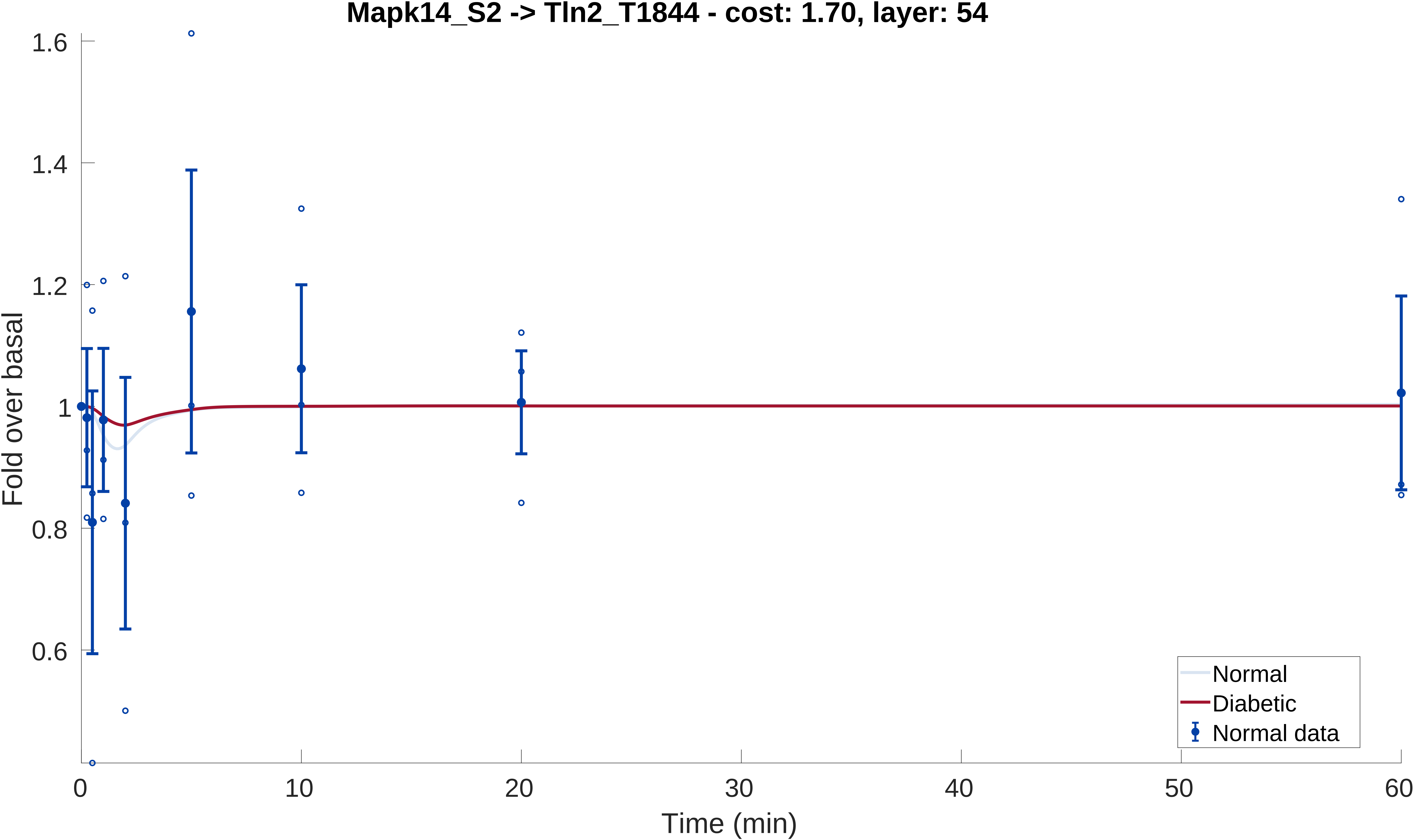
Mapk14_S2 -> Tln2_T1844 - cost: 1.70, layer: 54
Fold over basal
1.6
1.4
1.2
1
0.8
0.6
0
10
20
30
40
50
60
Time (min)
Normal
Diabetic
Normal data

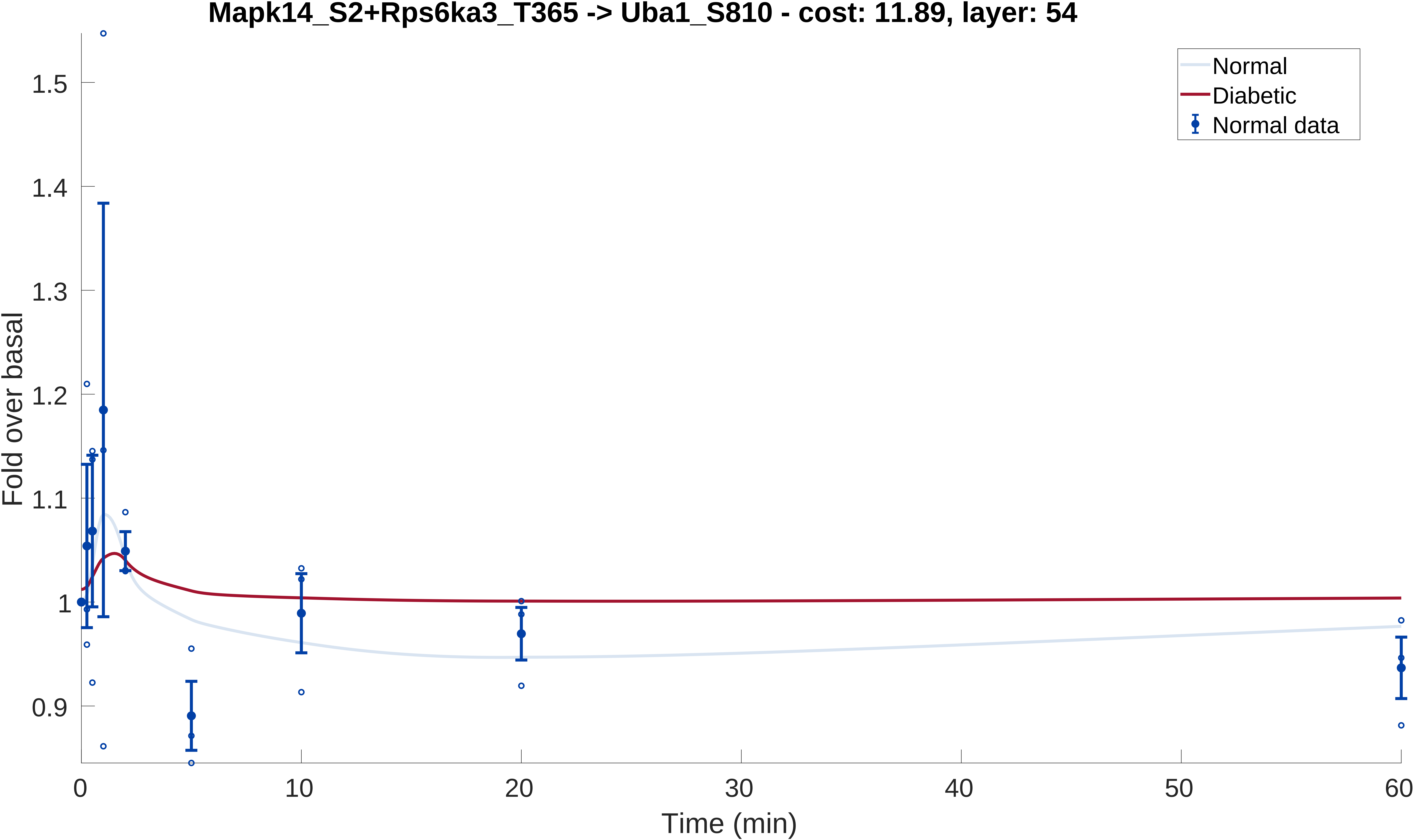
Mapk14_S2+Rps6ka3_T365 -> Uba1_S810 - cost: 11.89, layer: 54
Normal
Diabetic
Normal data
Fold over basal
1.5
1.4
1.3
1.2
1.1
1
0.9
0
10
20
30
40
50
60
Time (min)

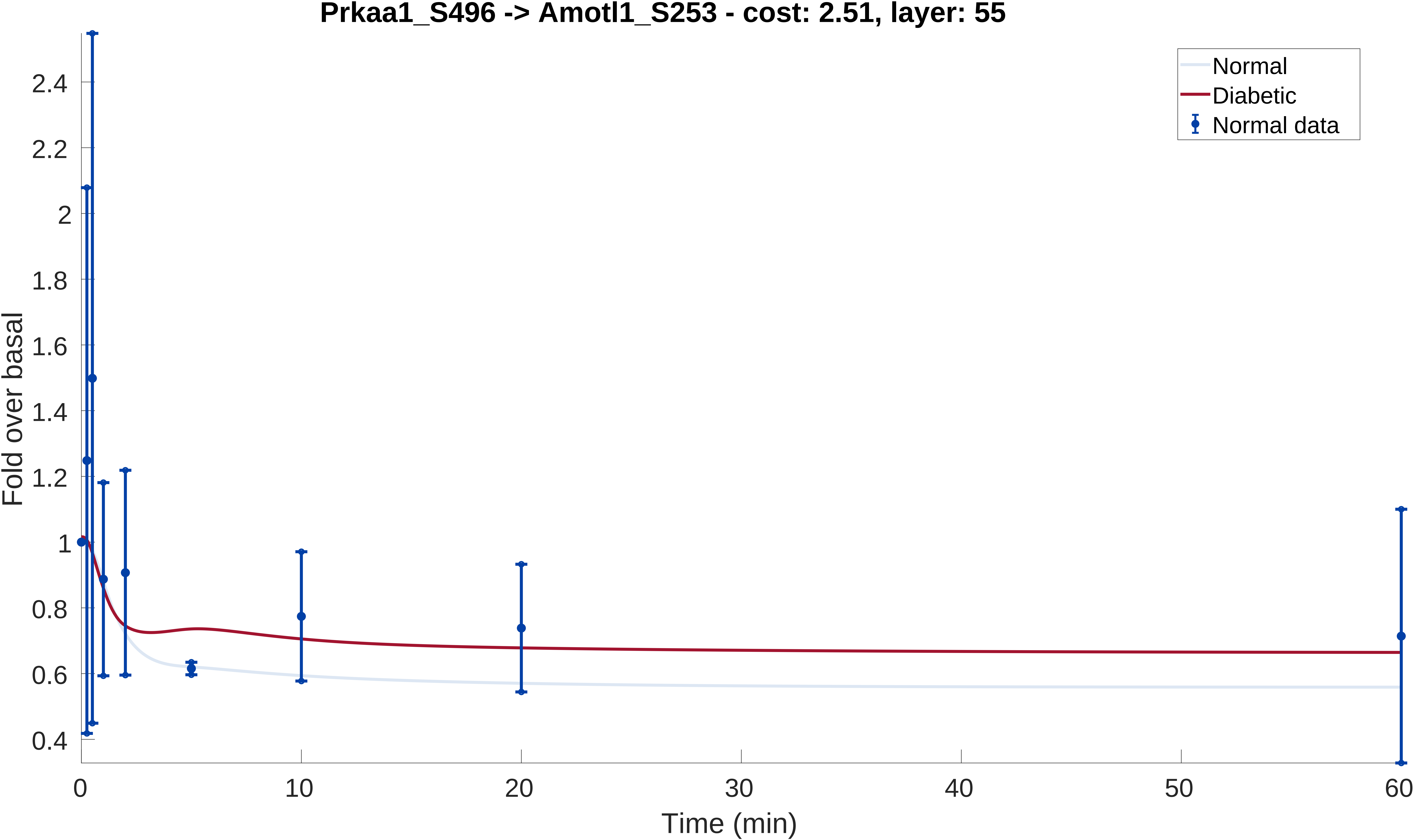

Prkaa1_S496 -> Amotl1_S253 - cost: 2.51, layer: 55
Normal
Diabetic
Normal data
Fold over basal
2.4
2.2
2
1.8
1.6
1.4
1.2
1
0.8
0.6
0.4
0
10
20
30
40
50
60
Time (min)

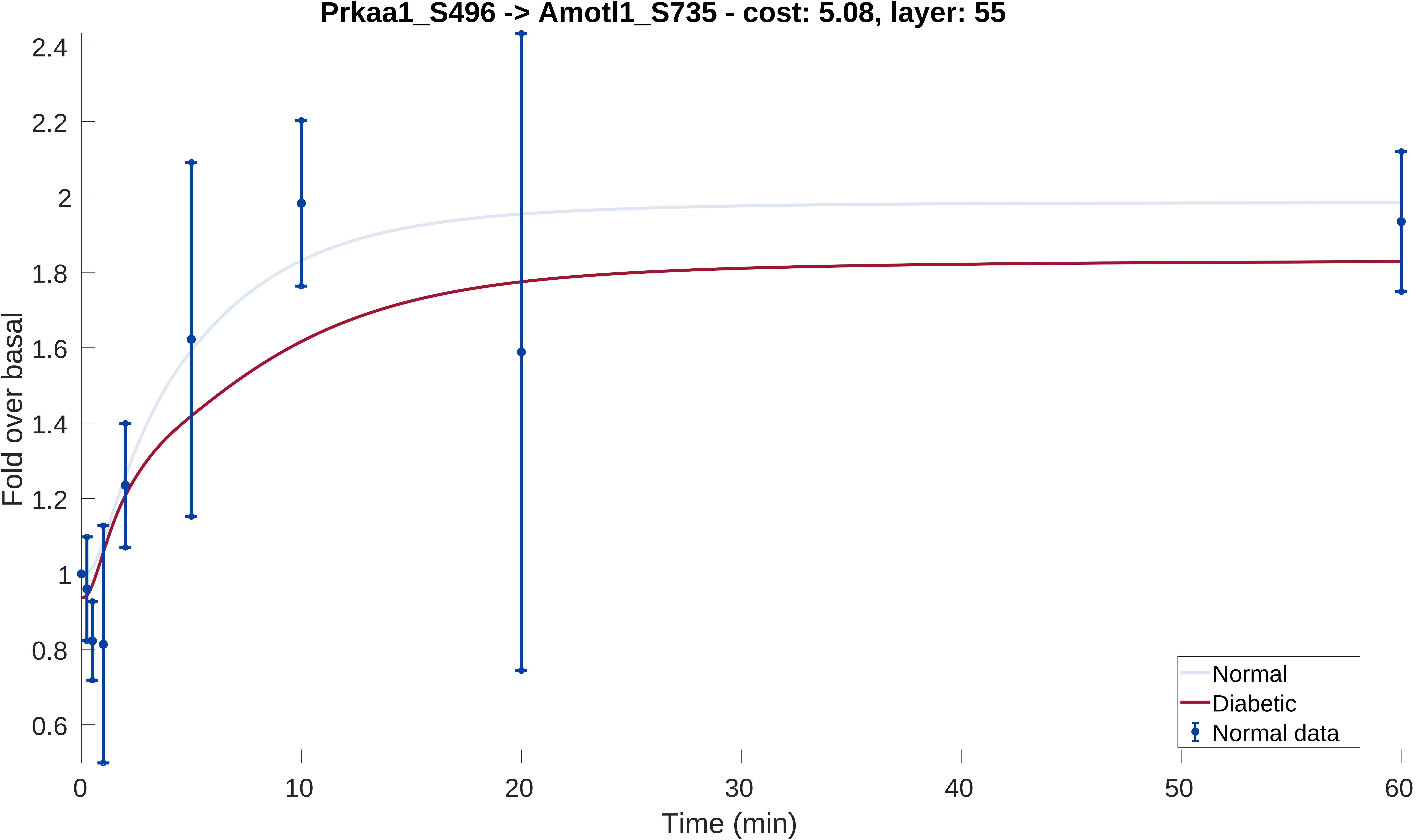
Prkaa1_S496 -> Amotl1_S735 - cost: 5.08, layer: 55
Fold over basal
2.4
2.2
2
1.8
1.6
1.4
1.2
1
0.8
0.6
0
10
20
30
40
50
60
Time (min)
Normal
Diabetic
Normal data

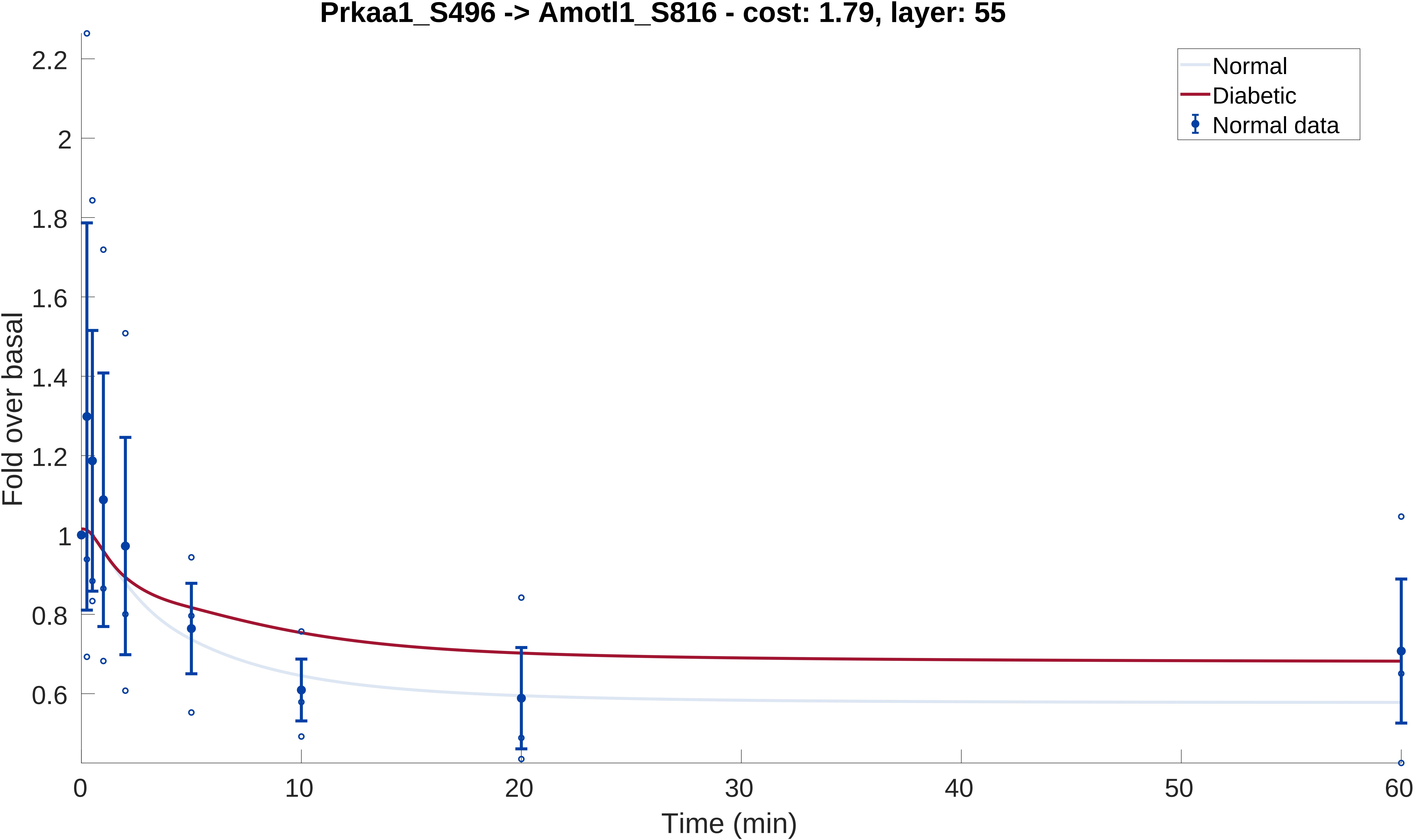
Prkaa1_S496 -> Amotl1_S816 - cost: 1.79, layer: 55
Normal
Diabetic
Normal data
Fold over basal
Time (min)
0.6
0.8
1
1.2
1.4
1.6
1.8
2
2.2
0
10
20
30
40
50
60

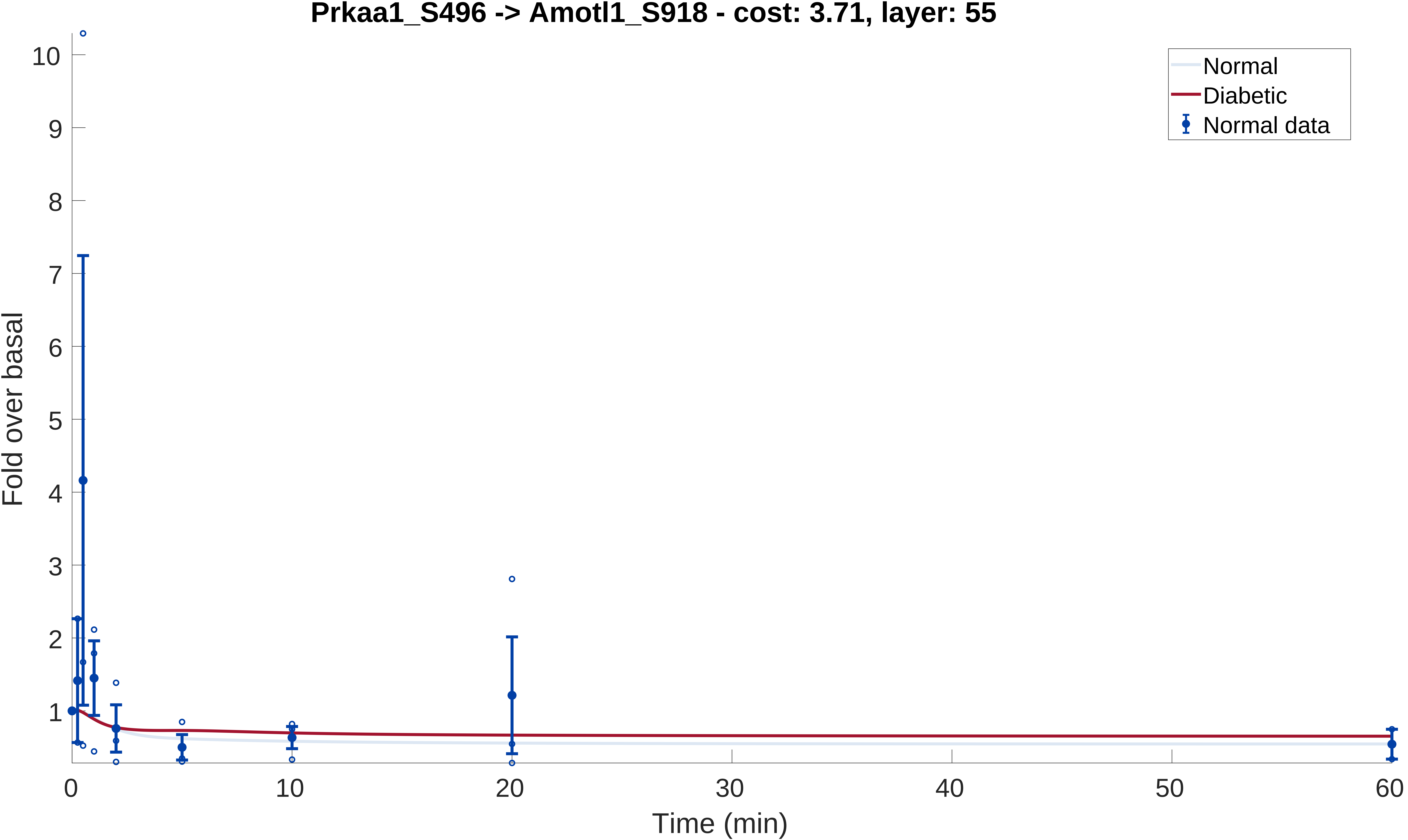

Prkaa1_S496 -> Amotl1_S918 - cost: 3.71, layer: 55
Normal
Diabetic
Normal data
Fold over basal
Time (min)
10
9
8
7
6
5
4
3
2
1
0
10
20
30
40
50
60

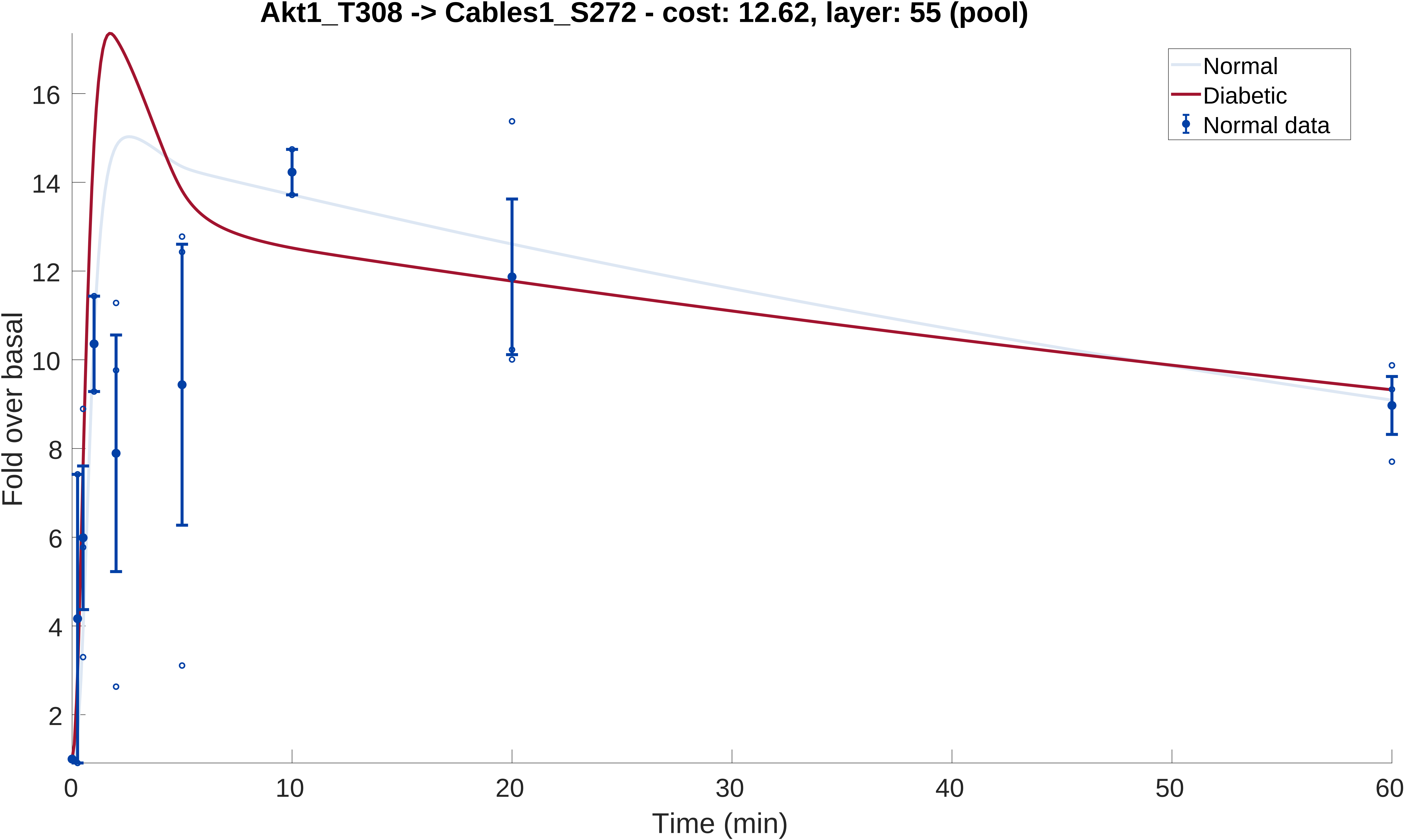

Akt1_T308 -> Cables1_S272 - cost: 12.62, layer: 55 (pool)
Normal
Diabetic
Normal data
Fold over basal
Time (min)
16
14
12
10
8
6
4
2
0
10
20
30
40
50
60

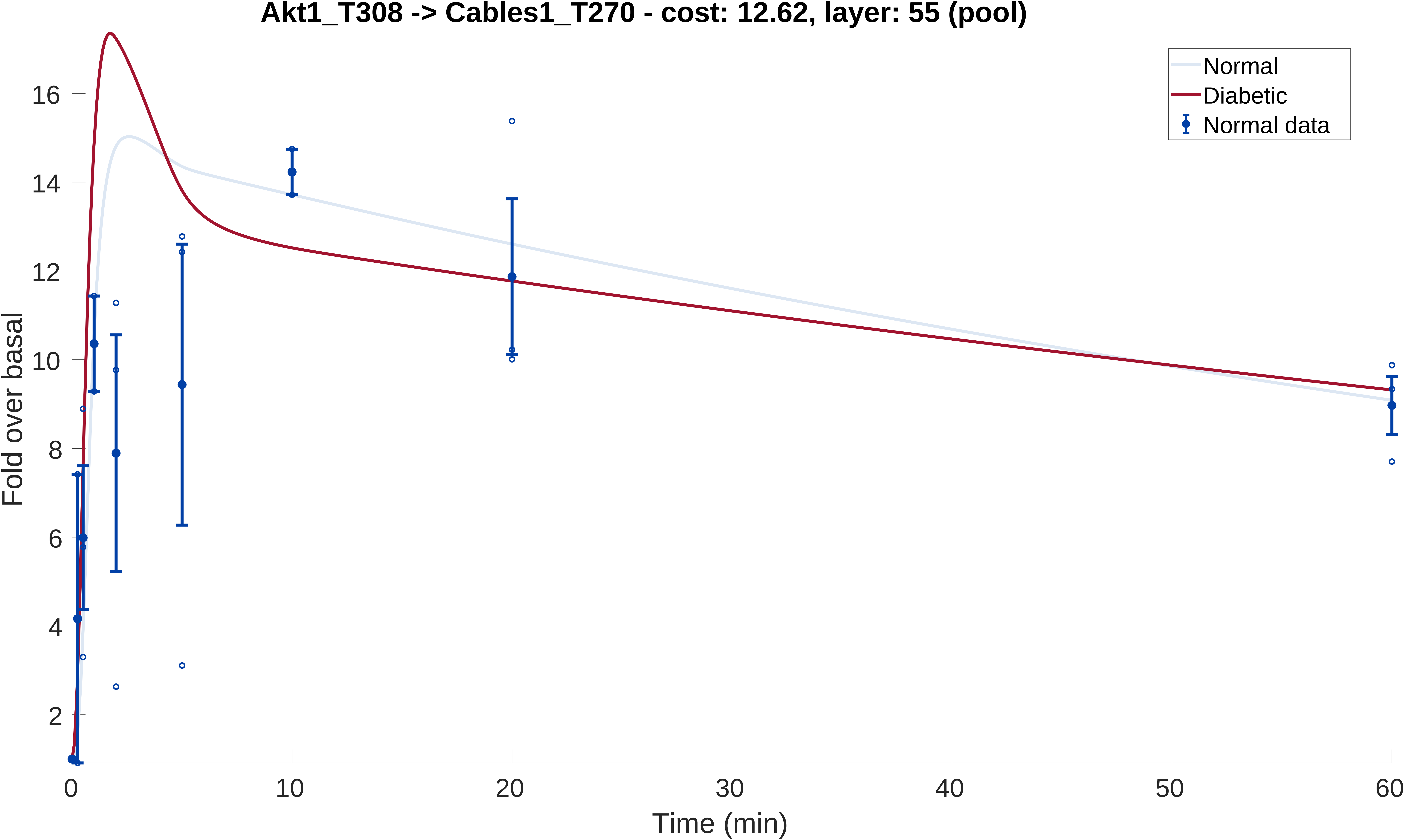

Akt1_T308 -> Cables1_T270 - cost: 12.62, layer: 55 (pool)
Fold over basal
Time (min)
Normal
Diabetic
Normal data
0
10
20
30
40
50
60
2
4
6
8
10
12
14
16

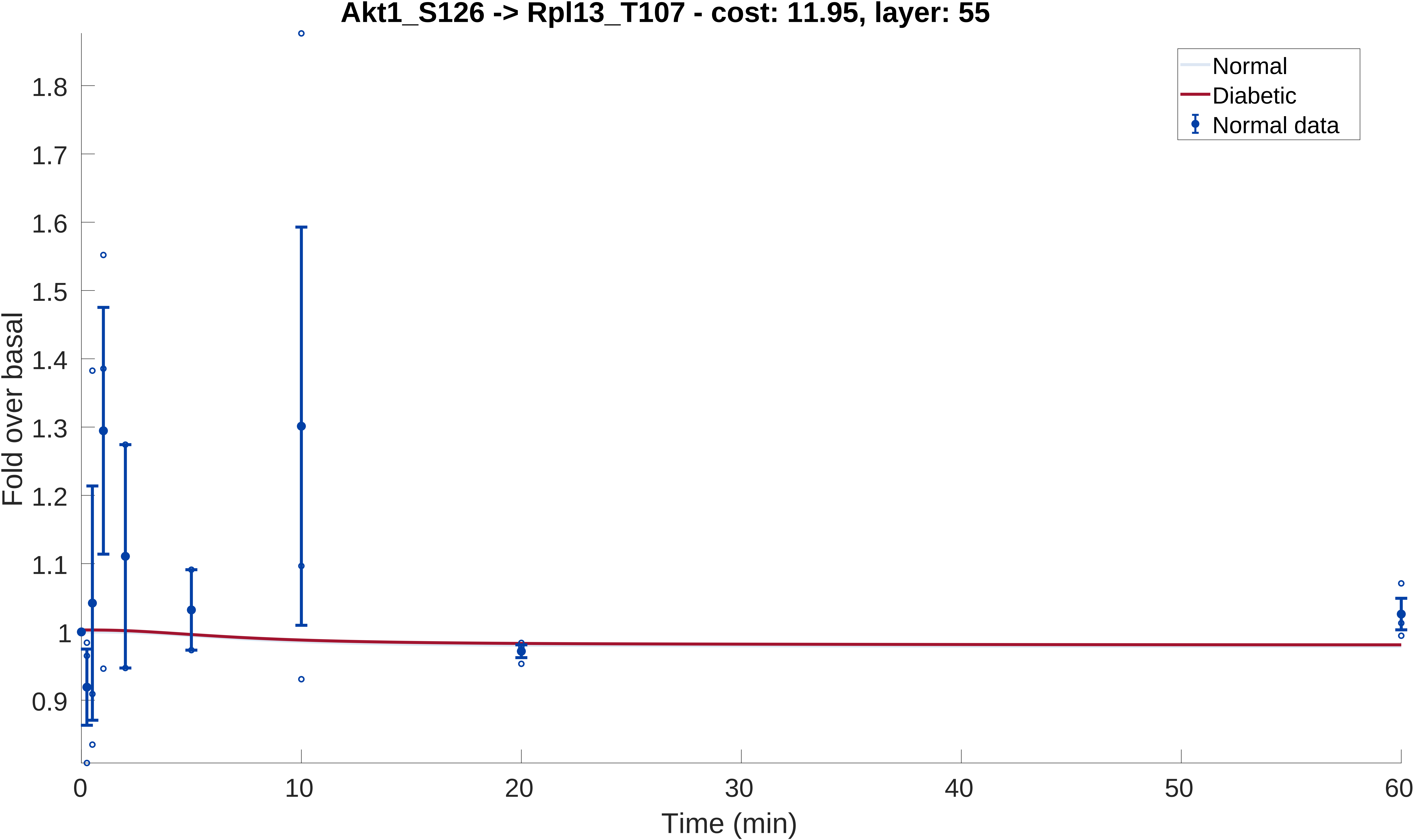

Akt1_S126 -> Rpl13_T107 - cost: 11.95, layer: 55
Normal
Diabetic
Normal data
Fold over basal
Time (min)
1.8
1.7
1.6
1.5
1.4
1.3
1.2
1.1
1
0.9
0
10
20
30
40
50
60

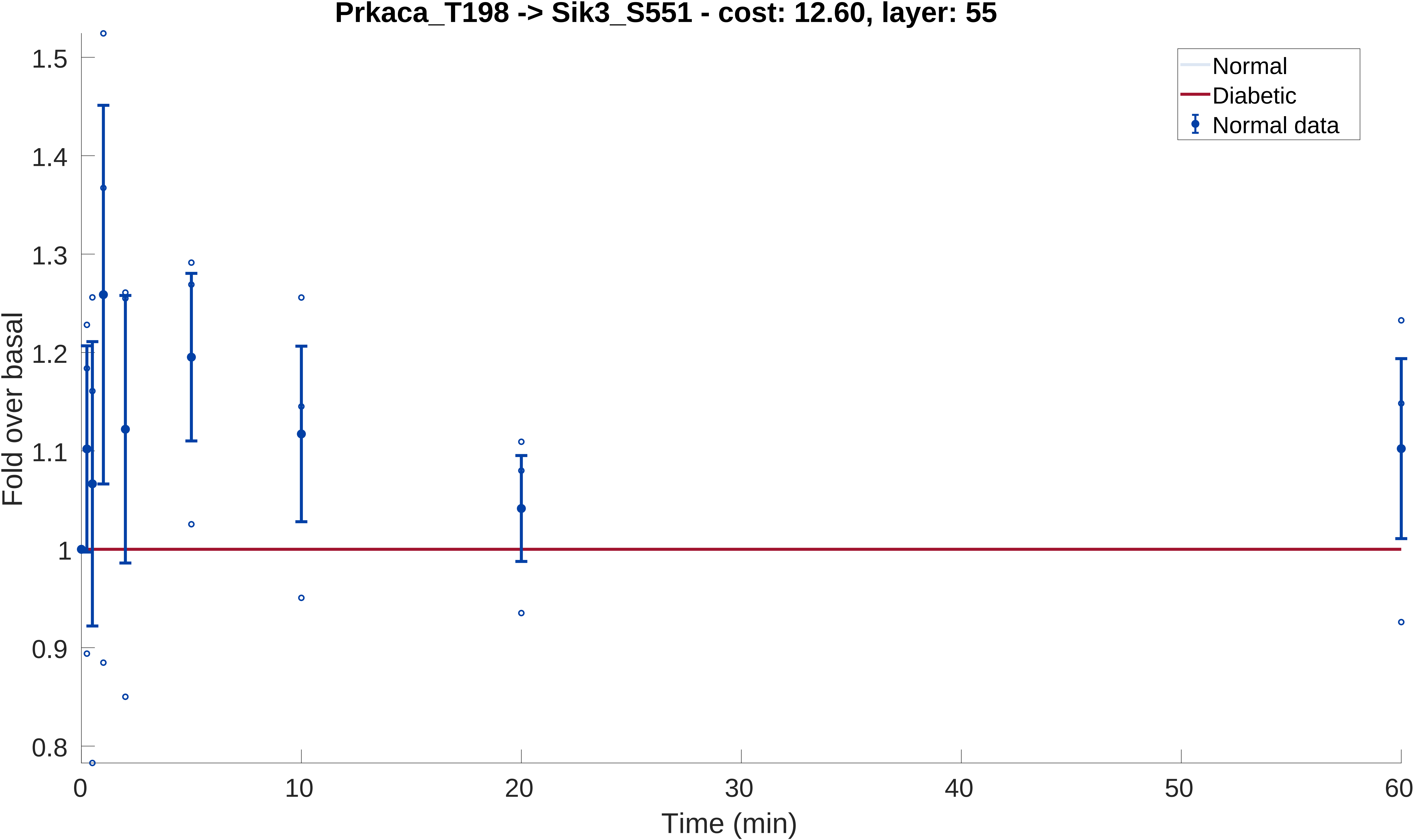

Prrkaca_T198 -> Sik3_S551 - cost: 12.60, layer: 55
Normal
Diabetic
Normal data
Fold over basal
Time (min)
0.8
0.9
1
1.1
1.2
1.3
1.4
1.5
0
10
20
30
40
50
60

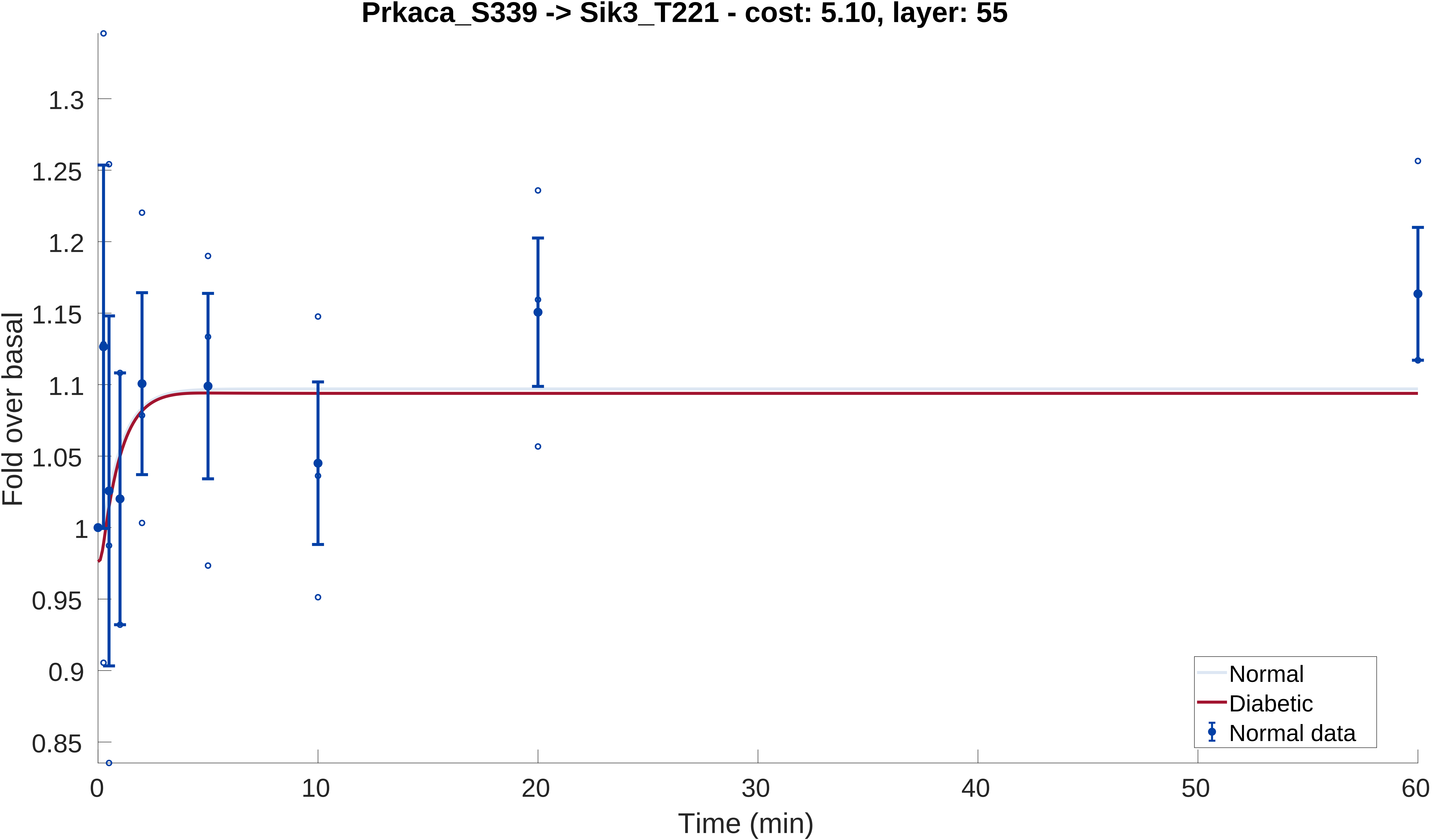

Prrkaca_S339 -> Sik3_T221 - cost: 5.10, layer: 55
Fold over basal
1.3
1.25
1.2
1.15
1.1
1.05
1
0.95
0.9
0.85
0
10
20
30
40
50
60
Time (min)
Normal
Diabetic
Normal data

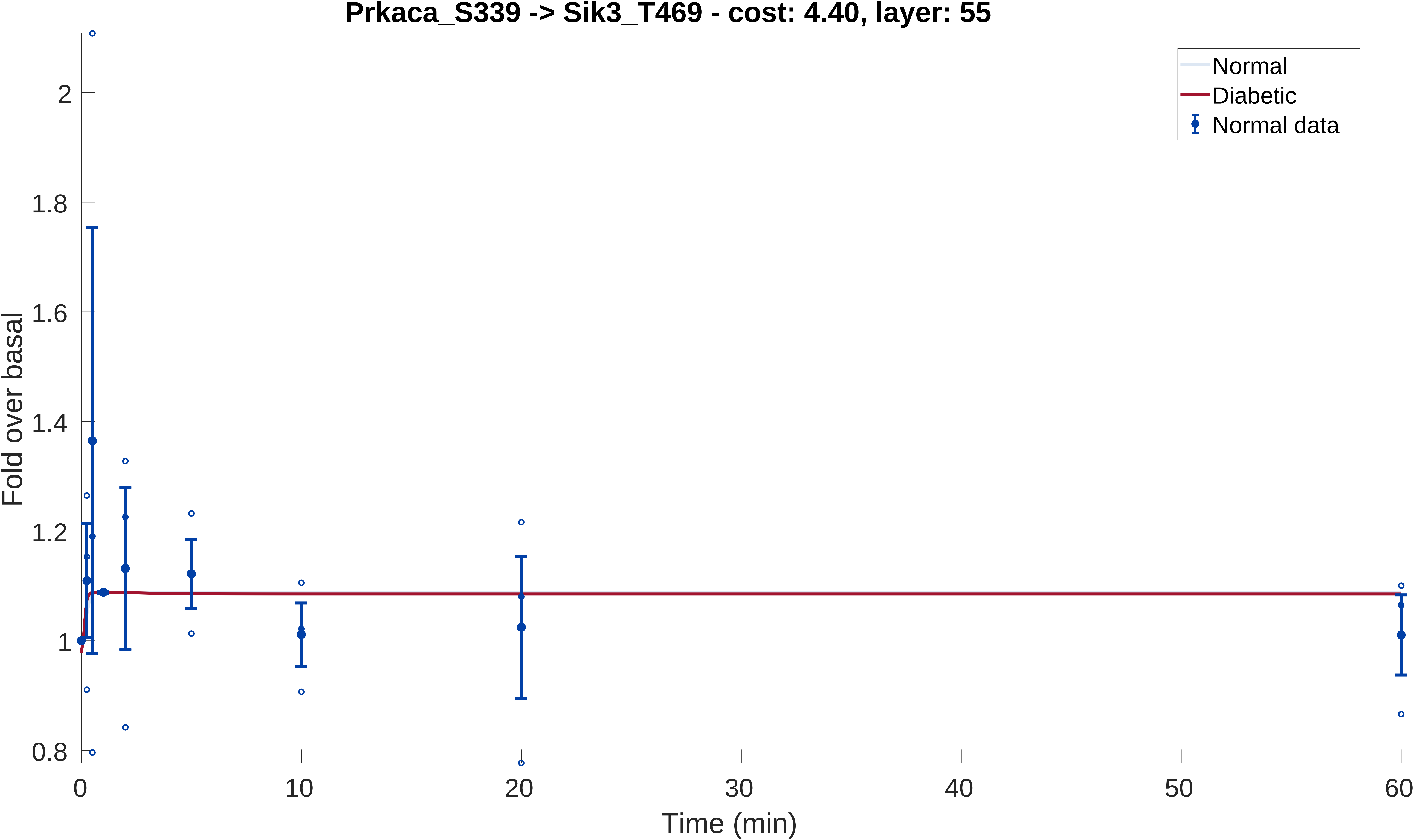

Prrkaca_S339 -> Sik3_T469 - cost: 4.40, layer: 55
Normal
Diabetic
Normal data
Fold over basal
2
1.8
1.6
1.4
1.2
1
0.8
0
10
20
30
40
50
60
Time (min)

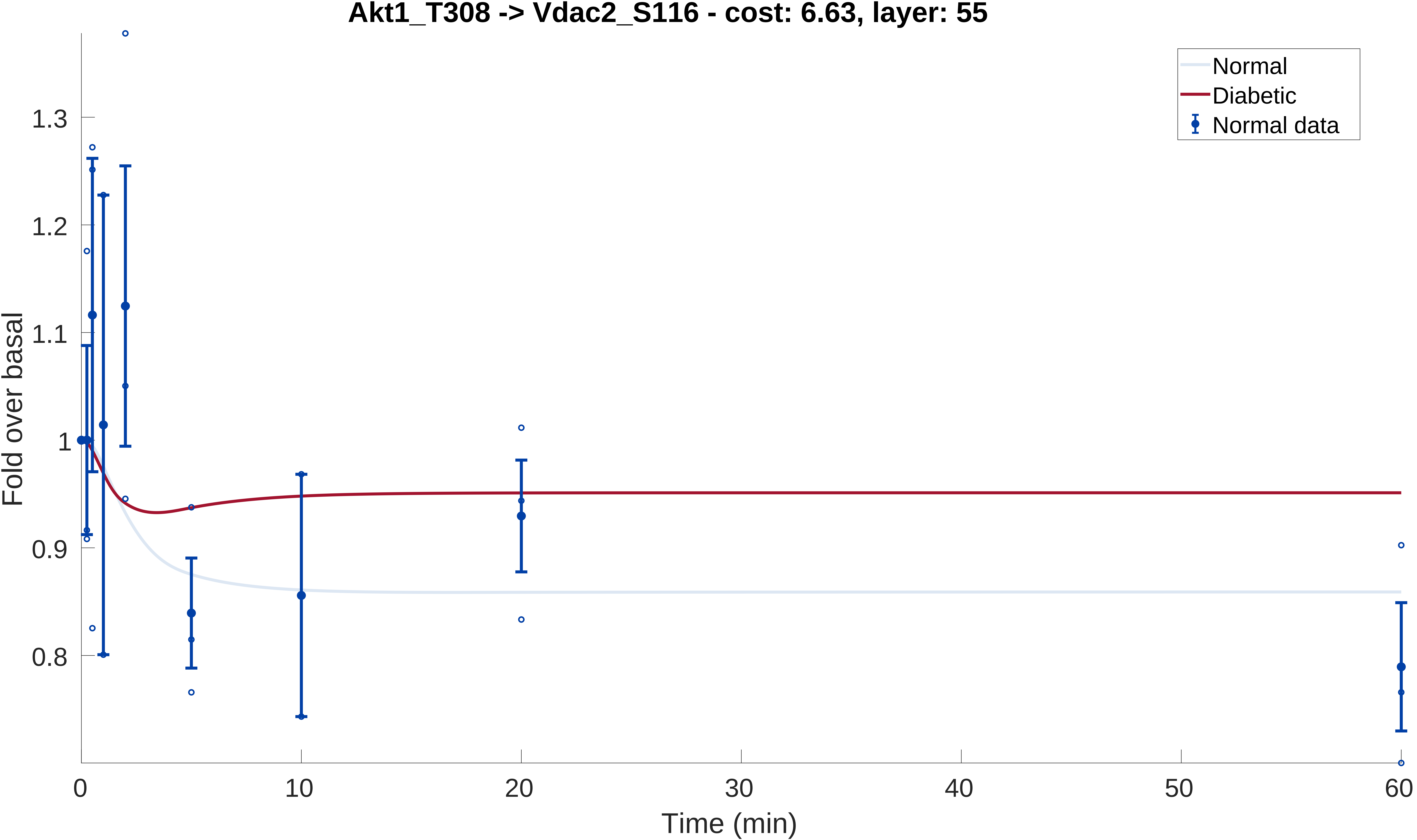
Akt1_T308 -> Vdac2_S116 - cost: 6.63, layer: 55
Normal
Diabetic
Normal data
Fold over basal
1.3
1.2
1.1
1
0.9
0.8
0
10
20
30
40
50
60
Time (min)

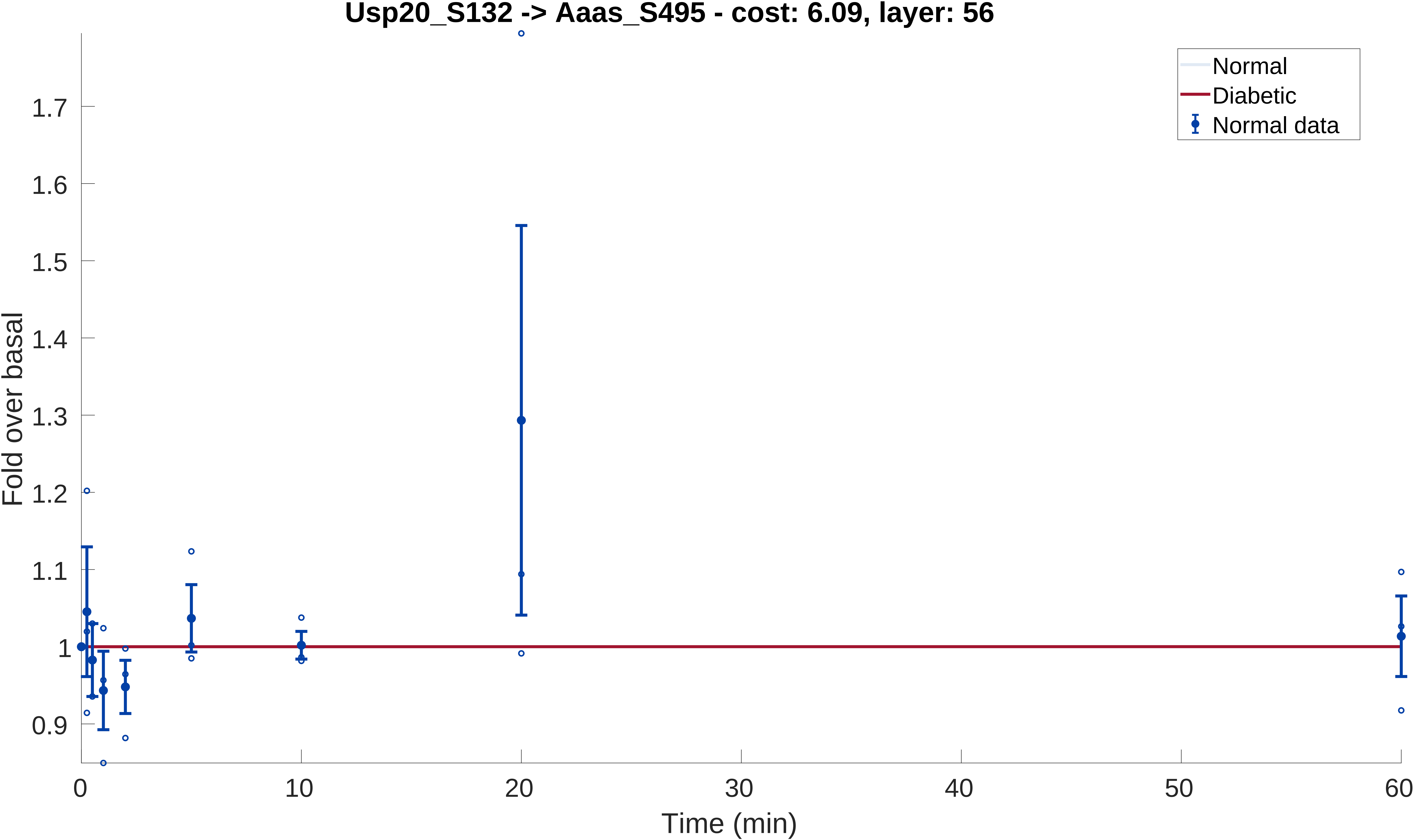

Usp20_S132 -> Aaas_S495 - cost: 6.09, layer: 56
Normal
Diabetic
Normal data
Fold over basal
Time (min)
0.9
1
1.1
1.2
1.3
1.4
1.5
1.6
1.7
0
10
20
30
40
50
60

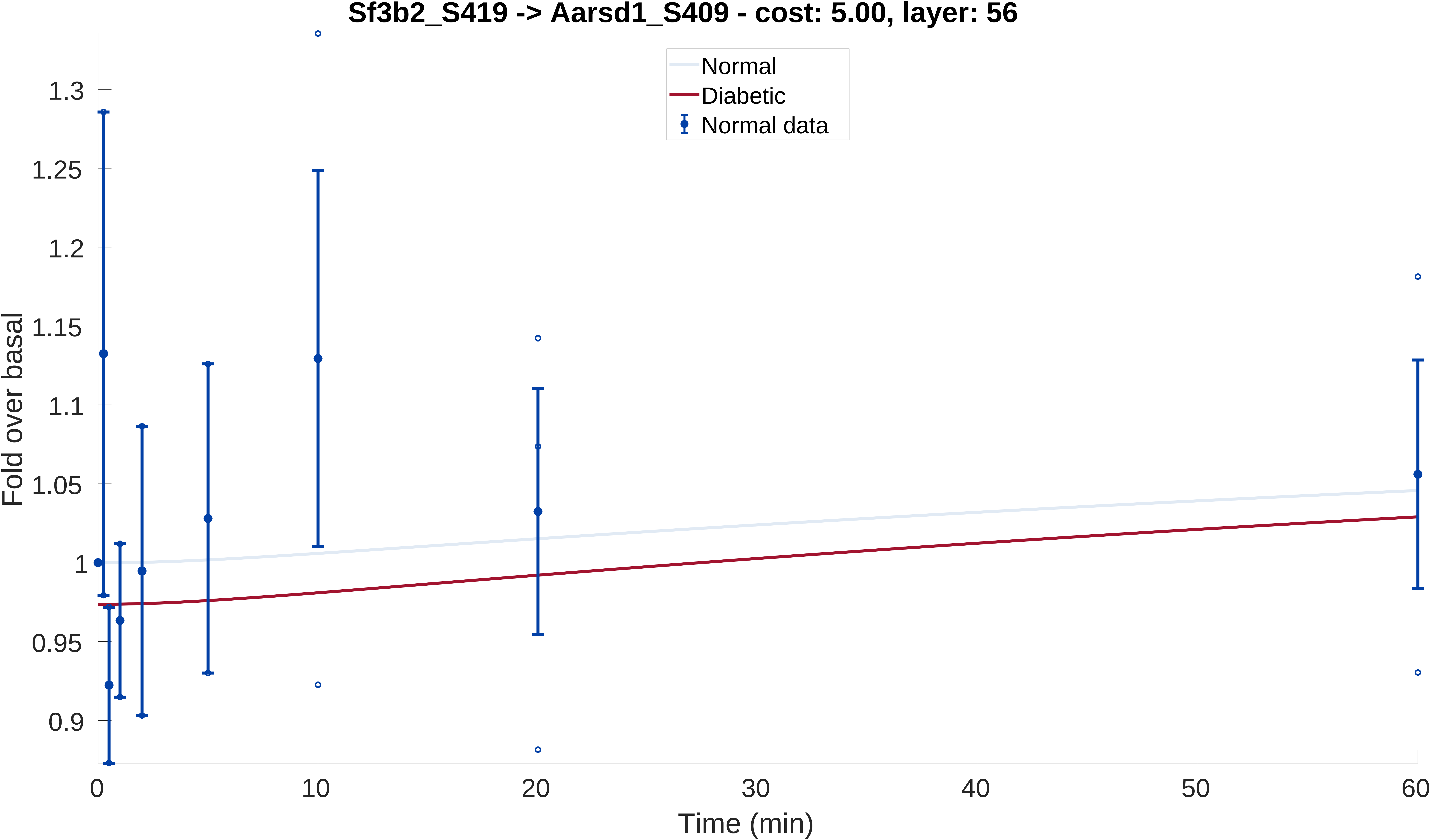

Sf3b2_S419 -> Aarsd1_S409 - cost: 5.00, layer: 56
Normal
Diabetic
Normal data
Fold over basal
1.3
1.25
1.2
1.15
1.1
1.05
1
0.95
0.9
0
10
20
30
40
50
60
Time (min)

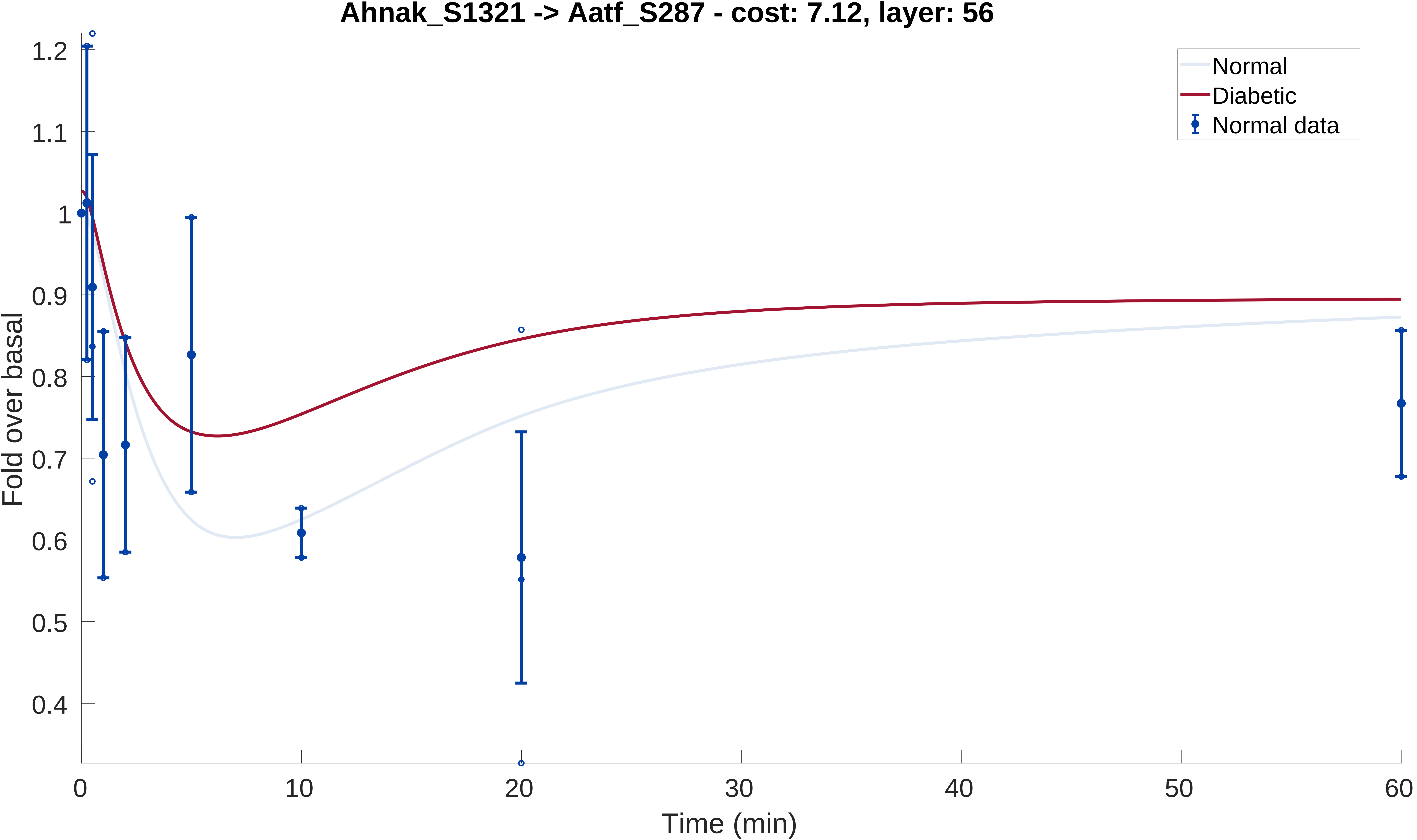

Ahnak_S1321 -> Aatf_S287 - cost: 7.12, layer: 56
Normal
Diabetic
Normal data
Fold over basal
Time (min)
1.2
1.1
1
0.9
0.8
0.7
0.6
0.5
0.4
0
10
20
30
40
50
60

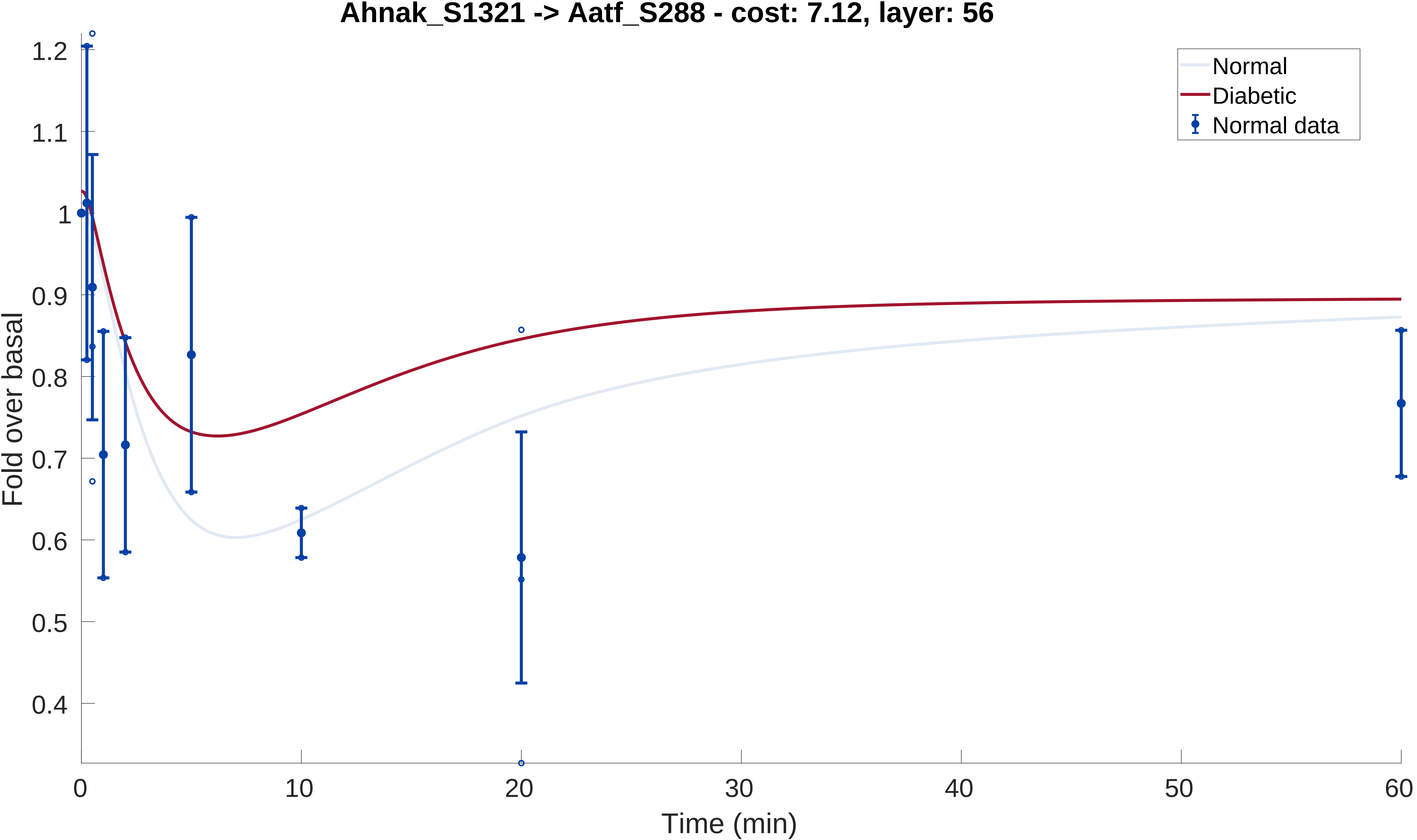

Ahnak_S1321 -> Aatf_S288 - cost: 7.12, layer: 56
Normal
Diabetic
Normal data
Fold over basal
Time (min)
0
10
20
30
40
50
60
0.4
0.5
0.6
0.7
0.8
0.9
1
1.1
1.2

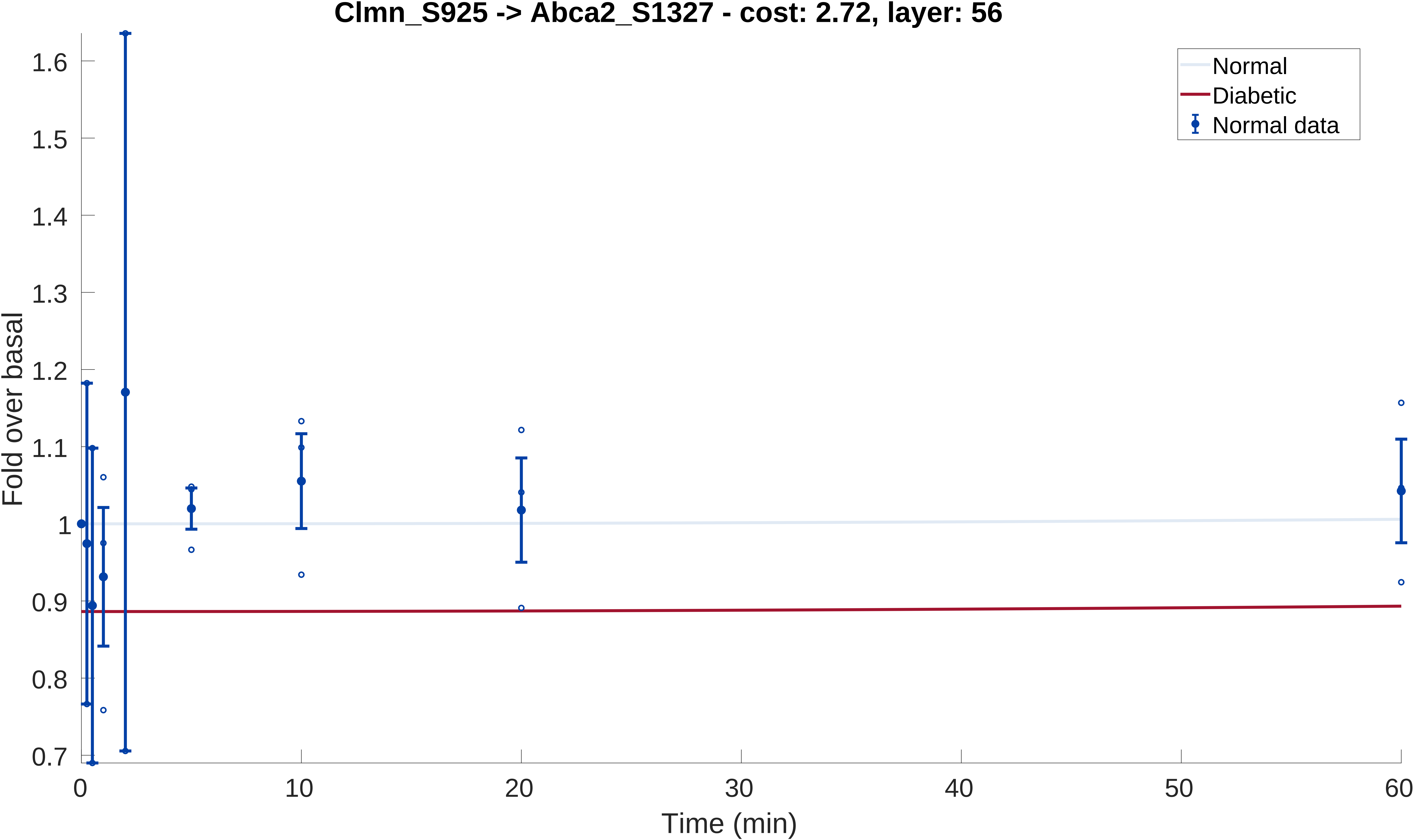

Clmn_S925 -> Abca2_S1327 - cost: 2.72, layer: 56
Normal
Diabetic
Normal data
Fold over basal
Time (min)
1.6
1.5
1.4
1.3
1.2
1.1
1
0.9
0.8
0.7
0
10
20
30
40
50
60

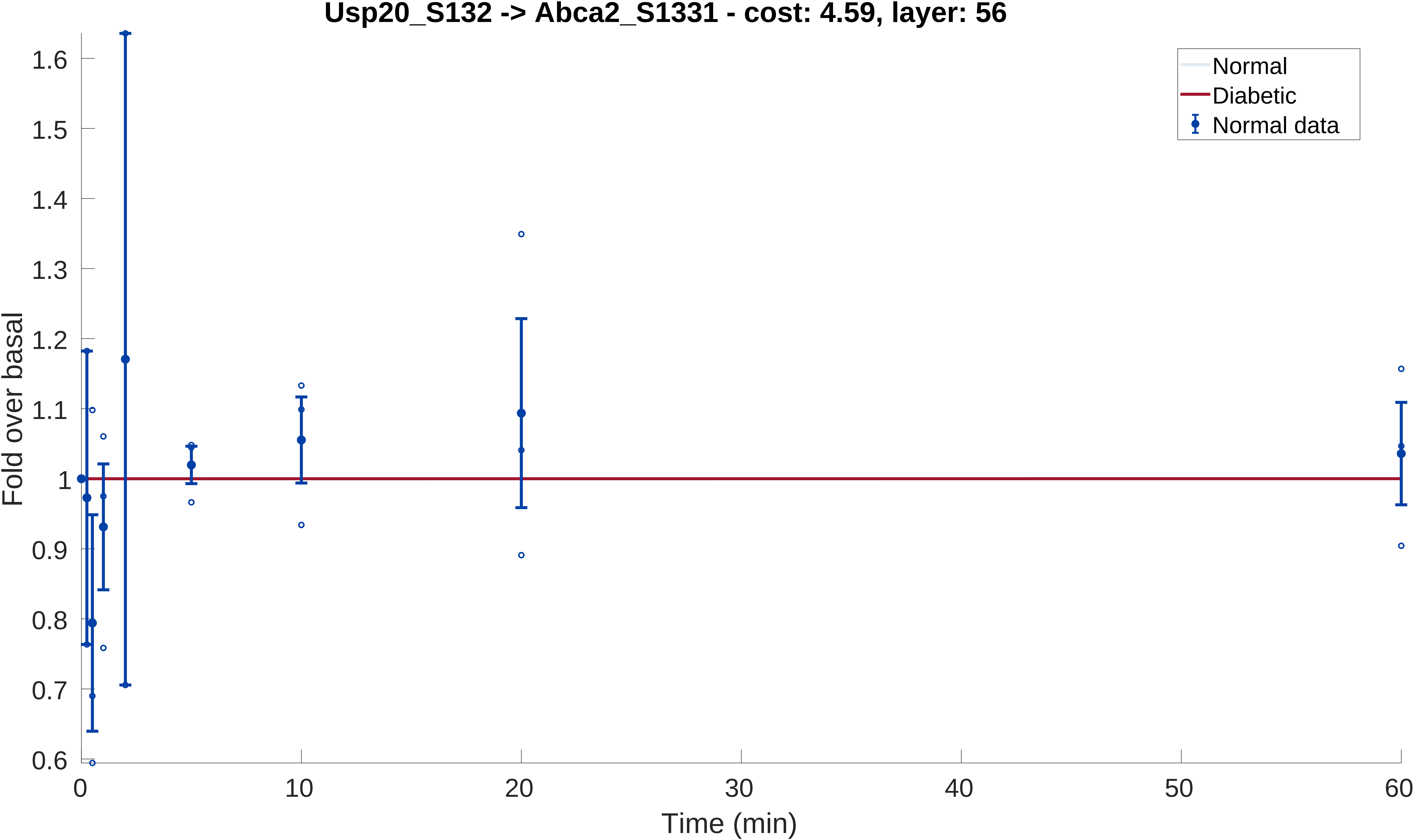

Usp20_S132 -> Abca2_S1331 - cost: 4.59, layer: 56
Normal
Diabetic
Normal data
Fold over basal
Time (min)
0.6
0.7
0.8
0.9
1
1.1
1.2
1.3
1.4
1.5
1.6
0
10
20
30
40
50
60

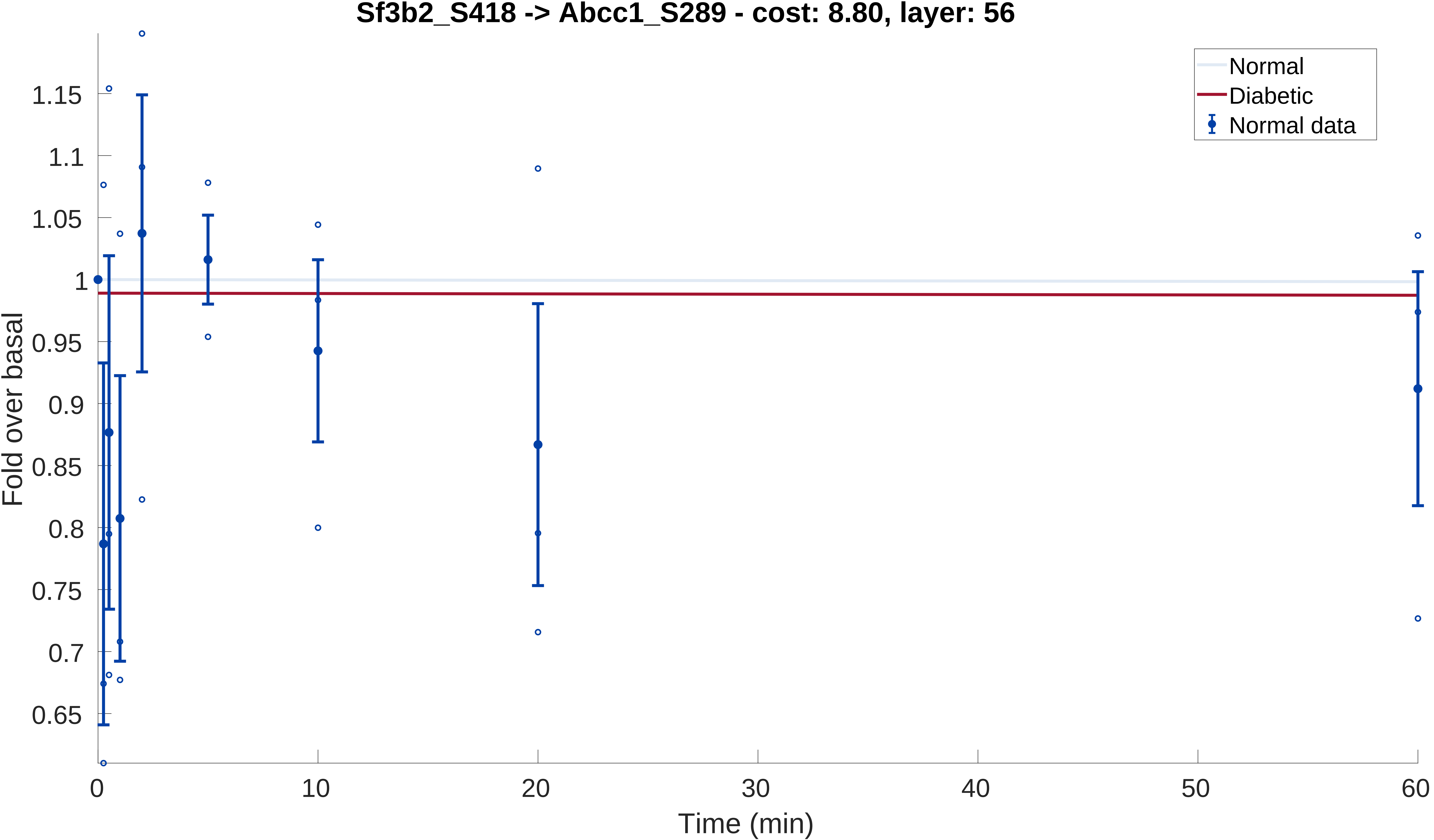

Sf3b2_S418 -> Abcc1_S289 - cost: 8.80, layer: 56
Normal
Diabetic
Normal data
1.15
1.1
1.05
1
0.95
0.9
0.85
0.8
0.75
0.7
0.65
Fold over basal
0
10
20
30
40
50
60
Time (min)

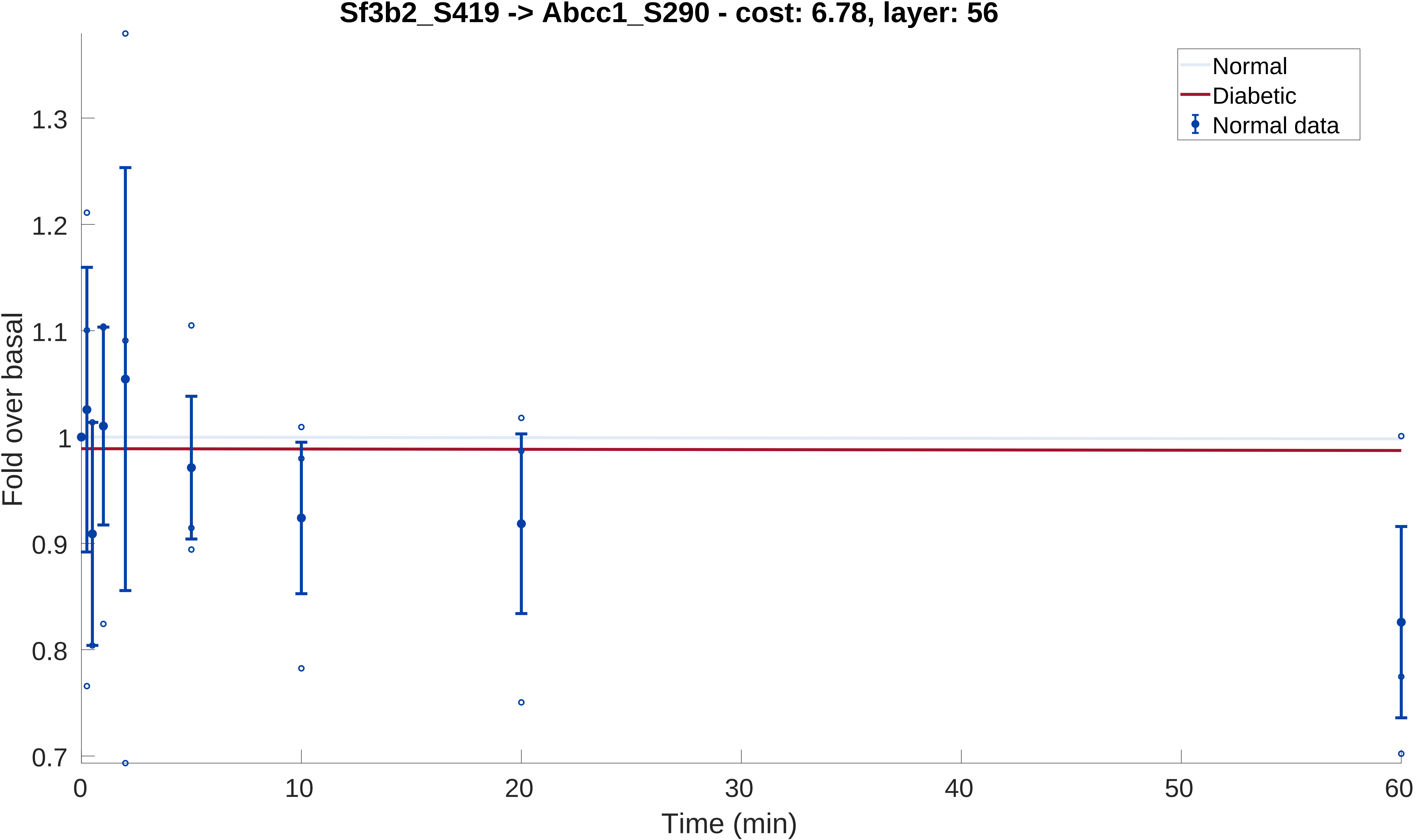
Sf3b2_S419 -> Abcc1_S290 - cost: 6.78, layer: 56
Normal
Diabetic
Normal data
Fold over basal
Time (min)
0.7
0.8
0.9
1
1.1
1.2
1.3
0
10
20
30
40
50
60

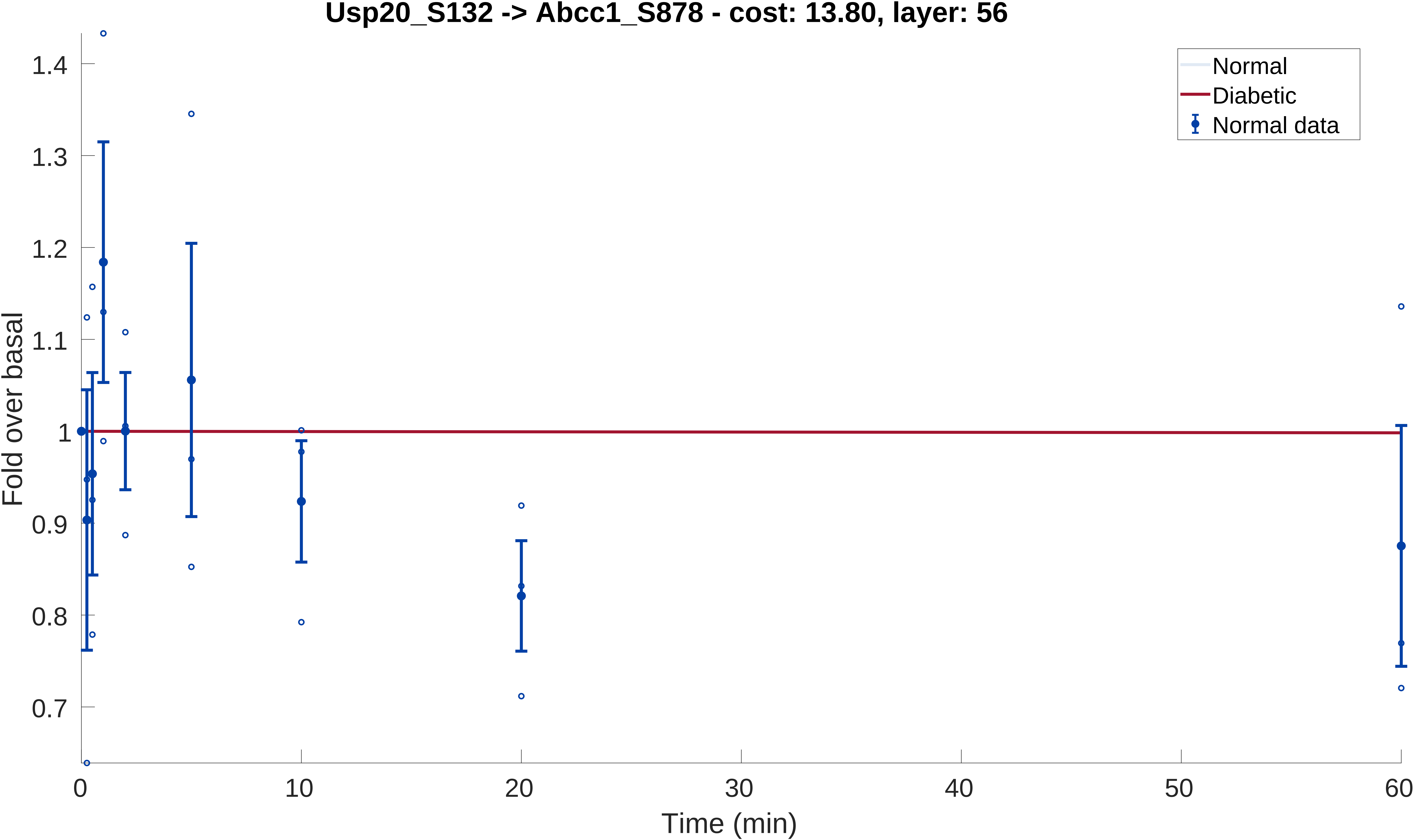

Usp20_S132 -> Abcc1_S878 - cost: 13.80, layer: 56
Normal
Diabetic
Normal data
Fold over basal
Time (min)
1.4
1.3
1.2
1.1
1
0.9
0.8
0.7
0
10
20
30
40
50
60

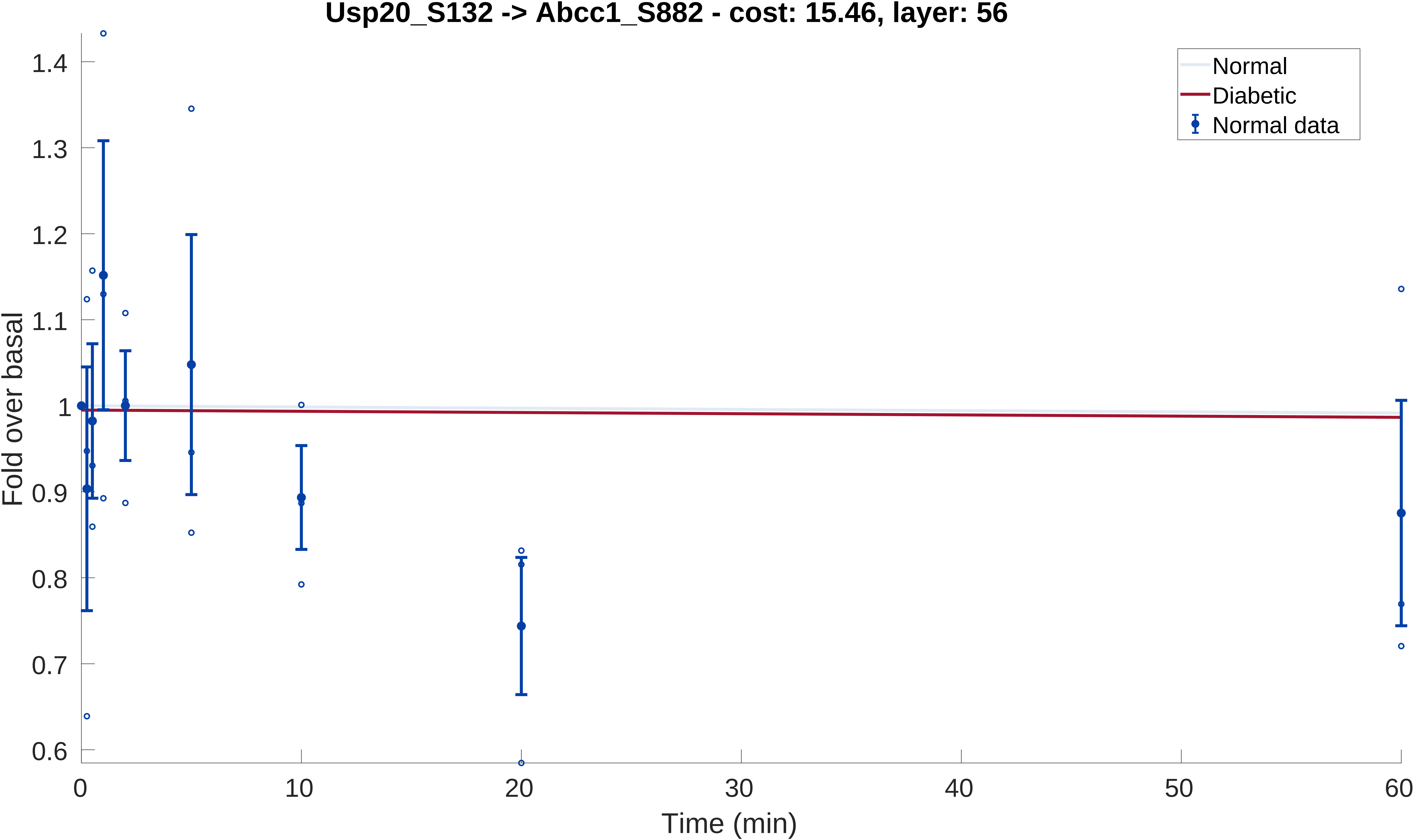

Usp20_S132 -> Abcc1_S882 - cost: 15.46, layer: 56
Normal
Diabetic
Normal data
Fold over basal
Time (min)
1.4
1.3
1.2
1.1
1
0.9
0.8
0.7
0.6
0
10
20
30
40
50
60

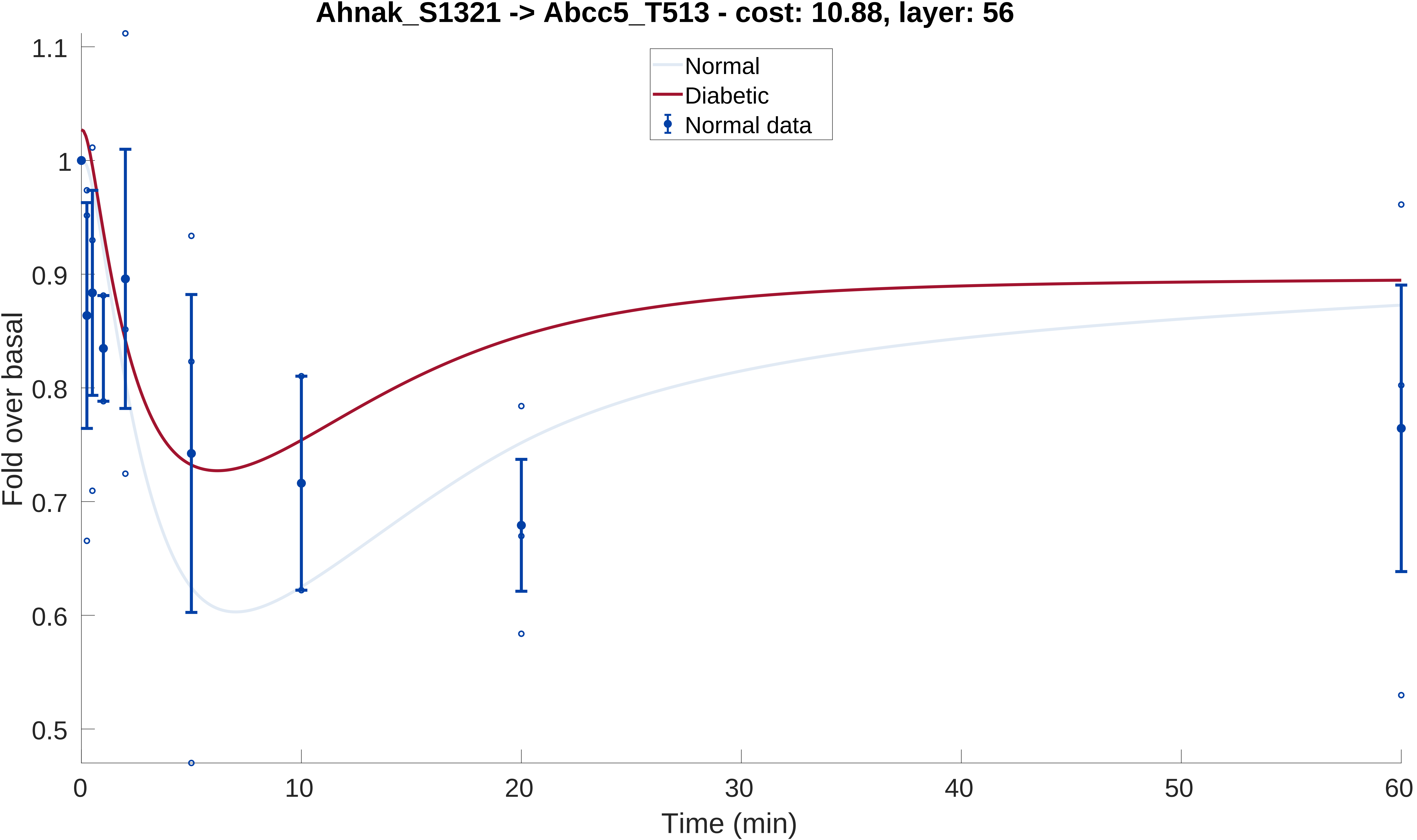

Ahnak_S1321 -> Abcc5_T513 - cost: 10.88, layer: 56
Normal
Diabetic
Normal data
Fold over basal
1.1
1
0.9
0.8
0.7
0.6
0.5
0
10
20
30
40
50
60
Time (min)

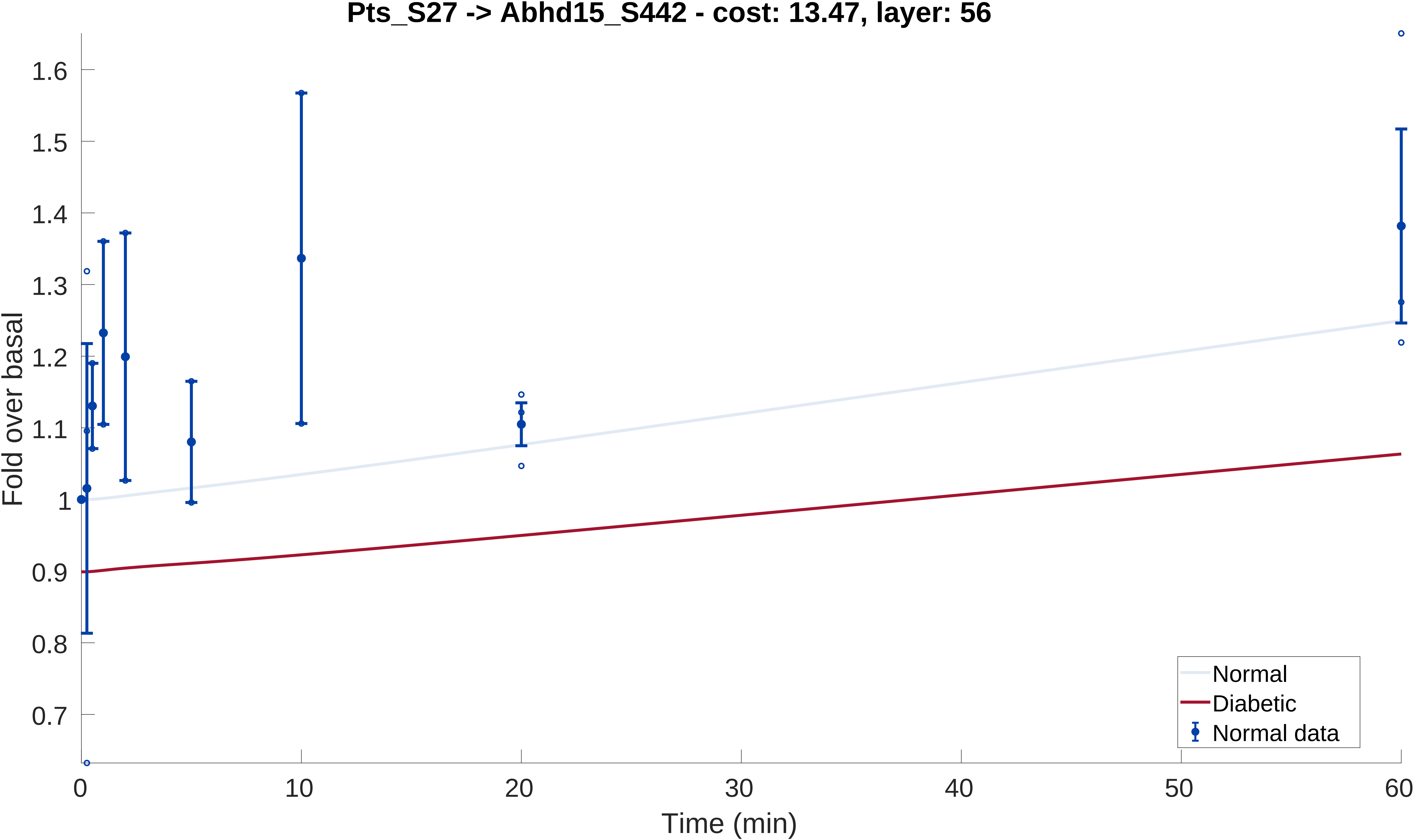

Pts_S27 -> Abhd15_S442 - cost: 13.47, layer: 56
Fold over basal
1.6
1.5
1.4
1.3
1.2
1.1
1
0.9
0.8
0.7
0
10
20
30
40
50
60
Time (min)
Normal
Diabetic
Normal data

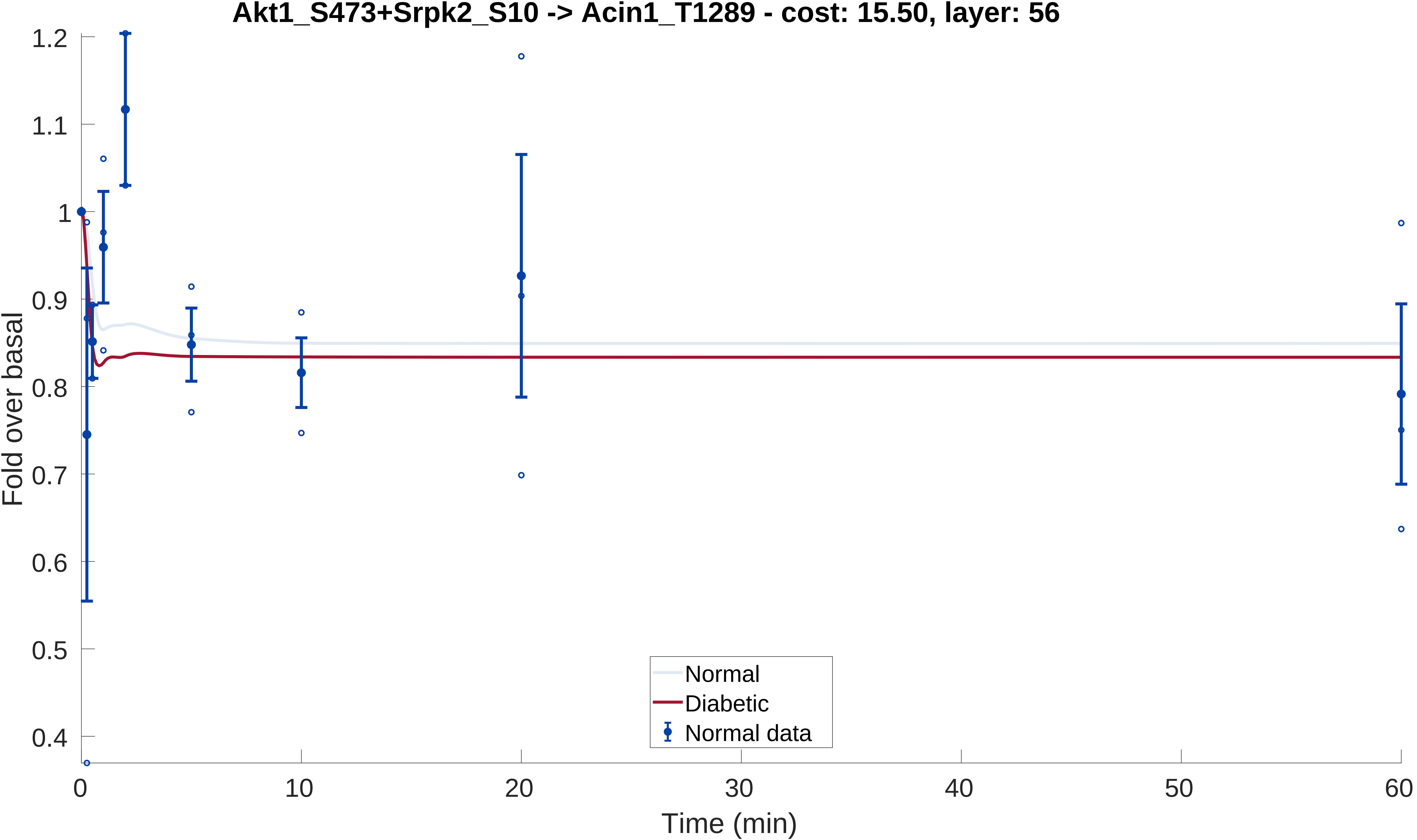
Akt1_S473+Srpk2_S10 -> Acin1_T1289 - cost: 15.50, layer: 56
Fold over basal
1.2
1.1
1
0.9
0.8
0.7
0.6
0.5
0.4
0
10
20
30
40
50
60
Time (min)
Normal
Diabetic
Normal data

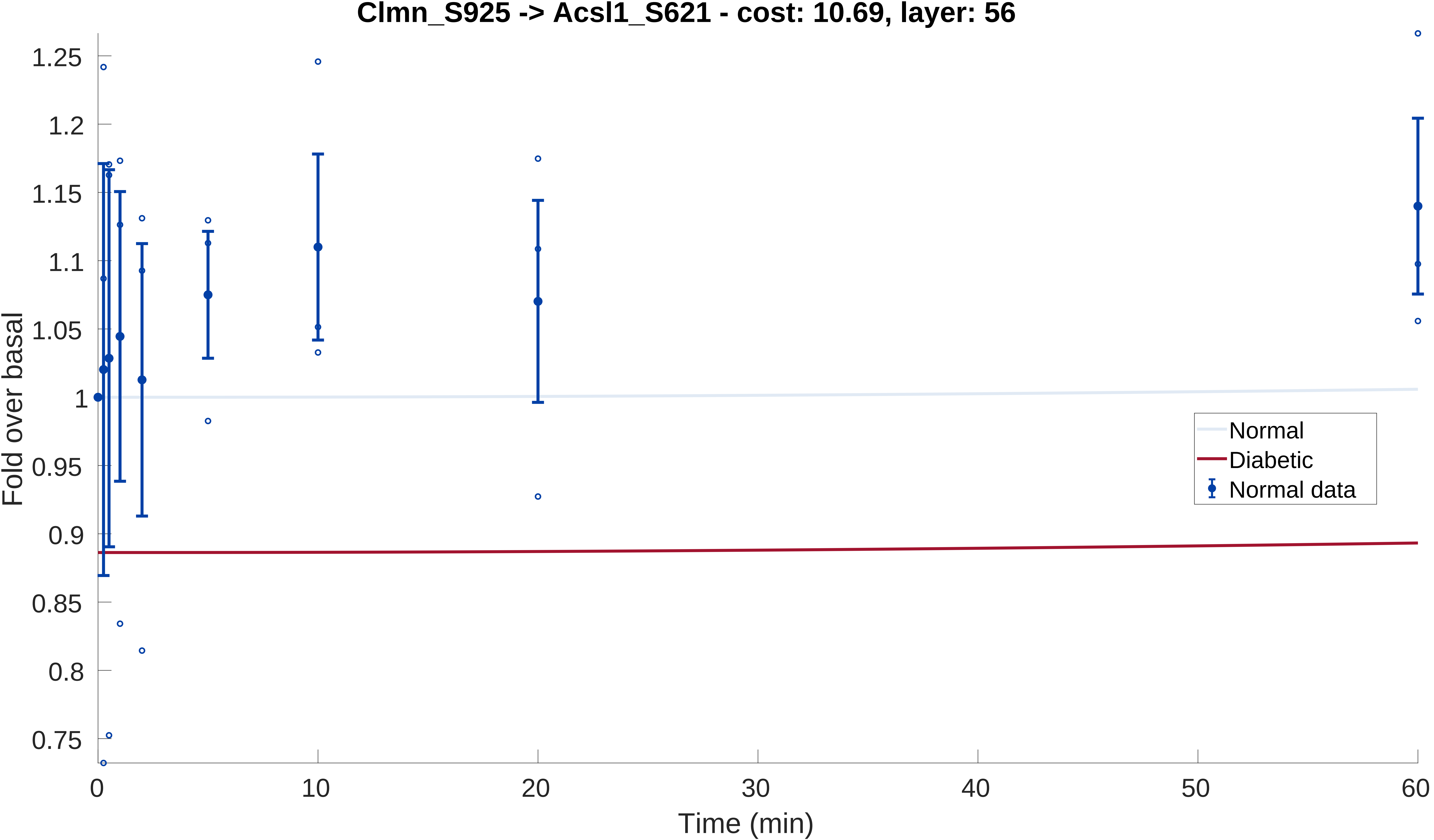
Clmn_S925 -> Acsl1_S621 - cost: 10.69, layer: 56
1.25
1.2
1.15
1.1
1.05
1
0.95
0.9
0.85
0.8
0.75
Fold over basal
0
10
20
30
40
50
60
Time (min)
Normal
Diabetic
Normal data

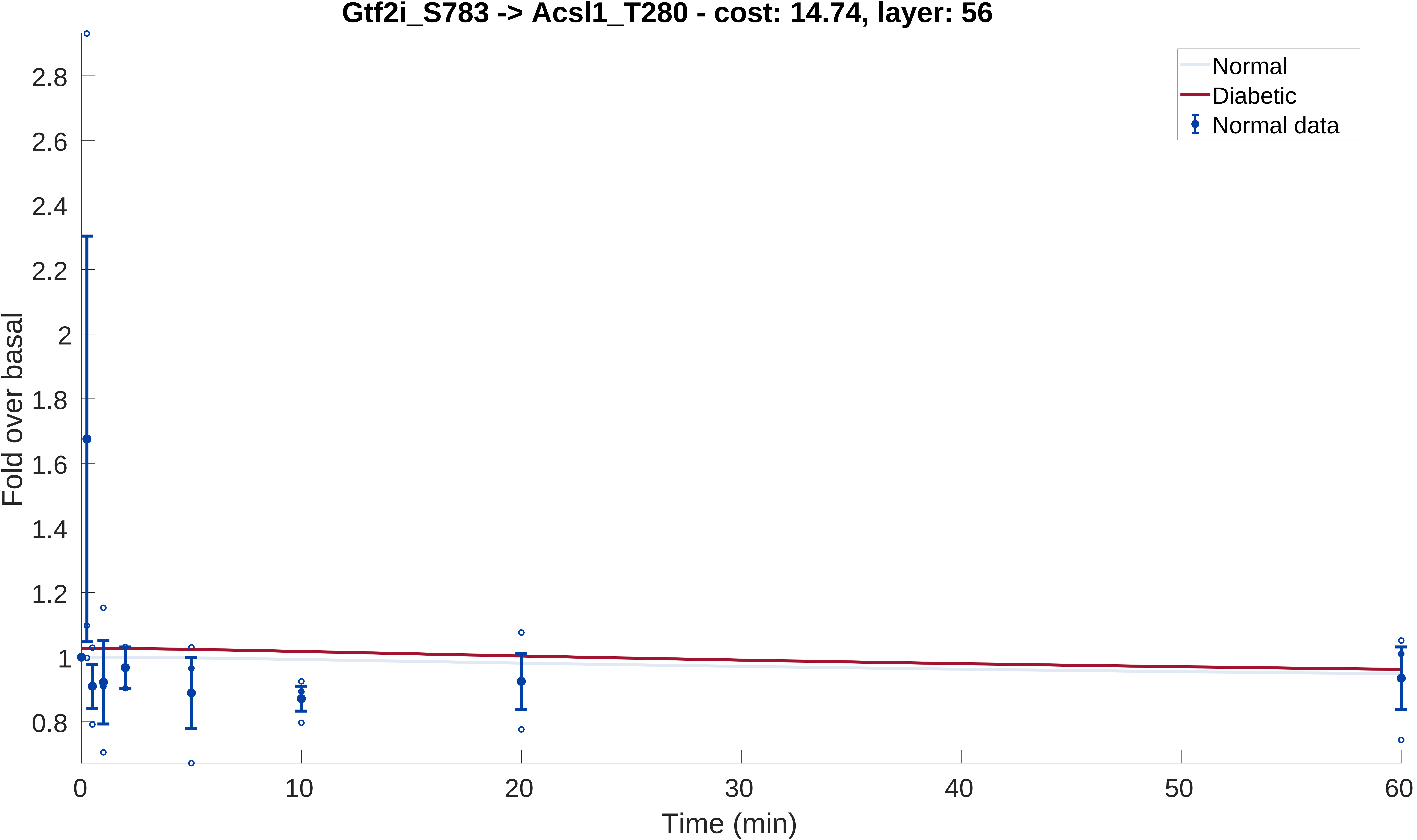

Gtf2i_S783 -> Acsl1_T280 - cost: 14.74, layer: 56
Normal
Diabetic
Normal data
Fold over basal
2.8
2.6
2.4
2.2
2
1.8
1.6
1.4
1.2
1
0.8
0
10
20
30
40
50
60
Time (min)

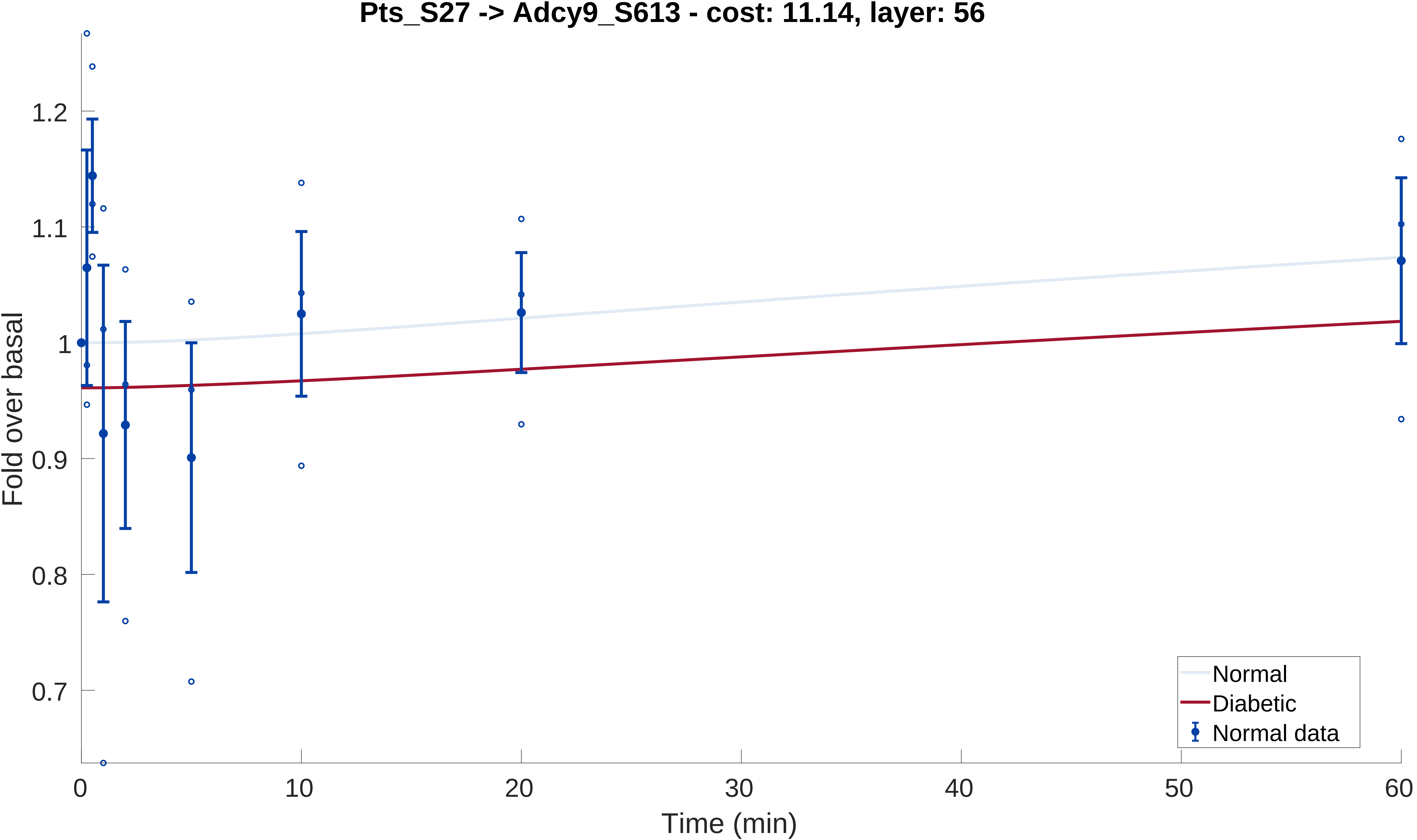

Pts_S27 -> Adcy9_S613 - cost: 11.14, layer: 56
Fold over basal
1.2
1.1
1
0.9
0.8
0.7
0
10
20
30
40
50
60
Time (min)
Normal
Diabetic
Normal data

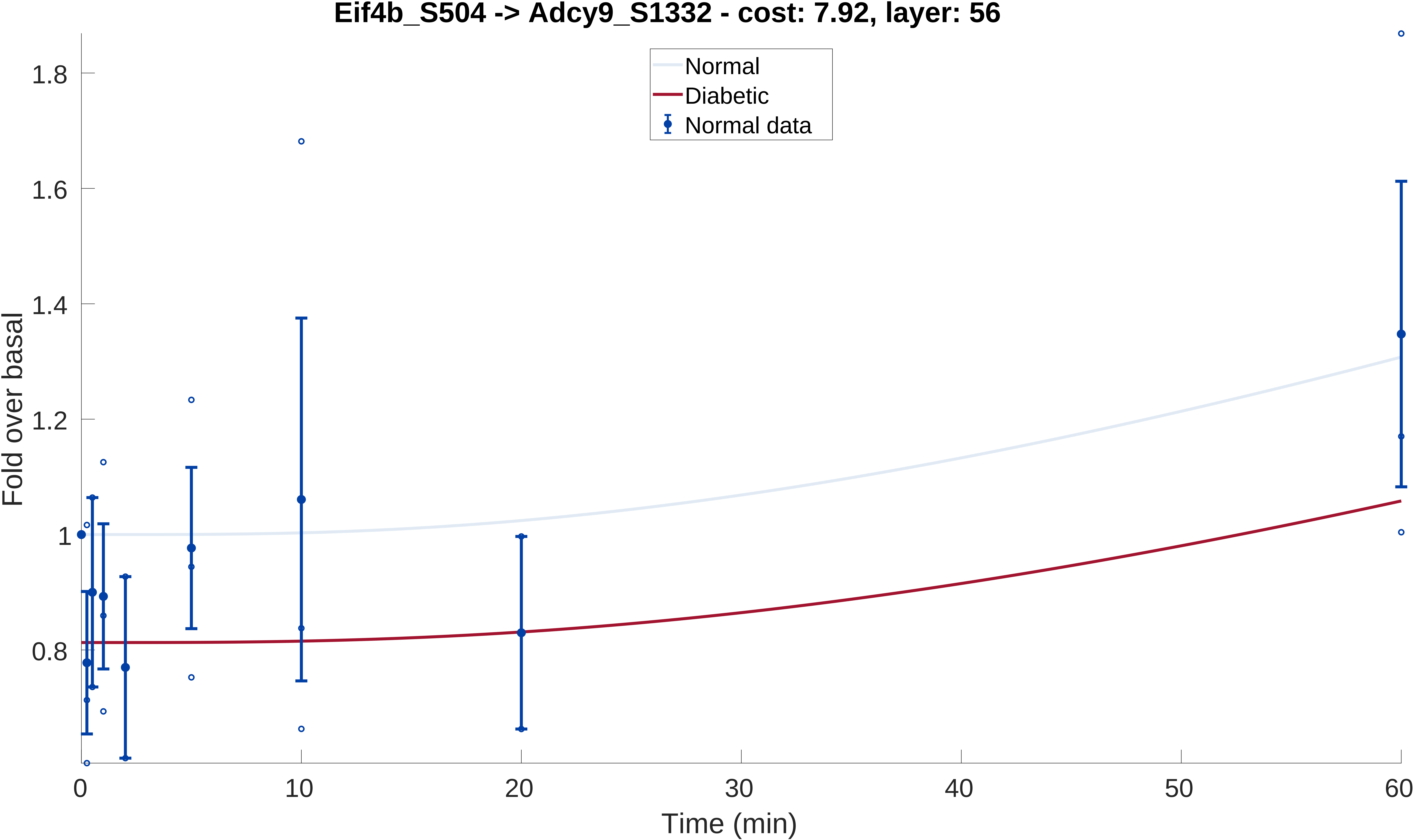
Eif4b_S504 -> Adcy9_S1332 - cost: 7.92, layer: 56
Normal
Diabetic
Normal data
Fold over basal
1.8
1.6
1.4
1.2
1
0.8
0
10
20
30
40
50
60
Time (min)

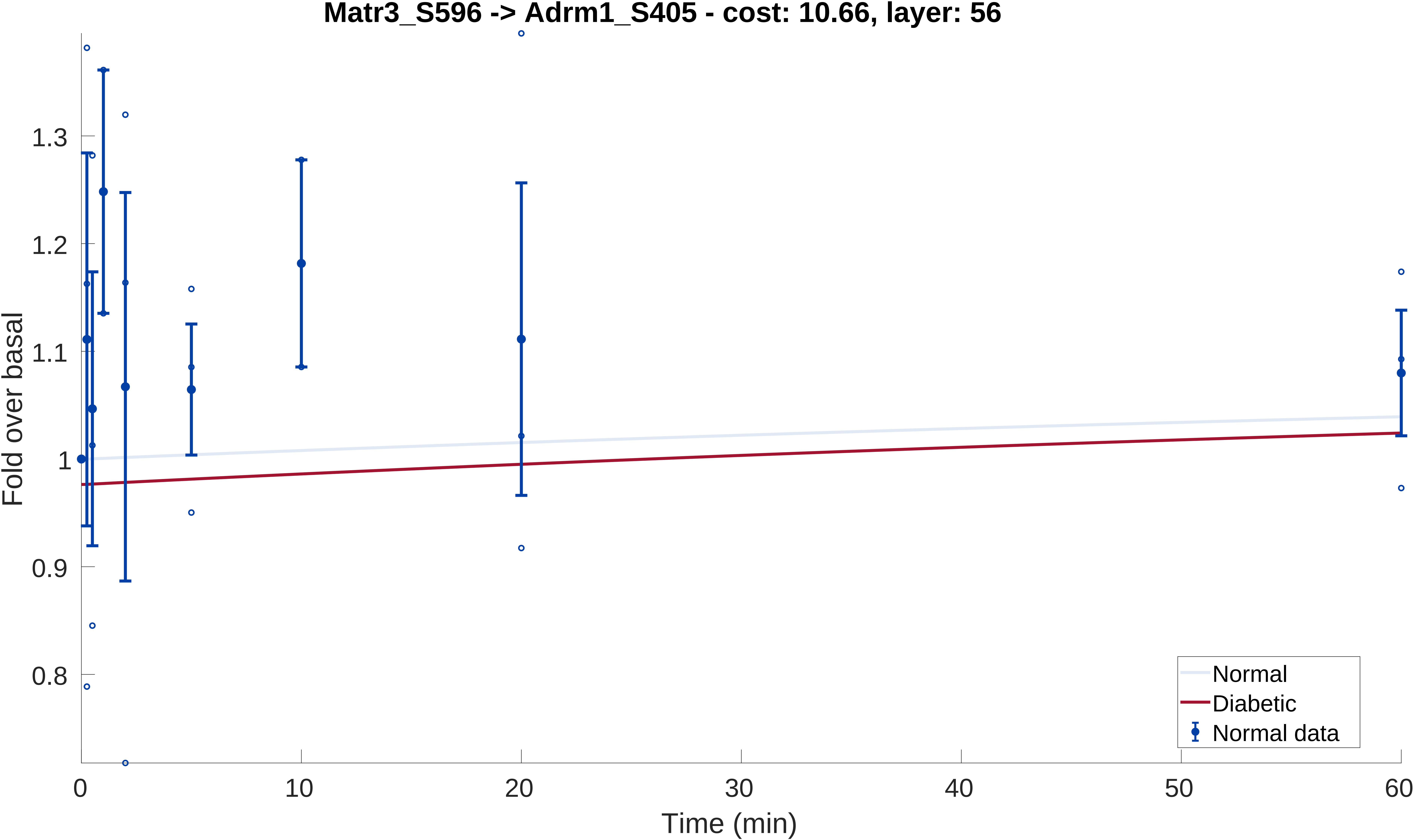
Matr3_S596 -> Adrm1_S405 - cost: 10.66, layer: 56
Fold over basal
Time (min)
0.8
0.9
1
1.1
1.2
1.3
0
10
20
30
40
50
60
Normal
Diabetic
Normal data

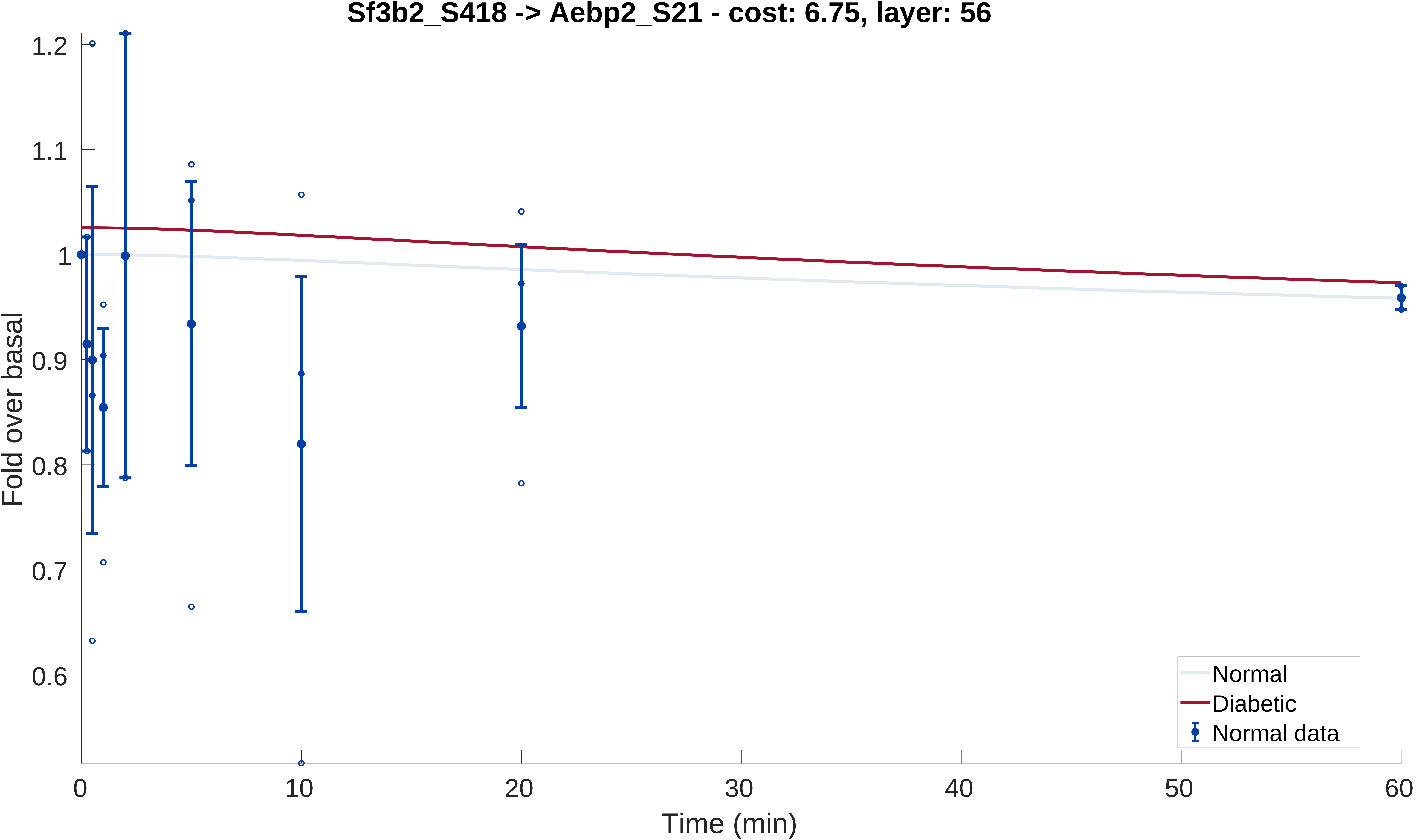

Sf3b2_S418 -> Aebp2_S21 - cost: 6.75, layer: 56
Fold over basal
1.2
1.1
1
0.9
0.8
0.7
0.6
0
10
20
30
40
50
60
Time (min)
Normal
Diabetic
Normal data

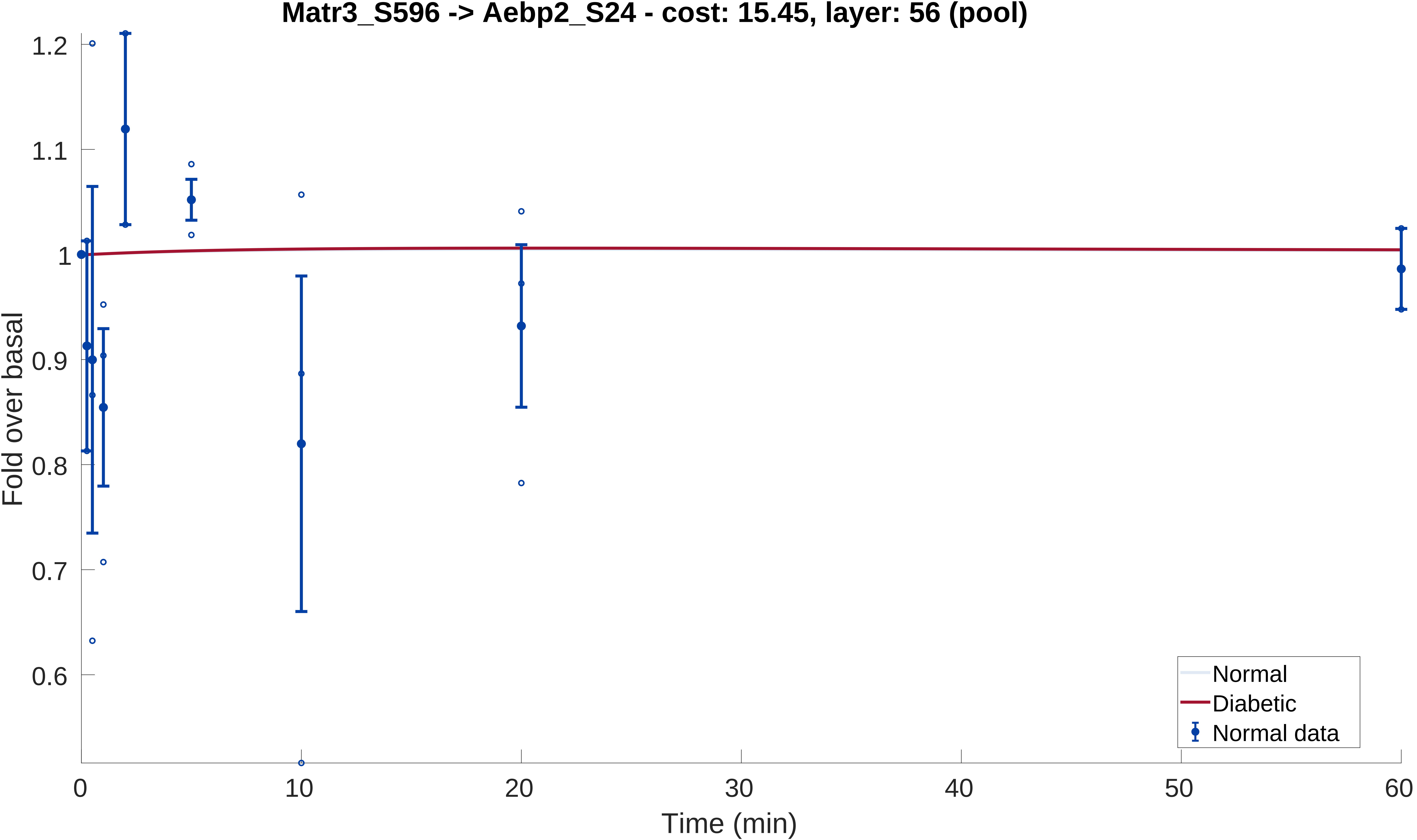
Matr3_S596 -> Aebp2_S24 - cost: 15.45, layer: 56 (pool)
Fold over basal
1.2
1.1
1
0.9
0.8
0.7
0.6
0
10
20
30
40
50
60
Time (min)
Normal
Diabetic
Normal data

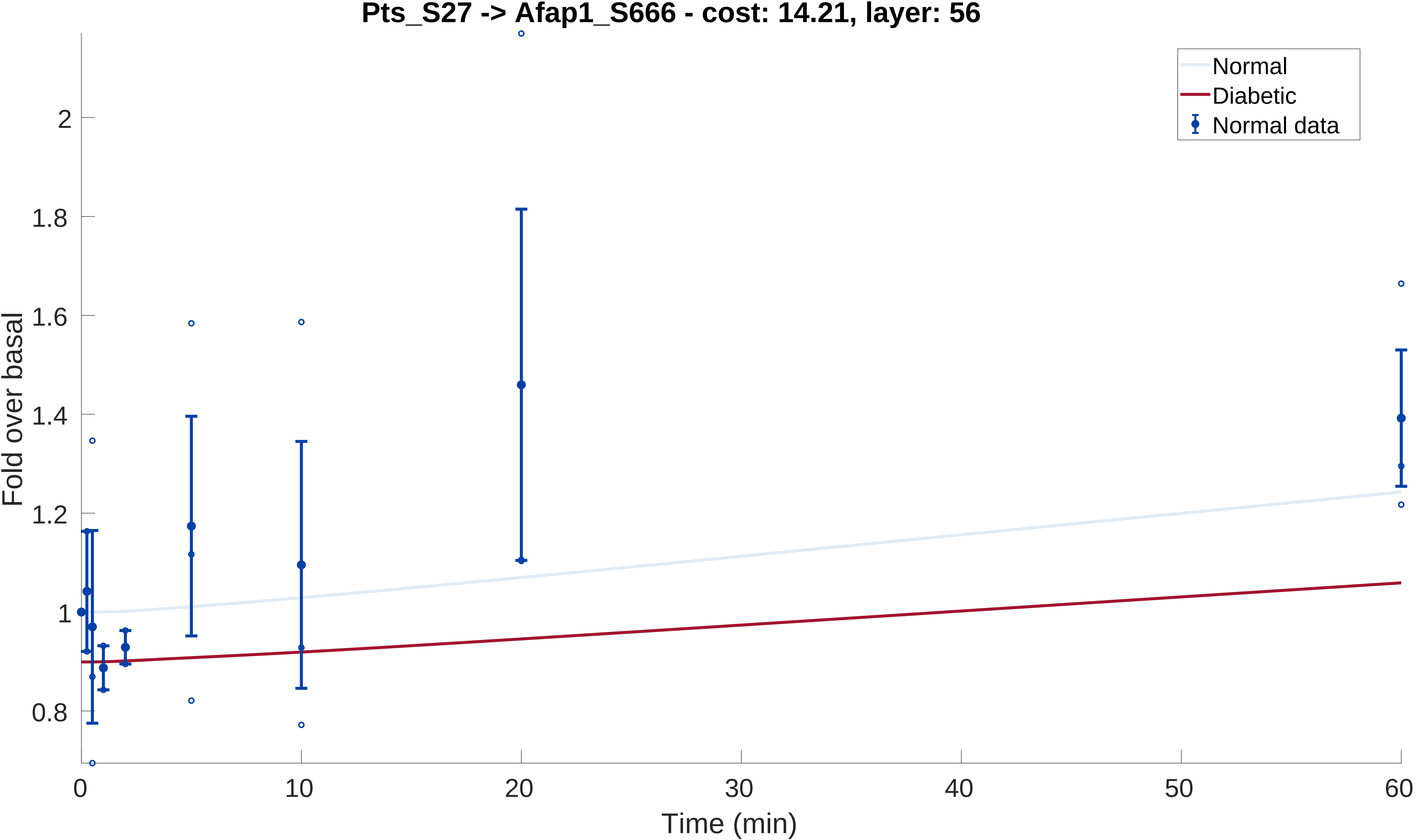
Pts_S27 -> Afap1_S666 - cost: 14.21, layer: 56
Normal
Diabetic
Normal data
Fold over basal
0.8
1
1.2
1.4
1.6
1.8
2
0
10
20
30
40
50
60
Time (min)

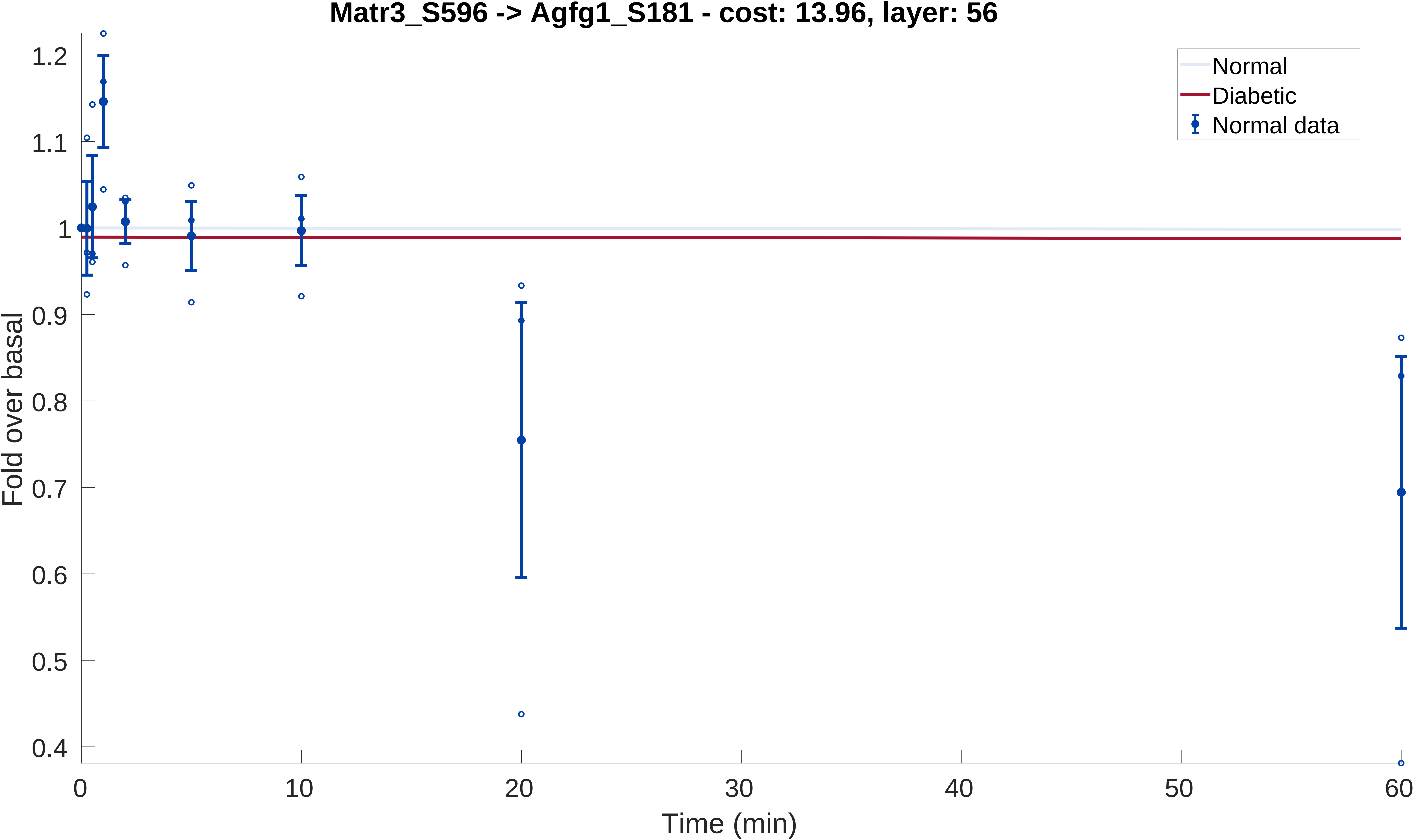

Matr3_S596 -> Agfg1_S181 - cost: 13.96, layer: 56
Normal
Diabetic
Normal data
Fold over basal
Time (min)
1.2
1.1
1
0.9
0.8
0.7
0.6
0.5
0.4
0
10
20
30
40
50
60

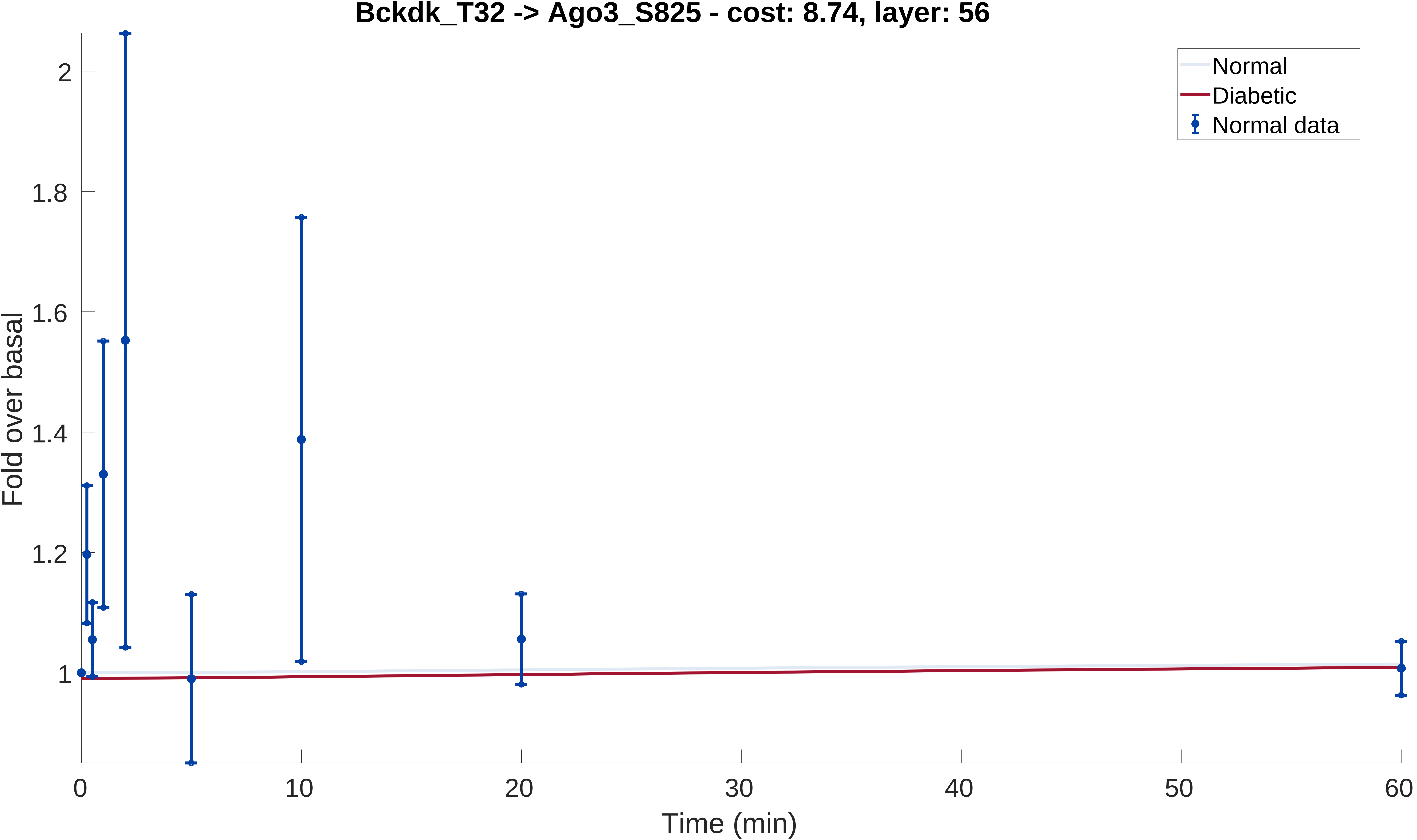
Bckdk_T32 -> Ago3_S825 - cost: 8.74, layer: 56
Fold over basal
Time (min)
Normal
Diabetic
Normal data
2
1.8
1.6
1.4
1.2
1
0
10
20
30
40
50
60

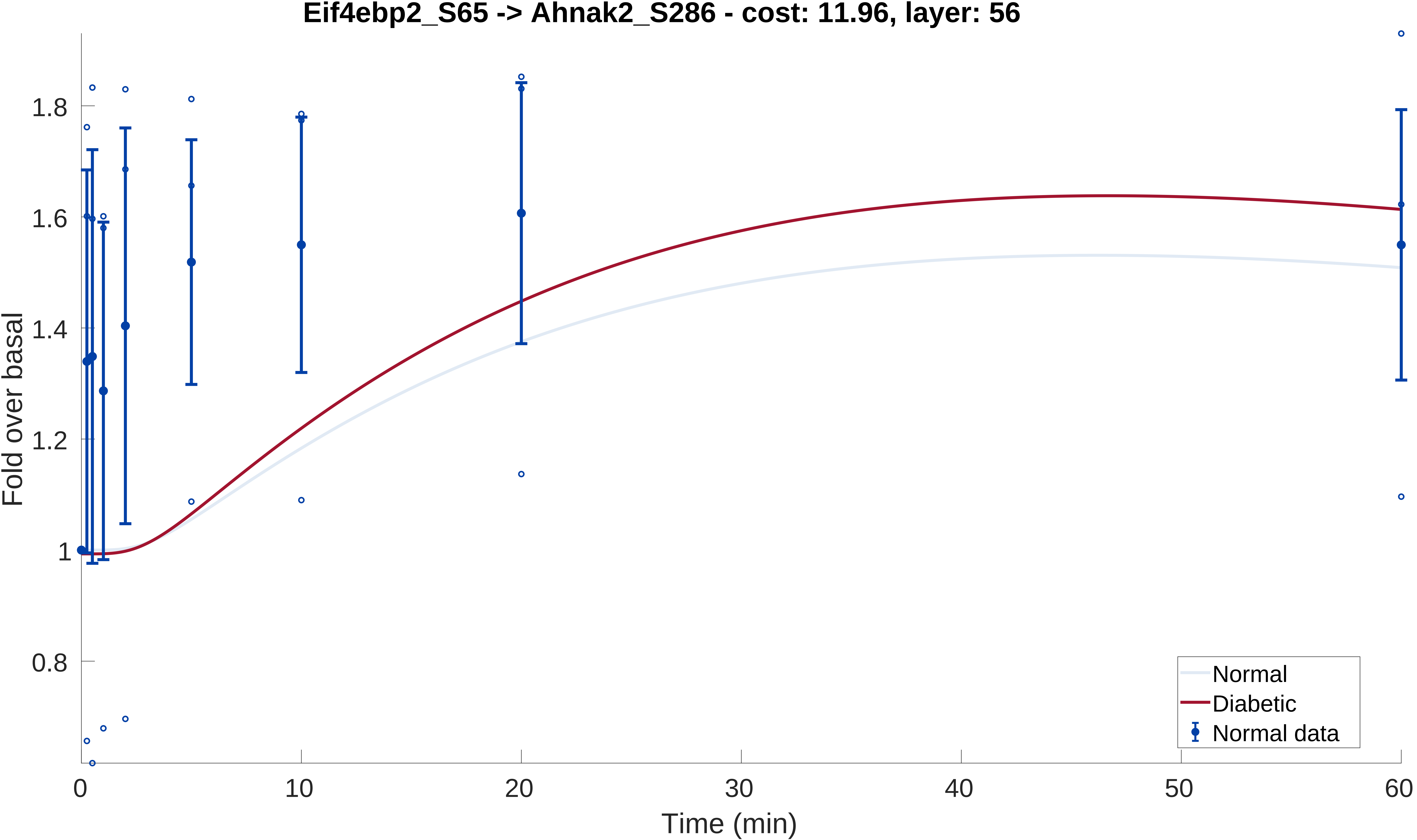

Eif4ebp2_S65 -> Ahnak2_S286 - cost: 11.96, layer: 56
Fold over basal
Time (min)
0.8
1
1.2
1.4
1.6
1.8
0
10
20
30
40
50
60
Normal
Diabetic
Normal data

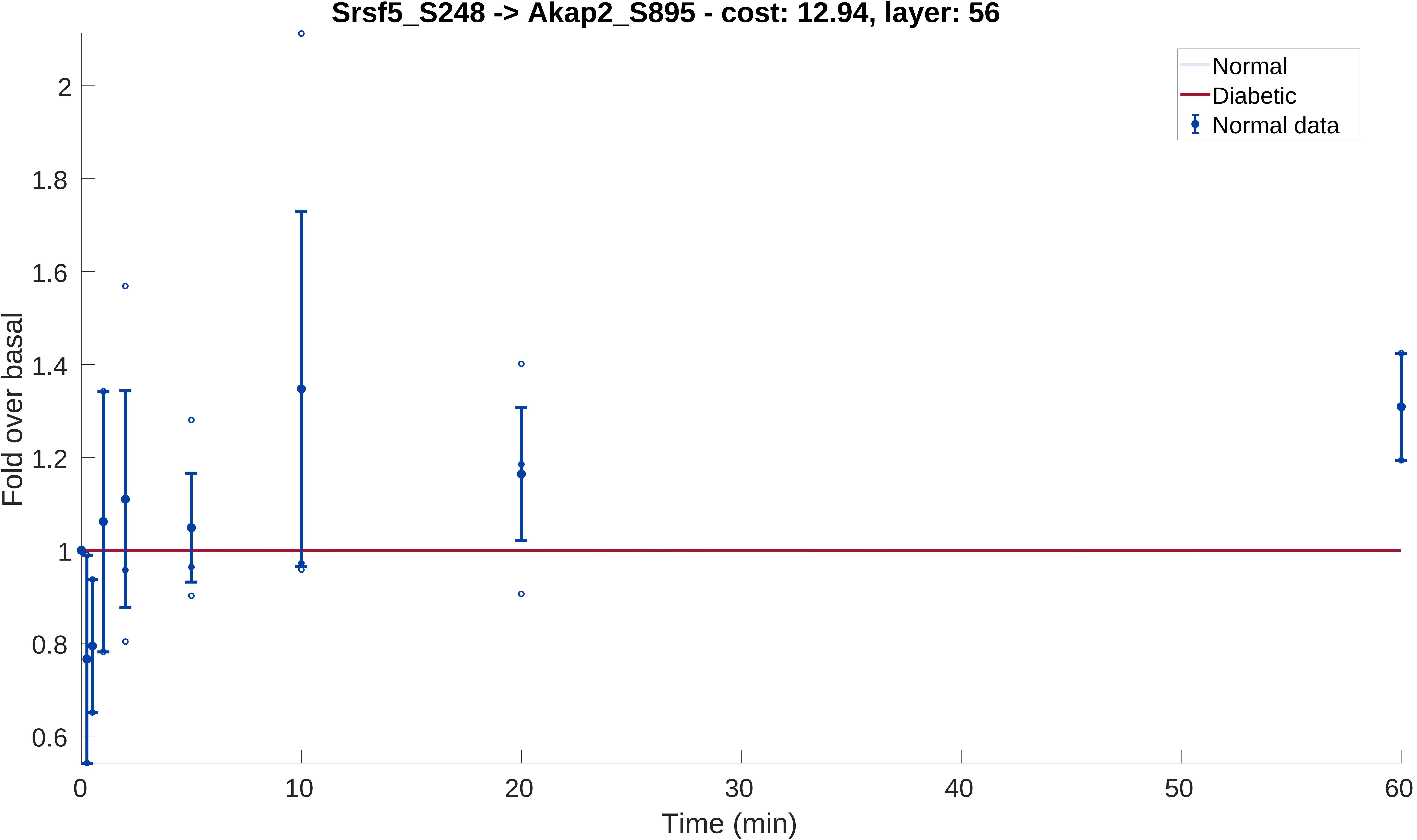

Srsf5_S248 -> Akap2_S895 - cost: 12.94, layer: 56
Normal
Diabetic
Normal data
Fold over basal
Time (min)
0.6
0.8
1
1.2
1.4
1.6
1.8
2
0
10
20
30
40
50
60

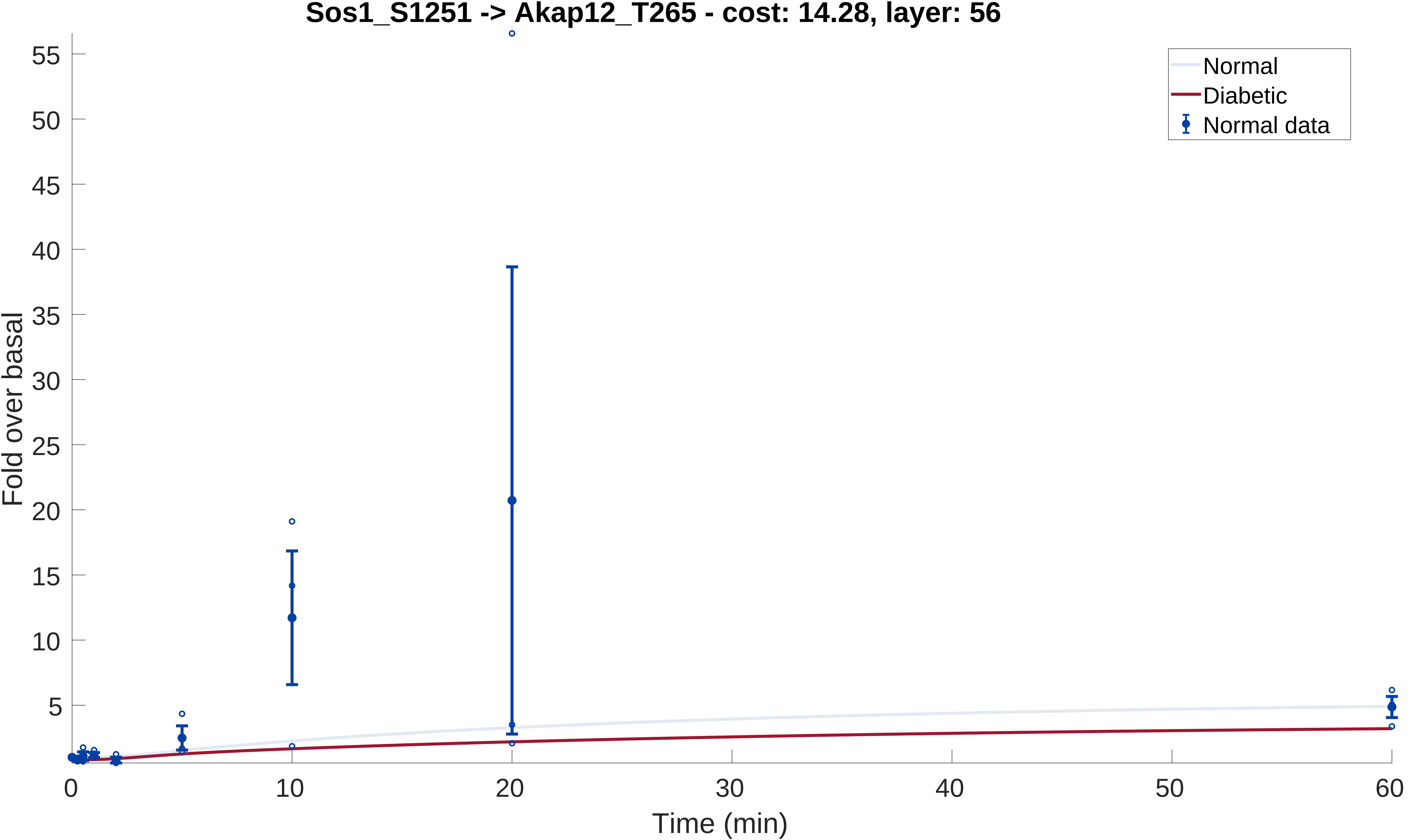

Sos1_S1251 -> Akap12_T265 - cost: 14.28, layer: 56
Normal
Diabetic
Normal data
Fold over basal
Time (min)
0
10
20
30
40
50
60
5
10
15
20
25
30
35
40
45
50
55

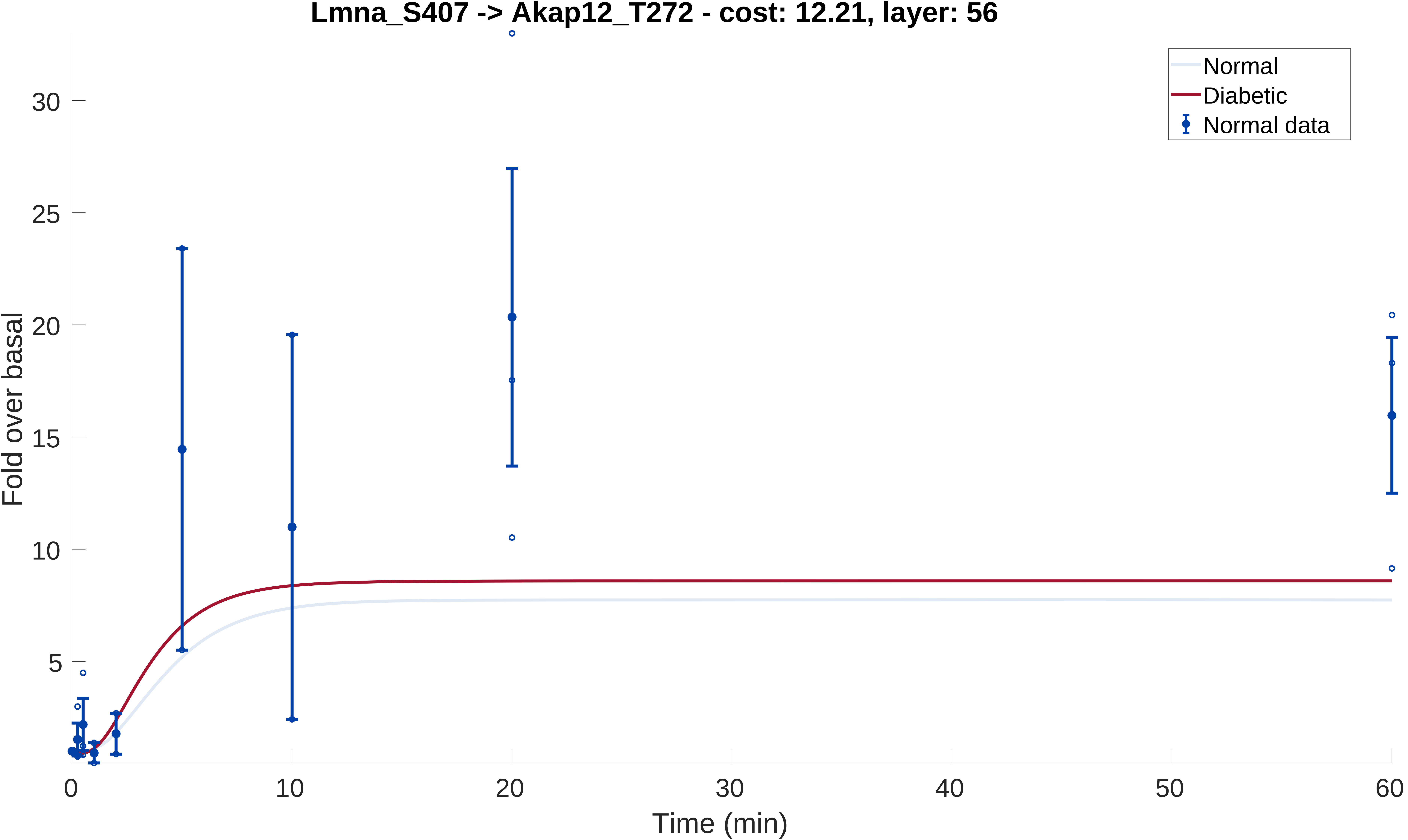
Lmna_S407 -> Akap12_T272 - cost: 12.21, layer: 56
Normal
Diabetic
Normal data
Fold over basal
Time (min)
0
10
20
30
40
50
60
5
10
15
20
25
30

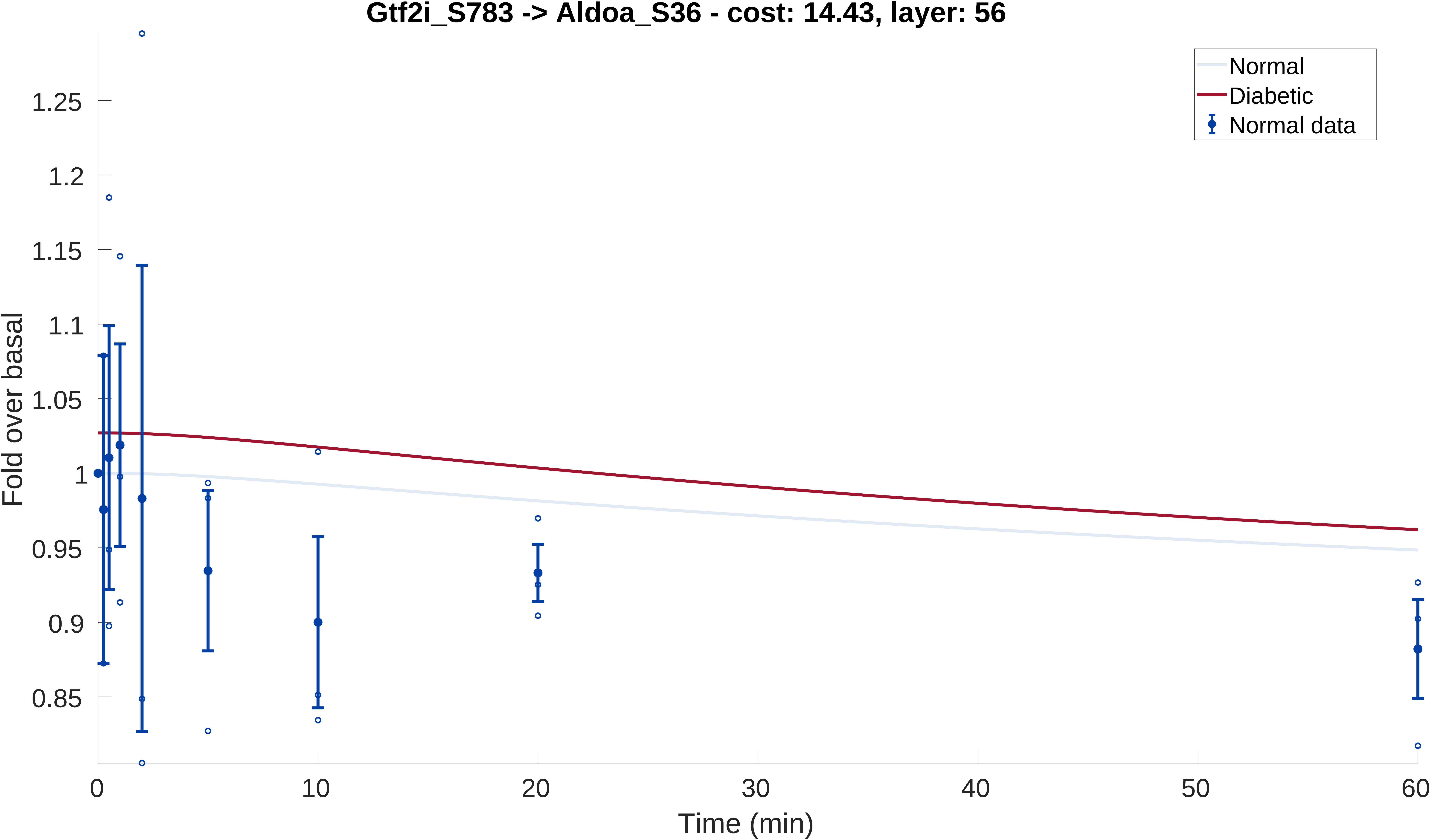

Gtf2i_S783 -> Aldoa_S36 - cost: 14.43, layer: 56
Normal
Diabetic
Normal data
Fold over basal
1.25
1.2
1.15
1.1
1.05
1
0.95
0.9
0.85
0
10
20
30
40
50
60
Time (min)

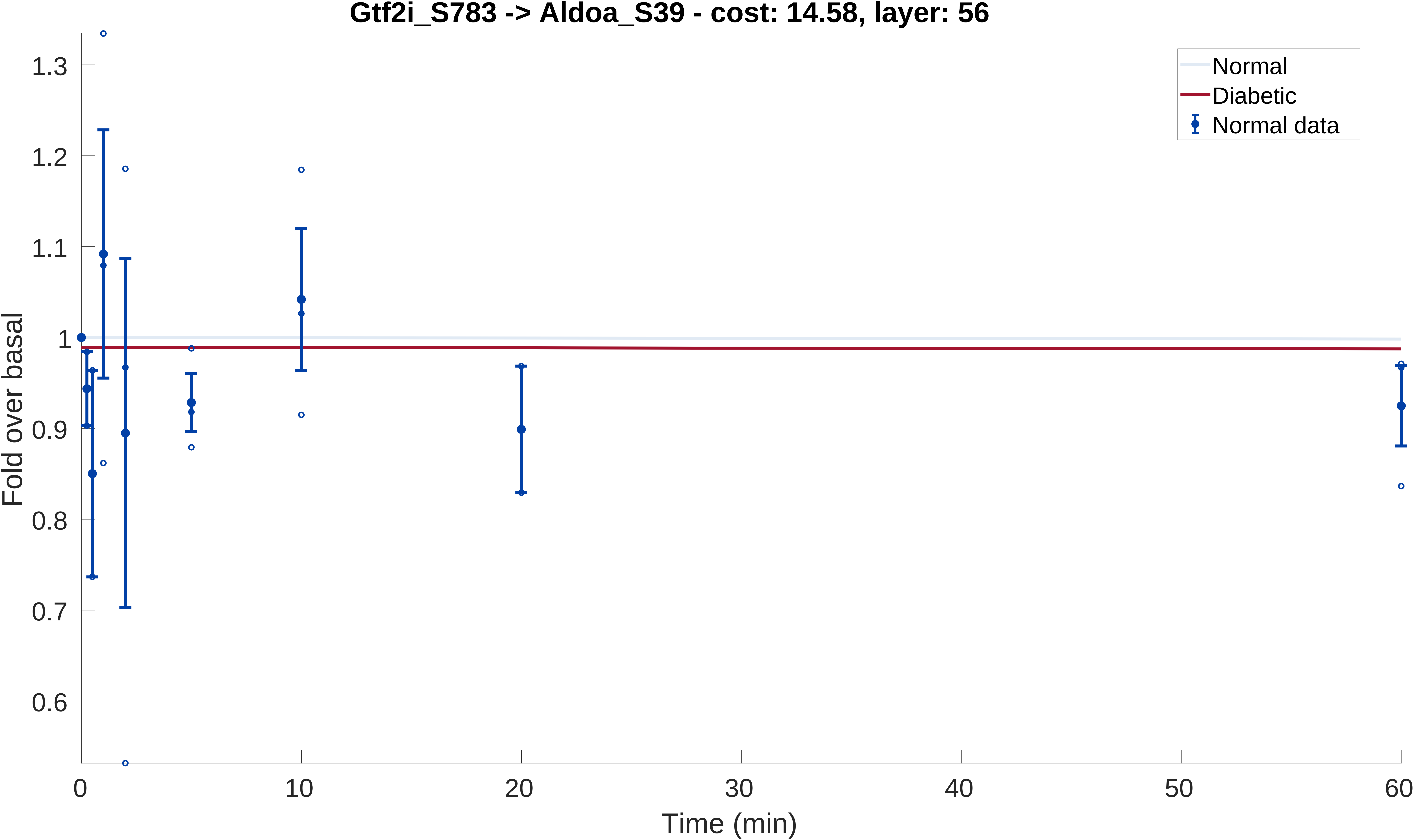
Gtf2i_S783 -> Aldoa_S39 - cost: 14.58, layer: 56
Normal
Diabetic
Normal data
Fold over basal
Time (min)
1.3
1.2
1.1
1
0.9
0.8
0.7
0.6
0
10
20
30
40
50
60

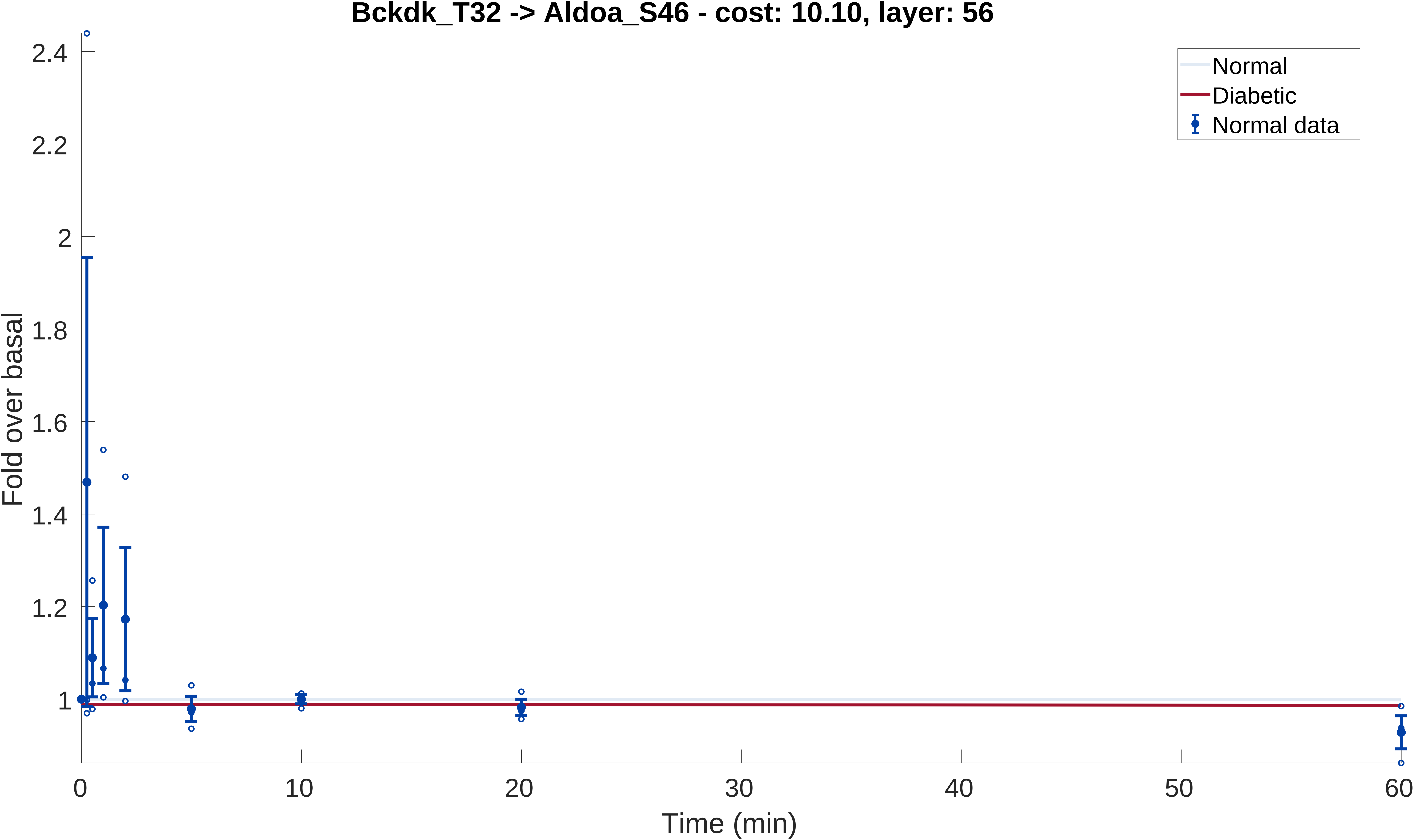
Bckdk_T32 -> Aldoa_S46 - cost: 10.10, layer: 56
Normal
Diabetic
Normal data
Fold over basal
2.4
2.2
2
1.8
1.6
1.4
1.2
1
0
10
20
30
40
50
60
Time (min)

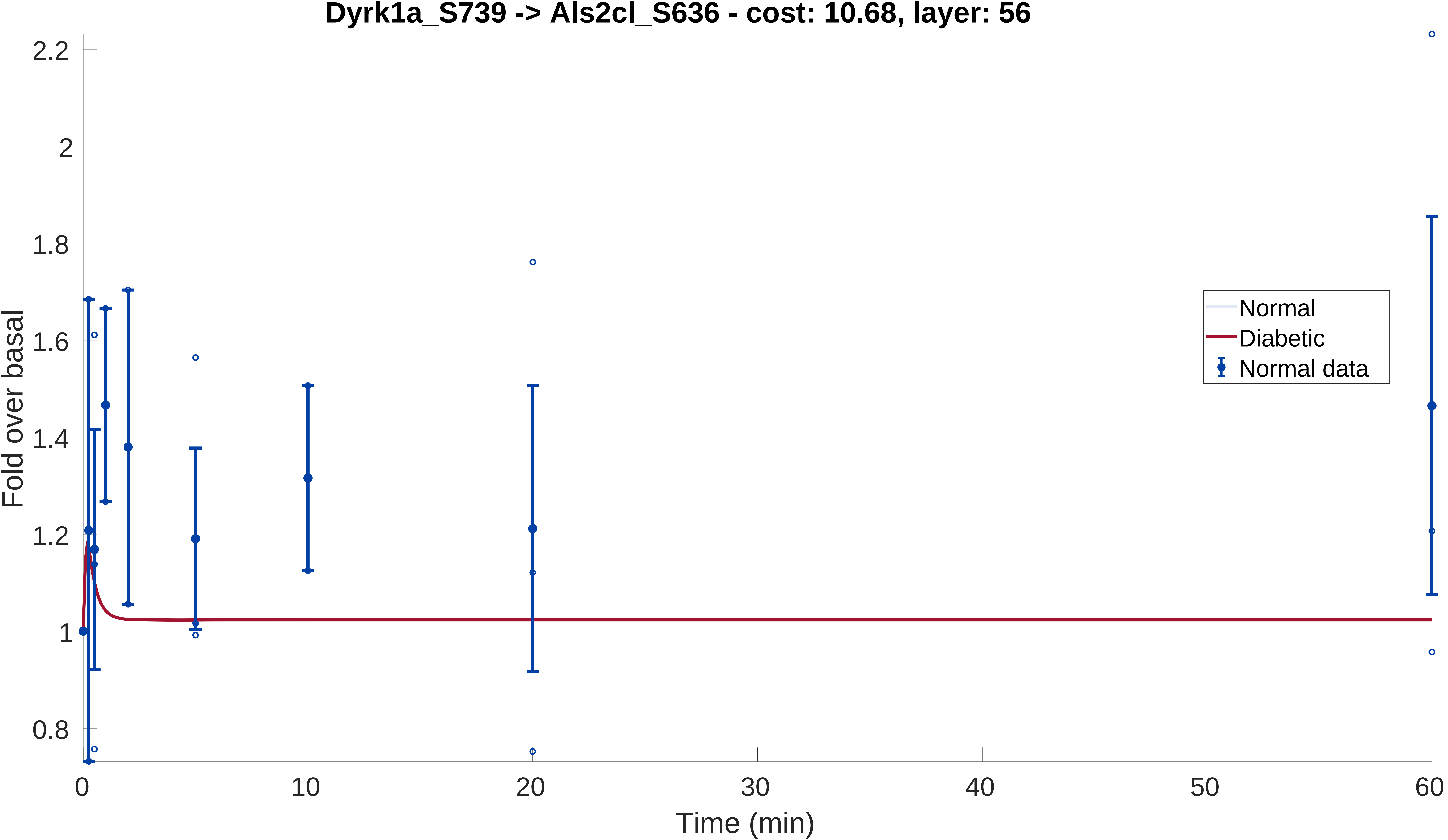

Dyrk1a_S739 -> Als2cl_S636 - cost: 10.68, layer: 56
Fold over basal
Time (min)
Normal
Diabetic
Normal data
2.2
2
1.8
1.6
1.4
1.2
1
0.8
0
10
20
30
40
50
60

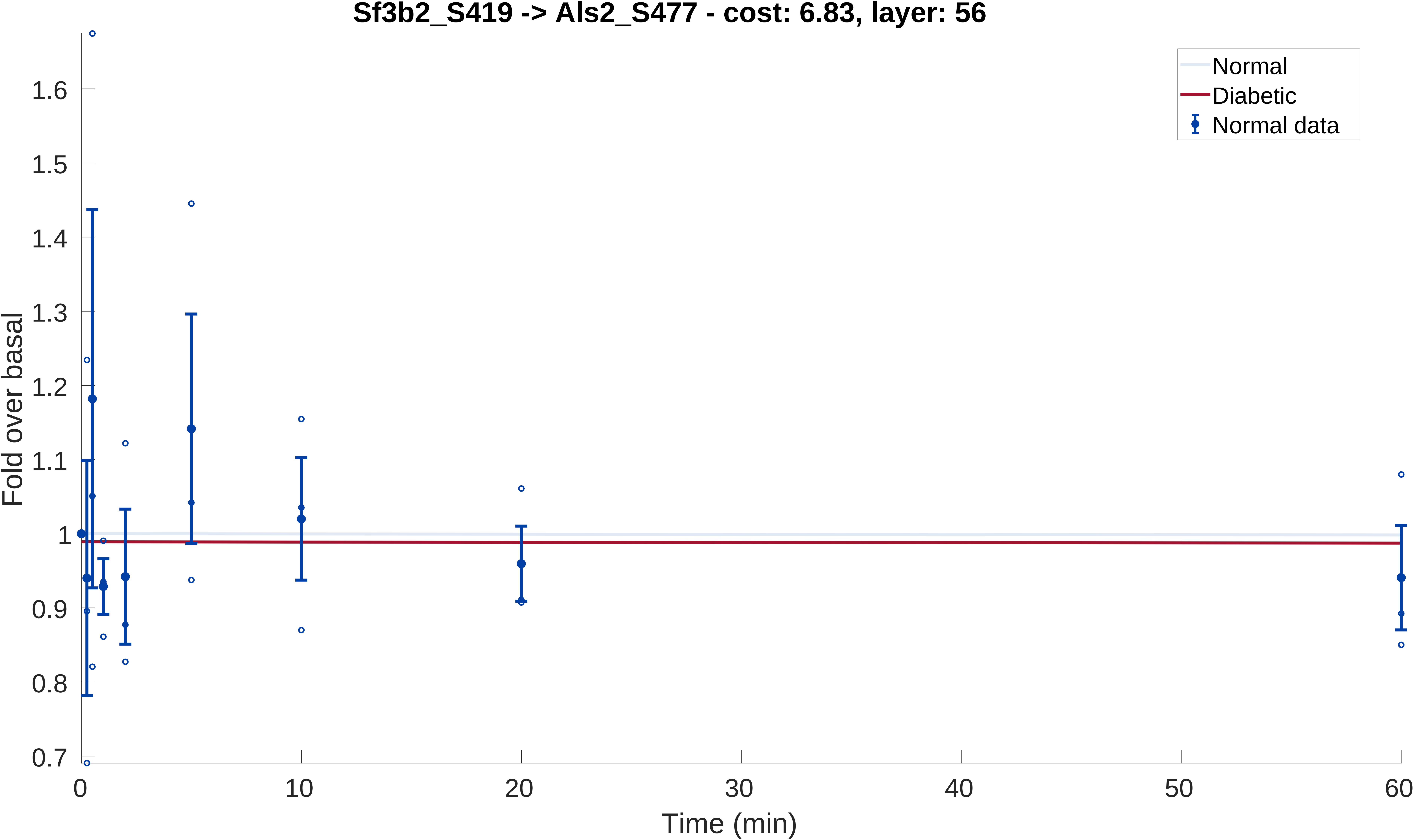

Sf3b2_S419 -> Als2_S477 - cost: 6.83, layer: 56
Normal
Diabetic
Normal data
Fold over basal
1.6
1.5
1.4
1.3
1.2
1.1
1
0.9
0.8
0.7
0
10
20
30
40
50
60
Time (min)

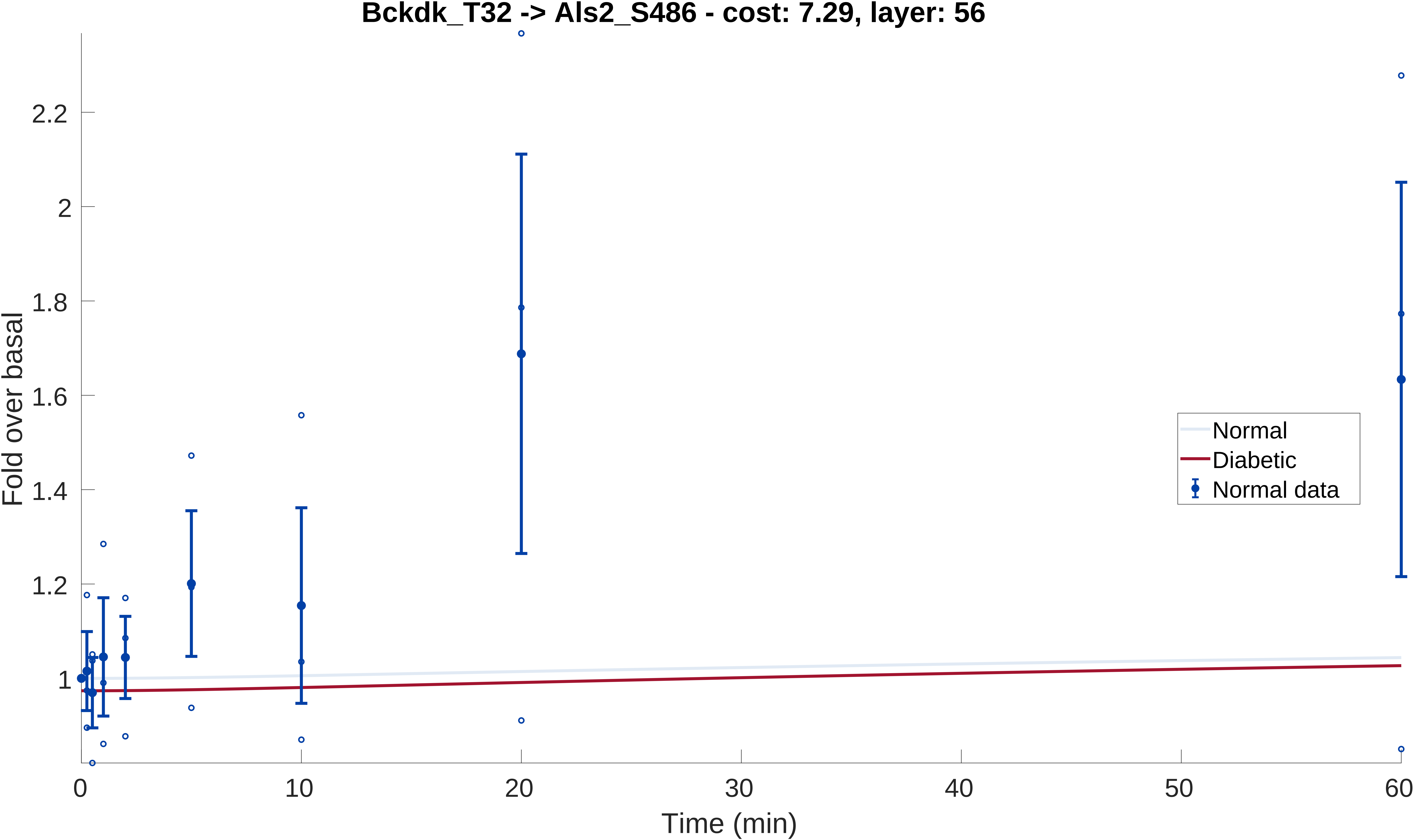
Bckdk_T32 -> Als2_S486 - cost: 7.29, layer: 56
Fold over basal
2.2
2
1.8
1.6
1.4
1.2
1
0
10
20
30
40
50
60
Time (min)
Normal
Diabetic
Normal data

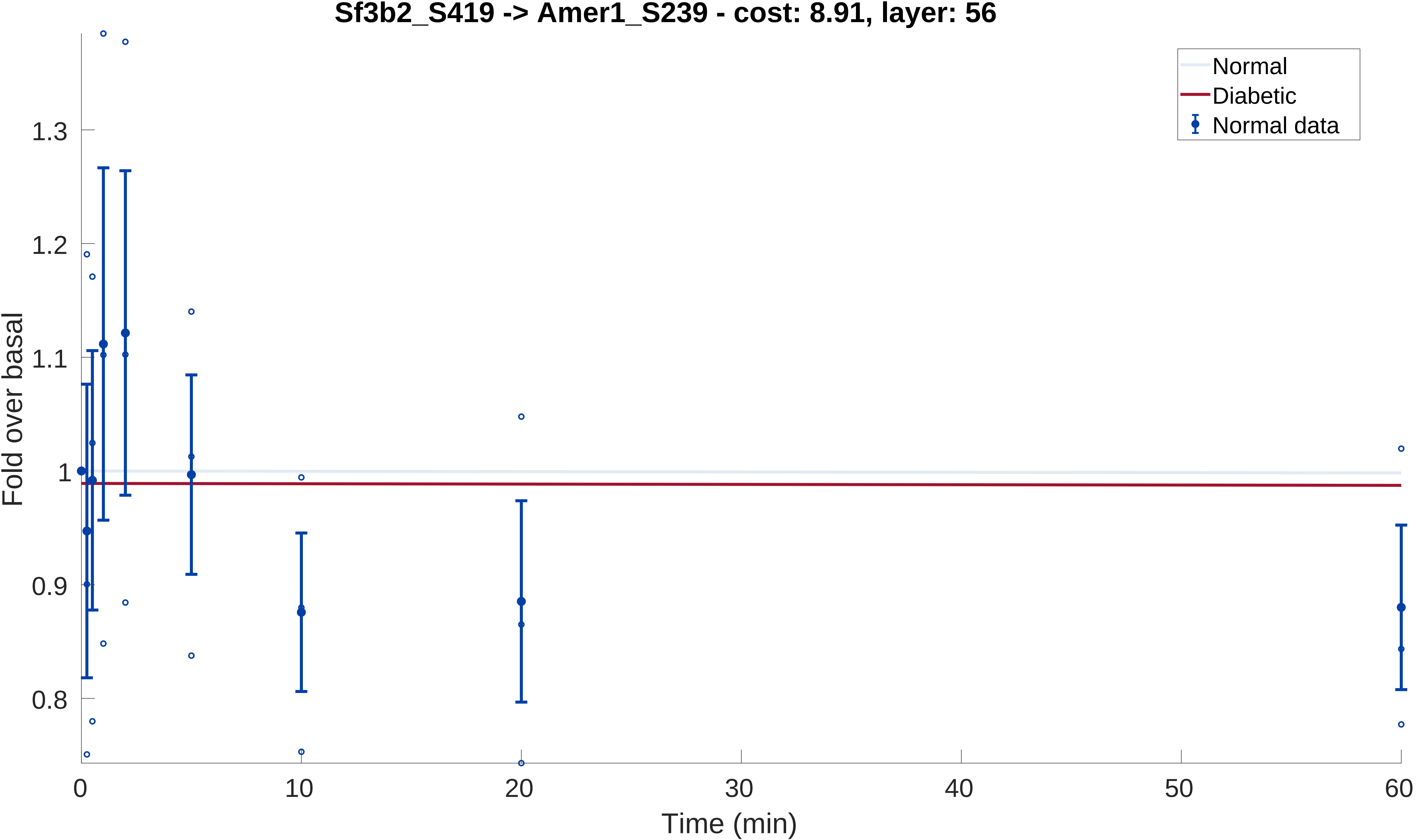

Sf3b2_S419 -> Amer1_S239 - cost: 8.91, layer: 56
Normal
Diabetic
Normal data
Fold over basal
Time (min)
0.8
0.9
1
1.1
1.2
1.3
0
10
20
30
40
50
60

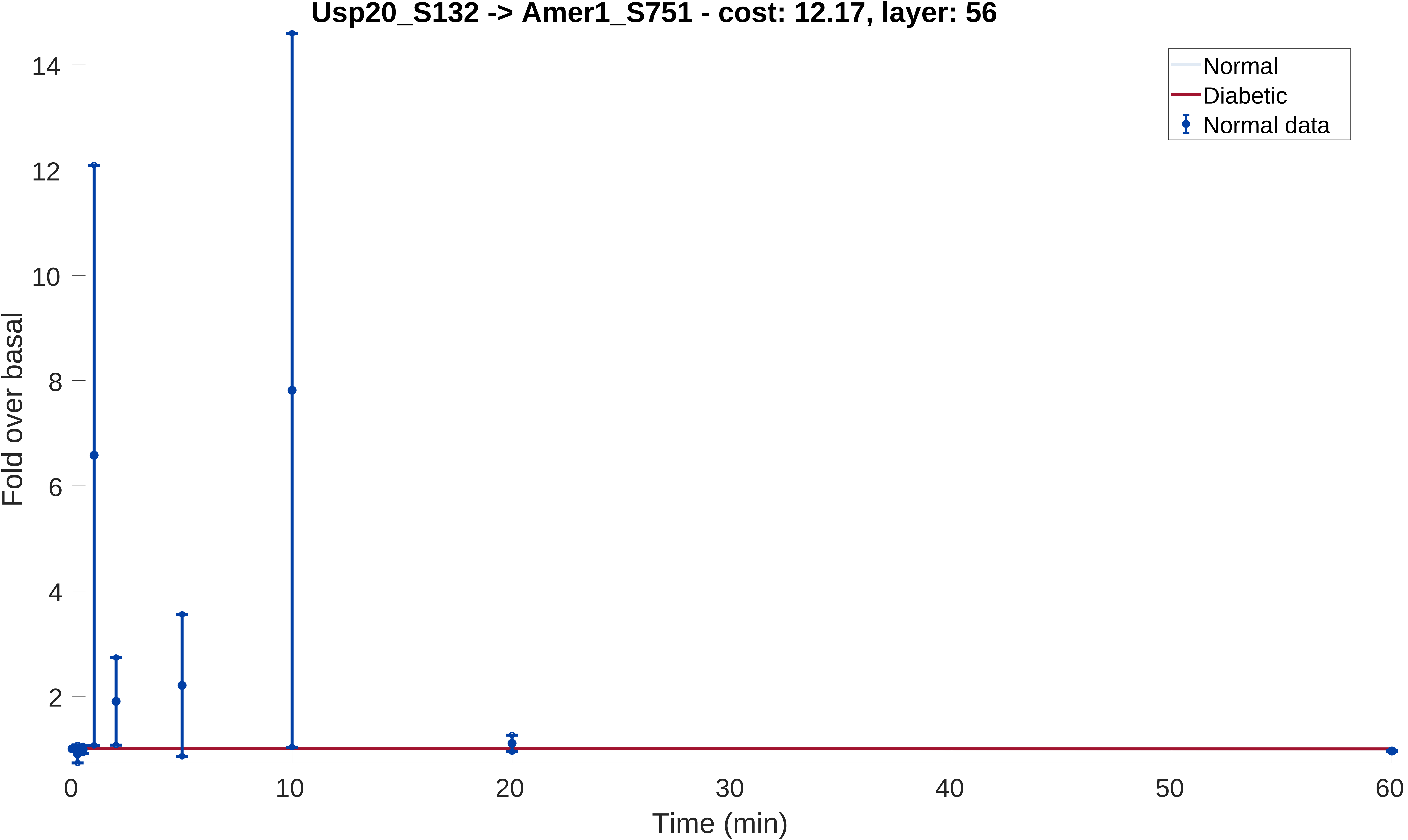

Usp20_S132 -> Amer1_S751 - cost: 12.17, layer: 56
Normal
Diabetic
Normal data
Fold over basal
Time (min)
0
10
20
30
40
50
60
2
4
6
8
10
12
14

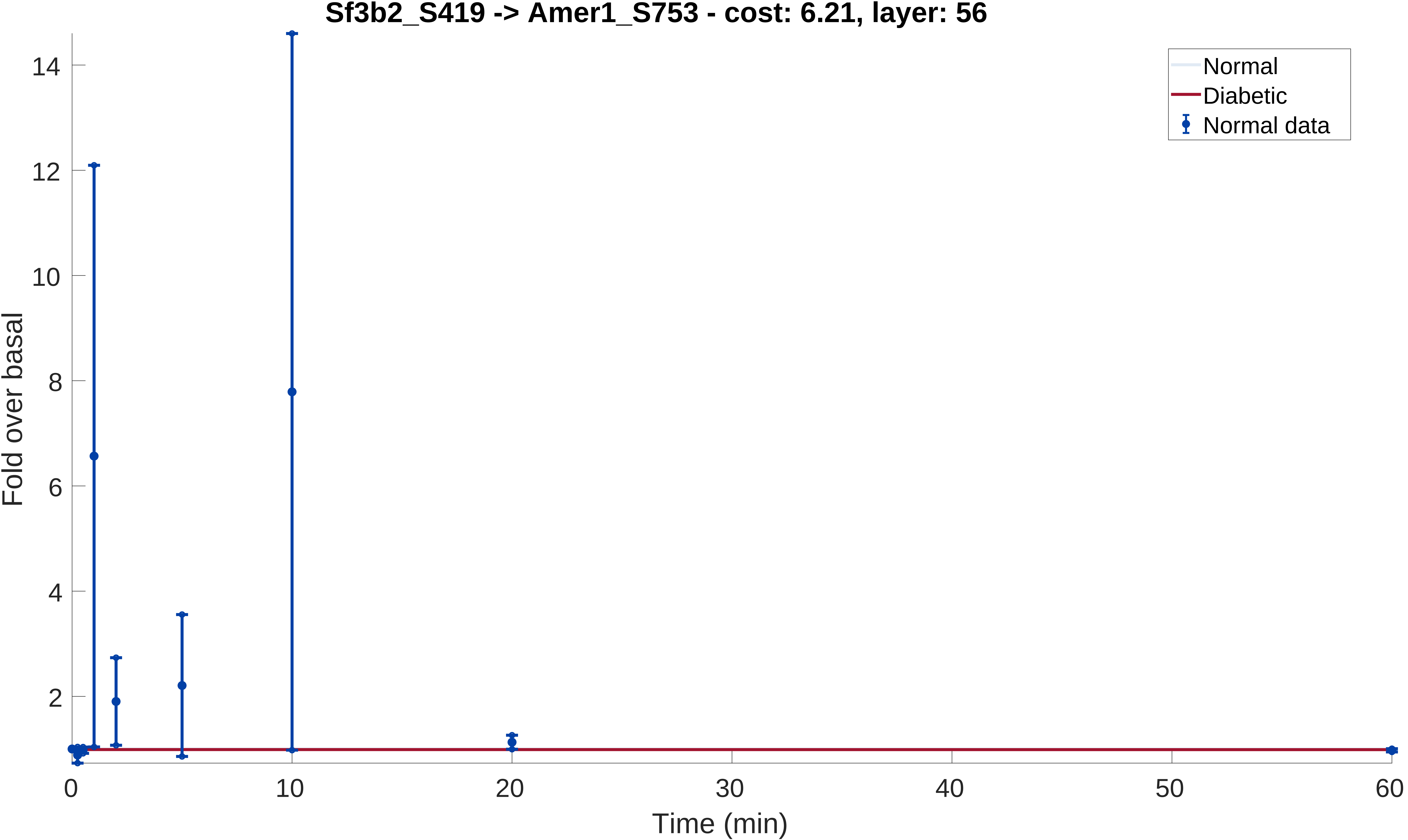

Sf3b2_S419 -> Amer1_S753 - cost: 6.21, layer: 56
Normal
Diabetic
Normal data
Fold over basal
Time (min)
14
12
10
8
6
4
2
0
10
20
30
40
50
60

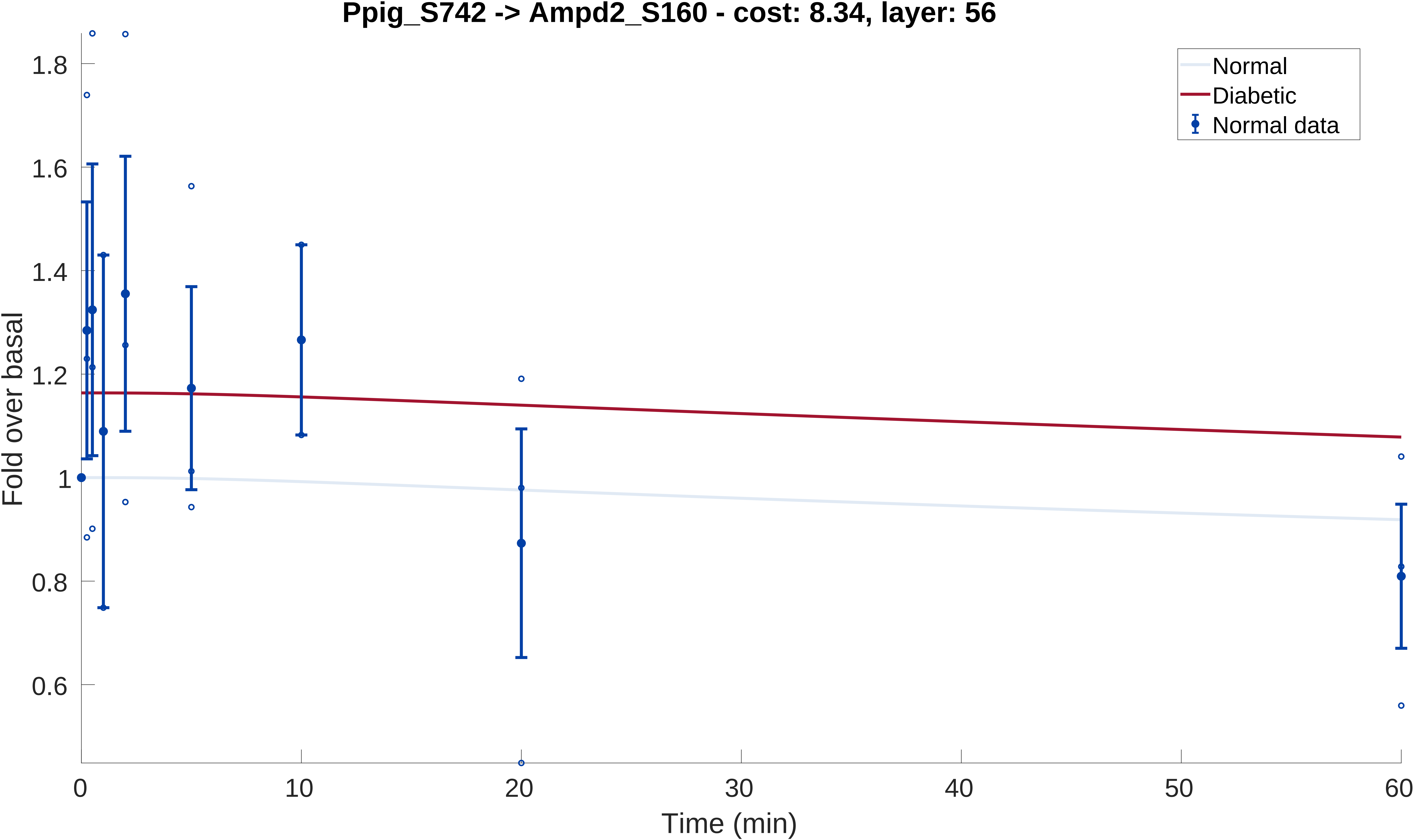

Ppig_S742 -> Ampd2_S160 - cost: 8.34, layer: 56
Normal
Diabetic
Normal data
Fold over basal
1.8
1.6
1.4
1.2
1
0.8
0.6
0
10
20
30
40
50
60
Time (min)

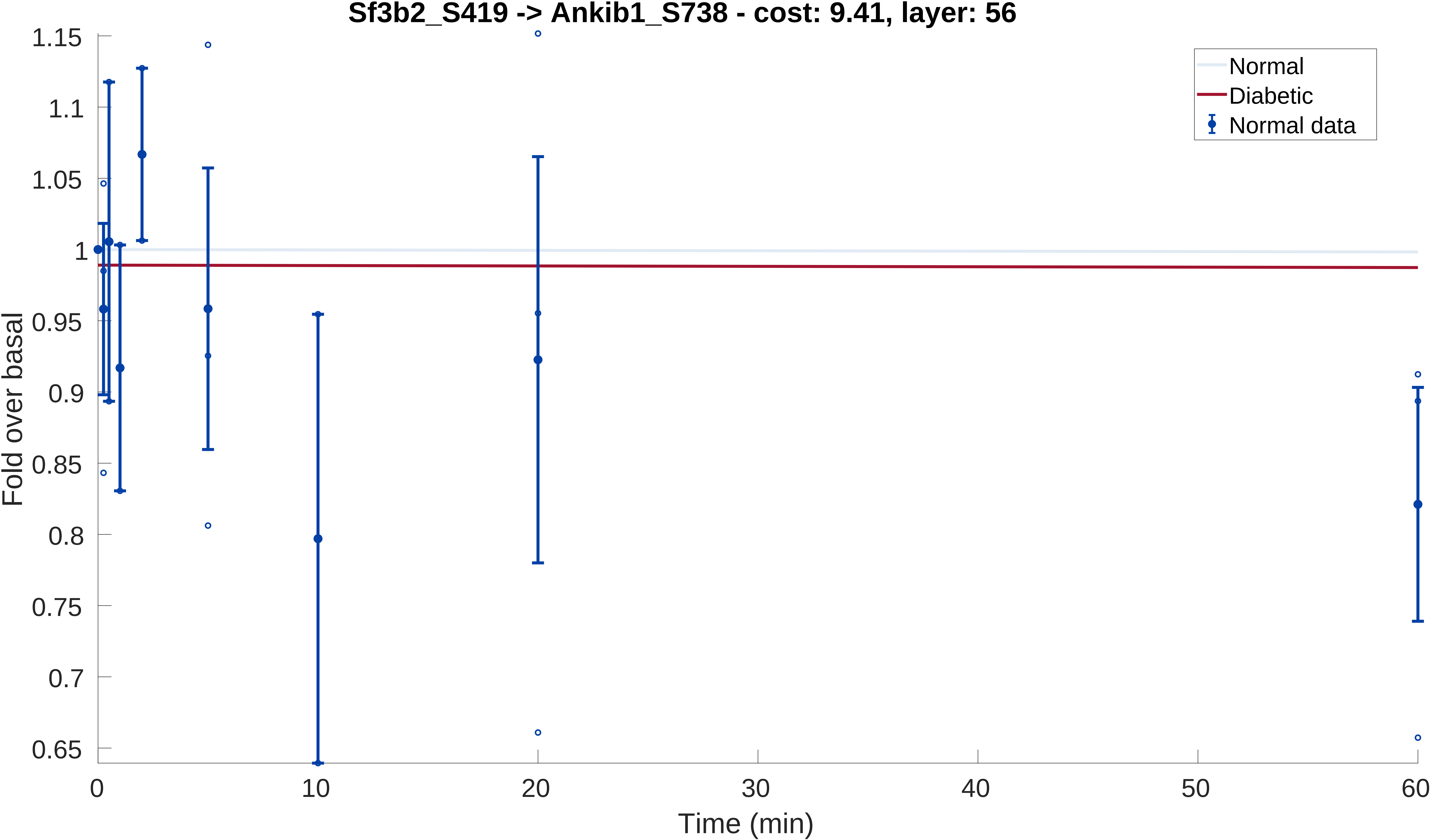

Sf3b2_S419 -> Ankib1_S738 - cost: 9.41, layer: 56
Normal
Diabetic
Normal data
Fold over basal
Time (min)
1.15
1.1
1.05
1
0.95
0.9
0.85
0.8
0.75
0.7
0.65
0
10
20
30
40
50
60

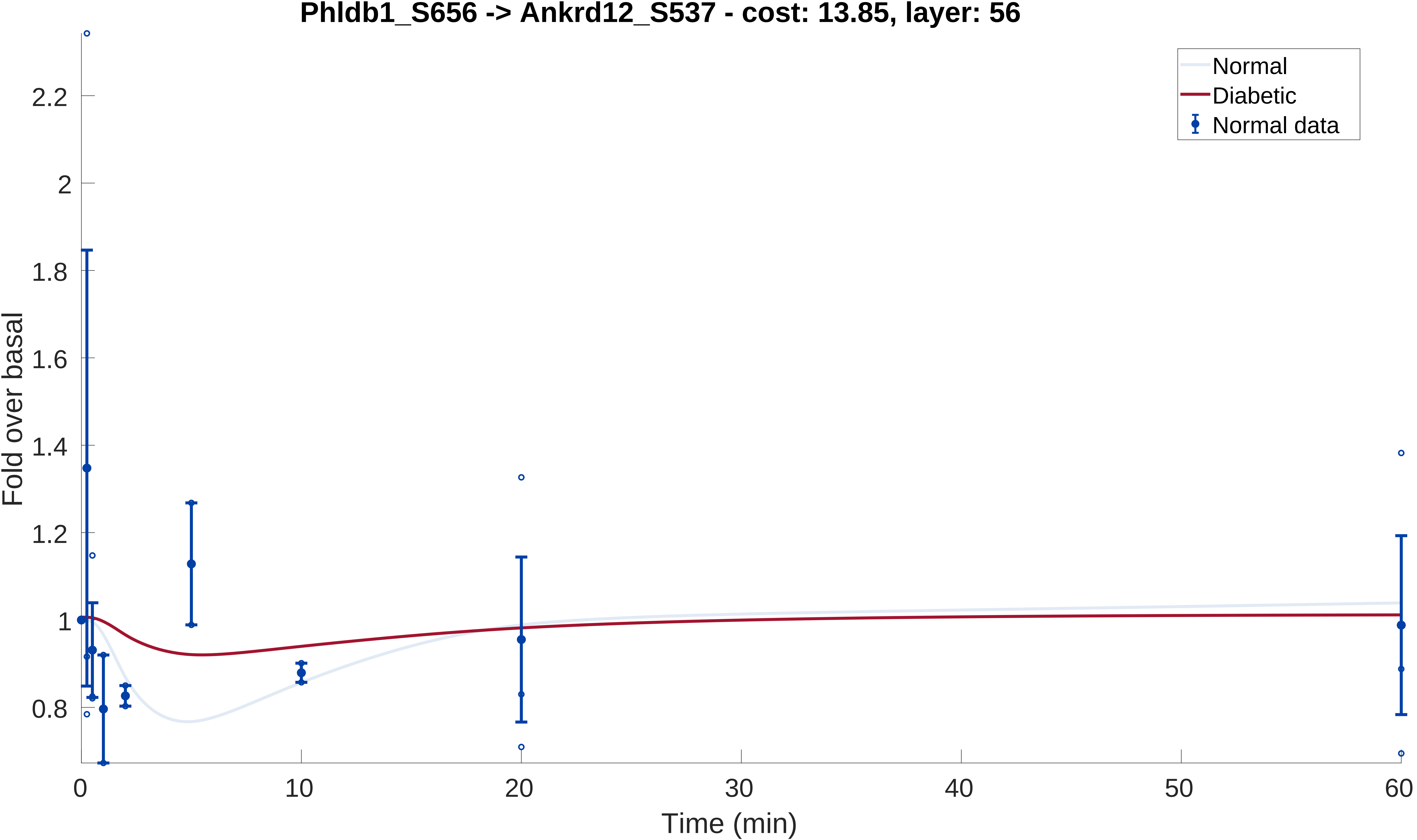

Phldb1_S656 -> Ankrd12_S537 - cost: 13.85, layer: 56
Normal
Diabetic
Normal data
Fold over basal
2.2
2
1.8
1.6
1.4
1.2
1
0.8
0
10
20
30
40
50
60
Time (min)

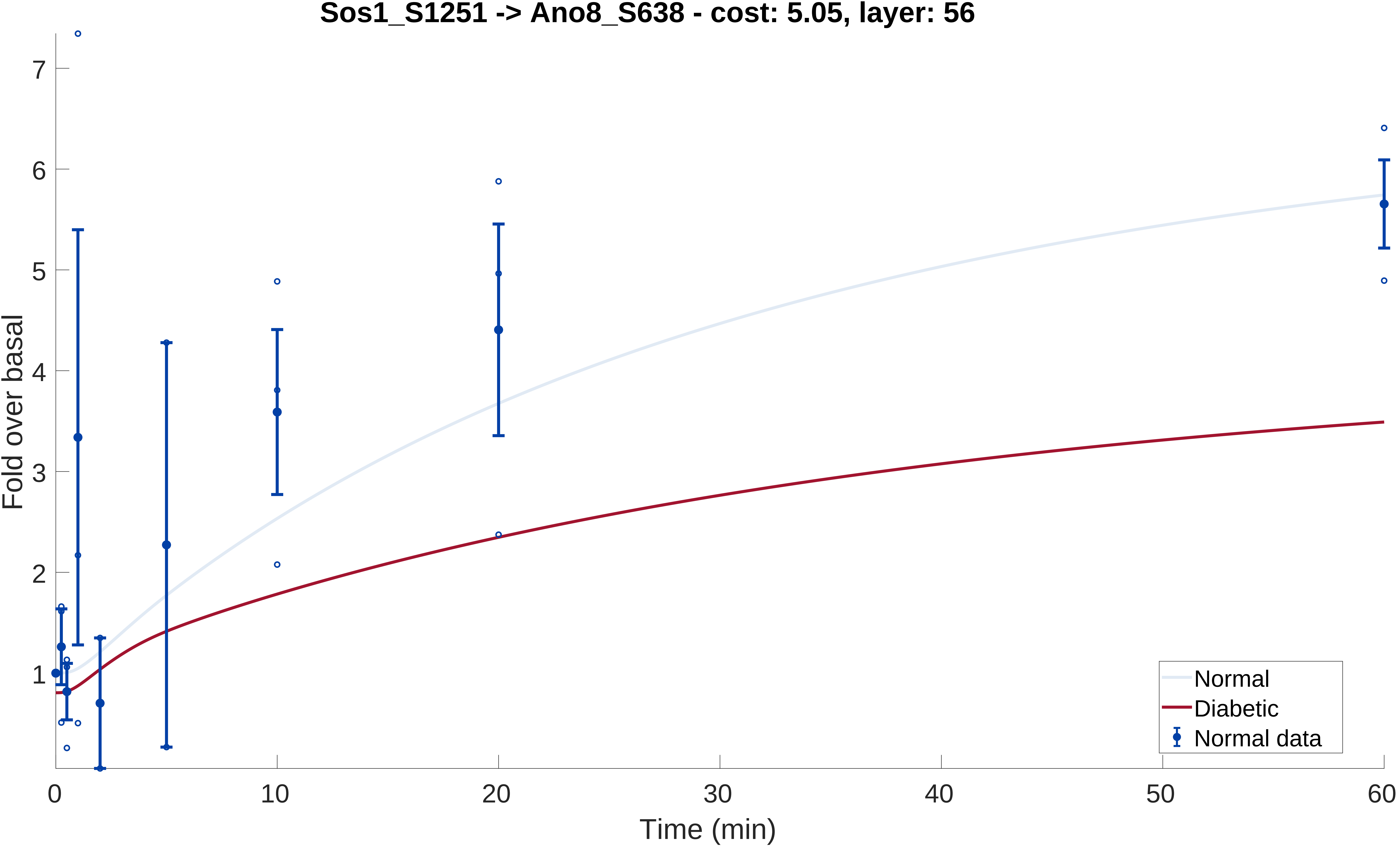

Sos1_S1251 -> Ano8_S638 - cost: 5.05, layer: 56
Fold over basal
Time (min)
Normal
Diabetic
Normal data
0
10
20
30
40
50
60
1
2
3
4
5
6
7

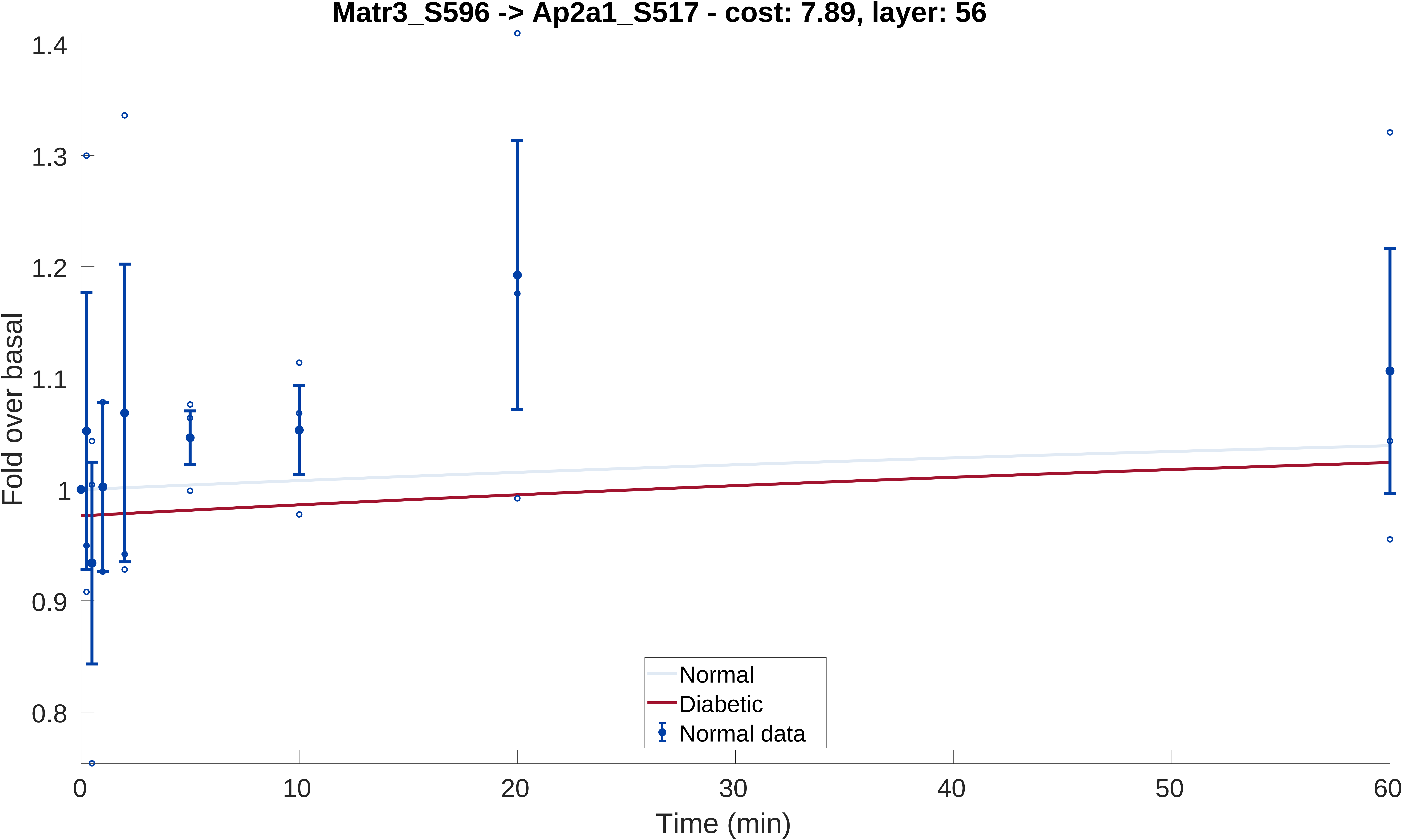
Matr3_S596 -> Ap2a1_S517 - cost: 7.89, layer: 56
Fold over basal
Time (min)
Normal
Diabetic
Normal data
1.4
1.3
1.2
1.1
1
0.9
0.8
0
10
20
30
40
50
60

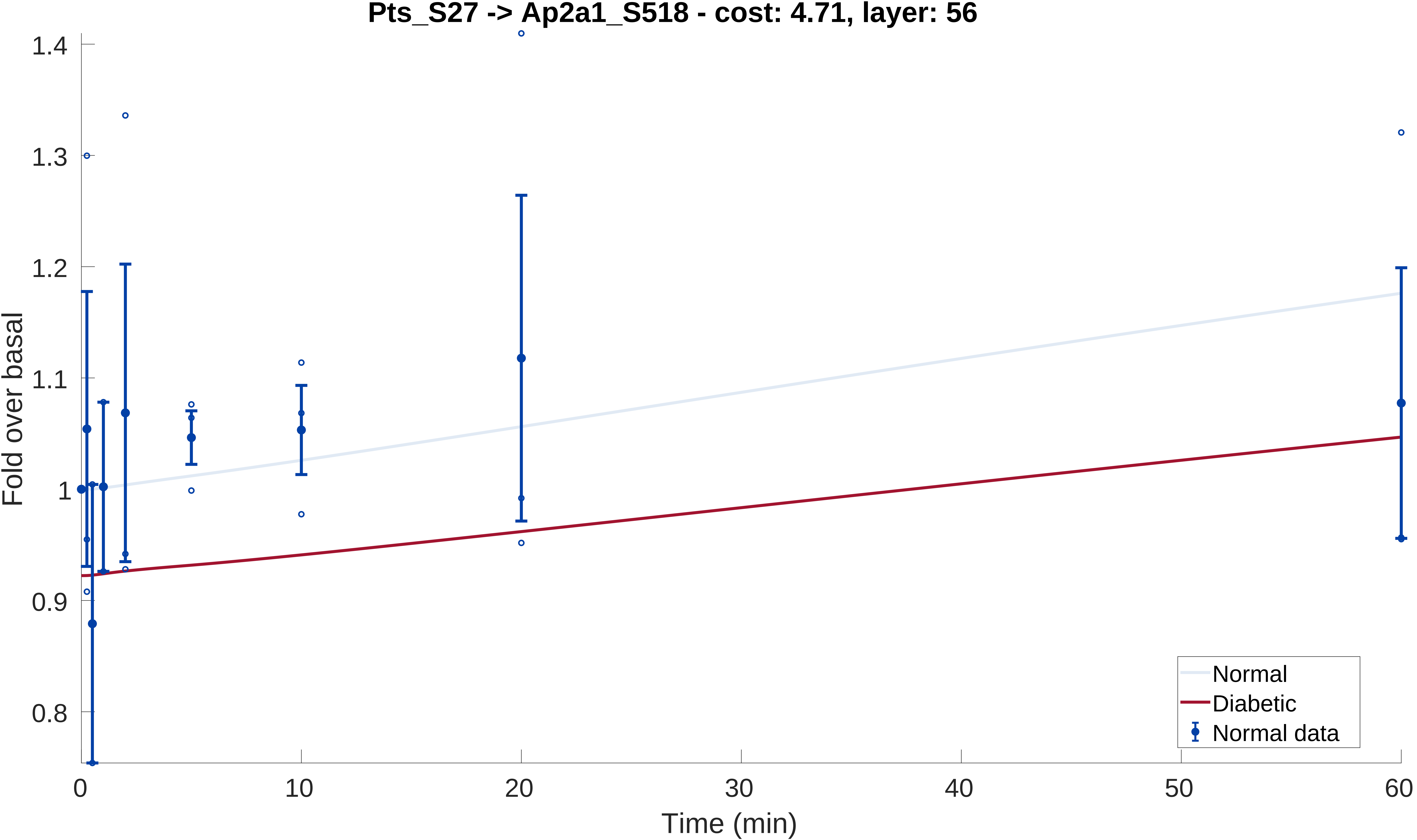
Pts_S27 -> Ap2a1_S518 - cost: 4.71, layer: 56
Fold over basal
1.4
1.3
1.2
1.1
1
0.9
0.8
Time (min)
0
10
20
30
40
50
60
Normal
Diabetic
Normal data

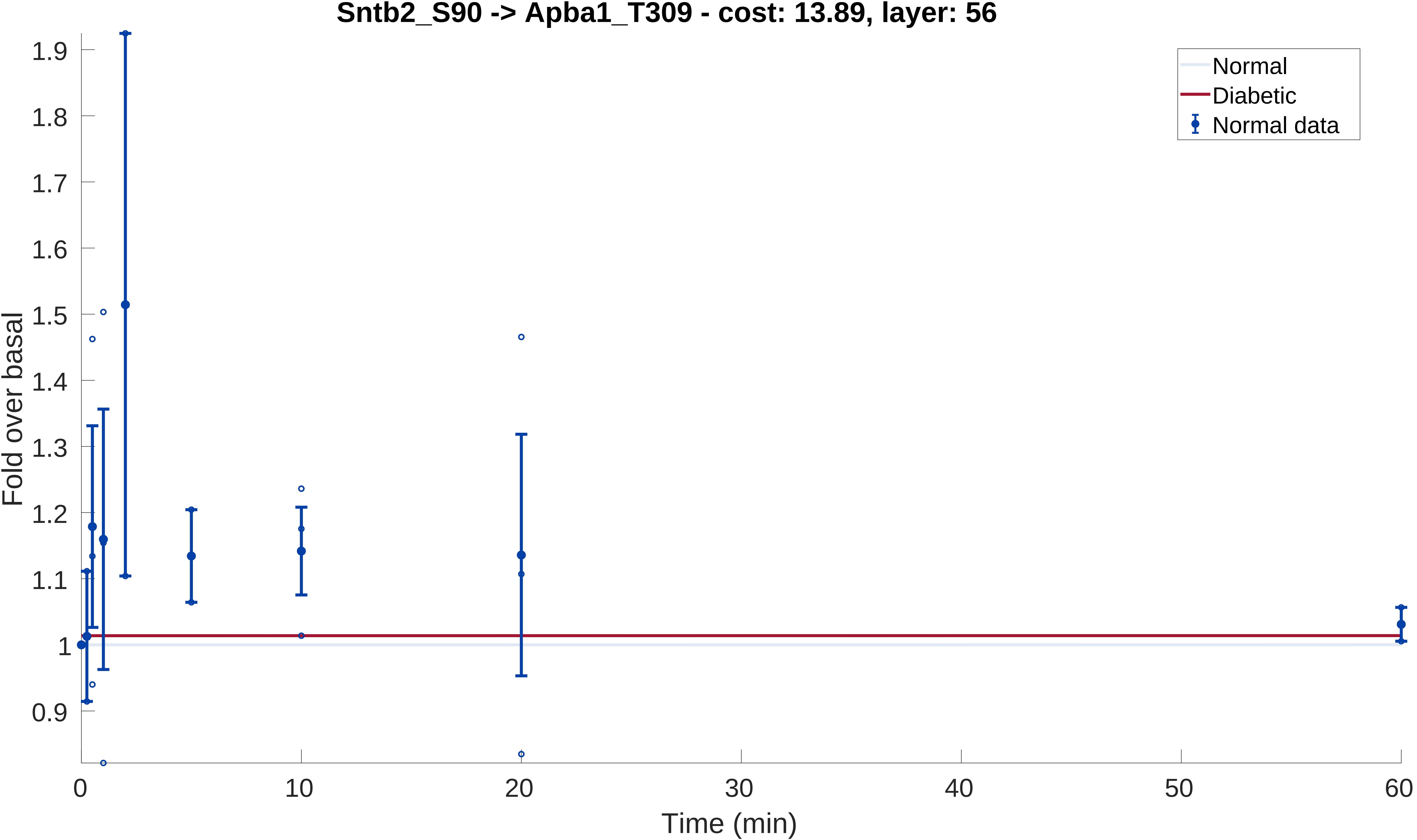

Sntb2_S90 -> Apba1_T309 - cost: 13.89, layer: 56
Normal
Diabetic
Normal data
Fold over basal
Time (min)
1.9
1.8
1.7
1.6
1.5
1.4
1.3
1.2
1.1
1
0.9
0
10
20
30
40
50
60

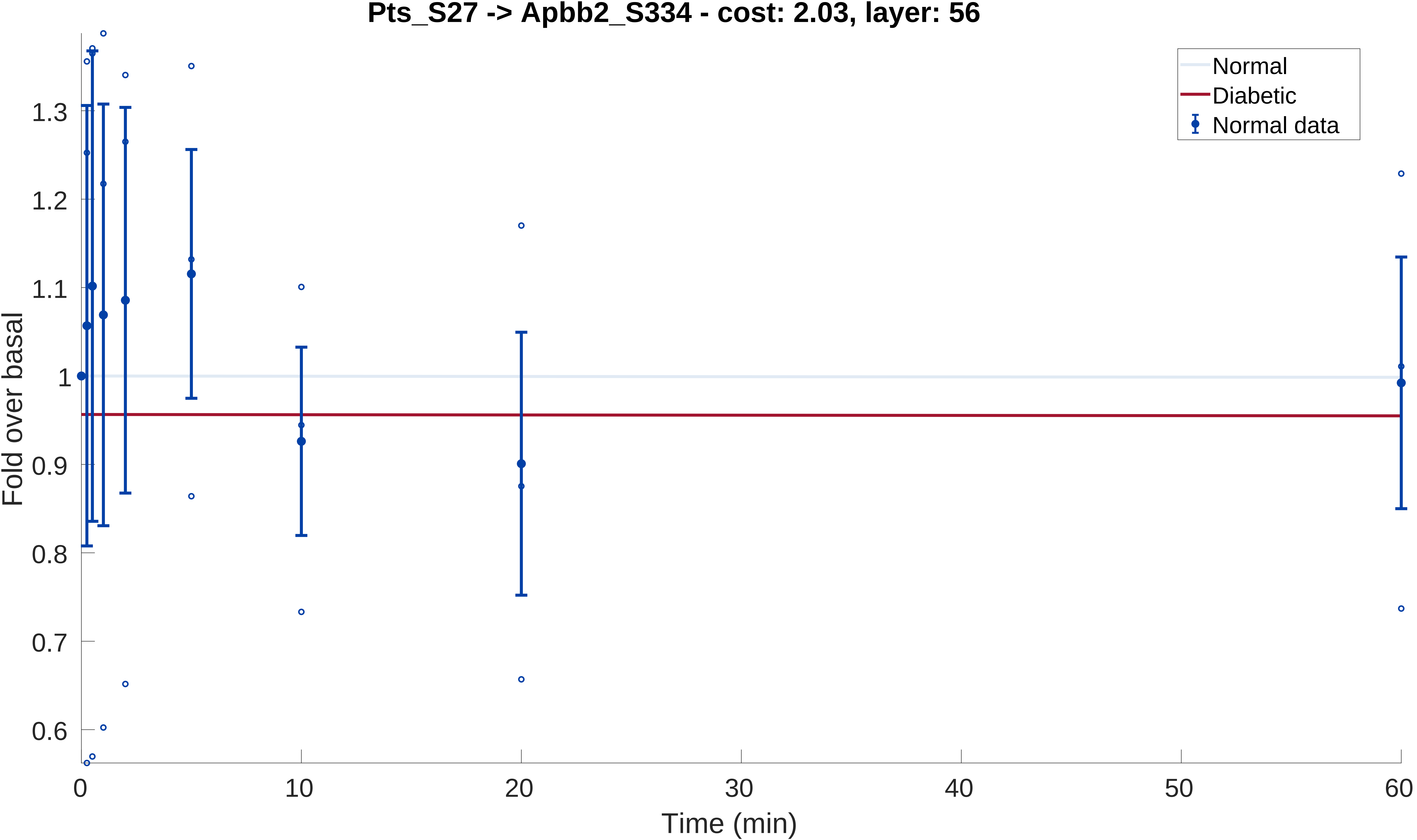

Pts_S27 -> Apbb2_S334 - cost: 2.03, layer: 56
Normal
Diabetic
Normal data
Fold over basal
1.3
1.2
1.1
1
0.9
0.8
0.7
0.6
0
10
20
30
40
50
60
Time (min)

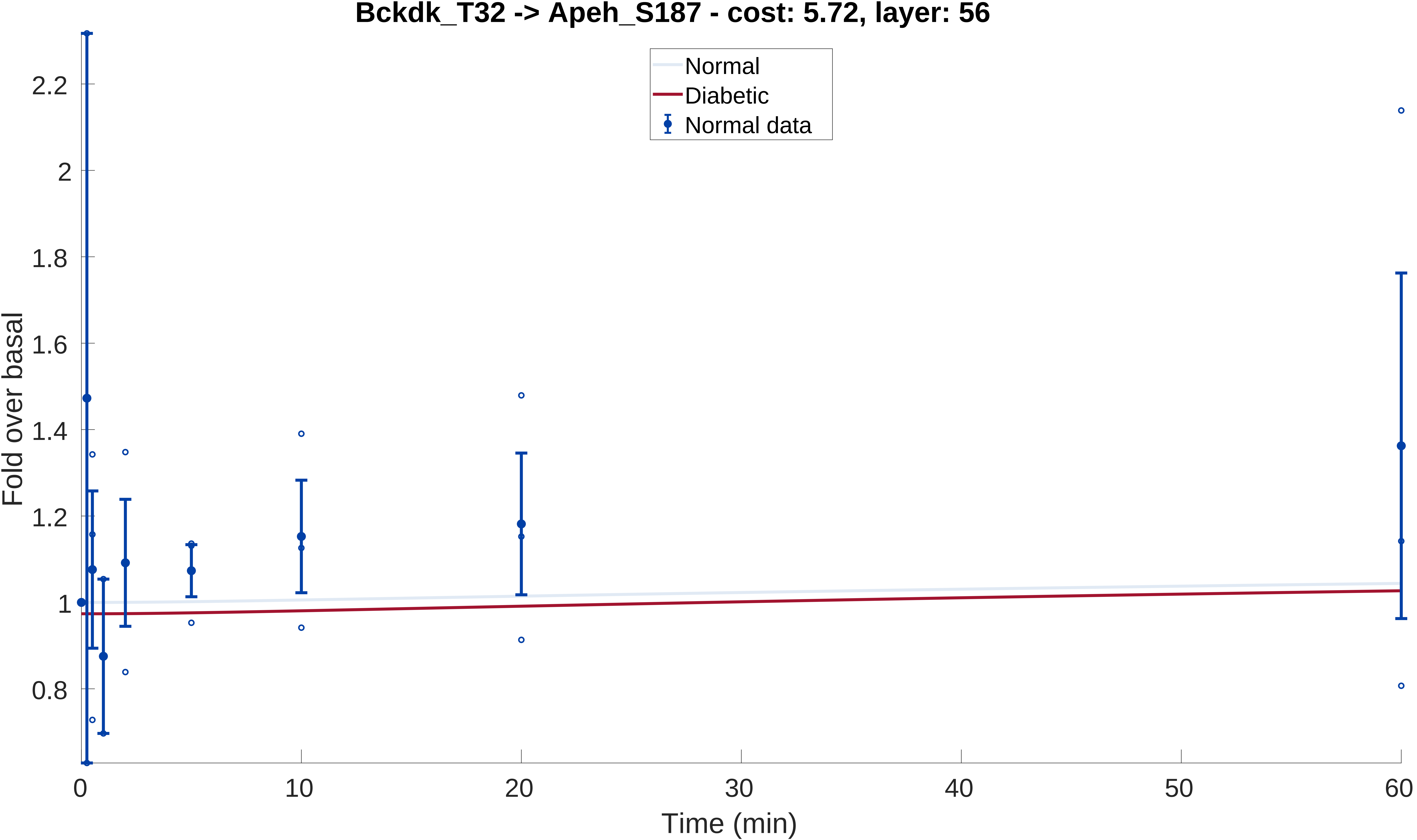

Bckdk_T32 -> Apeh_S187 - cost: 5.72, layer: 56
Normal
Diabetic
Normal data
Fold over basal
2.2
2
1.8
1.6
1.4
1.2
1
0.8
0
10
20
30
40
50
60
Time (min)

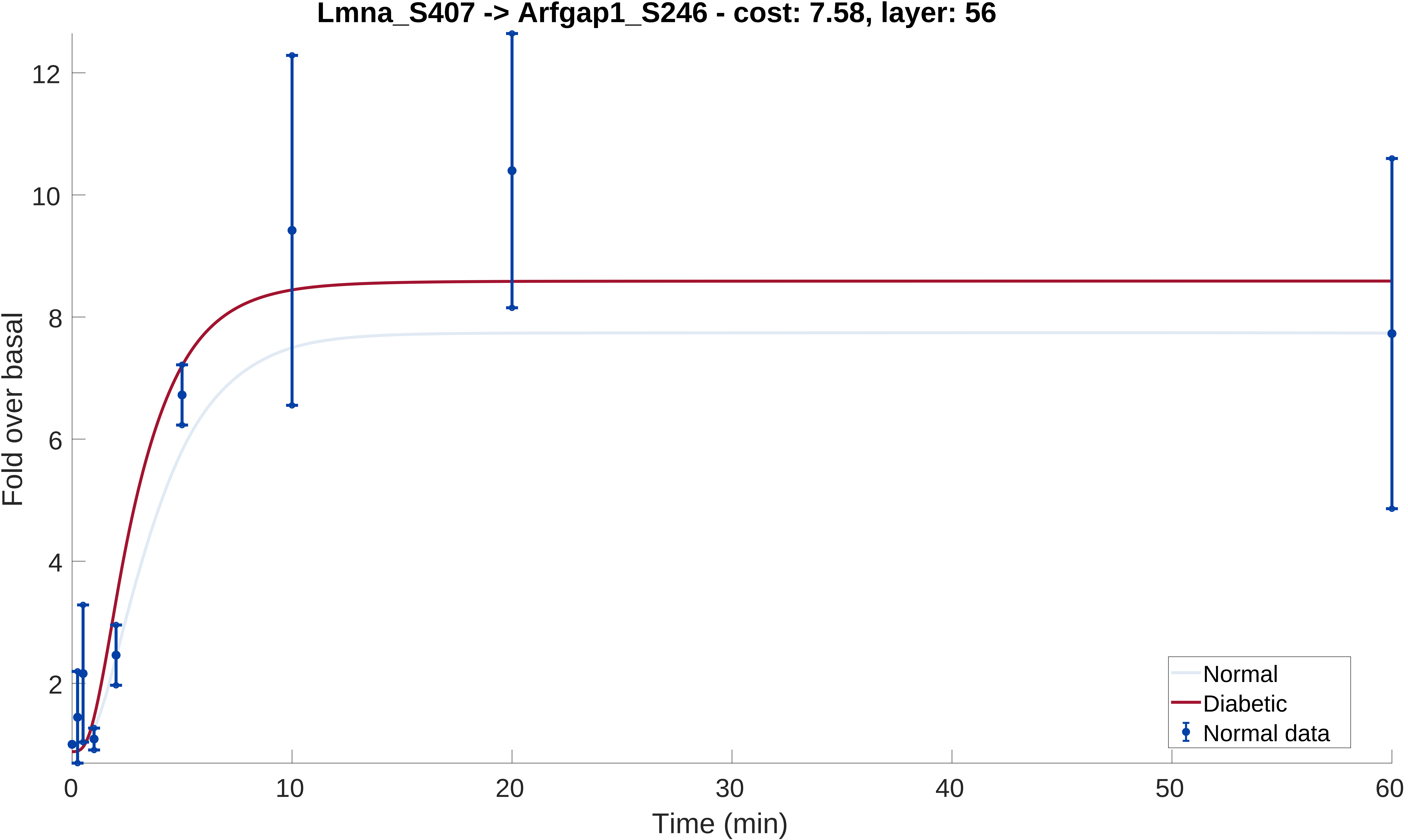

Lmna_S407 -> Arfgap1_S246 - cost: 7.58, layer: 56
Fold over basal
Time (min)
0
2
4
6
8
10
12
0
10
20
30
40
50
60
Normal
Diabetic
Normal data

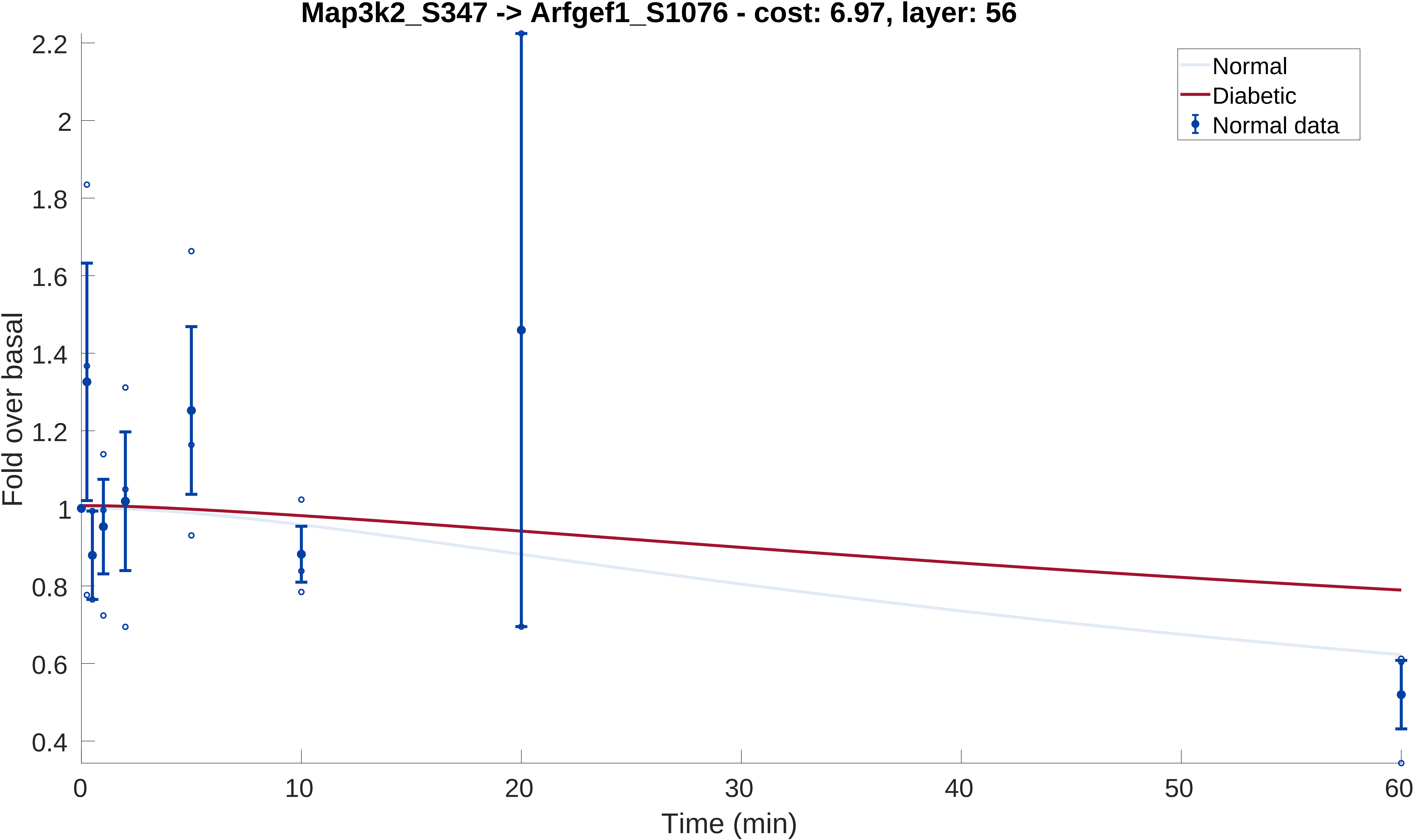
Map3k2_S347 -> Arfgef1_S1076 - cost: 6.97, layer: 56
Normal
Diabetic
Normal data
Fold over basal
Time (min)
0.4
0.6
0.8
1
1.2
1.4
1.6
1.8
2
2.2
0
10
20
30
40
50
60

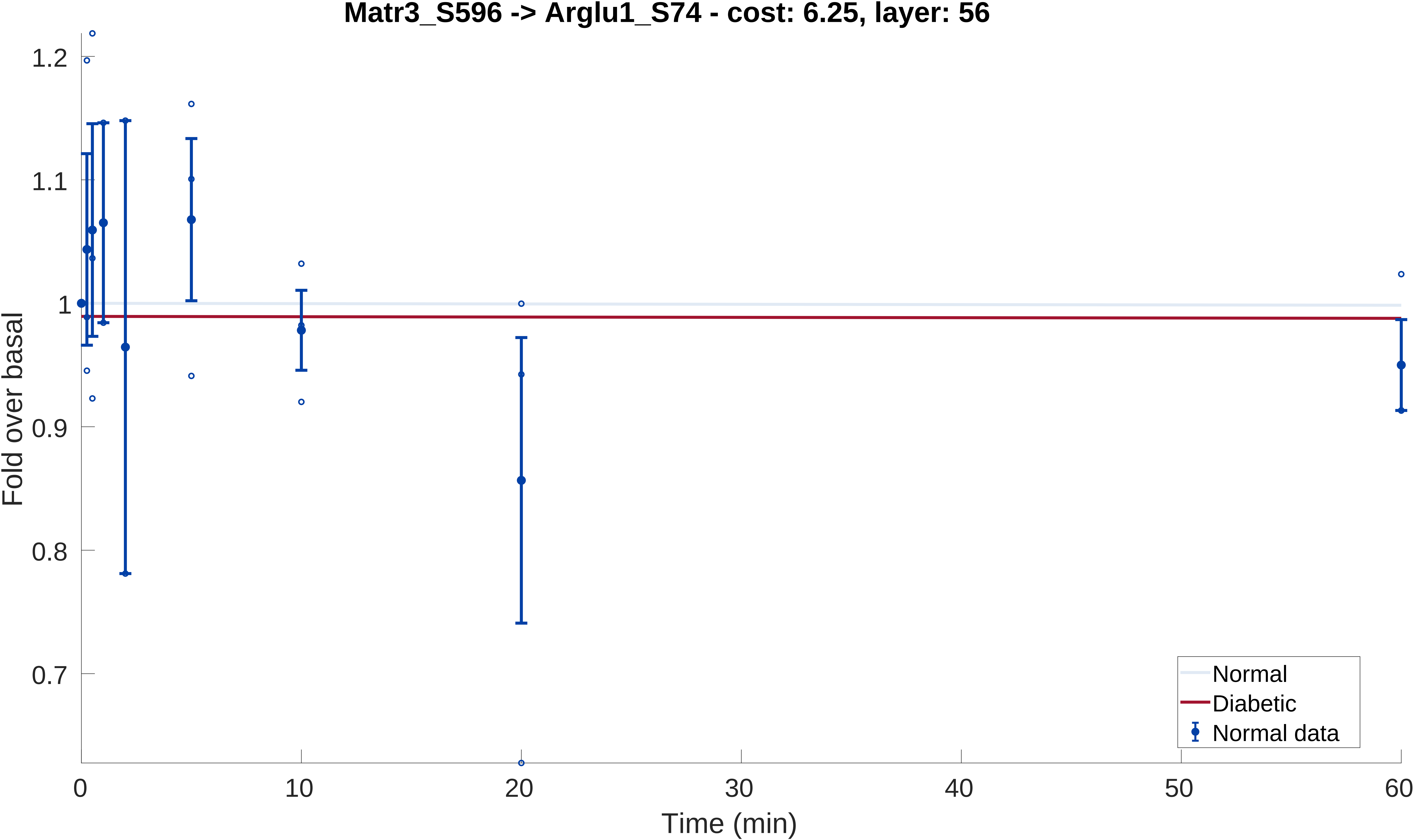
Matr3_S596 -> Arglu1_S74 - cost: 6.25, layer: 56
Fold over basal
1.2
1.1
1
0.9
0.8
0.7
0
10
20
30
40
50
60
Time (min)
Normal
Diabetic
Normal data

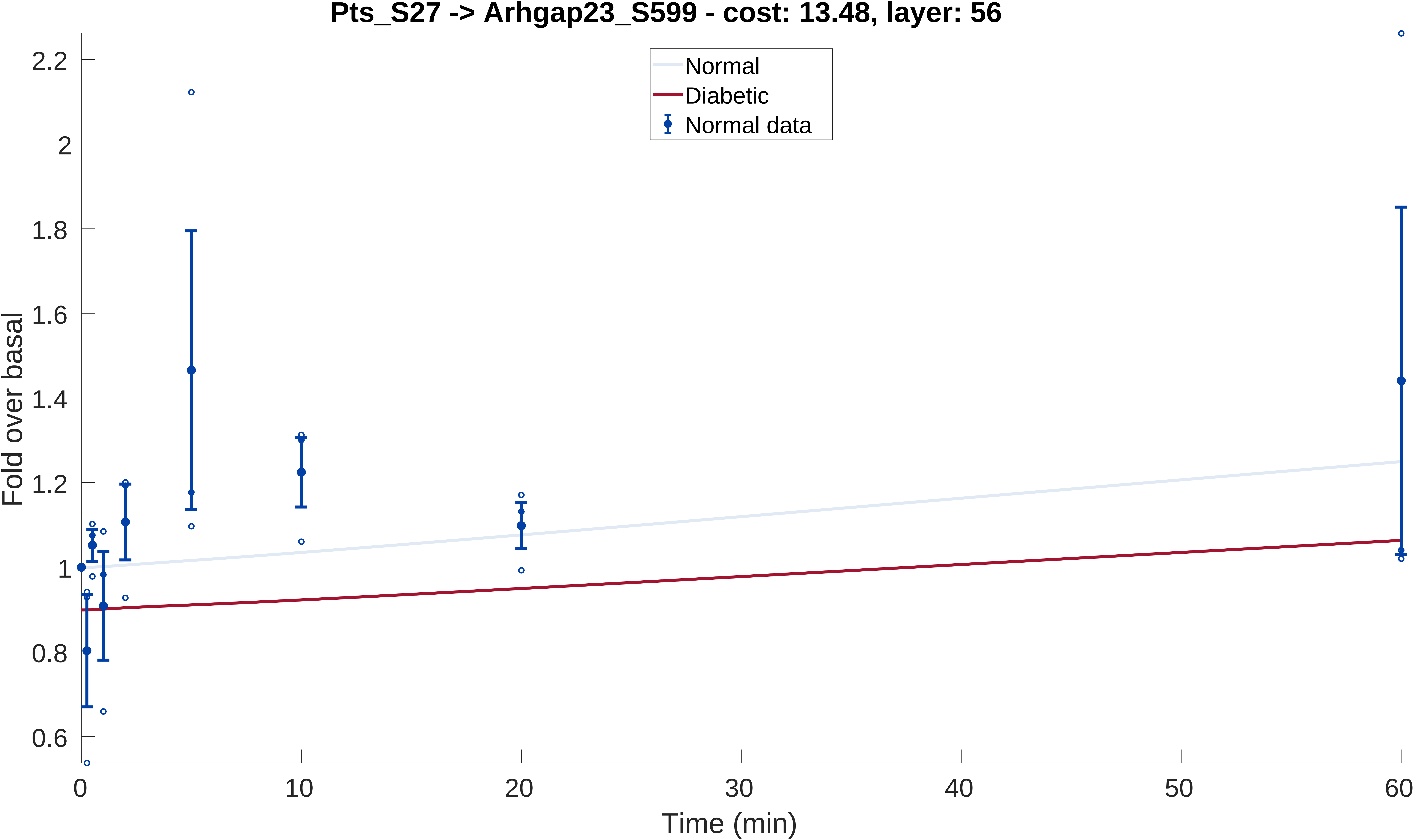
Pts_S27 -> Arhgap23_S599 - cost: 13.48, layer: 56
Normal
Diabetic
Normal data
Fold over basal
Time (min)
0.6
0.8
1
1.2
1.4
1.6
1.8
2
2.2
0
10
20
30
40
50
60

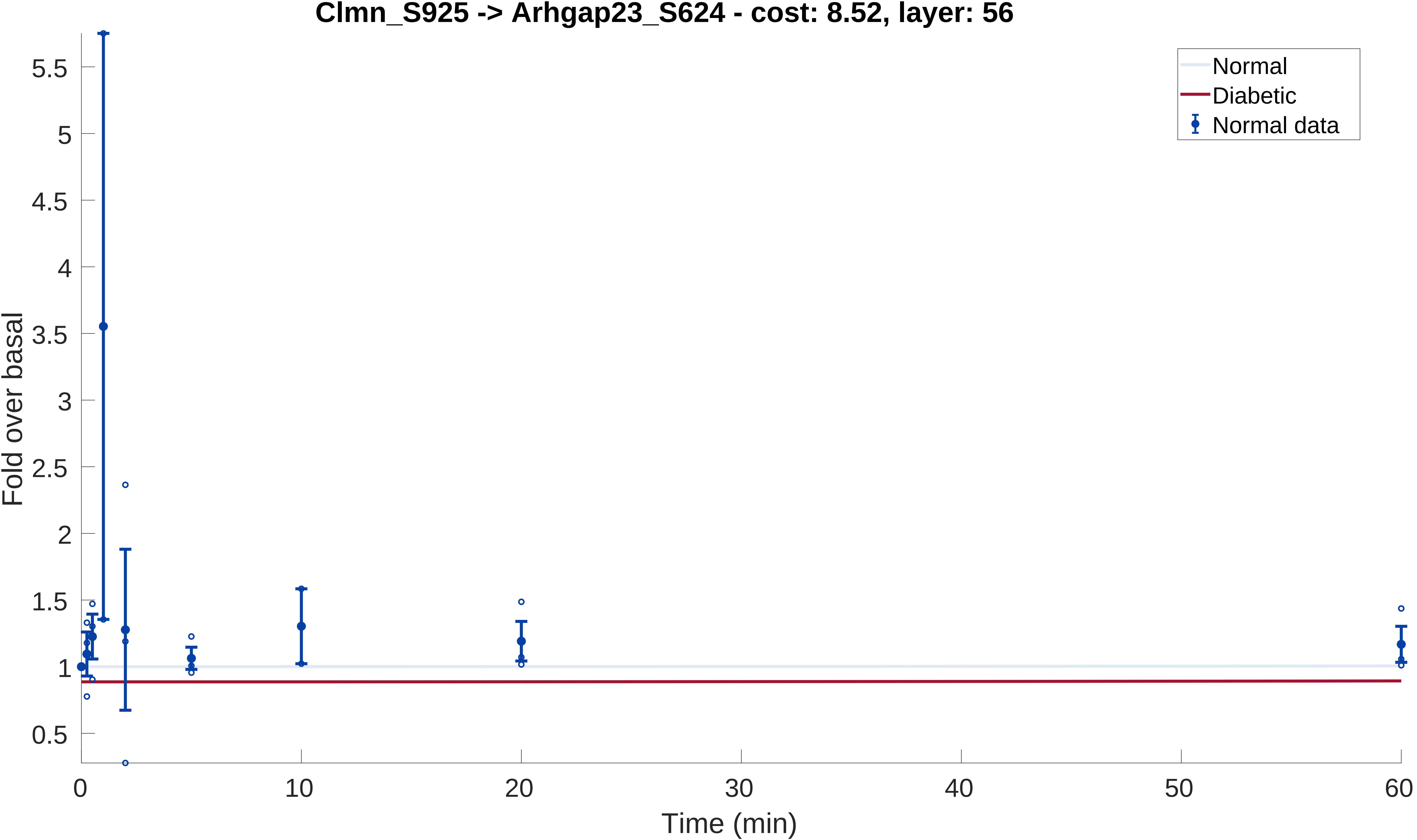
Clmn_S925 -> Arhgap23_S624 - cost: 8.52, layer: 56
Normal
Diabetic
Normal data
Fold over basal
0.5
1
1.5
2
2.5
3
3.5
4
4.5
5
5.5
0
10
20
30
40
50
60
Time (min)

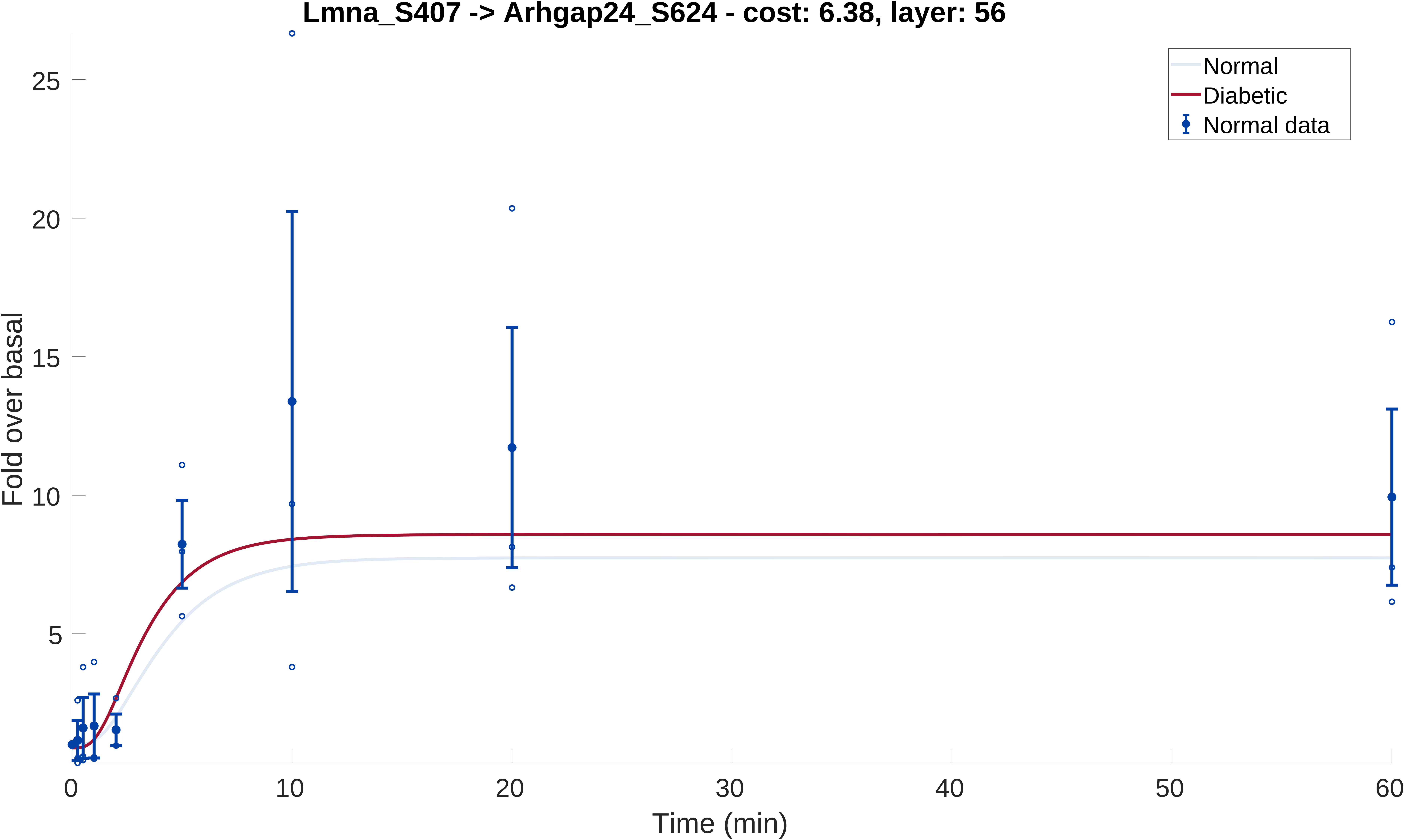

Lmna_S407 -> Arhgap24_S624 - cost: 6.38, layer: 56
Normal
Diabetic
Normal data
Fold over basal
Time (min)
0
10
20
30
40
50
60
5
10
15
20
25

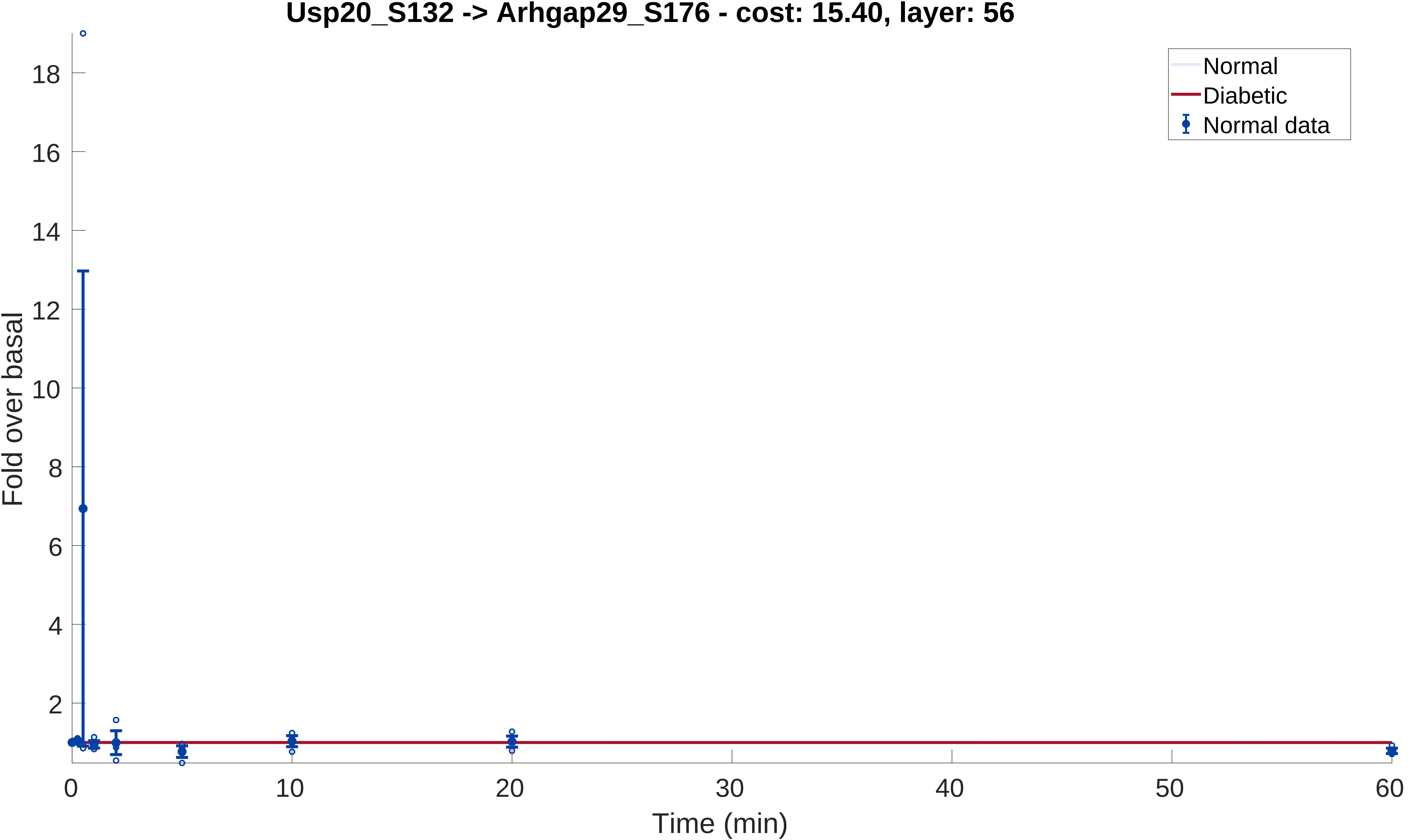

Usp20_S132 -> Arhgap29_S176 - cost: 15.40, layer: 56
Normal
Diabetic
Normal data
Fold over basal
18
16
14
12
10
8
6
4
2
0
10
20
30
40
50
60
Time (min)

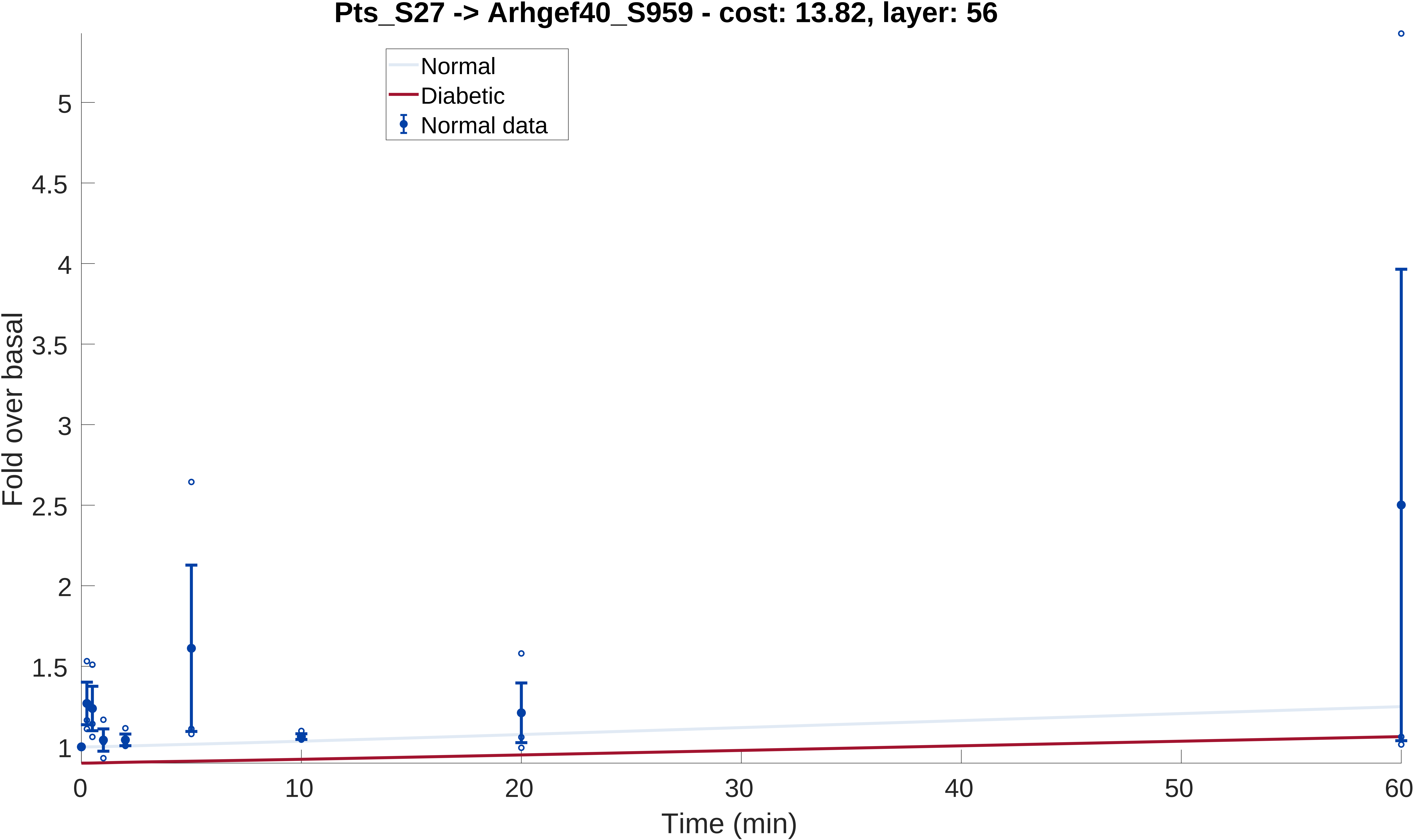

Pts_S27 -> Arhgef40_S959 - cost: 13.82, layer: 56
Normal
Diabetic
Normal data
Fold over basal
Time (min)
0
10
20
30
40
50
60
1
1.5
2
2.5
3
3.5
4
4.5
5

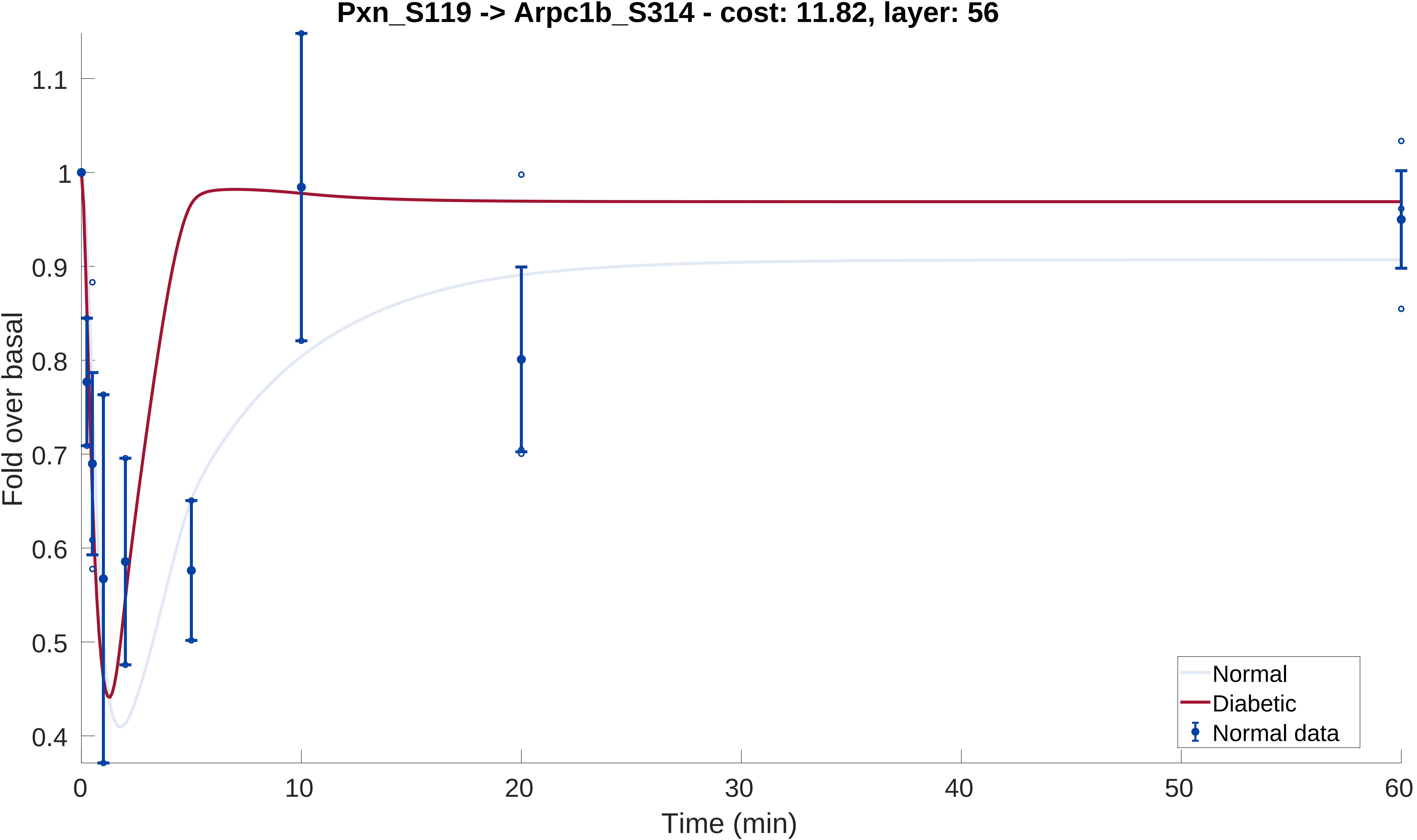

Pxn_S119 -> Arpc1b_S314 - cost: 11.82, layer: 56
Fold over basal
Time (min)
Normal
Diabetic
Normal data
1.1
1
0.9
0.8
0.7
0.6
0.5
0.4
0
10
20
30
40
50
60

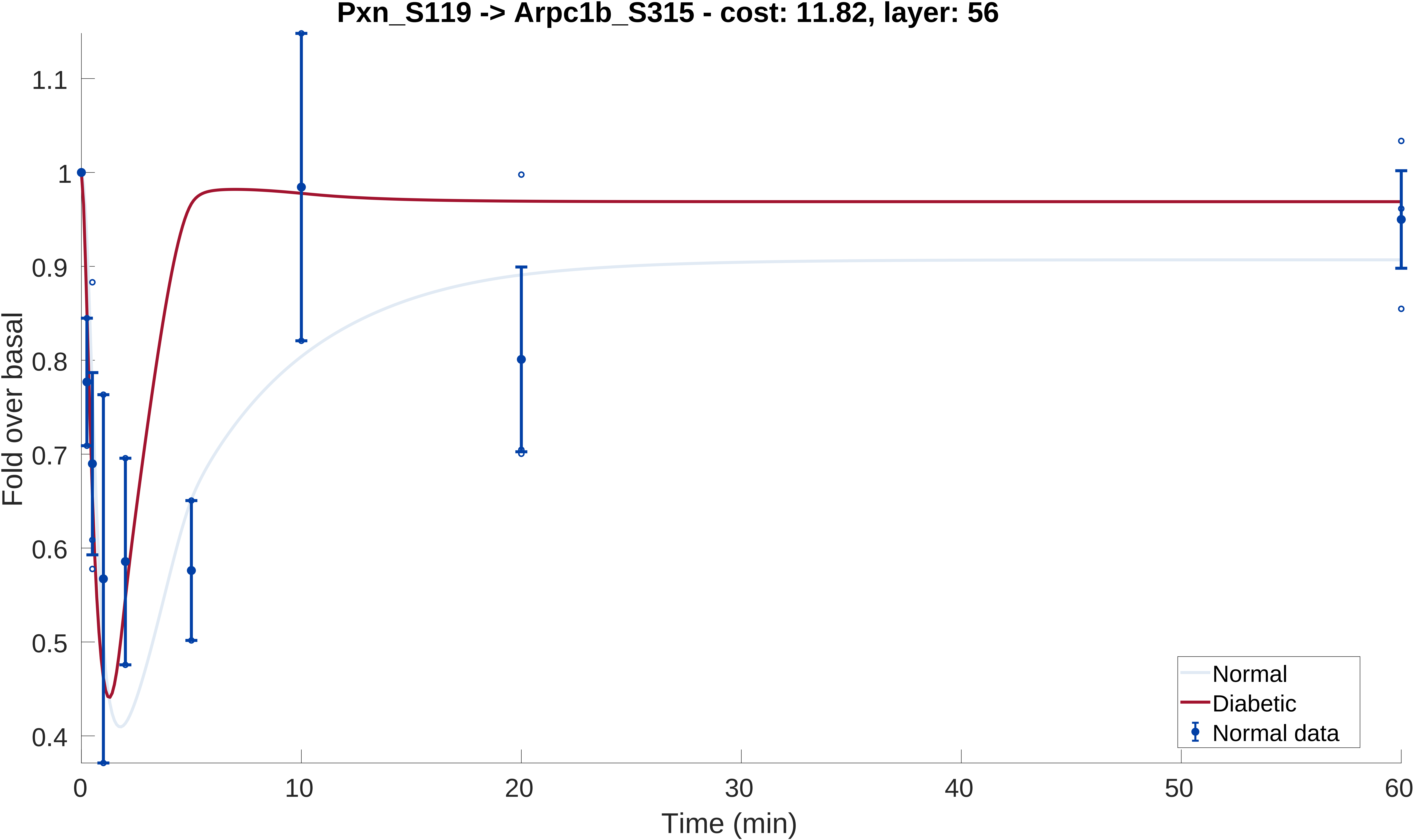
Pxn_S119 -> Arpc1b_S315 - cost: 11.82, layer: 56
Fold over basal
1.1
1
0.9
0.8
0.7
0.6
0.5
0.4
0
10
20
30
40
50
60
Time (min)
Normal
Diabetic
Normal data

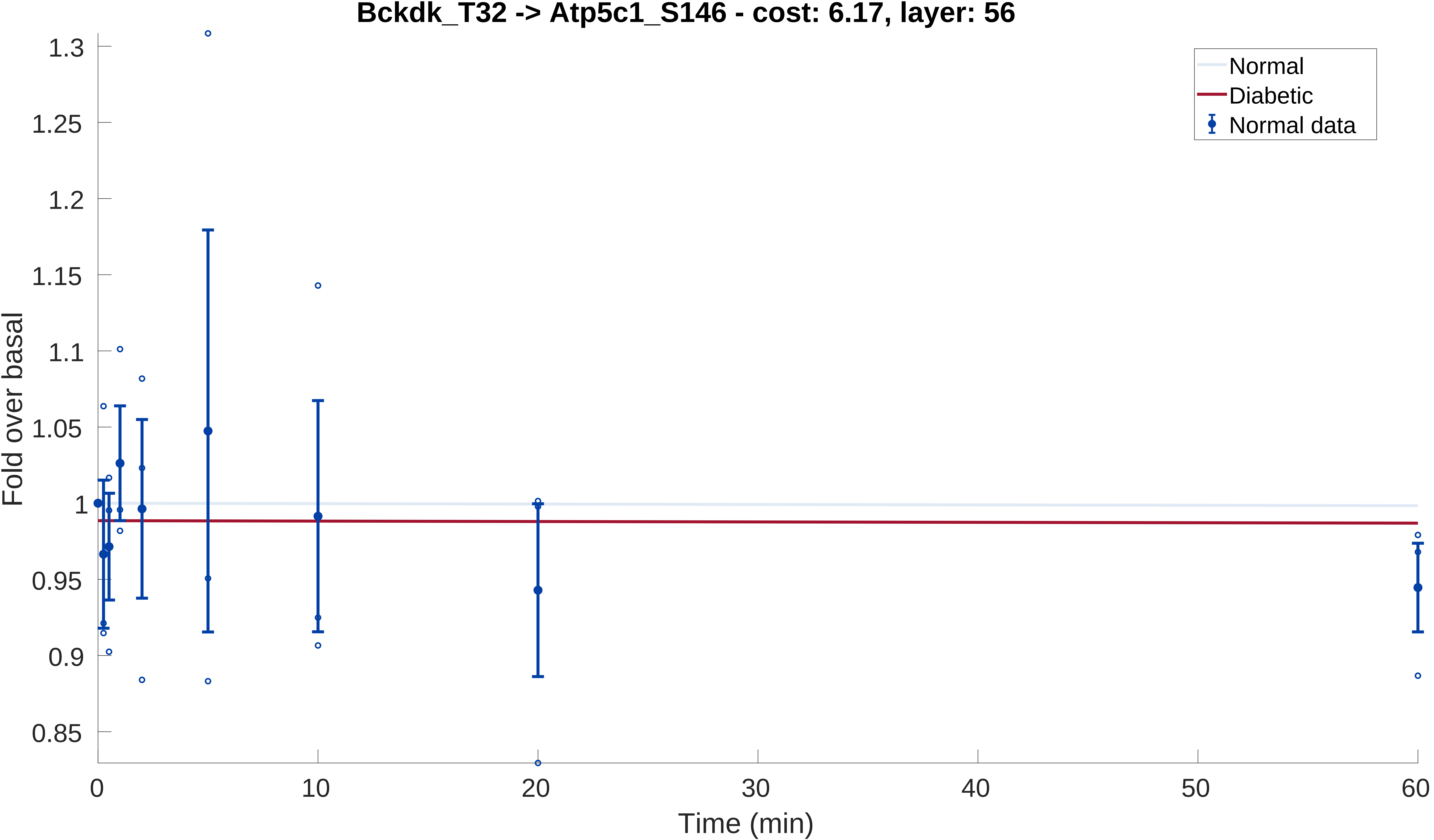

Bckdk_T32 -> Atp5c1_S146 - cost: 6.17, layer: 56
Normal
Diabetic
Normal data
Fold over basal
Time (min)
1.3
1.25
1.2
1.15
1.1
1.05
1
0.95
0.9
0.85
0
10
20
30
40
50
60

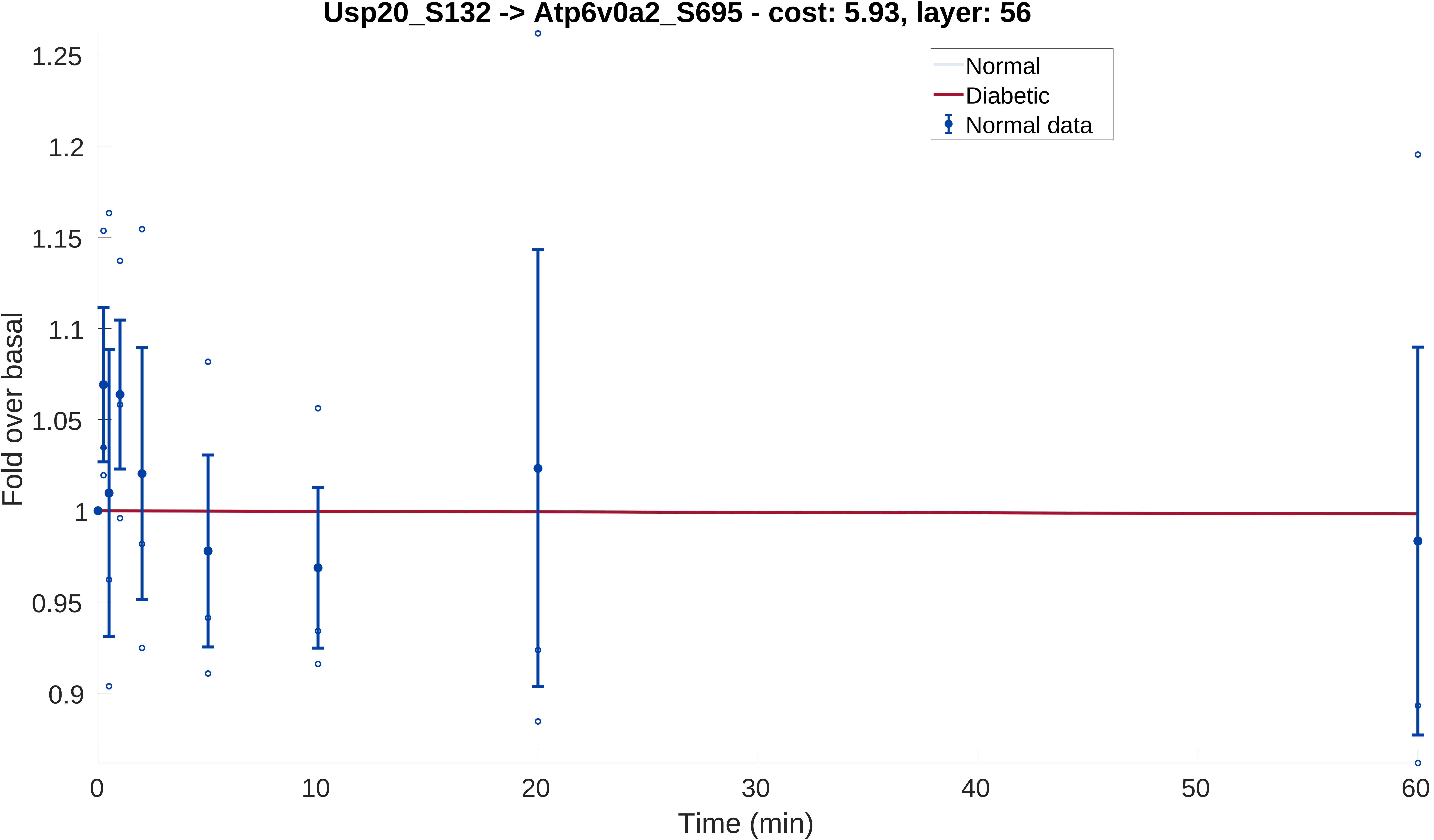

Usp20_S132 -> Atp6v0a2_S695 - cost: 5.93, layer: 56
Normal
Diabetic
Normal data
Fold over basal
1.25
1.2
1.15
1.1
1.05
1
0.95
0.9
0
10
20
30
40
50
60
Time (min)

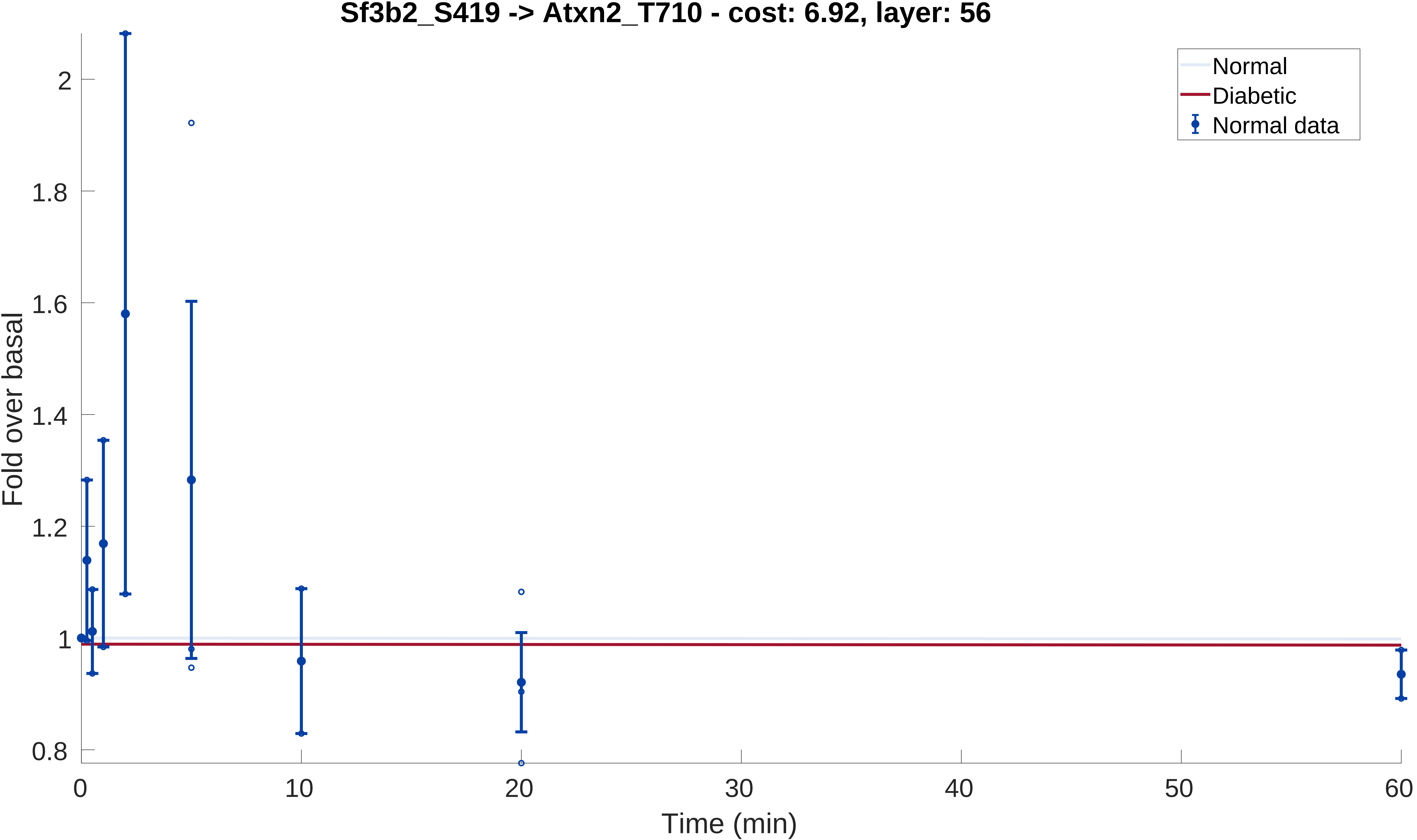
Sf3b2_S419 -> Atxn2_T710 - cost: 6.92, layer: 56
Normal
Diabetic
Normal data
Fold over basal
Time (min)
0.8
1
1.2
1.4
1.6
1.8
2
0
10
20
30
40
50
60

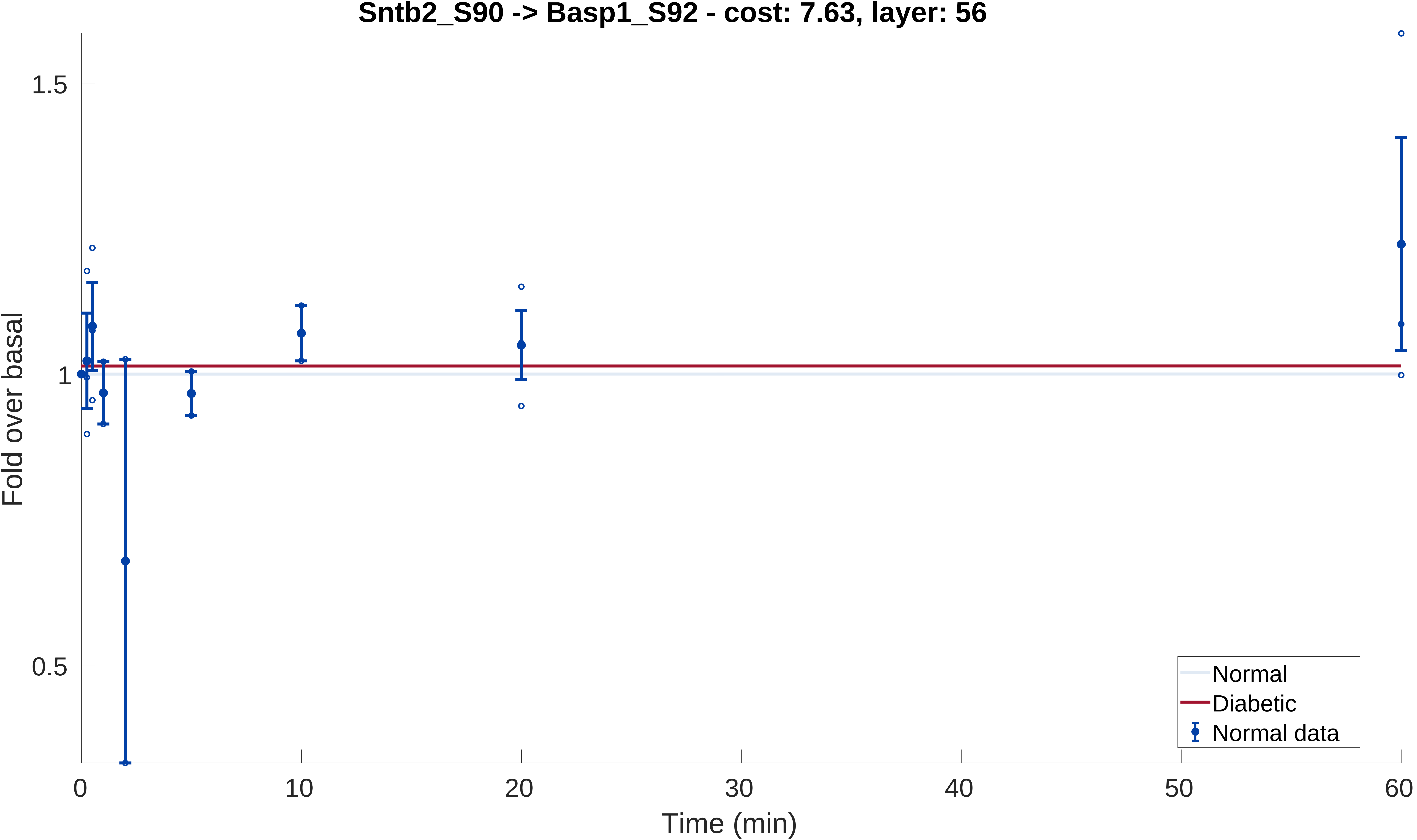
Sntb2_S90 -> Basp1_S92 - cost: 7.63, layer: 56
Fold over basal
1.5
1
0.5
0
10
20
30
40
50
60
Time (min)
Normal
Diabetic
Normal data

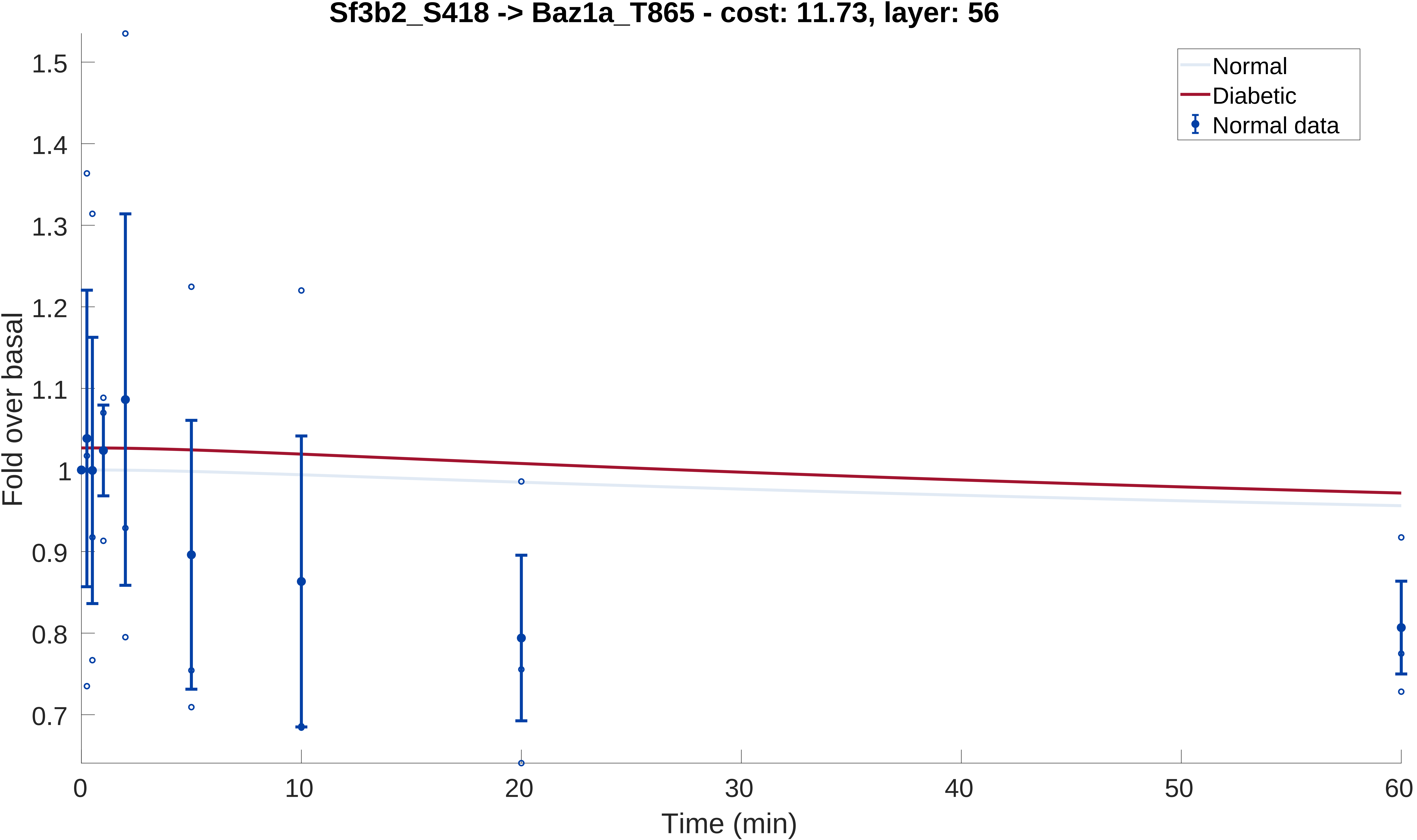

Sf3b2_S418 -> Baz1a_T865 - cost: 11.73, layer: 56
Normal
Diabetic
Normal data
Fold over basal
Time (min)
1.5
1.4
1.3
1.2
1.1
1
0.9
0.8
0.7
0
10
20
30
40
50
60

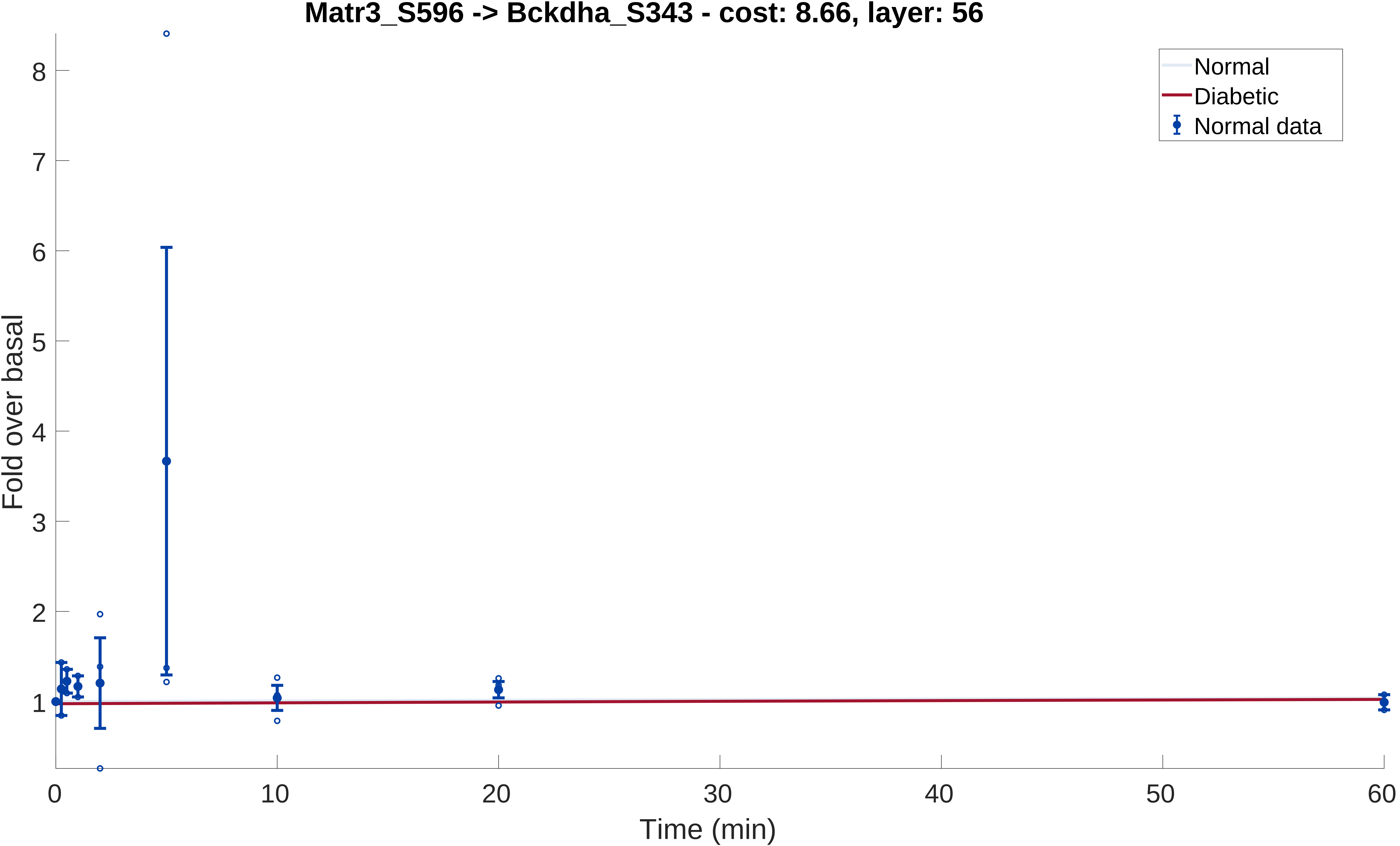
Matr3_S596 -> Bckdha_S343 - cost: 8.66, layer: 56
Normal
Diabetic
Normal data
Fold over basal
Time (min)
0
10
20
30
40
50
60
1
2
3
4
5
6
7
8

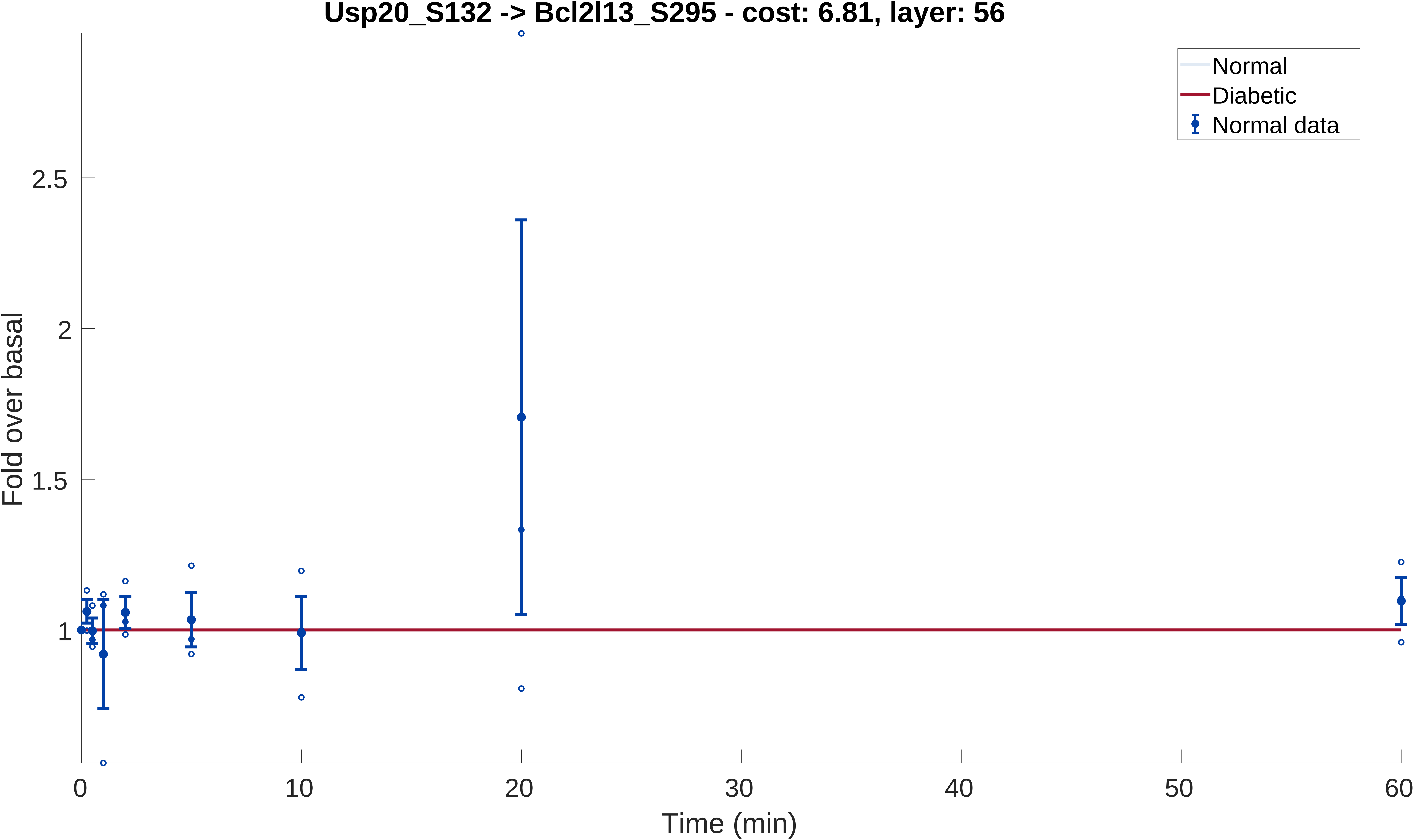

Usp20_S132 -> Bcl2l13_S295 - cost: 6.81, layer: 56
Normal
Diabetic
Normal data
Fold over basal
2.5
2
1.5
1
0
10
20
30
40
50
60
Time (min)

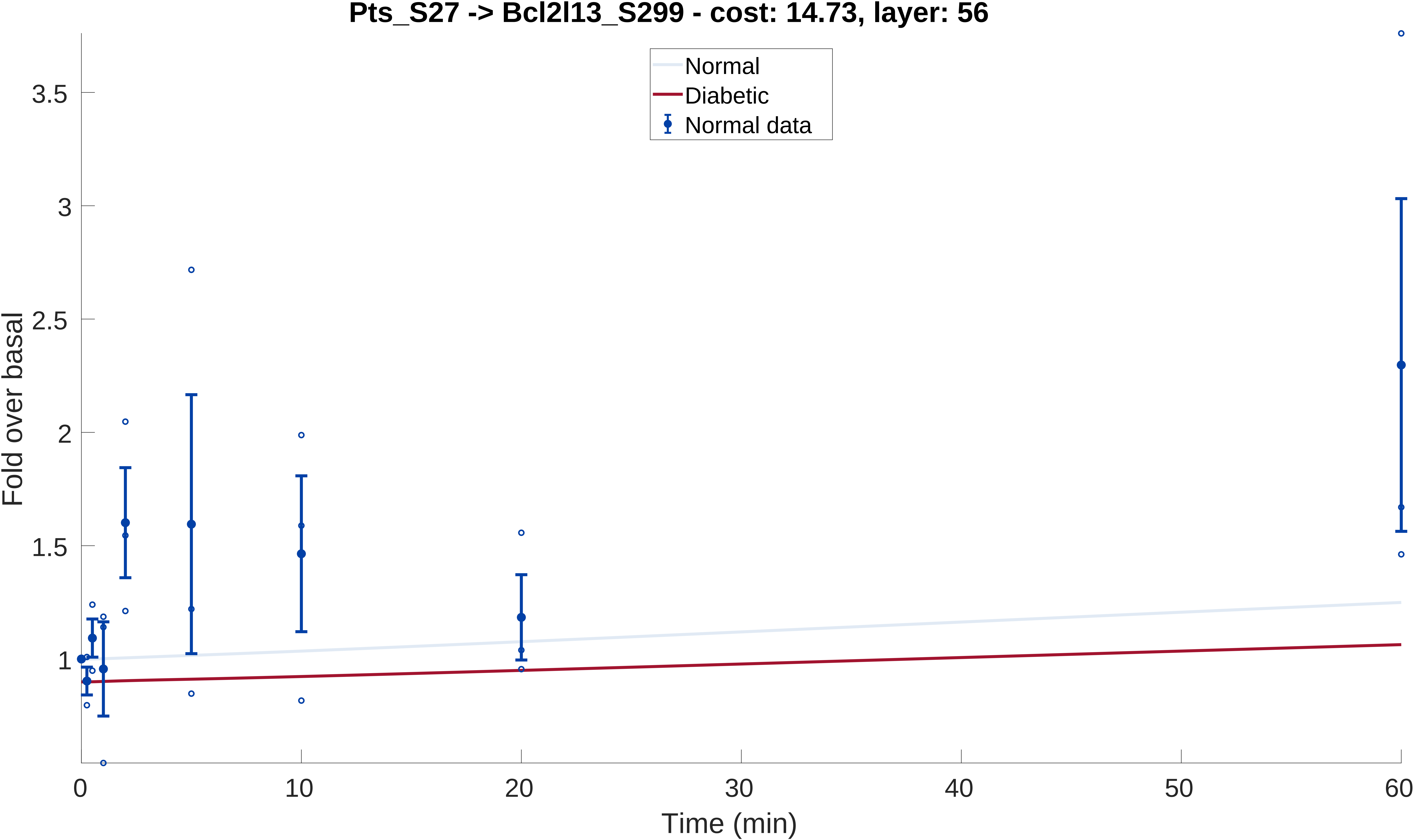

Pts_S27 -> Bcl2l13_S299 - cost: 14.73, layer: 56
Normal
Diabetic
Normal data
3.5
3
2.5
2
1.5
1
0
10
20
30
40
50
60
Fold over basal
Time (min)

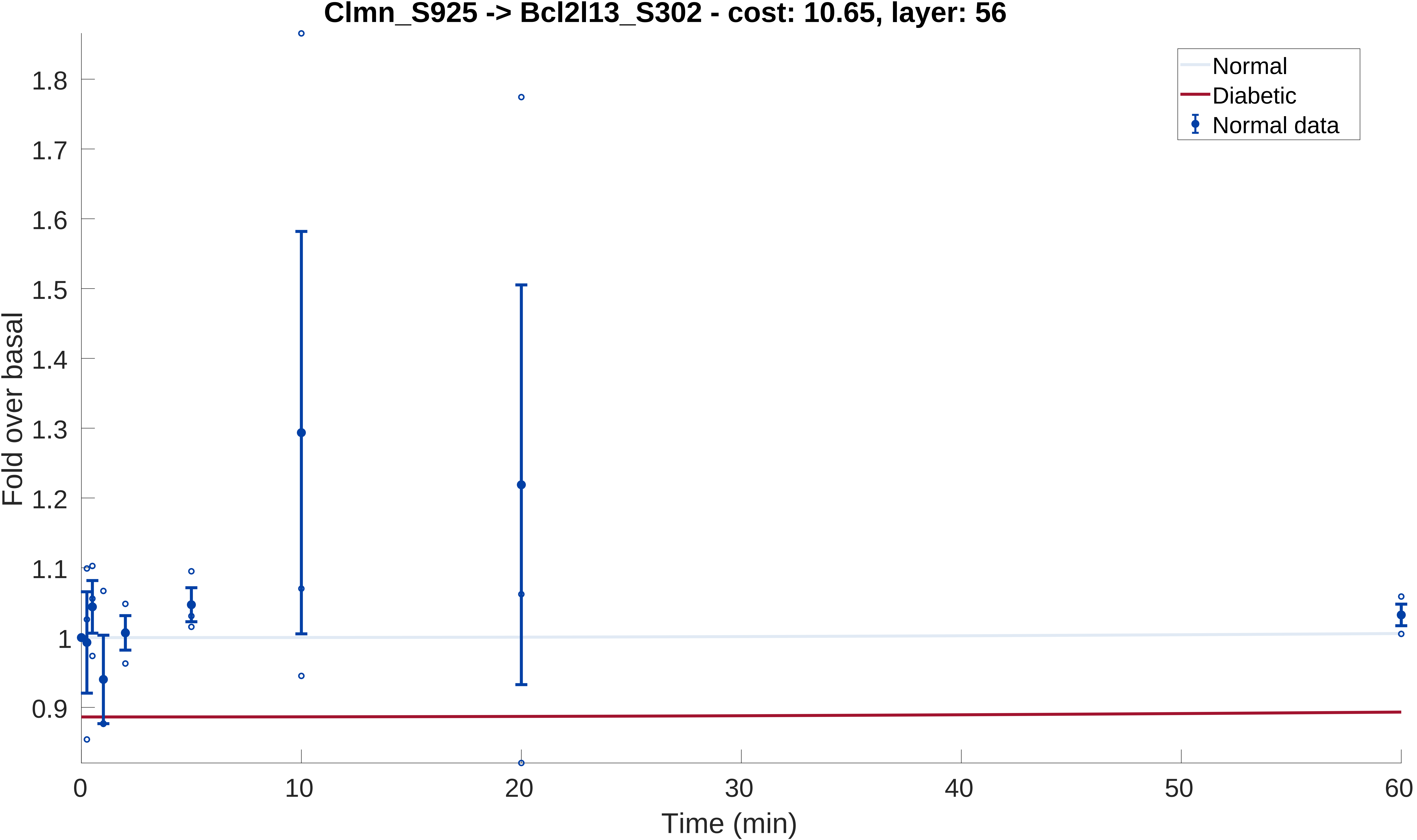

Clmn_S925 -> Bcl2l13_S302 - cost: 10.65, layer: 56
Normal
Diabetic
Normal data
Fold over basal
Time (min)
1.8
1.7
1.6
1.5
1.4
1.3
1.2
1.1
1
0.9
0
10
20
30
40
50
60

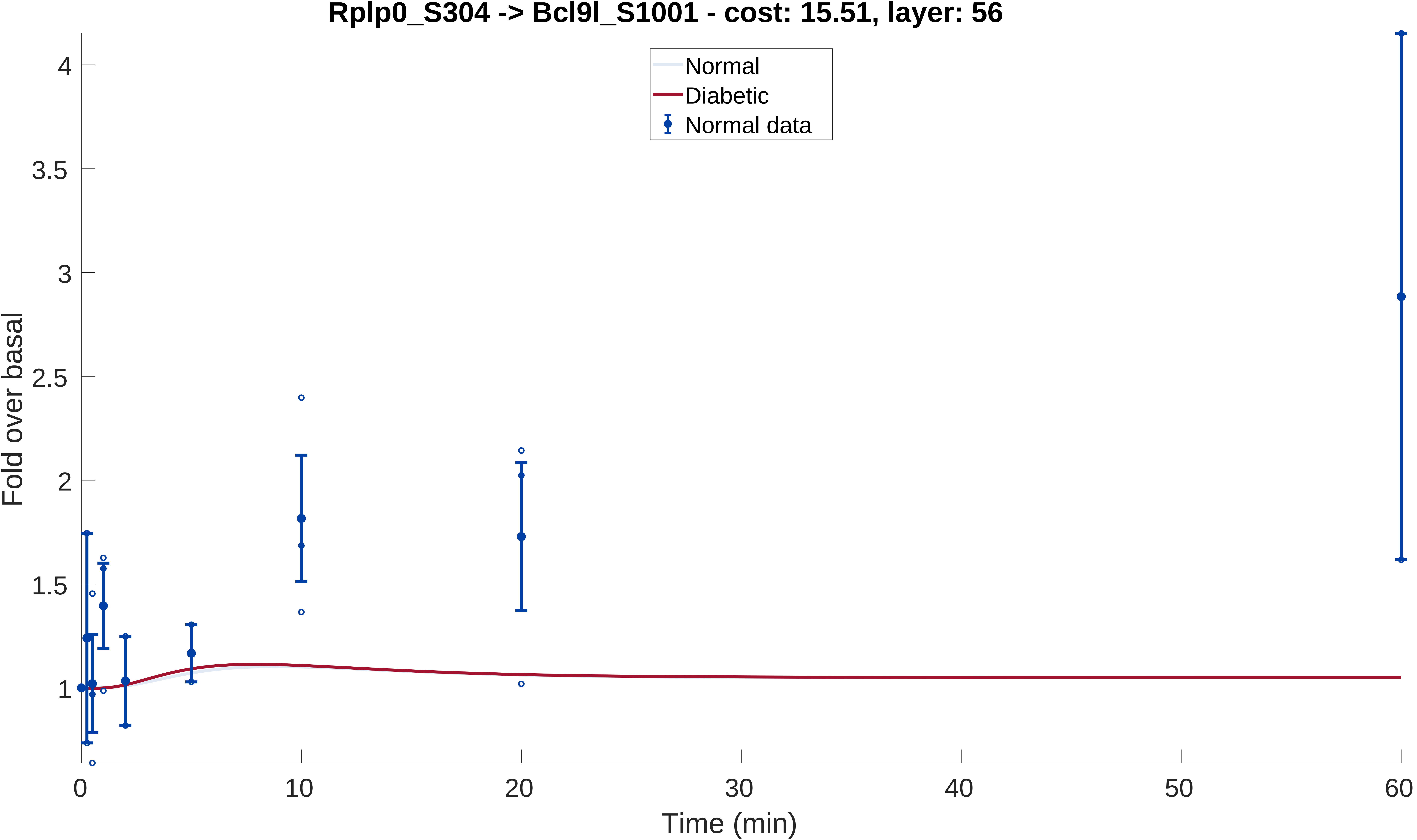

Rplp0_S304 -> Bcl9l_S1001 - cost: 15.51, layer: 56
Normal
Diabetic
Normal data
Fold over basal
Time (min)
4
3.5
3
2.5
2
1.5
1
0
10
20
30
40
50
60

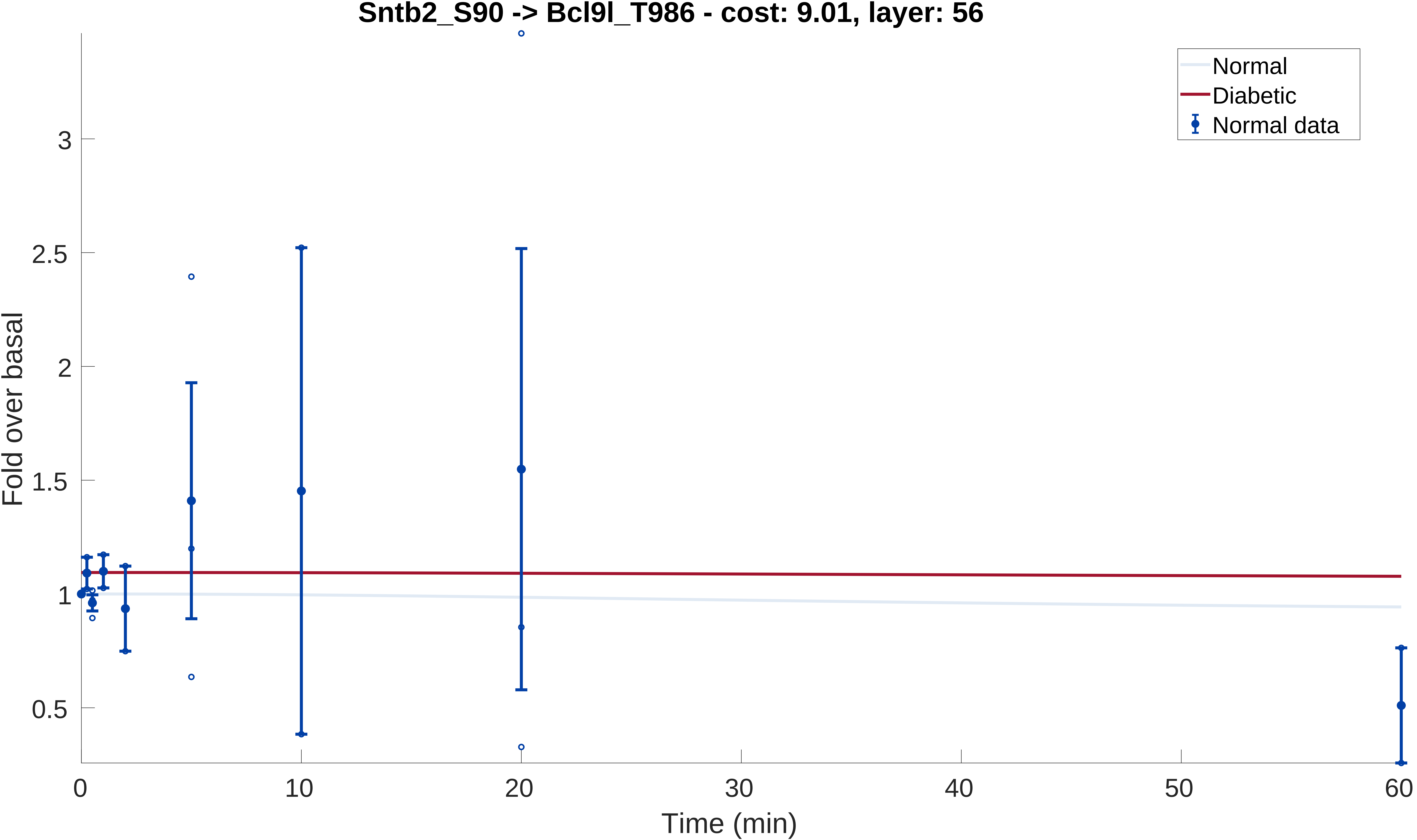

Sntb2_S90 -> Bcl9l_T986 - cost: 9.01, layer: 56
Normal
Diabetic
Normal data
Fold over basal
Time (min)
0.5
1
1.5
2
2.5
3
0
10
20
30
40
50
60

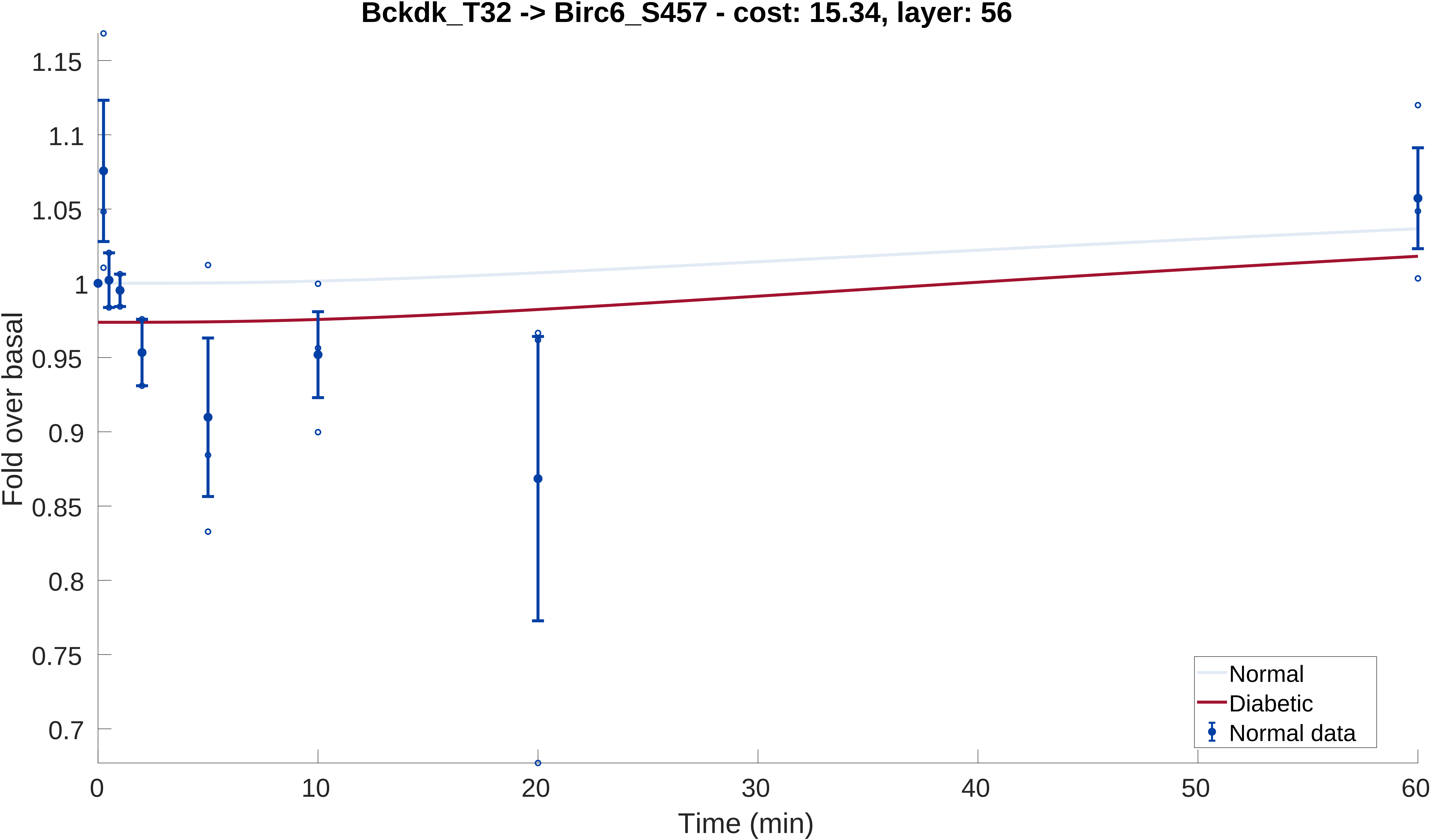
Bckdk_T32 -> Birc6_S457 - cost: 15.34, layer: 56
Fold over basal
Time (min)
1.15
1.1
1.05
1
0.95
0.9
0.85
0.8
0.75
0.7
0
10
20
30
40
50
60
Normal
Diabetic
Normal data

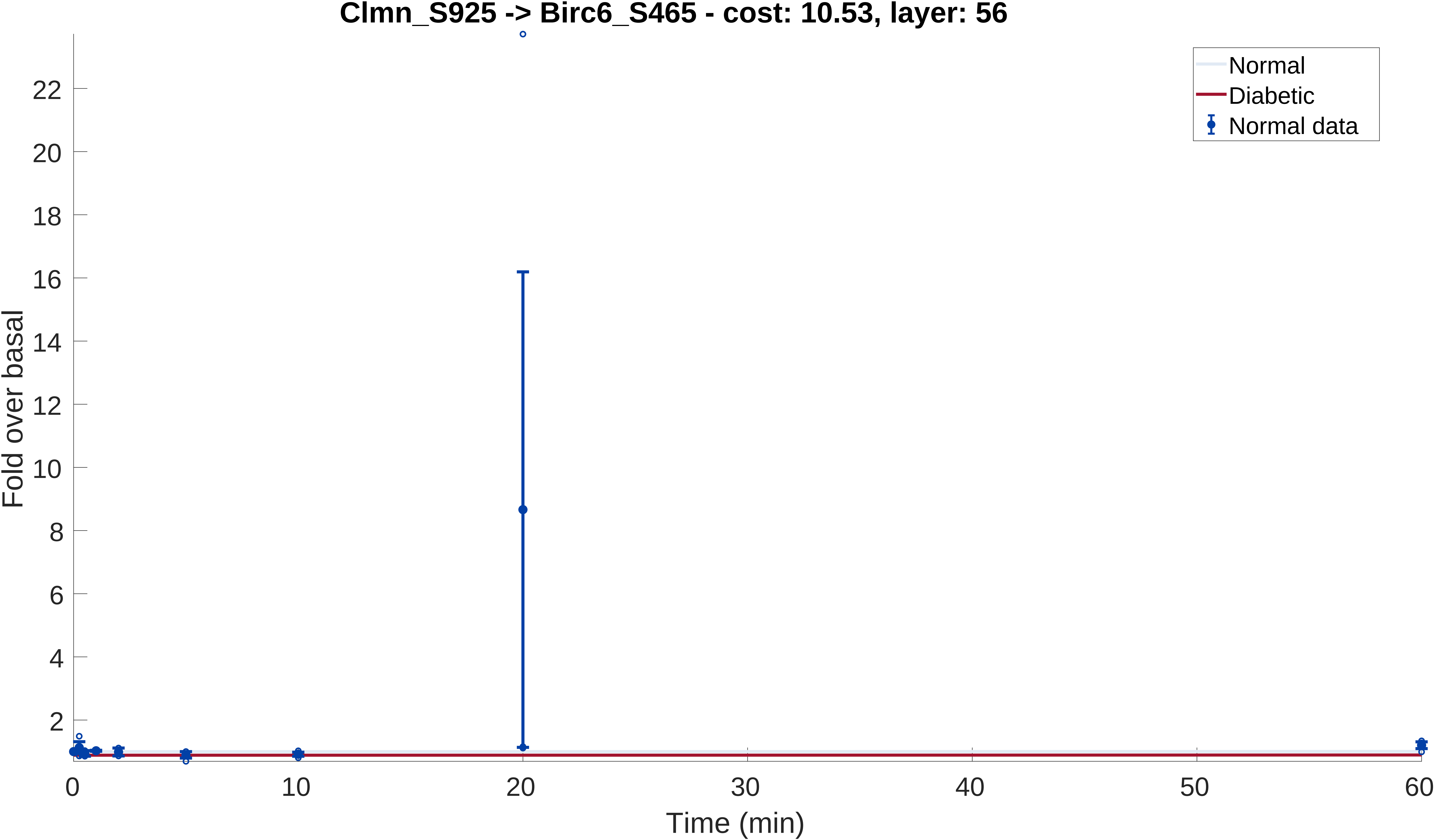

Clmn_S925 -> Birc6_S465 - cost: 10.53, layer: 56
Normal
Diabetic
Normal data
Fold over basal
Time (min)
0
10
20
30
40
50
60
2
4
6
8
10
12
14
16
18
20
22

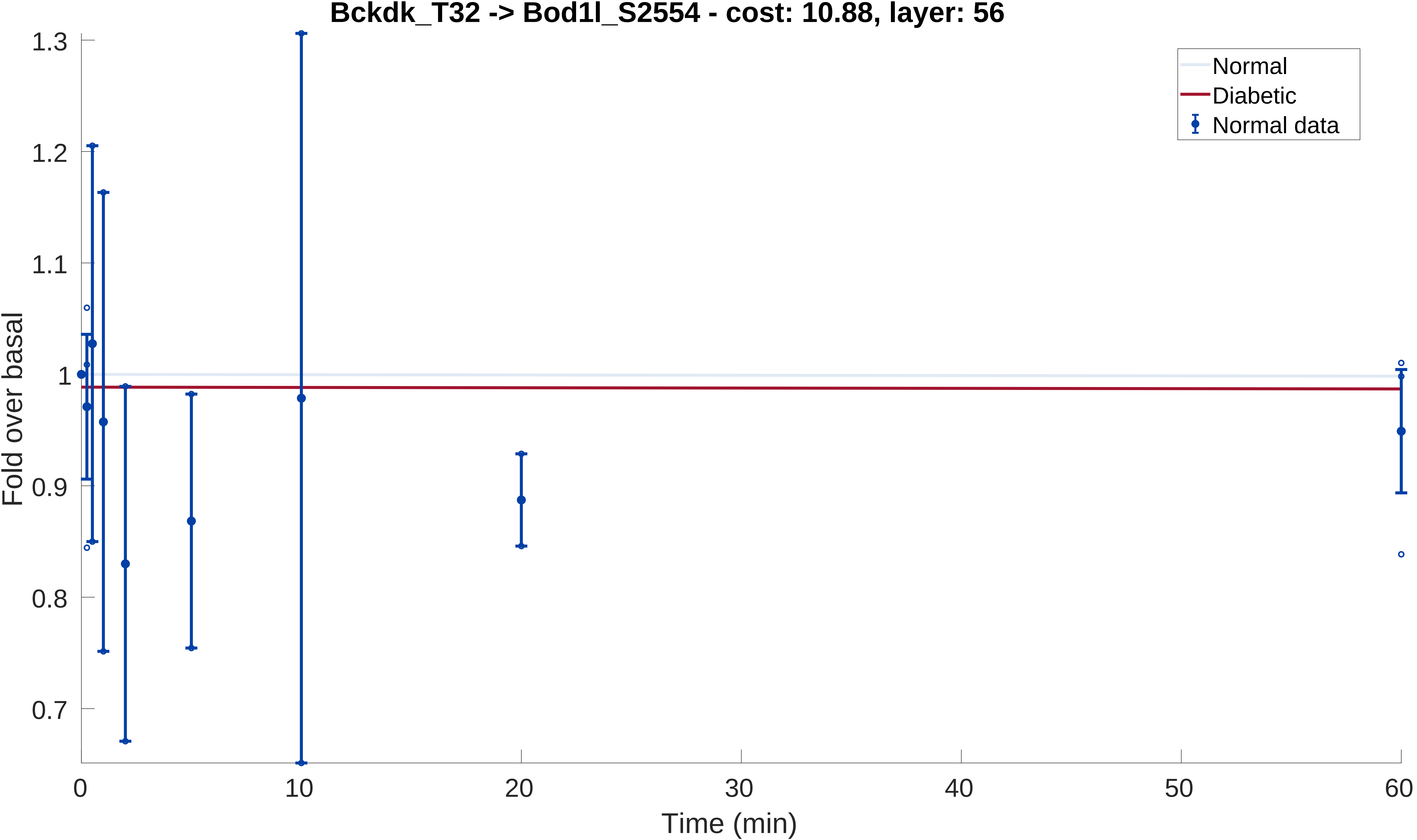

Bckdk_T32 -> Bod1l_S2554 - cost: 10.88, layer: 56
Normal
Diabetic
Normal data
Fold over basal
1.3
1.2
1.1
1
0.9
0.8
0.7
0
10
20
30
40
50
60
Time (min)

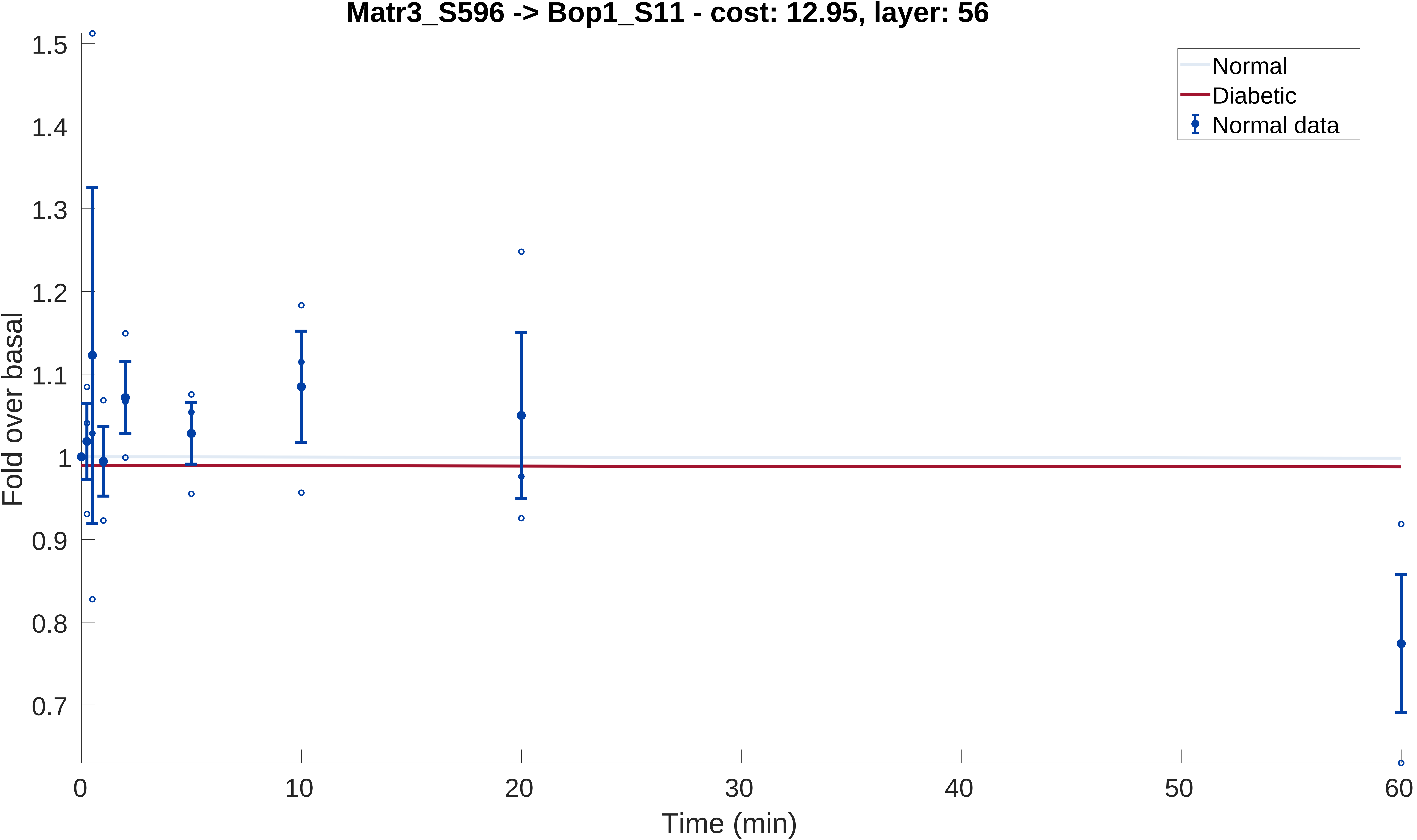

Matr3_S596 -> Bop1_S11 - cost: 12.95, layer: 56
Normal
Diabetic
Normal data
Fold over basal
1.5
1.4
1.3
1.2
1.1
1
0.9
0.8
0.7
0
10
20
30
40
50
60
Time (min)

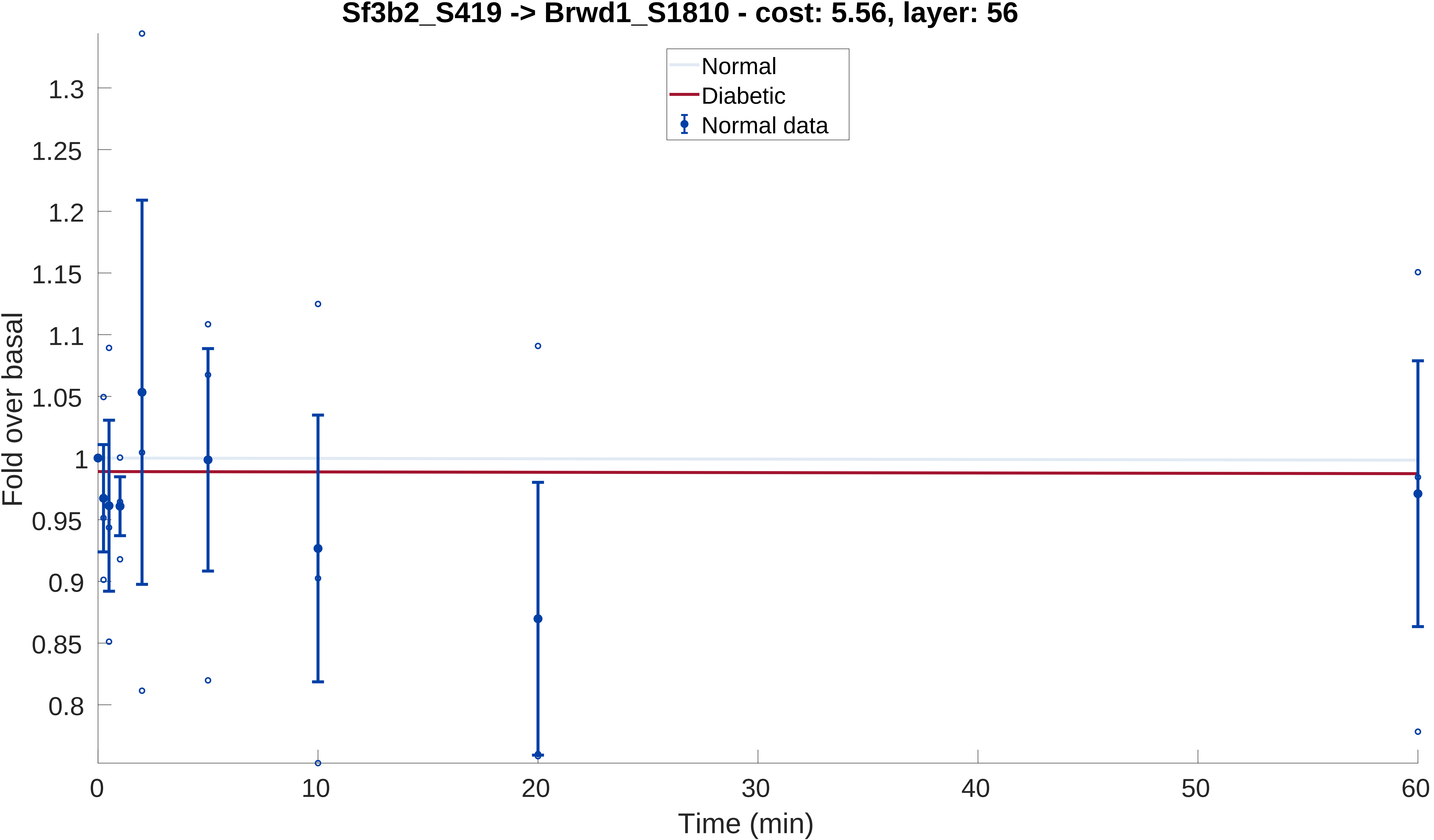
Sf3b2_S419 -> Brwd1_S1810 - cost: 5.56, layer: 56
Normal
Diabetic
Normal data
Fold over basal
1.3
1.25
1.2
1.15
1.1
1.05
1
0.95
0.9
0.85
0.8
0
10
20
30
40
50
60
Time (min)

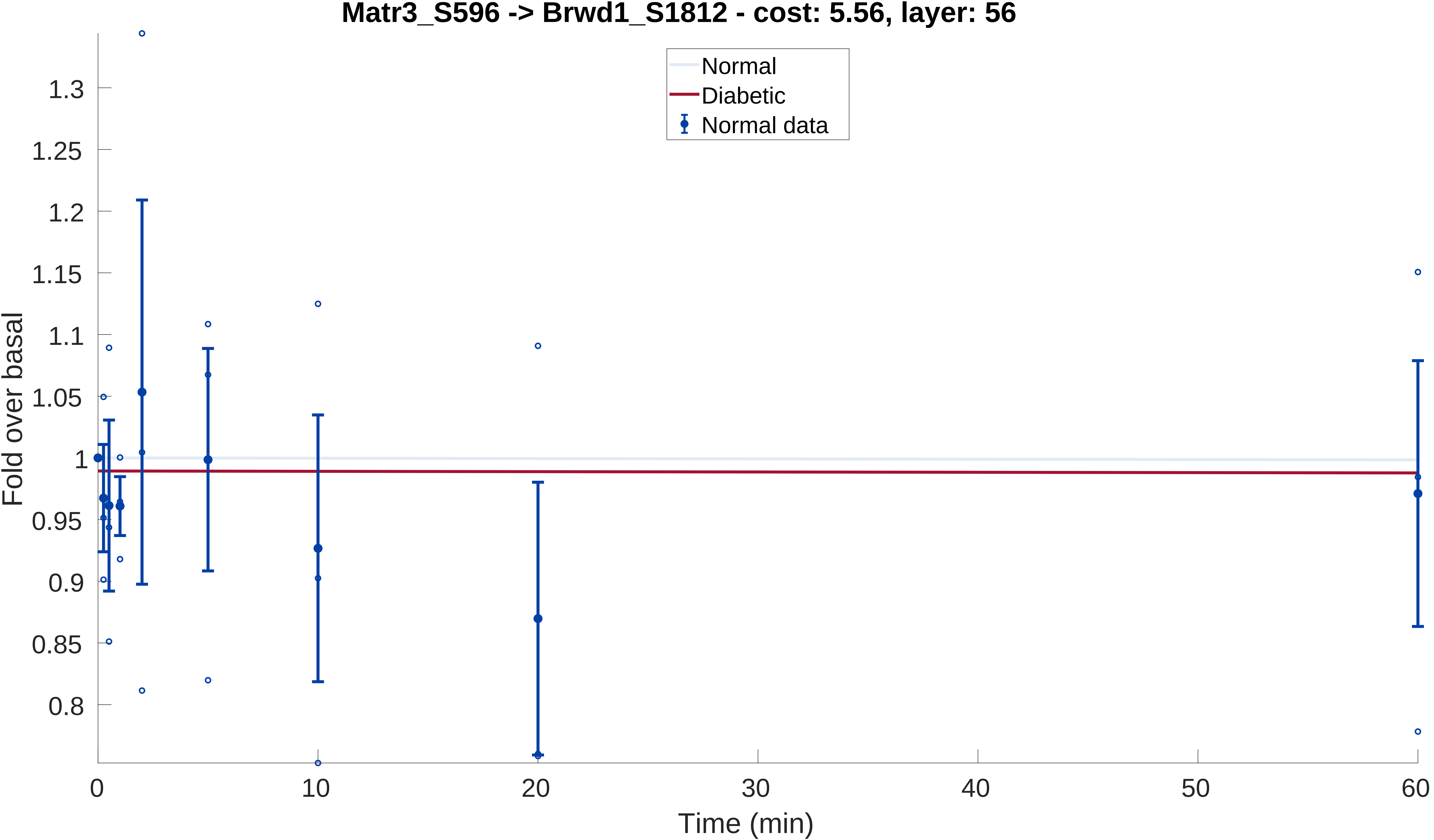

Matr3_S596 -> Brwd1_S1812 - cost: 5.56, layer: 56
Normal
Diabetic
Normal data
Fold over basal
Time (min)
1.3
1.25
1.2
1.15
1.1
1.05
1
0.95
0.9
0.85
0.8
0
10
20
30
40
50
60

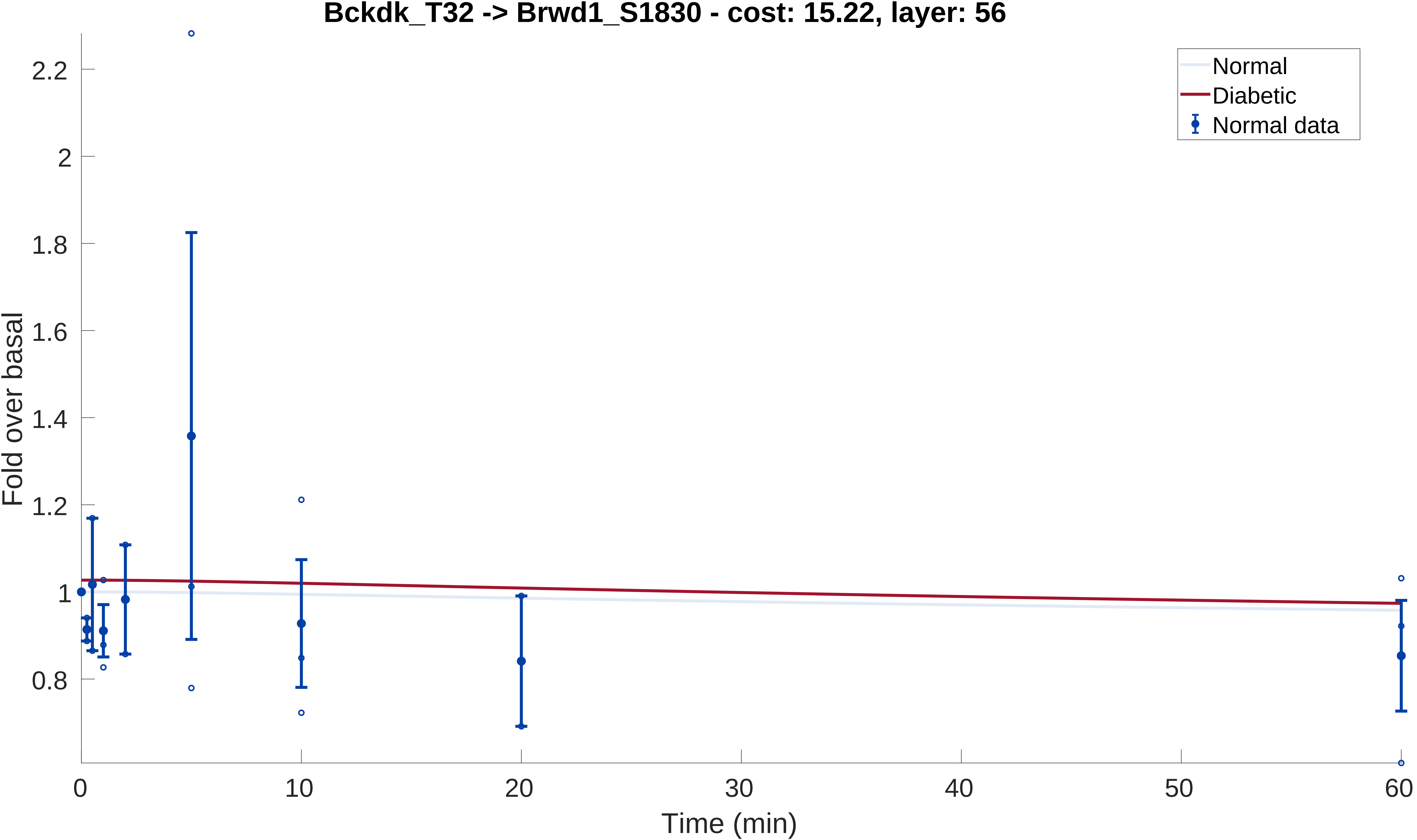

Bckdk_T32 -> Brwd1_S1830 - cost: 15.22, layer: 56
Normal
Diabetic
Normal data
Fold over basal
2.2
2
1.8
1.6
1.4
1.2
1
0.8
0
10
20
30
40
50
60
Time (min)

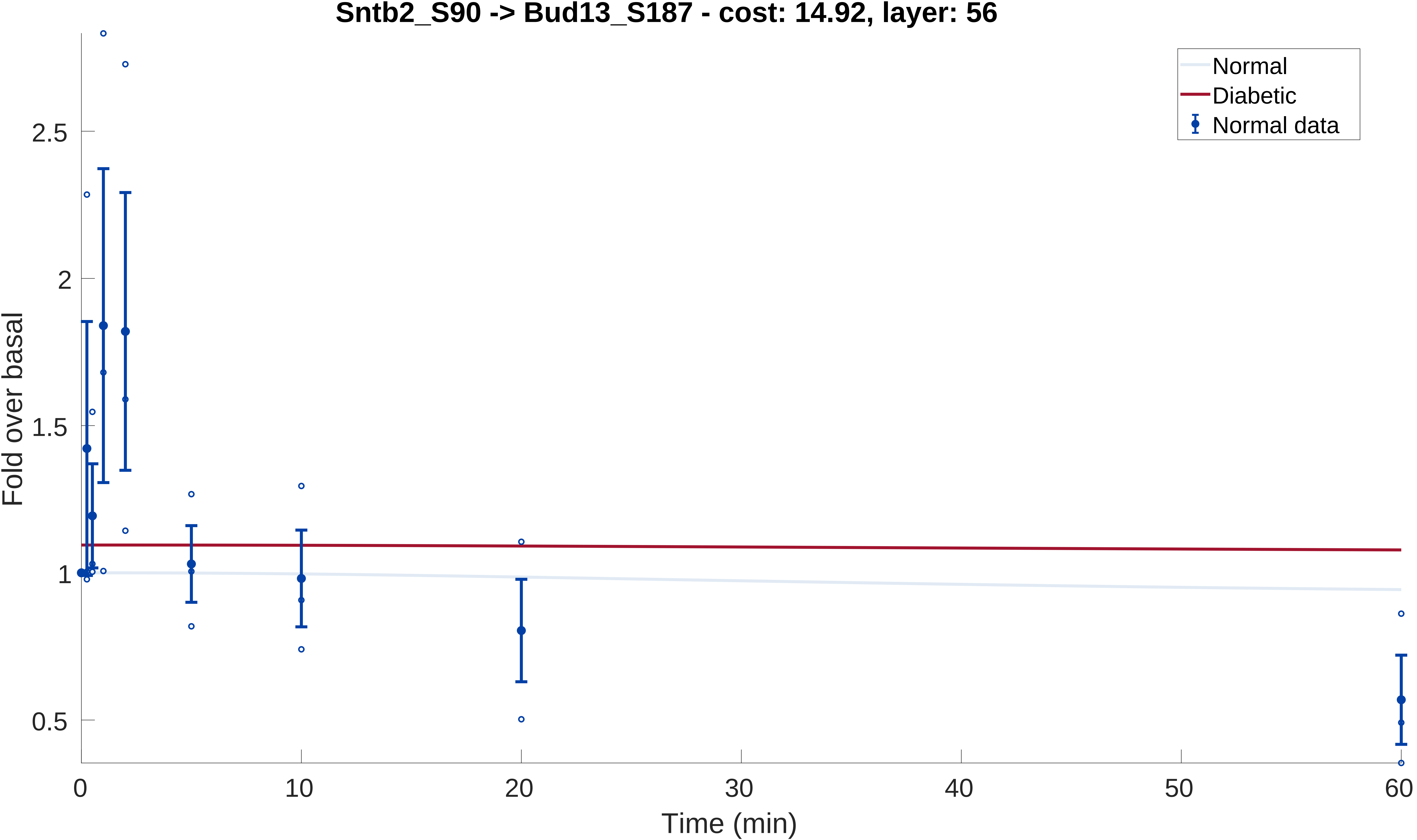
Sntb2_S90 -> Bud13_S187 - cost: 14.92, layer: 56
Normal
Diabetic
Normal data
Fold over basal
0.5
1
1.5
2
2.5
0
10
20
30
40
50
60
Time (min)

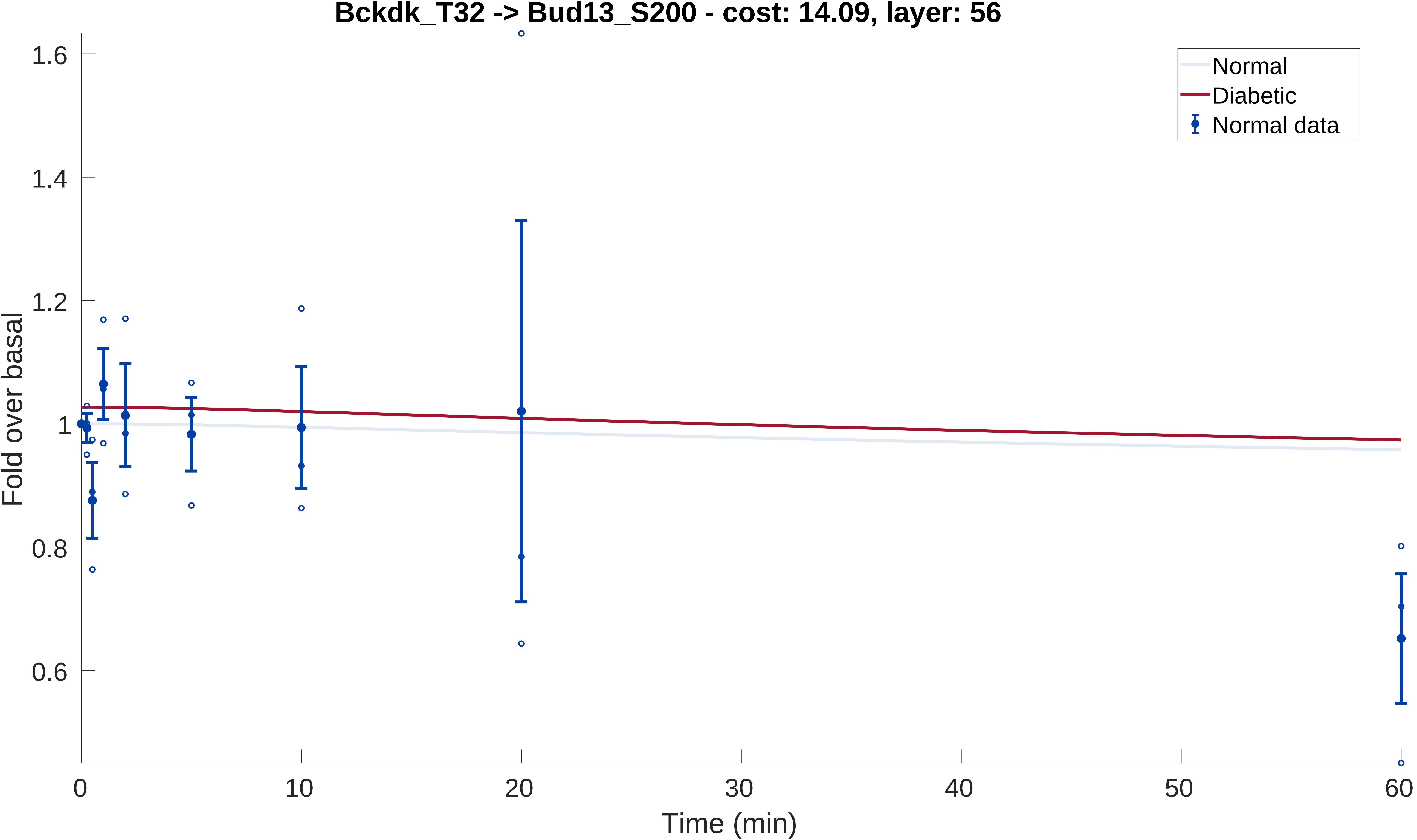

Bckdk_T32 -> Bud13_S200 - cost: 14.09, layer: 56
Fold over basal
1.6
1.4
1.2
1
0.8
0.6
Normal
Diabetic
Normal data
0
10
20
30
40
50
60
Time (min)

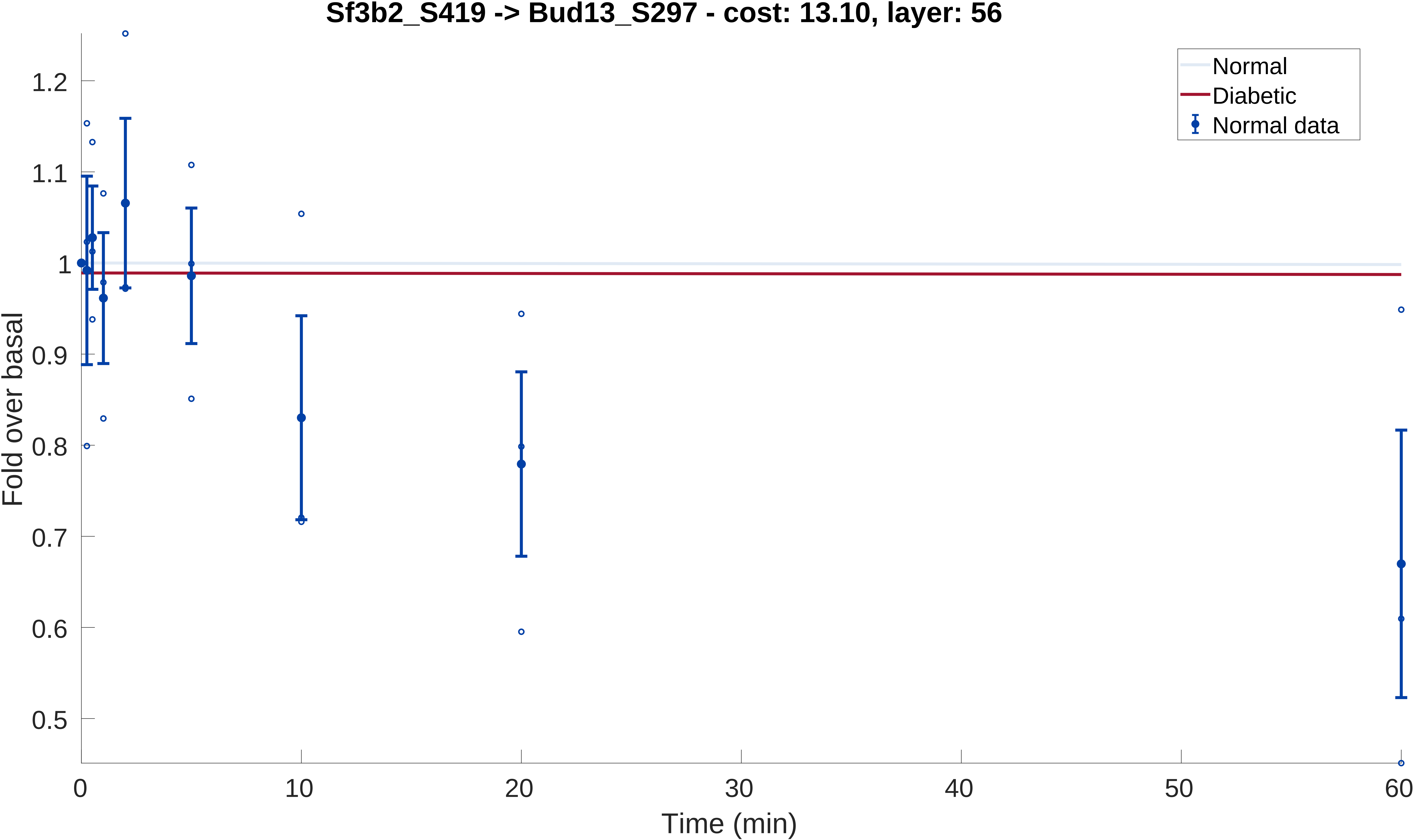
Sf3b2_S419 -> Bud13_S297 - cost: 13.10, layer: 56
Normal
Diabetic
Normal data
Fold over basal
Time (min)
1.2
1.1
1
0.9
0.8
0.7
0.6
0.5
0
10
20
30
40
50
60

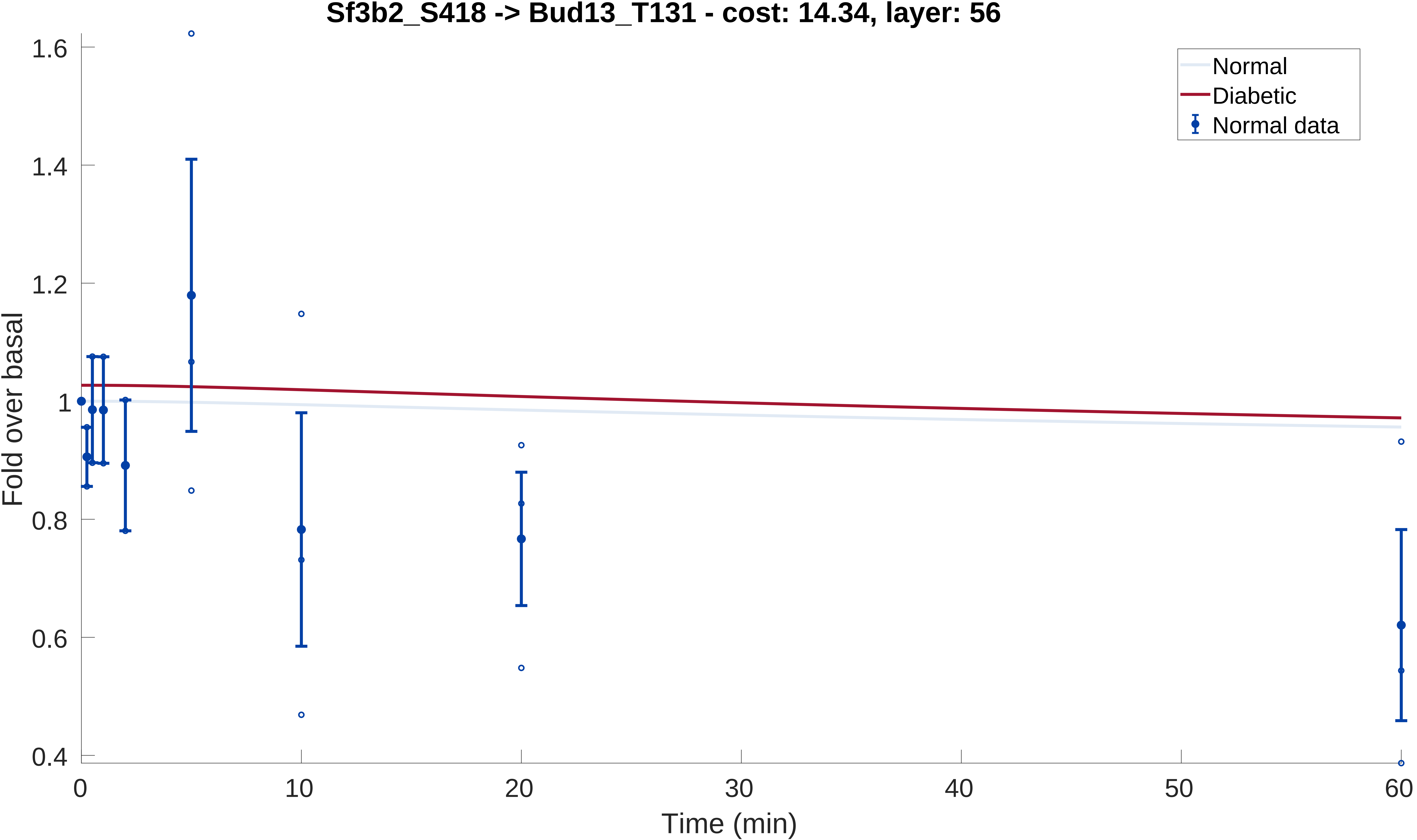

Sf3b2_S418 -> Bud13_T131 - cost: 14.34, layer: 56
Normal
Diabetic
Normal data
Fold over basal
Time (min)
1.6
1.4
1.2
1
0.8
0.6
0.4
0
10
20
30
40
50
60

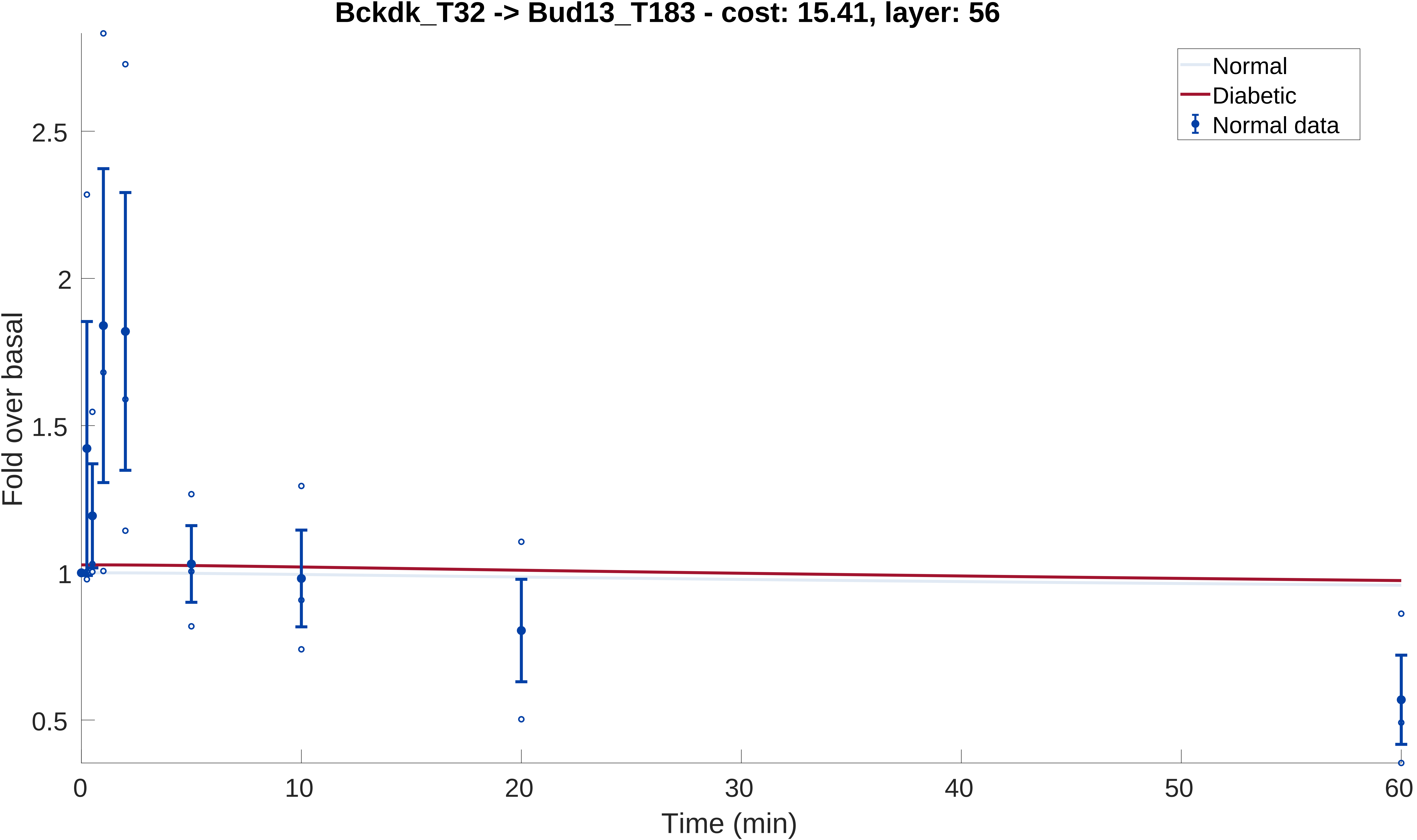

Bckdk_T32 -> Bud13_T183 - cost: 15.41, layer: 56
Normal
Diabetic
Normal data
2.5
2
1.5
1
0.5
Fold over basal
0
10
20
30
40
50
60
Time (min)

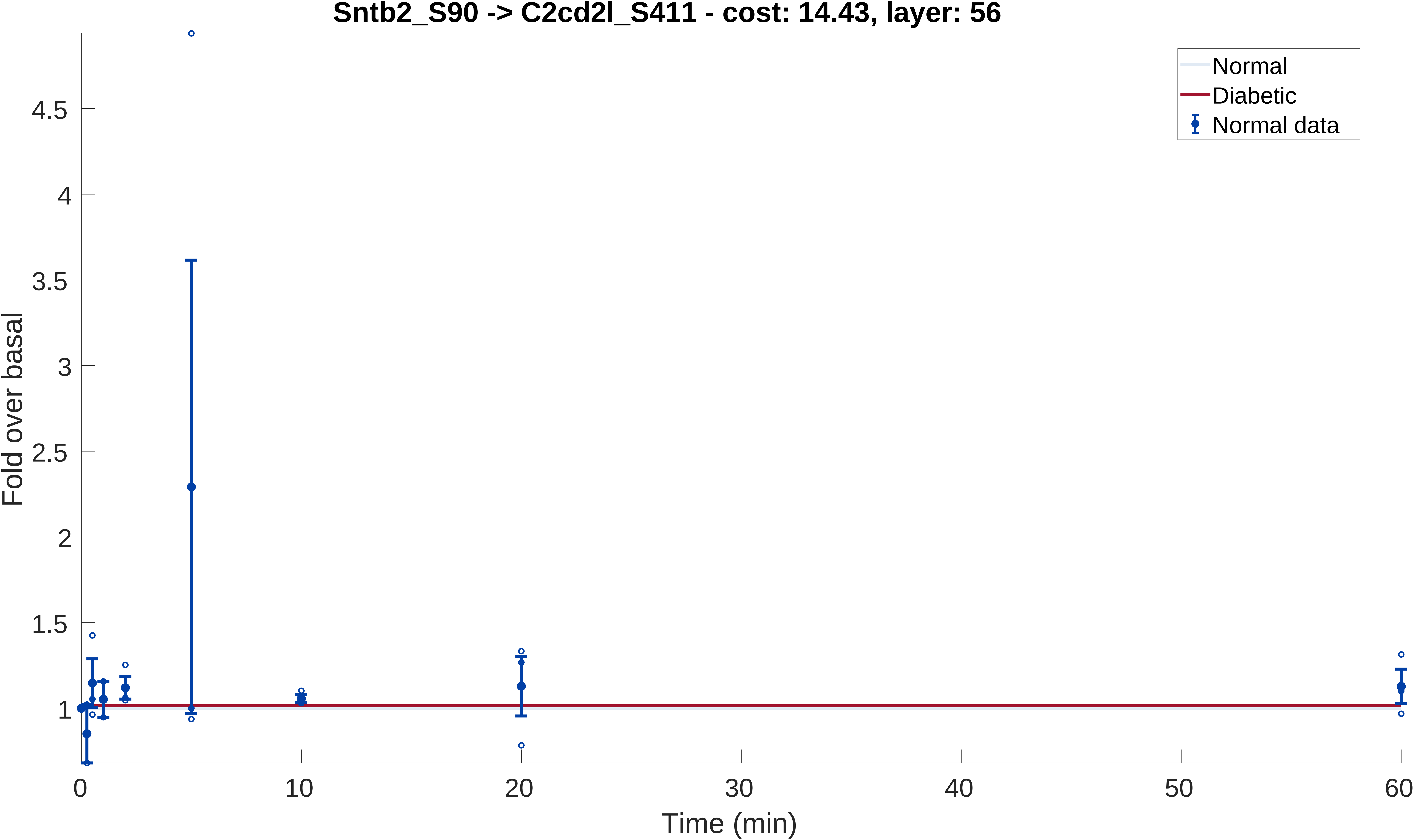

Sntb2_S90 -> C2cd2l_S411 - cost: 14.43, layer: 56
Normal
Diabetic
Normal data
Fold over basal
4.5
4
3.5
3
2.5
2
1.5
1
0
10
20
30
40
50
60
Time (min)

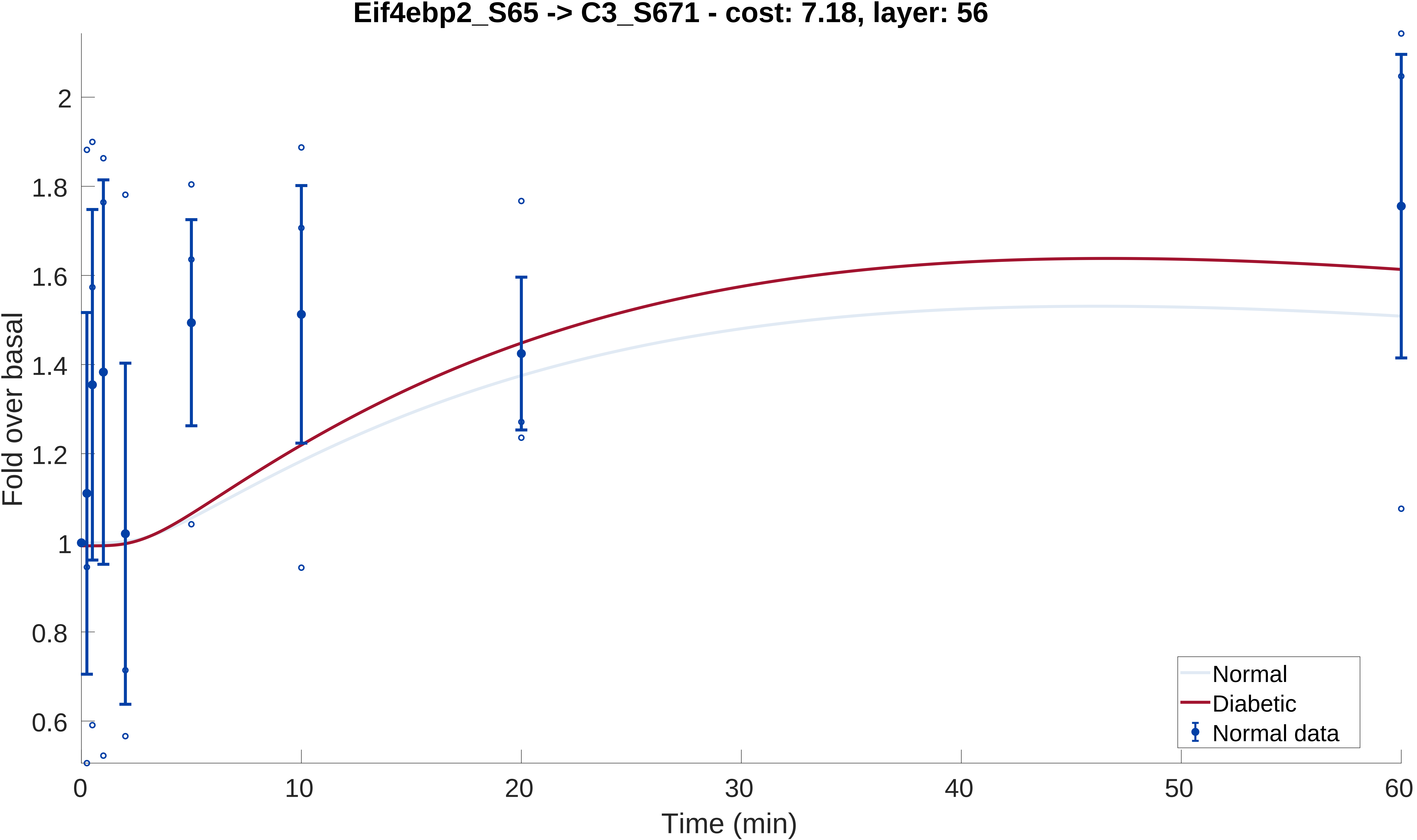

Eif4ebp2_S65 -> C3_S671 - cost: 7.18, layer: 56
Fold over basal
2
1.8
1.6
1.4
1.2
1
0.8
0.6
0
10
20
30
40
50
60
Time (min)
Normal
Diabetic
Normal data

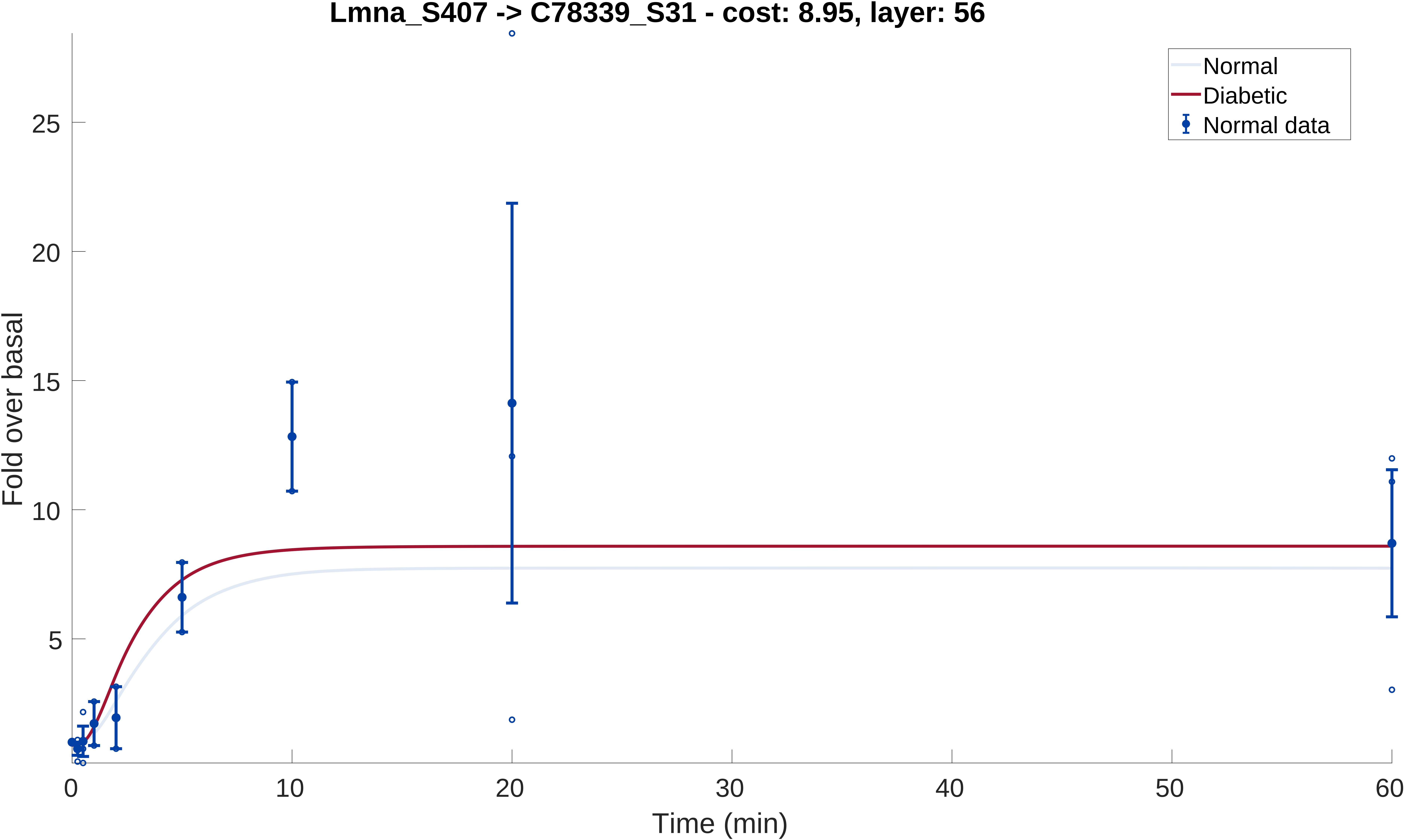

Lmna_S407 -> C78339_S31 - cost: 8.95, layer: 56
Normal
Diabetic
Normal data
Fold over basal
Time (min)
0
10
20
30
40
50
60
5
10
15
20
25

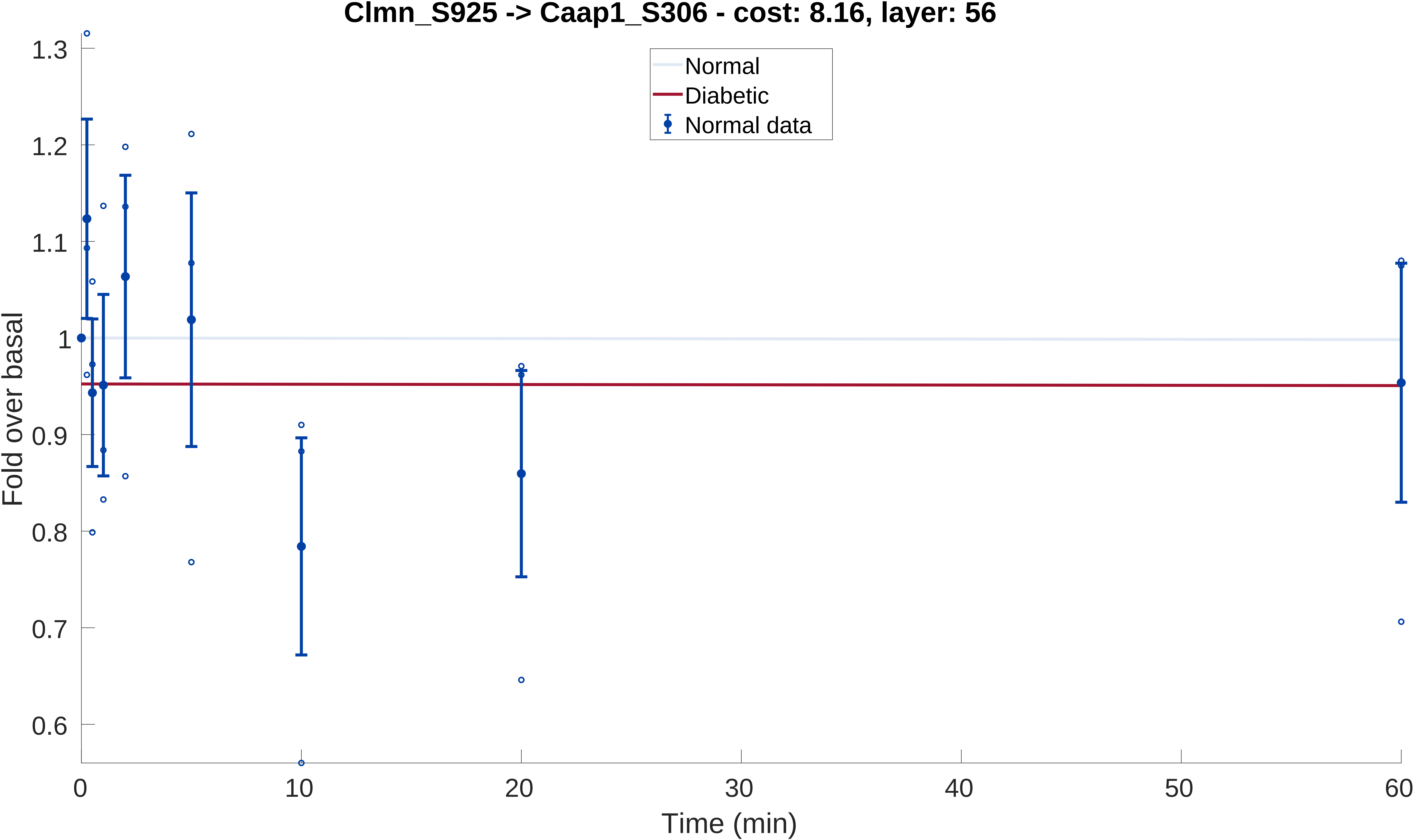

Clmn_S925 -> Caap1_S306 - cost: 8.16, layer: 56
Normal
Diabetic
Normal data
Fold over basal
Time (min)
1.3
1.2
1.1
1
0.9
0.8
0.7
0.6
0
10
20
30
40
50
60

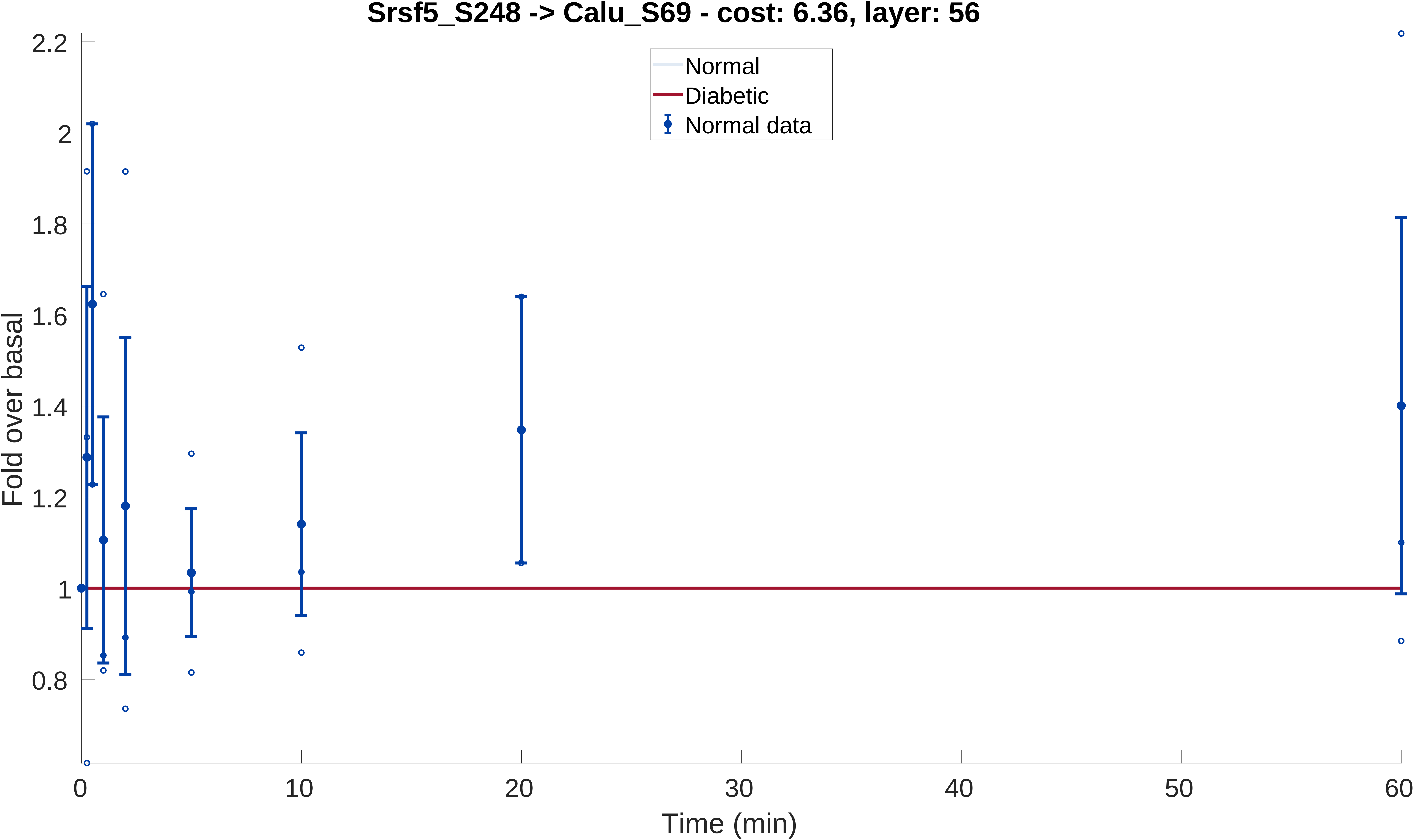

Srsf5_S248 -> Calu_S69 - cost: 6.36, layer: 56
Normal
Diabetic
Normal data
Fold over basal
Time (min)
2.2
2
1.8
1.6
1.4
1.2
1
0.8
0
10
20
30
40
50
60

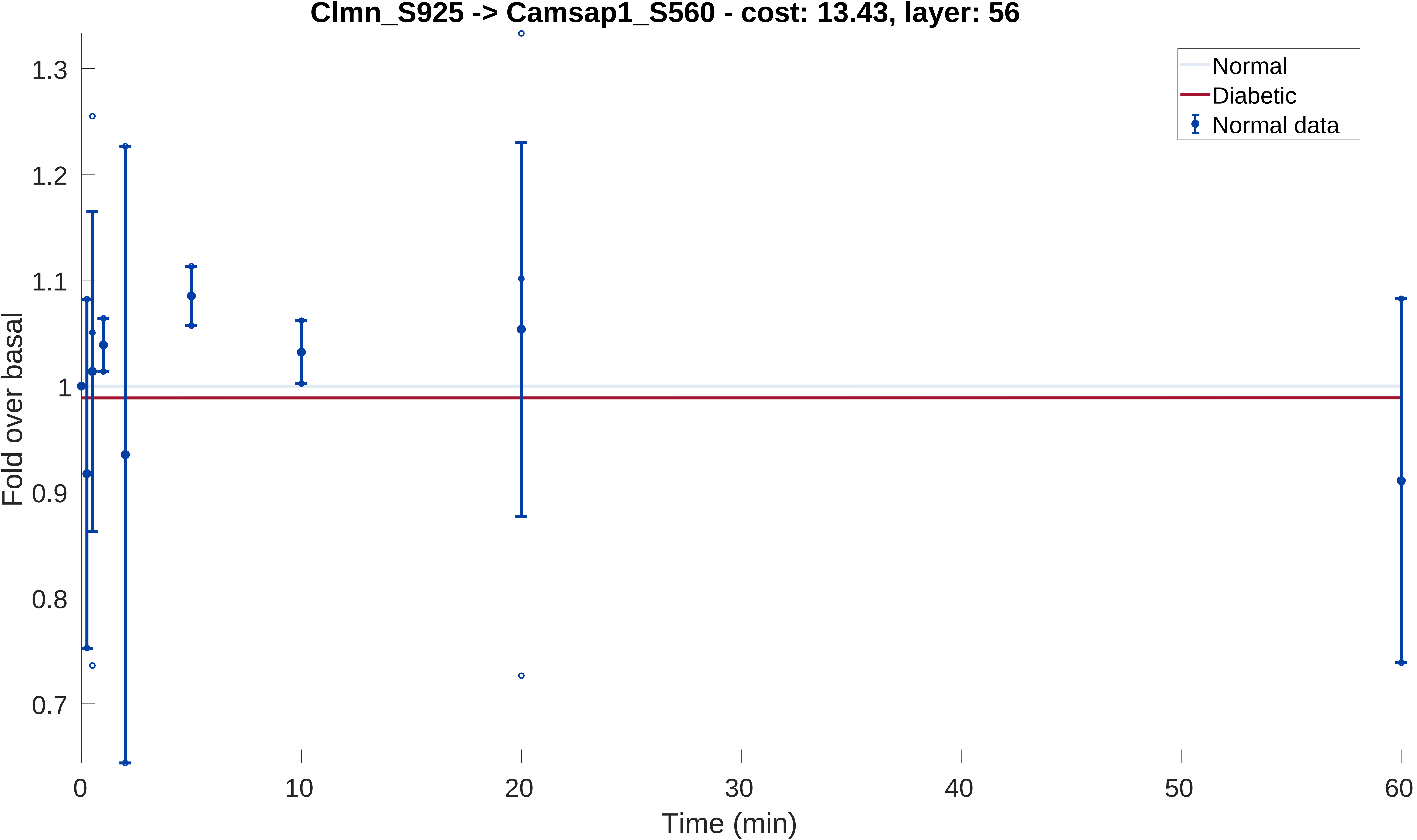
Clmn_S925 -> Camsap1_S560 - cost: 13.43, layer: 56
Normal
Diabetic
Normal data
Fold over basal
Time (min)
1.3
1.2
1.1
1
0.9
0.8
0.7
0
10
20
30
40
50
60

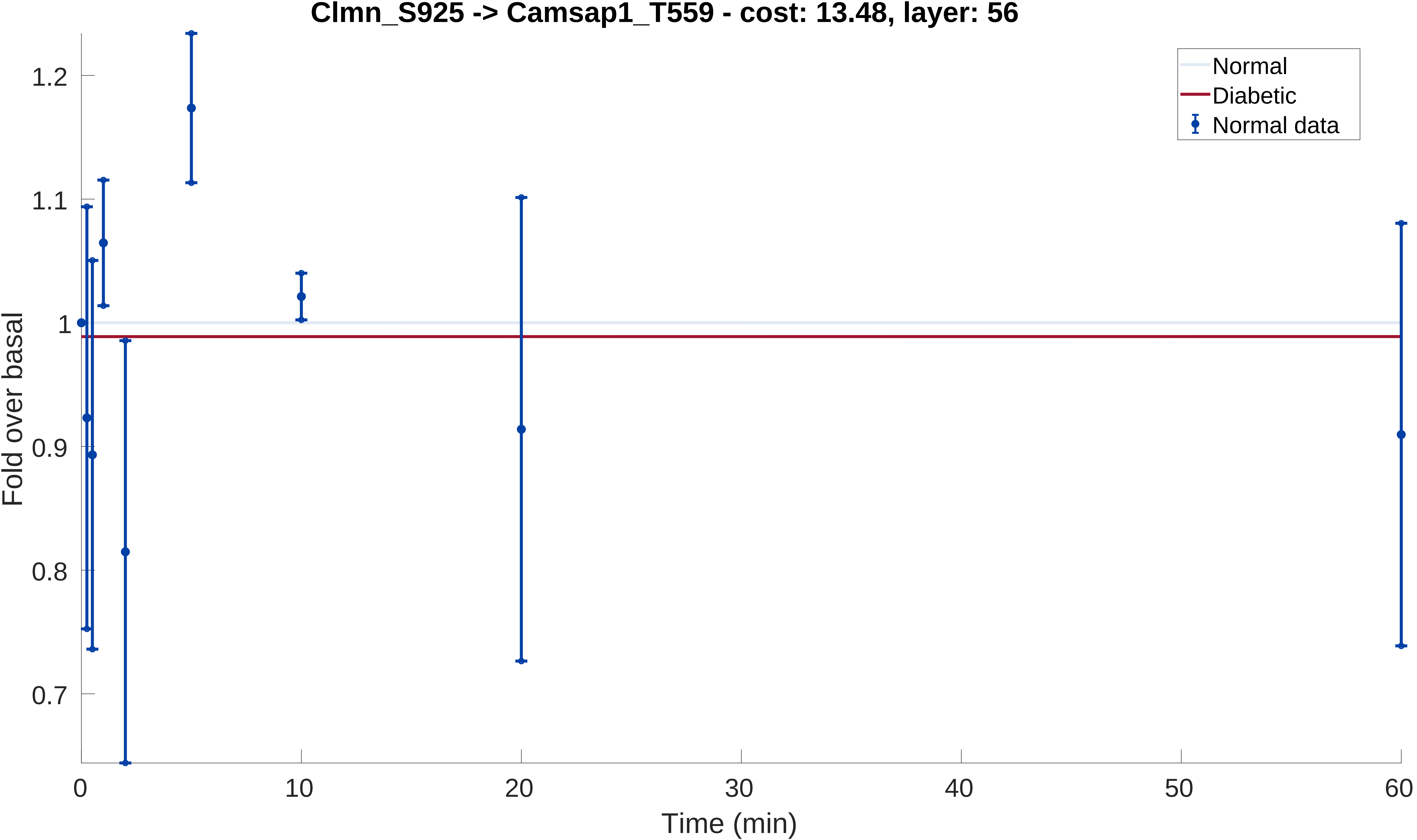
Clmn_S925 -> Camsap1_T559 - cost: 13.48, layer: 56
Normal
Diabetic
Normal data
Fold over basal
1.2
1.1
1
0.9
0.8
0.7
0
10
20
30
40
50
60
Time (min)

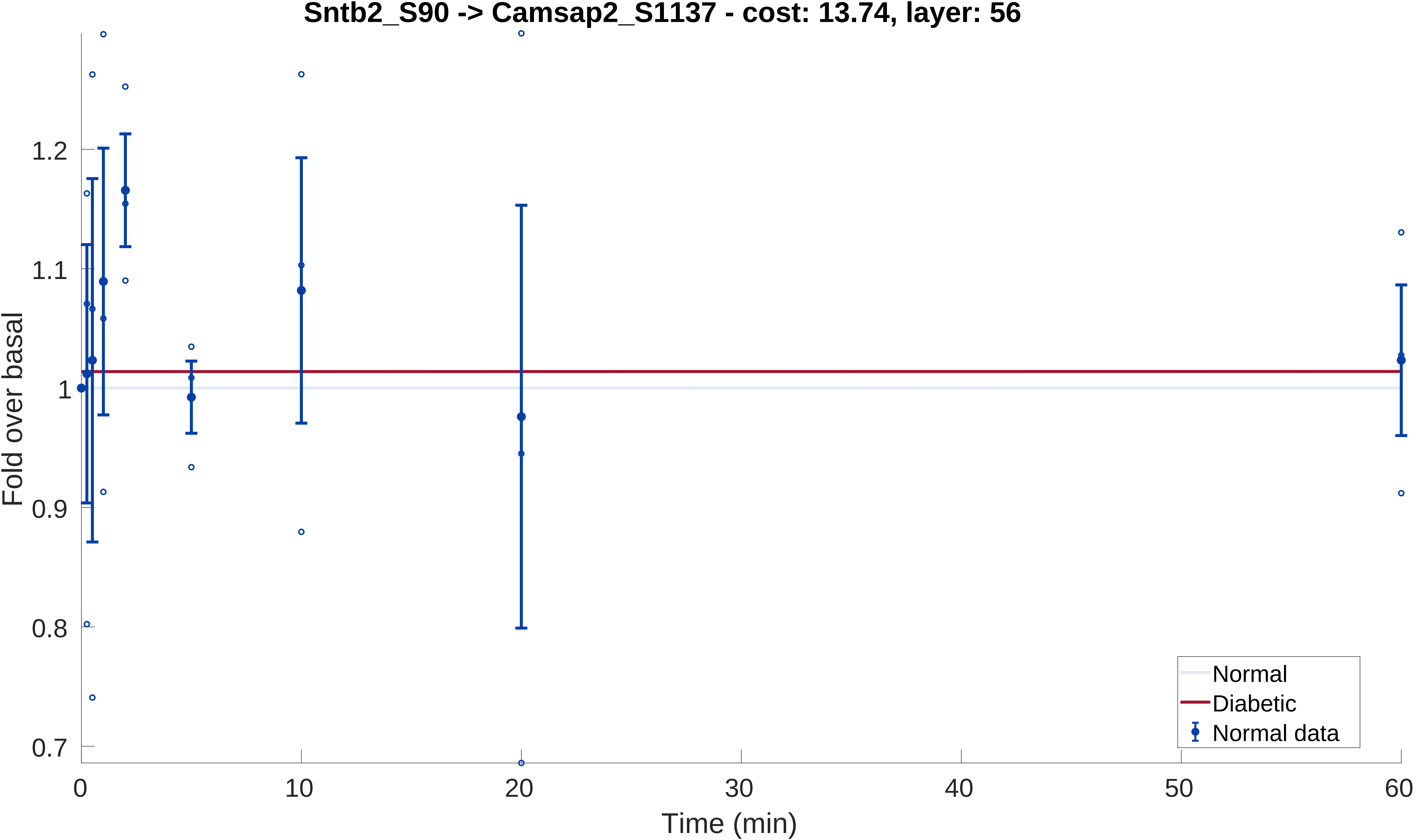

Sntb2_S90 -> Camsap2_S1137 - cost: 13.74, layer: 56
Fold over basal
Time (min)
1.2
1.1
1
0.9
0.8
0.7
0
10
20
30
40
50
60
Normal
Diabetic
Normal data

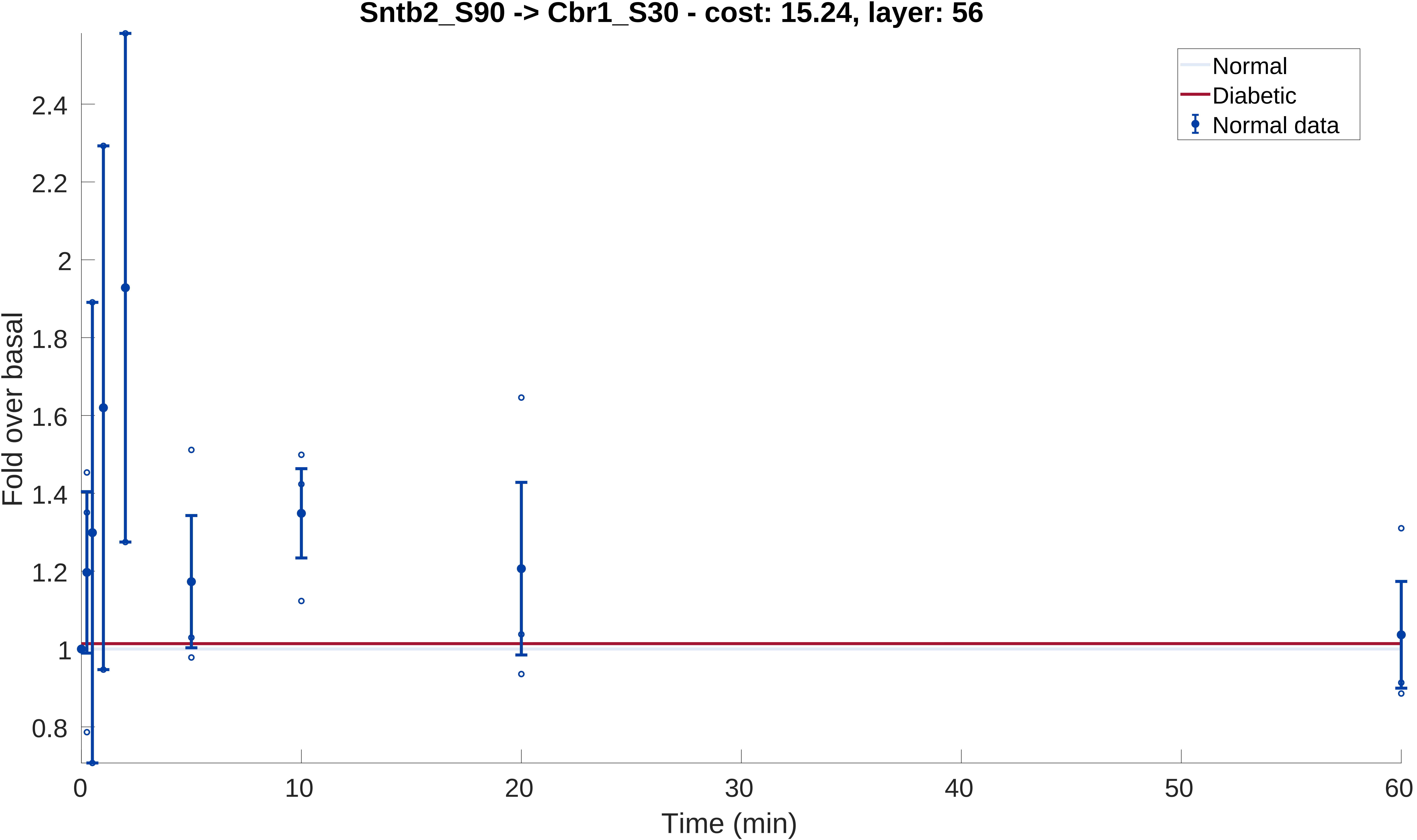

Sntb2_S90 -> Cbr1_S30 - cost: 15.24, layer: 56
Normal
Diabetic
Normal data
Fold over basal
2.4
2.2
2
1.8
1.6
1.4
1.2
1
0.8
0
10
20
30
40
50
60
Time (min)

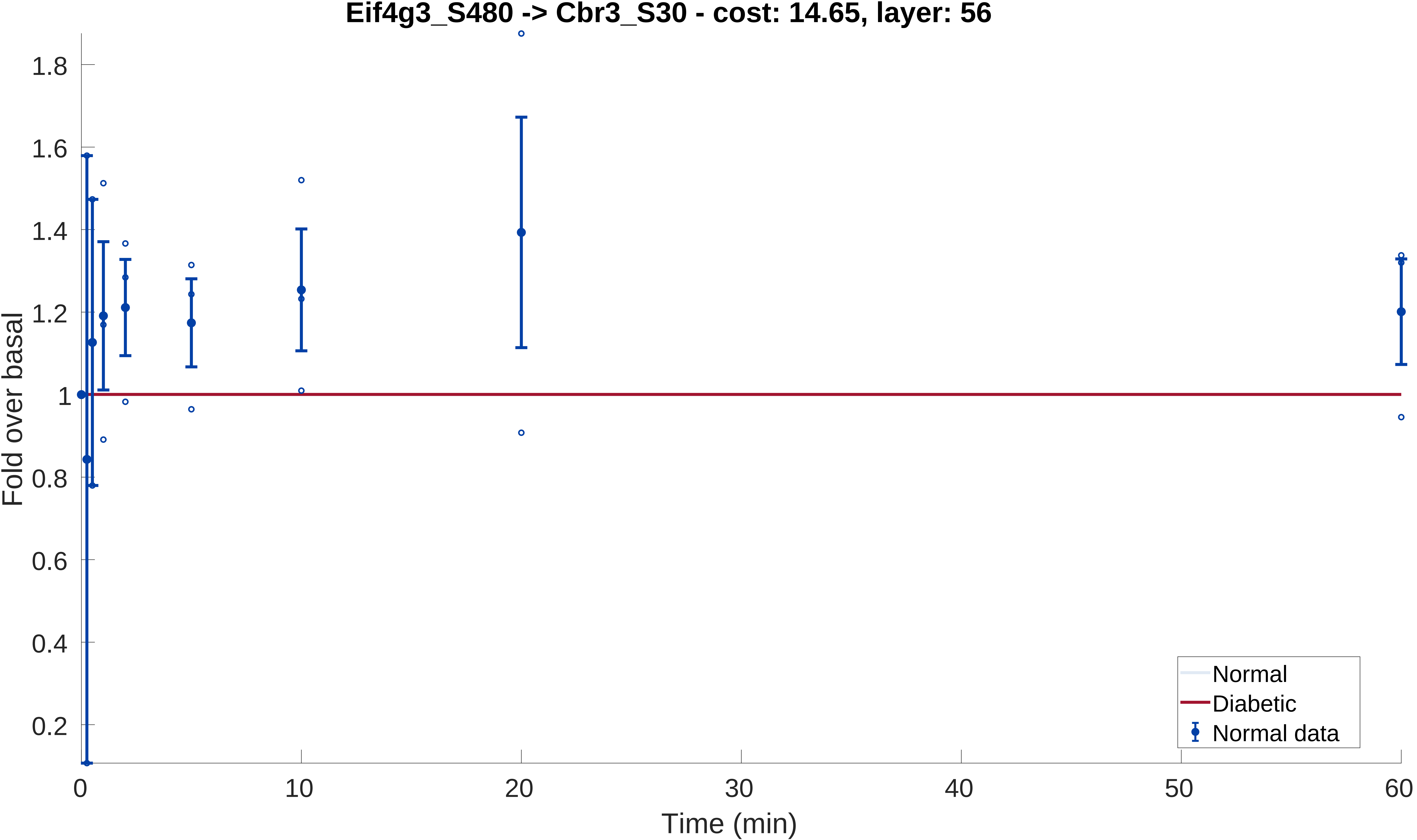

Eif4g3_S480 -> Cbr3_S30 - cost: 14.65, layer: 56
Fold over basal
0.2
0.4
0.6
0.8
1
1.2
1.4
1.6
1.8
Time (min)
0
10
20
30
40
50
60
Normal
Diabetic
Normal data

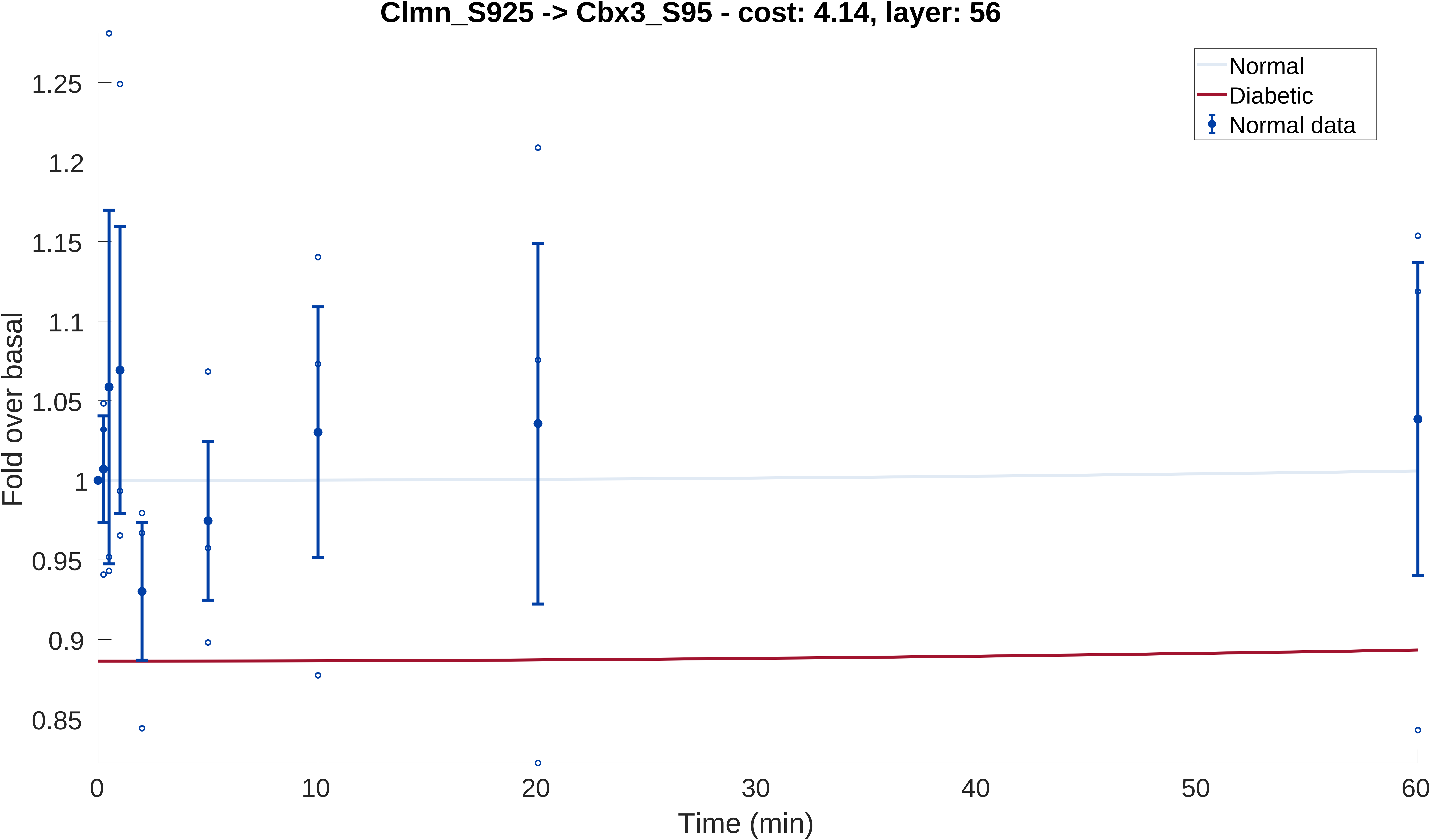

Clmn_S925 -> Cbx3_S95 - cost: 4.14, layer: 56
Normal
Diabetic
Normal data
Fold over basal
1.25
1.2
1.15
1.1
1.05
1
0.95
0.9
0.85
0
10
20
30
40
50
60
Time (min)

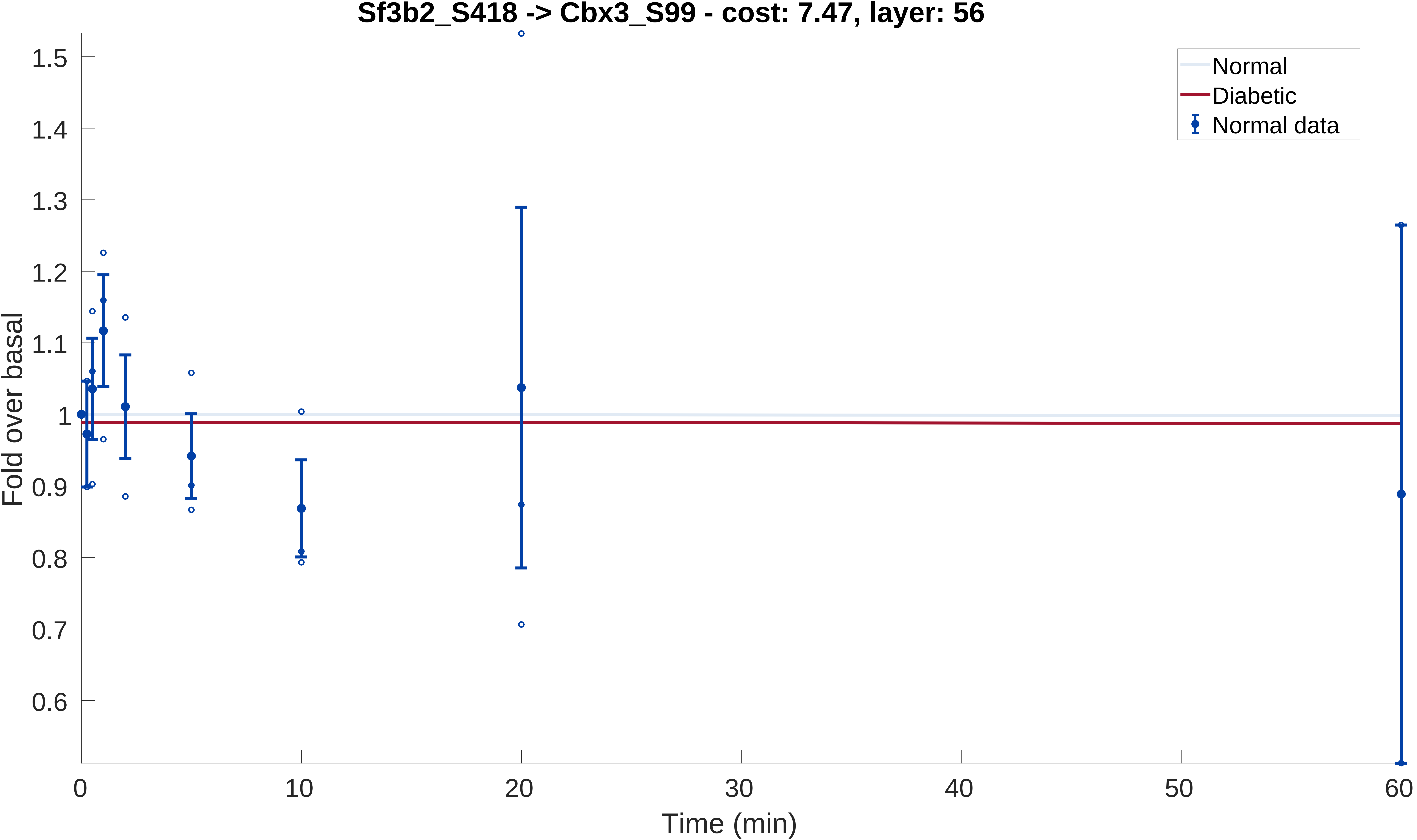

Sf3b2_S418 -> Cbx3_S99 - cost: 7.47, layer: 56
Normal
Diabetic
Normal data
Fold over basal
1.5
1.4
1.3
1.2
1.1
1
0.9
0.8
0.7
0.6
0
10
20
30
40
50
60
Time (min)

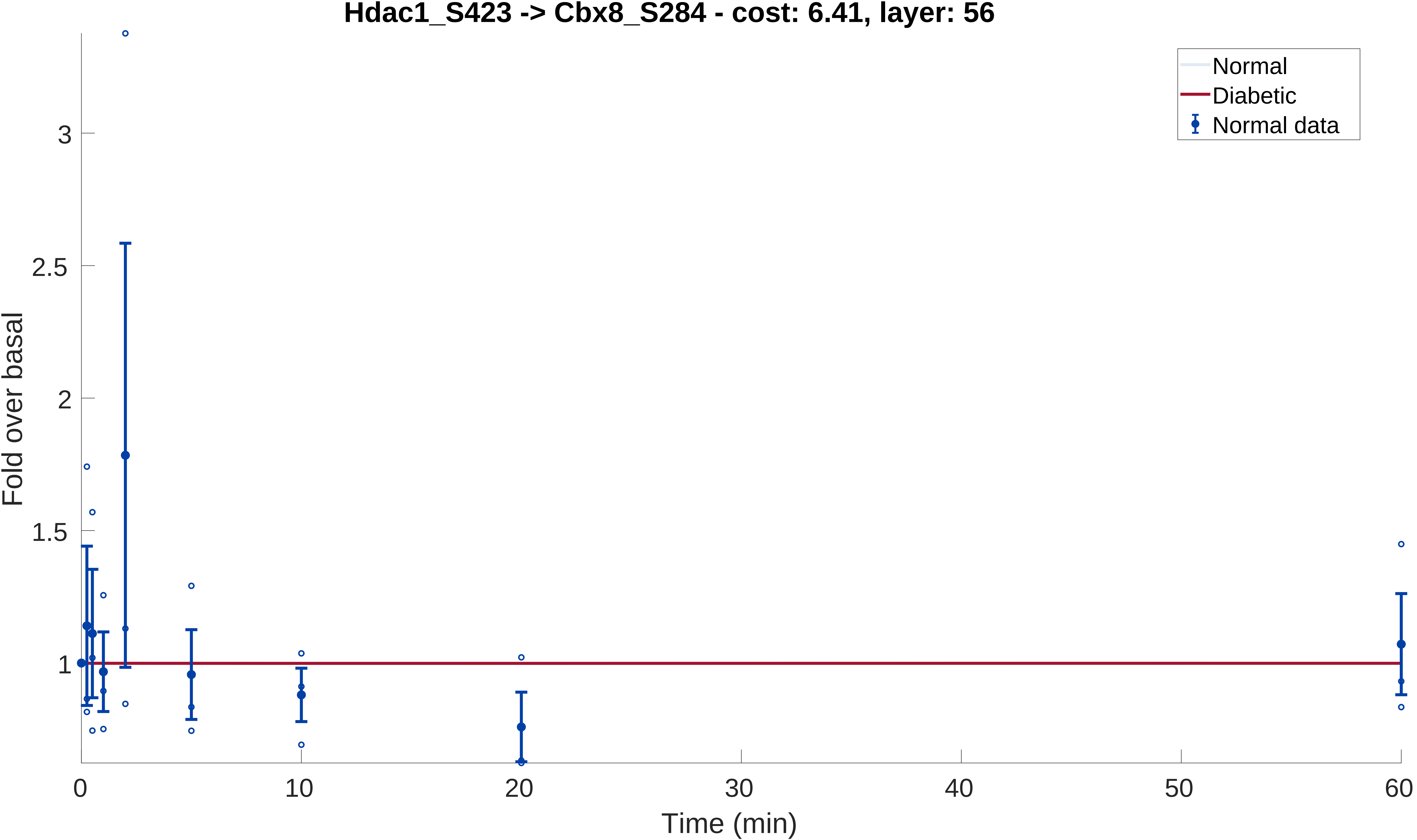

Hdac1_S423 -> Cbx8_S284 - cost: 6.41, layer: 56
Normal
Diabetic
Normal data
Fold over basal
3
2.5
2
1.5
1
0
10
20
30
40
50
60
Time (min)

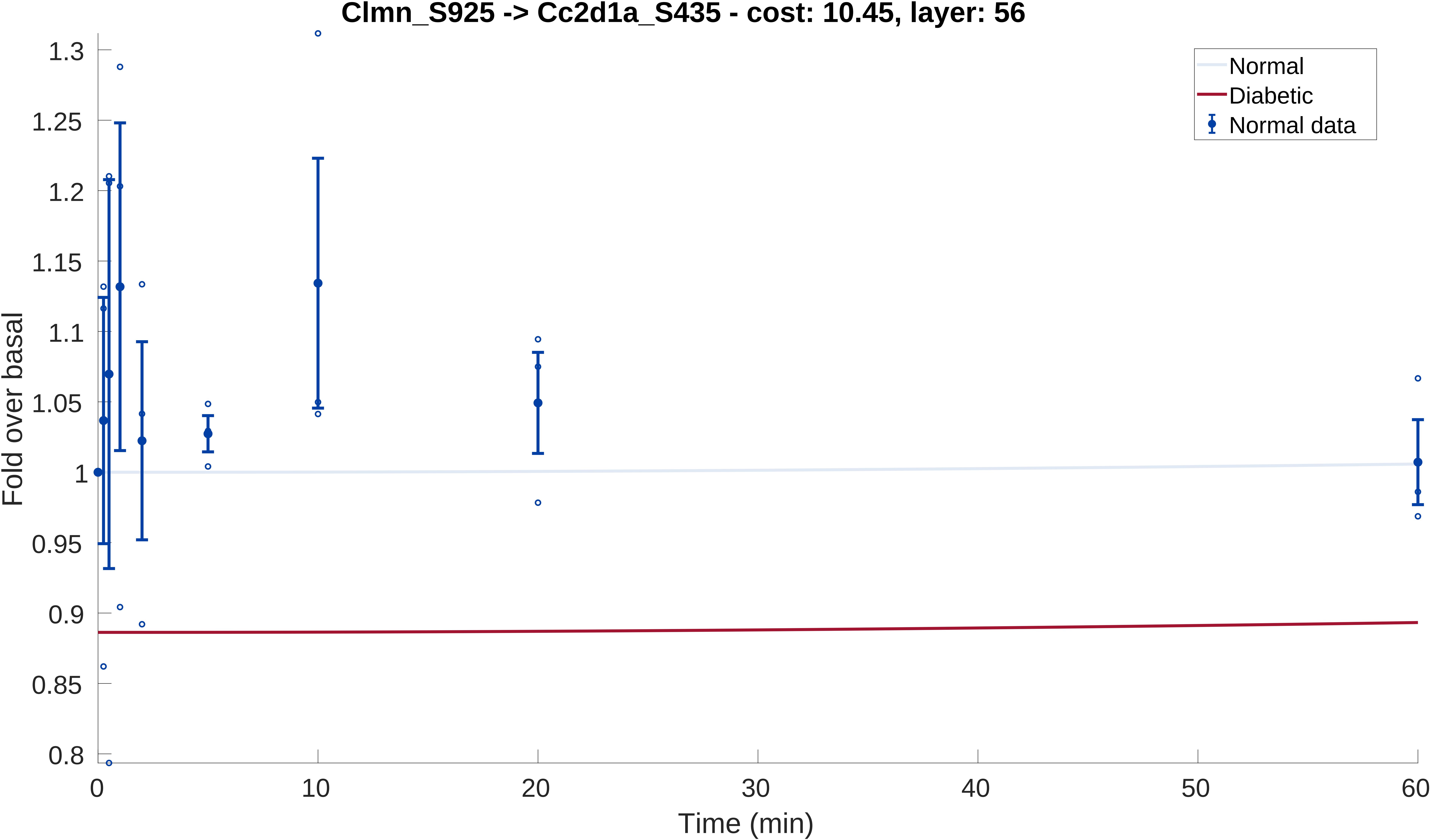
Clmn_S925 -> Cc2d1a_S435 - cost: 10.45, layer: 56
Normal
Diabetic
Normal data
Fold over basal
1.3
1.25
1.2
1.15
1.1
1.05
1
0.95
0.9
0.85
0.8
0
10
20
30
40
50
60
Time (min)

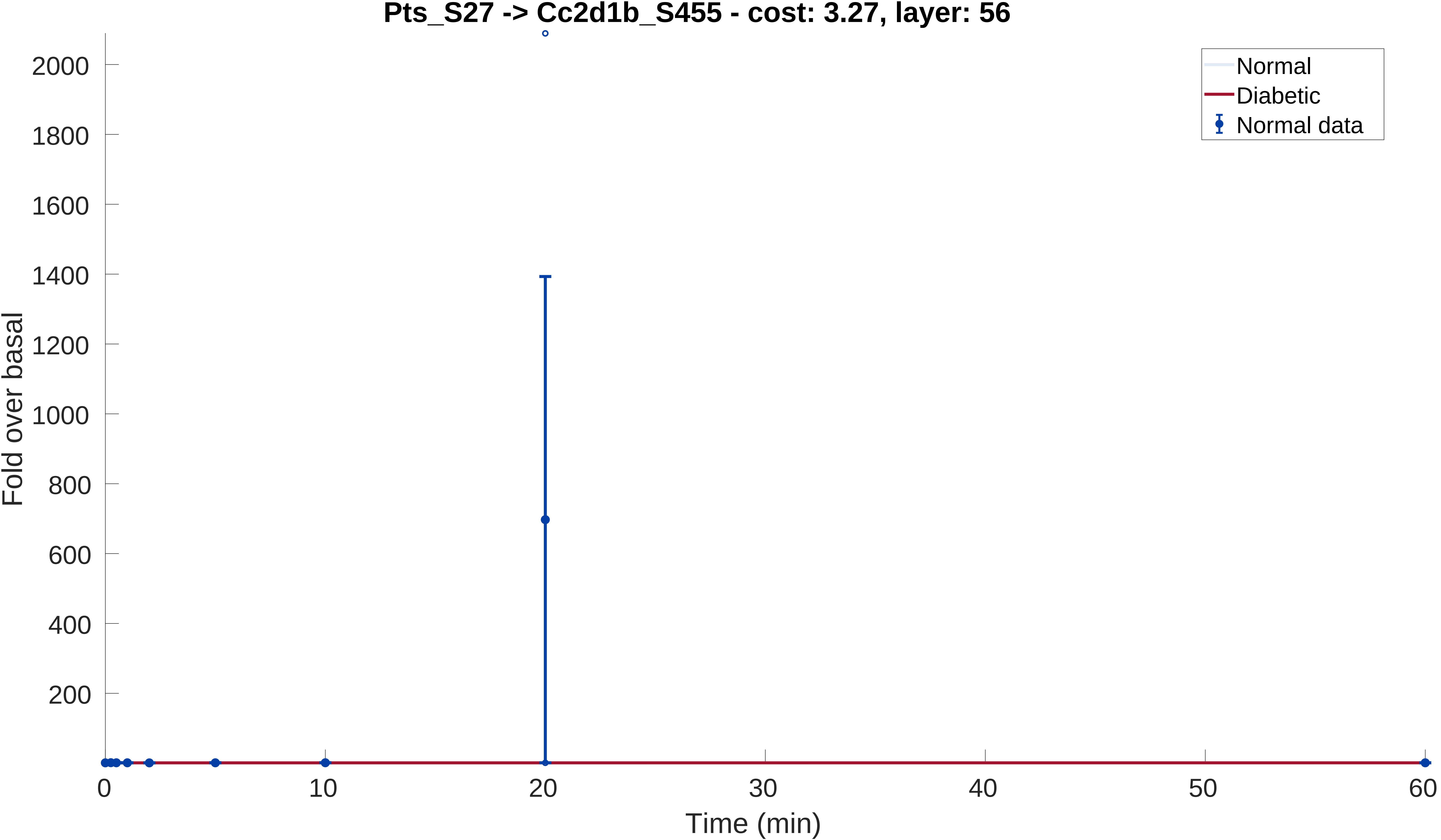

Pts_S27 -> Cc2d1b_S455 - cost: 3.27, layer: 56
Fold over basal
2000
1800
1600
1400
1200
1000
800
600
400
200
0
10
20
30
40
50
60
Time (min)
Normal
Diabetic
Normal data

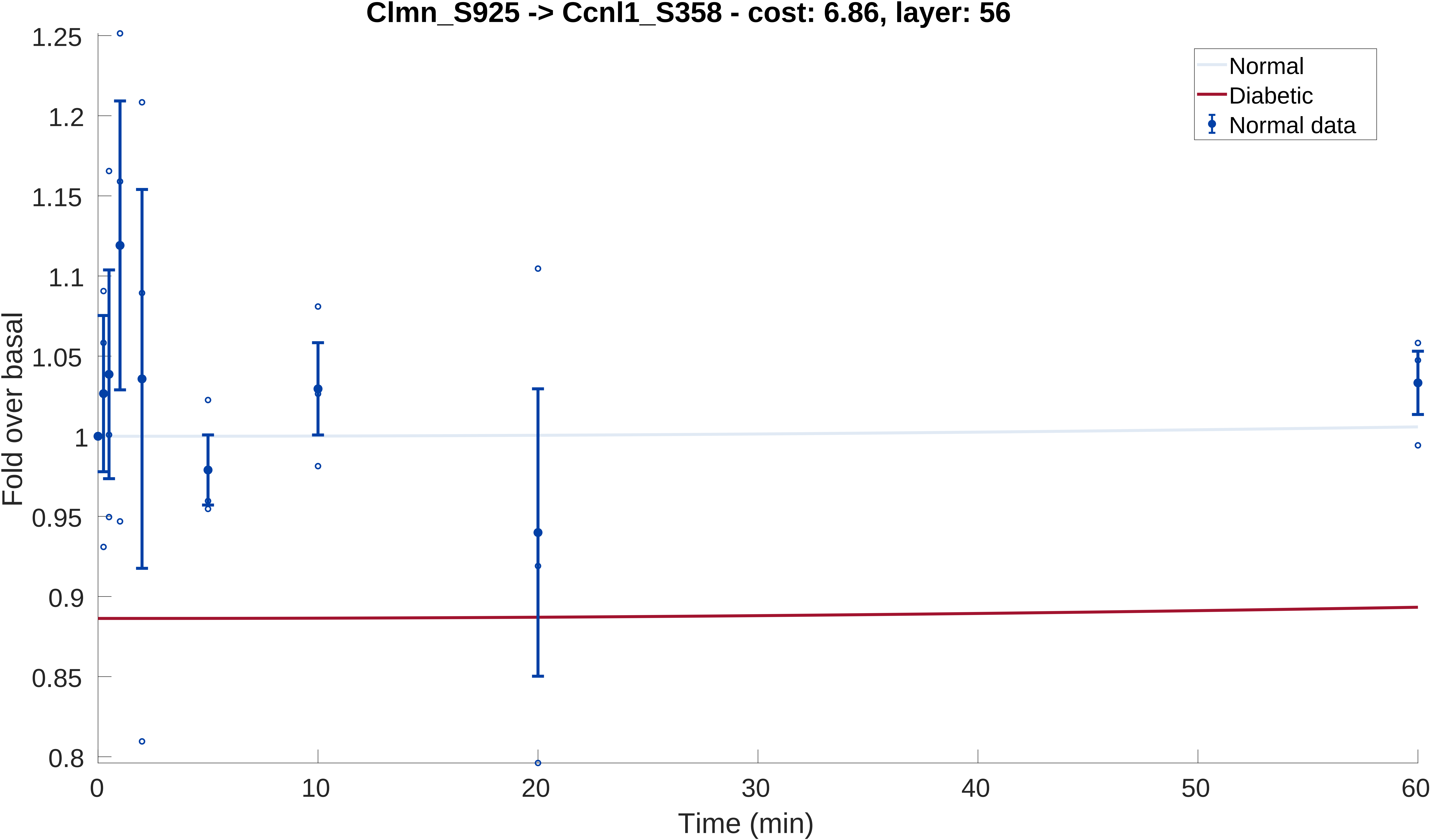

Clmn_S925 -> Ccnl1_S358 - cost: 6.86, layer: 56
Fold over basal
Time (min)
Normal
Diabetic
Normal data
1.25
1.2
1.15
1.1
1.05
1
0.95
0.9
0.85
0.8
0
10
20
30
40
50
60

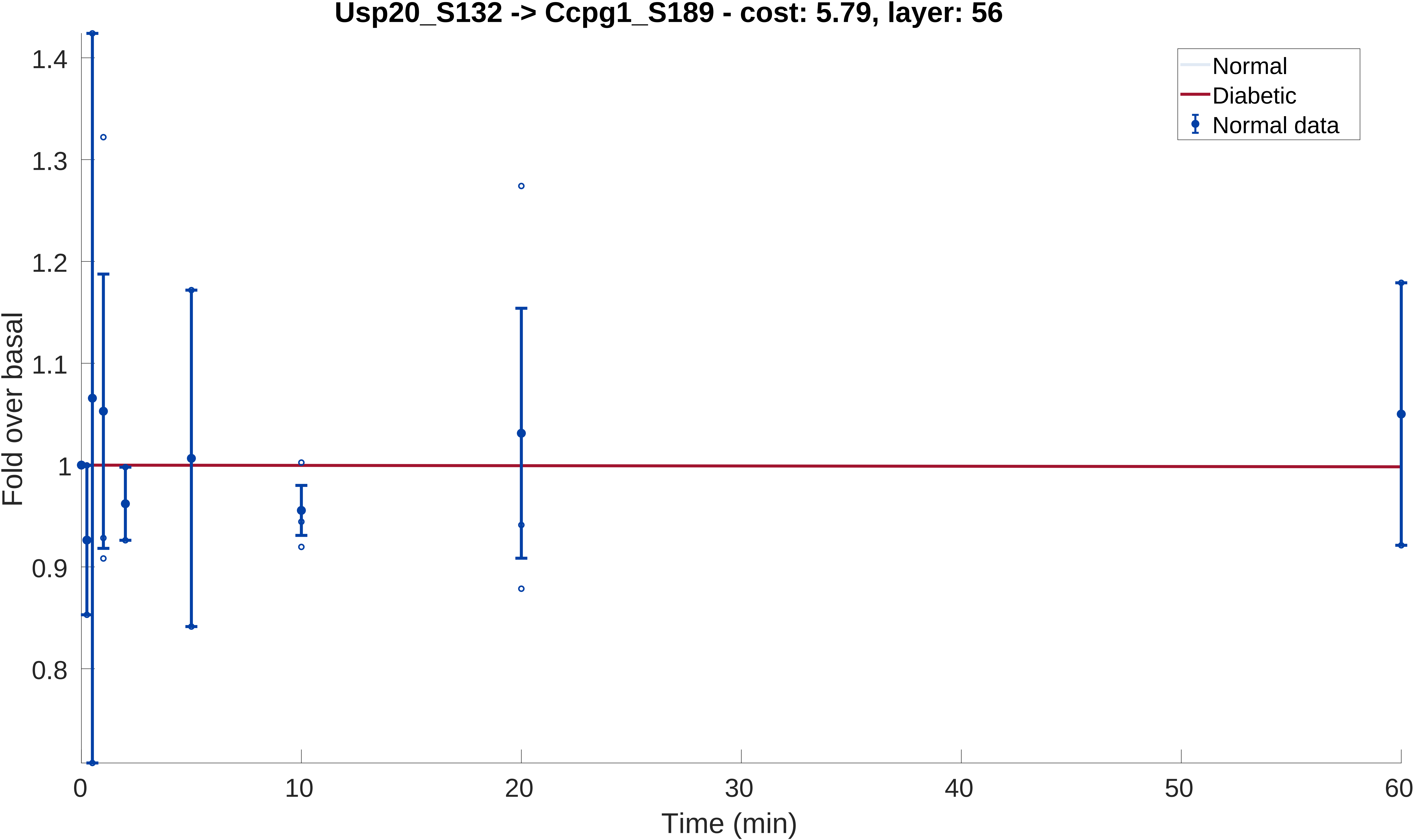
Usp20_S132 -> Ccpg1_S189 - cost: 5.79, layer: 56
Normal
Diabetic
Normal data
Fold over basal
1.4
1.3
1.2
1.1
1
0.9
0.8
0
10
20
30
40
50
60
Time (min)

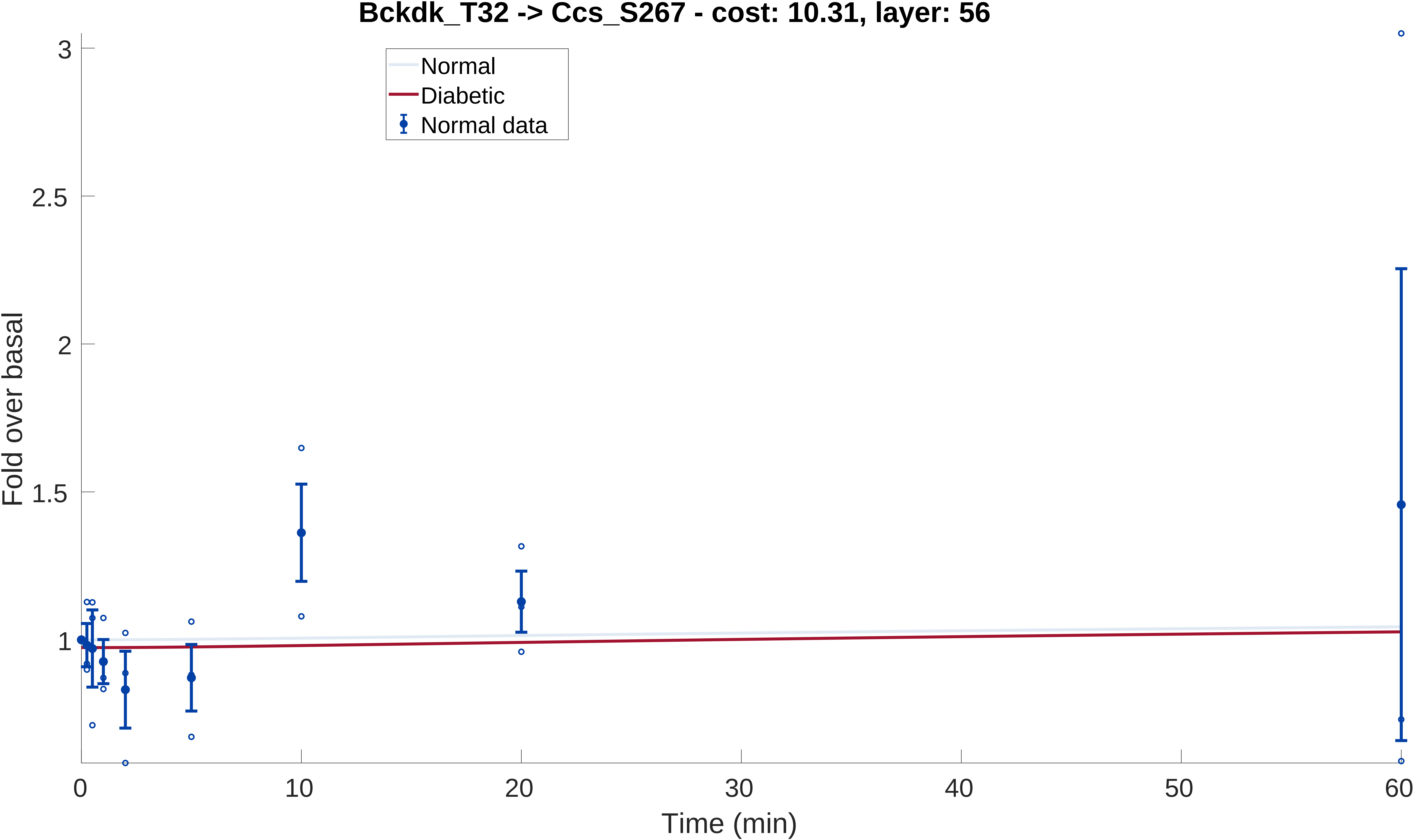

Bckdk_T32 -> Ccs_S267 - cost: 10.31, layer: 56
Normal
Diabetic
Normal data
Fold over basal
Time (min)
3
2.5
2
1.5
1
0
10
20
30
40
50
60

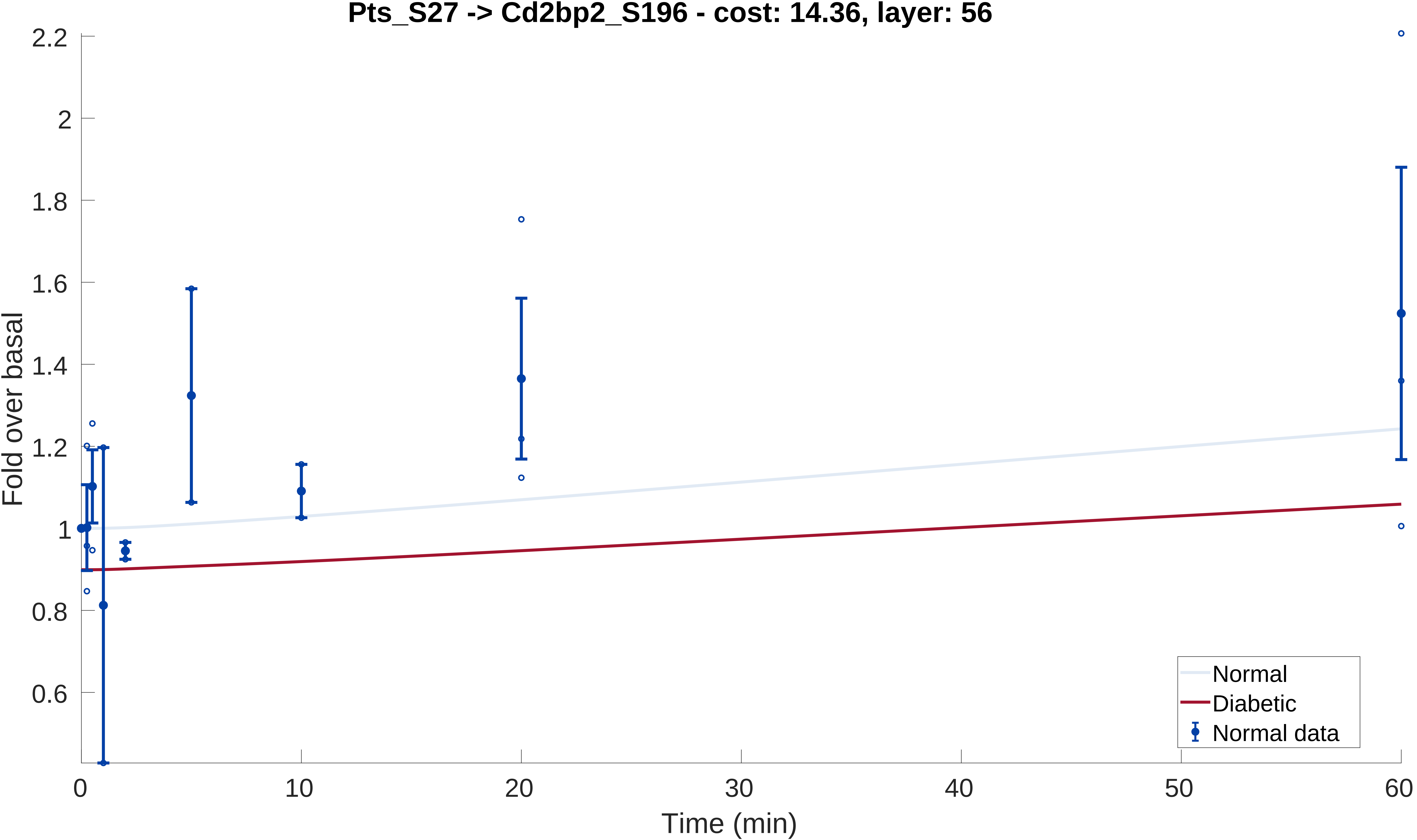
Pts_S27 -> Cd2bp2_S196 - cost: 14.36, layer: 56
Fold over basal
2.2
2
1.8
1.6
1.4
1.2
1
0.8
0.6
0
10
20
30
40
50
60
Time (min)
Normal
Diabetic
Normal data

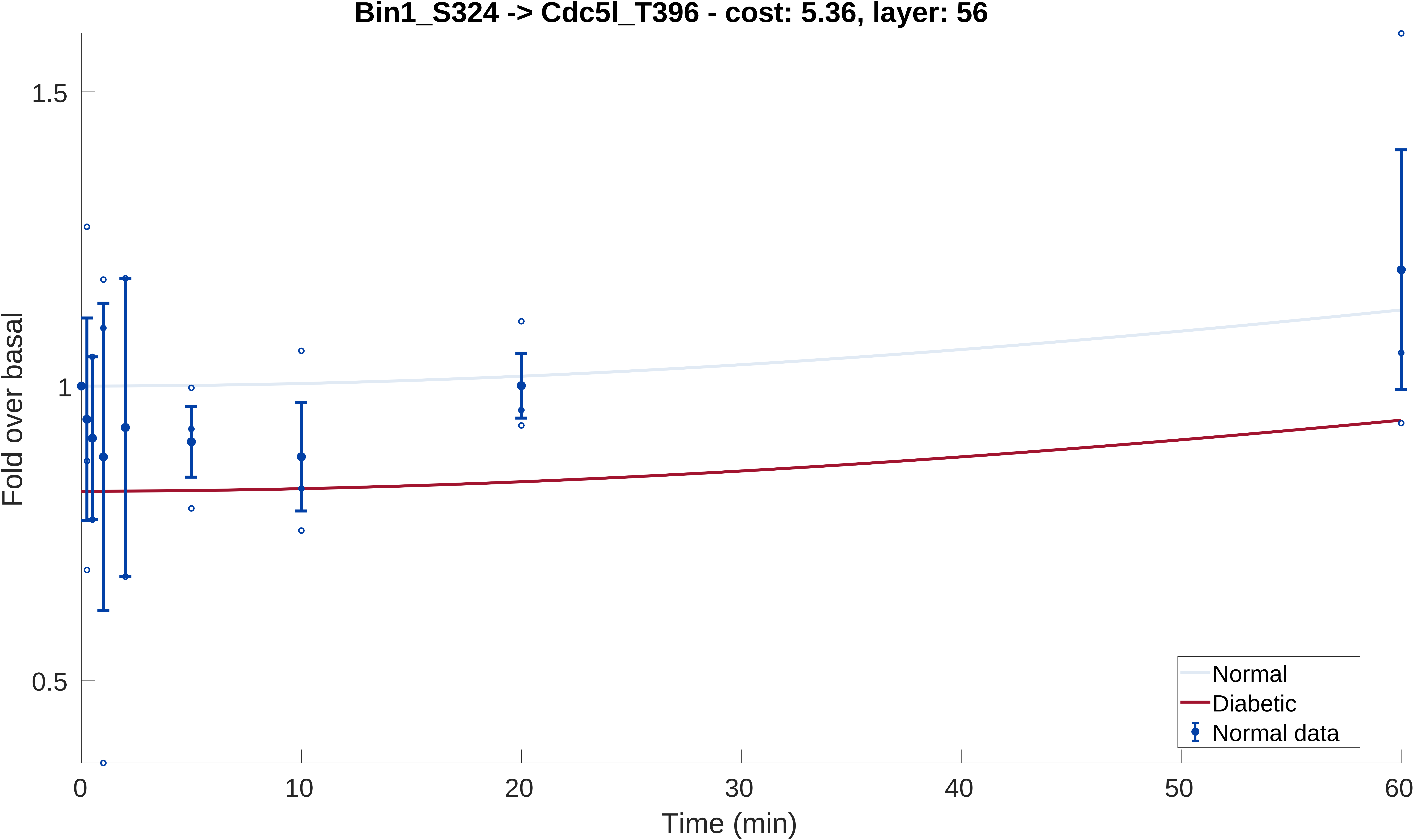

Bin1_S324 -> Cdc5l_T396 - cost: 5.36, layer: 56
Fold over basal
1.5
1
0.5
0
10
20
30
40
50
60
Time (min)
Normal
Diabetic
Normal data

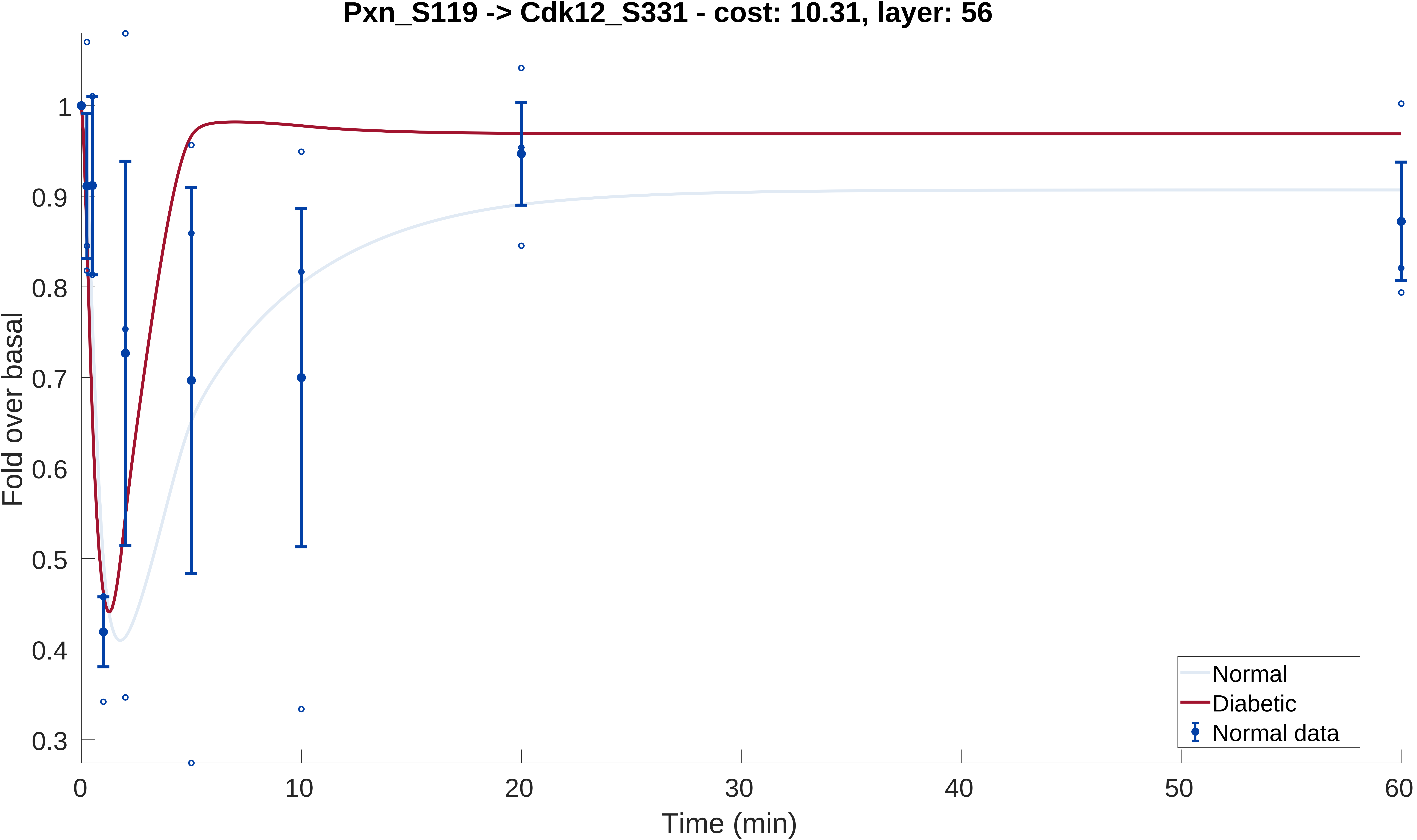

Pxn_S119 -> Cdk12_S331 - cost: 10.31, layer: 56
Fold over basal
Time (min)
0.3
0.4
0.5
0.6
0.7
0.8
0.9
1
0
10
20
30
40
50
60
Normal
Diabetic
Normal data

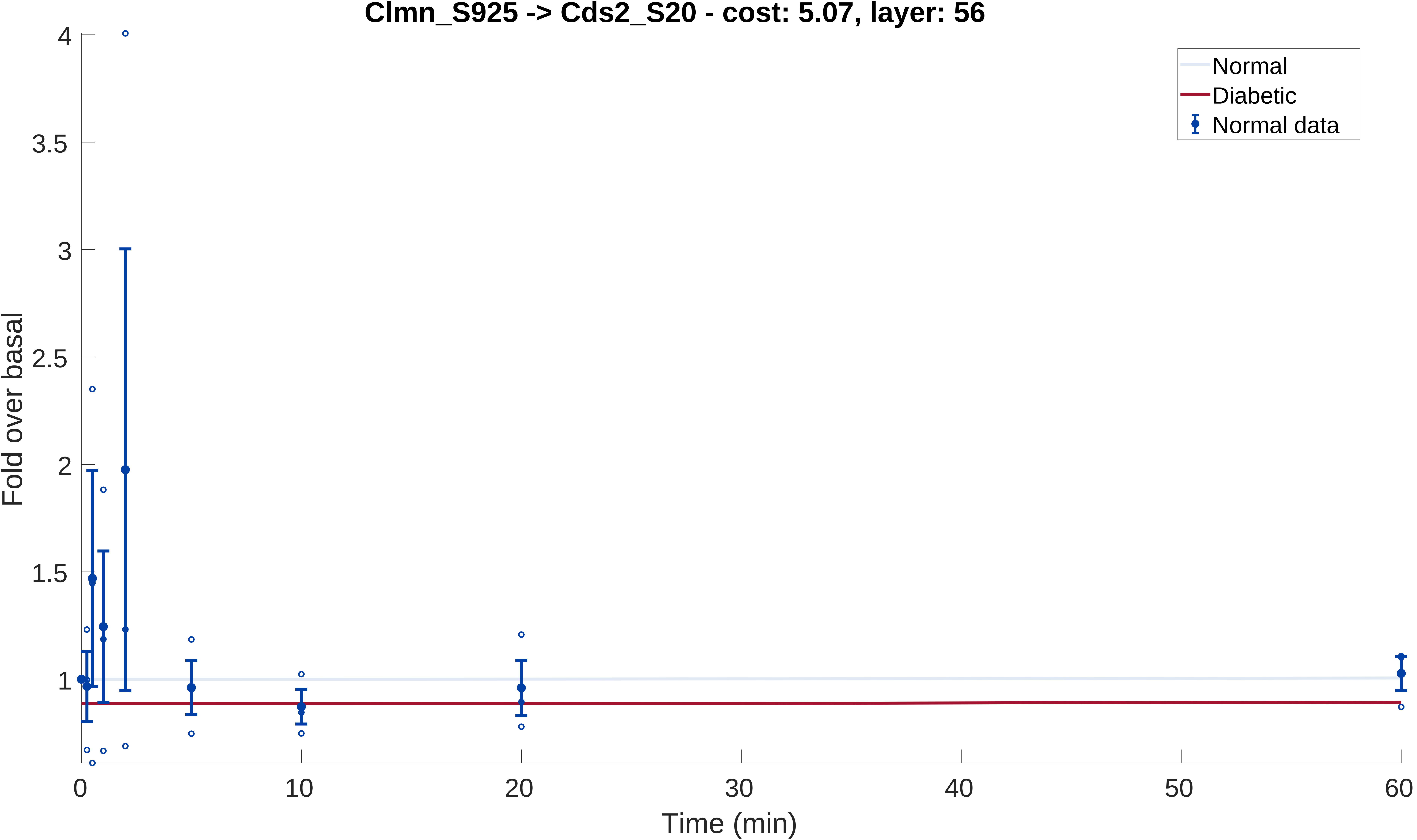
Clmn_S925 -> Cds2_S20 - cost: 5.07, layer: 56
Fold over basal
Time (min)
Normal
Diabetic
Normal data
4
3.5
3
2.5
2
1.5
1
0
10
20
30
40
50
60

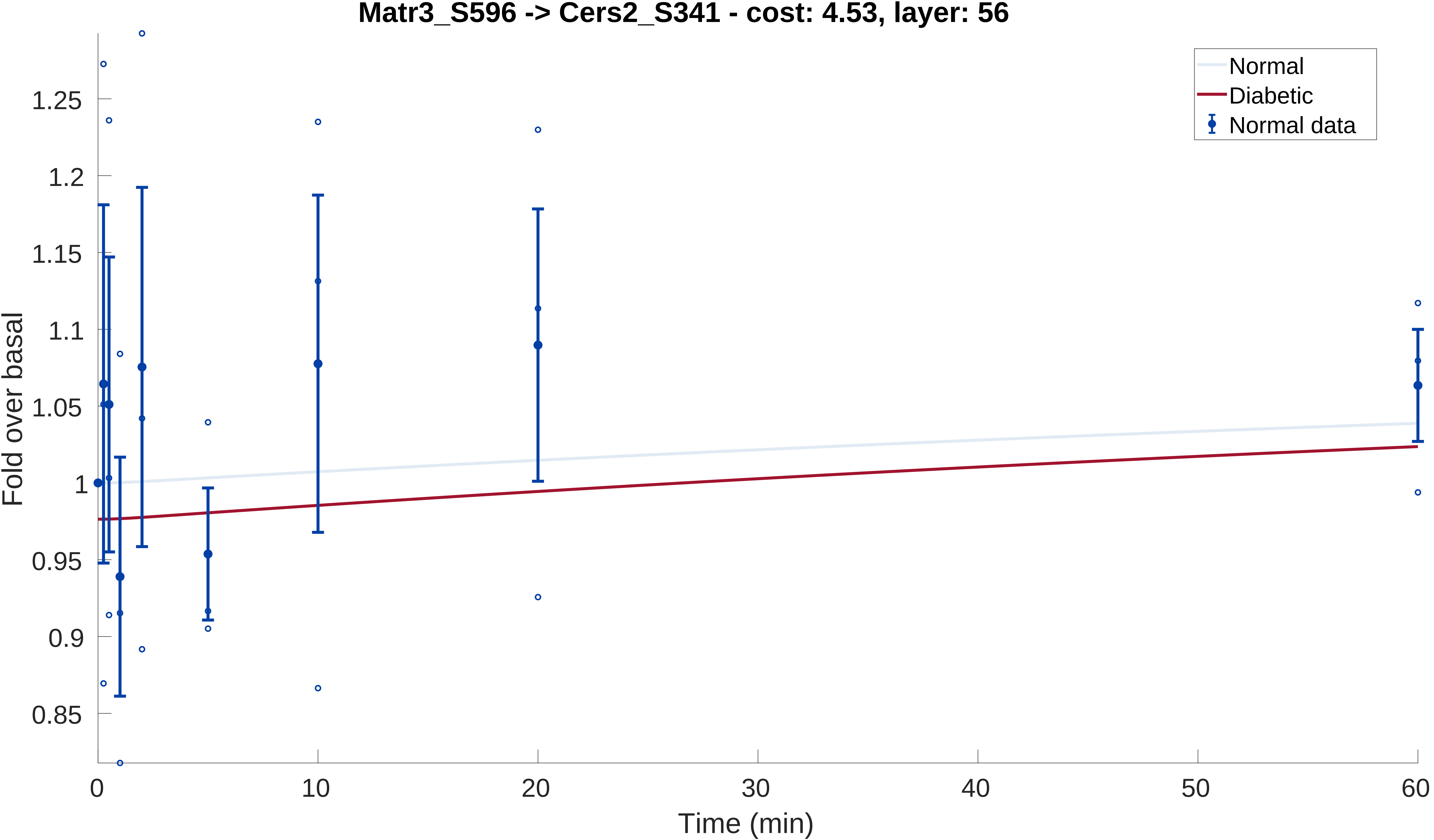

Matr3_S596 -> Cers2_S341 - cost: 4.53, layer: 56
Normal
Diabetic
Normal data
Fold over basal
Time (min)
1.25
1.2
1.15
1.1
1.05
1
0.95
0.9
0.85
0
10
20
30
40
50
60

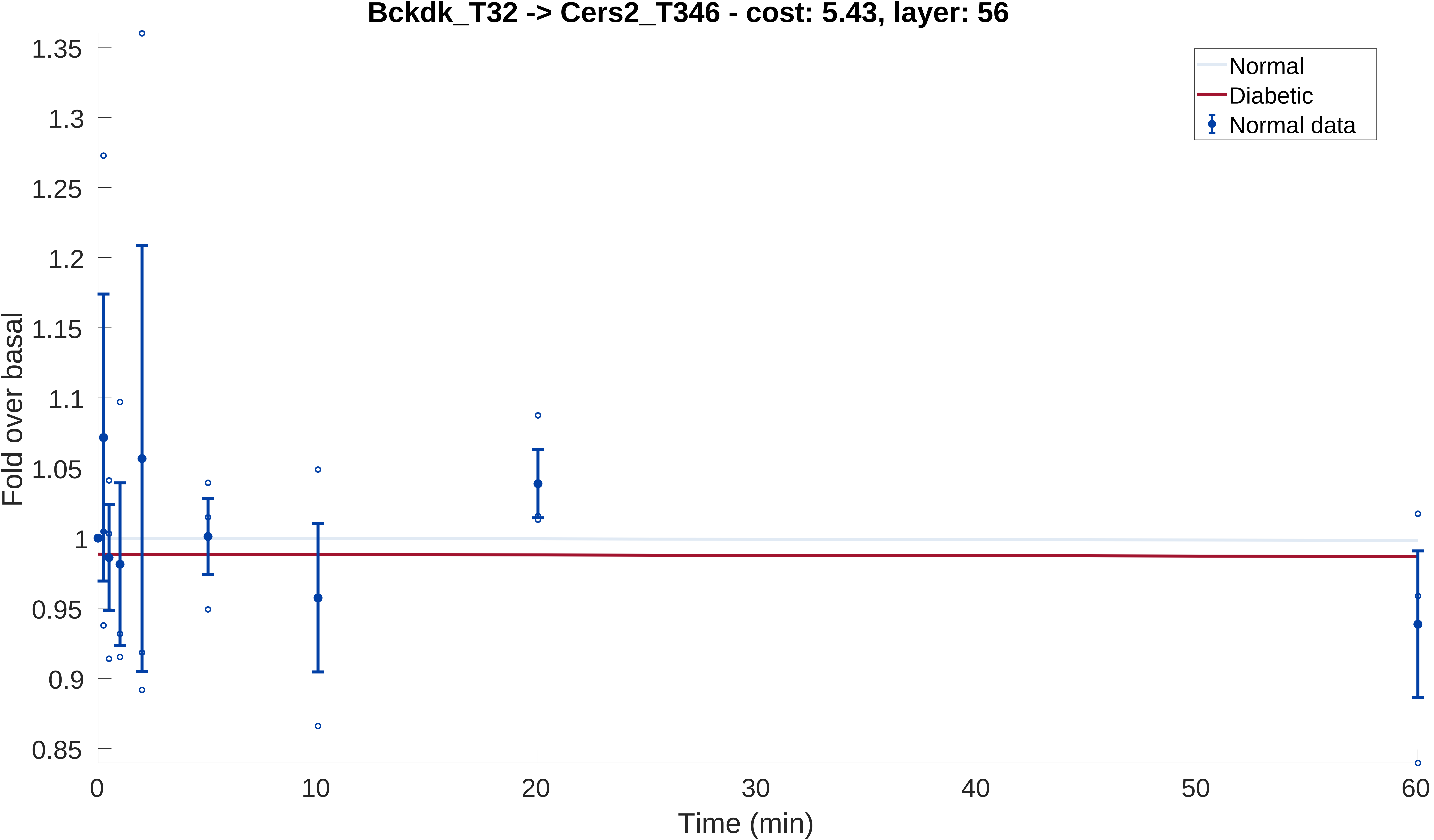
Bckdk_T32 -> Cers2_T346 - cost: 5.43, layer: 56
Normal
Diabetic
Normal data
Fold over basal
1.35
1.3
1.25
1.2
1.15
1.1
1.05
1
0.95
0.9
0.85
0
10
20
30
40
50
60
Time (min)

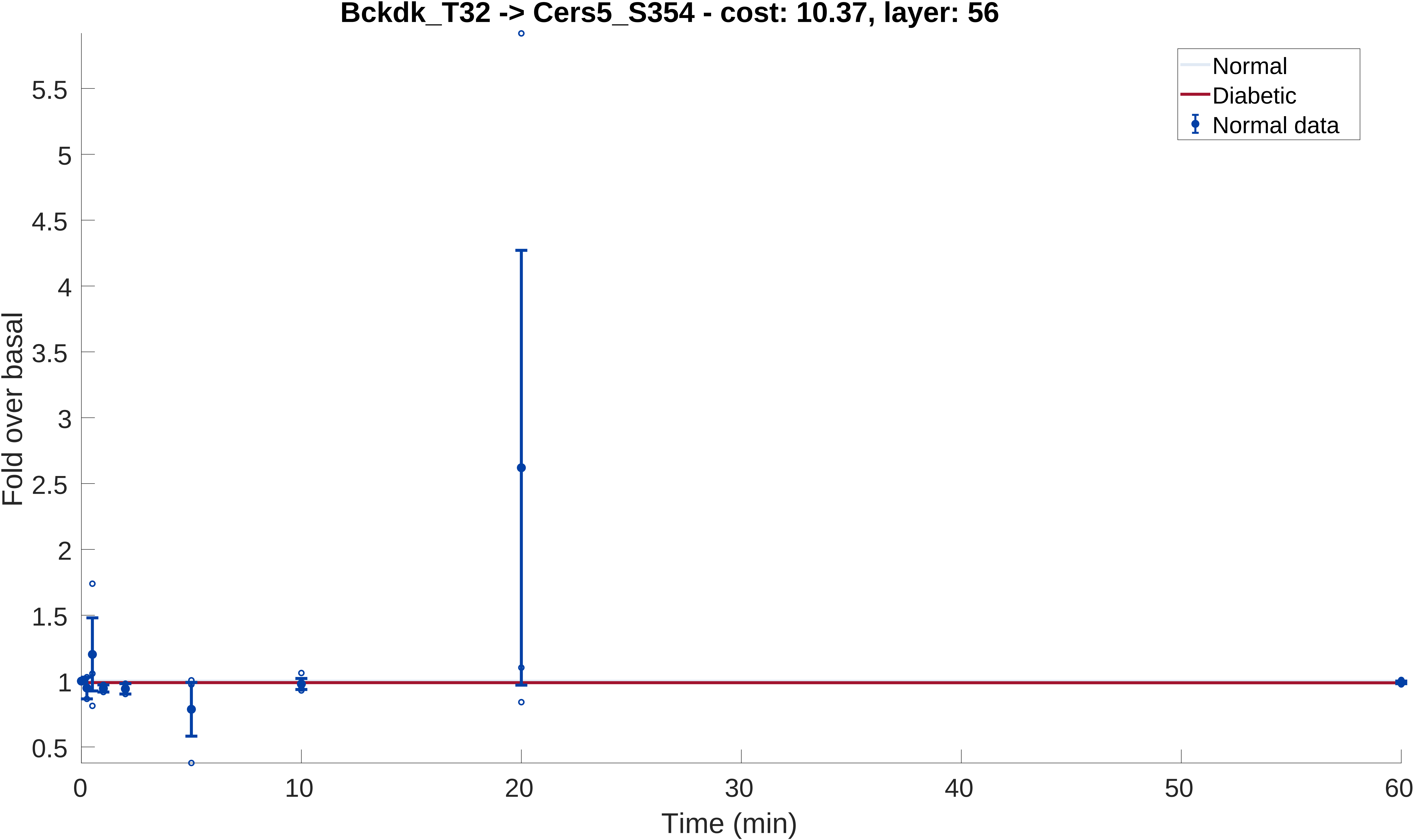

Bckdk_T32 -> Cers5_S354 - cost: 10.37, layer: 56
Normal
Diabetic
Normal data
Fold over basal
Time (min)
0.5
1
1.5
2
2.5
3
3.5
4
4.5
5
5.5
0
10
20
30
40
50
60

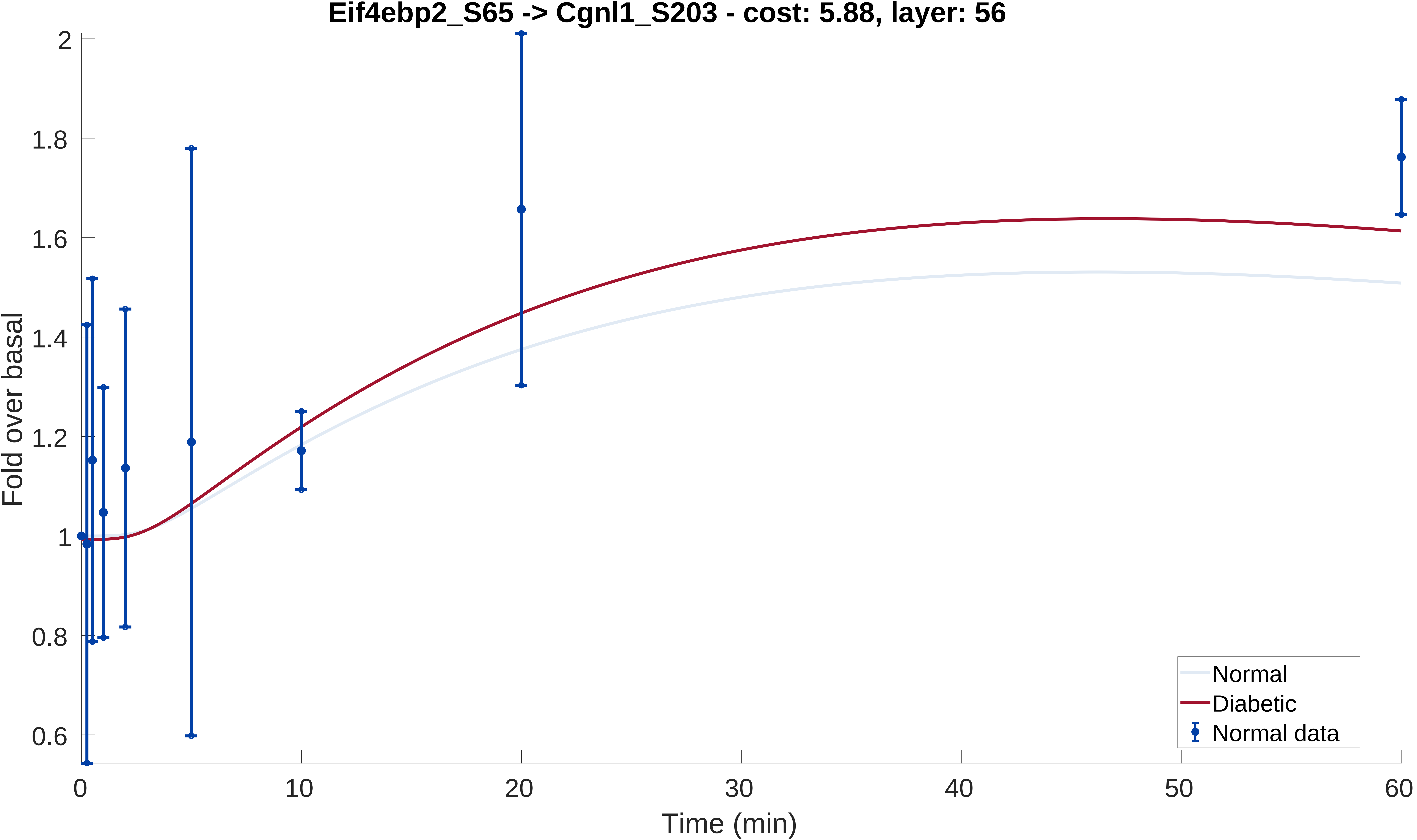
Eif4ebp2_S65 -> Cgnl1_S203 - cost: 5.88, layer: 56
Fold over basal
Time (min)
0.6
0.8
1
1.2
1.4
1.6
1.8
2
0
10
20
30
40
50
60
Normal
Diabetic
Normal data

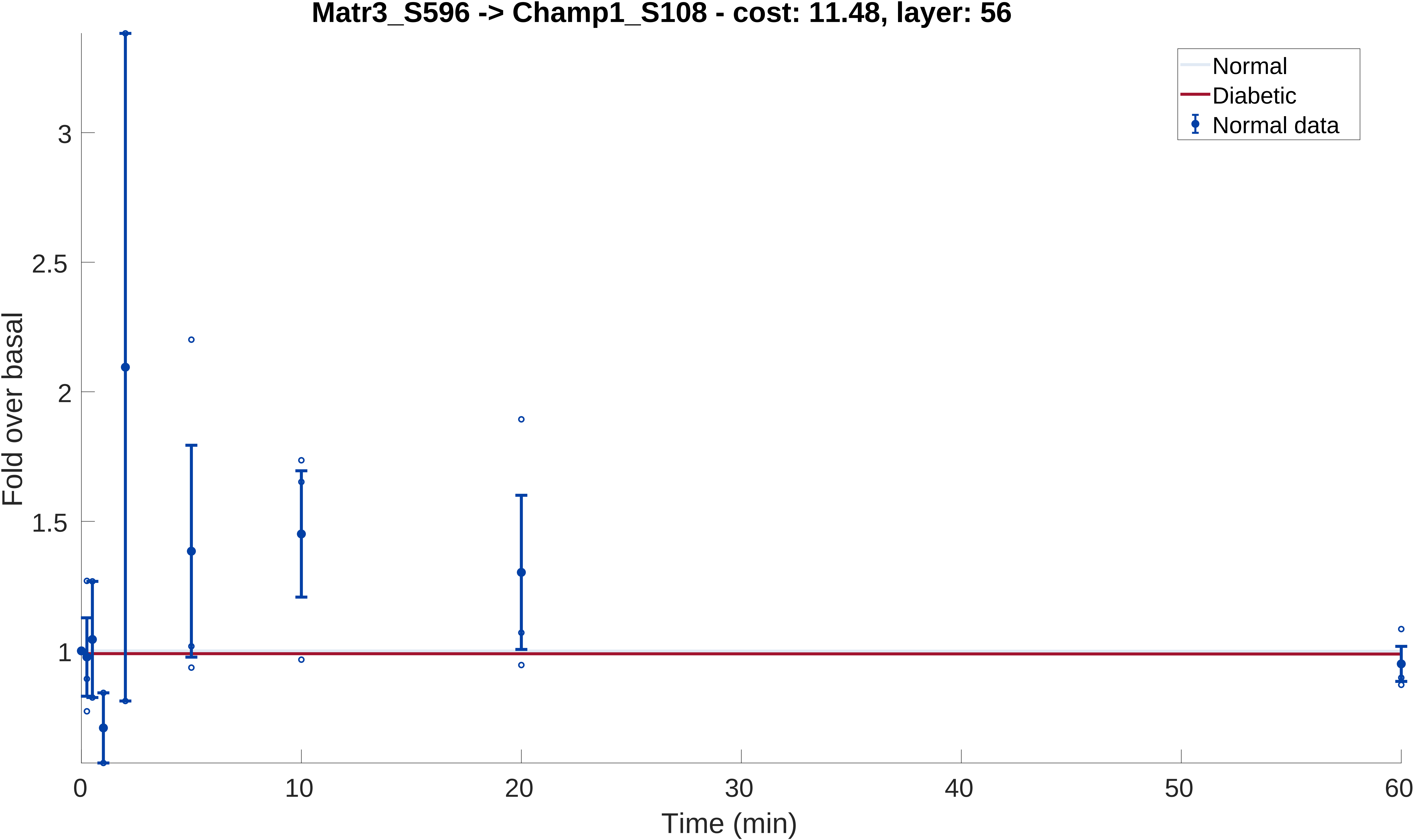

Matr3_S596 -> Champ1_S108 - cost: 11.48, layer: 56
Normal
Diabetic
Normal data
Fold over basal
Time (min)
3
2.5
2
1.5
1
0
10
20
30
40
50
60

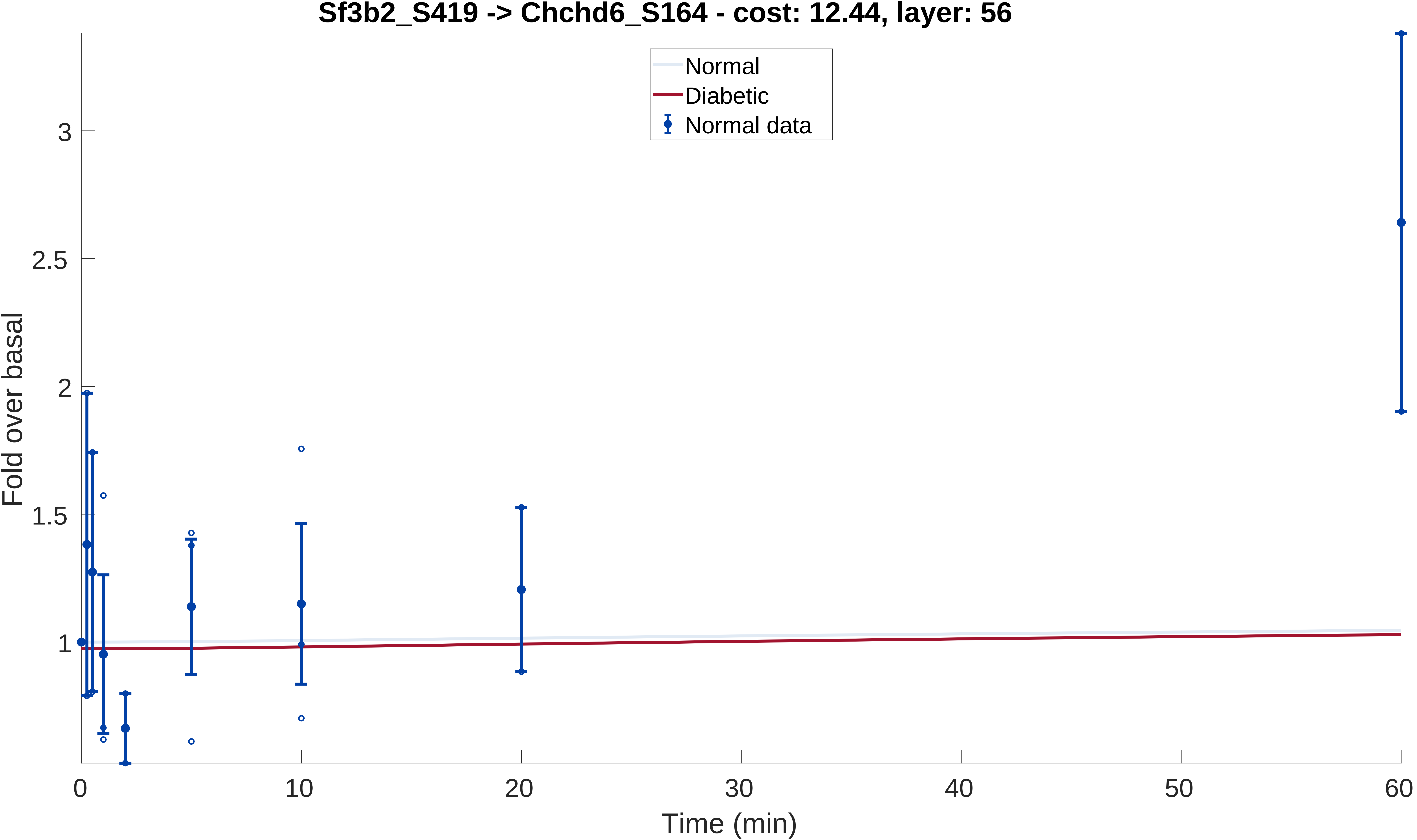
Sf3b2_S419 -> Chchd6_S164 - cost: 12.44, layer: 56
Normal
Diabetic
Normal data
Fold over basal
3
2.5
2
1.5
1
0
10
20
30
40
50
60
Time (min)

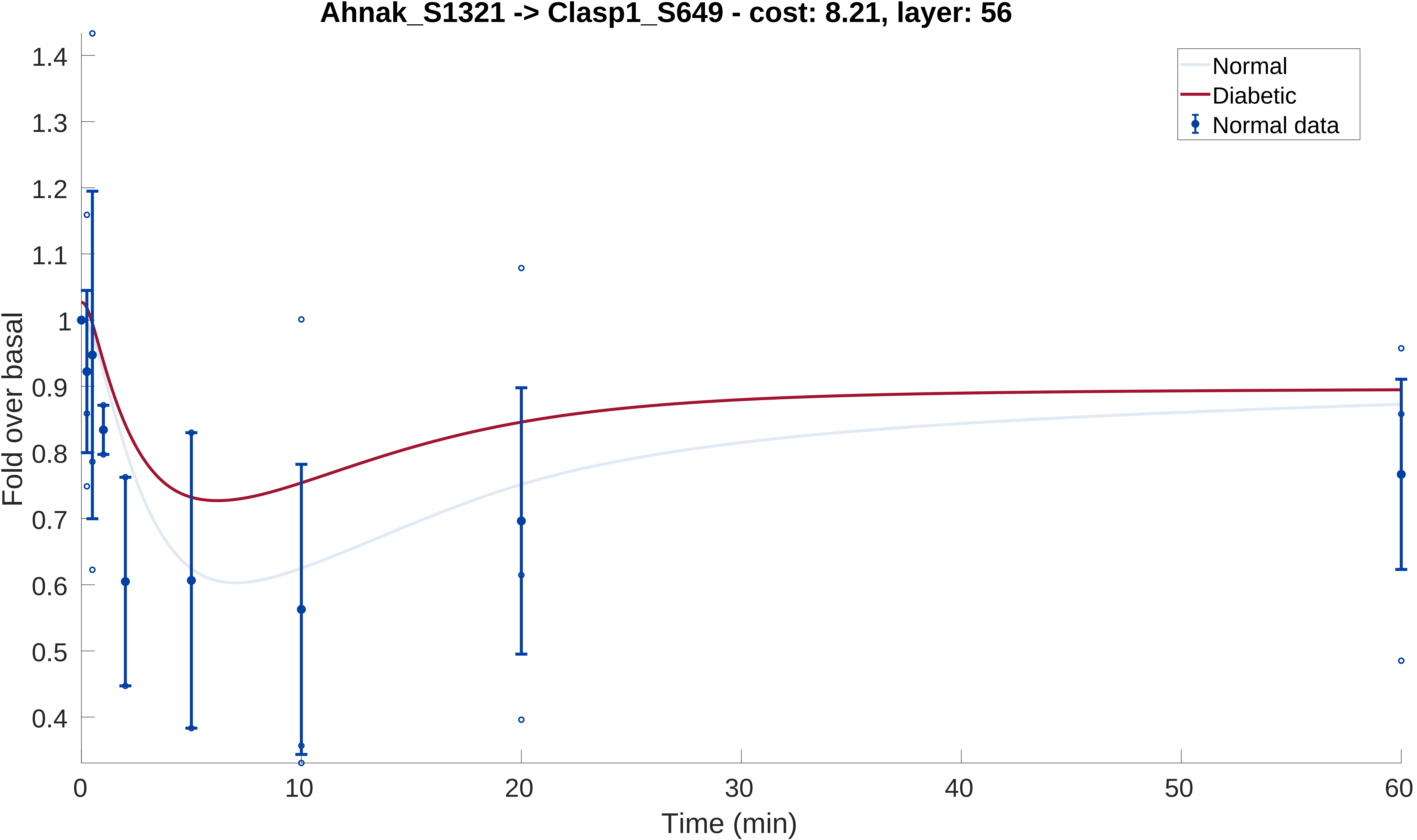

Ahnak_S1321 -> Clasp1_S649 - cost: 8.21, layer: 56
Normal
Diabetic
Normal data
Fold over basal
Time (min)
1.4
1.3
1.2
1.1
1
0.9
0.8
0.7
0.6
0.5
0.4
0
10
20
30
40
50
60

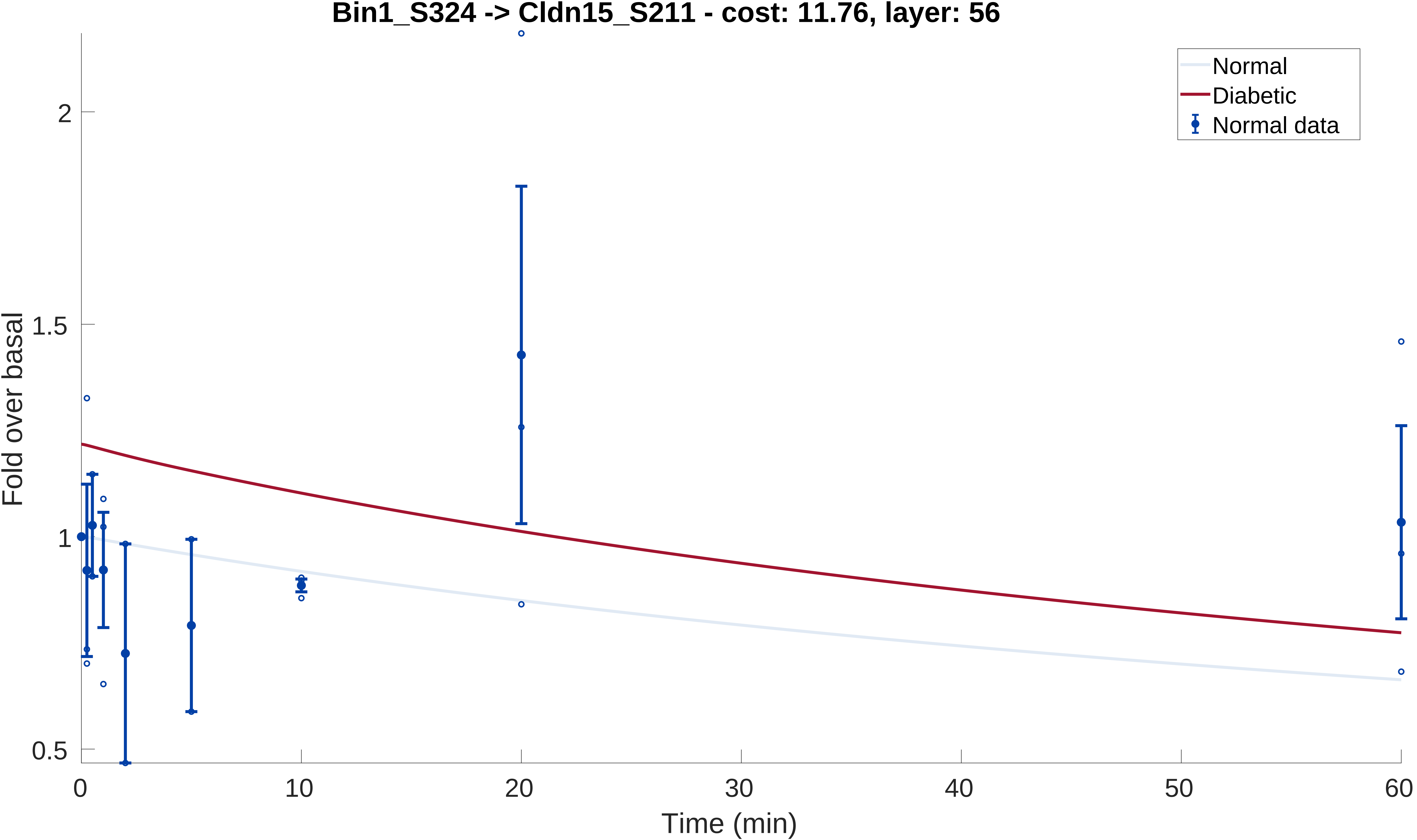

Bin1_S324 -> Cldn15_S211 - cost: 11.76, layer: 56
Normal
Diabetic
Normal data
Fold over basal
2
1.5
1
0.5
0
10
20
30
40
50
60
Time (min)

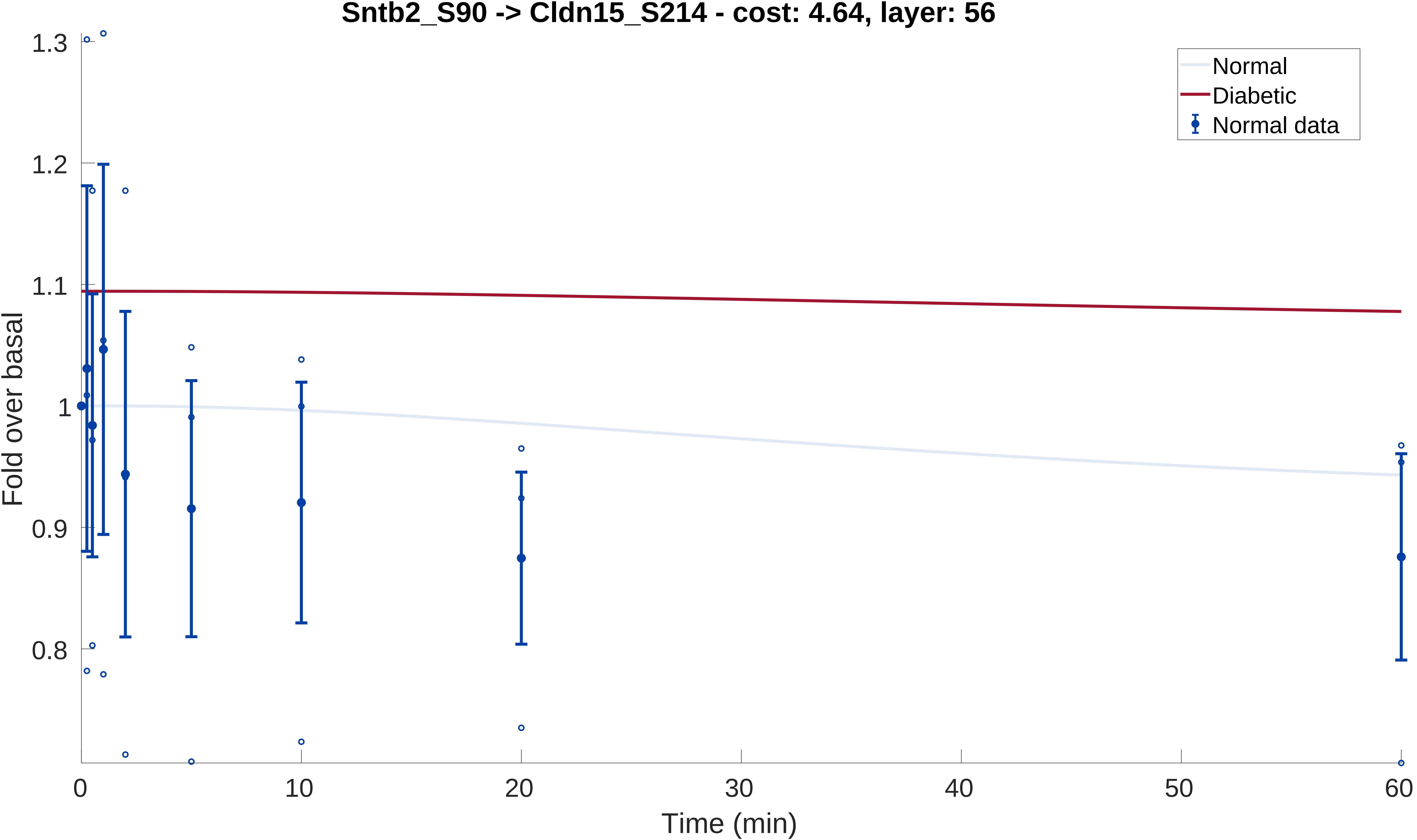

Sntb2_S90 -> Cldn15_S214 - cost: 4.64, layer: 56
Normal
Diabetic
Normal data
Fold over basal
Time (min)
1.3
1.2
1.1
1
0.9
0.8
0
10
20
30
40
50
60

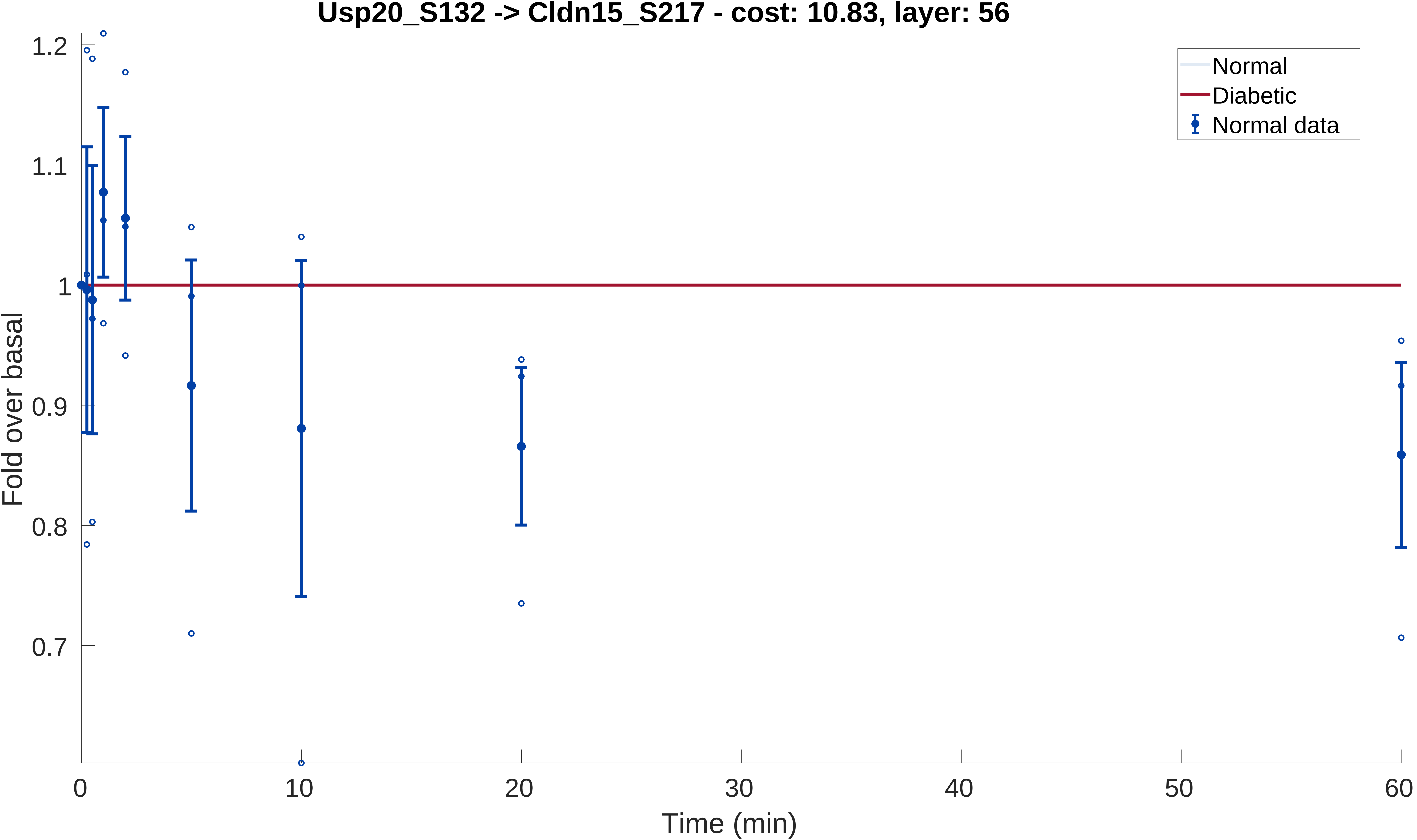

Usp20_S132 -> Cldn15_S217 - cost: 10.83, layer: 56
Normal
Diabetic
Normal data
Fold over basal
Time (min)
1.2
1.1
1
0.9
0.8
0.7
0
10
20
30
40
50
60

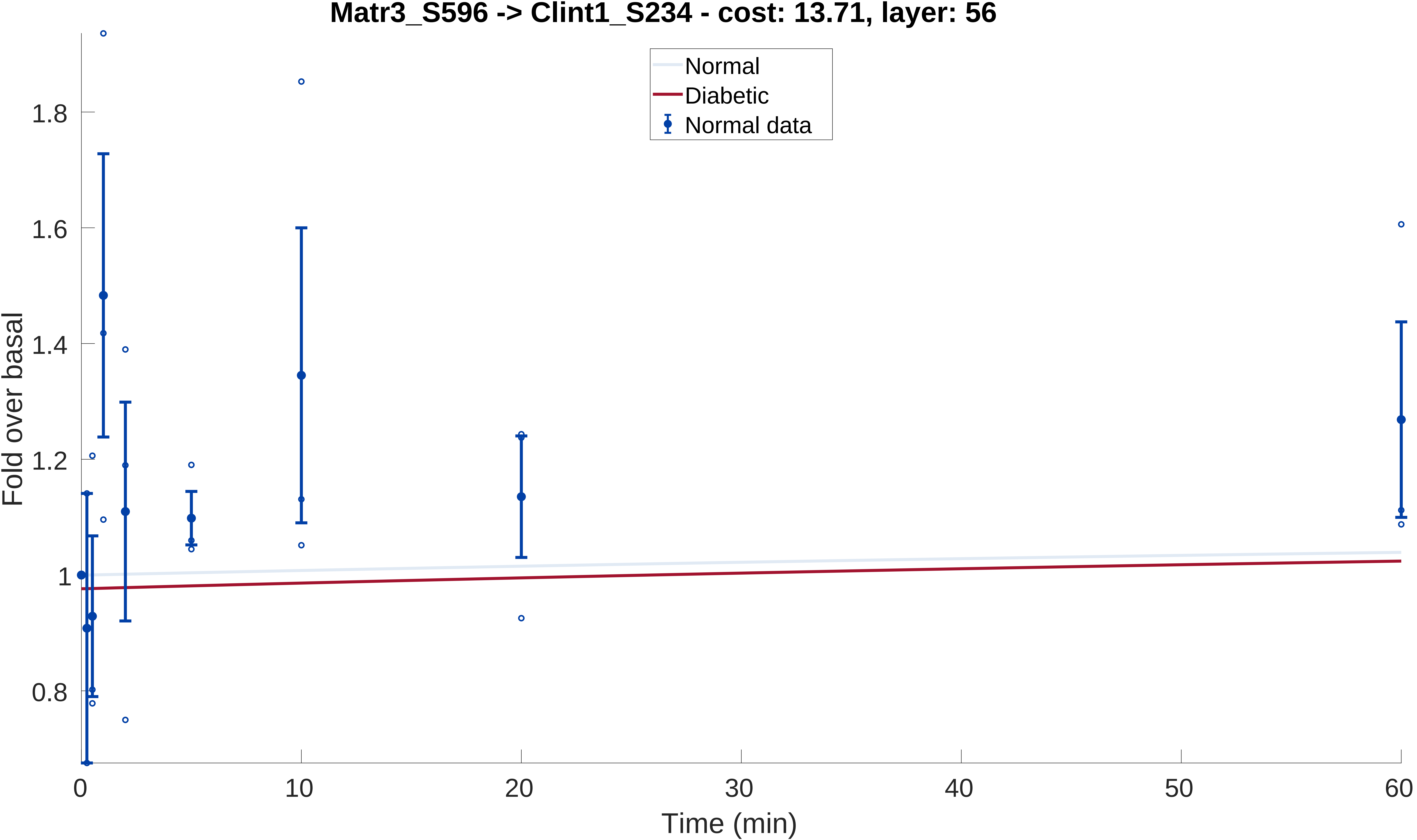
Matr3_S596 -> Clint1_S234 - cost: 13.71, layer: 56
Normal
Diabetic
Normal data
Fold over basal
0.8
1
1.2
1.4
1.6
1.8
Time (min)
0
10
20
30
40
50
60

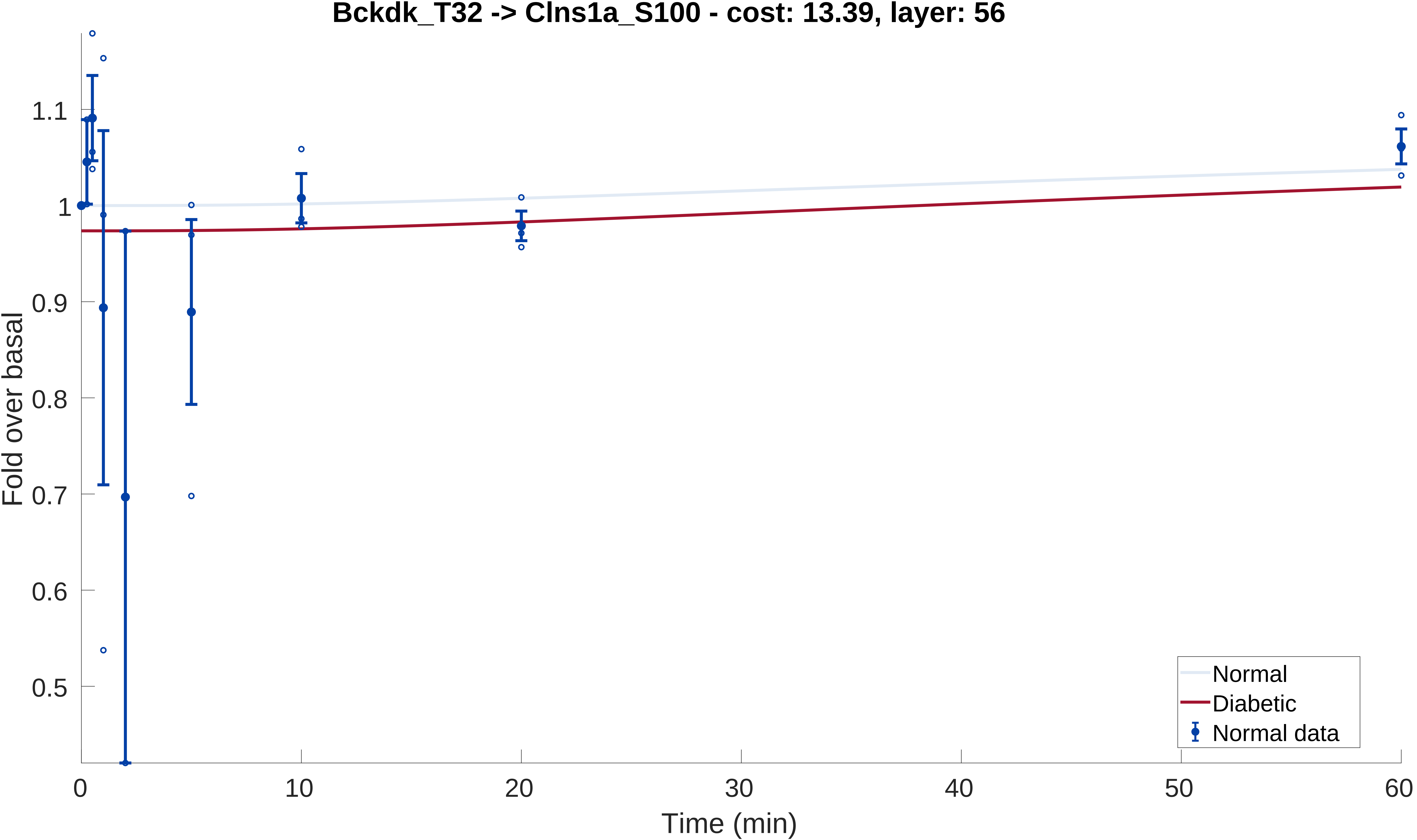

Bckdk_T32 -> Clns1a_S100 - cost: 13.39, layer: 56
Fold over basal
1.1
1
0.9
0.8
0.7
0.6
0.5
0
10
20
30
40
50
60
Time (min)
Normal
Diabetic
Normal data

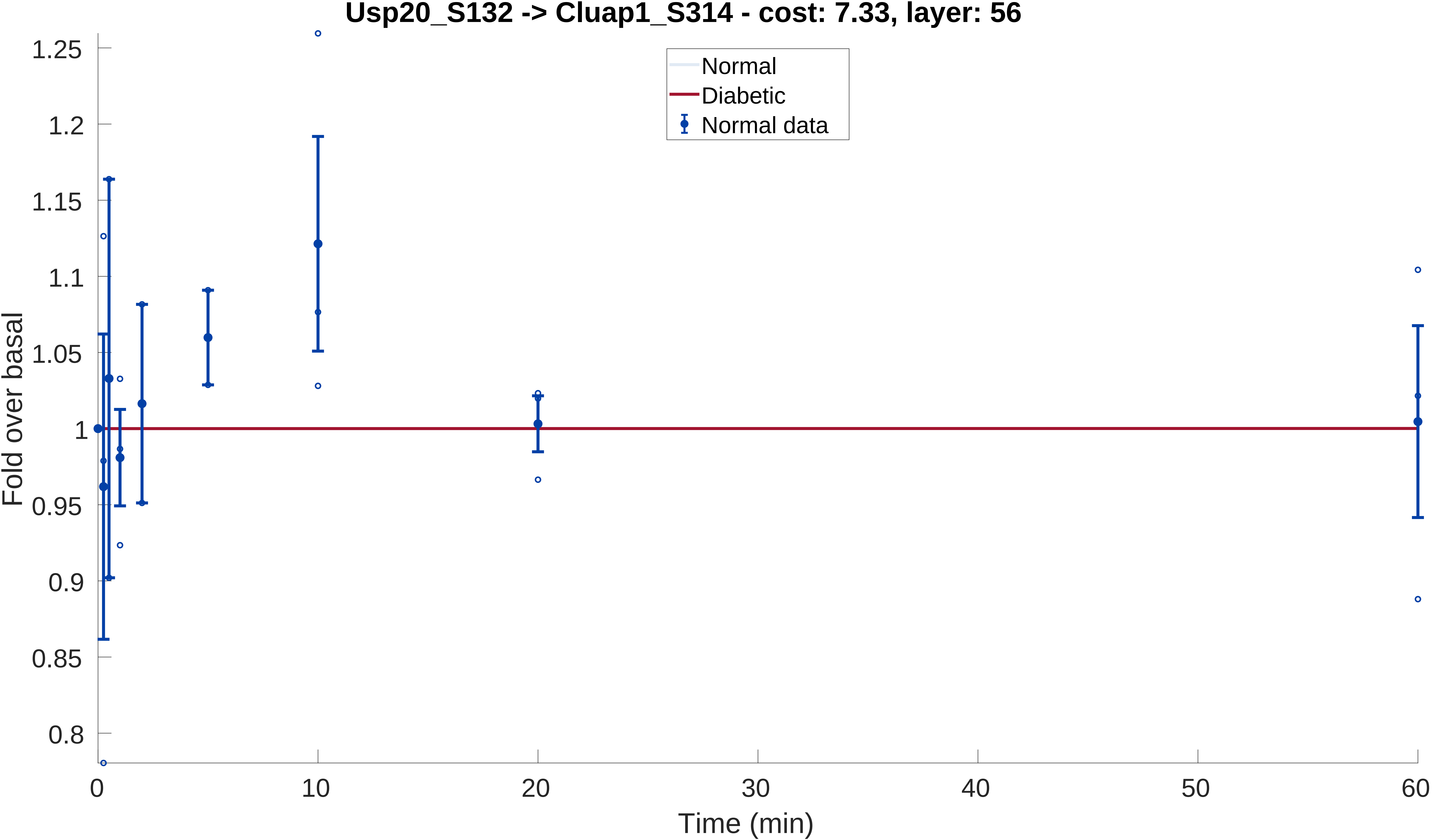

Usp20_S132 -> Cluap1_S314 - cost: 7.33, layer: 56
Normal
Diabetic
Normal data
Fold over basal
1.25
1.2
1.15
1.1
1.05
1
0.95
0.9
0.85
0.8
0
10
20
30
40
50
60
Time (min)

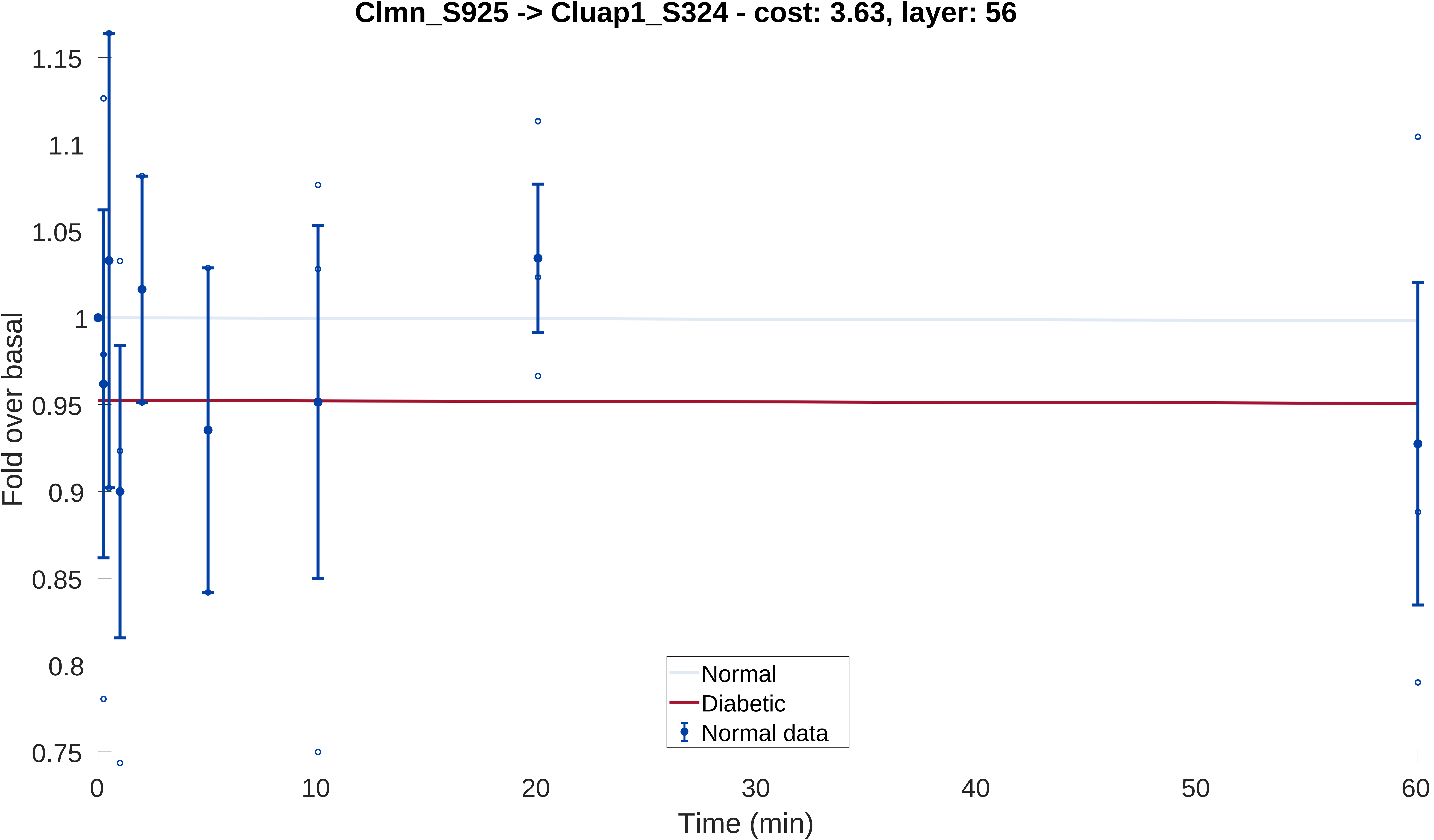

Clmn_S925 -> Cluap1_S324 - cost: 3.63, layer: 56
Fold over basal
1.15
1.1
1.05
1
0.95
0.9
0.85
0.8
0.75
0
10
20
30
40
50
60
Time (min)
Normal
Diabetic
Normal data

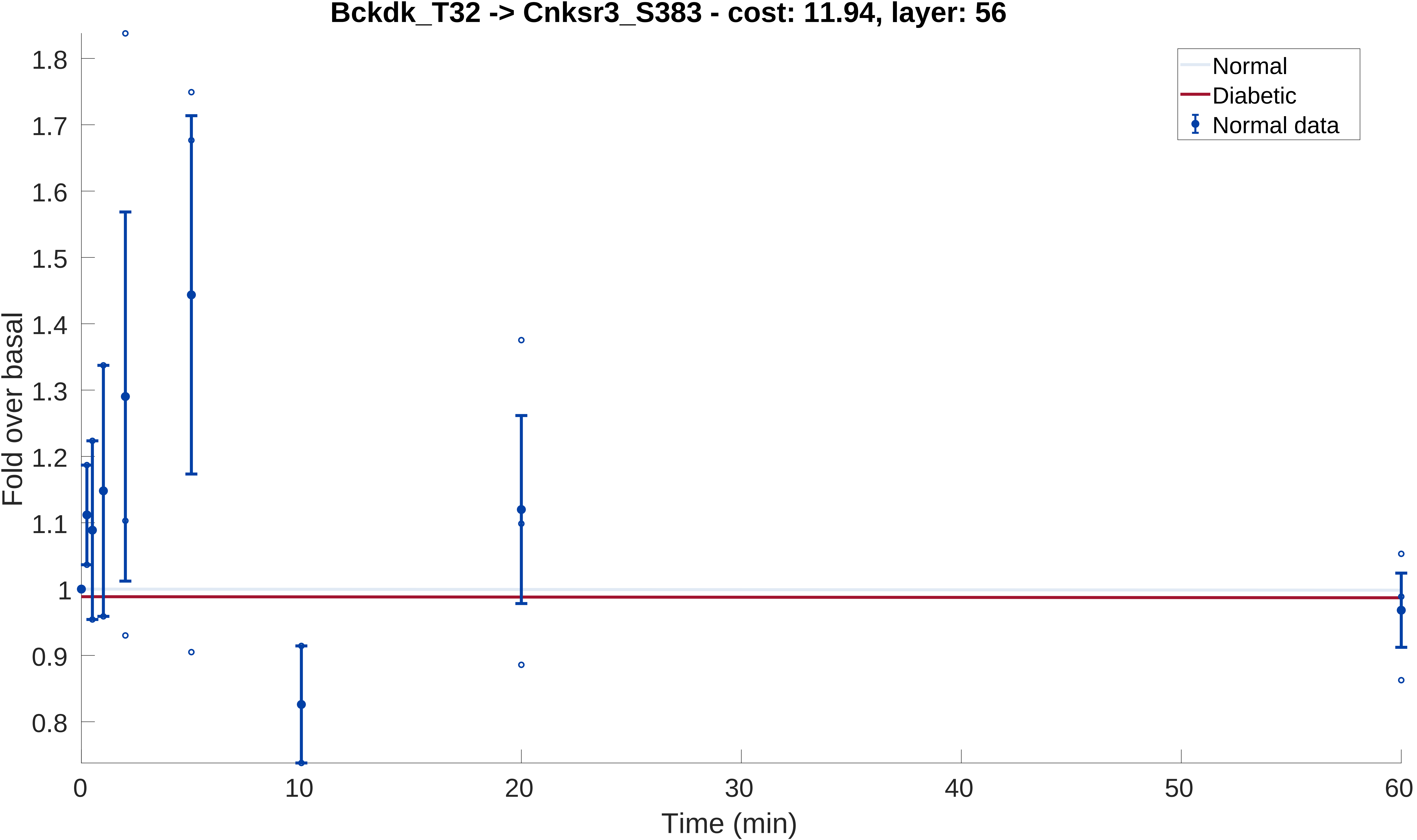

Bckdk_T32 -> Cnksr3_S383 - cost: 11.94, layer: 56
Normal
Diabetic
Normal data
Fold over basal
1.8
1.7
1.6
1.5
1.4
1.3
1.2
1.1
1
0.9
0.8
0
10
20
30
40
50
60
Time (min)

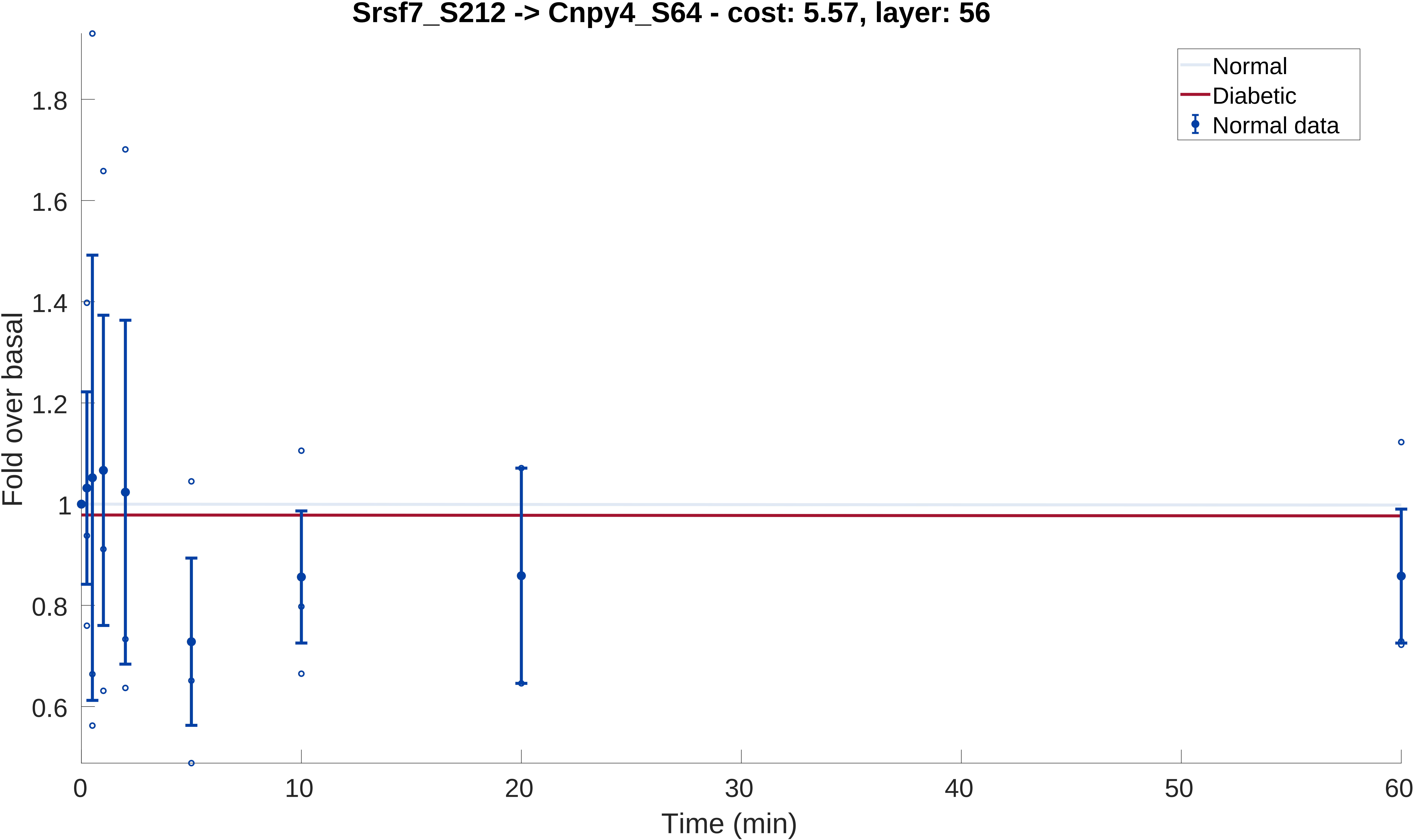
Srsf7_S212 -> Cnpy4_S64 - cost: 5.57, layer: 56
Normal
Diabetic
Normal data
Fold over basal
1.8
1.6
1.4
1.2
1
0.8
0.6
0
10
20
30
40
50
60
Time (min)

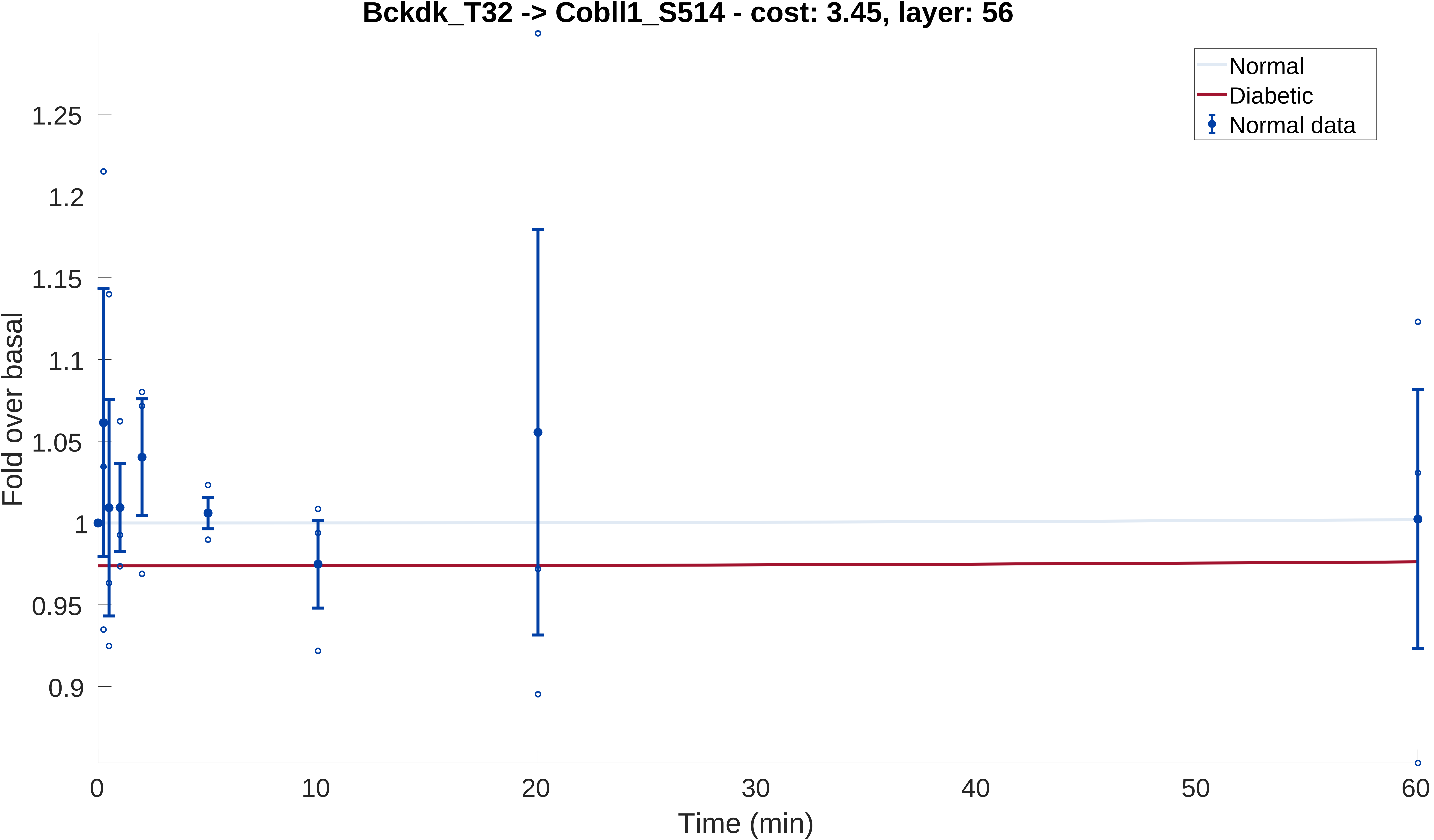

Bckdk_T32 -> Cobll1_S514 - cost: 3.45, layer: 56
Normal
Diabetic
Normal data
Fold over basal
Time (min)
1.25
1.2
1.15
1.1
1.05
1
0.95
0.9
0
10
20
30
40
50
60

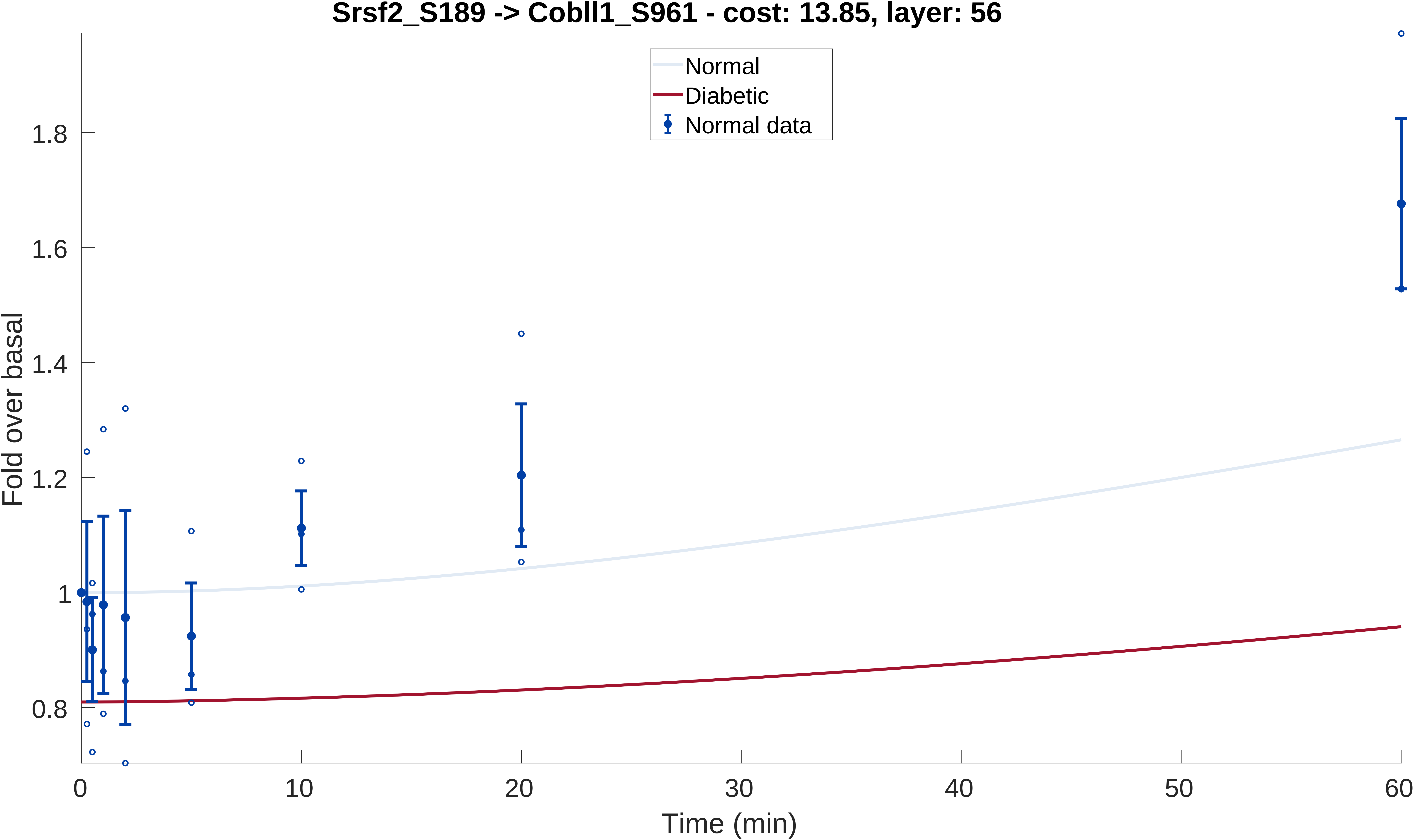

Srsf2_S189 -> Cobll1_S961 - cost: 13.85, layer: 56
Normal
Diabetic
Normal data
Fold over basal
0.8
1
1.2
1.4
1.6
1.8
0
10
20
30
40
50
60
Time (min)

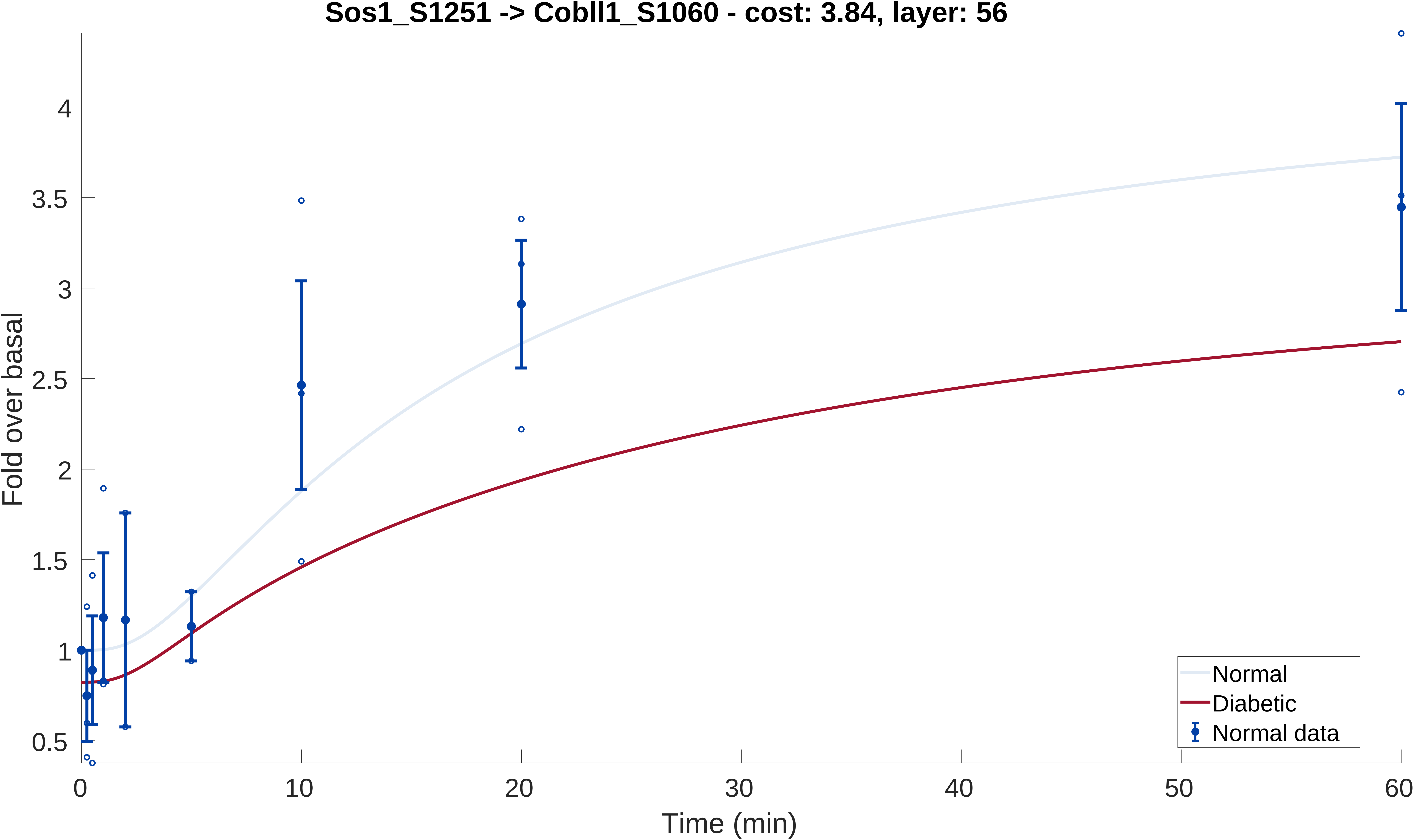

Sos1_S1251 -> Cobll1_S1060 - cost: 3.84, layer: 56
Fold over basal
Time (min)
0
10
20
30
40
50
60
0.5
1
1.5
2
2.5
3
3.5
4
Normal
Diabetic
Normal data

Sos1_S1251 -> Cobll1_T1059 - cost: 3.27, layer: 56

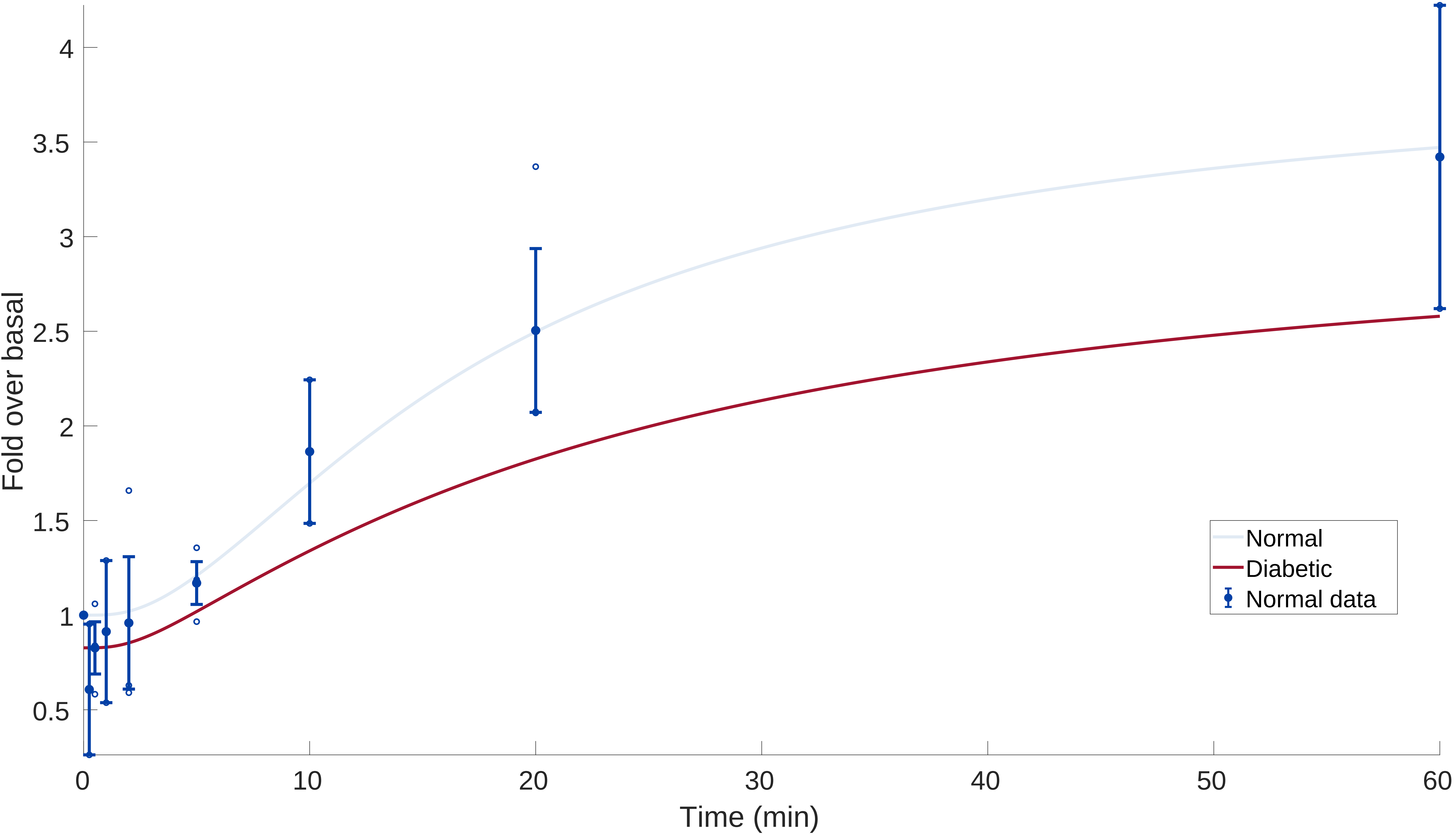

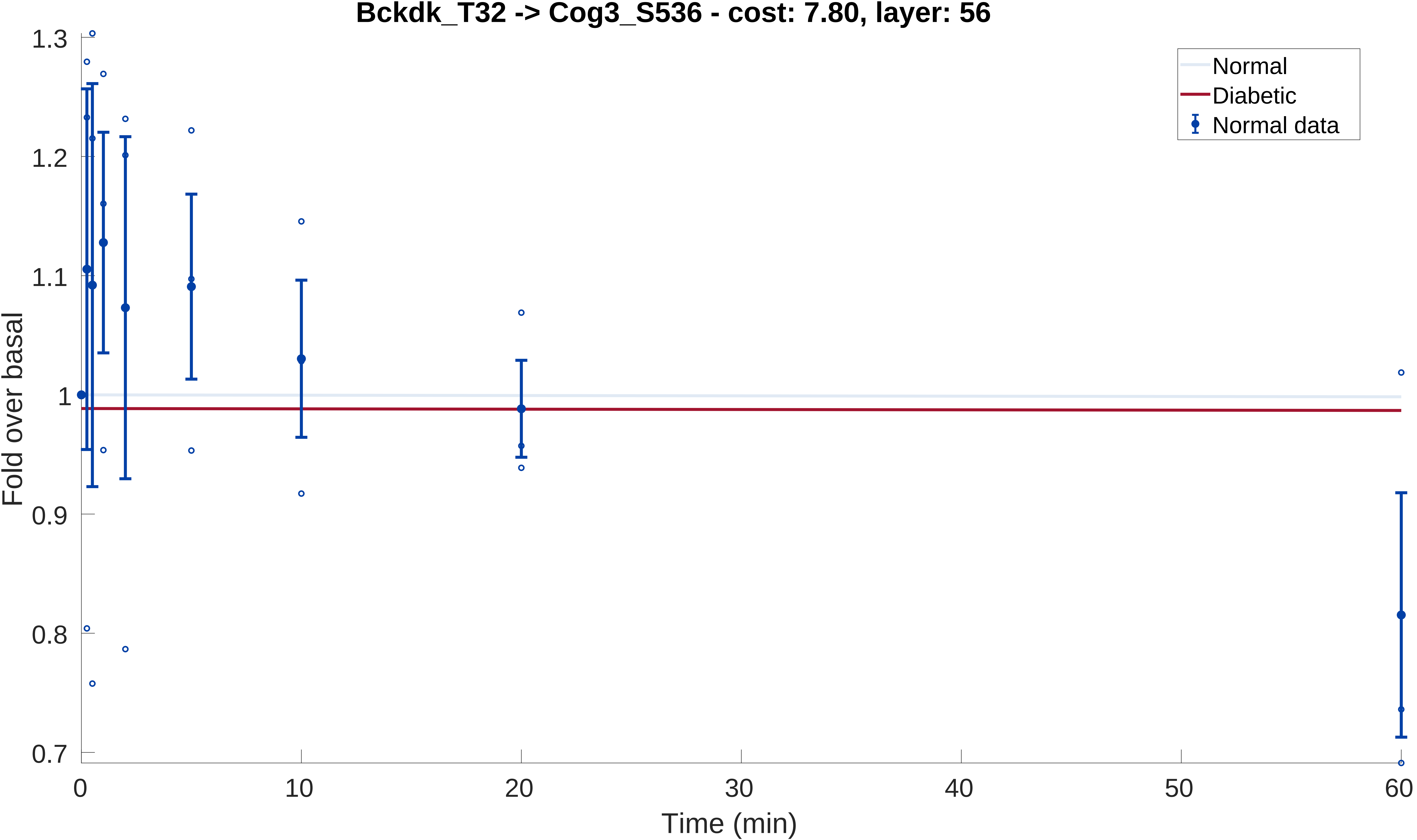

Bckdk_T32 -> Cog3_S536 - cost: 7.80, layer: 56
Normal
Diabetic
Normal data
Fold over basal
Time (min)
1.3
1.2
1.1
1
0.9
0.8
0.7
0
10
20
30
40
50
60

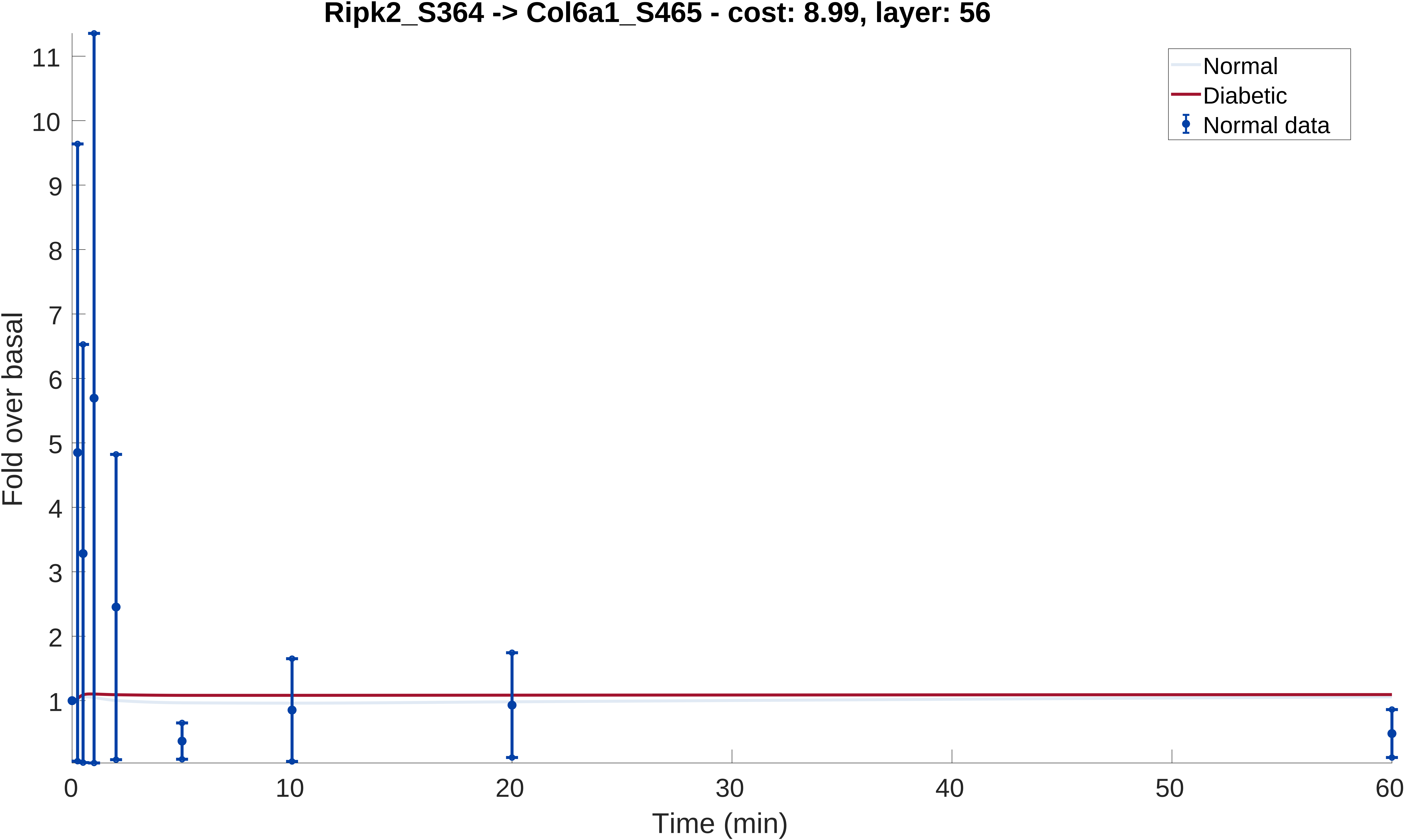

Ripk2_S364 -> Col6a1_S465 - cost: 8.99, layer: 56
Normal
Diabetic
Normal data
Fold over basal
Time (min)
0
10
20
30
40
50
60
1
2
3
4
5
6
7
8
9
10
11

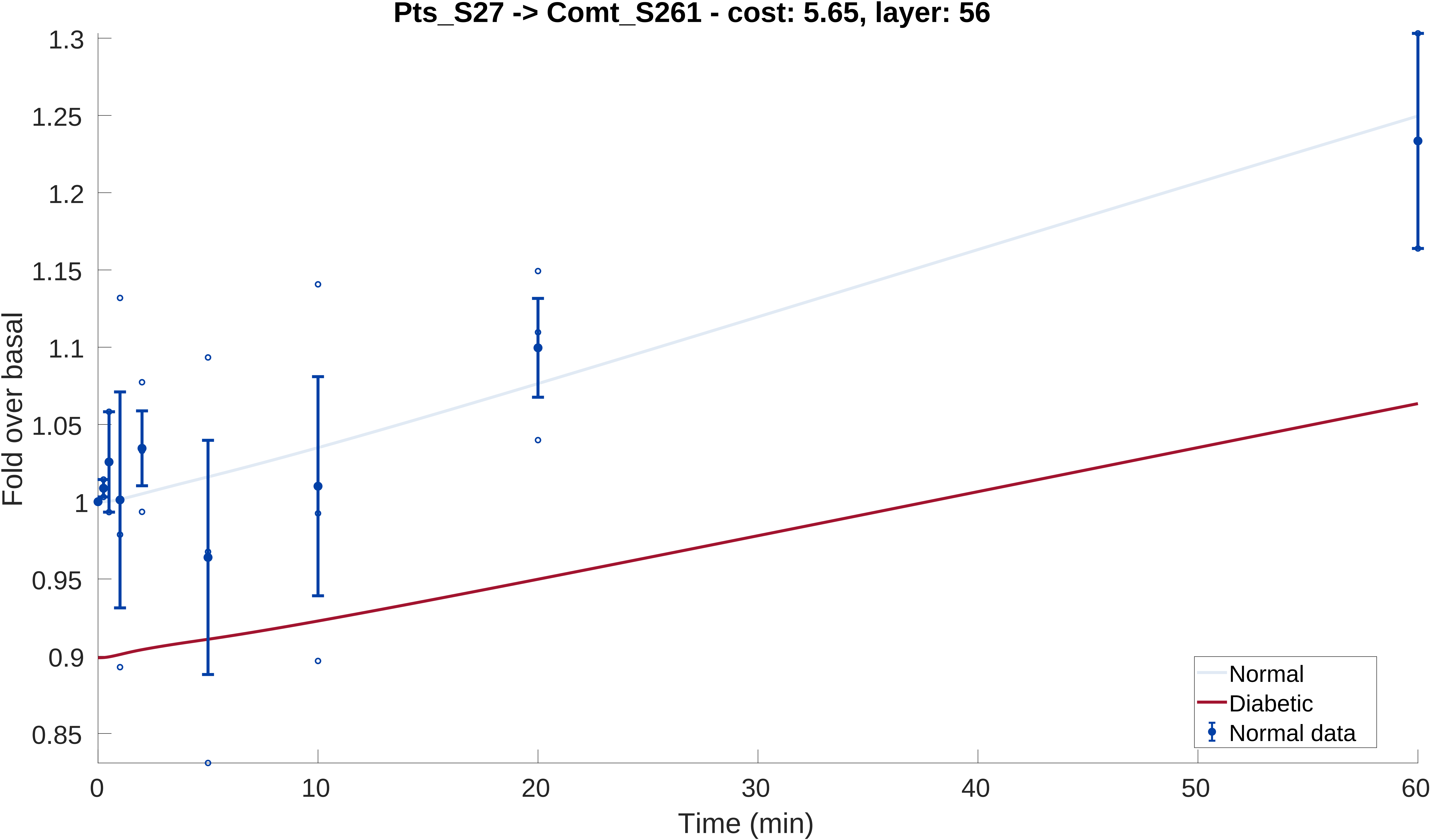

Pts_S27 -> Comt_S261 - cost: 5.65, layer: 56
Fold over basal
Time (min)
Normal
Diabetic
Normal data
1.3
1.25
1.2
1.15
1.1
1.05
1
0.95
0.9
0.85
0
10
20
30
40
50
60

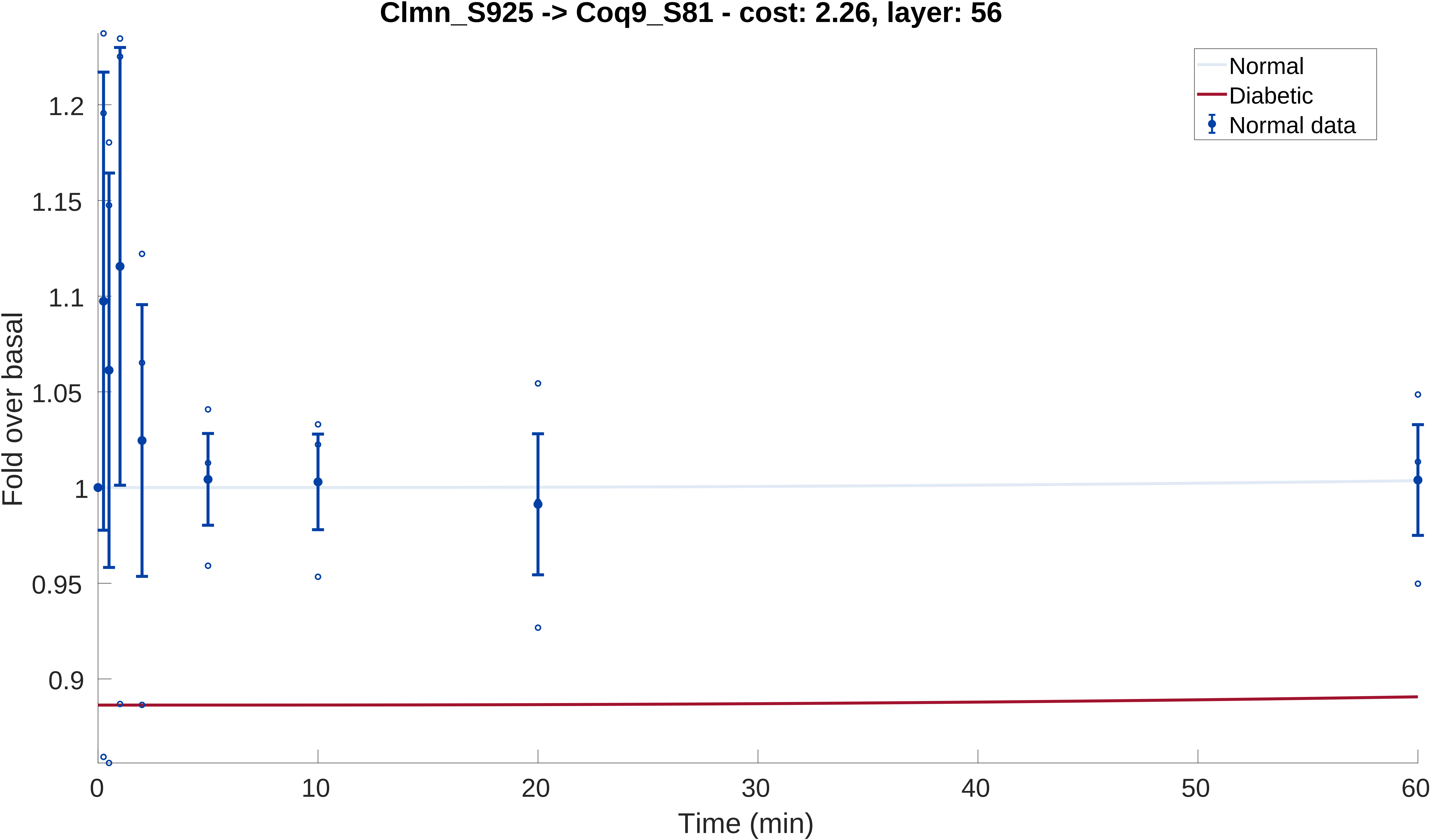

Clmn_S925 -> Coq9_S81 - cost: 2.26, layer: 56
Normal
Diabetic
Normal data
Fold over basal
1.2
1.15
1.1
1.05
1
0.95
0.9
0
10
20
30
40
50
60
Time (min)

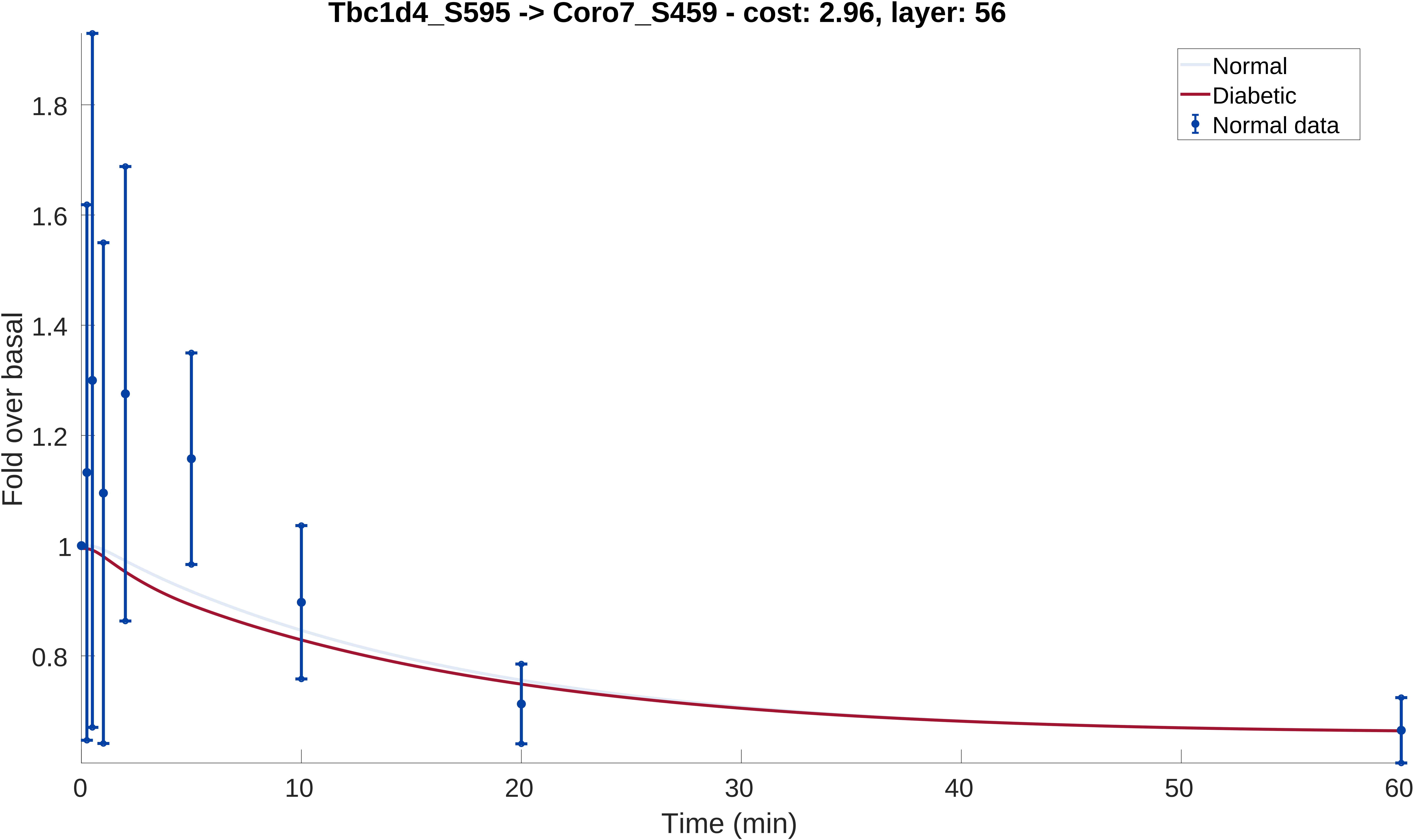

Tbc1d4_S595 -> Coro7_S459 - cost: 2.96, layer: 56
Normal
Diabetic
Normal data
Fold over basal
1.8
1.6
1.4
1.2
1
0.8
0
10
20
30
40
50
60
Time (min)

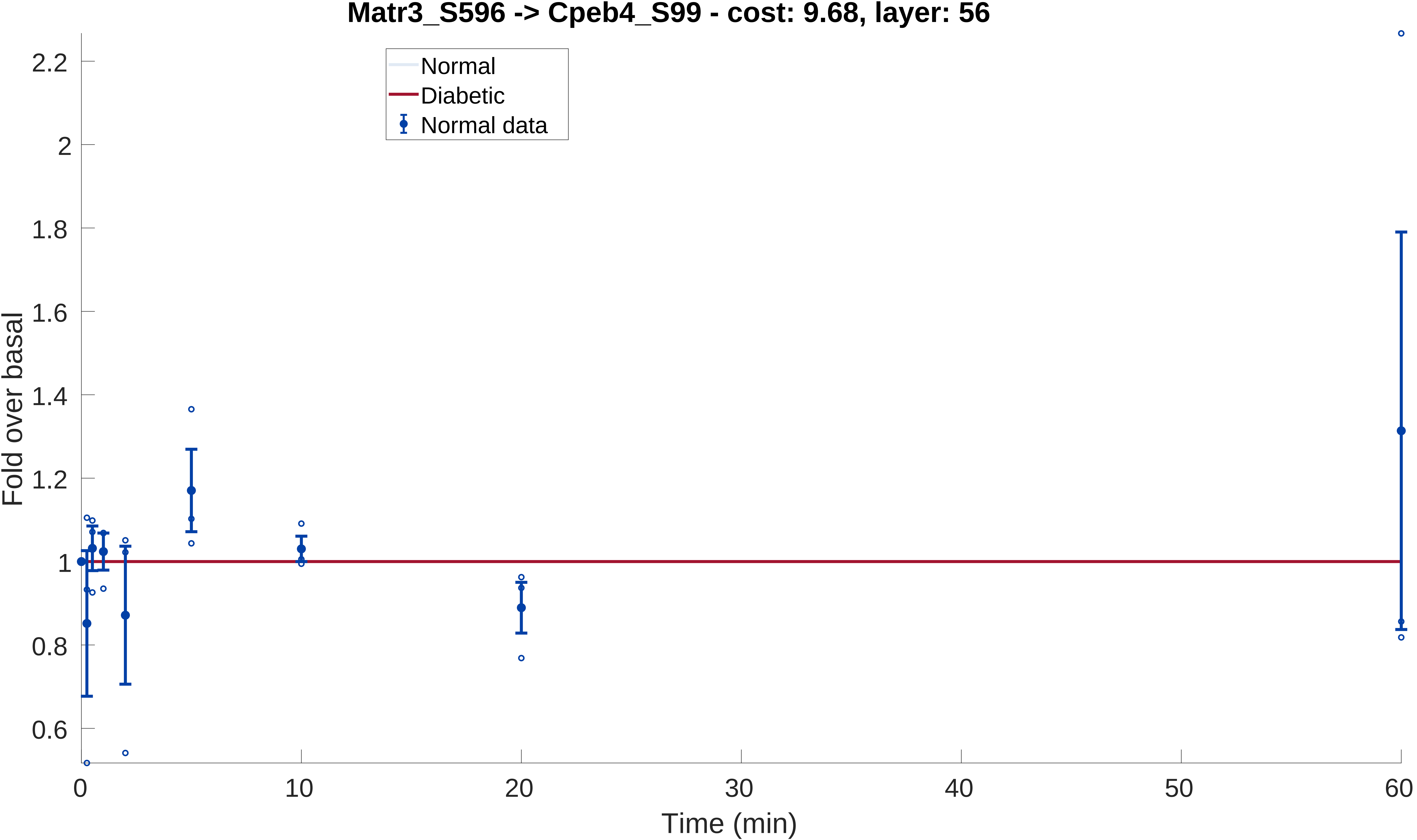
Matr3_S596 -> Cpeb4_S99 - cost: 9.68, layer: 56
Normal
Diabetic
Normal data
Fold over basal
Time (min)
0.6
0.8
1
1.2
1.4
1.6
1.8
2
2.2
0
10
20
30
40
50
60

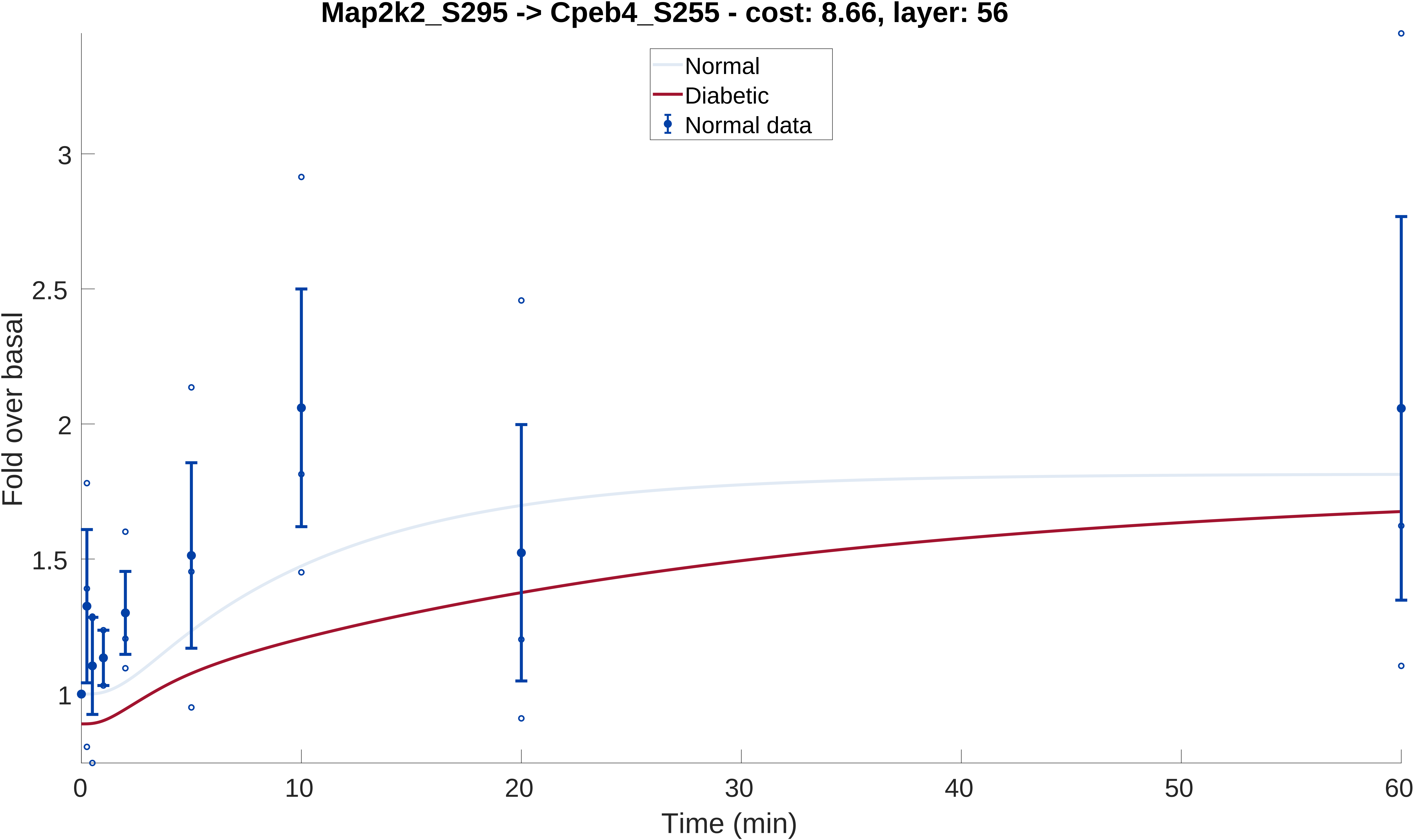

Map2k2_S295 -> Cpeb4_S255 - cost: 8.66, layer: 56
Normal
Diabetic
Normal data
Fold over basal
Time (min)
1
1.5
2
2.5
3
0
10
20
30
40
50
60

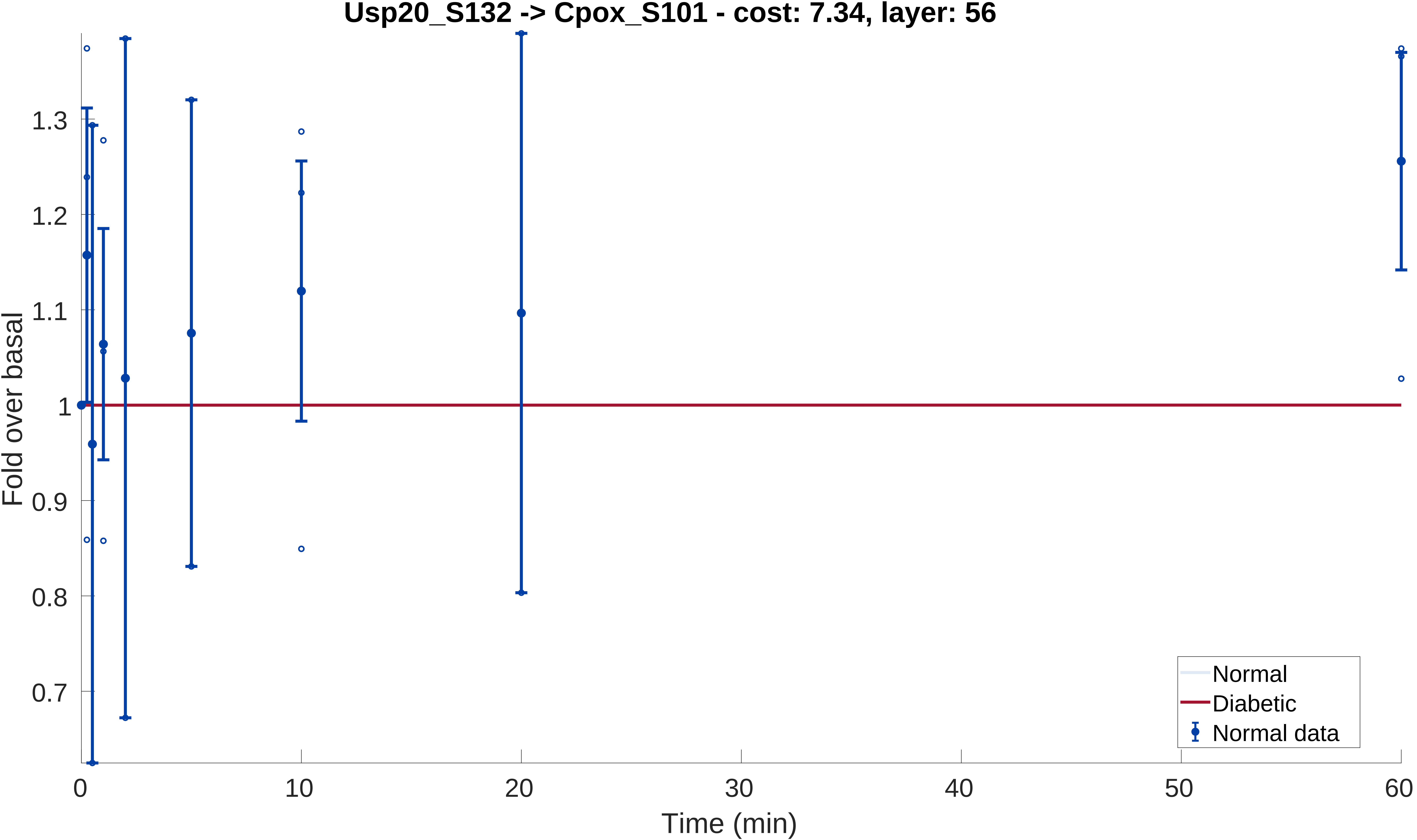

Usp20_S132 -> Cpox_S101 - cost: 7.34, layer: 56
Fold over basal
1.3
1.2
1.1
1
0.9
0.8
0.7
0
10
20
30
40
50
60
Time (min)
Normal
Diabetic
Normal data

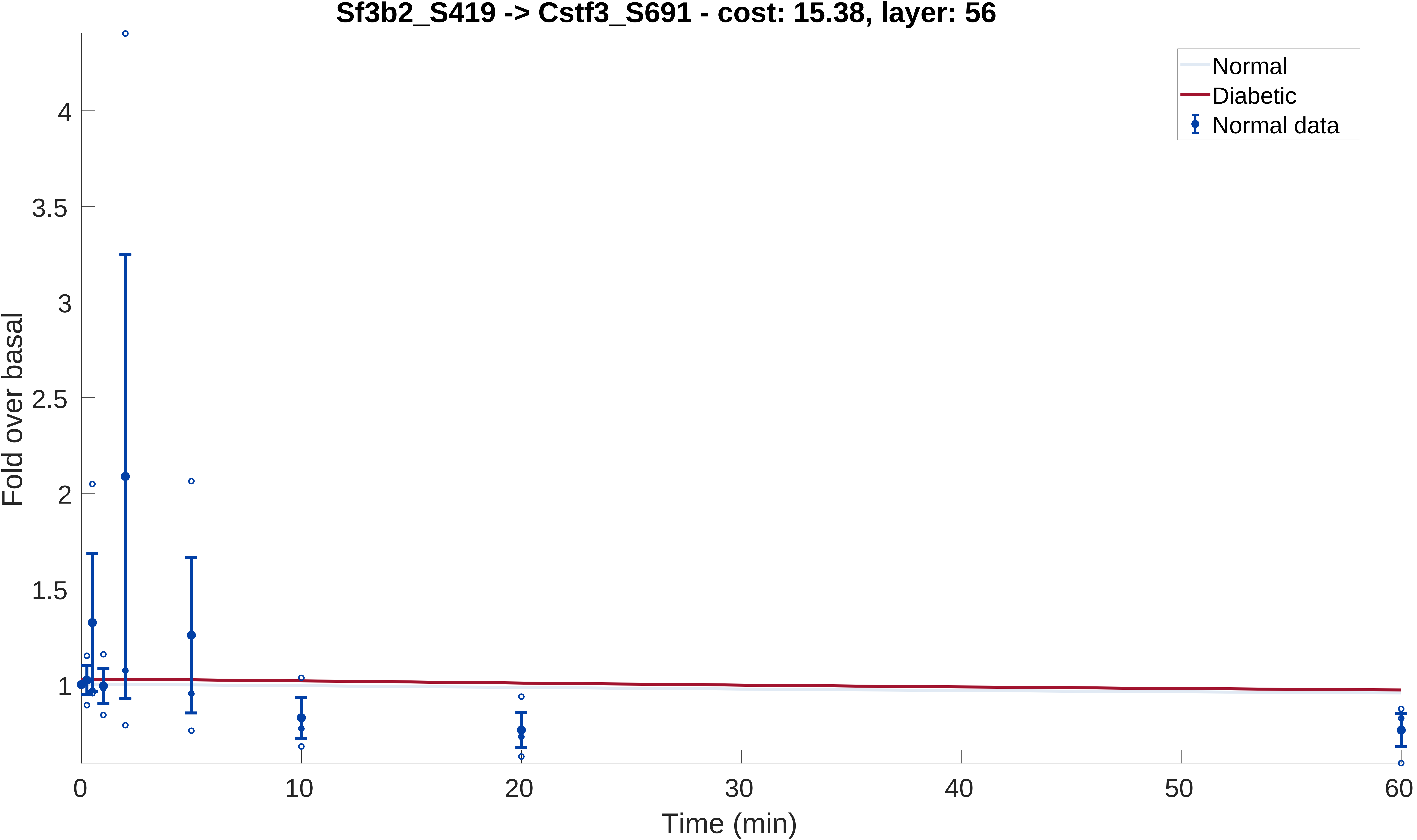
Sf3b2_S419 -> Cstf3_S691 - cost: 15.38, layer: 56
Normal
Diabetic
Normal data
Fold over basal
4
3.5
3
2.5
2
1.5
1
0
10
20
30
40
50
60
Time (min)

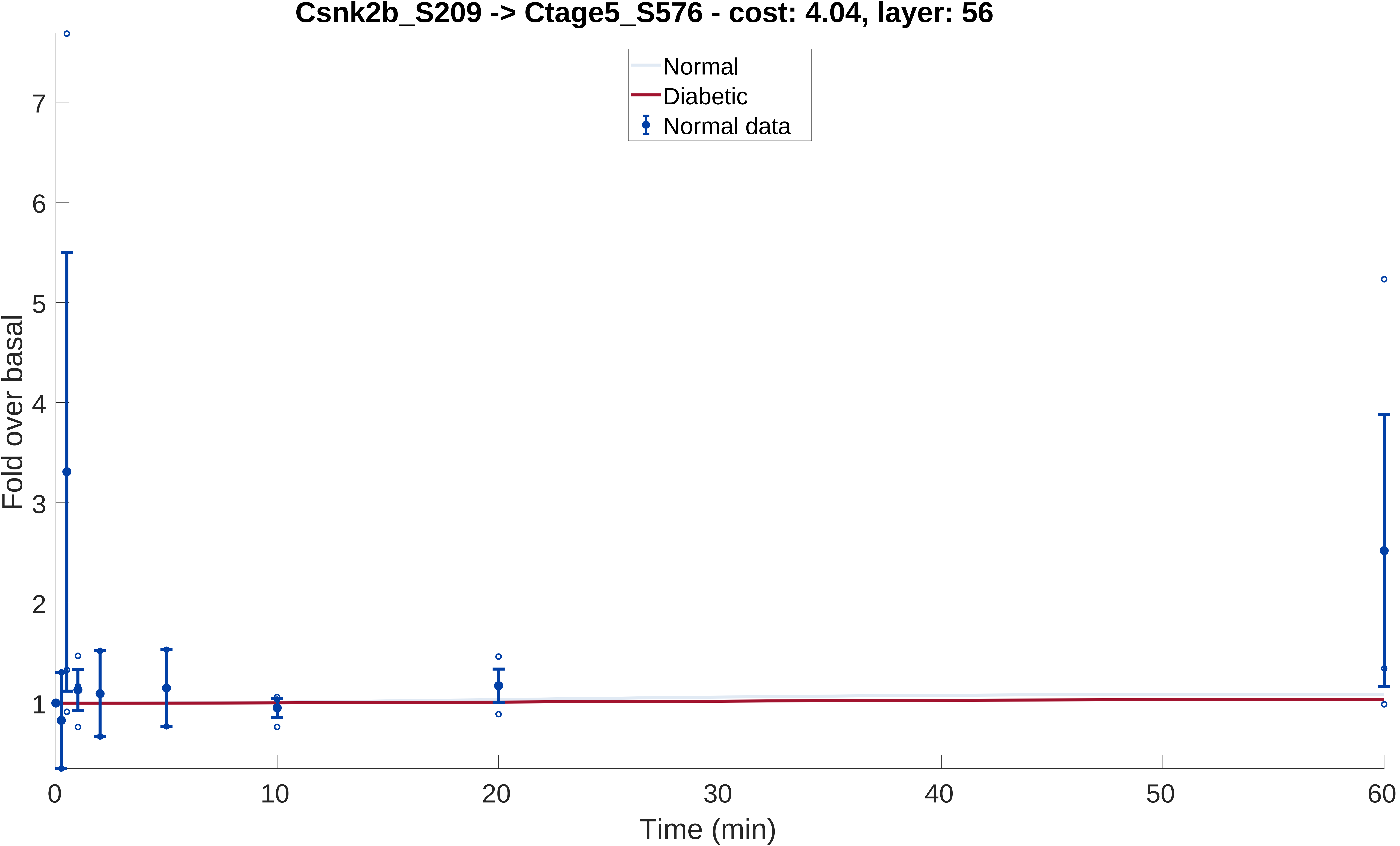

Csnk2b_S209 -> Ctage5_S576 - cost: 4.04, layer: 56
Normal
Diabetic
Normal data
Fold over basal
Time (min)
1
2
3
4
5
6
7
0
10
20
30
40
50
60

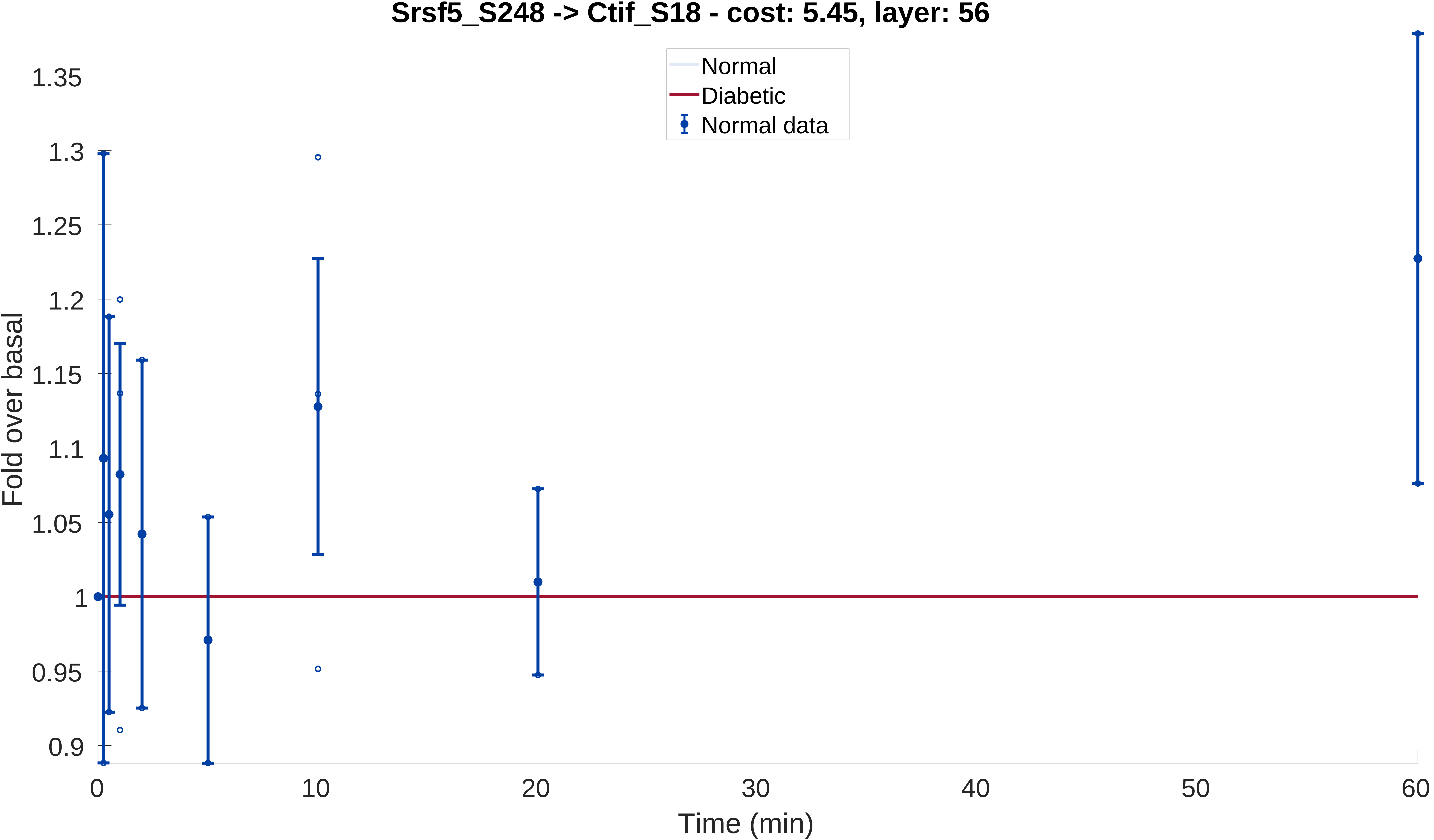

Srsf5_S248 -> Ctif_S18 - cost: 5.45, layer: 56
Normal
Diabetic
Normal data
Fold over basal
Time (min)
1.35
1.3
1.25
1.2
1.15
1.1
1.05
1
0.95
0.9
0
10
20
30
40
50
60

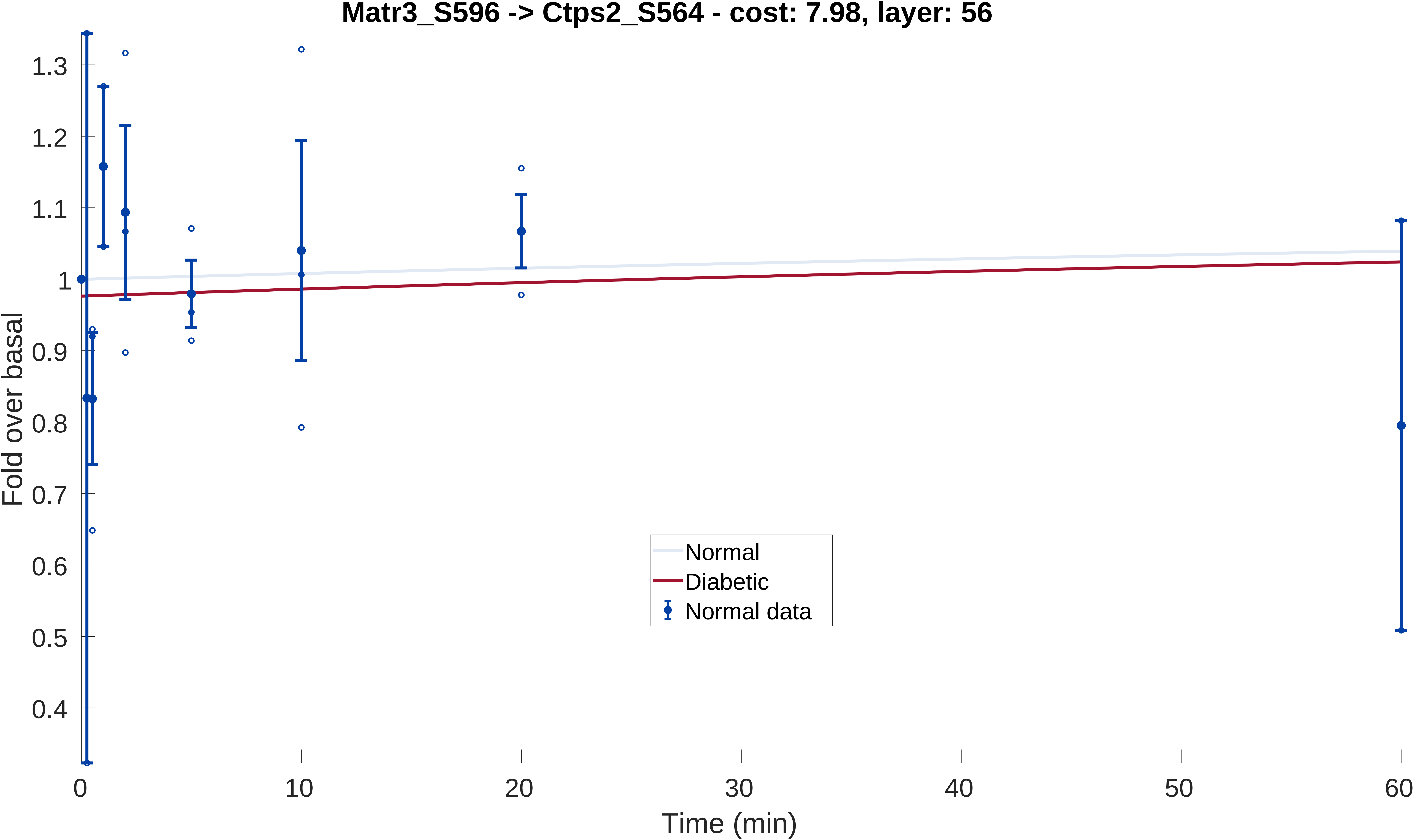

Matr3_S596 -> Ctps2_S564 - cost: 7.98, layer: 56
Fold over basal
Time (min)
1.3
1.2
1.1
1
0.9
0.8
0.7
0.6
0.5
0.4
0
10
20
30
40
50
60
Normal
Diabetic
Normal data

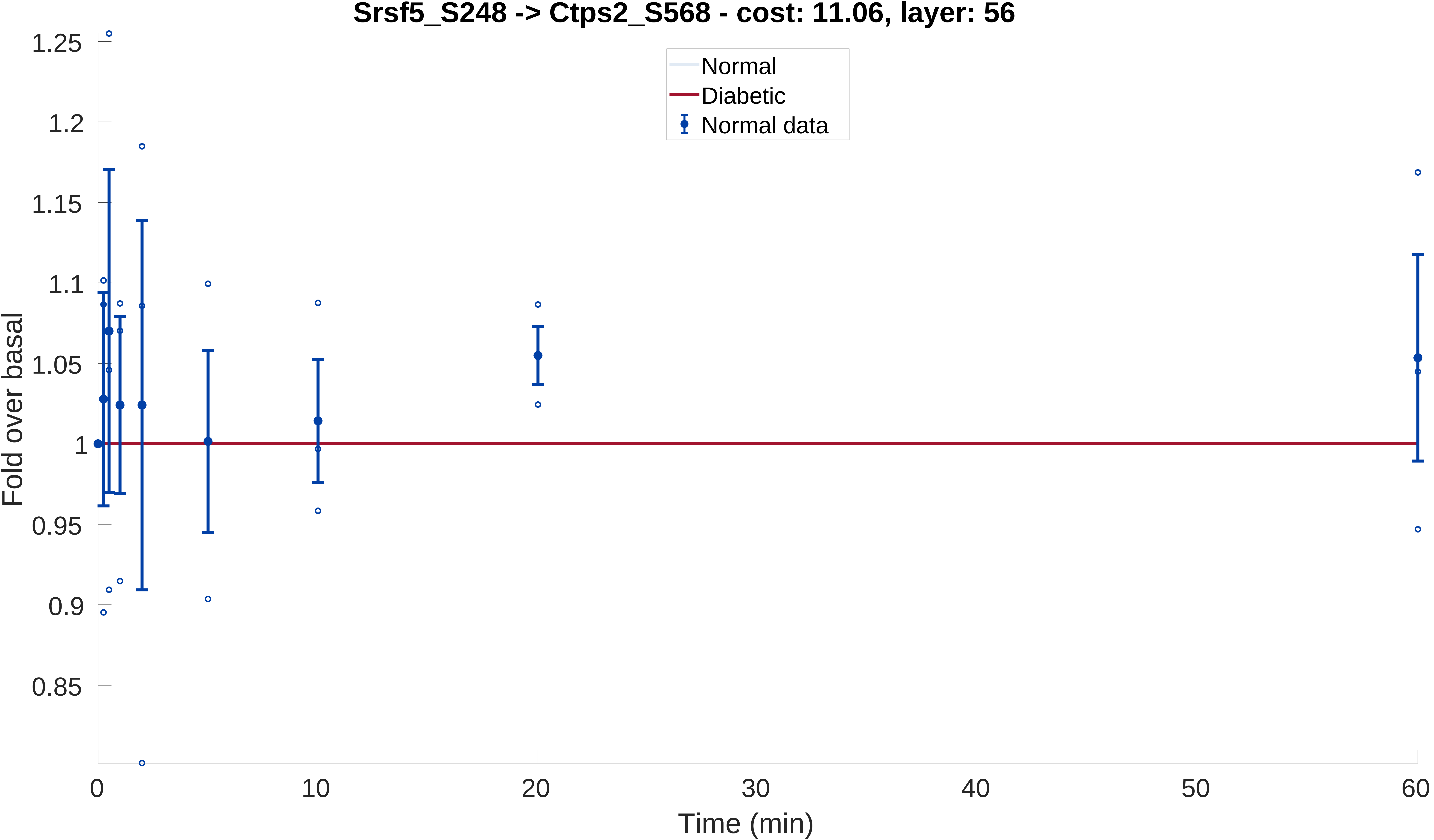
Srsf5_S248 -> Ctps2_S568 - cost: 11.06, layer: 56
Normal
Diabetic
Normal data
Fold over basal
1.25
1.2
1.15
1.1
1.05
1
0.95
0.9
0.85
0
10
20
30
40
50
60
Time (min)

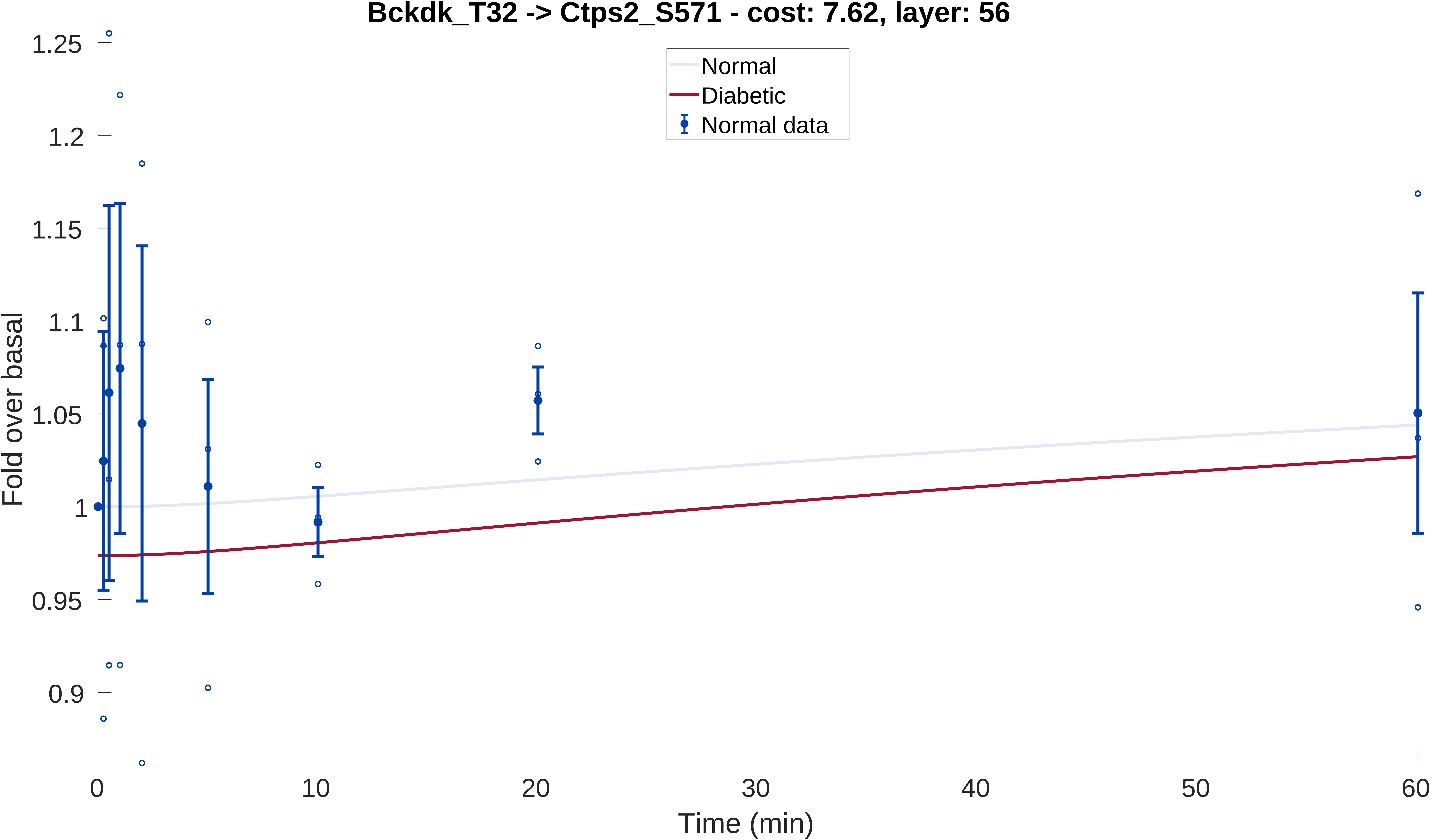

Bckdk_T32 -> Ctps2_S571 - cost: 7.62, layer: 56
Normal
Diabetic
Normal data
Fold over basal
1.25
1.2
1.15
1.1
1.05
1
0.95
0.9
0
10
20
30
40
50
60
Time (min)

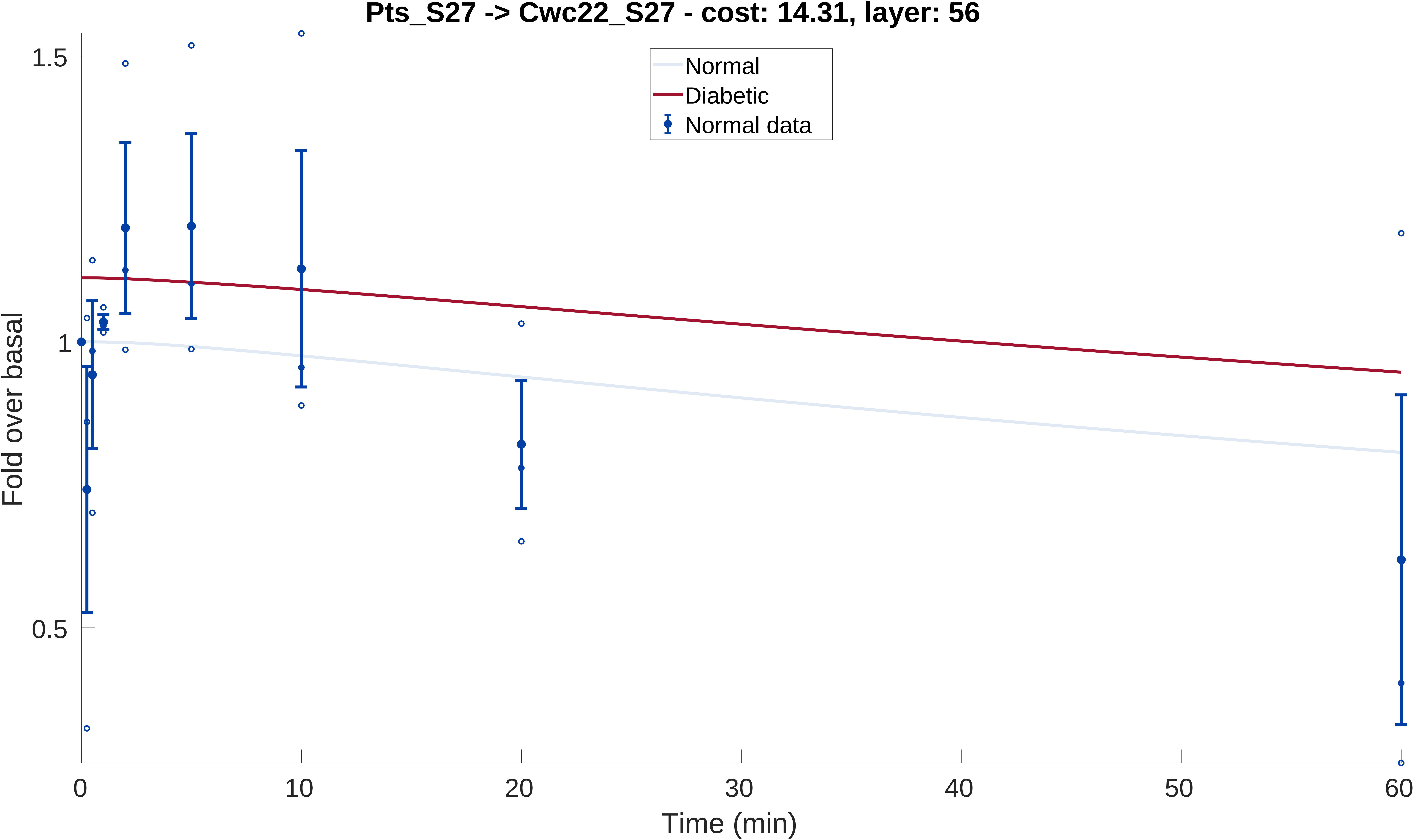
Pts_S27 -> Cwc22_S27 - cost: 14.31, layer: 56
Normal
Diabetic
Normal data
Fold over basal
1.5
1
0.5
0
10
20
30
40
50
60
Time (min)

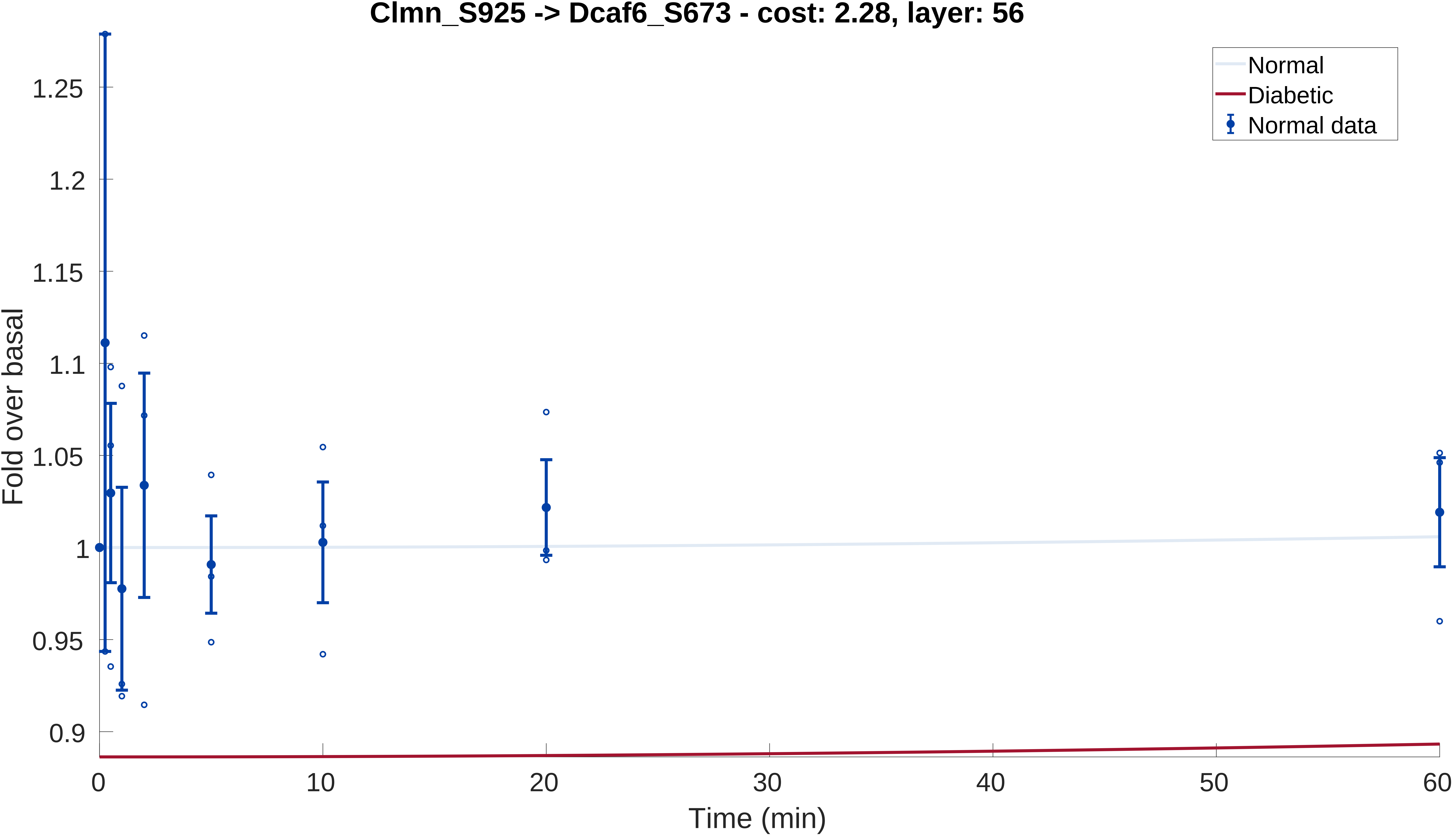
Clmn_S925 -> Dcaf6_S673 - cost: 2.28, layer: 56
Normal
Diabetic
Normal data
Fold over basal
Time (min)
1.25
1.2
1.15
1.1
1.05
1
0.95
0.9
0
10
20
30
40
50
60

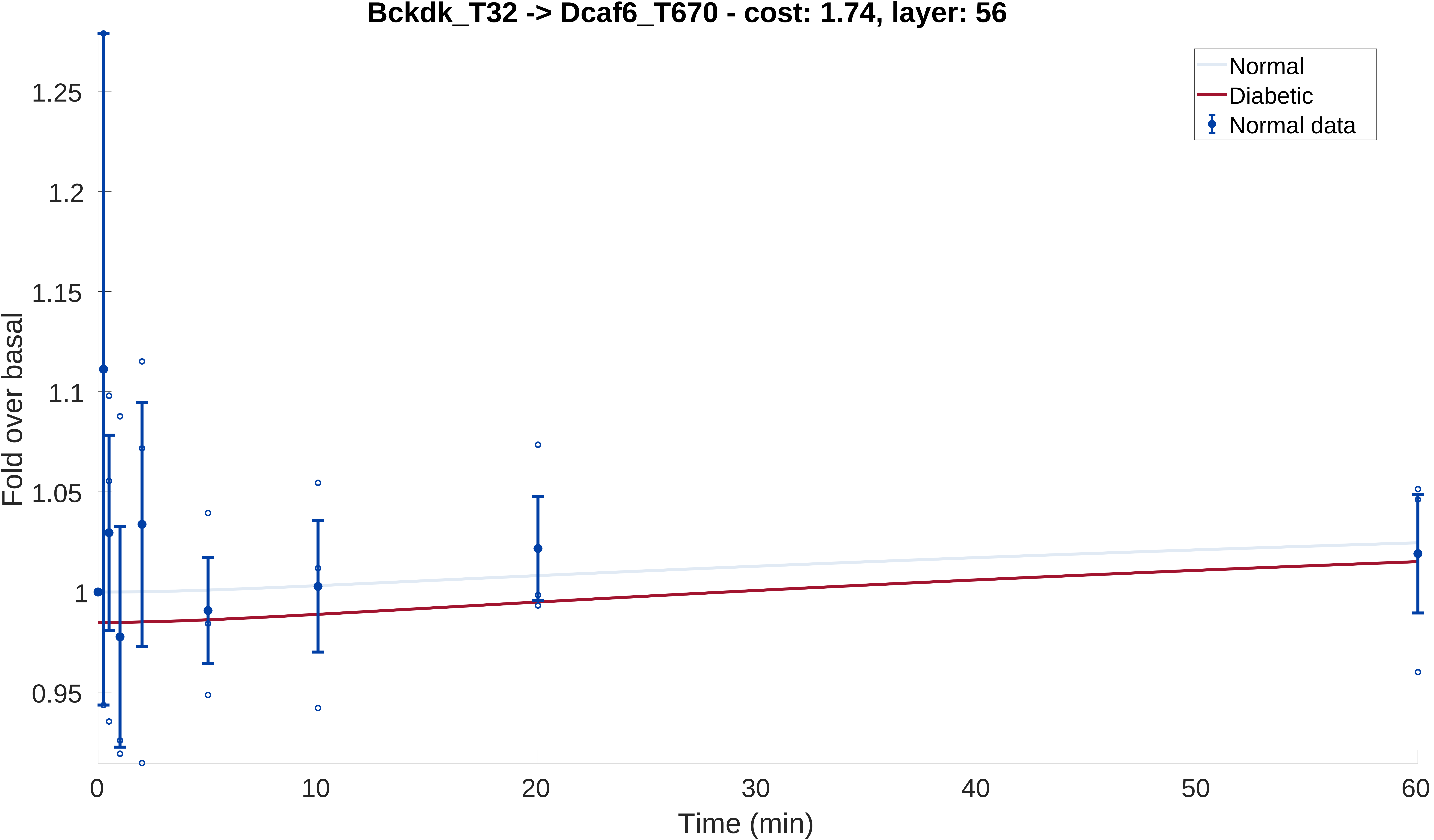

Bckdk_T32 -> Dcaf6_T670 - cost: 1.74, layer: 56
Normal
Diabetic
Normal data
Fold over basal
1.25
1.2
1.15
1.1
1.05
1
0.95
0
10
20
30
40
50
60
Time (min)

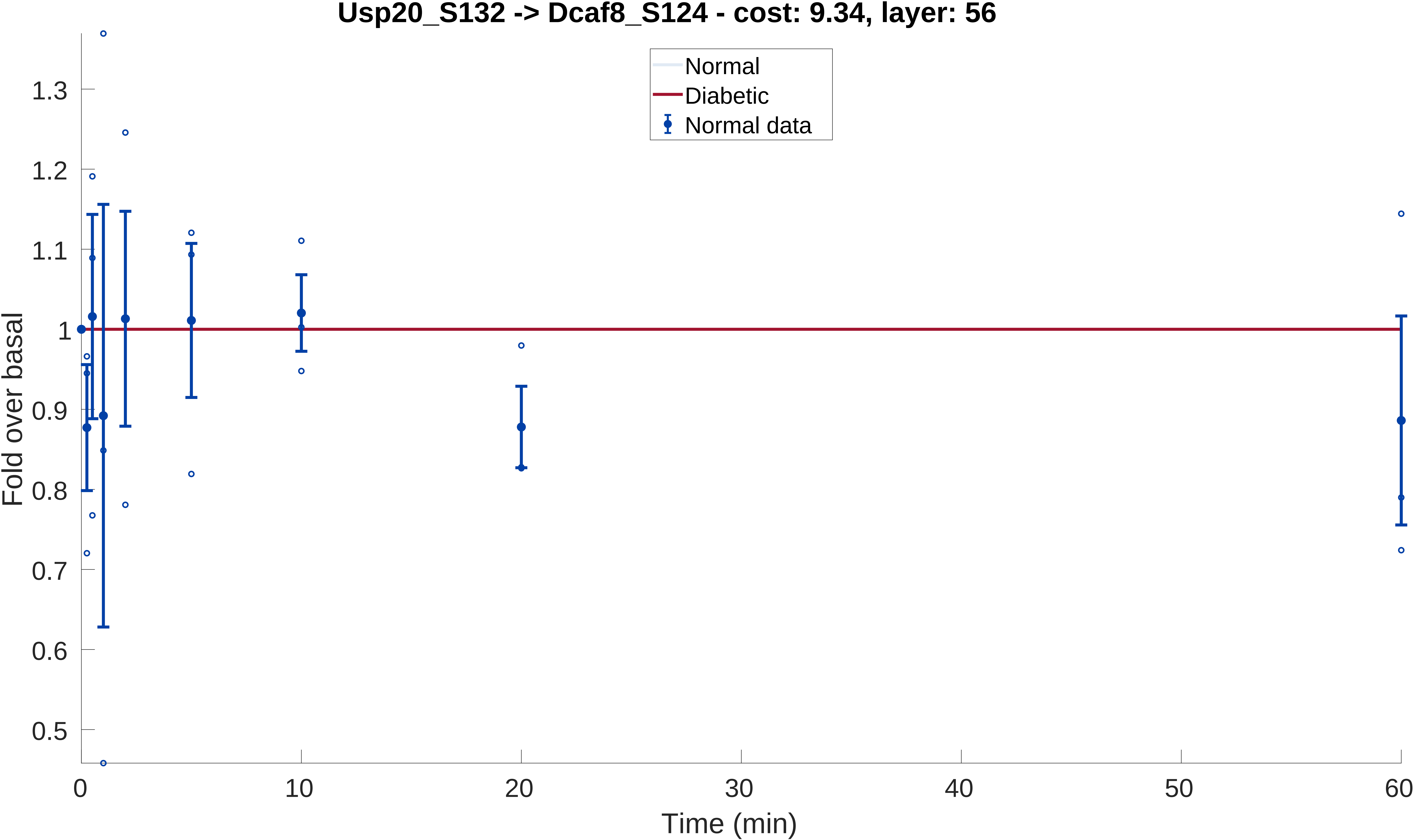

Usp20_S132 -> Dcaf8_S124 - cost: 9.34, layer: 56
Normal
Diabetic
Normal data
Fold over basal
Time (min)
1.3
1.2
1.1
1
0.9
0.8
0.7
0.6
0.5
0
10
20
30
40
50
60

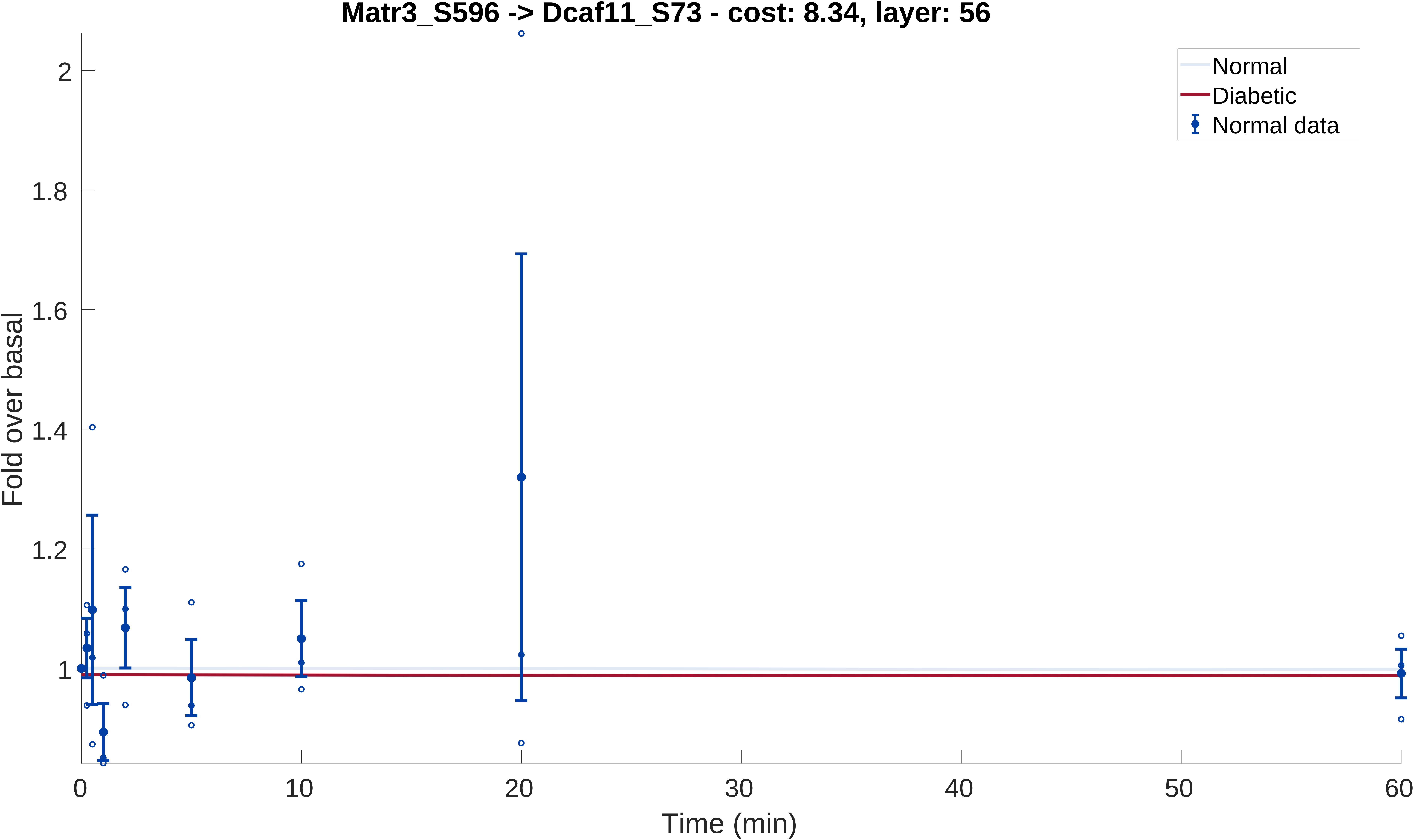

Matr3_S596 -> Dcaf11_S73 - cost: 8.34, layer: 56
Normal
Diabetic
Normal data
Fold over basal
2
1.8
1.6
1.4
1.2
1
0
10
20
30
40
50
60
Time (min)

Pts_S27 -> Ddx3x_S131 - cost: 10.06, layer: 56

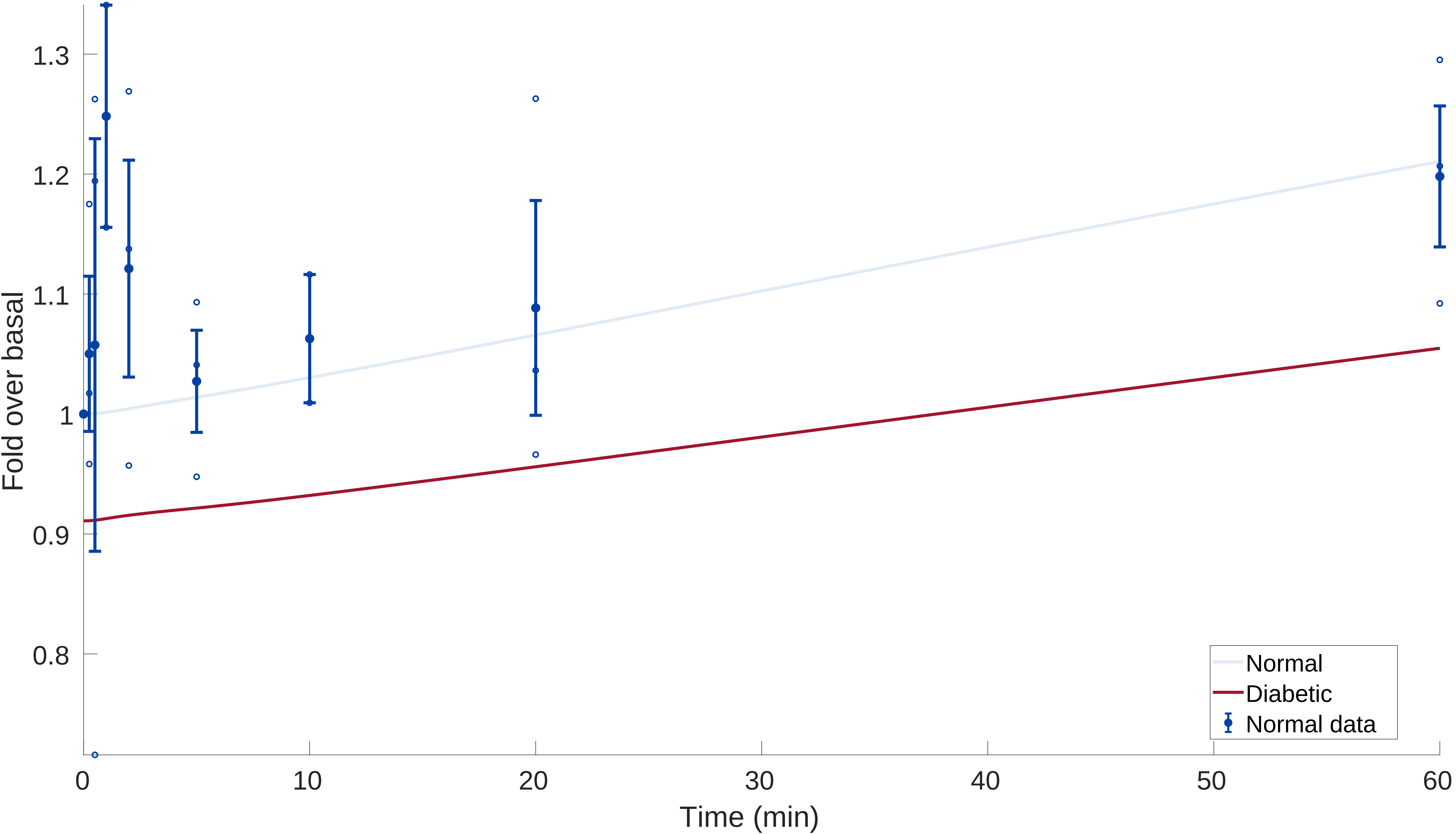

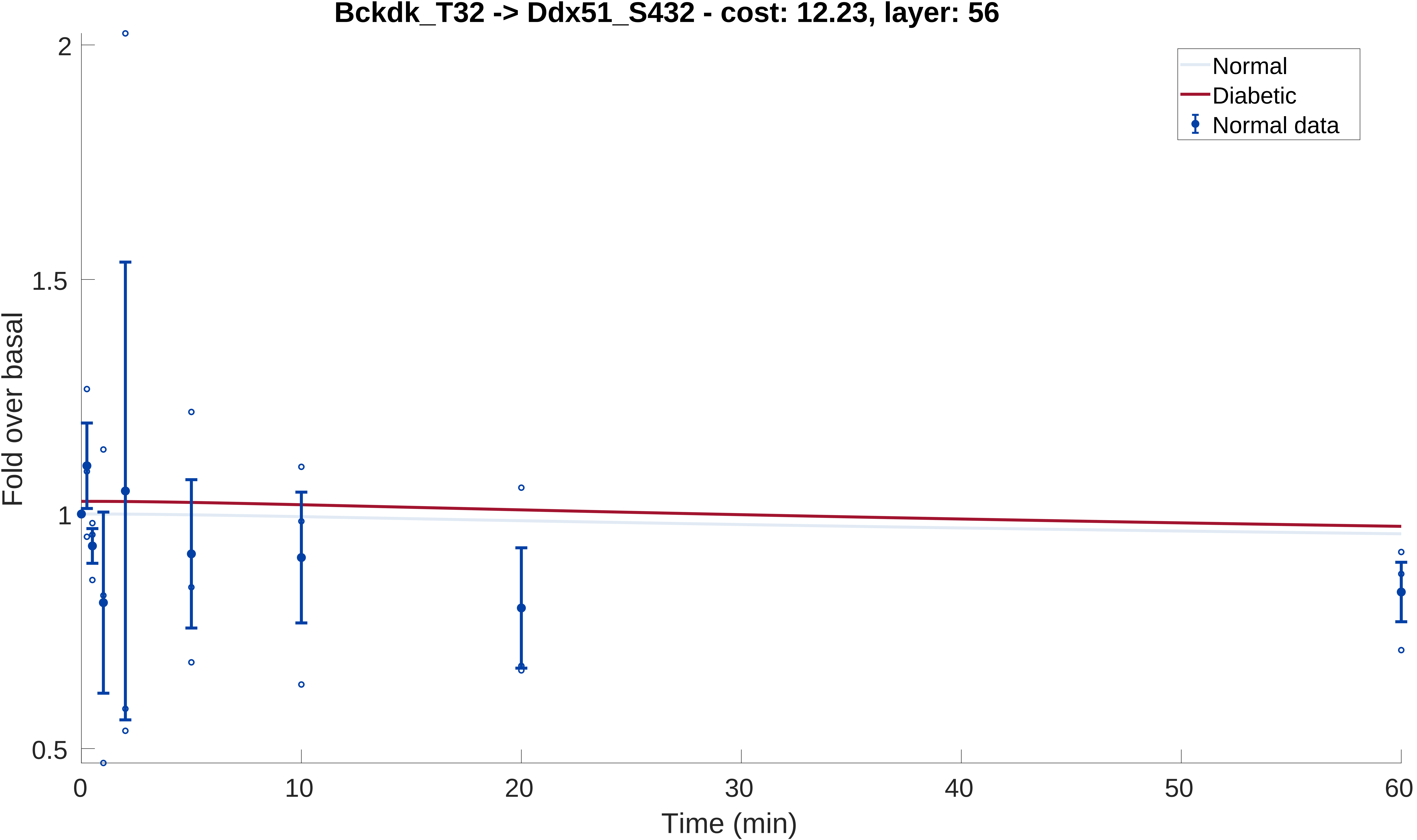

Bckdk_T32 -> Ddx51_S432 - cost: 12.23, layer: 56
Normal
Diabetic
Normal data
Fold over basal
Time (min)
0.5
1
1.5
2
0
10
20
30
40
50
60

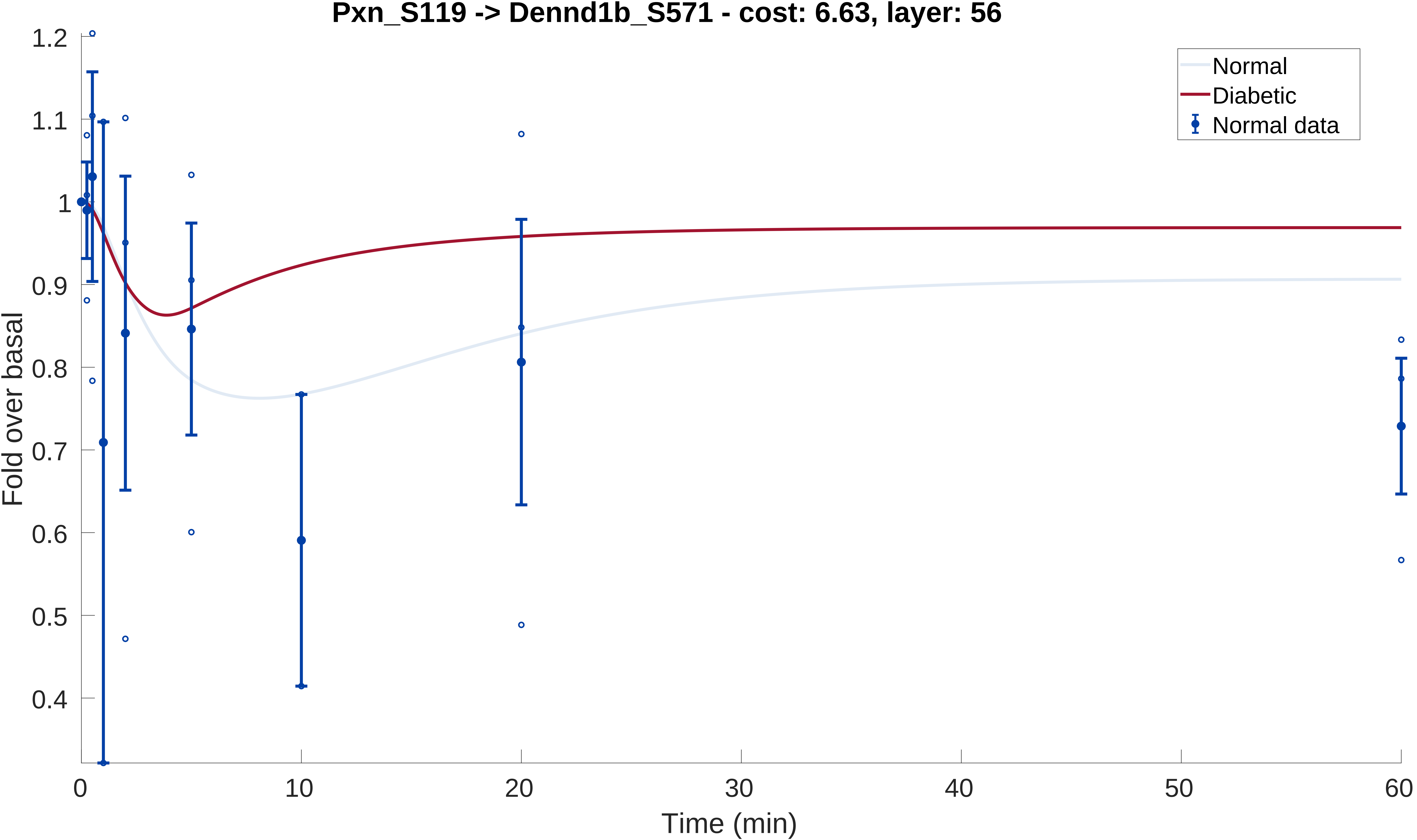

Pxn_S119 -> Dennd1b_S571 - cost: 6.63, layer: 56
Normal
Diabetic
Normal data
Fold over basal
Time (min)
1.2
1.1
1
0.9
0.8
0.7
0.6
0.5
0.4
0
10
20
30
40
50
60

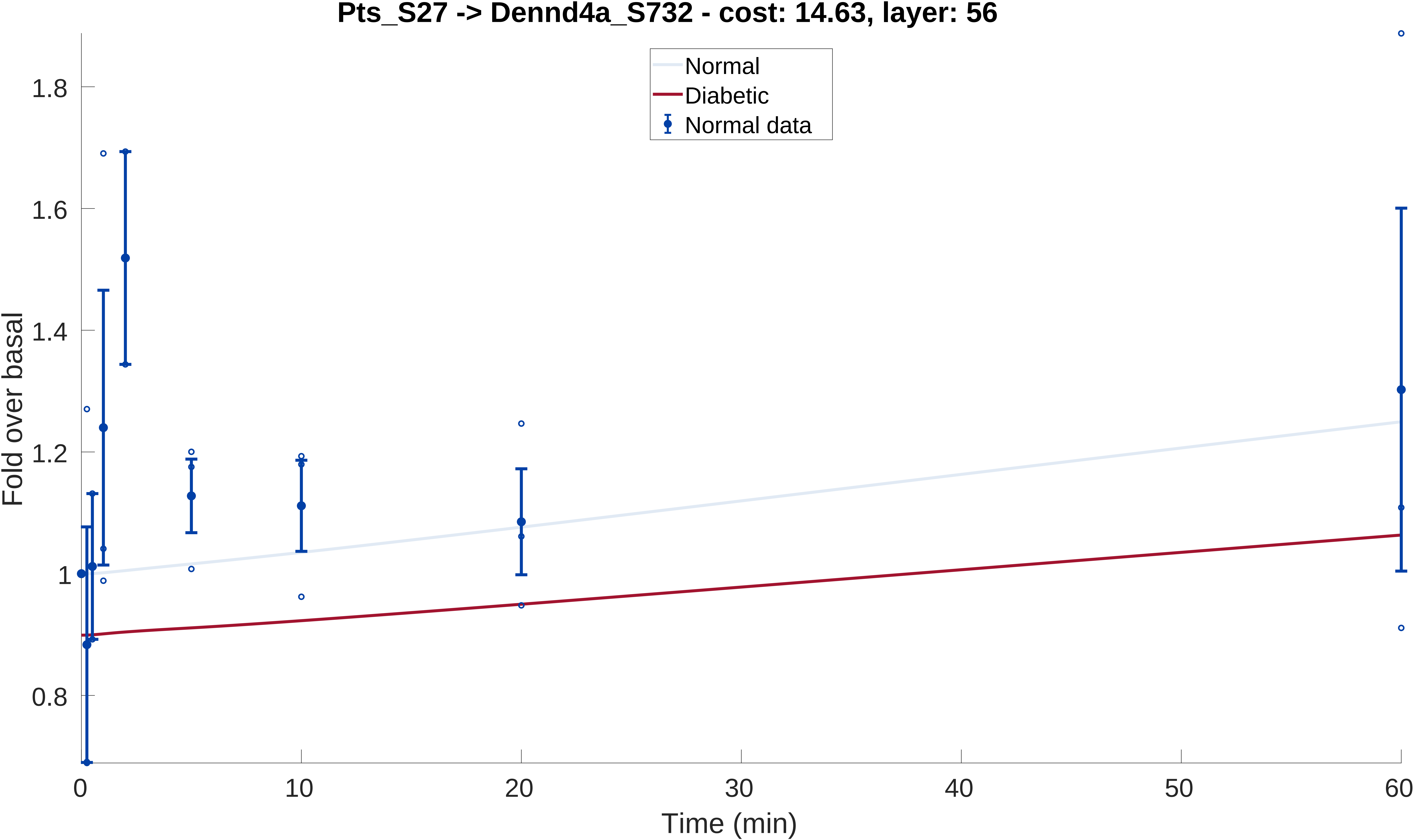

Pts_S27 -> Dennd4a_S732 - cost: 14.63, layer: 56
Normal
Diabetic
Normal data
Fold over basal
Time (min)
0.8
1
1.2
1.4
1.6
1.8
0
10
20
30
40
50
60

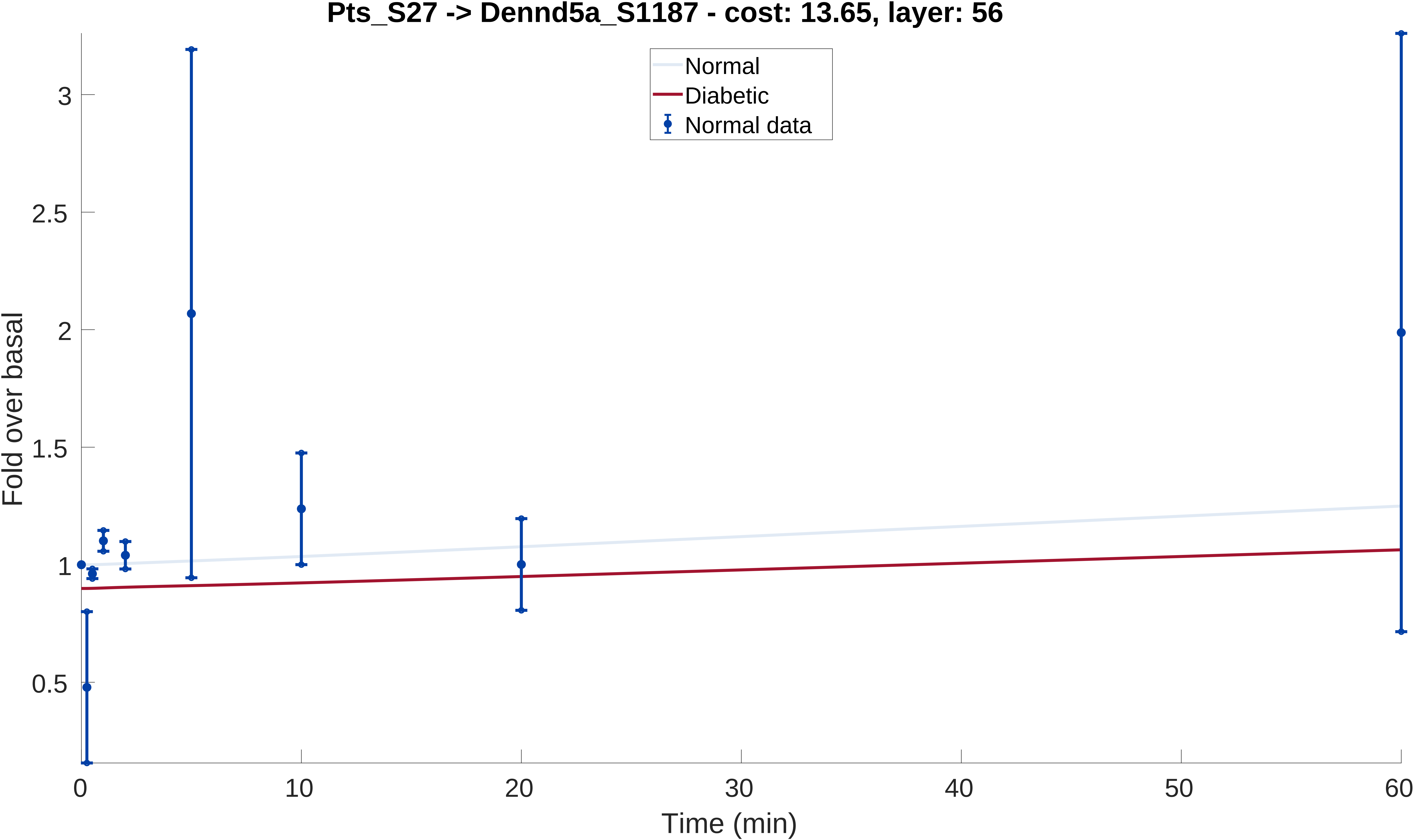

Pts_S27 -> Dennd5a_S1187 - cost: 13.65, layer: 56
Normal
Diabetic
Normal data
Fold over basal
0.5
1
1.5
2
2.5
3
0
10
20
30
40
50
60
Time (min)

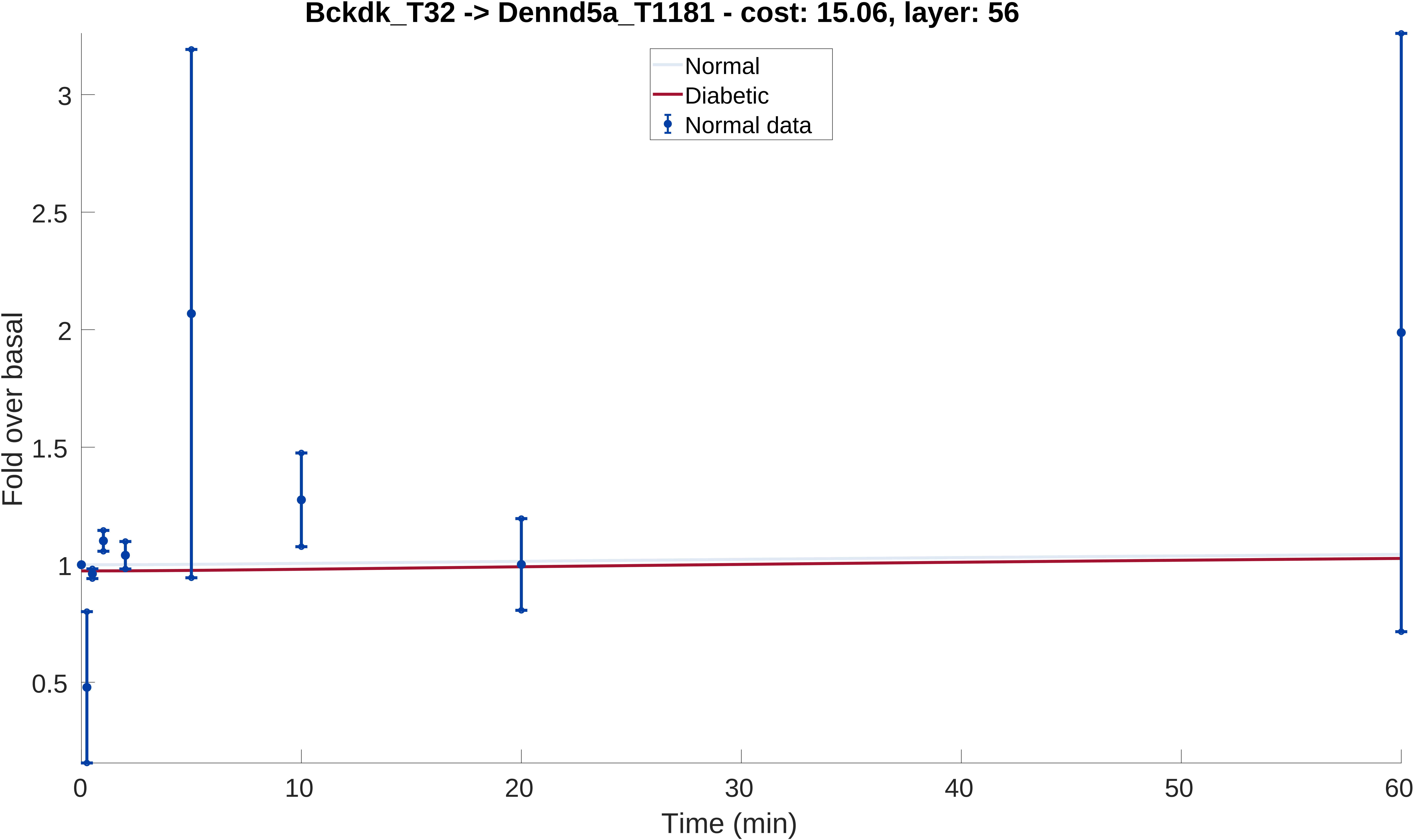
Bckdk_T32 -> Dennd5a_T1181 - cost: 15.06, layer: 56
Normal
Diabetic
Normal data
Fold over basal
Time (min)
0.5
1
1.5
2
2.5
3
0
10
20
30
40
50
60

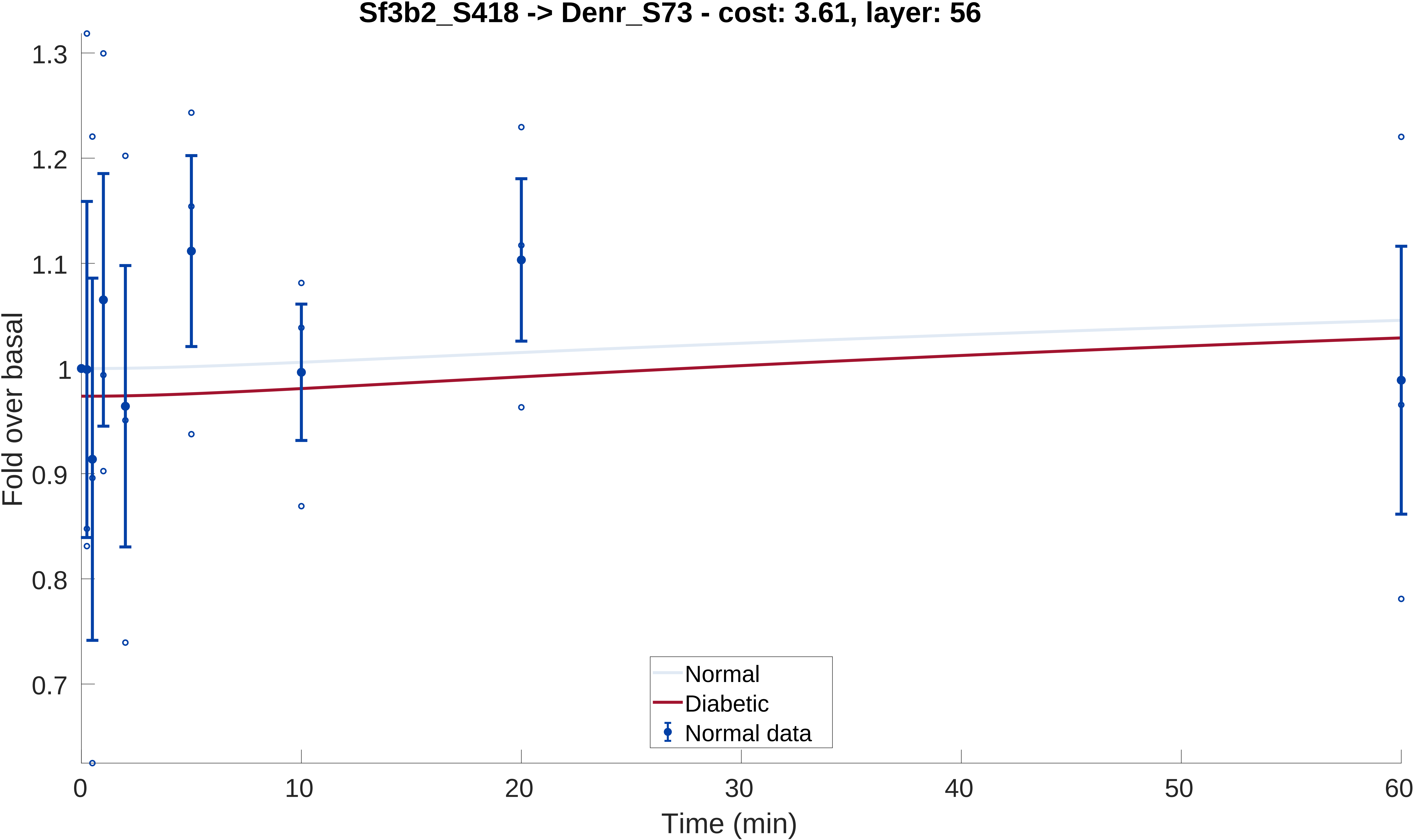

Sf3b2_S418 -> Denr_S73 - cost: 3.61, layer: 56
Fold over basal
Time (min)
Normal
Diabetic
Normal data
1.3
1.2
1.1
1
0.9
0.8
0.7
0
10
20
30
40
50
60

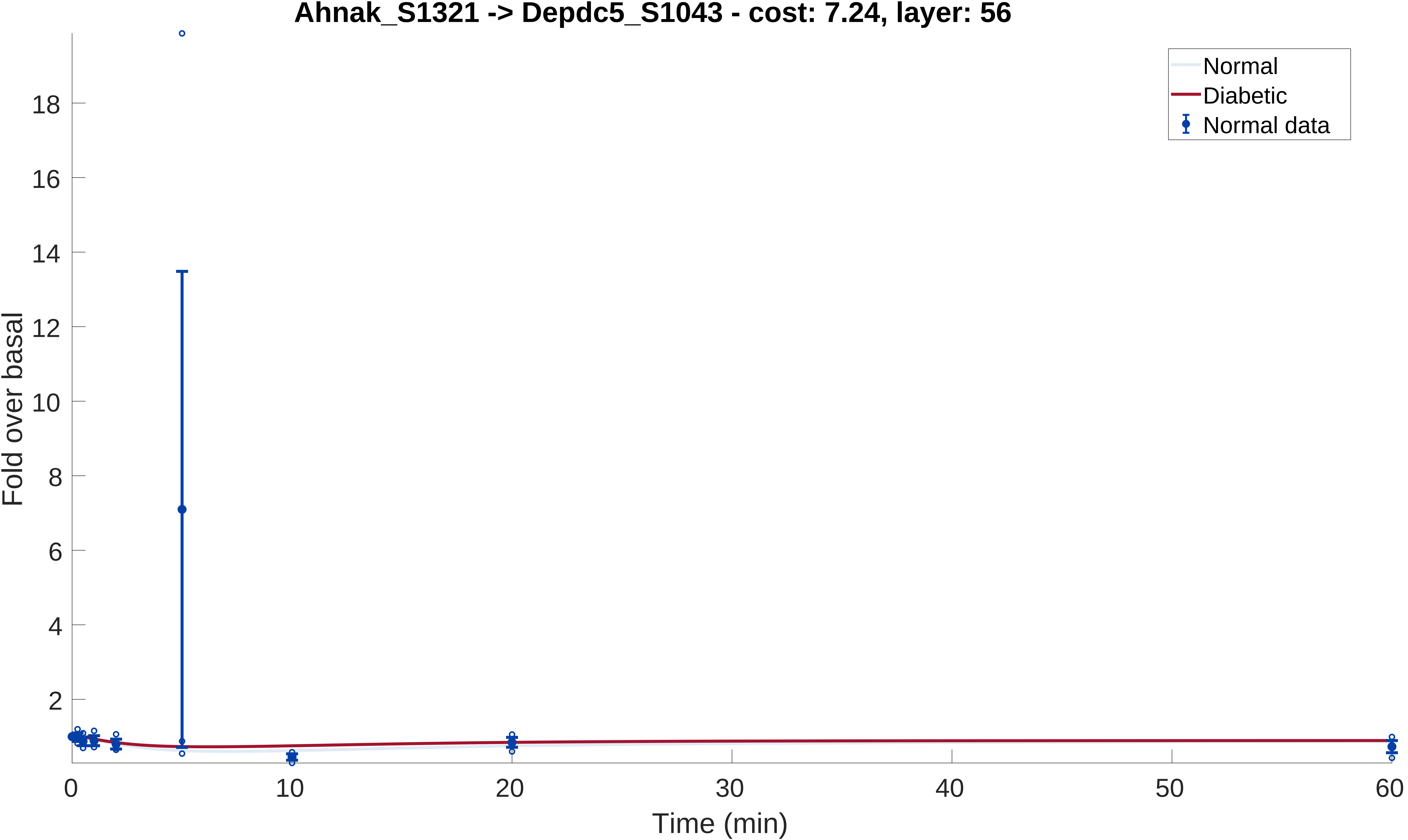

Ahnak_S1321 -> Depdc5_S1043 - cost: 7.24, layer: 56
Normal
Diabetic
Normal data
Fold over basal
Time (min)
0
10
20
30
40
50
60
2
4
6
8
10
12
14
16
18

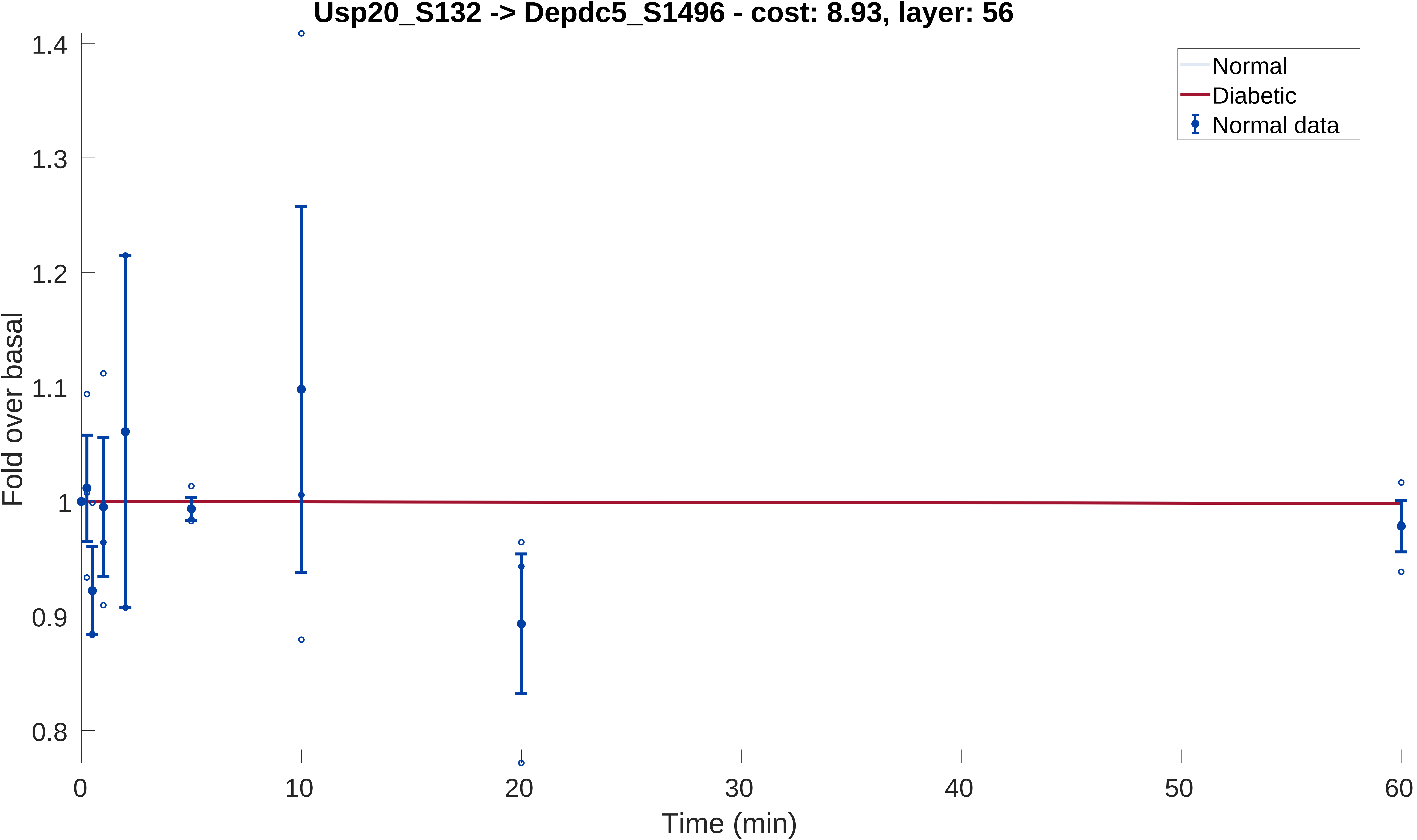

Usp20_S132 -> Depdc5_S1496 - cost: 8.93, layer: 56
Fold over basal
Time (min)
Normal
Diabetic
Normal data
1.4
1.3
1.2
1.1
1
0.9
0.8
0
10
20
30
40
50
60

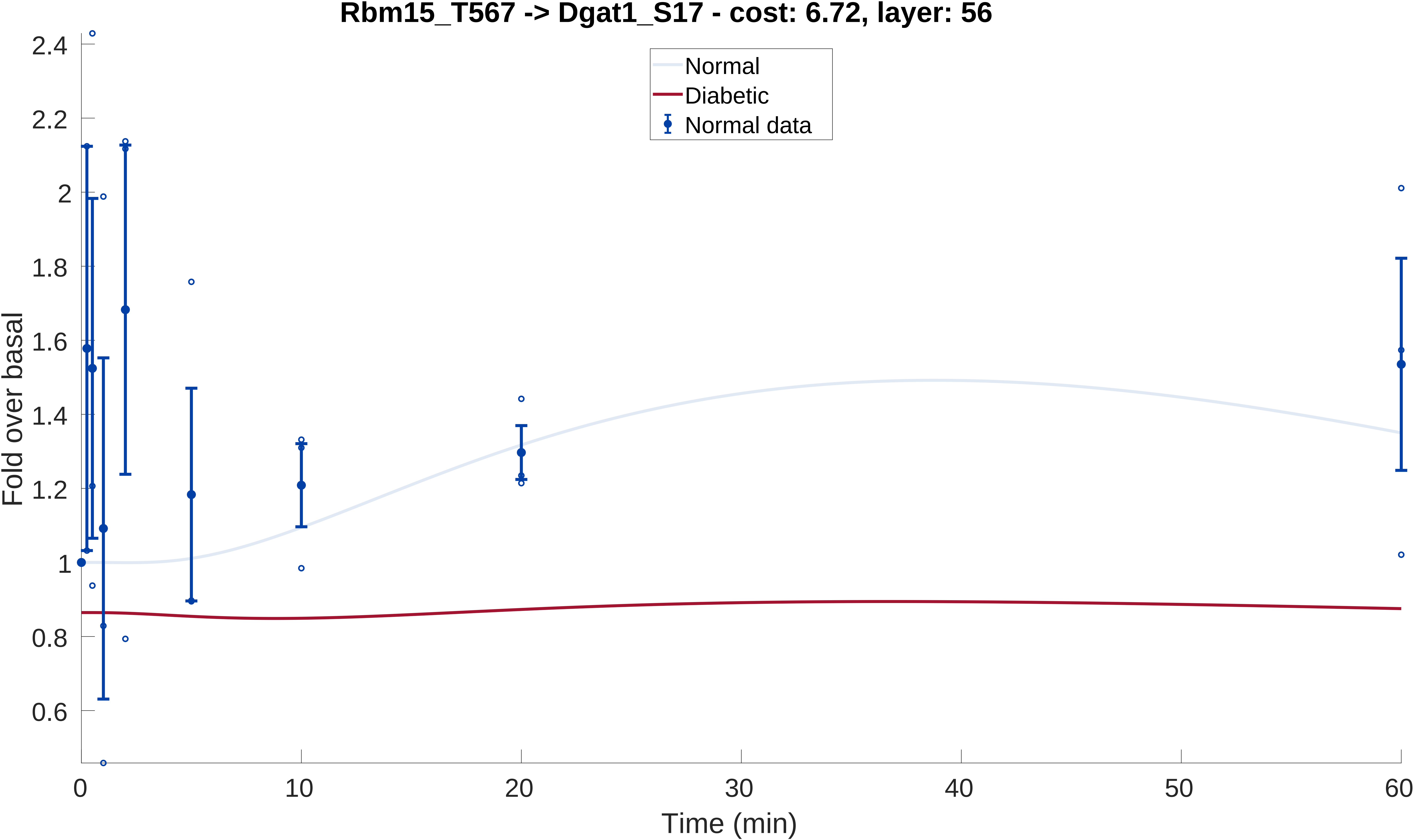

Rbm15_T567 -> Dgat1_S17 - cost: 6.72, layer: 56
Normal
Diabetic
Normal data
Fold over basal
Time (min)
2.4
2.2
2
1.8
1.6
1.4
1.2
1
0.8
0.6
0
10
20
30
40
50
60

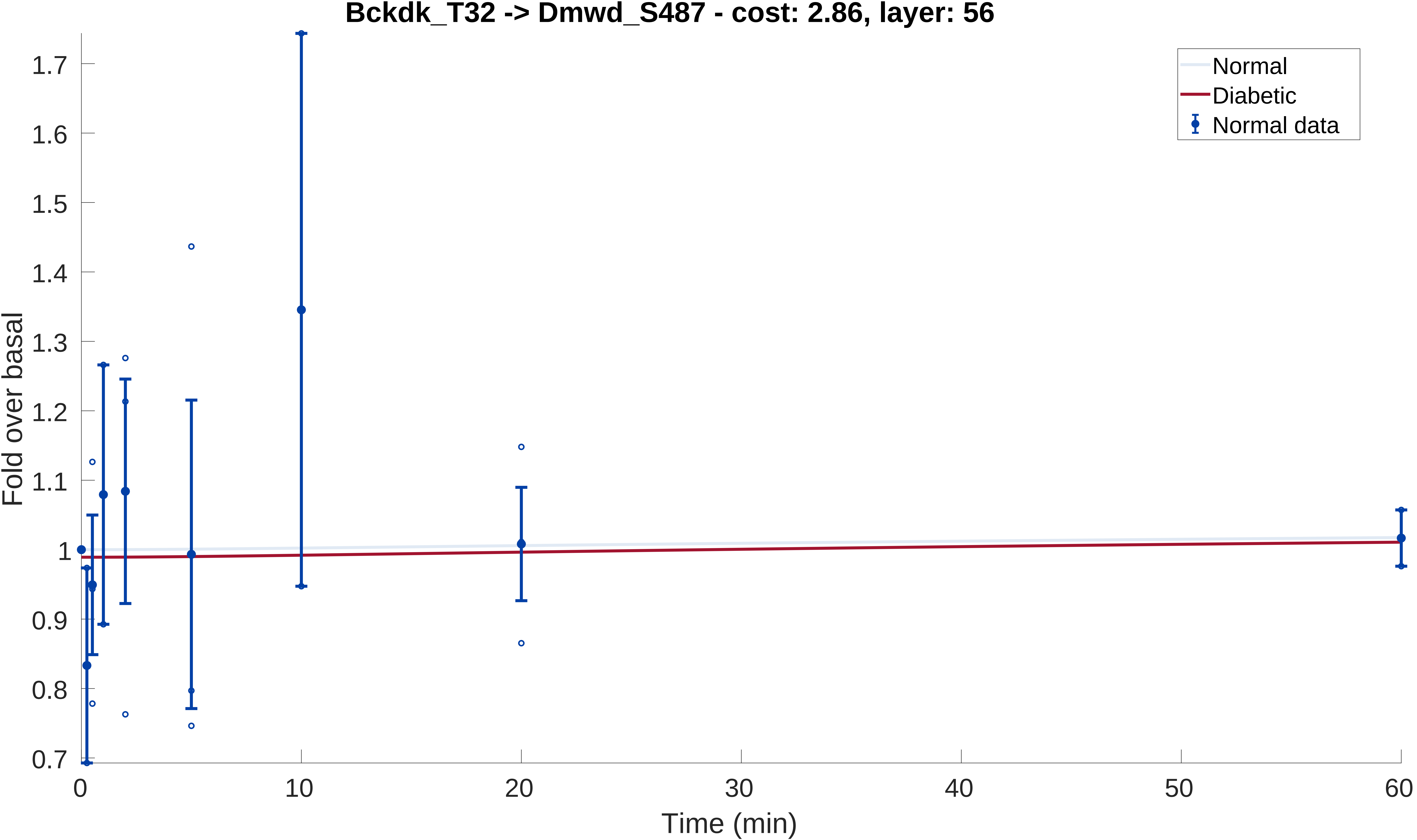
Bckdk_T32 -> Dmwd_S487 - cost: 2.86, layer: 56
Normal
Diabetic
Normal data
Fold over basal
Time (min)
1.7
1.6
1.5
1.4
1.3
1.2
1.1
1
0.9
0.8
0.7
0
10
20
30
40
50
60

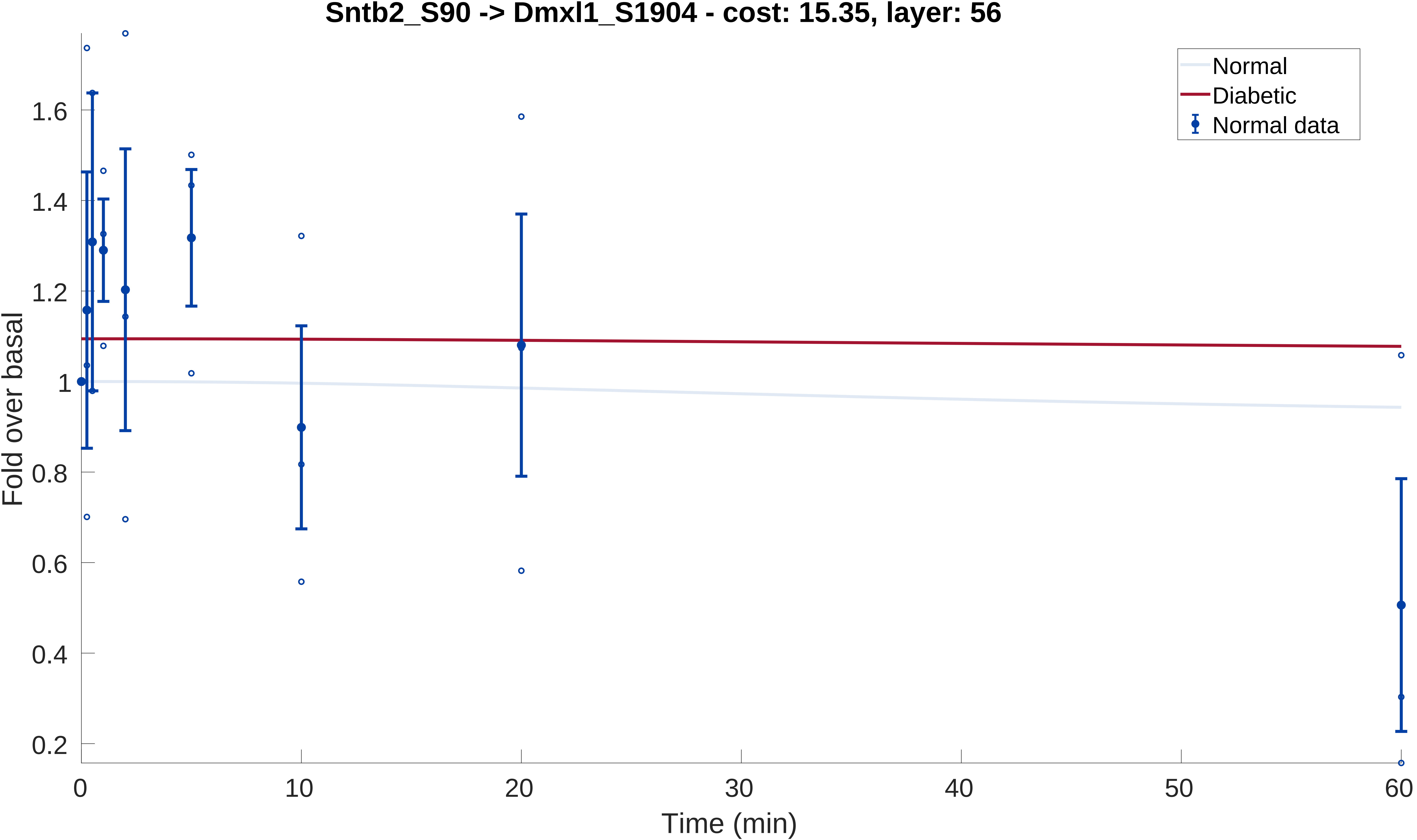
Sntb2_S90 -> Dmxl1_S1904 - cost: 15.35, layer: 56
Normal
Diabetic
Normal data
Fold over basal
Time (min)
0.2
0.4
0.6
0.8
1
1.2
1.4
1.6
0
10
20
30
40
50
60

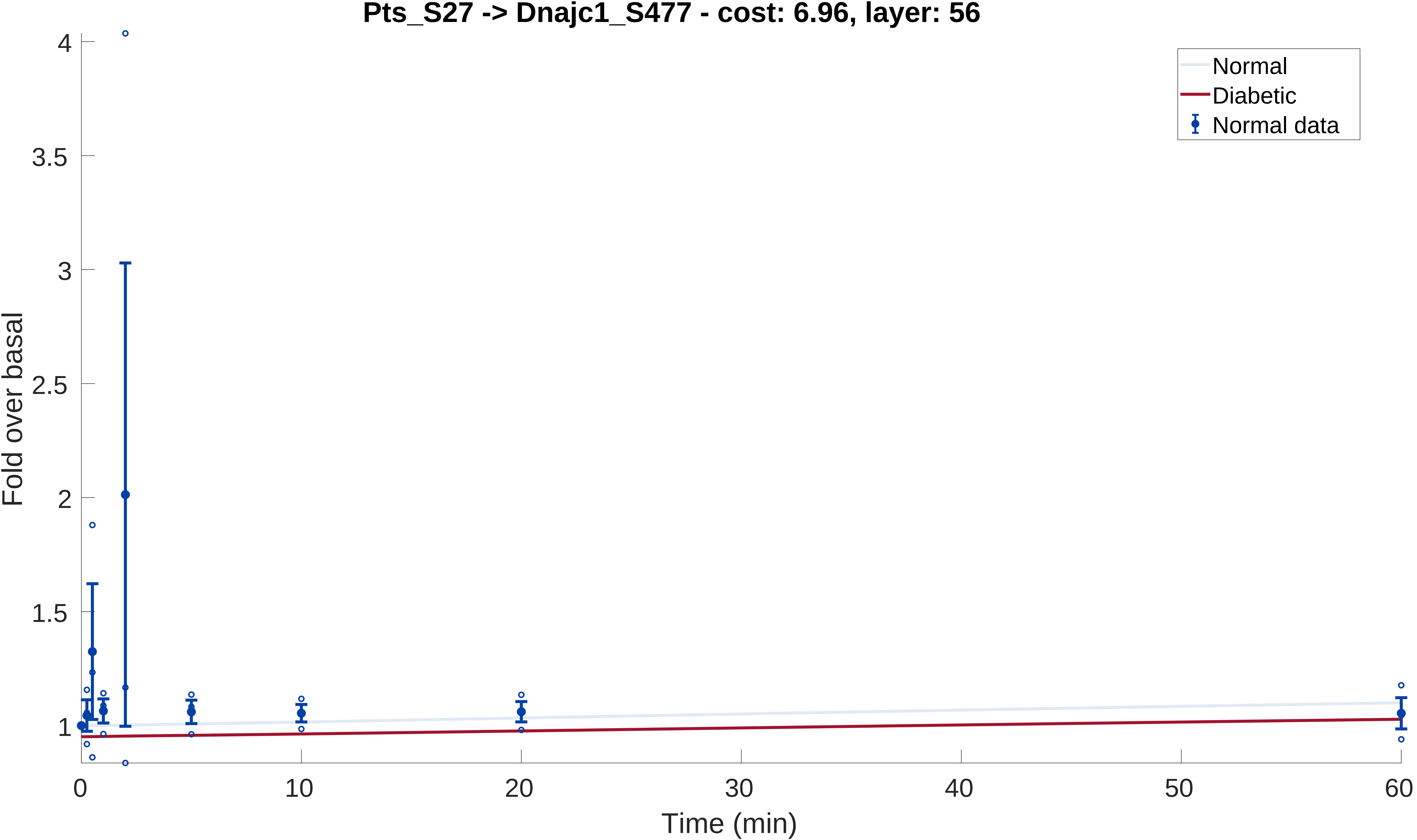
Pts_S27 -> Dnajc1_S477 - cost: 6.96, layer: 56
Normal
Diabetic
Normal data
Fold over basal
Time (min)
4
3.5
3
2.5
2
1.5
1
0
10
20
30
40
50
60

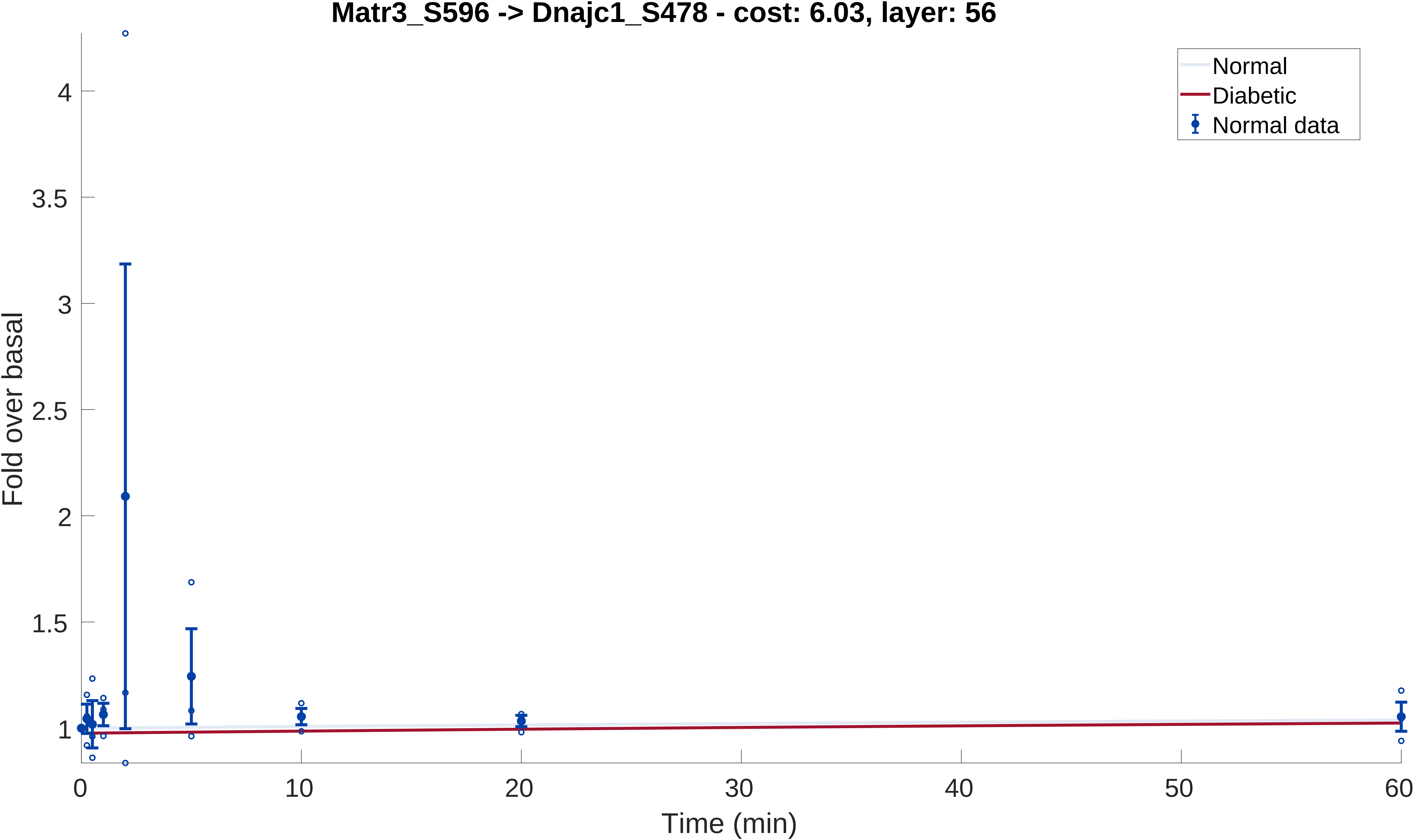

Matr3_S596 -> Dnajc1_S478 - cost: 6.03, layer: 56
Normal
Diabetic
Normal data
Fold over basal
Time (min)
4
3.5
3
2.5
2
1.5
1
0
10
20
30
40
50
60

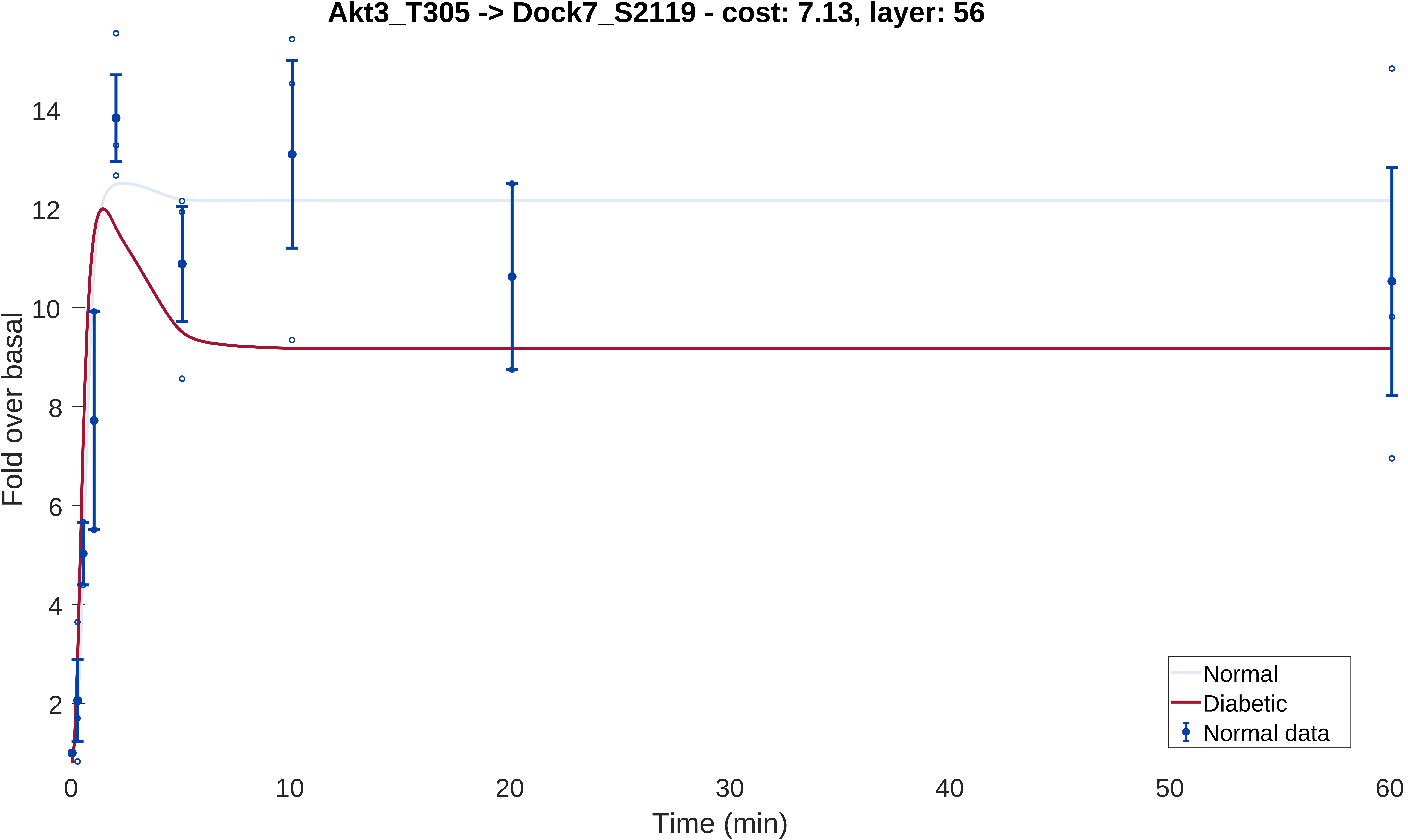

Akt3_T305 -> Dock7_S2119 - cost: 7.13, layer: 56
Fold over basal
Time (min)
0
10
20
30
40
50
60
2
4
6
8
10
12
14
Normal
Diabetic
Normal data

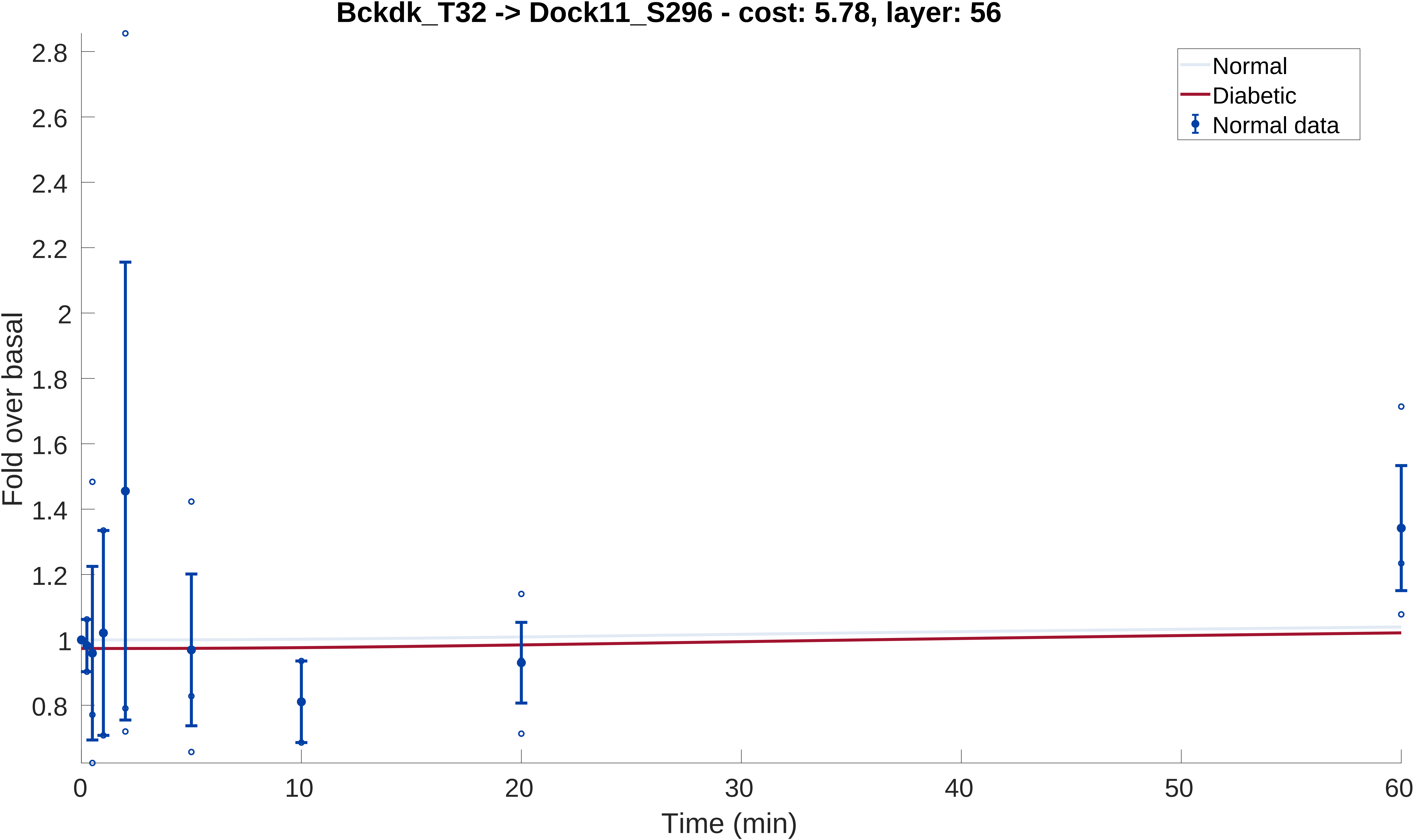

Bckdk_T32 -> Dock11_S296 - cost: 5.78, layer: 56
Normal
Diabetic
Normal data
Fold over basal
Time (min)
2.8
2.6
2.4
2.2
2
1.8
1.6
1.4
1.2
1
0.8
0
10
20
30
40
50
60

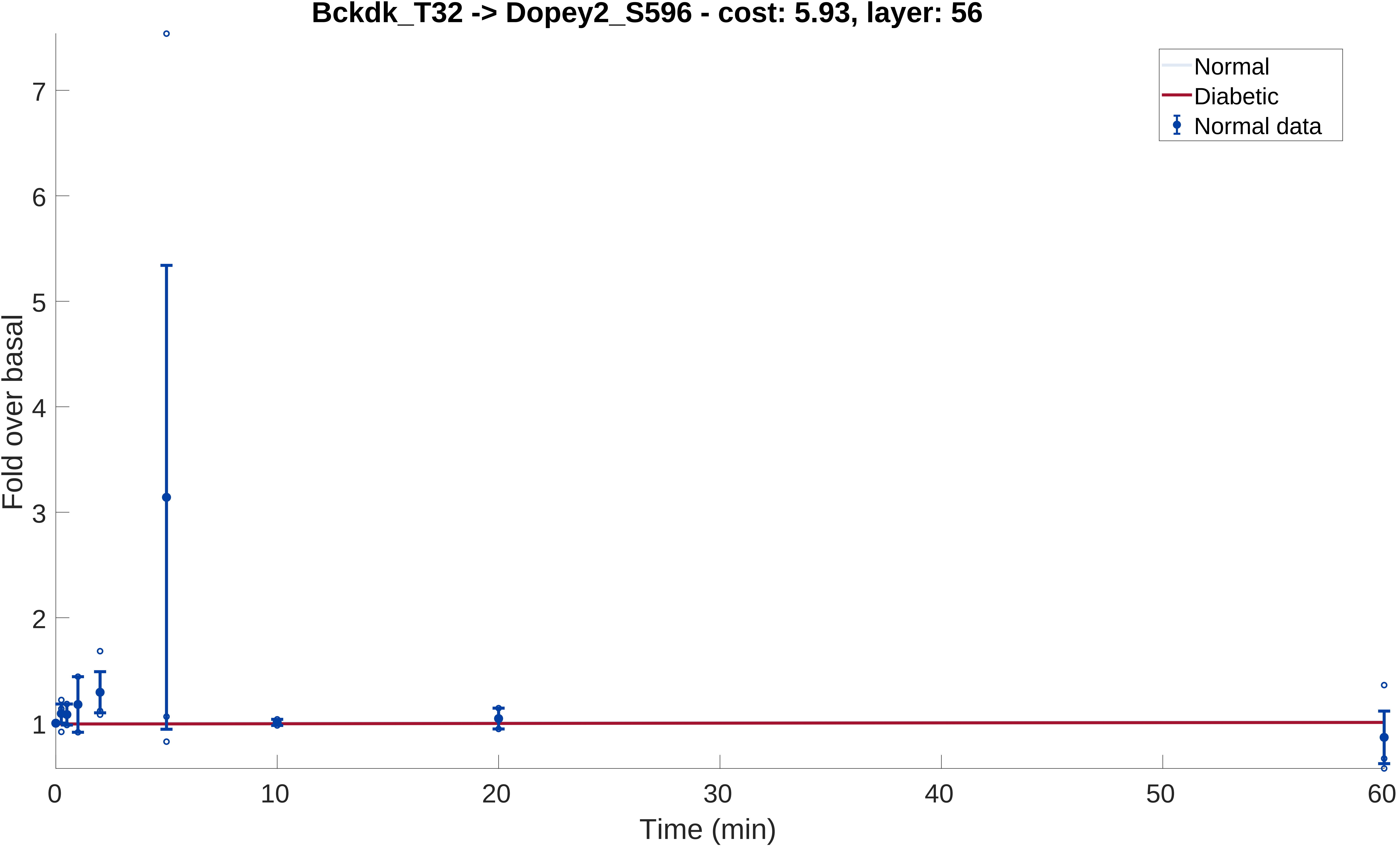

Bckdk_T32 -> Dopey2_S596 - cost: 5.93, layer: 56
Normal
Diabetic
Normal data
Fold over basal
Time (min)
7
6
5
4
3
2
1
0
10
20
30
40
50
60

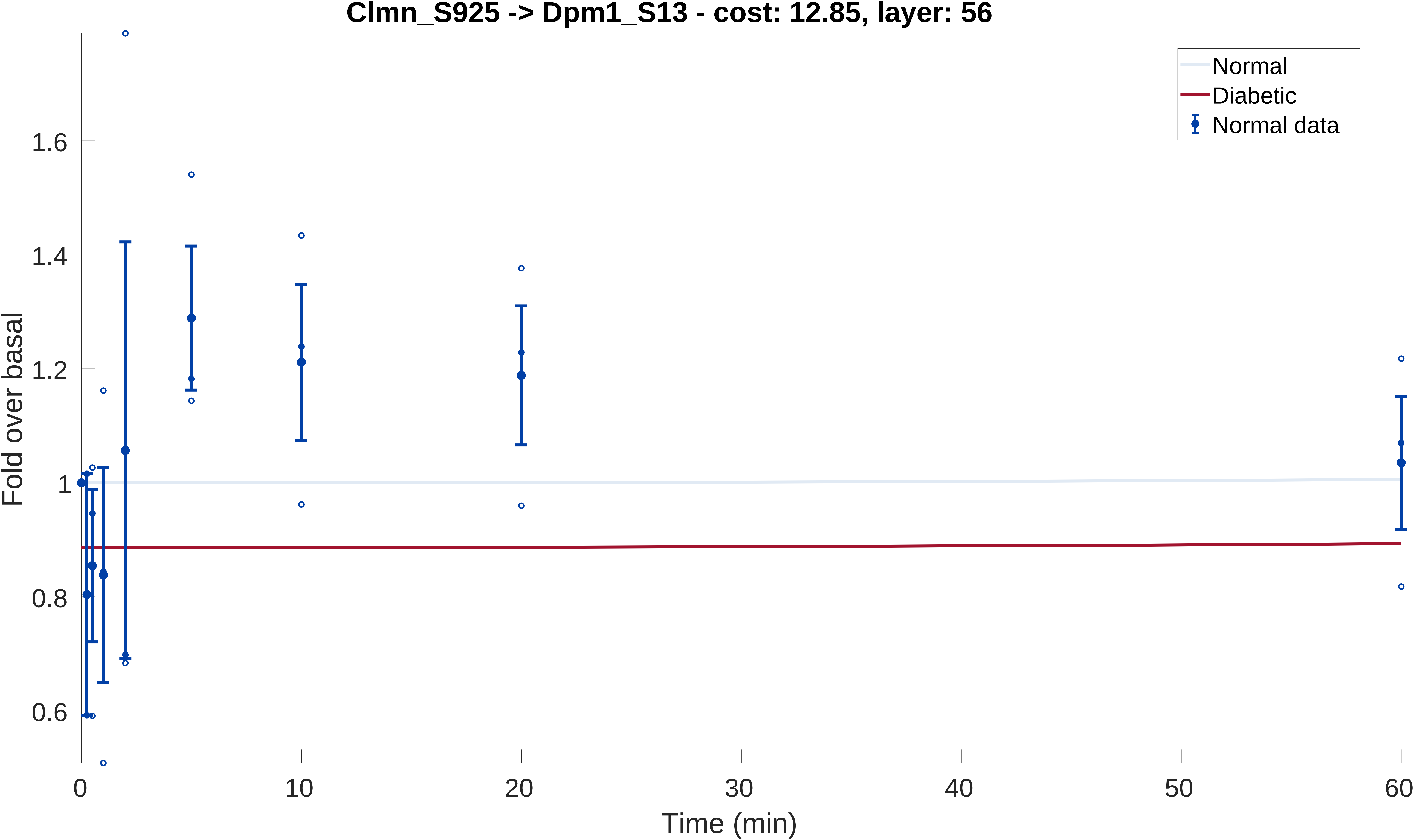

Clmn_S925 -> Dpm1_S13 - cost: 12.85, layer: 56
Normal
Diabetic
Normal data
Fold over basal
1.6
1.4
1.2
1
0.8
0.6
0
10
20
30
40
50
60
Time (min)

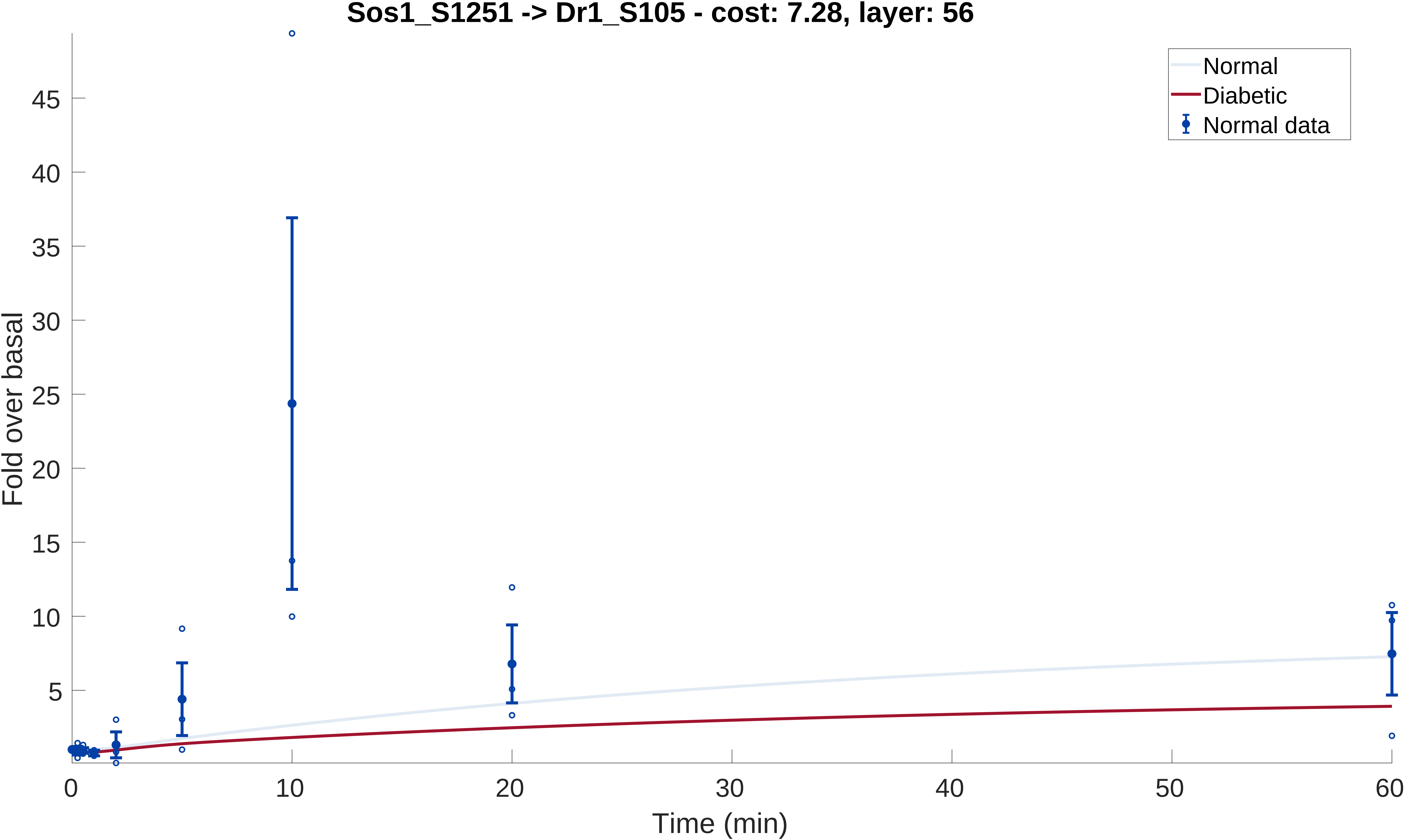

Sos1_S1251 -> Dr1_S105 - cost: 7.28, layer: 56
Normal
Diabetic
Normal data
Fold over basal
45
40
35
30
25
20
15
10
5
0
10
20
30
40
50
60
Time (min)

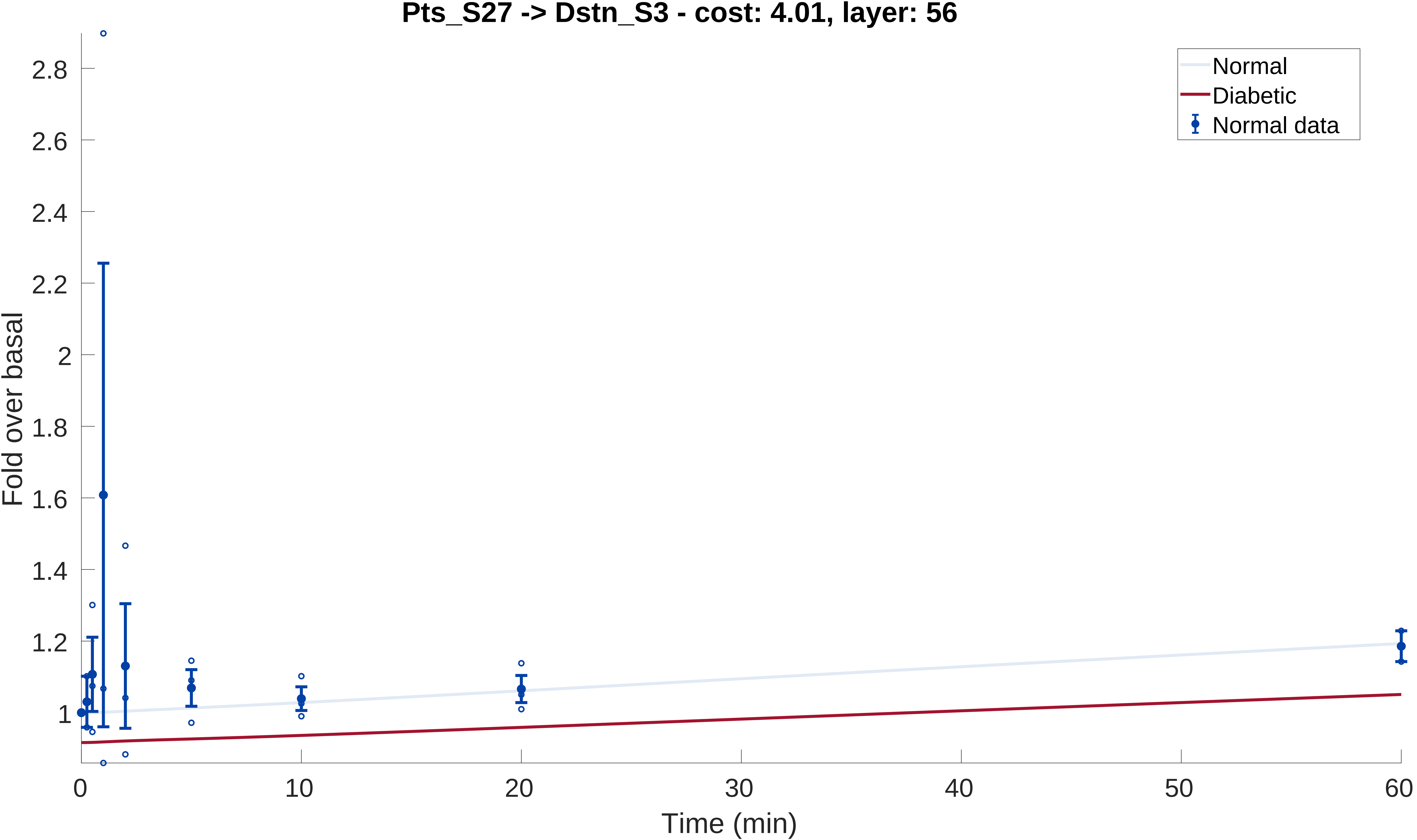

Pts_S27 -> Dstn_S3 - cost: 4.01, layer: 56
Normal
Diabetic
Normal data
Fold over basal
Time (min)
2.8
2.6
2.4
2.2
2
1.8
1.6
1.4
1.2
1
0
10
20
30
40
50
60

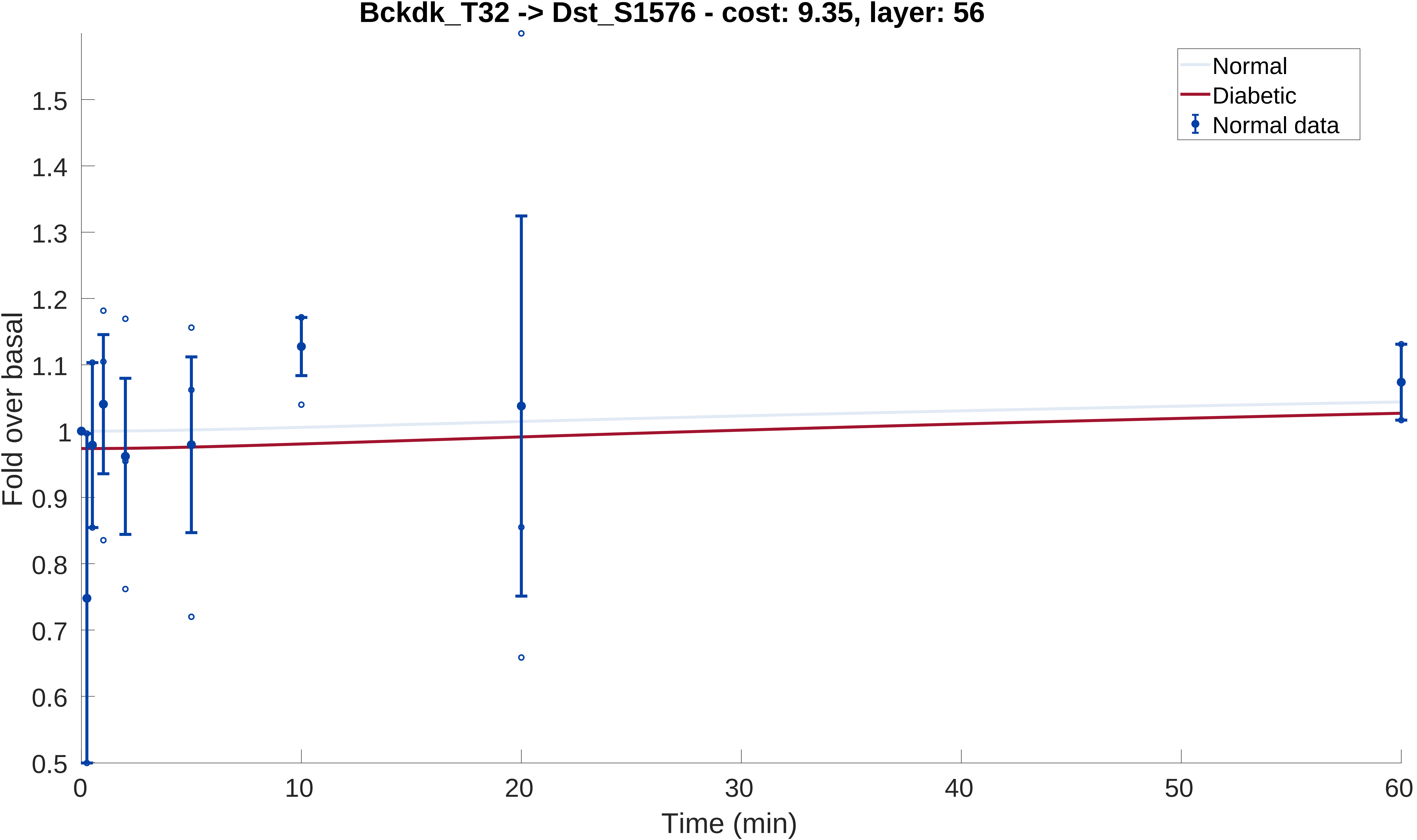
Bckdk_T32 -> Dst_S1576 - cost: 9.35, layer: 56
Normal
Diabetic
Normal data
Fold over basal
1.5
1.4
1.3
1.2
1.1
1
0.9
0.8
0.7
0.6
0.5
0
10
20
30
40
50
60
Time (min)

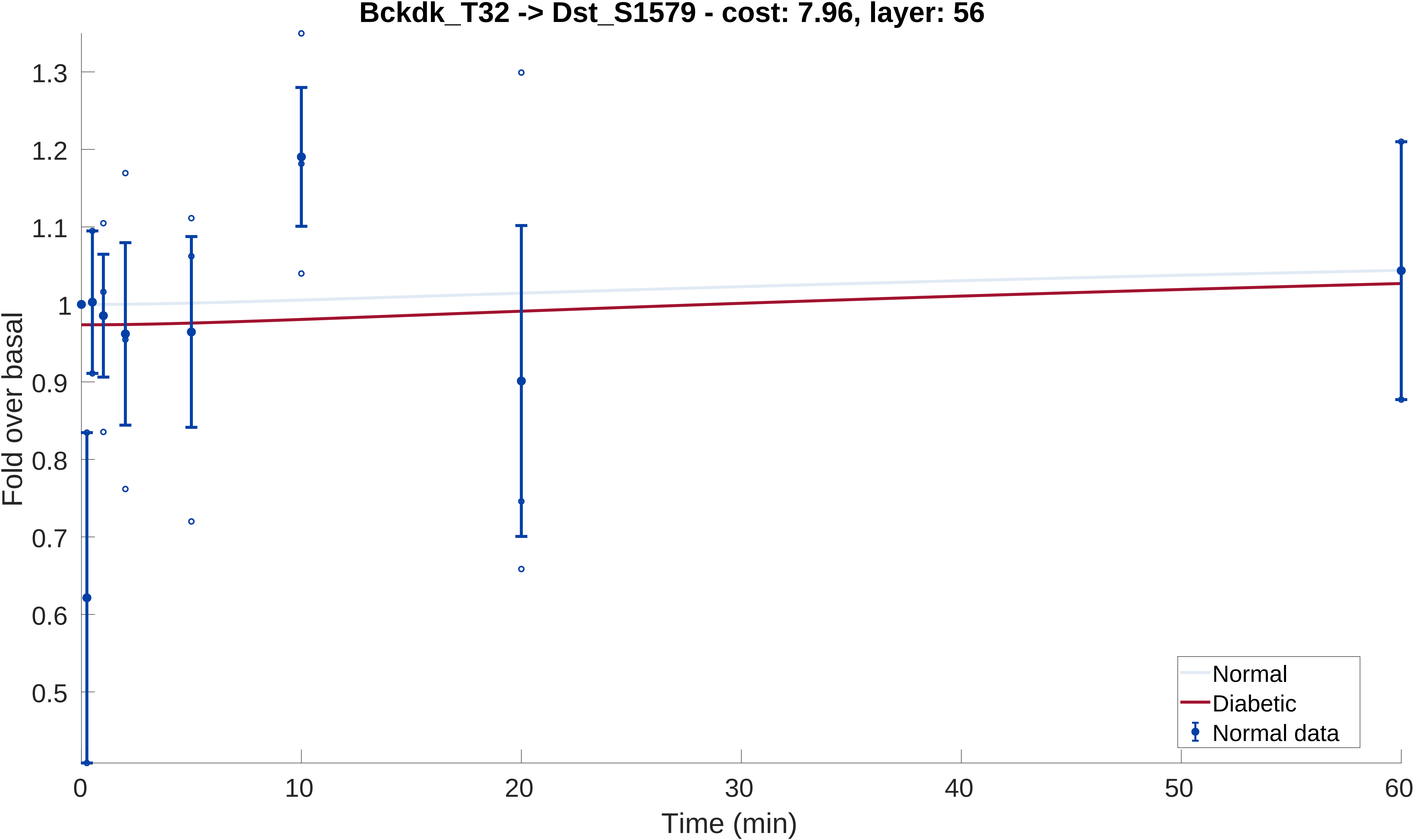

Bckdk_T32 -> Dst_S1579 - cost: 7.96, layer: 56
1.3
1.2
1.1
1
0.9
0.8
0.7
0.6
0.5
Fold over basal
0
10
20
30
40
50
60
Time (min)
Normal
Diabetic
Normal data

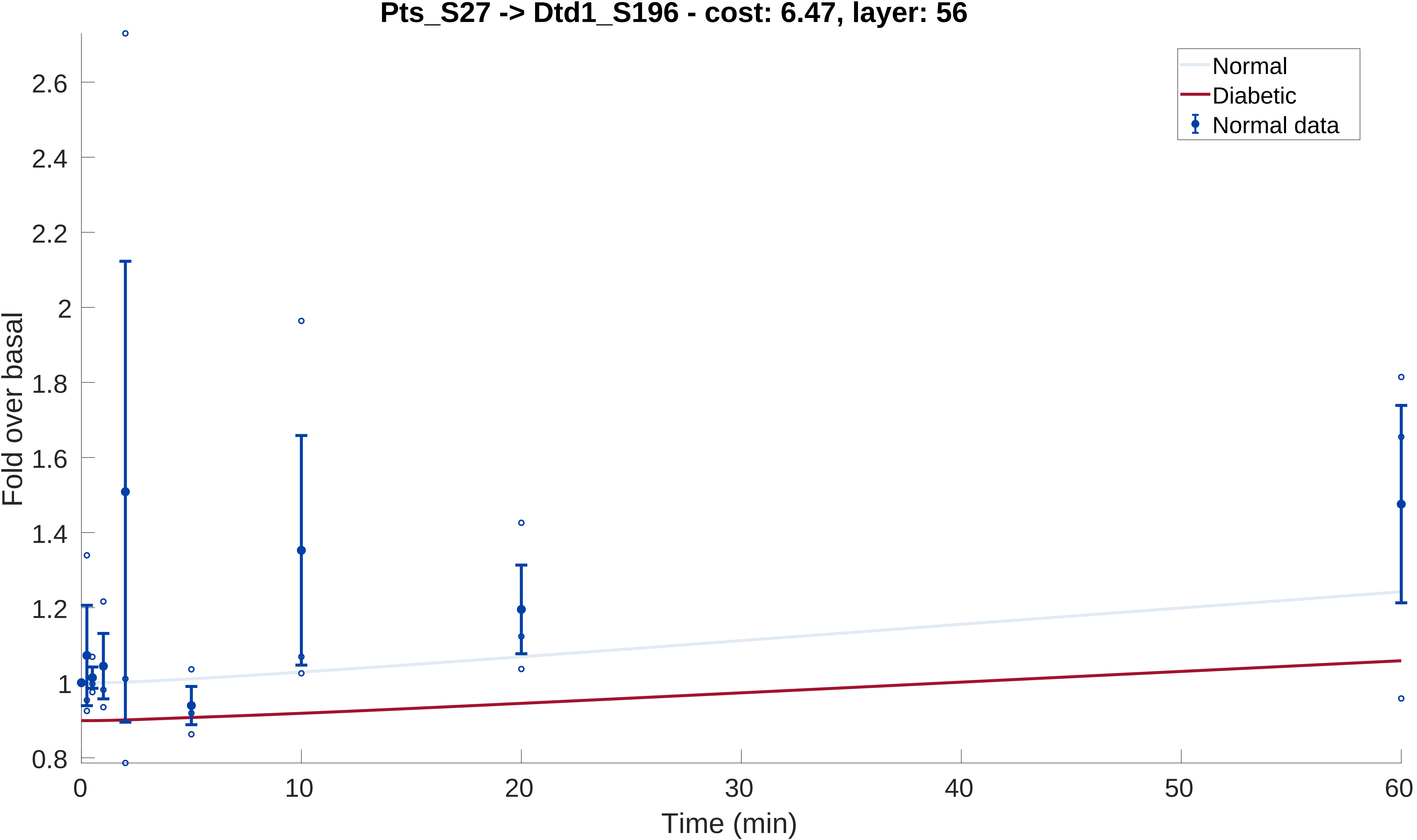

Pts_S27 -> Dtd1_S196 - cost: 6.47, layer: 56
Normal
Diabetic
Normal data
Fold over basal
Time (min)
0.8
1
1.2
1.4
1.6
1.8
2
2.2
2.4
2.6
0
10
20
30
40
50
60

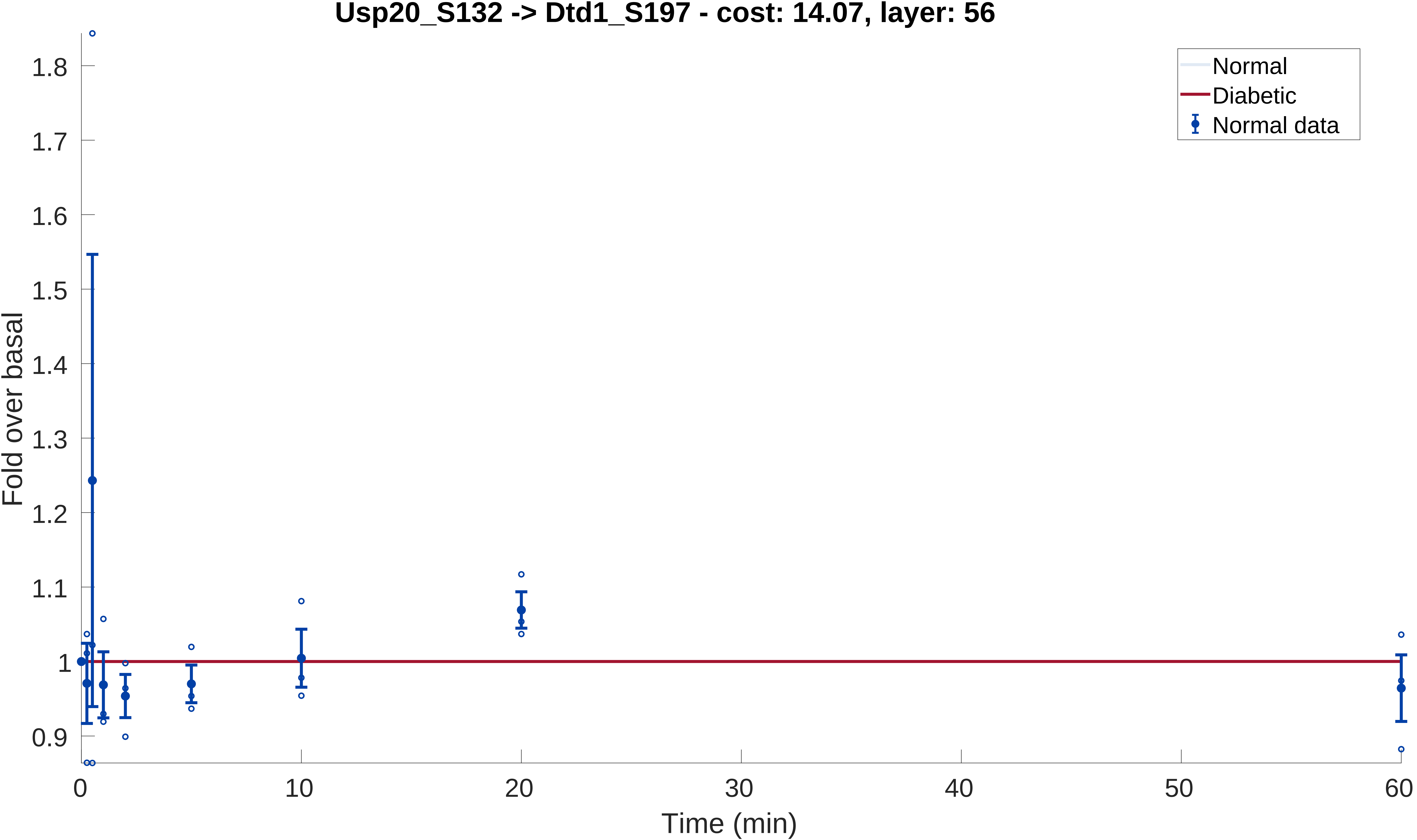

Usp20_S132 -> Dtd1_S197 - cost: 14.07, layer: 56
Normal
Diabetic
Normal data
Fold over basal
1.8
1.7
1.6
1.5
1.4
1.3
1.2
1.1
1
0.9
0
10
20
30
40
50
60
Time (min)

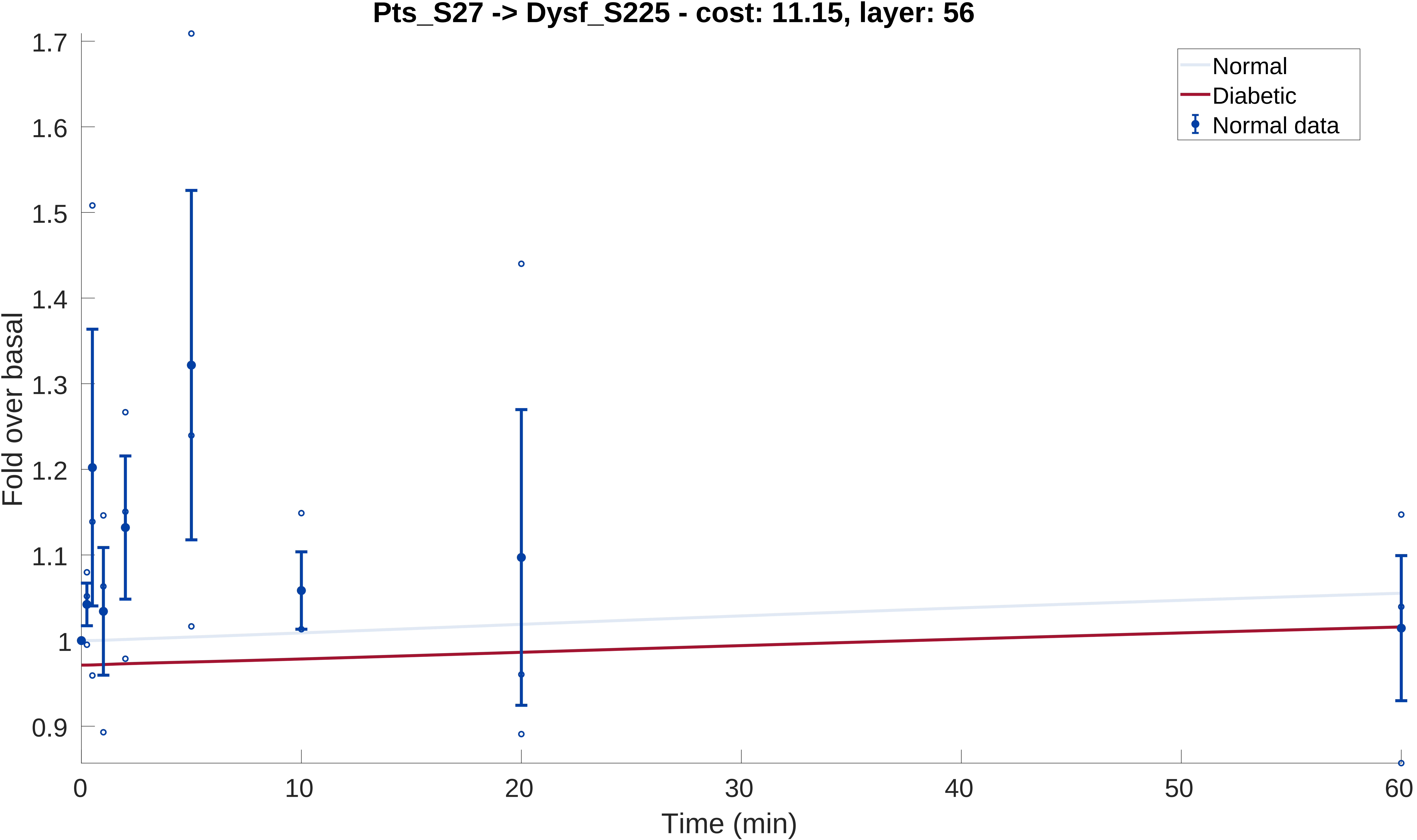
Pts_S27 -> Dysf_S225 - cost: 11.15, layer: 56
Normal
Diabetic
Normal data
Fold over basal
Time (min)
0.9
1
1.1
1.2
1.3
1.4
1.5
1.6
1.7
0
10
20
30
40
50
60

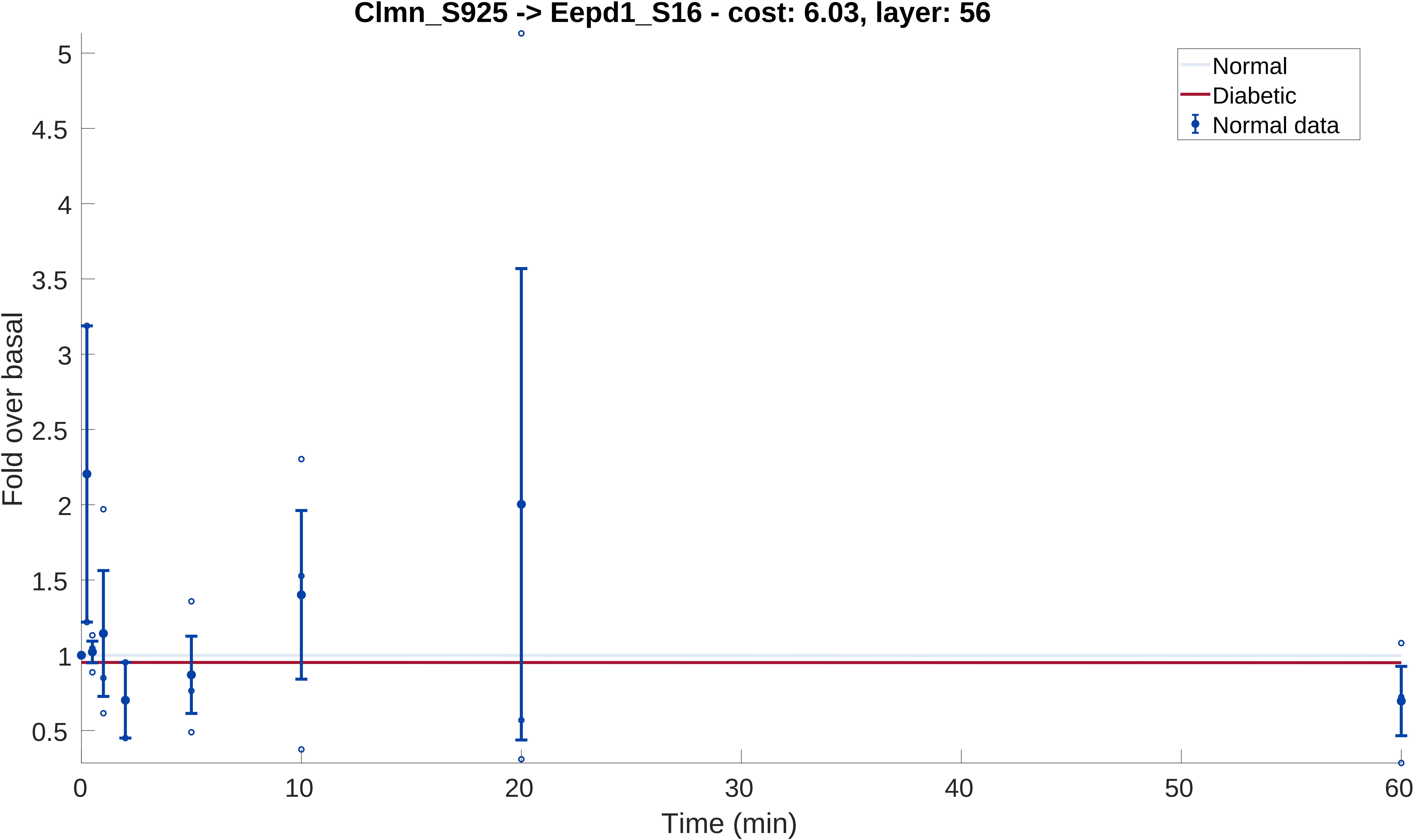

Clmn_S925 -> Eepd1_S16 - cost: 6.03, layer: 56
Normal
Diabetic
Normal data
Fold over basal
Time (min)
0
10
20
30
40
50
60
0.5
1
1.5
2
2.5
3
3.5
4
4.5
5

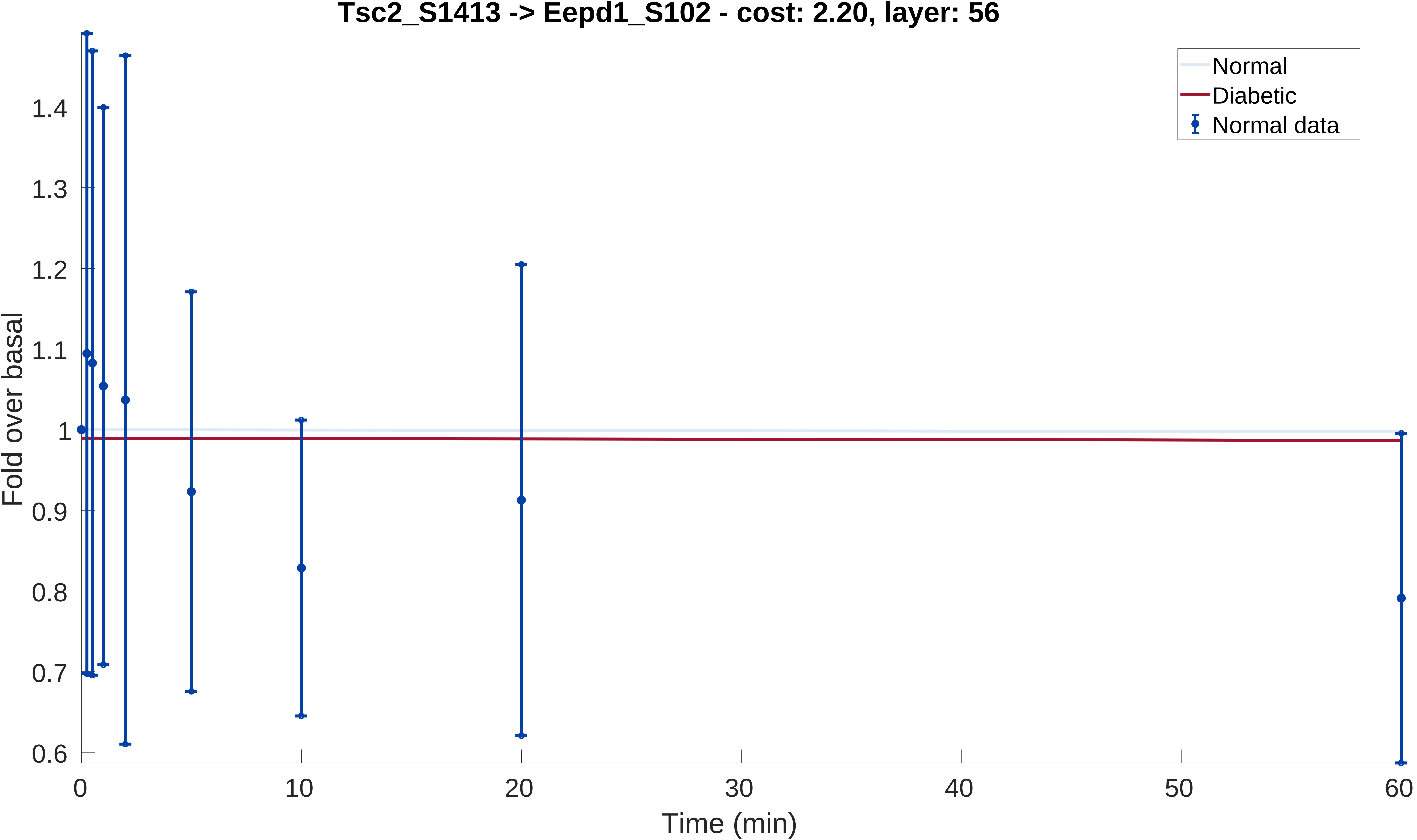
Tsc2_S1413 -> Eepd1_S102 - cost: 2.20, layer: 56
Normal
Diabetic
Normal data
Fold over basal
Time (min)
0.6
0.7
0.8
0.9
1
1.1
1.2
1.3
1.4
0
10
20
30
40
50
60

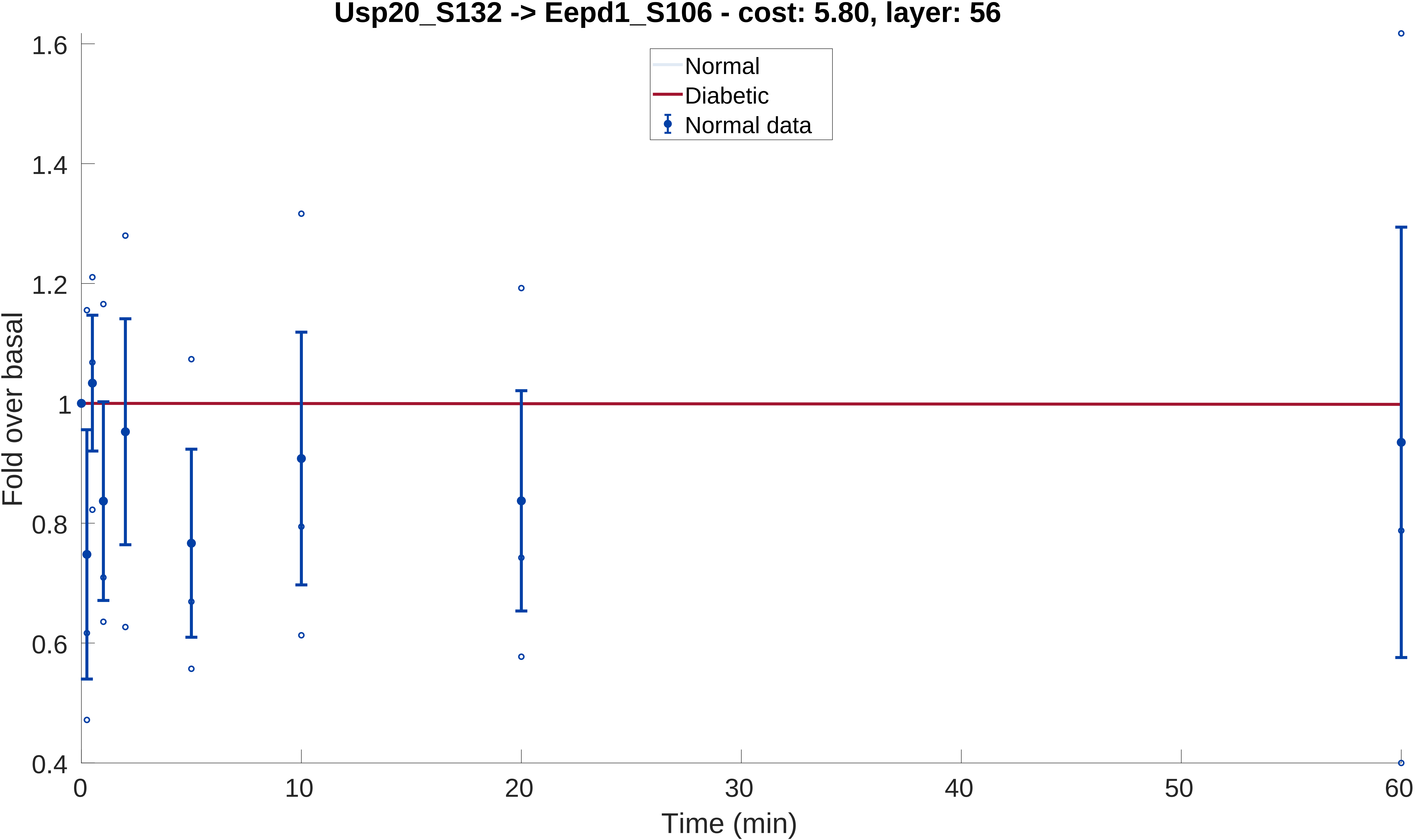

Usp20_S132 -> Eepd1_S106 - cost: 5.80, layer: 56
Normal
Diabetic
Normal data
Fold over basal
Time (min)
1.6
1.4
1.2
1
0.8
0.6
0.4
0
10
20
30
40
50
60

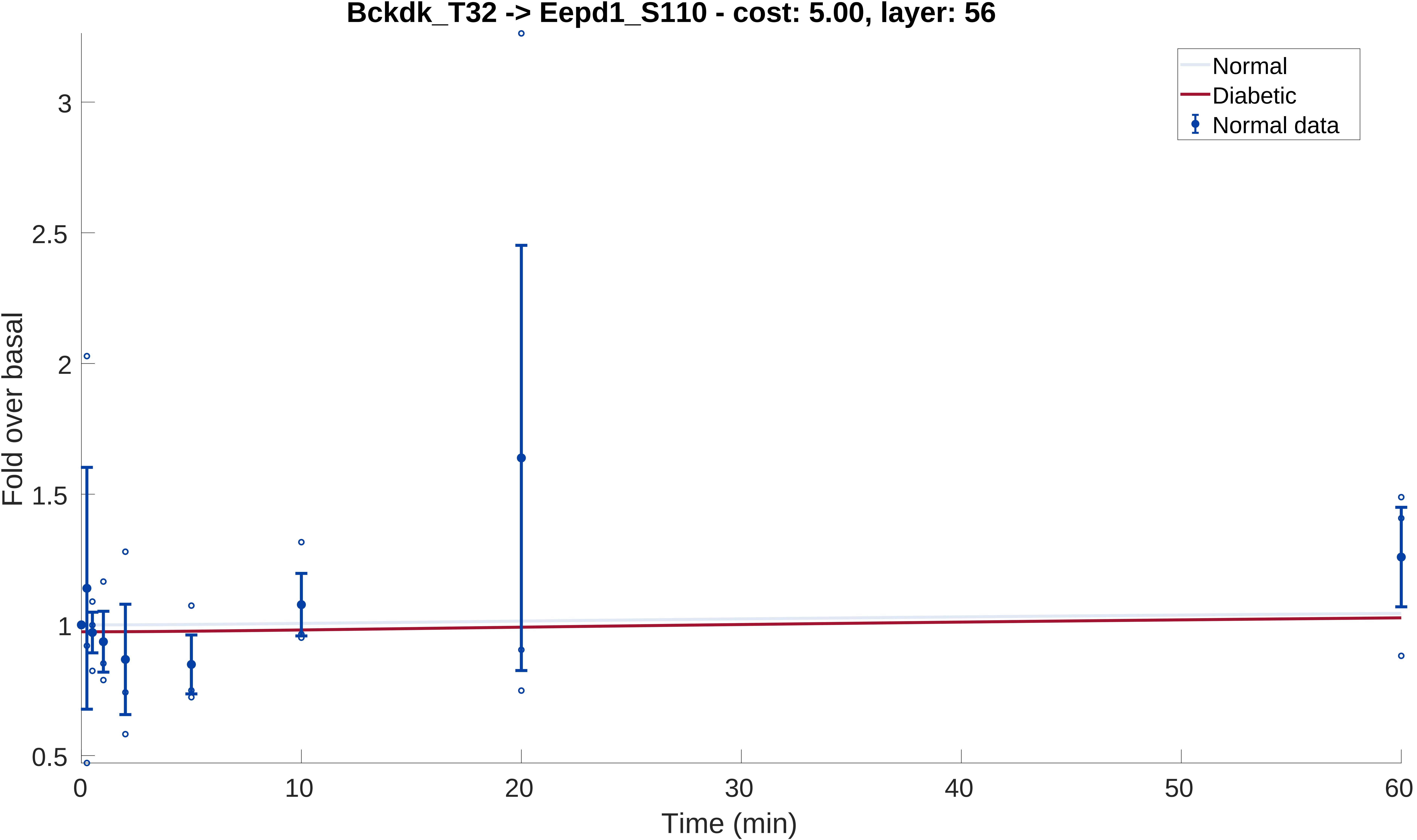

Bckdk_T32 -> Eepd1_S110 - cost: 5.00, layer: 56
Normal
Diabetic
Normal data
Fold over basal
0.5
1
1.5
2
2.5
3
0
10
20
30
40
50
60
Time (min)

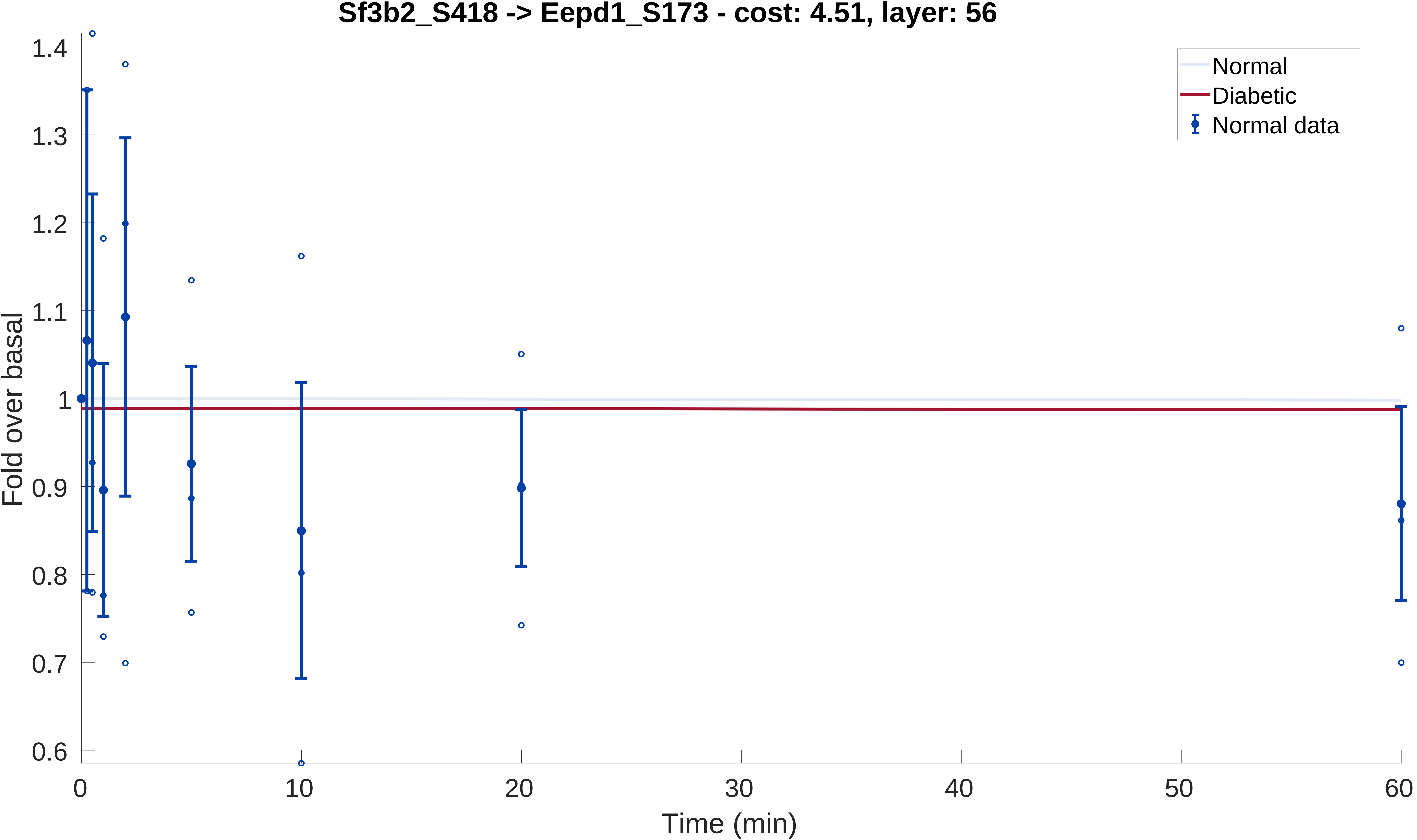

Sf3b2_S418 -> Eepd1_S173 - cost: 4.51, layer: 56
Normal
Diabetic
Normal data
Fold over basal
Time (min)
1.4
1.3
1.2
1.1
1
0.9
0.8
0.7
0.6
0
10
20
30
40
50
60

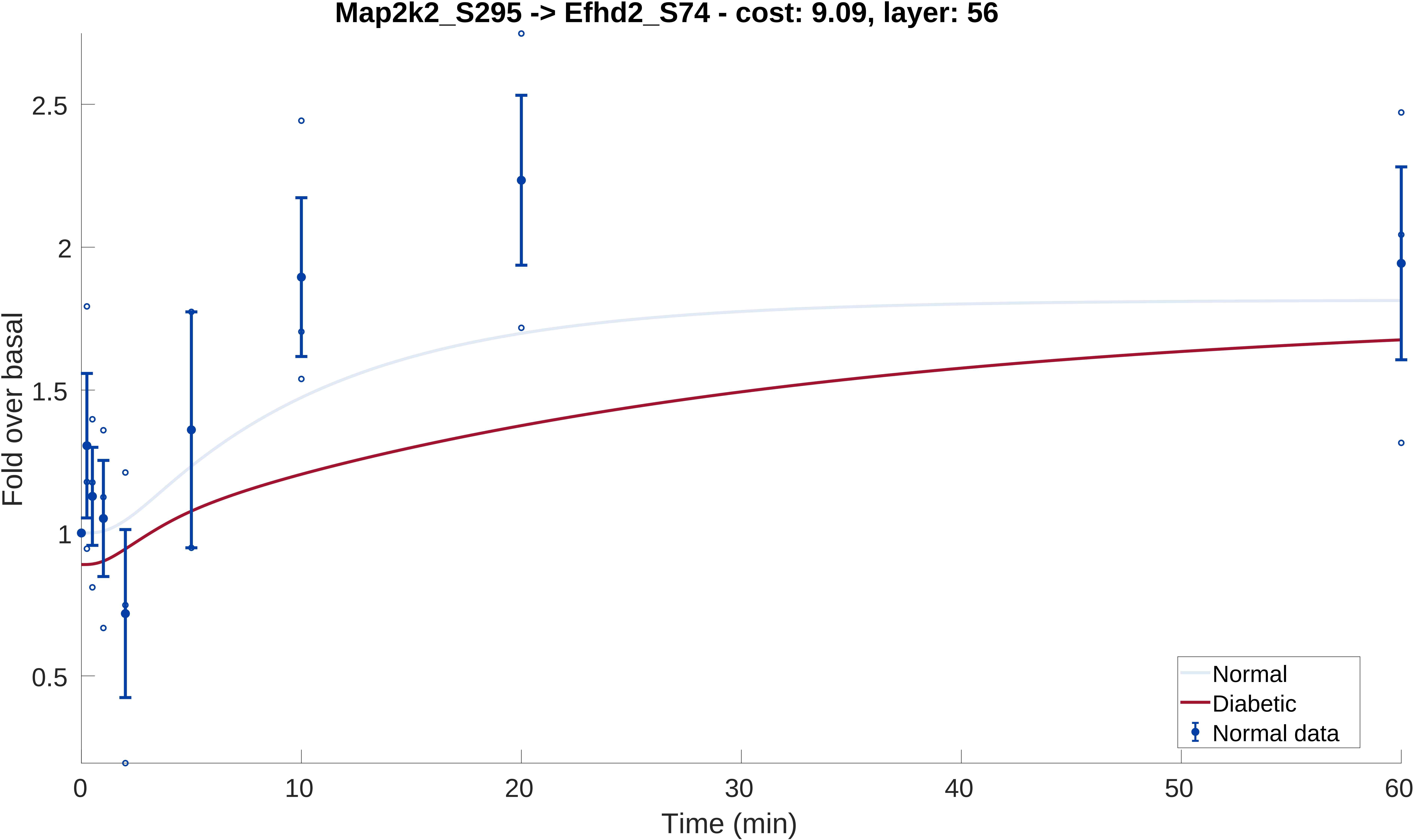

Map2k2_S295 -> Efhd2_S74 - cost: 9.09, layer: 56
Fold over basal
2.5
2
1.5
1
0.5
0
10
20
30
40
50
60
Time (min)
Normal
Diabetic
Normal data

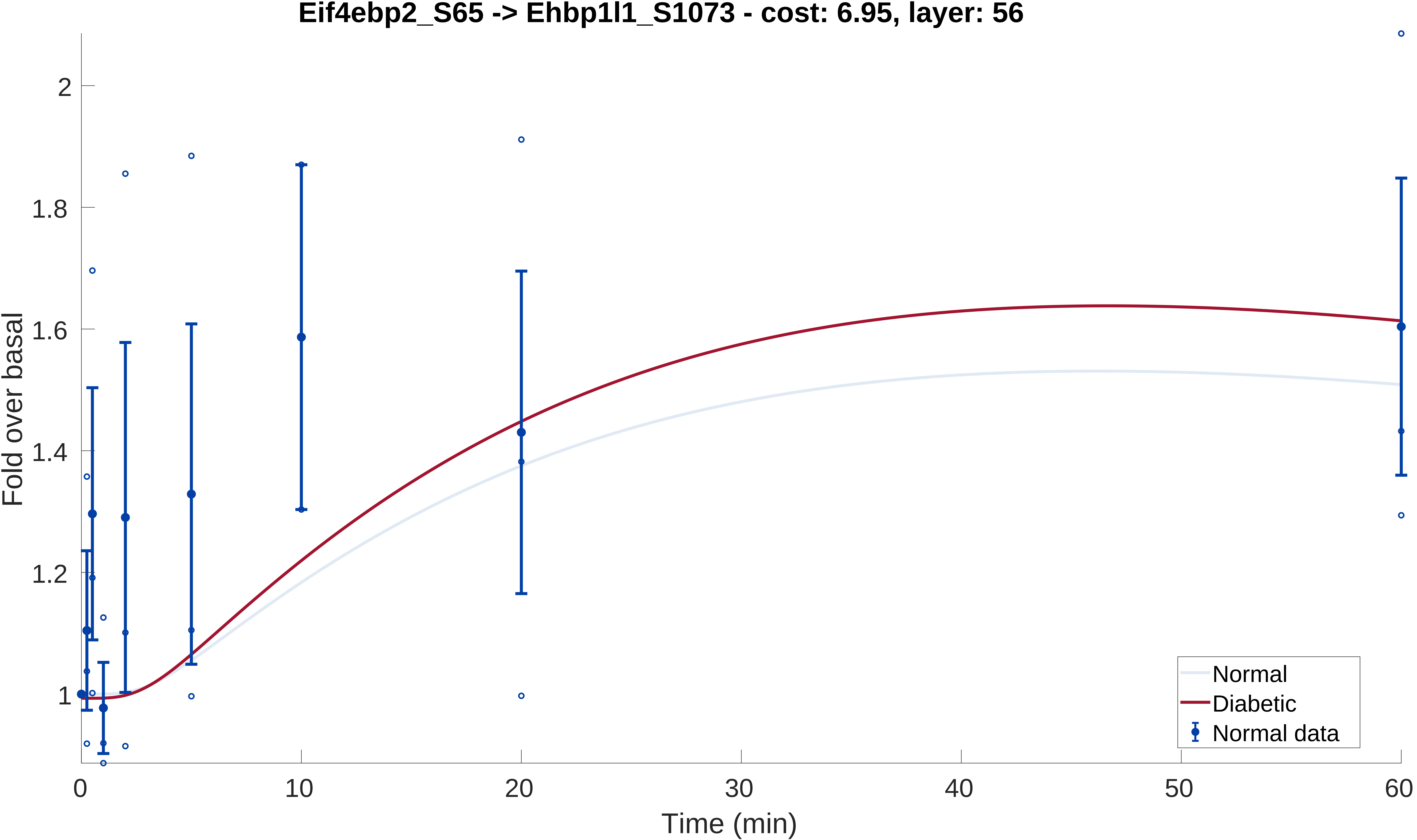

Eif4ebp2_S65 -> Ehbp1l1_S1073 - cost: 6.95, layer: 56
Fold over basal
2
1.8
1.6
1.4
1.2
1
0
10
20
30
40
50
60
Time (min)
Normal
Diabetic
Normal data

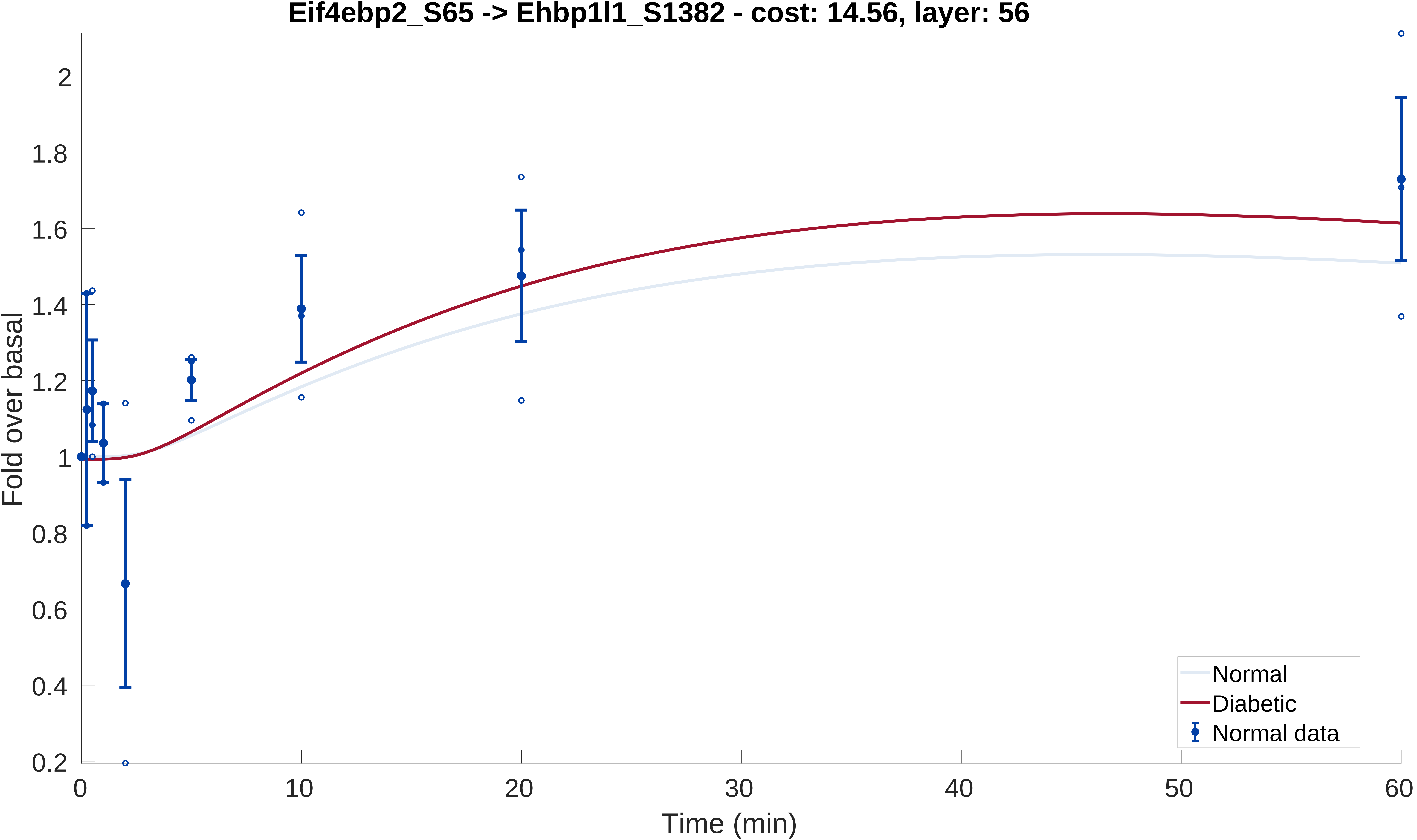

Eif4ebp2_S65 -> Ehbp1l1_S1382 - cost: 14.56, layer: 56
Fold over basal
2
1.8
1.6
1.4
1.2
1
0.8
0.6
0.4
0.2
0
10
20
30
40
50
60
Time (min)
Normal
Diabetic
Normal data

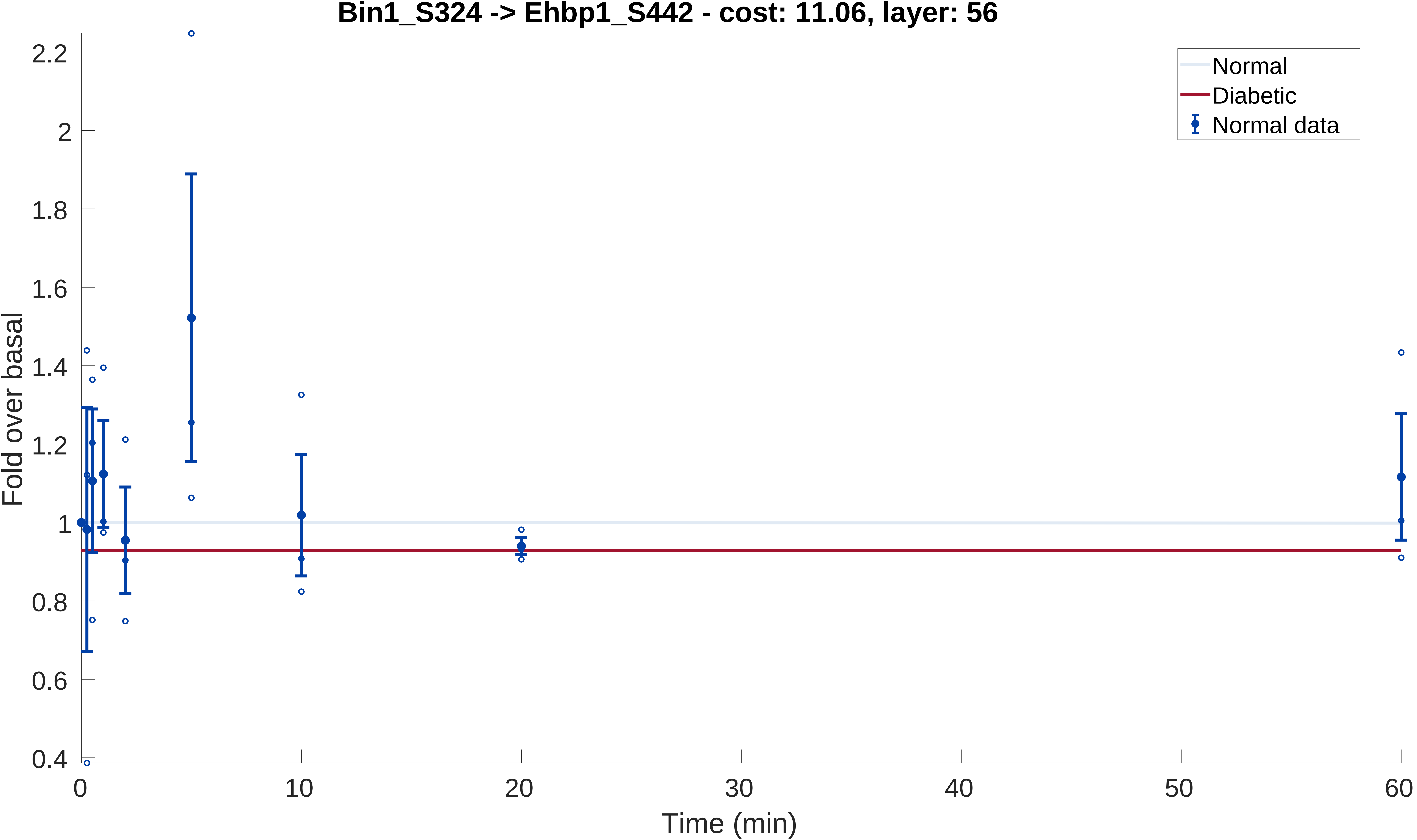

Bin1_S324 -> Ehbp1_S442 - cost: 11.06, layer: 56
Normal
Diabetic
Normal data
Fold over basal
2.2
2
1.8
1.6
1.4
1.2
1
0.8
0.6
0.4
0
10
20
30
40
50
60
Time (min)

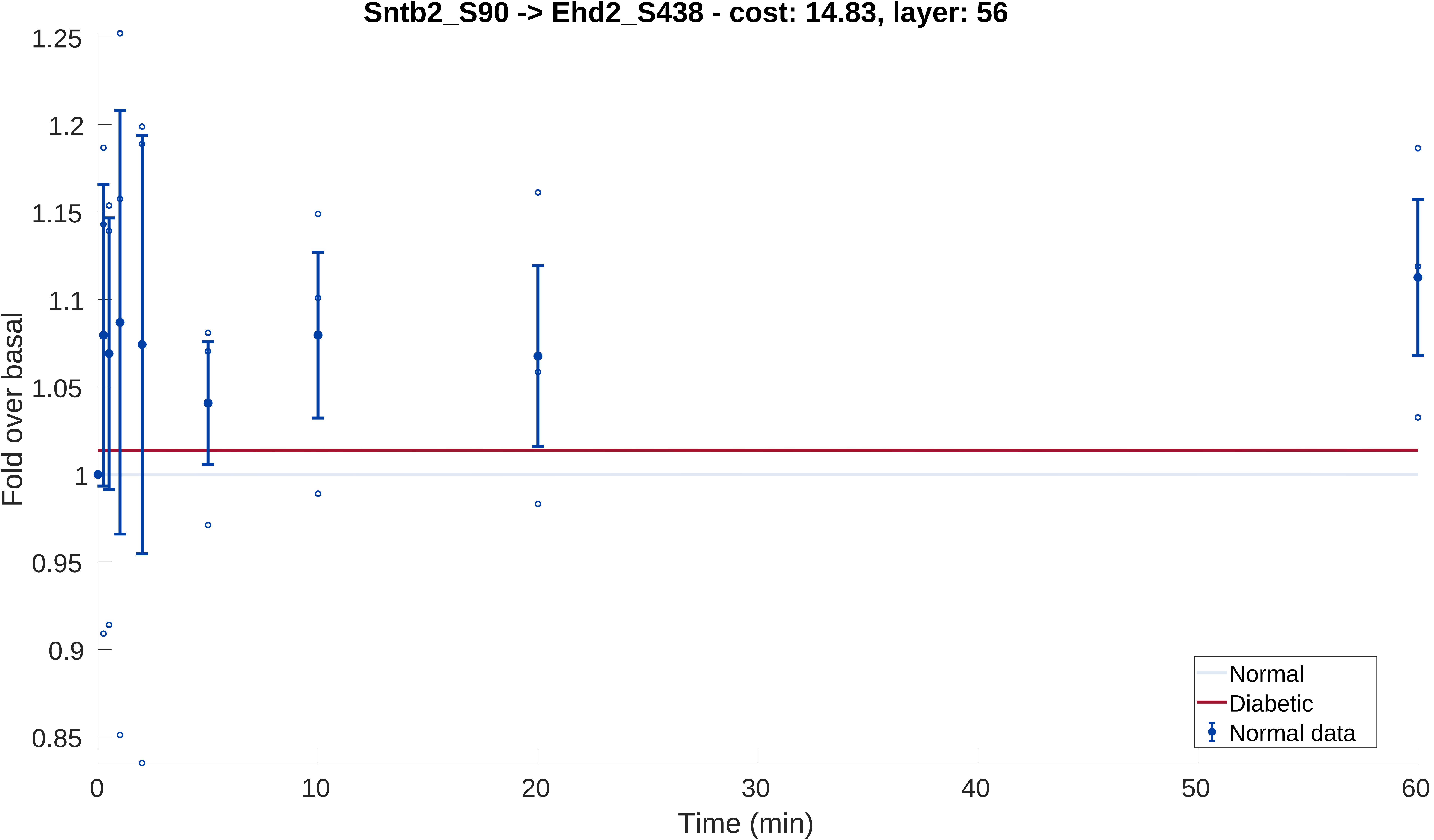

Sntb2_S90 -> Ehd2_S438 - cost: 14.83, layer: 56
Fold over basal
1.25
1.2
1.15
1.1
1.05
1
0.95
0.9
0.85
0
10
20
30
40
50
60
Time (min)
Normal
Diabetic
Normal data

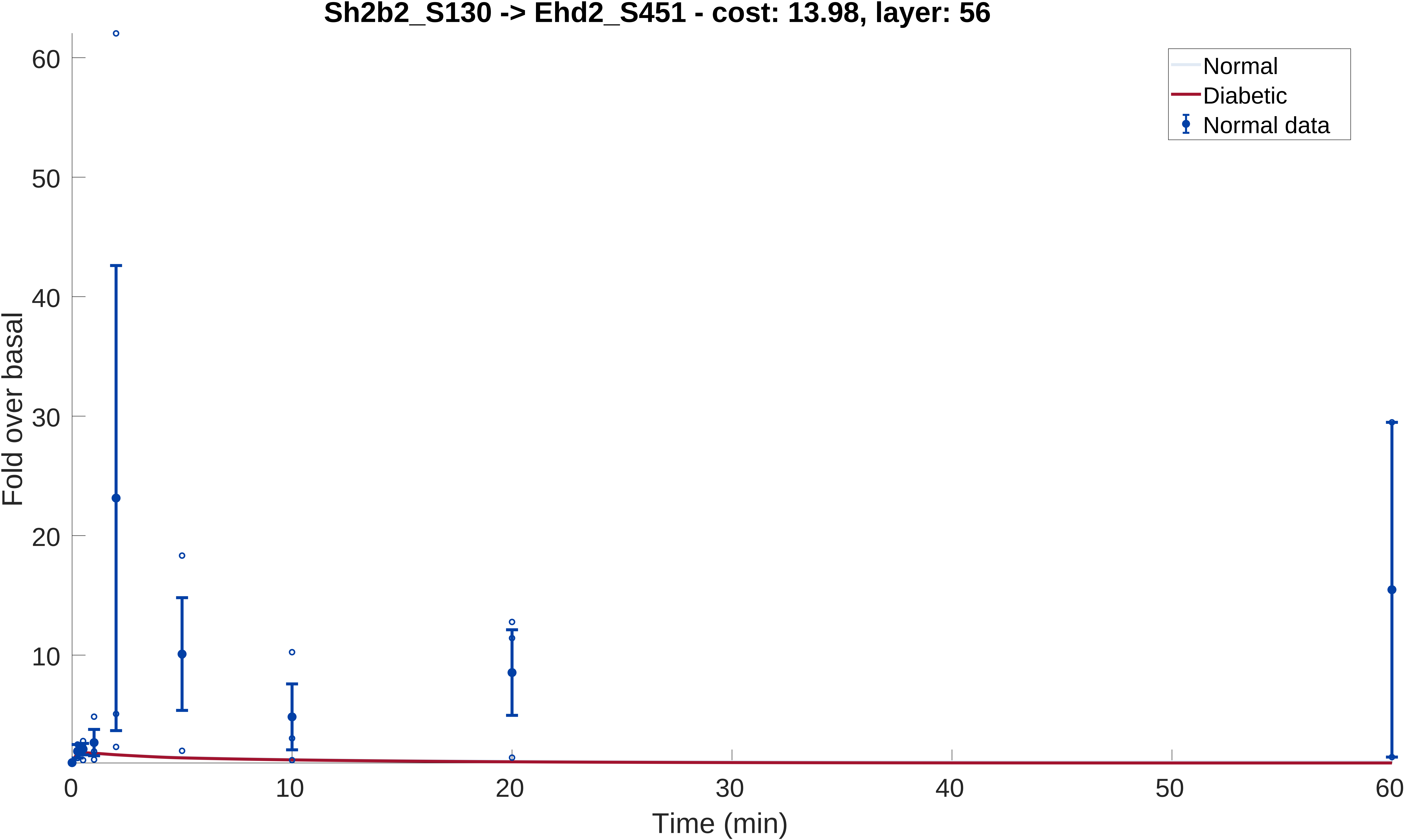

Sh2b2_S130 -> Ehd2_S451 - cost: 13.98, layer: 56
Normal
Diabetic
Normal data
Fold over basal
Time (min)
0
10
20
30
40
50
60

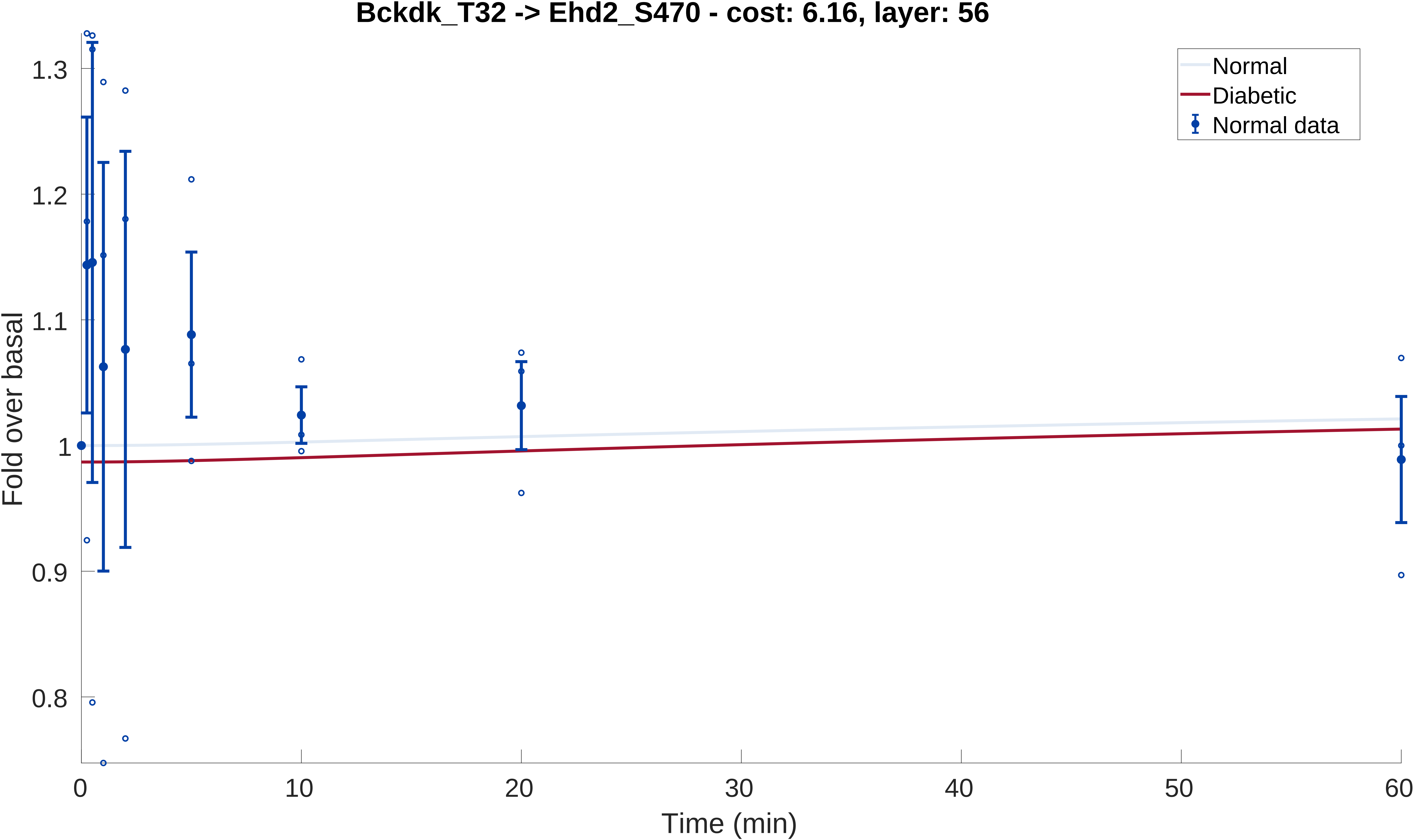

Bckdk_T32 -> Ehd2_S470 - cost: 6.16, layer: 56
Normal
Diabetic
Normal data
Fold over basal
1.3
1.2
1.1
1
0.9
0.8
0
10
20
30
40
50
60
Time (min)

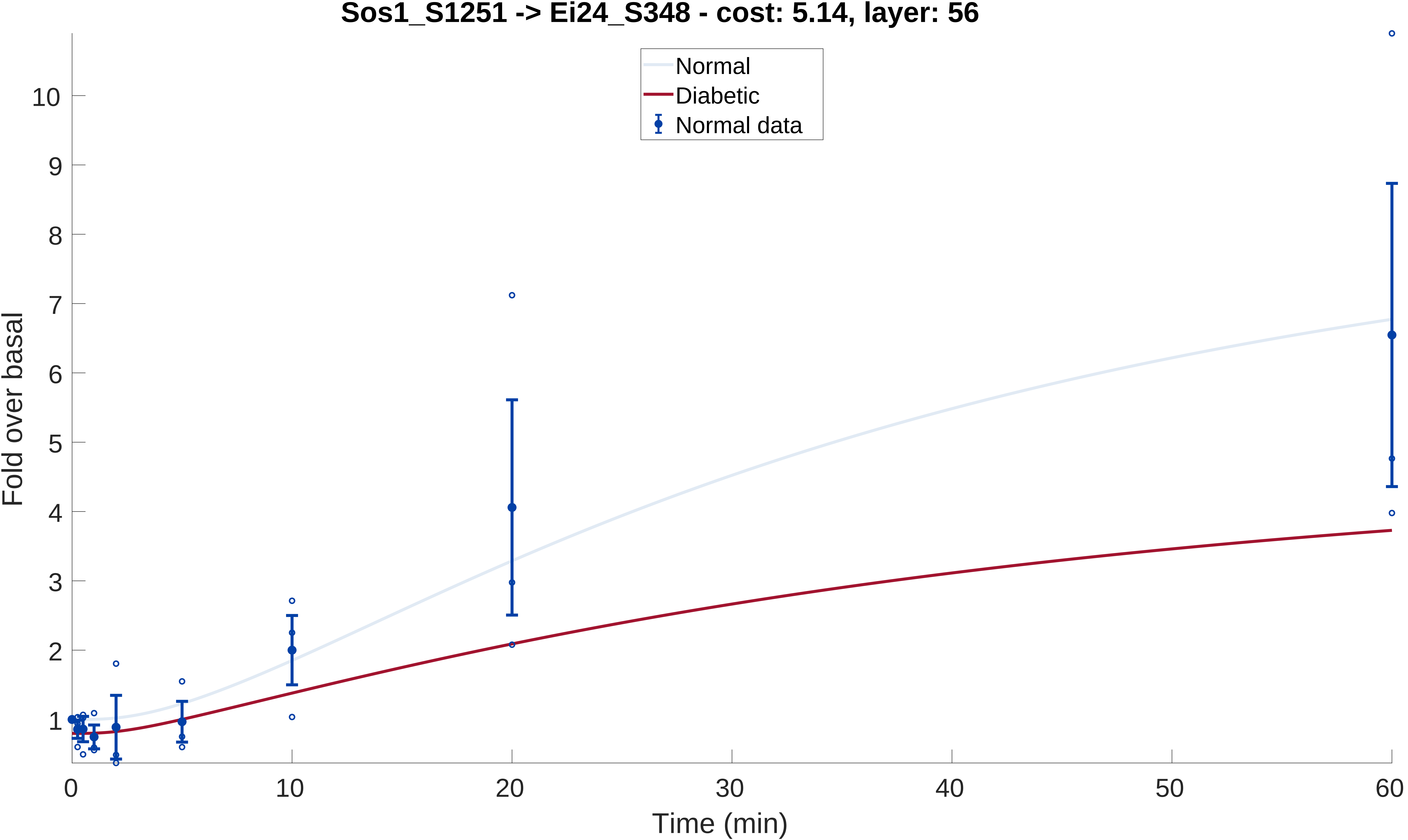
Sos1_S1251 -> Ei24_S348 - cost: 5.14, layer: 56
Normal
Diabetic
Normal data
Fold over basal
Time (min)
0
10
20
30
40
50
60
1
2
3
4
5
6
7
8
9
10

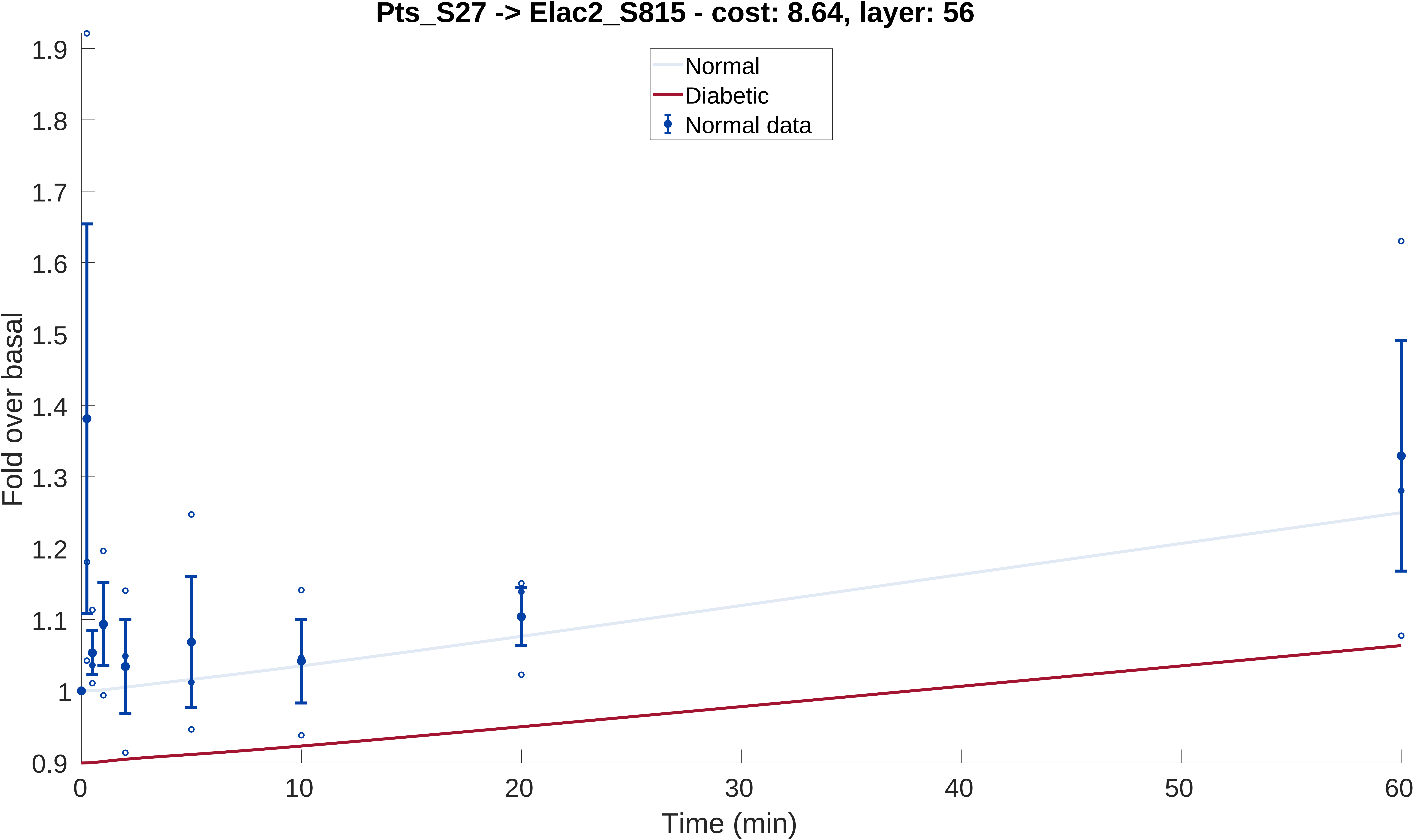

Pts_S27 -> Elac2_S815 - cost: 8.64, layer: 56
Normal
Diabetic
Normal data
Fold over basal
Time (min)
0.9
1
1.1
1.2
1.3
1.4
1.5
1.6
1.7
1.8
1.9
0
10
20
30
40
50
60

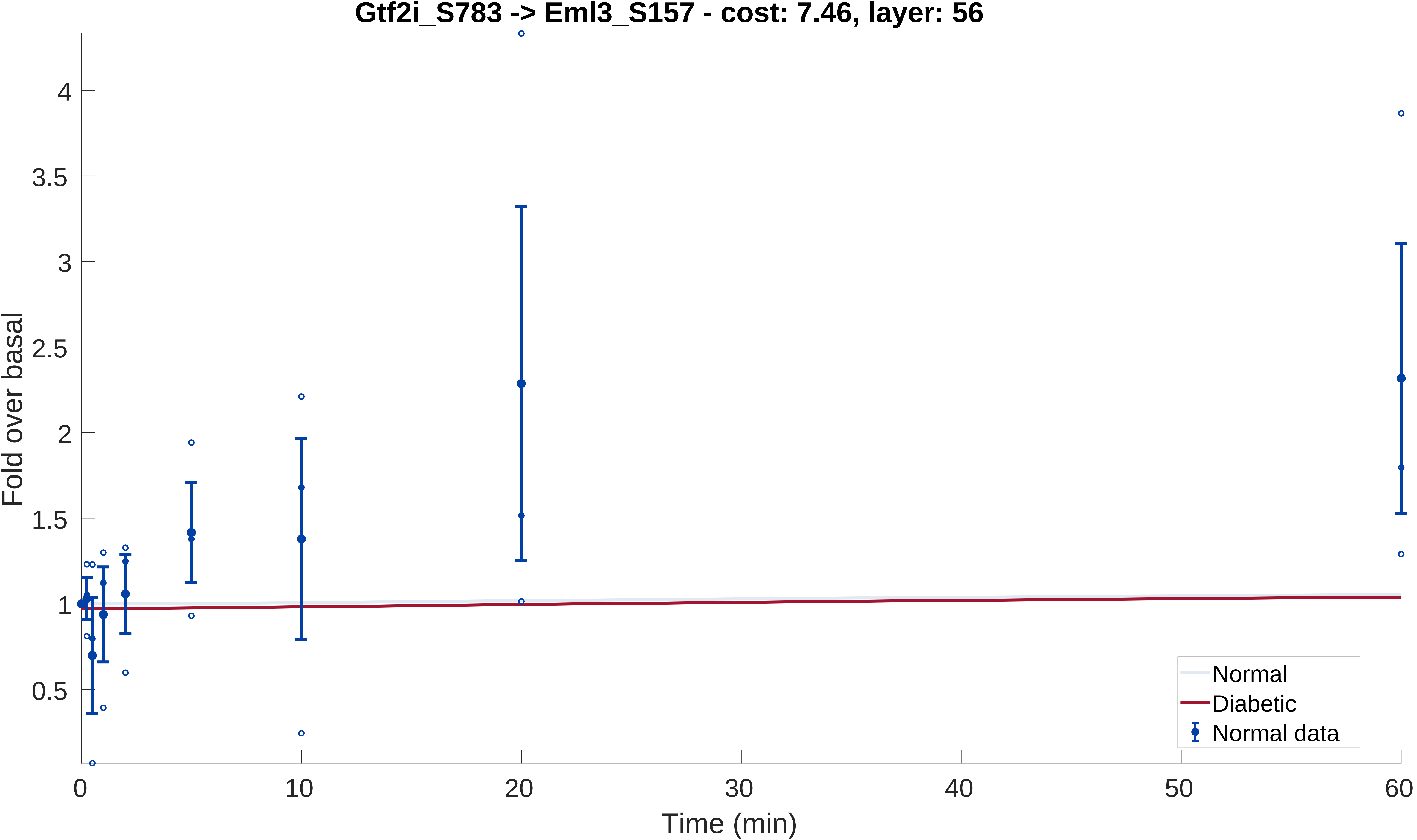

Gtf2i_S783 -> Eml3_S157 - cost: 7.46, layer: 56
Fold over basal
Time (min)
0.5
1
1.5
2
2.5
3
3.5
4
0
10
20
30
40
50
60
Normal
Diabetic
Normal data

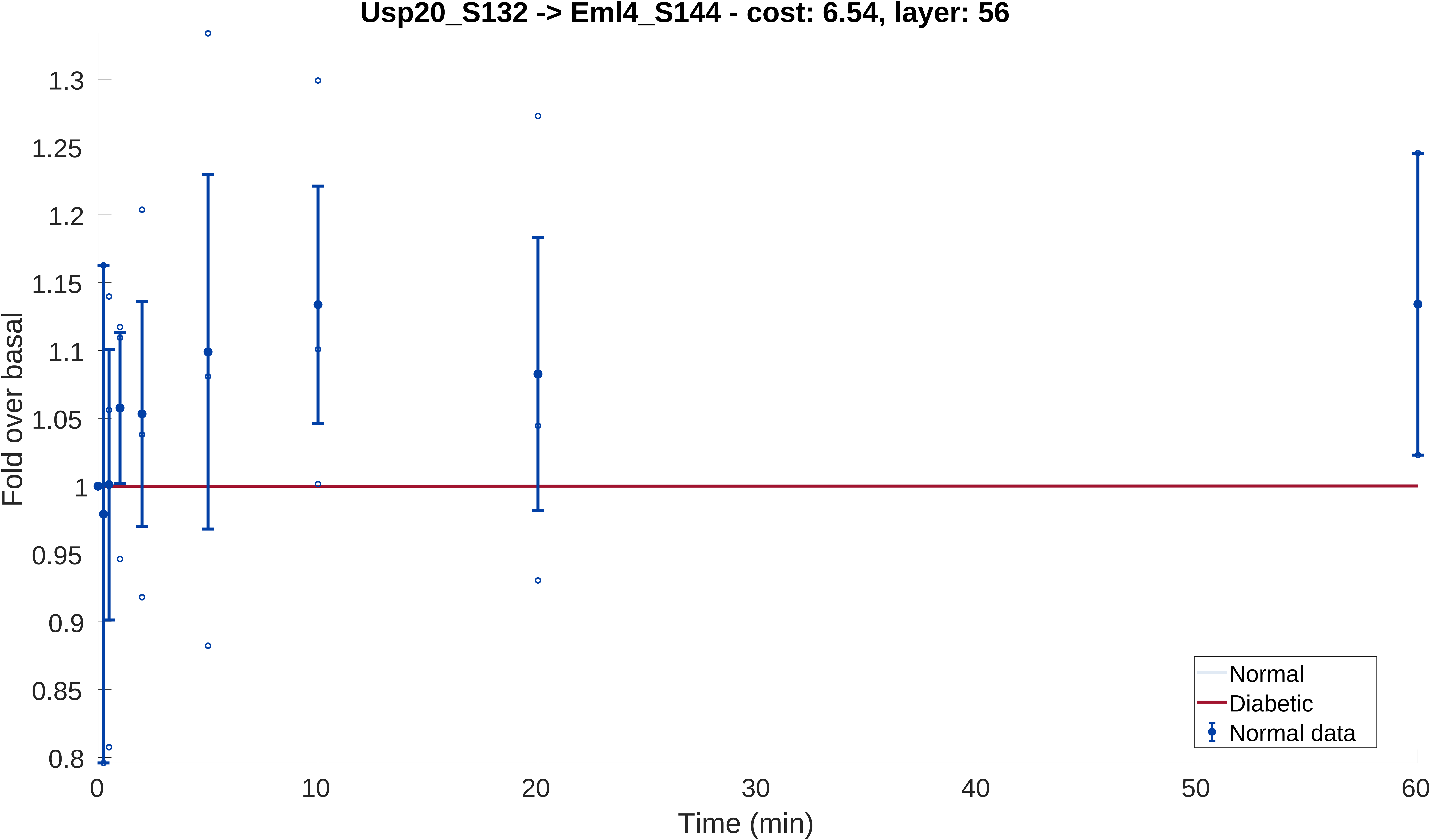

Usp20_S132 -> Eml4_S144 - cost: 6.54, layer: 56
Fold over basal
1.3
1.25
1.2
1.15
1.1
1.05
1
0.95
0.9
0.85
0.8
0
10
20
30
40
50
60
Time (min)
Normal
Diabetic
Normal data

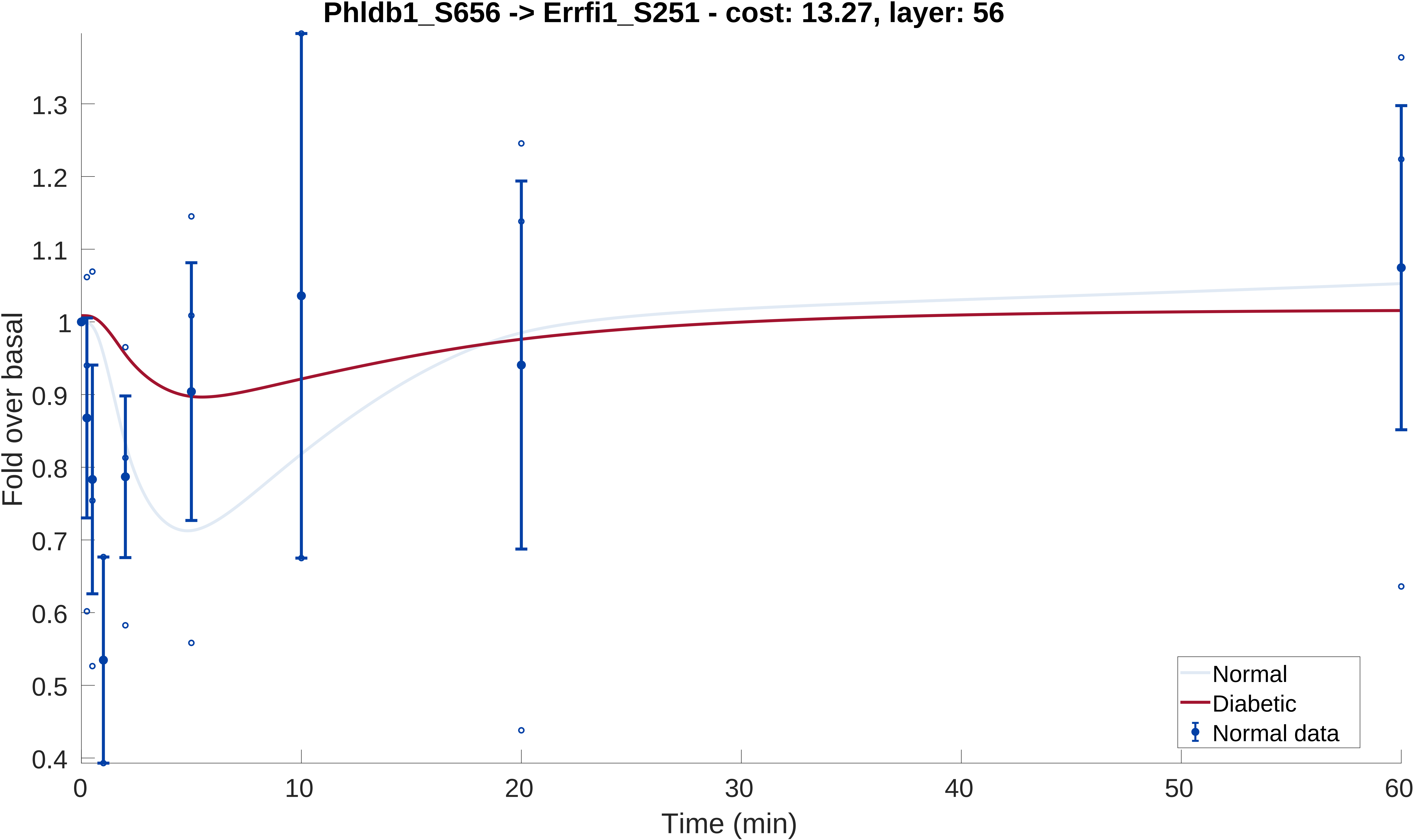

Phldb1_S656 -> Errfi1_S251 - cost: 13.27, layer: 56
Fold over basal
1.3
1.2
1.1
1
0.9
0.8
0.7
0.6
0.5
0.4
0
10
20
30
40
50
60
Time (min)
Normal
Diabetic
Normal data

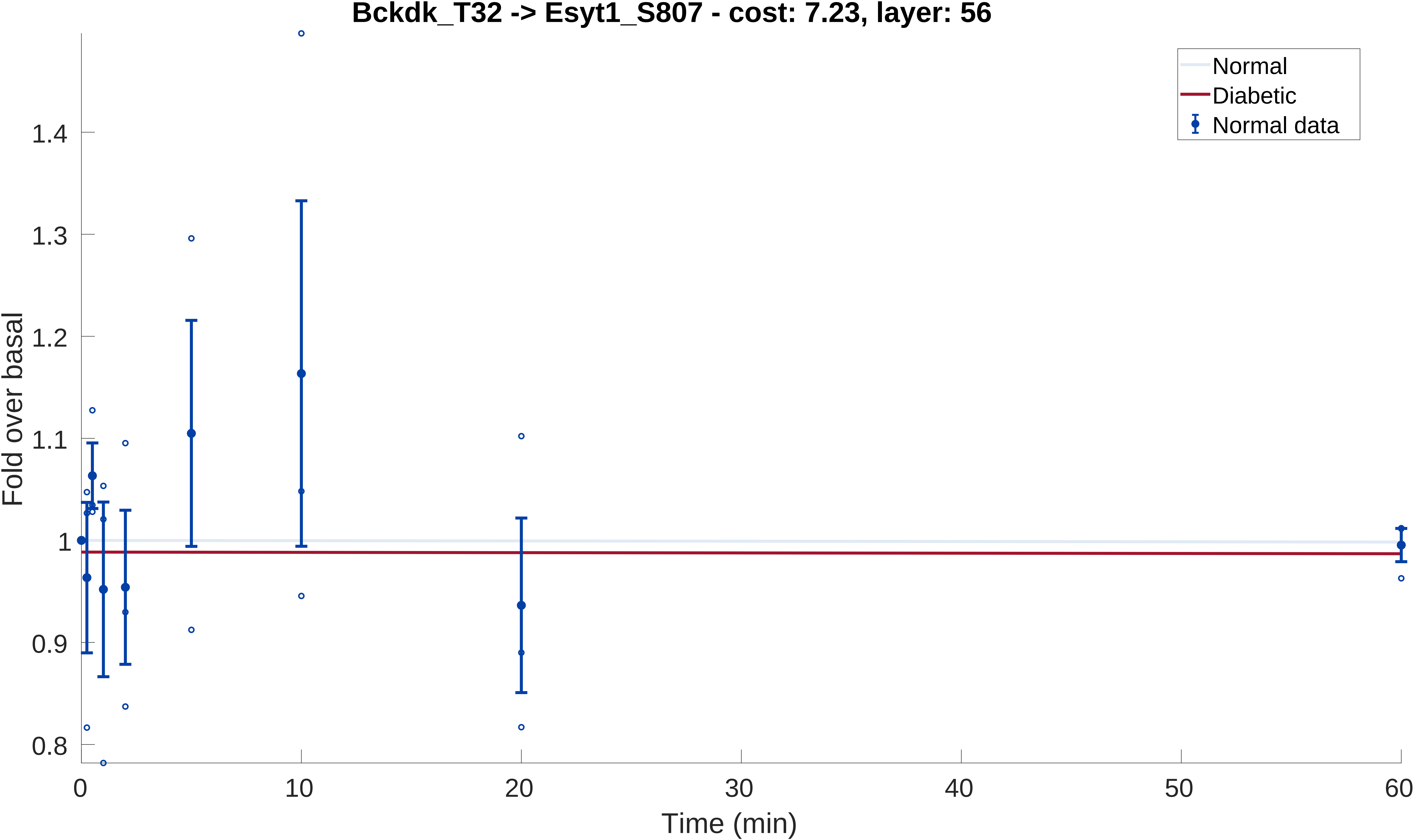

Bckdk_T32 -> Esyt1_S807 - cost: 7.23, layer: 56
Normal
Diabetic
Normal data
Fold over basal
1.4
1.3
1.2
1.1
1
0.9
0.8
0
10
20
30
40
50
60
Time (min)

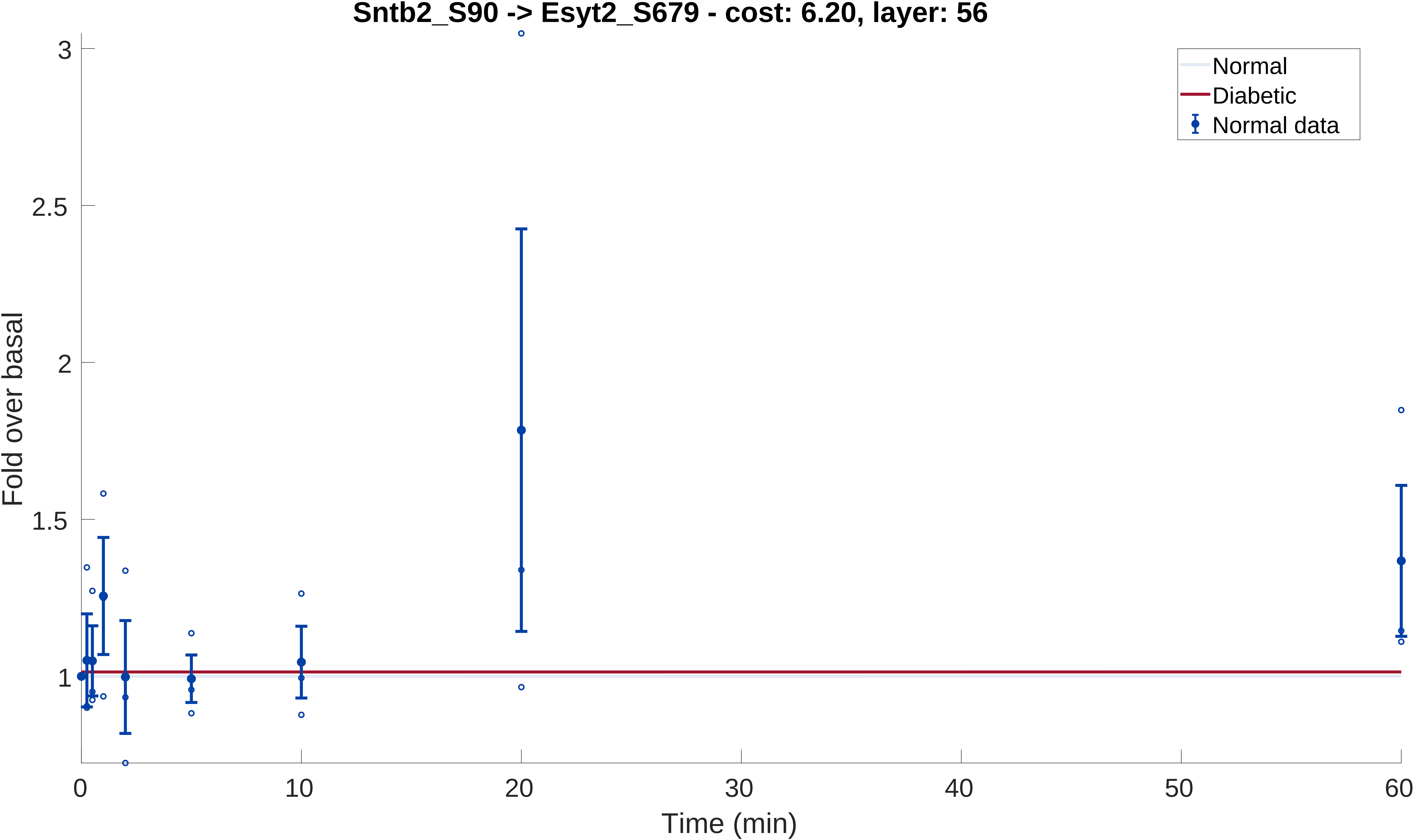

Sntb2_S90 -> Esyt2_S679 - cost: 6.20, layer: 56
Normal
Diabetic
Normal data
Fold over basal
Time (min)
3
2.5
2
1.5
1
0
10
20
30
40
50
60

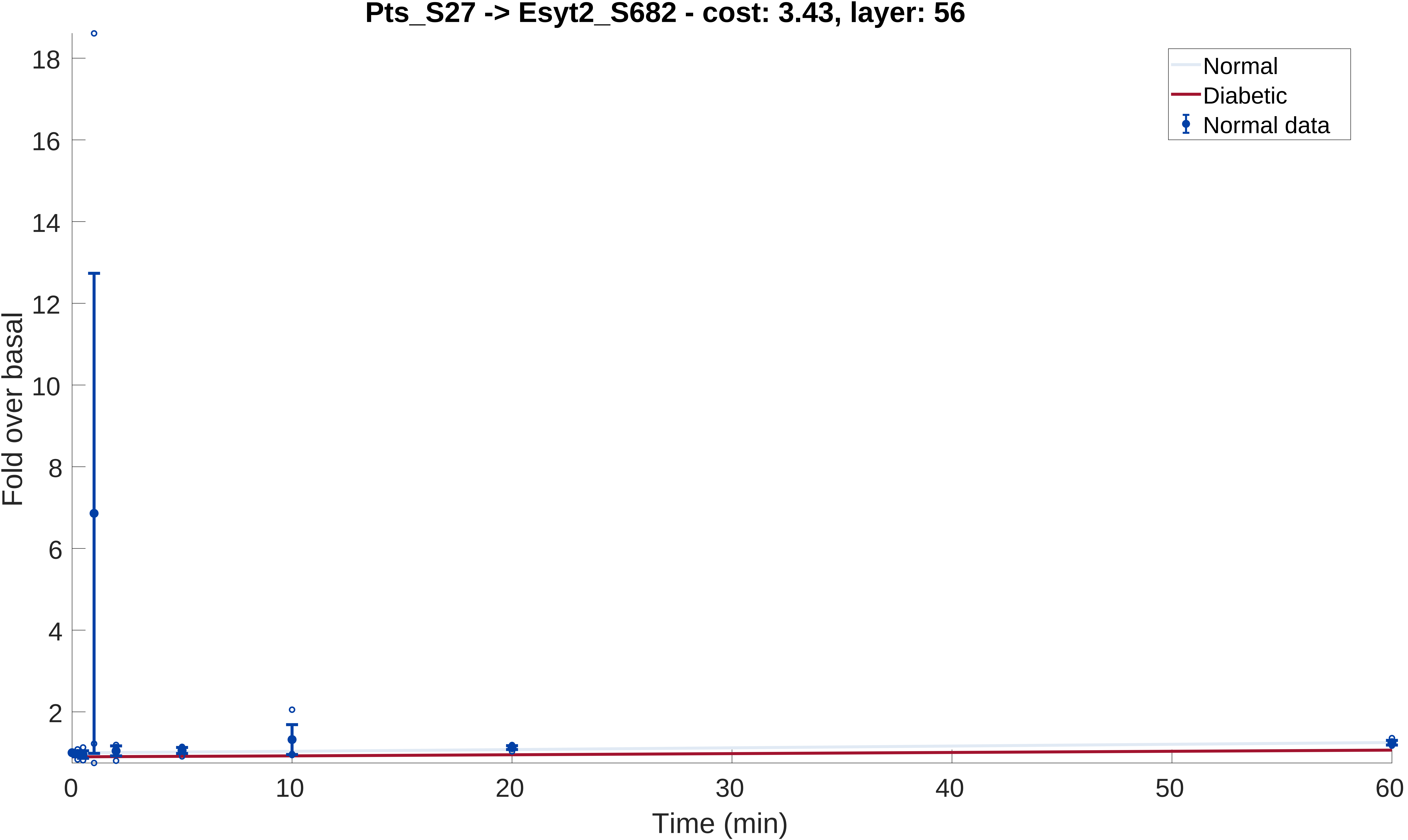

Pts_S27 -> Esyt2_S682 - cost: 3.43, layer: 56
Normal
Diabetic
Normal data
Fold over basal
Time (min)
0
10
20
30
40
50
60
2
4
6
8
10
12
14
16
18

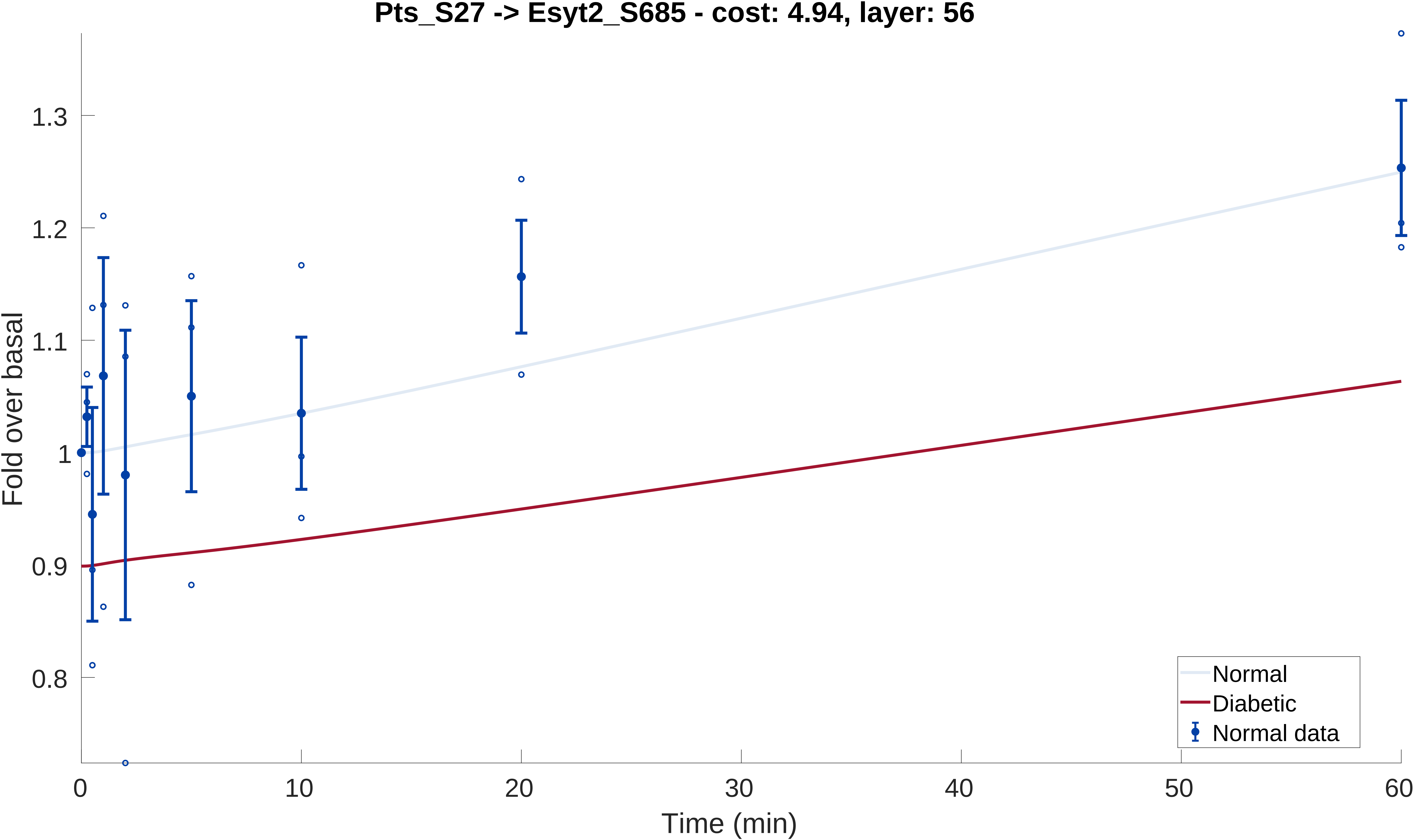

Pts_S27 -> Esyt2_S685 - cost: 4.94, layer: 56
Fold over basal
Time (min)
1.3
1.2
1.1
1
0.9
0.8
0
10
20
30
40
50
60
Normal
Diabetic
Normal data

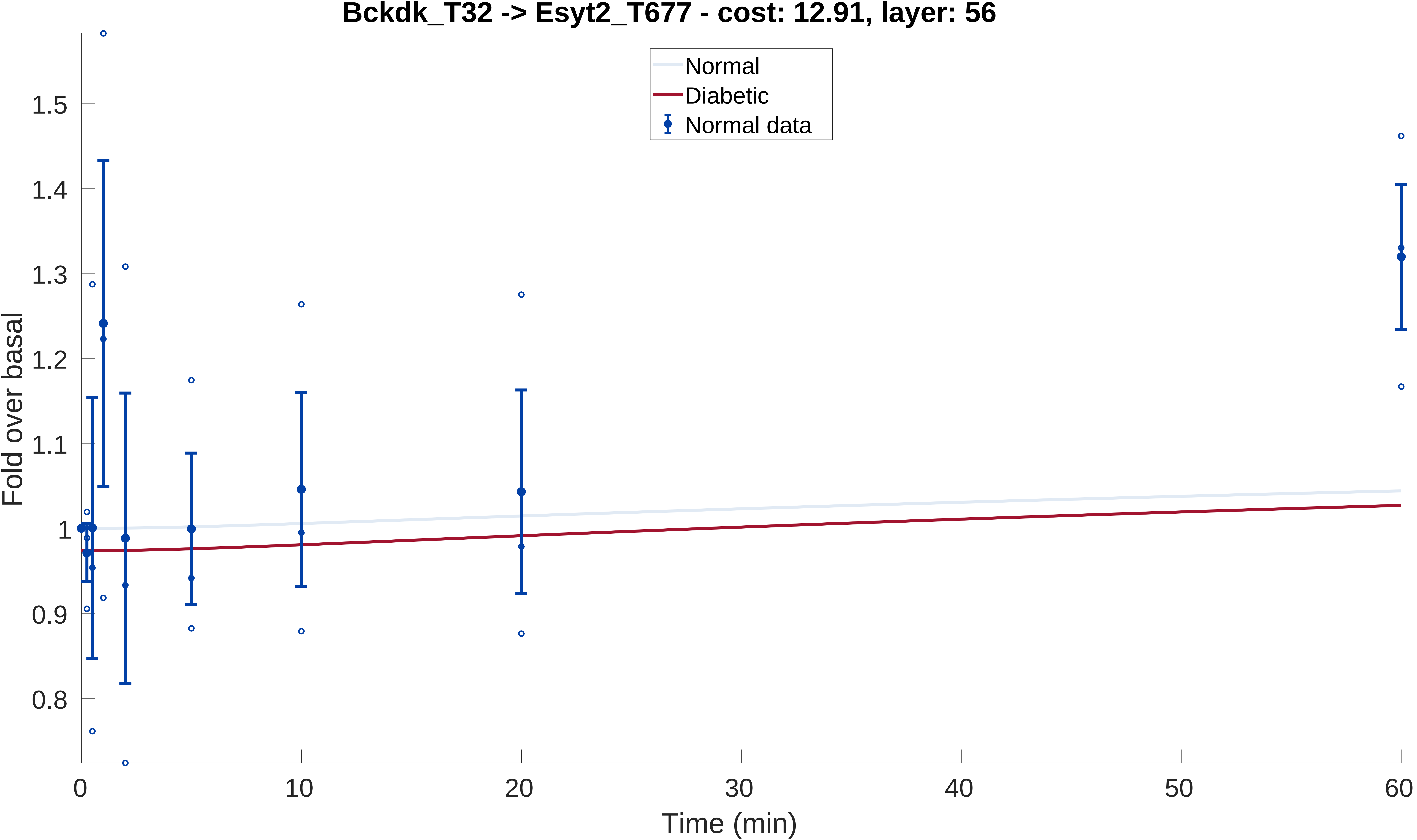

Bckdk_T32 -> Esyt2_T677 - cost: 12.91, layer: 56
Normal
Diabetic
Normal data
Fold over basal
Time (min)
1.5
1.4
1.3
1.2
1.1
1
0.9
0.8
0
10
20
30
40
50
60

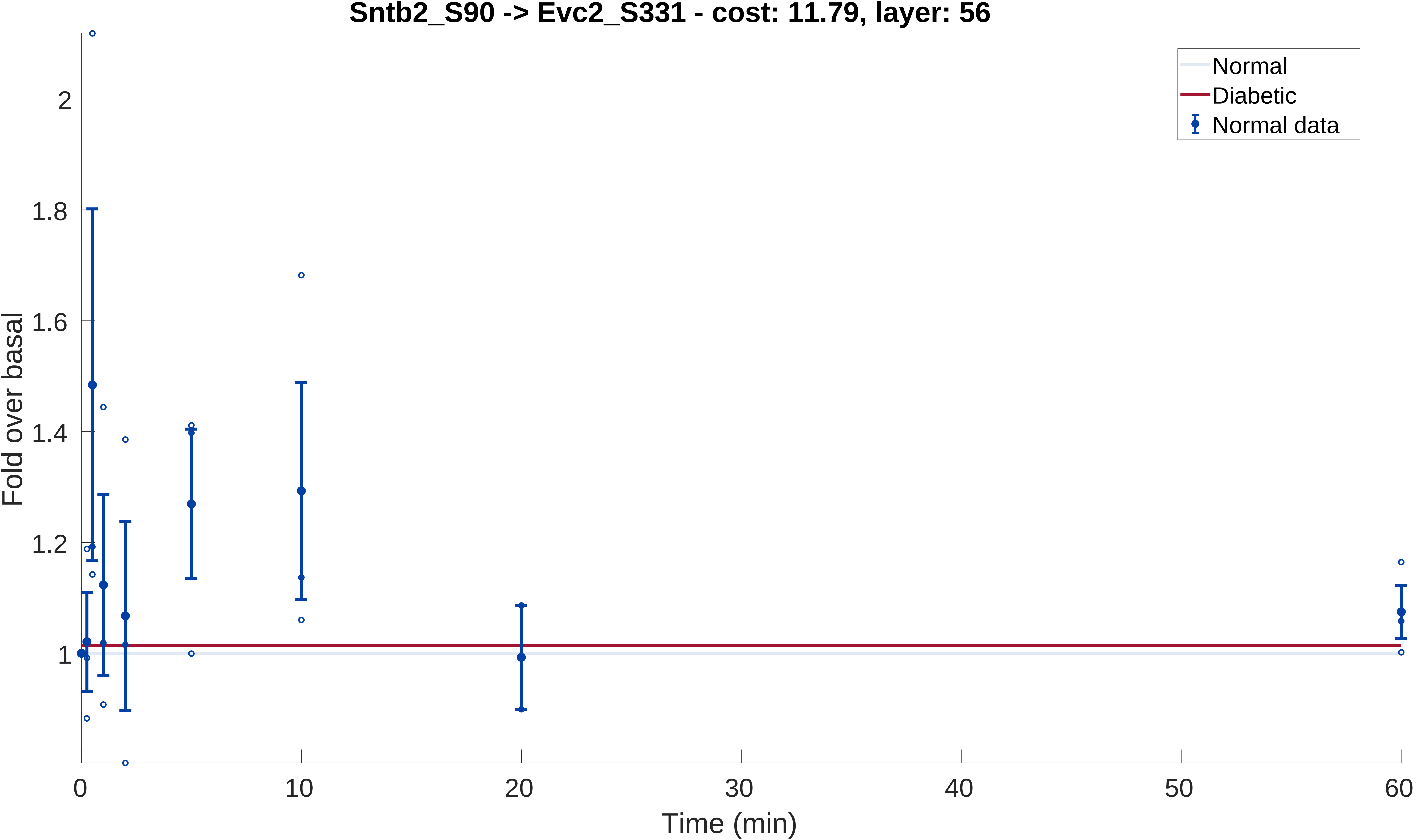

Sntb2_S90 -> Evc2_S331 - cost: 11.79, layer: 56
Normal
Diabetic
Normal data
Fold over basal
Time (min)
2
1.8
1.6
1.4
1.2
1
0
10
20
30
40
50
60

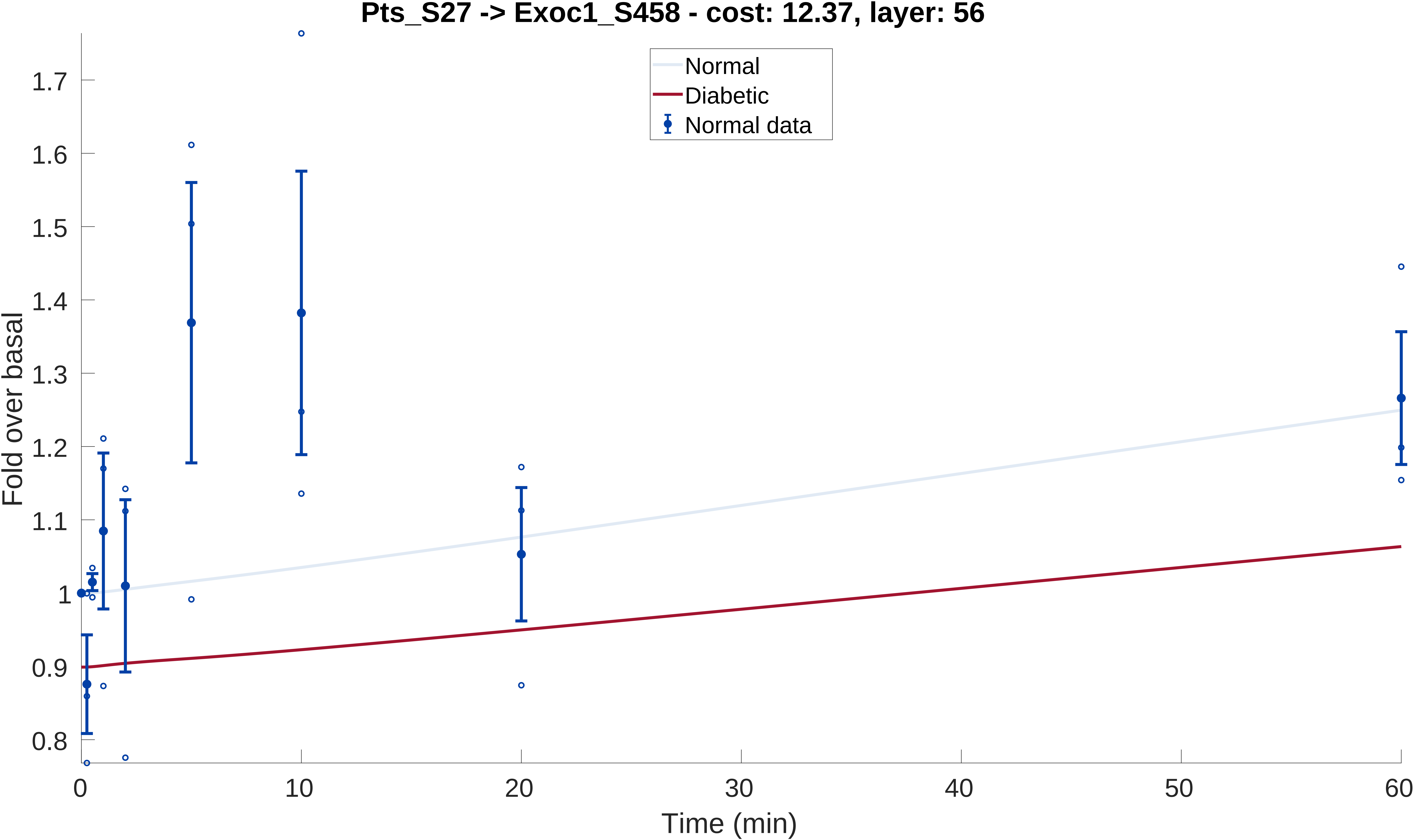

Pts_S27 -> Exoc1_S458 - cost: 12.37, layer: 56
Normal
Diabetic
Normal data
Fold over basal
1.7
1.6
1.5
1.4
1.3
1.2
1.1
1
0.9
0.8
0
10
20
30
40
50
60
Time (min)

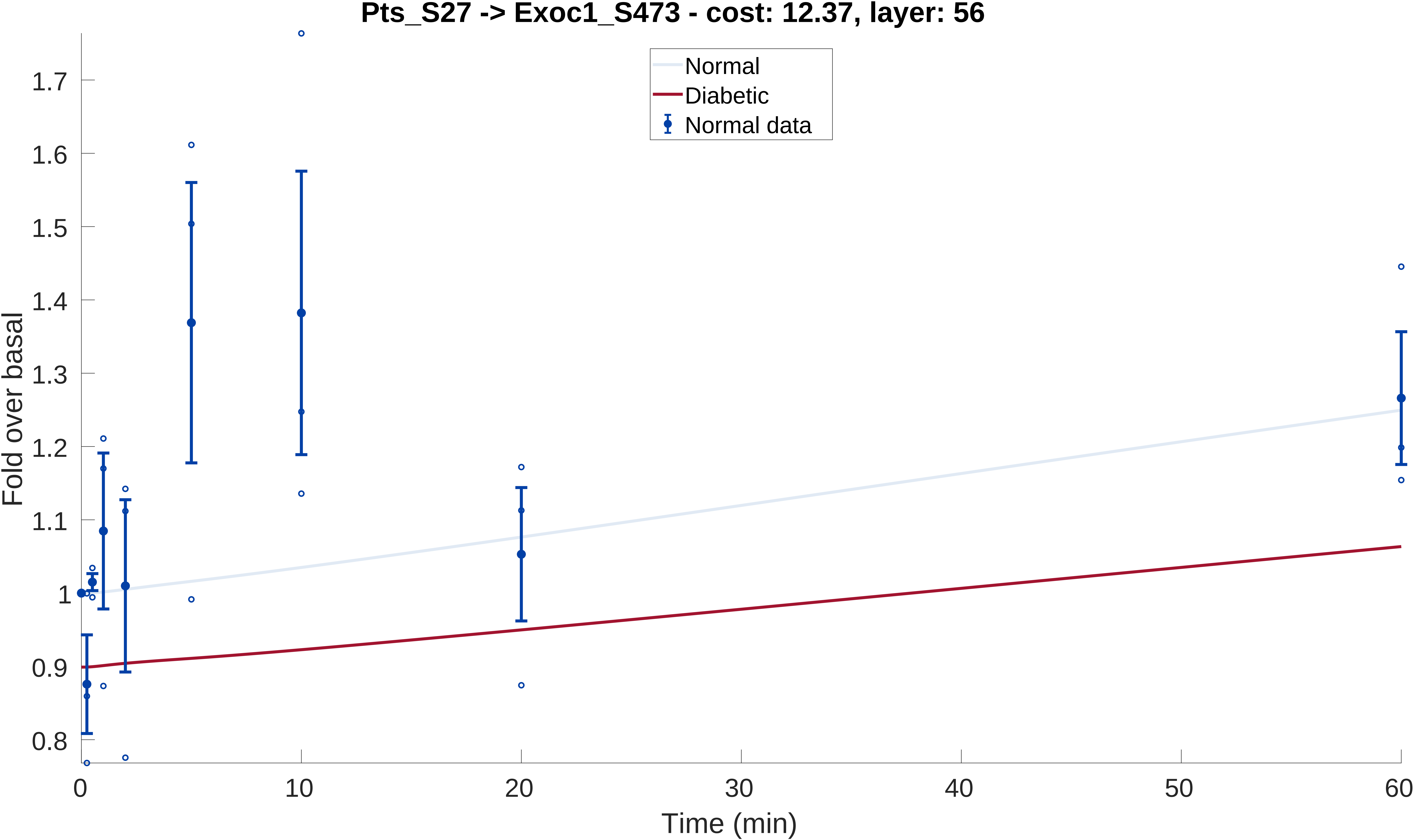

Pts_S27 -> Exoc1_S473 - cost: 12.37, layer: 56
Normal
Diabetic
Normal data
Fold over basal
1.7
1.6
1.5
1.4
1.3
1.2
1.1
1
0.9
0.8
0
10
20
30
40
50
60
Time (min)

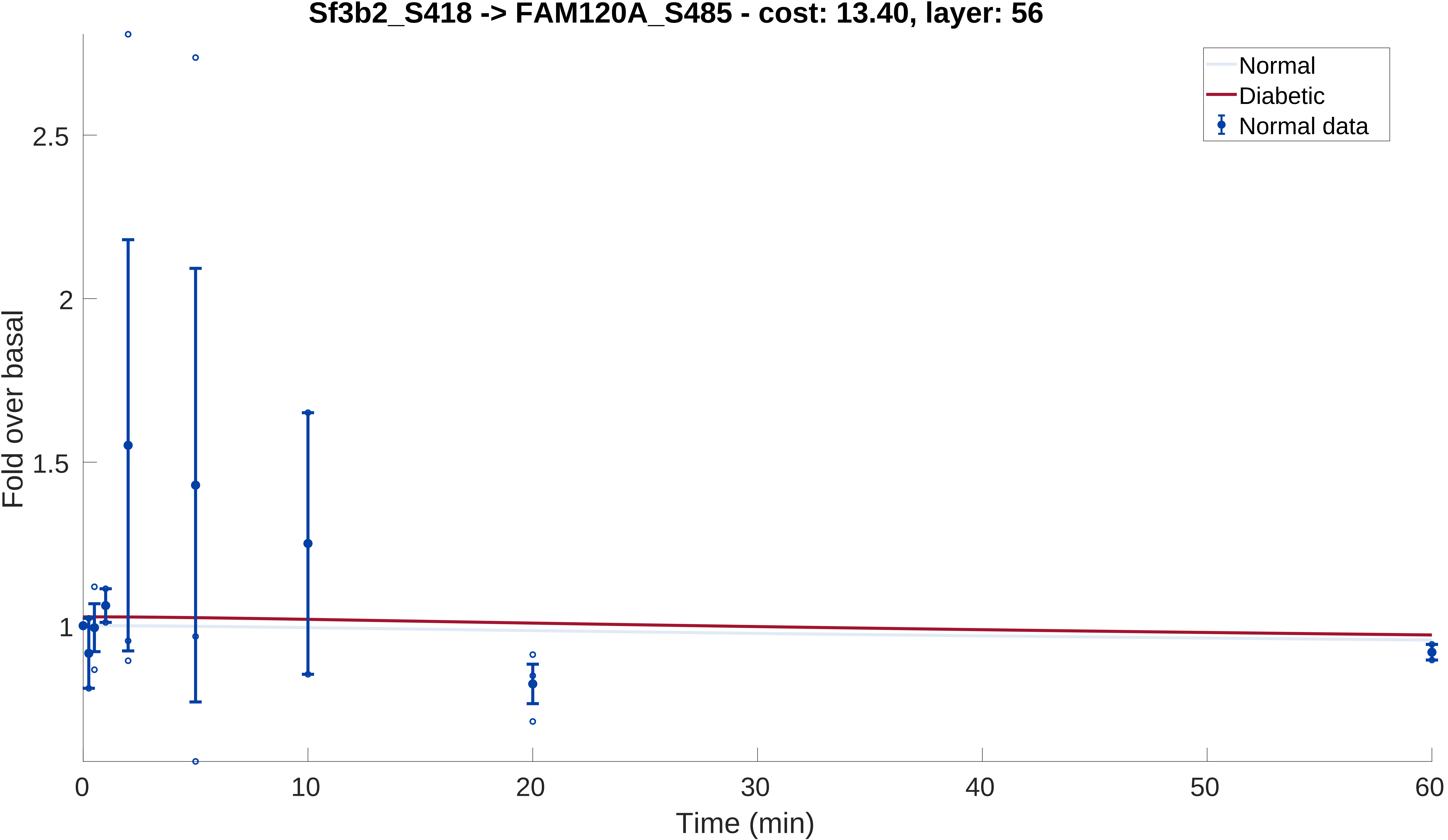

Sf3b2_S418 -> FAM120A_S485 - cost: 13.40, layer: 56
Normal
Diabetic
Normal data
Fold over basal
2.5
2
1.5
1
0
10
20
30
40
50
60
Time (min)

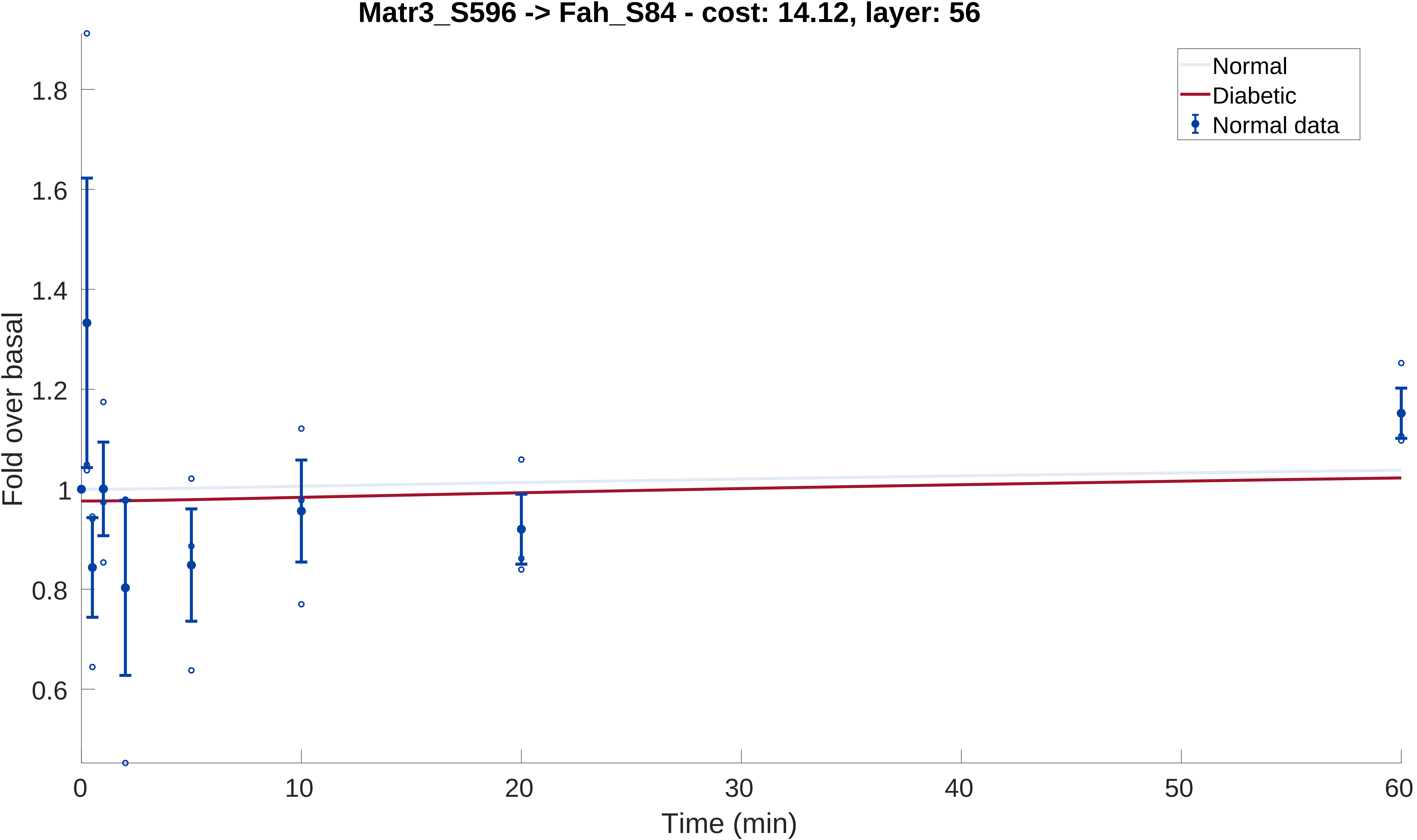

Matr3_S596 -> Fah_S84 - cost: 14.12, layer: 56
Normal
Diabetic
Normal data
1.8
1.6
1.4
1.2
1
0.8
0.6
Fold over basal
0
10
20
30
40
50
60
Time (min)

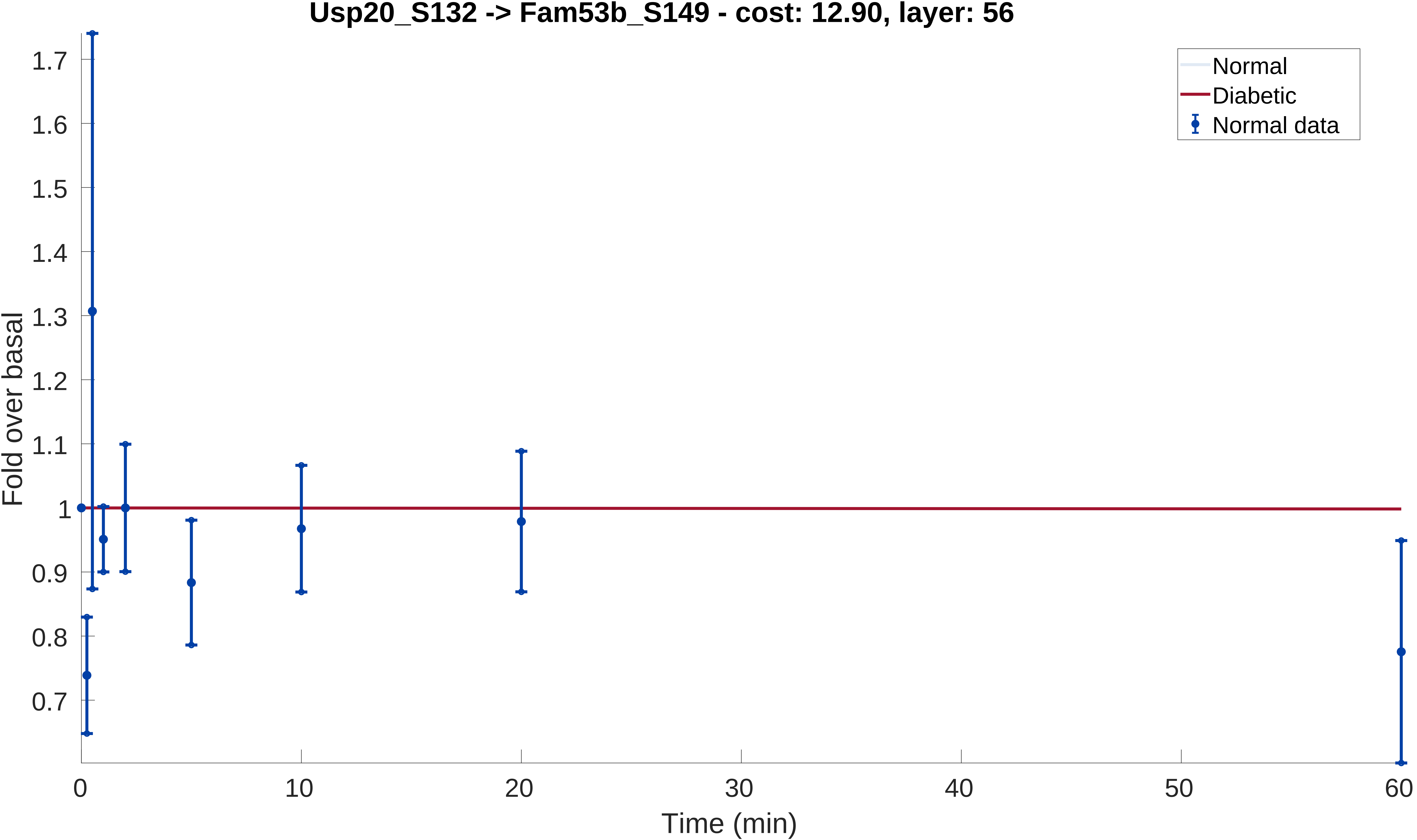

Usp20_S132 -> Fam53b_S149 - cost: 12.90, layer: 56
Normal
Diabetic
Normal data
Fold over basal
Time (min)
0.7
0.8
0.9
1
1.1
1.2
1.3
1.4
1.5
1.6
1.7
0
10
20
30
40
50
60

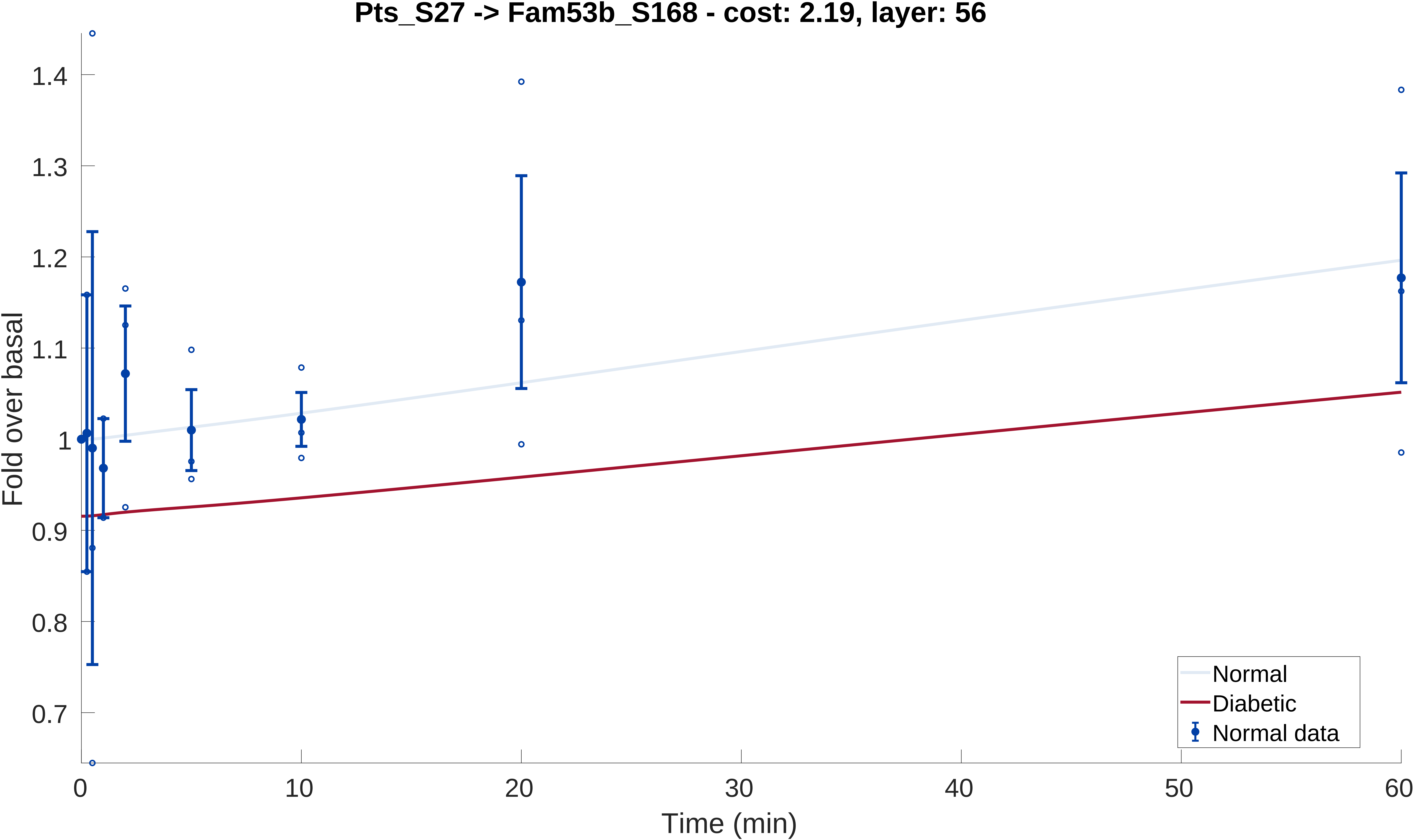

Pts_S27 -> Fam53b_S168 - cost: 2.19, layer: 56
Fold over basal
1.4
1.3
1.2
1.1
1
0.9
0.8
0.7
0
10
20
30
40
50
60
Time (min)
Normal
Diabetic
Normal data

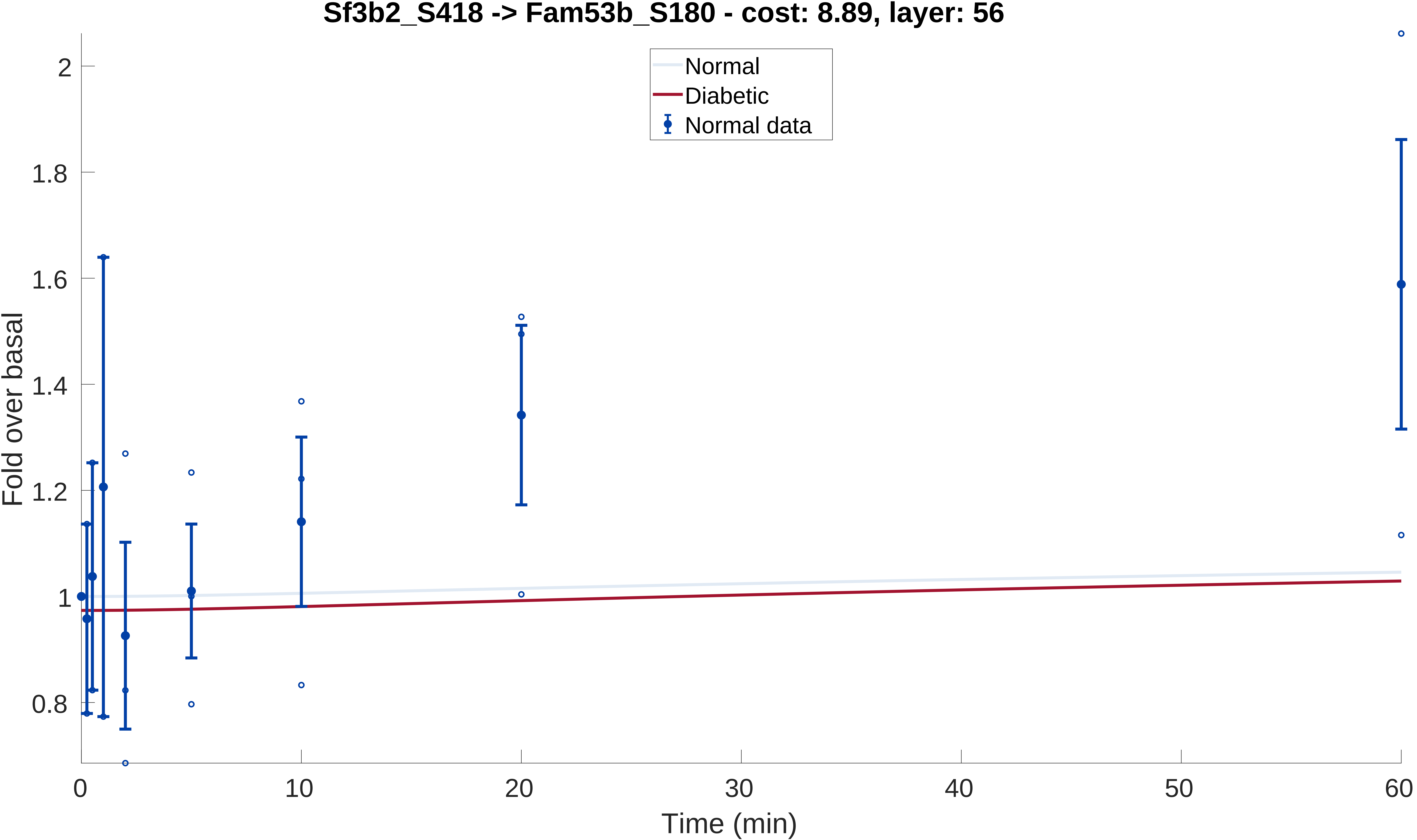

Sf3b2_S418 -> Fam53b_S180 - cost: 8.89, layer: 56
Normal
Diabetic
Normal data
Fold over basal
2
1.8
1.6
1.4
1.2
1
0.8
0
10
20
30
40
50
60
Time (min)

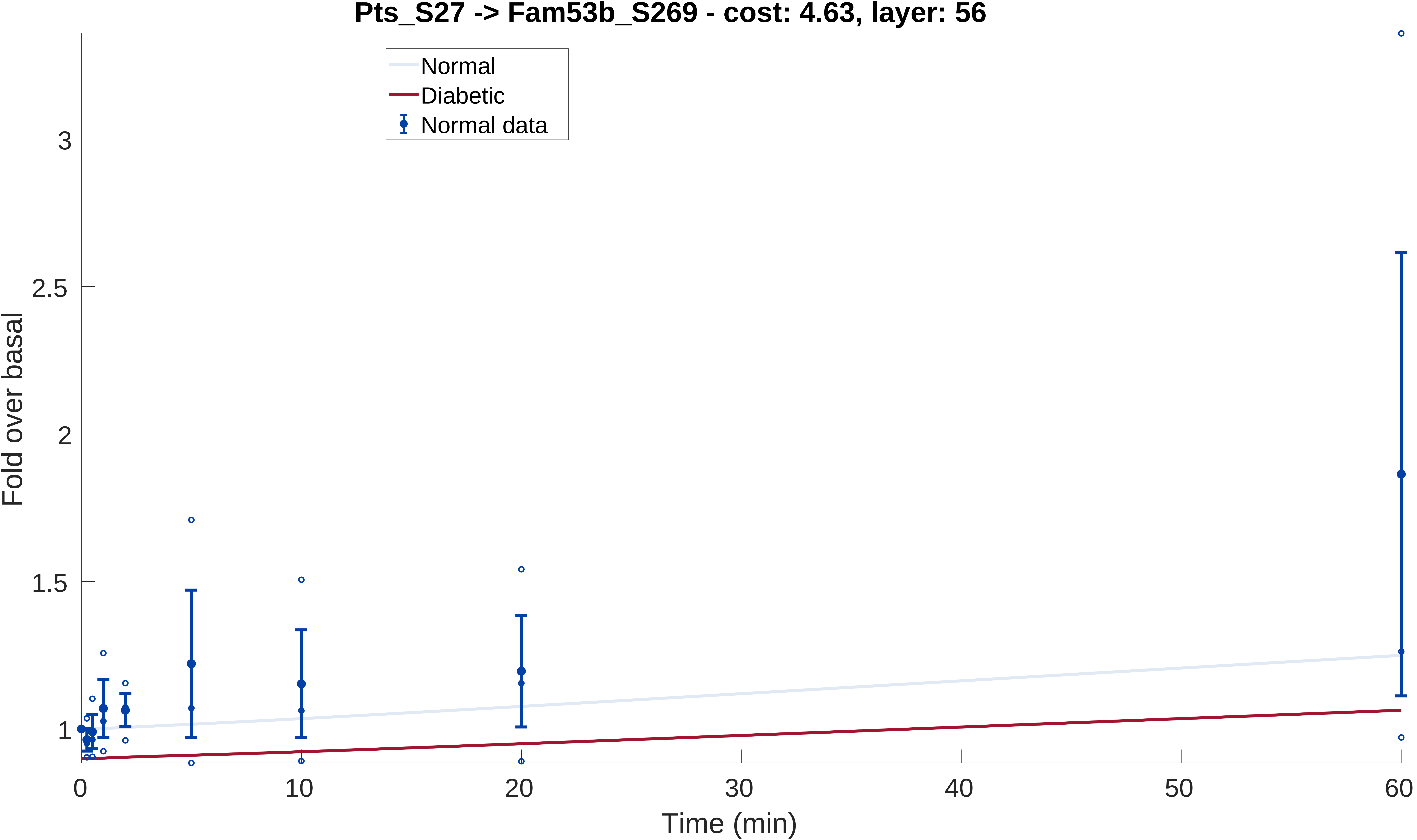

Pts_S27 -> Fam53b_S269 - cost: 4.63, layer: 56
Normal
Diabetic
Normal data
Fold over basal
3
2.5
2
1.5
1
0
10
20
30
40
50
60
Time (min)

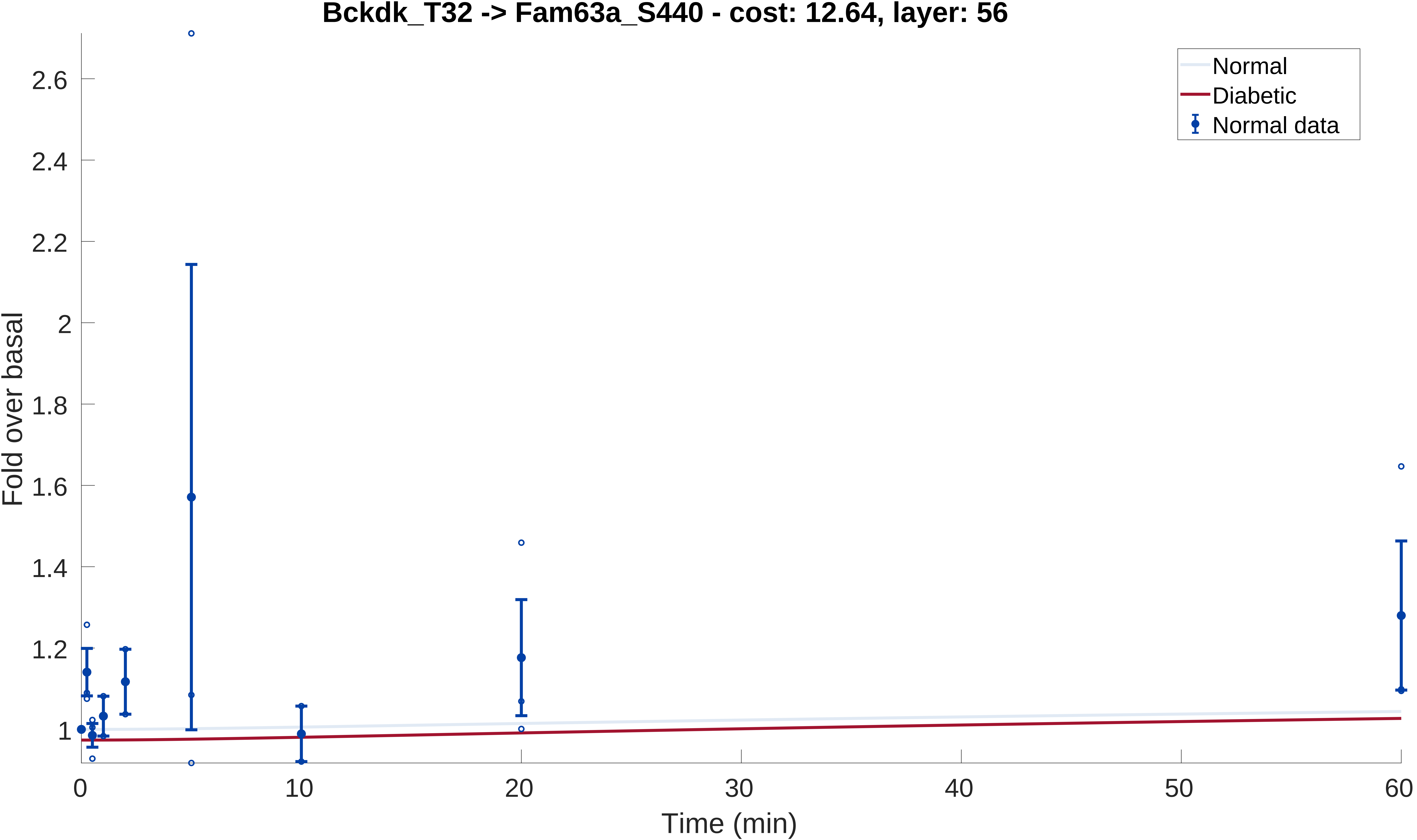

Bckdk_T32 -> Fam63a_S440 - cost: 12.64, layer: 56
Normal
Diabetic
Normal data
Fold over basal
Time (min)
2.6
2.4
2.2
2
1.8
1.6
1.4
1.2
1
0
10
20
30
40
50
60

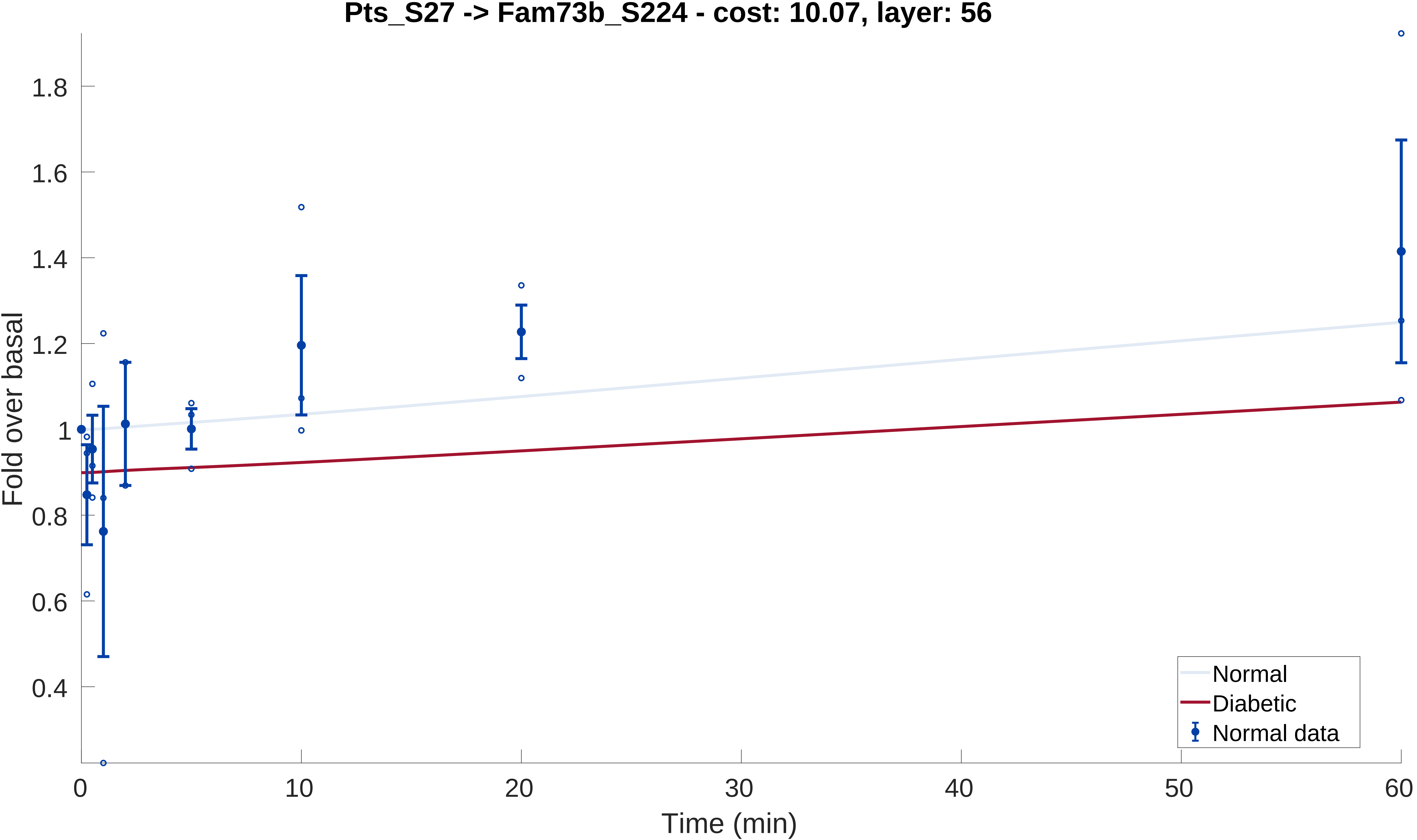

Pts_S27 -> Fam73b_S224 - cost: 10.07, layer: 56
Fold over basal
1.8
1.6
1.4
1.2
1
0.8
0.6
0.4
0
10
20
30
40
50
60
Time (min)
Normal
Diabetic
Normal data

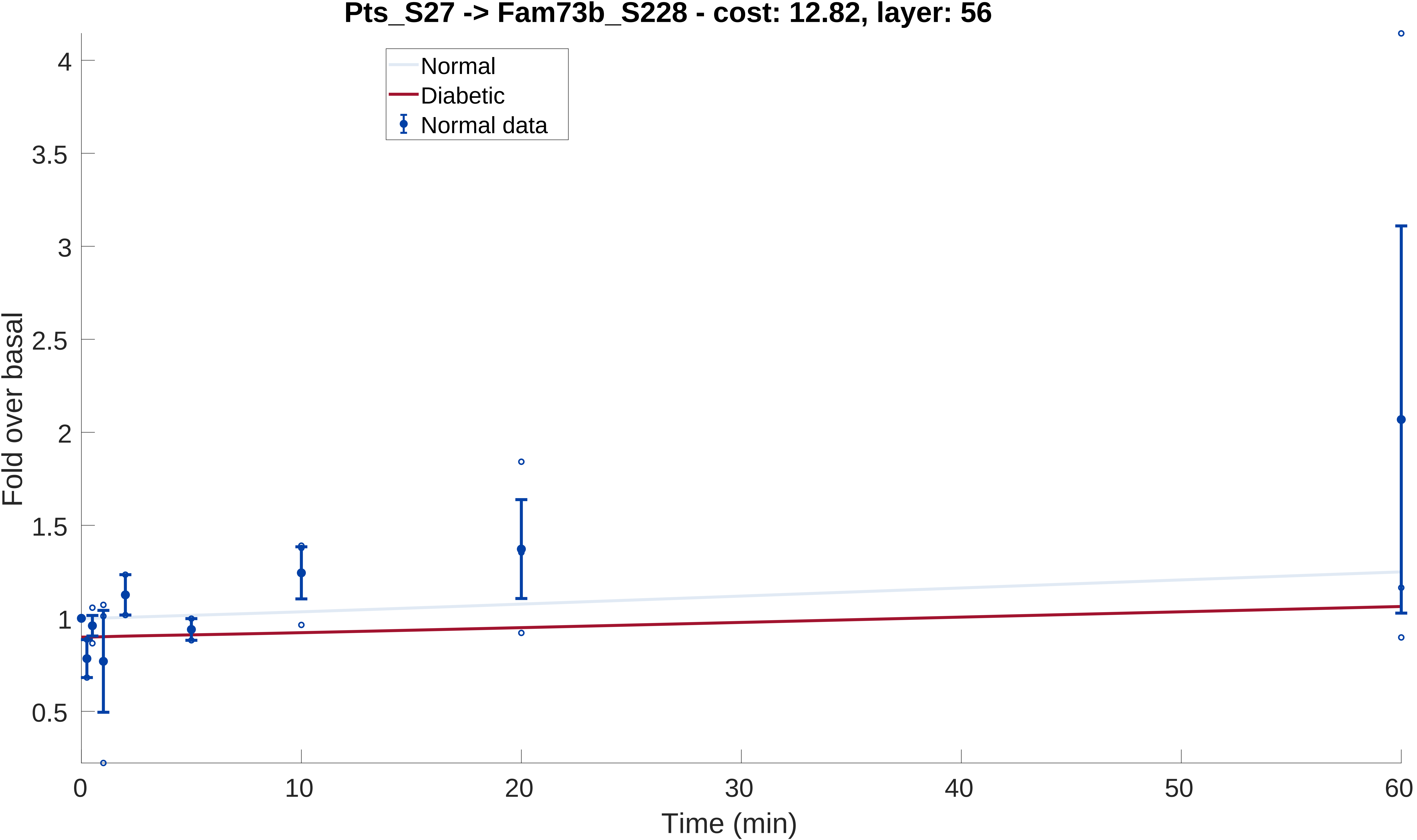
Pts_S27 -> Fam73b_S228 - cost: 12.82, layer: 56
Normal
Diabetic
Normal data
Fold over basal
Time (min)
0.5
1
1.5
2
2.5
3
3.5
4
0
10
20
30
40
50
60

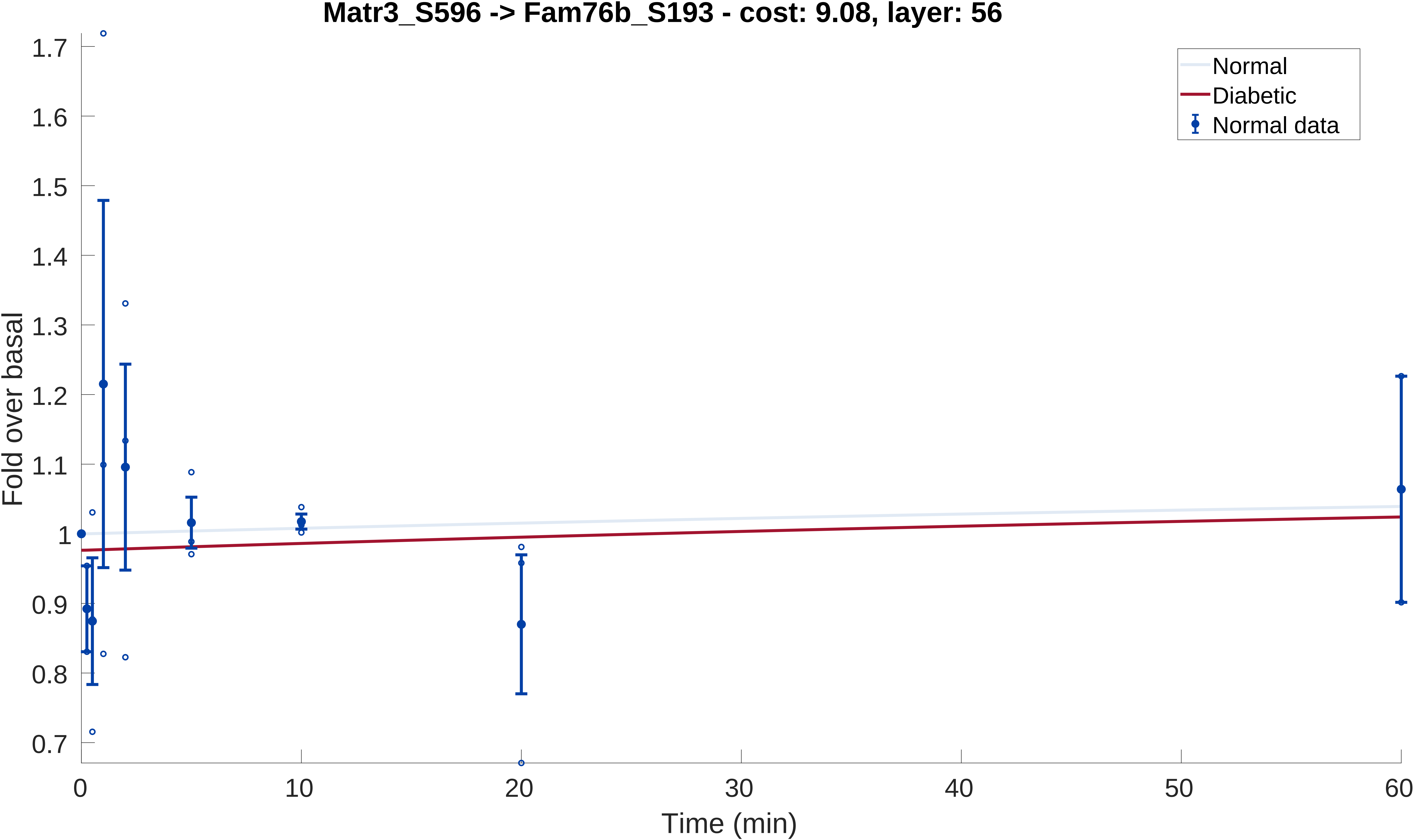

Matr3_S596 -> Fam76b_S193 - cost: 9.08, layer: 56
Normal
Diabetic
Normal data
Fold over basal
Time (min)
1.7
1.6
1.5
1.4
1.3
1.2
1.1
1
0.9
0.8
0.7
0
10
20
30
40
50
60

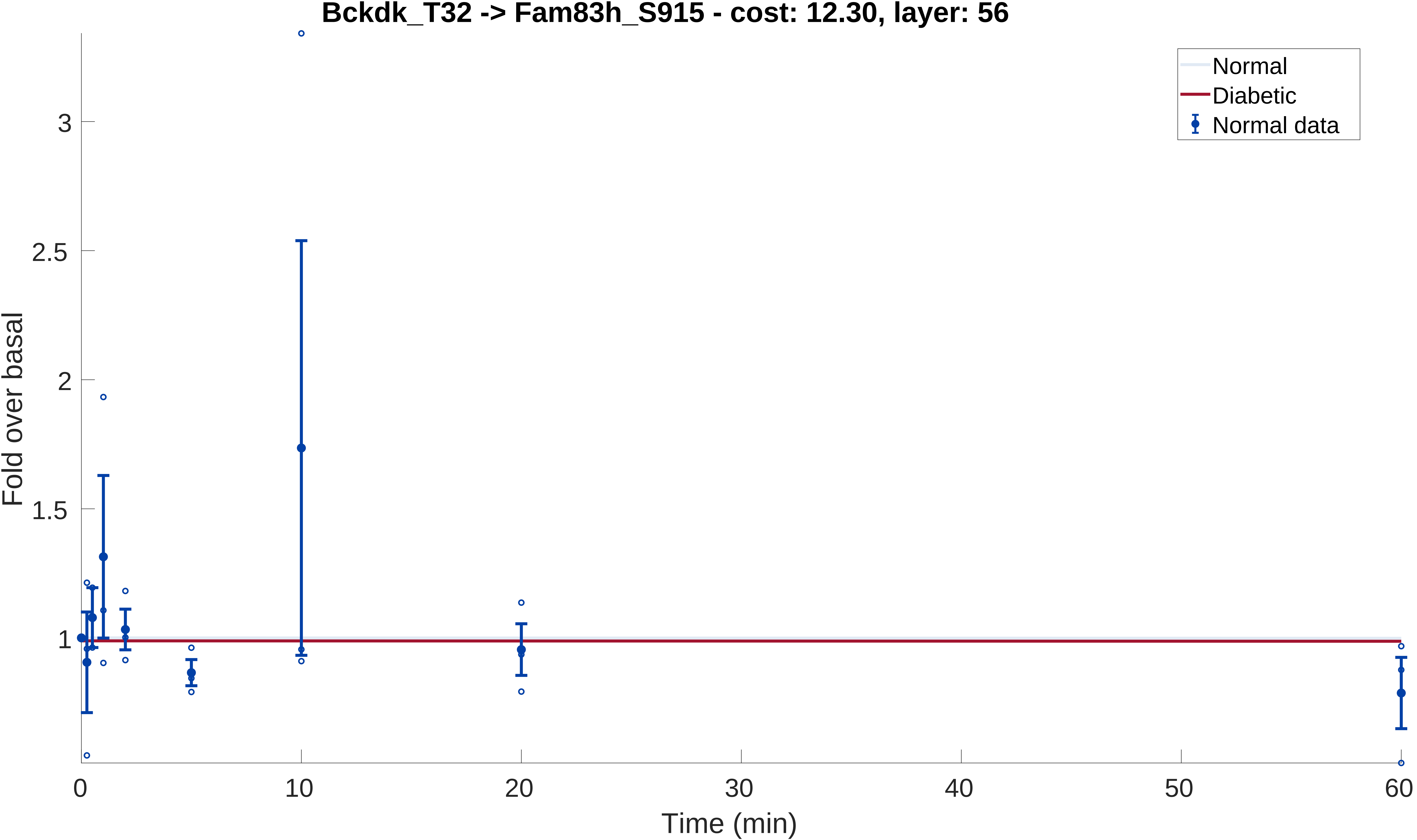

Bckdk_T32 -> Fam83h_S915 - cost: 12.30, layer: 56
Normal
Diabetic
Normal data
Fold over basal
3
2.5
2
1.5
1
0
10
20
30
40
50
60
Time (min)

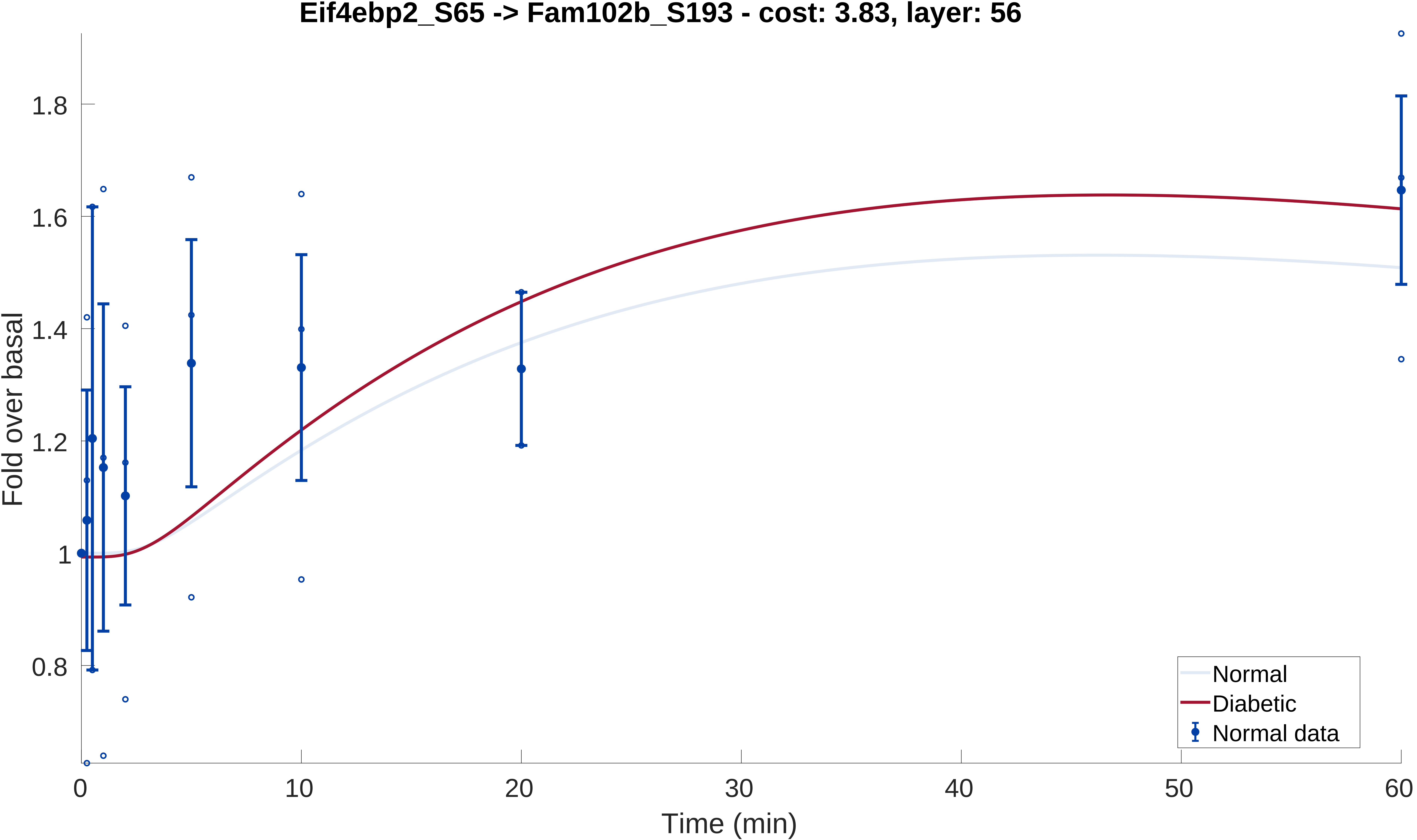

Eif4ebp2_S65 -> Fam102b_S193 - cost: 3.83, layer: 56
Fold over basal
Time (min)
1.8
1.6
1.4
1.2
1
0.8
0
10
20
30
40
50
60
Normal
Diabetic
Normal data

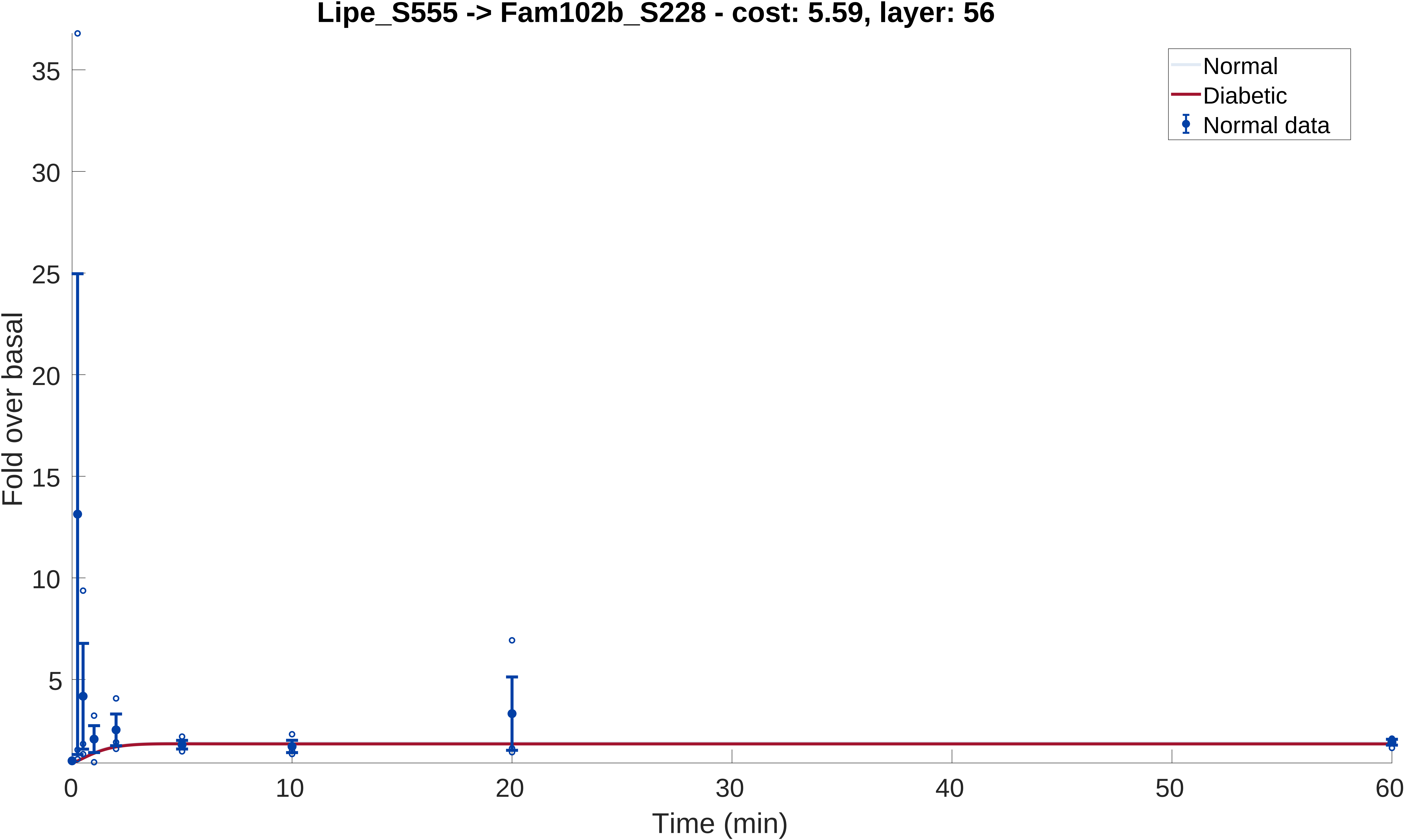

Lipe_S555 -> Fam102b_S228 - cost: 5.59, layer: 56
Normal
Diabetic
Normal data
Fold over basal
Time (min)
0
10
20
30
40
50
60
5
10
15
20
25
30
35

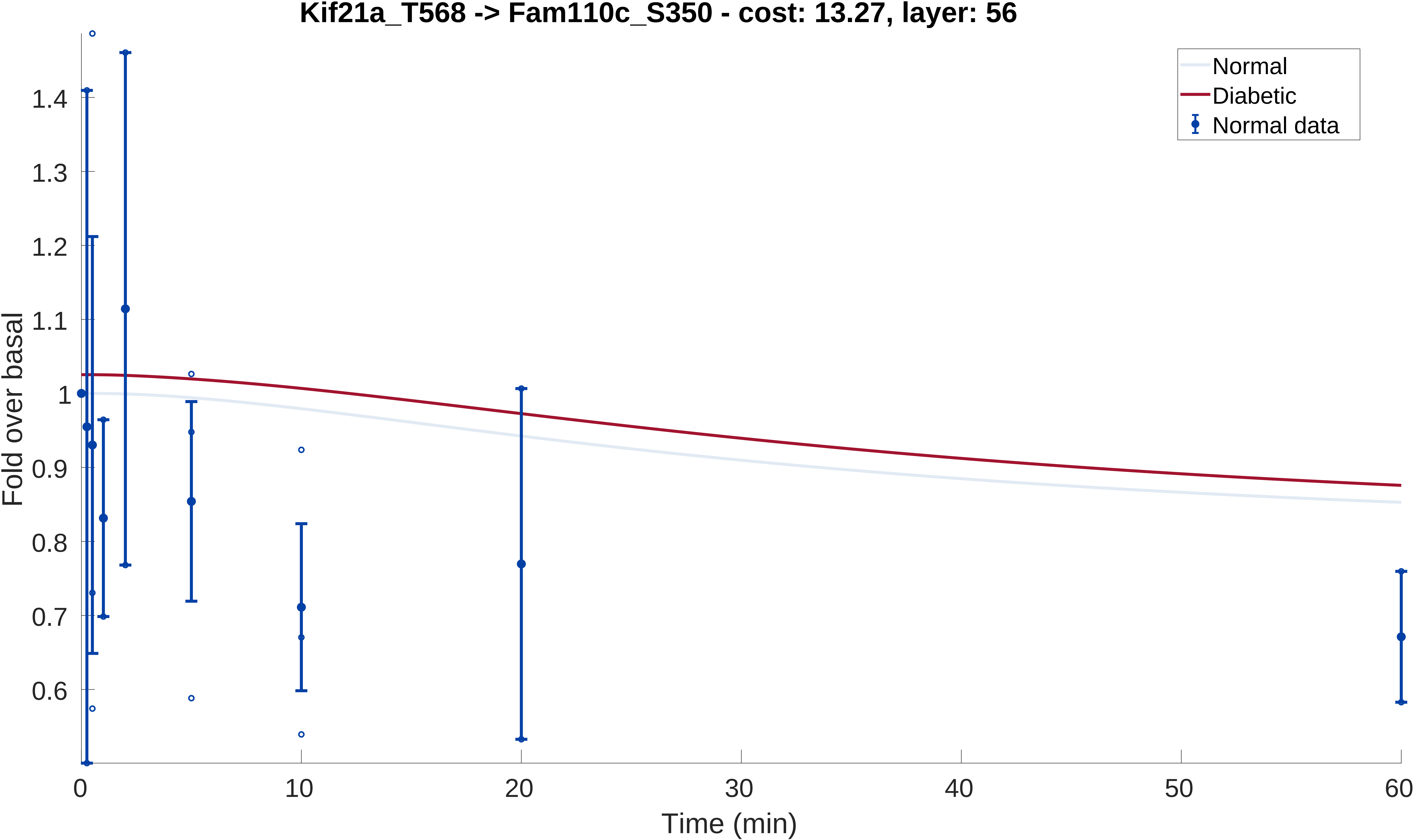

Kif21a_T568 -> Fam110c_S350 - cost: 13.27, layer: 56
Normal
Diabetic
Normal data
Fold over basal
1.4
1.3
1.2
1.1
1
0.9
0.8
0.7
0.6
0
10
20
30
40
50
60
Time (min)

Srsf2_S189 -> Fam122a_S144 - cost: 14.51, layer: 56

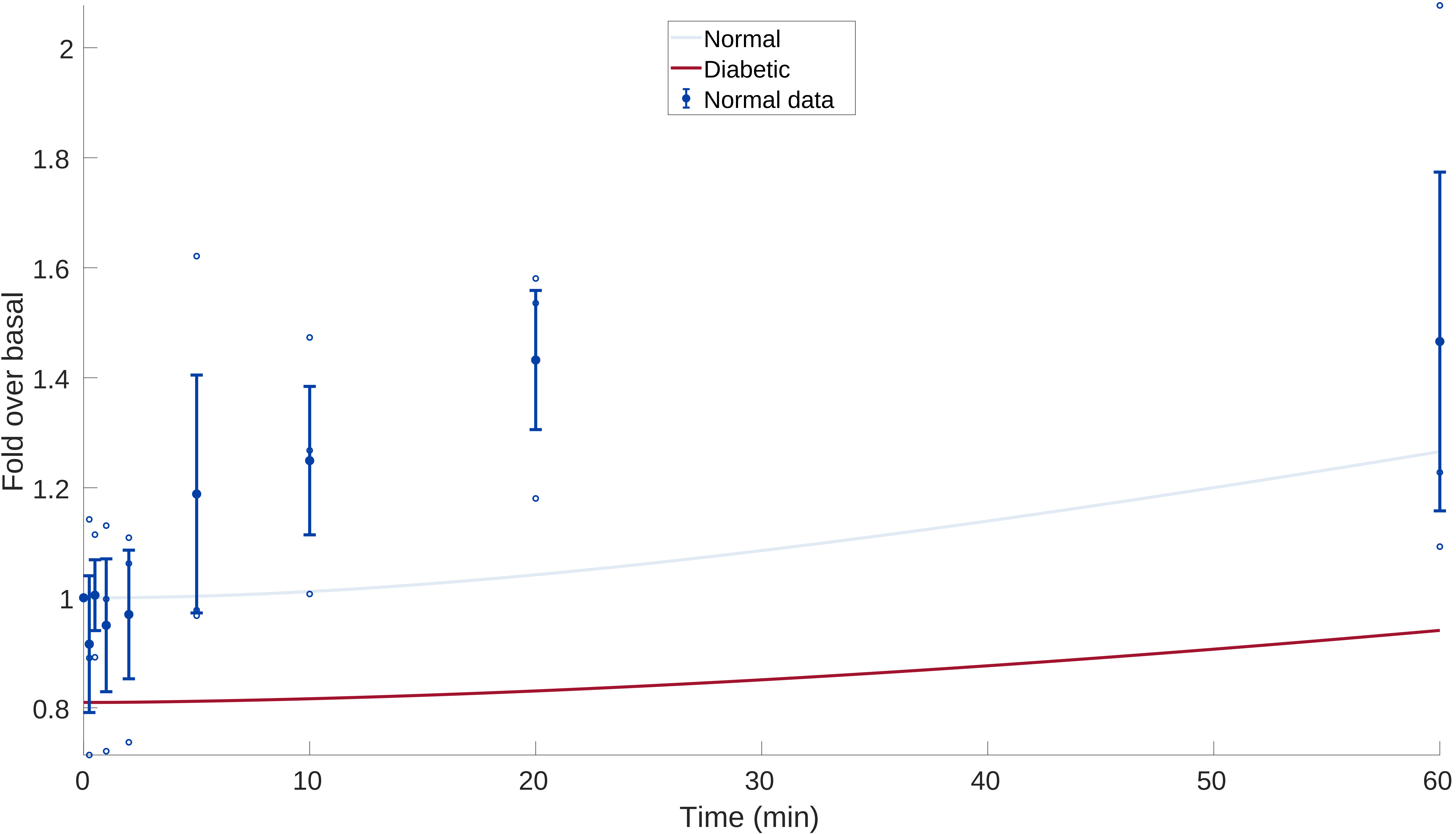

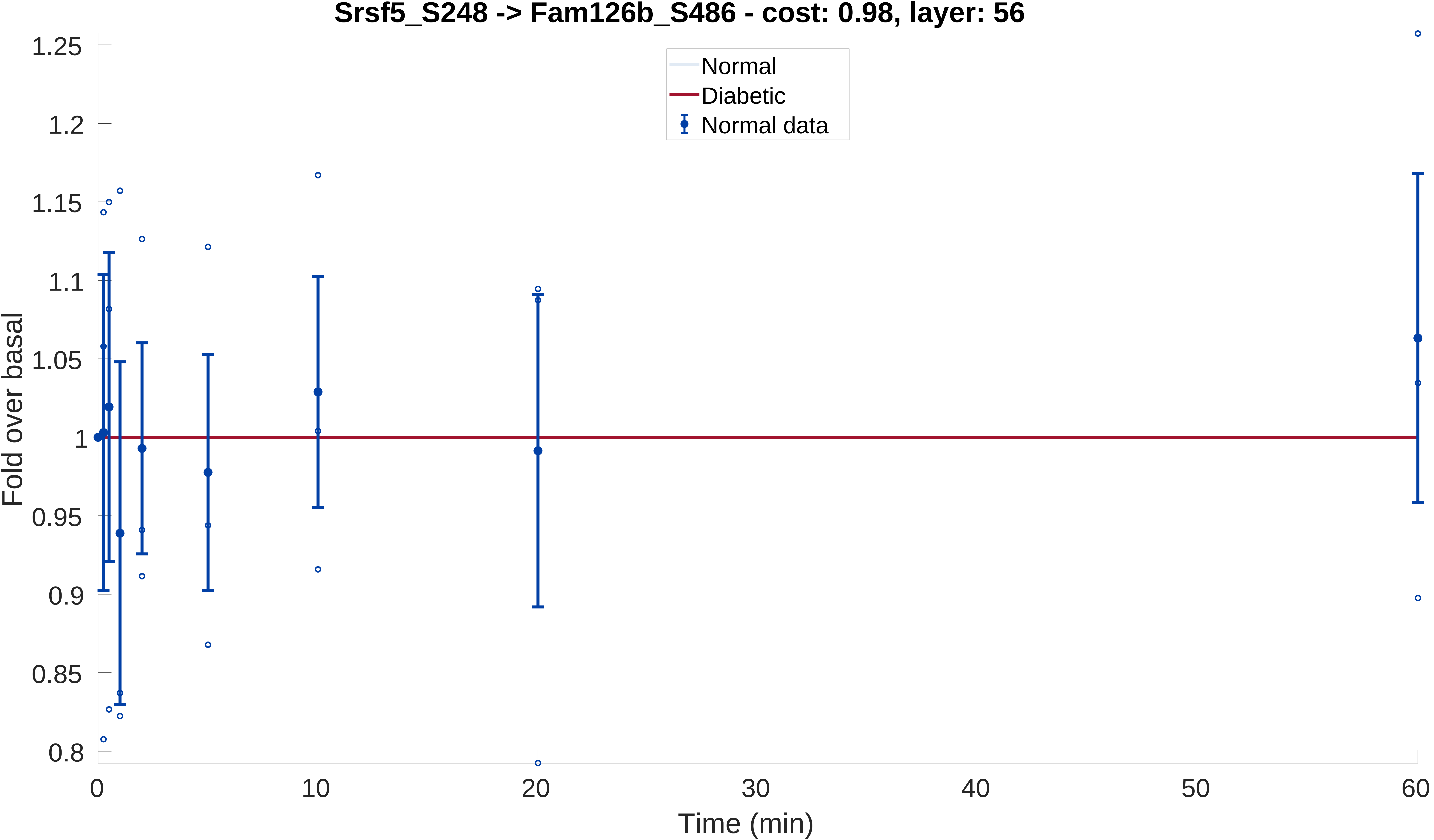
Srsf5_S248 -> Fam126b_S486 - cost: 0.98, layer: 56
Normal
Diabetic
Normal data
Fold over basal
Time (min)
1.25
1.2
1.15
1.1
1.05
1
0.95
0.9
0.85
0.8
0
10
20
30
40
50
60

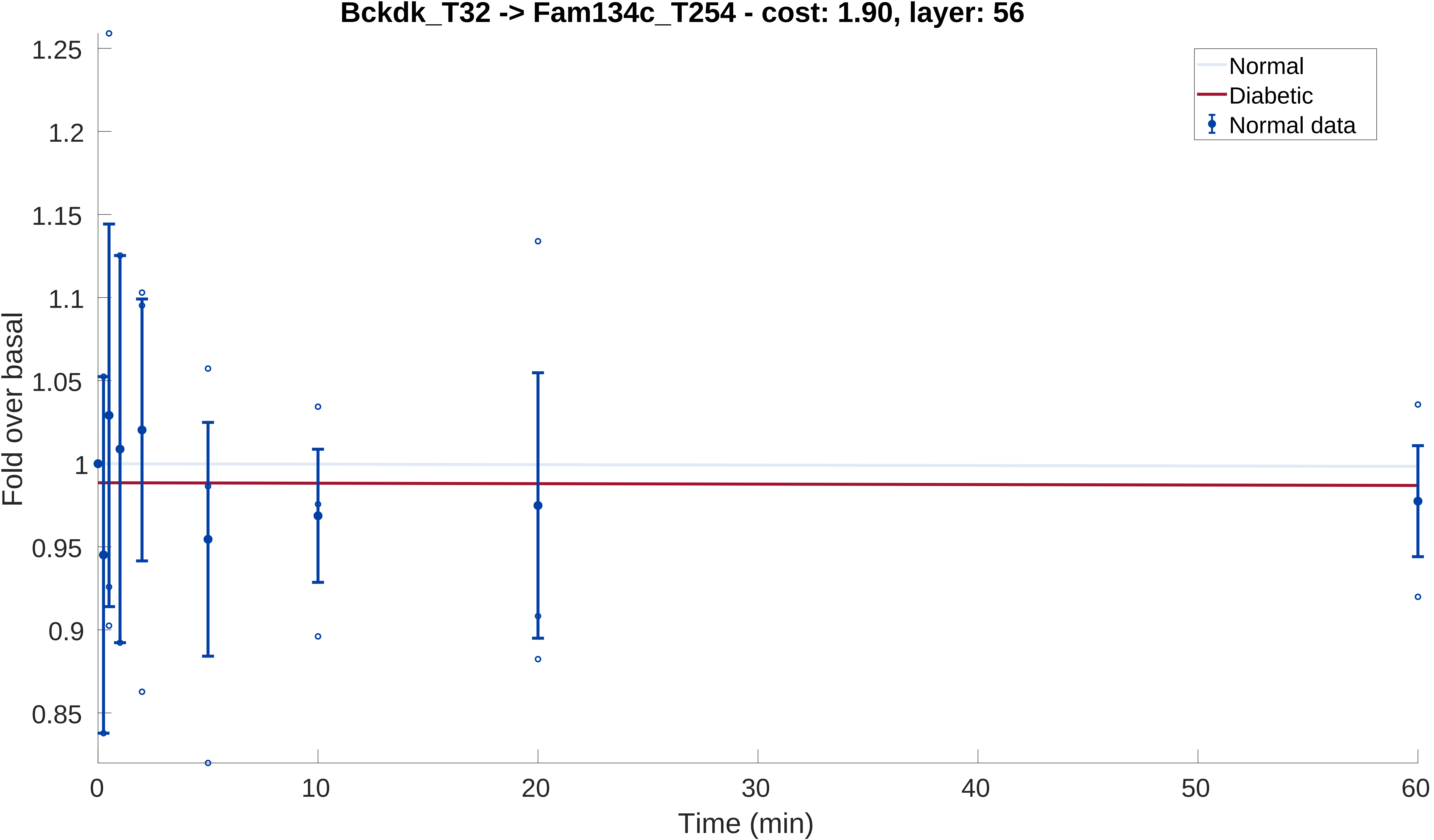

Bckdk_T32 -> Fam134c_T254 - cost: 1.90, layer: 56
Normal
Diabetic
Normal data
Fold over basal
1.25
1.2
1.15
1.1
1.05
1
0.95
0.9
0.85
0
10
20
30
40
50
60
Time (min)

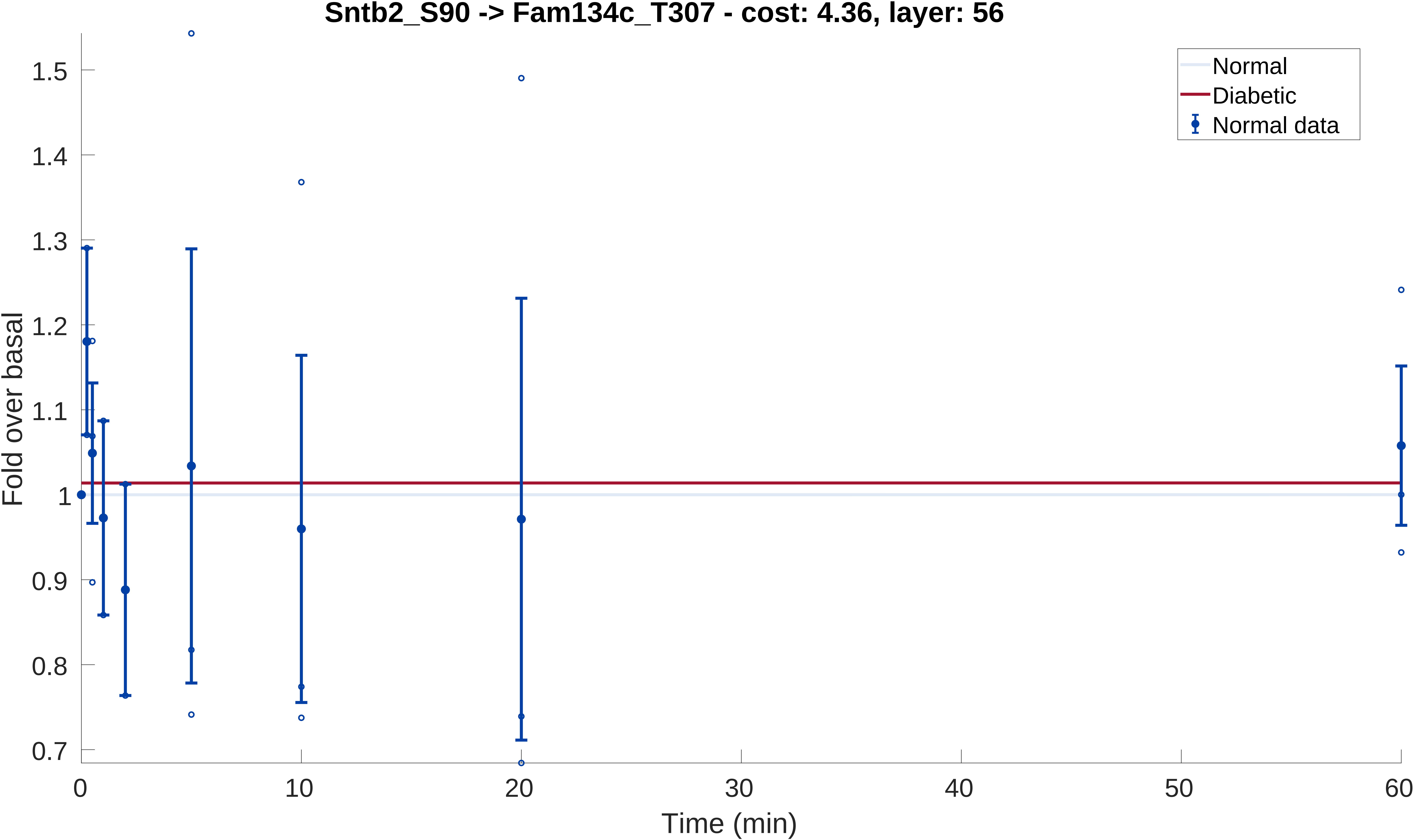
Sntb2_S90 -> Fam134c_T307 - cost: 4.36, layer: 56
Normal
Diabetic
Normal data
Fold over basal
1.5
1.4
1.3
1.2
1.1
1
0.9
0.8
0.7
0
10
20
30
40
50
60
Time (min)

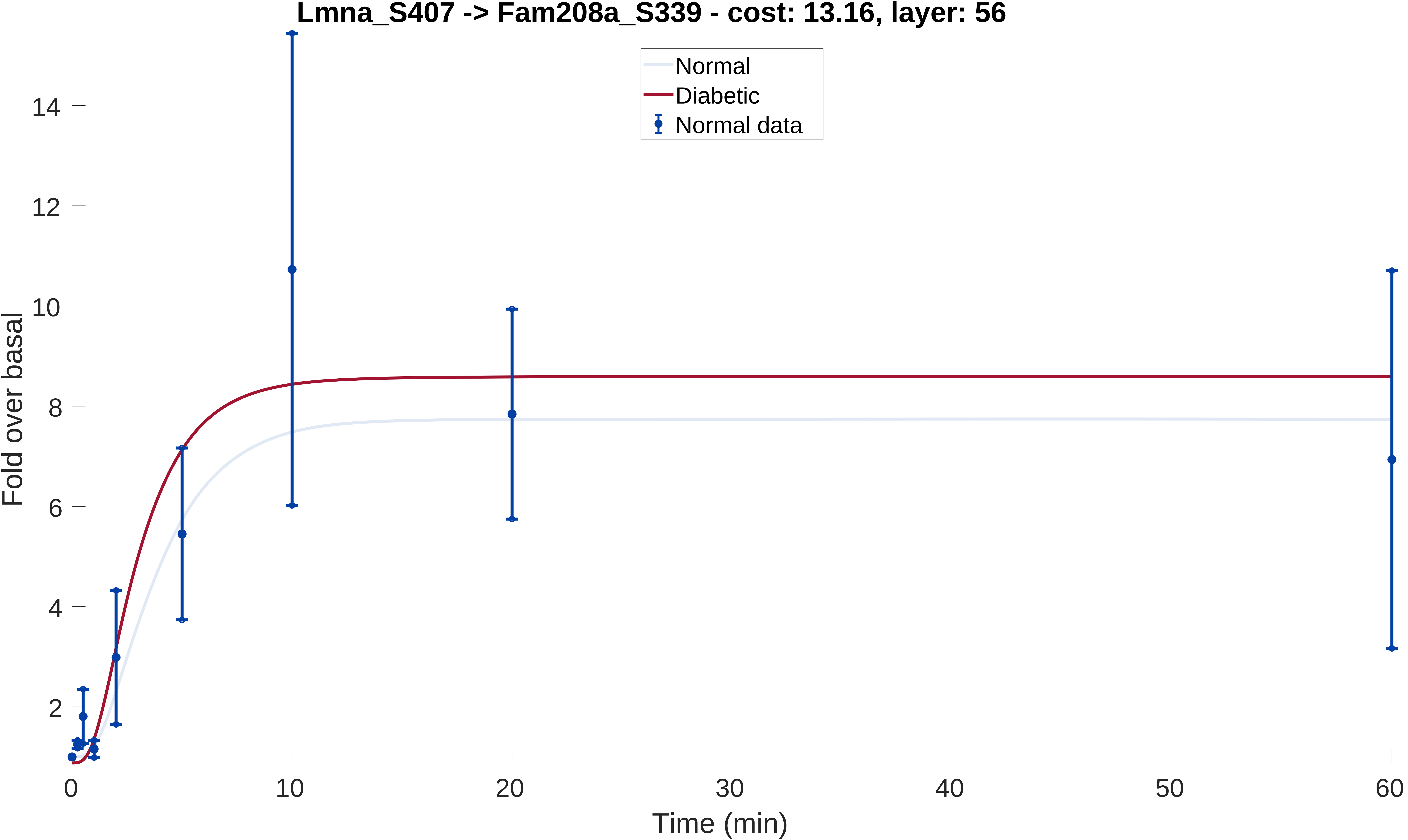

Lmna_S407 -> Fam208a_S339 - cost: 13.16, layer: 56
Normal
Diabetic
Normal data
Fold over basal
Time (min)
2
4
6
8
10
12
14
0
10
20
30
40
50
60

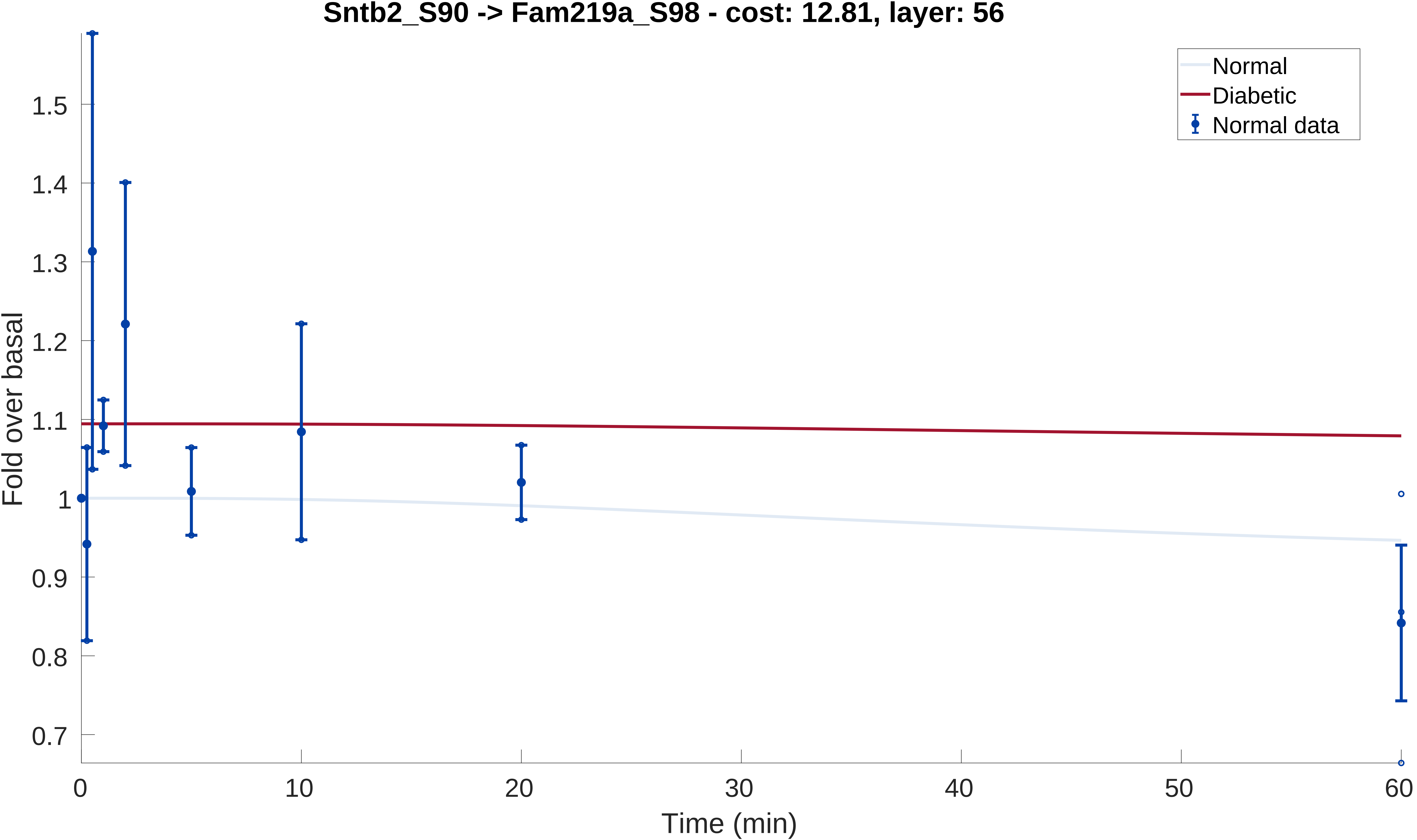

Sntb2_S90 -> Fam219a_S98 - cost: 12.81, layer: 56
Normal
Diabetic
Normal data
Fold over basal
Time (min)
0.7
0.8
0.9
1
1.1
1.2
1.3
1.4
1.5
0
10
20
30
40
50
60

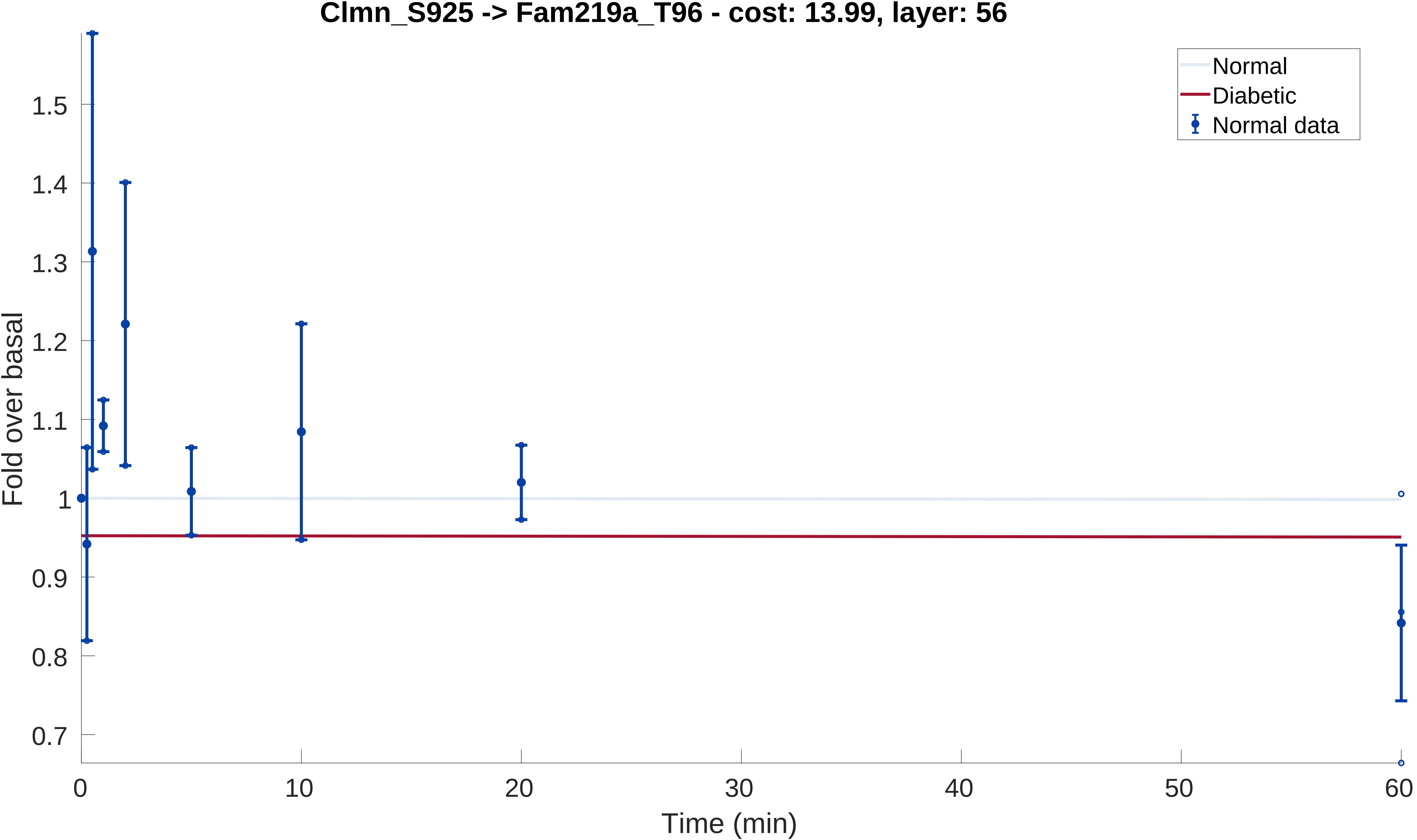

Clmn_S925 -> Fam219a_T96 - cost: 13.99, layer: 56
Normal
Diabetic
Normal data
Fold over basal
1.5
1.4
1.3
1.2
1.1
1
0.9
0.8
0.7
0
10
20
30
40
50
60
Time (min)

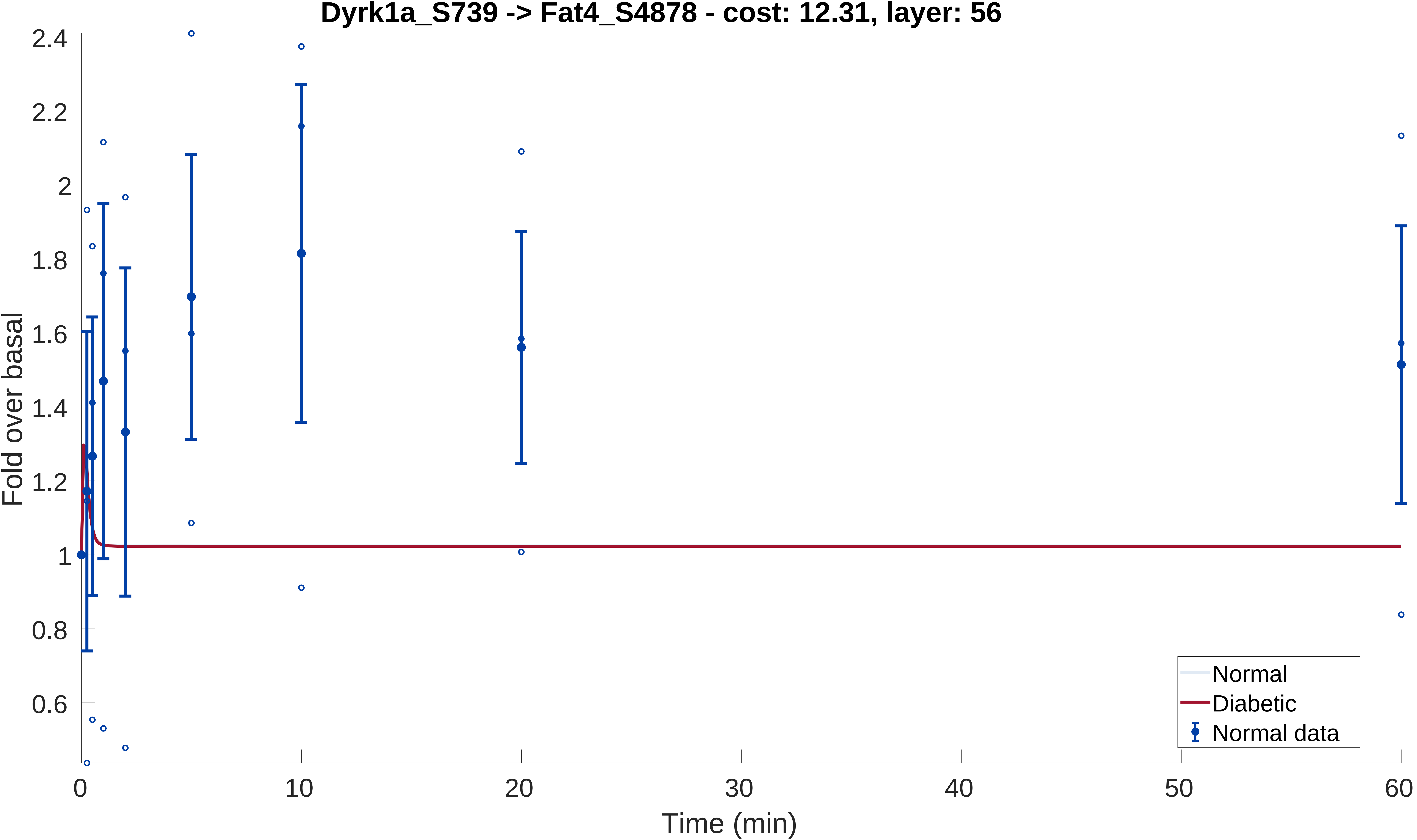

Dyrk1a_S739 -> Fat4_S4878 - cost: 12.31, layer: 56
Fold over basal
Time (min)
2.4
2.2
2
1.8
1.6
1.4
1.2
1
0.8
0.6
0
10
20
30
40
50
60
Normal
Diabetic
Normal data

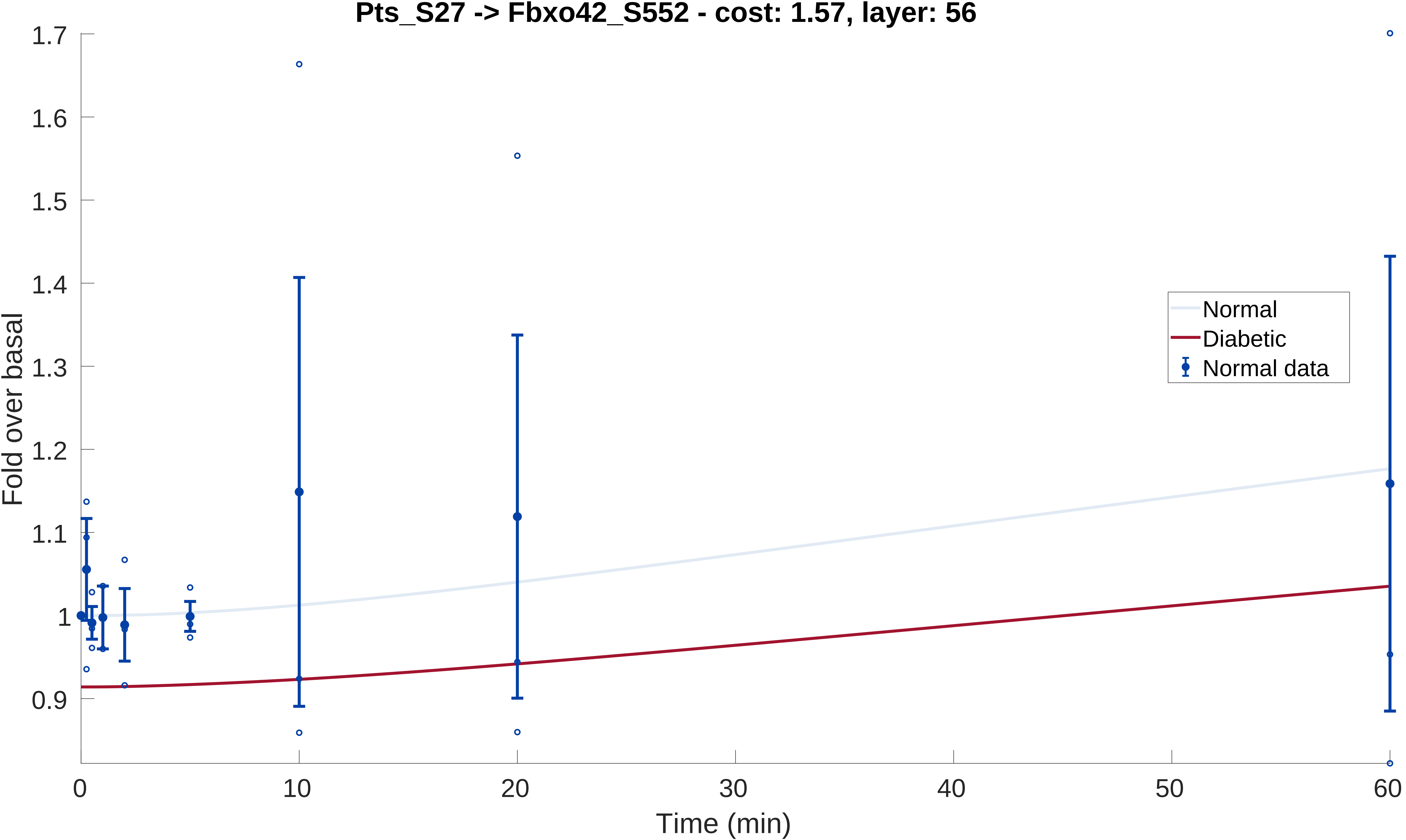

Pts_S27 -> Fbxo42_S552 - cost: 1.57, layer: 56
Fold over basal
Time (min)
Normal
Diabetic
Normal data
1.7
1.6
1.5
1.4
1.3
1.2
1.1
1
0.9
0
10
20
30
40
50
60

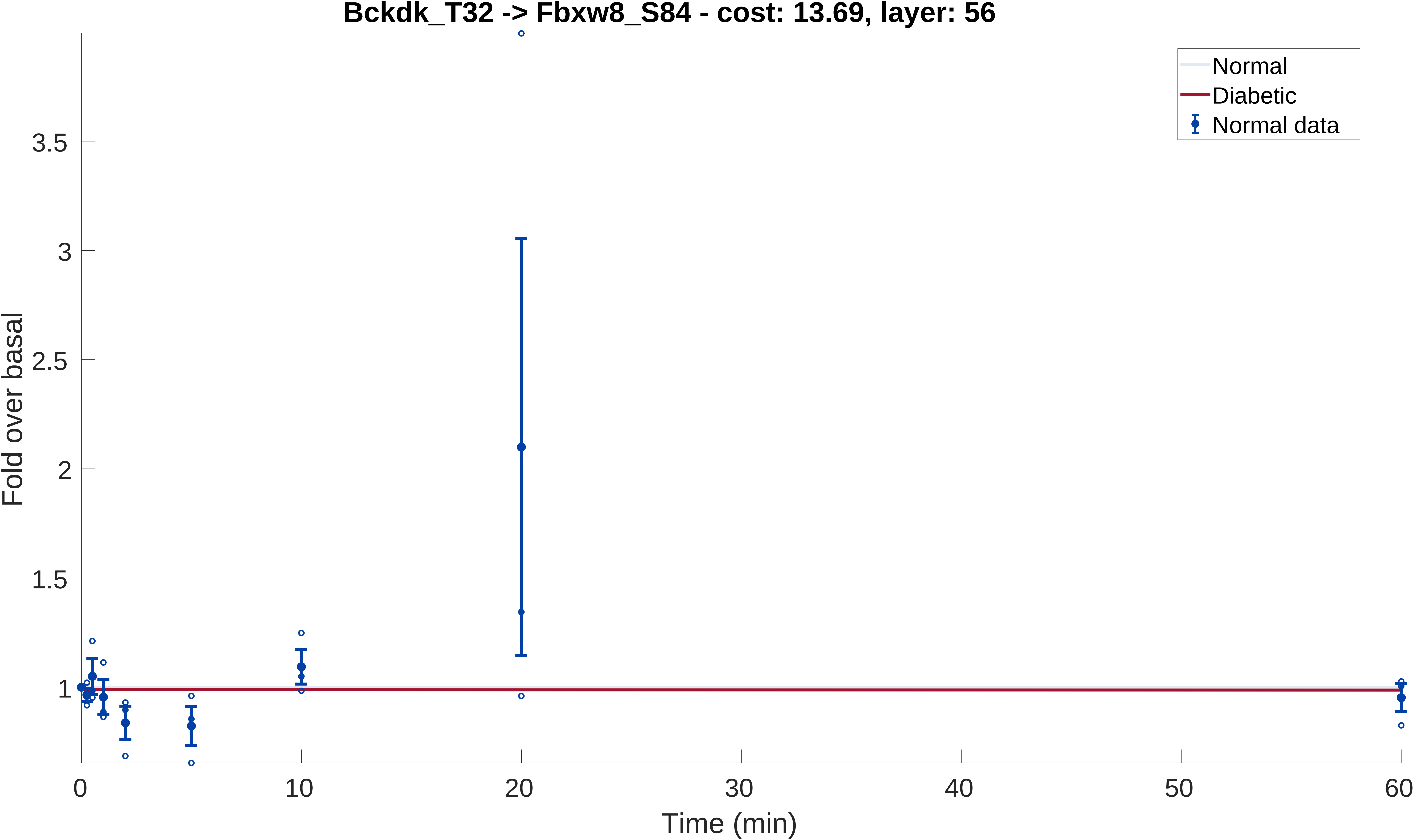

Bckdk_T32 -> Fbxw8_S84 - cost: 13.69, layer: 56
Normal
Diabetic
Normal data
Fold over basal
Time (min)
3.5
3
2.5
2
1.5
1
0
10
20
30
40
50
60

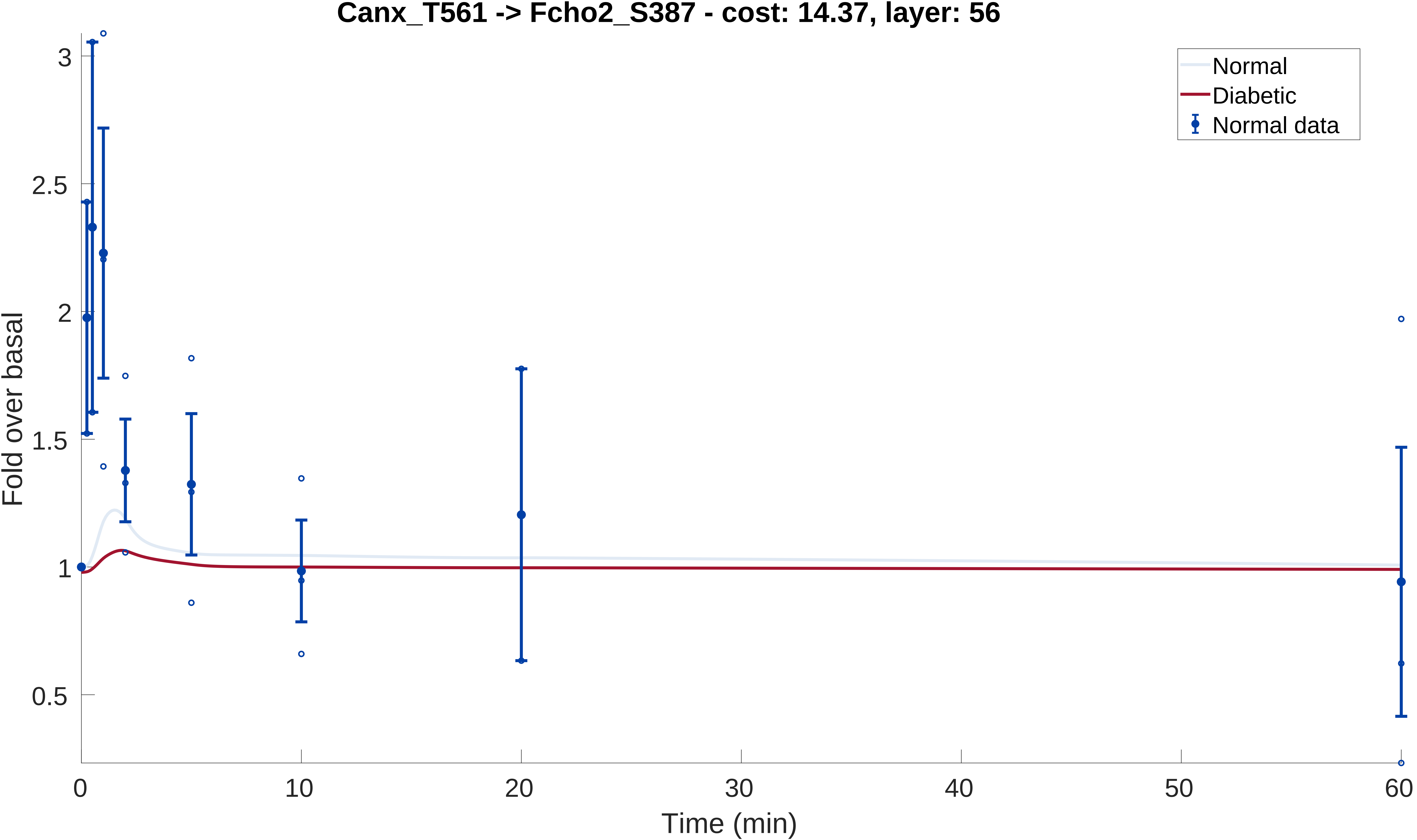

Canx_T561 -> Fcho2_S387 - cost: 14.37, layer: 56
Normal
Diabetic
Normal data
Fold over basal
Time (min)
0
10
20
30
40
50
60
0.5
1
1.5
2
2.5
3

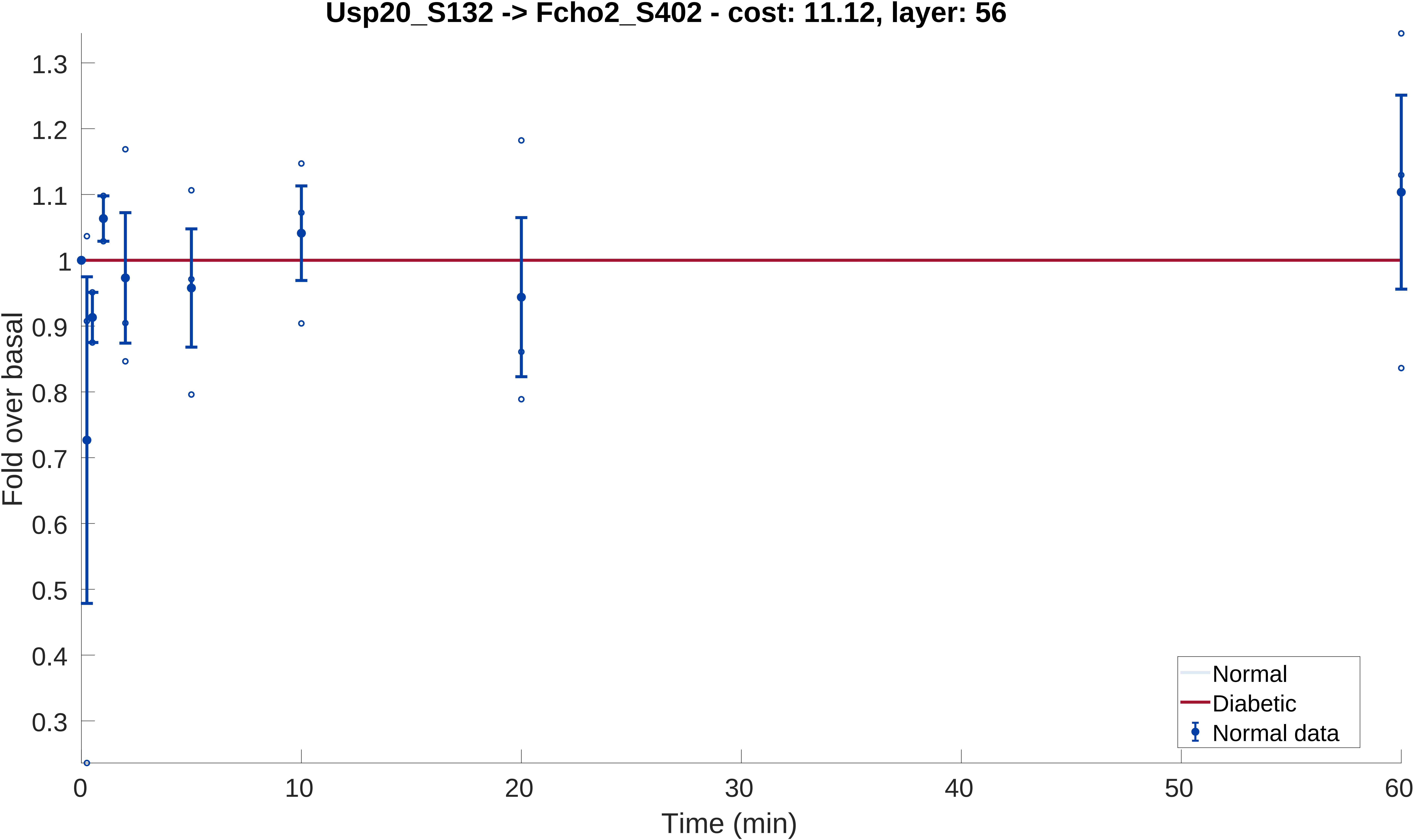

Usp20_S132 -> Fcho2_S402 - cost: 11.12, layer: 56
Fold over basal
1.3
1.2
1.1
1
0.9
0.8
0.7
0.6
0.5
0.4
0.3
0
10
20
30
40
50
60
Time (min)
Normal
Diabetic
Normal data

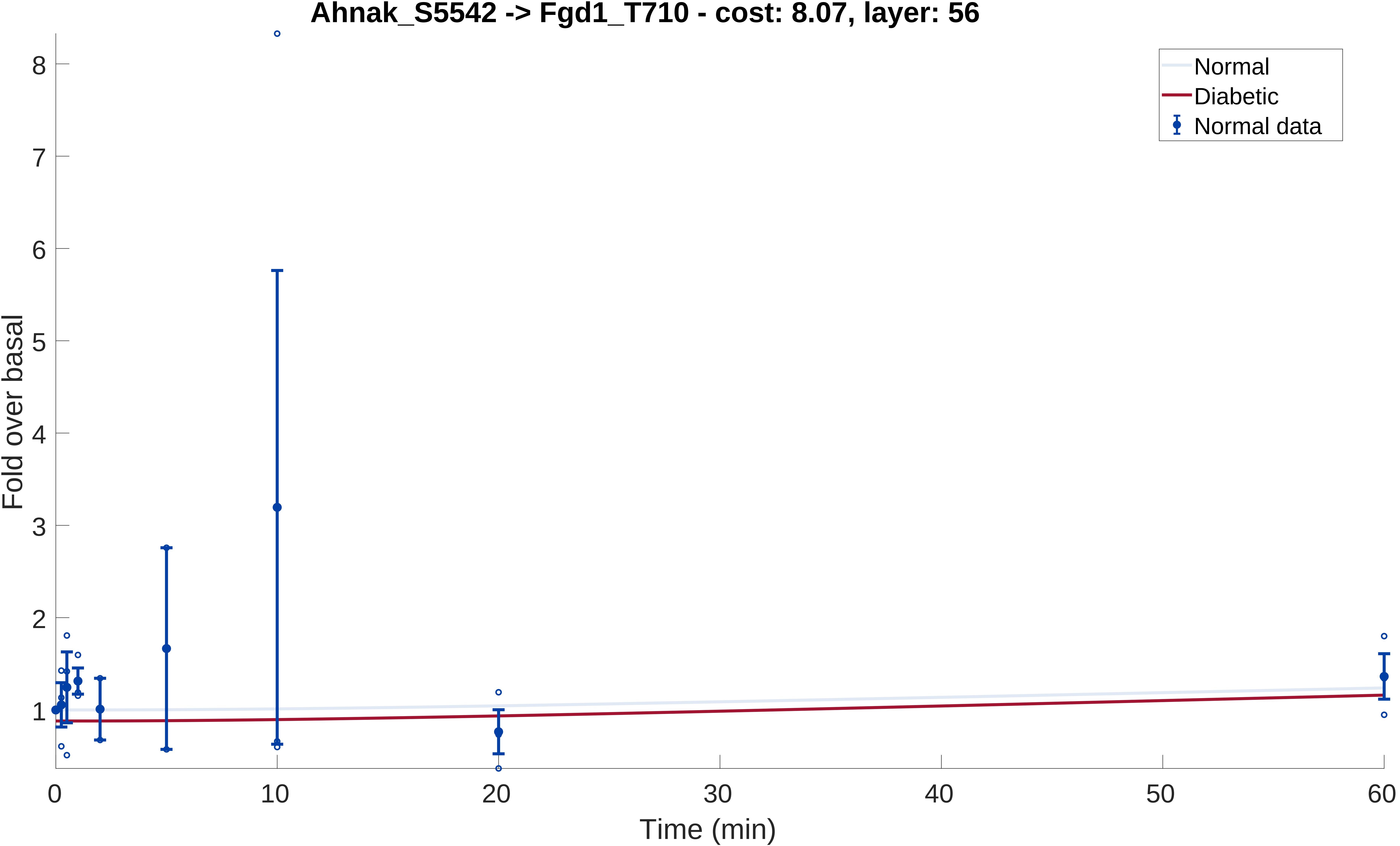

Ahnak_S5542 -> Fgd1_T710 - cost: 8.07, layer: 56
Normal
Diabetic
Normal data
Fold over basal
Time (min)
1
2
3
4
5
6
7
8
0
10
20
30
40
50
60

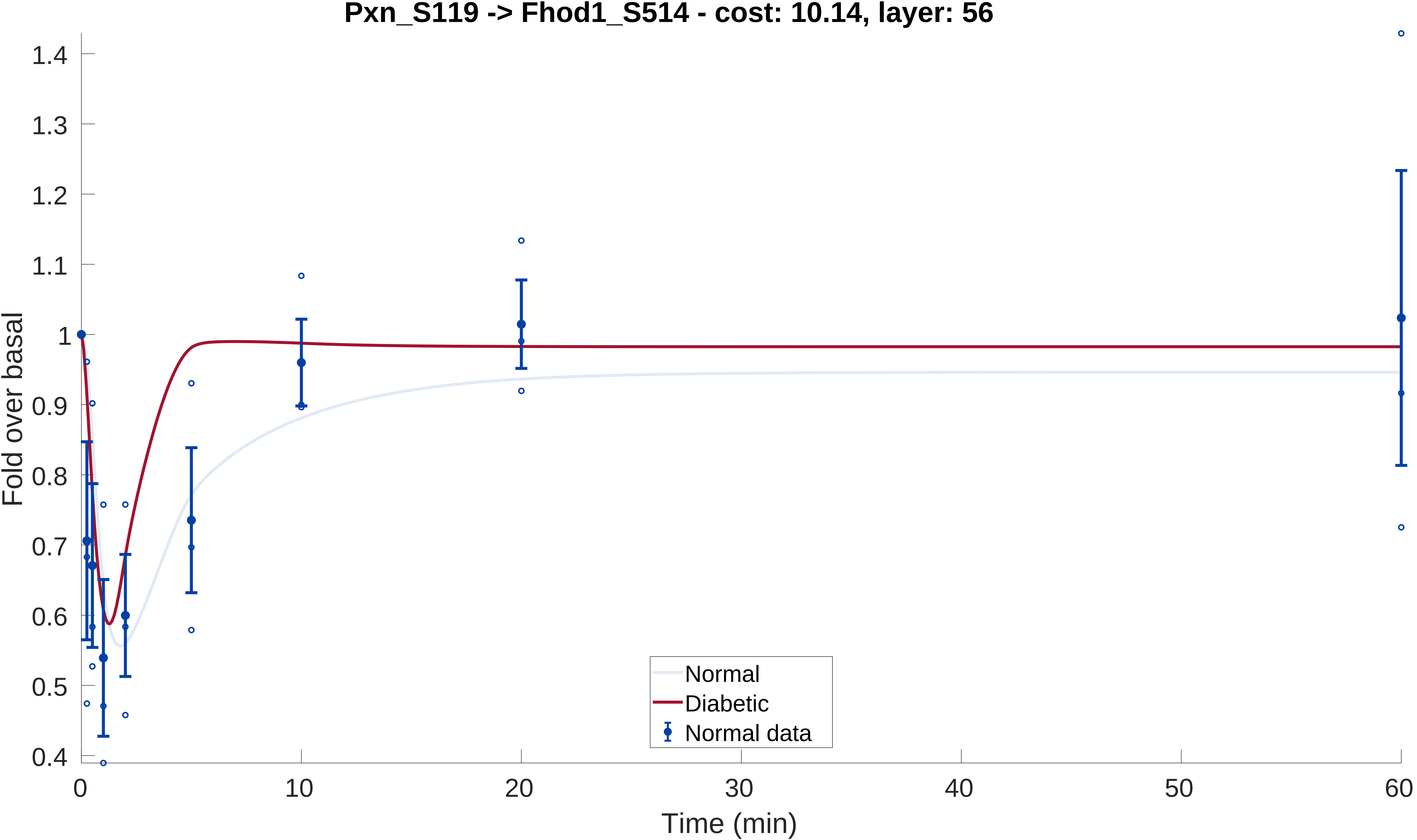

Pxn_S119 -> Fhod1_S514 - cost: 10.14, layer: 56
Fold over basal
Time (min)
1.4
1.3
1.2
1.1
1
0.9
0.8
0.7
0.6
0.5
0.4
0
10
20
30
40
50
60
Normal
Diabetic
Normal data

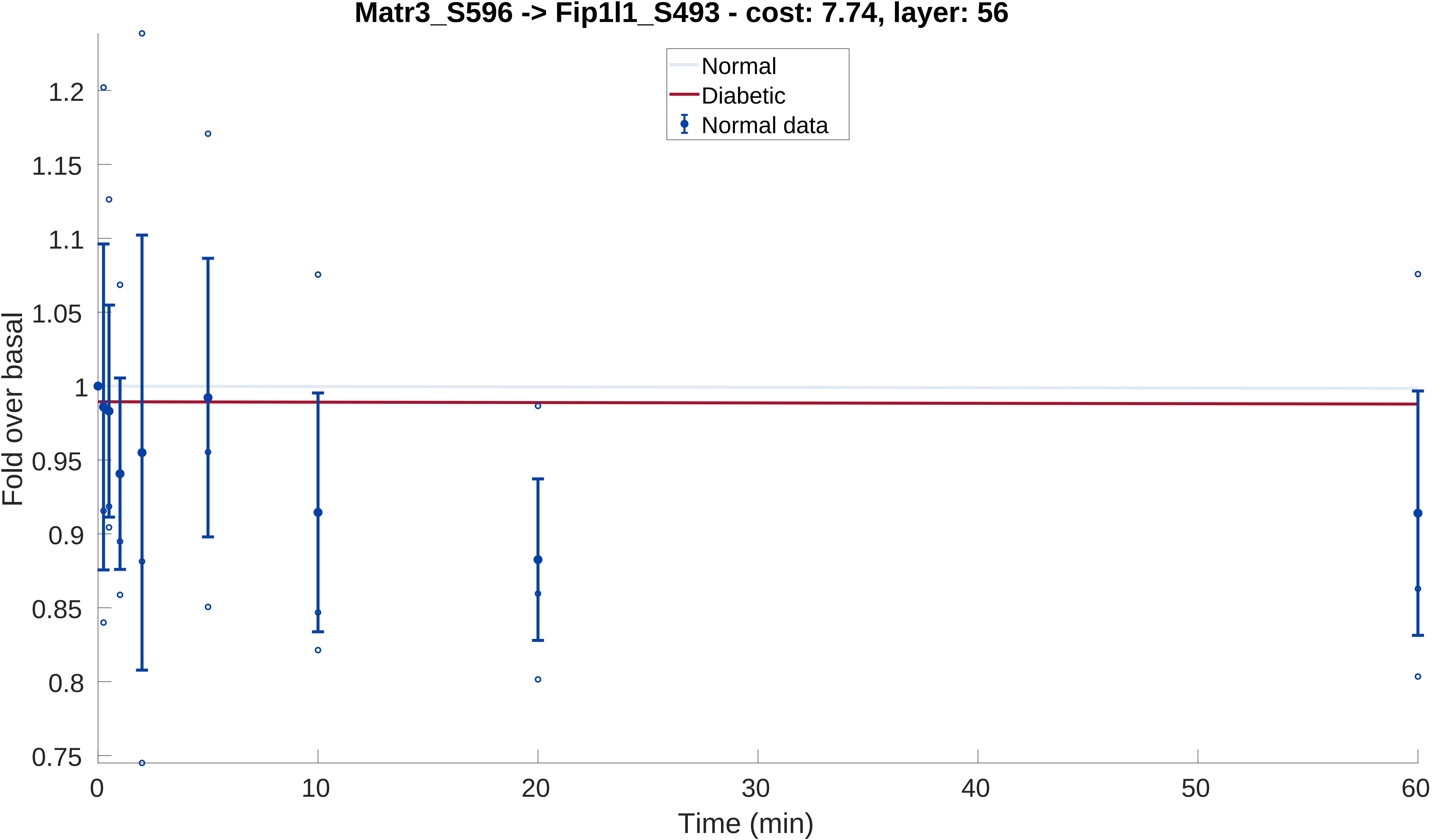
Matr3_S596 -> Fip1l1_S493 - cost: 7.74, layer: 56
Normal
Diabetic
Normal data
Fold over basal
Time (min)
0.75
0.8
0.85
0.9
0.95
1
1.05
1.1
1.15
1.2
0
10
20
30
40
50
60

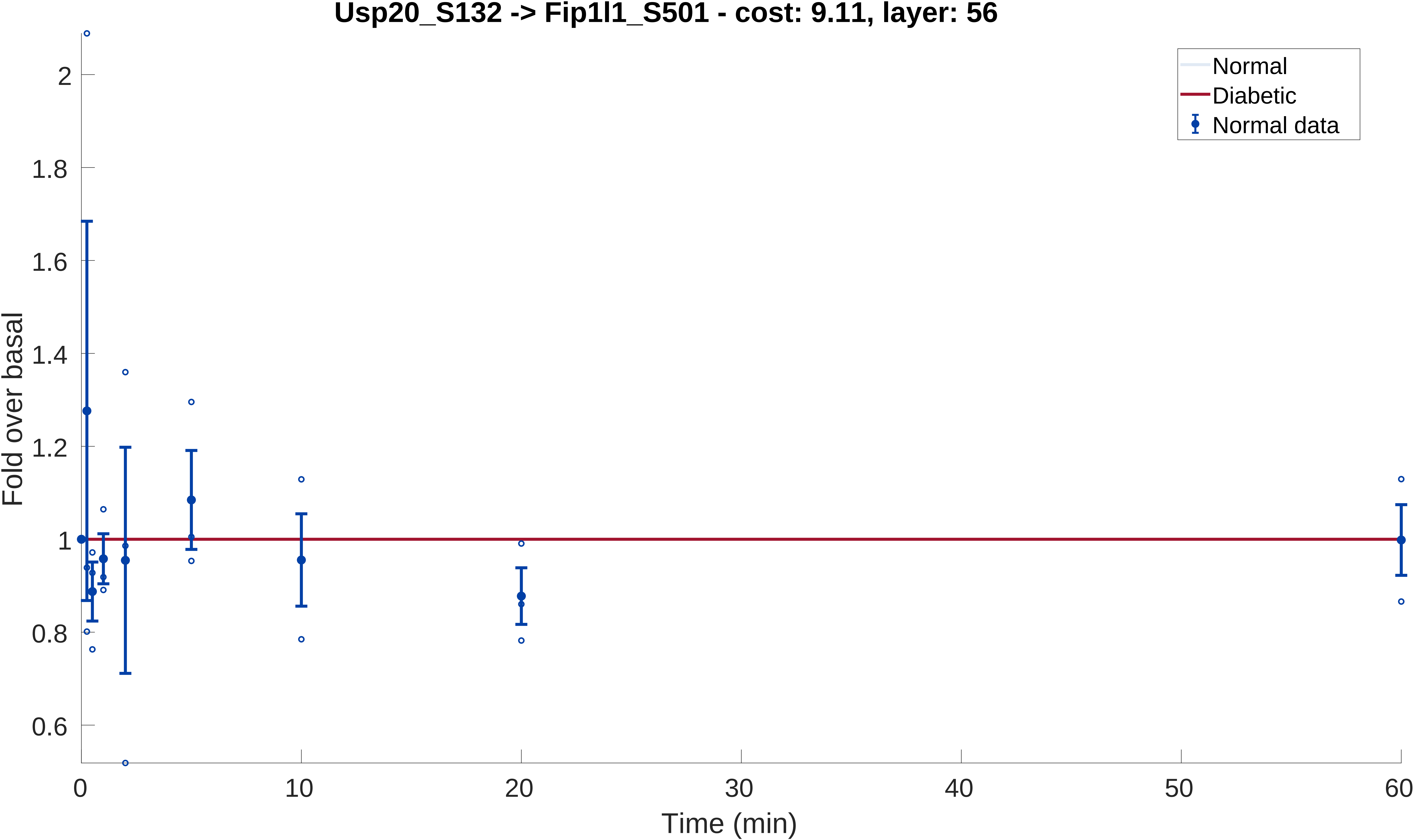

Usp20_S132 -> Fip1l1_S501 - cost: 9.11, layer: 56
Normal
Diabetic
Normal data
Fold over basal
Time (min)
2
1.8
1.6
1.4
1.2
1
0.8
0.6
0
10
20
30
40
50
60

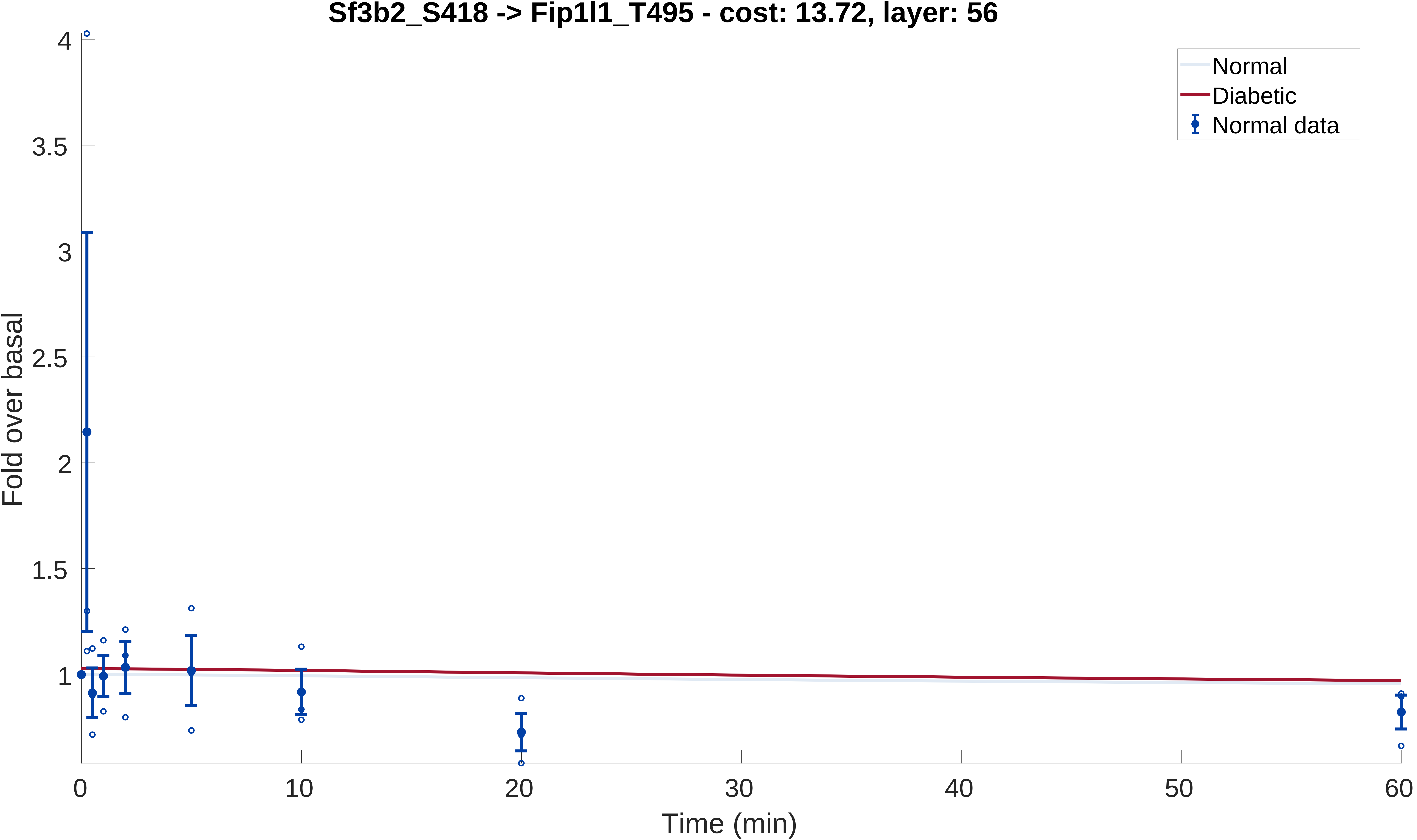
Sf3b2_S418 -> Fip1l1_T495 - cost: 13.72, layer: 56
Normal
Diabetic
Normal data
Fold over basal
Time (min)
4
3.5
3
2.5
2
1.5
1
0
10
20
30
40
50
60

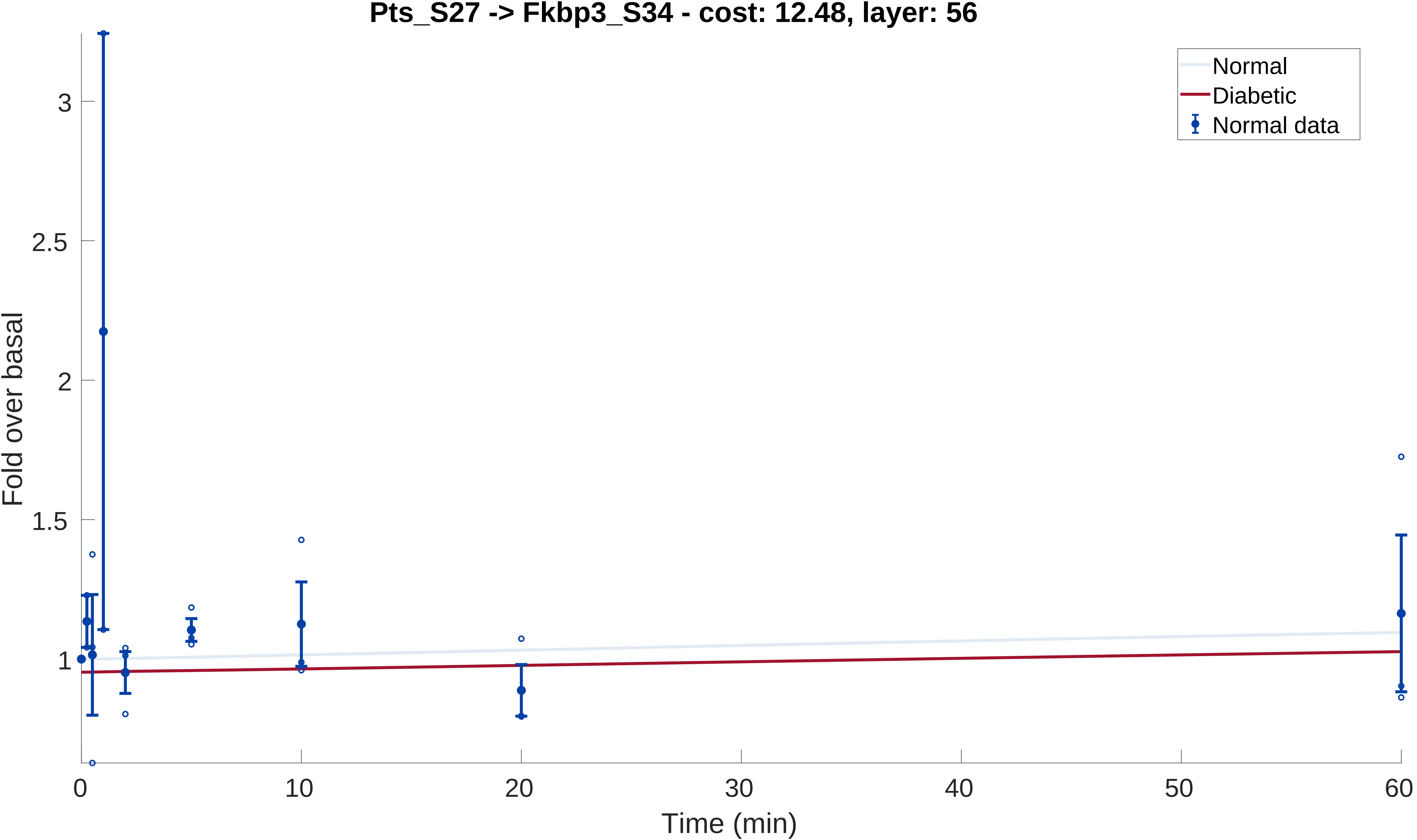
Pts_S27 -> Fkbp3_S34 - cost: 12.48, layer: 56
Normal
Diabetic
Normal data
Fold over basal
Time (min)
3
2.5
2
1.5
1
0
10
20
30
40
50
60

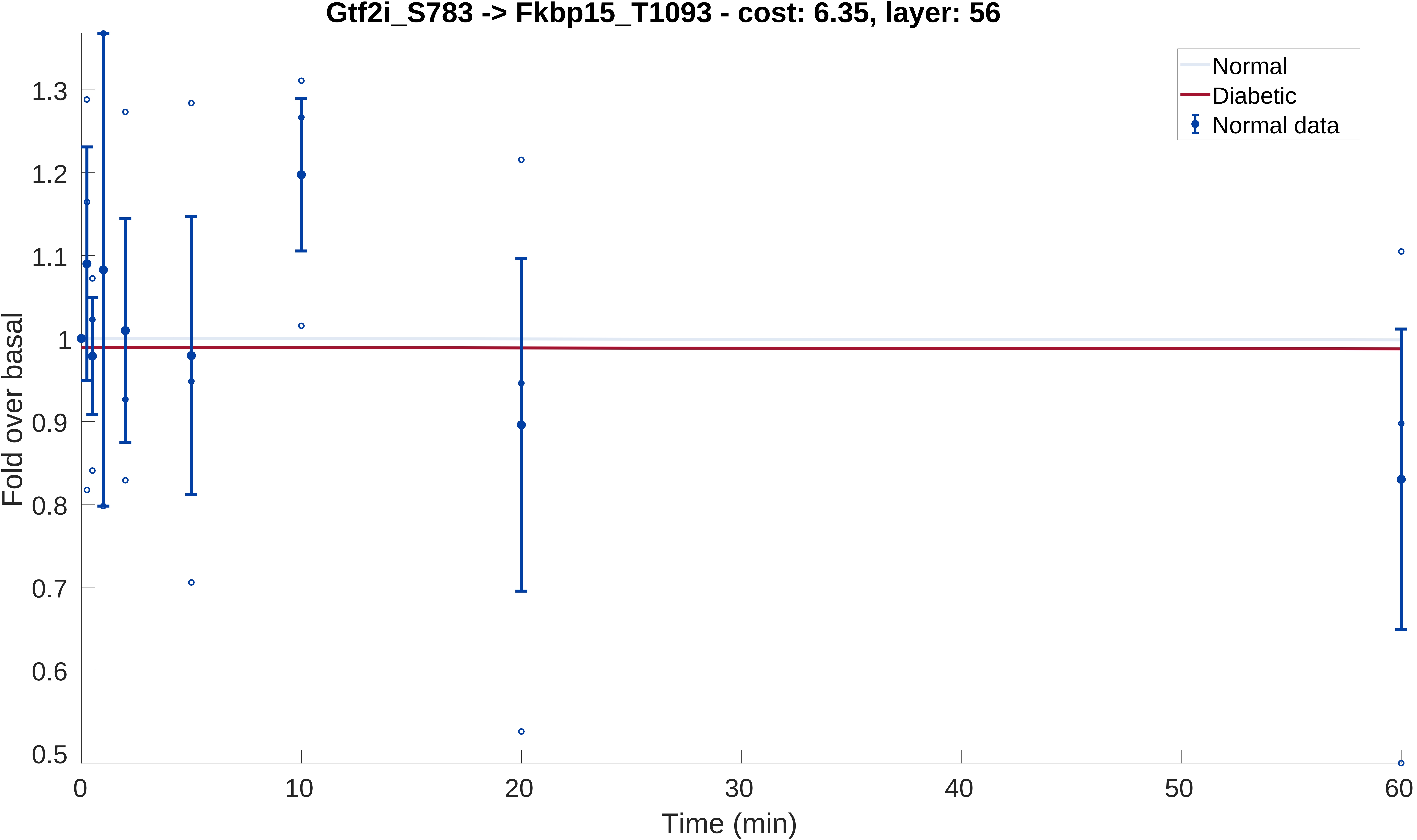

Gtf2i_S783 -> Fkbp15_T1093 - cost: 6.35, layer: 56
Normal
Diabetic
Normal data
Fold over basal
Time (min)
1.3
1.2
1.1
1
0.9
0.8
0.7
0.6
0.5
0
10
20
30
40
50
60

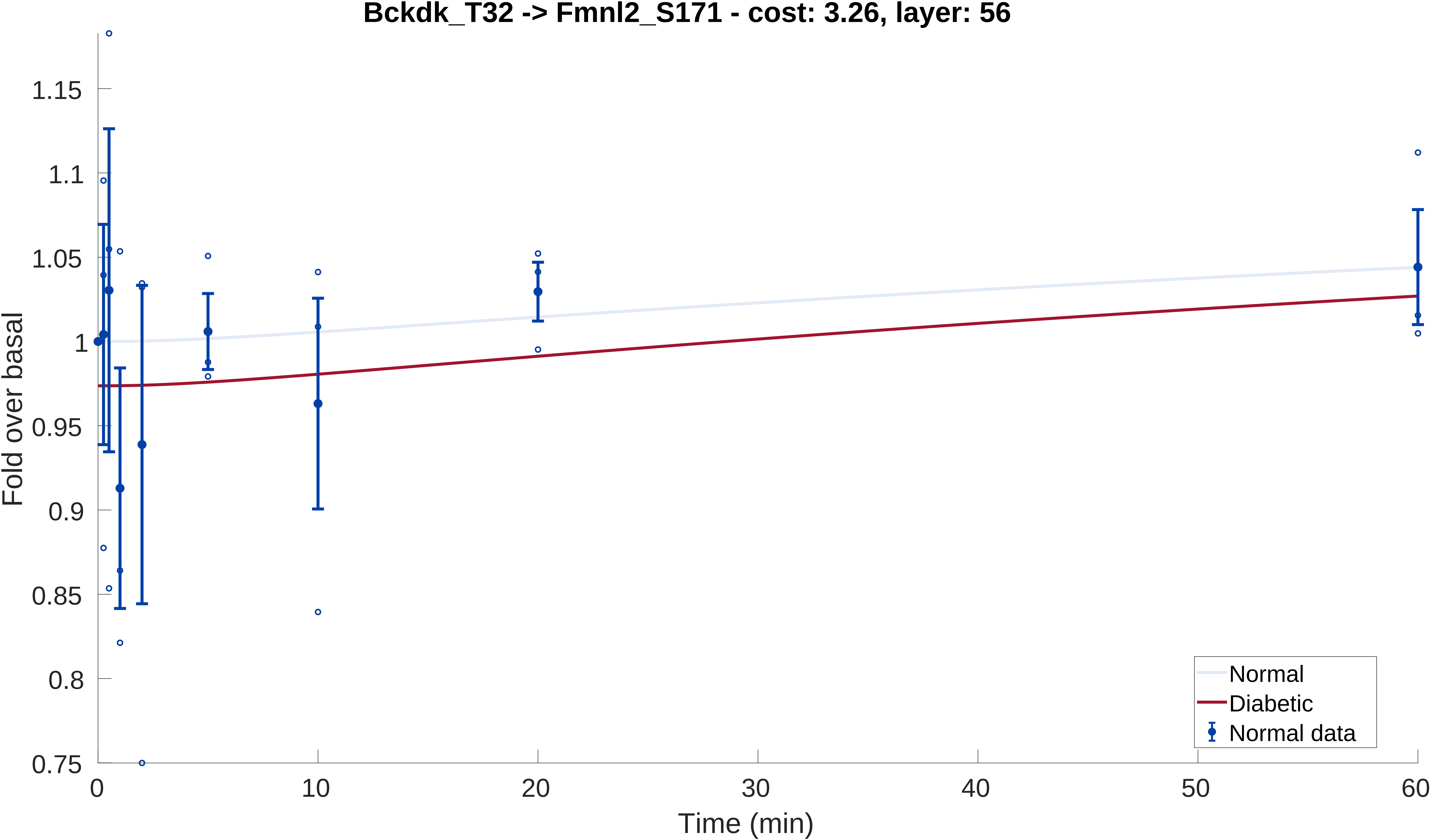
Bckdk_T32 -> Fmnl2_S171 - cost: 3.26, layer: 56
Fold over basal
1.15
1.1
1.05
1
0.95
0.9
0.85
0.8
0.75
0
10
20
30
40
50
60
Time (min)
Normal
Diabetic
Normal data

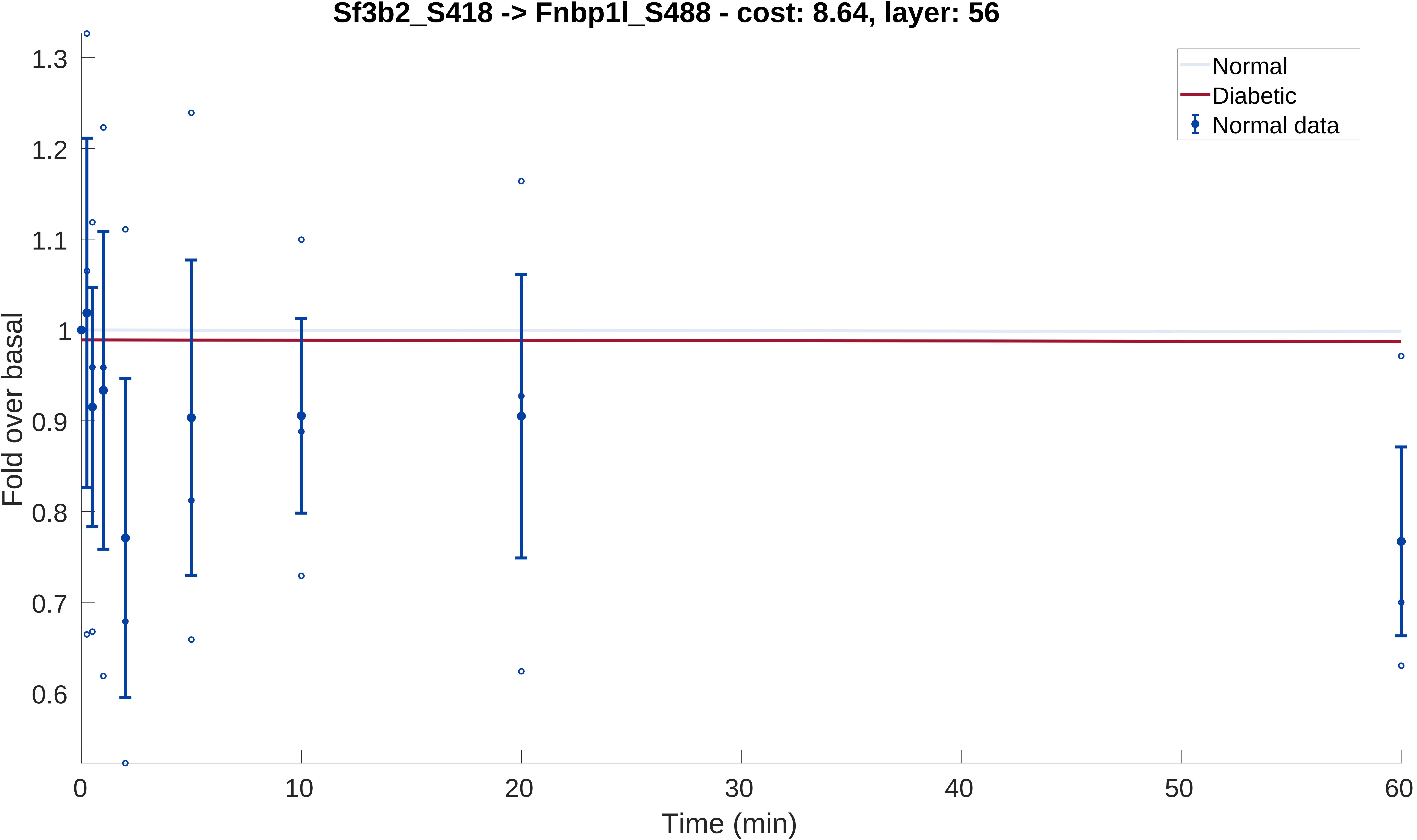

Sf3b2_S418 -> Fnbp1l_S488 - cost: 8.64, layer: 56
Normal
Diabetic
Normal data
Fold over basal
Time (min)
1.3
1.2
1.1
1
0.9
0.8
0.7
0.6
0
10
20
30
40
50
60

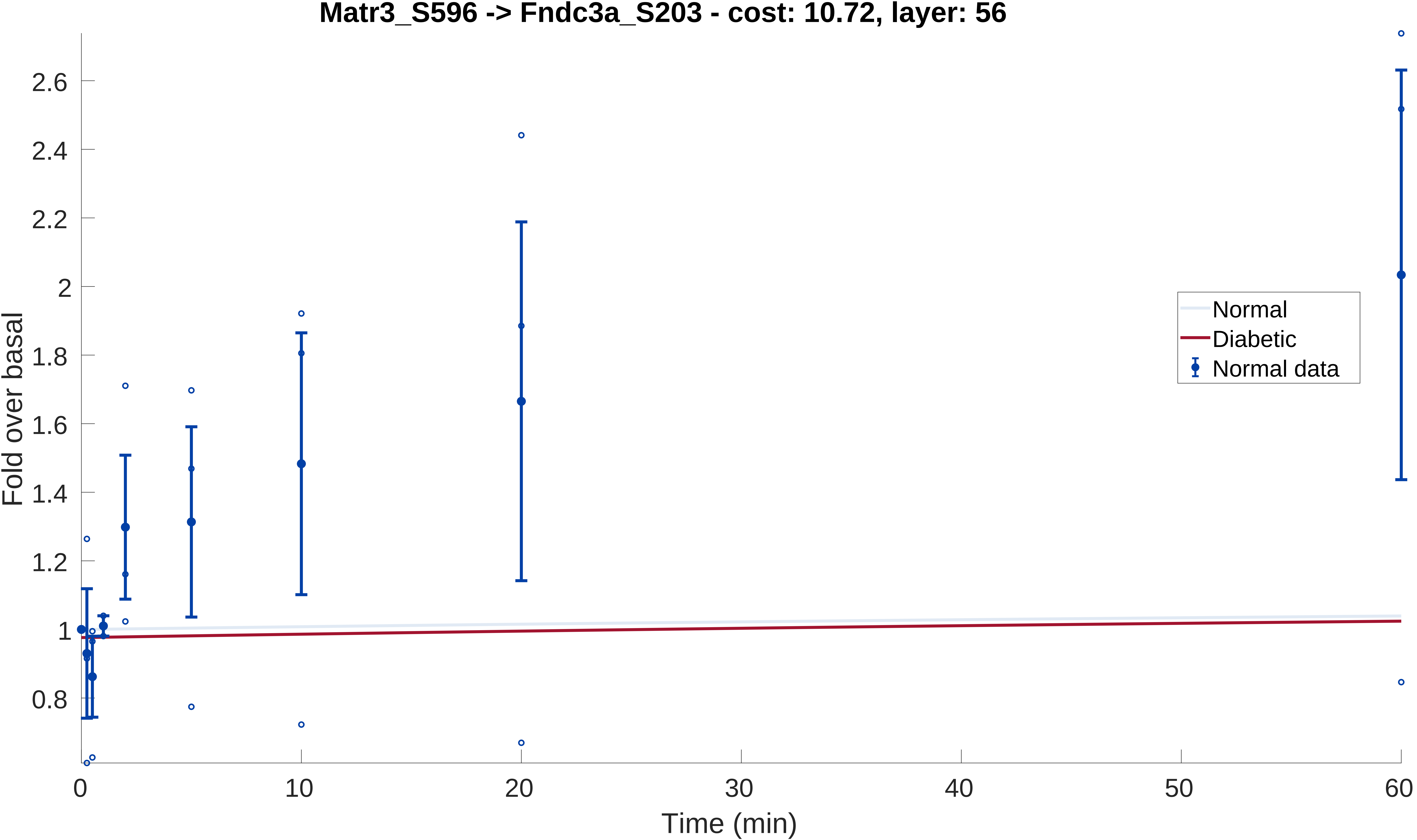
Matr3_S596 -> Fndc3a_S203 - cost: 10.72, layer: 56
Fold over basal
Time (min)
Normal
Diabetic
Normal data
0.8
1
1.2
1.4
1.6
1.8
2
2.2
2.4
2.6
0
10
20
30
40
50
60

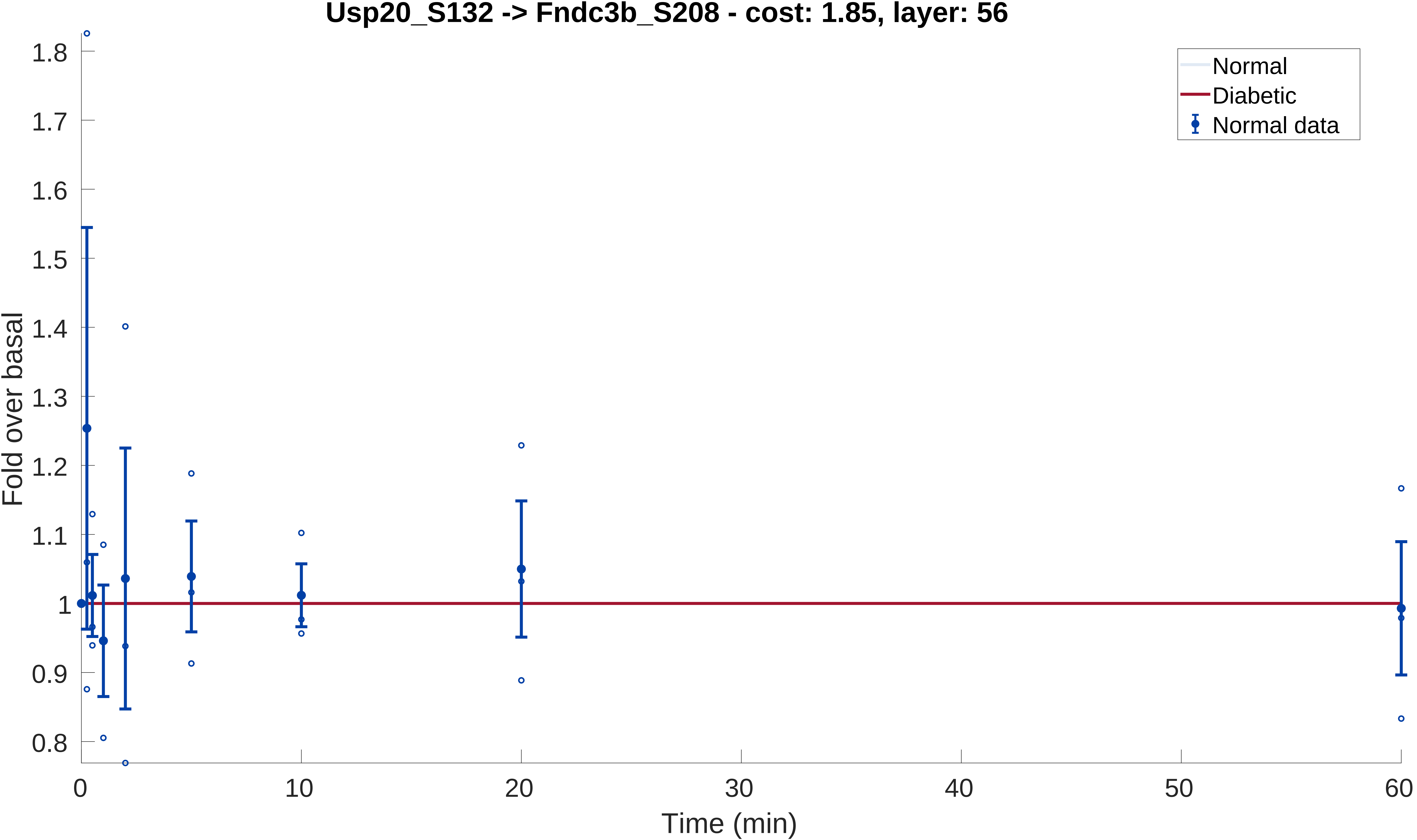

Usp20_S132 -> Fndc3b_S208 - cost: 1.85, layer: 56
Fold over basal
Time (min)
Normal
Diabetic
Normal data
1.8
1.7
1.6
1.5
1.4
1.3
1.2
1.1
1
0.9
0.8
0
10
20
30
40
50
60

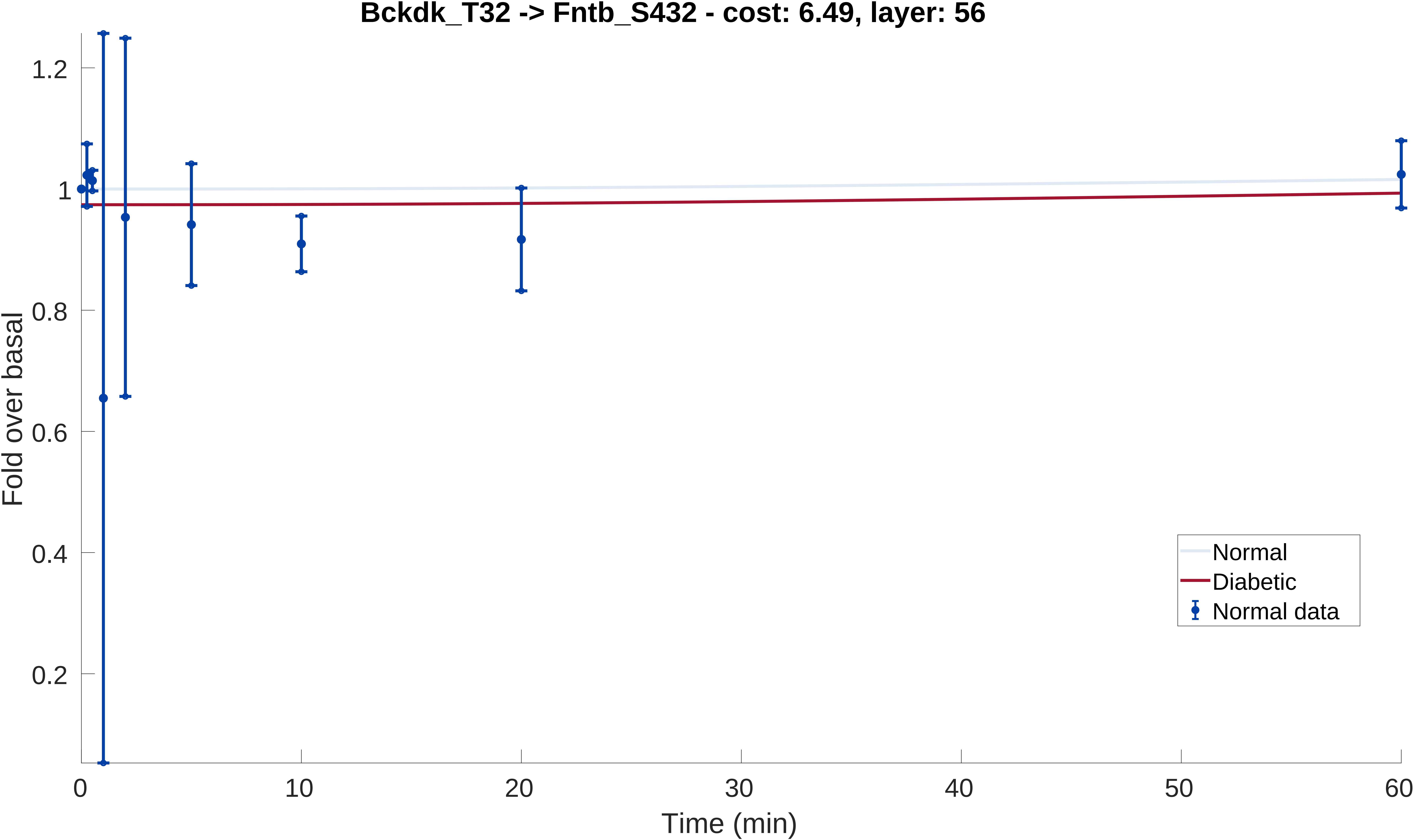

Bckdk_T32 -> Fntb_S432 - cost: 6.49, layer: 56
Fold over basal
1.2
1
0.8
0.6
0.4
0.2
0
10
20
30
40
50
60
Time (min)
Normal
Diabetic
Normal data

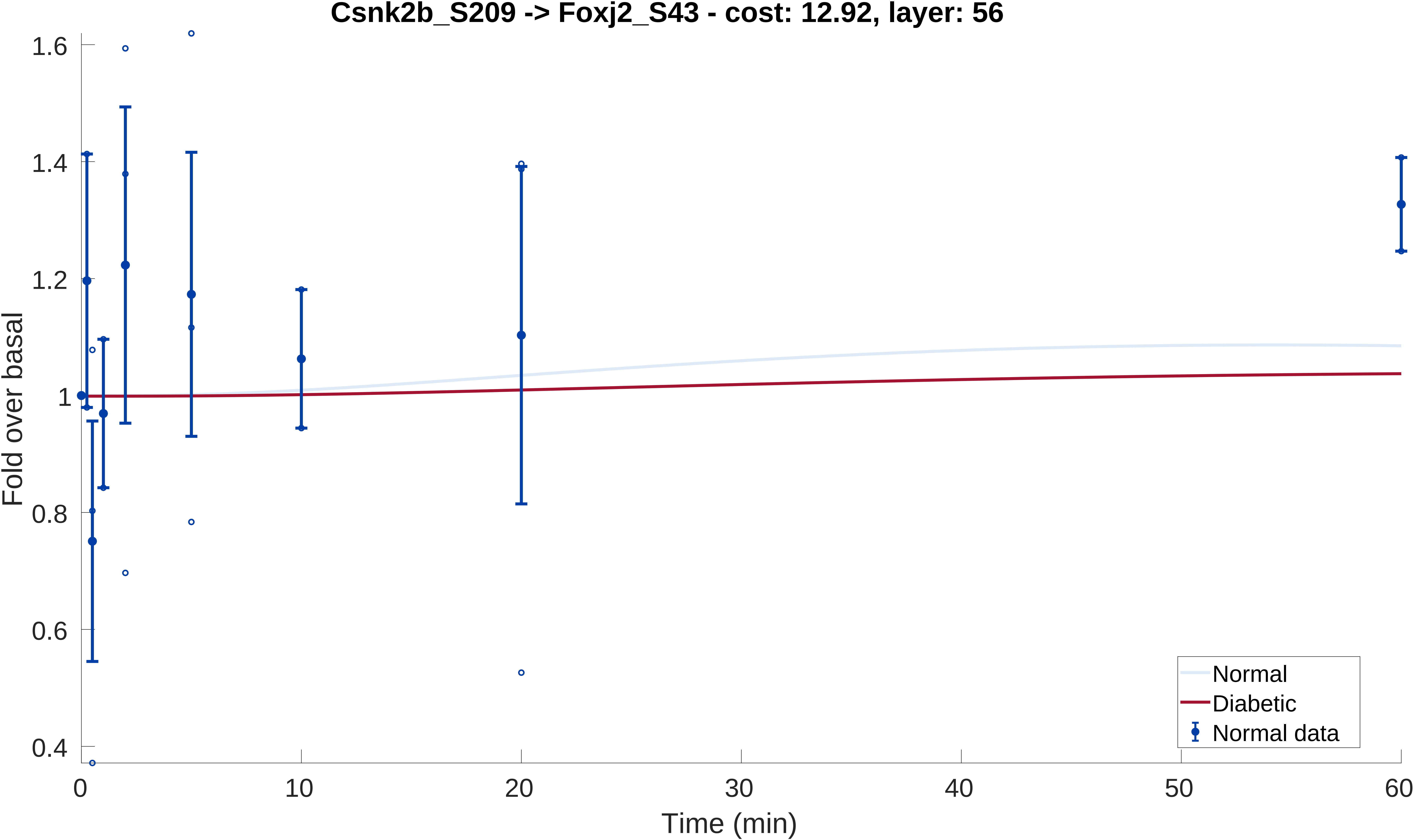

Csnk2b_S209 -> Foxj2_S43 - cost: 12.92, layer: 56
Fold over basal
1.6
1.4
1.2
1
0.8
0.6
0.4
0
10
20
30
40
50
60
Time (min)
Normal
Diabetic
Normal data

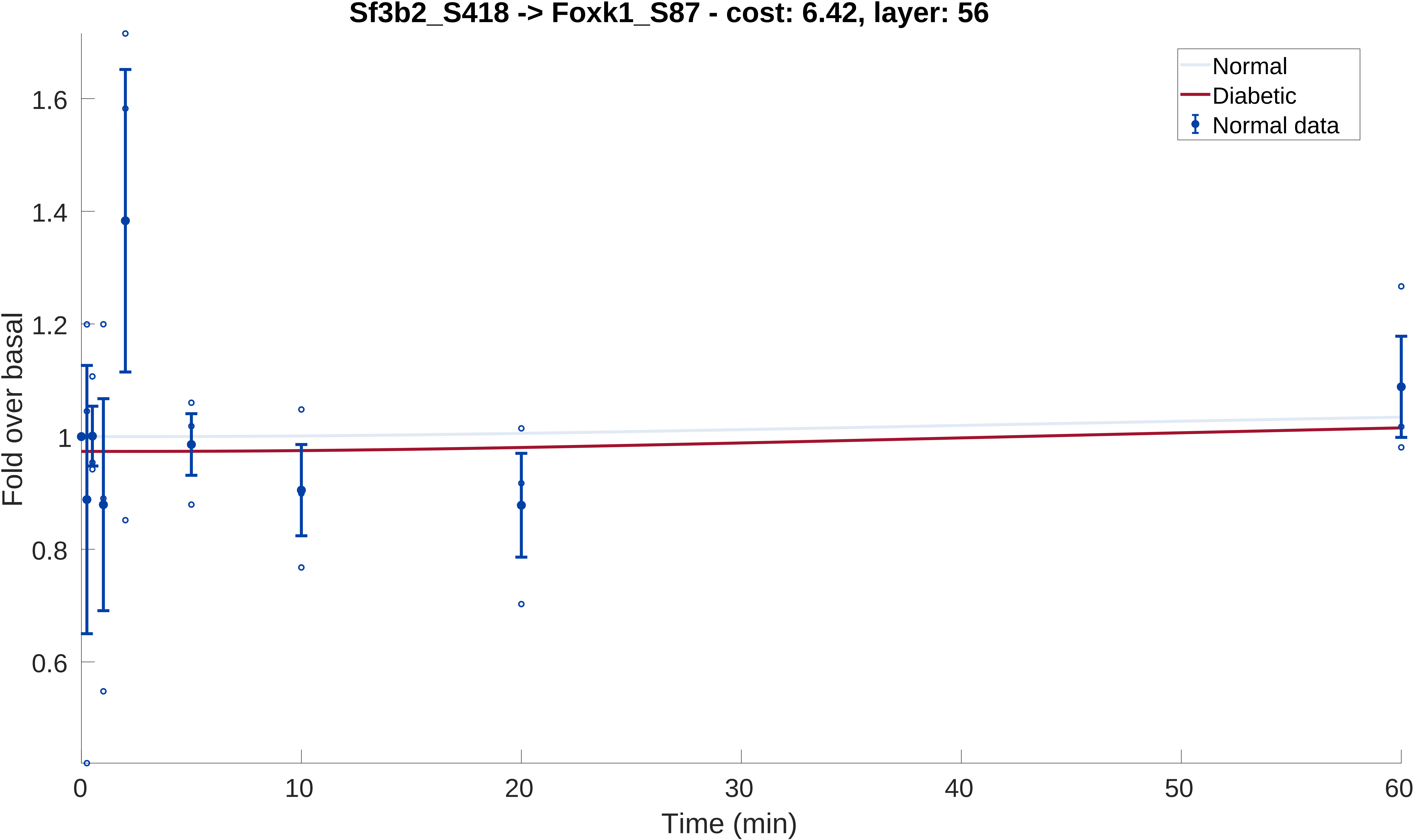
Sf3b2_S418 -> Foxk1_S87 - cost: 6.42, layer: 56
Normal
Diabetic
Normal data
Fold over basal
Time (min)
1.6
1.4
1.2
1
0.8
0.6
0
10
20
30
40
50
60

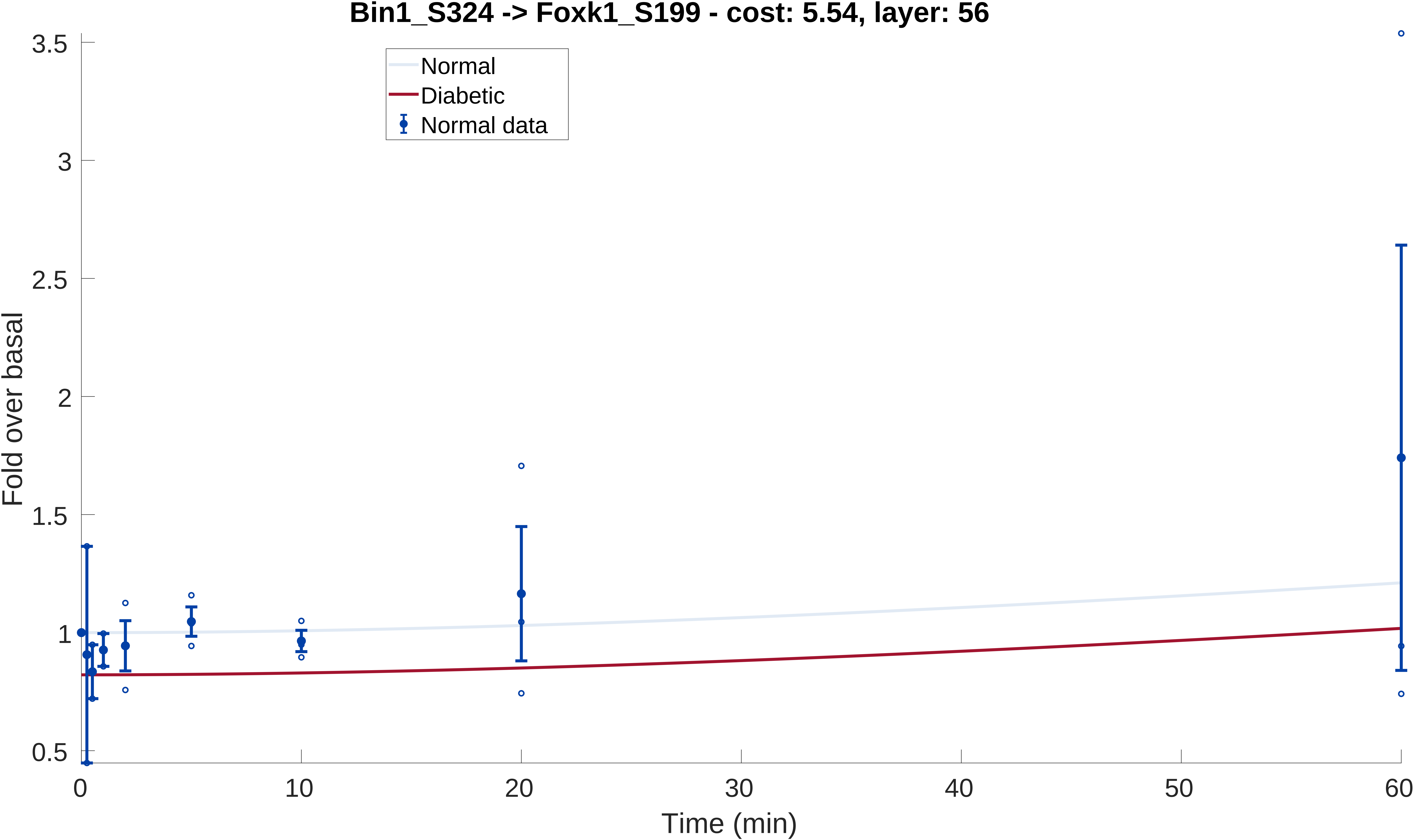

Bin1_S324 -> Foxk1_S199 - cost: 5.54, layer: 56
Normal
Diabetic
Normal data
Fold over basal
Time (min)
3.5
3
2.5
2
1.5
1
0.5
0
10
20
30
40
50
60

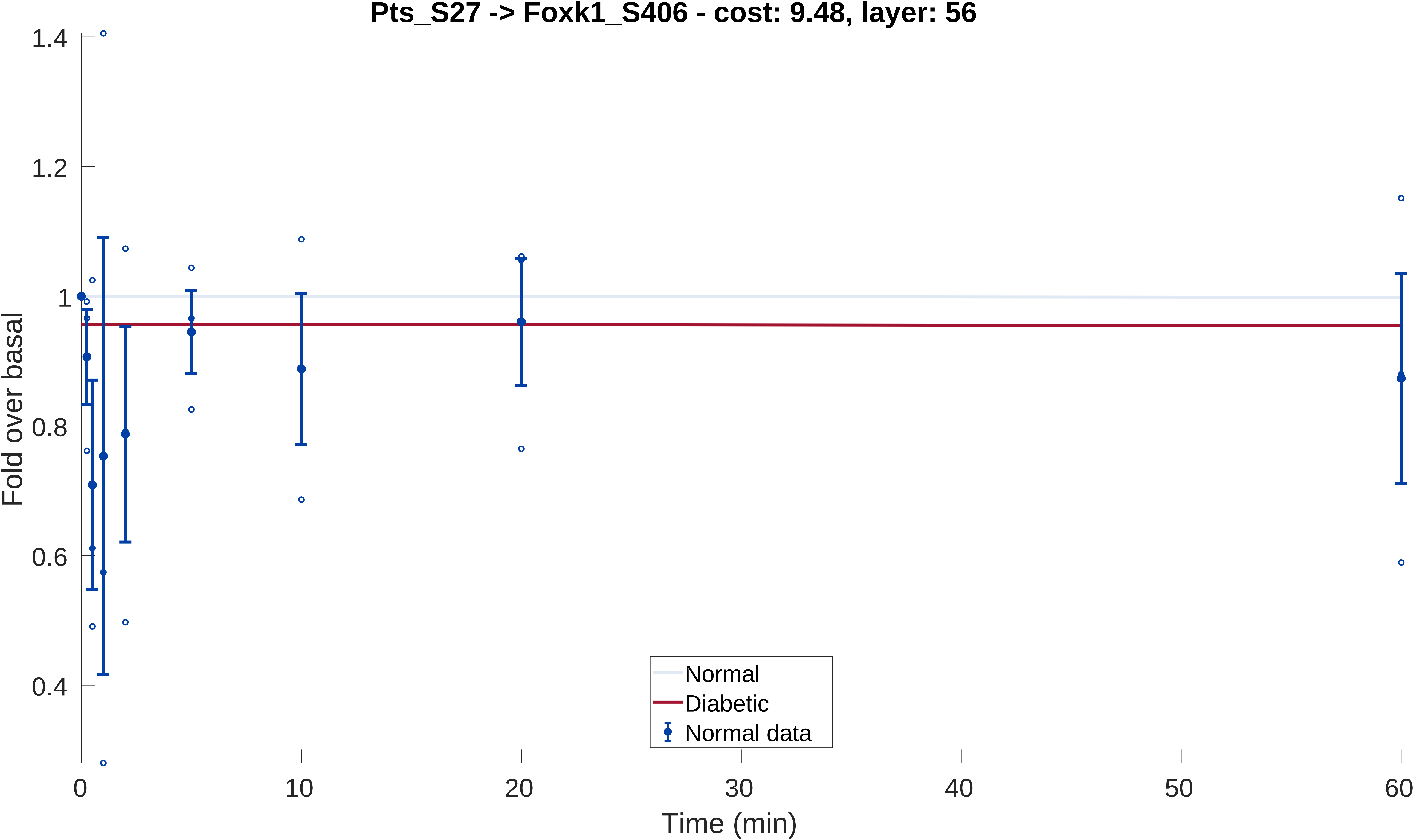

Pts_S27 -> Foxk1_S406 - cost: 9.48, layer: 56
1.4
1.2
1
0.8
0.6
0.4
Fold over basal
0
10
20
30
40
50
60
Time (min)
Normal
Diabetic
Normal data

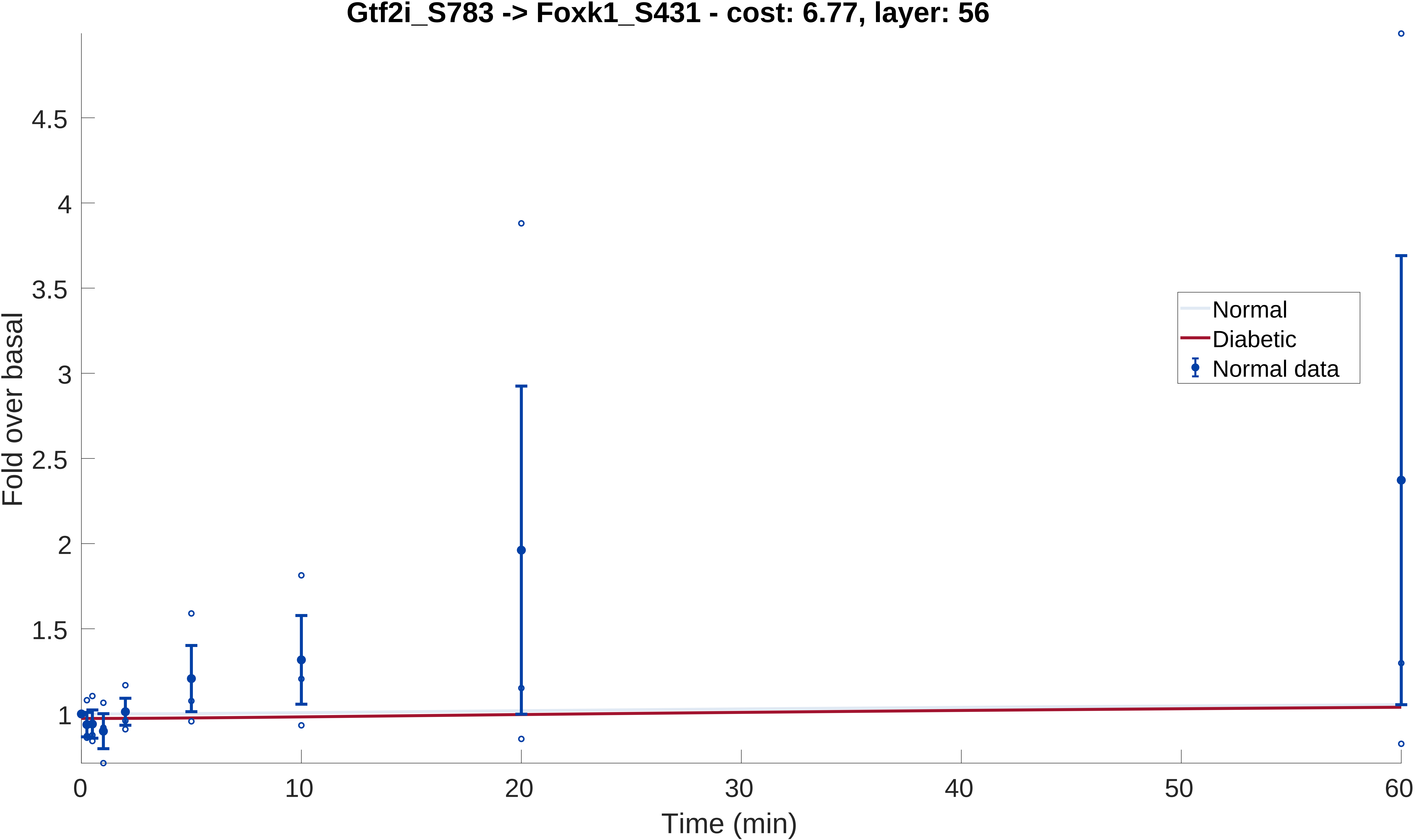

Gtf2i_S783 -> Foxk1_S431 - cost: 6.77, layer: 56
Fold over basal
4.5
4
3.5
3
2.5
2
1.5
1
0
10
20
30
40
50
60
Time (min)
Normal
Diabetic
Normal data

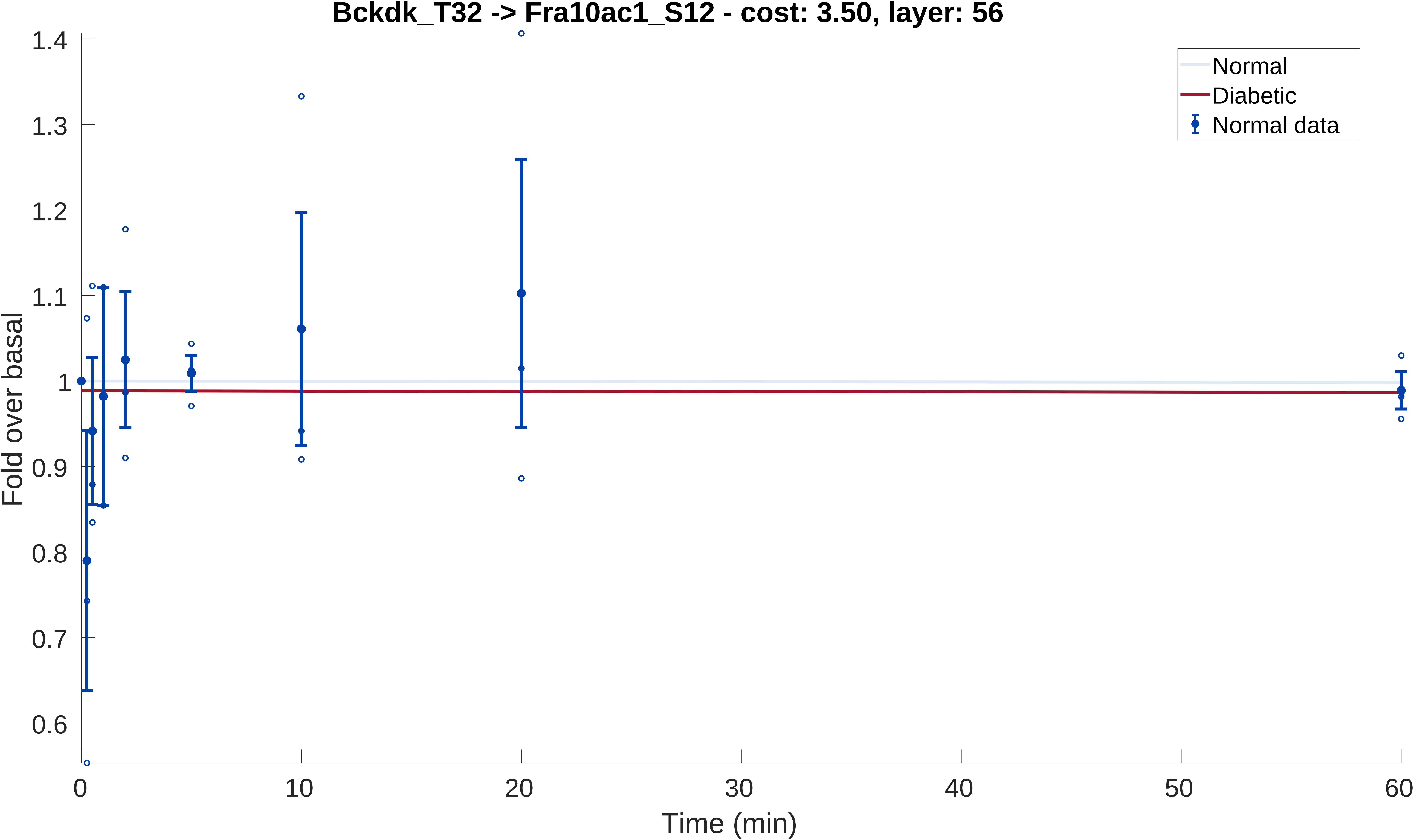

Bckdk_T32 -> Fra10ac1_S12 - cost: 3.50, layer: 56
Fold over basal
1.4
1.3
1.2
1.1
1
0.9
0.8
0.7
0.6
0
10
20
30
40
50
60
Time (min)
Normal
Diabetic
Normal data

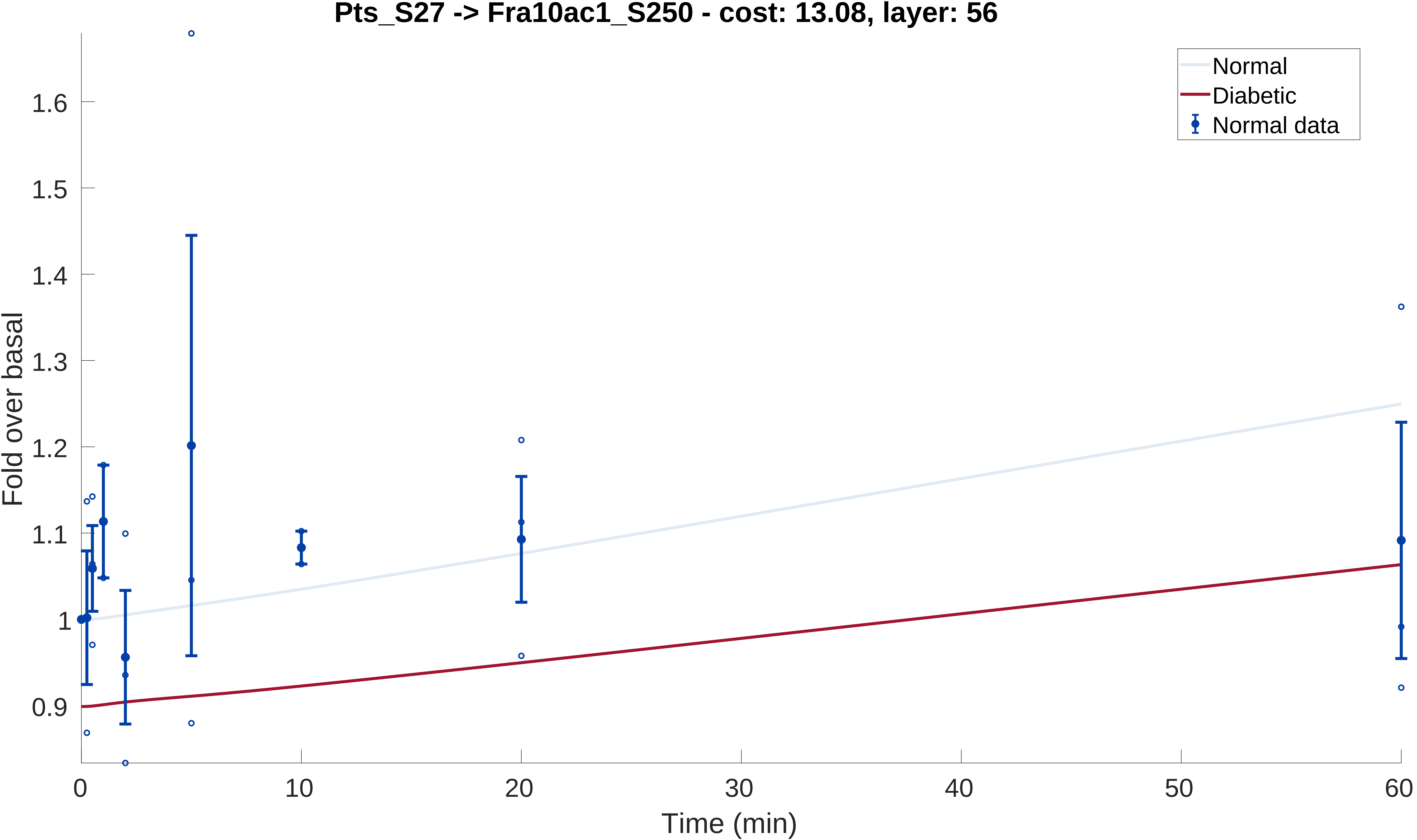

Pts_S27 -> Fra10ac1_S250 - cost: 13.08, layer: 56
Normal
Diabetic
Normal data
Fold over basal
1.6
1.5
1.4
1.3
1.2
1.1
1
0.9
0
10
20
30
40
50
60
Time (min)

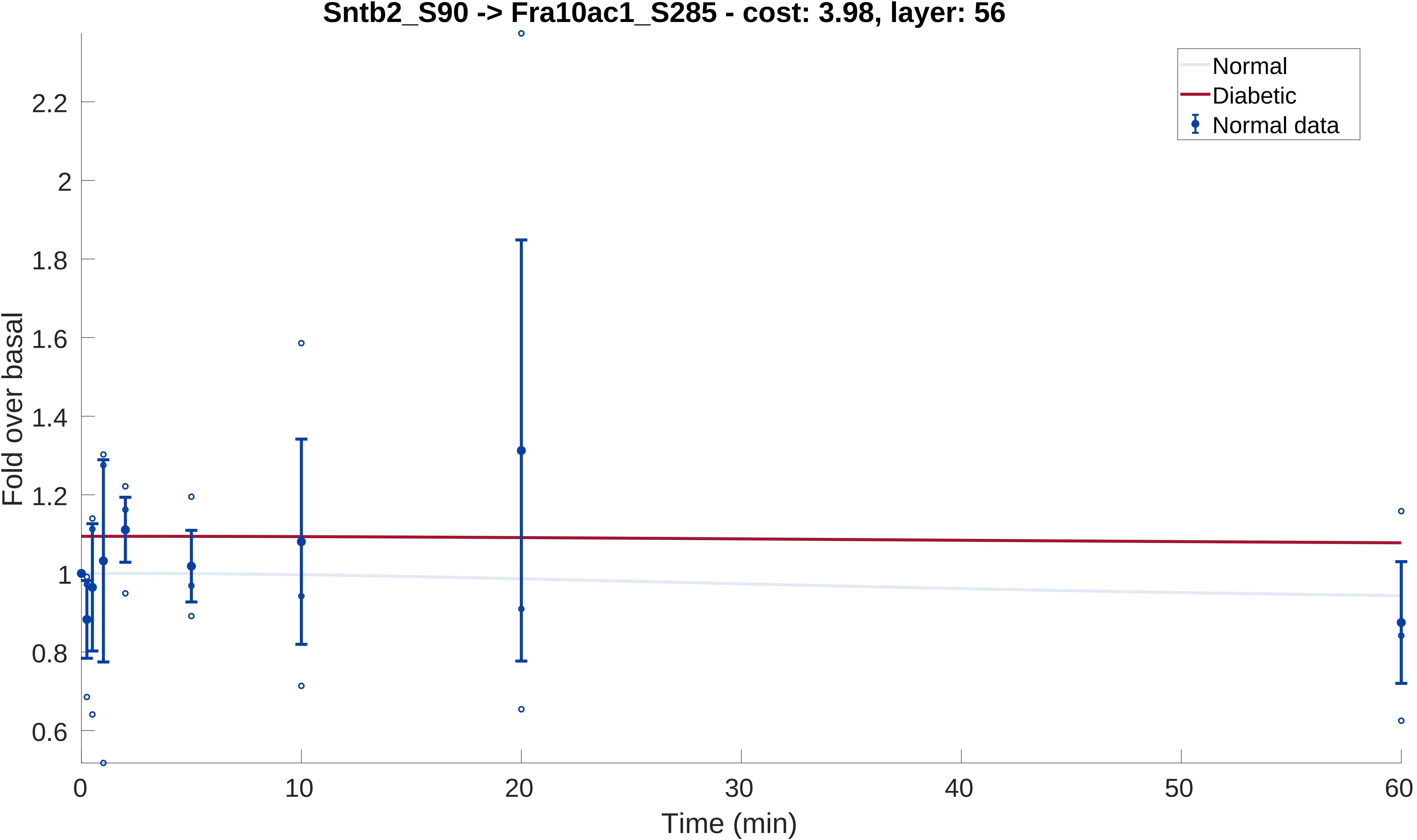

Sntb2_S90 -> Fra10ac1_S285 - cost: 3.98, layer: 56
Normal
Diabetic
Normal data
Fold over basal
Time (min)
0.6
0.8
1
1.2
1.4
1.6
1.8
2
2.2
0
10
20
30
40
50
60

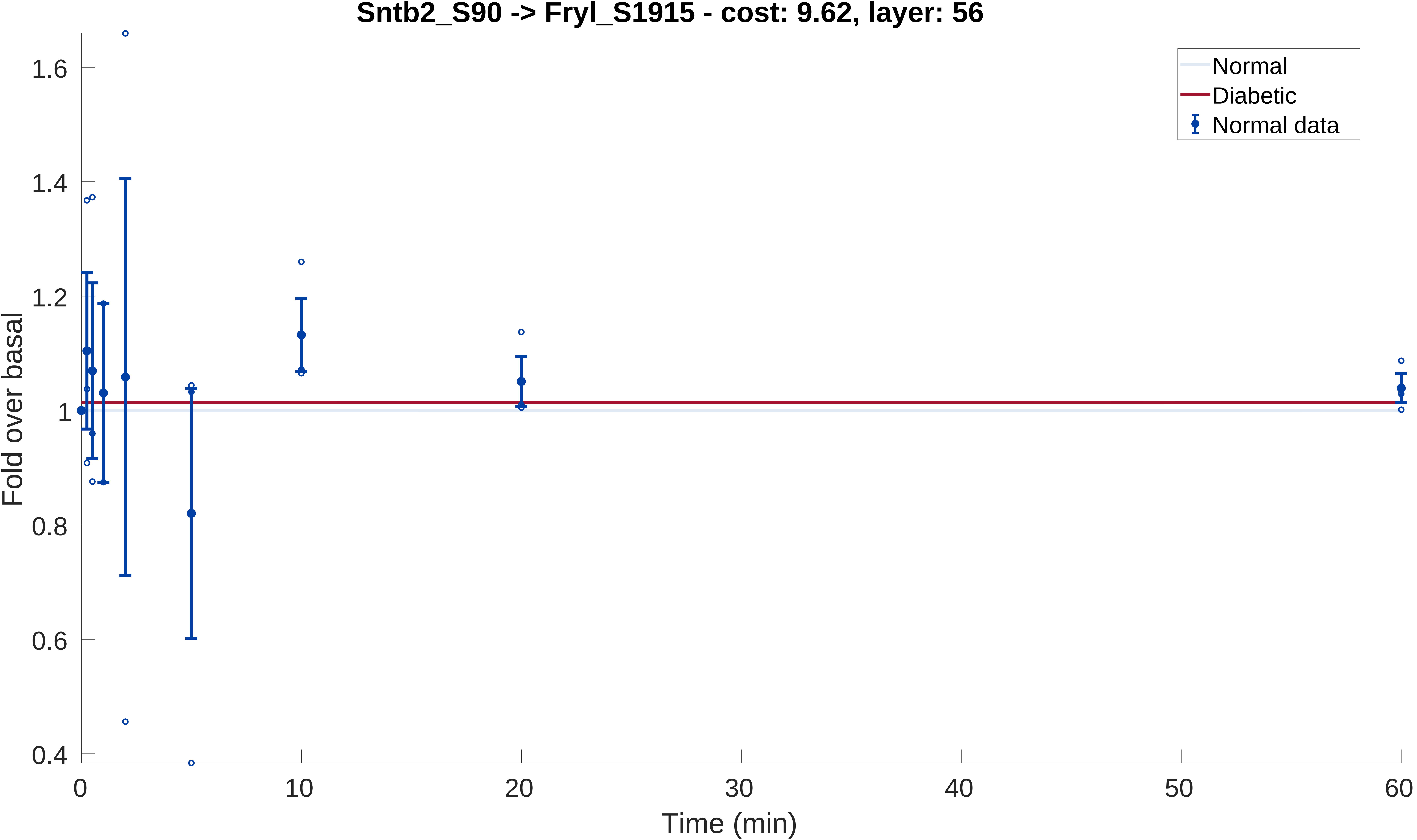

Sntb2_S90 -> Fryl_S1915 - cost: 9.62, layer: 56
Normal
Diabetic
Normal data
Fold over basal
1.6
1.4
1.2
1
0.8
0.6
0.4
0
10
20
30
40
50
60
Time (min)

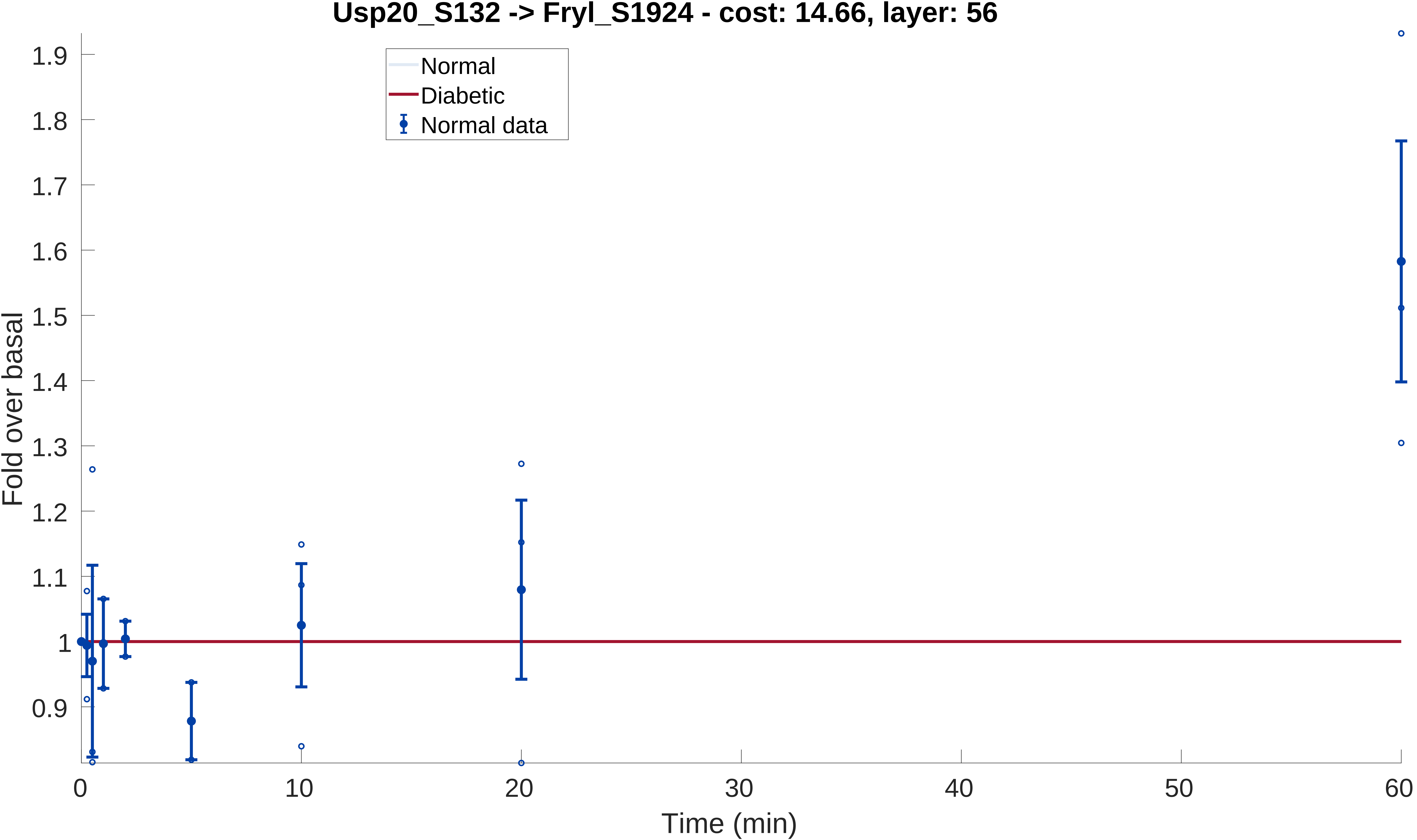

Usp20_S132 -> Fryl_S1924 - cost: 14.66, layer: 56
Normal
Diabetic
Normal data
Fold over basal
Time (min)
1.9
1.8
1.7
1.6
1.5
1.4
1.3
1.2
1.1
1
0.9
0
10
20
30
40
50
60

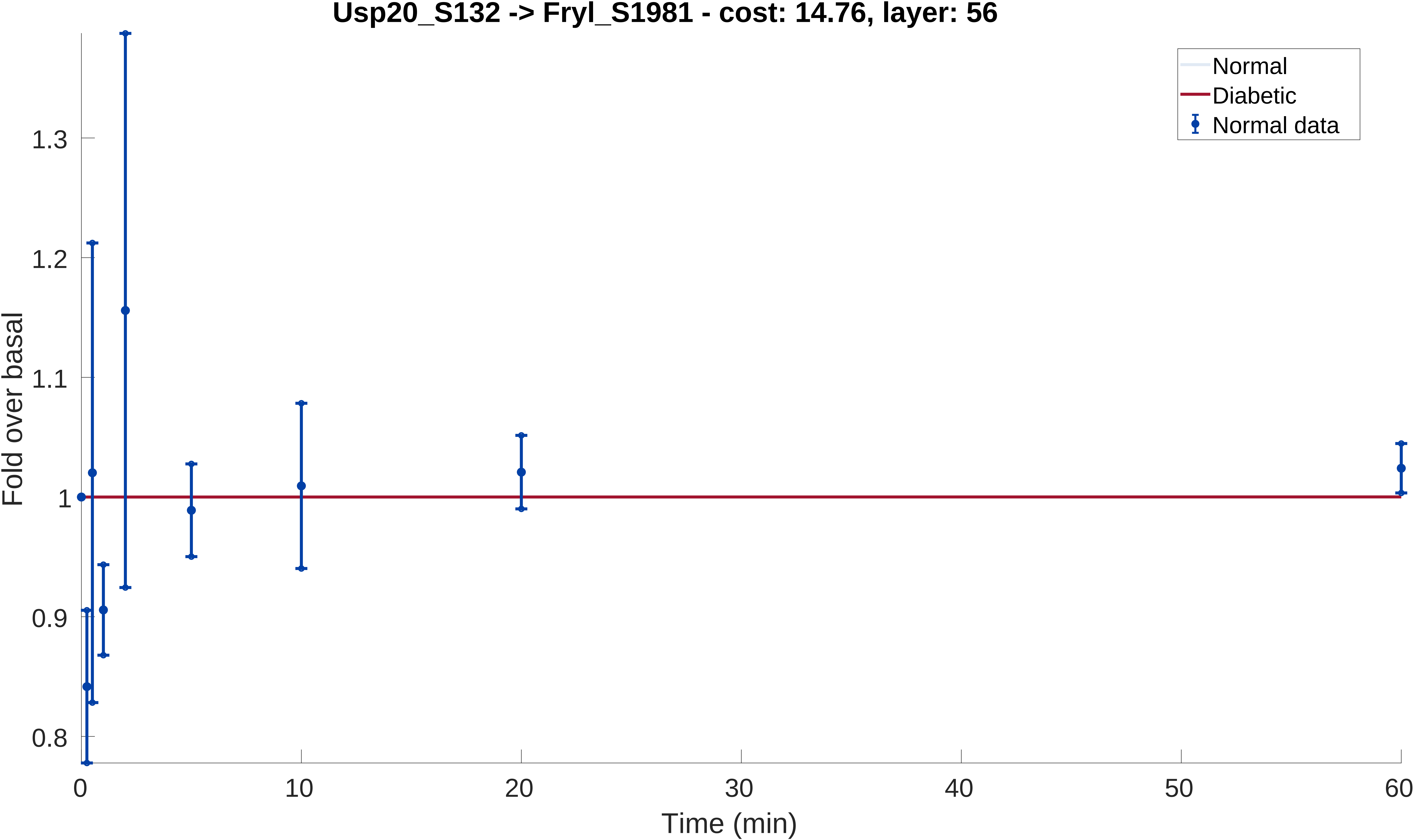

Usp20_S132 -> Fryl_S1981 - cost: 14.76, layer: 56
Normal
Diabetic
Normal data
Fold over basal
1.3
1.2
1.1
1
0.9
0.8
0
10
20
30
40
50
60
Time (min)

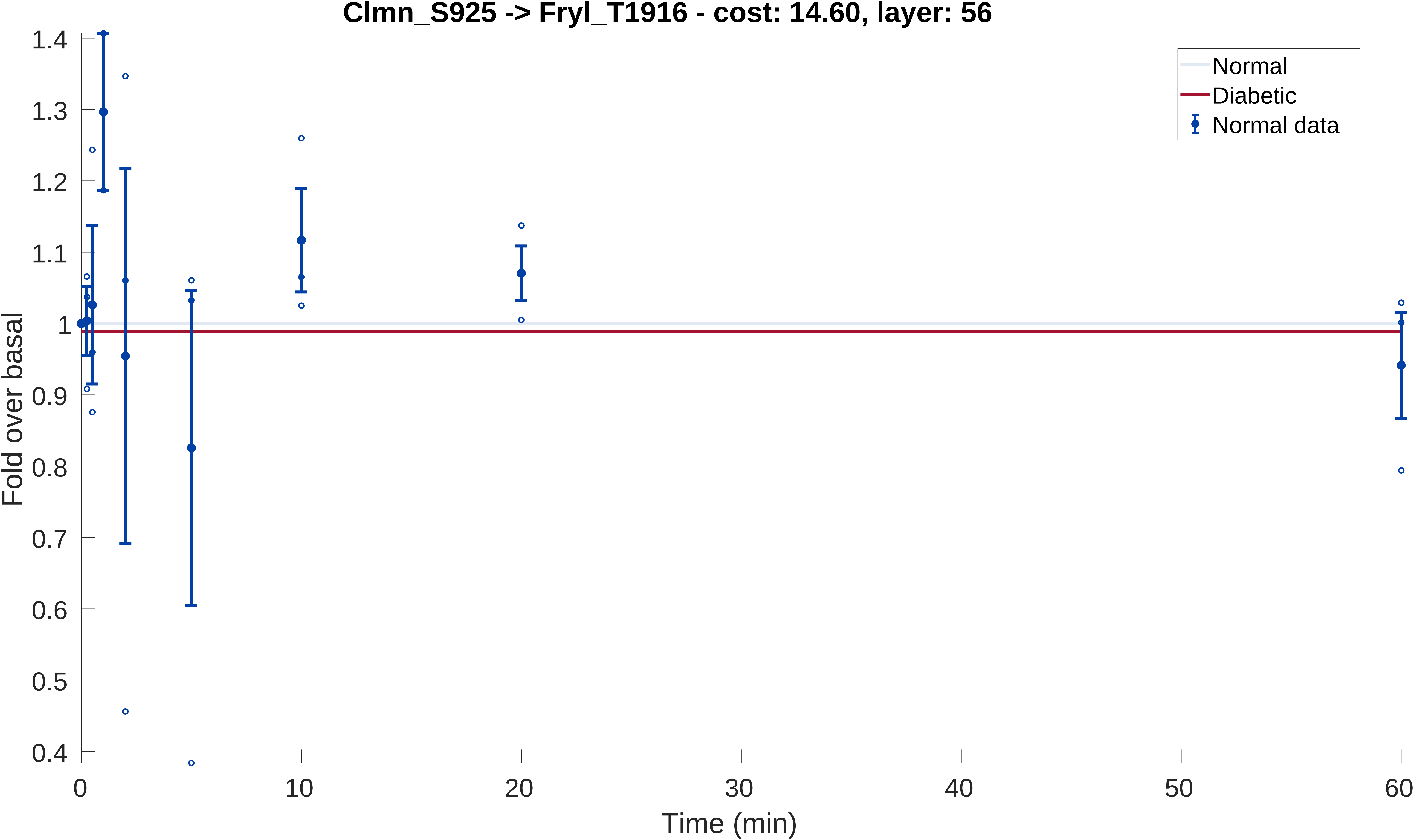

Clmn_S925 -> Fryl_T1916 - cost: 14.60, layer: 56
Fold over basal
Time (min)
Normal
Diabetic
Normal data
1.4
1.3
1.2
1.1
1
0.9
0.8
0.7
0.6
0.5
0.4
0
10
20
30
40
50
60

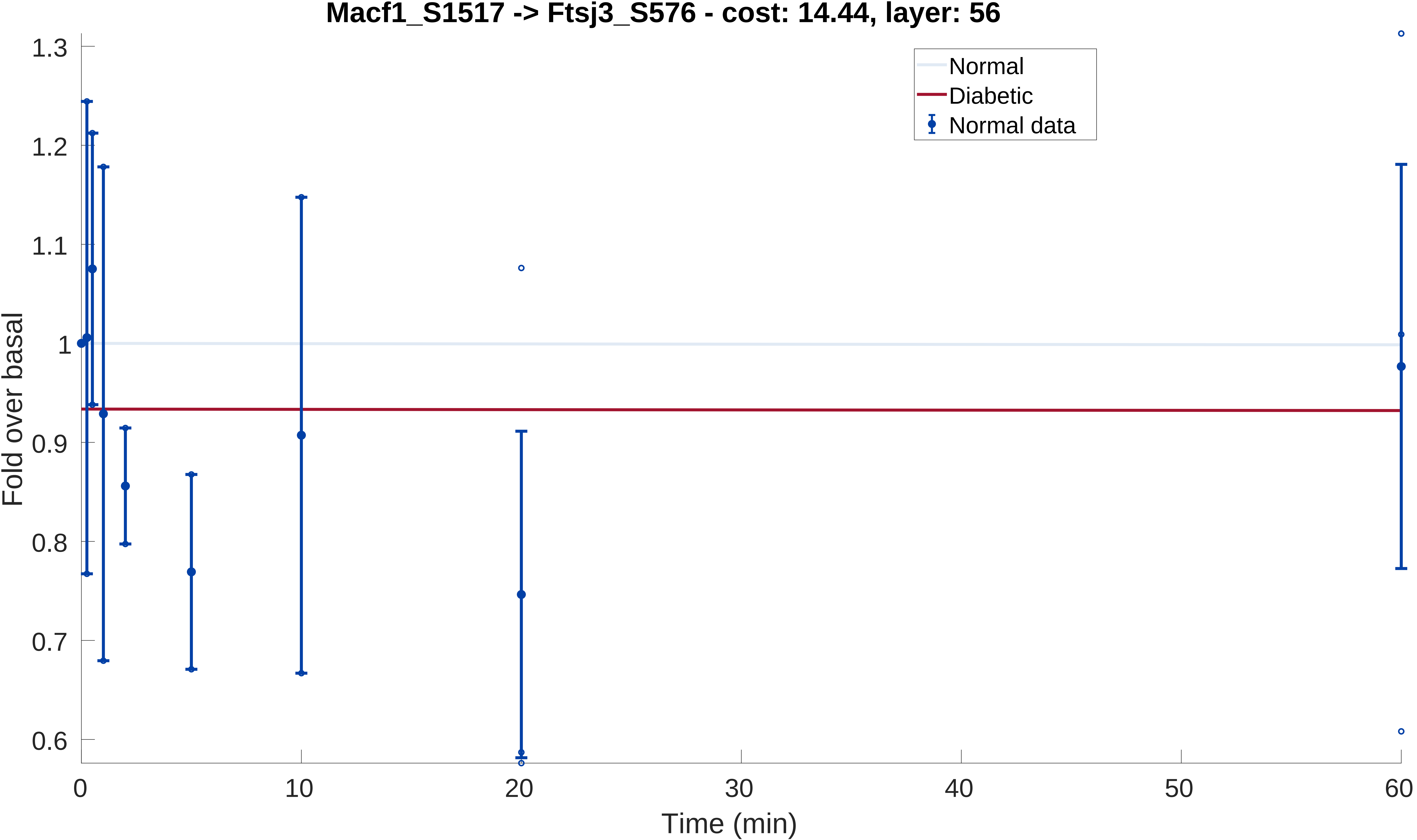

Macf1_S1517 -> Ftsj3_S576 - cost: 14.44, layer: 56
Normal
Diabetic
Normal data
Fold over basal
Time (min)
1.3
1.2
1.1
1
0.9
0.8
0.7
0.6
0
10
20
30
40
50
60

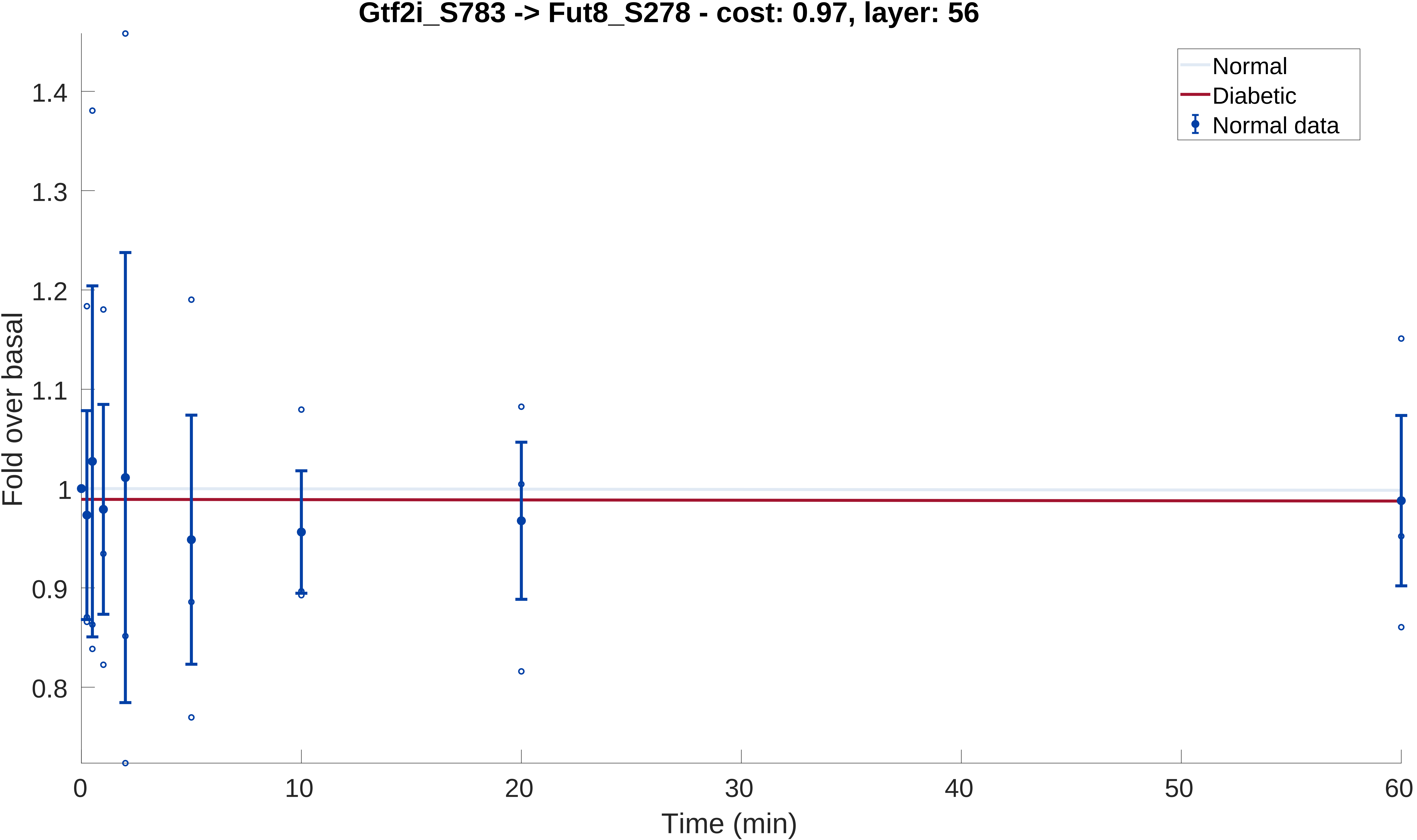

Gtf2i_S783 -> Fut8_S278 - cost: 0.97, layer: 56
Normal
Diabetic
Normal data
Fold over basal
Time (min)
0.8
0.9
1
1.1
1.2
1.3
1.4
0
10
20
30
40
50
60

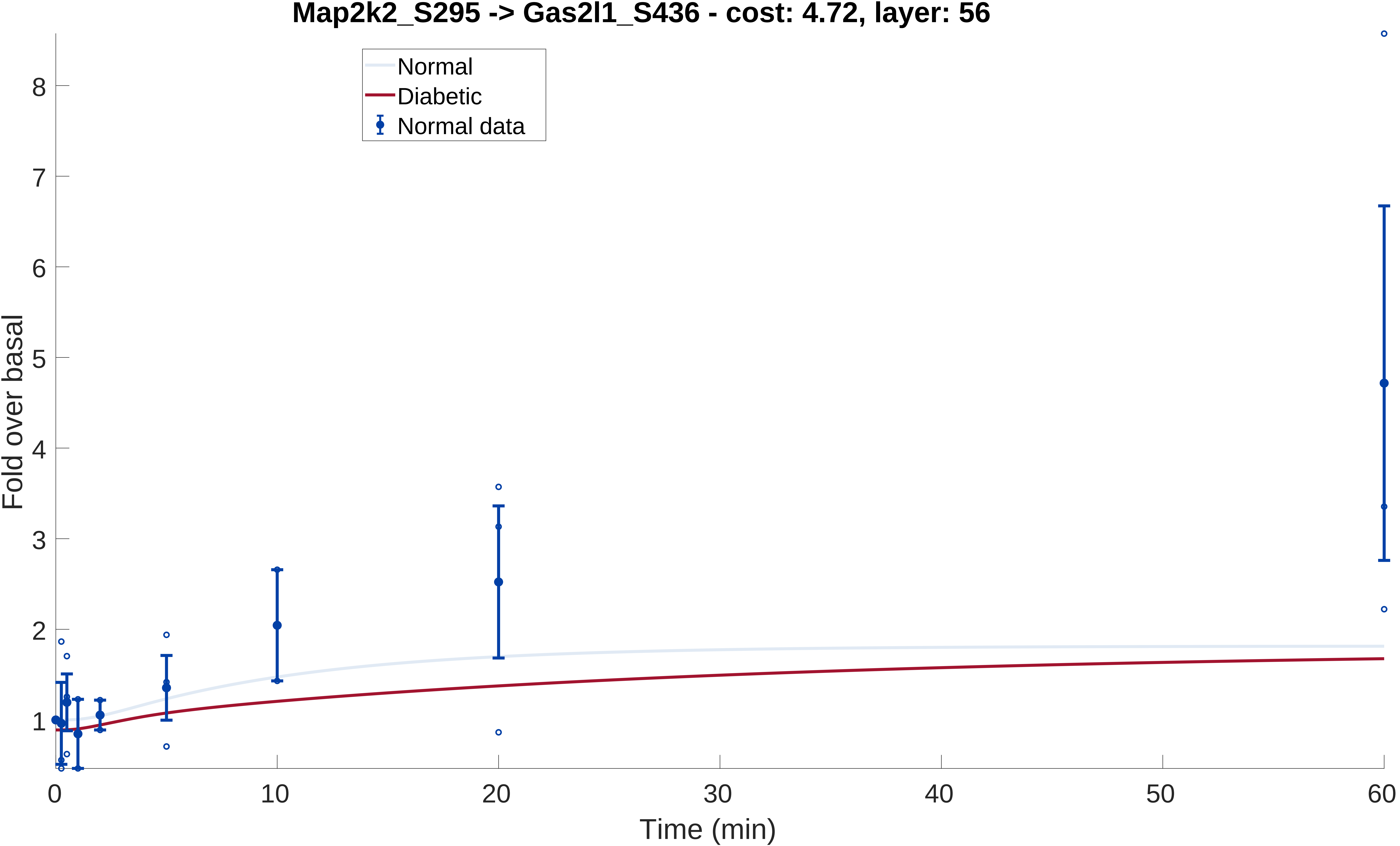
Map2k2_S295 -> Gas2l1_S436 - cost: 4.72, layer: 56
Normal
Diabetic
Normal data
Fold over basal
Time (min)
0
10
20
30
40
50
60
1
2
3
4
5
6
7
8

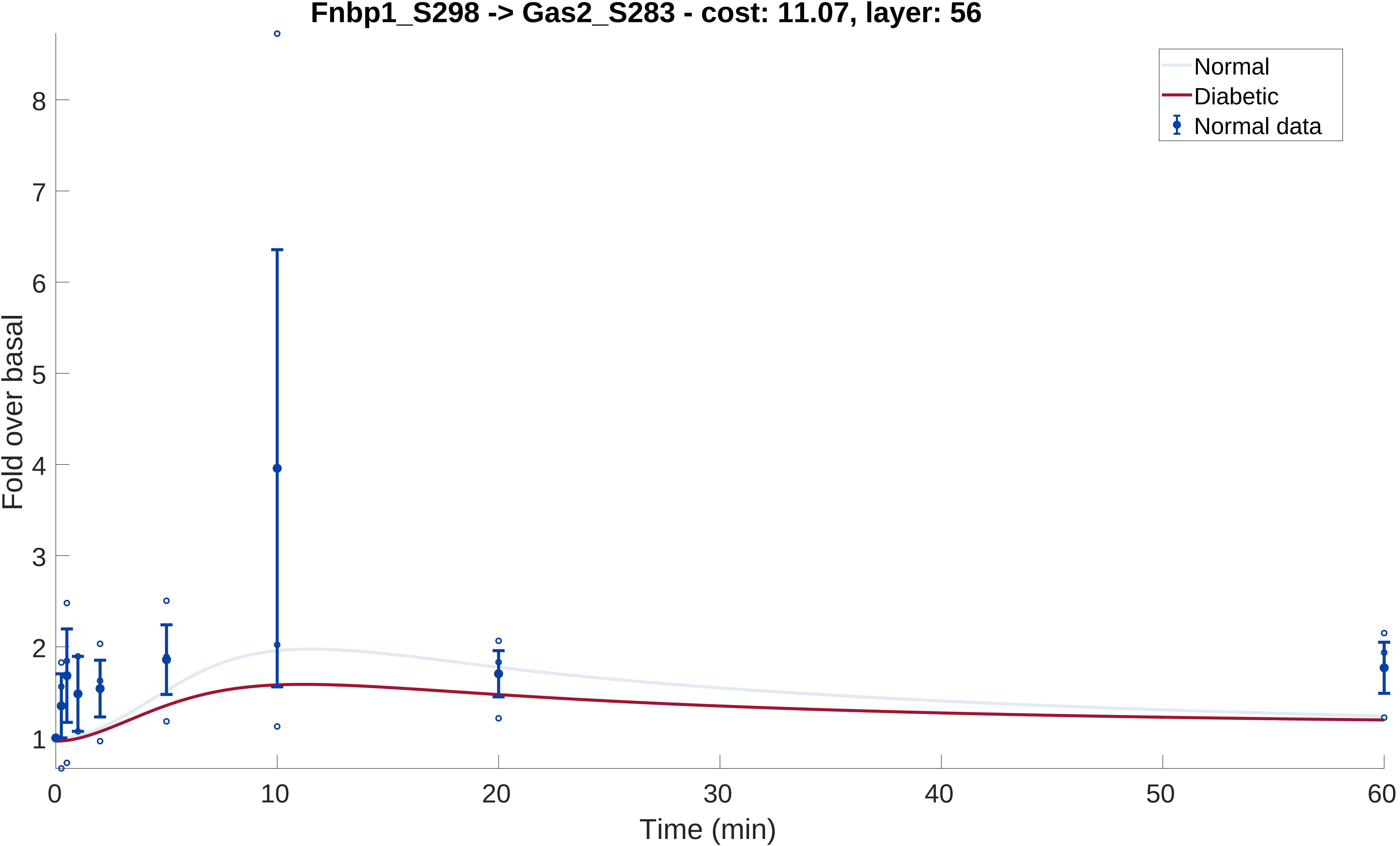

Fnbp1_S298 -> Gas2_S283 - cost: 11.07, layer: 56
Normal
Diabetic
Normal data
Fold over basal
Time (min)
1
2
3
4
5
6
7
8
0
10
20
30
40
50
60

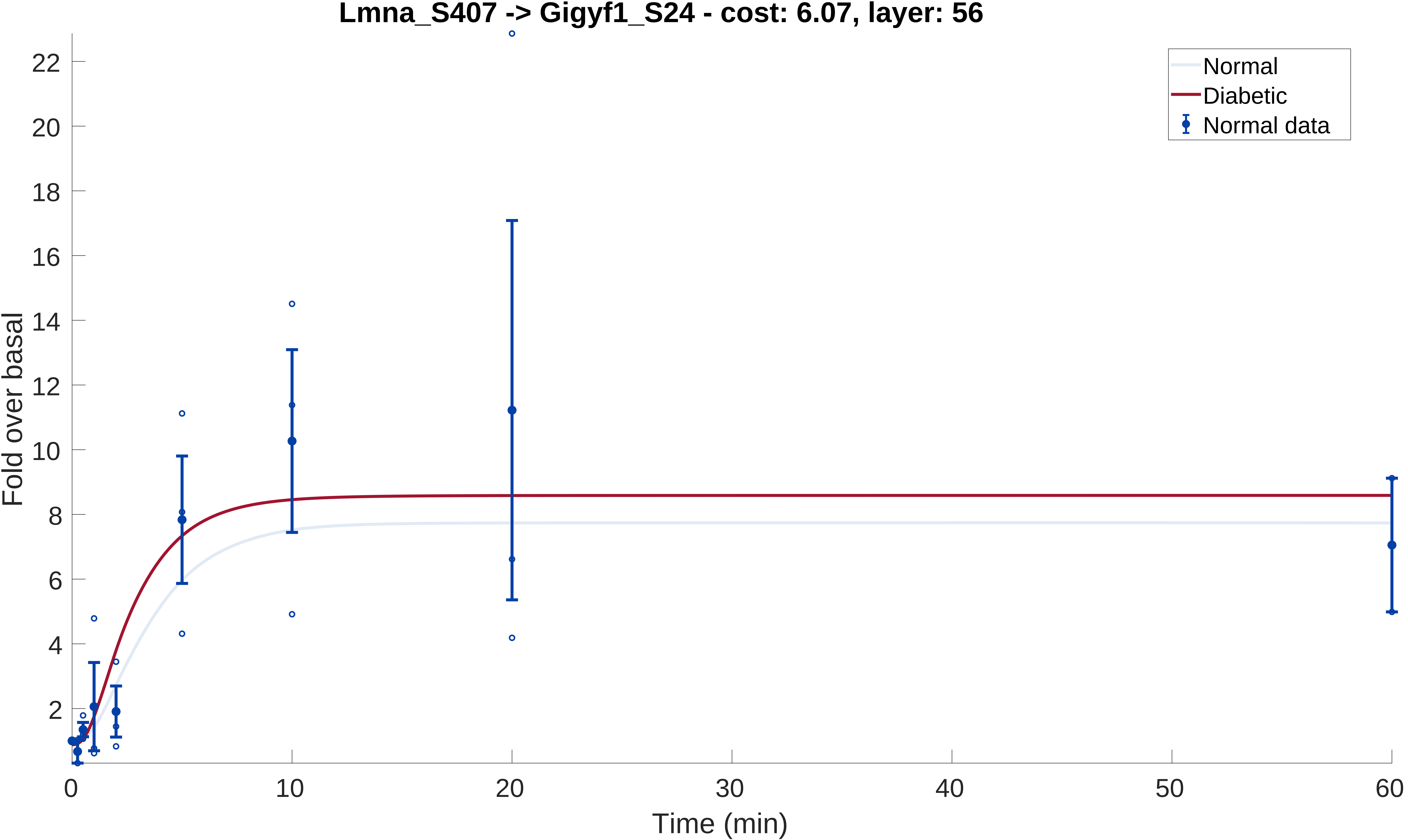

Lmna_S407 -> Gigyf1_S24 - cost: 6.07, layer: 56
Normal
Diabetic
Normal data
Fold over basal
Time (min)
0
10
20
30
40
50
60
2
4
6
8
10
12
14
16
18
20
22

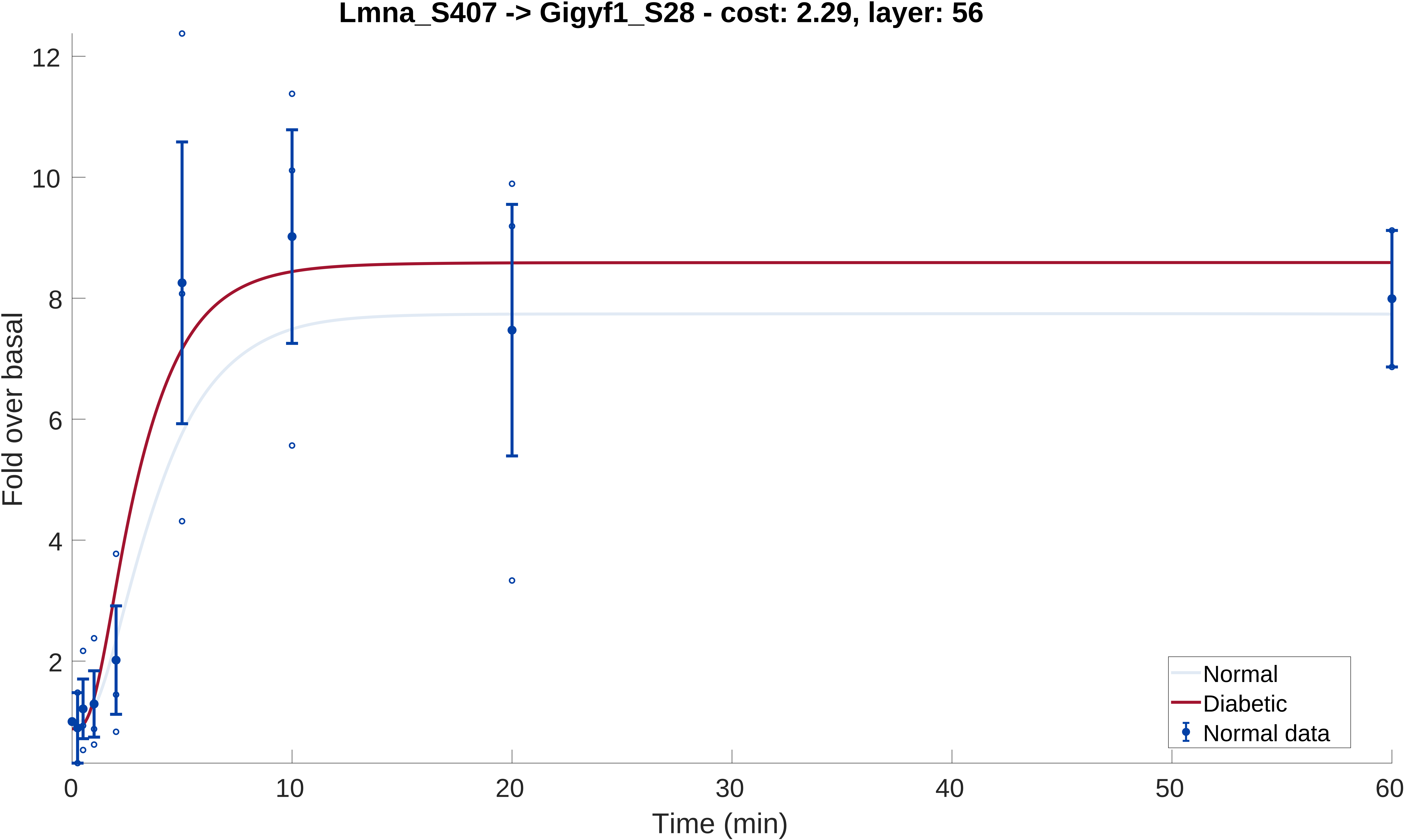
Lmna_S407 -> Gigyf1_S28 - cost: 2.29, layer: 56
Fold over basal
Time (min)
0
10
20
30
40
50
60
2
4
6
8
10
12
Normal
Diabetic
Normal data

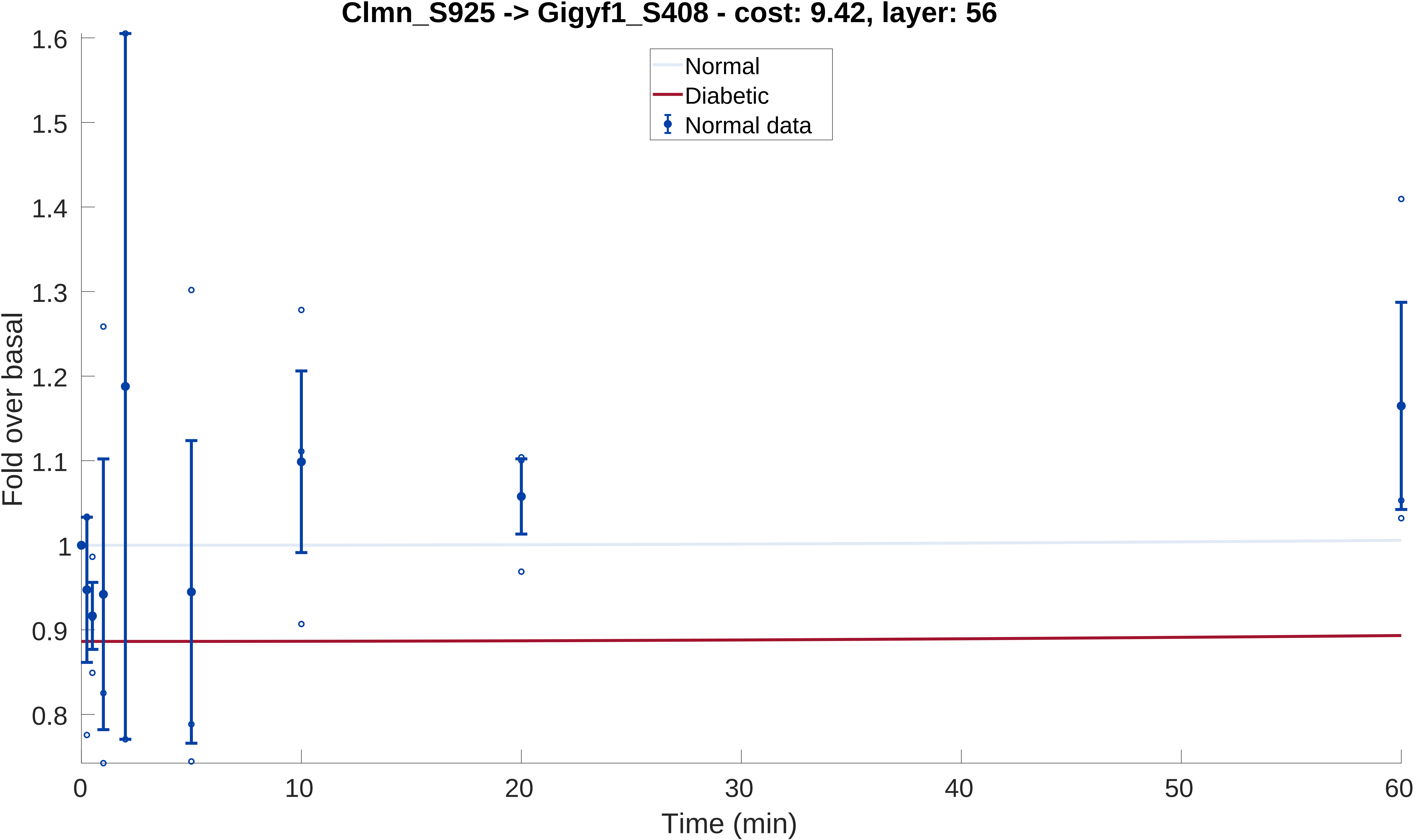

Clmn_S925 -> Gigyf1_S408 - cost: 9.42, layer: 56
Normal
Diabetic
Normal data
Fold over basal
1.6
1.5
1.4
1.3
1.2
1.1
1
0.9
0.8
0
10
20
30
40
50
60
Time (min)

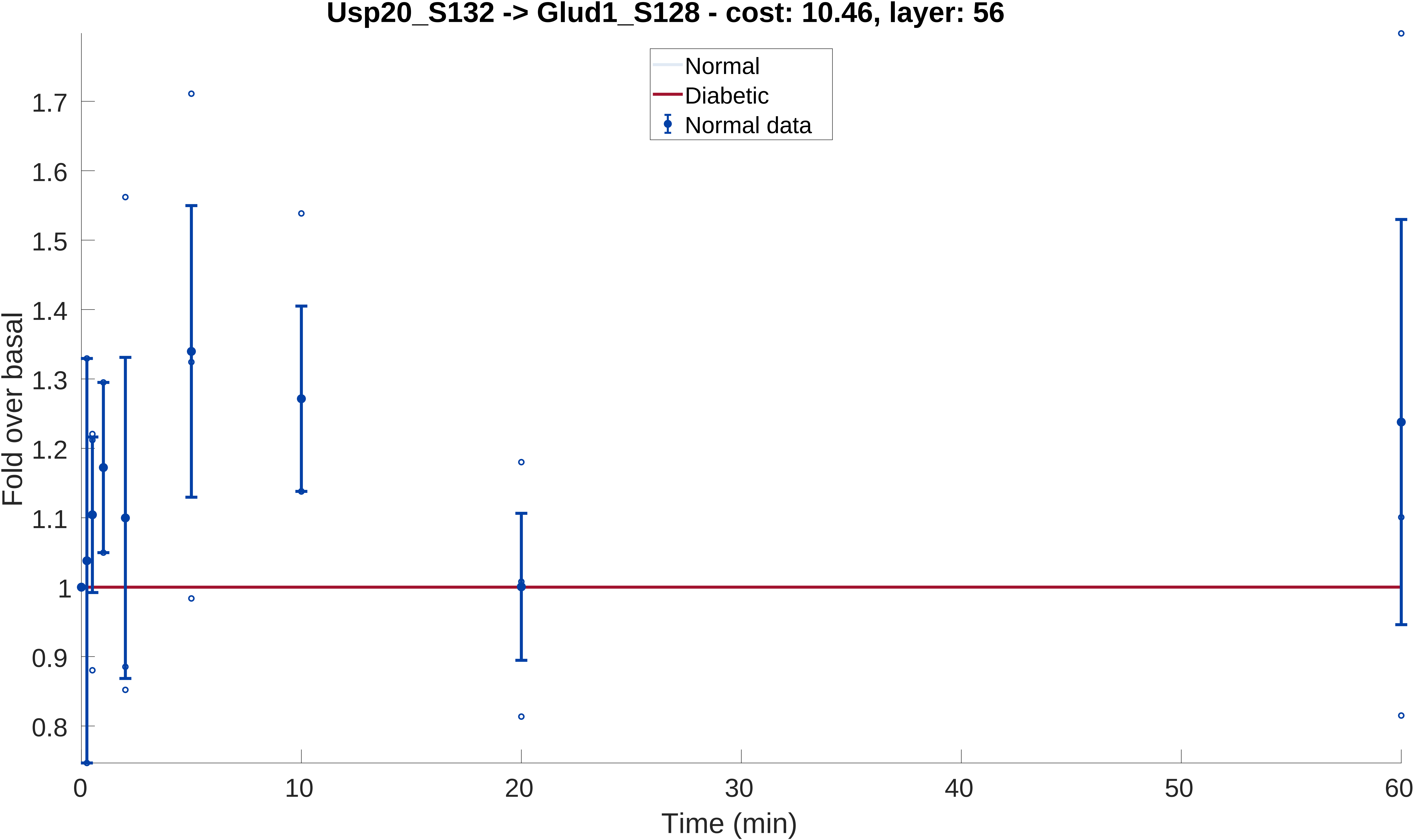

Usp20_S132 -> Glud1_S128 - cost: 10.46, layer: 56
Normal
Diabetic
Normal data
Fold over basal
1.7
1.6
1.5
1.4
1.3
1.2
1.1
1
0.9
0.8
0
10
20
30
40
50
60
Time (min)

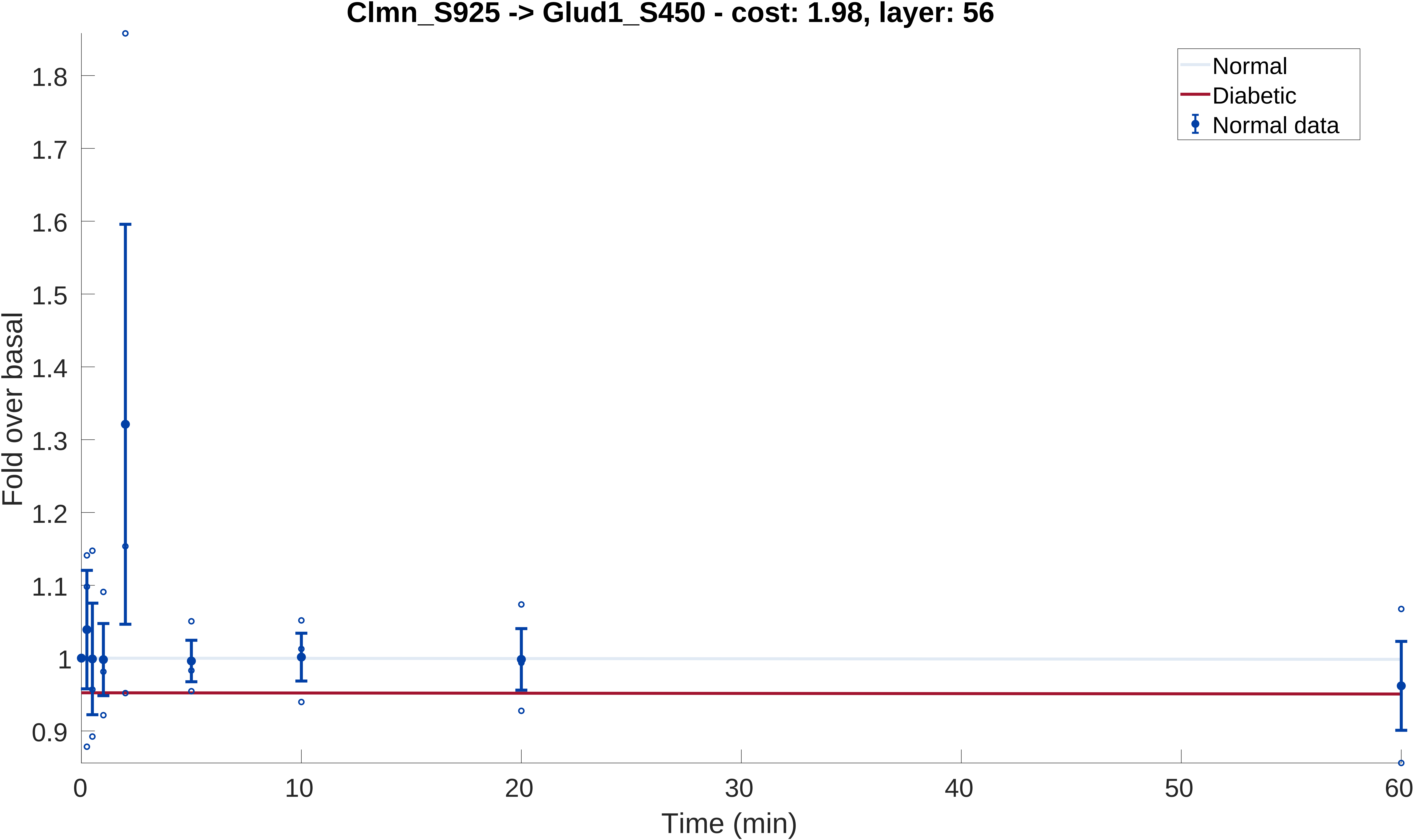
Clmn_S925 -> Glud1_S450 - cost: 1.98, layer: 56
Normal
Diabetic
Normal data
Fold over basal
Time (min)
0.9
1
1.1
1.2
1.3
1.4
1.5
1.6
1.7
1.8
0
10
20
30
40
50
60

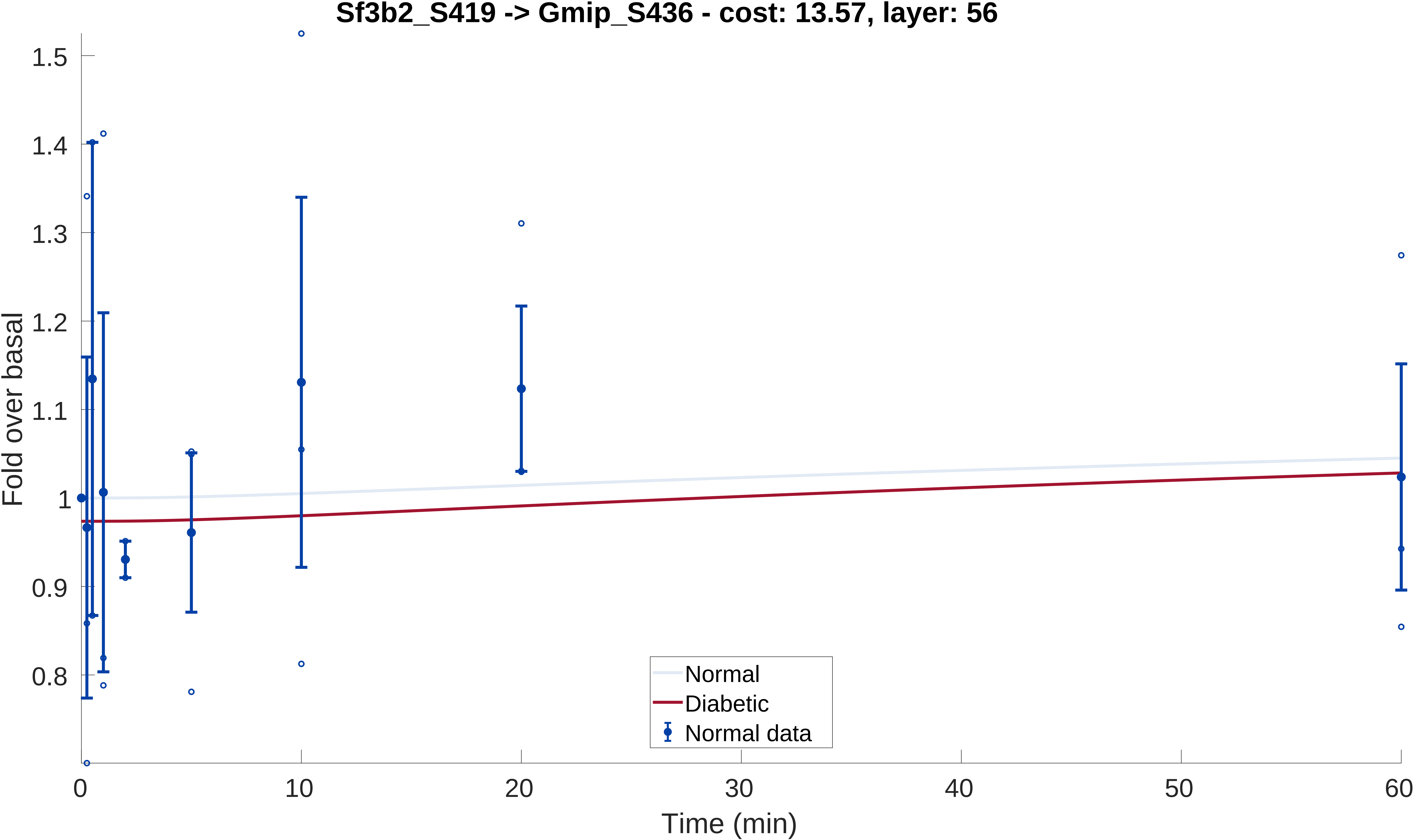

Sf3b2_S419 -> Gmip_S436 - cost: 13.57, layer: 56
Fold over basal
1.5
1.4
1.3
1.2
1.1
1
0.9
0.8
0
10
20
30
40
50
60
Time (min)
Normal
Diabetic
Normal data

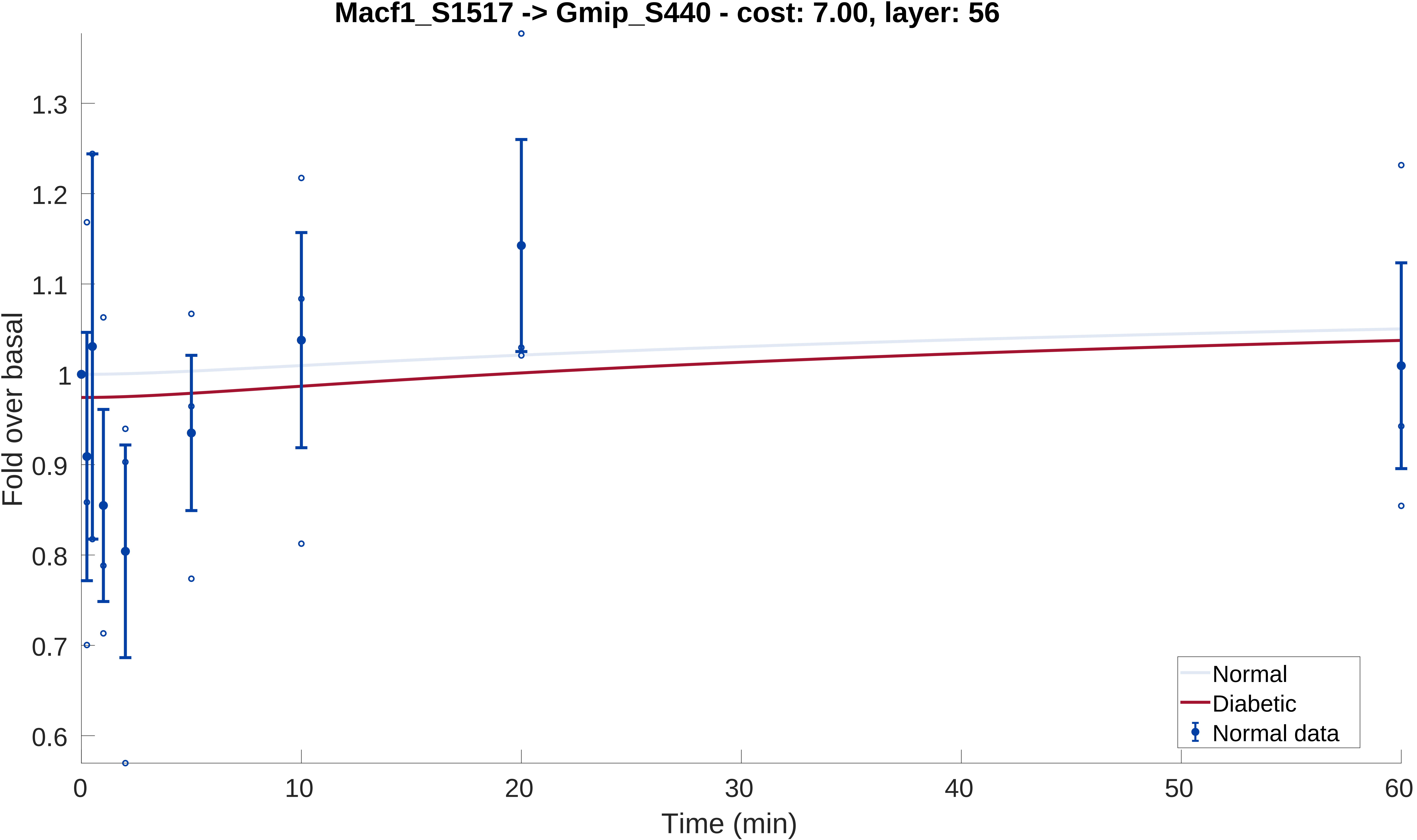
Macf1_S1517 -> Gmip_S440 - cost: 7.00, layer: 56
Fold over basal
1.3
1.2
1.1
1
0.9
0.8
0.7
0.6
0
10
20
30
40
50
60
Time (min)
Normal
Diabetic
Normal data

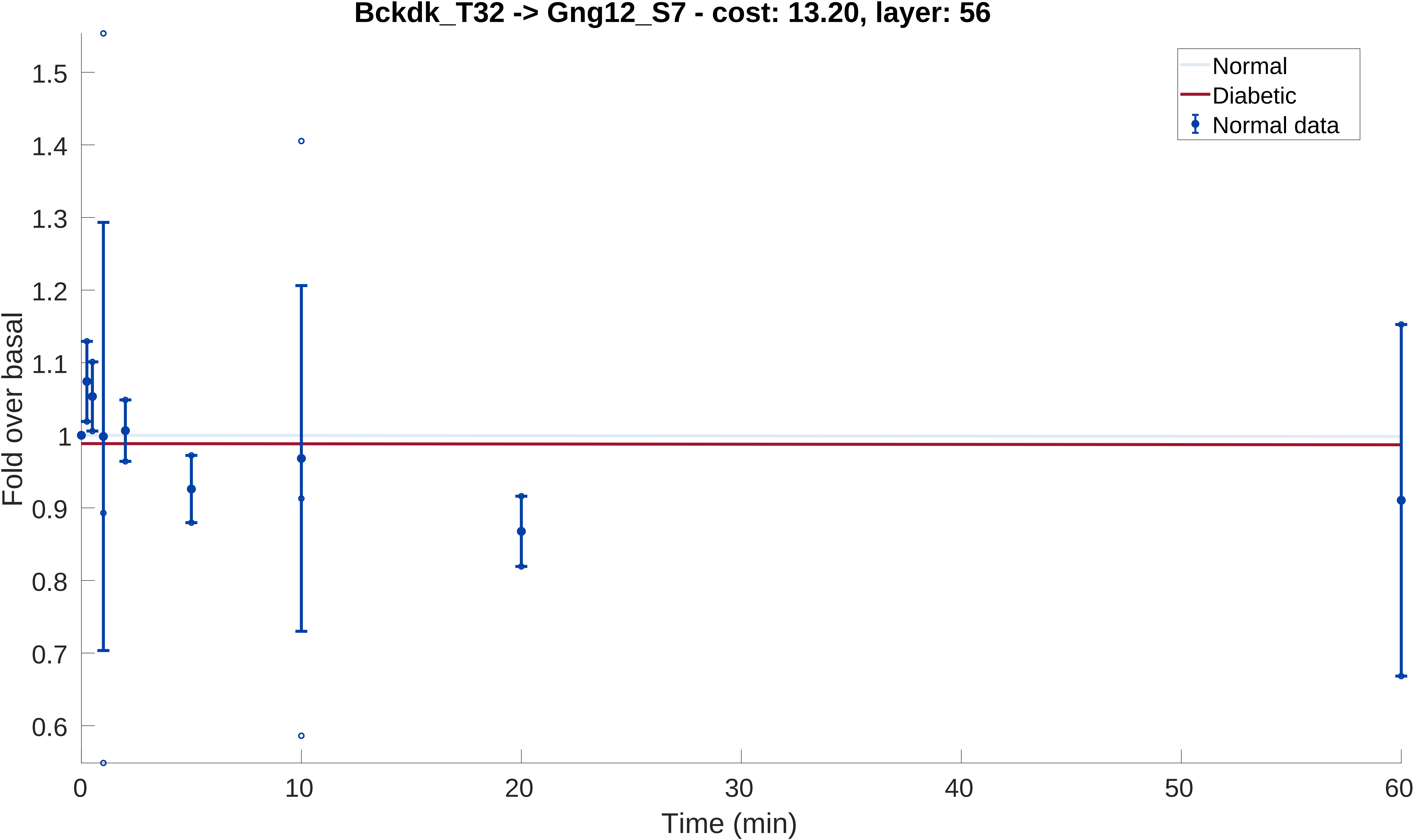

Bckdk_T32 -> Gng12_S7 - cost: 13.20, layer: 56
Normal
Diabetic
Normal data
Fold over basal
Time (min)
1.5
1.4
1.3
1.2
1.1
1
0.9
0.8
0.7
0.6
0
10
20
30
40
50
60

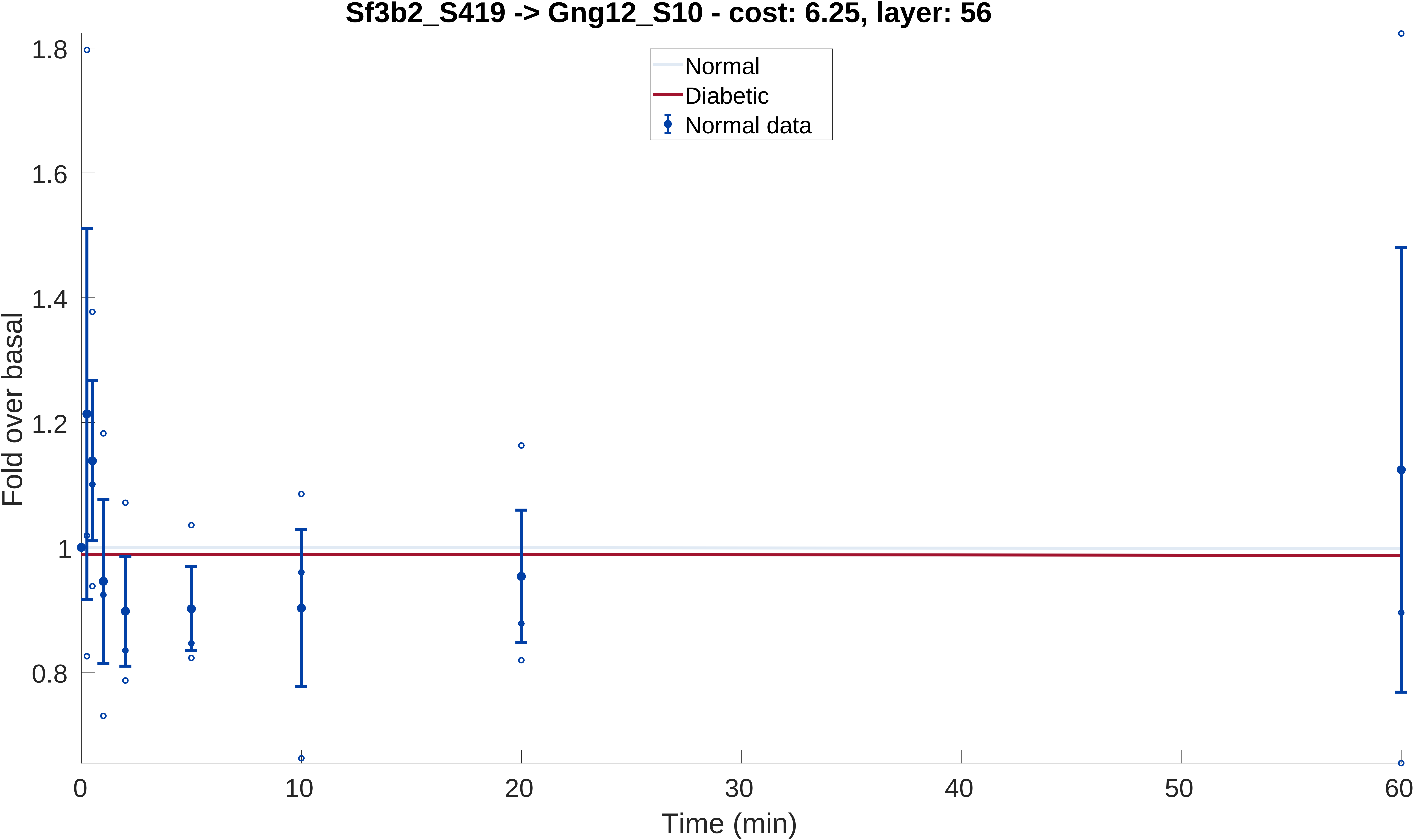

Sf3b2_S419 -> Gng12_S10 - cost: 6.25, layer: 56
Normal
Diabetic
Normal data
Fold over basal
1.8
1.6
1.4
1.2
1
0.8
0
10
20
30
40
50
60
Time (min)

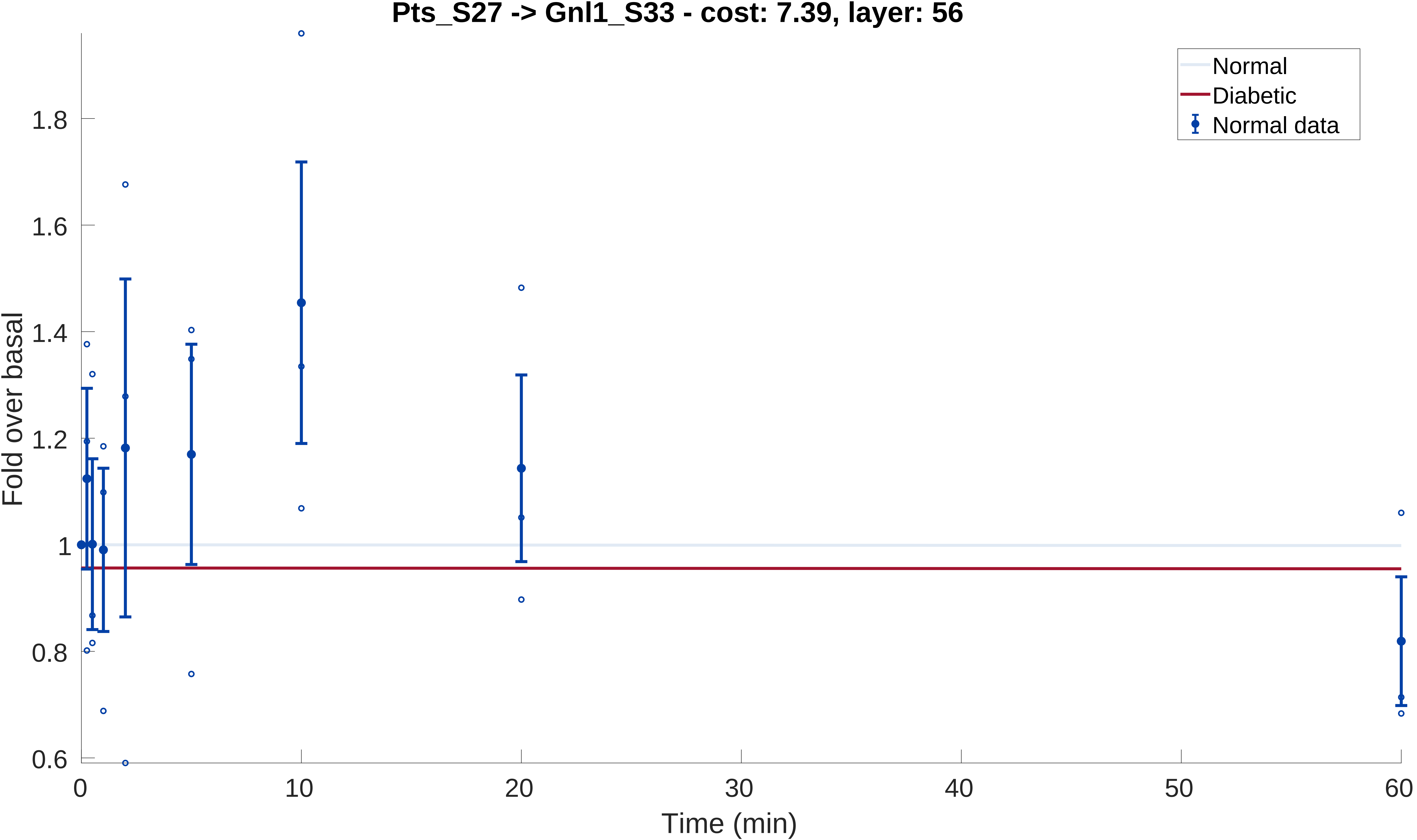

Pts_S27 -> Gnl1_S33 - cost: 7.39, layer: 56
Normal
Diabetic
Normal data
Fold over basal
1.8
1.6
1.4
1.2
1
0.8
0.6
0
10
20
30
40
50
60
Time (min)

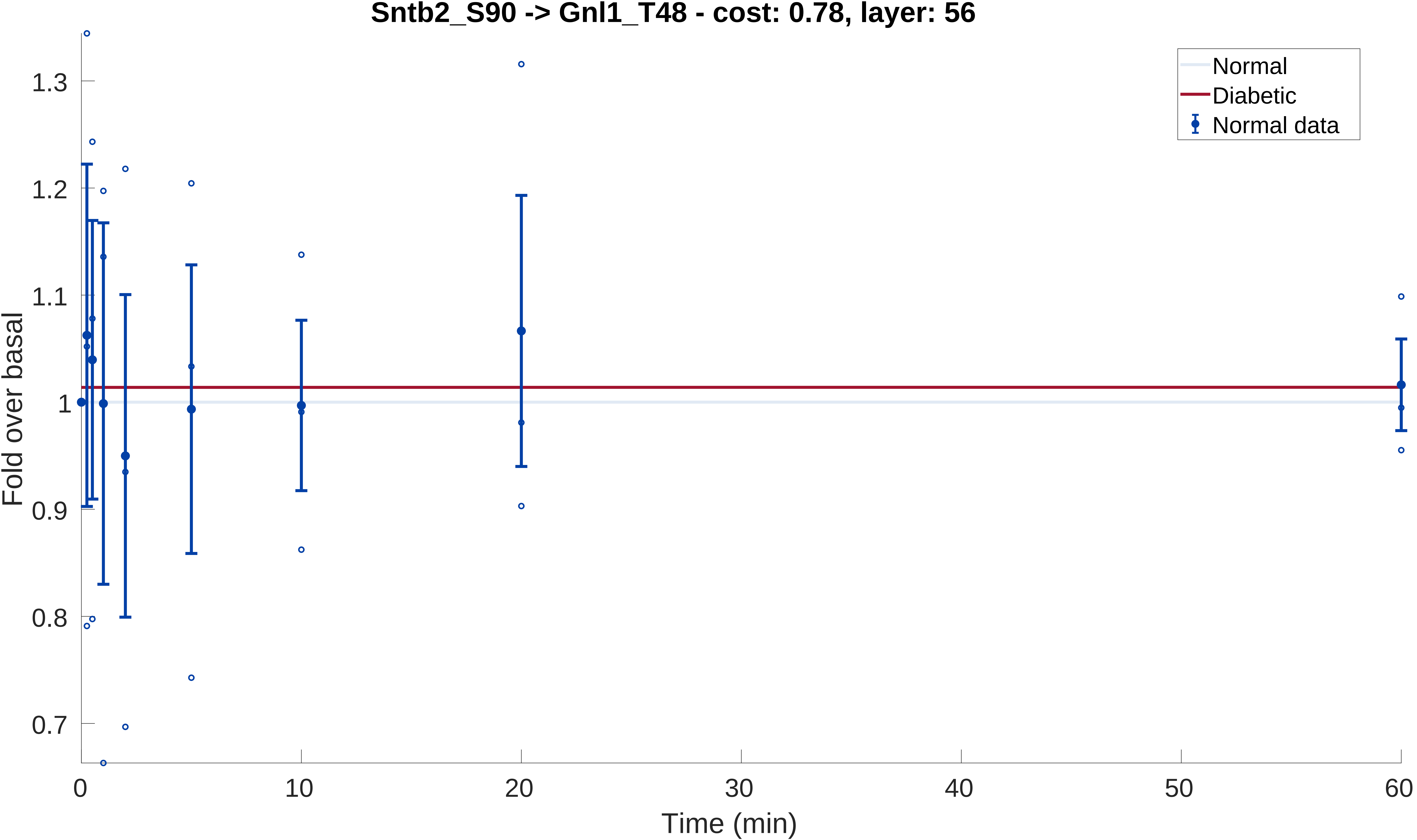

Sntb2_S90 -> Gnl1_T48 - cost: 0.78, layer: 56
Normal
Diabetic
Normal data
Fold over basal
1.3
1.2
1.1
1
0.9
0.8
0.7
0
10
20
30
40
50
60
Time (min)

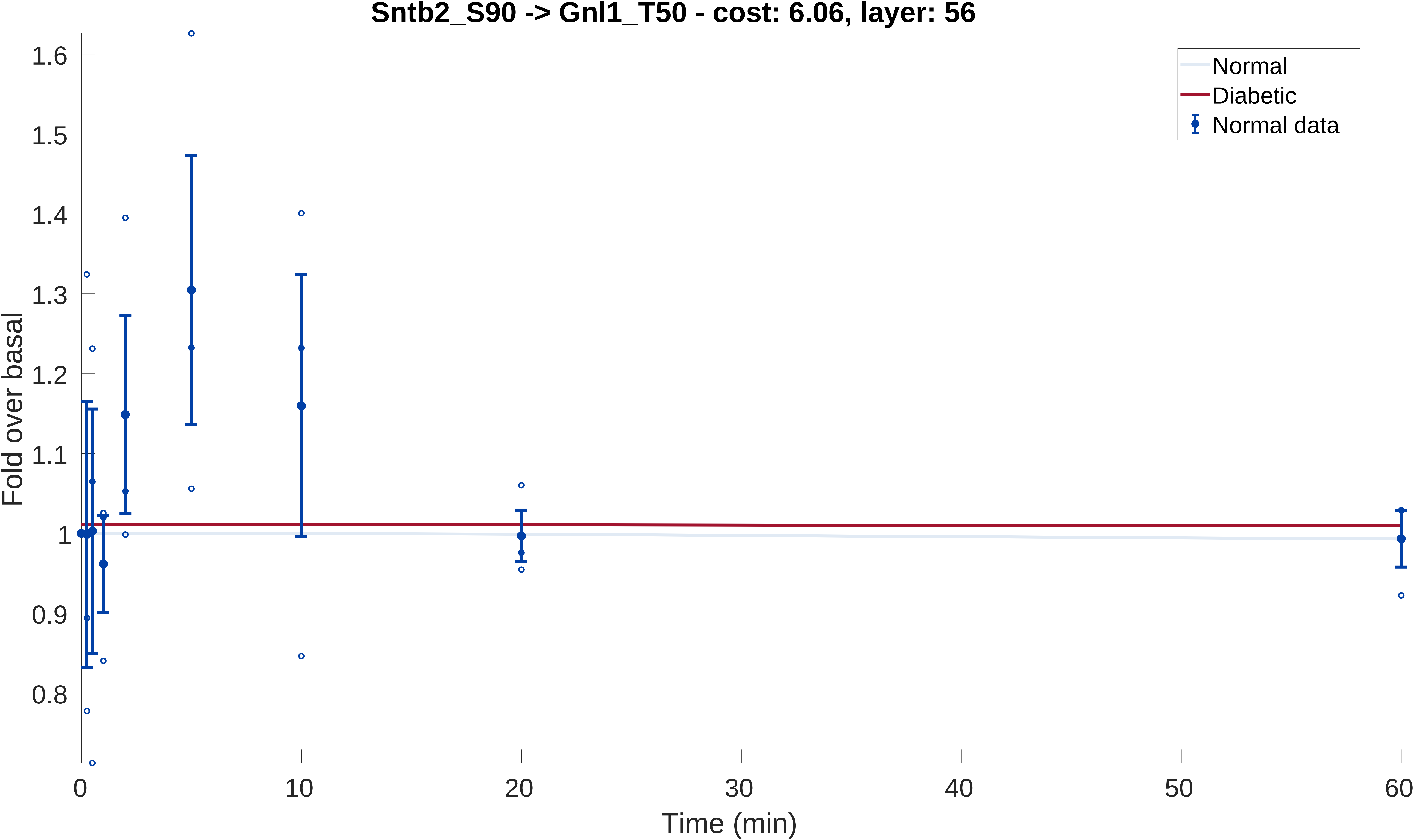

Sntb2_S90 -> Gnl1_T50 - cost: 6.06, layer: 56
Normal
Diabetic
Normal data
Fold over basal
Time (min)
1.6
1.5
1.4
1.3
1.2
1.1
1
0.9
0.8
0
10
20
30
40
50
60

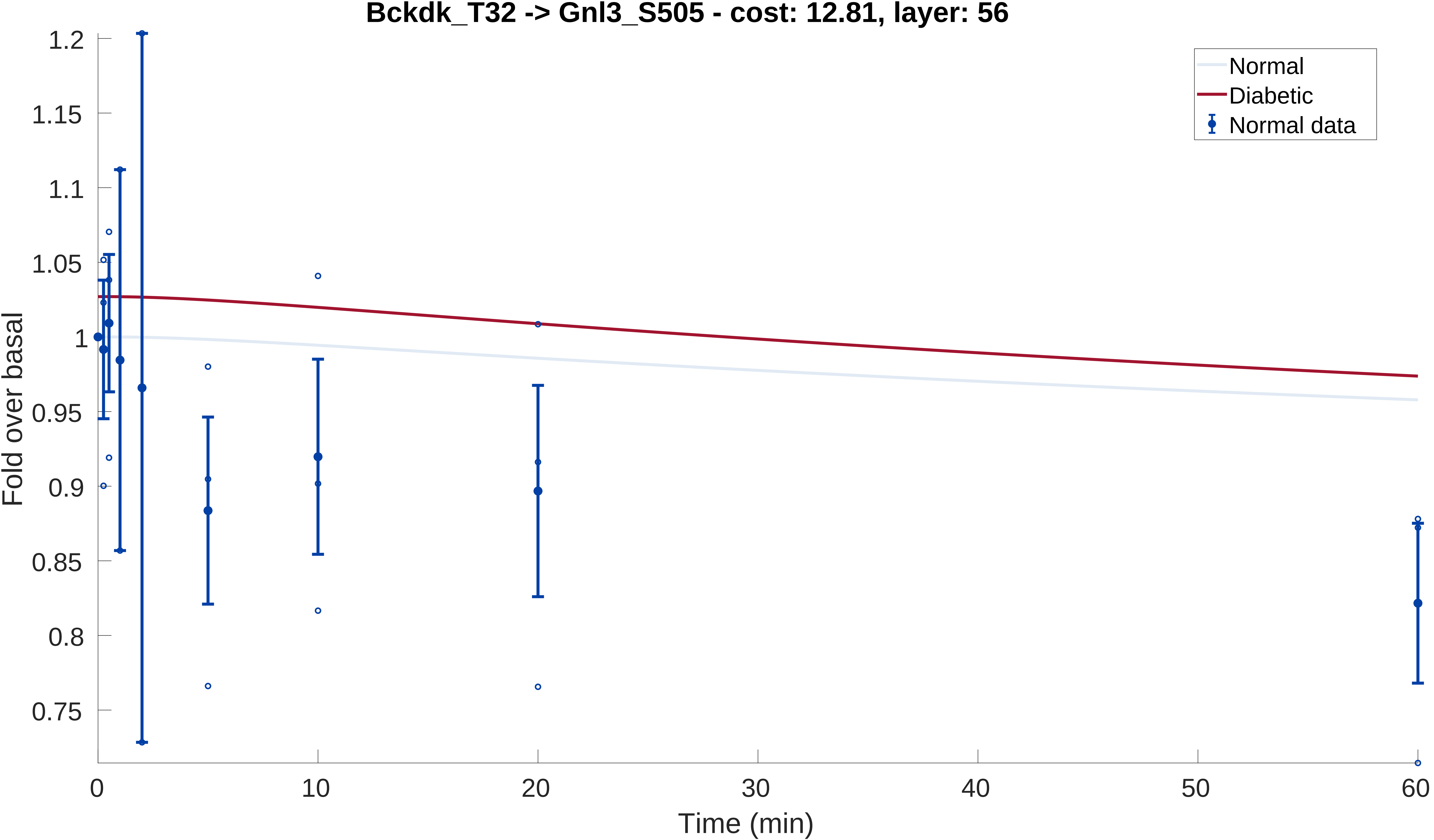

Bckdk_T32 -> Gnl3_S505 - cost: 12.81, layer: 56
Normal
Diabetic
Normal data
Fold over basal
Time (min)
1.2
1.15
1.1
1.05
1
0.95
0.9
0.85
0.8
0.75
0
10
20
30
40
50
60

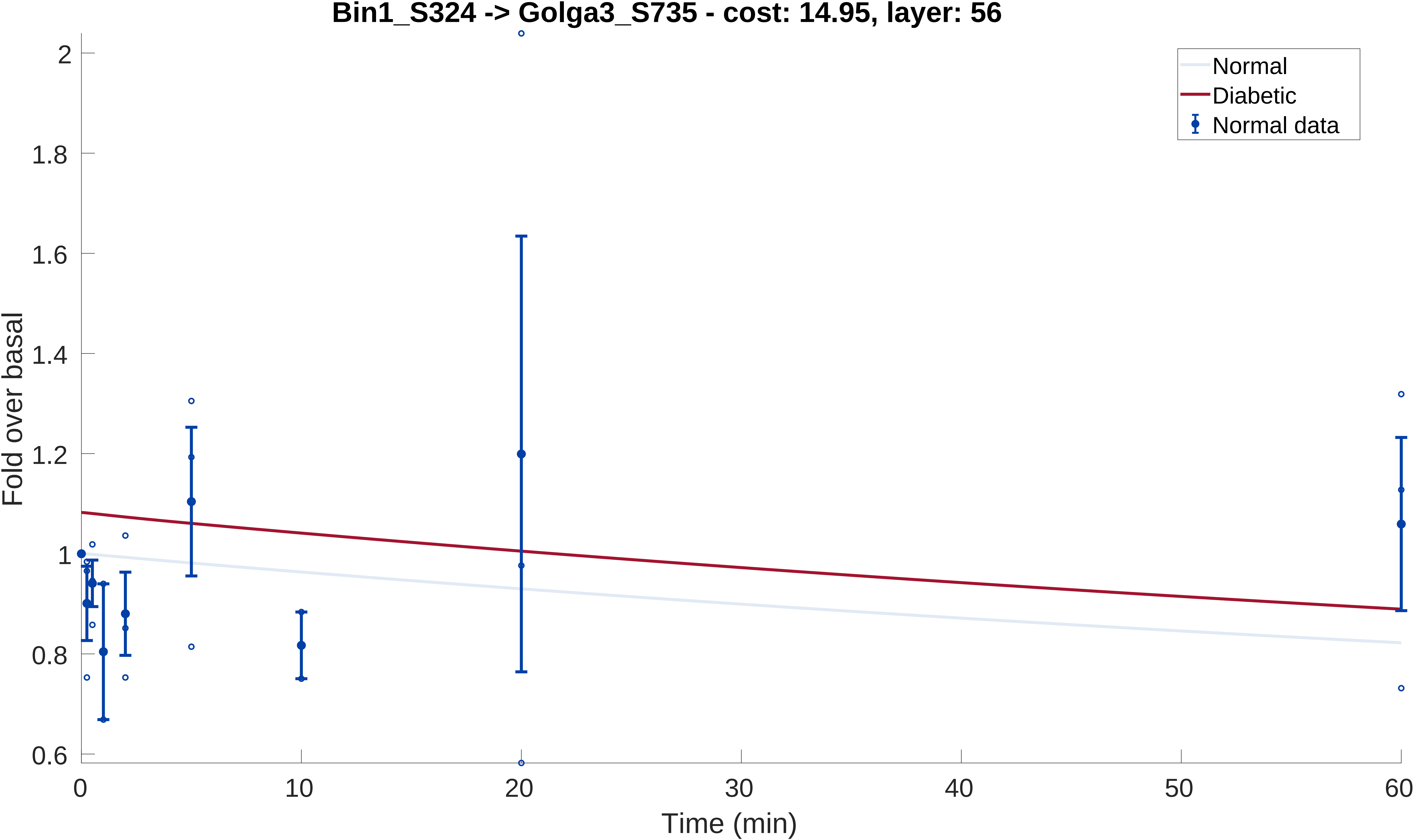

Bin1_S324 -> Golga3_S735 - cost: 14.95, layer: 56
Normal
Diabetic
Normal data
Fold over basal
2
1.8
1.6
1.4
1.2
1
0.8
0.6
0
10
20
30
40
50
60
Time (min)

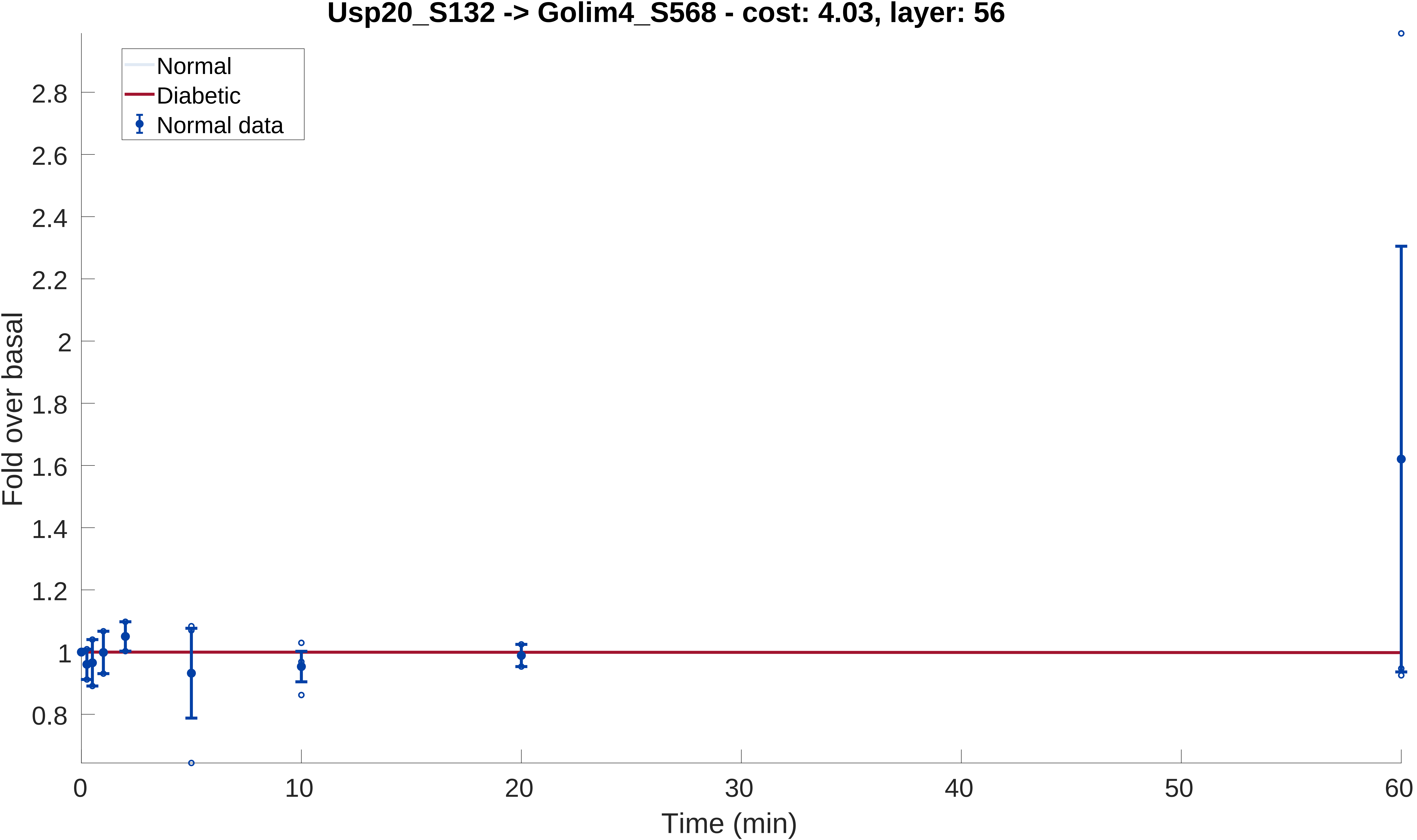

Usp20_S132 -> Golim4_S568 - cost: 4.03, layer: 56
Normal
Diabetic
Normal data
Fold over basal
Time (min)
0.8
1
1.2
1.4
1.6
1.8
2
2.2
2.4
2.6
2.8
0
10
20
30
40
50
60

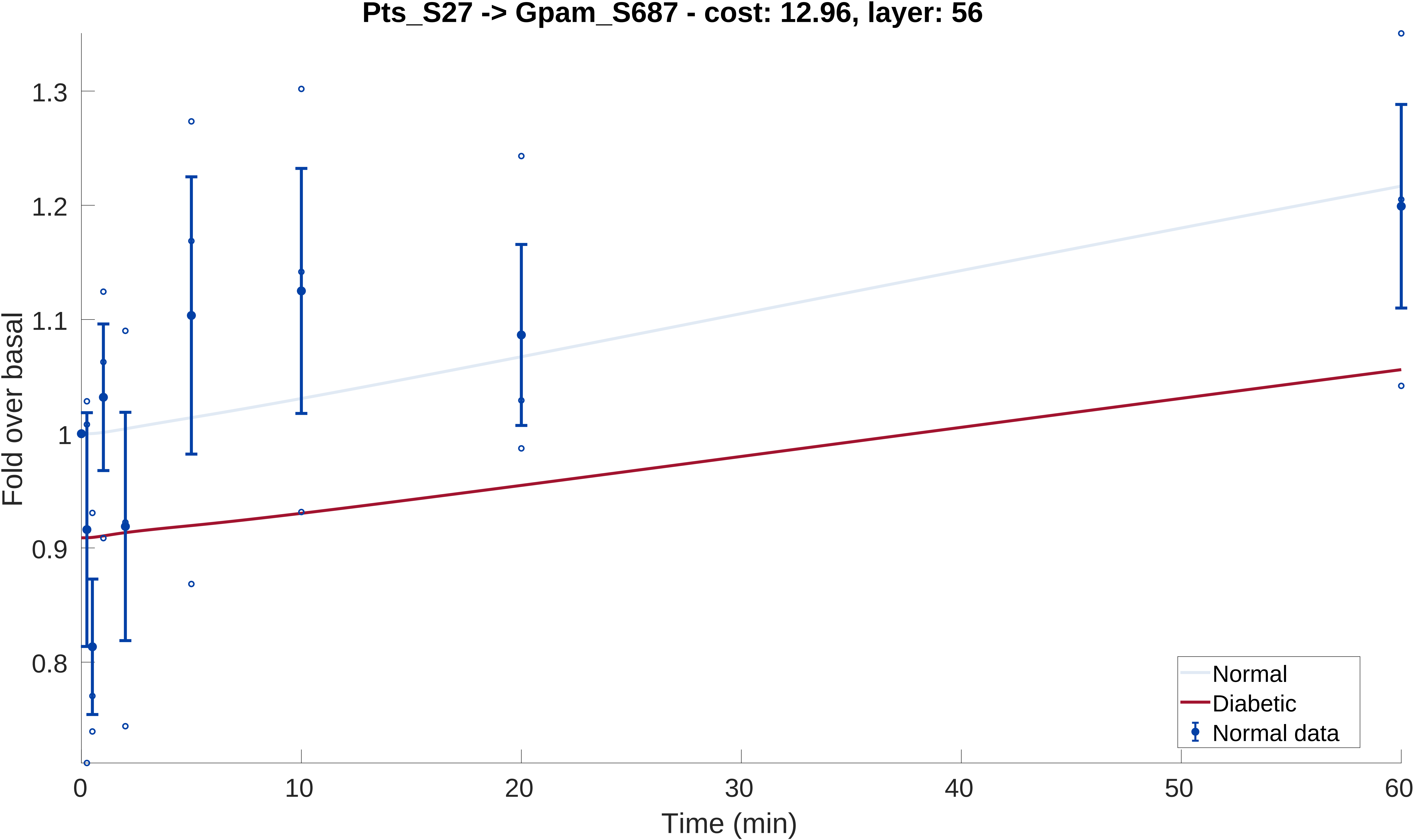
Pts_S27 -> Gpam_S687 - cost: 12.96, layer: 56
Fold over basal
1.3
1.2
1.1
1
0.9
0.8
0
10
20
30
40
50
60
Time (min)
Normal
Diabetic
Normal data

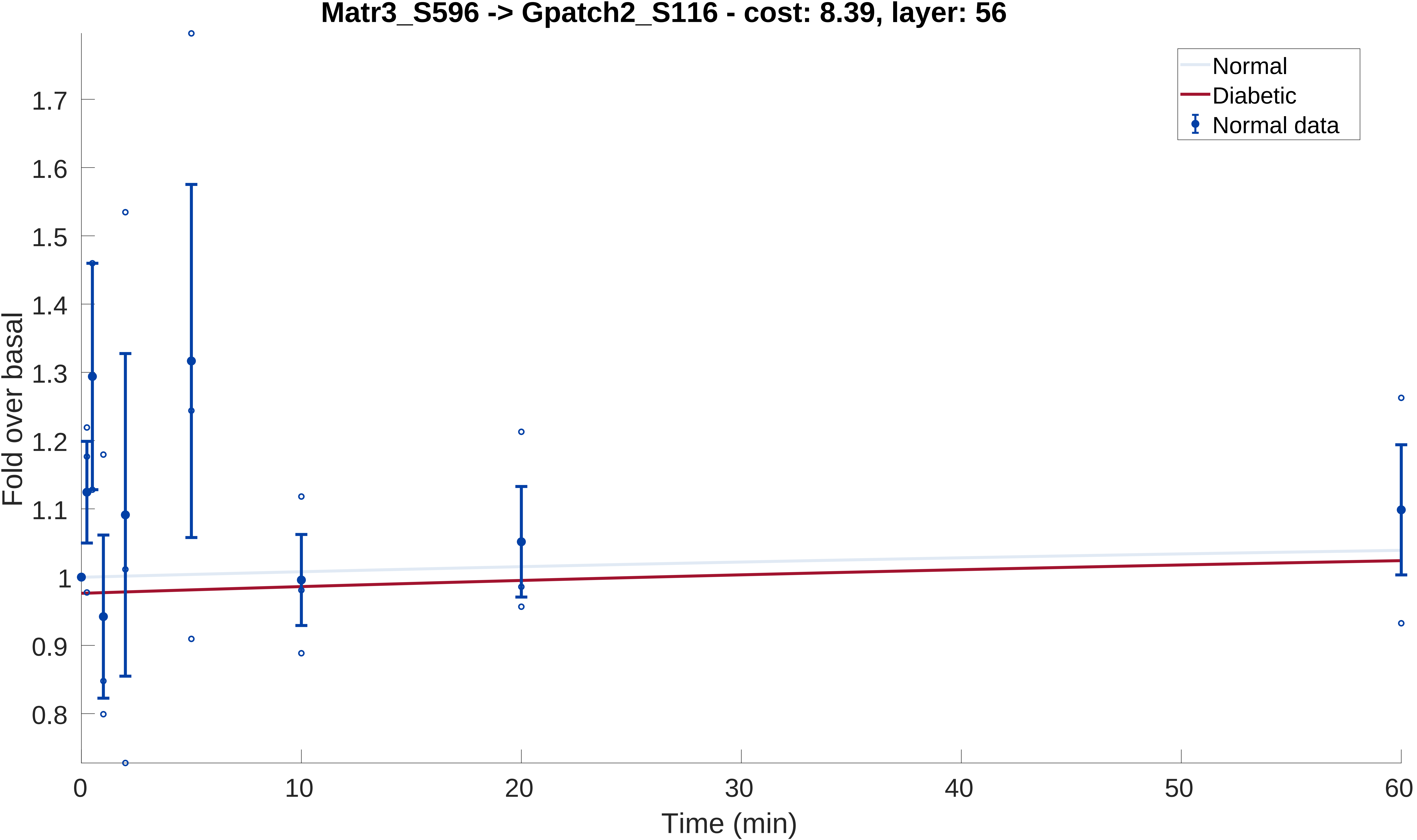
Matr3_S596 -> Gpatch2_S116 - cost: 8.39, layer: 56
Normal
Diabetic
Normal data
Fold over basal
Time (min)
0.8
0.9
1
1.1
1.2
1.3
1.4
1.5
1.6
1.7
0
10
20
30
40
50
60

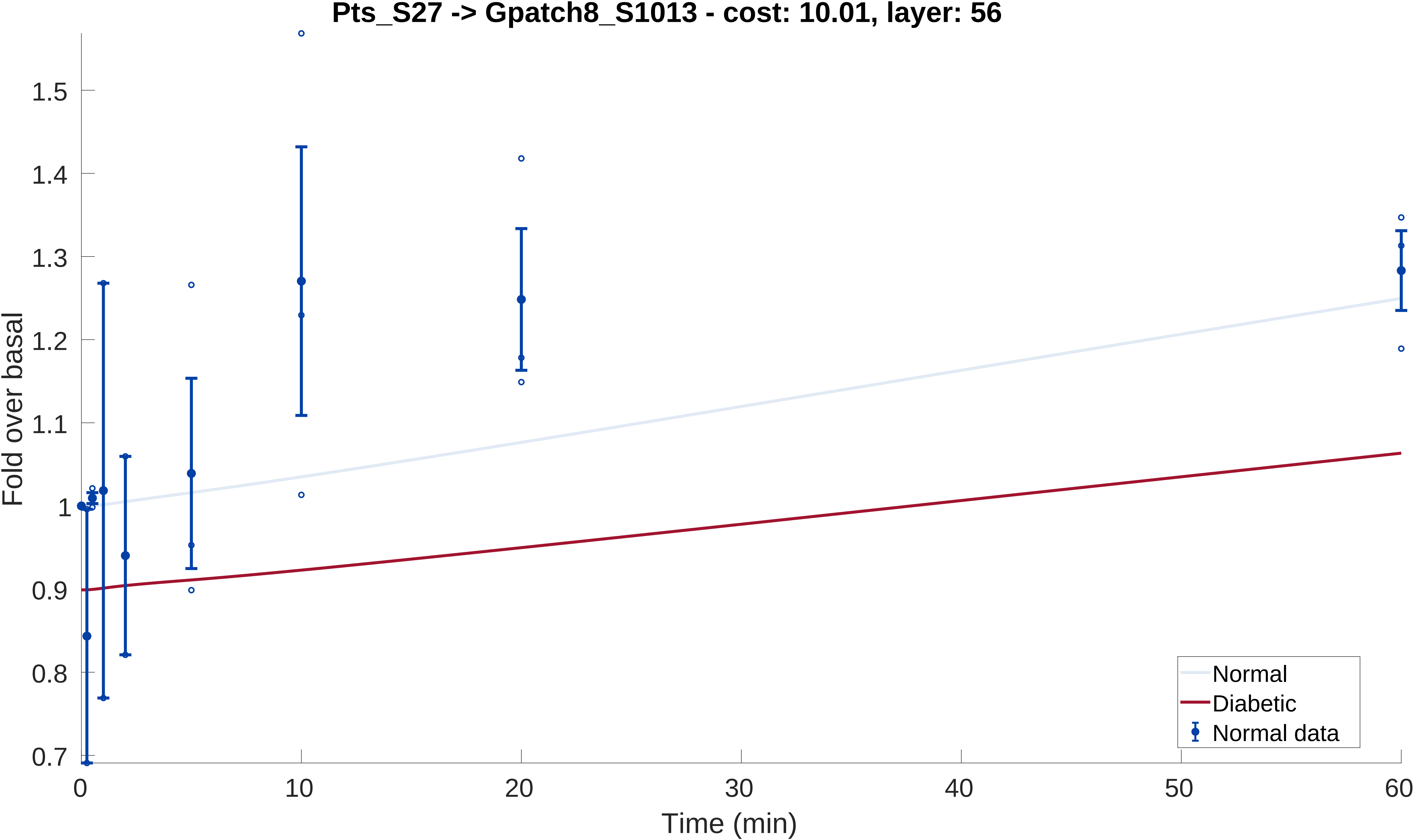
Pts_S27 -> Gpatch8_S1013 - cost: 10.01, layer: 56
Fold over basal
Time (min)
0.7
0.8
0.9
1
1.1
1.2
1.3
1.4
1.5
0
10
20
30
40
50
60
Normal
Diabetic
Normal data

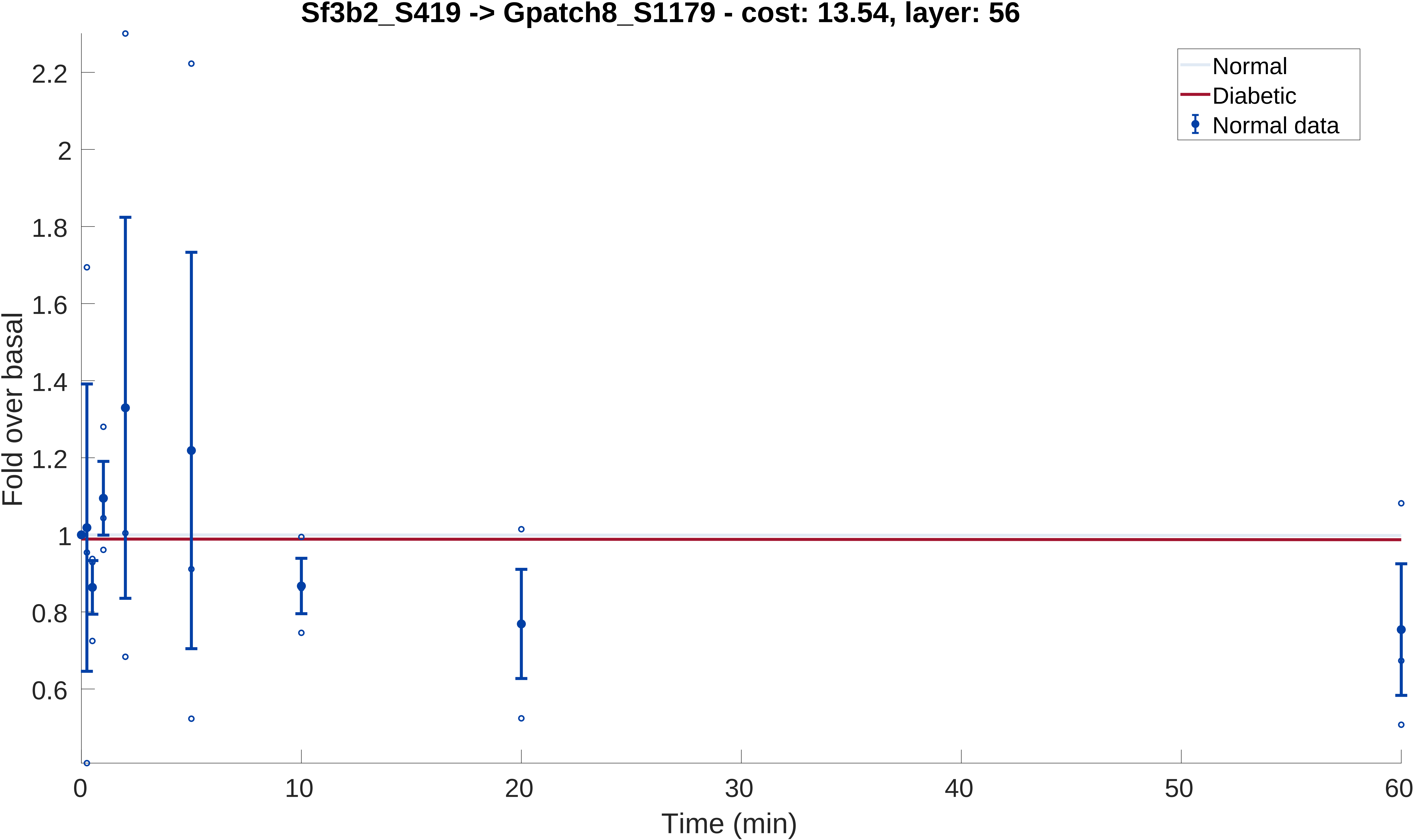
Sf3b2_S419 -> Gpatch8_S1179 - cost: 13.54, layer: 56
Normal
Diabetic
Normal data
Fold over basal
Time (min)
0.6
0.8
1
1.2
1.4
1.6
1.8
2
2.2
0
10
20
30
40
50
60

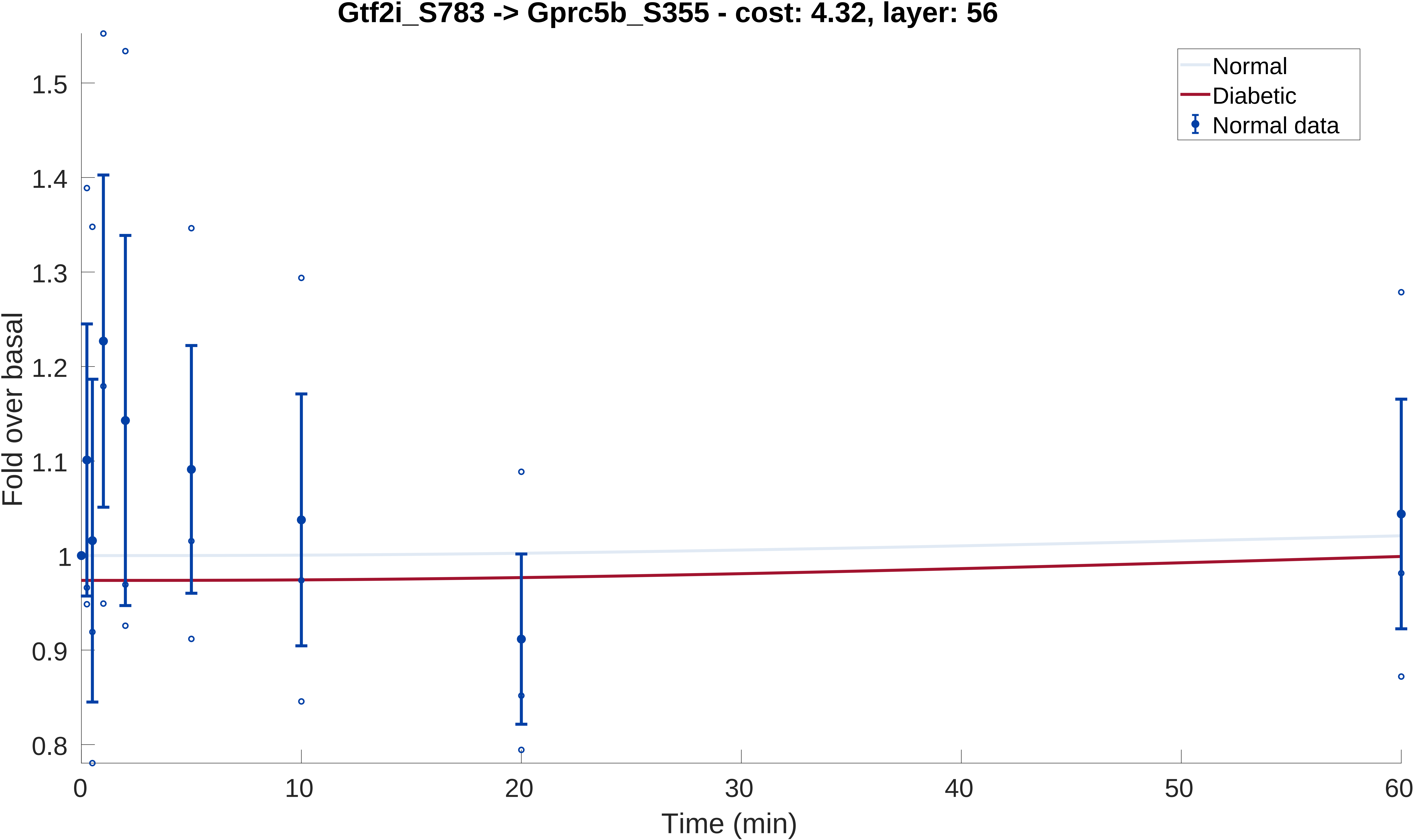

Gtf2i_S783 -> Gprc5b_S355 - cost: 4.32, layer: 56
Normal
Diabetic
Normal data
Fold over basal
1.5
1.4
1.3
1.2
1.1
1
0.9
0.8
0
10
20
30
40
50
60
Time (min)

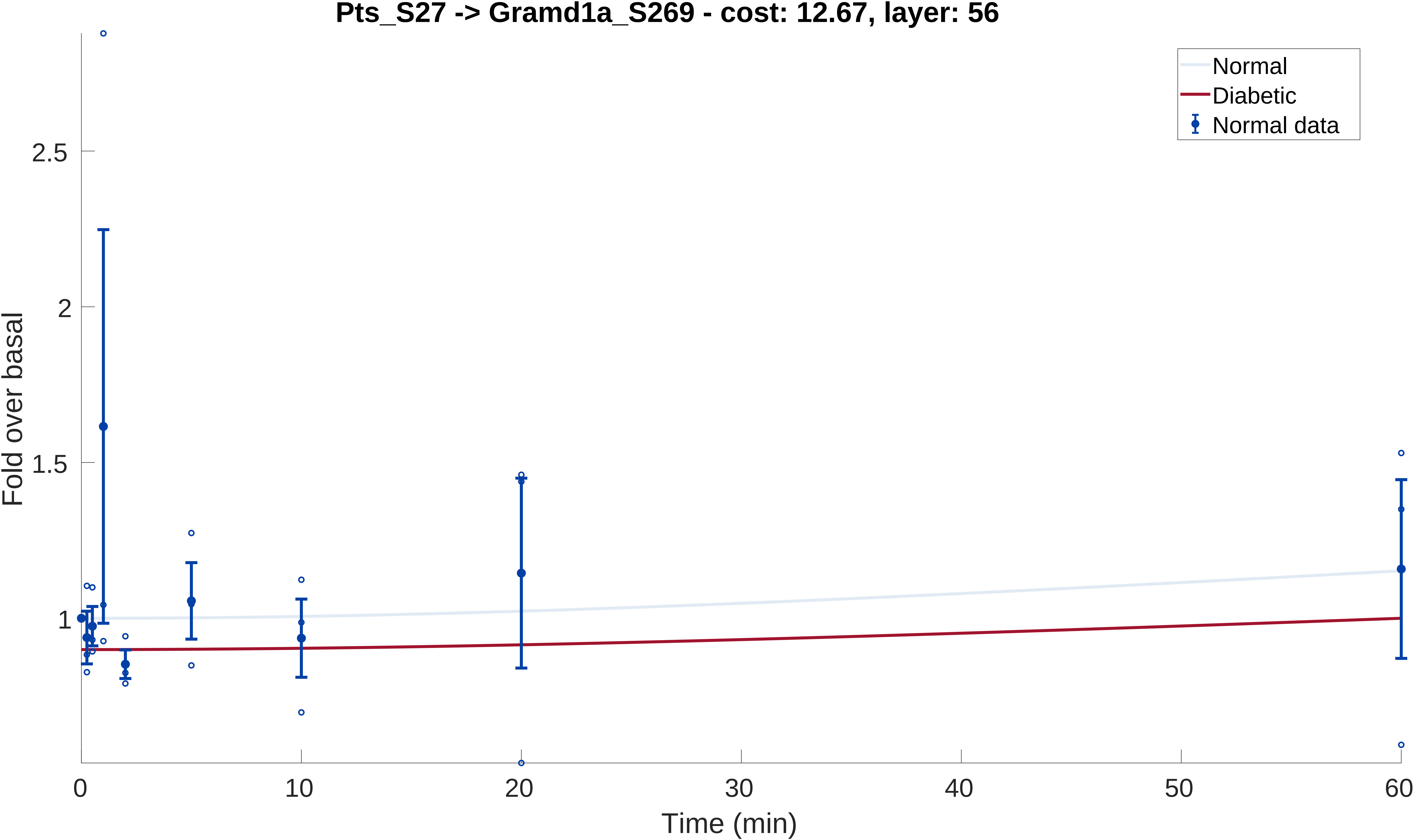

Pts_S27 -> Gramd1a_S269 - cost: 12.67, layer: 56
Normal
Diabetic
Normal data
Fold over basal
2.5
2
1.5
1
0
10
20
30
40
50
60
Time (min)

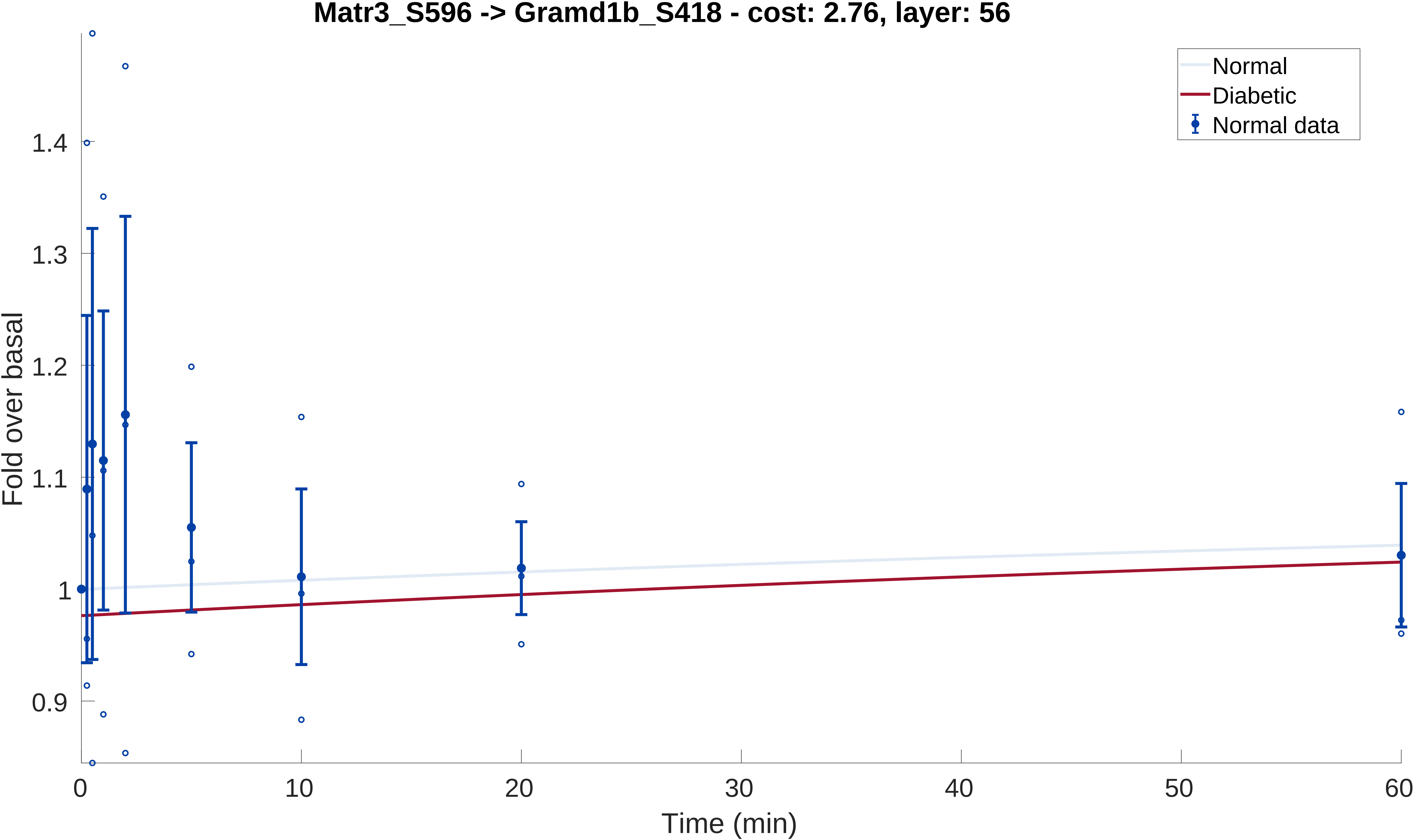

Matr3_S596 -> Gramd1b_S418 - cost: 2.76, layer: 56
Normal
Diabetic
Normal data
Fold over basal
Time (min)
1.4
1.3
1.2
1.1
1
0.9
0
10
20
30
40
50
60

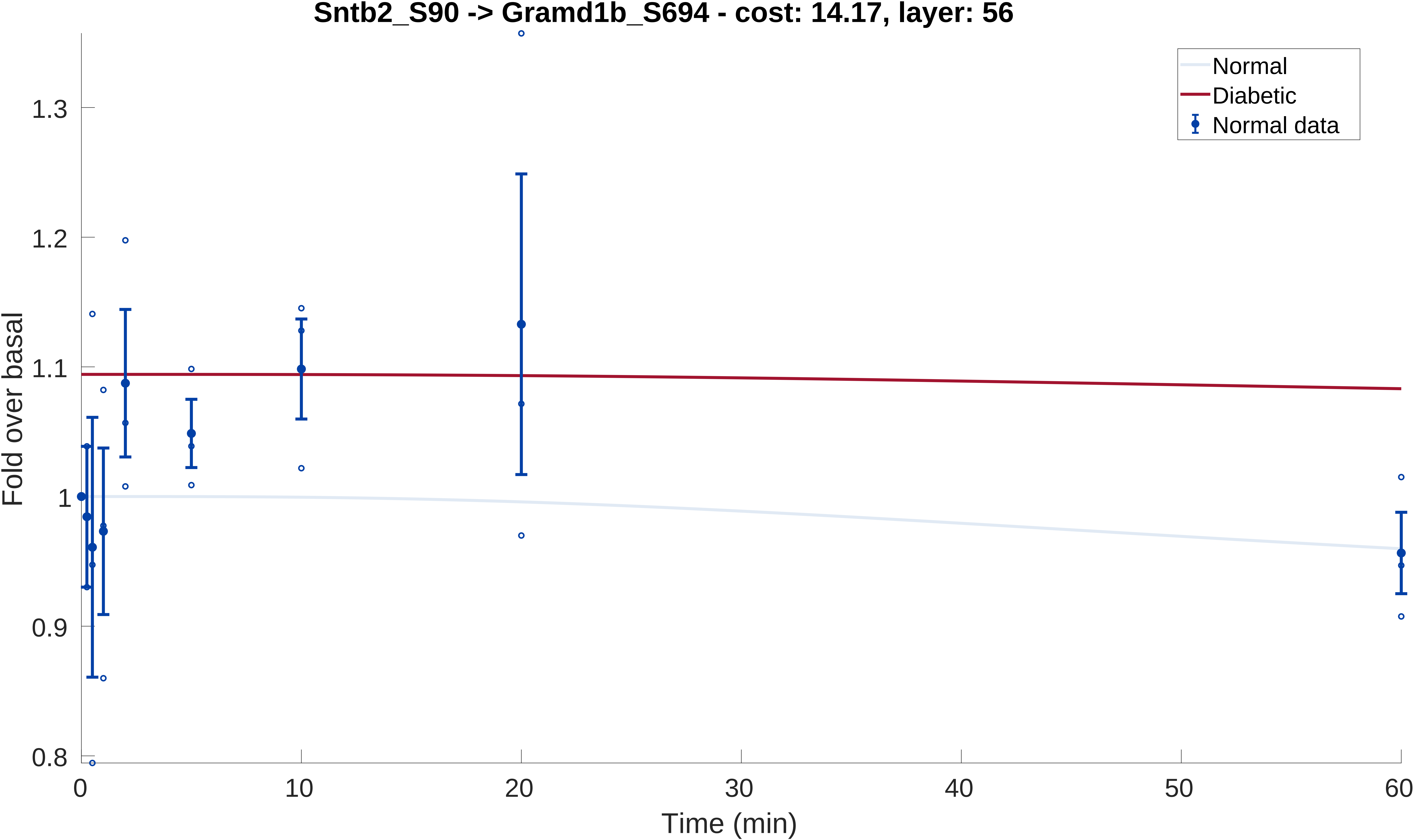

Sntb2_S90 -> Gramd1b_S694 - cost: 14.17, layer: 56
Normal
Diabetic
Normal data
Fold over basal
1.3
1.2
1.1
1
0.9
0.8
0
10
20
30
40
50
60
Time (min)

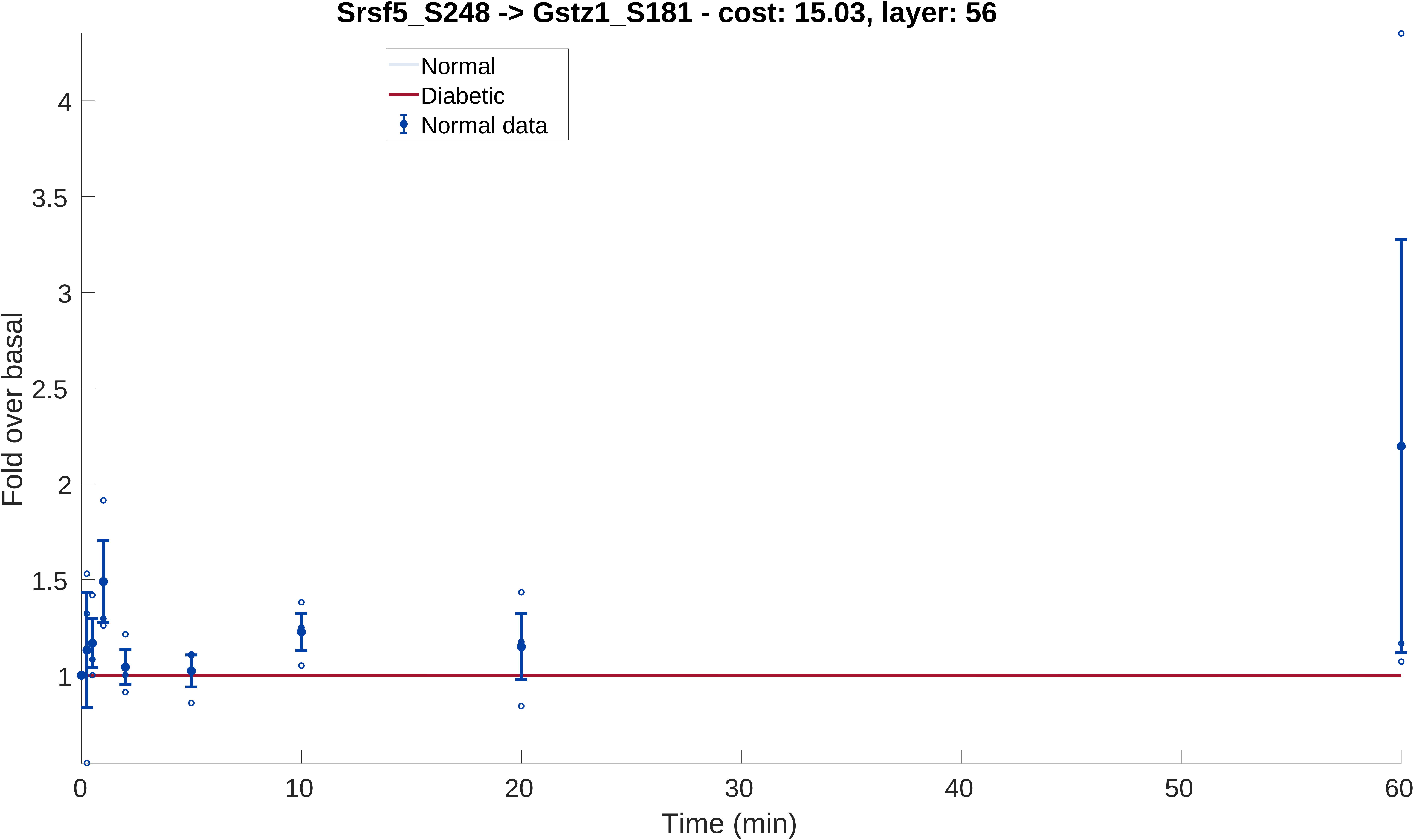

Srsf5_S248 -> Gstz1_S181 - cost: 15.03, layer: 56
Normal
Diabetic
Normal data
Fold over basal
4
3.5
3
2.5
2
1.5
1
0
10
20
30
40
50
60
Time (min)

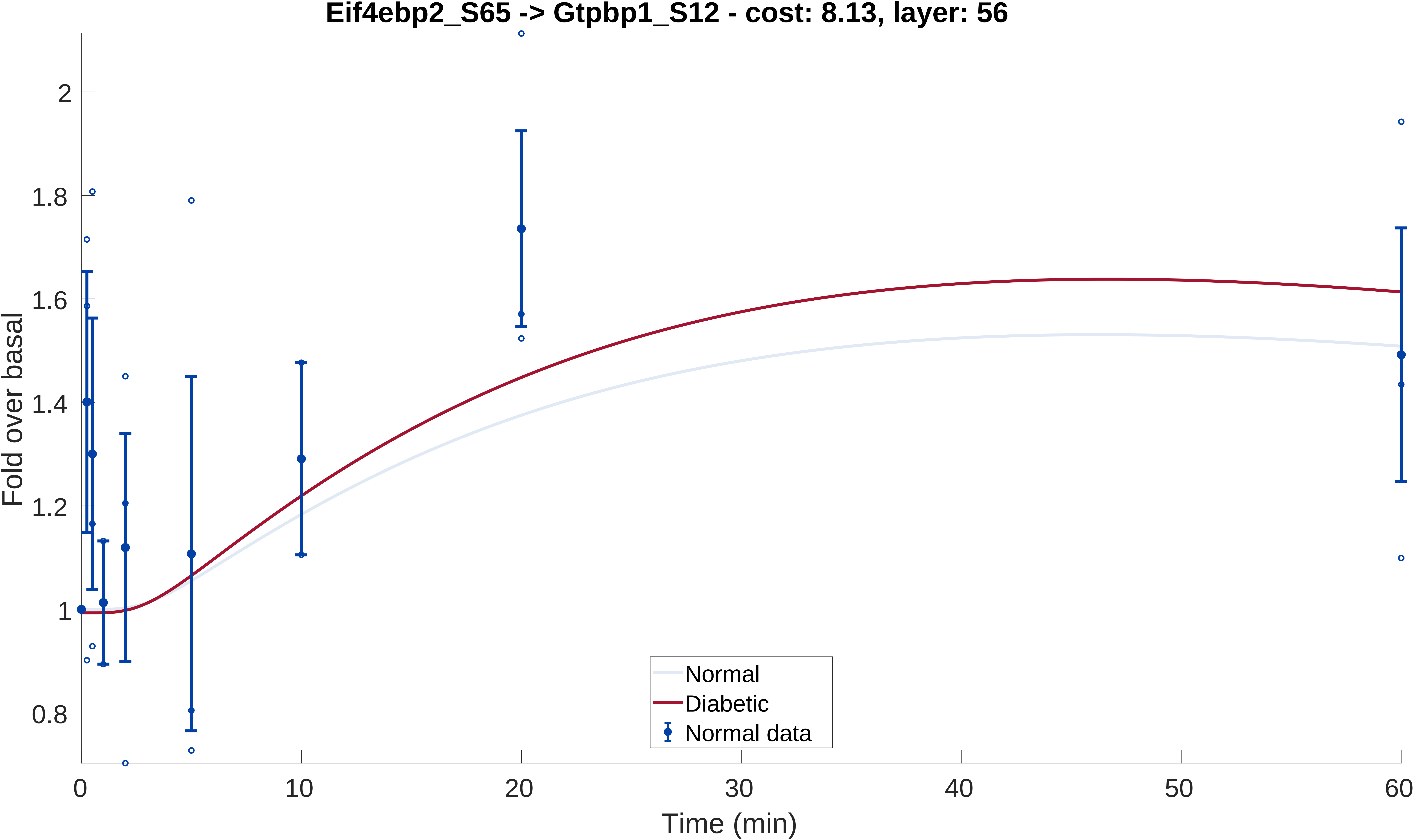

Eif4ebp2_S65 -> Gtpbp1_S12 - cost: 8.13, layer: 56
Fold over basal
2
1.8
1.6
1.4
1.2
1
0.8
0
10
20
30
40
50
60
Time (min)
Normal
Diabetic
Normal data

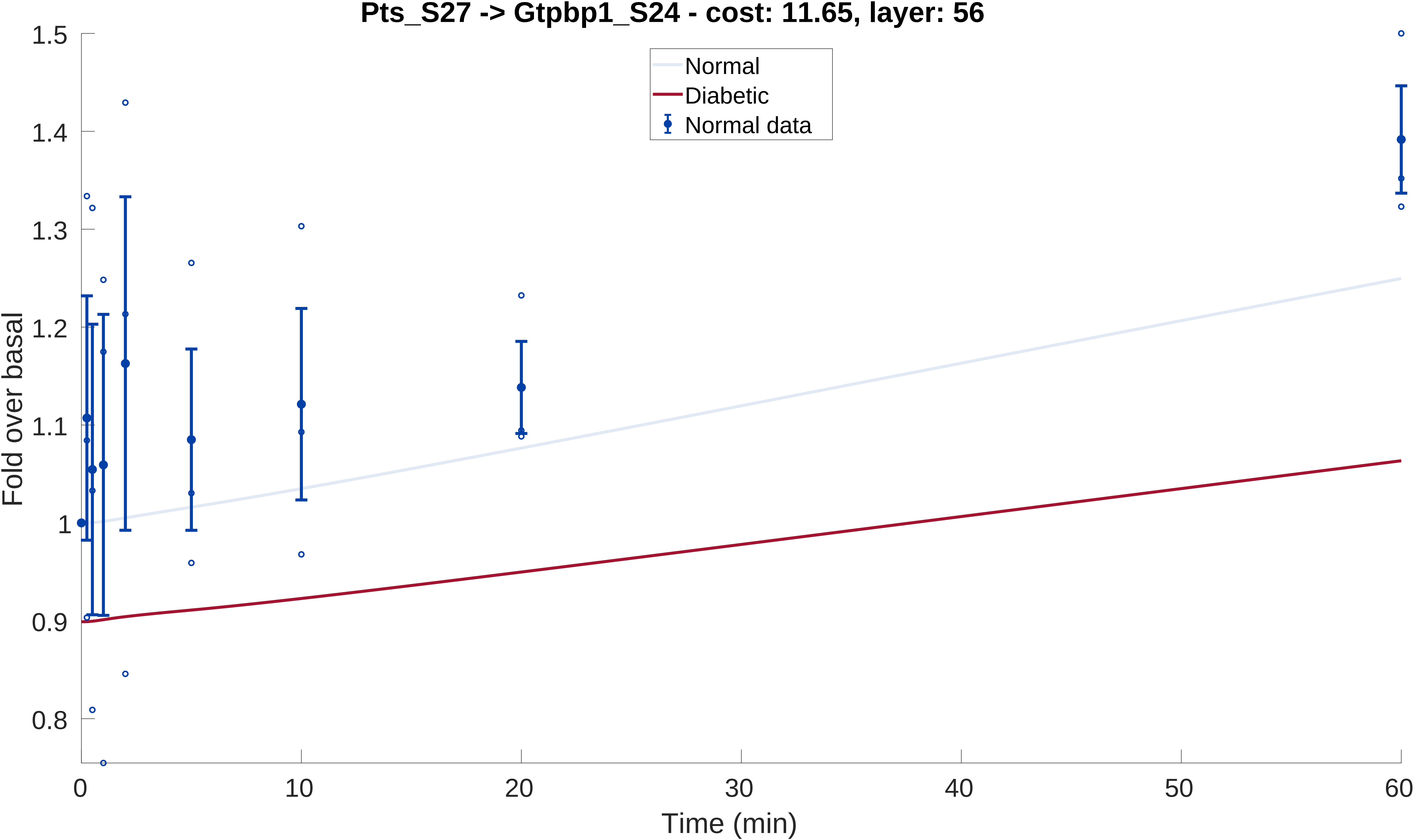
Pts_S27 -> Gtpbp1_S24 - cost: 11.65, layer: 56
Normal
Diabetic
Normal data
Fold over basal
Time (min)
1.5
1.4
1.3
1.2
1.1
1
0.9
0.8
0
10
20
30
40
50
60

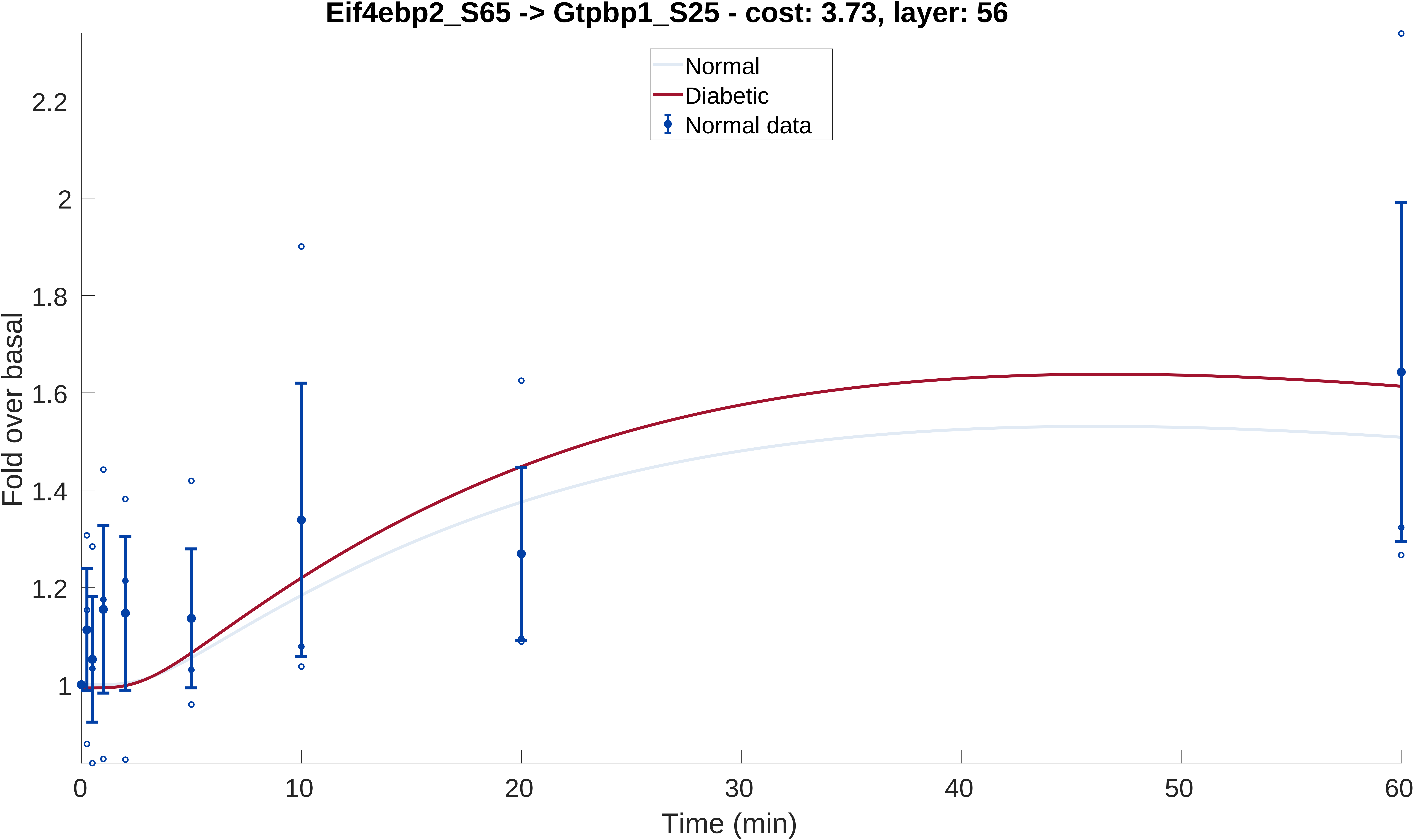

Eif4ebp2_S65 -> Gtpbp1_S25 - cost: 3.73, layer: 56
Normal
Diabetic
Normal data
Fold over basal
2.2
2
1.8
1.6
1.4
1.2
1
0
10
20
30
40
50
60
Time (min)

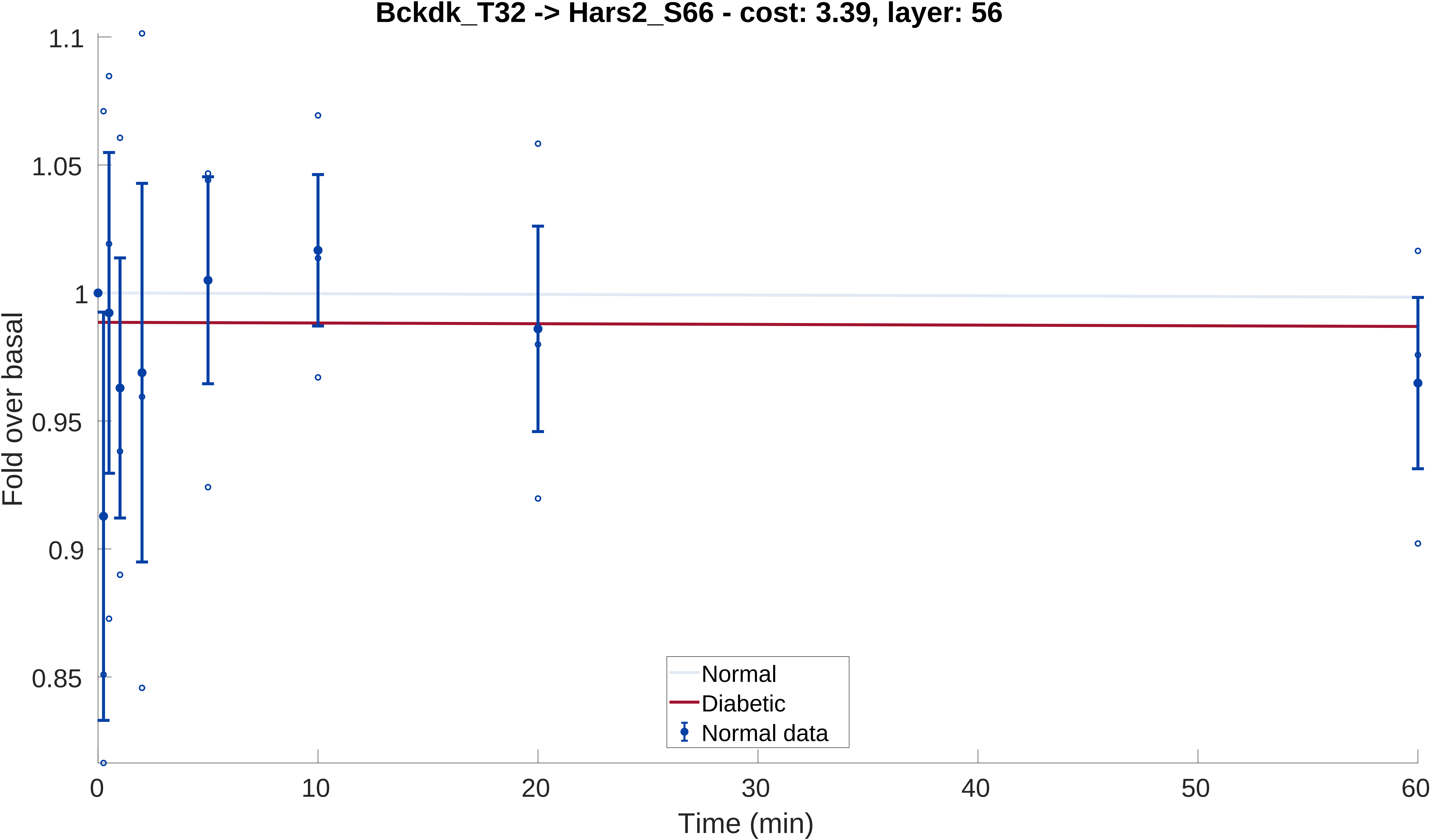

Bckdk_T32 -> Hars2_S66 - cost: 3.39, layer: 56
Fold over basal
1.1
1.05
1
0.95
0.9
0.85
0
10
20
30
40
50
60
Time (min)
Normal
Diabetic
Normal data

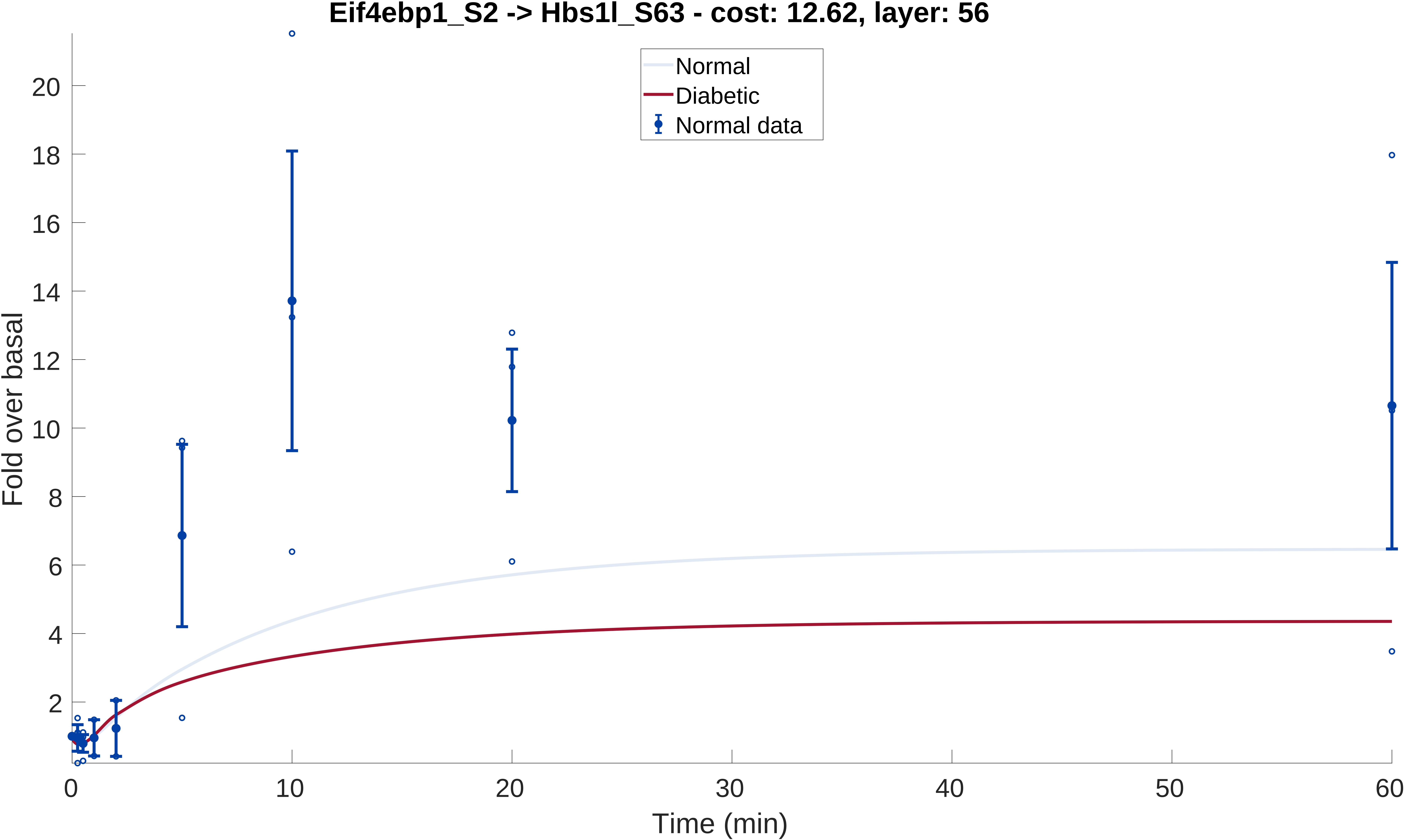

Eif4ebp1_S2 -> Hbs1l_S63 - cost: 12.62, layer: 56
Normal
Diabetic
Normal data
Fold over basal
Time (min)
0
10
20
30
40
50
60
2
4
6
8
10
12
14
16
18
20

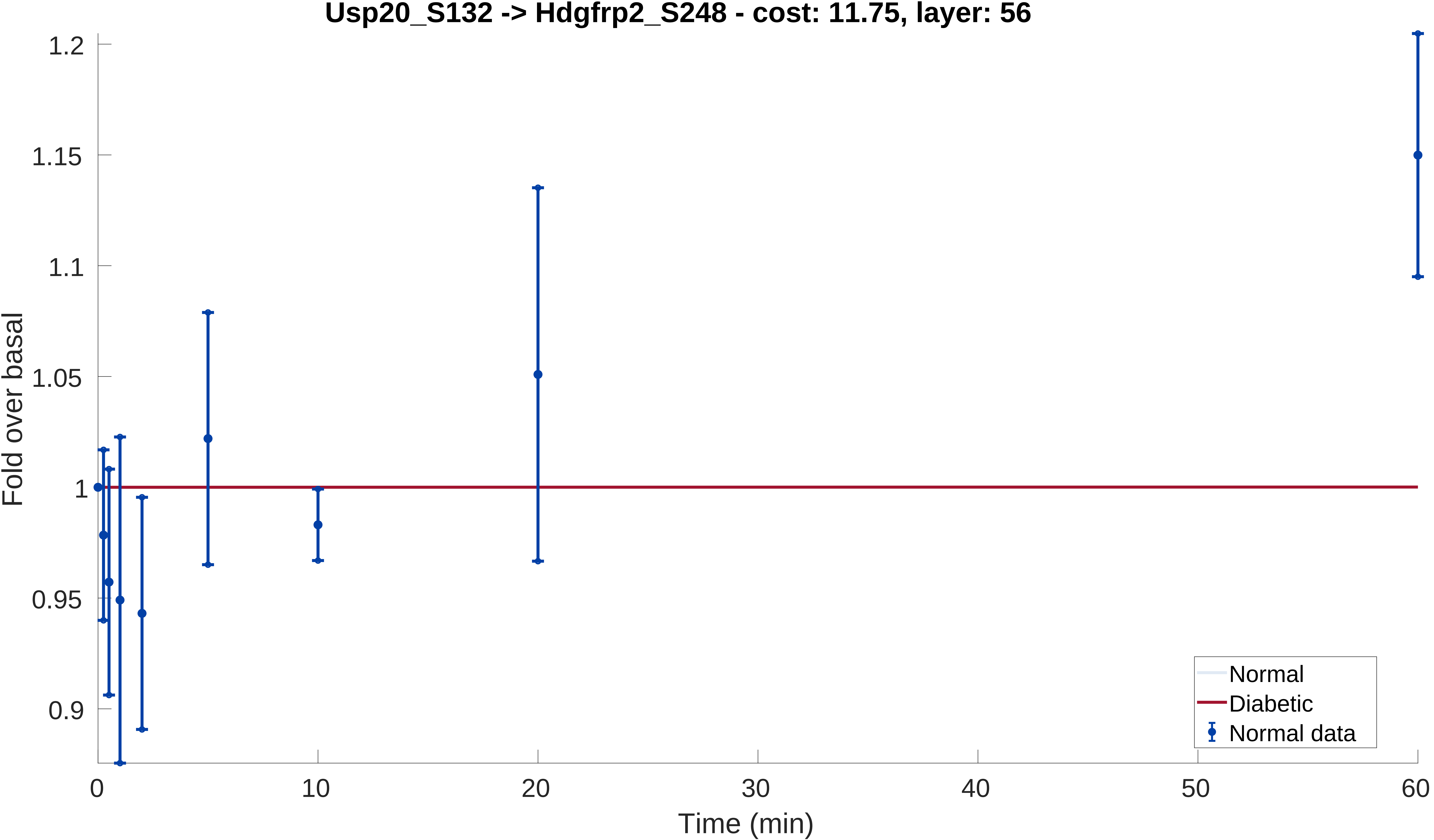

Usp20_S132 -> Hdgfrp2_S248 - cost: 11.75, layer: 56
Fold over basal
1.2
1.15
1.1
1.05
1
0.95
0.9
0
10
20
30
40
50
60
Time (min)
Normal
Diabetic
Normal data

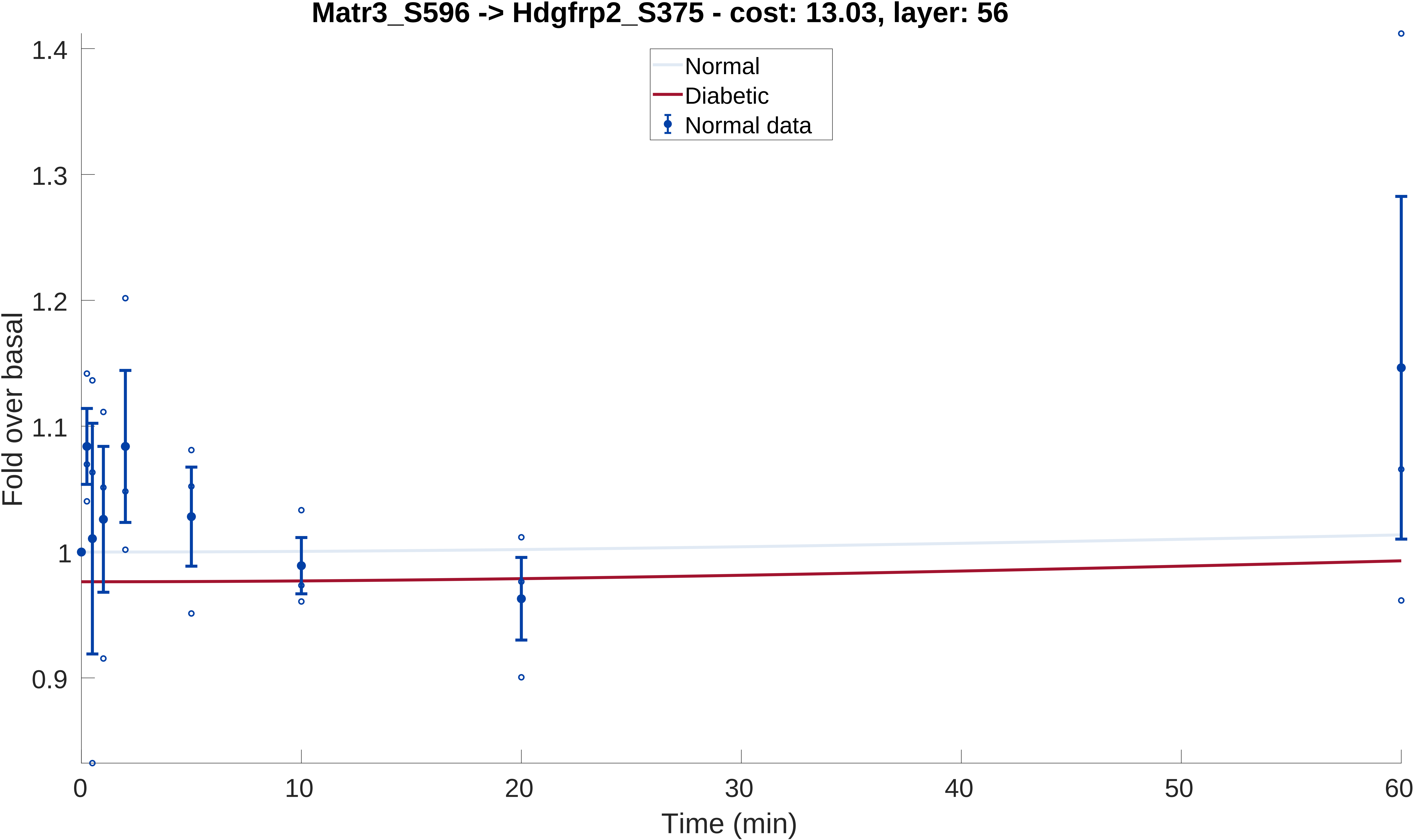

Matr3_S596 -> Hdgfrp2_S375 - cost: 13.03, layer: 56
Normal
Diabetic
Normal data
Fold over basal
Time (min)
0.9
1
1.1
1.2
1.3
1.4
0
10
20
30
40
50
60

Clmn_S925 -> Hdgfrp2_S376 - cost: 13.03, layer: 56

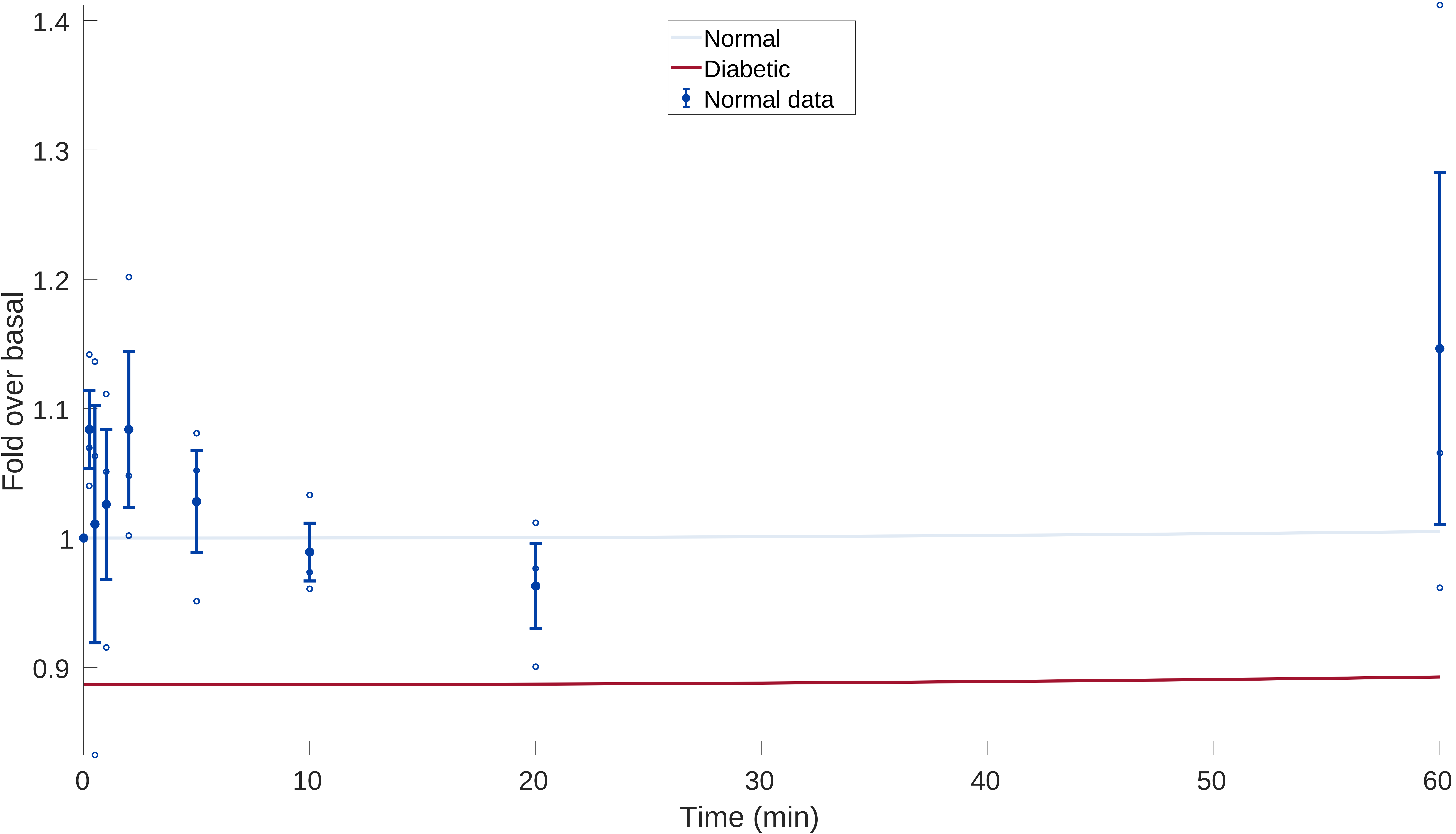

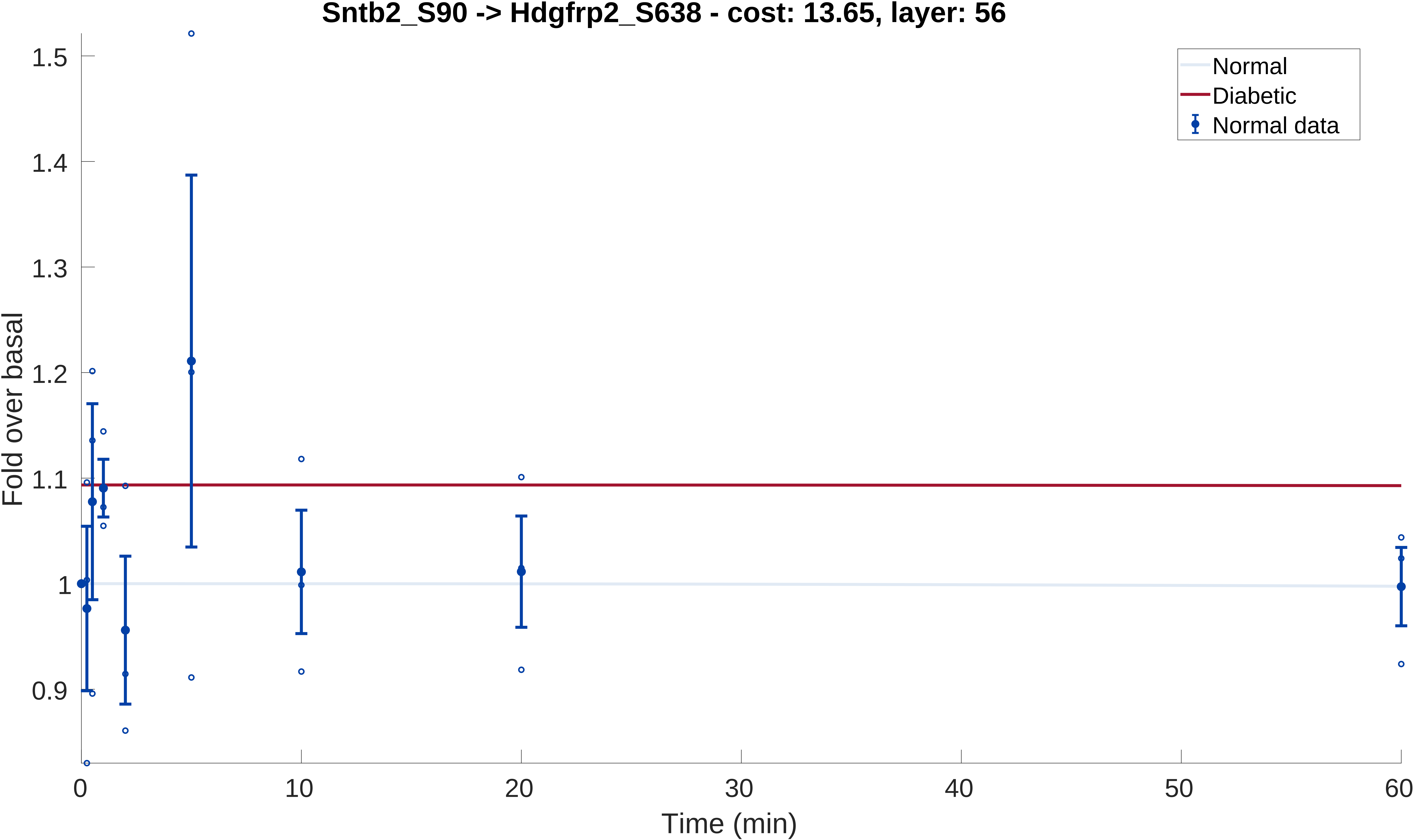

Sntb2_S90 -> Hdgfrp2_S638 - cost: 13.65, layer: 56
Normal
Diabetic
Normal data
Fold over basal
Time (min)
0.9
1
1.1
1.2
1.3
1.4
1.5
0
10
20
30
40
50
60

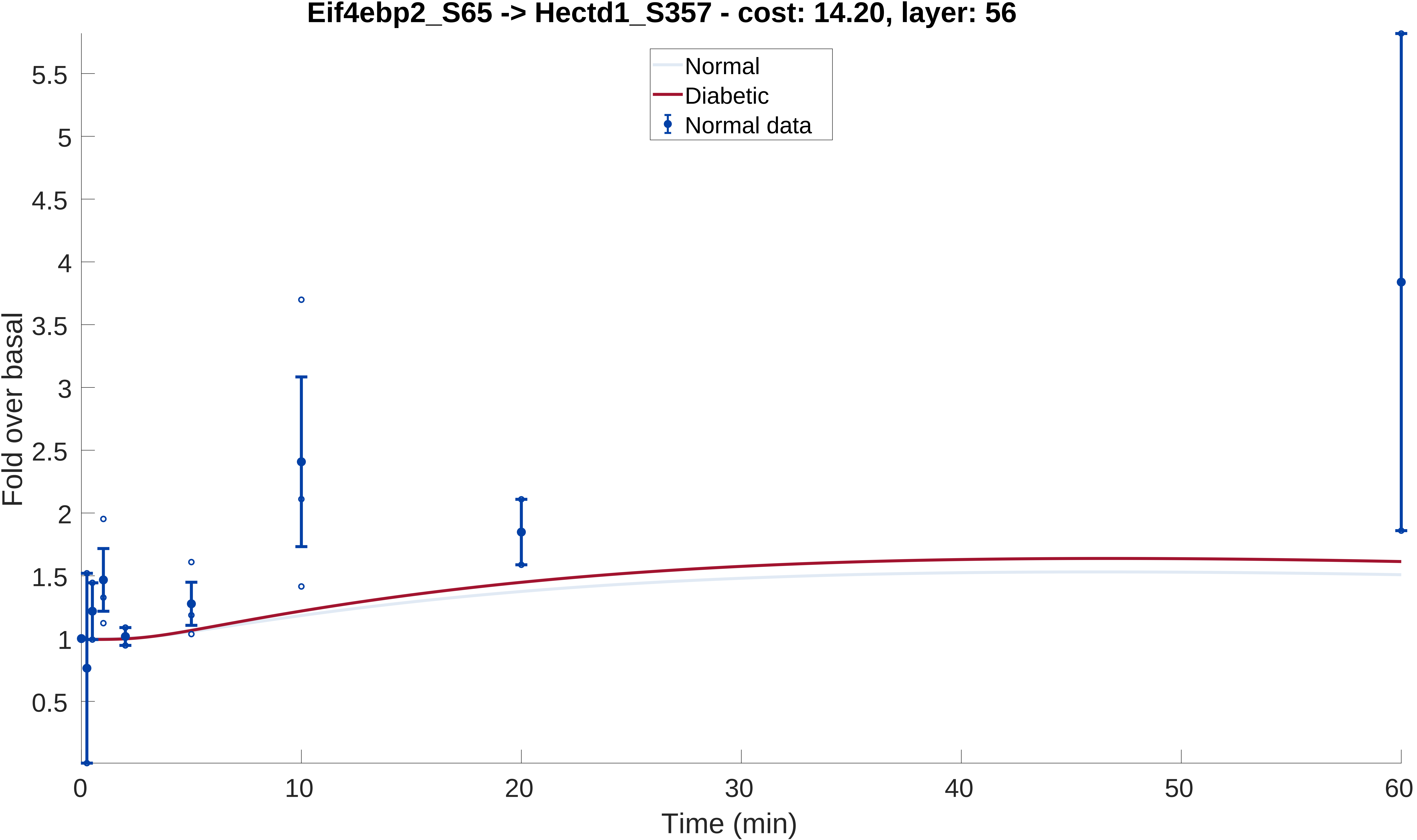

Eif4ebp2_S65 -> Hectd1_S357 - cost: 14.20, layer: 56
Normal
Diabetic
Normal data
Fold over basal
Time (min)
0.5
1
1.5
2
2.5
3
3.5
4
4.5
5
5.5
0
10
20
30
40
50
60

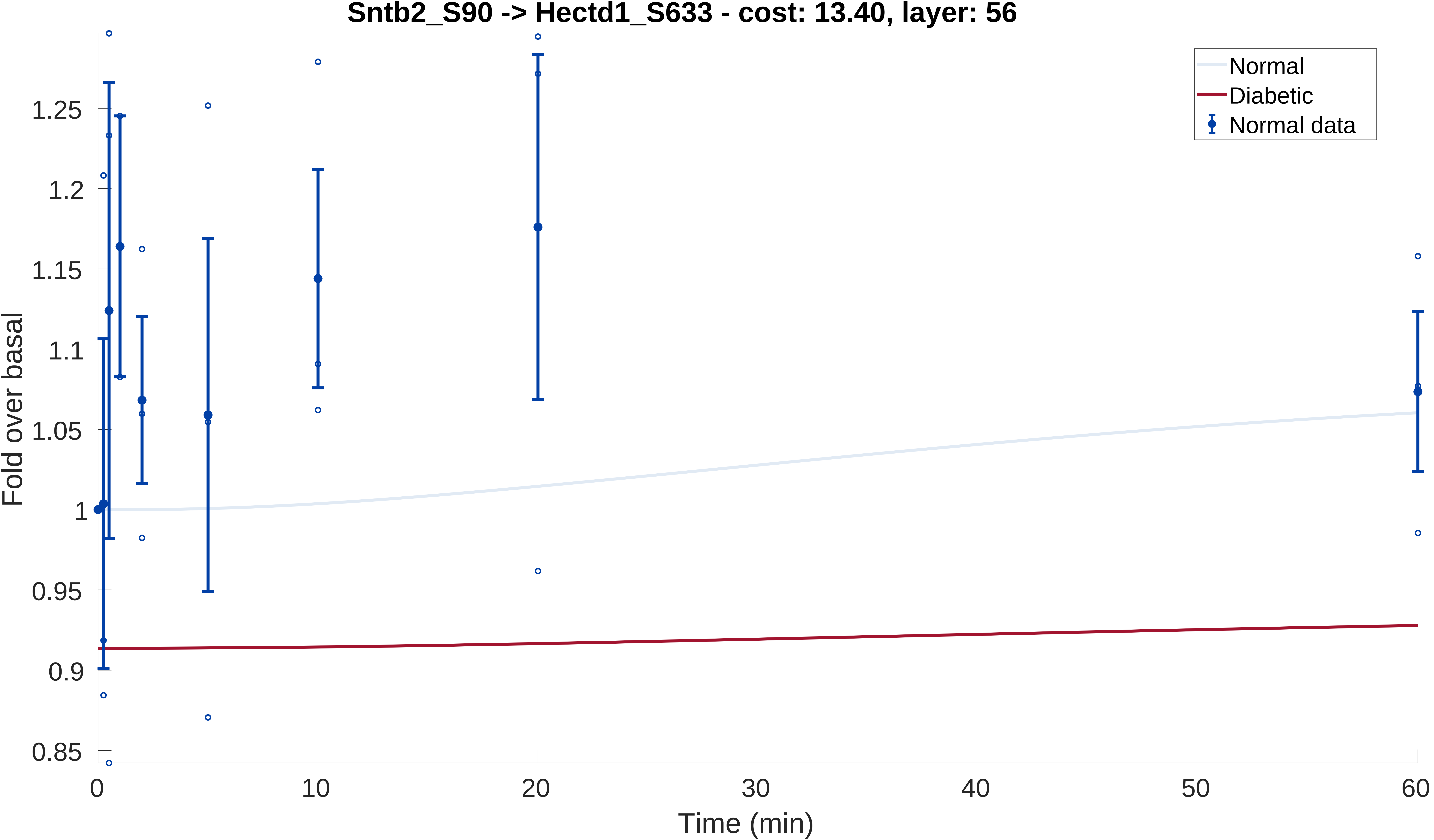
Sntb2_S90 -> Hectd1_S633 - cost: 13.40, layer: 56
Normal
Diabetic
Normal data
Fold over basal
Time (min)
1.25
1.2
1.15
1.1
1.05
1
0.95
0.9
0.85
0
10
20
30
40
50
60

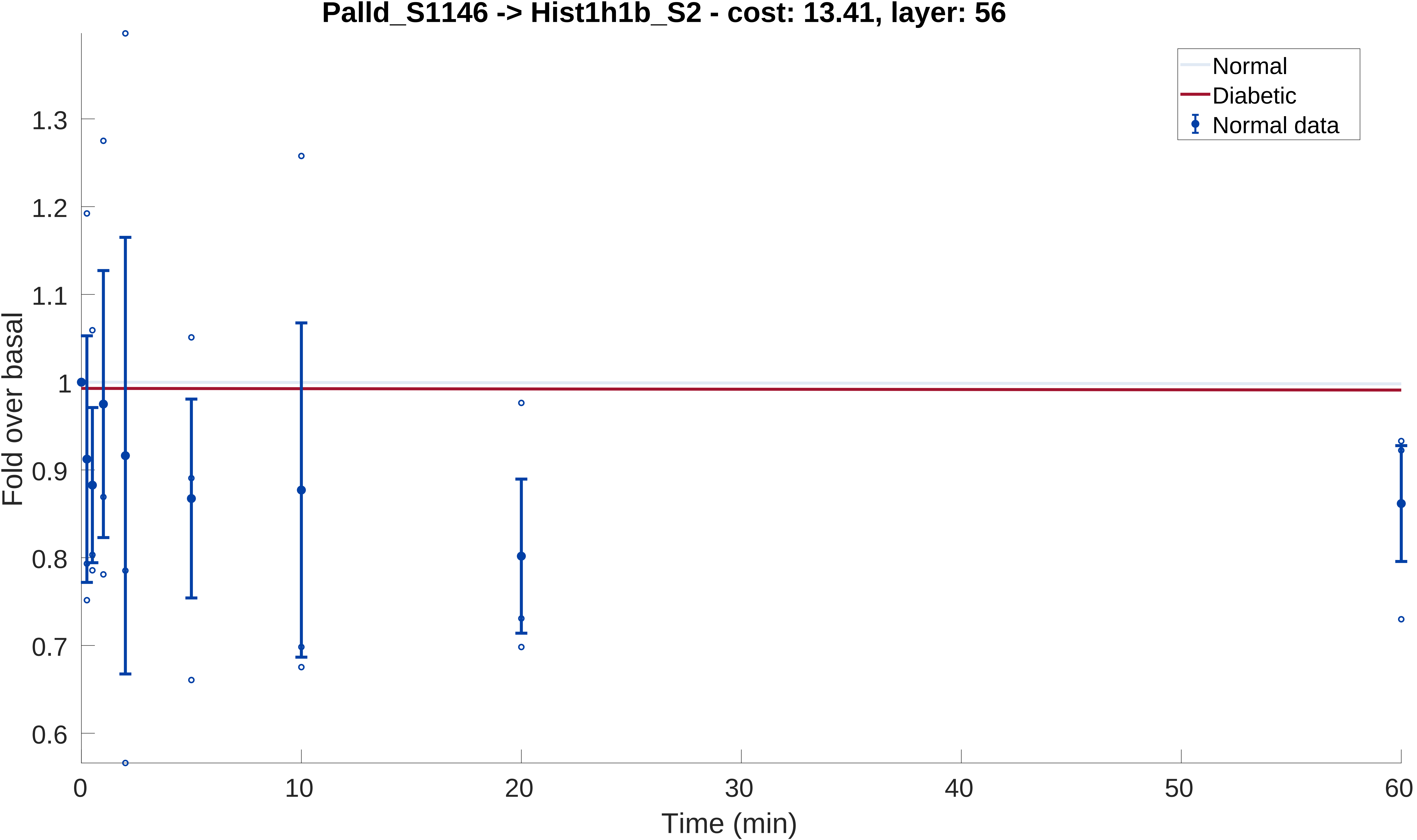

Palld_S1146 -> Hist1h1b_S2 - cost: 13.41, layer: 56
Normal
Diabetic
Normal data
Fold over basal
Time (min)
1.3
1.2
1.1
1
0.9
0.8
0.7
0.6
0
10
20
30
40
50
60

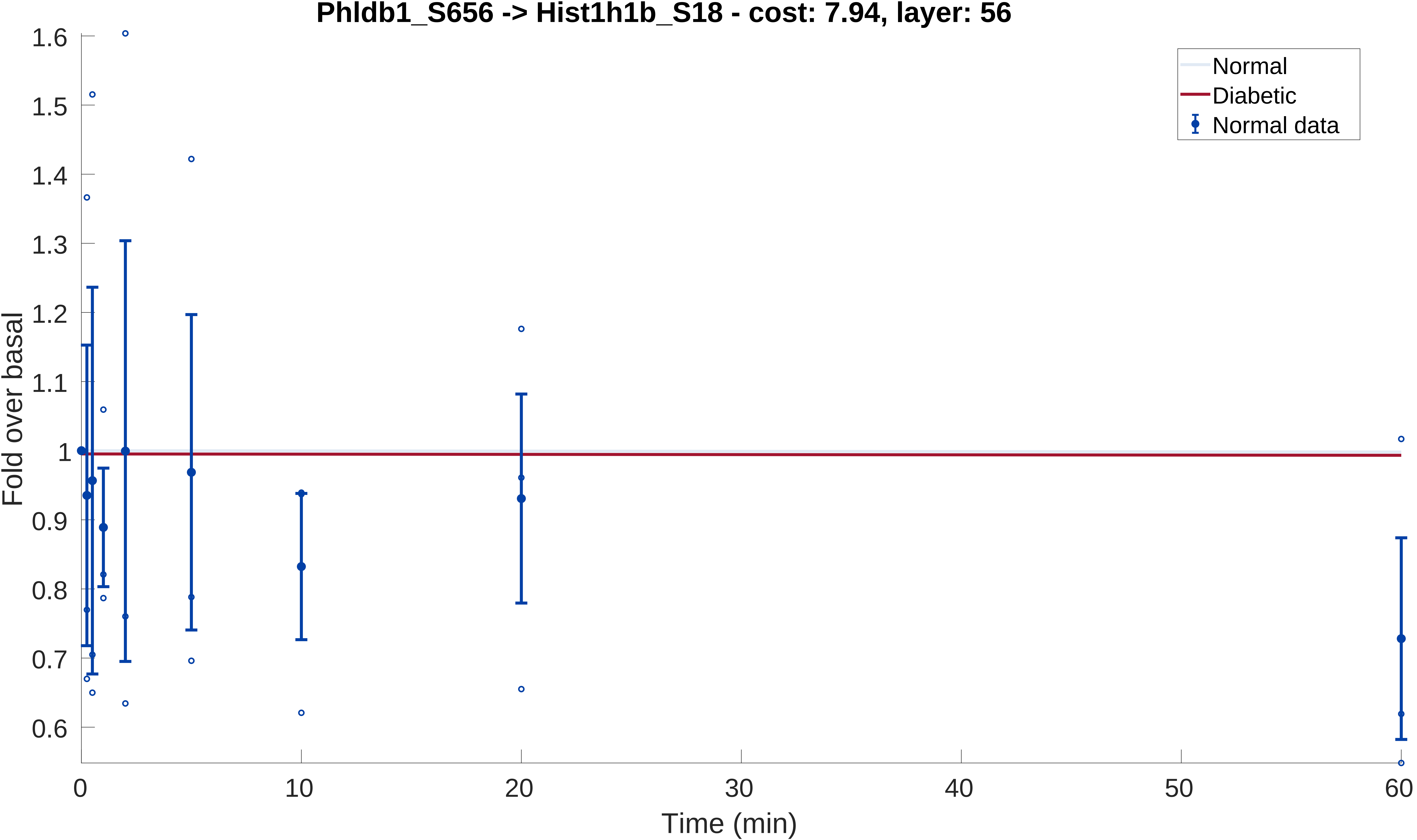

Phldb1_S656 -> Hist1h1b_S18 - cost: 7.94, layer: 56
Normal
Diabetic
Normal data
Fold over basal
Time (min)
1.6
1.5
1.4
1.3
1.2
1.1
1
0.9
0.8
0.7
0.6
0
10
20
30
40
50
60

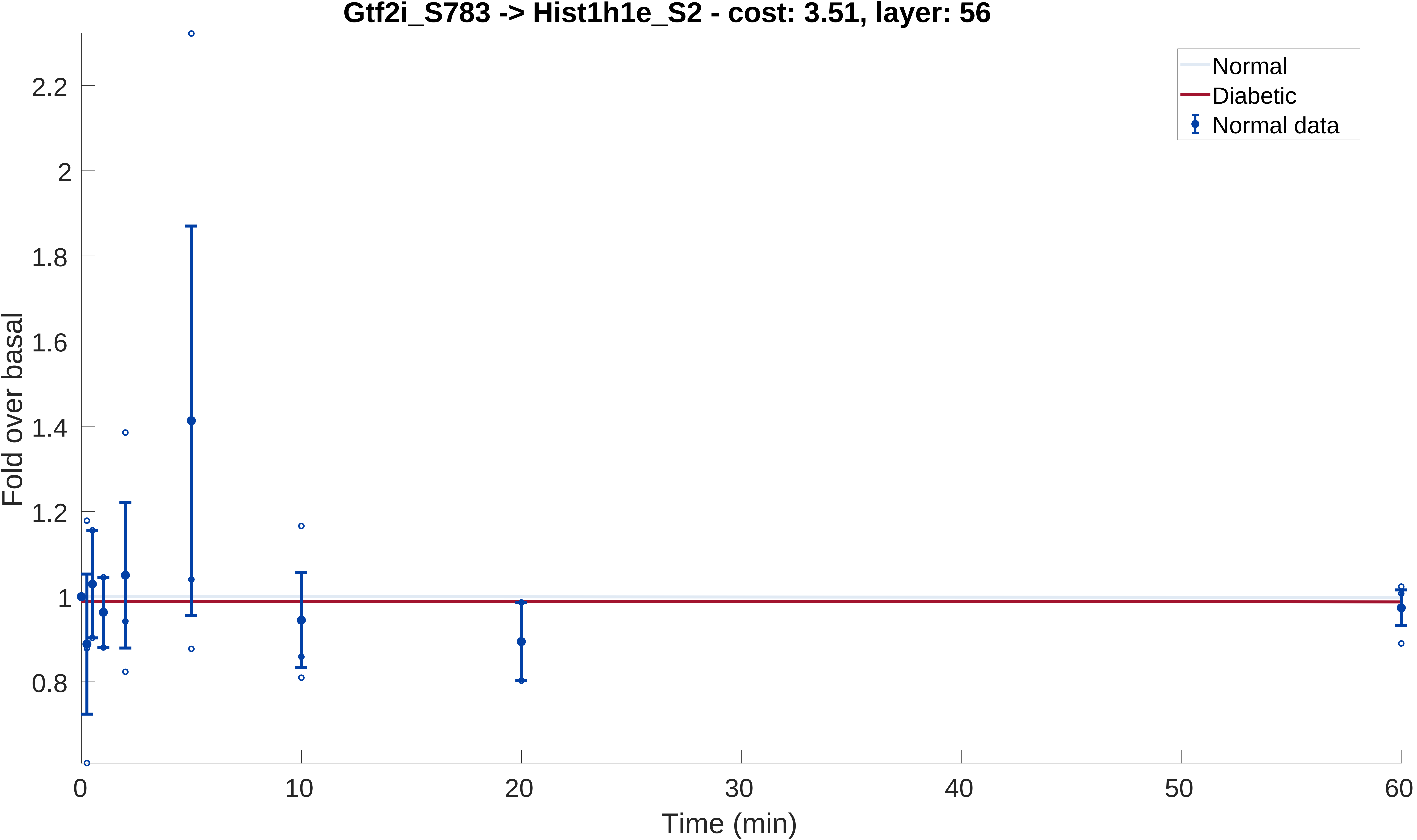
Gtf2i_S783 -> Hist1h1e_S2 - cost: 3.51, layer: 56
Normal
Diabetic
Normal data
Fold over basal
2.2
2
1.8
1.6
1.4
1.2
1
0.8
0
10
20
30
40
50
60
Time (min)

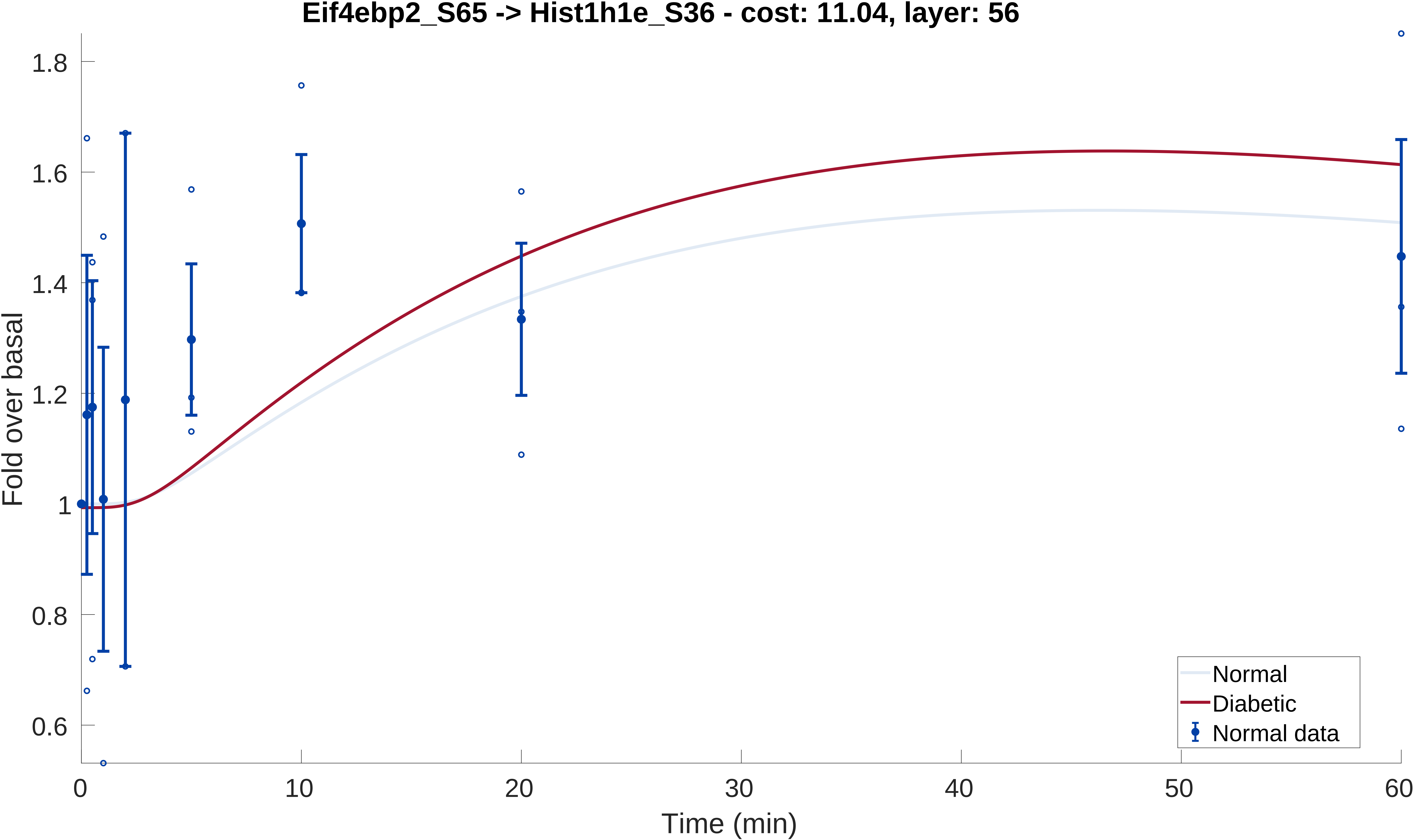

Eif4ebp2_S65 -> Hist1h1e_S36 - cost: 11.04, layer: 56
Fold over basal
1.8
1.6
1.4
1.2
1
0.8
0.6
0
10
20
30
40
50
60
Time (min)
Normal
Diabetic
Normal data

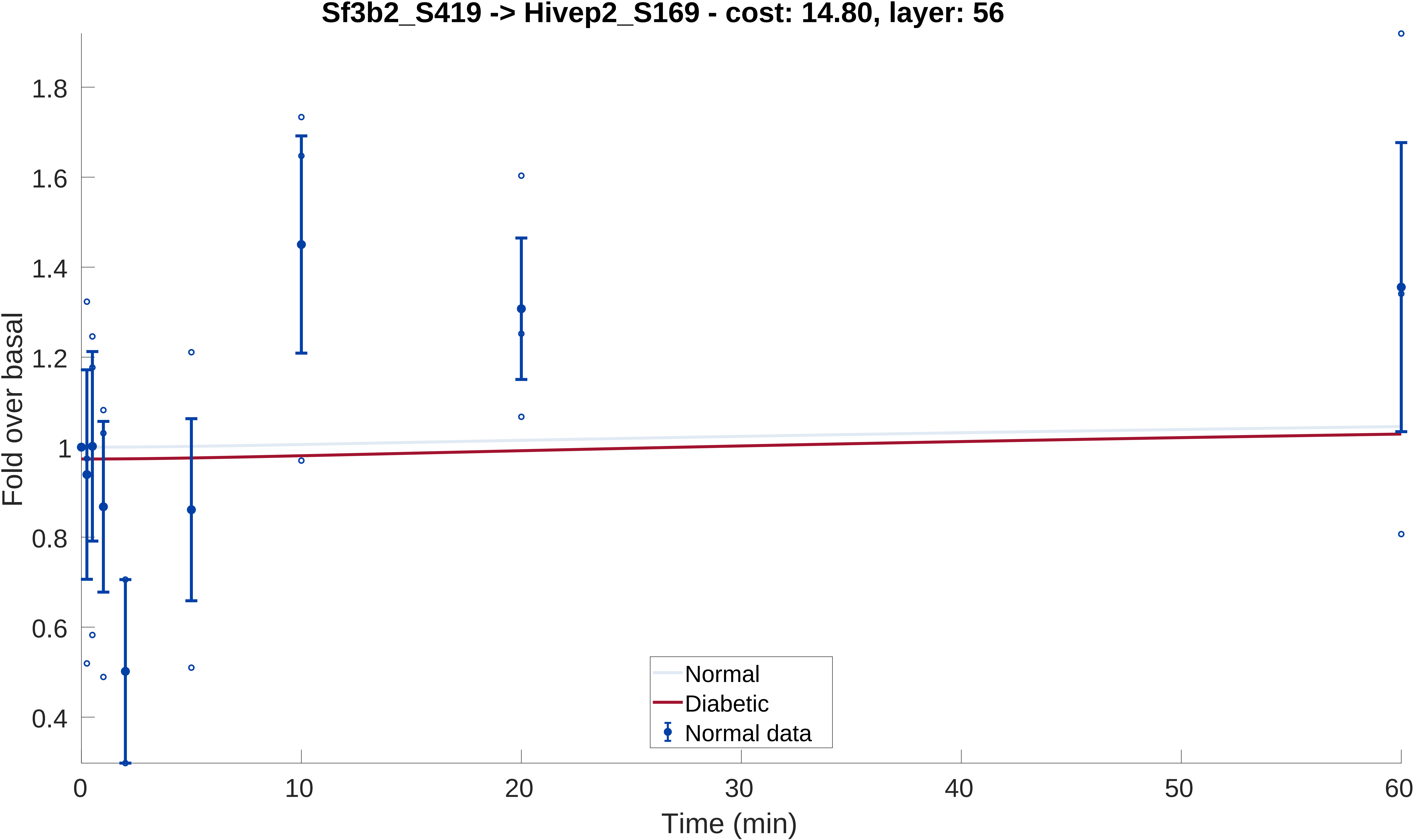

Sf3b2_S419 -> Hivep2_S169 - cost: 14.80, layer: 56
Fold over basal
1.8
1.6
1.4
1.2
1
0.8
0.6
0.4
0
10
20
30
40
50
60
Time (min)
Normal
Diabetic
Normal data

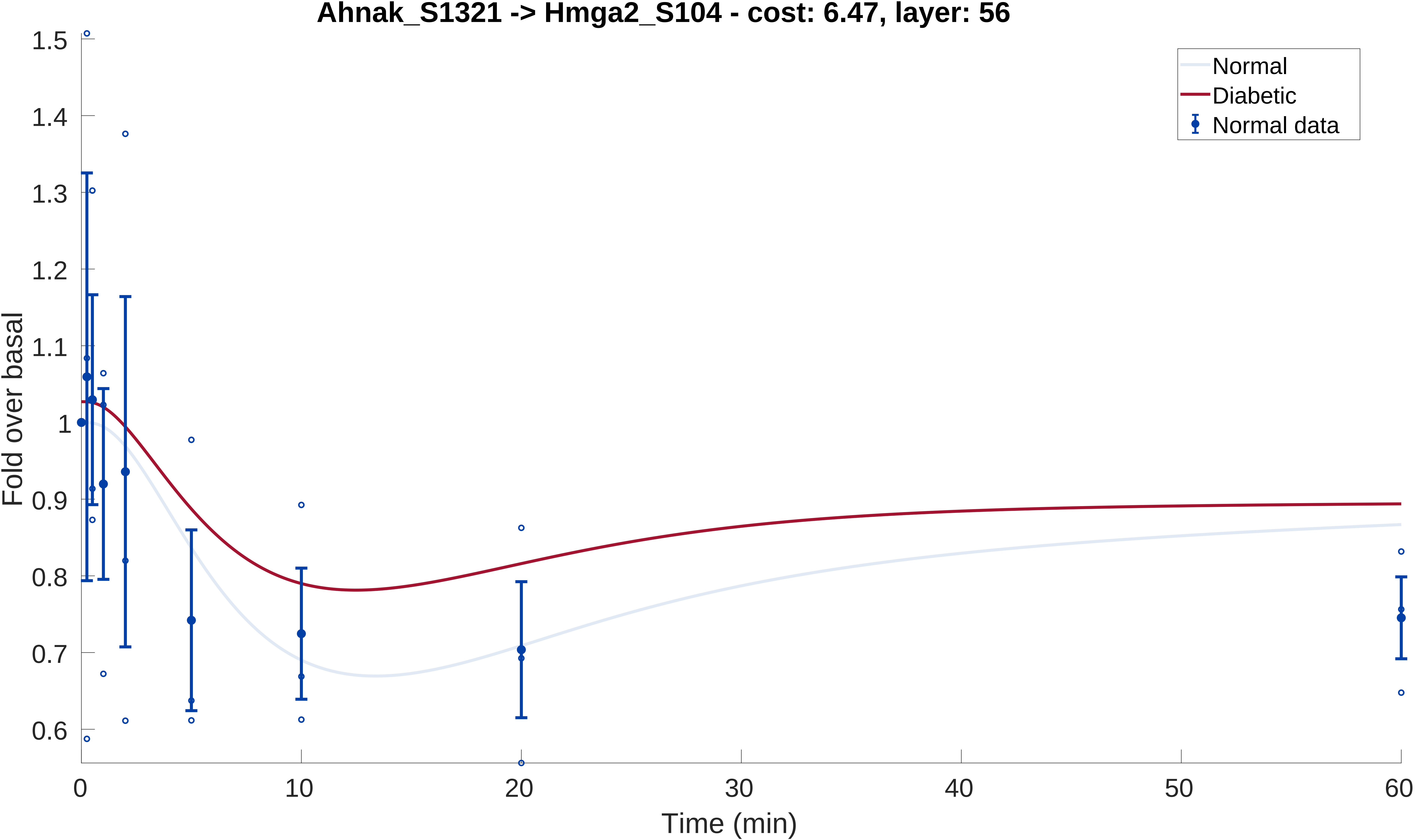
Ahnak_S1321 -> Hmga2_S104 - cost: 6.47, layer: 56
Normal
Diabetic
Normal data
Fold over basal
Time (min)
1.5
1.4
1.3
1.2
1.1
1
0.9
0.8
0.7
0.6
0
10
20
30
40
50
60

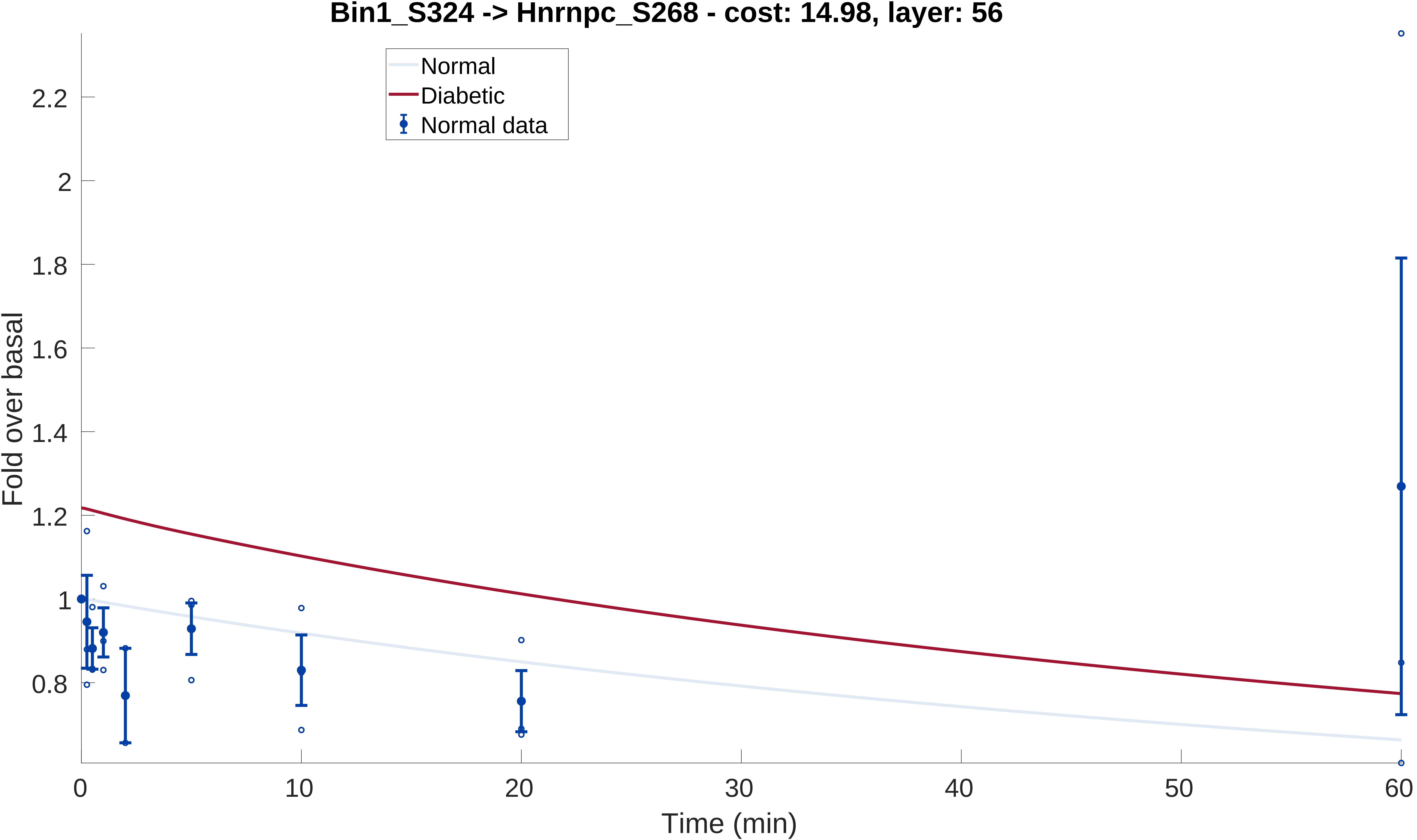

Bin1_S324 -> Hnrnpc_S268 - cost: 14.98, layer: 56
Normal
Diabetic
Normal data
Fold over basal
Time (min)
0
10
20
30
40
50
60
0.8
1
1.2
1.4
1.6
1.8
2
2.2

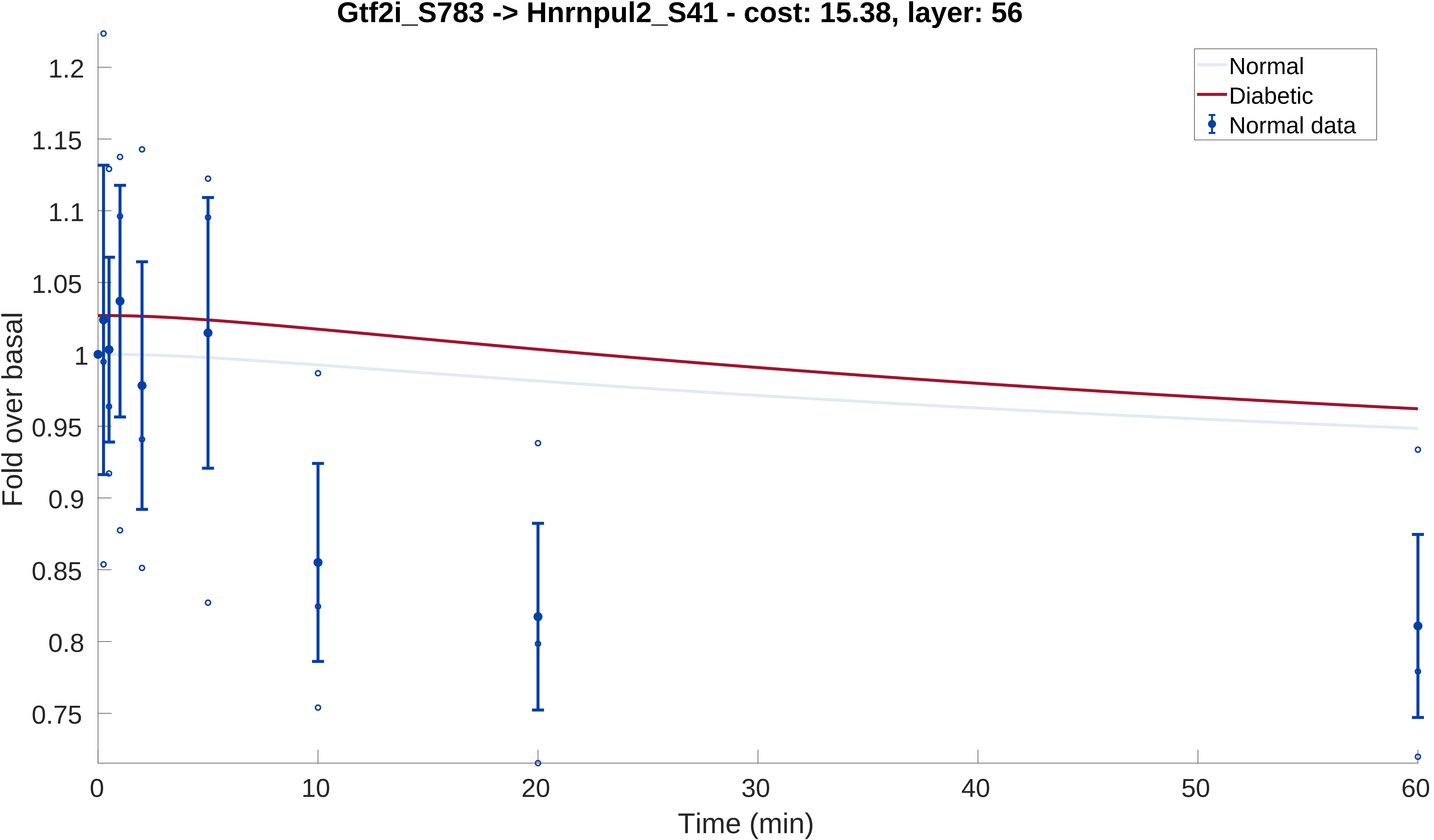

Gtf2i_S783 -> Hnrnpul2_S41 - cost: 15.38, layer: 56
Normal
Diabetic
Normal data
Fold over basal
1.2
1.15
1.1
1.05
1
0.95
0.9
0.85
0.8
0.75
0
10
20
30
40
50
60
Time (min)

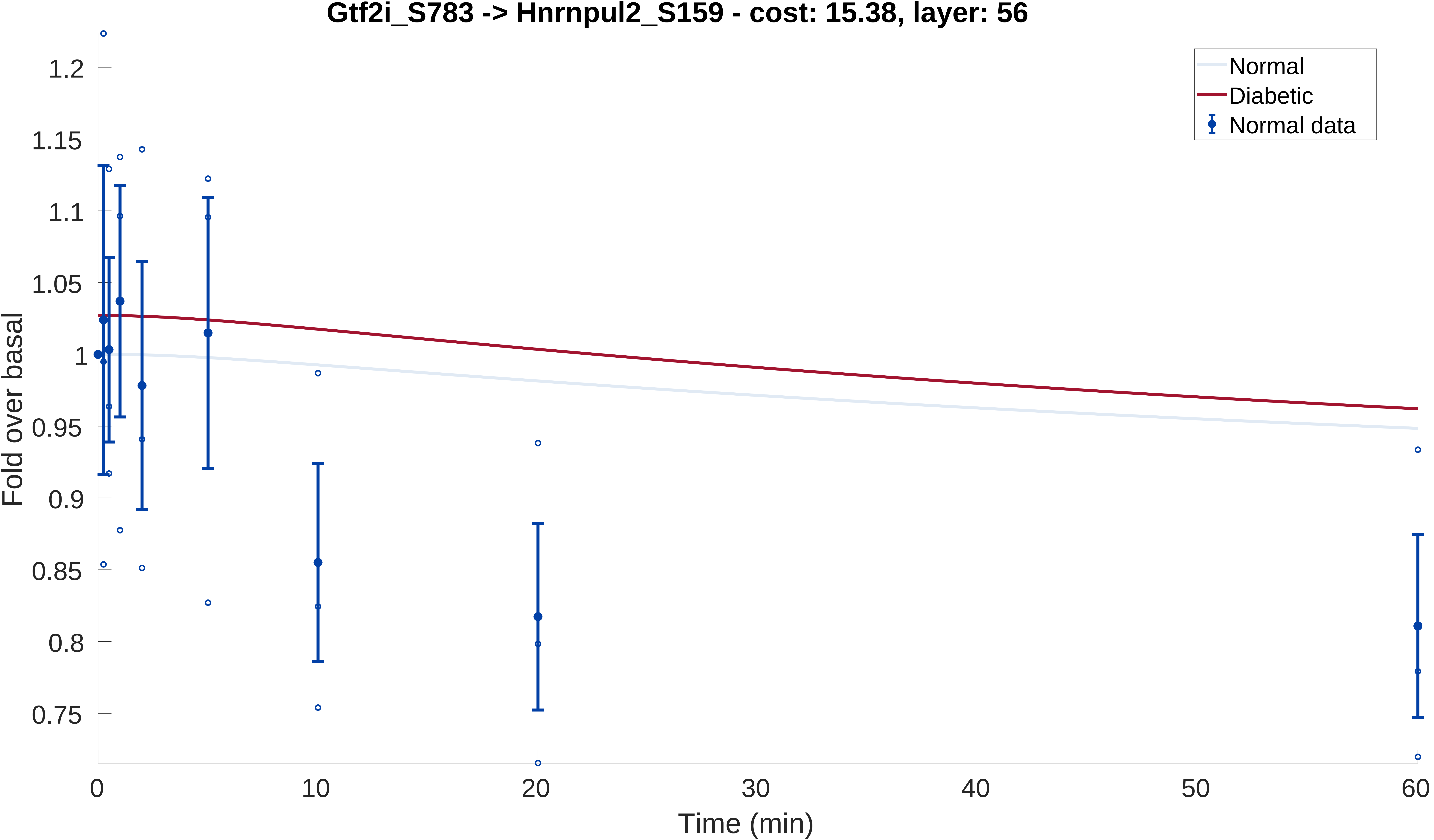

Gtf2i_S783 -> Hnrnpul2_S159 - cost: 15.38, layer: 56
Normal
Diabetic
Normal data
Fold over basal
Time (min)
1.2
1.15
1.1
1.05
1
0.95
0.9
0.85
0.8
0.75
0
10
20
30
40
50
60

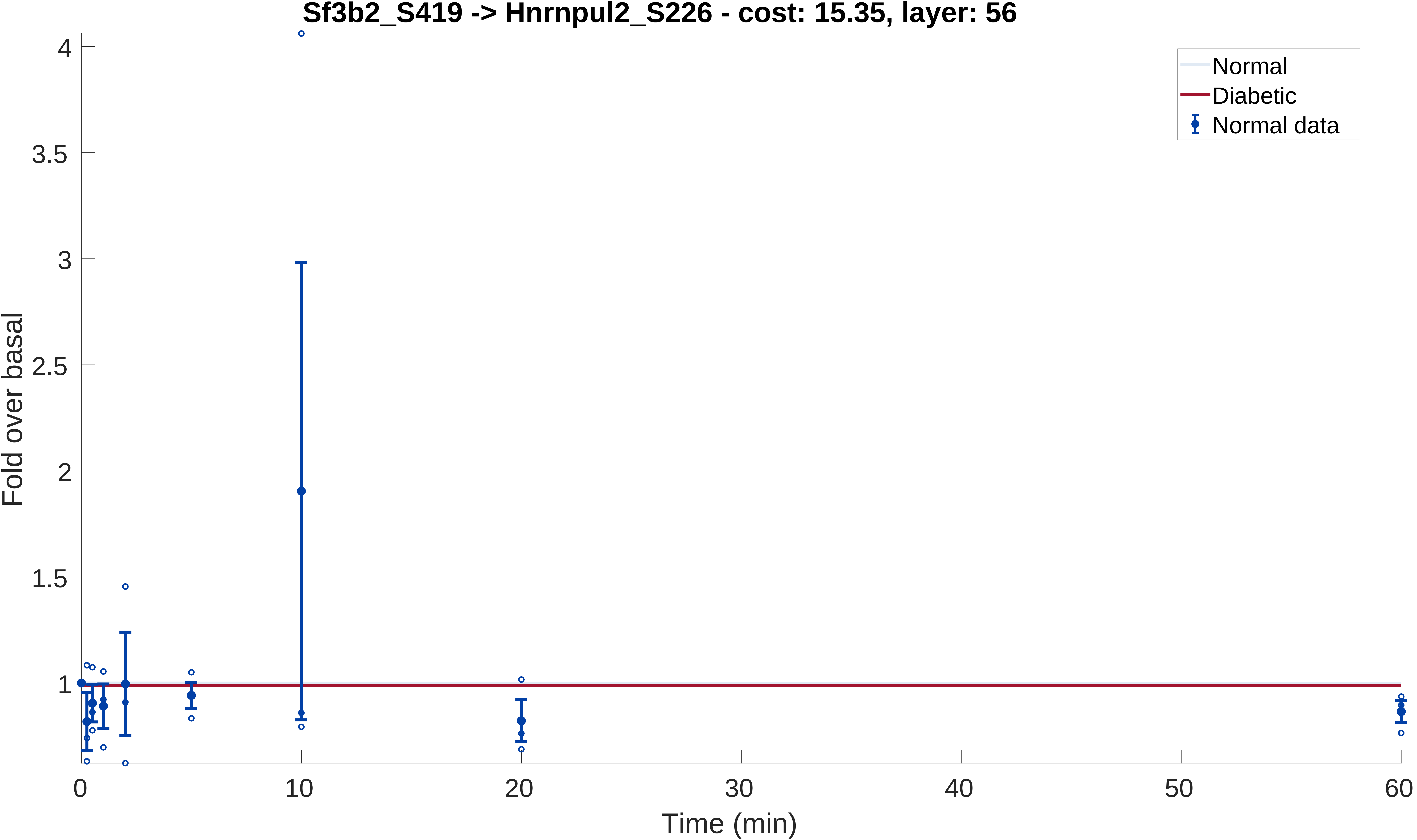

Sf3b2_S419 -> Hnrnpul2_S226 - cost: 15.35, layer: 56
Normal
Diabetic
Normal data
Fold over basal
Time (min)
4
3.5
3
2.5
2
1.5
1
0
10
20
30
40
50
60

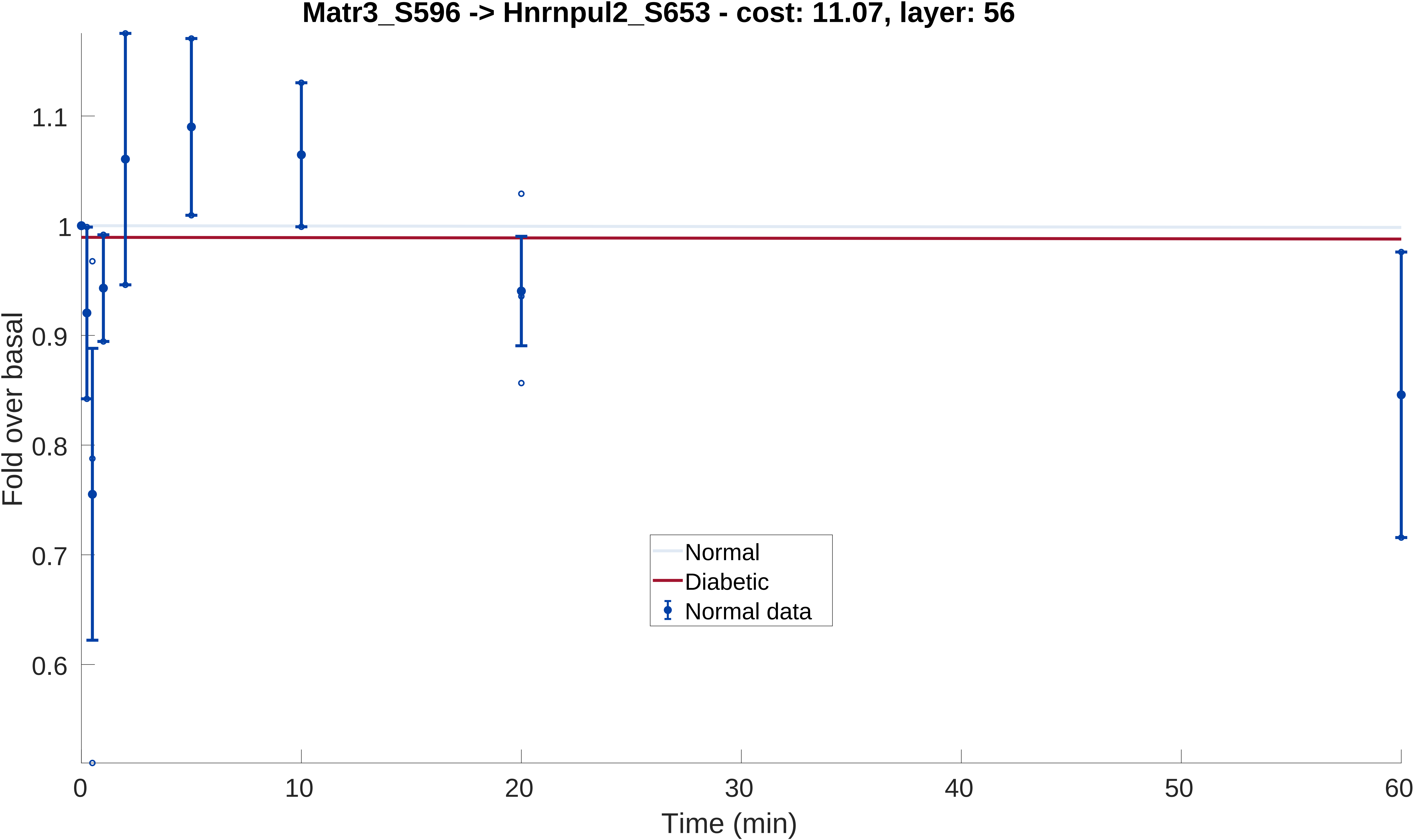

Matr3_S596 -> Hnrnpul2_S653 - cost: 11.07, layer: 56
Fold over basal
1.1
1
0.9
0.8
0.7
0.6
0
10
20
30
40
50
60
Time (min)
Normal
Diabetic
Normal data

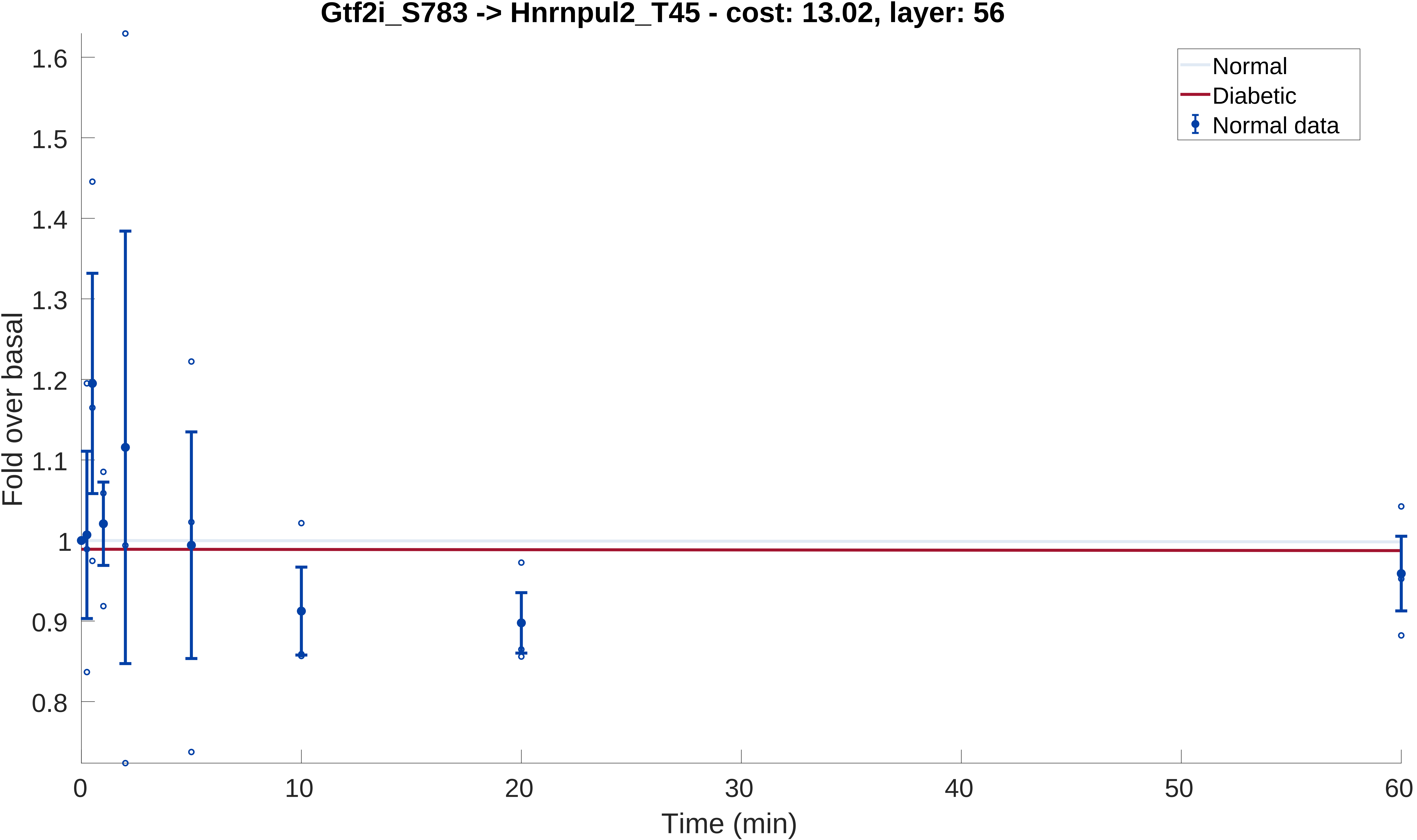

Gtf2i_S783 -> Hnrnpul2_T45 - cost: 13.02, layer: 56
Normal
Diabetic
Normal data
Fold over basal
Time (min)
1.6
1.5
1.4
1.3
1.2
1.1
1
0.9
0.8
0
10
20
30
40
50
60

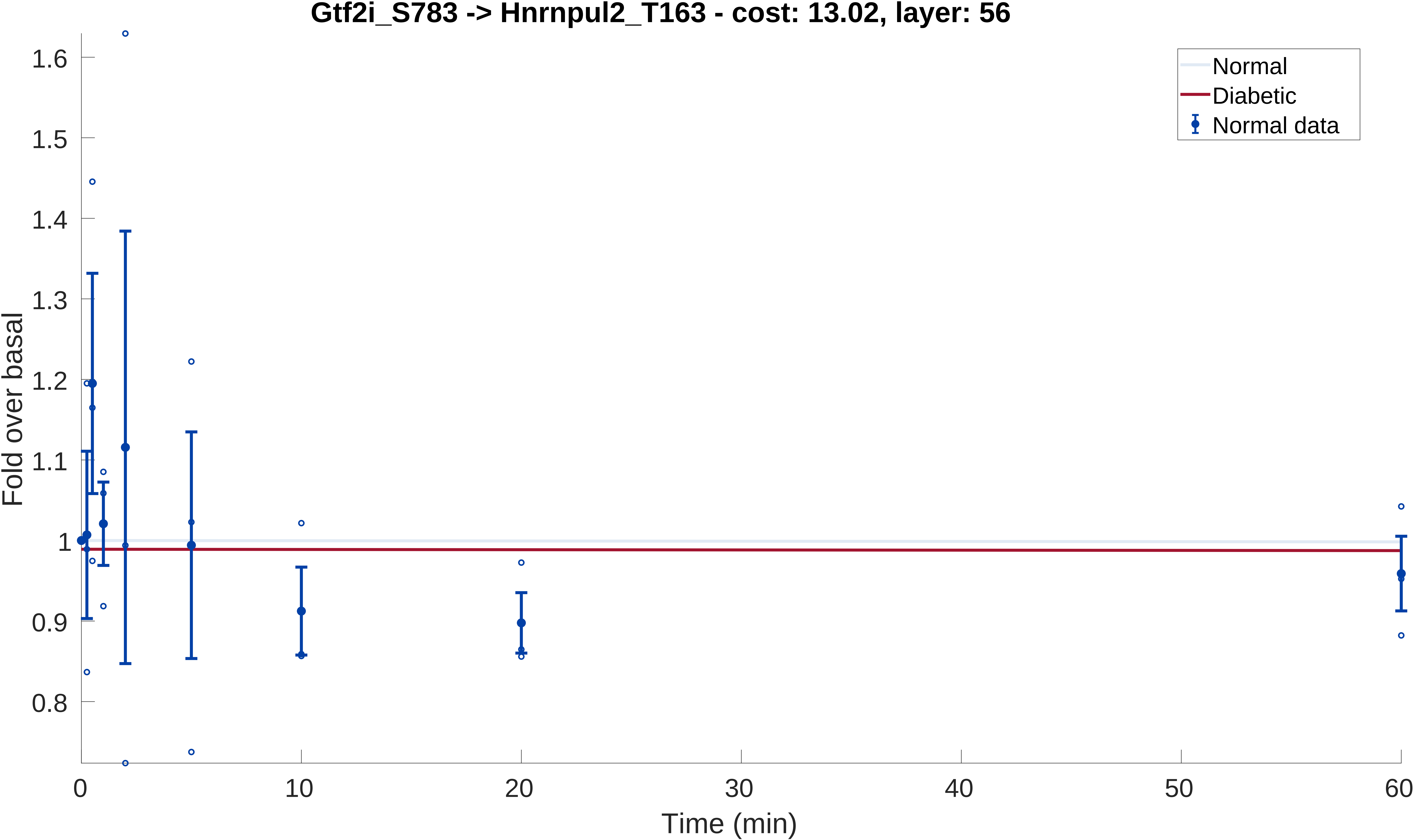
Gtf2i_S783 -> Hnrnpul2_T163 - cost: 13.02, layer: 56
Normal
Diabetic
Normal data
Fold over basal
Time (min)
1.6
1.5
1.4
1.3
1.2
1.1
1
0.9
0.8
0
10
20
30
40
50
60

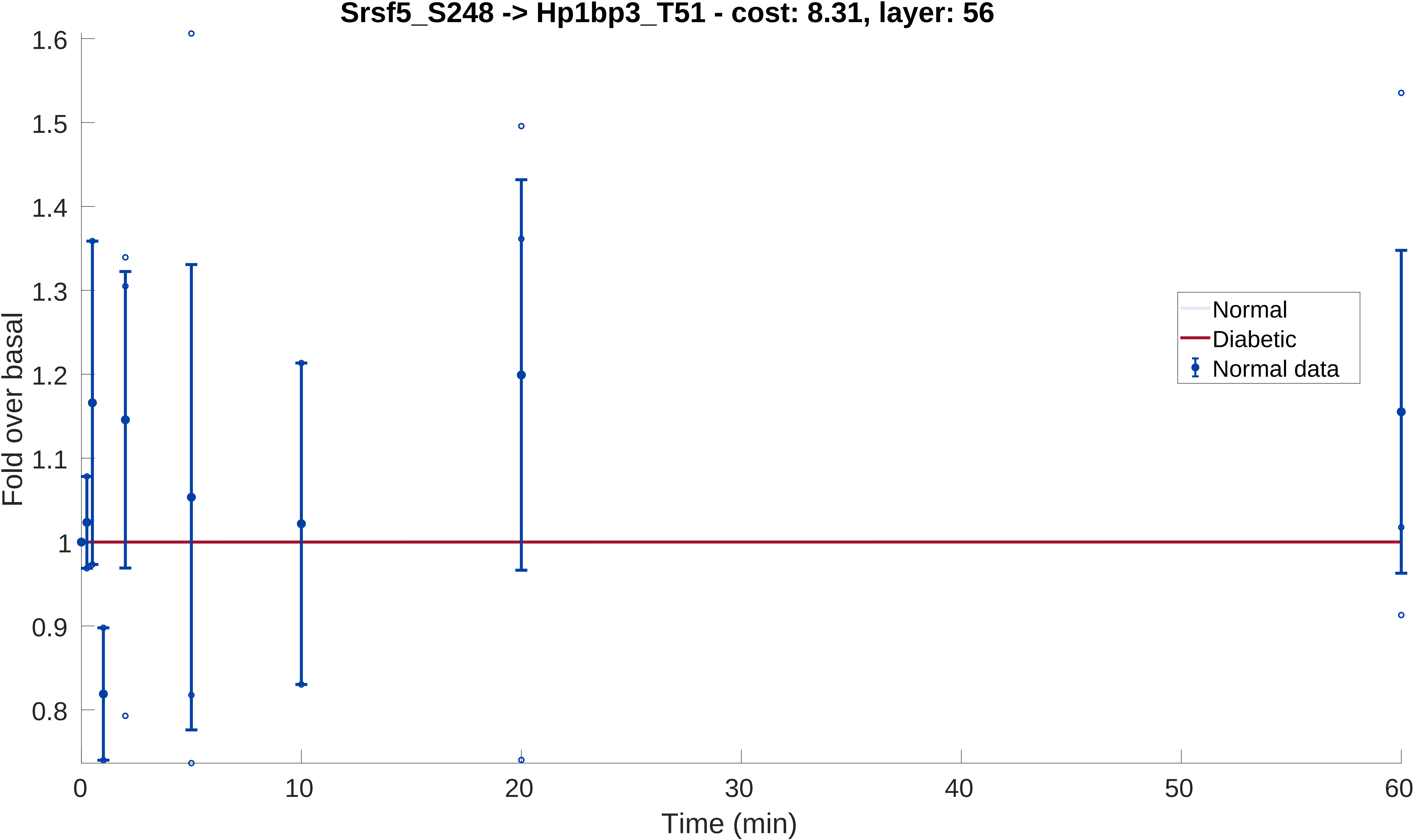

Srsf5_S248 -> Hp1bp3_T51 - cost: 8.31, layer: 56
Fold over basal
Time (min)
Normal
Diabetic
Normal data
1.6
1.5
1.4
1.3
1.2
1.1
1
0.9
0.8
0
10
20
30
40
50
60

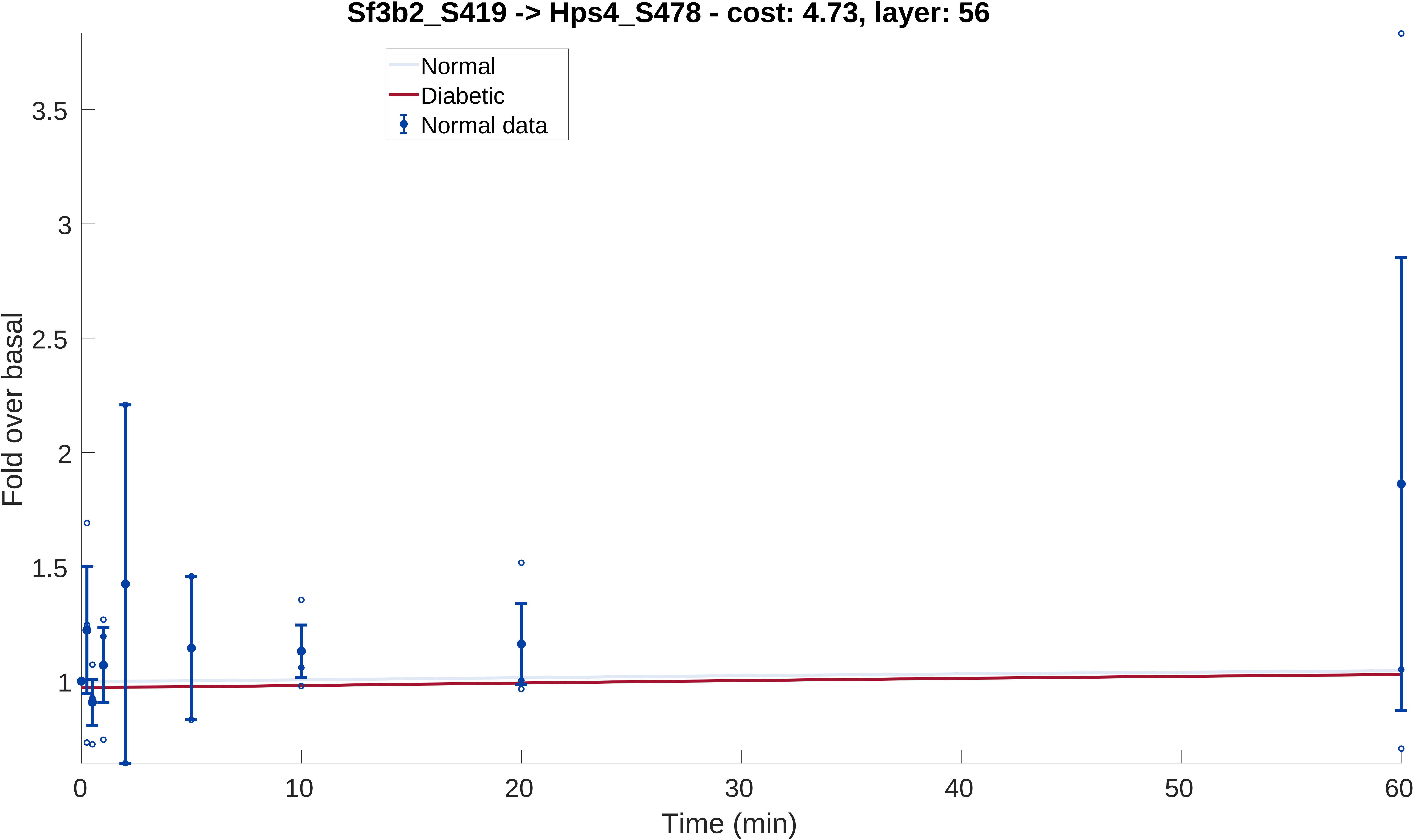

Sf3b2_S419 -> Hps4_S478 - cost: 4.73, layer: 56
Normal
Diabetic
Normal data
Fold over basal
3.5
3
2.5
2
1.5
1
0
10
20
30
40
50
60
Time (min)

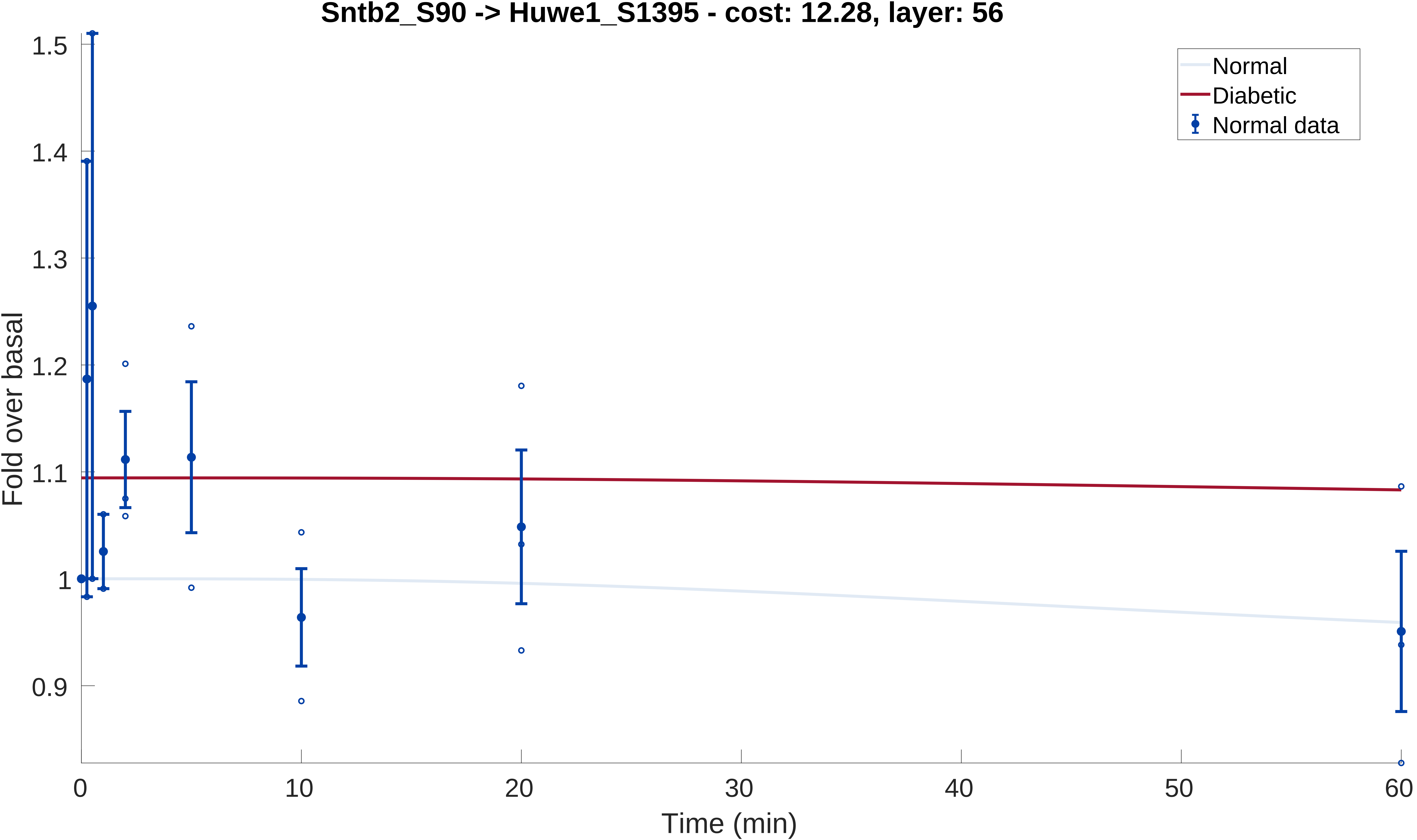

Sntb2_S90 -> Huwe1_S1395 - cost: 12.28, layer: 56
Normal
Diabetic
Normal data
Fold over basal
1.5
1.4
1.3
1.2
1.1
1
0.9
0
10
20
30
40
50
60
Time (min)

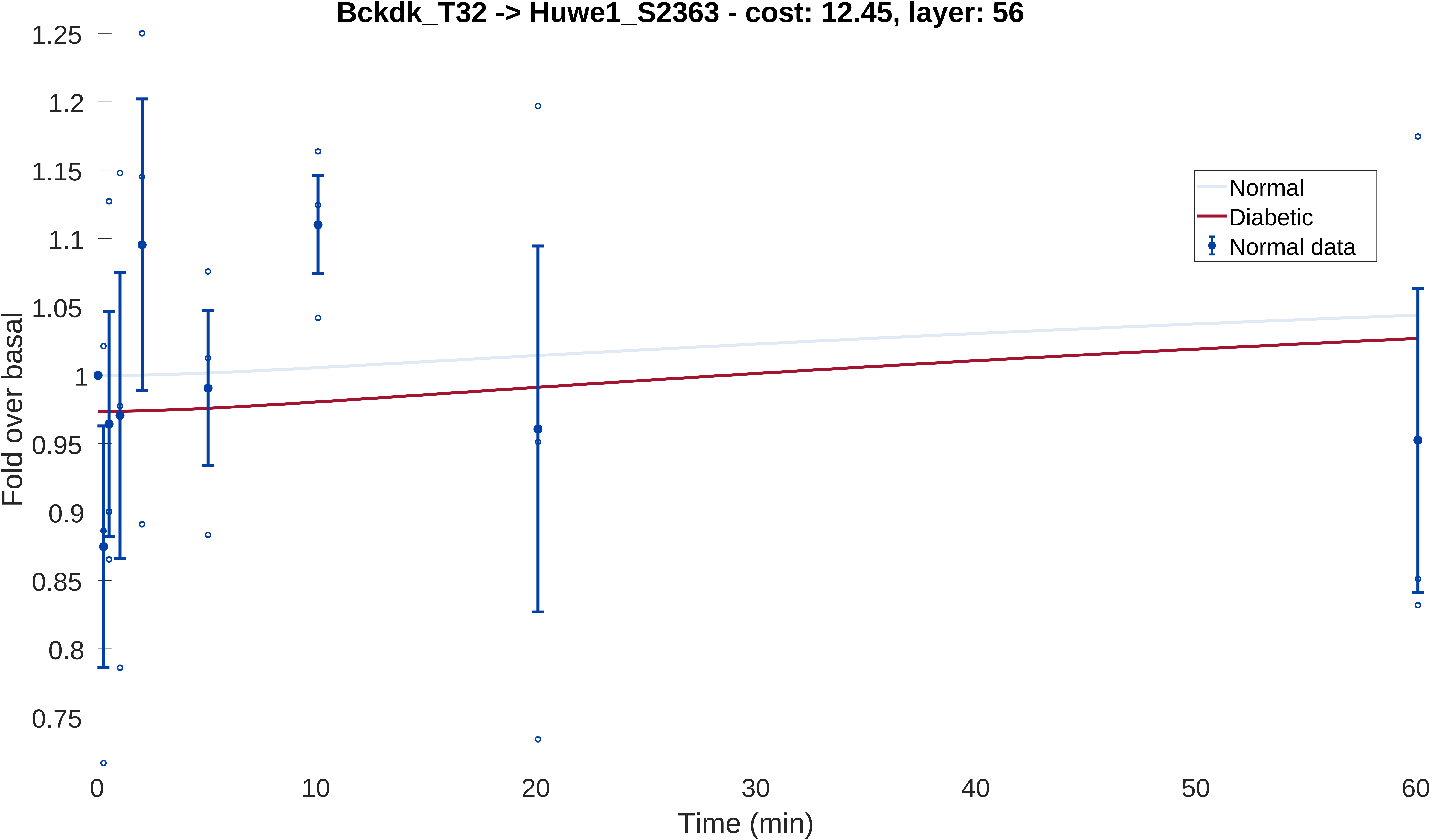

Bckdk_T32 -> Huwe1_S2363 - cost: 12.45, layer: 56
1.25
1.2
1.15
1.1
1.05
1
0.95
0.9
0.85
0.8
0.75
Fold over basal
0
10
20
30
40
50
60
Time (min)
Normal
Diabetic
Normal data

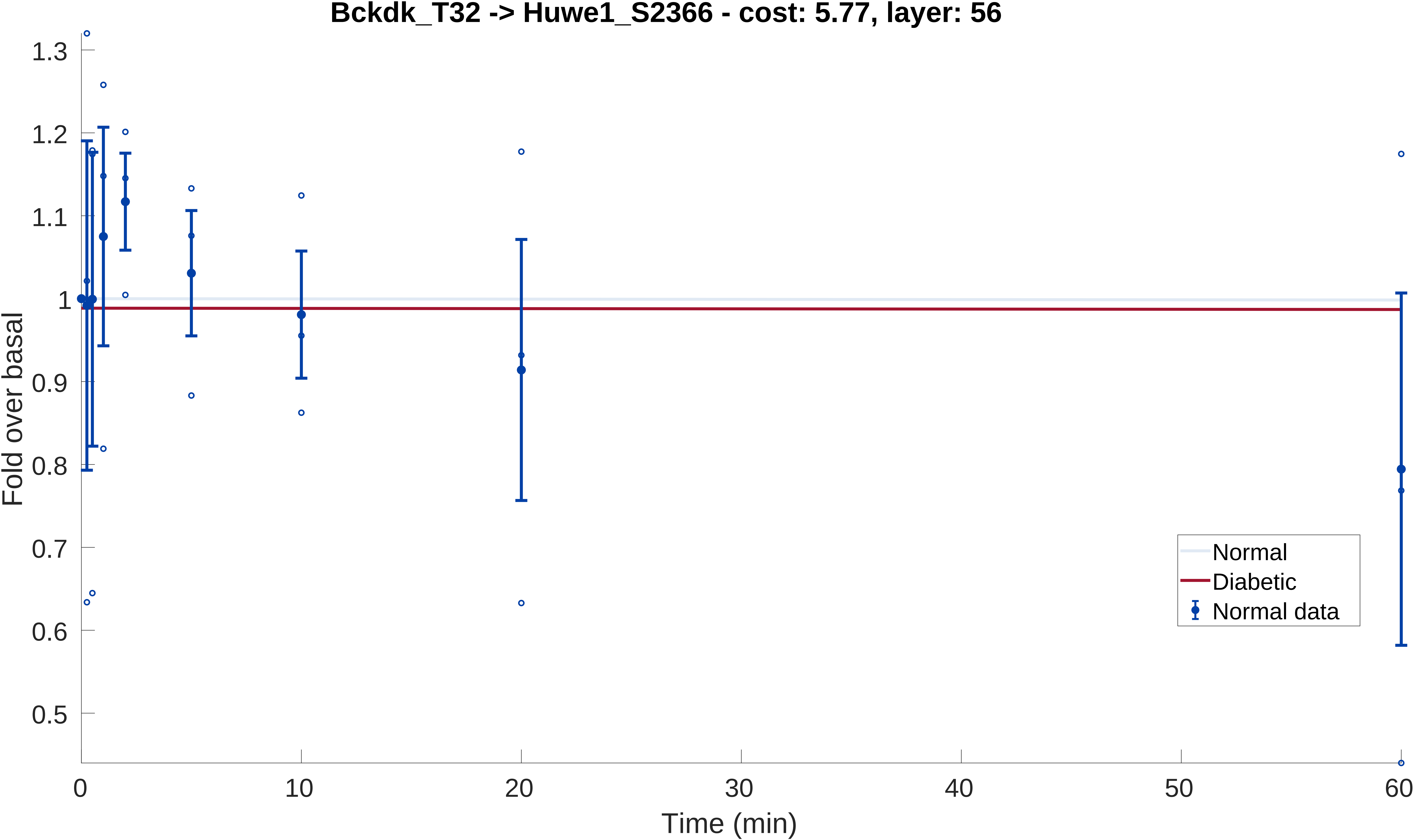

Bckdk_T32 -> Huwe1_S2366 - cost: 5.77, layer: 56
Fold over basal
Time (min)
1.3
1.2
1.1
1
0.9
0.8
0.7
0.6
0.5
0
10
20
30
40
50
60
Normal
Diabetic
Normal data

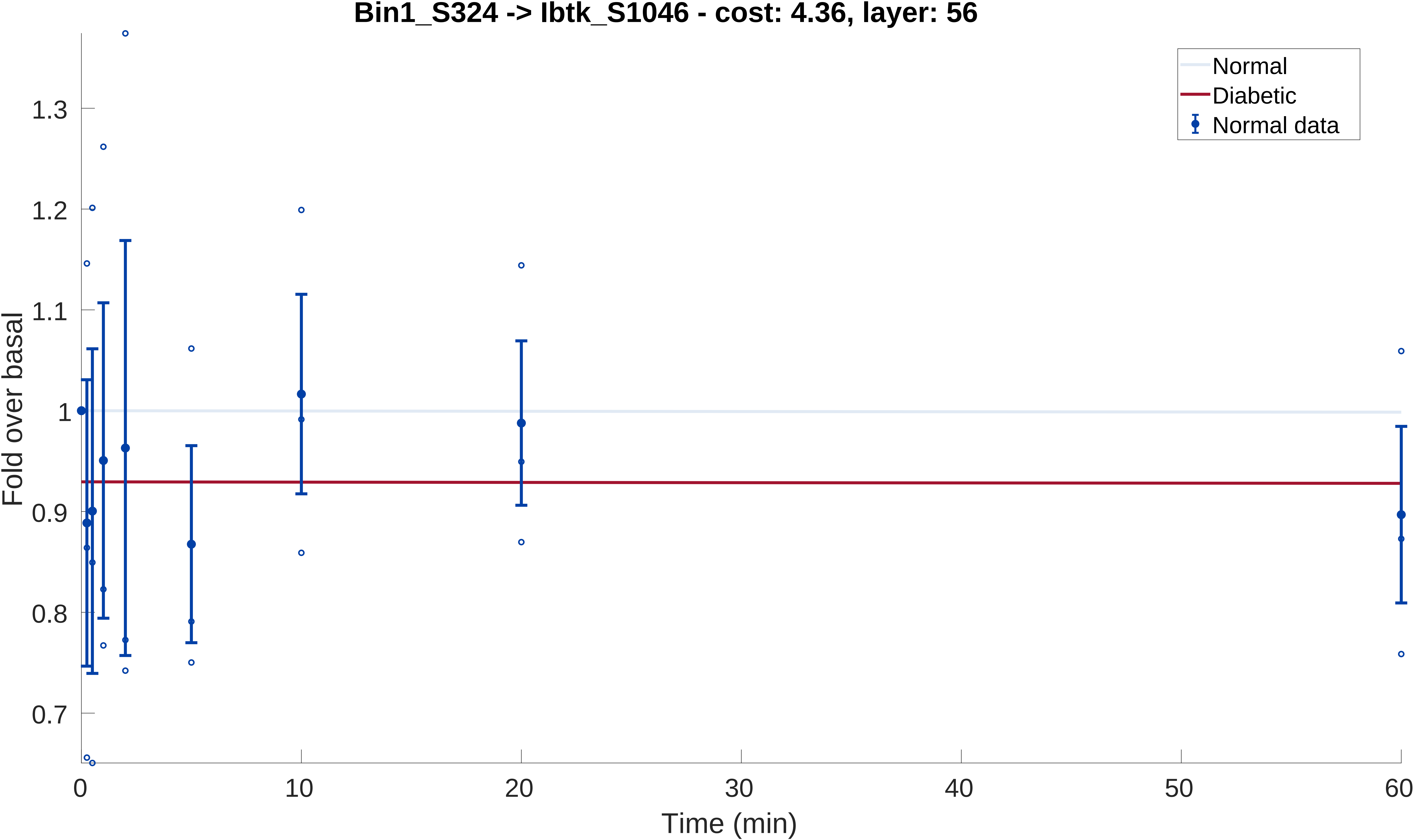

Bin1_S324 -> Ibtk_S1046 - cost: 4.36, layer: 56
Normal
Diabetic
Normal data
Fold over basal
0.7
0.8
0.9
1
1.1
1.2
1.3
0
10
20
30
40
50
60
Time (min)

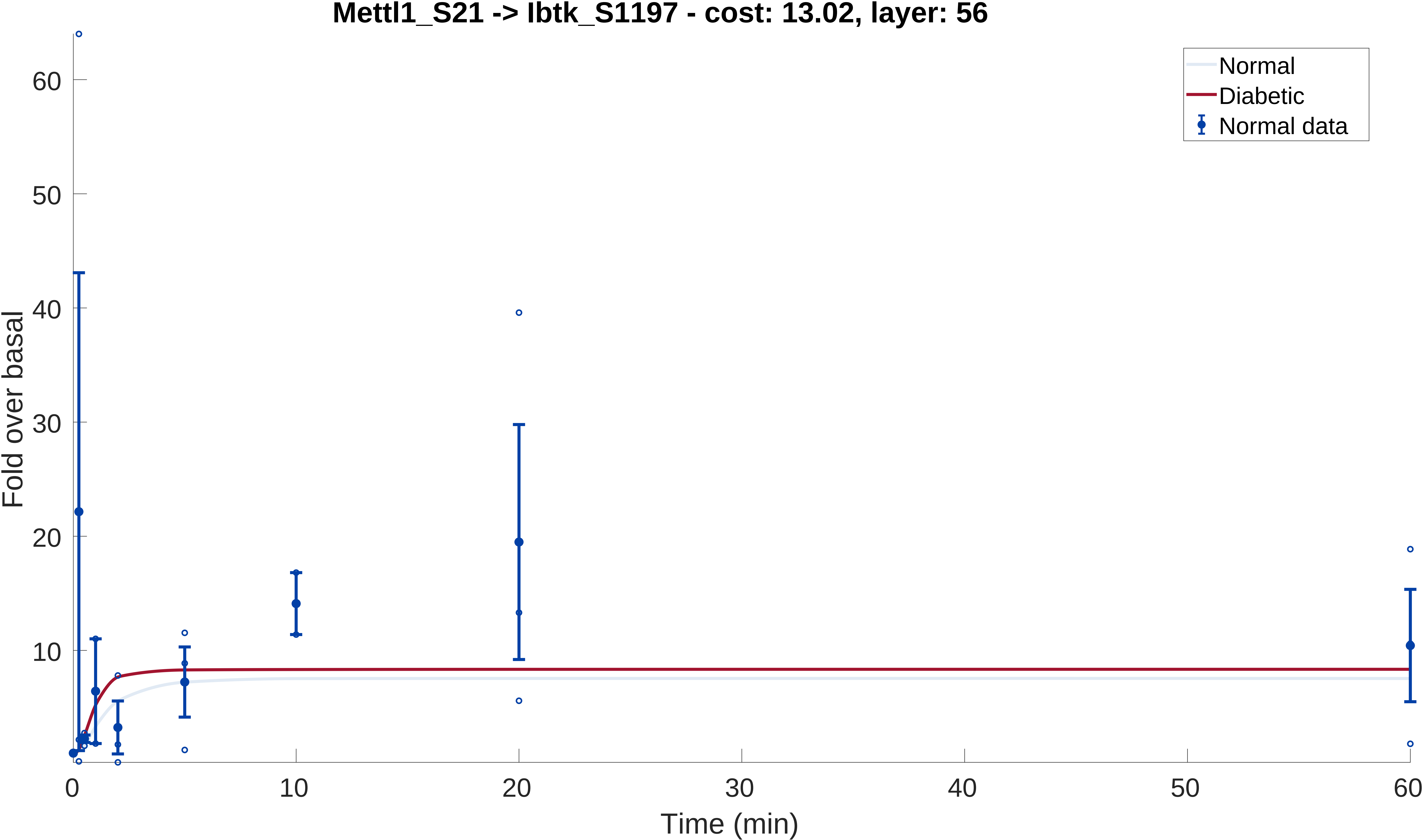

Mettl1_S21 -> Ibtk_S1197 - cost: 13.02, layer: 56
Normal
Diabetic
Normal data
Fold over basal
60
50
40
30
20
10
0
10
20
30
40
50
60
Time (min)

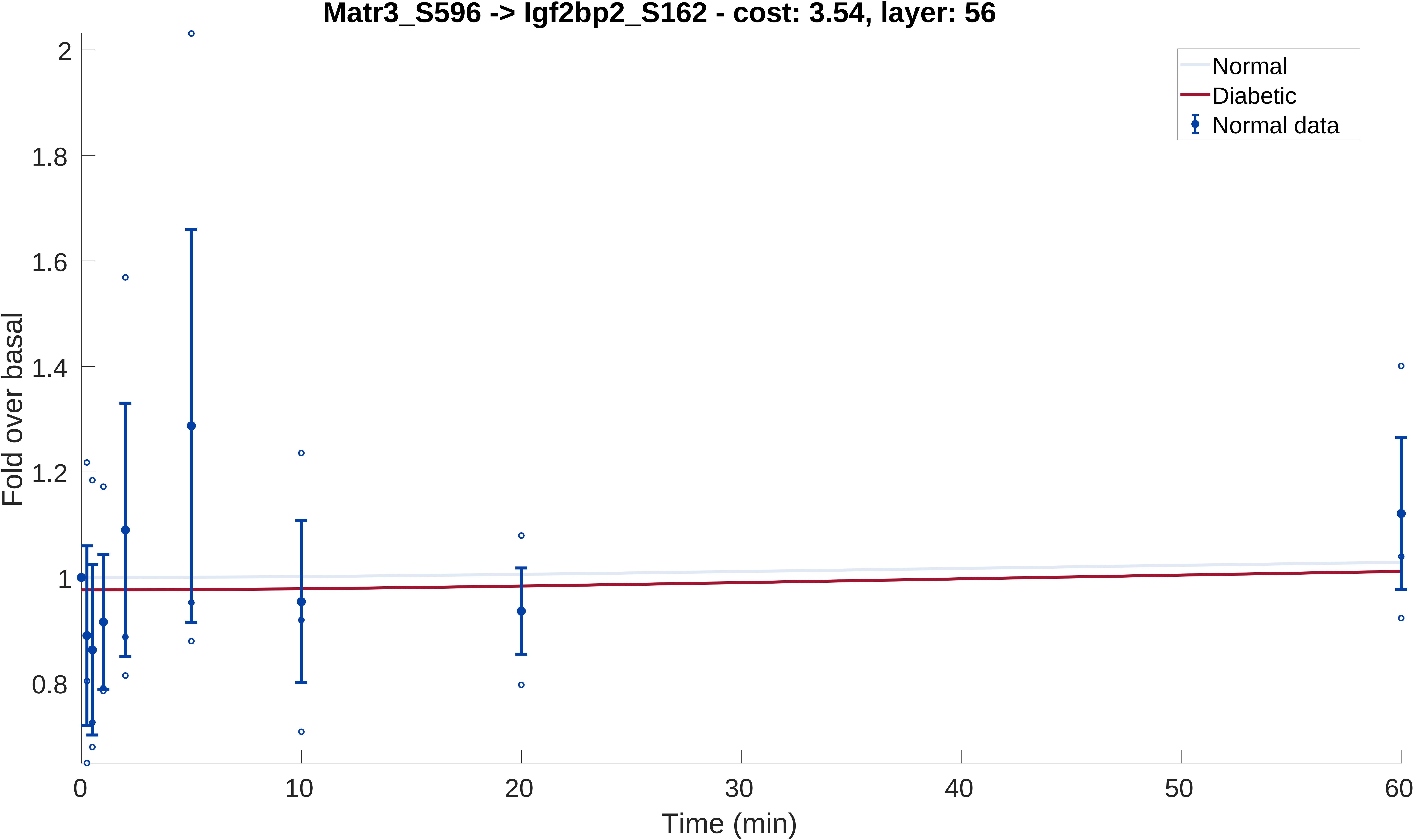

Matr3_S596 -> Igf2bp2_S162 - cost: 3.54, layer: 56
Normal
Diabetic
Normal data
Fold over basal
Time (min)
0.8
1
1.2
1.4
1.6
1.8
2
0
10
20
30
40
50
60

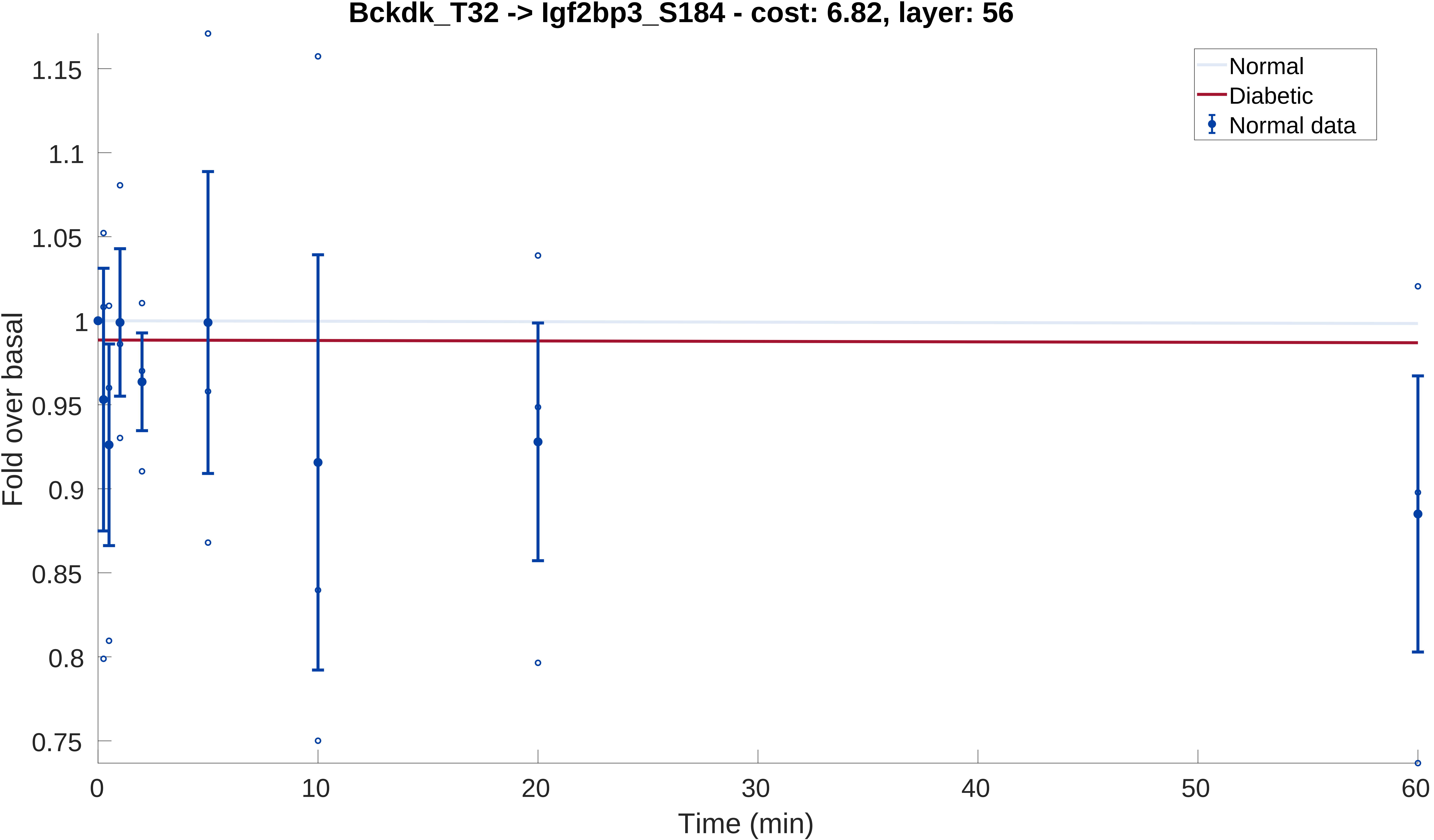

Bckdk_T32 -> Igf2bp3_S184 - cost: 6.82, layer: 56
Normal
Diabetic
Normal data
Fold over basal
Time (min)
1.15
1.1
1.05
1
0.95
0.9
0.85
0.8
0.75
0
10
20
30
40
50
60

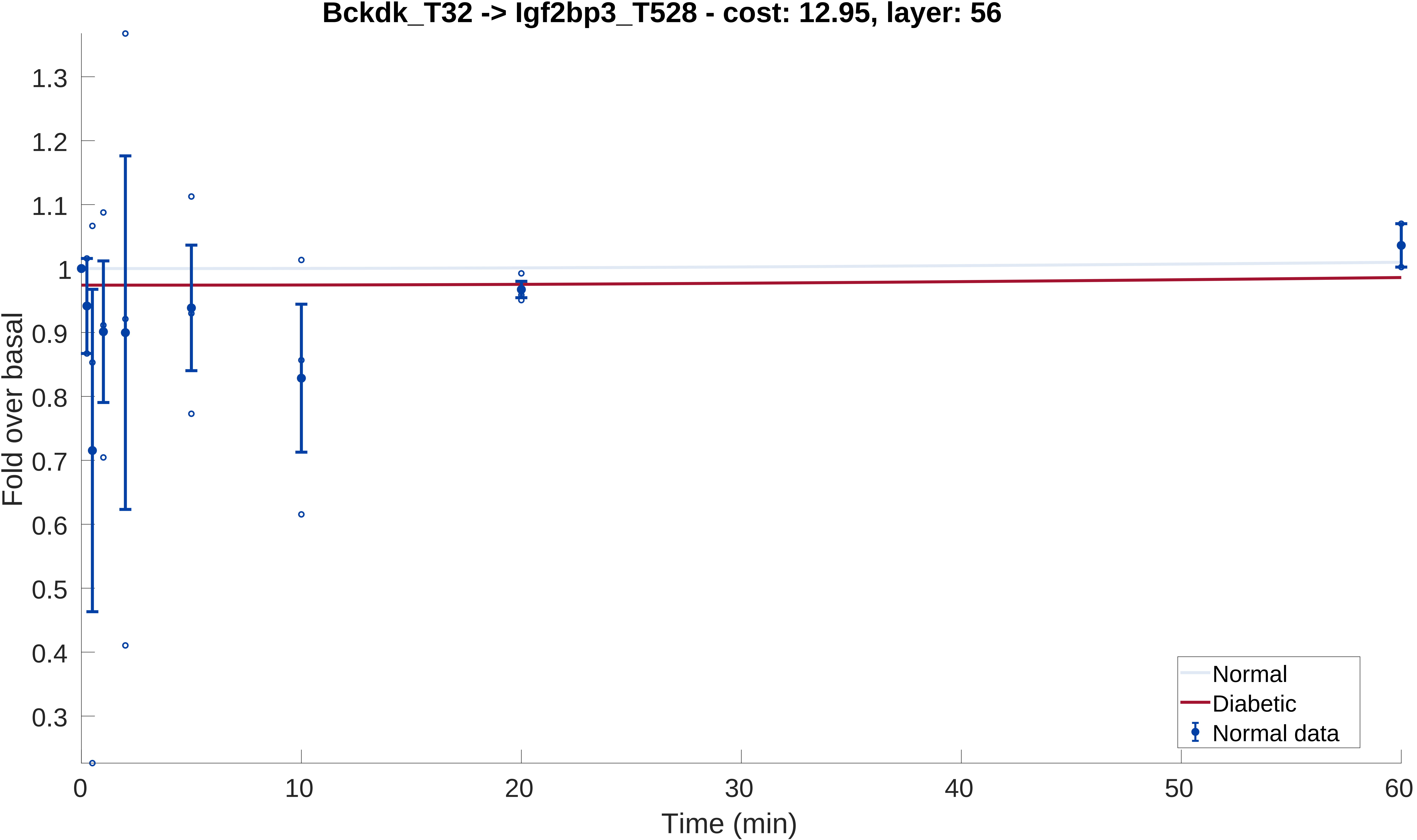

Bckdk_T32 -> Igf2bp3_T528 - cost: 12.95, layer: 56
Fold over basal
Time (min)
1.3
1.2
1.1
1
0.9
0.8
0.7
0.6
0.5
0.4
0.3
0
10
20
30
40
50
60
Normal
Diabetic
Normal data

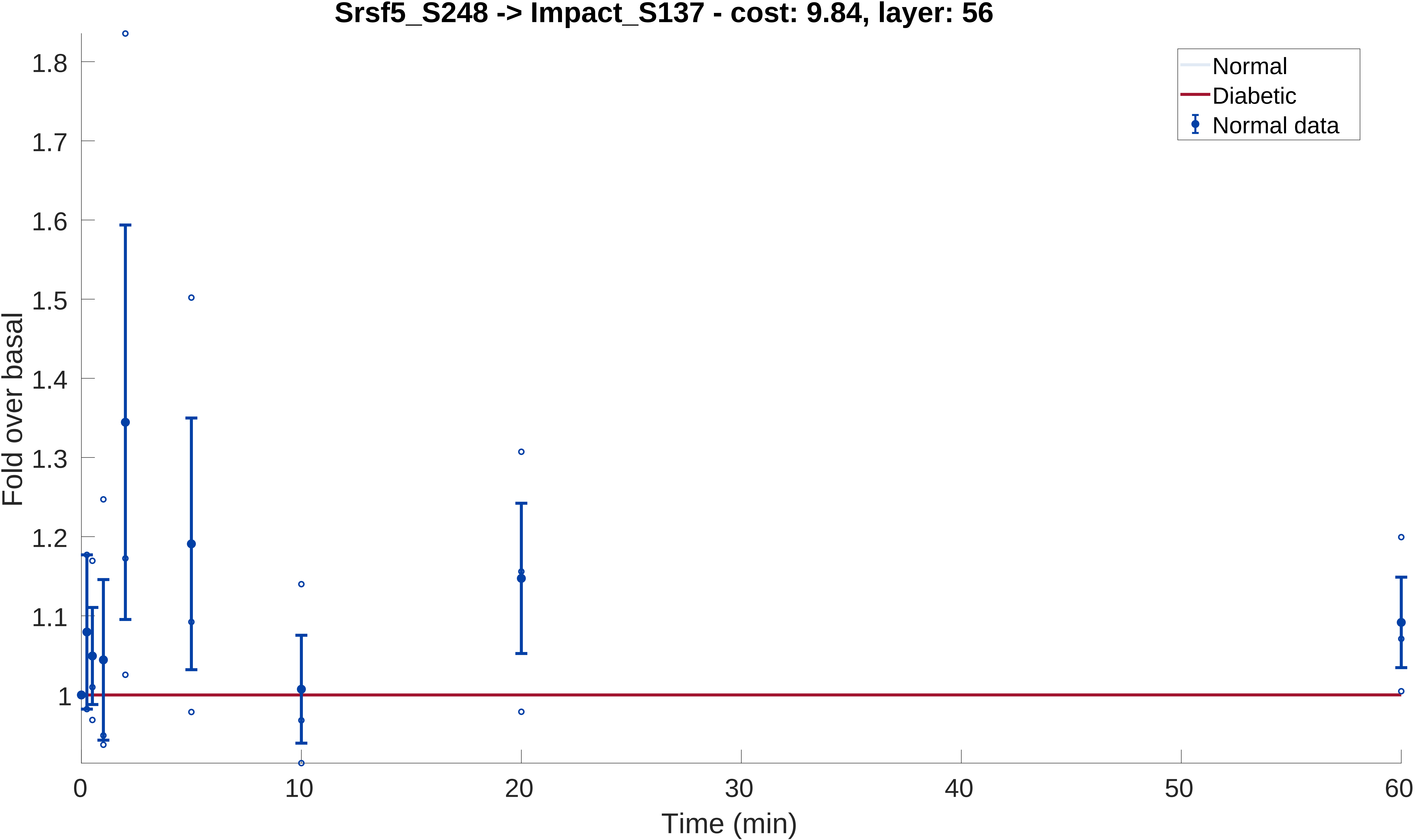

Srsf5_S248 -> Impact_S137 - cost: 9.84, layer: 56
Fold over basal
1.8
1.7
1.6
1.5
1.4
1.3
1.2
1.1
1
0
10
20
30
40
50
60
Time (min)
Normal
Diabetic
Normal data

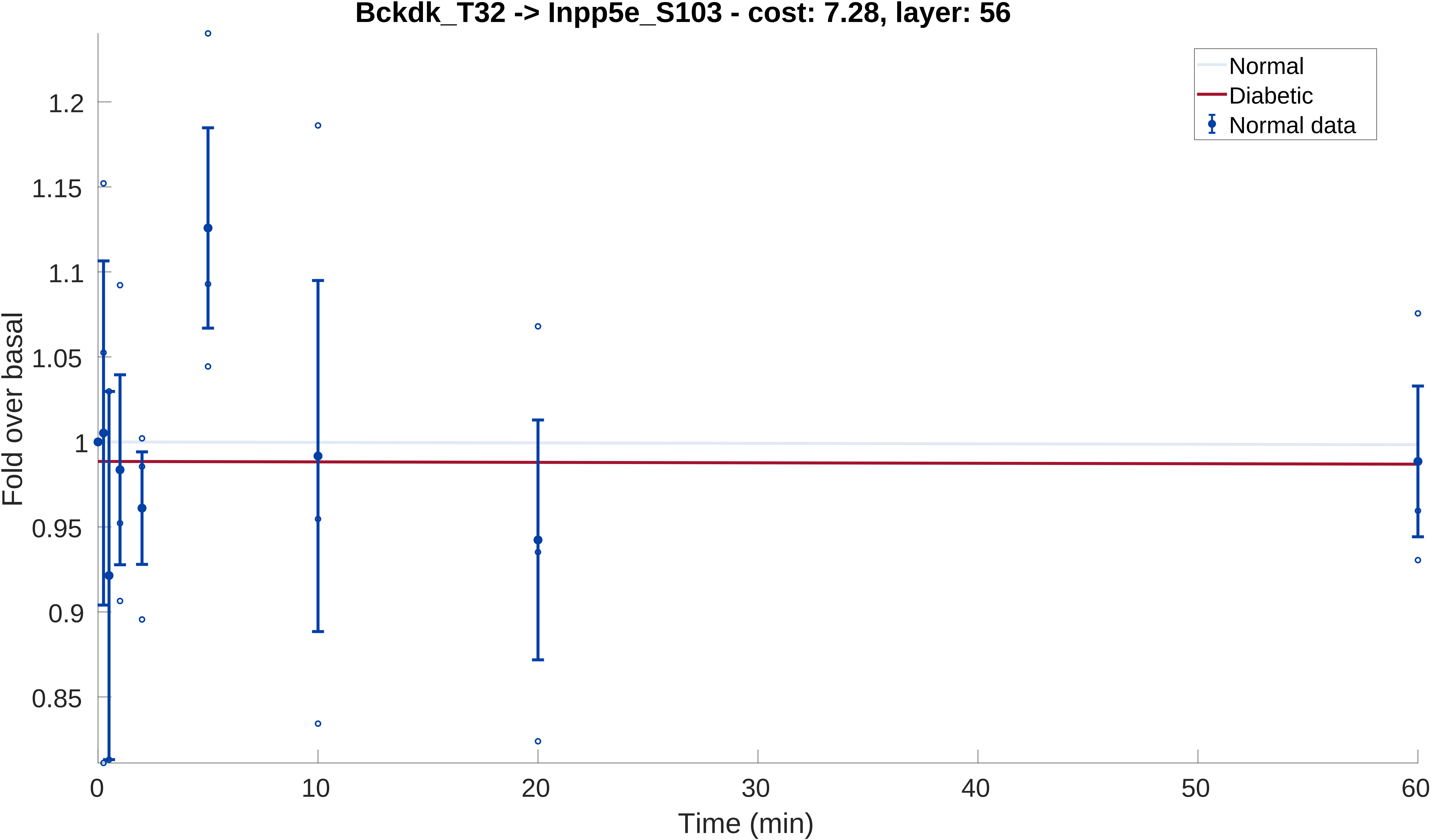

Bckdk_T32 -> Inpp5e_S103 - cost: 7.28, layer: 56
Normal
Diabetic
Normal data
Fold over basal
1.2
1.15
1.1
1.05
1
0.95
0.9
0.85
0
10
20
30
40
50
60
Time (min)

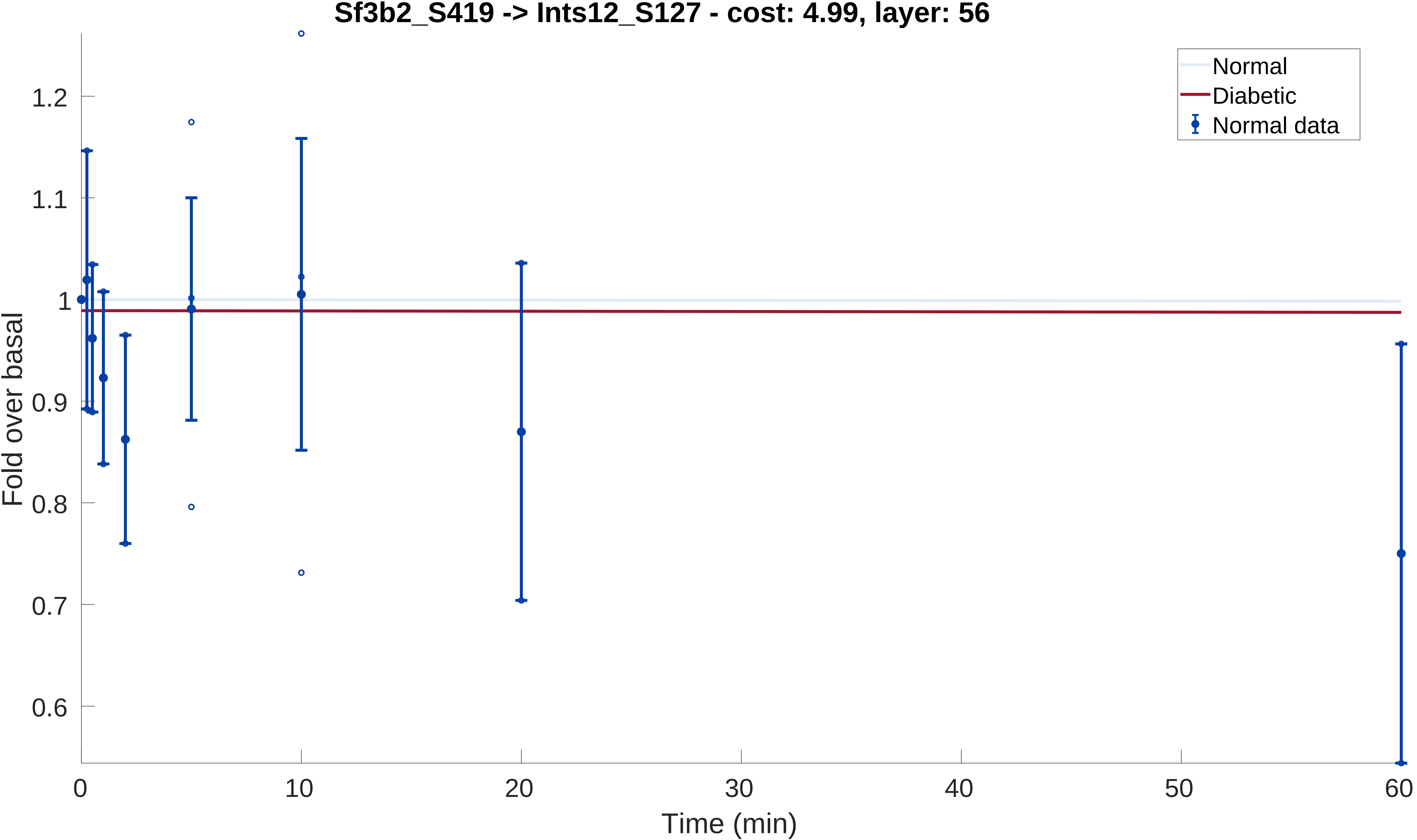

Sf3b2_S419 -> Ints12_S127 - cost: 4.99, layer: 56
Normal
Diabetic
Normal data
Fold over basal
1.2
1.1
1
0.9
0.8
0.7
0.6
0
10
20
30
40
50
60
Time (min)

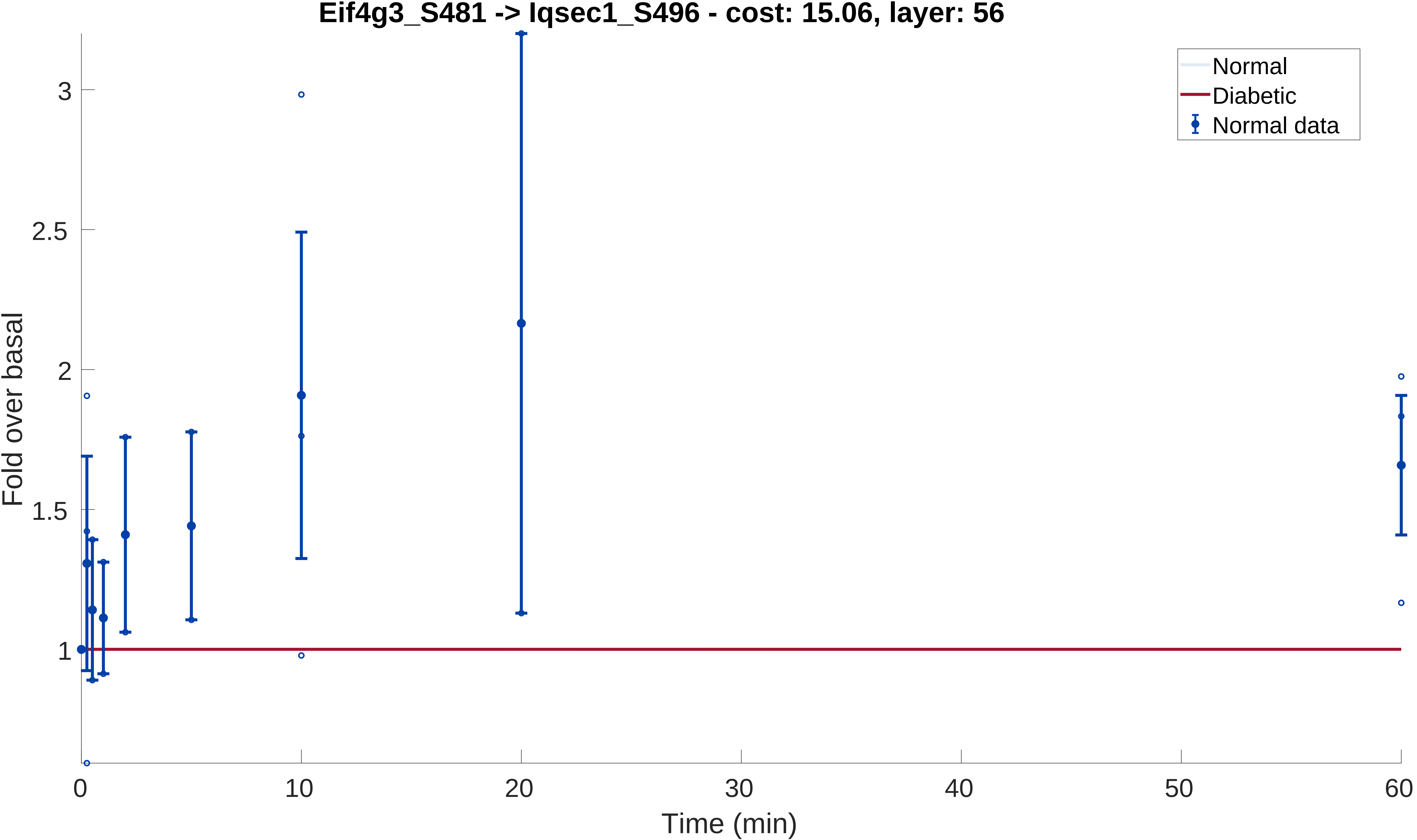

Eif4g3_S481 -> Iqsec1_S496 - cost: 15.06, layer: 56
Normal
Diabetic
Normal data
Fold over basal
3
2.5
2
1.5
1
0
10
20
30
40
50
60
Time (min)

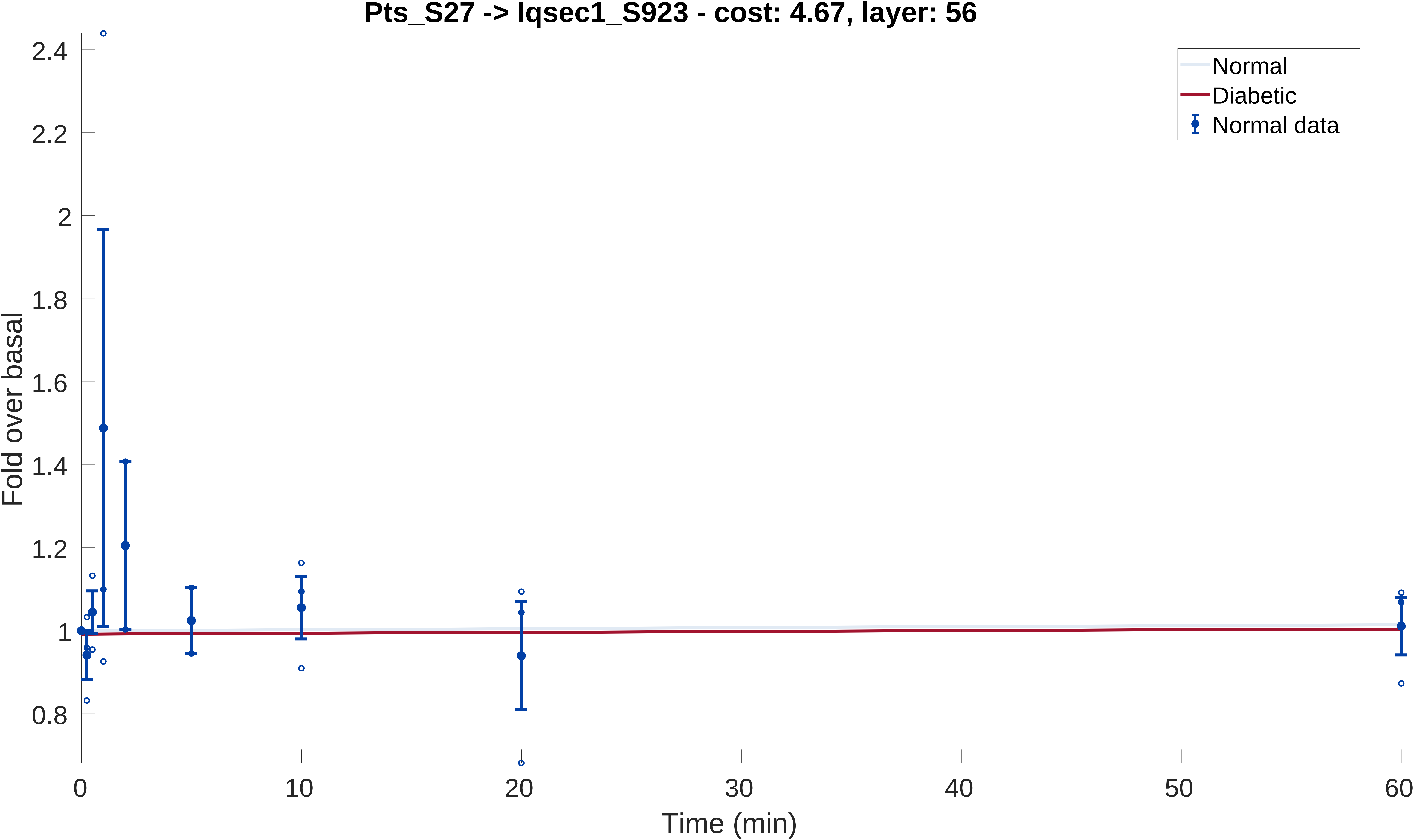
Pts_S27 -> Iqsec1_S923 - cost: 4.67, layer: 56
Normal
Diabetic
Normal data
Fold over basal
Time (min)
2.4
2.2
2
1.8
1.6
1.4
1.2
1
0.8
0
10
20
30
40
50
60

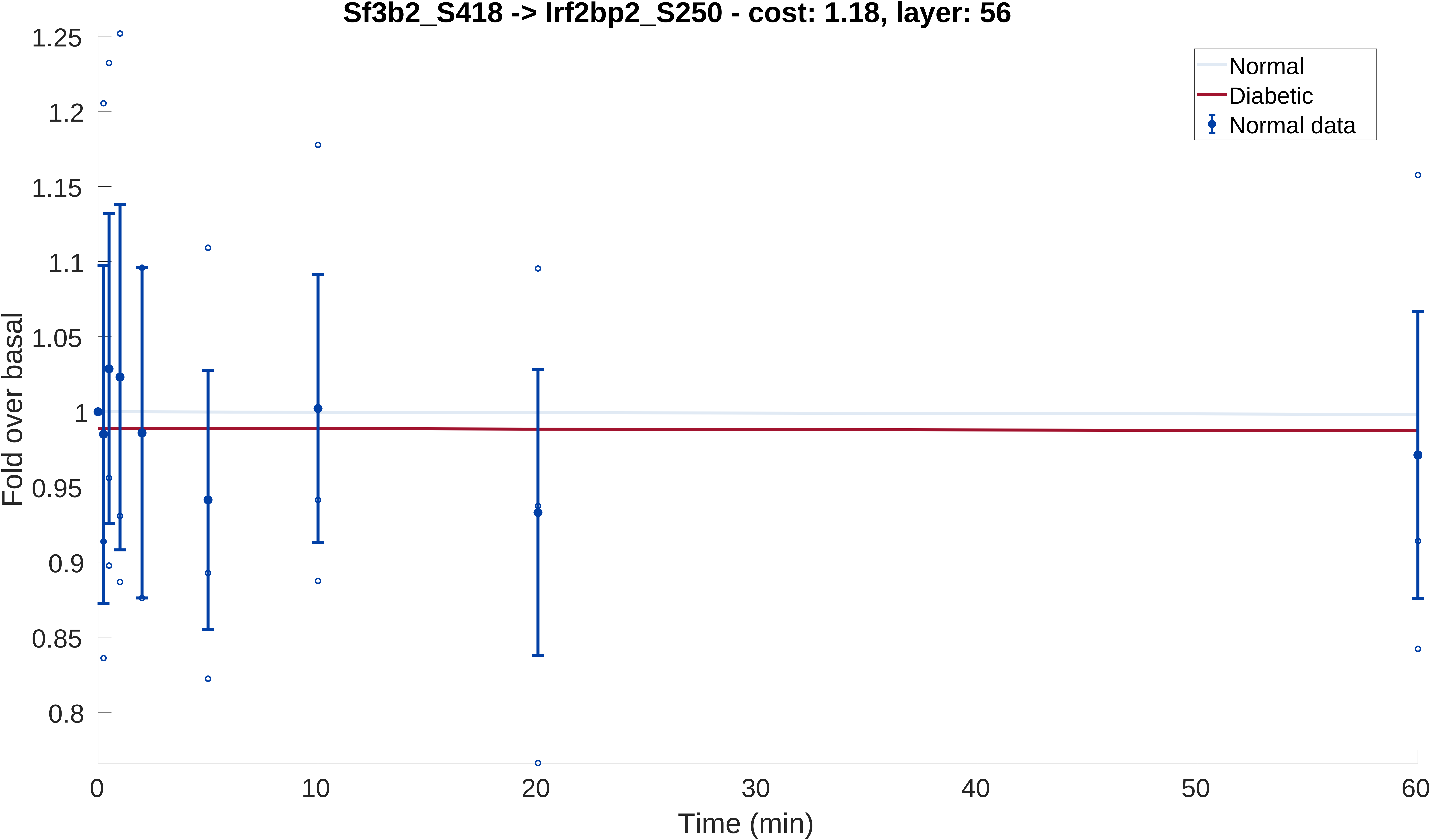

Sf3b2_S418 -> Irf2bp2_S250 - cost: 1.18, layer: 56
Normal
Diabetic
Normal data
Fold over basal
1.25
1.2
1.15
1.1
1.05
1
0.95
0.9
0.85
0.8
0
10
20
30
40
50
60
Time (min)

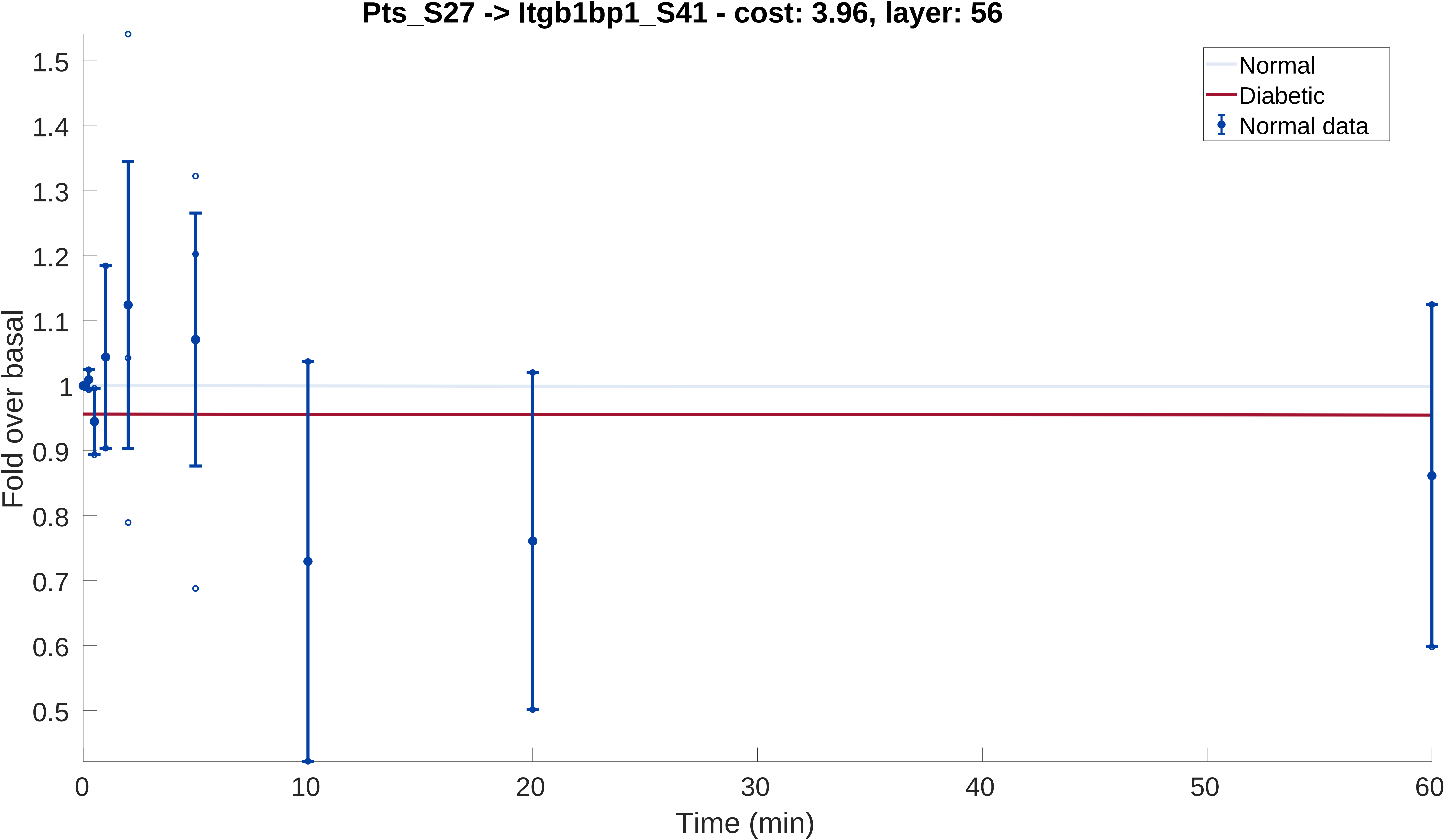

Pts_S27 -> Itgb1bp1_S41 - cost: 3.96, layer: 56
Normal
Diabetic
Normal data
Fold over basal
Time (min)
1.5
1.4
1.3
1.2
1.1
1
0.9
0.8
0.7
0.6
0.5
0
10
20
30
40
50
60

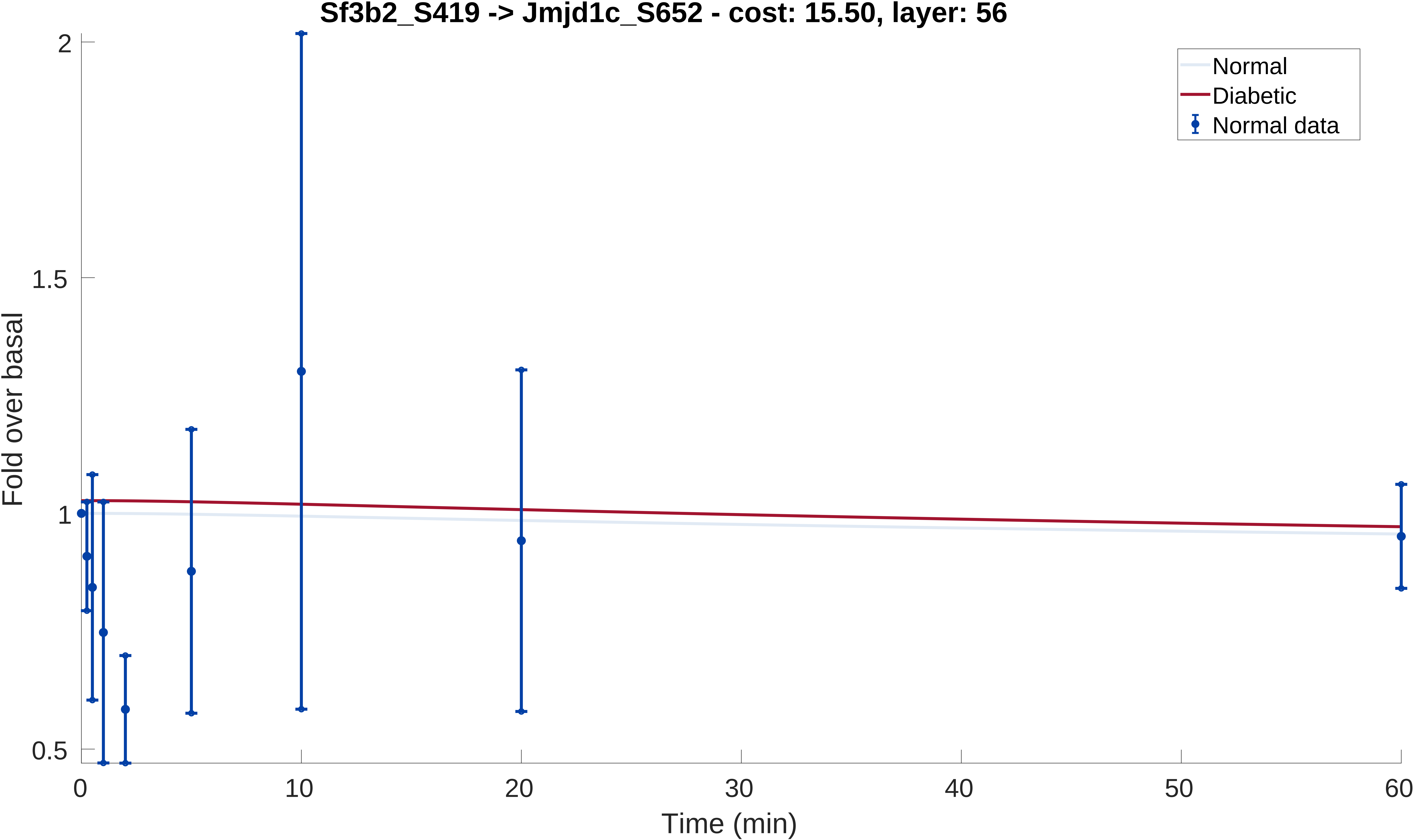
Sf3b2_S419 -> Jmjd1c_S652 - cost: 15.50, layer: 56
Normal
Diabetic
Normal data
Fold over basal
Time (min)
2
1.5
1
0.5
0
10
20
30
40
50
60

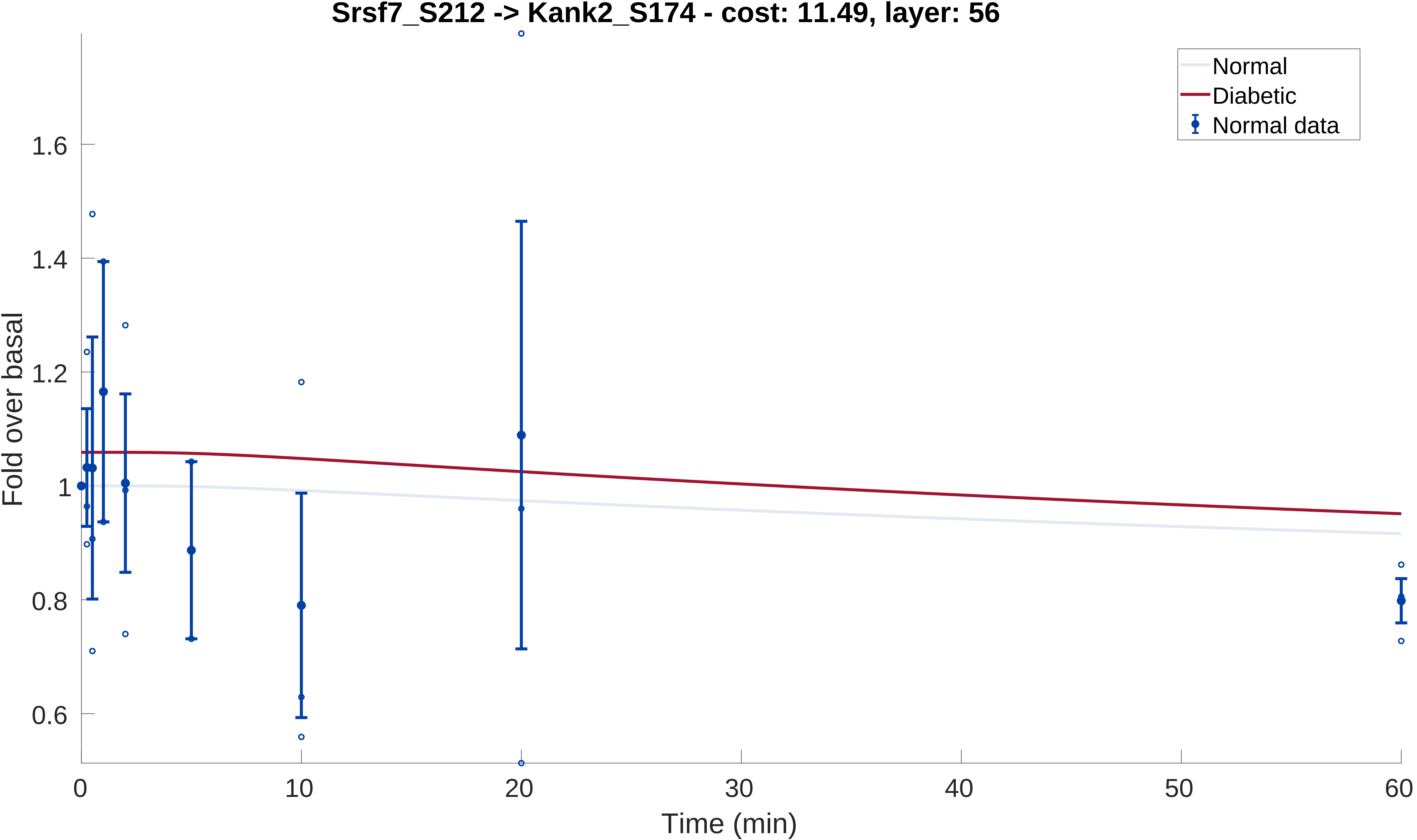

Srsf7_S212 -> Kank2_S174 - cost: 11.49, layer: 56
Normal
Diabetic
Normal data
Fold over basal
Time (min)
1.6
1.4
1.2
1
0.8
0.6
0
10
20
30
40
50
60

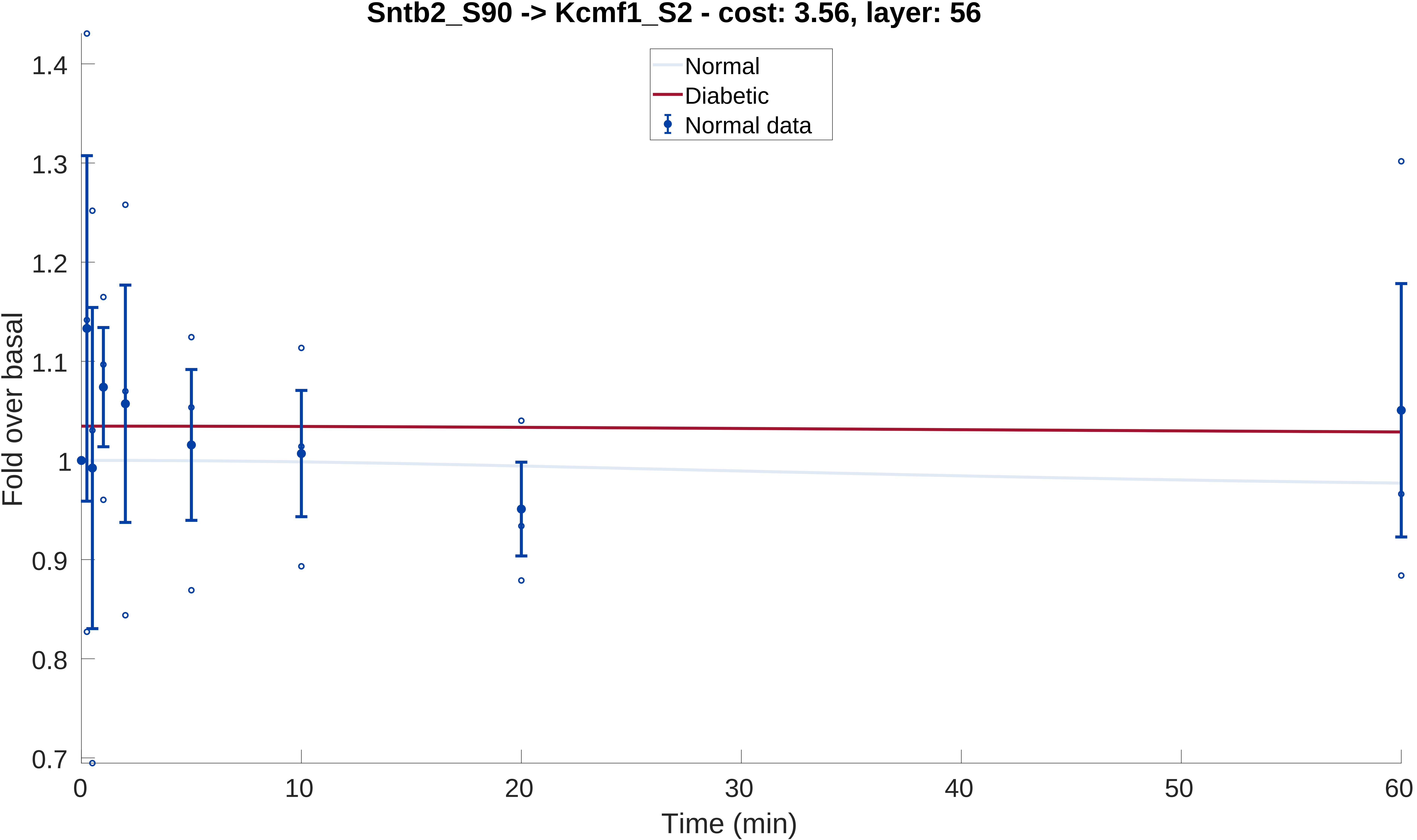
Sntb2_S90 -> Kcmf1_S2 - cost: 3.56, layer: 56
Normal
Diabetic
Normal data
Fold over basal
Time (min)
1.4
1.3
1.2
1.1
1
0.9
0.8
0.7
0
10
20
30
40
50
60

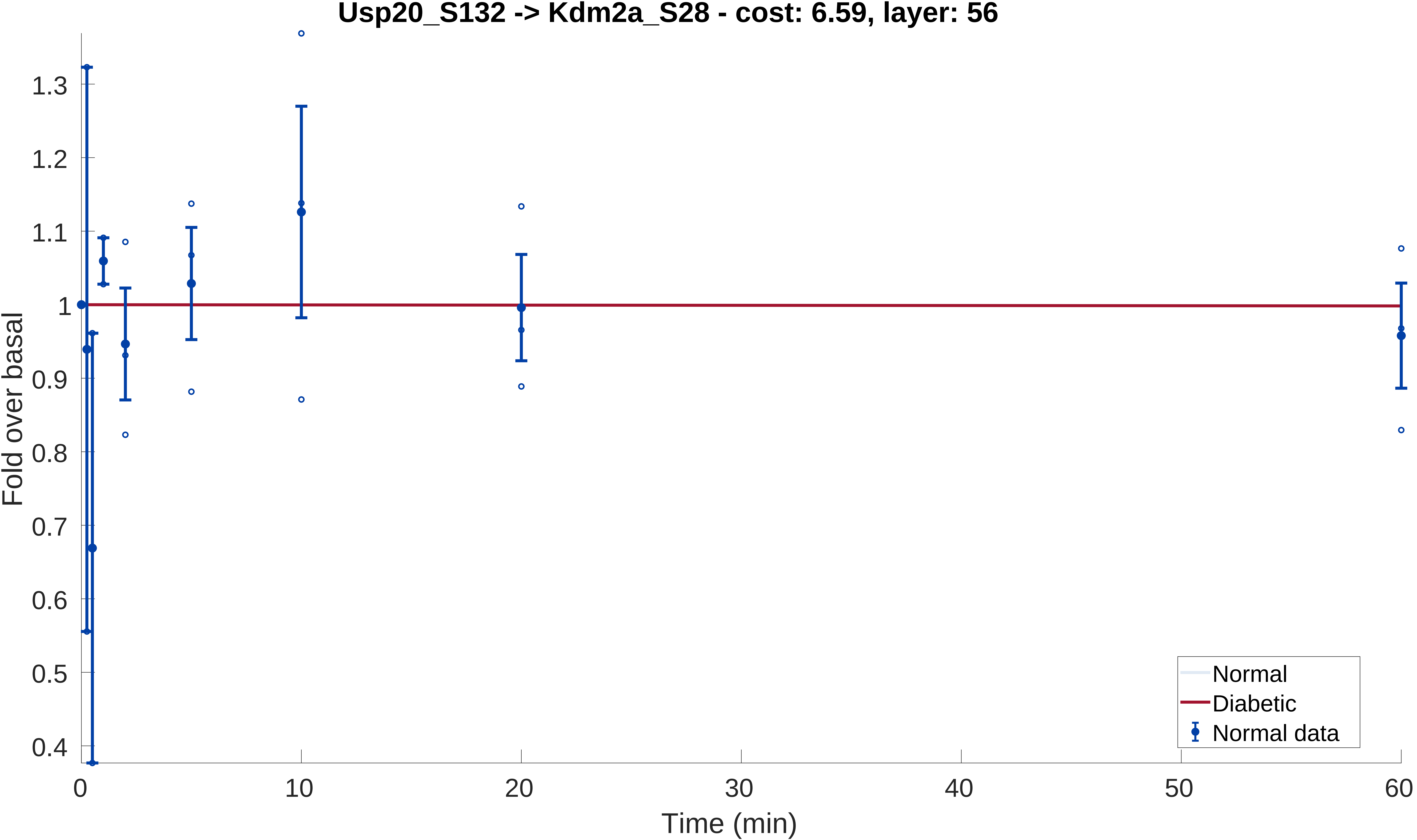

Usp20_S132 -> Kdm2a_S28 - cost: 6.59, layer: 56
Fold over basal
1.3
1.2
1.1
1
0.9
0.8
0.7
0.6
0.5
0.4
0
10
20
30
40
50
60
Time (min)
Normal
Diabetic
Normal data

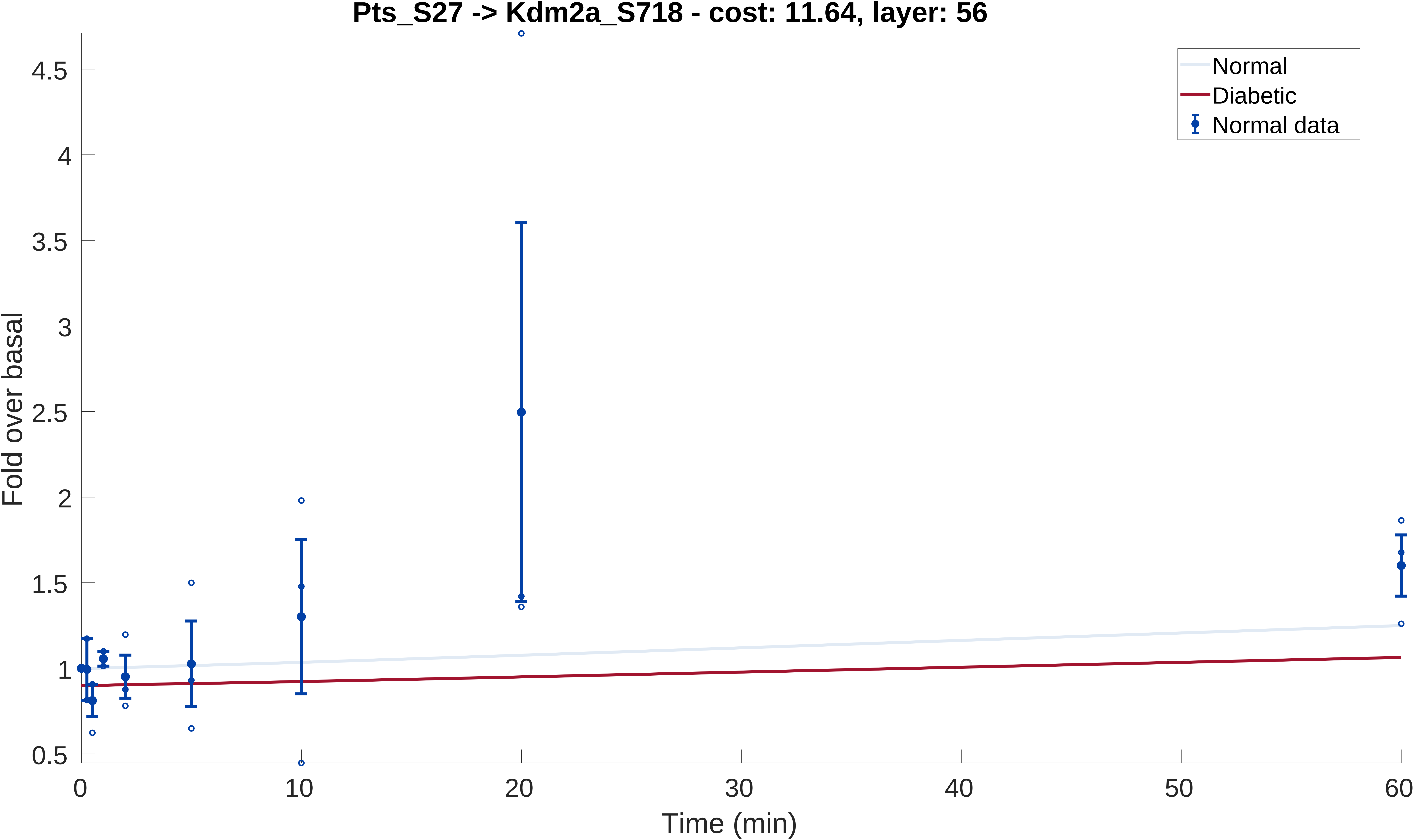

Pts_S27 -> Kdm2a_S718 - cost: 11.64, layer: 56
Normal
Diabetic
Normal data
Fold over basal
Time (min)
0.5
1
1.5
2
2.5
3
3.5
4
4.5
0
10
20
30
40
50
60

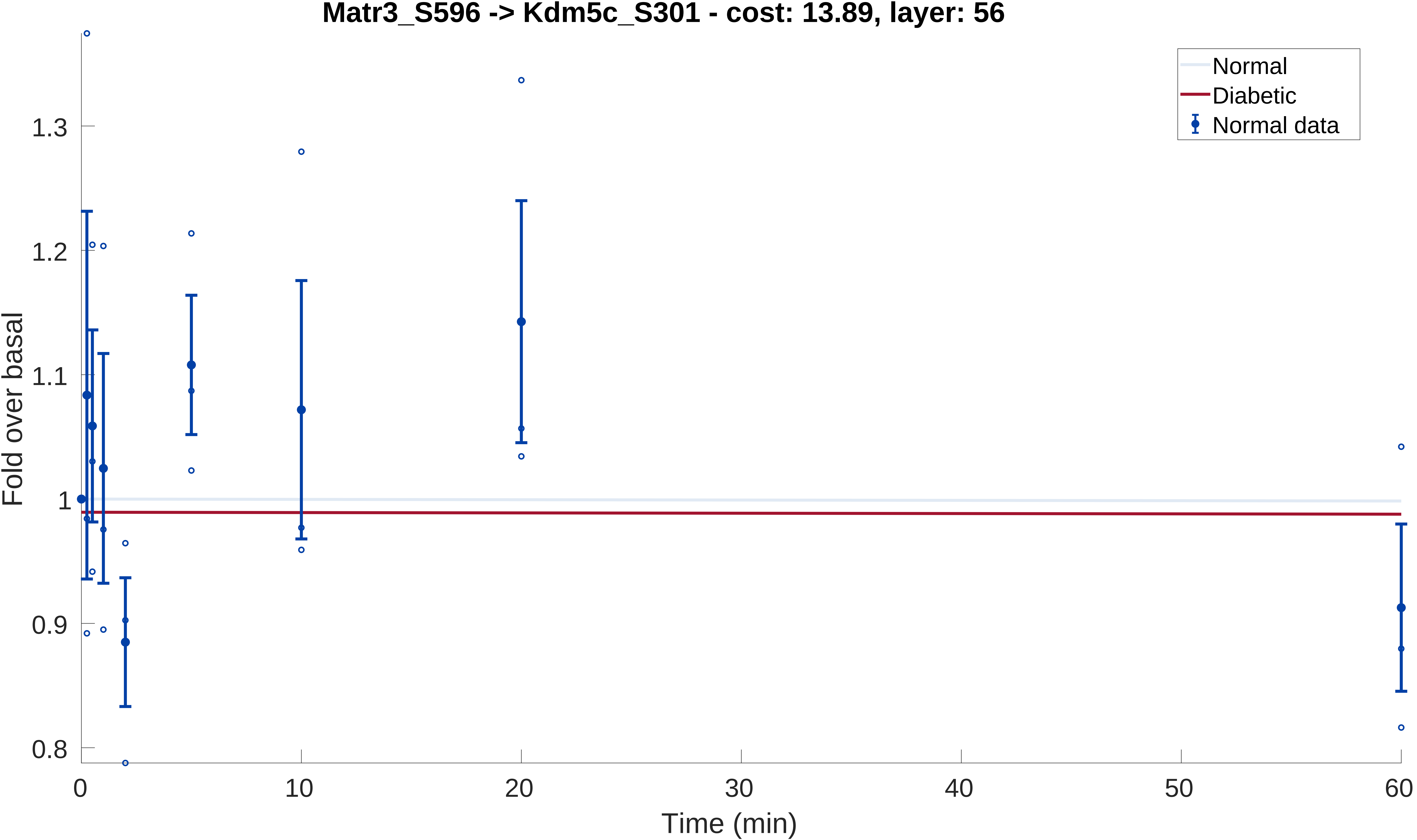

Matr3_S596 -> Kdm5c_S301 - cost: 13.89, layer: 56
Normal
Diabetic
Normal data
Fold over basal
1.3
1.2
1.1
1
0.9
0.8
0
10
20
30
40
50
60
Time (min)

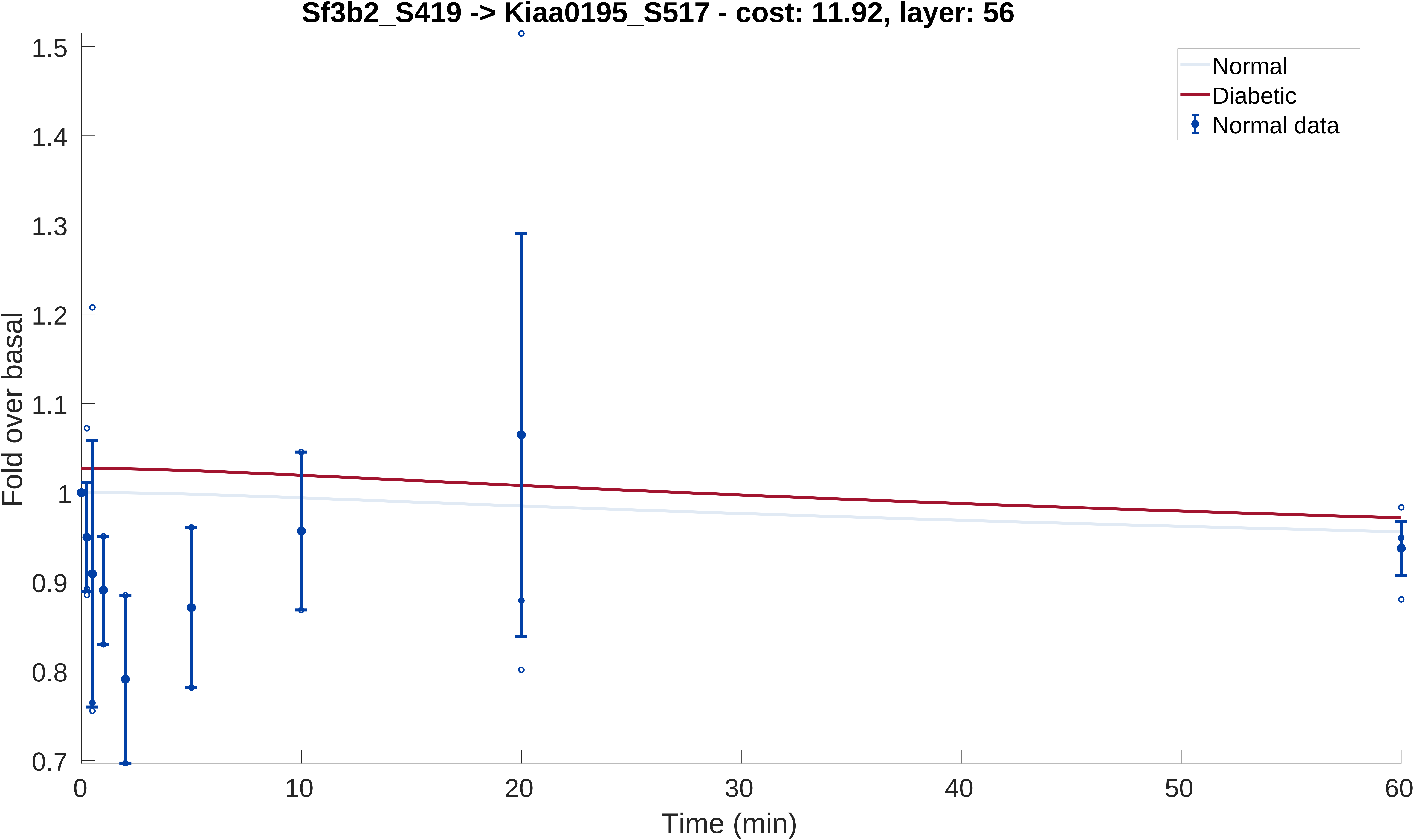

Sf3b2_S419 -> Kiaa0195_S517 - cost: 11.92, layer: 56
Normal
Diabetic
Normal data
Fold over basal
Time (min)
1.5
1.4
1.3
1.2
1.1
1
0.9
0.8
0.7
0
10
20
30
40
50
60

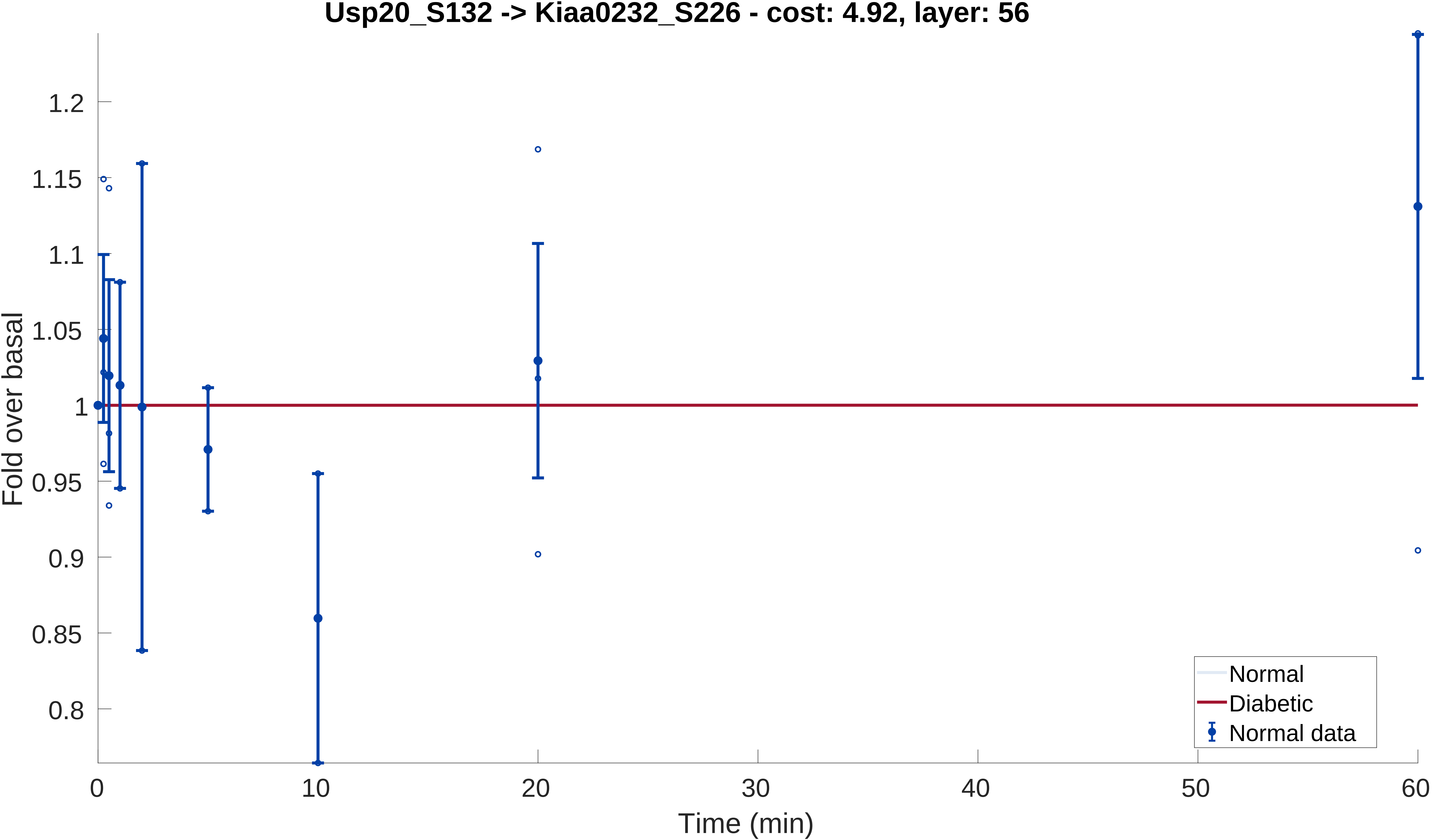
Usp20_S132 -> Kiaa0232_S226 - cost: 4.92, layer: 56
Fold over basal
1.2
1.15
1.1
1.05
1
0.95
0.9
0.85
0.8
0
10
20
30
40
50
60
Time (min)
Normal
Diabetic
Normal data

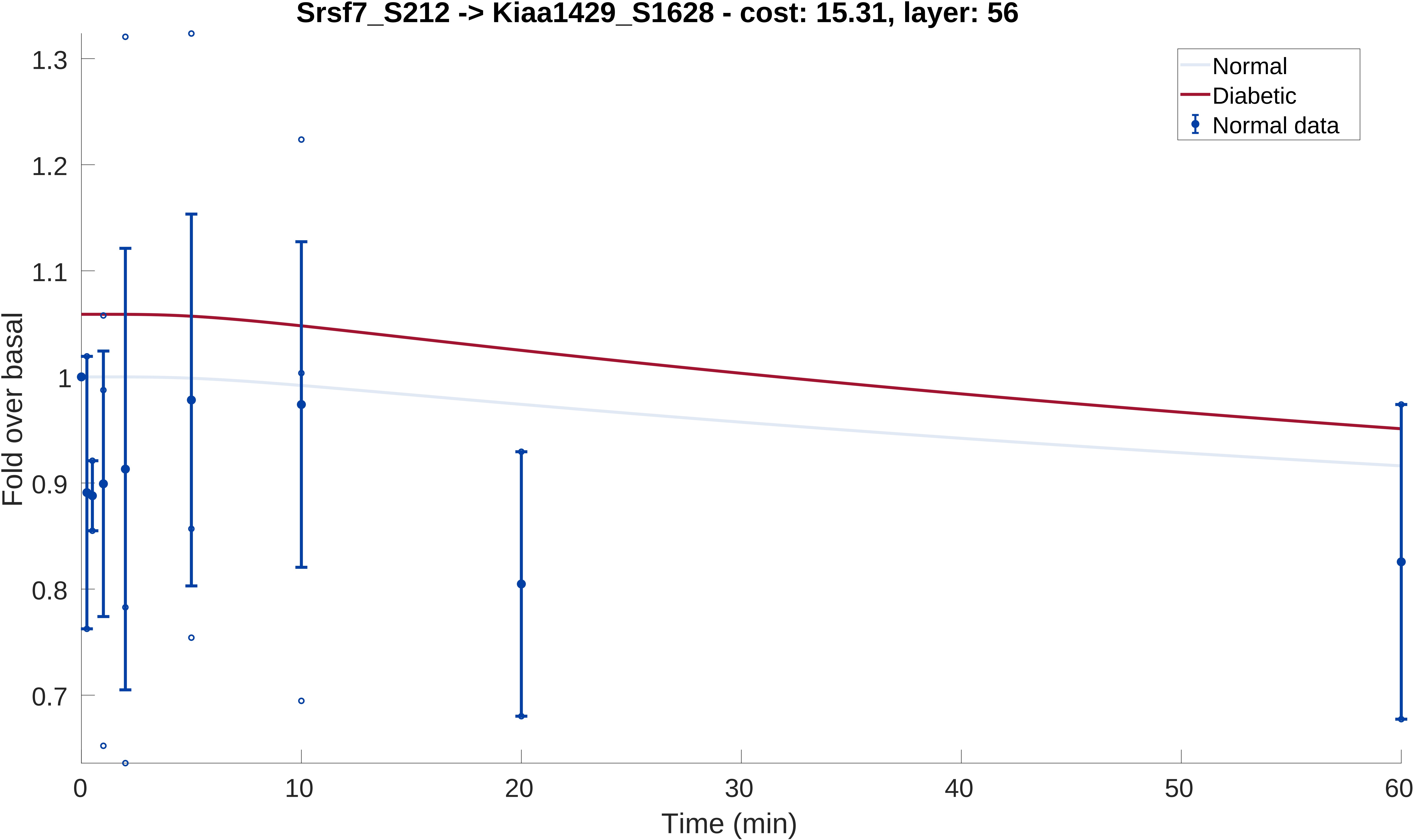

Srsf7_S212 -> Kiaa1429_S1628 - cost: 15.31, layer: 56
Normal
Diabetic
Normal data
Fold over basal
1.3
1.2
1.1
1
0.9
0.8
0.7
0
10
20
30
40
50
60
Time (min)

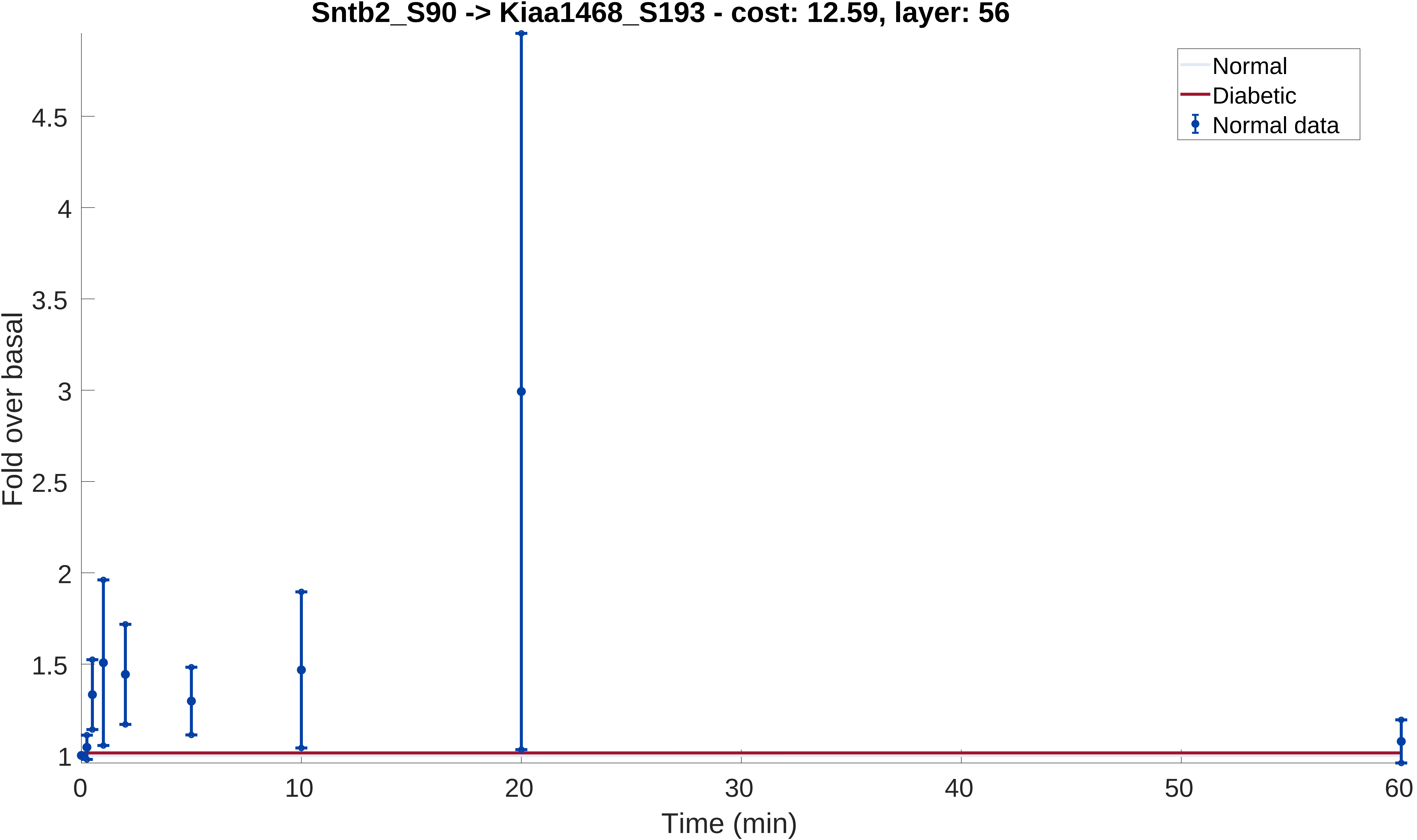

Sntb2_S90 -> Kiaa1468_S193 - cost: 12.59, layer: 56
Normal
Diabetic
Normal data
Fold over basal
Time (min)
4.5
4
3.5
3
2.5
2
1.5
1
0
10
20
30
40
50
60

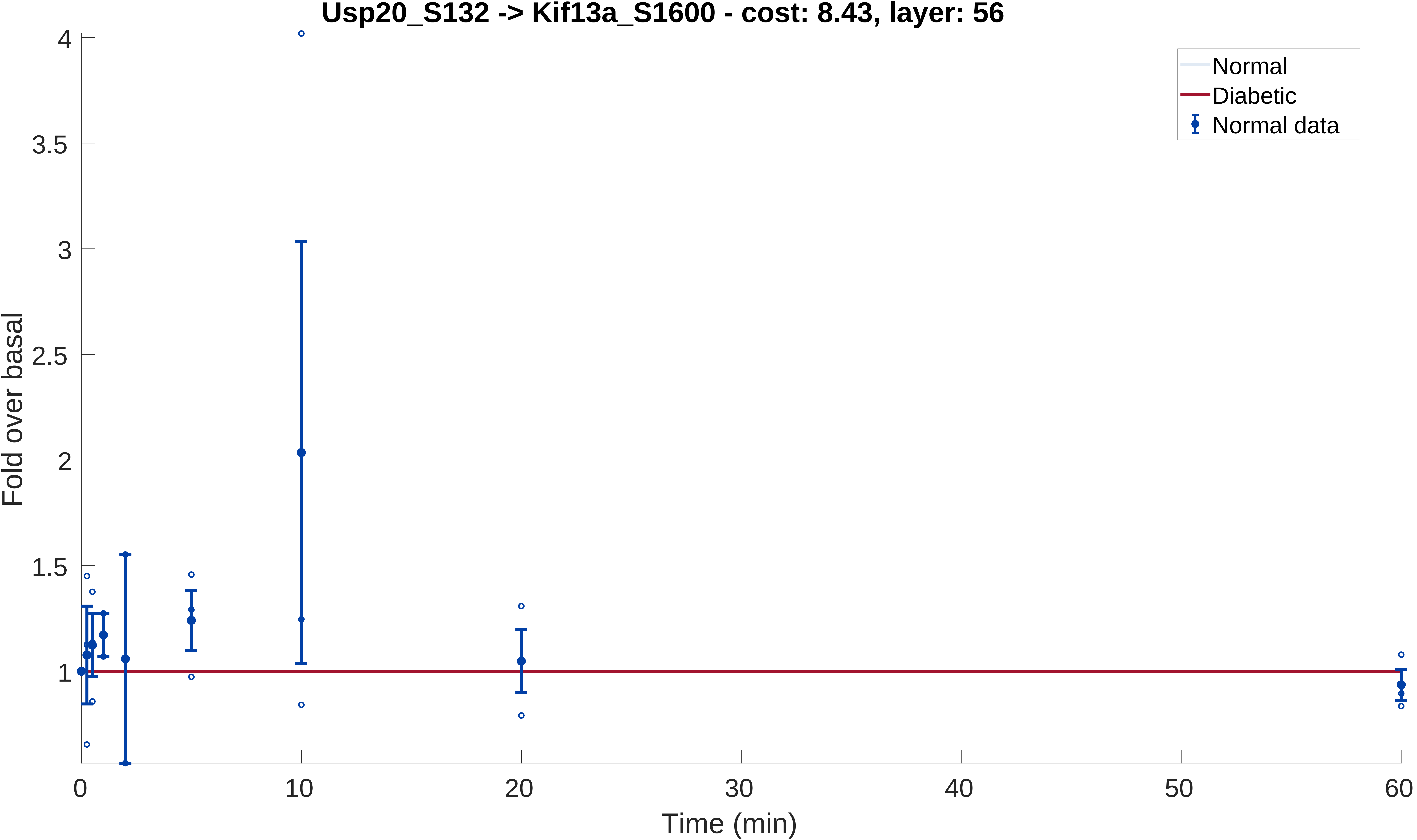
Usp20_S132 -> Kif13a_S1600 - cost: 8.43, layer: 56
Fold over basal
Time (min)
Normal
Diabetic
Normal data
0
10
20
30
40
50
60
1
1.5
2
2.5
3
3.5
4

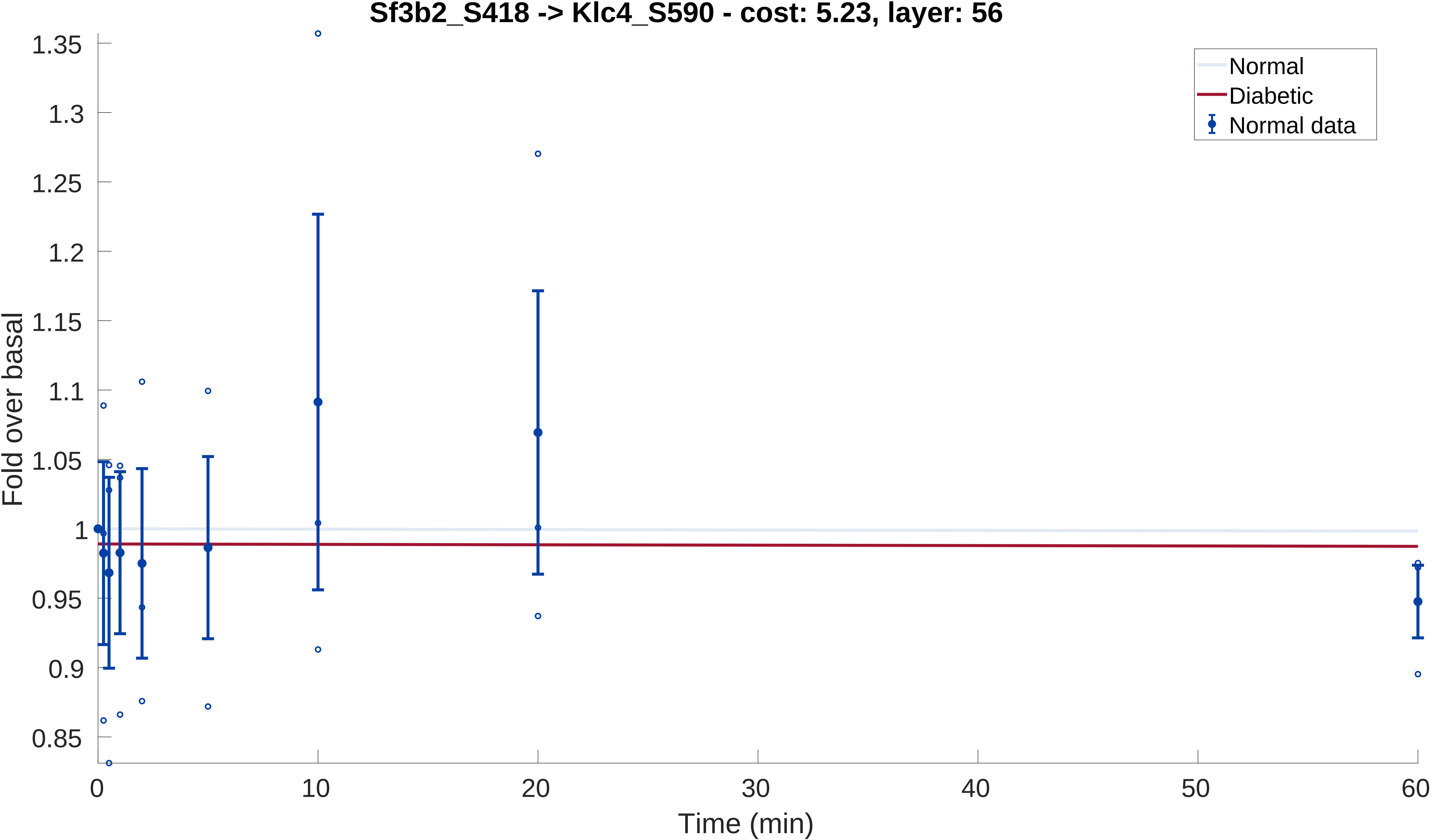

Sf3b2_S418 -> Klc4_S590 - cost: 5.23, layer: 56
Normal
Diabetic
Normal data
Fold over basal
Time (min)
1.35
1.3
1.25
1.2
1.15
1.1
1.05
1
0.95
0.9
0.85
0
10
20
30
40
50
60

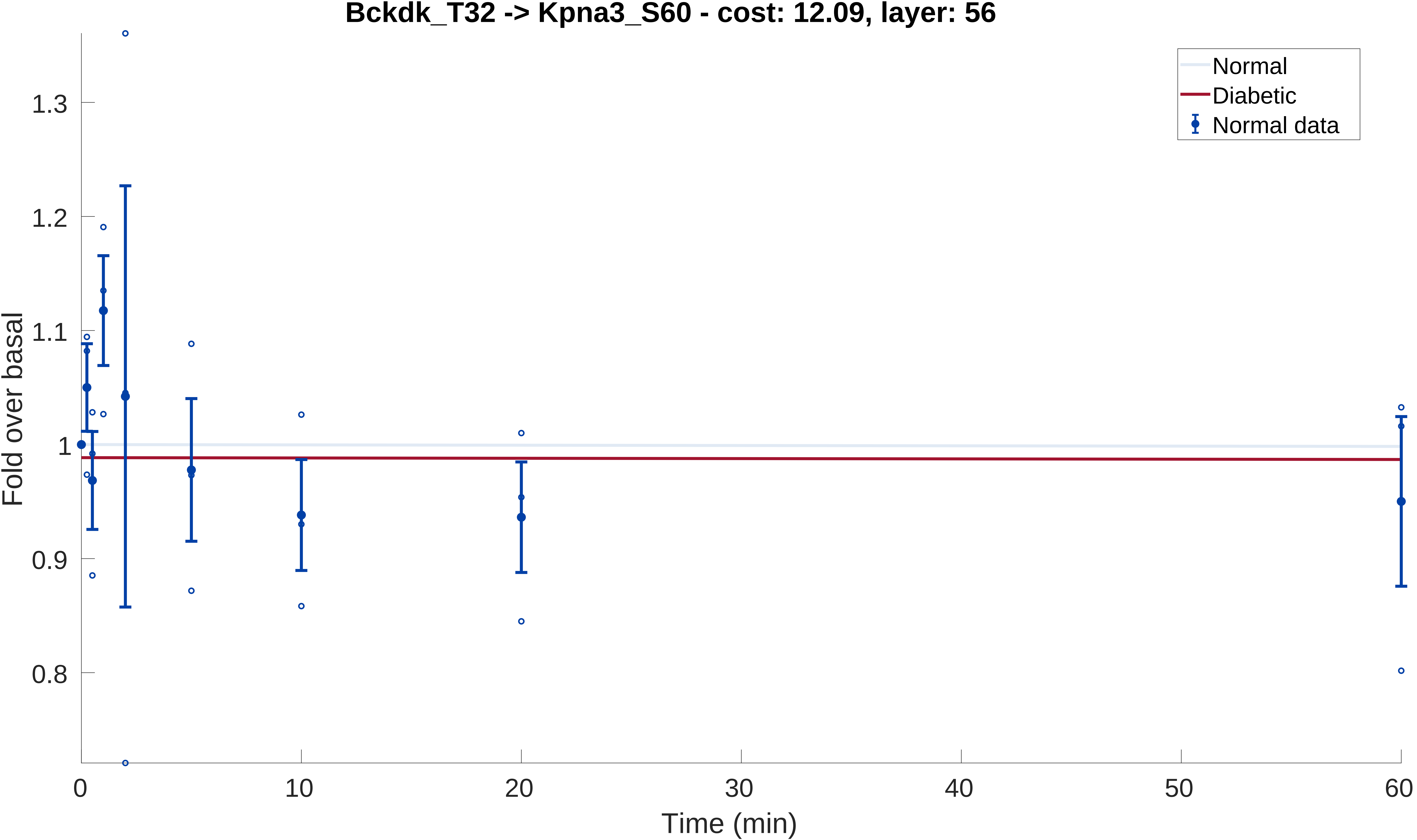

Bckdk_T32 -> Kpna3_S60 - cost: 12.09, layer: 56
Normal
Diabetic
Normal data
Fold over basal
1.3
1.2
1.1
1
0.9
0.8
0
10
20
30
40
50
60
Time (min)

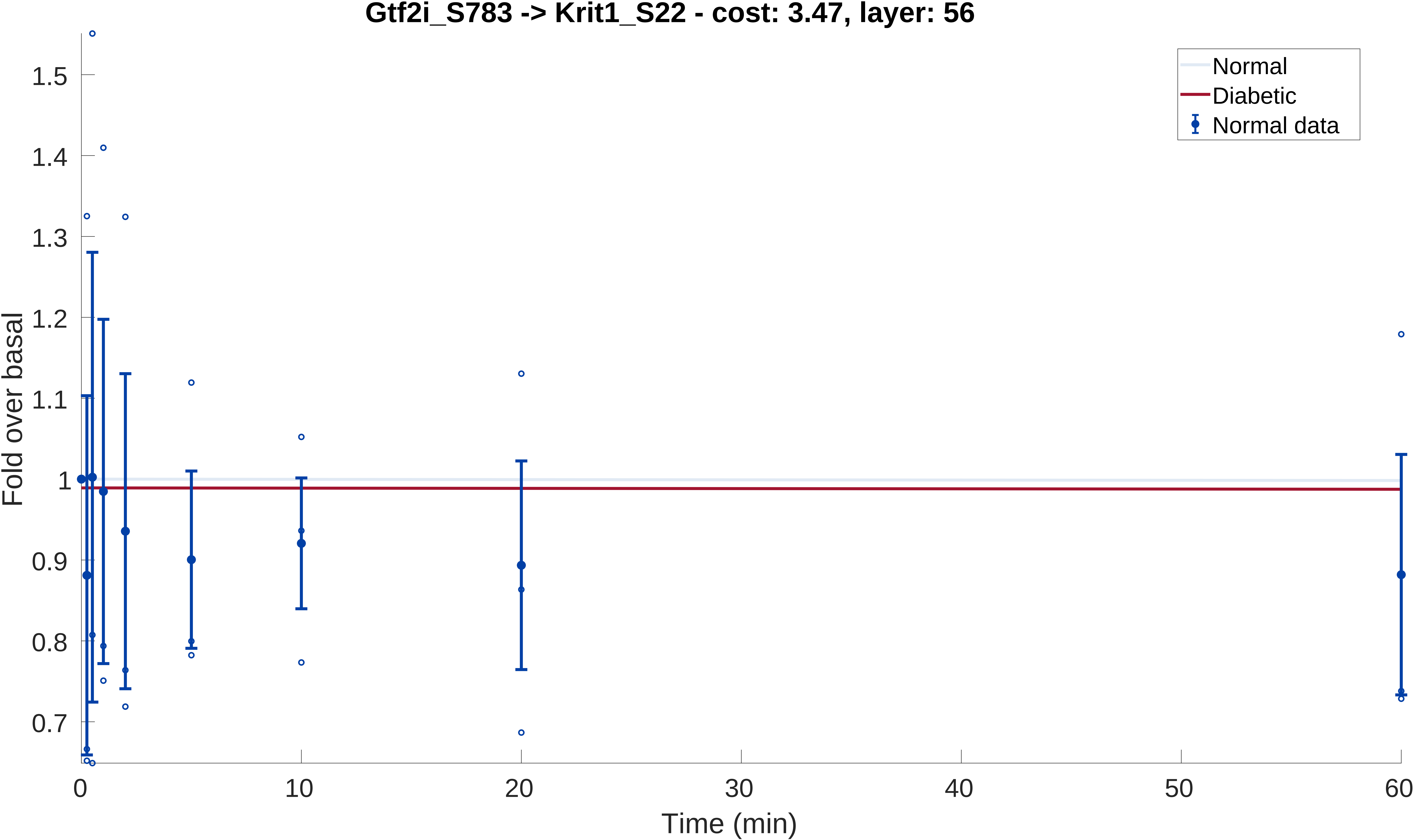

Gtf2i_S783 -> Krit1_S22 - cost: 3.47, layer: 56
Normal
Diabetic
Normal data
Fold over basal
Time (min)
1.5
1.4
1.3
1.2
1.1
1
0.9
0.8
0.7
0
10
20
30
40
50
60

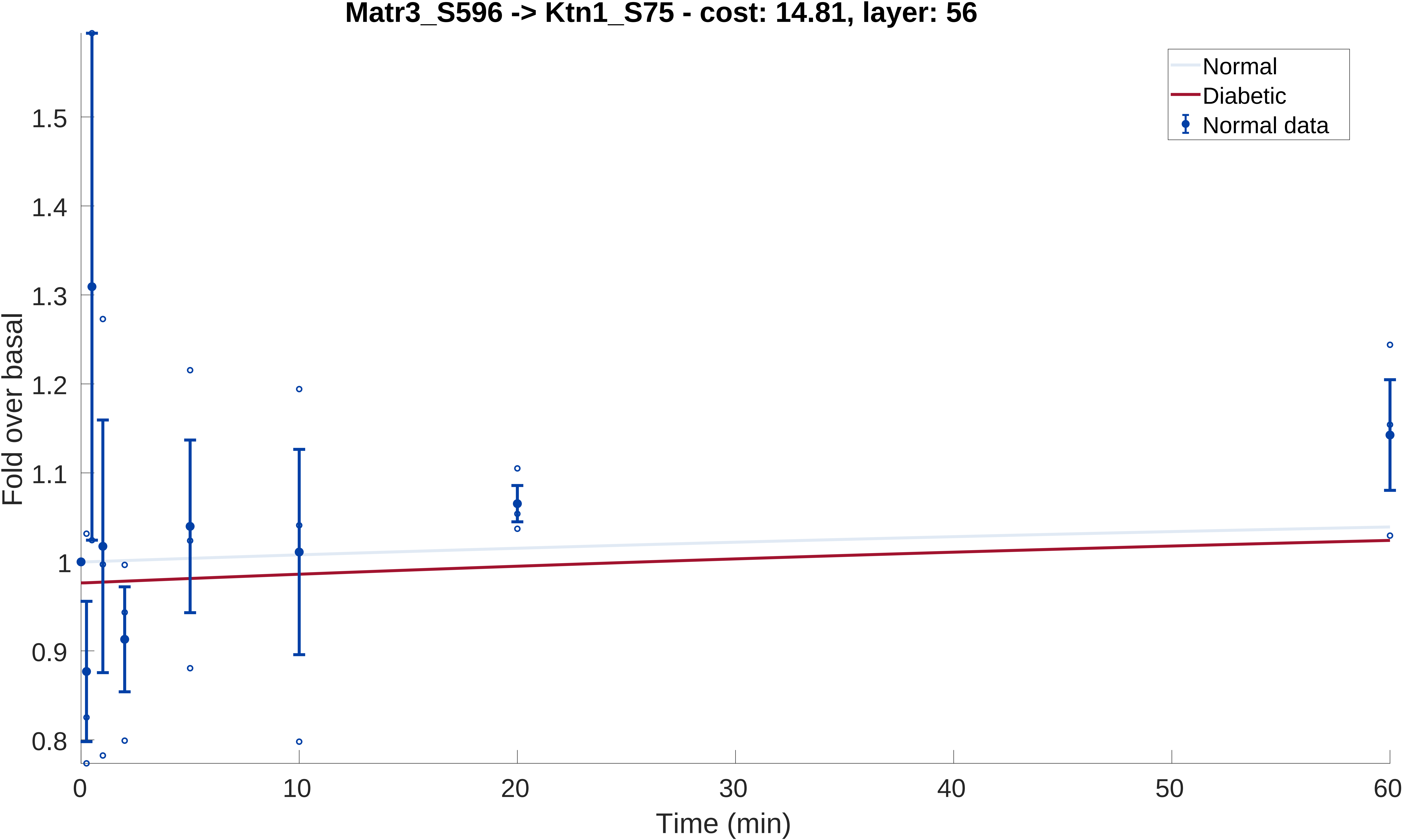
Matr3_S596 -> Ktn1_S75 - cost: 14.81, layer: 56
Normal
Diabetic
Normal data
Fold over basal
0.8
0.9
1
1.1
1.2
1.3
1.4
1.5
0
10
20
30
40
50
60
Time (min)

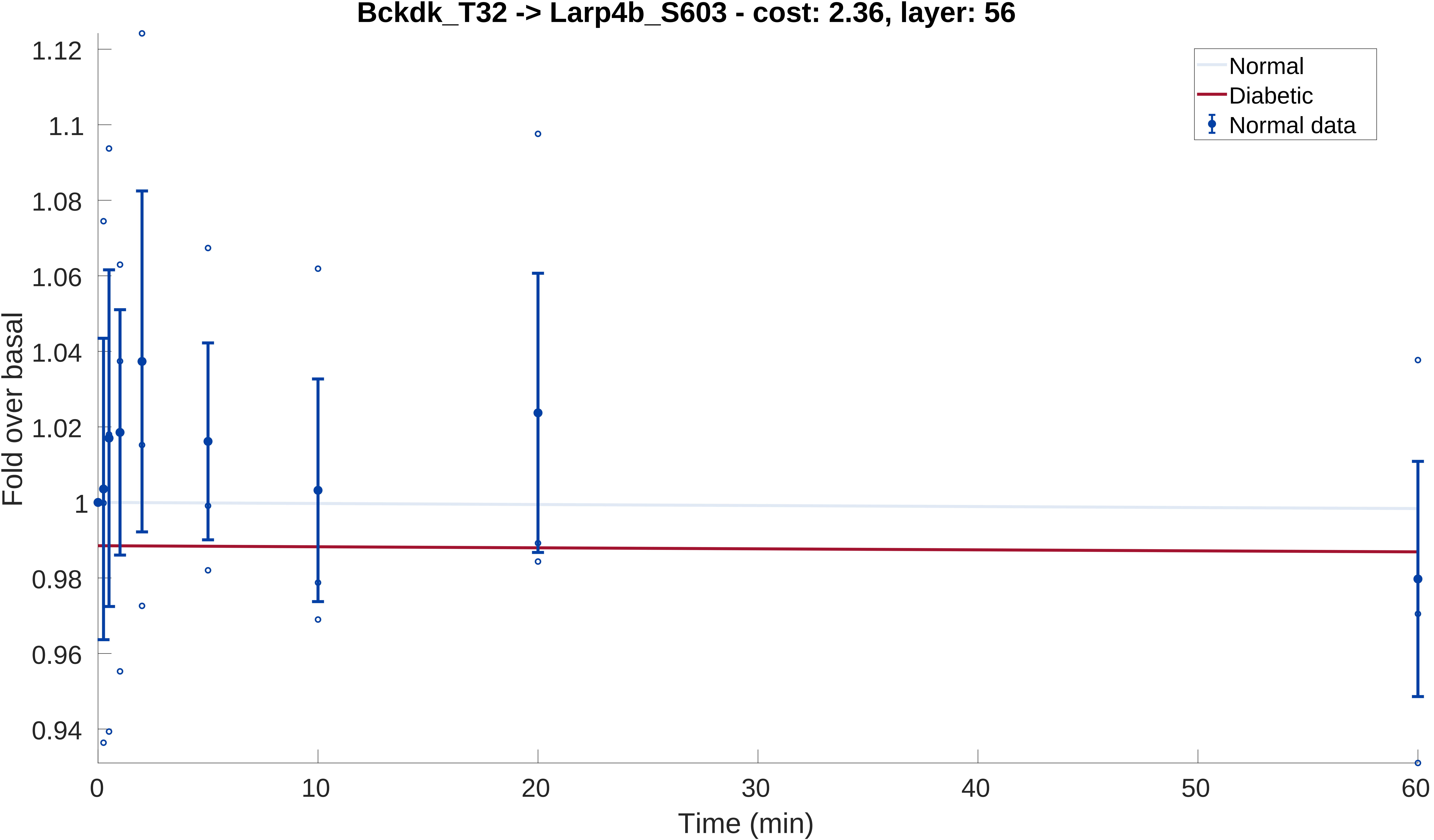

Bckdk_T32 -> Larp4b_S603 - cost: 2.36, layer: 56
Fold over basal
1.12
1.1
1.08
1.06
1.04
1.02
1
0.98
0.96
0.94
0
10
20
30
40
50
60
Time (min)
Normal
Diabetic
Normal data

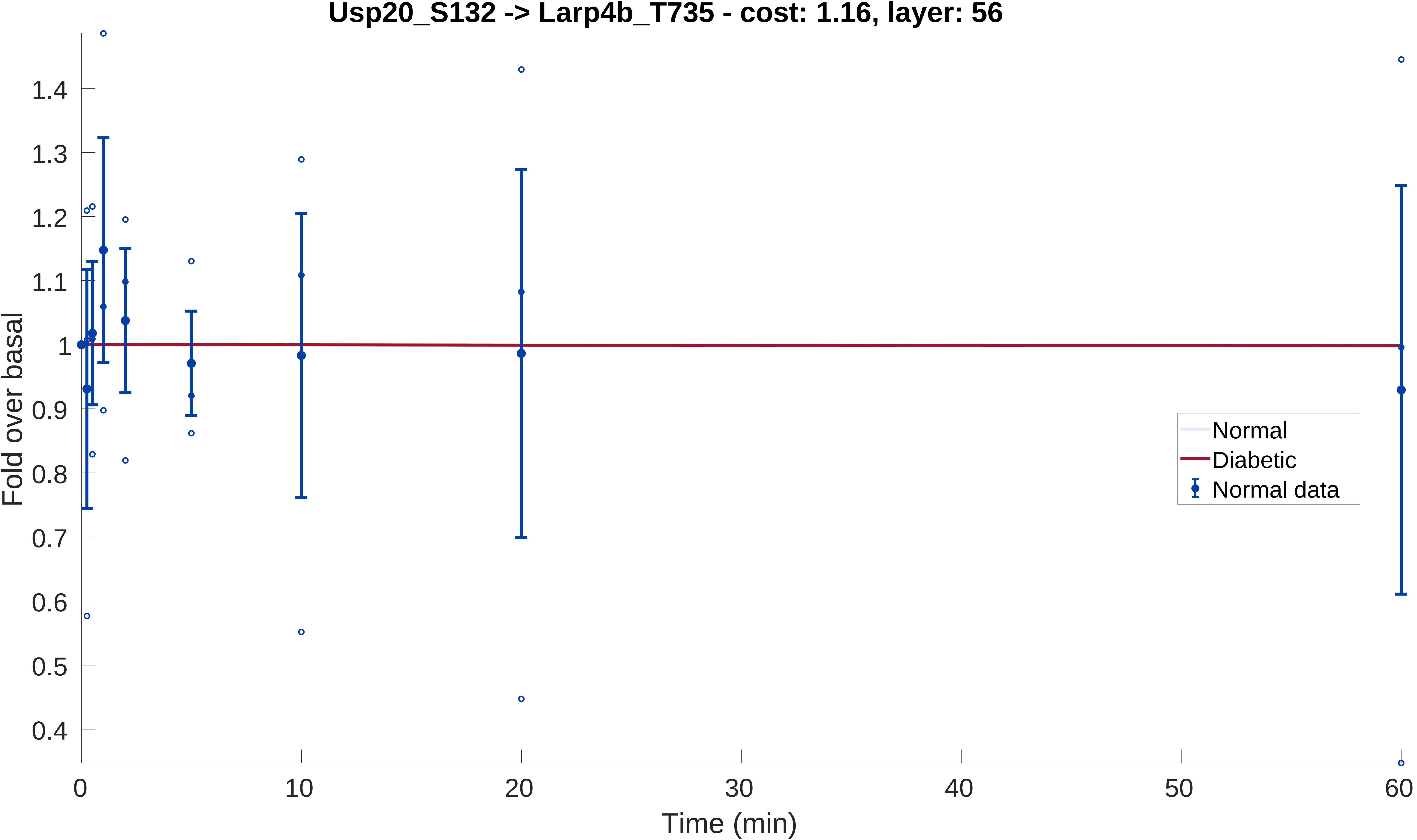
Usp20_S132 -> Larp4b_T735 - cost: 1.16, layer: 56
Fold over basal
Time (min)
1.4
1.3
1.2
1.1
1
0.9
0.8
0.7
0.6
0.5
0.4
0
10
20
30
40
50
60
Normal
Diabetic
Normal data

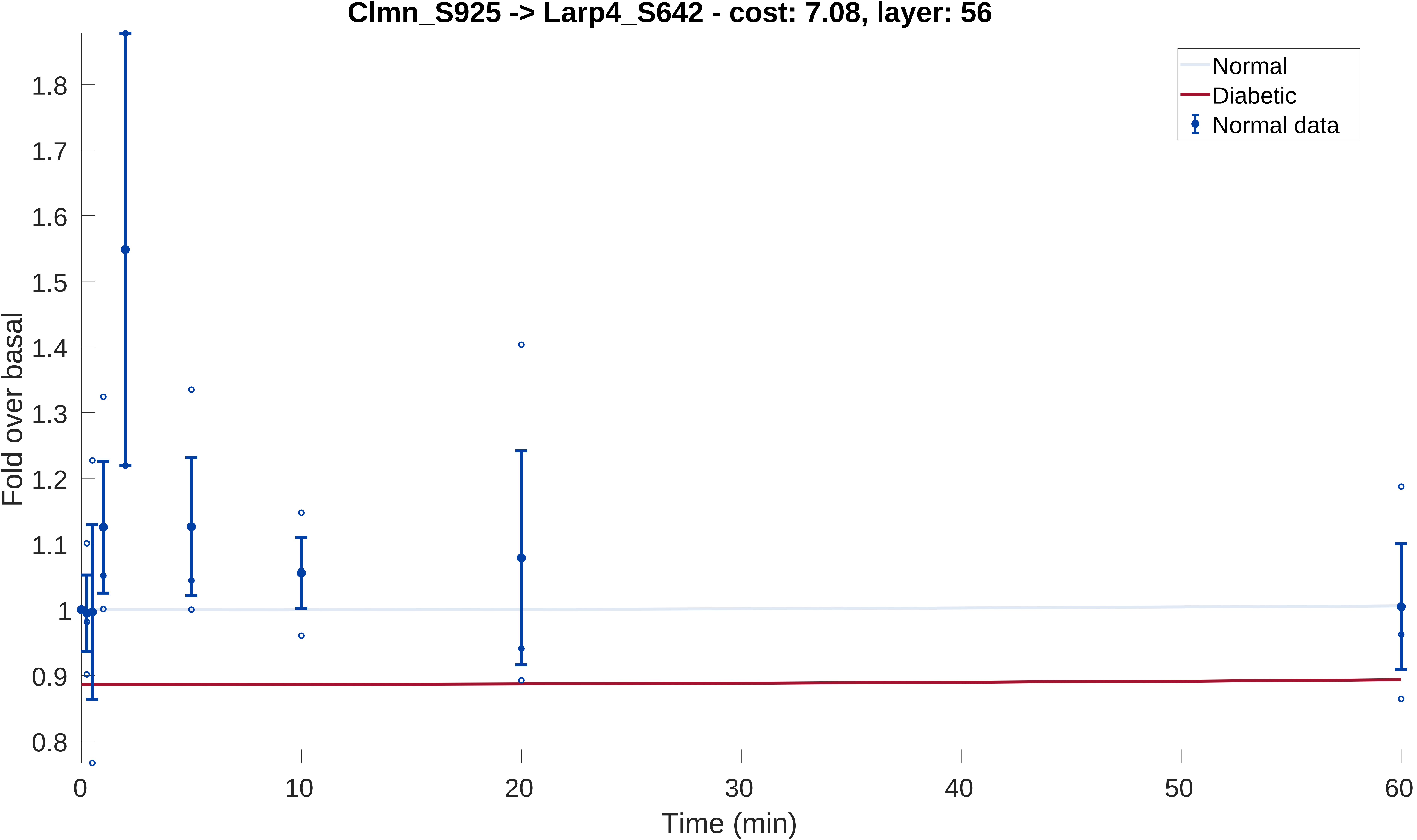

Clmn_S925 -> Larp4_S642 - cost: 7.08, layer: 56
Normal
Diabetic
Normal data
Fold over basal
Time (min)
0.8
0.9
1
1.1
1.2
1.3
1.4
1.5
1.6
1.7
1.8
0
10
20
30
40
50
60

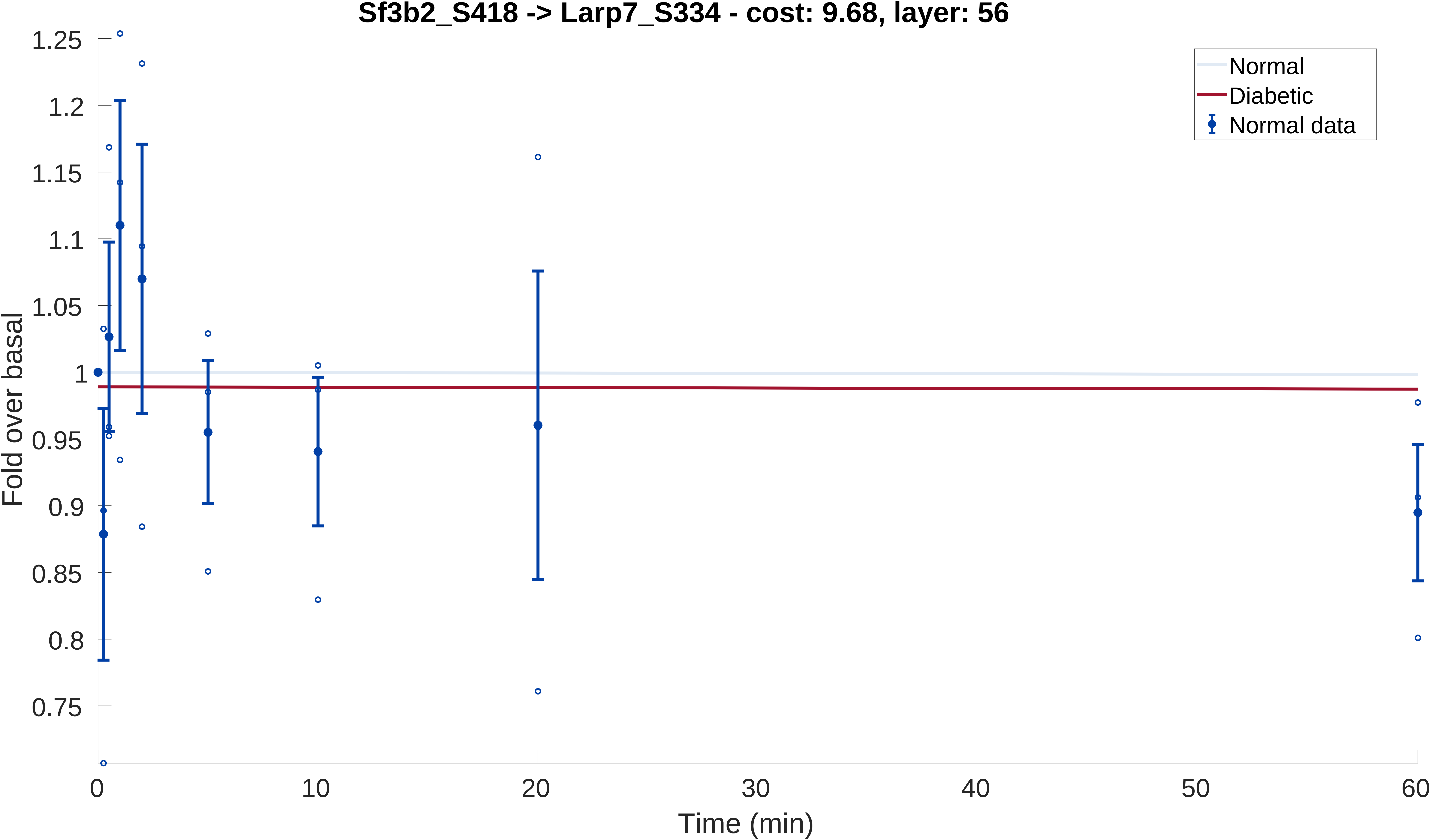
Sf3b2_S418 -> Larp7_S334 - cost: 9.68, layer: 56
Normal
Diabetic
Normal data
Fold over basal
1.25
1.2
1.15
1.1
1.05
1
0.95
0.9
0.85
0.8
0.75
0
10
20
30
40
50
60
Time (min)

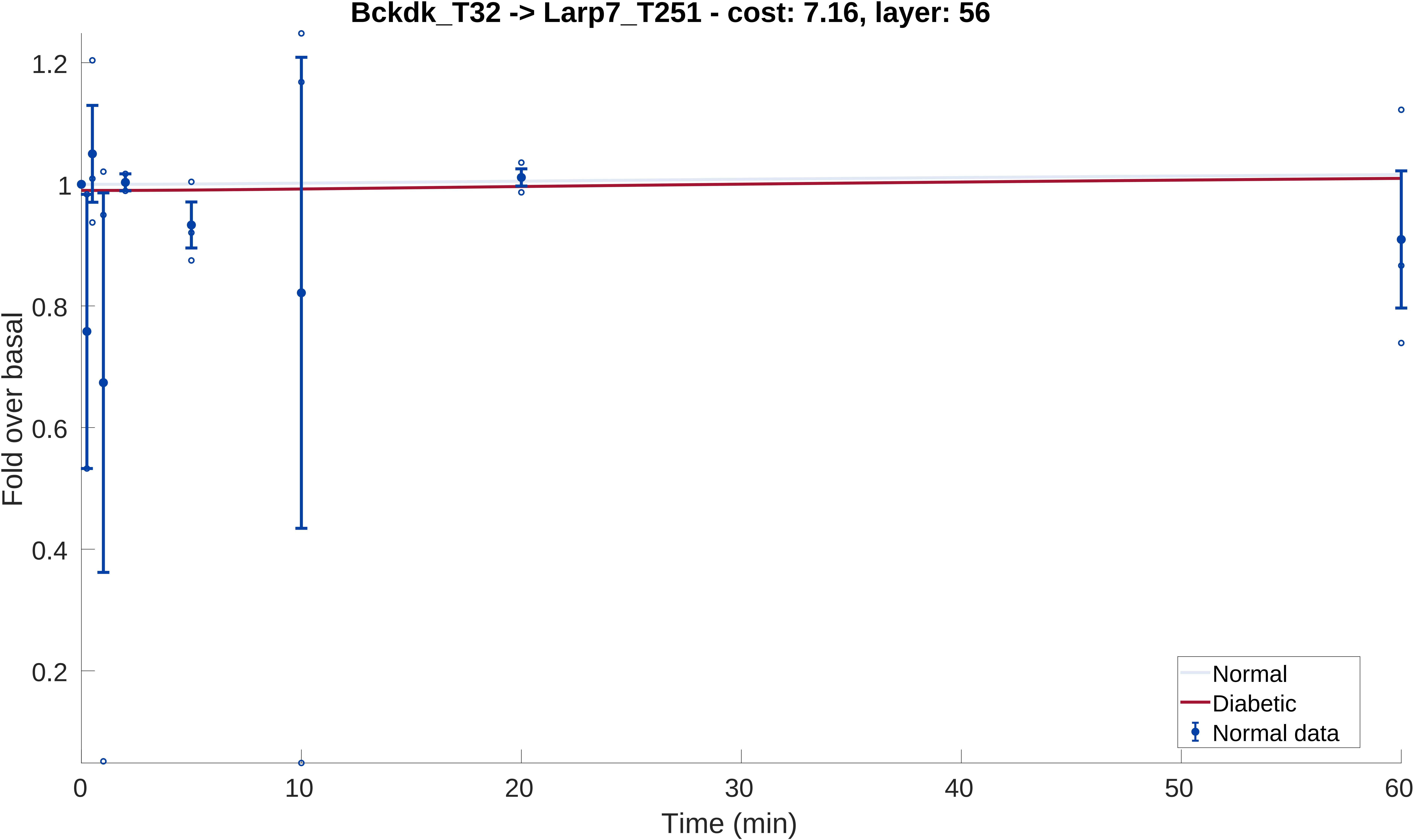
Bckdk_T32 -> Larp7_T251 - cost: 7.16, layer: 56
Fold over basal
1.2
1
0.8
0.6
0.4
0.2
0
10
20
30
40
50
60
Time (min)
Normal
Diabetic
Normal data

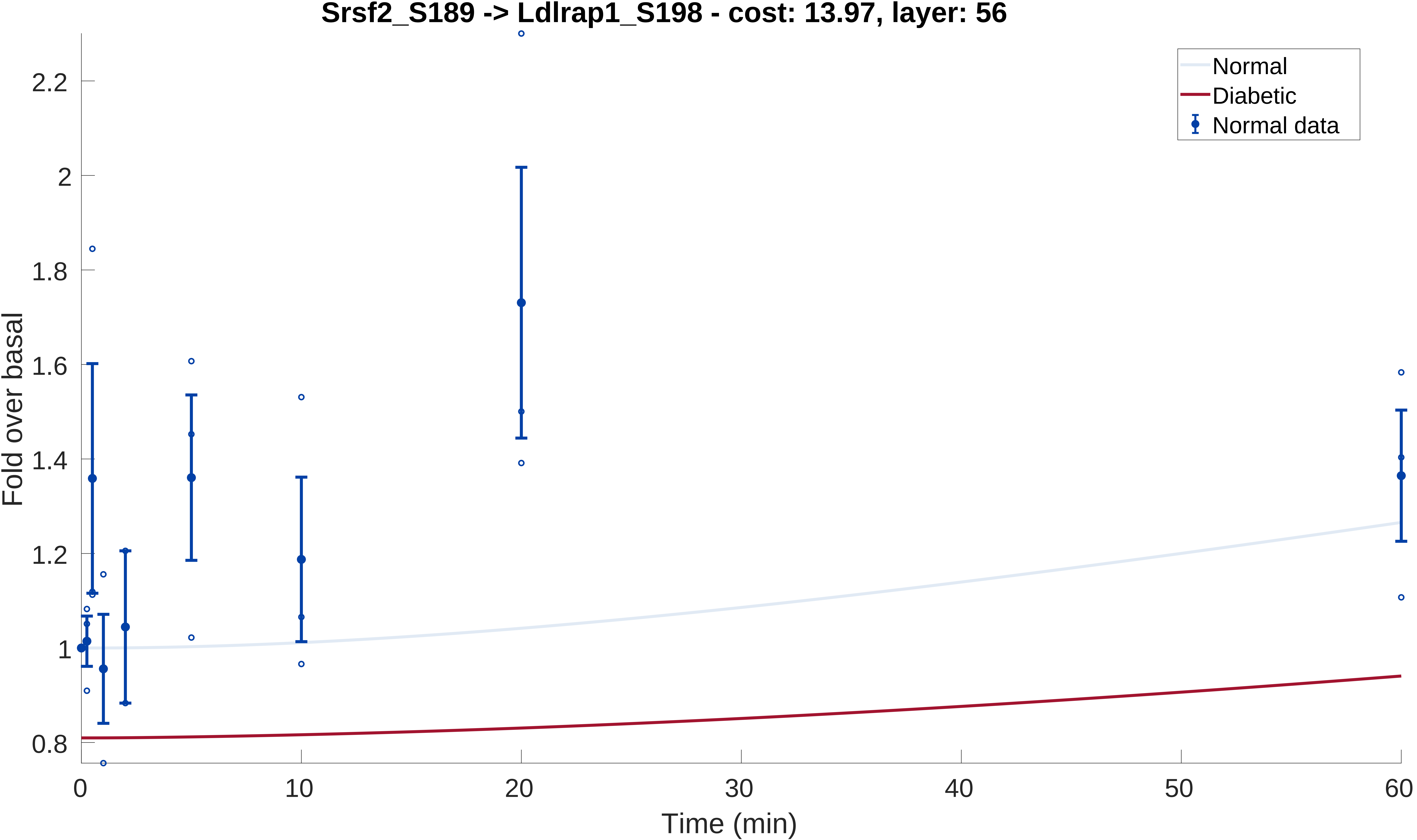

Srsf2_S189 -> Ldlrap1_S198 - cost: 13.97, layer: 56
Normal
Diabetic
Normal data
Fold over basal
Time (min)
0.8
1
1.2
1.4
1.6
1.8
2
2.2
0
10
20
30
40
50
60

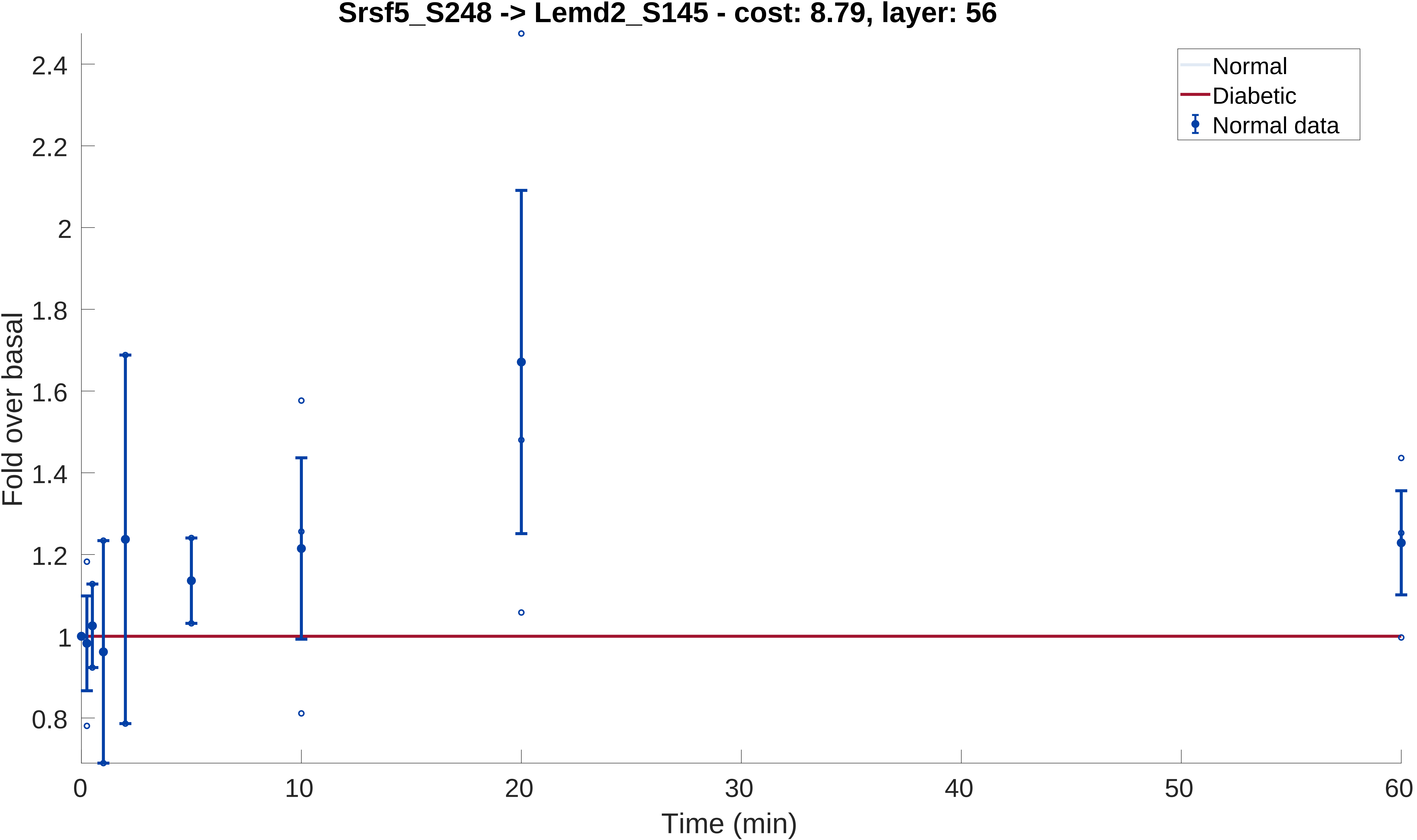

Srsf5_S248 -> Lemd2_S145 - cost: 8.79, layer: 56
Normal
Diabetic
Normal data
Fold over basal
Time (min)
2.4
2.2
2
1.8
1.6
1.4
1.2
1
0.8
0
10
20
30
40
50
60

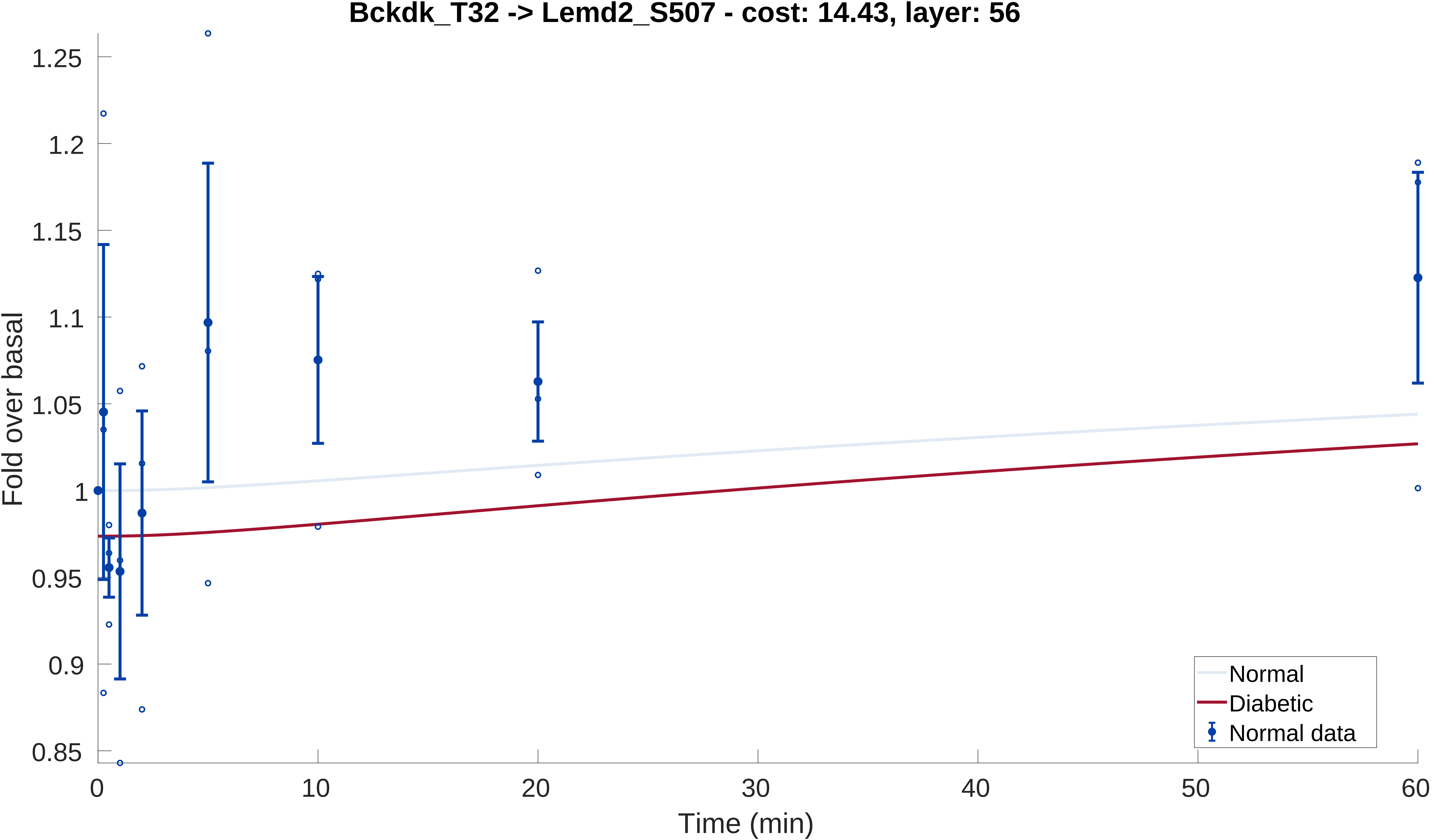

Bckdk_T32 -> Lemd2_S507 - cost: 14.43, layer: 56
Fold over basal
1.25
1.2
1.15
1.1
1.05
1
0.95
0.9
0.85
0
10
20
30
40
50
60
Time (min)
Normal
Diabetic
Normal data

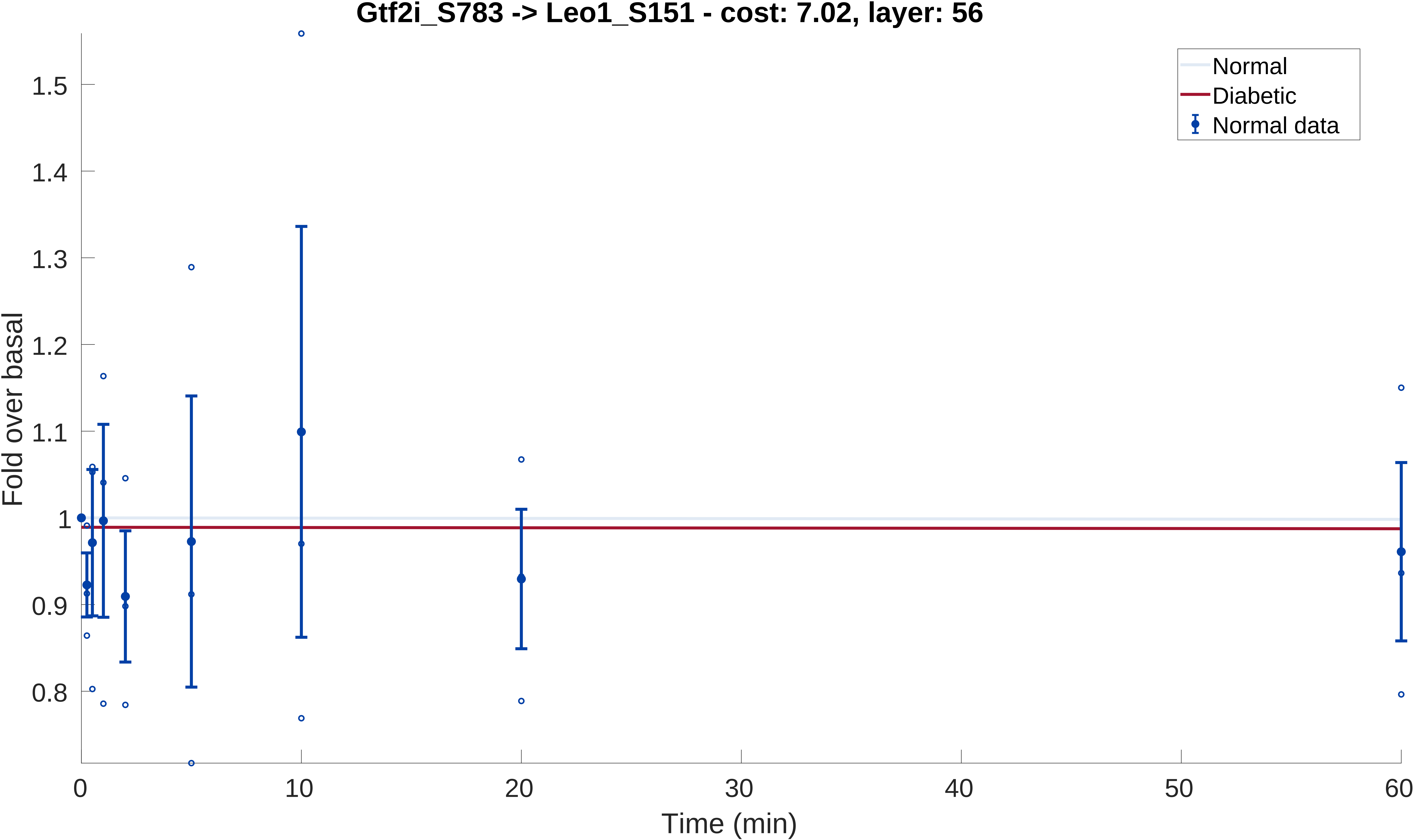
Gtf2i_S783 -> Leo1_S151 - cost: 7.02, layer: 56
Normal
Diabetic
Normal data
Fold over basal
1.5
1.4
1.3
1.2
1.1
1
0.9
0.8
0
10
20
30
40
50
60
Time (min)

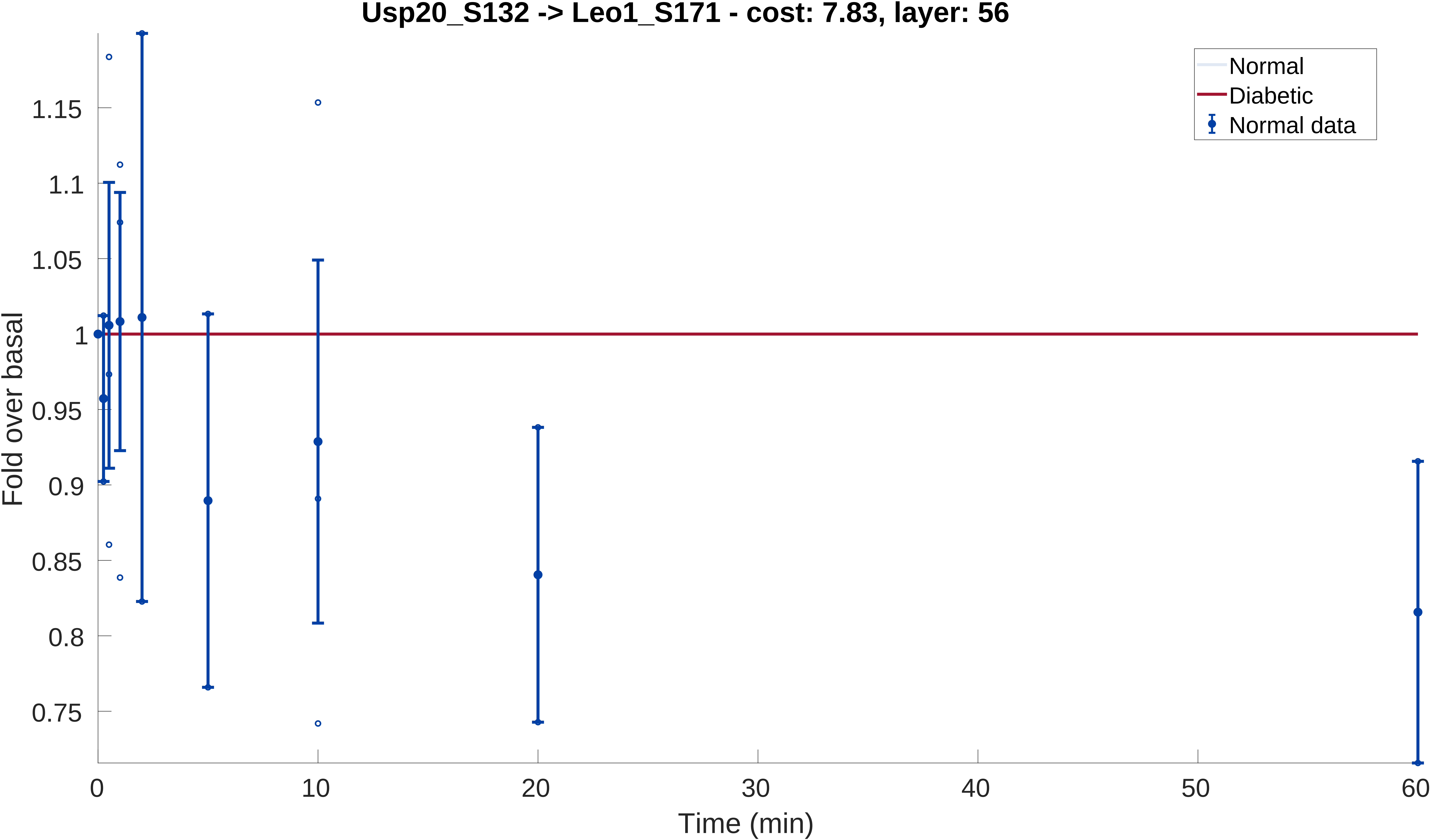

Usp20_S132 -> Leo1_S171 - cost: 7.83, layer: 56
Normal
Diabetic
Normal data
Fold over basal
Time (min)
1.15
1.1
1.05
1
0.95
0.9
0.85
0.8
0.75
0
10
20
30
40
50
60

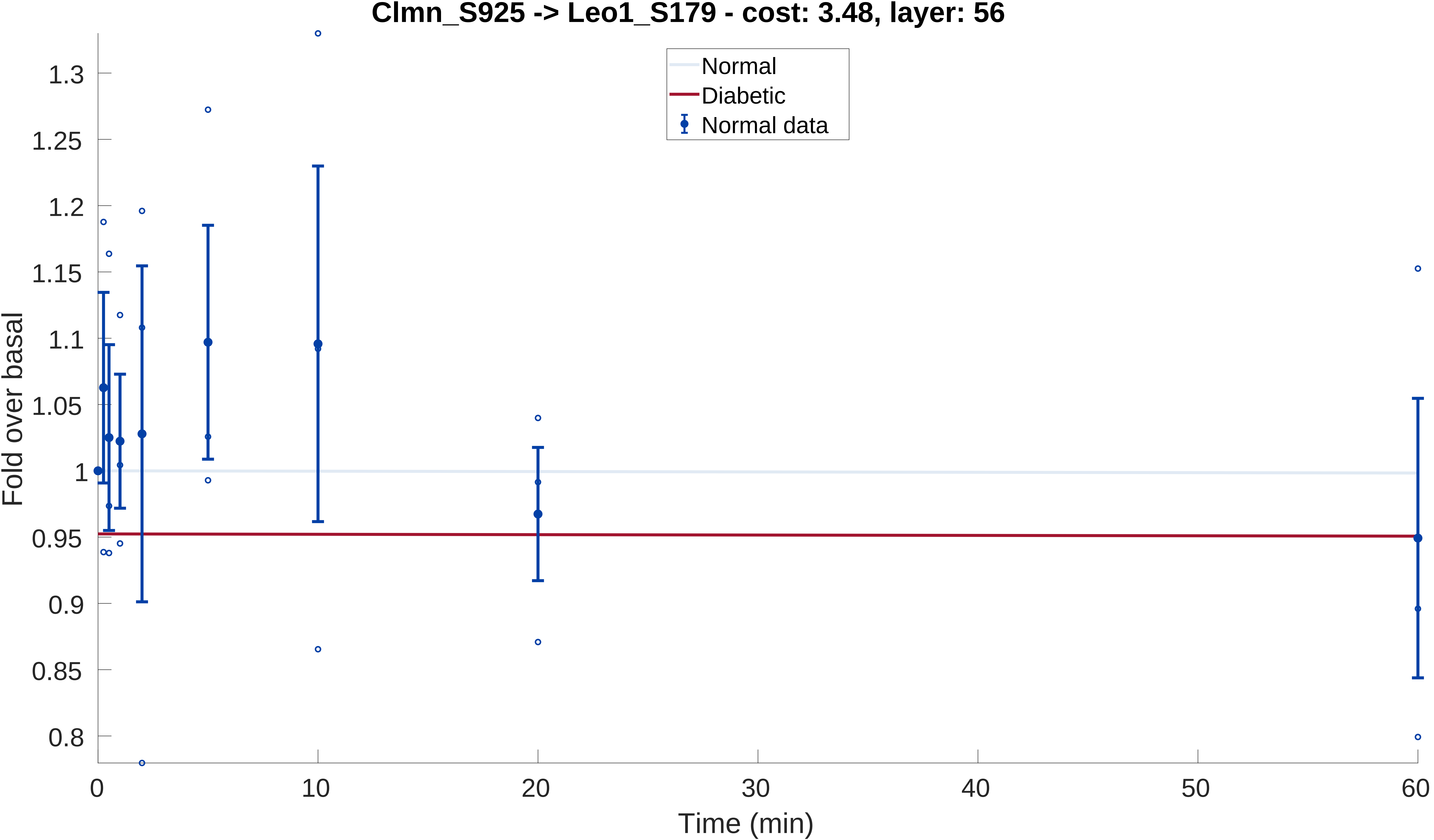

Clmn_S925 -> Leo1_S179 - cost: 3.48, layer: 56
Normal
Diabetic
Normal data
Fold over basal
1.3
1.25
1.2
1.15
1.1
1.05
1
0.95
0.9
0.85
0.8
0
10
20
30
40
50
60
Time (min)

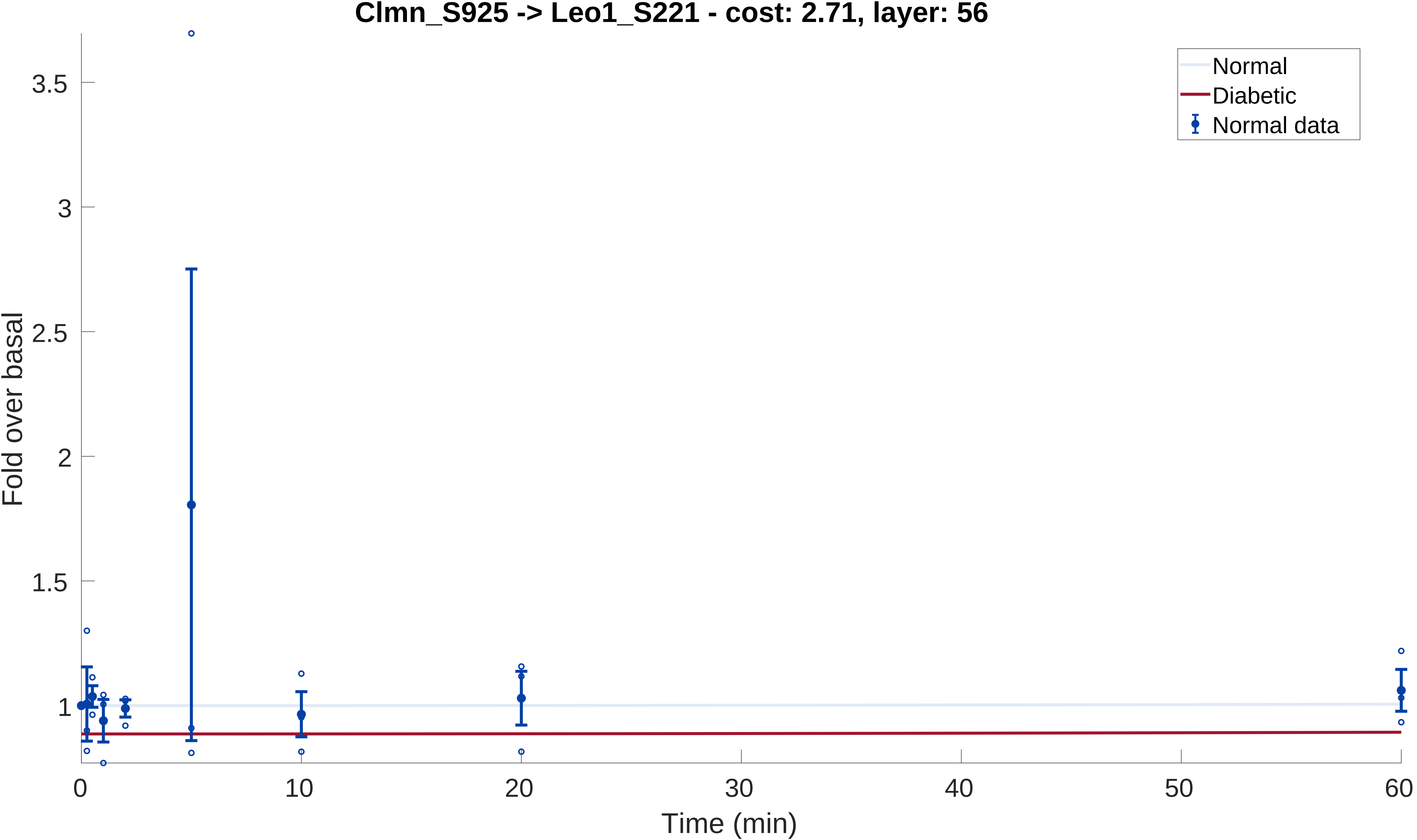
Clmn_S925 -> Leo1_S221 - cost: 2.71, layer: 56
Normal
Diabetic
Normal data
Fold over basal
Time (min)
3.5
3
2.5
2
1.5
1
0
10
20
30
40
50
60

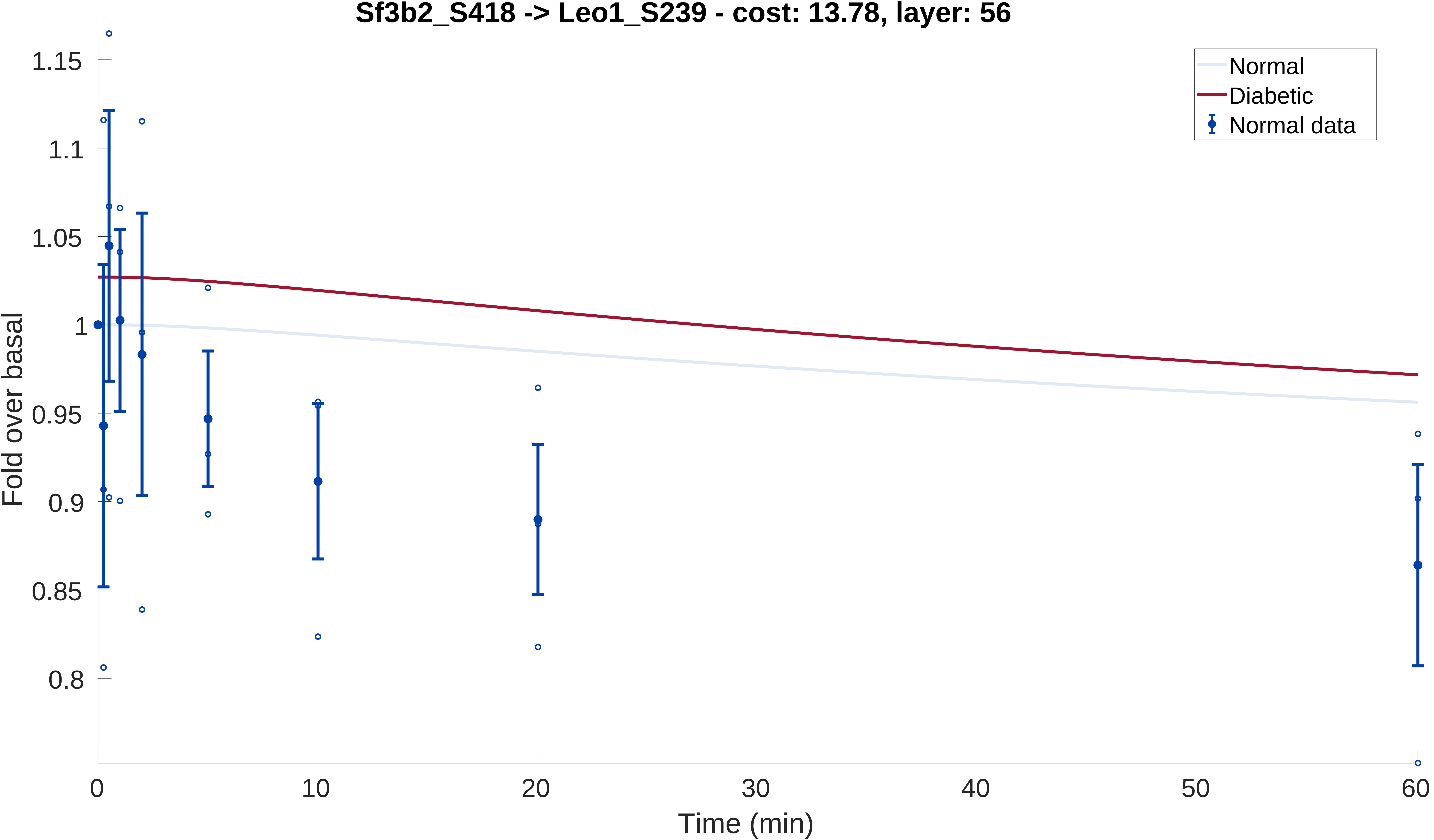

Sf3b2_S418 -> Leo1_S239 - cost: 13.78, layer: 56
Normal
Diabetic
Normal data
Fold over basal
1.15
1.1
1.05
1
0.95
0.9
0.85
0.8
0
10
20
30
40
50
60
Time (min)

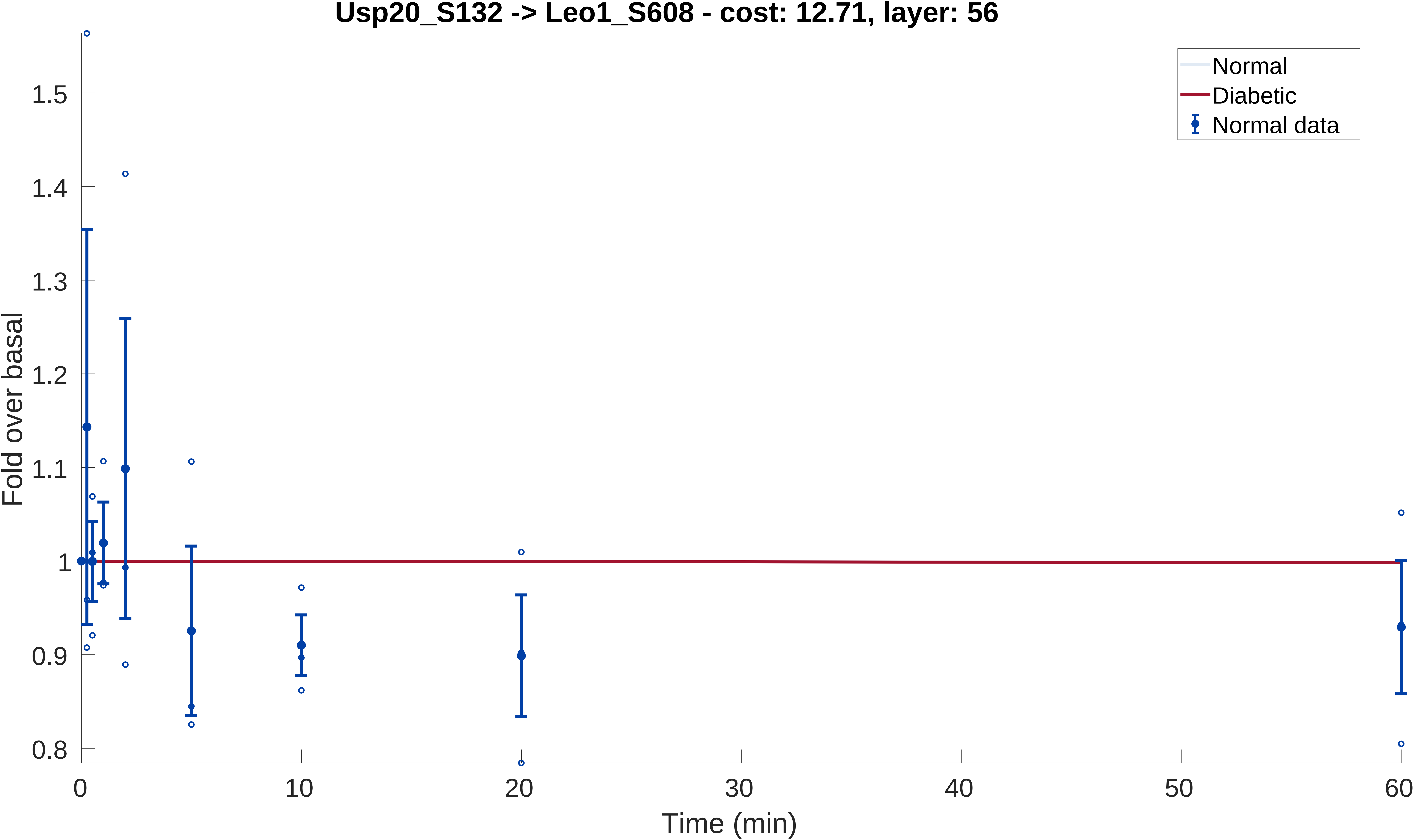

Usp20_S132 -> Leo1_S608 - cost: 12.71, layer: 56
Normal
Diabetic
Normal data
Fold over basal
Time (min)
1.5
1.4
1.3
1.2
1.1
1
0.9
0.8
0
10
20
30
40
50
60

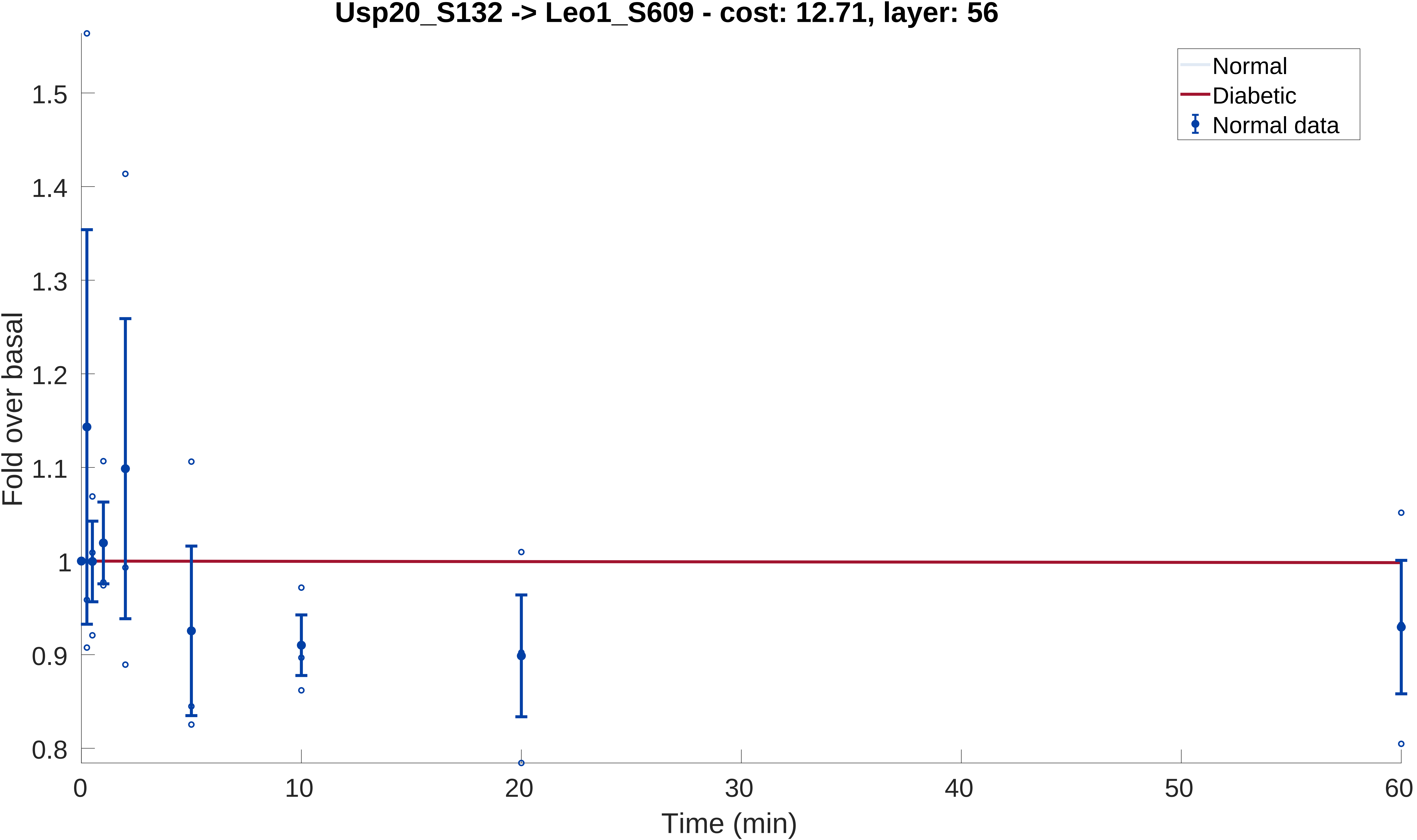

Usp20_S132 -> Leo1_S609 - cost: 12.71, layer: 56
Normal
Diabetic
Normal data
Fold over basal
Time (min)
1.5
1.4
1.3
1.2
1.1
1
0.9
0.8
0
10
20
30
40
50
60

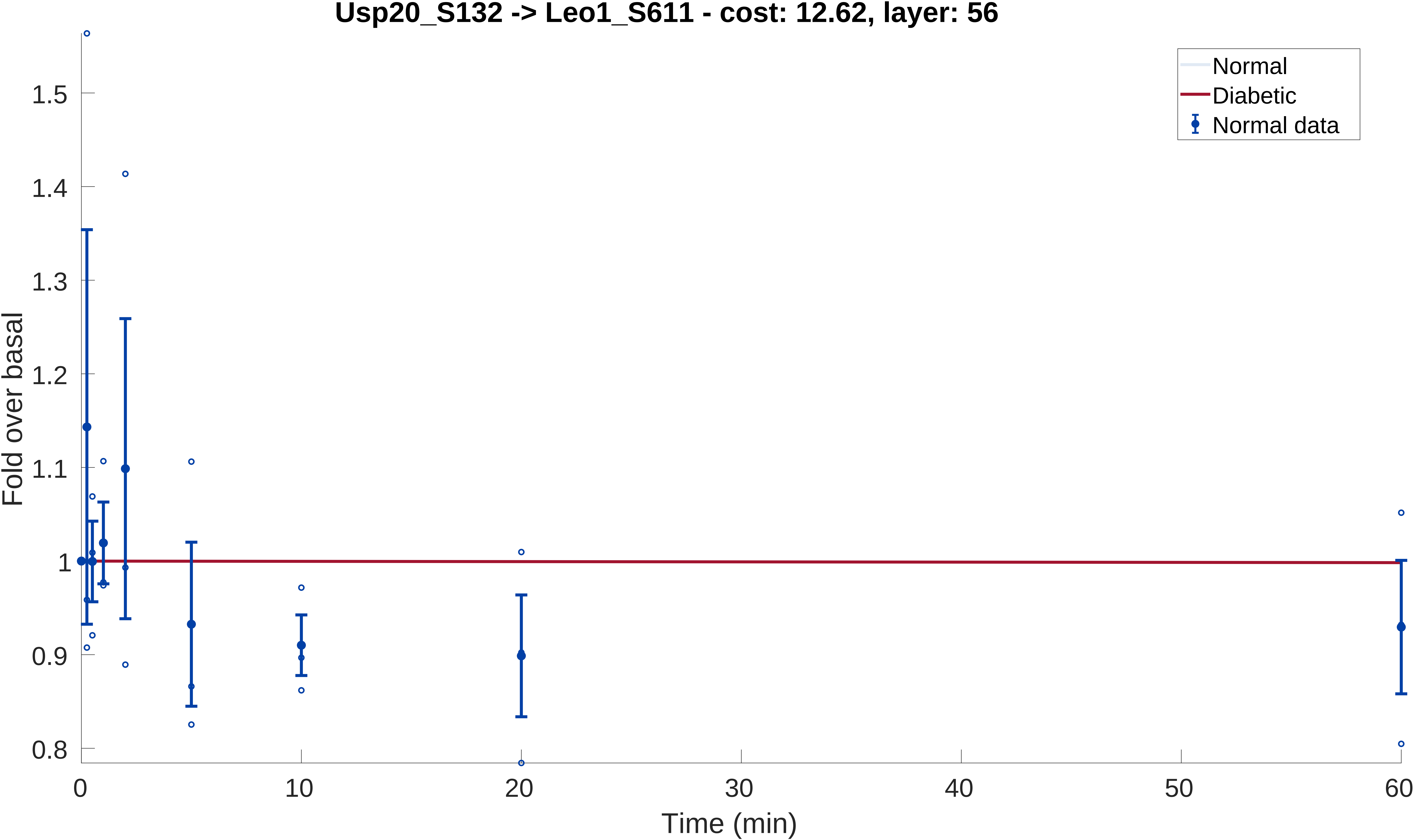

Usp20_S132 -> Leo1_S611 - cost: 12.62, layer: 56
Normal
Diabetic
Normal data
Fold over basal
1.5
1.4
1.3
1.2
1.1
1
0.9
0.8
0
10
20
30
40
50
60
Time (min)

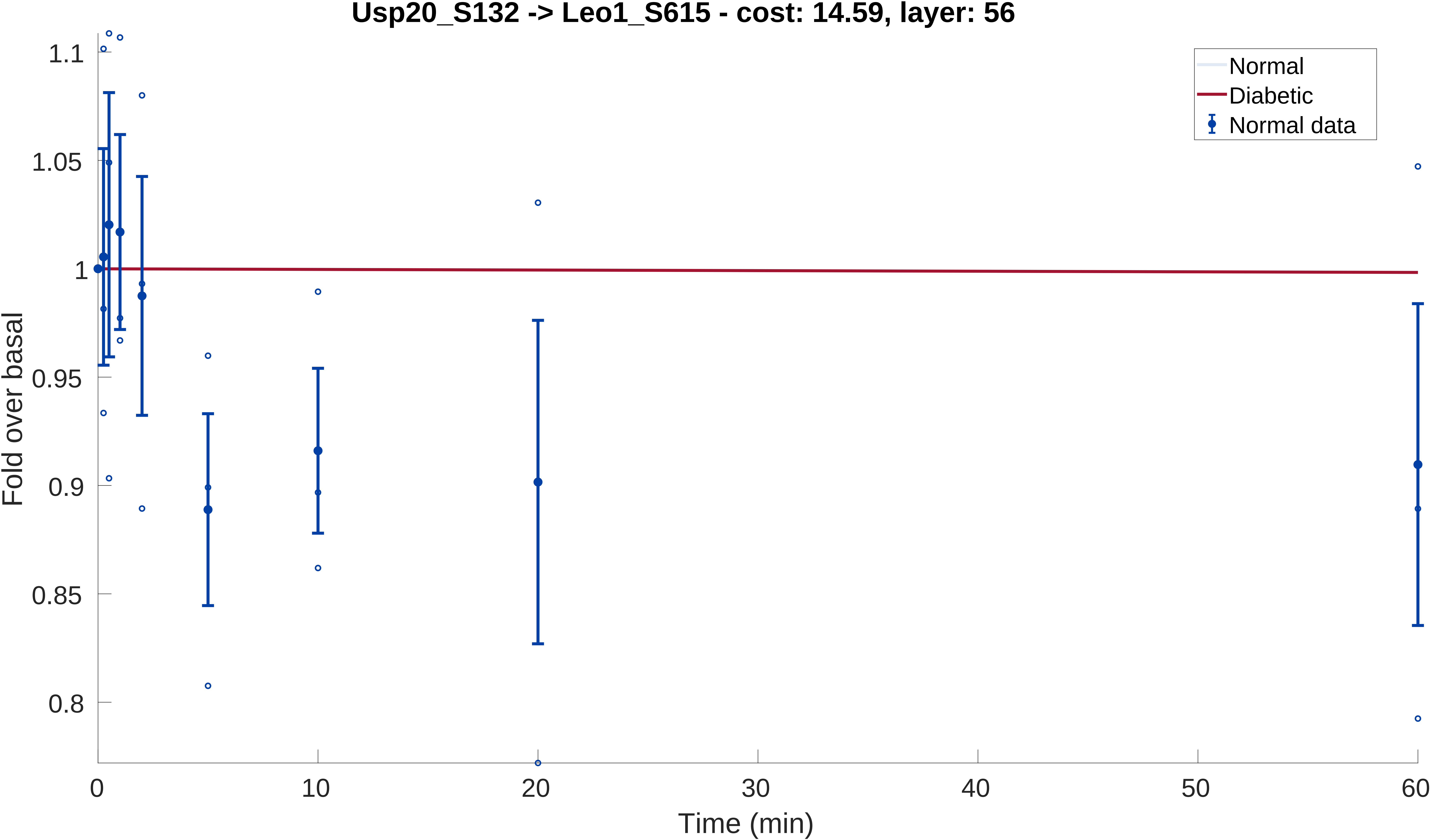

Usp20_S132 -> Leo1_S615 - cost: 14.59, layer: 56
Normal
Diabetic
Normal data
Fold over basal
Time (min)
1.1
1.05
1
0.95
0.9
0.85
0.8
0
10
20
30
40
50
60

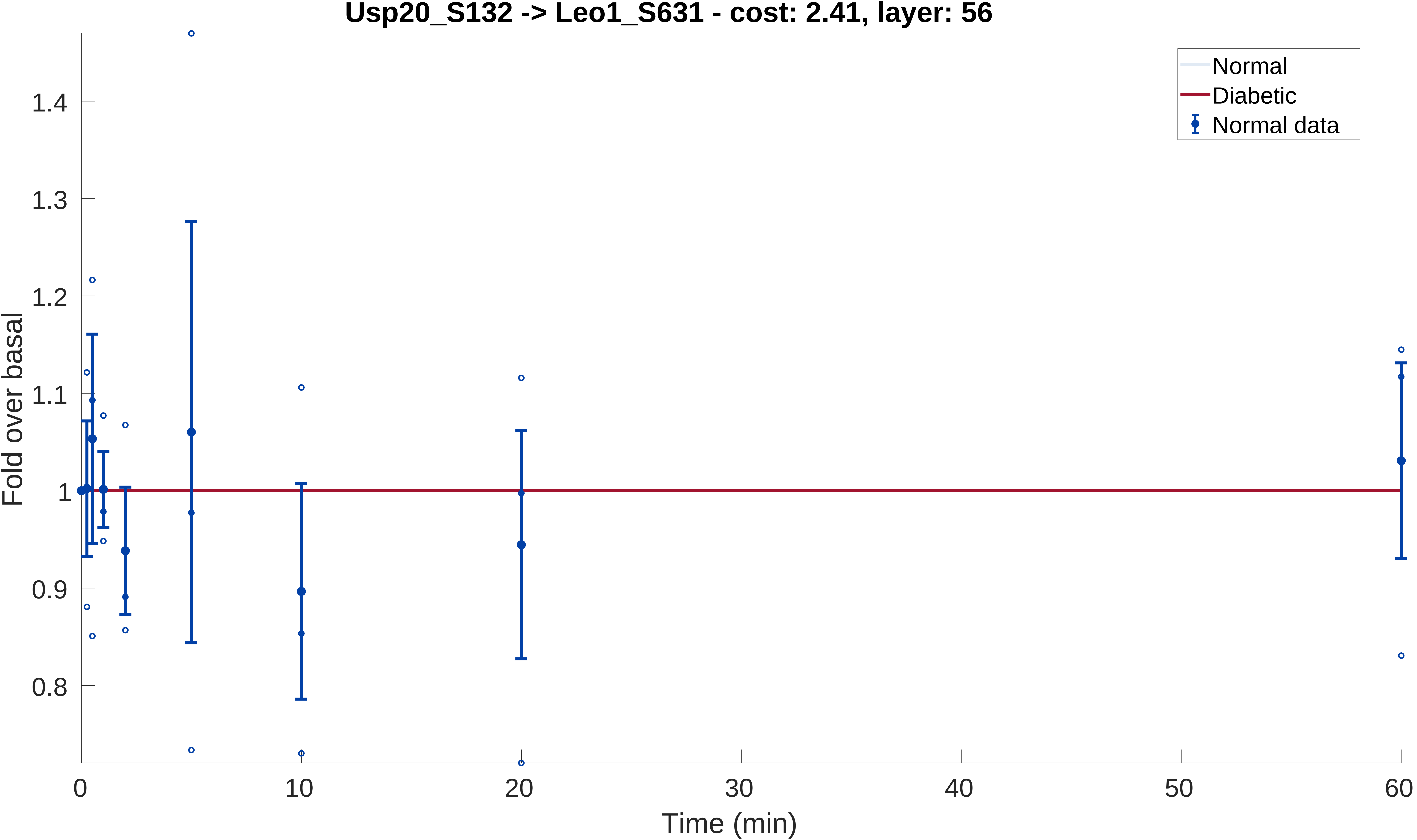
Usp20_S132 -> Leo1_S631 - cost: 2.41, layer: 56
Fold over basal
1.4
1.3
1.2
1.1
1
0.9
0.8
0
10
20
30
40
50
60
Time (min)
Normal
Diabetic
Normal data

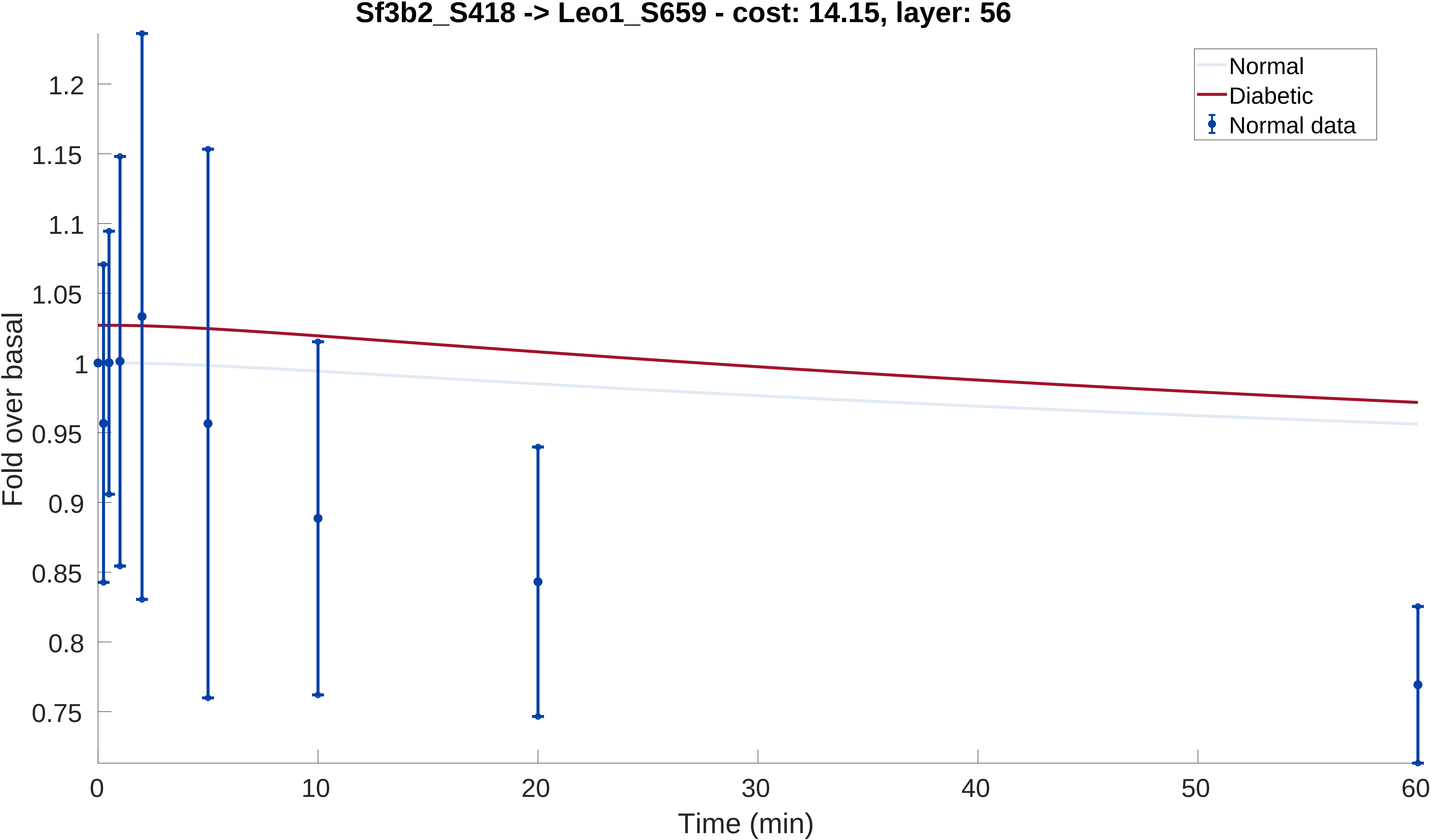
Sf3b2_S418 -> Leo1_S659 - cost: 14.15, layer: 56
Normal
Diabetic
Normal data
Fold over basal
1.2
1.15
1.1
1.05
1
0.95
0.9
0.85
0.8
0.75
0
10
20
30
40
50
60
Time (min)

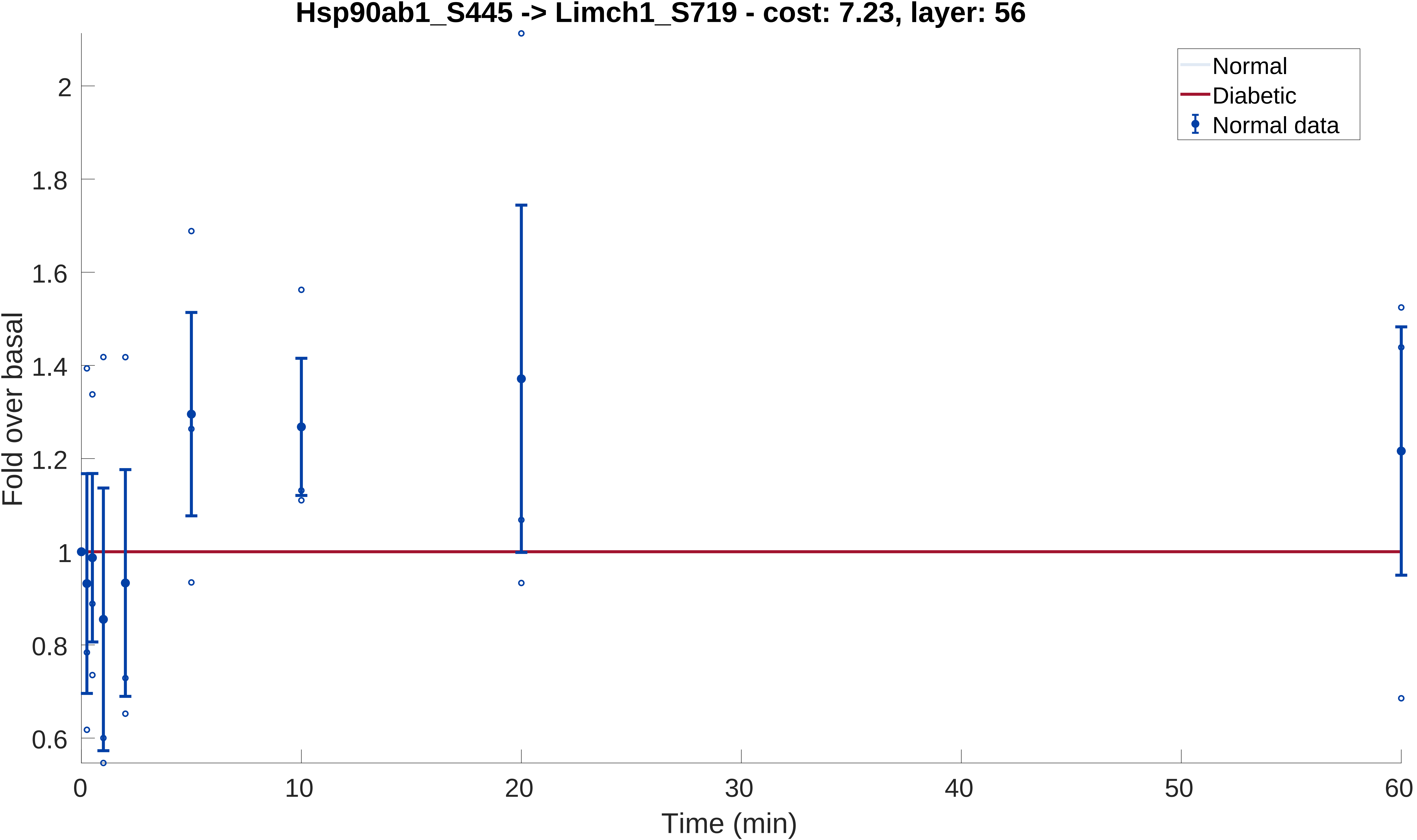

Hsp90ab1_S445 -> Limch1_S719 - cost: 7.23, layer: 56
Normal
Diabetic
Normal data
Fold over basal
Time (min)
0.6
0.8
1
1.2
1.4
1.6
1.8
2
0
10
20
30
40
50
60

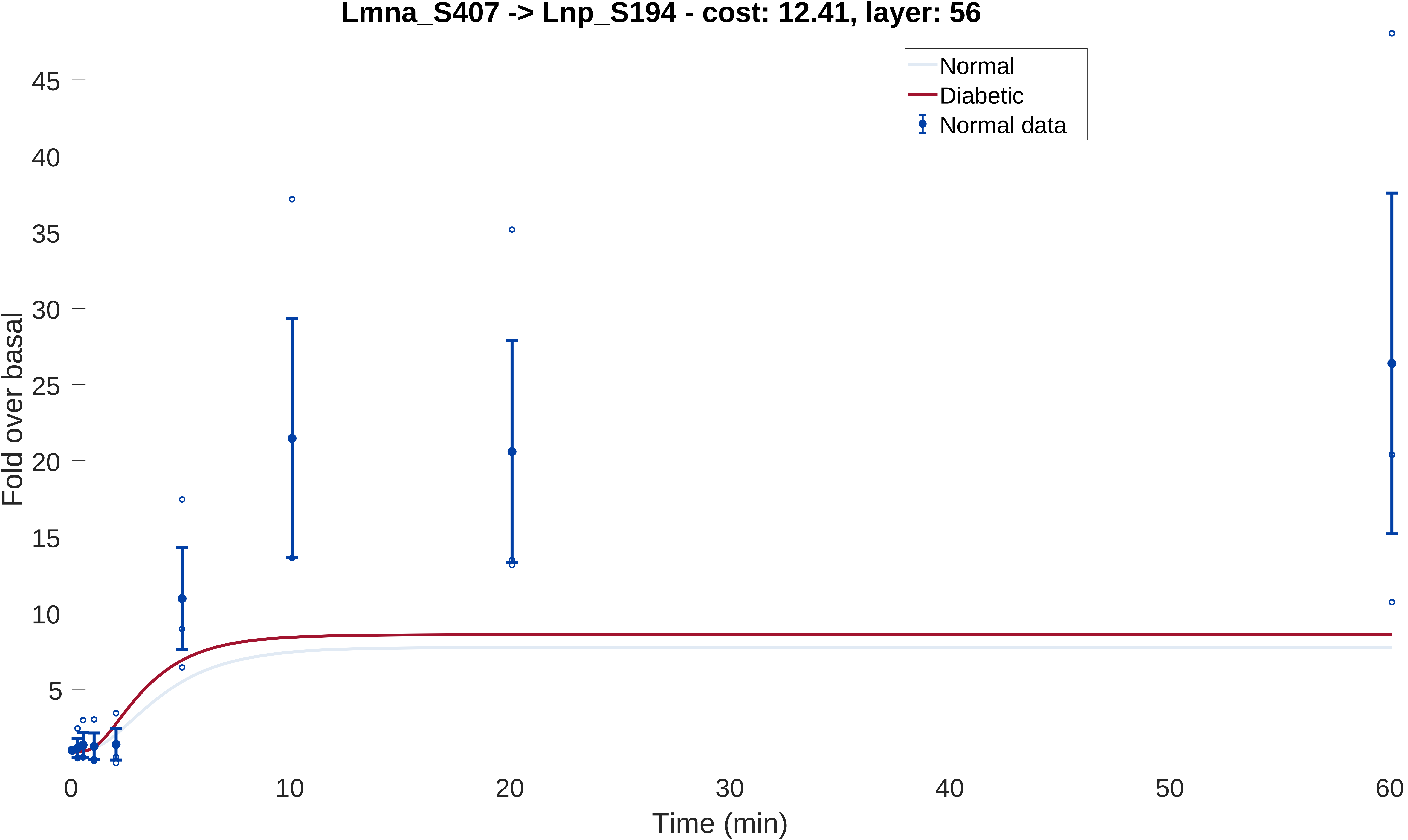

Lmna_S407 -> Lnp_S194 - cost: 12.41, layer: 56
Normal
Diabetic
Normal data
Fold over basal
Time (min)
0
10
20
30
40
50
60
5
10
15
20
25
30
35
40
45

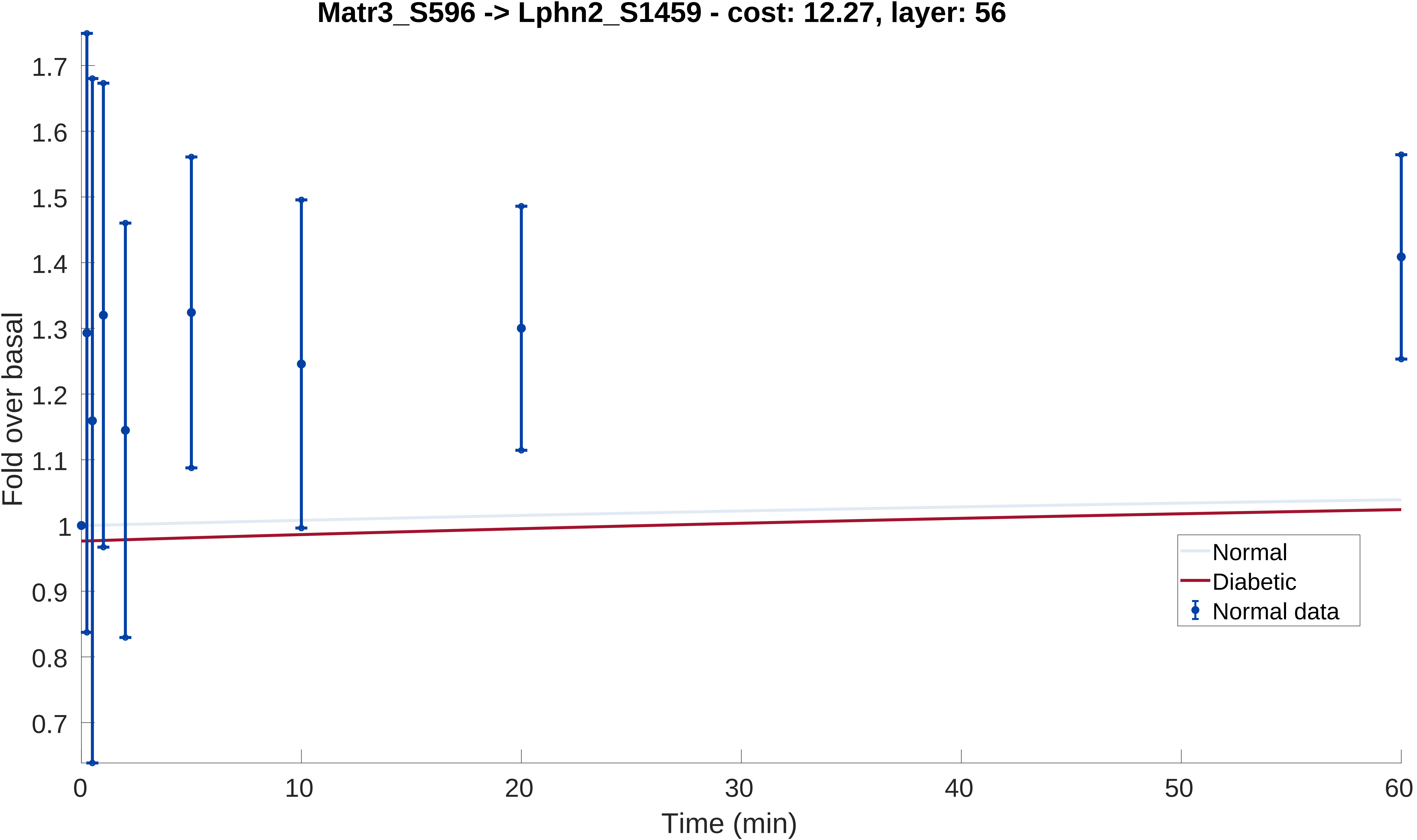

Matr3_S596 -> Lphn2_S1459 - cost: 12.27, layer: 56
Fold over basal
1.7
1.6
1.5
1.4
1.3
1.2
1.1
1
0.9
0.8
0.7
0
10
20
30
40
50
60
Time (min)
Normal
Diabetic
Normal data

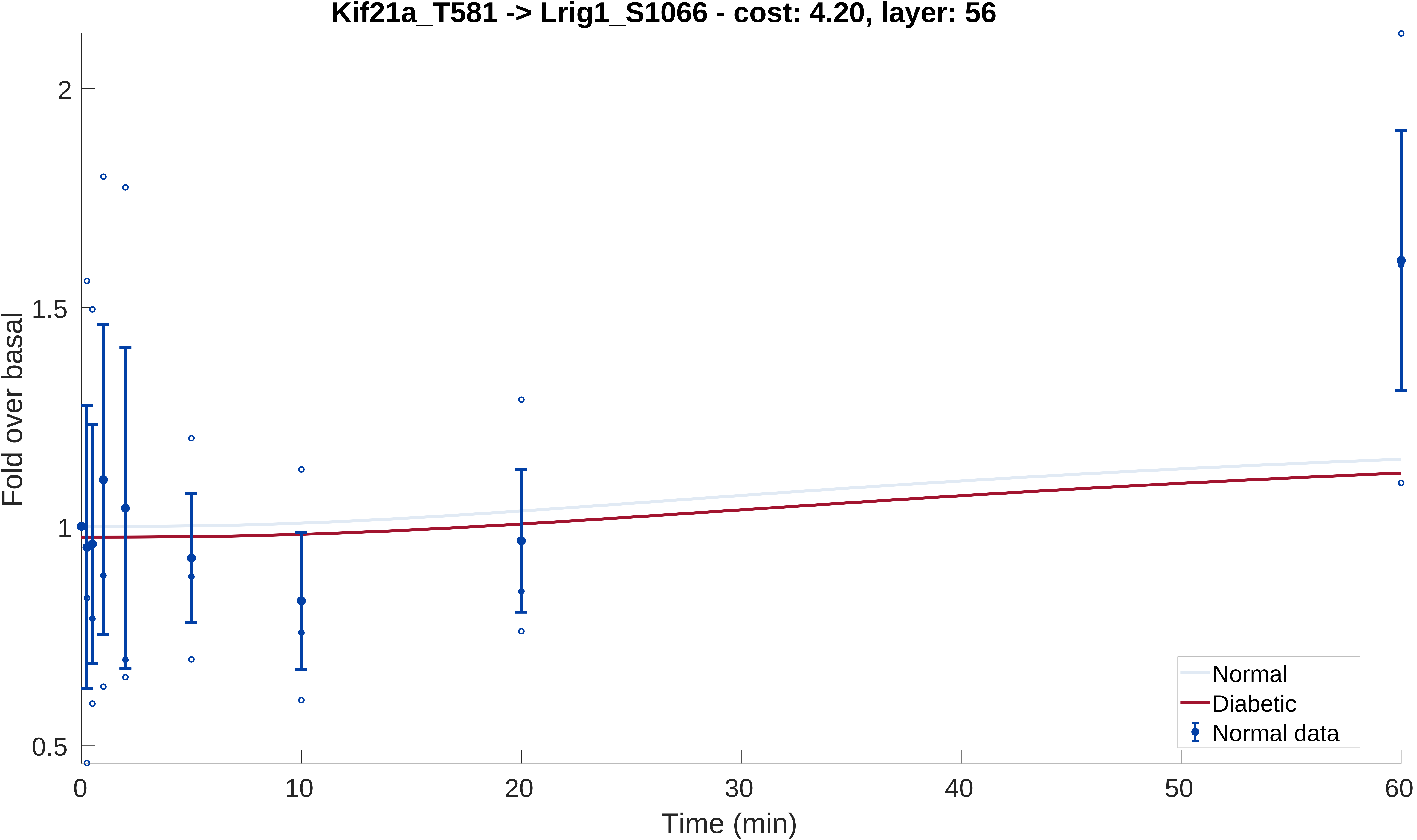

Kif21a_T581 -> Lrig1_S1066 - cost: 4.20, layer: 56
Fold over basal
Time (min)
0.5
1
1.5
2
0
10
20
30
40
50
60
Normal
Diabetic
Normal data

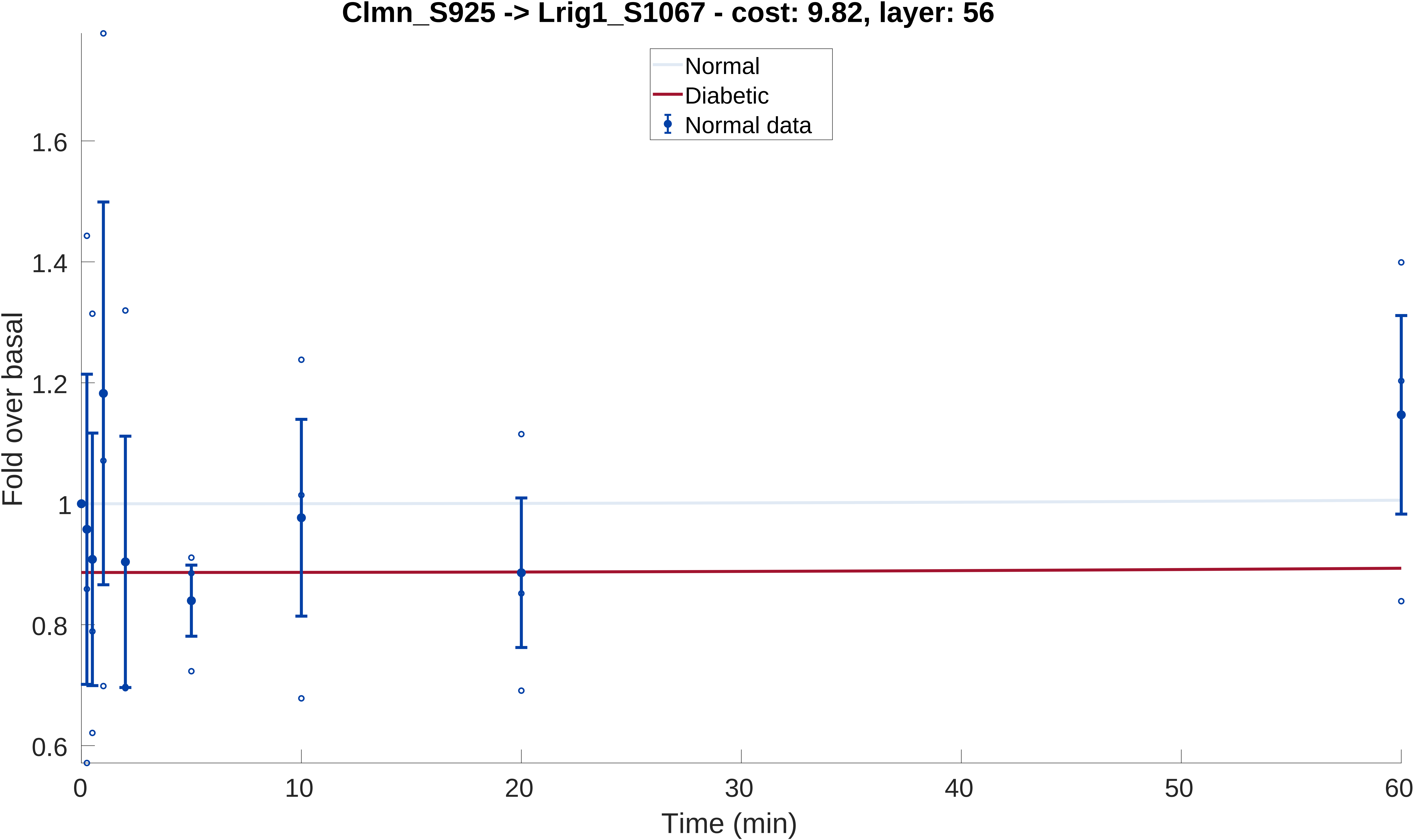

Clmn_S925 -> Lrig1_S1067 - cost: 9.82, layer: 56
Normal
Diabetic
Normal data
Fold over basal
1.6
1.4
1.2
1
0.8
0.6
0
10
20
30
40
50
60
Time (min)

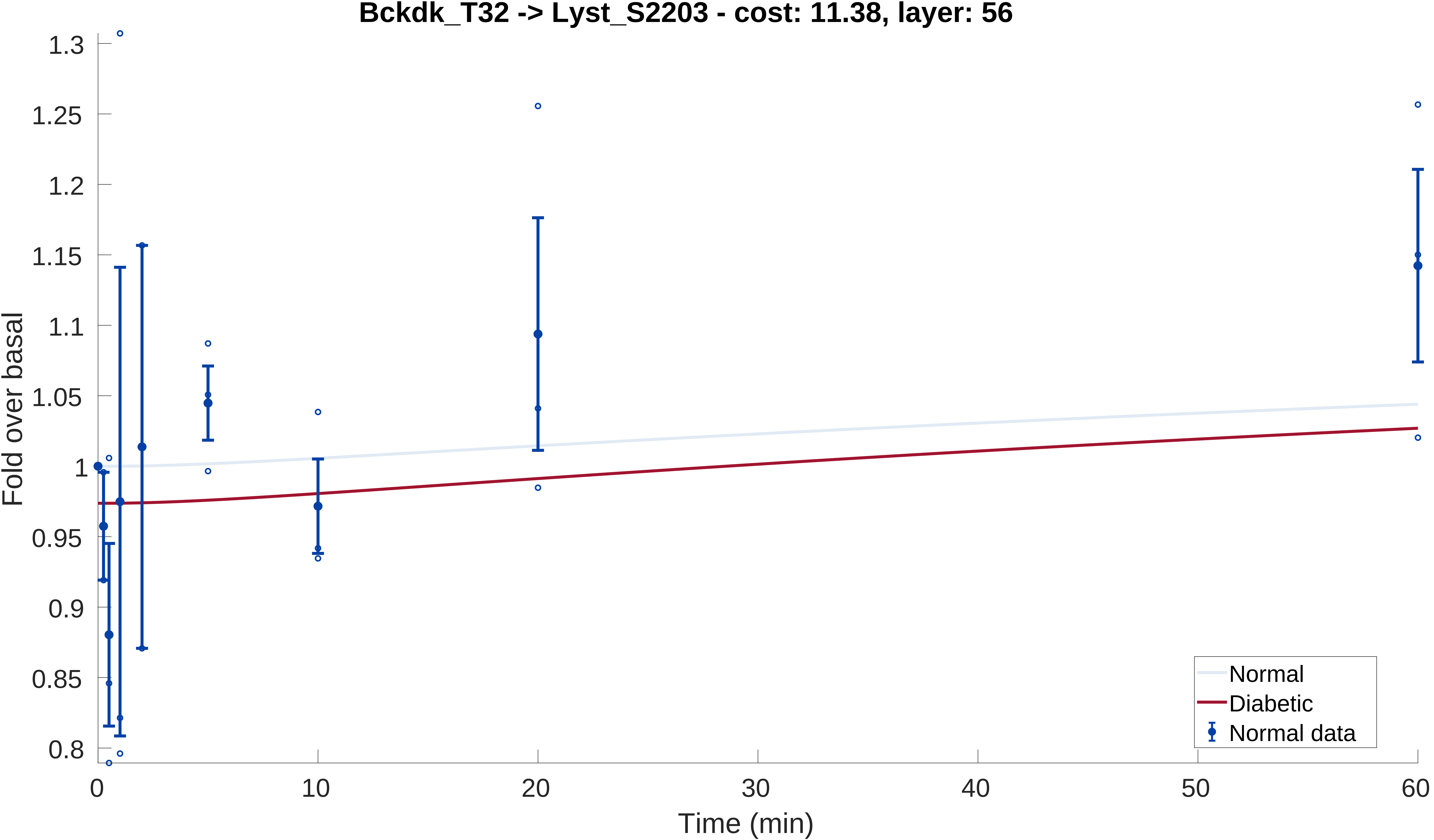

Bckdk_T32 -> Lyst_S2203 - cost: 11.38, layer: 56
Fold over basal
1.3
1.25
1.2
1.15
1.1
1.05
1
0.95
0.9
0.85
0.8
0
10
20
30
40
50
60
Time (min)
Normal
Diabetic
Normal data

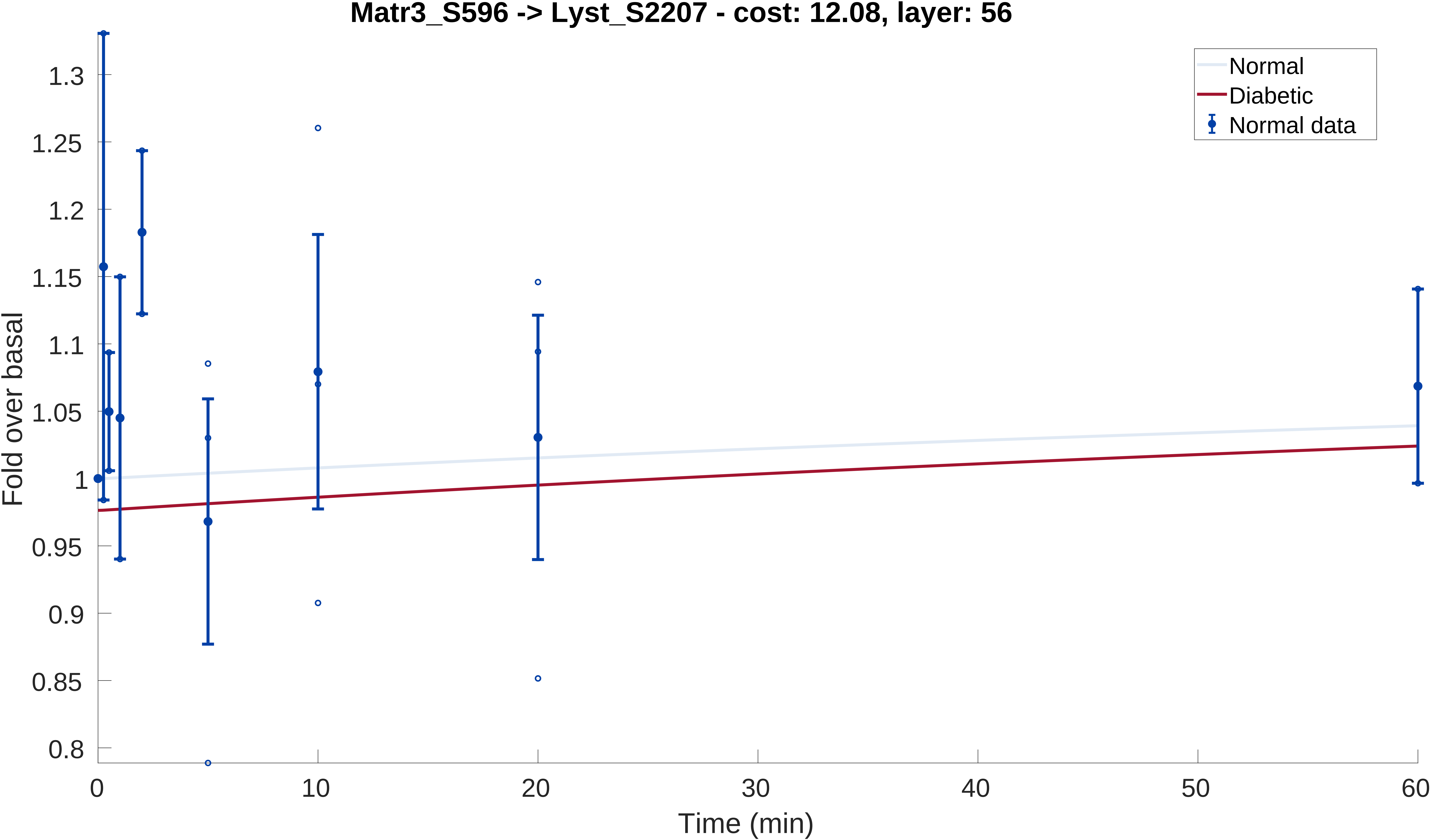

Matr3_S596 -> Lyst_S2207 - cost: 12.08, layer: 56
Normal
Diabetic
Normal data
Fold over basal
1.3
1.25
1.2
1.15
1.1
1.05
1
0.95
0.9
0.85
0.8
0
10
20
30
40
50
60
Time (min)

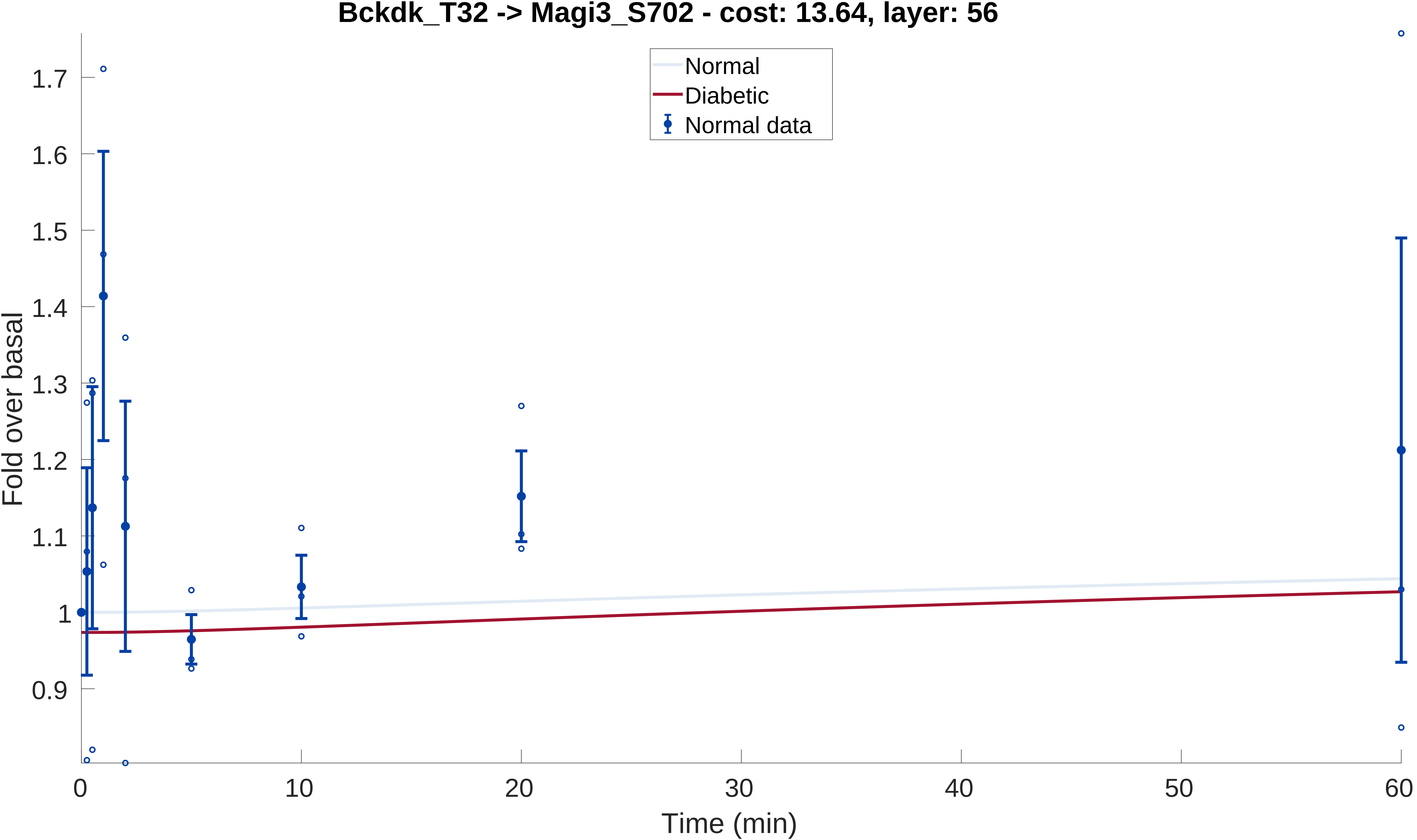

Bckdk_T32 -> Magi3_S702 - cost: 13.64, layer: 56
Normal
Diabetic
Normal data
Fold over basal
1.7
1.6
1.5
1.4
1.3
1.2
1.1
1
0.9
0
10
20
30
40
50
60
Time (min)

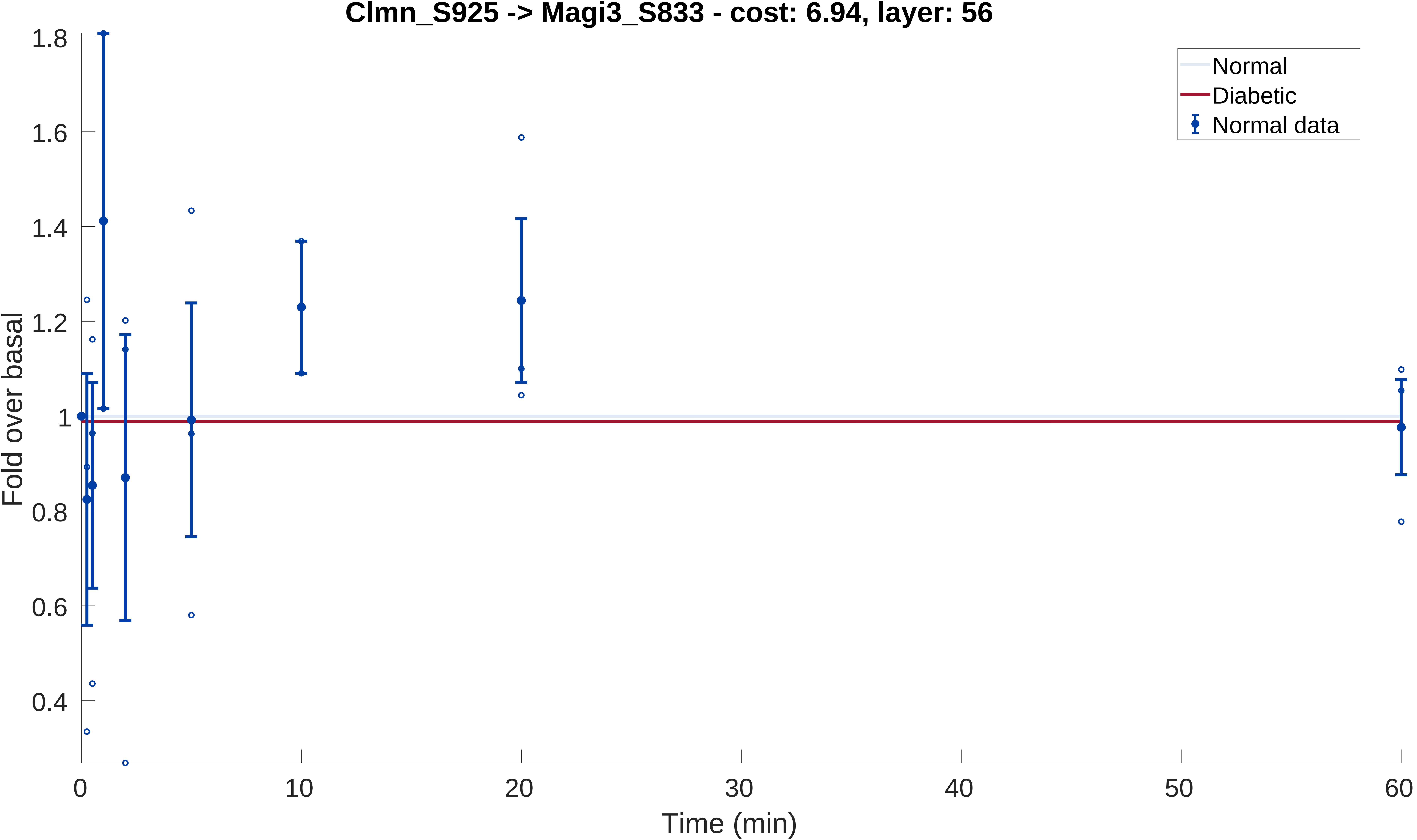

Clmn_S925 -> Magi3_S833 - cost: 6.94, layer: 56
Normal
Diabetic
Normal data
Fold over basal
1.8
1.6
1.4
1.2
1
0.8
0.6
0.4
0
10
20
30
40
50
60
Time (min)

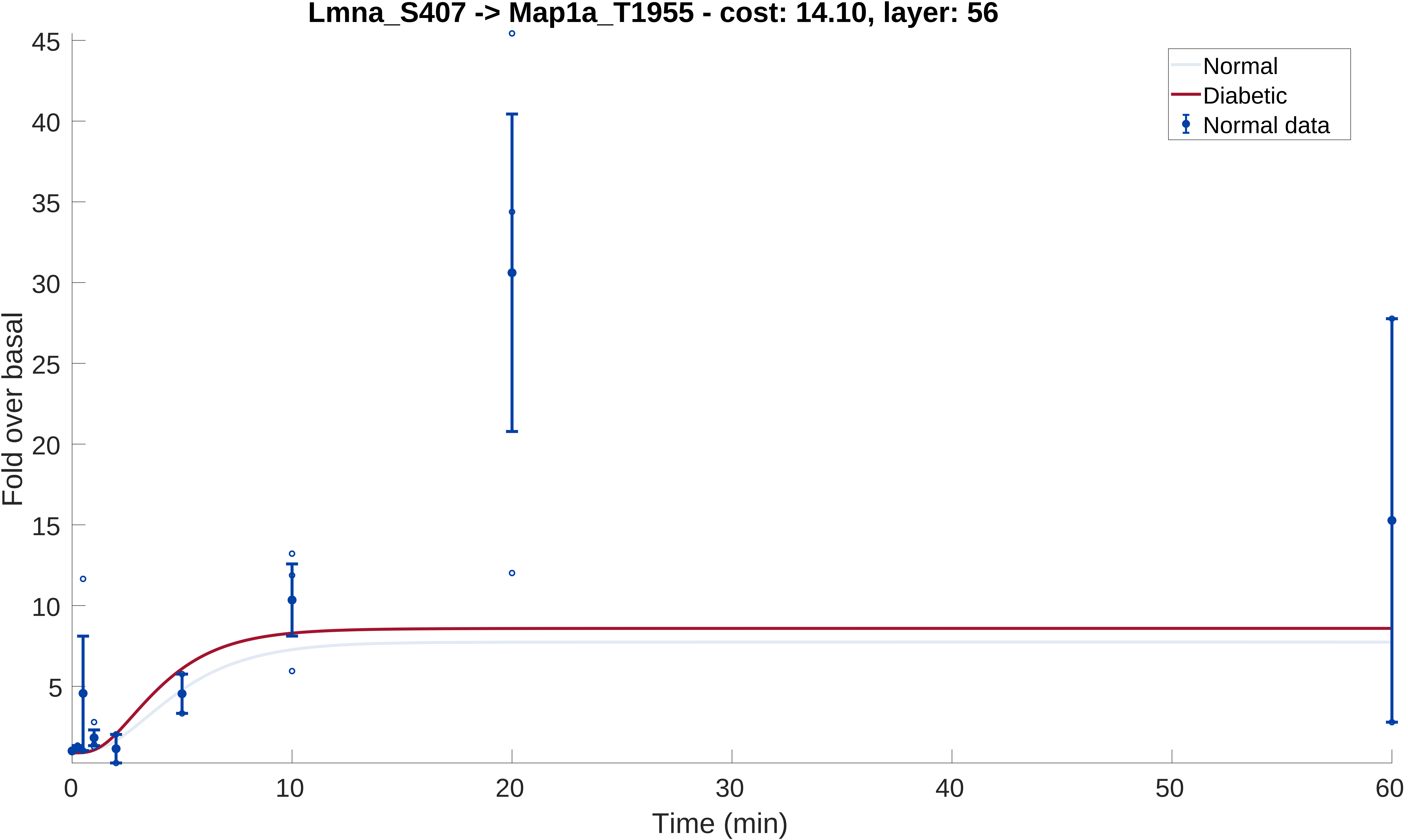
Lmna_S407 -> Map1a_T1955 - cost: 14.10, layer: 56
Normal
Diabetic
Normal data
Fold over basal
Time (min)
0
10
20
30
40
50
60
5
10
15
20
25
30
35
40
45

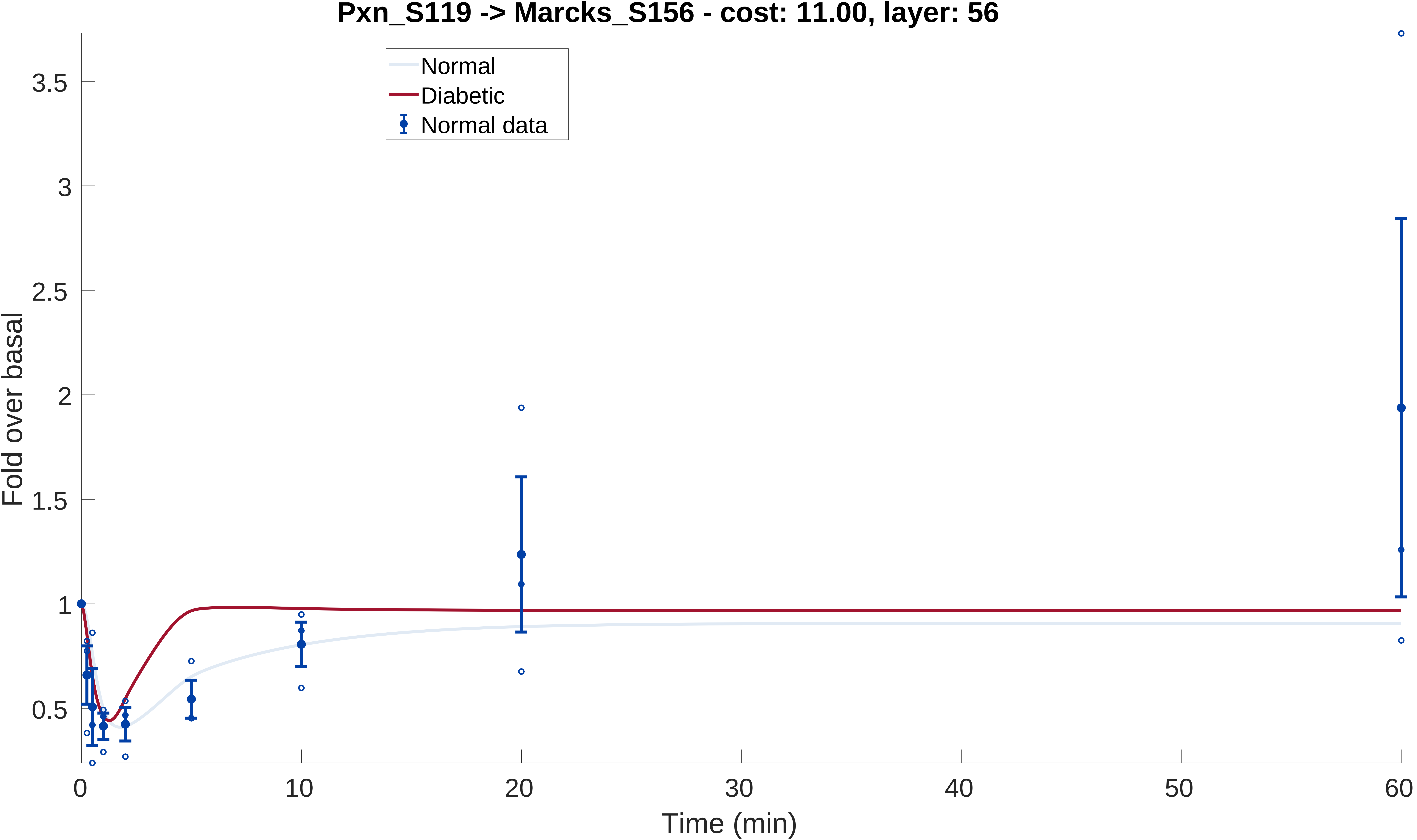
Pxn_S119 -> Marcks_S156 - cost: 11.00, layer: 56
Normal
Diabetic
Normal data
Fold over basal
Time (min)
0.5
1
1.5
2
2.5
3
3.5
0
10
20
30
40
50
60

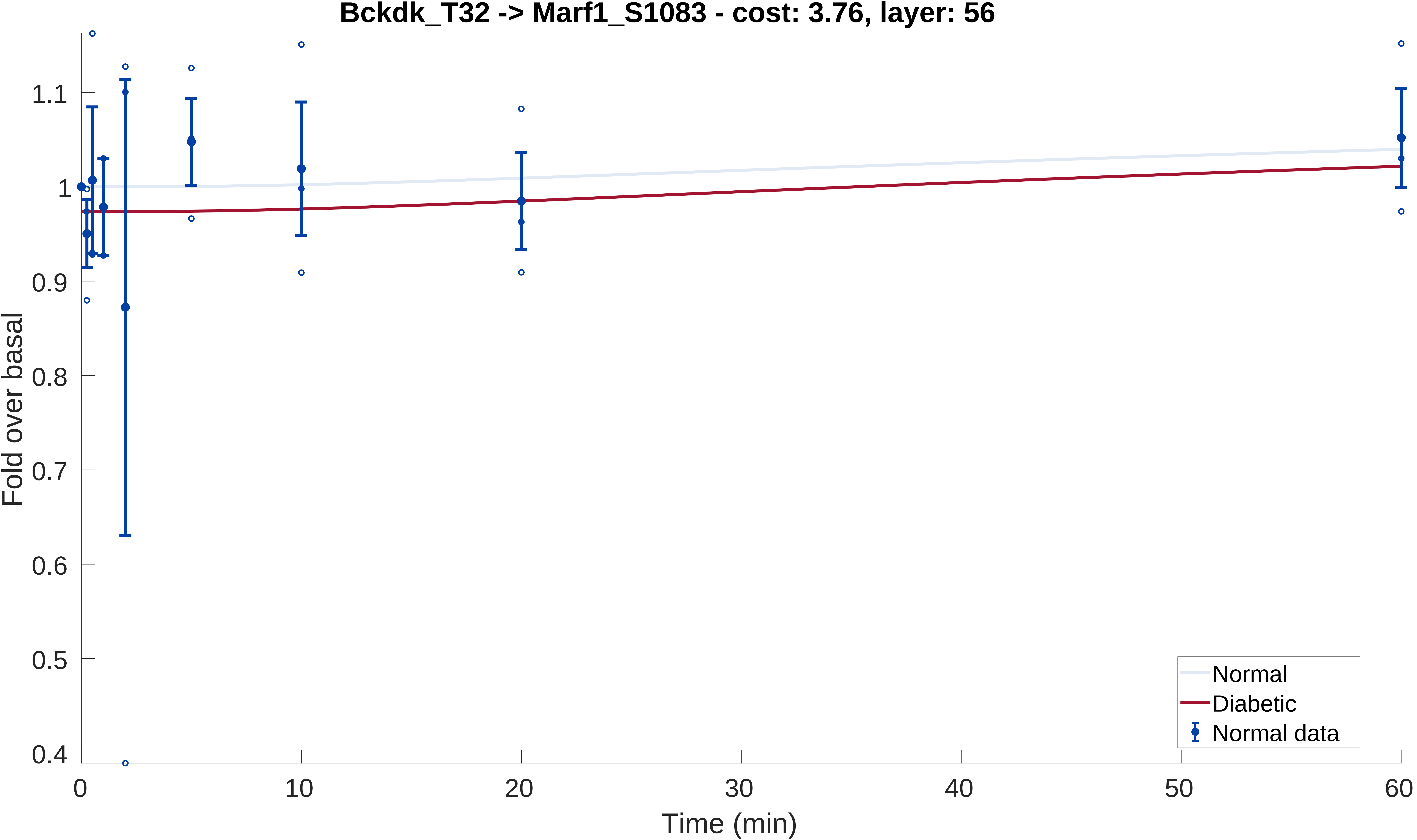
Bckdk_T32 -> Marf1_S1083 - cost: 3.76, layer: 56
Fold over basal
1.1
1
0.9
0.8
0.7
0.6
0.5
0.4
0
10
20
30
40
50
60
Time (min)
Normal
Diabetic
Normal data

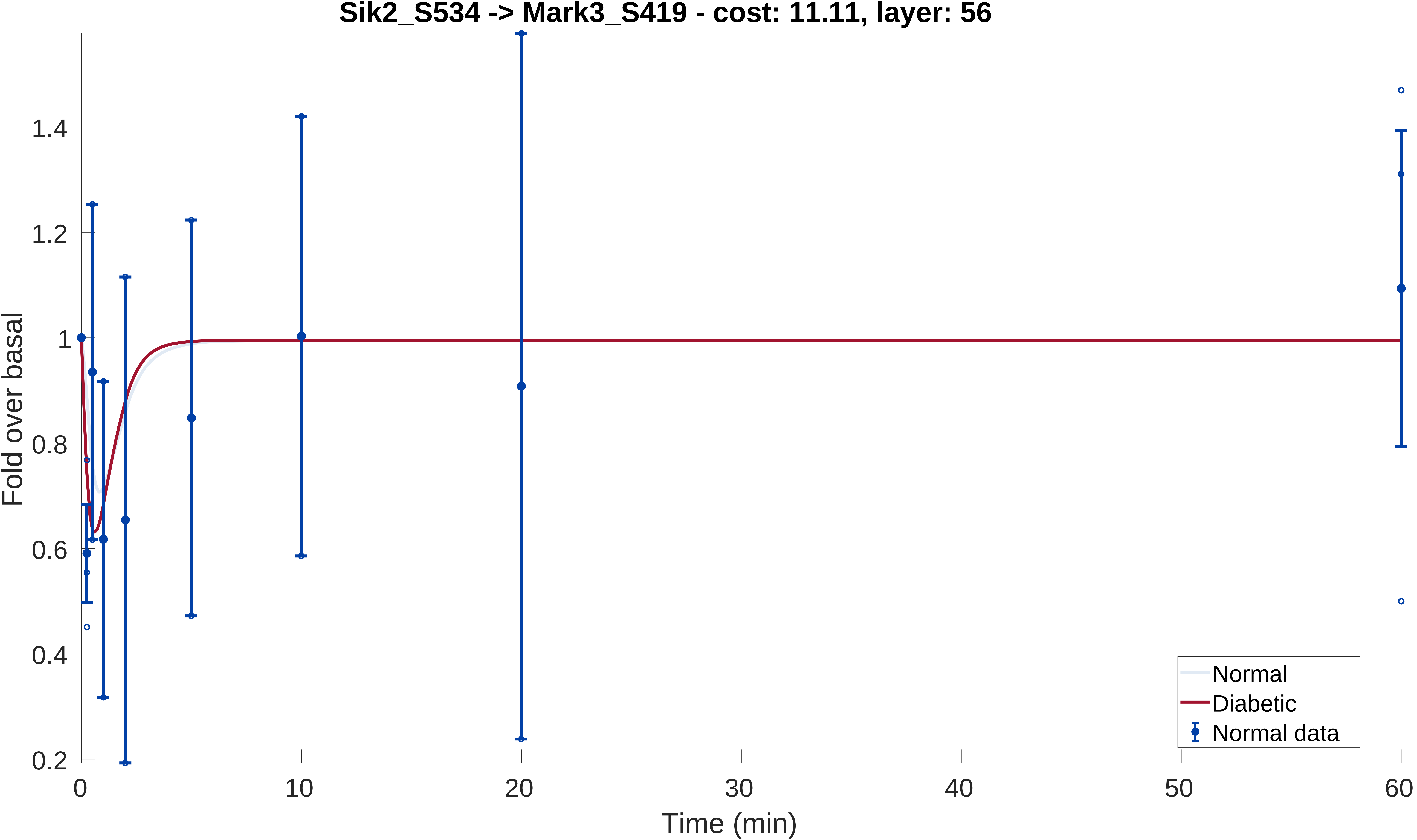
Sik2_S534 -> Mark3_S419 - cost: 11.11, layer: 56
Fold over basal
0.2
0.4
0.6
0.8
1
1.2
1.4
0
10
20
30
40
50
60
Time (min)
Normal
Diabetic
Normal data

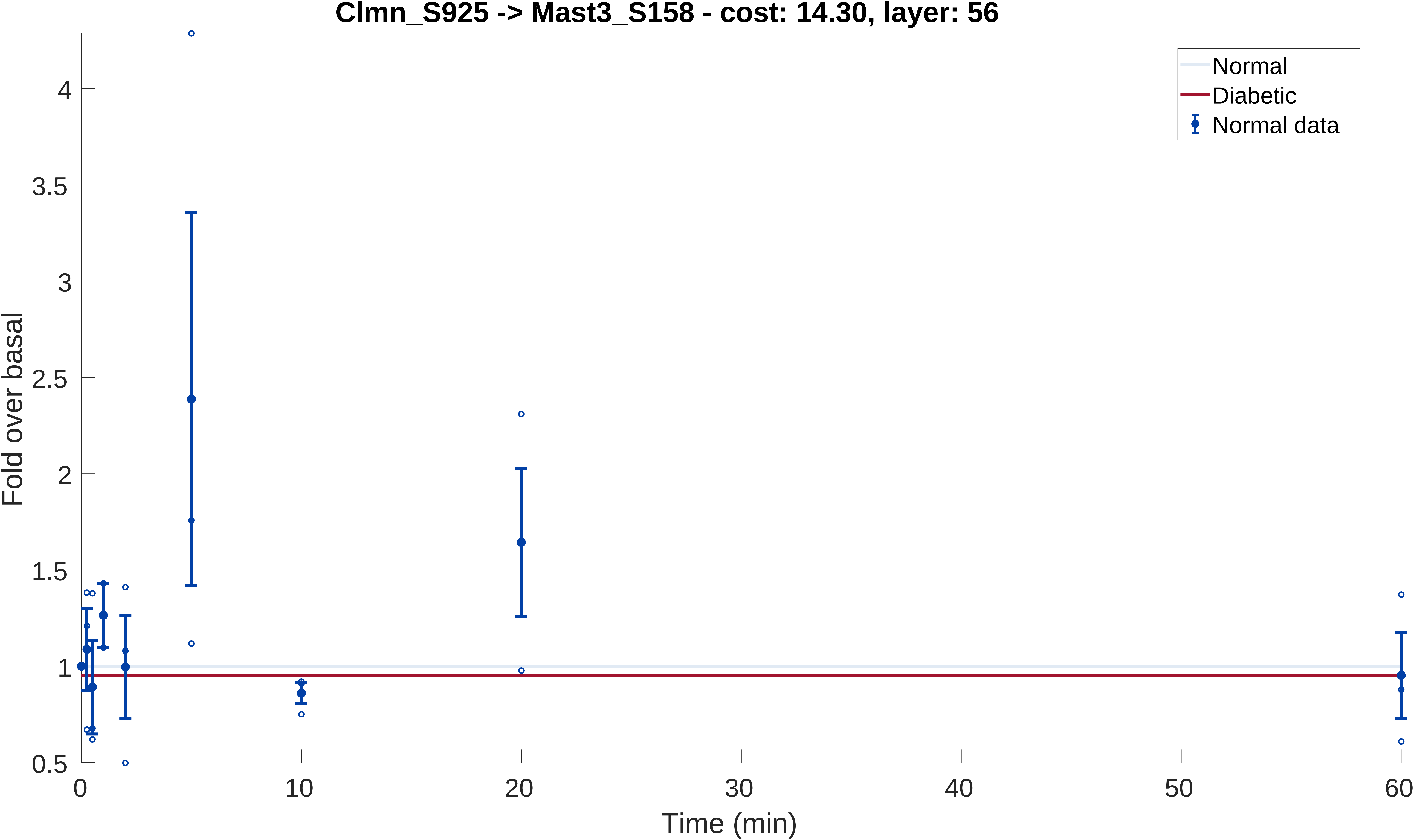
Clmn_S925 -> Mast3_S158 - cost: 14.30, layer: 56
Normal
Diabetic
Normal data
Fold over basal
Time (min)
0.5
1
1.5
2
2.5
3
3.5
4
0
10
20
30
40
50
60

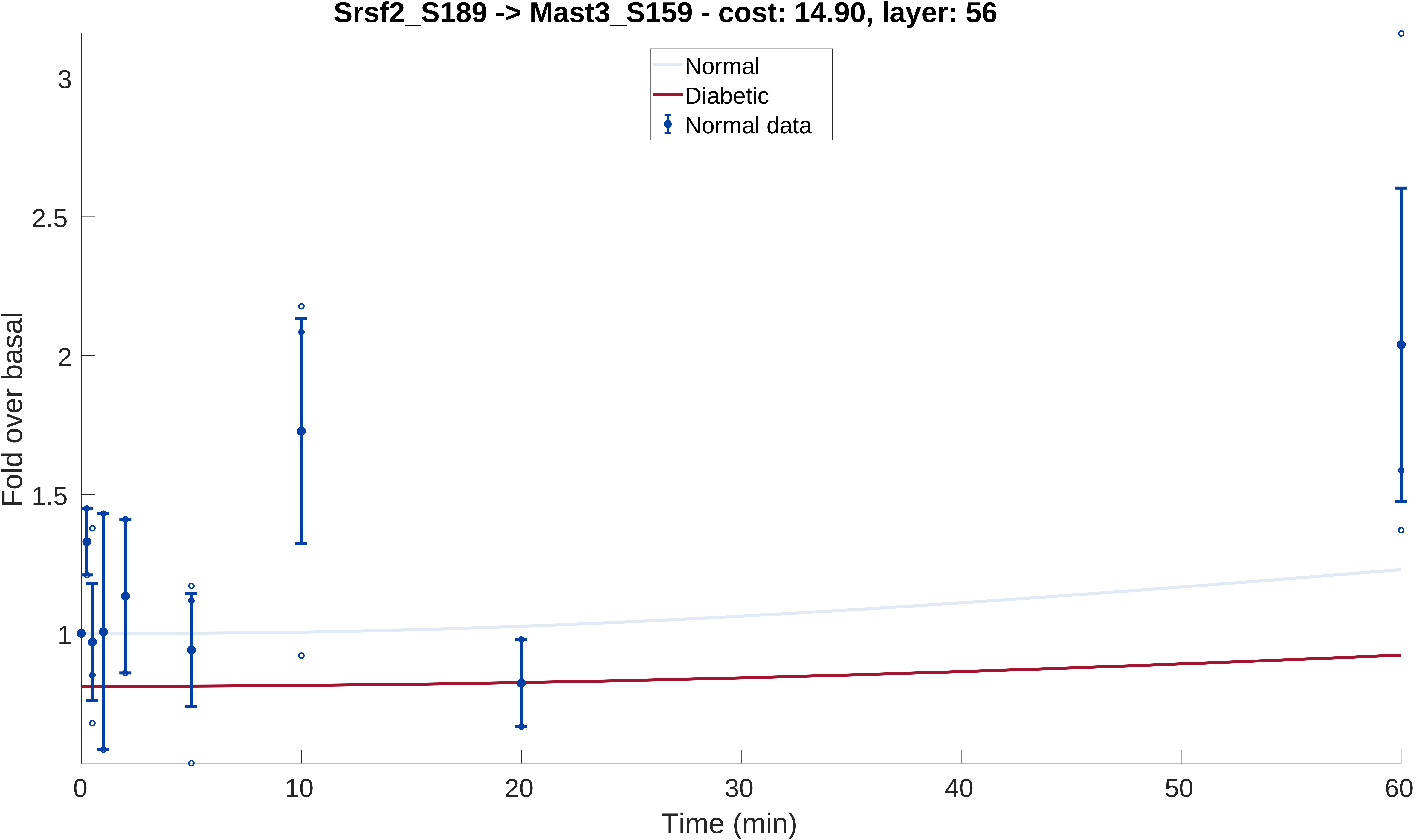

Srsf2_S189 -> Mast3_S159 - cost: 14.90, layer: 56
Normal
Diabetic
Normal data
Fold over basal
3
2.5
2
1.5
1
0
10
20
30
40
50
60
Time (min)

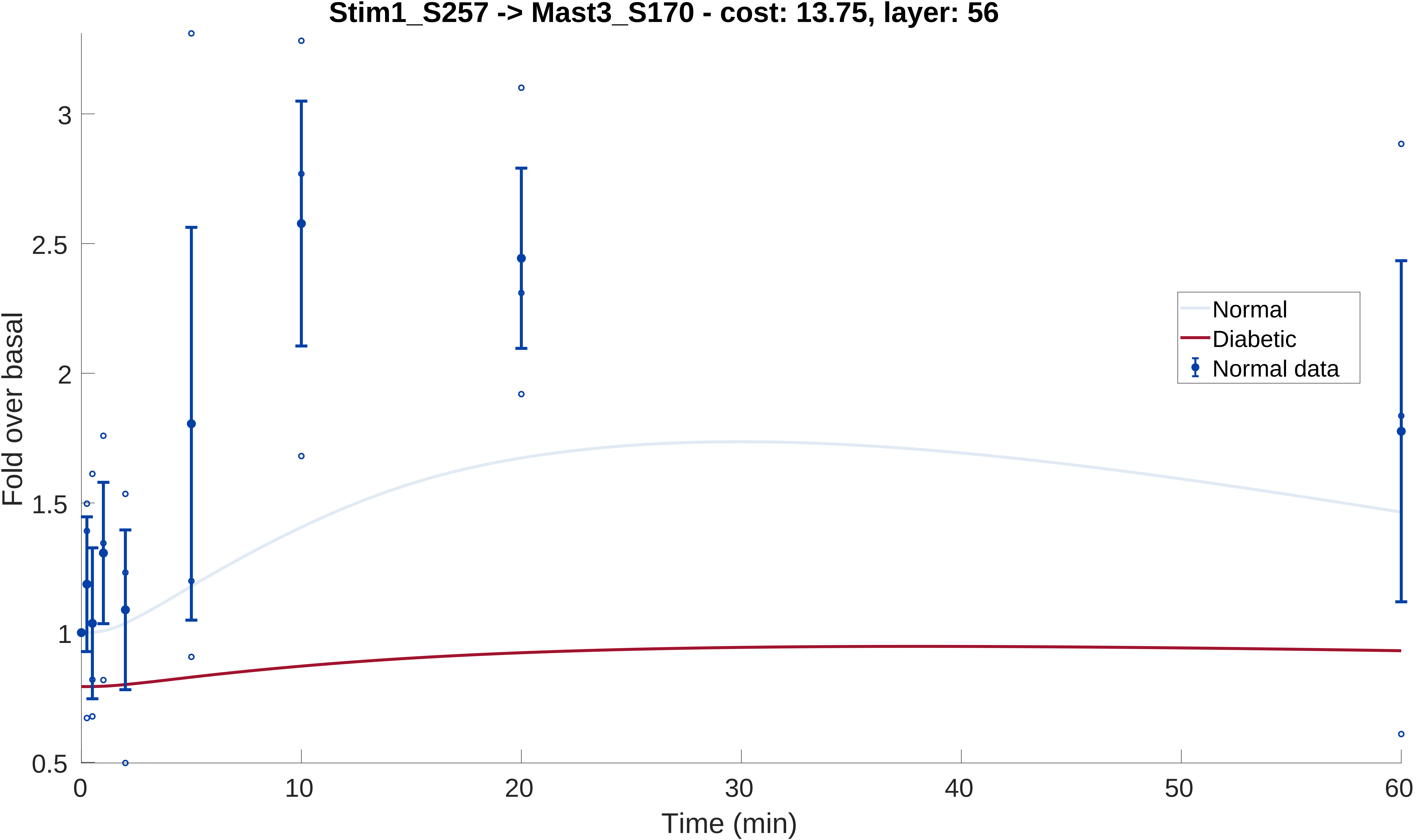

Stim1_S257 -> Mast3_S170 - cost: 13.75, layer: 56
Fold over basal
3
2.5
2
1.5
1
0.5
0
10
20
30
40
50
60
Time (min)
Normal
Diabetic
Normal data

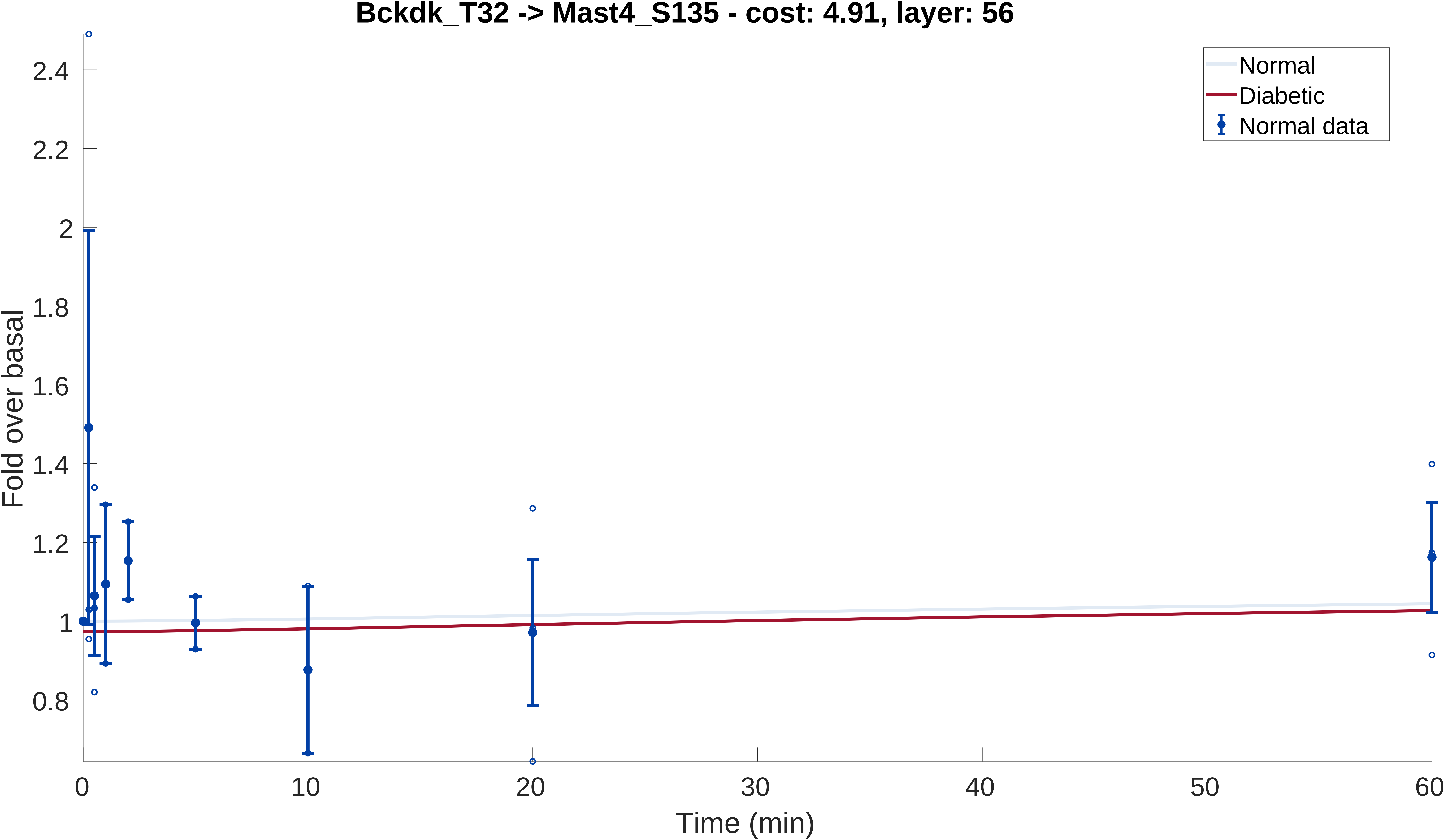
Bckdk_T32 -> Mast4_S135 - cost: 4.91, layer: 56
Fold over basal
Time (min)
Normal
Diabetic
Normal data
2.4
2.2
2
1.8
1.6
1.4
1.2
1
0.8
0
10
20
30
40
50
60

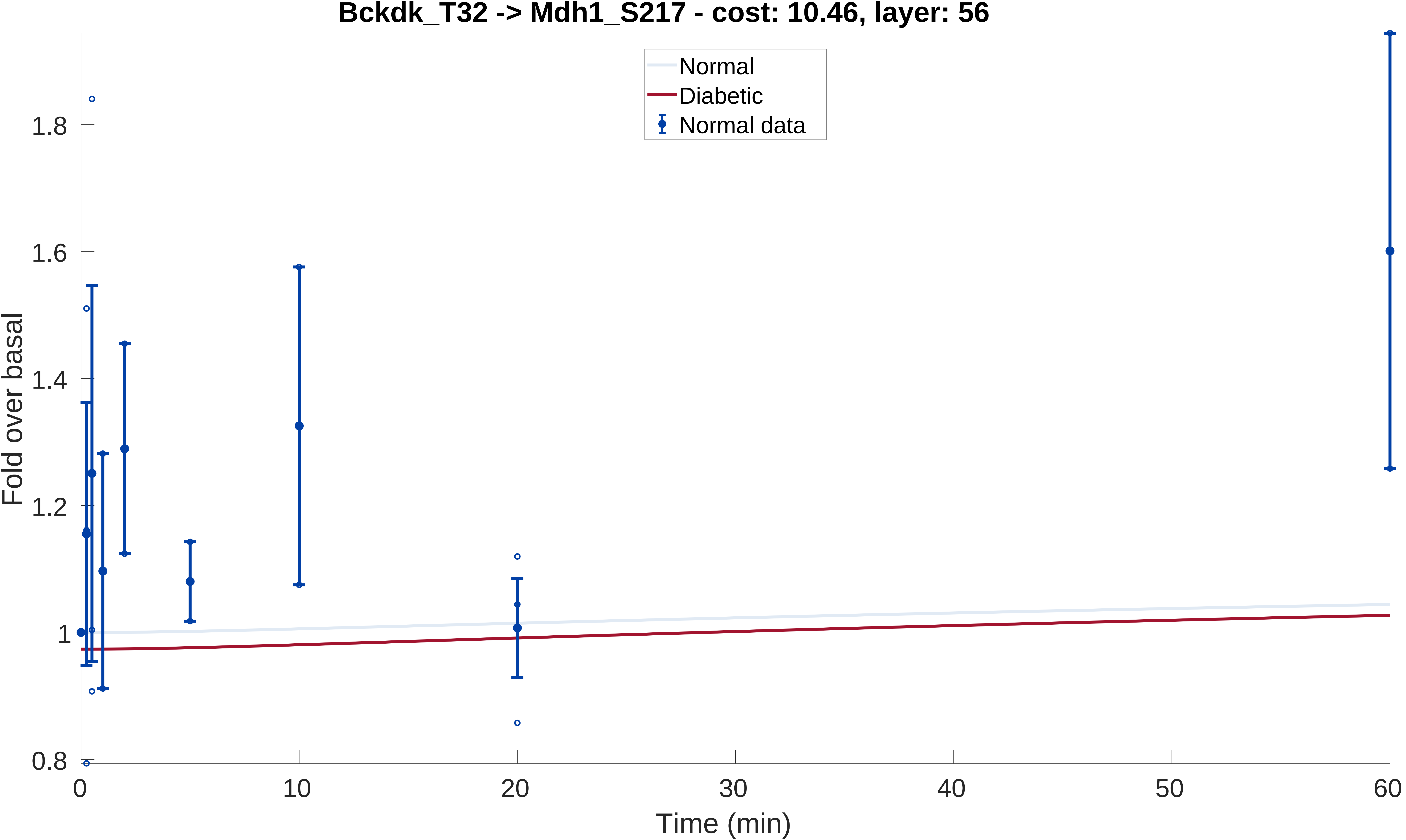

Bckdk_T32 -> Mdh1_S217 - cost: 10.46, layer: 56
Normal
Diabetic
Normal data
Fold over basal
1.8
1.6
1.4
1.2
1
0.8
0
10
20
30
40
50
60
Time (min)

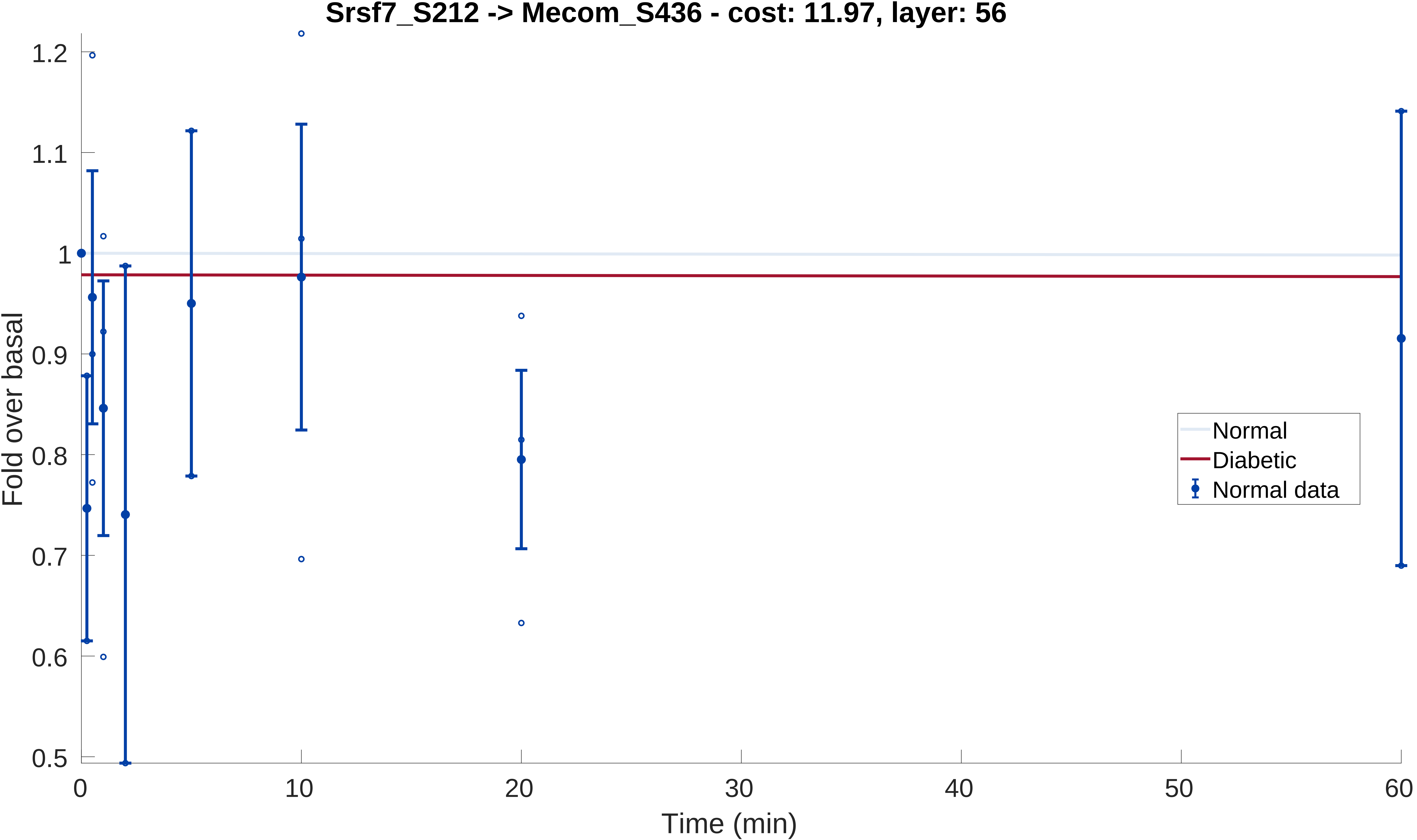

Srsf7_S212 -> Mecom_S436 - cost: 11.97, layer: 56
Fold over basal
1.2
1.1
1
0.9
0.8
0.7
0.6
0.5
0
10
20
30
40
50
60
Time (min)
Normal
Diabetic
Normal data

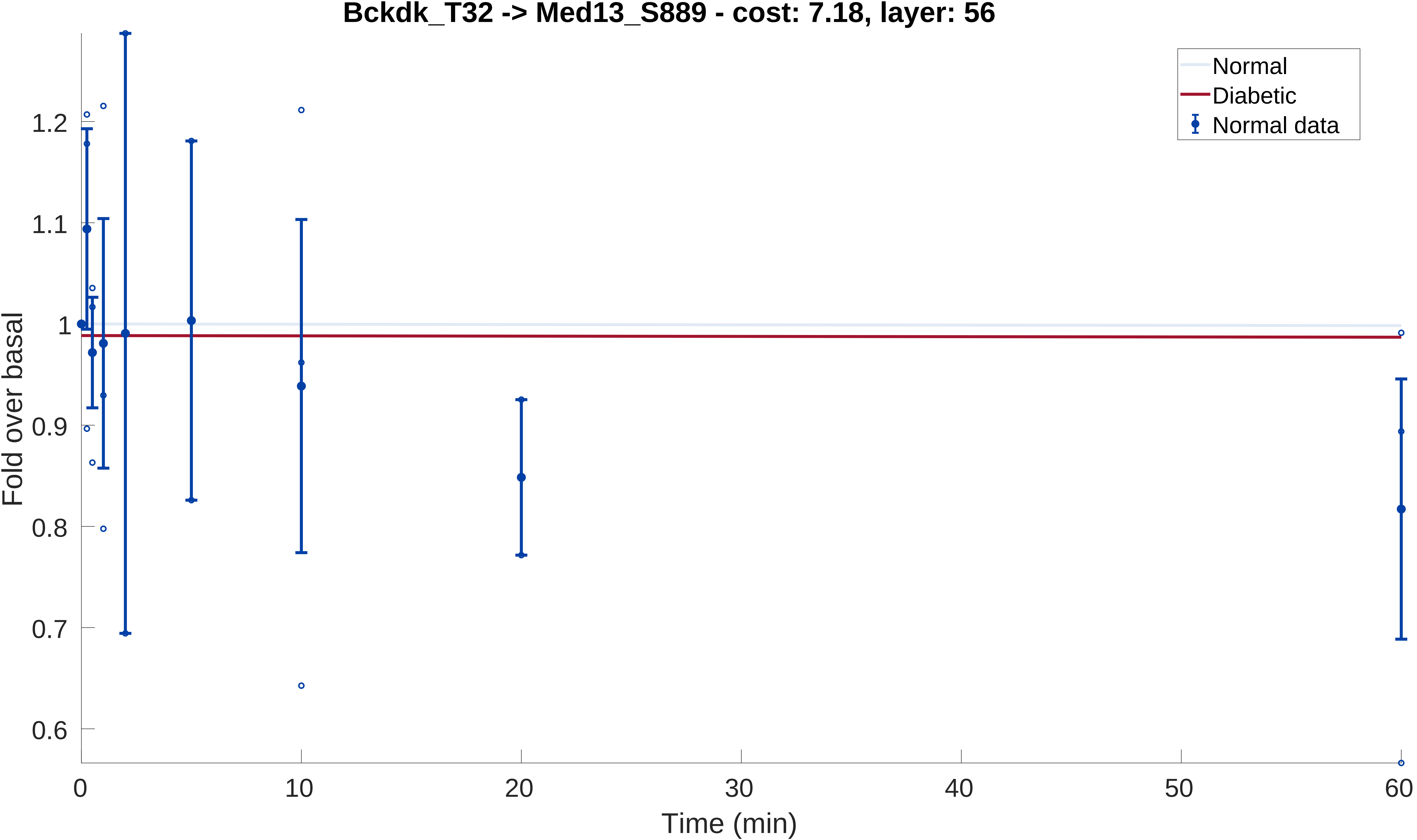

Bckdk_T32 -> Med13_S889 - cost: 7.18, layer: 56
Normal
Diabetic
Normal data
Fold over basal
1.2
1.1
1
0.9
0.8
0.7
0.6
0
10
20
30
40
50
60
Time (min)

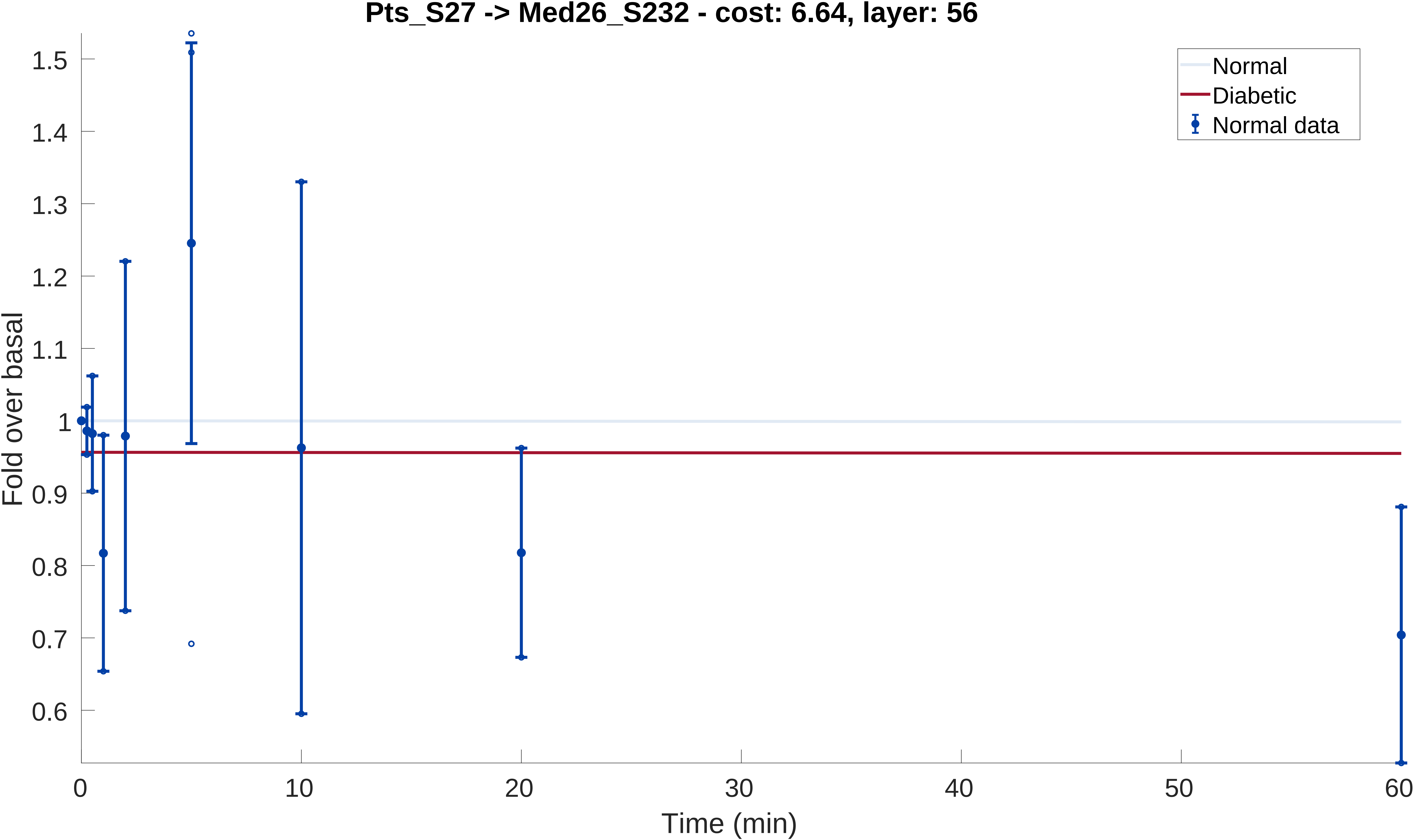
Pts_S27 -> Med26_S232 - cost: 6.64, layer: 56
Normal
Diabetic
Normal data
Fold over basal
1.5
1.4
1.3
1.2
1.1
1
0.9
0.8
0.7
0.6
0
10
20
30
40
50
60
Time (min)

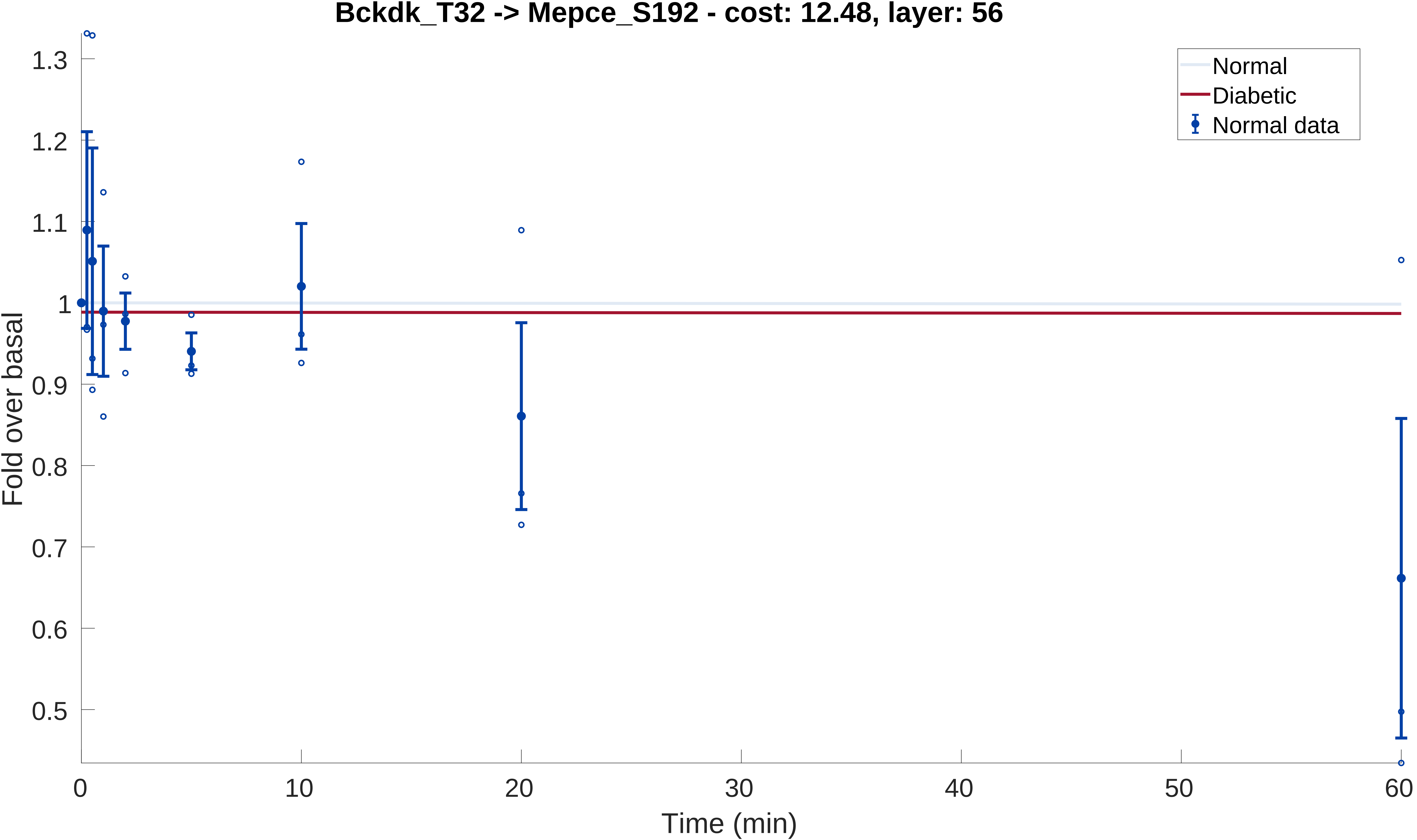
Bckdk_T32 -> Mepce_S192 - cost: 12.48, layer: 56
Normal
Diabetic
Normal data
Fold over basal
1.3
1.2
1.1
1
0.9
0.8
0.7
0.6
0.5
0
10
20
30
40
50
60
Time (min)

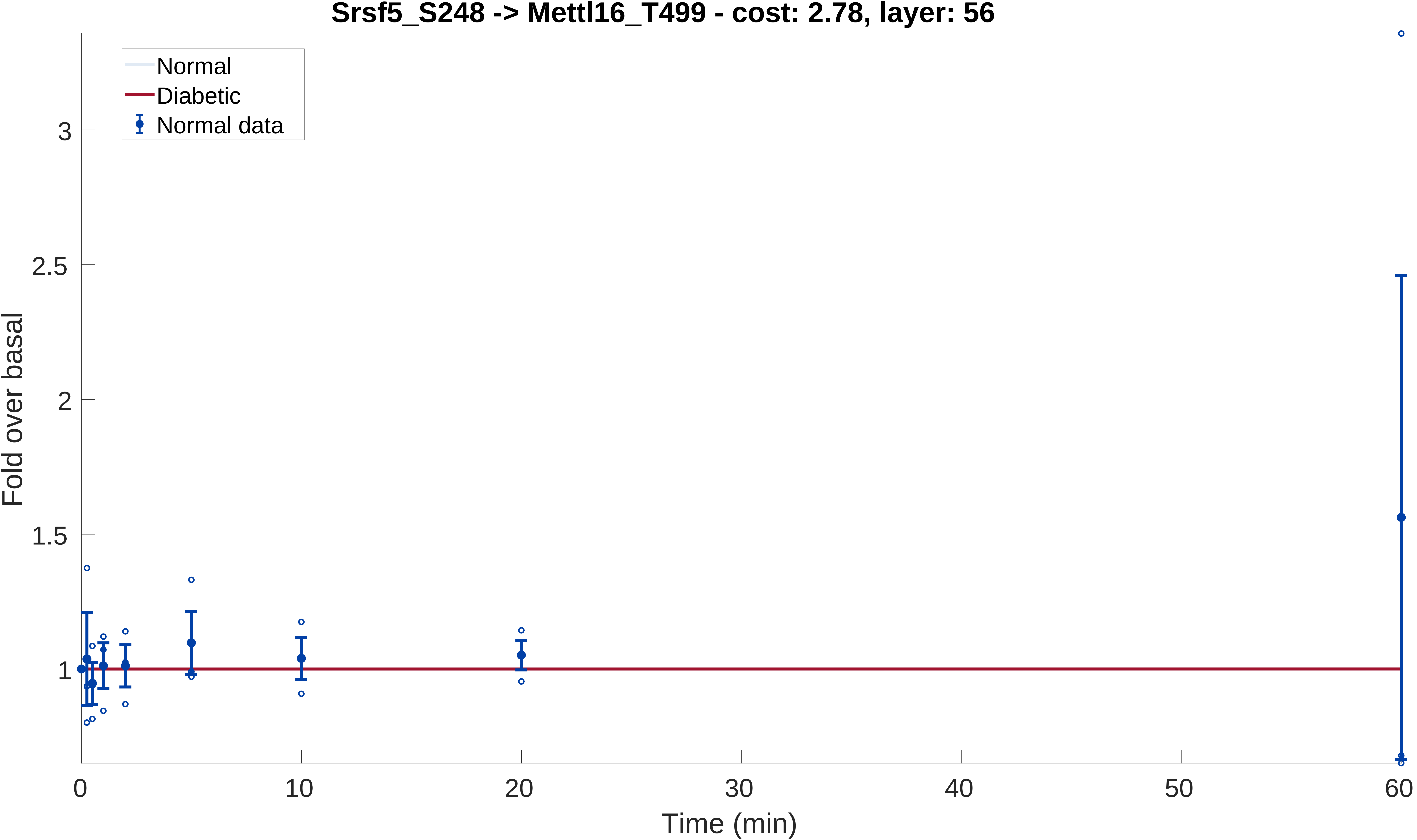

Srsf5_S248 -> Mettl16_T499 - cost: 2.78, layer: 56
Normal
Diabetic
Normal data
Fold over basal
Time (min)
0
10
20
30
40
50
60
1
1.5
2
2.5
3

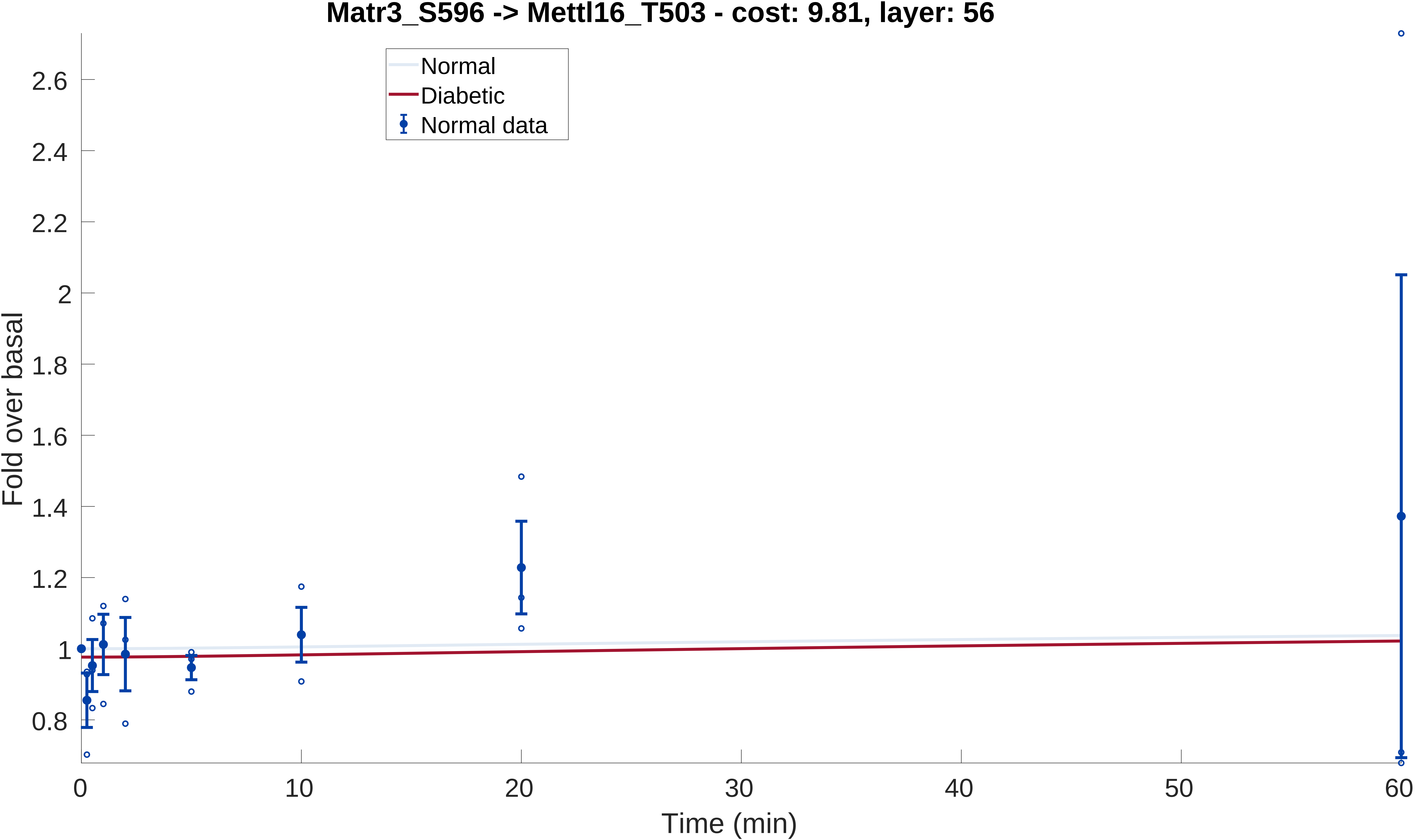

Matr3_S596 -> Mettl16_T503 - cost: 9.81, layer: 56
Normal
Diabetic
Normal data
Fold over basal
2.6
2.4
2.2
2
1.8
1.6
1.4
1.2
1
0.8
0
10
20
30
40
50
60
Time (min)

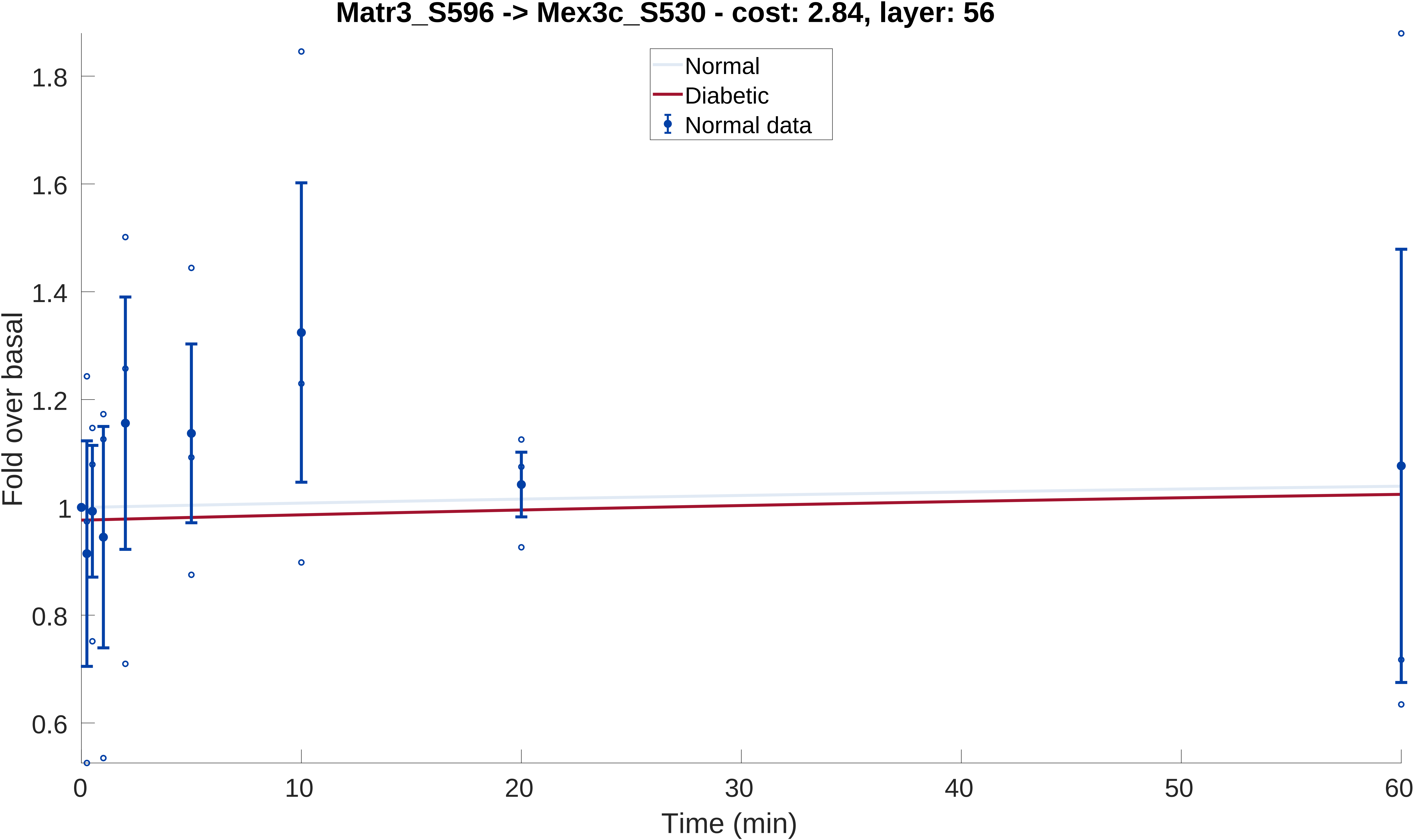
Matr3_S596 -> Mex3c_S530 - cost: 2.84, layer: 56
Normal
Diabetic
Normal data
Fold over basal
Time (min)
1.8
1.6
1.4
1.2
1
0.8
0.6
0
10
20
30
40
50
60

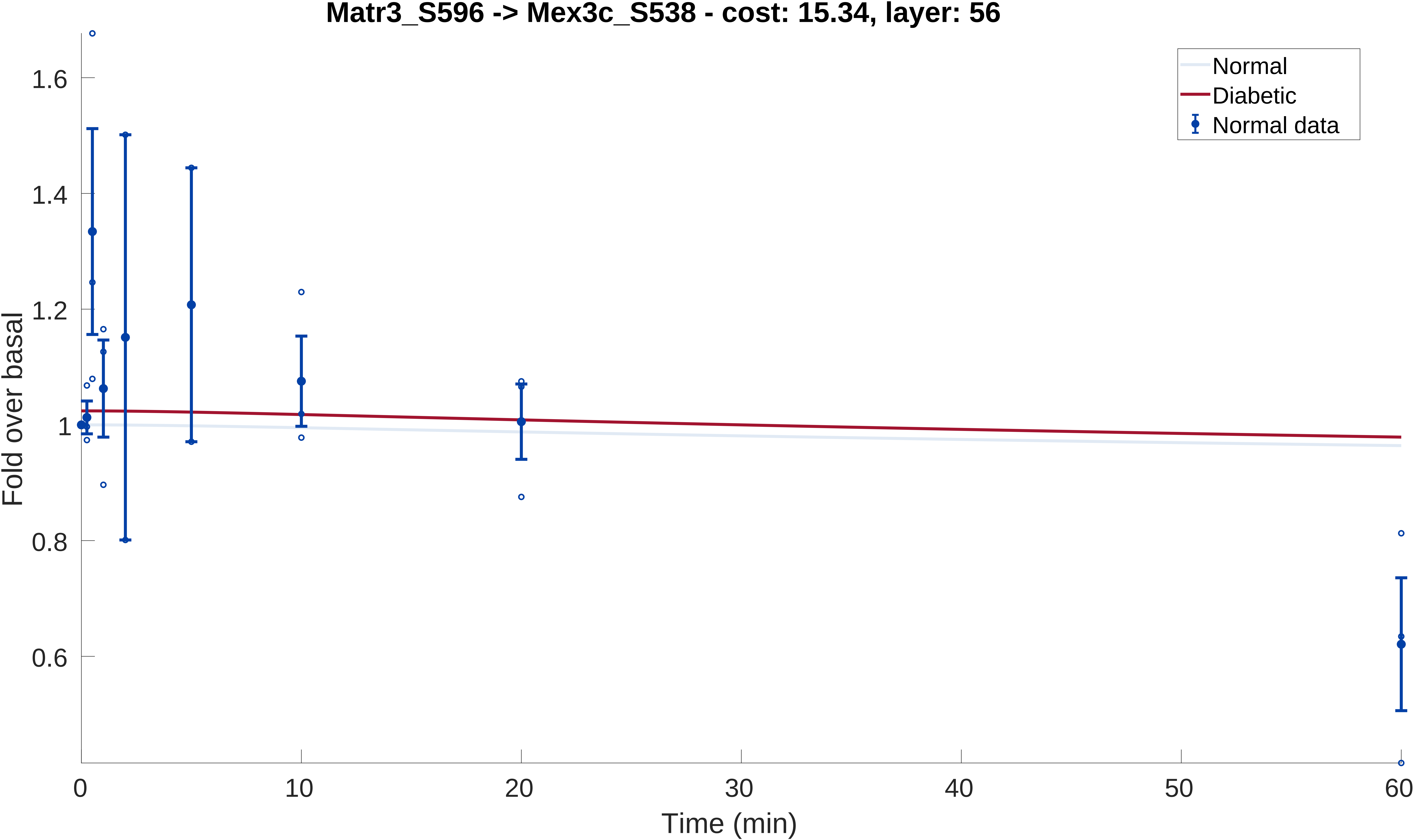

Matr3_S596 -> Mex3c_S538 - cost: 15.34, layer: 56
Normal
Diabetic
Normal data
Fold over basal
Time (min)
1.6
1.4
1.2
1
0.8
0.6
0
10
20
30
40
50
60

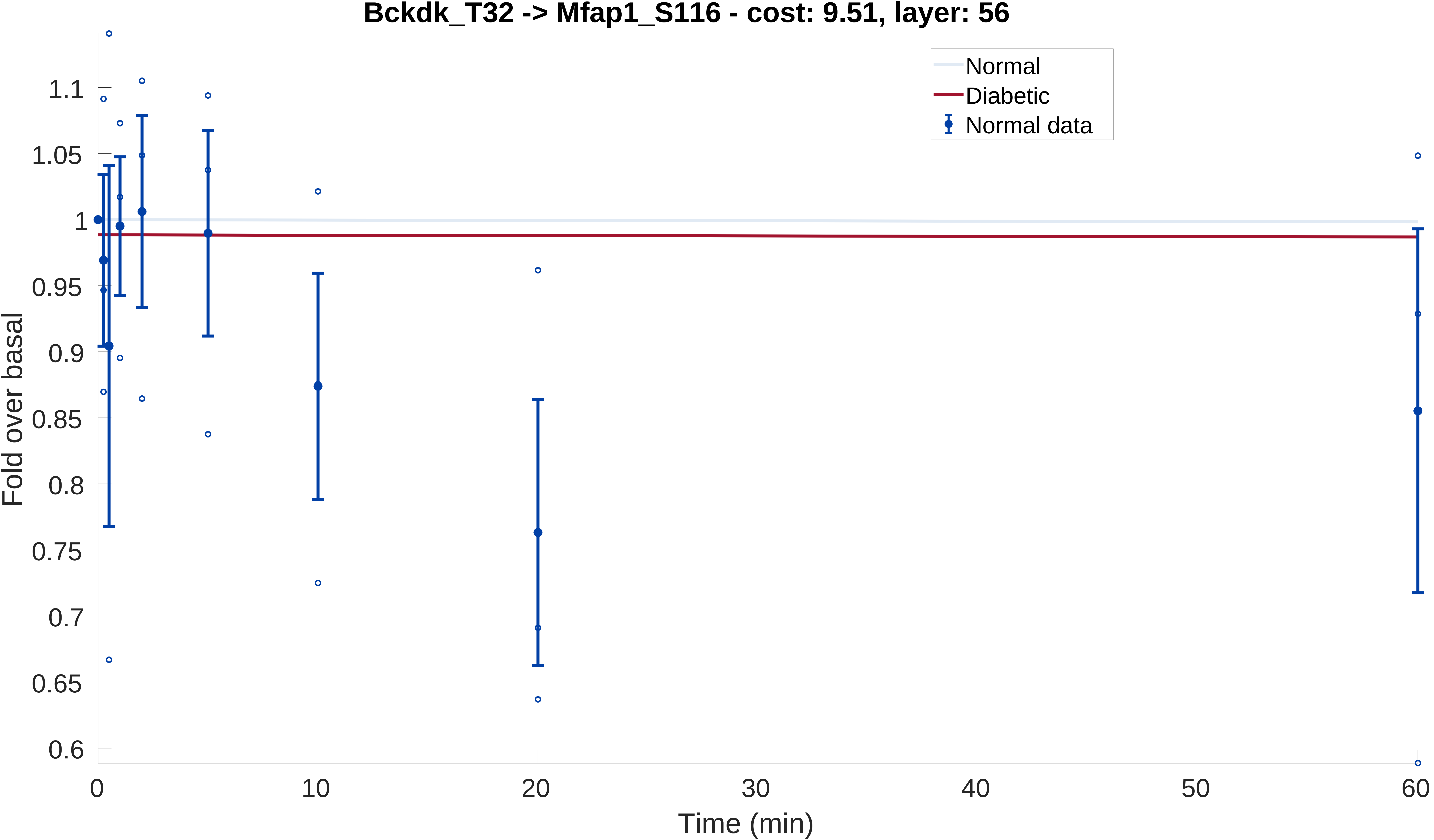
Bckdk_T32 -> Mfap1_S116 - cost: 9.51, layer: 56
Normal
Diabetic
Normal data
Fold over basal
Time (min)
0
10
20
30
40
50
60
0.6
0.65
0.7
0.75
0.8
0.85
0.9
0.95
1
1.05
1.1

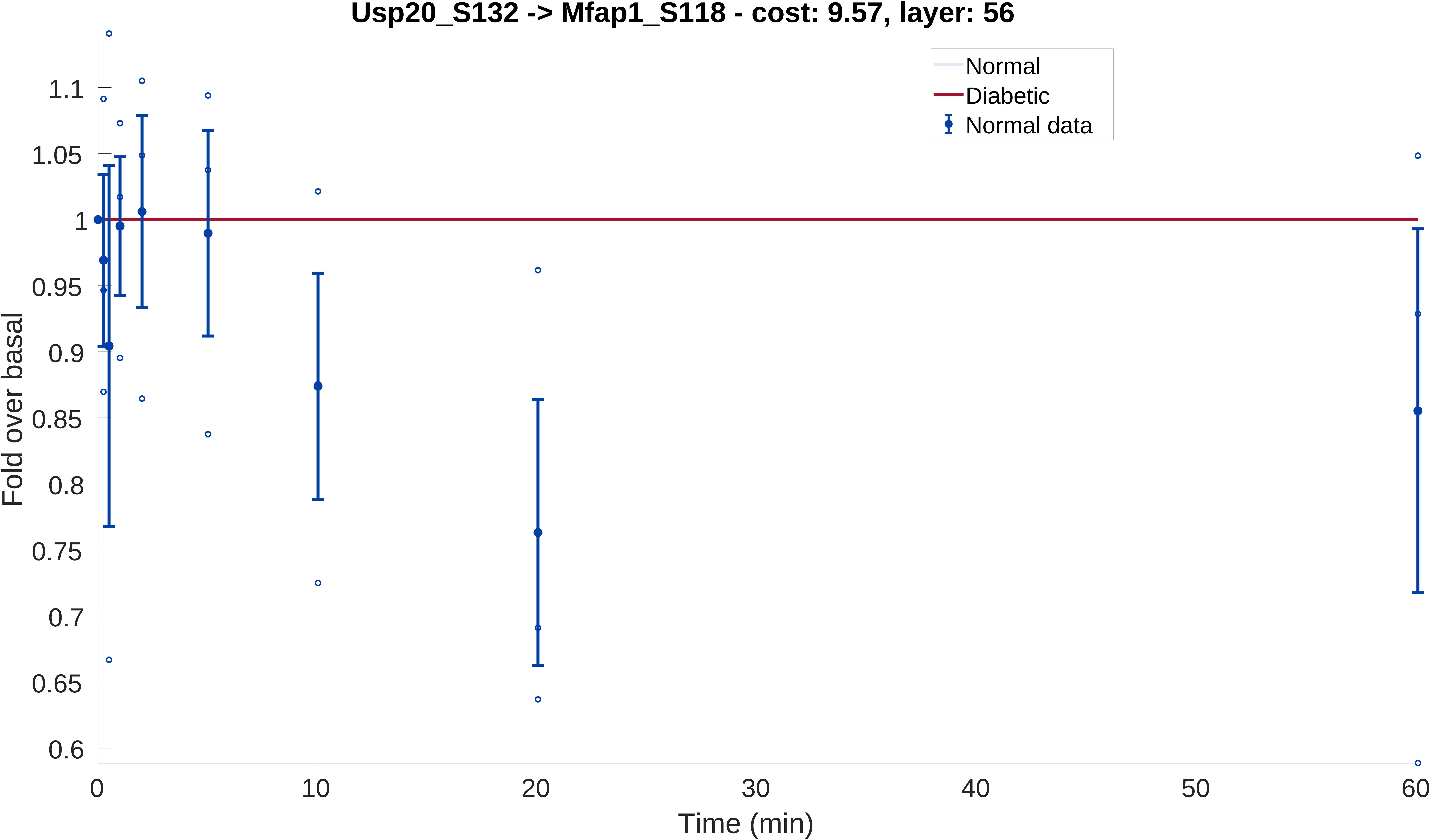

Usp20_S132 -> Mfap1_S118 - cost: 9.57, layer: 56
Normal
Diabetic
Normal data
Fold over basal
1.1
1.05
1
0.95
0.9
0.85
0.8
0.75
0.7
0.65
0.6
0
10
20
30
40
50
60
Time (min)

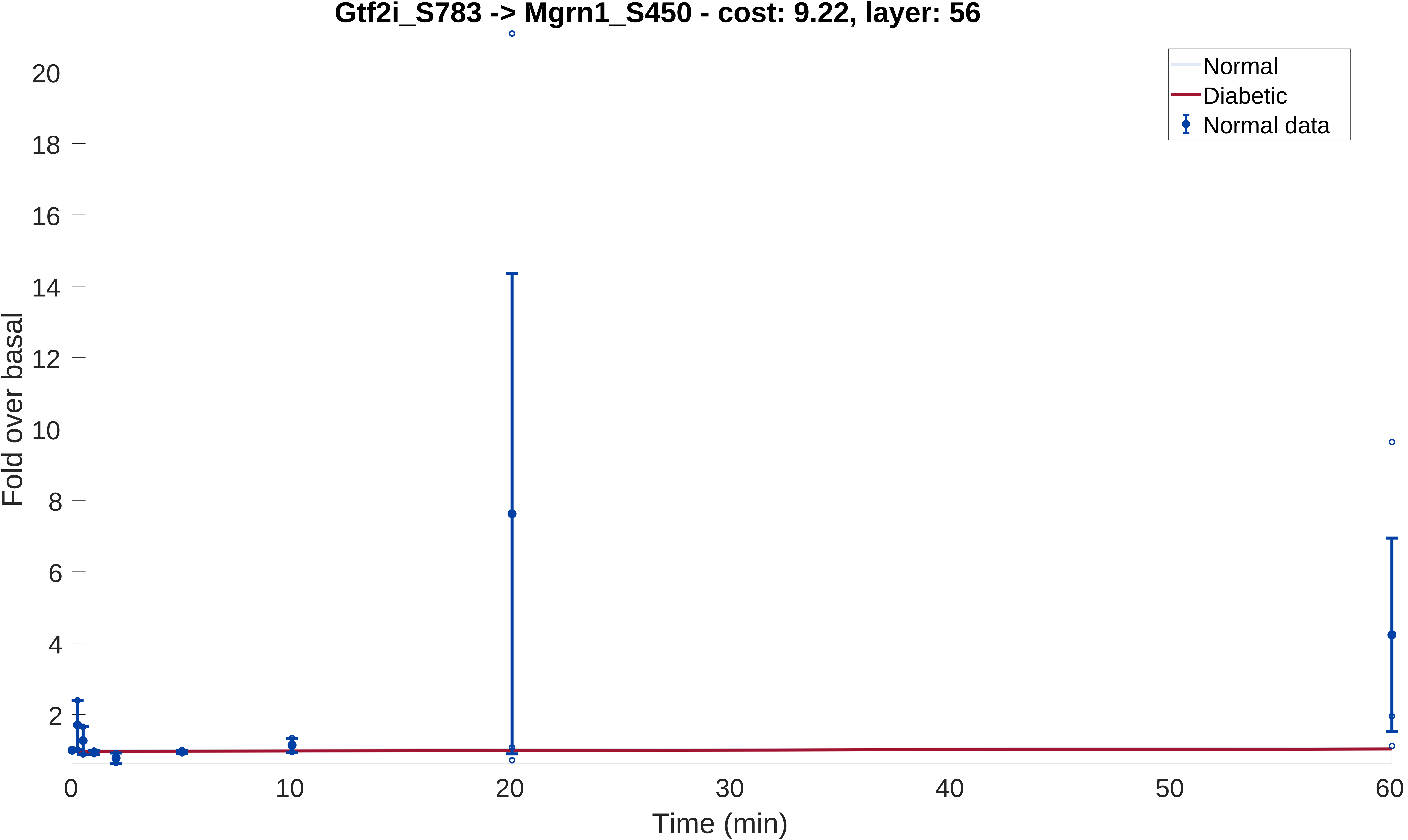

Gtf2i_S783 -> Mgrn1_S450 - cost: 9.22, layer: 56
Normal
Diabetic
Normal data
Fold over basal
Time (min)
0
10
20
30
40
50
60
2
4
6
8
10
12
14
16
18
20

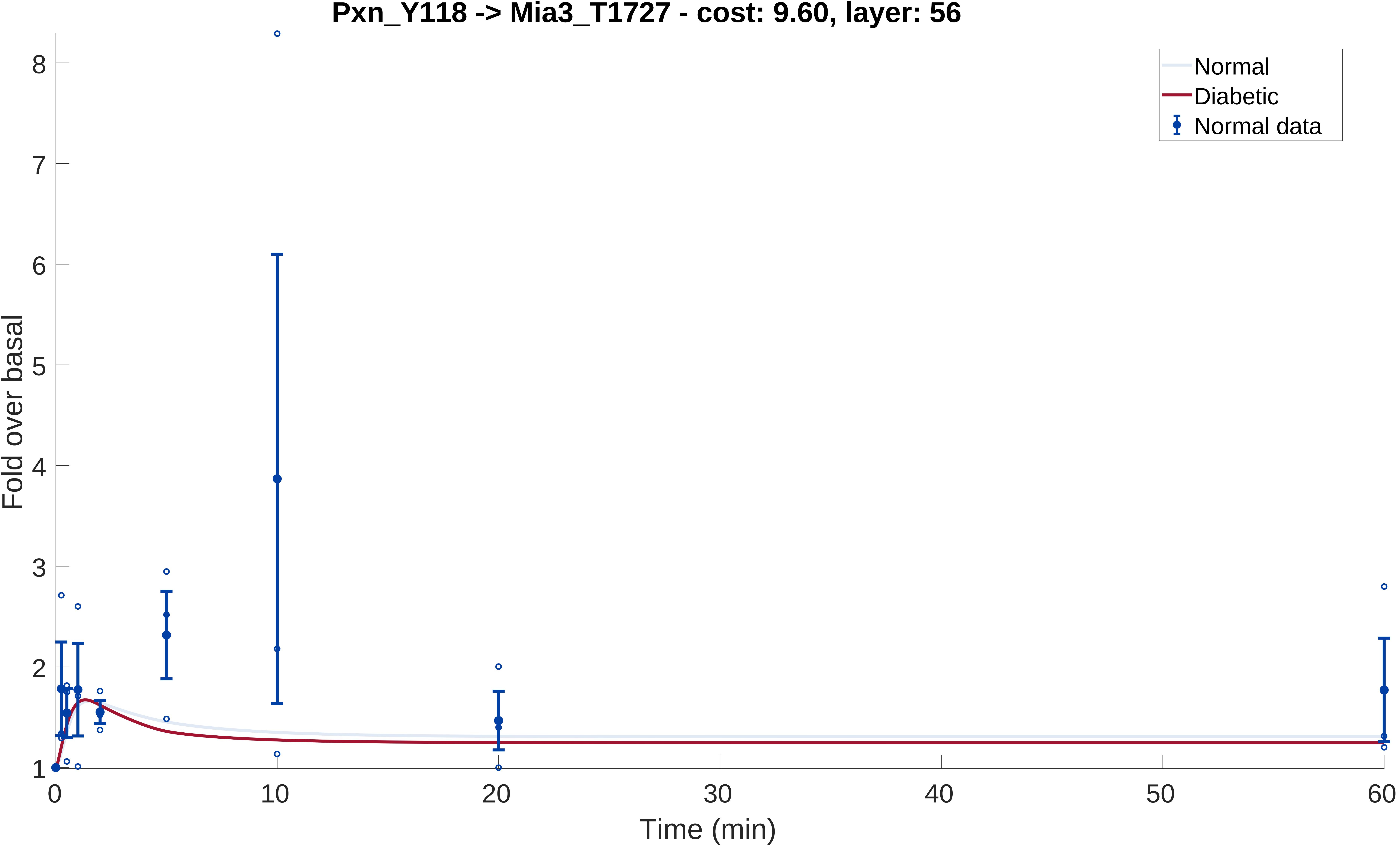

Pxn_Y118 -> Mia3_T1727 - cost: 9.60, layer: 56
Normal
Diabetic
Normal data
Fold over basal
Time (min)
1
2
3
4
5
6
7
8
0
10
20
30
40
50
60

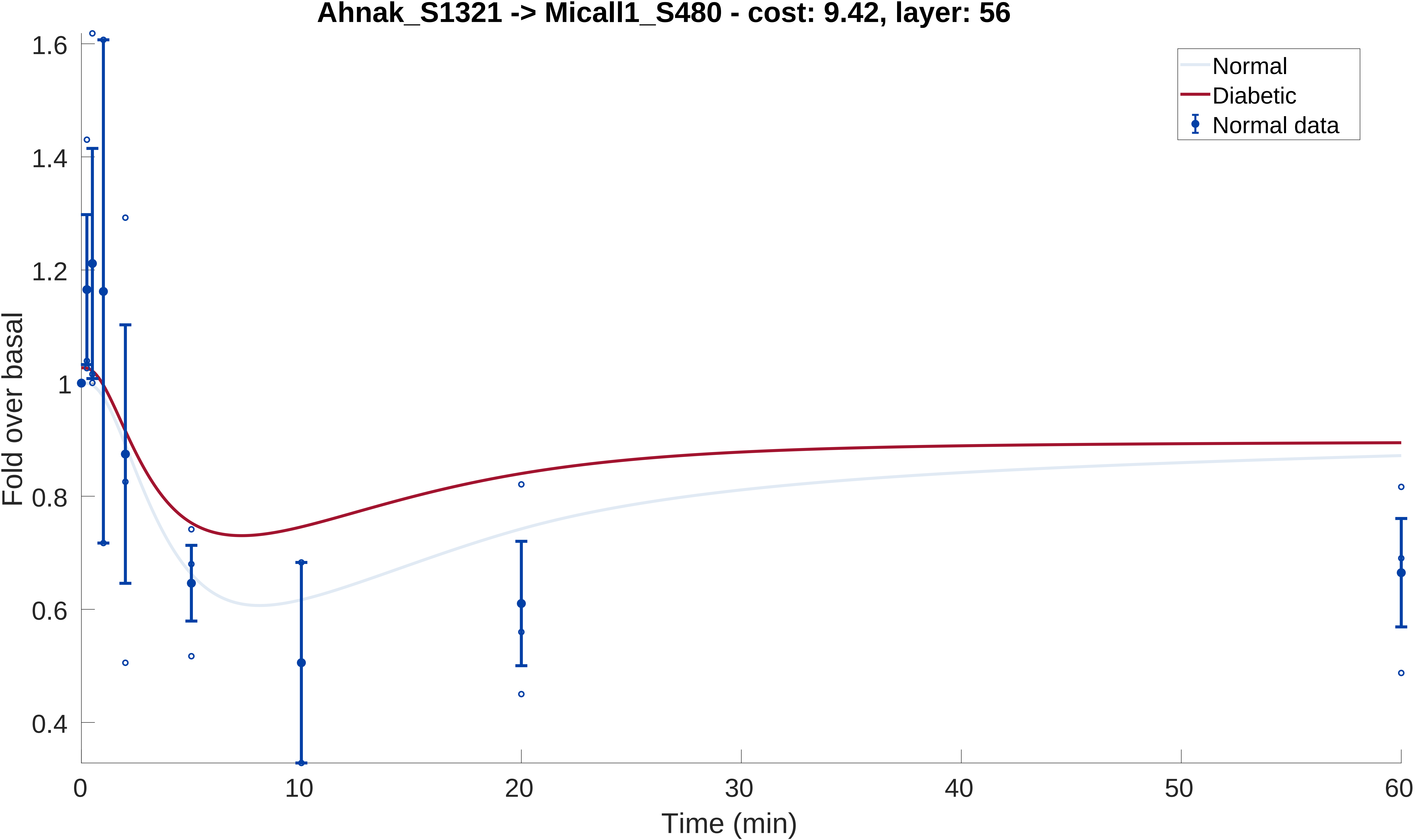

Ahnak_S1321 -> Micall1_S480 - cost: 9.42, layer: 56
Normal
Diabetic
Normal data
Fold over basal
Time (min)
1.6
1.4
1.2
1
0.8
0.6
0.4
0
10
20
30
40
50
60

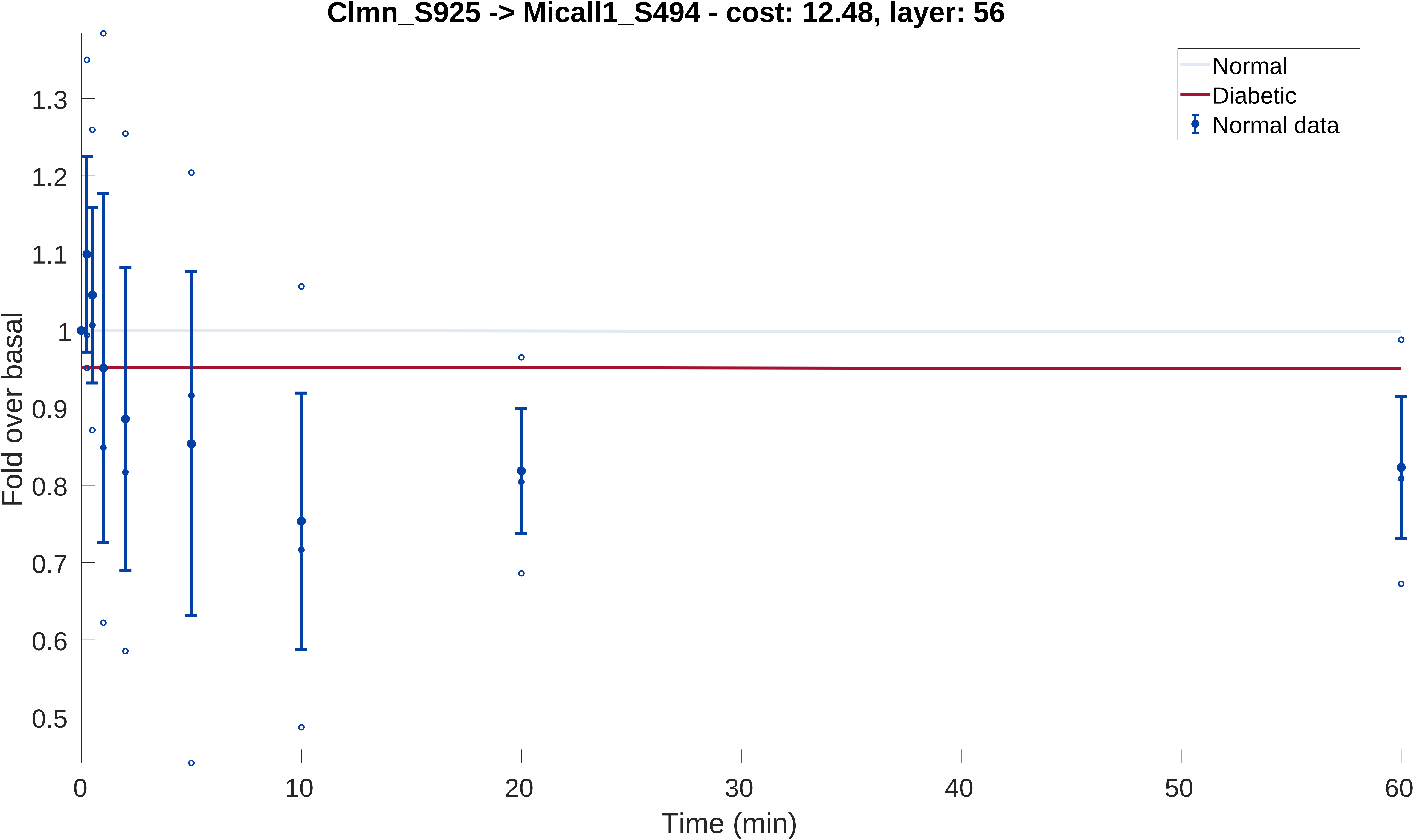
Clmn_S925 -> Micall1_S494 - cost: 12.48, layer: 56
Normal
Diabetic
Normal data
Fold over basal
Time (min)
1.3
1.2
1.1
1
0.9
0.8
0.7
0.6
0.5
0
10
20
30
40
50
60

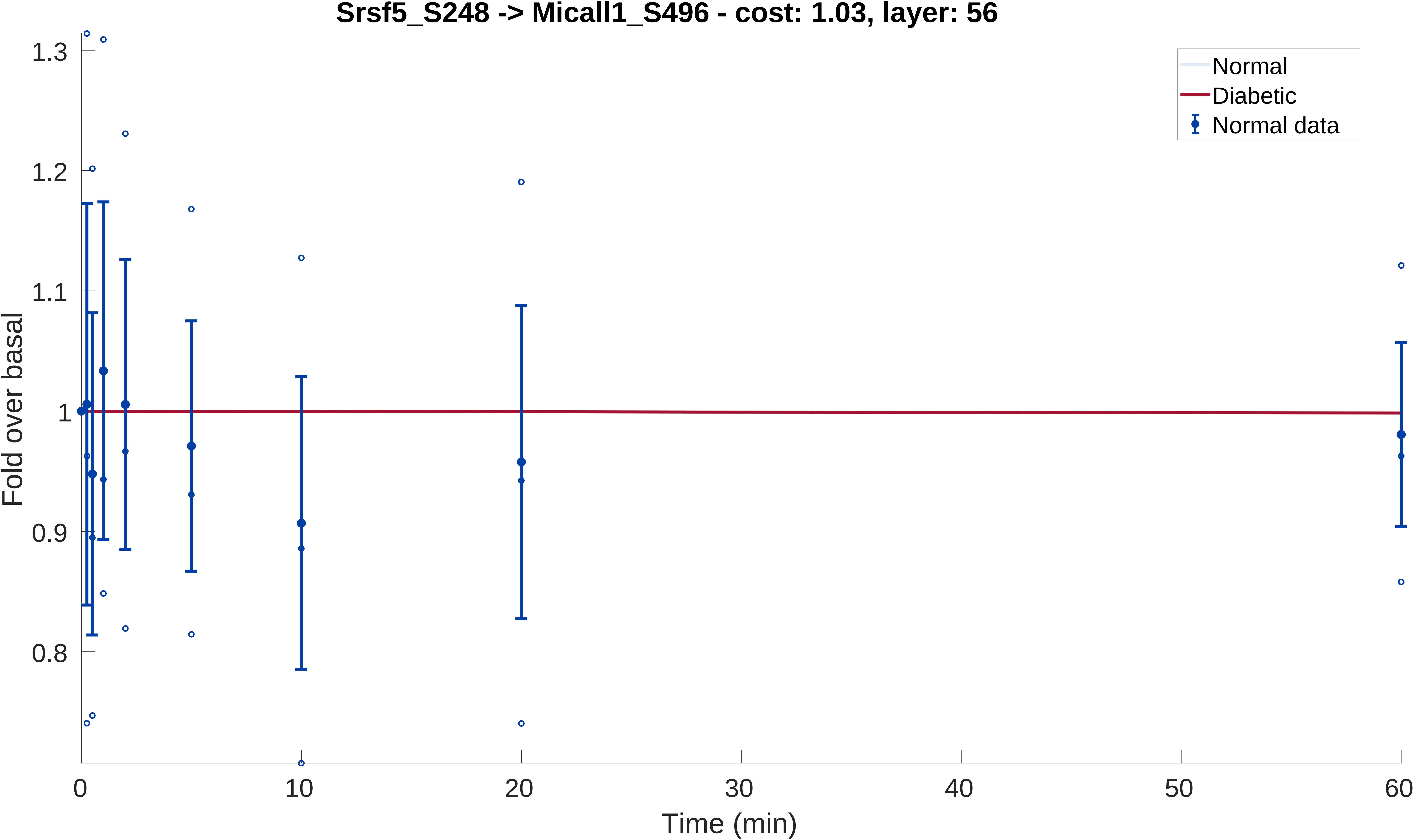

Srsf5_S248 -> Micall1_S496 - cost: 1.03, layer: 56
Normal
Diabetic
Normal data
Fold over basal
1.3
1.2
1.1
1
0.9
0.8
0
10
20
30
40
50
60
Time (min)

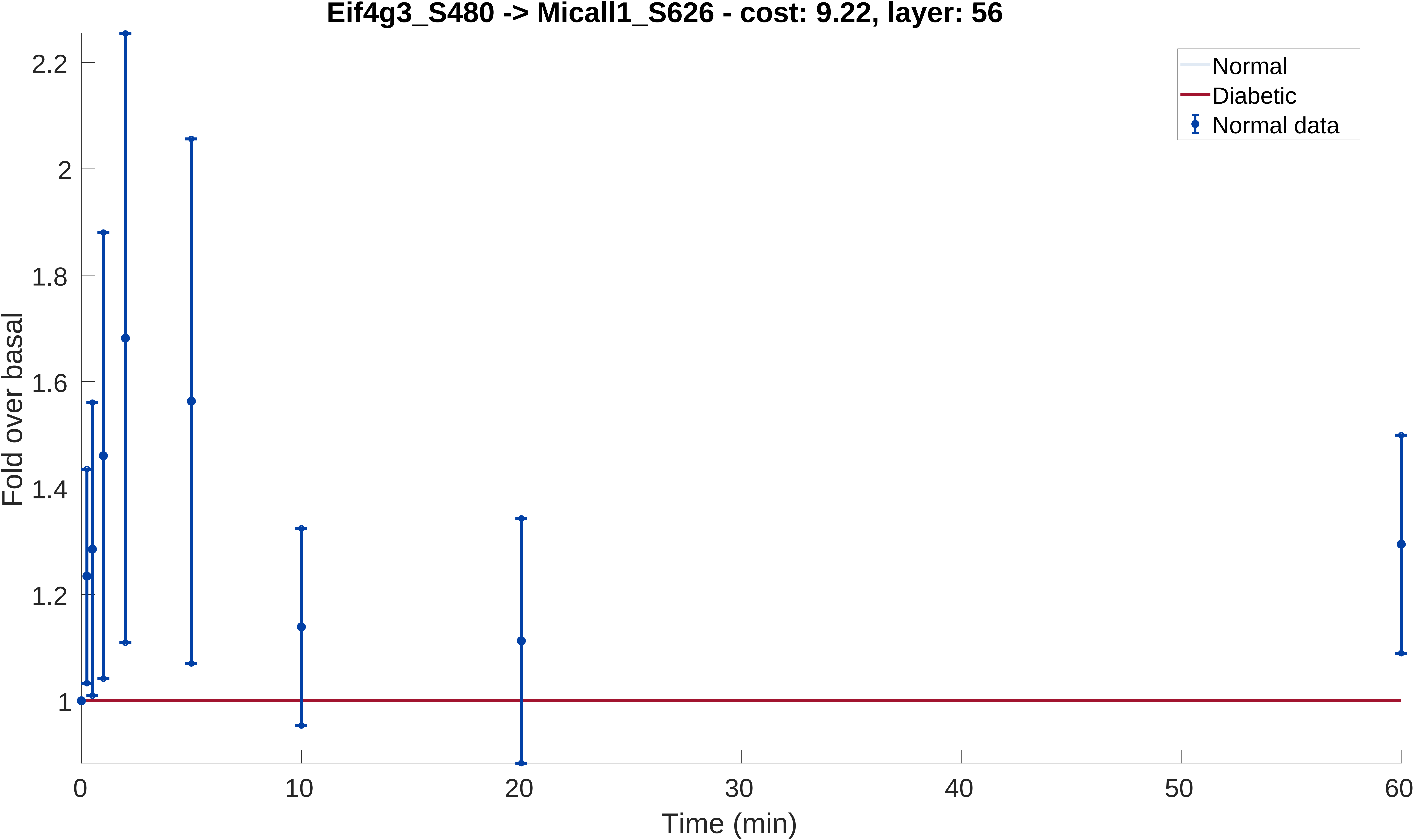

Eif4g3_S480 -> Micall1_S626 - cost: 9.22, layer: 56
Normal
Diabetic
Normal data
Fold over basal
2.2
2
1.8
1.6
1.4
1.2
1
0
10
20
30
40
50
60
Time (min)

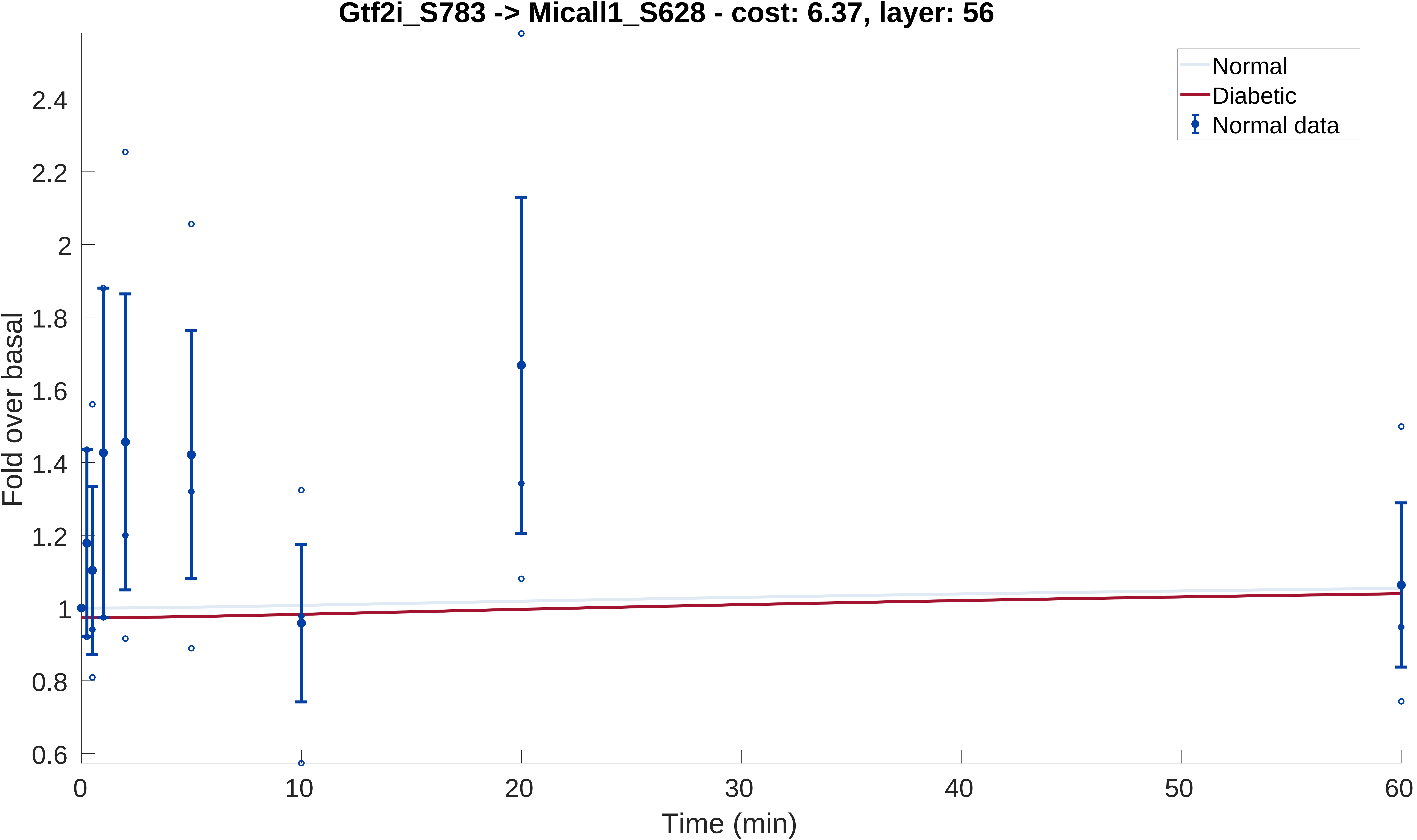

Gtf2i_S783 -> Micall1_S628 - cost: 6.37, layer: 56
Normal
Diabetic
Normal data
Fold over basal
Time (min)
0.6
0.8
1
1.2
1.4
1.6
1.8
2
2.2
2.4
0
10
20
30
40
50
60

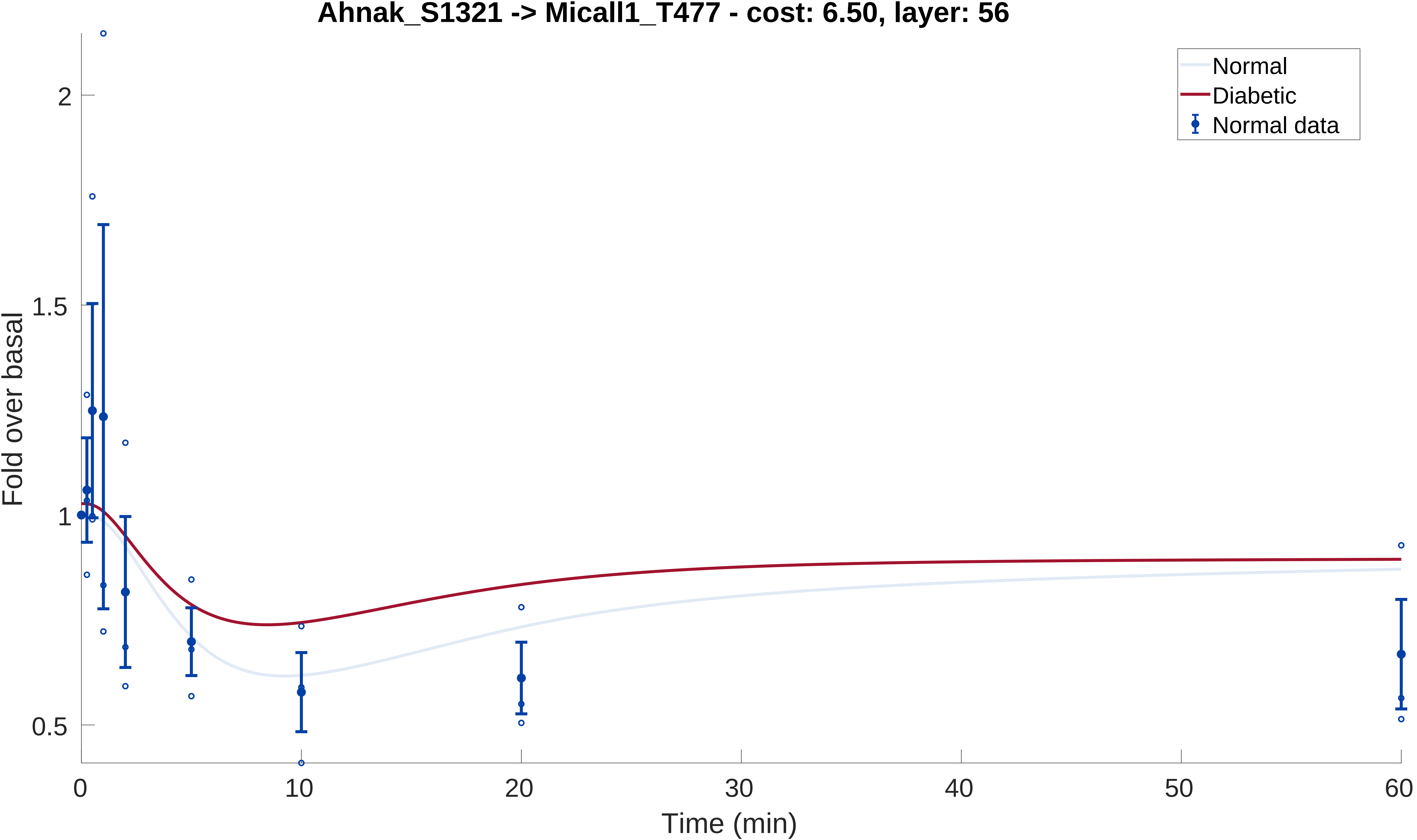

Ahnak_S1321 -> Micall1_T477 - cost: 6.50, layer: 56
Normal
Diabetic
Normal data
Fold over basal
2
1.5
1
0.5
0
10
20
30
40
50
60
Time (min)

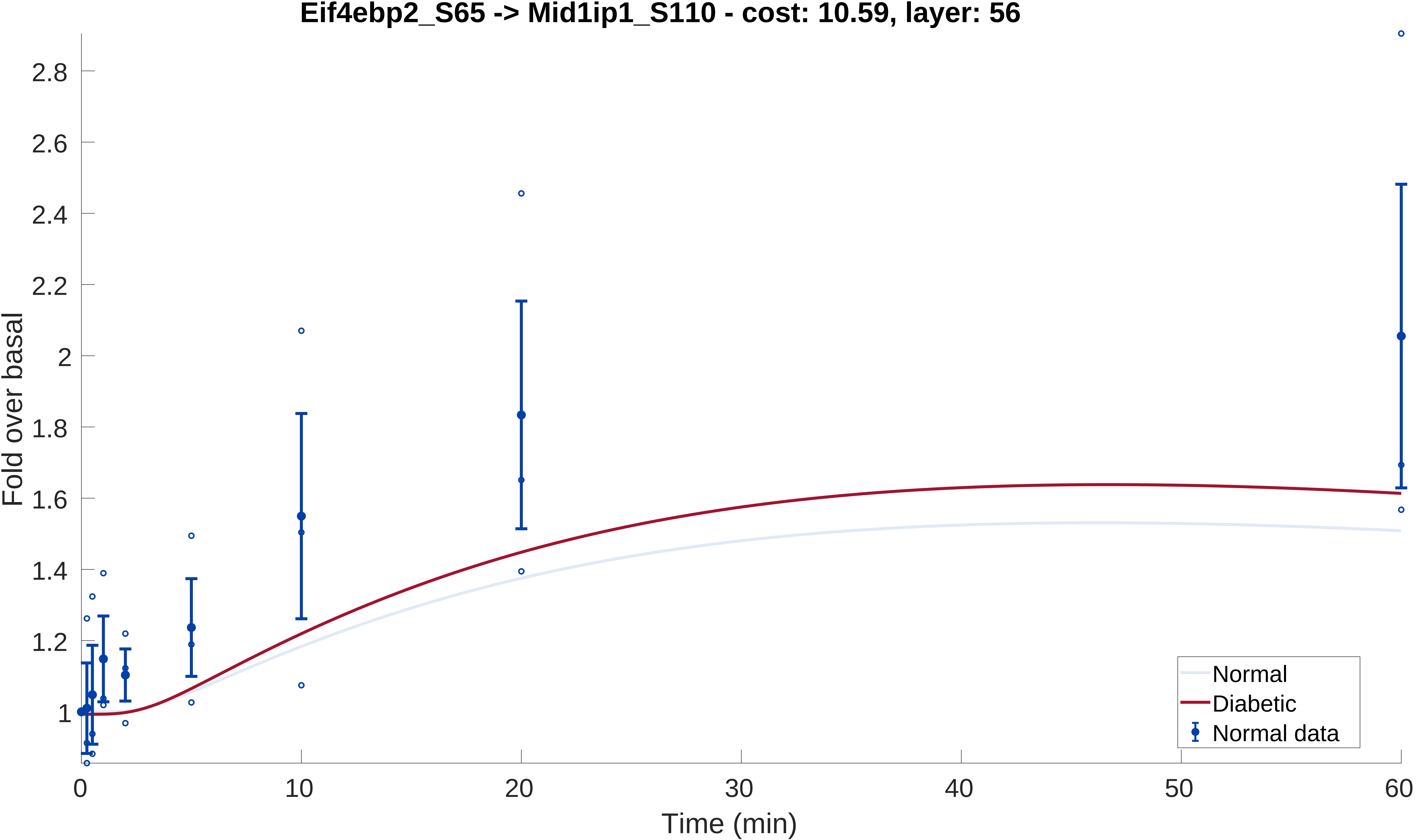

Eif4ebp2_S65 -> Mid1ip1_S110 - cost: 10.59, layer: 56
Fold over basal
2.8
2.6
2.4
2.2
2
1.8
1.6
1.4
1.2
1
0
10
20
30
40
50
60
Time (min)
Normal
Diabetic
Normal data

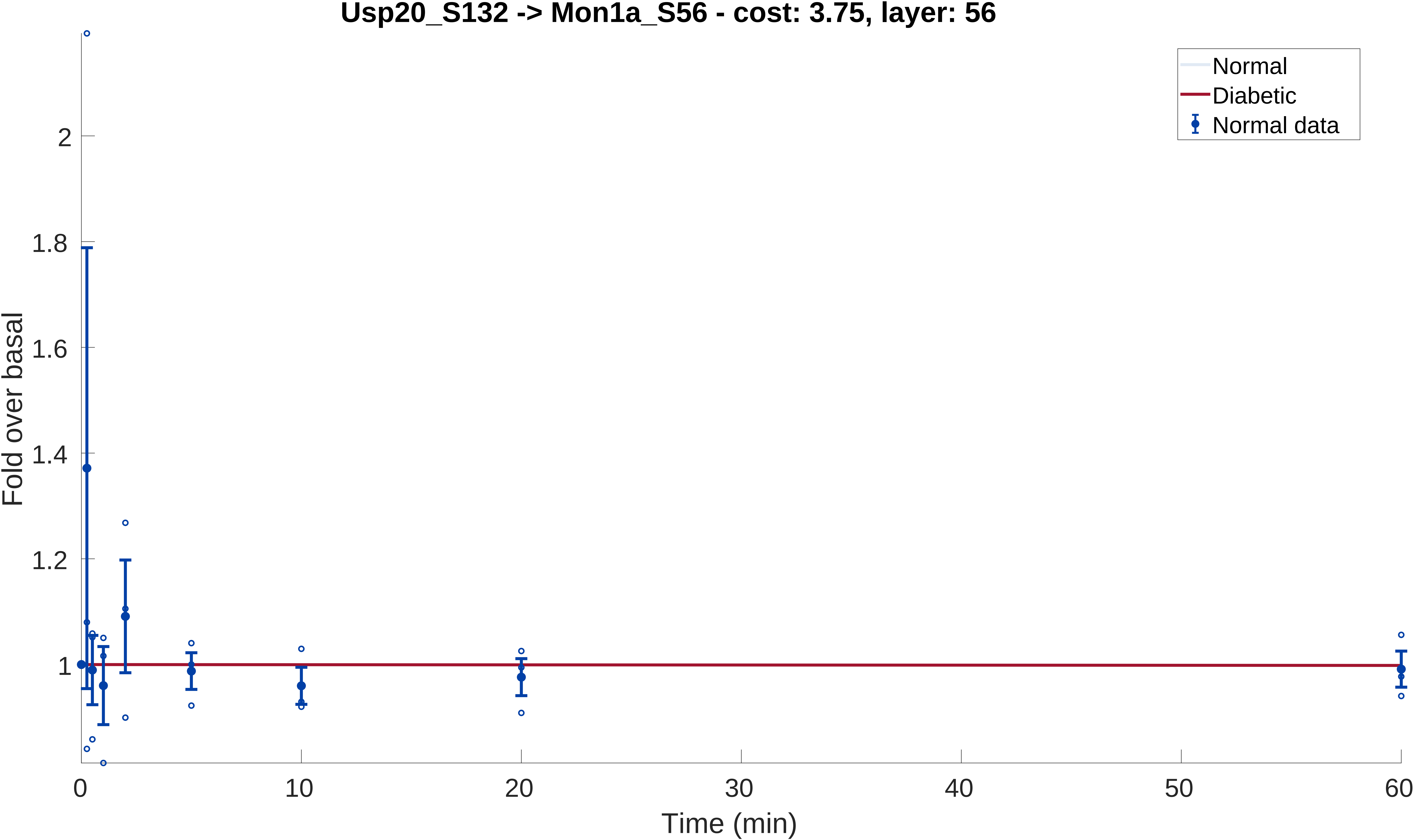
Usp20_S132 -> Mon1a_S56 - cost: 3.75, layer: 56
Normal
Diabetic
Normal data
Fold over basal
Time (min)
2
1.8
1.6
1.4
1.2
1
0
10
20
30
40
50
60

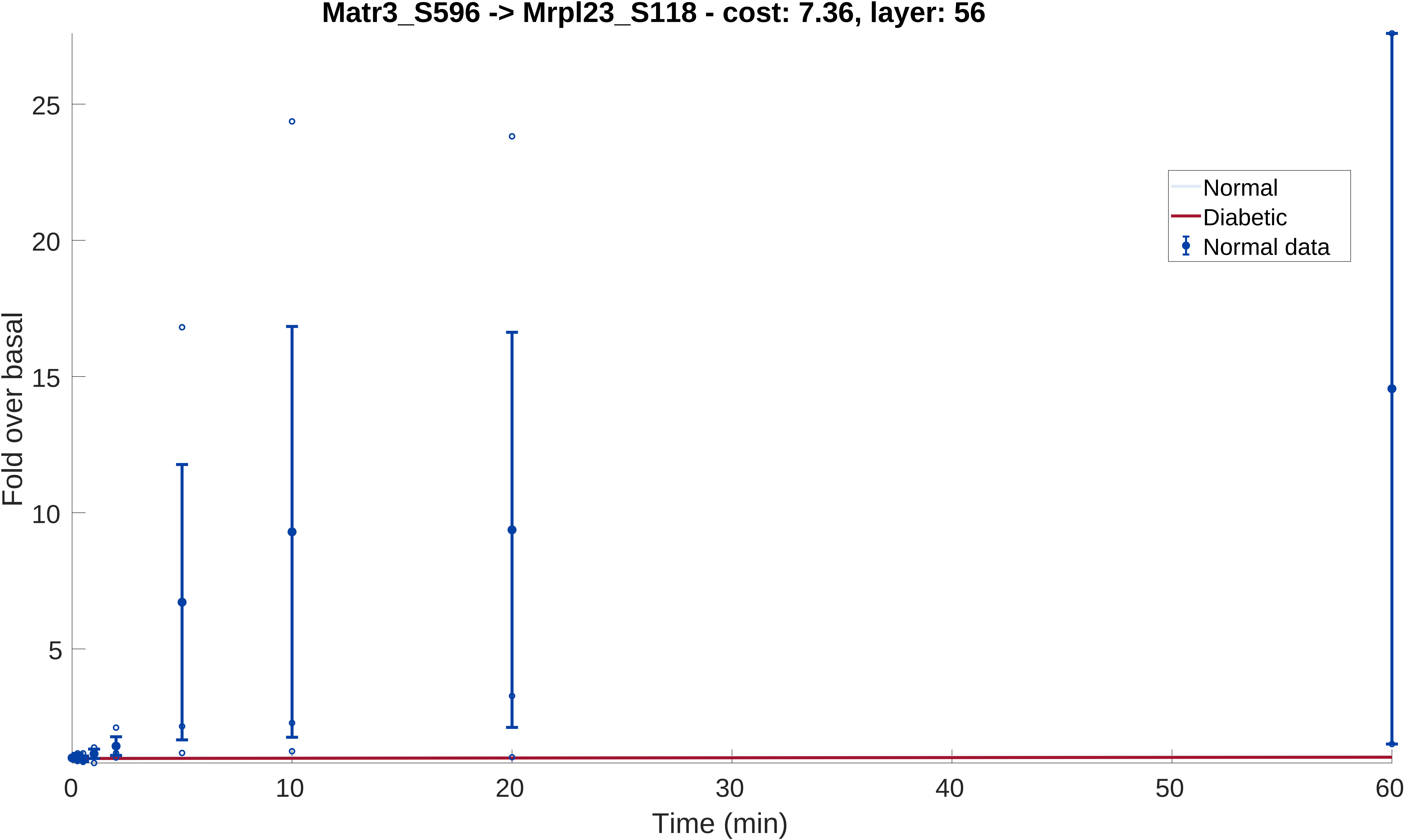

Matr3_S596 -> Mrpl23_S118 - cost: 7.36, layer: 56
Fold over basal
Time (min)
0
10
20
30
40
50
60
5
10
15
20
25
Normal
Diabetic
Normal data

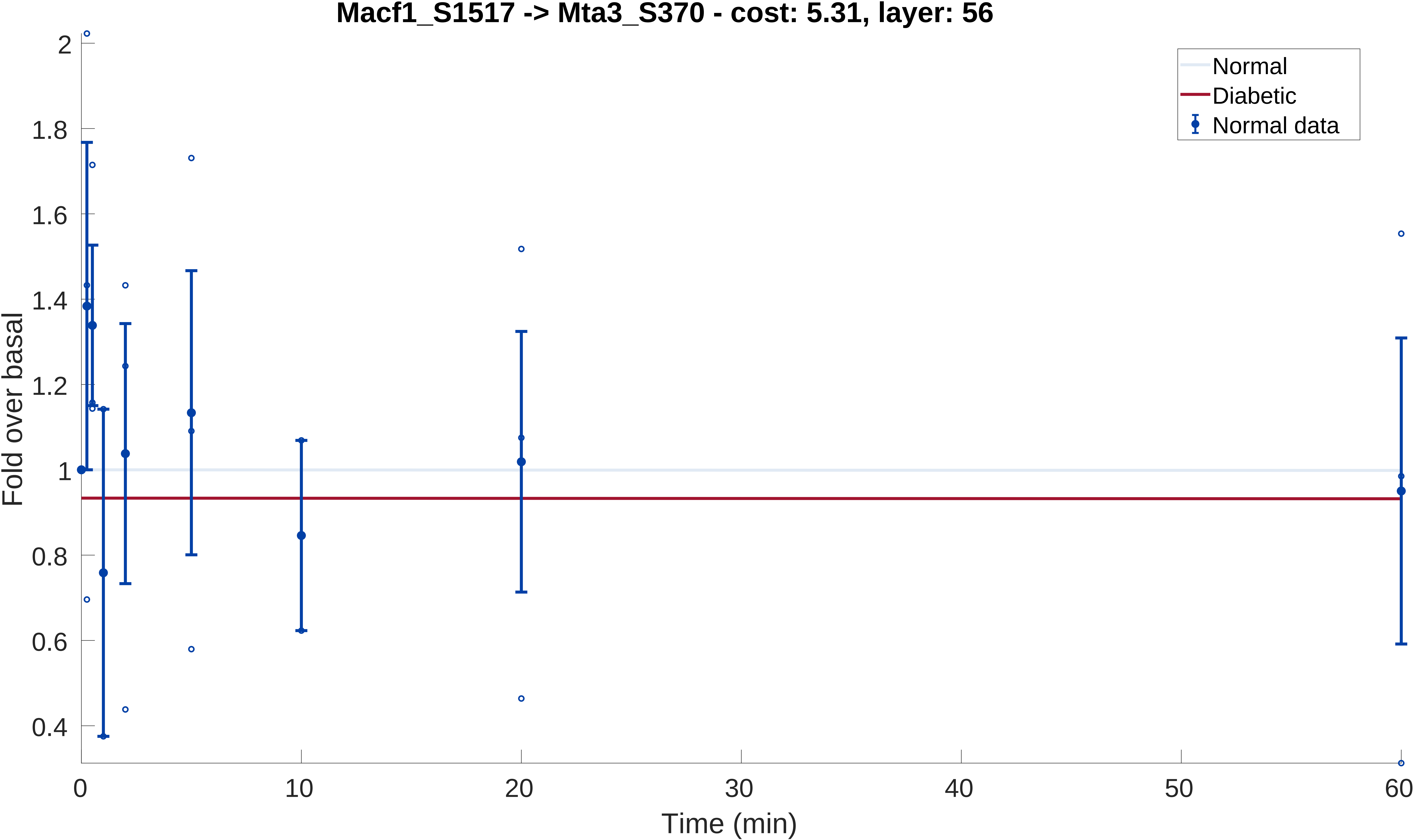

Macf1_S1517 -> Mta3_S370 - cost: 5.31, layer: 56
Normal
Diabetic
Normal data
Fold over basal
Time (min)
0.4
0.6
0.8
1
1.2
1.4
1.6
1.8
2
0
10
20
30
40
50
60

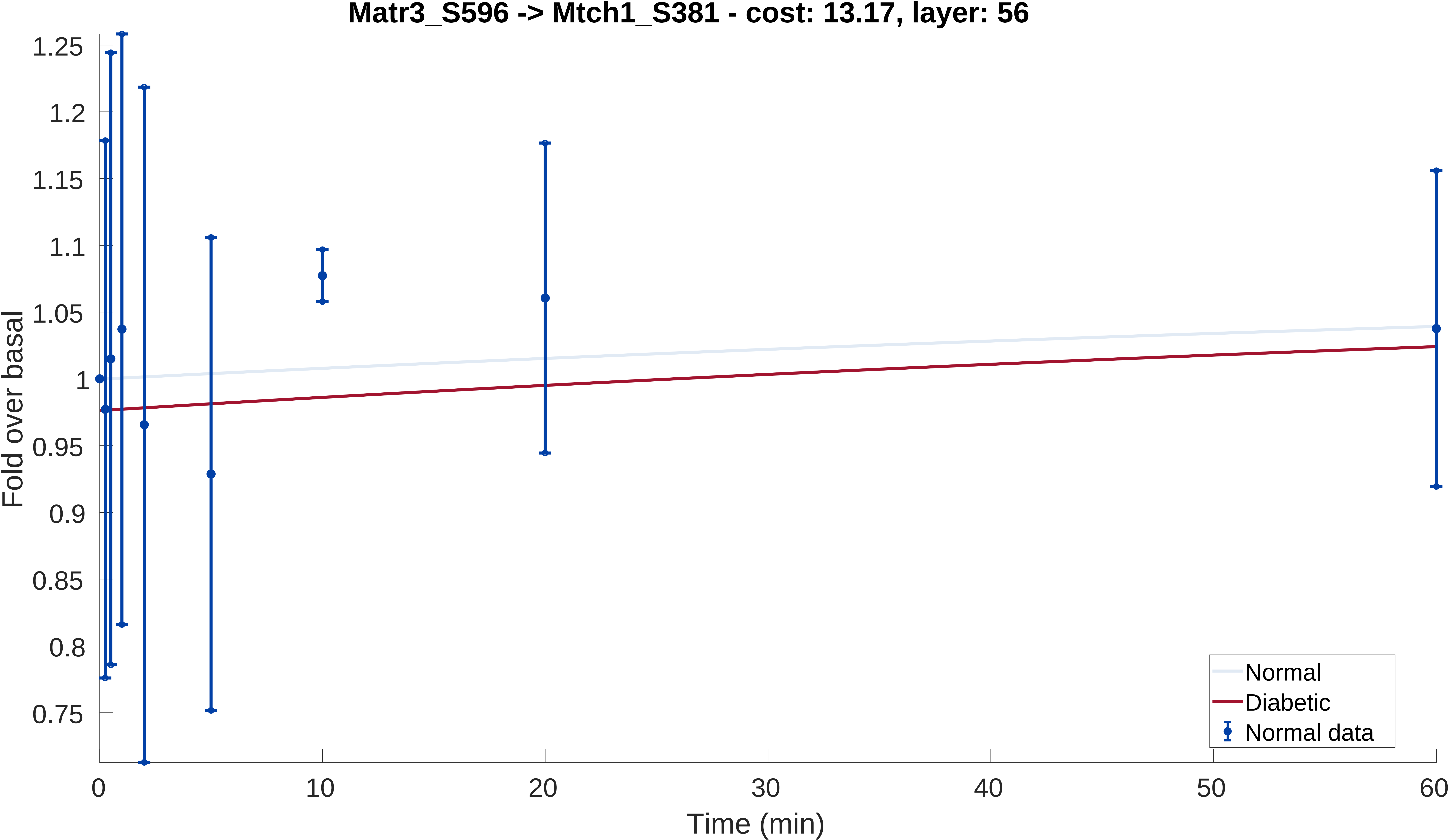

Matr3_S596 -> Mtch1_S381 - cost: 13.17, layer: 56
Fold over basal
1.25
1.2
1.15
1.1
1.05
1
0.95
0.9
0.85
0.8
0.75
0
10
20
30
40
50
60
Time (min)
Normal
Diabetic
Normal data

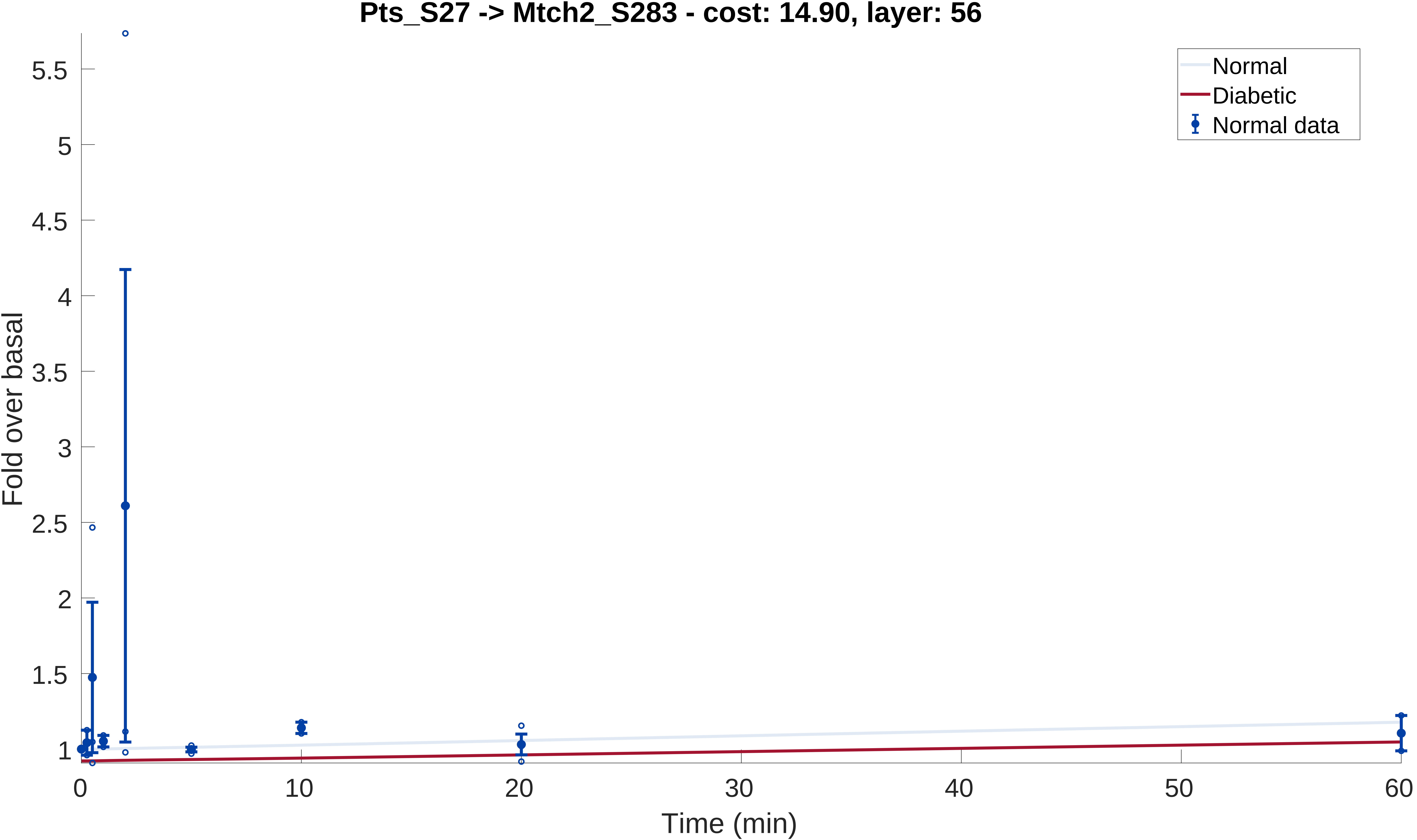

Pts_S27 -> Mtch2_S283 - cost: 14.90, layer: 56
Normal
Diabetic
Normal data
Fold over basal
Time (min)
5.5
5
4.5
4
3.5
3
2.5
2
1.5
1
0
10
20
30
40
50
60

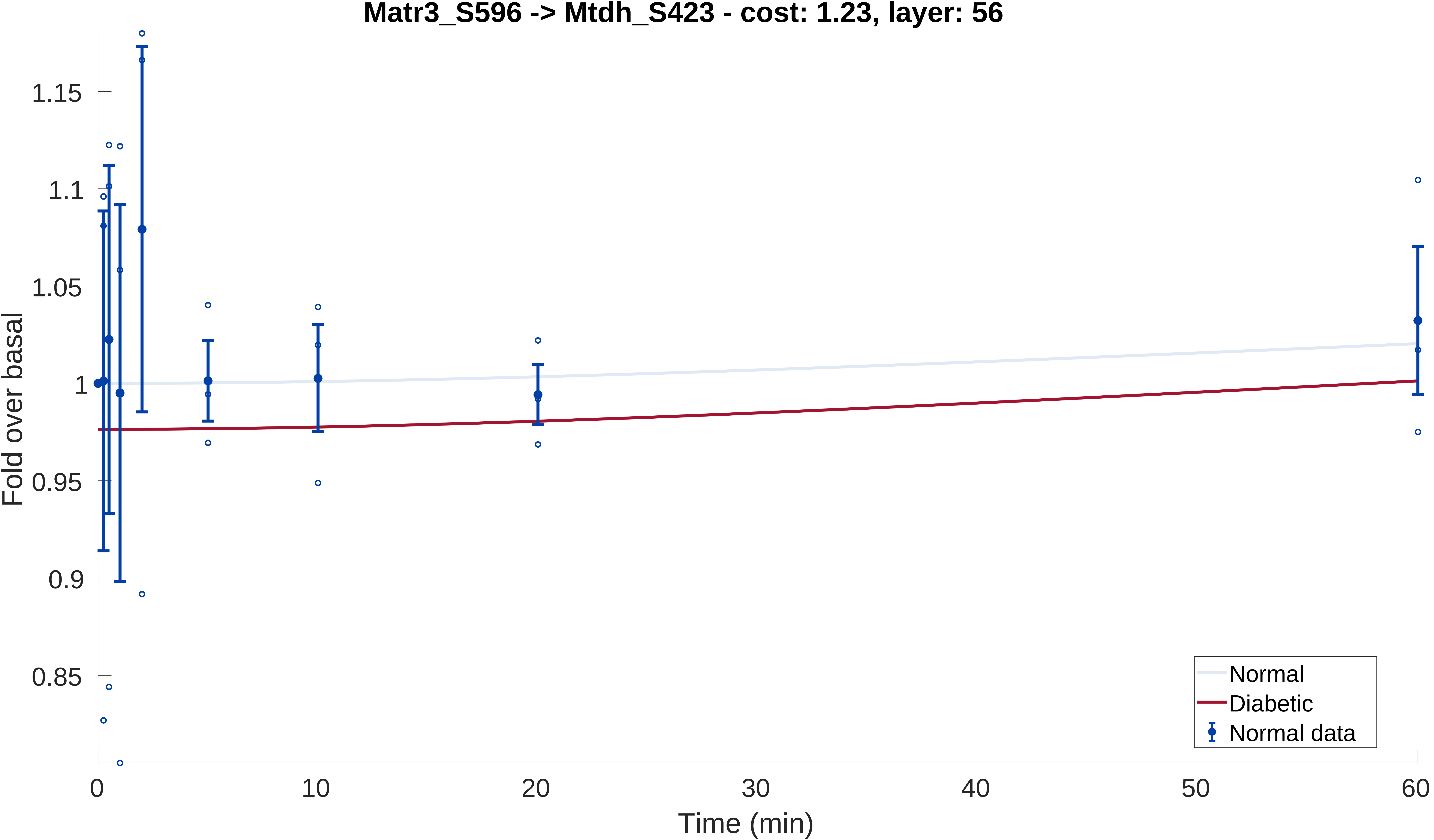
Matr3_S596 -> Mtdh_S423 - cost: 1.23, layer: 56
Fold over basal
1.15
1.1
1.05
1
0.95
0.9
0.85
0
10
20
30
40
50
60
Time (min)
Normal
Diabetic
Normal data

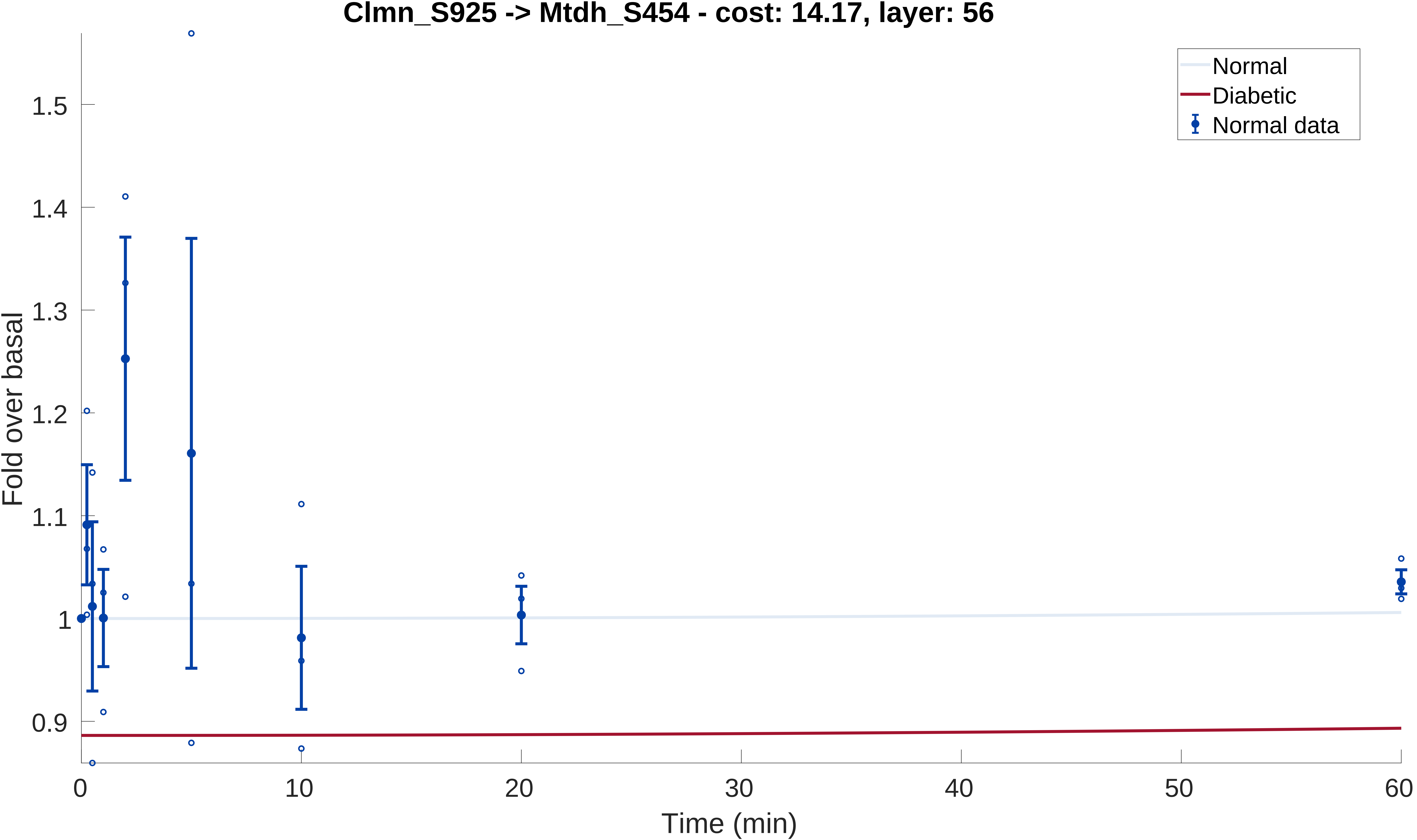

Clmn_S925 -> Mtdh_S454 - cost: 14.17, layer: 56
Normal
Diabetic
Normal data
Fold over basal
1.5
1.4
1.3
1.2
1.1
1
0.9
0
10
20
30
40
50
60
Time (min)

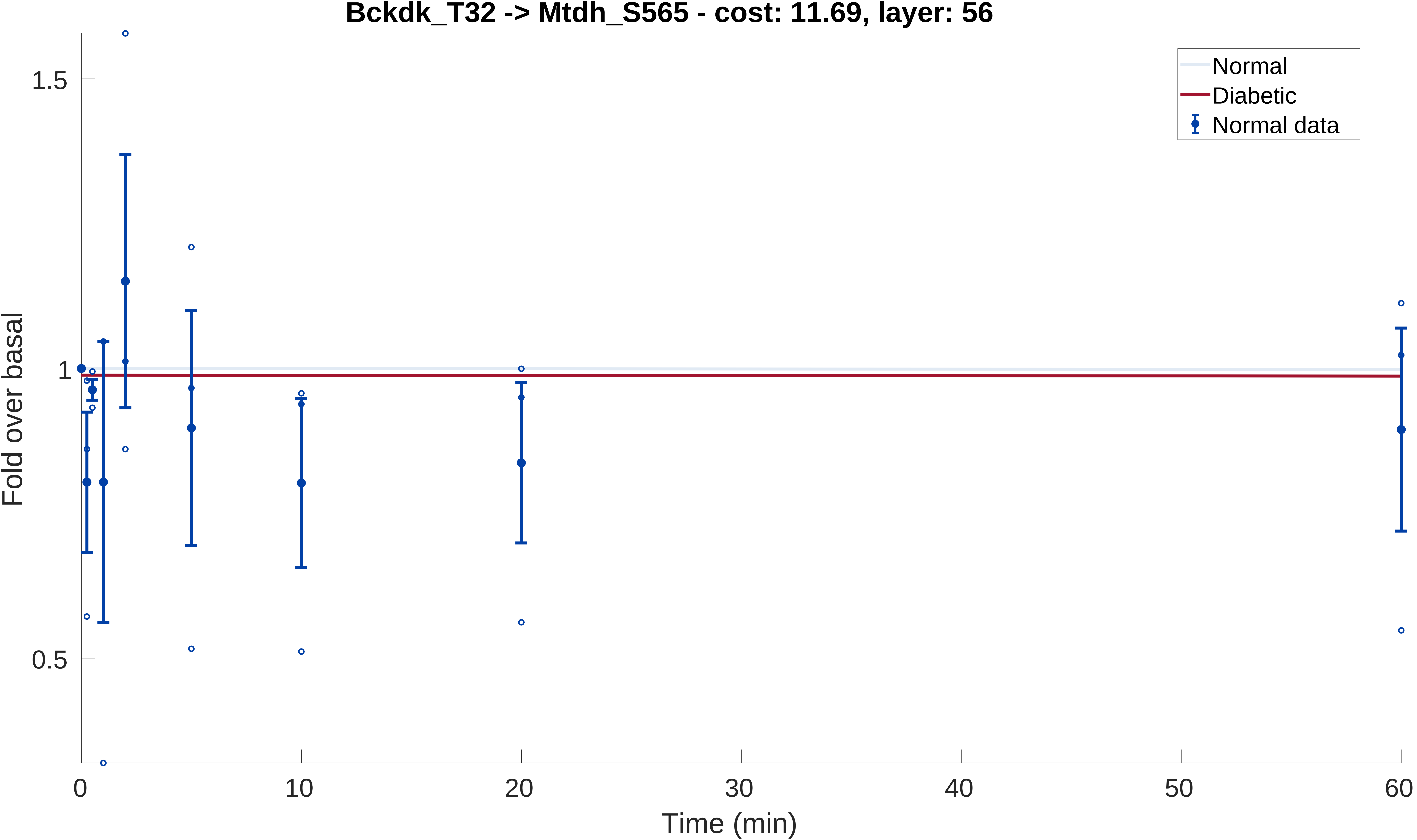

Bckdk_T32 -> Mtdh_S565 - cost: 11.69, layer: 56
Normal
Diabetic
Normal data
1.5
1
0.5
Fold over basal
0
10
20
30
40
50
60
Time (min)

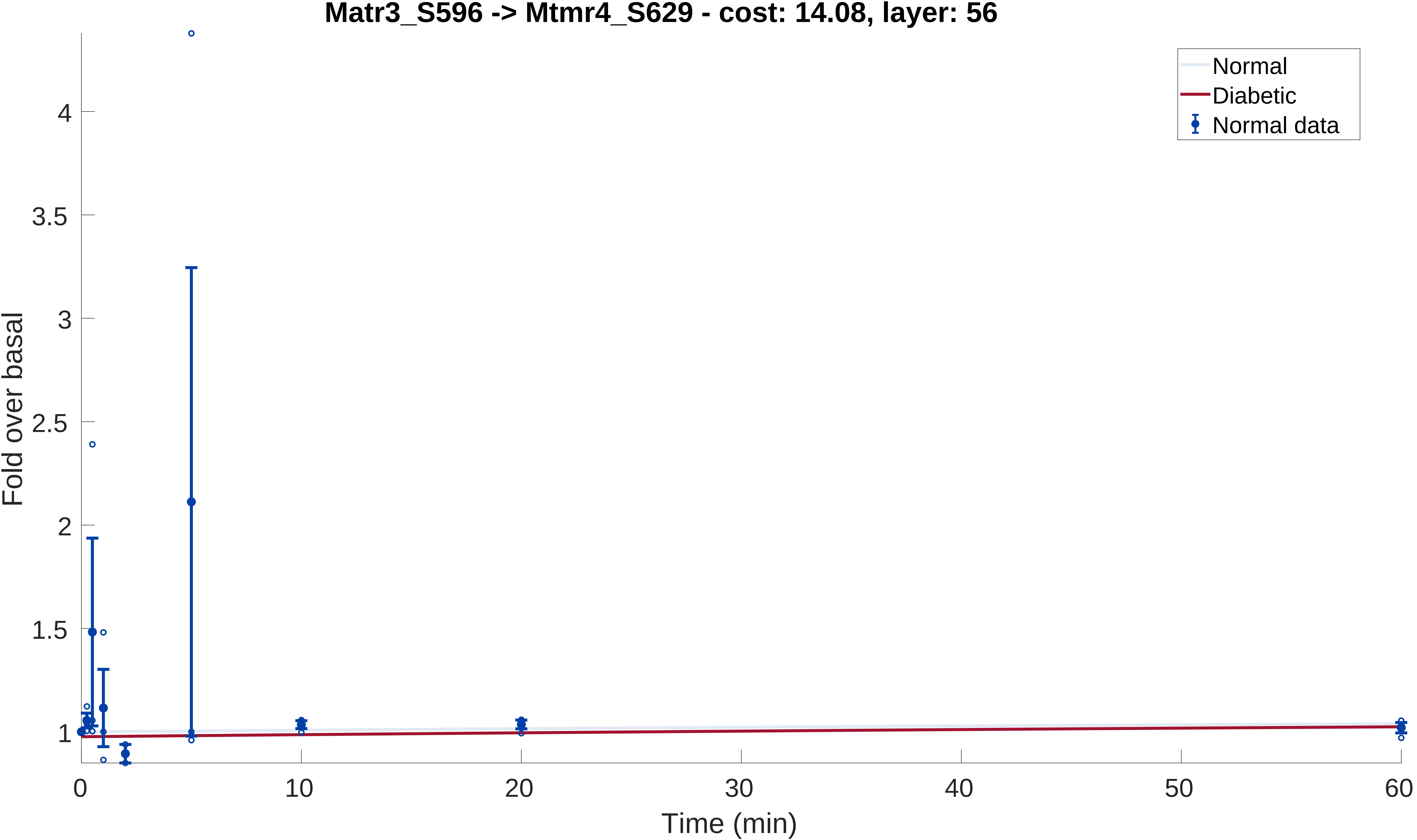

Matr3_S596 -> Mtmr4_S629 - cost: 14.08, layer: 56
Normal
Diabetic
Normal data
Fold over basal
Time (min)
4
3.5
3
2.5
2
1.5
1
0
10
20
30
40
50
60

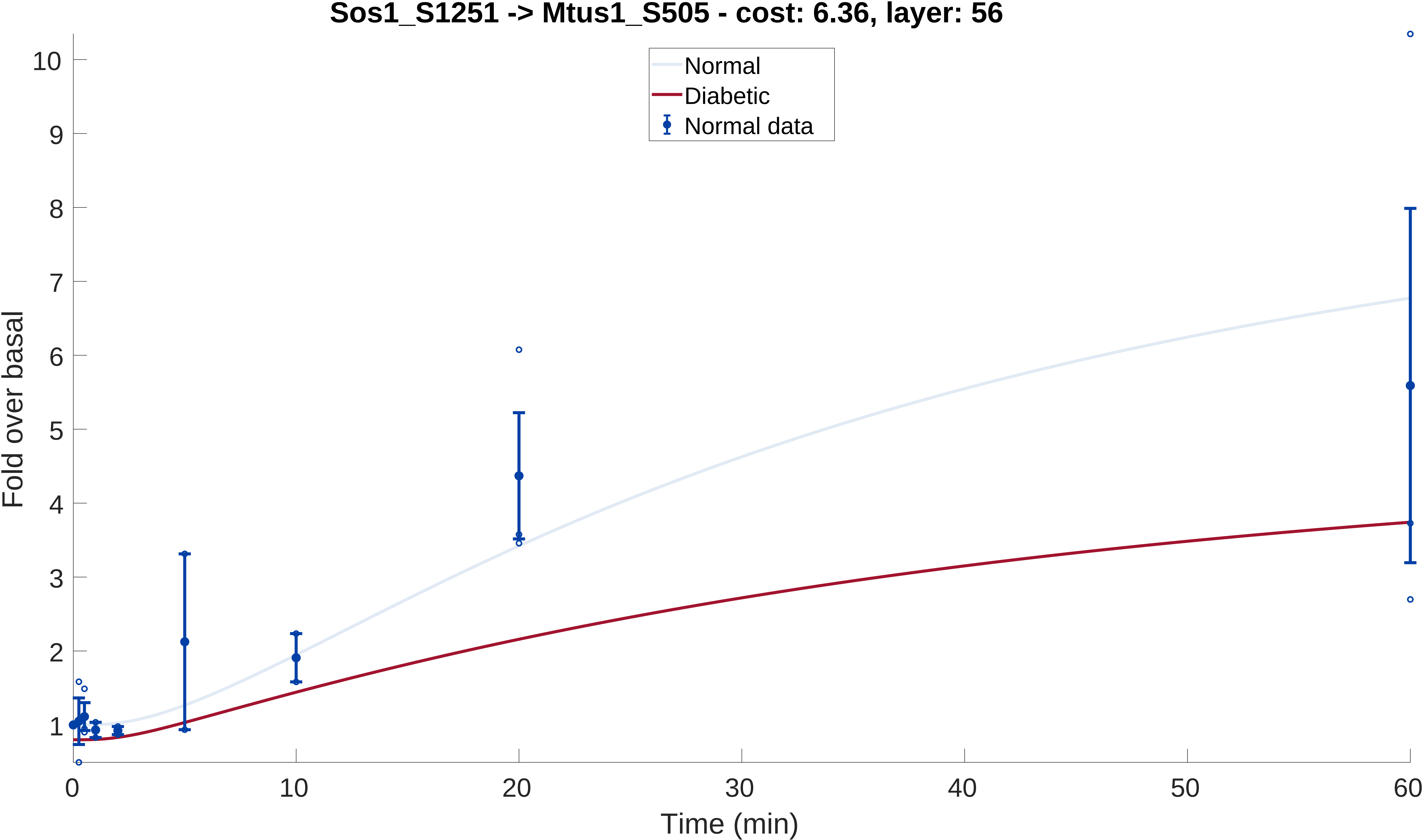

Sos1_S1251 -> Mtus1_S505 - cost: 6.36, layer: 56
Normal
Diabetic
Normal data
Fold over basal
Time (min)
1
2
3
4
5
6
7
8
9
10
0
10
20
30
40
50
60

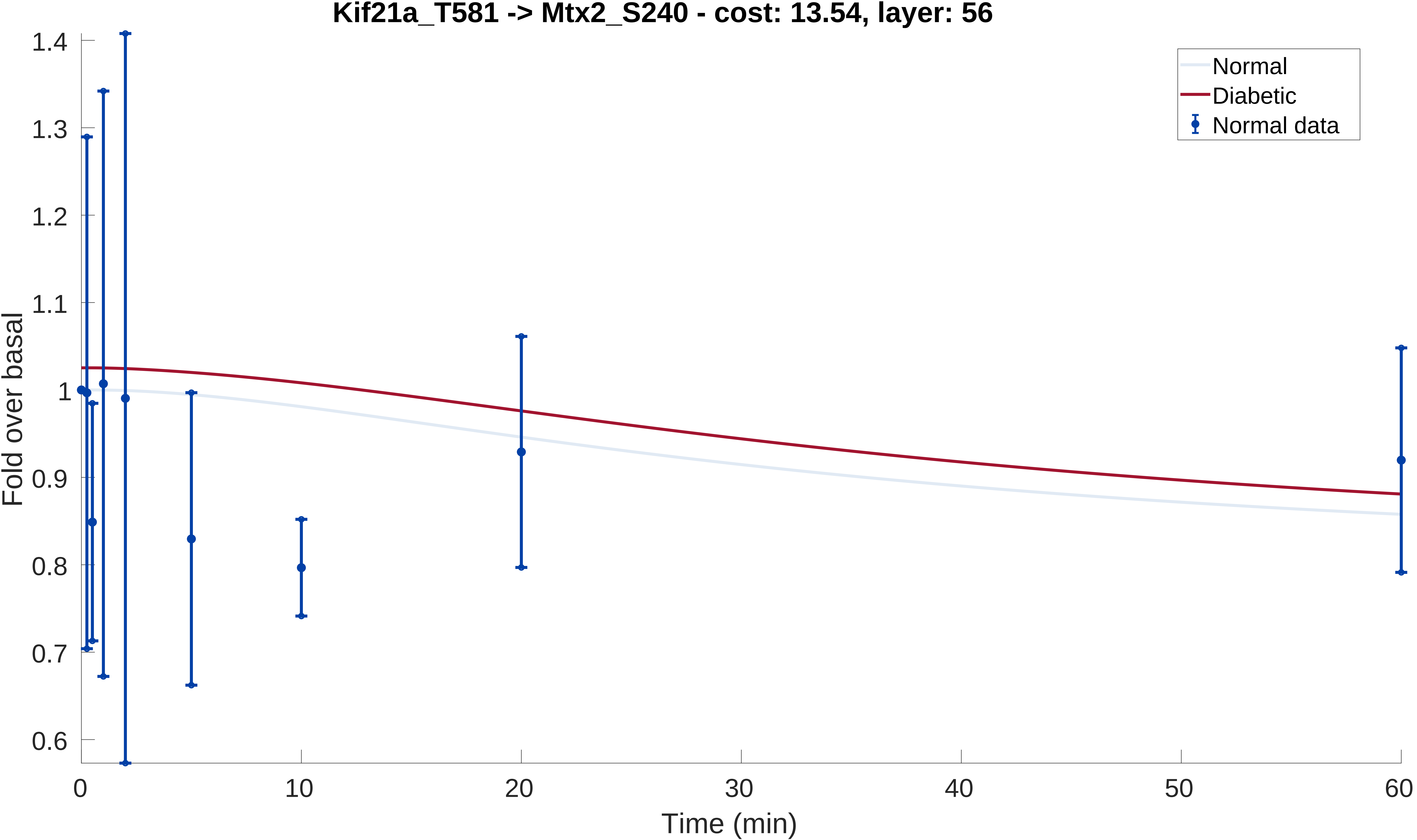

Kif21a_T581 -> Mtx2_S240 - cost: 13.54, layer: 56
Normal
Diabetic
Normal data
Fold over basal
1.4
1.3
1.2
1.1
1
0.9
0.8
0.7
0.6
0
10
20
30
40
50
60
Time (min)

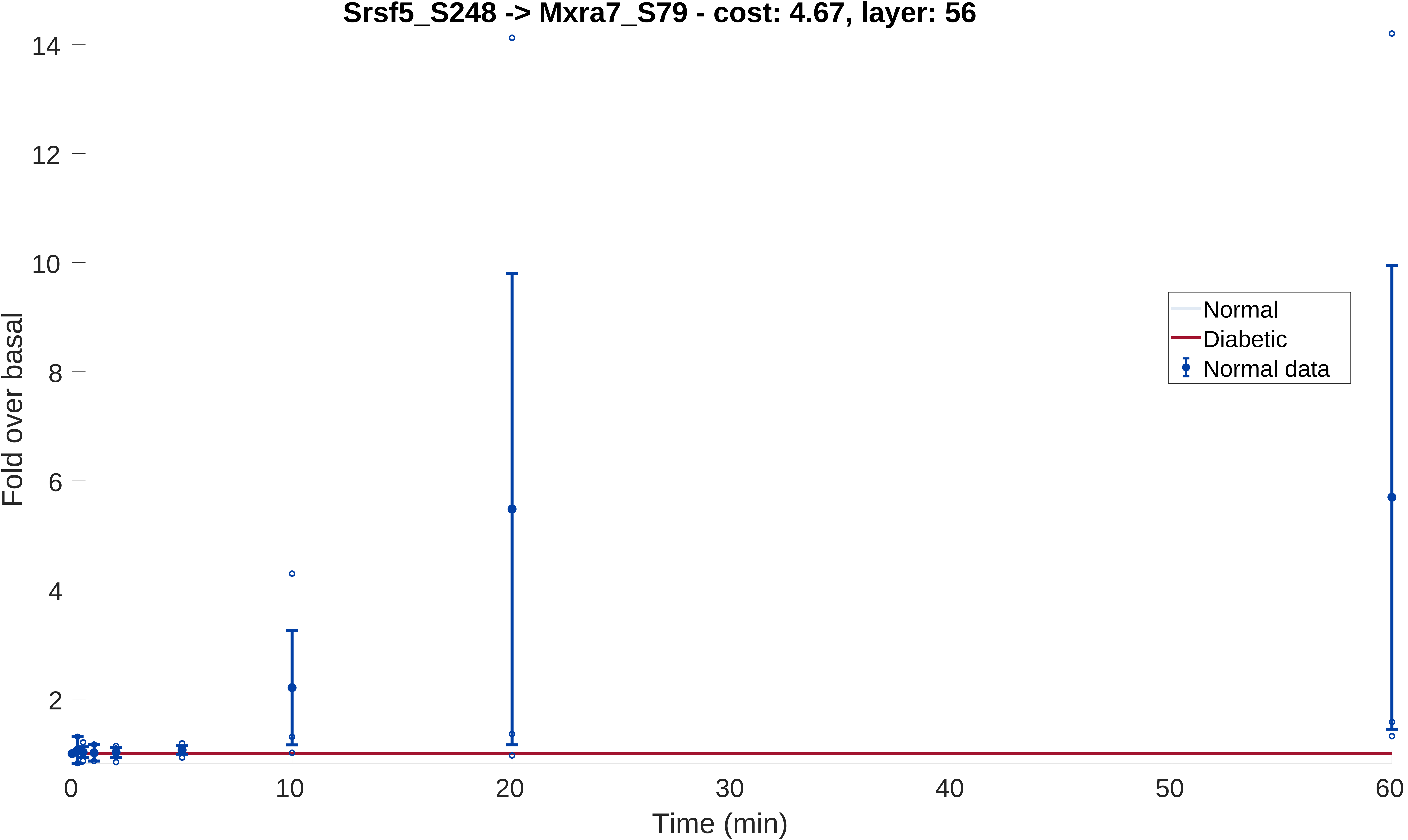

Srsf5_S248 -> Mxra7_S79 - cost: 4.67, layer: 56
Fold over basal
Time (min)
Normal
Diabetic
Normal data
0
10
20
30
40
50
60
2
4
6
8
10
12
14

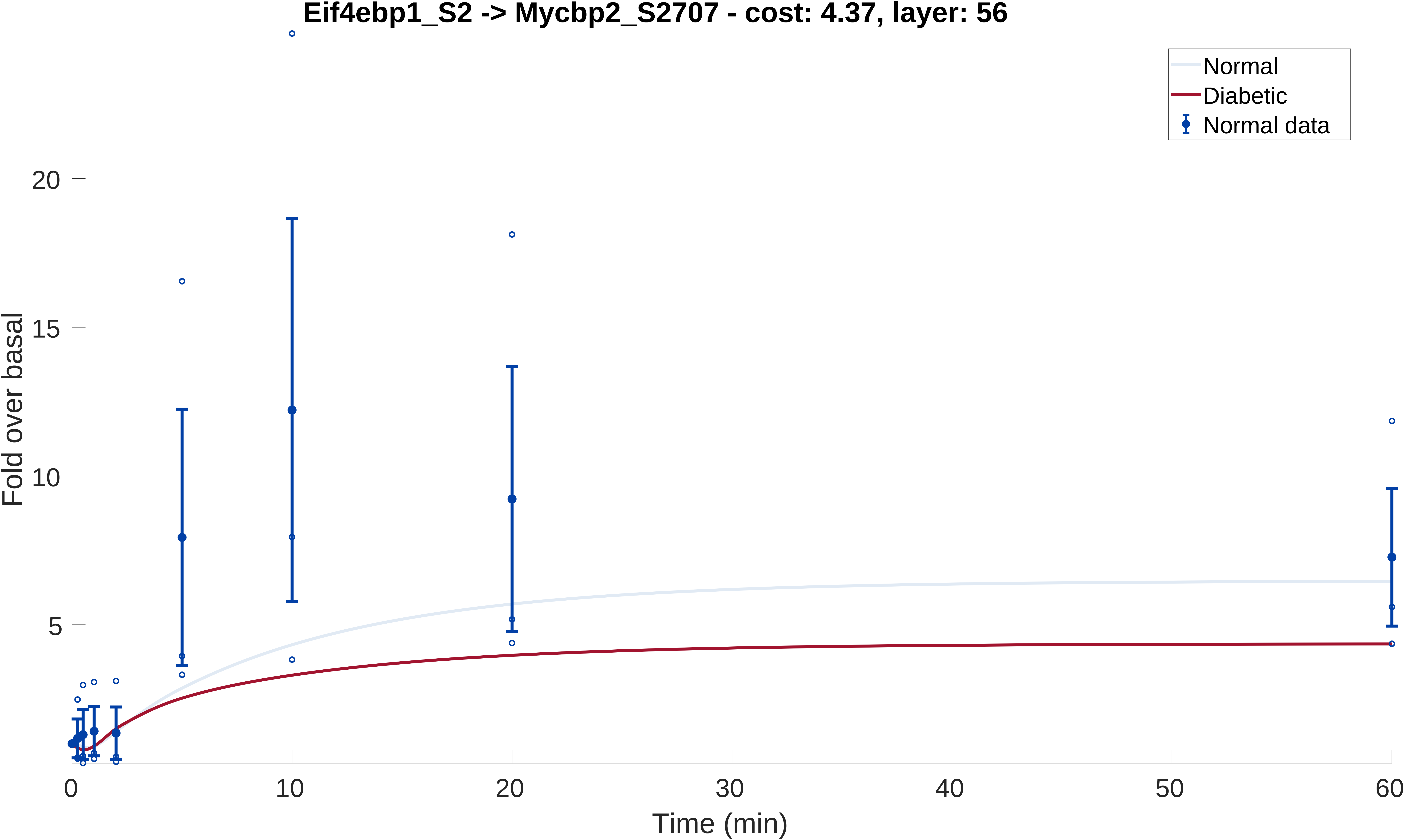

Eif4ebp1_S2 -> Mycbp2_S2707 - cost: 4.37, layer: 56
Normal
Diabetic
Normal data
Fold over basal
Time (min)
0
10
20
30
40
50
60
5
10
15
20

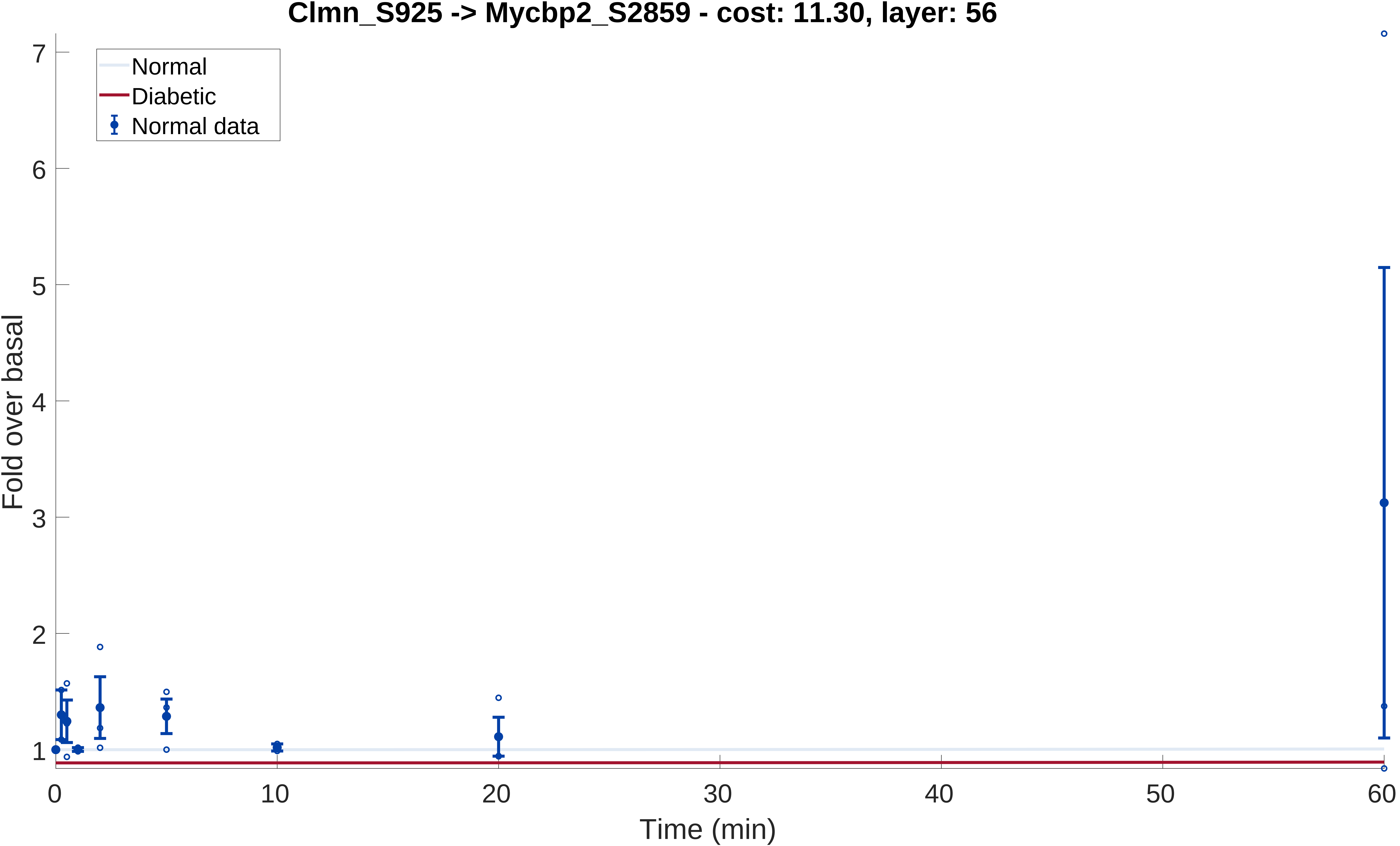

Clmn_S925 -> Mycbp2_S2859 - cost: 11.30, layer: 56
Normal
Diabetic
Normal data
Fold over basal
Time (min)
1
2
3
4
5
6
7
0
10
20
30
40
50
60

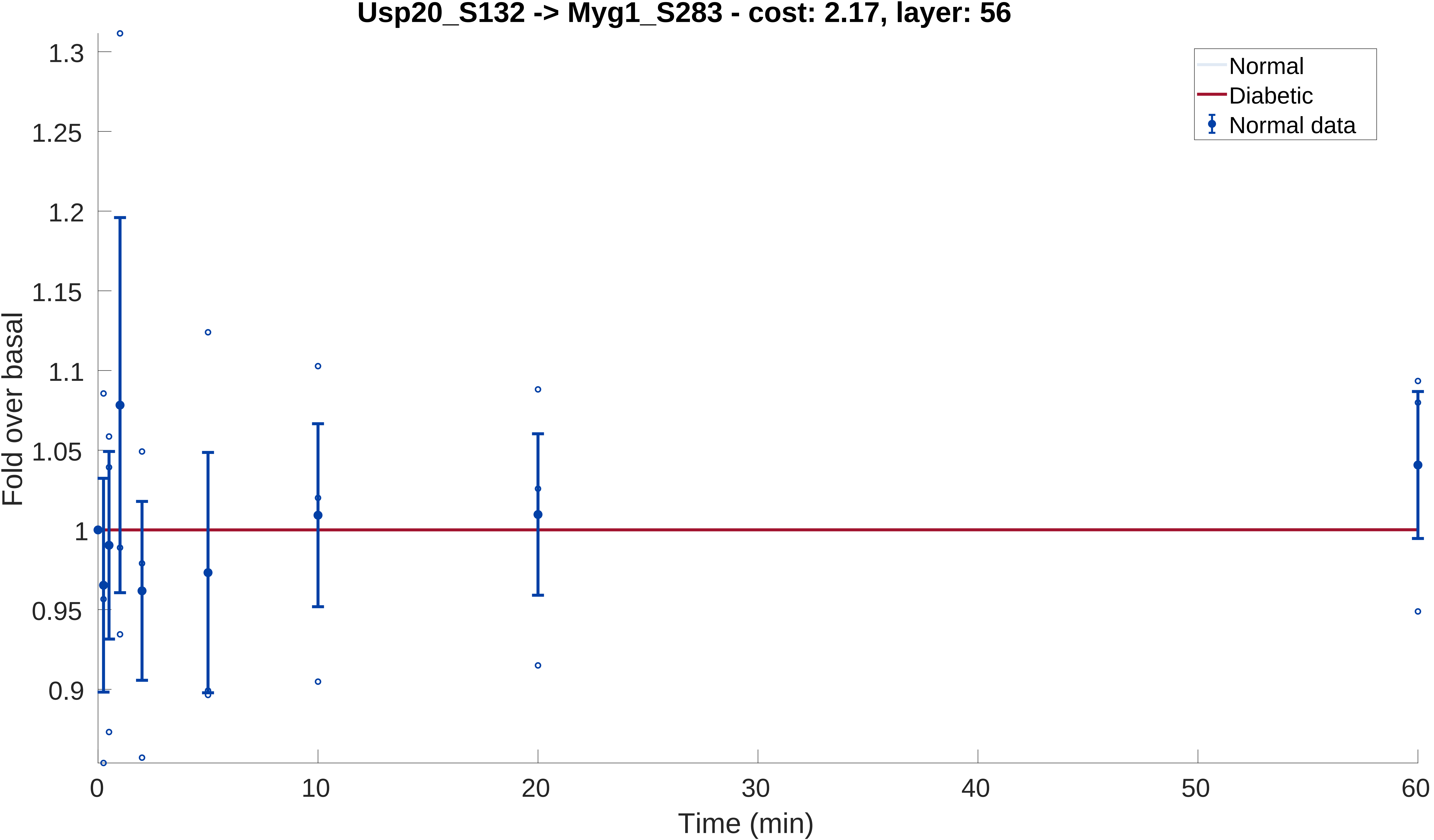

Usp20_S132 -> Myg1_S283 - cost: 2.17, layer: 56
Normal
Diabetic
Normal data
Fold over basal
1.3
1.25
1.2
1.15
1.1
1.05
1
0.95
0.9
0
10
20
30
40
50
60
Time (min)

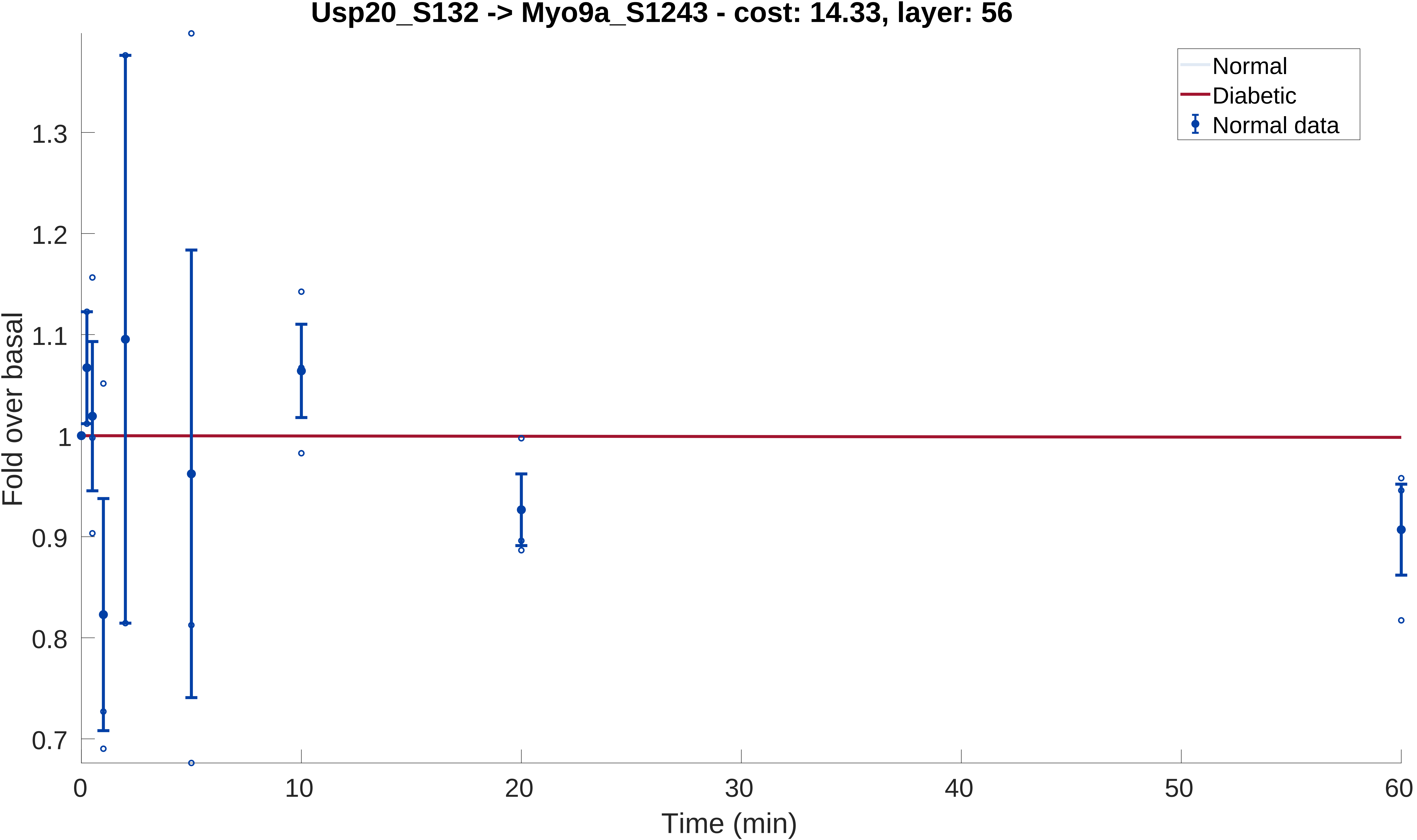

Usp20_S132 -> Myo9a_S1243 - cost: 14.33, layer: 56
Normal
Diabetic
Normal data
Fold over basal
1.3
1.2
1.1
1
0.9
0.8
0.7
0
10
20
30
40
50
60
Time (min)

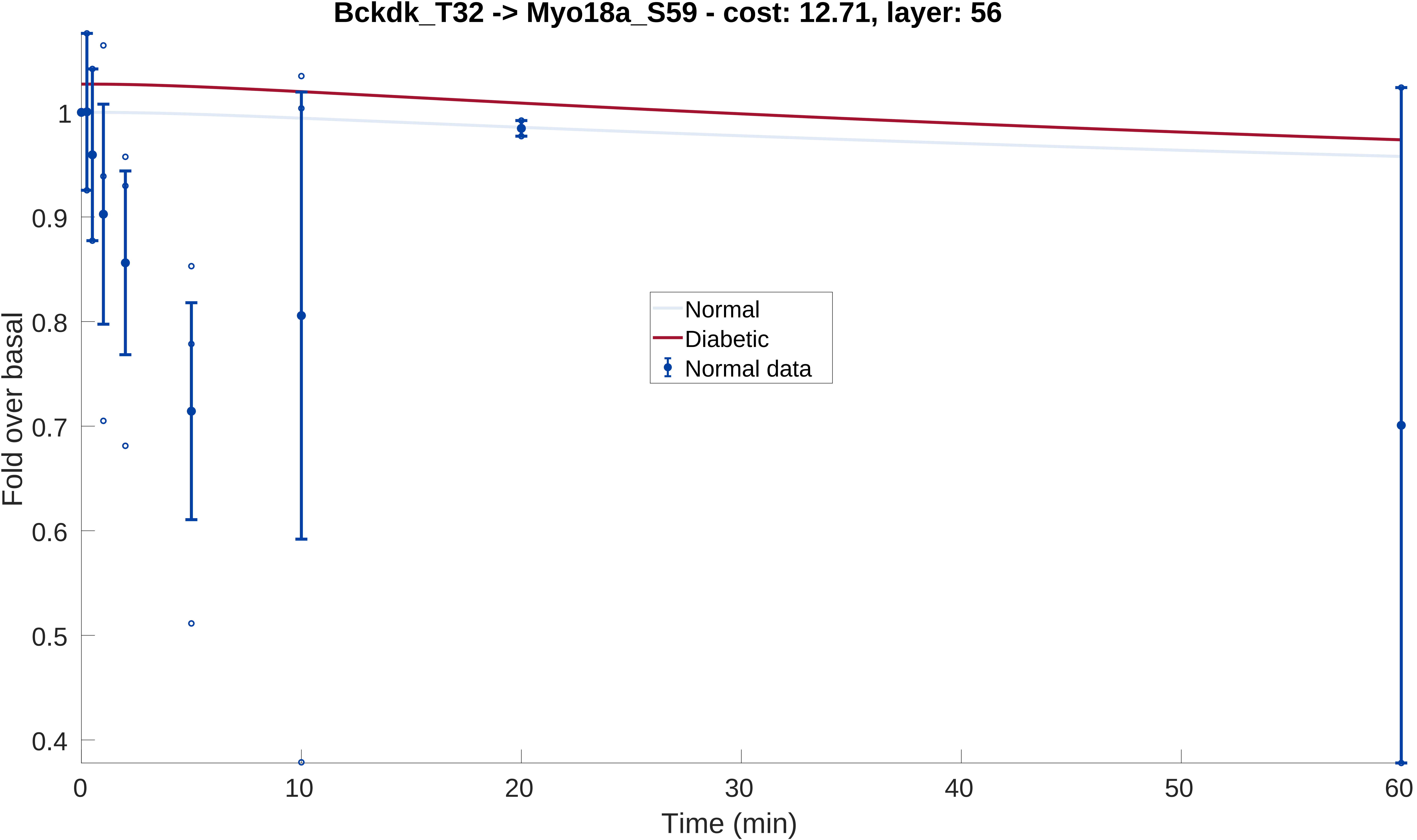

Bckdk_T32 -> Myo18a_S59 - cost: 12.71, layer: 56
Fold over basal
1
0.9
0.8
0.7
0.6
0.5
0.4
0
10
20
30
40
50
60
Time (min)
Normal
Diabetic
Normal data

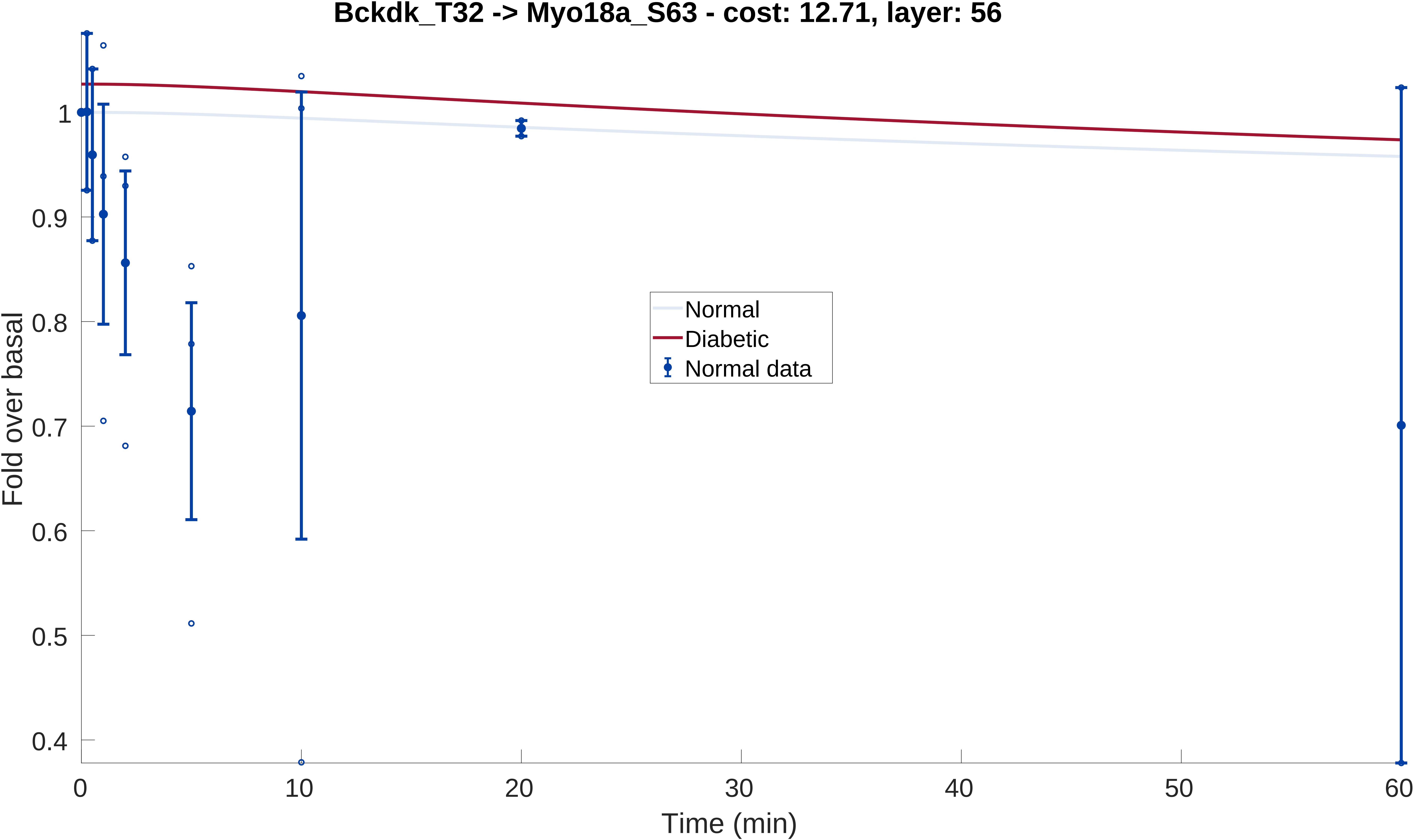

Bckdk_T32 -> Myo18a_S63 - cost: 12.71, layer: 56
Fold over basal
Time (min)
Normal
Diabetic
Normal data
0.4
0.5
0.6
0.7
0.8
0.9
1
0
10
20
30
40
50
60

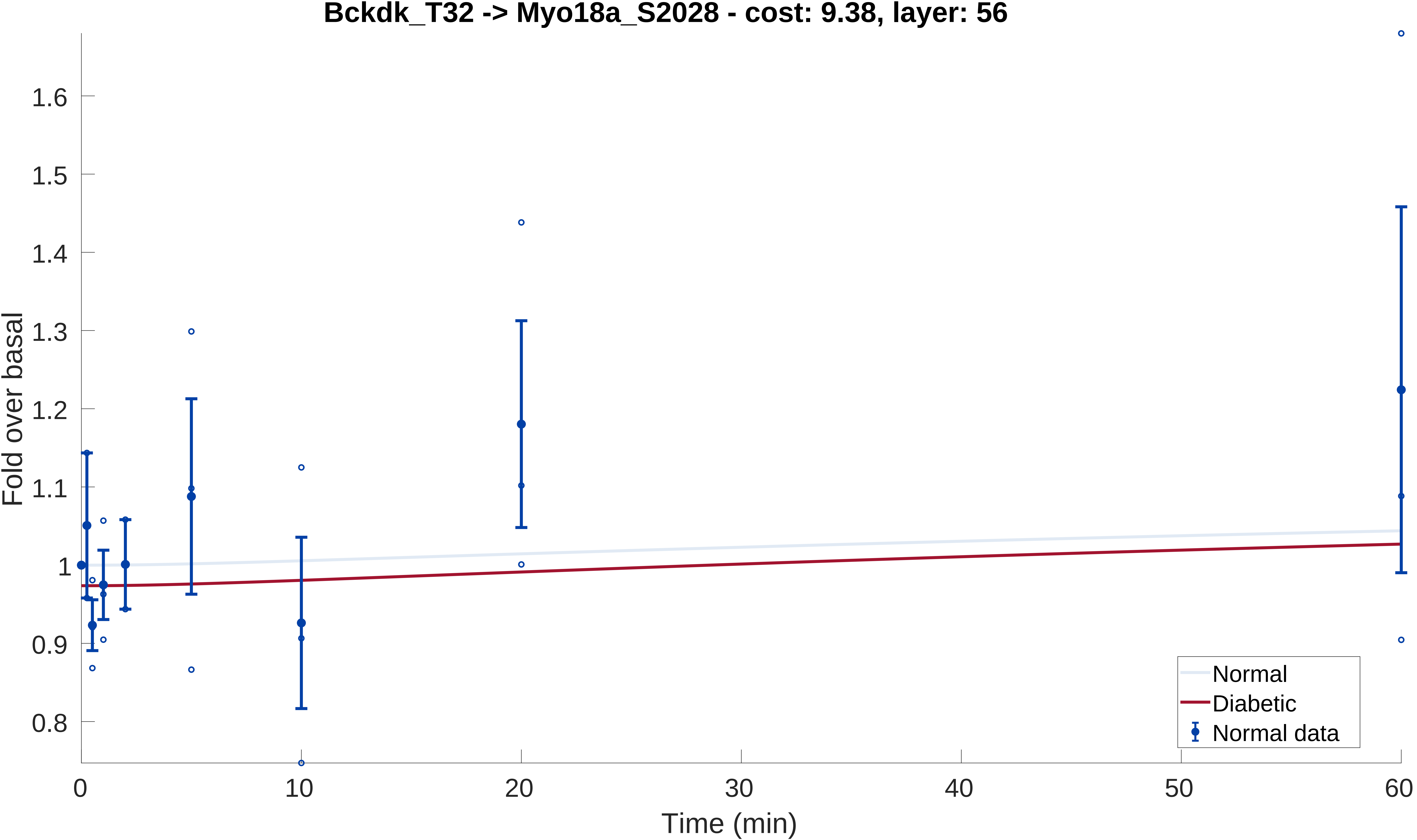

Bckdk_T32 -> Myo18a_S2028 - cost: 9.38, layer: 56
Fold over basal
1.6
1.5
1.4
1.3
1.2
1.1
1
0.9
0.8
0
10
20
30
40
50
60
Time (min)
Normal
Diabetic
Normal data

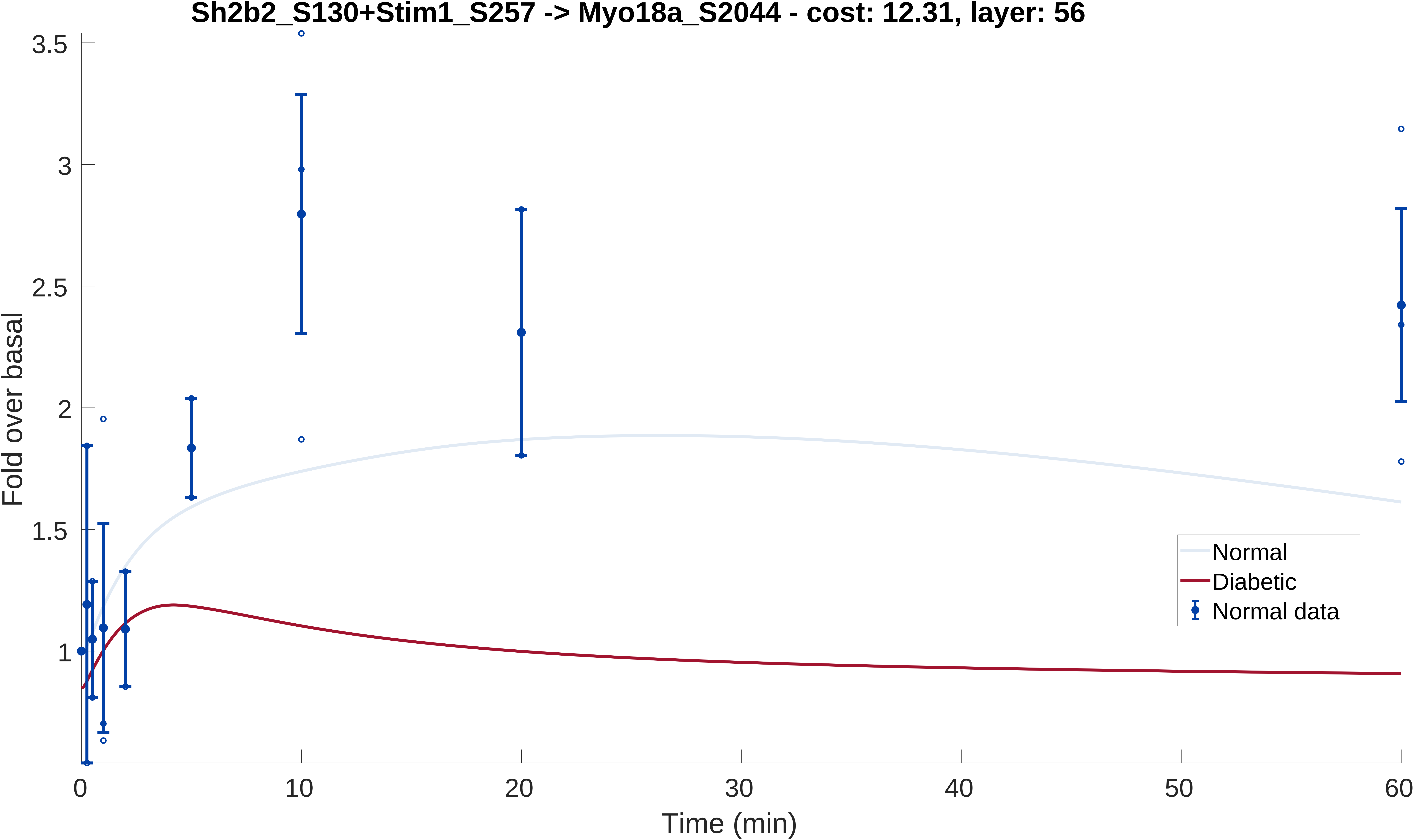
Sh2b2_S130+Stim1_S257 -> Myo18a_S2044 - cost: 12.31, layer: 56
Fold over basal
Time (min)
3.5
3
2.5
2
1.5
1
0
10
20
30
40
50
60
Normal
Diabetic
Normal data

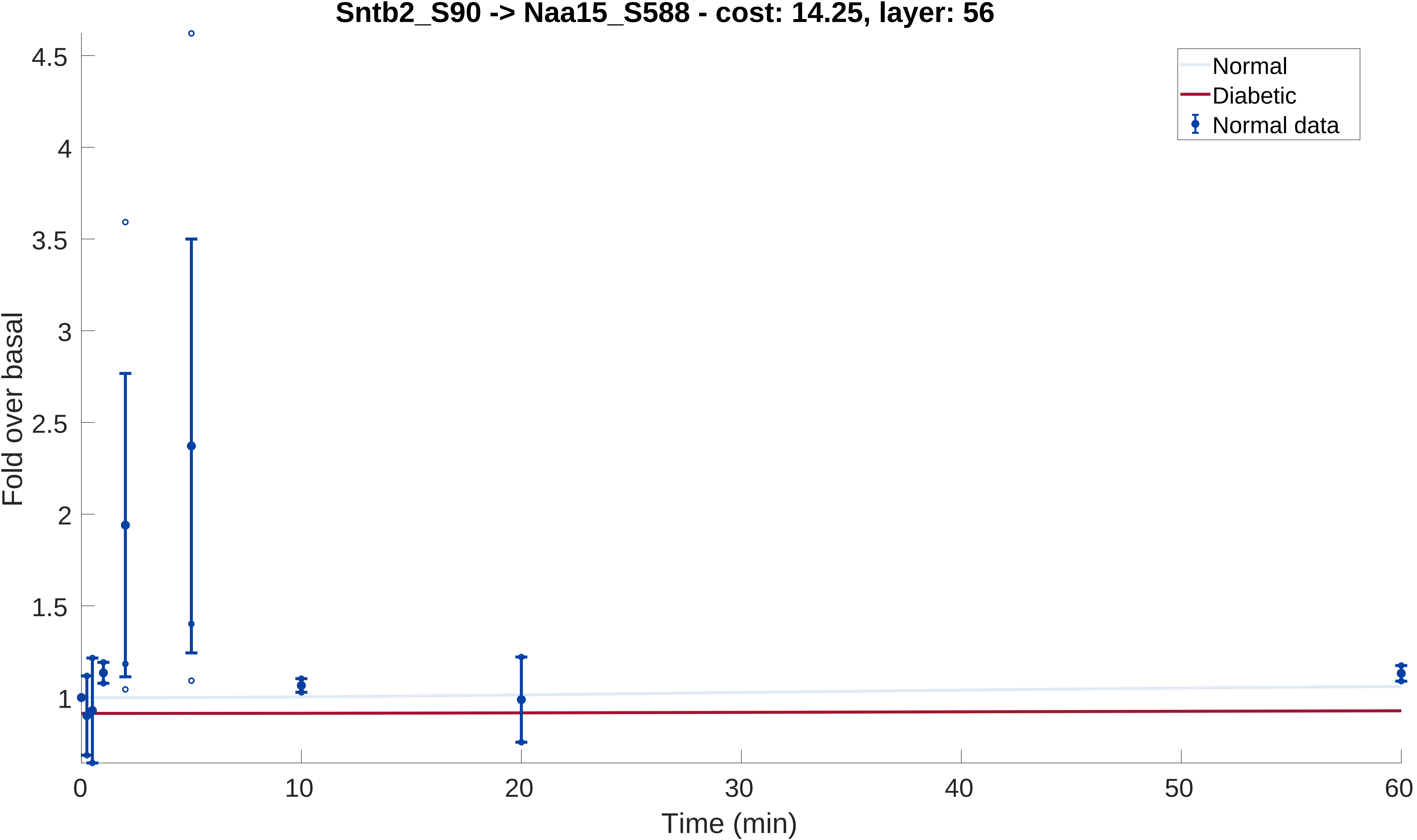

Sntb2_S90 -> Naa15_S588 - cost: 14.25, layer: 56
Normal
Diabetic
Normal data
Fold over basal
Time (min)
4.5
4
3.5
3
2.5
2
1.5
1
0
10
20
30
40
50
60

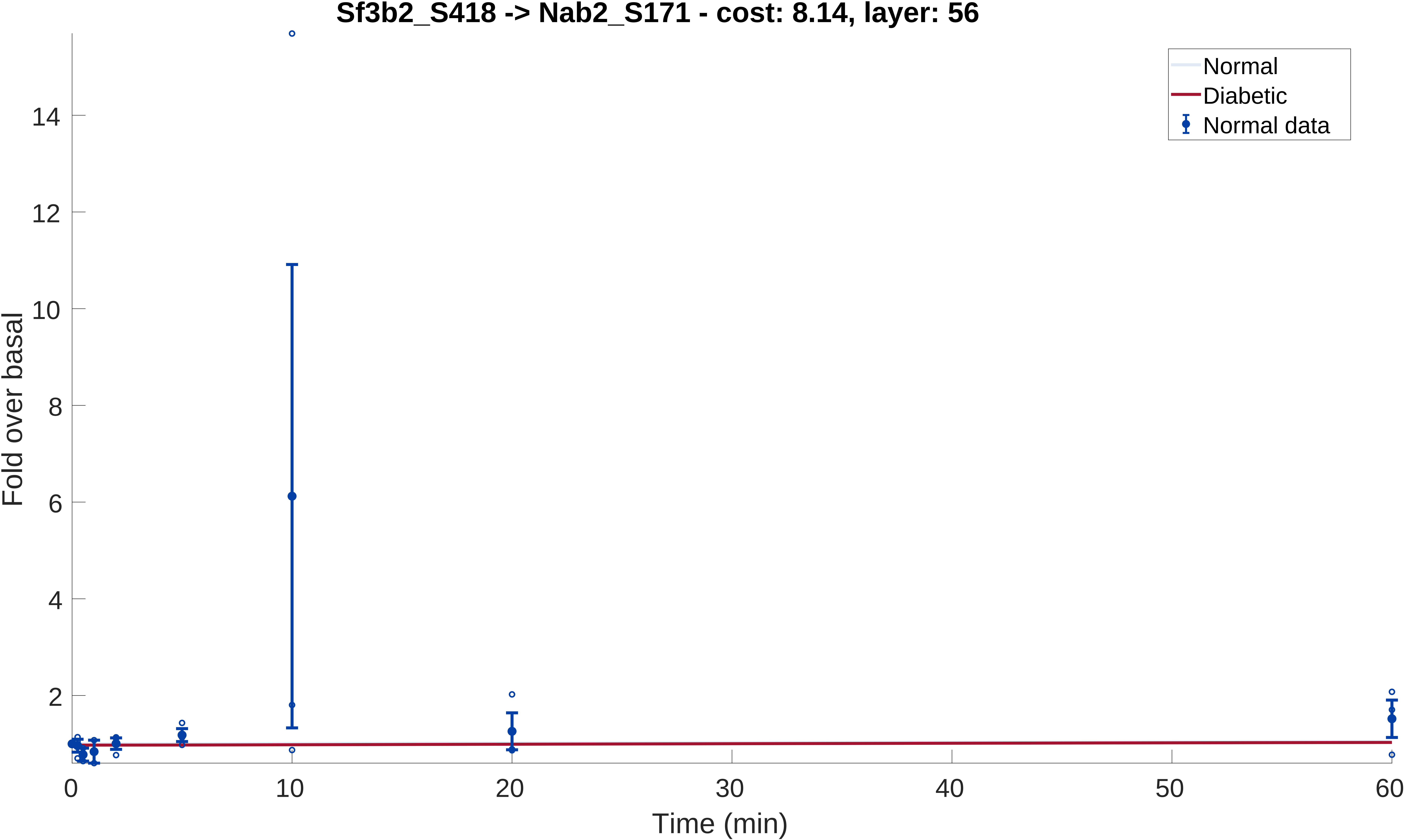

Sf3b2_S418 -> Nab2_S171 - cost: 8.14, layer: 56
Normal
Diabetic
Normal data
Fold over basal
14
12
10
8
6
4
2
0
10
20
30
40
50
60
Time (min)

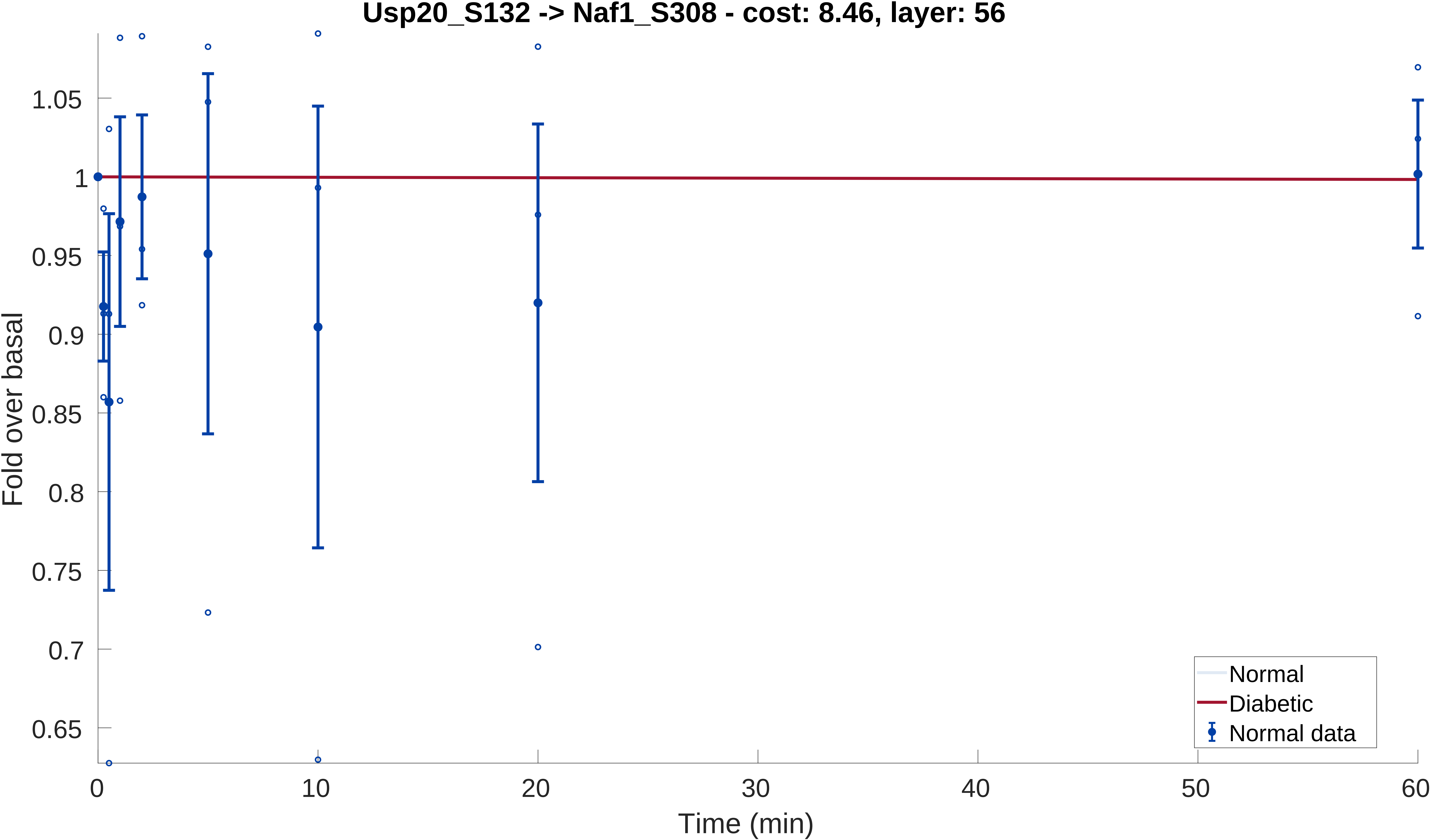

Usp20_S132 -> Naf1_S308 - cost: 8.46, layer: 56
Fold over basal
Time (min)
1.05
1
0.95
0.9
0.85
0.8
0.75
0.7
0.65
0
10
20
30
40
50
60
Normal
Diabetic
Normal data

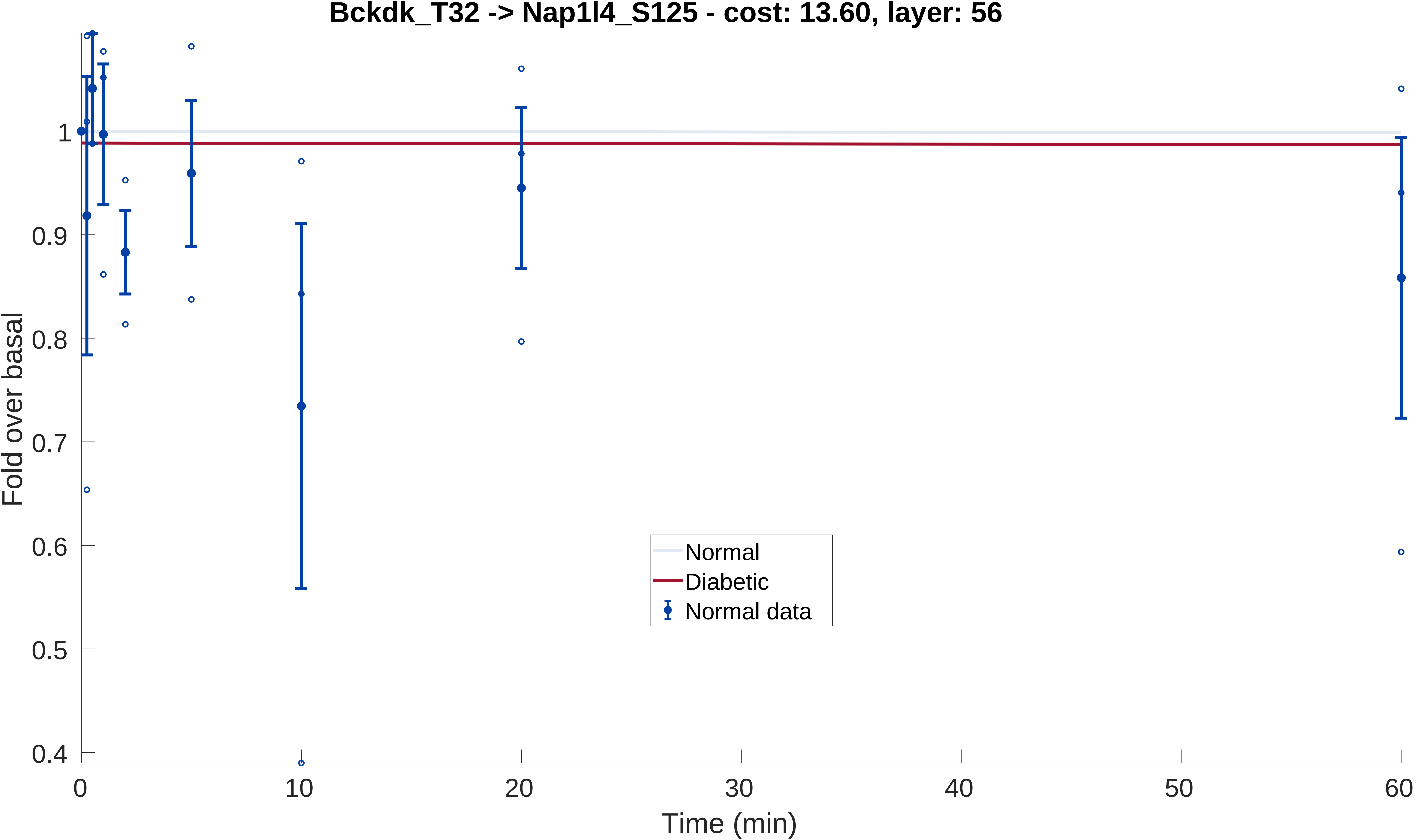

Bckdk_T32 -> Nap1l4_S125 - cost: 13.60, layer: 56
Fold over basal
Time (min)
0.4
0.5
0.6
0.7
0.8
0.9
1
0
10
20
30
40
50
60
Normal
Diabetic
Normal data

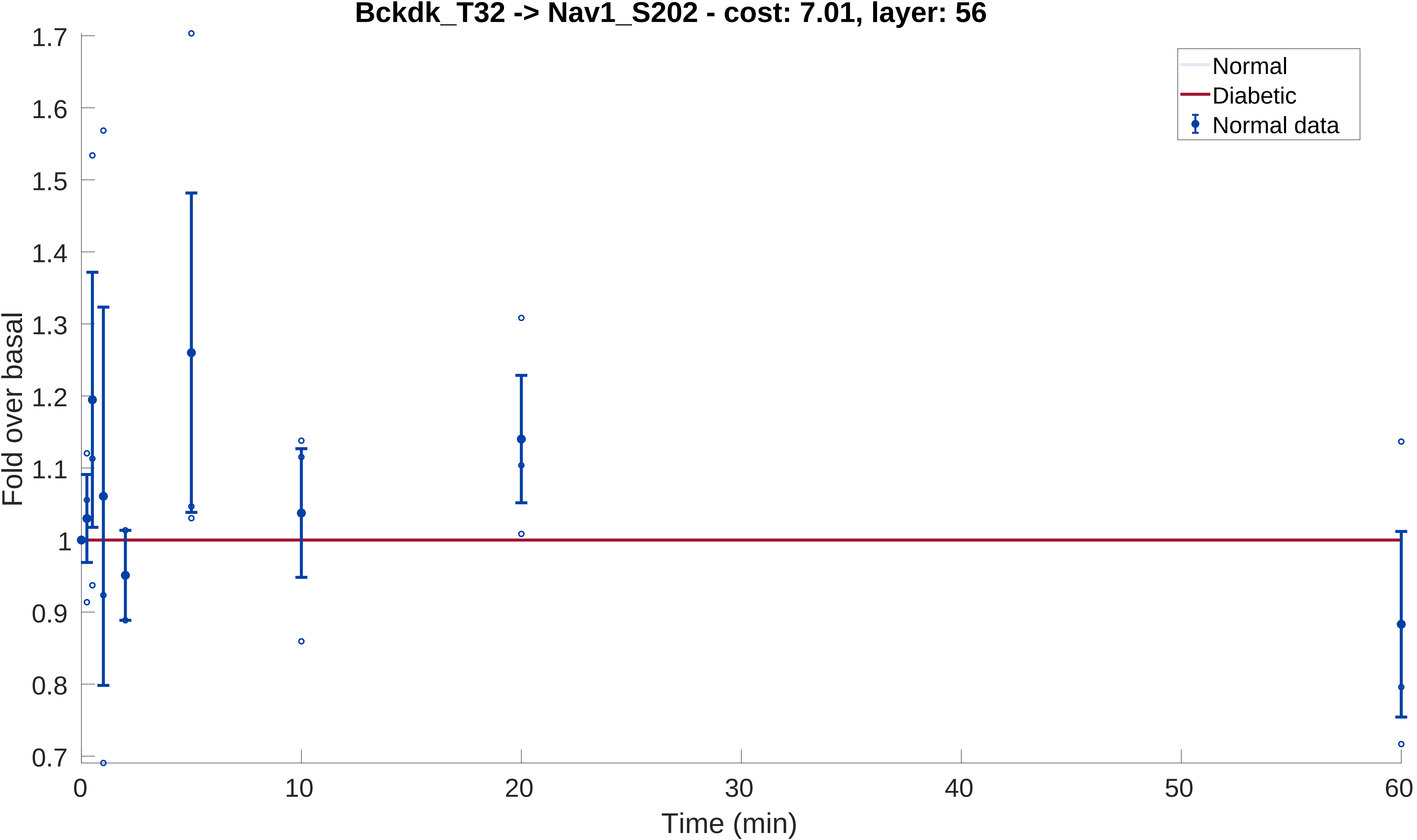

Bckdk_T32 -> Nav1_S202 - cost: 7.01, layer: 56
Normal
Diabetic
Normal data
Fold over basal
Time (min)
1.7
1.6
1.5
1.4
1.3
1.2
1.1
1
0.9
0.8
0.7
0
10
20
30
40
50
60

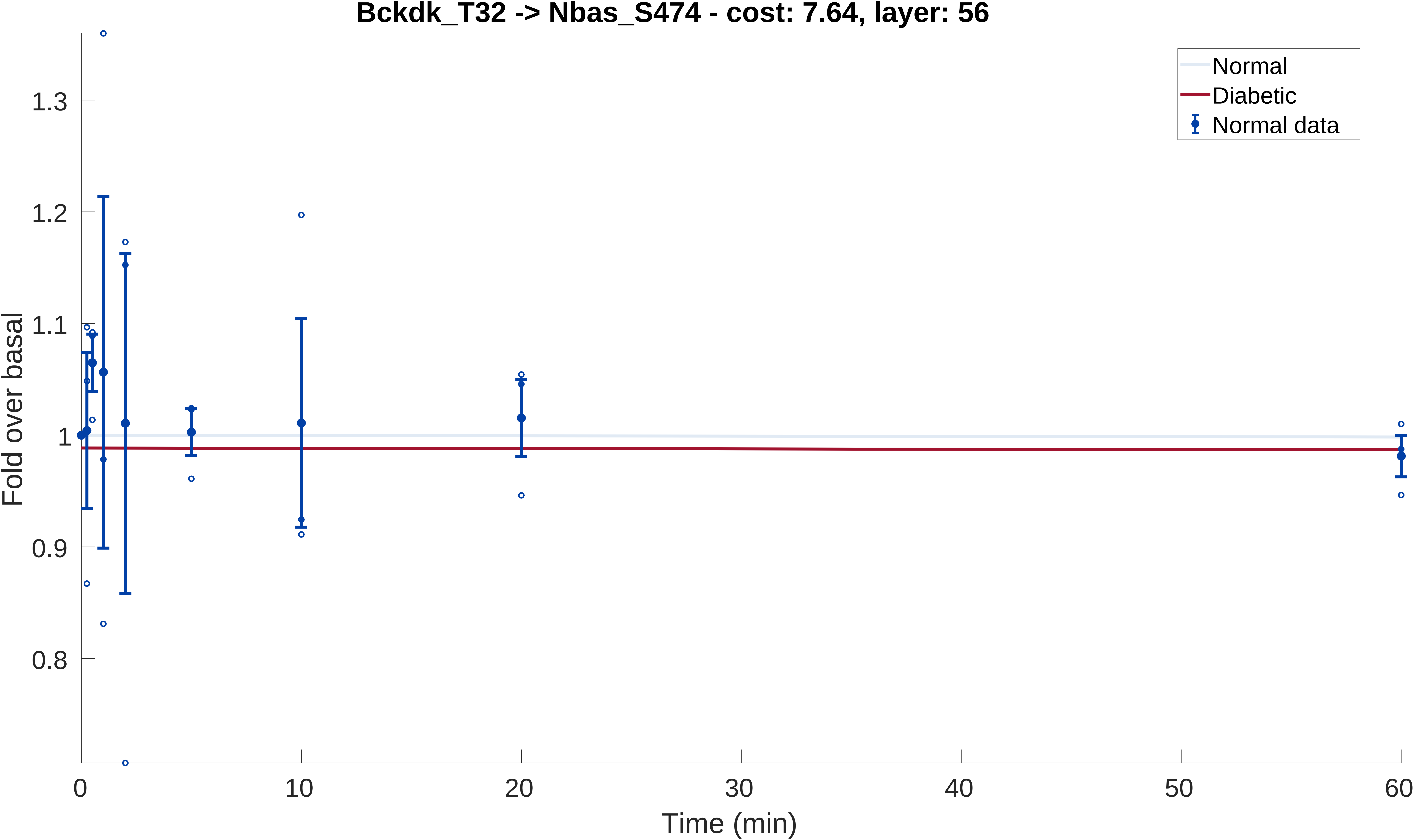
Bckdk_T32 -> Nbas_S474 - cost: 7.64, layer: 56
Normal
Diabetic
Normal data
Fold over basal
1.3
1.2
1.1
1
0.9
0.8
0
10
20
30
40
50
60
Time (min)

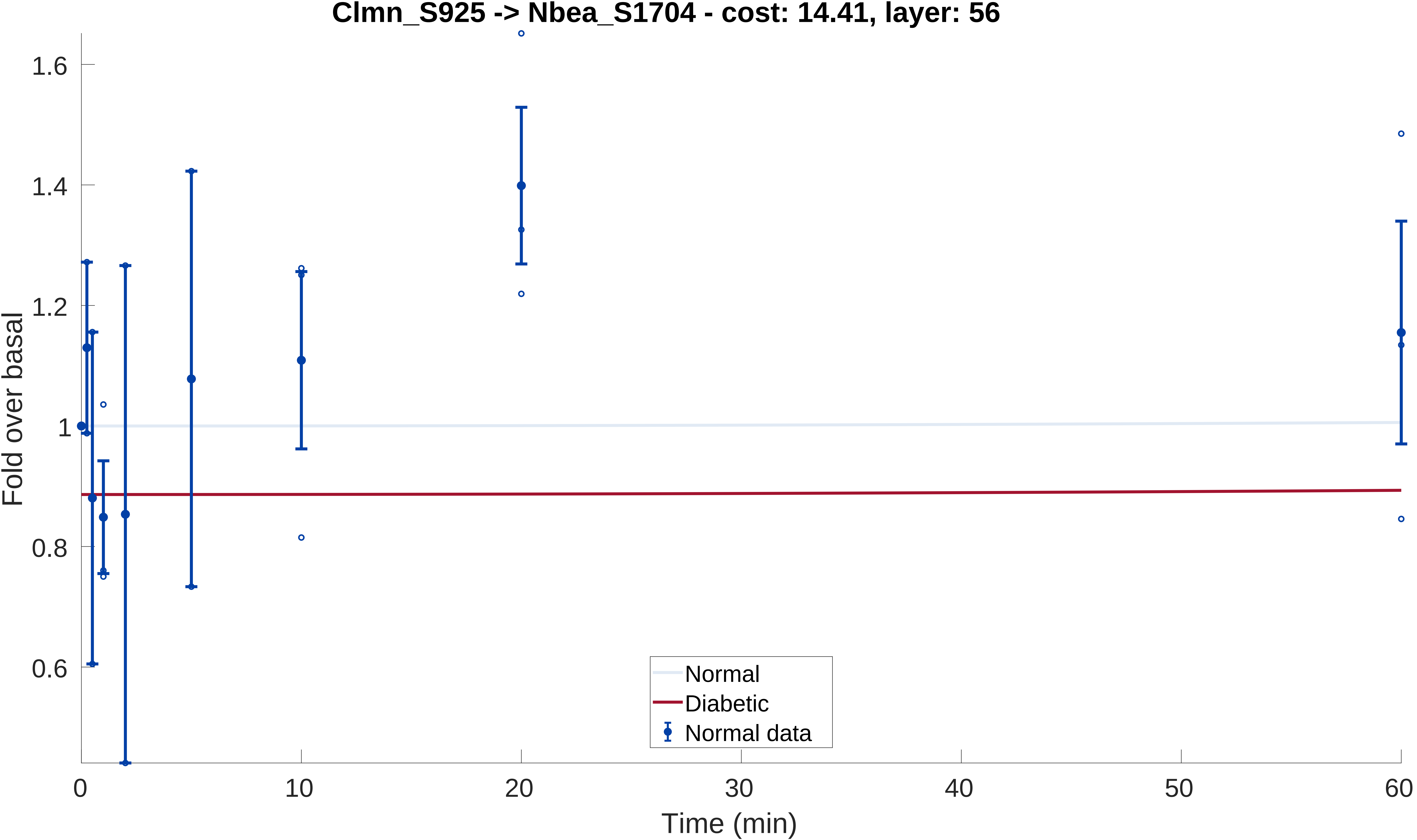

Clmn_S925 -> Nbea_S1704 - cost: 14.41, layer: 56
Fold over basal
1.6
1.4
1.2
1
0.8
0.6
0
10
20
30
40
50
60
Time (min)
Normal
Diabetic
Normal data

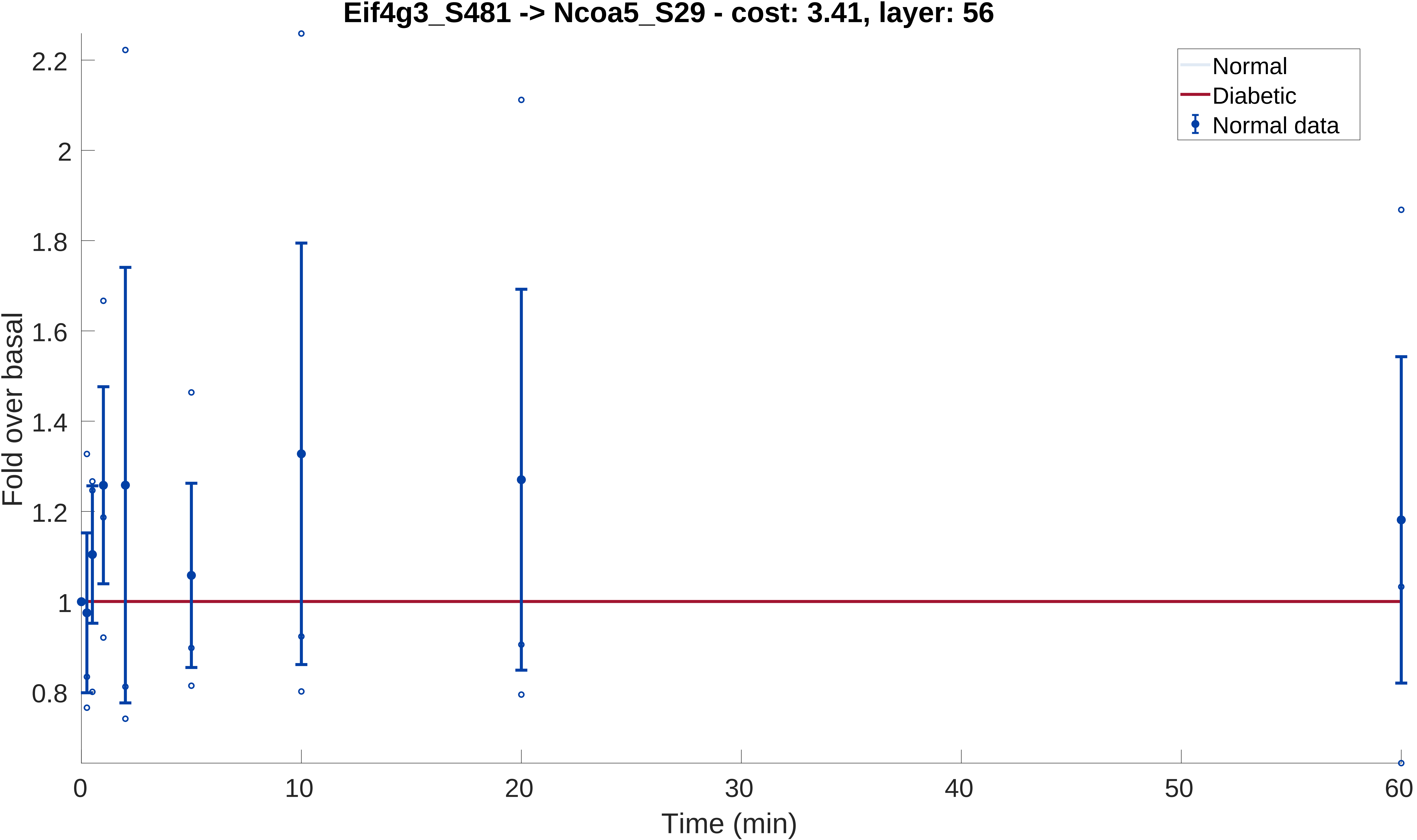

Eif4g3_S481 -> Ncoa5_S29 - cost: 3.41, layer: 56
Normal
Diabetic
Normal data
Fold over basal
2.2
2
1.8
1.6
1.4
1.2
1
0.8
0
10
20
30
40
50
60
Time (min)

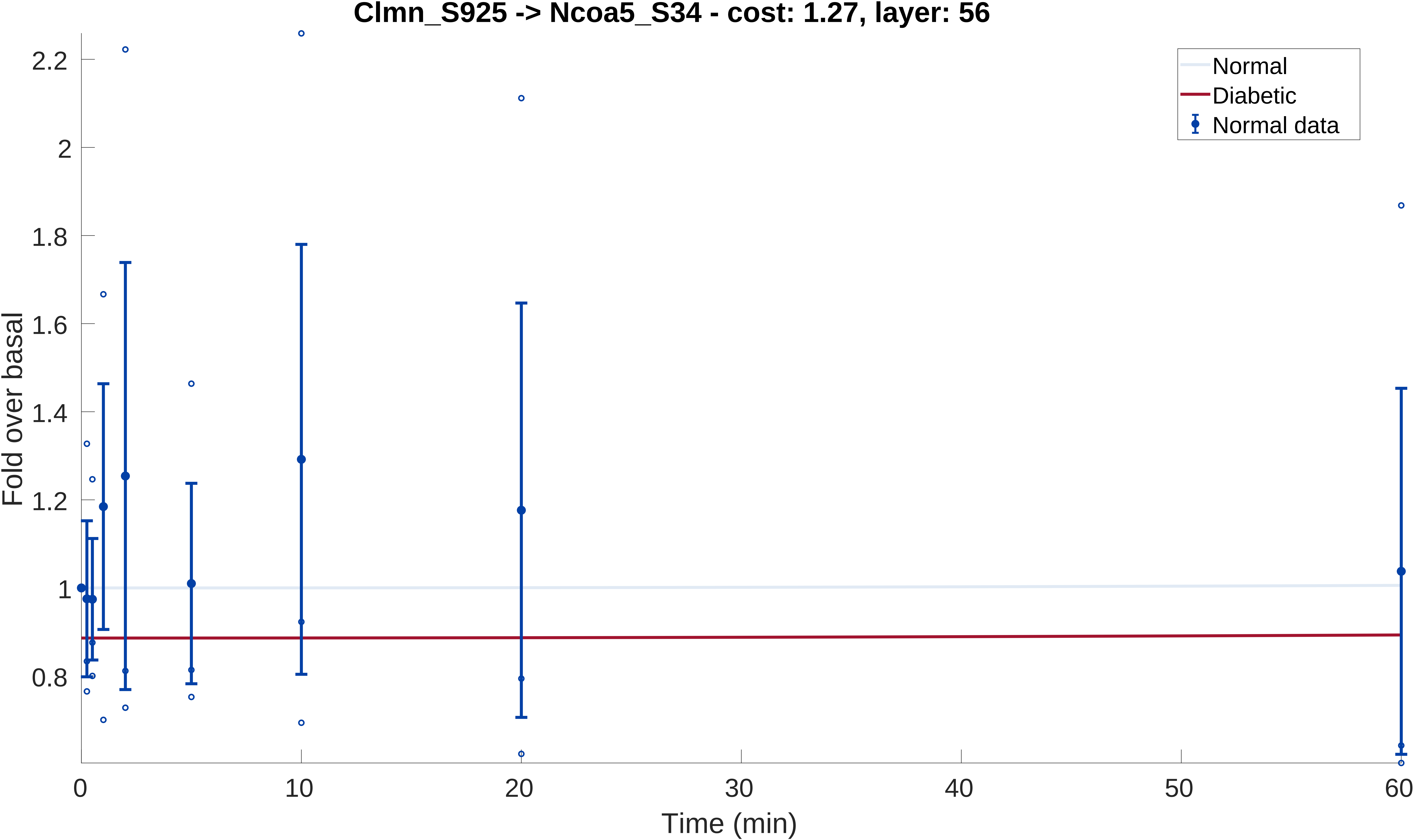
Clmn_S925 -> Ncoa5_S34 - cost: 1.27, layer: 56
Normal
Diabetic
Normal data
Fold over basal
2.2
2
1.8
1.6
1.4
1.2
1
0.8
0
10
20
30
40
50
60
Time (min)

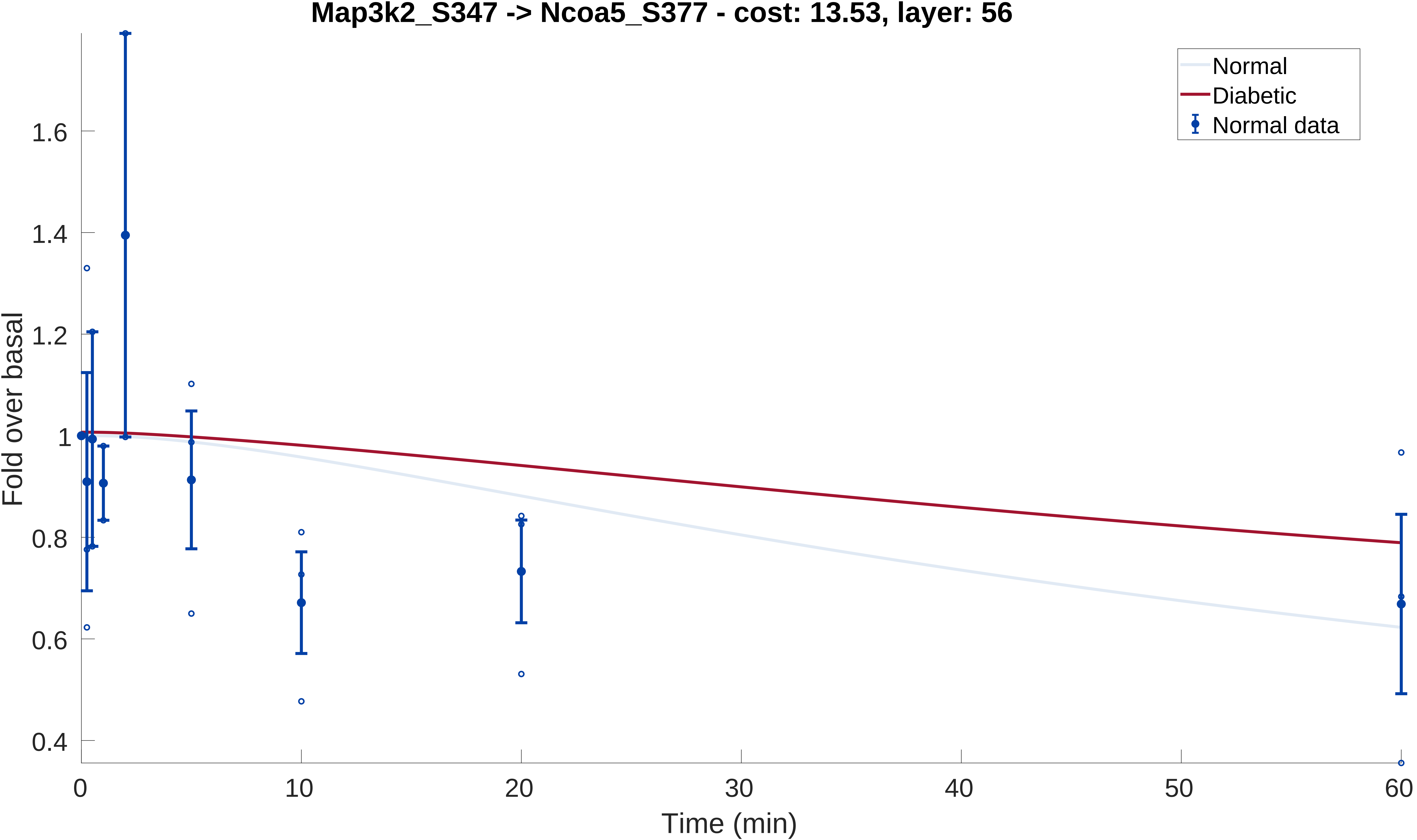

Map3k2_S347 -> Ncoa5_S377 - cost: 13.53, layer: 56
Normal
Diabetic
Normal data
Fold over basal
1.6
1.4
1.2
1
0.8
0.6
0.4
0
10
20
30
40
50
60
Time (min)

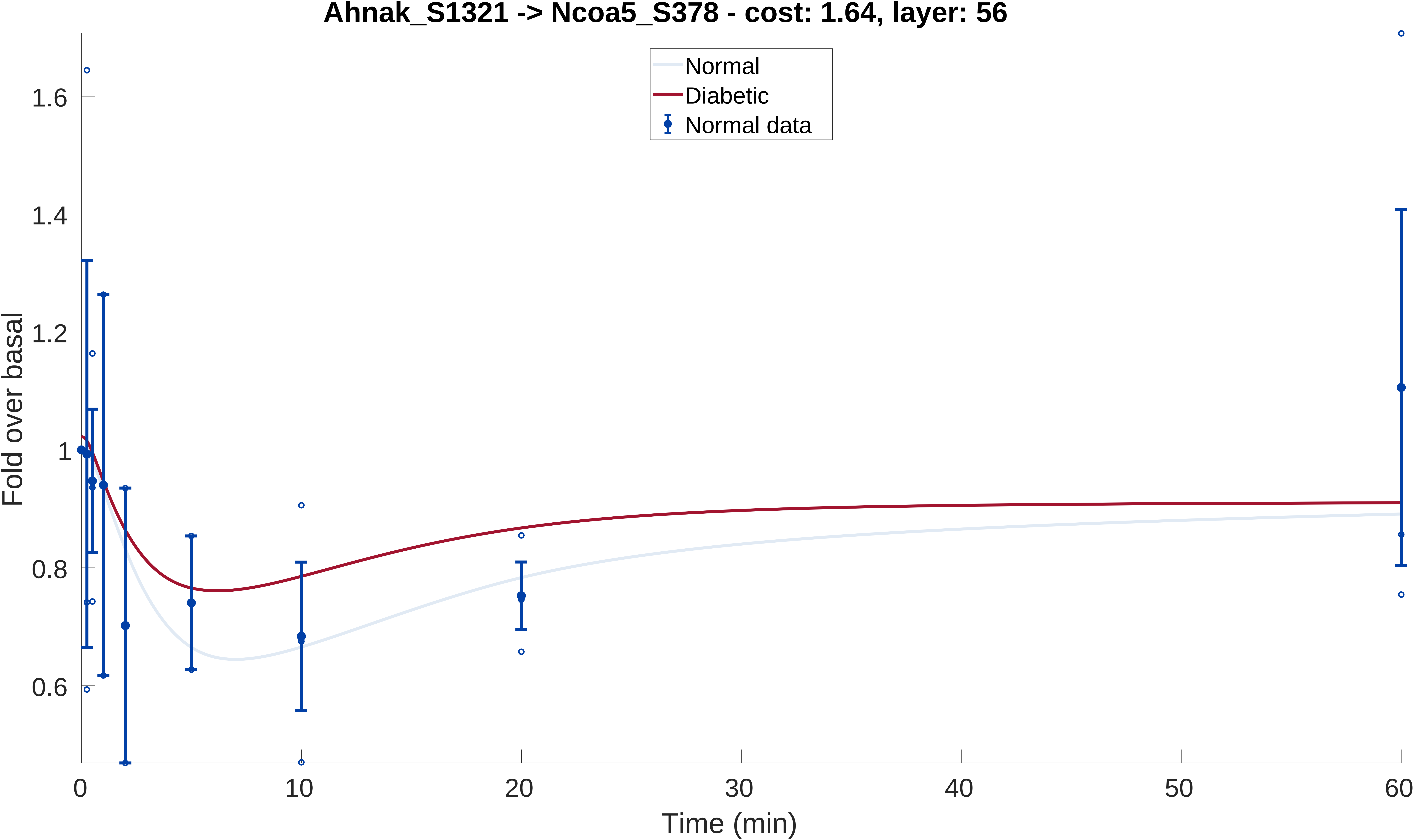

Ahnak_S1321 -> Ncoa5_S378 - cost: 1.64, layer: 56
Normal
Diabetic
Normal data
Fold over basal
1.6
1.4
1.2
1
0.8
0.6
0
10
20
30
40
50
60
Time (min)

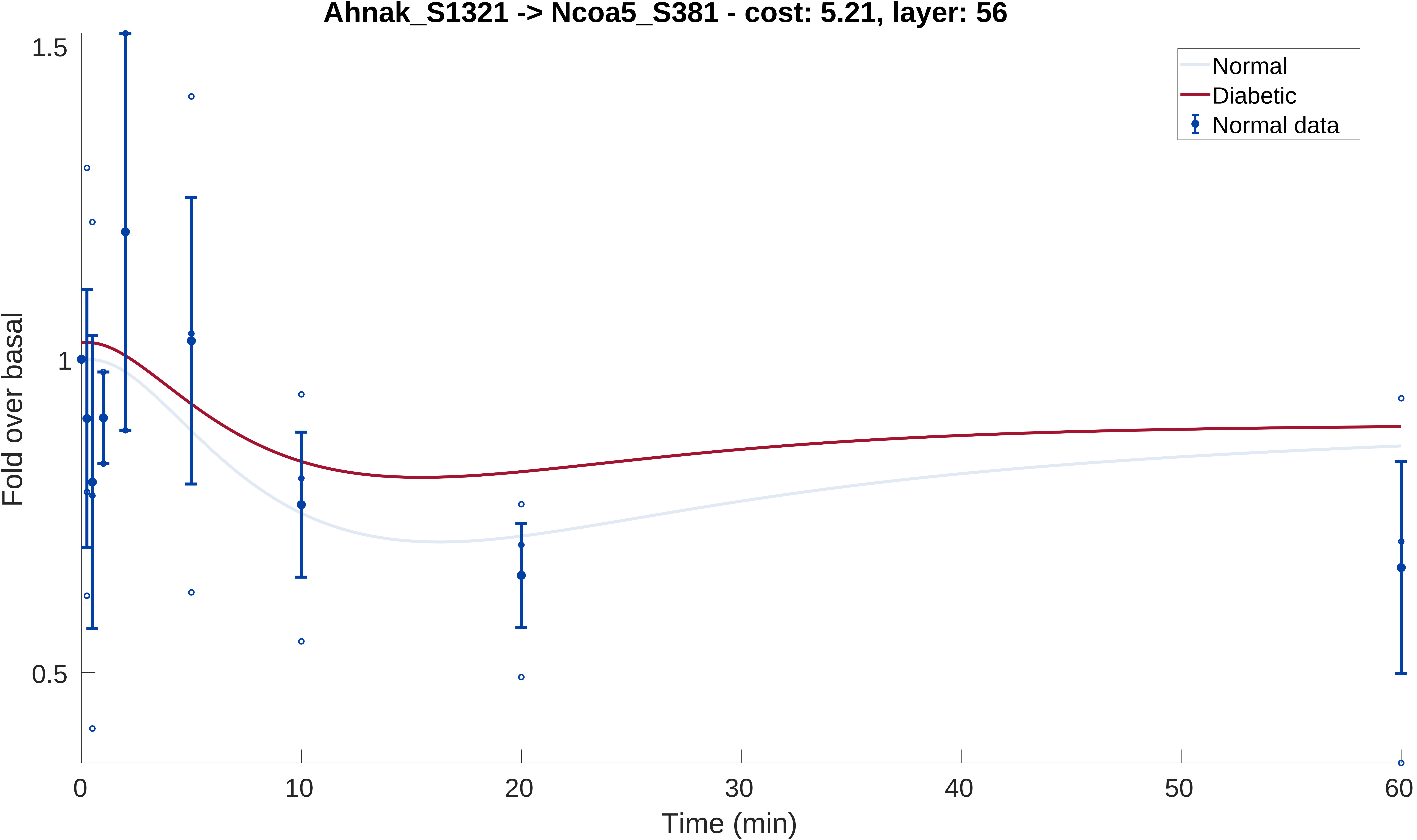

Ahnak_S1321 -> Ncoa5_S381 - cost: 5.21, layer: 56
Fold over basal
1.5
1
0.5
0
10
20
30
40
50
60
Time (min)
Normal
Diabetic
Normal data

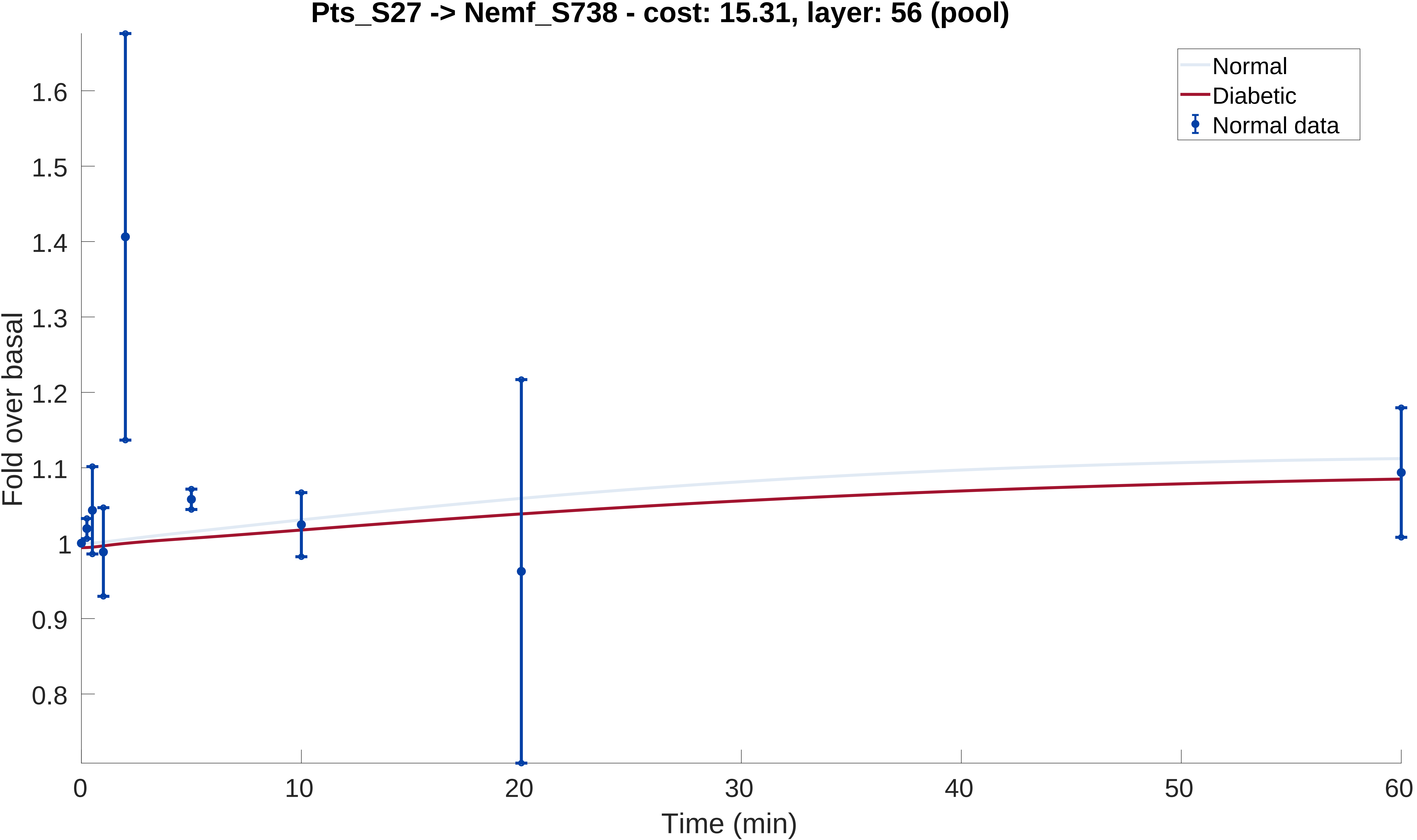
Pts_S27 -> Nemf_S738 - cost: 15.31, layer: 56 (pool)
Normal
Diabetic
Normal data
Fold over basal
1.6
1.5
1.4
1.3
1.2
1.1
1
0.9
0.8
0
10
20
30
40
50
60
Time (min)

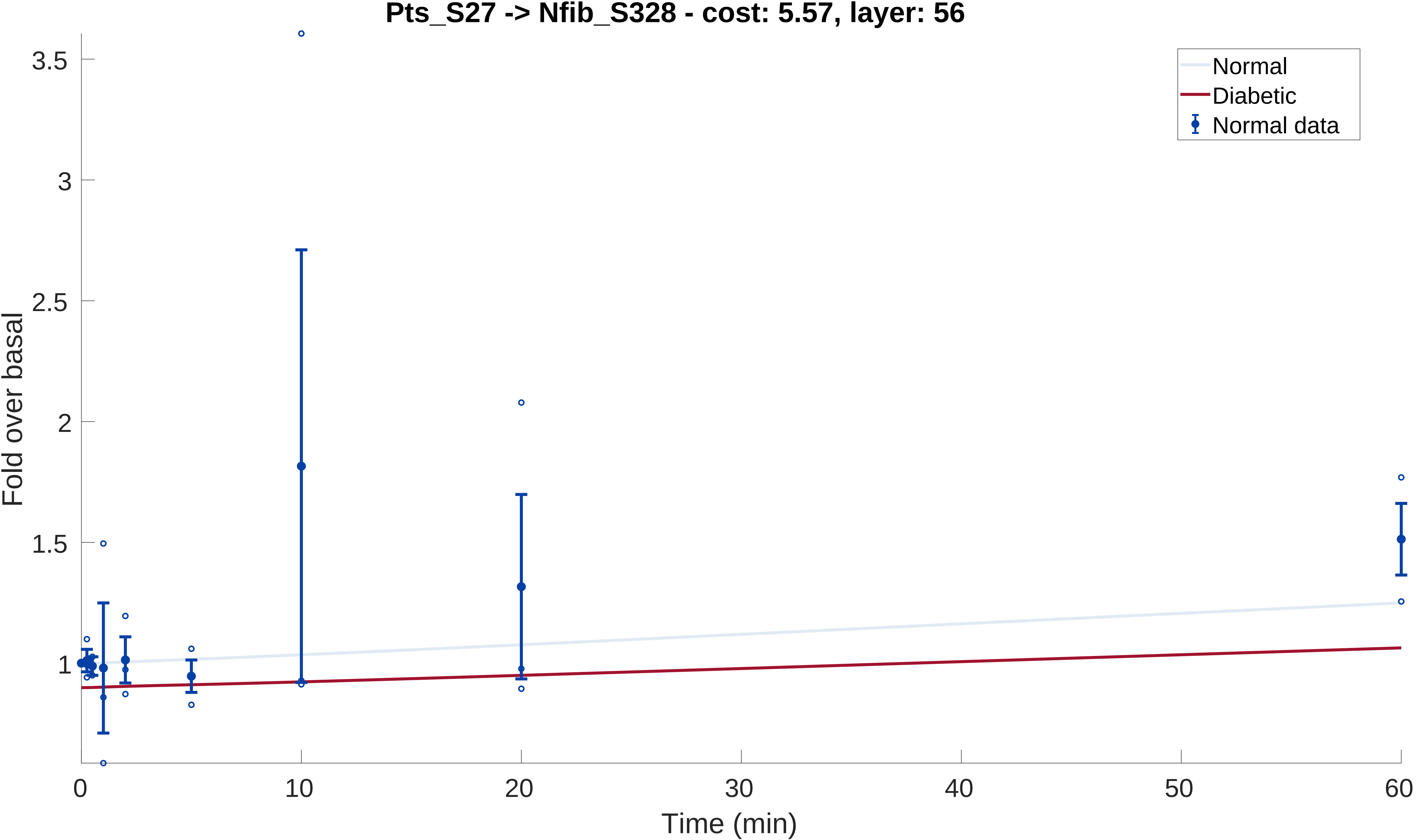
Pts_S27 -> Nfib_S328 - cost: 5.57, layer: 56
Fold over basal
3.5
3
2.5
2
1.5
1
0
10
20
30
40
50
60
Time (min)
Normal
Diabetic
Normal data

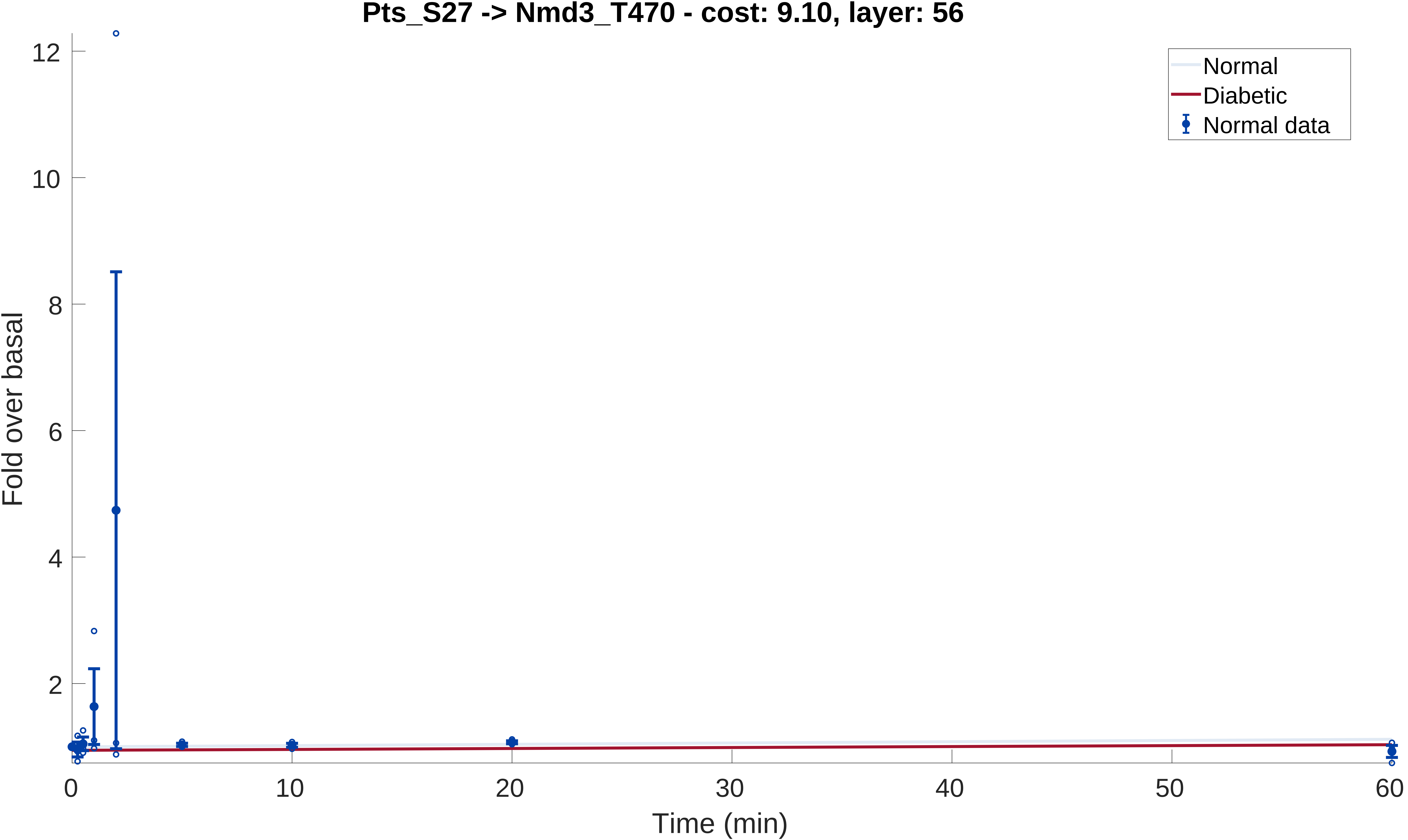

Pts_S27 -> Nmd3_T470 - cost: 9.10, layer: 56
Fold over basal
Time (min)
0
2
4
6
8
10
12
0
10
20
30
40
50
60
Normal
Diabetic
Normal data

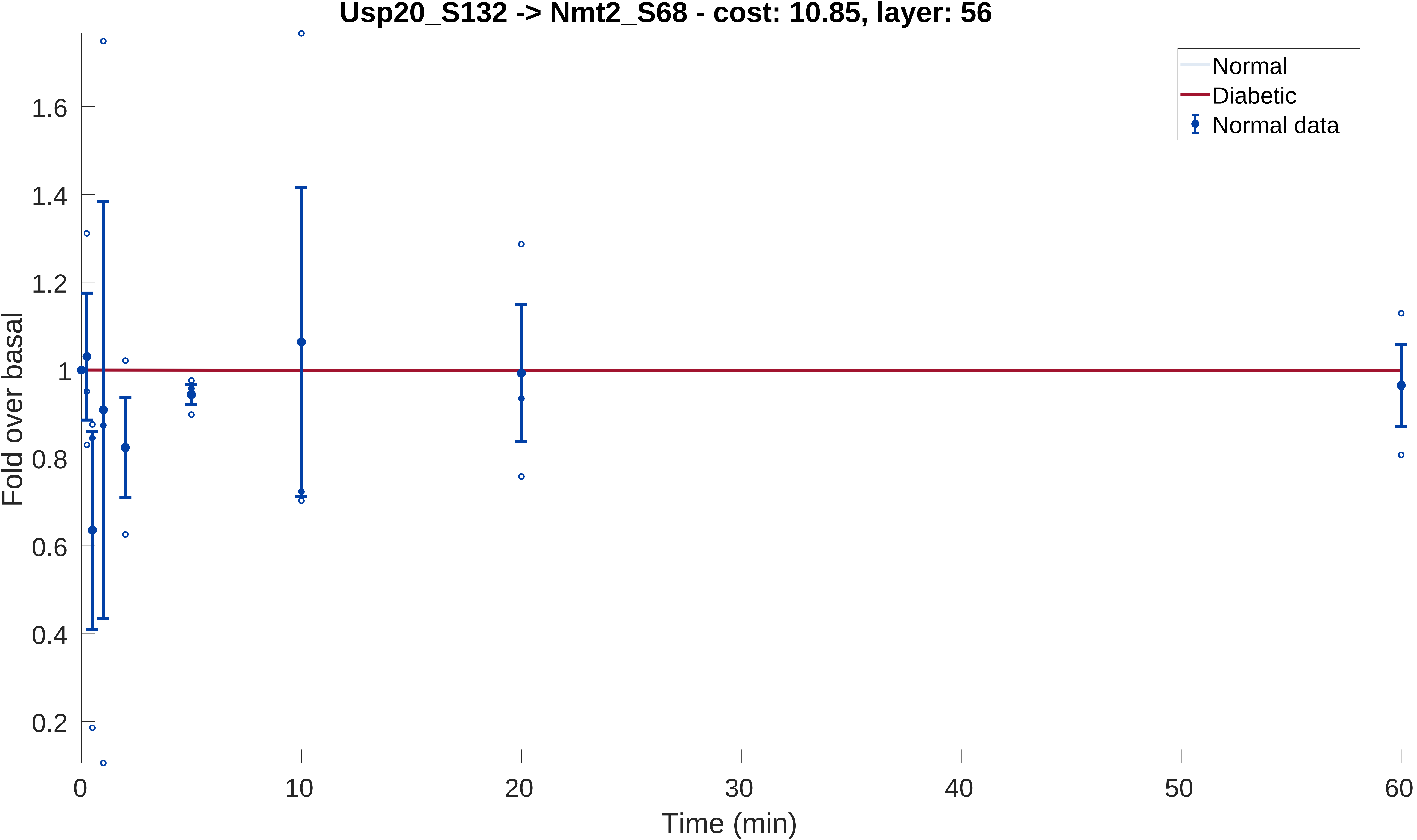

Usp20_S132 -> Nmt2_S68 - cost: 10.85, layer: 56
Normal
Diabetic
Normal data
Fold over basal
Time (min)
0.2
0.4
0.6
0.8
1
1.2
1.4
1.6
0
10
20
30
40
50
60

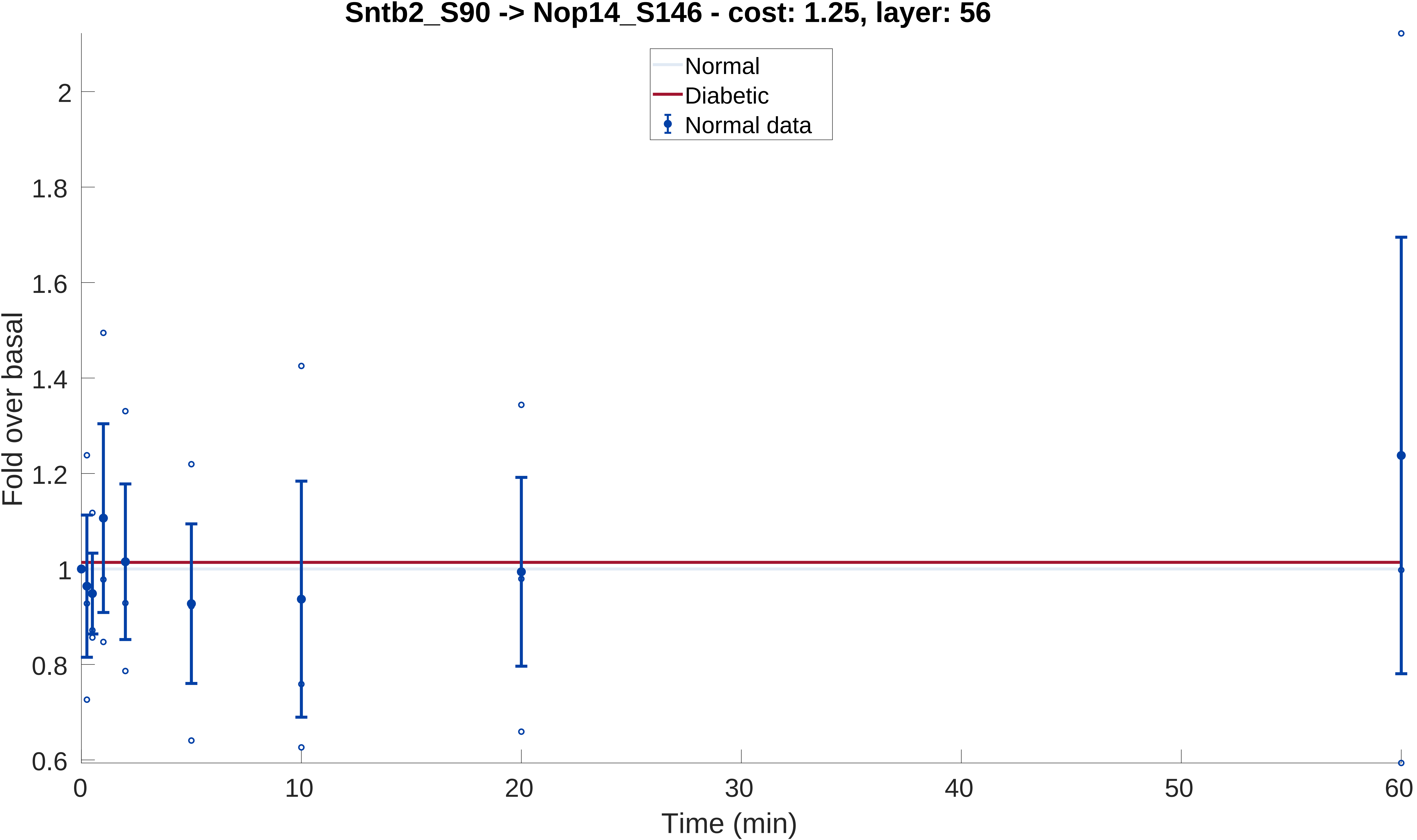
Sntb2_S90 -> Nop14_S146 - cost: 1.25, layer: 56
Normal
Diabetic
Normal data
Fold over basal
0.6
0.8
1
1.2
1.4
1.6
1.8
2
0
10
20
30
40
50
60
Time (min)

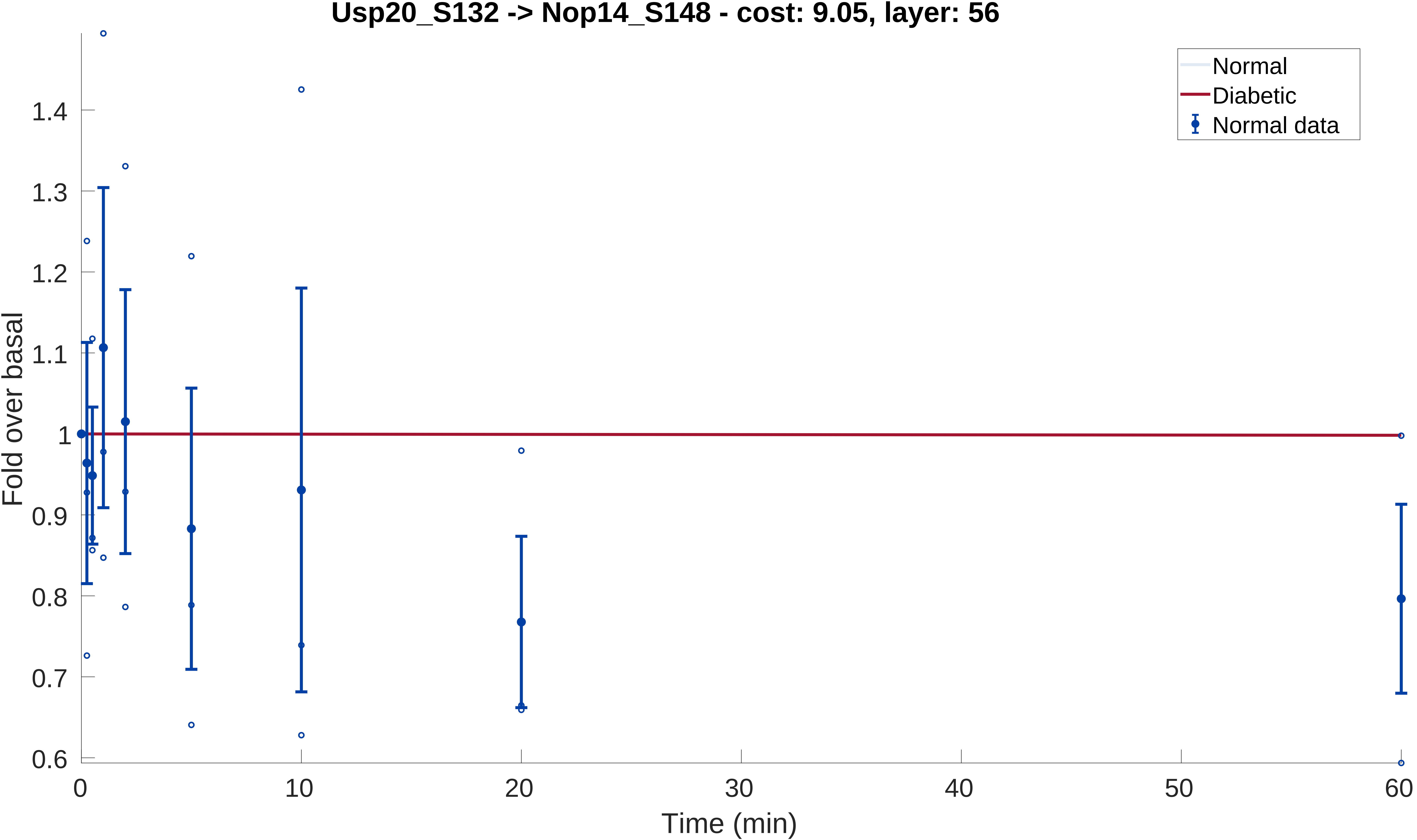
Usp20_S132 -> Nop14_S148 - cost: 9.05, layer: 56
Normal
Diabetic
Normal data
Fold over basal
Time (min)
0.6
0.7
0.8
0.9
1
1.1
1.2
1.3
1.4
0
10
20
30
40
50
60

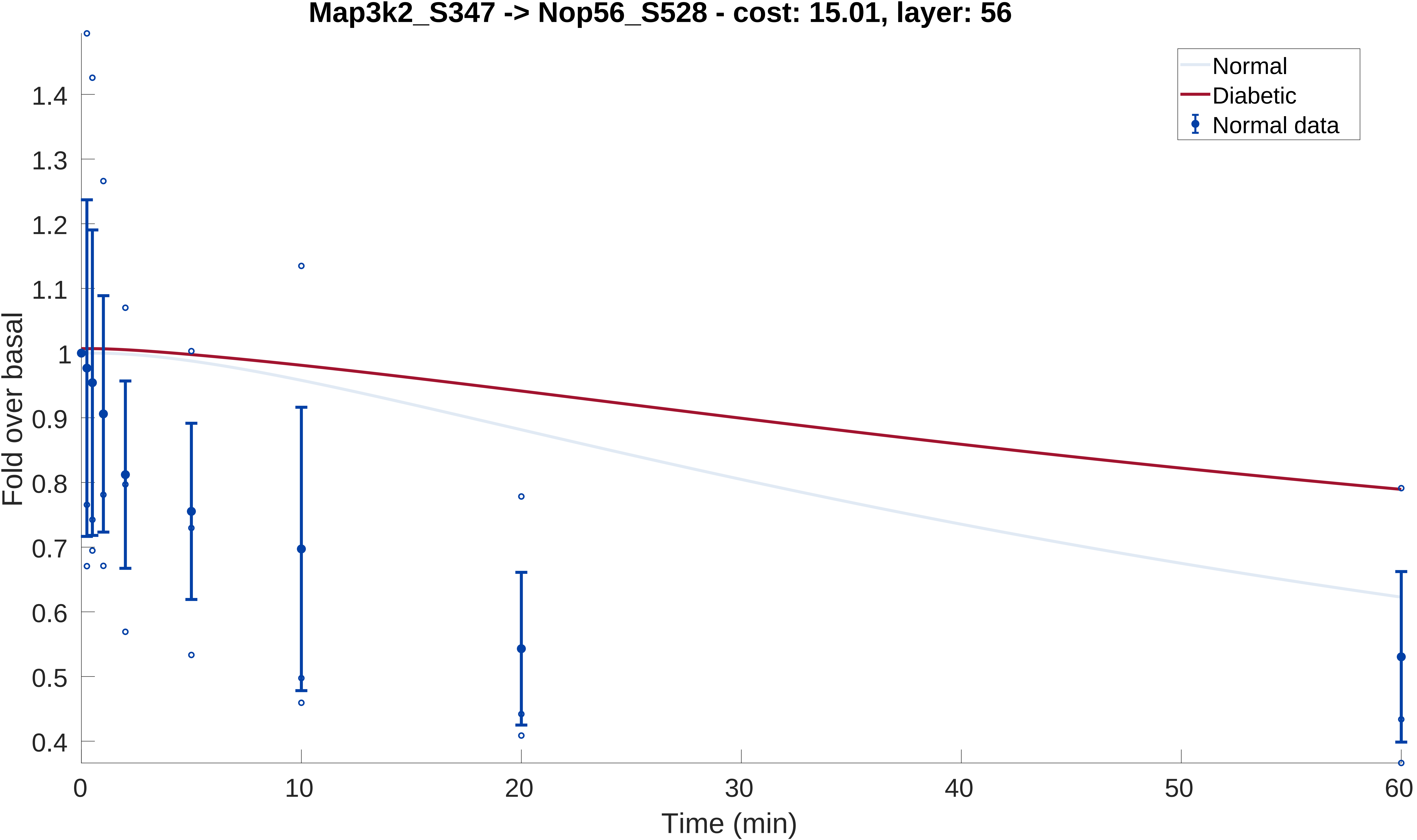
Map3k2_S347 -> Nop56_S528 - cost: 15.01, layer: 56
Normal
Diabetic
Normal data
Fold over basal
1.4
1.3
1.2
1.1
1
0.9
0.8
0.7
0.6
0.5
0.4
0
10
20
30
40
50
60
Time (min)

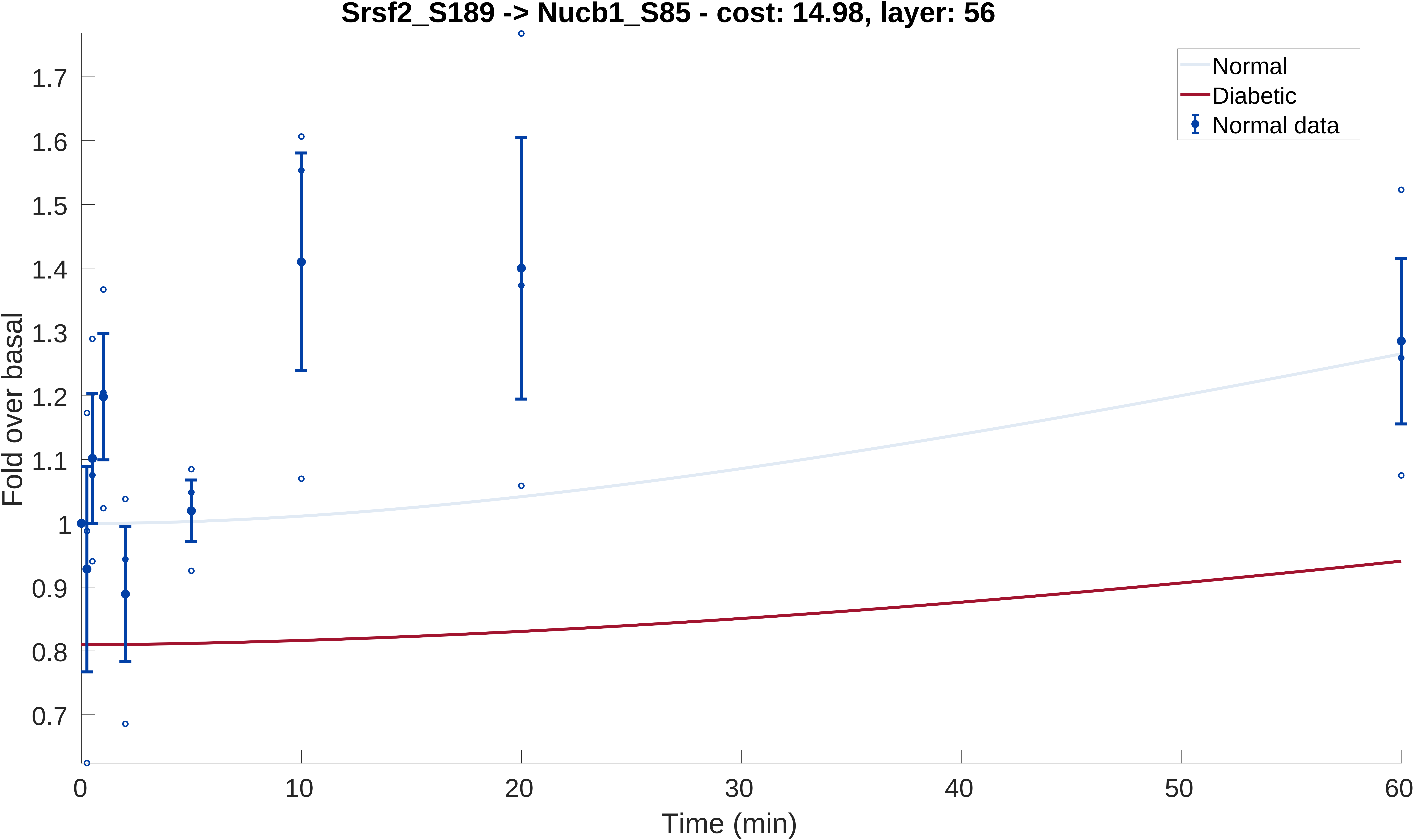

Srsf2_S189 -> Nucb1_S85 - cost: 14.98, layer: 56
Normal
Diabetic
Normal data
Fold over basal
1.7
1.6
1.5
1.4
1.3
1.2
1.1
1
0.9
0.8
0.7
0
10
20
30
40
50
60
Time (min)

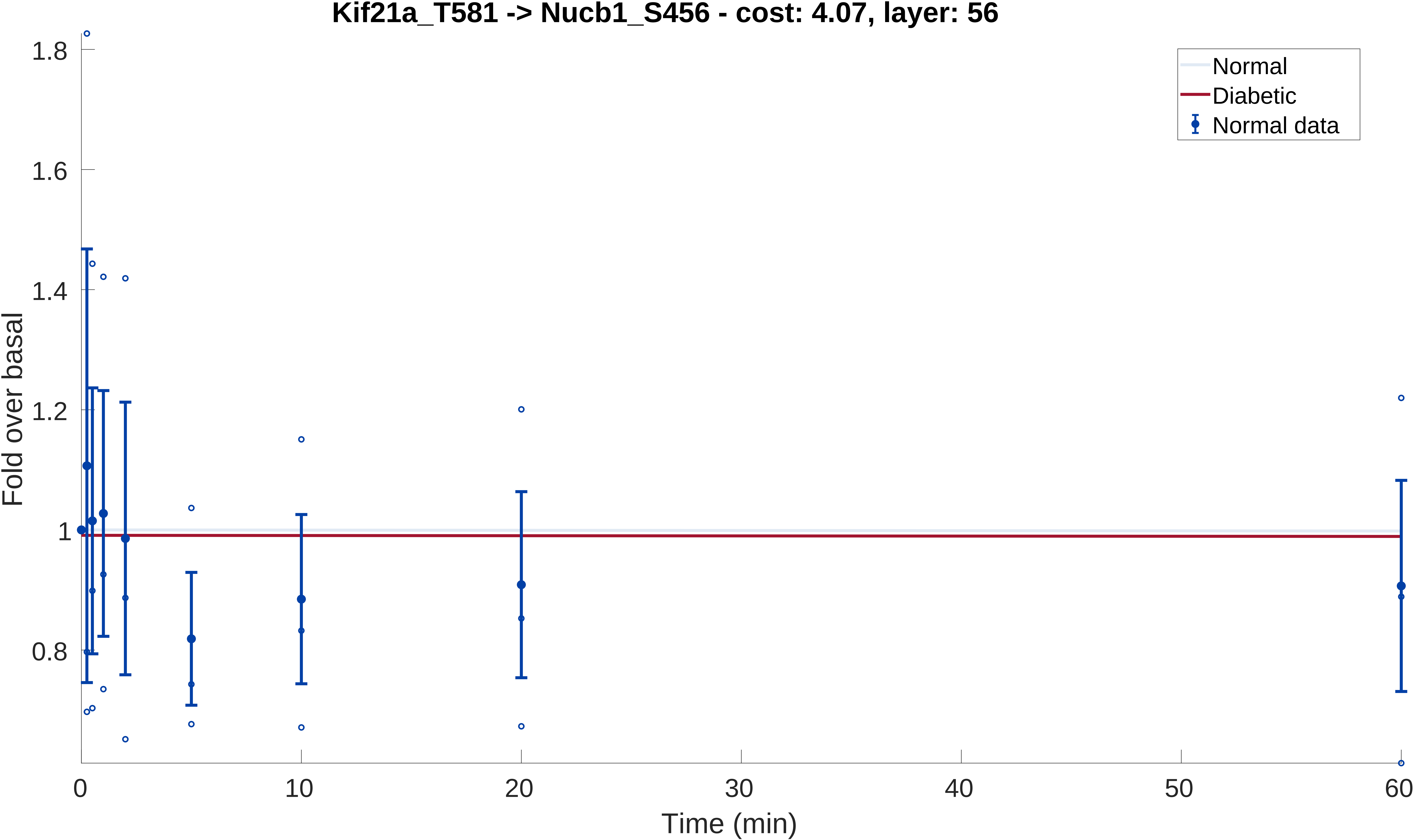

Kif21a_T581 -> Nucb1_S456 - cost: 4.07, layer: 56
Normal
Diabetic
Normal data
Fold over basal
Time (min)
1.8
1.6
1.4
1.2
1
0.8
0
10
20
30
40
50
60

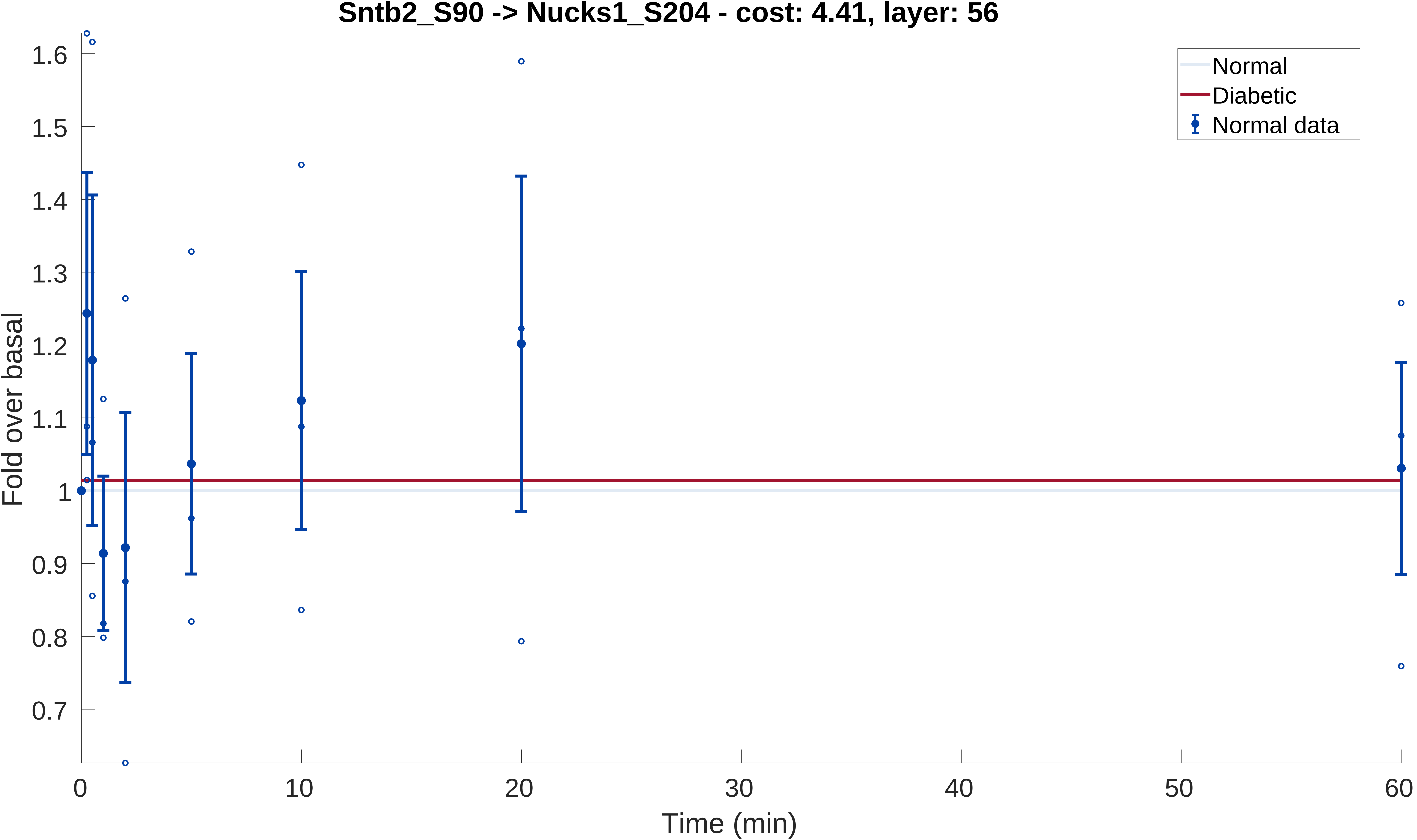

Sntb2_S90 -> Nucks1_S204 - cost: 4.41, layer: 56
Normal
Diabetic
Normal data
Fold over basal
Time (min)
1.6
1.5
1.4
1.3
1.2
1.1
1
0.9
0.8
0.7
0
10
20
30
40
50
60

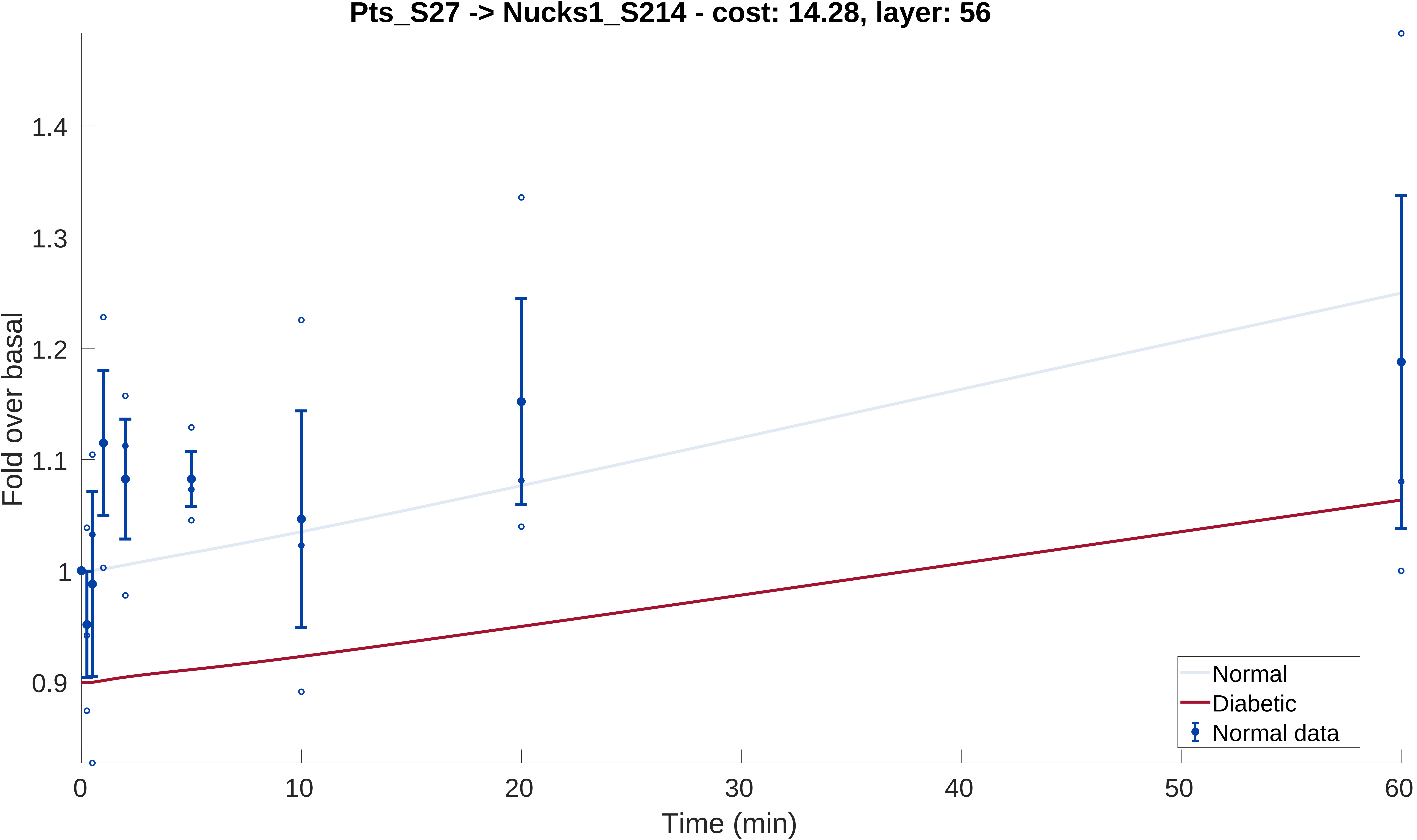

Pts_S27 -> Nucks1_S214 - cost: 14.28, layer: 56
Fold over basal
1.4
1.3
1.2
1.1
1
0.9
0
10
20
30
40
50
60
Time (min)
Normal
Diabetic
Normal data

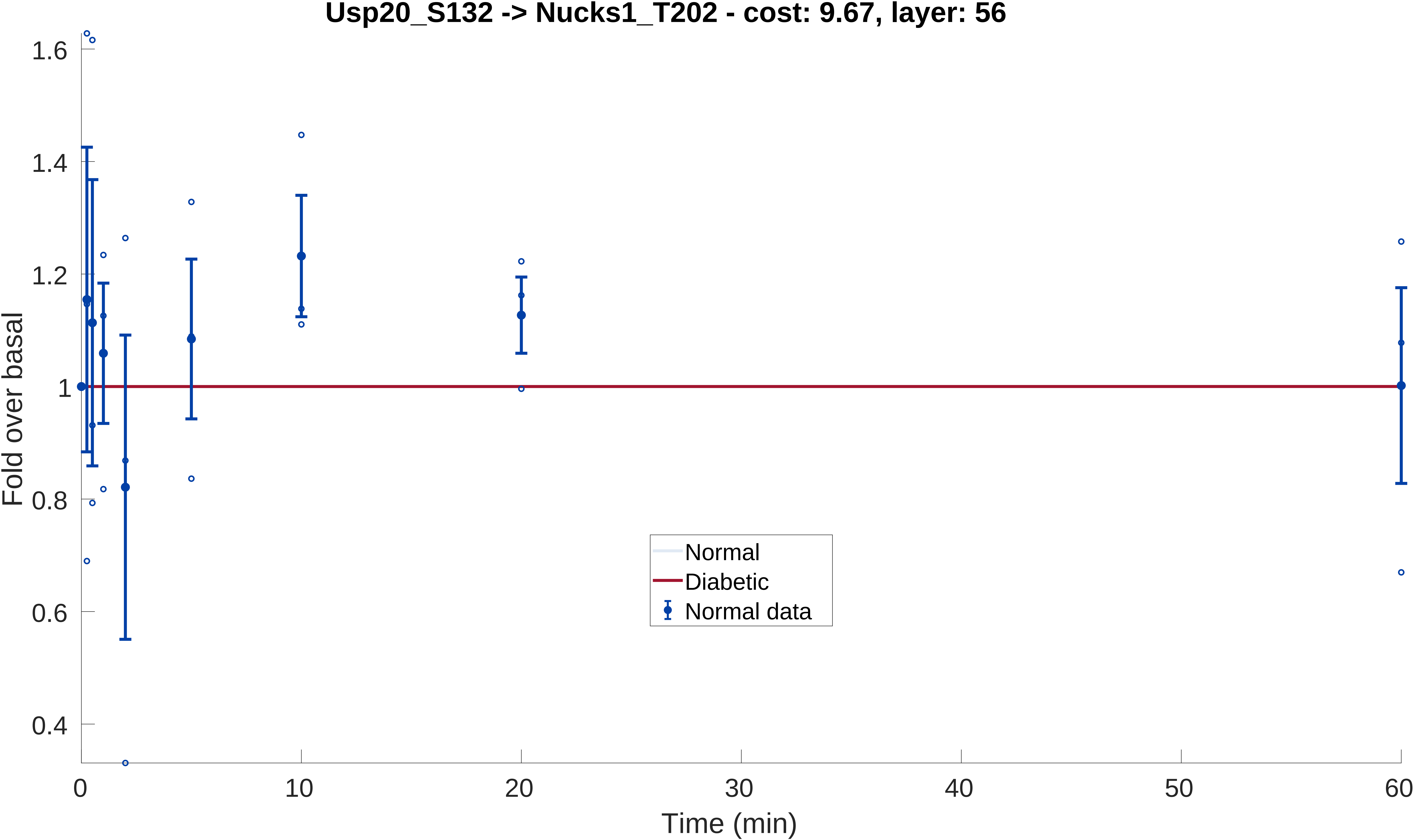

Usp20_S132 -> Nucks1_T202 - cost: 9.67, layer: 56
Fold over basal
Time (min)
1.6
1.4
1.2
1
0.8
0.6
0.4
0
10
20
30
40
50
60
Normal
Diabetic
Normal data

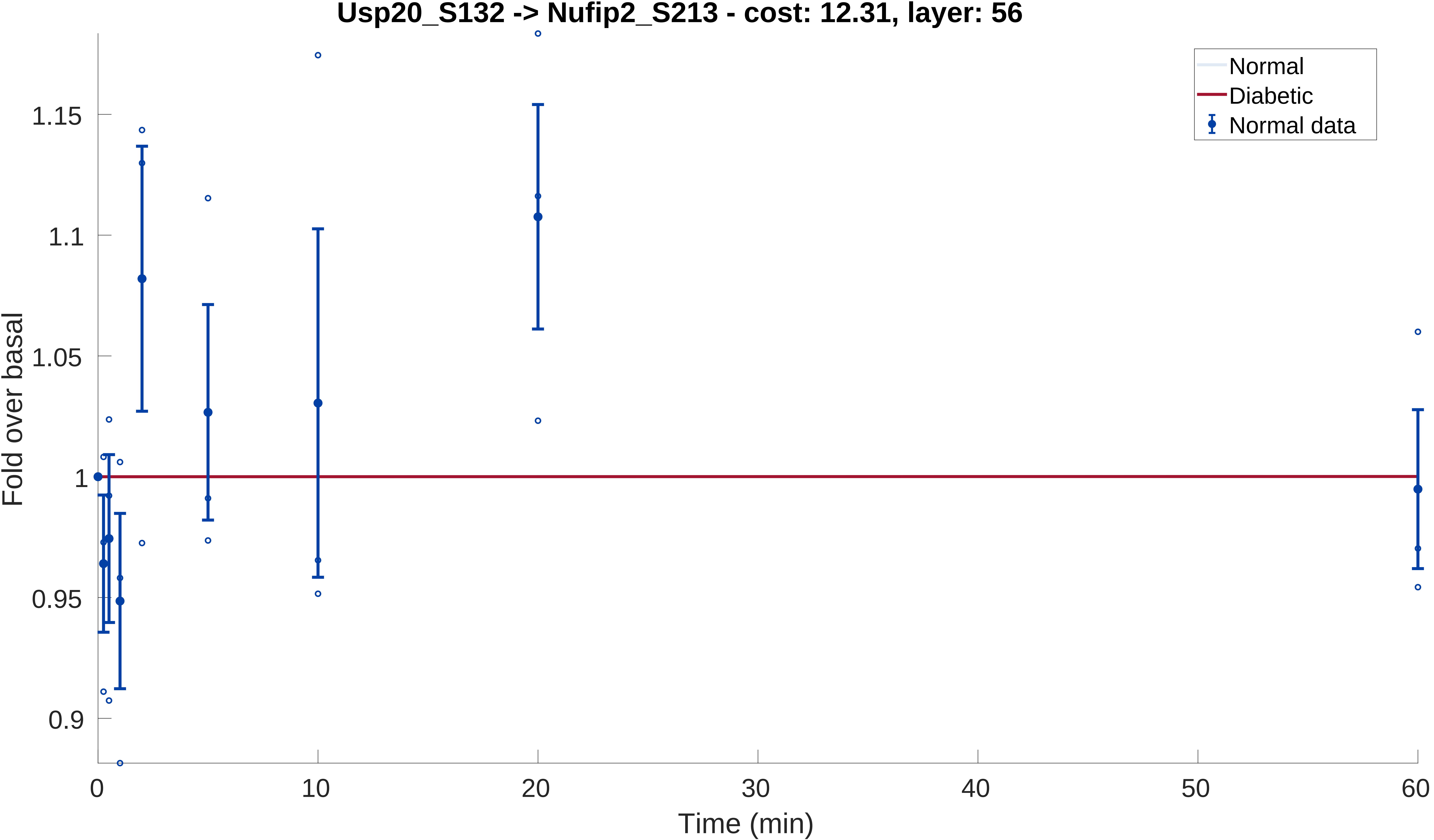

Usp20_S132 -> Nufip2_S213 - cost: 12.31, layer: 56
Normal
Diabetic
Normal data
Fold over basal
1.15
1.1
1.05
1
0.95
0.9
0
10
20
30
40
50
60
Time (min)

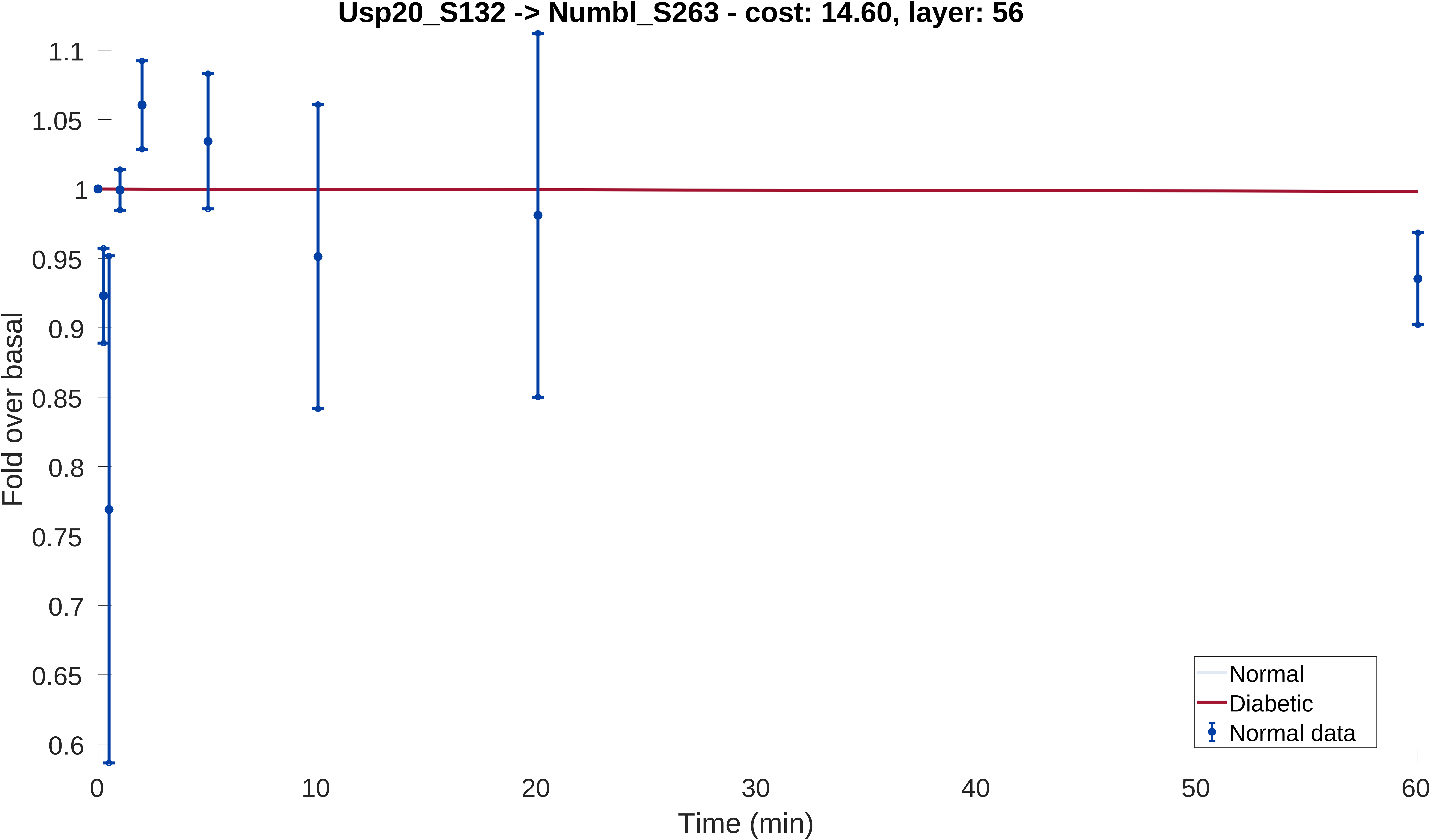

Usp20_S132 -> Numbl_S263 - cost: 14.60, layer: 56
Fold over basal
1.1
1.05
1
0.95
0.9
0.85
0.8
0.75
0.7
0.65
0.6
0
10
20
30
40
50
60
Time (min)
Normal
Diabetic
Normal data

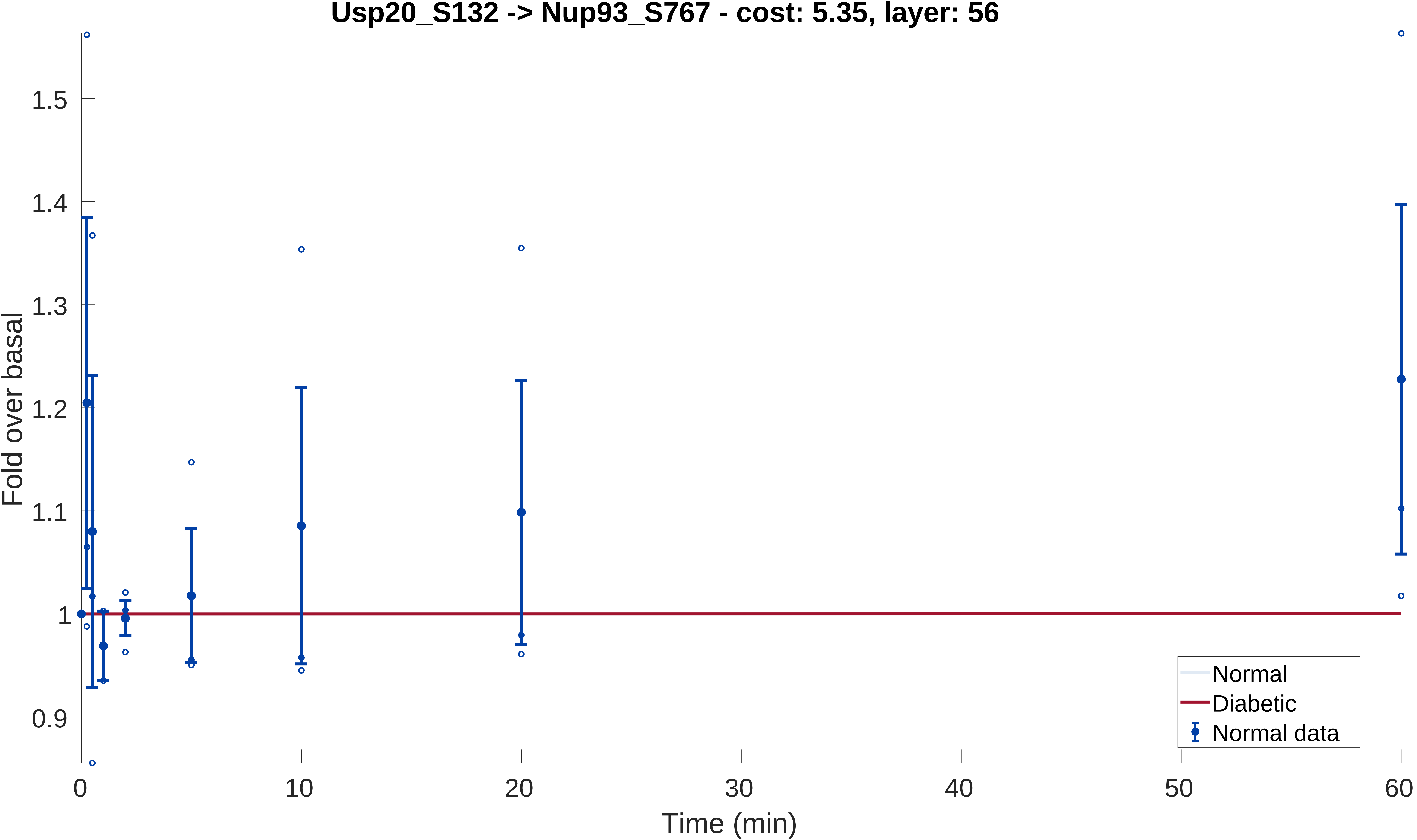

Usp20_S132 -> Nup93_S767 - cost: 5.35, layer: 56
Fold over basal
1.5
1.4
1.3
1.2
1.1
1
0.9
0
10
20
30
40
50
60
Time (min)
Normal
Diabetic
Normal data

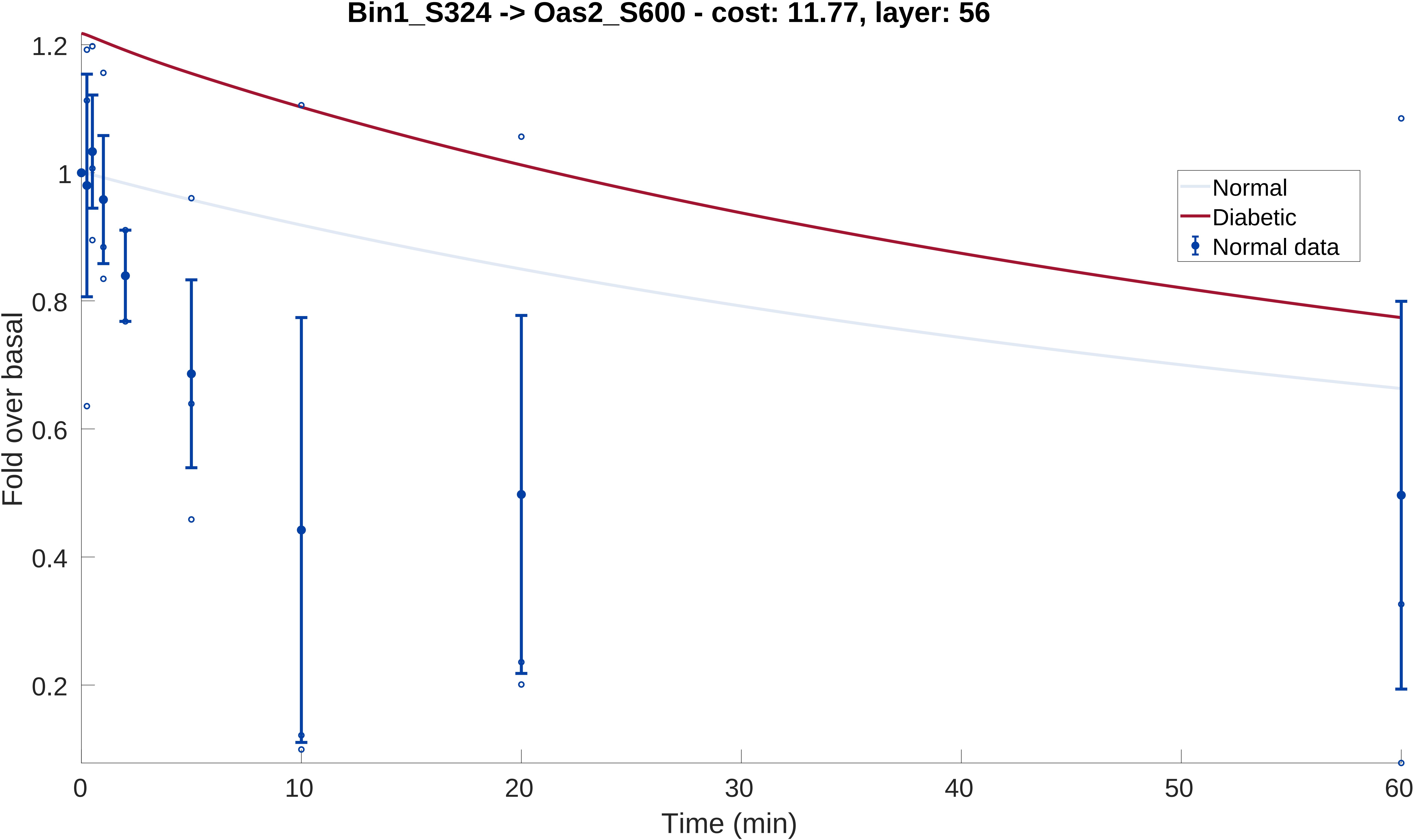

Bin1_S324 -> Oas2_S600 - cost: 11.77, layer: 56
Fold over basal
1.2
1
0.8
0.6
0.4
0.2
0
10
20
30
40
50
60
Time (min)
Normal
Diabetic
Normal data

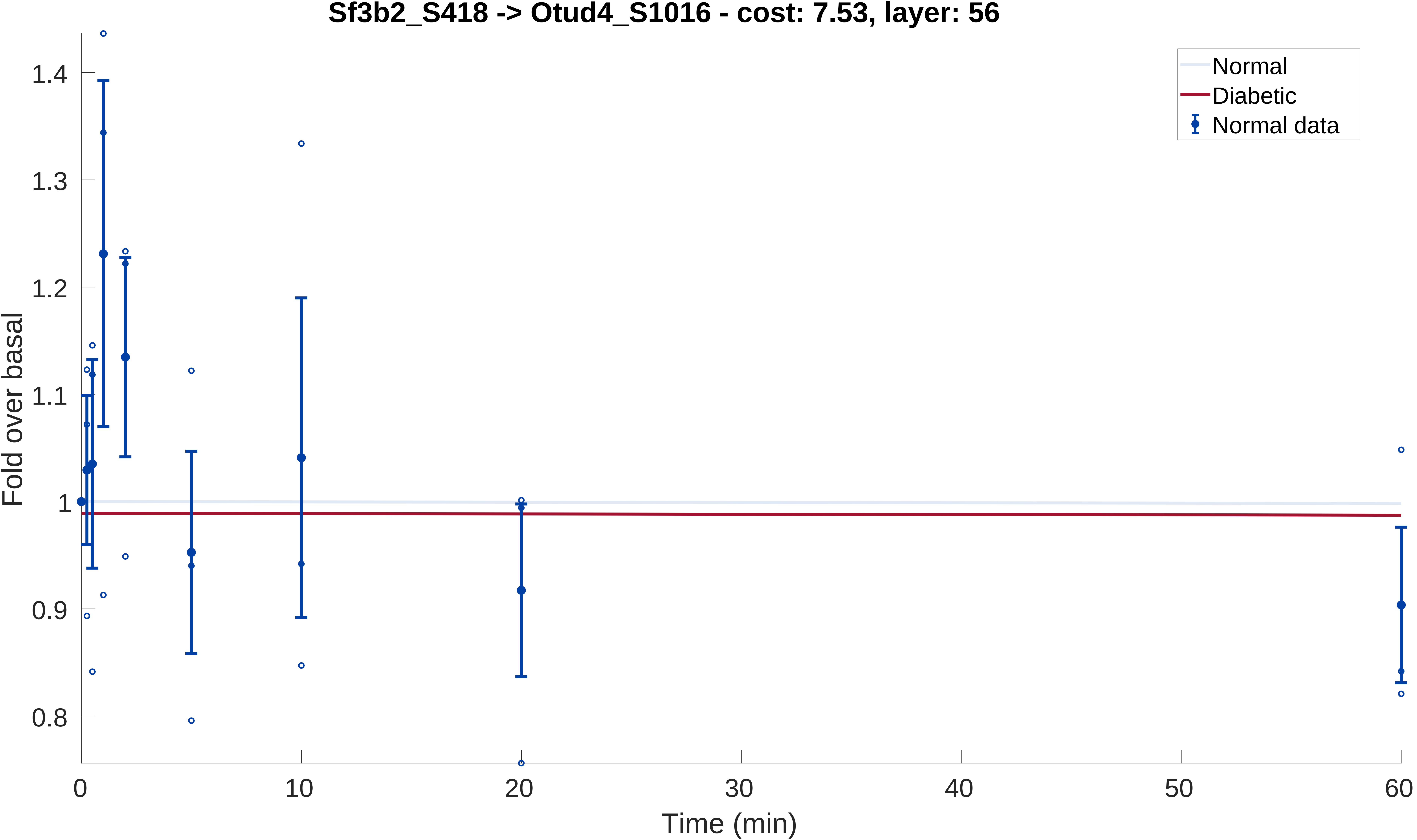

Sf3b2_S418 -> Otud4_S1016 - cost: 7.53, layer: 56
Normal
Diabetic
Normal data
Fold over basal
1.4
1.3
1.2
1.1
1
0.9
0.8
0
10
20
30
40
50
60
Time (min)

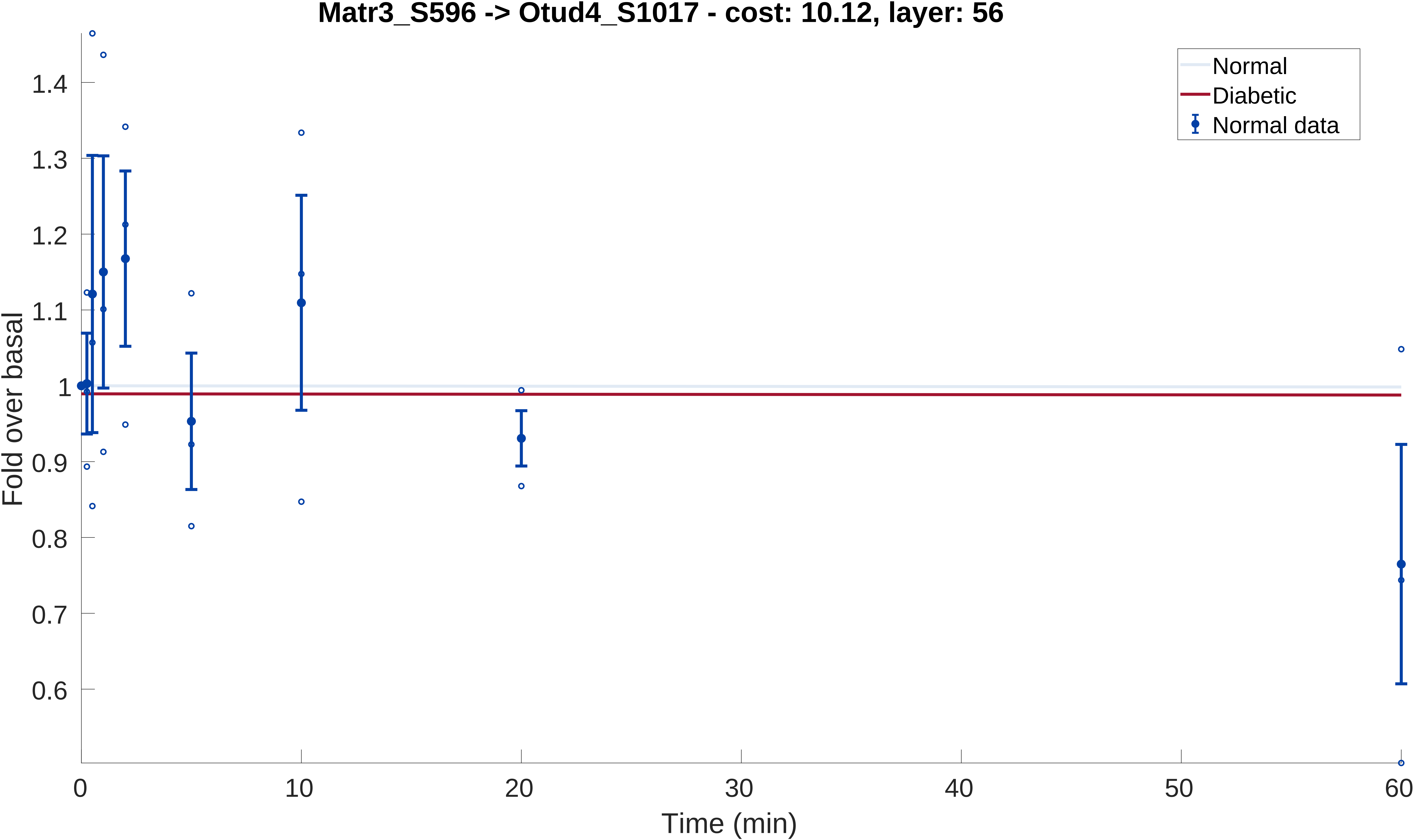

Matr3_S596 -> Otud4_S1017 - cost: 10.12, layer: 56
Normal
Diabetic
Normal data
Fold over basal
Time (min)
1.4
1.3
1.2
1.1
1
0.9
0.8
0.7
0.6
0
10
20
30
40
50
60

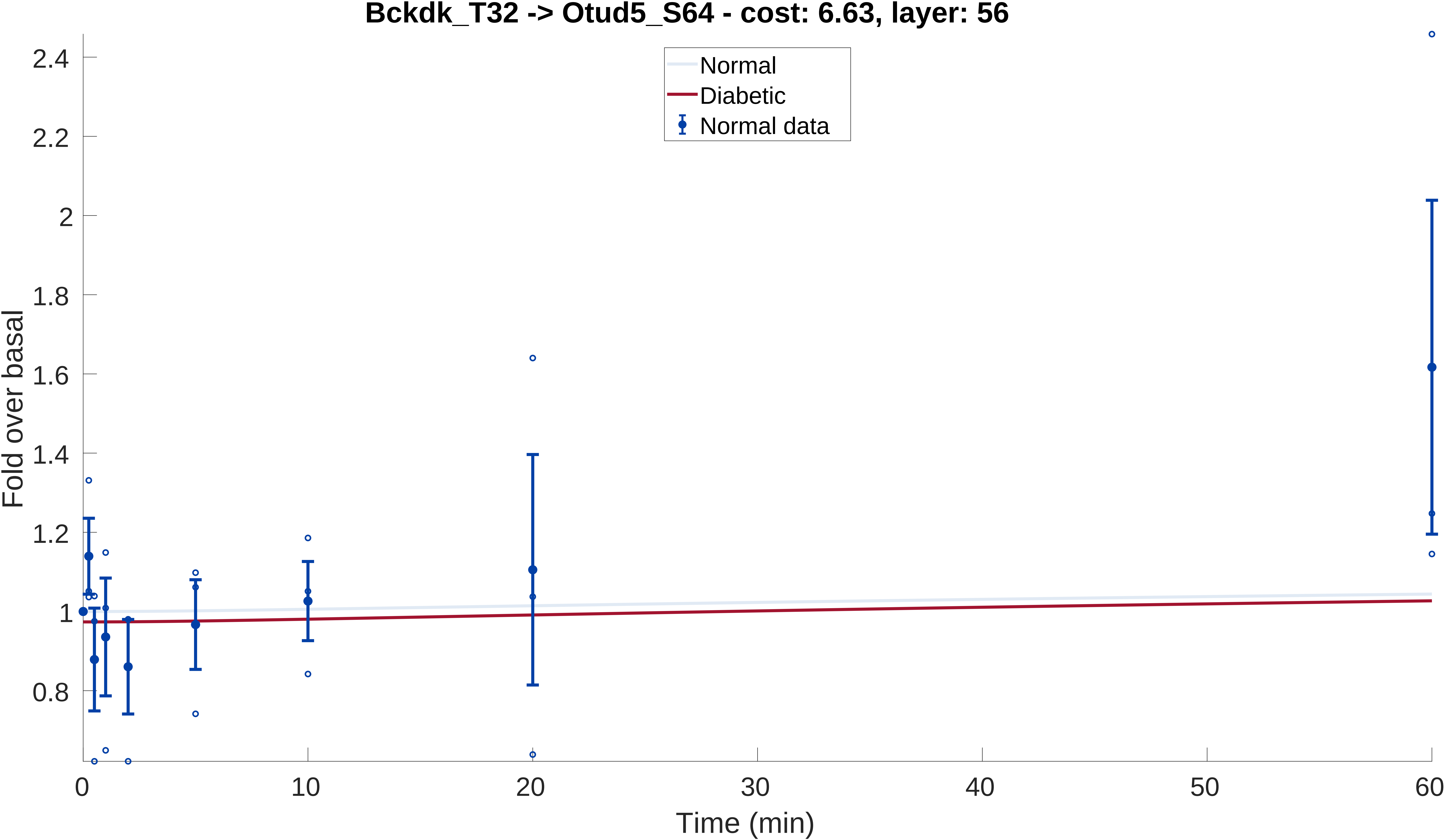

Bckdk_T32 -> Otud5_S64 - cost: 6.63, layer: 56
Normal
Diabetic
Normal data
Fold over basal
Time (min)
2.4
2.2
2
1.8
1.6
1.4
1.2
1
0.8
0
10
20
30
40
50
60

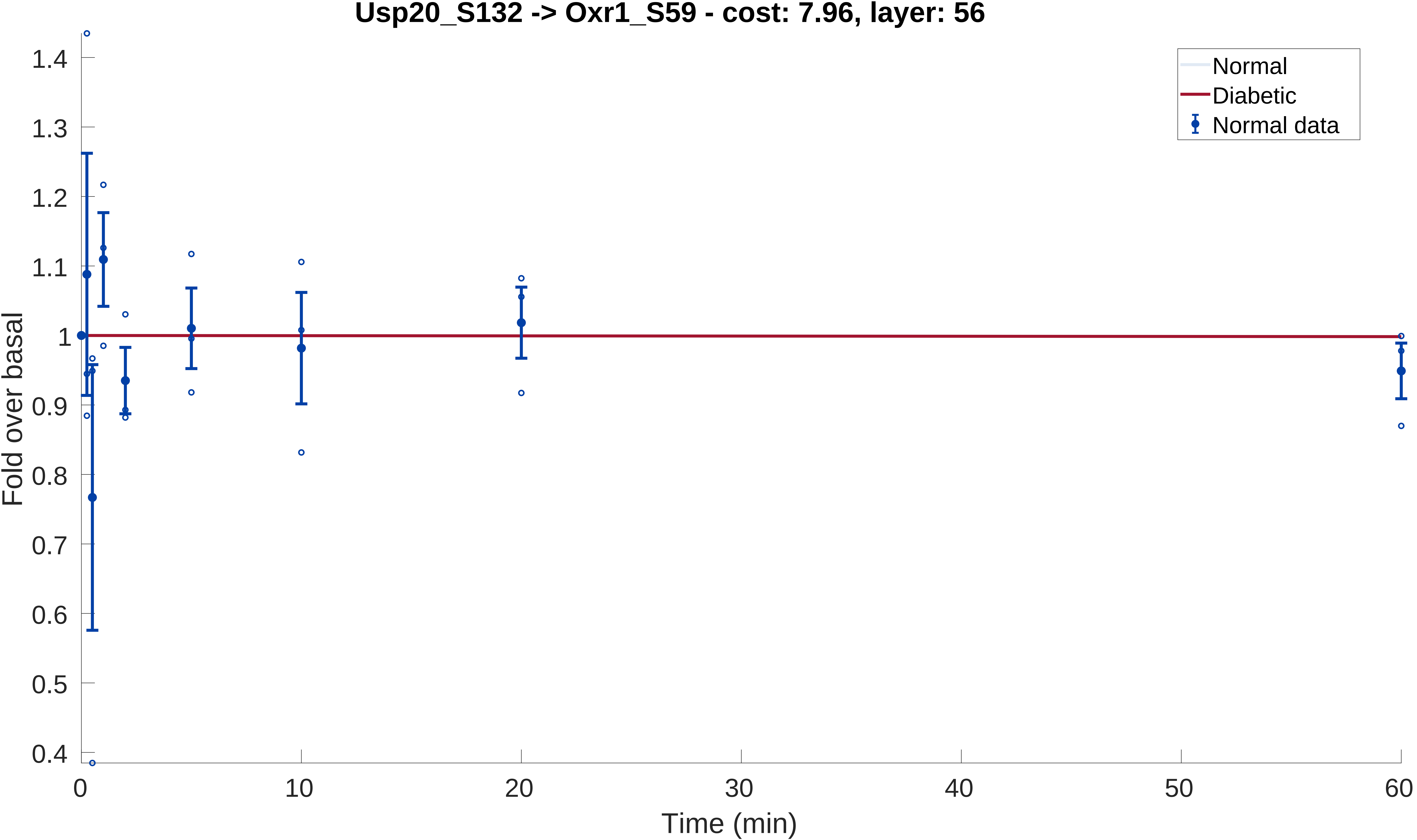

Usp20_S132 -> Oxr1_S59 - cost: 7.96, layer: 56
Normal
Diabetic
Normal data
Fold over basal
Time (min)
1.4
1.3
1.2
1.1
1
0.9
0.8
0.7
0.6
0.5
0.4
0
10
20
30
40
50
60

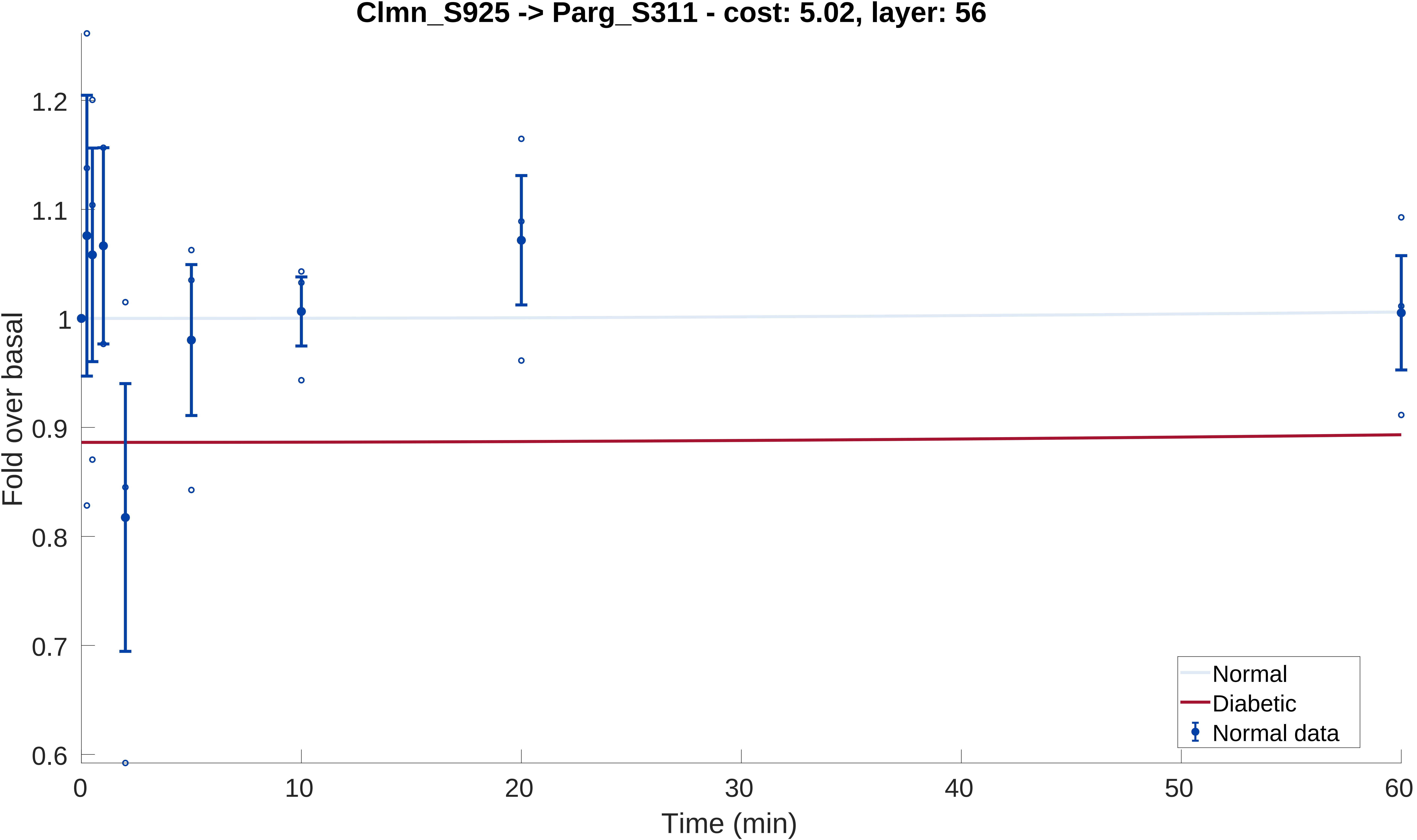

Clmn_S925 -> Parg_S311 - cost: 5.02, layer: 56
Fold over basal
1.2
1.1
1
0.9
0.8
0.7
0.6
0
10
20
30
40
50
60
Time (min)
Normal
Diabetic
Normal data

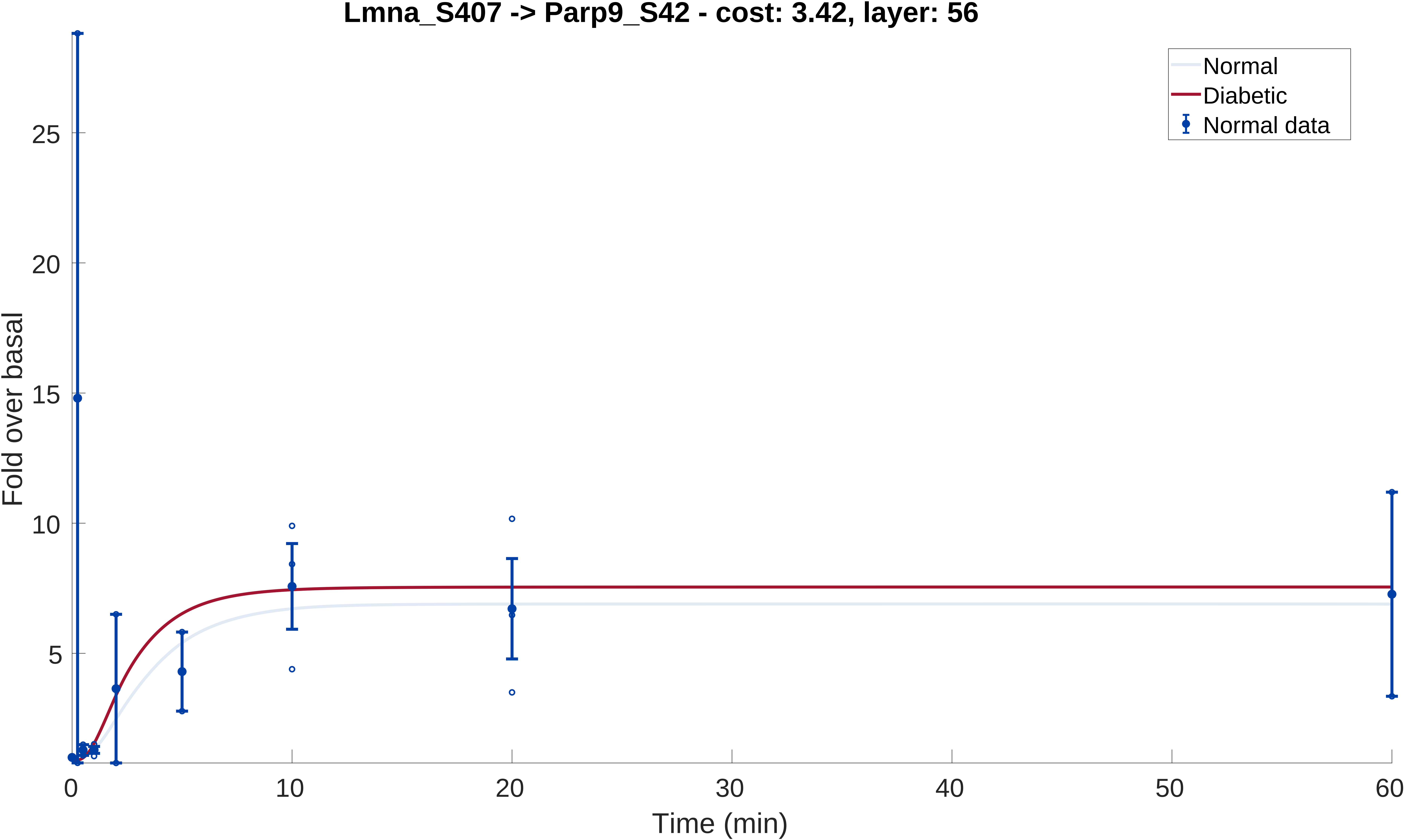
Lmna_S407 -> Parp9_S42 - cost: 3.42, layer: 56
Normal
Diabetic
Normal data
Fold over basal
Time (min)
5
10
15
20
25
0
10
20
30
40
50
60

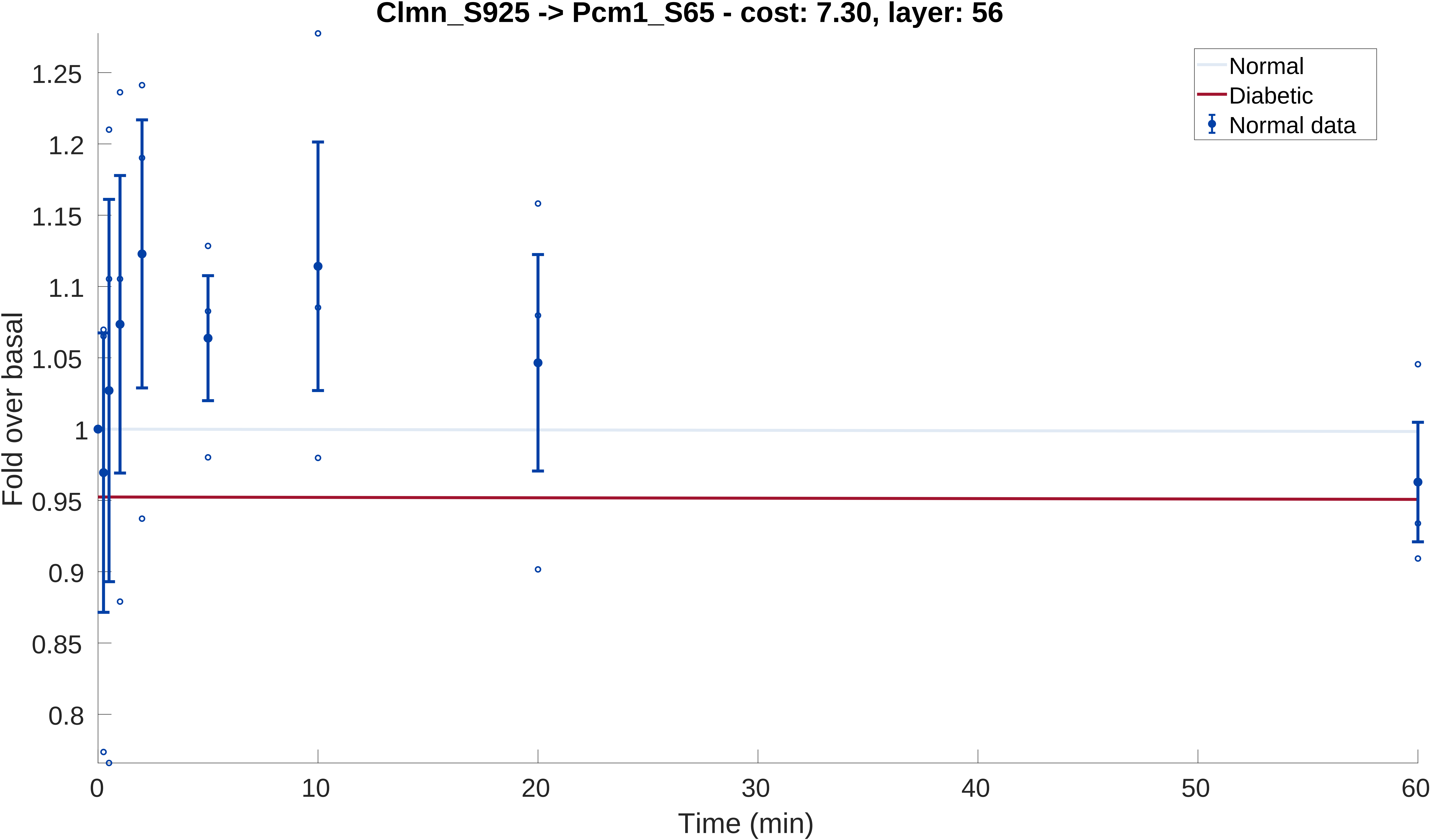

Clmn_S925 -> Pcm1_S65 - cost: 7.30, layer: 56
Normal
Diabetic
Normal data
Fold over basal
1.25
1.2
1.15
1.1
1.05
1
0.95
0.9
0.85
0.8
0
10
20
30
40
50
60
Time (min)

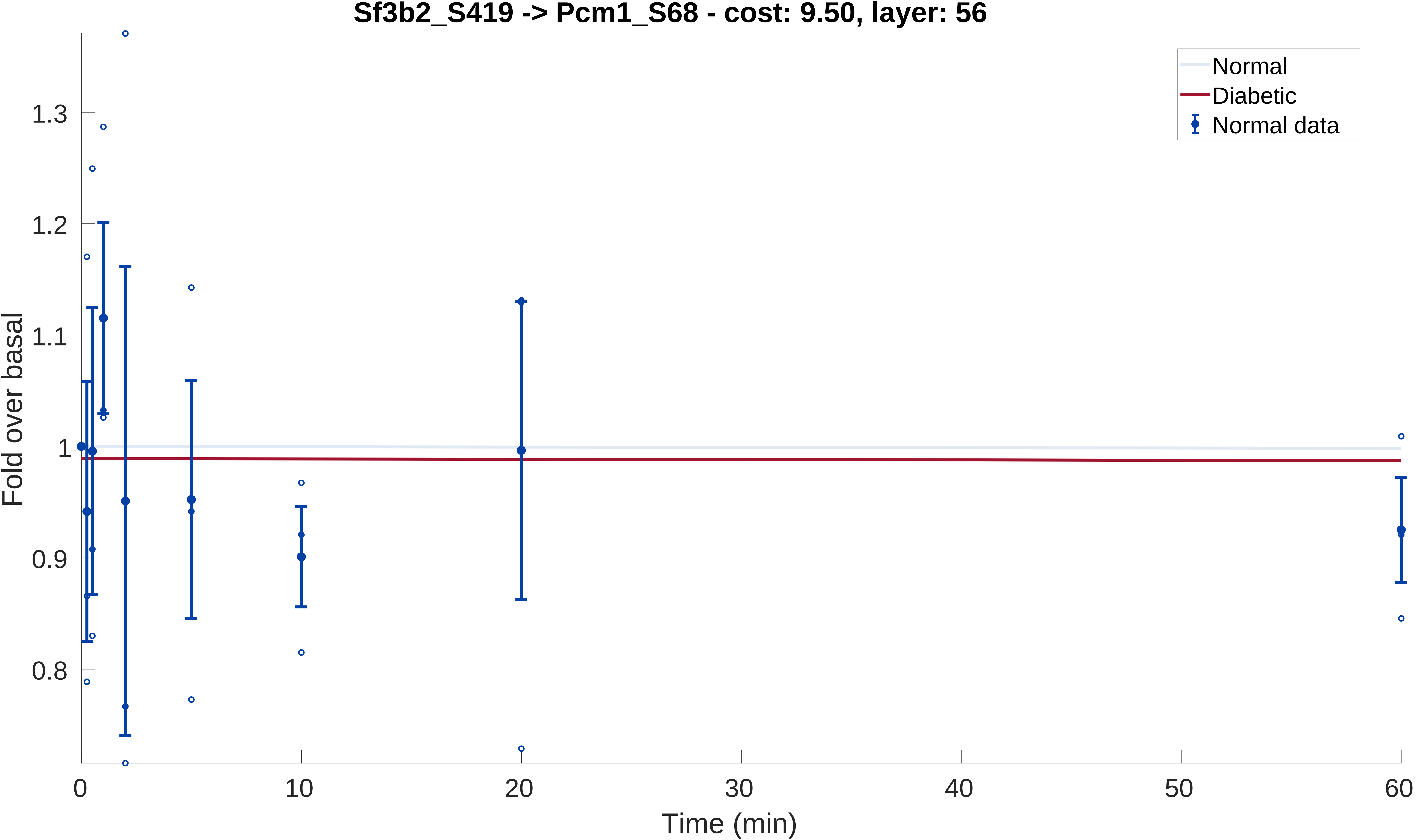

Sf3b2_S419 -> Pcm1_S68 - cost: 9.50, layer: 56
Normal
Diabetic
Normal data
Fold over basal
1.3
1.2
1.1
1
0.9
0.8
0
10
20
30
40
50
60
Time (min)

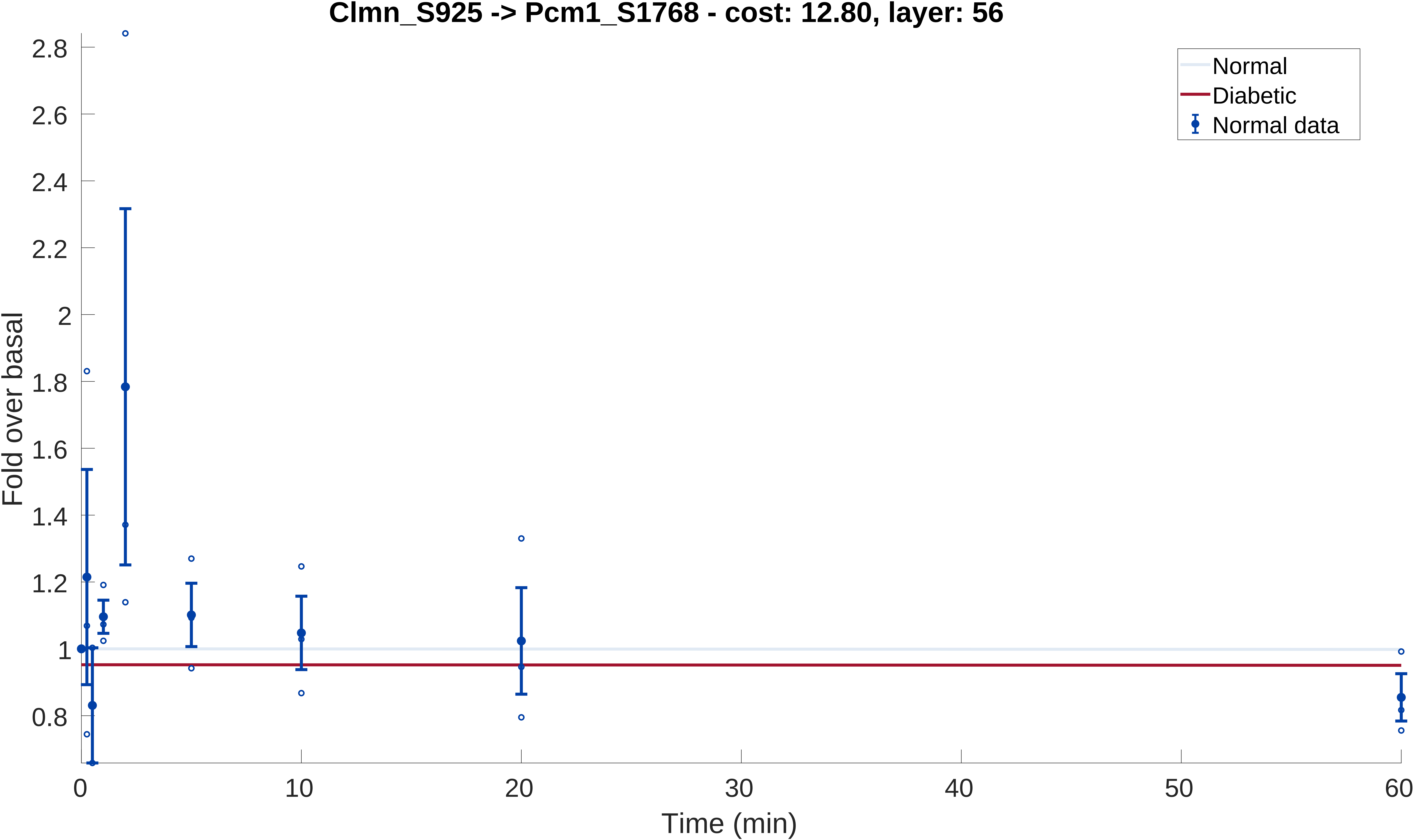
Clmn_S925 -> Pcm1_S1768 - cost: 12.80, layer: 56
Normal
Diabetic
Normal data
Fold over basal
2.8
2.6
2.4
2.2
2
1.8
1.6
1.4
1.2
1
0.8
0
10
20
30
40
50
60
Time (min)

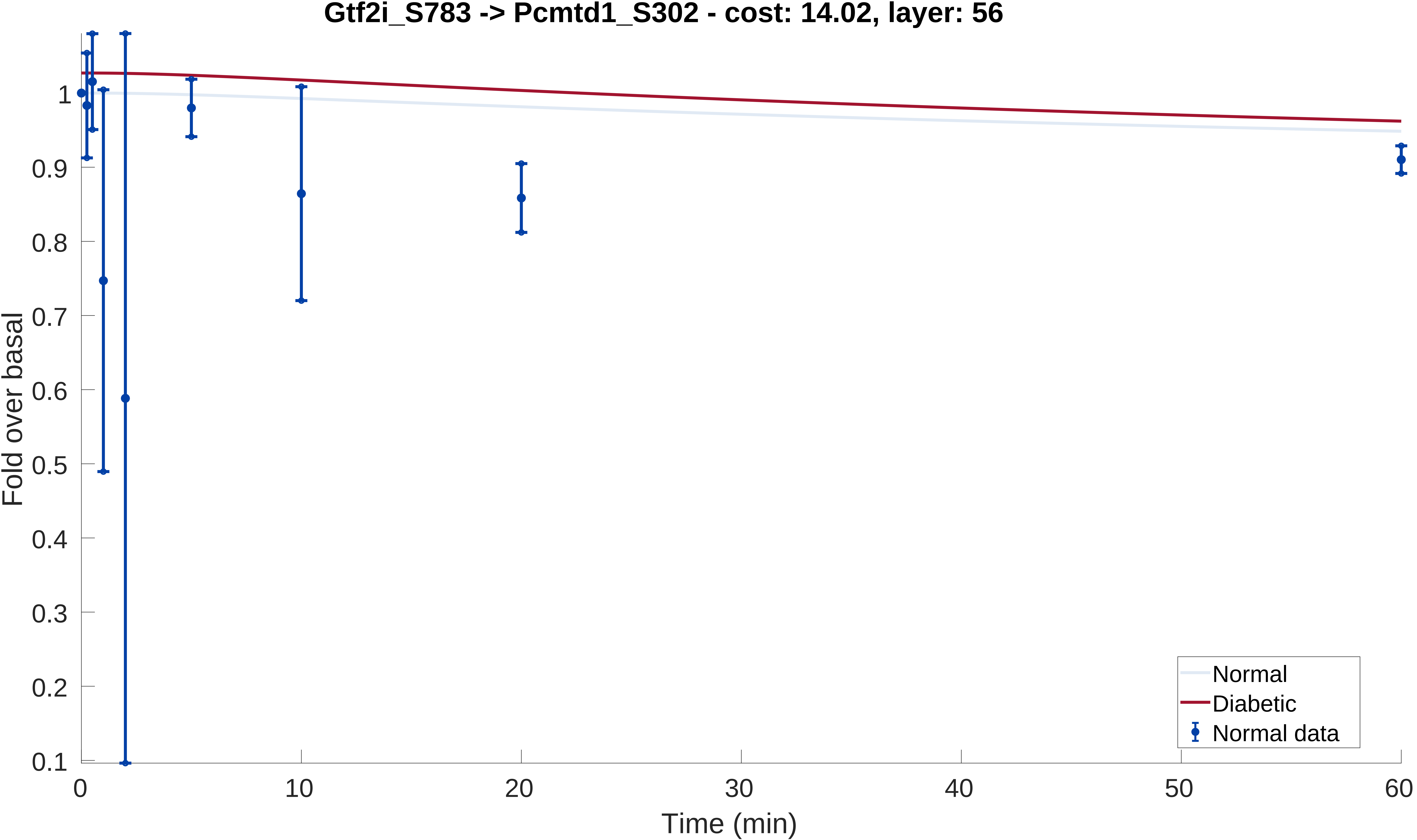
Gtf2i_S783 -> Pcmtd1_S302 - cost: 14.02, layer: 56
Fold over basal
0.1
0.2
0.3
0.4
0.5
0.6
0.7
0.8
0.9
1
Time (min)
0
10
20
30
40
50
60
Normal
Diabetic
Normal data

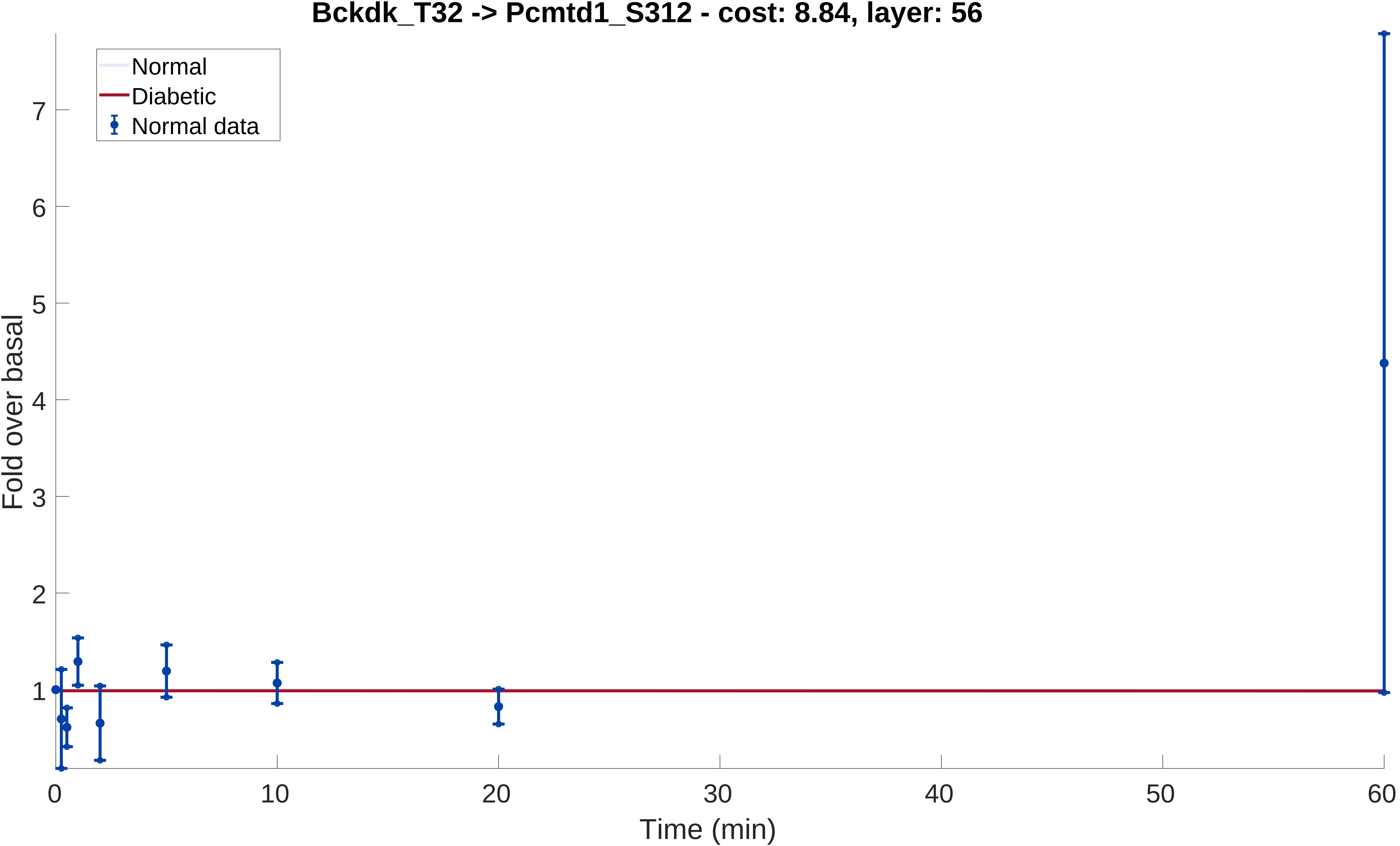
Bckdk_T32 -> Pcmtd1_S312 - cost: 8.84, layer: 56
Normal
Diabetic
Normal data
Fold over basal
Time (min)
0
10
20
30
40
50
60
1
2
3
4
5
6
7

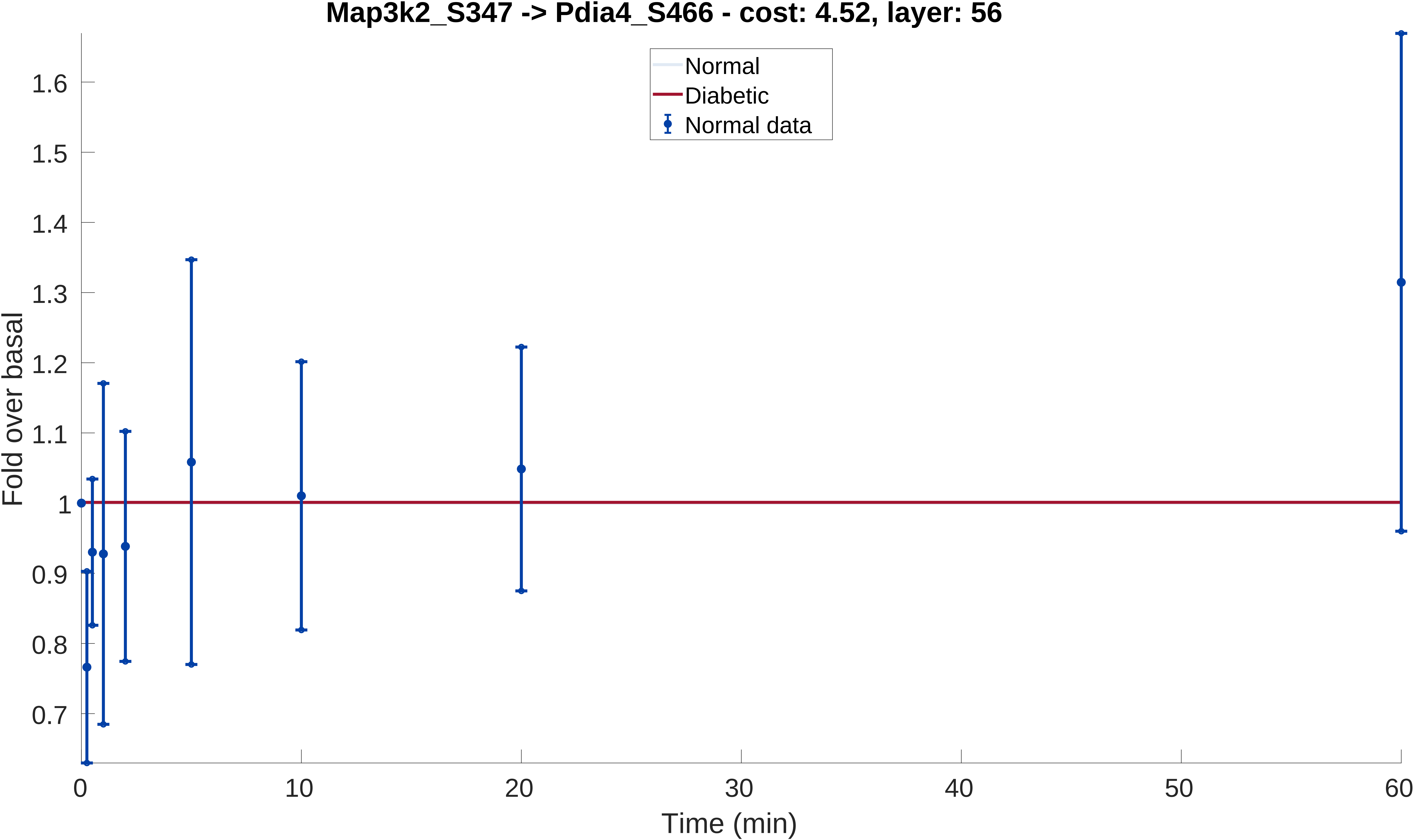

Map3k2_S347 -> Pdia4_S466 - cost: 4.52, layer: 56
Normal
Diabetic
Normal data
Fold over basal
1.6
1.5
1.4
1.3
1.2
1.1
1
0.9
0.8
0.7
0
10
20
30
40
50
60
Time (min)

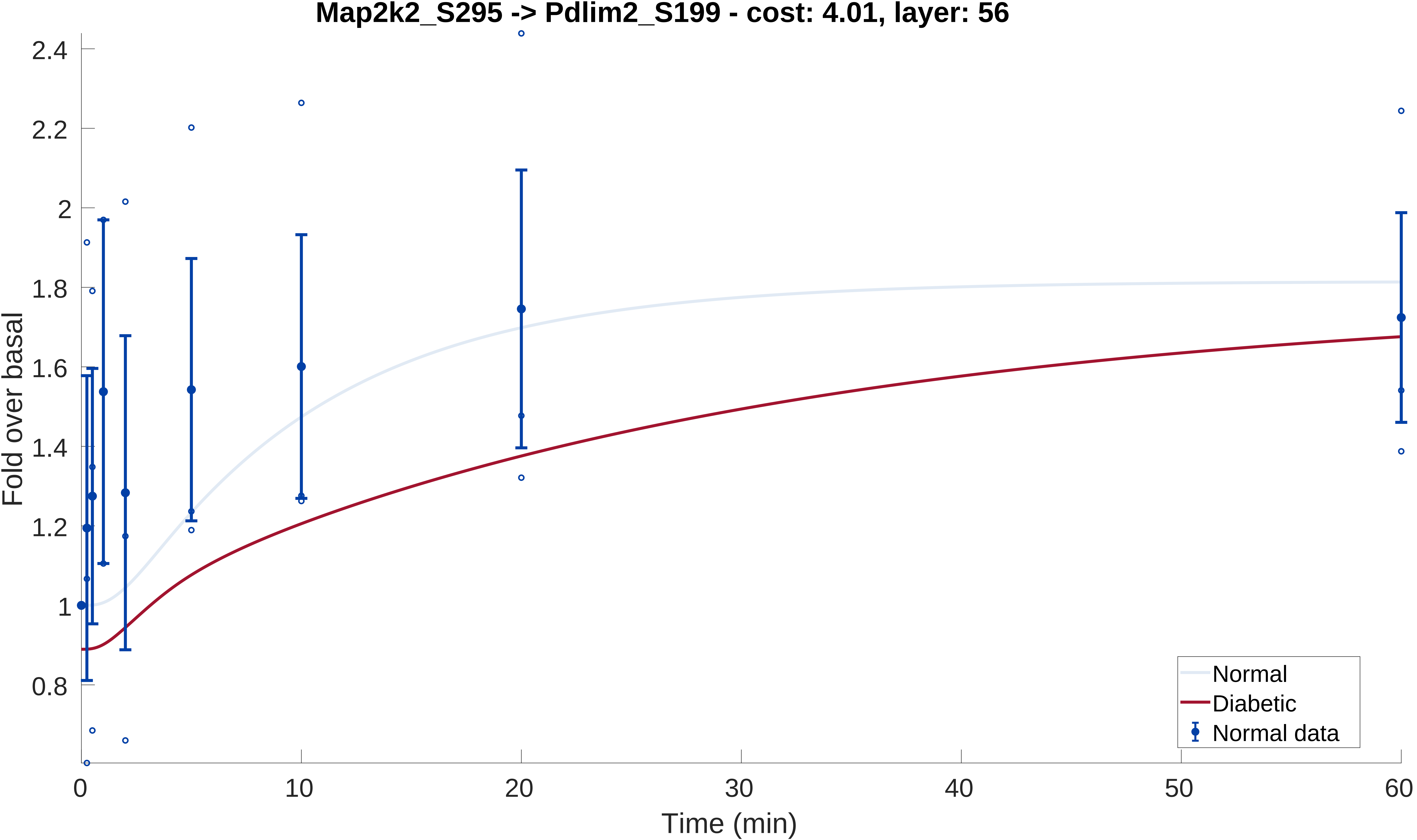
Map2k2_S295 -> Pdlim2_S199 - cost: 4.01, layer: 56
Fold over basal
Time (min)
0.8
1
1.2
1.4
1.6
1.8
2
2.2
2.4
0
10
20
30
40
50
60
Normal
Diabetic
Normal data

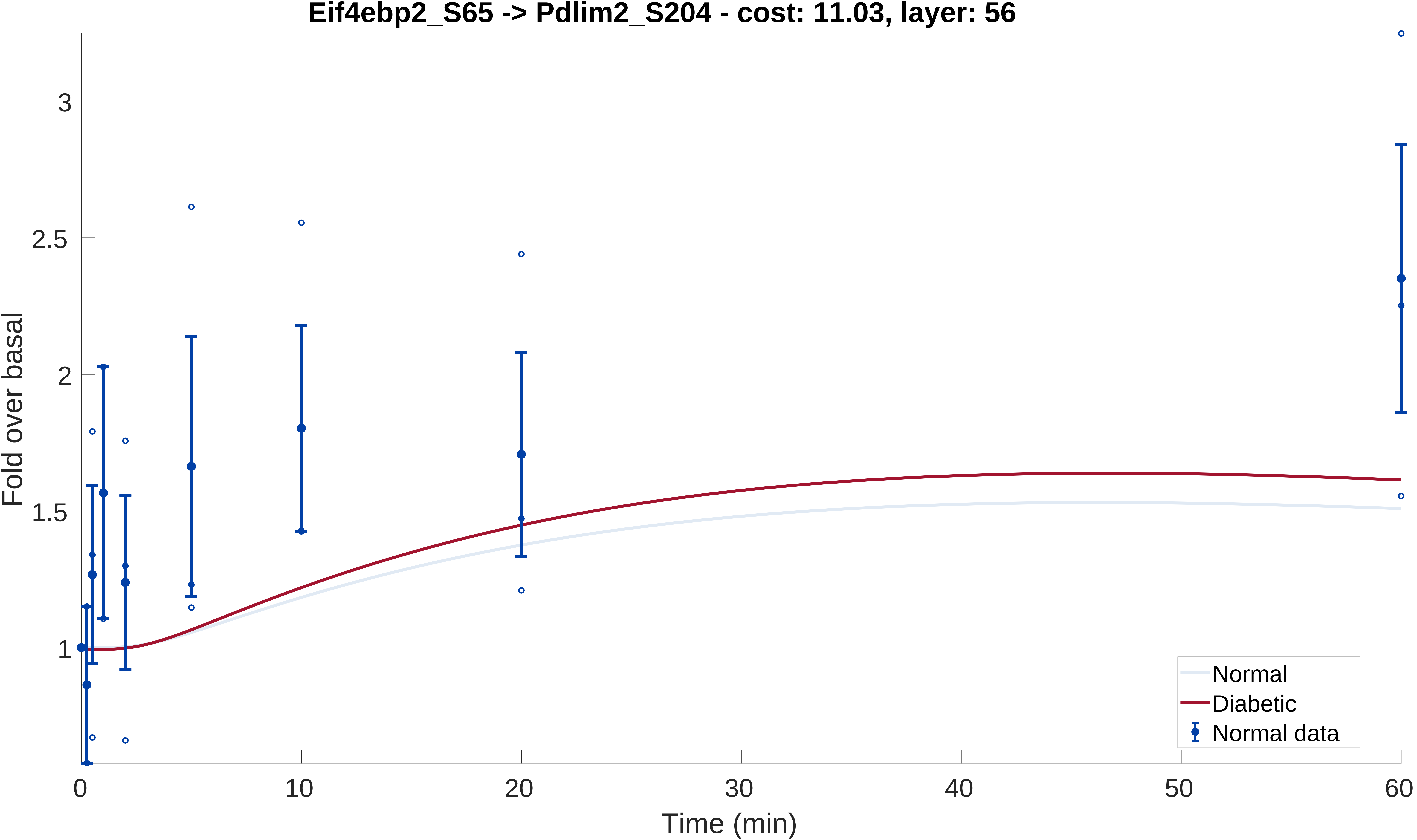

Eif4ebp2_S65 -> Pdlim2_S204 - cost: 11.03, layer: 56
Fold over basal
3
2.5
2
1.5
1
0
10
20
30
40
50
60
Time (min)
Normal
Diabetic
Normal data

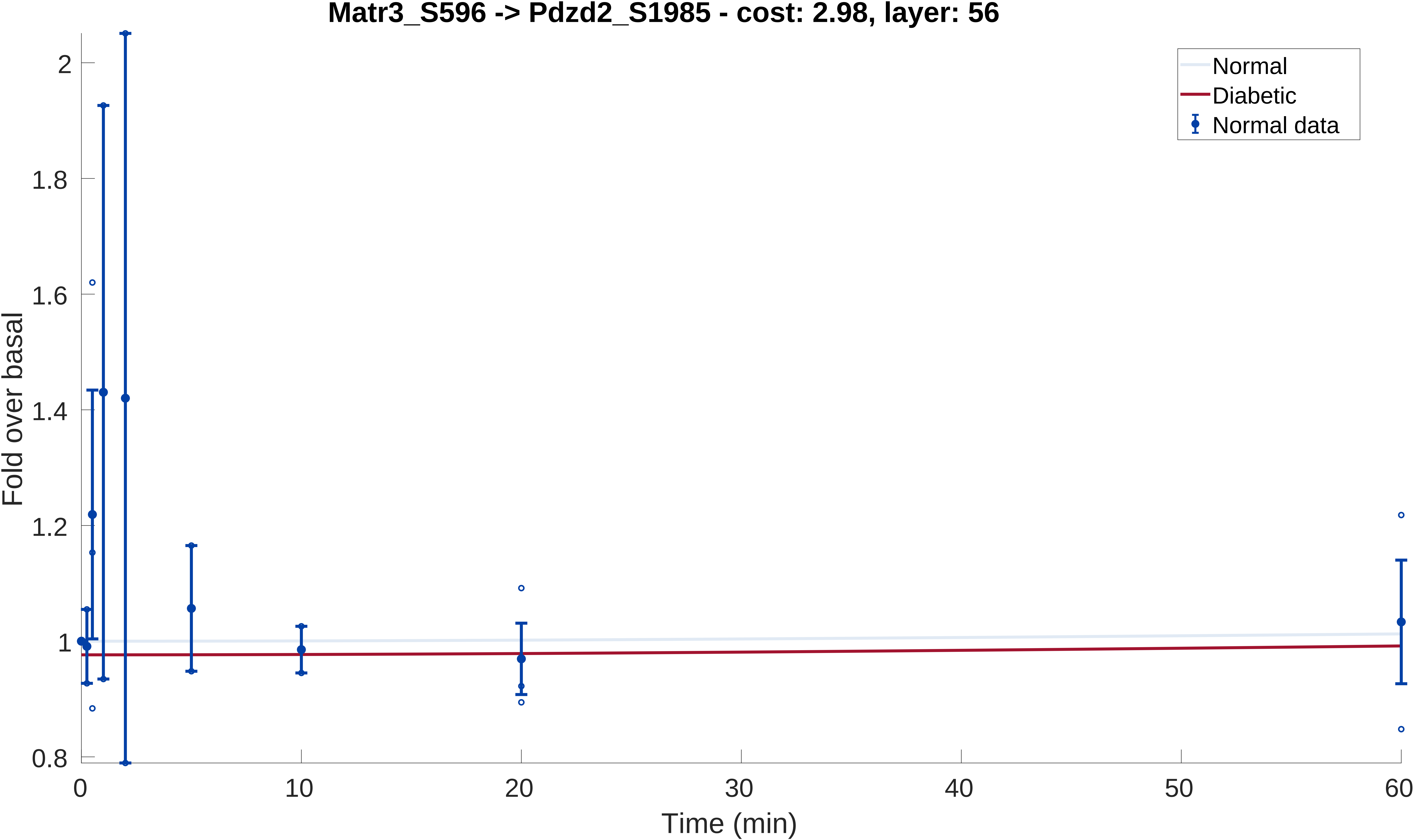
Matr3_S596 -> Pdzd2_S1985 - cost: 2.98, layer: 56
Normal
Diabetic
Normal data
Fold over basal
Time (min)
0.8
1
1.2
1.4
1.6
1.8
2
0
10
20
30
40
50
60

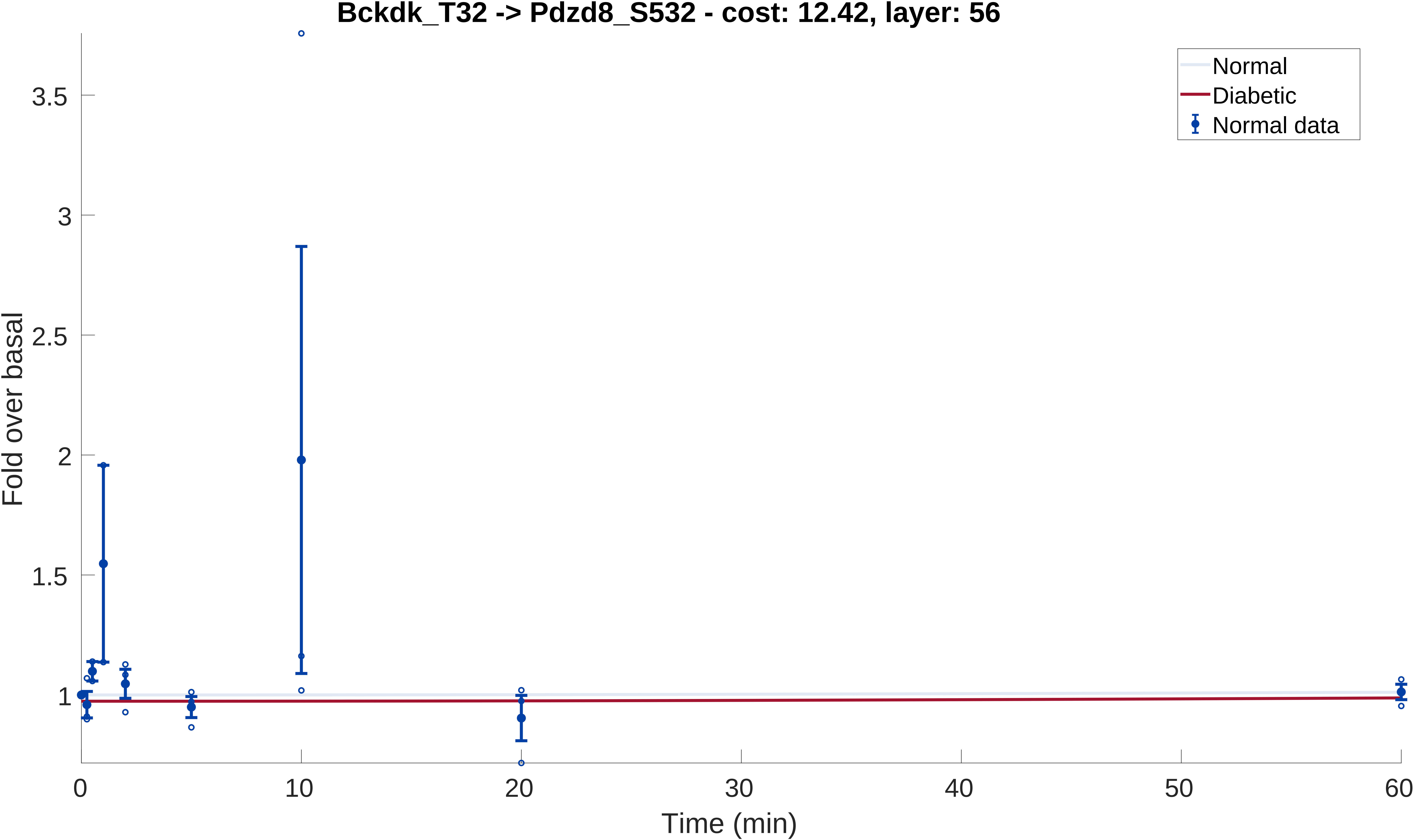
Bckdk_T32 -> Pdzd8_S532 - cost: 12.42, layer: 56
Normal
Diabetic
Normal data
3.5
3
2.5
2
1.5
1
0
10
20
30
40
50
60
Fold over basal
Time (min)

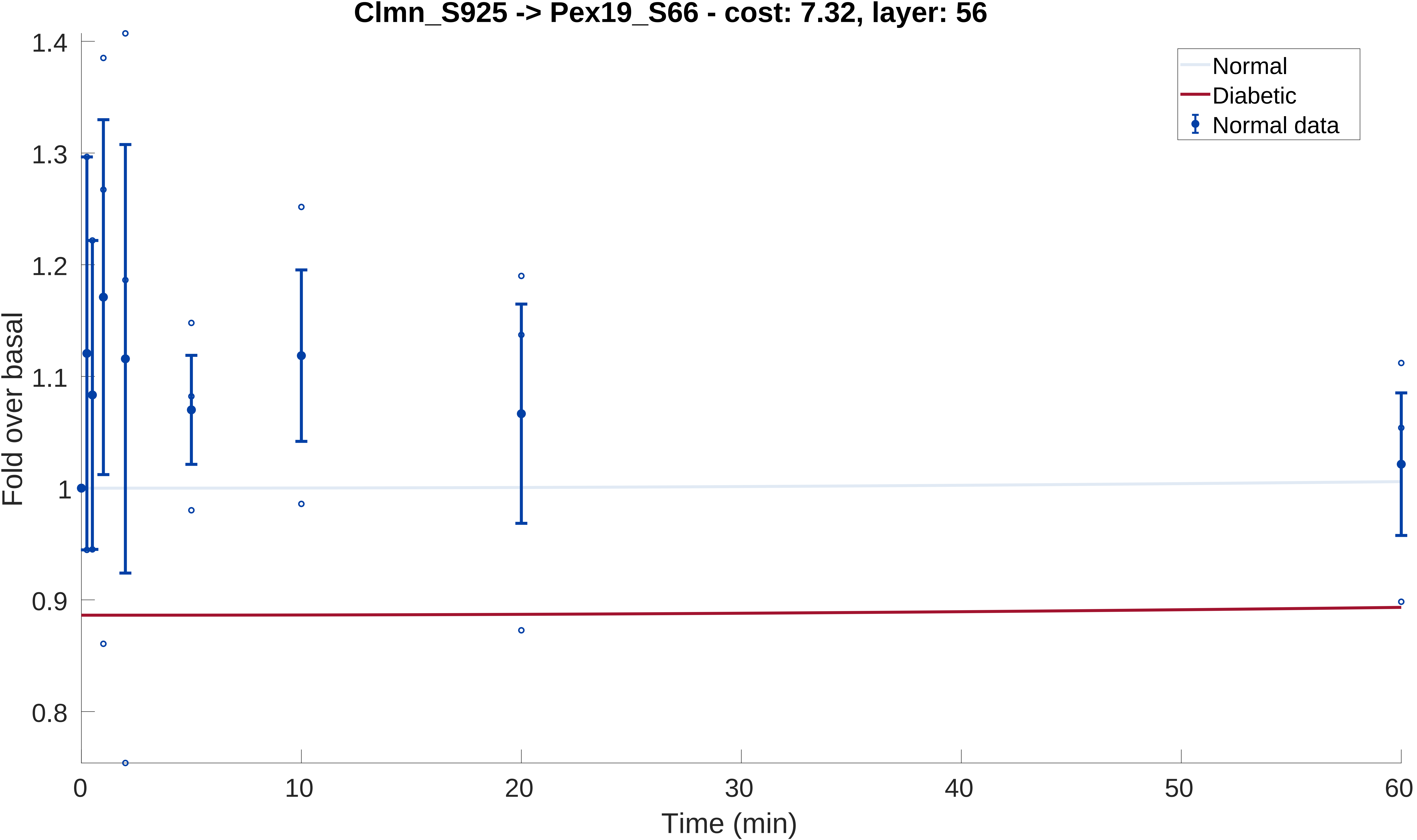

Clmn_S925 -> Pex19_S66 - cost: 7.32, layer: 56
Fold over basal
Time (min)
Normal
Diabetic
Normal data
1.4
1.3
1.2
1.1
1
0.9
0.8
0
10
20
30
40
50
60

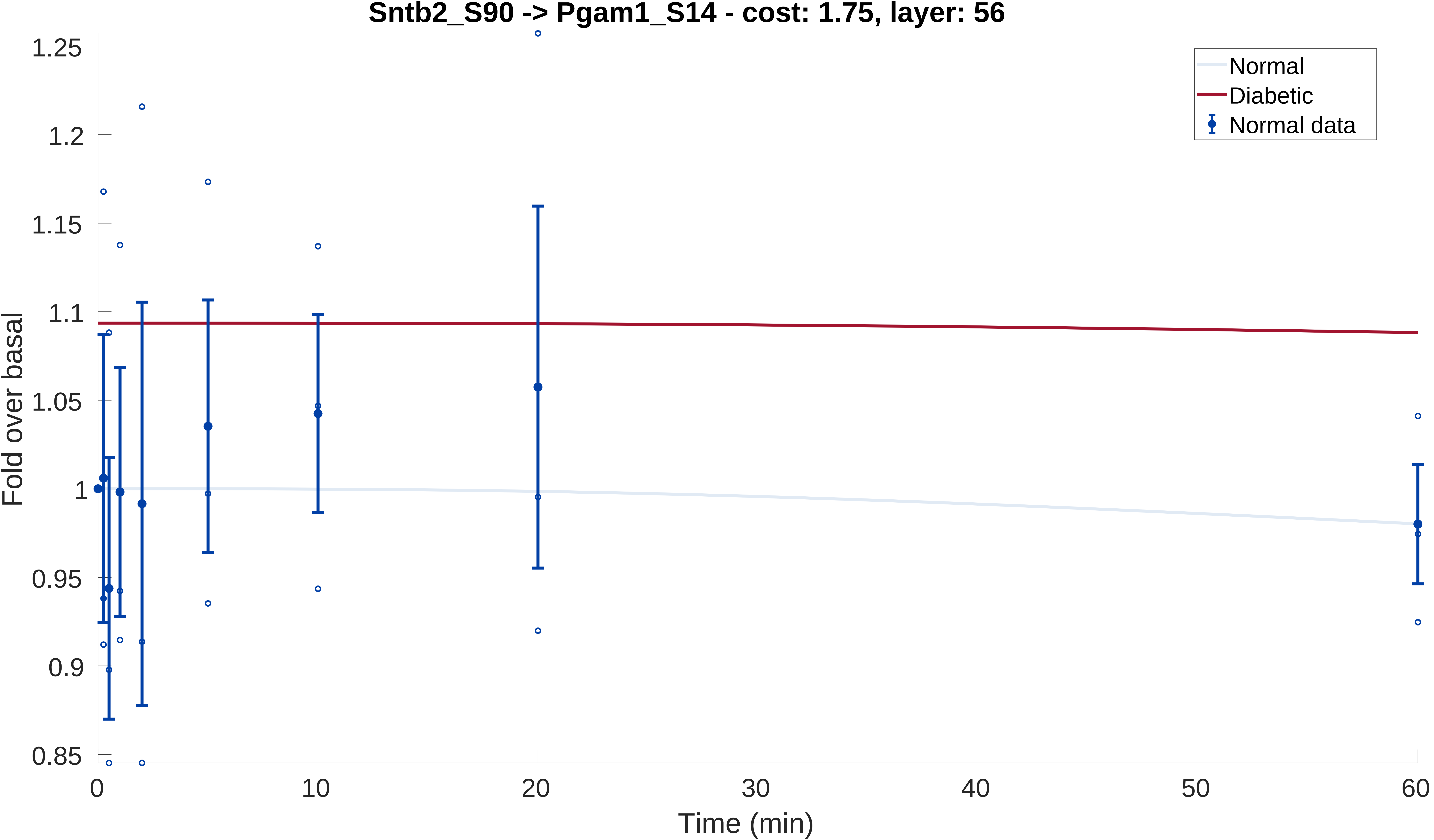

Sntb2_S90 -> Pgam1_S14 - cost: 1.75, layer: 56
Normal
Diabetic
Normal data
Fold over basal
Time (min)
0.85
0.9
0.95
1
1.05
1.1
1.15
1.2
1.25
0
10
20
30
40
50
60

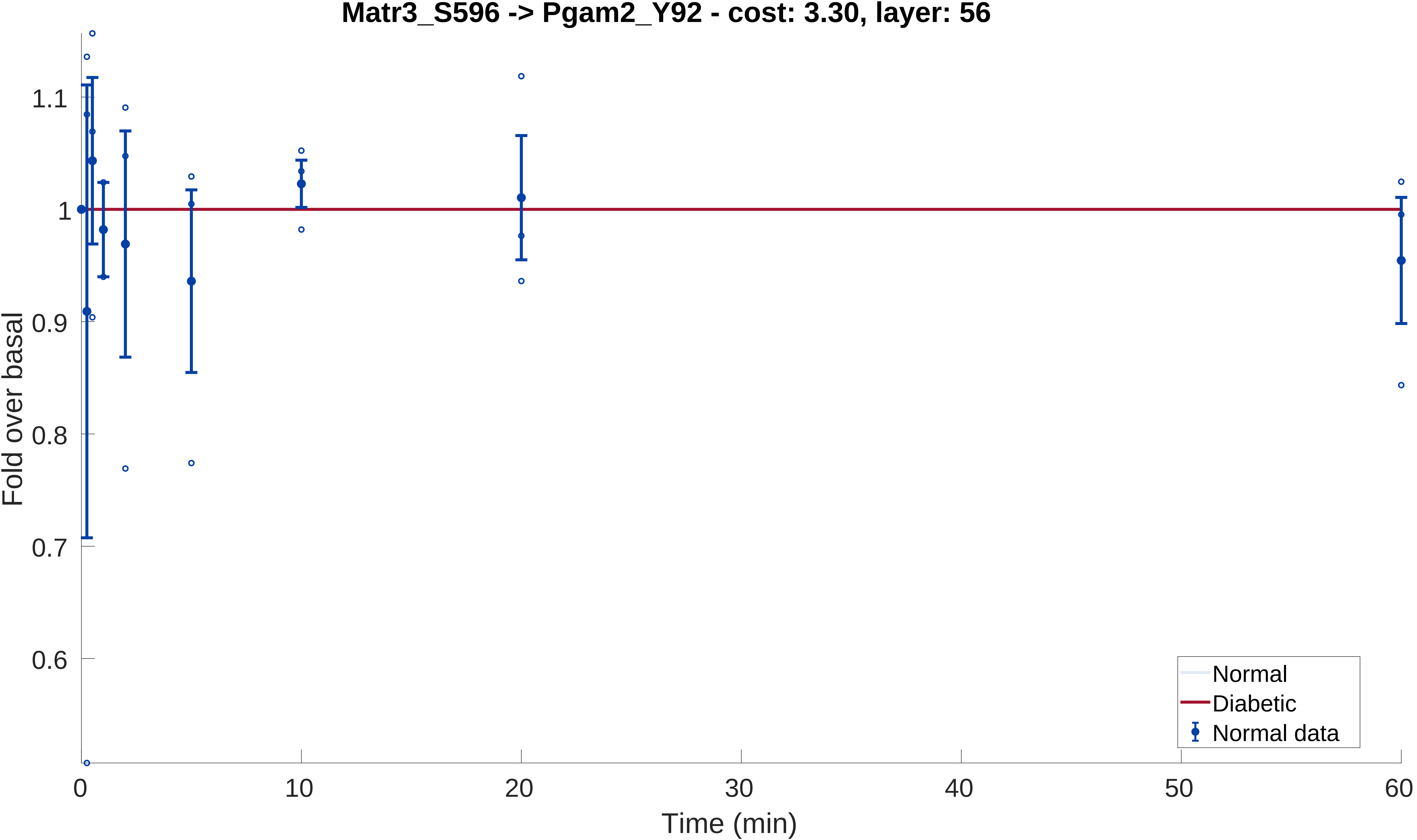

Matr3_S596 -> Pgam2_Y92 - cost: 3.30, layer: 56
Fold over basal
Time (min)
0.6
0.7
0.8
0.9
1
1.1
0
10
20
30
40
50
60
Normal
Diabetic
Normal data

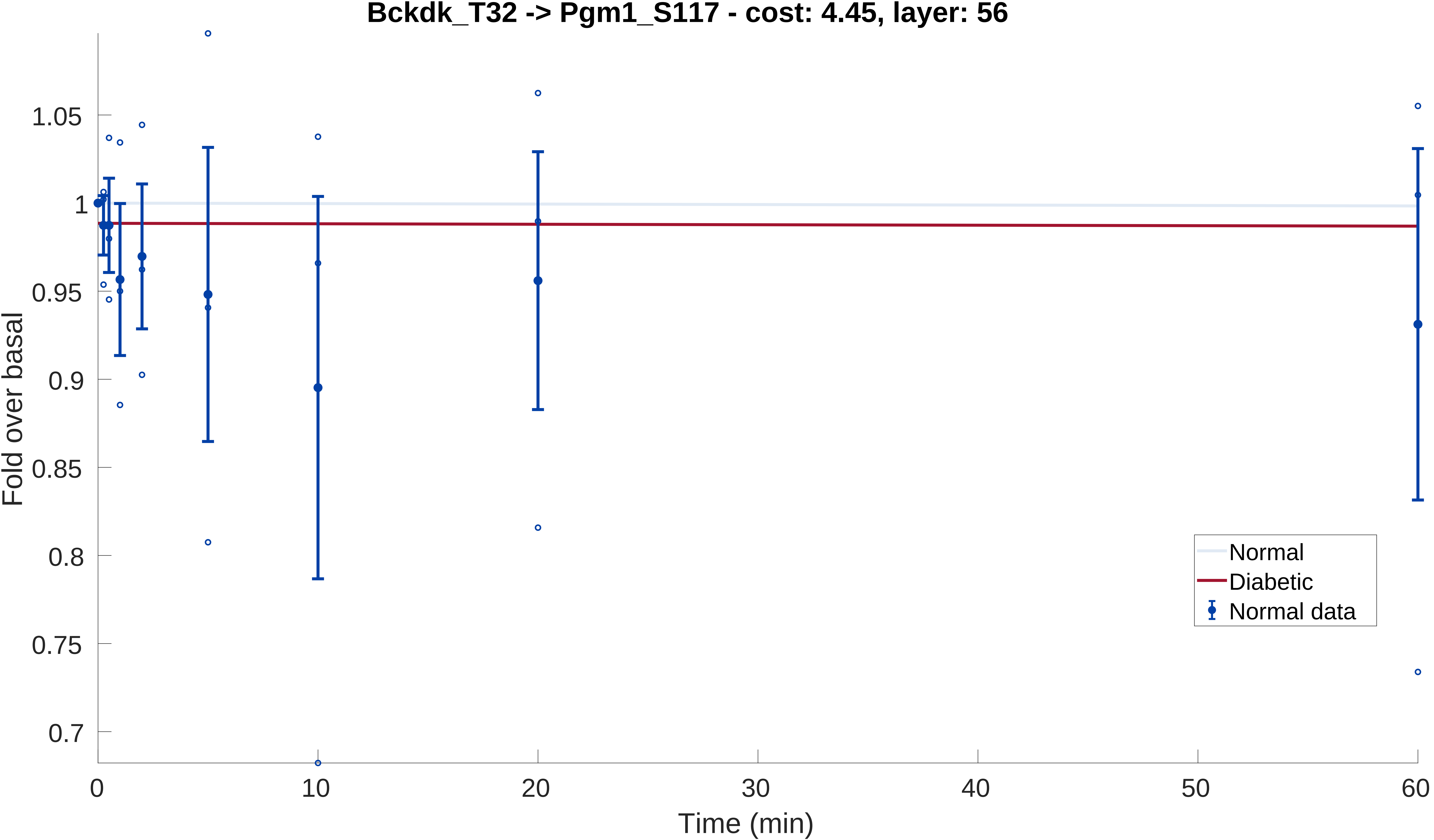
Bckdk_T32 -> Pgm1_S117 - cost: 4.45, layer: 56
Fold over basal
Time (min)
1.05
1
0.95
0.9
0.85
0.8
0.75
0.7
0
10
20
30
40
50
60
Normal
Diabetic
Normal data

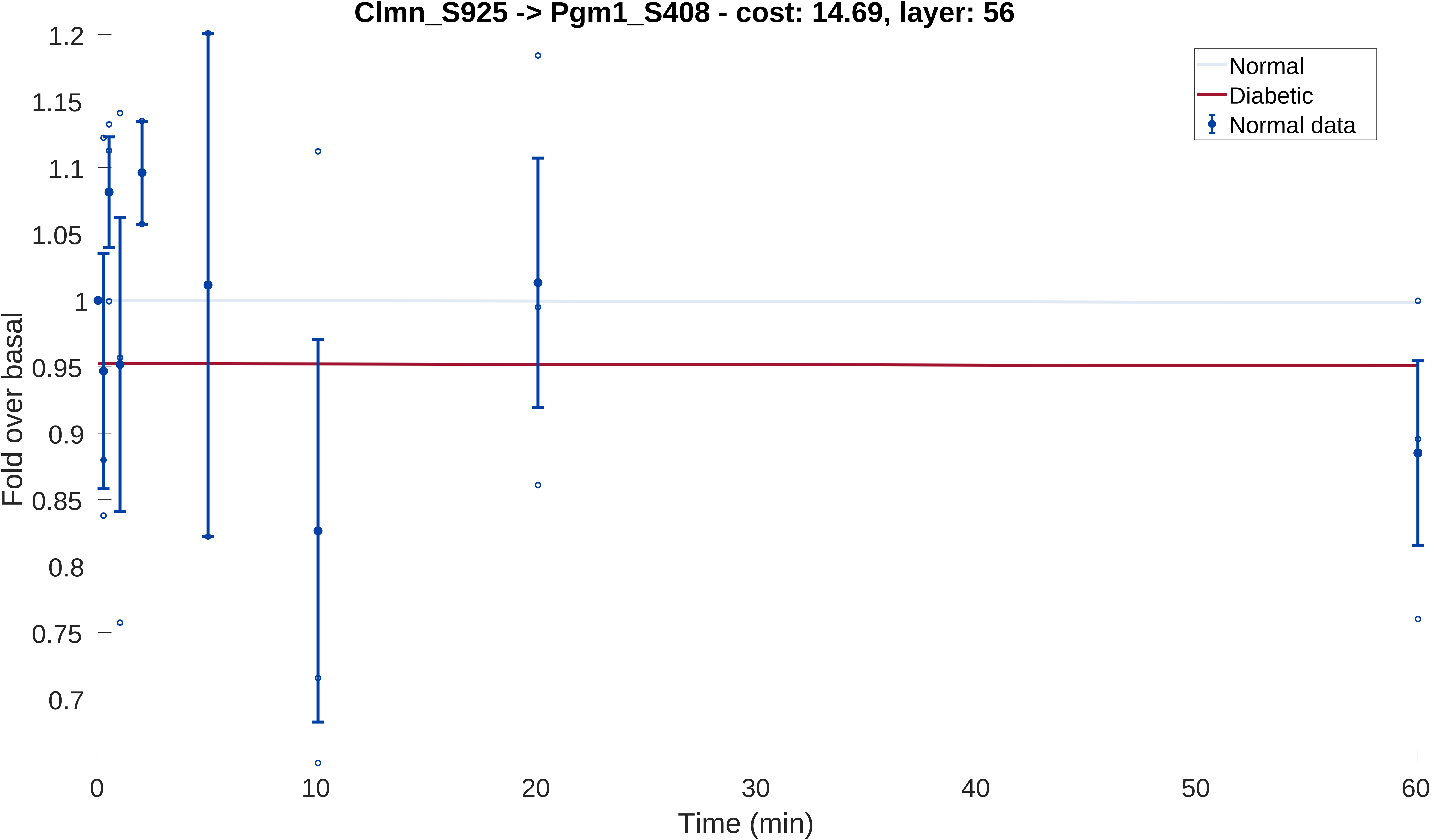

Clmn_S925 -> Pgm1_S408 - cost: 14.69, layer: 56
Normal
Diabetic
Normal data
Fold over basal
Time (min)
1.2
1.15
1.1
1.05
1
0.95
0.9
0.85
0.8
0.75
0.7
0
10
20
30
40
50
60

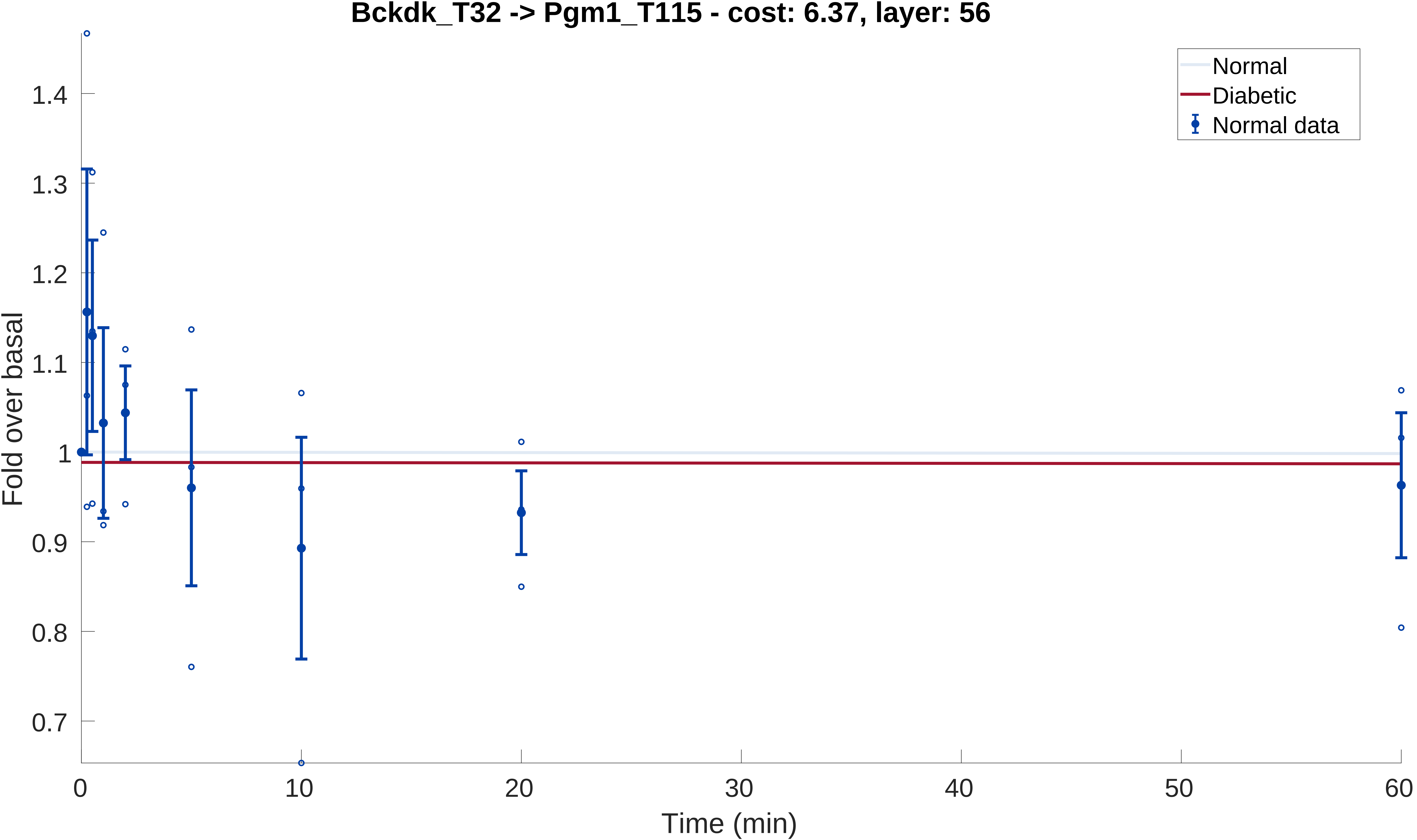

Bckdk_T32 -> Pgm1_T115 - cost: 6.37, layer: 56
Normal
Diabetic
Normal data
Fold over basal
Time (min)
1.4
1.3
1.2
1.1
1
0.9
0.8
0.7
0
10
20
30
40
50
60

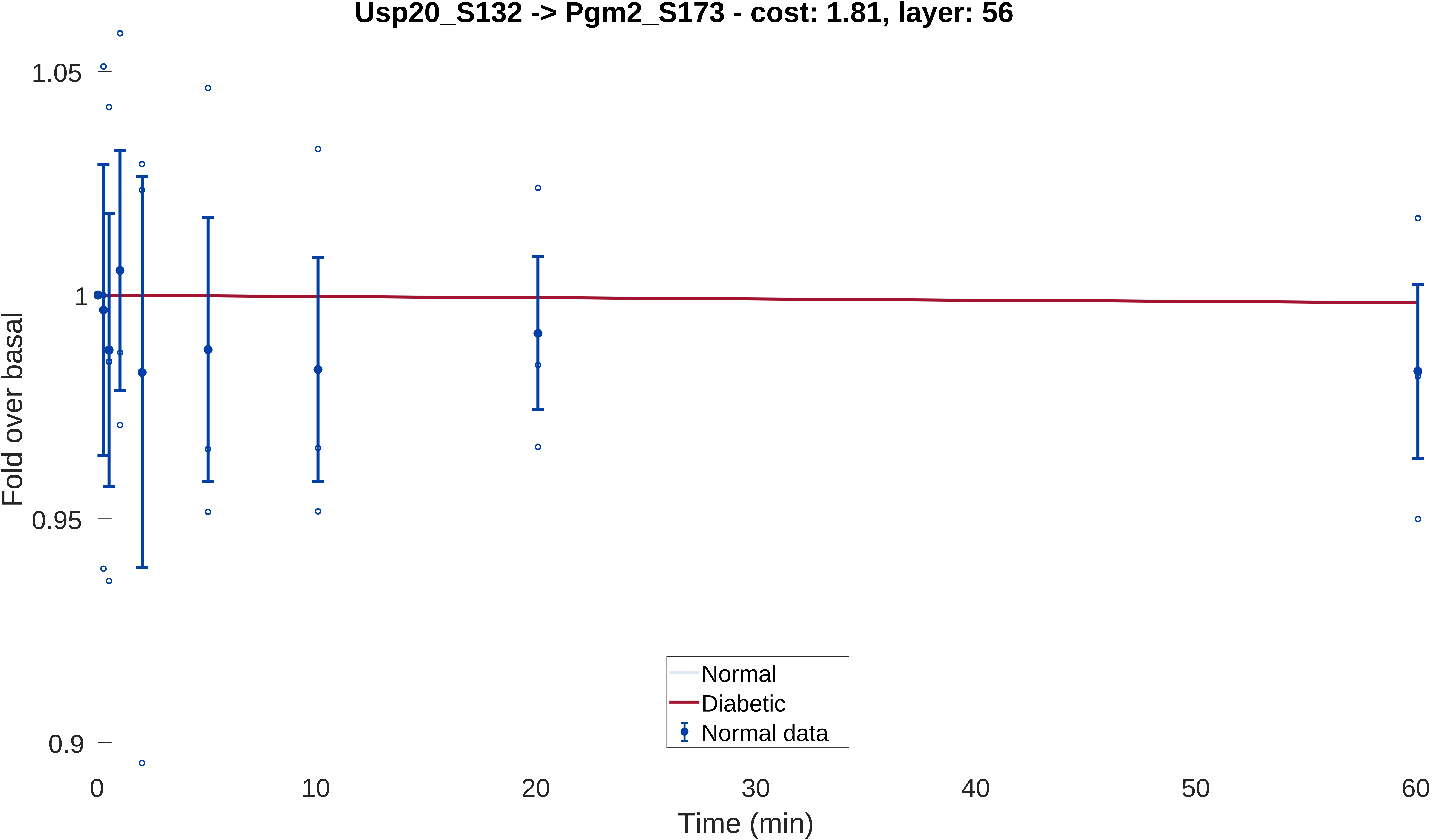

Usp20_S132 -> Pgm2_S173 - cost: 1.81, layer: 56
Fold over basal
1.05
1
0.95
0.9
0
10
20
30
40
50
60
Time (min)
Normal
Diabetic
Normal data

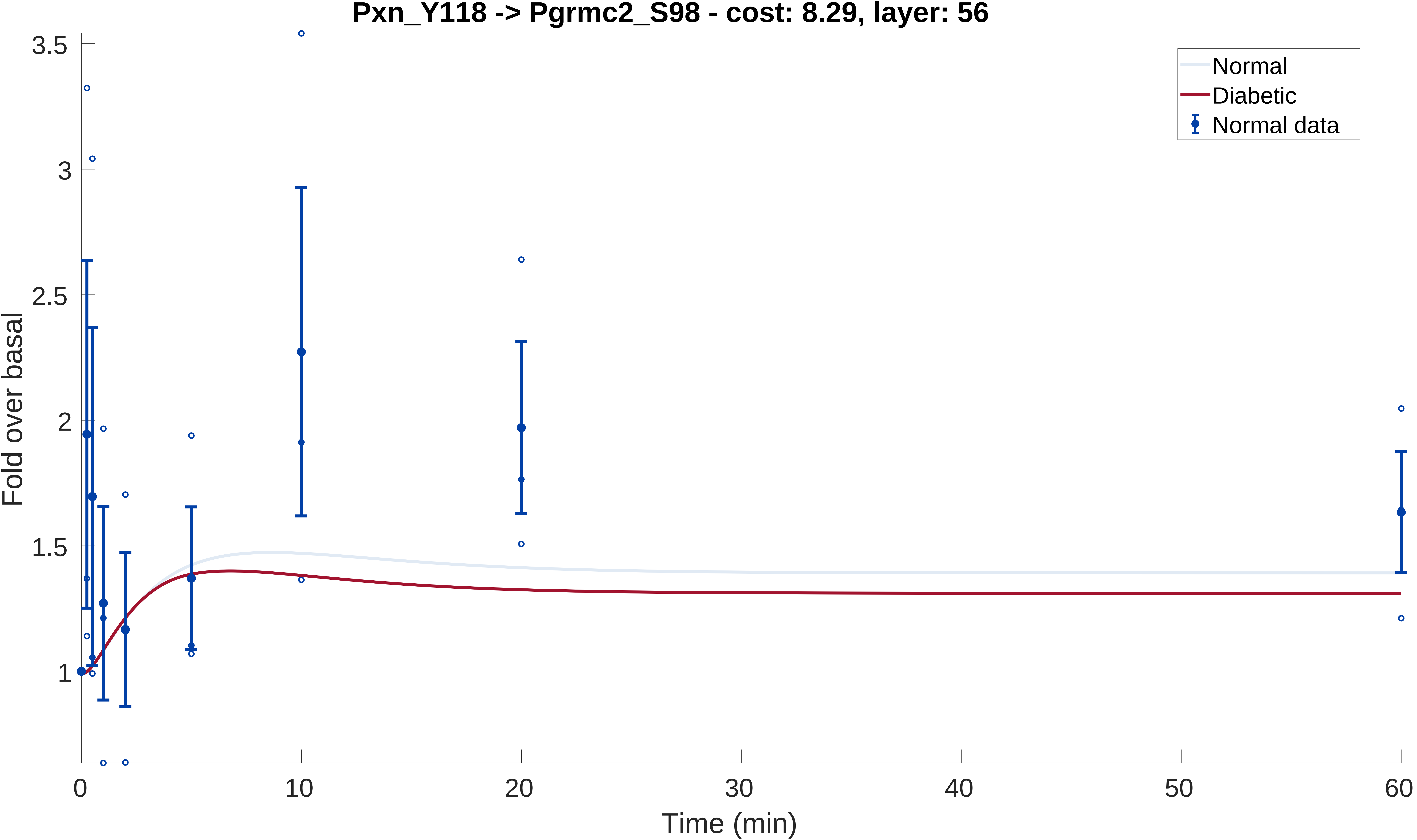

Pxn_Y118 -> Pgrmc2_S98 - cost: 8.29, layer: 56
Normal
Diabetic
Normal data
Fold over basal
3.5
3
2.5
2
1.5
1
0
10
20
30
40
50
60
Time (min)

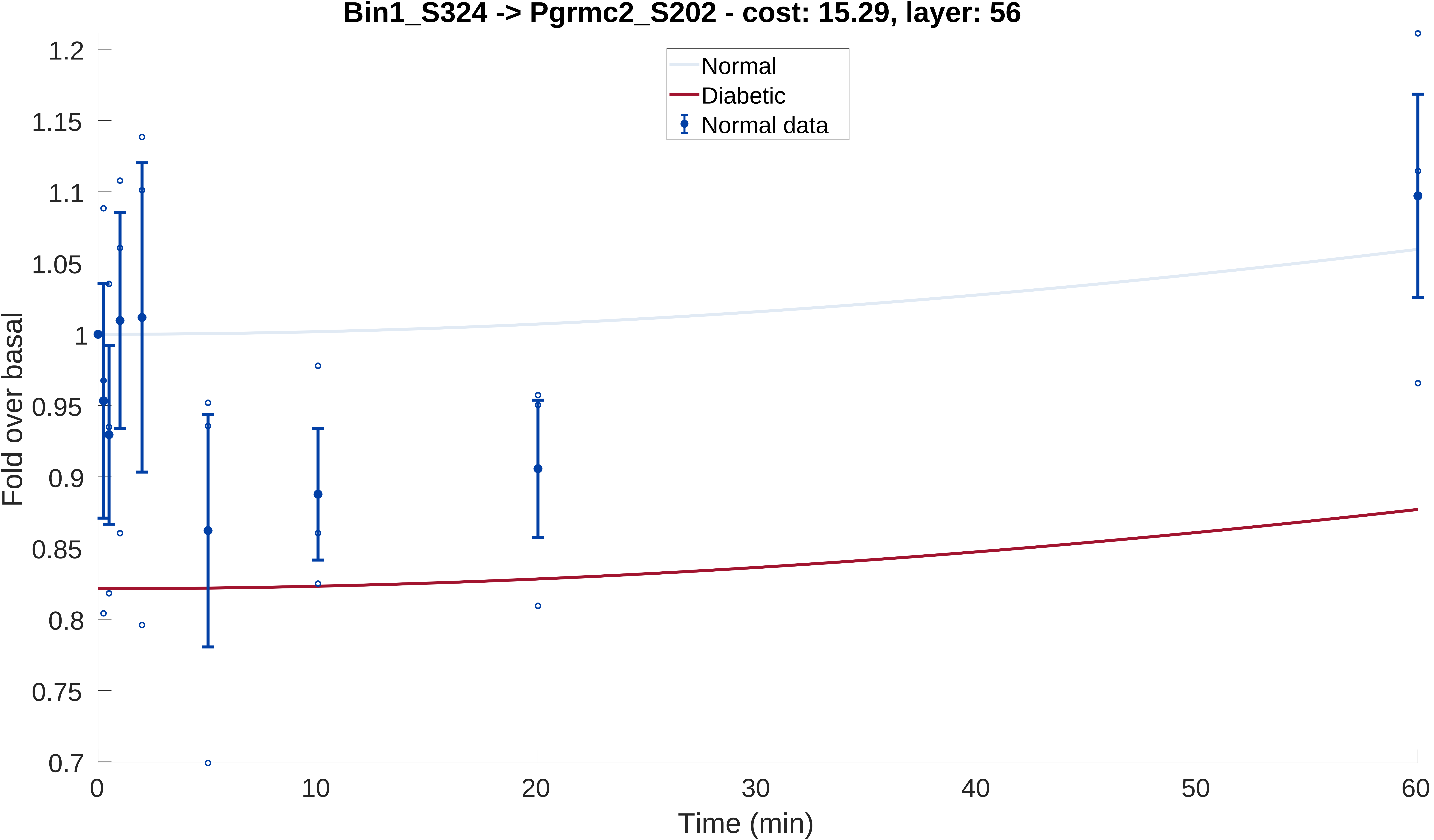

Bin1_S324 -> Pgrmc2_S202 - cost: 15.29, layer: 56
Normal
Diabetic
Normal data
Fold over basal
Time (min)
1.2
1.15
1.1
1.05
1
0.95
0.9
0.85
0.8
0.75
0.7
0
10
20
30
40
50
60

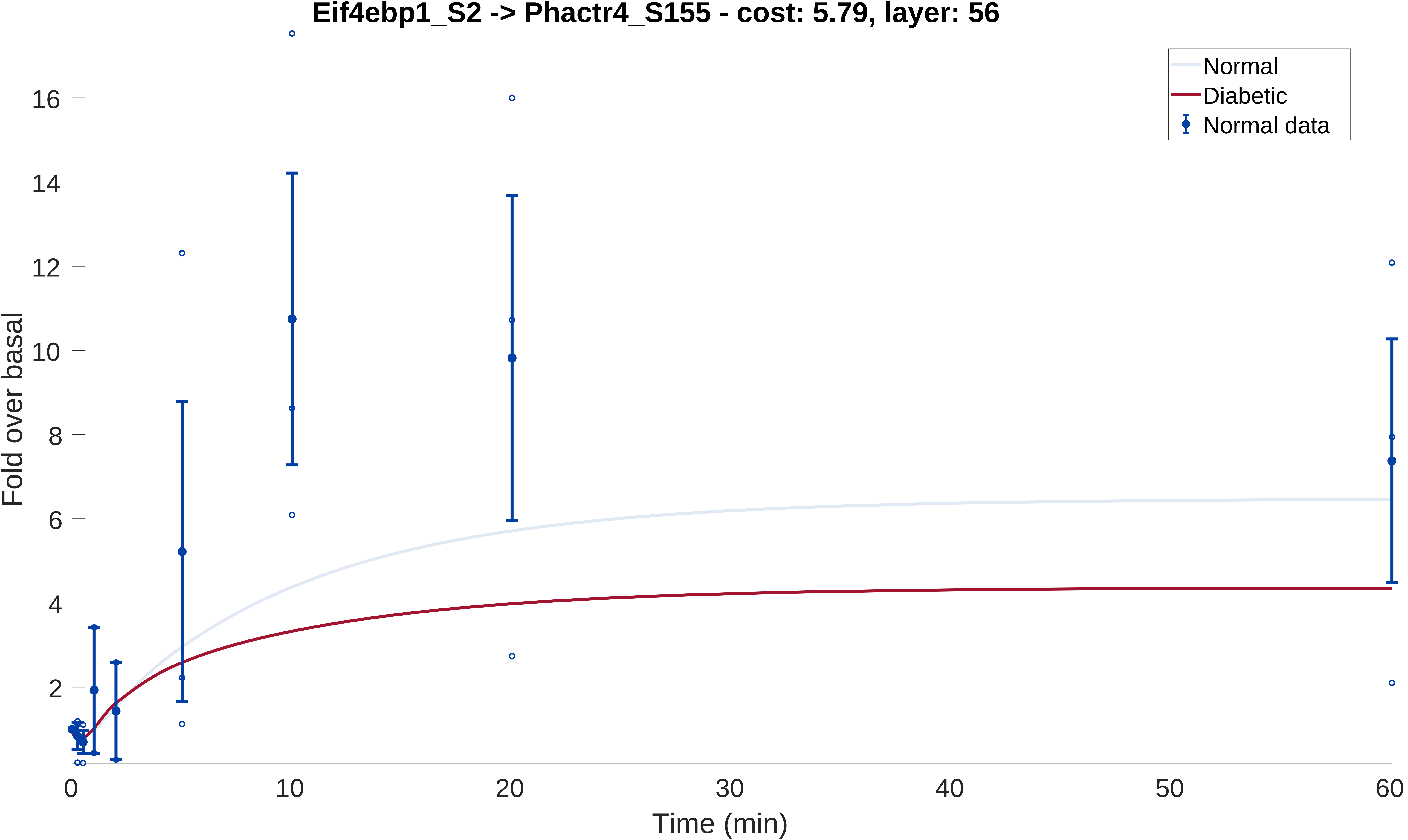

Eif4ebp1_S2 -> Phactr4_S155 - cost: 5.79, layer: 56
Normal
Diabetic
Normal data
Fold over basal
Time (min)
16
14
12
10
8
6
4
2
0
10
20
30
40
50
60

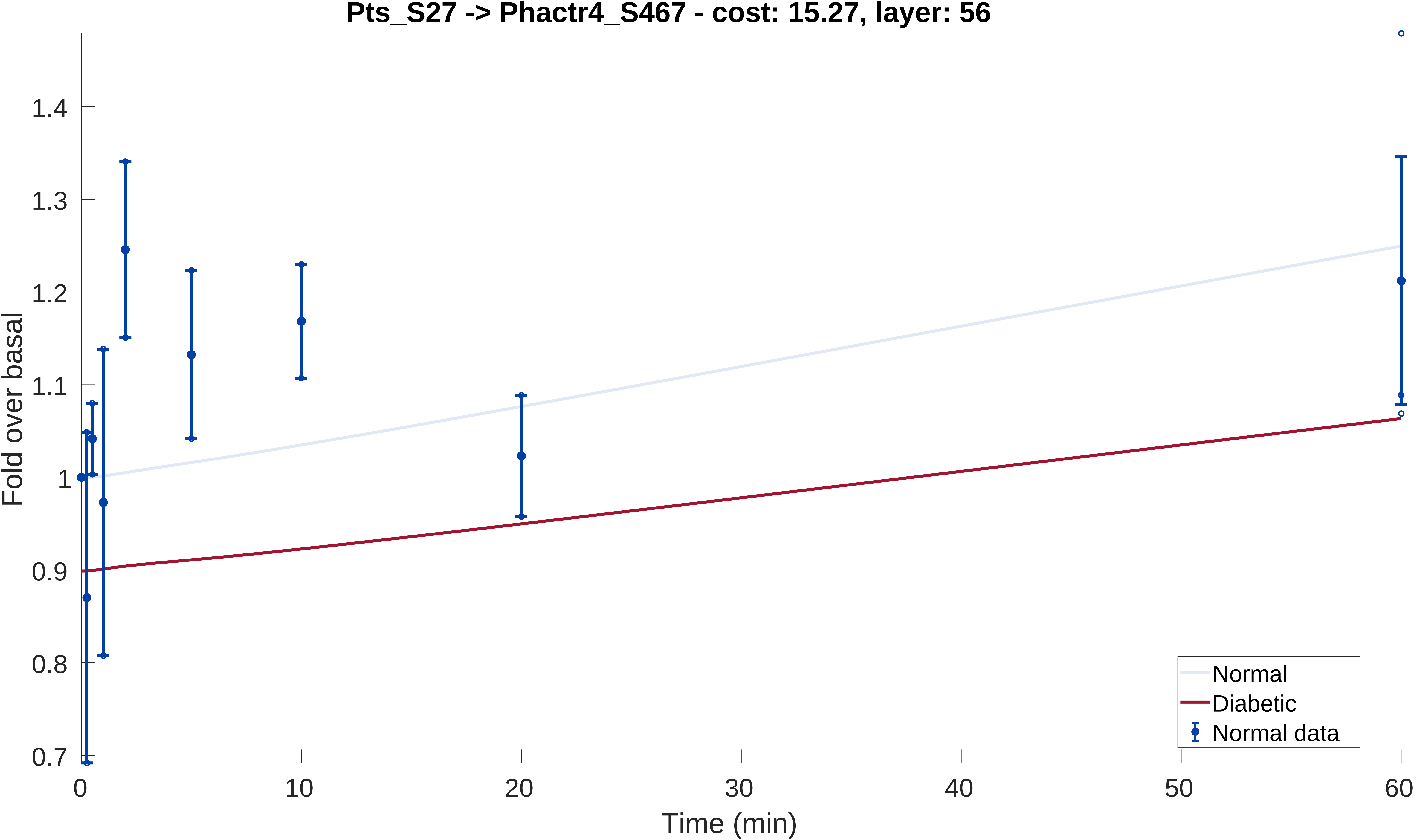
Pts_S27 -> Phactr4_S467 - cost: 15.27, layer: 56
Fold over basal
0.7
0.8
0.9
1
1.1
1.2
1.3
1.4
Time (min)
0
10
20
30
40
50
60
Normal
Diabetic
Normal data

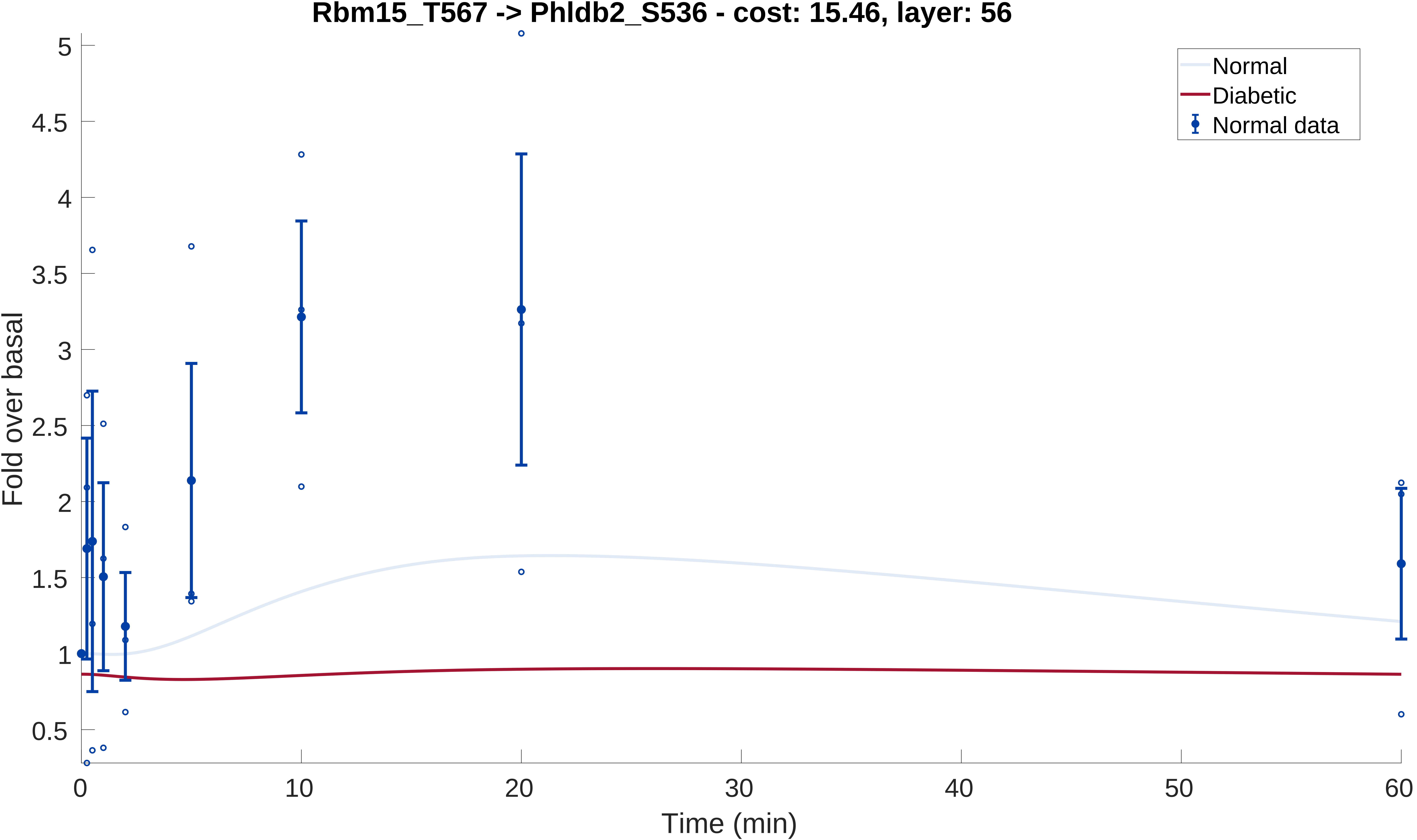

Rbm15_T567 -> Phldb2_S536 - cost: 15.46, layer: 56
Fold over basal
Time (min)
Normal
Diabetic
Normal data
0
10
20
30
40
50
60
0.5
1
1.5
2
2.5
3
3.5
4
4.5
5

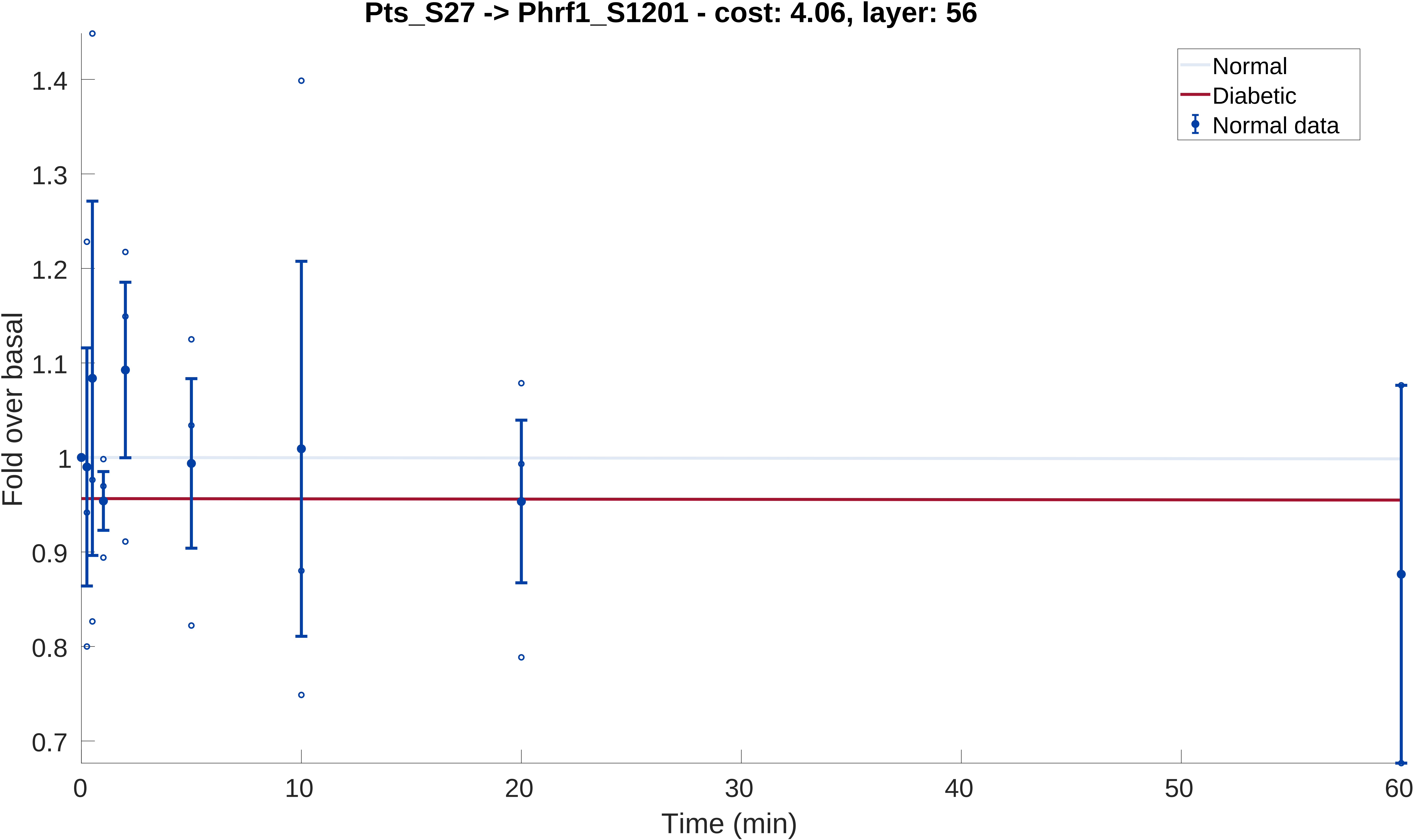
Pts_S27 -> Phrf1_S1201 - cost: 4.06, layer: 56
Normal
Diabetic
Normal data
Fold over basal
1.4
1.3
1.2
1.1
1
0.9
0.8
0.7
0
10
20
30
40
50
60
Time (min)

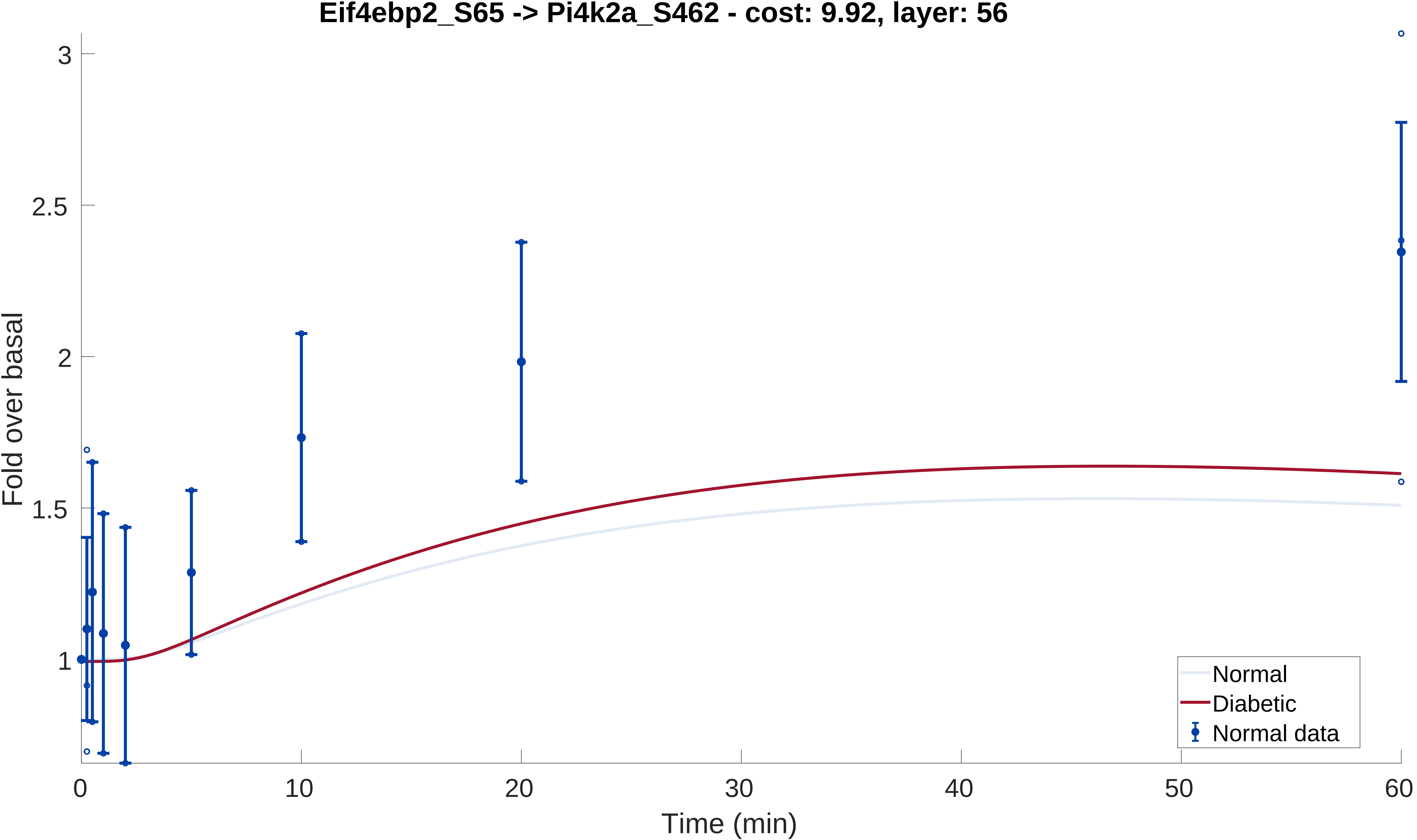
Eif4ebp2_S65 -> Pi4k2a_S462 - cost: 9.92, layer: 56
Fold over basal
Time (min)
0
10
20
30
40
50
60
1
1.5
2
2.5
3
Normal
Diabetic
Normal data

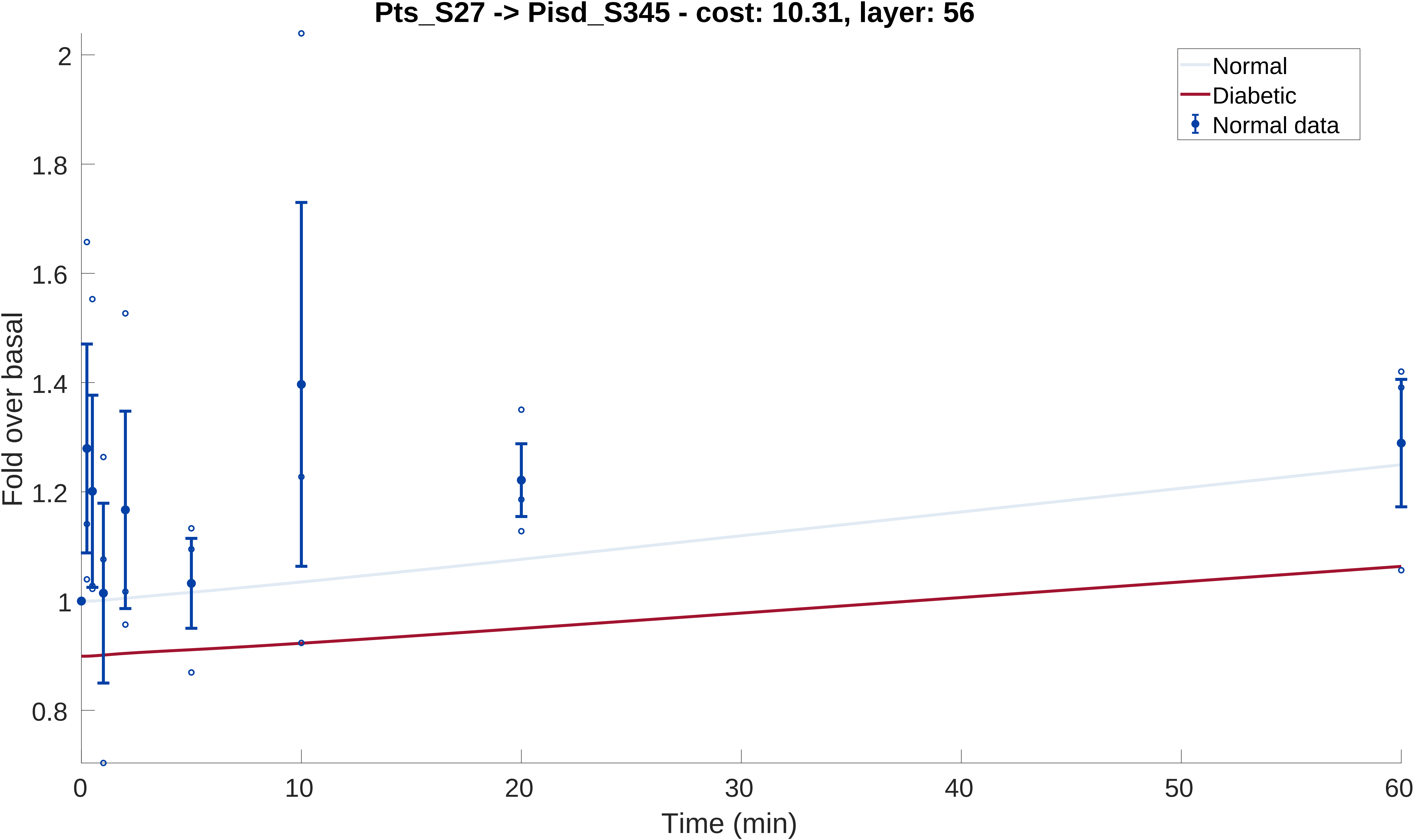
Pts_S27 -> Pisd_S345 - cost: 10.31, layer: 56
Normal
Diabetic
Normal data
Fold over basal
Time (min)
0.8
1
1.2
1.4
1.6
1.8
2
0
10
20
30
40
50
60

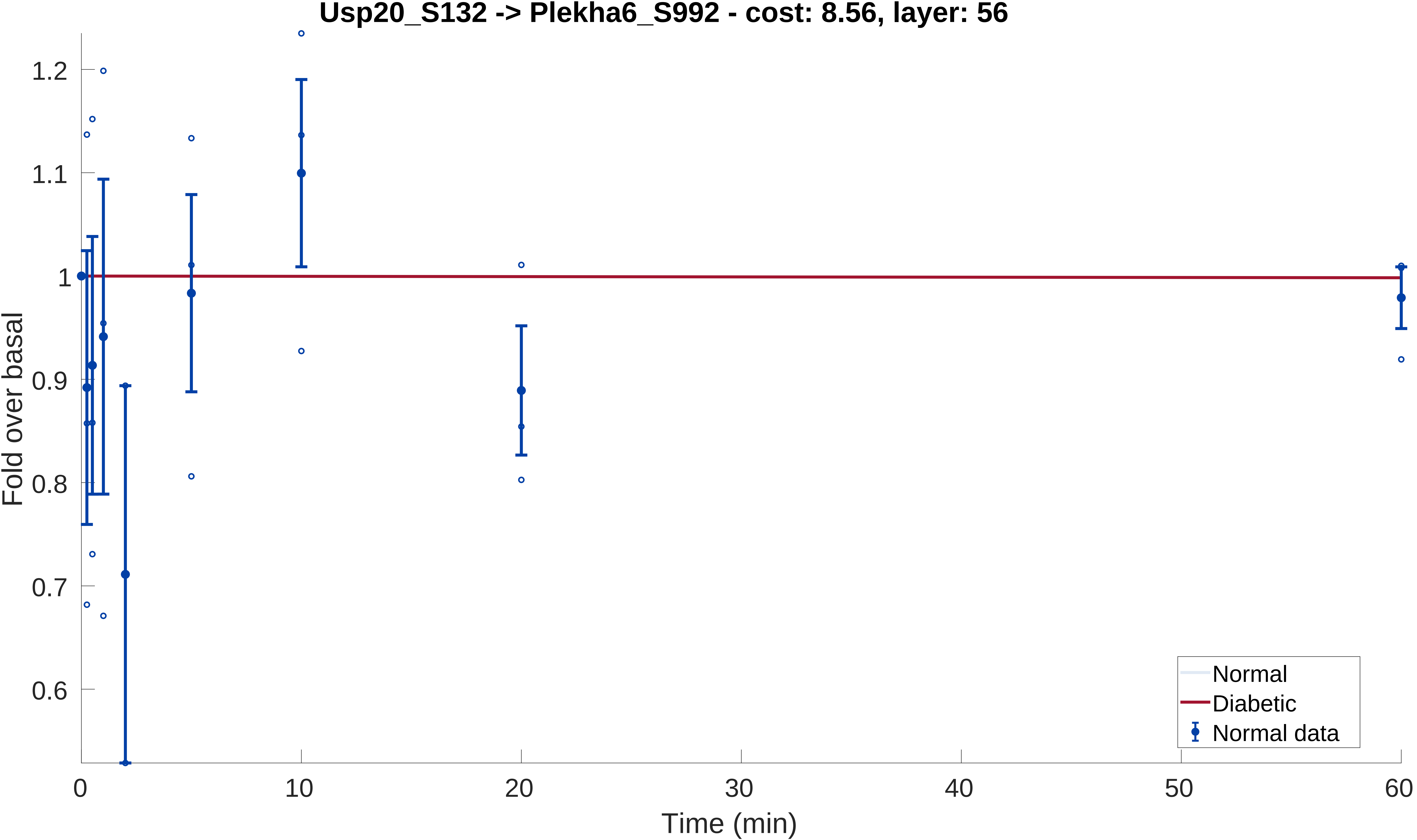
Usp20_S132 -> Plekha6_S992 - cost: 8.56, layer: 56
Fold over basal
1.2
1.1
1
0.9
0.8
0.7
0.6
0
10
20
30
40
50
60
Time (min)
Normal
Diabetic
Normal data

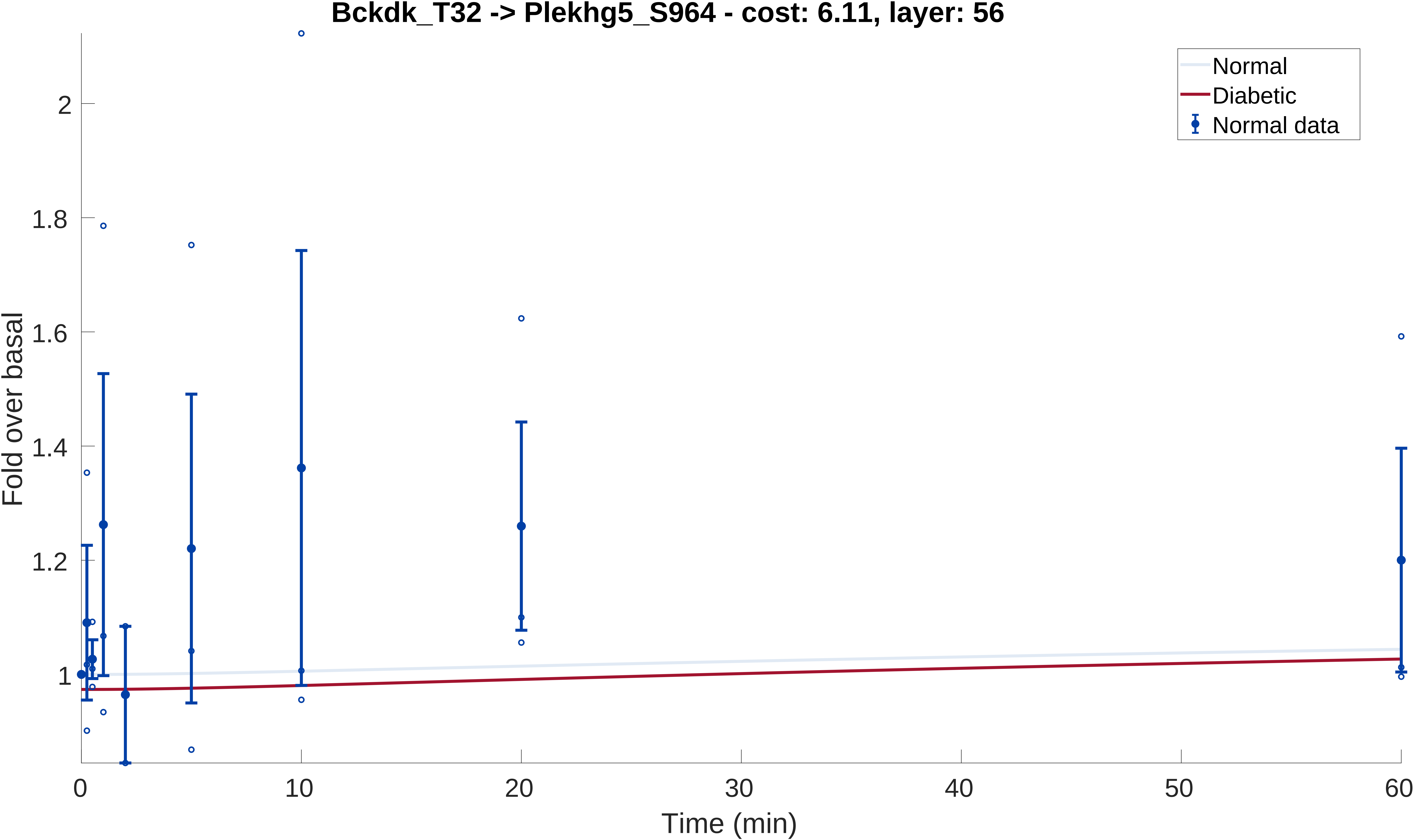
Bckdk_T32 -> Plekhg5_S964 - cost: 6.11, layer: 56
Normal
Diabetic
Normal data
Fold over basal
2
1.8
1.6
1.4
1.2
1
0
10
20
30
40
50
60
Time (min)

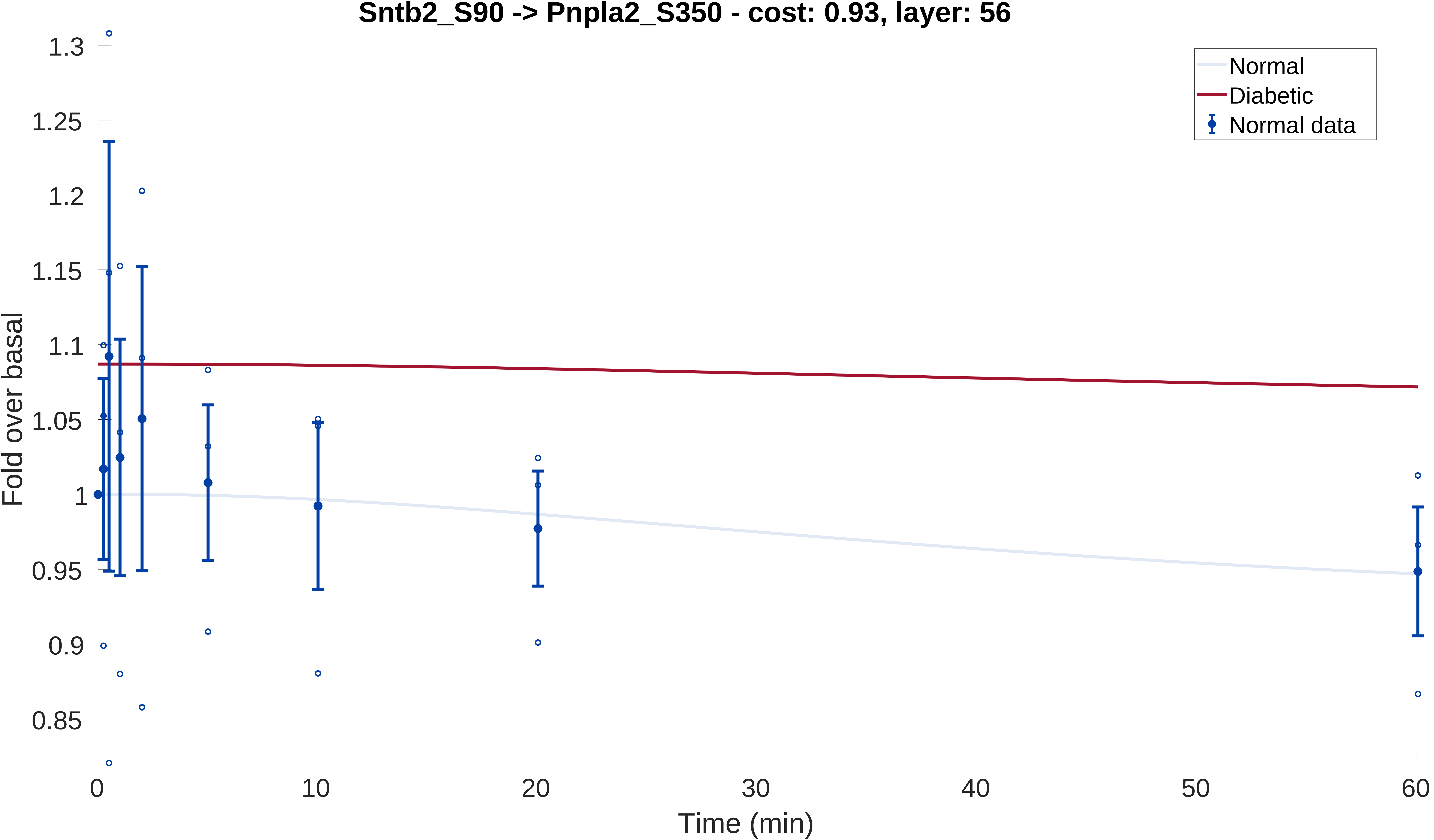

Sntb2_S90 -> Pnpla2_S350 - cost: 0.93, layer: 56
Normal
Diabetic
Normal data
Fold over basal
Time (min)
1.3
1.25
1.2
1.15
1.1
1.05
1
0.95
0.9
0.85
0
10
20
30
40
50
60

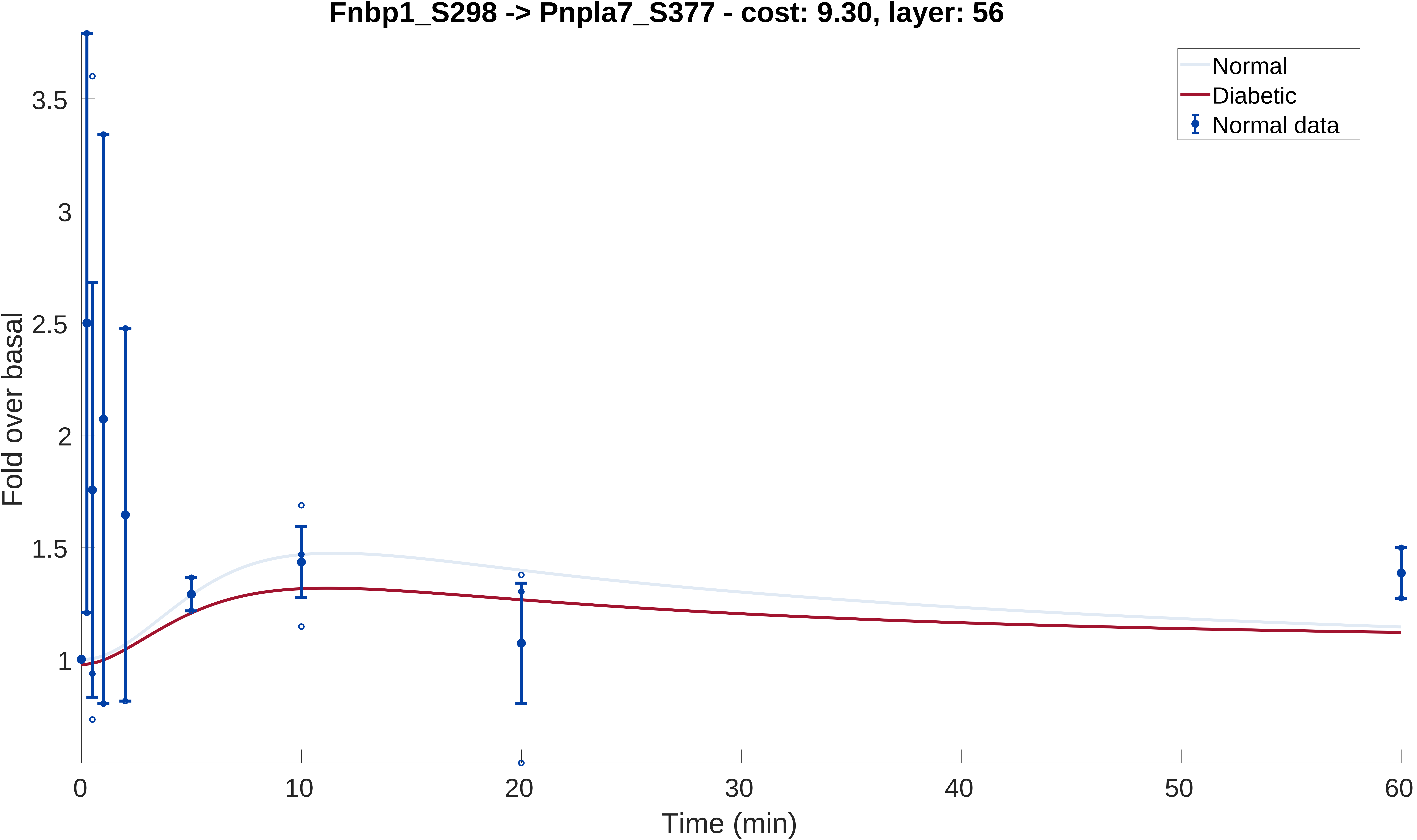

Fnbp1_S298 -> Pnpla7_S377 - cost: 9.30, layer: 56
Normal
Diabetic
Normal data
Fold over basal
3.5
3
2.5
2
1.5
1
0
10
20
30
40
50
60
Time (min)

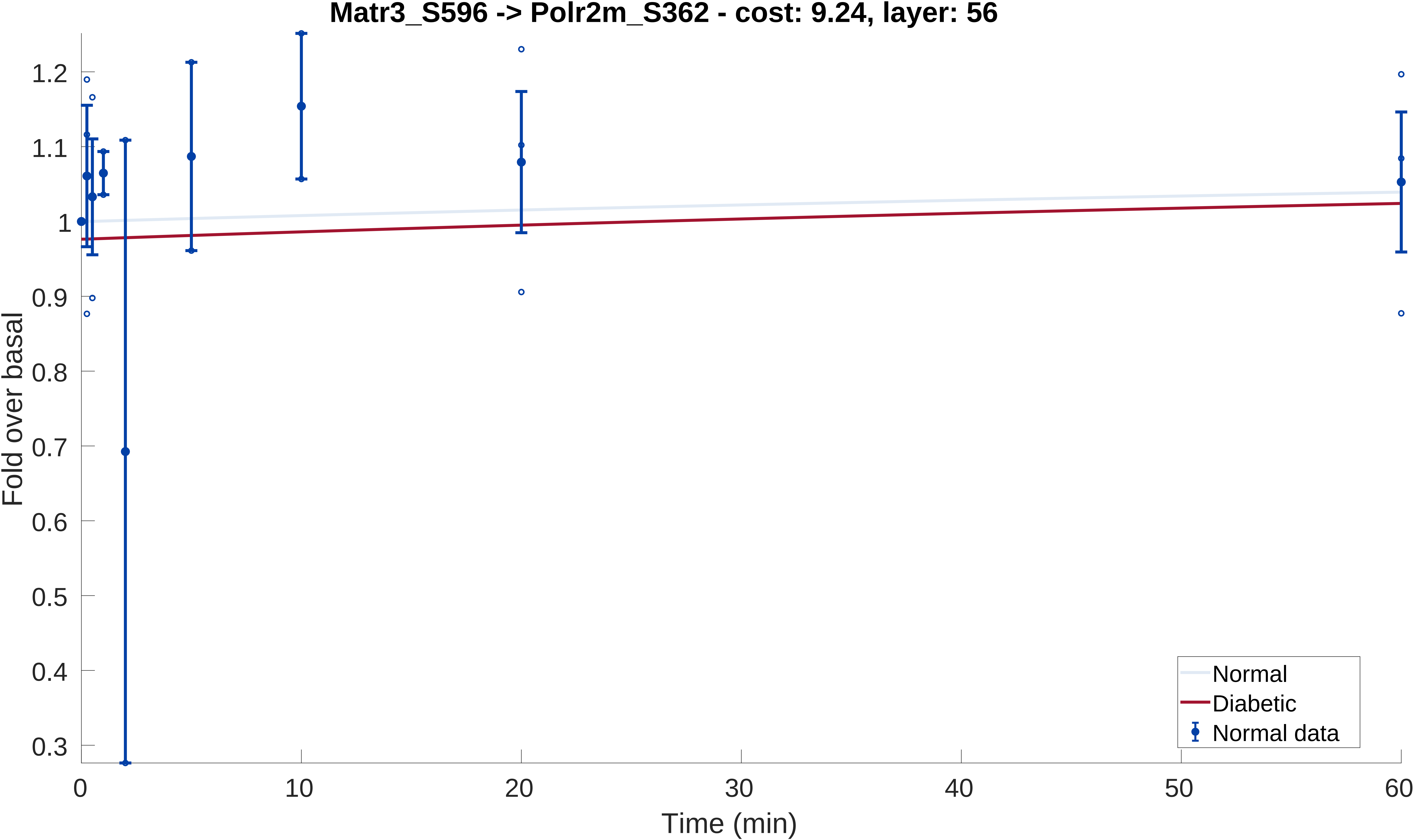

Matr3_S596 -> Polr2m_S362 - cost: 9.24, layer: 56
Fold over basal
1.2
1.1
1
0.9
0.8
0.7
0.6
0.5
0.4
0.3
0
10
20
30
40
50
60
Time (min)
Normal
Diabetic
Normal data

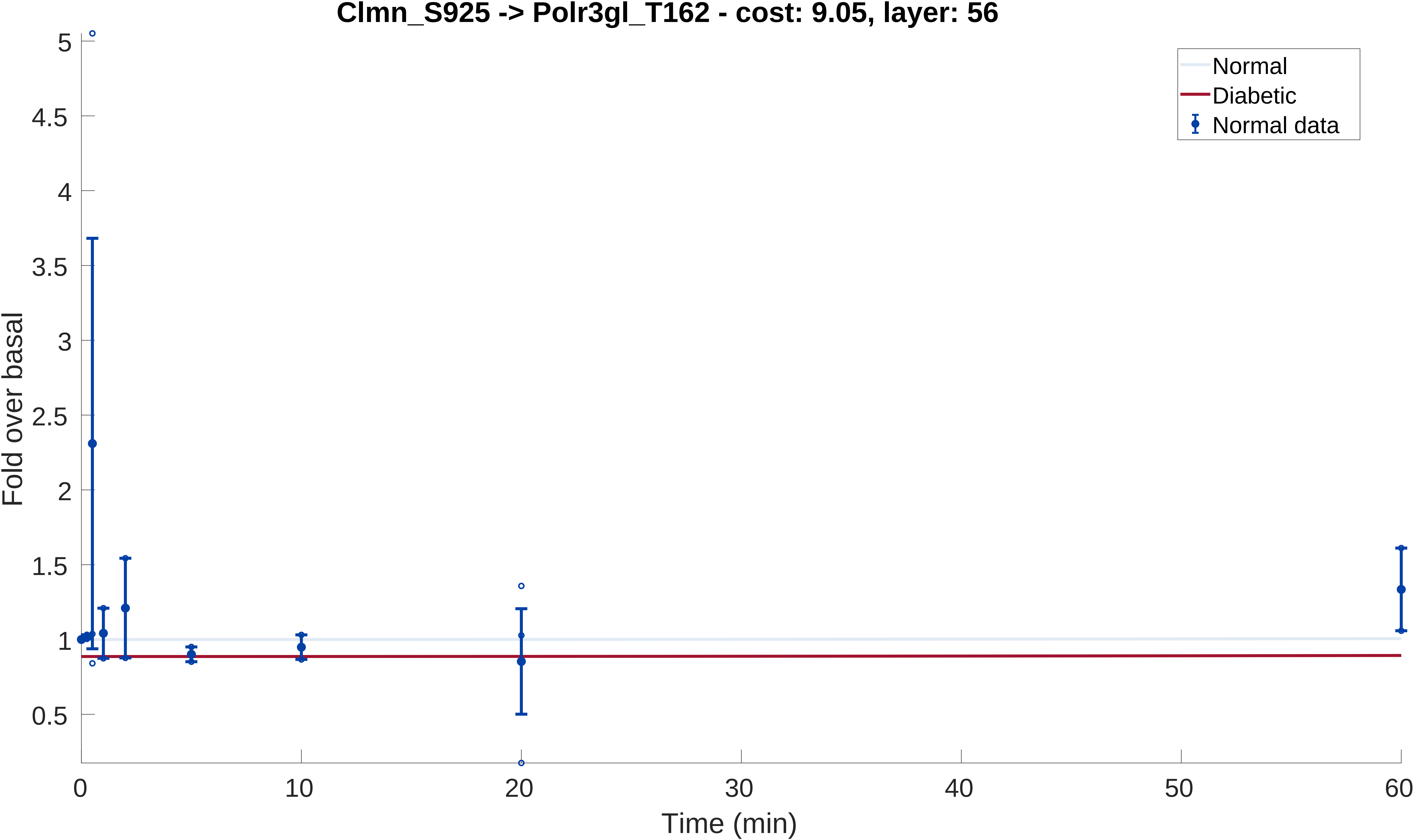
Clmn_S925 -> Polr3gl_T162 - cost: 9.05, layer: 56
Normal
Diabetic
Normal data
Fold over basal
Time (min)
0
10
20
30
40
50
60
0.5
1
1.5
2
2.5
3
3.5
4
4.5
5

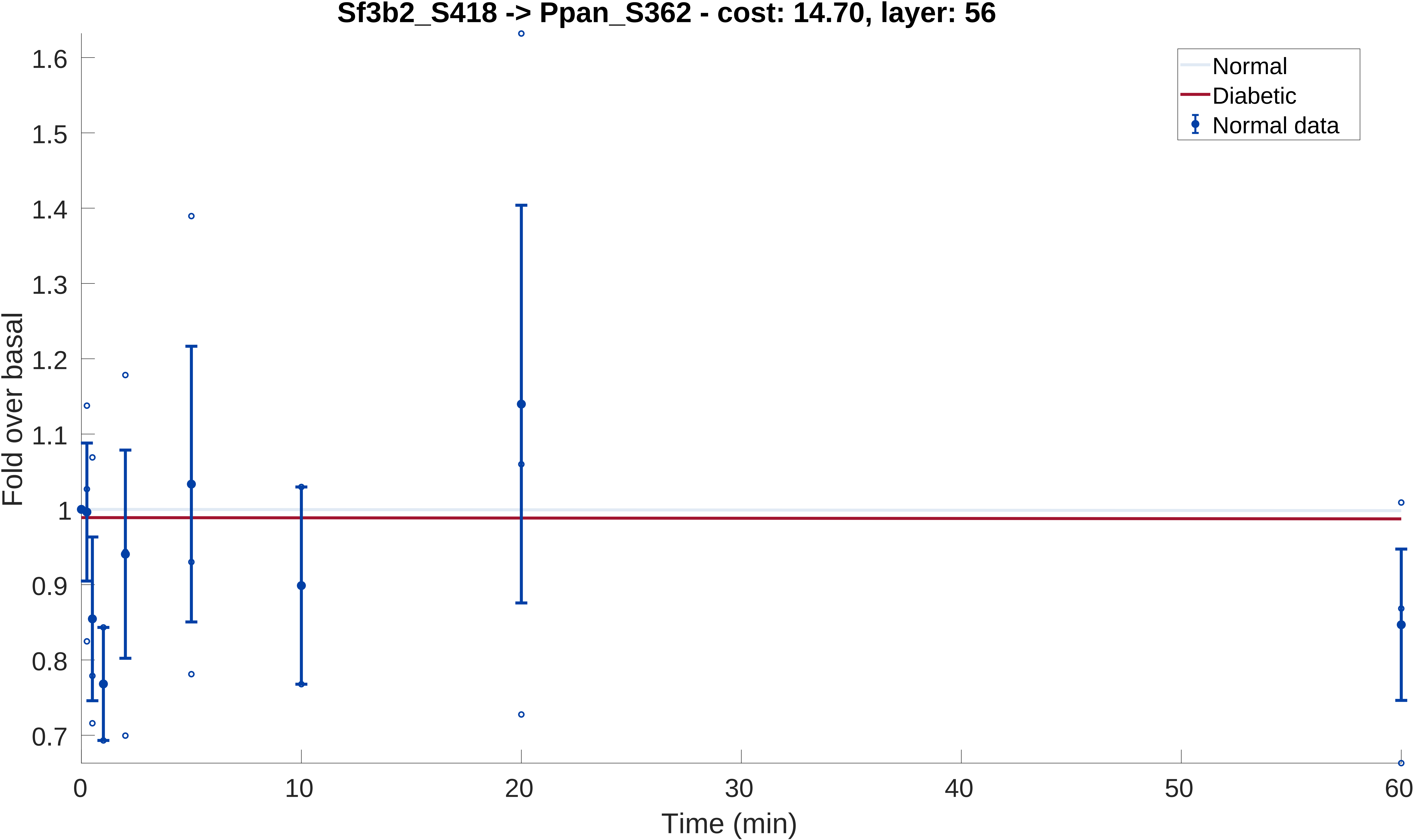
Sf3b2_S418 -> Ppan_S362 - cost: 14.70, layer: 56
Normal
Diabetic
Normal data
Fold over basal
Time (min)
1.6
1.5
1.4
1.3
1.2
1.1
1
0.9
0.8
0.7
0
10
20
30
40
50
60

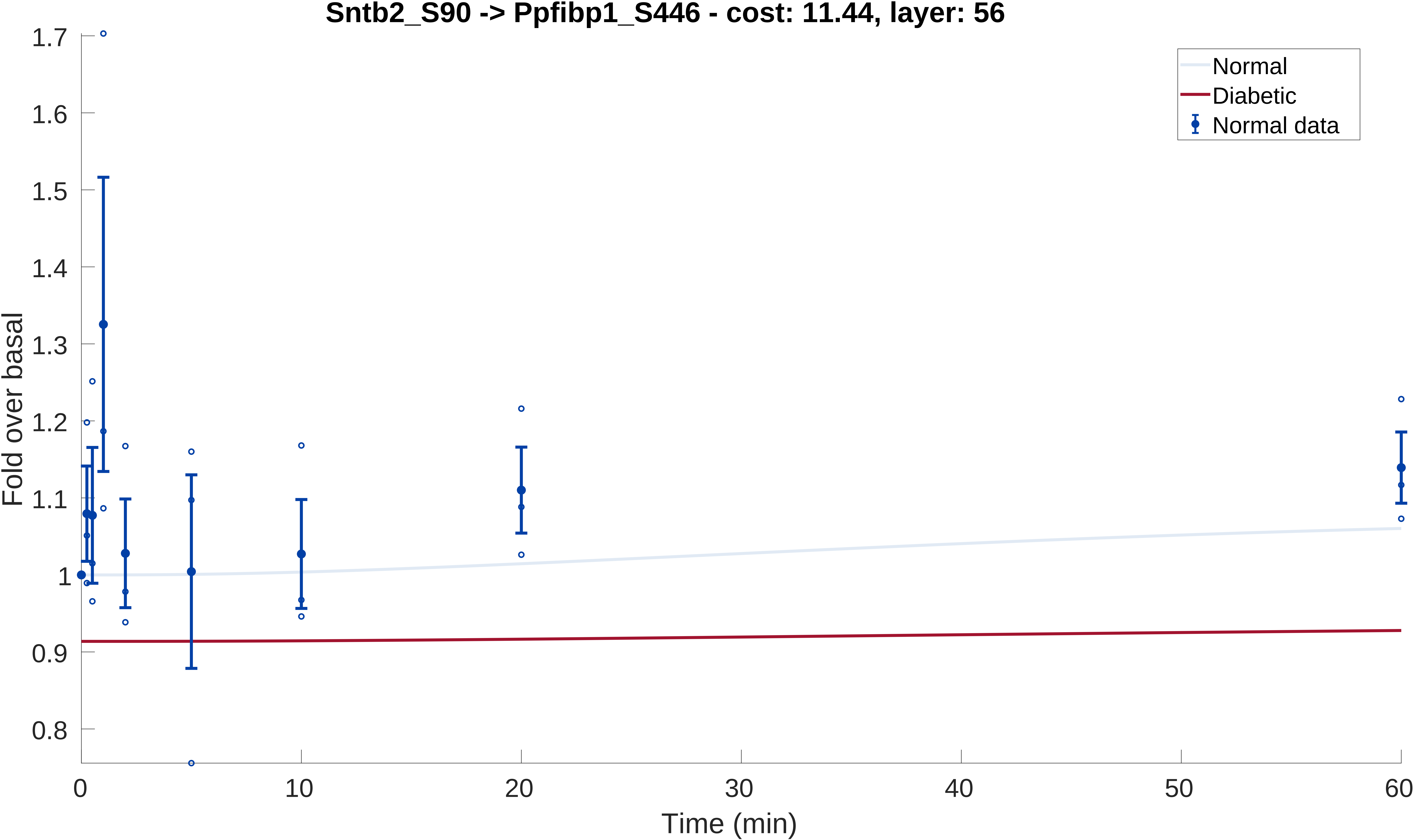
Sntb2_S90 -> Ppfibp1_S446 - cost: 11.44, layer: 56
Normal
Diabetic
Normal data
Fold over basal
Time (min)
0.8
0.9
1
1.1
1.2
1.3
1.4
1.5
1.6
1.7
0
10
20
30
40
50
60

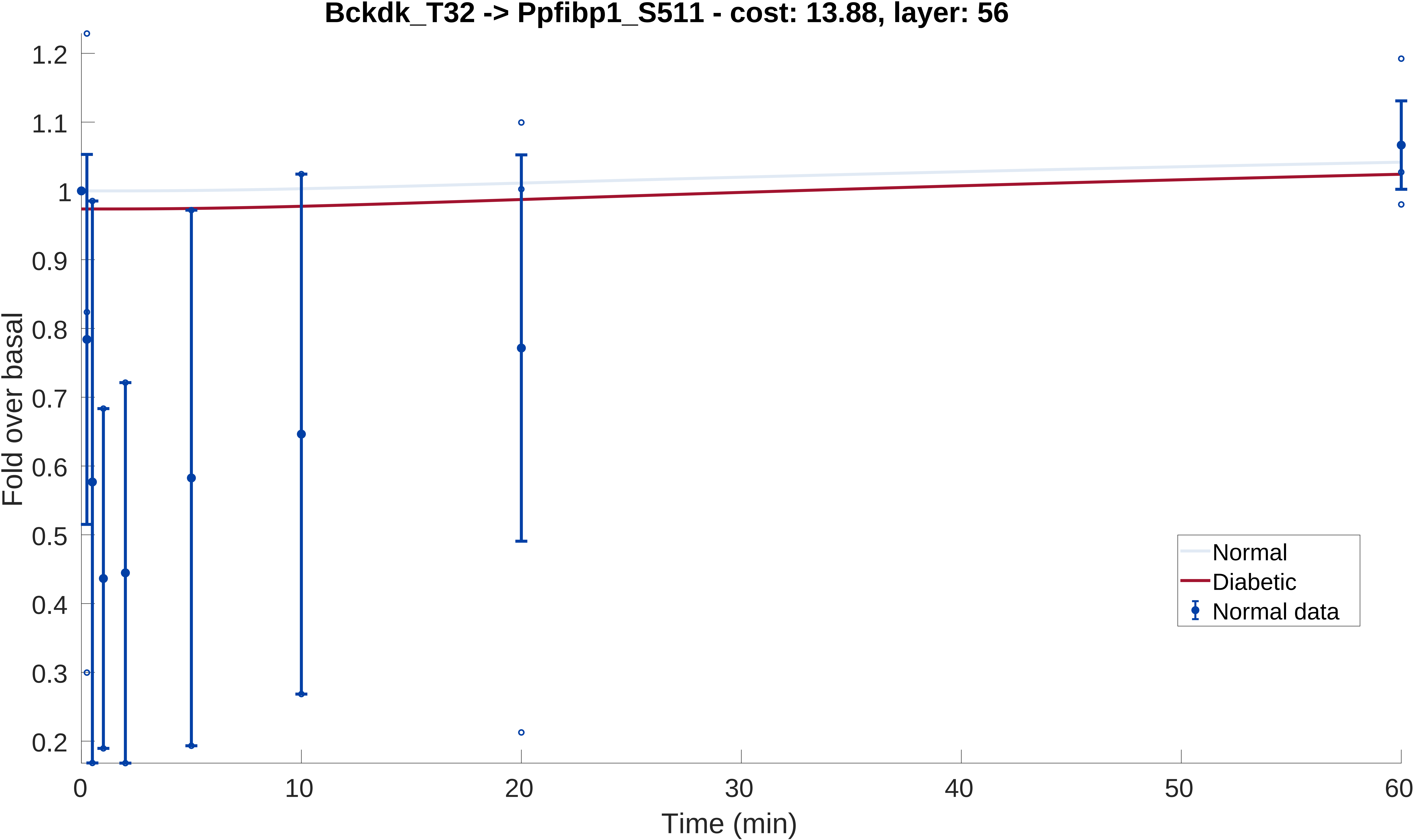

Bckdk_T32 -> Ppfibp1_S511 - cost: 13.88, layer: 56
Fold over basal
1.2
1.1
1
0.9
0.8
0.7
0.6
0.5
0.4
0.3
0.2
0
10
20
30
40
50
60
Time (min)
Normal
Diabetic
Normal data

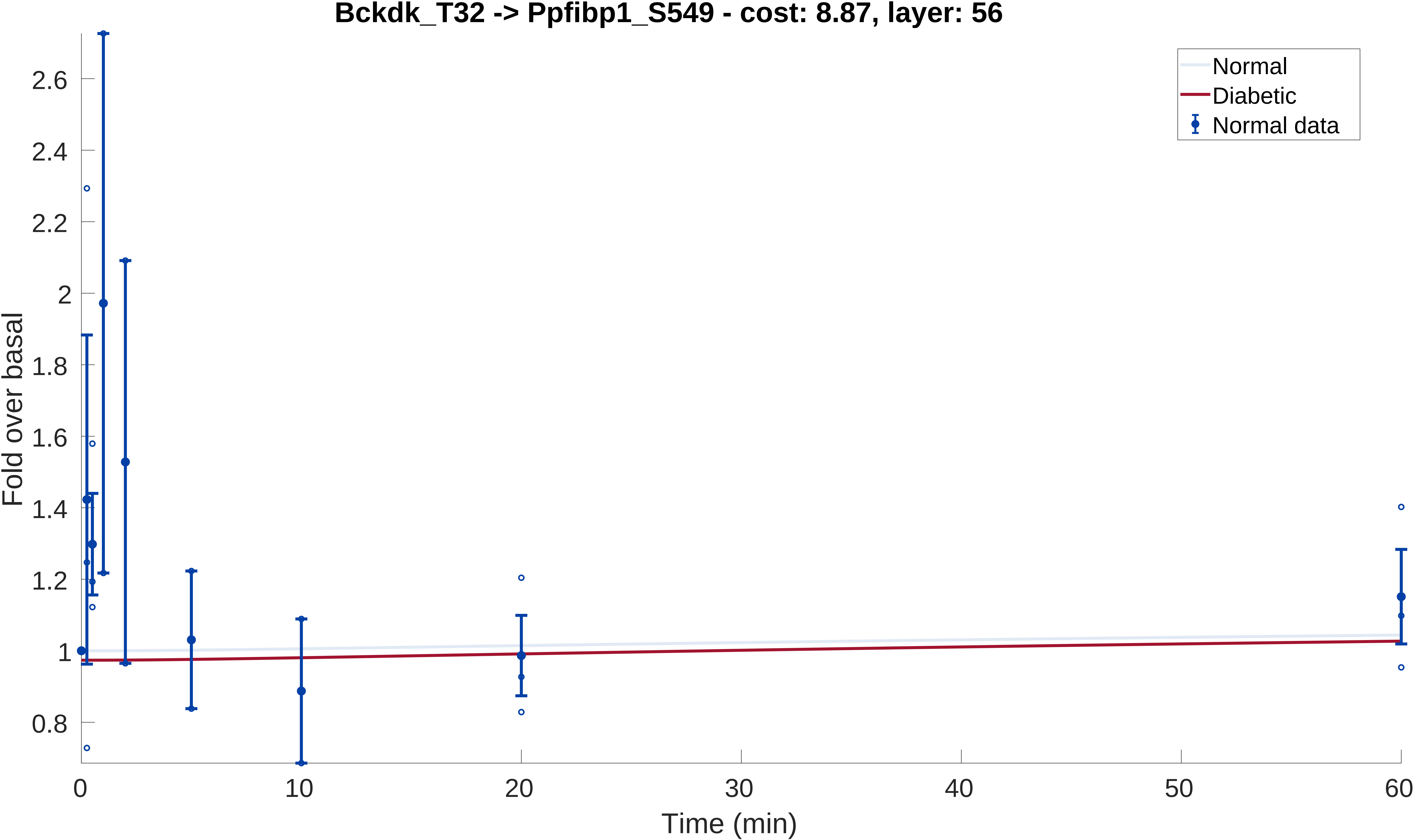
Bckdk_T32 -> Ppfibp1_S549 - cost: 8.87, layer: 56
Normal
Diabetic
Normal data
Fold over basal
Time (min)
0.8
1
1.2
1.4
1.6
1.8
2
2.2
2.4
2.6
0
10
20
30
40
50
60

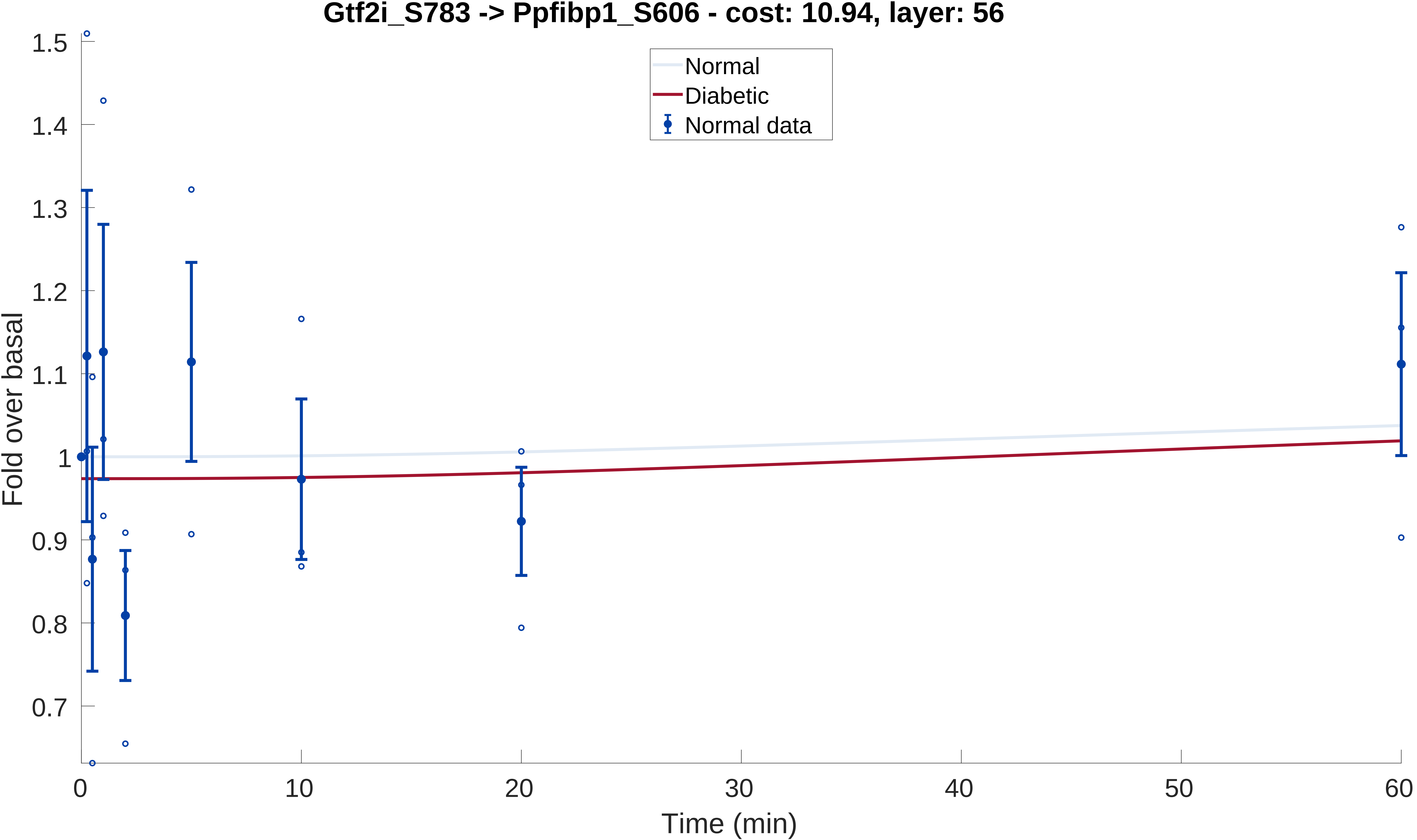

Gtf2i_S783 -> Ppfibp1_S606 - cost: 10.94, layer: 56
Normal
Diabetic
Normal data
Fold over basal
Time (min)
1.5
1.4
1.3
1.2
1.1
1
0.9
0.8
0.7
0
10
20
30
40
50
60

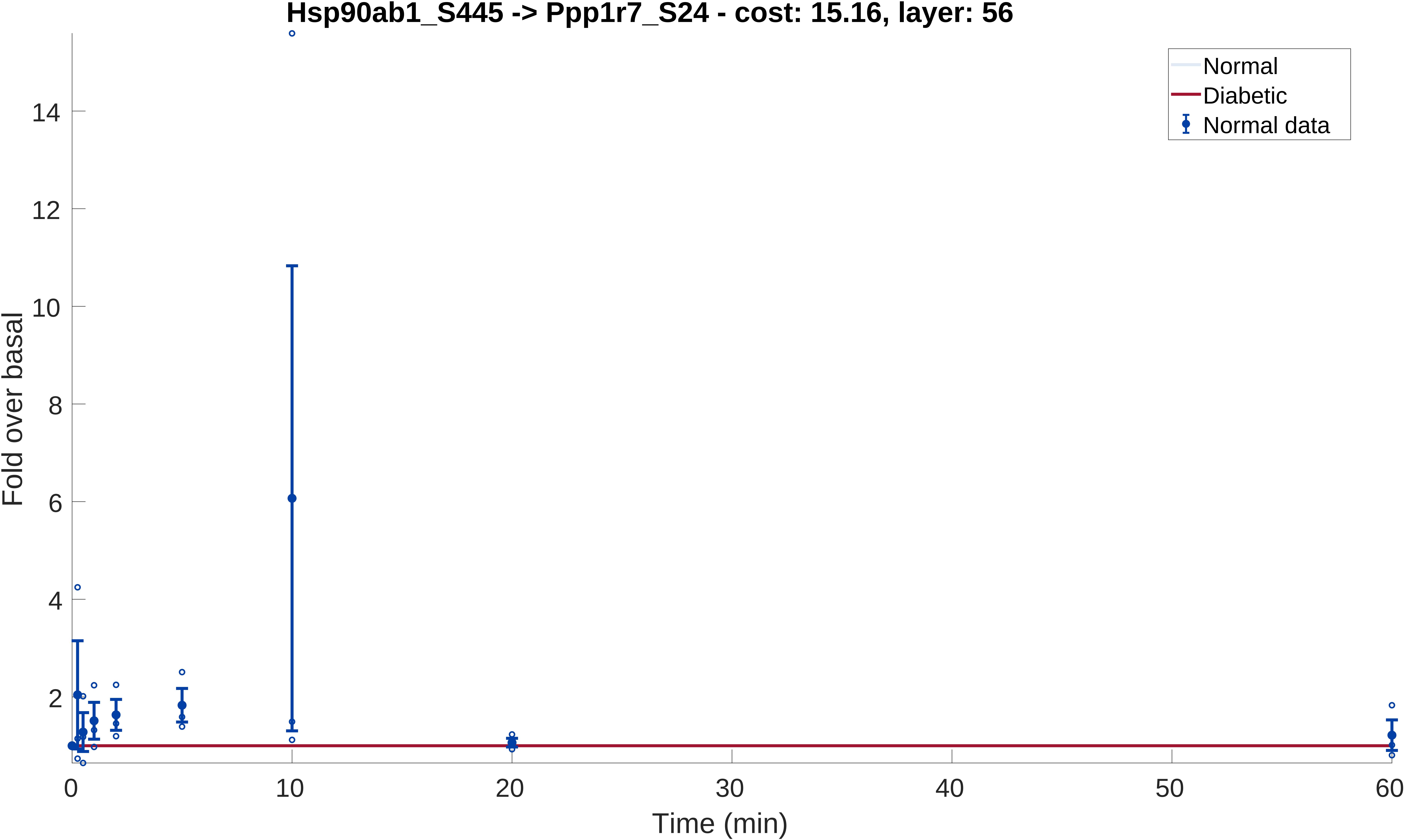

Hsp90ab1_S445 -> Ppp1r7_S24 - cost: 15.16, layer: 56
Normal
Diabetic
Normal data
Fold over basal
Time (min)
0
10
20
30
40
50
60
2
4
6
8
10
12
14

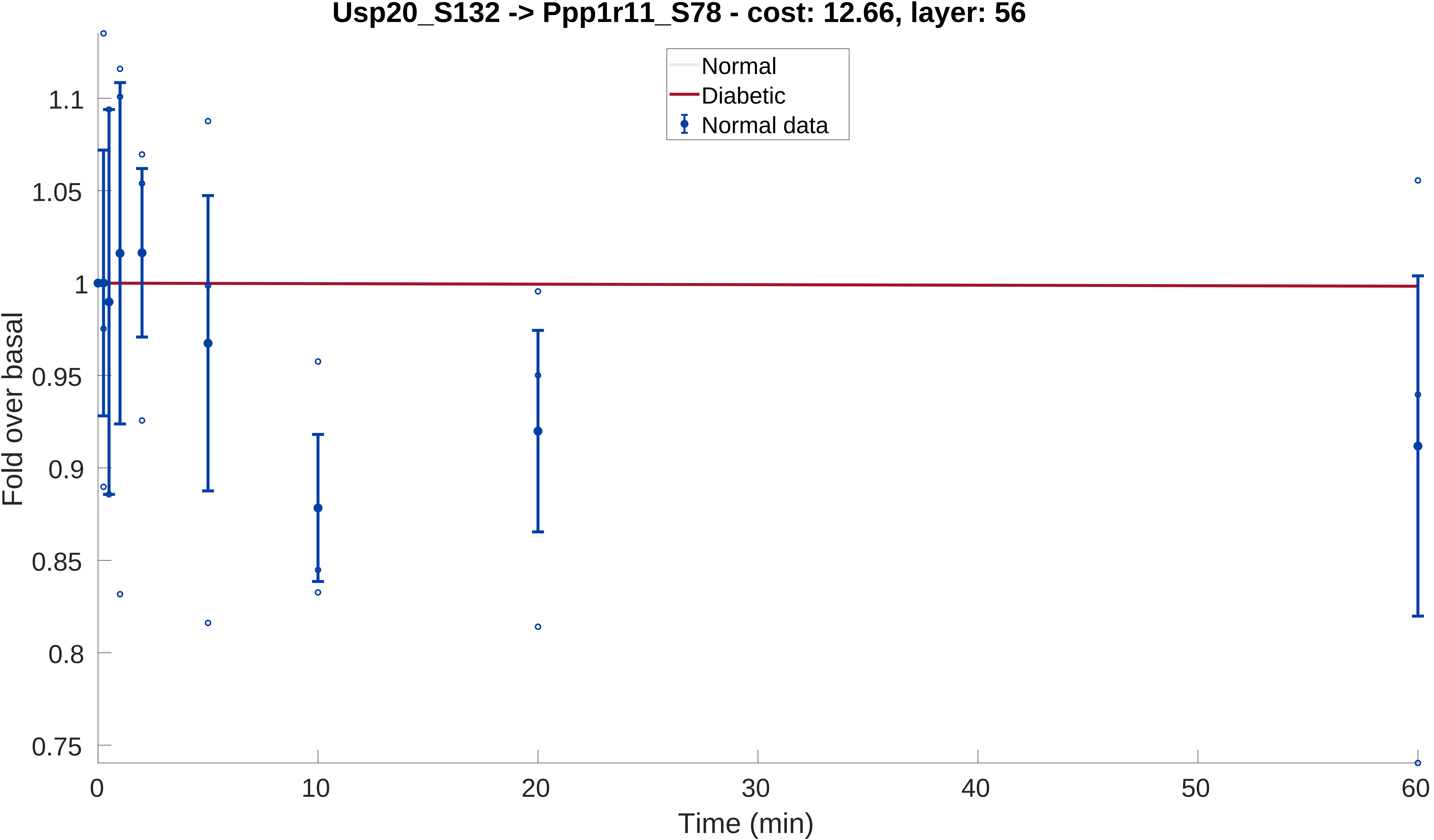
Usp20_S132 -> Ppp1r11_S78 - cost: 12.66, layer: 56
Normal
Diabetic
Normal data
Fold over basal
1.1
1.05
1
0.95
0.9
0.85
0.8
0.75
0
10
20
30
40
50
60
Time (min)

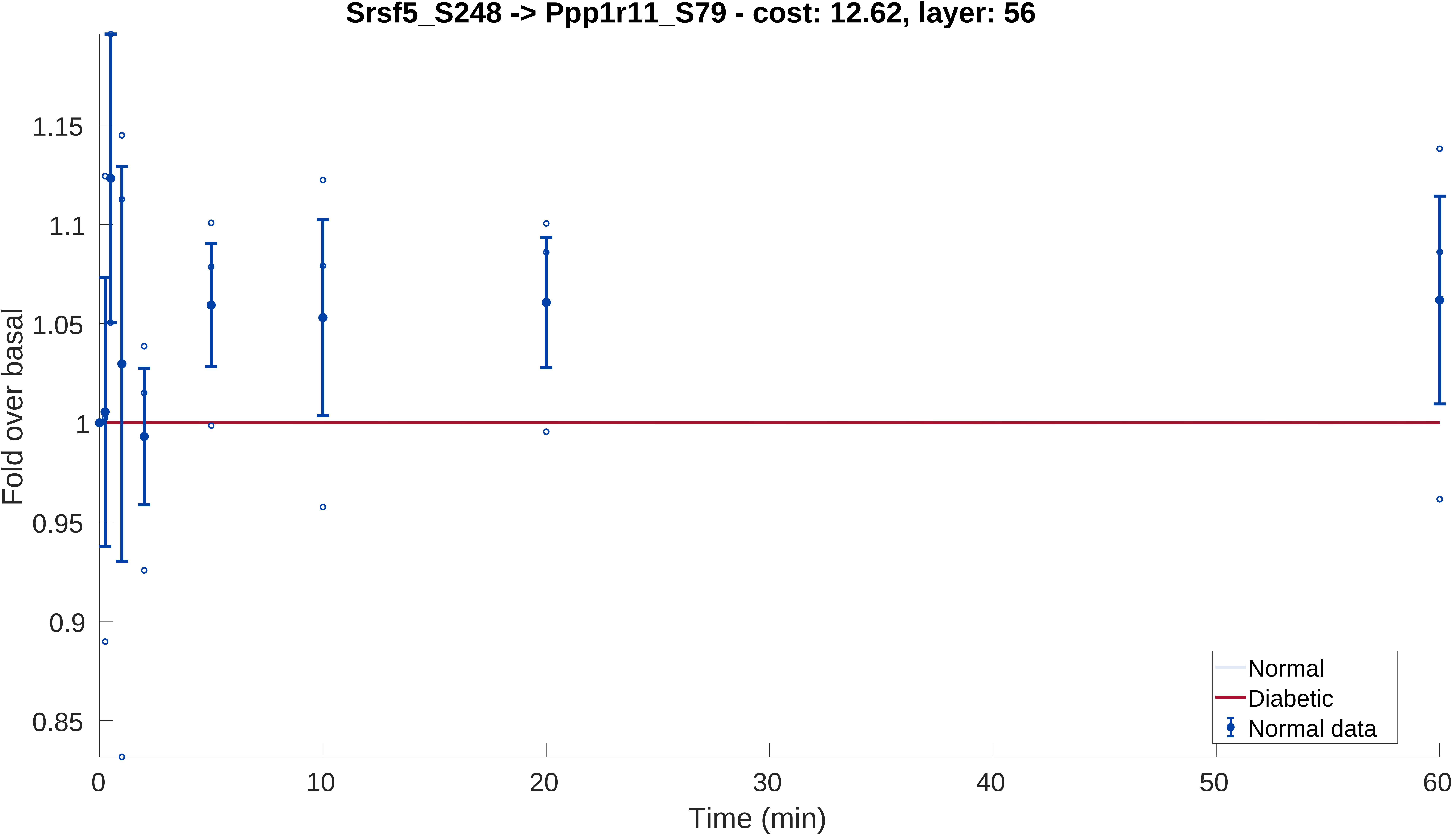

Srsf5_S248 -> Ppp1r11_S79 - cost: 12.62, layer: 56
Fold over basal
1.15
1.1
1.05
1
0.95
0.9
0.85
0
10
20
30
40
50
60
Time (min)
Normal
Diabetic
Normal data

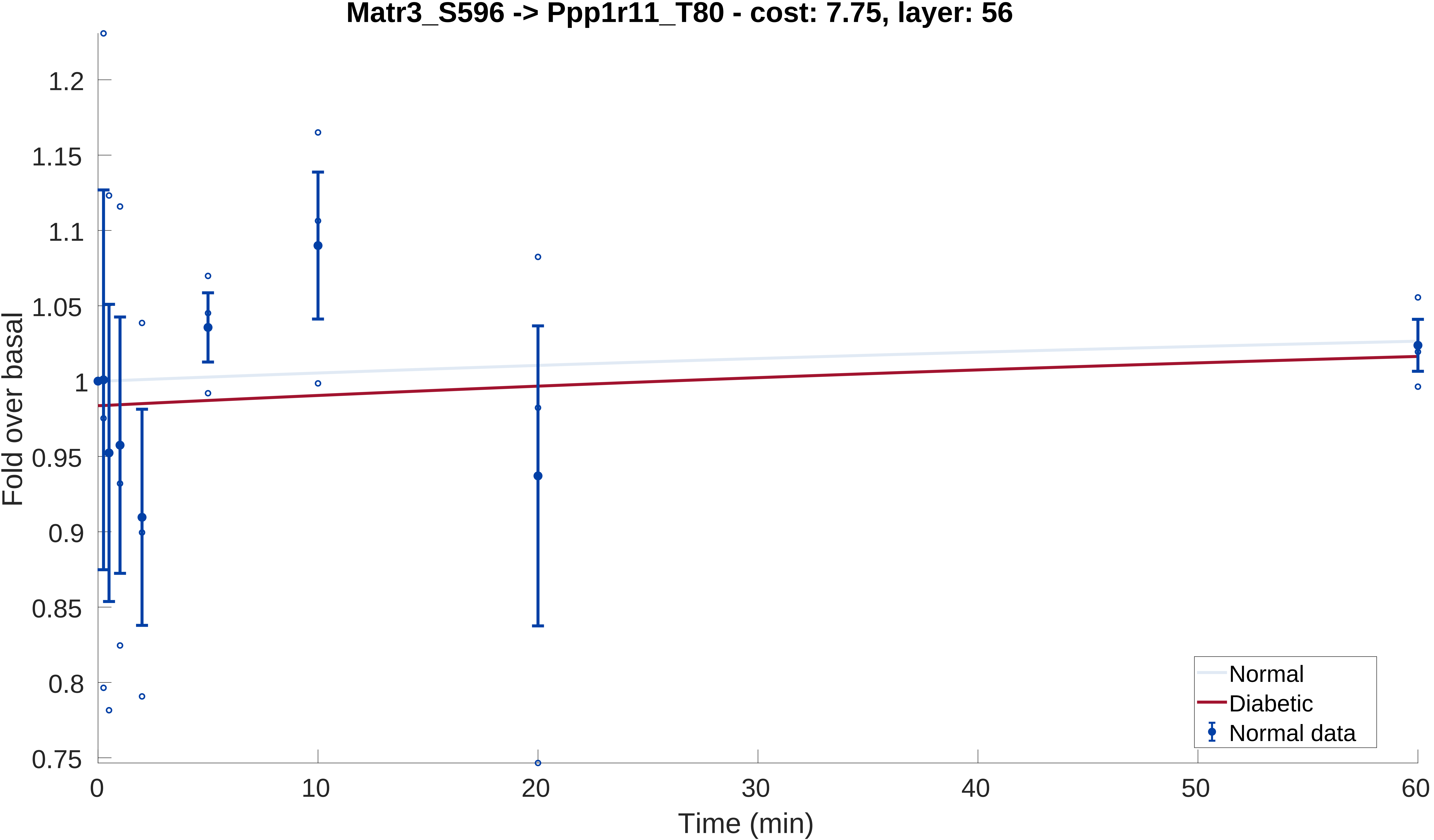

Matr3_S596 -> Ppp1r11_T80 - cost: 7.75, layer: 56
Fold over basal
Time (min)
1.2
1.15
1.1
1.05
1
0.95
0.9
0.85
0.8
0.75
0
10
20
30
40
50
60
Normal
Diabetic
Normal data

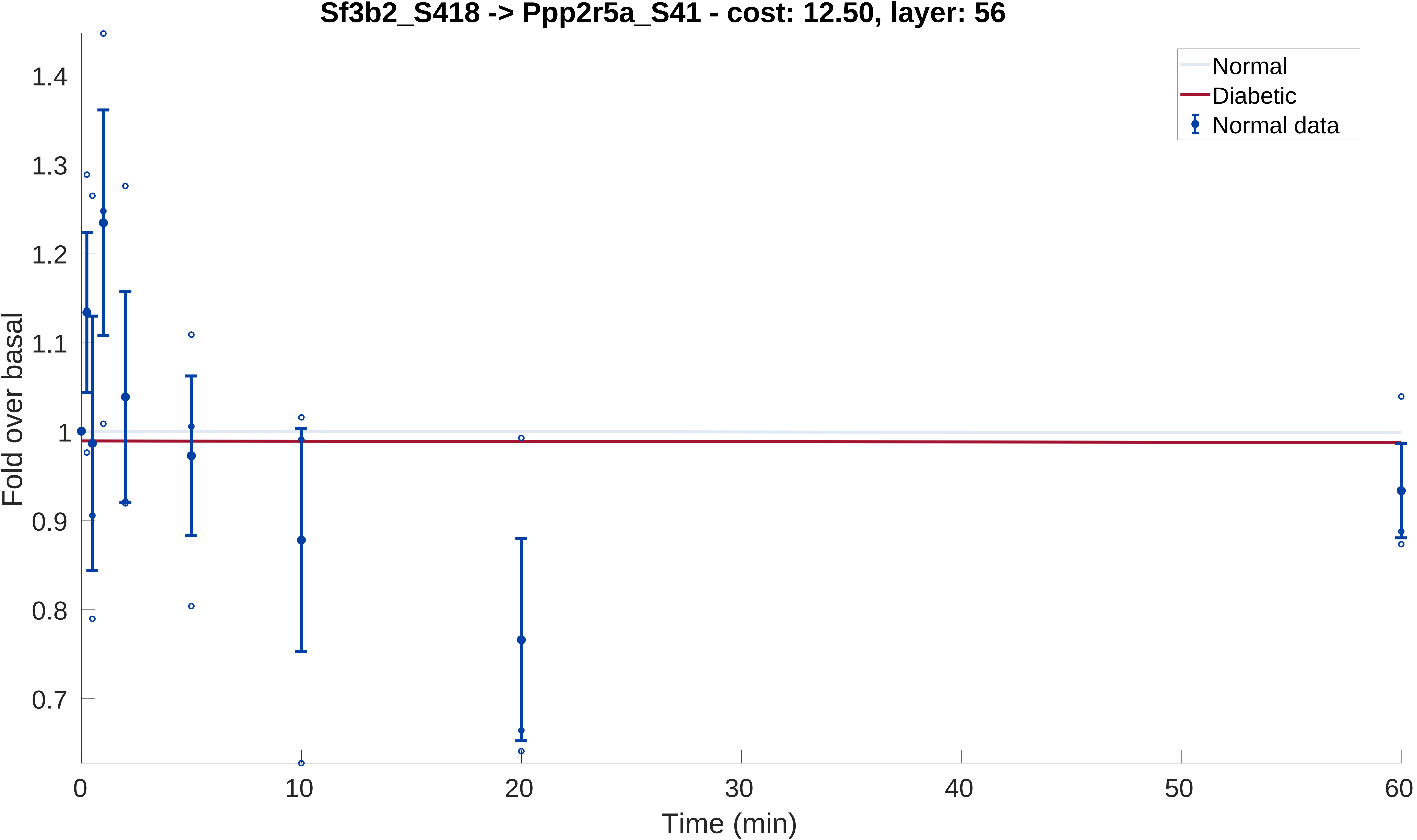

Sf3b2_S418 -> Ppp2r5a_S41 - cost: 12.50, layer: 56
Normal
Diabetic
Normal data
Fold over basal
Time (min)
1.4
1.3
1.2
1.1
1
0.9
0.8
0.7
0
10
20
30
40
50
60

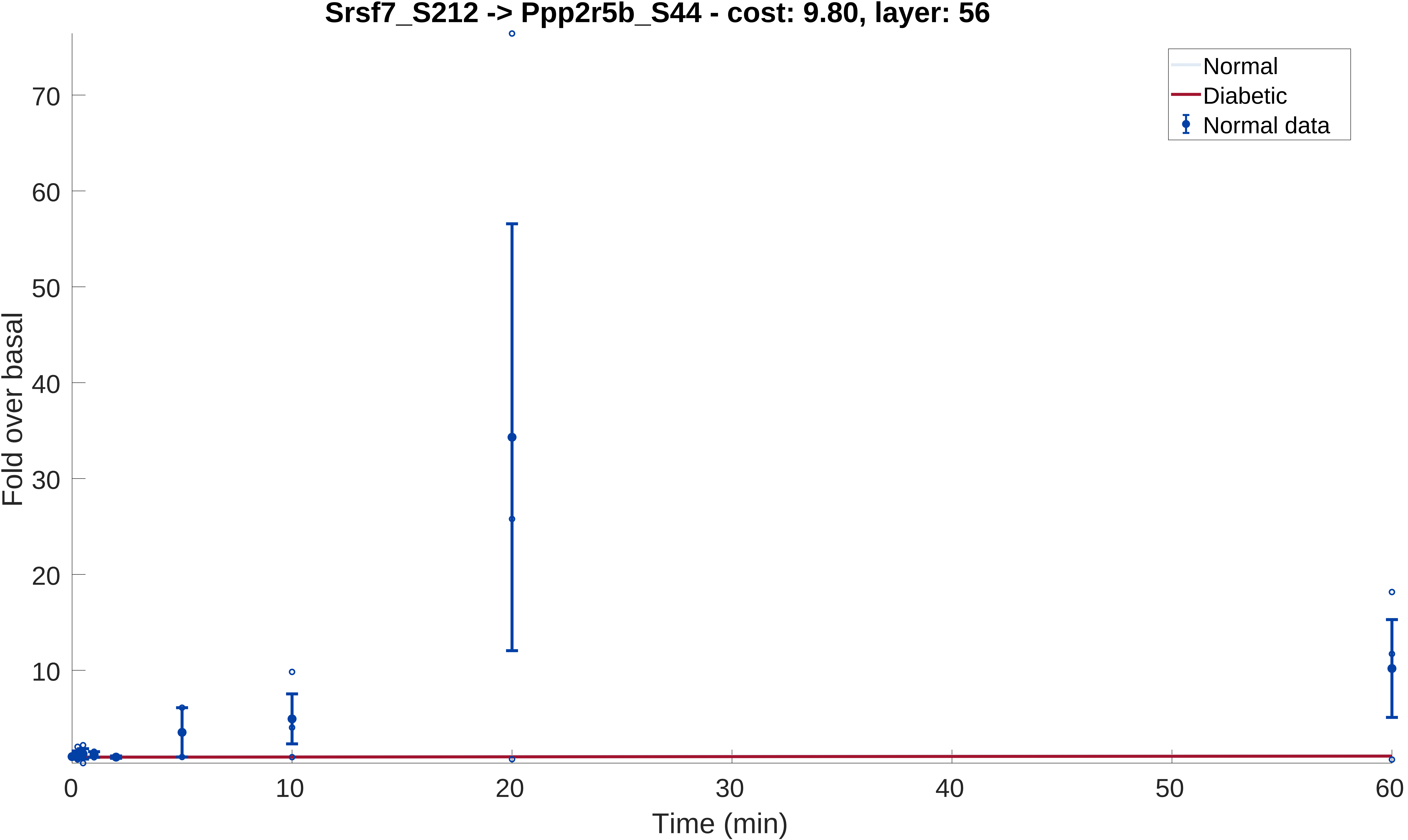

Srsf7_S212 -> Ppp2r5b_S44 - cost: 9.80, layer: 56
Normal
Diabetic
Normal data
Fold over basal
Time (min)
0
10
20
30
40
50
60
70

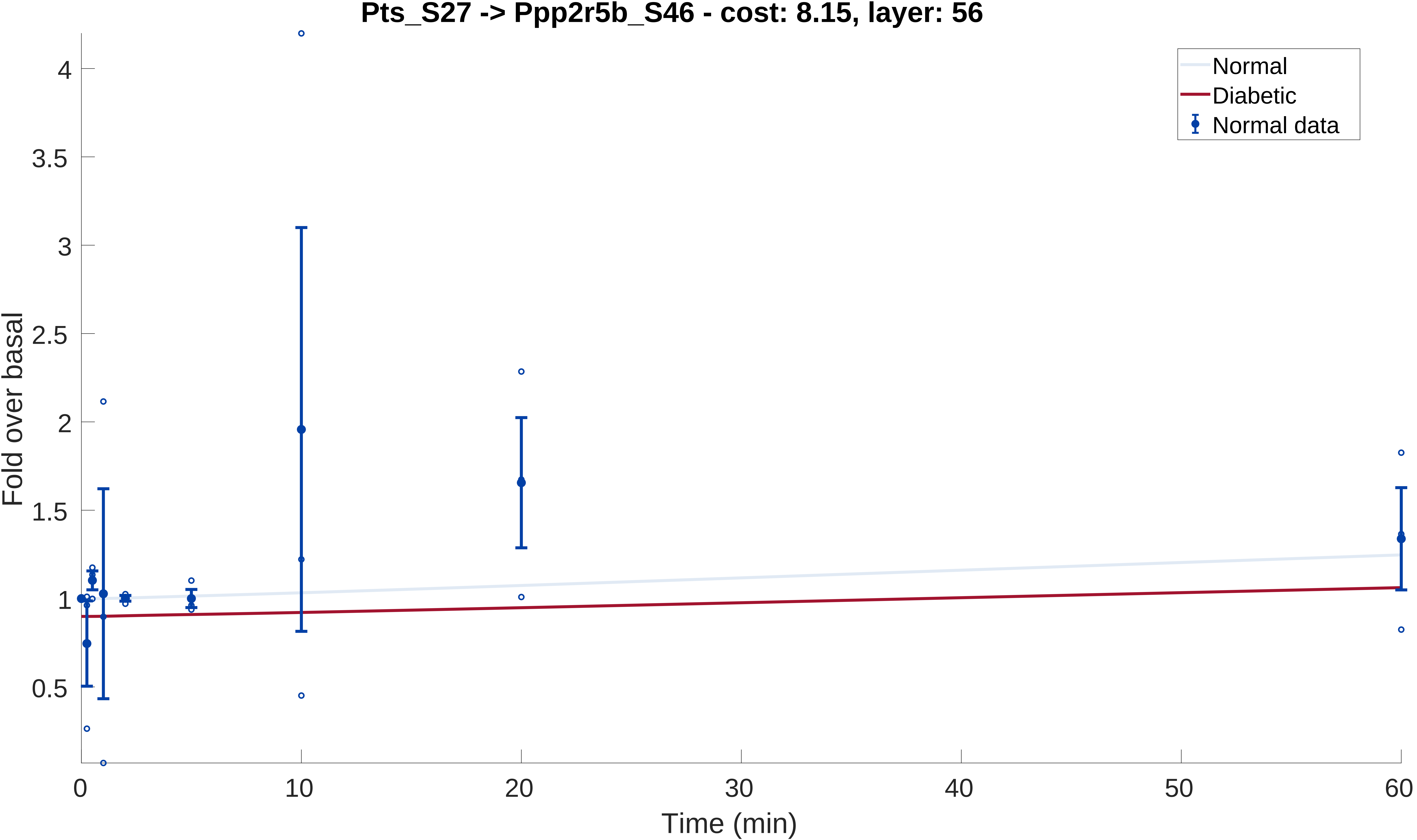
Pts_S27 -> Ppp2r5b_S46 - cost: 8.15, layer: 56
Normal
Diabetic
Normal data
Fold over basal
4
3.5
3
2.5
2
1.5
1
0.5
0
10
20
30
40
50
60
Time (min)

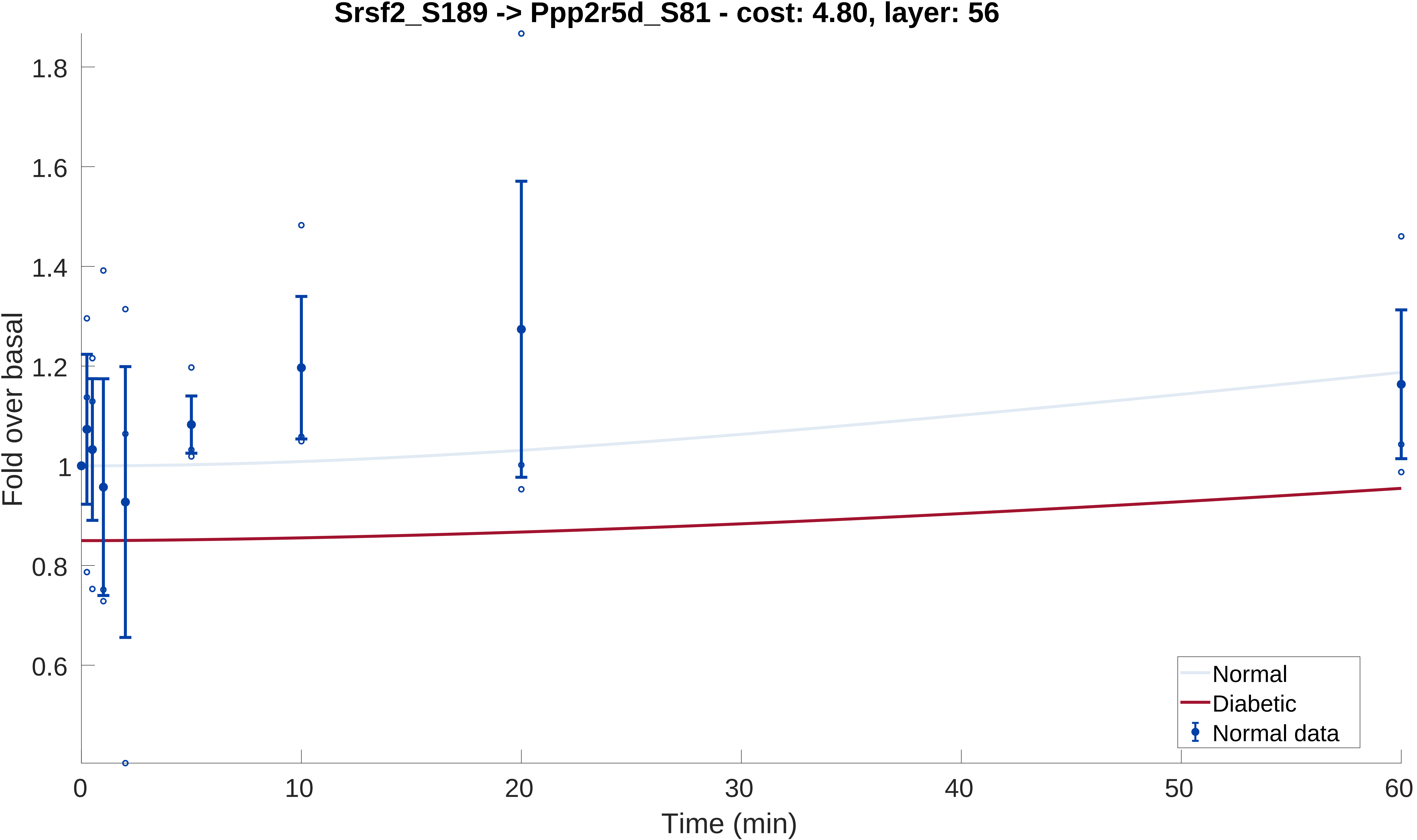

Srsf2_S189 -> Ppp2r5d_S81 - cost: 4.80, layer: 56
Fold over basal
1.8
1.6
1.4
1.2
1
0.8
0.6
0
10
20
30
40
50
60
Time (min)
Normal
Diabetic
Normal data

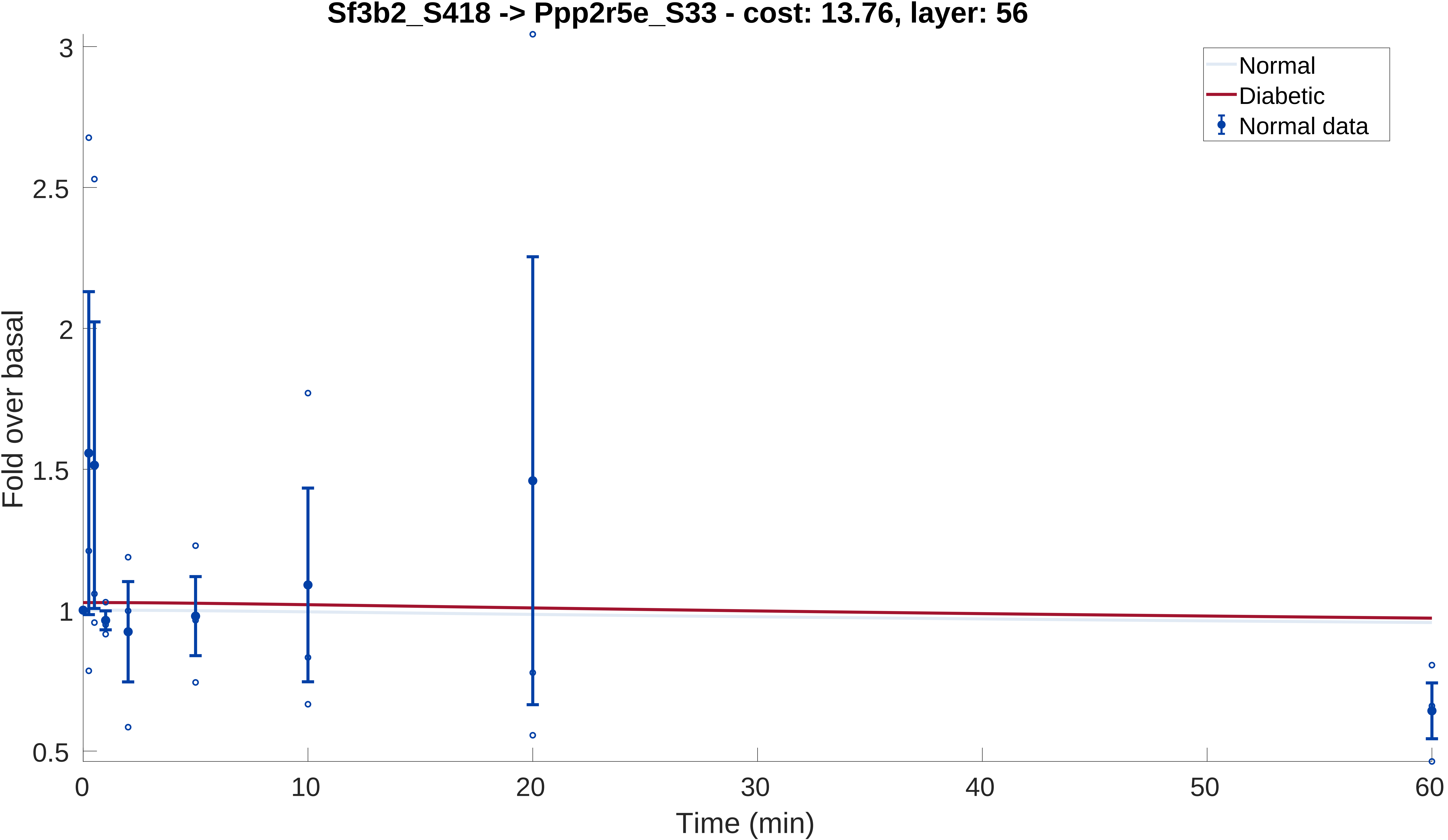

Sf3b2_S418 -> Ppp2r5e_S33 - cost: 13.76, layer: 56
Normal
Diabetic
Normal data
Fold over basal
Time (min)
0.5
1
1.5
2
2.5
3
0
10
20
30
40
50
60

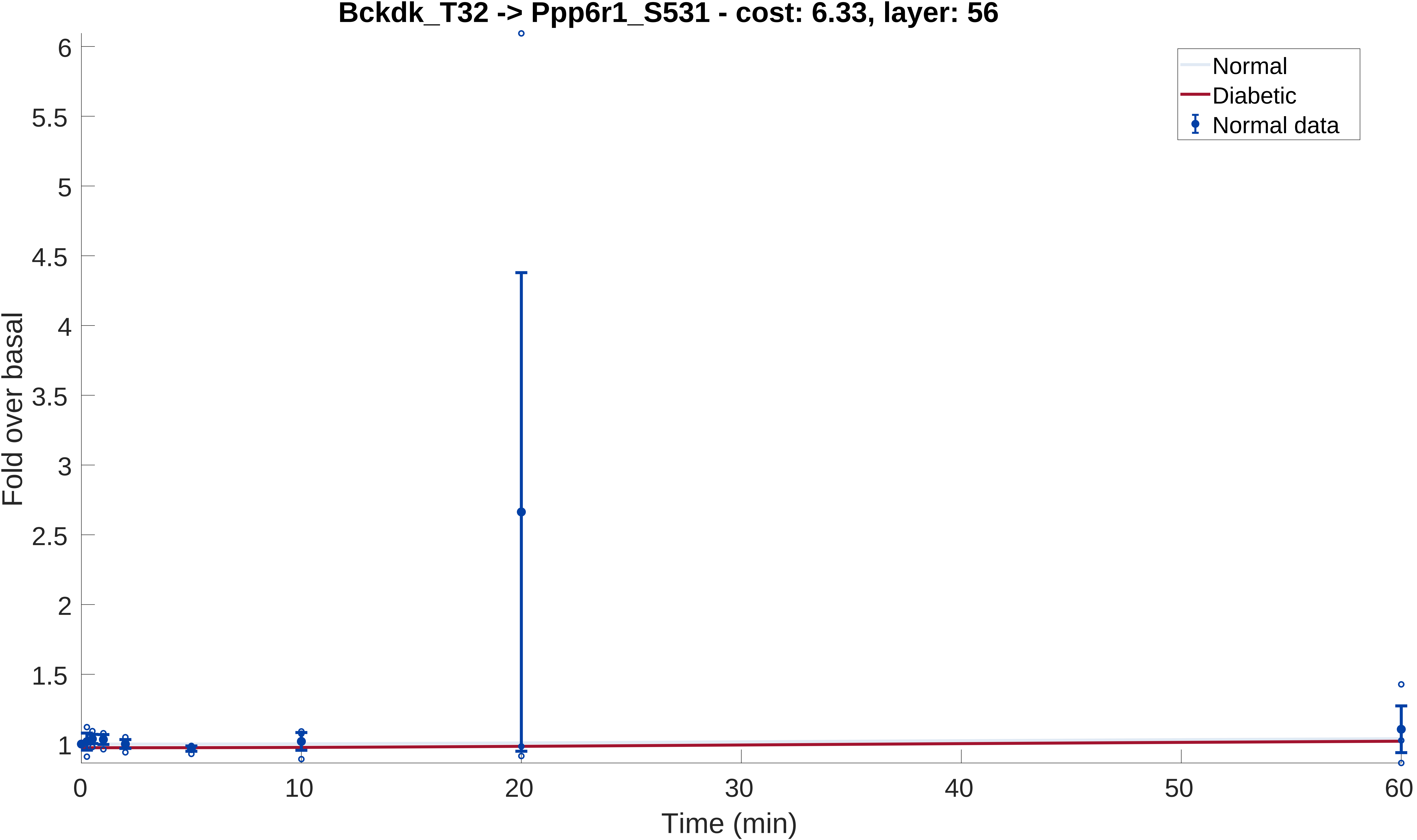

Bckdk_T32 -> Ppp6r1_S531 - cost: 6.33, layer: 56
Normal
Diabetic
Normal data
Fold over basal
Time (min)
6
5.5
5
4.5
4
3.5
3
2.5
2
1.5
1
0
10
20
30
40
50
60

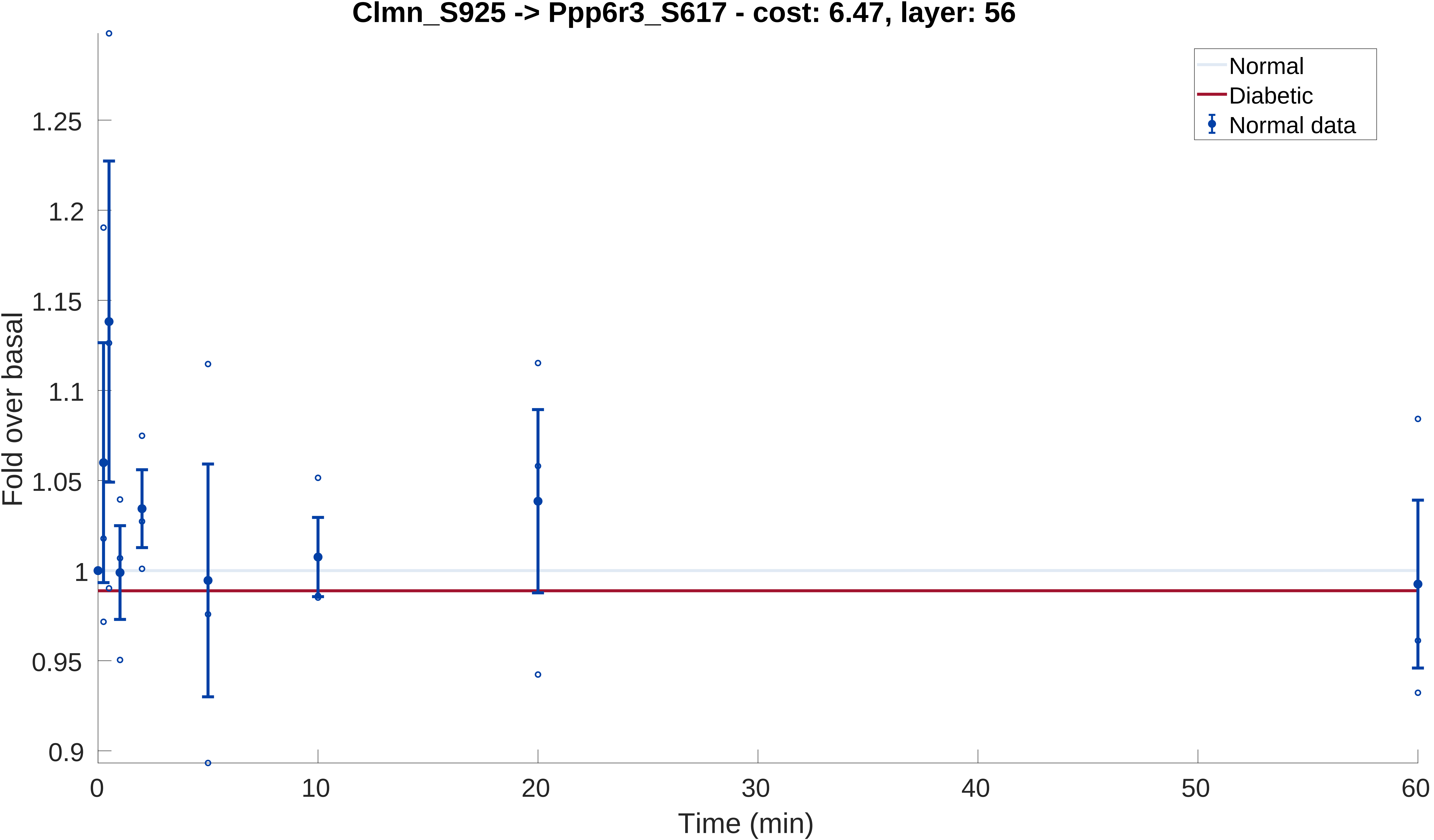

Clmn_S925 -> Ppp6r3_S617 - cost: 6.47, layer: 56
Normal
Diabetic
Normal data
Fold over basal
1.25
1.2
1.15
1.1
1.05
1
0.95
0.9
0
10
20
30
40
50
60
Time (min)

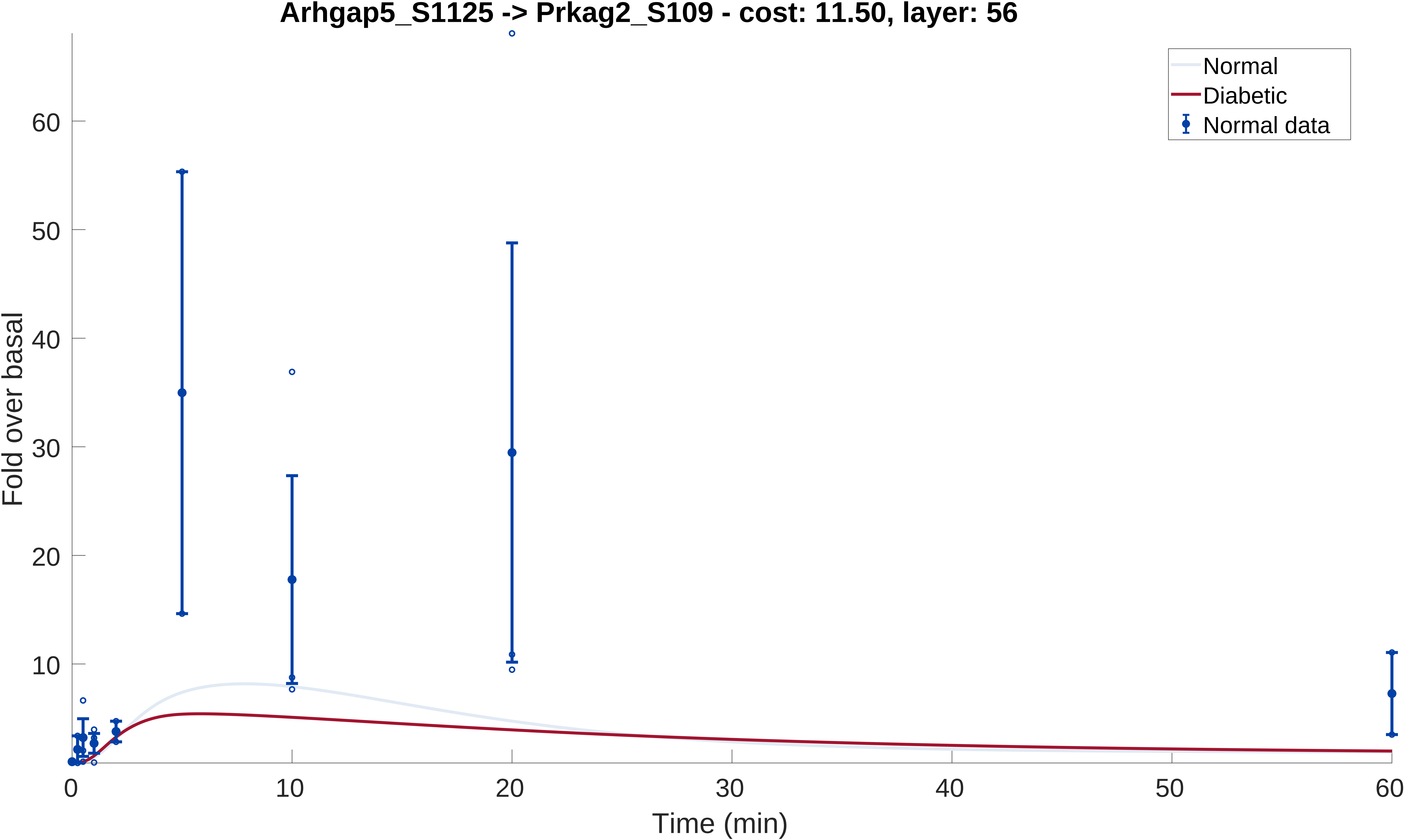
Arhgap5_S1125 -> Prkag2_S109 - cost: 11.50, layer: 56
Normal
Diabetic
Normal data
Fold over basal
Time (min)
0
10
20
30
40
50
60
10
20
30
40
50
60

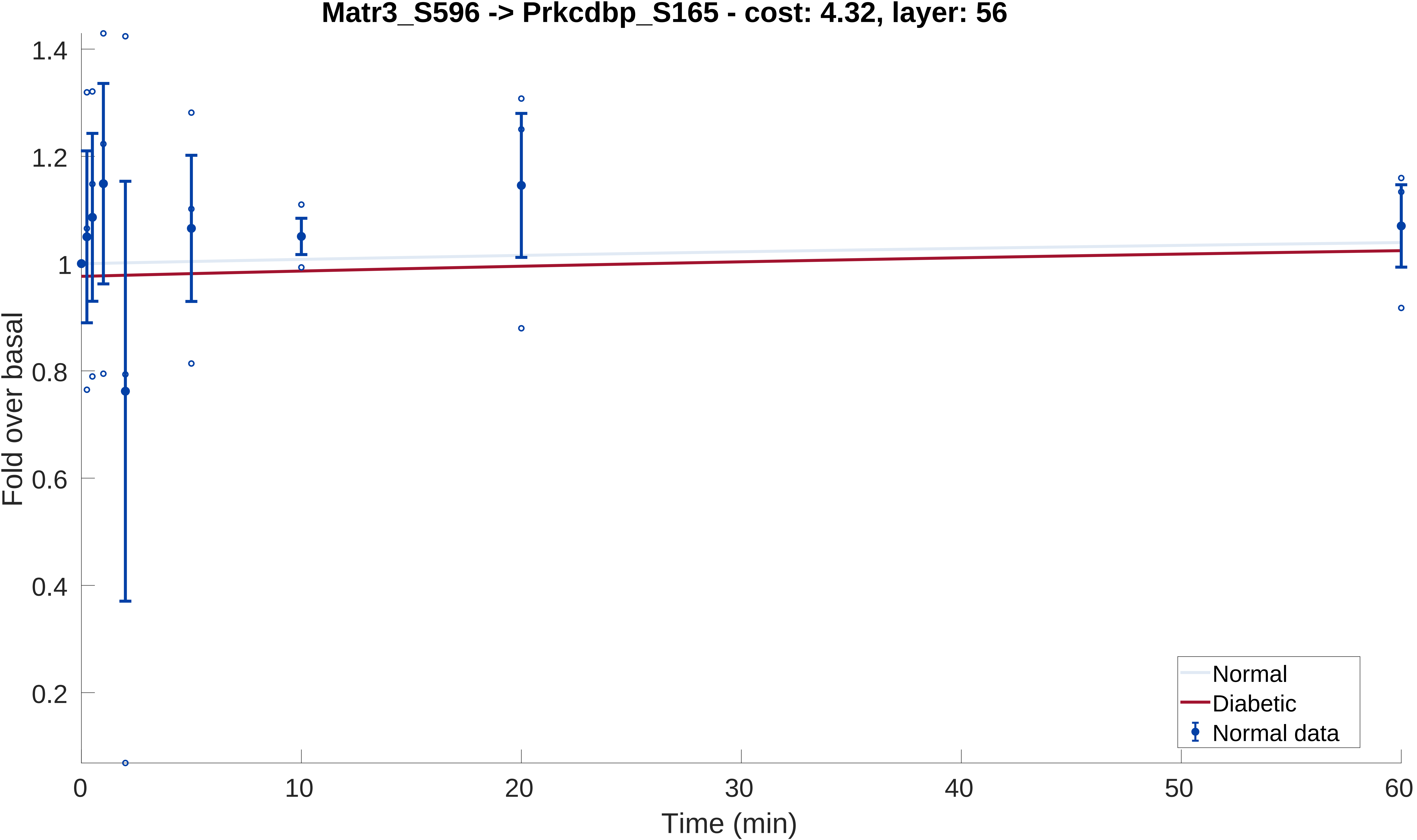

Matr3_S596 -> Prkcdbp_S165 - cost: 4.32, layer: 56
Fold over basal
1.4
1.2
1
0.8
0.6
0.4
0.2
0
10
20
30
40
50
60
Time (min)
Normal
Diabetic
Normal data

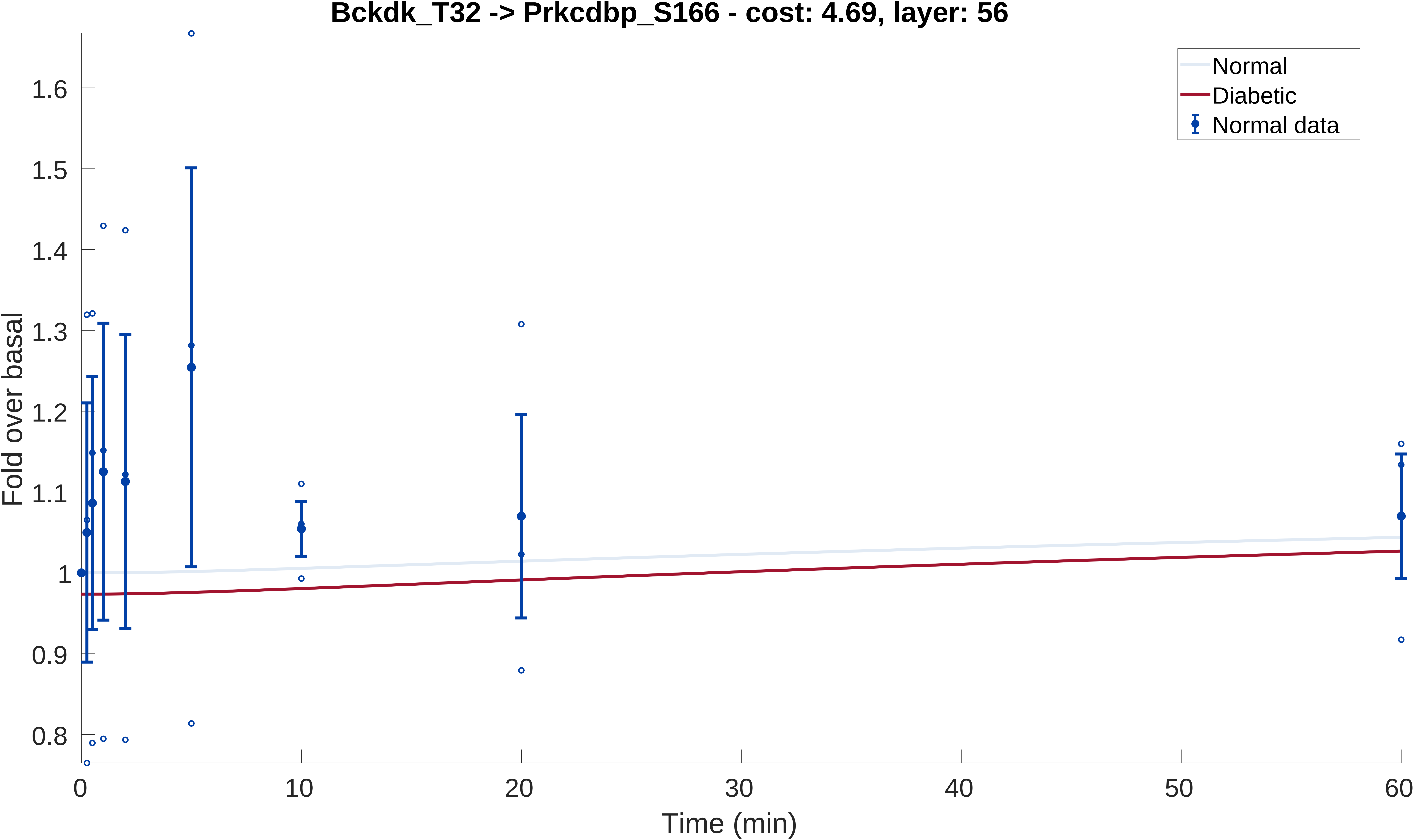
Bckdk_T32 -> Prkcdbp_S166 - cost: 4.69, layer: 56
Normal
Diabetic
Normal data
Fold over basal
Time (min)
1.6
1.5
1.4
1.3
1.2
1.1
1
0.9
0.8
0
10
20
30
40
50
60

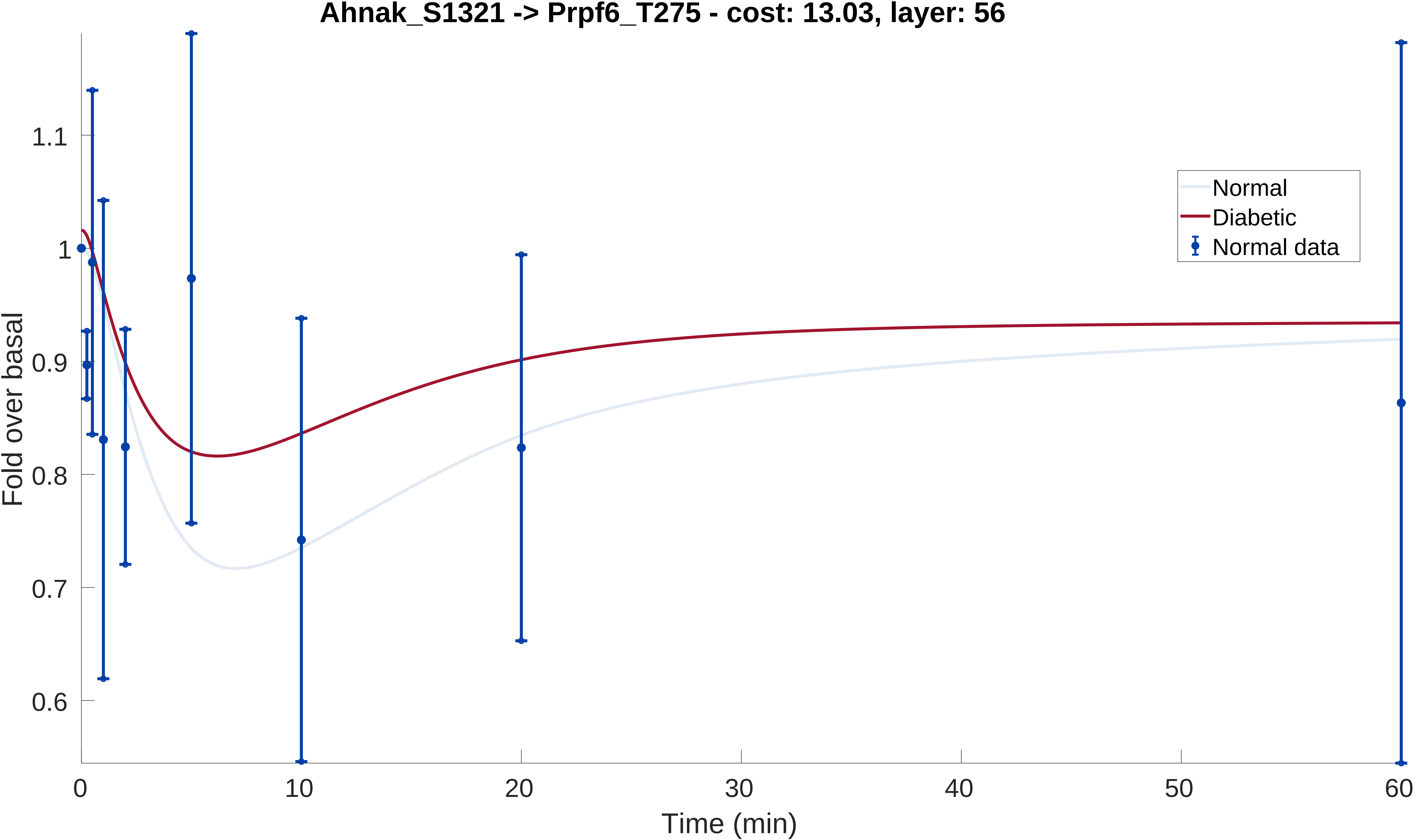

Ahnak_S1321 -> Prpf6_T275 - cost: 13.03, layer: 56
Fold over basal
1.1
1
0.9
0.8
0.7
0.6
0
10
20
30
40
50
60
Time (min)
Normal
Diabetic
Normal data

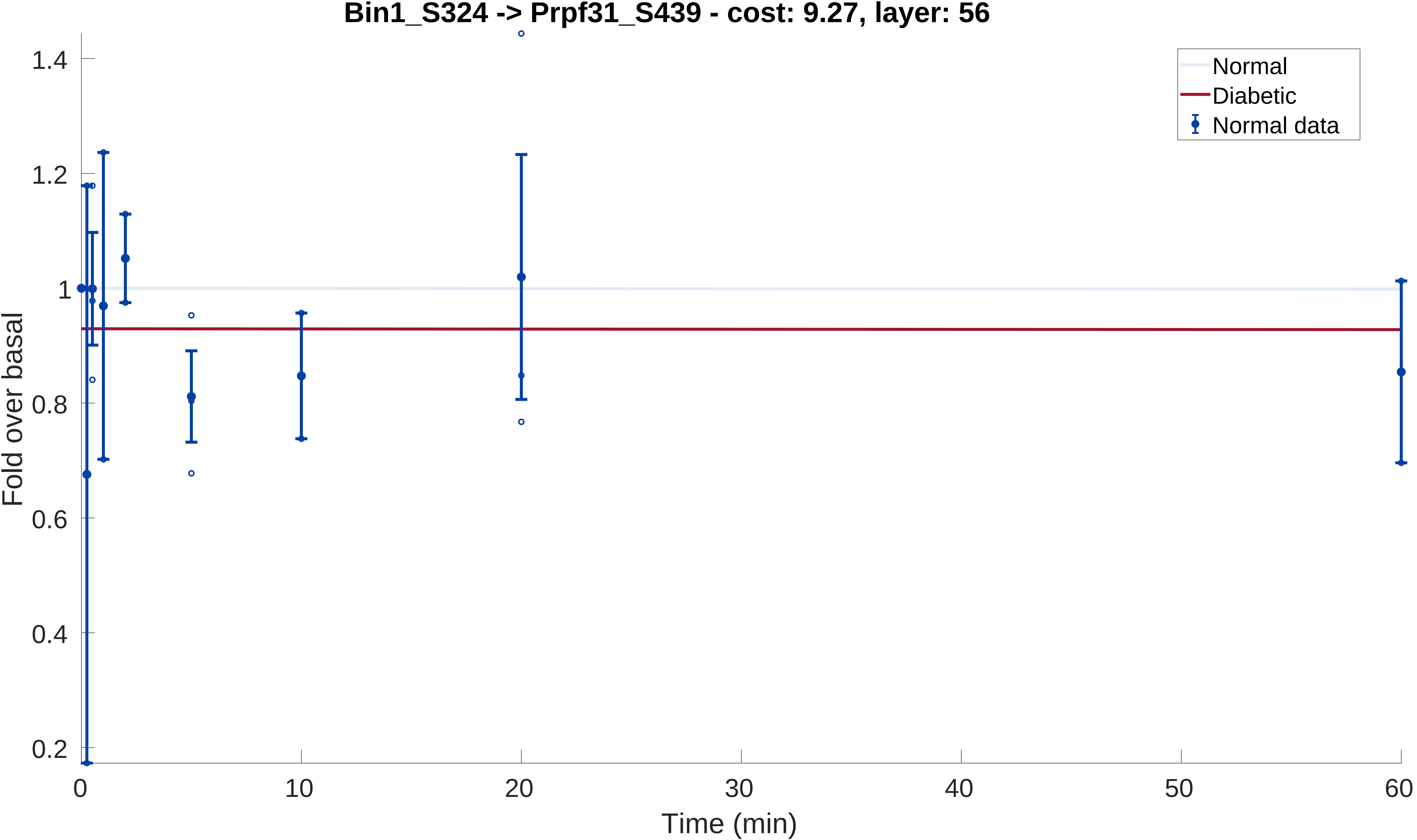

Bin1_S324 -> Prpf31_S439 - cost: 9.27, layer: 56
Normal
Diabetic
Normal data
Fold over basal
Time (min)
1.4
1.2
1
0.8
0.6
0.4
0.2
0
10
20
30
40
50
60

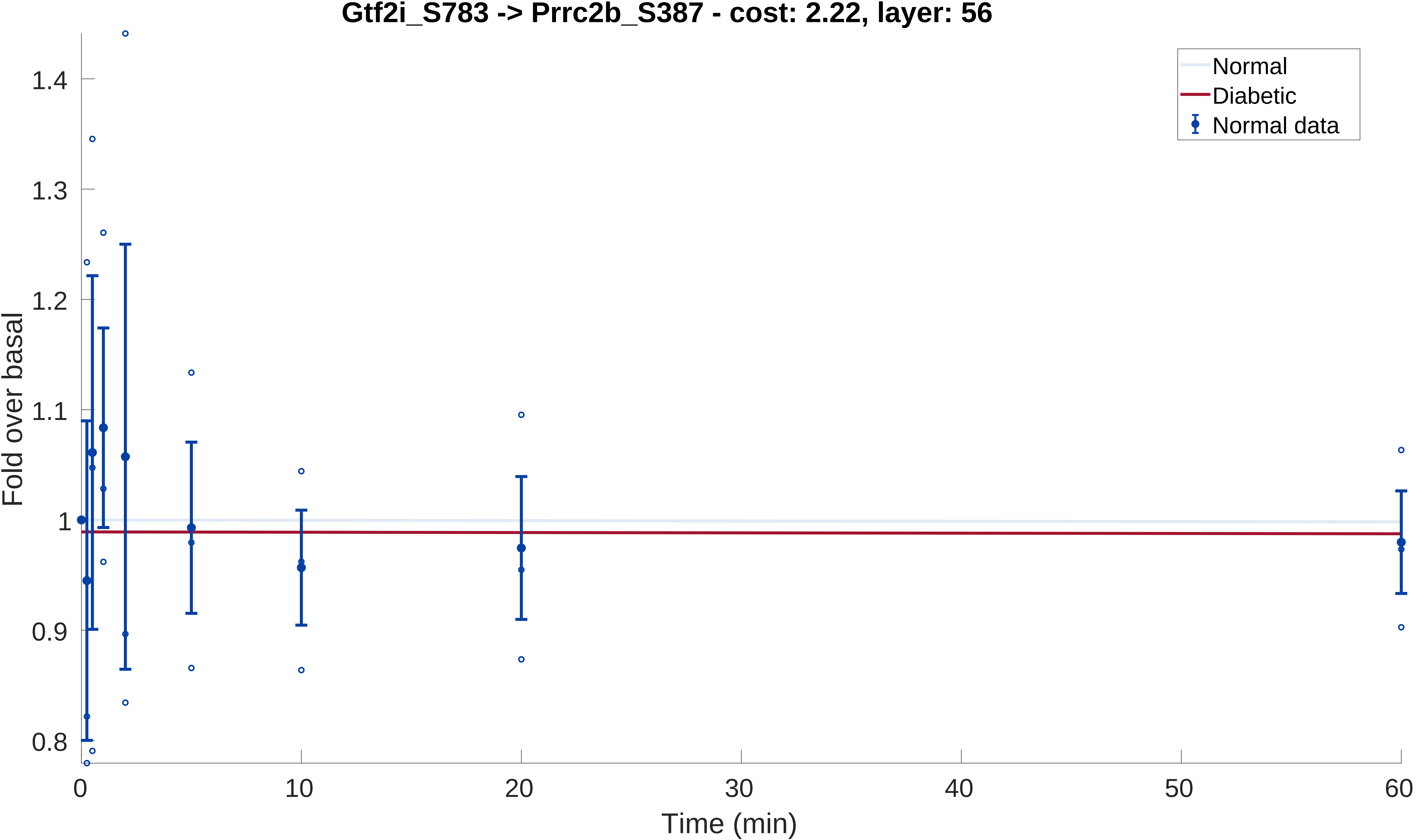

Gtf2i_S783 -> Prrc2b_S387 - cost: 2.22, layer: 56
Normal
Diabetic
Normal data
Fold over basal
Time (min)
1.4
1.3
1.2
1.1
1
0.9
0.8
0
10
20
30
40
50
60

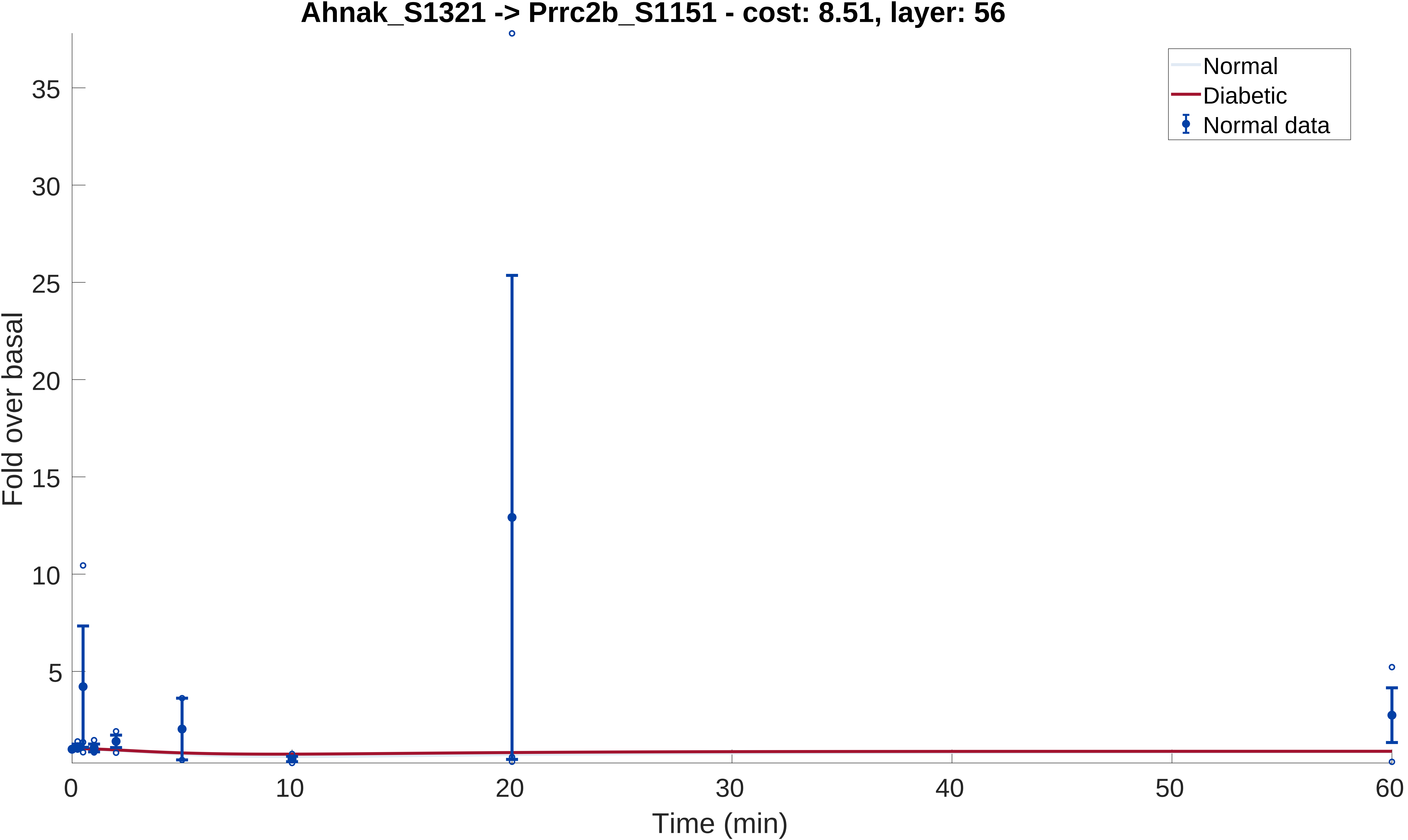

Ahnak_S1321 -> Prrc2b_S1151 - cost: 8.51, layer: 56
Normal
Diabetic
Normal data
Fold over basal
Time (min)
0
10
20
30
40
50
60
5
10
15
20
25
30
35

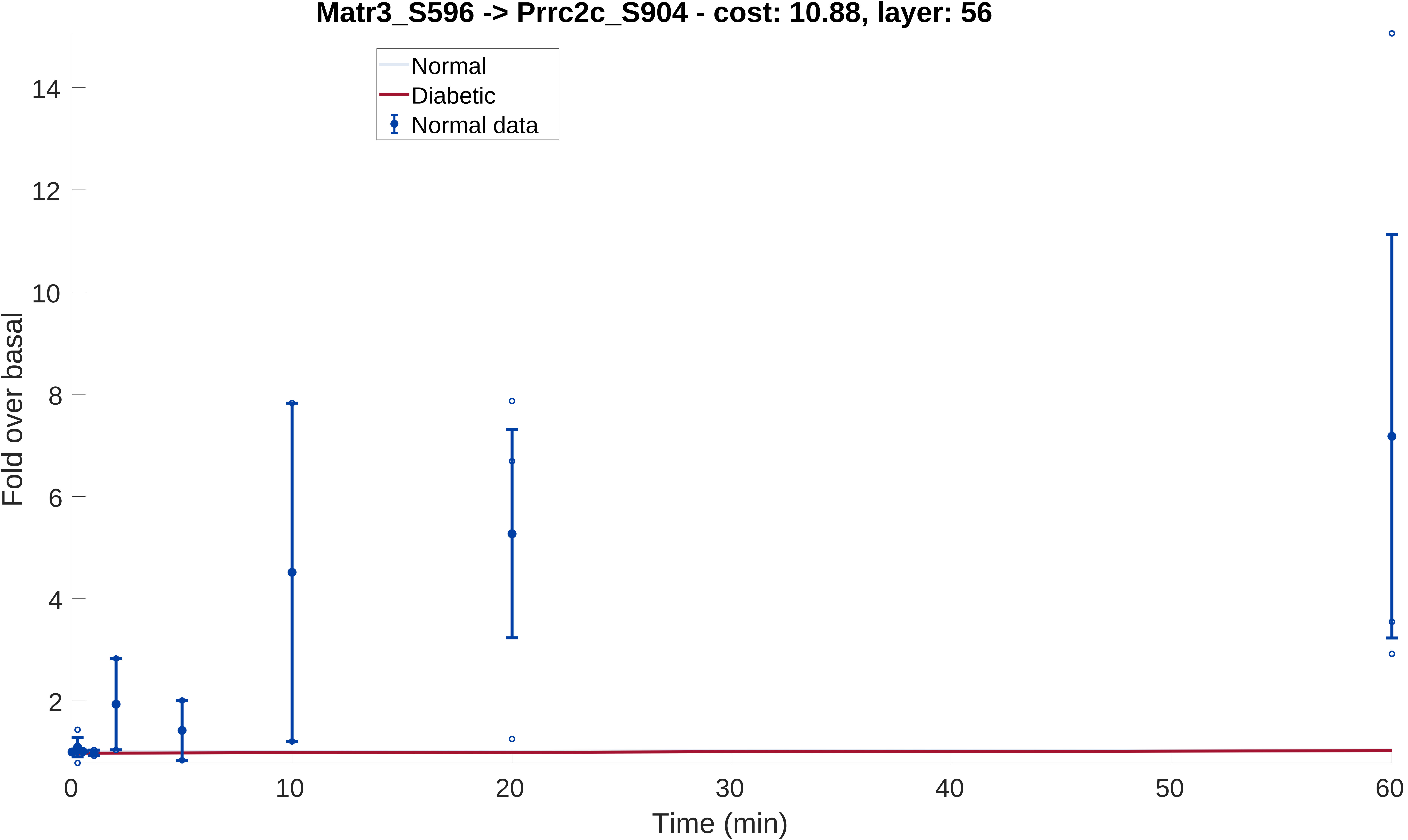
Matr3_S596 -> Prrc2c_S904 - cost: 10.88, layer: 56
Normal
Diabetic
Normal data
Fold over basal
Time (min)
0
10
20
30
40
50
60
2
4
6
8
10
12
14

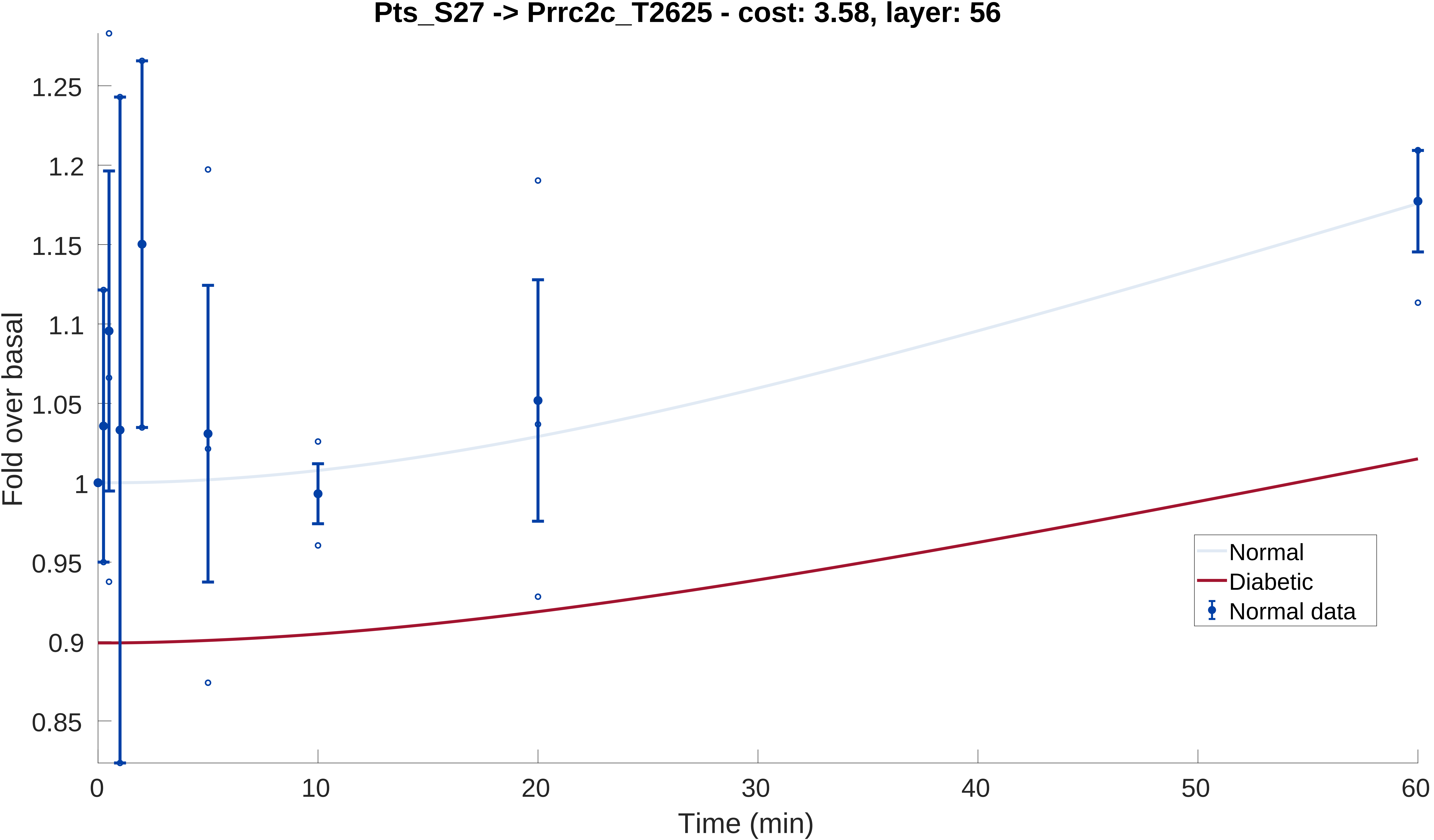

Pts_S27 -> Prrc2c_T2625 - cost: 3.58, layer: 56
Fold over basal
1.25
1.2
1.15
1.1
1.05
1
0.95
0.9
0.85
0
10
20
30
40
50
60
Time (min)
Normal
Diabetic
Normal data

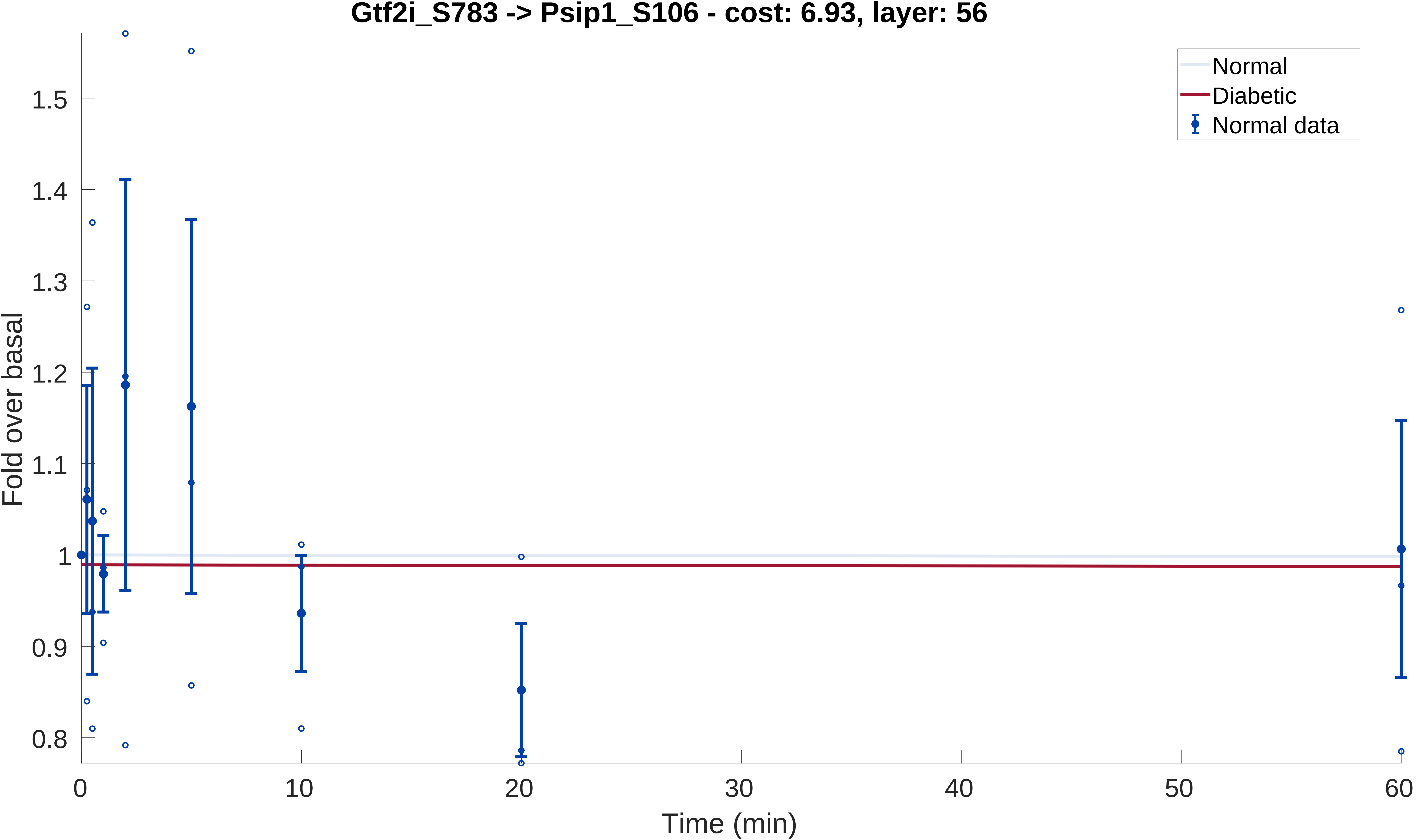

Gtf2i_S783 -> Psip1_S106 - cost: 6.93, layer: 56
Normal
Diabetic
Normal data
Fold over basal
1.5
1.4
1.3
1.2
1.1
1
0.9
0.8
0
10
20
30
40
50
60
Time (min)

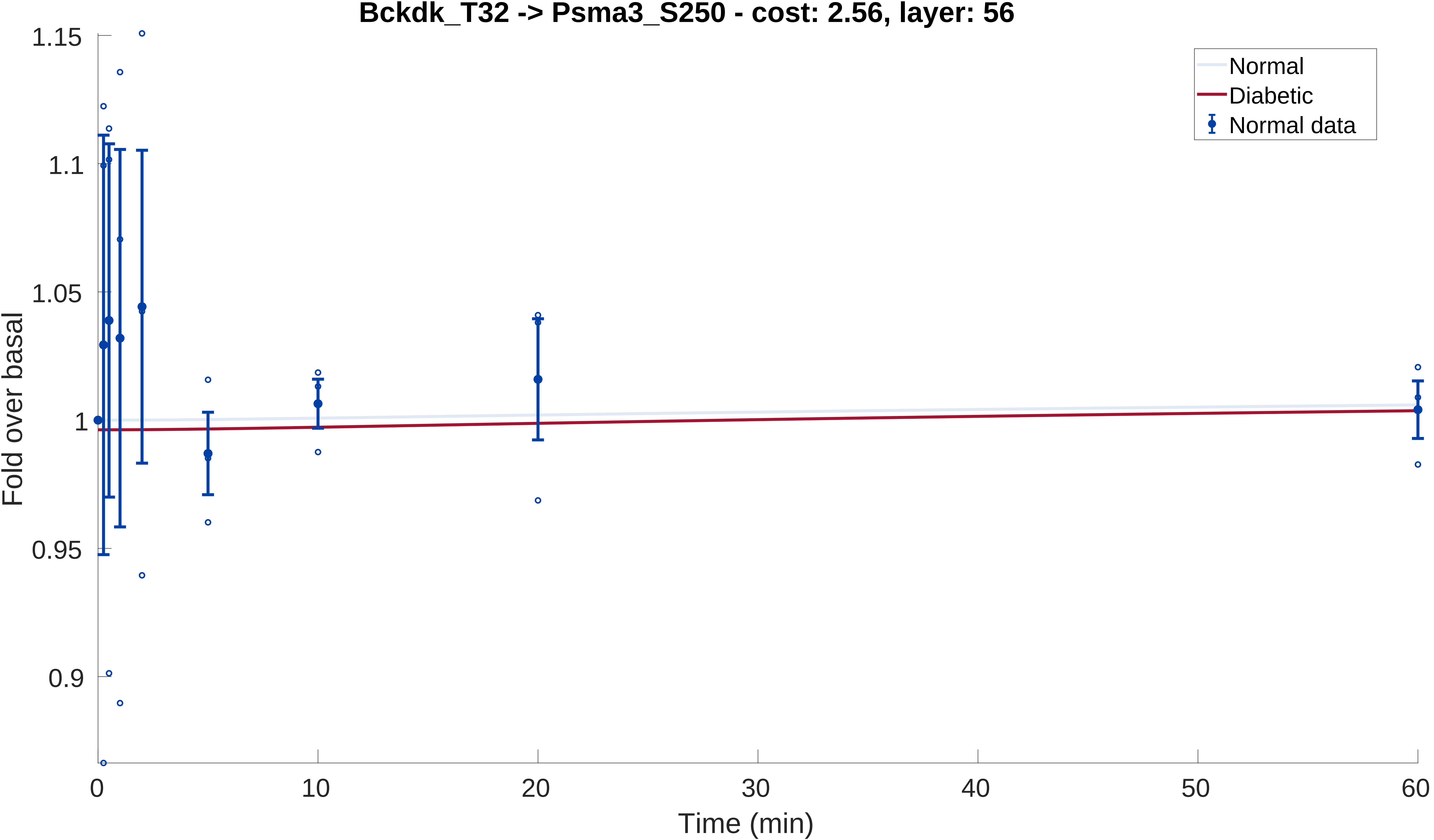

Bckdk_T32 -> Psma3_S250 - cost: 2.56, layer: 56
Normal
Diabetic
Normal data
Fold over basal
1.15
1.1
1.05
1
0.95
0.9
0
10
20
30
40
50
60
Time (min)

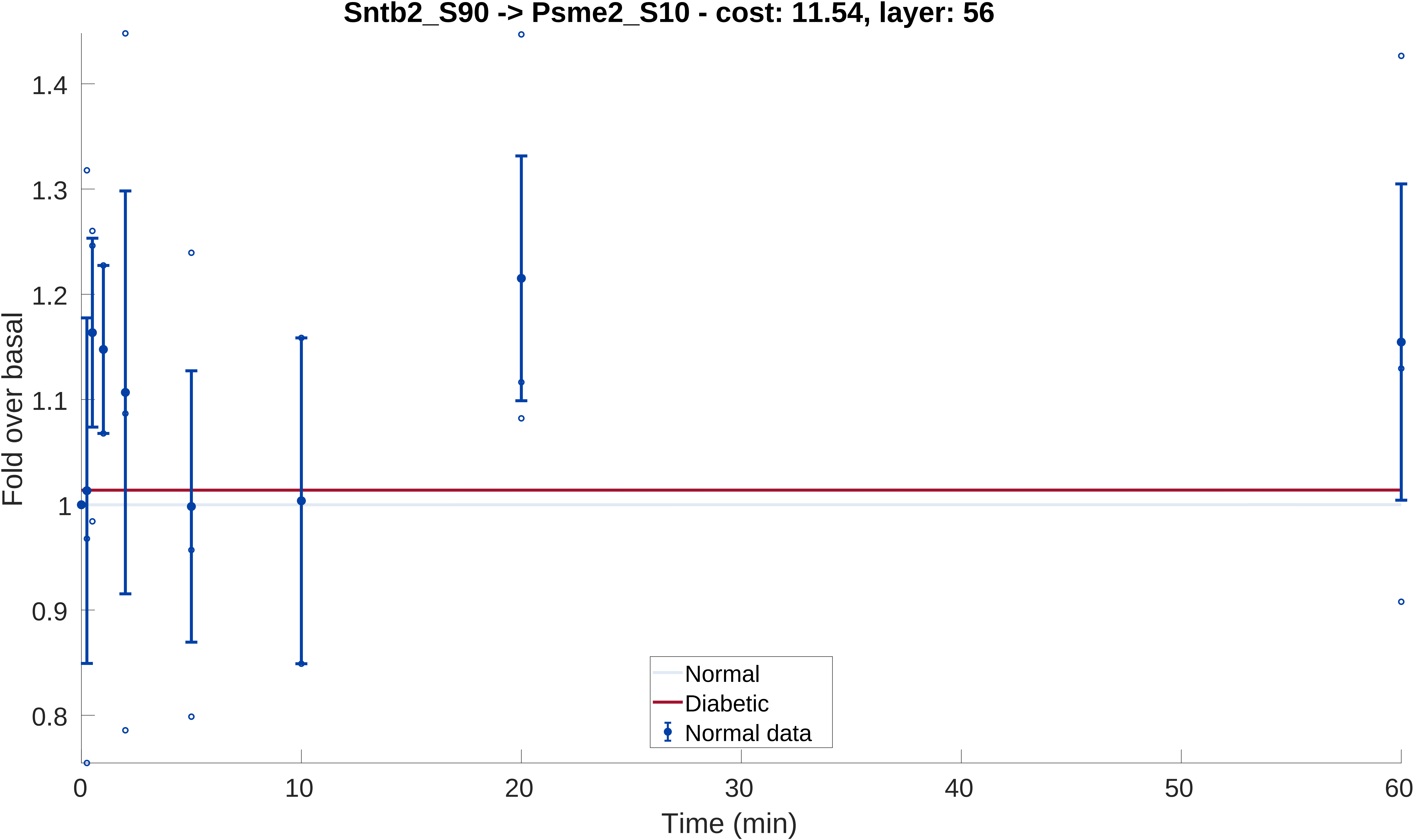

Sntb2_S90 -> Psme2_S10 - cost: 11.54, layer: 56
Fold over basal
1.4
1.3
1.2
1.1
1
0.9
0.8
0
10
20
30
40
50
60
Time (min)
Normal
Diabetic
Normal data

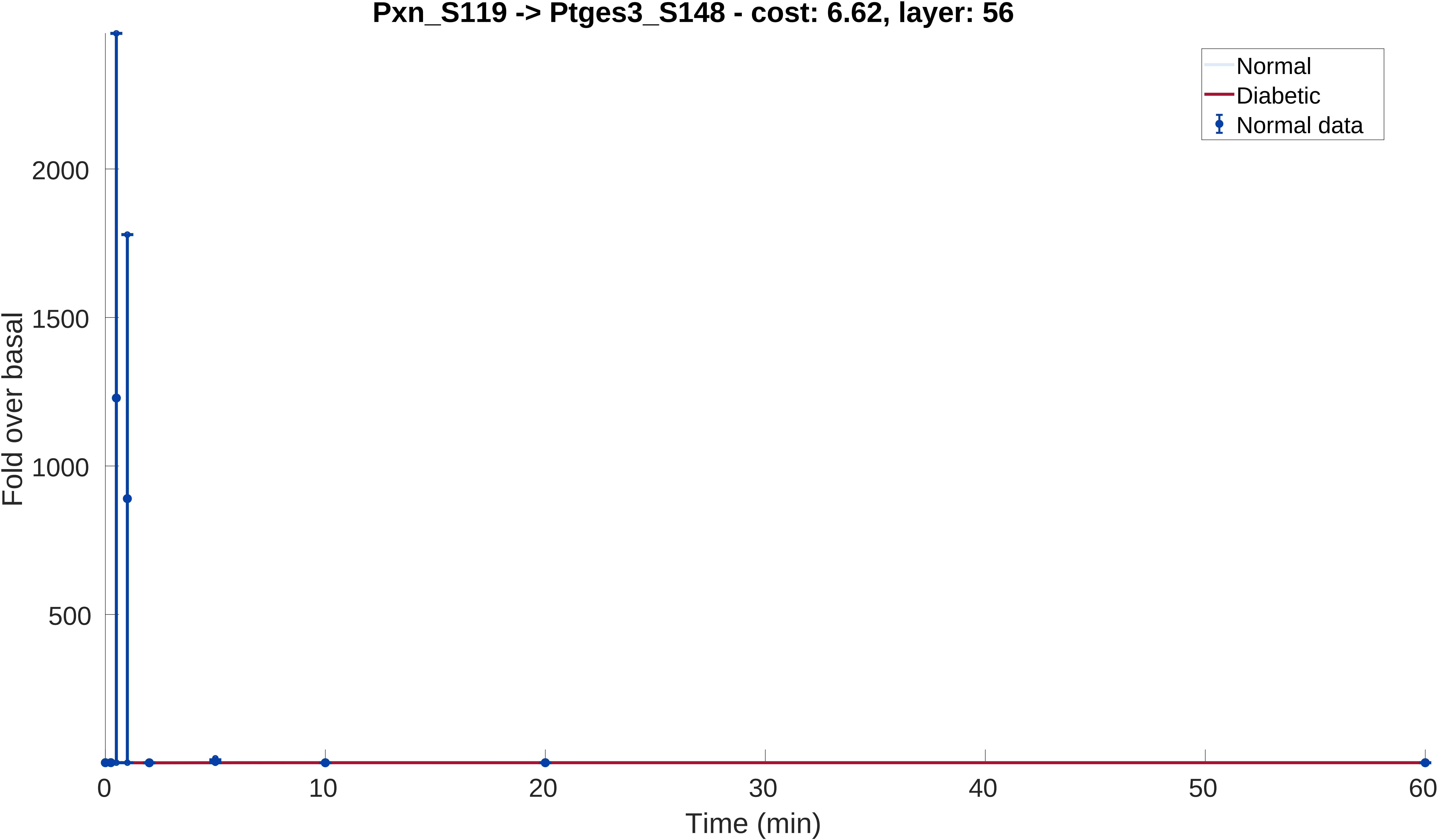

Pxn_S119 -> Ptges3_S148 - cost: 6.62, layer: 56
Normal
Diabetic
Normal data
Fold over basal
2000
1500
1000
500
0
10
20
30
40
50
60
Time (min)

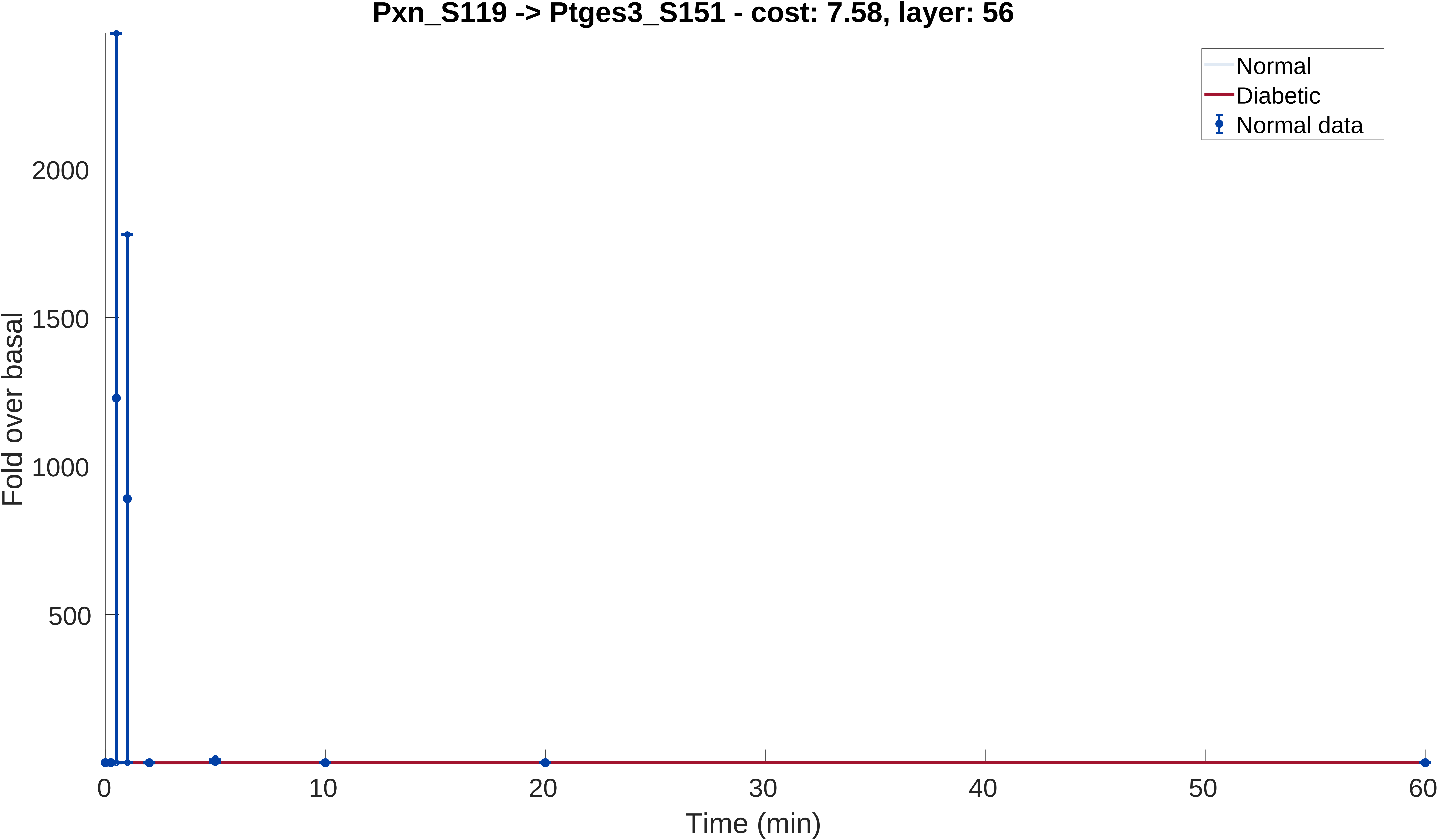

Pxn_S119 -> Ptges3_S151 - cost: 7.58, layer: 56
Normal
Diabetic
Normal data
Fold over basal
2000
1500
1000
500
0
10
20
30
40
50
60
Time (min)

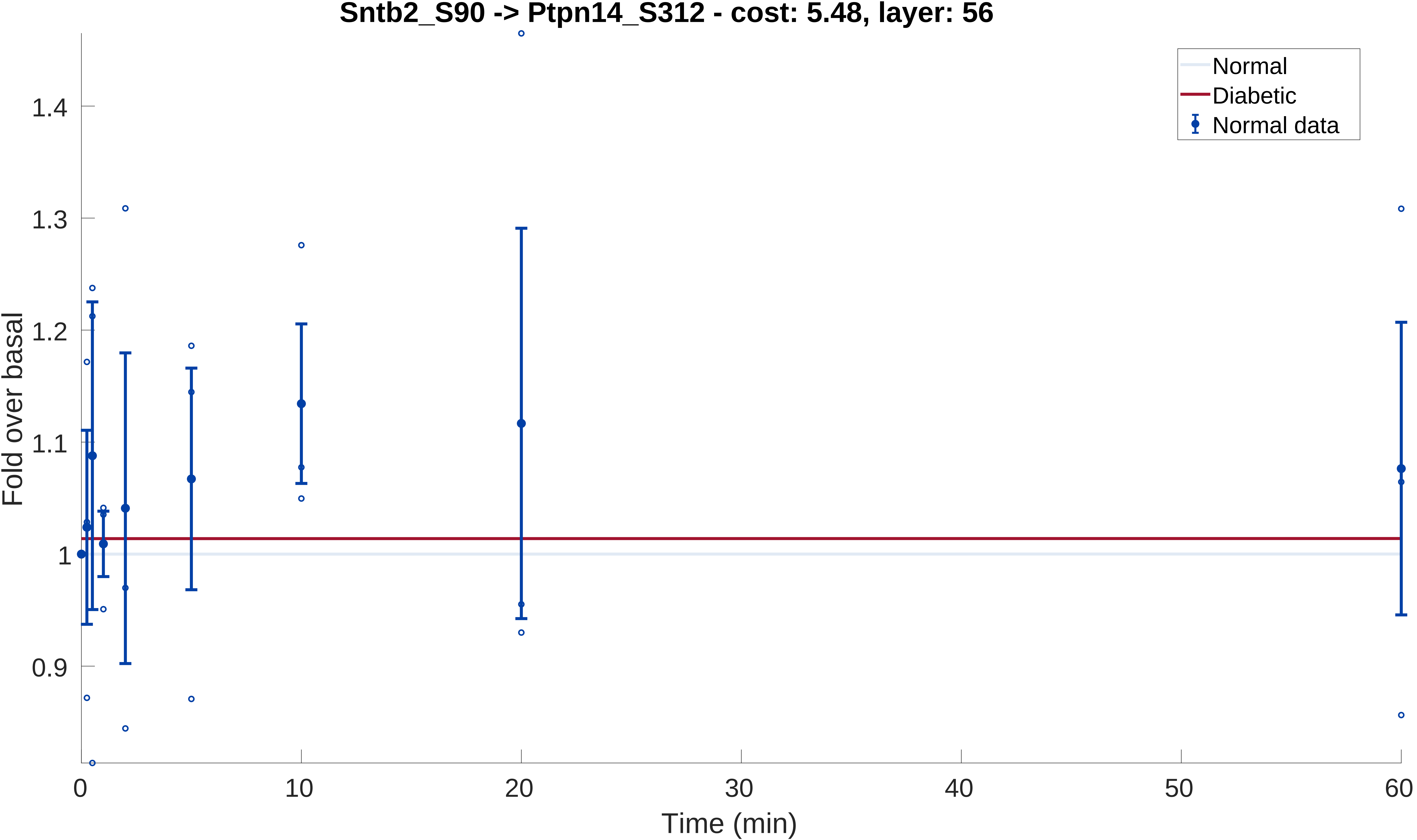

Sntb2_S90 -> Ptpn14_S312 - cost: 5.48, layer: 56
Normal
Diabetic
Normal data
Fold over basal
Time (min)
1.4
1.3
1.2
1.1
1
0.9
0
10
20
30
40
50
60

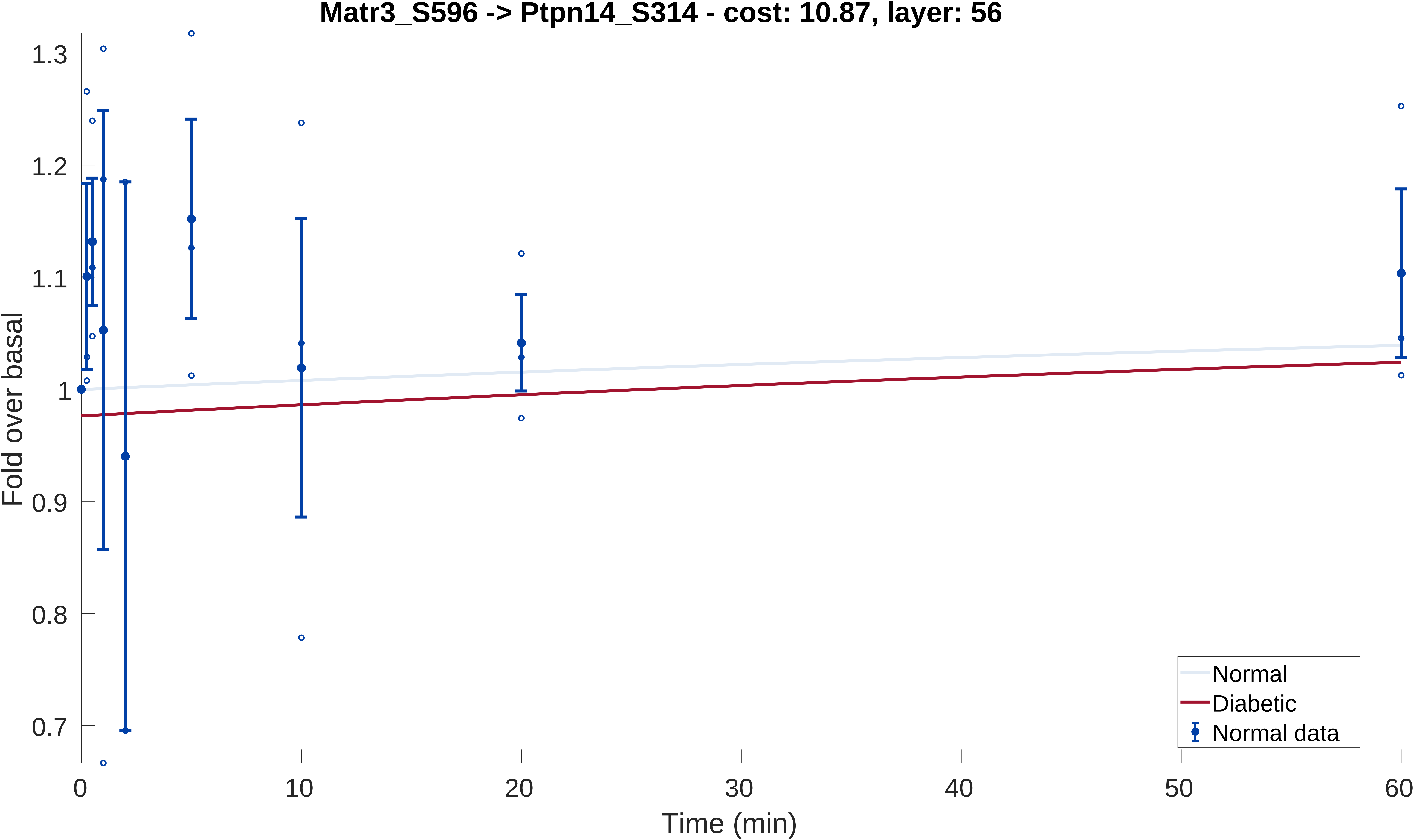

Matr3_S596 -> Ptpn14_S314 - cost: 10.87, layer: 56
Fold over basal
1.3
1.2
1.1
1
0.9
0.8
0.7
0
10
20
30
40
50
60
Time (min)
Normal
Diabetic
Normal data

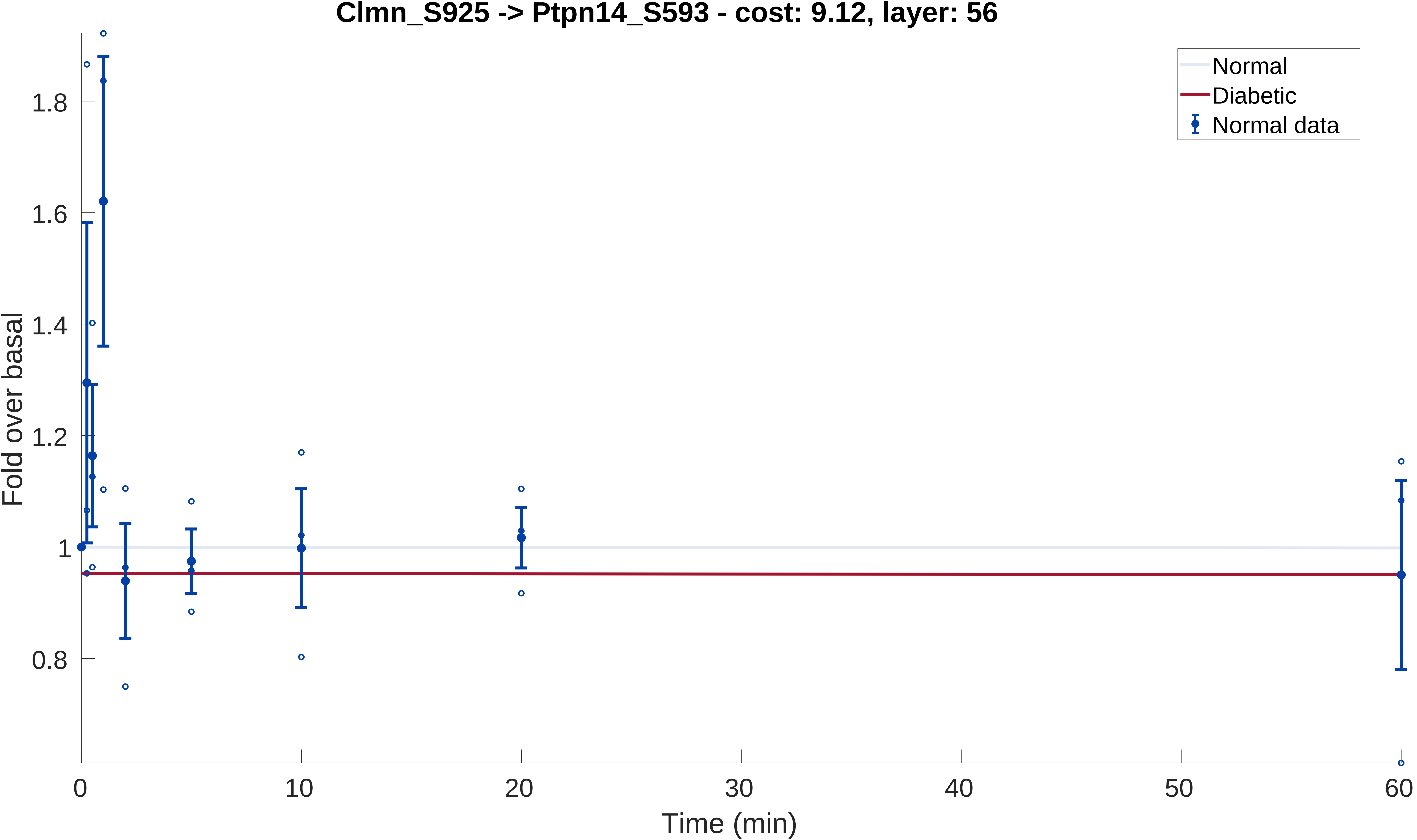
Clmn_S925 -> Ptpn14_S593 - cost: 9.12, layer: 56
Normal
Diabetic
Normal data
Fold over basal
Time (min)
1.8
1.6
1.4
1.2
1
0.8
0
10
20
30
40
50
60

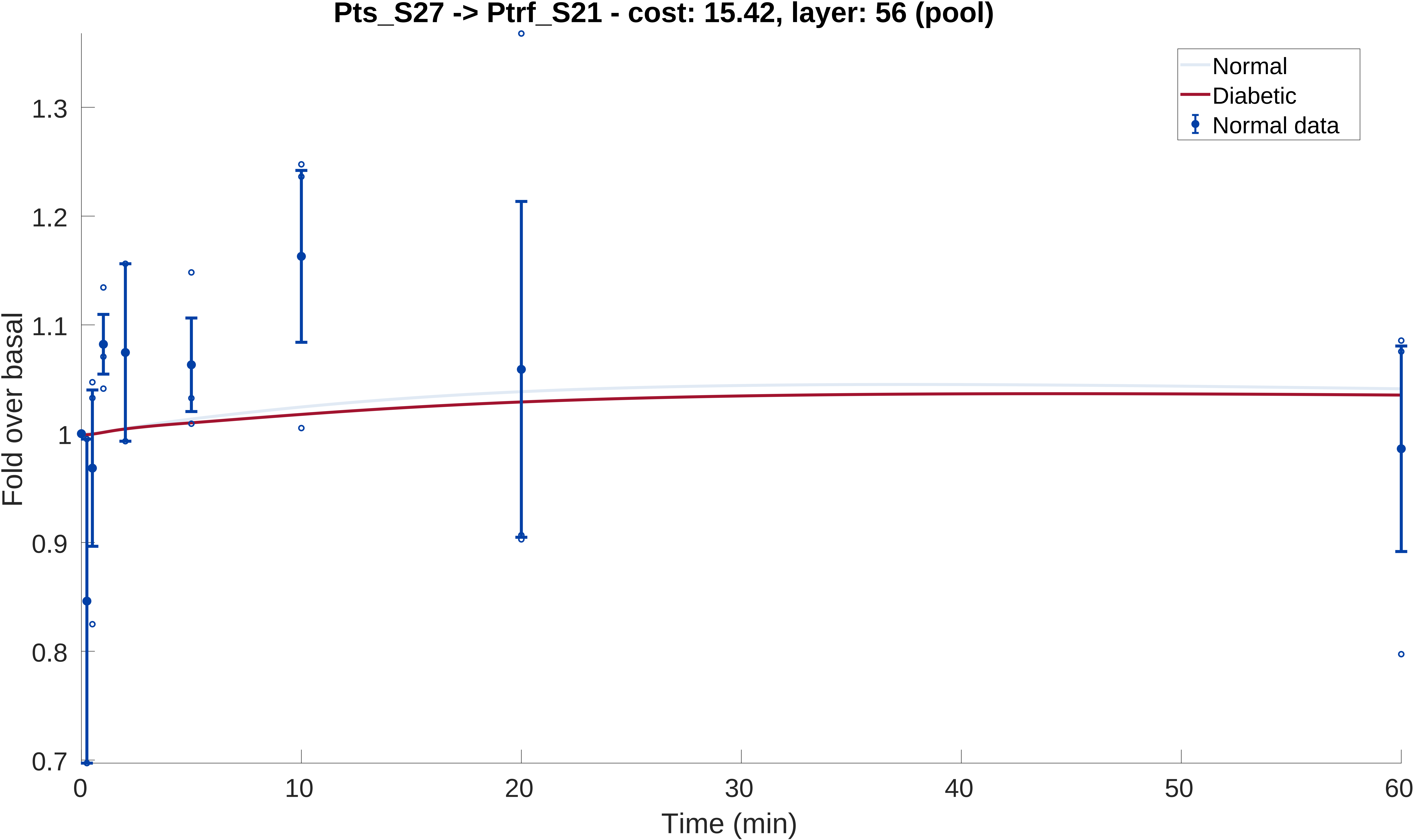
Pts_S27 -> Ptrf_S21 - cost: 15.42, layer: 56 (pool)
Normal
Diabetic
Normal data
Fold over basal
1.3
1.2
1.1
1
0.9
0.8
0.7
0
10
20
30
40
50
60
Time (min)

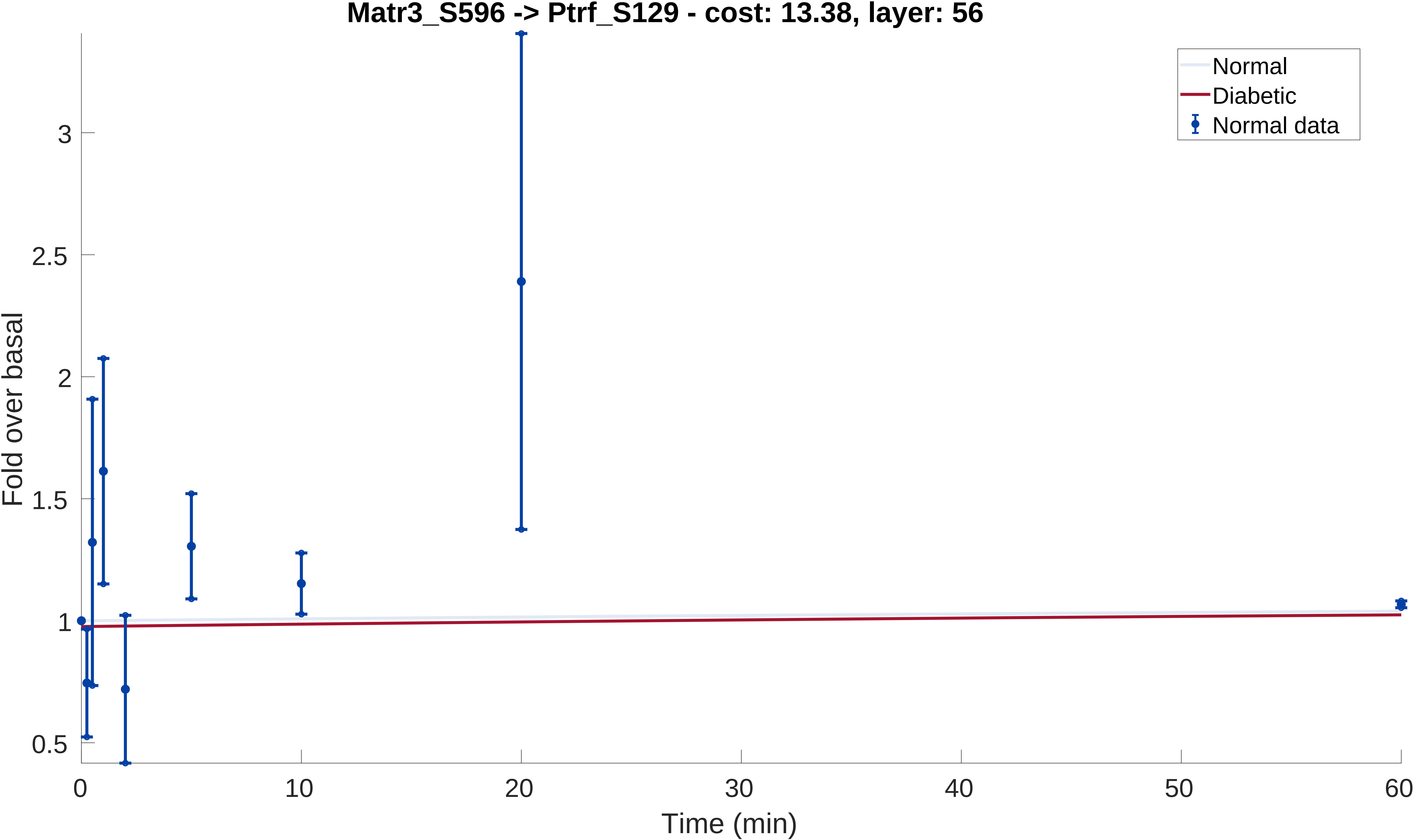
Matr3_S596 -> Ptrf_S129 - cost: 13.38, layer: 56
Normal
Diabetic
Normal data
Fold over basal
0.5
1
1.5
2
2.5
3
0
10
20
30
40
50
60
Time (min)

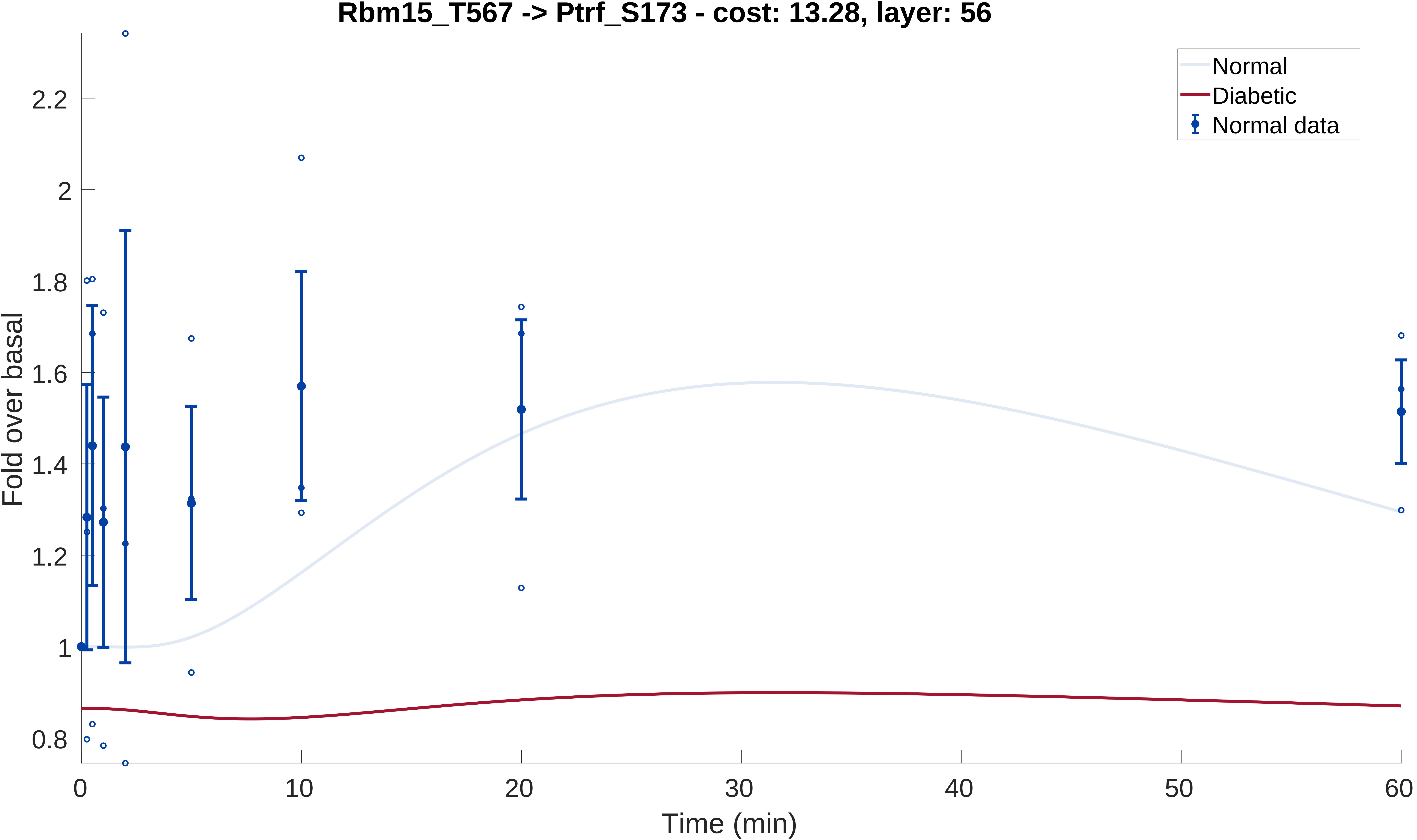
Rbm15_T567 -> Ptrf_S173 - cost: 13.28, layer: 56
Normal
Diabetic
Normal data
Fold over basal
Time (min)
0.8
1
1.2
1.4
1.6
1.8
2
2.2
0
10
20
30
40
50
60

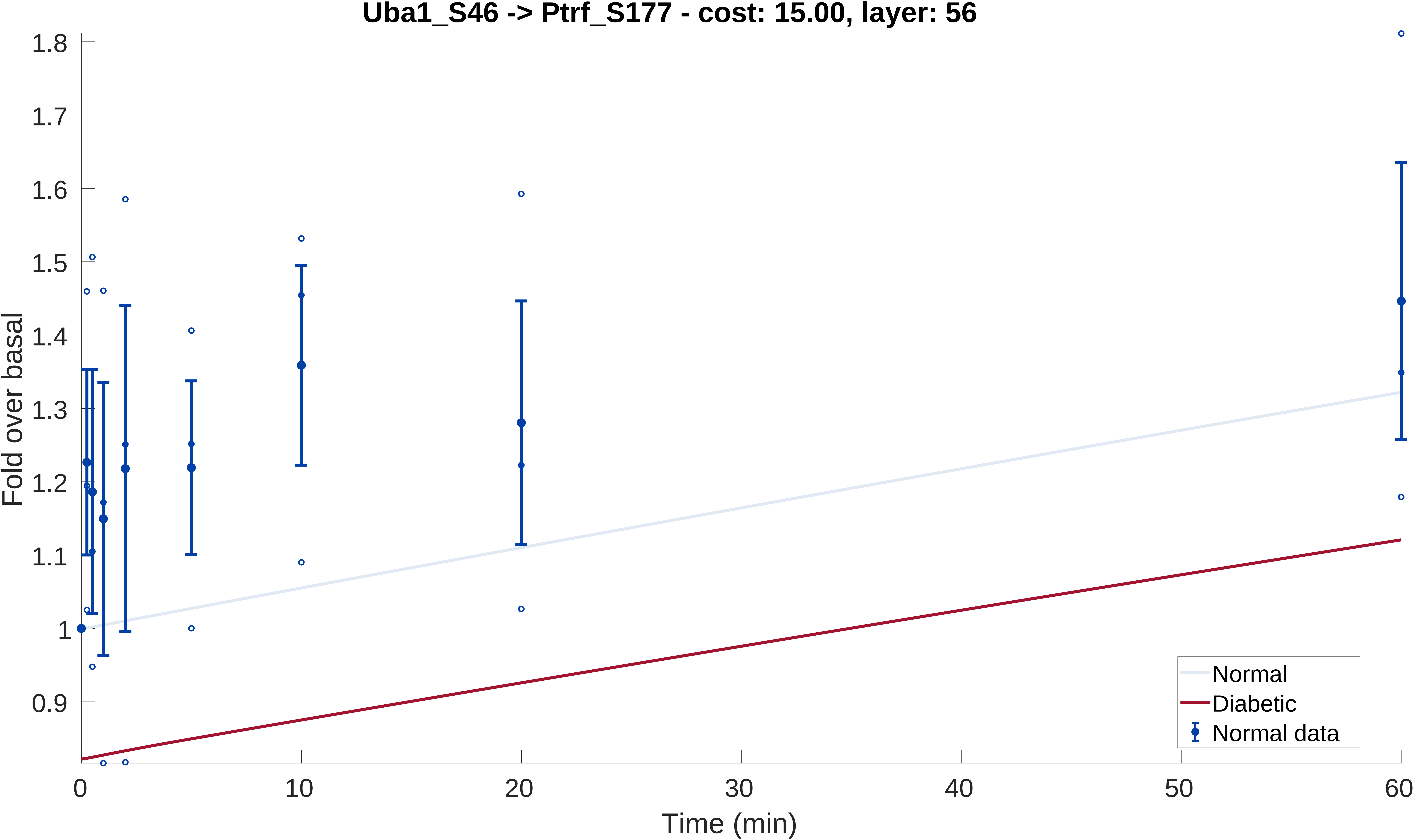

Uba1_S46 -> Ptrf_S177 - cost: 15.00, layer: 56
1.8
1.7
1.6
1.5
1.4
1.3
1.2
1.1
1
0.9
Fold over basal
0
10
20
30
40
50
60
Time (min)
Normal
Diabetic
Normal data

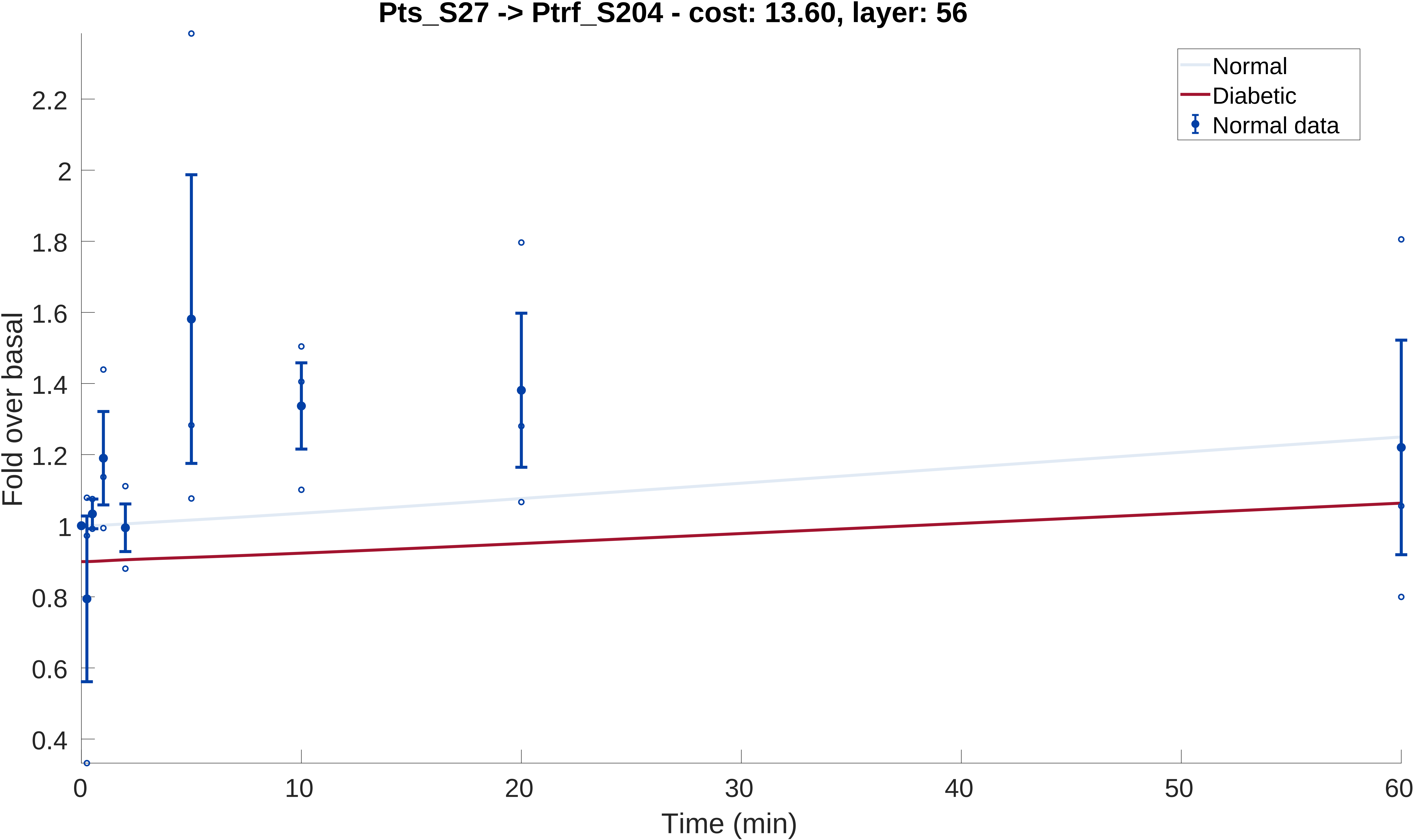
Pts_S27 -> Ptrf_S204 - cost: 13.60, layer: 56
Normal
Diabetic
Normal data
Fold over basal
Time (min)
2.2
2
1.8
1.6
1.4
1.2
1
0.8
0.6
0.4
0
10
20
30
40
50
60

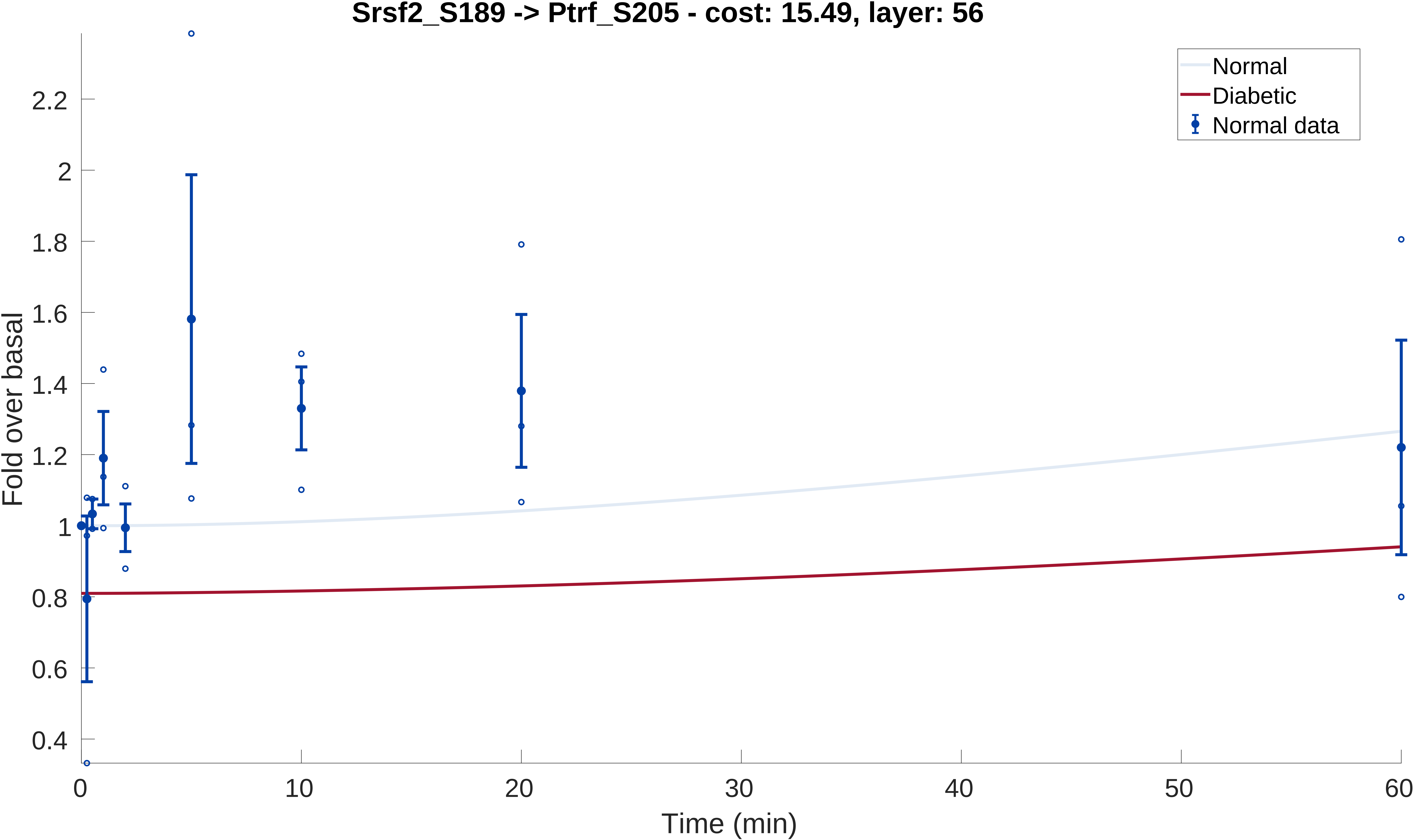
Srsf2_S189 -> Ptrf_S205 - cost: 15.49, layer: 56
Normal
Diabetic
Normal data
Fold over basal
Time (min)
0.4
0.6
0.8
1
1.2
1.4
1.6
1.8
2
2.2
0
10
20
30
40
50
60

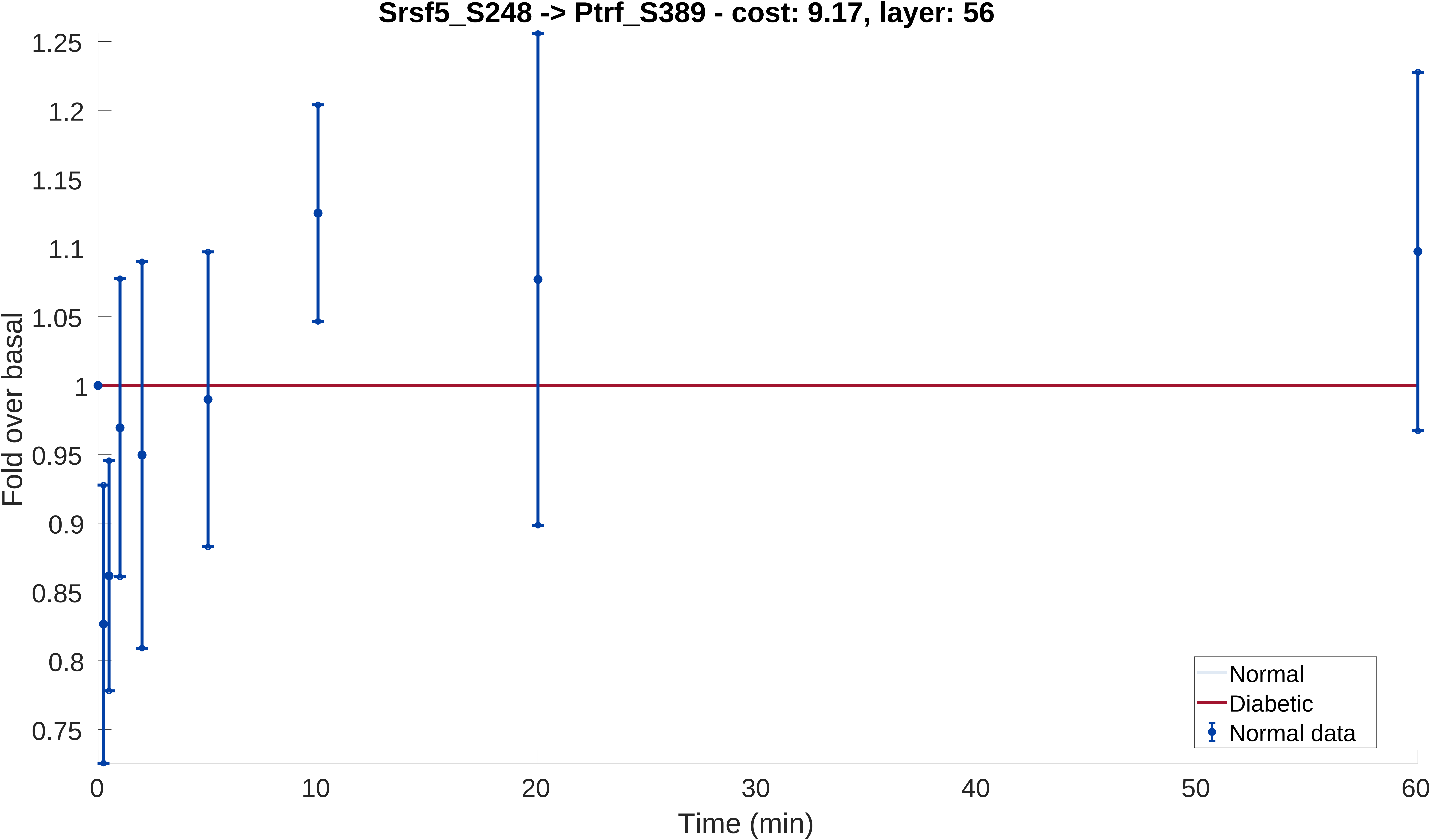

Srsf5_S248 -> Ptrf_S389 - cost: 9.17, layer: 56
Fold over basal
1.25
1.2
1.15
1.1
1.05
1
0.95
0.9
0.85
0.8
0.75
0
10
20
30
40
50
60
Time (min)
Normal
Diabetic
Normal data

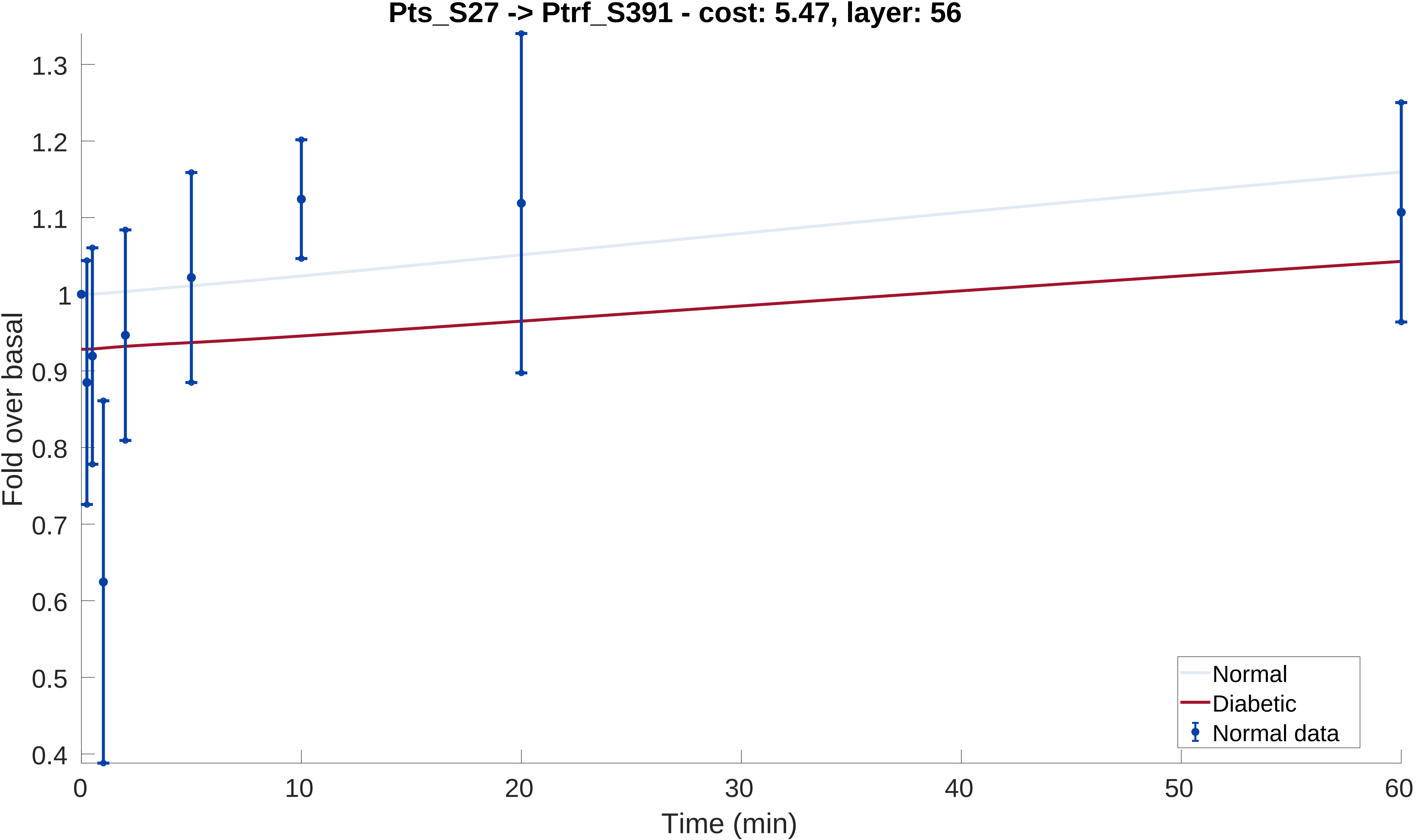

Pts_S27 -> Ptrf_S391 - cost: 5.47, layer: 56
Fold over basal
Time (min)
1.3
1.2
1.1
1
0.9
0.8
0.7
0.6
0.5
0.4
0
10
20
30
40
50
60
Normal
Diabetic
Normal data

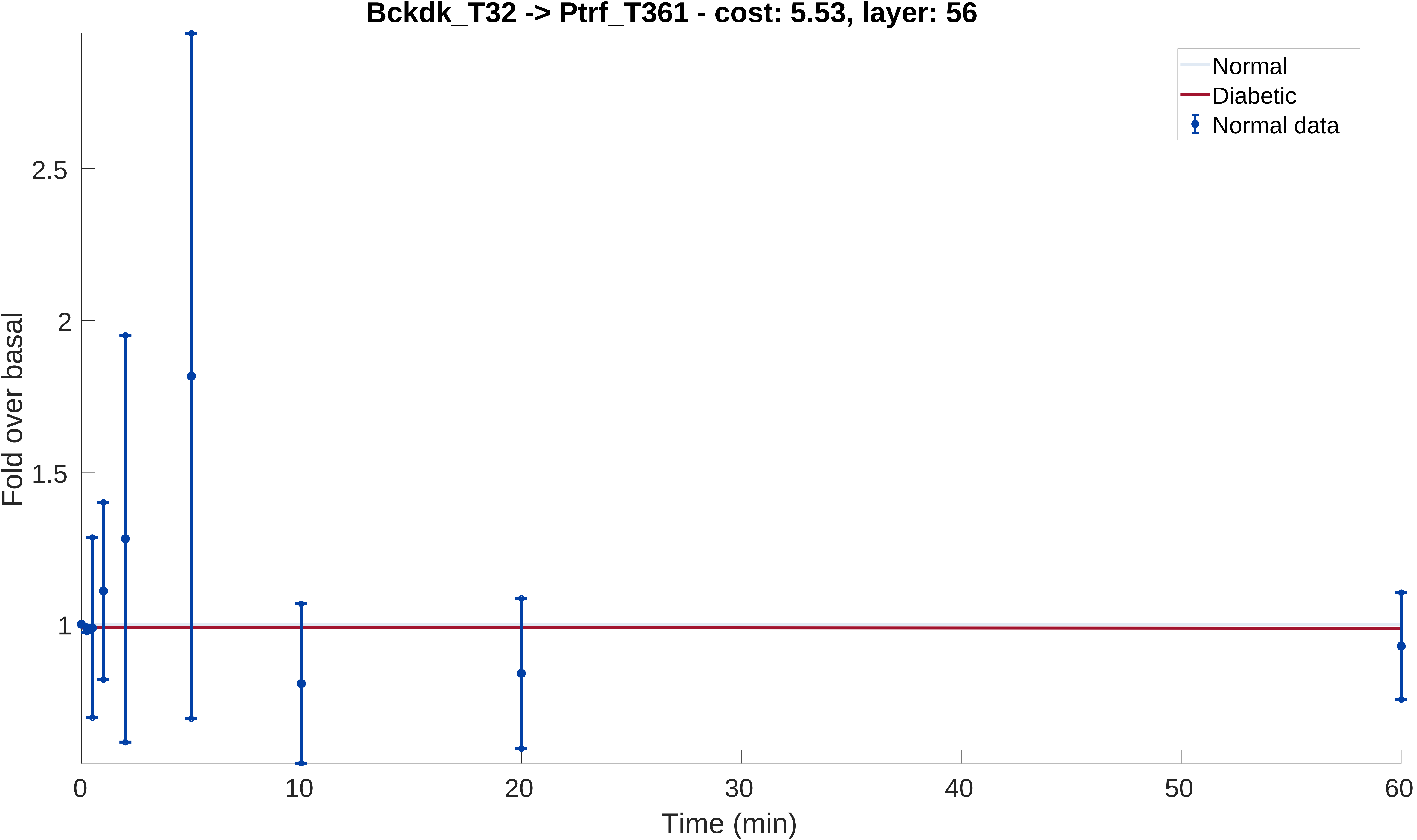
Bckdk_T32 -> Ptrf_T361 - cost: 5.53, layer: 56
Normal
Diabetic
Normal data
Fold over basal
2.5
2
1.5
1
0
10
20
30
40
50
60
Time (min)

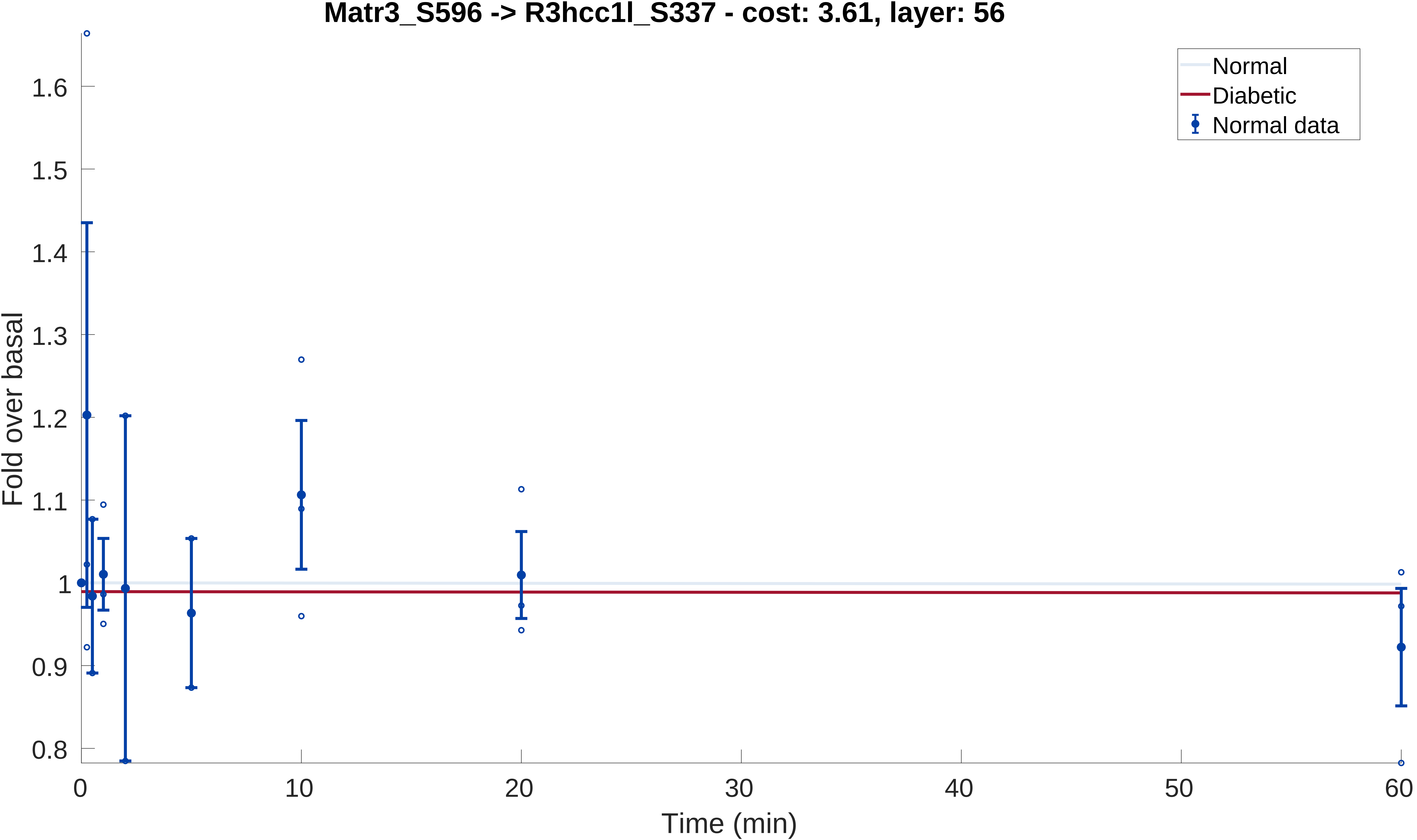

Matr3_S596 -> R3hcc1l_S337 - cost: 3.61, layer: 56
Normal
Diabetic
Normal data
Fold over basal
Time (min)
0.8
0.9
1
1.1
1.2
1.3
1.4
1.5
1.6
0
10
20
30
40
50
60

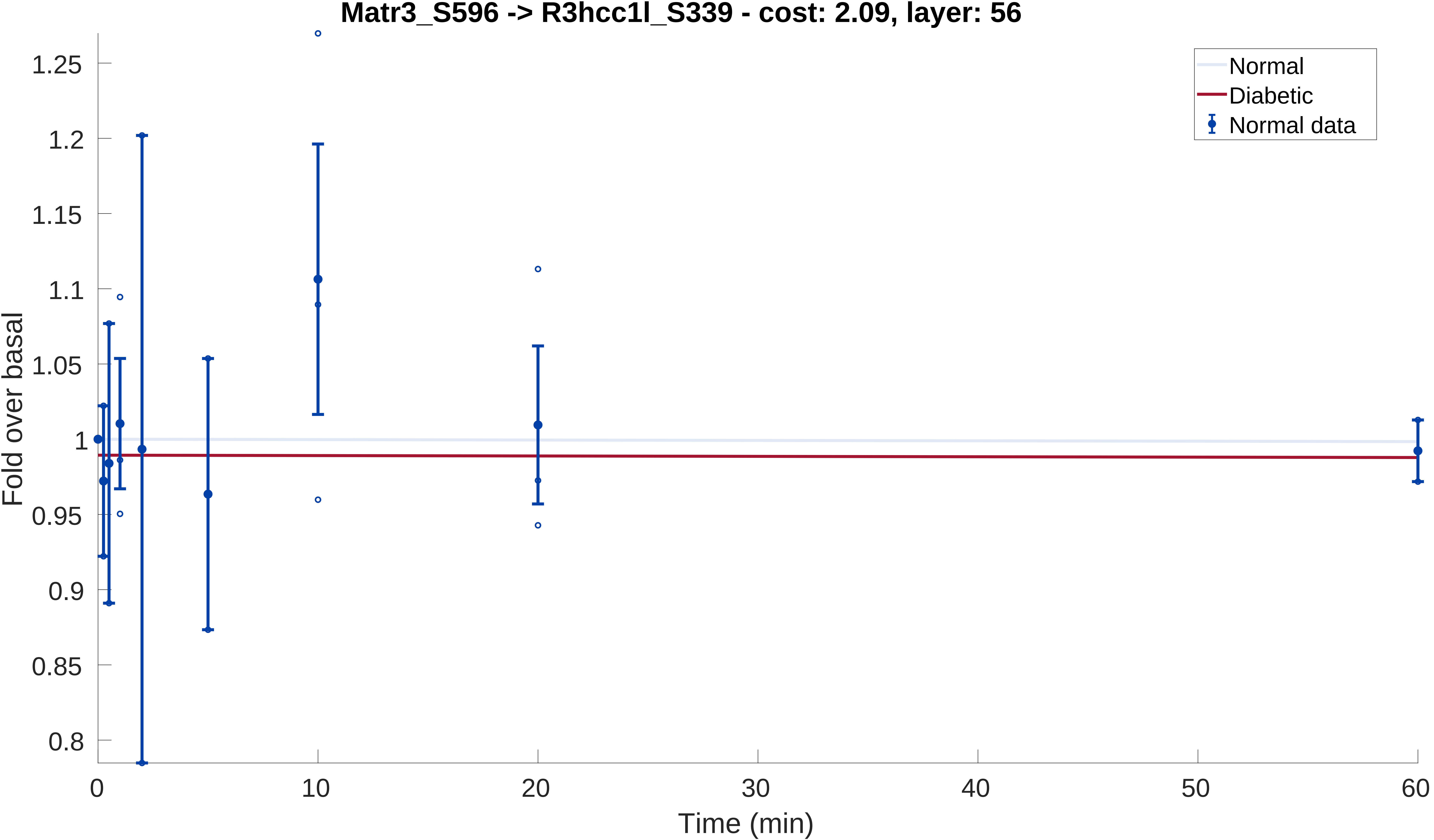

Matr3_S596 -> R3hcc1l_S339 - cost: 2.09, layer: 56
Normal
Diabetic
Normal data
Fold over basal
1.25
1.2
1.15
1.1
1.05
1
0.95
0.9
0.85
0.8
0
10
20
30
40
50
60
Time (min)

Srsf5_S248 -> R3hcc1l_S340 - cost: 3.61, layer: 56

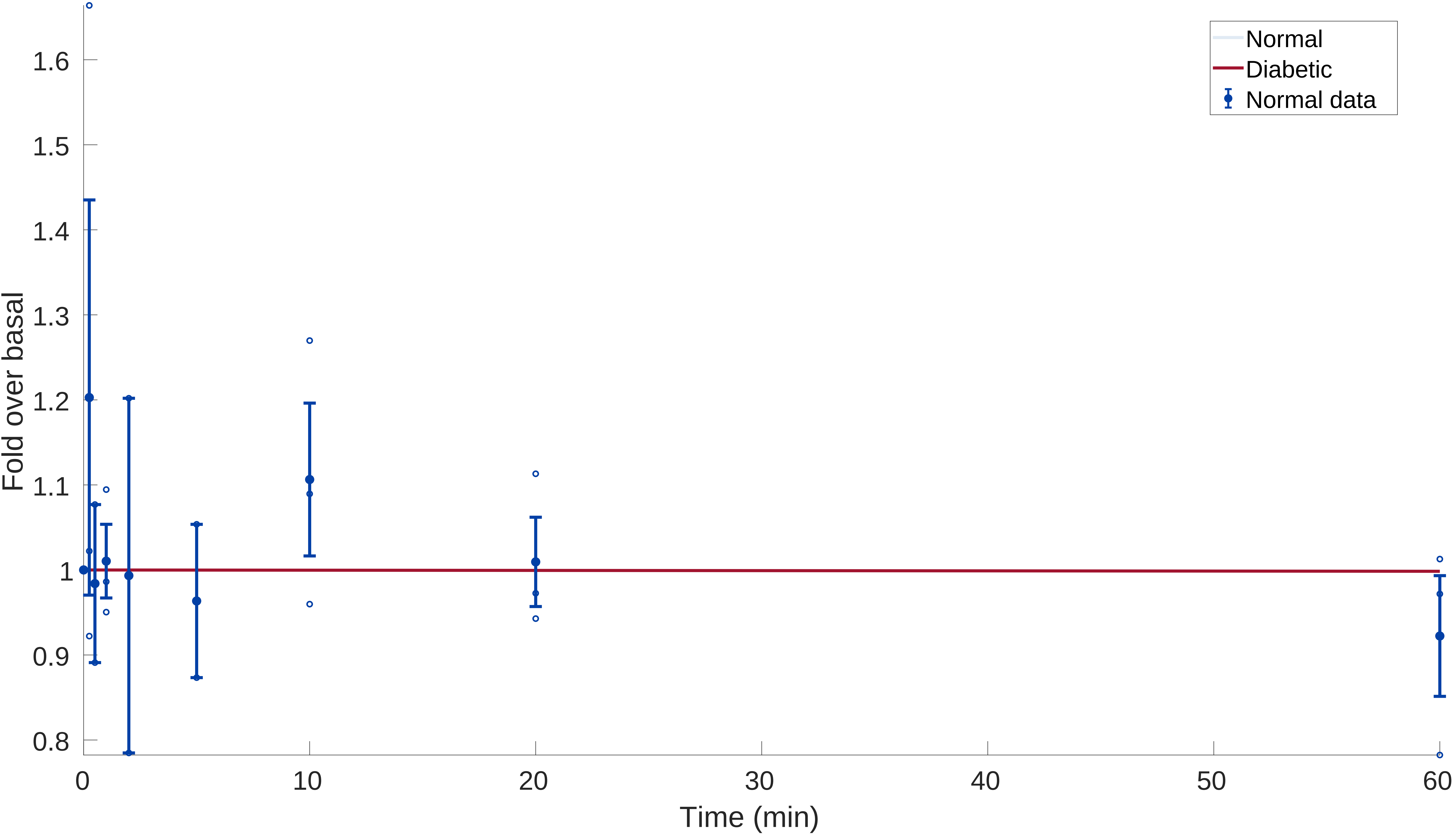

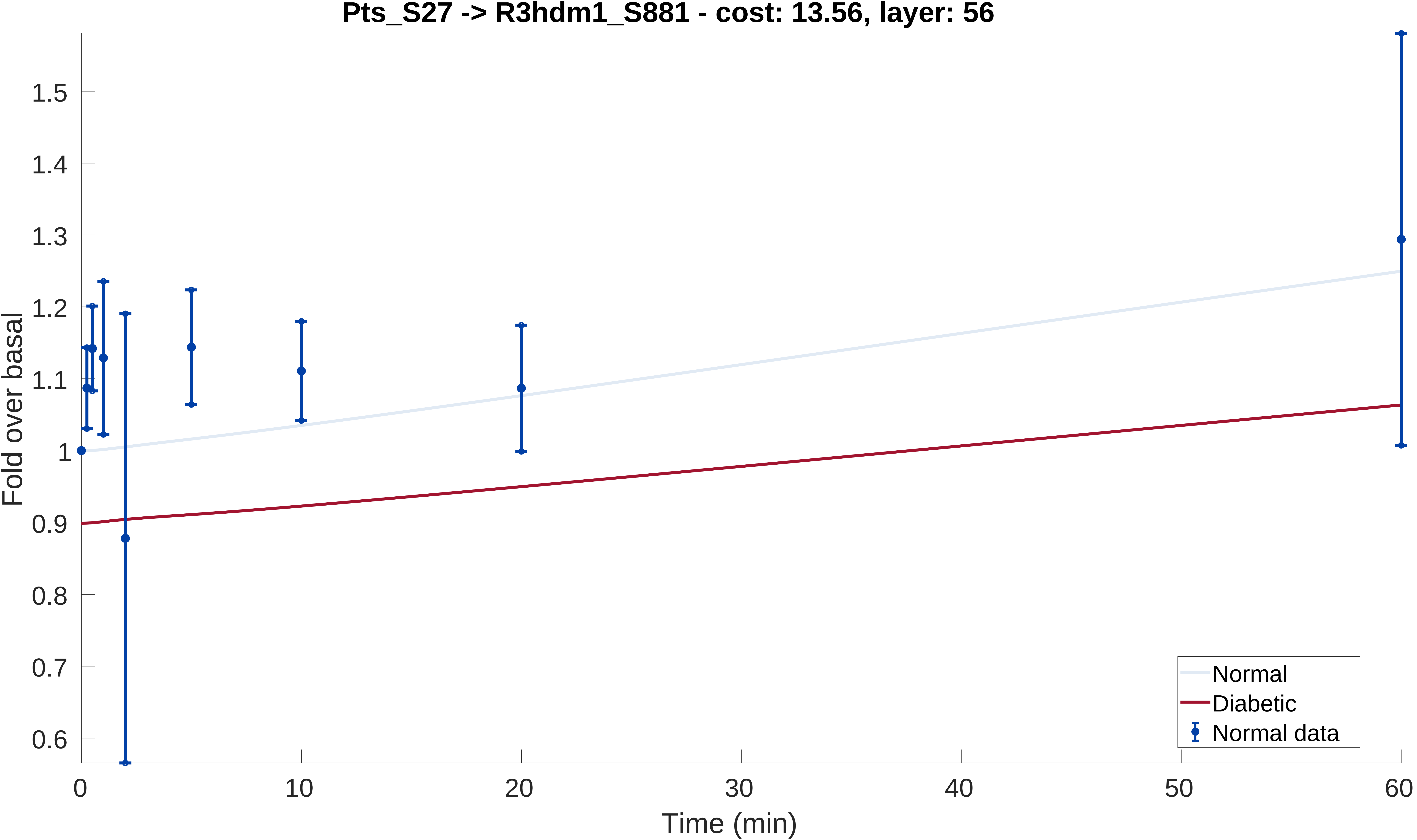

Pts_S27 -> R3hdm1_S881 - cost: 13.56, layer: 56
Fold over basal
1.5
1.4
1.3
1.2
1.1
1
0.9
0.8
0.7
0.6
0
10
20
30
40
50
60
Time (min)
Normal
Diabetic
Normal data

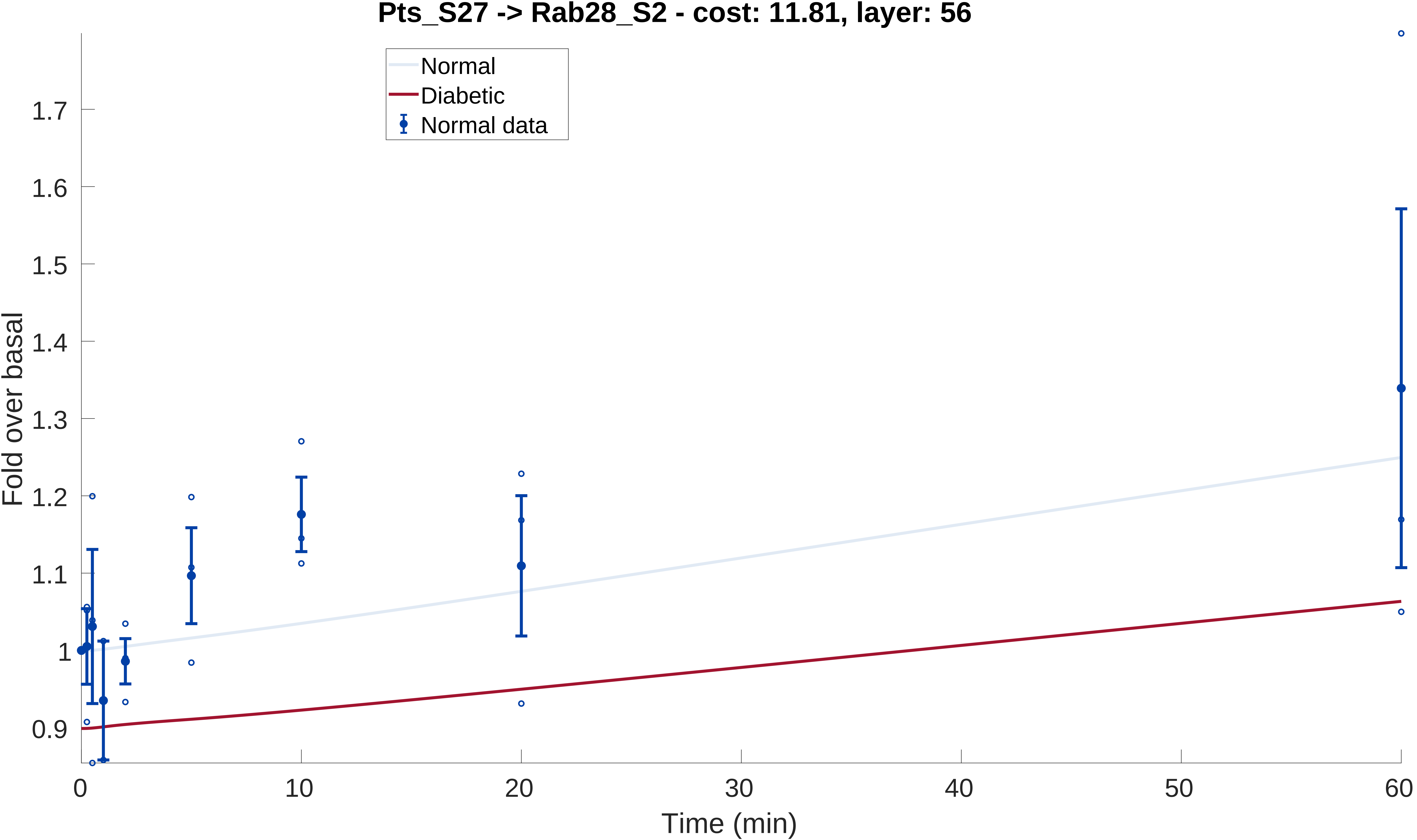

Pts_S27 -> Rab28_S2 - cost: 11.81, layer: 56
Normal
Diabetic
Normal data
Fold over basal
1.7
1.6
1.5
1.4
1.3
1.2
1.1
1
0.9
0
10
20
30
40
50
60
Time (min)

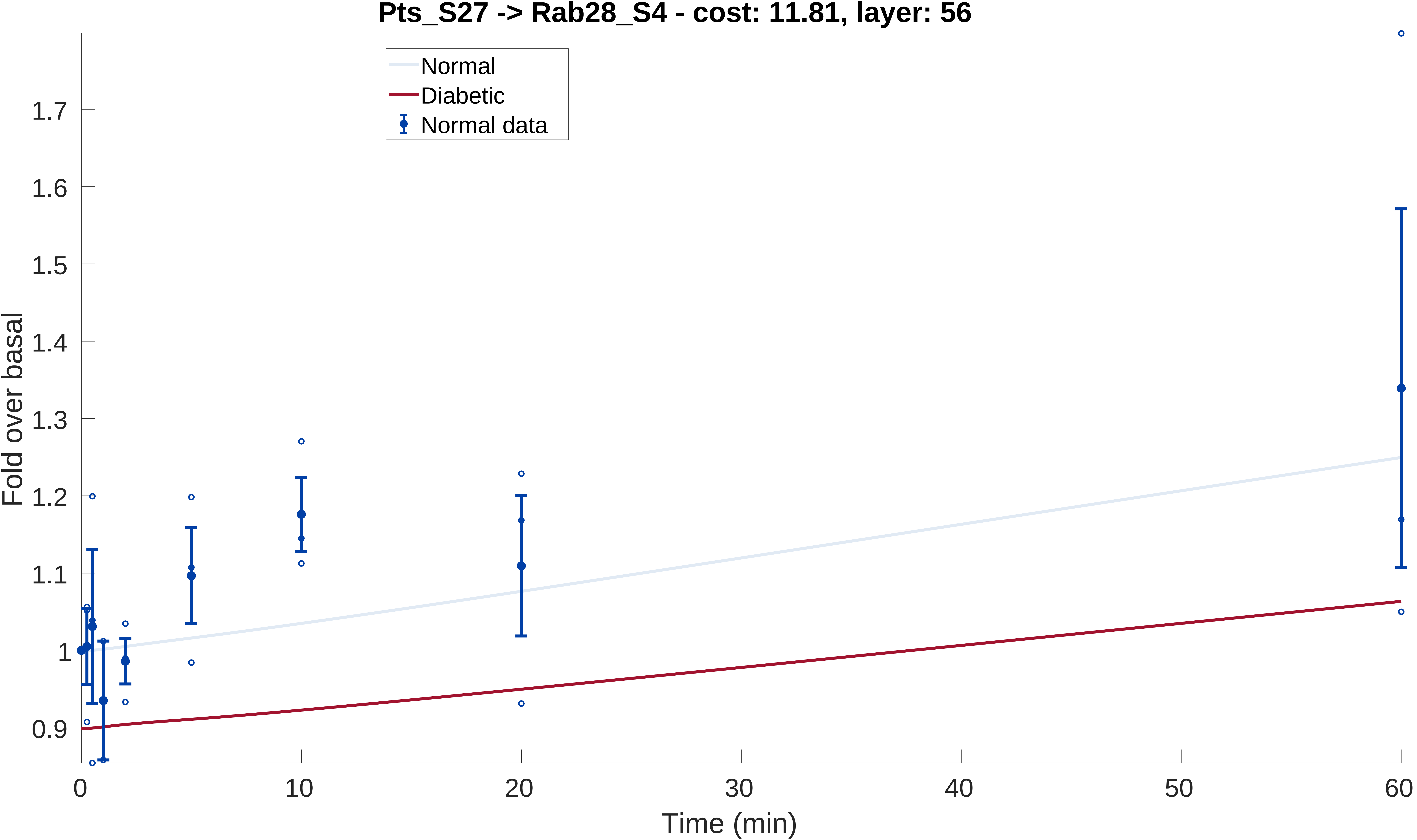
Pts_S27 -> Rab28_S4 - cost: 11.81, layer: 56
Normal
Diabetic
Normal data
Fold over basal
1.7
1.6
1.5
1.4
1.3
1.2
1.1
1
0.9
0
10
20
30
40
50
60
Time (min)

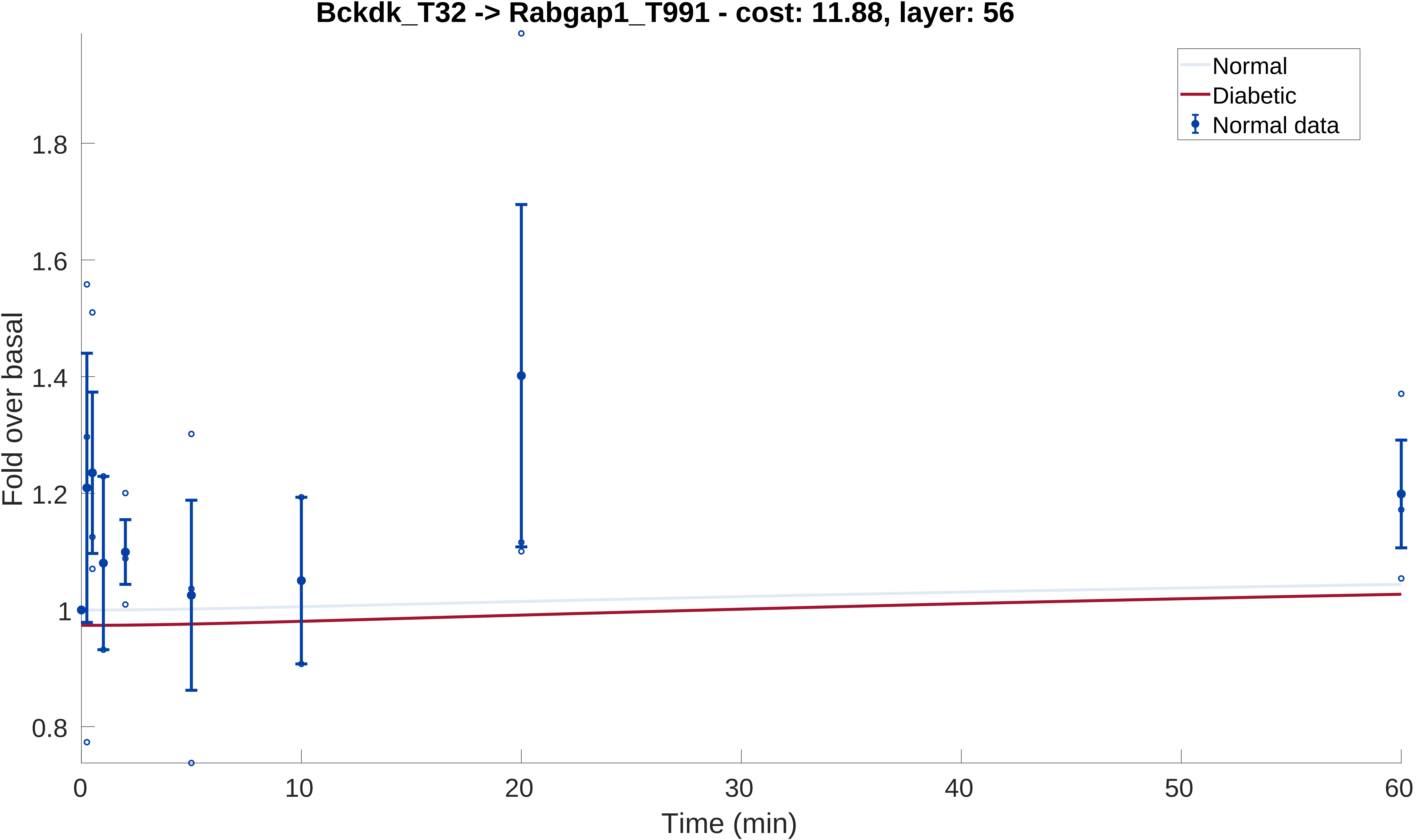

Bckdk_T32 -> Rabgap1_T991 - cost: 11.88, layer: 56
Normal
Diabetic
Normal data
Fold over basal
1.8
1.6
1.4
1.2
1
0.8
0
10
20
30
40
50
60
Time (min)

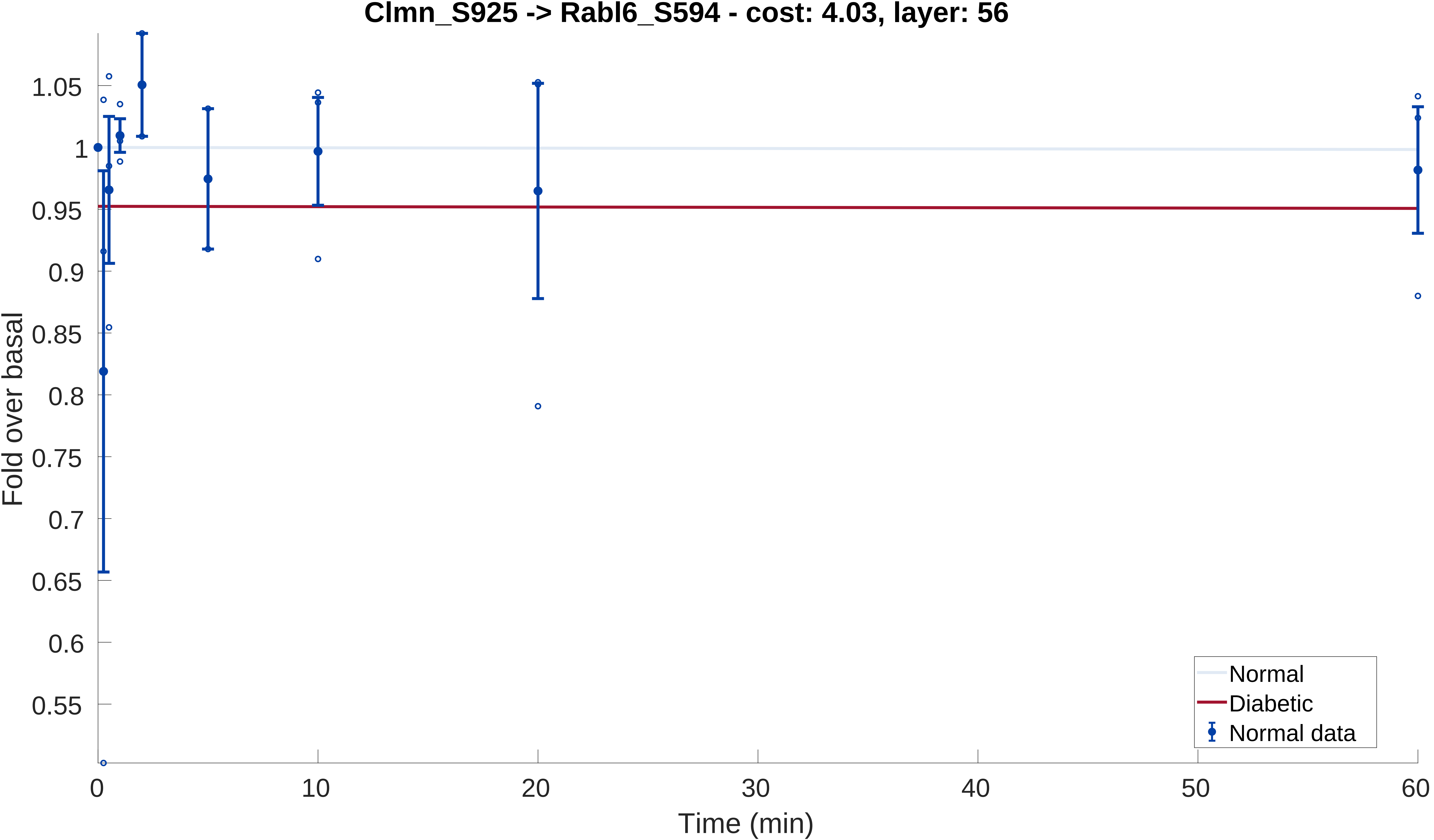

Clmn_S925 -> Rabl6_S594 - cost: 4.03, layer: 56
Fold over basal
1.05
1
0.95
0.9
0.85
0.8
0.75
0.7
0.65
0.6
0.55
0
10
20
30
40
50
60
Time (min)
Normal
Diabetic
Normal data

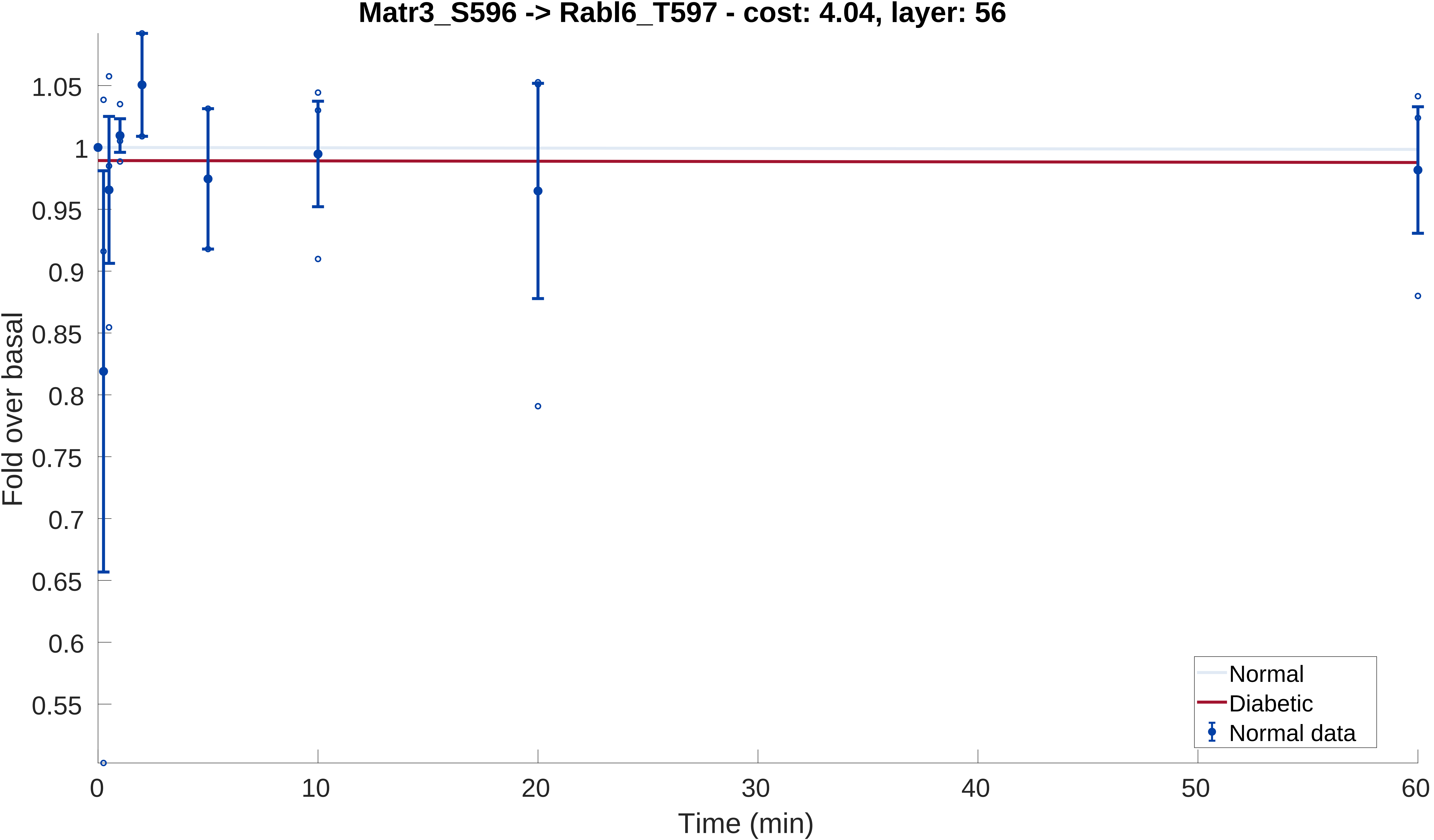

Matr3_S596 -> Rabl6_T597 - cost: 4.04, layer: 56
Fold over basal
Time (min)
1.05
1
0.95
0.9
0.85
0.8
0.75
0.7
0.65
0.6
0.55
0
10
20
30
40
50
60
Normal
Diabetic
Normal data

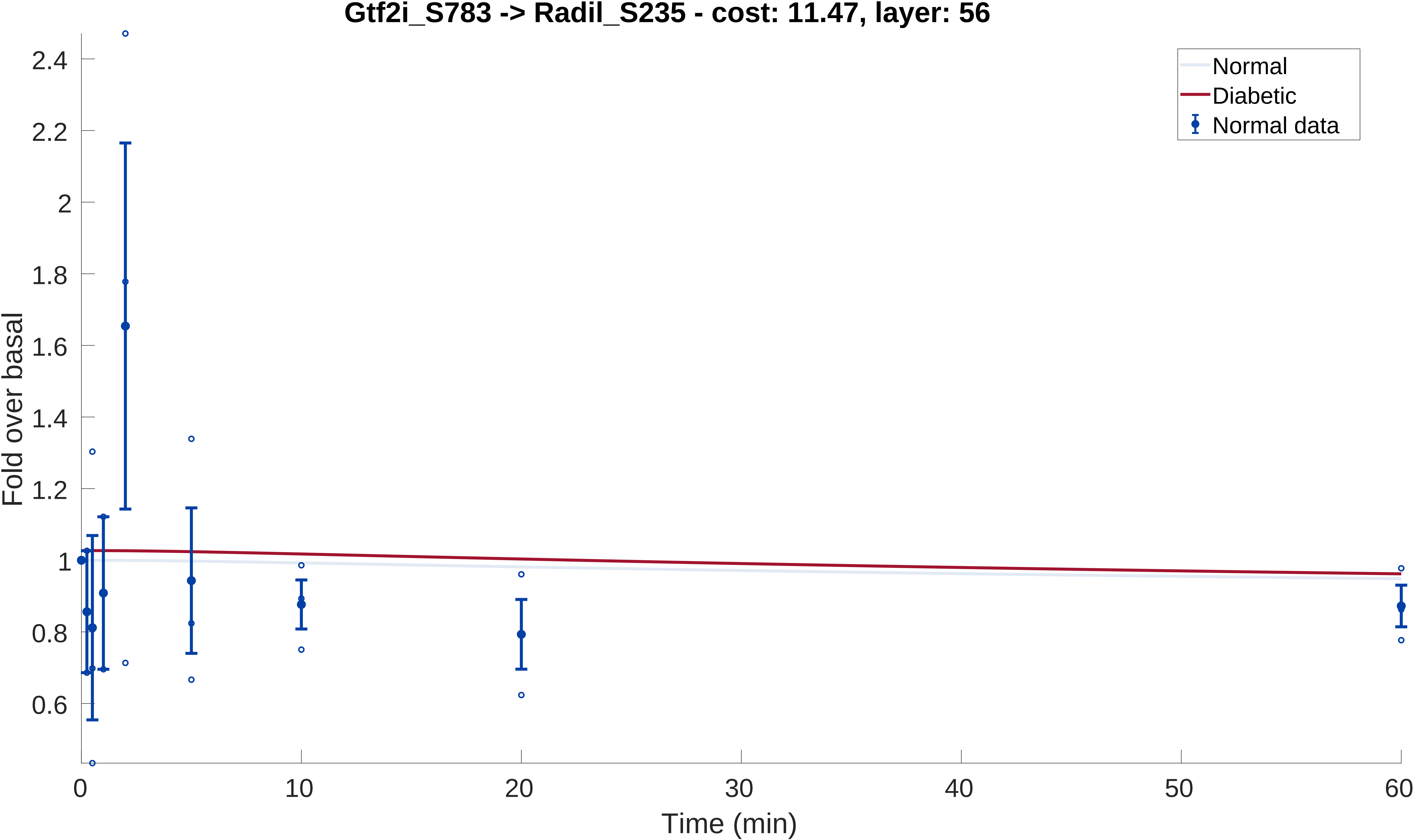

Gtf2i_S783 -> Radil_S235 - cost: 11.47, layer: 56
Normal
Diabetic
Normal data
Fold over basal
2.4
2.2
2
1.8
1.6
1.4
1.2
1
0.8
0.6
0
10
20
30
40
50
60
Time (min)

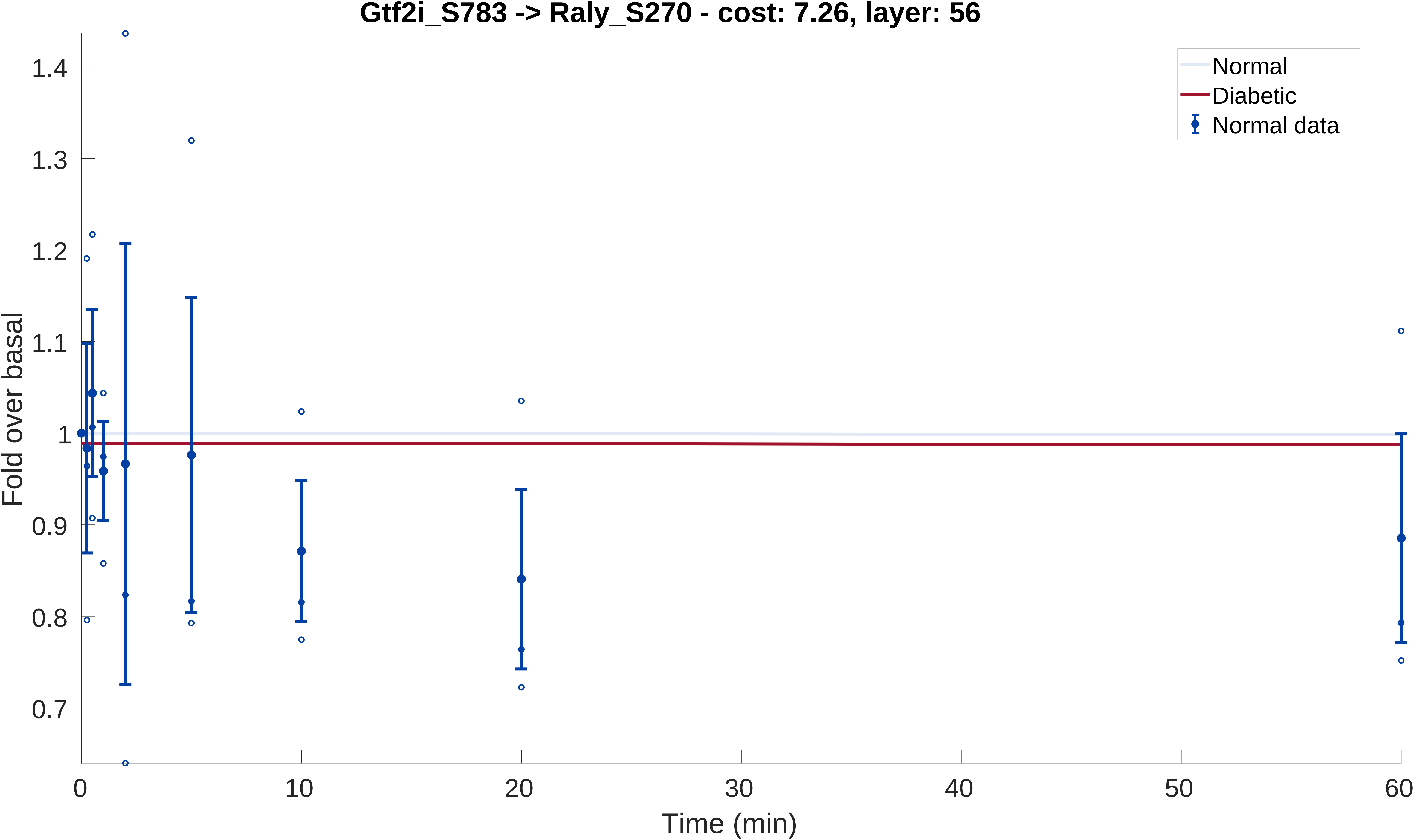
Gtf2i_S783 -> Raly_S270 - cost: 7.26, layer: 56
Normal
Diabetic
Normal data
1.4
1.3
1.2
1.1
1
0.9
0.8
0.7
Fold over basal
0
10
20
30
40
50
60
Time (min)

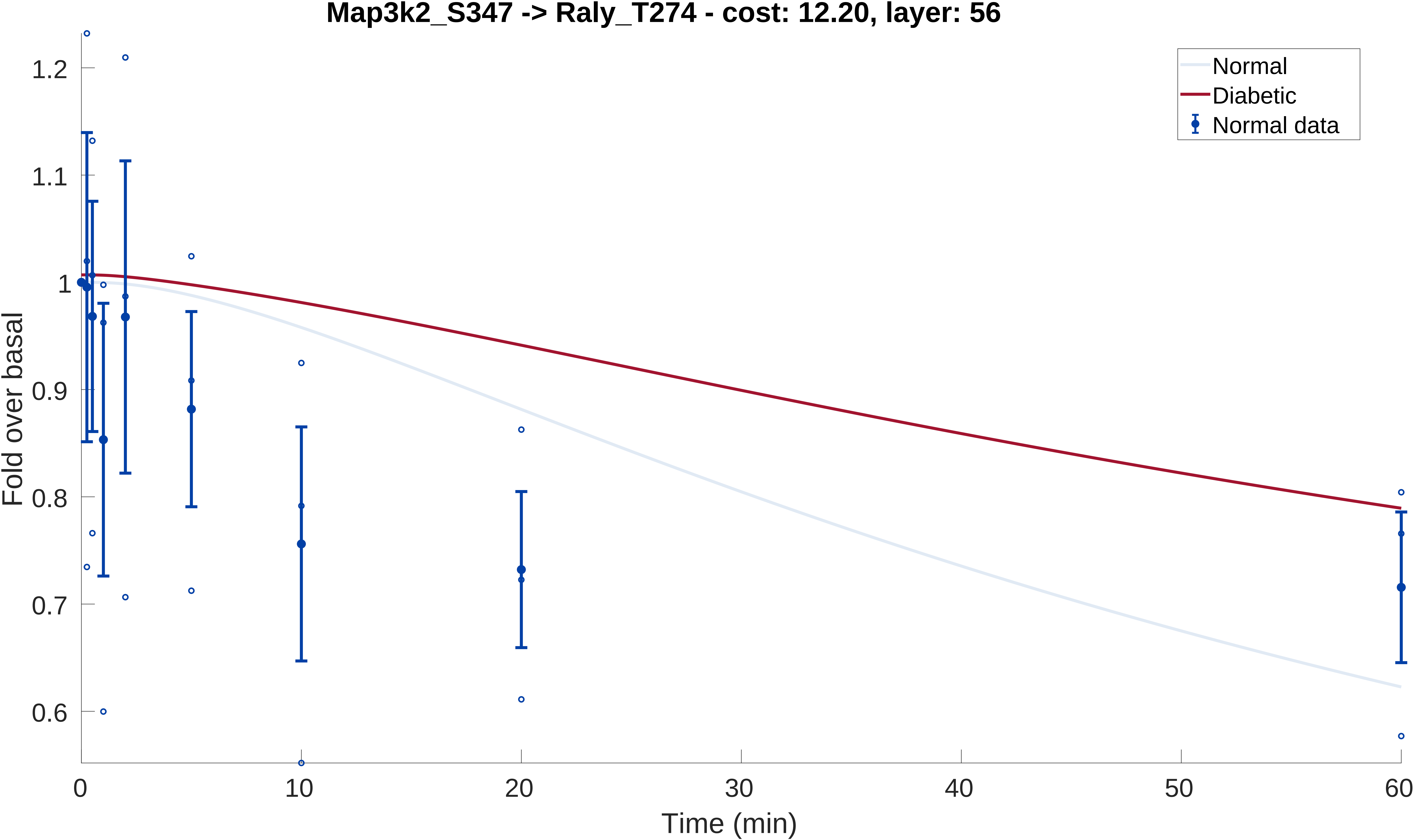

Map3k2_S347 -> Raly_T274 - cost: 12.20, layer: 56
Normal
Diabetic
Normal data
Fold over basal
Time (min)
1.2
1.1
1
0.9
0.8
0.7
0.6
0
10
20
30
40
50
60

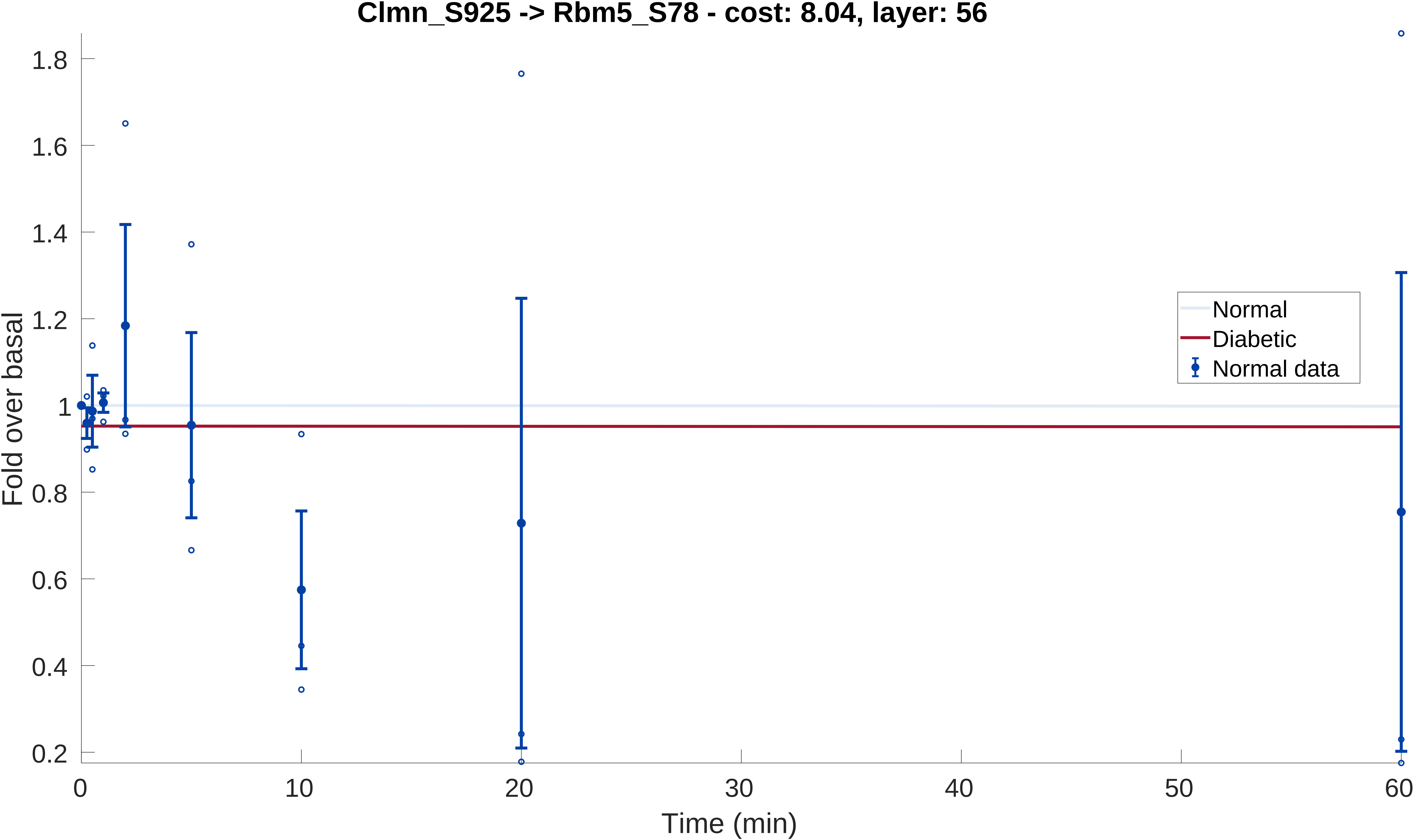
Clmn_S925 -> Rbm5_S78 - cost: 8.04, layer: 56
Fold over basal
Time (min)
Normal
Diabetic
Normal data
0.2
0.4
0.6
0.8
1
1.2
1.4
1.6
1.8
0
10
20
30
40
50
60

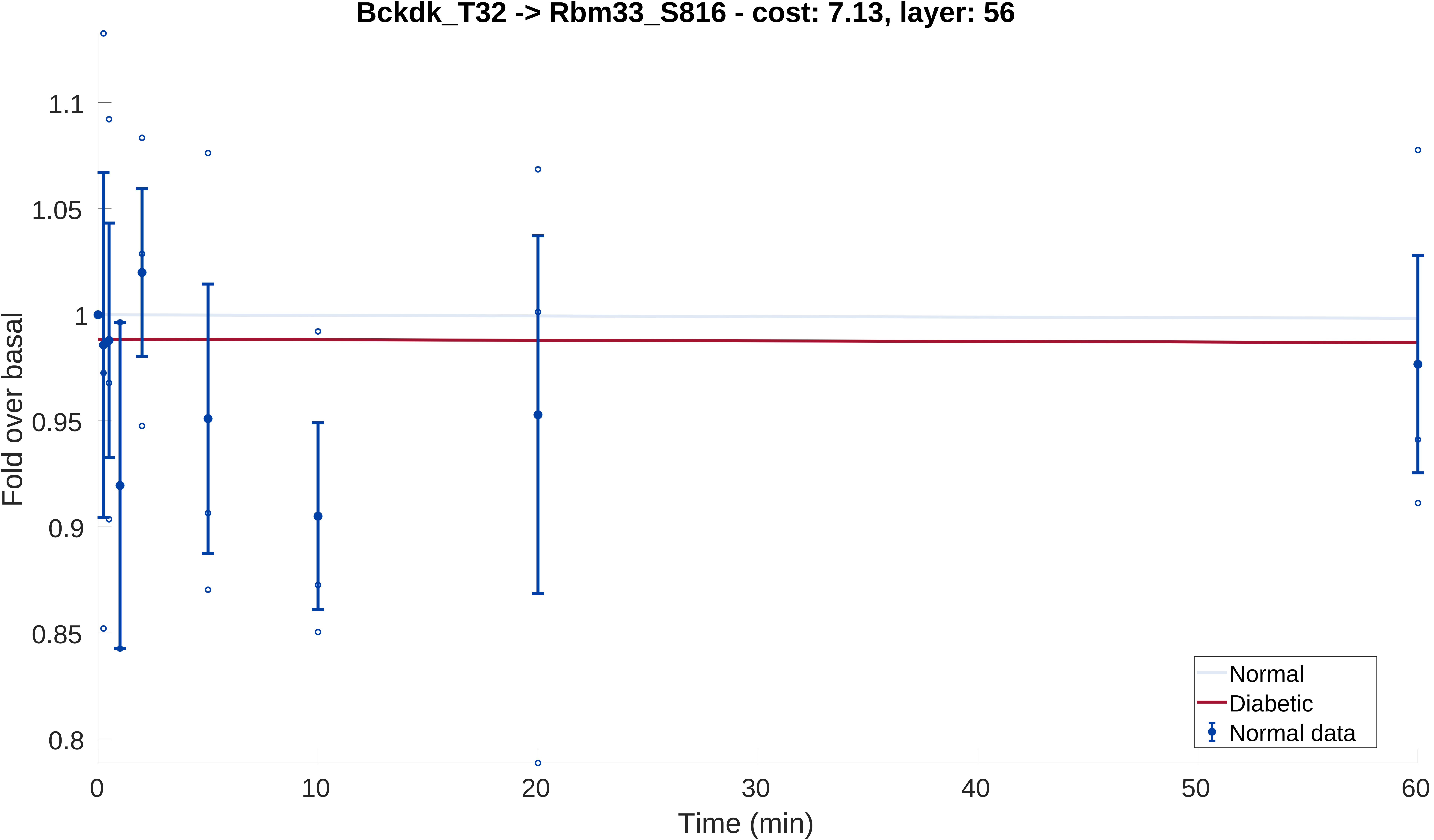
Bckdk_T32 -> Rbm33_S816 - cost: 7.13, layer: 56
Fold over basal
1.1
1.05
1
0.95
0.9
0.85
0.8
0
10
20
30
40
50
60
Time (min)
Normal
Diabetic
Normal data

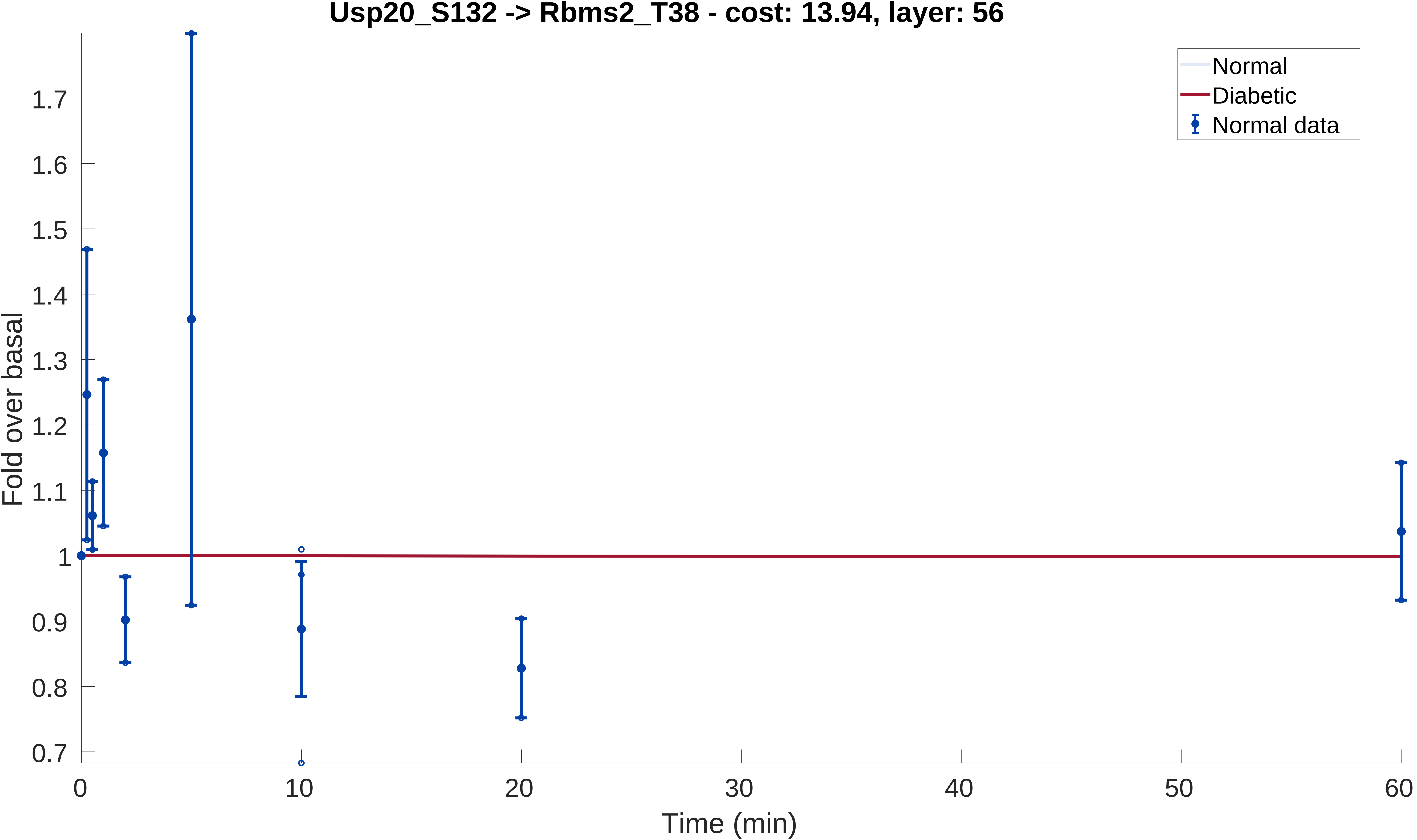
Usp20_S132 -> Rbms2_T38 - cost: 13.94, layer: 56
Normal
Diabetic
Normal data
Fold over basal
Time (min)
0.7
0.8
0.9
1
1.1
1.2
1.3
1.4
1.5
1.6
1.7
0
10
20
30
40
50
60

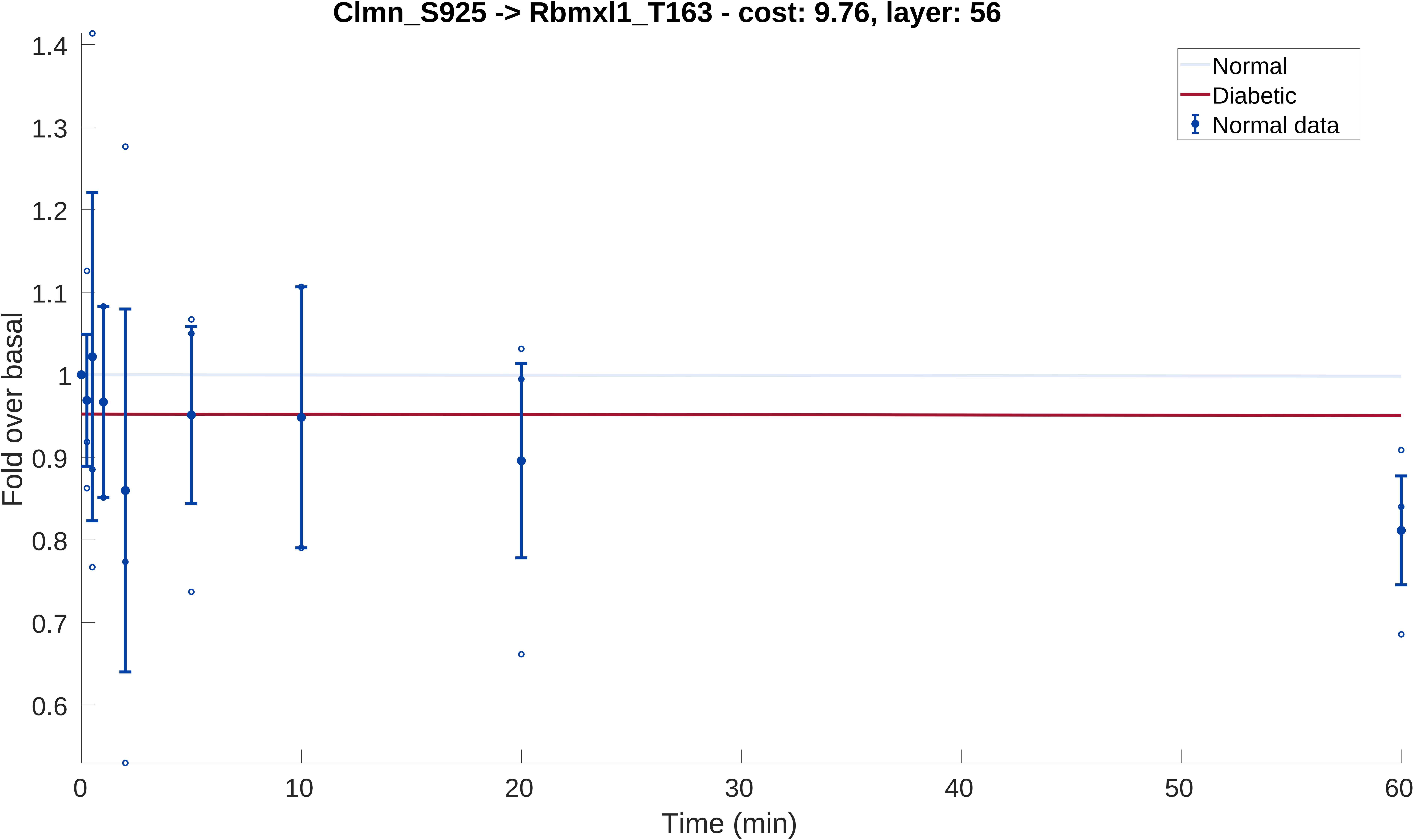

Clmn_S925 -> Rbmxl1_T163 - cost: 9.76, layer: 56
Normal
Diabetic
Normal data
Fold over basal
1.4
1.3
1.2
1.1
1
0.9
0.8
0.7
0.6
0
10
20
30
40
50
60
Time (min)

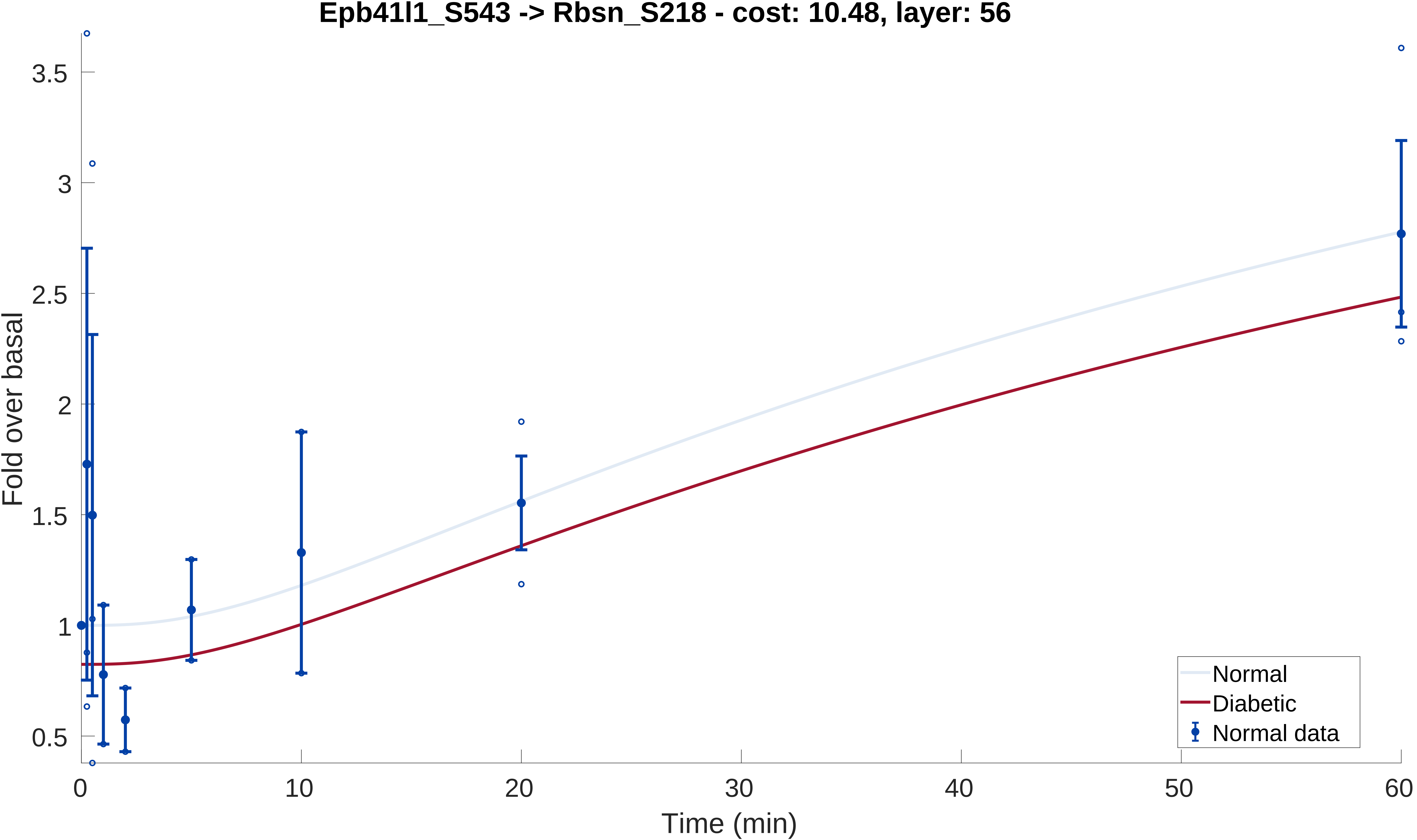
Epb41l1_S543 -> Rbsn_S218 - cost: 10.48, layer: 56
Fold over basal
3.5
3
2.5
2
1.5
1
0.5
0
10
20
30
40
50
60
Time (min)
Normal
Diabetic
Normal data

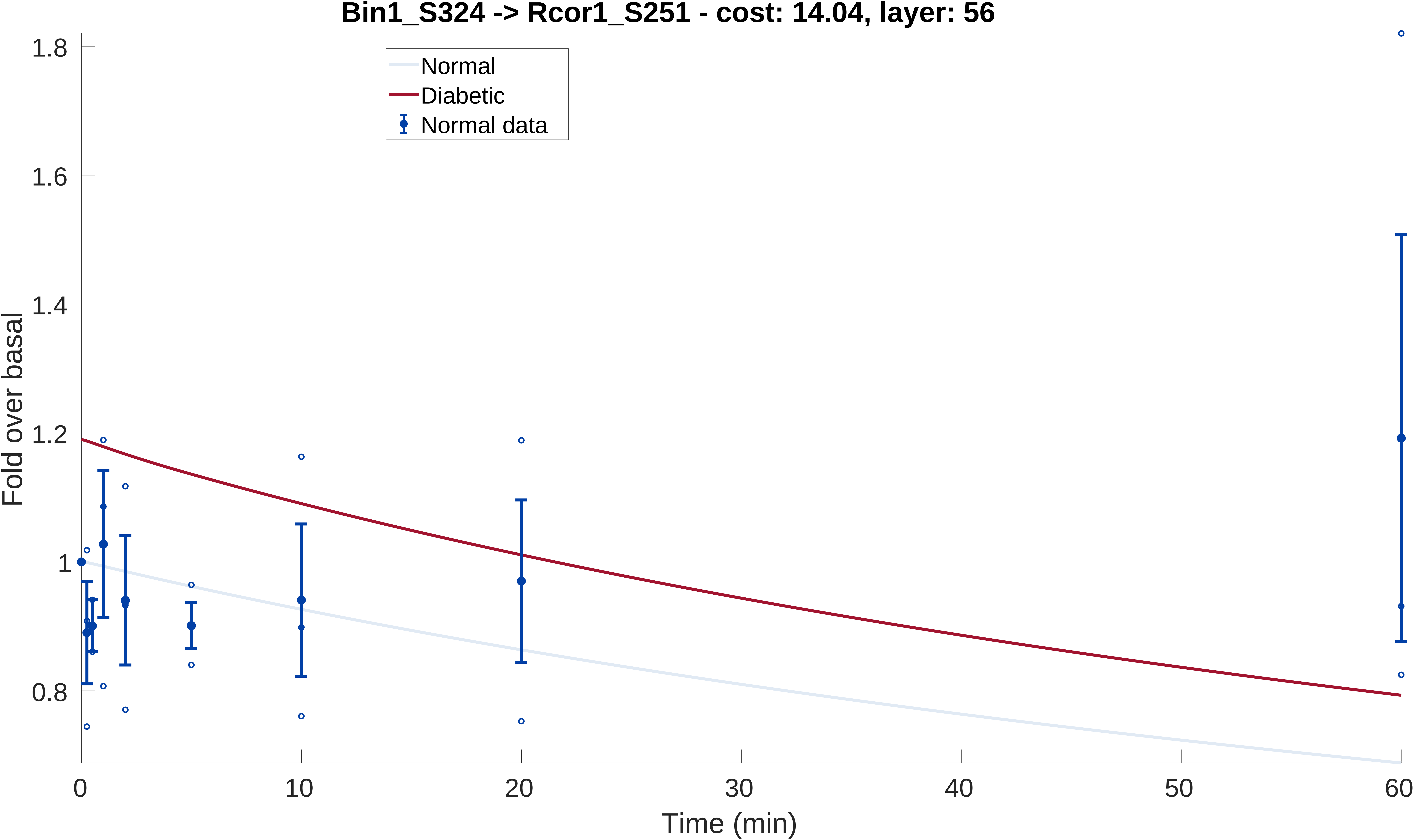

Bin1_S324 -> Rcor1_S251 - cost: 14.04, layer: 56
Normal
Diabetic
Normal data
1.8
1.6
1.4
1.2
1
0.8
Fold over basal
0
10
20
30
40
50
60
Time (min)

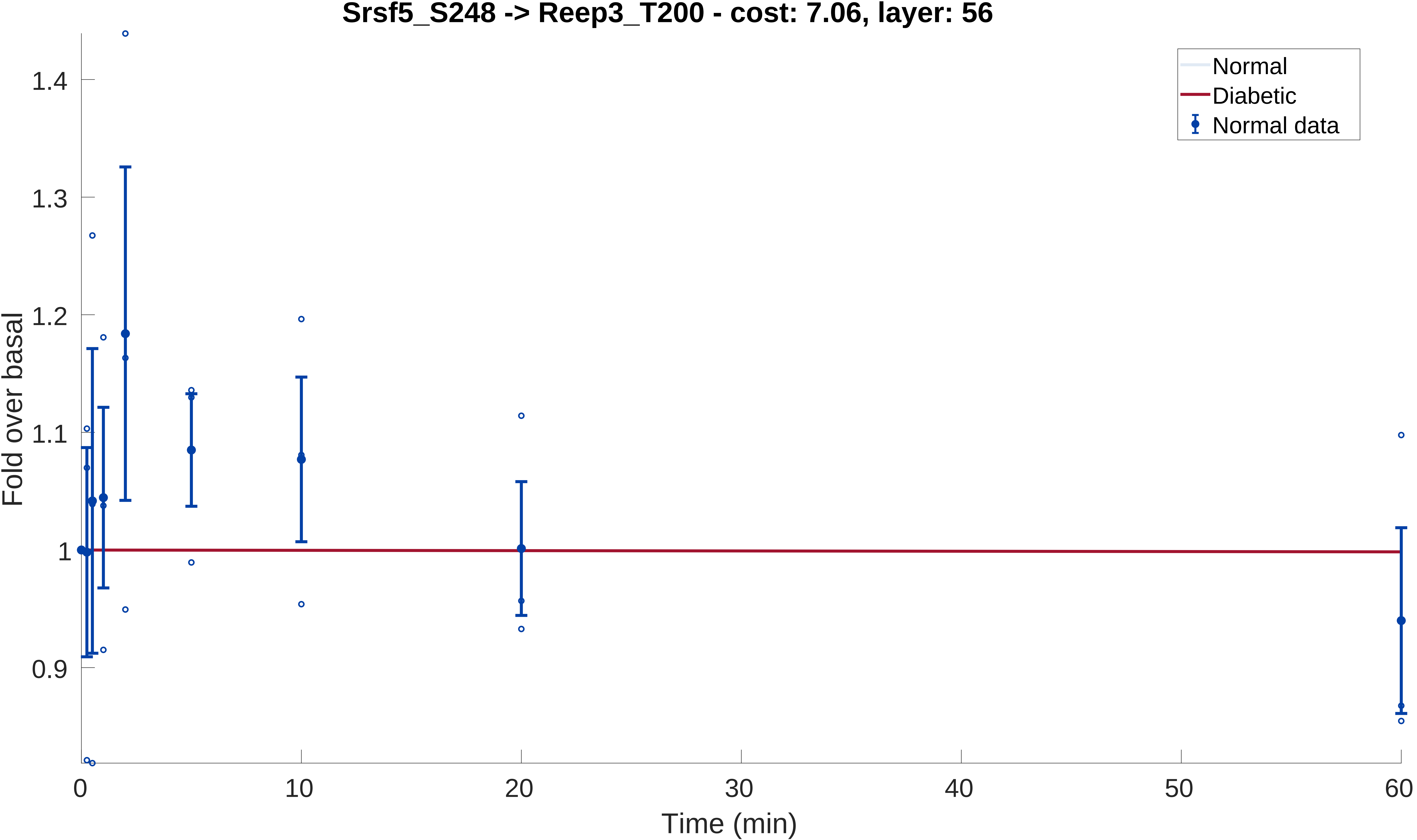

Srsf5_S248 -> Reep3_T200 - cost: 7.06, layer: 56
Normal
Diabetic
Normal data
Fold over basal
1.4
1.3
1.2
1.1
1
0.9
0
10
20
30
40
50
60
Time (min)

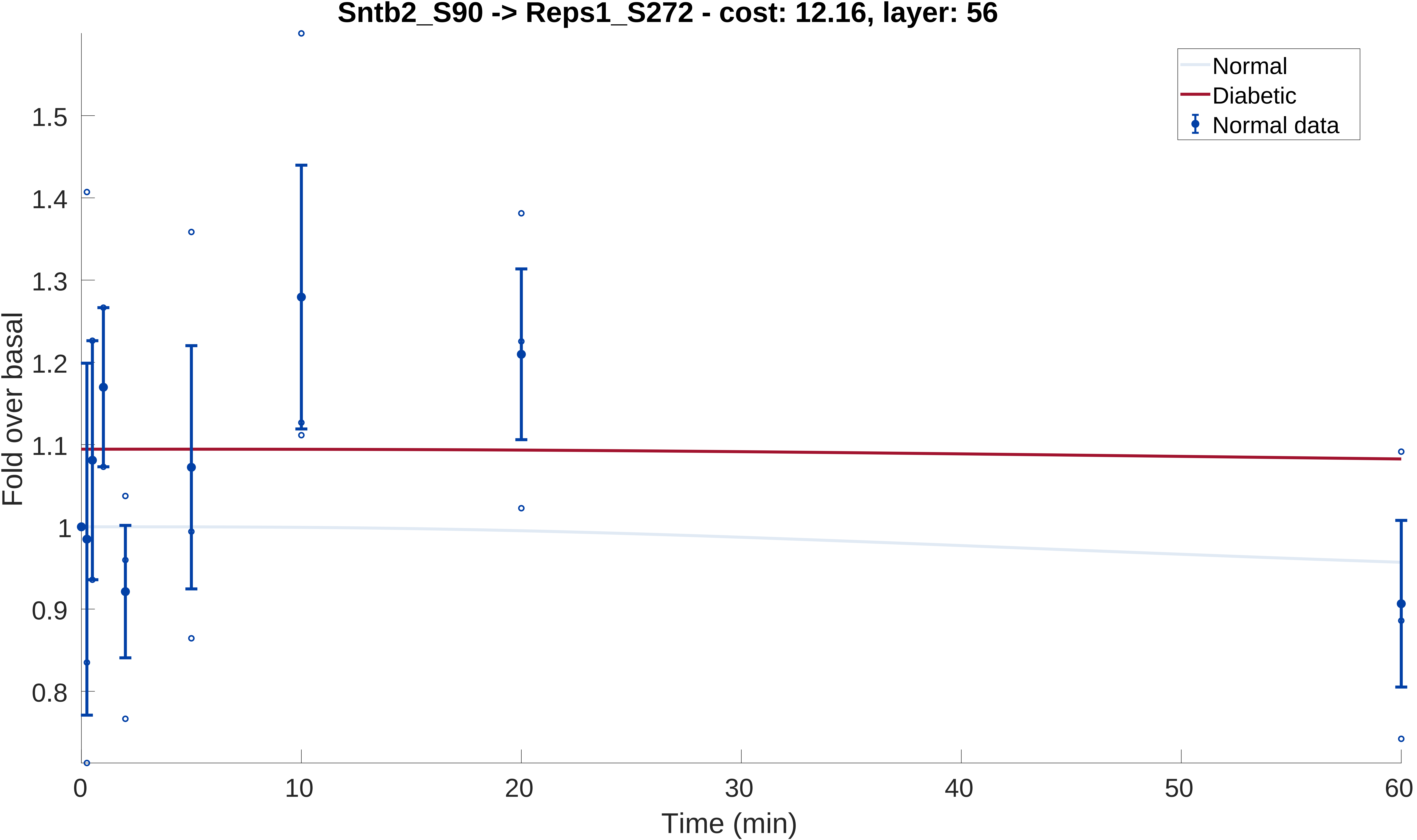
Sntb2_S90 -> Reps1_S272 - cost: 12.16, layer: 56
Normal
Diabetic
Normal data
Fold over basal
1.5
1.4
1.3
1.2
1.1
1
0.9
0.8
0
10
20
30
40
50
60
Time (min)

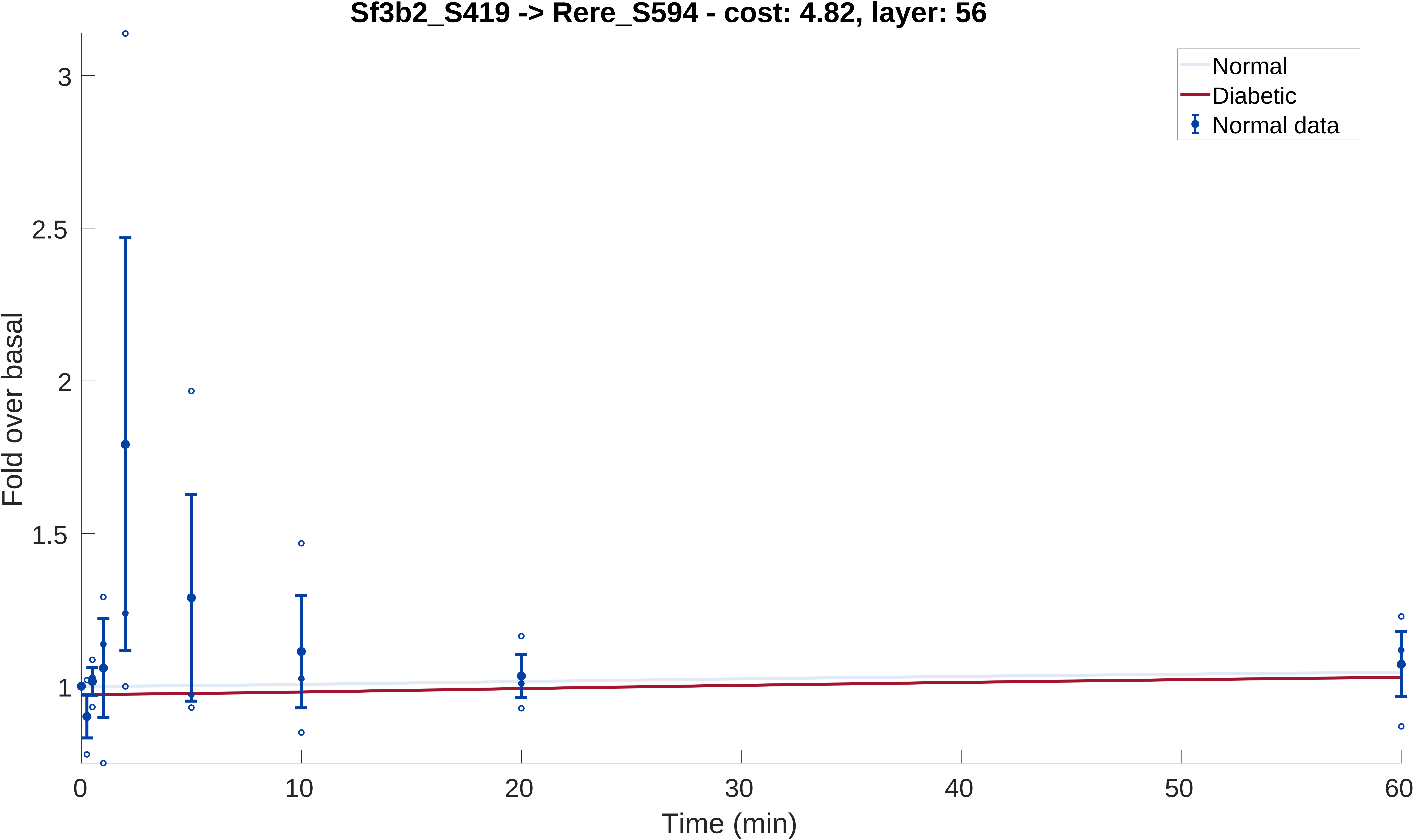

Sf3b2_S419 -> Rere_S594 - cost: 4.82, layer: 56
Normal
Diabetic
Normal data
Fold over basal
3
2.5
2
1.5
1
0
10
20
30
40
50
60
Time (min)

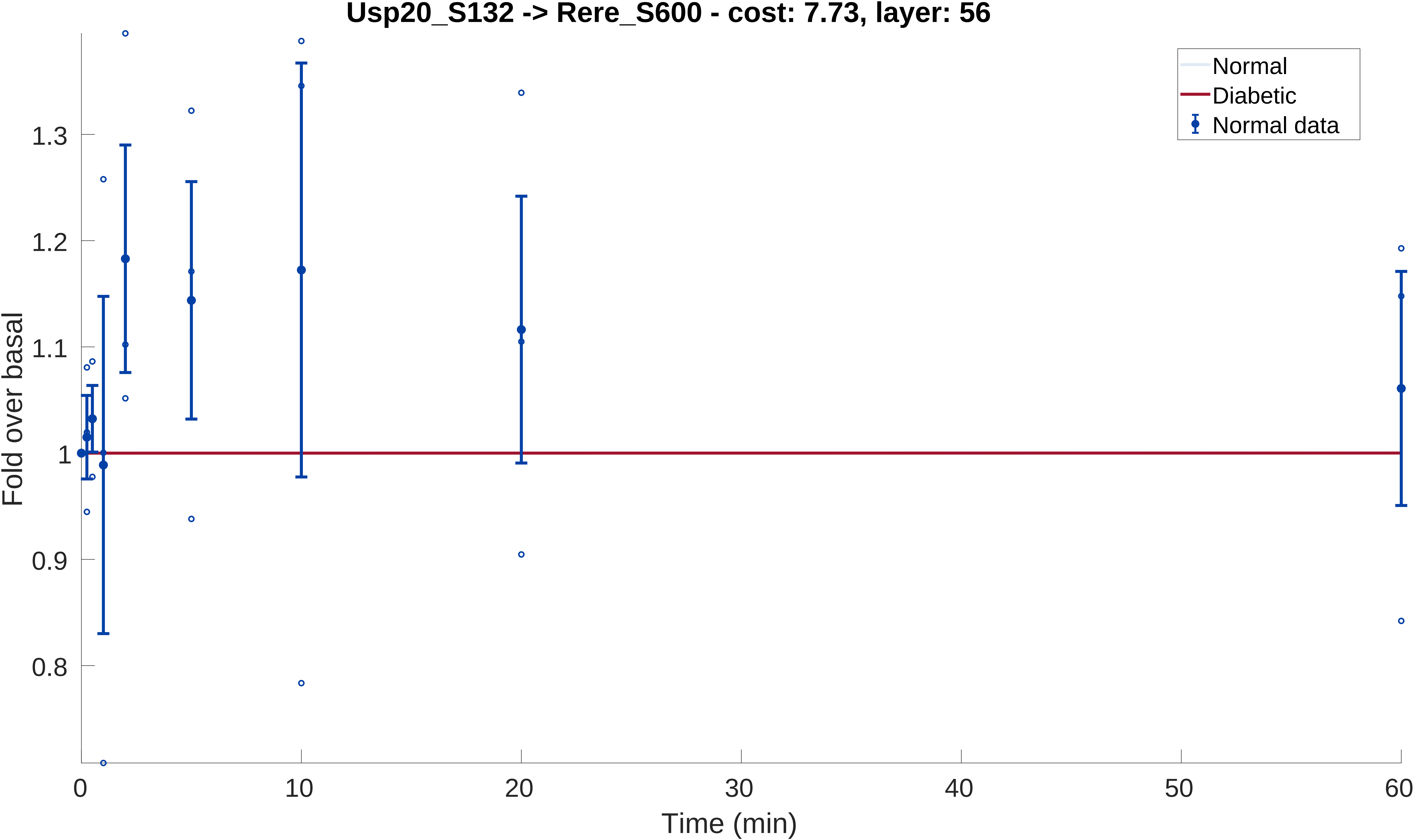
Usp20_S132 -> Rere_S600 - cost: 7.73, layer: 56
Normal
Diabetic
Normal data
Fold over basal
1.3
1.2
1.1
1
0.9
0.8
0
10
20
30
40
50
60
Time (min)

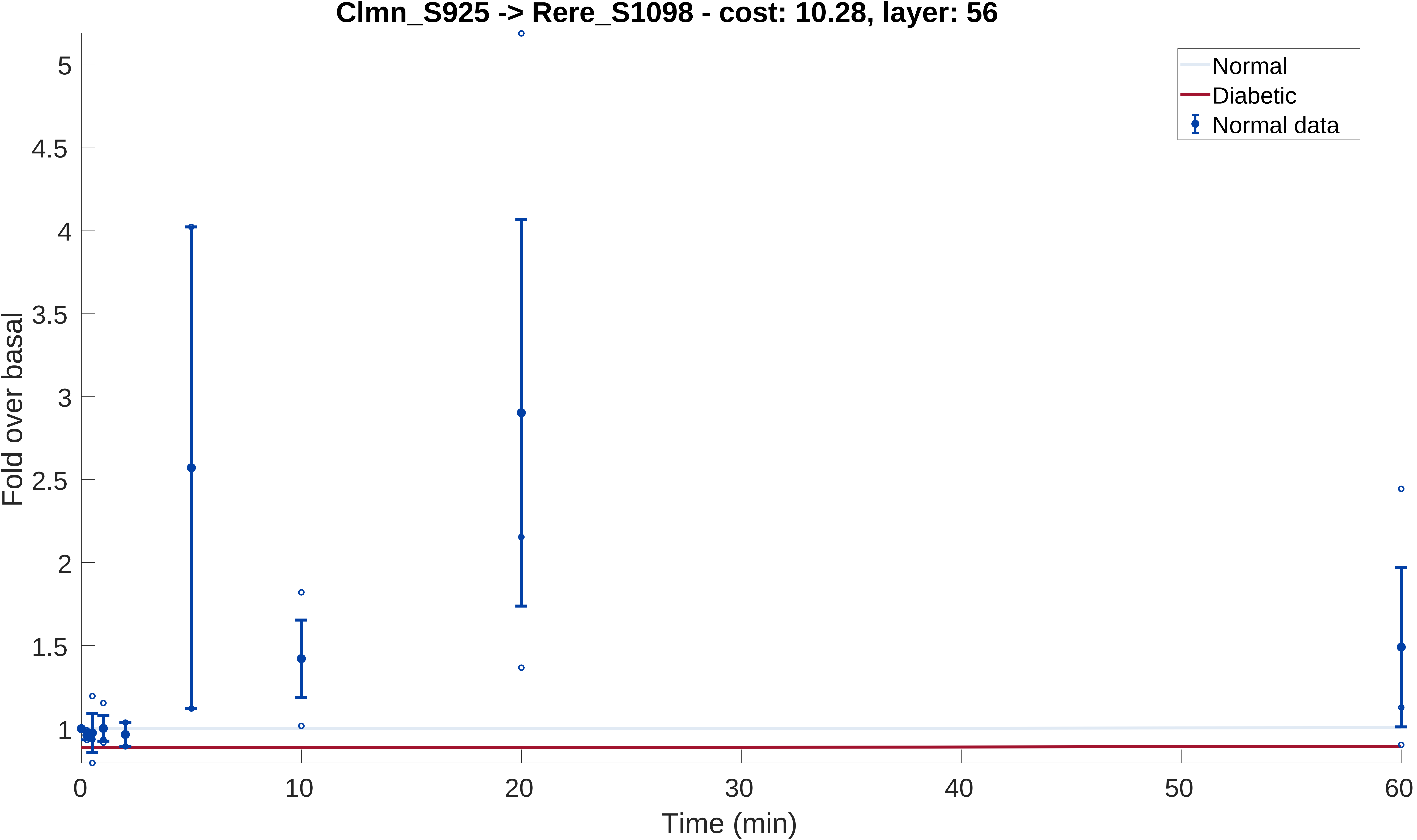

Clmn_S925 -> Rere_S1098 - cost: 10.28, layer: 56
Normal
Diabetic
Normal data
Fold over basal
5
4.5
4
3.5
3
2.5
2
1.5
1
0
10
20
30
40
50
60
Time (min)

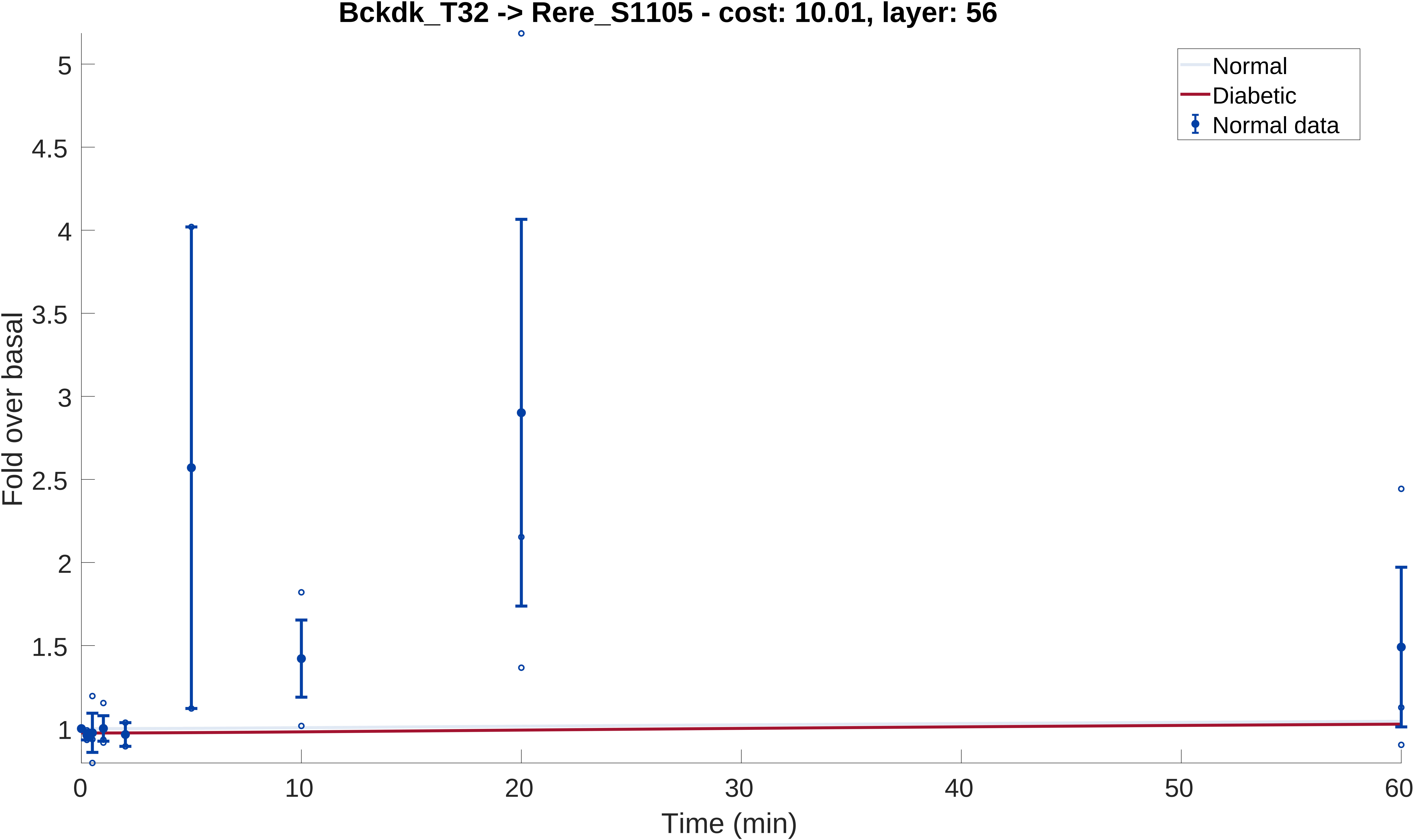

Bckdk_T32 -> Rere_S1105 - cost: 10.01, layer: 56
Normal
Diabetic
Normal data
Fold over basal
Time (min)
5
4.5
4
3.5
3
2.5
2
1.5
1
0
10
20
30
40
50
60

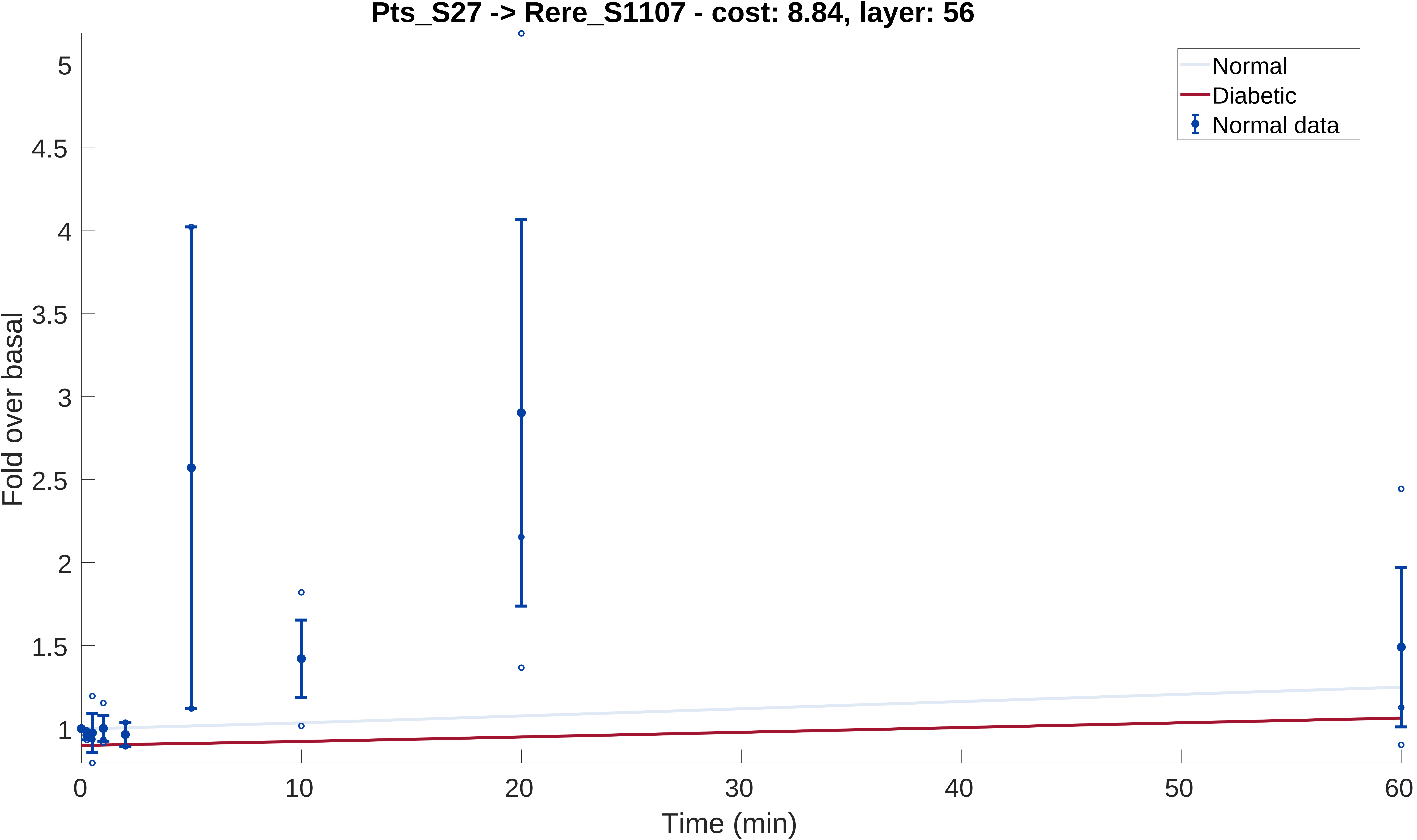

Pts_S27 -> Rere_S1107 - cost: 8.84, layer: 56
Normal
Diabetic
Normal data
Fold over basal
Time (min)
0
10
20
30
40
50
60
1
1.5
2
2.5
3
3.5
4
4.5
5

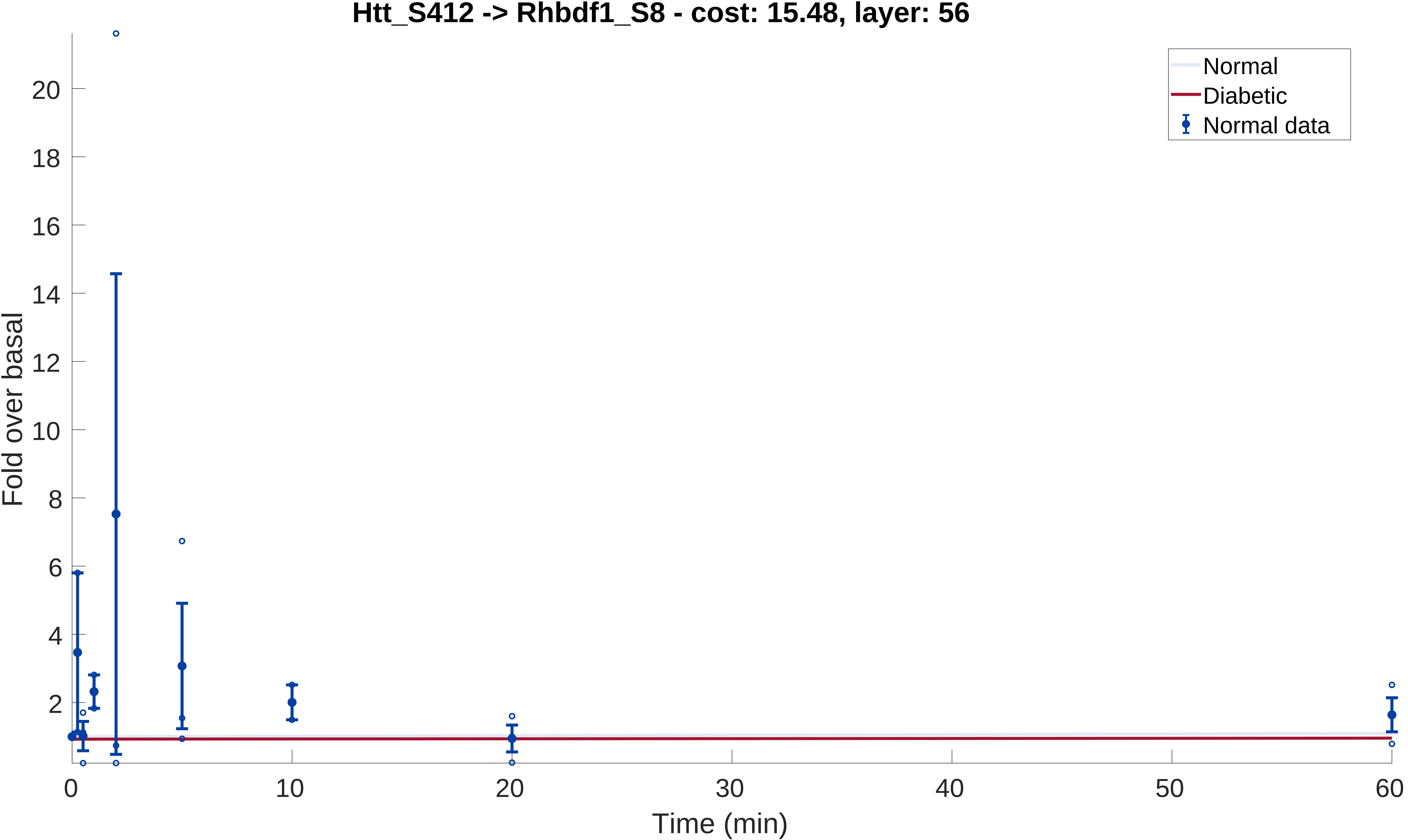
Htt_S412 -> Rhbdf1_S8 - cost: 15.48, layer: 56
Normal
Diabetic
Normal data
Fold over basal
Time (min)
0
10
20
30
40
50
60
2
4
6
8
10
12
14
16
18
20

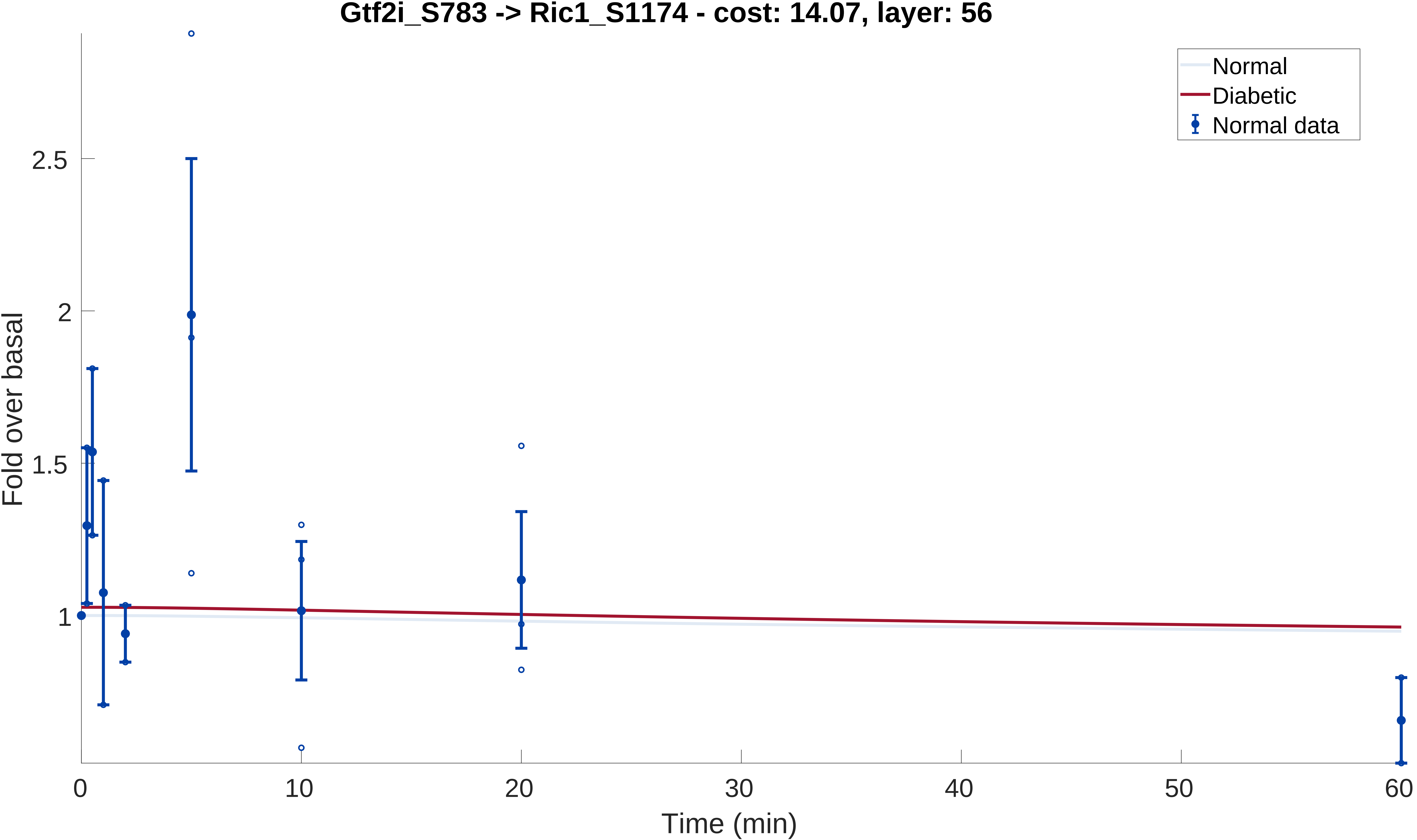

Gtf2i_S783 -> Ric1_S1174 - cost: 14.07, layer: 56
Normal
Diabetic
Normal data
2.5
2
1.5
1
Fold over basal
0
10
20
30
40
50
60
Time (min)

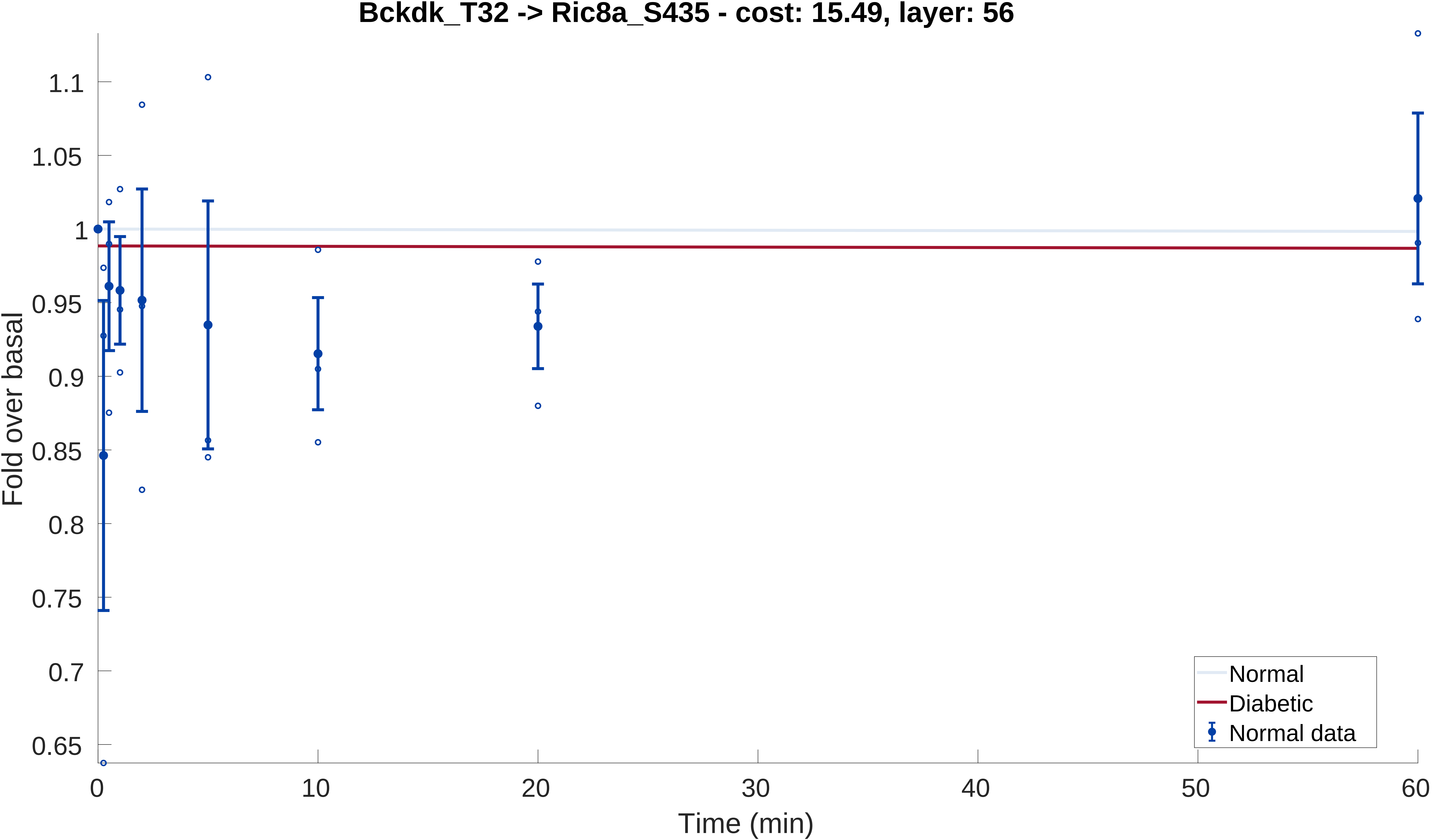
Bckdk_T32 -> Ric8a_S435 - cost: 15.49, layer: 56
Fold over basal
Time (min)
1.1
1.05
1
0.95
0.9
0.85
0.8
0.75
0.7
0.65
0
10
20
30
40
50
60
Normal
Diabetic
Normal data

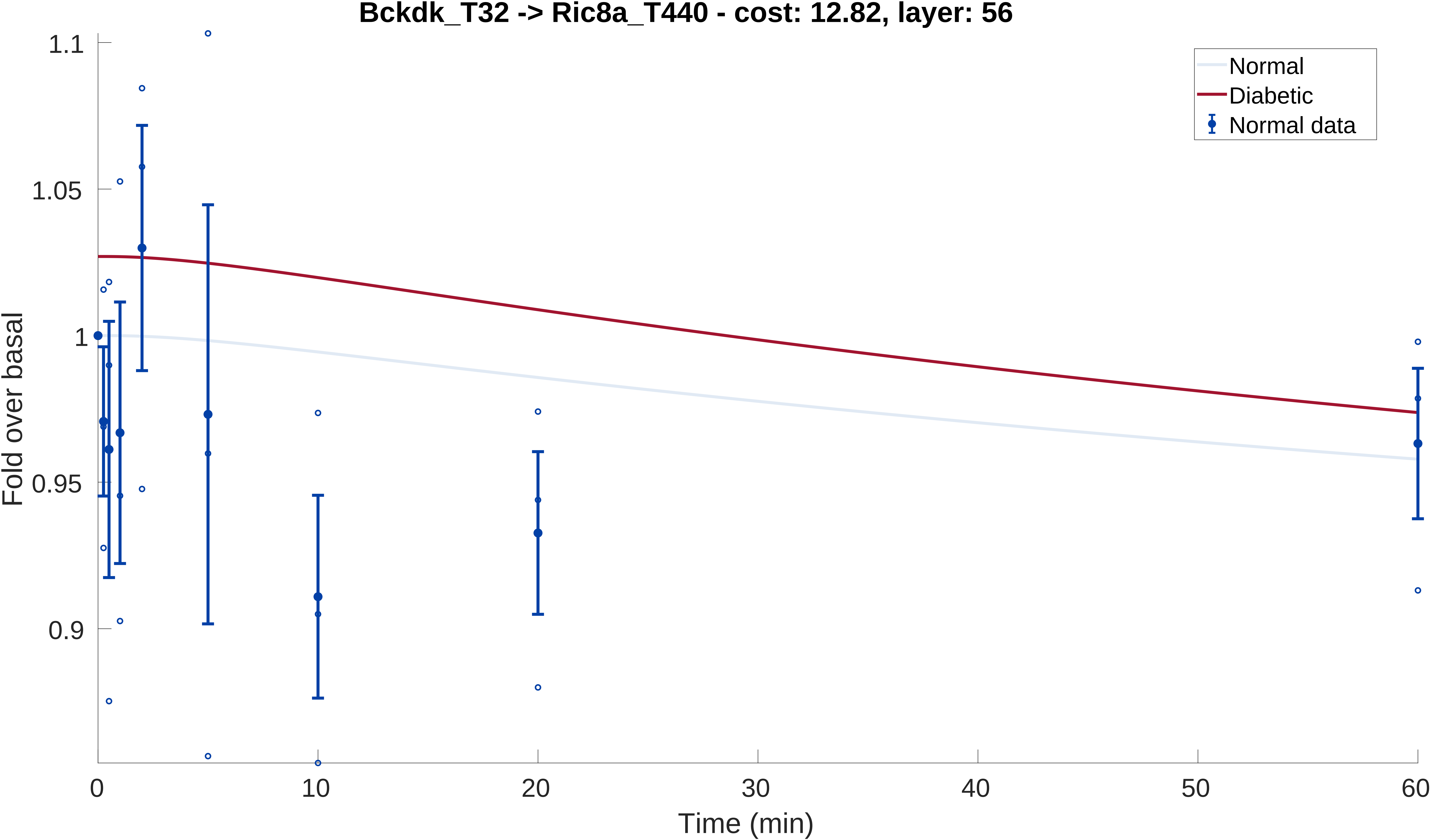

Bckdk_T32 -> Ric8a_T440 - cost: 12.82, layer: 56
Normal
Diabetic
Normal data
Fold over basal
Time (min)
1.1
1.05
1
0.95
0.9
0
10
20
30
40
50
60

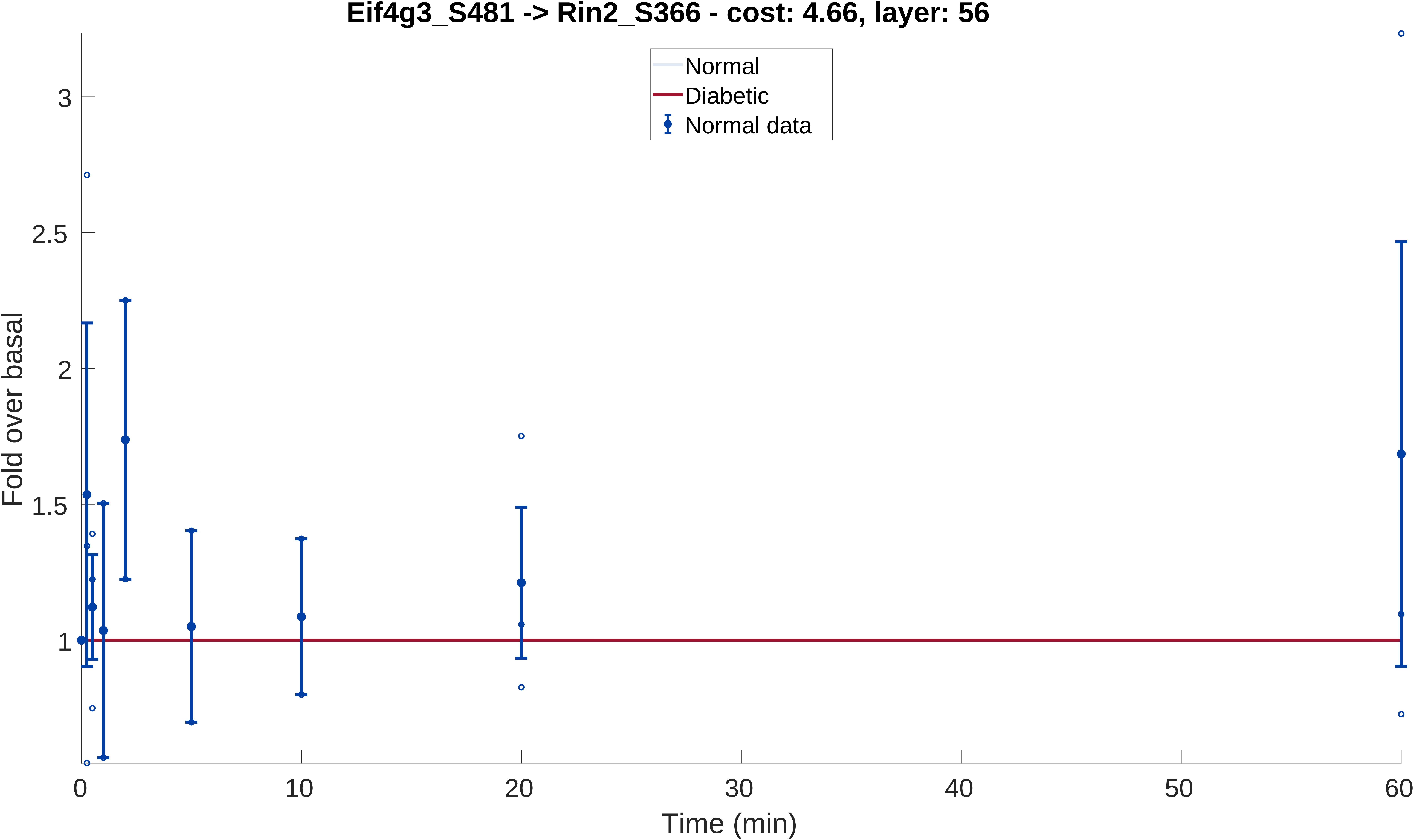

Eif4g3_S481 -> Rin2_S366 - cost: 4.66, layer: 56
Normal
Diabetic
Normal data
Fold over basal
Time (min)
3
2.5
2
1.5
1
0
10
20
30
40
50
60

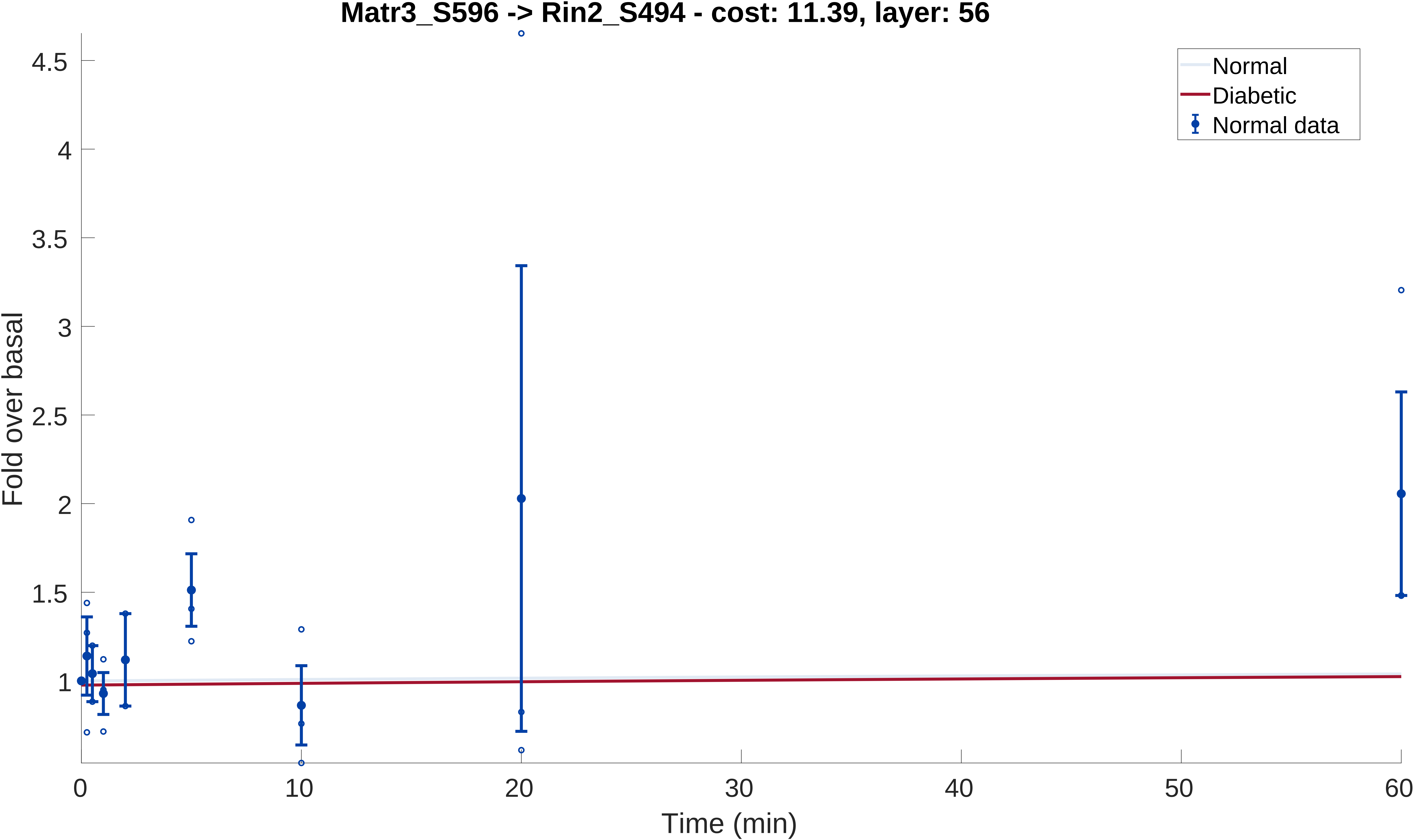

Matr3_S596 -> Rin2_S494 - cost: 11.39, layer: 56
Fold over basal
Time (min)
4.5
4
3.5
3
2.5
2
1.5
1
0
10
20
30
40
50
60
Normal
Diabetic
Normal data

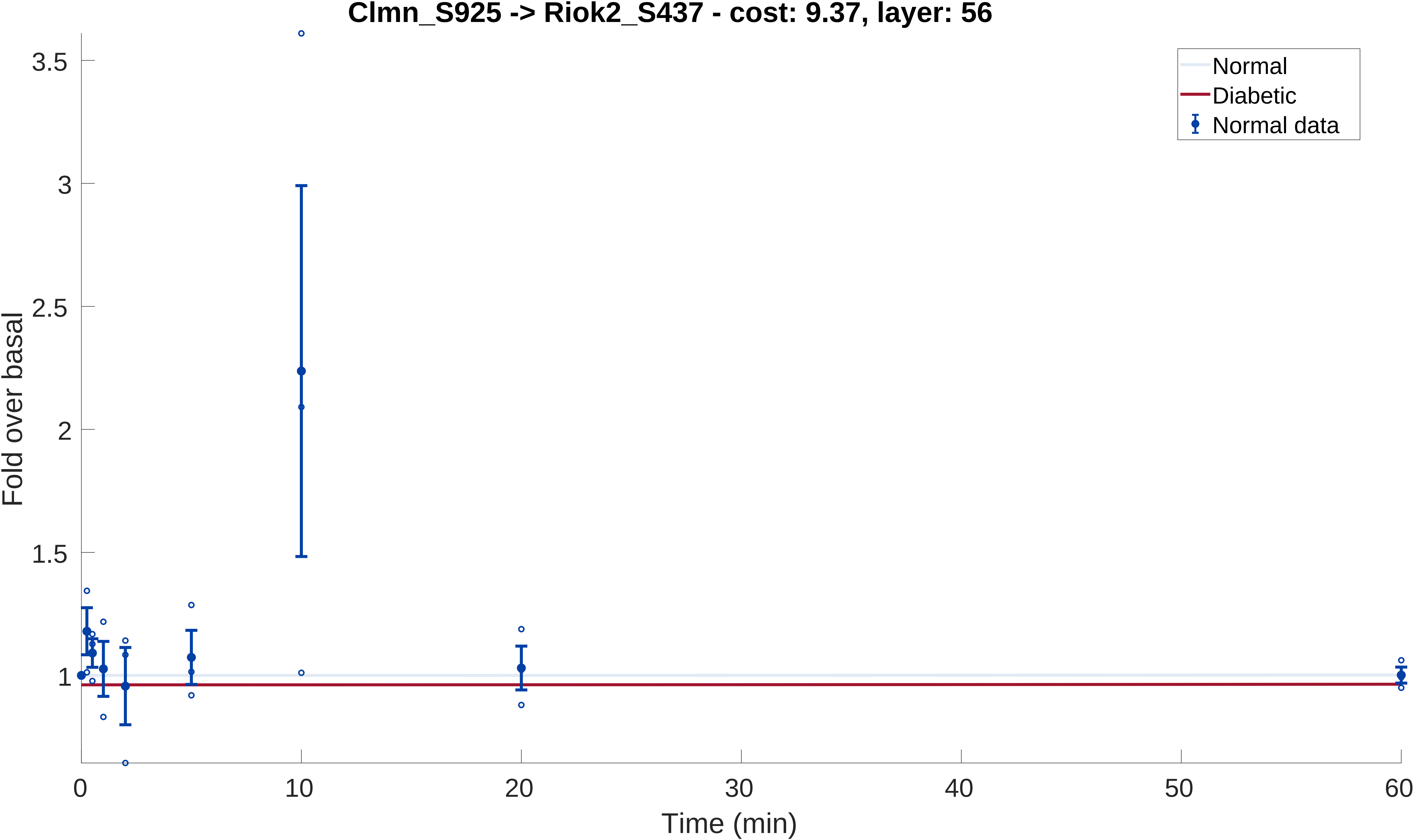

Clmn_S925 -> Riok2_S437 - cost: 9.37, layer: 56
Normal
Diabetic
Normal data
Fold over basal
3.5
3
2.5
2
1.5
1
0
10
20
30
40
50
60
Time (min)

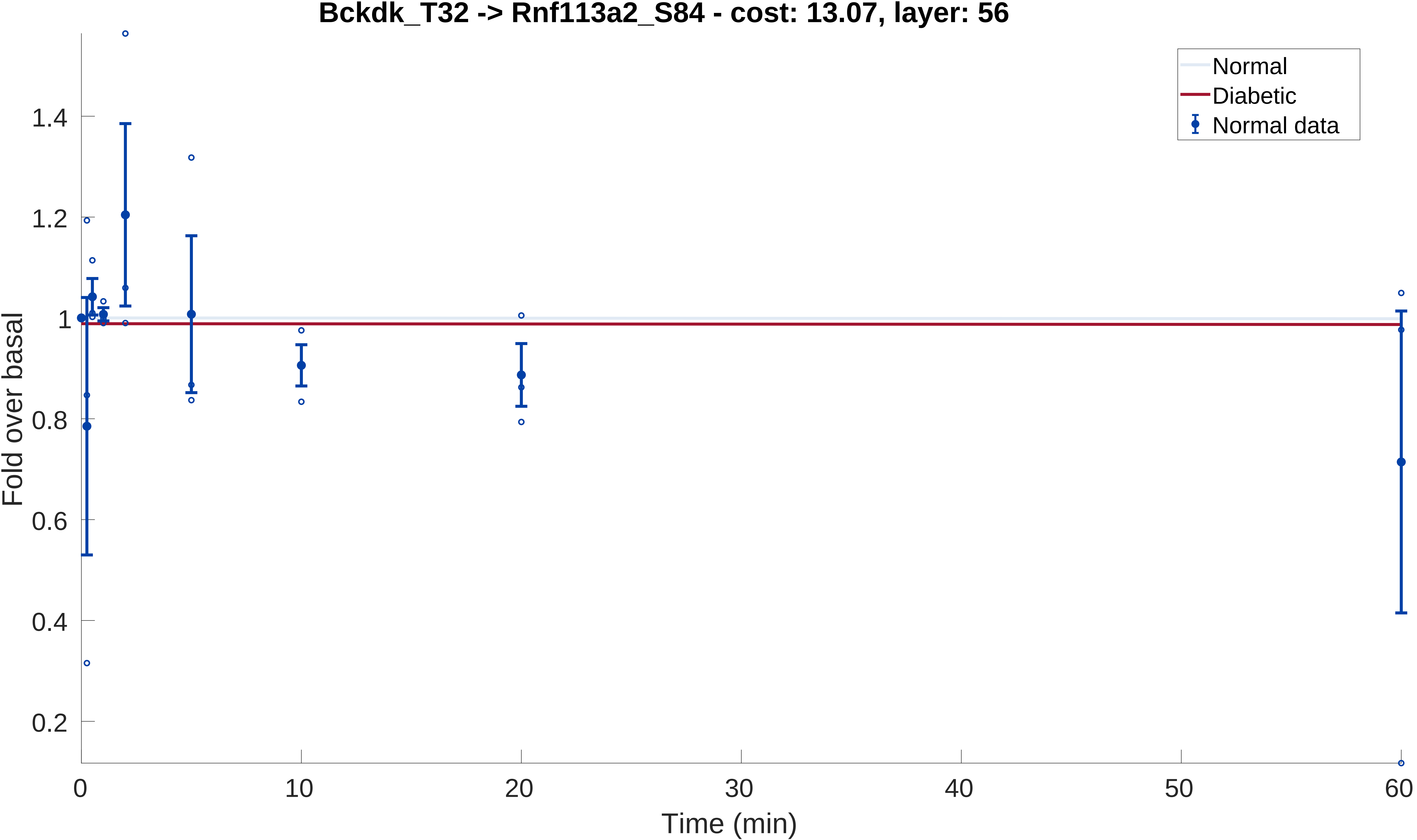

Bckdk_T32 -> Rnf113a2_S84 - cost: 13.07, layer: 56
Normal
Diabetic
Normal data
Fold over basal
1.4
1.2
1
0.8
0.6
0.4
0.2
0
10
20
30
40
50
60
Time (min)

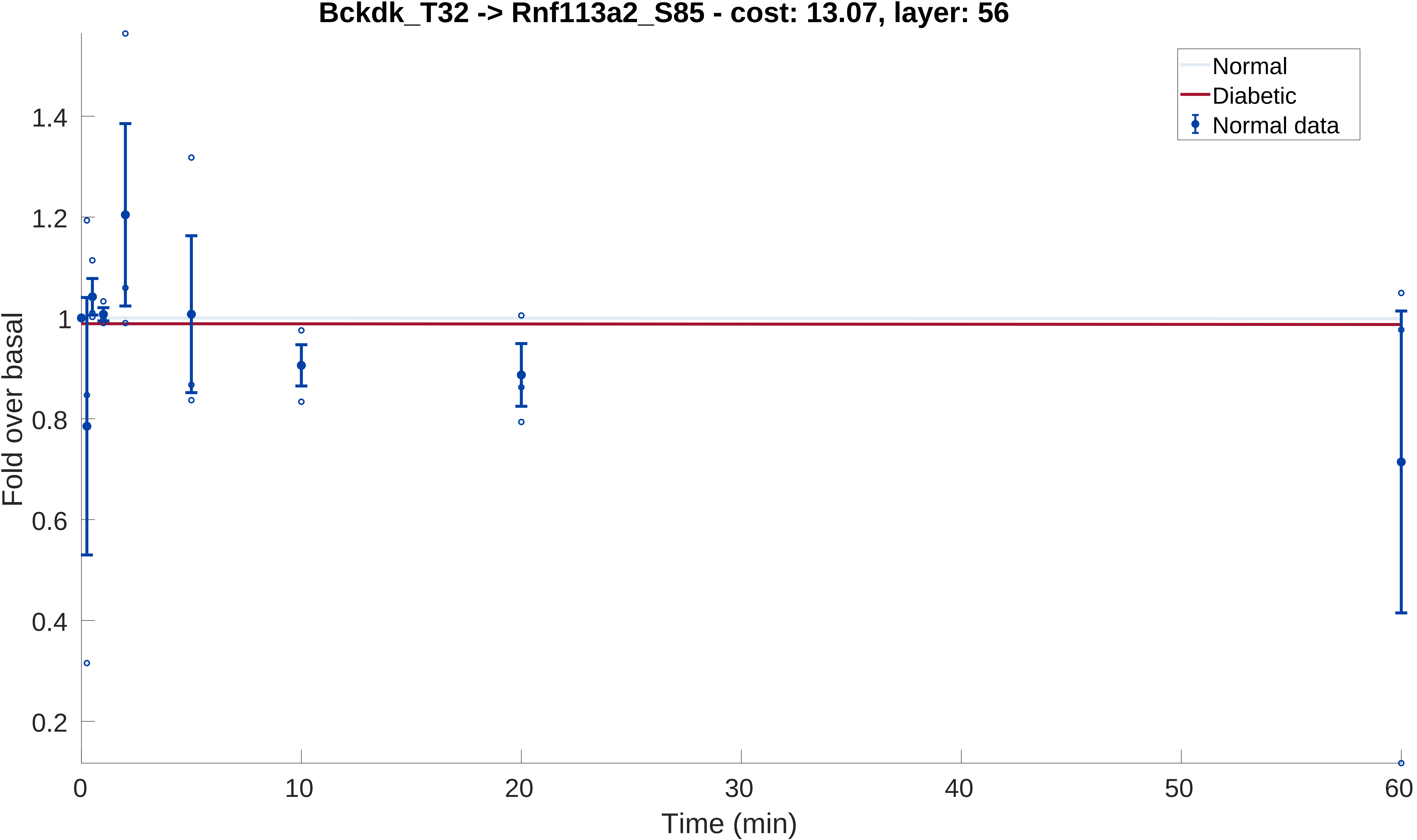
Bckdk_T32 -> Rnf113a2_S85 - cost: 13.07, layer: 56
Normal
Diabetic
Normal data
Fold over basal
1.4
1.2
1
0.8
0.6
0.4
0.2
0
10
20
30
40
50
60
Time (min)

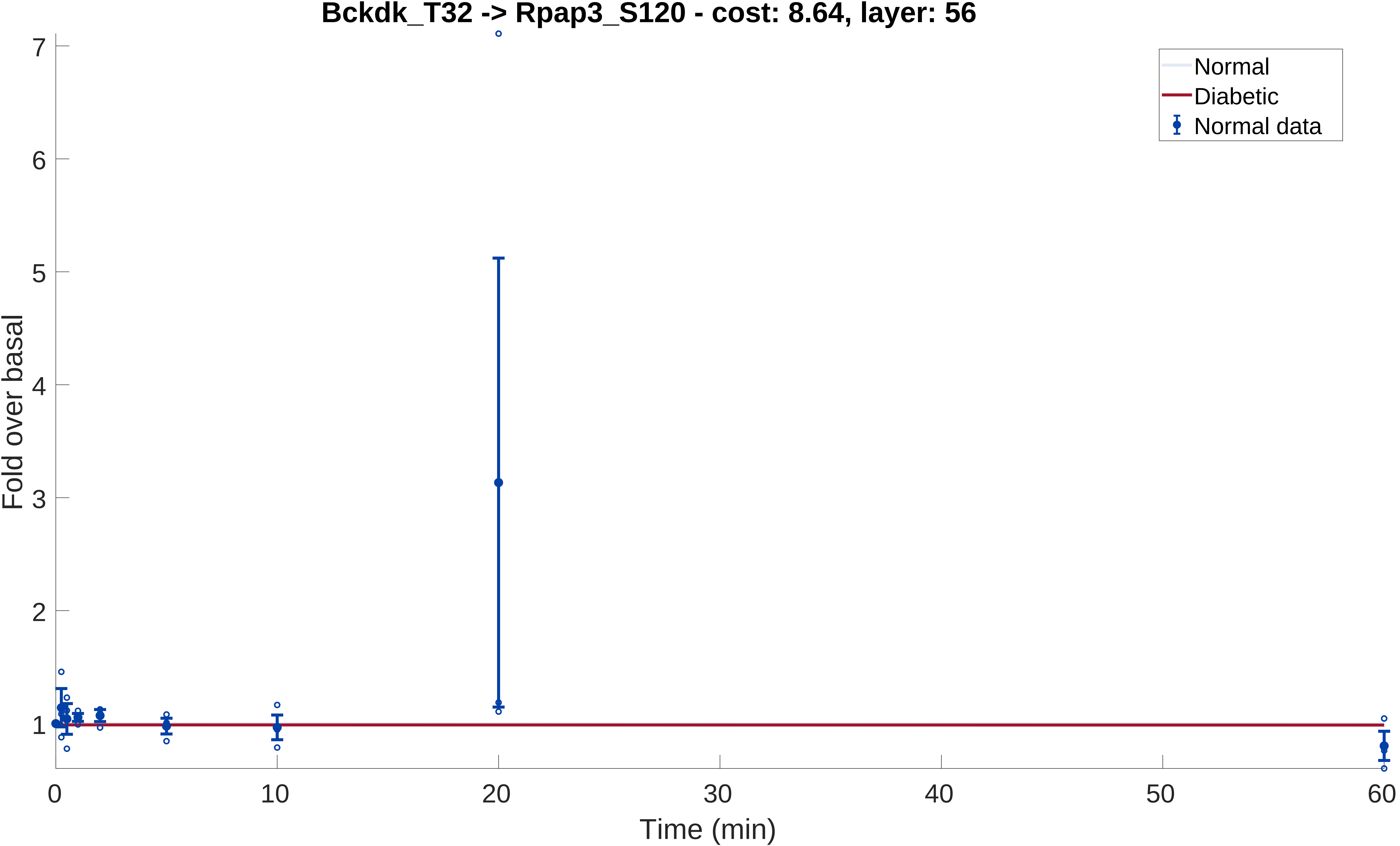

Bckdk_T32 -> Rpap3_S120 - cost: 8.64, layer: 56
Normal
Diabetic
Normal data
Fold over basal
Time (min)
0
10
20
30
40
50
60
1
2
3
4
5
6
7

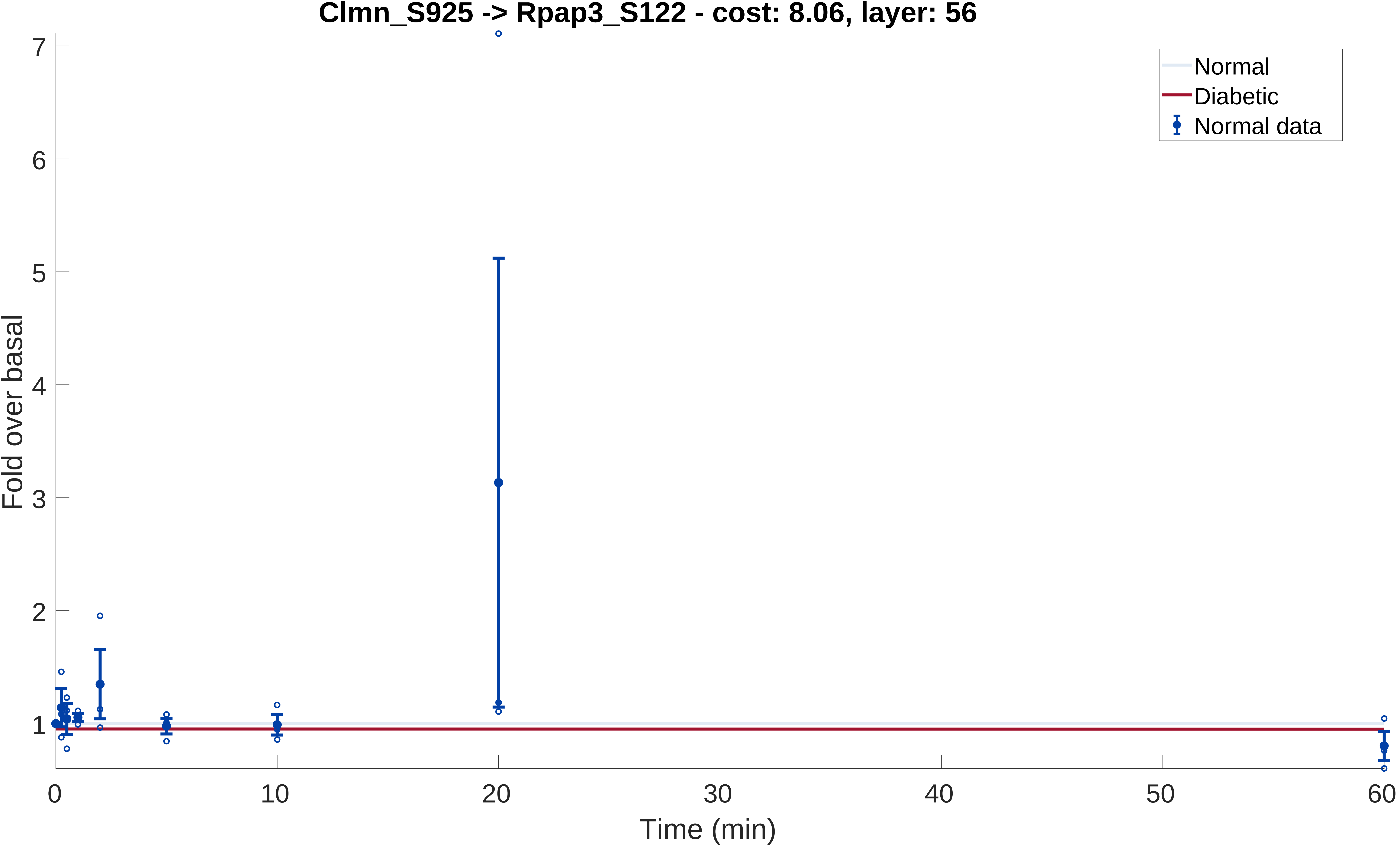

Clmn_S925 -> Rpap3_S122 - cost: 8.06, layer: 56
Normal
Diabetic
Normal data
Fold over basal
1
2
3
4
5
6
7
0
10
20
30
40
50
60
Time (min)

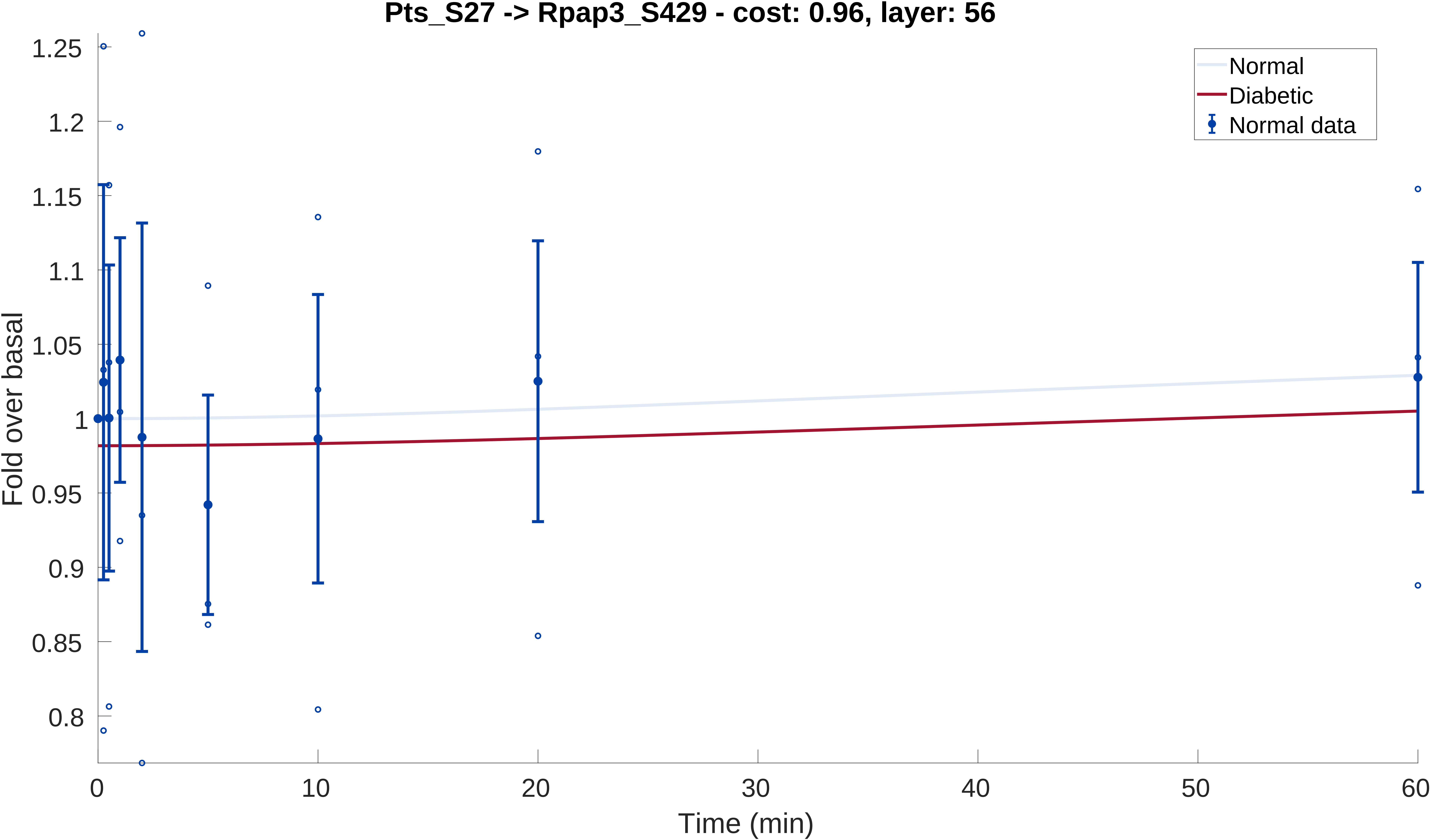

Pts_S27 -> Rpap3_S429 - cost: 0.96, layer: 56
Normal
Diabetic
Normal data
Fold over basal
1.25
1.2
1.15
1.1
1.05
1
0.95
0.9
0.85
0.8
0
10
20
30
40
50
60
Time (min)

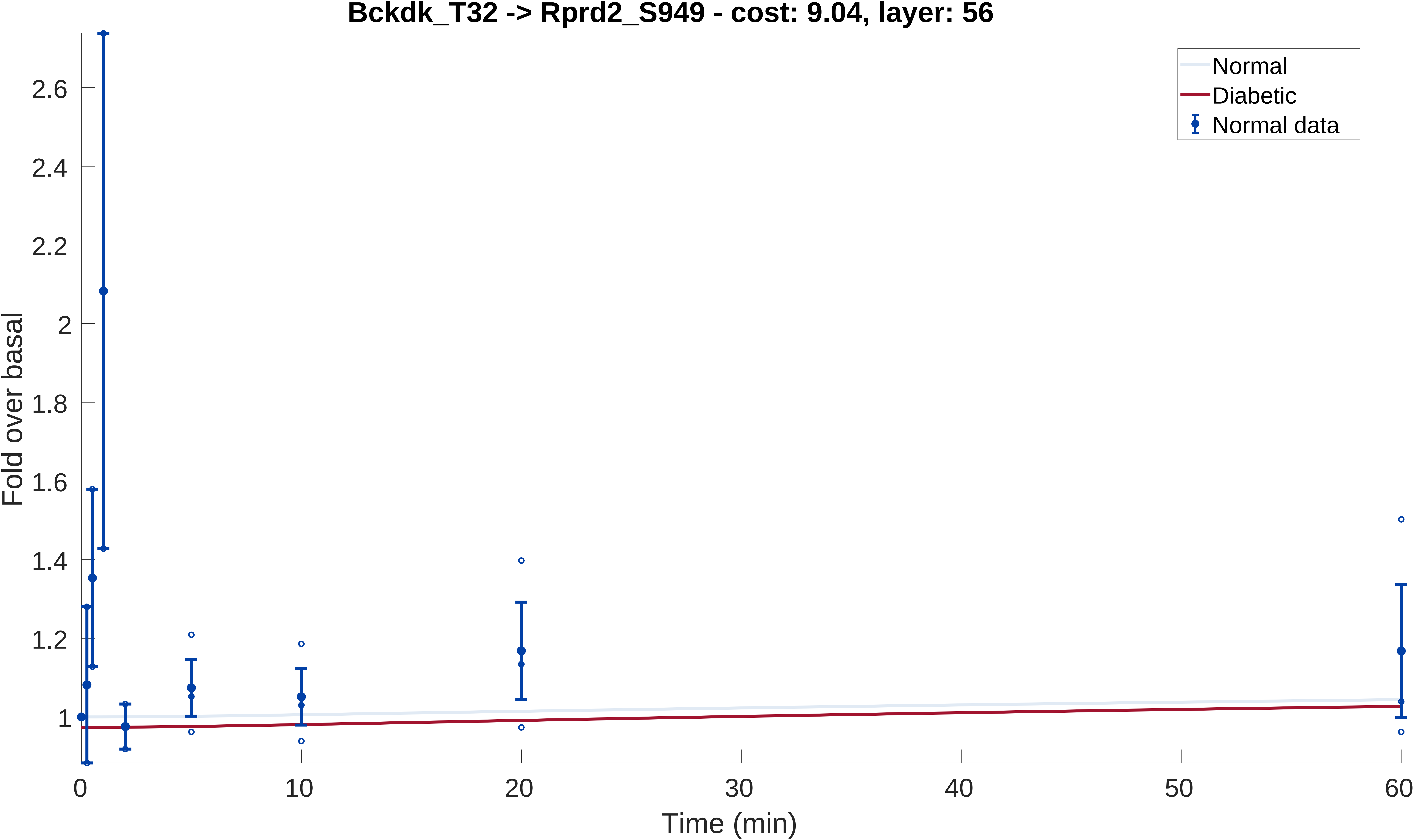

Bckdk_T32 -> Rprd2_S949 - cost: 9.04, layer: 56
Normal
Diabetic
Normal data
Fold over basal
2.6
2.4
2.2
2
1.8
1.6
1.4
1.2
1
0
10
20
30
40
50
60
Time (min)

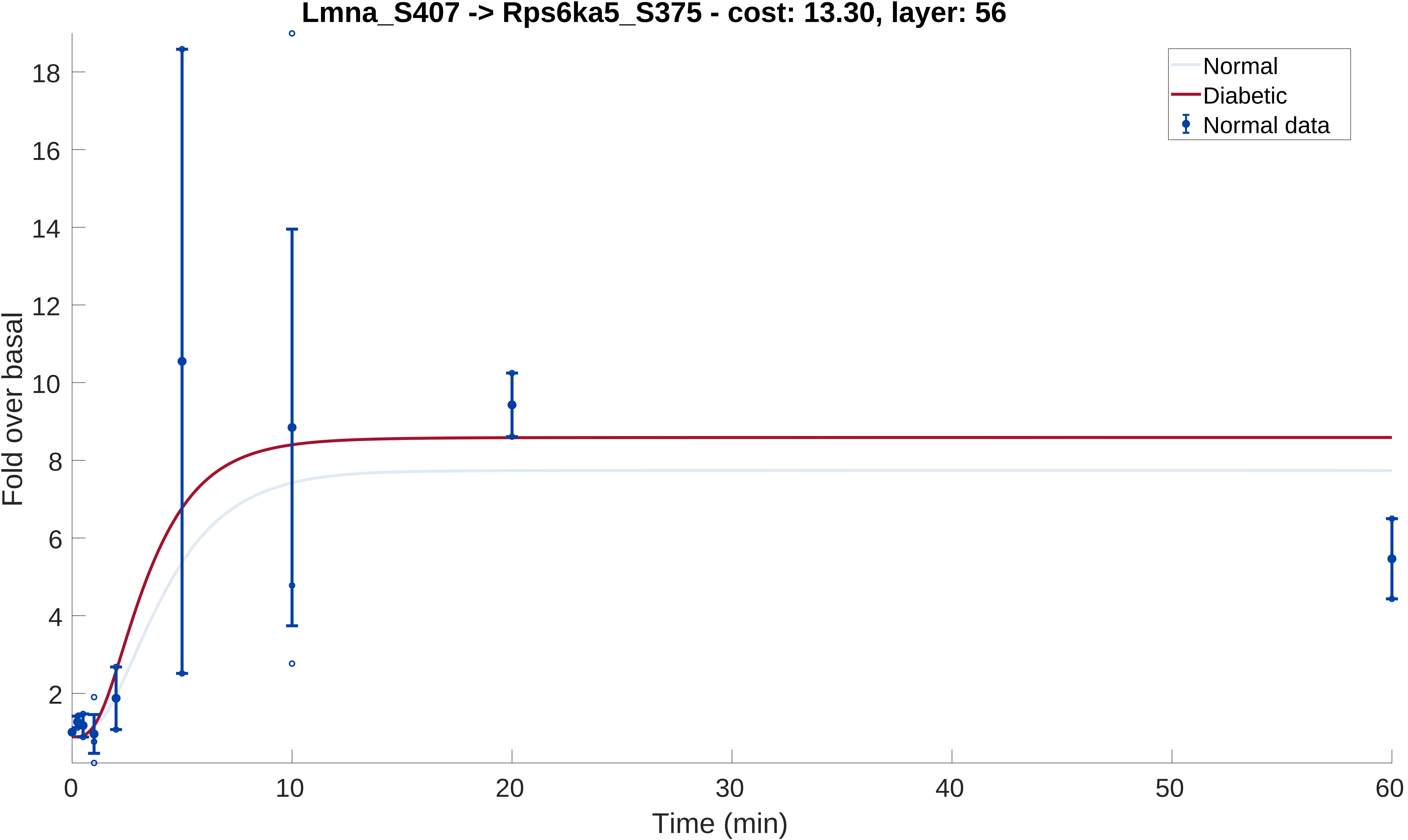
Lmna_S407 -> Rps6ka5_S375 - cost: 13.30, layer: 56
Fold over basal
Time (min)
Normal
Diabetic
Normal data
0
10
20
30
40
50
60
2
4
6
8
10
12
14
16
18

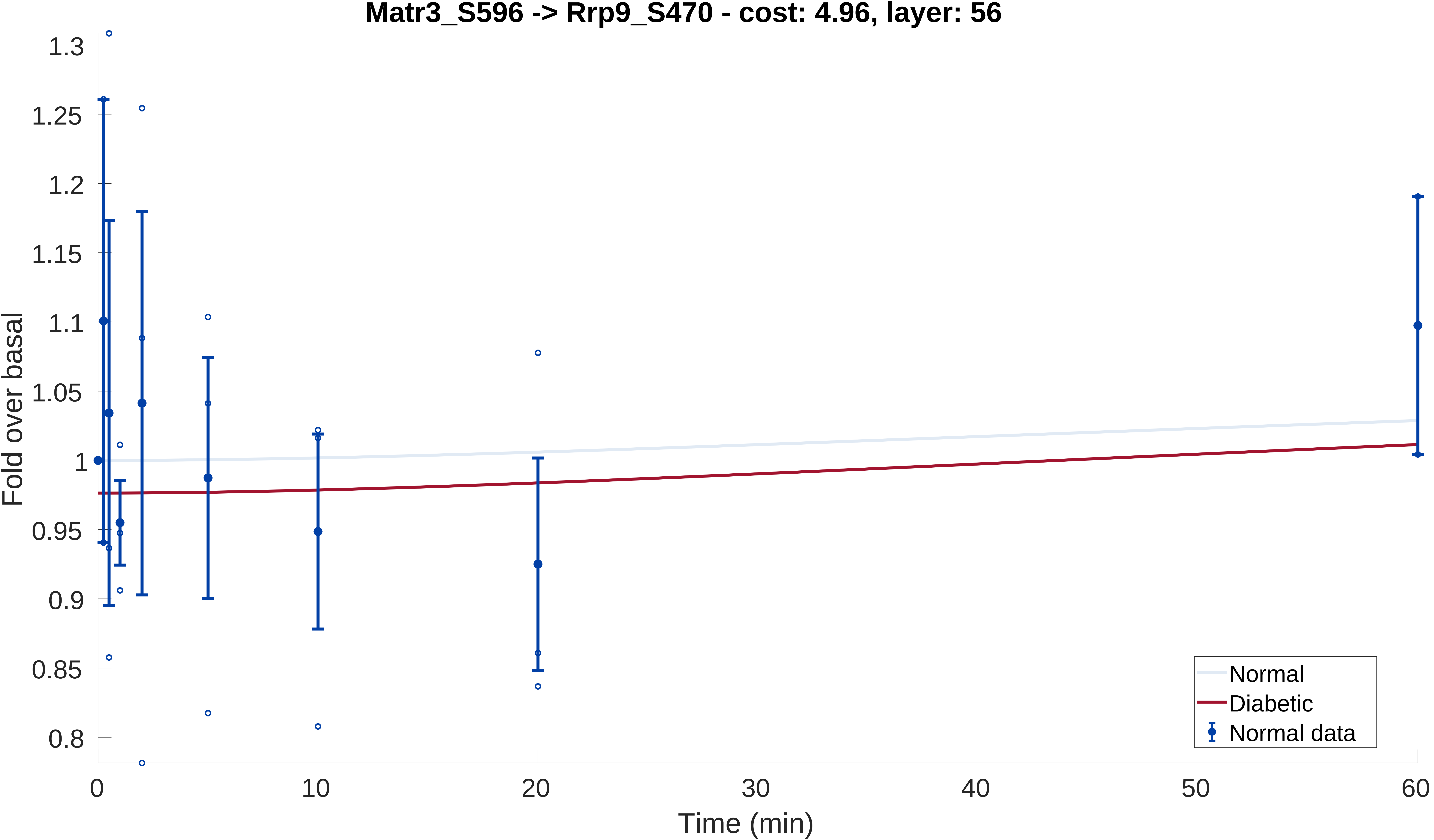
Matr3_S596 -> Rrp9_S470 - cost: 4.96, layer: 56
Fold over basal
1.3
1.25
1.2
1.15
1.1
1.05
1
0.95
0.9
0.85
0.8
0
10
20
30
40
50
60
Time (min)
Normal
Diabetic
Normal data

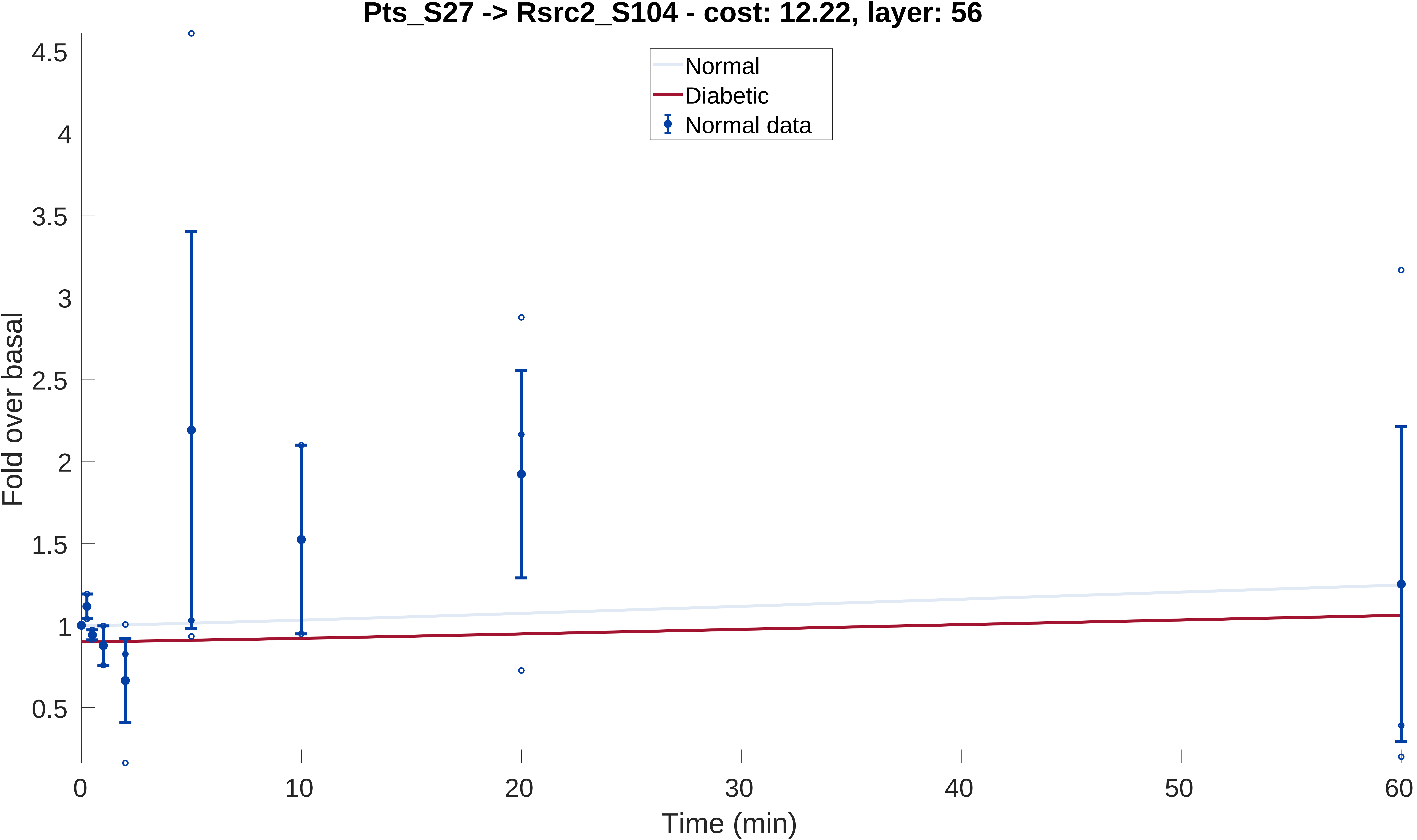

Pts_S27 -> Rsrc2_S104 - cost: 12.22, layer: 56
Normal
Diabetic
Normal data
4.5
4
3.5
3
2.5
2
1.5
1
0.5
Fold over basal
0
10
20
30
40
50
60
Time (min)

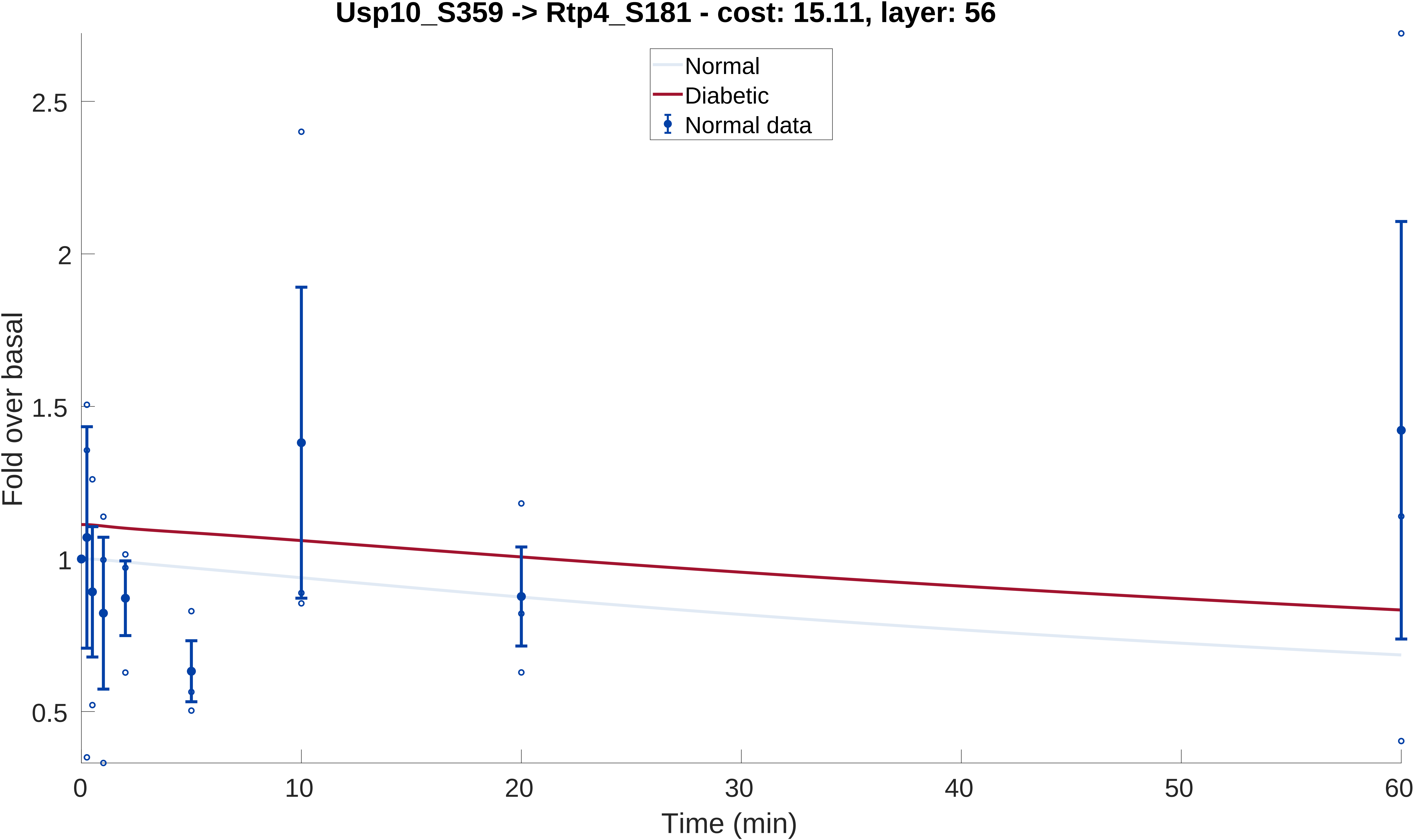
Usp10_S359 -> Rtp4_S181 - cost: 15.11, layer: 56
Normal
Diabetic
Normal data
Fold over basal
2.5
2
1.5
1
0.5
0
10
20
30
40
50
60
Time (min)

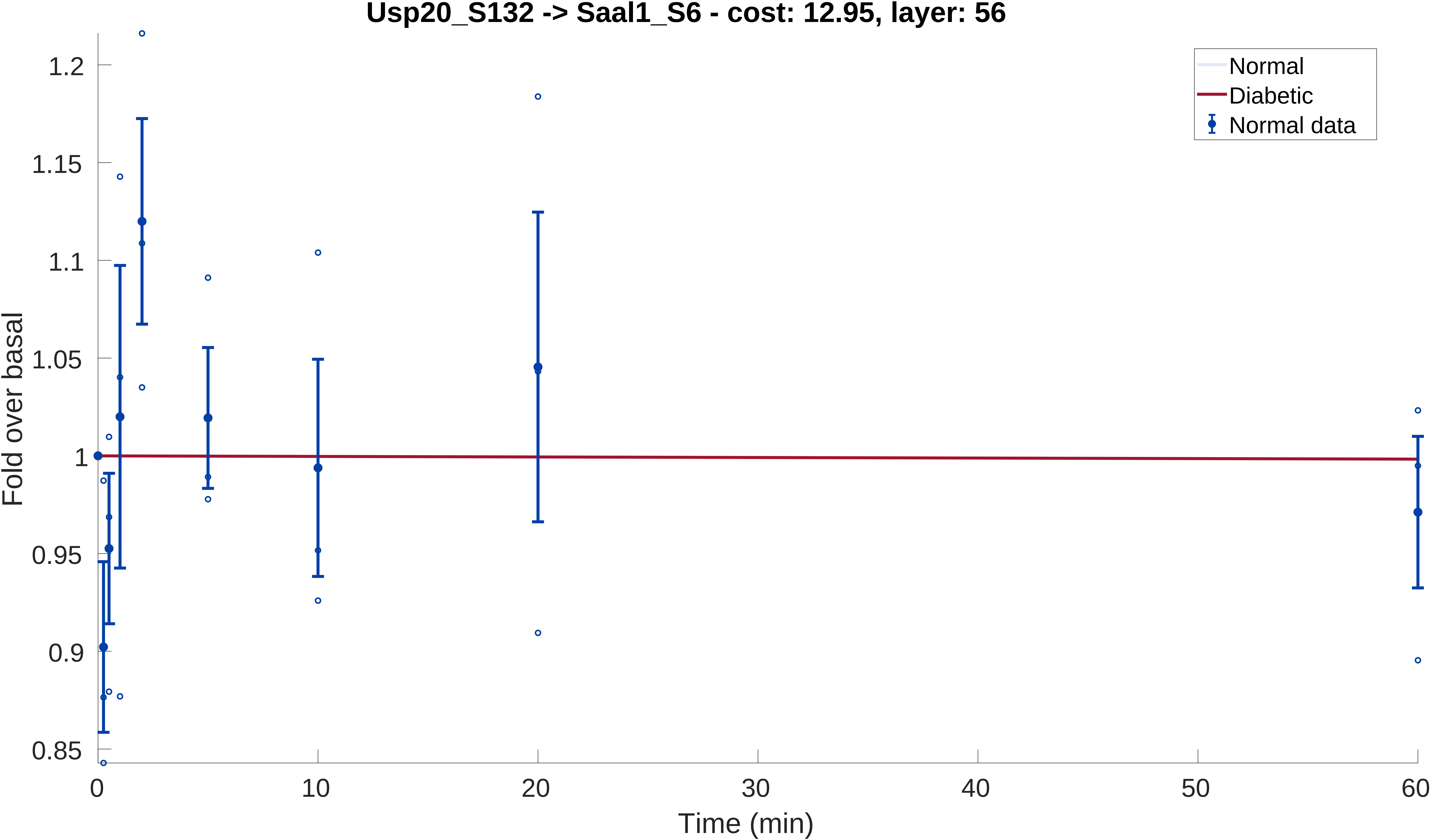
Usp20_S132 -> Saal1_S6 - cost: 12.95, layer: 56
Normal
Diabetic
Normal data
Fold over basal
1.2
1.15
1.1
1.05
1
0.95
0.9
0.85
0
10
20
30
40
50
60
Time (min)

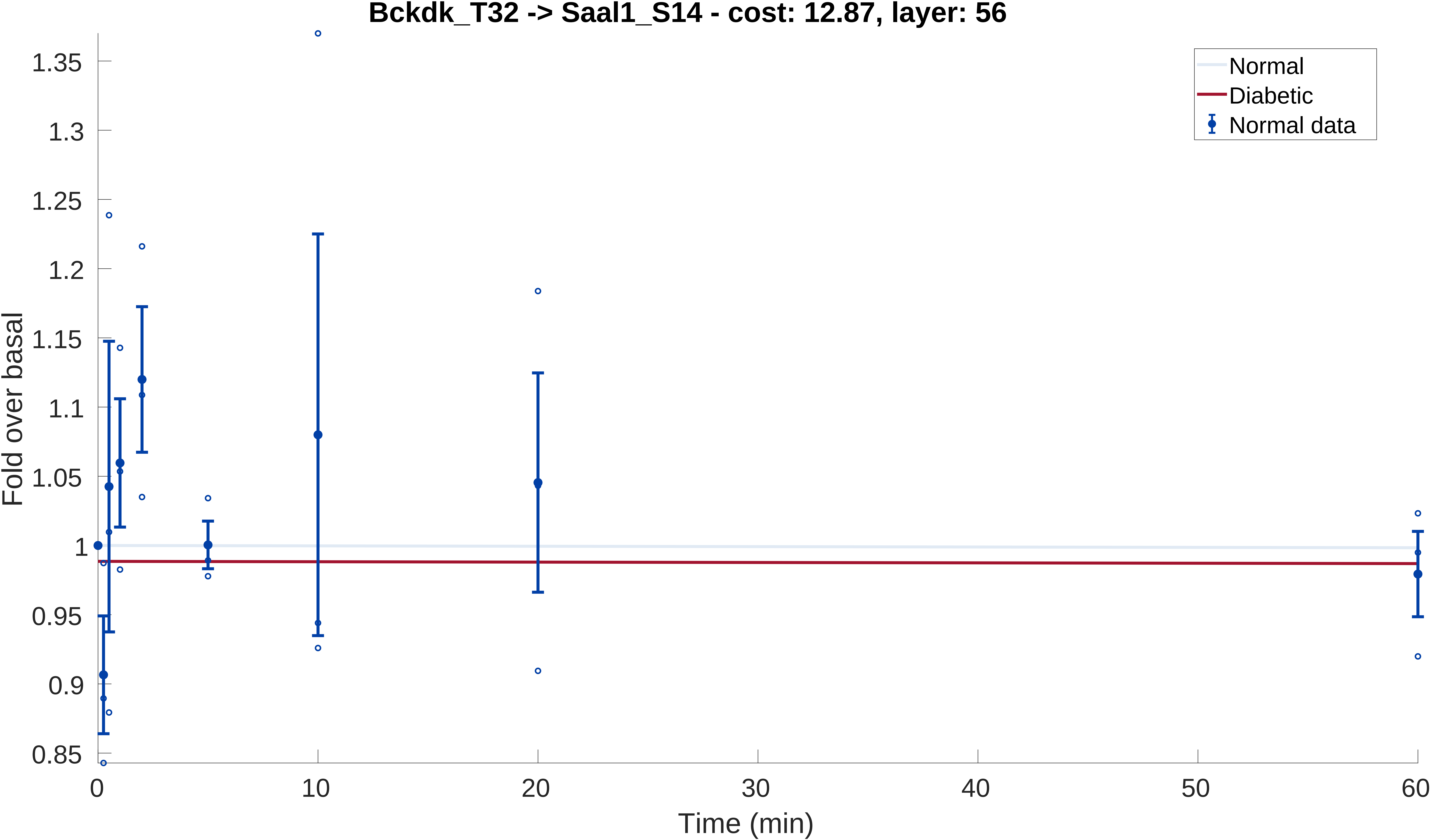

Bckdk_T32 -> Saal1_S14 - cost: 12.87, layer: 56
Fold over basal
1.35
1.3
1.25
1.2
1.15
1.1
1.05
1
0.95
0.9
0.85
0
10
20
30
40
50
60
Time (min)
Normal
Diabetic
Normal data

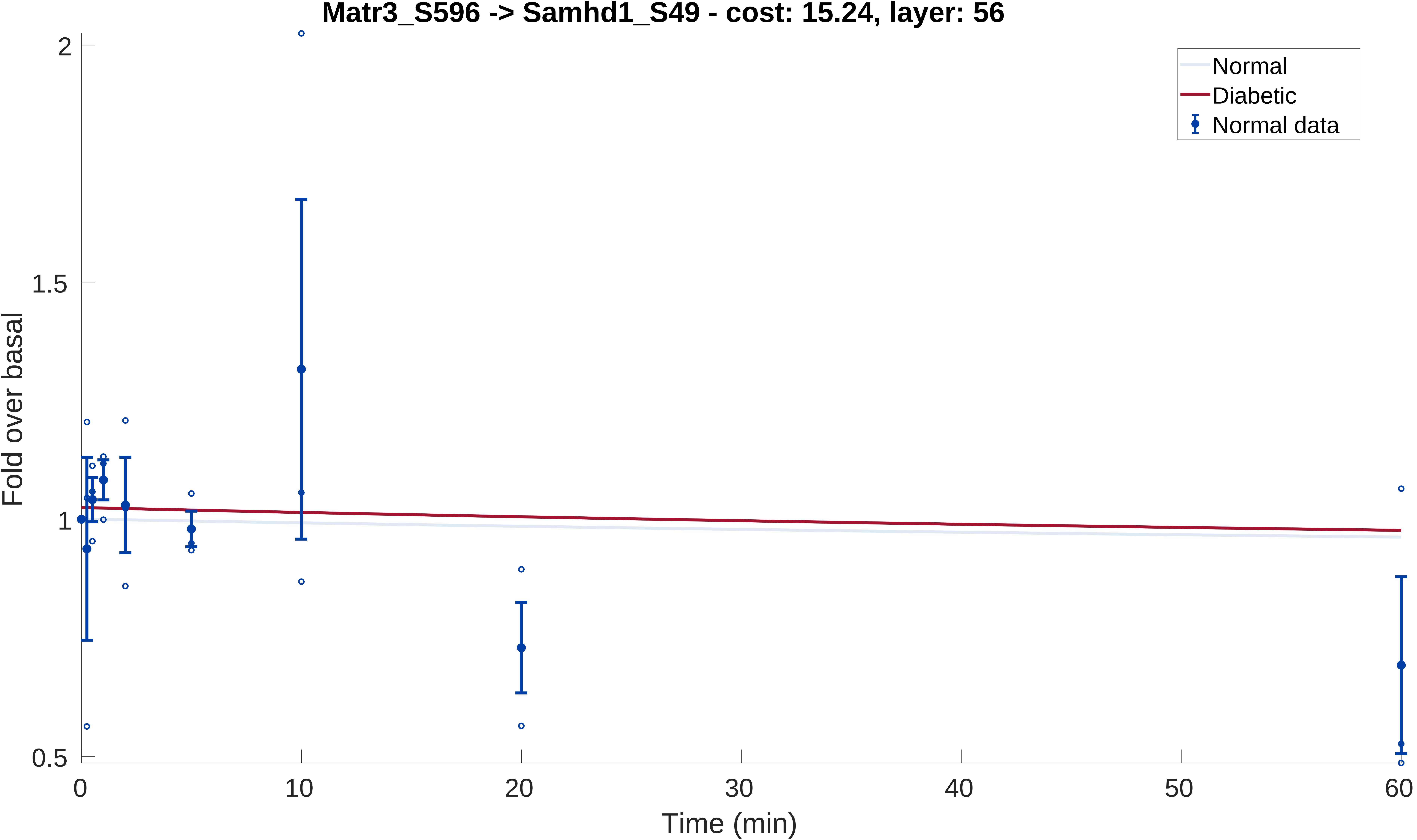
Matr3_S596 -> Samhd1_S49 - cost: 15.24, layer: 56
Fold over basal
Time (min)
Normal
Diabetic
Normal data
2
1.5
1
0.5
0
10
20
30
40
50
60

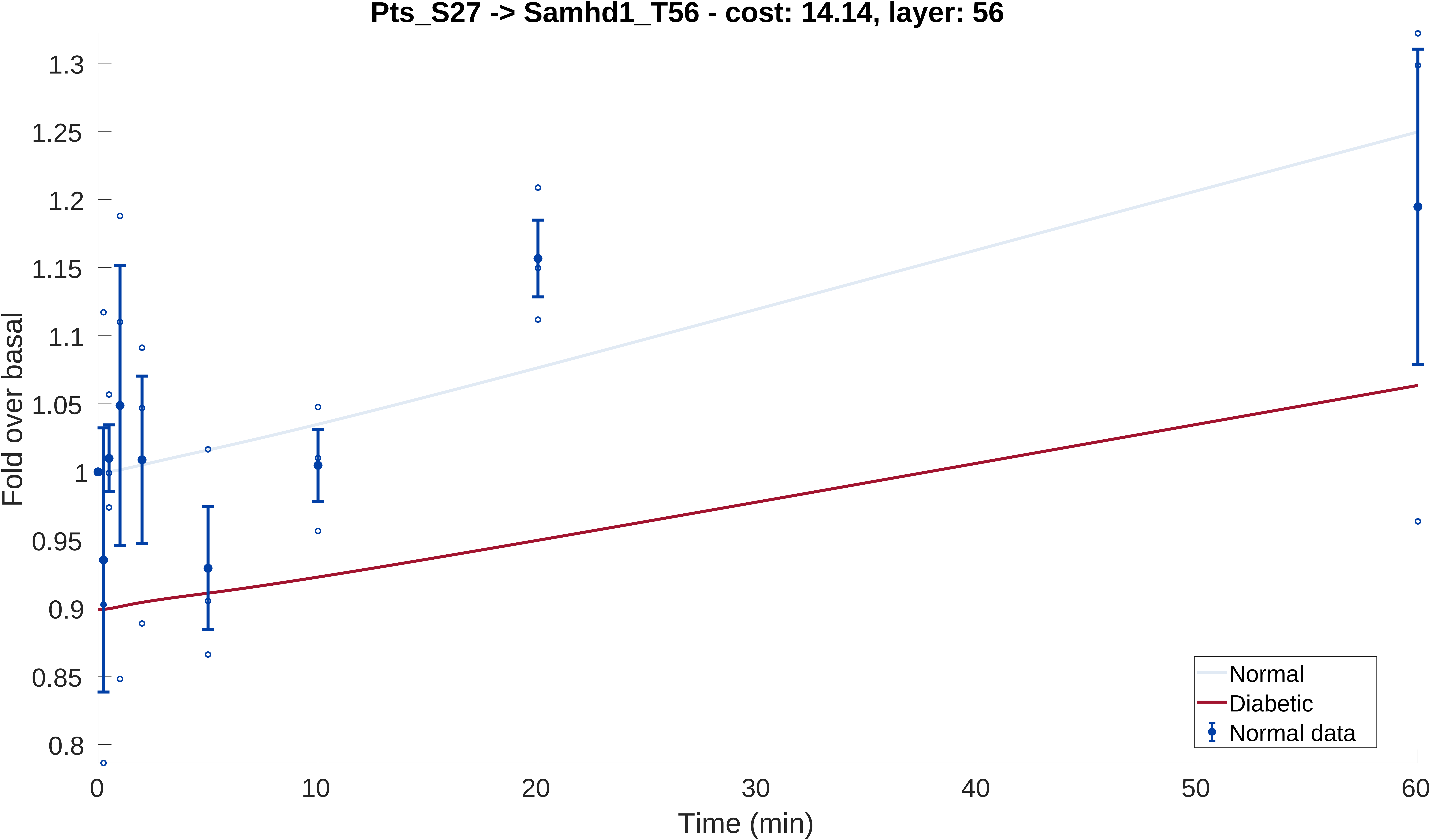

Pts_S27 -> Samhd1_T56 - cost: 14.14, layer: 56
Fold over basal
1.3
1.25
1.2
1.15
1.1
1.05
1
0.95
0.9
0.85
0.8
0
10
20
30
40
50
60
Time (min)
Normal
Diabetic
Normal data

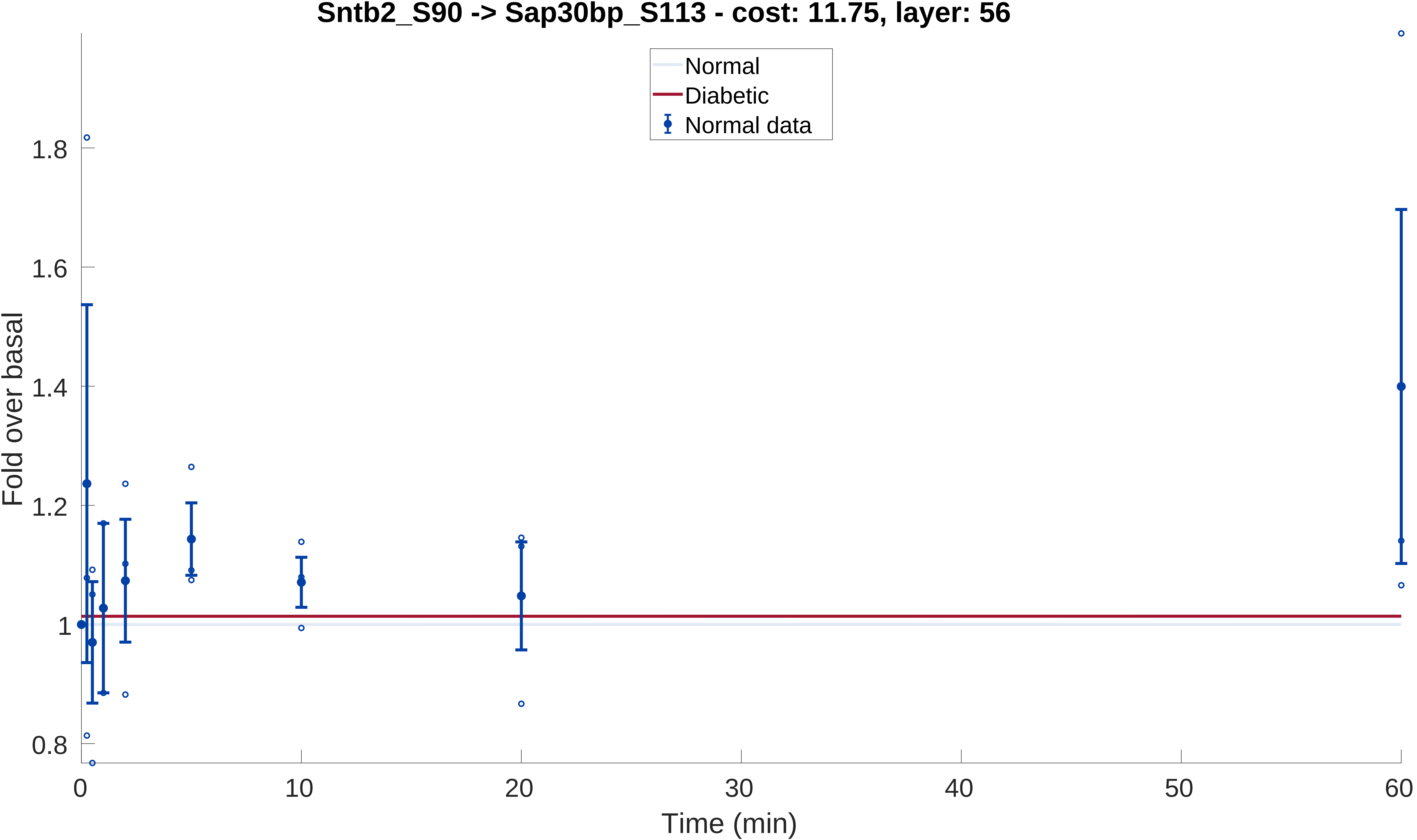

Sntb2_S90 -> Sap30bp_S113 - cost: 11.75, layer: 56
Normal
Diabetic
Normal data
Fold over basal
Time (min)
1.8
1.6
1.4
1.2
1
0.8
0
10
20
30
40
50
60

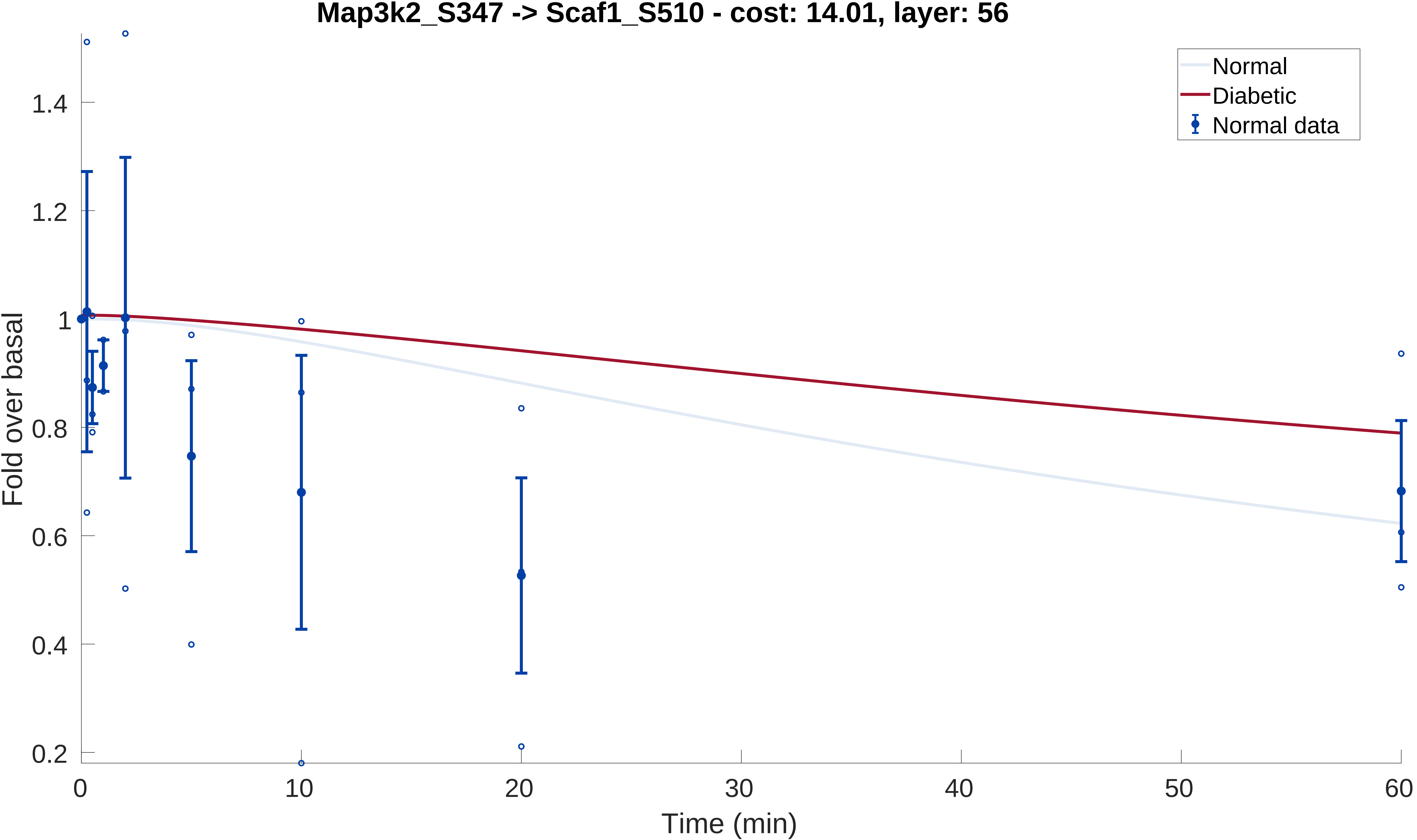

Map3k2_S347 -> Scaf1_S510 - cost: 14.01, layer: 56
Normal
Diabetic
Normal data
Fold over basal
Time (min)
0.2
0.4
0.6
0.8
1
1.2
1.4
0
10
20
30
40
50
60

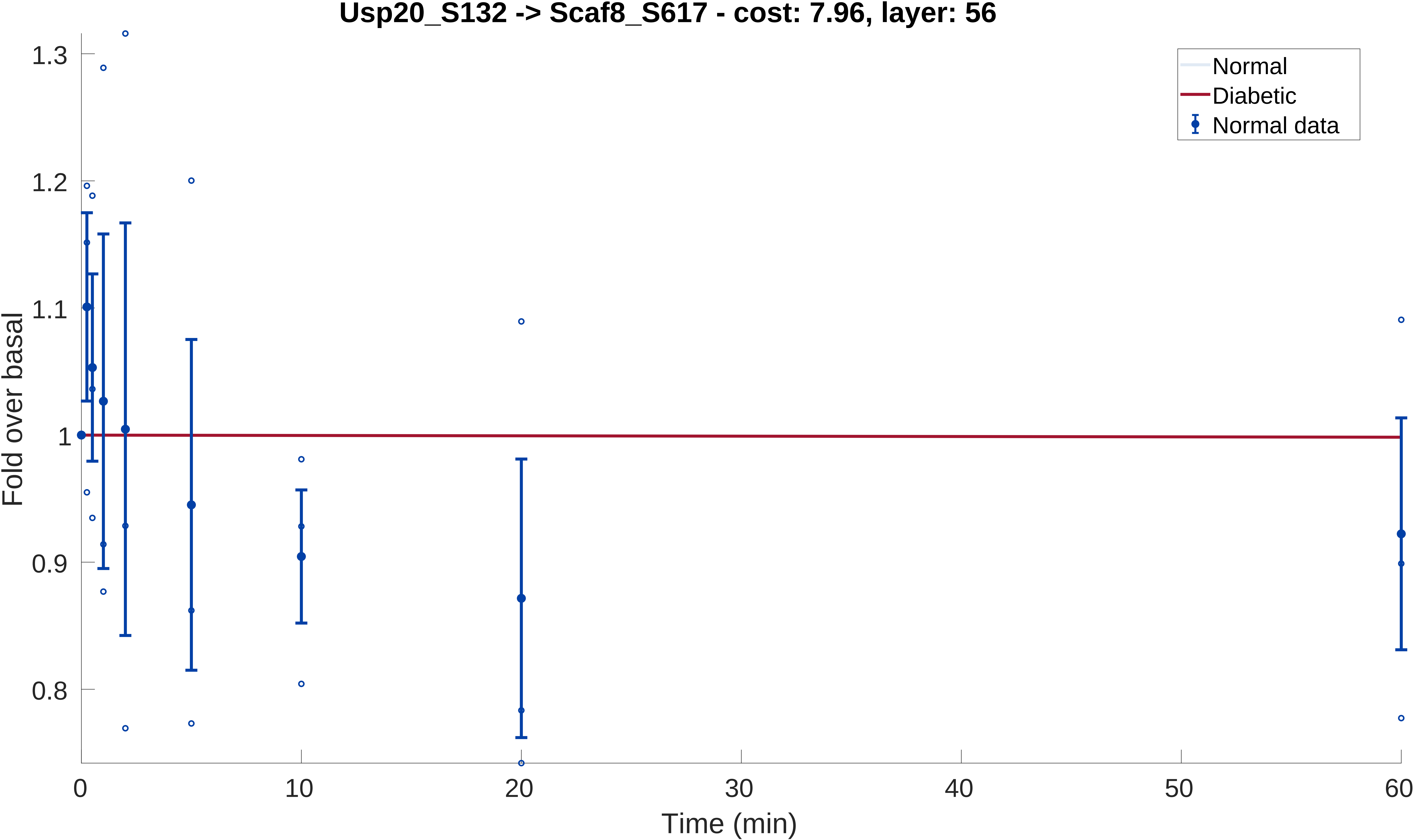
Usp20_S132 -> Scaf8_S617 - cost: 7.96, layer: 56
Normal
Diabetic
Normal data
Fold over basal
Time (min)
1.3
1.2
1.1
1
0.9
0.8
0
10
20
30
40
50
60

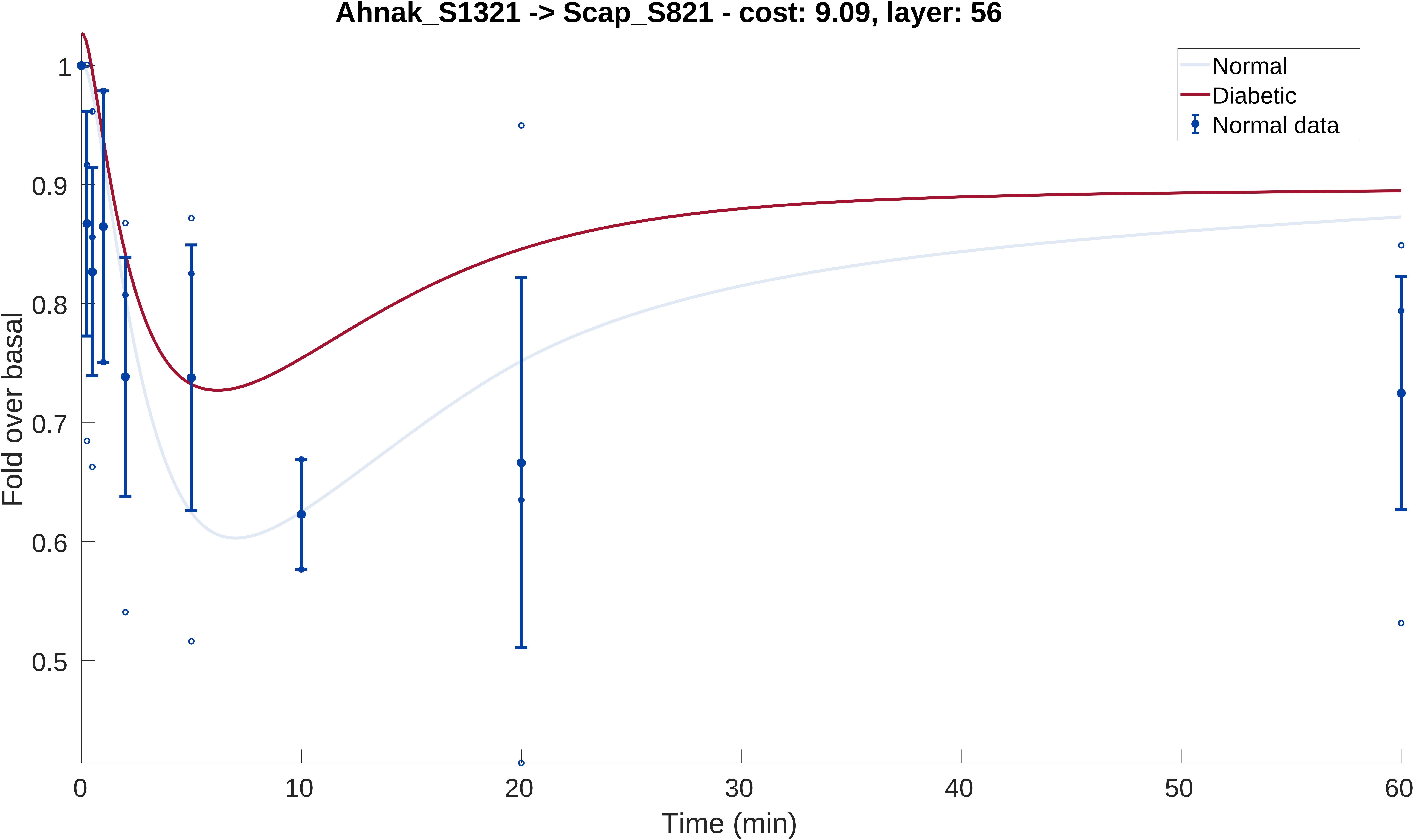

Ahnak_S1321 -> Scap_S821 - cost: 9.09, layer: 56
Normal
Diabetic
Normal data
Fold over basal
Time (min)
1
0.9
0.8
0.7
0.6
0.5
0
10
20
30
40
50
60

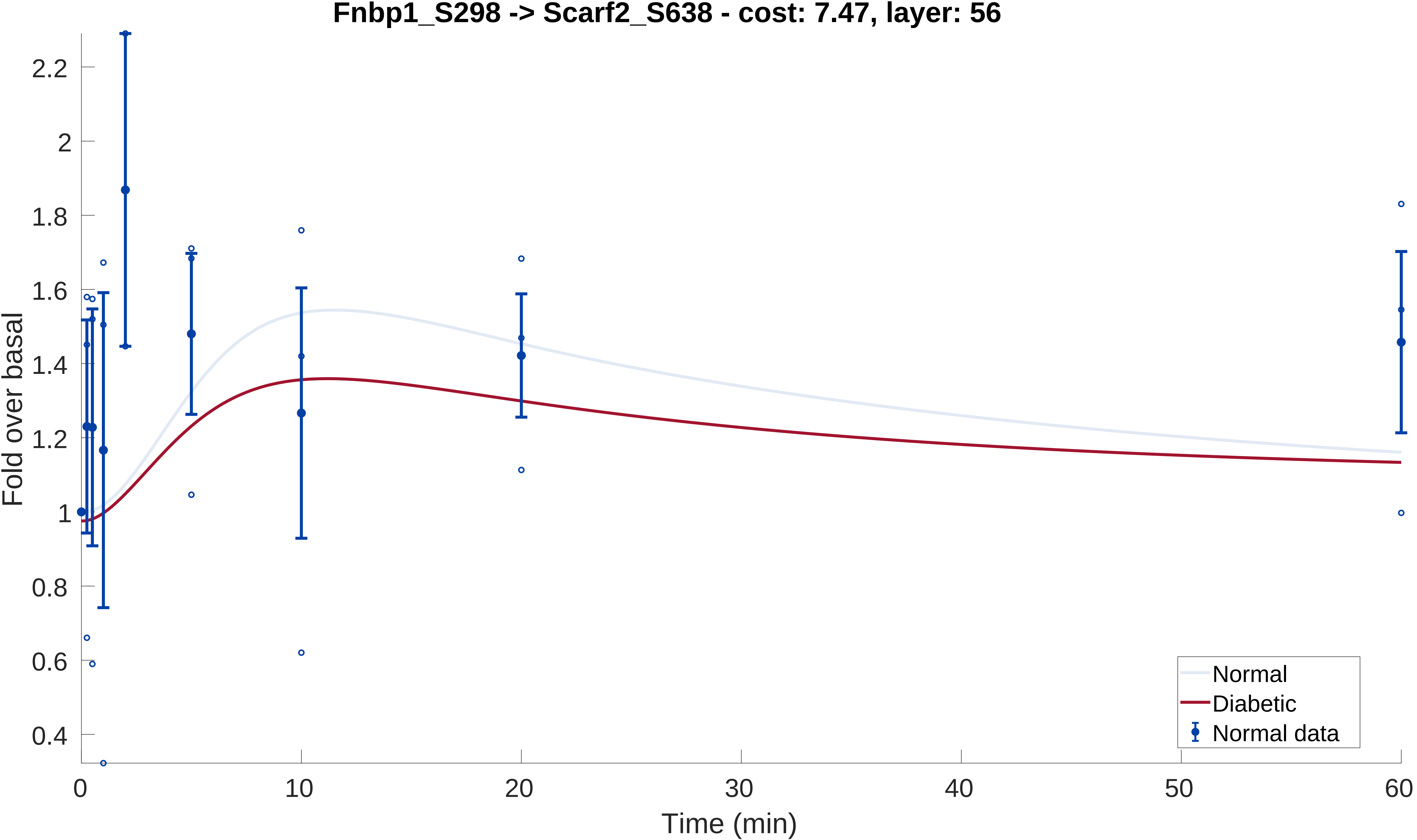
Fnbp1_S298 -> Scarf2_S638 - cost: 7.47, layer: 56
Fold over basal
Time (min)
Normal
Diabetic
Normal data
0.4
0.6
0.8
1
1.2
1.4
1.6
1.8
2
2.2
0
10
20
30
40
50
60

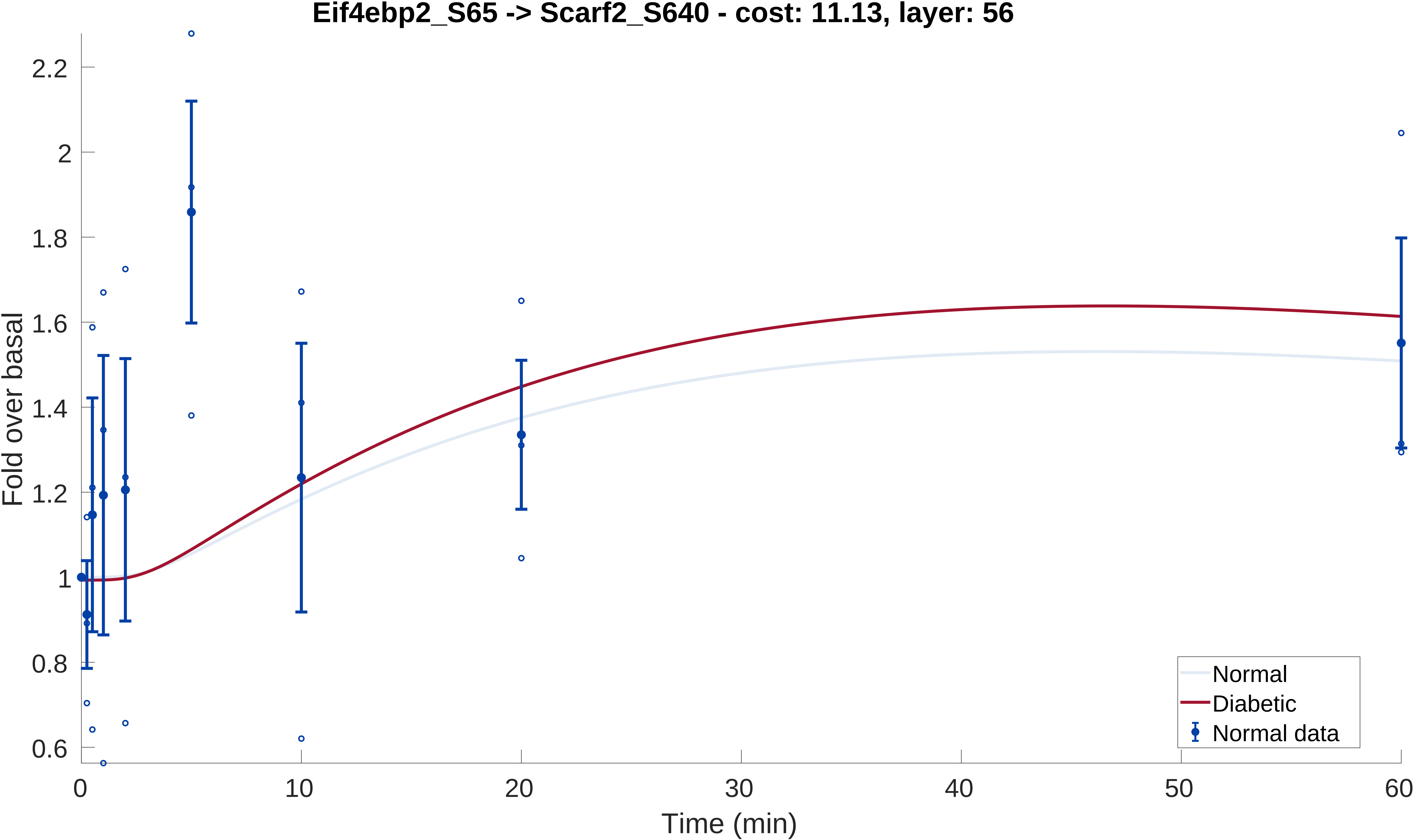

Eif4ebp2_S65 -> Scarf2_S640 - cost: 11.13, layer: 56
Fold over basal
2.2
2
1.8
1.6
1.4
1.2
1
0.8
0.6
0
10
20
30
40
50
60
Time (min)
Normal
Diabetic
Normal data

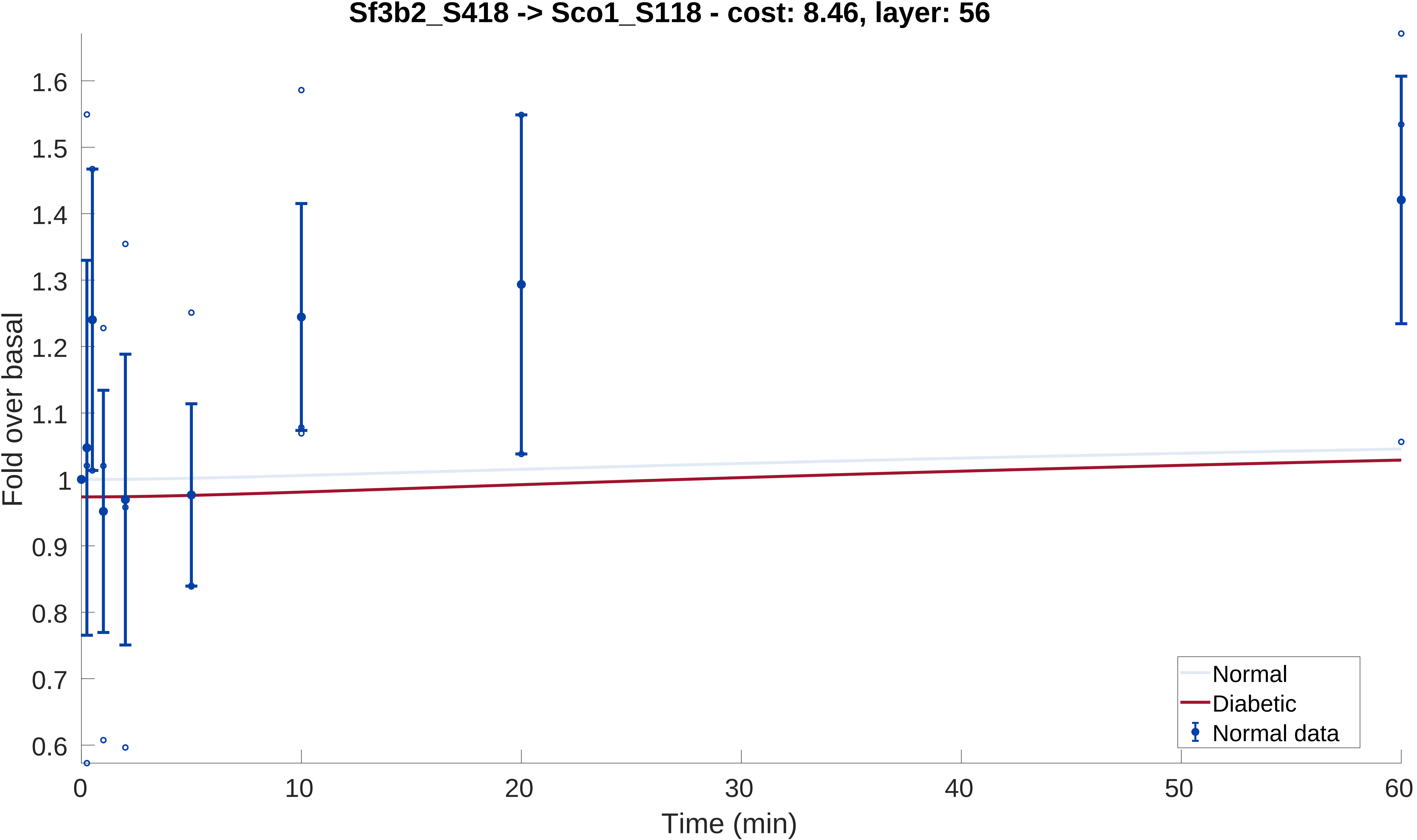
Sf3b2_S418 -> Sco1_S118 - cost: 8.46, layer: 56
Fold over basal
1.6
1.5
1.4
1.3
1.2
1.1
1
0.9
0.8
0.7
0.6
0
10
20
30
40
50
60
Time (min)
Normal
Diabetic
Normal data

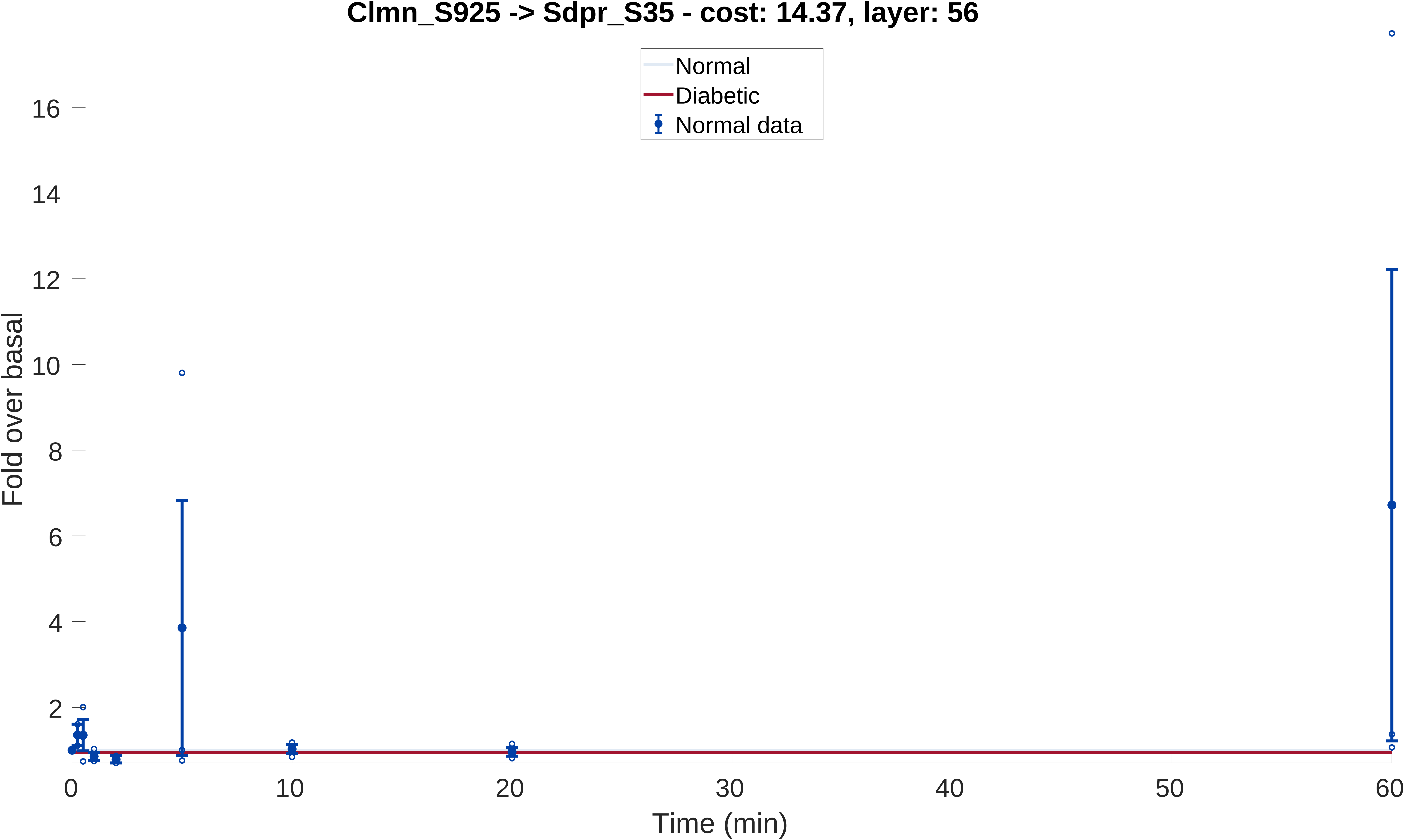

Clmn_S925 -> Sdpr_S35 - cost: 14.37, layer: 56
Normal
Diabetic
Normal data
Fold over basal
Time (min)
16
14
12
10
8
6
4
2
0
10
20
30
40
50
60

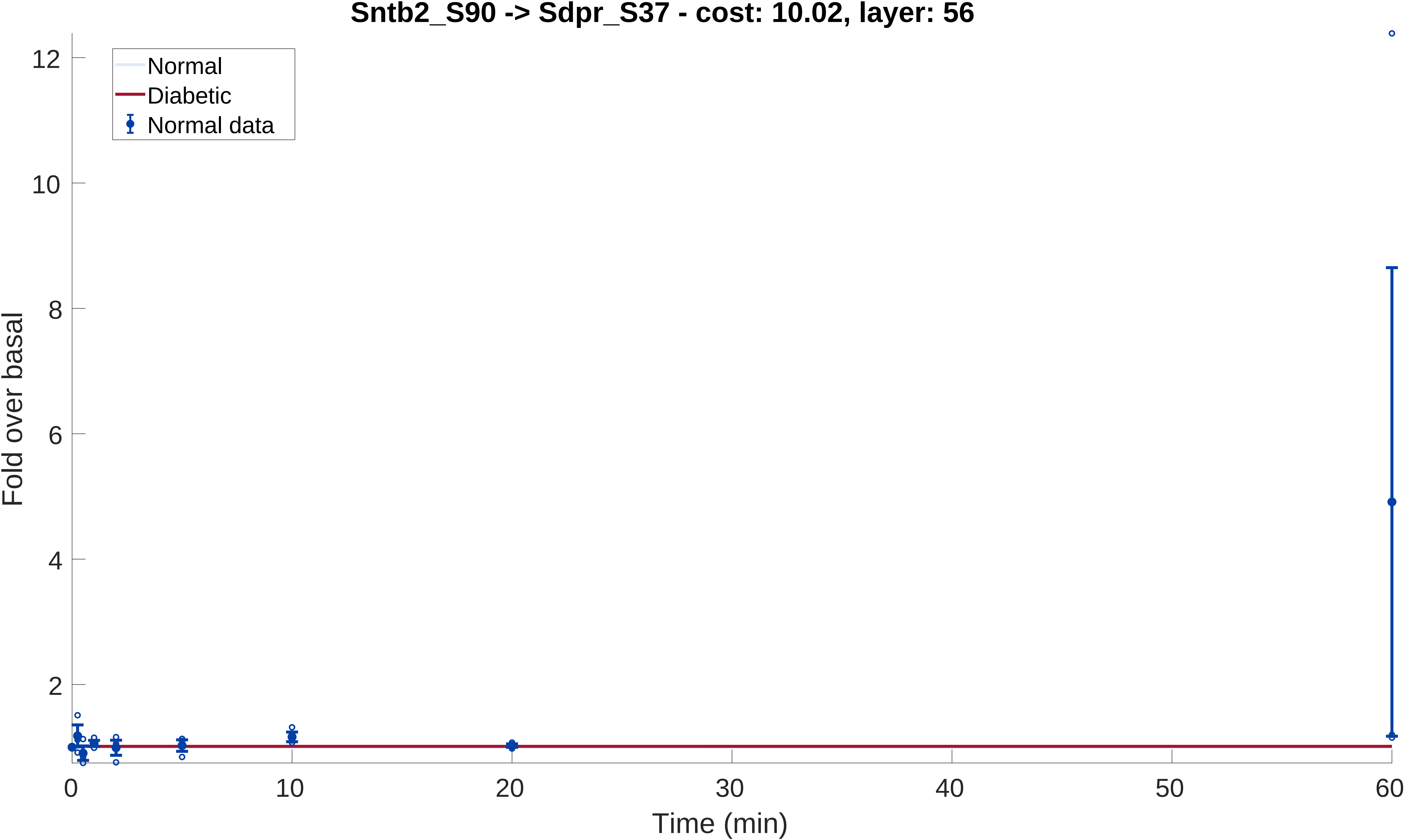

Sntb2_S90 -> Sdpr_S37 - cost: 10.02, layer: 56
Normal
Diabetic
Normal data
Fold over basal
Time (min)
0
10
20
30
40
50
60
2
4
6
8
10
12

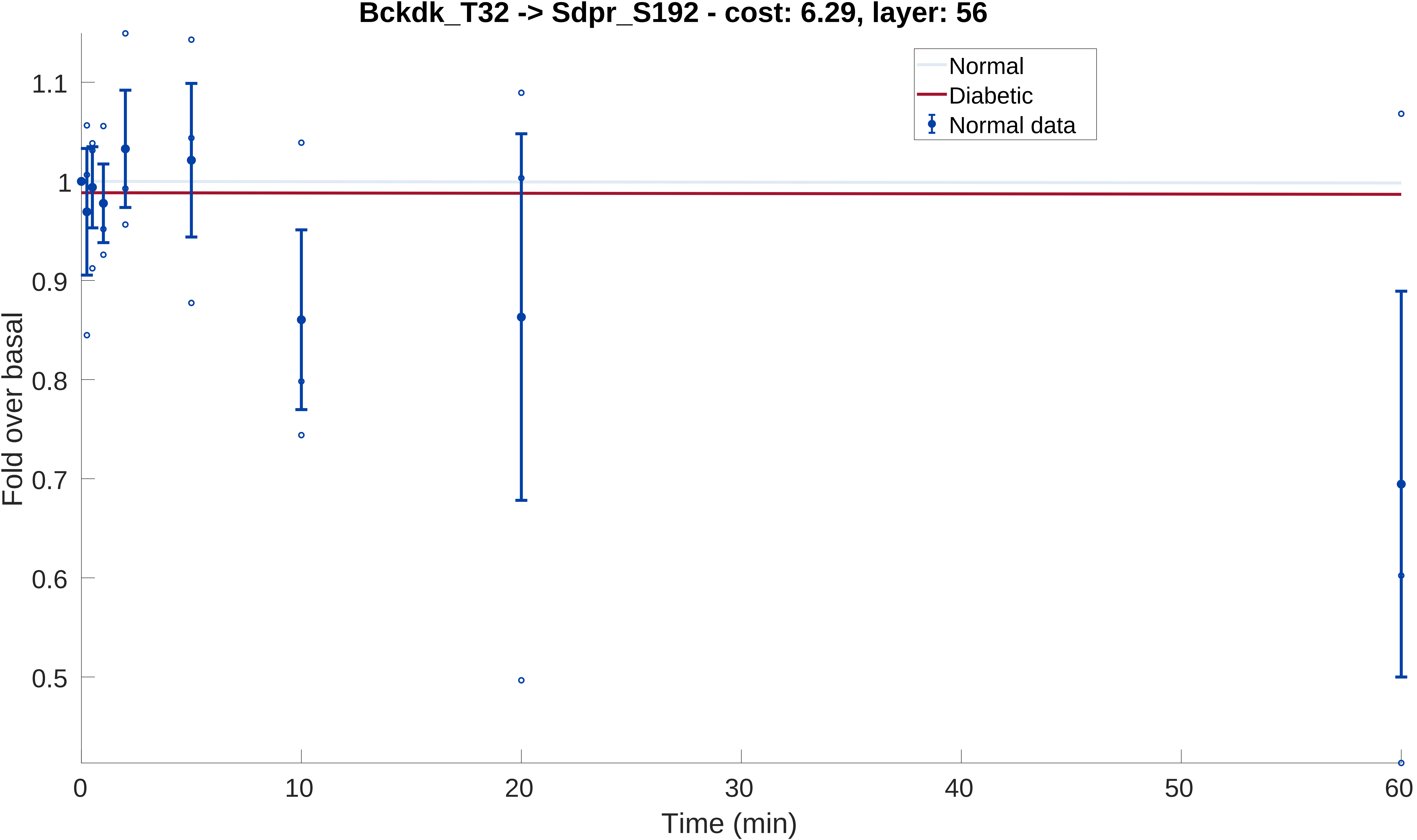

Bckdk_T32 -> Sdpr_S192 - cost: 6.29, layer: 56
Normal
Diabetic
Normal data
Fold over basal
1.1
1
0.9
0.8
0.7
0.6
0.5
0
10
20
30
40
50
60
Time (min)

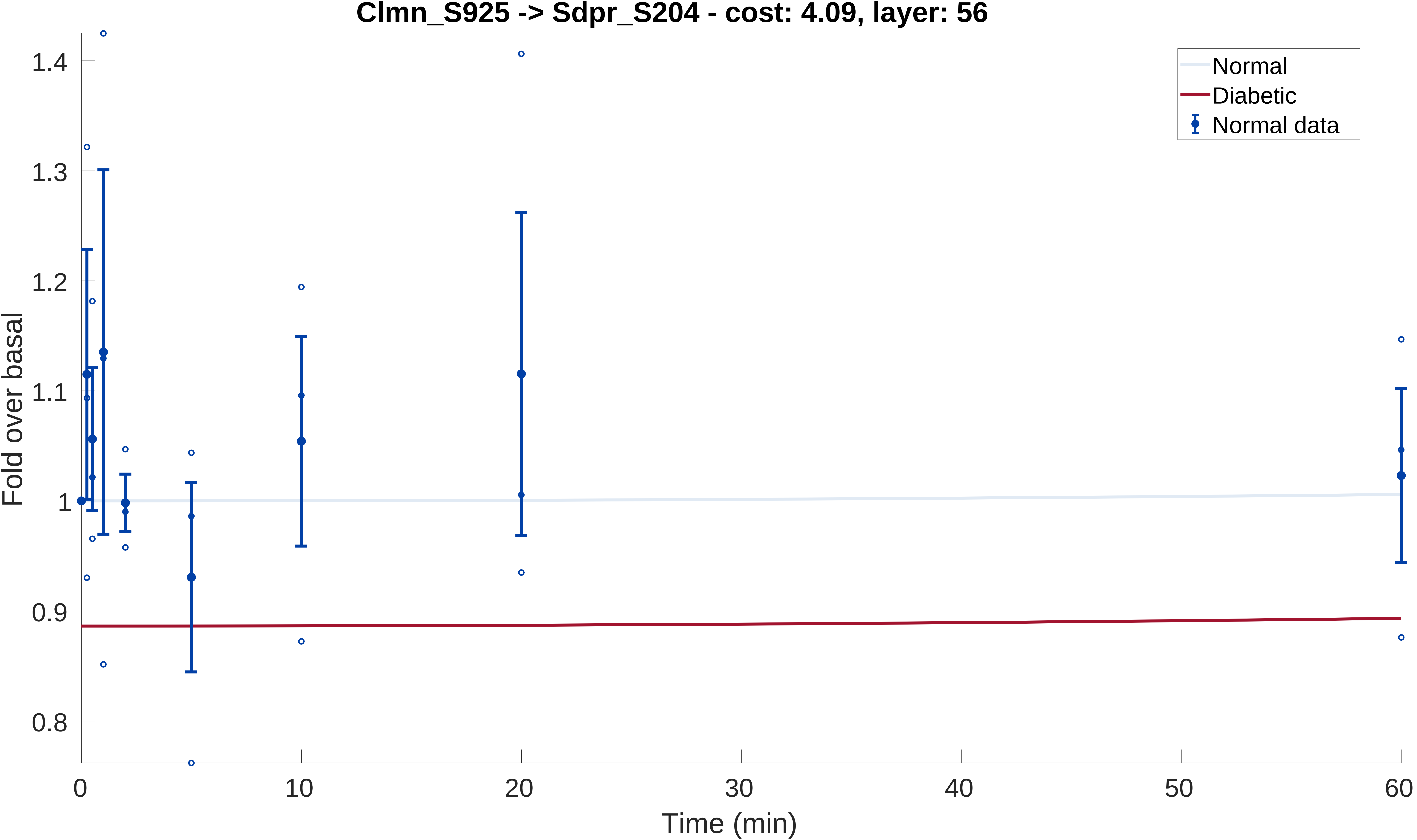

Clmn_S925 -> Sdpr_S204 - cost: 4.09, layer: 56
Normal
Diabetic
Normal data
Fold over basal
Time (min)
0.8
0.9
1
1.1
1.2
1.3
1.4
0
10
20
30
40
50
60

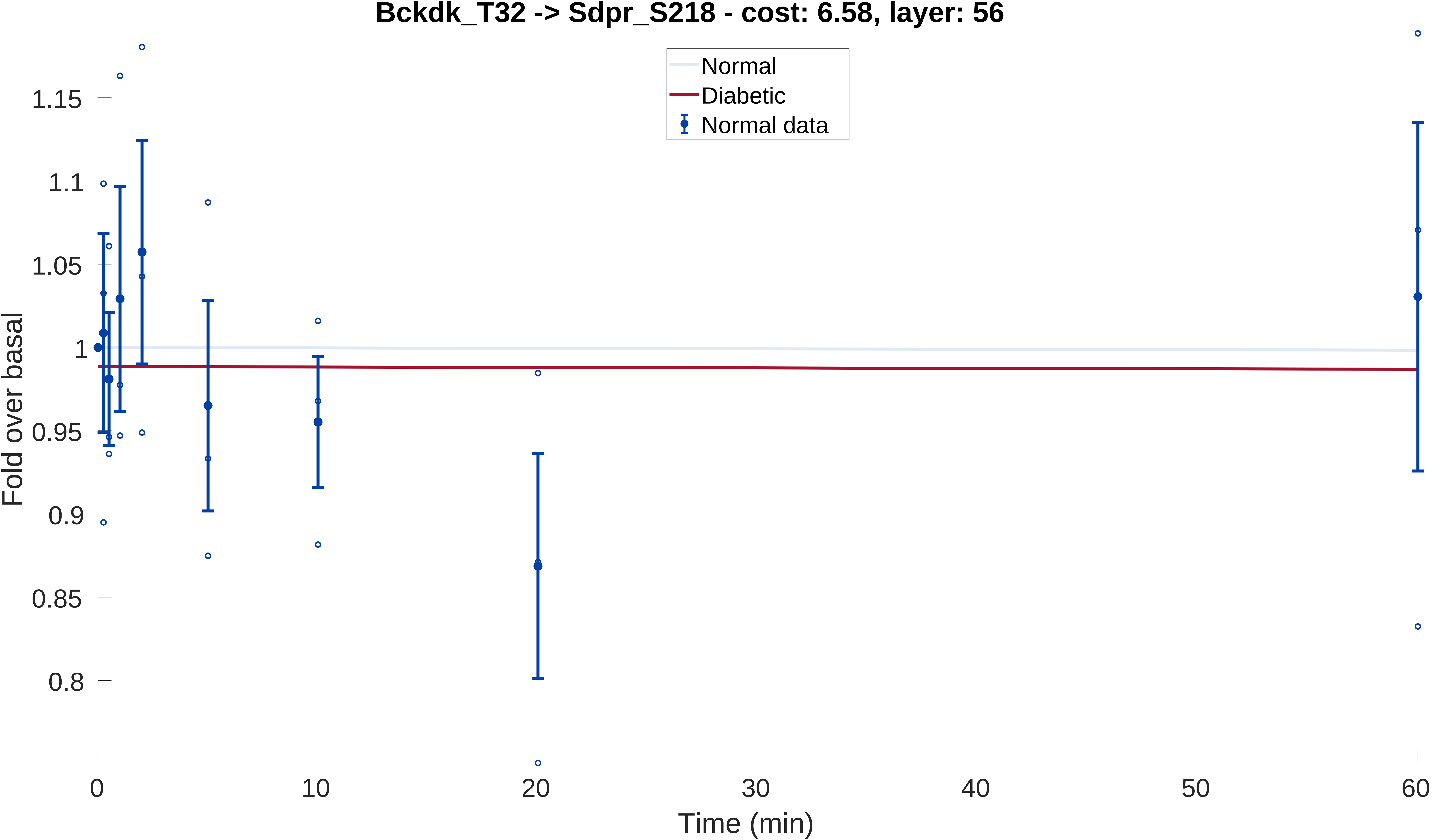

Bckdk_T32 -> Sdpr_S218 - cost: 6.58, layer: 56
Normal
Diabetic
Normal data
Fold over basal
Time (min)
1.15
1.1
1.05
1
0.95
0.9
0.85
0.8
0
10
20
30
40
50
60

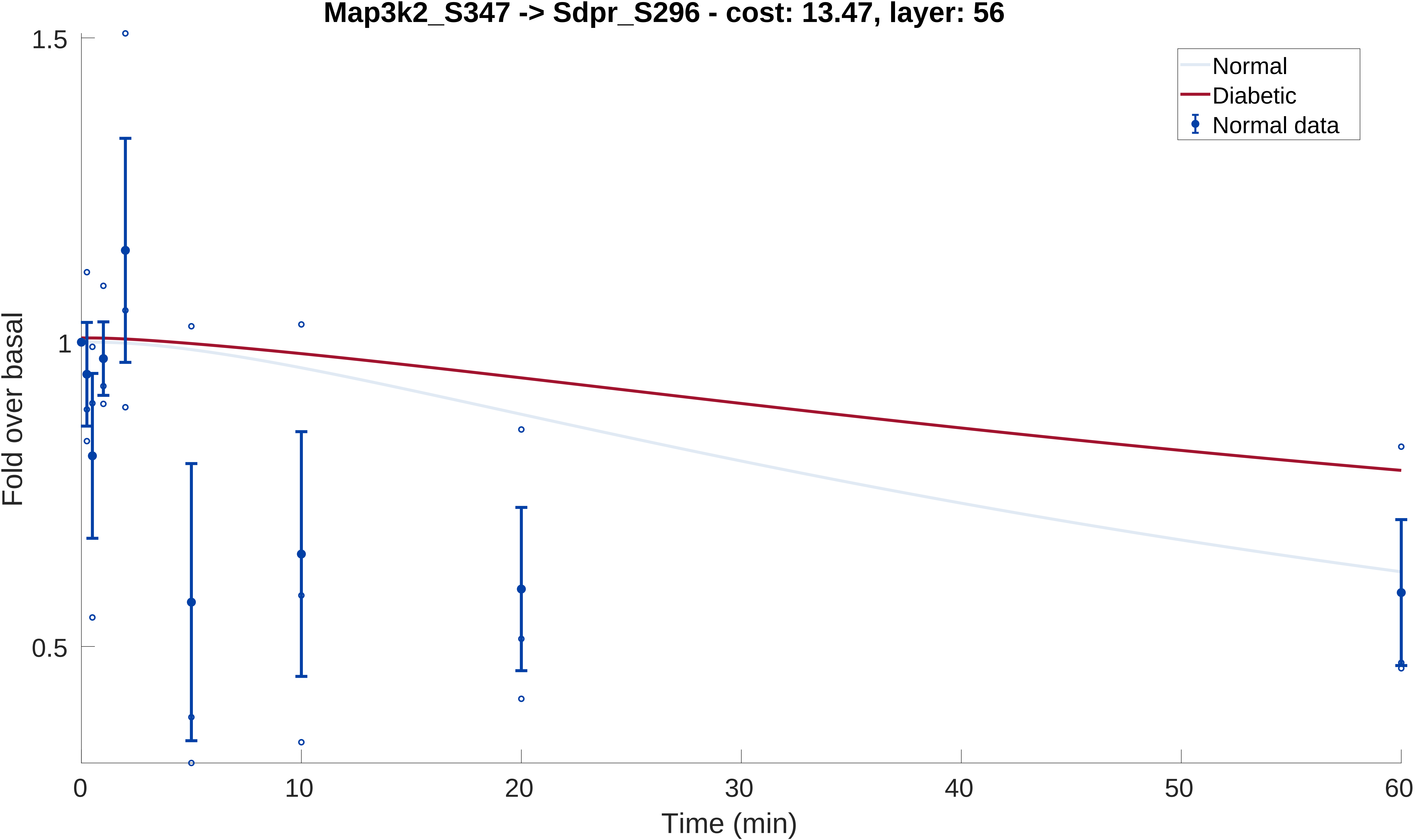

Map3k2_S347 -> Sdpr_S296 - cost: 13.47, layer: 56
Normal
Diabetic
Normal data
Fold over basal
Time (min)
1.5
1
0.5
0
10
20
30
40
50
60

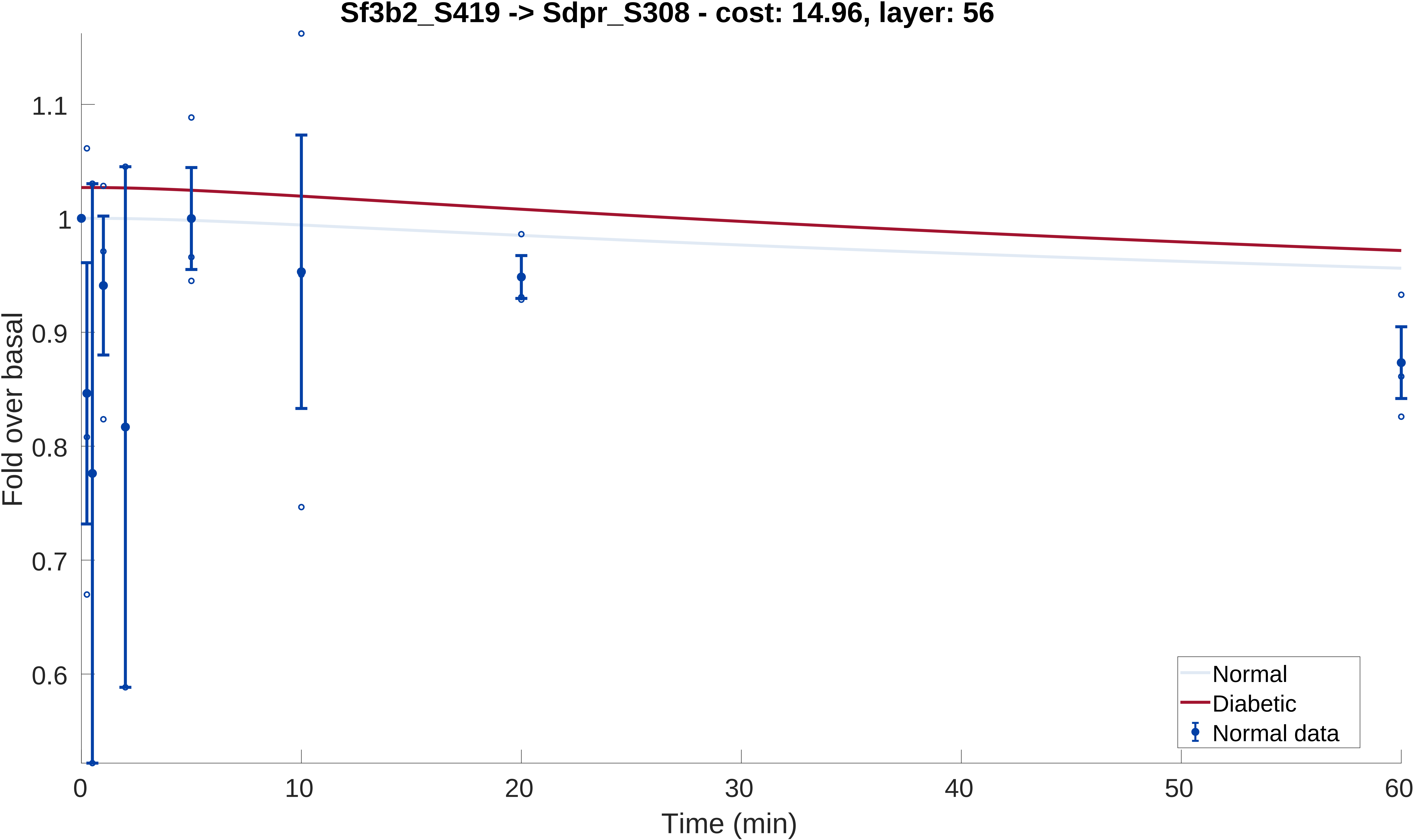
Sf3b2_S419 -> Sdpr_S308 - cost: 14.96, layer: 56
Fold over basal
1.1
1
0.9
0.8
0.7
0.6
0
10
20
30
40
50
60
Time (min)
Normal
Diabetic
Normal data

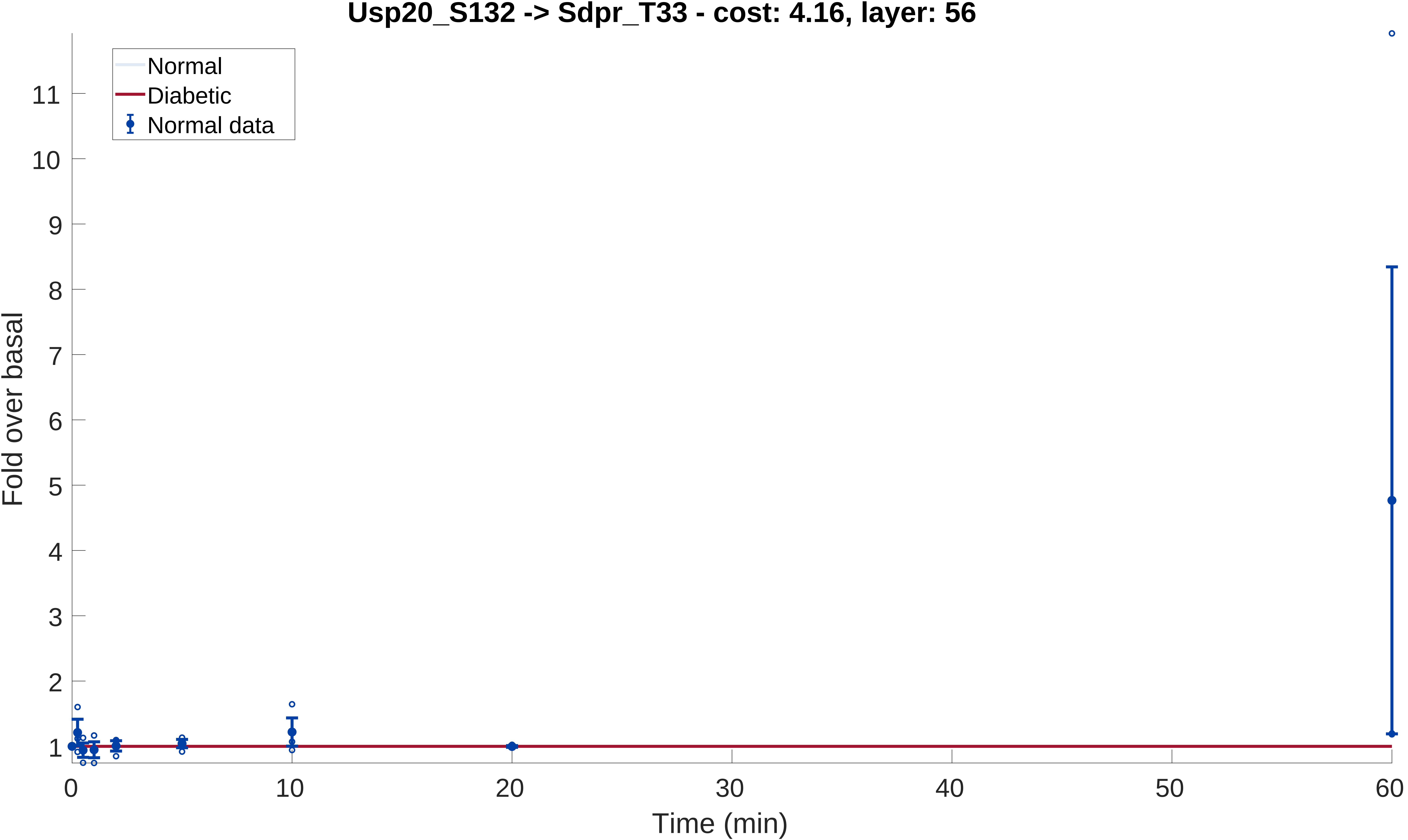

Usp20_S132 -> Sdpr_T33 - cost: 4.16, layer: 56
Normal
Diabetic
Normal data
Fold over basal
Time (min)
1
2
3
4
5
6
7
8
9
10
11
0
10
20
30
40
50
60

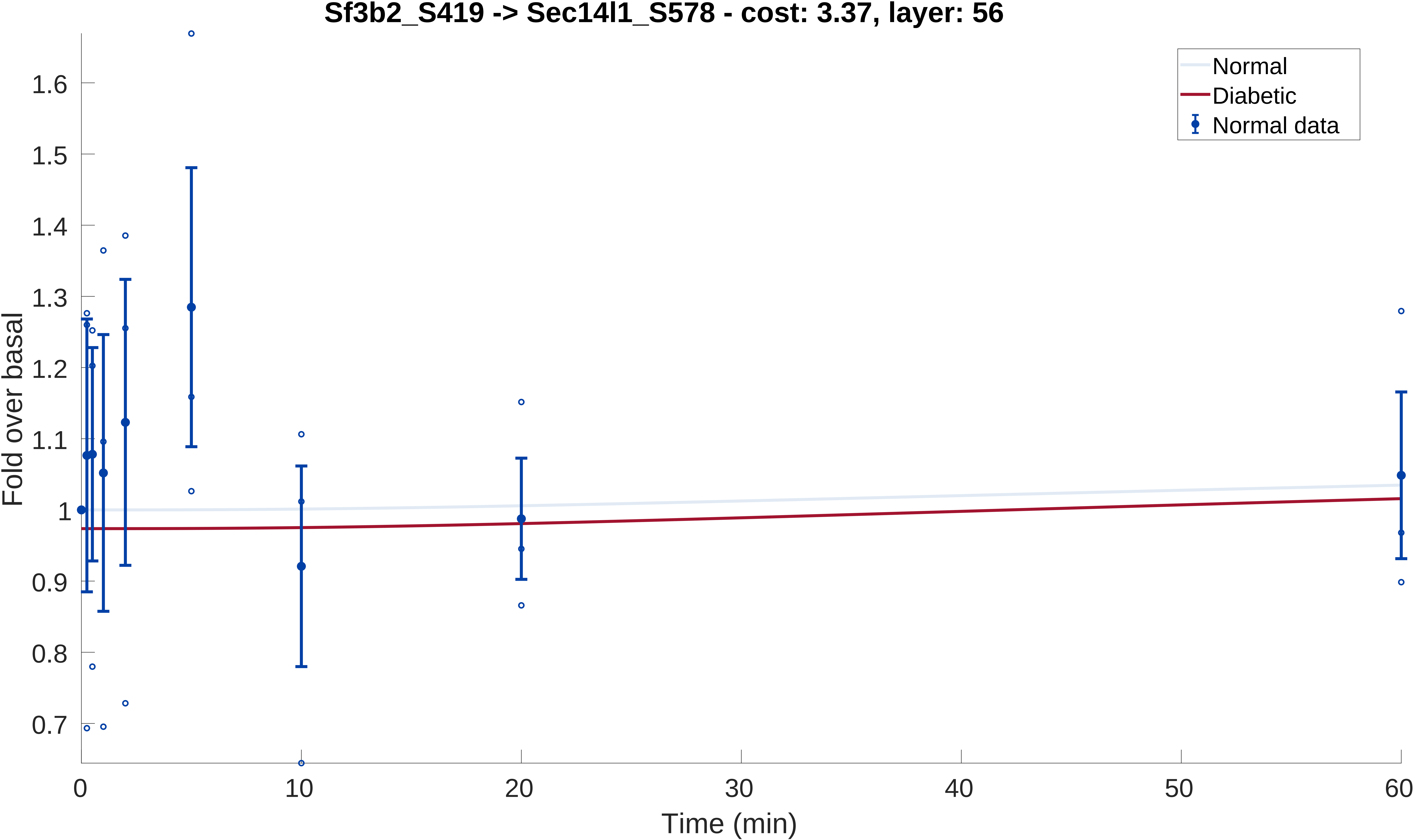

Sf3b2_S419 -> Sec14l1_S578 - cost: 3.37, layer: 56
Normal
Diabetic
Normal data
Fold over basal
1.6
1.5
1.4
1.3
1.2
1.1
1
0.9
0.8
0.7
0
10
20
30
40
50
60
Time (min)

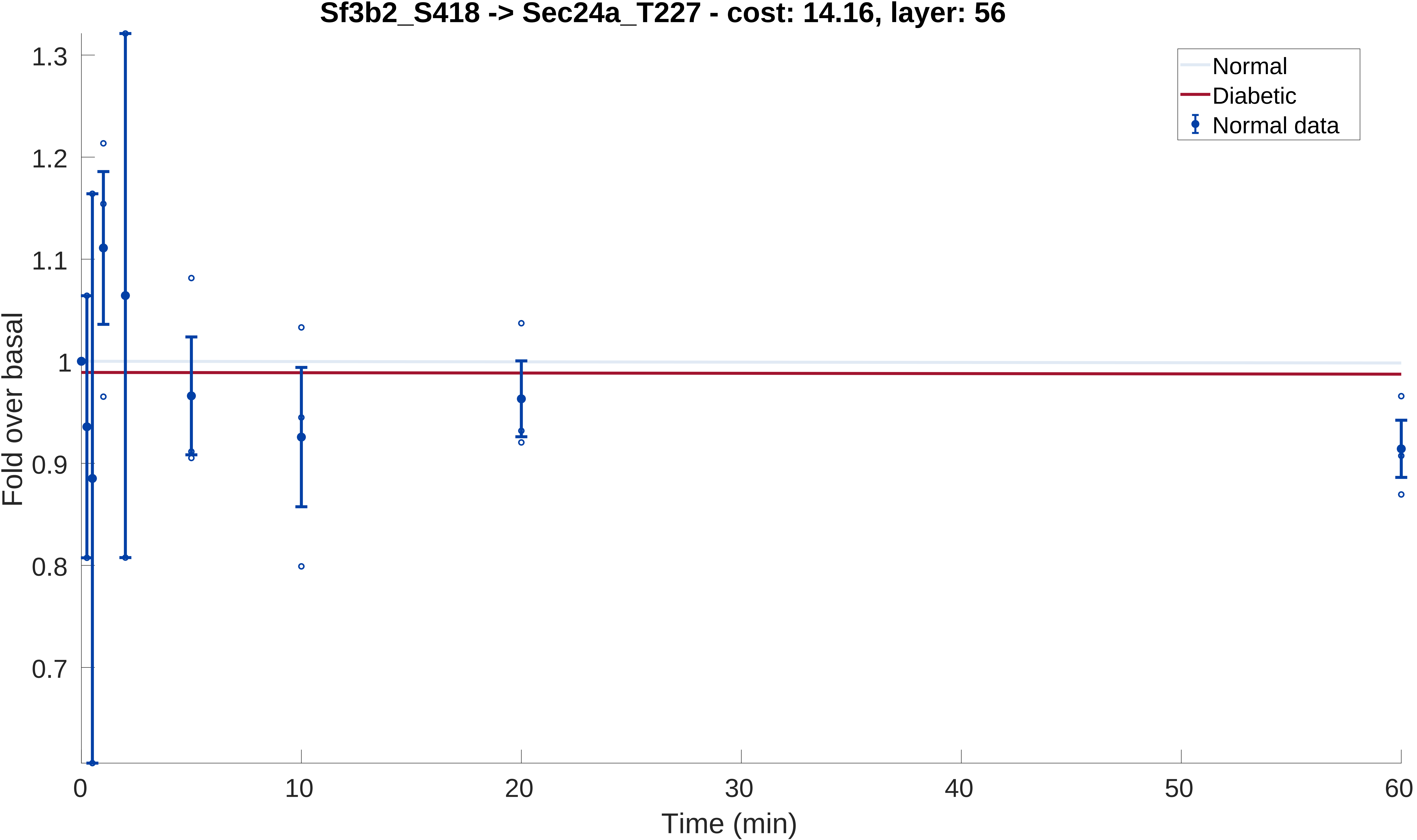

Sf3b2_S418 -> Sec24a_T227 - cost: 14.16, layer: 56
Normal
Diabetic
Normal data
Fold over basal
Time (min)
1.3
1.2
1.1
1
0.9
0.8
0.7
0
10
20
30
40
50
60

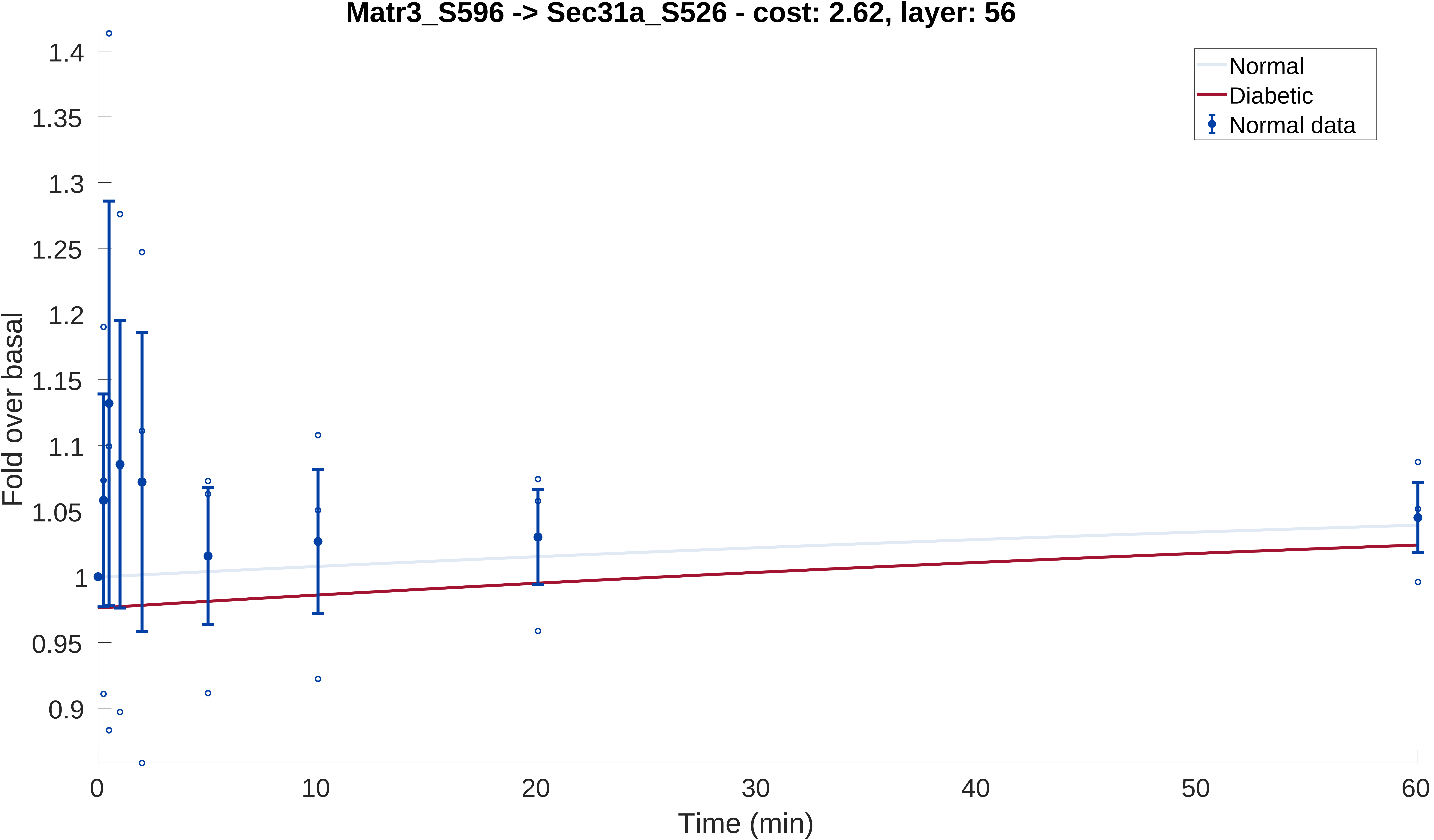

Matr3_S596 -> Sec31a_S526 - cost: 2.62, layer: 56
Normal
Diabetic
Normal data
Fold over basal
1.4
1.35
1.3
1.25
1.2
1.15
1.1
1.05
1
0.95
0.9
0
10
20
30
40
50
60
Time (min)

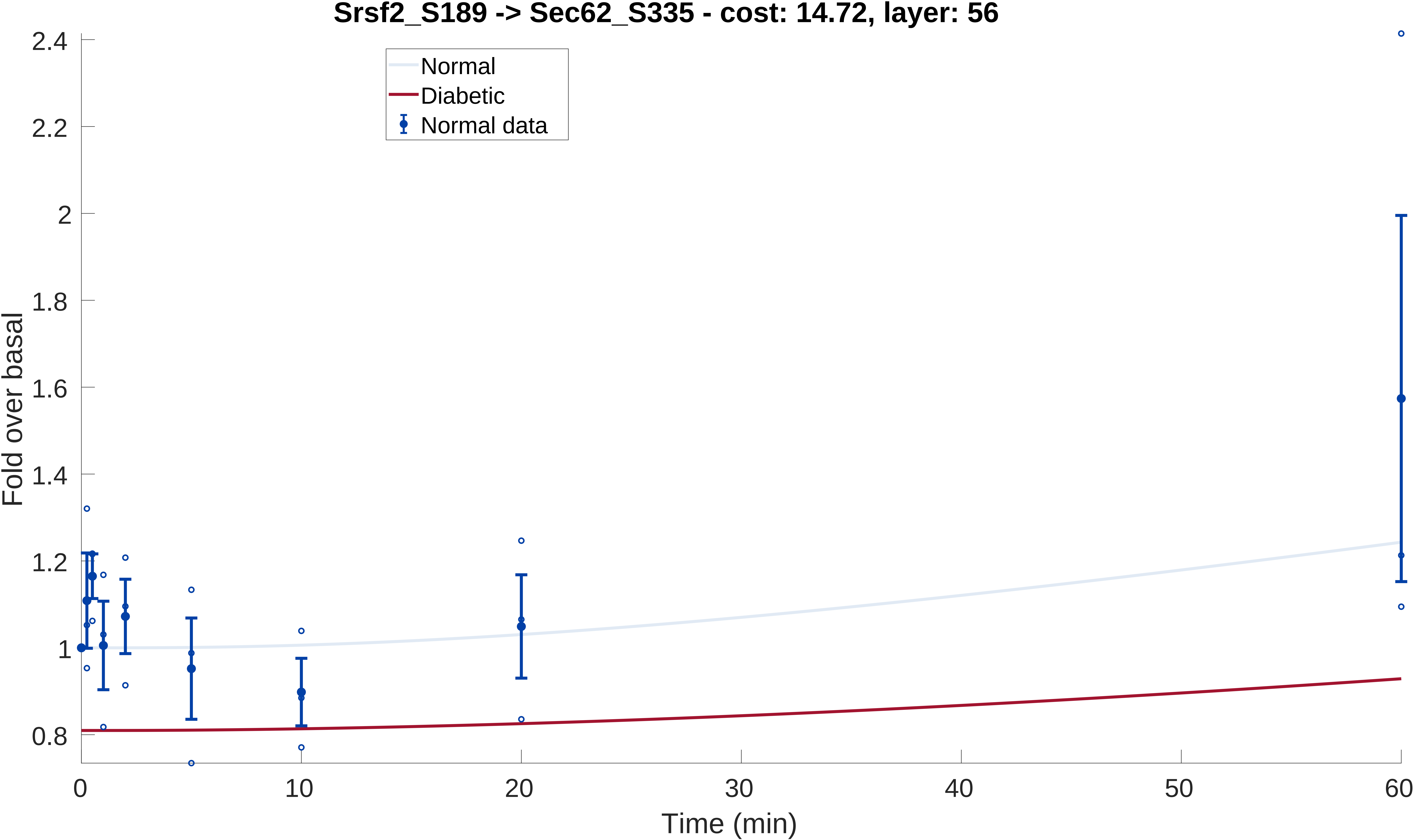

Srsf2_S189 -> Sec62_S335 - cost: 14.72, layer: 56
Normal
Diabetic
Normal data
Fold over basal
Time (min)
0.8
1
1.2
1.4
1.6
1.8
2
2.2
2.4
0
10
20
30
40
50
60

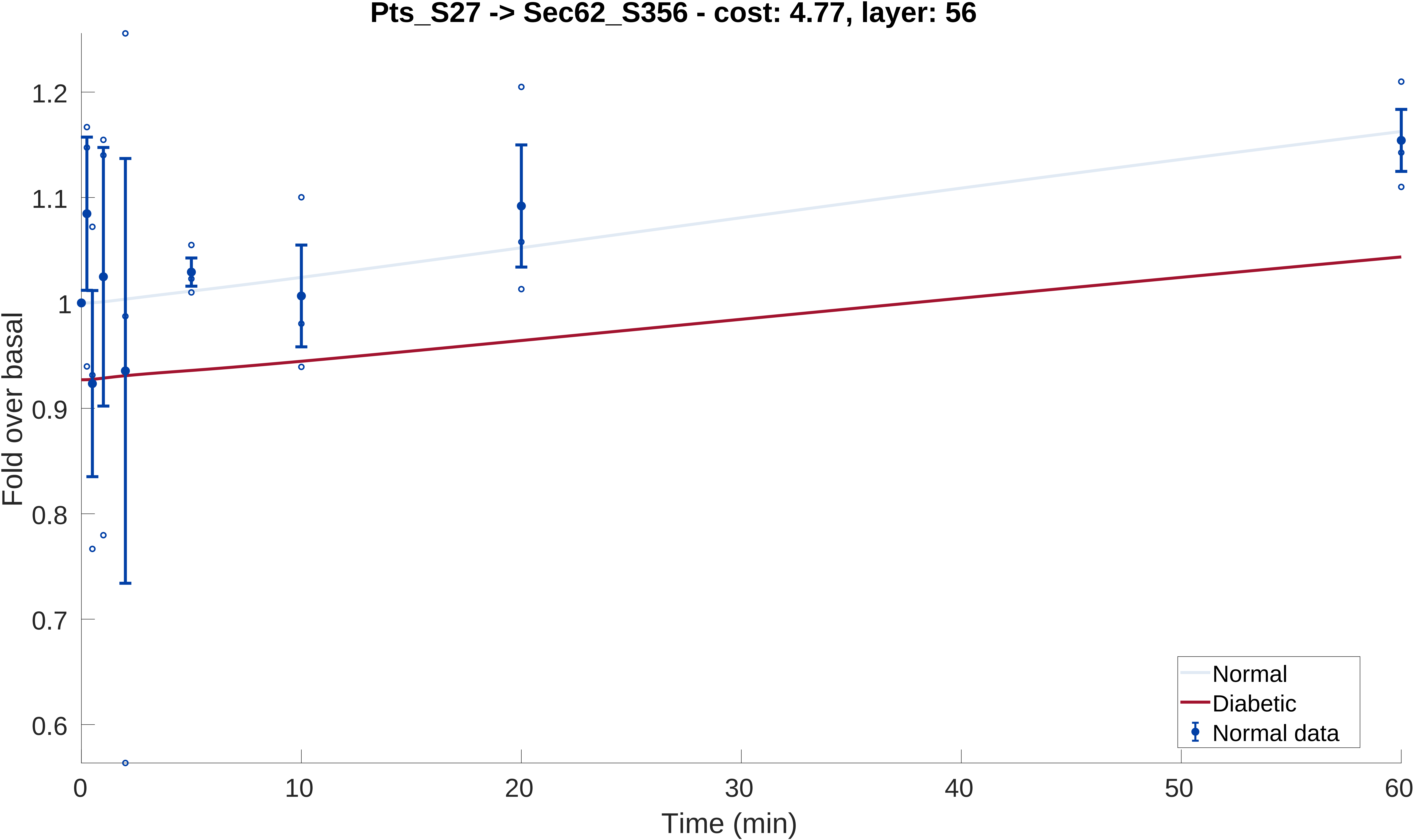
Pts_S27 -> Sec62_S356 - cost: 4.77, layer: 56
Fold over basal
Time (min)
1.2
1.1
1
0.9
0.8
0.7
0.6
0
10
20
30
40
50
60
Normal
Diabetic
Normal data

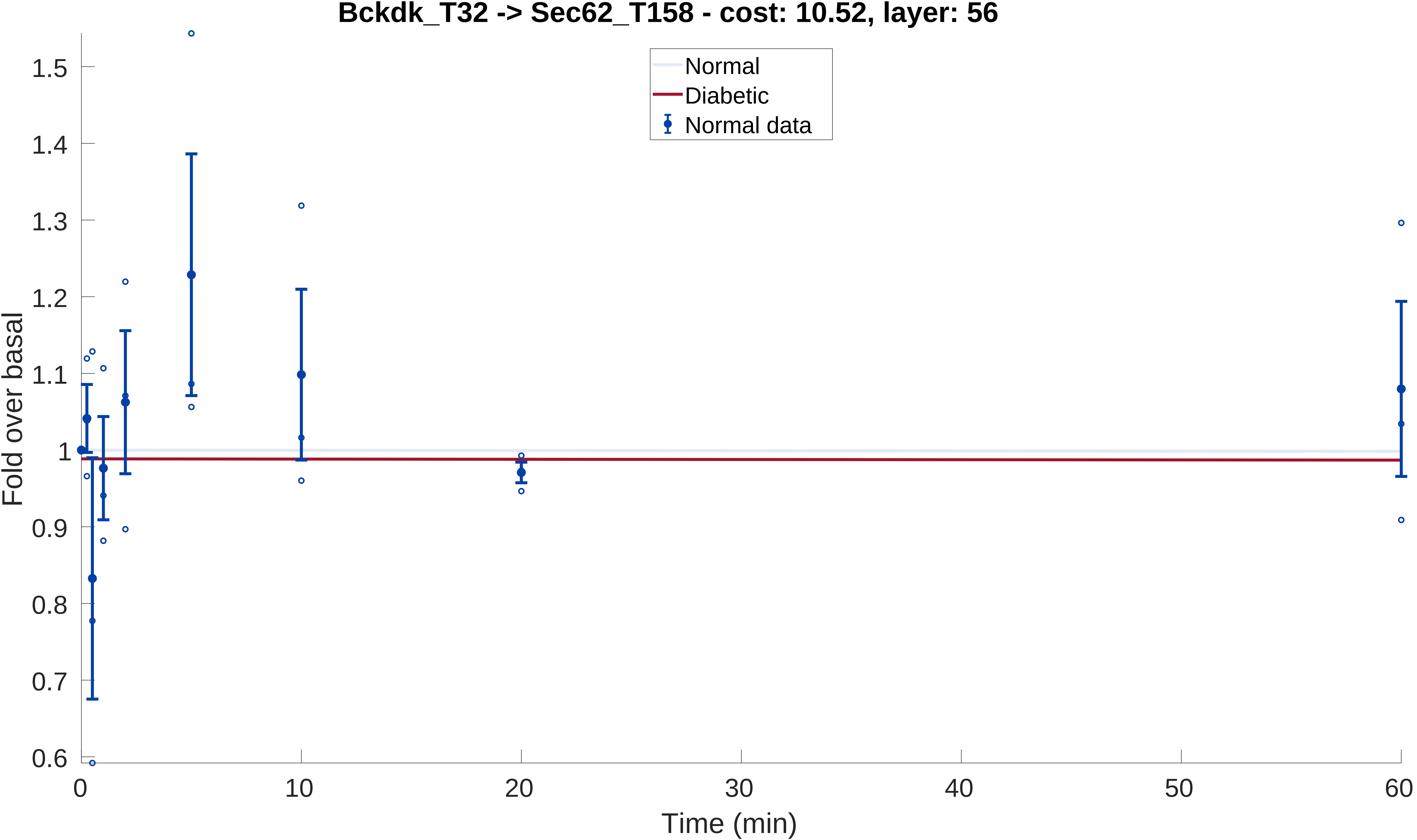

Bckdk_T32 -> Sec62_T158 - cost: 10.52, layer: 56
Normal
Diabetic
Normal data
Fold over basal
Time (min)
1.5
1.4
1.3
1.2
1.1
1
0.9
0.8
0.7
0.6
0
10
20
30
40
50
60

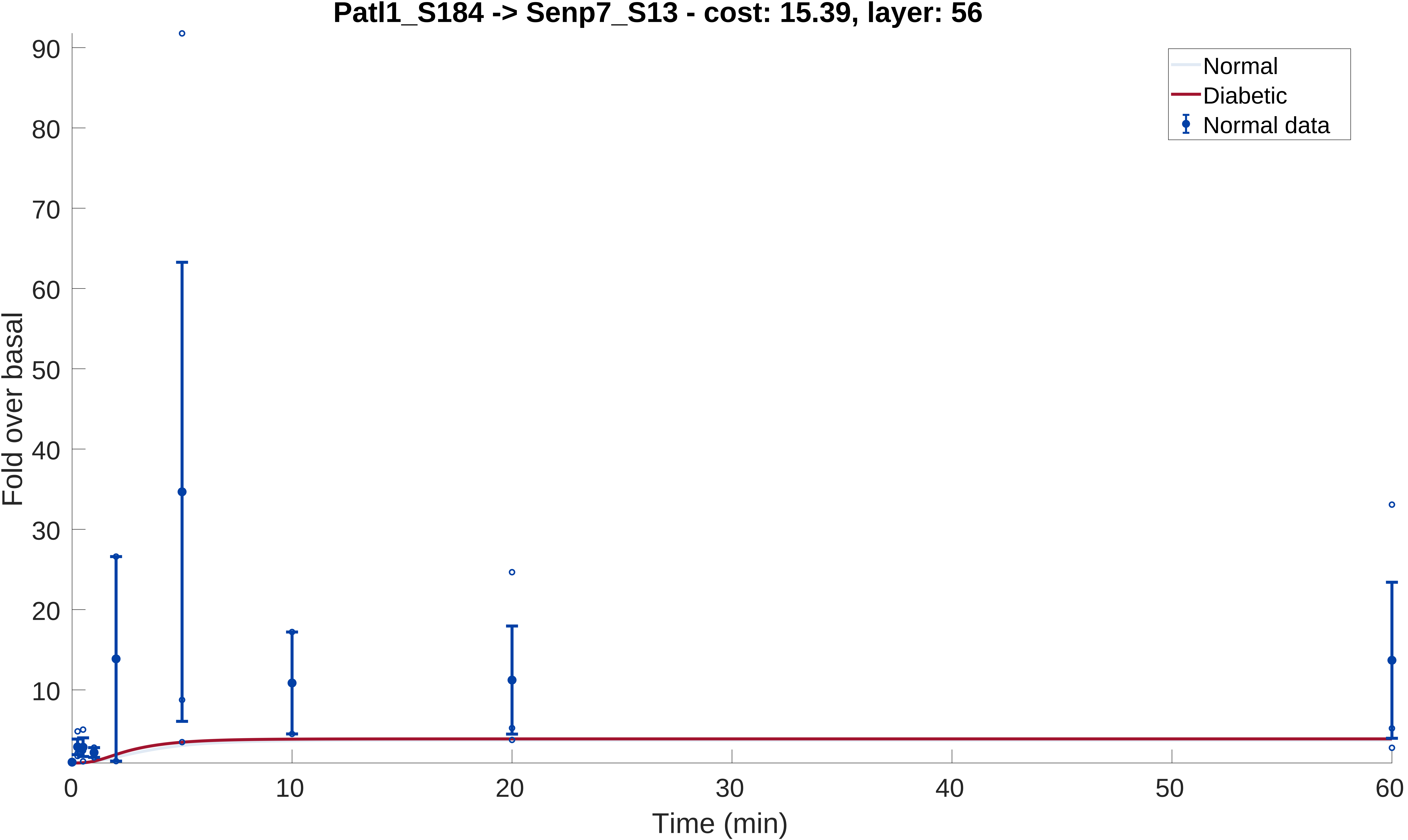

Patl1_S184 -> Senp7_S13 - cost: 15.39, layer: 56
Normal
Diabetic
Normal data
Fold over basal
Time (min)
0
10
20
30
40
50
60
70
80
90

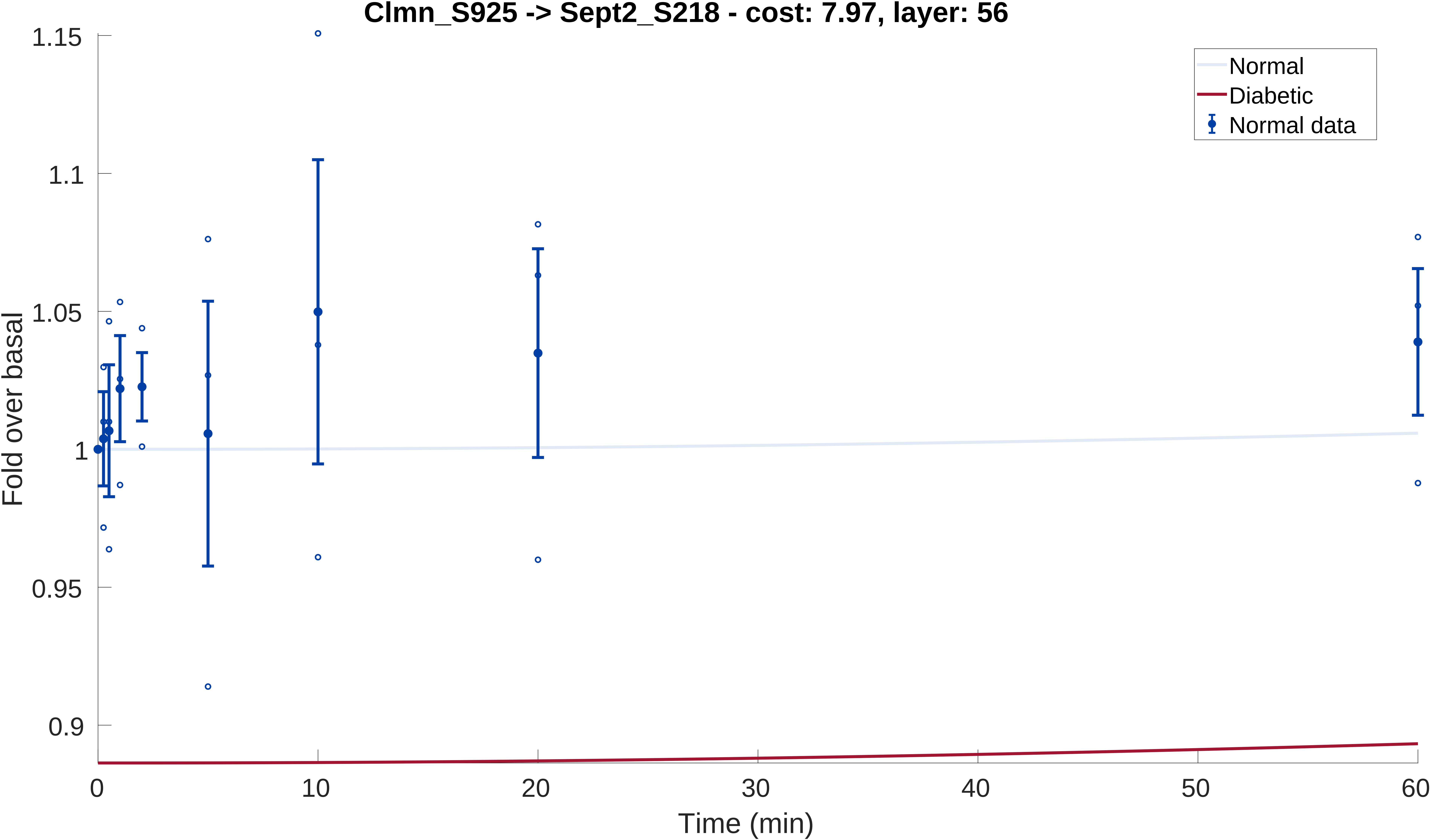

Clmn_S925 -> Sept2_S218 - cost: 7.97, layer: 56
Normal
Diabetic
Normal data
Fold over basal
1.15
1.1
1.05
1
0.95
0.9
0
10
20
30
40
50
60
Time (min)

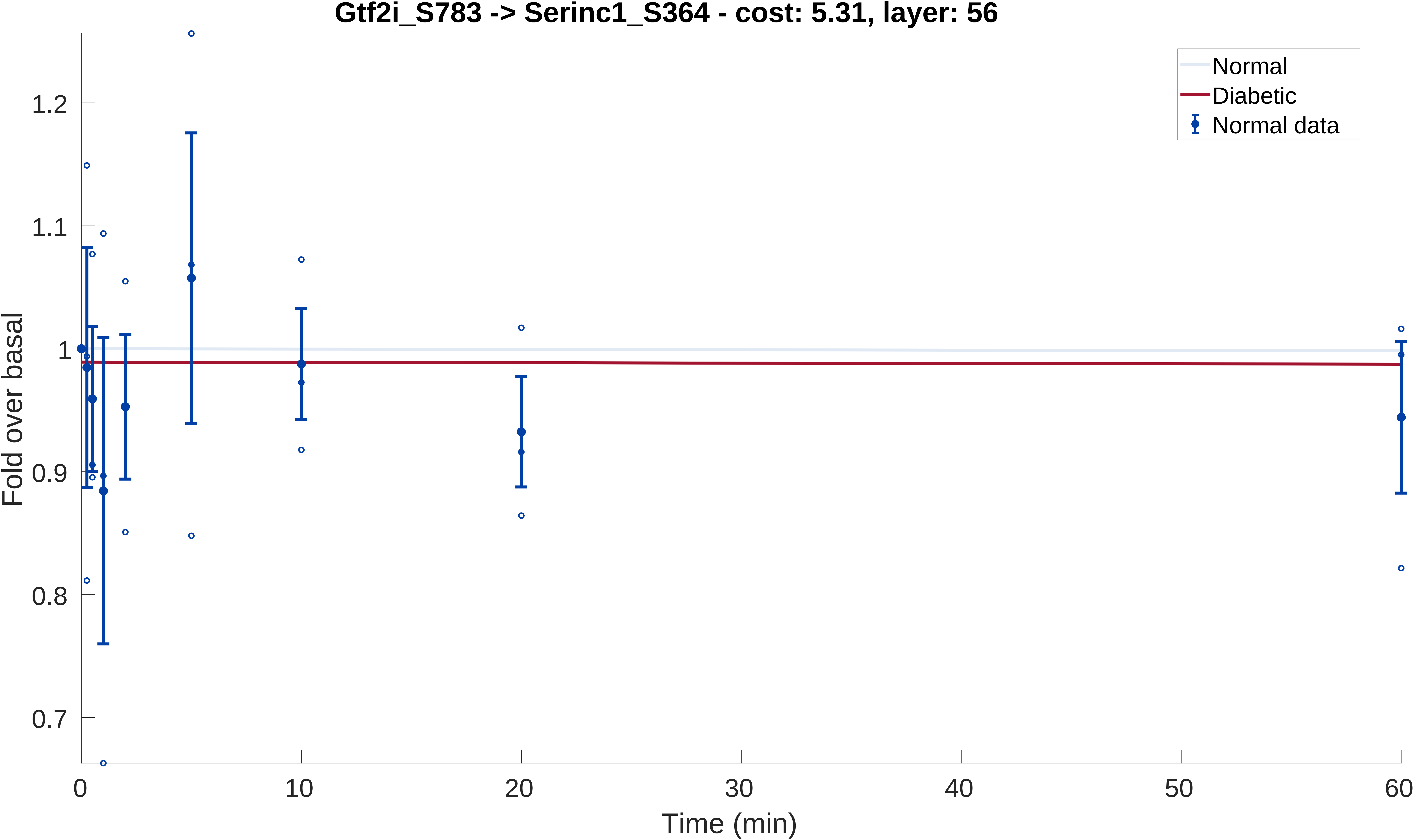

Gtf2i_S783 -> Serinc1_S364 - cost: 5.31, layer: 56
Normal
Diabetic
Normal data
Fold over basal
Time (min)
1.2
1.1
1
0.9
0.8
0.7
0
10
20
30
40
50
60

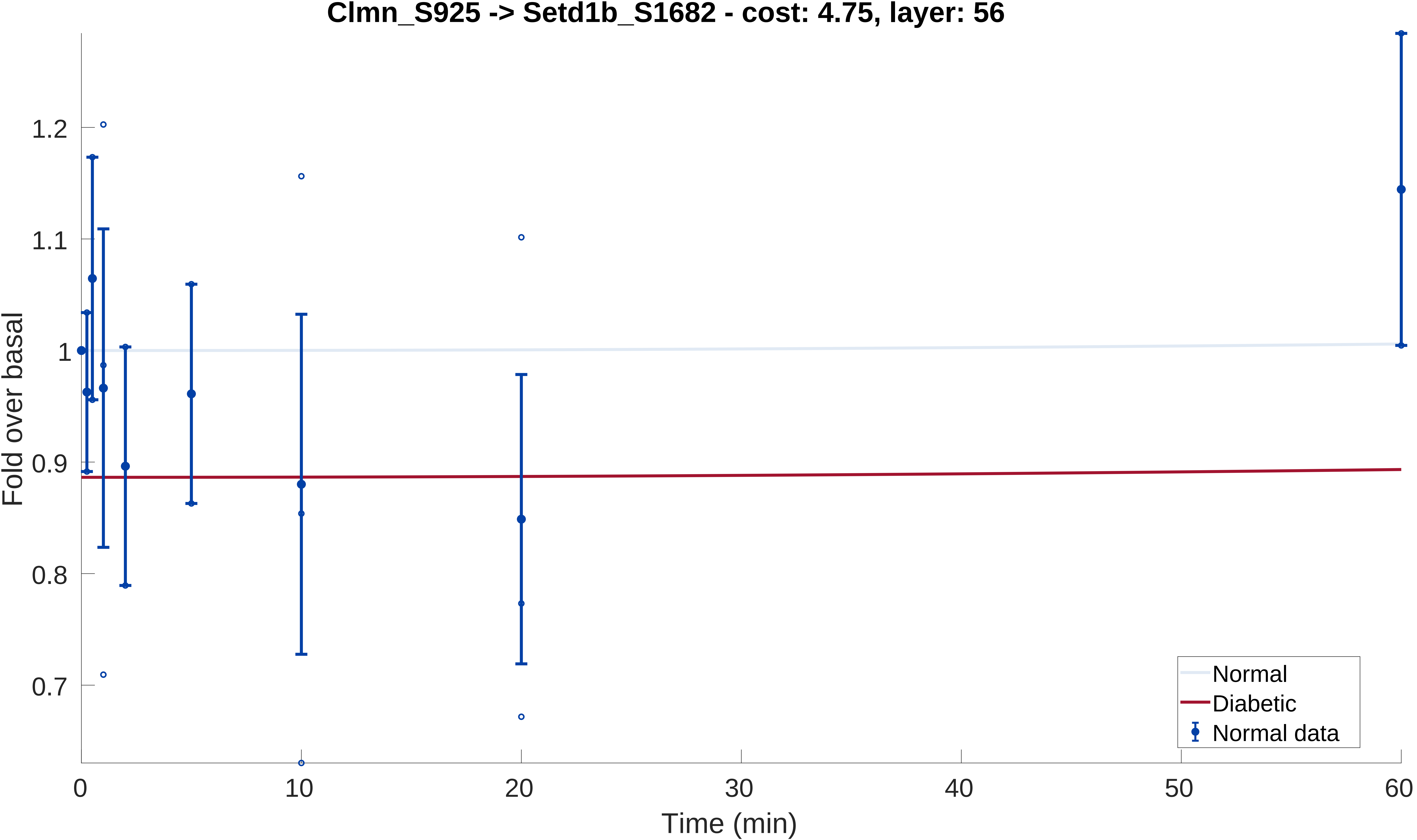
Clmn_S925 -> Setd1b_S1682 - cost: 4.75, layer: 56
Fold over basal
1.2
1.1
1
0.9
0.8
0.7
0
10
20
30
40
50
60
Time (min)
Normal
Diabetic
Normal data

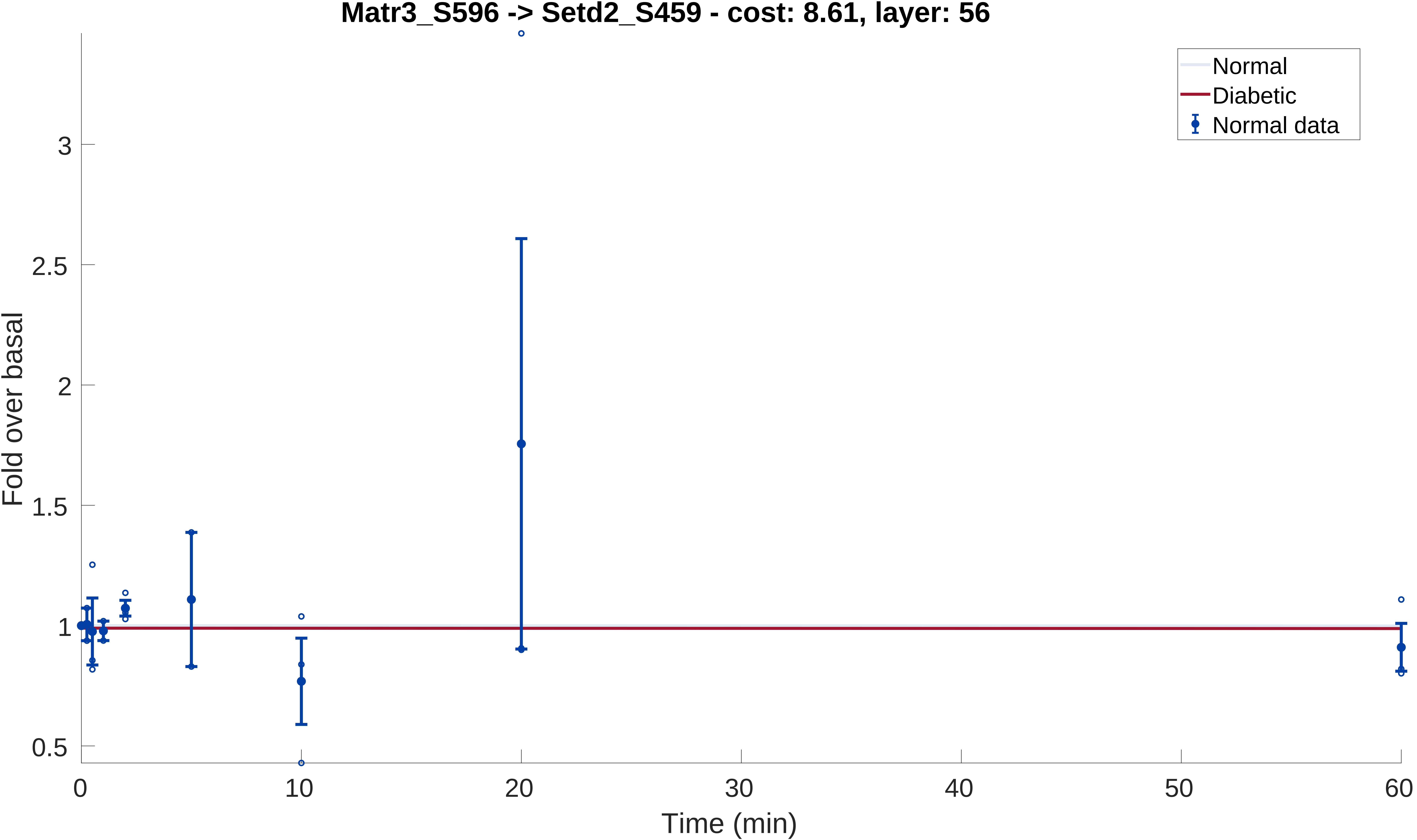
Matr3_S596 -> Setd2_S459 - cost: 8.61, layer: 56
Normal
Diabetic
Normal data
Fold over basal
3
2.5
2
1.5
1
0.5
0
10
20
30
40
50
60
Time (min)

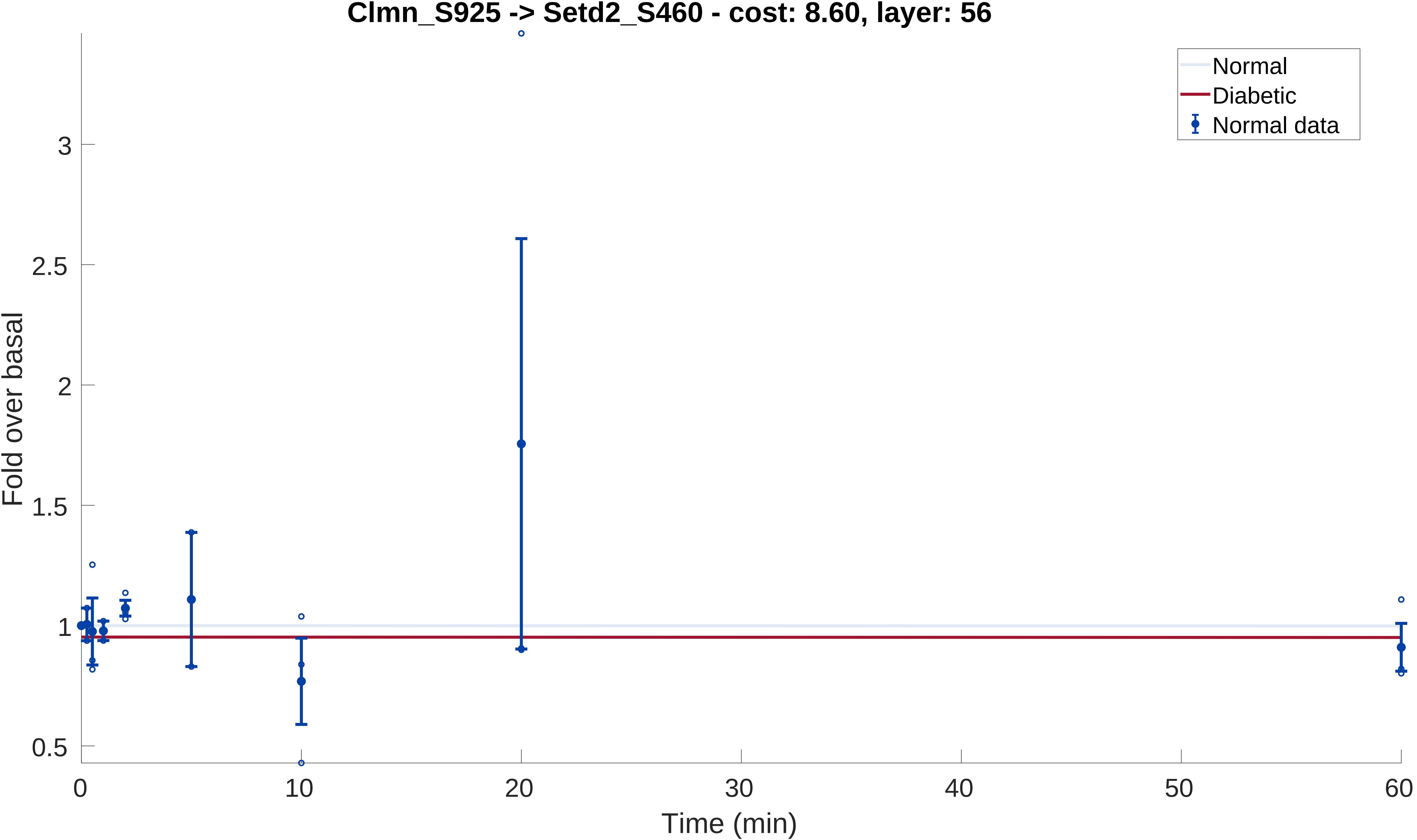
Clmn_S925 -> Setd2_S460 - cost: 8.60, layer: 56
Normal
Diabetic
Normal data
Fold over basal
Time (min)
0.5
1
1.5
2
2.5
3
0
10
20
30
40
50
60

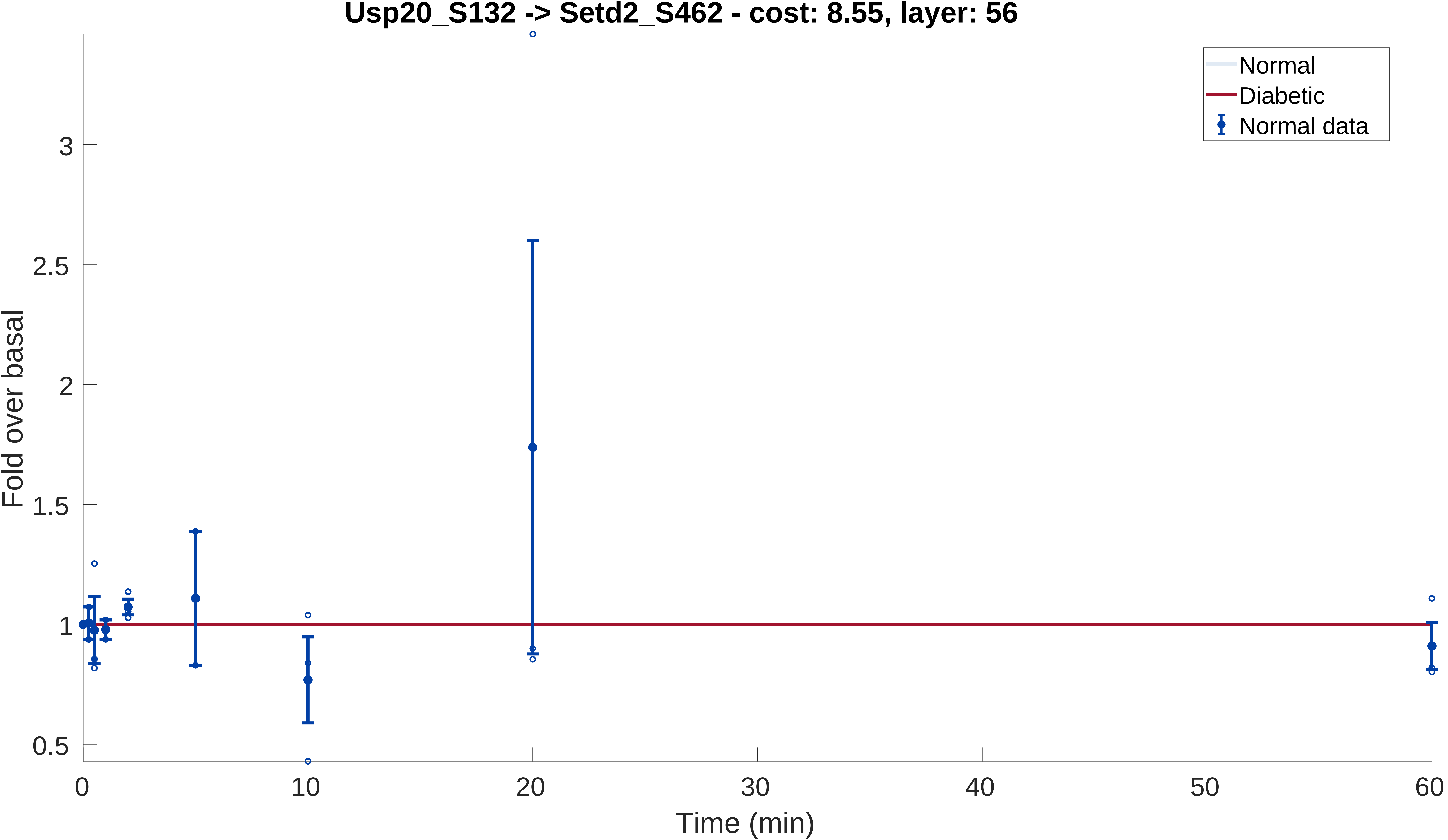

Usp20_S132 -> Setd2_S462 - cost: 8.55, layer: 56
Normal
Diabetic
Normal data
Fold over basal
0.5
1
1.5
2
2.5
3
0
10
20
30
40
50
60
Time (min)

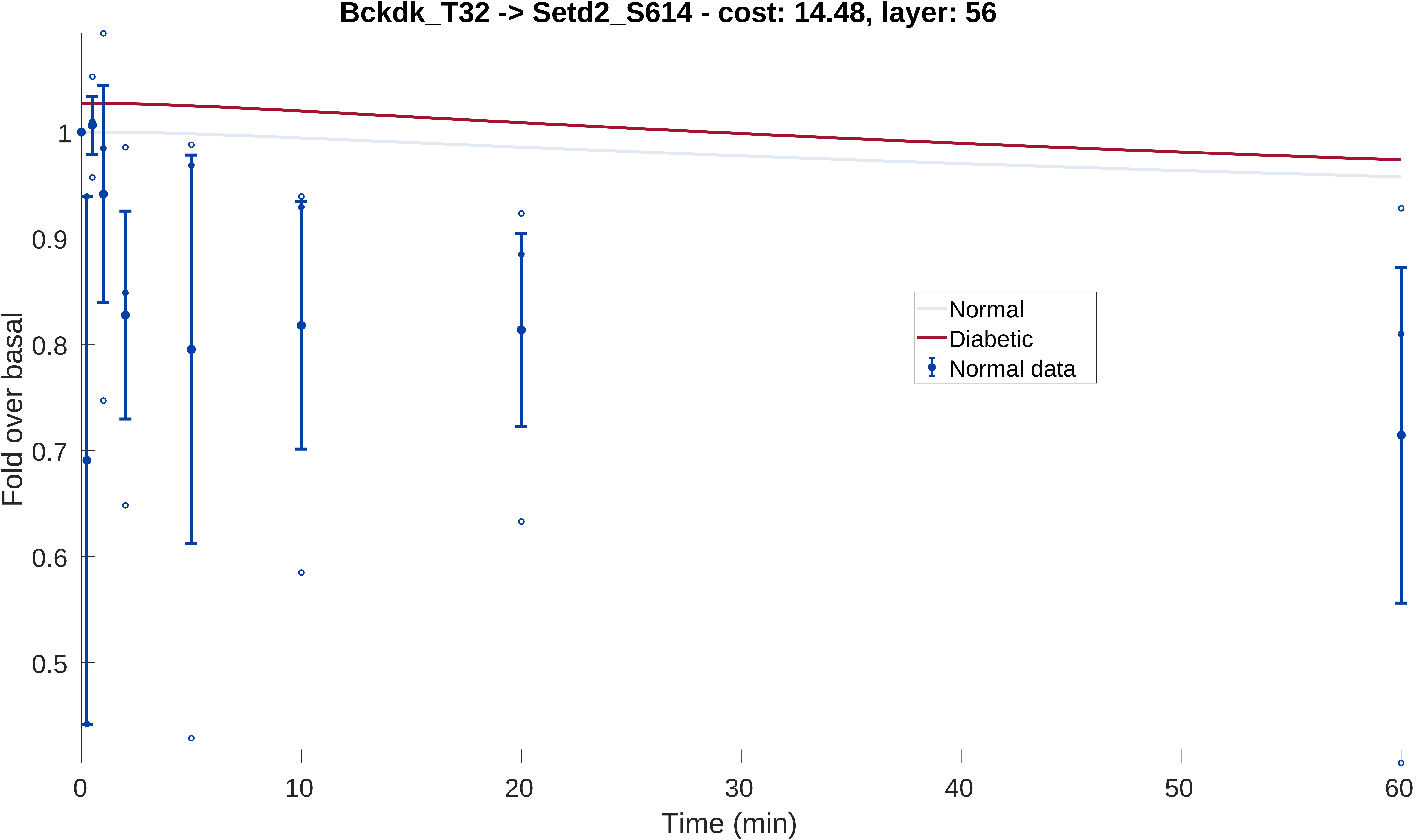

Bckdk_T32 -> Setd2_S614 - cost: 14.48, layer: 56
Fold over basal
Time (min)
Normal
Diabetic
Normal data
0.5
0.6
0.7
0.8
0.9
1
0
10
20
30
40
50
60

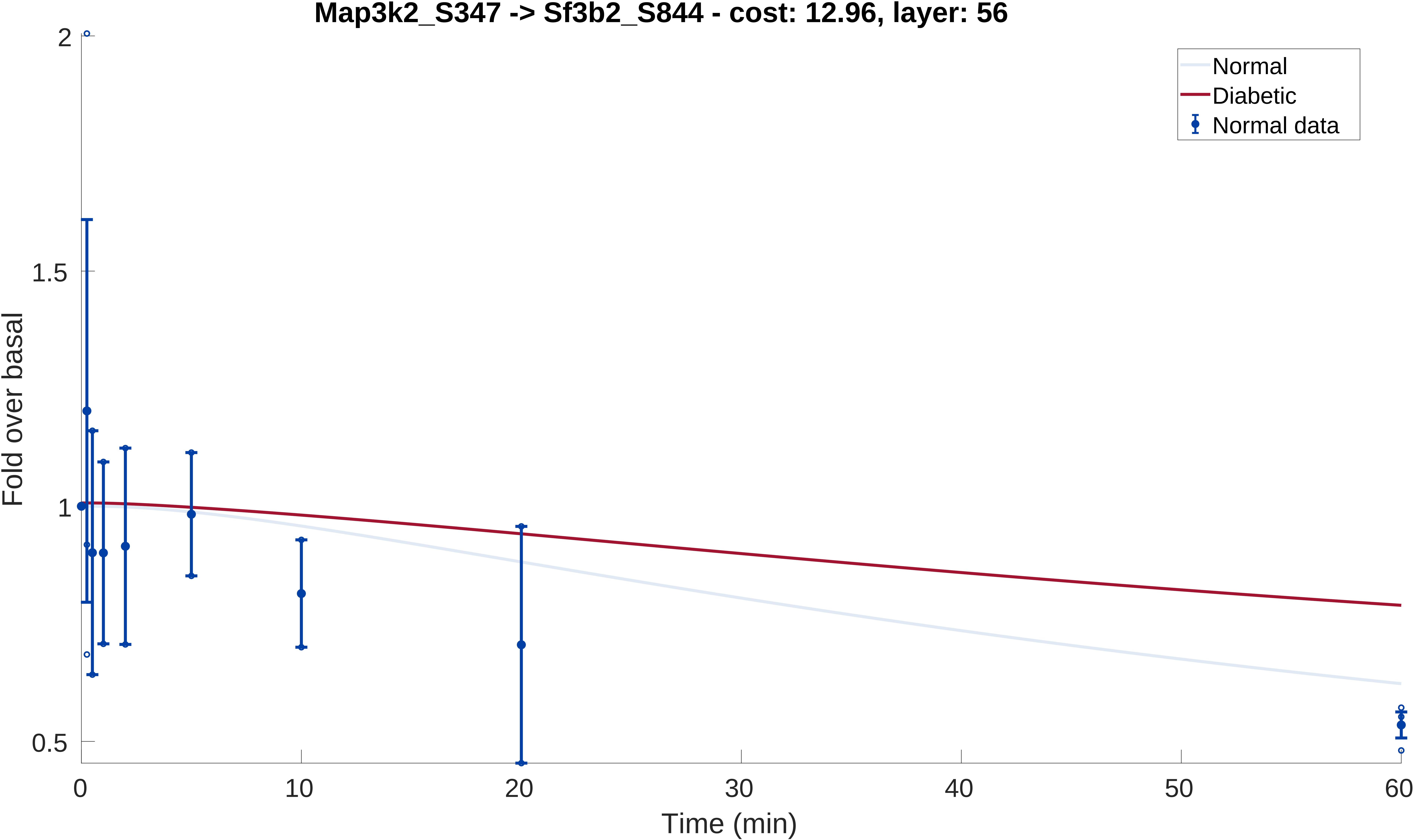

Map3k2_S347 -> Sf3b2_S844 - cost: 12.96, layer: 56
Normal
Diabetic
Normal data
Fold over basal
Time (min)
0.5
1
1.5
2
0
10
20
30
40
50
60

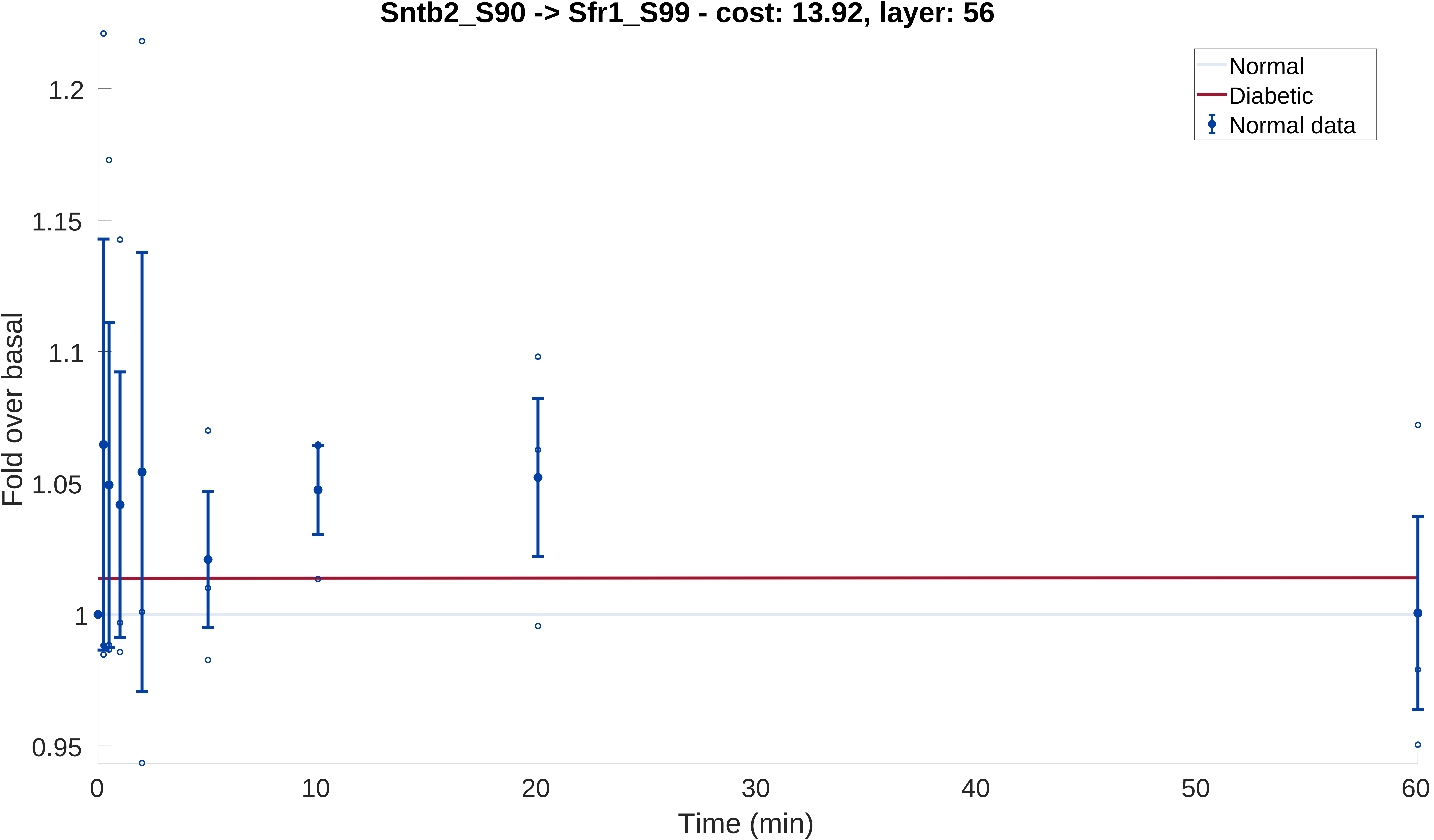

Sntb2_S90 -> Sfr1_S99 - cost: 13.92, layer: 56
Normal
Diabetic
Normal data
Fold over basal
1.2
1.15
1.1
1.05
1
0.95
0
10
20
30
40
50
60
Time (min)

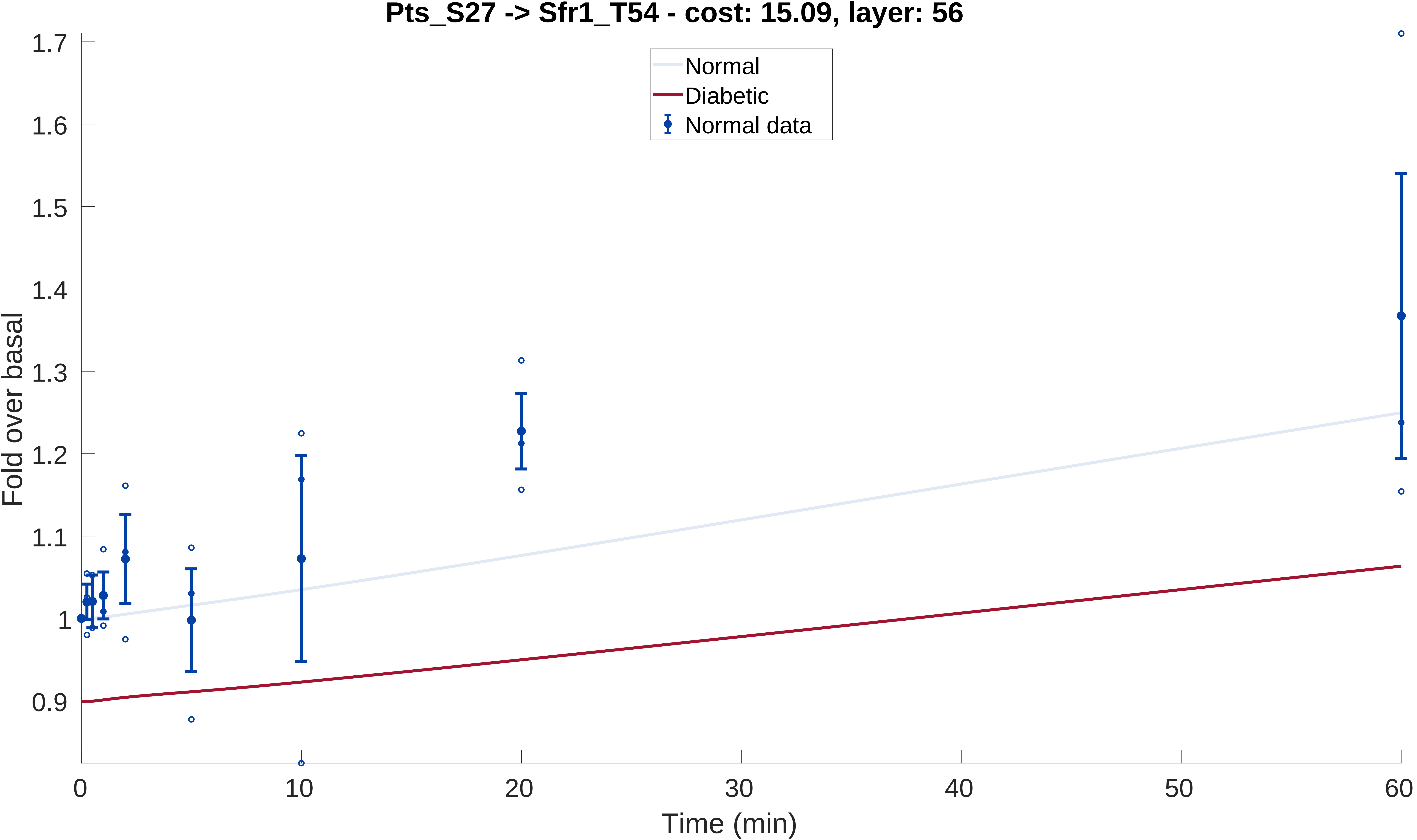

Pts_S27 -> Sfr1_T54 - cost: 15.09, layer: 56
Normal
Diabetic
Normal data
Fold over basal
1.7
1.6
1.5
1.4
1.3
1.2
1.1
1
0.9
0
10
20
30
40
50
60
Time (min)

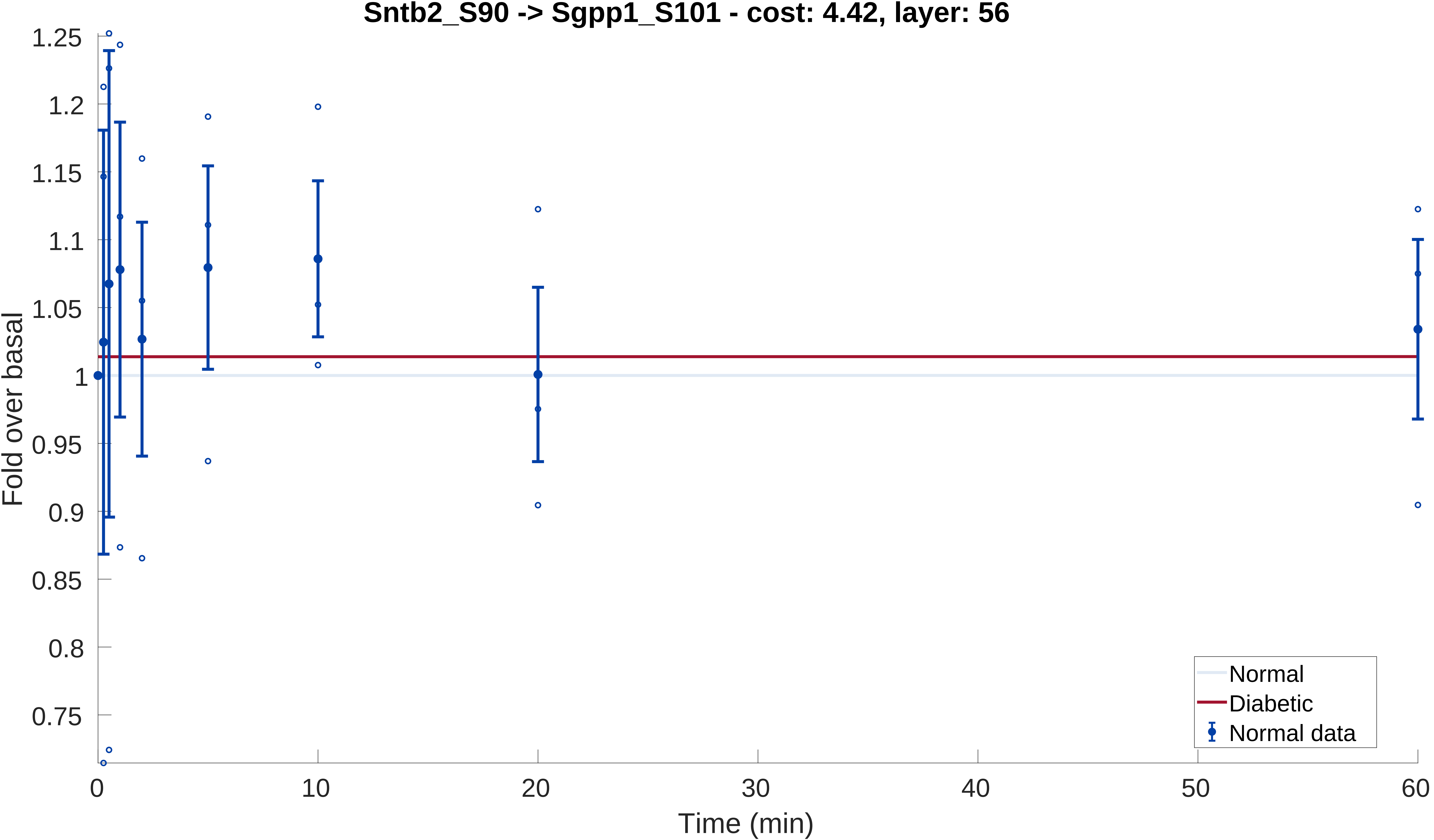

Sntb2_S90 -> Sgpp1_S101 - cost: 4.42, layer: 56
1.25
1.2
1.15
1.1
1.05
1
0.95
0.9
0.85
0.8
0.75
Fold over basal
0
10
20
30
40
50
60
Time (min)
Normal
Diabetic
Normal data

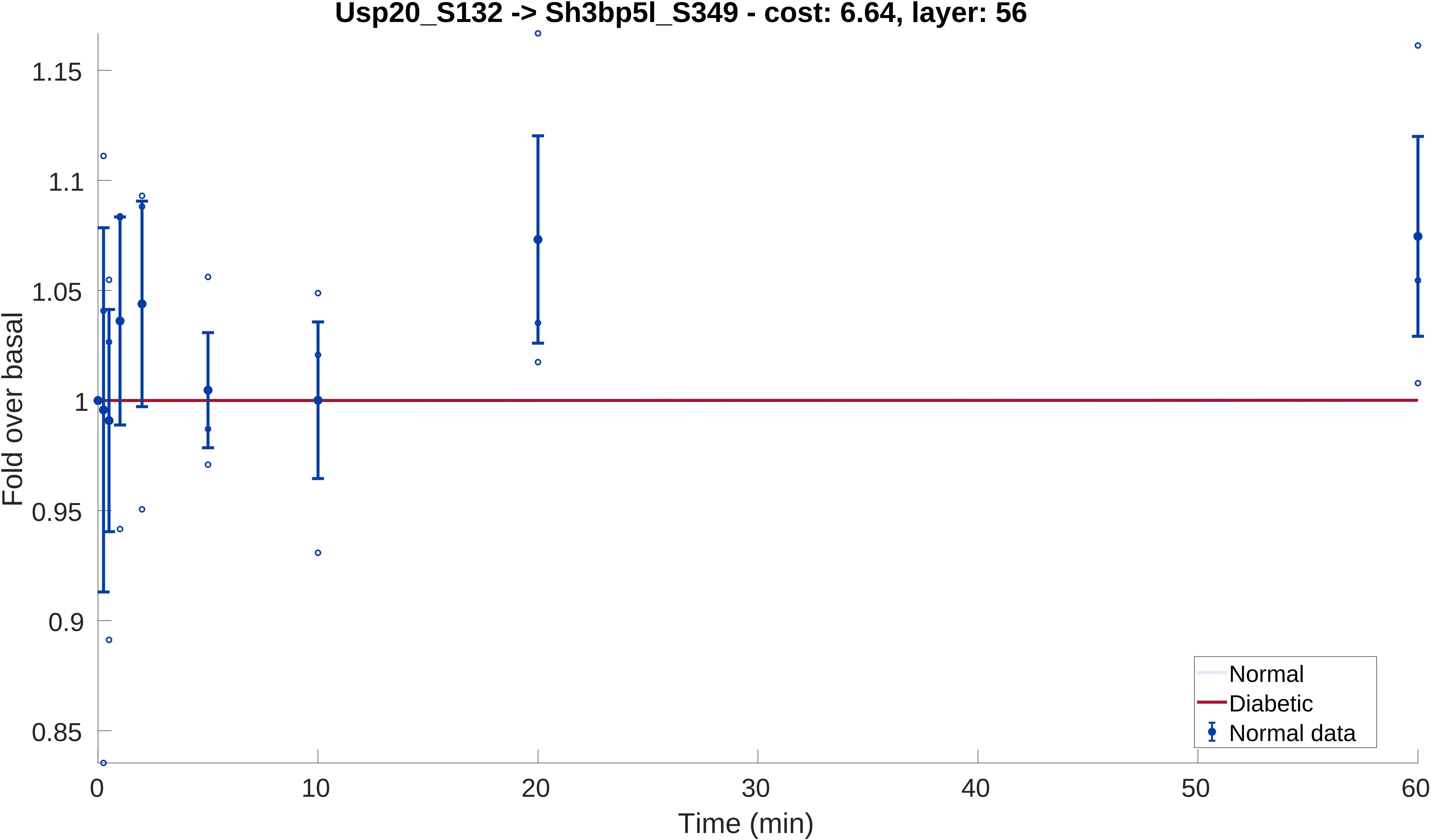

Usp20_S132 -> Sh3bp5l_S349 - cost: 6.64, layer: 56
Fold over basal
1.15
1.1
1.05
1
0.95
0.9
0.85
0
10
20
30
40
50
60
Time (min)
Normal
Diabetic
Normal data

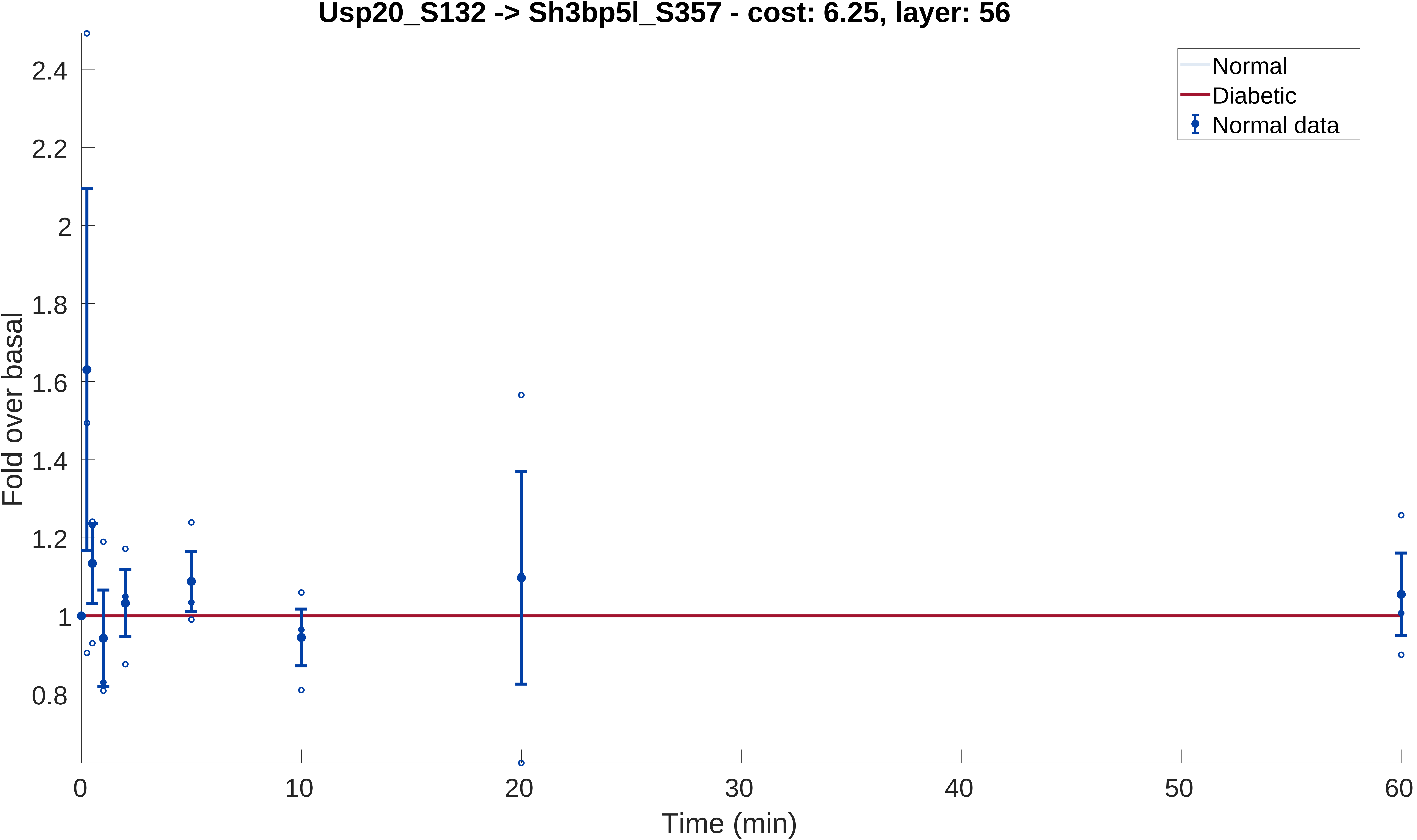
Usp20_S132 -> Sh3bp5l_S357 - cost: 6.25, layer: 56
Normal
Diabetic
Normal data
Fold over basal
Time (min)
0.8
1
1.2
1.4
1.6
1.8
2
2.2
2.4
0
10
20
30
40
50
60

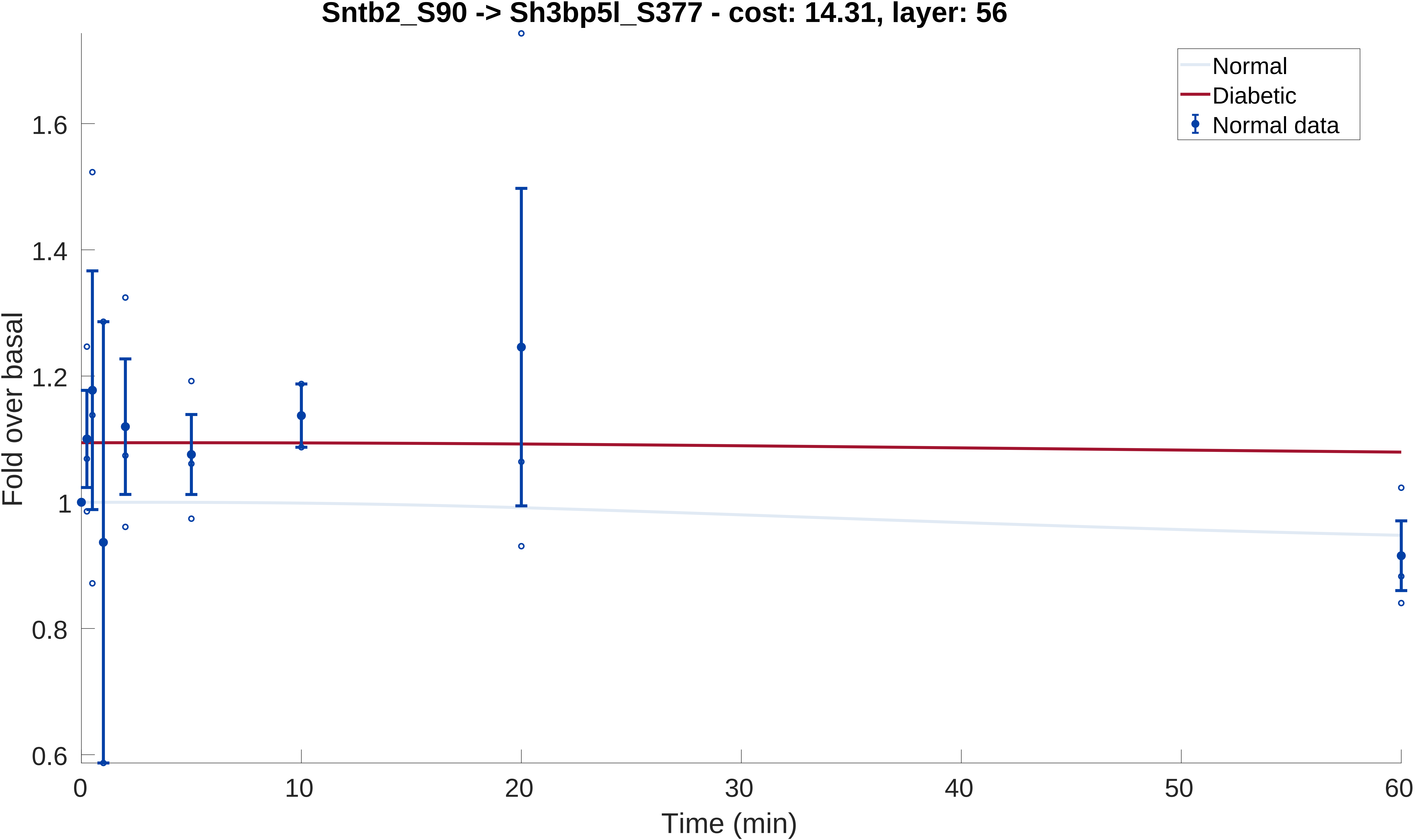
Sntb2_S90 -> Sh3bp5l_S377 - cost: 14.31, layer: 56
Normal
Diabetic
Normal data
Fold over basal
1.6
1.4
1.2
1
0.8
0.6
0
10
20
30
40
50
60
Time (min)

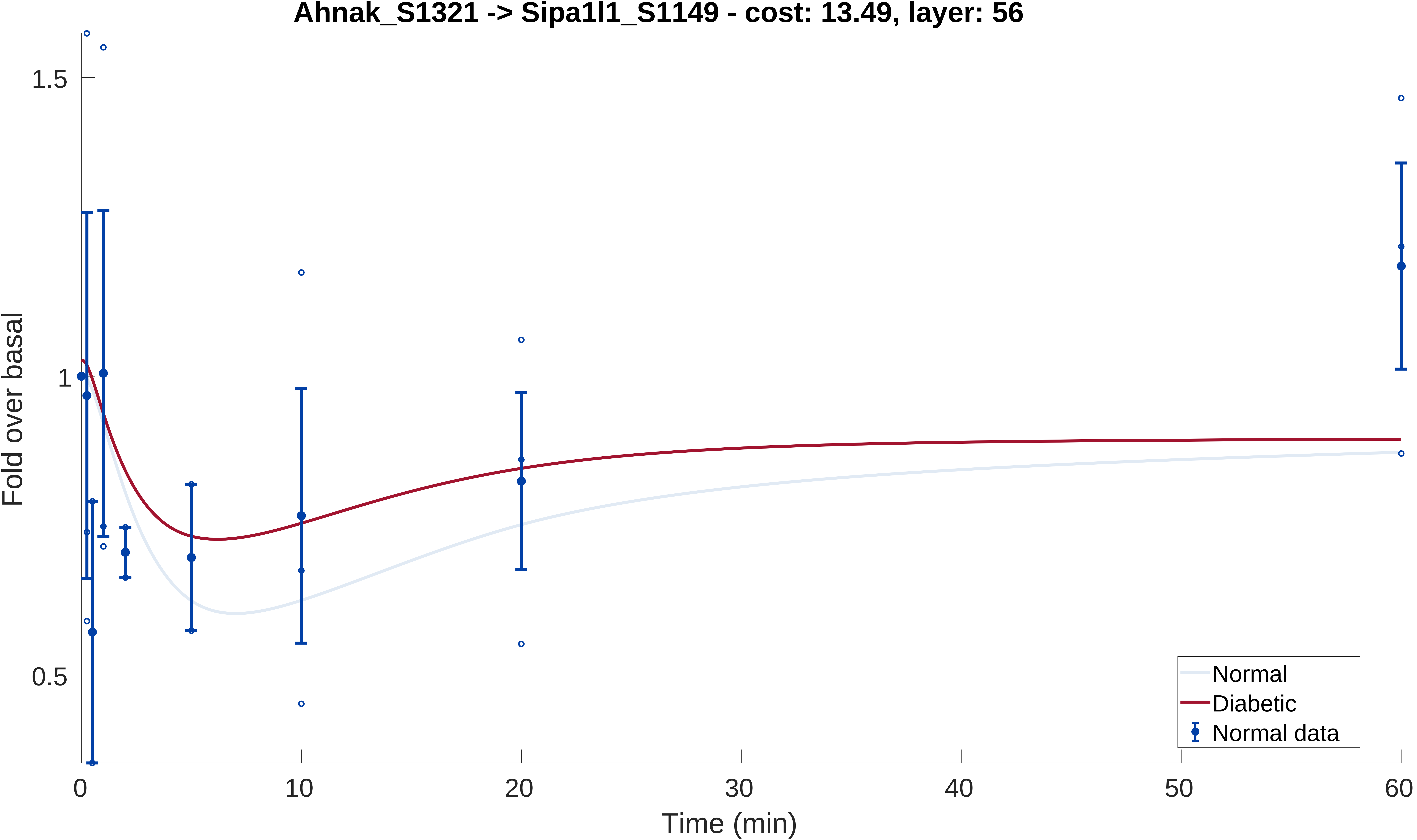

Ahnak_S1321 -> Sipa1l1_S1149 - cost: 13.49, layer: 56
Fold over basal
1.5
1
0.5
0
10
20
30
40
50
60
Time (min)
Normal
Diabetic
Normal data

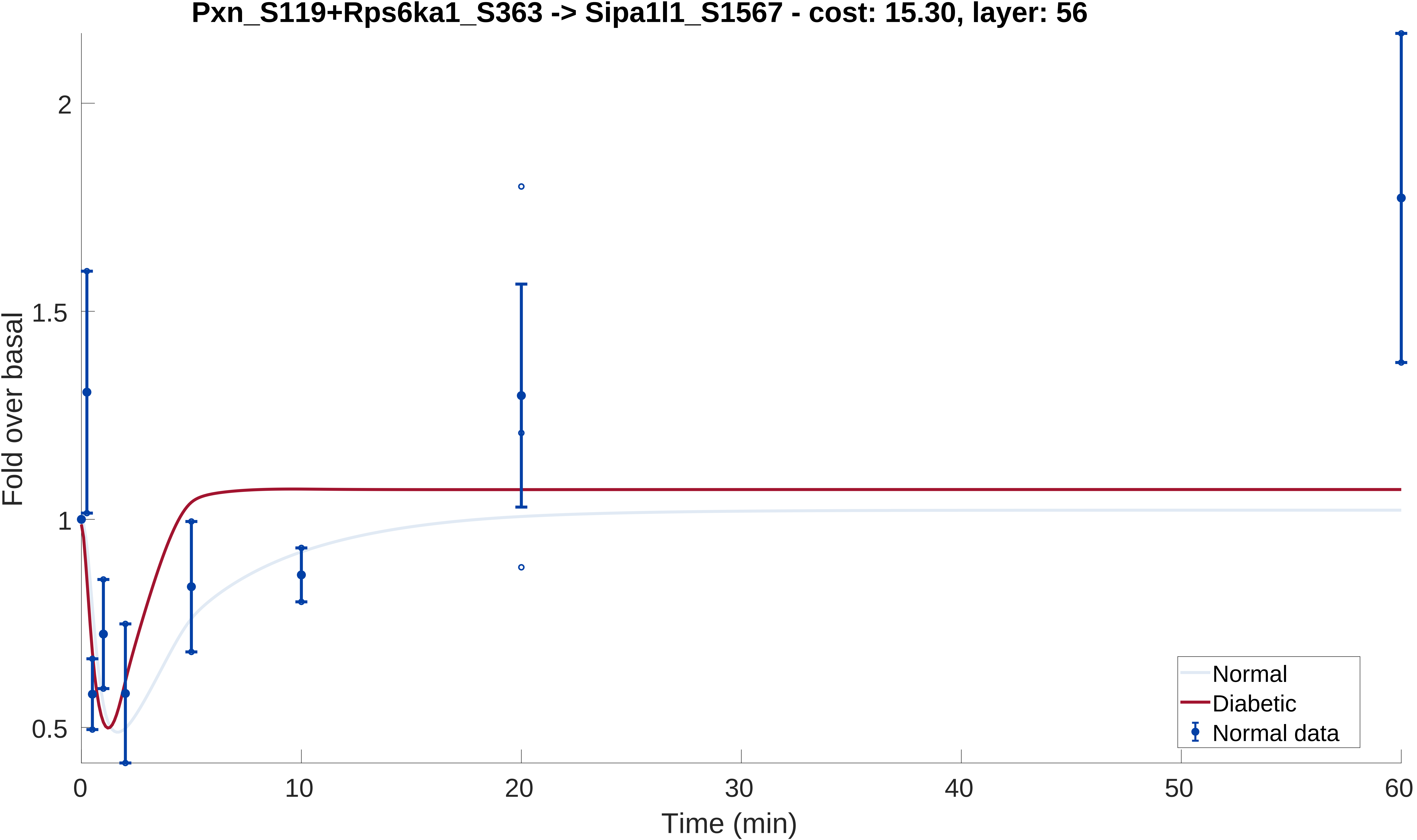

Pxn_S119+Rps6ka1_S363 -> Sipa1l1_S1567 - cost: 15.30, layer: 56
Fold over basal
Time (min)
0.5
1
1.5
2
0
10
20
30
40
50
60
Normal
Diabetic
Normal data

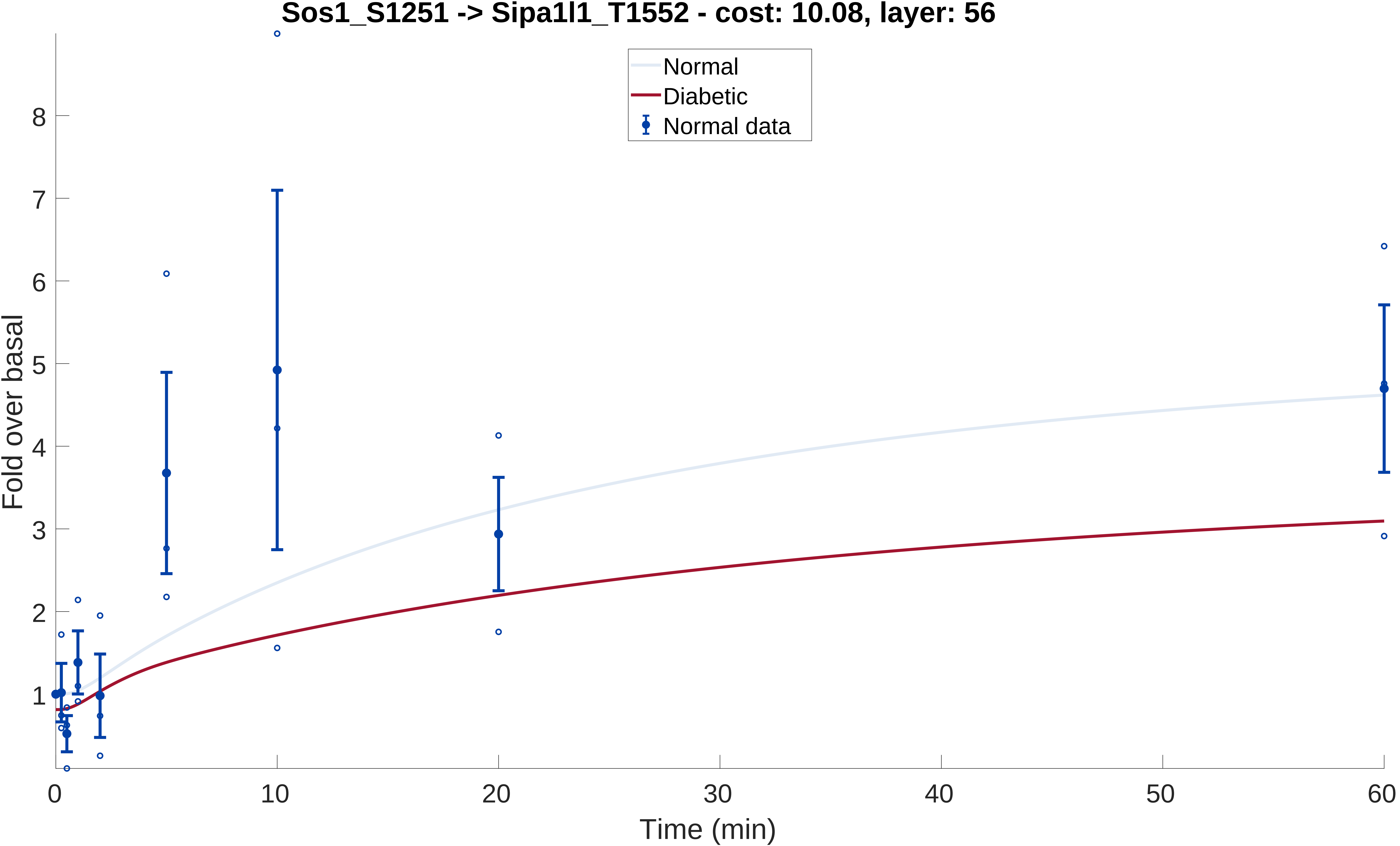
Sos1_S1251 -> Sipa1l1_T1552 - cost: 10.08, layer: 56
Normal
Diabetic
Normal data
Fold over basal
Time (min)
1
2
3
4
5
6
7
8
0
10
20
30
40
50
60

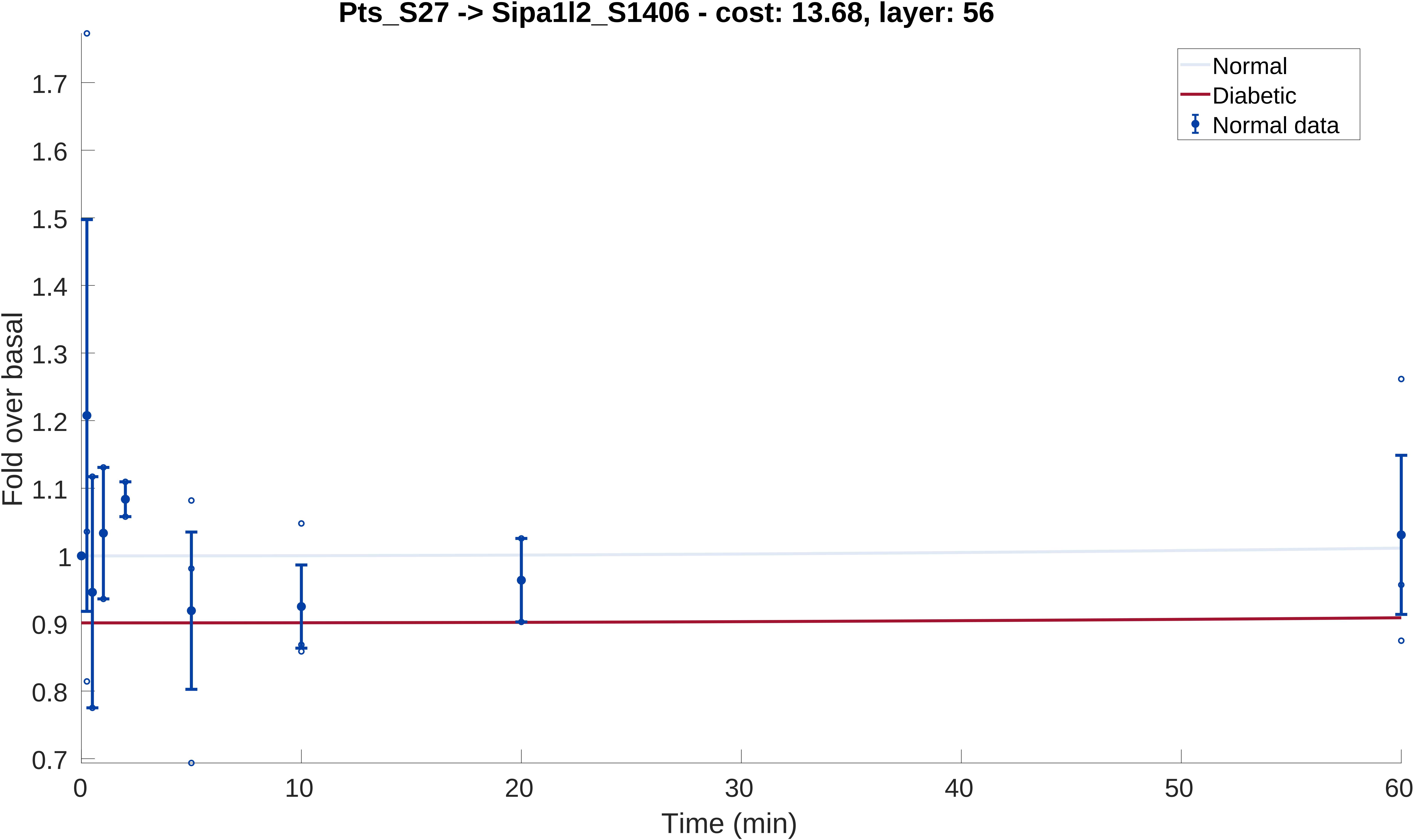

Pts_S27 -> Sipa1l2_S1406 - cost: 13.68, layer: 56
Normal
Diabetic
Normal data
Fold over basal
1.7
1.6
1.5
1.4
1.3
1.2
1.1
1
0.9
0.8
0.7
0
10
20
30
40
50
60
Time (min)

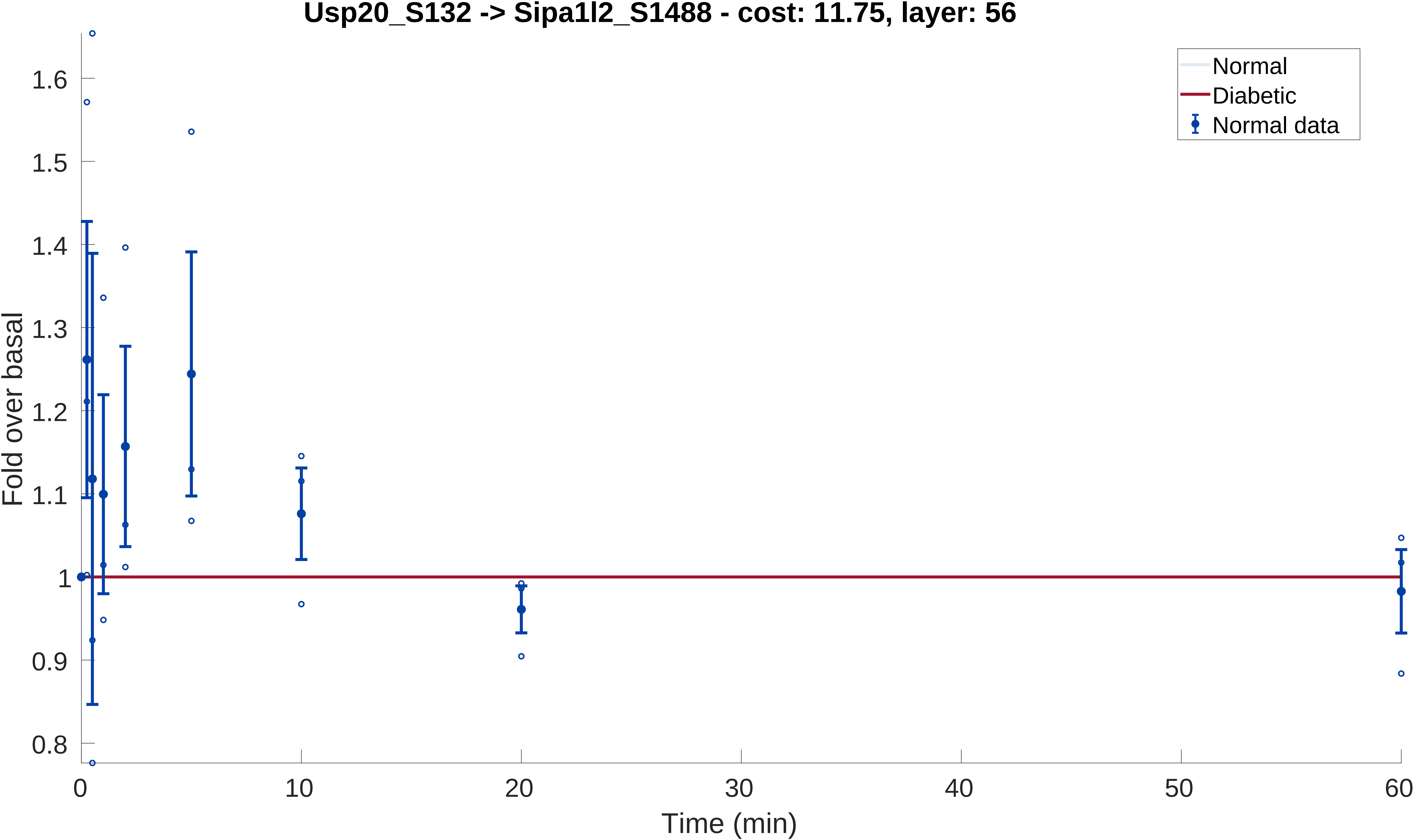

Usp20_S132 -> Sipa1l2_S1488 - cost: 11.75, layer: 56
Normal
Diabetic
Normal data
Fold over basal
1.6
1.5
1.4
1.3
1.2
1.1
1
0.9
0.8
0
10
20
30
40
50
60
Time (min)

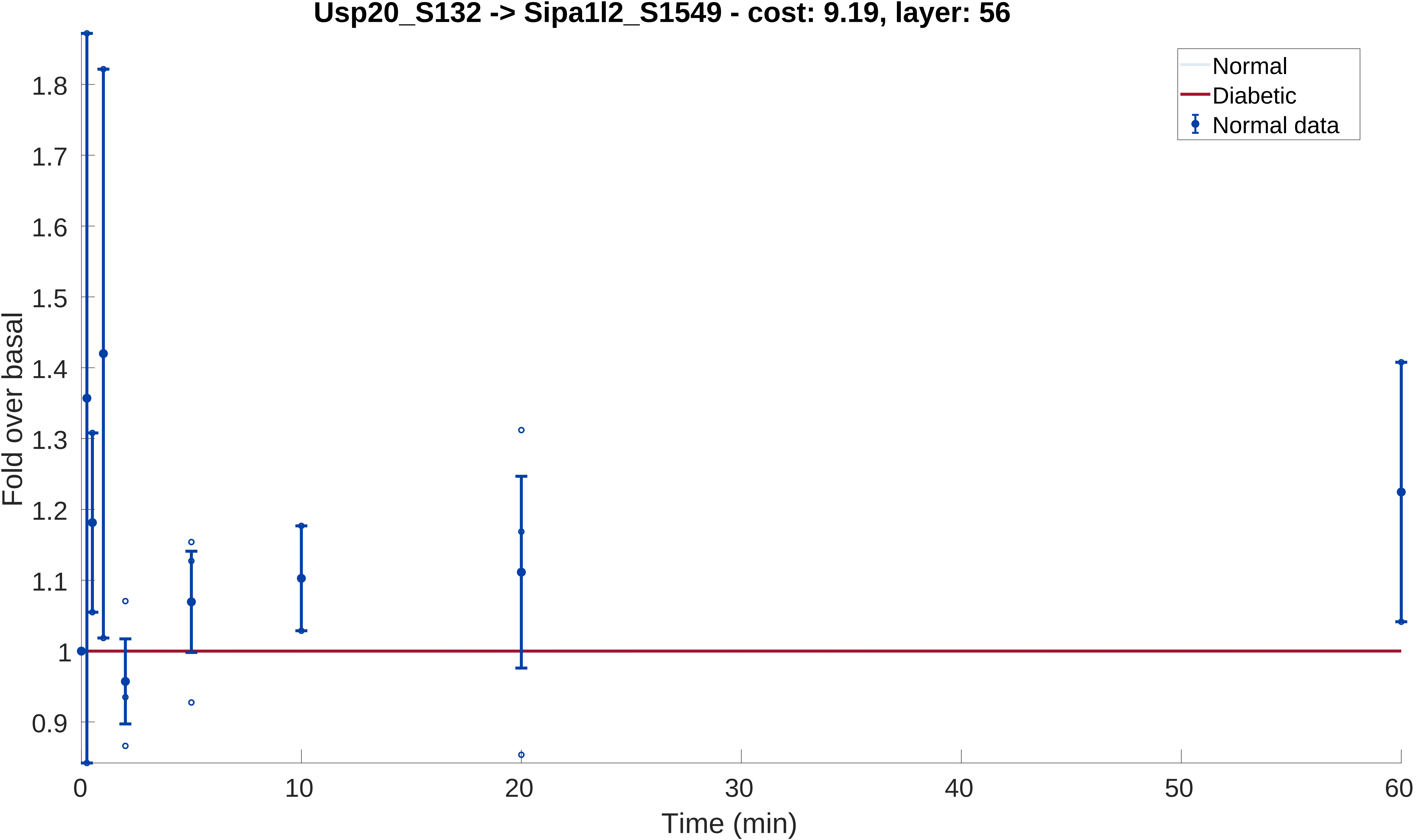
Usp20_S132 -> Sipa1l2_S1549 - cost: 9.19, layer: 56
Normal
Diabetic
Normal data
Fold over basal
Time (min)
0.9
1
1.1
1.2
1.3
1.4
1.5
1.6
1.7
1.8
0
10
20
30
40
50
60

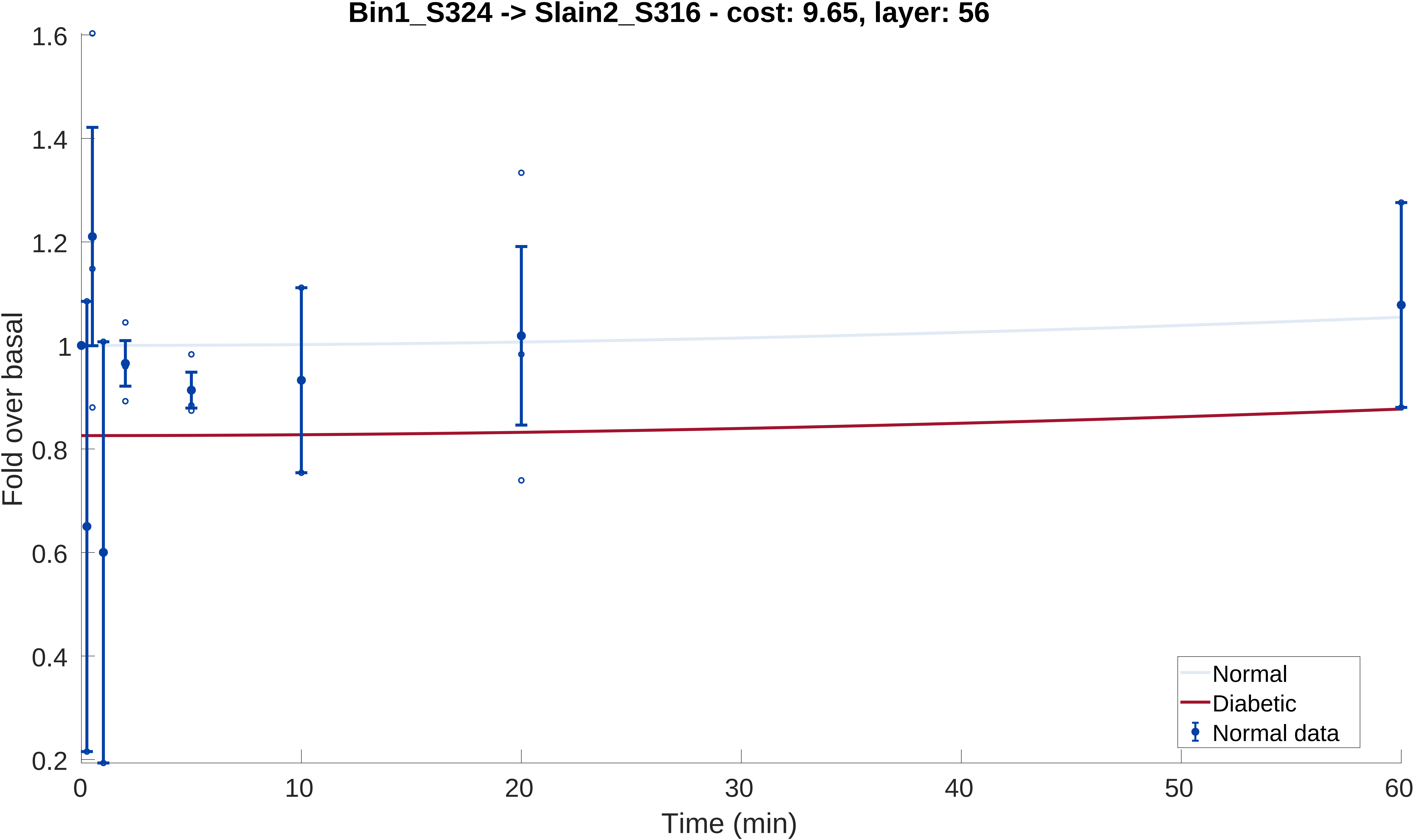

Bin1_S324 -> Slain2_S316 - cost: 9.65, layer: 56
Fold over basal
1.6
1.4
1.2
1
0.8
0.6
0.4
0.2
0
10
20
30
40
50
60
Time (min)
Normal
Diabetic
Normal data

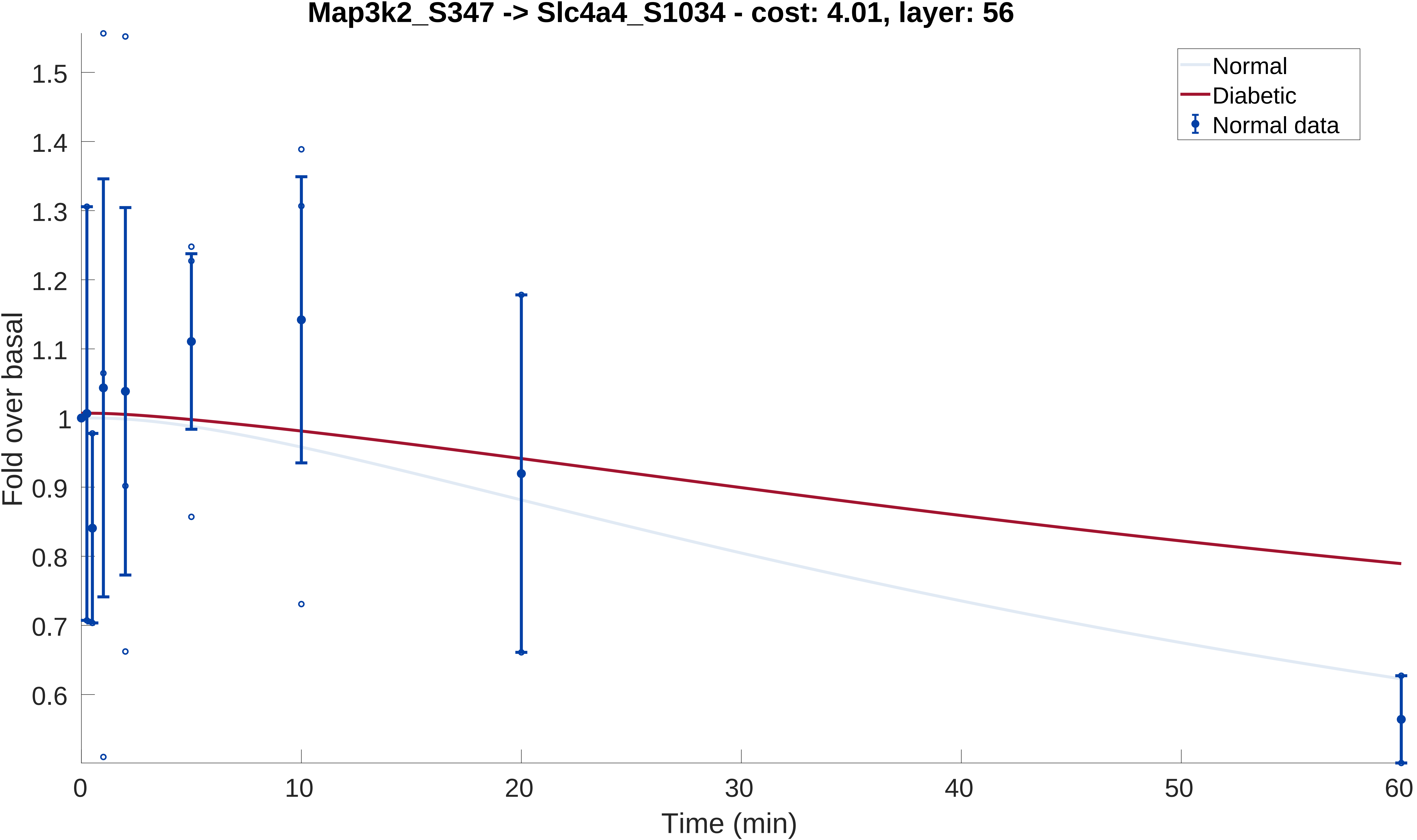
Map3k2_S347 -> Slc4a4_S1034 - cost: 4.01, layer: 56
Normal
Diabetic
Normal data
Fold over basal
1.5
1.4
1.3
1.2
1.1
1
0.9
0.8
0.7
0.6
0
10
20
30
40
50
60
Time (min)

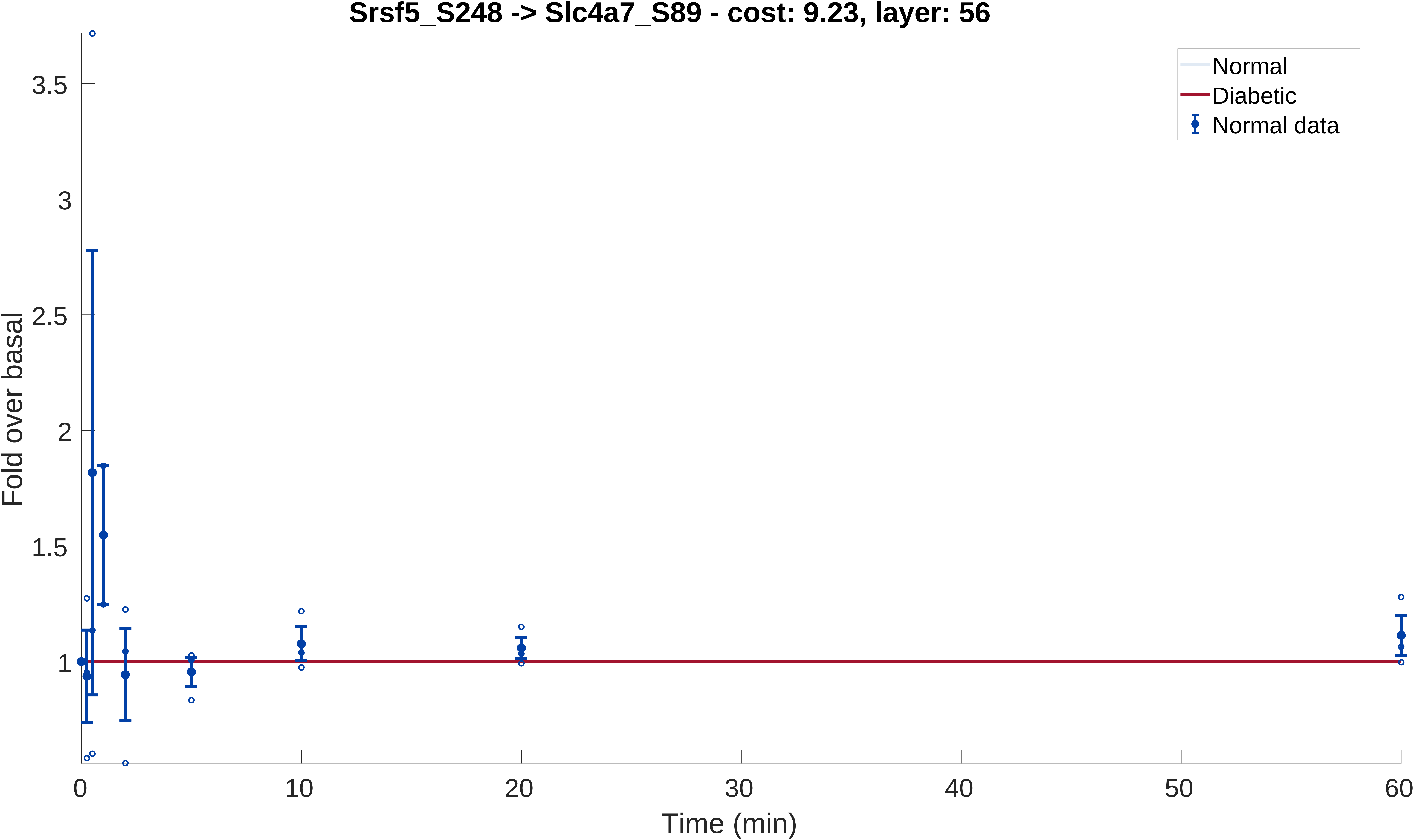

Srsf5_S248 -> Slc4a7_S89 - cost: 9.23, layer: 56
Normal
Diabetic
Normal data
Fold over basal
Time (min)
3.5
3
2.5
2
1.5
1
0
10
20
30
40
50
60

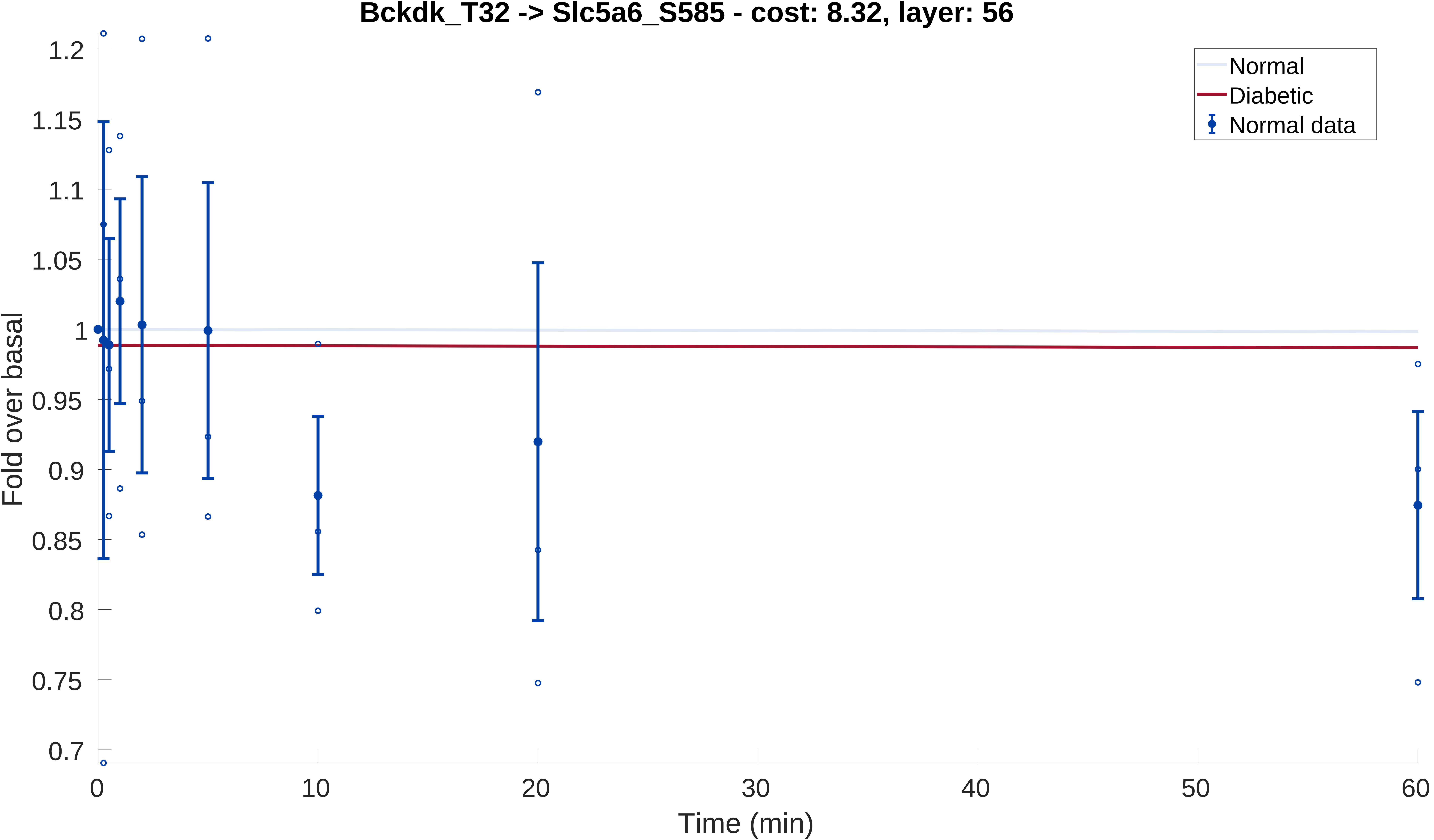
Bckdk_T32 -> Slc5a6_S585 - cost: 8.32, layer: 56
Normal
Diabetic
Normal data
Fold over basal
Time (min)
1.2
1.15
1.1
1.05
1
0.95
0.9
0.85
0.8
0.75
0.7
0
10
20
30
40
50
60

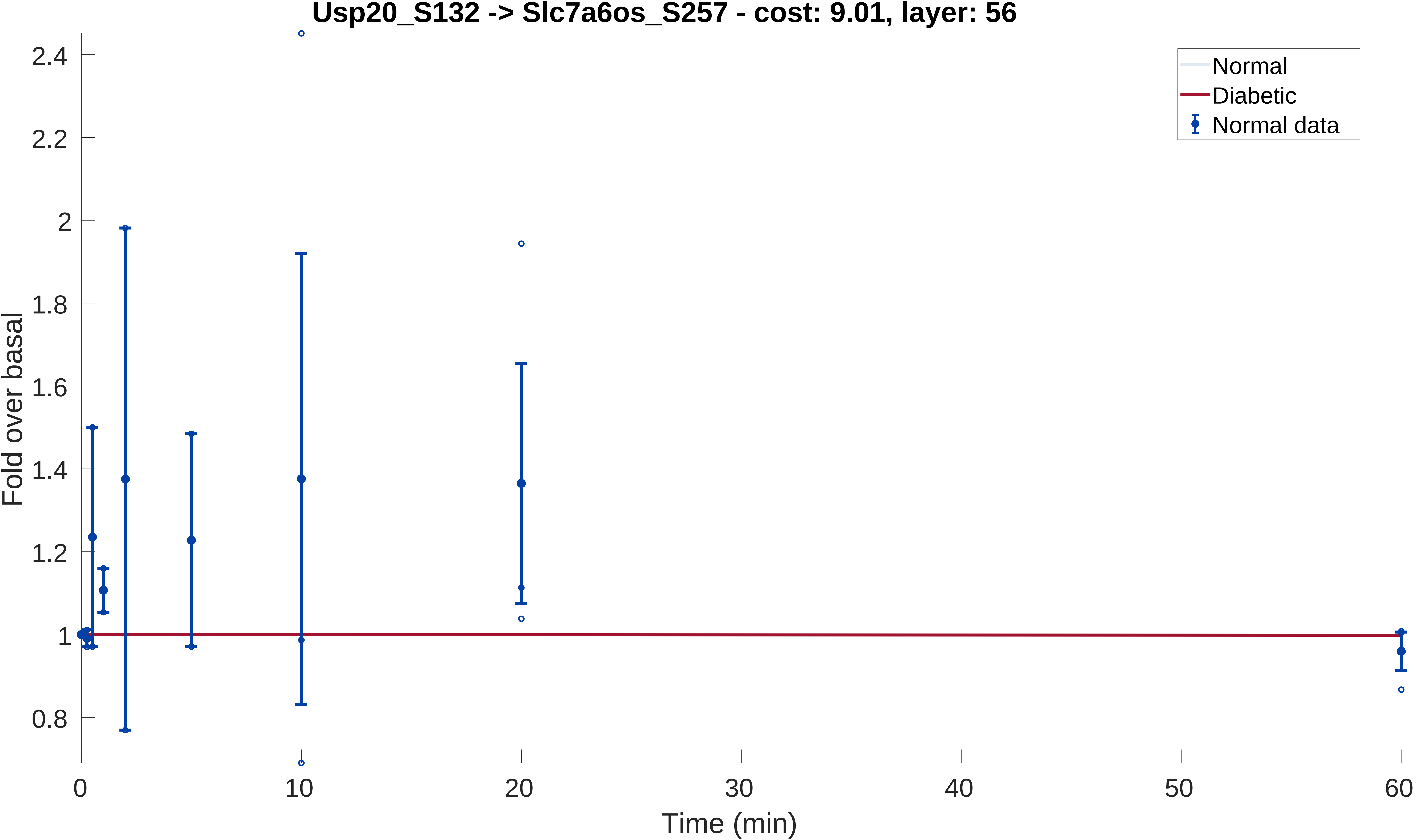

Usp20_S132 -> Slc7a6os_S257 - cost: 9.01, layer: 56
Normal
Diabetic
Normal data
Fold over basal
Time (min)
2.4
2.2
2
1.8
1.6
1.4
1.2
1
0.8
0
10
20
30
40
50
60

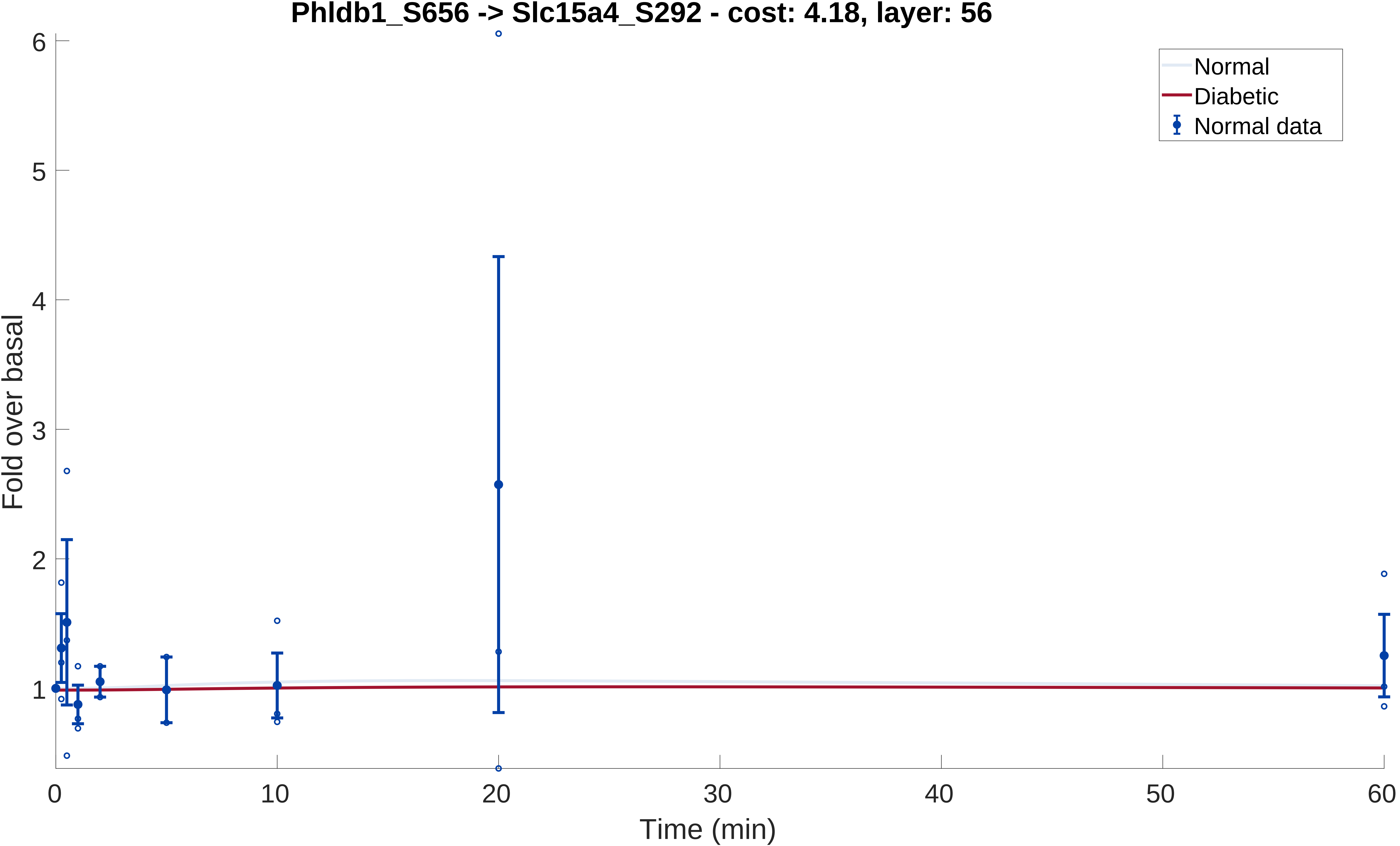

Phldb1_S656 -> Slc15a4_S292 - cost: 4.18, layer: 56
Normal
Diabetic
Normal data
Fold over basal
Time (min)
0
10
20
30
40
50
60
1
2
3
4
5
6

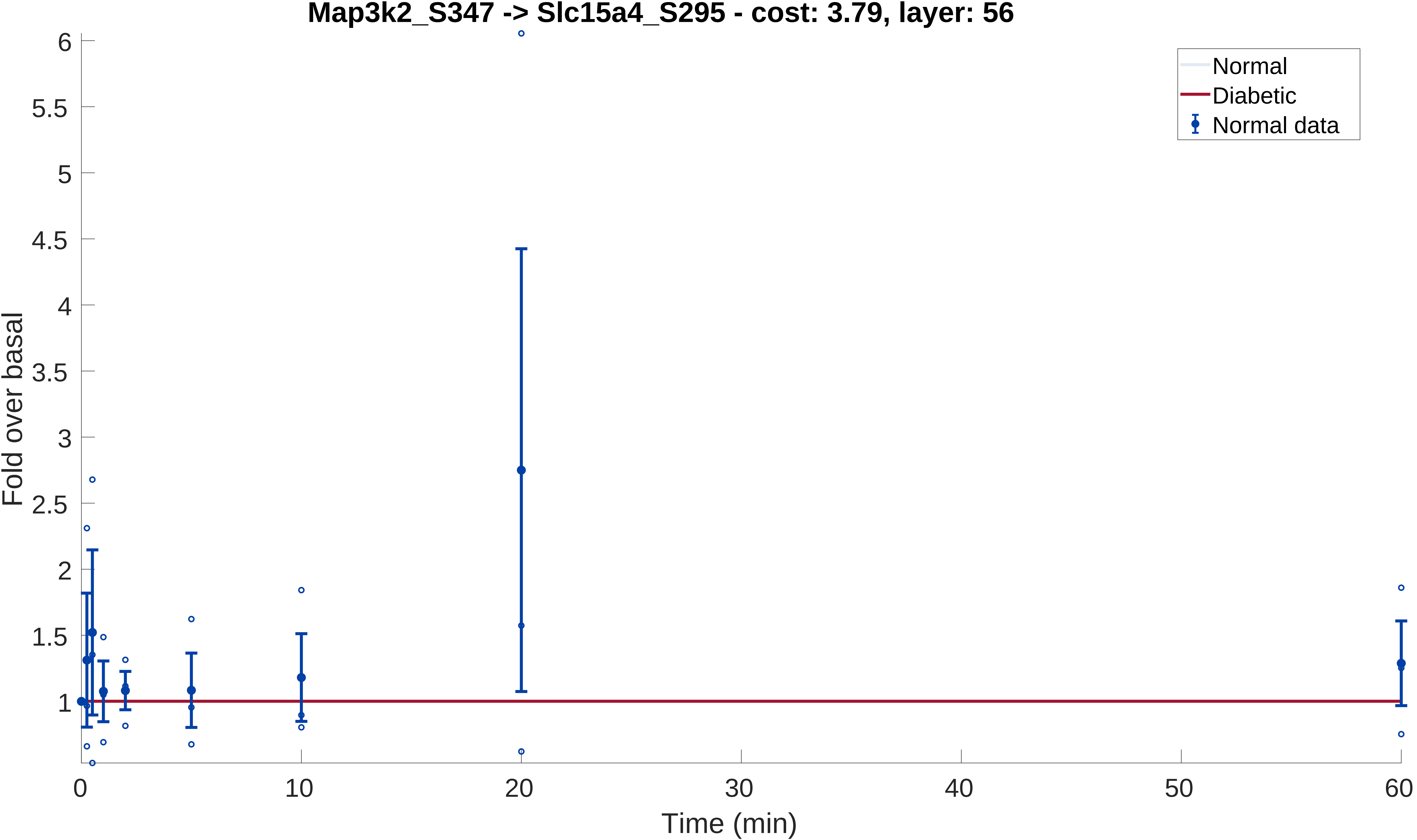

Map3k2_S347 -> Slc15a4_S295 - cost: 3.79, layer: 56
Fold over basal
Time (min)
Normal
Diabetic
Normal data
6
5.5
5
4.5
4
3.5
3
2.5
2
1.5
1
0
10
20
30
40
50
60

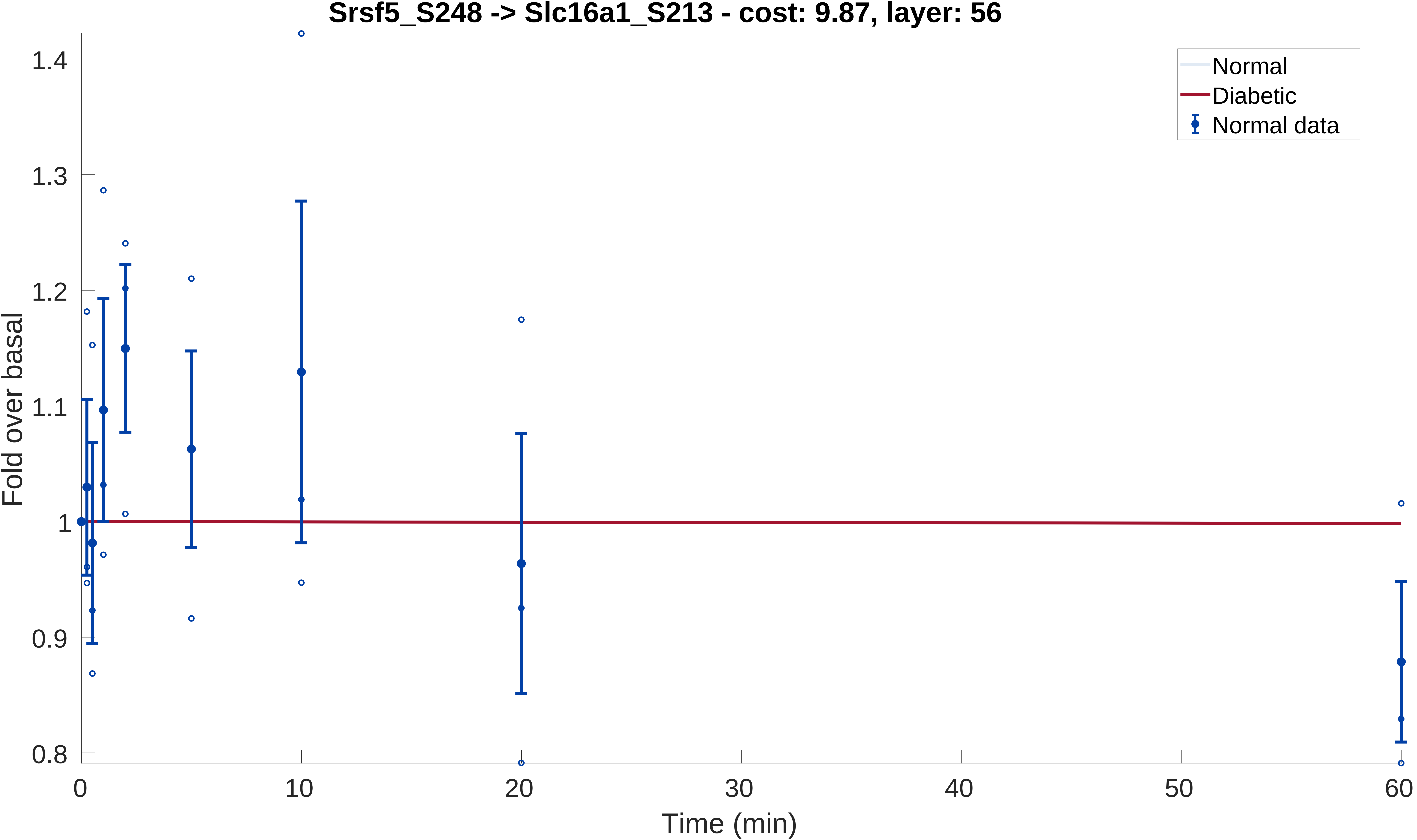
Srsf5_S248 -> Slc16a1_S213 - cost: 9.87, layer: 56
Normal
Diabetic
Normal data
Fold over basal
1.4
1.3
1.2
1.1
1
0.9
0.8
0
10
20
30
40
50
60
Time (min)

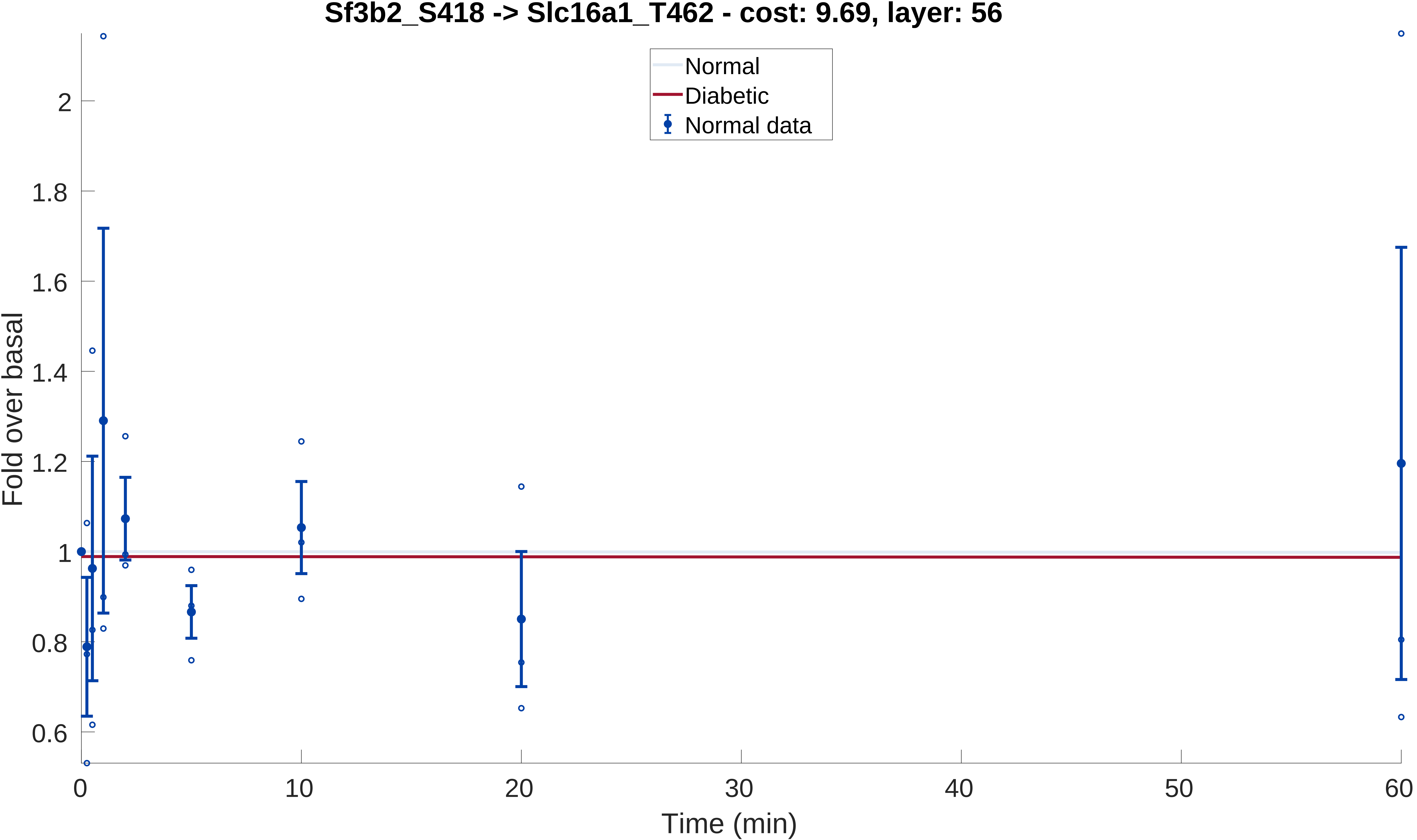

Sf3b2_S418 -> Slc16a1_T462 - cost: 9.69, layer: 56
Normal
Diabetic
Normal data
Fold over basal
Time (min)
0.6
0.8
1
1.2
1.4
1.6
1.8
2
0
10
20
30
40
50
60

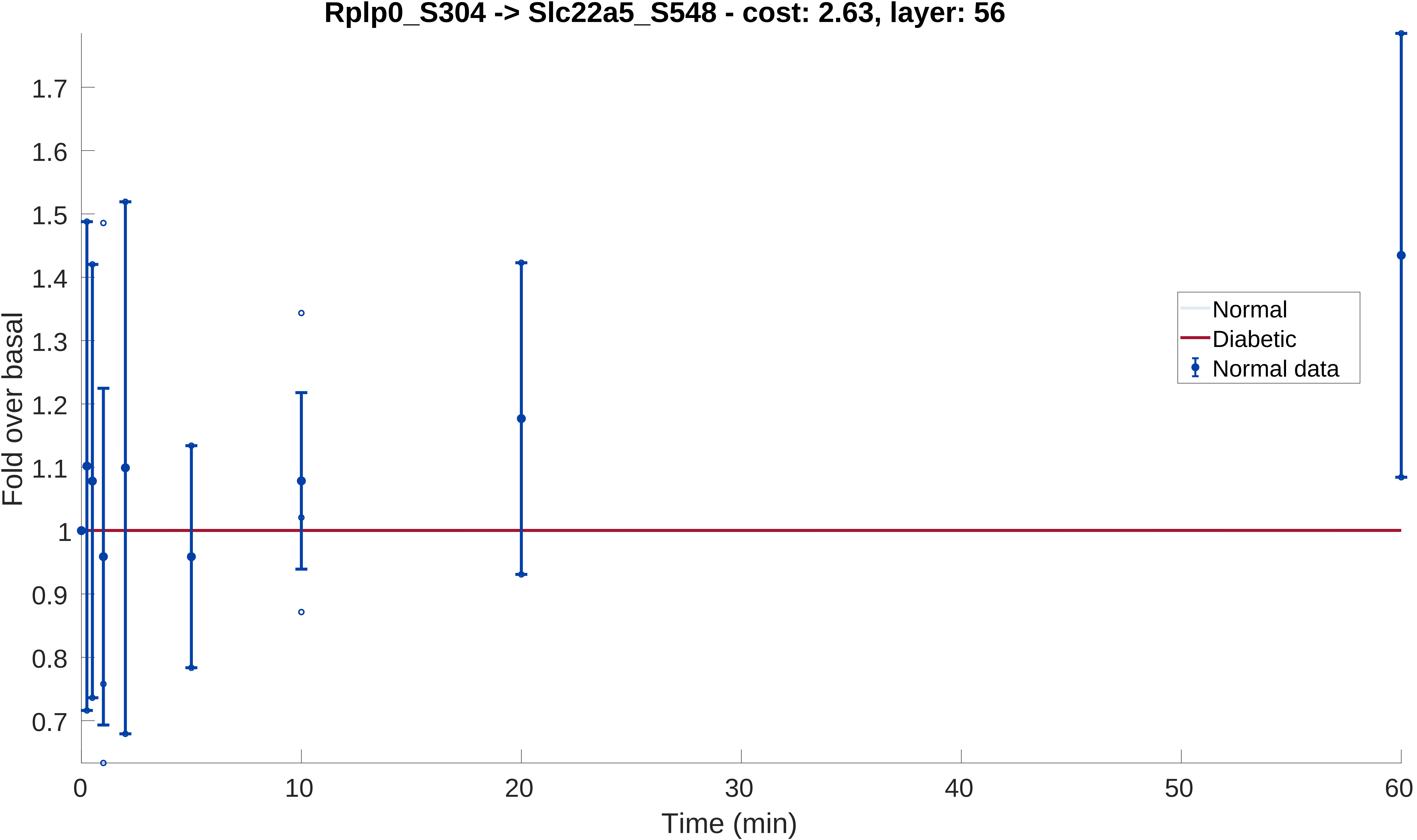
Rplp0_S304 -> Slc22a5_S548 - cost: 2.63, layer: 56
Fold over basal
1.7
1.6
1.5
1.4
1.3
1.2
1.1
1
0.9
0.8
0.7
0
10
20
30
40
50
60
Time (min)
Normal
Diabetic
Normal data

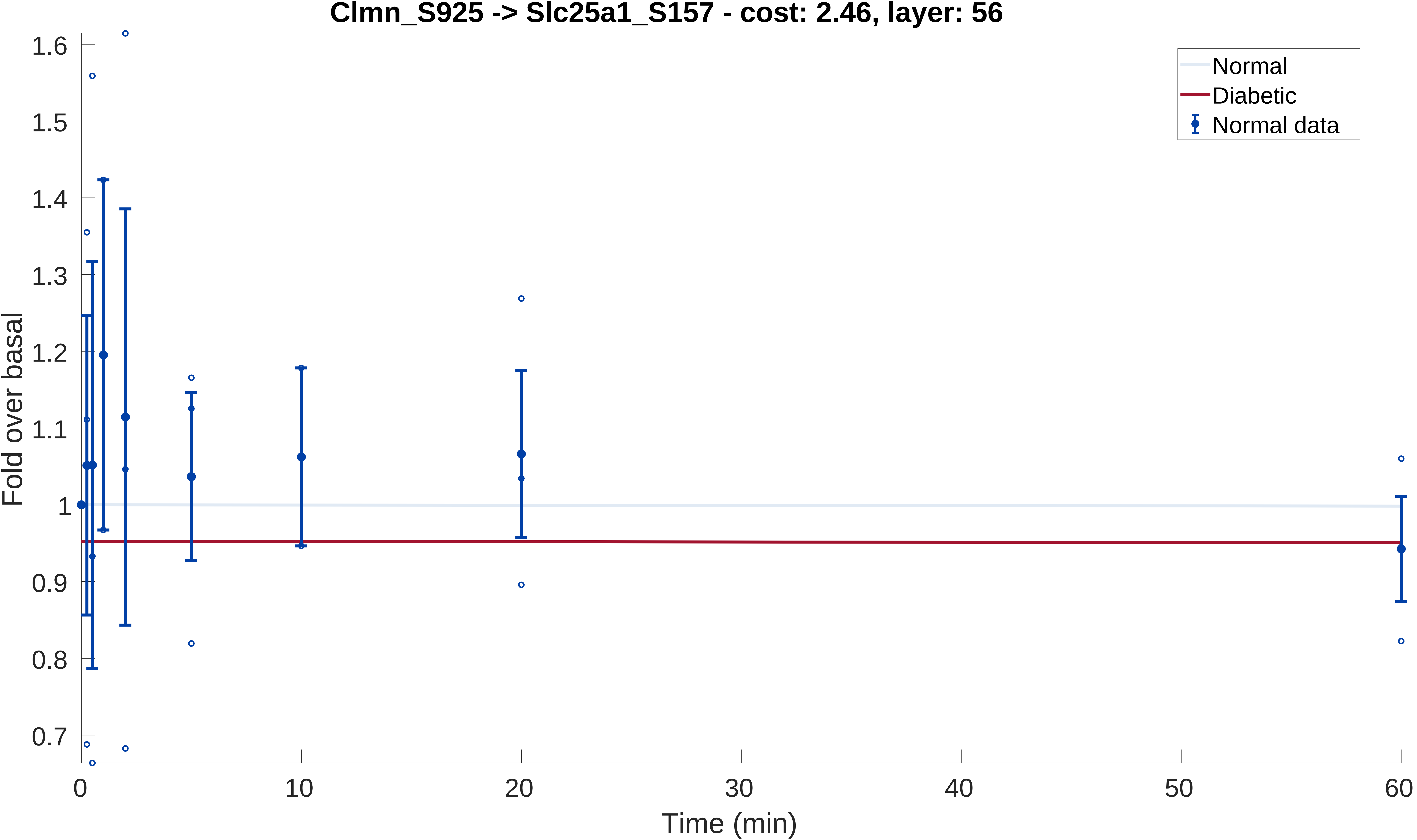

Clmn_S925 -> Slc25a1_S157 - cost: 2.46, layer: 56
Normal
Diabetic
Normal data
Fold over basal
Time (min)
1.6
1.5
1.4
1.3
1.2
1.1
1
0.9
0.8
0.7
0
10
20
30
40
50
60

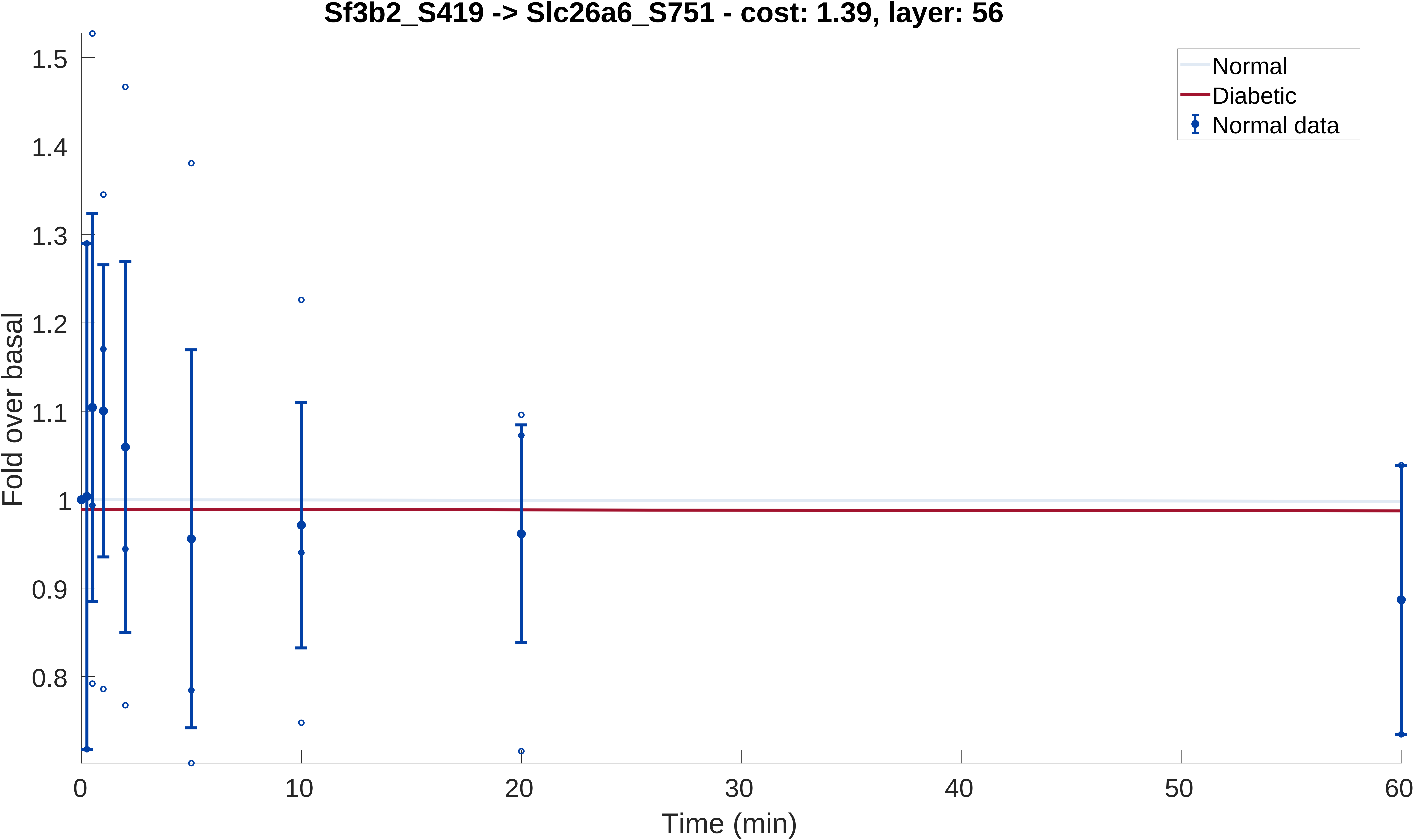

Sf3b2_S419 -> Slc26a6_S751 - cost: 1.39, layer: 56
Normal
Diabetic
Normal data
Fold over basal
Time (min)
1.5
1.4
1.3
1.2
1.1
1
0.9
0.8
0
10
20
30
40
50
60

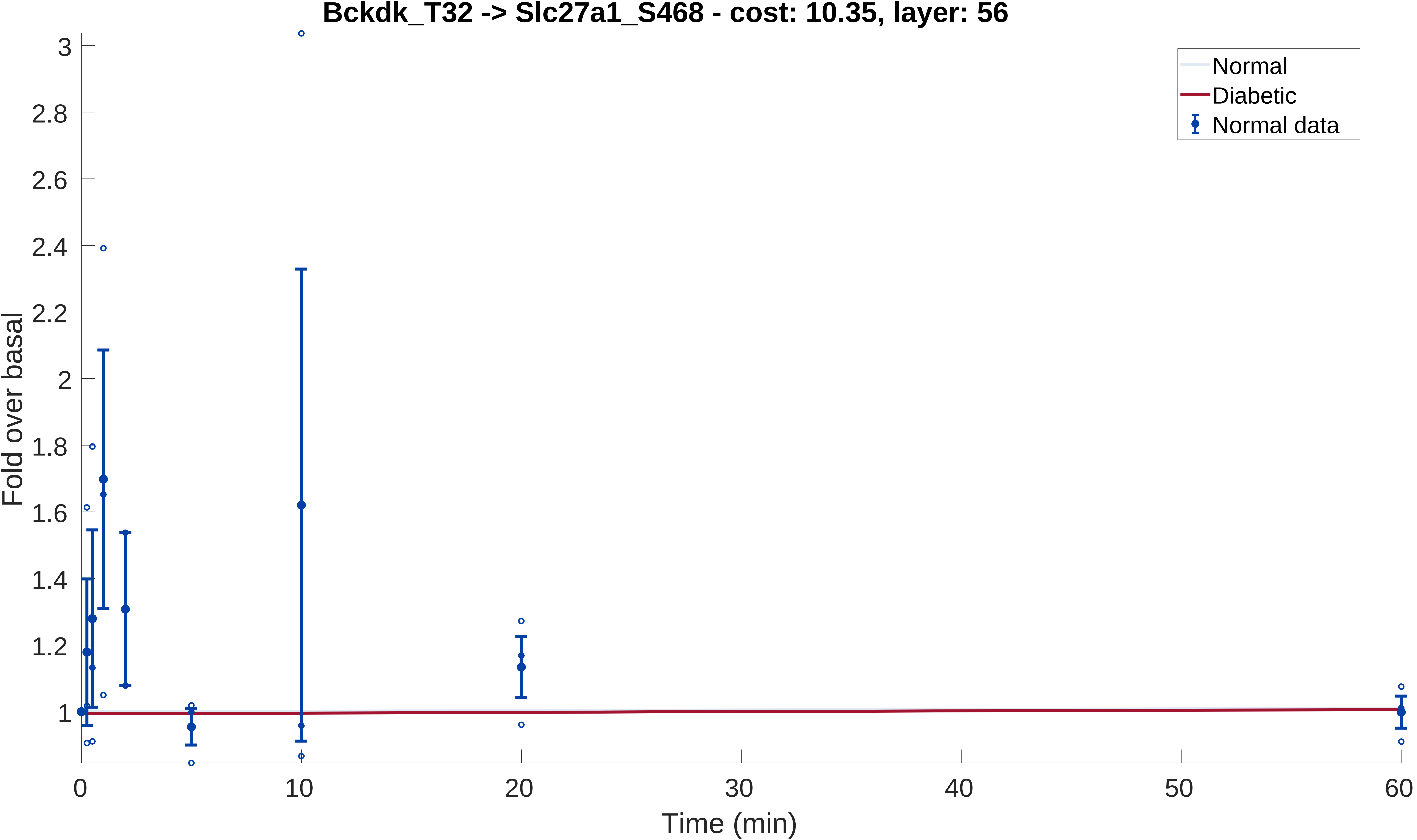

Bckdk_T32 -> Slc27a1_S468 - cost: 10.35, layer: 56
Normal
Diabetic
Normal data
Fold over basal
Time (min)
3
2.8
2.6
2.4
2.2
2
1.8
1.6
1.4
1.2
1
0
10
20
30
40
50
60

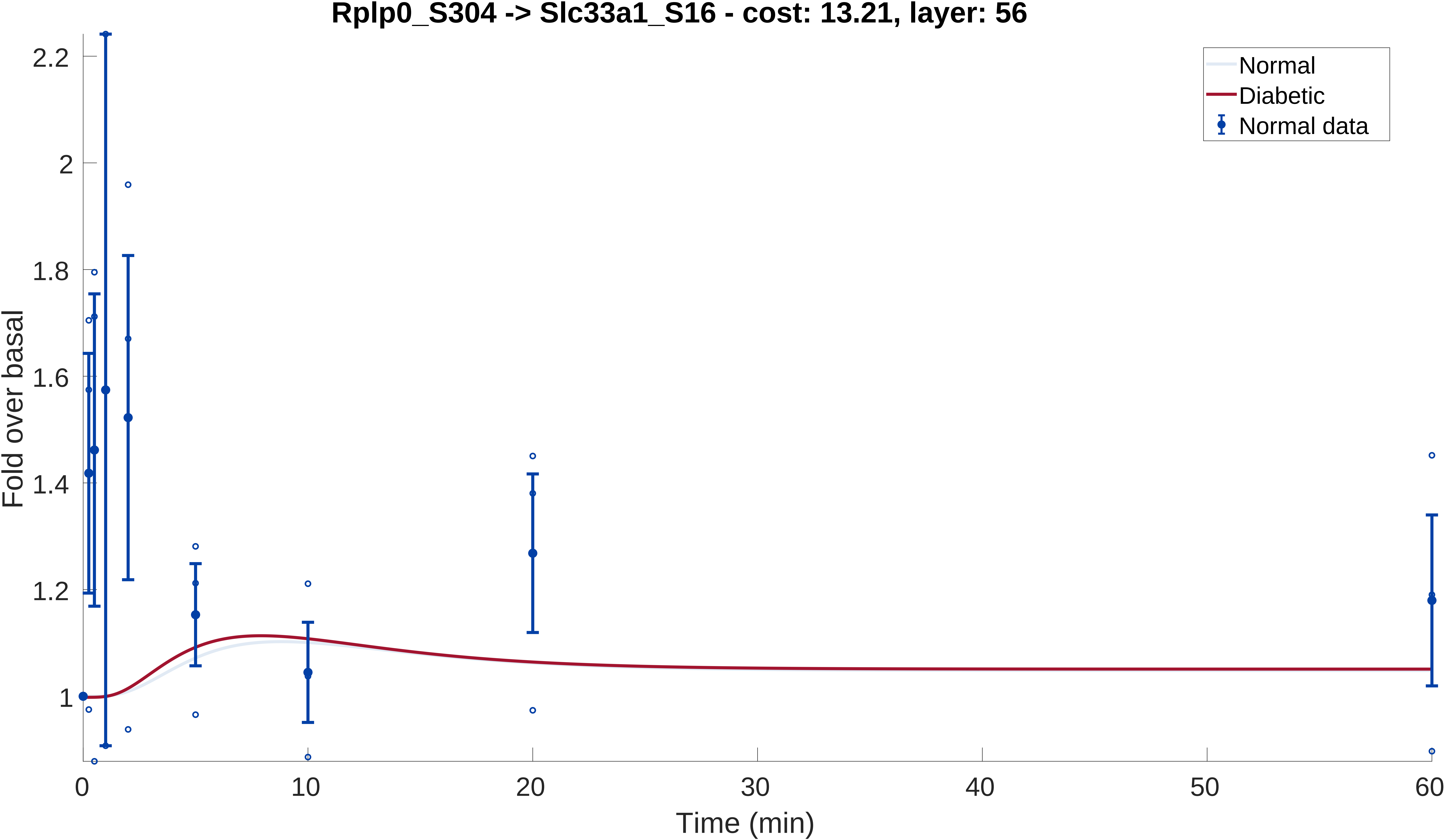
Rplp0_S304 -> Slc33a1_S16 - cost: 13.21, layer: 56
Normal
Diabetic
Normal data
Fold over basal
2.2
2
1.8
1.6
1.4
1.2
1
0
10
20
30
40
50
60
Time (min)

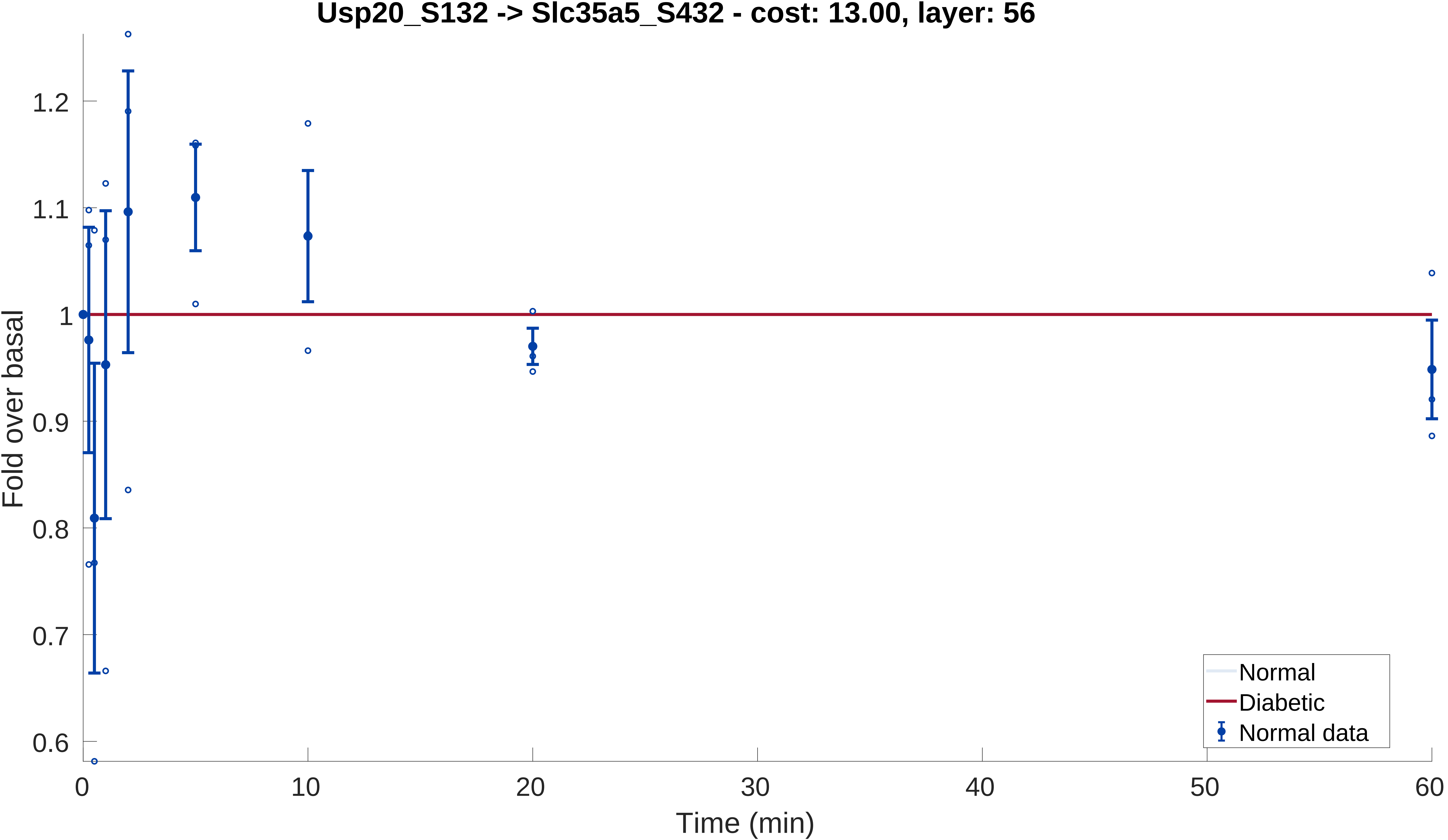

Usp20_S132 -> Slc35a5_S432 - cost: 13.00, layer: 56
Fold over basal
1.2
1.1
1
0.9
0.8
0.7
0.6
0
10
20
30
40
50
60
Time (min)
Normal
Diabetic
Normal data

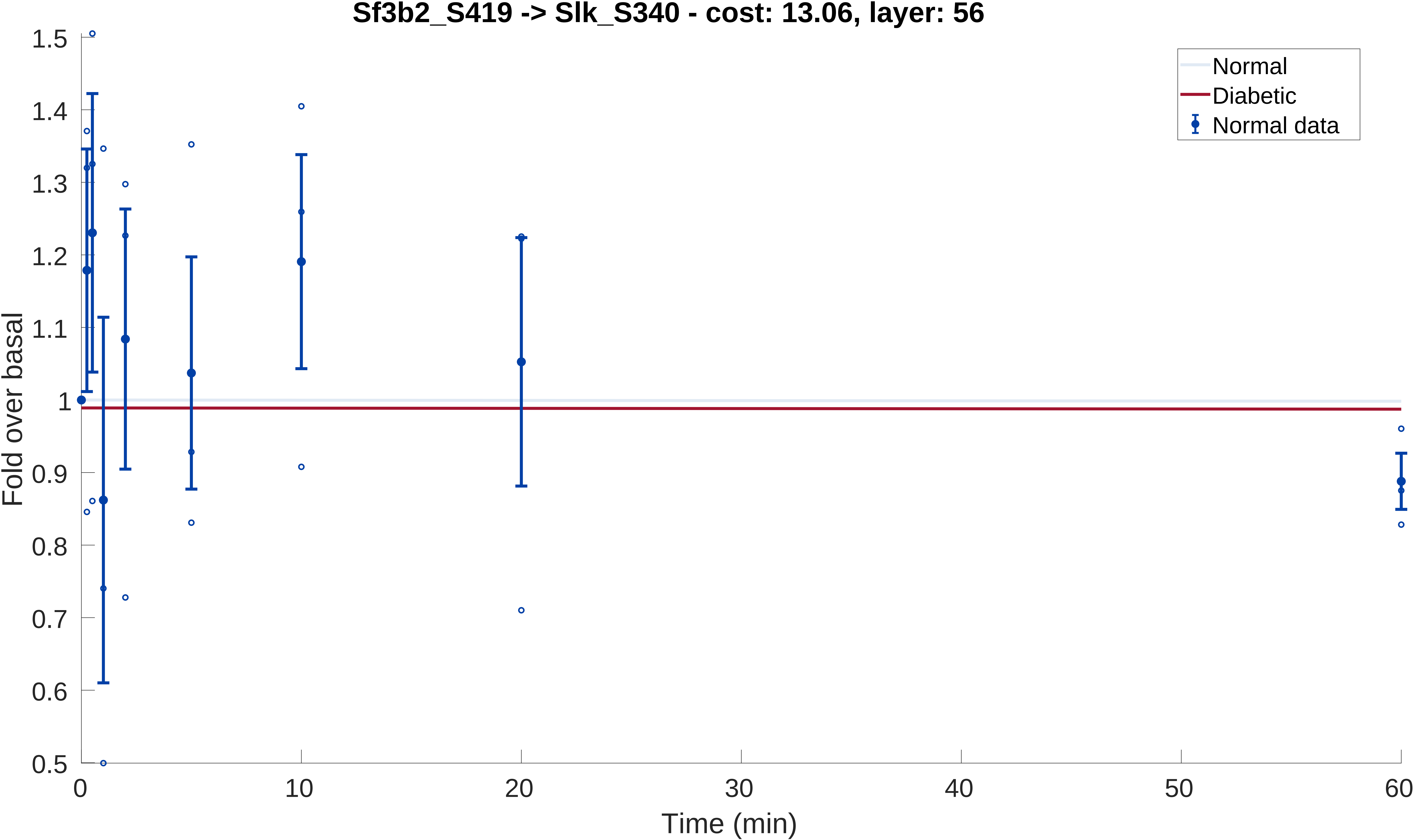

Sf3b2_S419 -> Slk_S340 - cost: 13.06, layer: 56
Fold over basal
1.5
1.4
1.3
1.2
1.1
1
0.9
0.8
0.7
0.6
0.5
0
10
20
30
40
50
60
Time (min)
Normal
Diabetic
Normal data

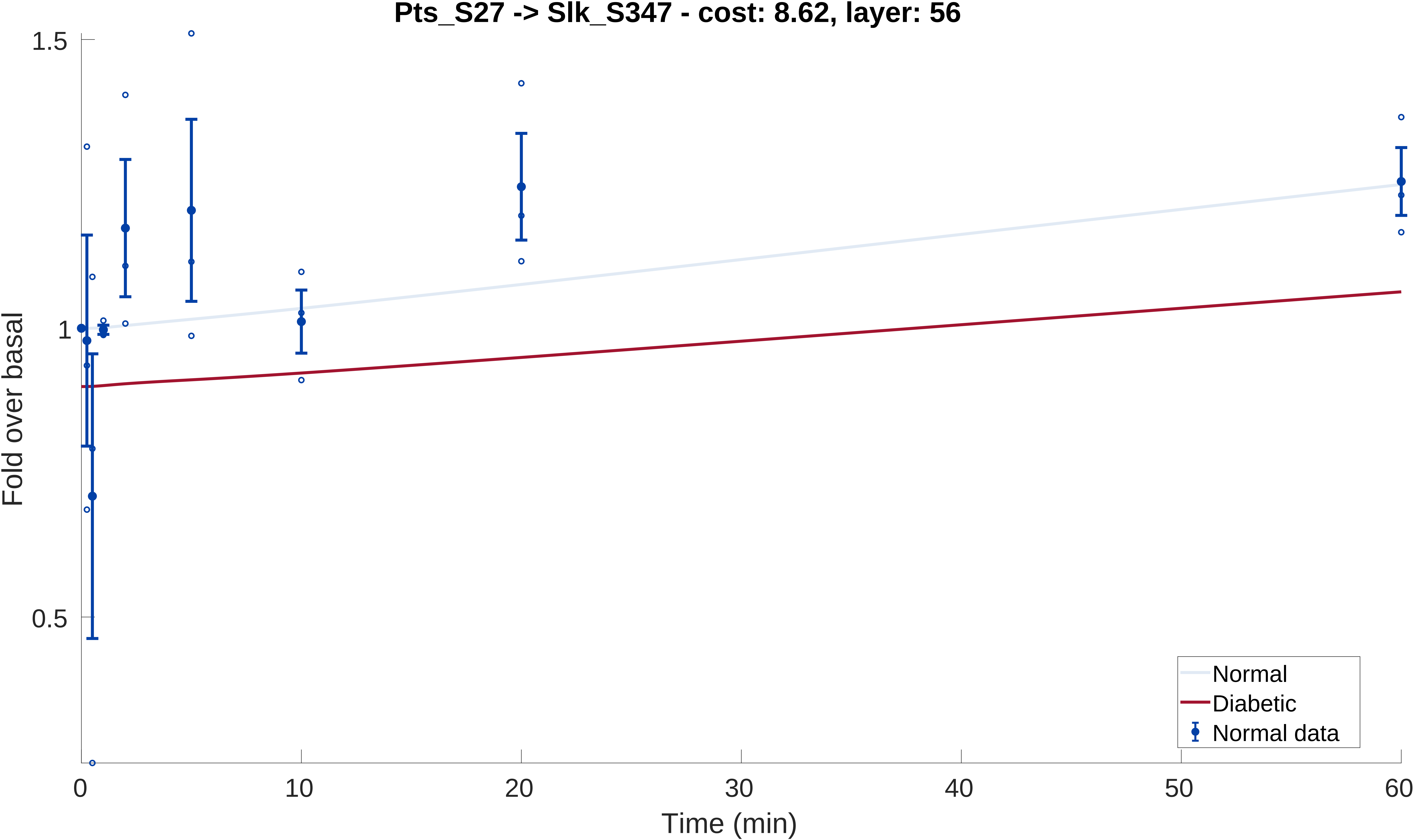
Pts_S27 -> Slk_S347 - cost: 8.62, layer: 56
1.5
1
0.5
Fold over basal
0
10
20
30
40
50
60
Time (min)
Normal
Diabetic
Normal data

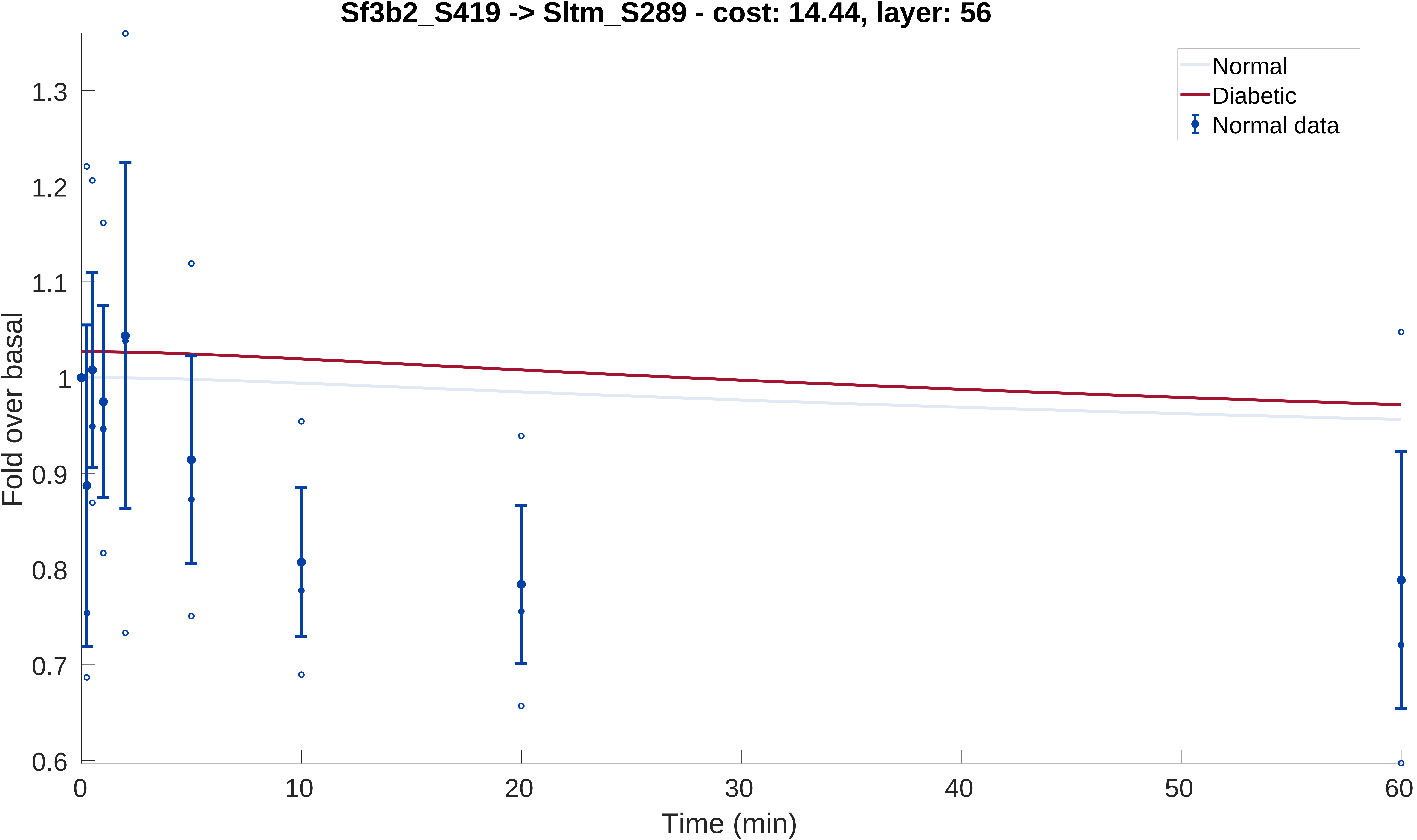

Sf3b2_S419 -> Sltm_S289 - cost: 14.44, layer: 56
Normal
Diabetic
Normal data
Fold over basal
1.3
1.2
1.1
1
0.9
0.8
0.7
0.6
0
10
20
30
40
50
60
Time (min)

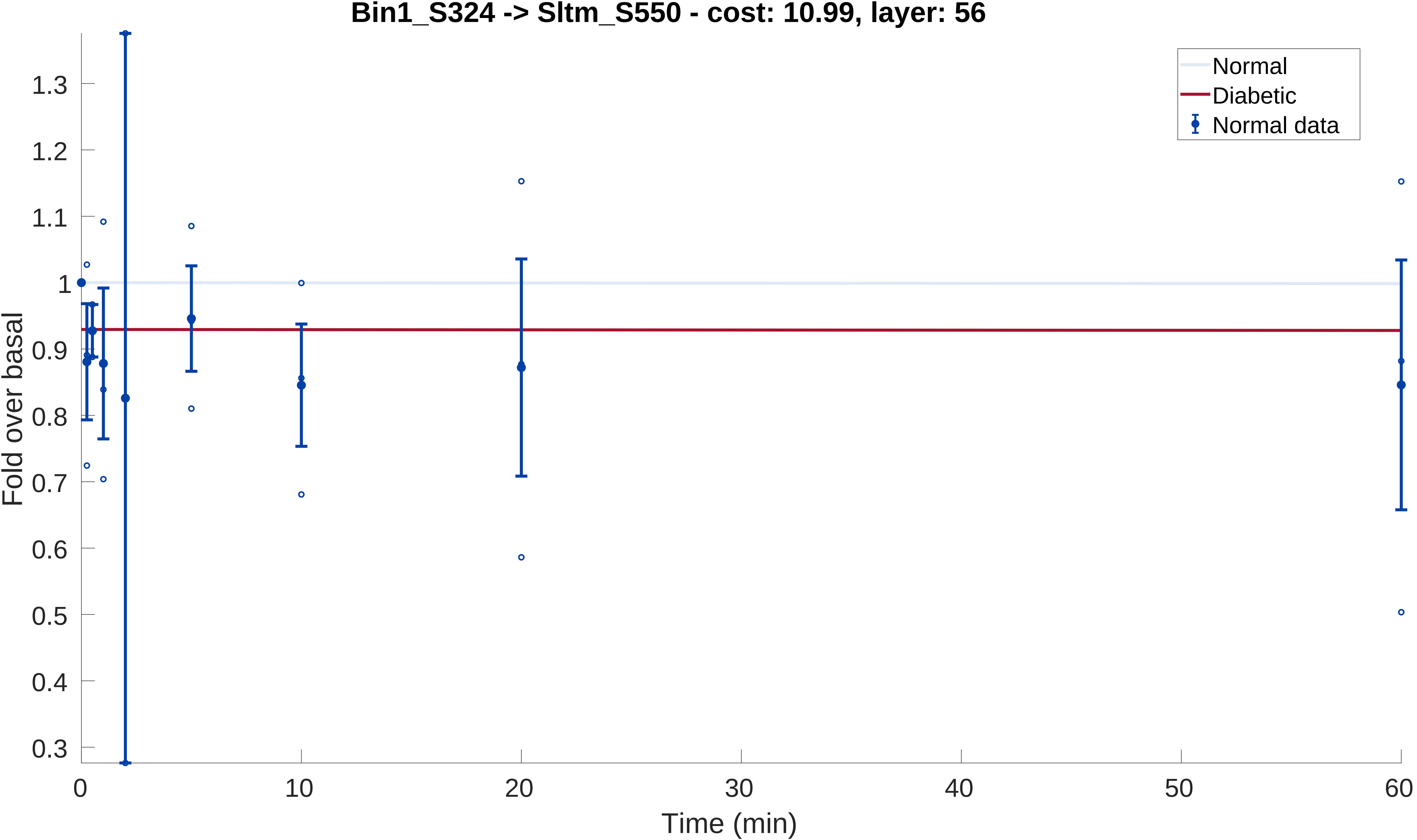
Bin1_S324 -> Sltm_S550 - cost: 10.99, layer: 56
Normal
Diabetic
Normal data
Fold over basal
Time (min)
1.3
1.2
1.1
1
0.9
0.8
0.7
0.6
0.5
0.4
0.3
0
10
20
30
40
50
60

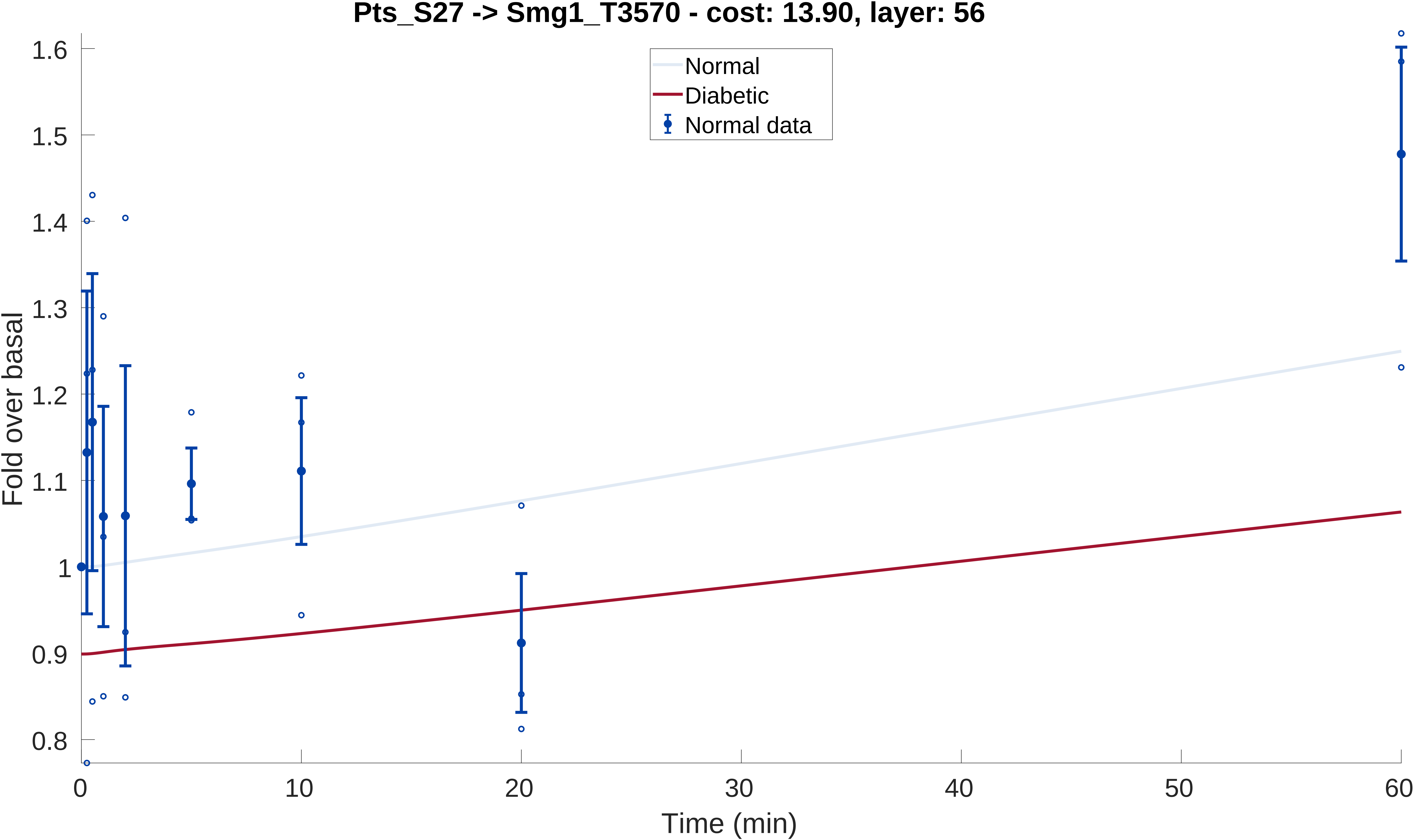

Pts_S27 -> Smg1_T3570 - cost: 13.90, layer: 56
Normal
Diabetic
Normal data
Fold over basal
1.6
1.5
1.4
1.3
1.2
1.1
1
0.9
0.8
0
10
20
30
40
50
60
Time (min)

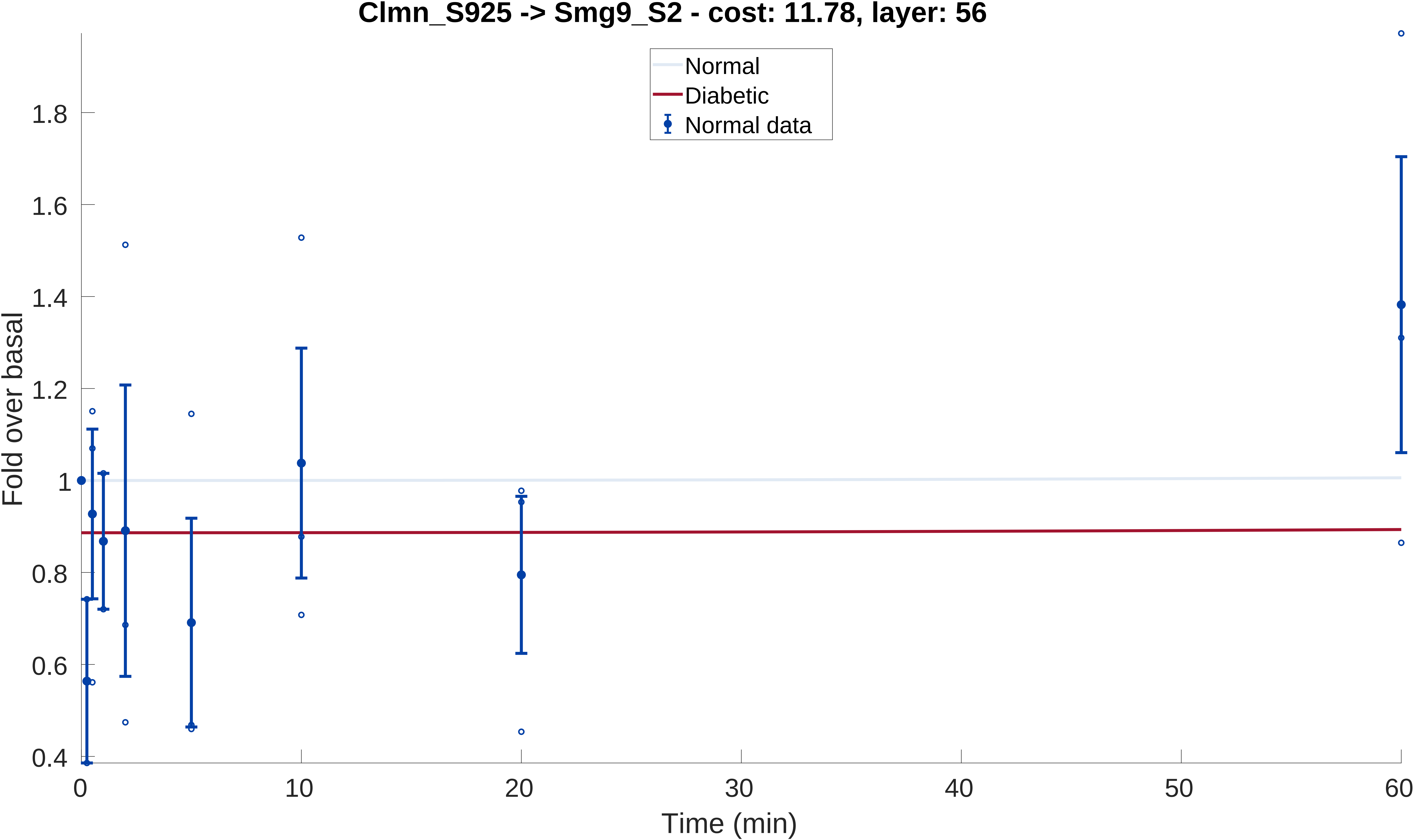

Clmn_S925 -> Smg9_S2 - cost: 11.78, layer: 56
Normal
Diabetic
Normal data
Fold over basal
1.8
1.6
1.4
1.2
1
0.8
0.6
0.4
0
10
20
30
40
50
60
Time (min)

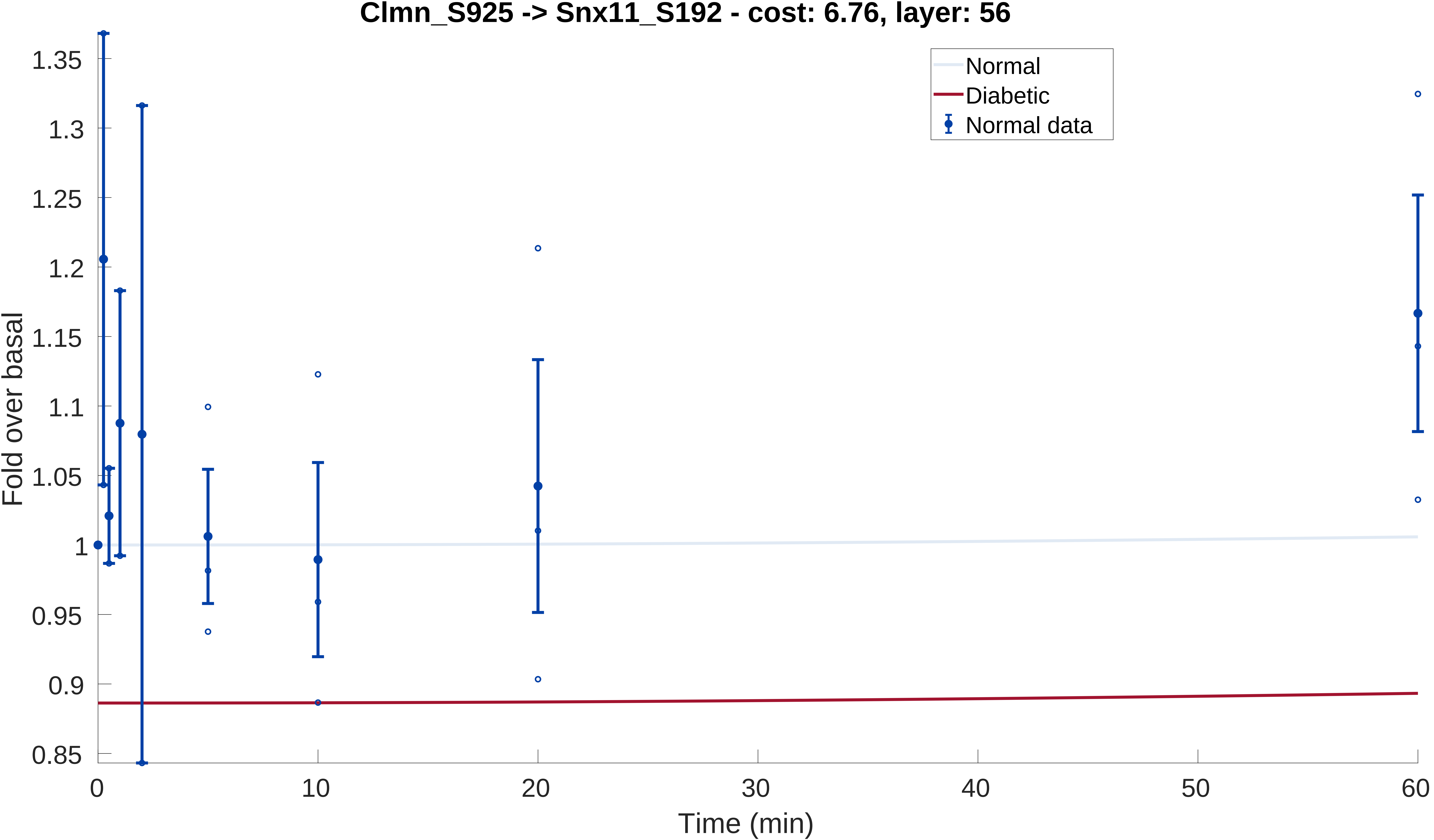

Clmn_S925 -> Snx11_S192 - cost: 6.76, layer: 56
Normal
Diabetic
Normal data
Fold over basal
Time (min)
1.35
1.3
1.25
1.2
1.15
1.1
1.05
1
0.95
0.9
0.85
0
10
20
30
40
50
60

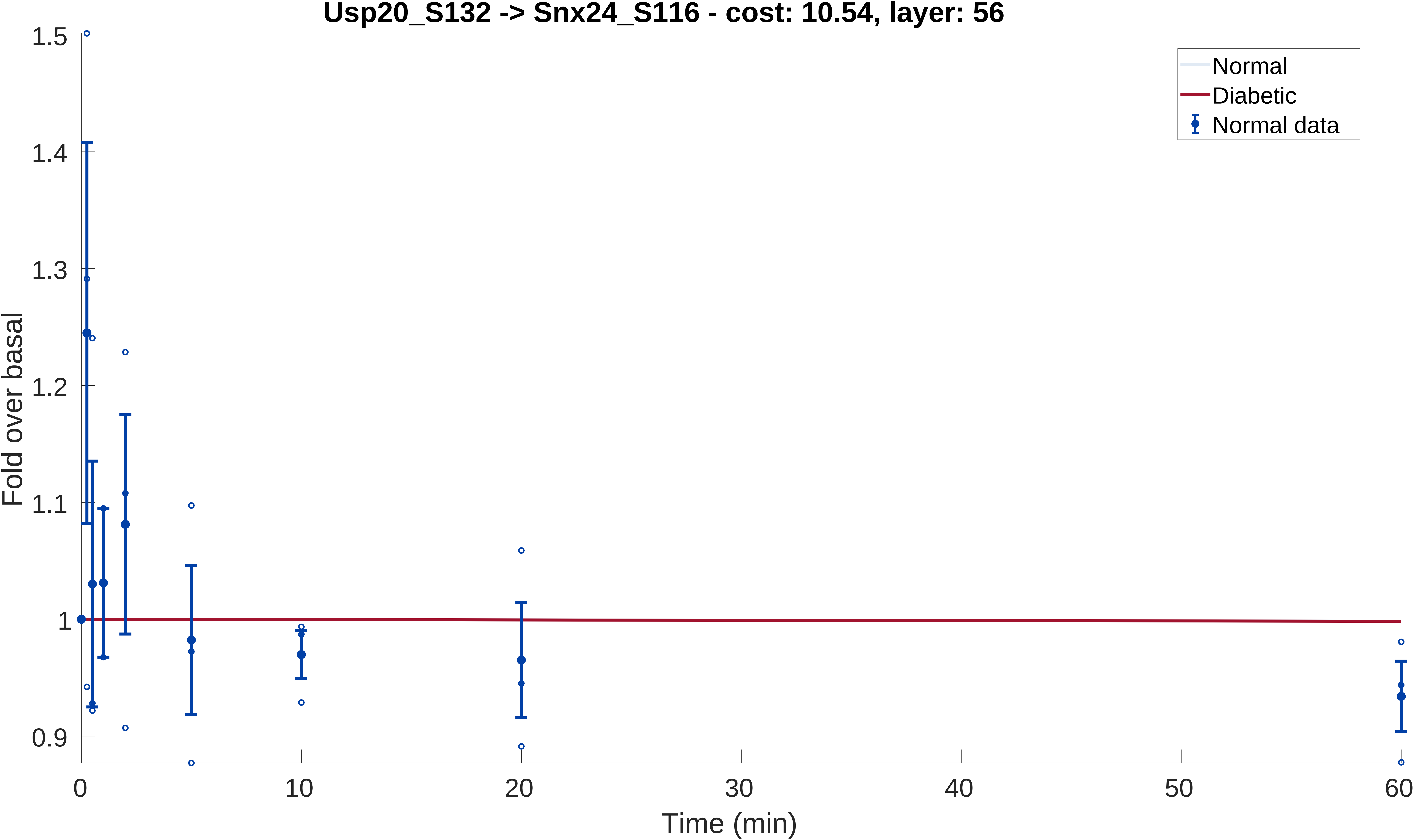

Usp20_S132 -> Snx24_S116 - cost: 10.54, layer: 56
Normal
Diabetic
Normal data
Fold over basal
1.5
1.4
1.3
1.2
1.1
1
0.9
0
10
20
30
40
50
60
Time (min)

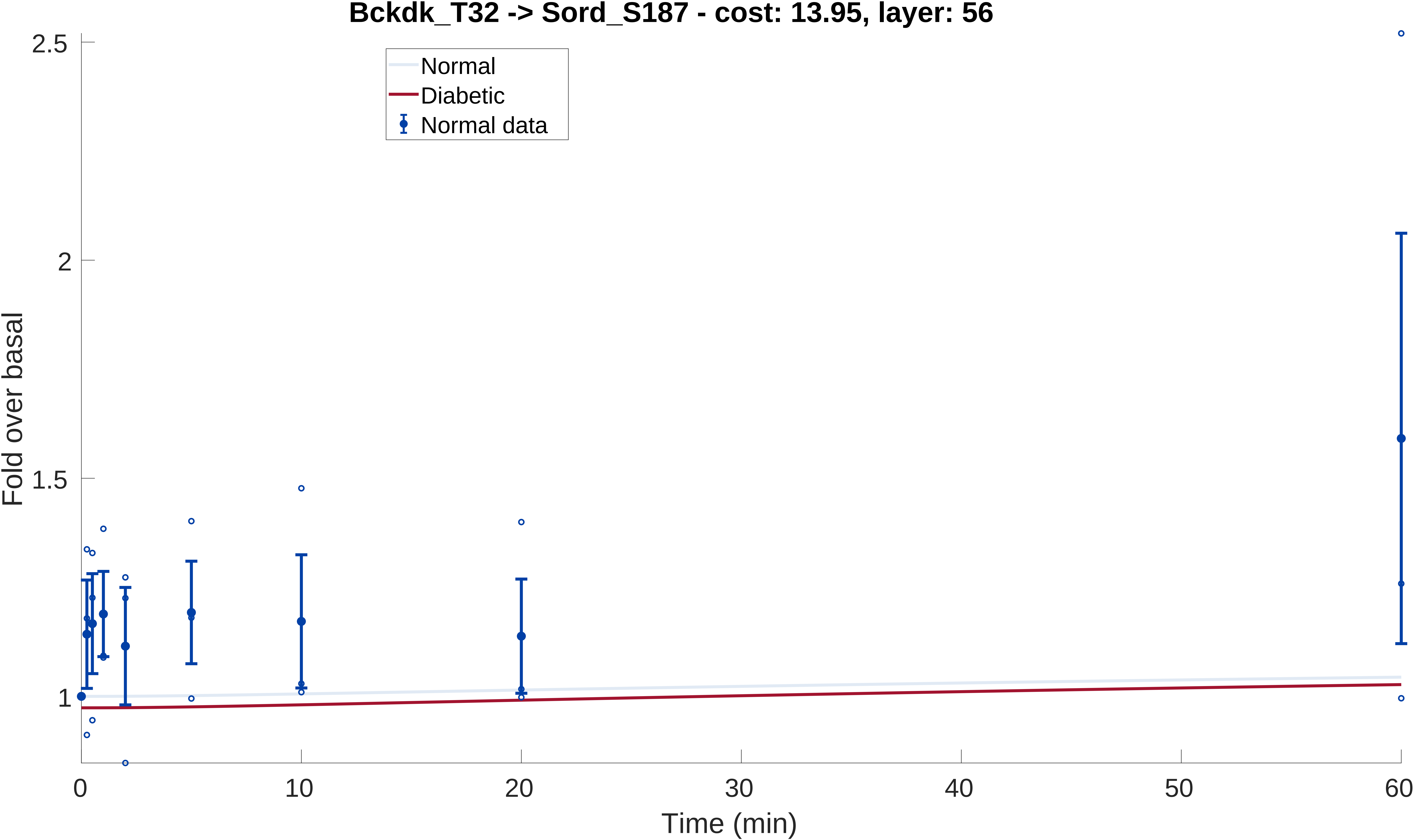

Bckdk_T32 -> Sord_S187 - cost: 13.95, layer: 56
Normal
Diabetic
Normal data
Fold over basal
2.5
2
1.5
1
Time (min)
0
10
20
30
40
50
60

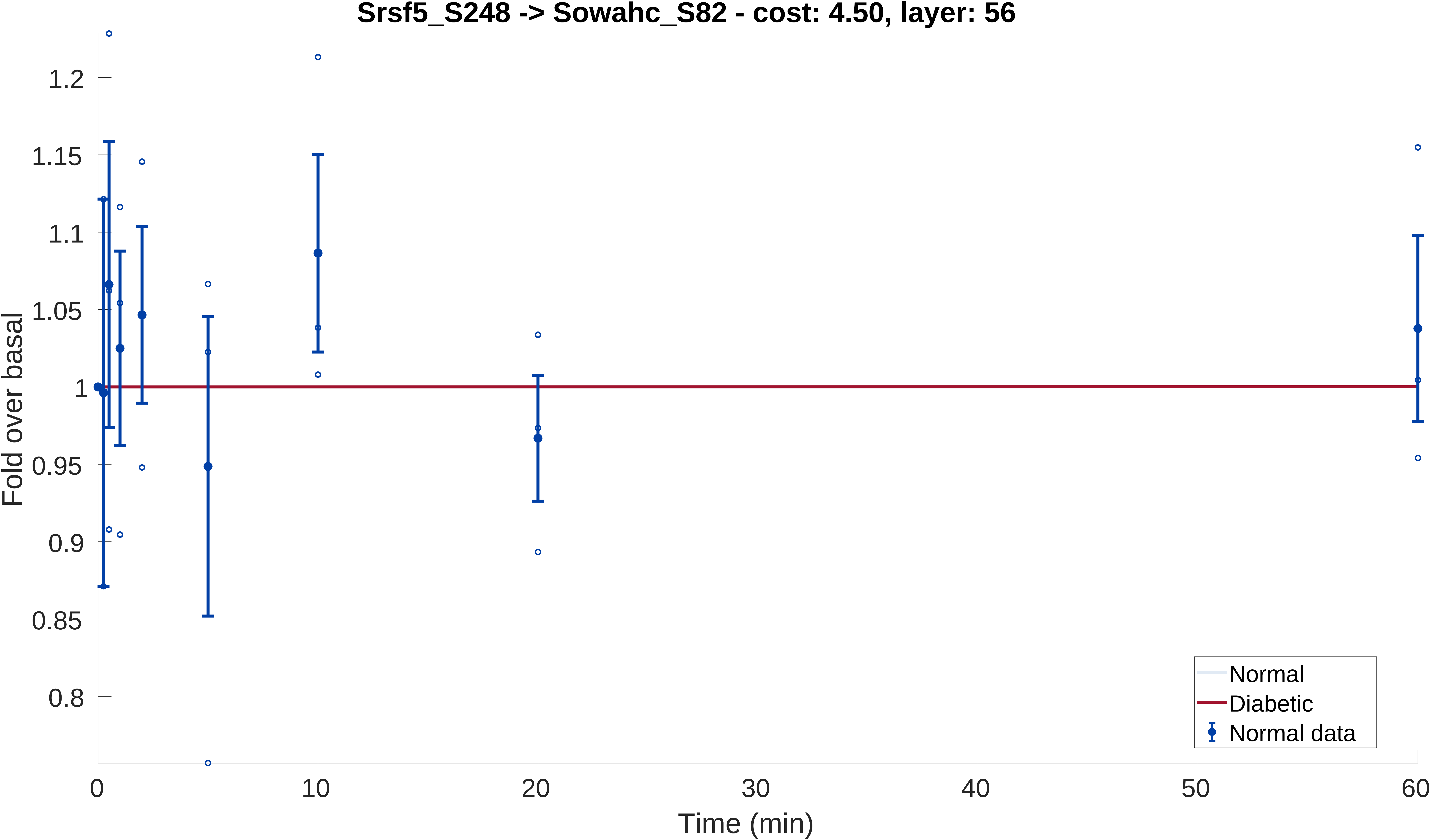
Srsf5_S248 -> Sowahc_S82 - cost: 4.50, layer: 56
Fold over basal
1.2
1.15
1.1
1.05
1
0.95
0.9
0.85
0.8
0
10
20
30
40
50
60
Time (min)
Normal
Diabetic
Normal data

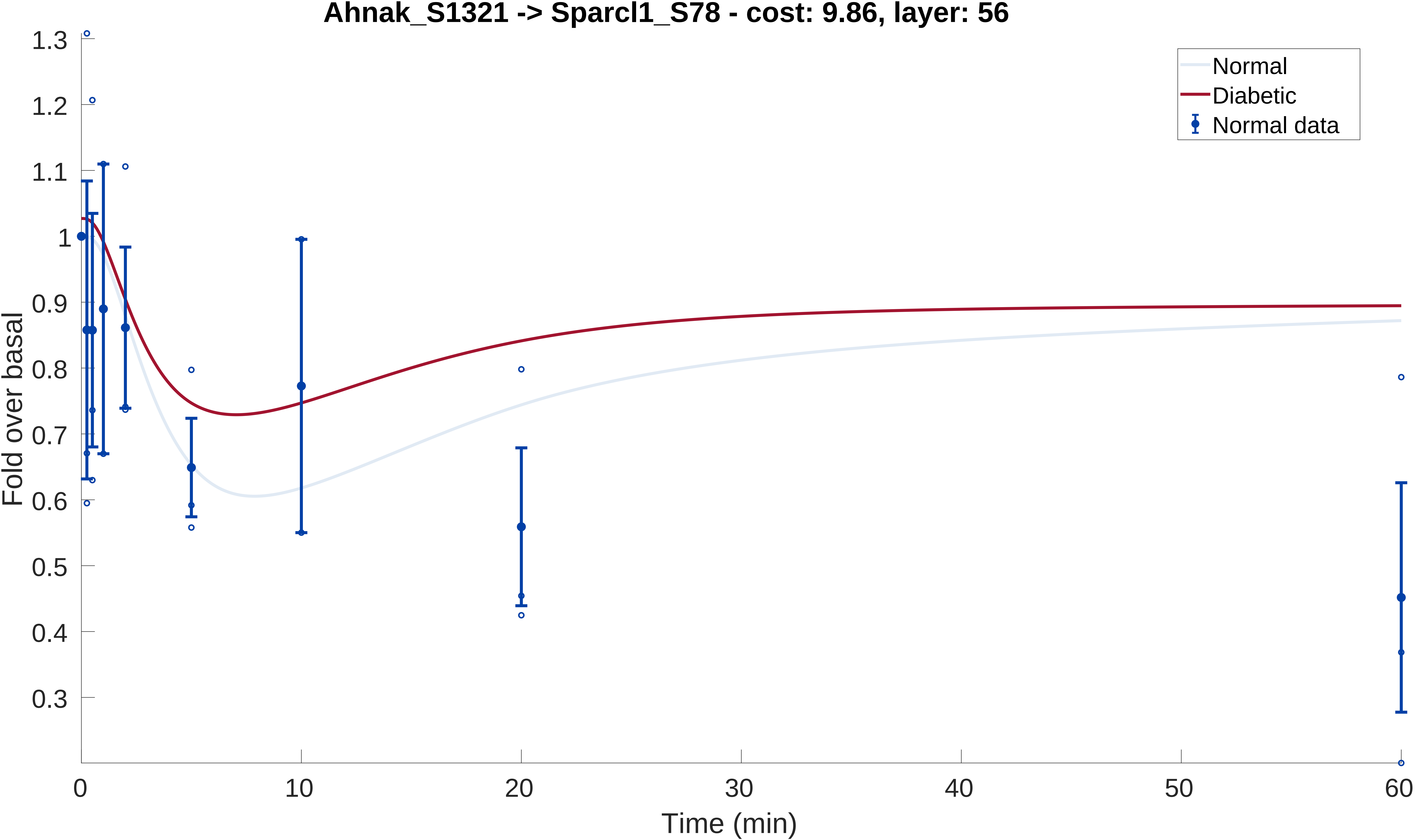

Ahnak_S1321 -> Sparcl1_S78 - cost: 9.86, layer: 56
Normal
Diabetic
Normal data
Fold over basal
Time (min)
1.3
1.2
1.1
1
0.9
0.8
0.7
0.6
0.5
0.4
0.3
0
10
20
30
40
50
60

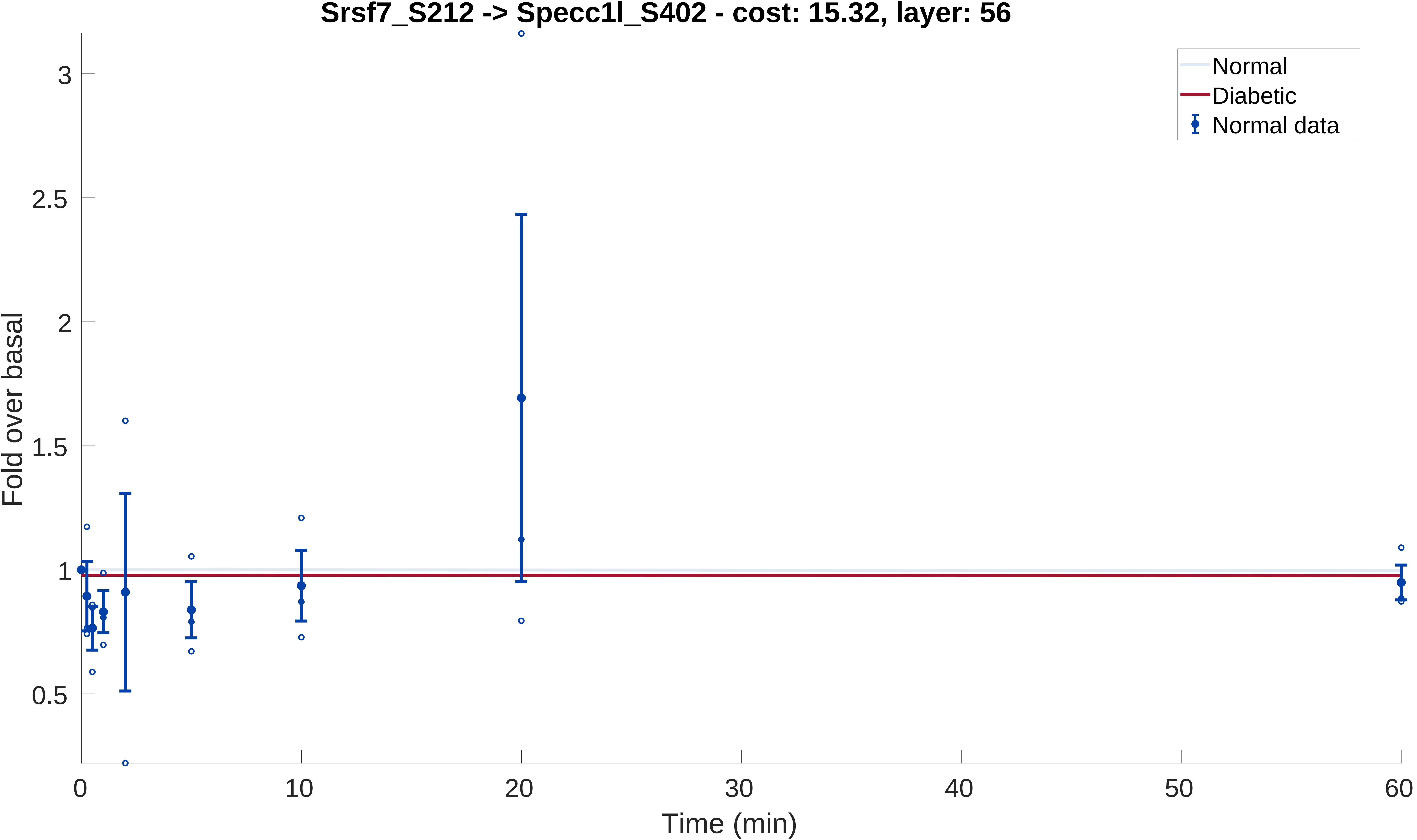
Srsf7_S212 -> Specc1l_S402 - cost: 15.32, layer: 56
Normal
Diabetic
Normal data
Fold over basal
Time (min)
0.5
1
1.5
2
2.5
3
0
10
20
30
40
50
60

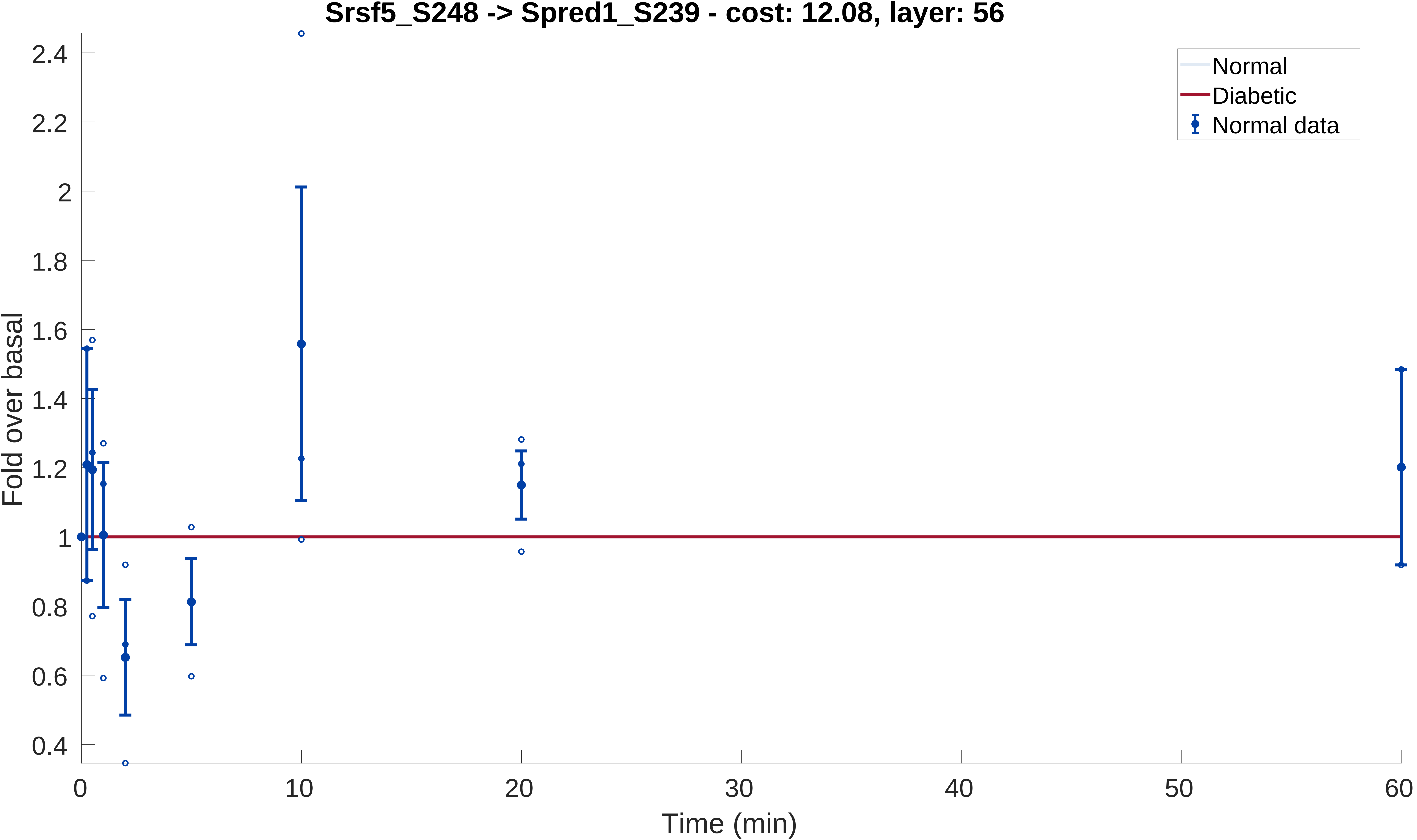

Srsf5_S248 -> Spred1_S239 - cost: 12.08, layer: 56
Fold over basal
Time (min)
Normal
Diabetic
Normal data

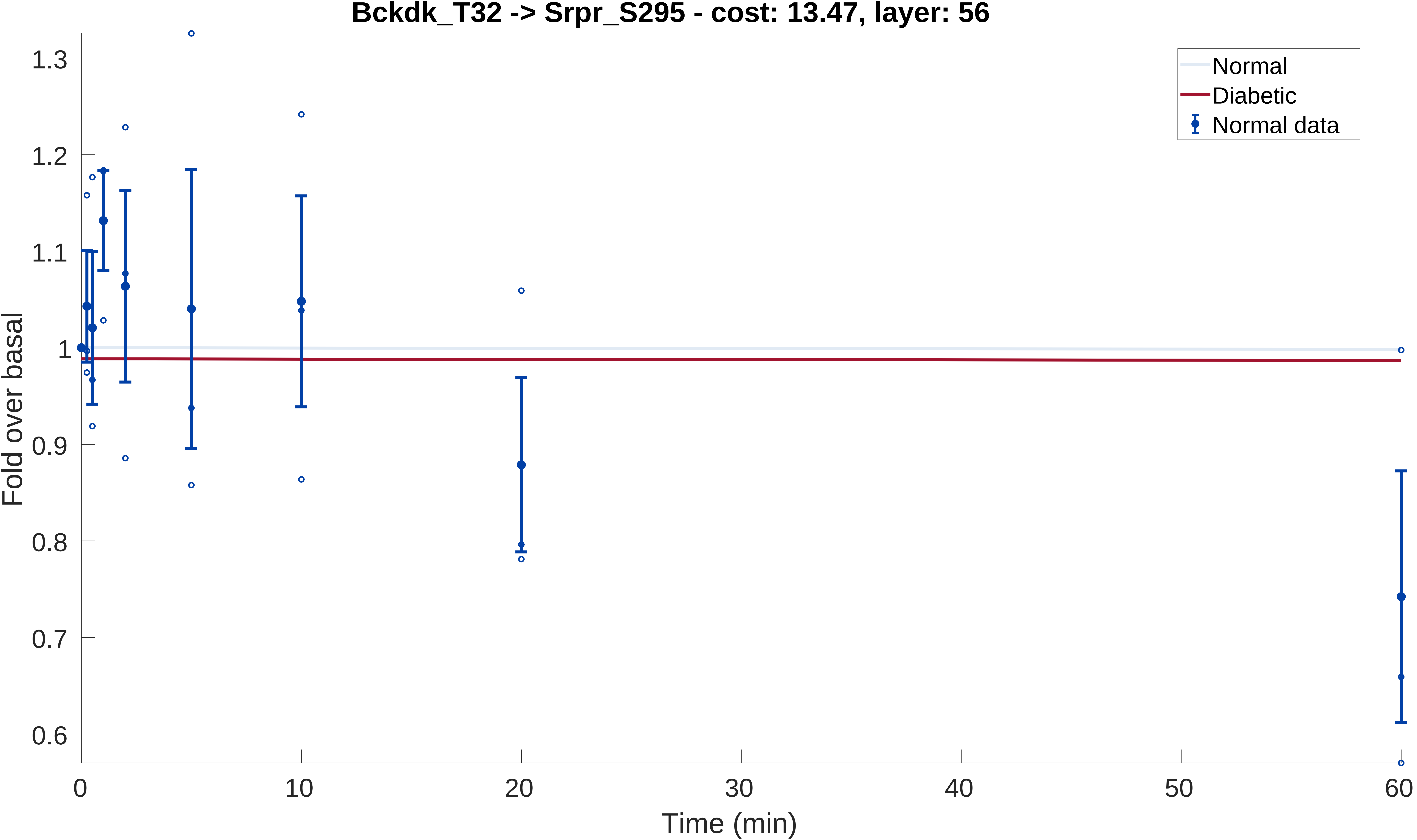
Bckdk_T32 -> Srpr_S295 - cost: 13.47, layer: 56
Normal
Diabetic
Normal data
Fold over basal
Time (min)
1.3
1.2
1.1
1
0.9
0.8
0.7
0.6
0
10
20
30
40
50
60

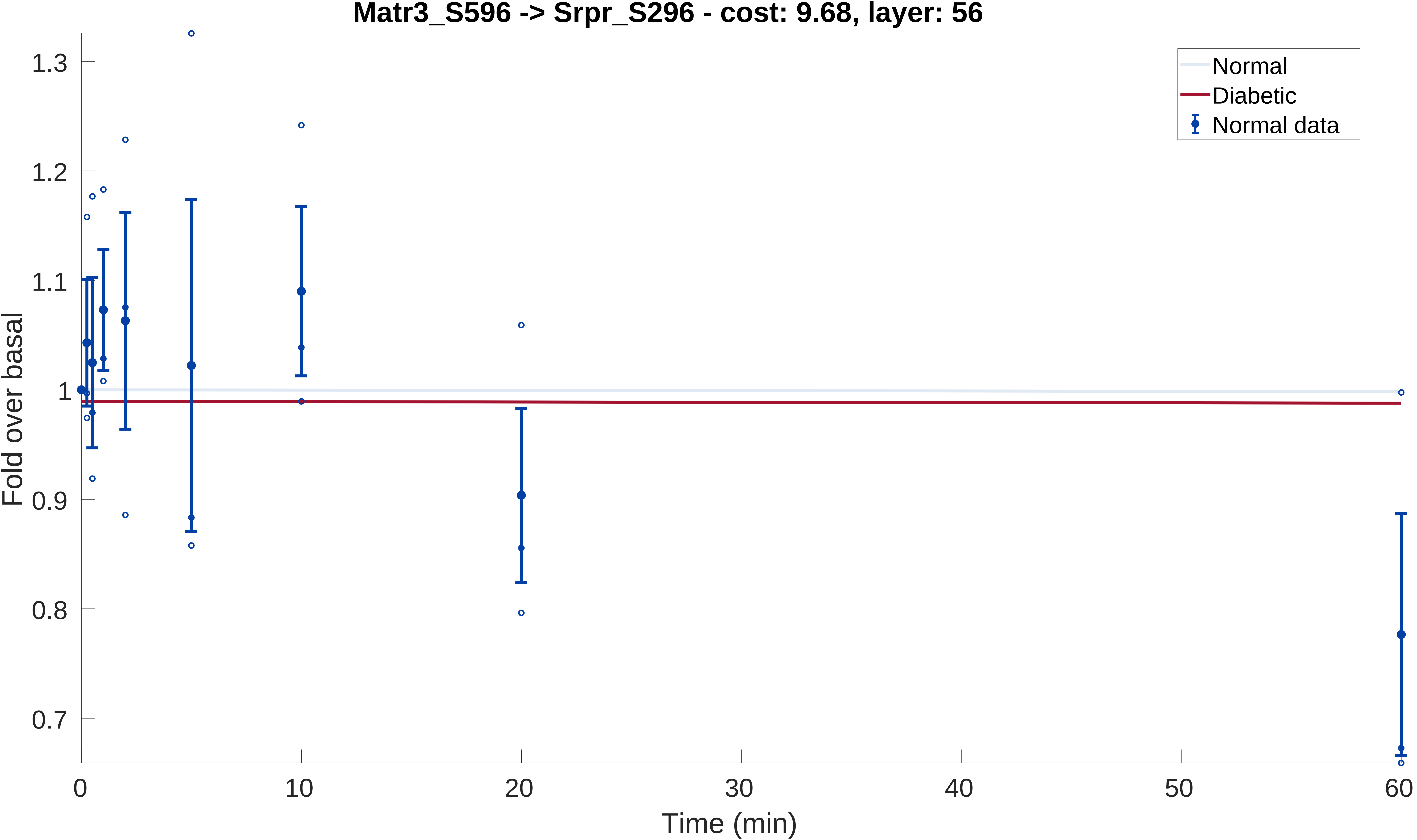

Matr3_S596 -> Srpr_S296 - cost: 9.68, layer: 56
Normal
Diabetic
Normal data
Fold over basal
1.3
1.2
1.1
1
0.9
0.8
0.7
0
10
20
30
40
50
60
Time (min)

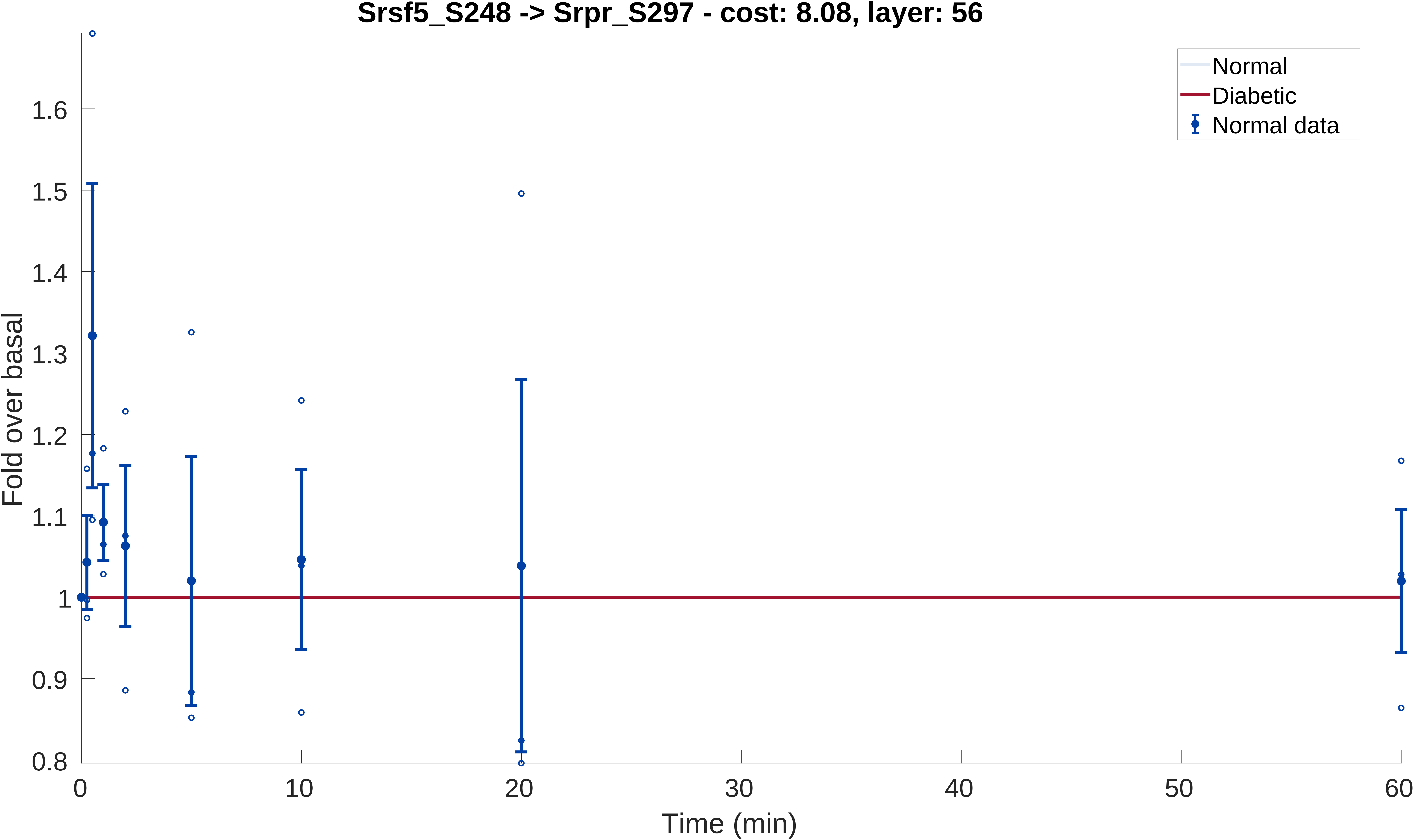

Srsf5_S248 -> Srpr_S297 - cost: 8.08, layer: 56
Normal
Diabetic
Normal data
Fold over basal
Time (min)
0.8
0.9
1
1.1
1.2
1.3
1.4
1.5
1.6
0
10
20
30
40
50
60

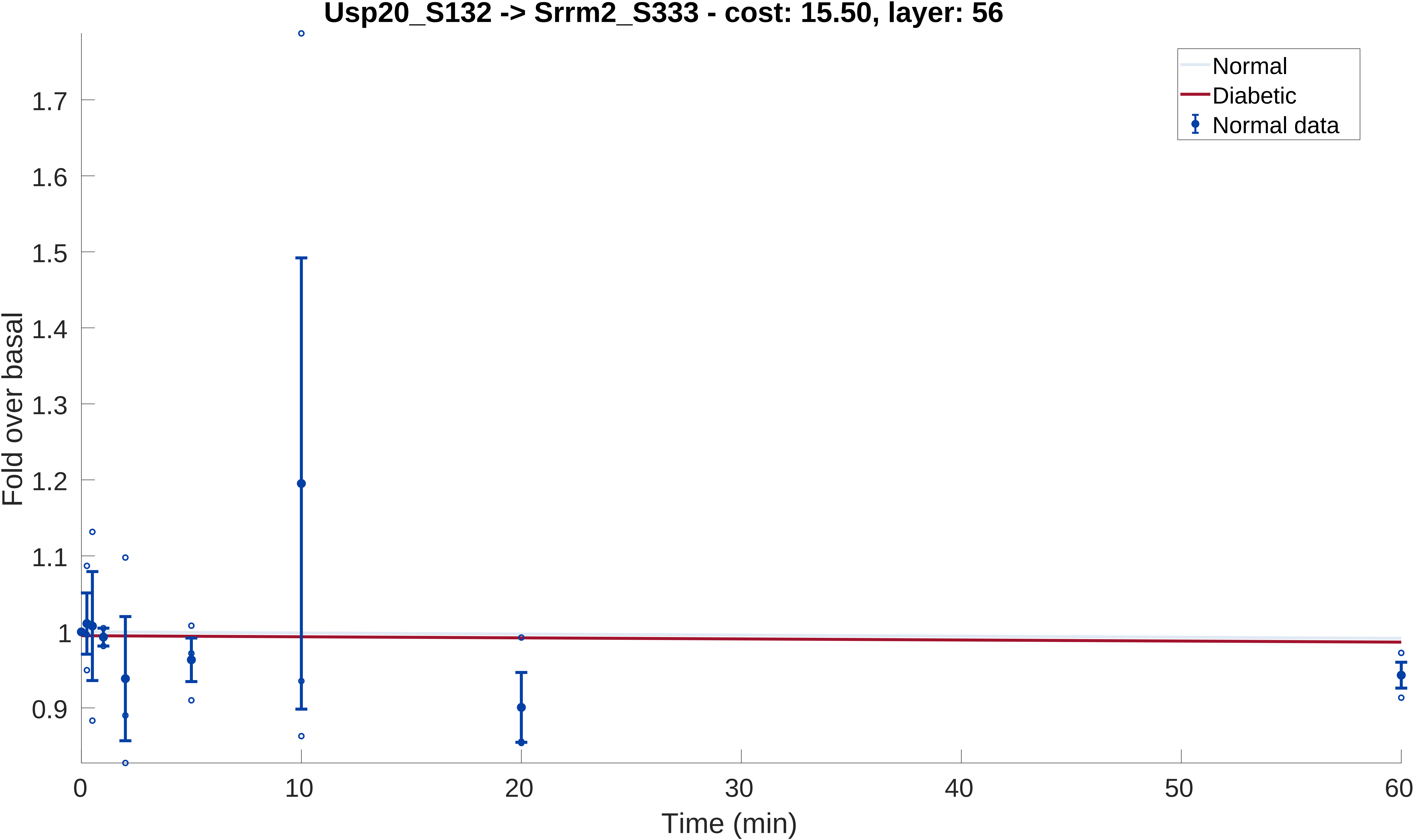
Usp20_S132 -> Srrm2_S333 - cost: 15.50, layer: 56
Normal
Diabetic
Normal data
Fold over basal
Time (min)
1.7
1.6
1.5
1.4
1.3
1.2
1.1
1
0.9
0
10
20
30
40
50
60

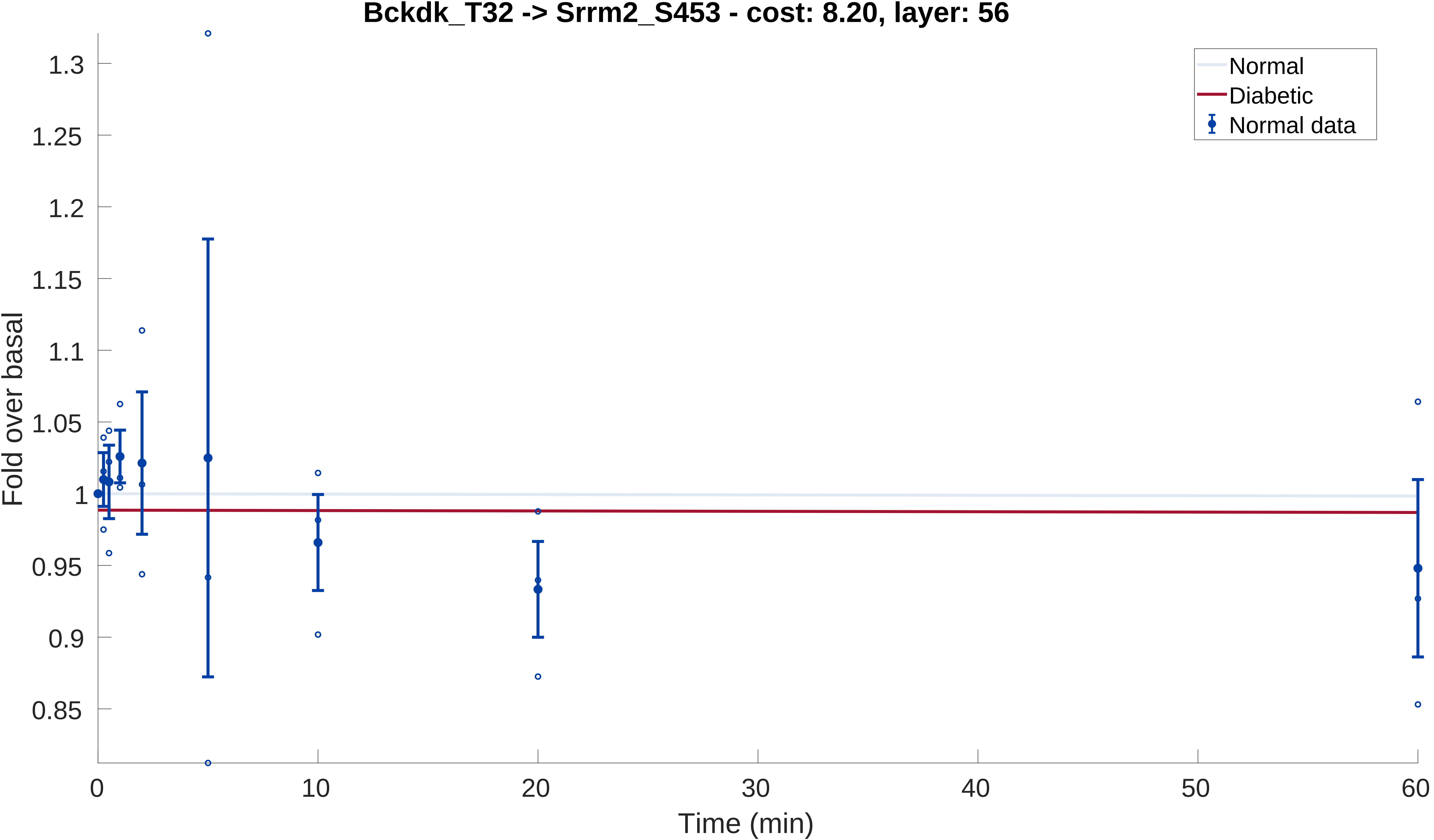
Bckdk_T32 -> Srrm2_S453 - cost: 8.20, layer: 56
Fold over basal
1.3
1.25
1.2
1.15
1.1
1.05
1
0.95
0.9
0.85
0
10
20
30
40
50
60
Time (min)
Normal
Diabetic
Normal data

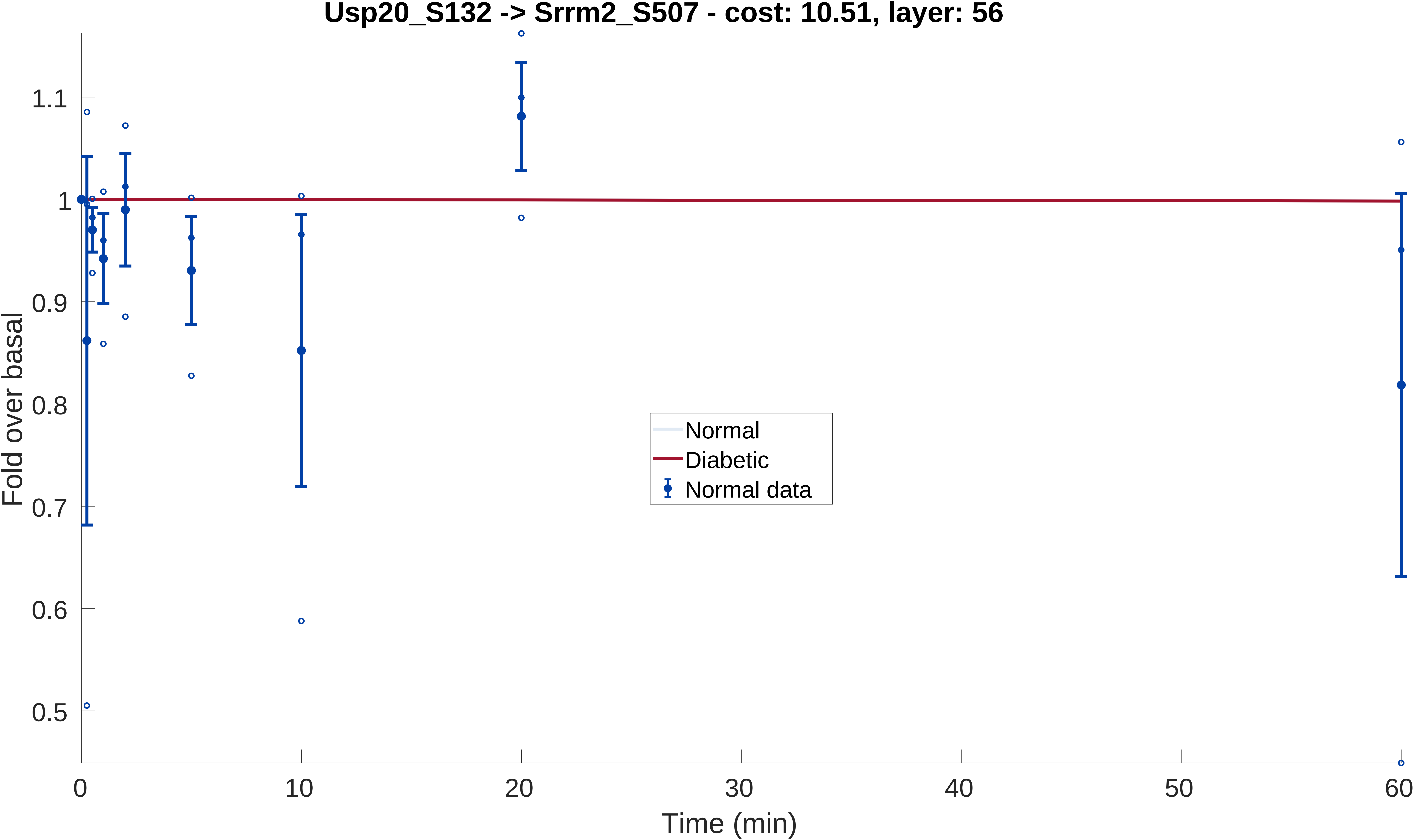

Usp20_S132 -> Srrm2_S507 - cost: 10.51, layer: 56
Fold over basal
1.1
1
0.9
0.8
0.7
0.6
0.5
0
10
20
30
40
50
60
Time (min)
Normal
Diabetic
Normal data

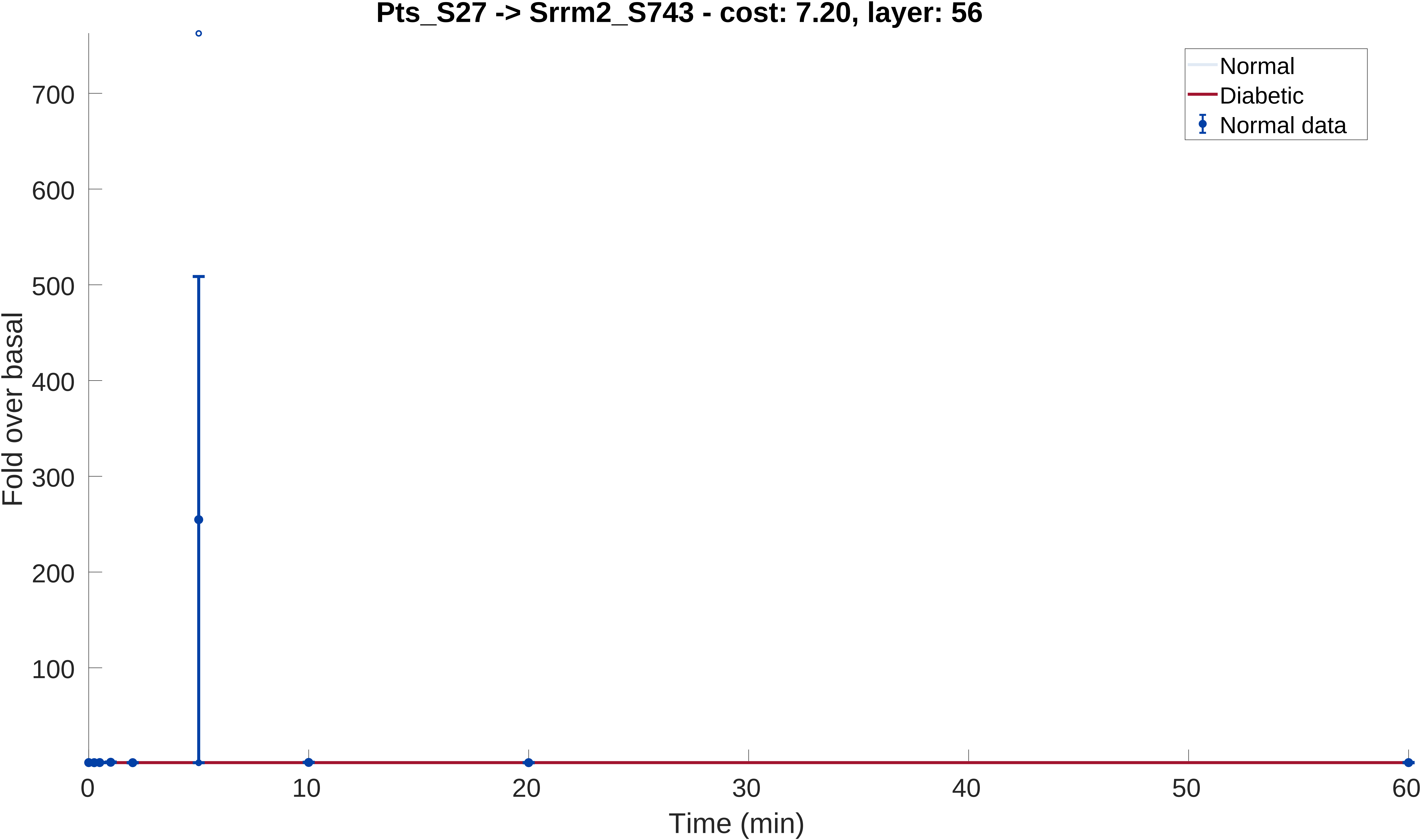
Pts_S27 -> Srrm2_S743 - cost: 7.20, layer: 56
Normal
Diabetic
Normal data
Fold over basal
100
200
300
400
500
600
700
0
10
20
30
40
50
60
Time (min)

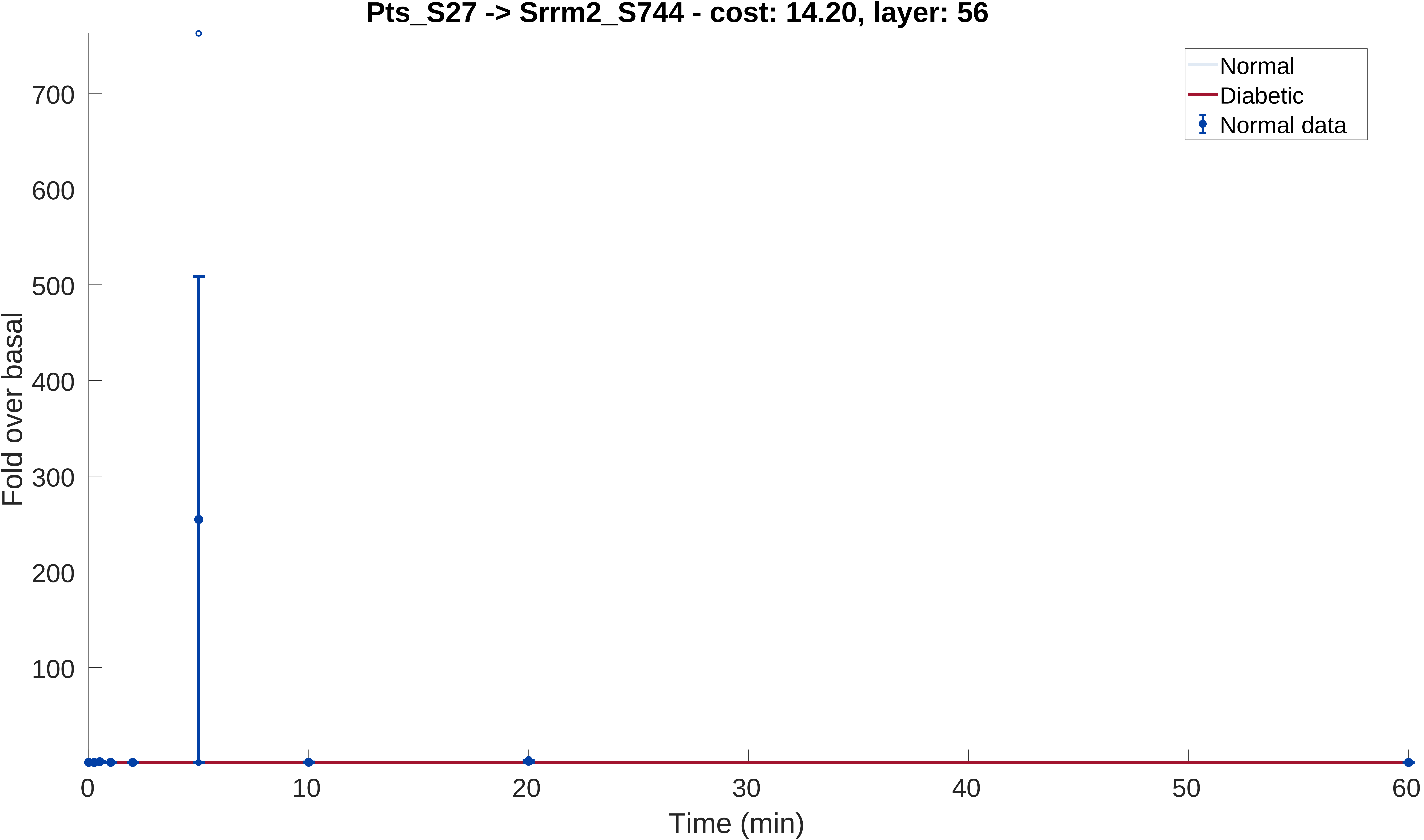

Pts_S27 -> Srrm2_S744 - cost: 14.20, layer: 56
Normal
Diabetic
Normal data
Fold over basal
700
600
500
400
300
200
100
0
10
20
30
40
50
60
Time (min)

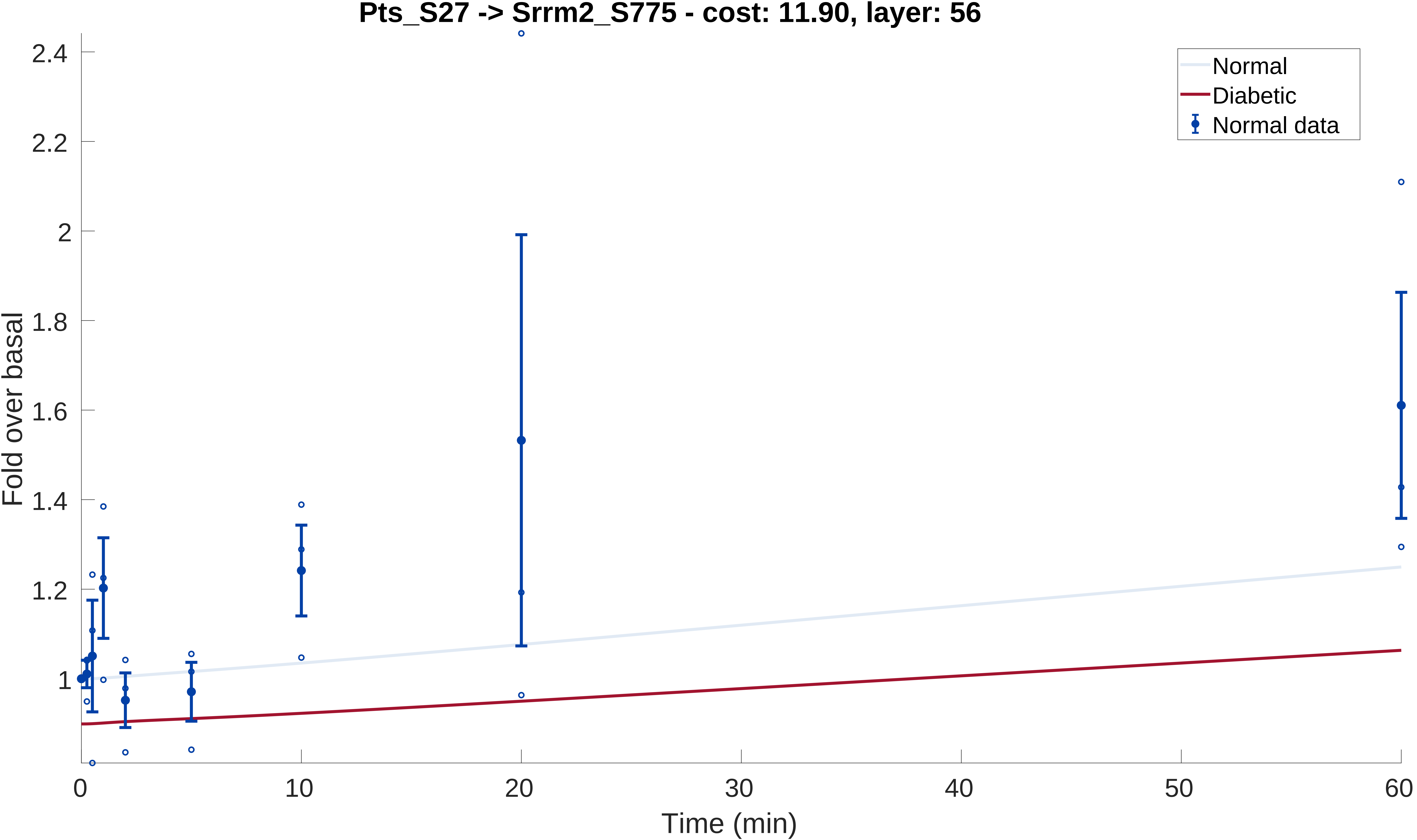
Pts_S27 -> Srrm2_S775 - cost: 11.90, layer: 56
Fold over basal
Time (min)
2.4
2.2
2
1.8
1.6
1.4
1.2
1
0
10
20
30
40
50
60
Normal
Diabetic
Normal data

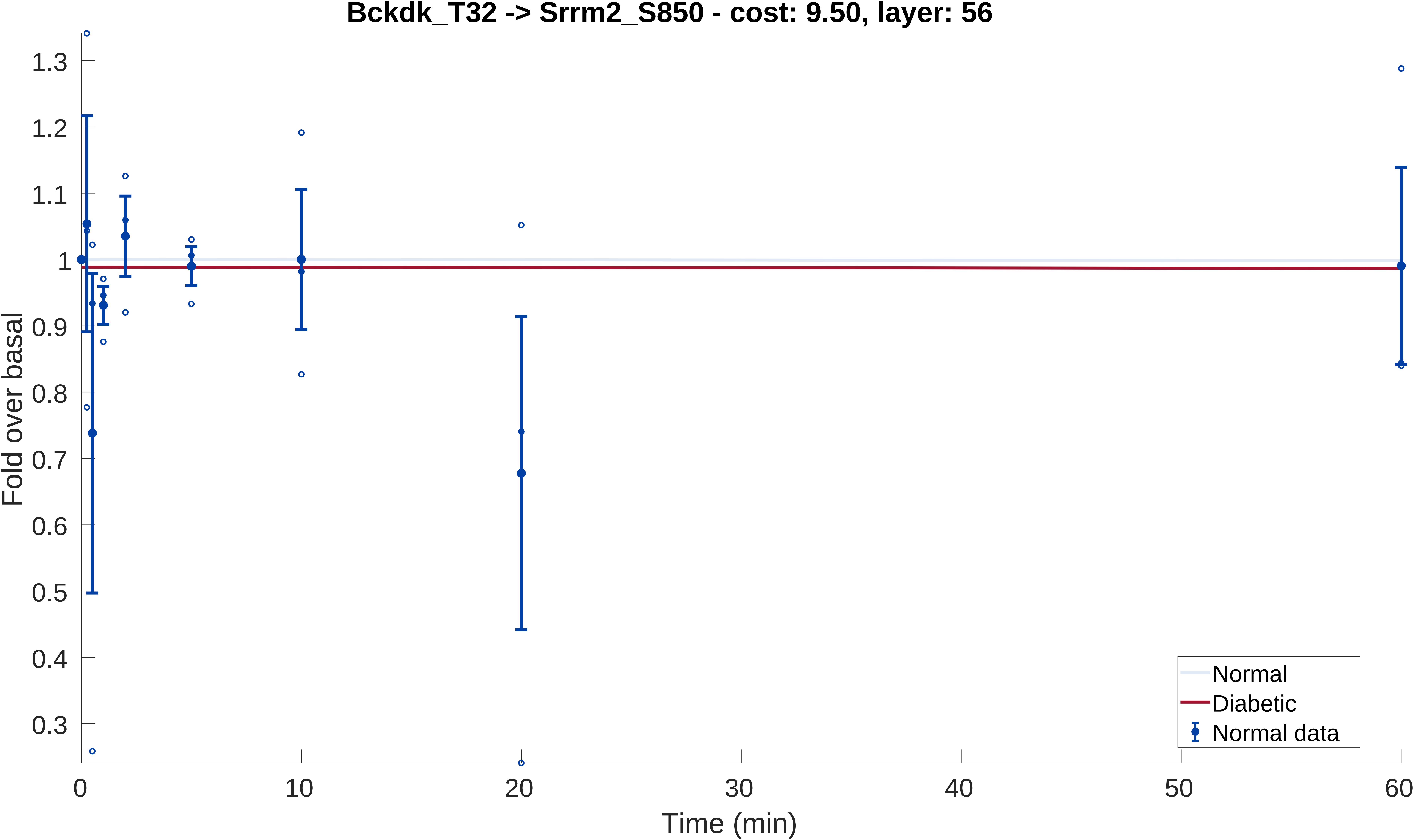

Bckdk_T32 -> Srrm2_S850 - cost: 9.50, layer: 56
Fold over basal
Time (min)
1.3
1.2
1.1
1
0.9
0.8
0.7
0.6
0.5
0.4
0.3
0
10
20
30
40
50
60
Normal
Diabetic
Normal data

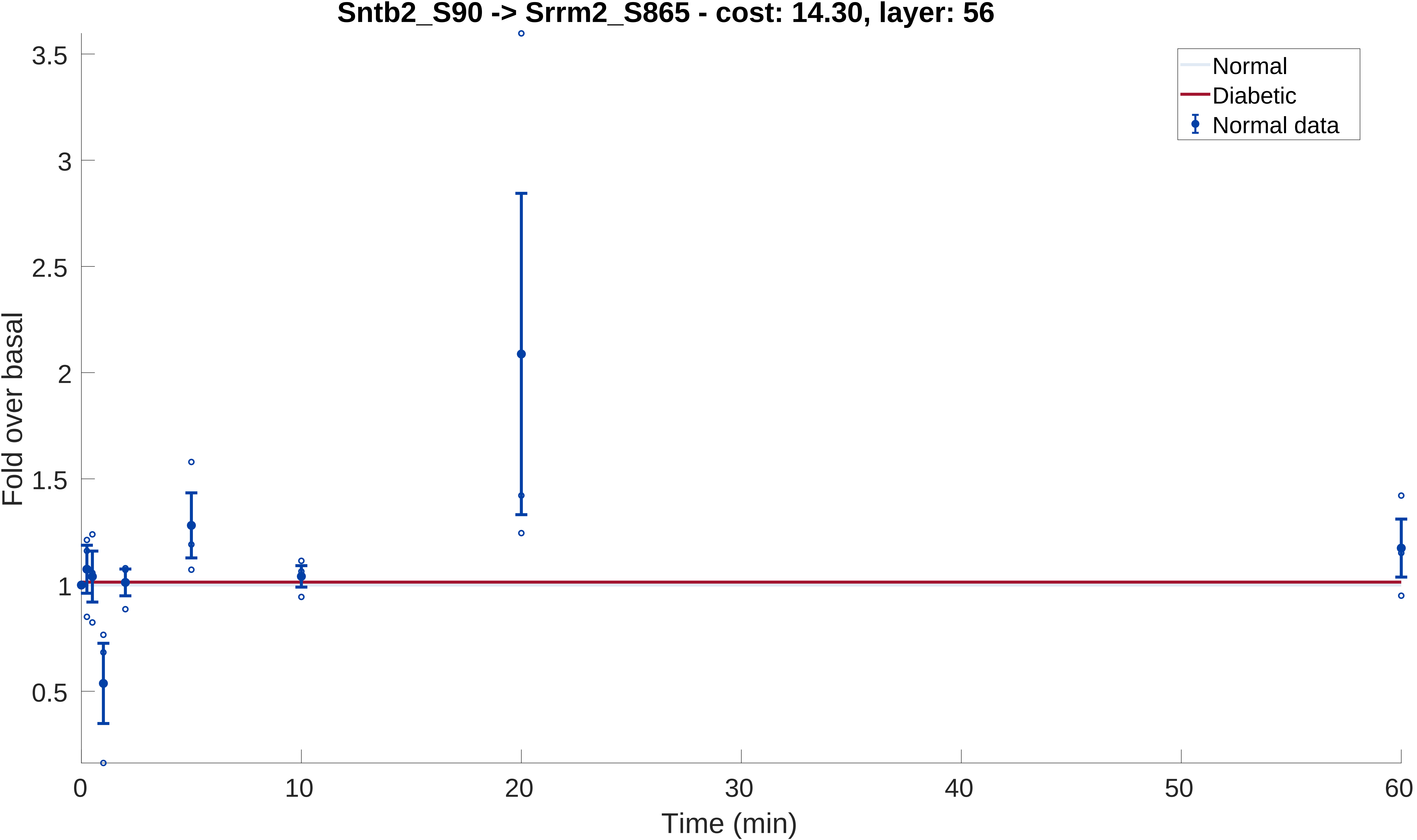

Sntb2_S90 -> Srrm2_S865 - cost: 14.30, layer: 56
Normal
Diabetic
Normal data
Fold over basal
Time (min)
3.5
3
2.5
2
1.5
1
0.5
0
10
20
30
40
50
60

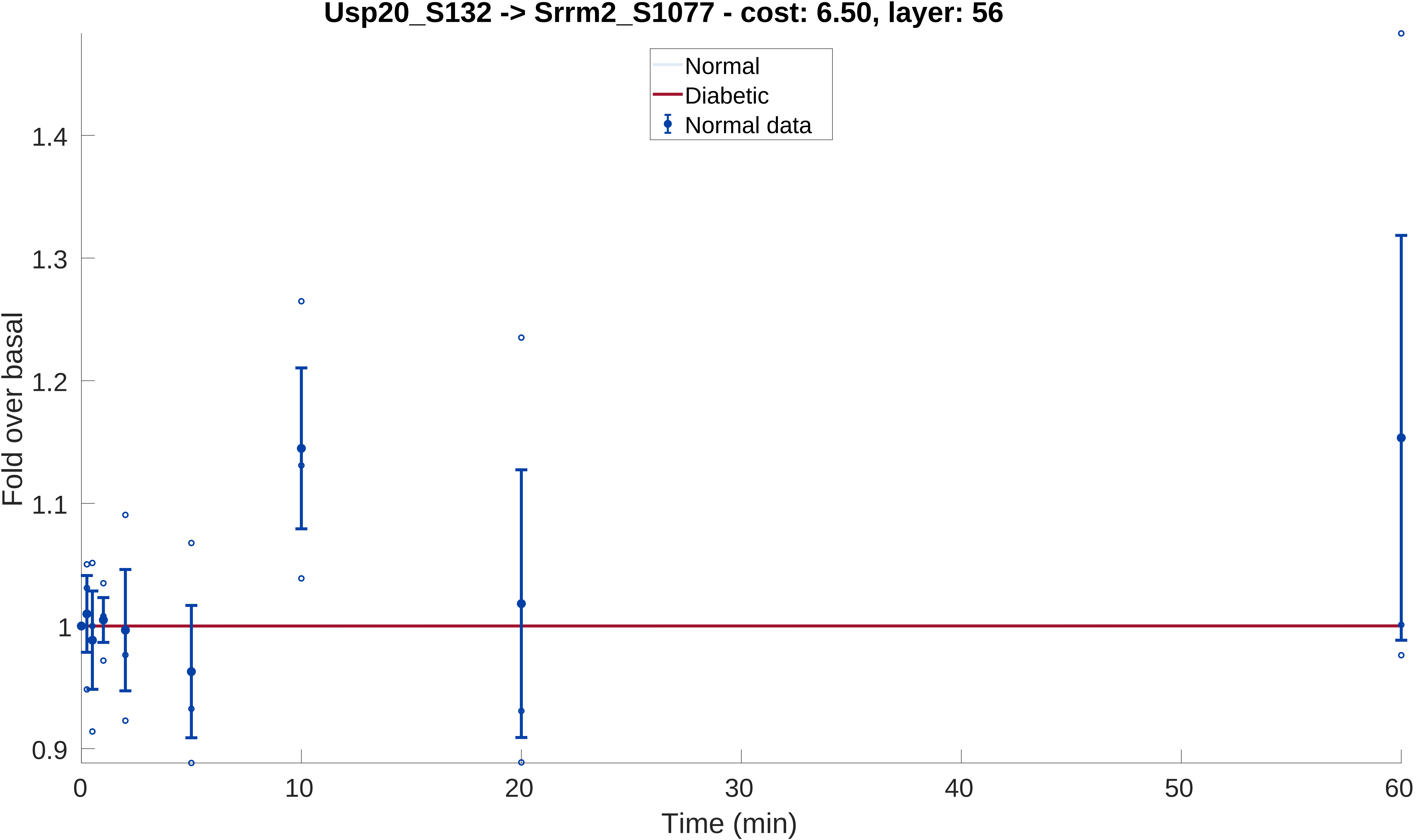
Usp20_S132 -> Srrm2_S1077 - cost: 6.50, layer: 56
Normal
Diabetic
Normal data
Fold over basal
1.4
1.3
1.2
1.1
1
0.9
0
10
20
30
40
50
60
Time (min)

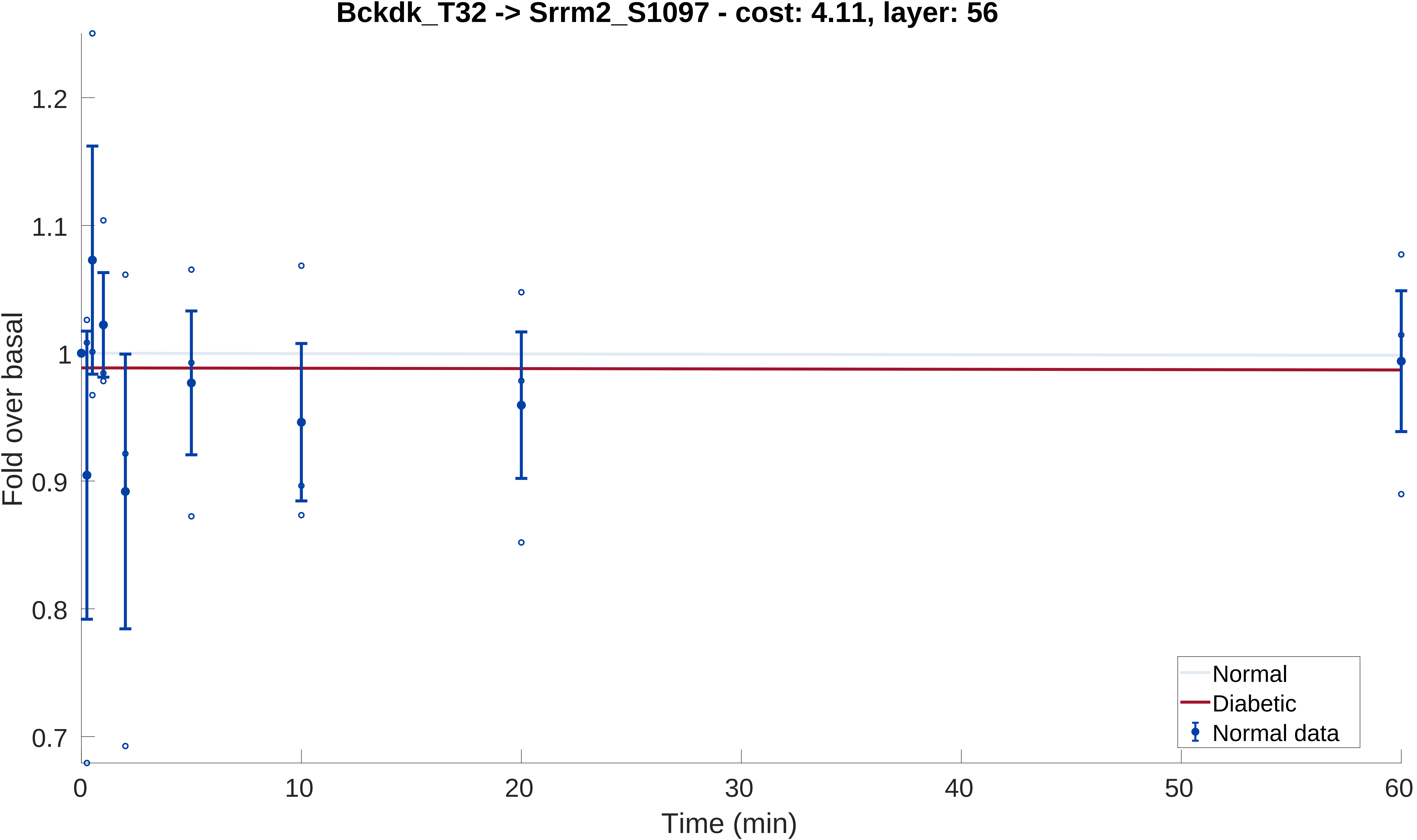
Bckdk_T32 -> Srrm2_S1097 - cost: 4.11, layer: 56
Fold over basal
1.2
1.1
1
0.9
0.8
0.7
0
10
20
30
40
50
60
Time (min)
Normal
Diabetic
Normal data

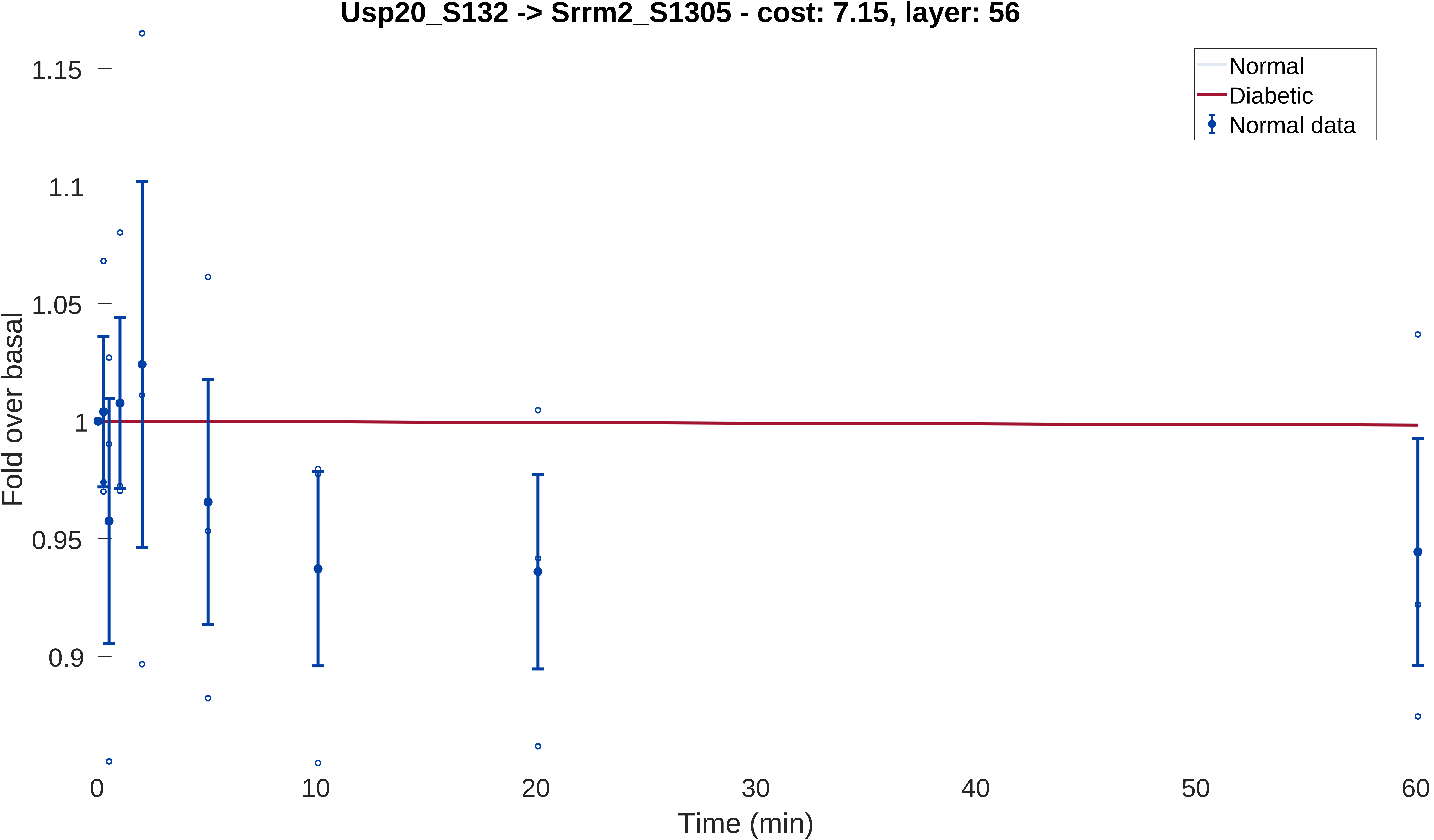

Usp20_S132 -> Srrm2_S1305 - cost: 7.15, layer: 56
Normal
Diabetic
Normal data
Fold over basal
1.15
1.1
1.05
1
0.95
0.9
0
10
20
30
40
50
60
Time (min)

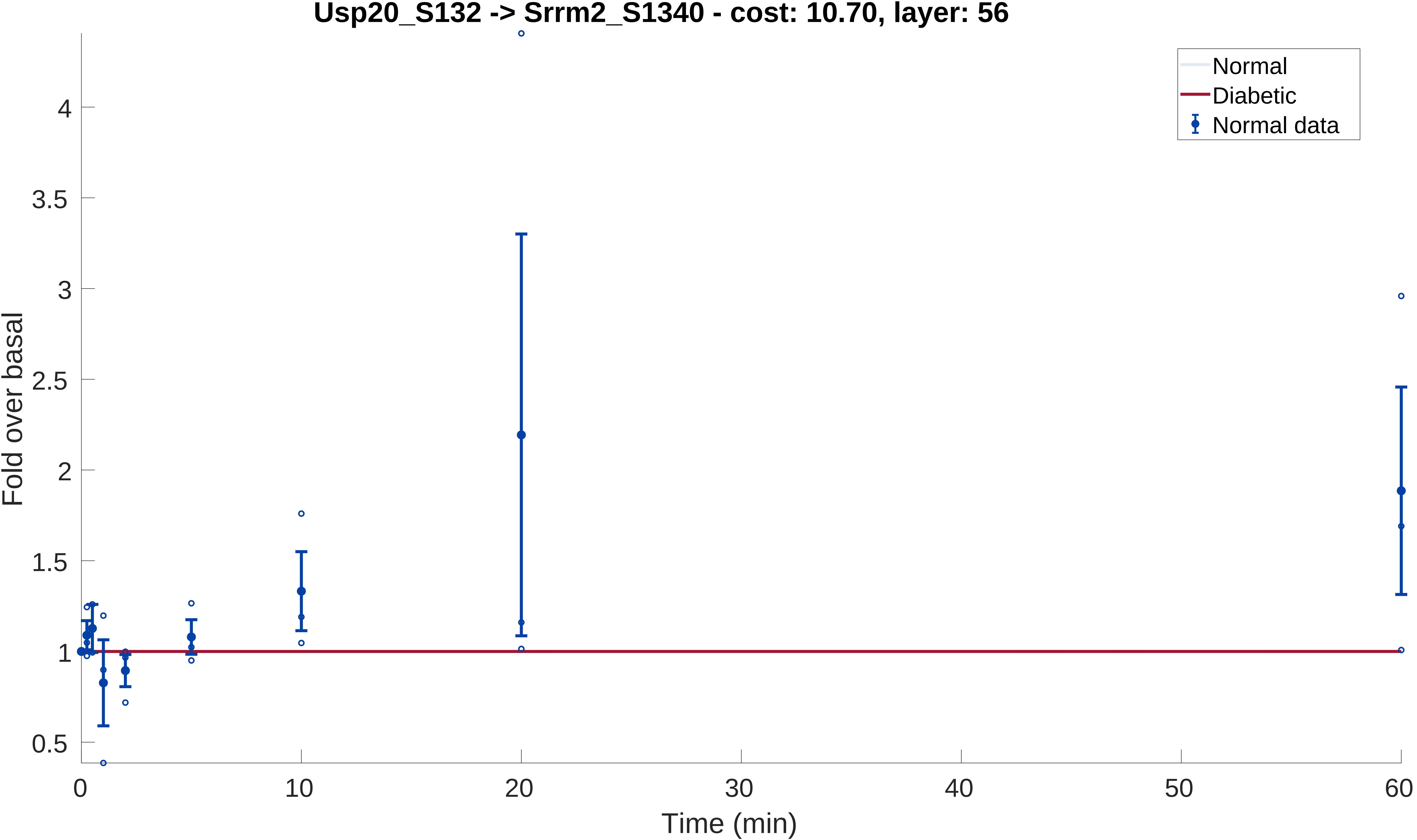

Usp20_S132 -> Srrm2_S1340 - cost: 10.70, layer: 56
Normal
Diabetic
Normal data
Fold over basal
4
3.5
3
2.5
2
1.5
1
0.5
0
10
20
30
40
50
60
Time (min)

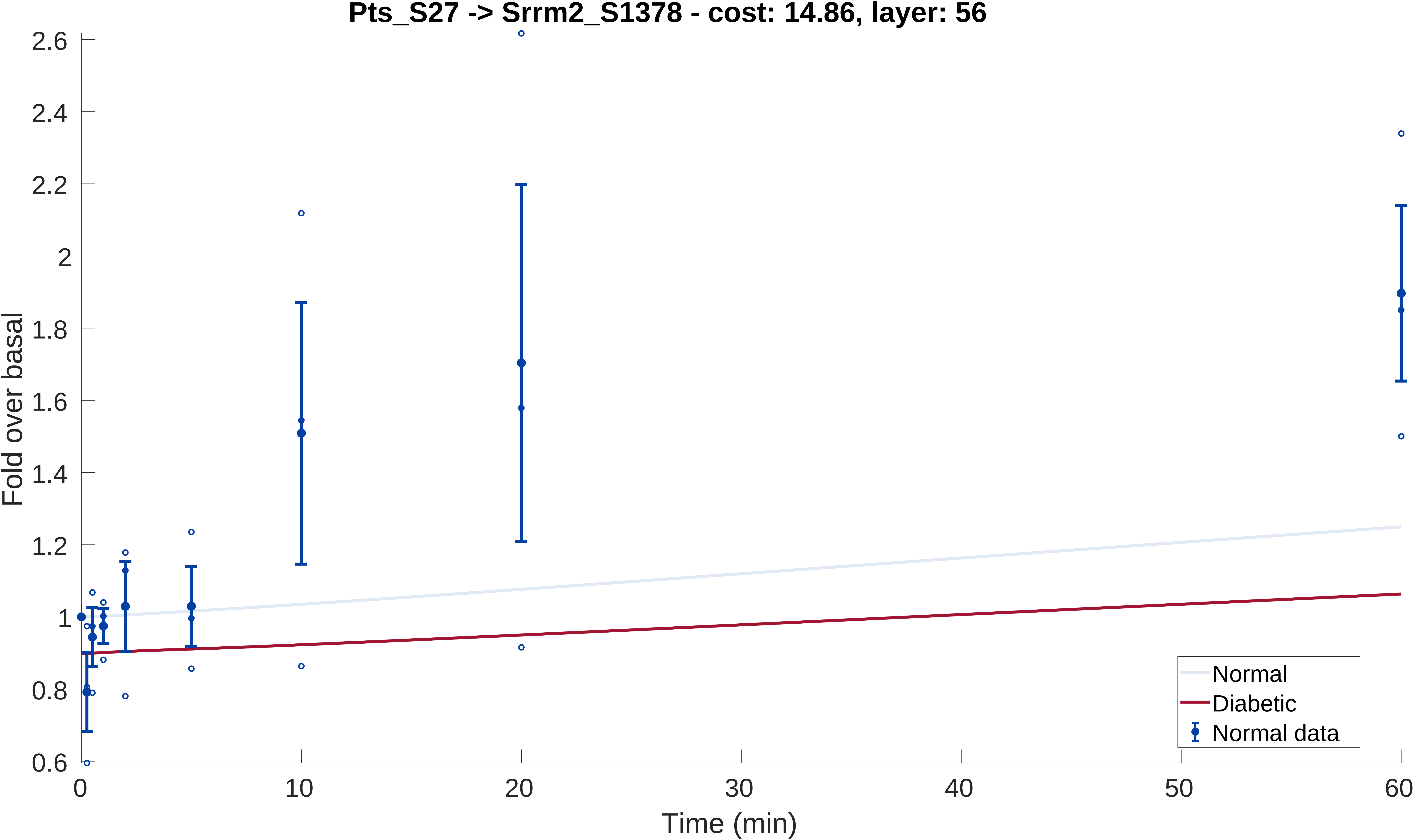
Pts_S27 -> Srrm2_S1378 - cost: 14.86, layer: 56
Fold over basal
2.6
2.4
2.2
2
1.8
1.6
1.4
1.2
1
0.8
0.6
0
10
20
30
40
50
60
Time (min)
Normal
Diabetic
Normal data

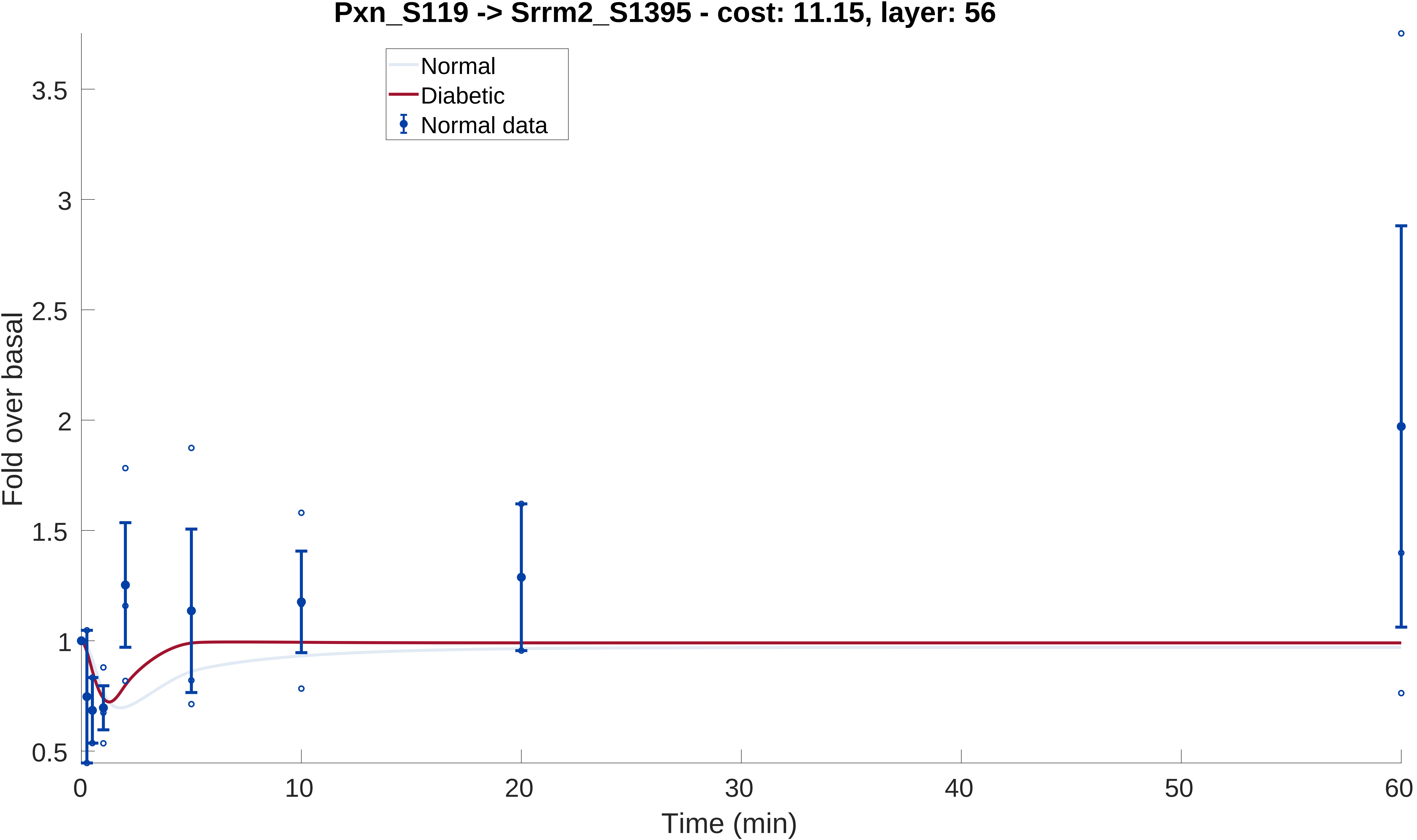

Pxn_S119 -> Srrm2_S1395 - cost: 11.15, layer: 56
Normal
Diabetic
Normal data
Fold over basal
Time (min)
3.5
3
2.5
2
1.5
1
0.5
0
10
20
30
40
50
60

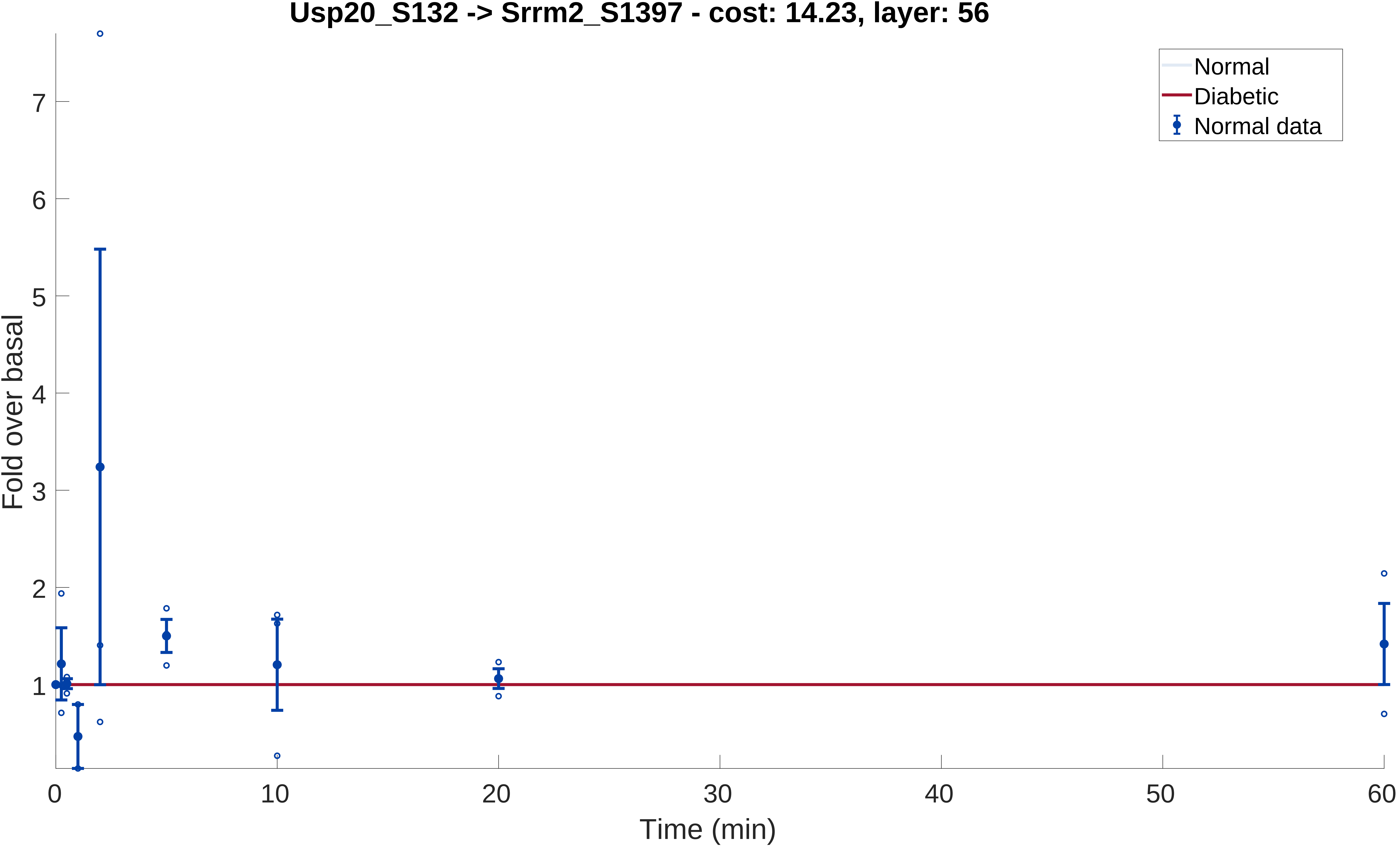

Usp20_S132 -> Srrm2_S1397 - cost: 14.23, layer: 56
Normal
Diabetic
Normal data
Fold over basal
1
2
3
4
5
6
7
0
10
20
30
40
50
60
Time (min)

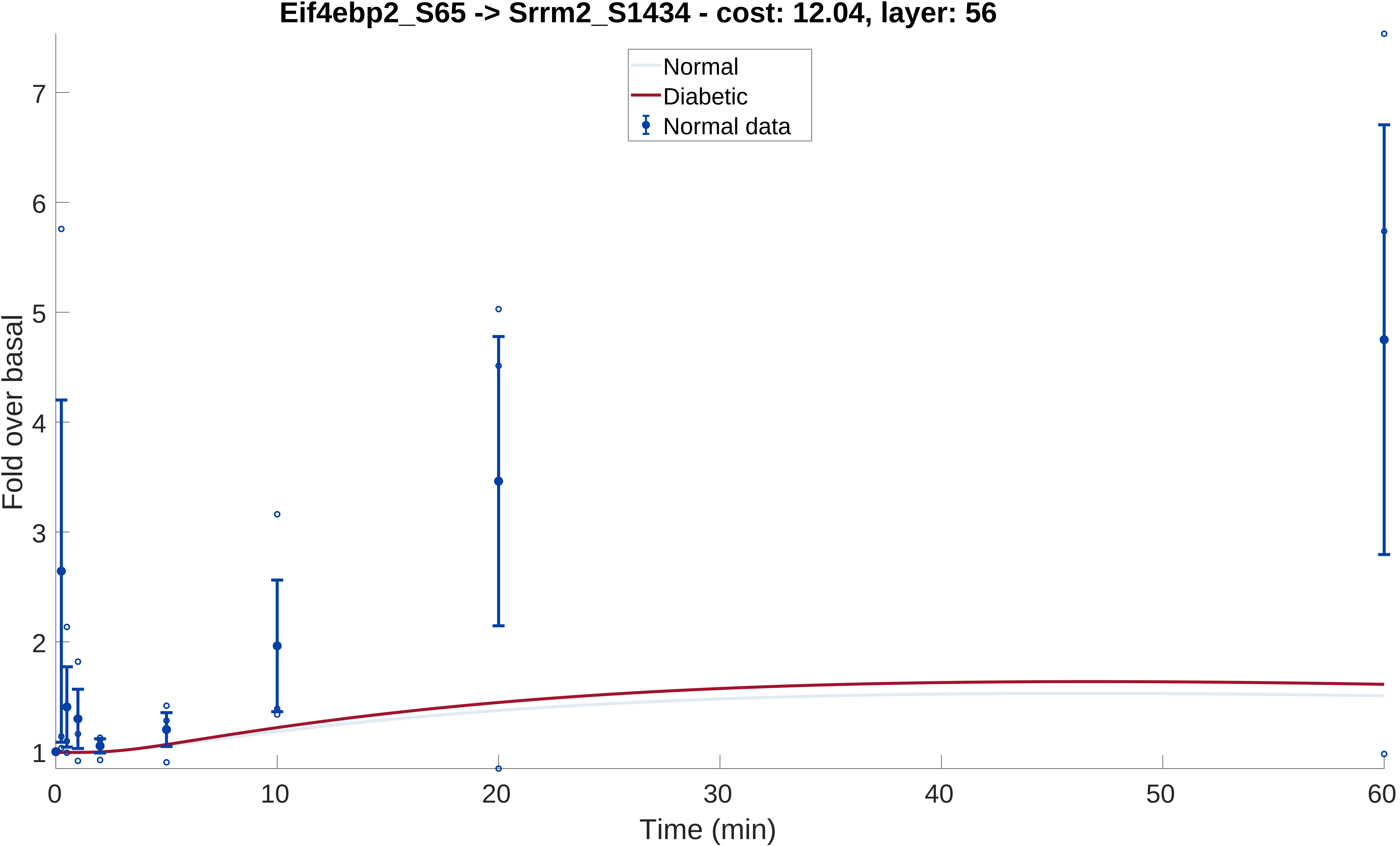
Eif4ebp2_S65 -> Srrm2_S1434 - cost: 12.04, layer: 56
Normal
Diabetic
Normal data
Fold over basal
Time (min)
1
2
3
4
5
6
7
0
10
20
30
40
50
60

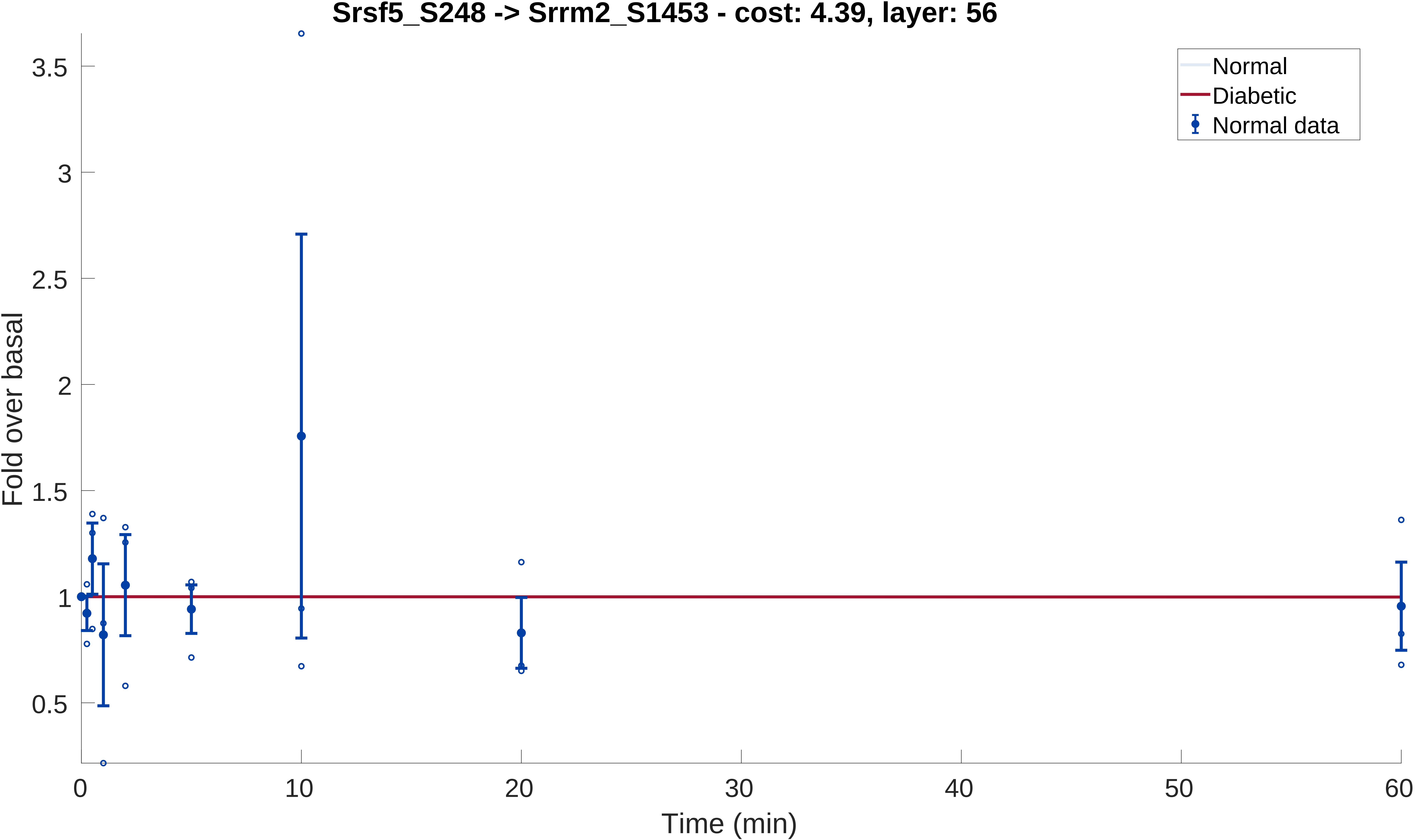
Srsf5_S248 -> Srrm2_S1453 - cost: 4.39, layer: 56
Fold over basal
Time (min)
Normal
Diabetic
Normal data
0.5
1
1.5
2
2.5
3
3.5
0
10
20
30
40
50
60

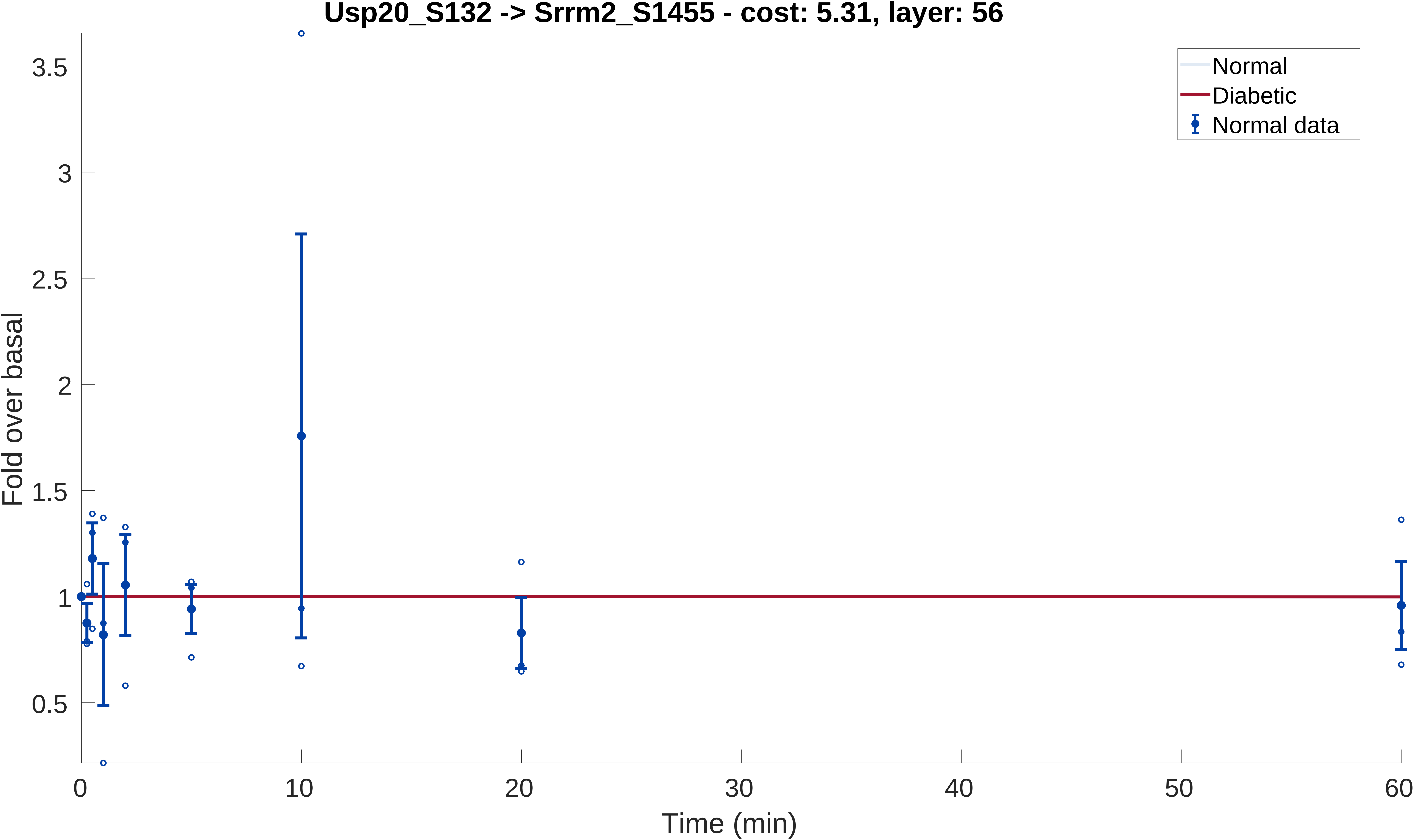

Usp20_S132 -> Srrm2_S1455 - cost: 5.31, layer: 56
Normal
Diabetic
Normal data
Fold over basal
Time (min)
0.5
1
1.5
2
2.5
3
3.5
0
10
20
30
40
50
60

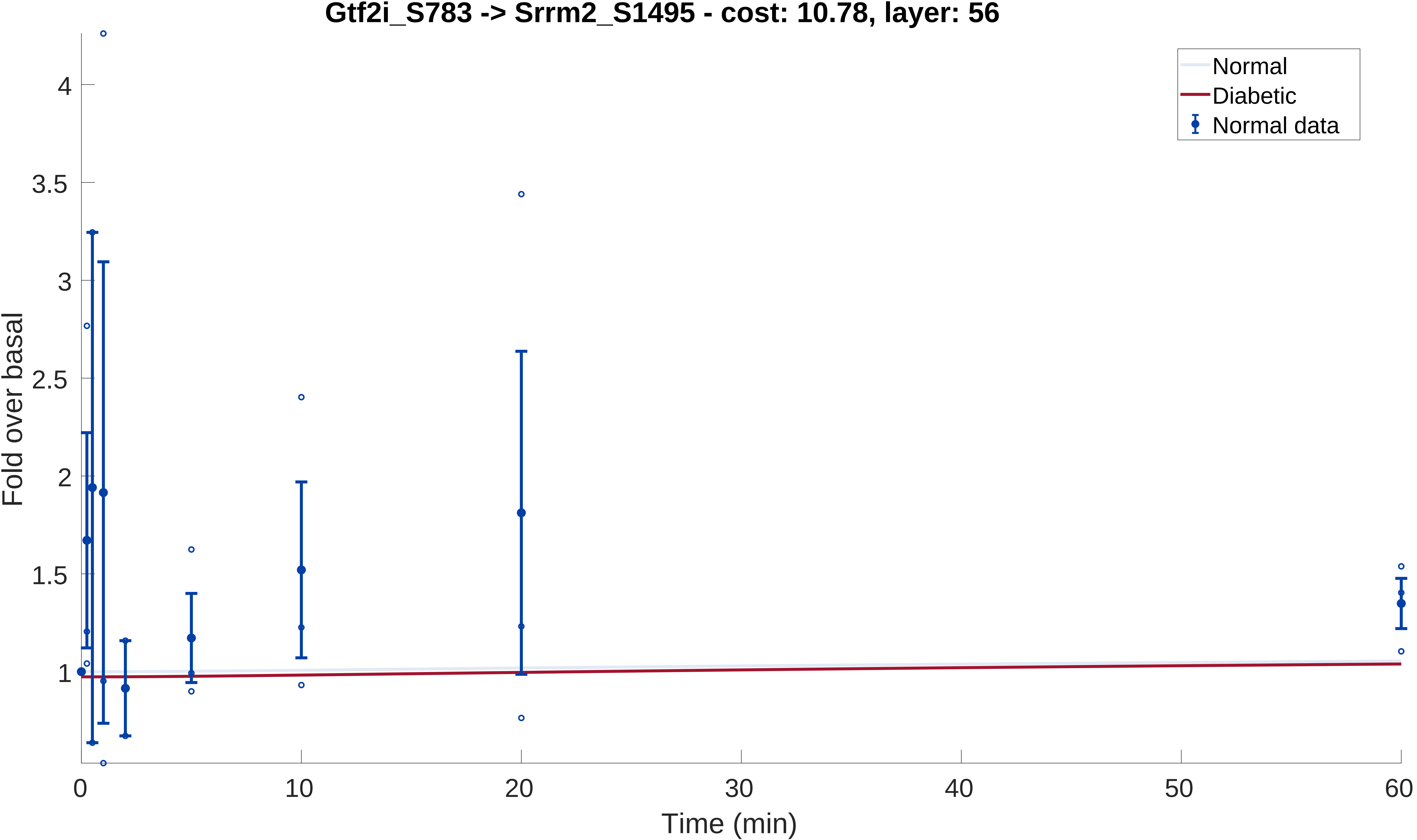

Gtf2i_S783 -> Srrm2_S1495 - cost: 10.78, layer: 56
Normal
Diabetic
Normal data
Fold over basal
Time (min)
4
3.5
3
2.5
2
1.5
1
0
10
20
30
40
50
60

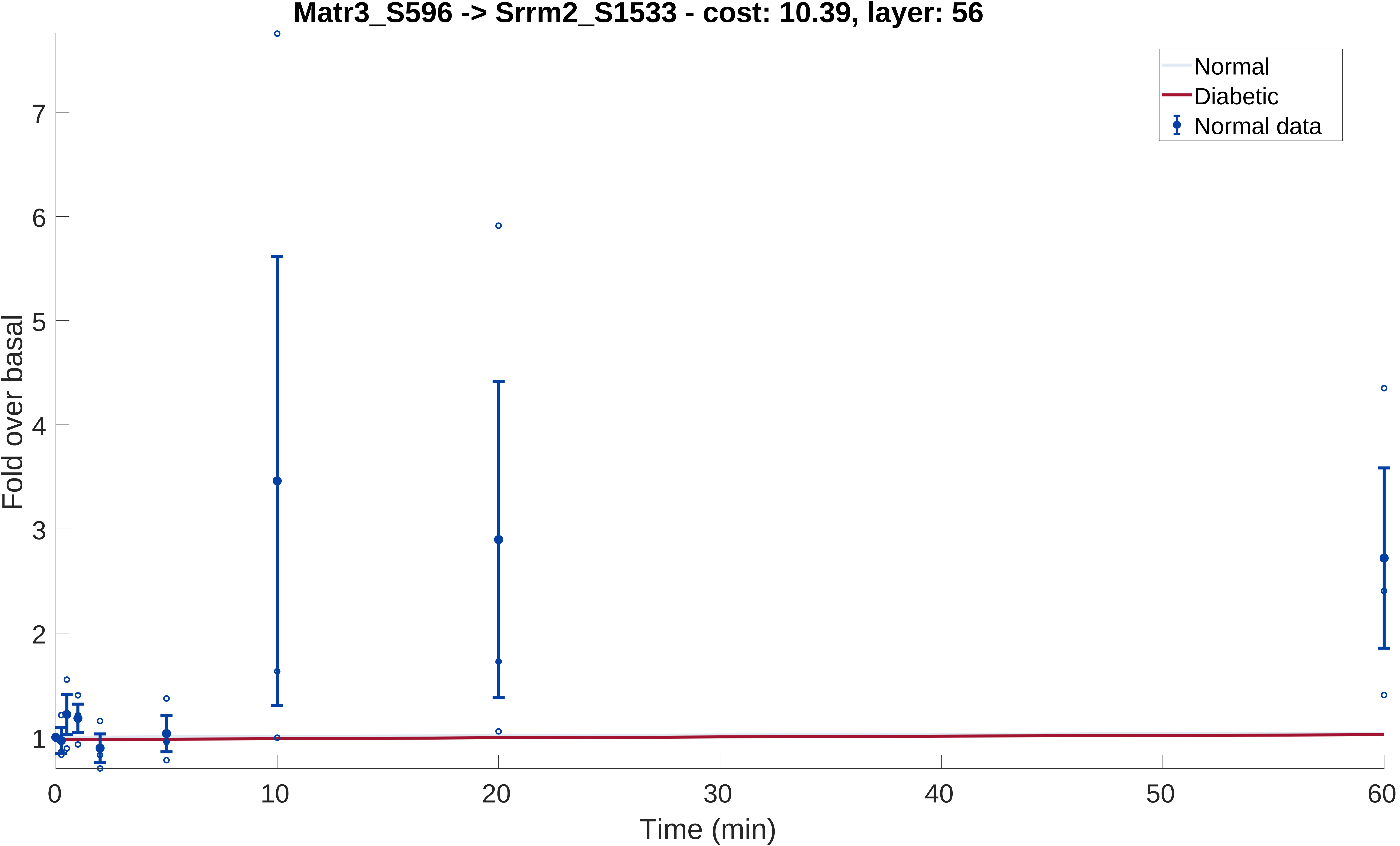

Matr3_S596 -> Srrm2_S1533 - cost: 10.39, layer: 56
Normal
Diabetic
Normal data
Fold over basal
Time (min)
0
10
20
30
40
50
60
1
2
3
4
5
6
7

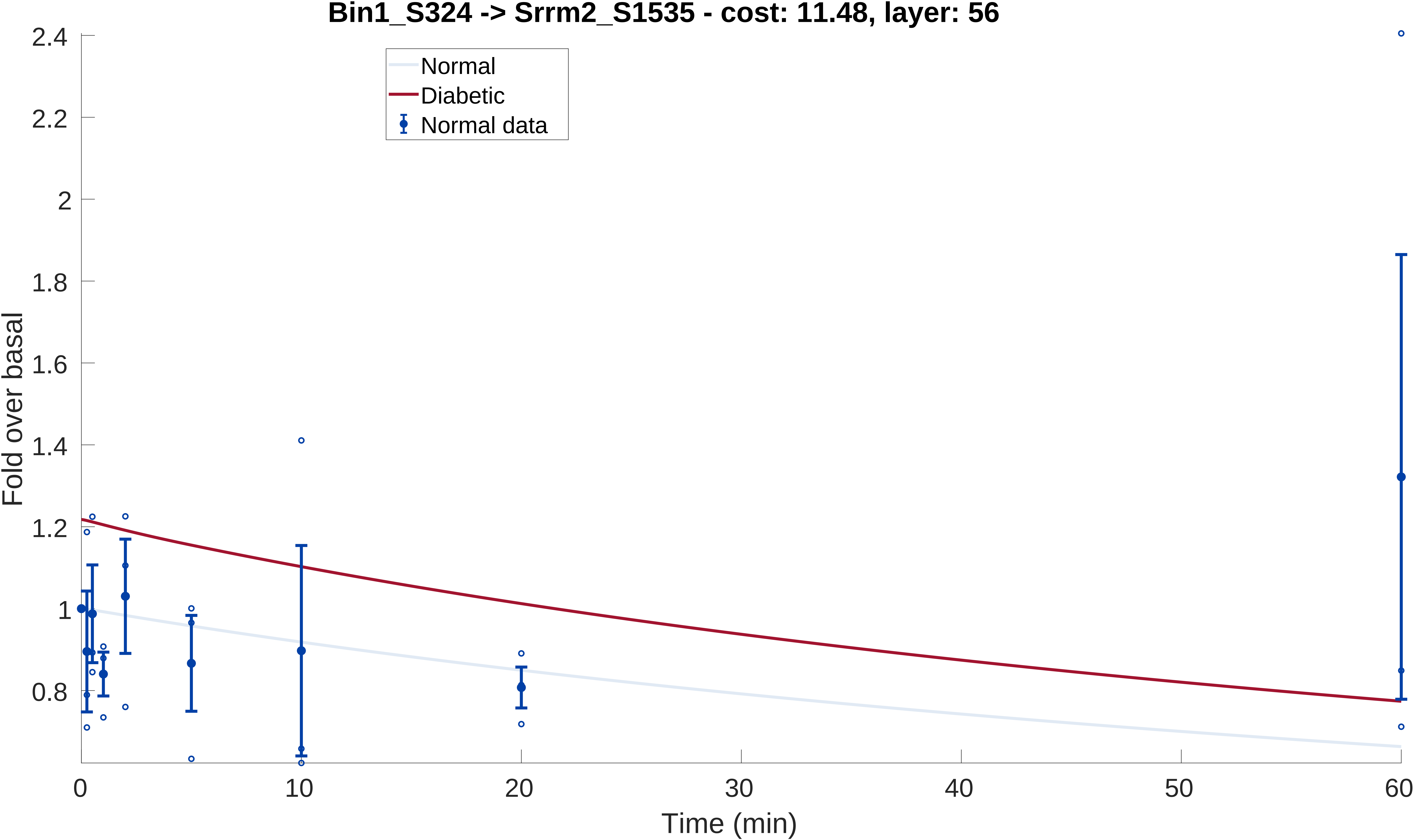

Bin1_S324 -> Srrm2_S1535 - cost: 11.48, layer: 56
Normal
Diabetic
Normal data
Fold over basal
Time (min)
2.4
2.2
2
1.8
1.6
1.4
1.2
1
0.8
0
10
20
30
40
50
60

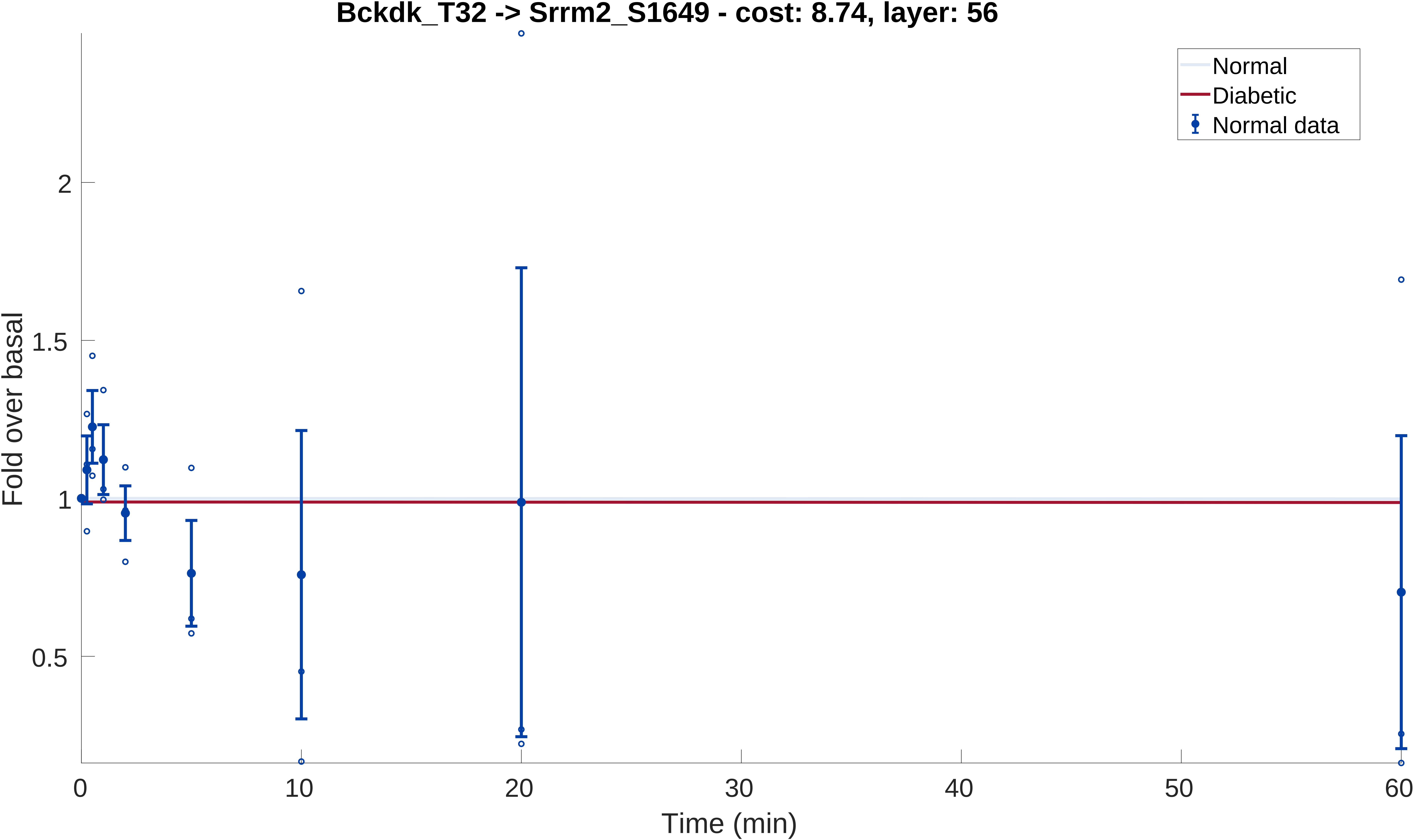

Bckdk_T32 -> Srrm2_S1649 - cost: 8.74, layer: 56
Normal
Diabetic
Normal data
Fold over basal
2
1.5
1
0.5
0
10
20
30
40
50
60
Time (min)

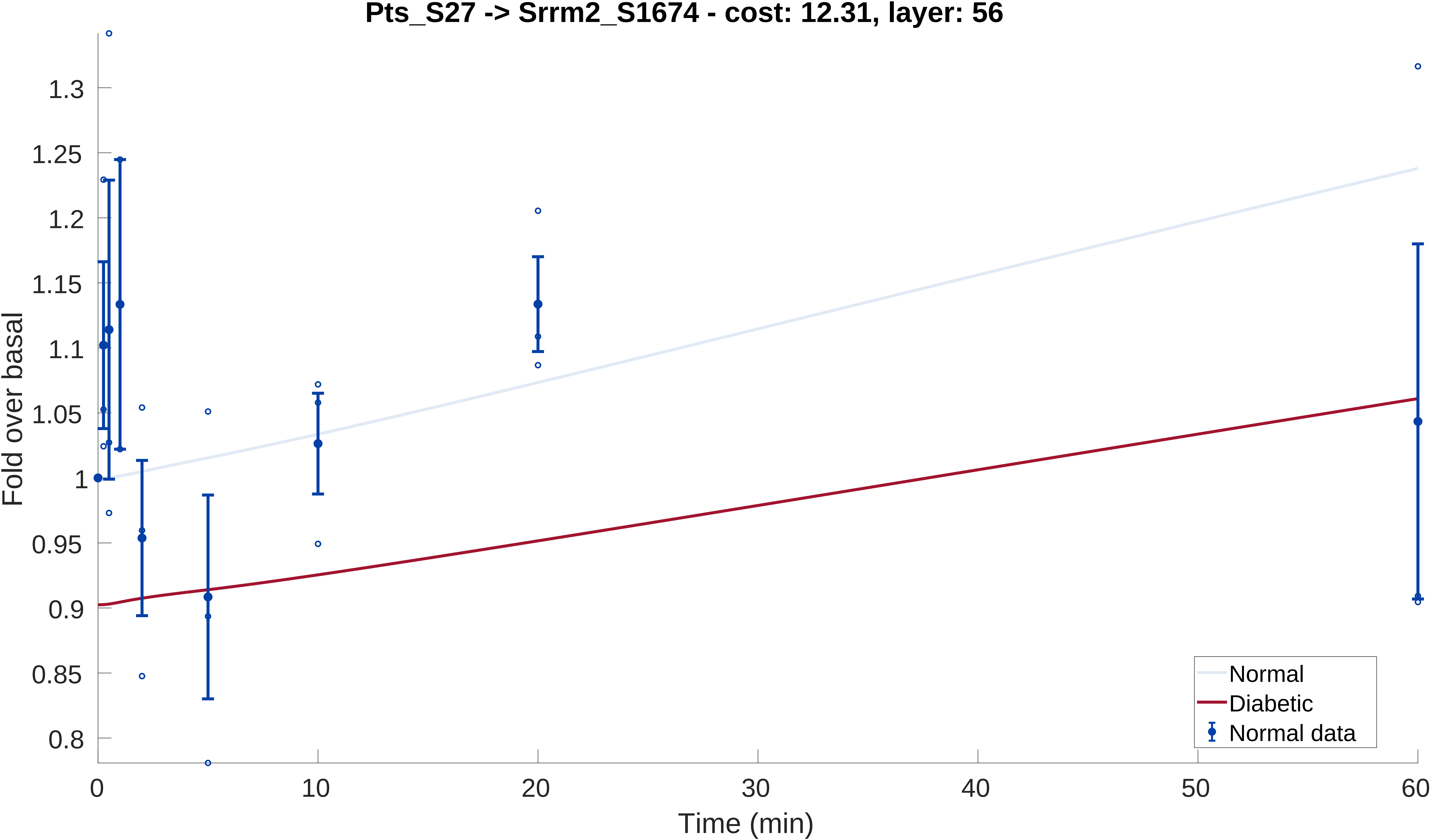

Pts_S27 -> Srrm2_S1674 - cost: 12.31, layer: 56
Fold over basal
1.3
1.25
1.2
1.15
1.1
1.05
1
0.95
0.9
0.85
0.8
0
10
20
30
40
50
60
Time (min)
Normal
Diabetic
Normal data

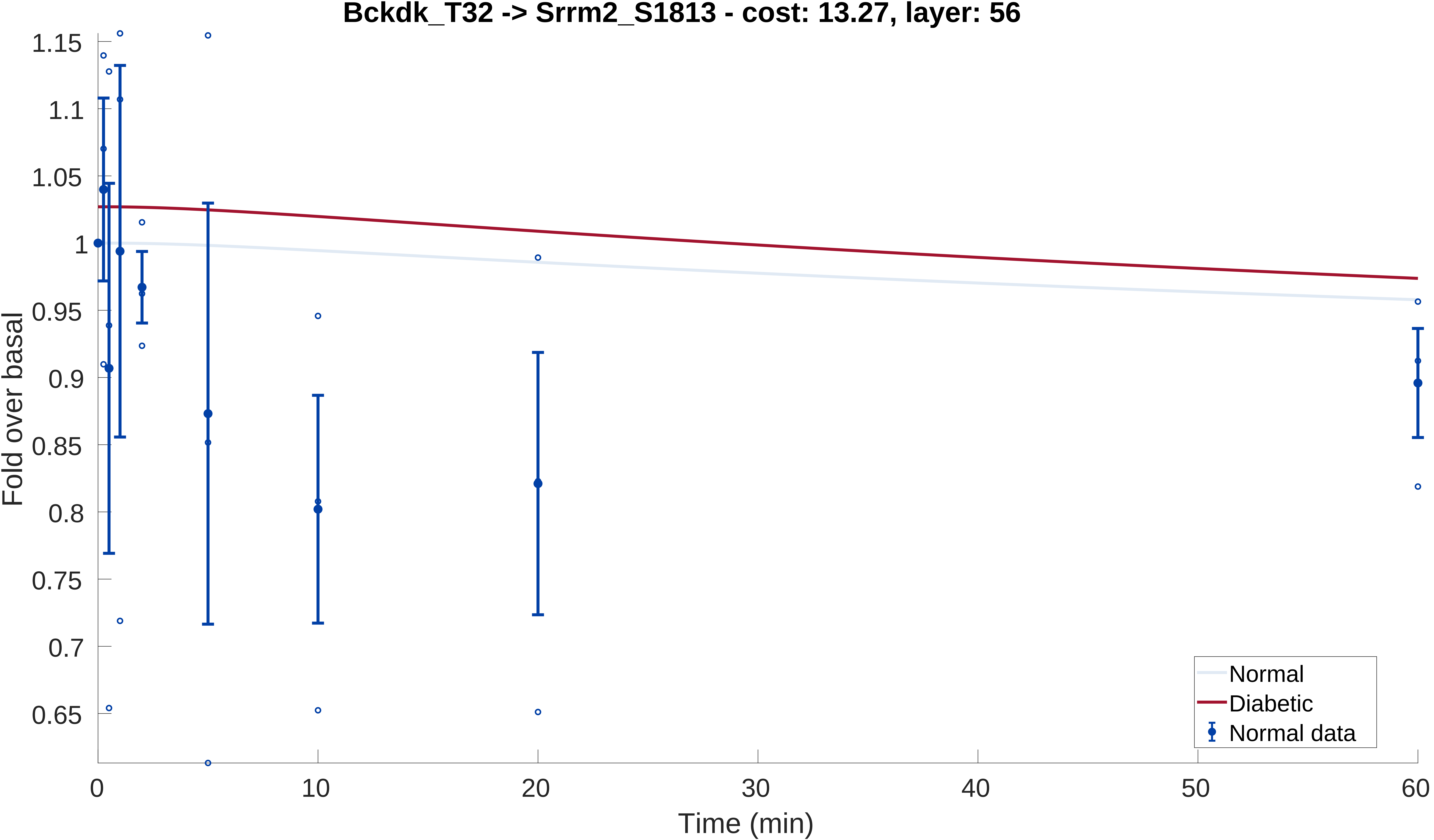

Bckdk_T32 -> Srrm2_S1813 - cost: 13.27, layer: 56
Fold over basal
1.15
1.1
1.05
1
0.95
0.9
0.85
0.8
0.75
0.7
0.65
0
10
20
30
40
50
60
Time (min)
Normal
Diabetic
Normal data

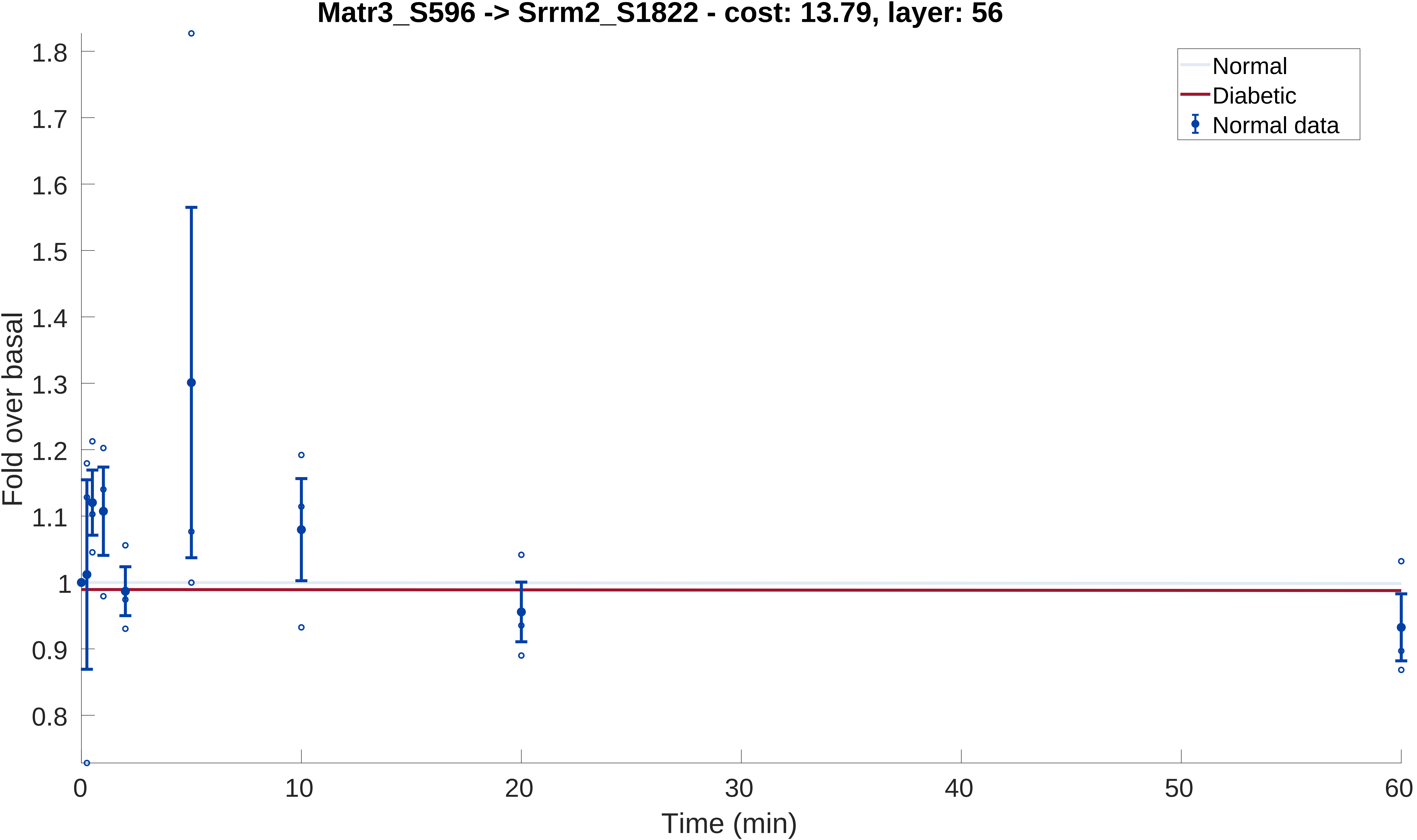
Matr3_S596 -> Srrm2_S1822 - cost: 13.79, layer: 56
Fold over basal
Time (min)
Normal
Diabetic
Normal data
1.8
1.7
1.6
1.5
1.4
1.3
1.2
1.1
1
0.9
0.8
0
10
20
30
40
50
60

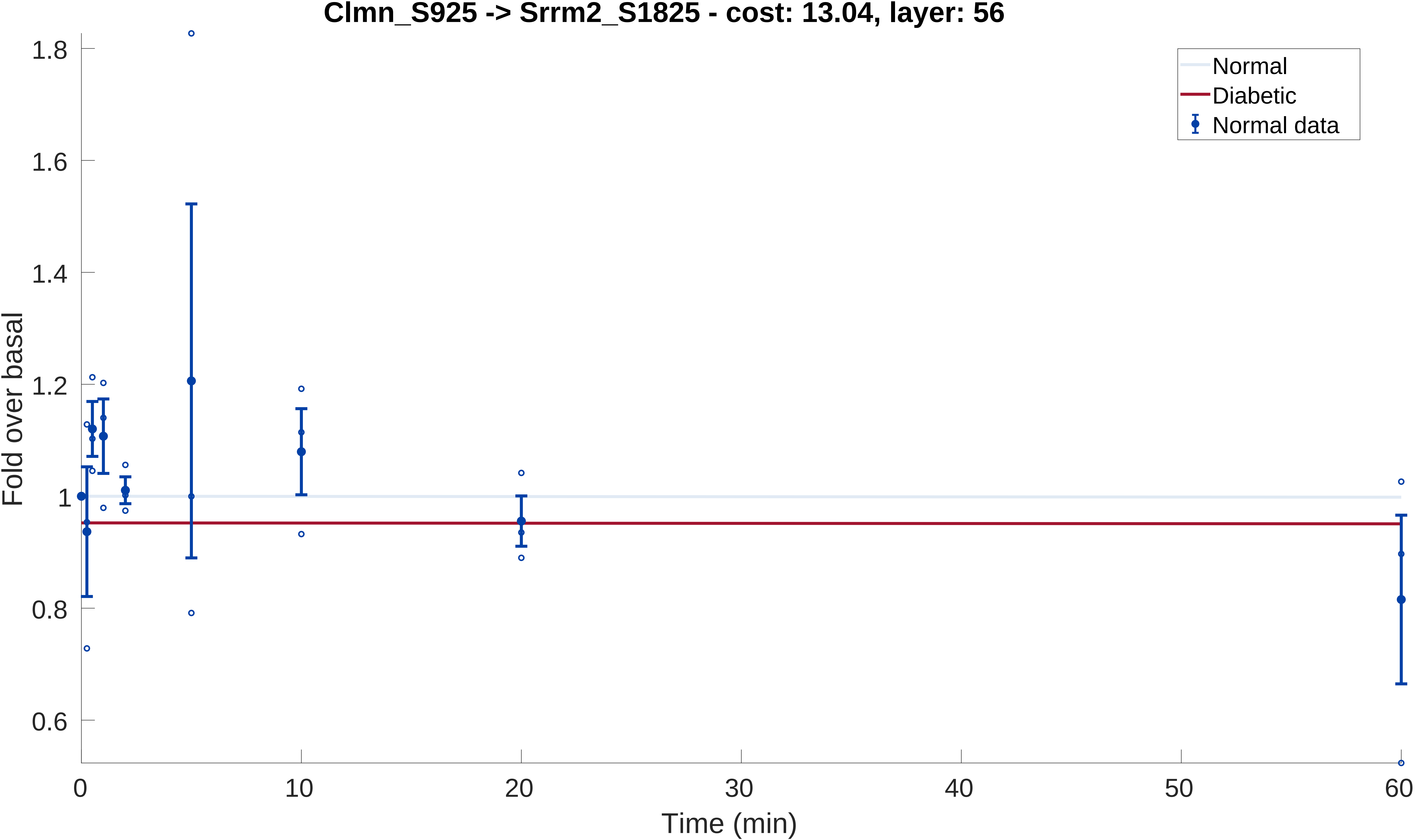
Clmn_S925 -> Srrm2_S1825 - cost: 13.04, layer: 56
Fold over basal
Time (min)
Normal
Diabetic
Normal data
1.8
1.6
1.4
1.2
1
0.8
0.6
0
10
20
30
40
50
60

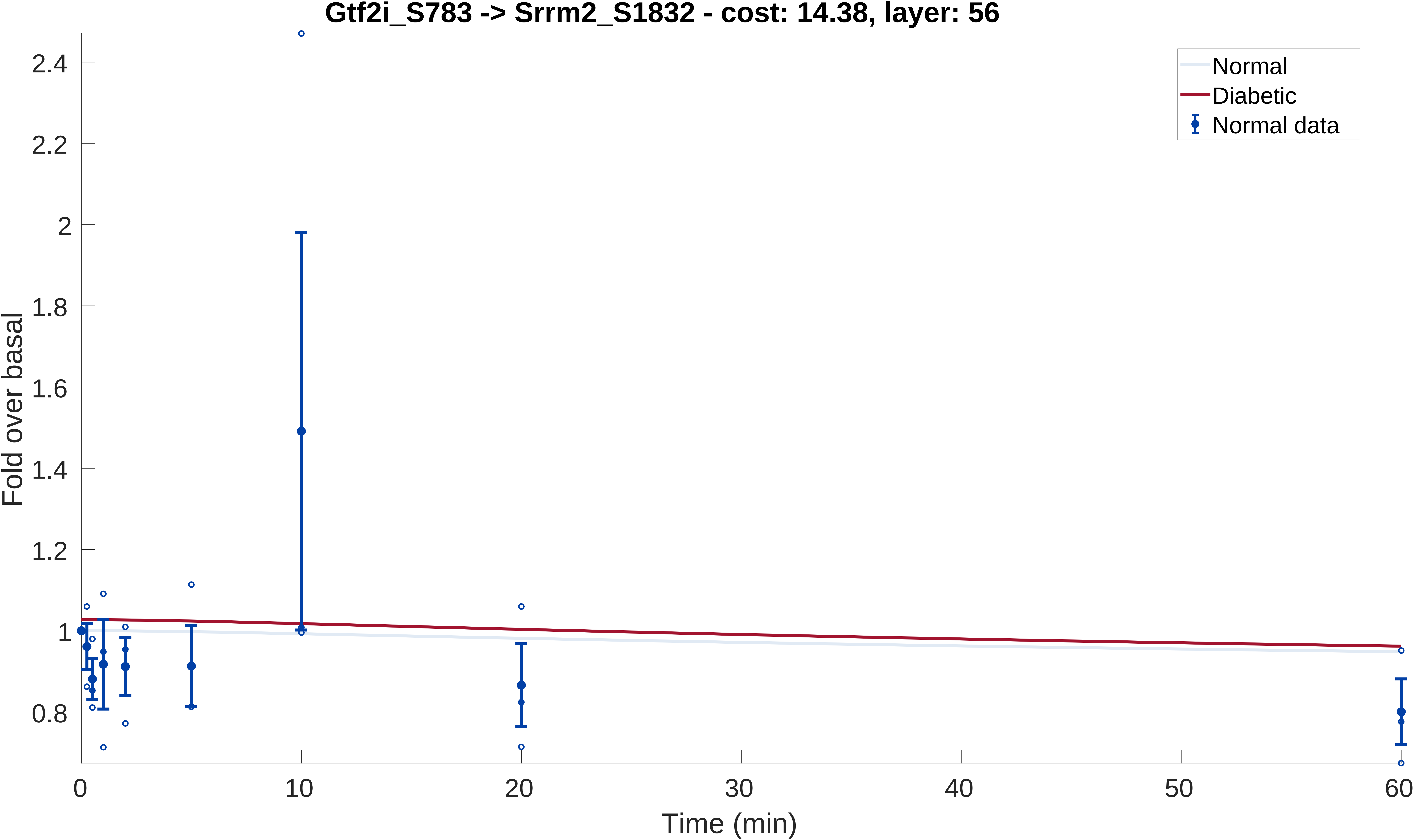

Gtf2i_S783 -> Srrm2_S1832 - cost: 14.38, layer: 56
Normal
Diabetic
Normal data
Fold over basal
Time (min)
2.4
2.2
2
1.8
1.6
1.4
1.2
1
0.8
0
10
20
30
40
50
60

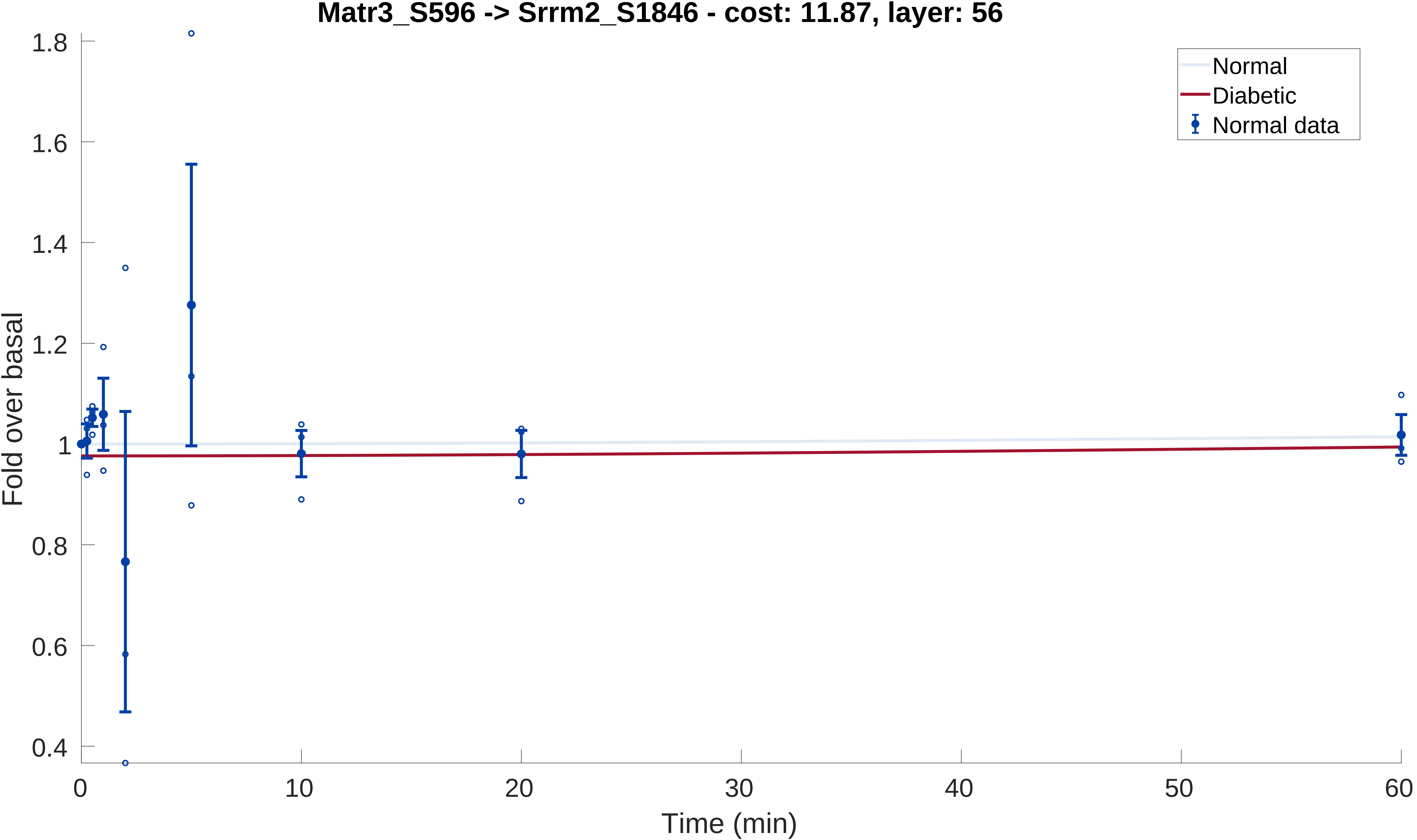

Matr3_S596 -> Srrm2_S1846 - cost: 11.87, layer: 56
Normal
Diabetic
Normal data
Fold over basal
Time (min)
1.8
1.6
1.4
1.2
1
0.8
0.6
0.4
0
10
20
30
40
50
60

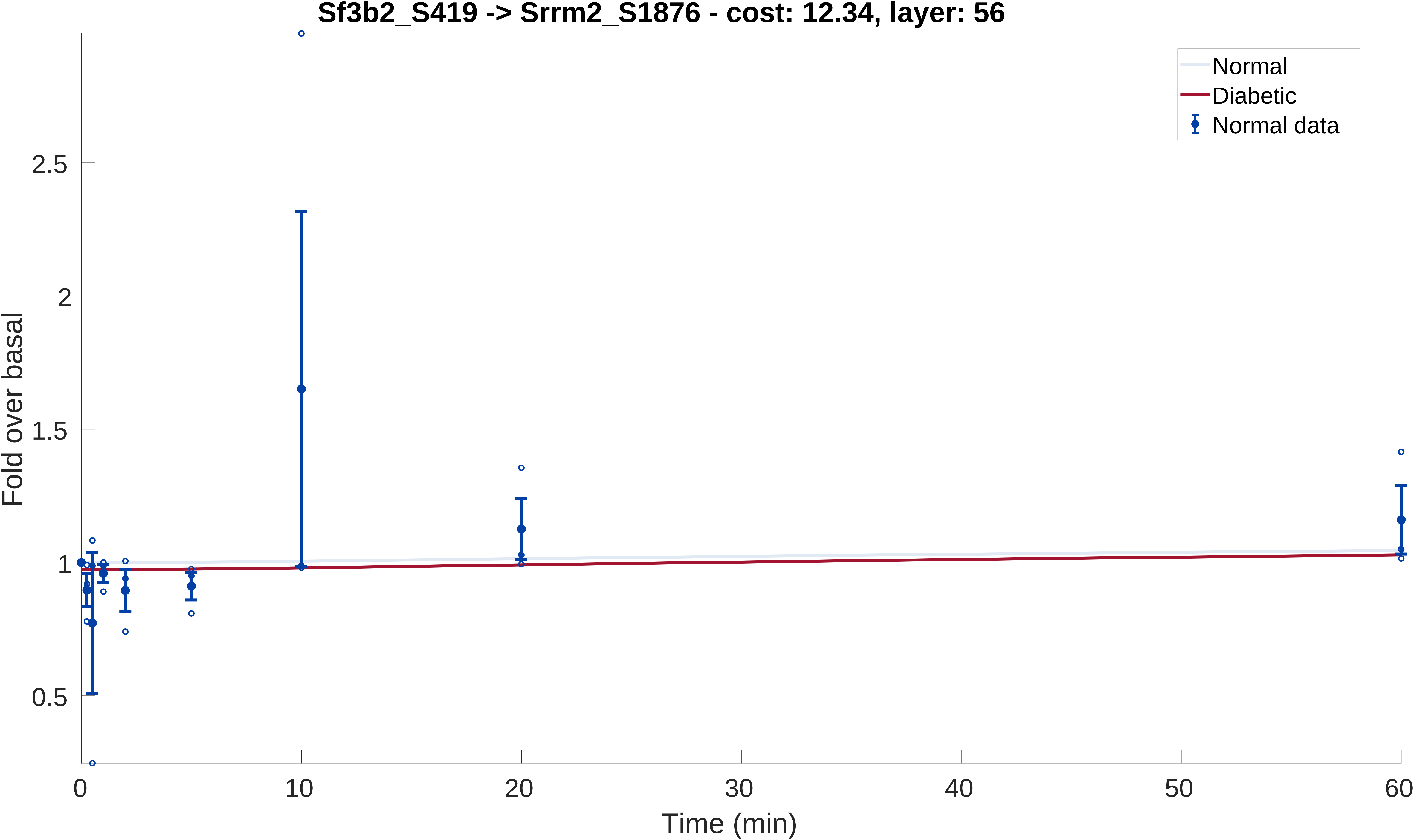

Sf3b2_S419 -> Srrm2_S1876 - cost: 12.34, layer: 56
Normal
Diabetic
Normal data
Fold over basal
0.5
1
1.5
2
2.5
0
10
20
30
40
50
60
Time (min)

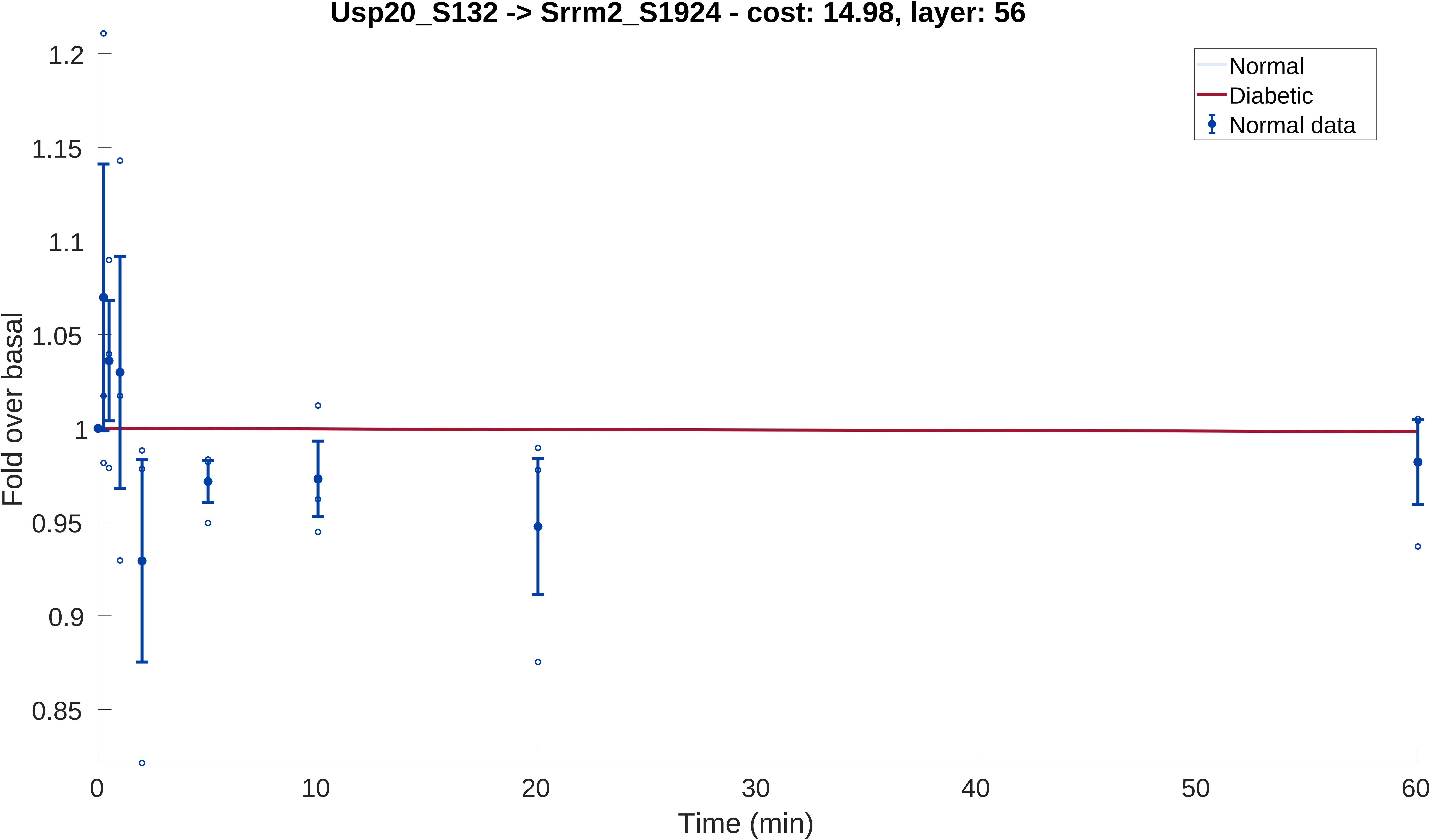

Usp20_S132 -> Srrm2_S1924 - cost: 14.98, layer: 56
Normal
Diabetic
Normal data
Fold over basal
Time (min)
1.2
1.15
1.1
1.05
1
0.95
0.9
0.85
0
10
20
30
40
50
60

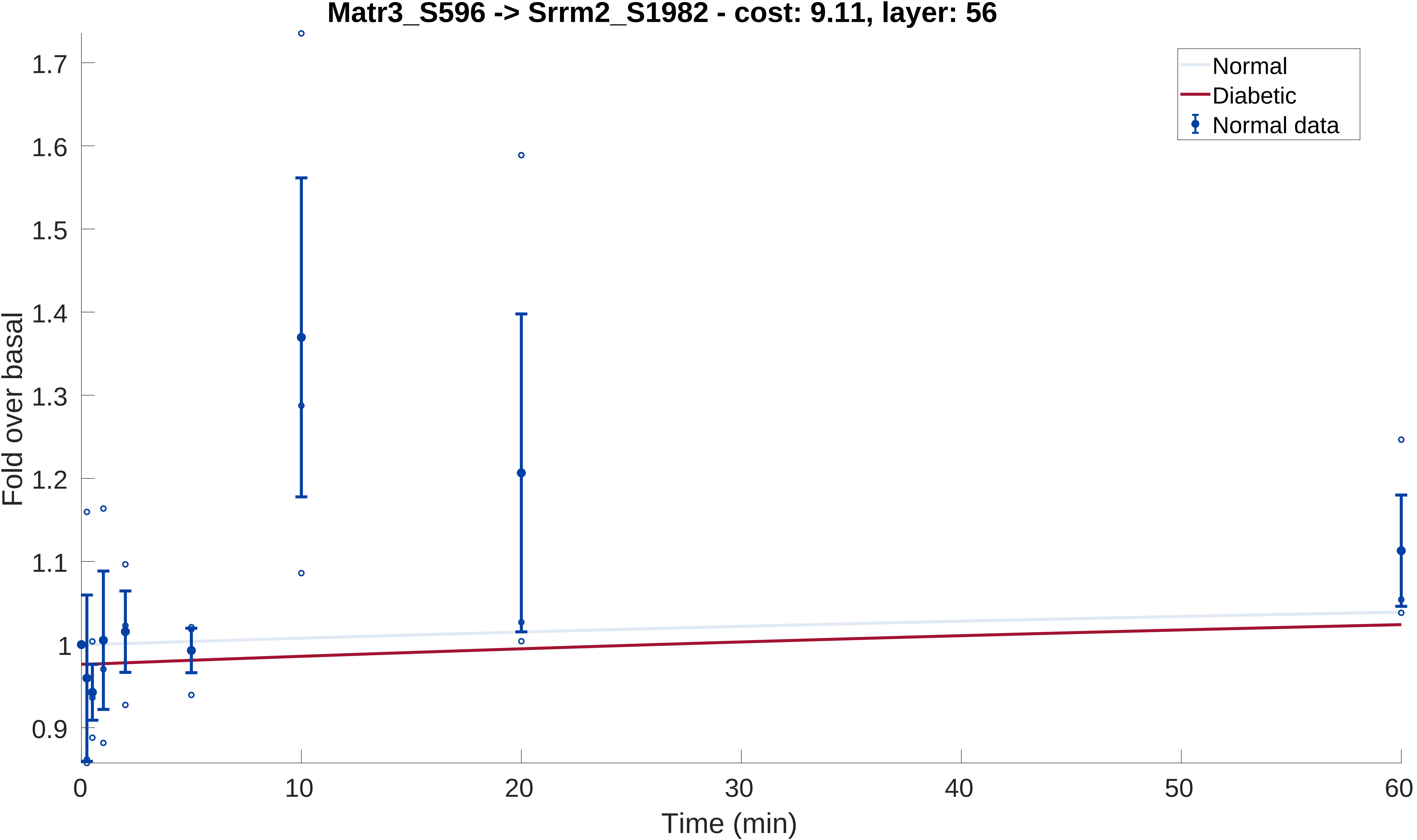

Matr3_S596 -> Srrm2_S1982 - cost: 9.11, layer: 56
Normal
Diabetic
Normal data
Fold over basal
Time (min)
0.9
1
1.1
1.2
1.3
1.4
1.5
1.6
1.7
0
10
20
30
40
50
60

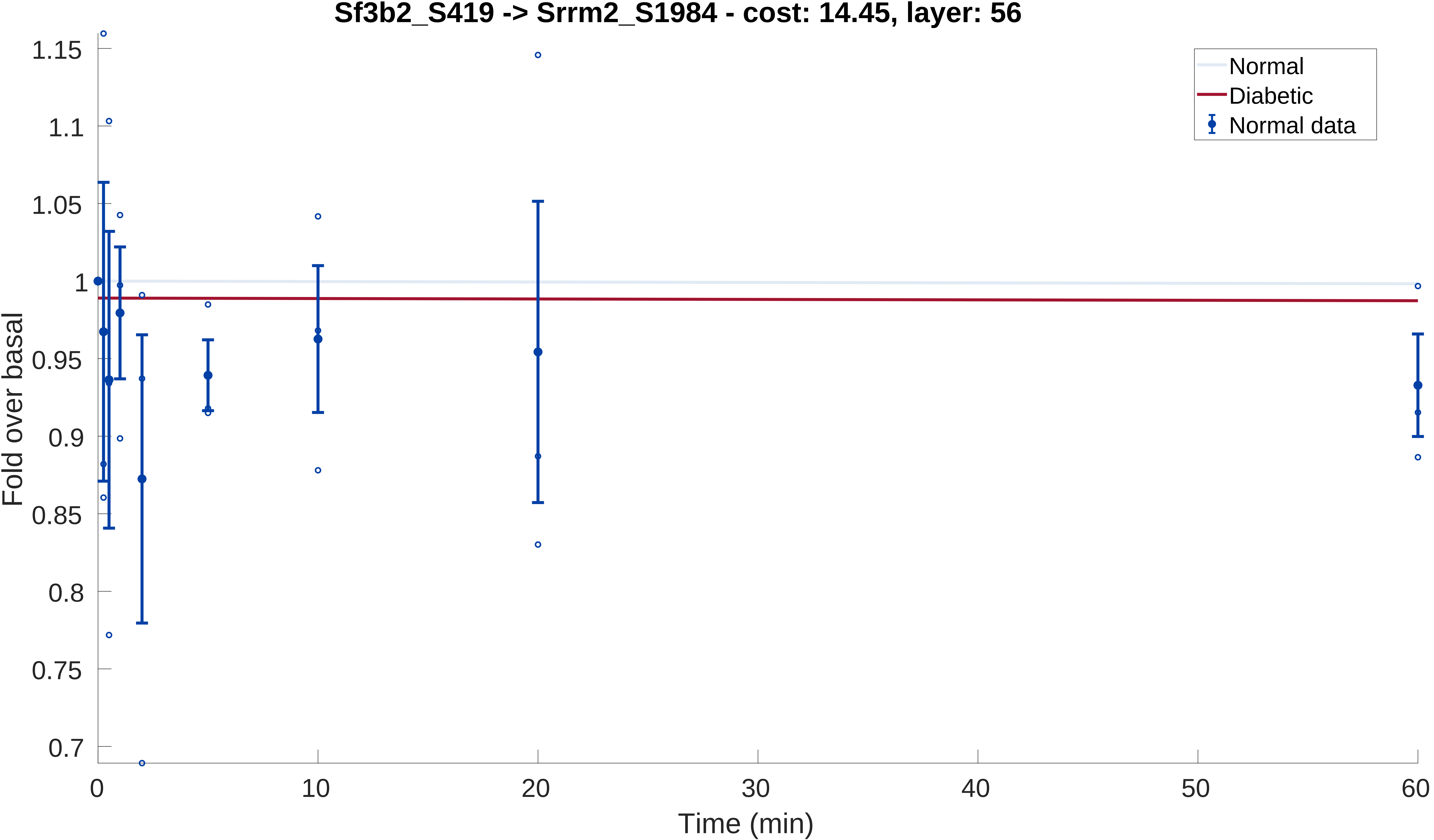

Sf3b2_S419 -> Srrm2_S1984 - cost: 14.45, layer: 56
Normal
Diabetic
Normal data
Fold over basal
Time (min)
1.15
1.1
1.05
1
0.95
0.9
0.85
0.8
0.75
0.7
0
10
20
30
40
50
60

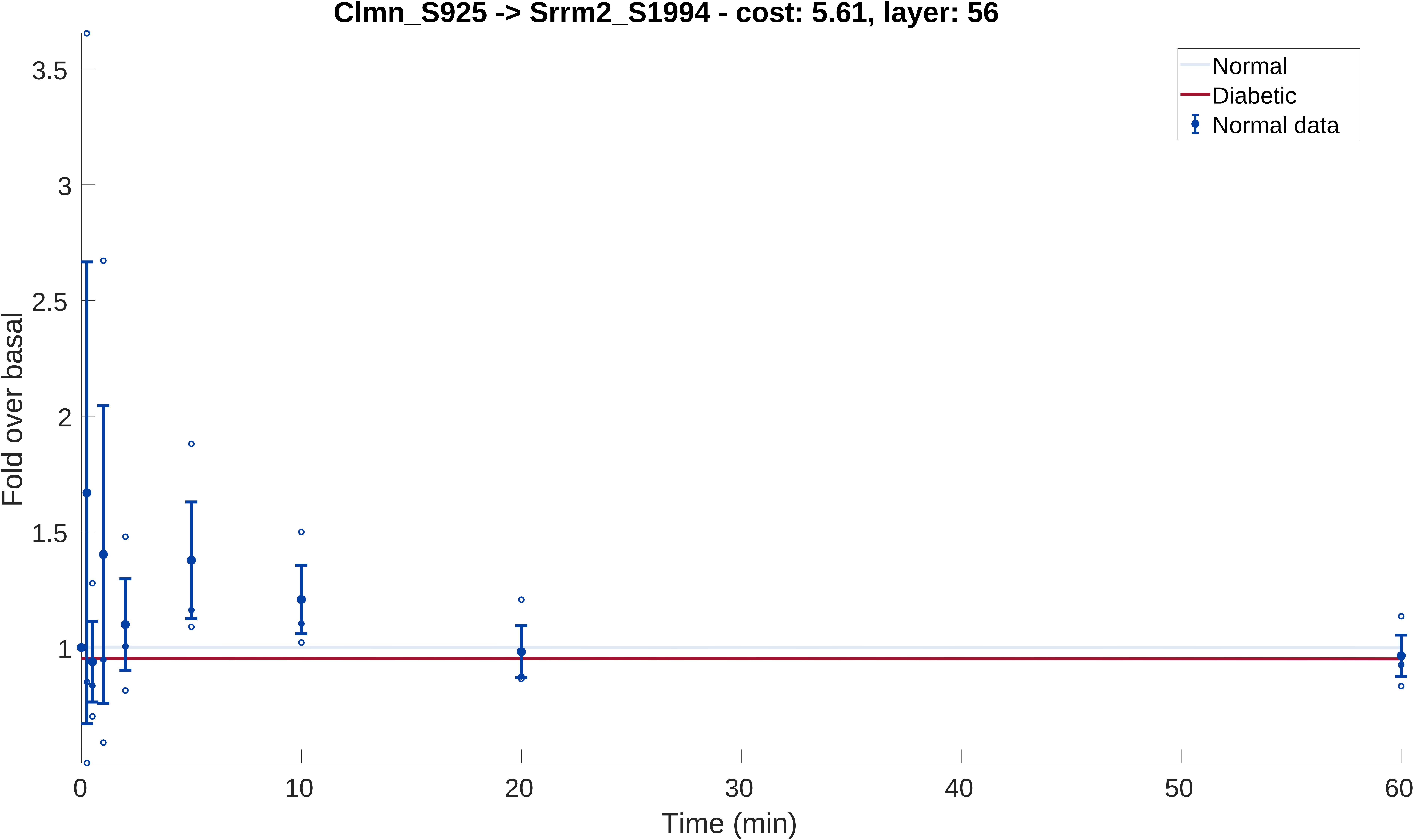
Clmn_S925 -> Srrm2_S1994 - cost: 5.61, layer: 56
Normal
Diabetic
Normal data
Fold over basal
3.5
3
2.5
2
1.5
1
0
10
20
30
40
50
60
Time (min)

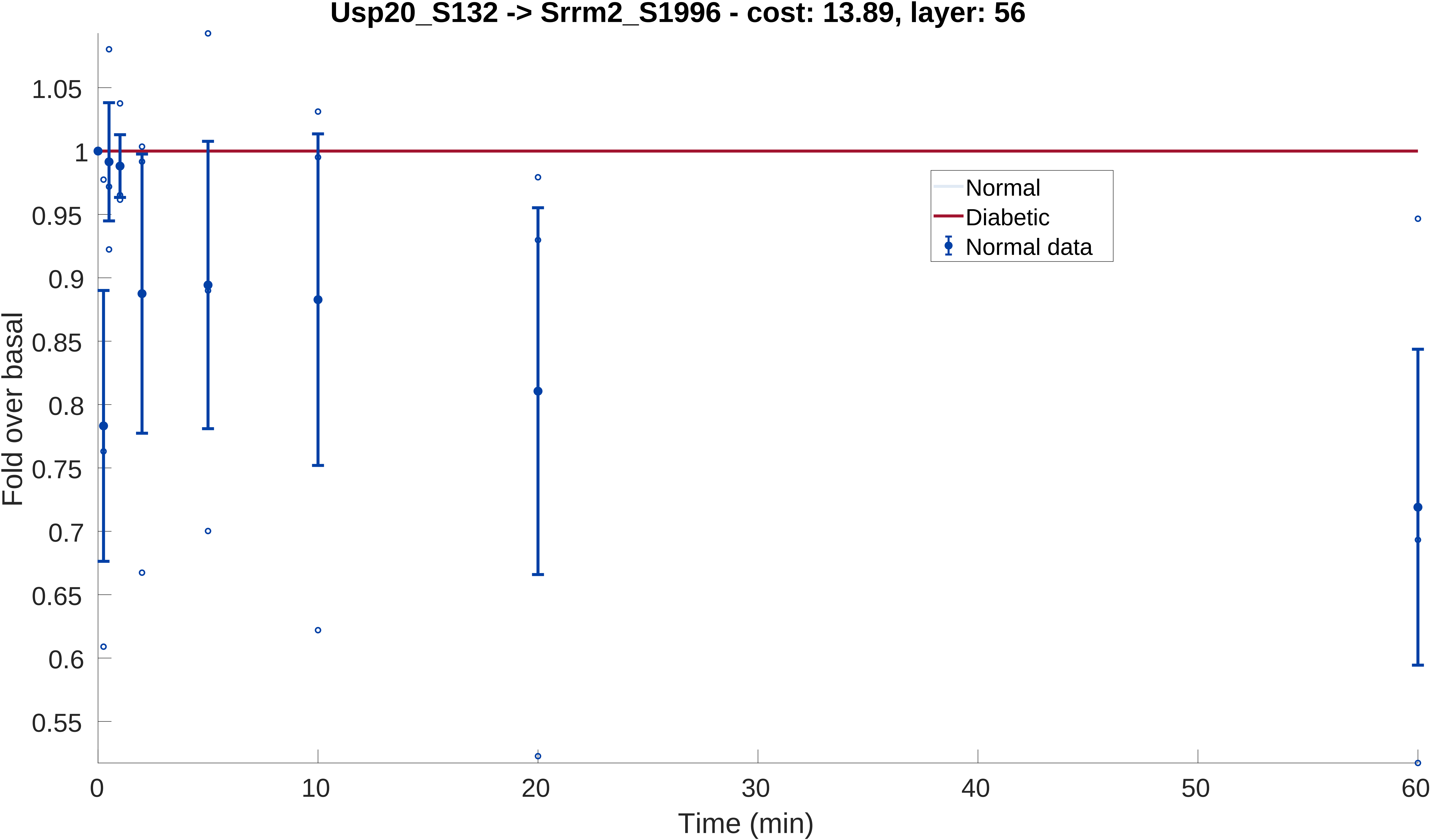

Usp20_S132 -> Srrm2_S1996 - cost: 13.89, layer: 56
Fold over basal
1.05
1
0.95
0.9
0.85
0.8
0.75
0.7
0.65
0.6
0.55
0
10
20
30
40
50
60
Time (min)
Normal
Diabetic
Normal data

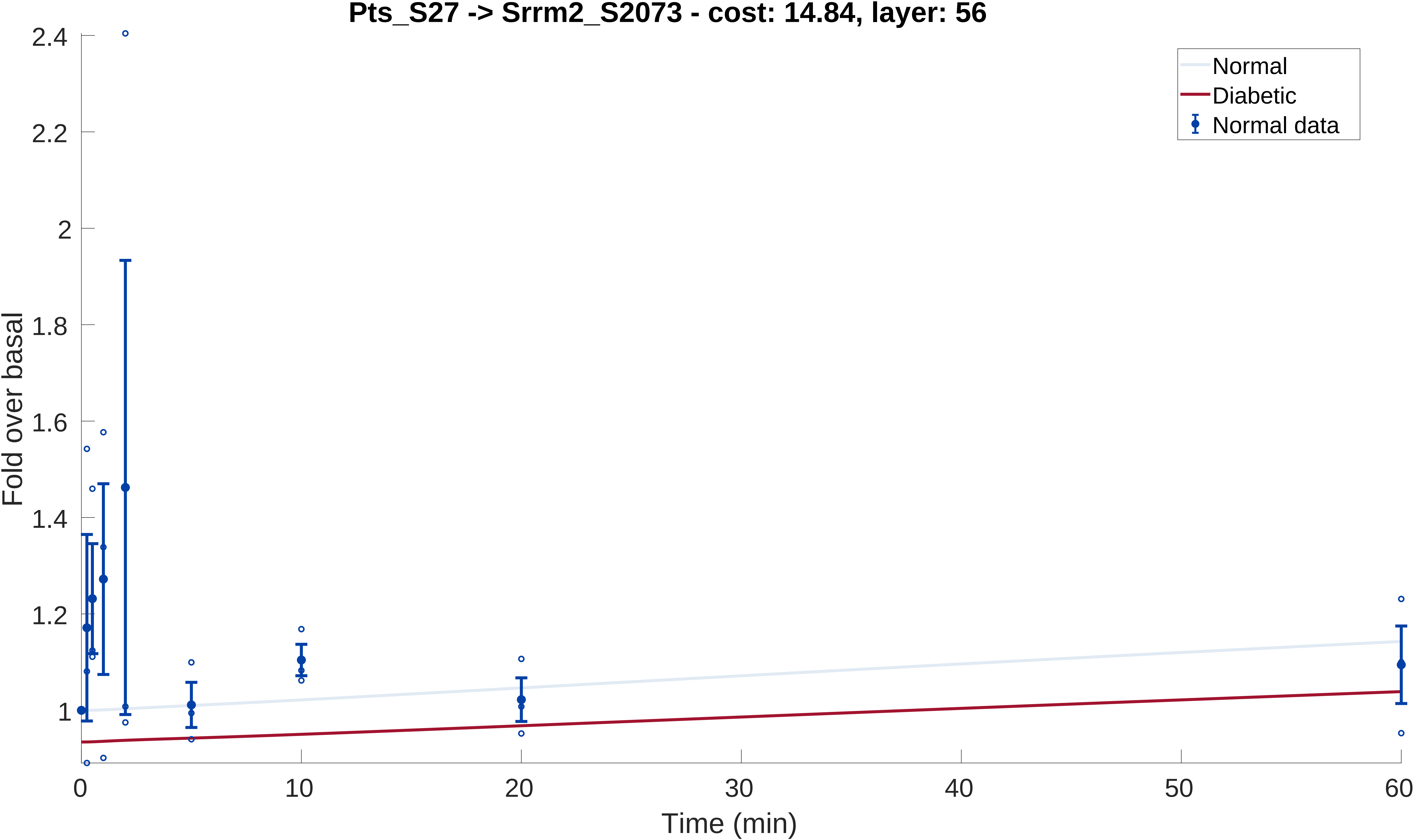

Pts_S27 -> Srrm2_S2073 - cost: 14.84, layer: 56
Normal
Diabetic
Normal data
Fold over basal
Time (min)
2.4
2.2
2
1.8
1.6
1.4
1.2
1
0
10
20
30
40
50
60

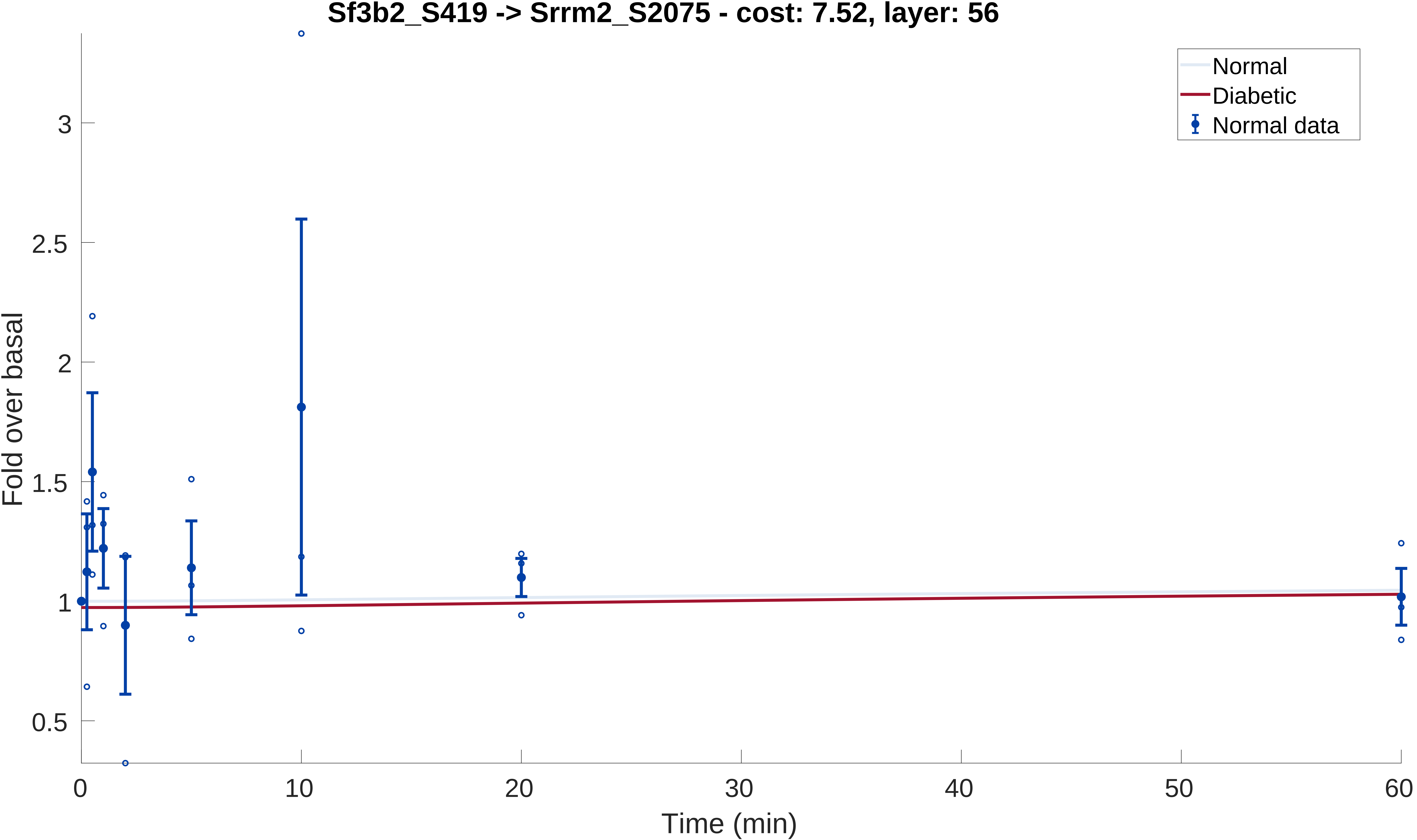

Sf3b2_S419 -> Srrm2_S2075 - cost: 7.52, layer: 56
Normal
Diabetic
Normal data
Fold over basal
0.5
1
1.5
2
2.5
3
0
10
20
30
40
50
60
Time (min)

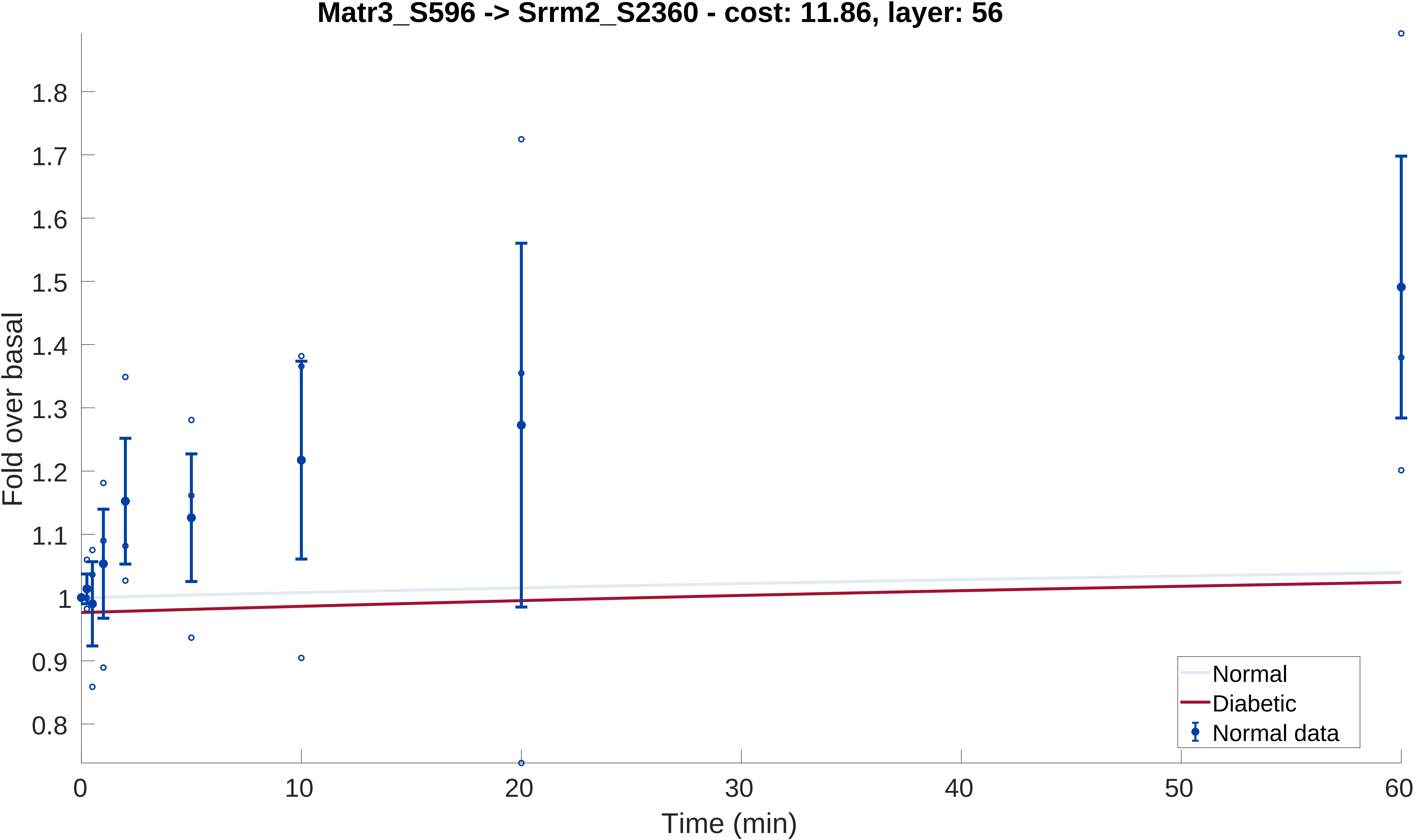

Matr3_S596 -> Srrm2_S2360 - cost: 11.86, layer: 56
Fold over basal
1.8
1.7
1.6
1.5
1.4
1.3
1.2
1.1
1
0.9
0.8
0
10
20
30
40
50
60
Time (min)
Normal
Diabetic
Normal data

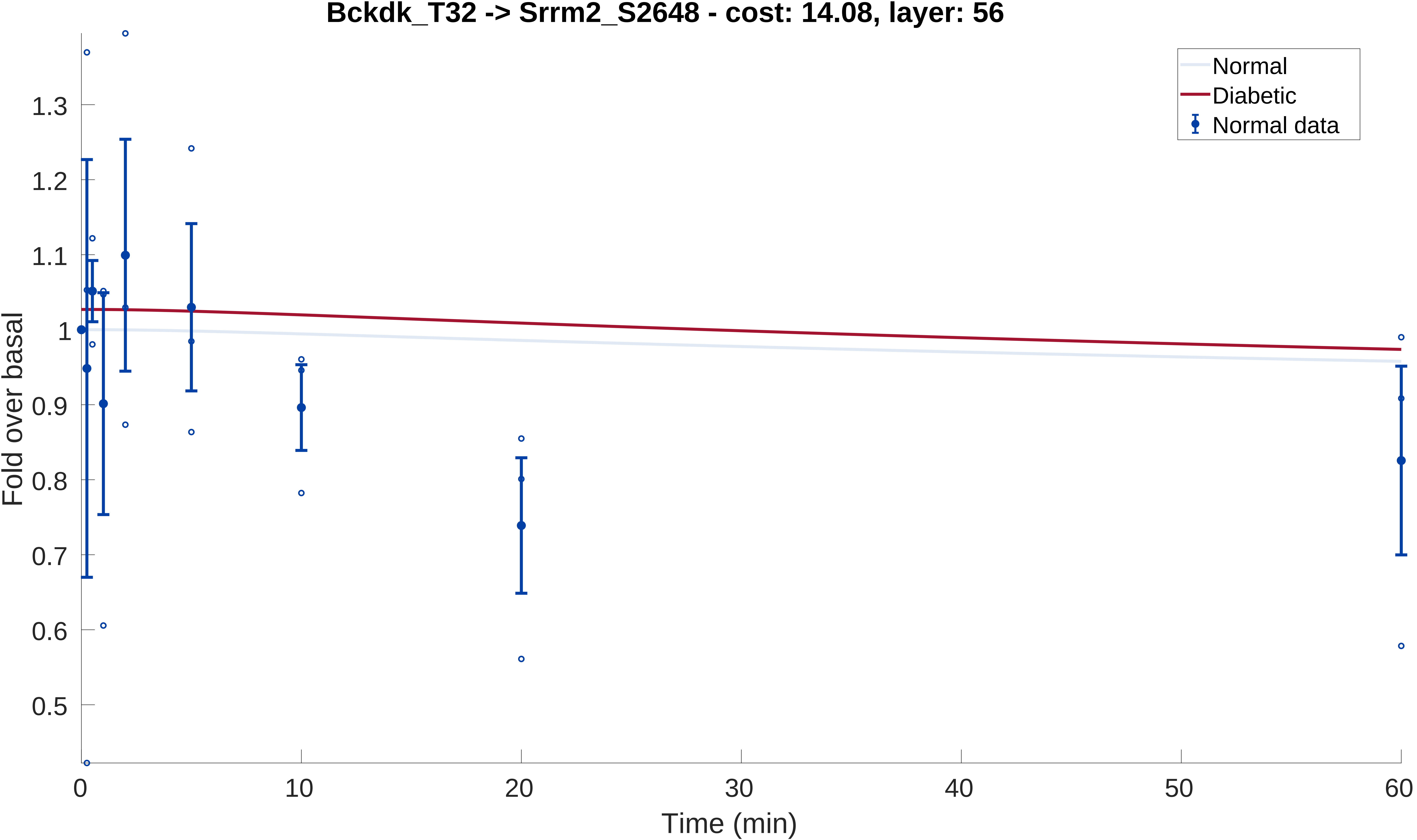

Bckdk_T32 -> Srrm2_S2648 - cost: 14.08, layer: 56
Normal
Diabetic
Normal data
Fold over basal
1.3
1.2
1.1
1
0.9
0.8
0.7
0.6
0.5
0
10
20
30
40
50
60
Time (min)

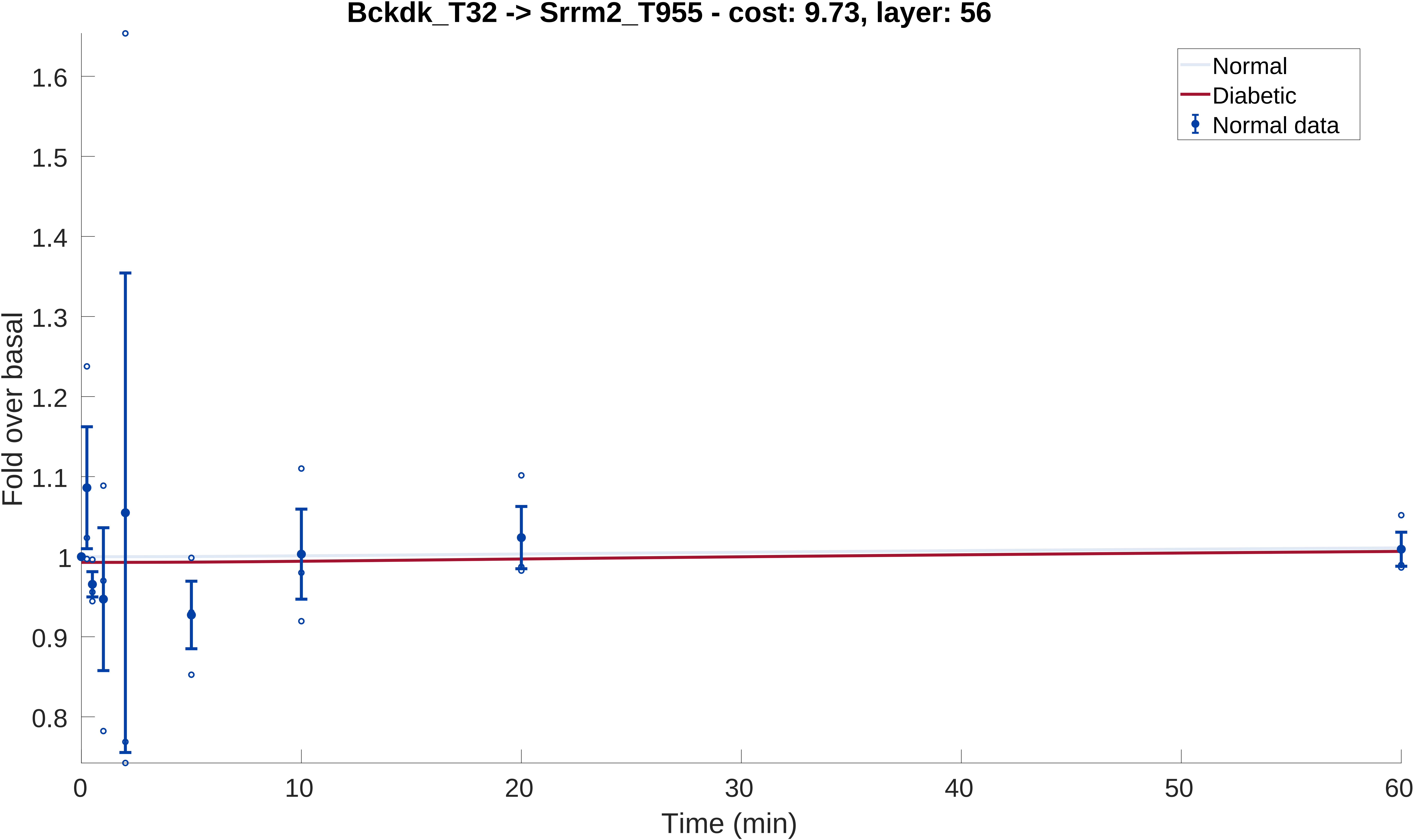
Bckdk_T32 -> Srrm2_T955 - cost: 9.73, layer: 56
Fold over basal
Time (min)
Normal
Diabetic
Normal data
0.8
0.9
1
1.1
1.2
1.3
1.4
1.5
1.6
0
10
20
30
40
50
60

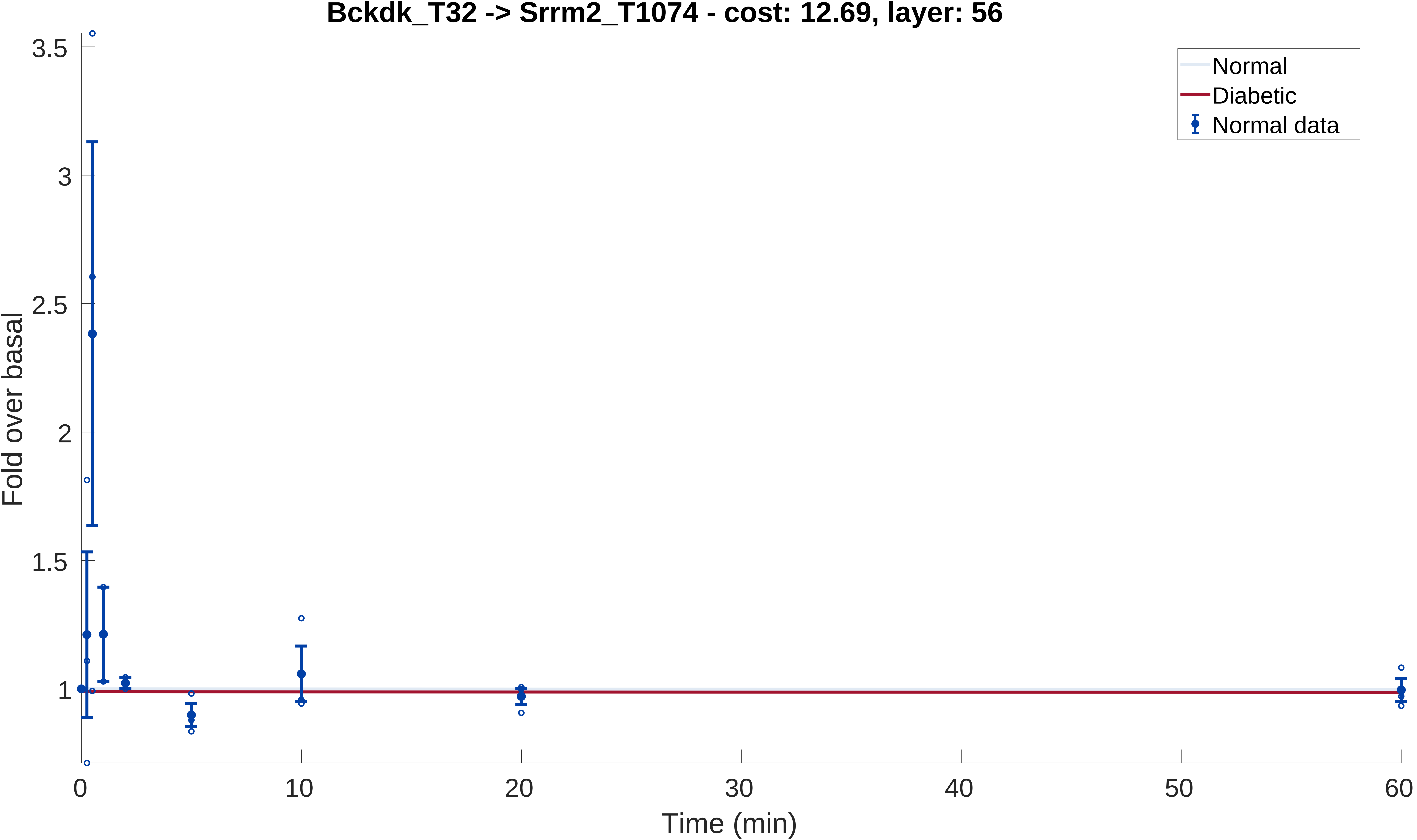

Bckdk_T32 -> Srrm2_T1074 - cost: 12.69, layer: 56
Fold over basal
Time (min)
Normal
Diabetic
Normal data
3.5
3
2.5
2
1.5
1
0
10
20
30
40
50
60

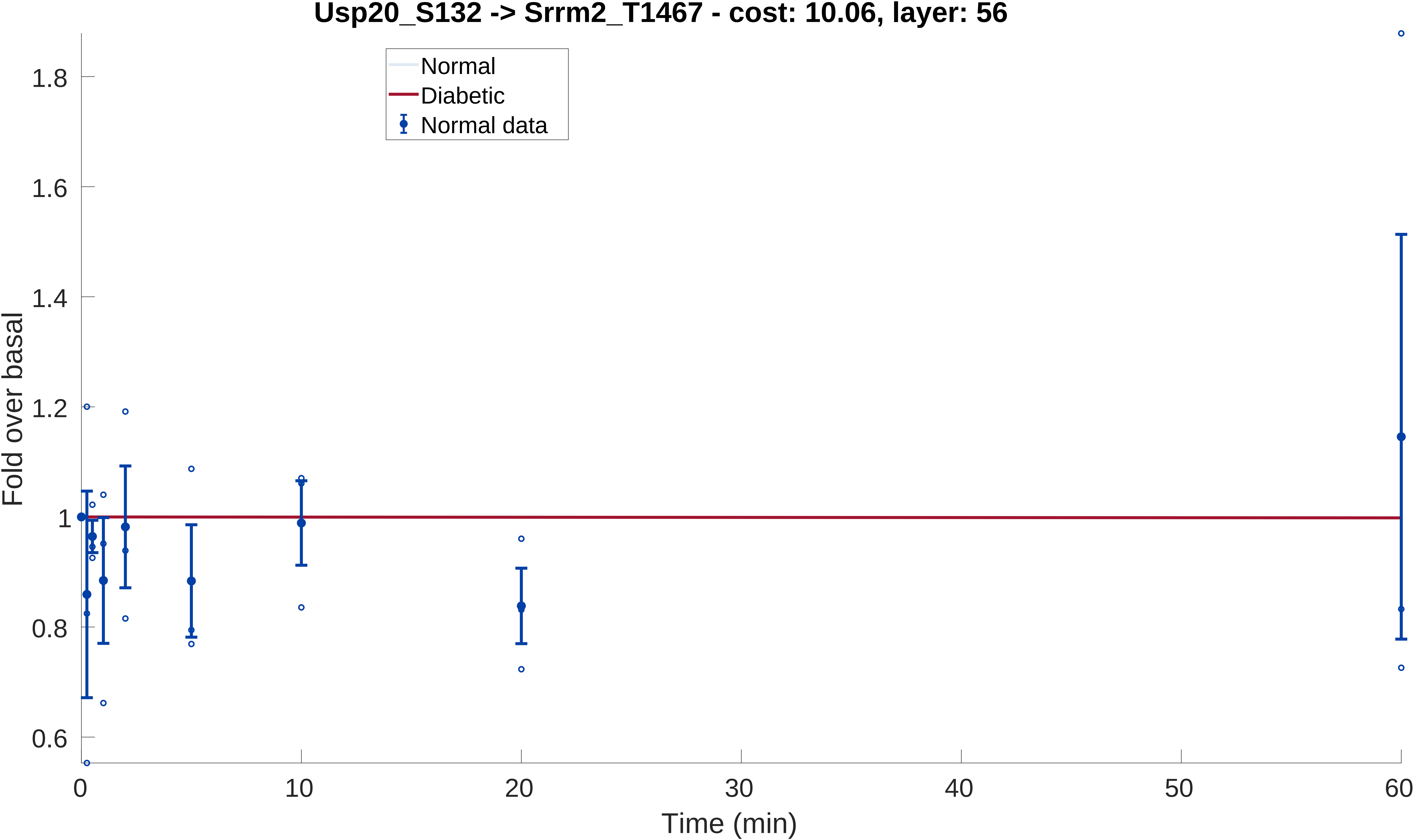

Usp20_S132 -> Srrm2_T1467 - cost: 10.06, layer: 56
Normal
Diabetic
Normal data
Fold over basal
1.8
1.6
1.4
1.2
1
0.8
0.6
0
10
20
30
40
50
60
Time (min)

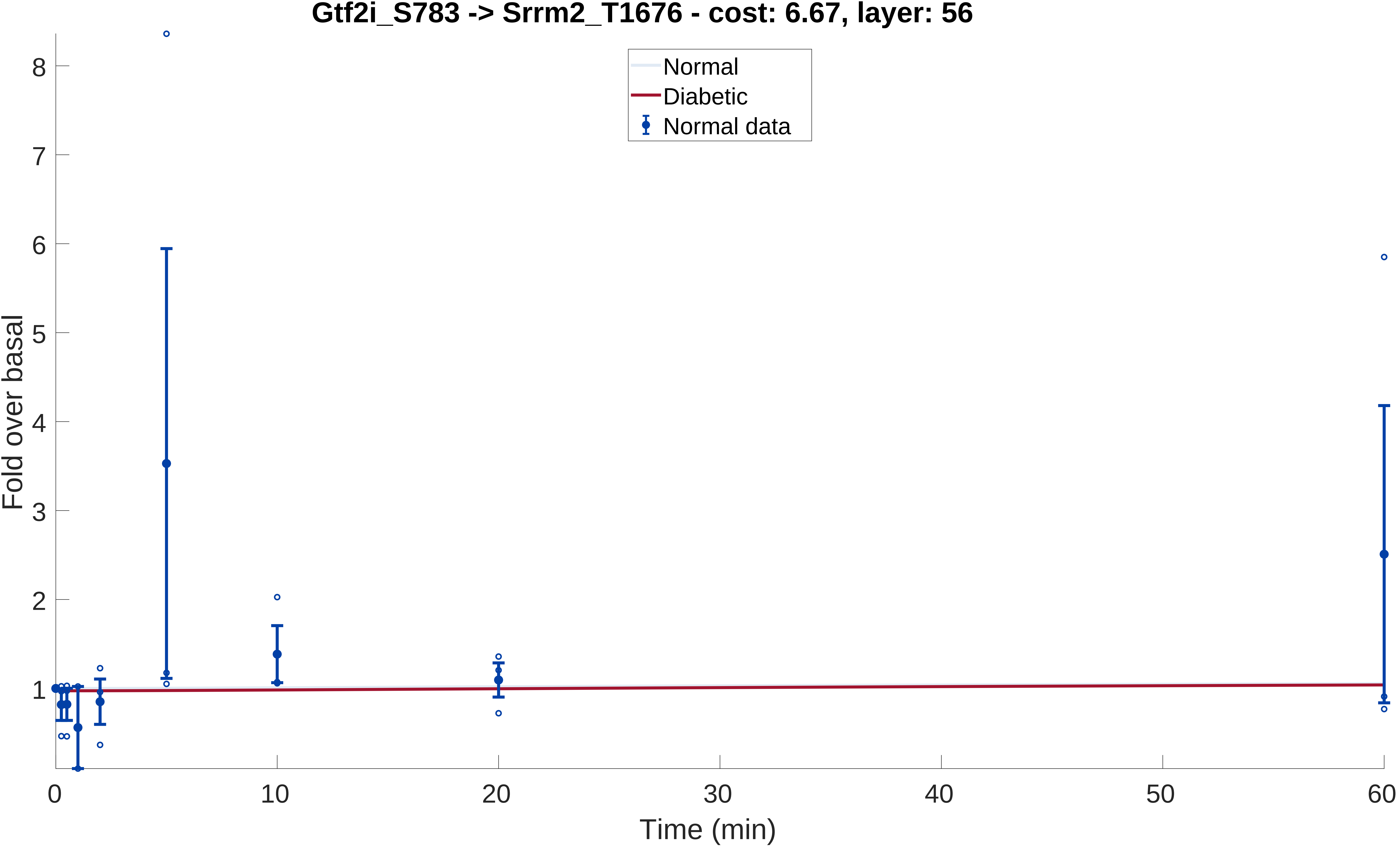
Gtf2i_S783 -> Srrm2_T1676 - cost: 6.67, layer: 56
Normal
Diabetic
Normal data
Fold over basal
Time (min)
0
10
20
30
40
50
60
1
2
3
4
5
6
7
8

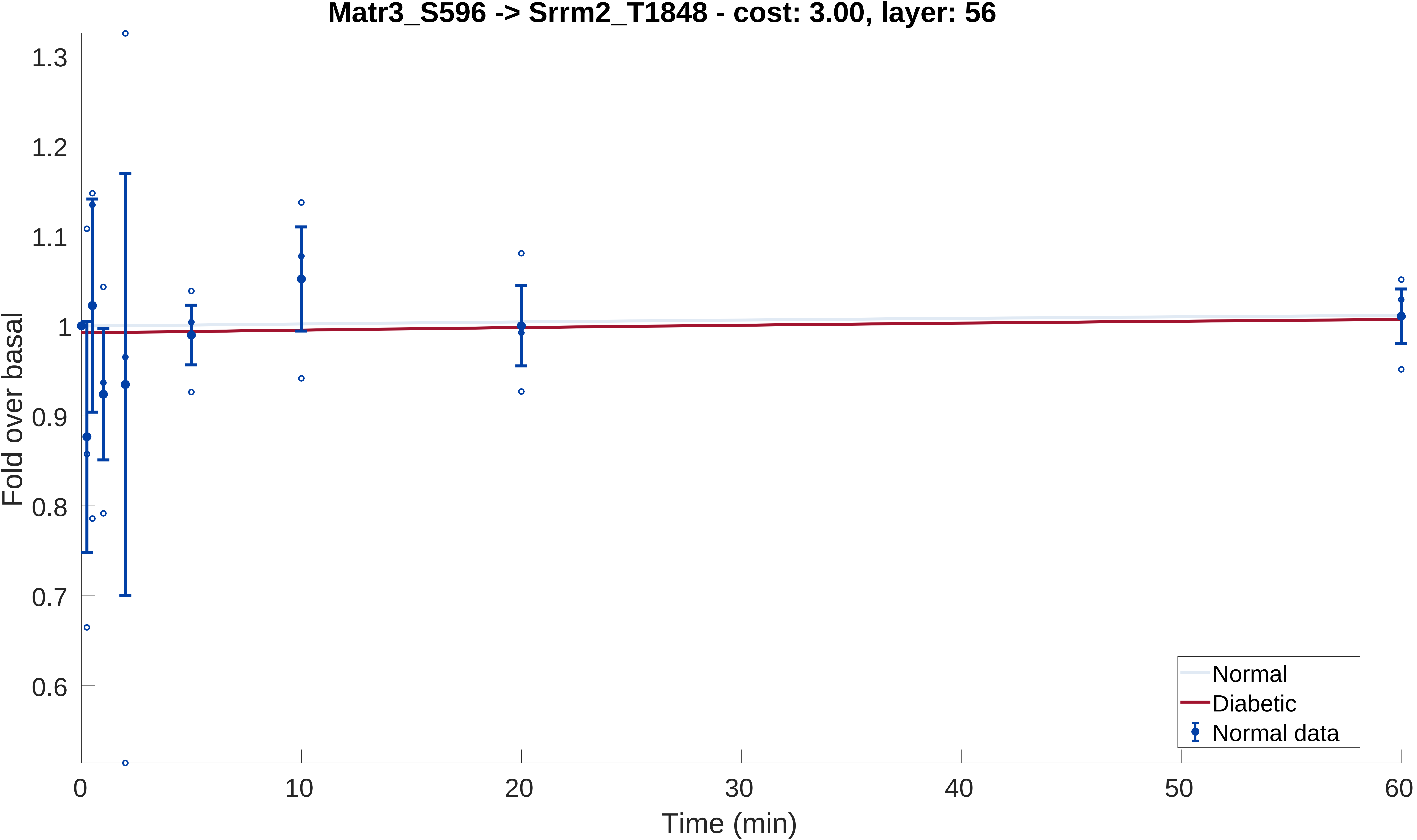

Matr3_S596 -> Srrm2_T1848 - cost: 3.00, layer: 56
Fold over basal
1.3
1.2
1.1
1
0.9
0.8
0.7
0.6
0
10
20
30
40
50
60
Time (min)
Normal
Diabetic
Normal data

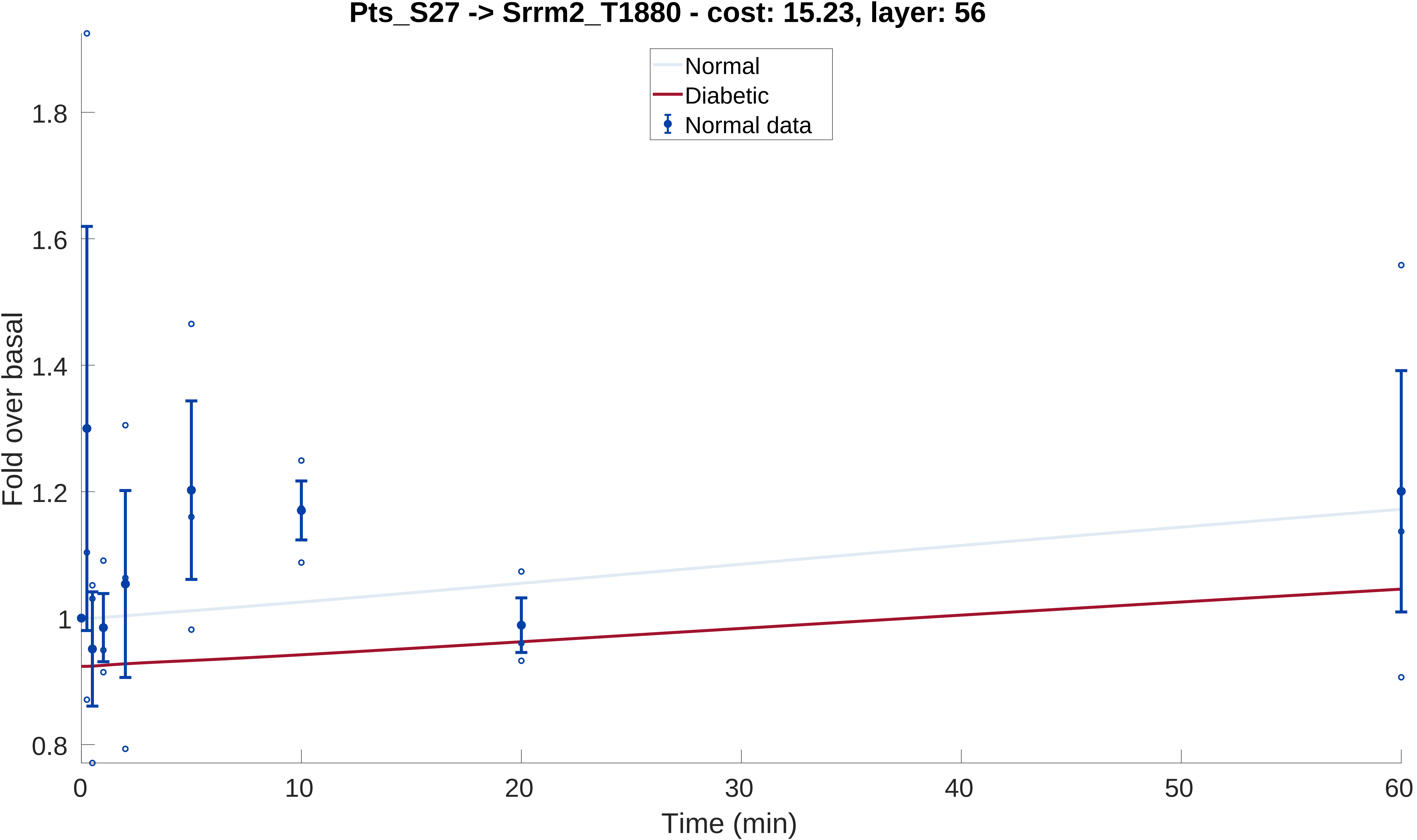

Pts_S27 -> Srrm2_T1880 - cost: 15.23, layer: 56
Normal
Diabetic
Normal data
Fold over basal
0.8
1
1.2
1.4
1.6
1.8
0
10
20
30
40
50
60
Time (min)

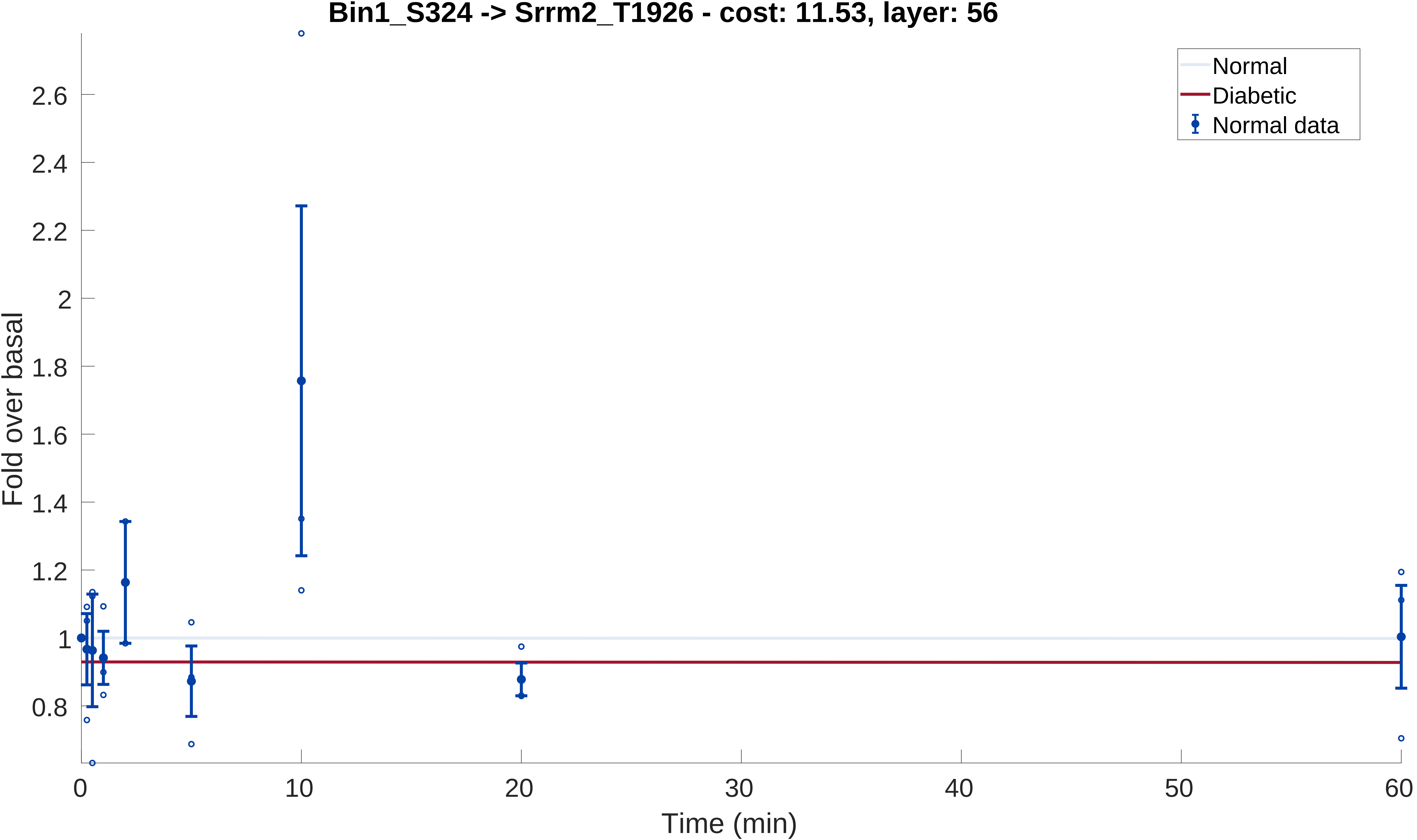
Bin1_S324 -> Srrm2_T1926 - cost: 11.53, layer: 56
Normal
Diabetic
Normal data
Fold over basal
Time (min)
0
10
20
30
40
50
60
0.8
1
1.2
1.4
1.6
1.8
2
2.2
2.4
2.6

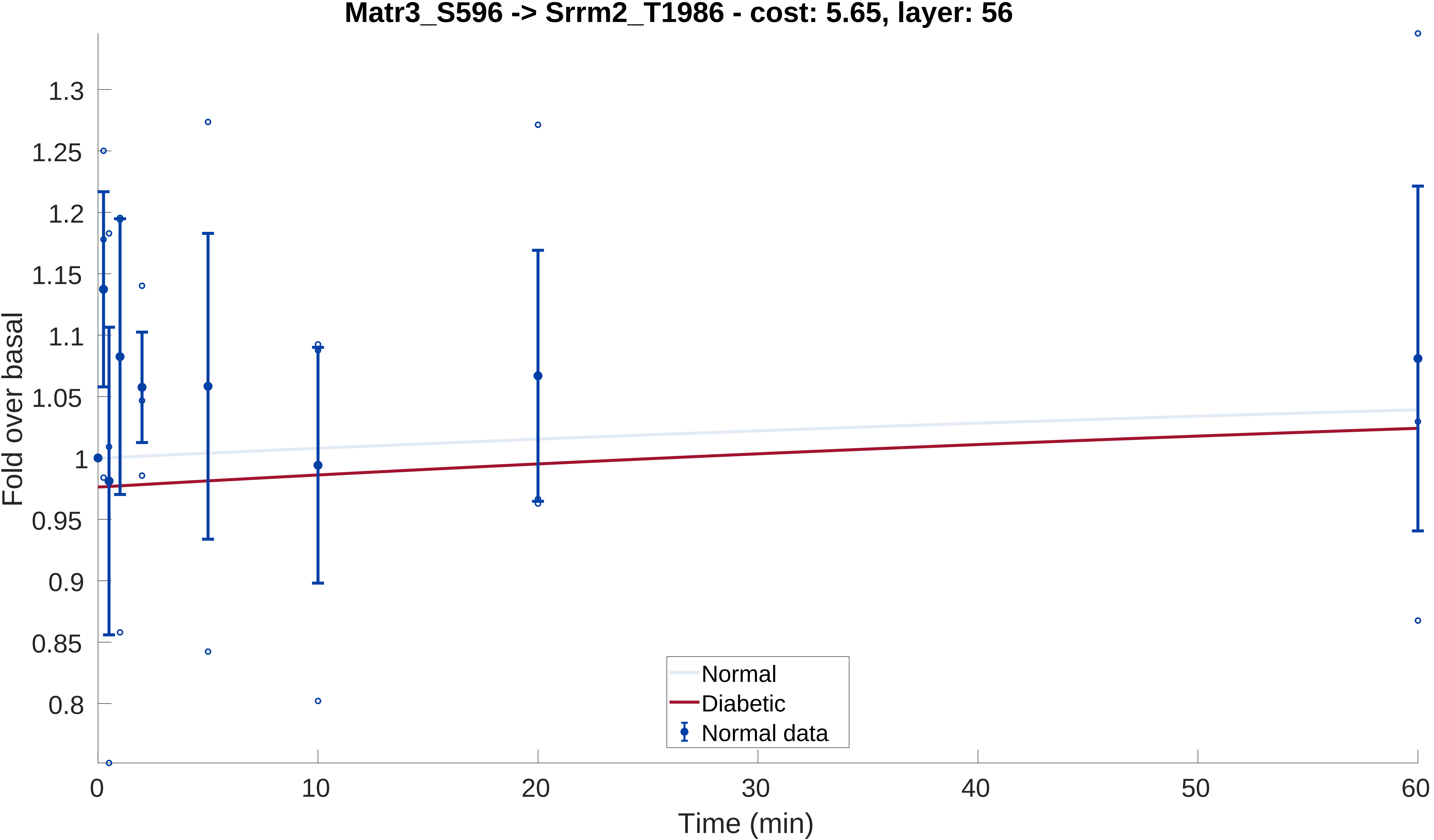

Matr3_S596 -> Srrm2_T1986 - cost: 5.65, layer: 56
Fold over basal
1.3
1.25
1.2
1.15
1.1
1.05
1
0.95
0.9
0.85
0.8
0
10
20
30
40
50
60
Time (min)
Normal
Diabetic
Normal data

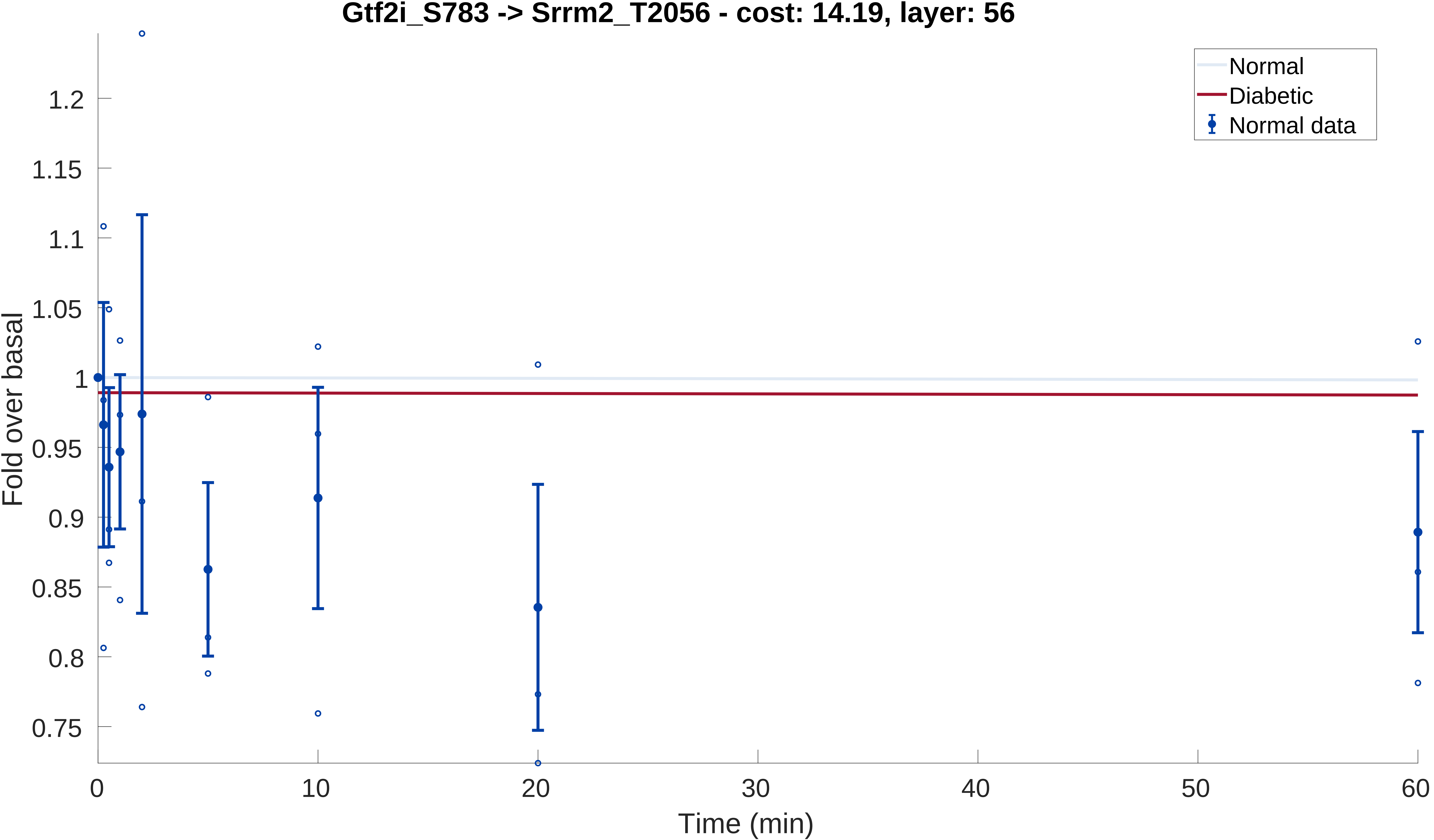
Gtf2i_S783 -> Srrm2_T2056 - cost: 14.19, layer: 56
Normal
Diabetic
Normal data
Fold over basal
1.2
1.15
1.1
1.05
1
0.95
0.9
0.85
0.8
0.75
0
10
20
30
40
50
60
Time (min)

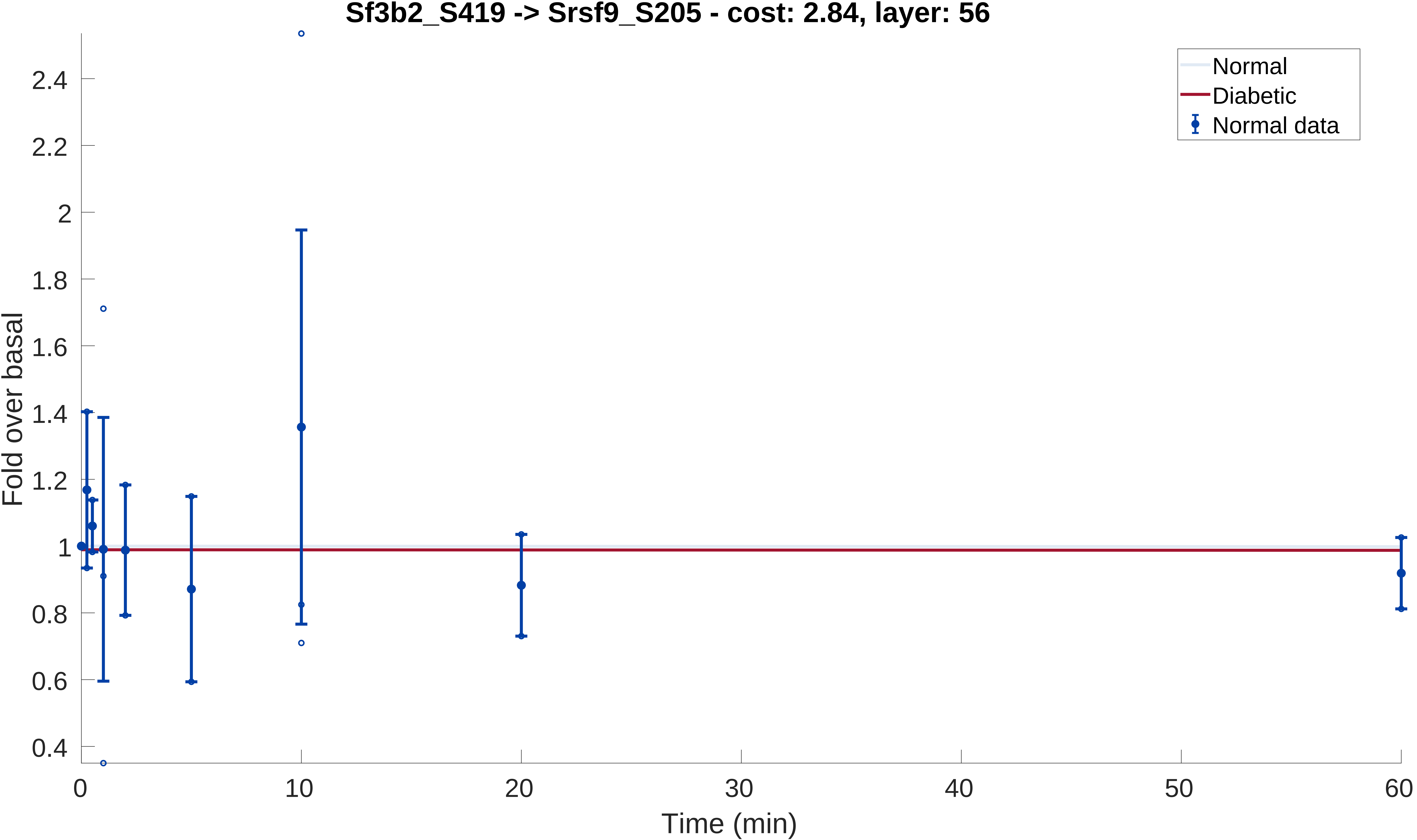

Sf3b2_S419 -> Srsf9_S205 - cost: 2.84, layer: 56
Normal
Diabetic
Normal data
Fold over basal
Time (min)
0.4
0.6
0.8
1
1.2
1.4
1.6
1.8
2
2.2
2.4
0
10
20
30
40
50
60

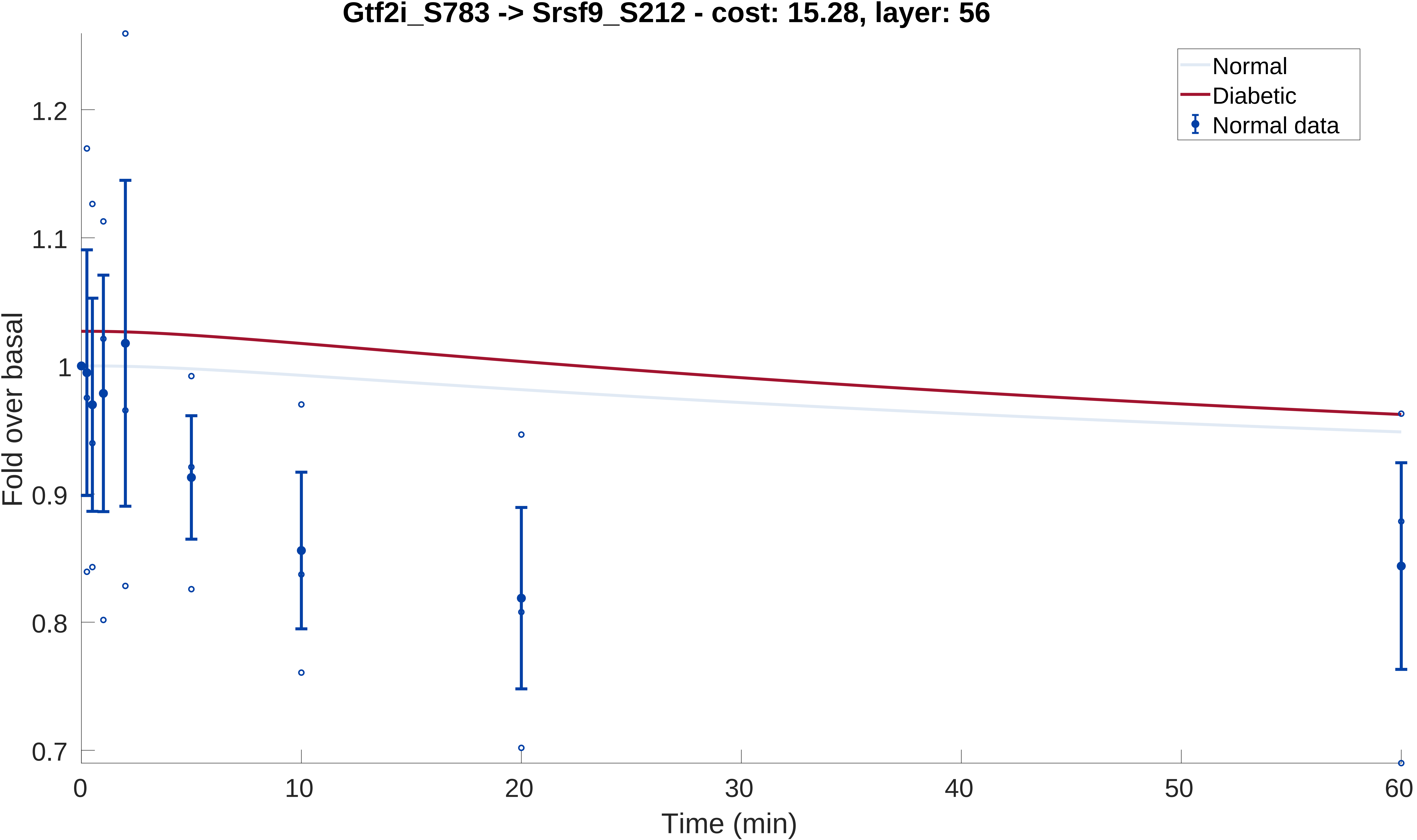

Gtf2i_S783 -> Srsf9_S212 - cost: 15.28, layer: 56
Normal
Diabetic
Normal data
Fold over basal
1.2
1.1
1
0.9
0.8
0.7
0
10
20
30
40
50
60
Time (min)

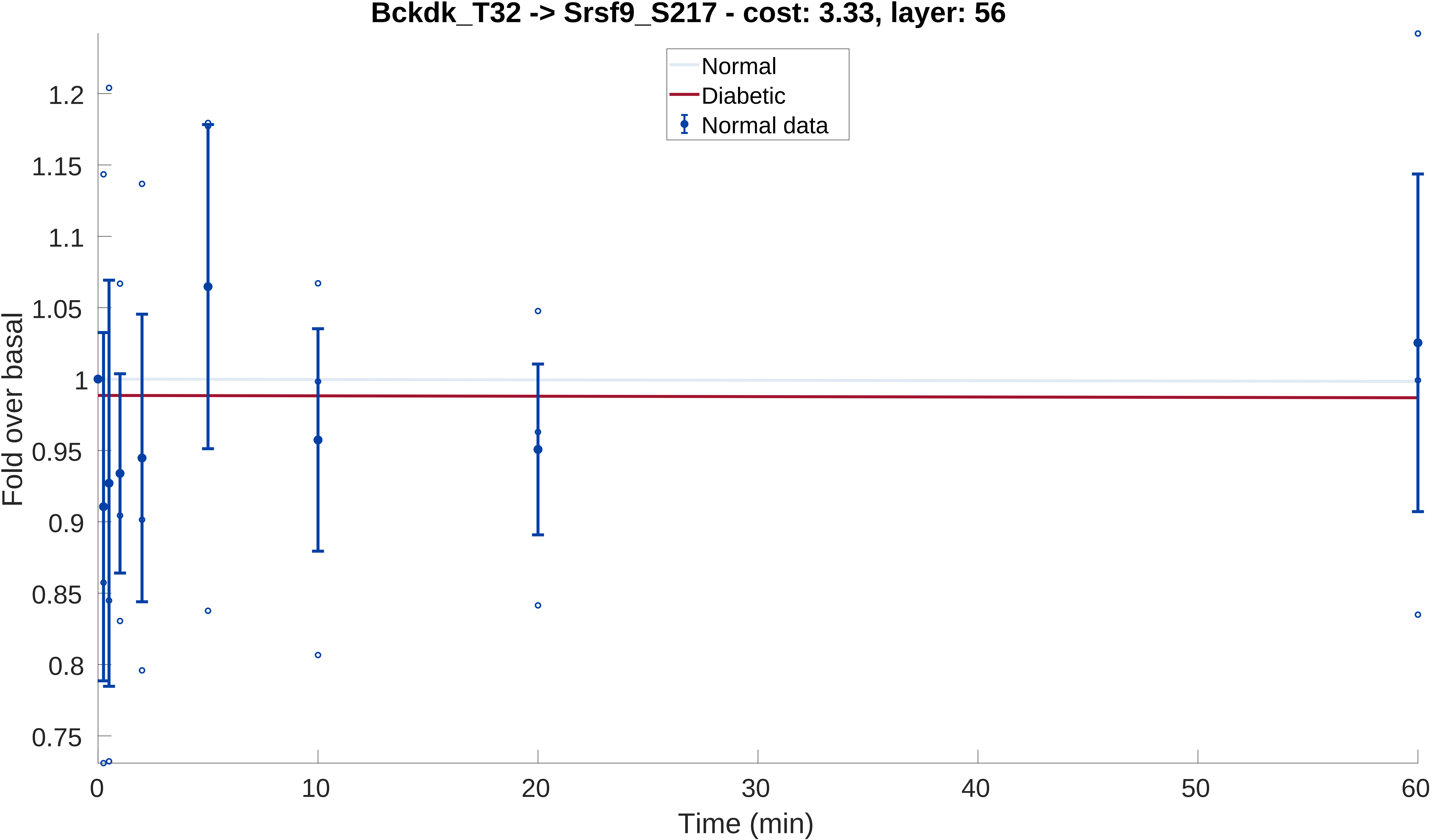

Bckdk_T32 -> Srsf9_S217 - cost: 3.33, layer: 56
Normal
Diabetic
Normal data
Fold over basal
Time (min)
0
10
20
30
40
50
60
0.75
0.8
0.85
0.9
0.95
1
1.05
1.1
1.15
1.2

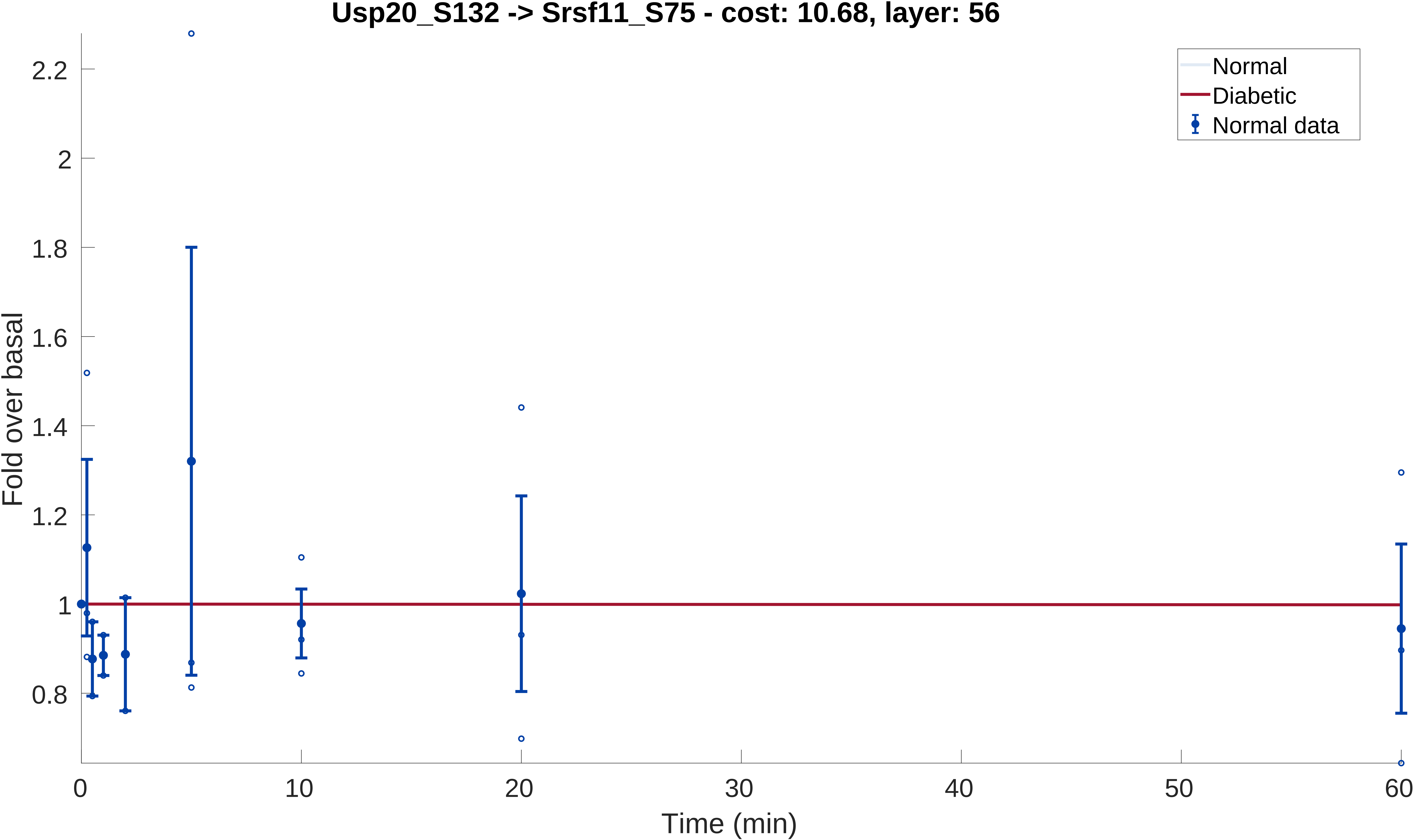

Usp20_S132 -> Srsf11_S75 - cost: 10.68, layer: 56
Normal
Diabetic
Normal data
Fold over basal
Time (min)
0.8
1
1.2
1.4
1.6
1.8
2
2.2
0
10
20
30
40
50
60

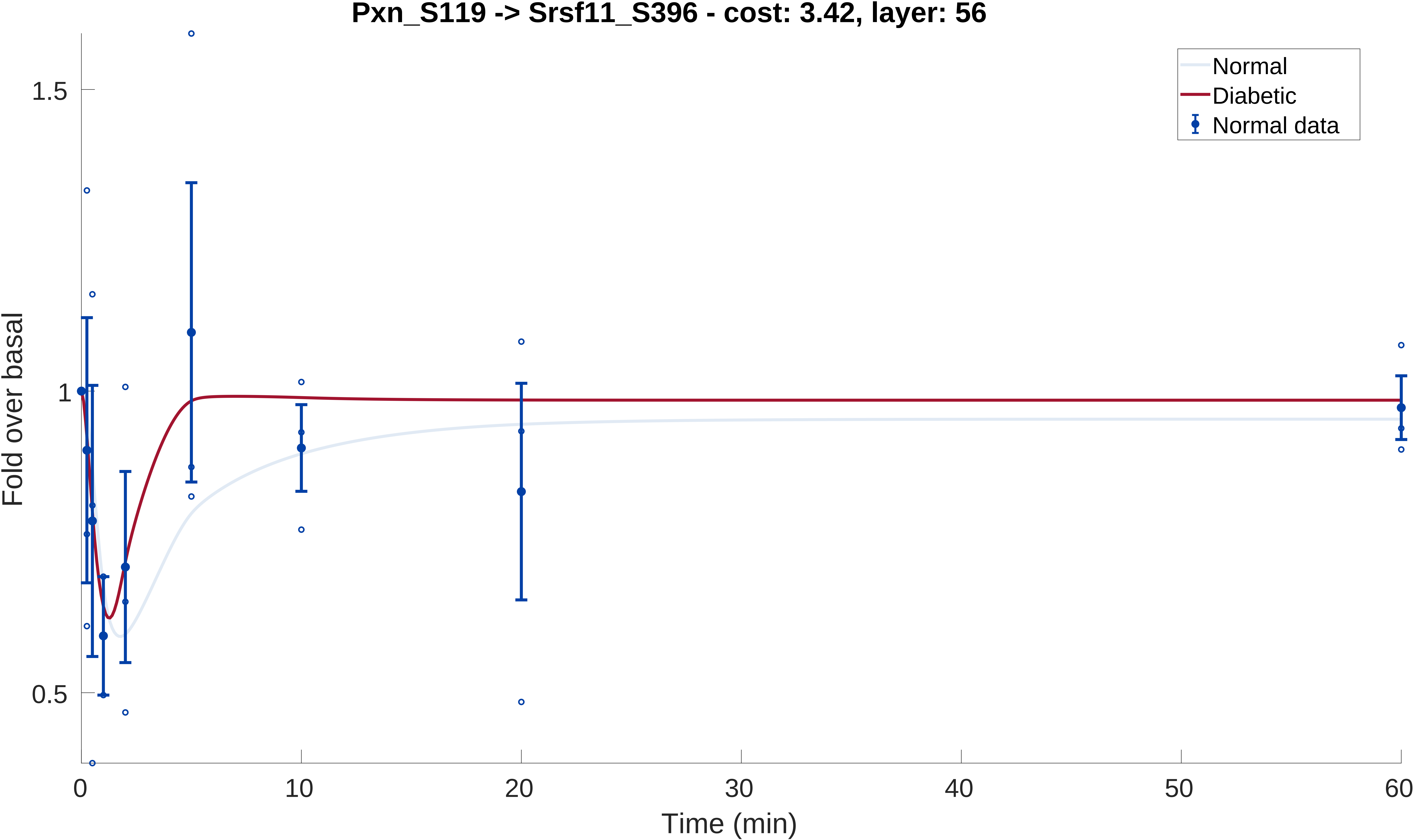

Pxn_S119 -> Srsf11_S396 - cost: 3.42, layer: 56
Normal
Diabetic
Normal data
1.5
1
0.5
Fold over basal
0
10
20
30
40
50
60
Time (min)

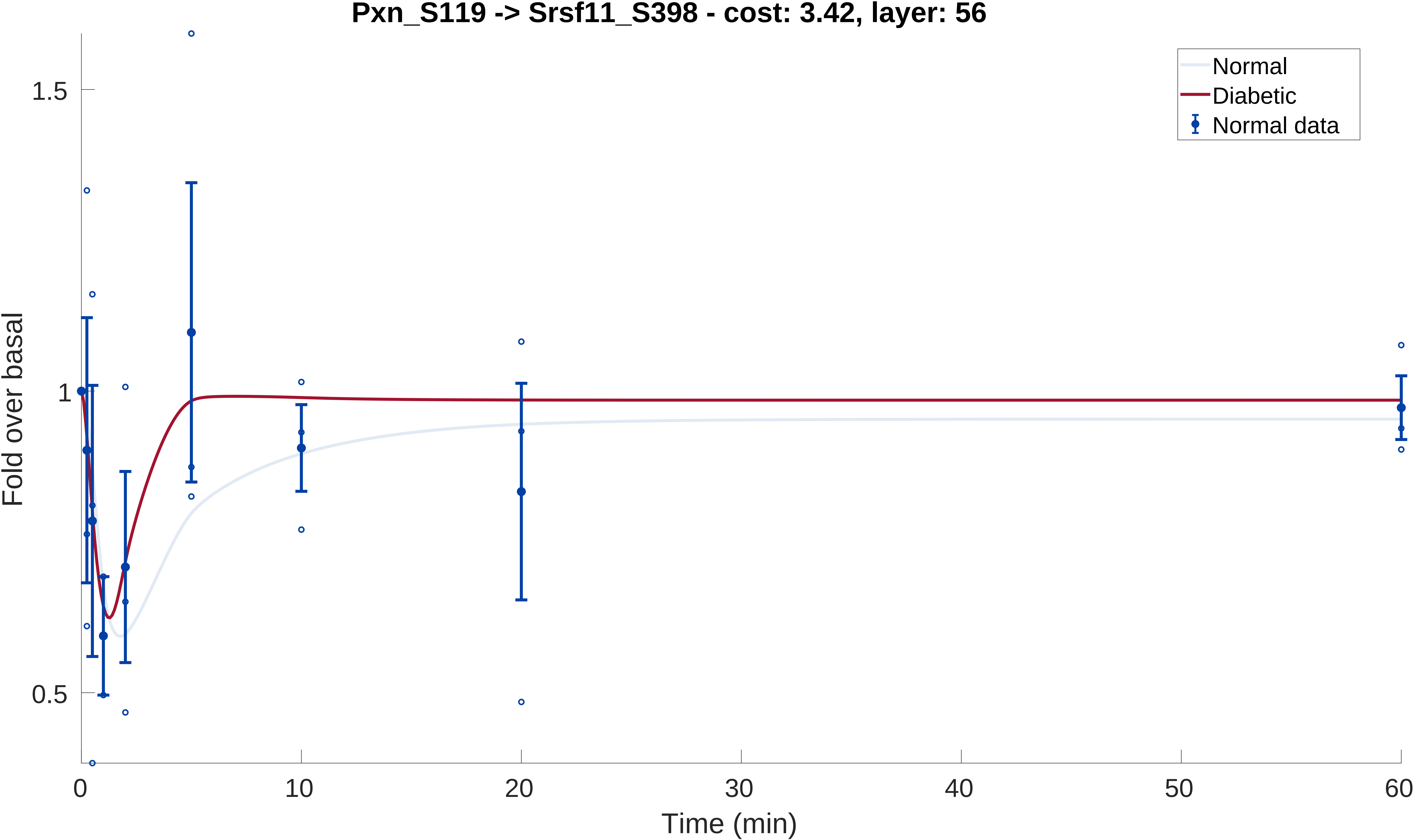

Pxn_S119 -> Srsf11_S398 - cost: 3.42, layer: 56
Normal
Diabetic
Normal data
1.5
1
0.5
0
10
20
30
40
50
60
Fold over basal
Time (min)

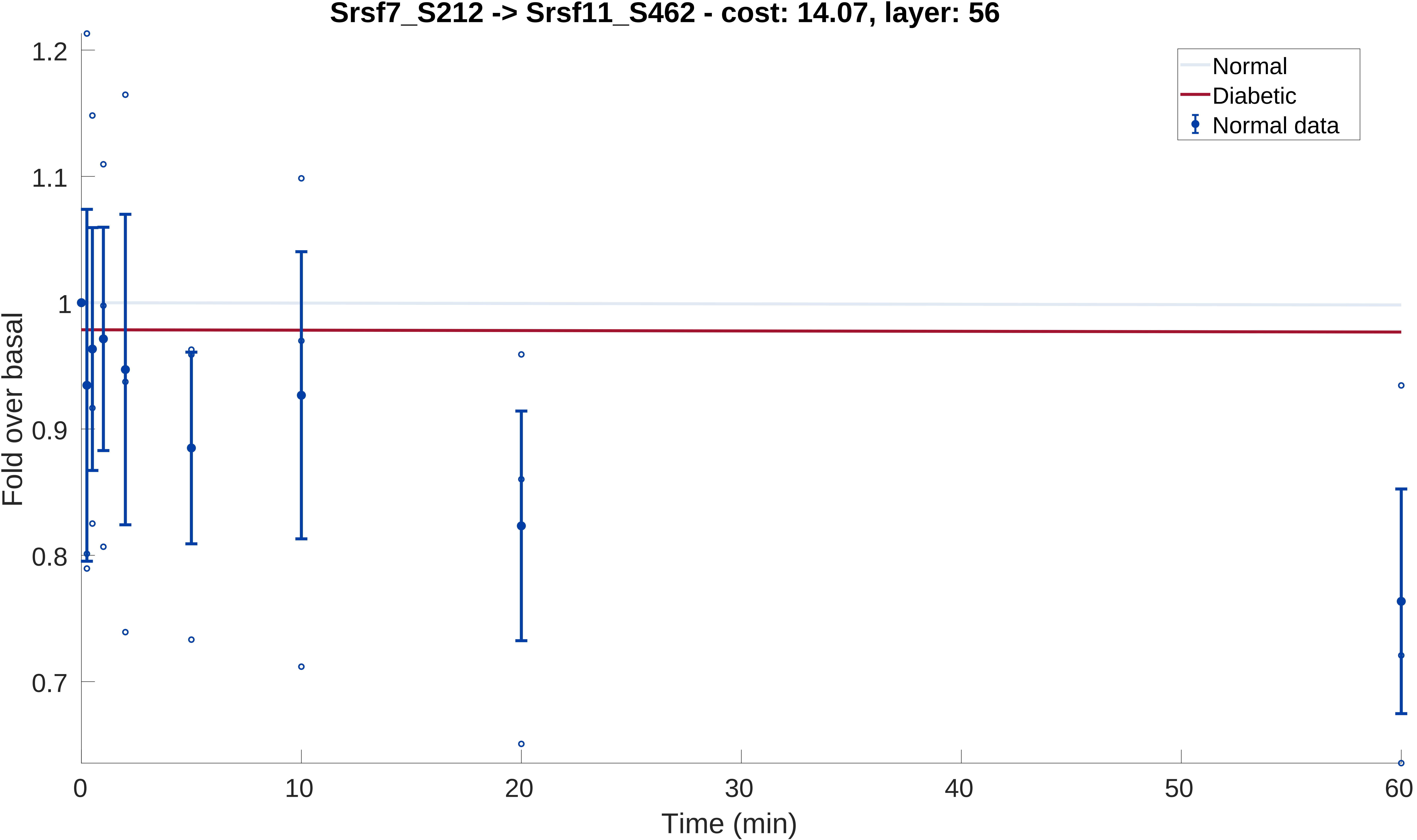

Srsf7_S212 -> Srsf11_S462 - cost: 14.07, layer: 56
Normal
Diabetic
Normal data
Fold over basal
1.2
1.1
1
0.9
0.8
0.7
0
10
20
30
40
50
60
Time (min)

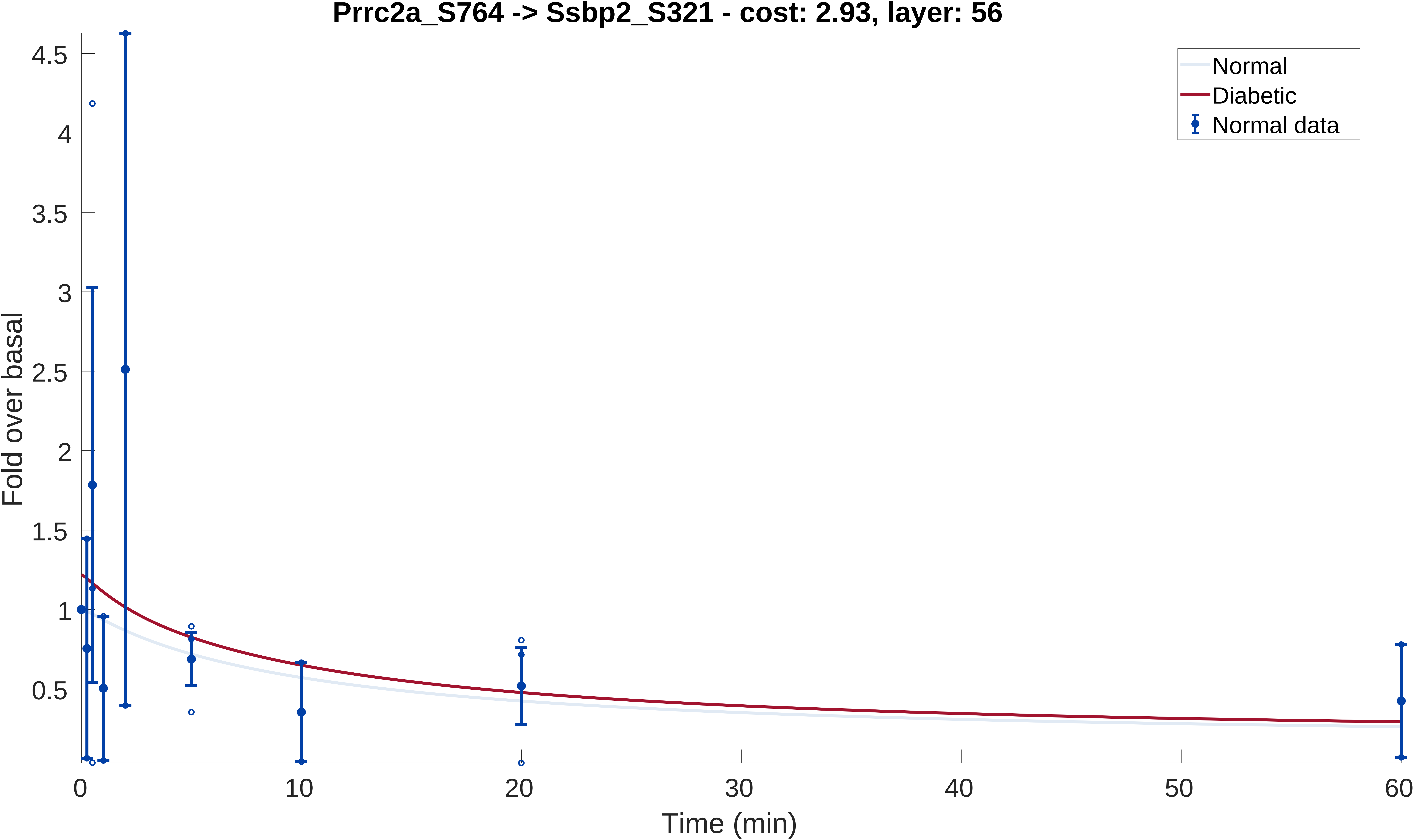

Prrc2a_S764 -> Ssbp2_S321 - cost: 2.93, layer: 56
Fold over basal
Time (min)
0
10
20
30
40
50
60
0.5
1
1.5
2
2.5
3
3.5
4
4.5
Normal
Diabetic
Normal data

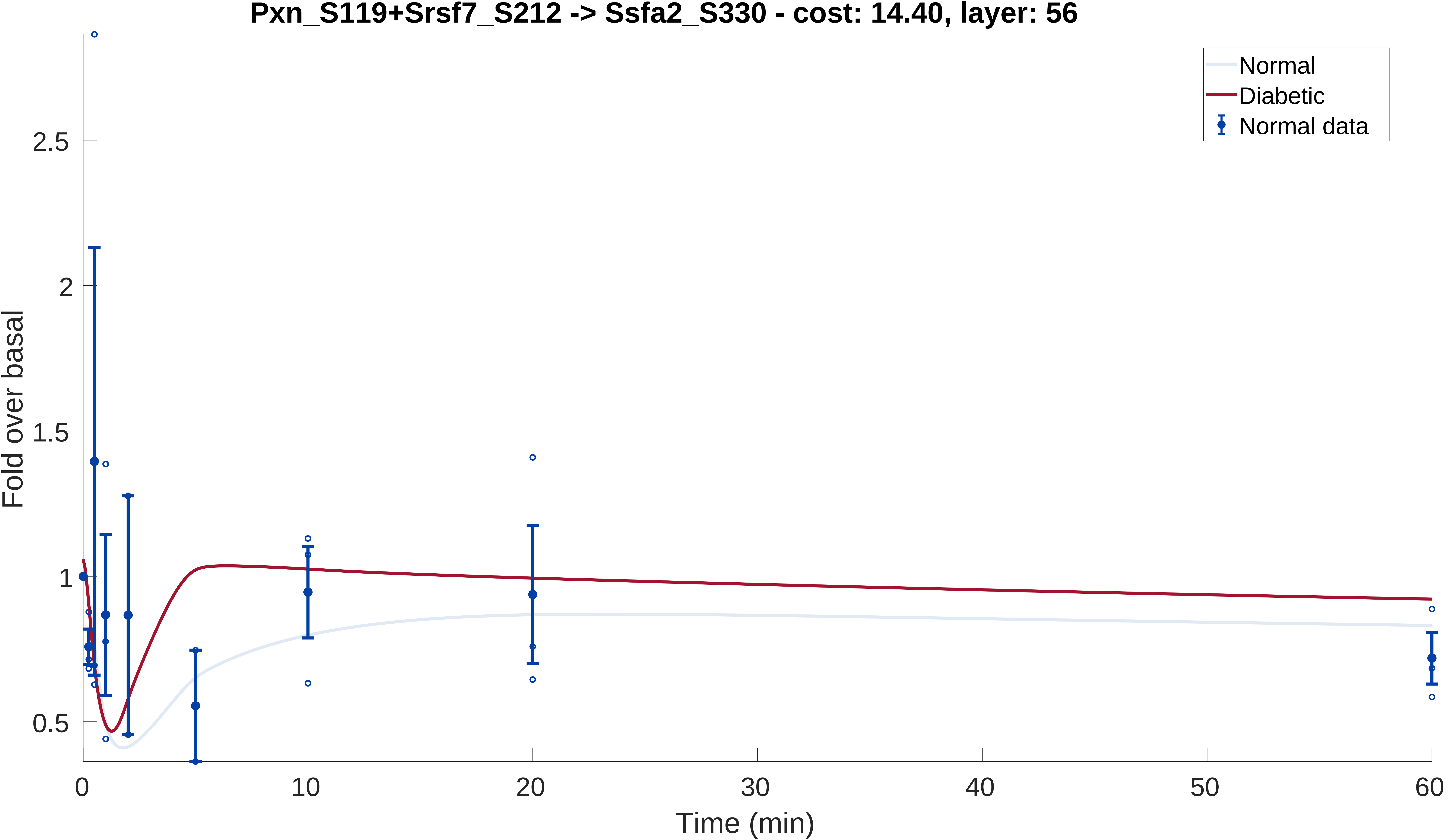
Pxn_S119+Srsf7_S212 -> Ssfa2_S330 - cost: 14.40, layer: 56
Normal
Diabetic
Normal data
Fold over basal
0.5
1
1.5
2
2.5
0
10
20
30
40
50
60
Time (min)

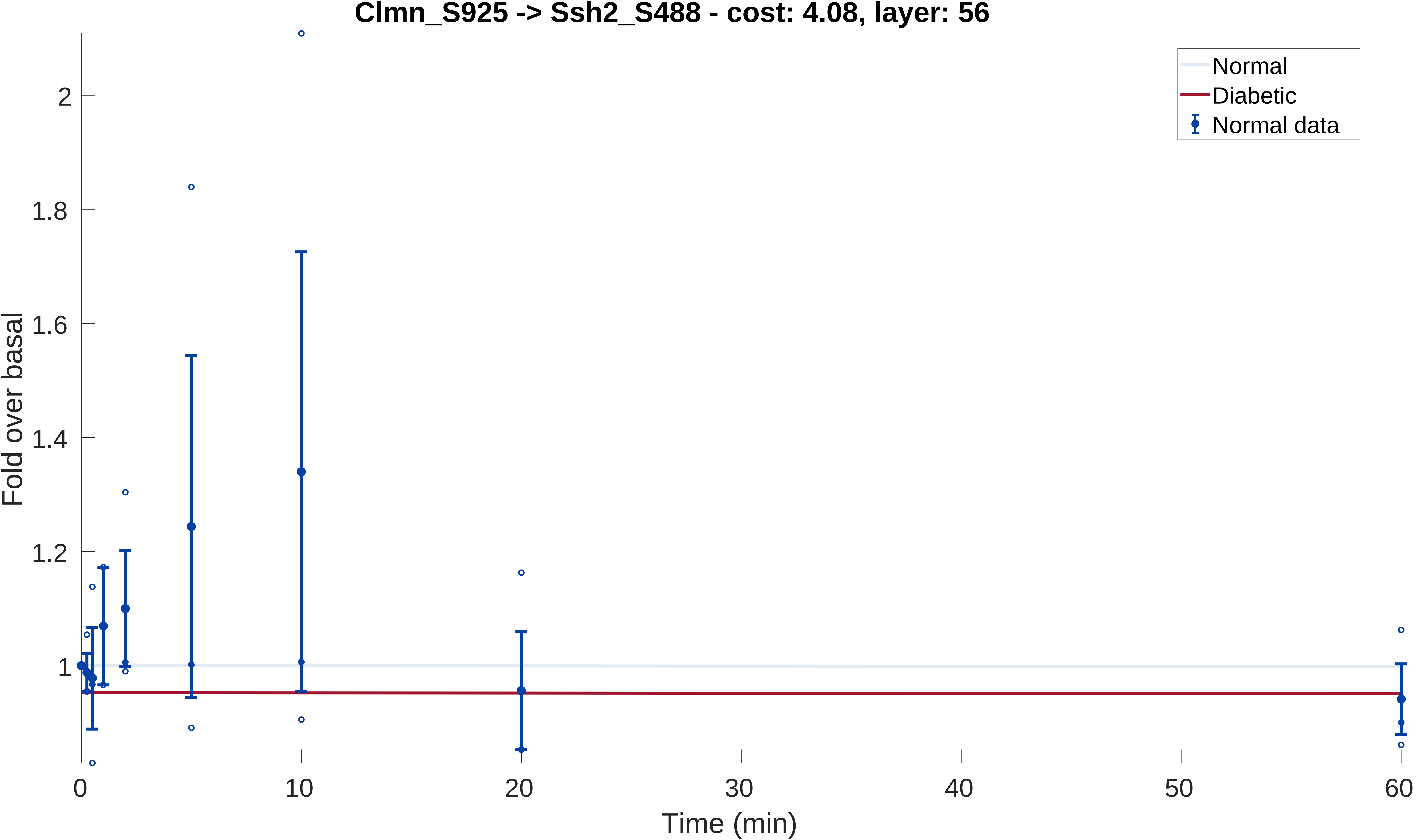

Clmn_S925 -> Ssh2_S488 - cost: 4.08, layer: 56
Normal
Diabetic
Normal data
Fold over basal
2
1.8
1.6
1.4
1.2
1
0
10
20
30
40
50
60
Time (min)

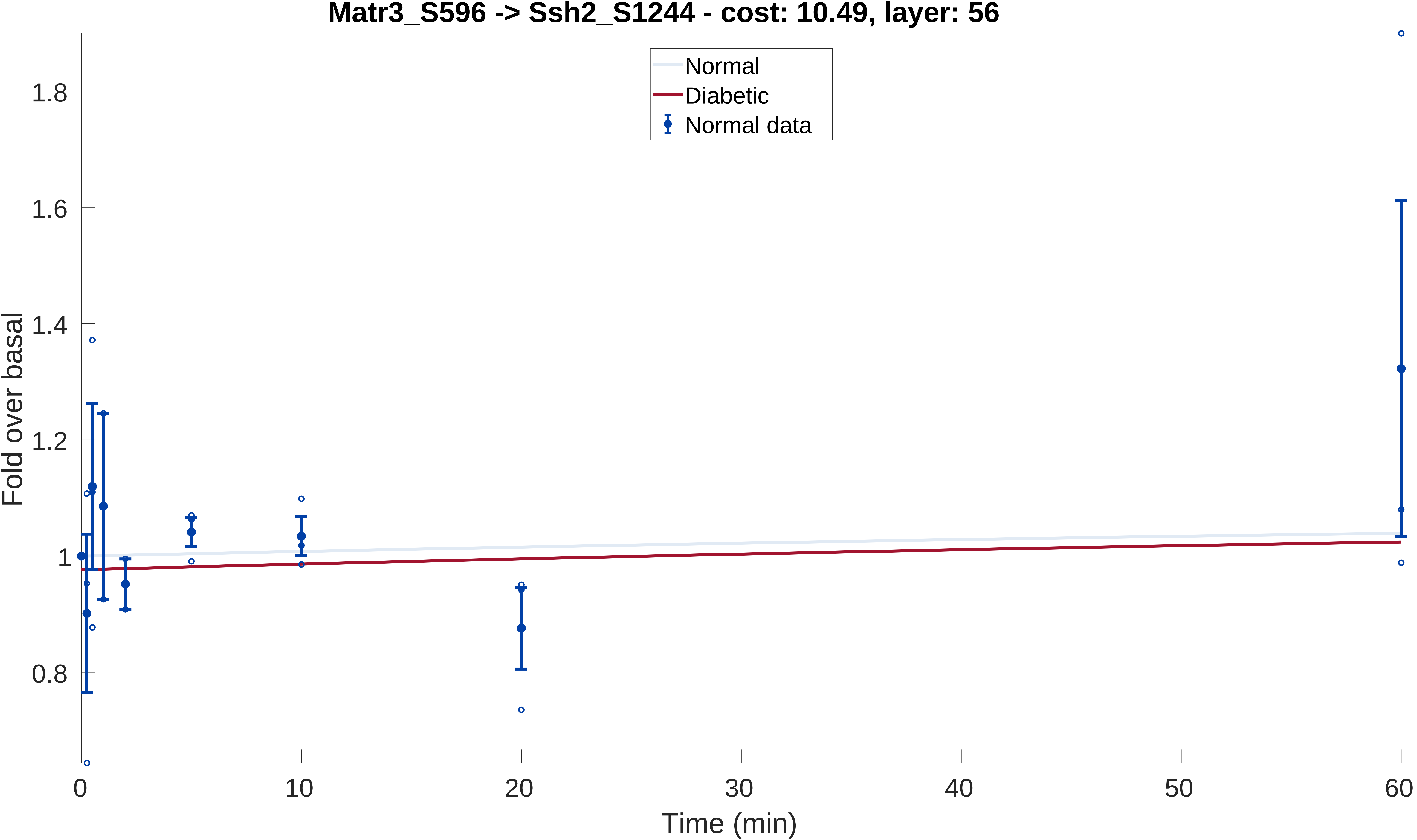

Matr3_S596 -> Ssh2_S1244 - cost: 10.49, layer: 56
Normal
Diabetic
Normal data
Fold over basal
Time (min)
0.8
1
1.2
1.4
1.6
1.8
0
10
20
30
40
50
60

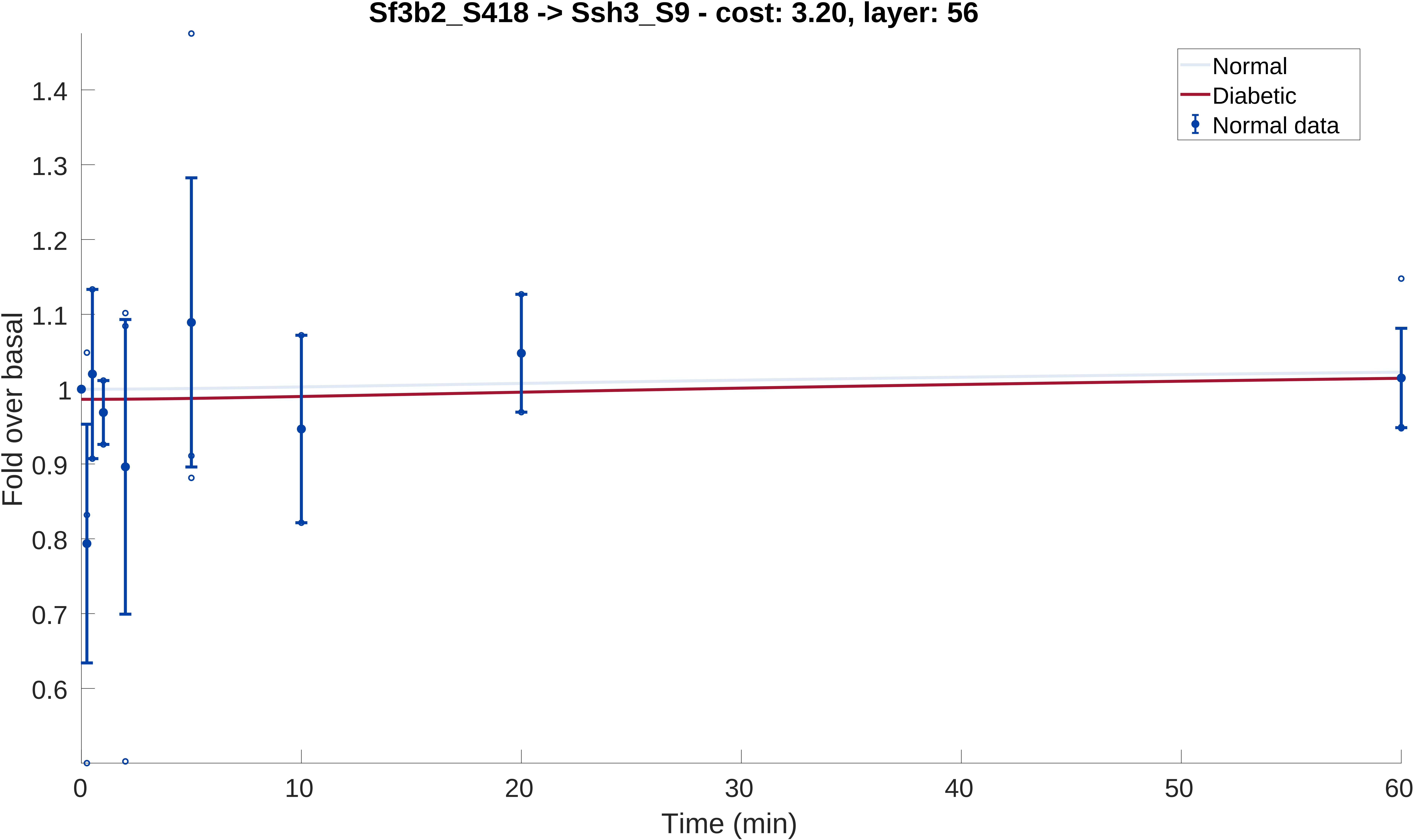

Sf3b2_S418 -> Ssh3_S9 - cost: 3.20, layer: 56
Normal
Diabetic
Normal data
Fold over basal
1.4
1.3
1.2
1.1
1
0.9
0.8
0.7
0.6
0
10
20
30
40
50
60
Time (min)

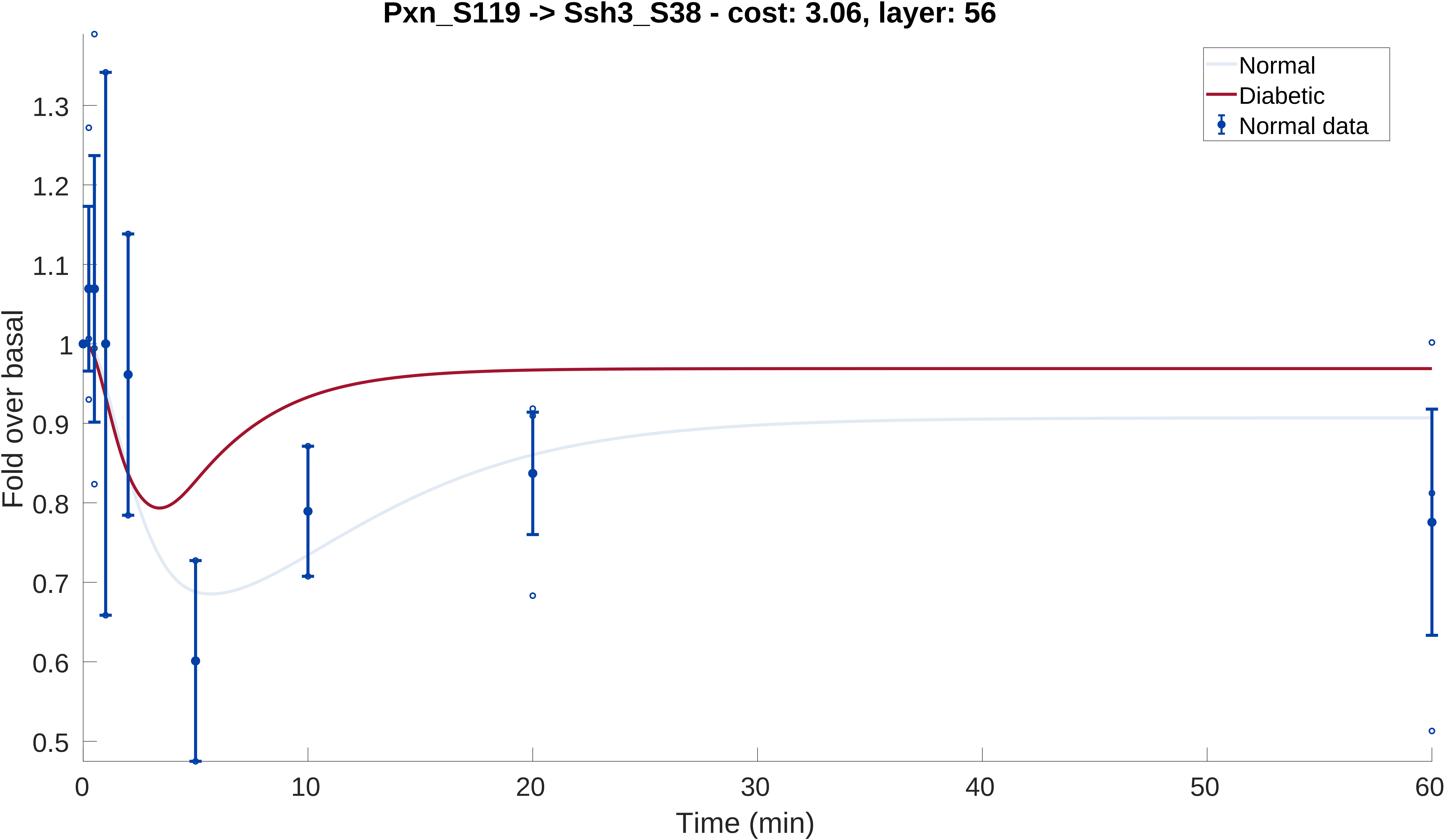

Pxn_S119 -> Ssh3_S38 - cost: 3.06, layer: 56
Normal
Diabetic
Normal data
Fold over basal
1.3
1.2
1.1
1
0.9
0.8
0.7
0.6
0.5
0
10
20
30
40
50
60
Time (min)

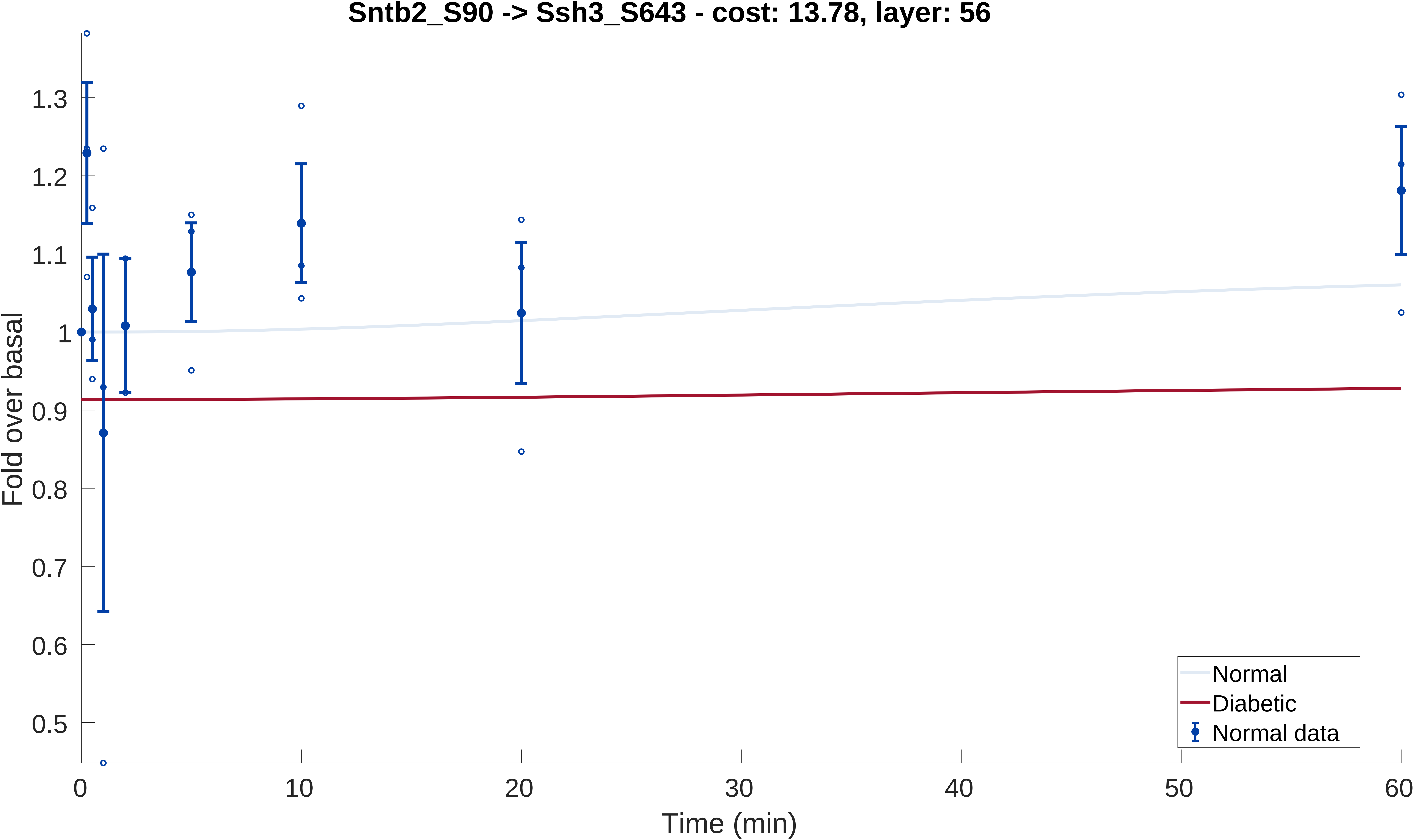

Sntb2_S90 -> Ssh3_S643 - cost: 13.78, layer: 56
Fold over basal
1.3
1.2
1.1
1
0.9
0.8
0.7
0.6
0.5
0
10
20
30
40
50
60
Time (min)
Normal
Diabetic
Normal data

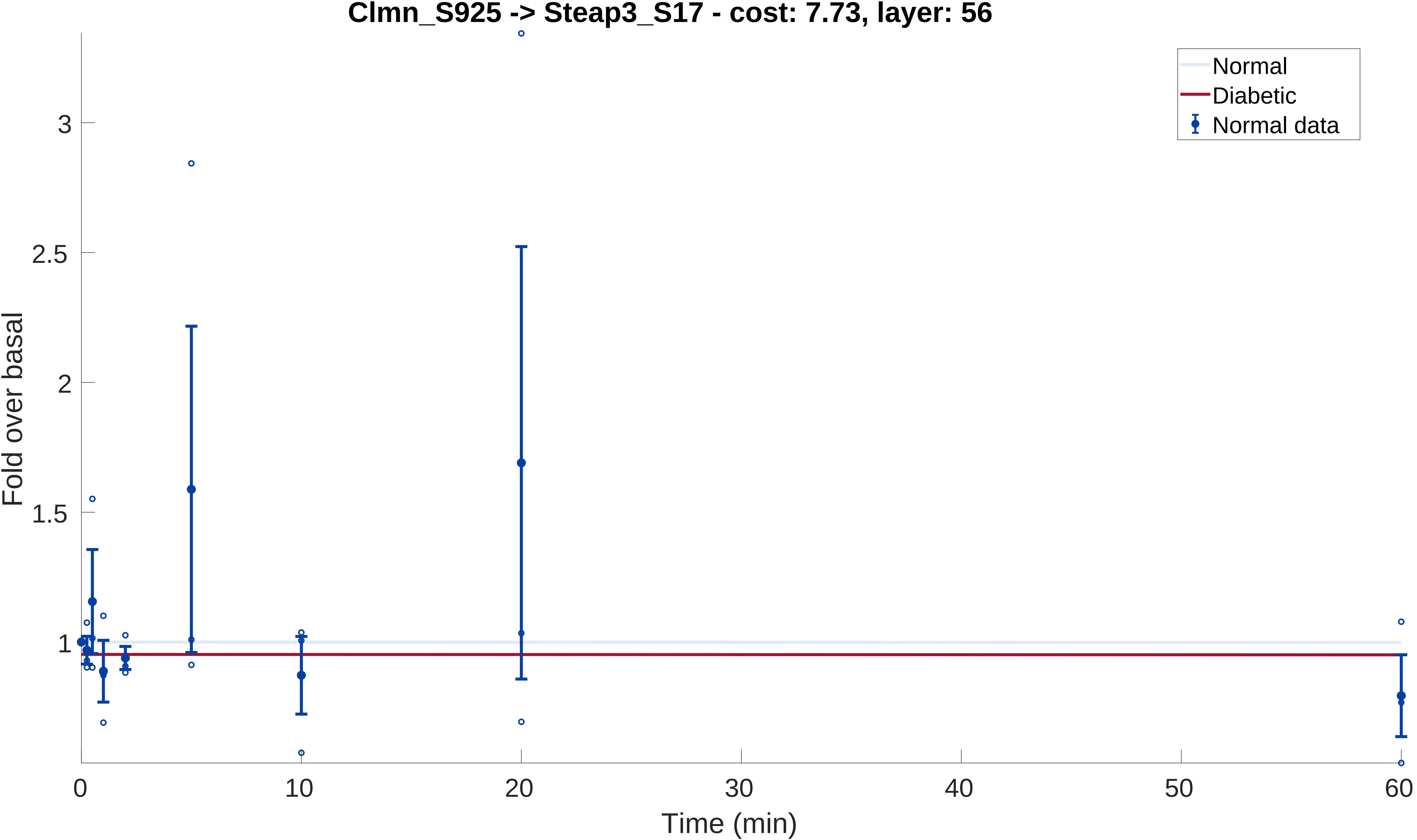

Clmn_S925 -> Steap3_S17 - cost: 7.73, layer: 56
Normal
Diabetic
Normal data
Fold over basal
3
2.5
2
1.5
1
0
10
20
30
40
50
60
Time (min)

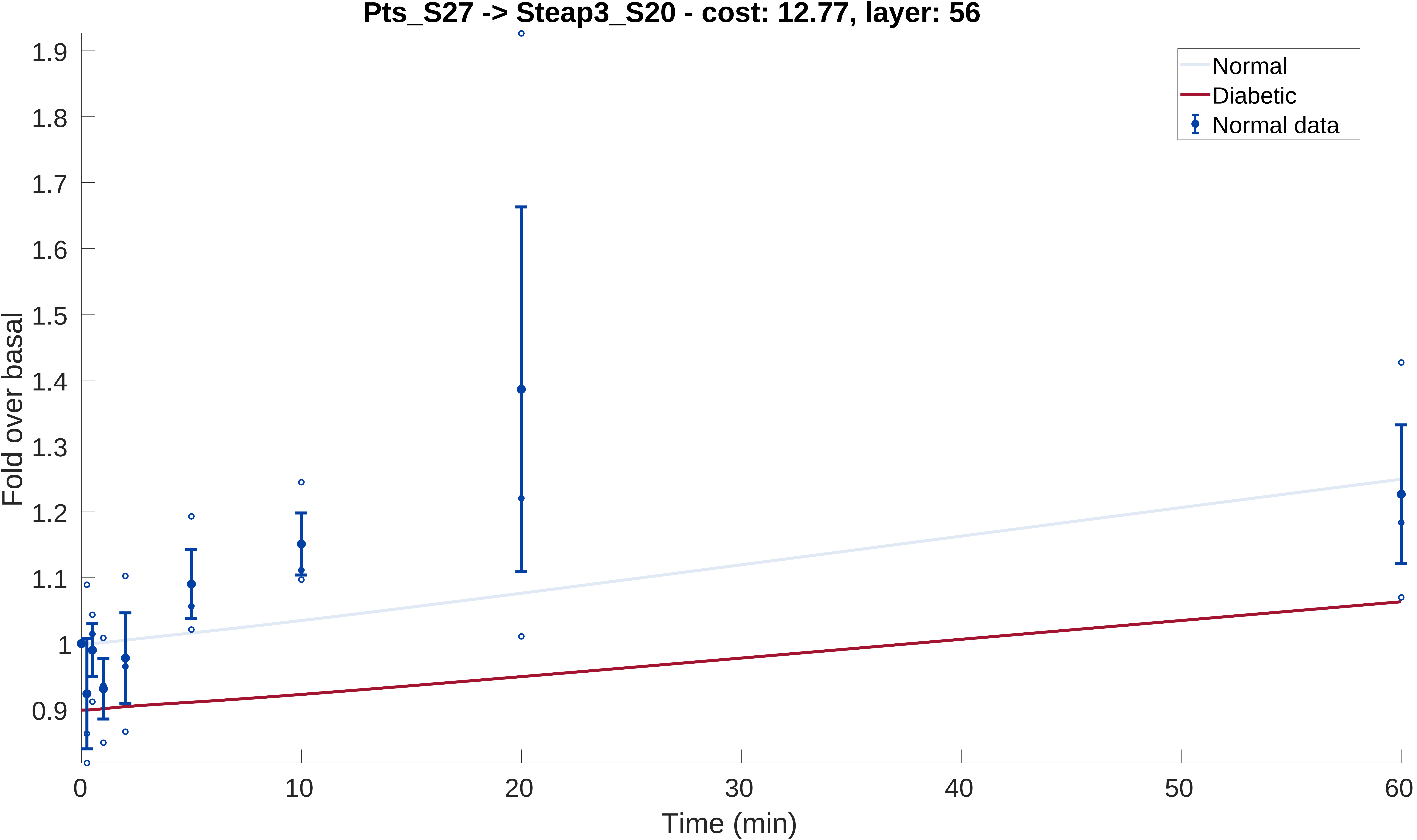

Pts_S27 -> Steap3_S20 - cost: 12.77, layer: 56
Normal
Diabetic
Normal data
Fold over basal
Time (min)
0.9
1
1.1
1.2
1.3
1.4
1.5
1.6
1.7
1.8
1.9
0
10
20
30
40
50
60

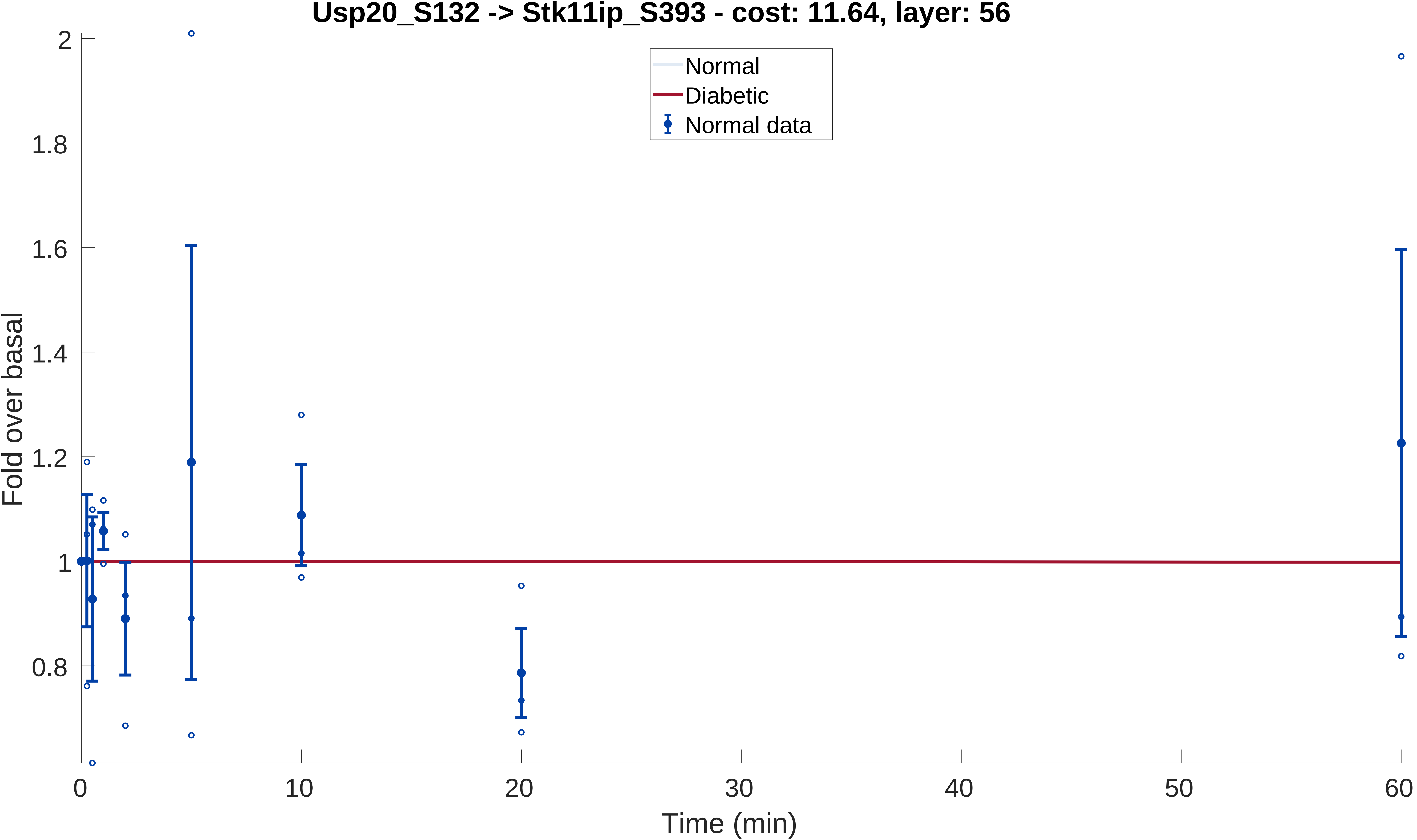

Usp20_S132 -> Stk11ip_S393 - cost: 11.64, layer: 56
Normal
Diabetic
Normal data
Fold over basal
2
1.8
1.6
1.4
1.2
1
0.8
0
10
20
30
40
50
60
Time (min)

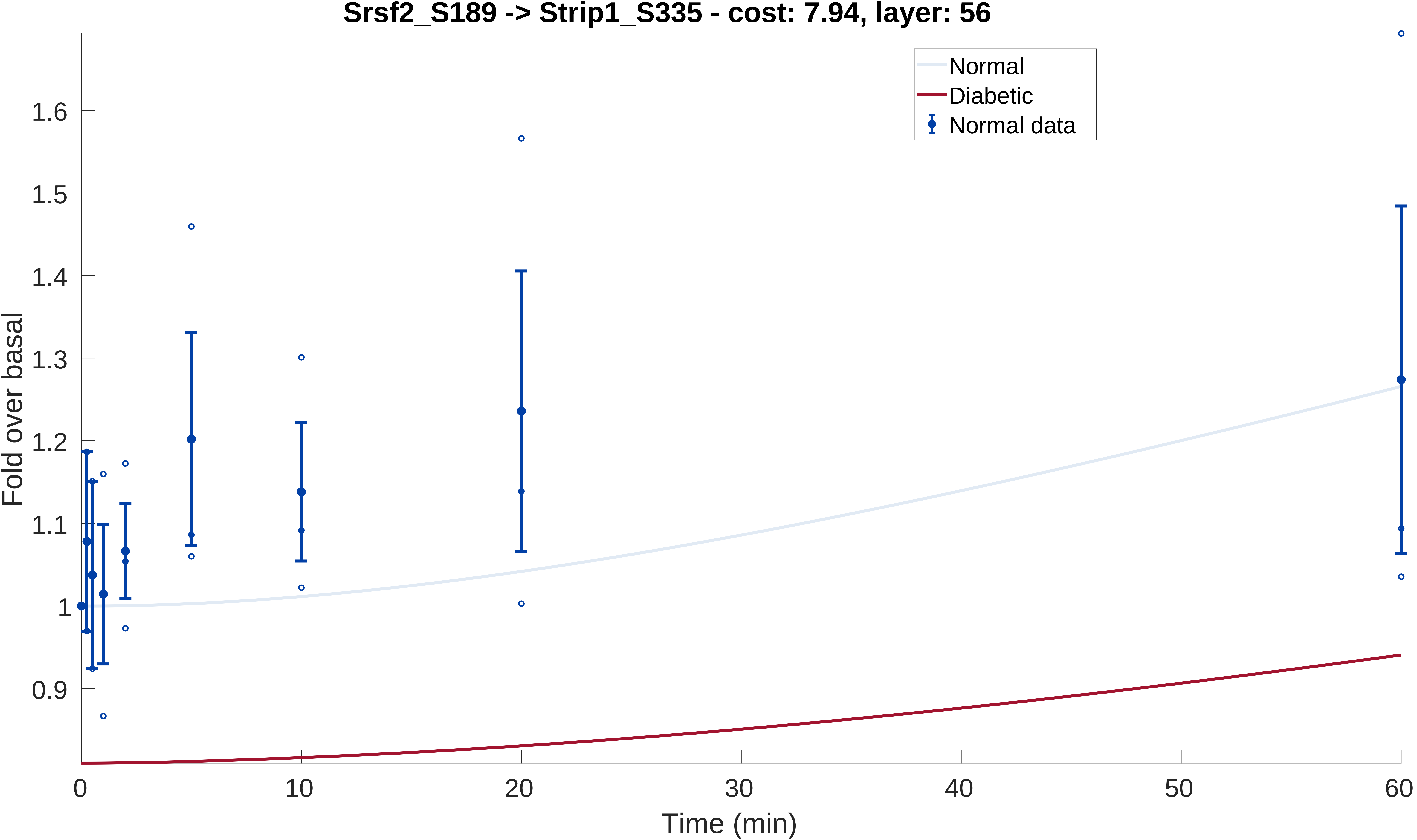

Srsf2_S189 -> Strip1_S335 - cost: 7.94, layer: 56
Normal
Diabetic
Normal data
Fold over basal
1.6
1.5
1.4
1.3
1.2
1.1
1
0.9
0
10
20
30
40
50
60
Time (min)

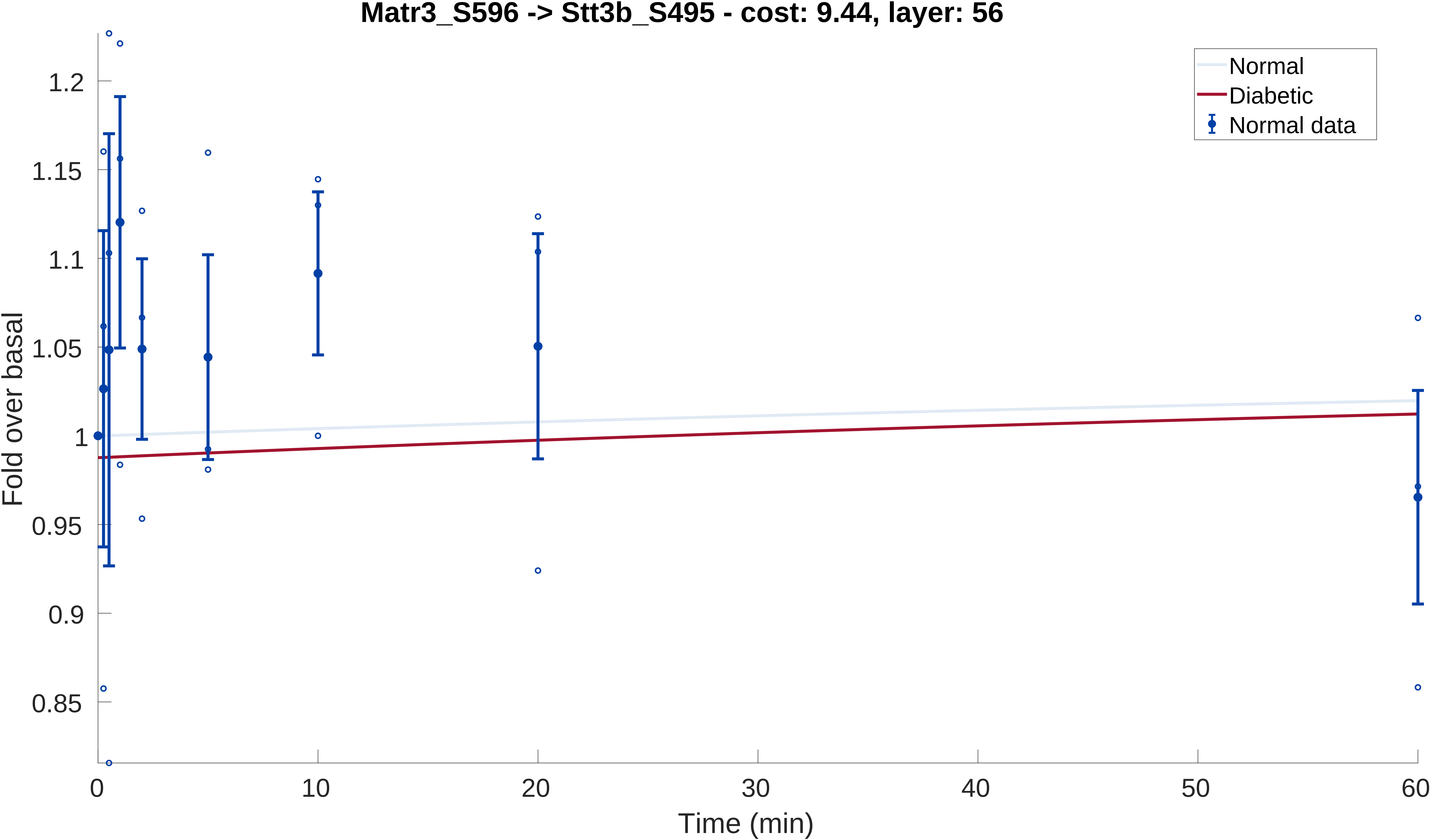

Matr3_S596 -> Stt3b_S495 - cost: 9.44, layer: 56
Normal
Diabetic
Normal data
Fold over basal
1.2
1.15
1.1
1.05
1
0.95
0.9
0.85
0
10
20
30
40
50
60
Time (min)

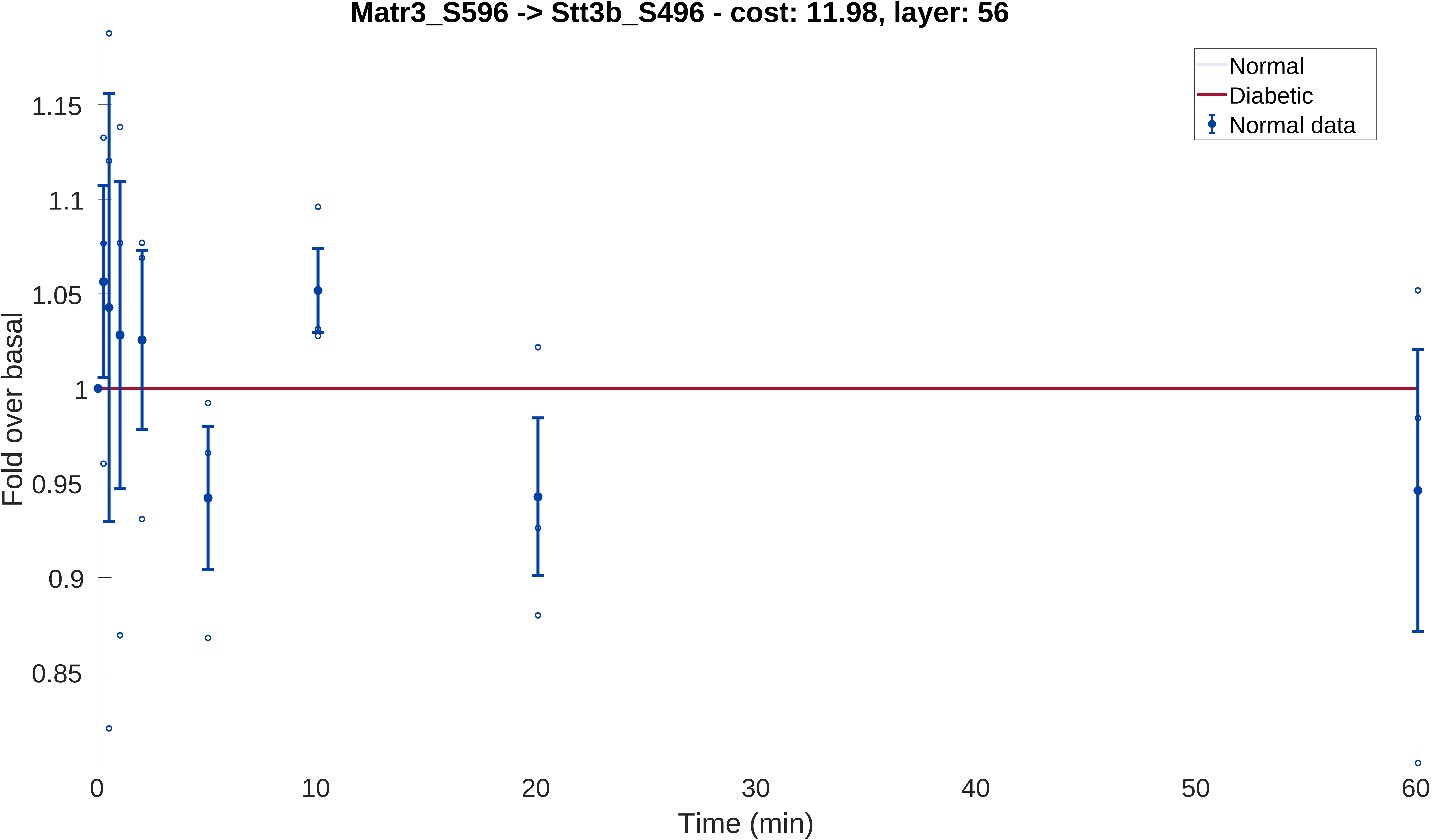

Matr3_S596 -> Stt3b_S496 - cost: 11.98, layer: 56
Normal
Diabetic
Normal data
1.15
1.1
1.05
1
0.95
0.9
0.85
Fold over basal
0
10
20
30
40
50
60
Time (min)

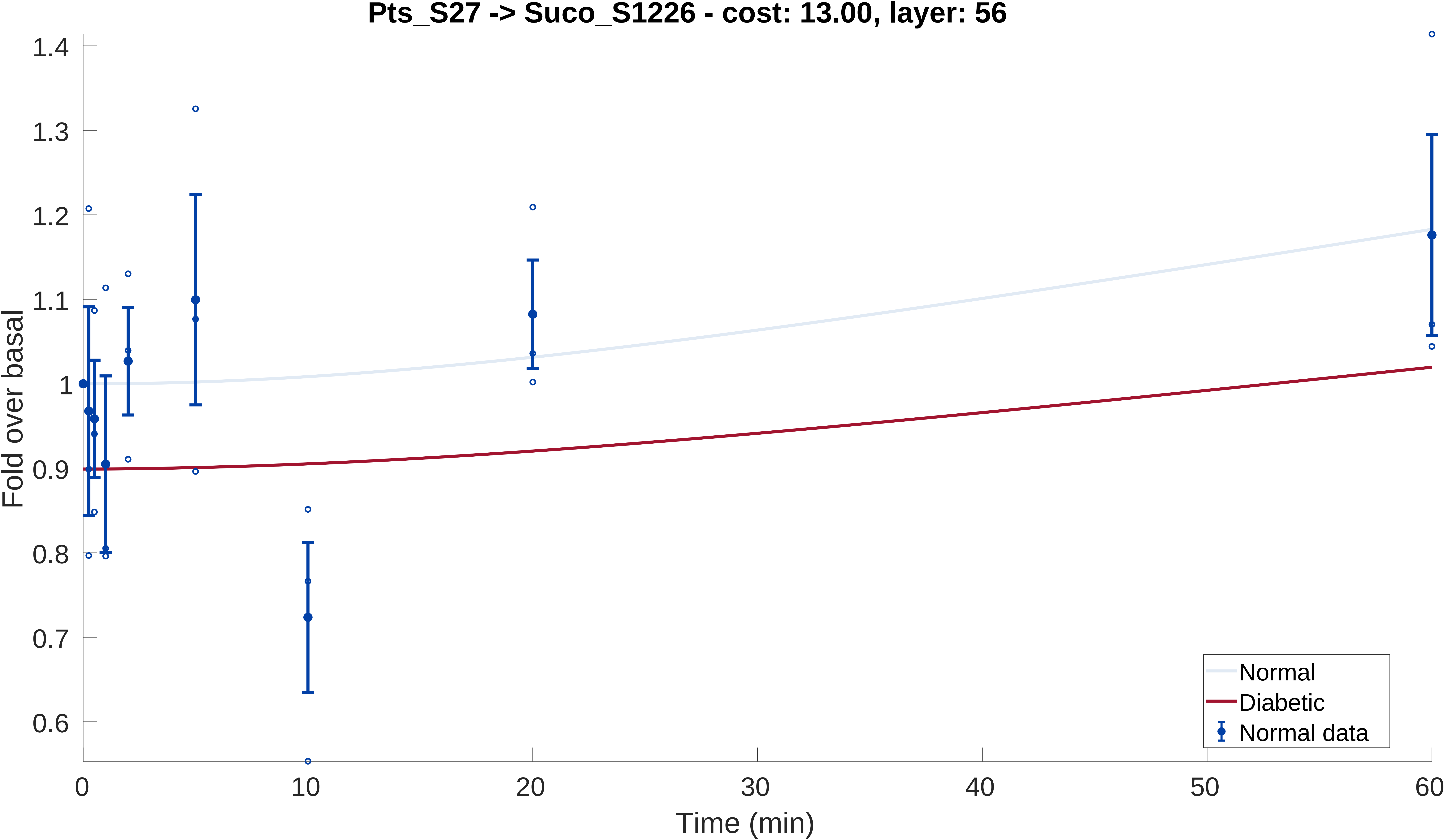

Pts_S27 -> Suco_S1226 - cost: 13.00, layer: 56
Fold over basal
1.4
1.3
1.2
1.1
1
0.9
0.8
0.7
0.6
0
10
20
30
40
50
60
Time (min)
Normal
Diabetic
Normal data

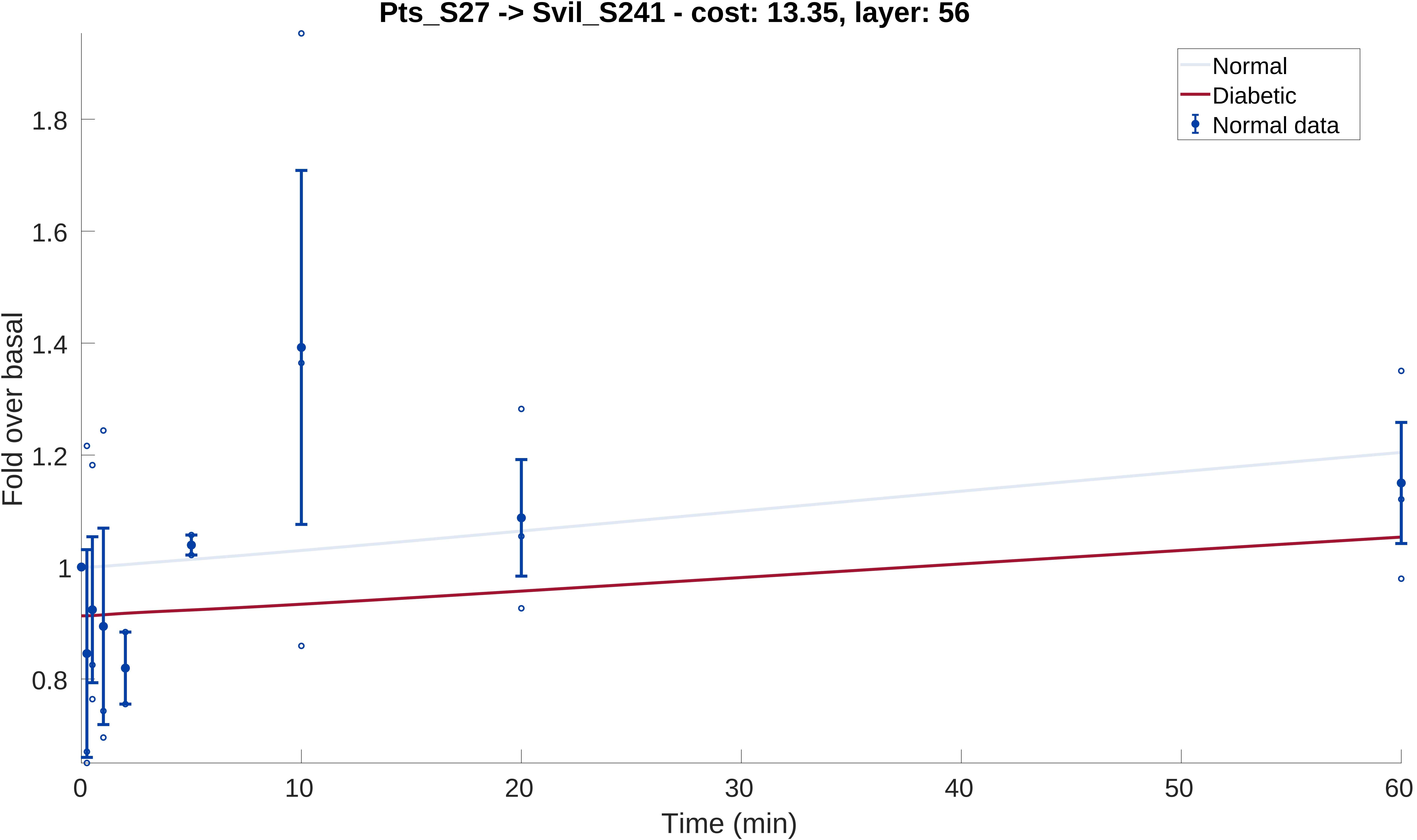

Pts_S27 -> Svil_S241 - cost: 13.35, layer: 56
Normal
Diabetic
Normal data
Fold over basal
Time (min)
1.8
1.6
1.4
1.2
1
0.8
0
10
20
30
40
50
60

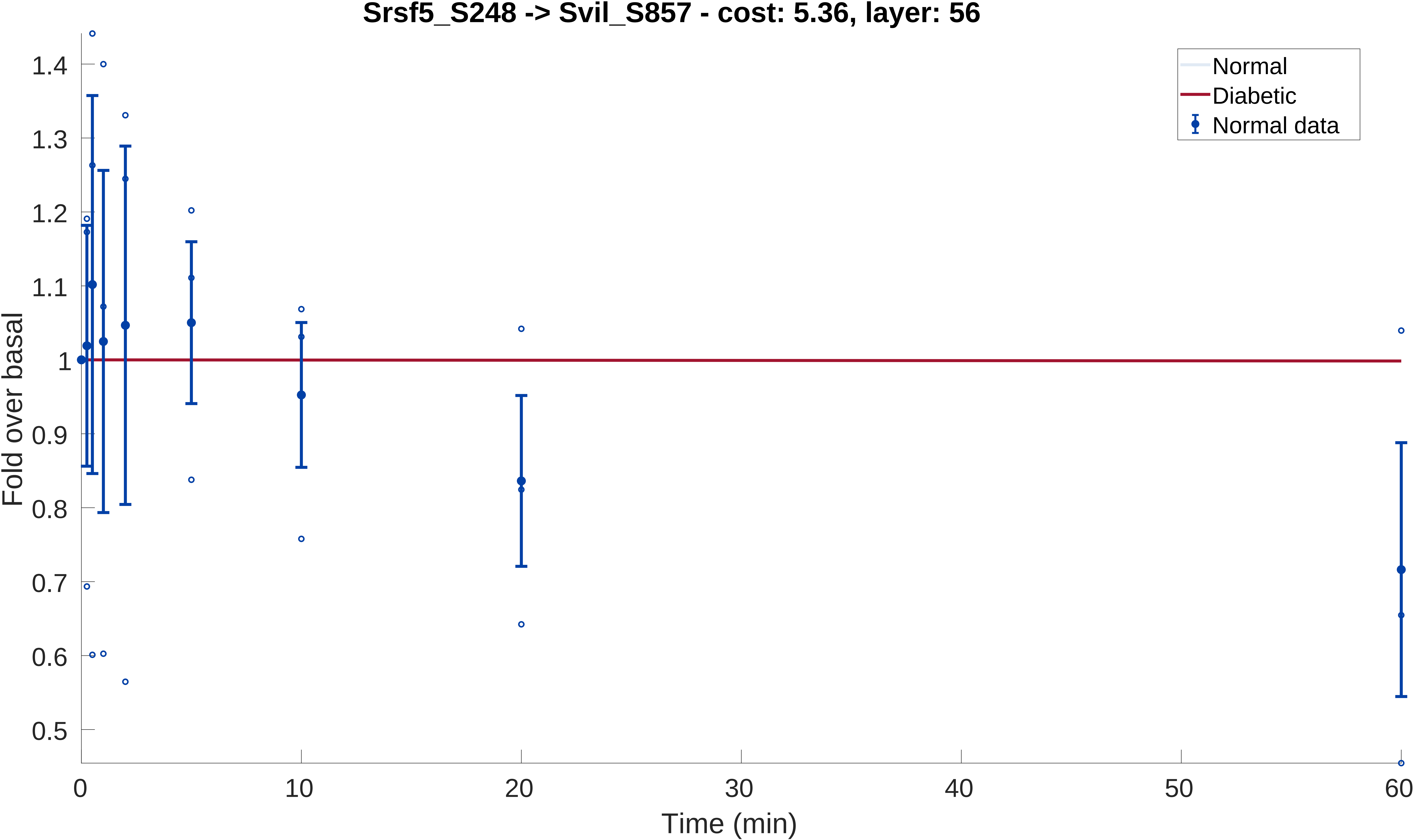

Srsf5_S248 -> Svil_S857 - cost: 5.36, layer: 56
Normal
Diabetic
Normal data
1.4
1.3
1.2
1.1
1
0.9
0.8
0.7
0.6
0.5
Fold over basal
0
10
20
30
40
50
60
Time (min)

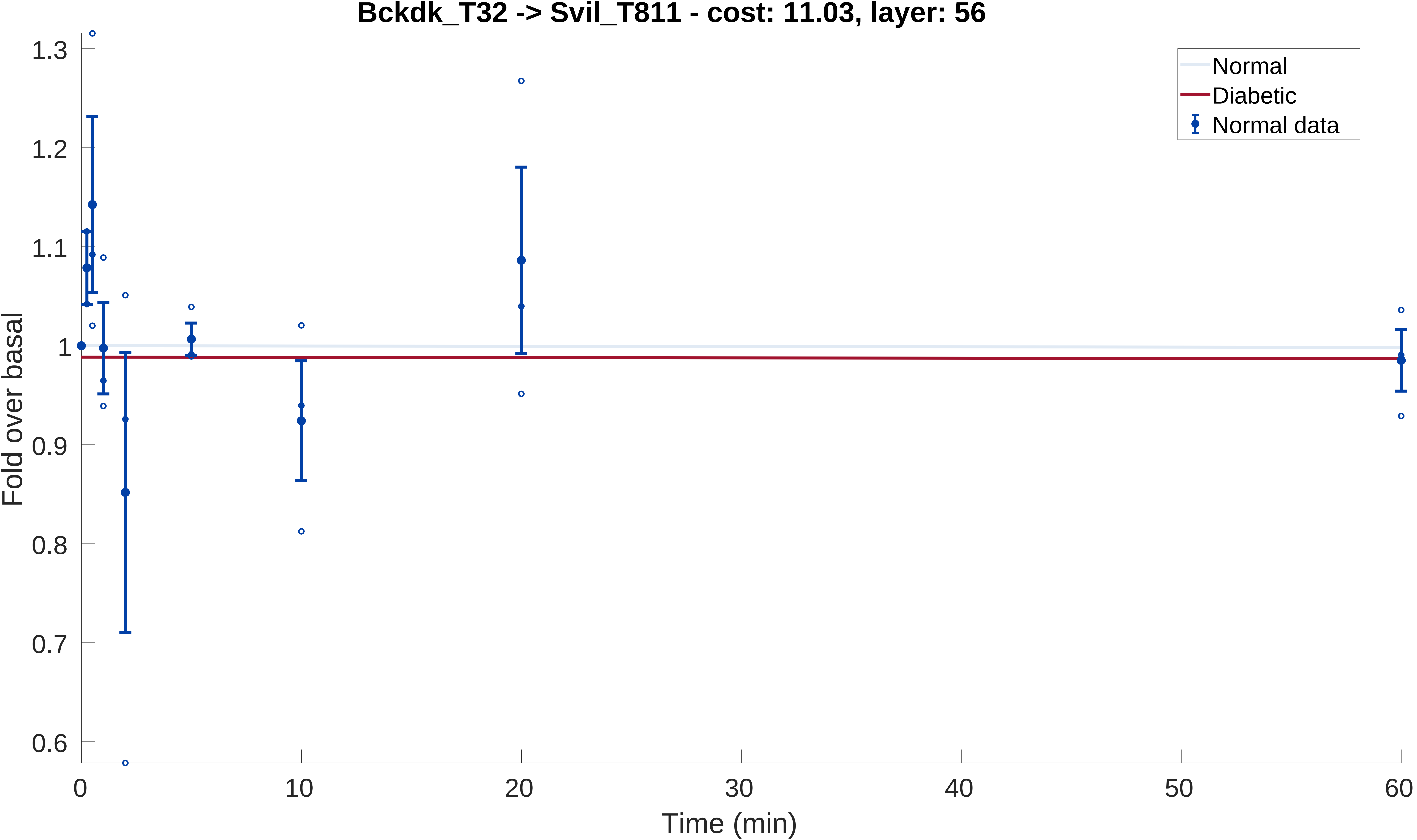

Bckdk_T32 -> Svil_T811 - cost: 11.03, layer: 56
Normal
Diabetic
Normal data
Fold over basal
Time (min)
1.3
1.2
1.1
1
0.9
0.8
0.7
0.6
0
10
20
30
40
50
60

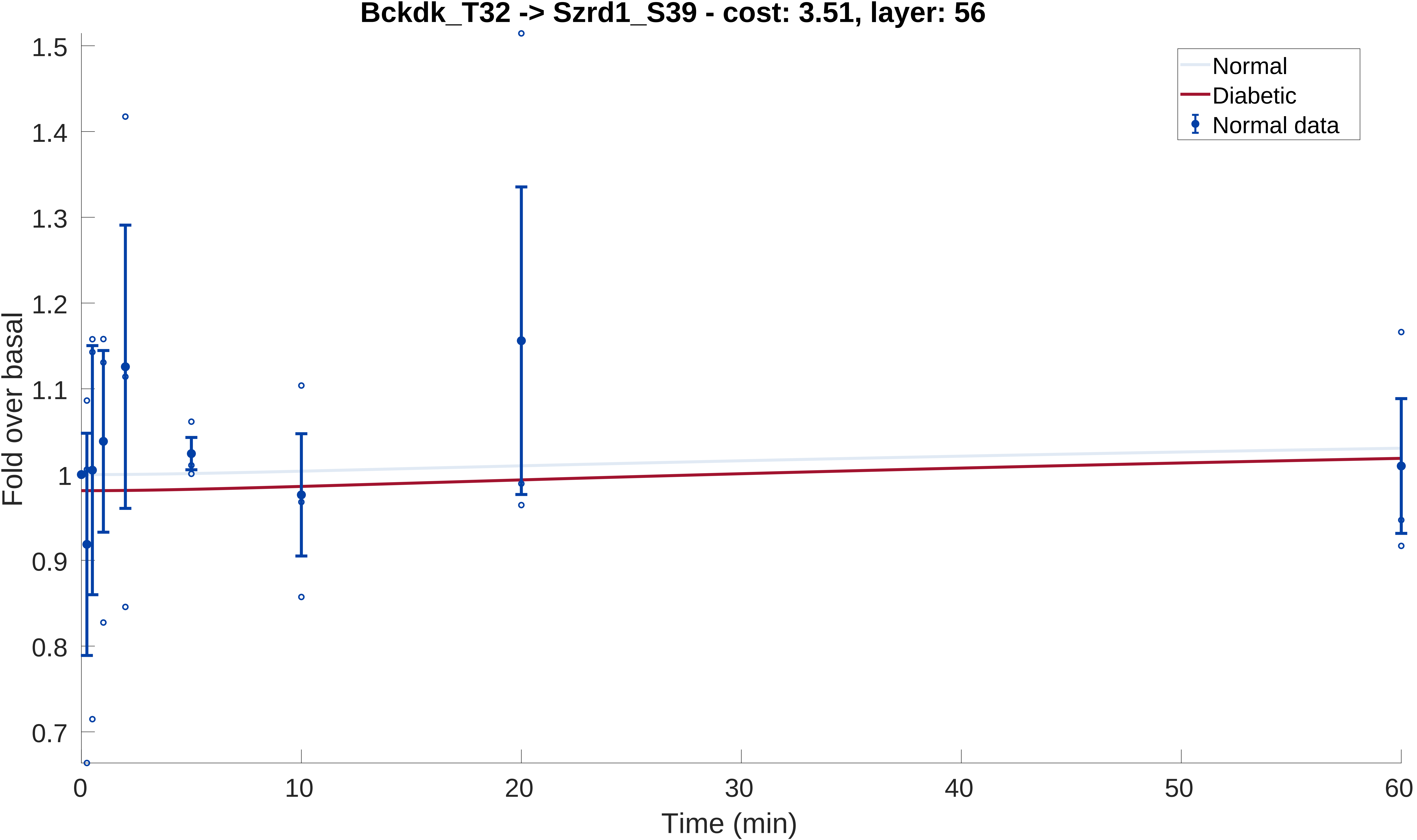

Bckdk_T32 -> Szrd1_S39 - cost: 3.51, layer: 56
Normal
Diabetic
Normal data
Fold over basal
Time (min)
1.5
1.4
1.3
1.2
1.1
1
0.9
0.8
0.7
0
10
20
30
40
50
60

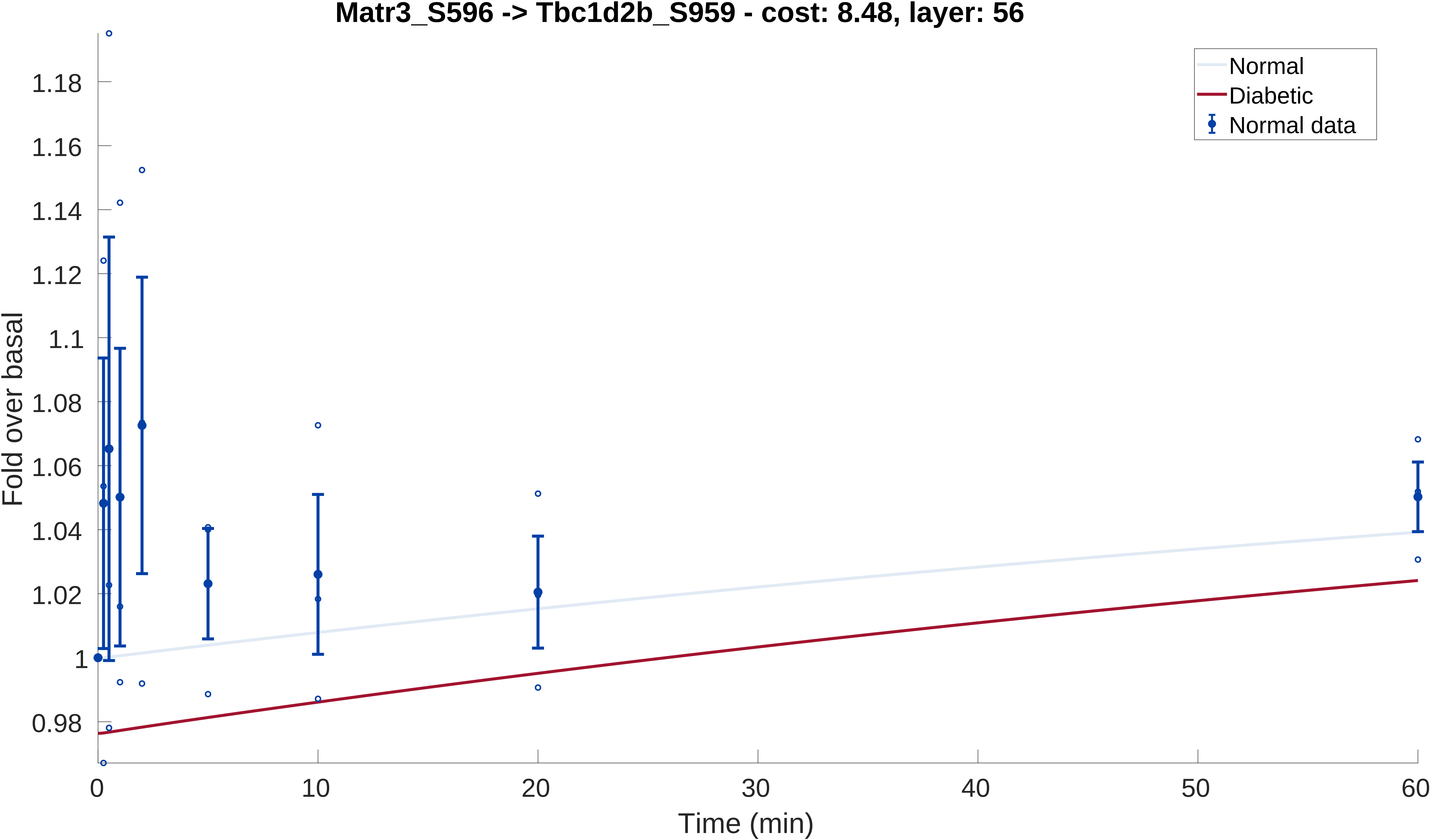
Matr3_S596 -> Tbc1d2b_S959 - cost: 8.48, layer: 56
Fold over basal
1.18
1.16
1.14
1.12
1.1
1.08
1.06
1.04
1.02
1
0.98
0
10
20
30
40
50
60
Time (min)
Normal
Diabetic
Normal data

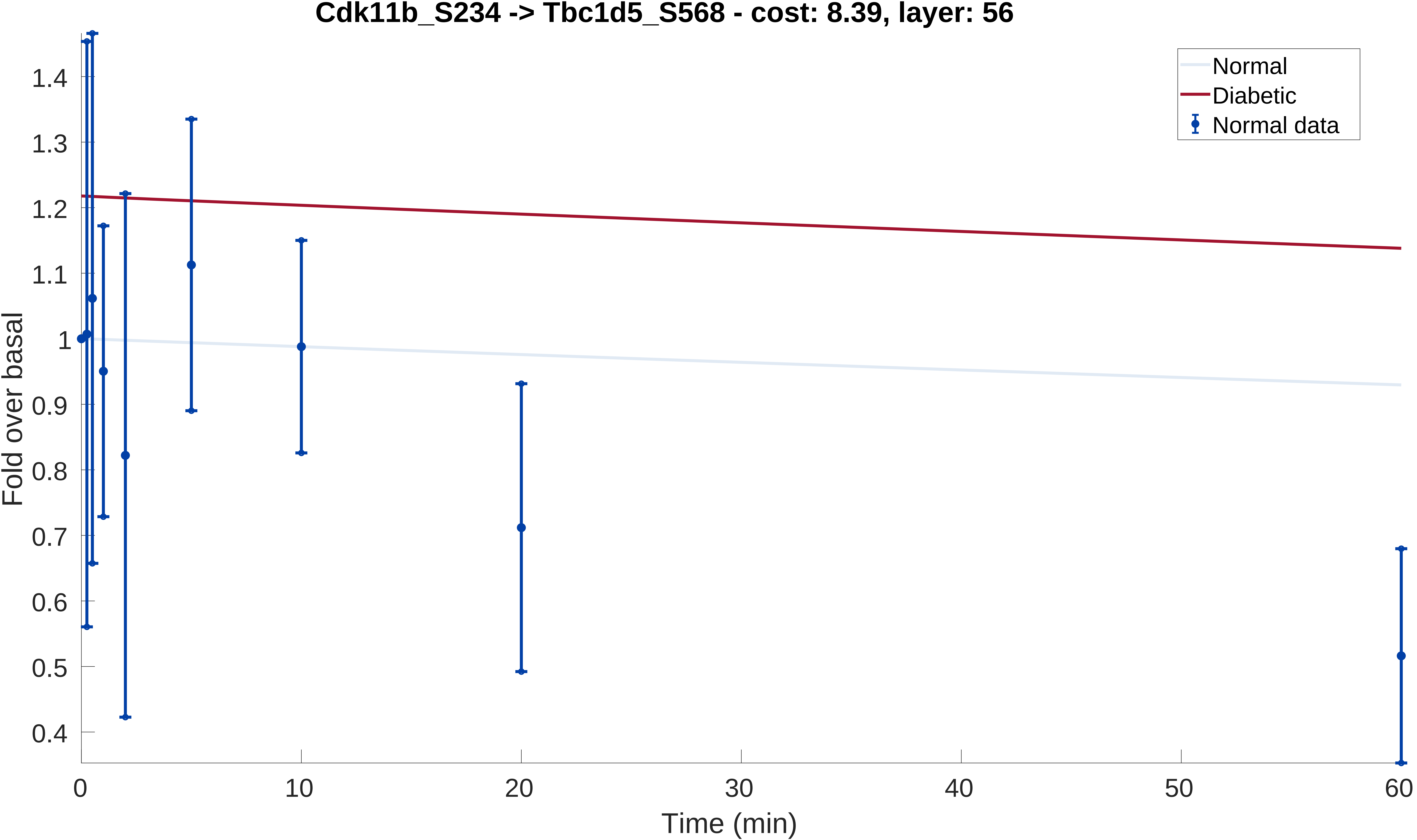

Cdk11b_S234 -> Tbc1d5_S568 - cost: 8.39, layer: 56
Normal
Diabetic
Normal data
Fold over basal
Time (min)
1.4
1.3
1.2
1.1
1
0.9
0.8
0.7
0.6
0.5
0.4
0
10
20
30
40
50
60

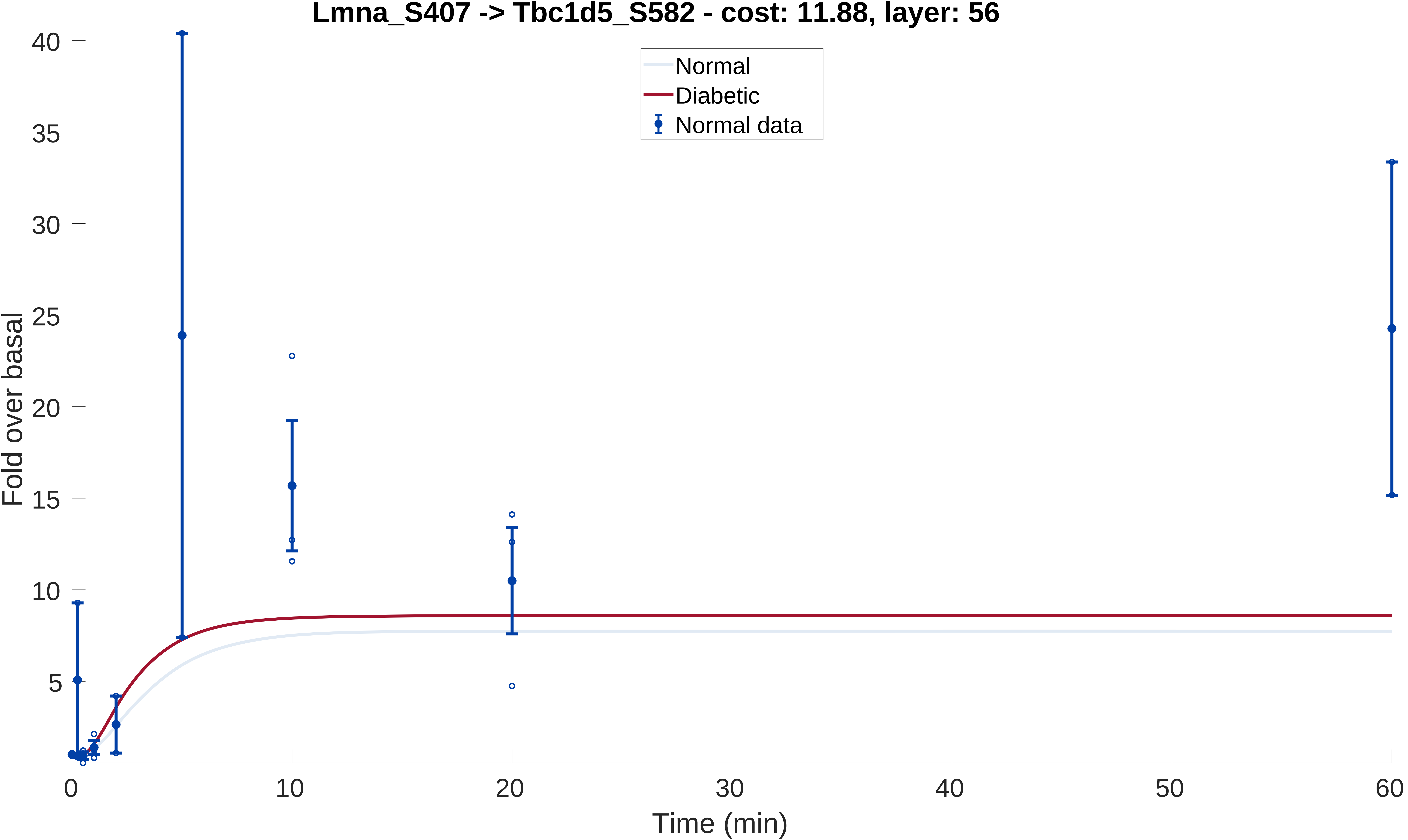
Lmna_S407 -> Tbc1d5_S582 - cost: 11.88, layer: 56
Normal
Diabetic
Normal data
Fold over basal
Time (min)
0
10
20
30
40
50
60
5
10
15
20
25
30
35
40

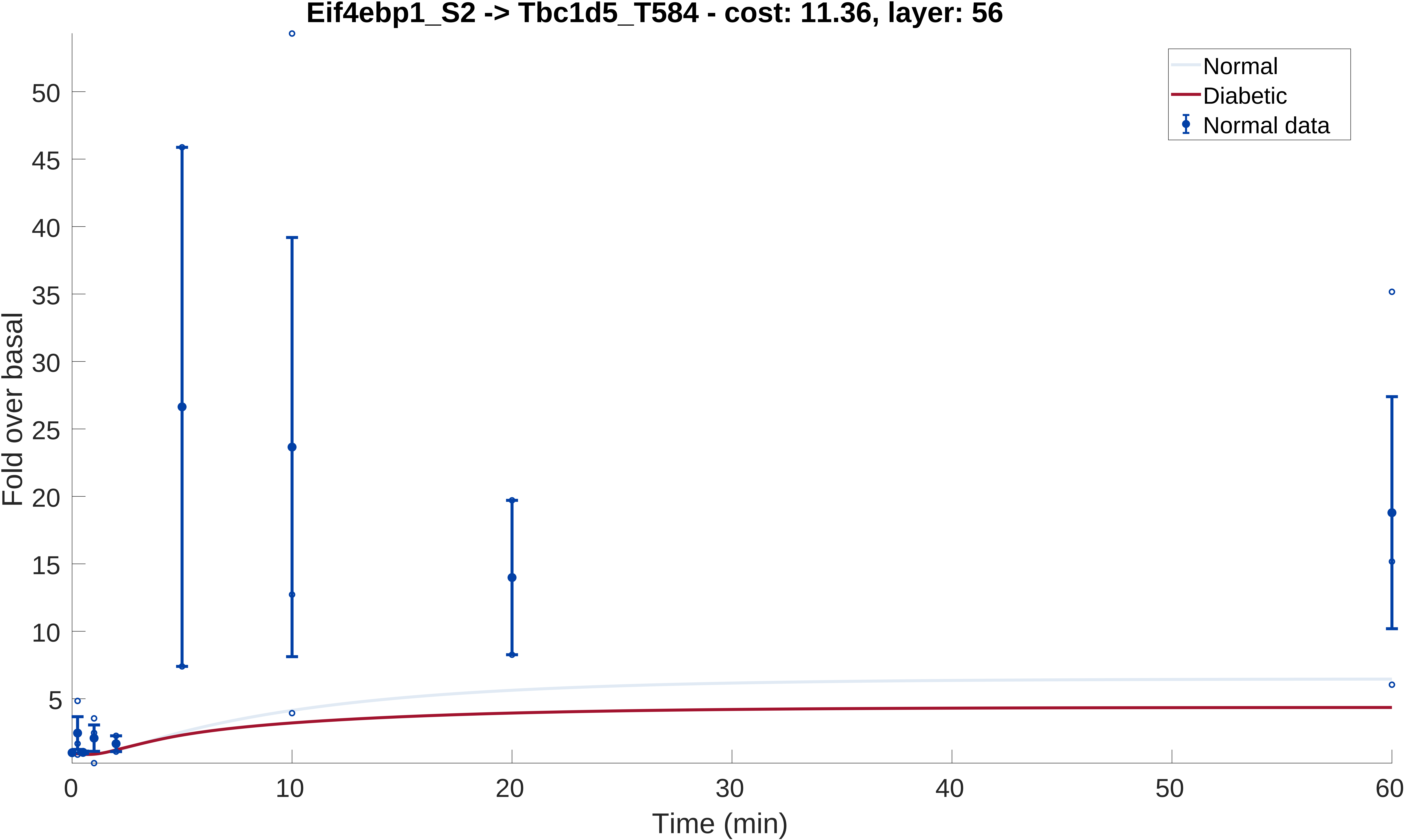

Eif4ebp1_S2 -> Tbc1d5_T584 - cost: 11.36, layer: 56
Normal
Diabetic
Normal data
Fold over basal
Time (min)
0
10
20
30
40
50
60
5
10
15
20
25
30
35
40
45
50

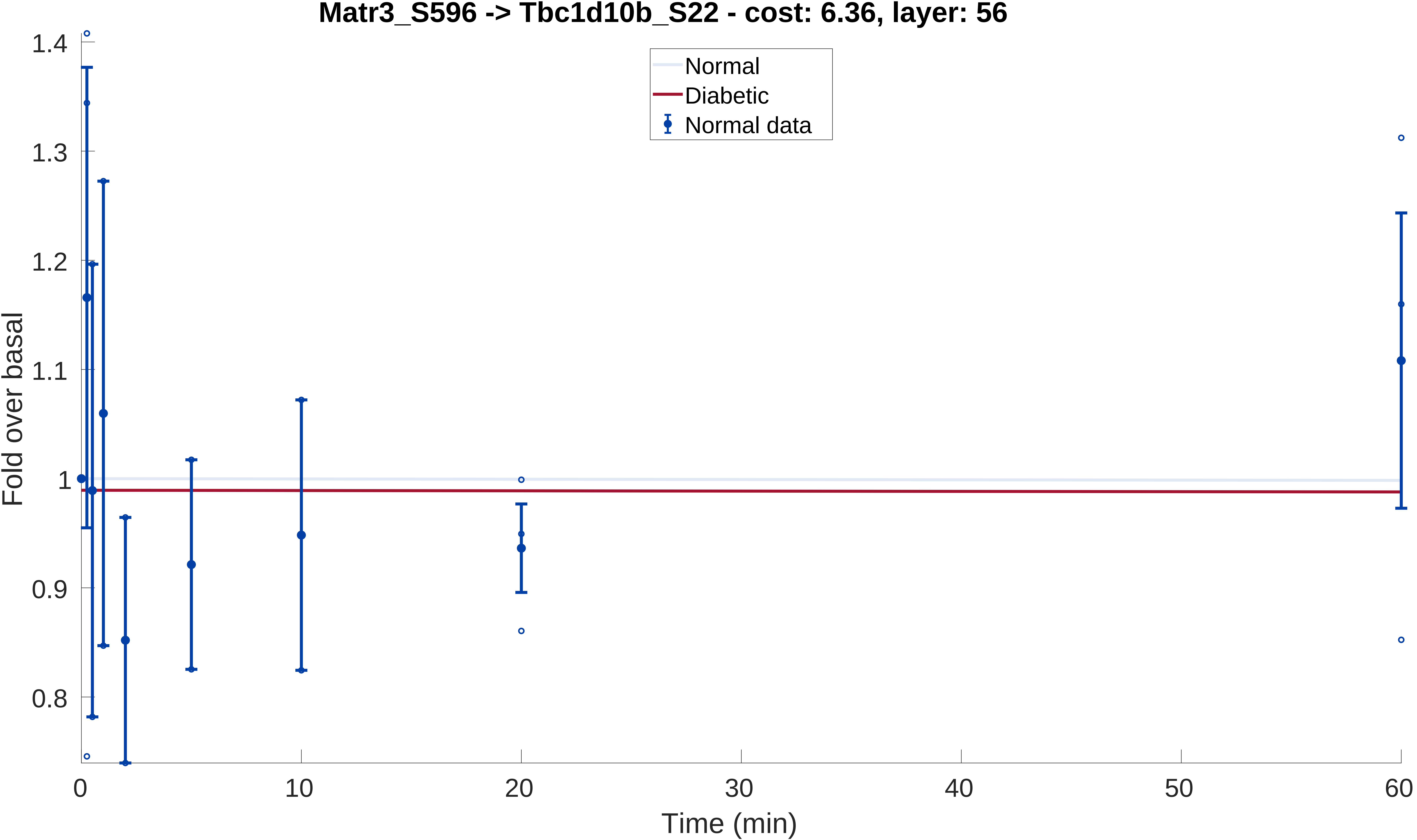

Matr3_S596 -> Tbc1d10b_S22 - cost: 6.36, layer: 56
Normal
Diabetic
Normal data
Fold over basal
1.4
1.3
1.2
1.1
1
0.9
0.8
0
10
20
30
40
50
60
Time (min)

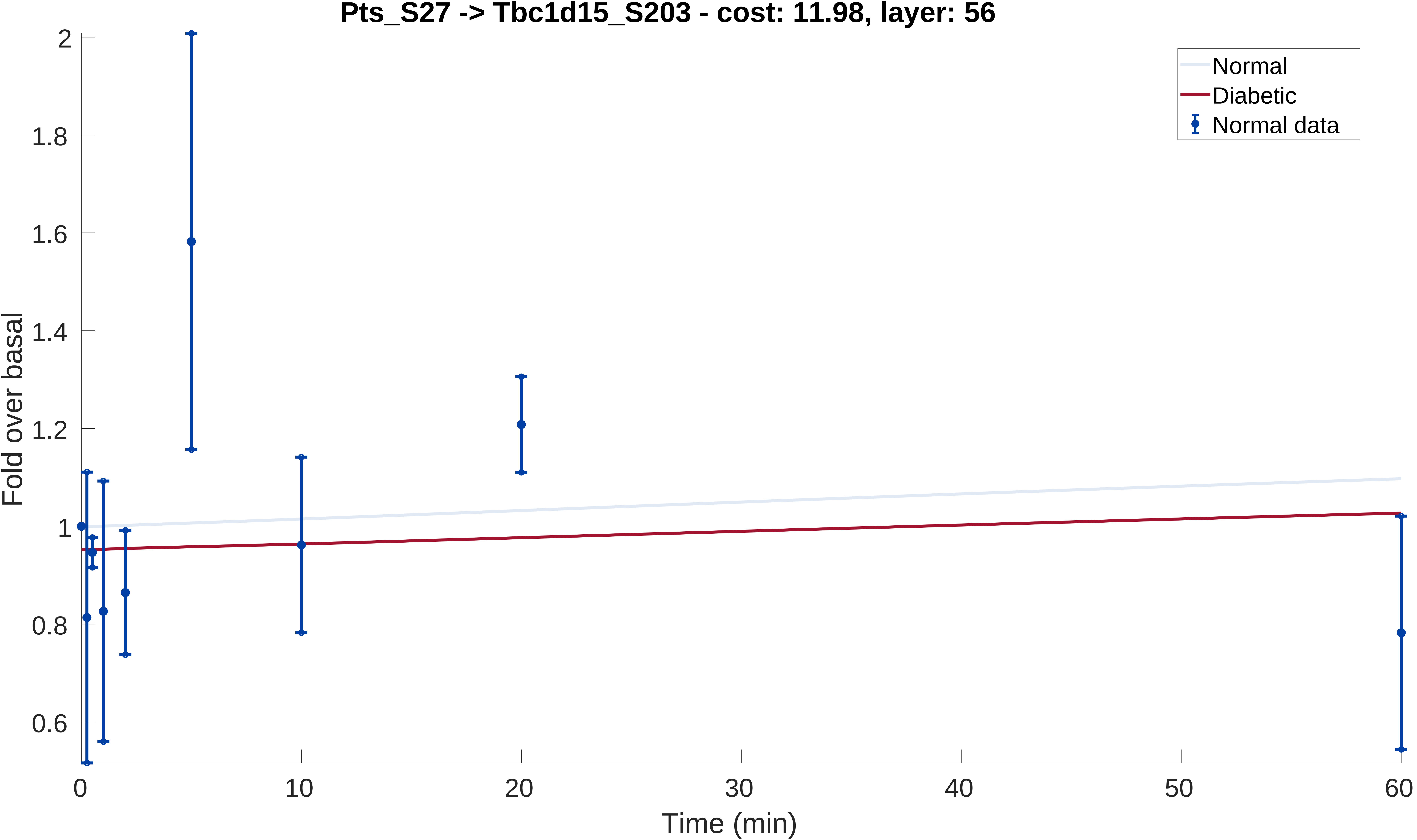

Pts_S27 -> Tbc1d15_S203 - cost: 11.98, layer: 56
Normal
Diabetic
Normal data
Fold over basal
Time (min)
2
1.8
1.6
1.4
1.2
1
0.8
0.6
0
10
20
30
40
50
60

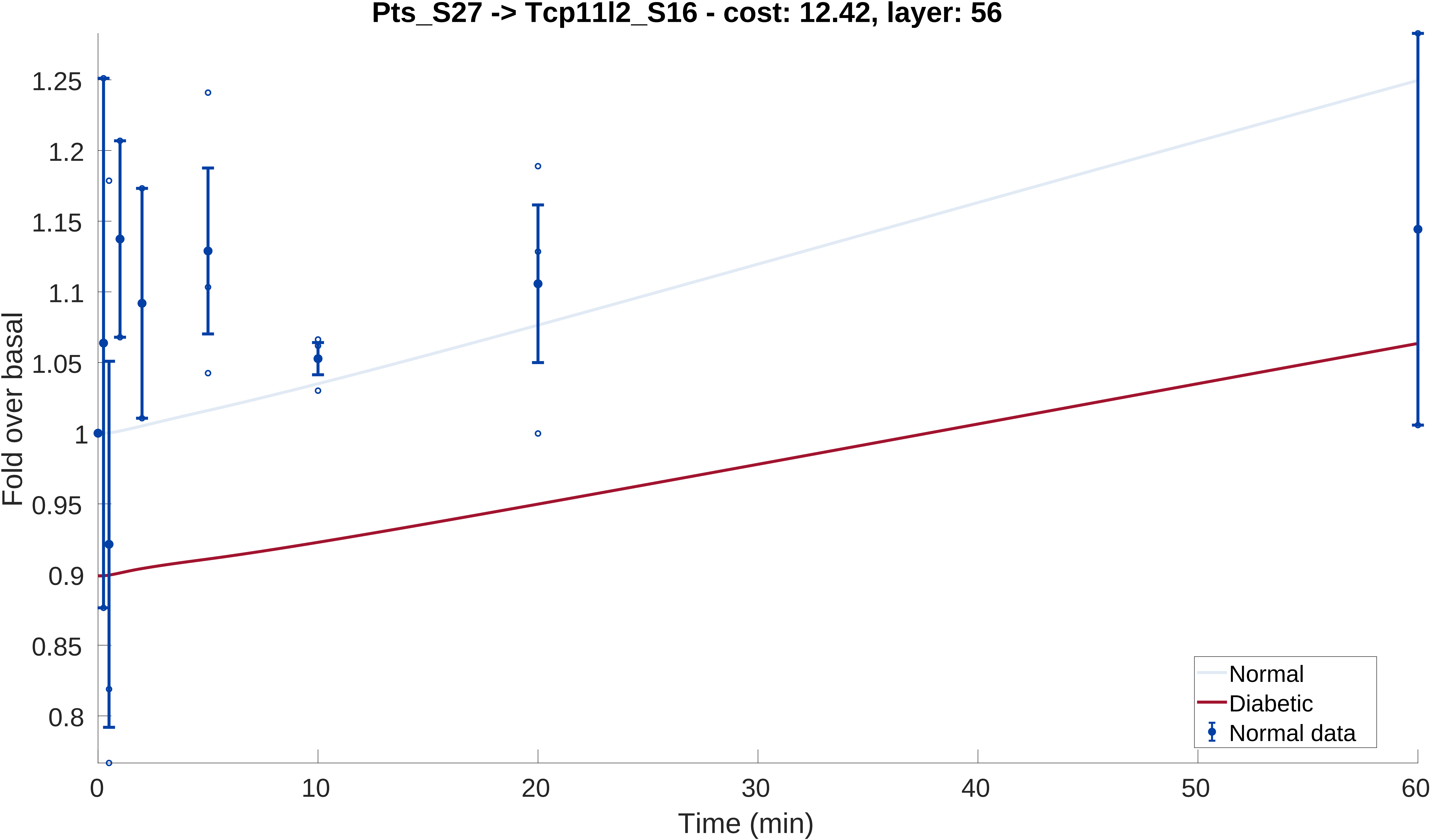

Pts_S27 -> Tcp11l2_S16 - cost: 12.42, layer: 56
Fold over basal
Time (min)
0
10
20
30
40
50
60
0.8
0.85
0.9
0.95
1
1.05
1.1
1.15
1.2
1.25
Normal
Diabetic
Normal data

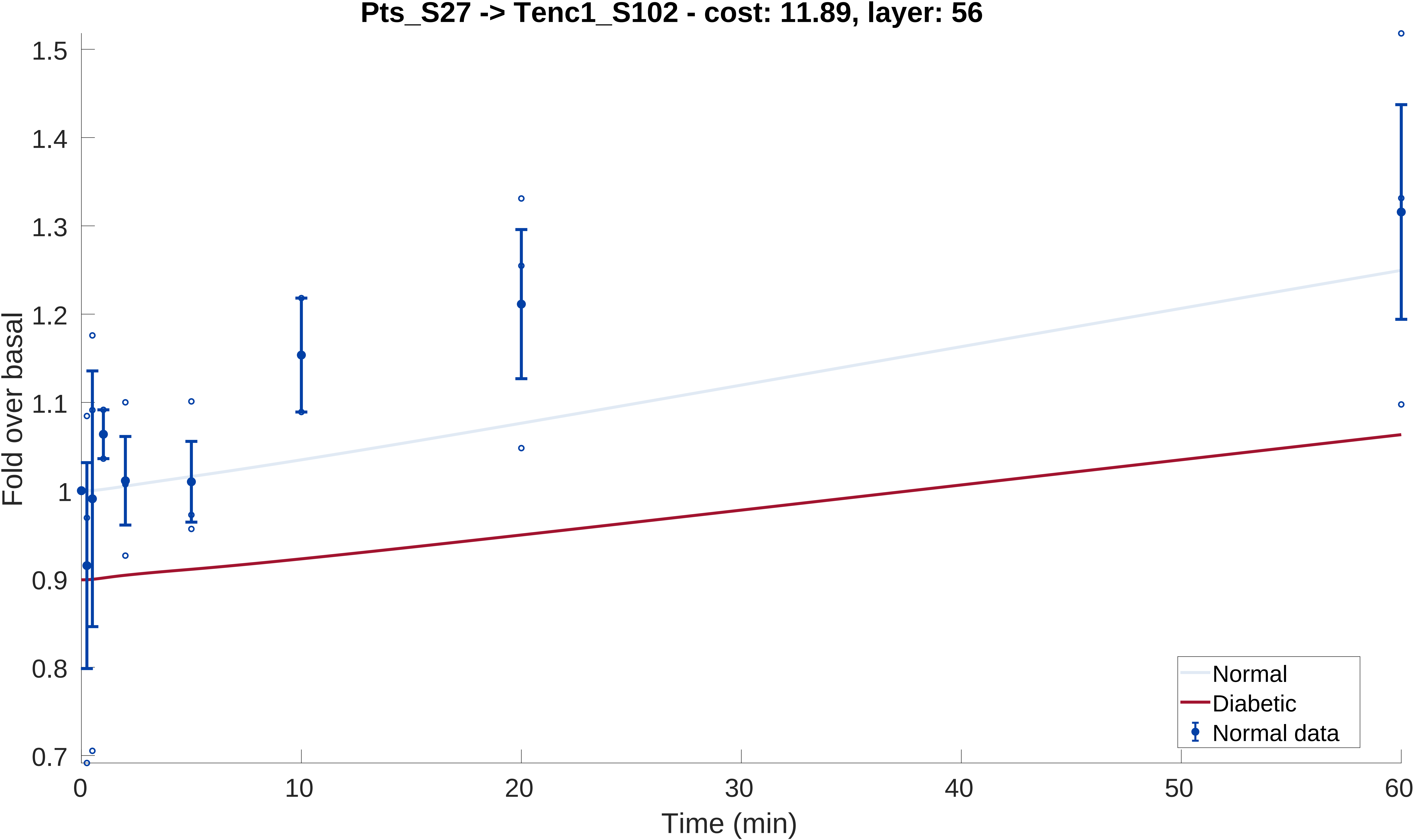

Pts_S27 -> Tenc1_S102 - cost: 11.89, layer: 56
Fold over basal
1.5
1.4
1.3
1.2
1.1
1
0.9
0.8
0.7
0
10
20
30
40
50
60
Time (min)
Normal
Diabetic
Normal data

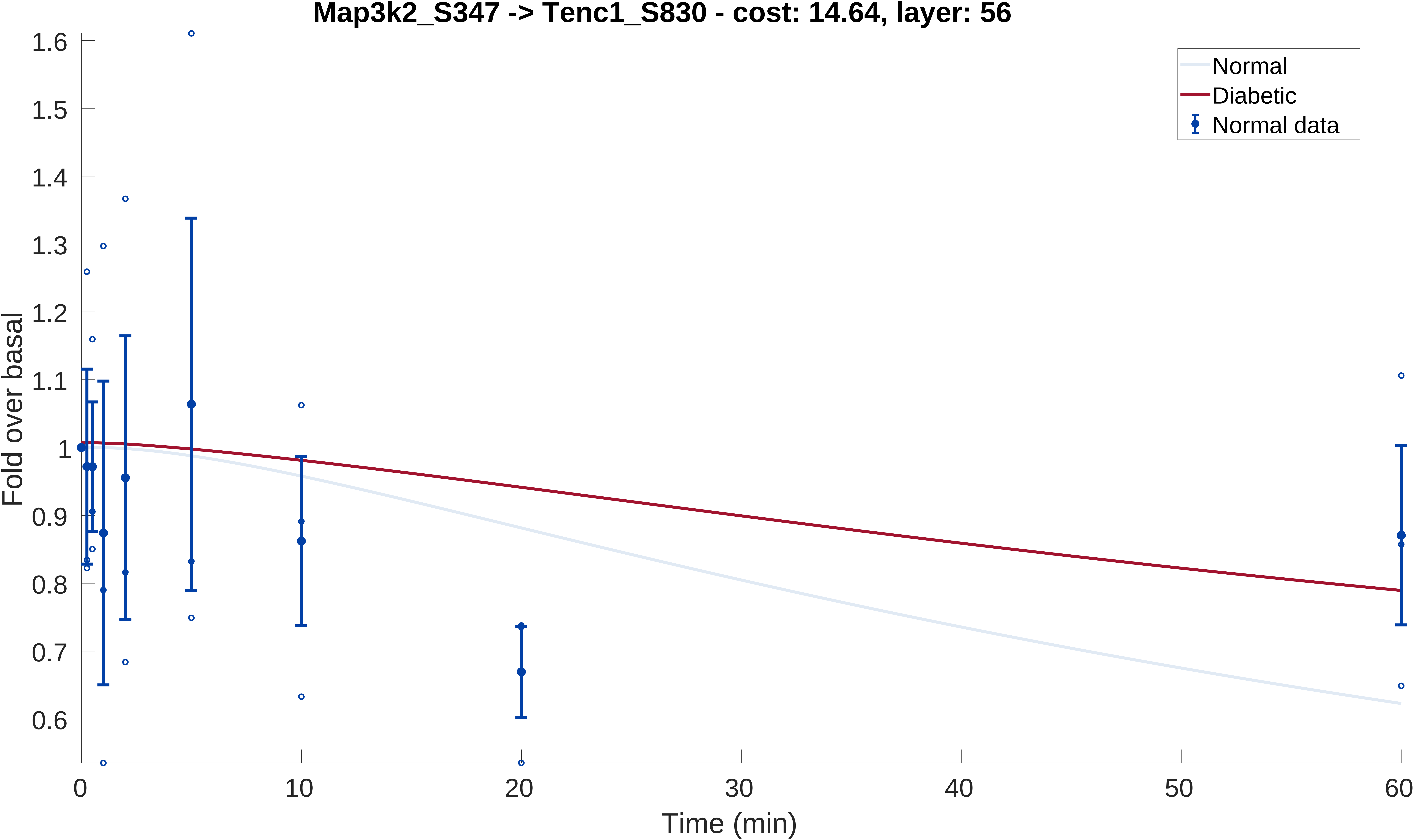

Map3k2_S347 -> Tenc1_S830 - cost: 14.64, layer: 56
Normal
Diabetic
Normal data
Fold over basal
Time (min)
1.6
1.5
1.4
1.3
1.2
1.1
1
0.9
0.8
0.7
0.6
0
10
20
30
40
50
60

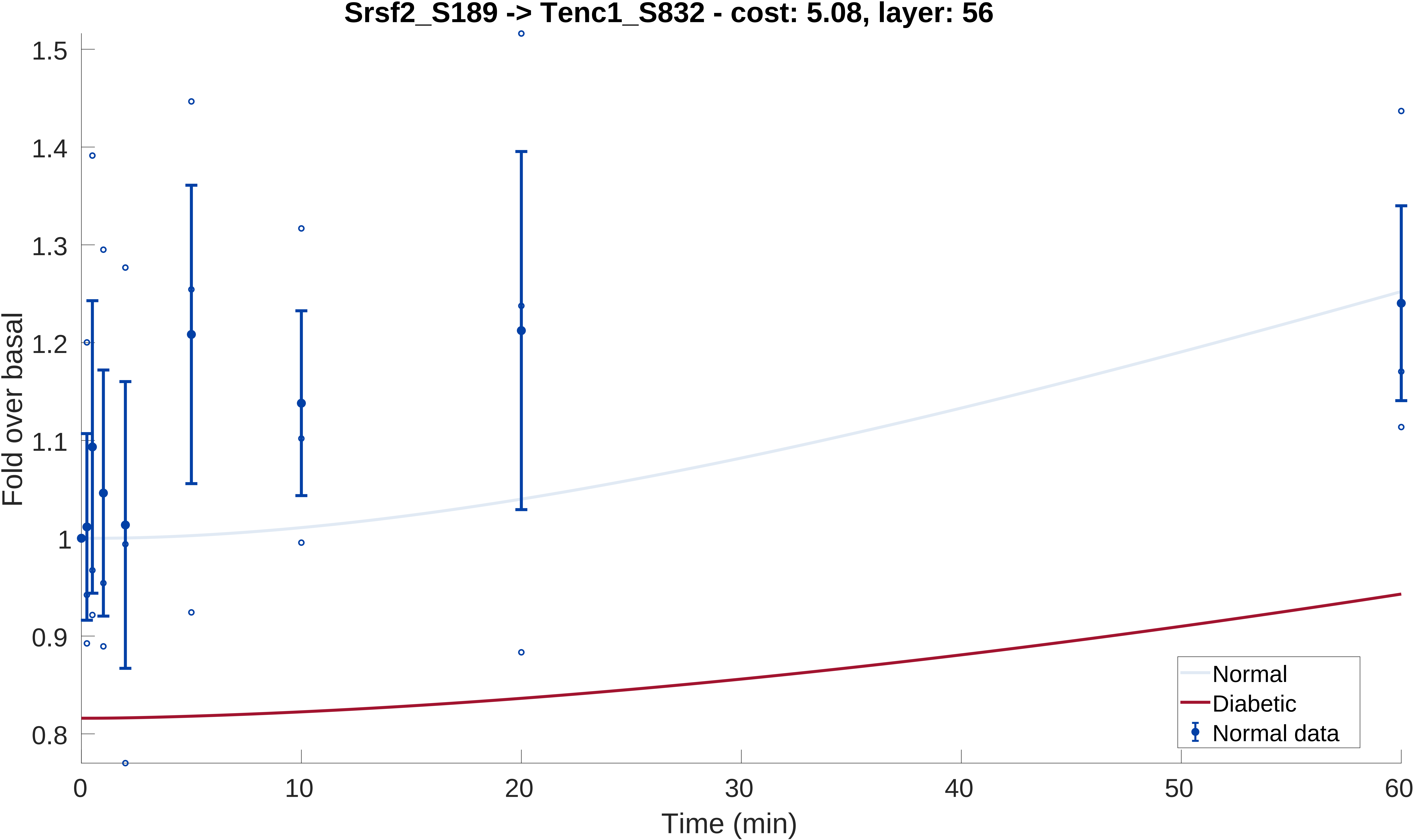

Srsf2_S189 -> Tenc1_S832 - cost: 5.08, layer: 56
Fold over basal
1.5
1.4
1.3
1.2
1.1
1
0.9
0.8
0
10
20
30
40
50
60
Time (min)
Normal
Diabetic
Normal data

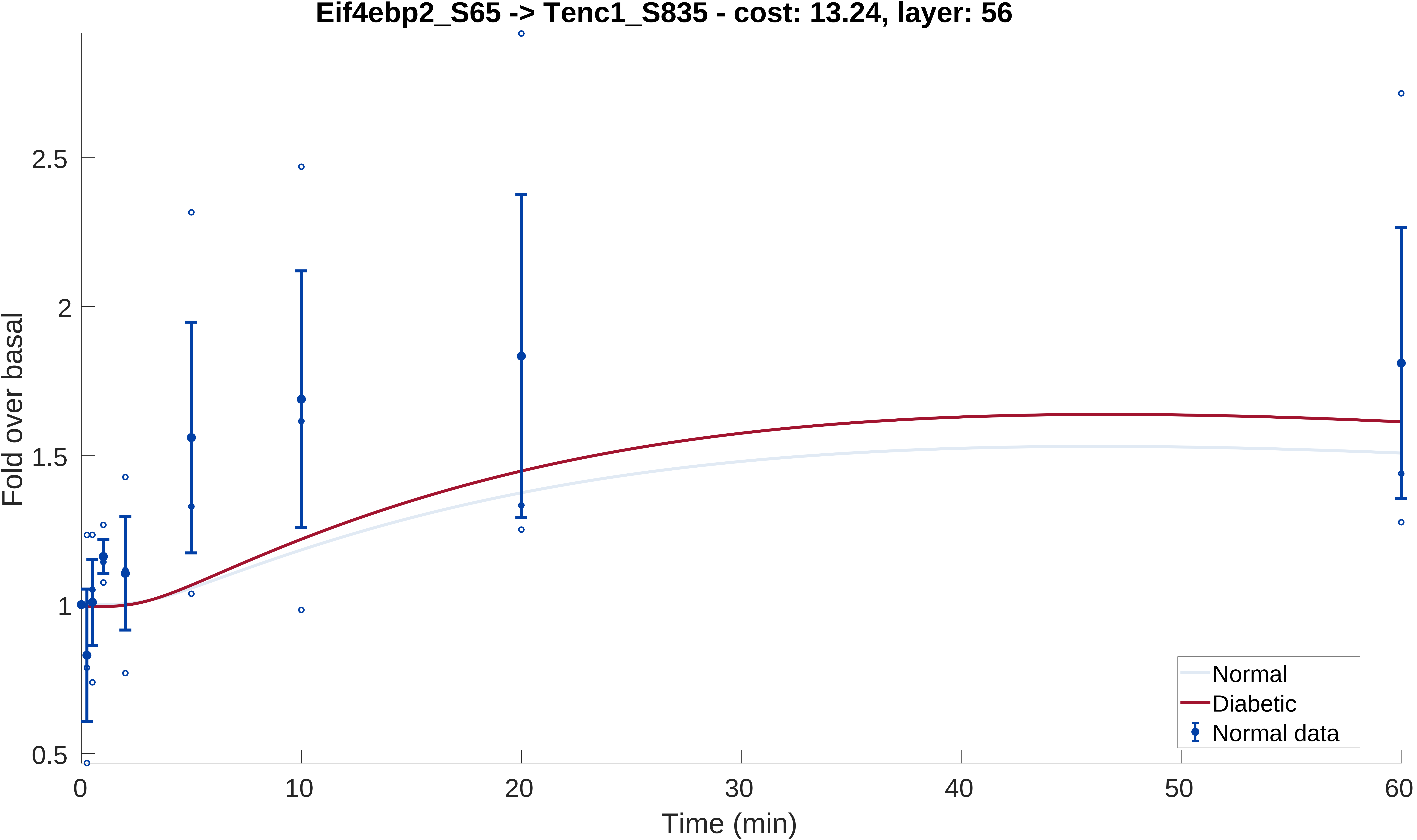

Eif4ebp2_S65 -> Tenc1_S835 - cost: 13.24, layer: 56
Fold over basal
0.5
1
1.5
2
2.5
0
10
20
30
40
50
60
Time (min)
Normal
Diabetic
Normal data

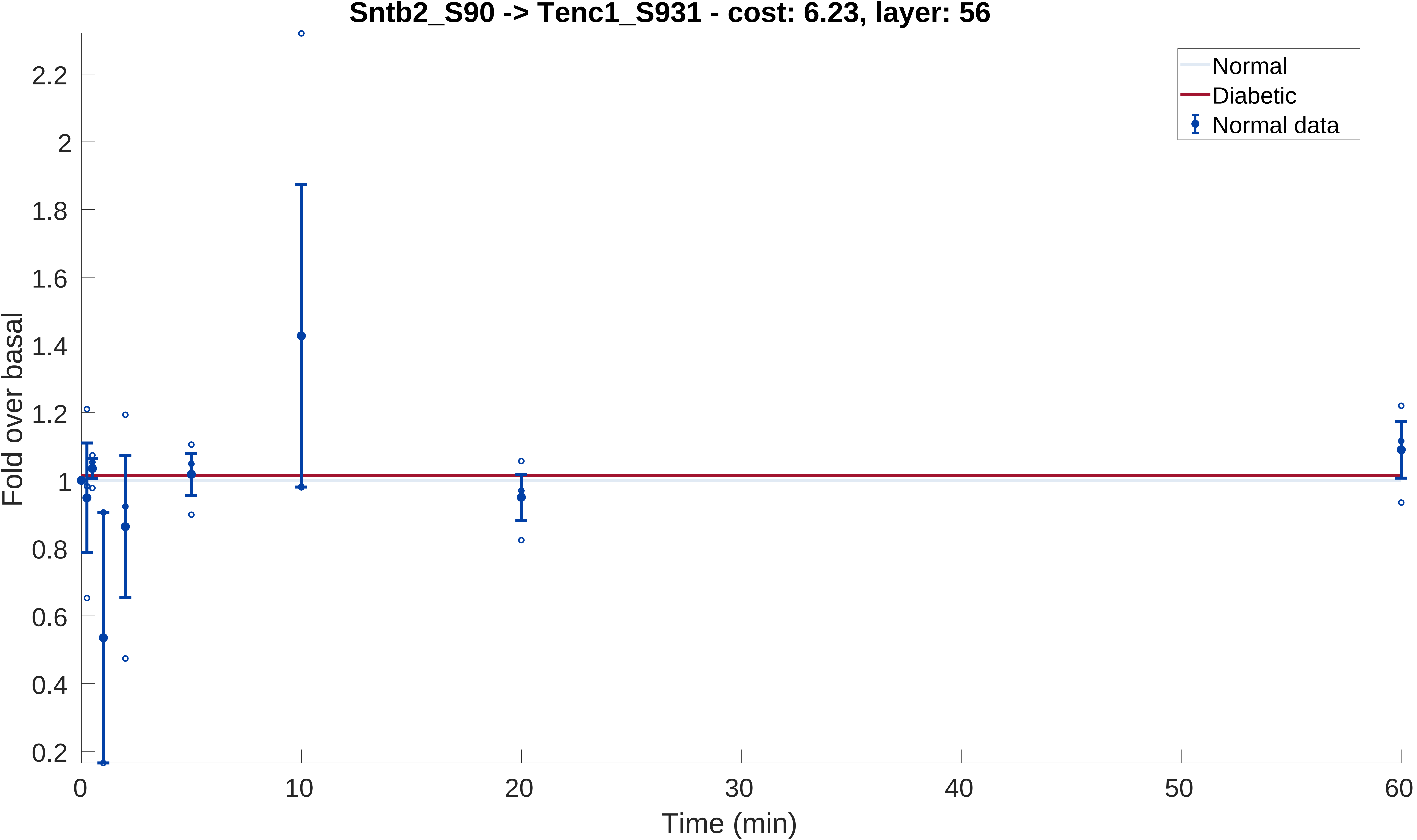

Sntb2_S90 -> Tenc1_S931 - cost: 6.23, layer: 56
Normal
Diabetic
Normal data
Fold over basal
Time (min)
0.2
0.4
0.6
0.8
1
1.2
1.4
1.6
1.8
2
2.2
0
10
20
30
40
50
60

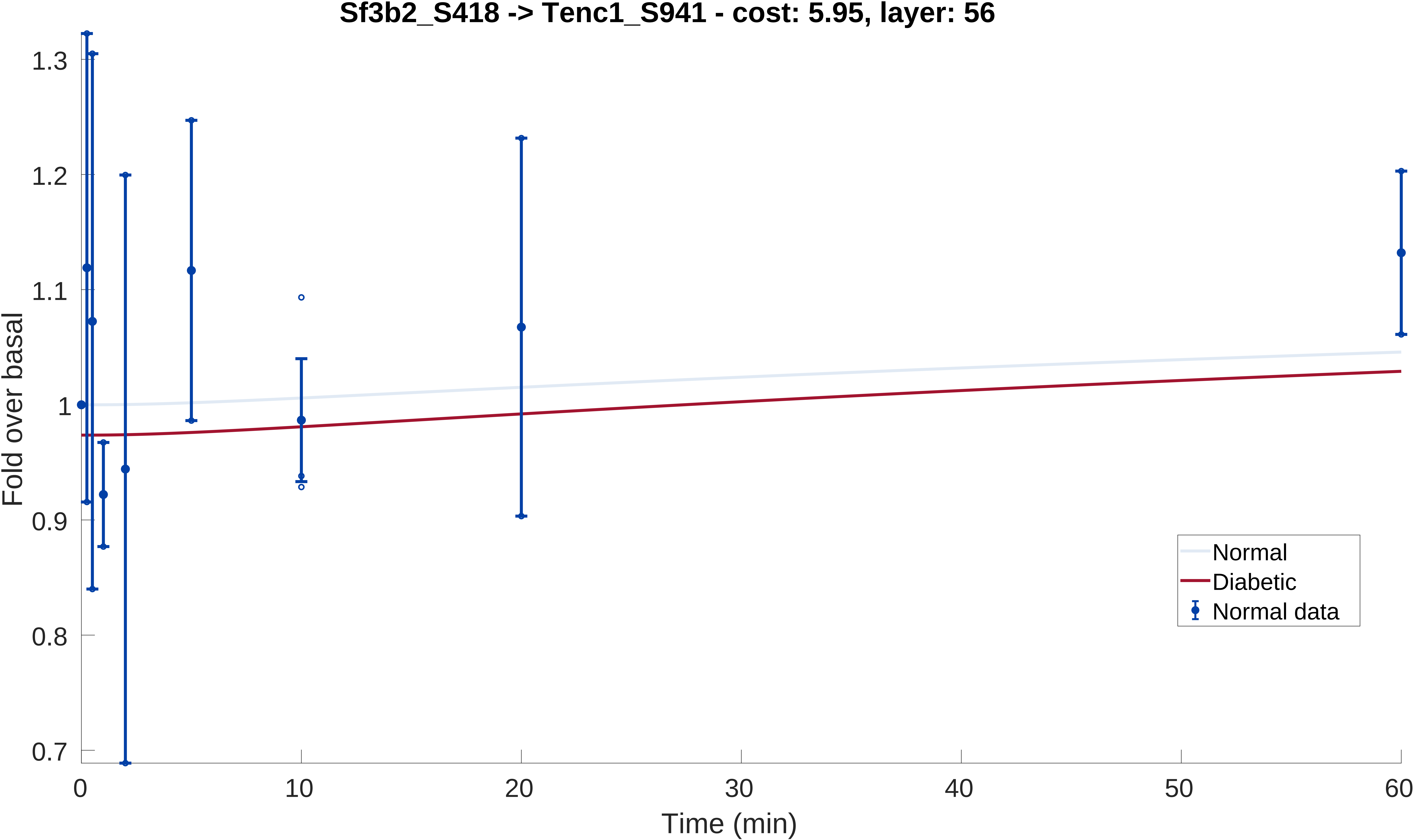

Sf3b2_S418 -> Tenc1_S941 - cost: 5.95, layer: 56
Fold over basal
1.3
1.2
1.1
1
0.9
0.8
0.7
0
10
20
30
40
50
60
Time (min)
Normal
Diabetic
Normal data

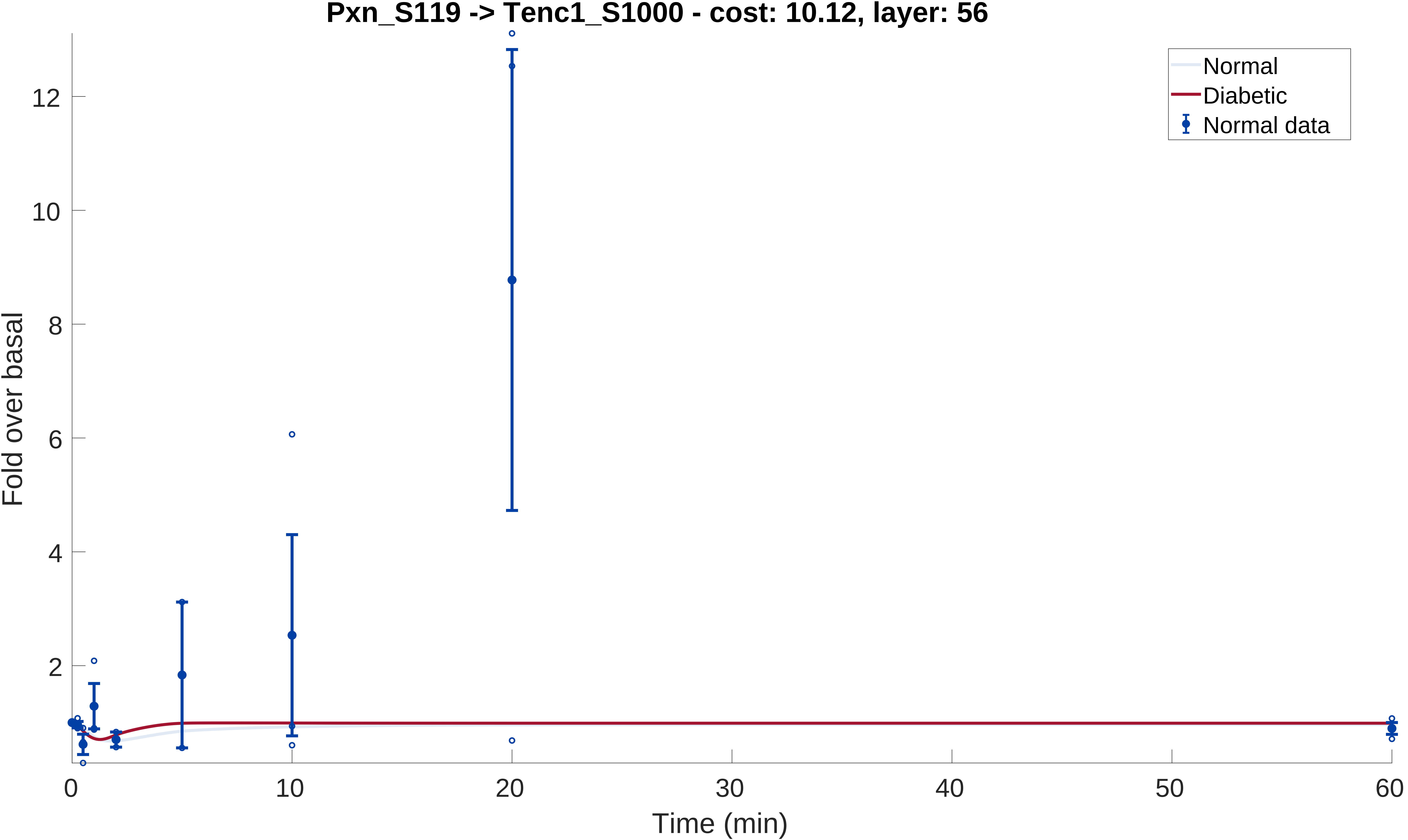

Pxn_S119 -> Tenc1_S1000 - cost: 10.12, layer: 56
Normal
Diabetic
Normal data
Fold over basal
Time (min)
0
10
20
30
40
50
60
2
4
6
8
10
12

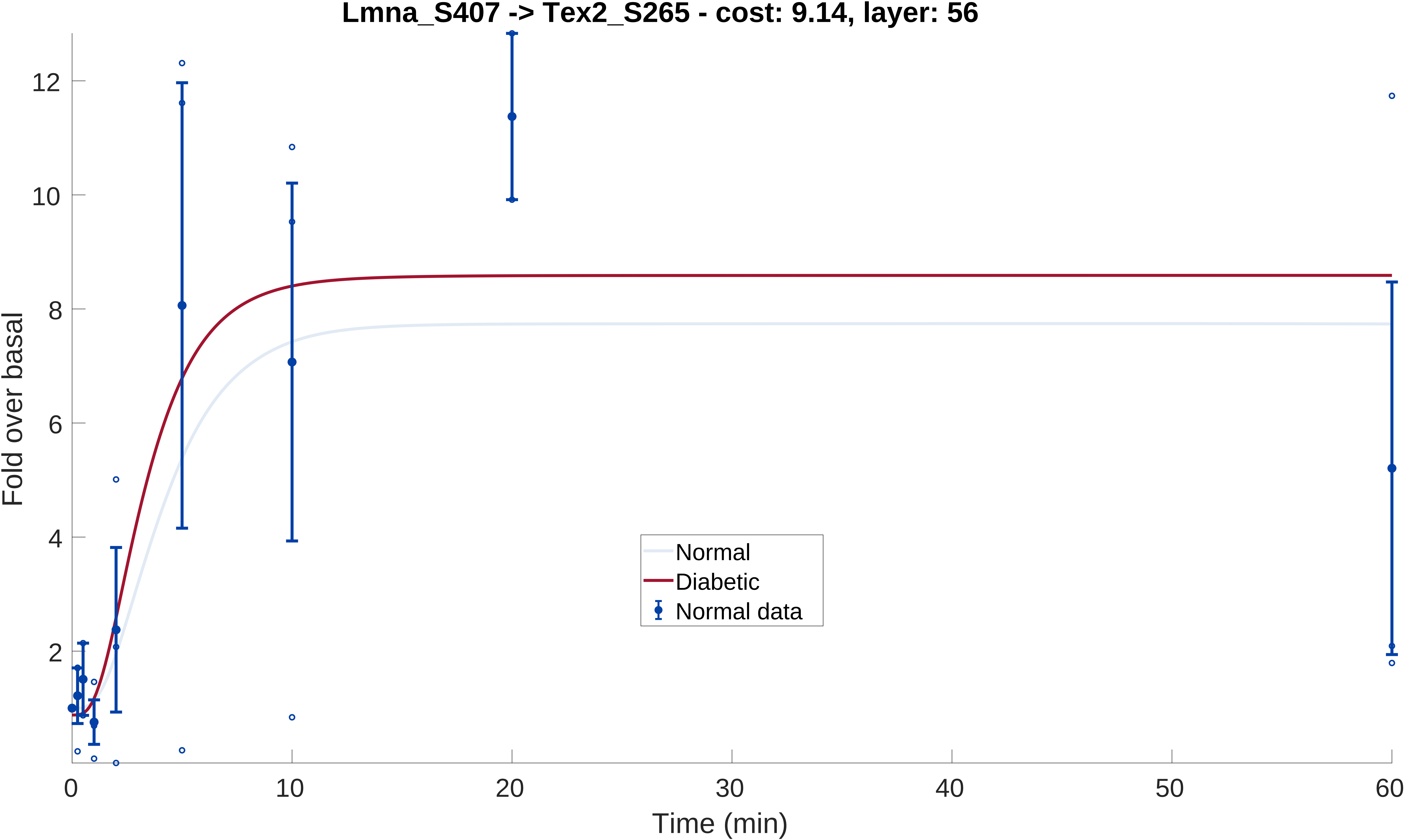

Lmna_S407 -> Tex2_S265 - cost: 9.14, layer: 56
Fold over basal
Time (min)
0
10
20
30
40
50
60
2
4
6
8
10
12
Normal
Diabetic
Normal data

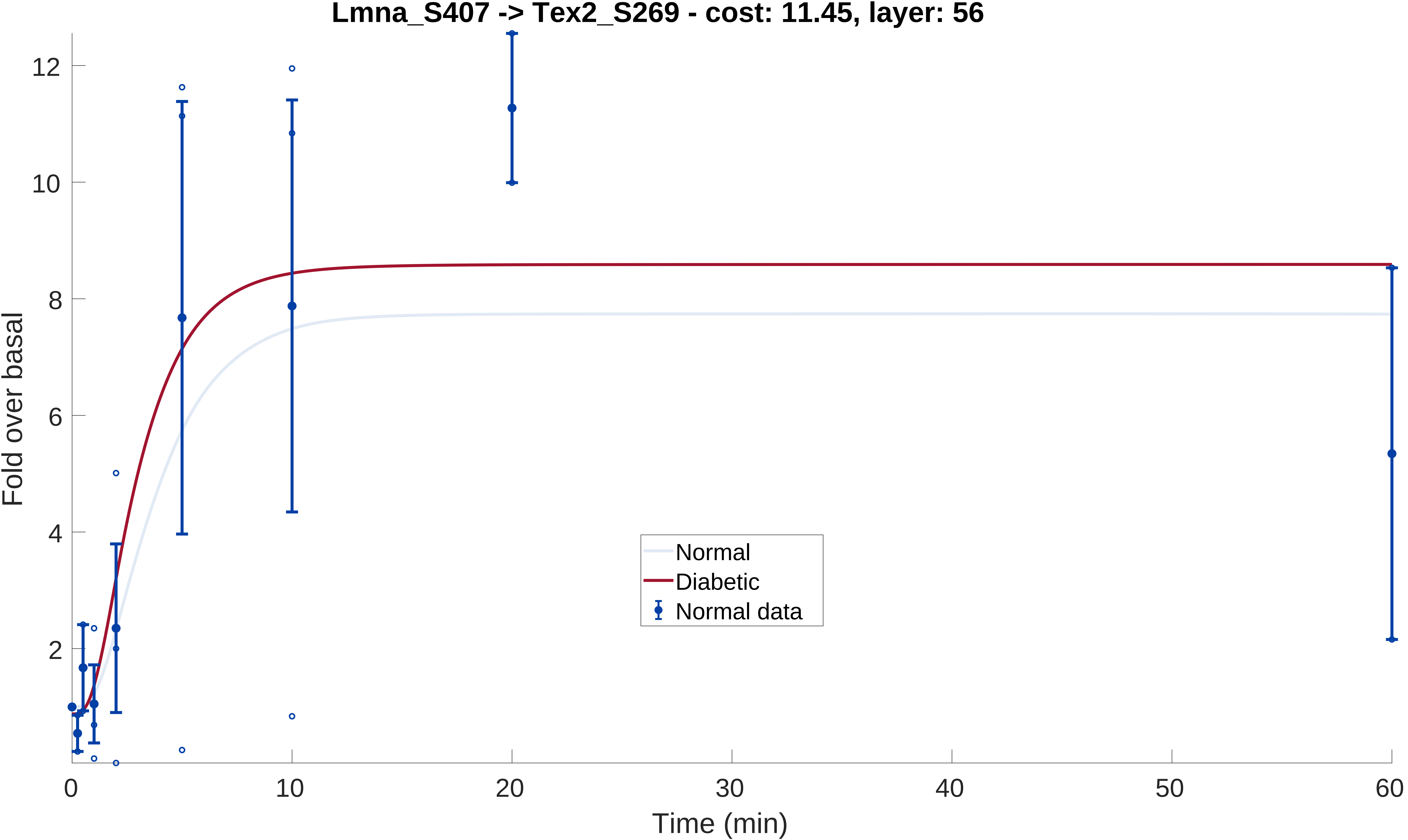

Lmna_S407 -> Tex2_S269 - cost: 11.45, layer: 56
Fold over basal
Time (min)
Normal
Diabetic
Normal data
0
10
20
30
40
50
60
2
4
6
8
10
12

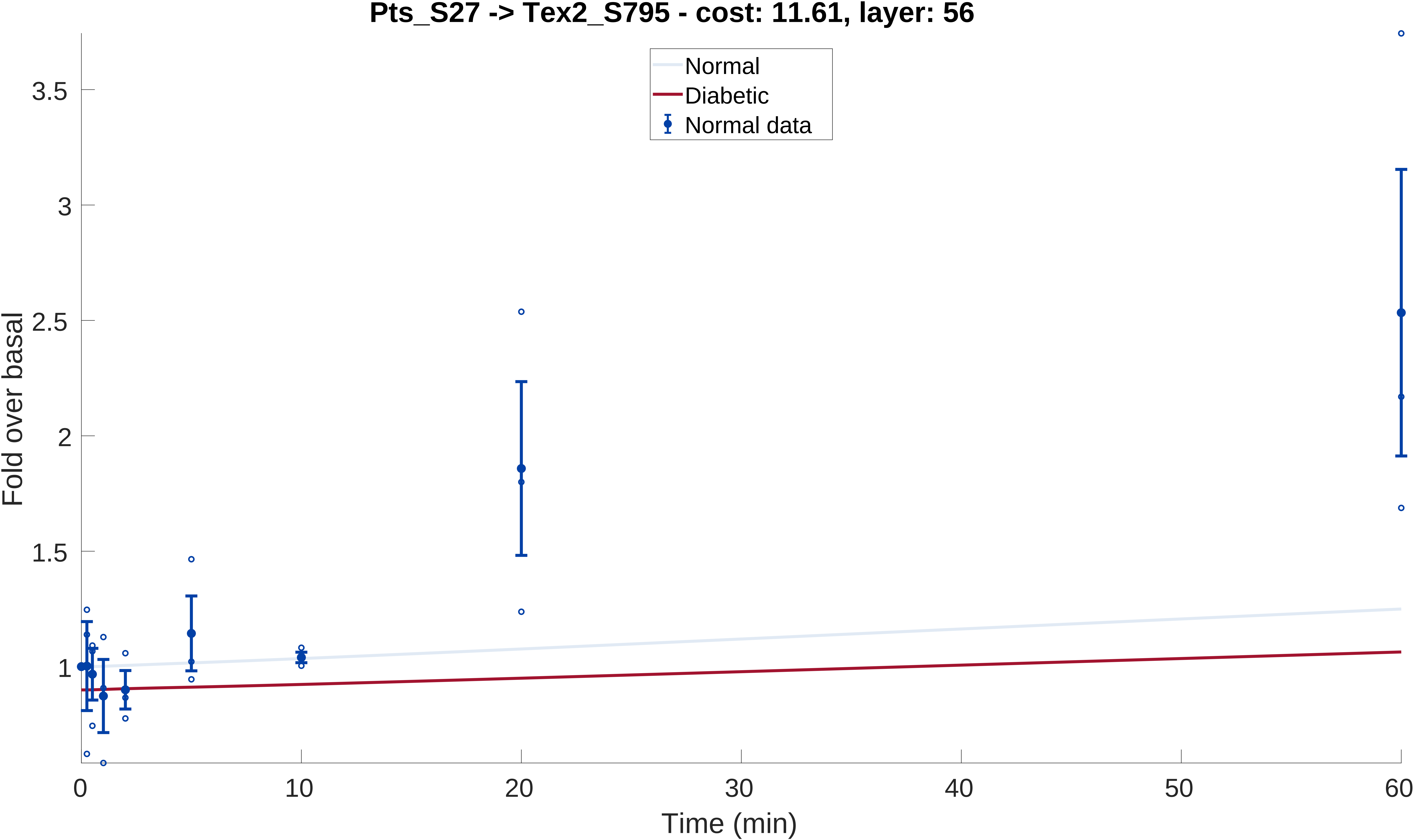

Pts_S27 -> Tex2_S795 - cost: 11.61, layer: 56
Normal
Diabetic
Normal data
Fold over basal
3.5
3
2.5
2
1.5
1
0
10
20
30
40
50
60
Time (min)

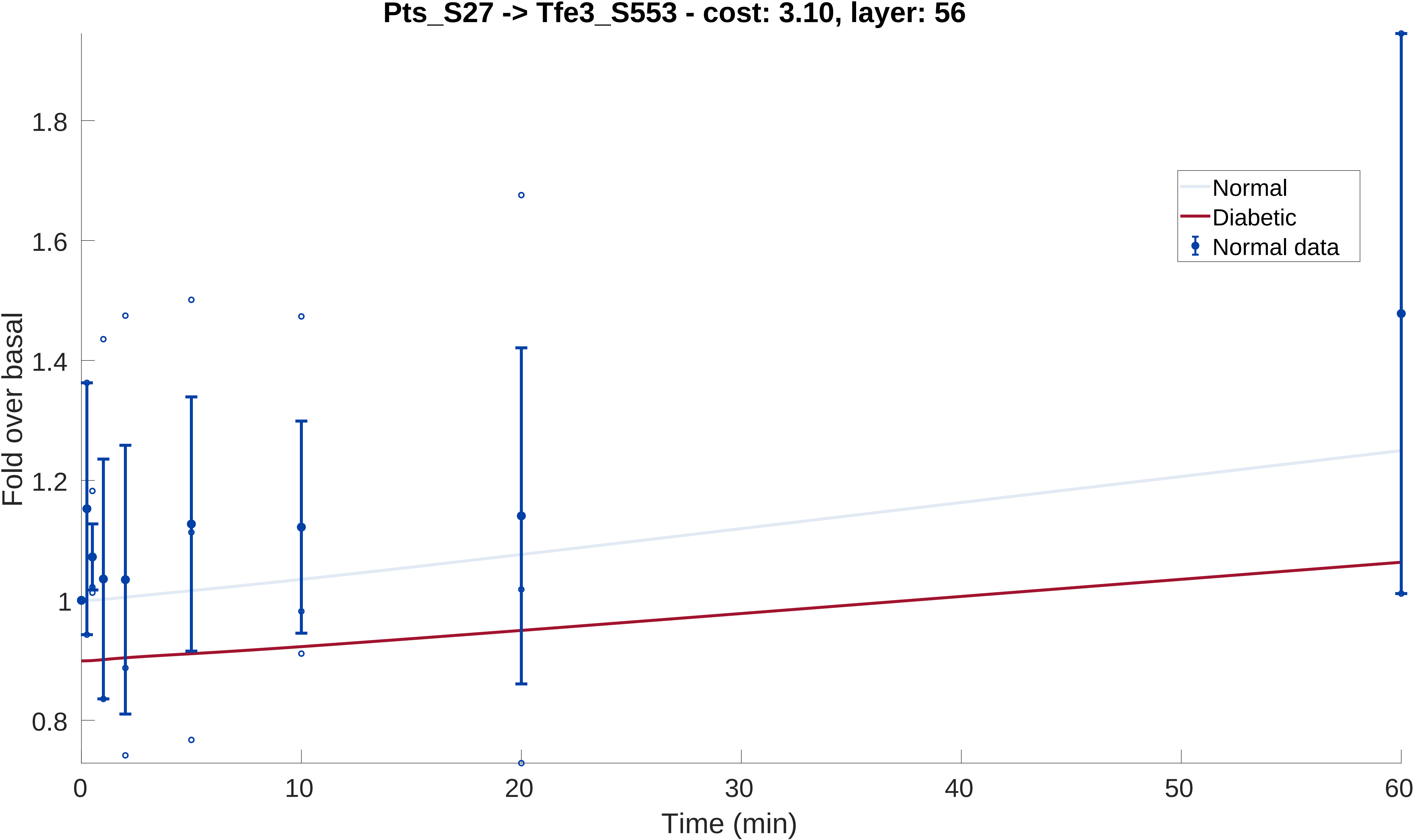

Pts_S27 -> Tfe3_S553 - cost: 3.10, layer: 56
Fold over basal
Time (min)
Normal
Diabetic
Normal data
0.8
1
1.2
1.4
1.6
1.8
0
10
20
30
40
50
60

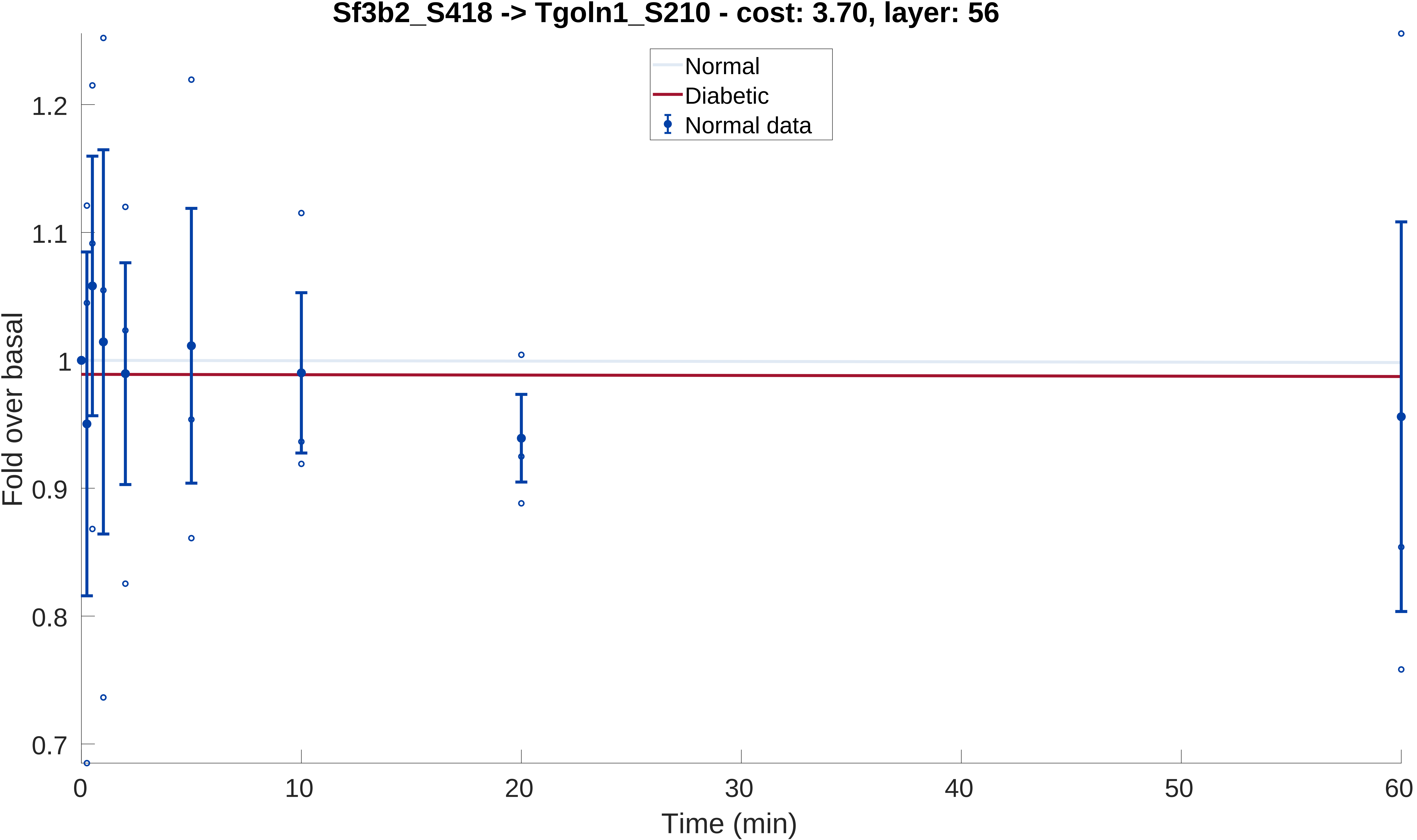

Sf3b2_S418 -> Tgoln1_S210 - cost: 3.70, layer: 56
Normal
Diabetic
Normal data
Fold over basal
1.2
1.1
1
0.9
0.8
0.7
0
10
20
30
40
50
60
Time (min)

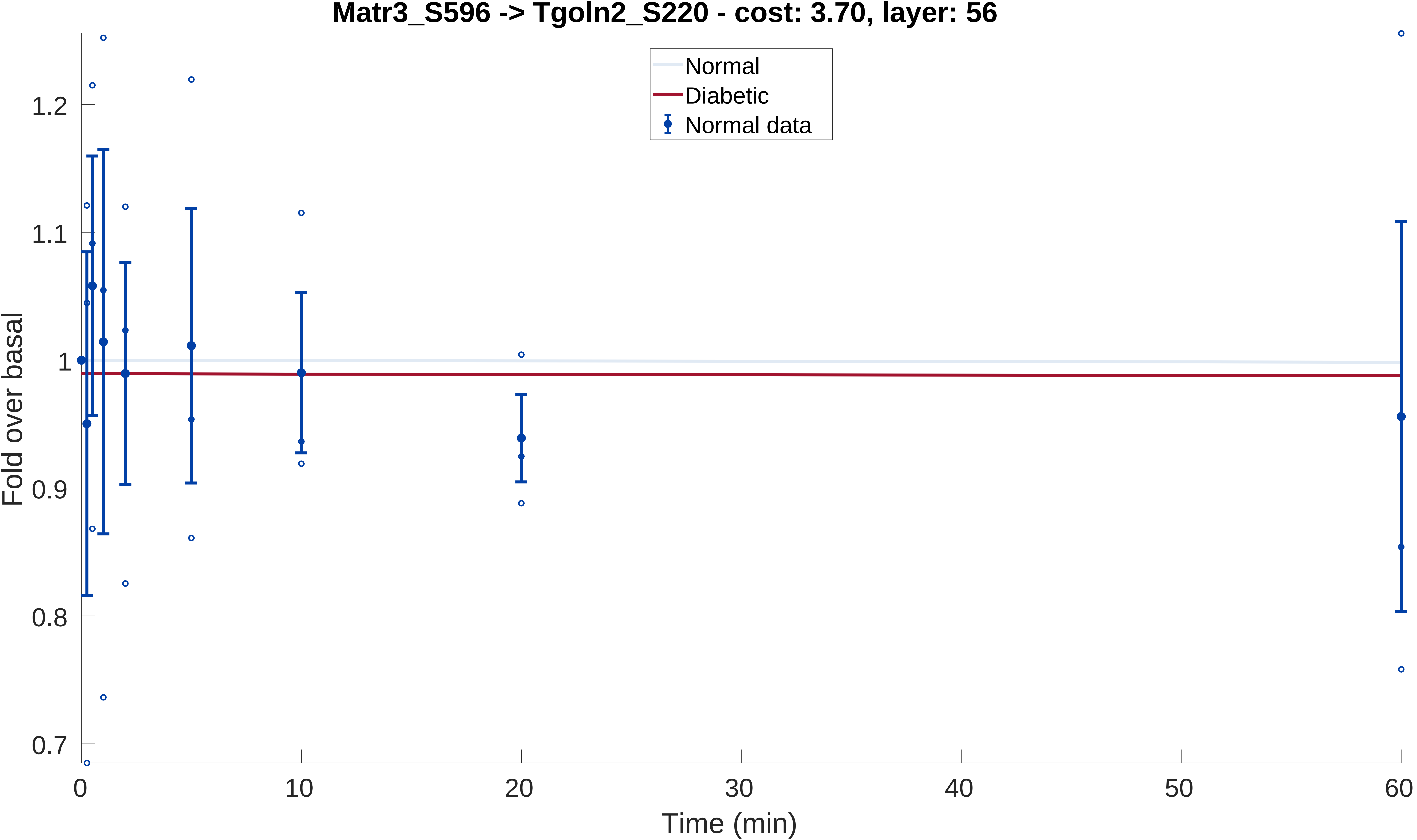

Matr3_S596 -> Tgoln2_S220 - cost: 3.70, layer: 56
Normal
Diabetic
Normal data
Fold over basal
1.2
1.1
1
0.9
0.8
0.7
0
10
20
30
40
50
60
Time (min)

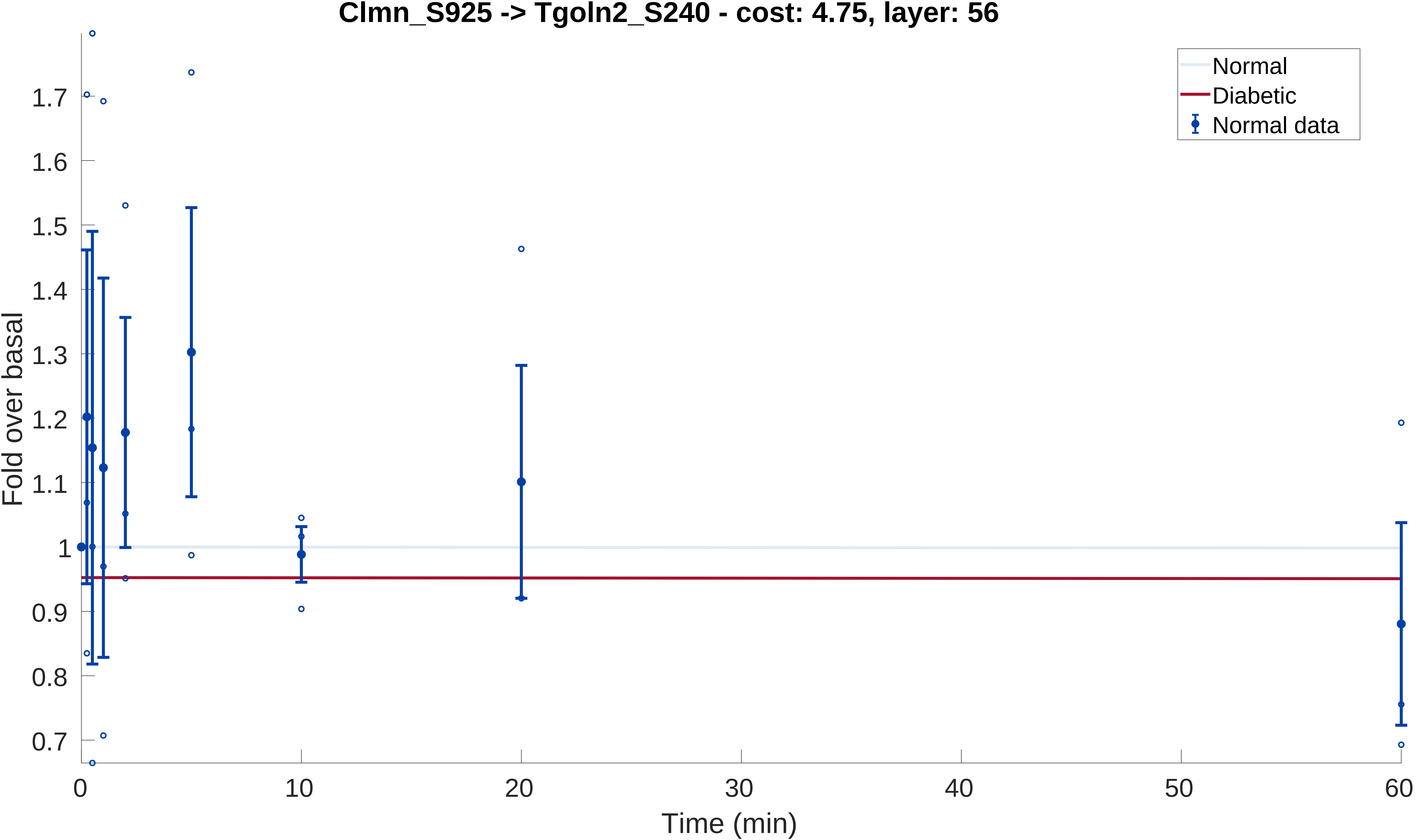

Clmn_S925 -> Tgoln2_S240 - cost: 4.75, layer: 56
Normal
Diabetic
Normal data
Fold over basal
Time (min)
1.7
1.6
1.5
1.4
1.3
1.2
1.1
1
0.9
0.8
0.7
0
10
20
30
40
50
60

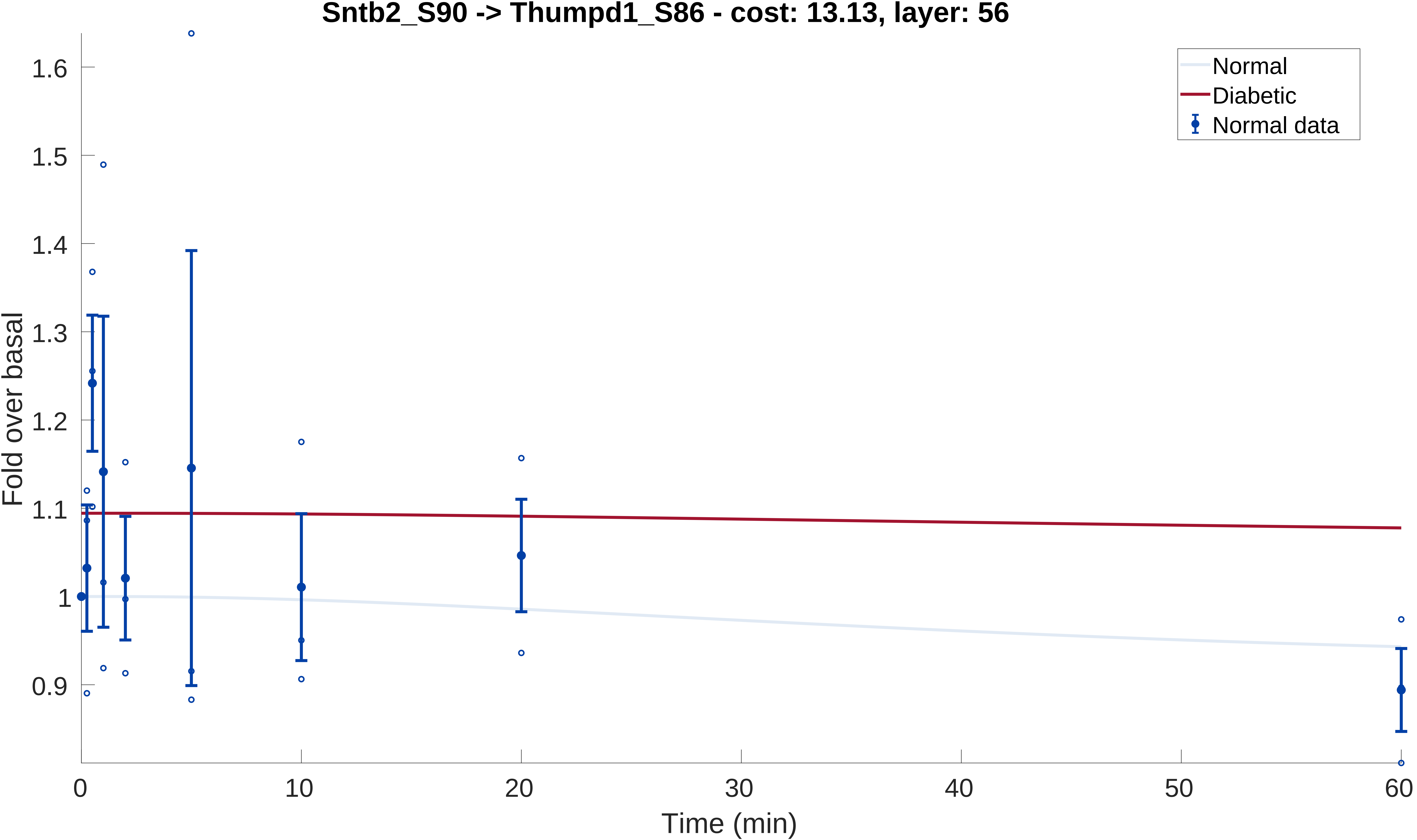

Sntb2_S90 -> Thumpd1_S86 - cost: 13.13, layer: 56
Normal
Diabetic
Normal data
Fold over basal
1.6
1.5
1.4
1.3
1.2
1.1
1
0.9
0
10
20
30
40
50
60
Time (min)

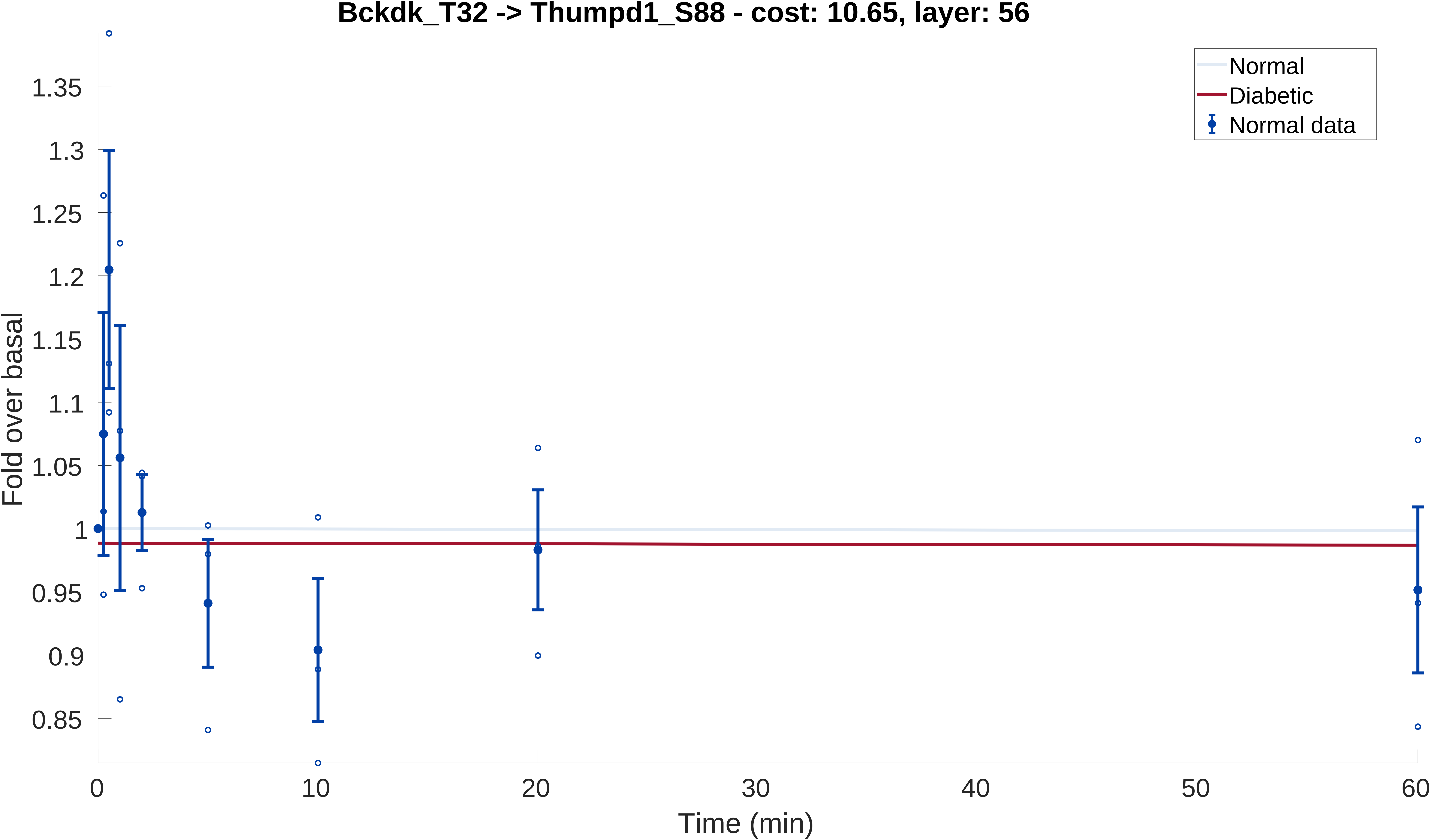

Bckdk_T32 -> Thumpd1_S88 - cost: 10.65, layer: 56
Normal
Diabetic
Normal data
Fold over basal
Time (min)
1.35
1.3
1.25
1.2
1.15
1.1
1.05
1
0.95
0.9
0.85
0
10
20
30
40
50
60

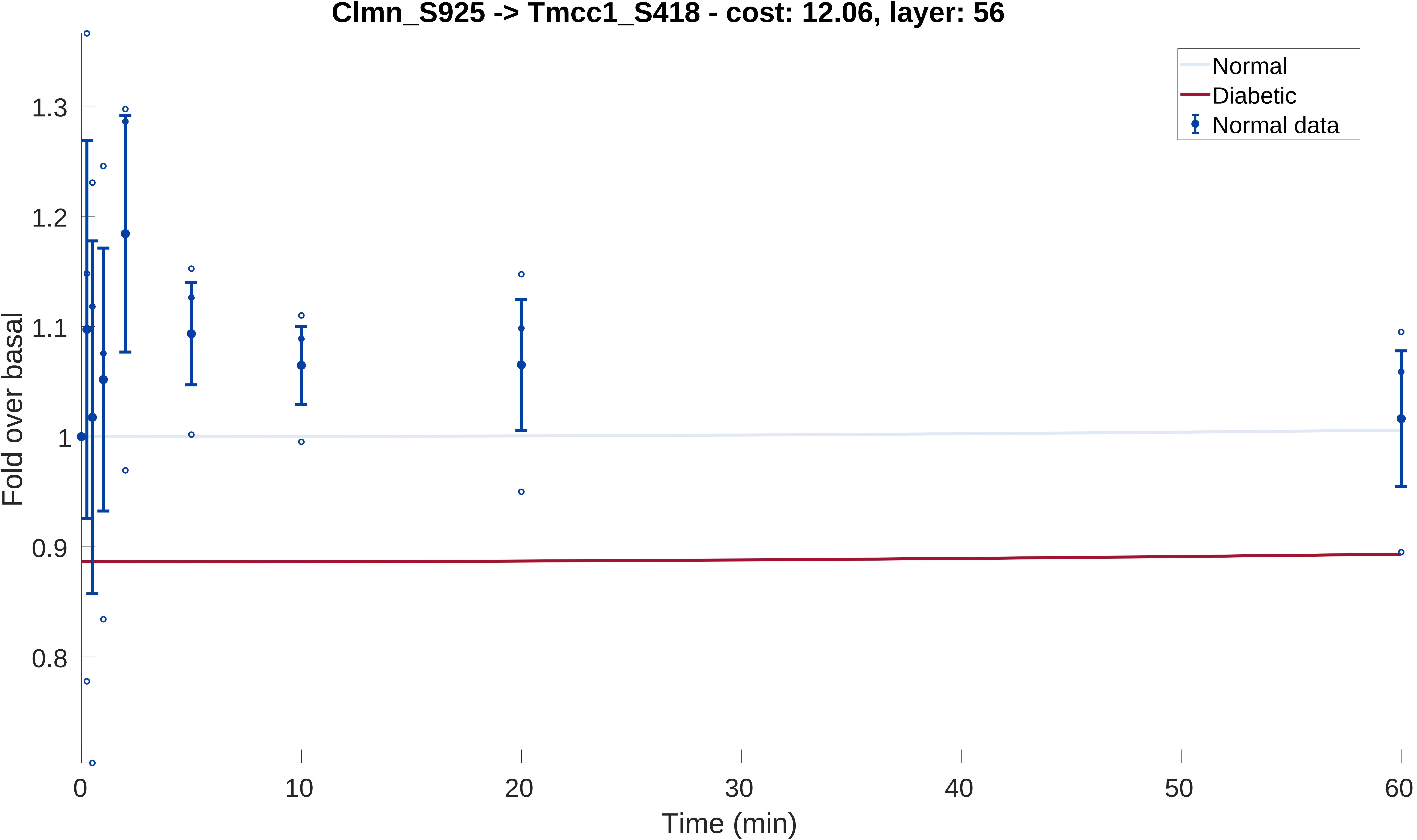

Clmn_S925 -> Tmcc1_S418 - cost: 12.06, layer: 56
Normal
Diabetic
Normal data
Fold over basal
1.3
1.2
1.1
1
0.9
0.8
0
10
20
30
40
50
60
Time (min)

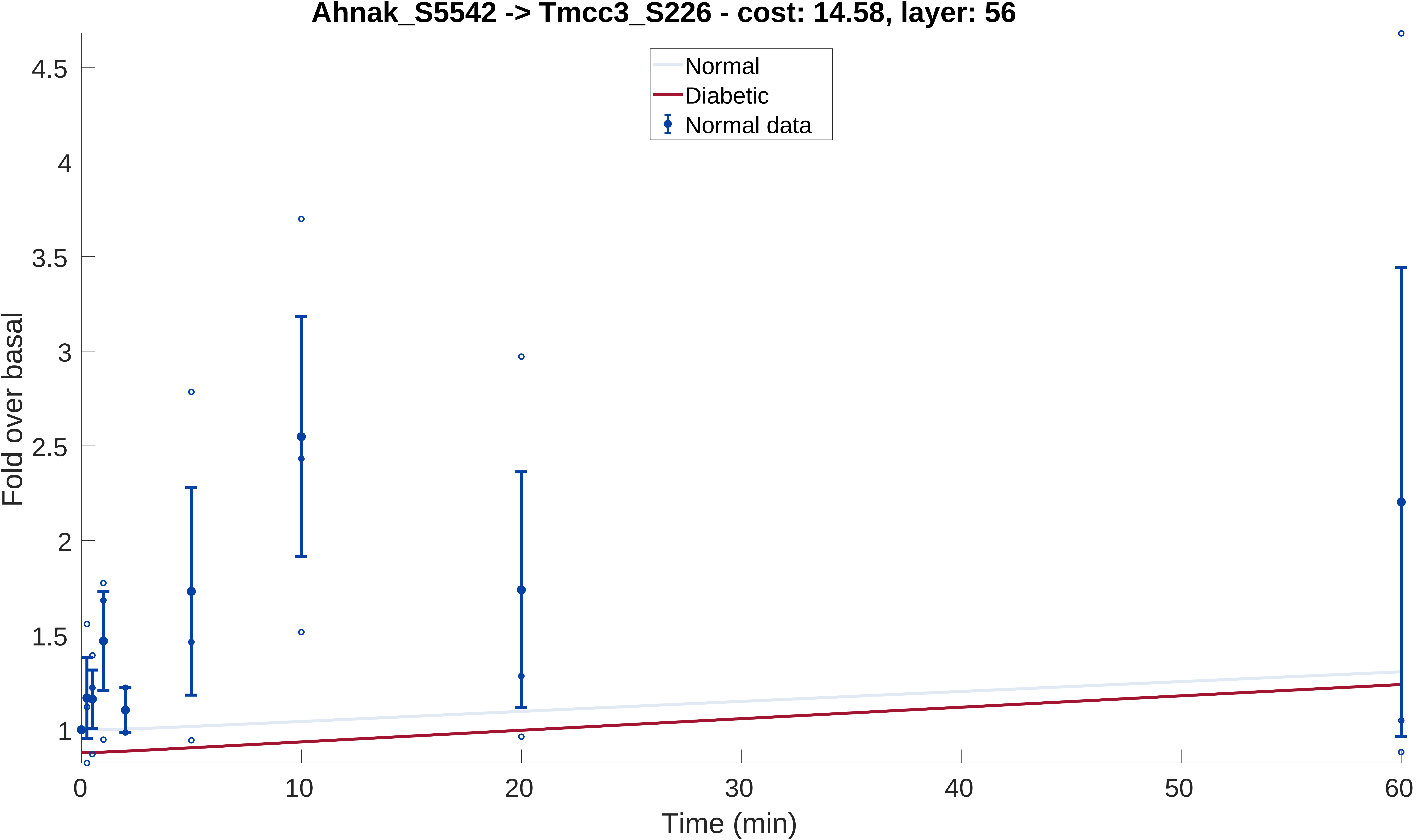

Ahnak_S5542 -> Tmcc3_S226 - cost: 14.58, layer: 56
Normal
Diabetic
Normal data
Fold over basal
Time (min)
4.5
4
3.5
3
2.5
2
1.5
1
0
10
20
30
40
50
60

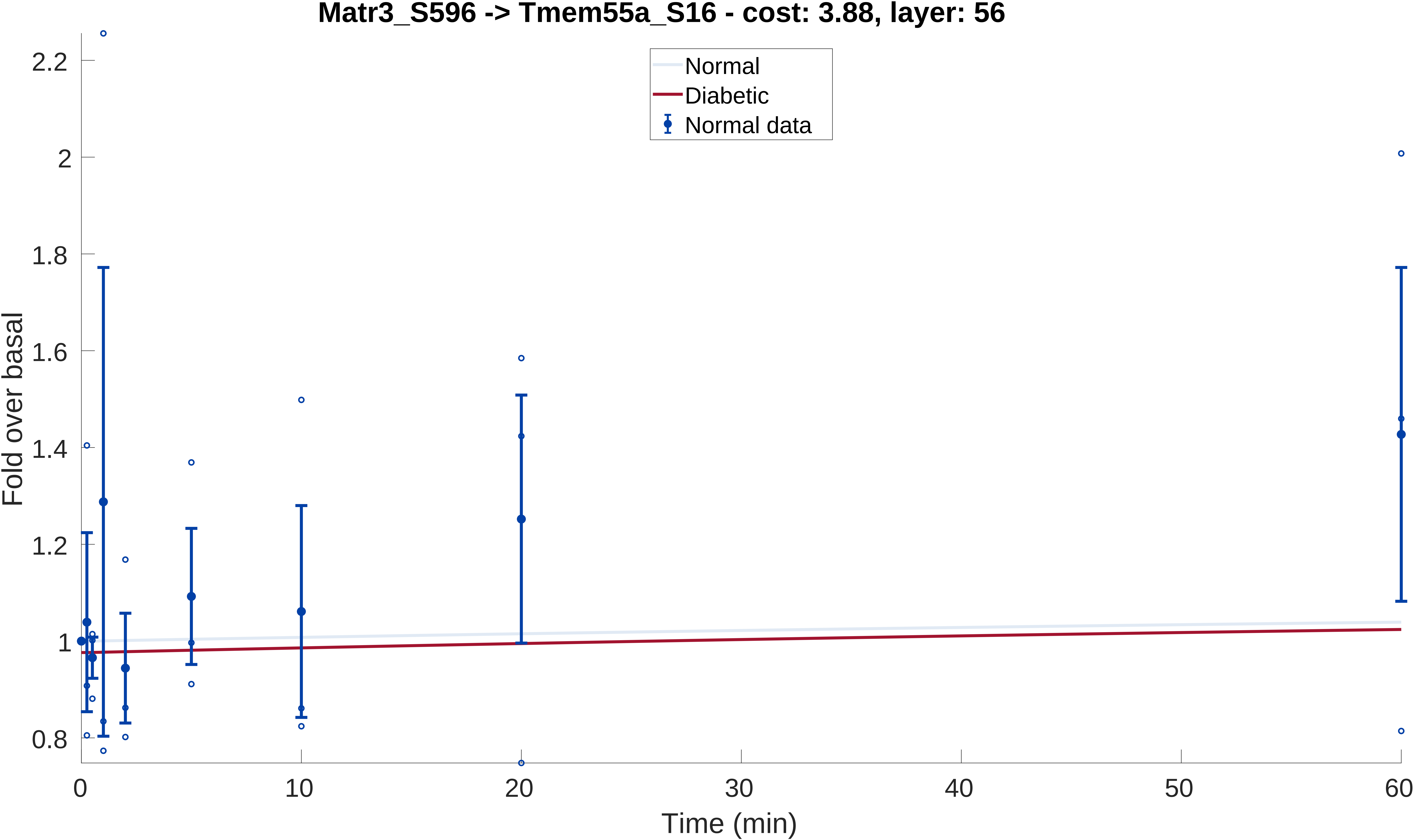

Matr3_S596 -> Tmem55a_S16 - cost: 3.88, layer: 56
Normal
Diabetic
Normal data
Fold over basal
Time (min)
0.8
1
1.2
1.4
1.6
1.8
2
2.2
0
10
20
30
40
50
60

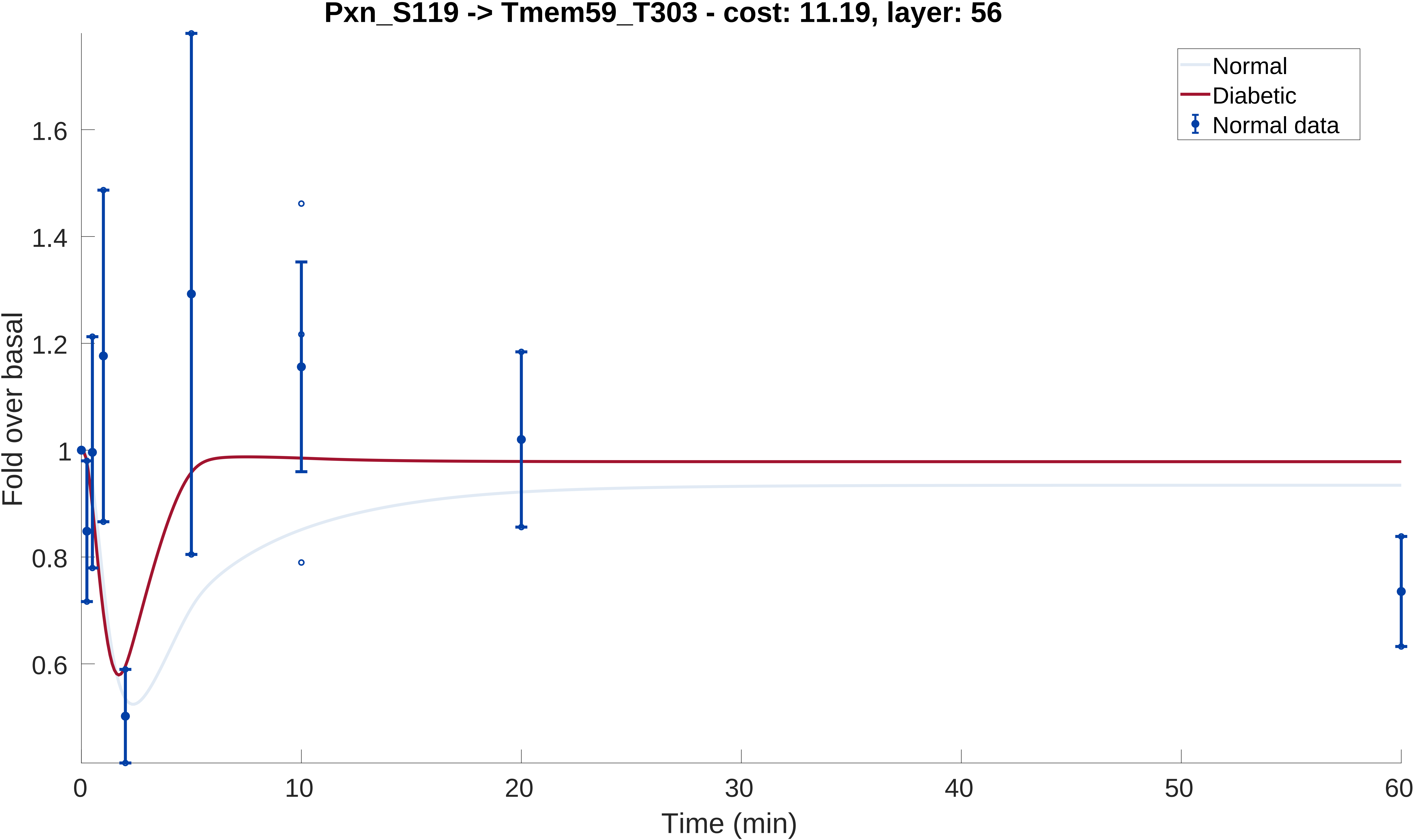
Pxn_S119 -> Tmem59_T303 - cost: 11.19, layer: 56
Normal
Diabetic
Normal data
Fold over basal
1.6
1.4
1.2
1
0.8
0.6
0
10
20
30
40
50
60
Time (min)

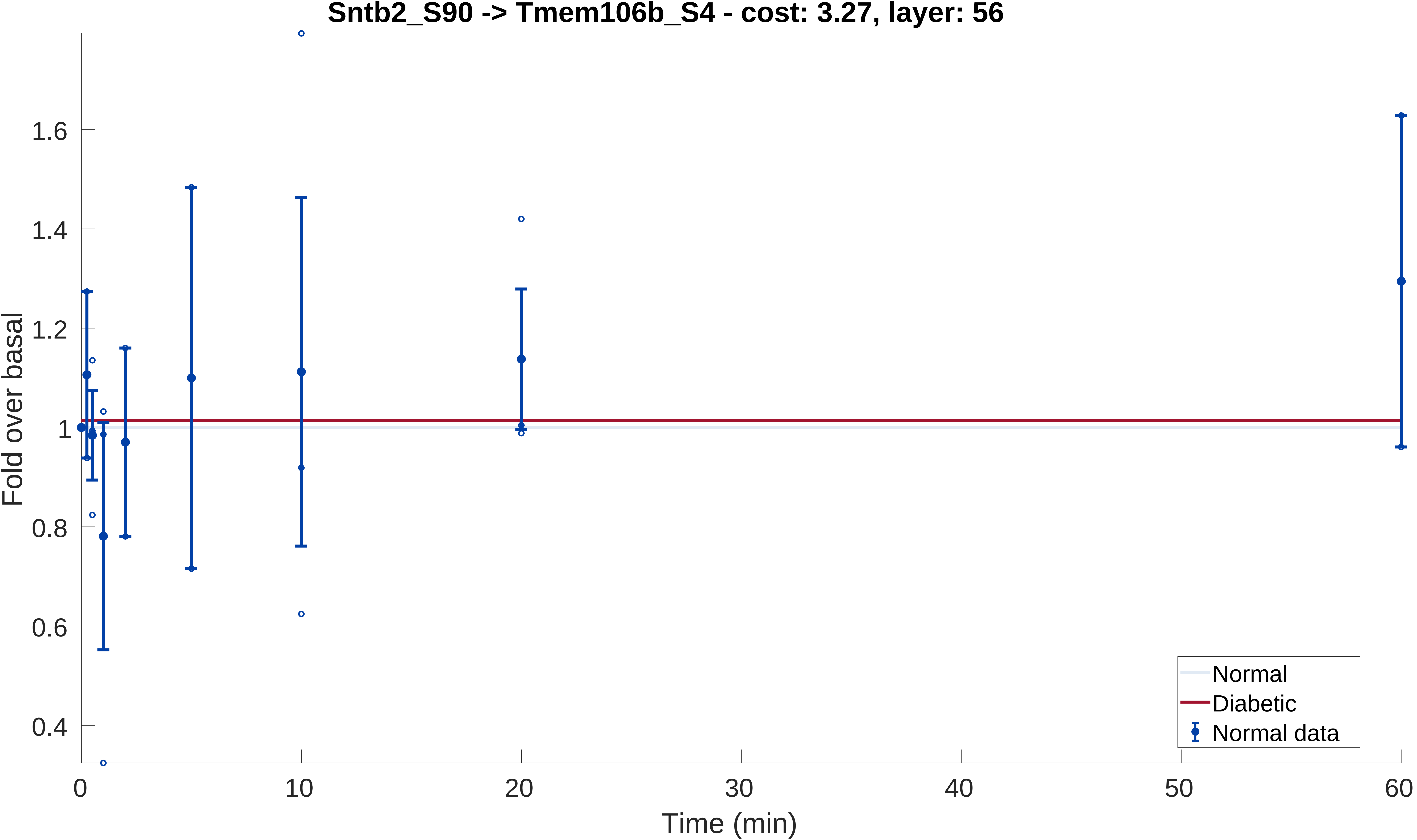

Sntb2_S90 -> Tmem106b_S4 - cost: 3.27, layer: 56
Fold over basal
1.6
1.4
1.2
1
0.8
0.6
0.4
0
10
20
30
40
50
60
Time (min)
Normal
Diabetic
Normal data

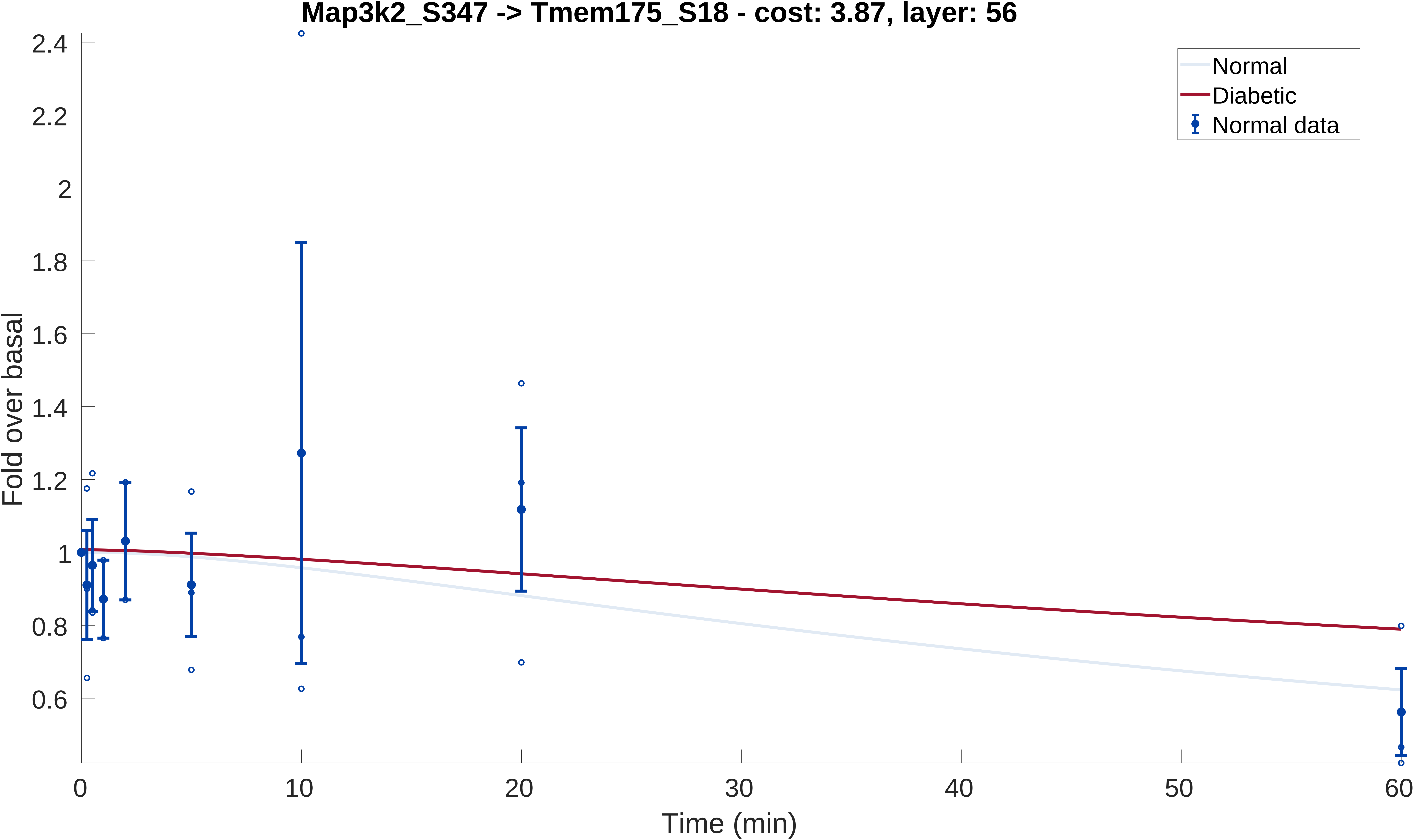
Map3k2_S347 -> Tmem175_S18 - cost: 3.87, layer: 56
Normal
Diabetic
Normal data
Fold over basal
Time (min)
2.4
2.2
2
1.8
1.6
1.4
1.2
1
0.8
0.6
0
10
20
30
40
50
60

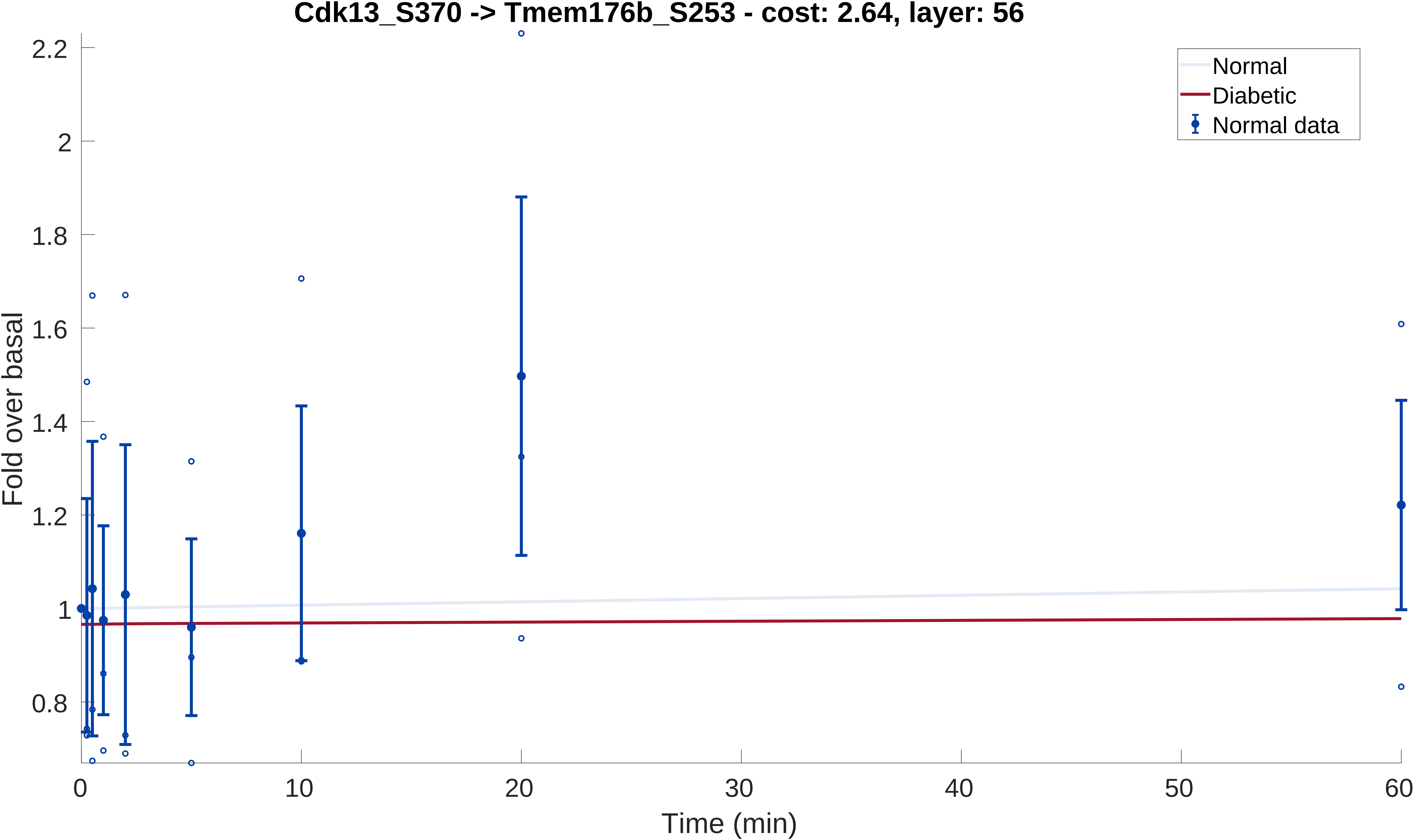
Cdk13_S370 -> Tmem176b_S253 - cost: 2.64, layer: 56
Normal
Diabetic
Normal data
Fold over basal
2.2
2
1.8
1.6
1.4
1.2
1
0.8
0
10
20
30
40
50
60
Time (min)

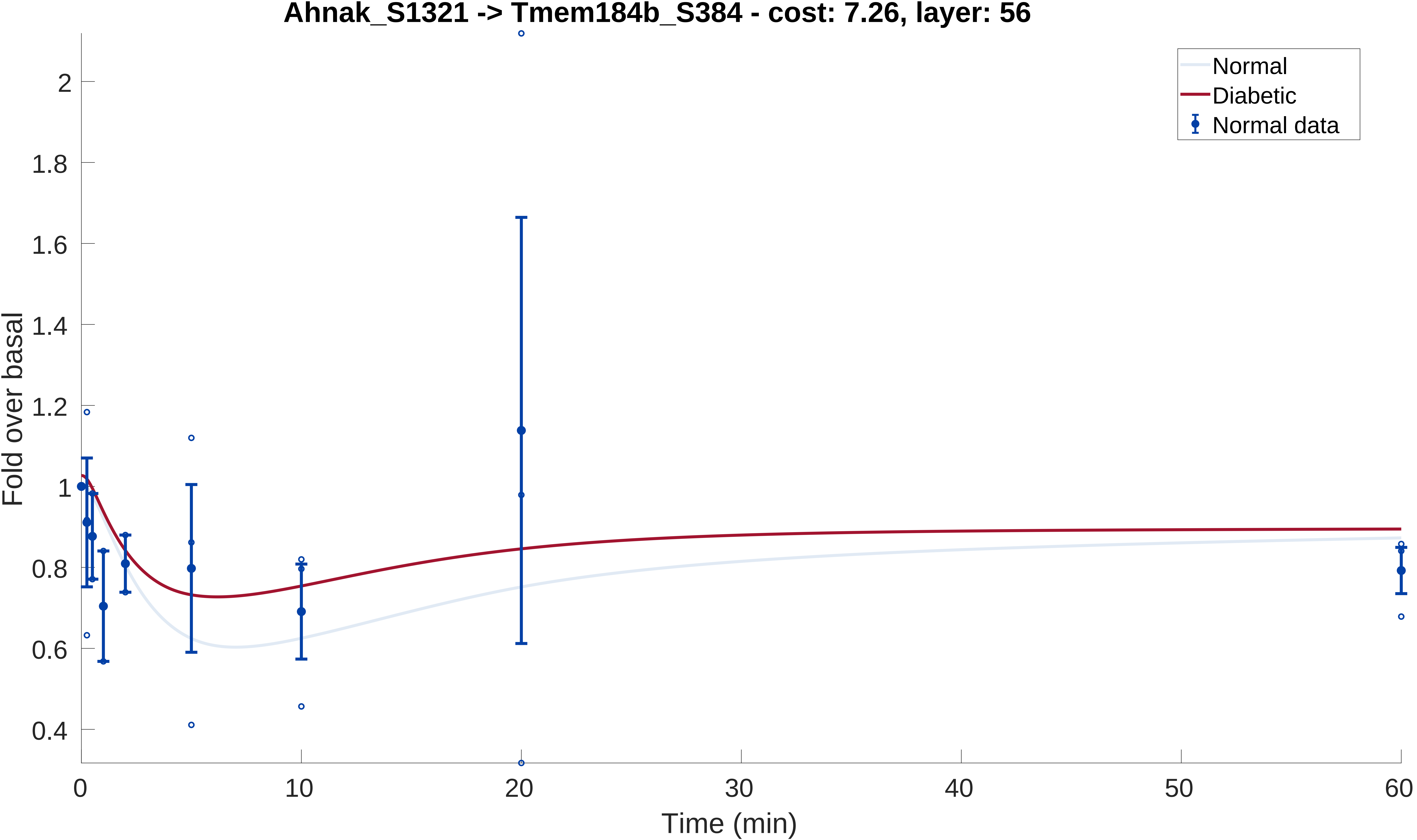

Ahnak_S1321 -> Tmem184b_S384 - cost: 7.26, layer: 56
Normal
Diabetic
Normal data
Fold over basal
Time (min)
0.4
0.6
0.8
1
1.2
1.4
1.6
1.8
2
0
10
20
30
40
50
60

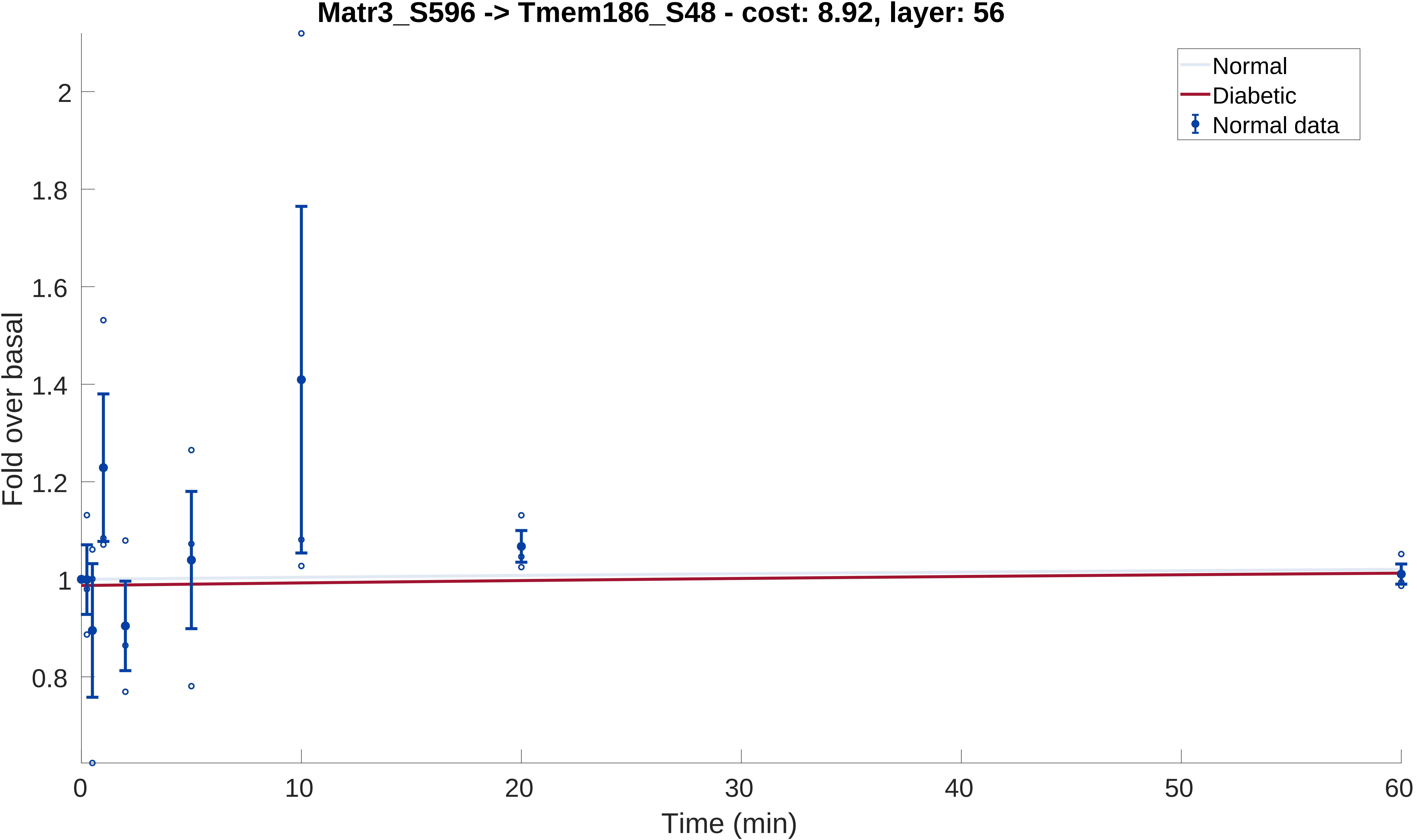
Matr3_S596 -> Tmem186_S48 - cost: 8.92, layer: 56
Normal
Diabetic
Normal data
Fold over basal
Time (min)
0.8
1
1.2
1.4
1.6
1.8
2
0
10
20
30
40
50
60

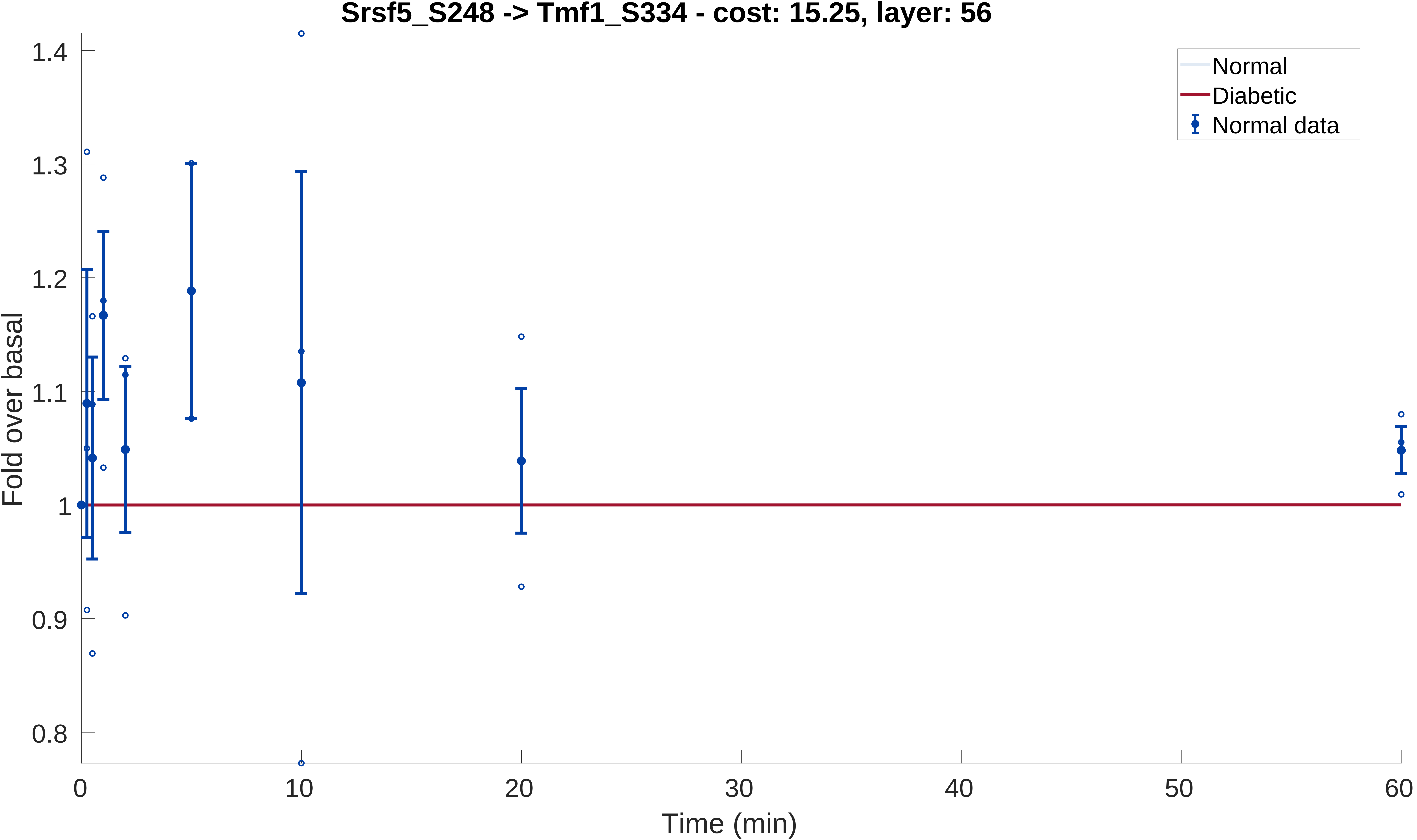
Srsf5_S248 -> Tmf1_S334 - cost: 15.25, layer: 56
Normal
Diabetic
Normal data
Fold over basal
1.4
1.3
1.2
1.1
1
0.9
0.8
0
10
20
30
40
50
60
Time (min)

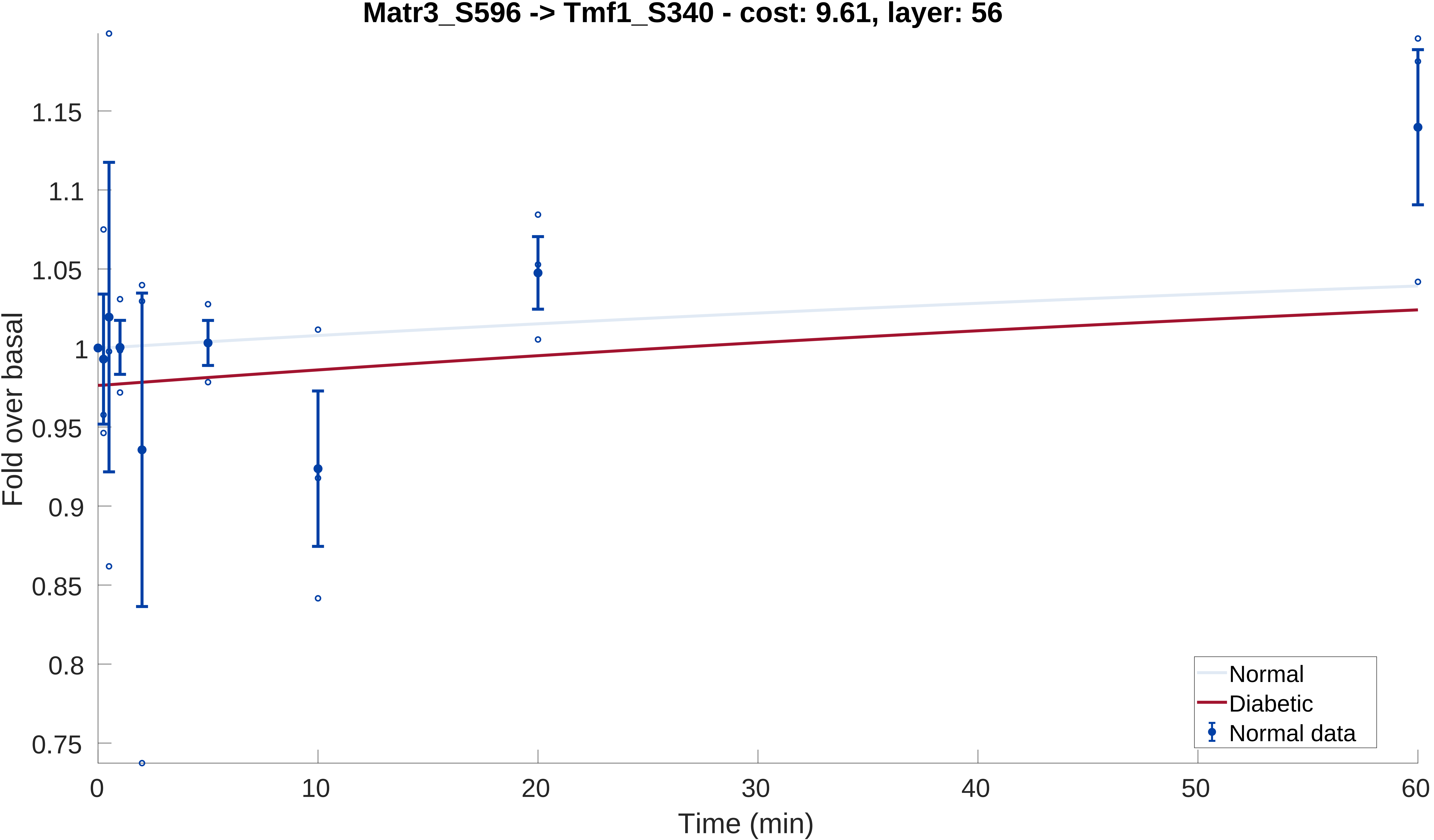

Matr3_S596 -> Tmf1_S340 - cost: 9.61, layer: 56
Fold over basal
1.15
1.1
1.05
1
0.95
0.9
0.85
0.8
0.75
0
10
20
30
40
50
60
Time (min)
Normal
Diabetic
Normal data

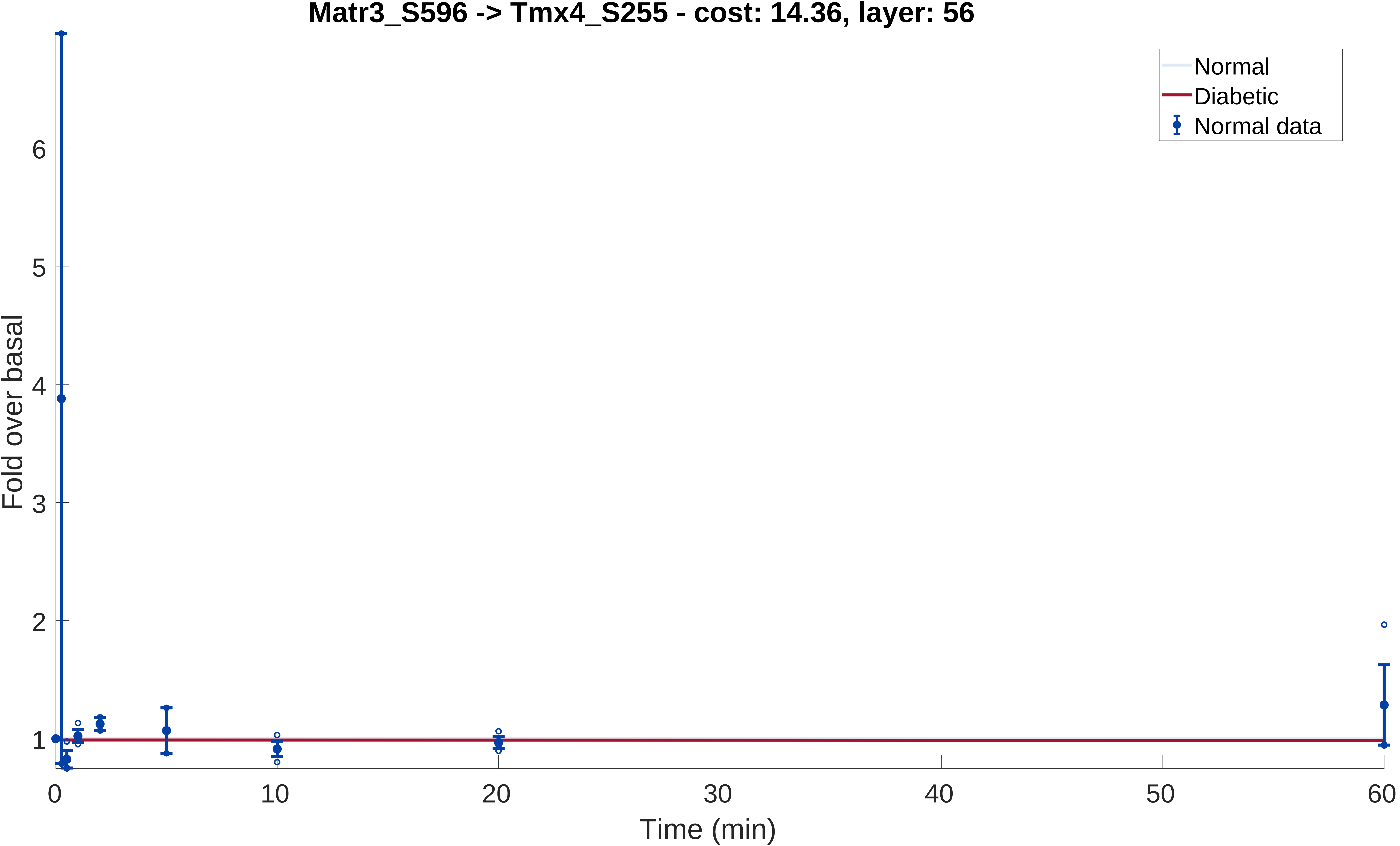

Matr3_S596 -> Tmx4_S255 - cost: 14.36, layer: 56
Normal
Diabetic
Normal data
Fold over basal
1
2
3
4
5
6
0
10
20
30
40
50
60
Time (min)

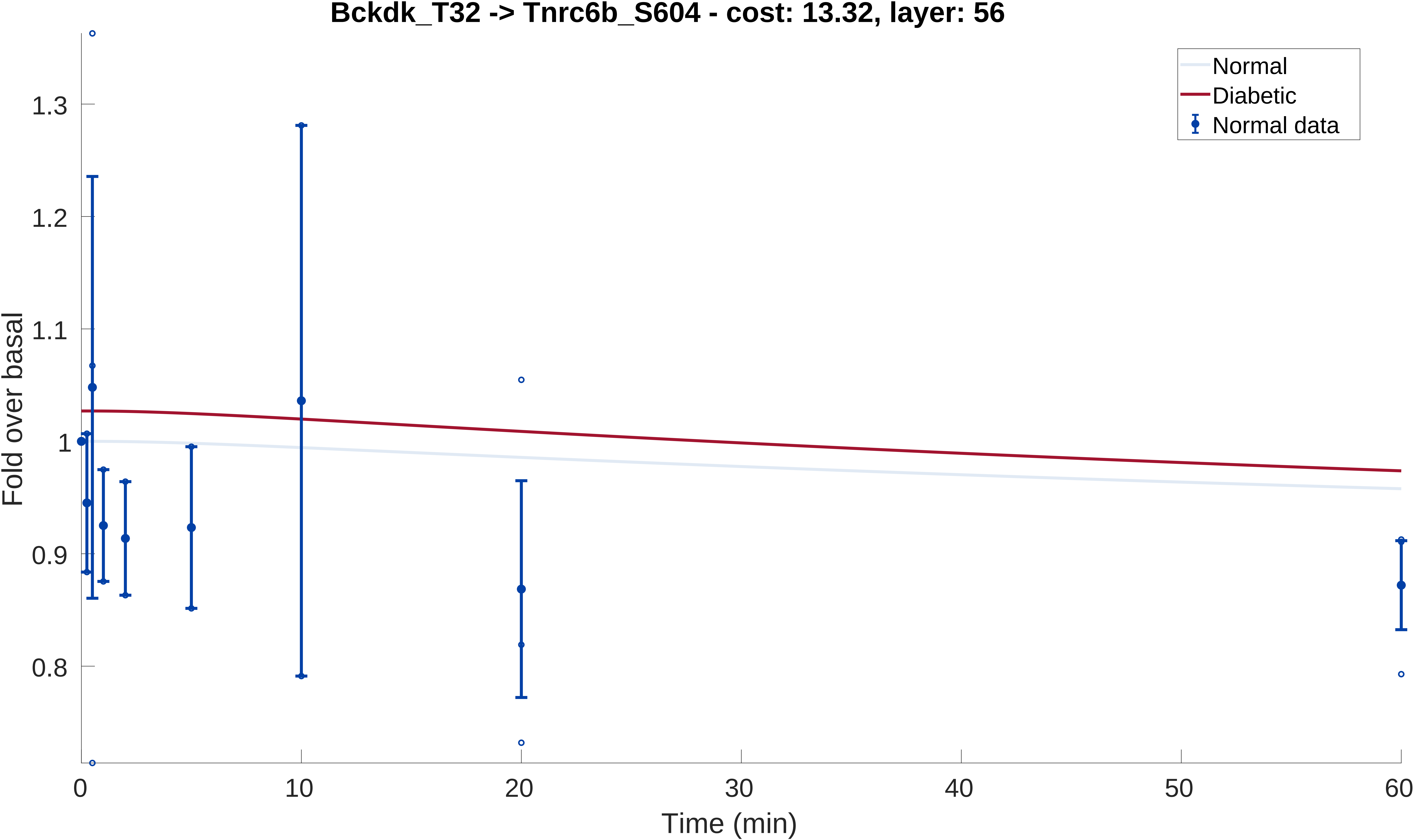
Bckdk_T32 -> Tnrc6b_S604 - cost: 13.32, layer: 56
Normal
Diabetic
Normal data
Fold over basal
1.3
1.2
1.1
1
0.9
0.8
0
10
20
30
40
50
60
Time (min)

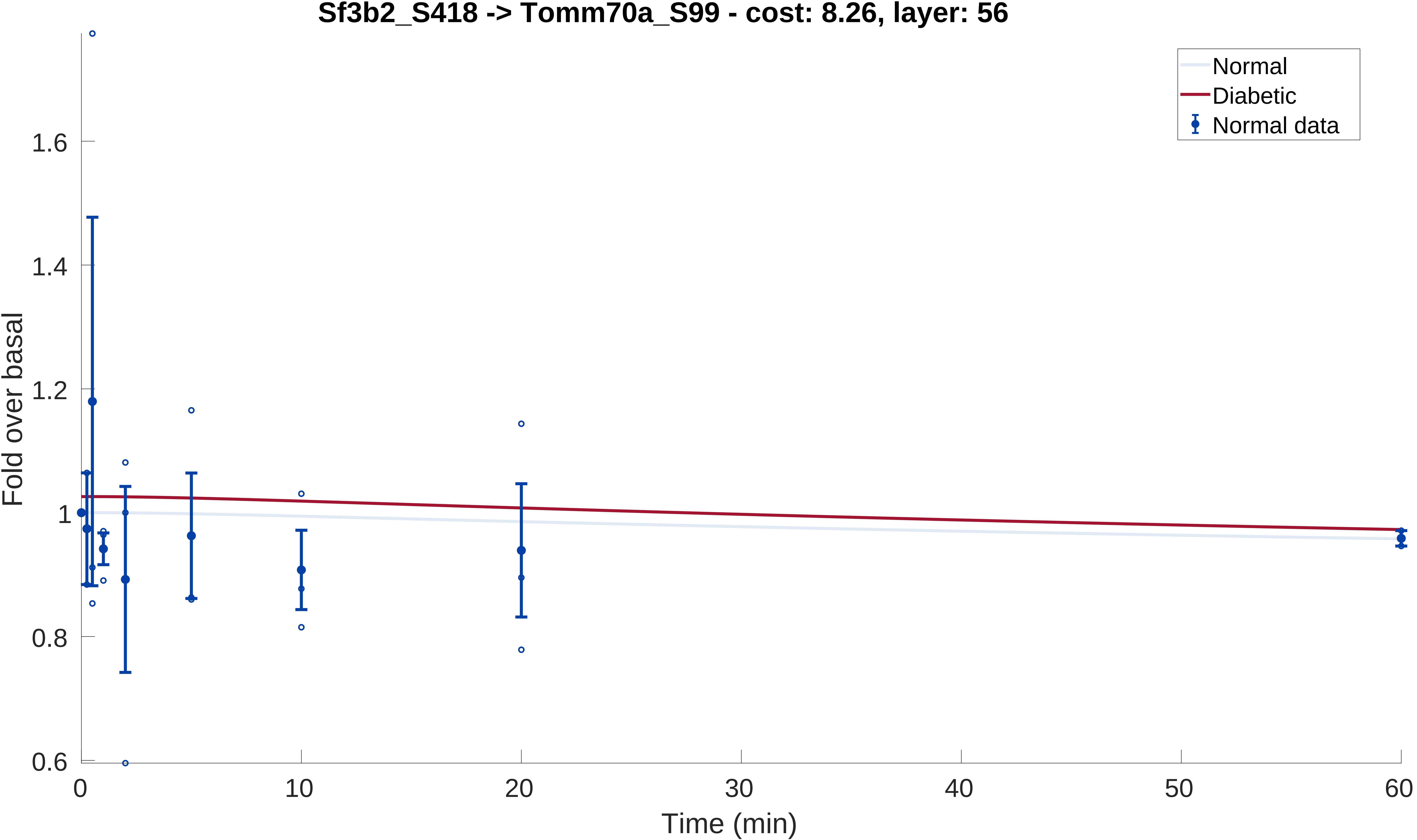

Sf3b2_S418 -> Tomm70a_S99 - cost: 8.26, layer: 56
Normal
Diabetic
Normal data
Fold over basal
Time (min)
0.6
0.8
1
1.2
1.4
1.6
0
10
20
30
40
50
60

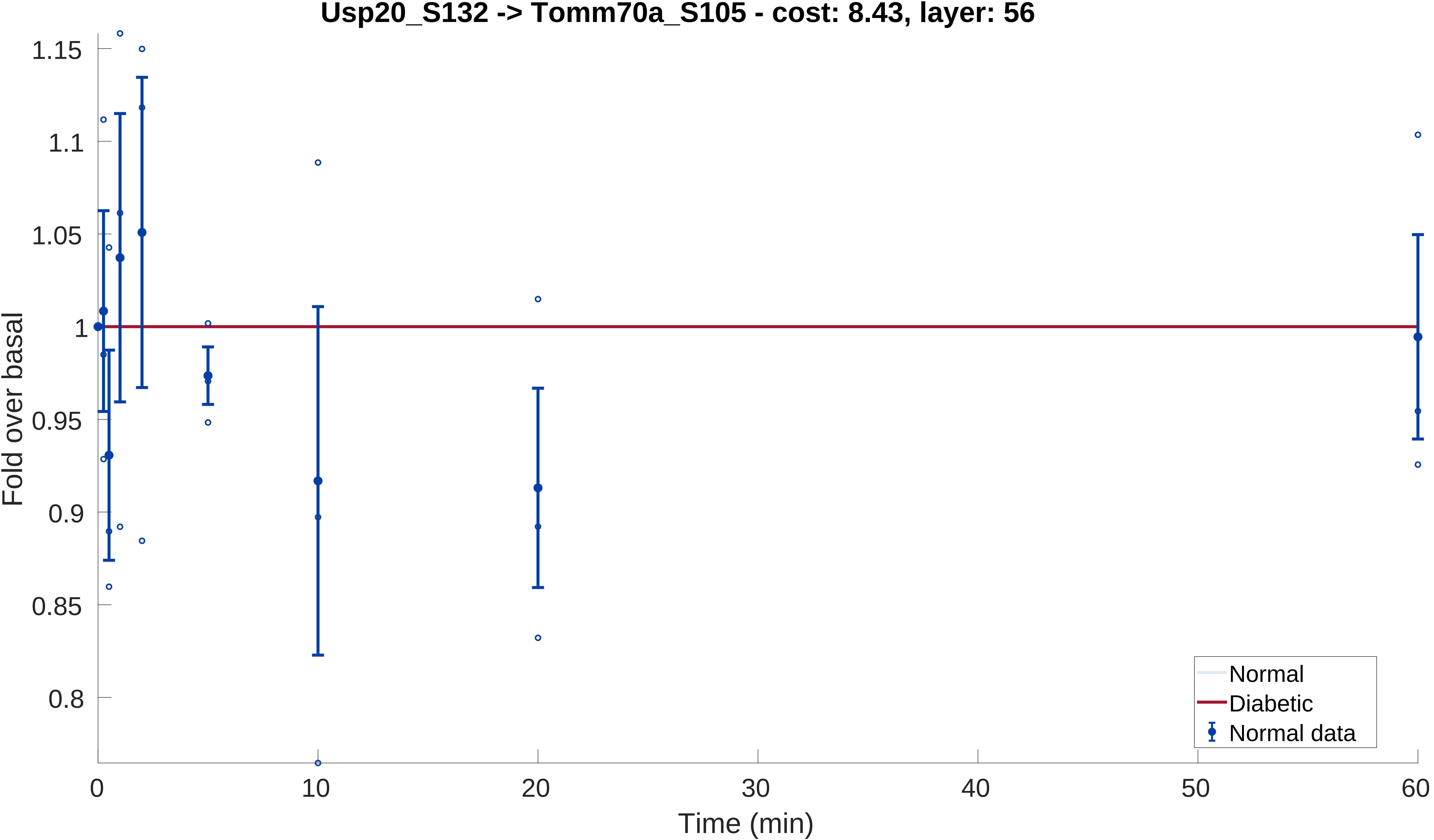

Usp20_S132 -> Tomm70a_S105 - cost: 8.43, layer: 56
Fold over basal
1.15
1.1
1.05
1
0.95
0.9
0.85
0.8
0
10
20
30
40
50
60
Time (min)
Normal
Diabetic
Normal data

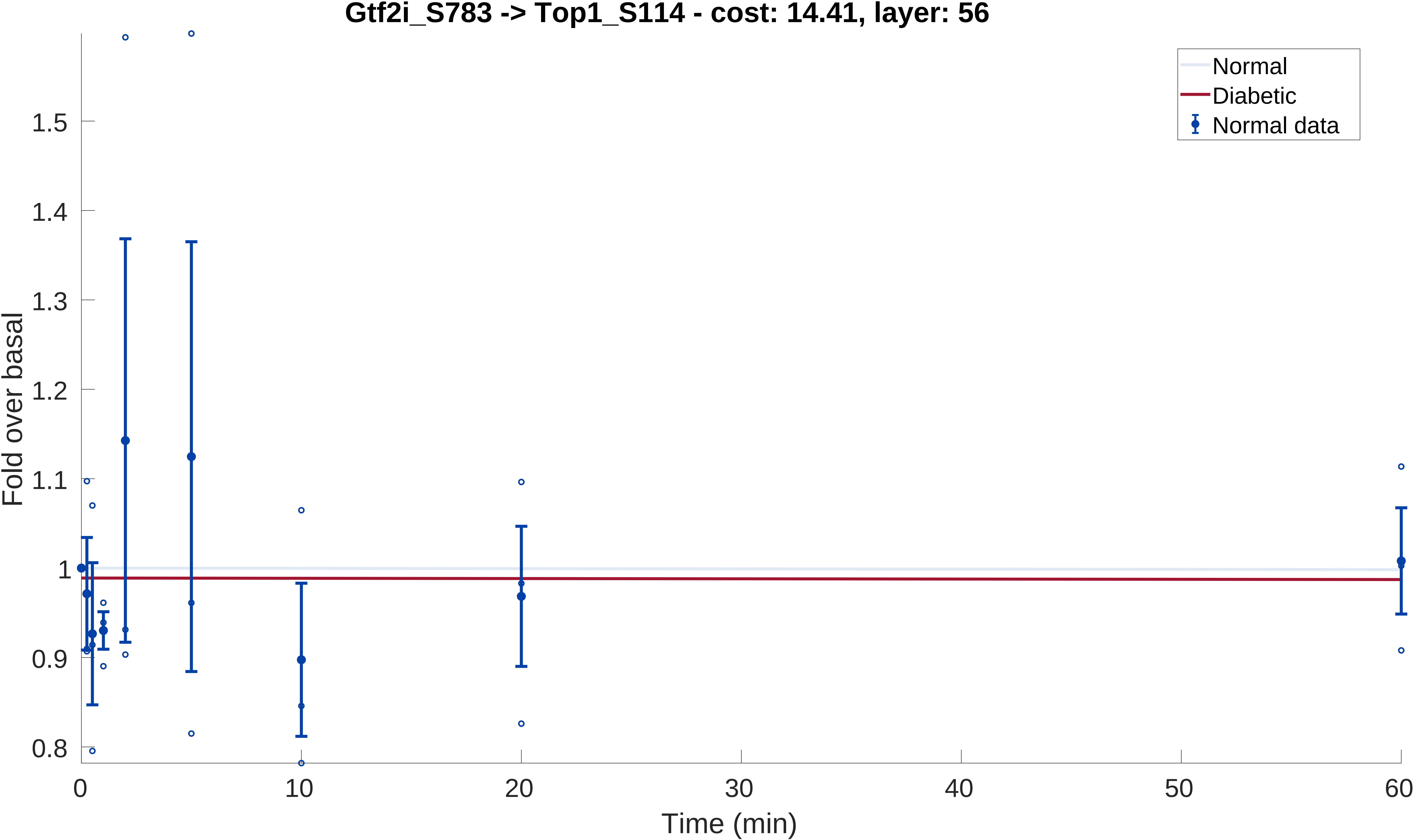

Gtf2i_S783 -> Top1_S114 - cost: 14.41, layer: 56
Normal
Diabetic
Normal data
Fold over basal
1.5
1.4
1.3
1.2
1.1
1
0.9
0.8
0
10
20
30
40
50
60
Time (min)

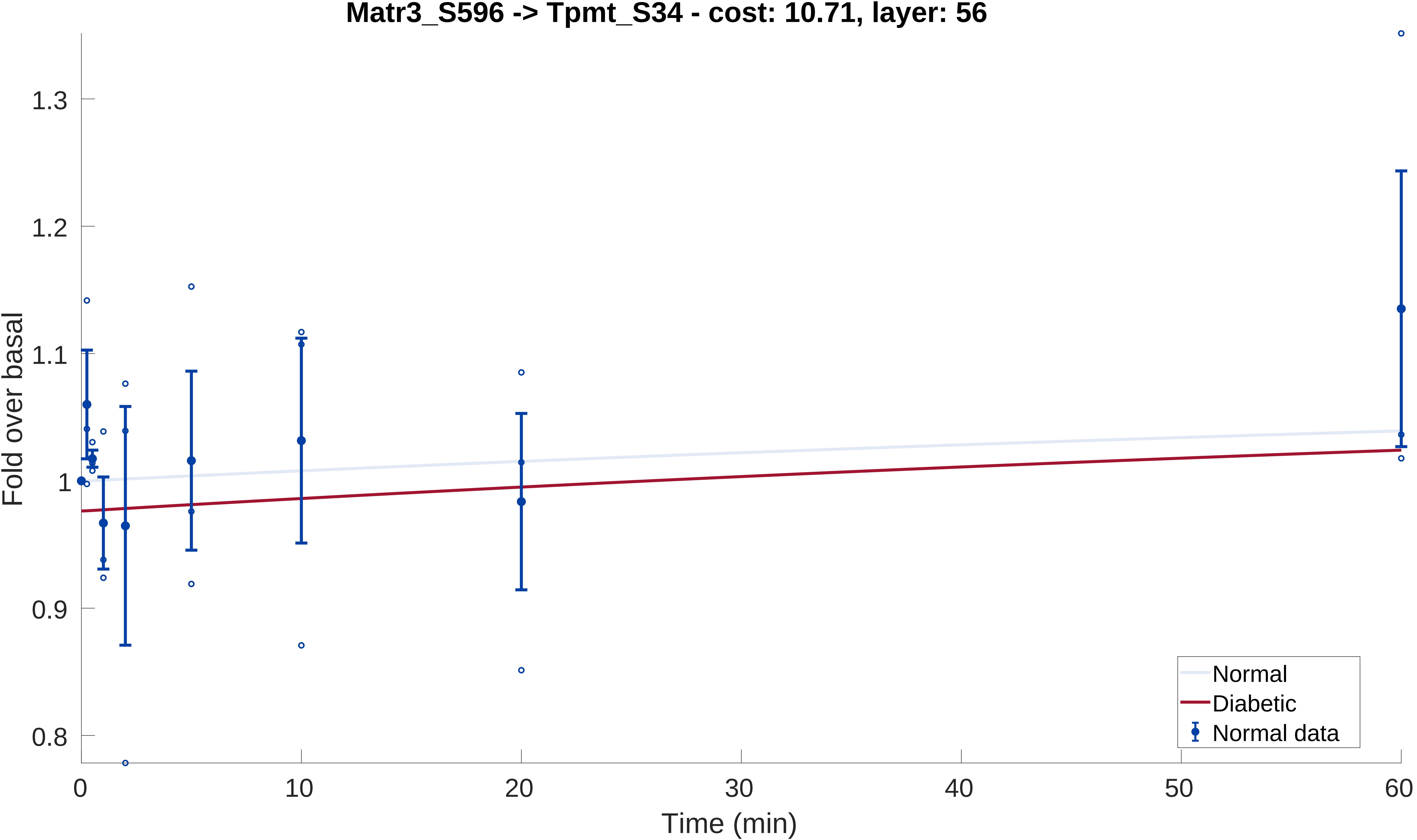
Matr3_S596 -> Tpmt_S34 - cost: 10.71, layer: 56
Fold over basal
1.3
1.2
1.1
1
0.9
0.8
0
10
20
30
40
50
60
Time (min)
Normal
Diabetic
Normal data

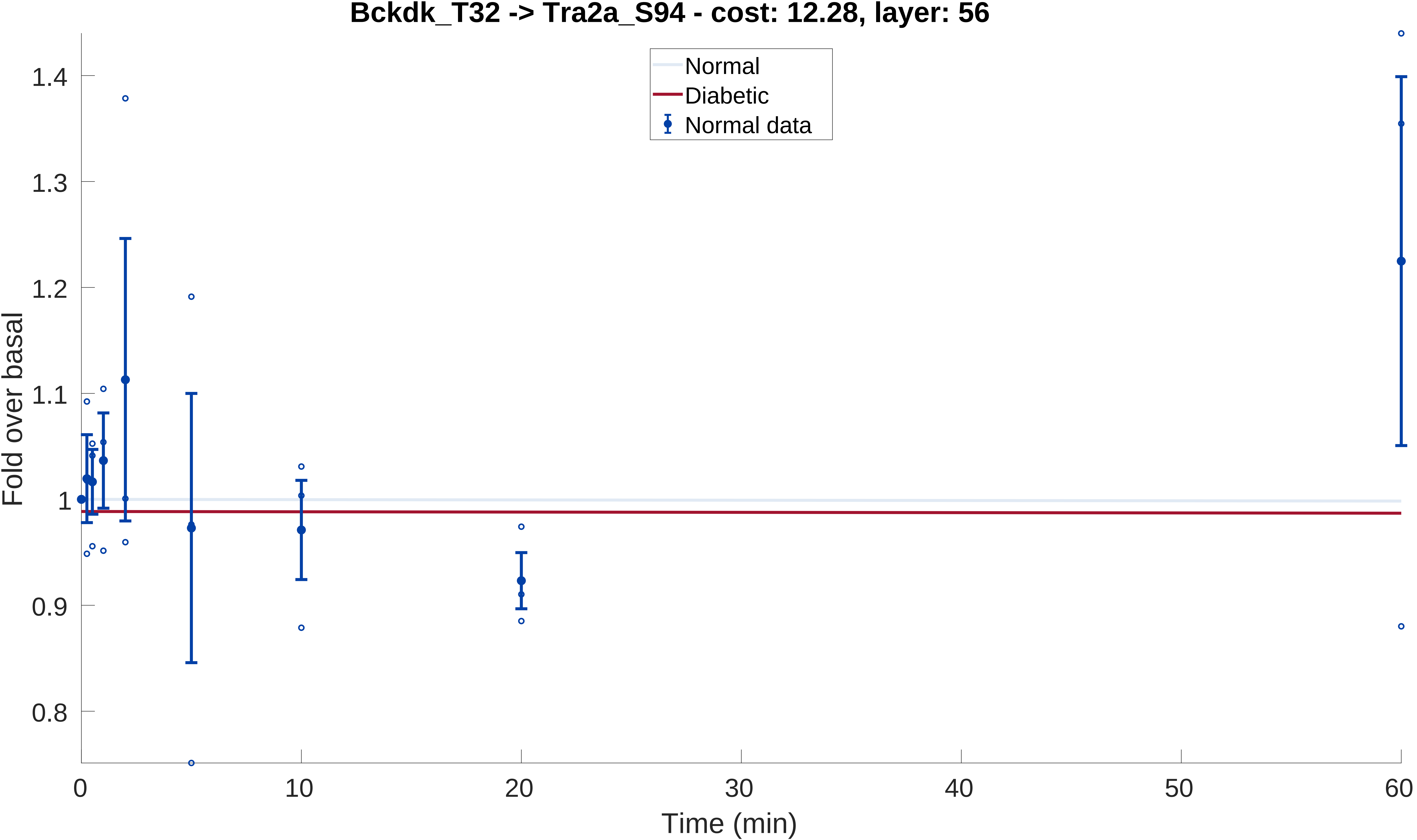

Bckdk_T32 -> Tra2a_S94 - cost: 12.28, layer: 56
Normal
Diabetic
Normal data
Fold over basal
1.4
1.3
1.2
1.1
1
0.9
0.8
0
10
20
30
40
50
60
Time (min)

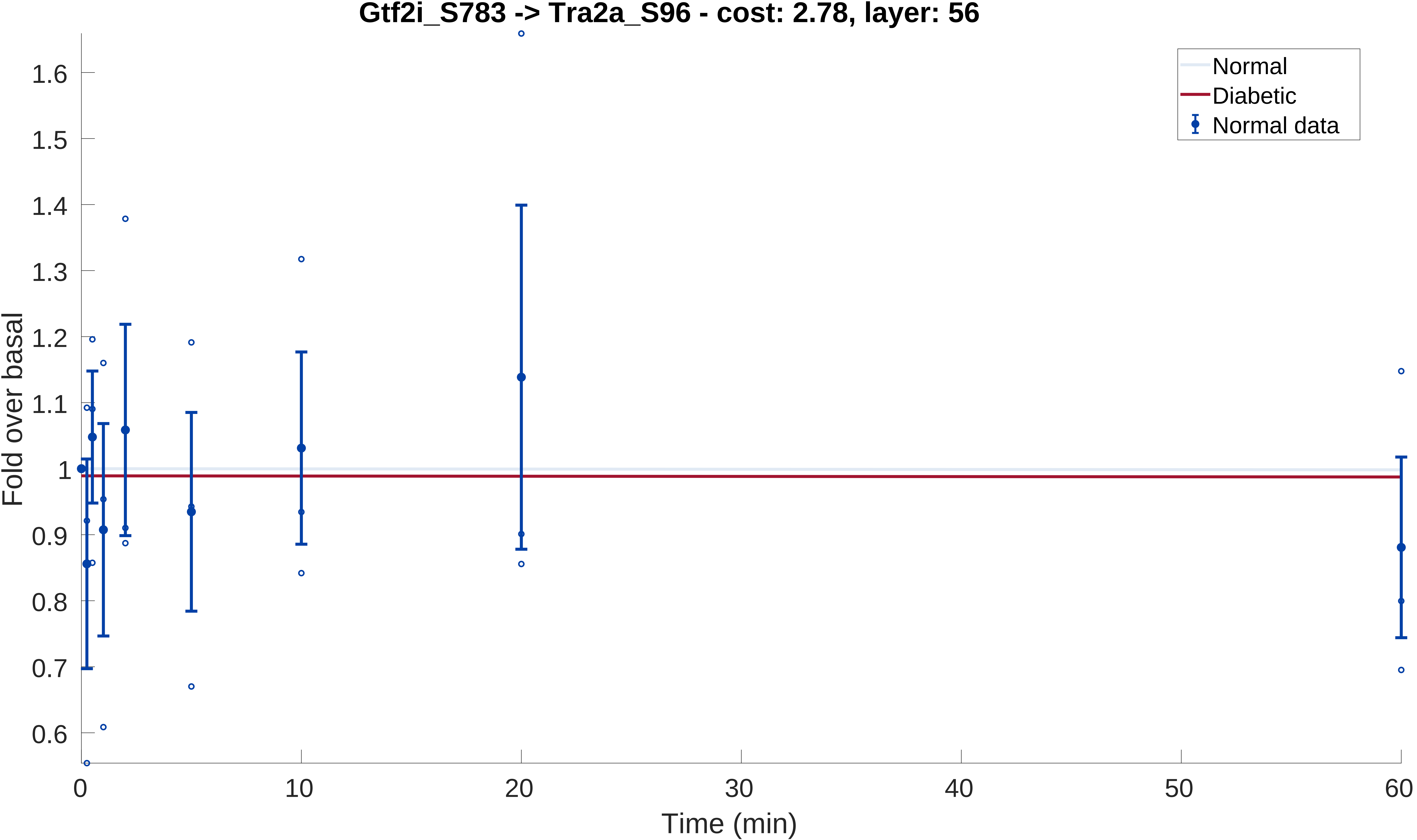

Gtf2i_S783 -> Tra2a_S96 - cost: 2.78, layer: 56
Normal
Diabetic
Normal data
Fold over basal
Time (min)
1.6
1.5
1.4
1.3
1.2
1.1
1
0.9
0.8
0.7
0.6
0
10
20
30
40
50
60

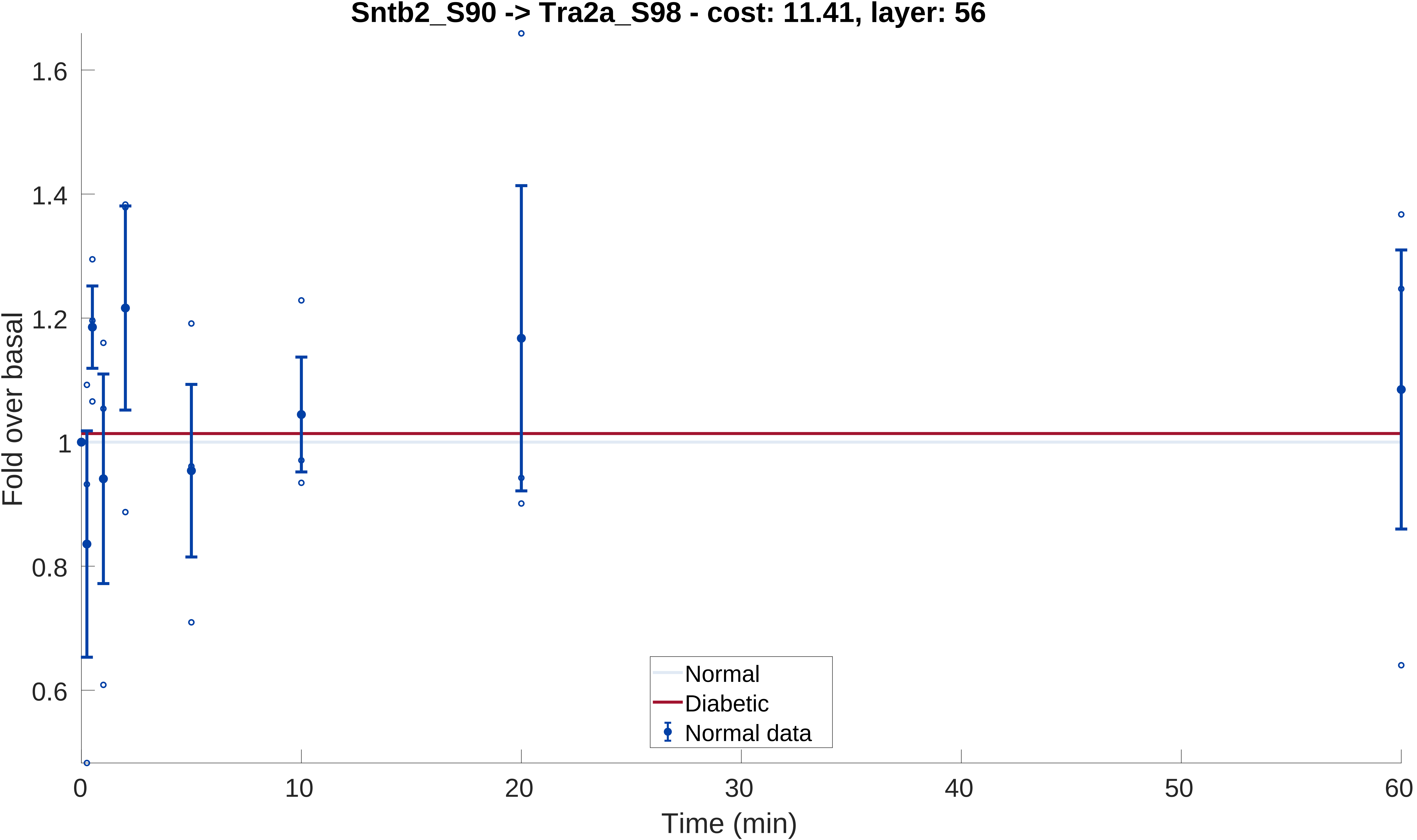
Sntb2_S90 -> Tra2a_S98 - cost: 11.41, layer: 56
Fold over basal
1.6
1.4
1.2
1
0.8
0.6
0
10
20
30
40
50
60
Time (min)
Normal
Diabetic
Normal data

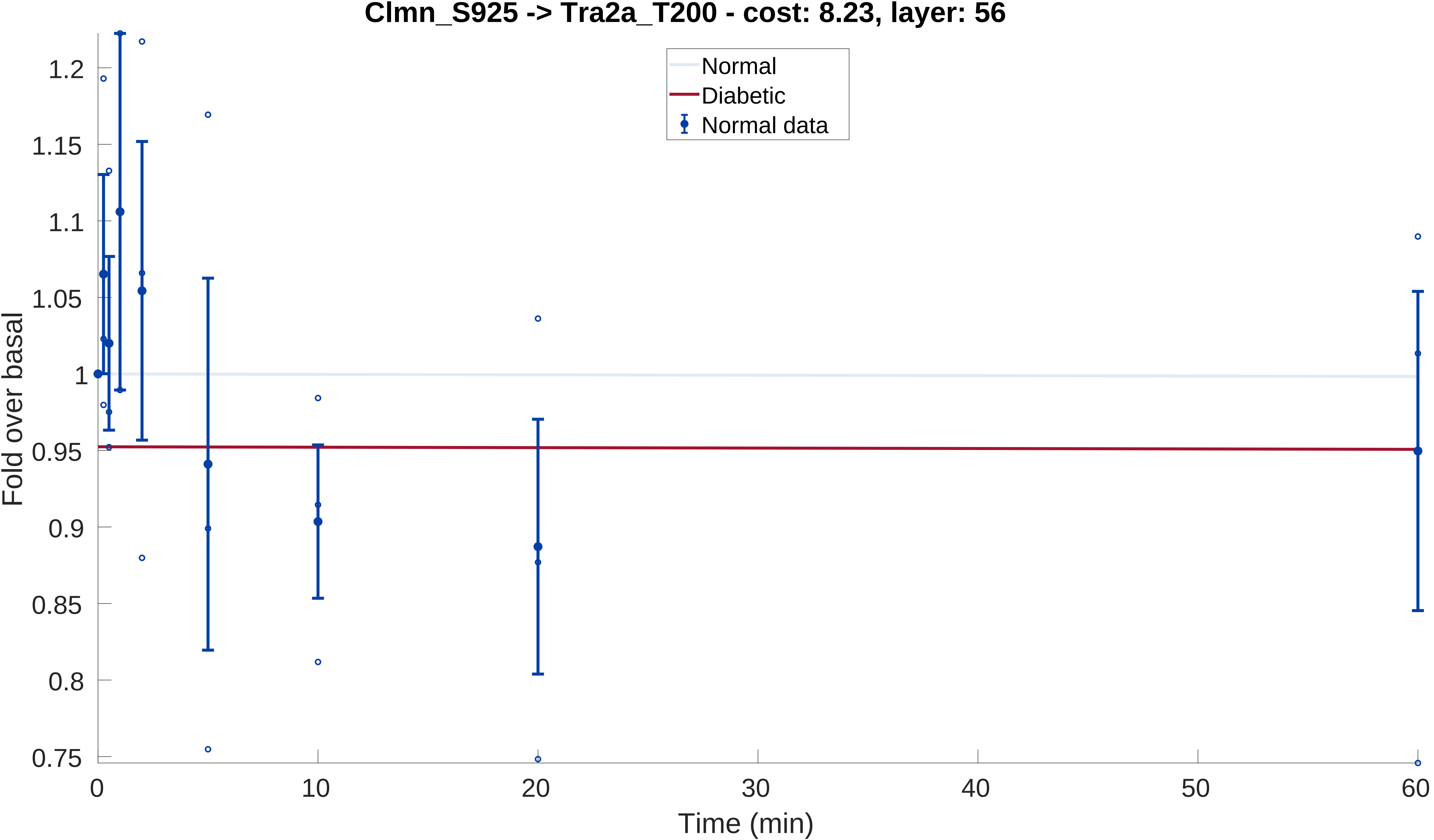

Clmn_S925 -> Tra2a_T200 - cost: 8.23, layer: 56
Normal
Diabetic
Normal data
Fold over basal
Time (min)
1.2
1.15
1.1
1.05
1
0.95
0.9
0.85
0.8
0.75
0
10
20
30
40
50
60

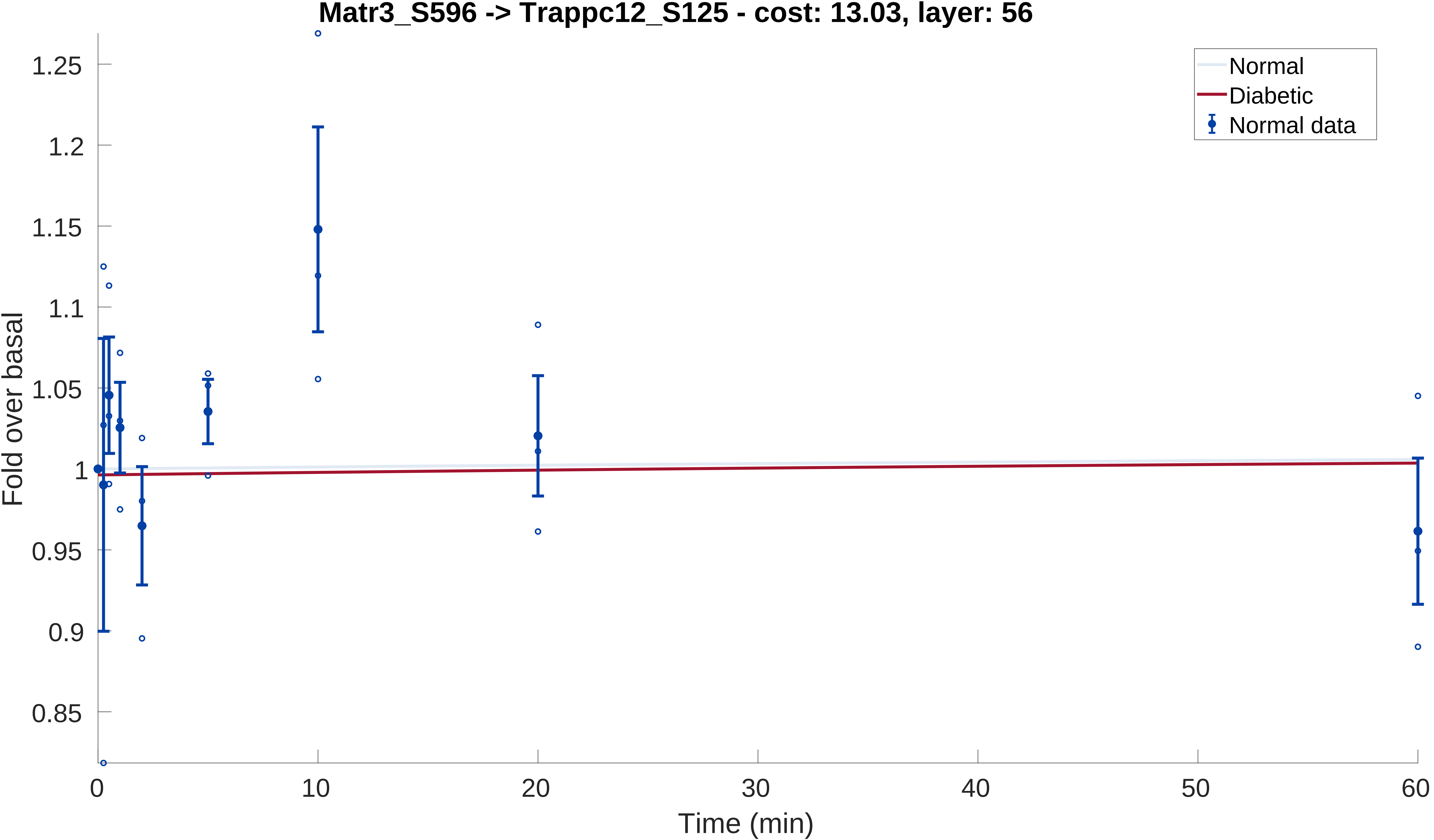
Matr3_S596 -> Trappc12_S125 - cost: 13.03, layer: 56
Normal
Diabetic
Normal data
Fold over basal
1.25
1.2
1.15
1.1
1.05
1
0.95
0.9
0.85
0
10
20
30
40
50
60
Time (min)

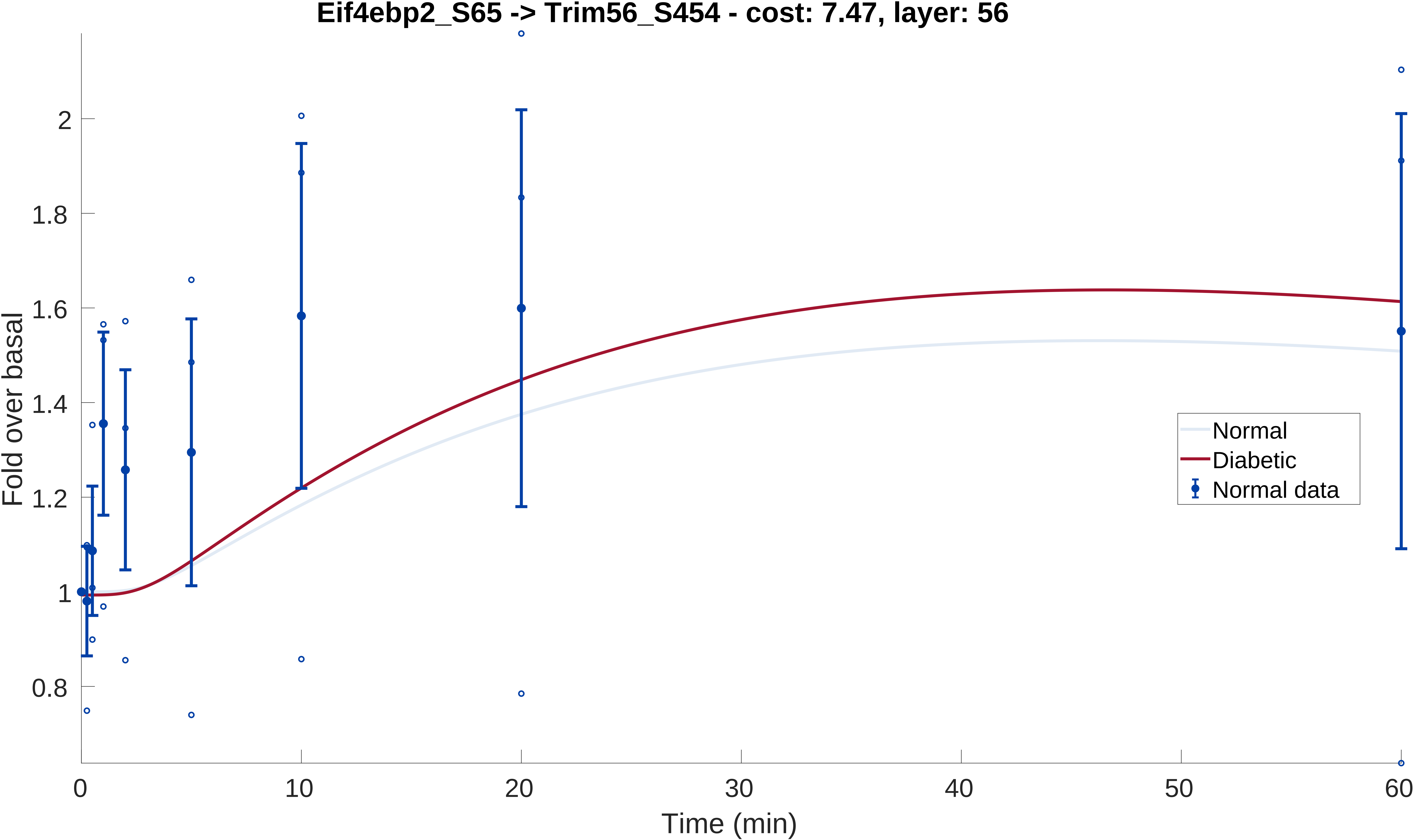

Eif4ebp2_S65 -> Trim56_S454 - cost: 7.47, layer: 56
Fold over basal
Time (min)
Normal
Diabetic
Normal data
0.8
1
1.2
1.4
1.6
1.8
2
0
10
20
30
40
50
60

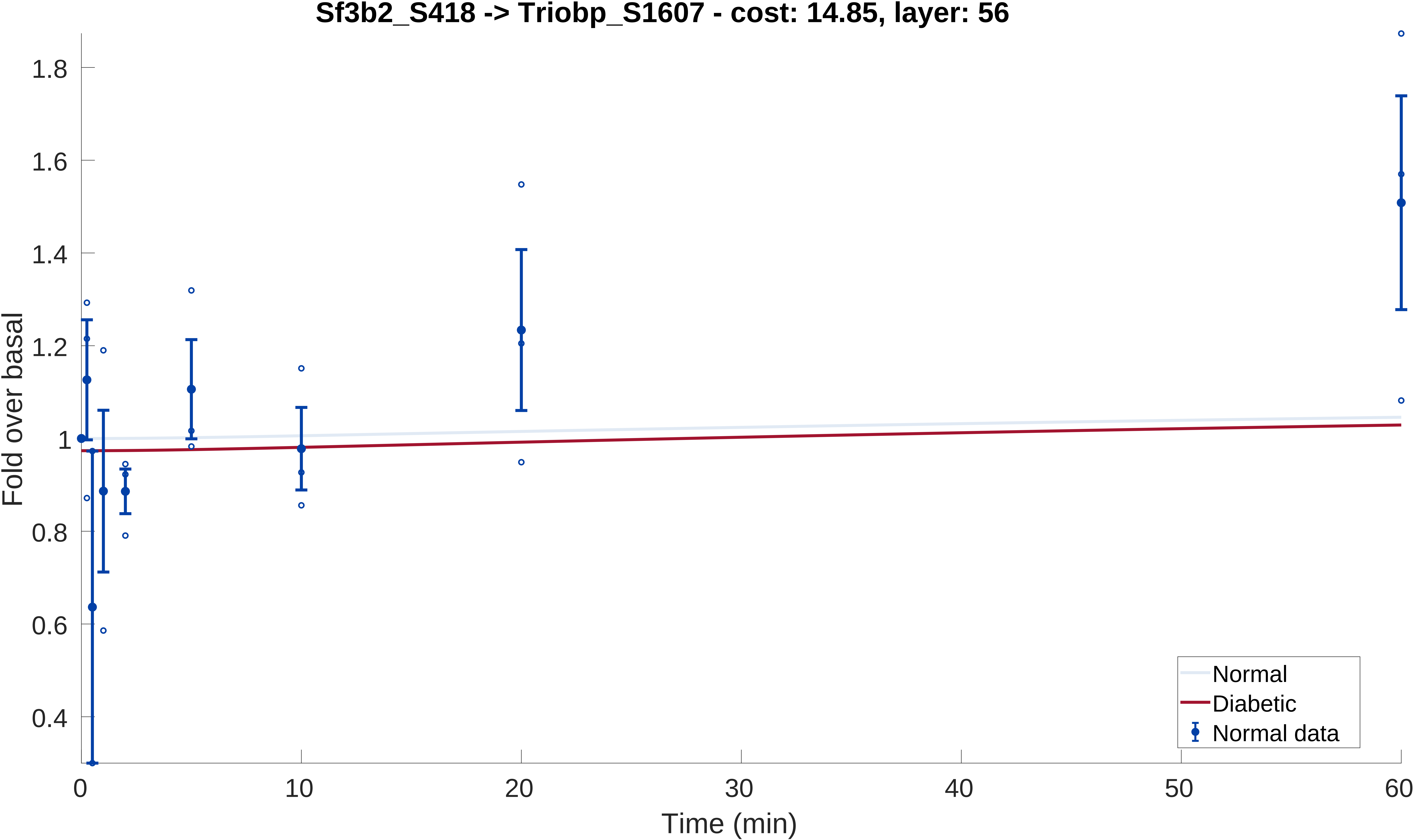

Sf3b2_S418 -> Triobp_S1607 - cost: 14.85, layer: 56
Fold over basal
1.8
1.6
1.4
1.2
1
0.8
0.6
0.4
0
10
20
30
40
50
60
Time (min)
Normal
Diabetic
Normal data

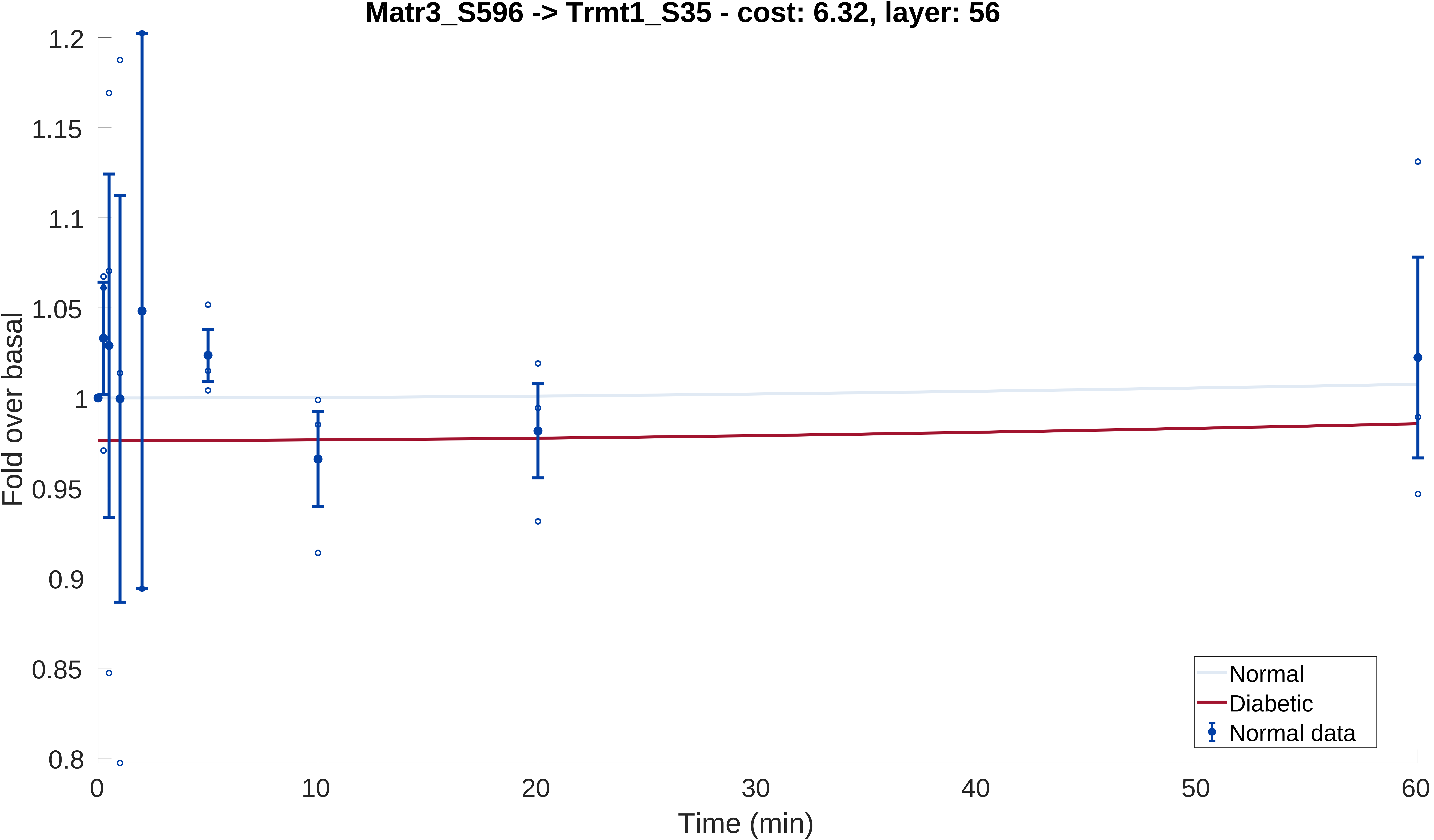
Matr3_S596 -> Trmt1_S35 - cost: 6.32, layer: 56
Fold over basal
Time (min)
1.2
1.15
1.1
1.05
1
0.95
0.9
0.85
0.8
0
10
20
30
40
50
60
Normal
Diabetic
Normal data

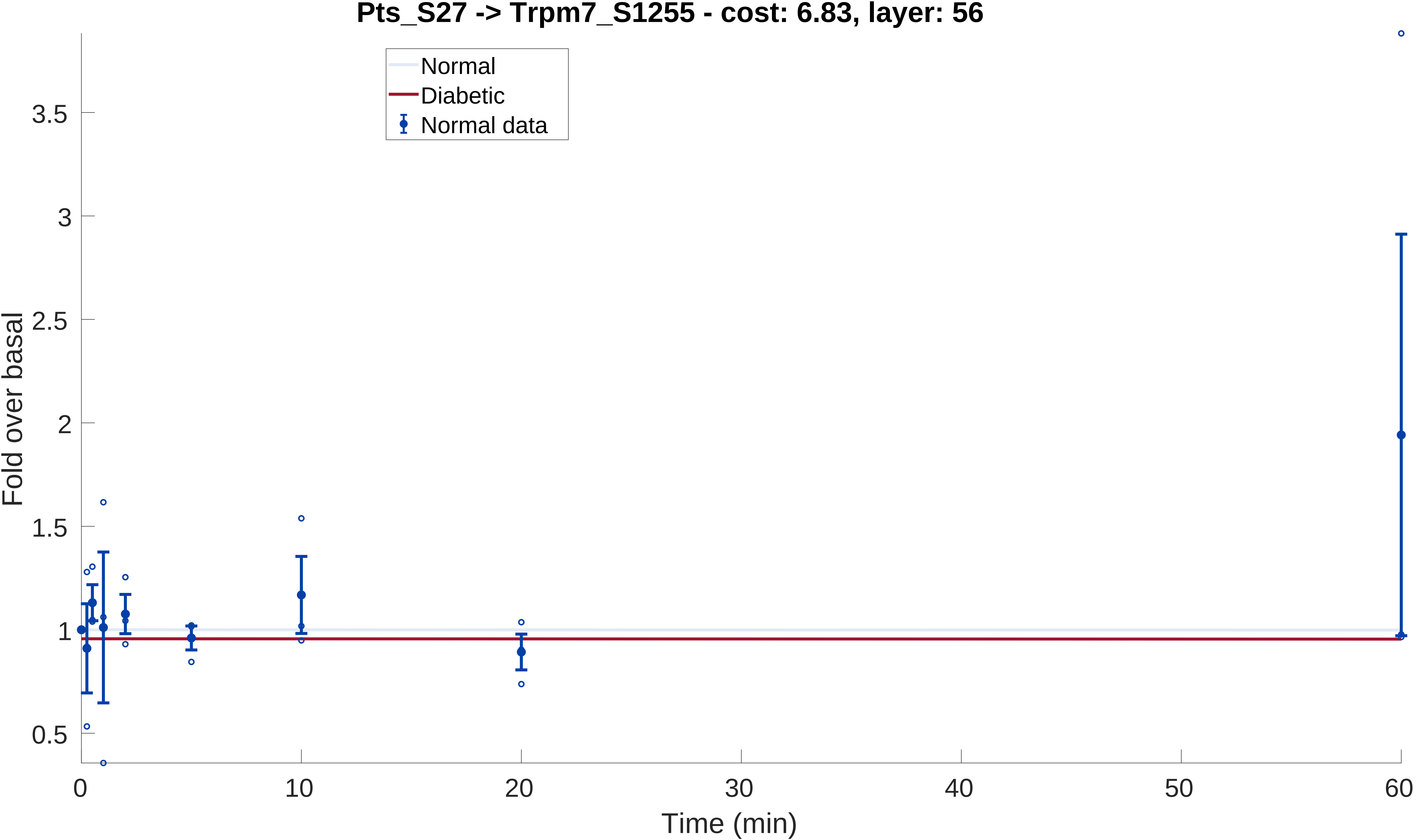

Pts_S27 -> Trpm7_S1255 - cost: 6.83, layer: 56
Normal
Diabetic
Normal data
Fold over basal
Time (min)
0.5
1
1.5
2
2.5
3
3.5
0
10
20
30
40
50
60

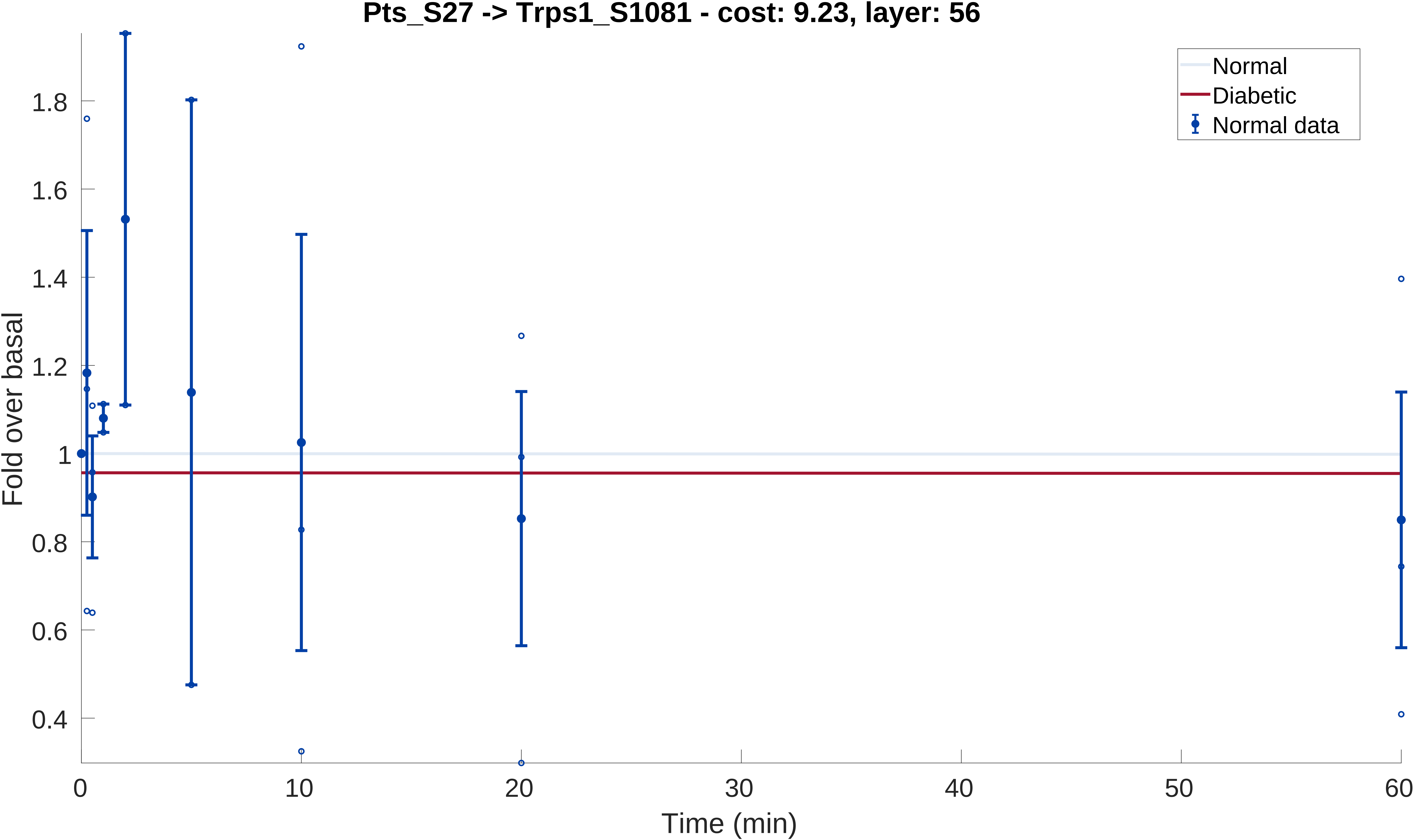

Pts_S27 -> Trps1_S1081 - cost: 9.23, layer: 56
Normal
Diabetic
Normal data
Fold over basal
Time (min)
1.8
1.6
1.4
1.2
1
0.8
0.6
0.4
0
10
20
30
40
50
60

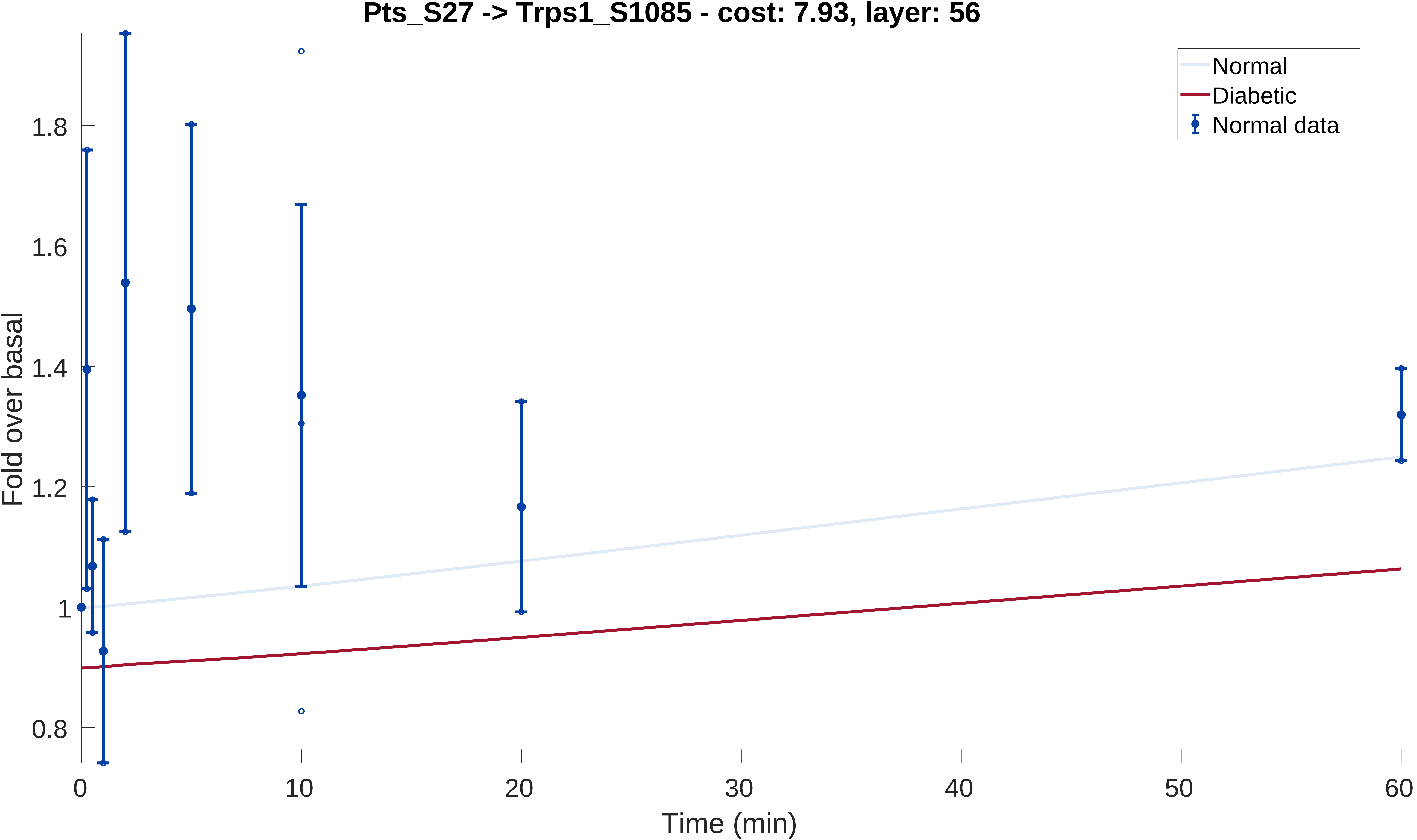
Pts_S27 -> Trps1_S1085 - cost: 7.93, layer: 56
Normal
Diabetic
Normal data
Fold over basal
1.8
1.6
1.4
1.2
1
0.8
0
10
20
30
40
50
60
Time (min)

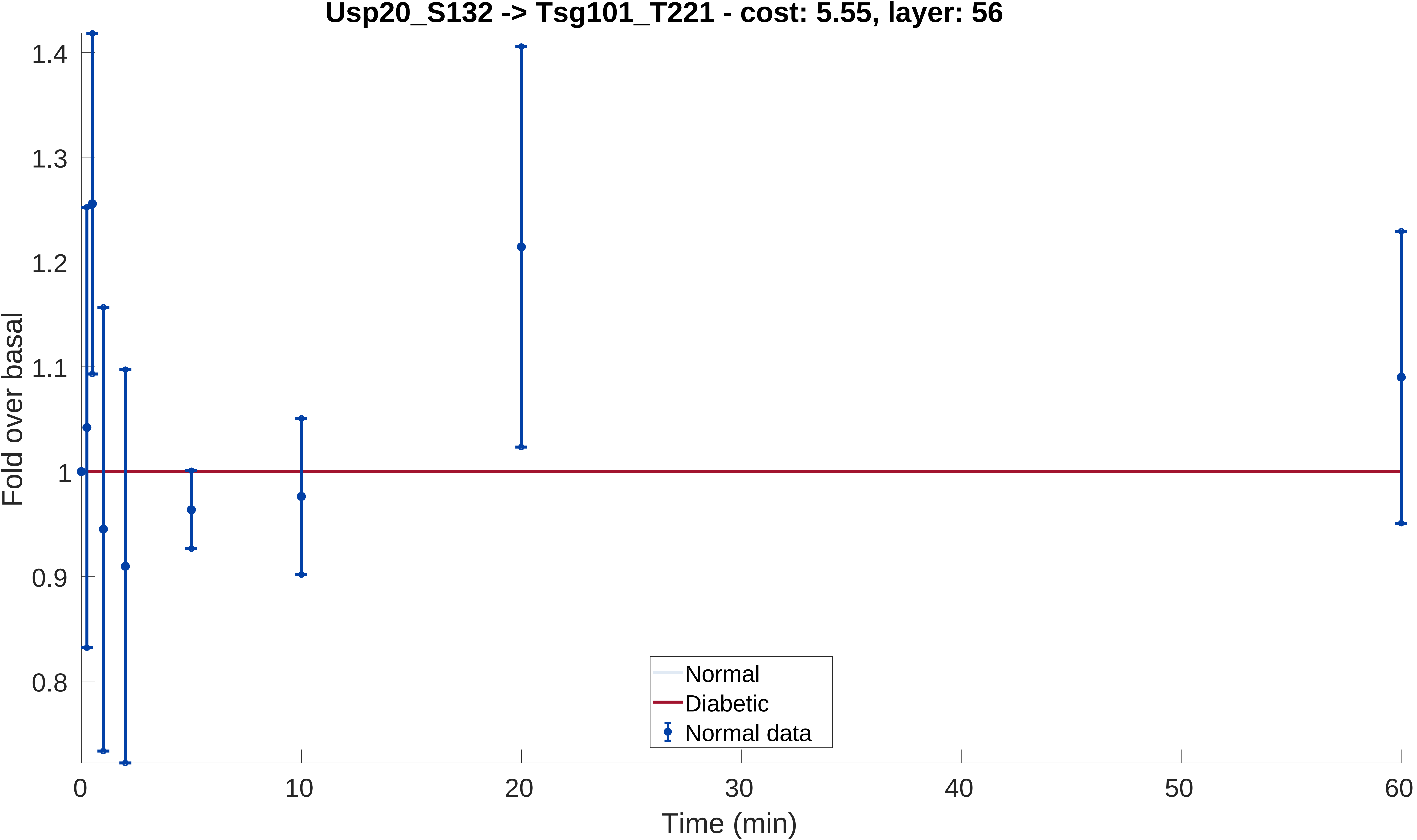
Usp20_S132 -> Tsg101_T221 - cost: 5.55, layer: 56
Fold over basal
1.4
1.3
1.2
1.1
1
0.9
0.8
0
10
20
30
40
50
60
Time (min)
Normal
Diabetic
Normal data

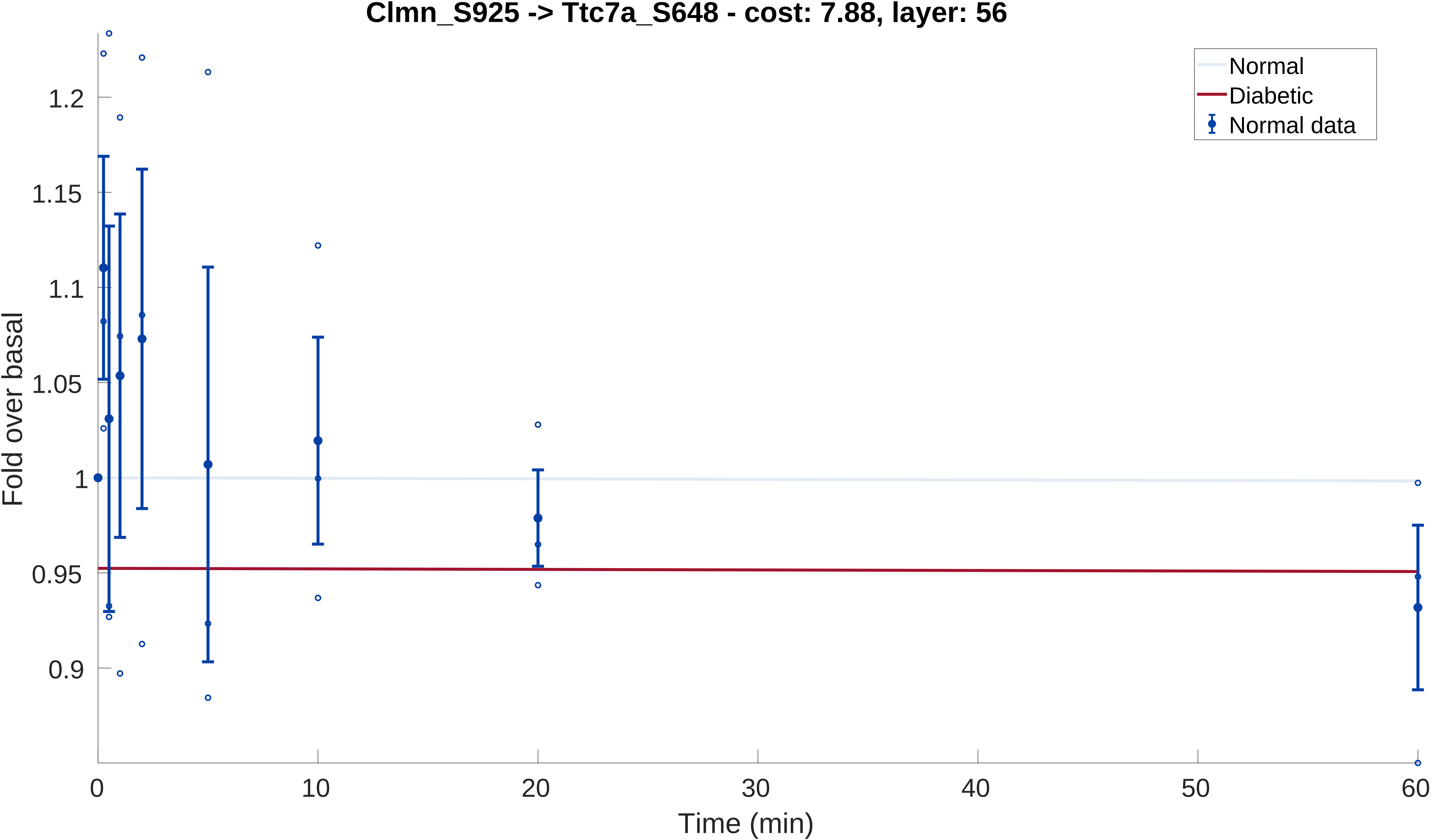
Clmn_S925 -> Ttc7a_S648 - cost: 7.88, layer: 56
Normal
Diabetic
Normal data
Fold over basal
1.2
1.15
1.1
1.05
1
0.95
0.9
0
10
20
30
40
50
60
Time (min)

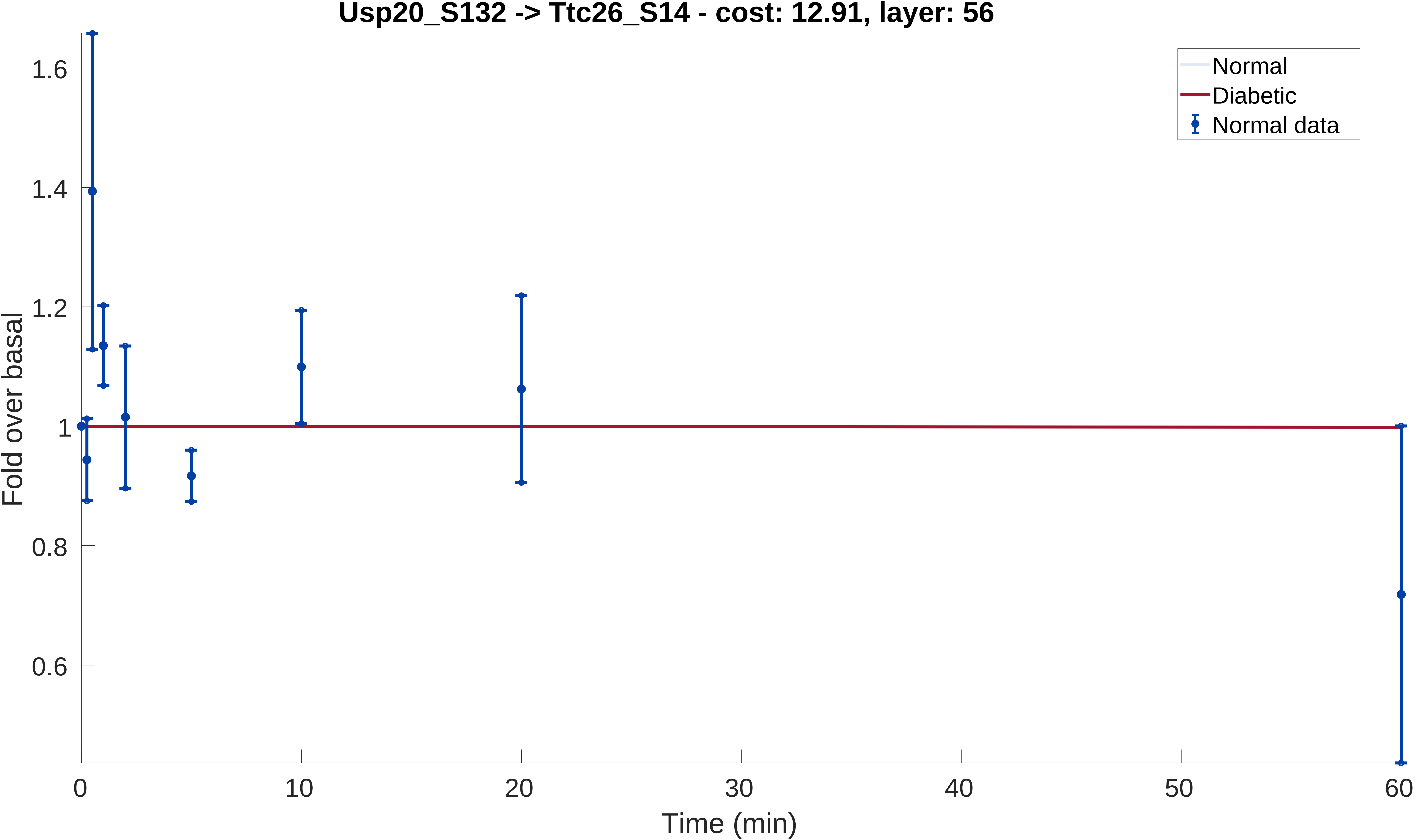

Usp20_S132 -> Ttc26_S14 - cost: 12.91, layer: 56
Normal
Diabetic
Normal data
Fold over basal
1.6
1.4
1.2
1
0.8
0.6
0
10
20
30
40
50
60
Time (min)

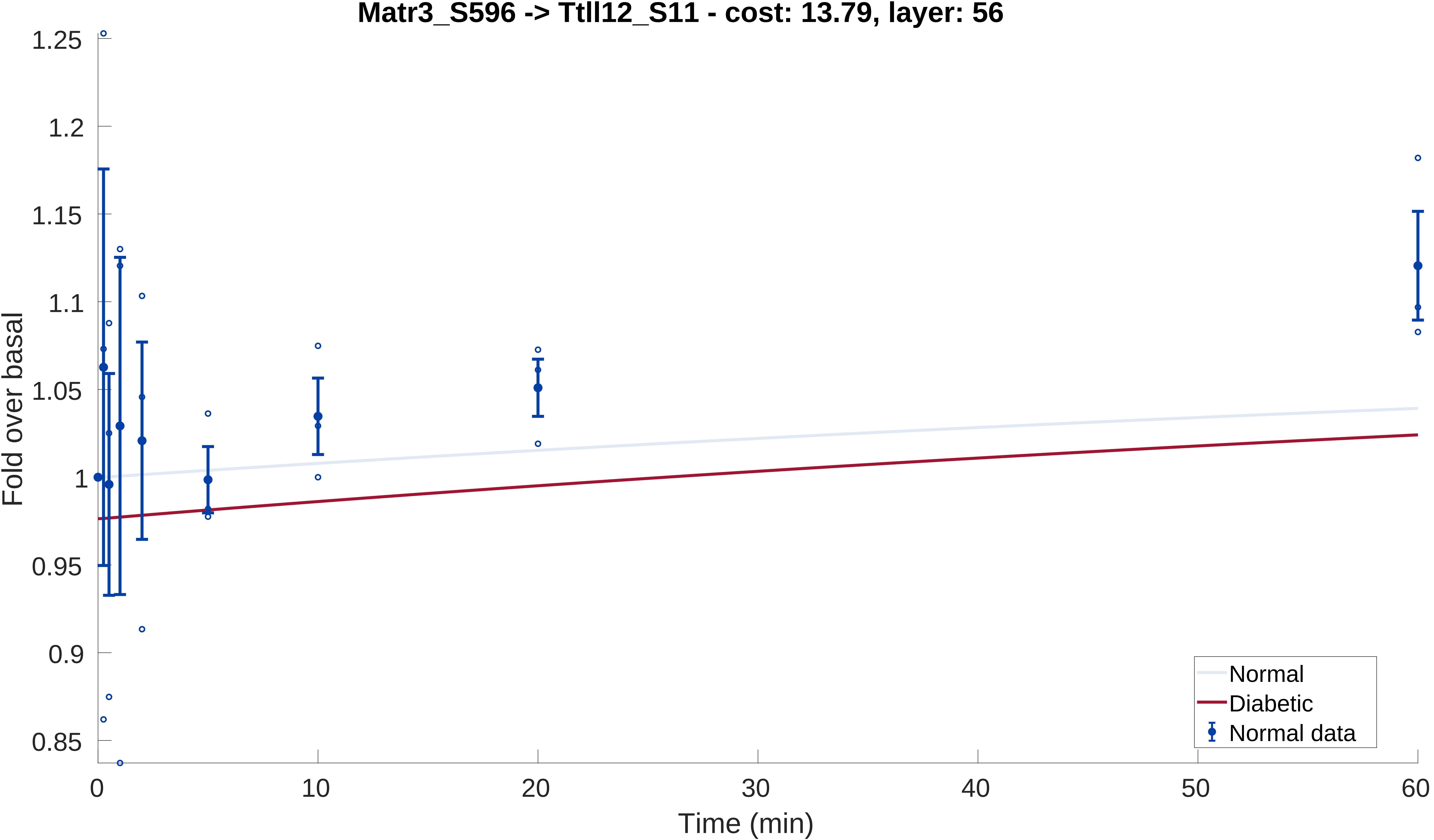
Matr3_S596 -> Ttll12_S11 - cost: 13.79, layer: 56
1.25
1.2
1.15
1.1
1.05
1
0.95
0.9
0.85
Fold over basal
0
10
20
30
40
50
60
Time (min)
Normal
Diabetic
Normal data

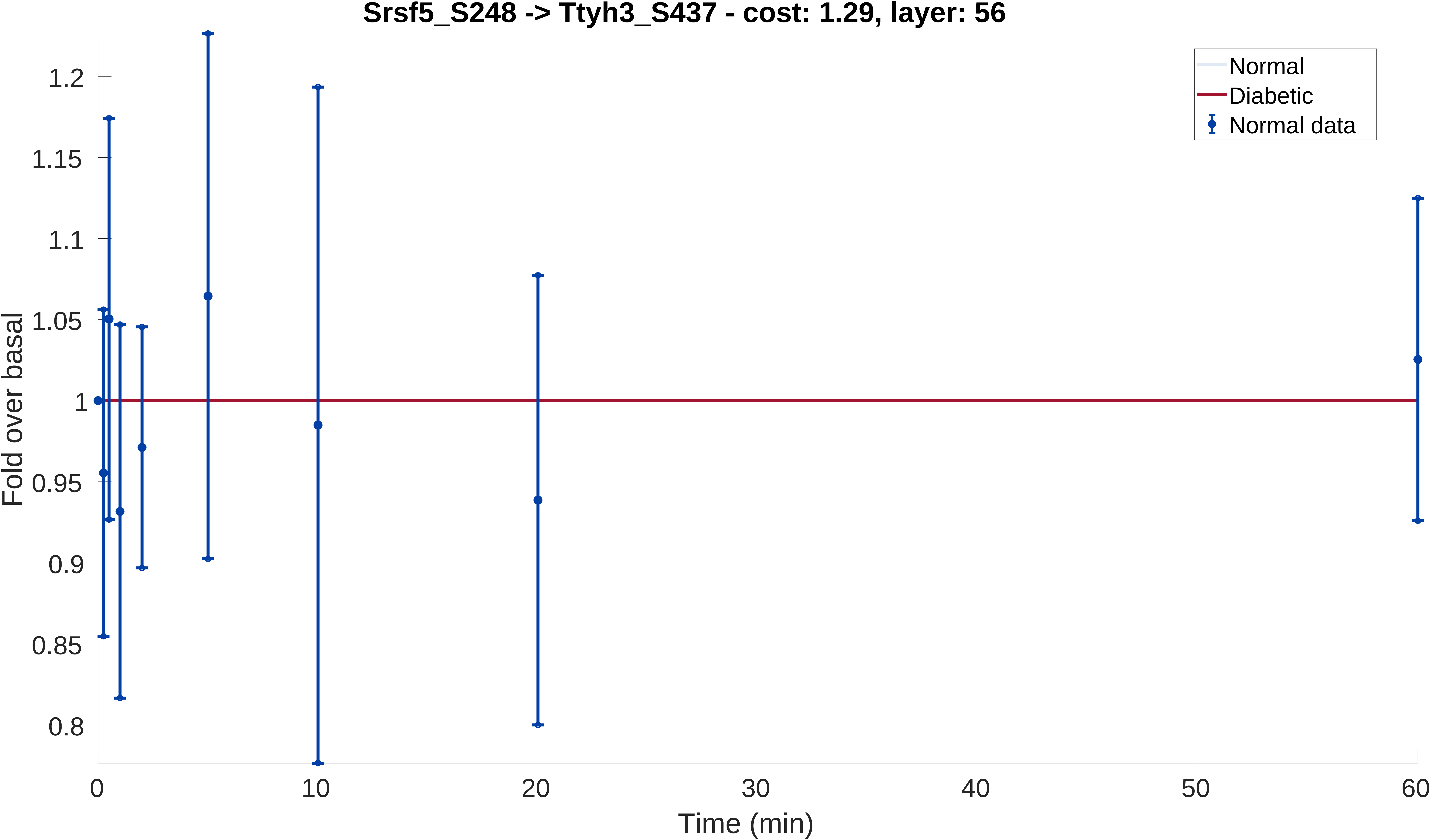

Srsf5_S248 -> Ttyh3_S437 - cost: 1.29, layer: 56
Normal
Diabetic
Normal data
Fold over basal
1.2
1.15
1.1
1.05
1
0.95
0.9
0.85
0.8
0
10
20
30
40
50
60
Time (min)

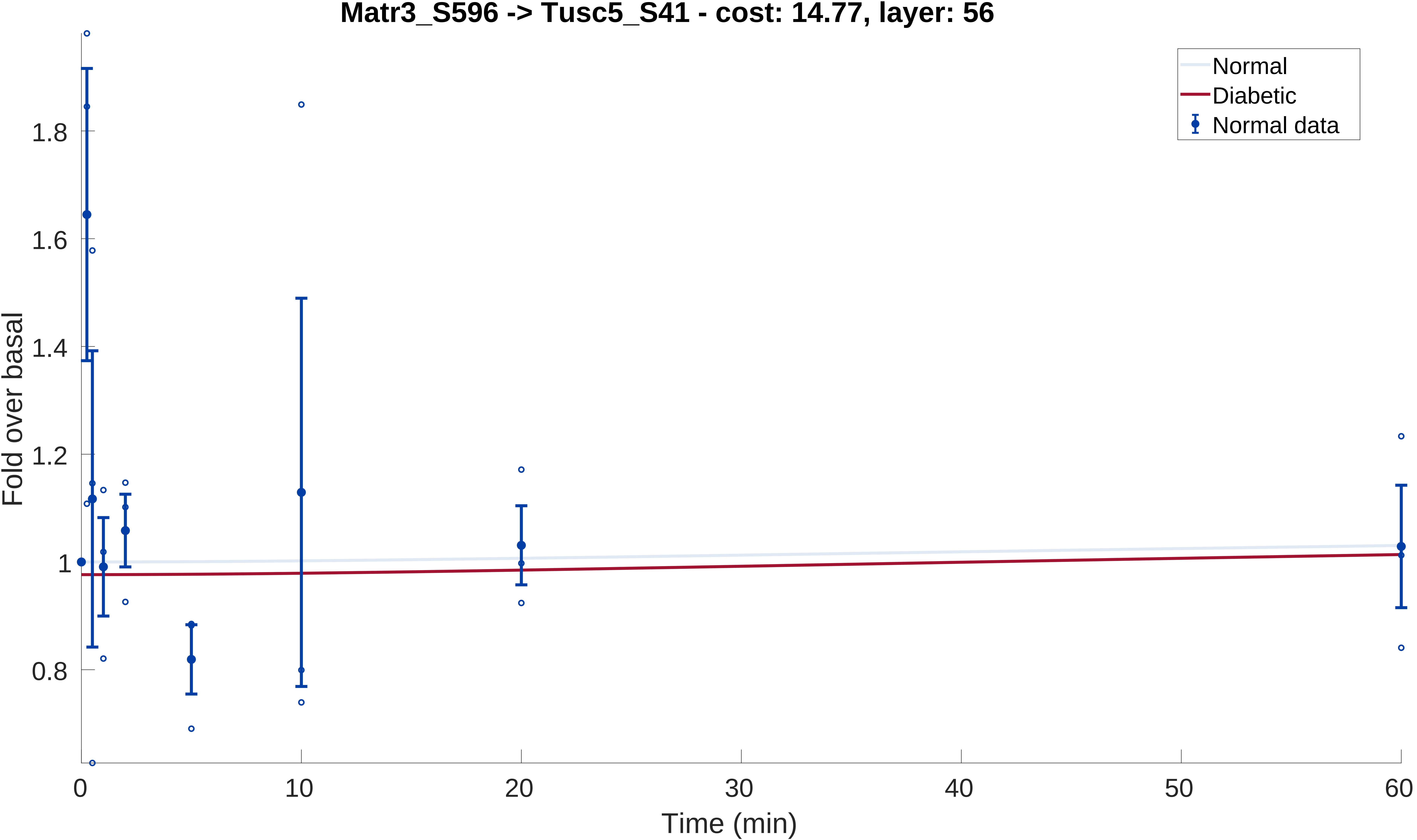

Matr3_S596 -> Tusc5_S41 - cost: 14.77, layer: 56
Normal
Diabetic
Normal data
Fold over basal
1.8
1.6
1.4
1.2
1
0.8
Time (min)
0
10
20
30
40
50
60

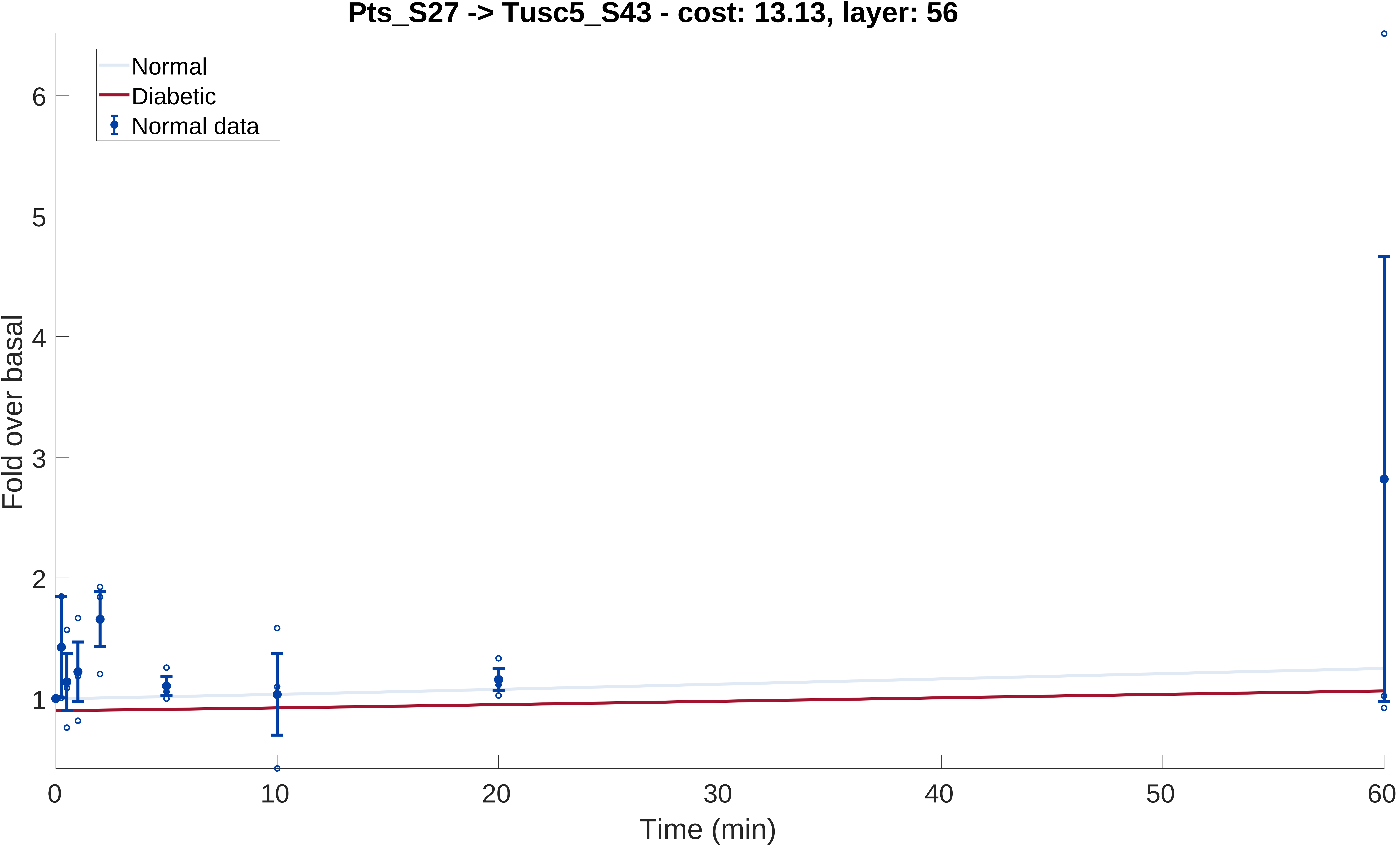

Pts_S27 -> Tusc5_S43 - cost: 13.13, layer: 56
Normal
Diabetic
Normal data
Fold over basal
Time (min)
0
10
20
30
40
50
60
1
2
3
4
5
6

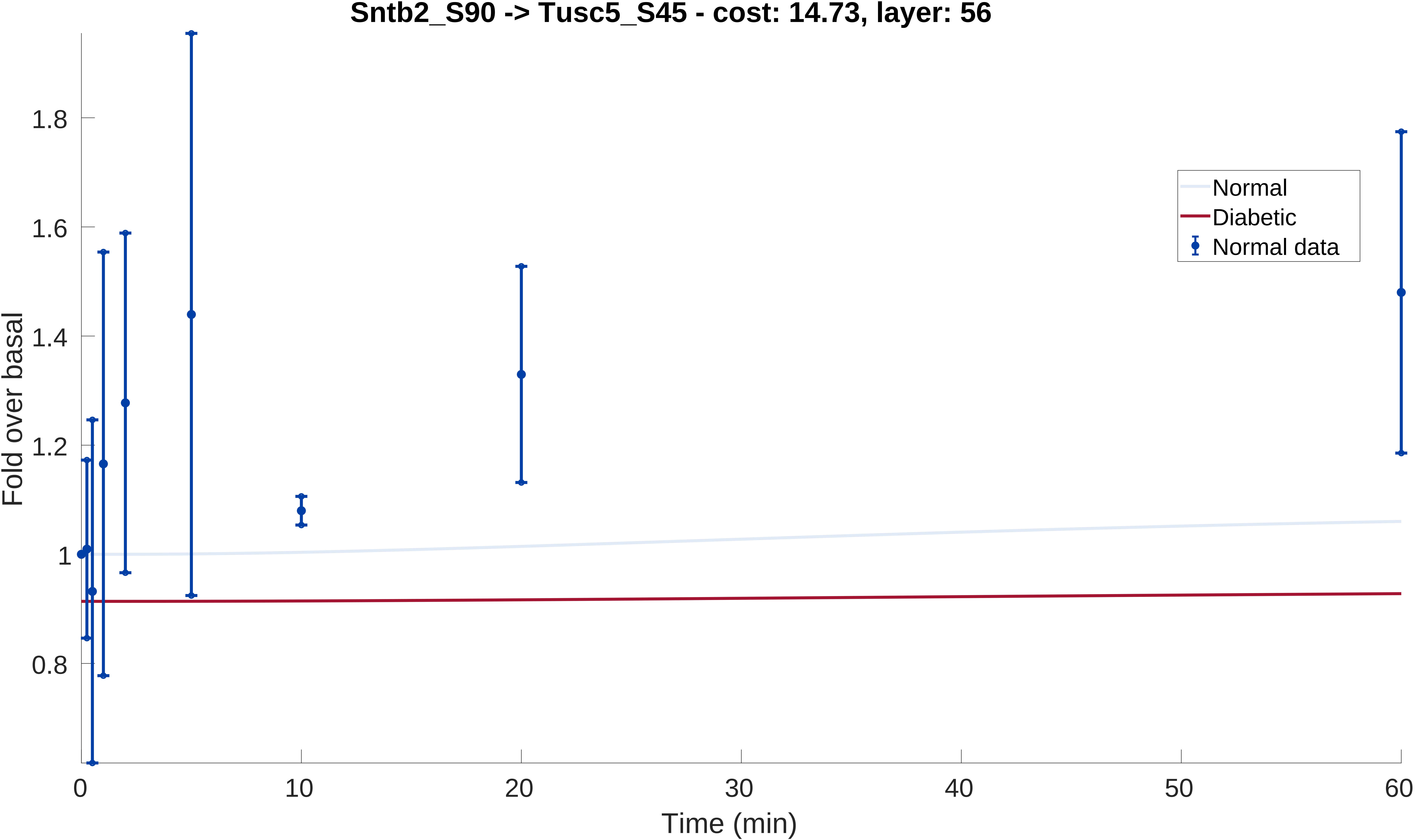
Sntb2_S90 -> Tusc5_S45 - cost: 14.73, layer: 56
Fold over basal
Time (min)
Normal
Diabetic
Normal data
1.8
1.6
1.4
1.2
1
0.8
0
10
20
30
40
50
60

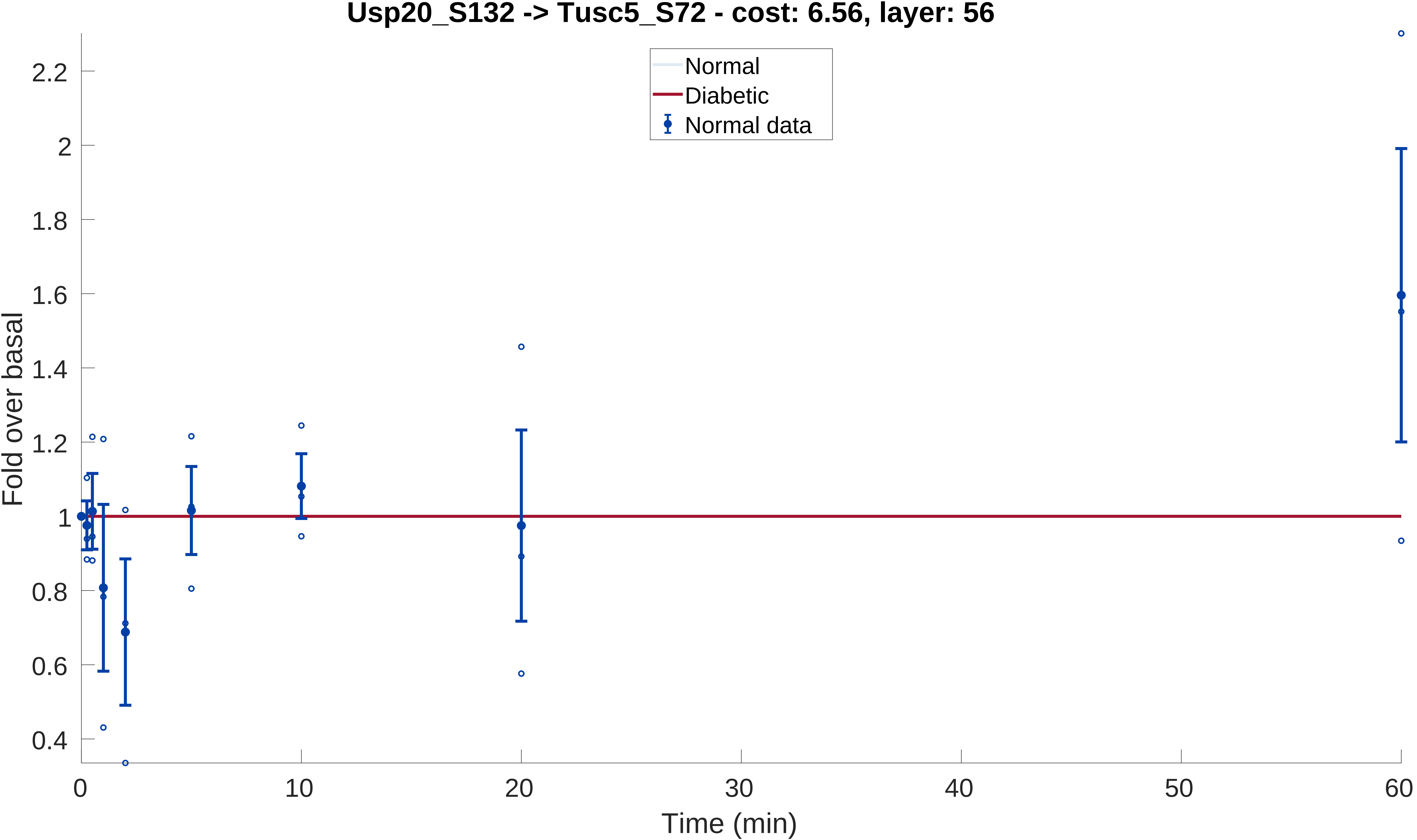

Usp20_S132 -> Tusc5_S72 - cost: 6.56, layer: 56
Normal
Diabetic
Normal data
Fold over basal
Time (min)
0.4
0.6
0.8
1
1.2
1.4
1.6
1.8
2
2.2
0
10
20
30
40
50
60

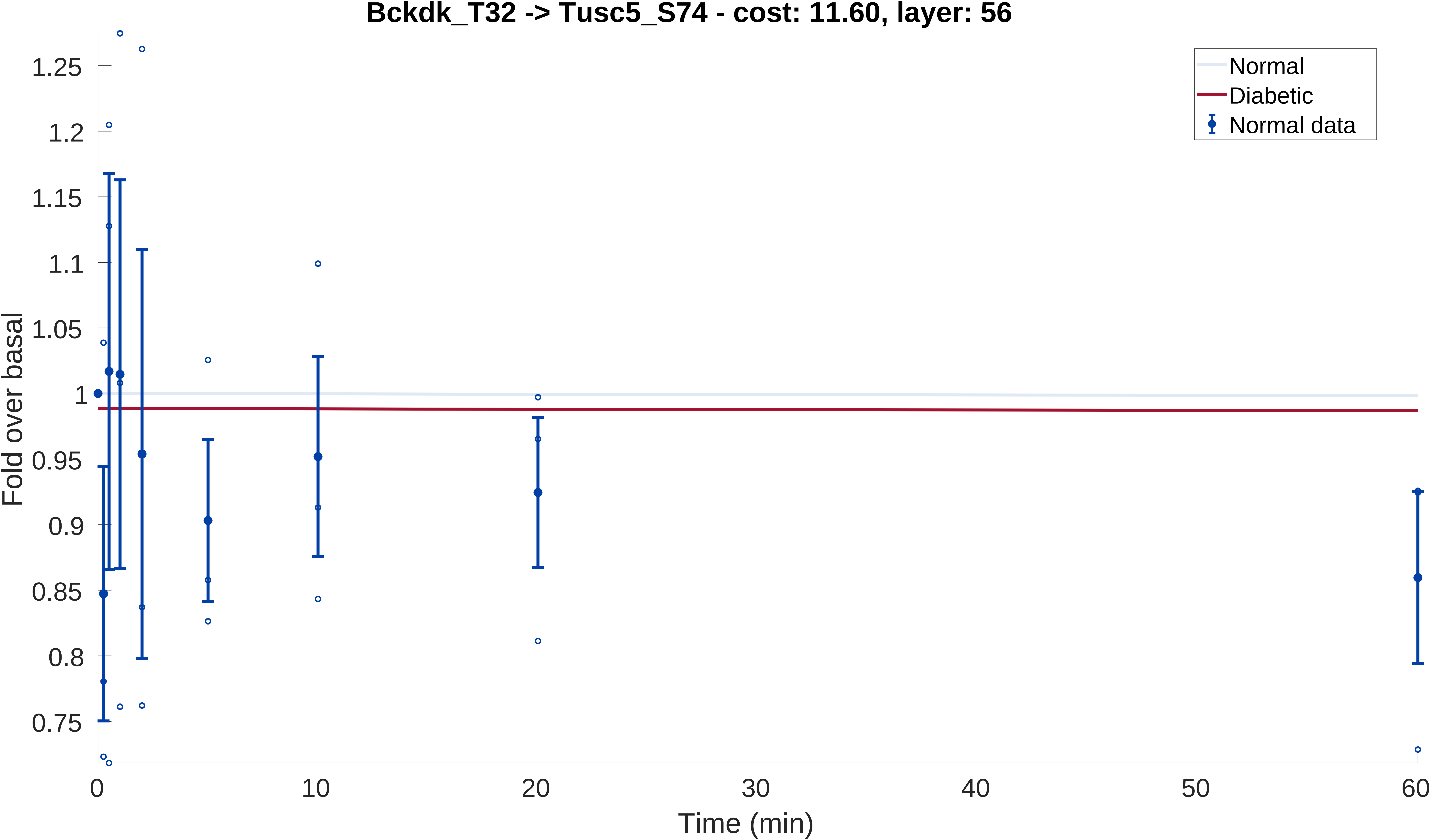

Bckdk_T32 -> Tusc5_S74 - cost: 11.60, layer: 56
Normal
Diabetic
Normal data
Fold over basal
1.25
1.2
1.15
1.1
1.05
1
0.95
0.9
0.85
0.8
0.75
0
10
20
30
40
50
60
Time (min)

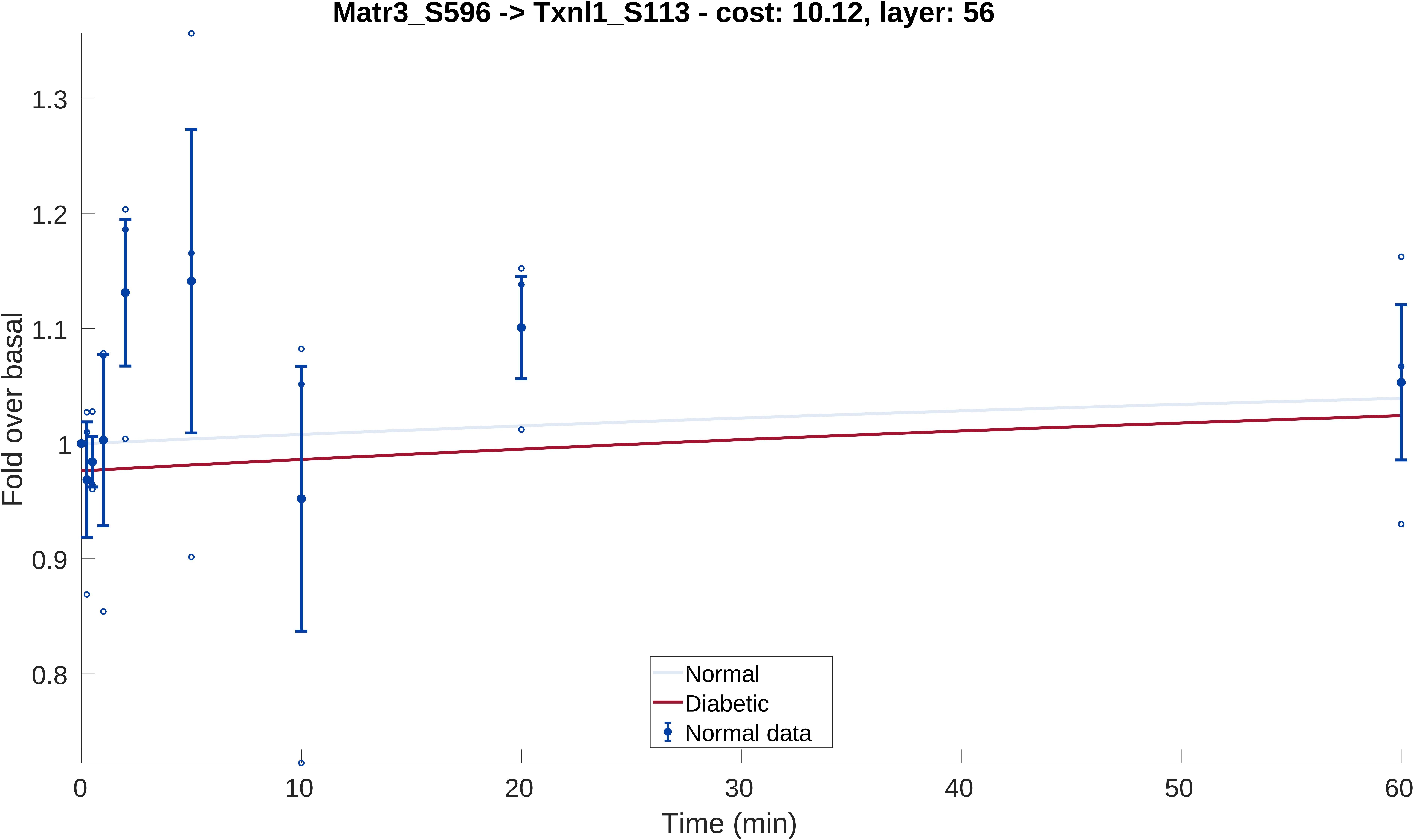
Matr3_S596 -> Txnl1_S113 - cost: 10.12, layer: 56
Fold over basal
1.3
1.2
1.1
1
0.9
0.8
0
10
20
30
40
50
60
Time (min)
Normal
Diabetic
Normal data

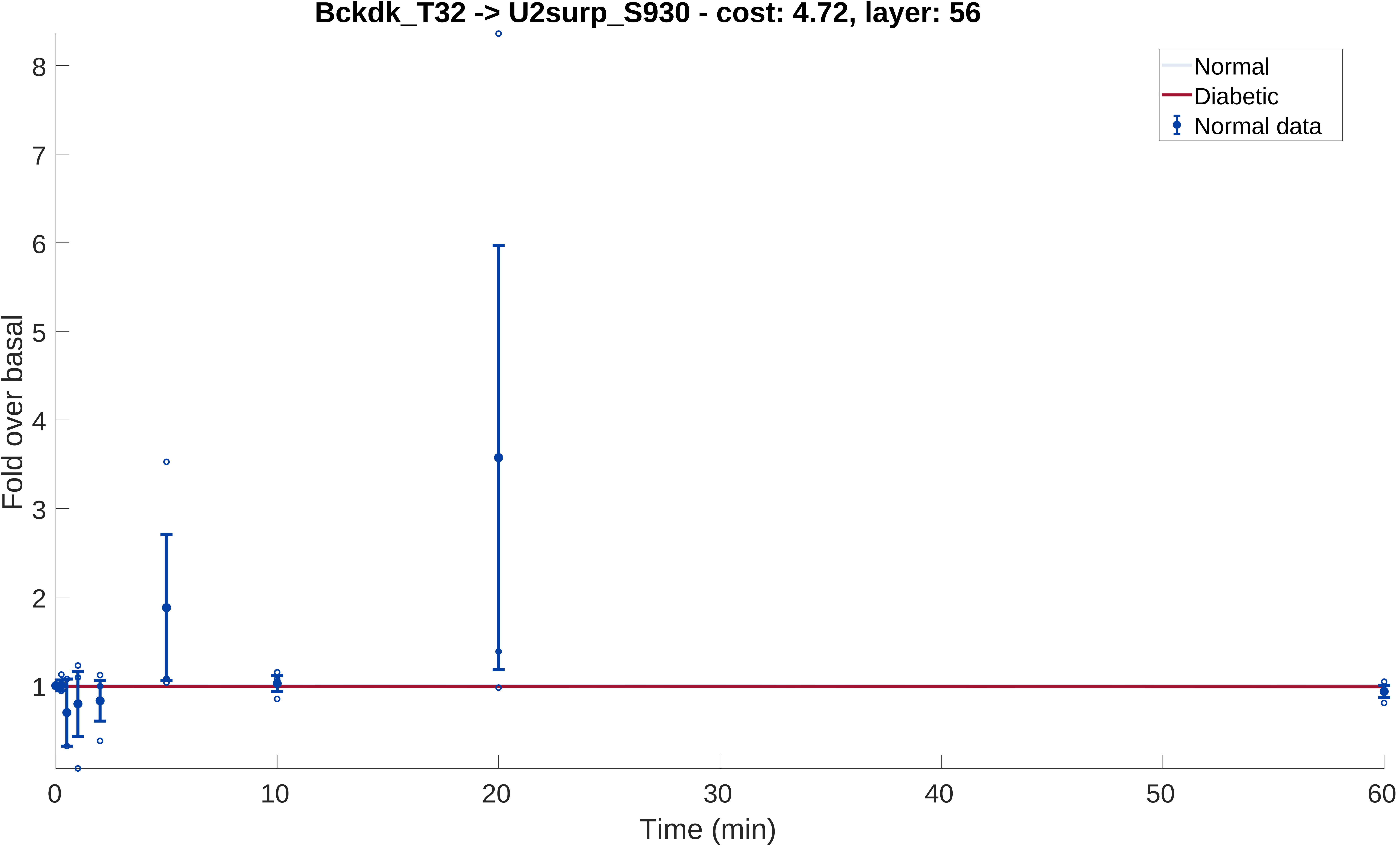

Bckdk_T32 -> U2surp_S930 - cost: 4.72, layer: 56
Normal
Diabetic
Normal data
Fold over basal
8
7
6
5
4
3
2
1
0
10
20
30
40
50
60
Time (min)

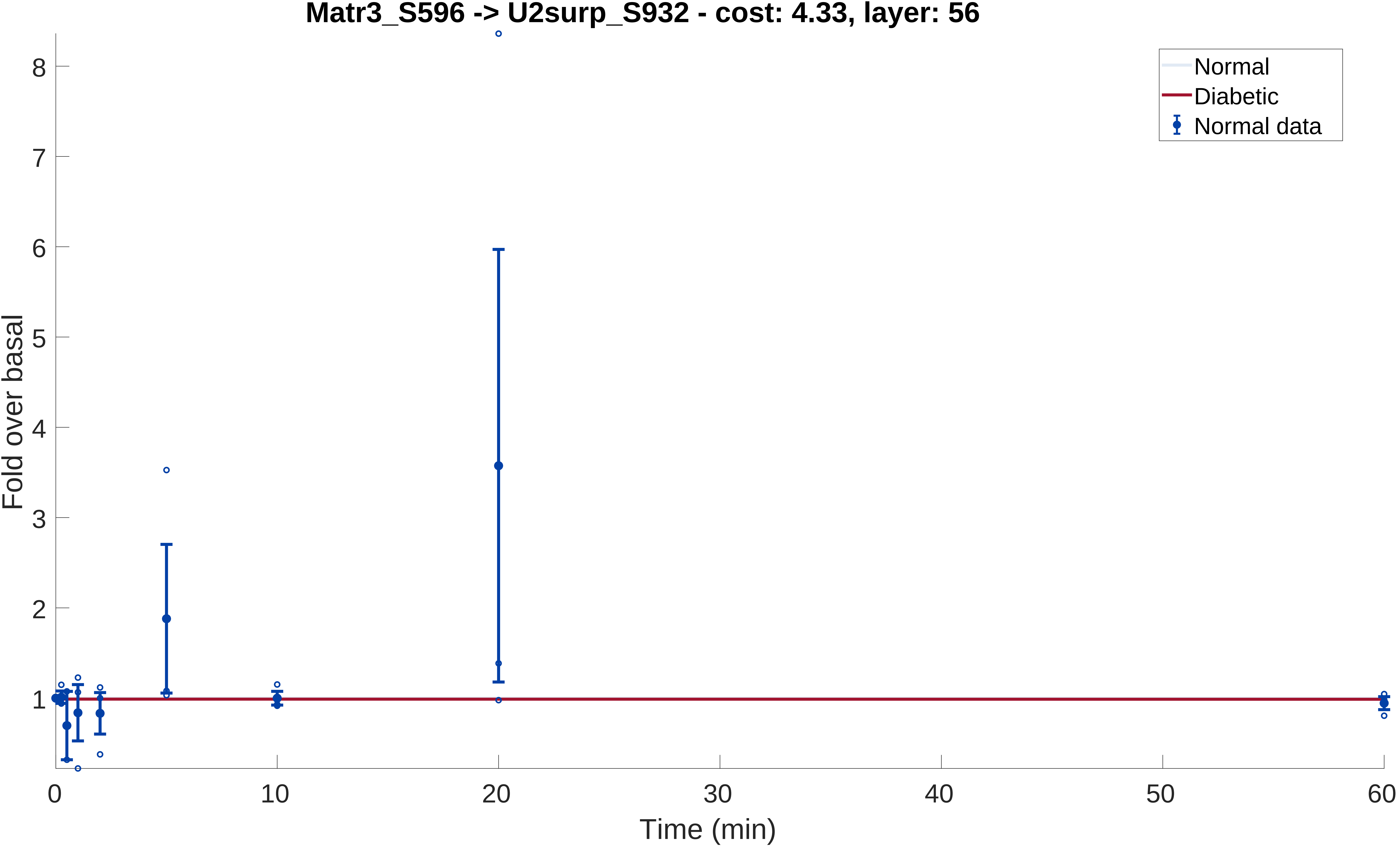

Matr3_S596 -> U2surp_S932 - cost: 4.33, layer: 56
Normal
Diabetic
Normal data
Fold over basal
Time (min)
0
10
20
30
40
50
60
1
2
3
4
5
6
7
8

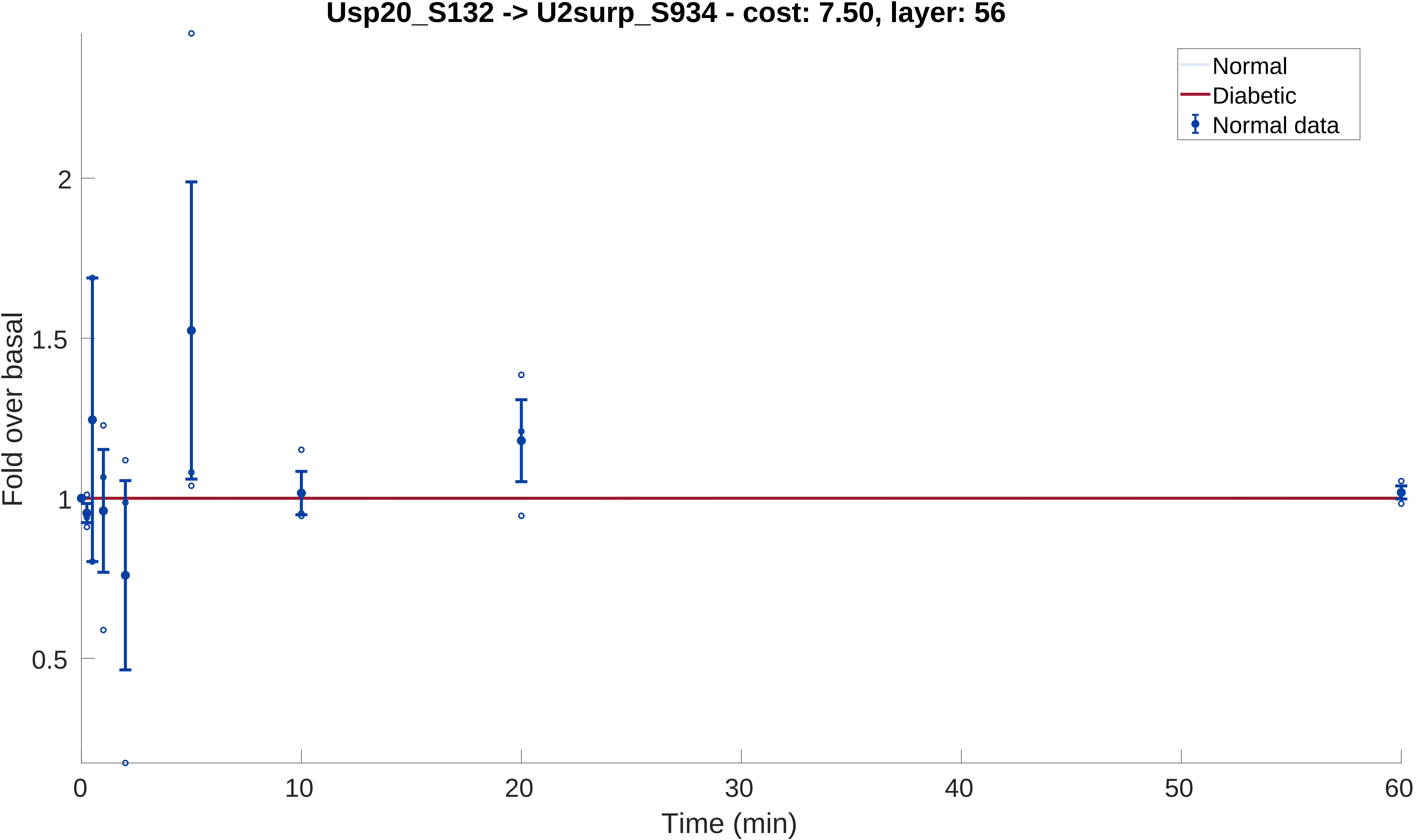

Usp20_S132 -> U2surp_S934 - cost: 7.50, layer: 56
Normal
Diabetic
Normal data
Fold over basal
2
1.5
1
0.5
0
10
20
30
40
50
60
Time (min)

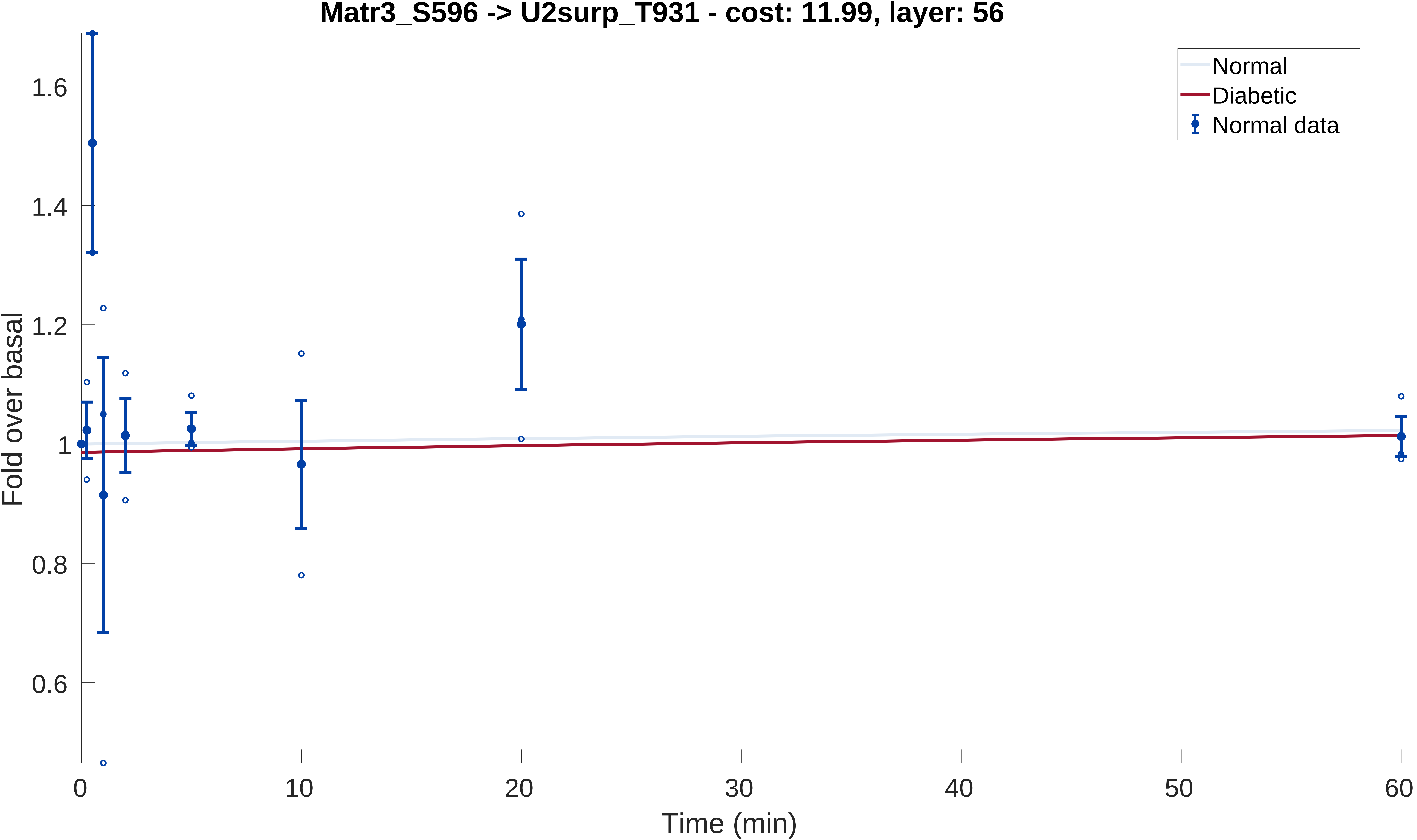

Matr3_S596 -> U2surp_T931 - cost: 11.99, layer: 56
Normal
Diabetic
Normal data
Fold over basal
1.6
1.4
1.2
1
0.8
0.6
0
10
20
30
40
50
60
Time (min)

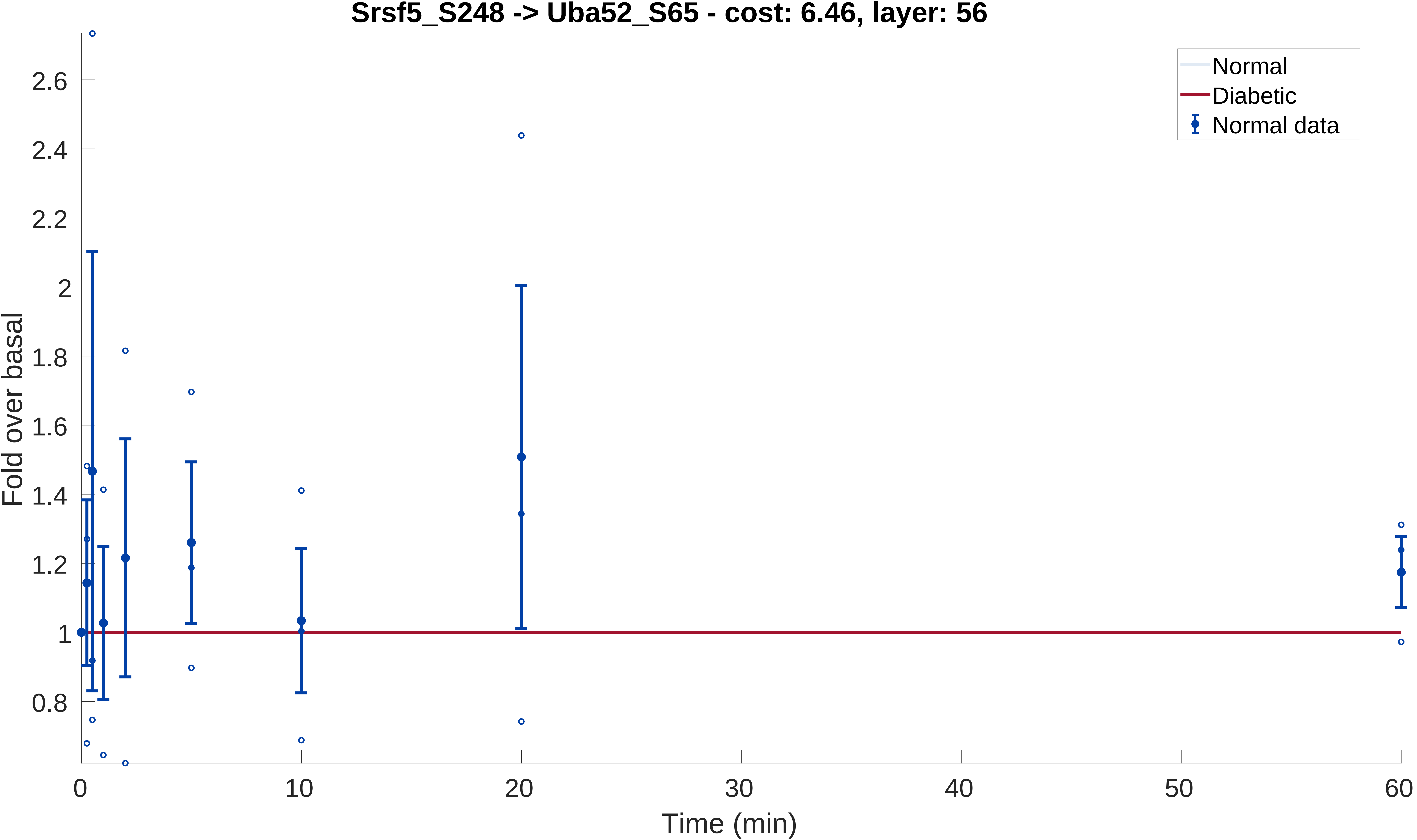

Srsf5_S248 -> Uba52_S65 - cost: 6.46, layer: 56
Normal
Diabetic
Normal data
Fold over basal
2.6
2.4
2.2
2
1.8
1.6
1.4
1.2
1
0.8
0
10
20
30
40
50
60
Time (min)

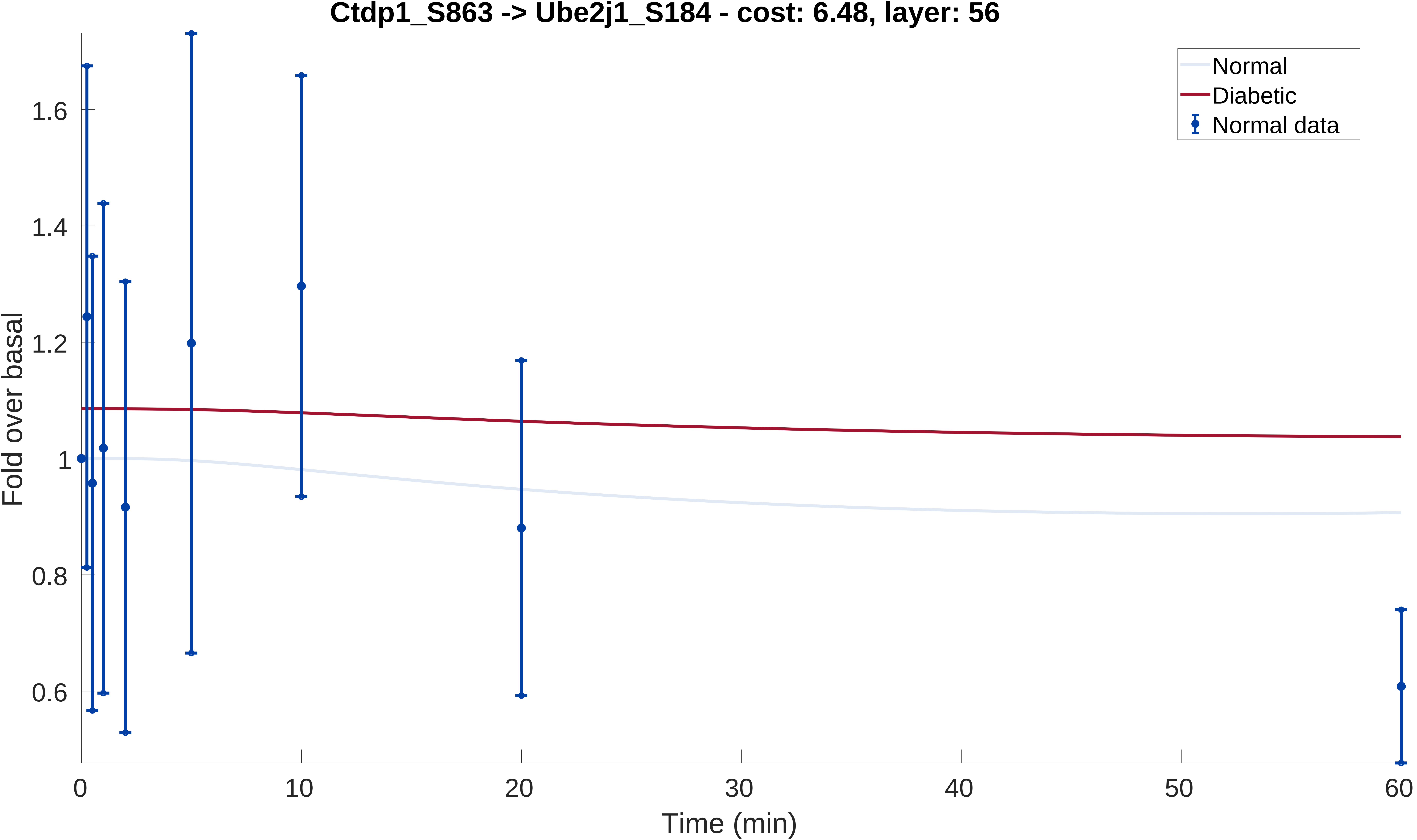

Ctdp1_S863 -> Ube2j1_S184 - cost: 6.48, layer: 56
Normal
Diabetic
Normal data
Fold over basal
1.6
1.4
1.2
1
0.8
0.6
0
10
20
30
40
50
60
Time (min)

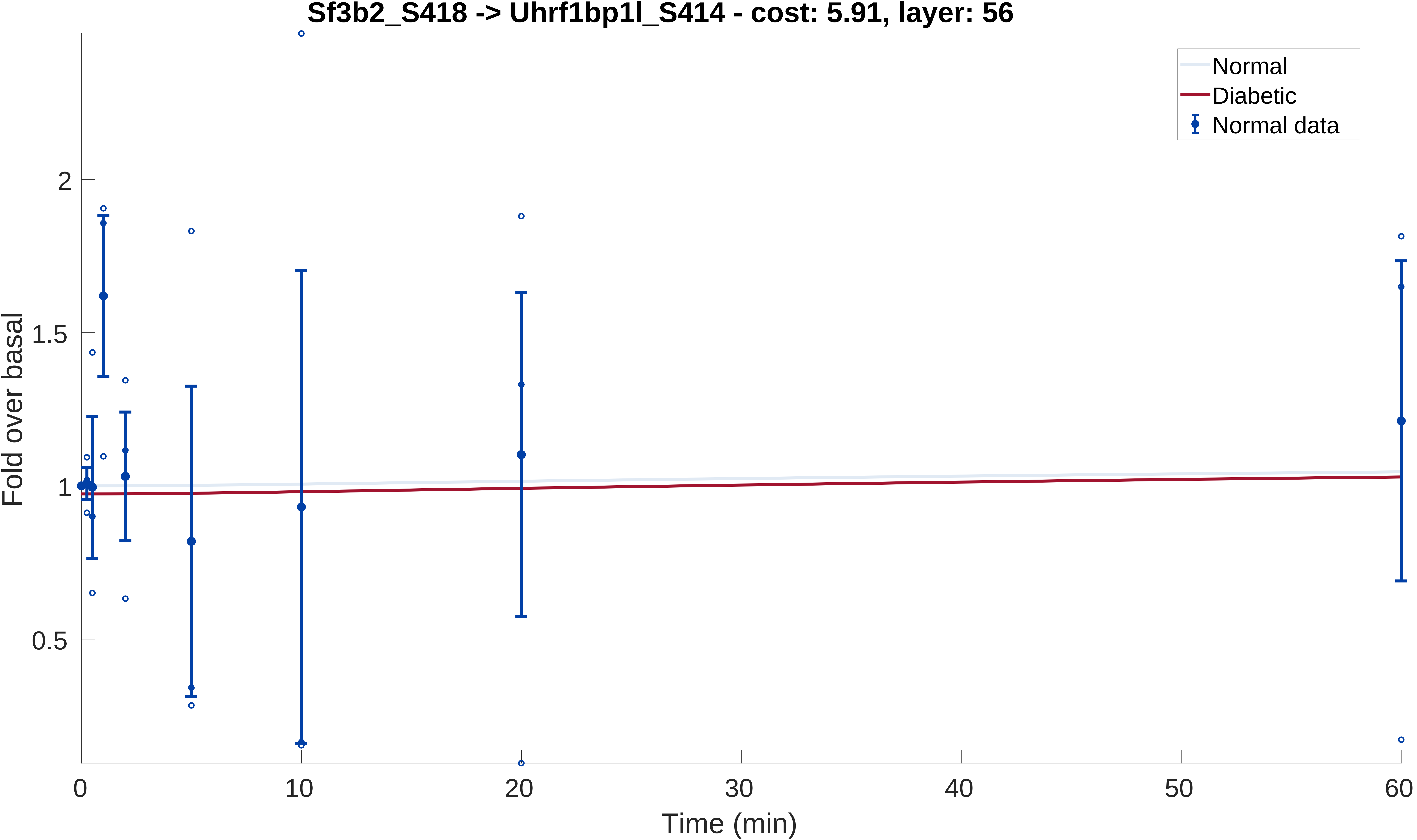

Sf3b2_S418 -> Uhrf1bp1l_S414 - cost: 5.91, layer: 56
Normal
Diabetic
Normal data
Fold over basal
2
1.5
1
0.5
0
10
20
30
40
50
60
Time (min)

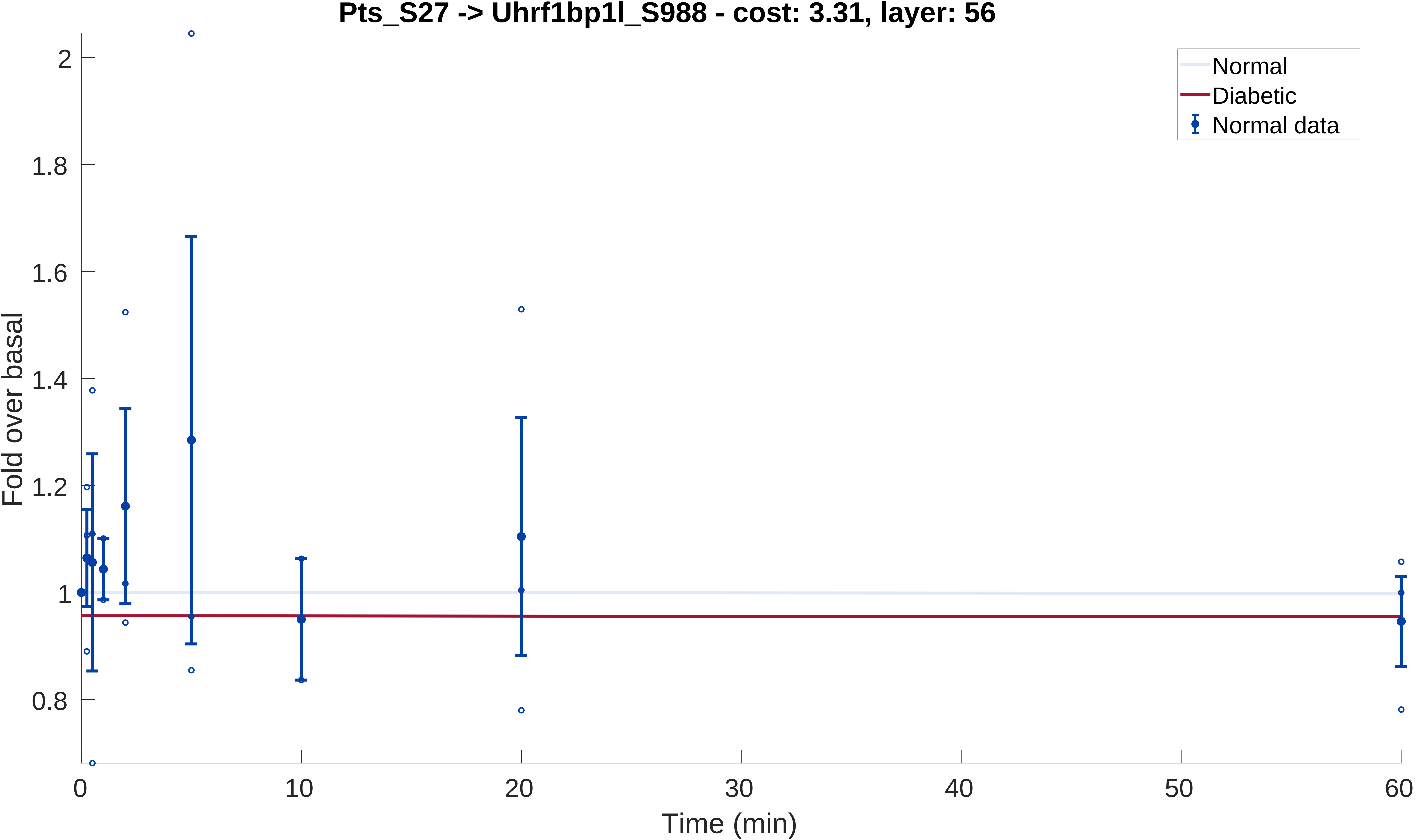

Pts_S27 -> Uhrf1bp1l_S988 - cost: 3.31, layer: 56
Normal
Diabetic
Normal data
Fold over basal
Time (min)
2
1.8
1.6
1.4
1.2
1
0.8
0
10
20
30
40
50
60

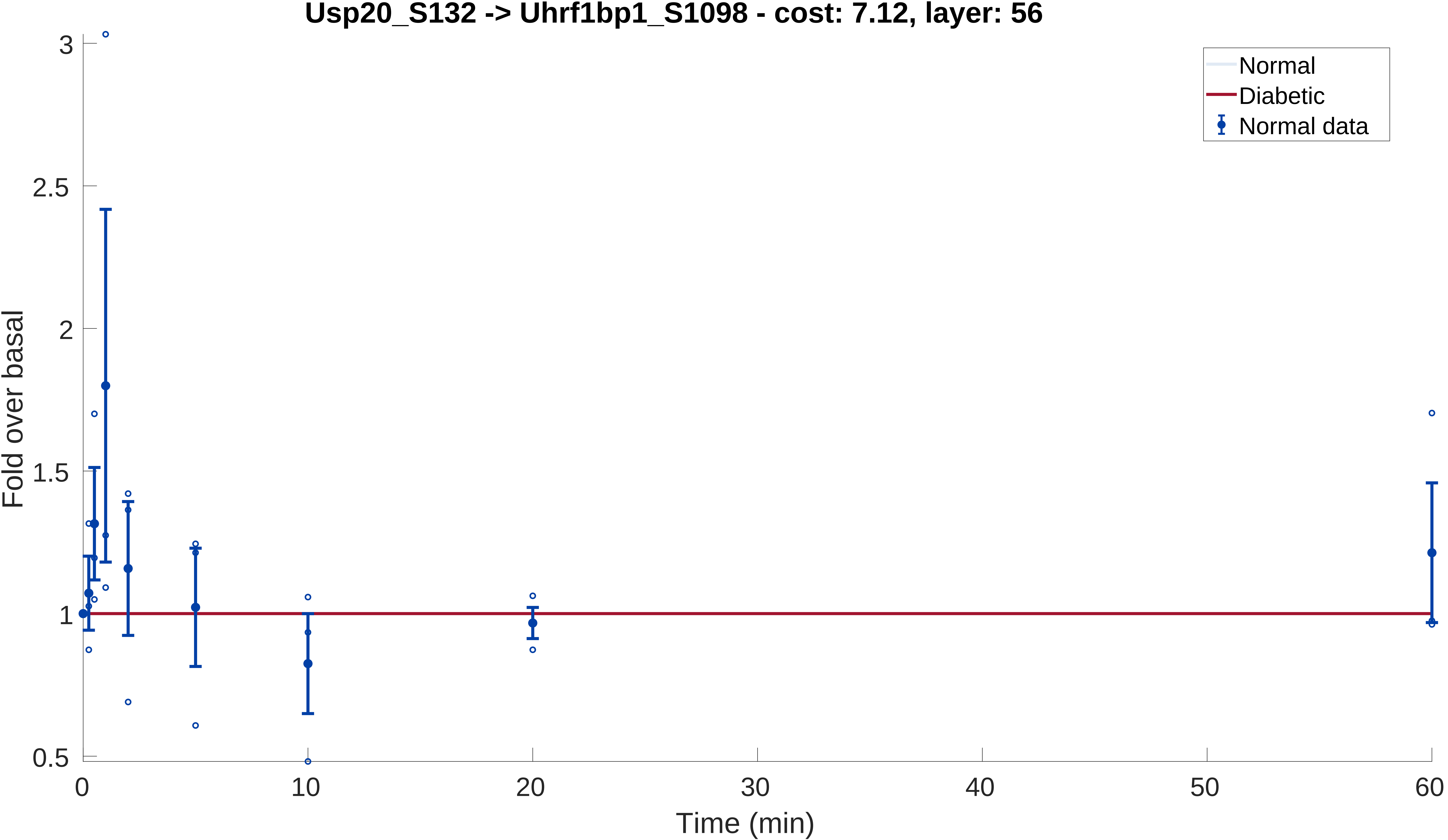

Usp20_S132 -> Uhrf1bp1_S1098 - cost: 7.12, layer: 56
Normal
Diabetic
Normal data
Fold over basal
0.5
1
1.5
2
2.5
3
0
10
20
30
40
50
60
Time (min)

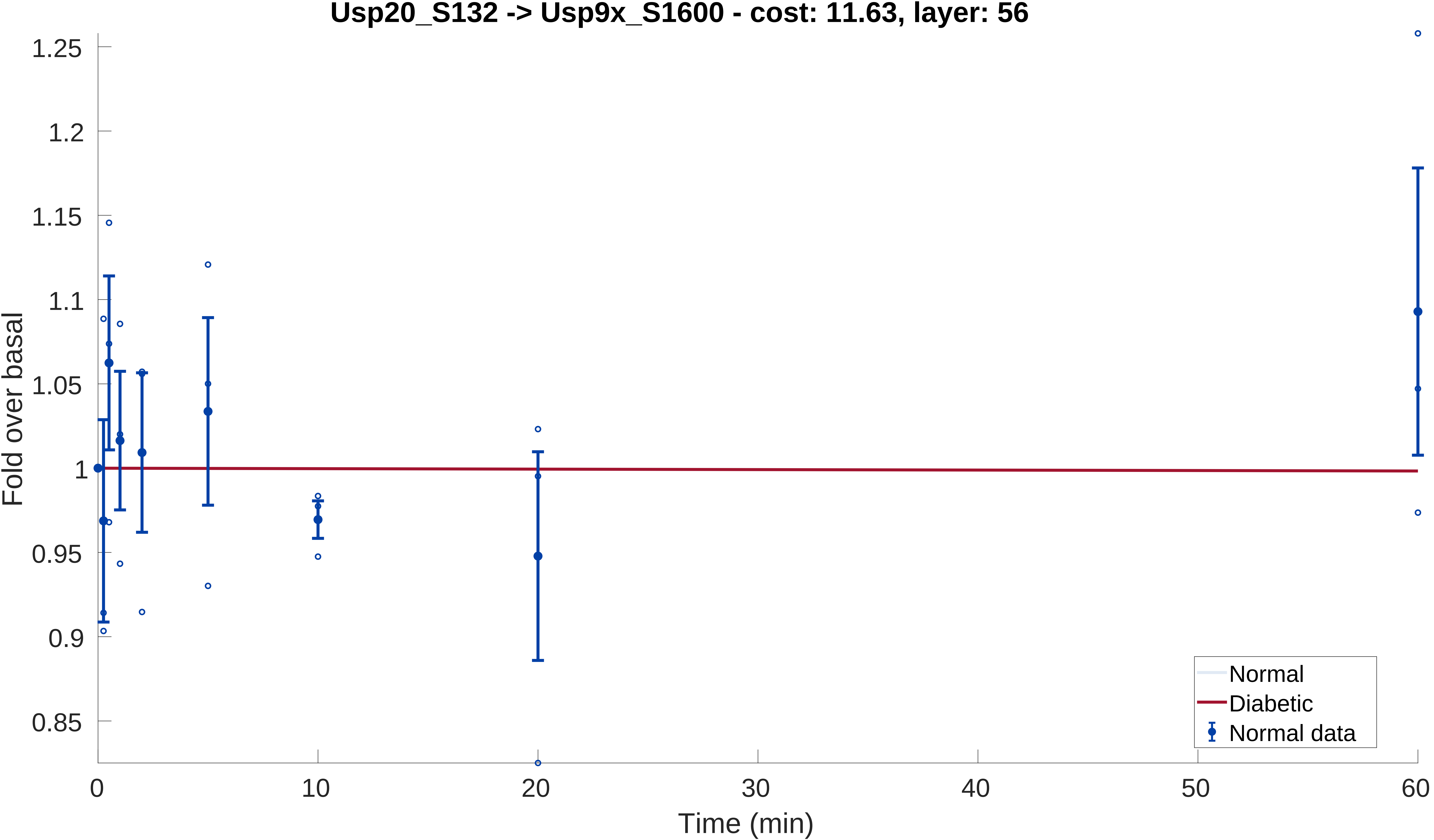
Usp20_S132 -> Usp9x_S1600 - cost: 11.63, layer: 56
Fold over basal
Time (min)
1.25
1.2
1.15
1.1
1.05
1
0.95
0.9
0.85
0
10
20
30
40
50
60
Normal
Diabetic
Normal data

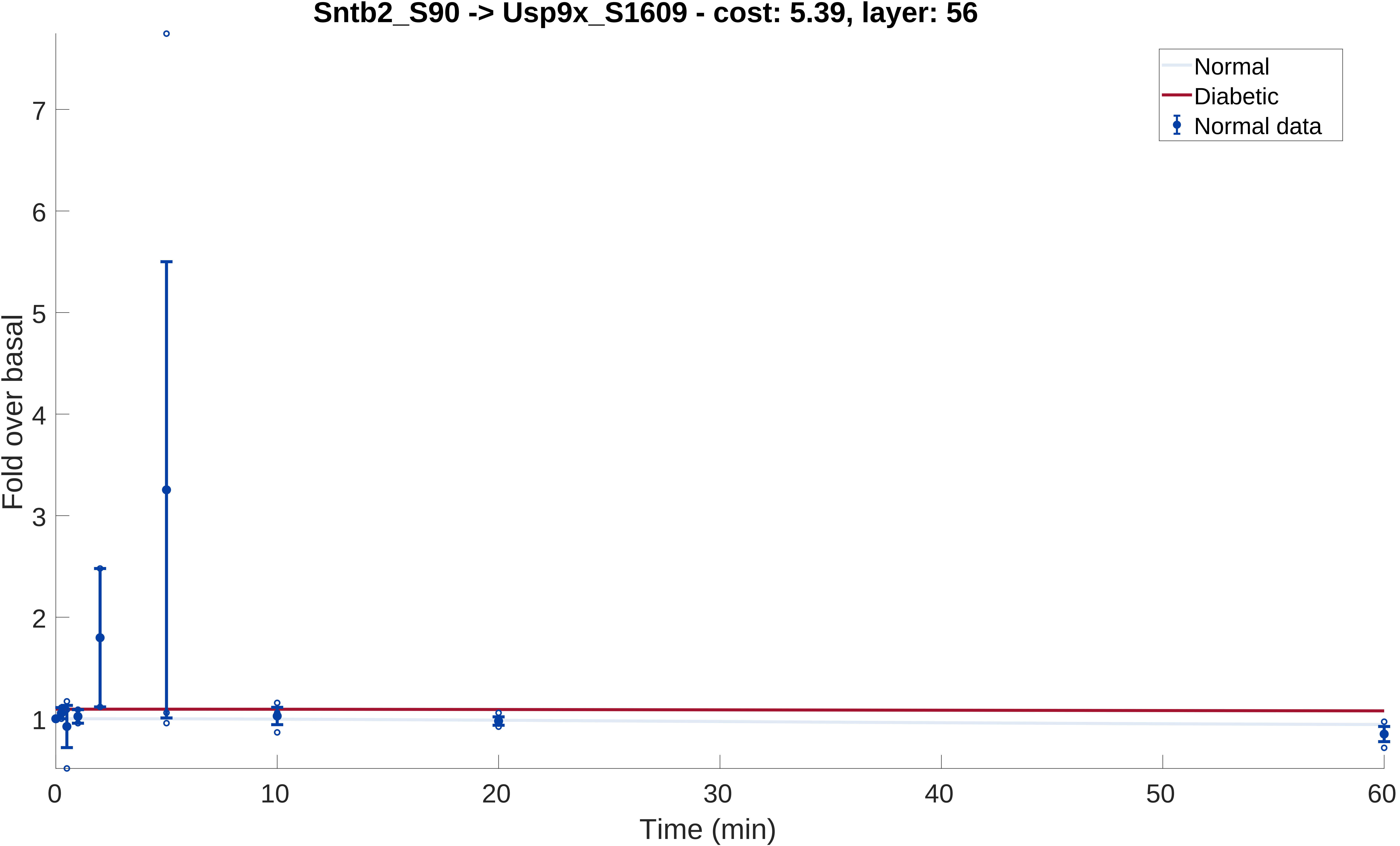

Sntb2_S90 -> Usp9x_S1609 - cost: 5.39, layer: 56
Fold over basal
Time (min)
Normal
Diabetic
Normal data
0
10
20
30
40
50
60
1
2
3
4
5
6
7

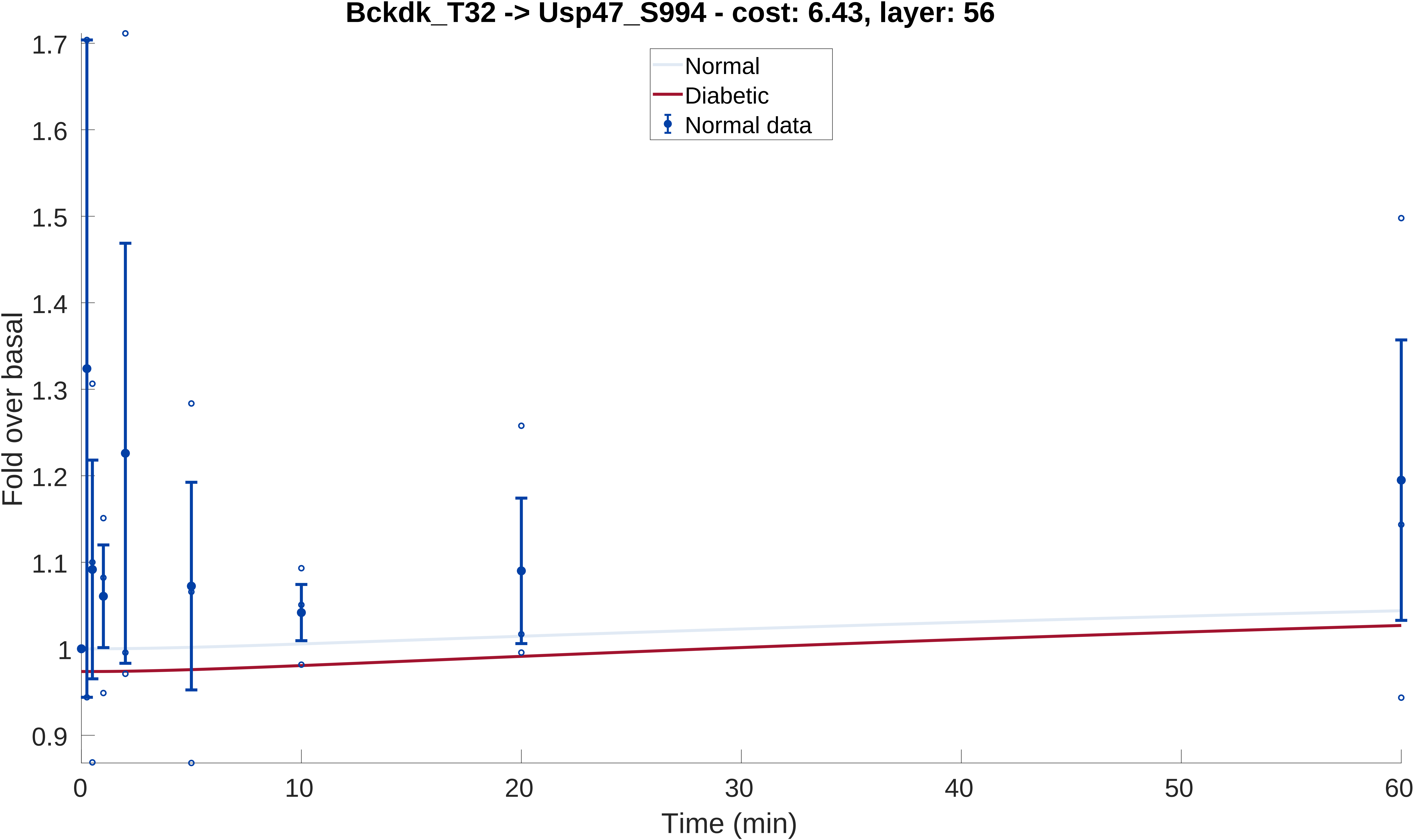

Bckdk_T32 -> Usp47_S994 - cost: 6.43, layer: 56
Normal
Diabetic
Normal data
Fold over basal
Time (min)
0.9
1
1.1
1.2
1.3
1.4
1.5
1.6
1.7
0
10
20
30
40
50
60

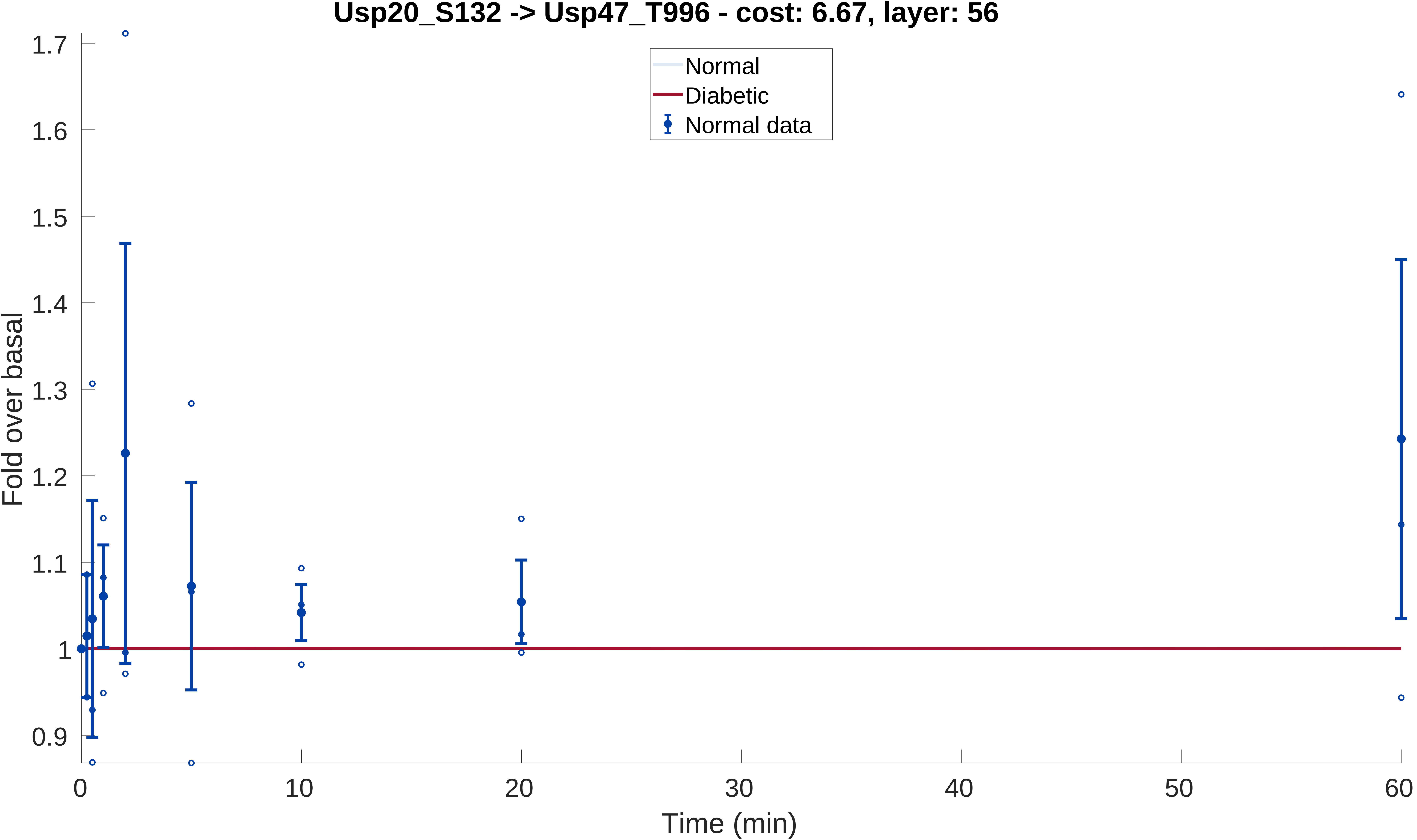

Usp20_S132 -> Usp47_T996 - cost: 6.67, layer: 56
Normal
Diabetic
Normal data
Fold over basal
Time (min)
1.7
1.6
1.5
1.4
1.3
1.2
1.1
1
0.9
0
10
20
30
40
50
60

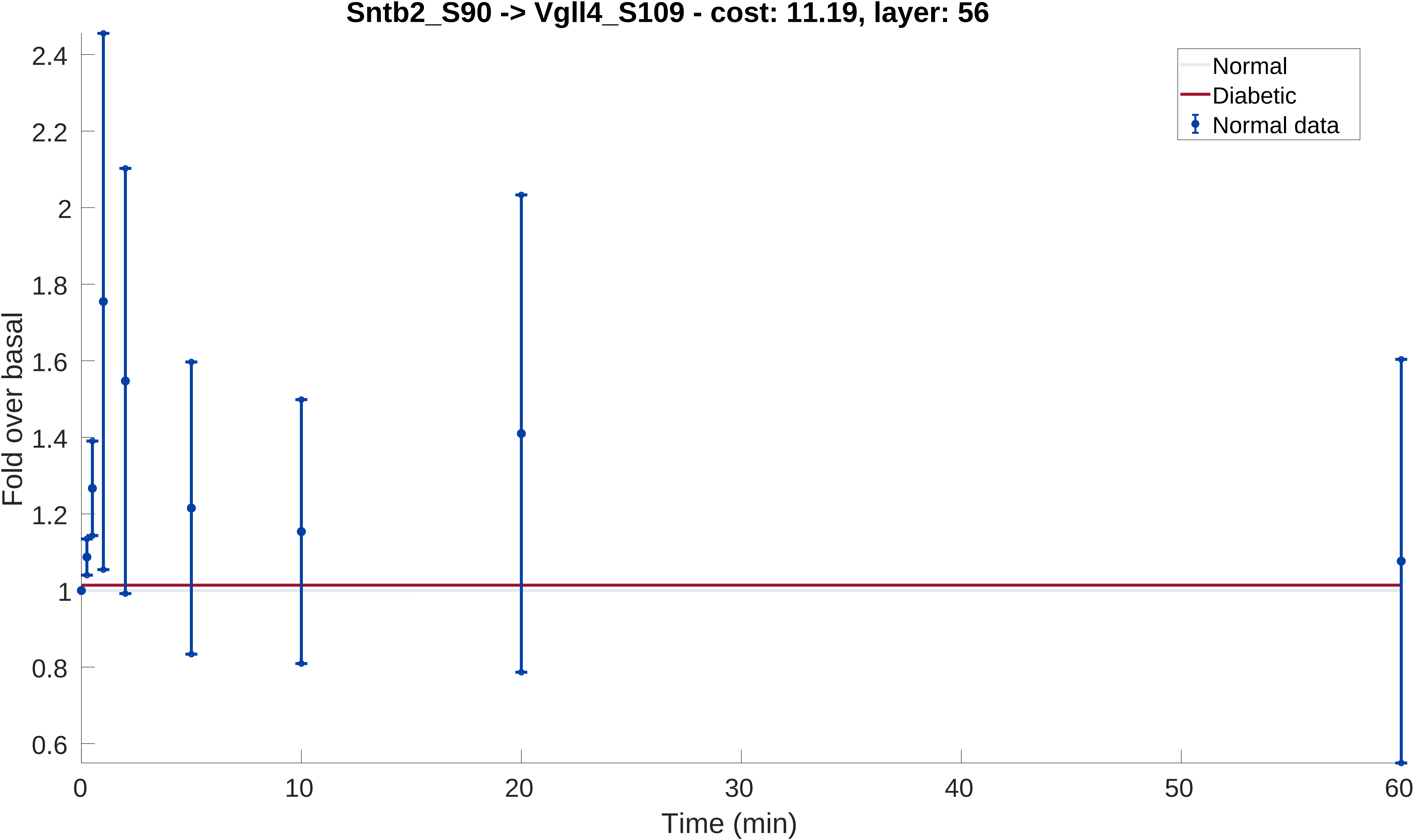
Sntb2_S90 -> Vgll4_S109 - cost: 11.19, layer: 56
Fold over basal
2.4
2.2
2
1.8
1.6
1.4
1.2
1
0.8
0.6
0
10
20
30
40
50
60
Time (min)
Normal
Diabetic
Normal data

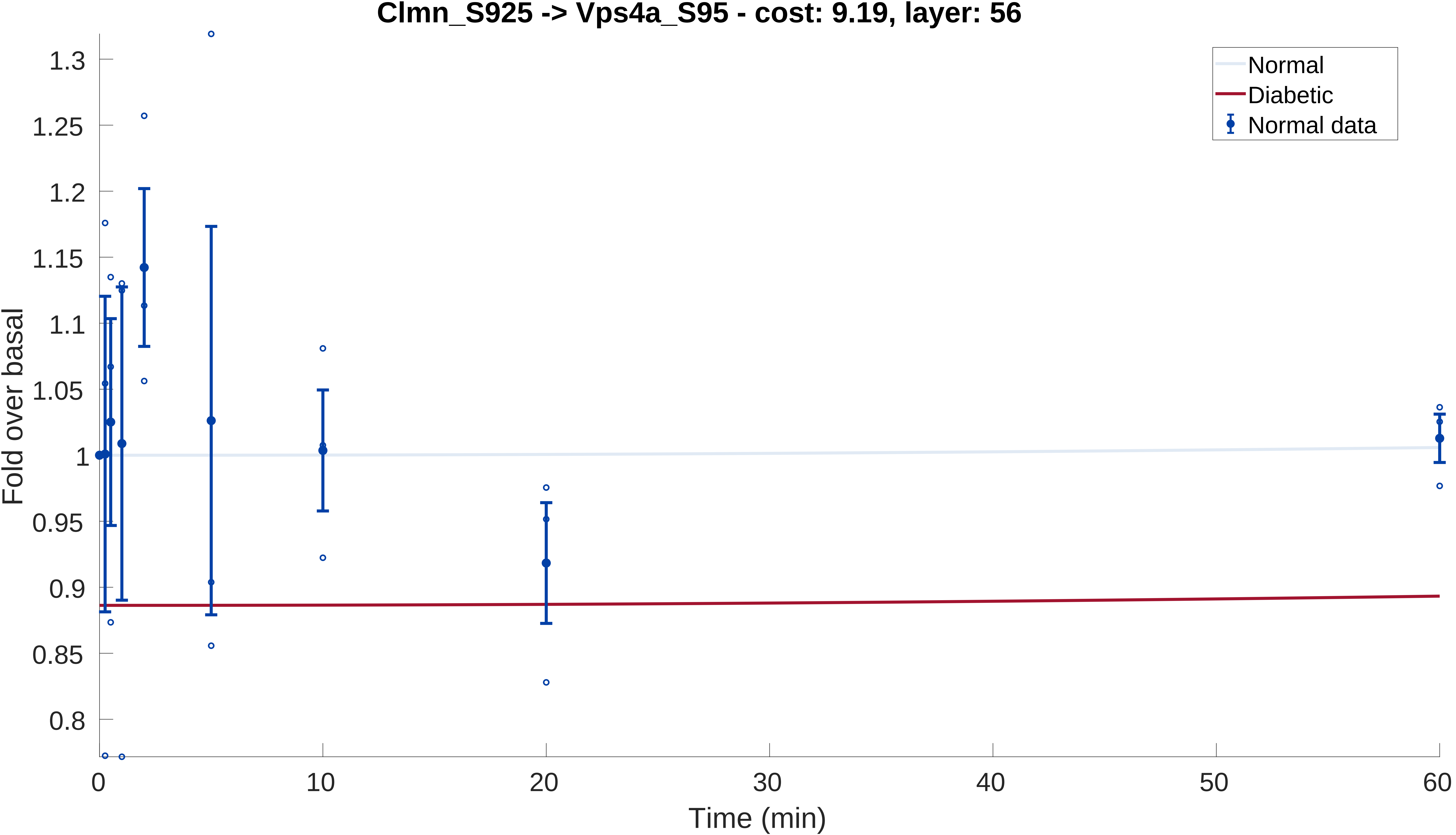

Clmn_S925 -> Vps4a_S95 - cost: 9.19, layer: 56
Normal
Diabetic
Normal data
Fold over basal
Time (min)
1.3
1.25
1.2
1.15
1.1
1.05
1
0.95
0.9
0.85
0.8
0
10
20
30
40
50
60

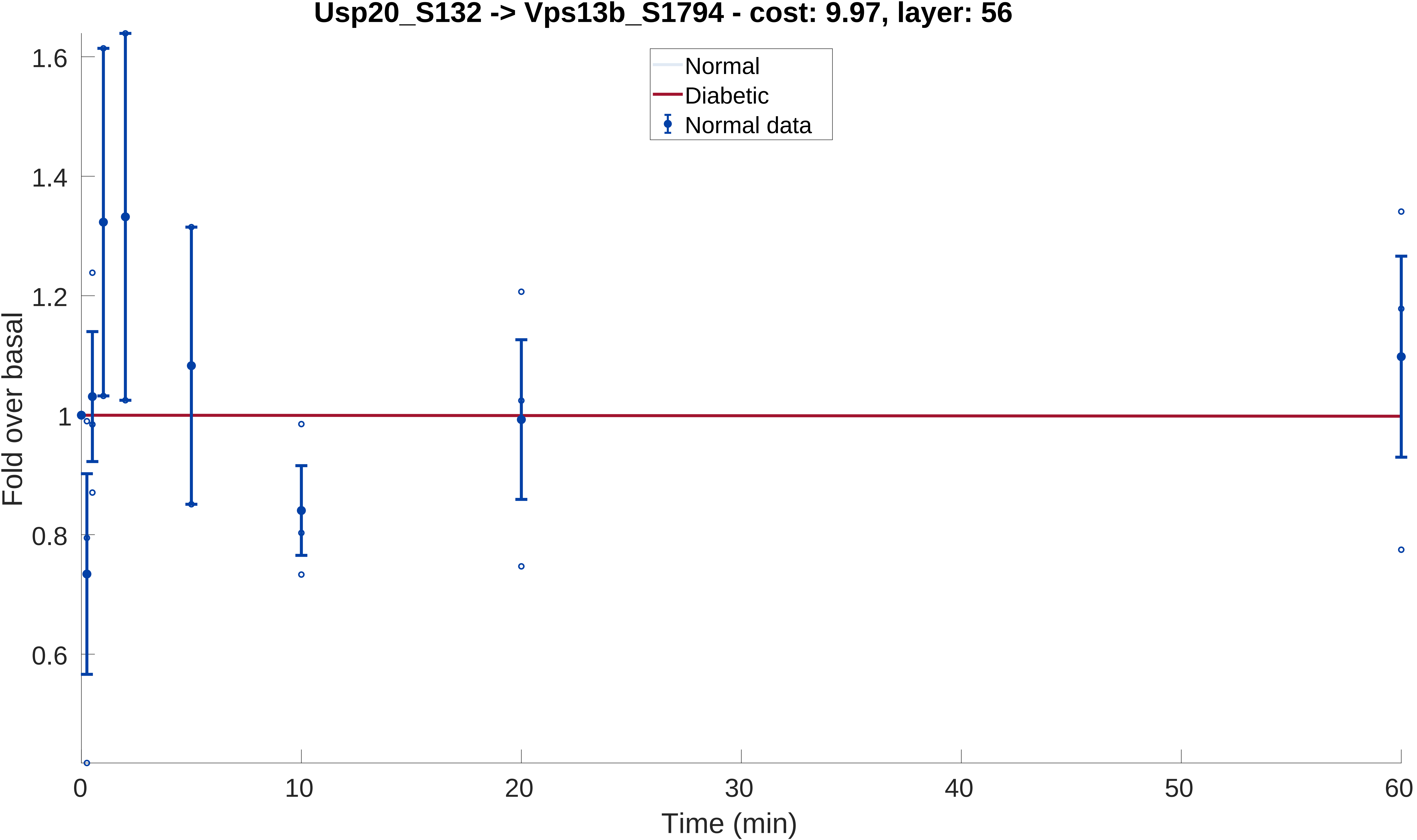

Usp20_S132 -> Vps13b_S1794 - cost: 9.97, layer: 56
Normal
Diabetic
Normal data
Fold over basal
1.6
1.4
1.2
1
0.8
0.6
0
10
20
30
40
50
60
Time (min)

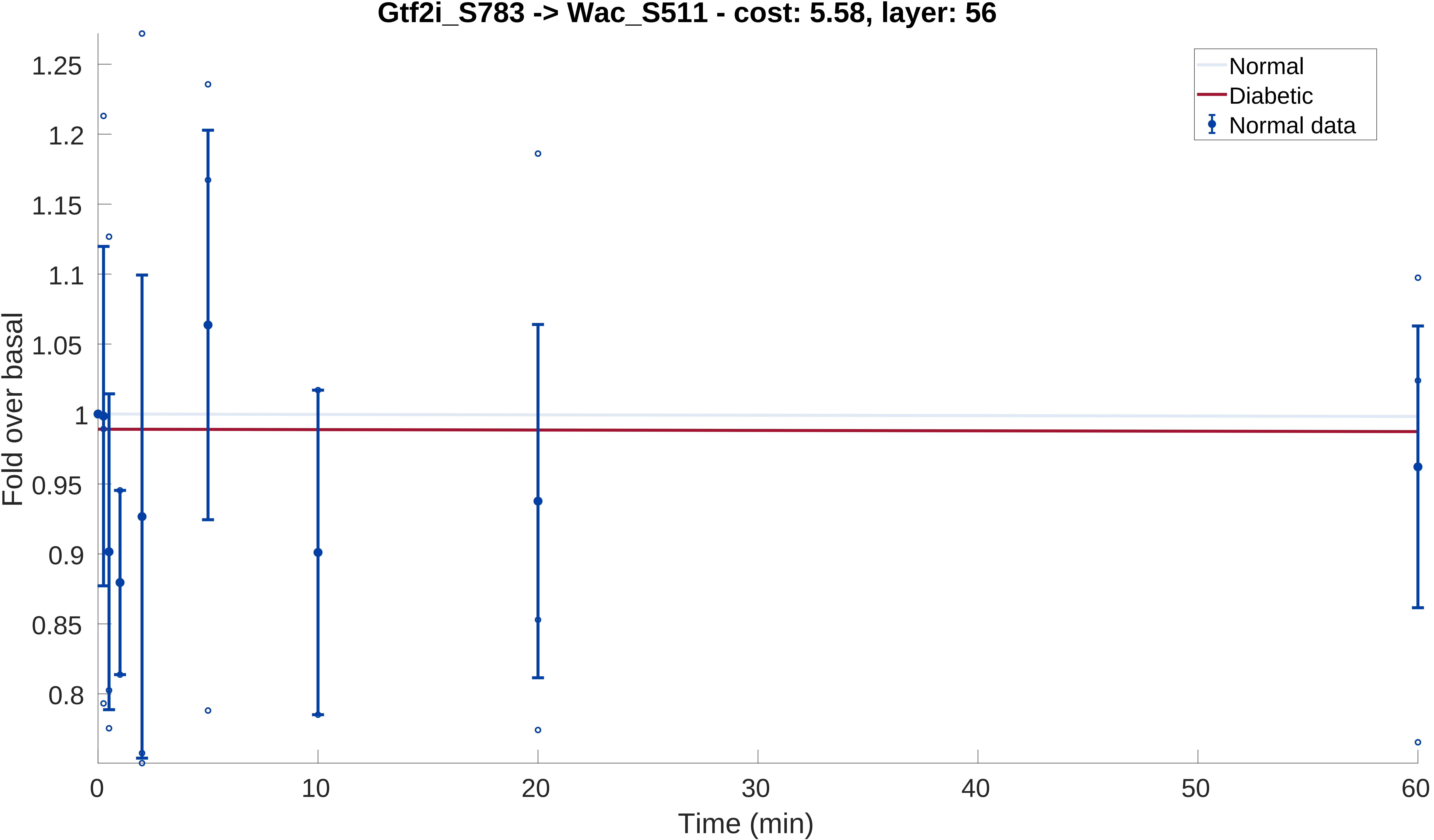
Gtf2i_S783 -> Wac_S511 - cost: 5.58, layer: 56
Normal
Diabetic
Normal data
Fold over basal
1.25
1.2
1.15
1.1
1.05
1
0.95
0.9
0.85
0.8
0
10
20
30
40
50
60
Time (min)

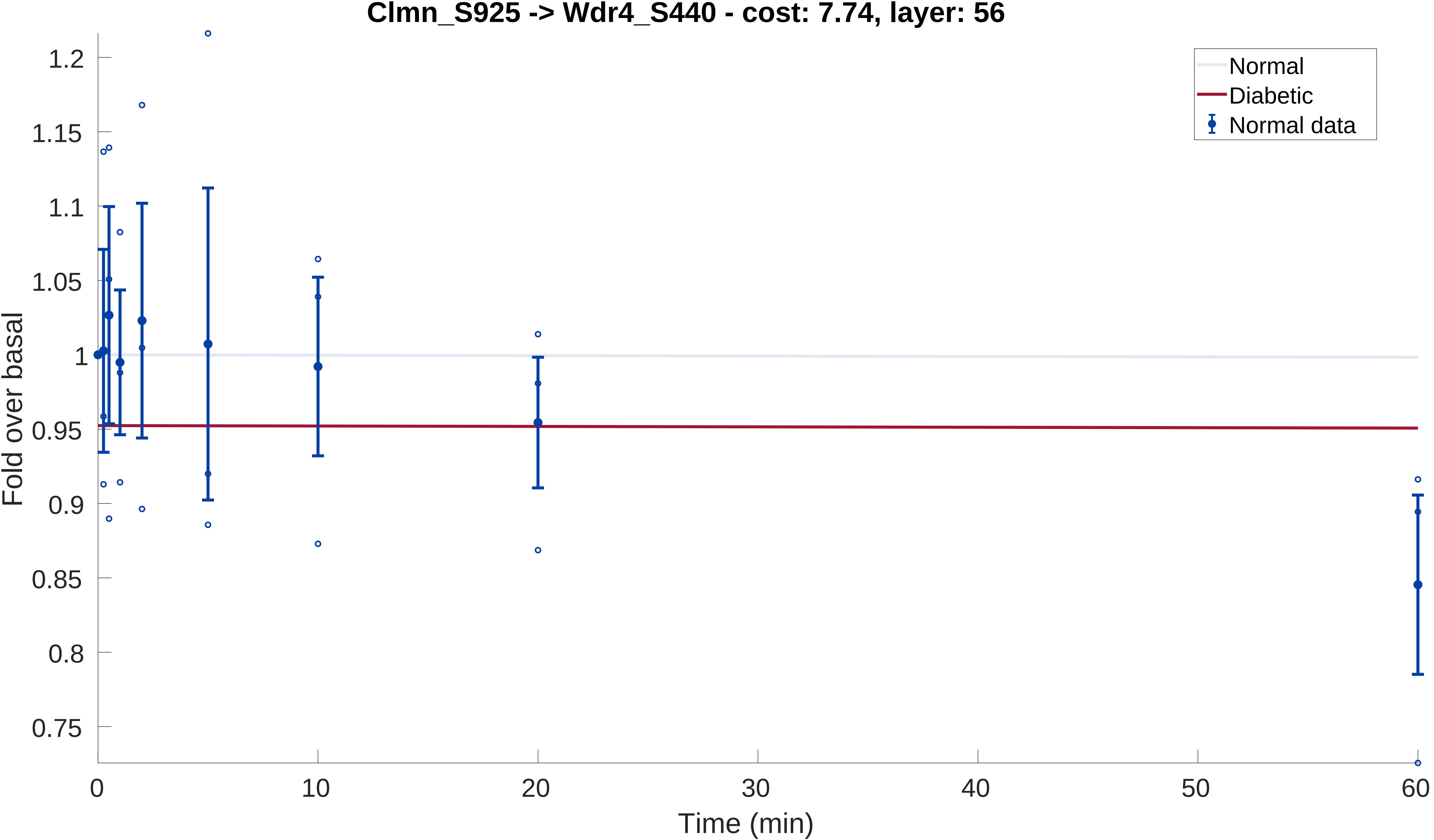
Clmn_S925 -> Wdr4_S440 - cost: 7.74, layer: 56
Normal
Diabetic
Normal data
Fold over basal
Time (min)
1.2
1.15
1.1
1.05
1
0.95
0.9
0.85
0.8
0.75
0
10
20
30
40
50
60

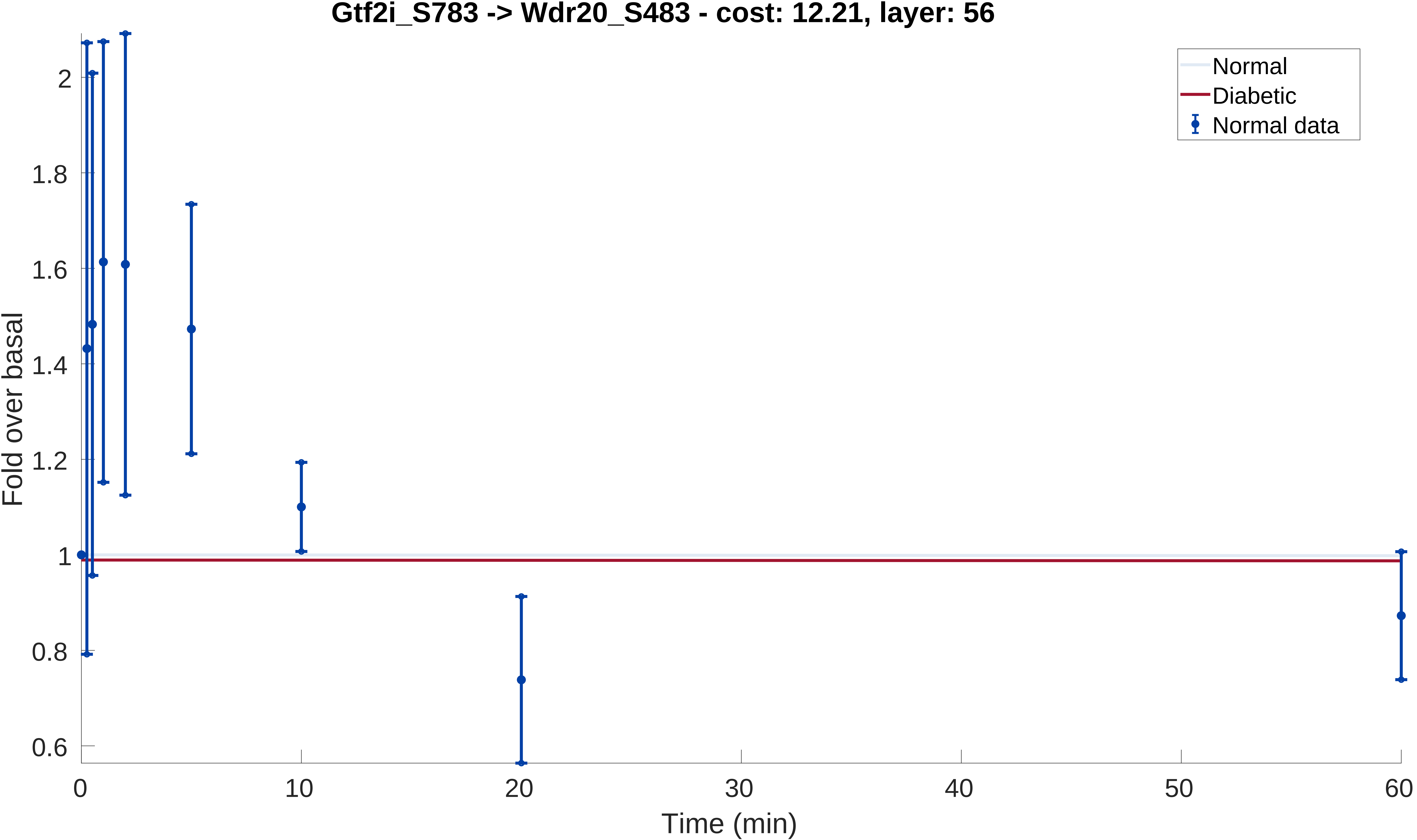
Gtf2i_S783 -> Wdr20_S483 - cost: 12.21, layer: 56
Fold over basal
Time (min)
Normal
Diabetic
Normal data
0.6
0.8
1
1.2
1.4
1.6
1.8
2
0
10
20
30
40
50
60

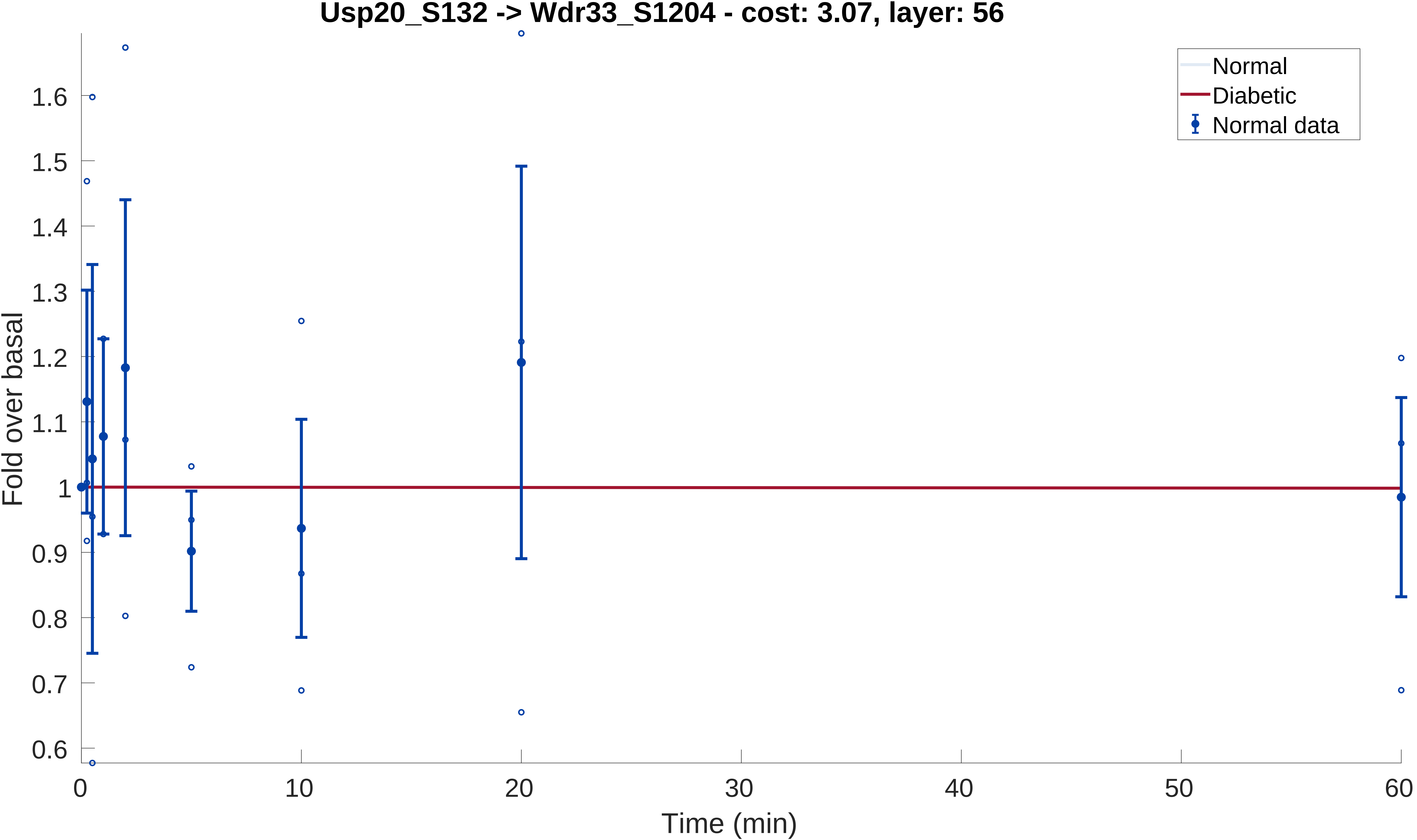

Usp20_S132 -> Wdr33_S1204 - cost: 3.07, layer: 56
Normal
Diabetic
Normal data
Fold over basal
Time (min)
1.6
1.5
1.4
1.3
1.2
1.1
1
0.9
0.8
0.7
0.6
0
10
20
30
40
50
60

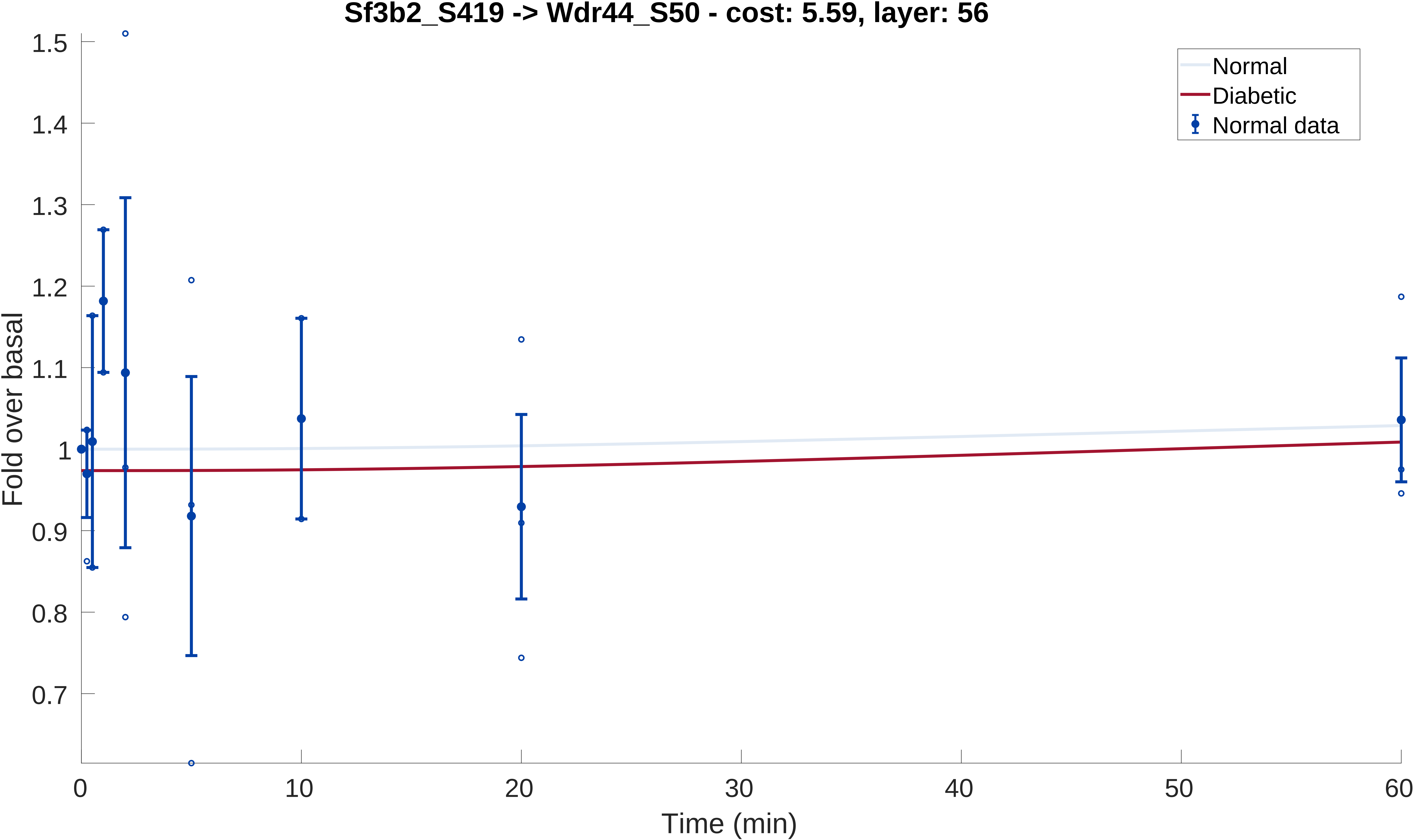

Sf3b2_S419 -> Wdr44_S50 - cost: 5.59, layer: 56
Normal
Diabetic
Normal data
Fold over basal
1.5
1.4
1.3
1.2
1.1
1
0.9
0.8
0.7
0
10
20
30
40
50
60
Time (min)

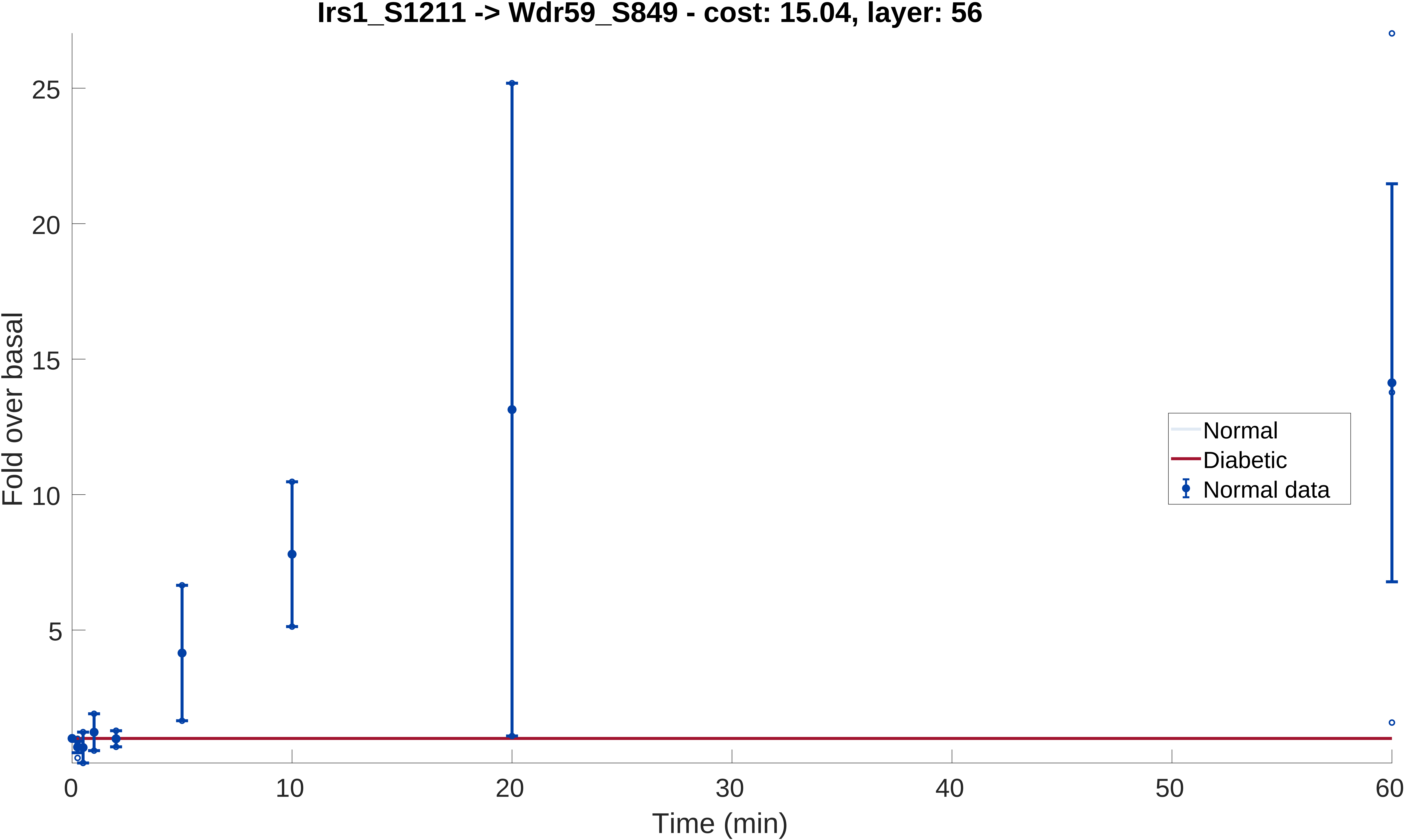

Irs1_S1211 -> Wdr59_S849 - cost: 15.04, layer: 56
Fold over basal
Time (min)
0
5
10
15
20
25
0
10
20
30
40
50
60
Normal
Diabetic
Normal data

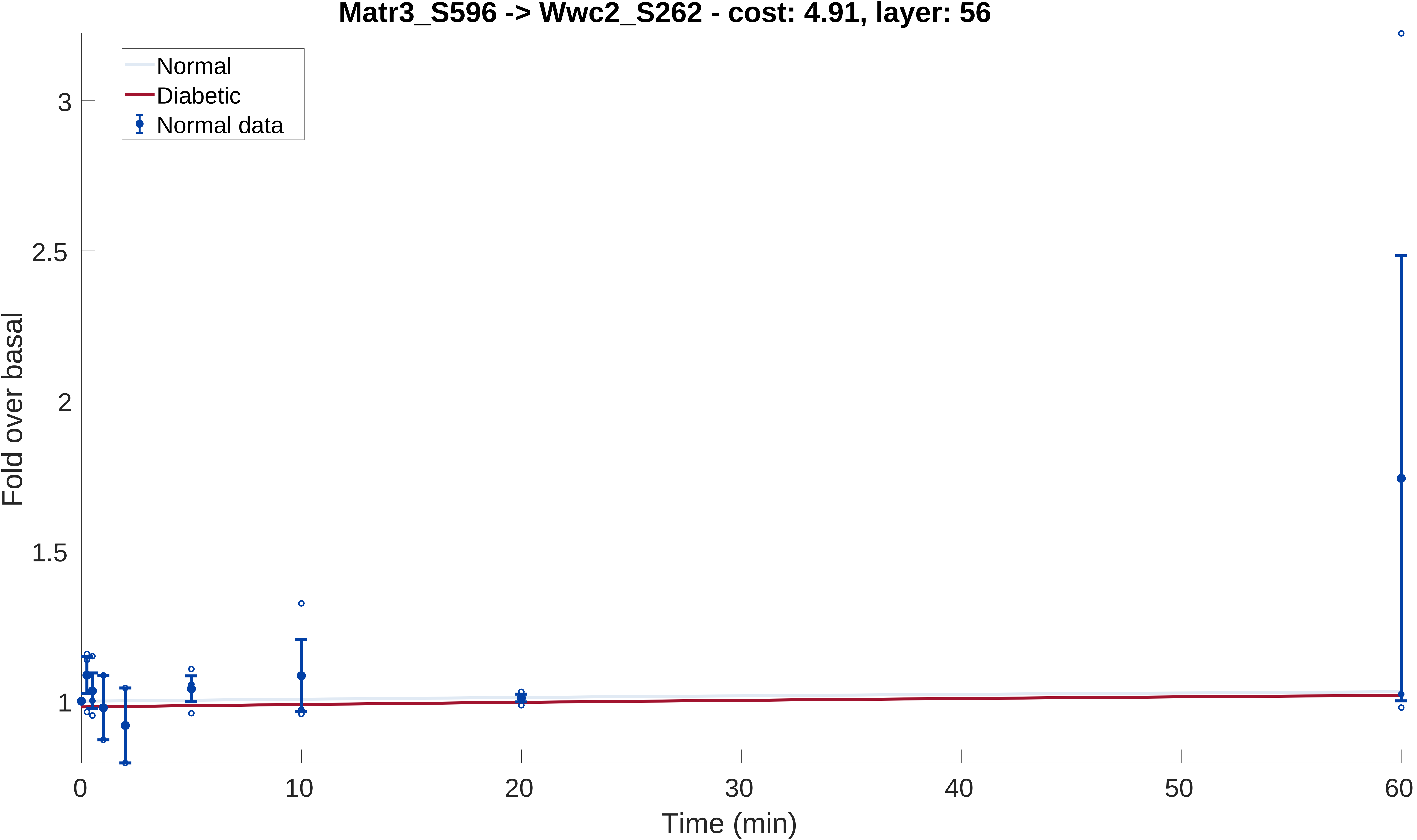

Matr3_S596 -> Wwc2_S262 - cost: 4.91, layer: 56
Normal
Diabetic
Normal data
Fold over basal
3
2.5
2
1.5
1
0
10
20
30
40
50
60
Time (min)

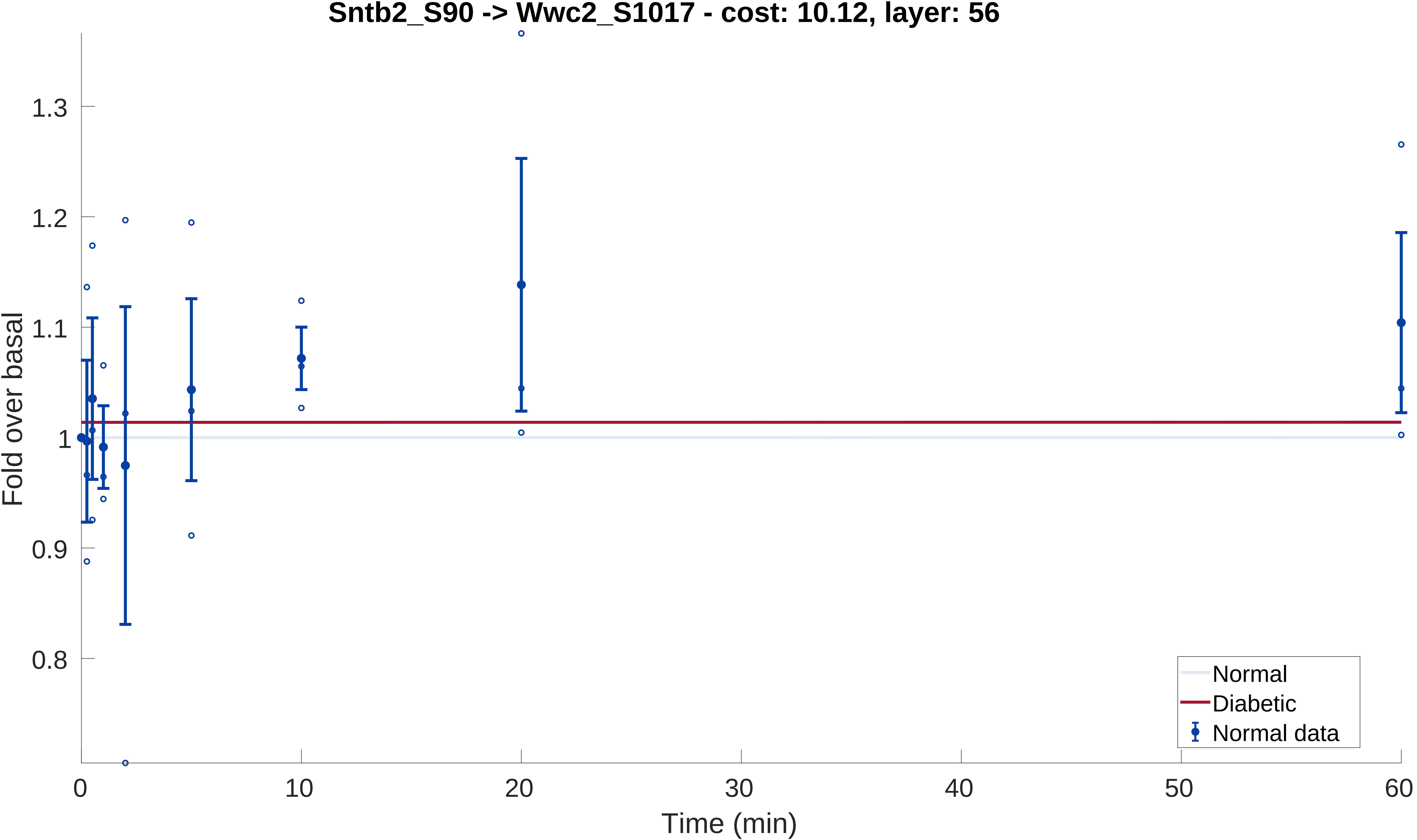

Sntb2_S90 -> Wwc2_S1017 - cost: 10.12, layer: 56
Fold over basal
1.3
1.2
1.1
1
0.9
0.8
0
10
20
30
40
50
60
Time (min)
Normal
Diabetic
Normal data

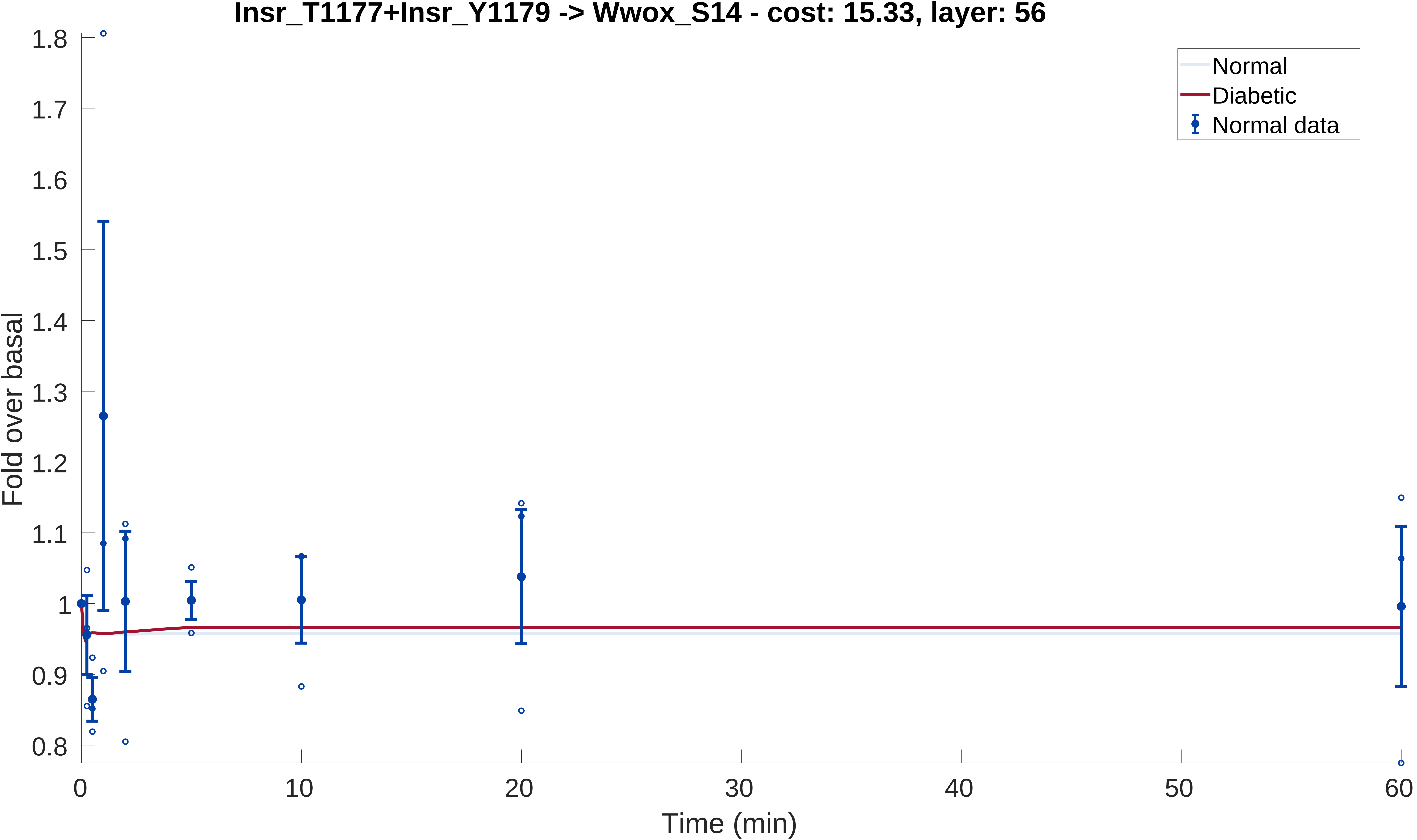

Insr_T1177+Insr_Y1179 -> Wwox_S14 - cost: 15.33, layer: 56
Fold over basal
1.8
1.7
1.6
1.5
1.4
1.3
1.2
1.1
1
0.9
0.8
0
10
20
30
40
50
60
Time (min)
Normal
Diabetic
Normal data

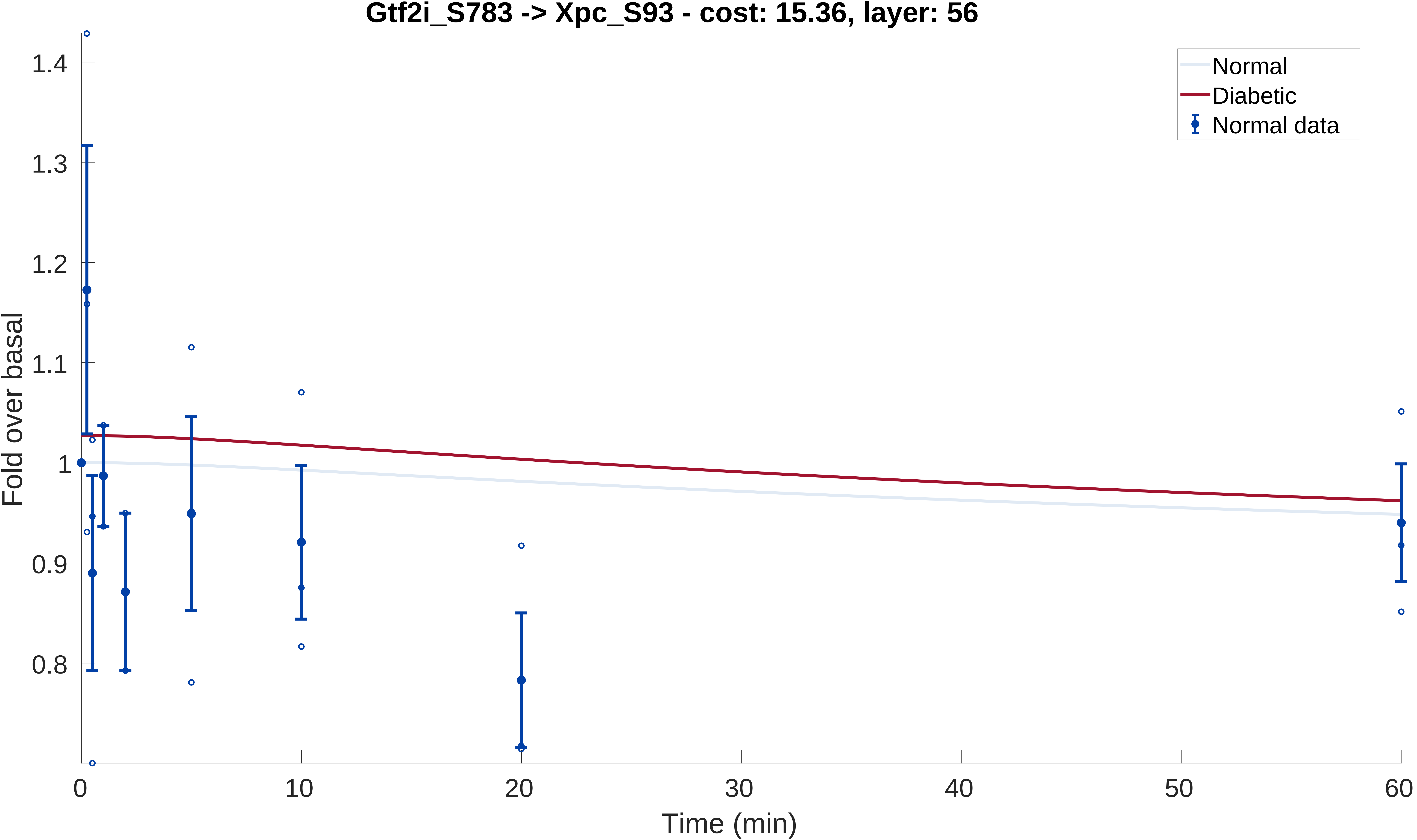

Gtf2i_S783 -> Xpc_S93 - cost: 15.36, layer: 56
Normal
Diabetic
Normal data
Fold over basal
1.4
1.3
1.2
1.1
1
0.9
0.8
0
10
20
30
40
50
60
Time (min)

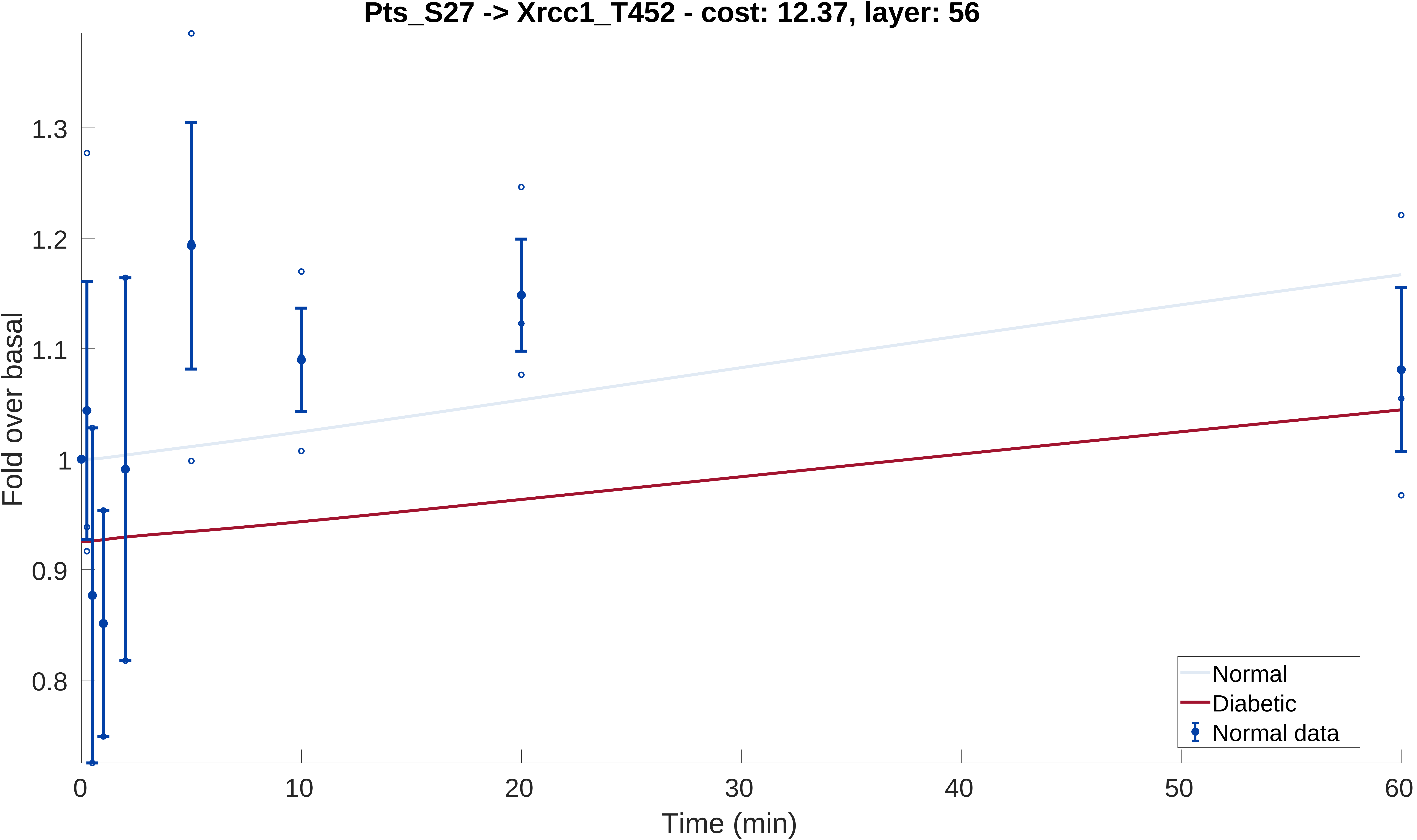

Pts_S27 -> Xrcc1_T452 - cost: 12.37, layer: 56
Fold over basal
1.3
1.2
1.1
1
0.9
0.8
0
10
20
30
40
50
60
Time (min)
Normal
Diabetic
Normal data

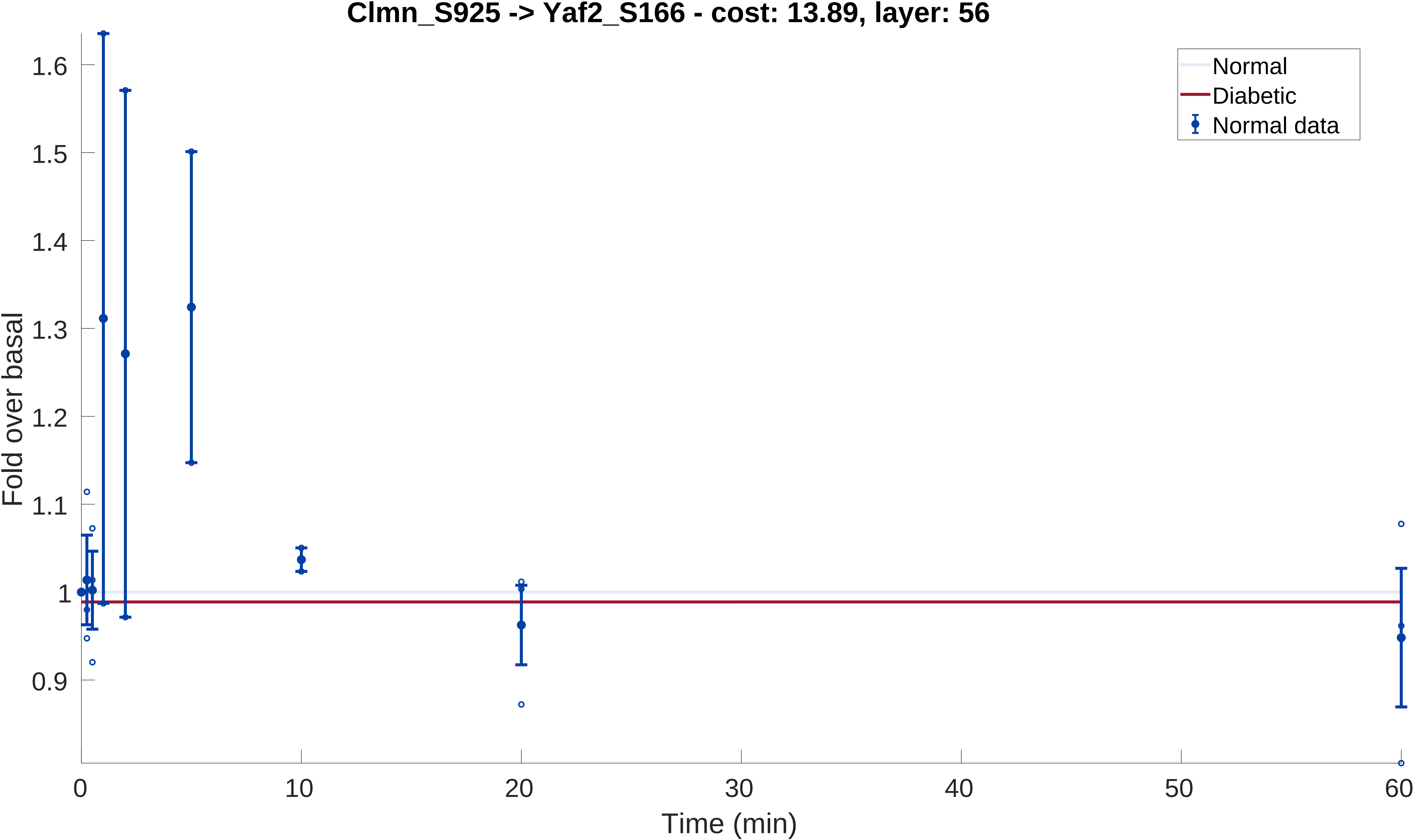

Clmn_S925 -> Yaf2_S166 - cost: 13.89, layer: 56
Normal
Diabetic
Normal data
Fold over basal
1.6
1.5
1.4
1.3
1.2
1.1
1
0.9
0
10
20
30
40
50
60
Time (min)

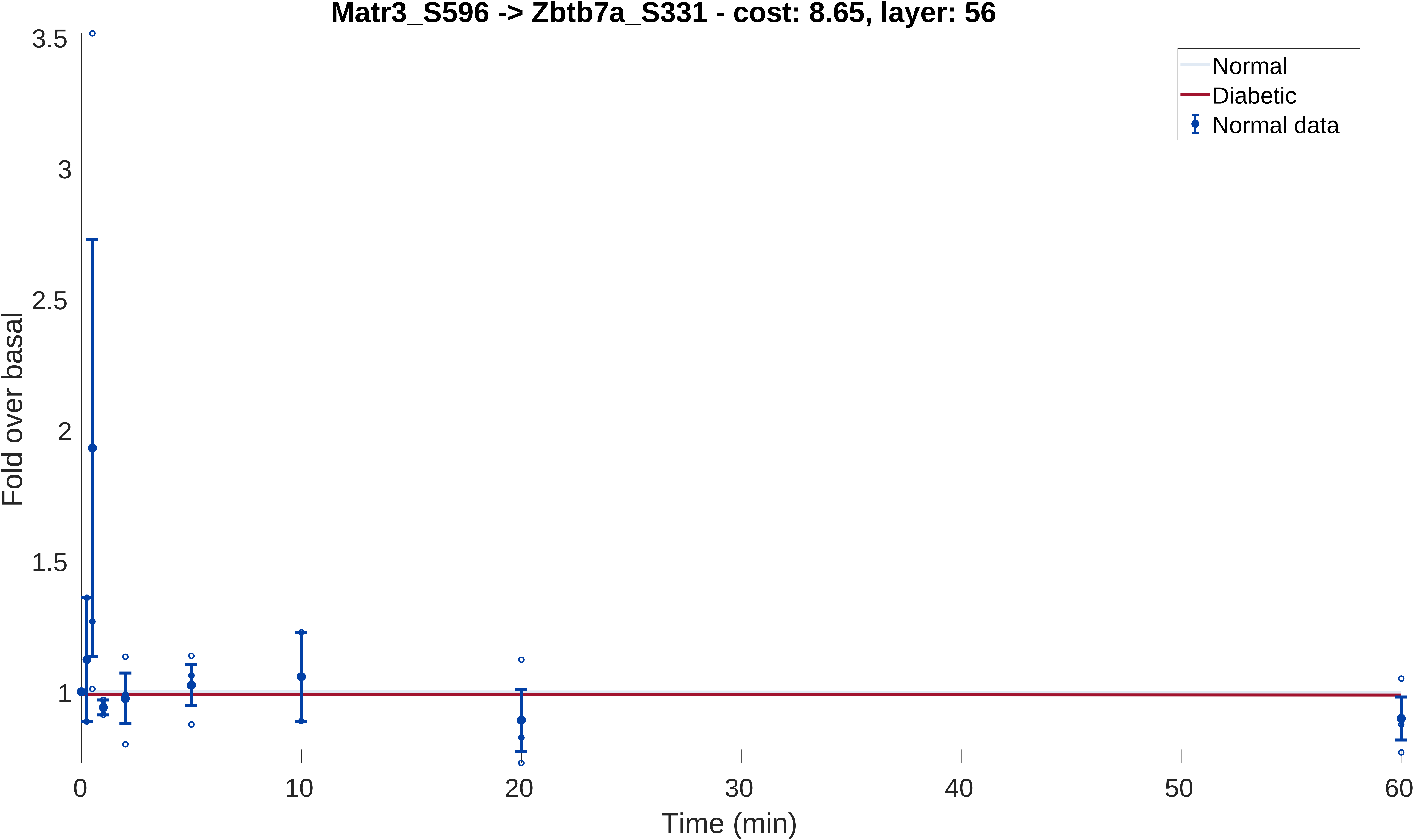
Matr3_S596 -> Zbtb7a_S331 - cost: 8.65, layer: 56
Normal
Diabetic
Normal data
Fold over basal
Time (min)
3.5
3
2.5
2
1.5
1
0
10
20
30
40
50
60

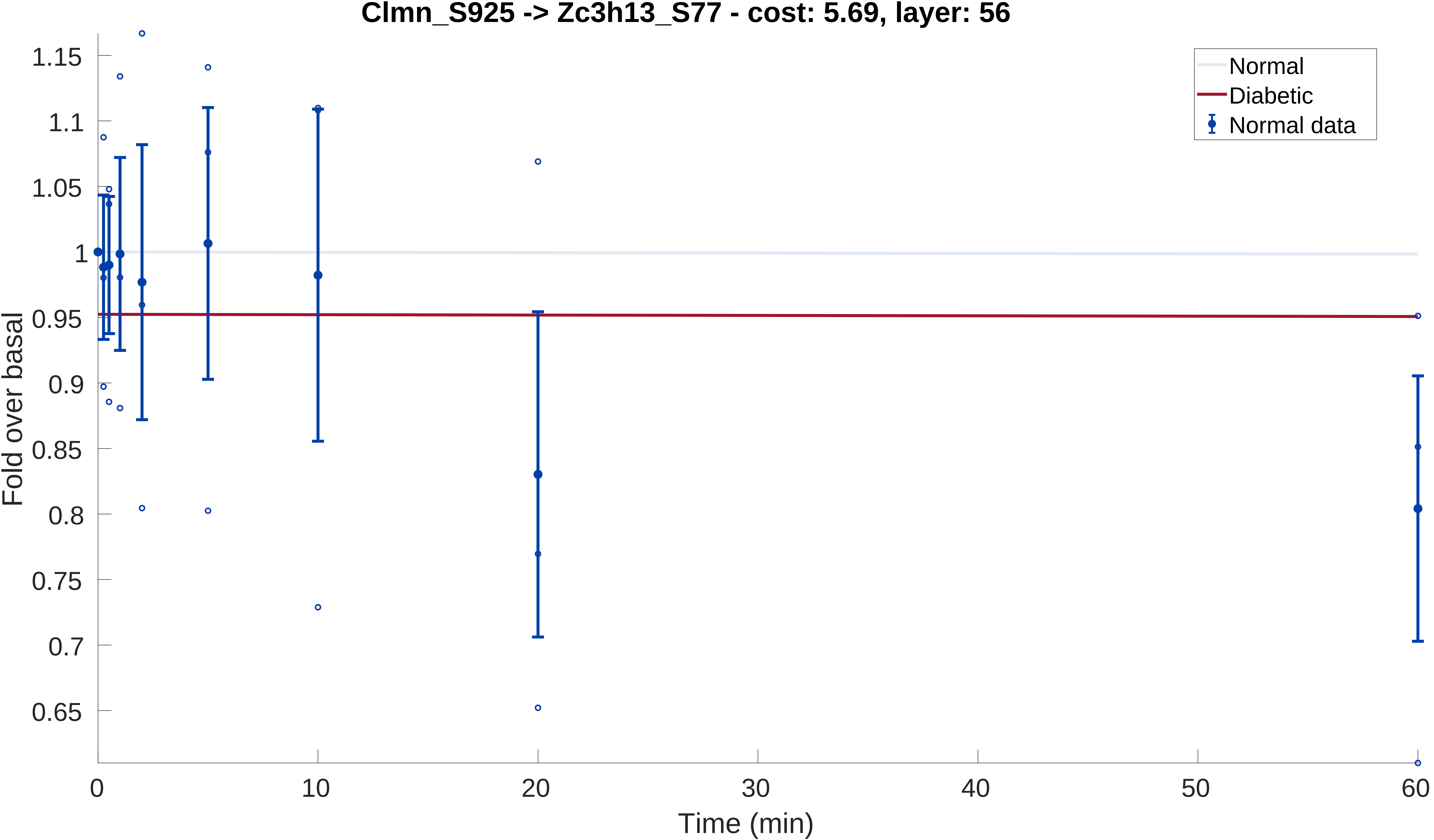
Clmn_S925 -> Zc3h13_S77 - cost: 5.69, layer: 56
Normal
Diabetic
Normal data
Fold over basal
Time (min)
1.15
1.1
1.05
1
0.95
0.9
0.85
0.8
0.75
0.7
0.65
0
10
20
30
40
50
60

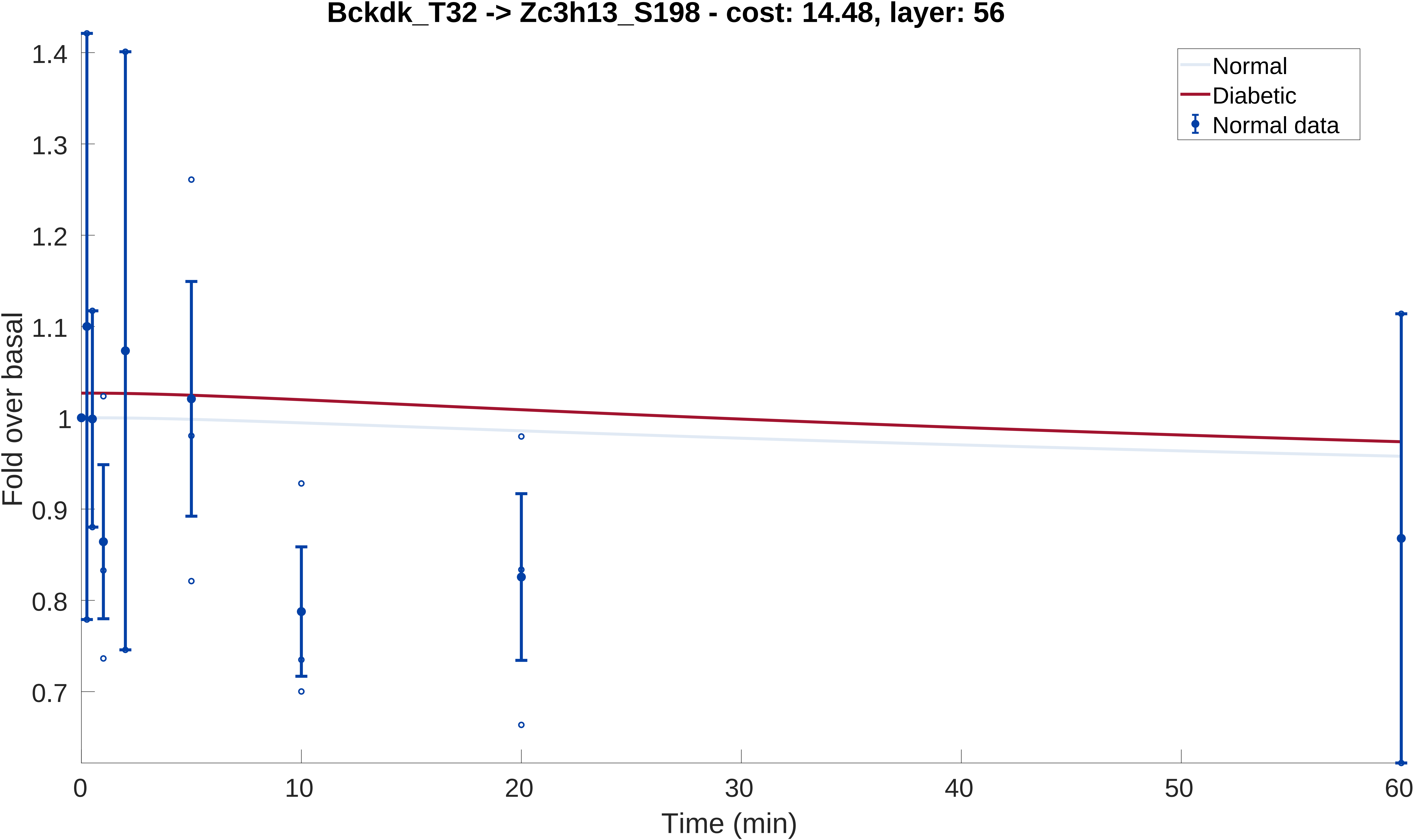

Bckdk_T32 -> Zc3h13_S198 - cost: 14.48, layer: 56
Normal
Diabetic
Normal data
Fold over basal
Time (min)
1.4
1.3
1.2
1.1
1
0.9
0.8
0.7
0
10
20
30
40
50
60

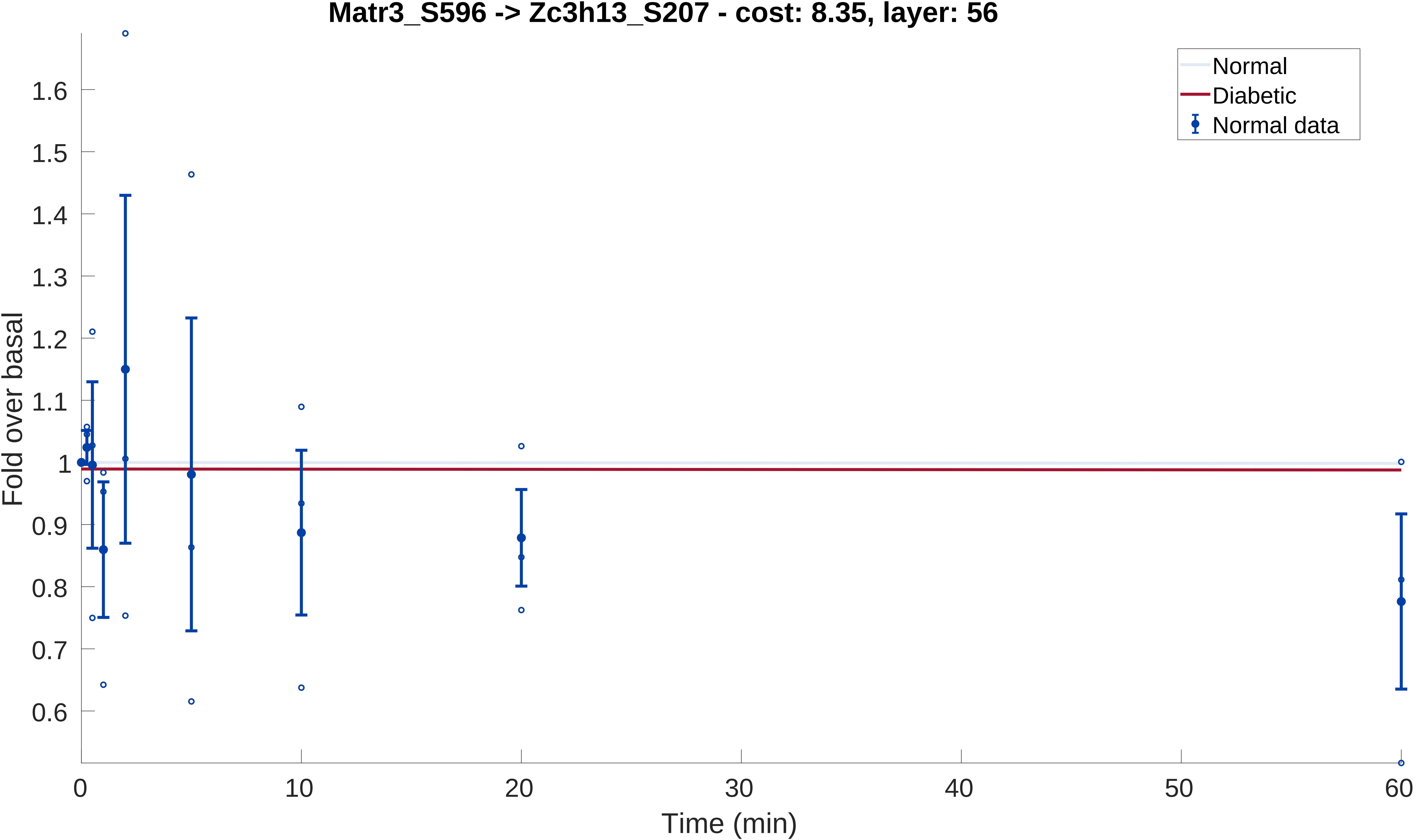

Matr3_S596 -> Zc3h13_S207 - cost: 8.35, layer: 56
Normal
Diabetic
Normal data
Fold over basal
1.6
1.5
1.4
1.3
1.2
1.1
1
0.9
0.8
0.7
0.6
0
10
20
30
40
50
60
Time (min)

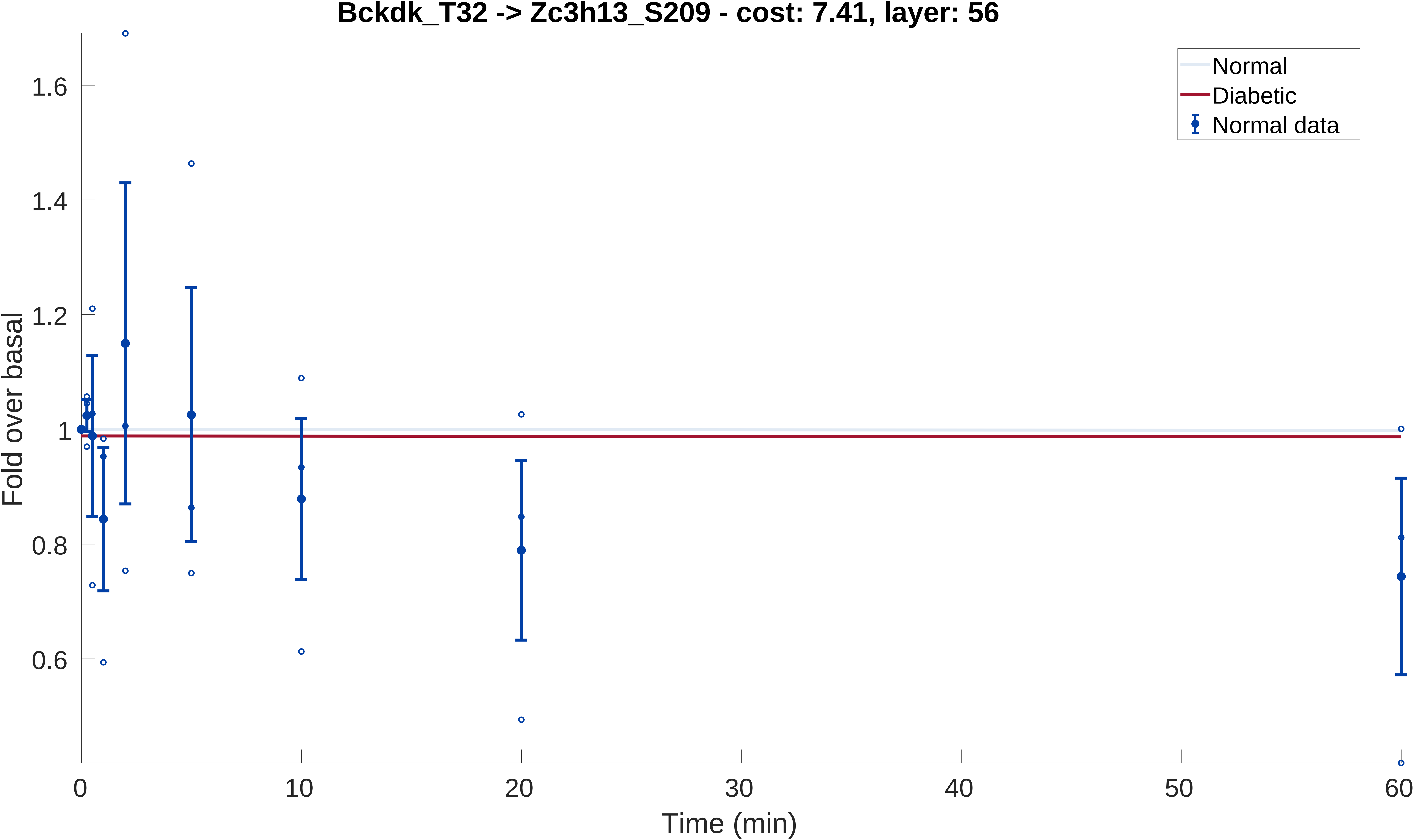

Bckdk_T32 -> Zc3h13_S209 - cost: 7.41, layer: 56
Normal
Diabetic
Normal data
Fold over basal
1.6
1.4
1.2
1
0.8
0.6
0
10
20
30
40
50
60
Time (min)

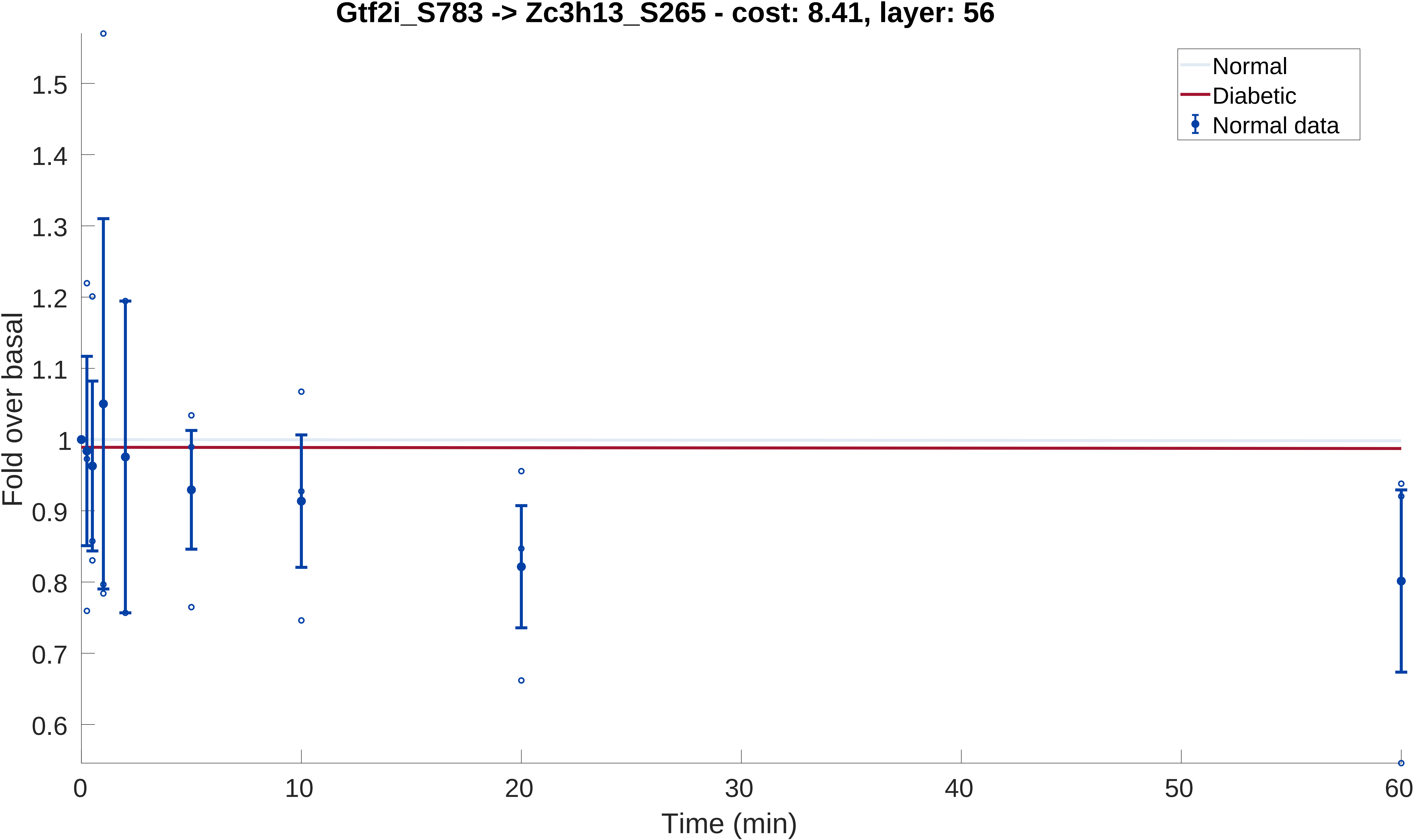
Gtf2i_S783 -> Zc3h13_S265 - cost: 8.41, layer: 56
Normal
Diabetic
Normal data
Fold over basal
1.5
1.4
1.3
1.2
1.1
1
0.9
0.8
0.7
0.6
0
10
20
30
40
50
60
Time (min)

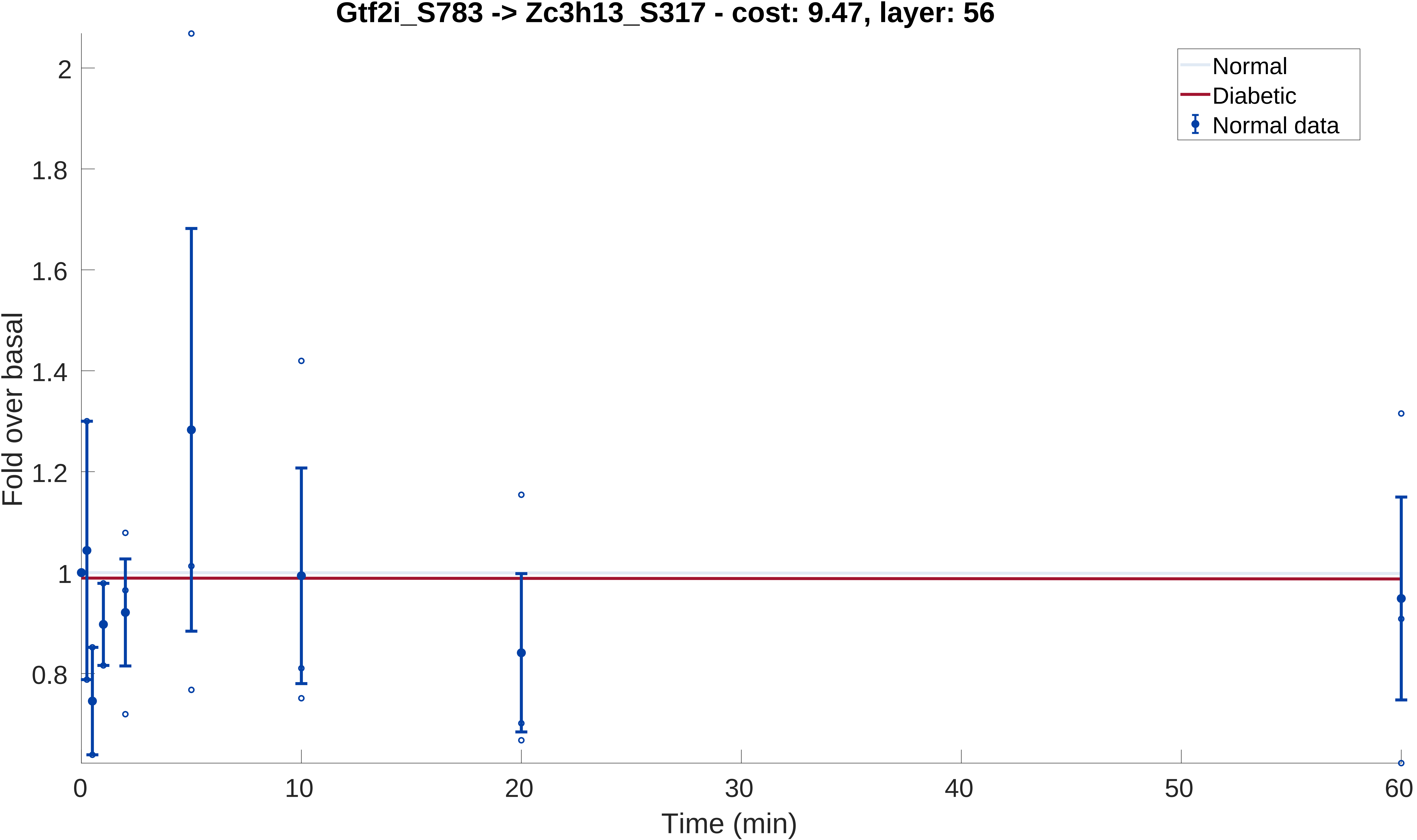

Gtf2i_S783 -> Zc3h13_S317 - cost: 9.47, layer: 56
Normal
Diabetic
Normal data
Fold over basal
Time (min)
0.8
1
1.2
1.4
1.6
1.8
2
0
10
20
30
40
50
60

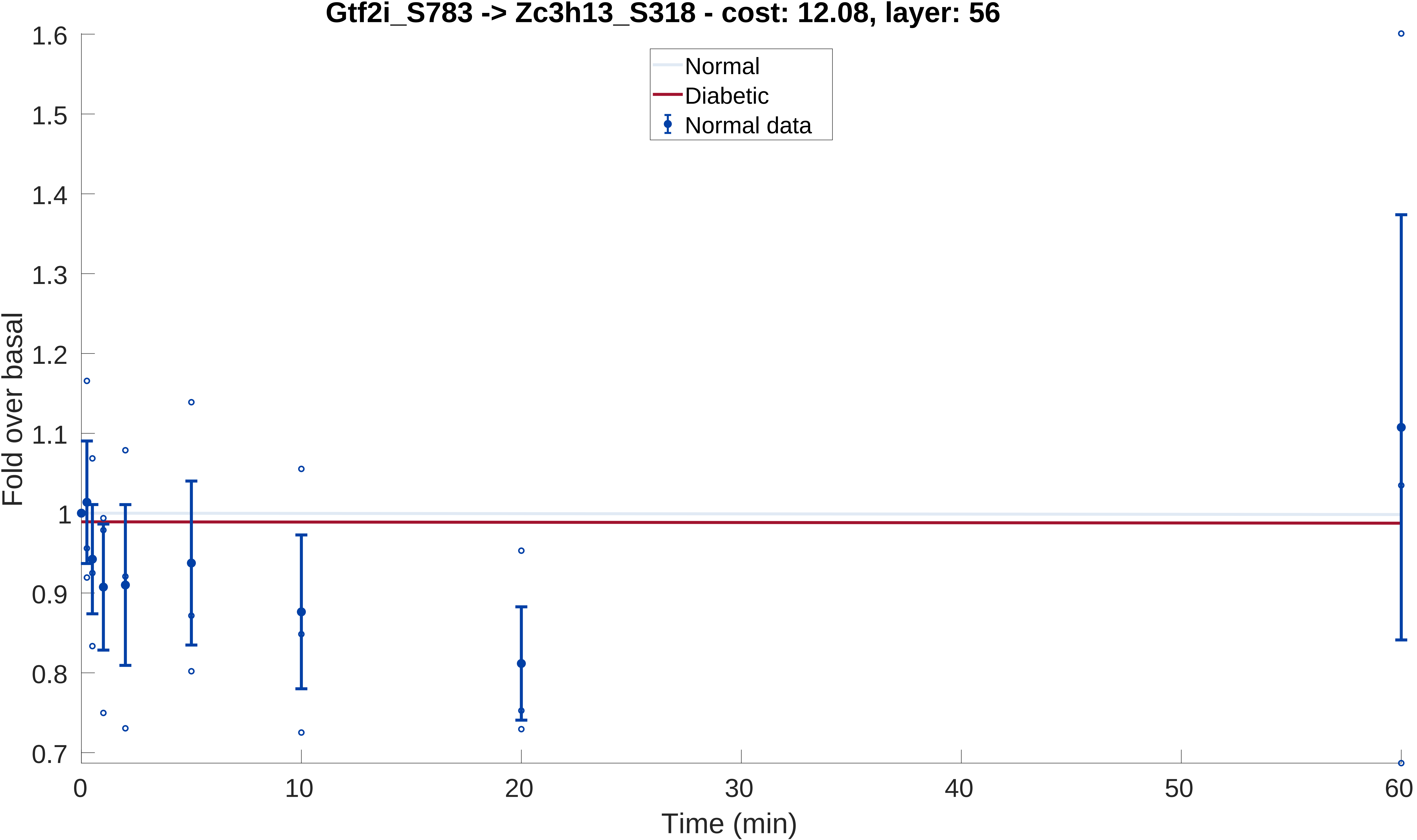

Gtf2i_S783 -> Zc3h13_S318 - cost: 12.08, layer: 56
Normal
Diabetic
Normal data
Fold over basal
1.6
1.5
1.4
1.3
1.2
1.1
1
0.9
0.8
0.7
0
10
20
30
40
50
60
Time (min)

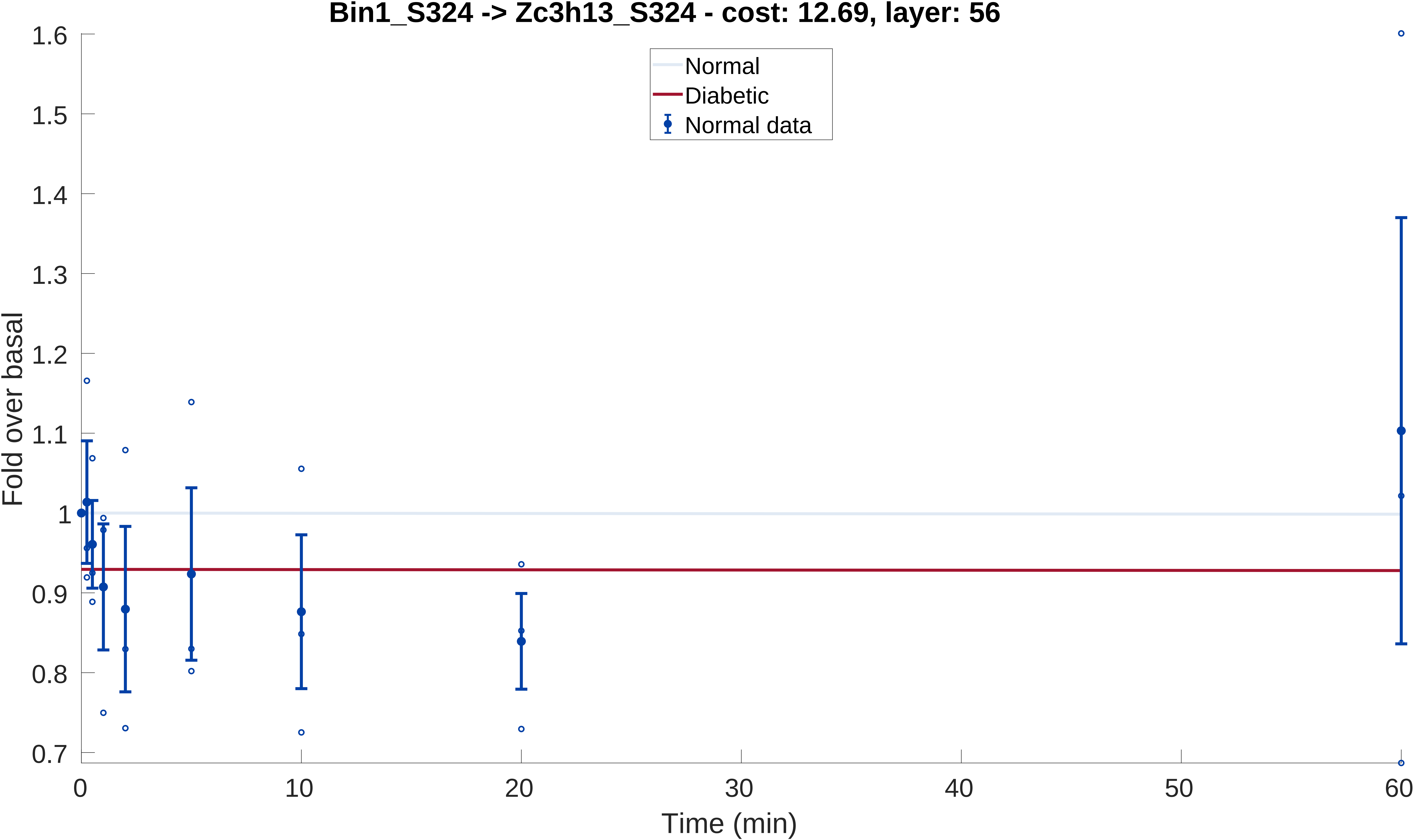

Bin1_S324 -> Zc3h13_S324 - cost: 12.69, layer: 56
Normal
Diabetic
Normal data
Fold over basal
Time (min)
1.6
1.5
1.4
1.3
1.2
1.1
1
0.9
0.8
0.7
0
10
20
30
40
50
60

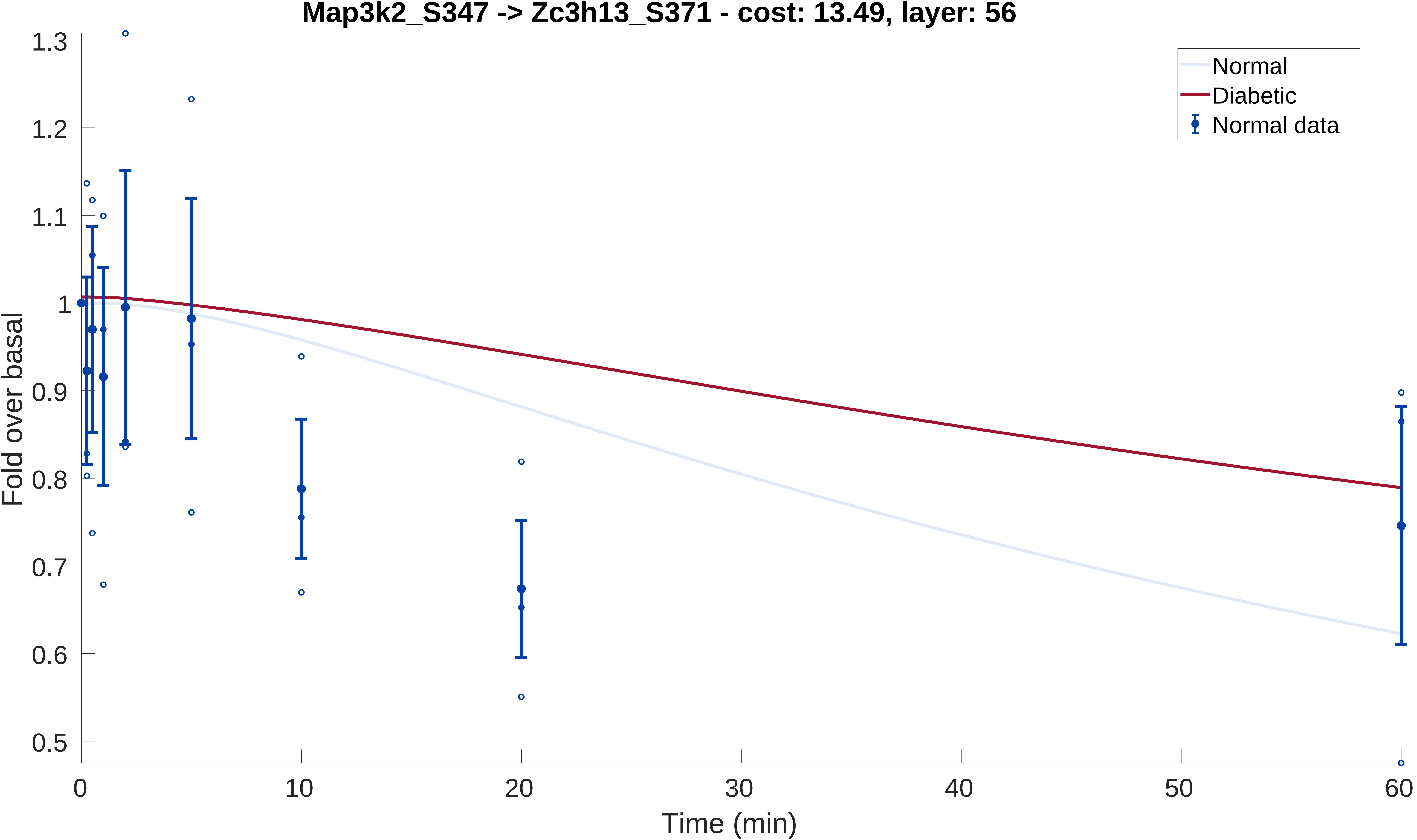

Map3k2_S347 -> Zc3h13_S371 - cost: 13.49, layer: 56
Normal
Diabetic
Normal data
Fold over basal
Time (min)
1.3
1.2
1.1
1
0.9
0.8
0.7
0.6
0.5
0
10
20
30
40
50
60

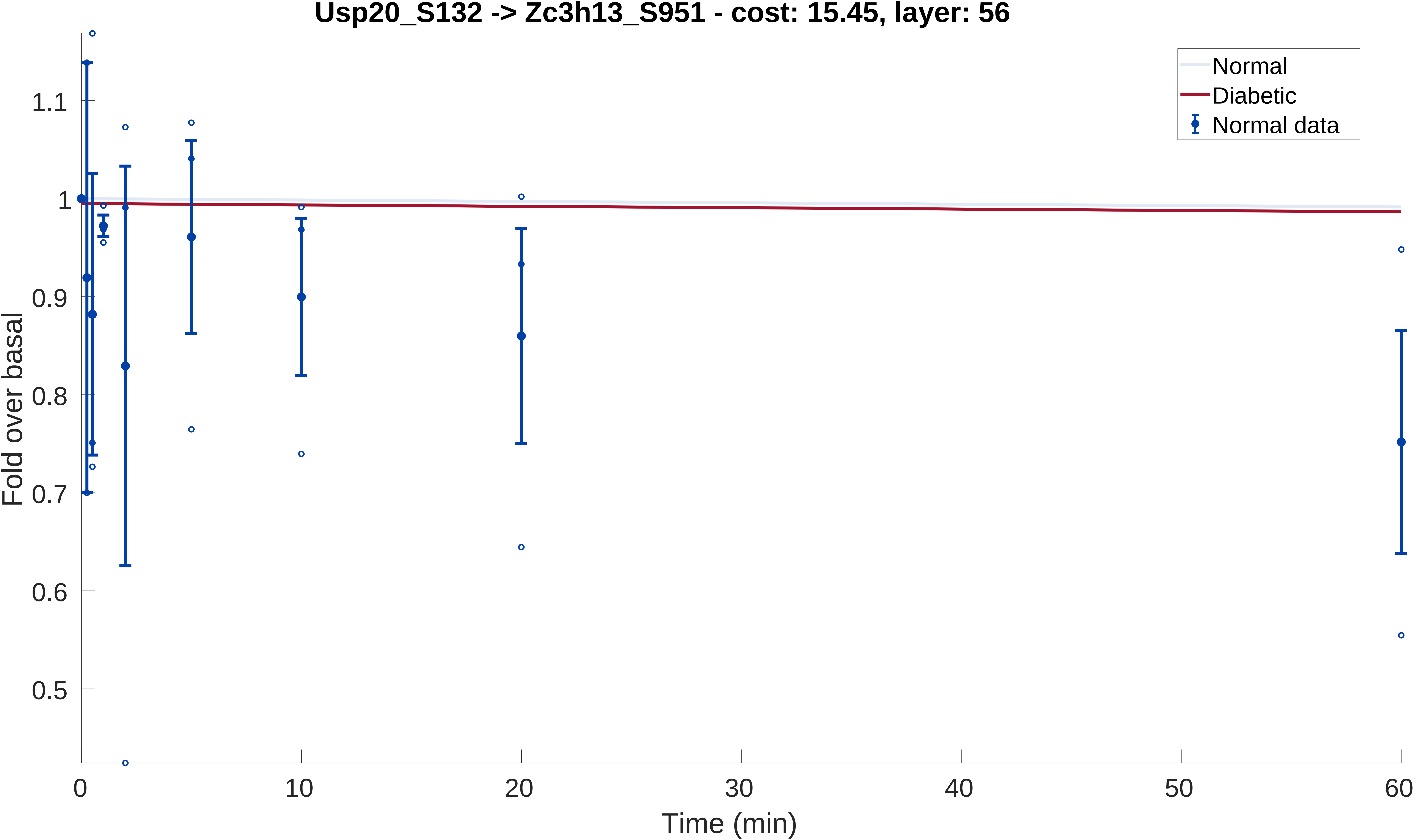

Usp20_S132 -> Zc3h13_S951 - cost: 15.45, layer: 56
Normal
Diabetic
Normal data
Fold over basal
1.1
1
0.9
0.8
0.7
0.6
0.5
0
10
20
30
40
50
60
Time (min)

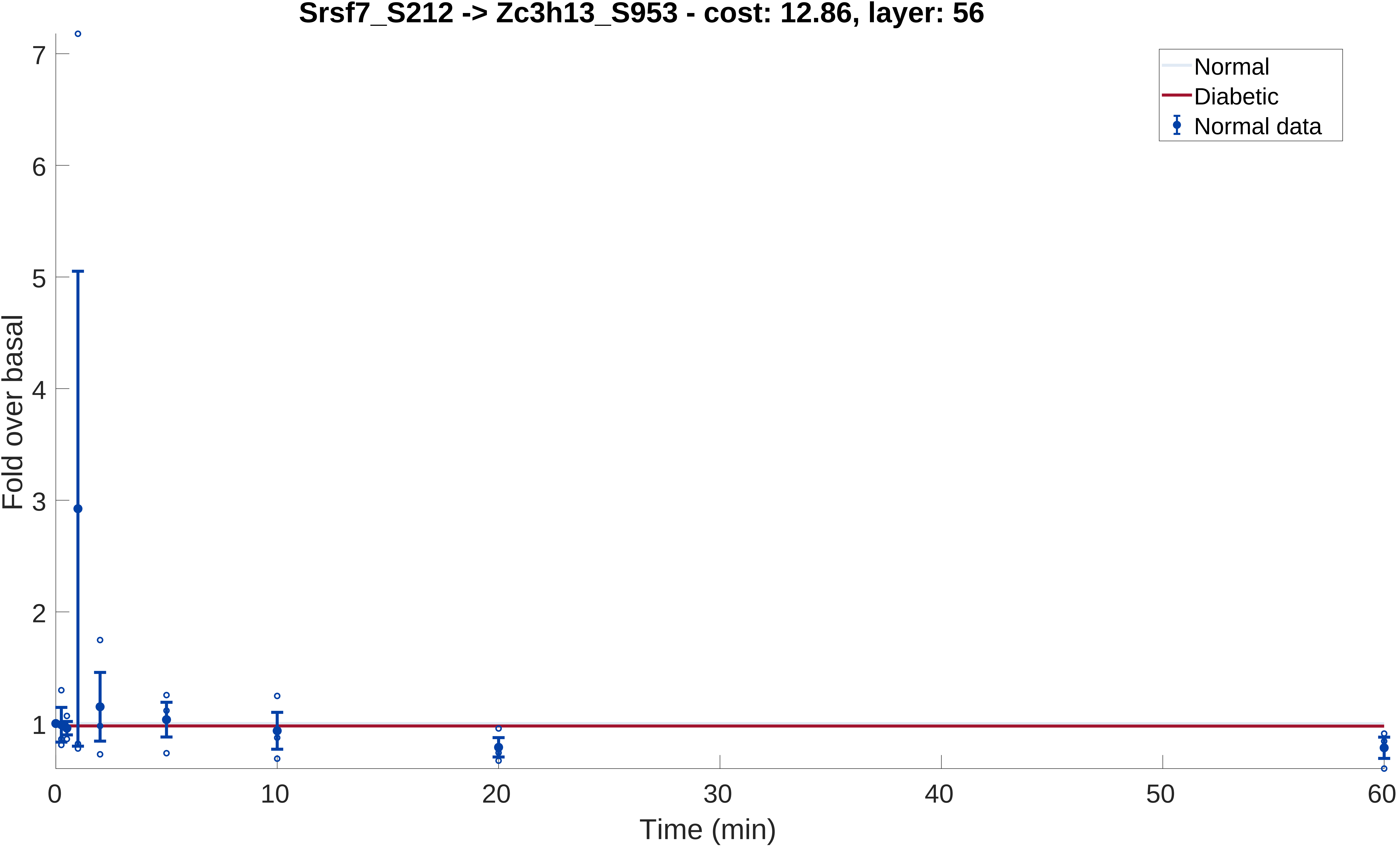

Srsf7_S212 -> Zc3h13_S953 - cost: 12.86, layer: 56
Normal
Diabetic
Normal data
Fold over basal
Time (min)
0
10
20
30
40
50
60
1
2
3
4
5
6
7

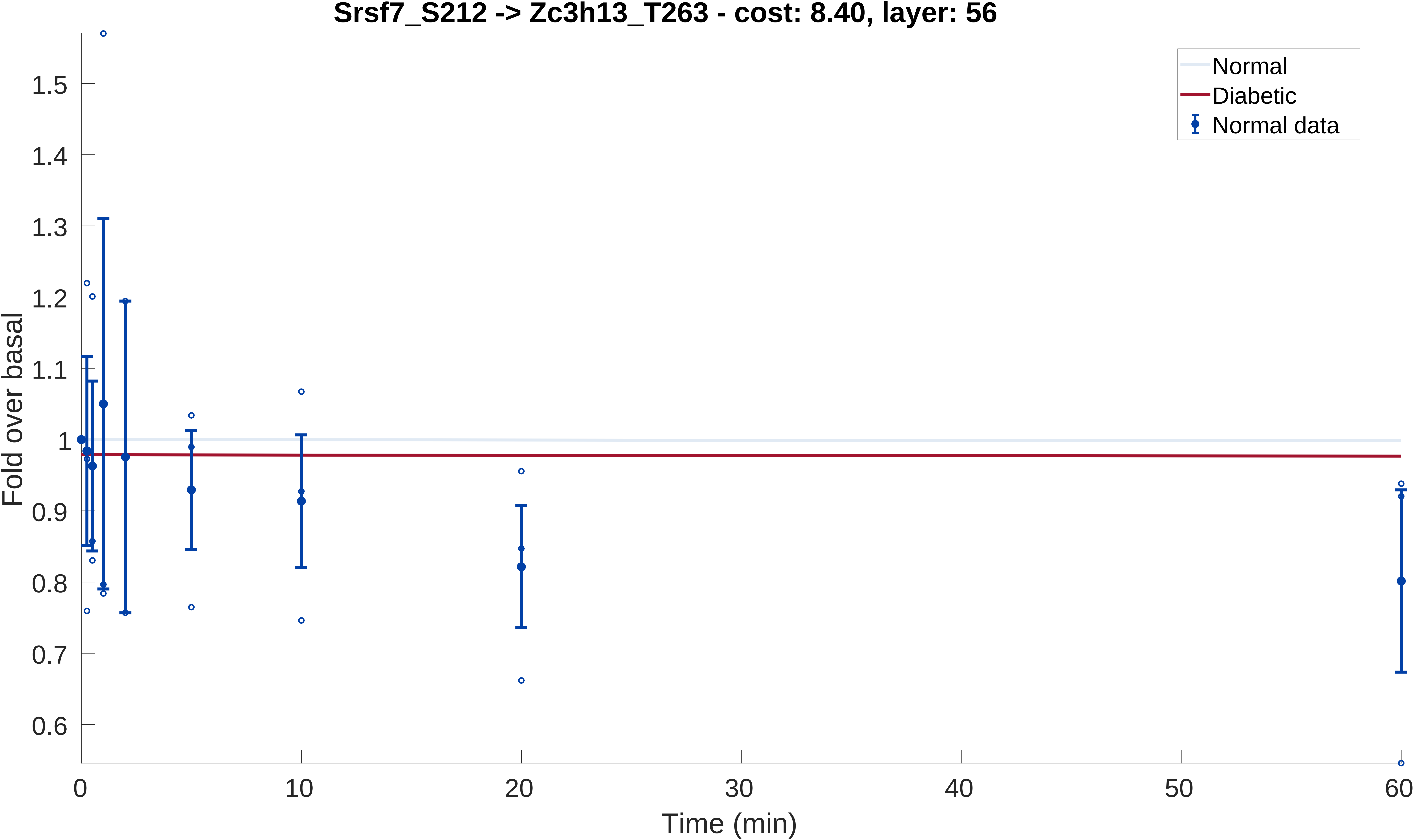

Srsf7_S212 -> Zc3h13_T263 - cost: 8.40, layer: 56
Normal
Diabetic
Normal data
Fold over basal
1.5
1.4
1.3
1.2
1.1
1
0.9
0.8
0.7
0.6
0
10
20
30
40
50
60
Time (min)

Gtf2i_S783 -> Zc3h14_S515 - cost: 10.56, layer: 56

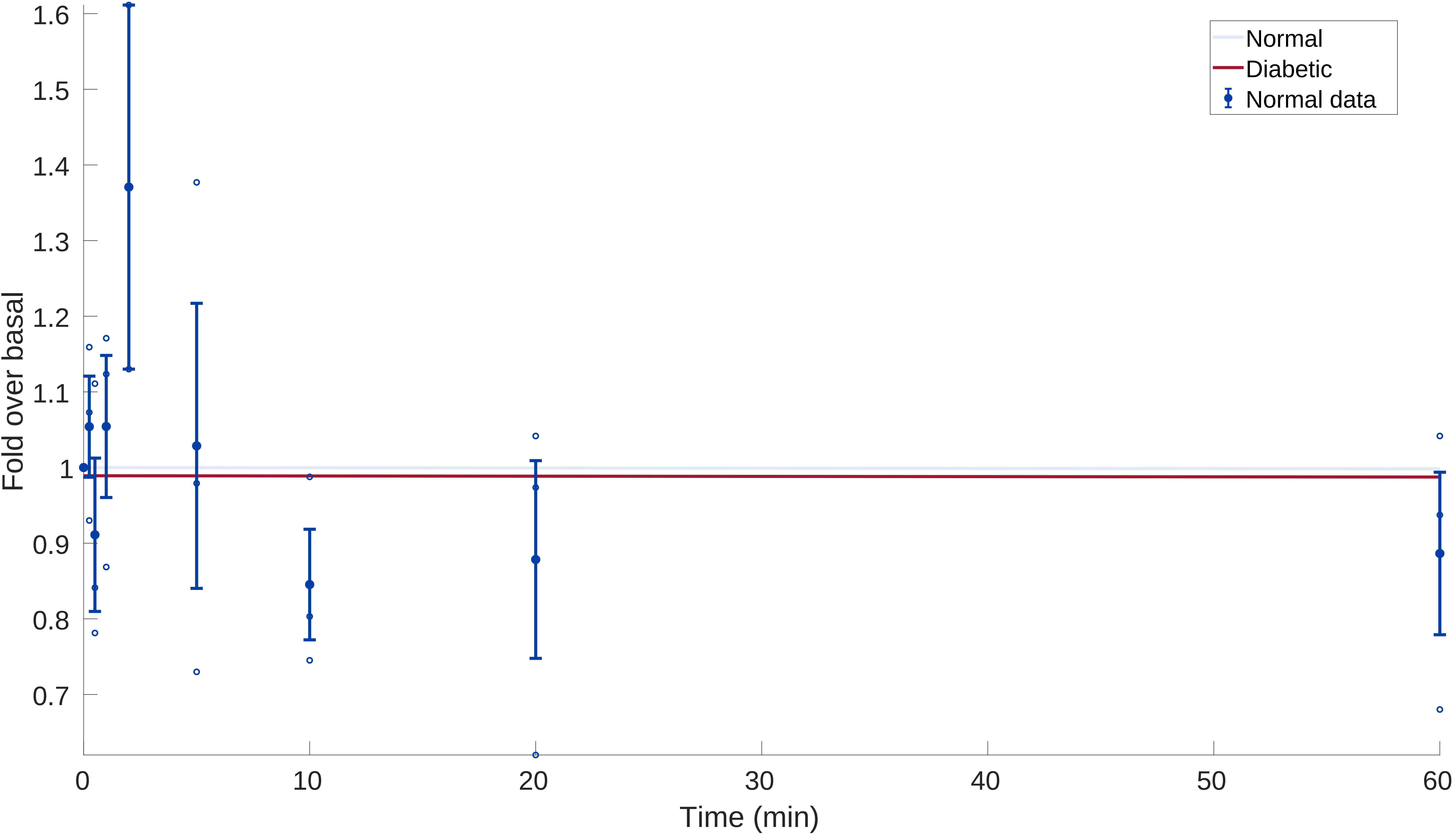

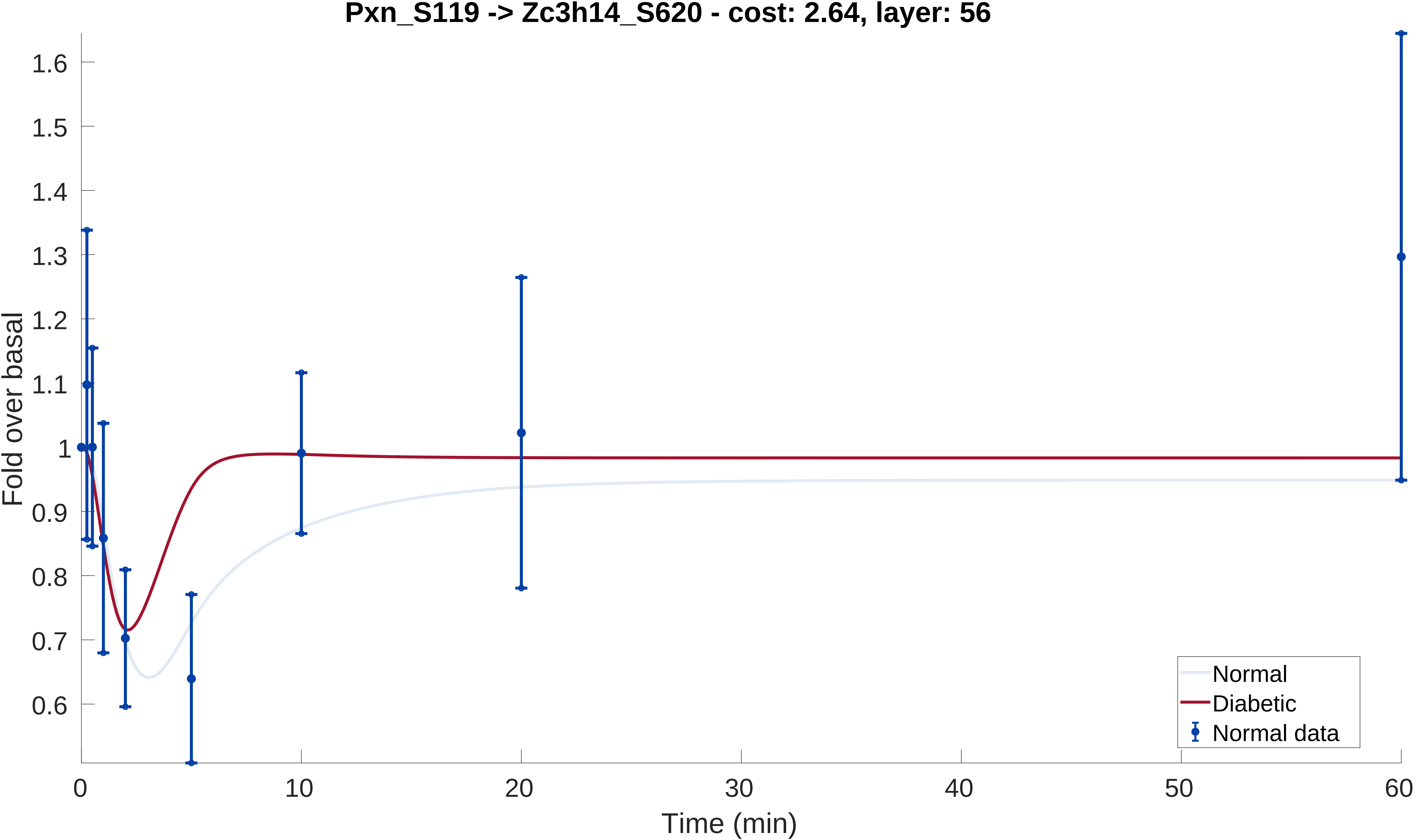

Pxn_S119 -> Zc3h14_S620 - cost: 2.64, layer: 56
Fold over basal
1.6
1.5
1.4
1.3
1.2
1.1
1
0.9
0.8
0.7
0.6
0
10
20
30
40
50
60
Time (min)
Normal
Diabetic
Normal data

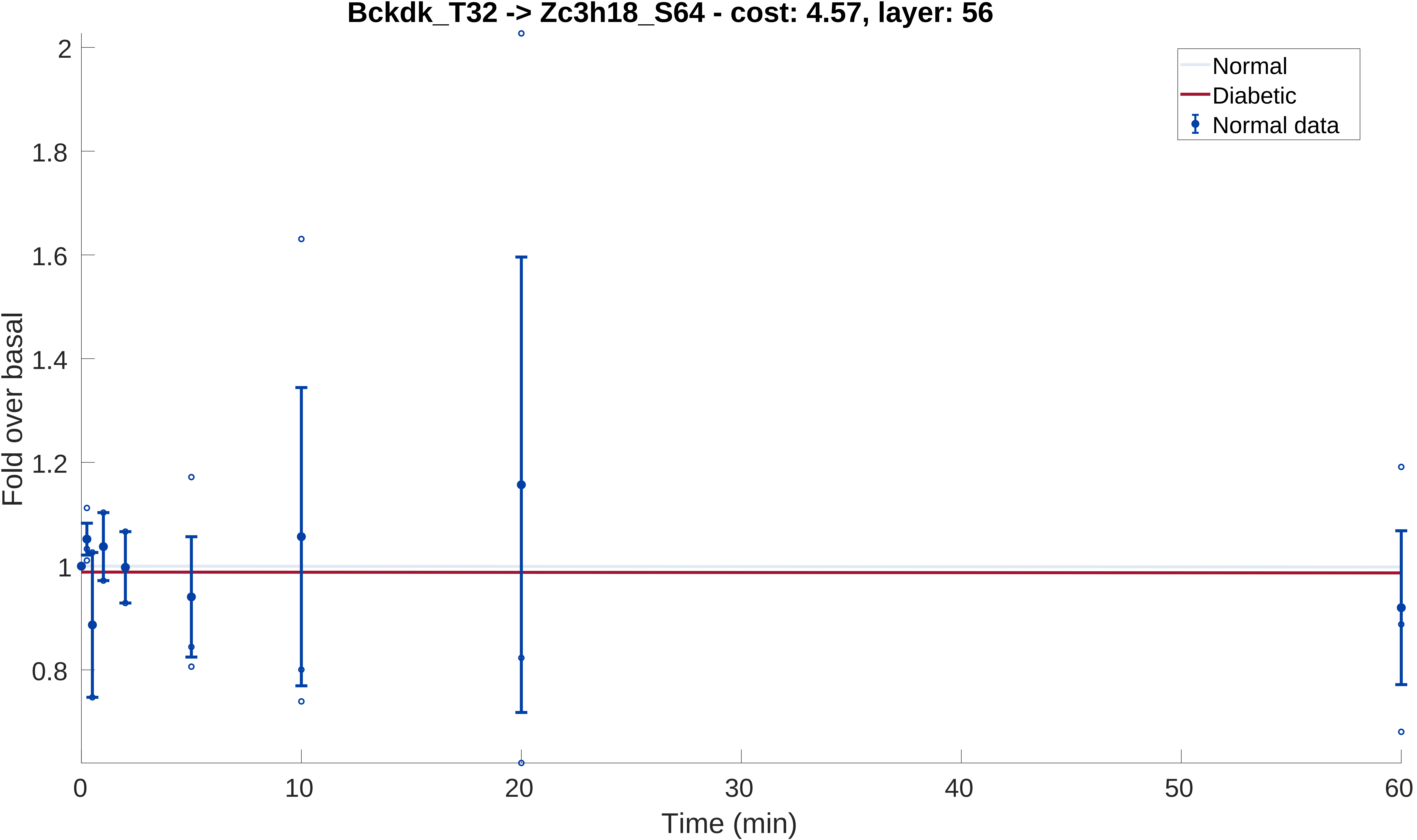

Bckdk_T32 -> Zc3h18_S64 - cost: 4.57, layer: 56
Normal
Diabetic
Normal data
Fold over basal
Time (min)
2
1.8
1.6
1.4
1.2
1
0.8
0
10
20
30
40
50
60

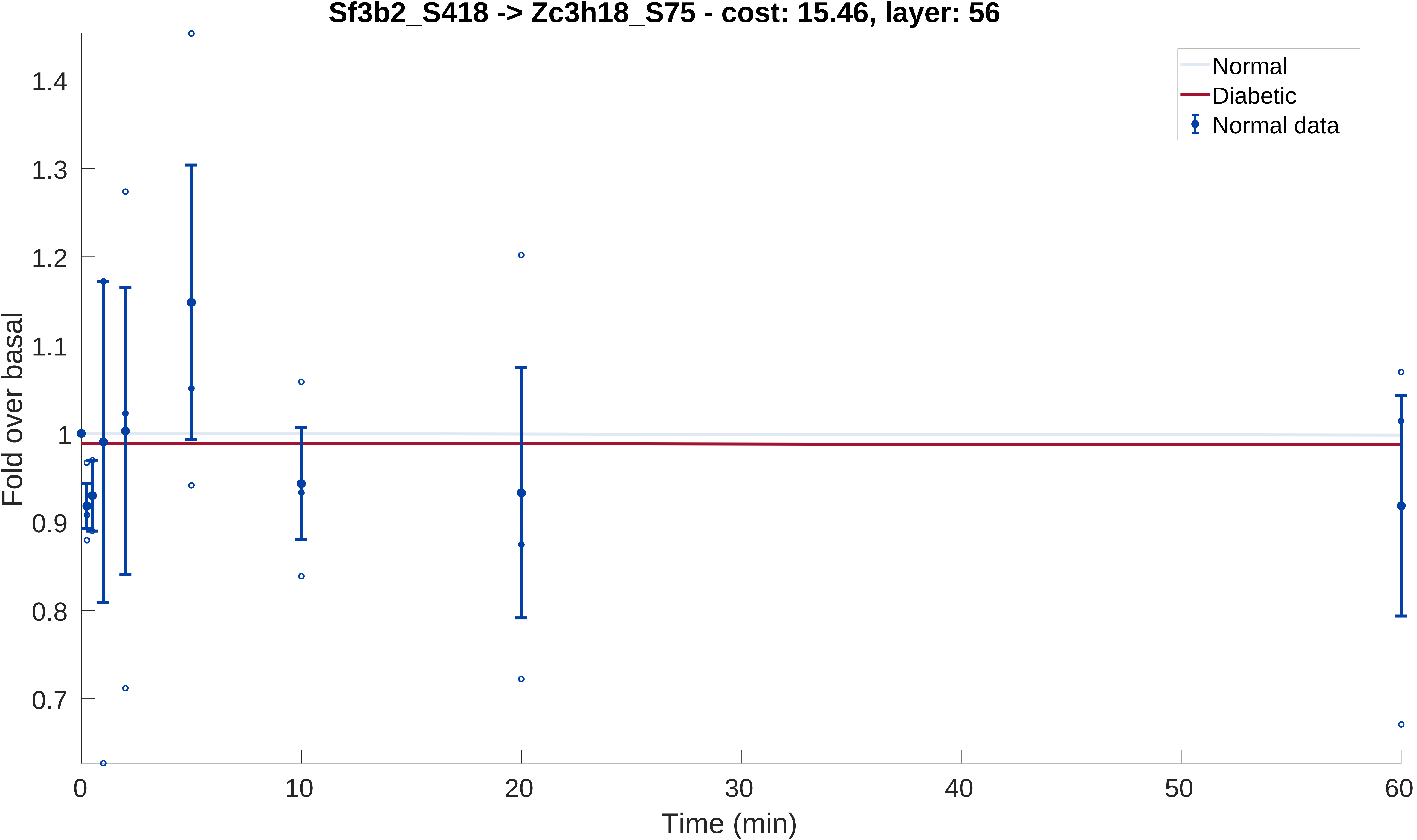

Sf3b2_S418 -> Zc3h18_S75 - cost: 15.46, layer: 56
Normal
Diabetic
Normal data
Fold over basal
1.4
1.3
1.2
1.1
1
0.9
0.8
0.7
0
10
20
30
40
50
60
Time (min)

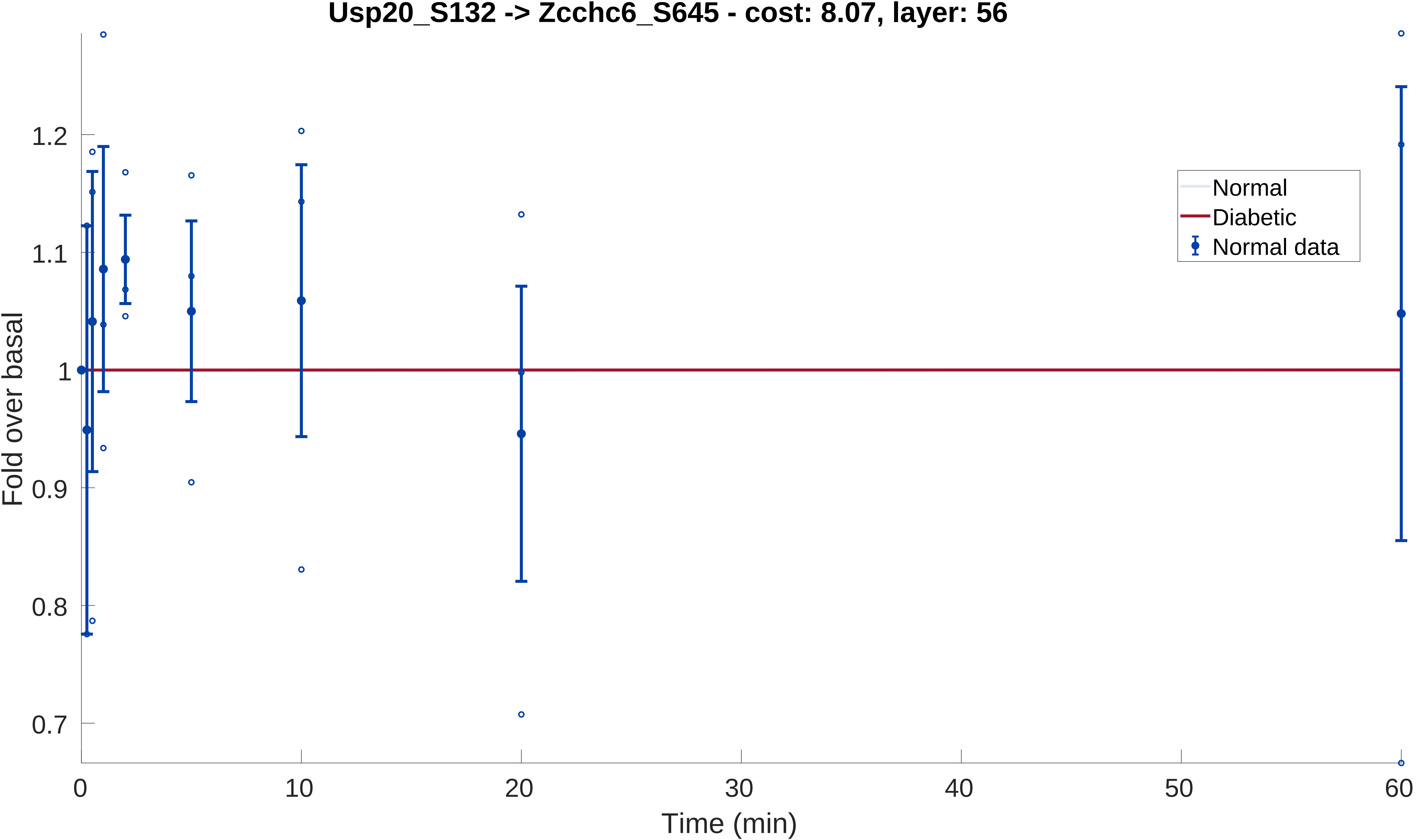

Usp20_S132 -> Zcchc6_S645 - cost: 8.07, layer: 56
Fold over basal
Time (min)
Normal
Diabetic
Normal data
0.7
0.8
0.9
1
1.1
1.2
0
10
20
30
40
50
60

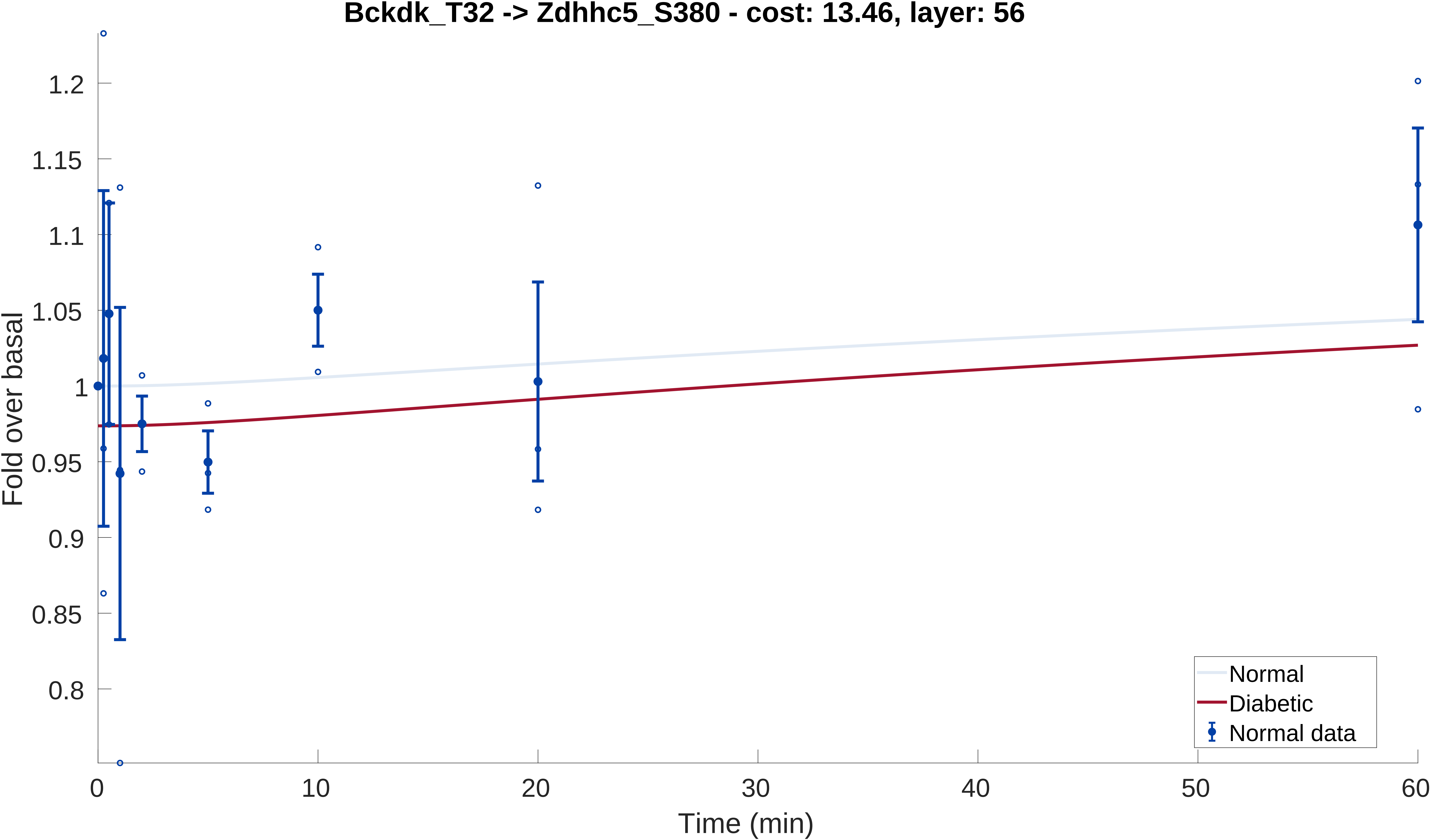

Bckdk_T32 -> Zdhhc5_S380 - cost: 13.46, layer: 56
Fold over basal
1.2
1.15
1.1
1.05
1
0.95
0.9
0.85
0.8
0
10
20
30
40
50
60
Time (min)
Normal
Diabetic
Normal data

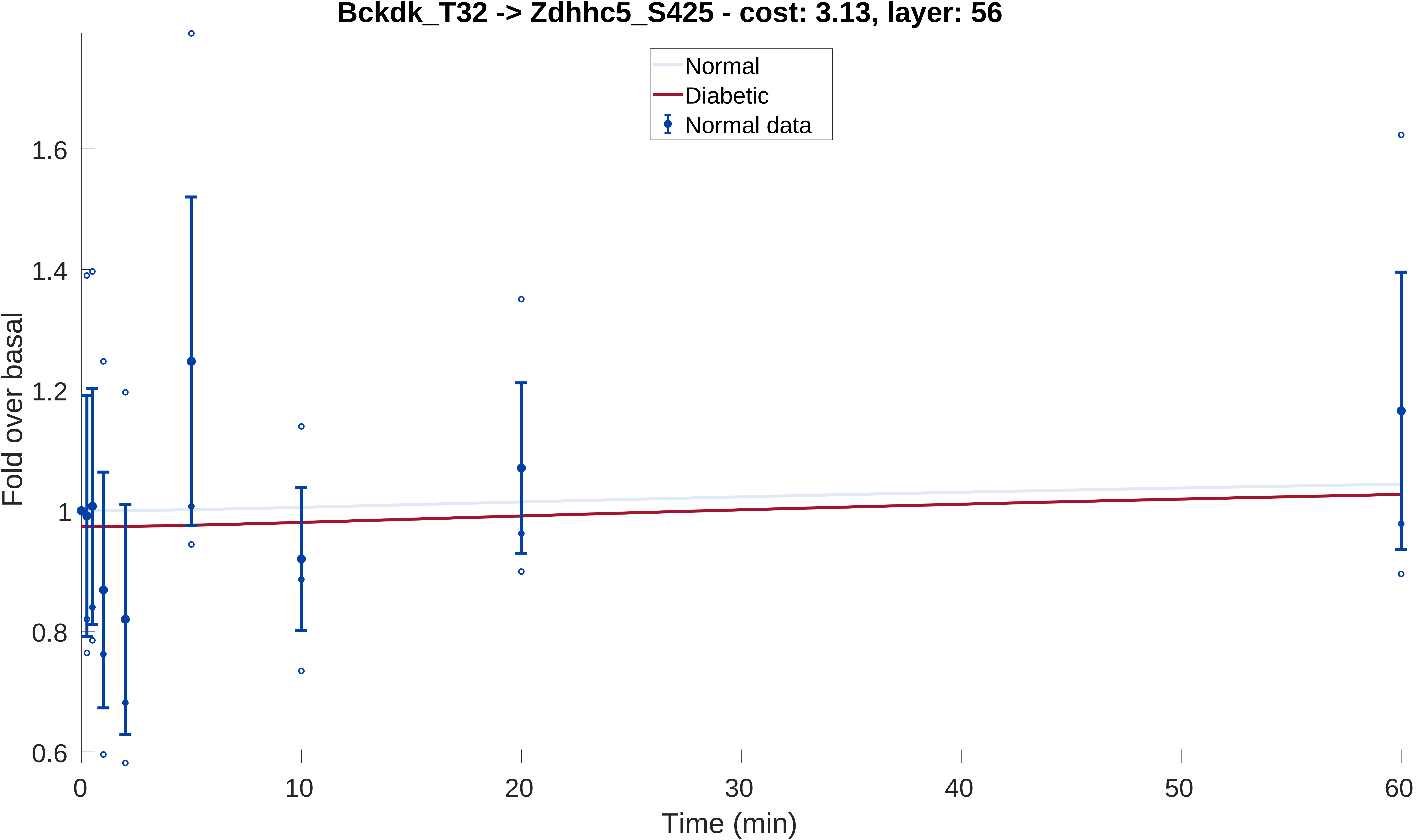

Bckdk_T32 -> Zdhhc5_S425 - cost: 3.13, layer: 56
Normal
Diabetic
Normal data
Fold over basal
1.6
1.4
1.2
1
0.8
0.6
0
10
20
30
40
50
60
Time (min)

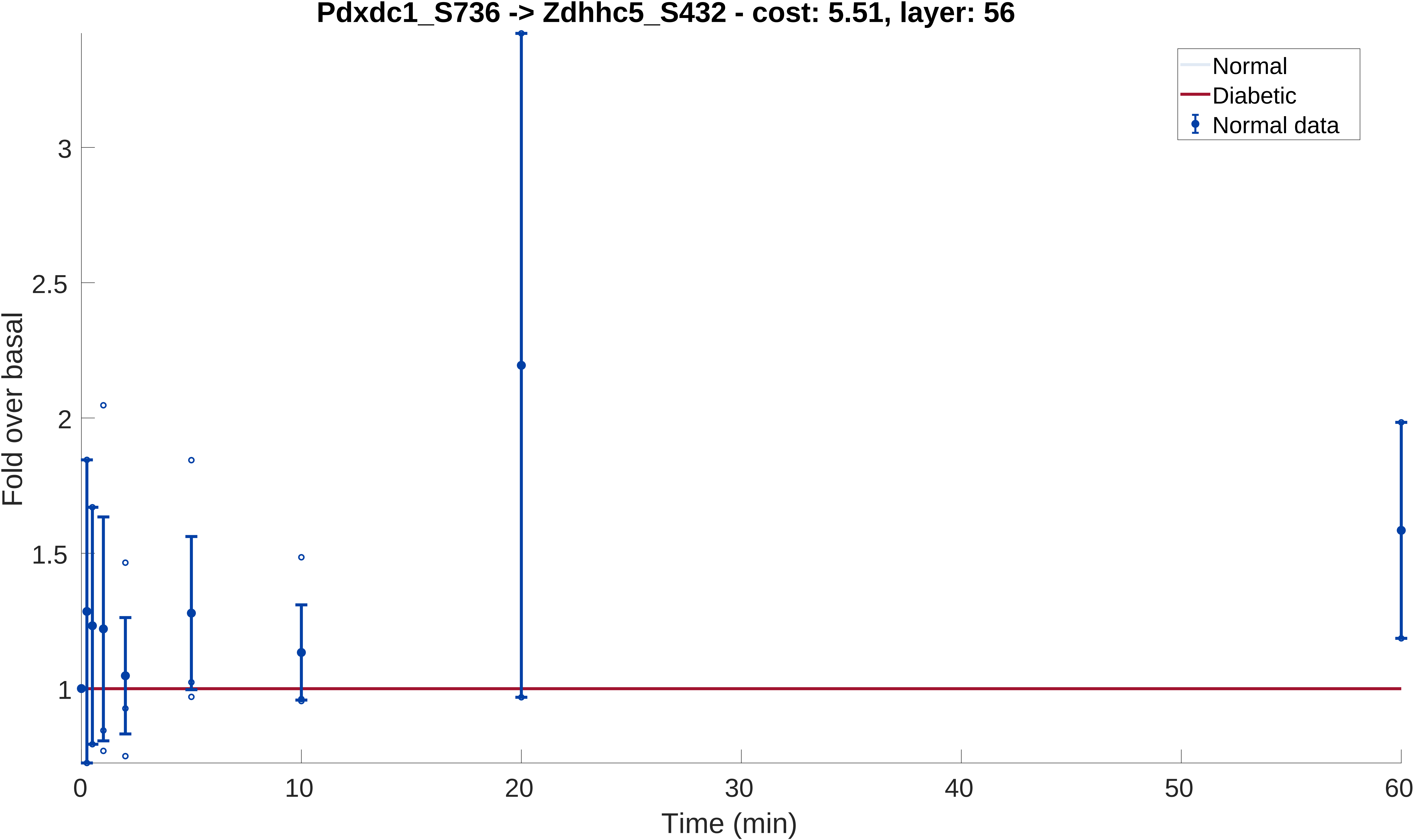

Pdxdc1_S736 -> Zdhhc5_S432 - cost: 5.51, layer: 56
Normal
Diabetic
Normal data
Fold over basal
3
2.5
2
1.5
1
0
10
20
30
40
50
60
Time (min)

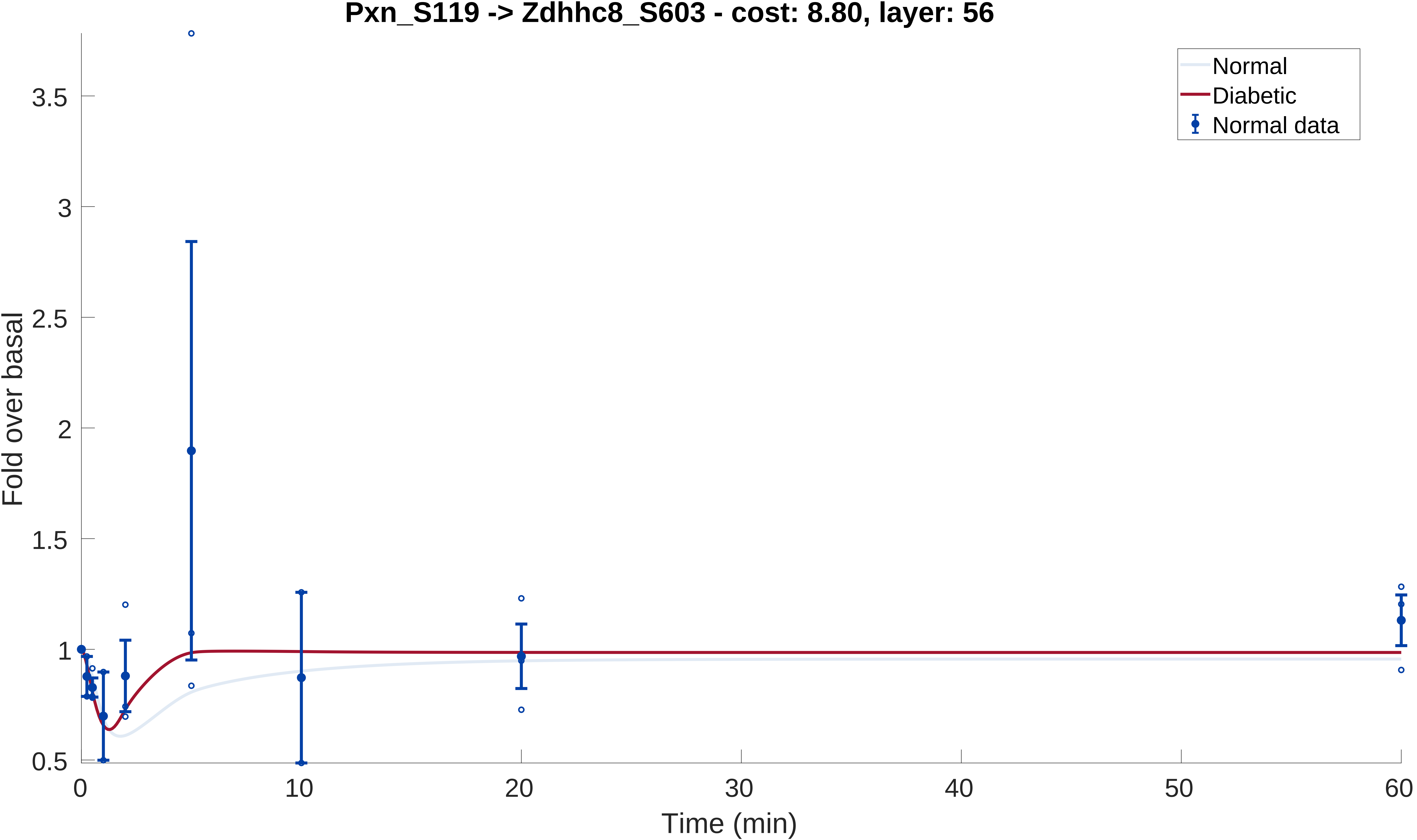

Pxn_S119 -> Zdhhc8_S603 - cost: 8.80, layer: 56
Normal
Diabetic
Normal data
Fold over basal
3.5
3
2.5
2
1.5
1
0.5
0
10
20
30
40
50
60
Time (min)

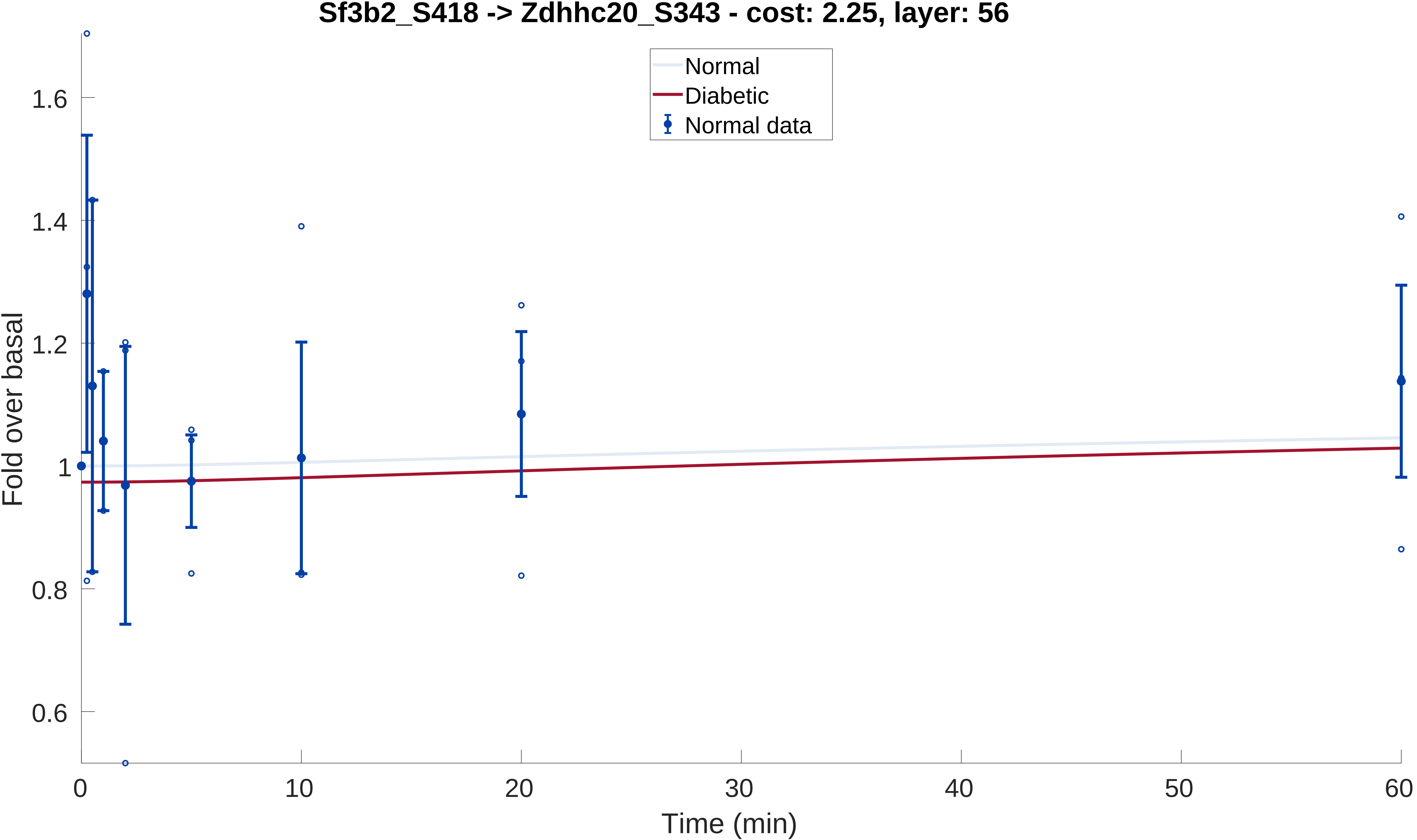

Sf3b2_S418 -> Zdhhc20_S343 - cost: 2.25, layer: 56
Normal
Diabetic
Normal data
Fold over basal
1.6
1.4
1.2
1
0.8
0.6
0
10
20
30
40
50
60
Time (min)

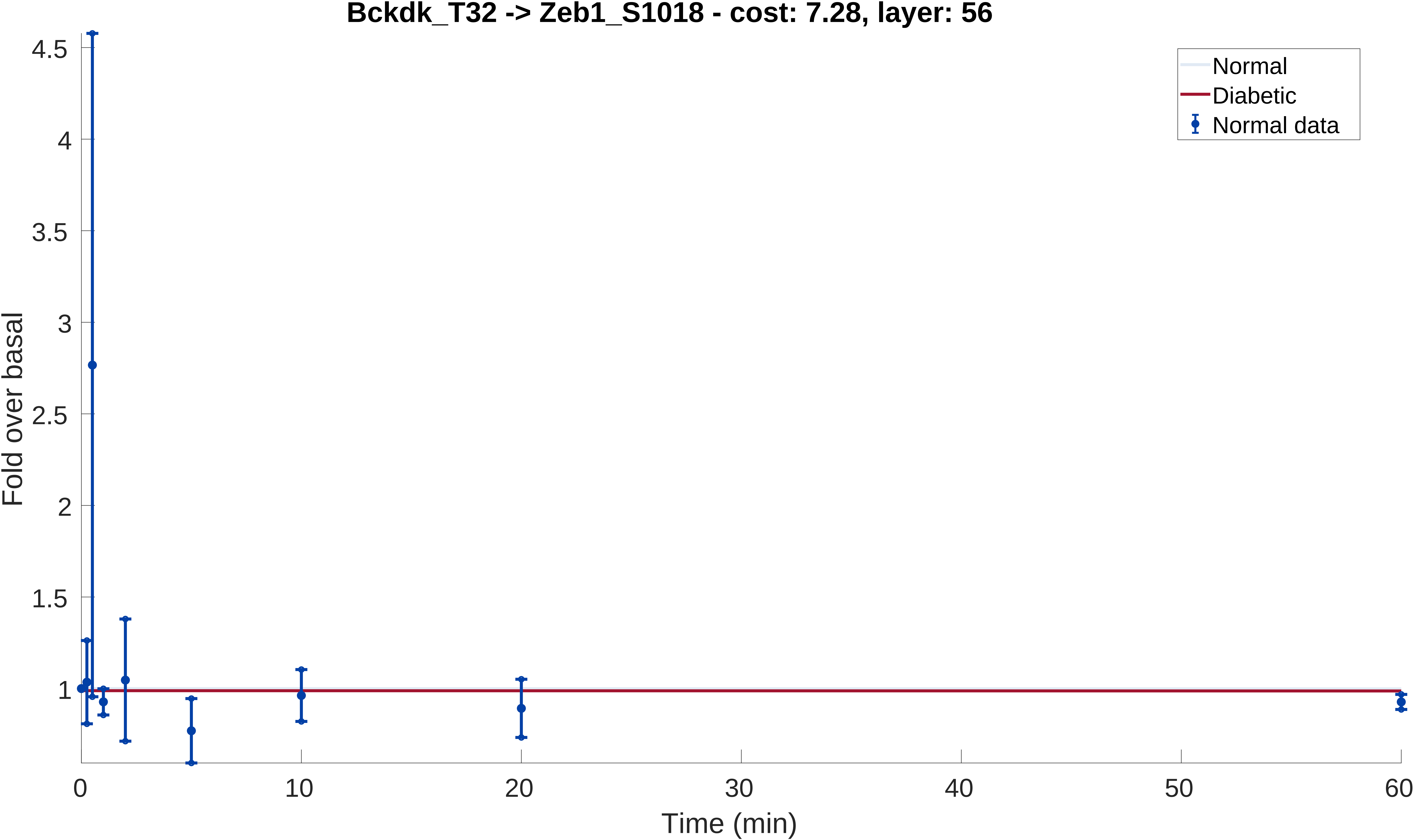

Bckdk_T32 -> Zeb1_S1018 - cost: 7.28, layer: 56
Fold over basal
Time (min)
Normal
Diabetic
Normal data
4.5
4
3.5
3
2.5
2
1.5
1
0
10
20
30
40
50
60

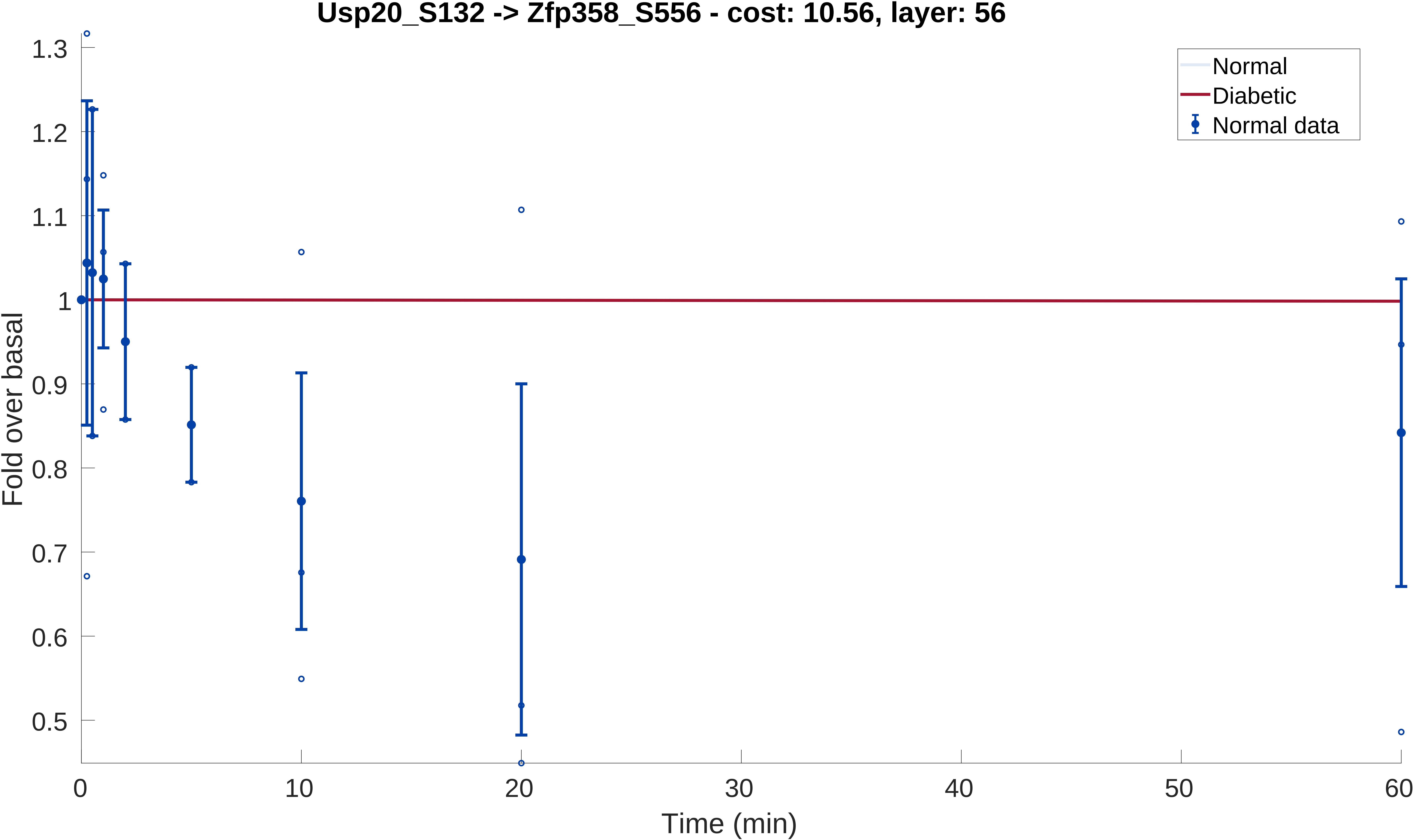

Usp20_S132 -> Zfp358_S556 - cost: 10.56, layer: 56
Normal
Diabetic
Normal data
Fold over basal
Time (min)
1.3
1.2
1.1
1
0.9
0.8
0.7
0.6
0.5
0
10
20
30
40
50
60

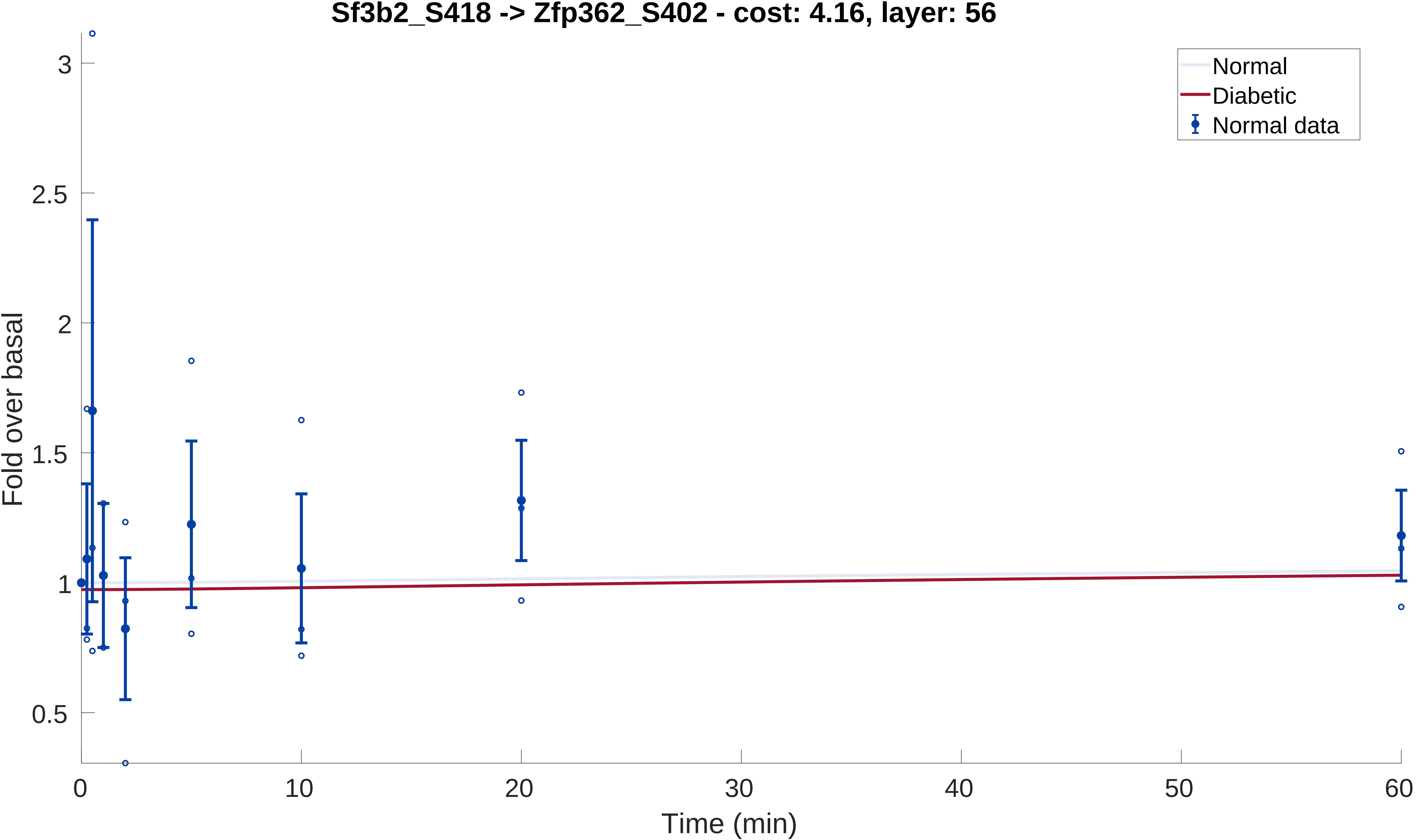

Sf3b2_S418 -> Zfp362_S402 - cost: 4.16, layer: 56
Normal
Diabetic
Normal data
Fold over basal
Time (min)
0.5
1
1.5
2
2.5
3
0
10
20
30
40
50
60

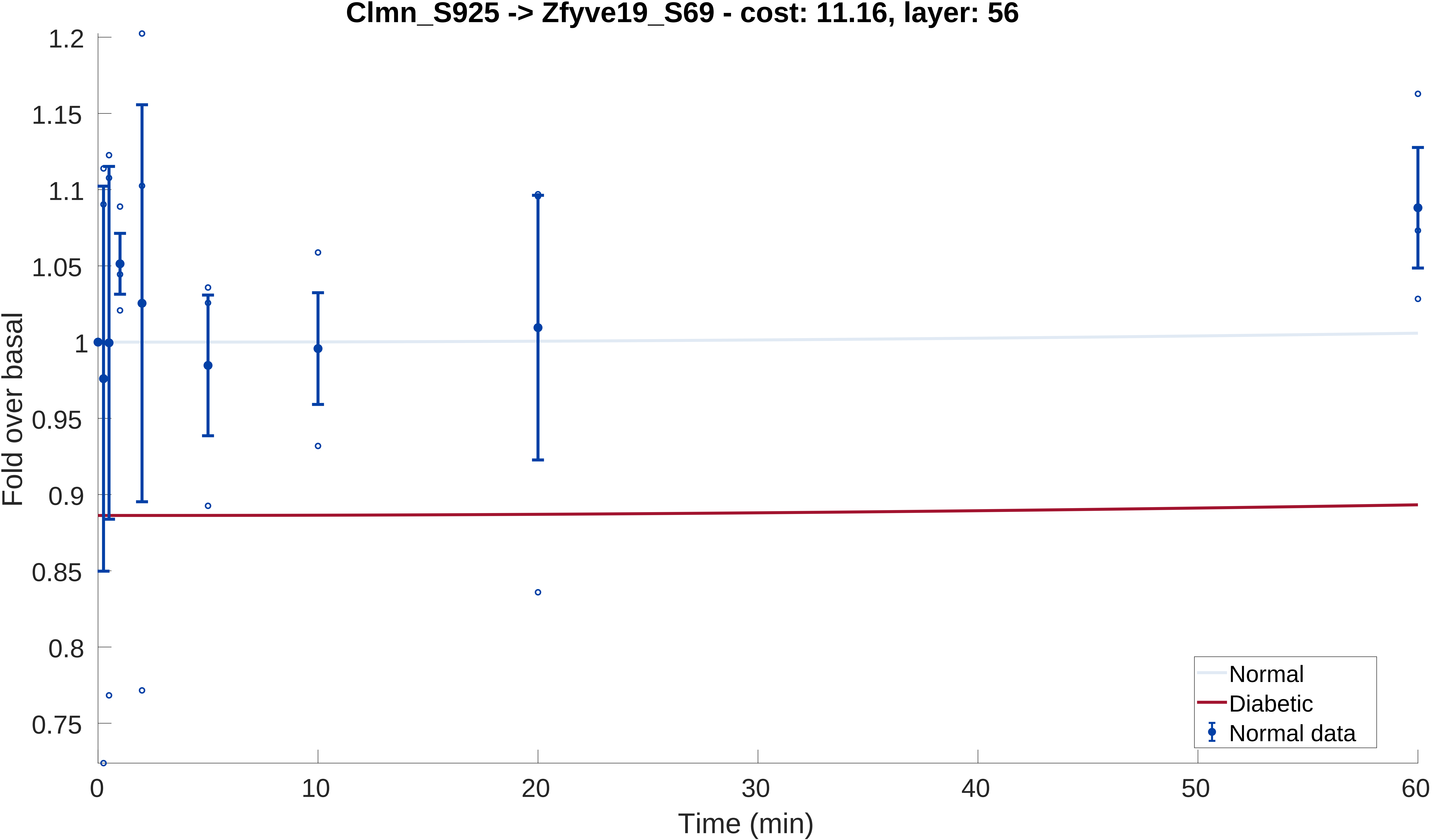

Clmn_S925 -> Zfyve19_S69 - cost: 11.16, layer: 56
Fold over basal
1.2
1.15
1.1
1.05
1
0.95
0.9
0.85
0.8
0.75
Time (min)
0
10
20
30
40
50
60
Normal
Diabetic
Normal data

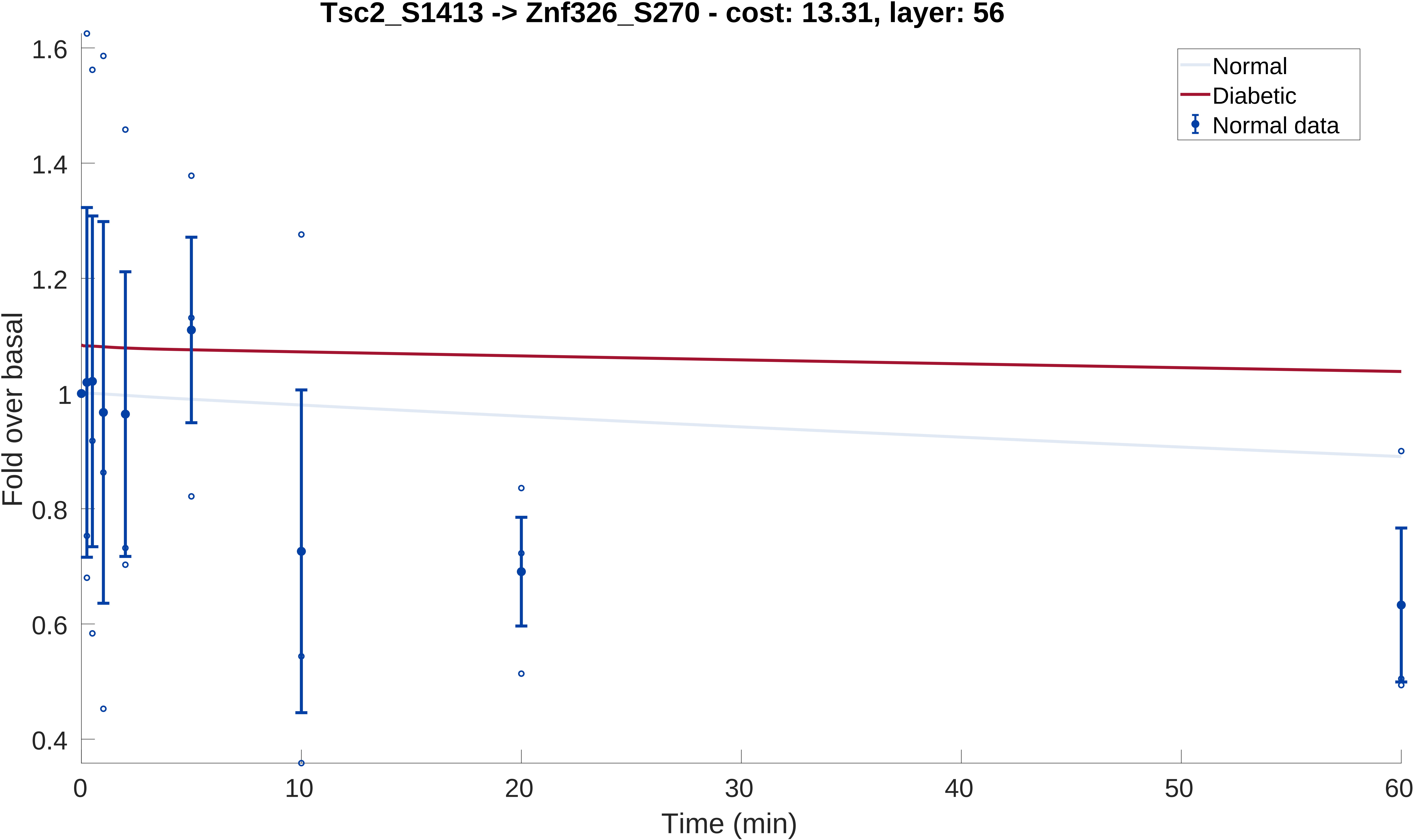
Tsc2_S1413 -> Znf326_S270 - cost: 13.31, layer: 56
Normal
Diabetic
Normal data
Fold over basal
Time (min)
1.6
1.4
1.2
1
0.8
0.6
0.4
0
10
20
30
40
50
60

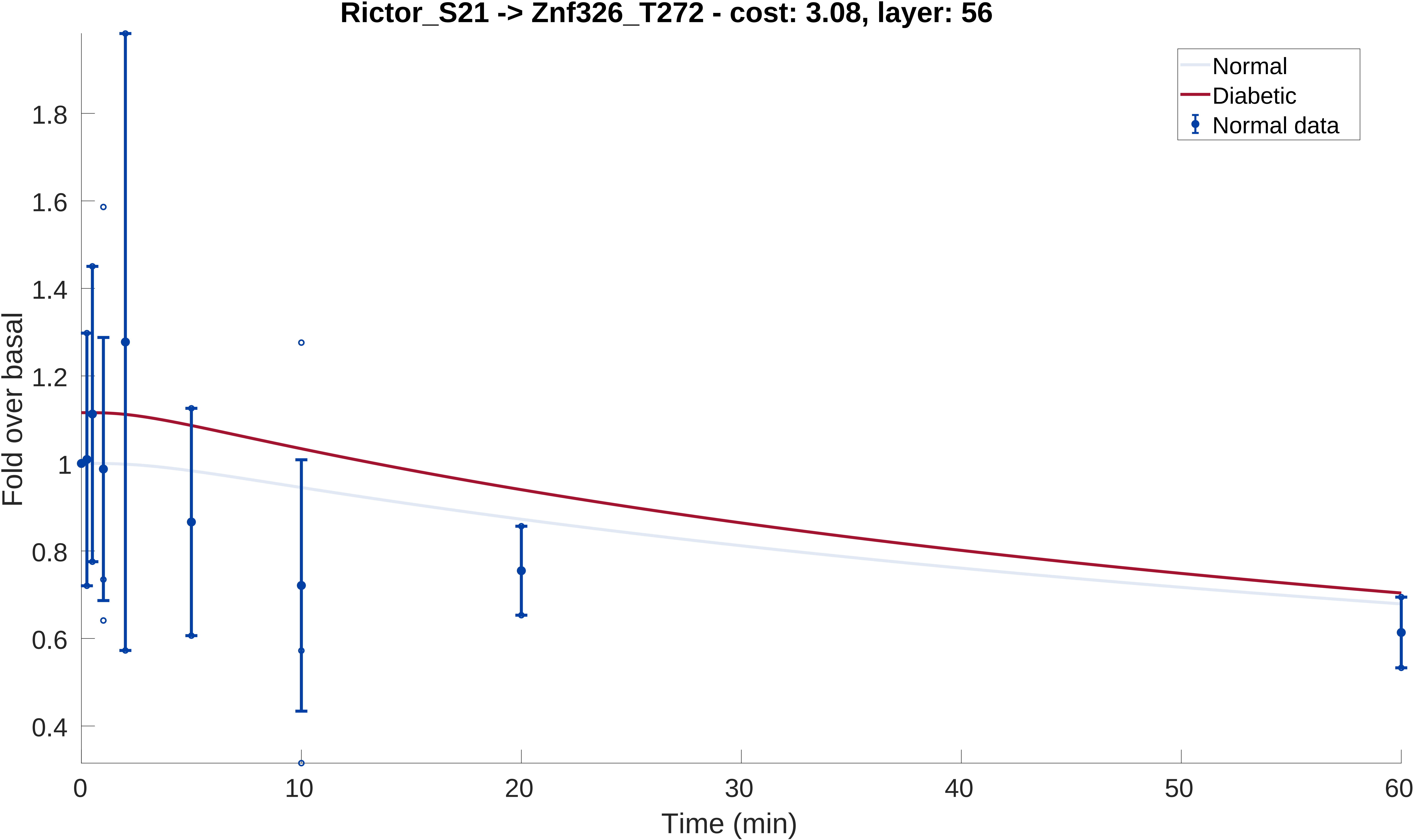

Rictor_S21 -> Znf326_T272 - cost: 3.08, layer: 56
Normal
Diabetic
Normal data
Fold over basal
Time (min)
1.8
1.6
1.4
1.2
1
0.8
0.6
0.4
0
10
20
30
40
50
60

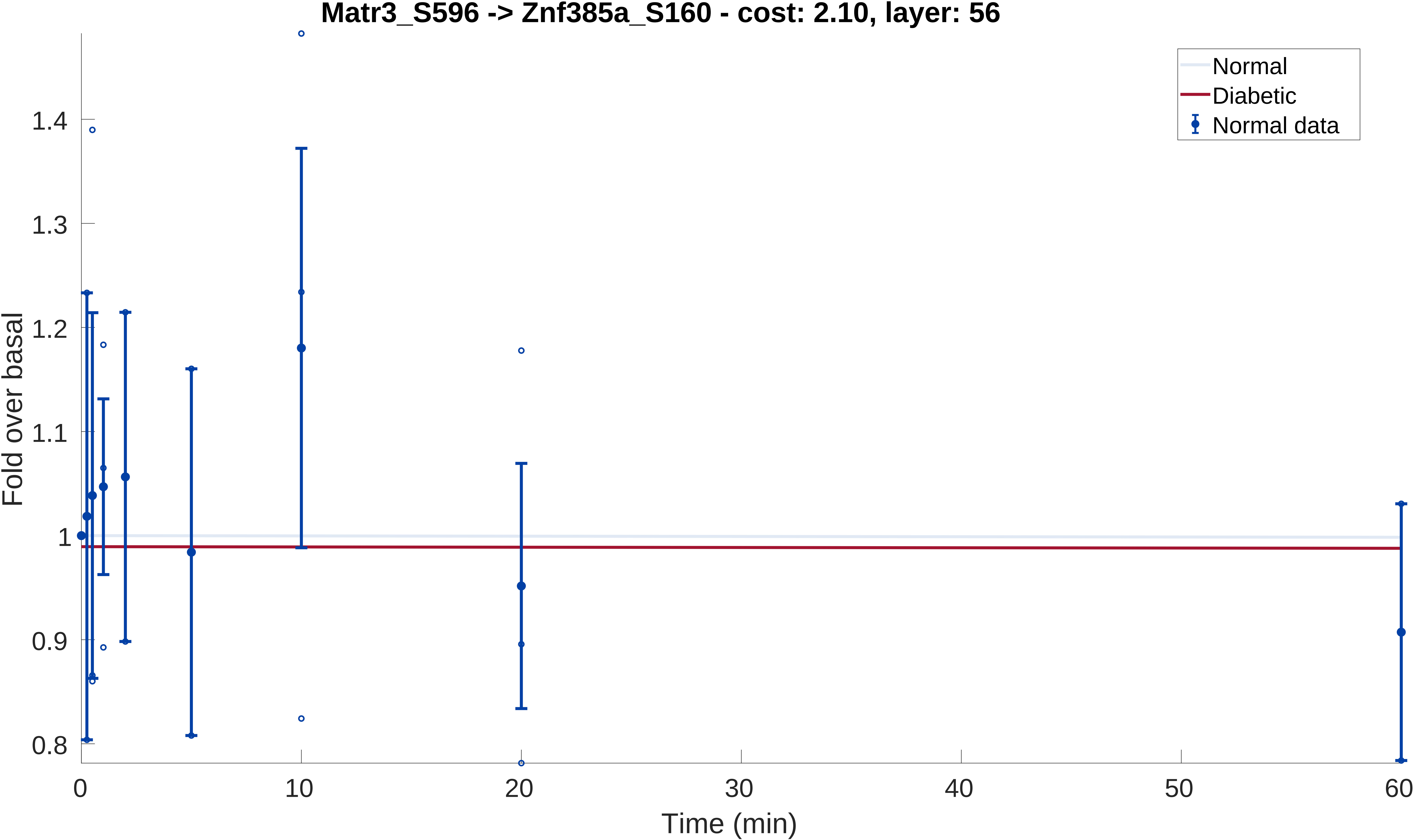

Matr3_S596 -> Znf385a_S160 - cost: 2.10, layer: 56
Normal
Diabetic
Normal data
Fold over basal
Time (min)
1.4
1.3
1.2
1.1
1
0.9
0.8
0
10
20
30
40
50
60

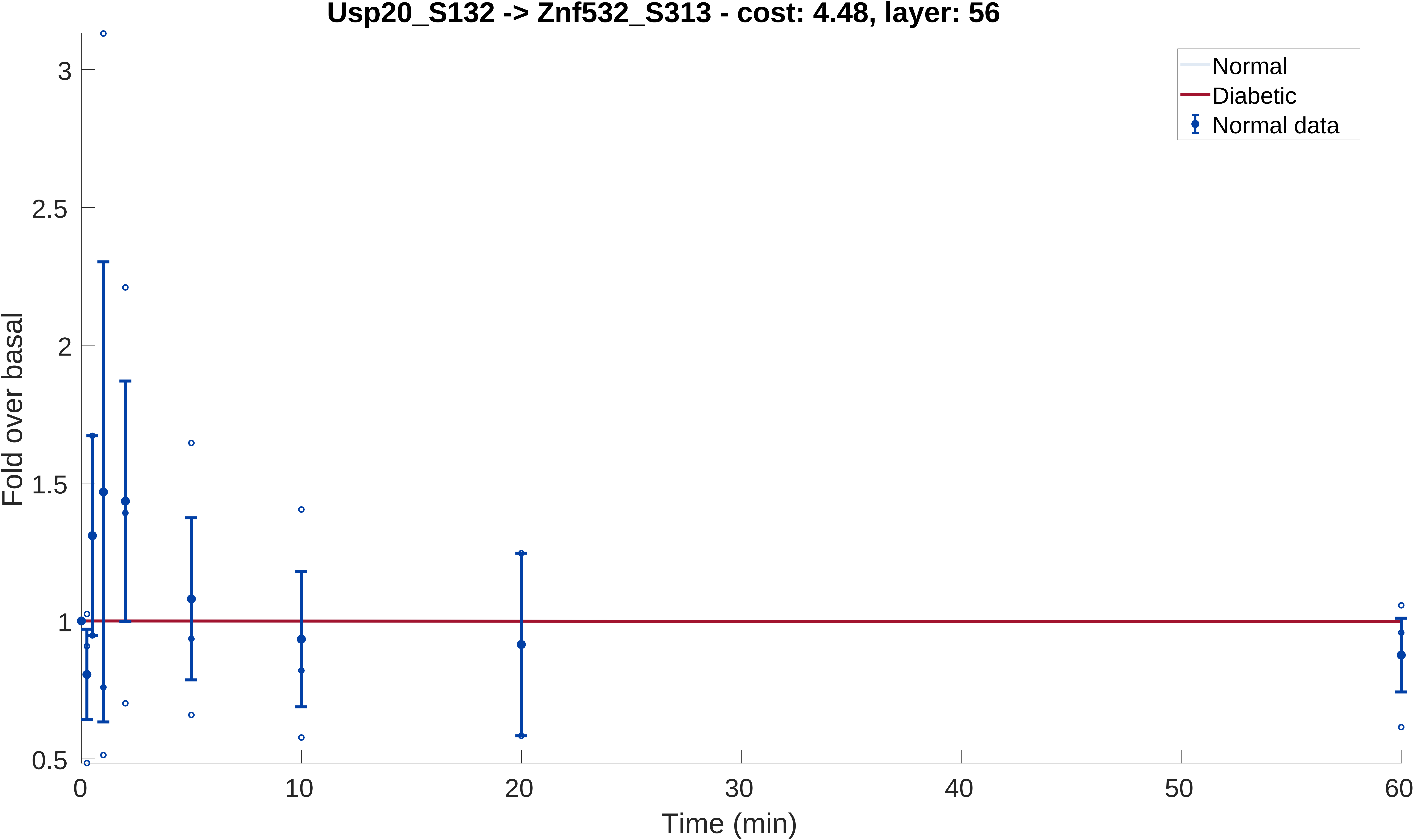

Usp20_S132 -> Znf532_S313 - cost: 4.48, layer: 56
Normal
Diabetic
Normal data
Fold over basal
Time (min)
0.5
1
1.5
2
2.5
3
0
10
20
30
40
50
60

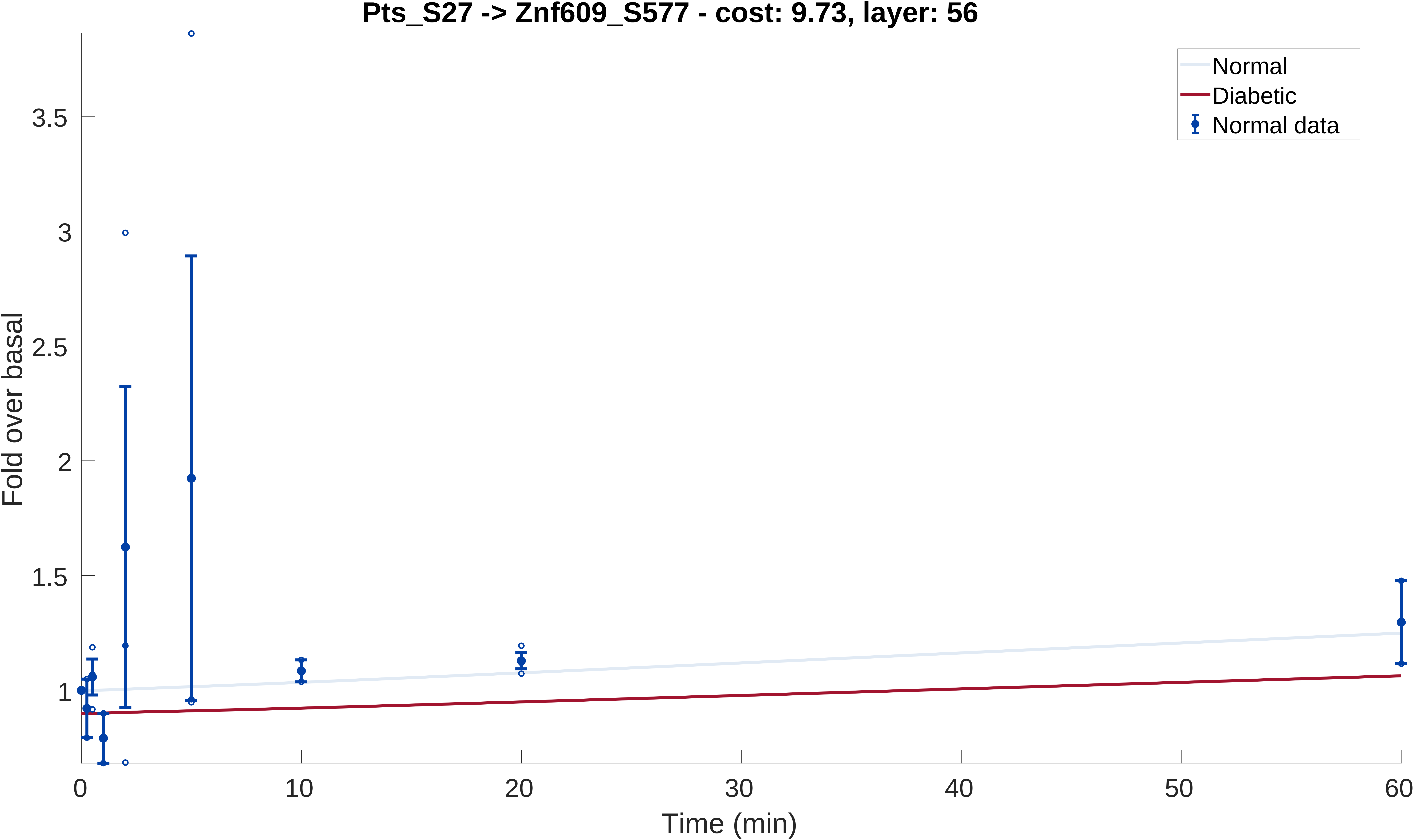

Pts_S27 -> Znf609_S577 - cost: 9.73, layer: 56
Normal
Diabetic
Normal data
Fold over basal
3.5
3
2.5
2
1.5
1
0
10
20
30
40
50
60
Time (min)

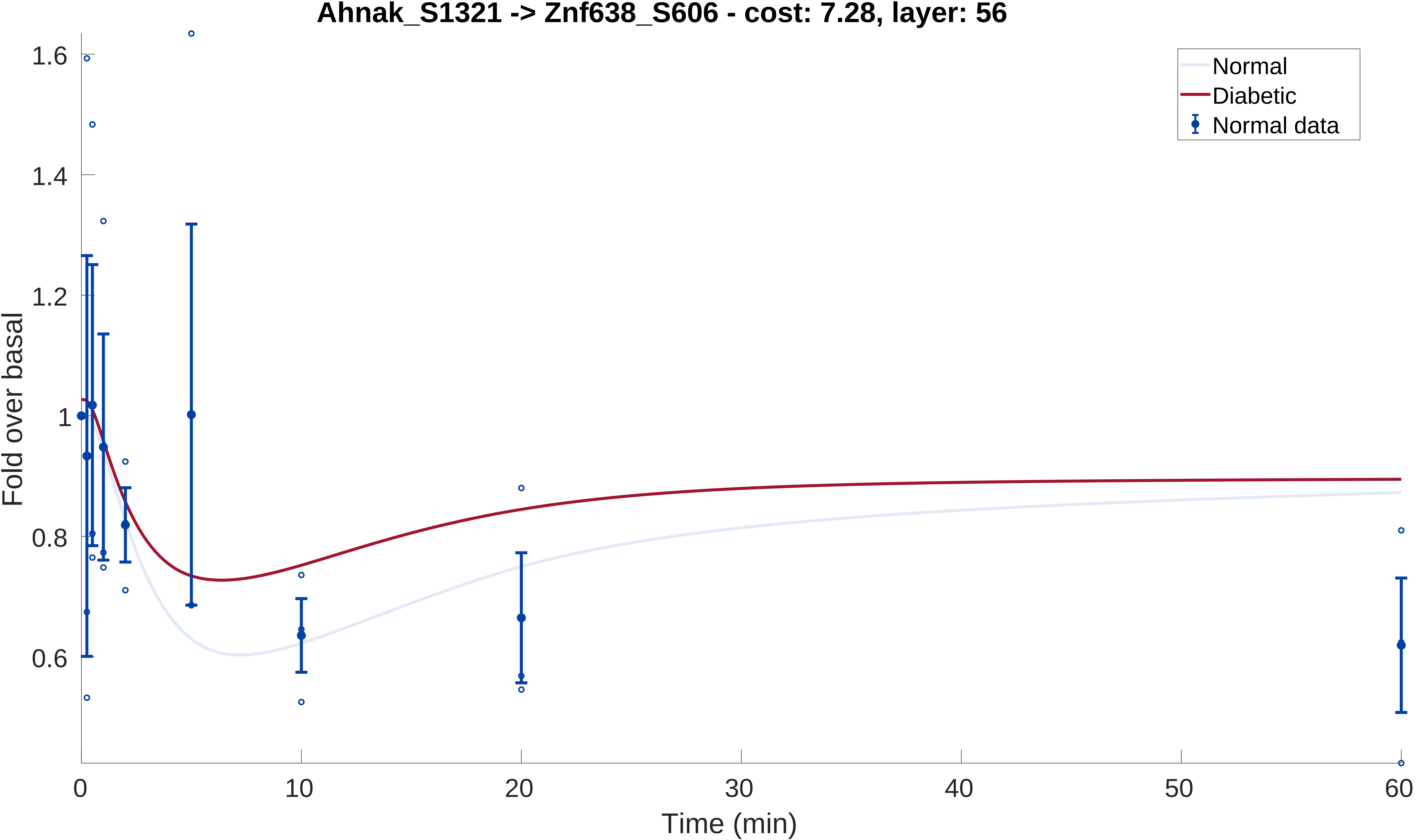
Ahnak_S1321 -> Znf638_S606 - cost: 7.28, layer: 56
Fold over basal
Time (min)
Normal
Diabetic
Normal data
1.6
1.4
1.2
1
0.8
0.6
0
10
20
30
40
50
60

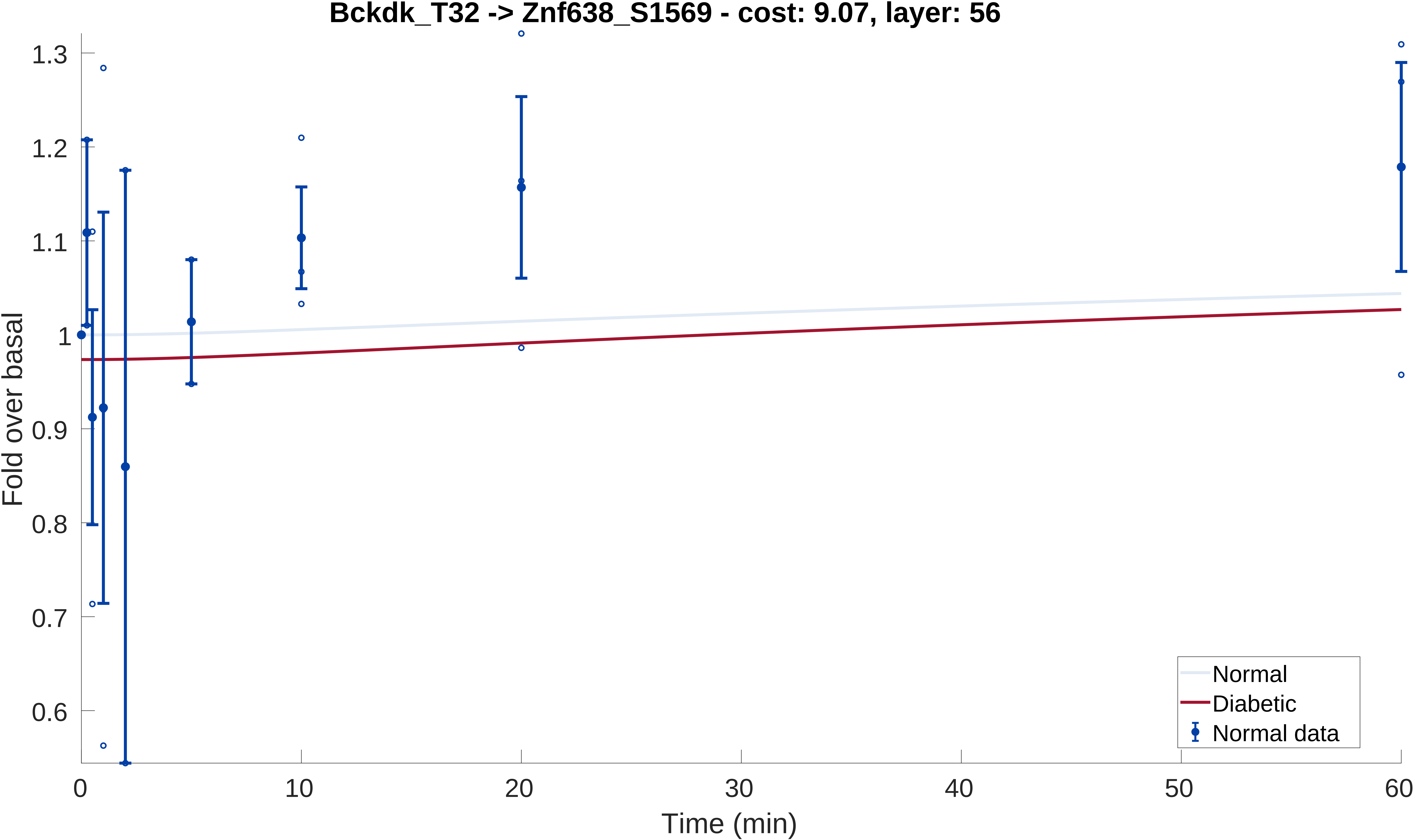

Bckdk_T32 -> Znf638_S1569 - cost: 9.07, layer: 56
Fold over basal
1.3
1.2
1.1
1
0.9
0.8
0.7
0.6
0
10
20
30
40
50
60
Time (min)
Normal
Diabetic
Normal data

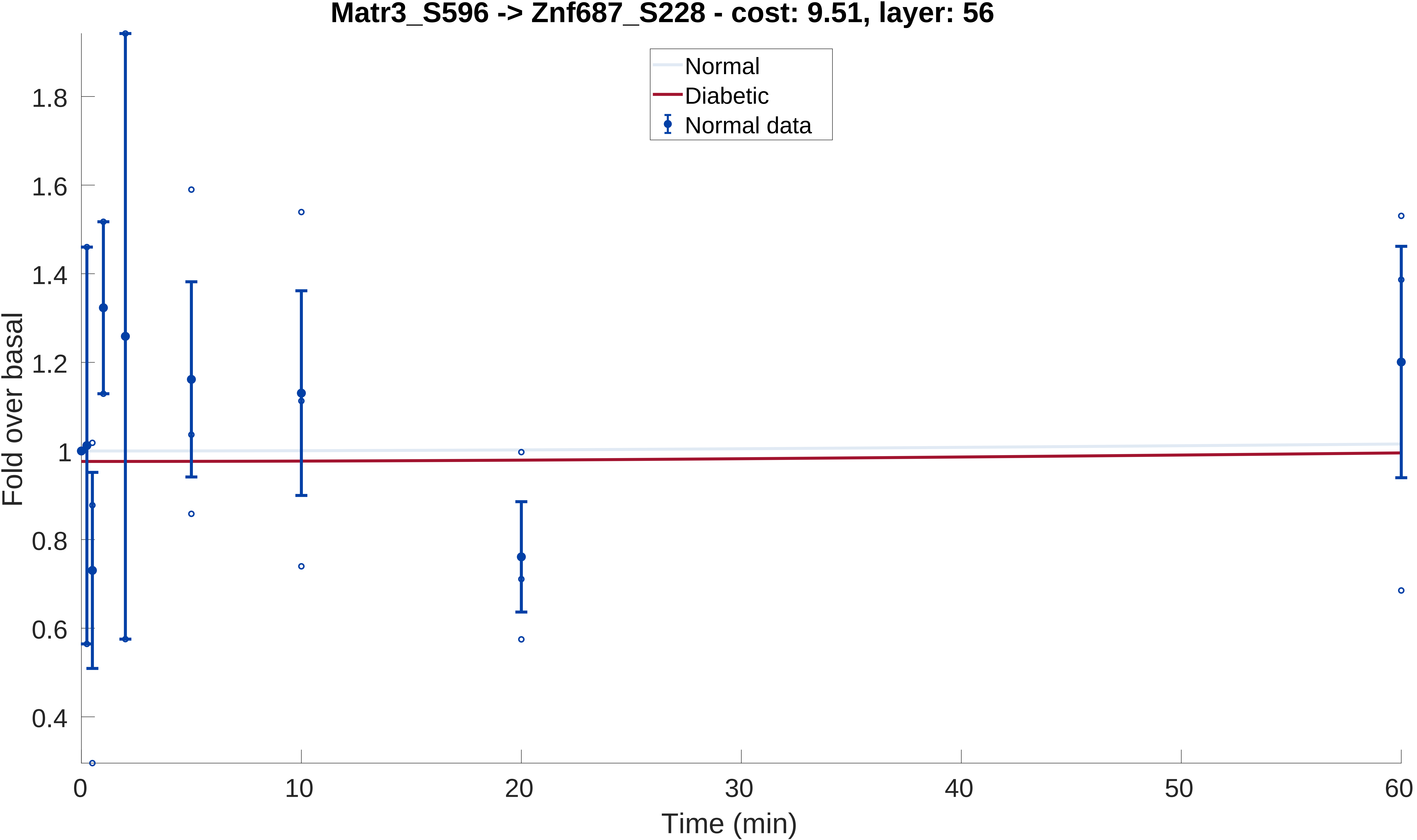
Matr3_S596 -> Znf687_S228 - cost: 9.51, layer: 56
Normal
Diabetic
Normal data
Fold over basal
1.8
1.6
1.4
1.2
1
0.8
0.6
0.4
0
10
20
30
40
50
60
Time (min)

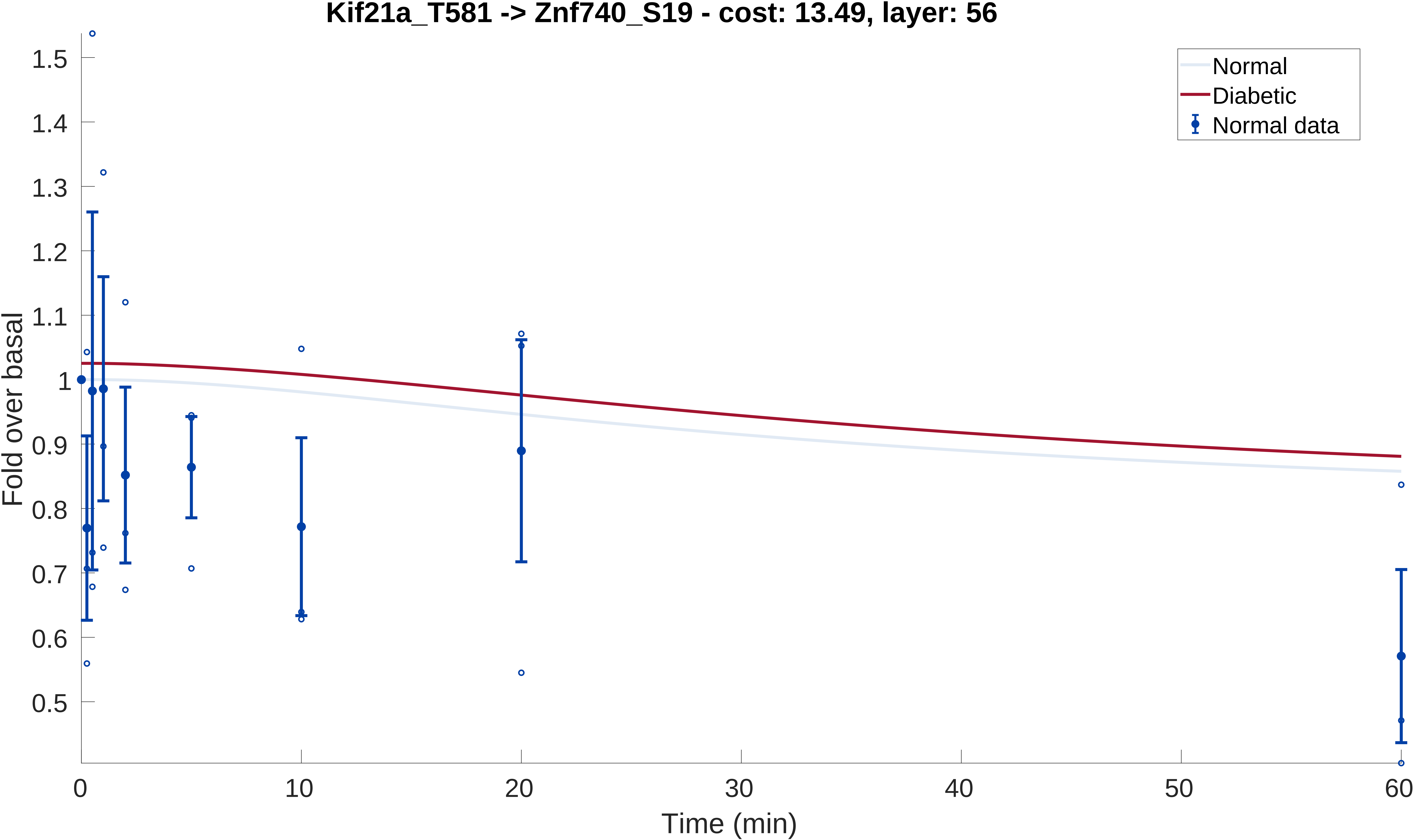
Kif21a_T581 -> Znf740_S19 - cost: 13.49, layer: 56
Normal
Diabetic
Normal data
Fold over basal
Time (min)
1.5
1.4
1.3
1.2
1.1
1
0.9
0.8
0.7
0.6
0.5
0
10
20
30
40
50
60

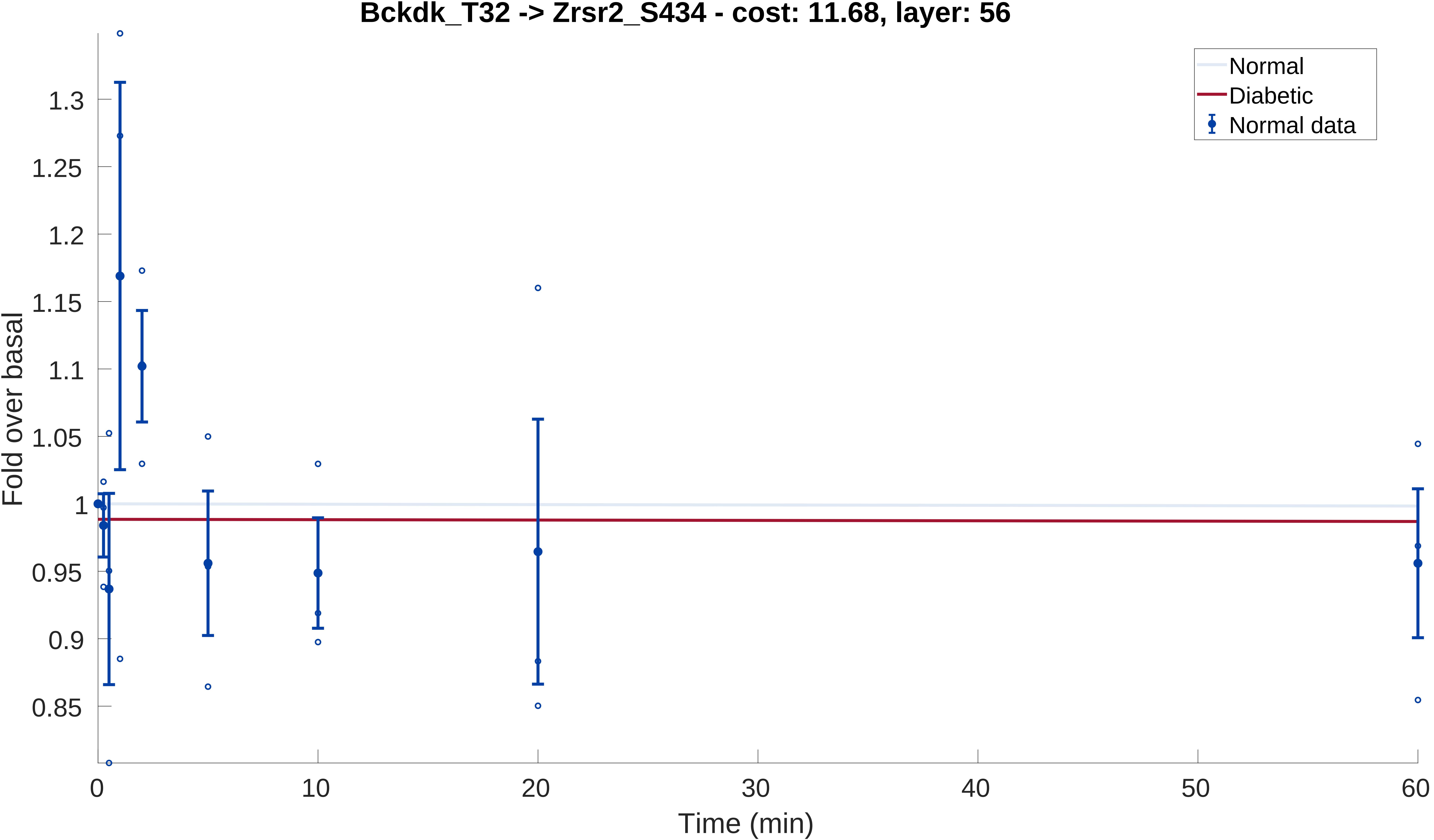
Bckdk_T32 -> Zrsr2_S434 - cost: 11.68, layer: 56
Normal
Diabetic
Normal data
Fold over basal
1.3
1.25
1.2
1.15
1.1
1.05
1
0.95
0.9
0.85
0
10
20
30
40
50
60
Time (min)

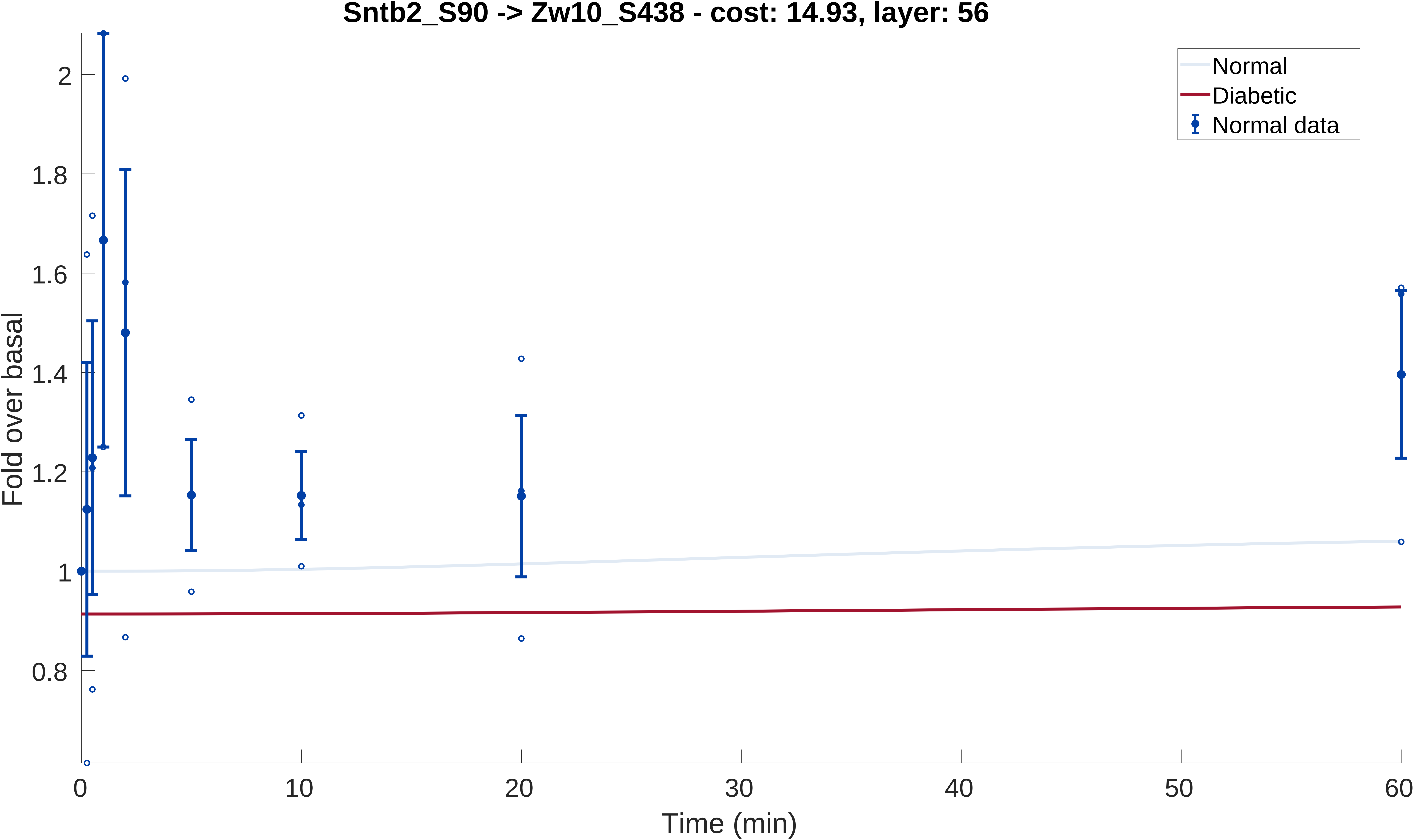

Sntb2_S90 -> Zw10_S438 - cost: 14.93, layer: 56
Normal
Diabetic
Normal data
Fold over basal
Time (min)
2
1.8
1.6
1.4
1.2
1
0.8
0
10
20
30
40
50
60

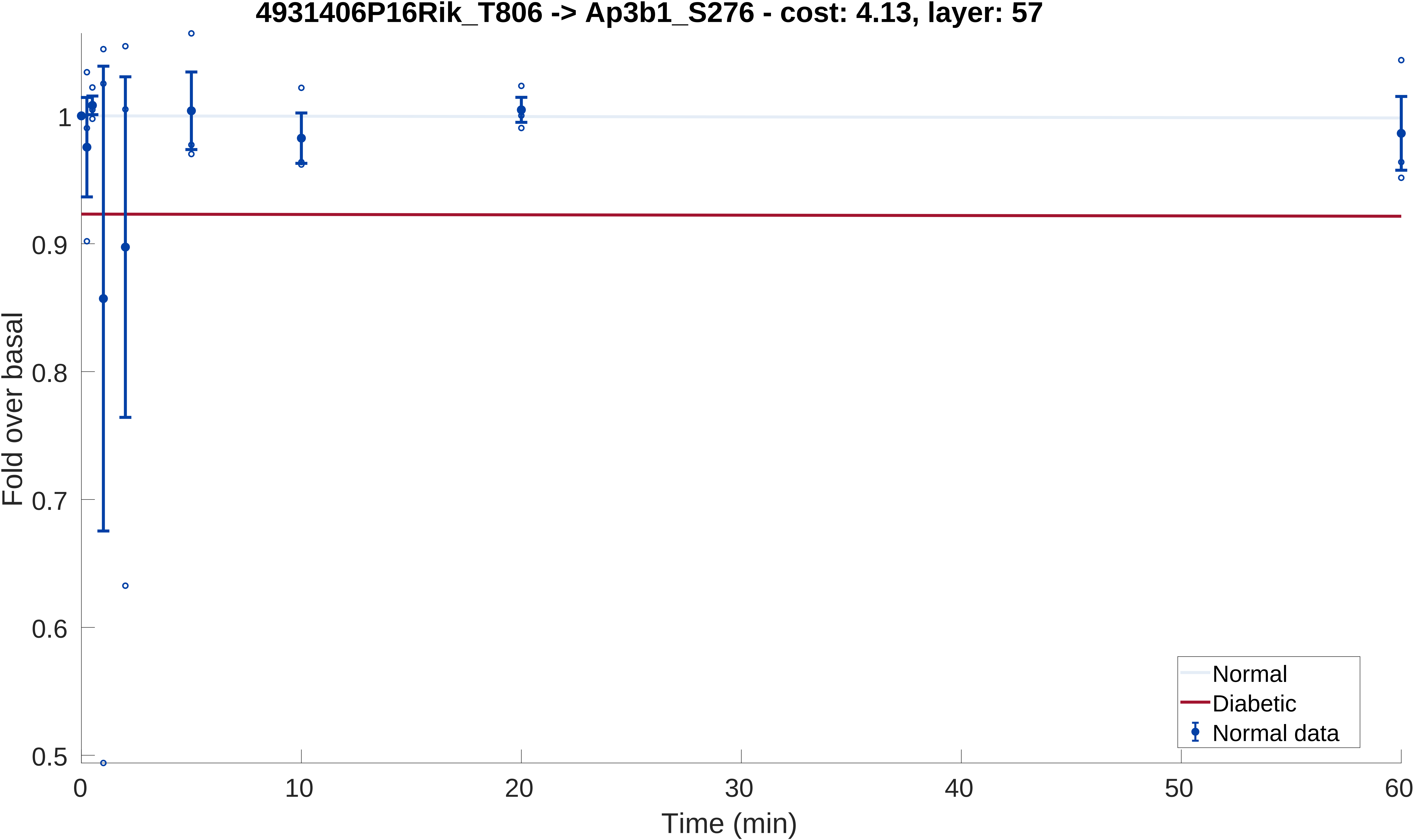

4931406P16Rik_T806 -> Ap3b1_S276 - cost: 4.13, layer: 57
Fold over basal
1
0.9
0.8
0.7
0.6
0.5
0
10
20
30
40
50
60
Time (min)
Normal
Diabetic
Normal data

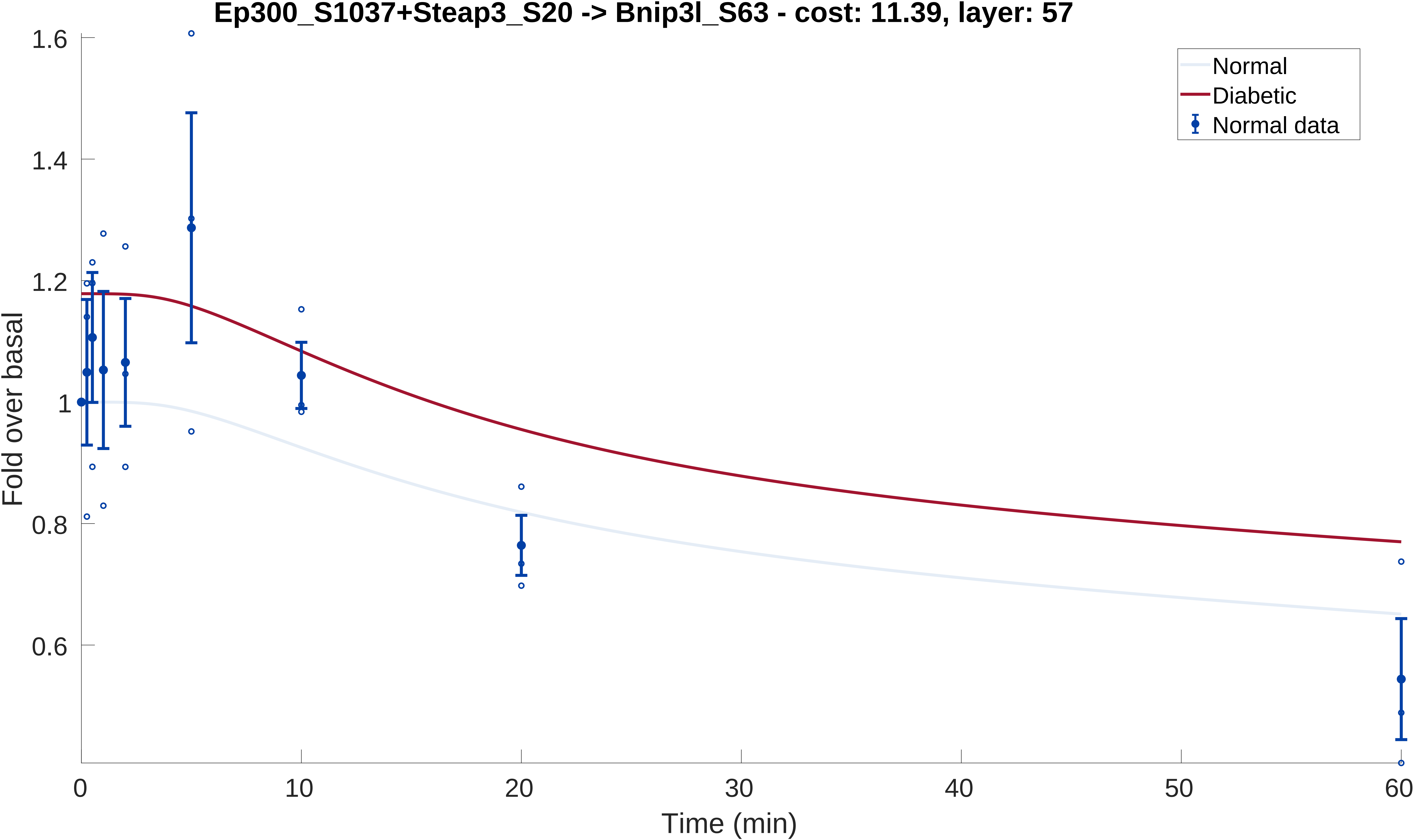
Ep300_S1037+Steap3_S20 -> Bnip3l_S63 - cost: 11.39, layer: 57
Normal
Diabetic
Normal data
Fold over basal
1.6
1.4
1.2
1
0.8
0.6
0
10
20
30
40
50
60
Time (min)

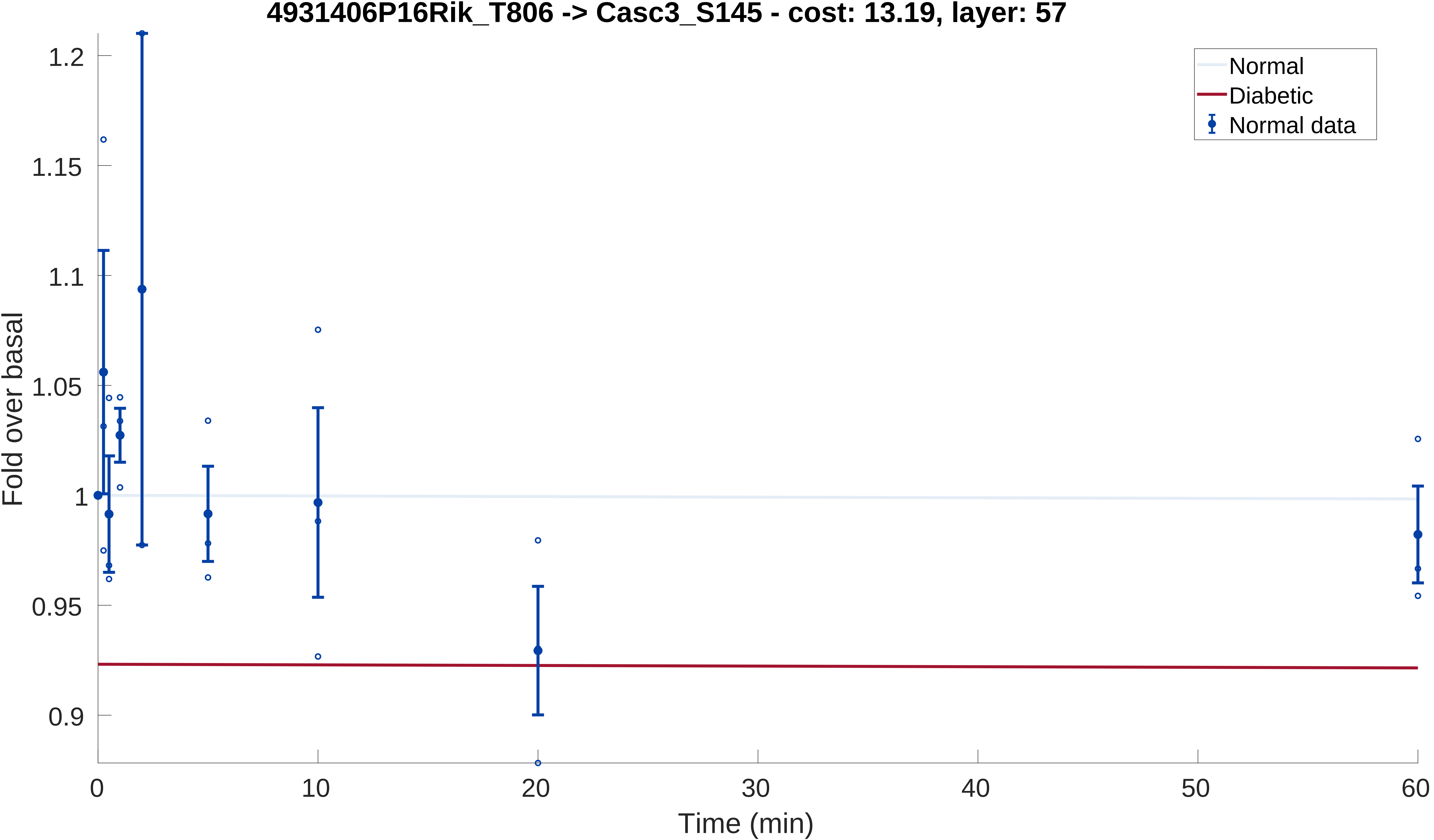

4931406P16Rik_T806 -> Casc3_S145 - cost: 13.19, layer: 57
Normal
Diabetic
Normal data
Fold over basal
1.2
1.15
1.1
1.05
1
0.95
0.9
0
10
20
30
40
50
60
Time (min)

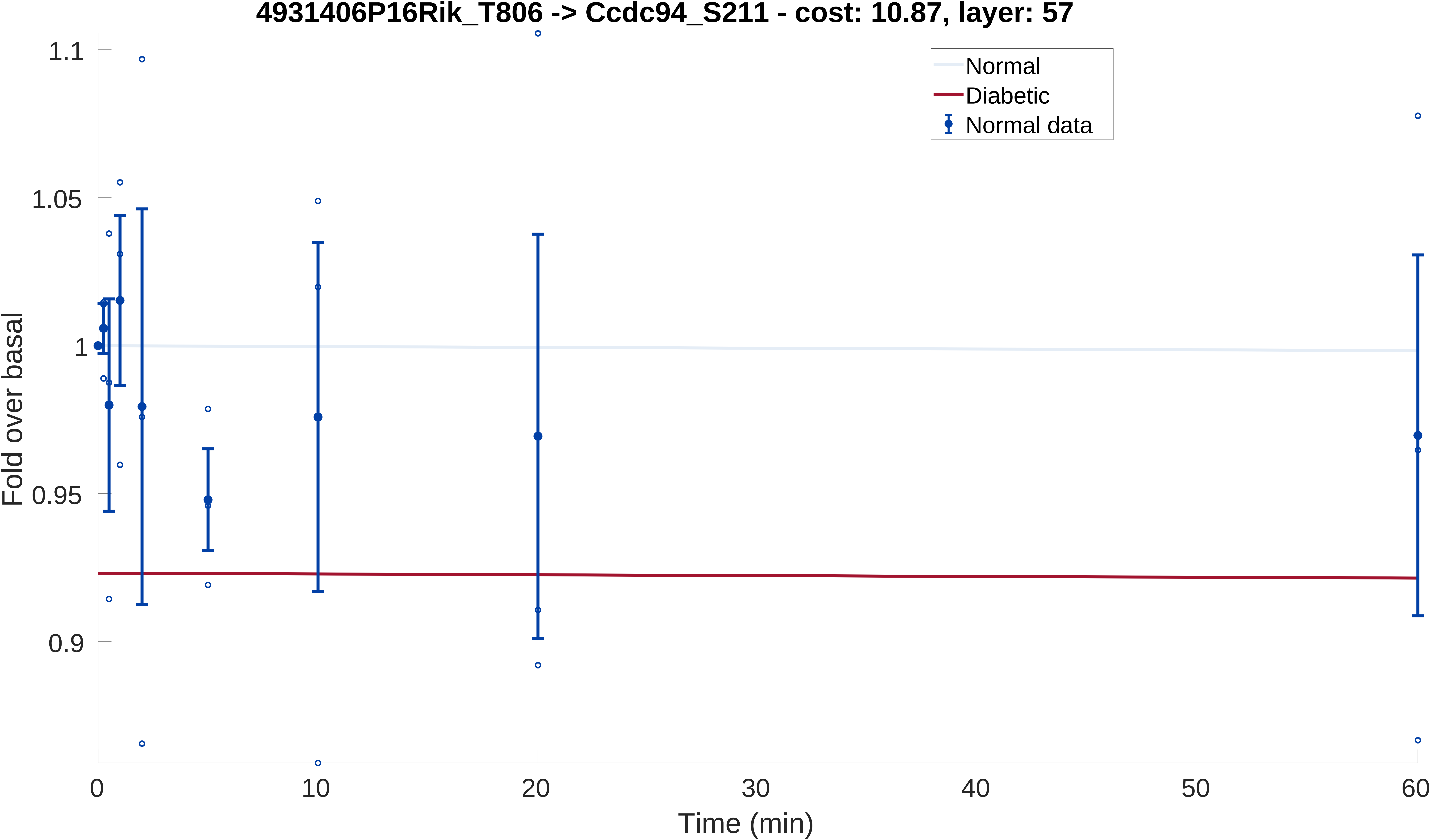

4931406P16Rik_T806 -> Ccdc94_S211 - cost: 10.87, layer: 57
Normal
Diabetic
Normal data
Fold over basal
1.1
1.05
1
0.95
0.9
0
10
20
30
40
50
60
Time (min)

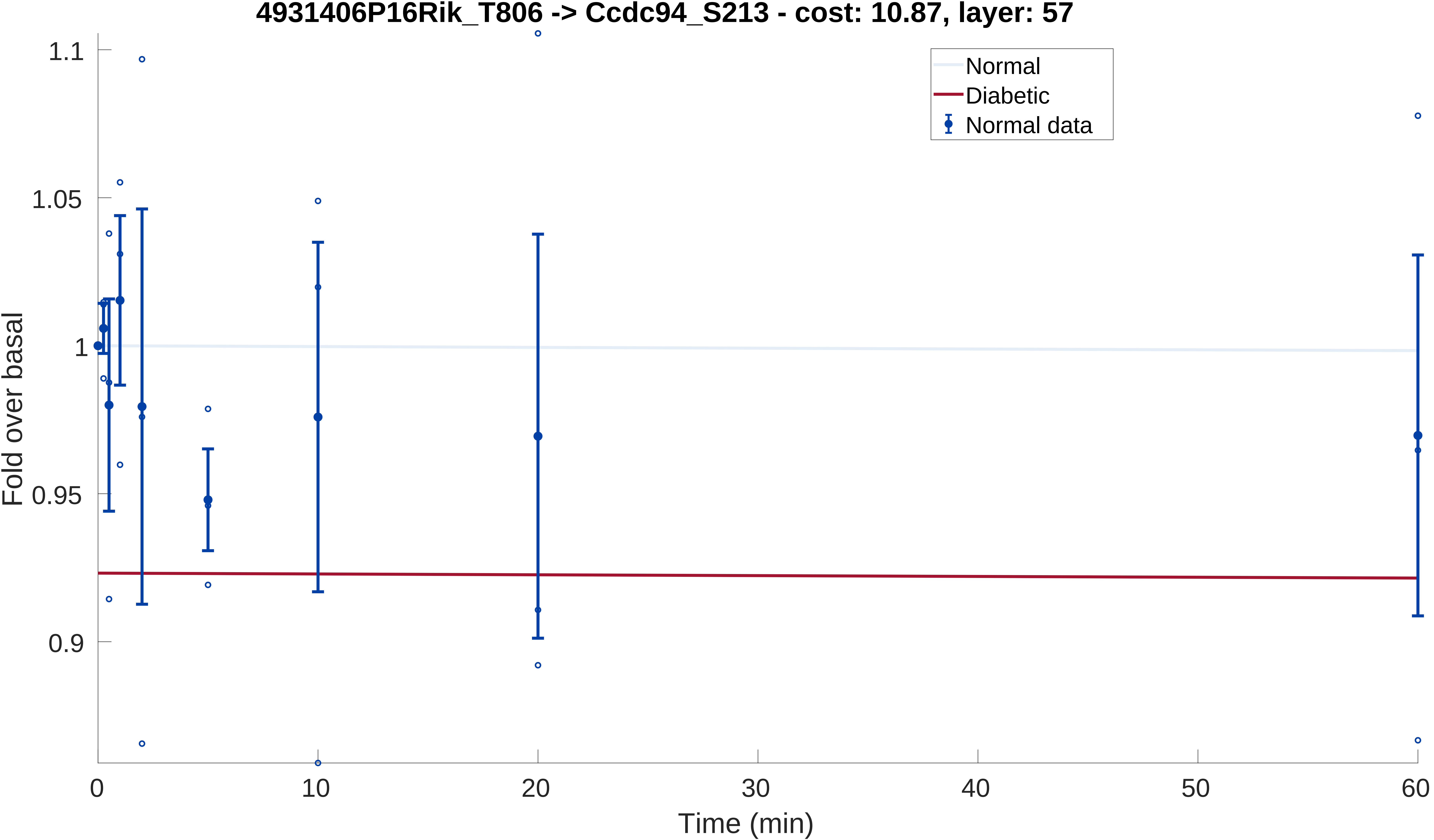

4931406P16Rik_T806 -> Ccdc94_S213 - cost: 10.87, layer: 57
Normal
Diabetic
Normal data
Fold over basal
1.1
1.05
1
0.95
0.9
0
10
20
30
40
50
60
Time (min)

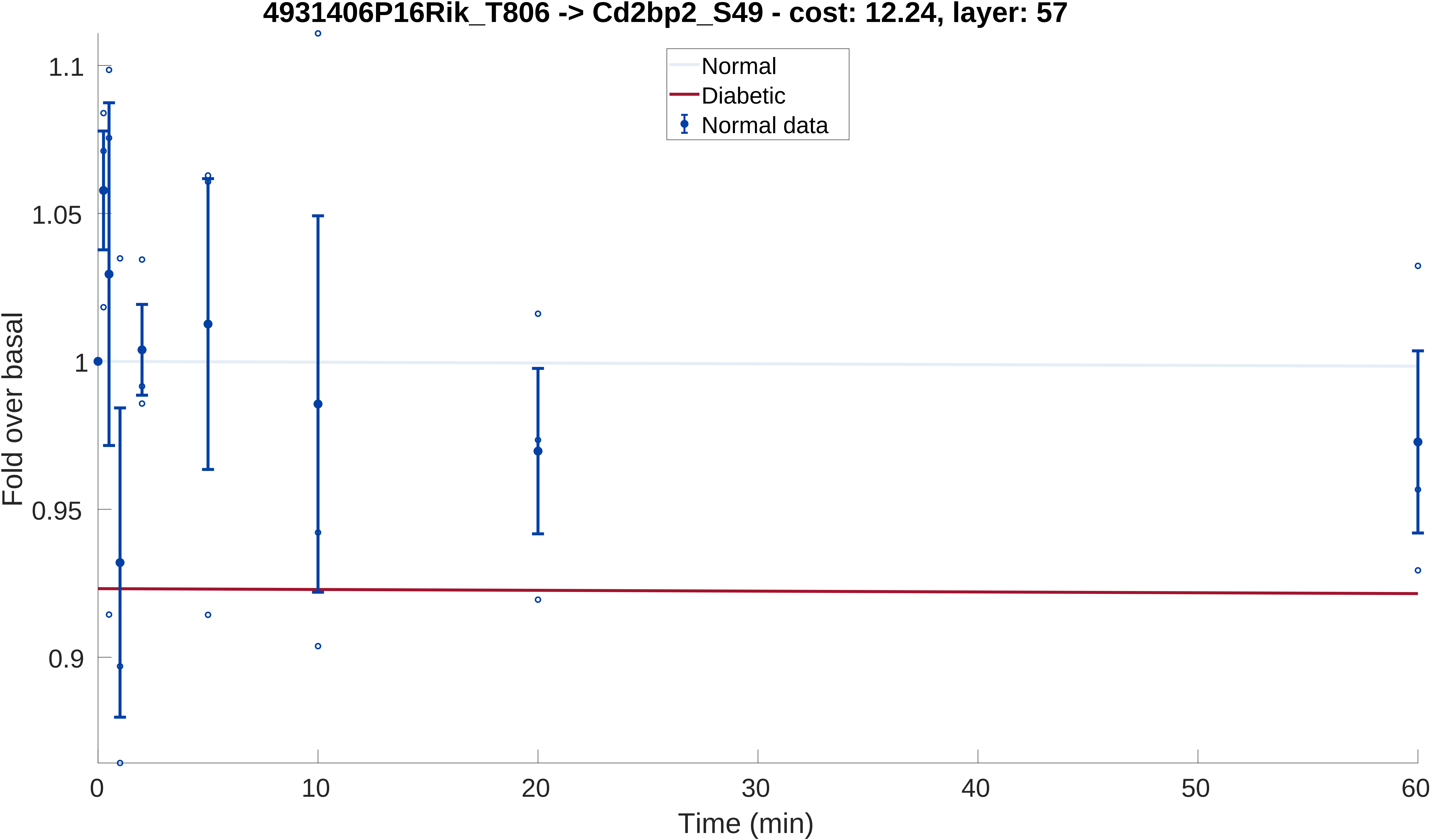

4931406P16Rik_T806 -> Cd2bp2_S49 - cost: 12.24, layer: 57
Normal
Diabetic
Normal data
1.1
1.05
1
0.95
0.9
Fold over basal
0
10
20
30
40
50
60
Time (min)

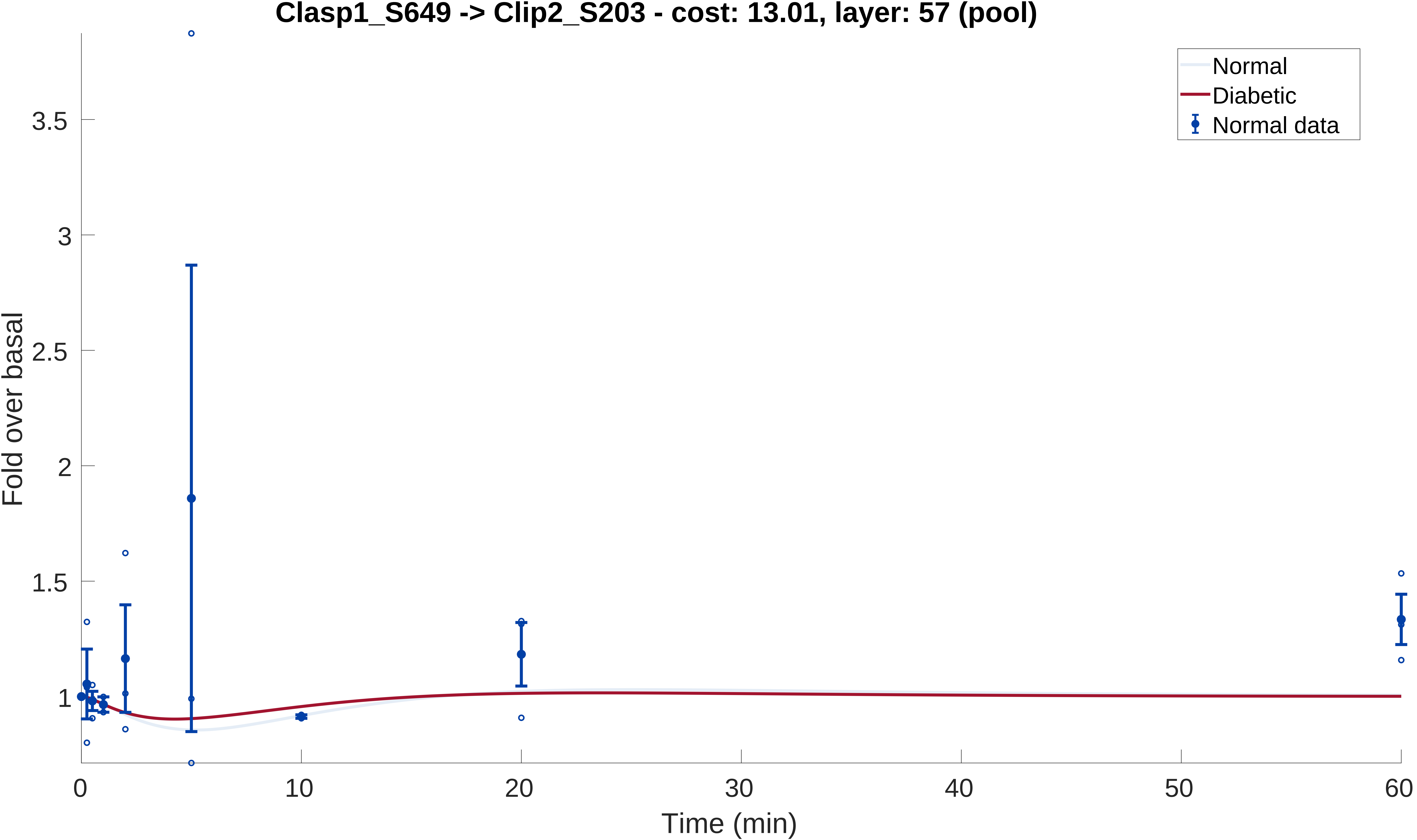

Clasp1_S649 -> Clip2_S203 - cost: 13.01, layer: 57 (pool)
Normal
Diabetic
Normal data
Fold over basal
3.5
3
2.5
2
1.5
1
0
10
20
30
40
50
60
Time (min)

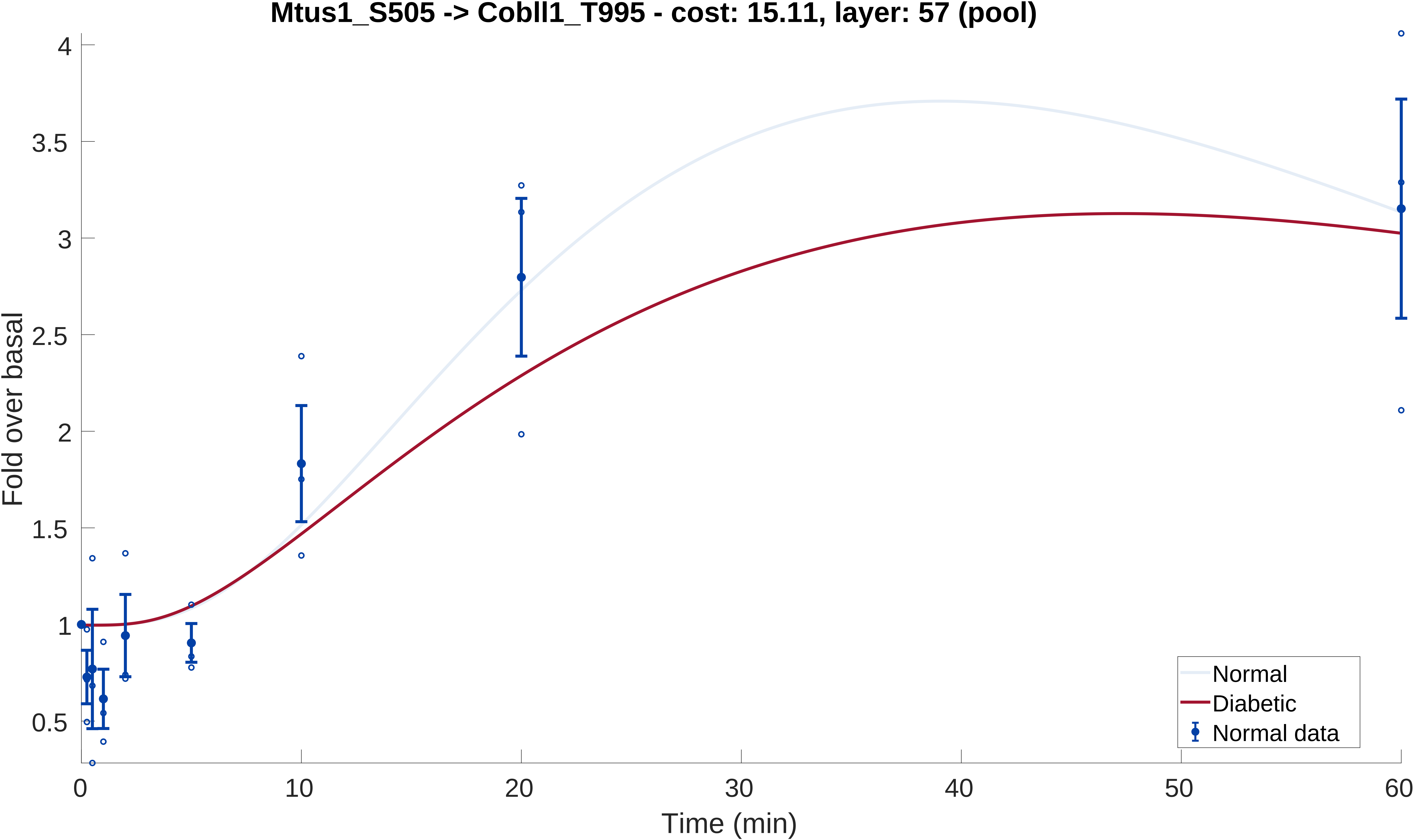
Mtus1_S505 -> Cobll1_T995 - cost: 15.11, layer: 57 (pool)
Fold over basal
Time (min)
4
3.5
3
2.5
2
1.5
1
0.5
0
10
20
30
40
50
60
Normal
Diabetic
Normal data

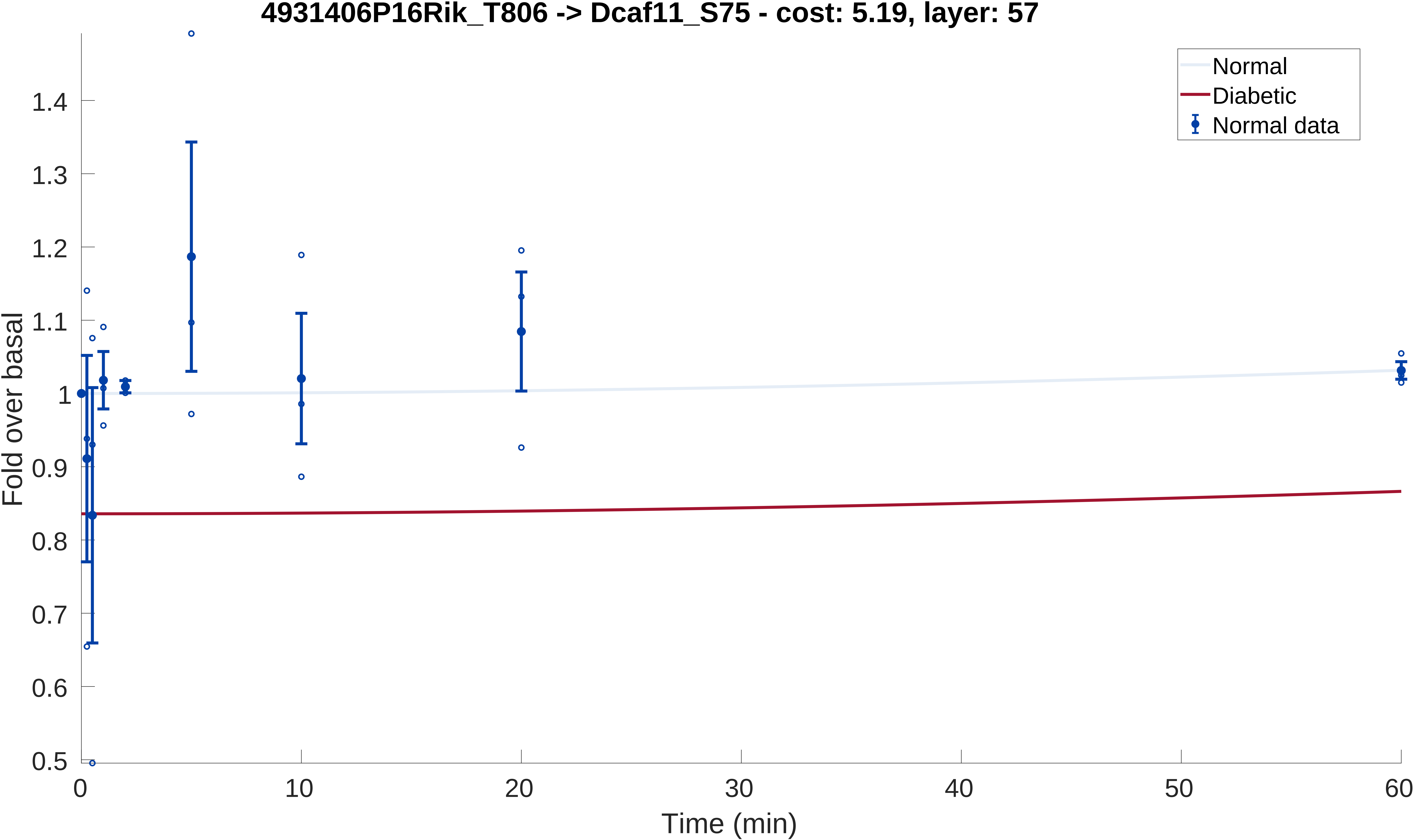
4931406P16Rik_T806 -> Dcaf11_S75 - cost: 5.19, layer: 57
Normal
Diabetic
Normal data
Fold over basal
1.4
1.3
1.2
1.1
1
0.9
0.8
0.7
0.6
0.5
0
10
20
30
40
50
60
Time (min)

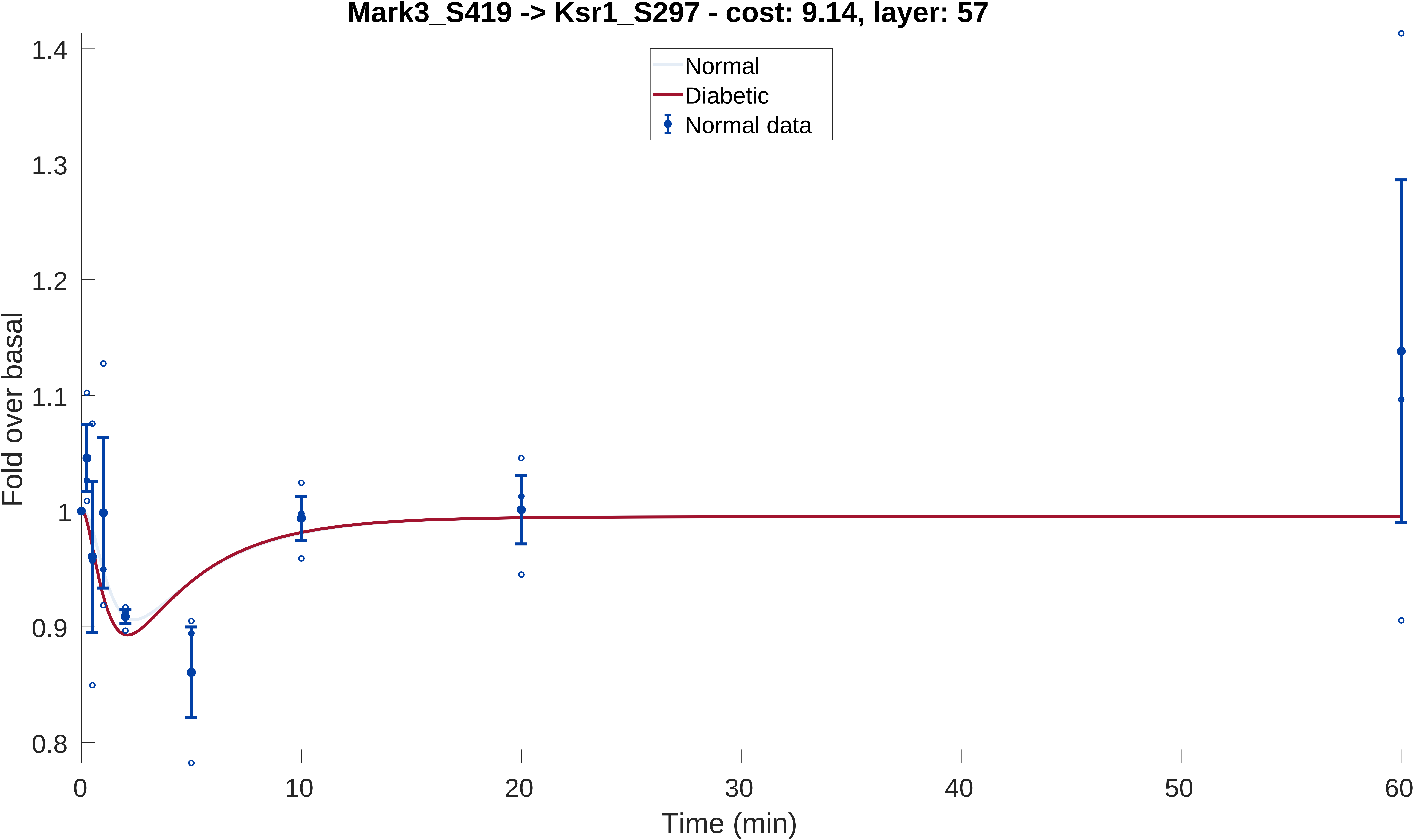

Mark3_S419 -> Ksr1_S297 - cost: 9.14, layer: 57
Normal
Diabetic
Normal data
Fold over basal
1.4
1.3
1.2
1.1
1
0.9
0.8
0
10
20
30
40
50
60
Time (min)

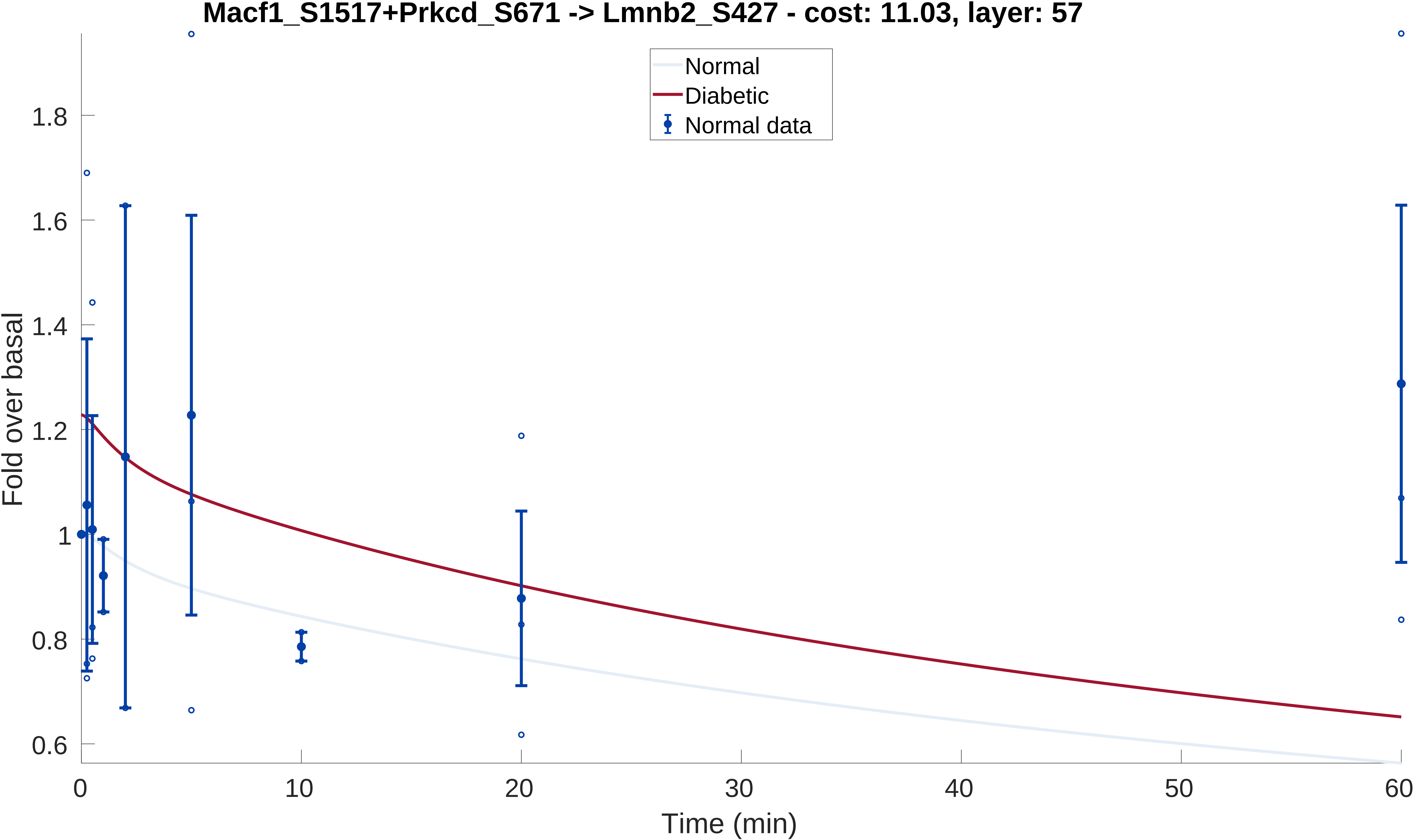

Macf1_S1517+Prkcd_S671 -> Lmnb2_S427 - cost: 11.03, layer: 57
Normal
Diabetic
Normal data
Fold over basal
Time (min)
0.6
0.8
1
1.2
1.4
1.6
1.8
0
10
20
30
40
50
60

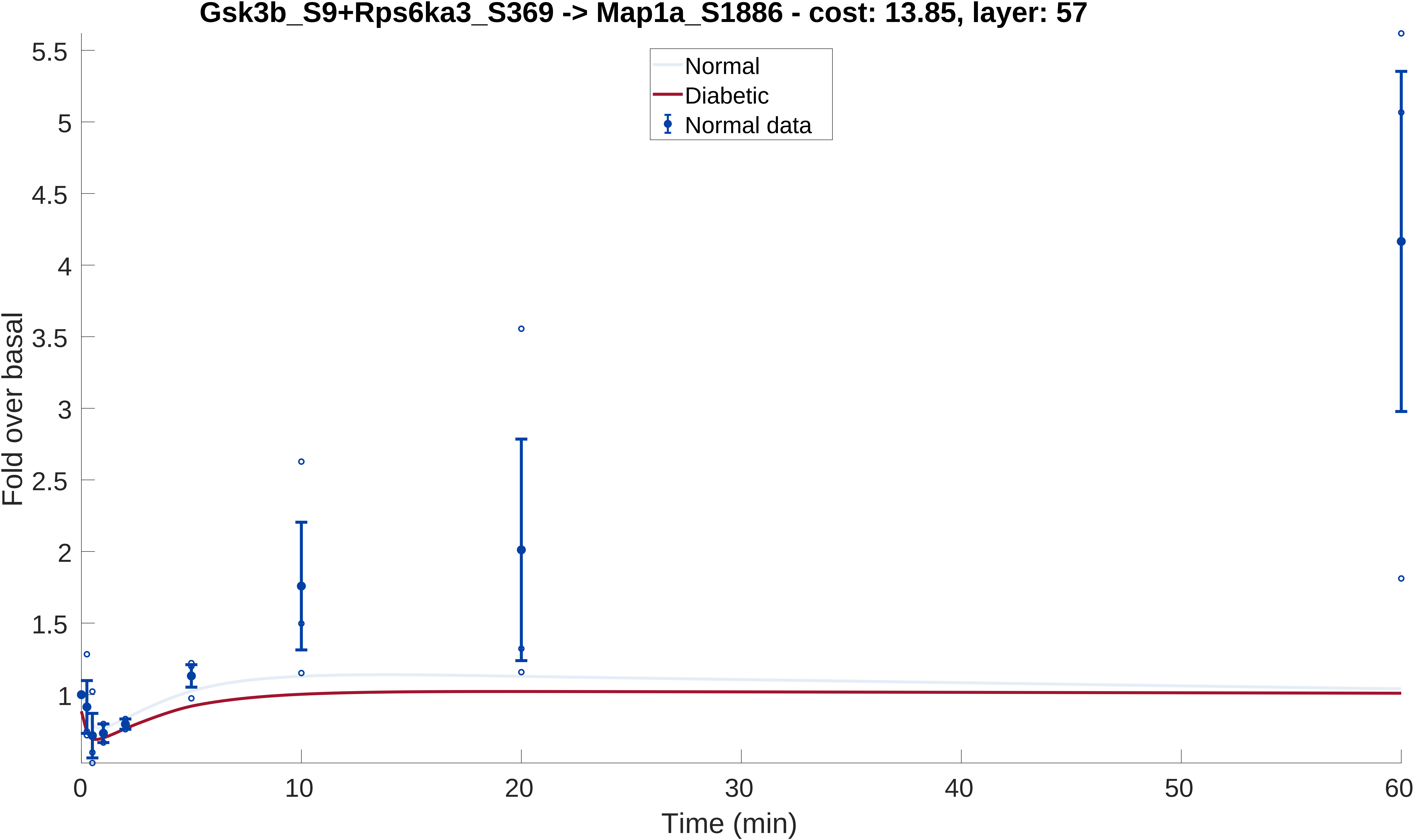
Gsk3b_S9+Rps6ka3_S369 -> Map1a_S1886 - cost: 13.85, layer: 57
Normal
Diabetic
Normal data
Fold over basal
5.5
5
4.5
4
3.5
3
2.5
2
1.5
1
0
10
20
30
40
50
60
Time (min)

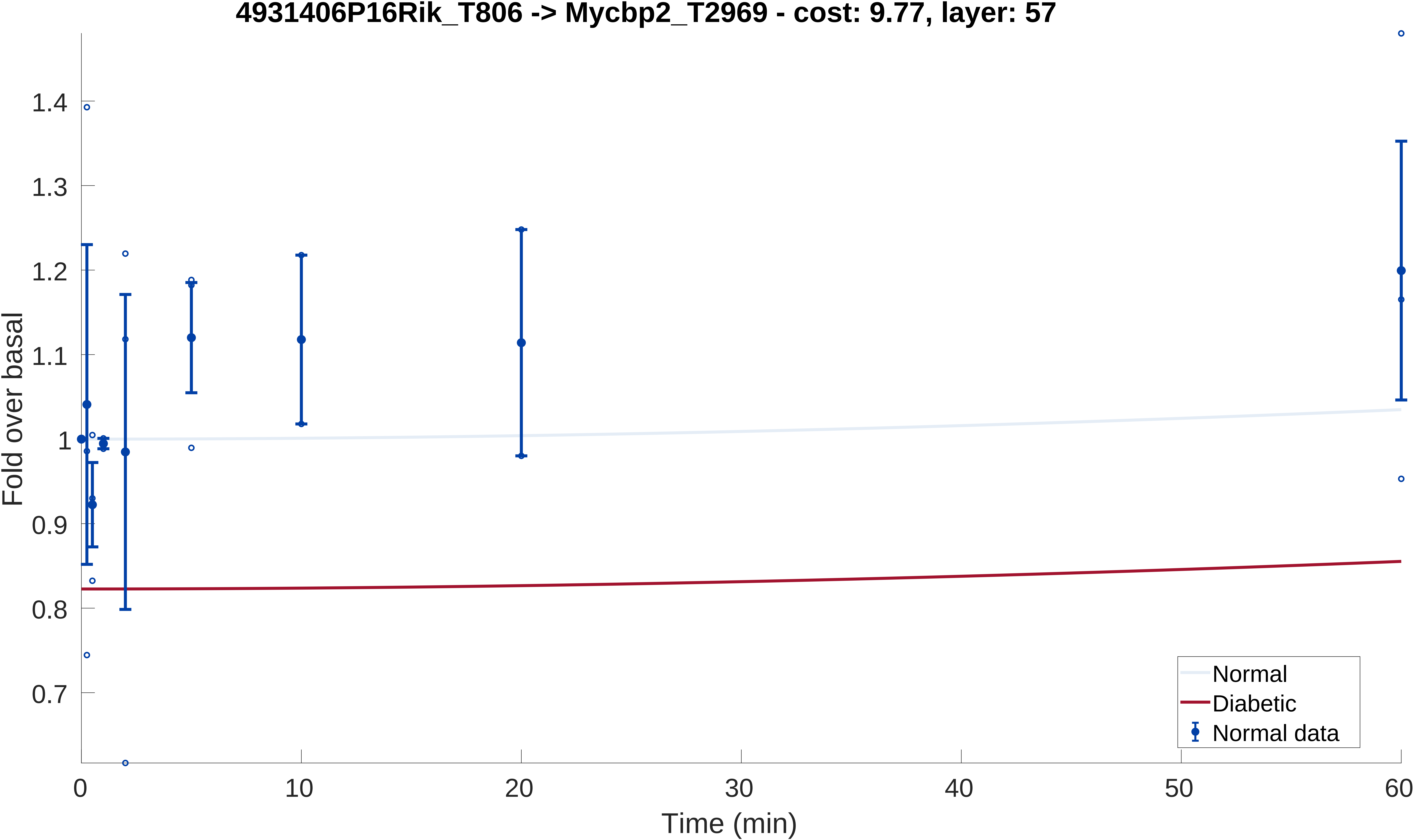

4931406P16Rik_T806 -> Mycbp2_T2969 - cost: 9.77, layer: 57
Fold over basal
1.4
1.3
1.2
1.1
1
0.9
0.8
0.7
0
10
20
30
40
50
60
Time (min)
Normal
Diabetic
Normal data

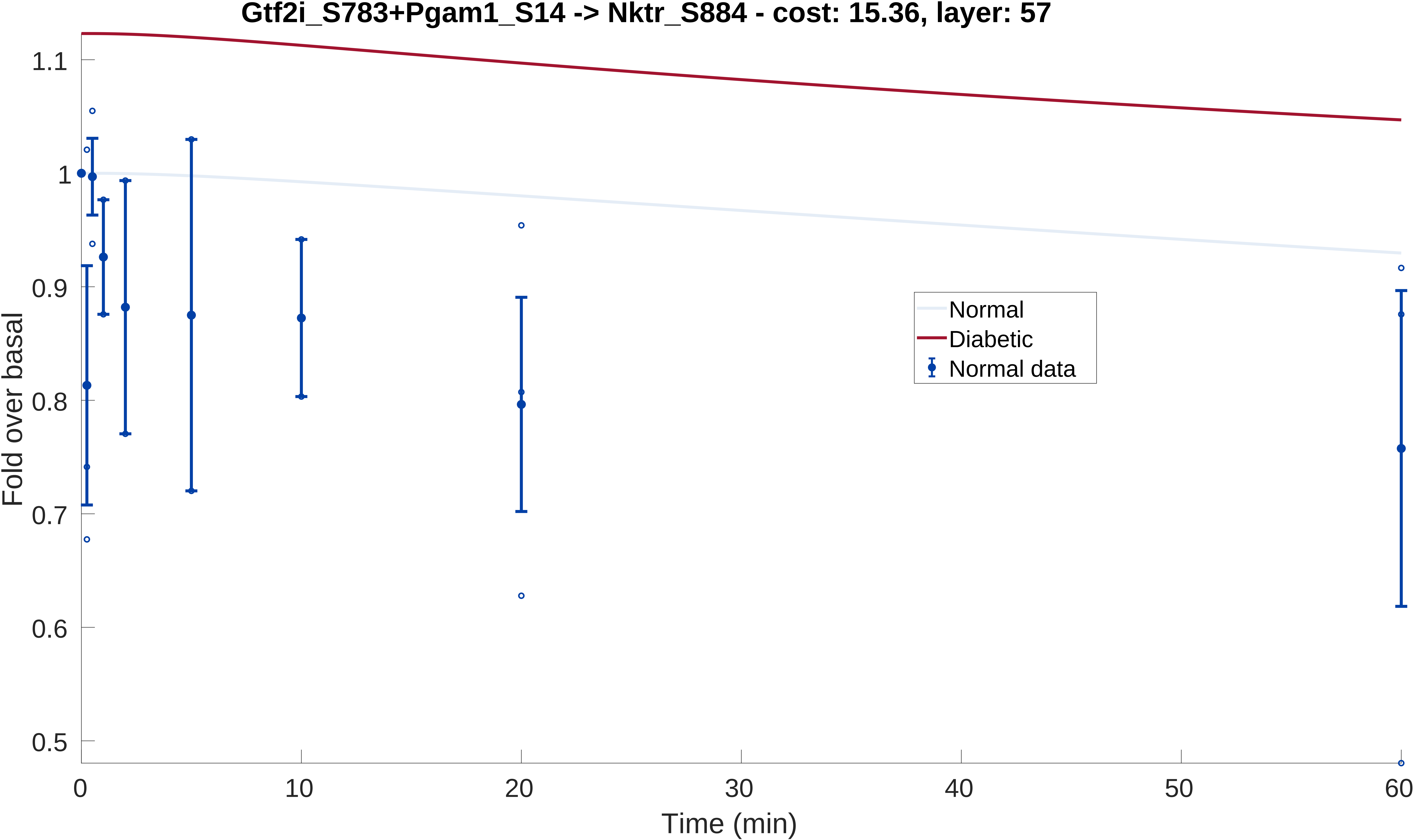

Gtf2i_S783+Pgam1_S14 -> Nktr_S884 - cost: 15.36, layer: 57
Fold over basal
1.1
1
0.9
0.8
0.7
0.6
0.5
0
10
20
30
40
50
60
Time (min)
Normal
Diabetic
Normal data

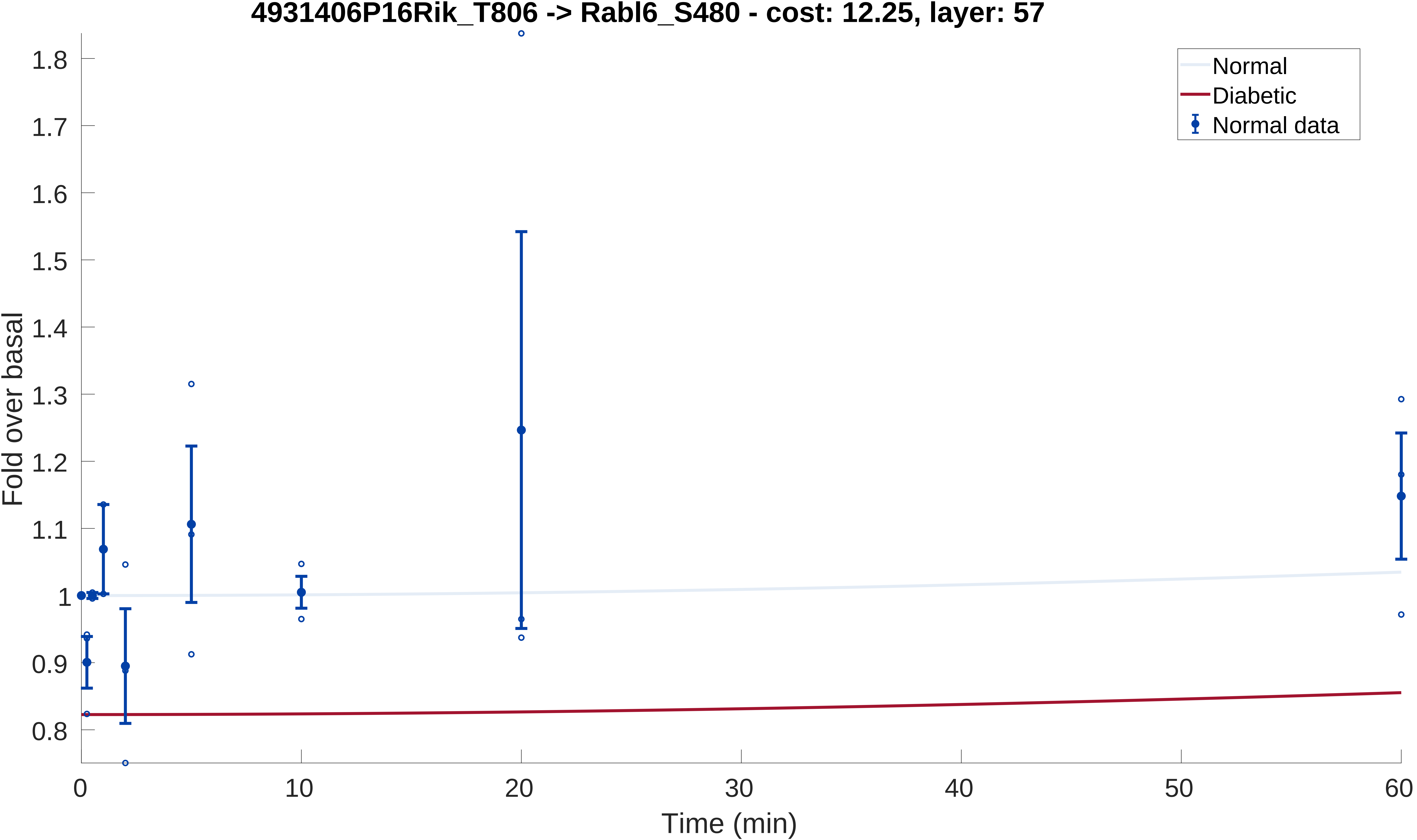
4931406P16Rik_T806 -> Rabl6_S480 - cost: 12.25, layer: 57
Normal
Diabetic
Normal data
Fold over basal
1.8
1.7
1.6
1.5
1.4
1.3
1.2
1.1
1
0.9
0.8
0
10
20
30
40
50
60
Time (min)

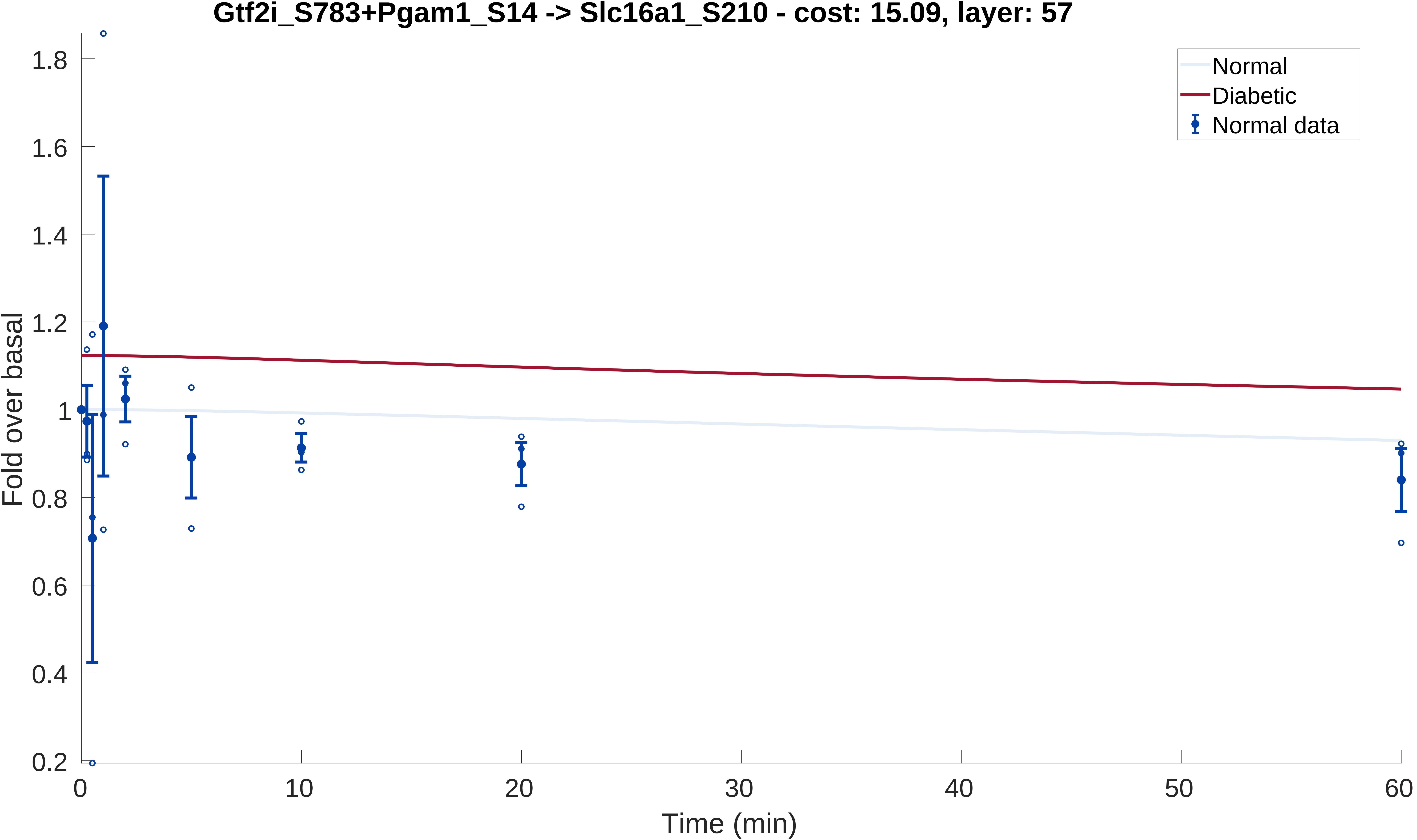

Gtf2i_S783+Pgam1_S14 -> Slc16a1_S210 - cost: 15.09, layer: 57
Normal
Diabetic
Normal data
Fold over basal
Time (min)
1.8
1.6
1.4
1.2
1
0.8
0.6
0.4
0.2
0
10
20
30
40
50
60

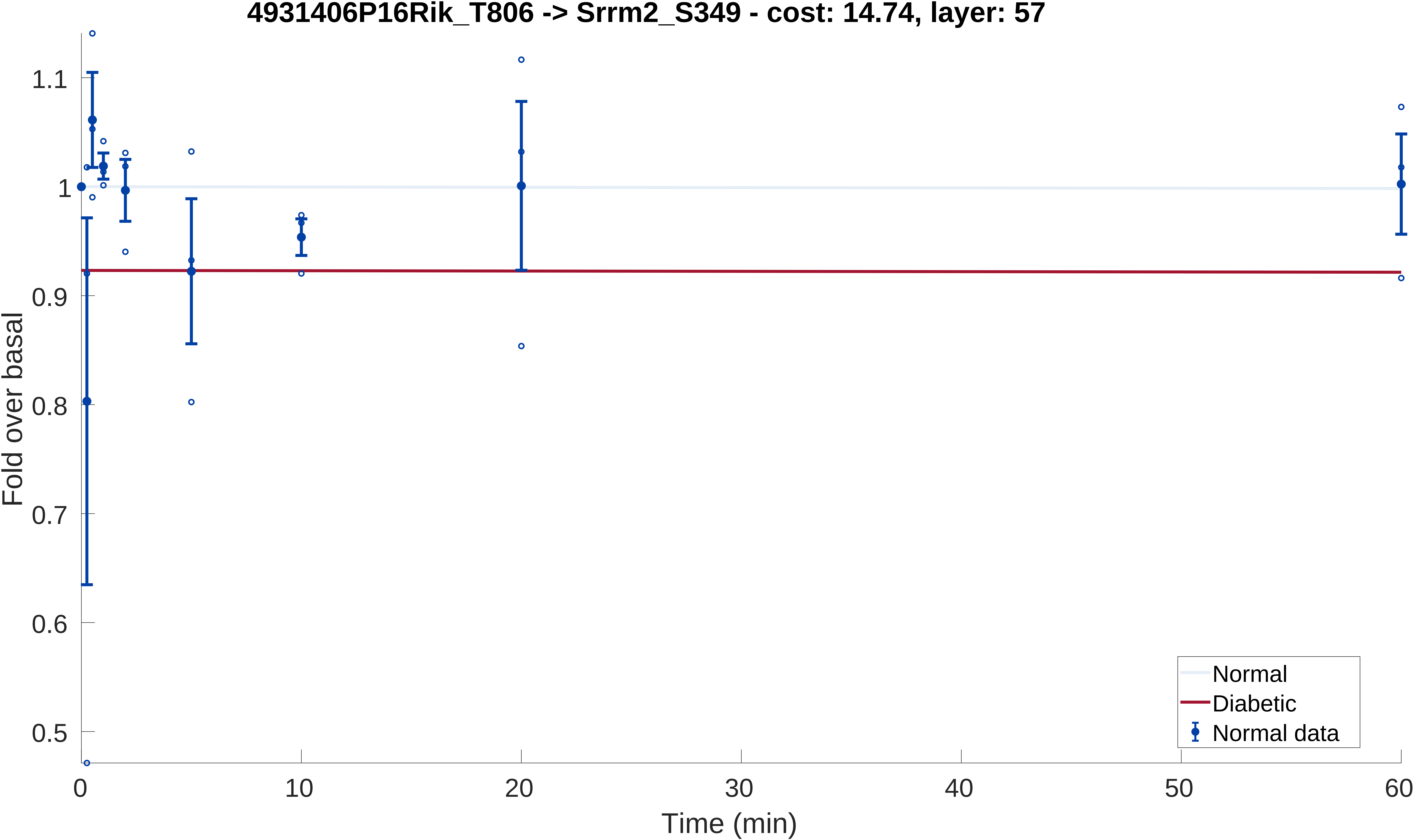

4931406P16Rik_T806 -> Srrm2_S349 - cost: 14.74, layer: 57
Fold over basal
Time (min)
1.1
1
0.9
0.8
0.7
0.6
0.5
0
10
20
30
40
50
60
Normal
Diabetic
Normal data

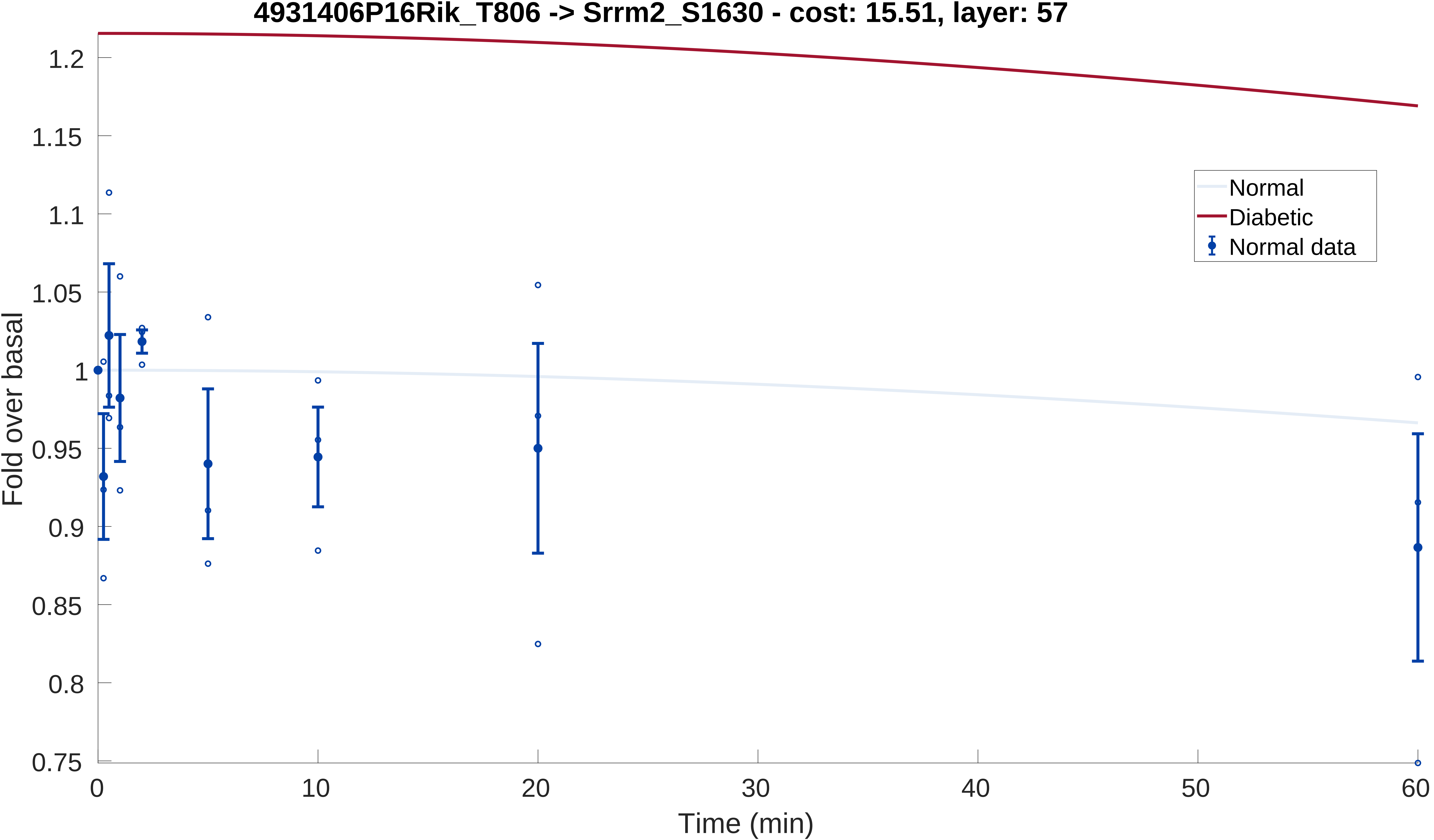

4931406P16Rik_T806 -> Srrm2_S1630 - cost: 15.51, layer: 57
Fold over basal
Time (min)
Normal
Diabetic
Normal data
1.2
1.15
1.1
1.05
1
0.95
0.9
0.85
0.8
0.75
0
10
20
30
40
50
60

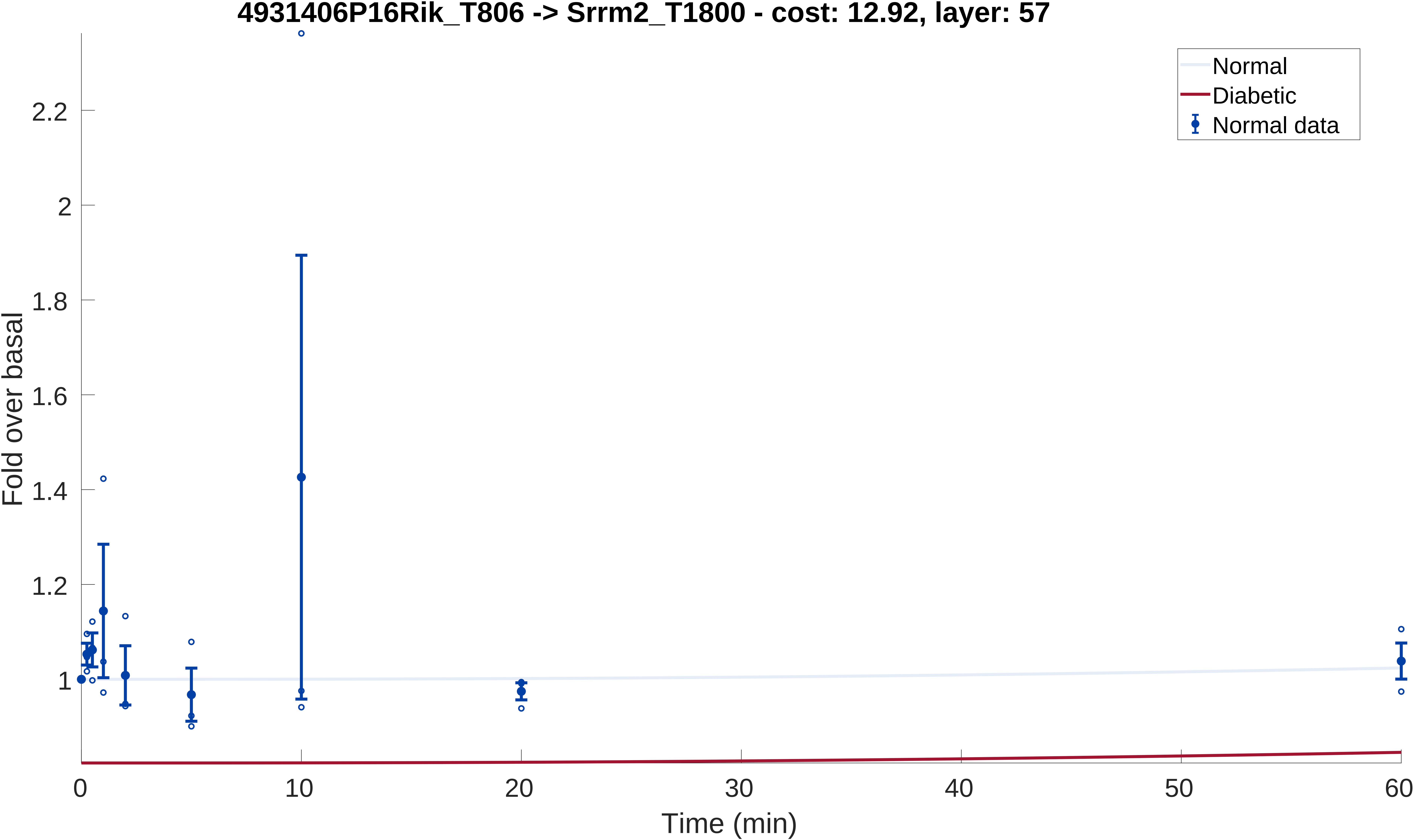
4931406P16Rik_T806 -> Srrm2_T1800 - cost: 12.92, layer: 57
Normal
Diabetic
Normal data
Fold over basal
2.2
2
1.8
1.6
1.4
1.2
1
0
10
20
30
40
50
60
Time (min)

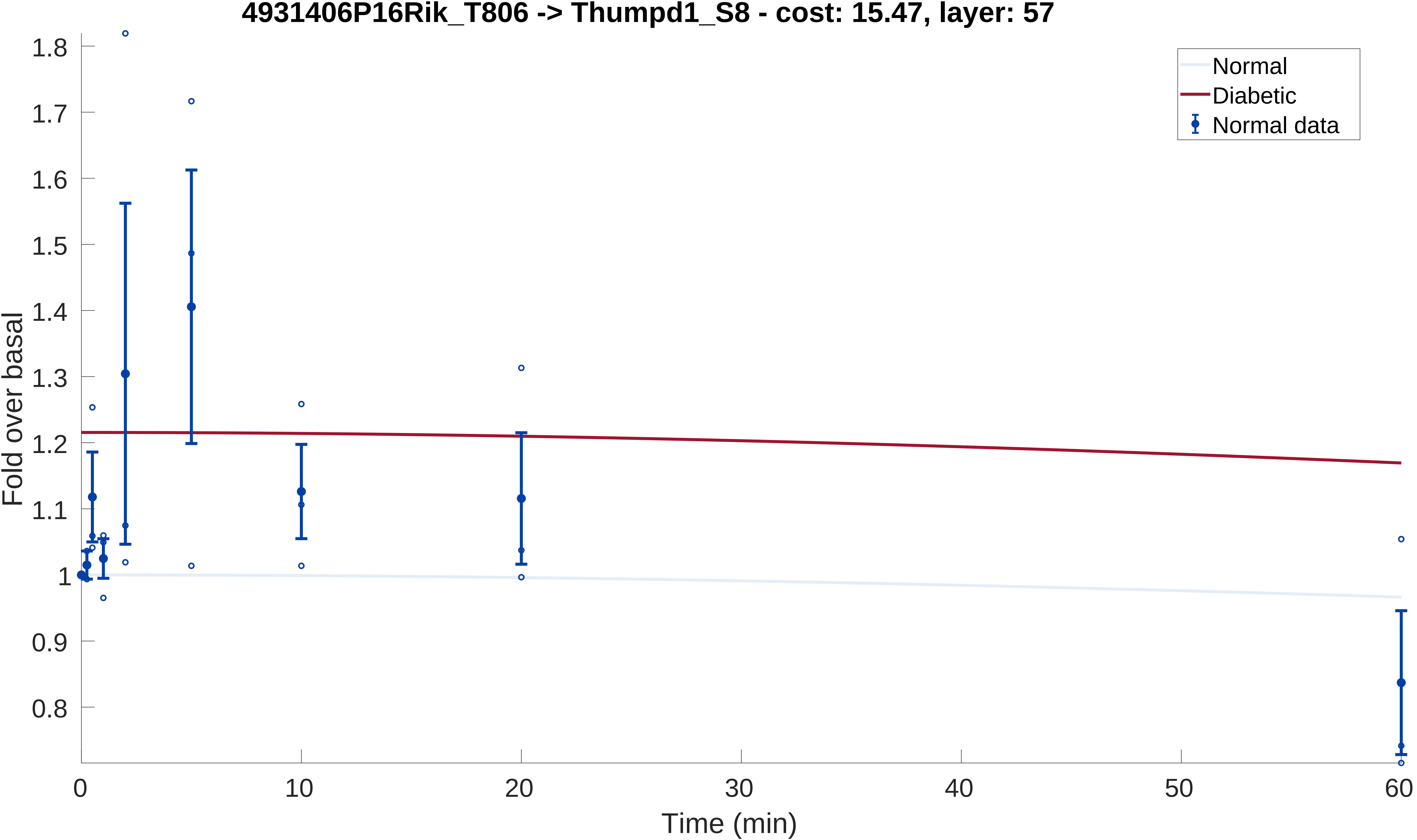

4931406P16Rik_T806 -> Thumpd1_S8 - cost: 15.47, layer: 57
Normal
Diabetic
Normal data
Fold over basal
Time (min)
1.8
1.7
1.6
1.5
1.4
1.3
1.2
1.1
1
0.9
0.8
0
10
20
30
40
50
60

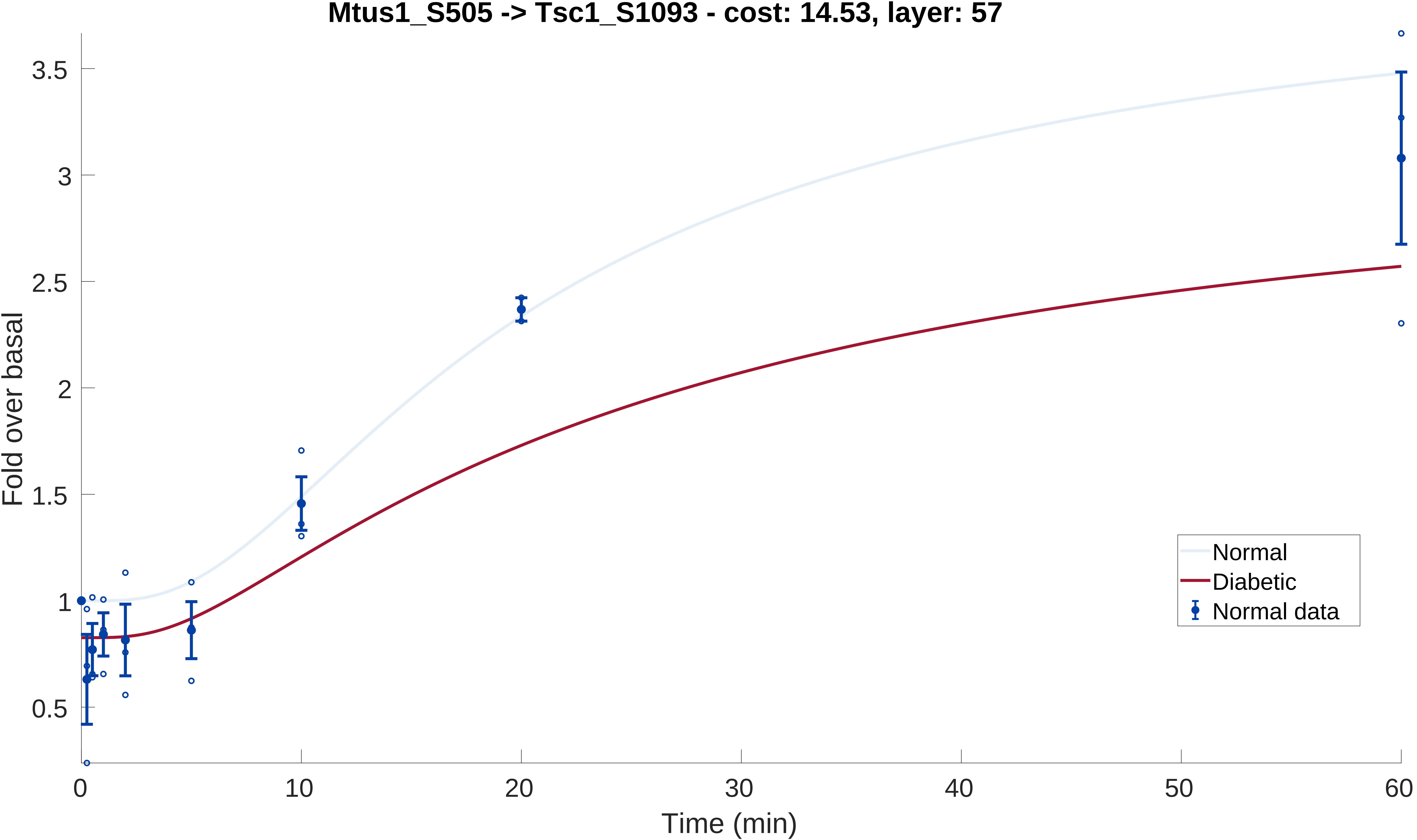
Mtus1_S505 -> Tsc1_S1093 - cost: 14.53, layer: 57
Fold over basal
3.5
3
2.5
2
1.5
1
0.5
0
10
20
30
40
50
60
Time (min)
Normal
Diabetic
Normal data

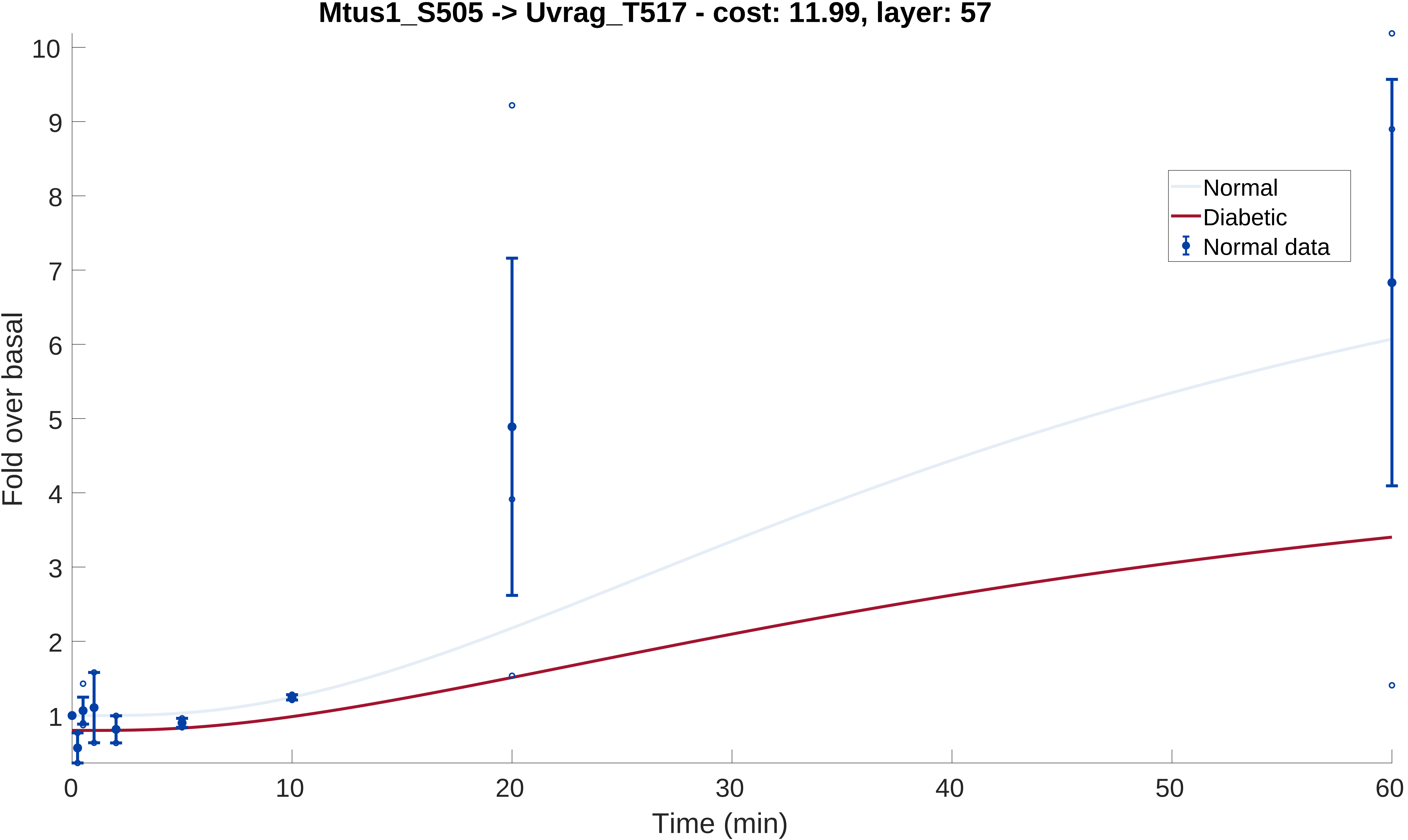

Mtus1_S505 -> Uvrag_T517 - cost: 11.99, layer: 57
Fold over basal
Time (min)
Normal
Diabetic
Normal data
0
10
20
30
40
50
60
1
2
3
4
5
6
7
8
9
10

3.3 Insulin stimulation, normal and with the PKB inhibitor

The following pages give all simulations of insulin stimulation normally and with the PKB inhibitor MK2206.

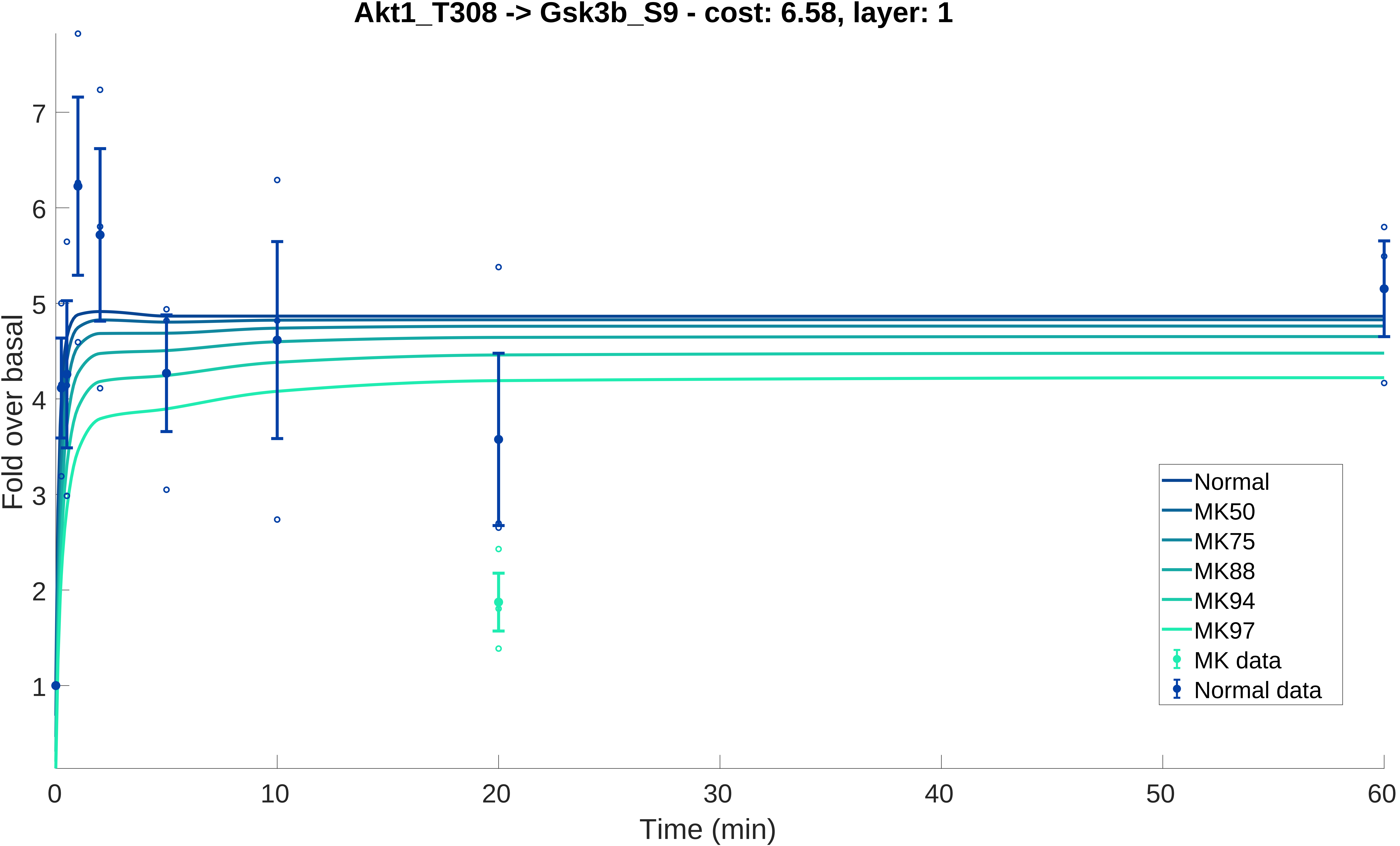

Akt1_T308 -> Gsk3b_S9 - cost: 6.58, layer: 1
Fold over basal
Time (min)
0
10
20
30
40
50
60
1
2
3
4
5
6
7
Normal
MK50
MK75
MK88
MK94
MK97
MK data
Normal data

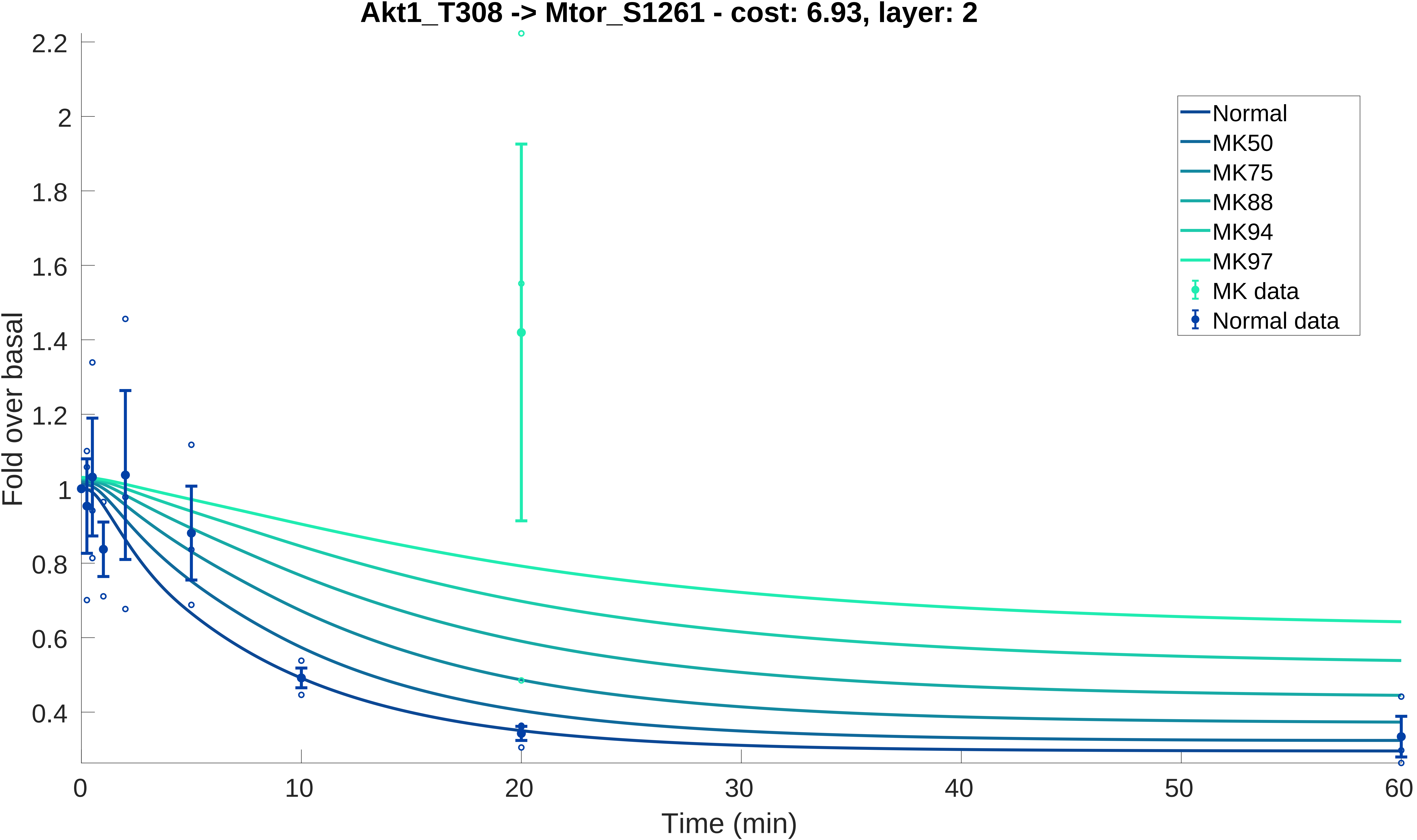

Akt1_T308 -> Mtor_S1261 - cost: 6.93, layer: 2
Fold over basal
2.2
2
1.8
1.6
1.4
1.2
1
0.8
0.6
0.4
0
10
20
30
40
50
60
Time (min)
Normal
MK50
MK75
MK88
MK94
MK97
MK data
Normal data

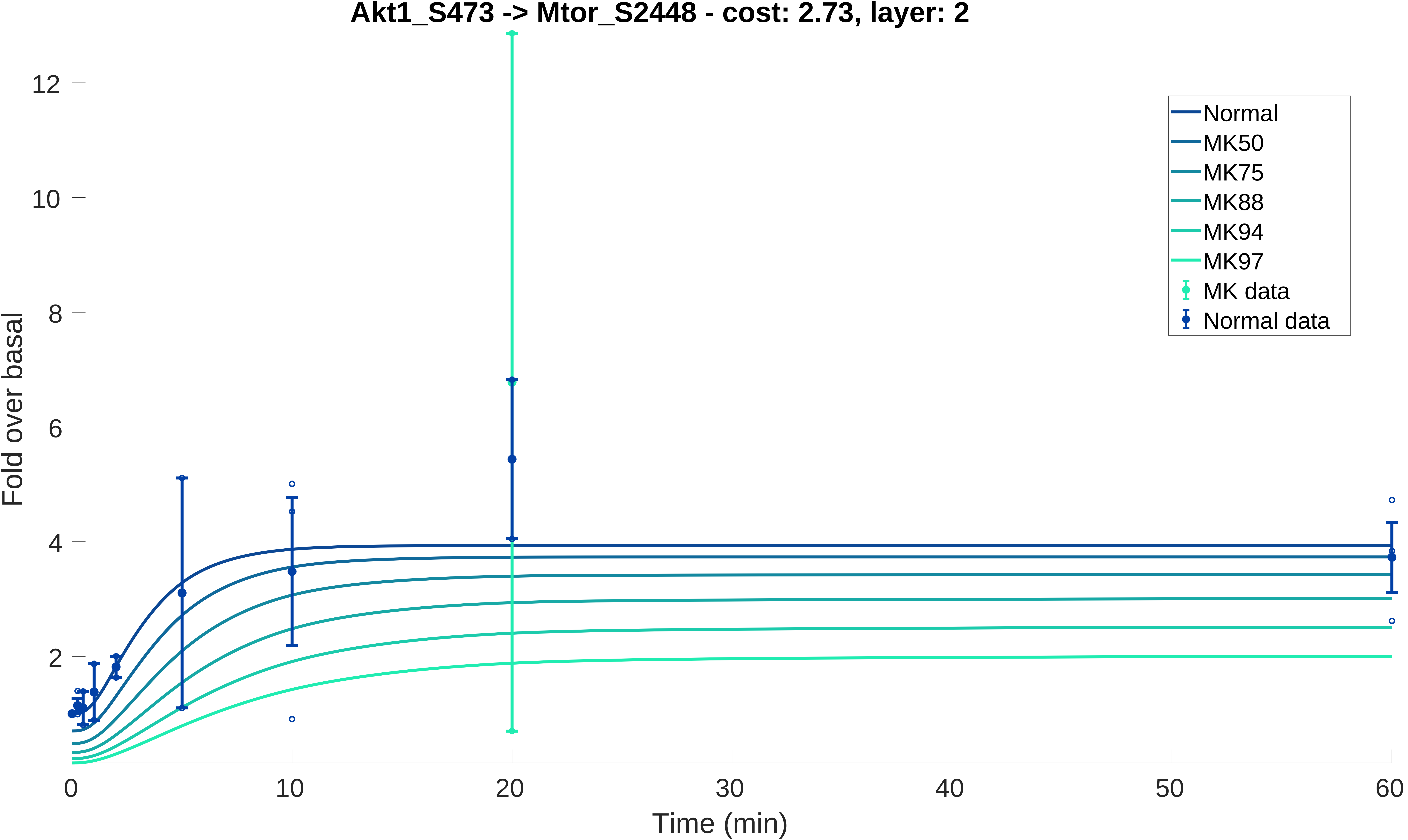

Akt1_S473 -> Mtor_S2448 - cost: 2.73, layer: 2
Fold over basal
Time (min)
0
10
20
30
40
50
60
2
4
6
8
10
12
Normal
MK50
MK75
MK88
MK94
MK97
MK data
Normal data

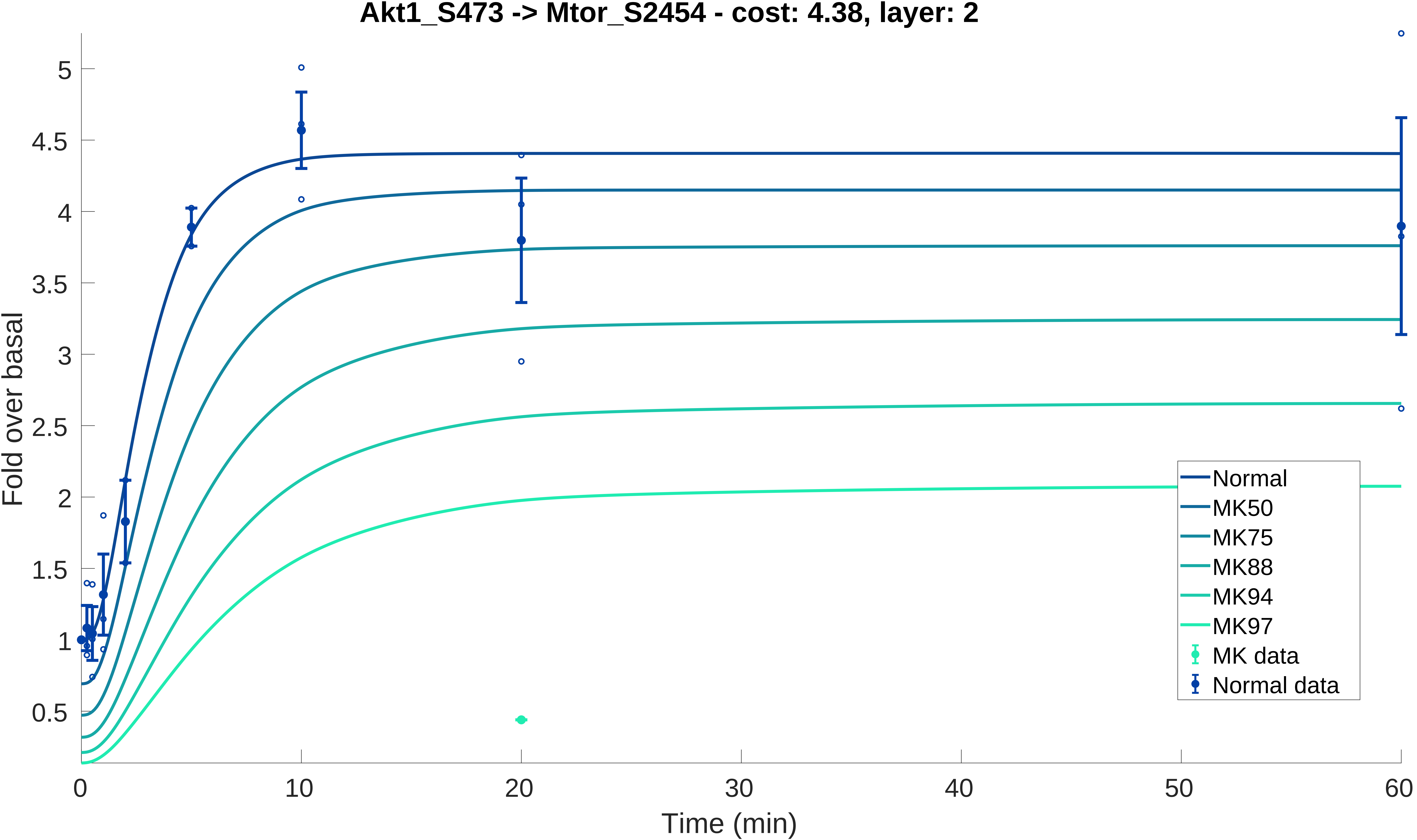
Akt1_S473 -> Mtor_S2454 - cost: 4.38, layer: 2
Fold over basal
Time (min)
0
10
20
30
40
50
60
0.5
1
1.5
2
2.5
3
3.5
4
4.5
5
Normal
MK50
MK75
MK88
MK94
MK97
MK data
Normal data

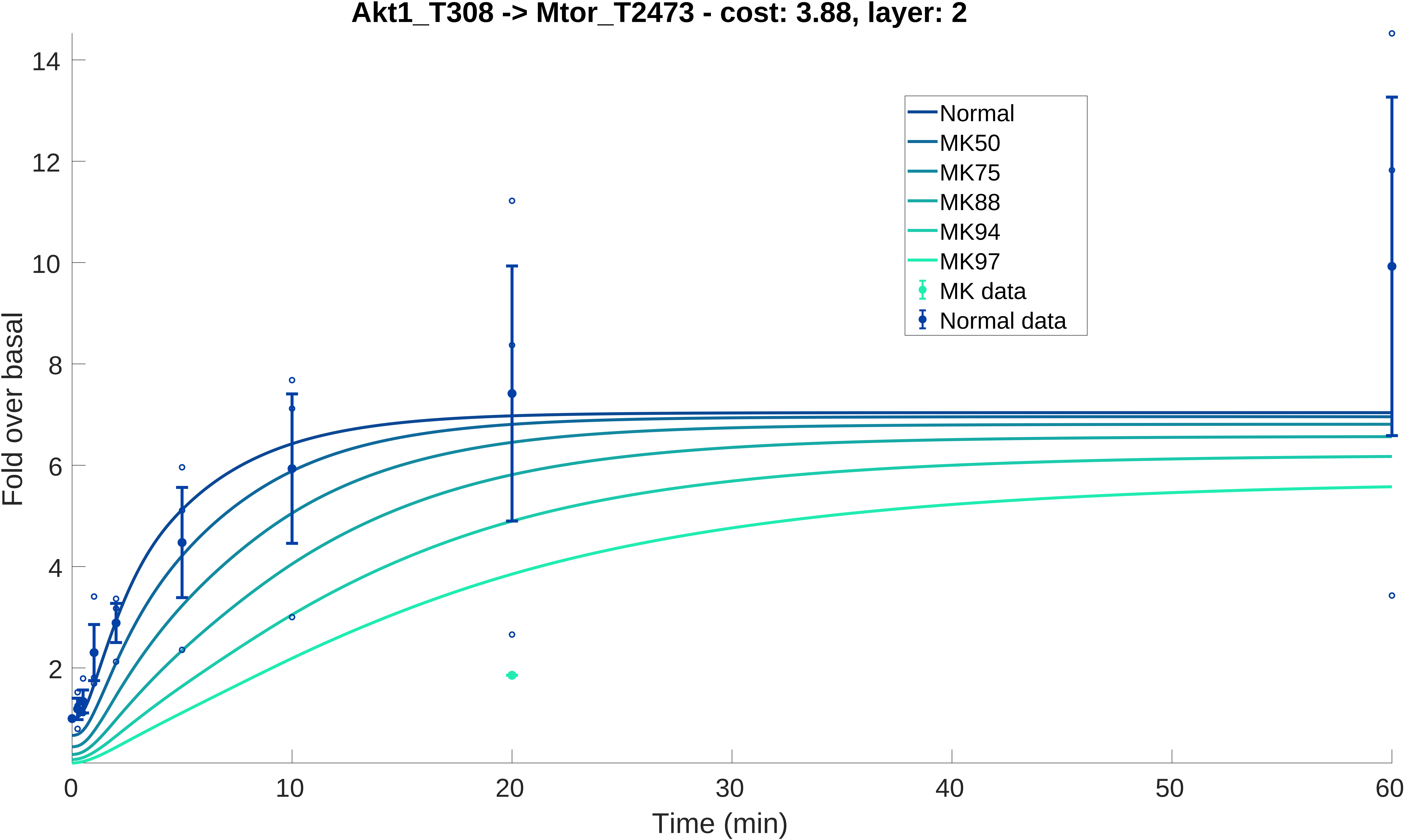

Akt1_T308 -> Mtor_T2473 - cost: 3.88, layer: 2
Fold over basal
Time (min)
0
10
20
30
40
50
60
2
4
6
8
10
12
14
Normal
MK50
MK75
MK88
MK94
MK97
MK data
Normal data

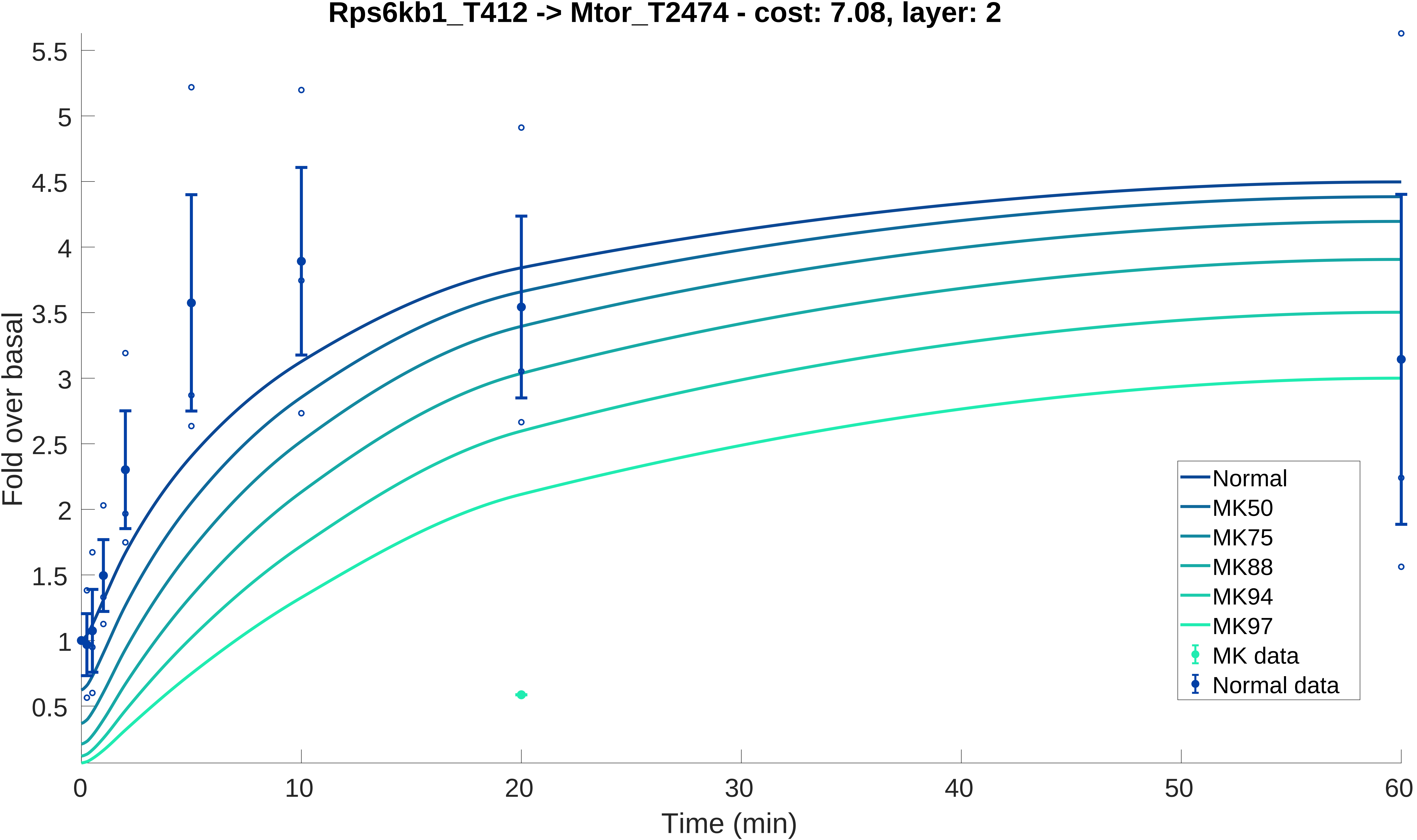

Rps6kb1_T412 -> Mtor_T2474 - cost: 7.08, layer: 2
Fold over basal
Time (min)
0.5
1
1.5
2
2.5
3
3.5
4
4.5
5
5.5
0
10
20
30
40
50
60
Normal
MK50
MK75
MK88
MK94
MK97
MK data
Normal data

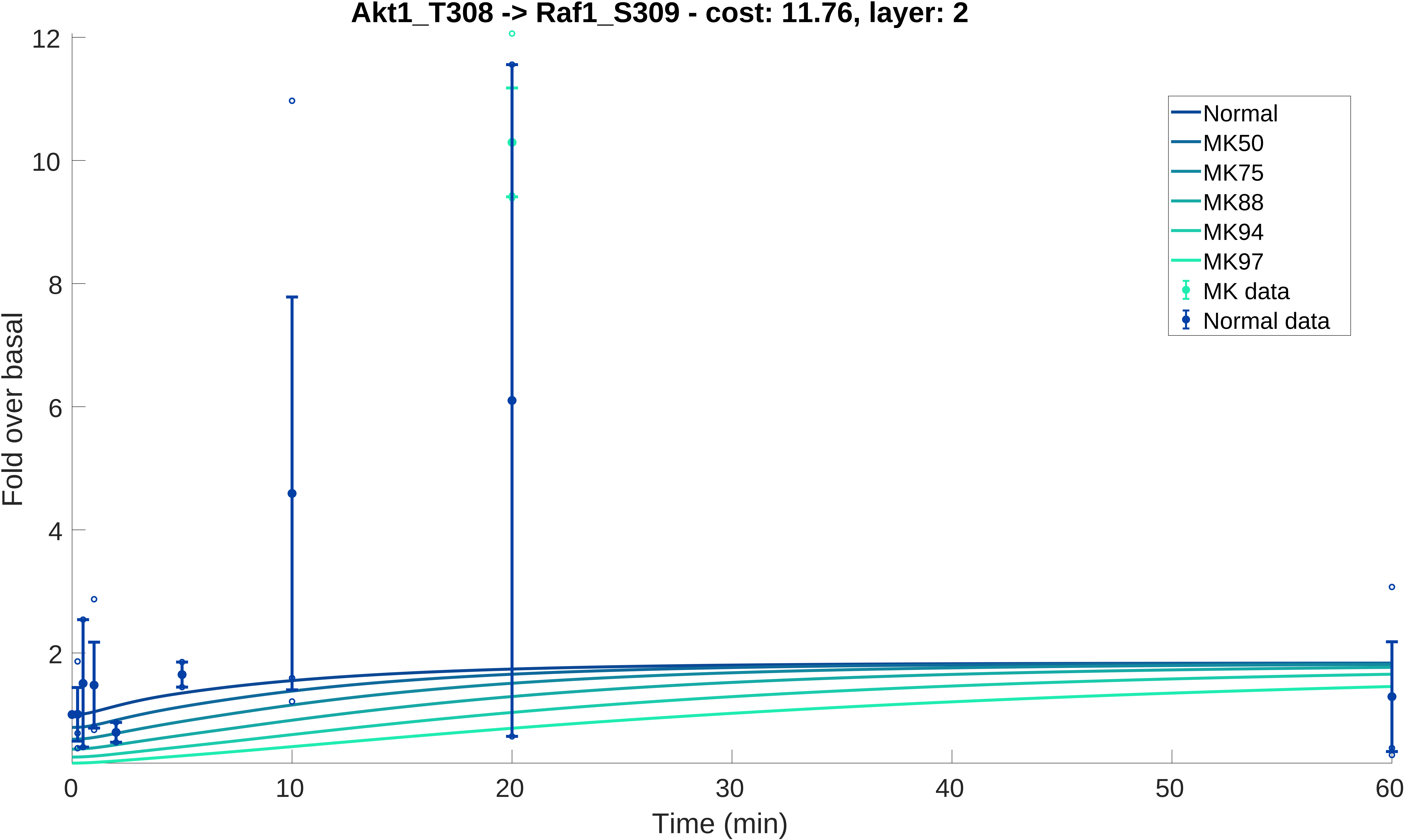

Akt1_T308 -> Raf1_S309 - cost: 11.76, layer: 2
Fold over basal
Time (min)
0
10
20
30
40
50
60
2
4
6
8
10
12
Normal
MK50
MK75
MK88
MK94
MK97
MK data
Normal data

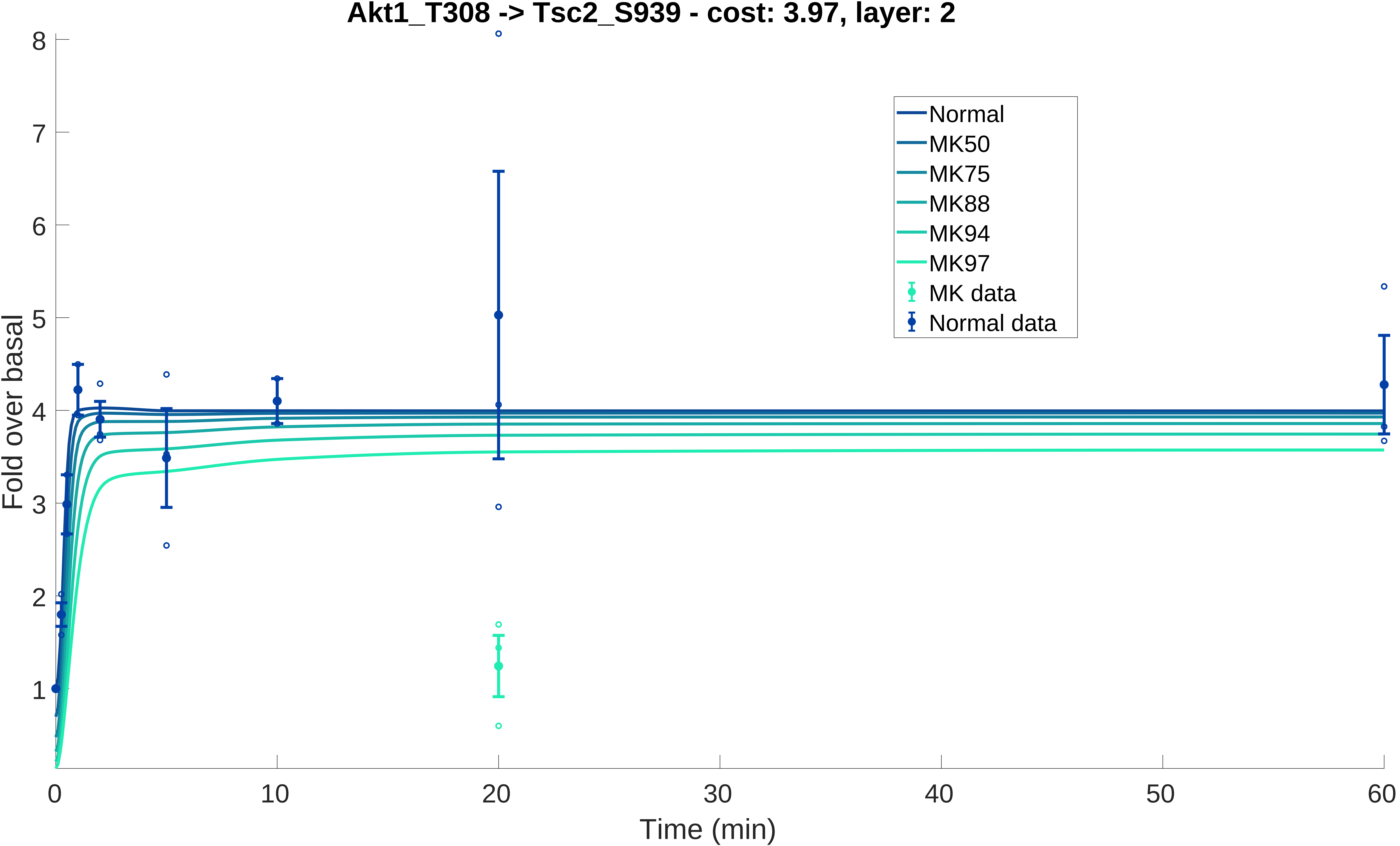

Akt1_T308 -> Tsc2_S939 - cost: 3.97, layer: 2
Fold over basal
Time (min)
0
10
20
30
40
50
60
1
2
3
4
5
6
7
8
Normal
MK50
MK75
MK88
MK94
MK97
MK data
Normal data

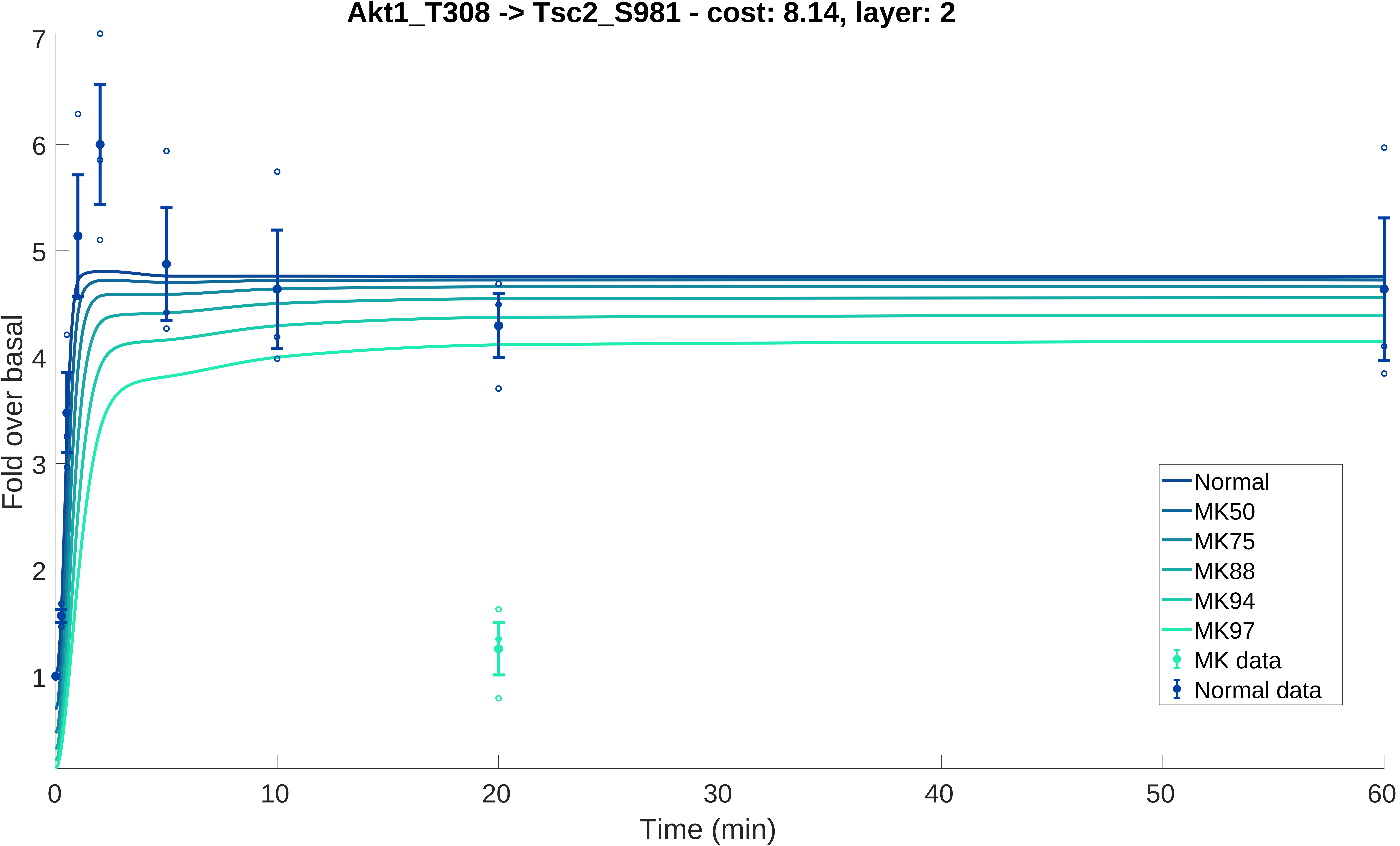

Akt1_T308 -> Tsc2_S981 - cost: 8.14, layer: 2
Fold over basal
Time (min)
0
10
20
30
40
50
60
1
2
3
4
5
6
7
Normal
MK50
MK75
MK88
MK94
MK97
MK data
Normal data

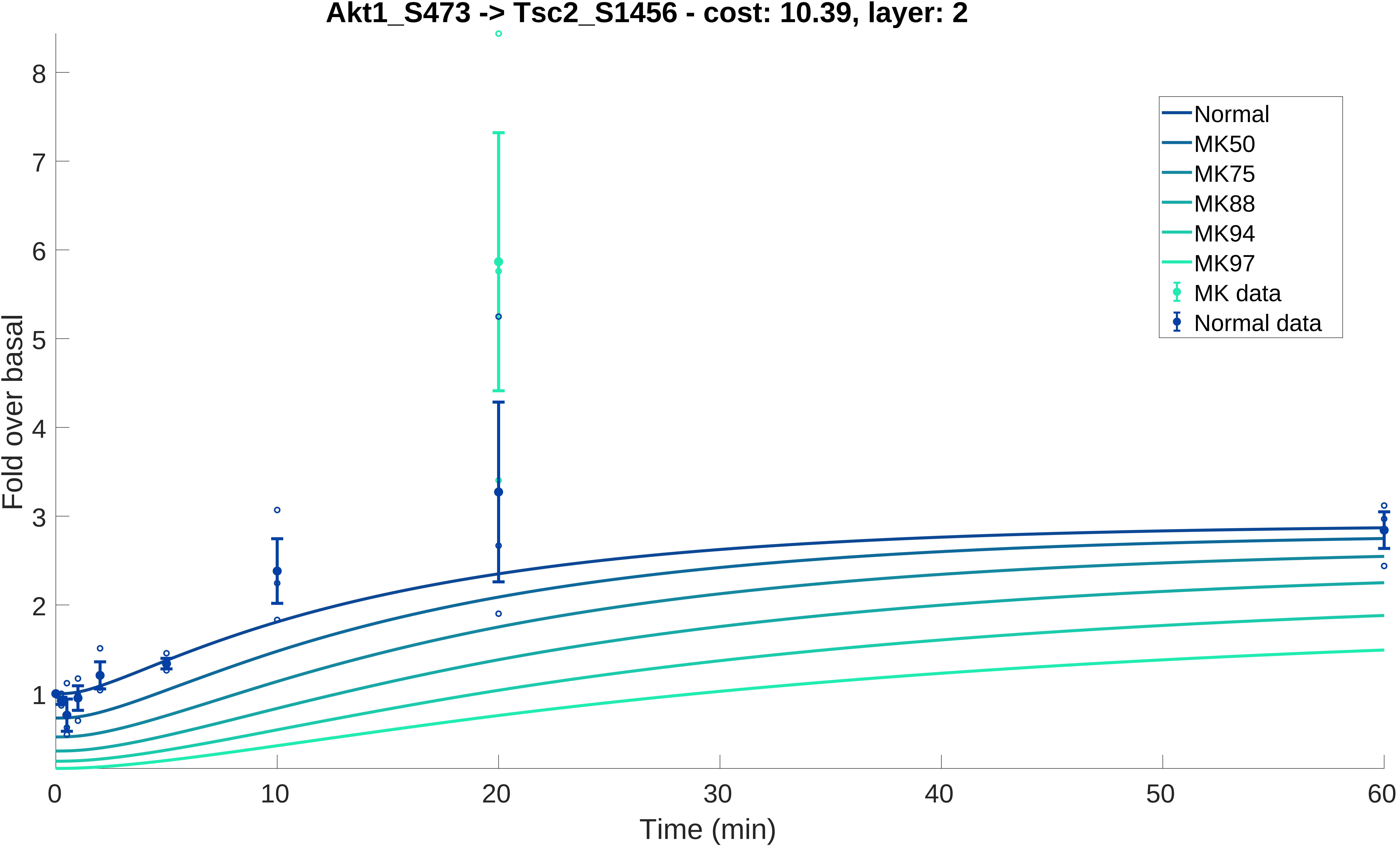

Akt1_S473 -> Tsc2_S1456 - cost: 10.39, layer: 2
Fold over basal
Time (min)
Normal
MK50
MK75
MK88
MK94
MK97
MK data
Normal data
0
10
20
30
40
50
60
1
2
3
4
5
6
7
8

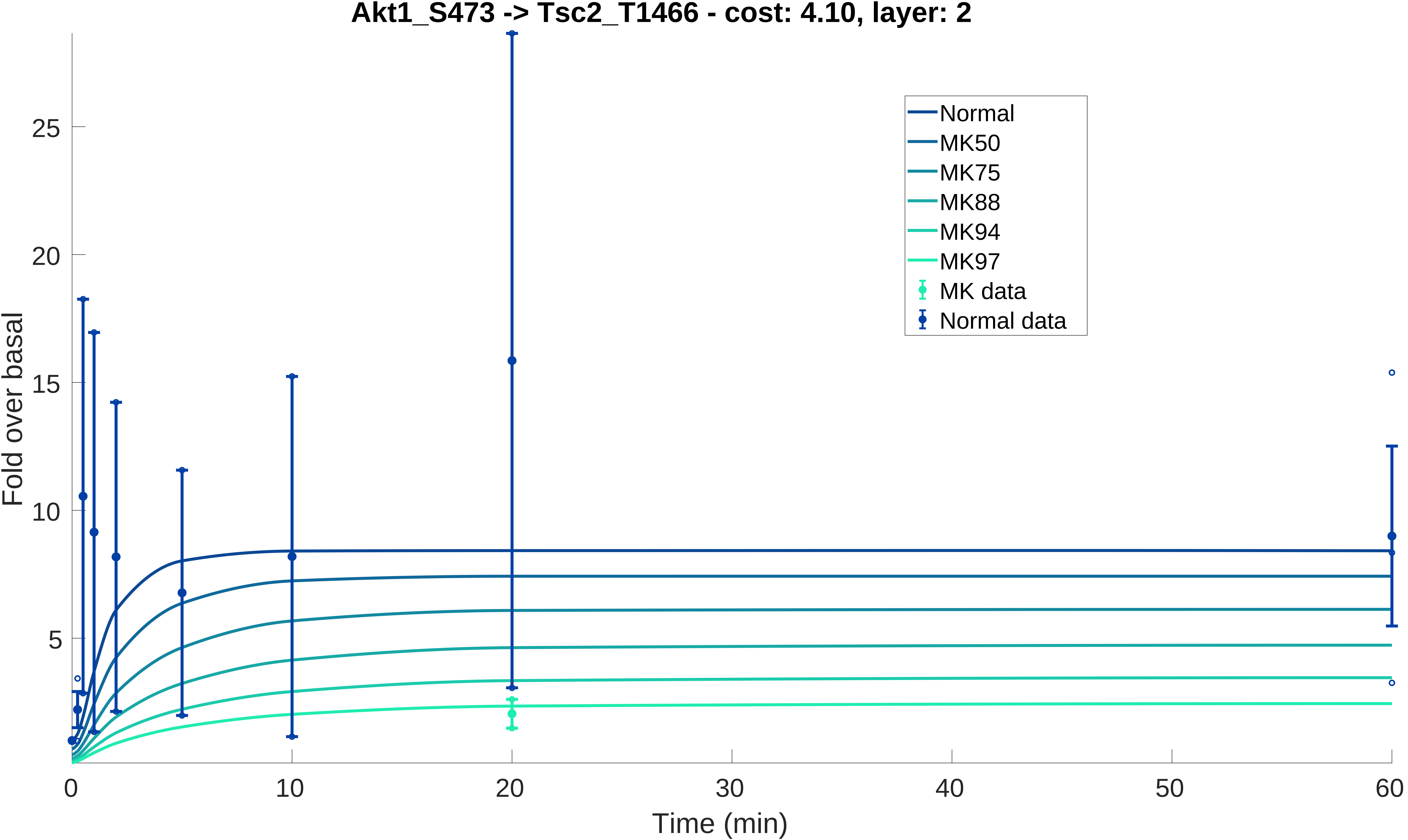
Akt1_S473 -> Tsc2_T1466 - cost: 4.10, layer: 2
Normal
MK50
MK75
MK88
MK94
MK97
MK data
Normal data
Fold over basal
25
20
15
10
5
0
10
20
30
40
50
60
Time (min)

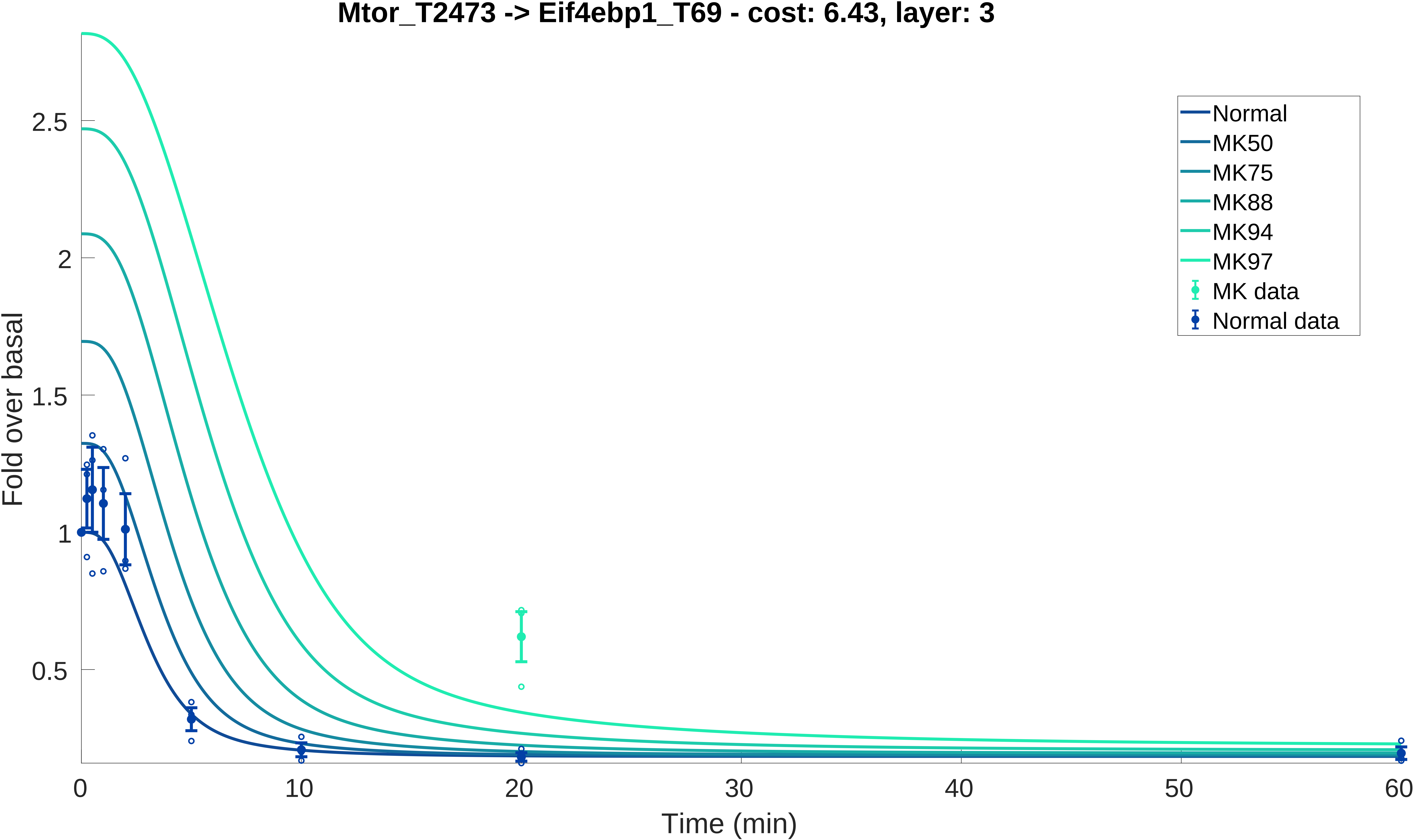

Mtor_T2473 -> Eif4ebp1_T69 - cost: 6.43, layer: 3
Fold over basal
2.5
2
1.5
1
0.5
0
10
20
30
40
50
60
Time (min)
Normal
MK50
MK75
MK88
MK94
MK97
MK data
Normal data

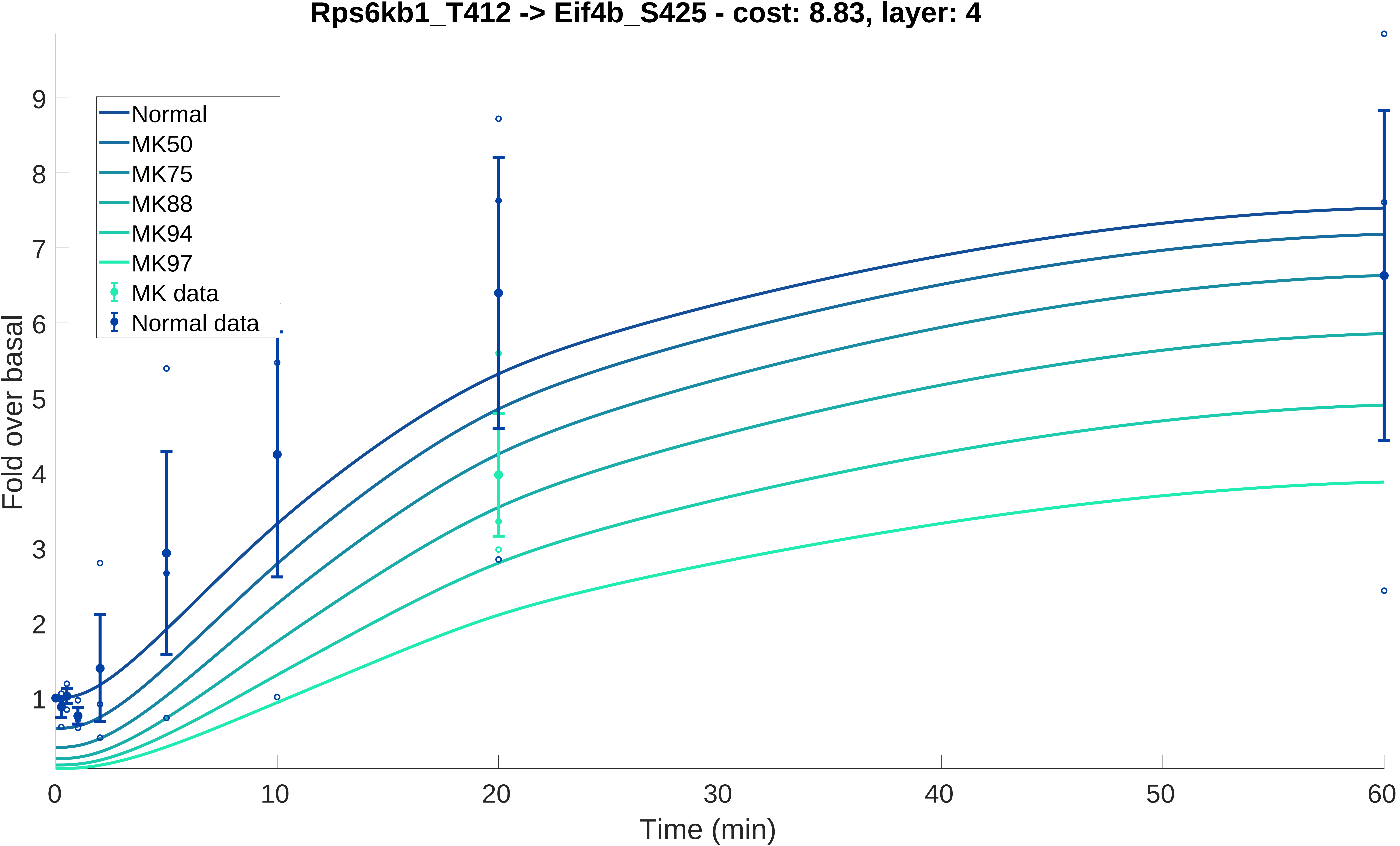
Rps6kb1_T412 -> Eif4b_S425 - cost: 8.83, layer: 4
Fold over basal
Time (min)
Normal
MK50
MK75
MK88
MK94
MK97
MK data
Normal data
0
10
20
30
40
50
60
1
2
3
4
5
6
7
8
9

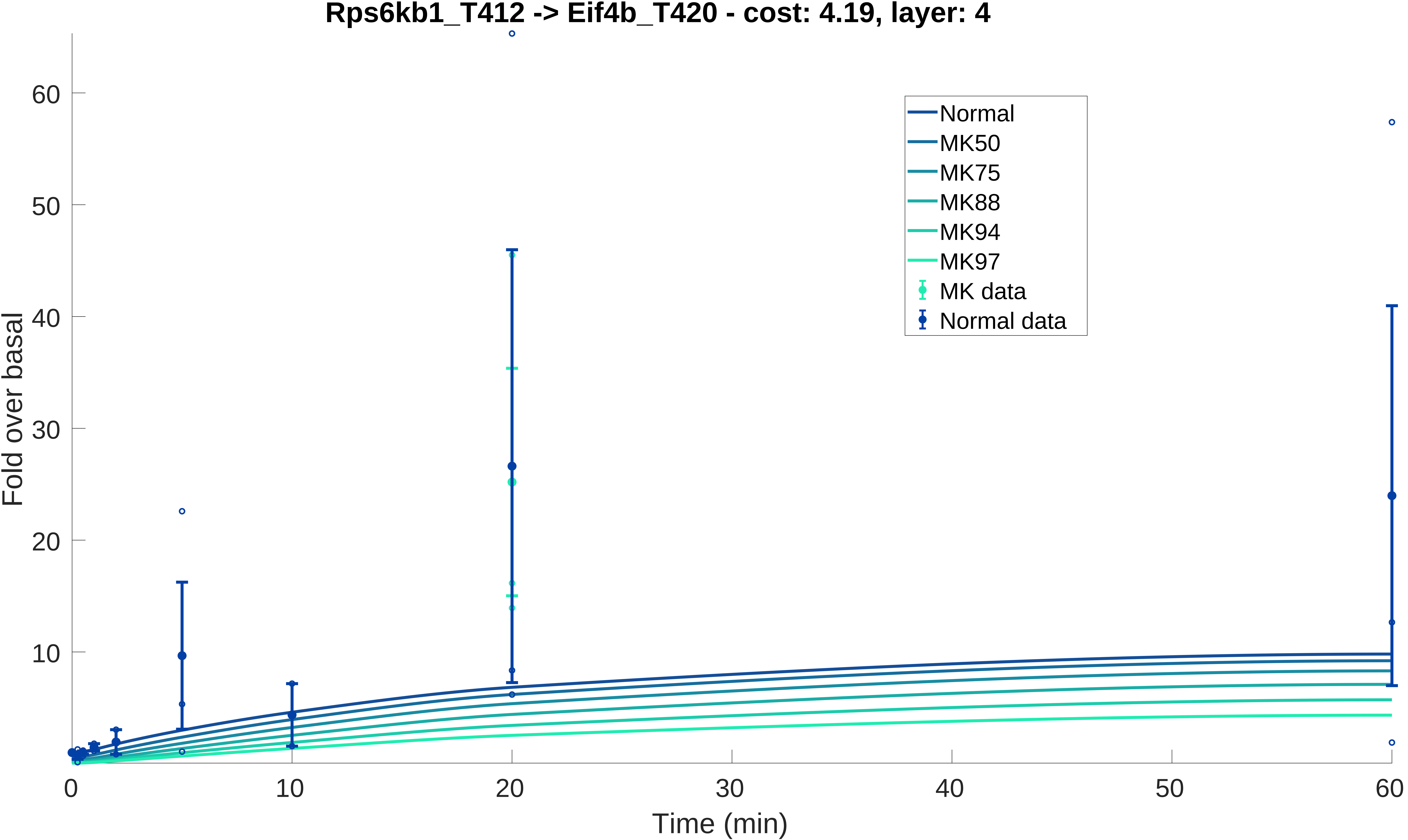

Rps6kb1_T412 -> Eif4b_T420 - cost: 4.19, layer: 4
Fold over basal
Time (min)
0
10
20
30
40
50
60
Normal
MK50
MK75
MK88
MK94
MK97
MK data
Normal data

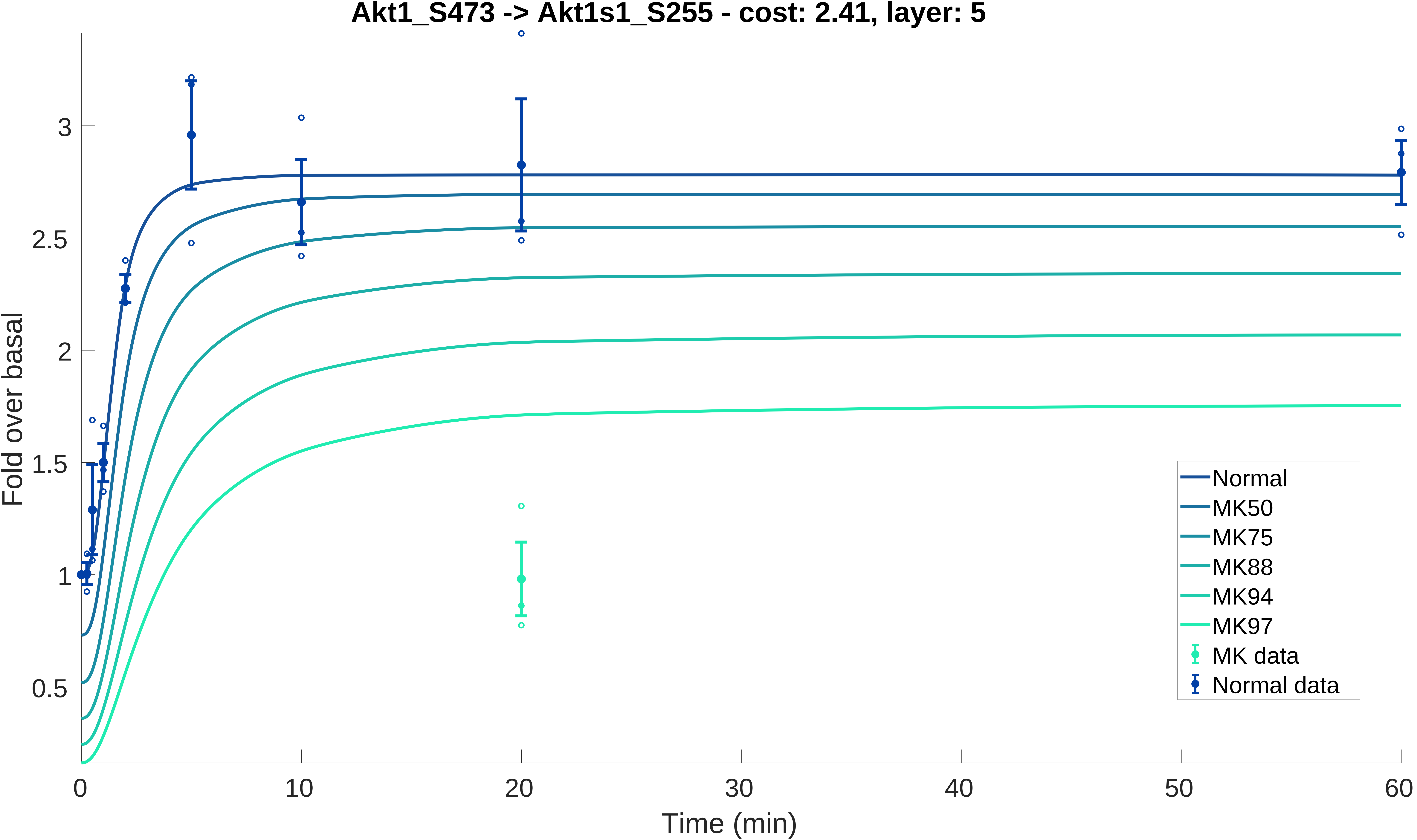
Akt1_S473 -> Akt1s1_S255 - cost: 2.41, layer: 5
Fold over basal
Time (min)
0
10
20
30
40
50
60
0.5
1
1.5
2
2.5
3
Normal
MK50
MK75
MK88
MK94
MK97
MK data
Normal data

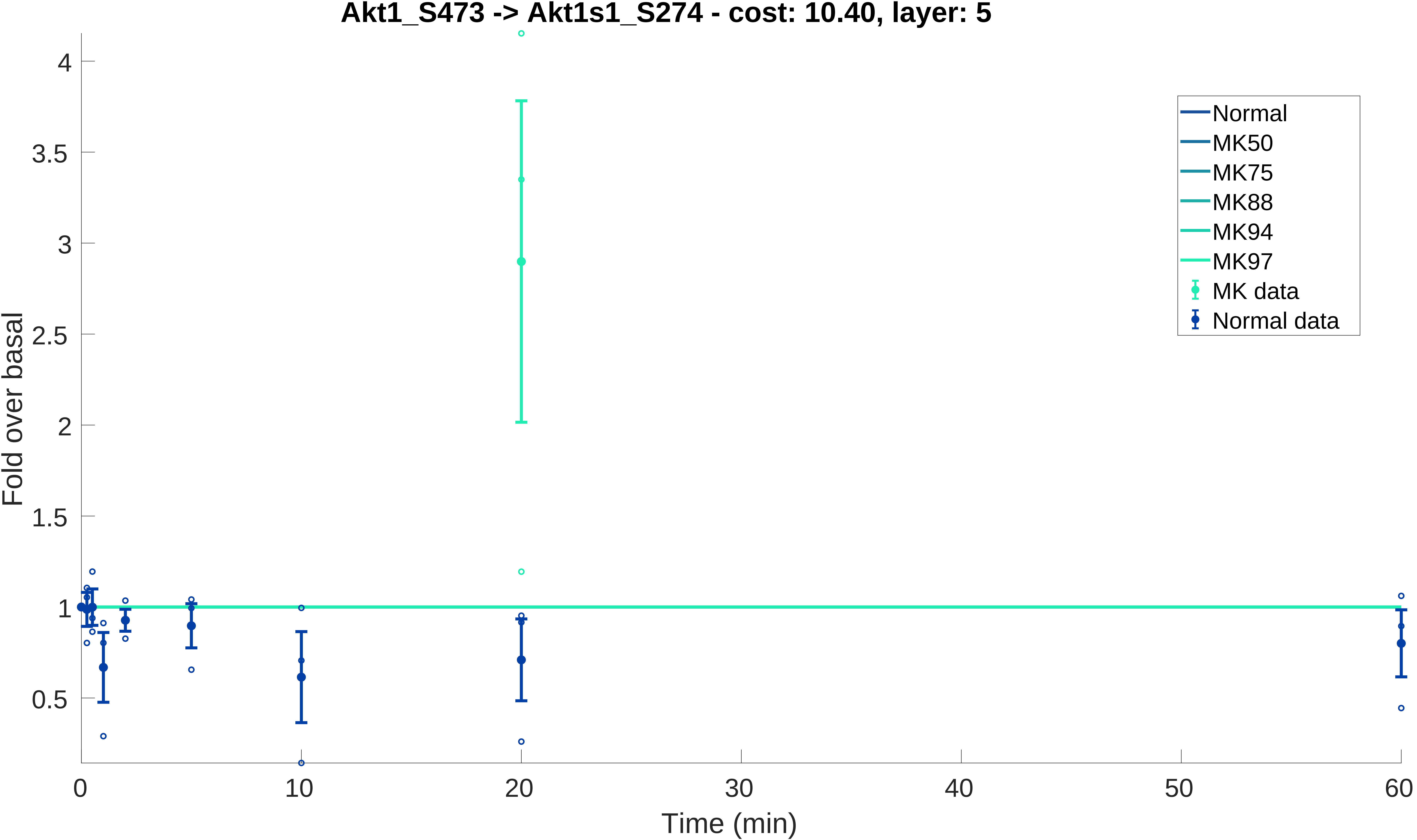

Akt1_S473 -> Akt1s1_S274 - cost: 10.40, layer: 5
Fold over basal
Time (min)
0.5
1
1.5
2
2.5
3
3.5
4
0
10
20
30
40
50
60
Normal
MK50
MK75
MK88
MK94
MK97
MK data
Normal data

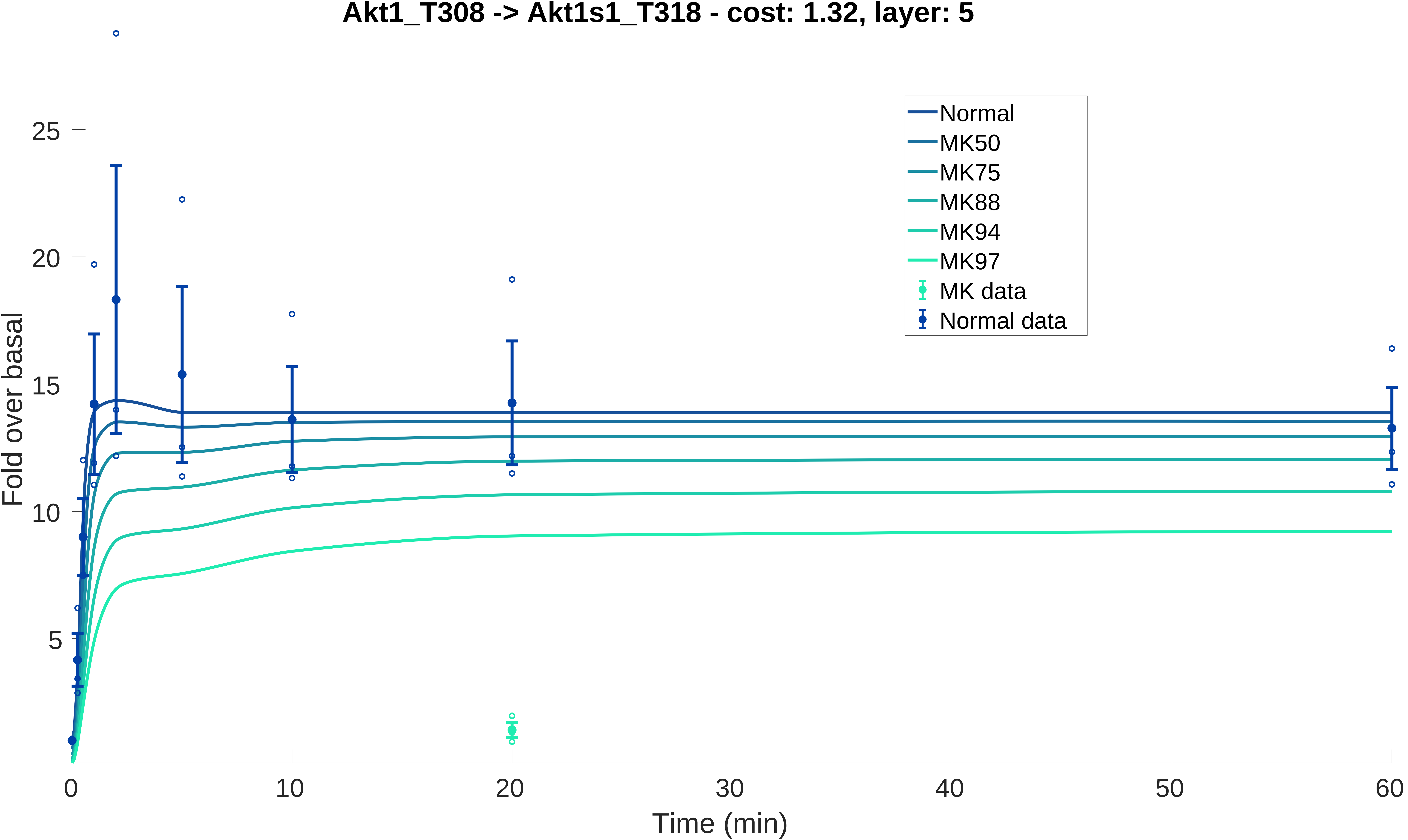

Akt1_T308 -> Akt1s1_T318 - cost: 1.32, layer: 5
Fold over basal
Time (min)
Normal
MK50
MK75
MK88
MK94
MK97
MK data
Normal data
0
10
20
30
40
50
60
5
10
15
20
25

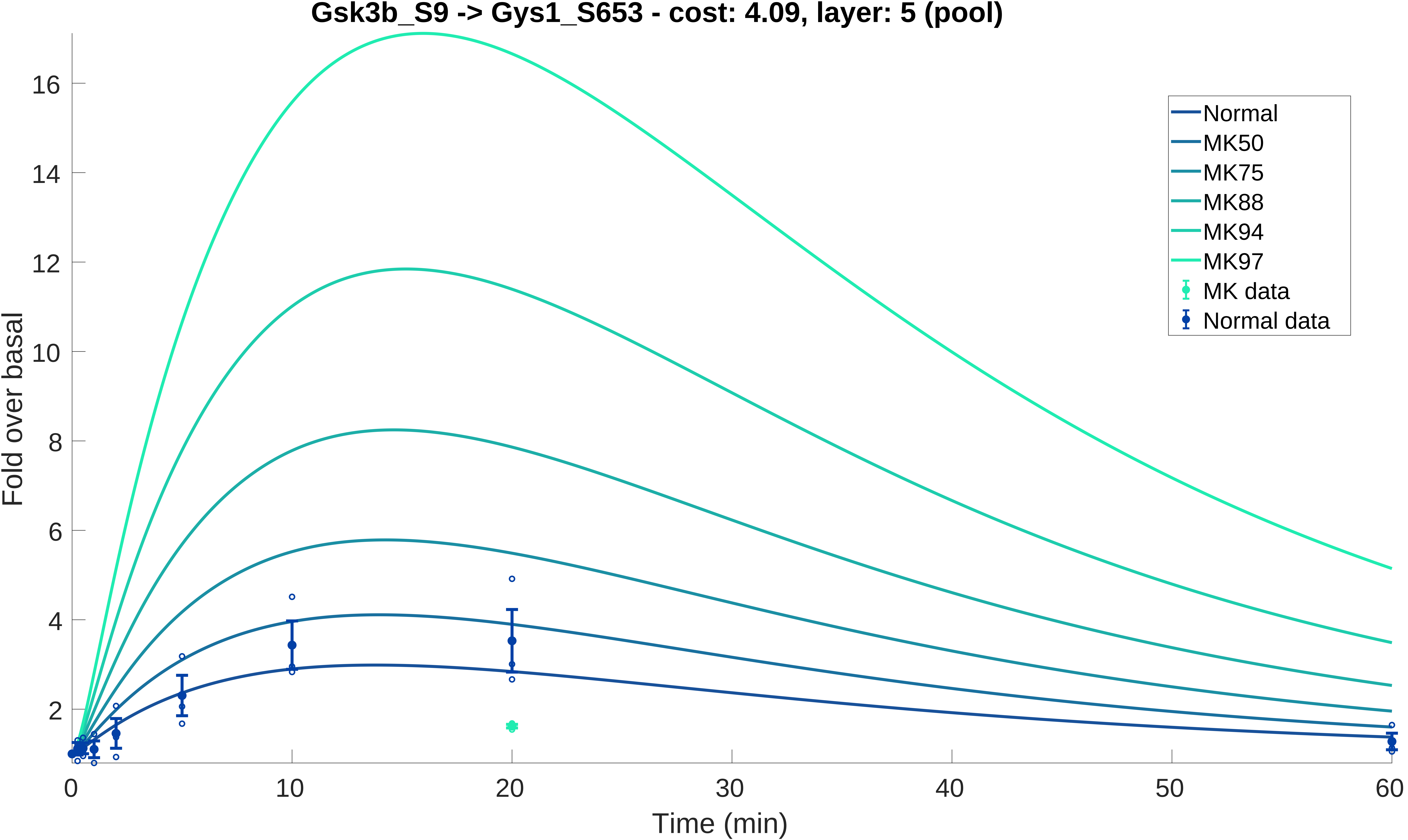

Gsk3b_S9 -> Gys1_S653 - cost: 4.09, layer: 5 (pool)
Fold over basal
Time (min)
0
10
20
30
40
50
60
2
4
6
8
10
12
14
16
Normal
MK50
MK75
MK88
MK94
MK97
MK data
Normal data

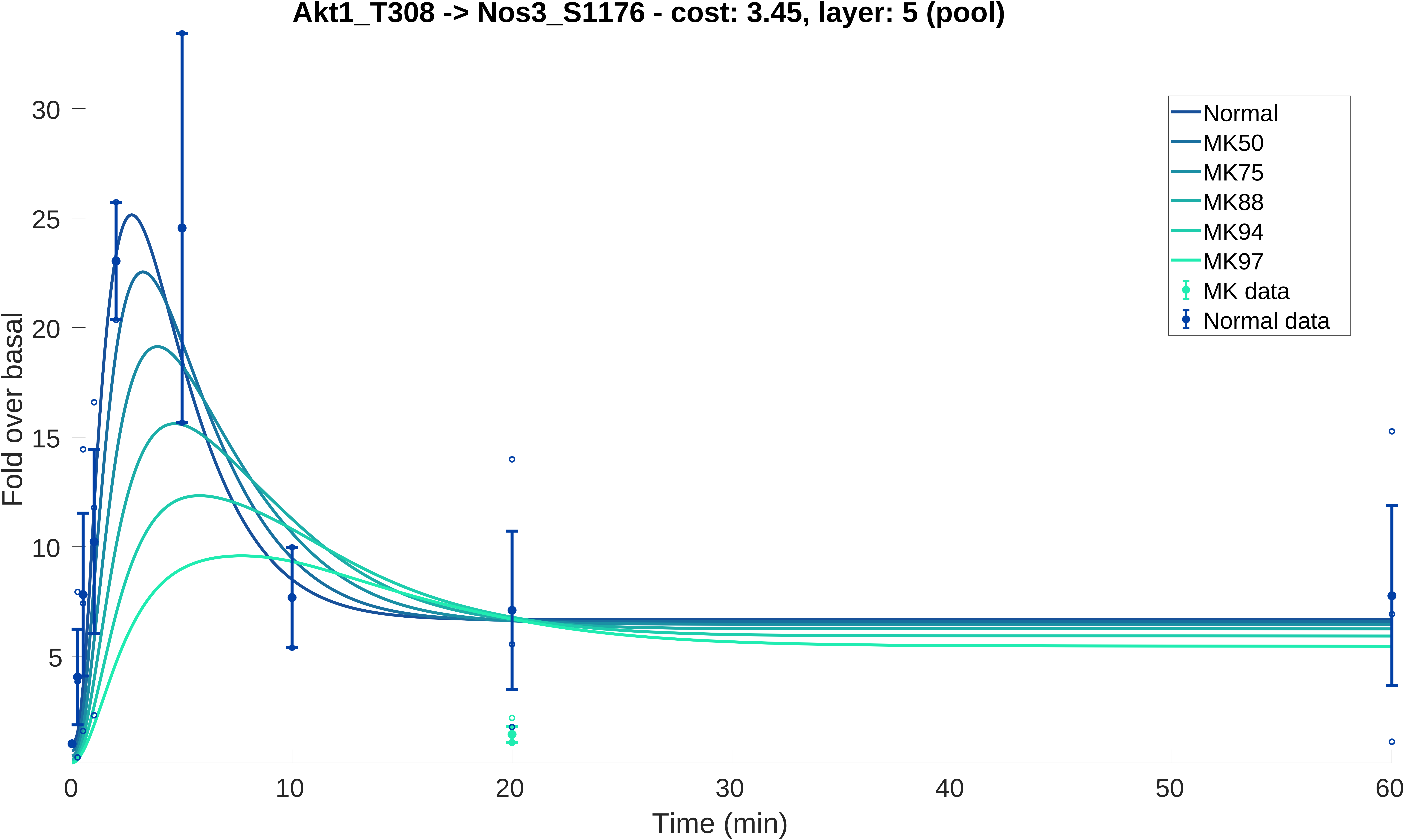

Akt1_T308 -> Nos3_S1176 - cost: 3.45, layer: 5 (pool)
Fold over basal
Time (min)
Normal
MK50
MK75
MK88
MK94
MK97
MK data
Normal data
0
10
20
30
40
50
60
5
10
15
20
25
30

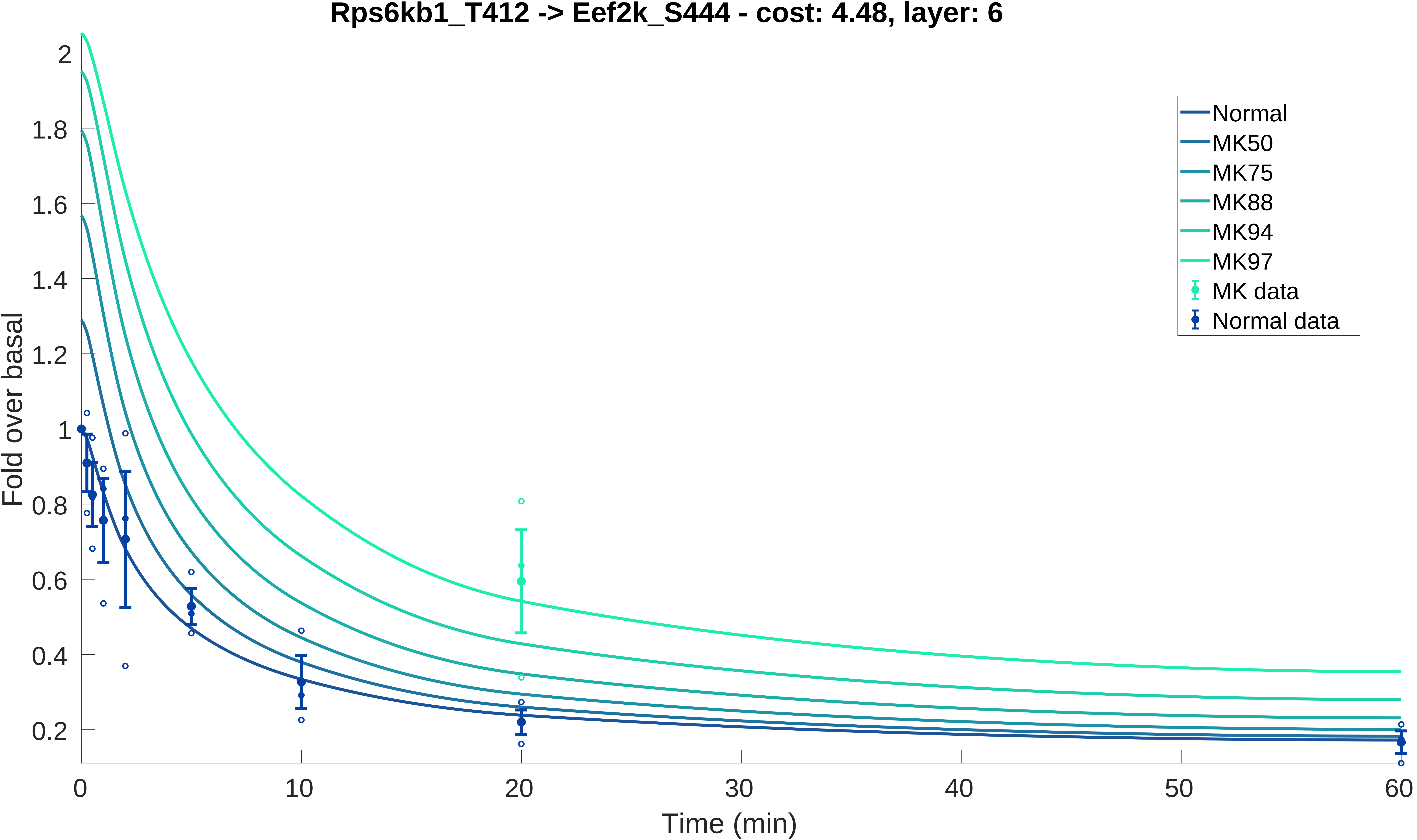

Rps6kb1_T412 -> Eef2k_S444 - cost: 4.48, layer: 6
Fold over basal
Time (min)
2
1.8
1.6
1.4
1.2
1
0.8
0.6
0.4
0.2
0
10
20
30
40
50
60
Normal
MK50
MK75
MK88
MK94
MK97
MK data
Normal data

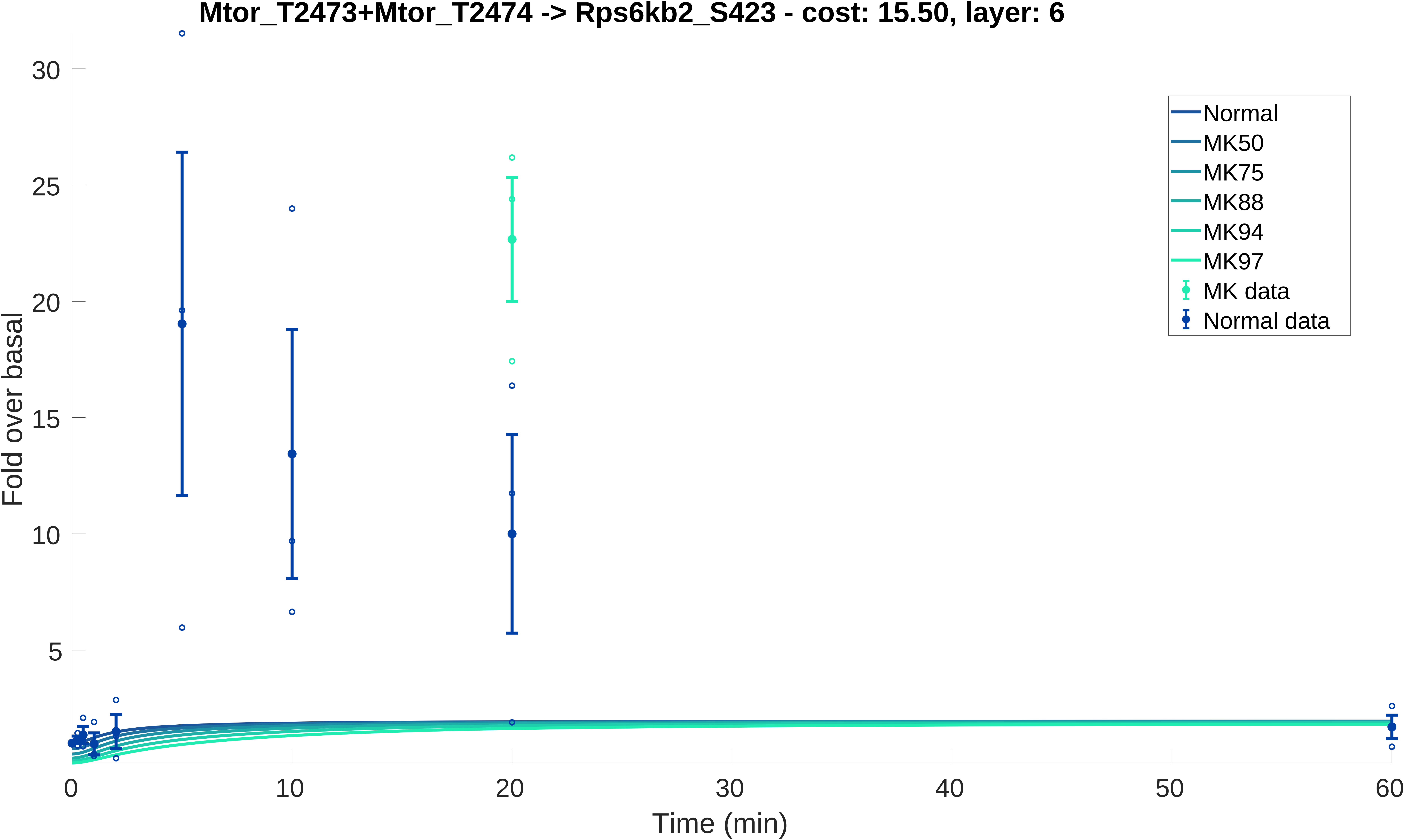

Mtor_T2473+Mtor_T2474 -> Rps6kb2_S423 - cost: 15.50, layer: 6
Fold over basal
30
25
20
15
10
5
0
10
20
30
40
50
60
Time (min)
Normal
MK50
MK75
MK88
MK94
MK97
MK data
Normal data

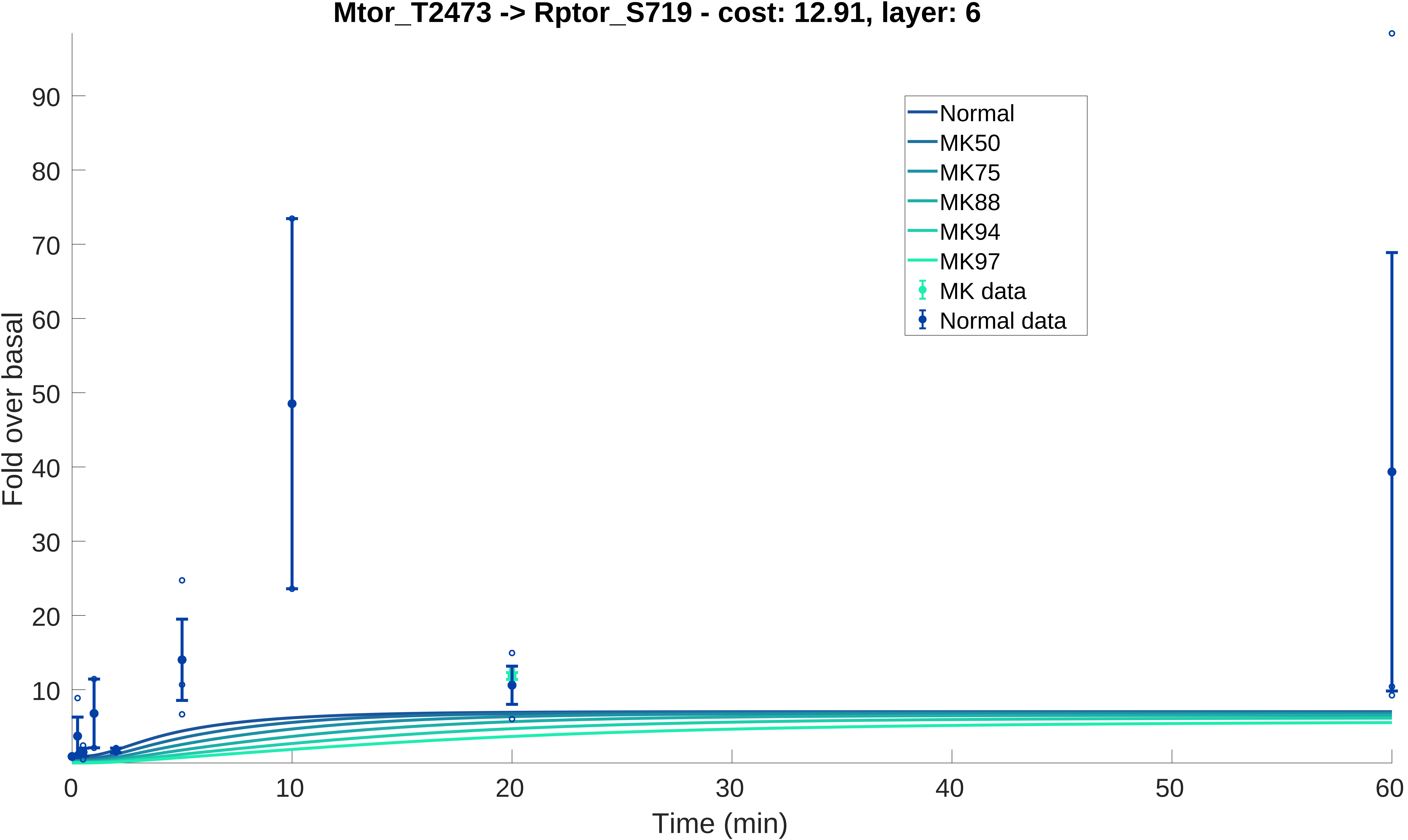

Mtor_T2473 -> Rptor_S719 - cost: 12.91, layer: 6
Fold over basal
Time (min)
0
10
20
30
40
50
60
70
80
90
Normal
MK50
MK75
MK88
MK94
MK97
MK data
Normal data

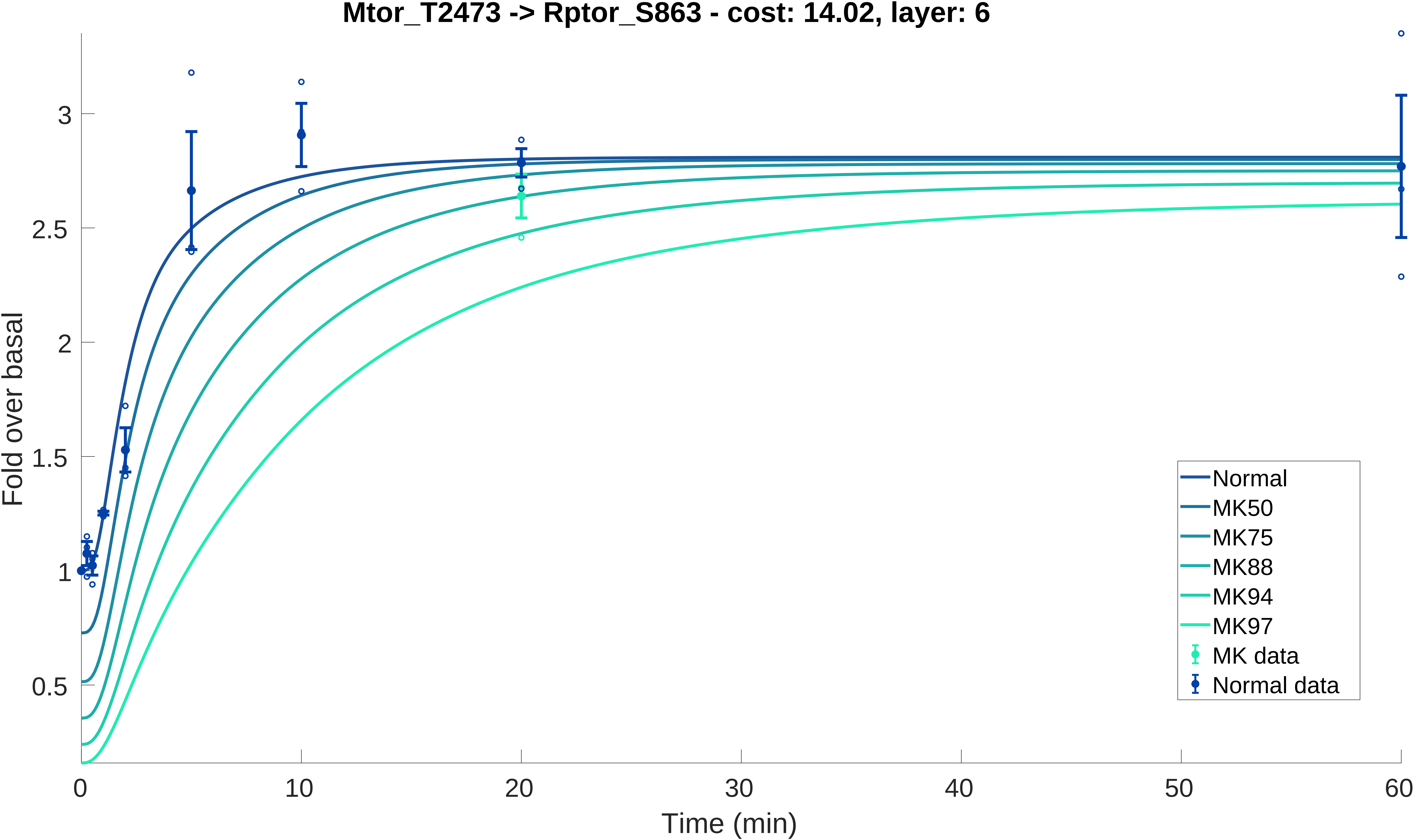
Mtor_T2473 -> Rptor_S863 - cost: 14.02, layer: 6
Fold over basal
Time (min)
0.5
1
1.5
2
2.5
3
0
10
20
30
40
50
60
Normal
MK50
MK75
MK88
MK94
MK97
MK data
Normal data

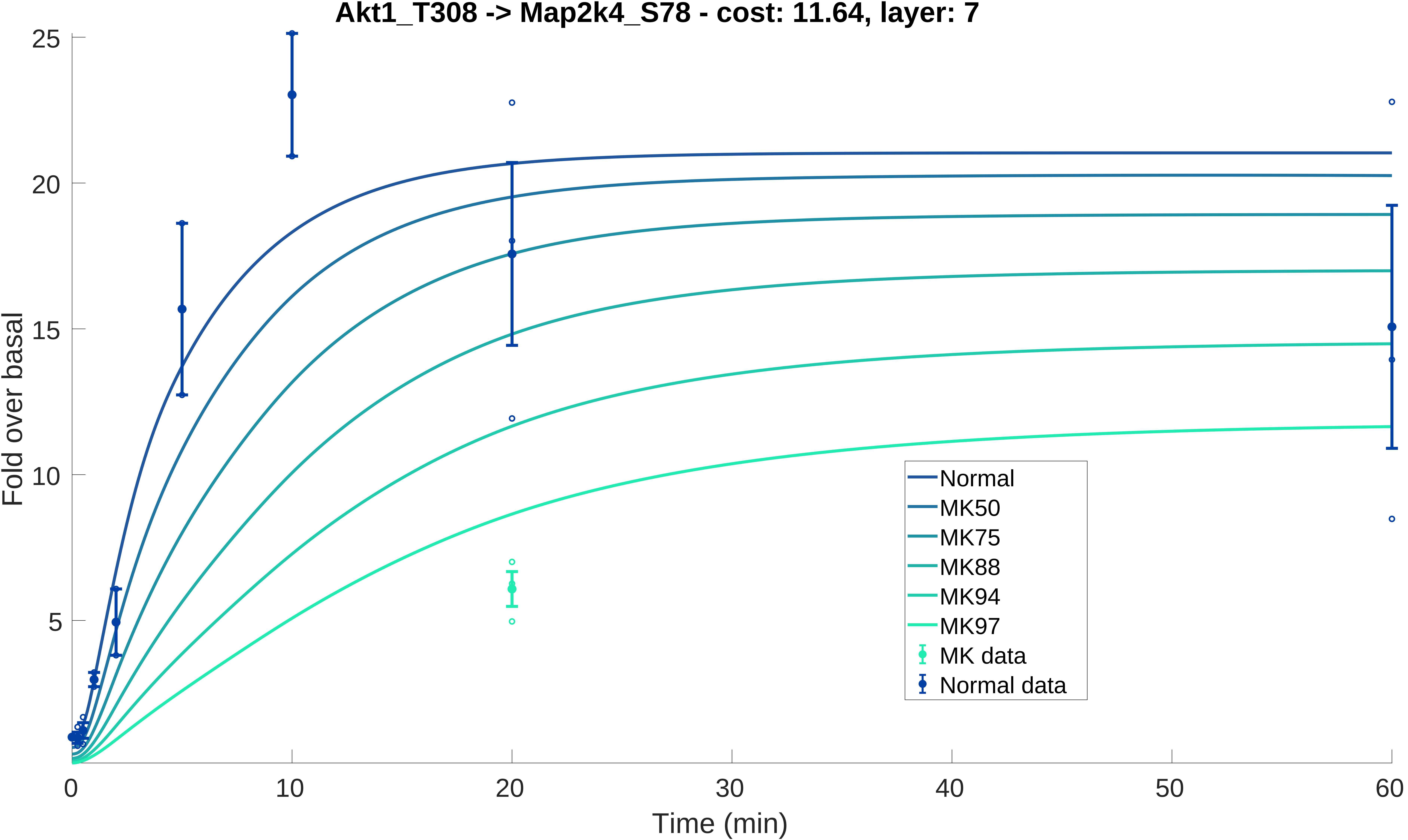

Akt1_T308 -> Map2k4_S78 - cost: 11.64, layer: 7
Fold over basal
Time (min)
0
5
10
15
20
25
30
40
50
60
Normal
MK50
MK75
MK88
MK94
MK97
MK data
Normal data

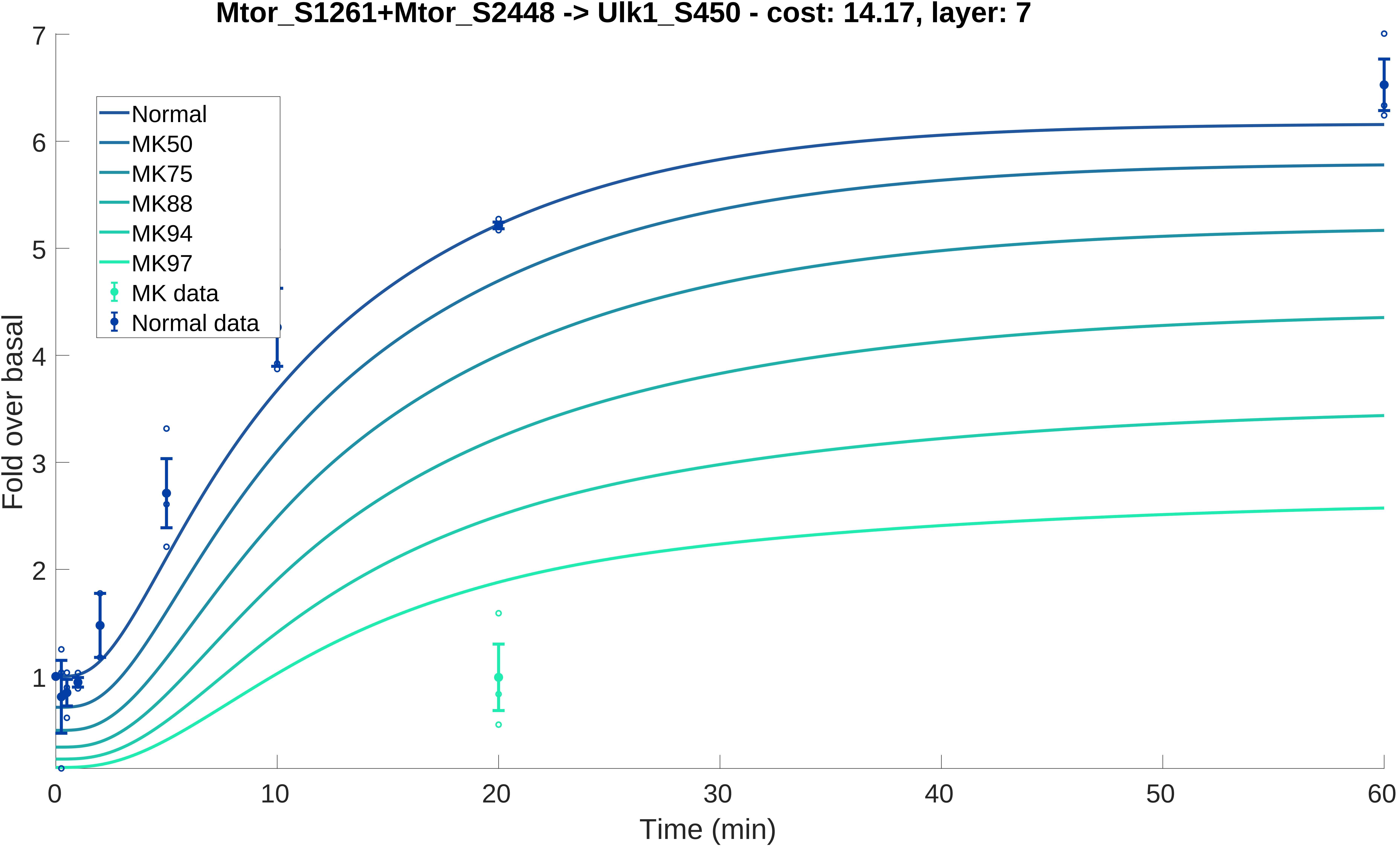

Mtor_S1261+Mtor_S2448 -> Ulk1_S450 - cost: 14.17, layer: 7
Fold over basal
Time (min)
Normal
MK50
MK75
MK88
MK94
MK97
MK data
Normal data
0
10
20
30
40
50
60
1
2
3
4
5
6
7

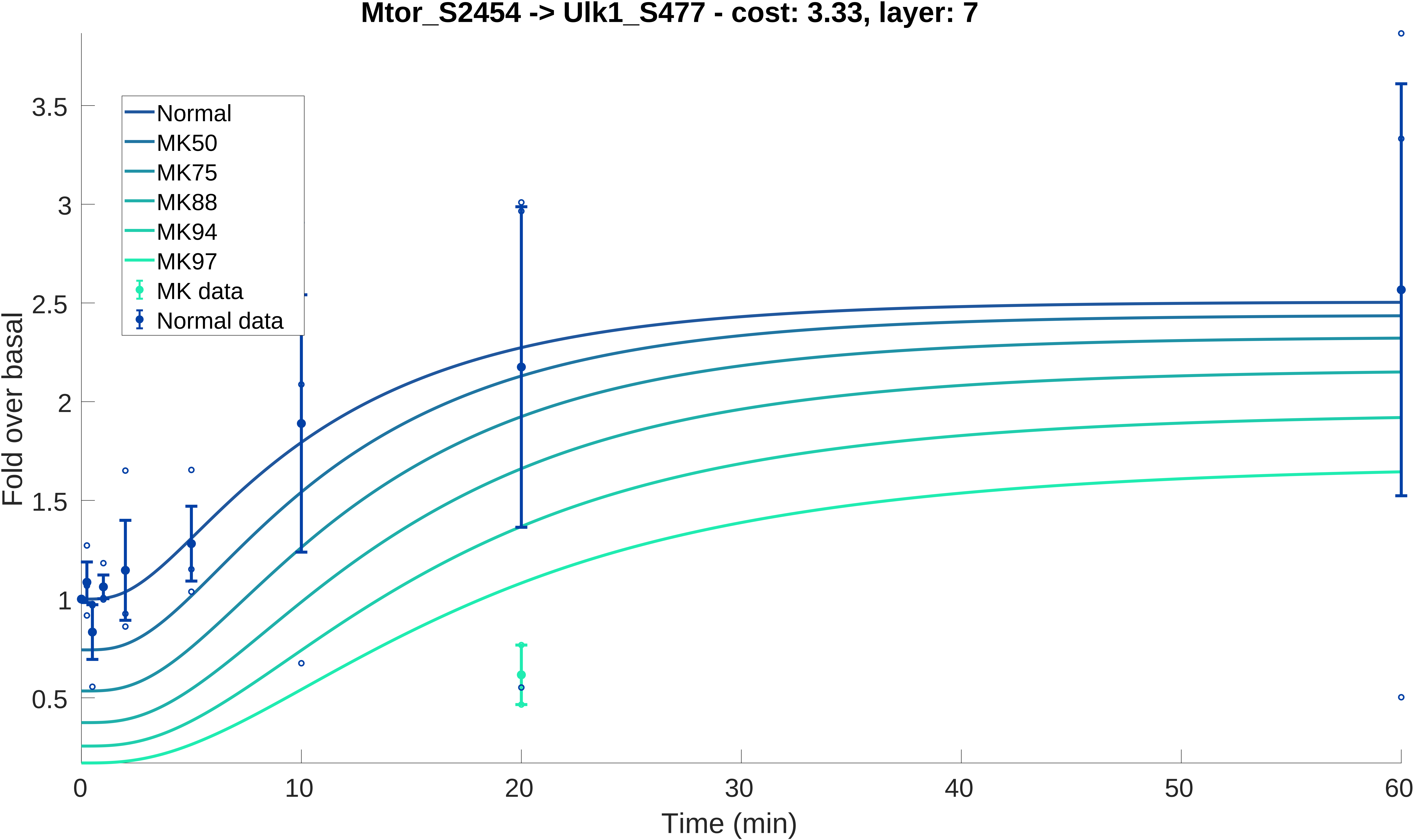

Mtor_S2454 -> Ulk1_S477 - cost: 3.33, layer: 7
Normal
MK50
MK75
MK88
MK94
MK97
MK data
Normal data
3.5
3
2.5
2
1.5
1
0.5
Fold over basal
0
10
20
30
40
50
60
Time (min)

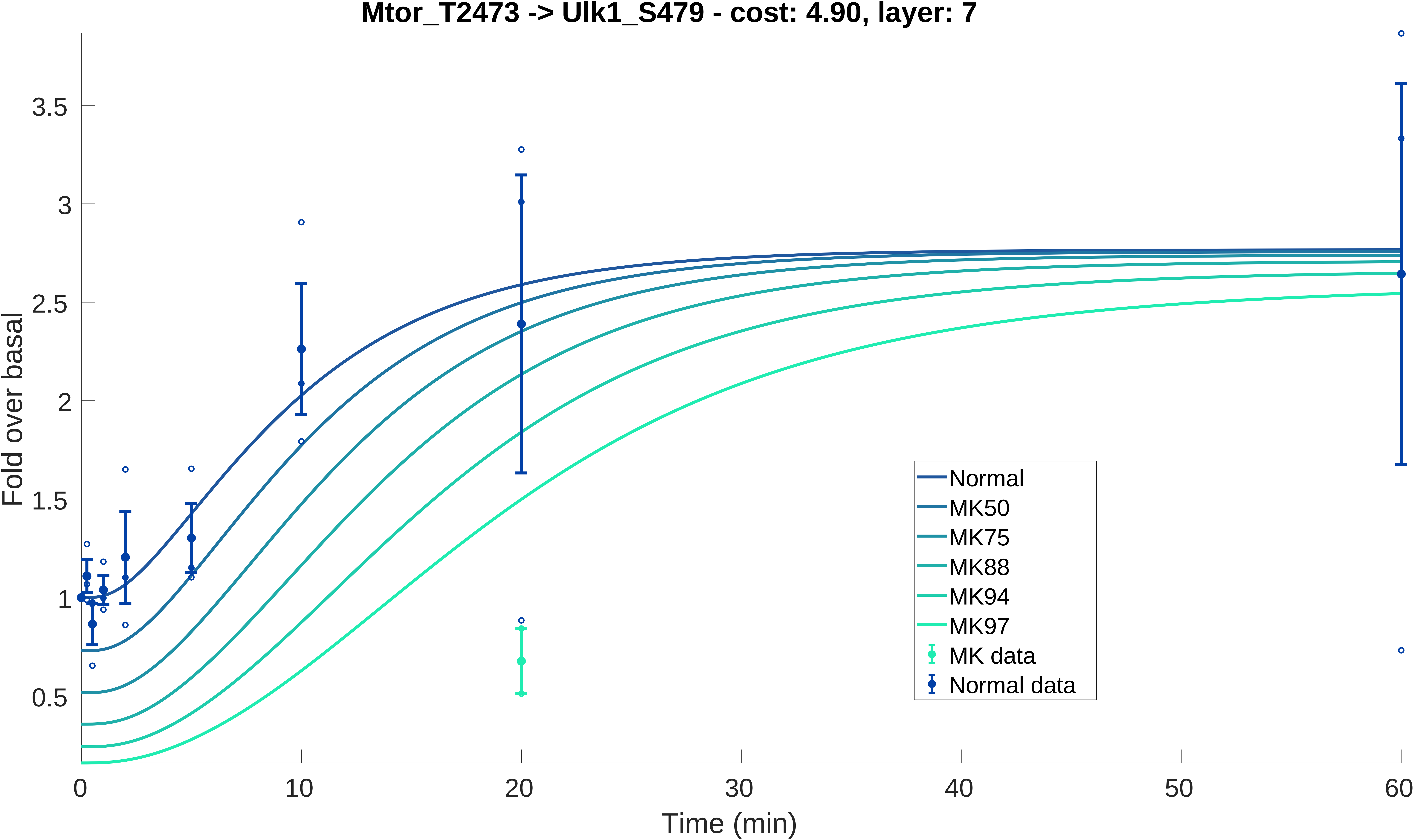

Mtor_T2473 -> Ulk1_S479 - cost: 4.90, layer: 7
Fold over basal
Time (min)
0.5
1
1.5
2
2.5
3
3.5
0
10
20
30
40
50
60
Normal
MK50
MK75
MK88
MK94
MK97
MK data
Normal data

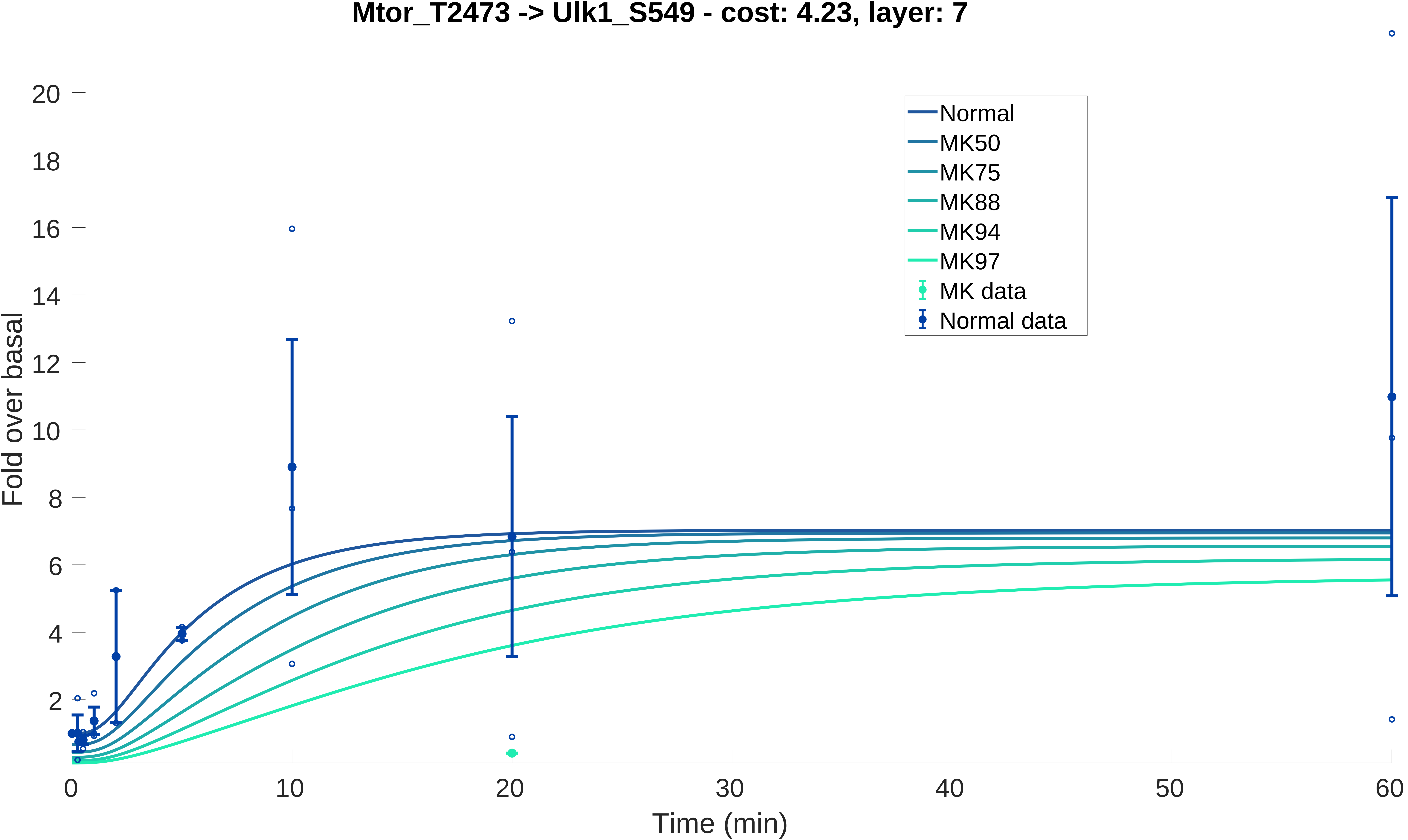

Mtor_T2473 -> Ulk1_S549 - cost: 4.23, layer: 7
Normal
MK50
MK75
MK88
MK94
MK97
MK data
Normal data
Fold over basal
Time (min)
0
10
20
30
40
50
60
2
4
6
8
10
12
14
16
18
20

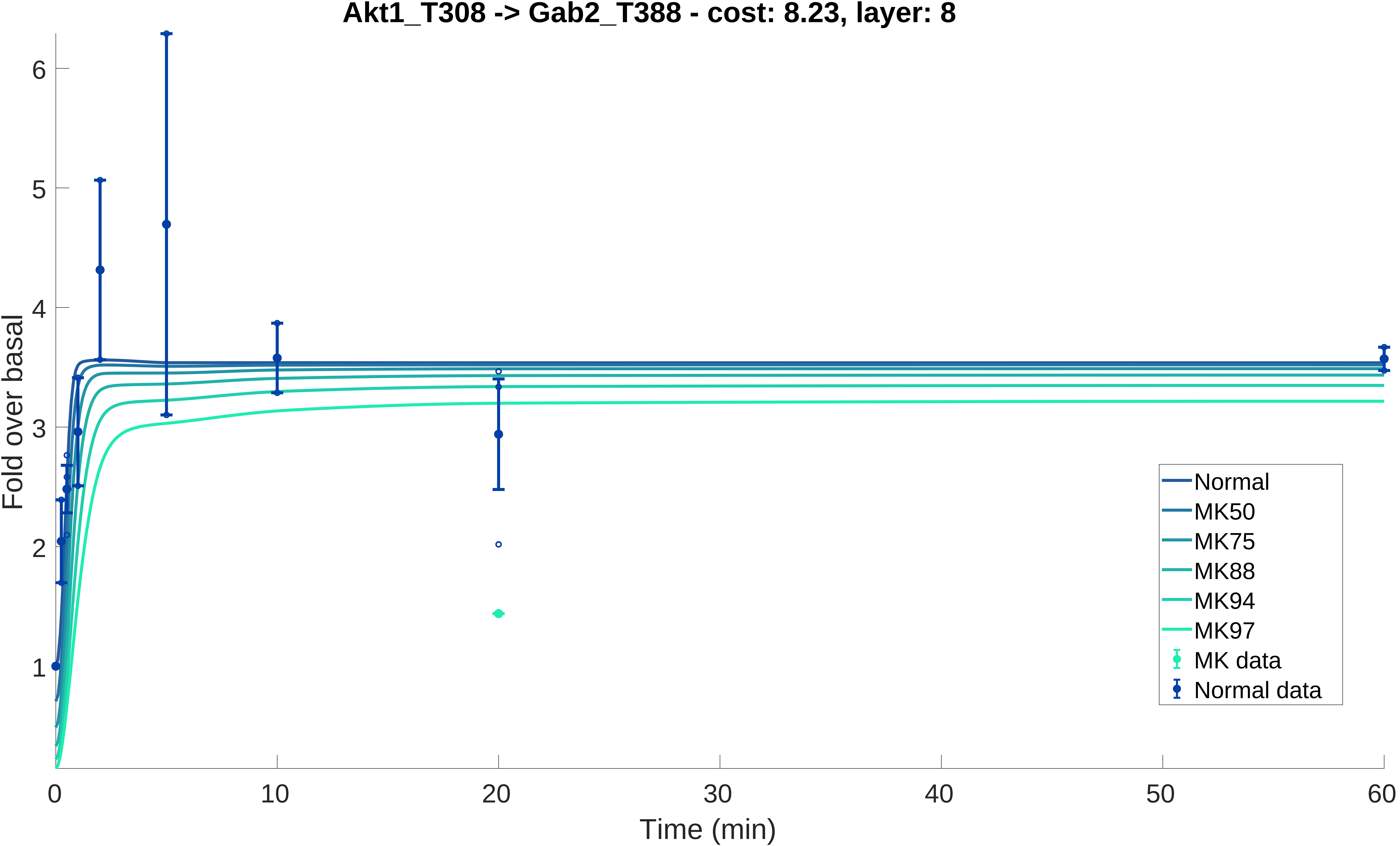
Akt1_T308 -> Gab2_T388 - cost: 8.23, layer: 8
Fold over basal
Time (min)
0
10
20
30
40
50
60
1
2
3
4
5
6
Normal
MK50
MK75
MK88
MK94
MK97
MK data
Normal data

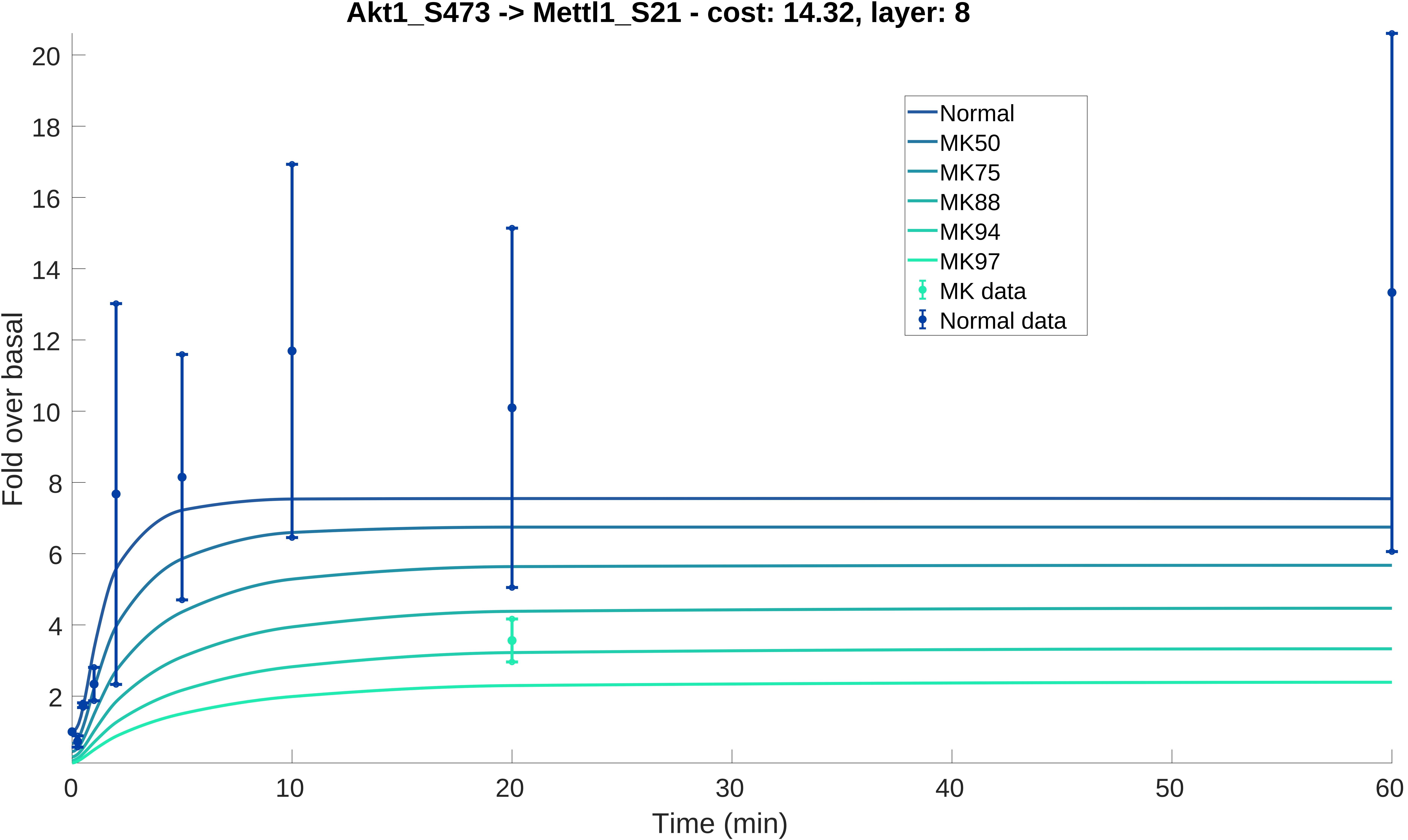

Akt1_S473 -> Mettl1_S21 - cost: 14.32, layer: 8
Fold over basal
Time (min)
0
10
20
30
40
50
60
2
4
6
8
10
12
14
16
18
20
Normal
MK50
MK75
MK88
MK94
MK97
MK data
Normal data

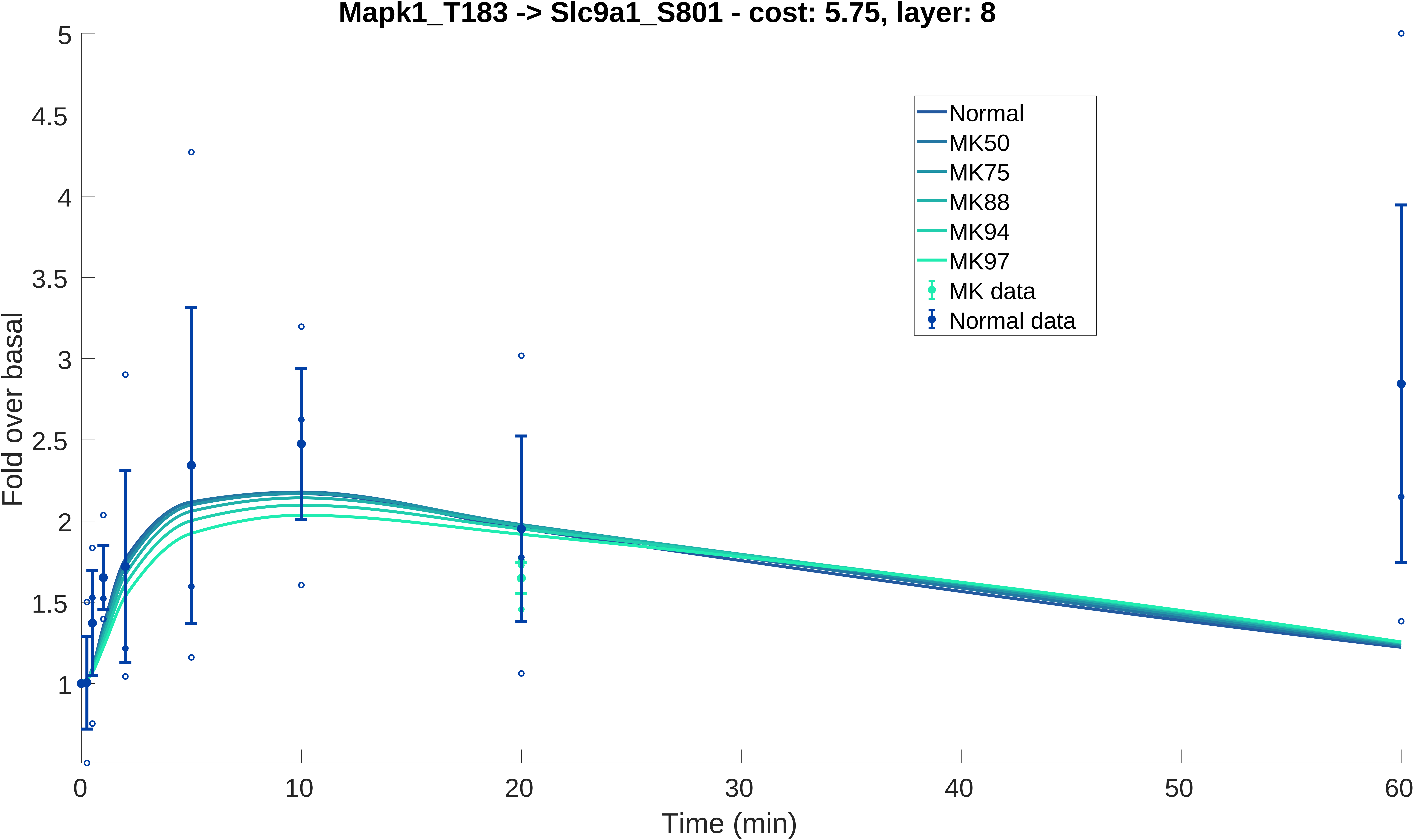

Mapk1_T183 -> Slc9a1_S801 - cost: 5.75, layer: 8
Fold over basal
Time (min)
0
10
20
30
40
50
60
1
1.5
2
2.5
3
3.5
4
4.5
5
Normal
MK50
MK75
MK88
MK94
MK97
MK data
Normal data

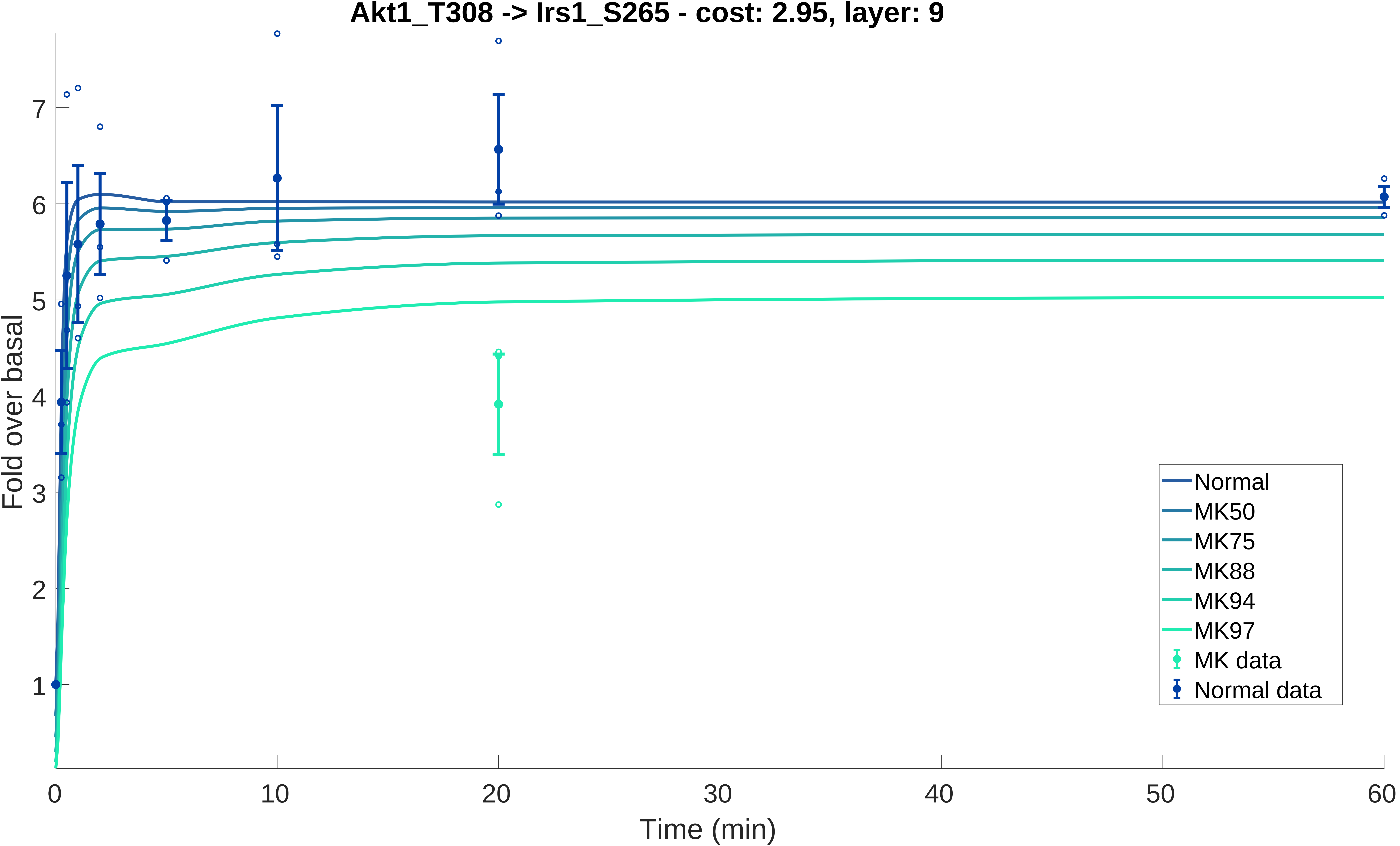

Akt1_T308 -> Irs1_S265 - cost: 2.95, layer: 9
Fold over basal
Time (min)
0
10
20
30
40
50
60
1
2
3
4
5
6
7
Normal
MK50
MK75
MK88
MK94
MK97
MK data
Normal data

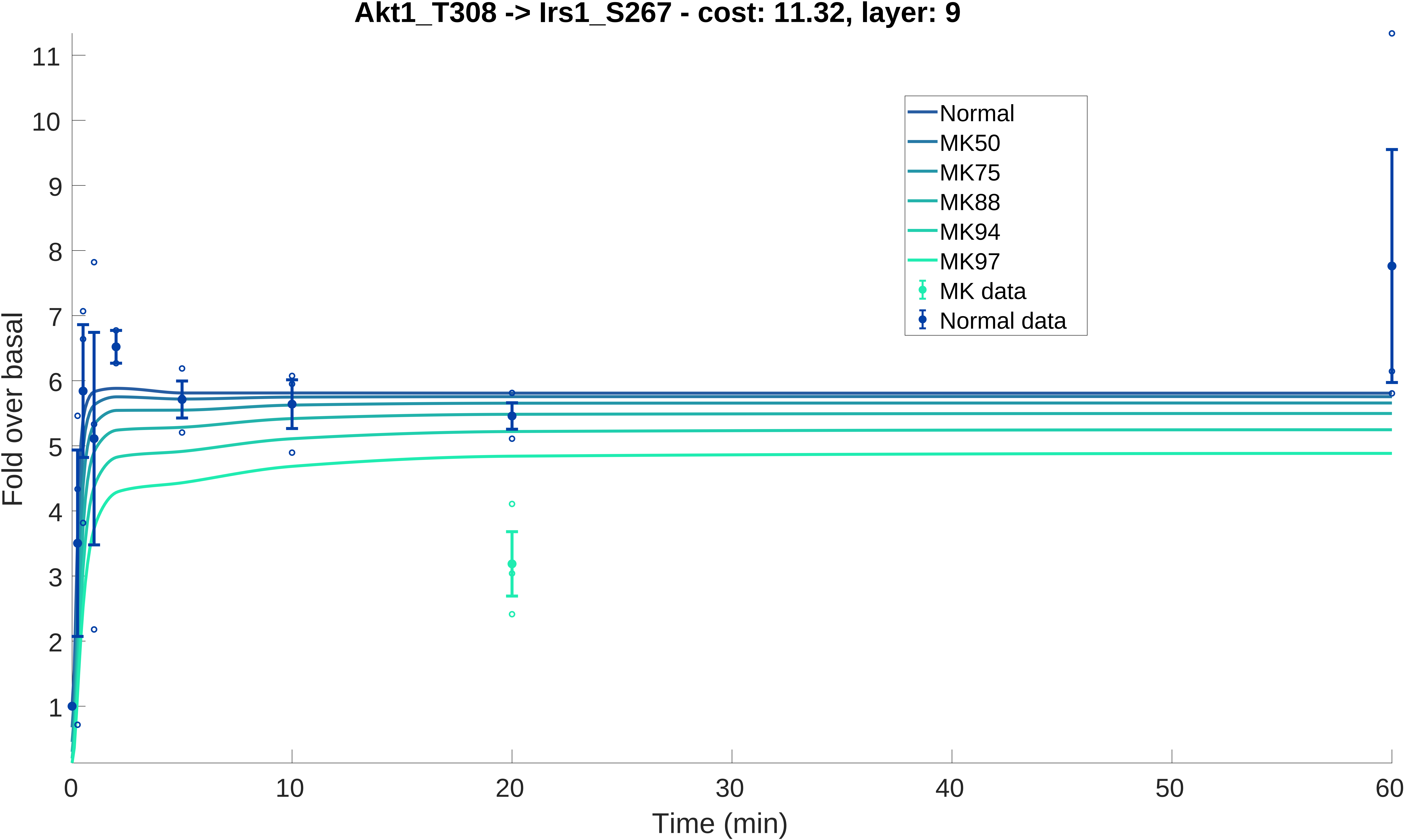

Akt1_T308 -> Irs1_S267 - cost: 11.32, layer: 9
Fold over basal
Time (min)
Normal
MK50
MK75
MK88
MK94
MK97
MK data
Normal data

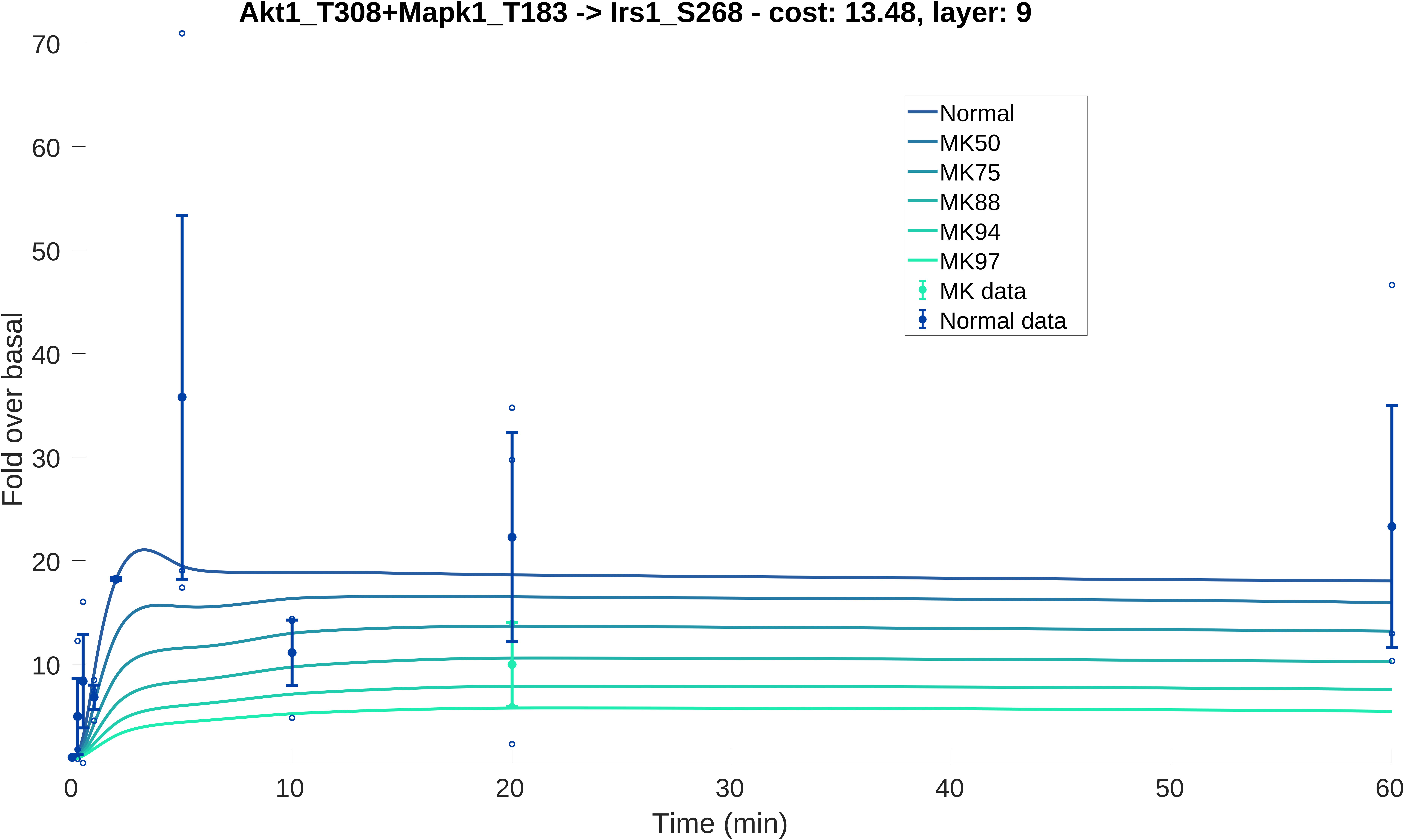
Akt1_T308+Mapk1_T183 -> Irs1_S268 - cost: 13.48, layer: 9
Normal
MK50
MK75
MK88
MK94
MK97
MK data
Normal data
Fold over basal
Time (min)
0
10
20
30
40
50
60
70

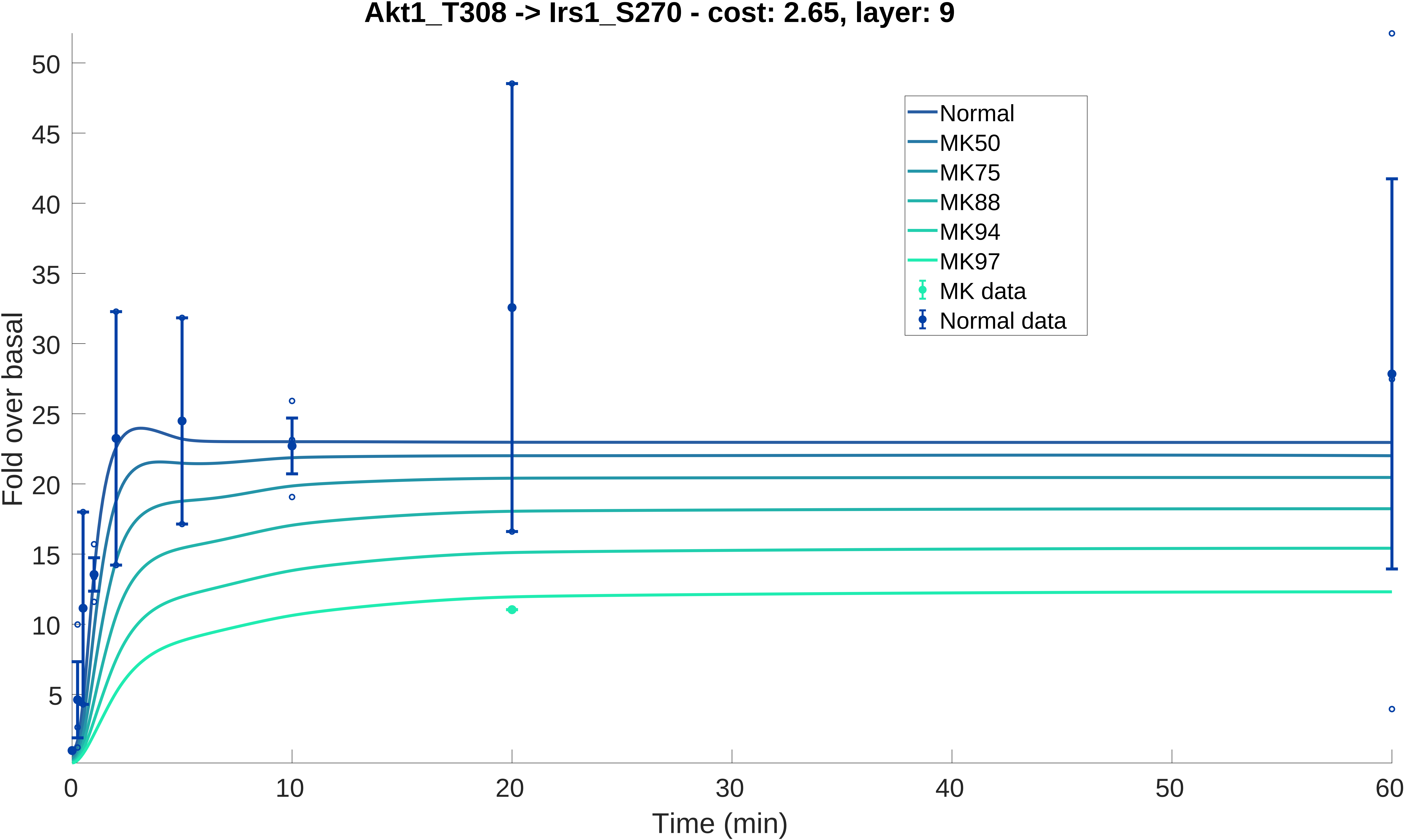

Akt1_T308 -> Irs1_S270 - cost: 2.65, layer: 9
Fold over basal
Time (min)
0
10
20
30
40
50
60
5
10
15
20
25
30
35
40
45
50
Normal
MK50
MK75
MK88
MK94
MK97
MK data
Normal data

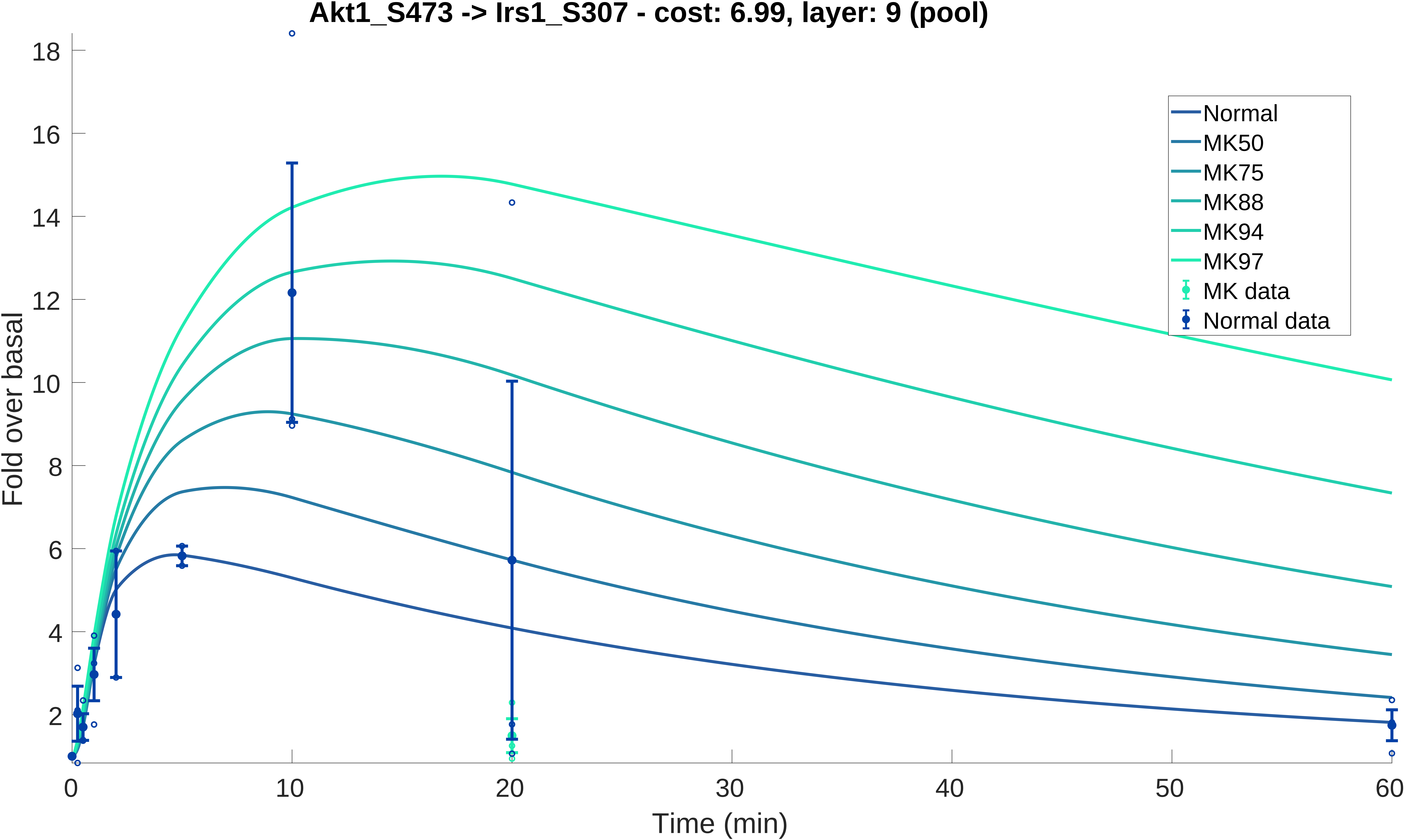

Akt1_S473 -> Irs1_S307 - cost: 6.99, layer: 9 (pool)
Fold over basal
Time (min)
0
10
20
30
40
50
60
2
4
6
8
10
12
14
16
18
Normal
MK50
MK75
MK88
MK94
MK97
MK data
Normal data

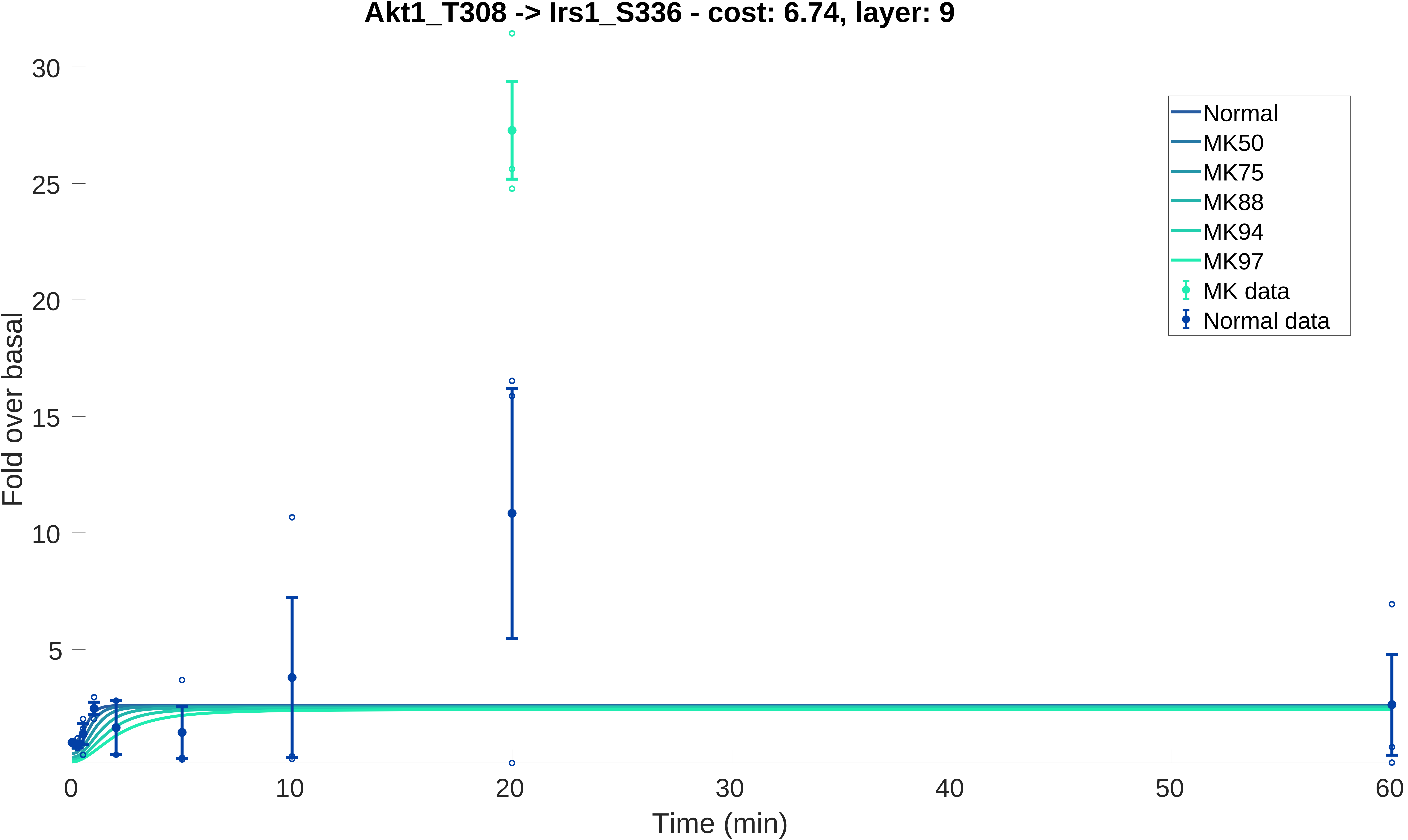

Akt1_T308 -> Irs1_S336 - cost: 6.74, layer: 9
Fold over basal
Time (min)
0
10
20
30
40
50
60
5
10
15
20
25
30
Normal
MK50
MK75
MK88
MK94
MK97
MK data
Normal data

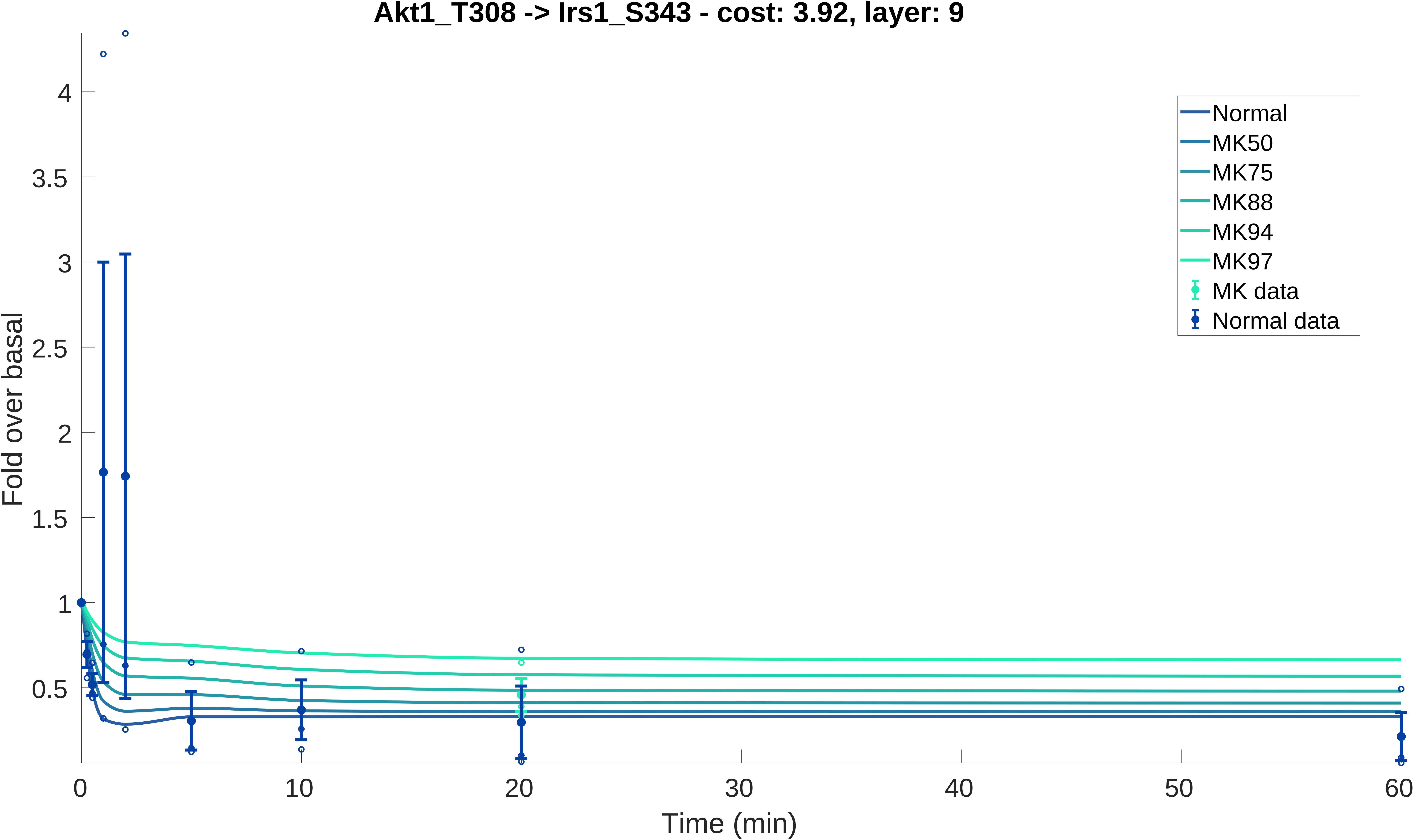
Akt1_T308 -> Irs1_S343 - cost: 3.92, layer: 9
Fold over basal
Time (min)
4
3.5
3
2.5
2
1.5
1
0.5
0
10
20
30
40
50
60
Normal
MK50
MK75
MK88
MK94
MK97
MK data
Normal data

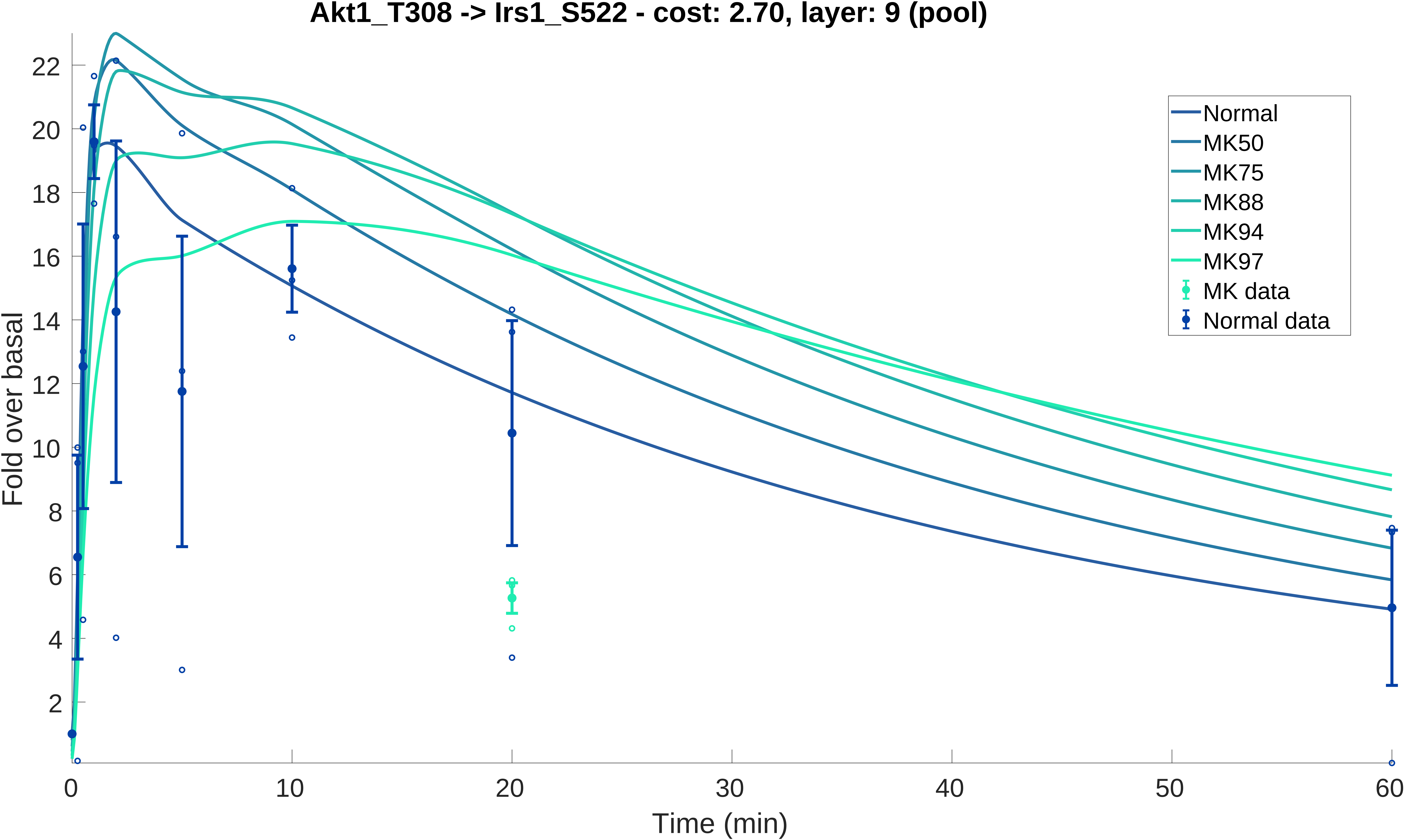

Akt1_T308 -> Irs1_S522 - cost: 2.70, layer: 9 (pool)
Fold over basal
Time (min)
Normal
MK50
MK75
MK88
MK94
MK97
MK data
Normal data
0
10
20
30
40
50
60
2
4
6
8
10
12
14
16
18
20
22

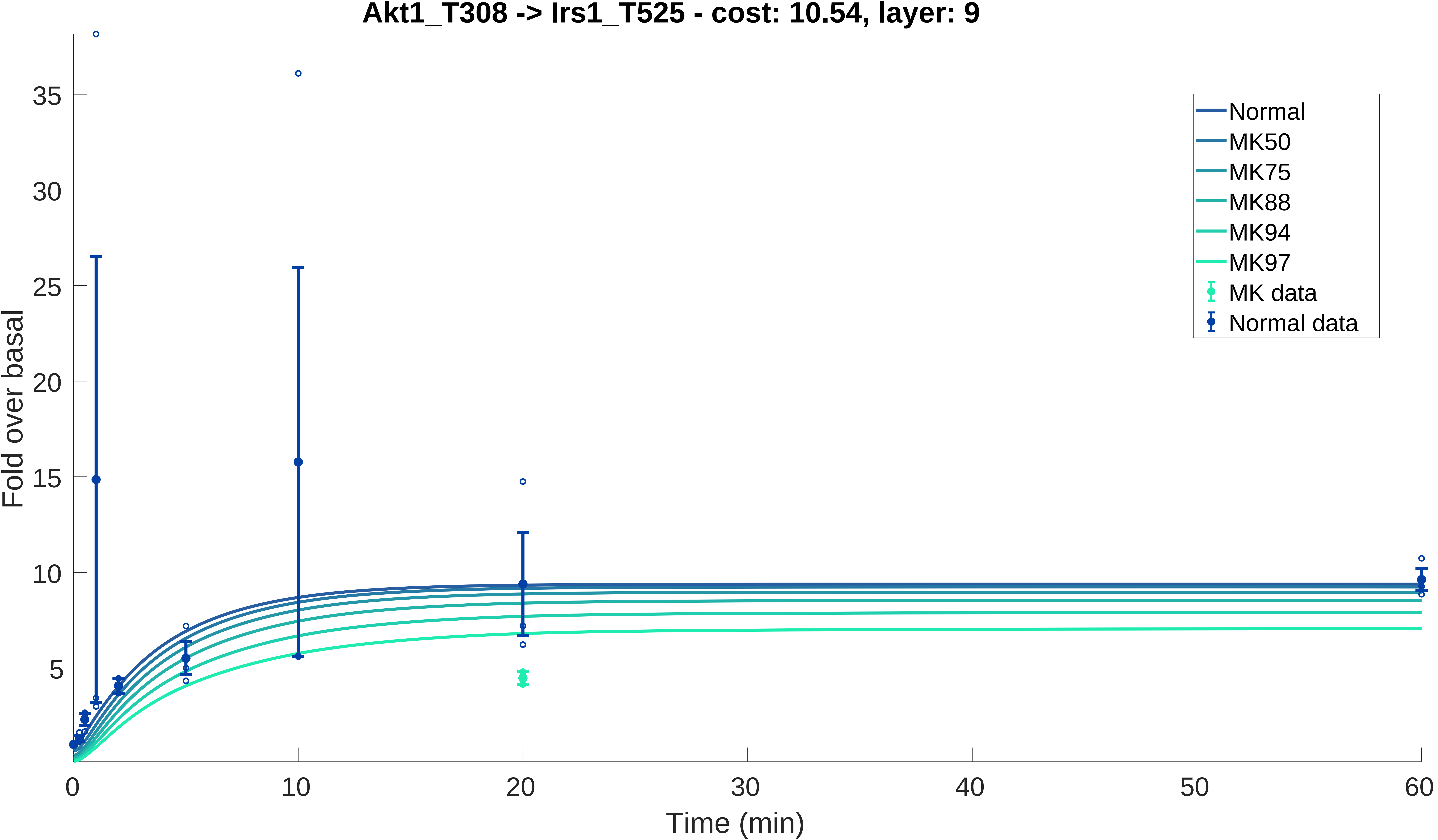
Akt1_T308 -> Irs1_T525 - cost: 10.54, layer: 9
Fold over basal
Time (min)
0
10
20
30
40
50
60
5
10
15
20
25
30
35
Normal
MK50
MK75
MK88
MK94
MK97
MK data
Normal data

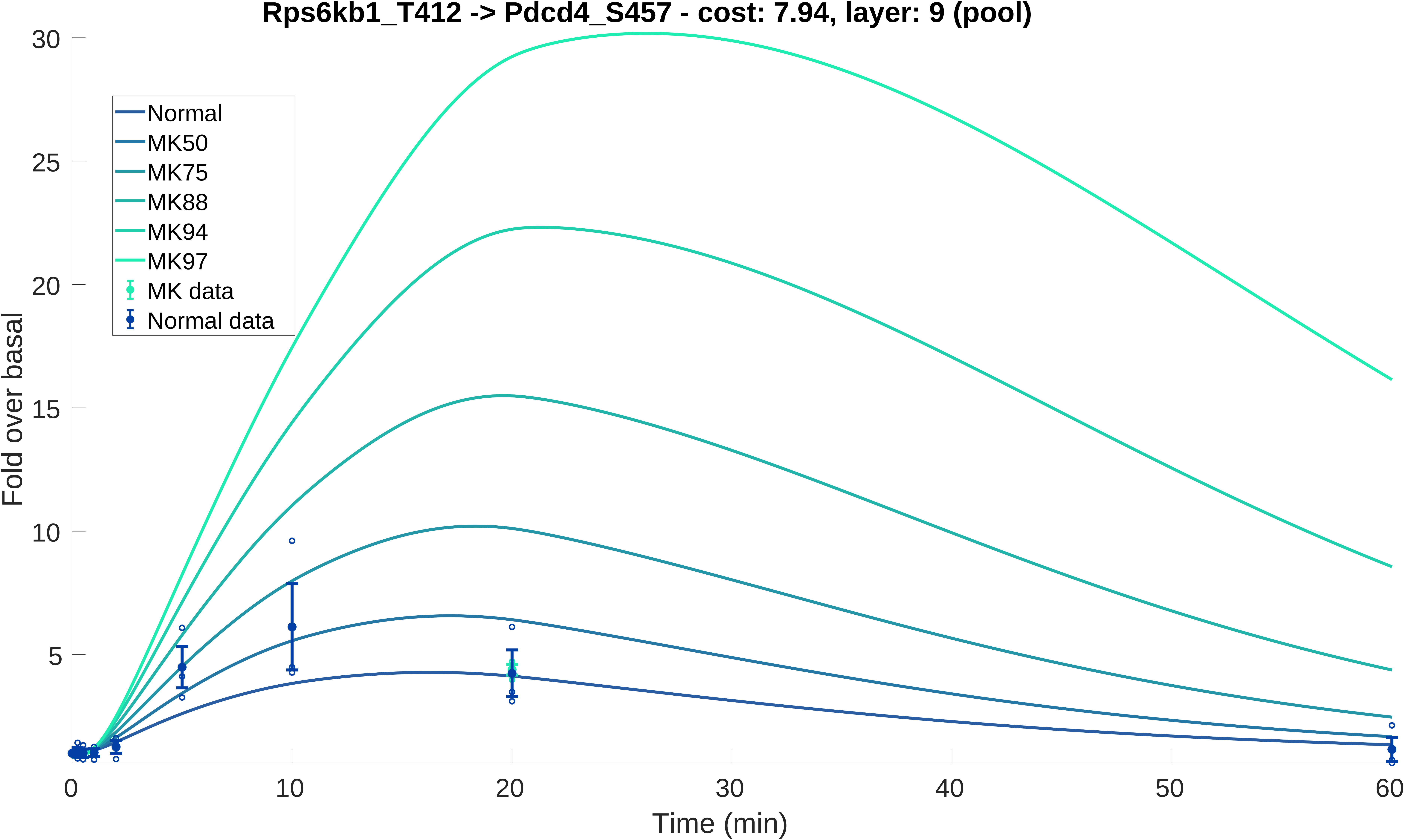

Rps6kb1_T412 -> Pdcd4_S457 - cost: 7.94, layer: 9 (pool)
Normal
MK50
MK75
MK88
MK94
MK97
MK data
Normal data
Fold over basal
Time (min)
30
25
20
15
10
5
0
10
20
30
40
50
60

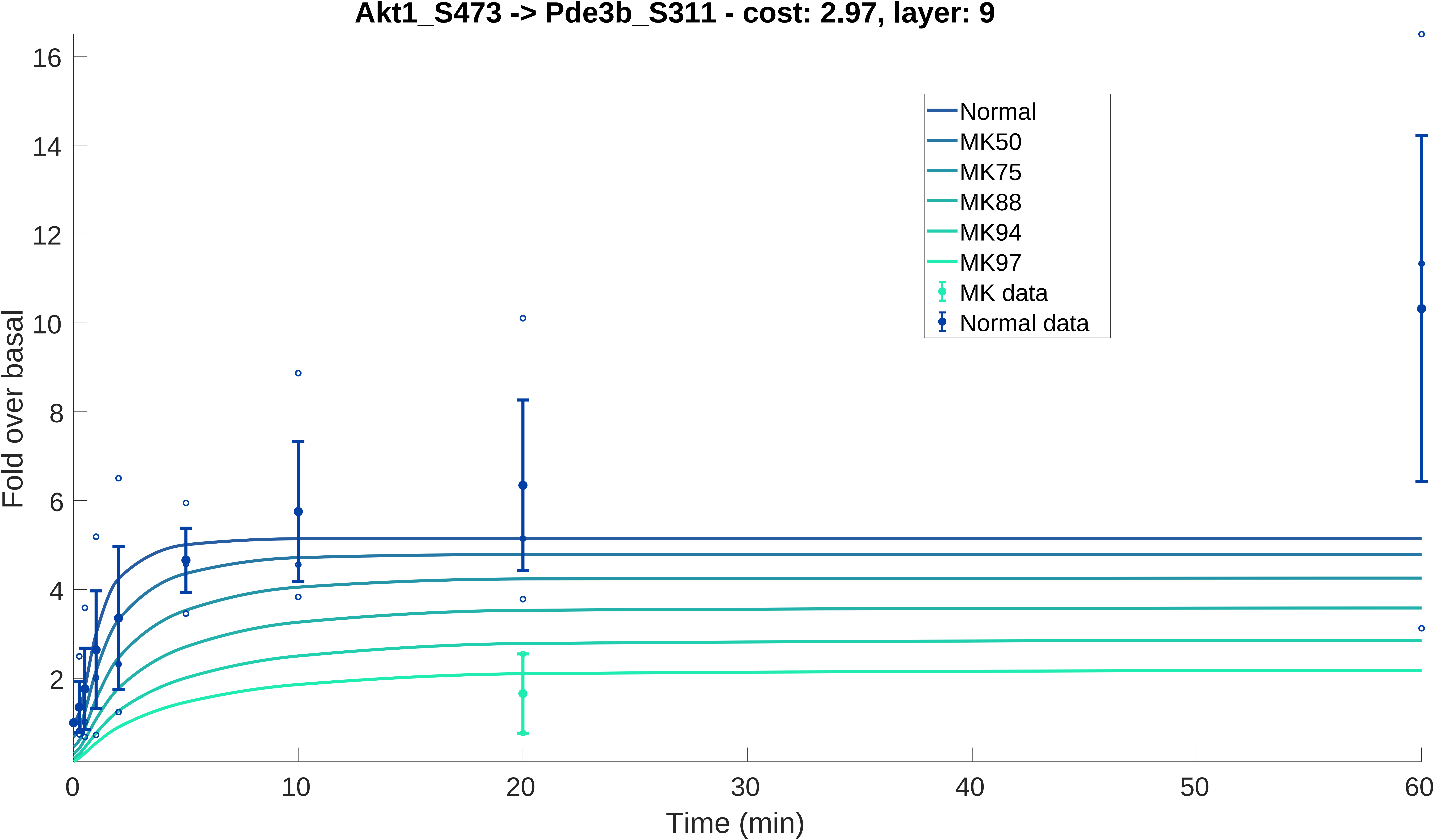

Akt1_S473 -> Pde3b_S311 - cost: 2.97, layer: 9
Fold over basal
Time (min)
0
10
20
30
40
50
60
2
4
6
8
10
12
14
16
Normal
MK50
MK75
MK88
MK94
MK97
MK data
Normal data

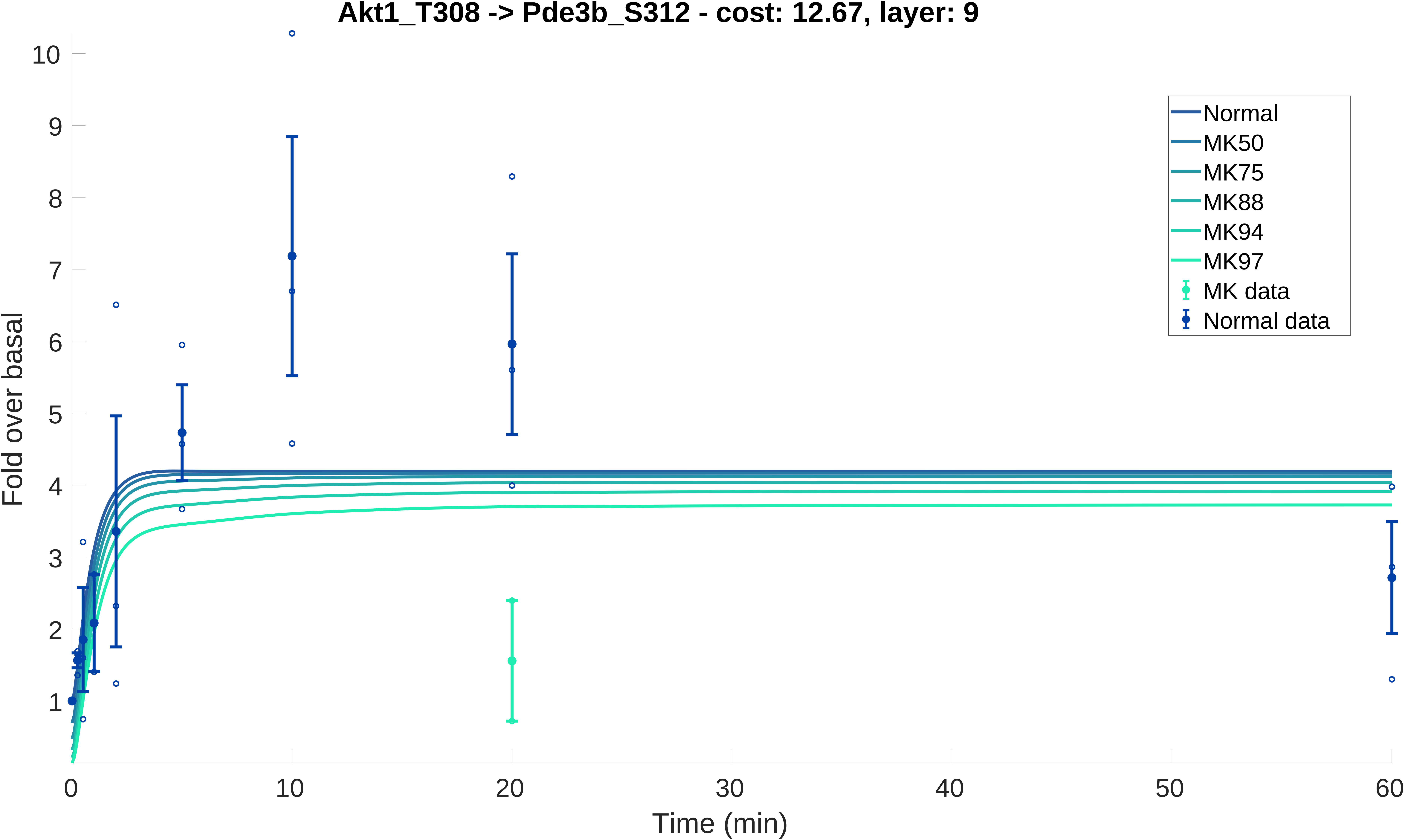

Akt1_T308 -> Pde3b_S312 - cost: 12.67, layer: 9
Fold over basal
Time (min)
0
10
20
30
40
50
60
1
2
3
4
5
6
7
8
9
10
Normal
MK50
MK75
MK88
MK94
MK97
MK data
Normal data

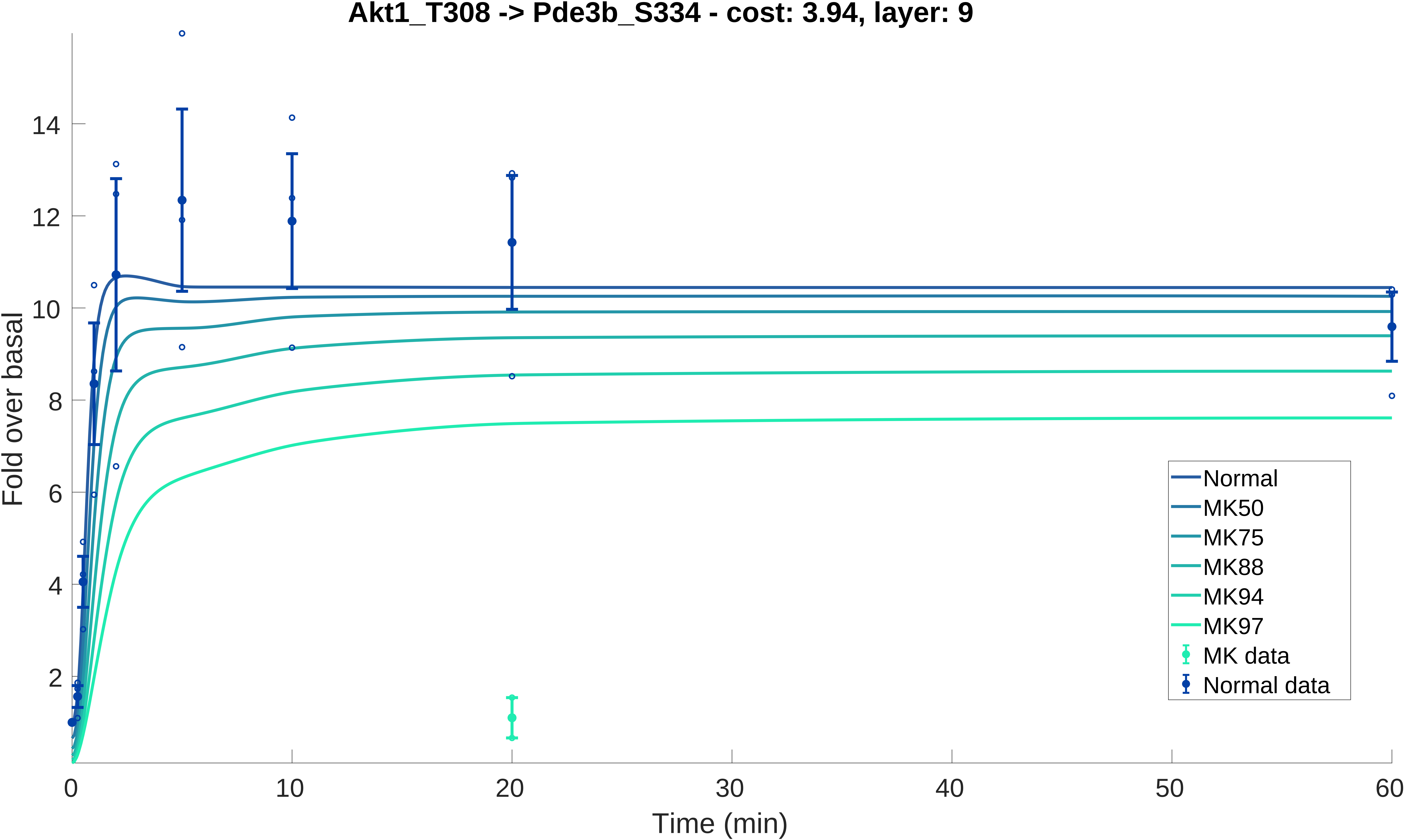
Akt1_T308 -> Pde3b_S334 - cost: 3.94, layer: 9
Fold over basal
Time (min)
0
10
20
30
40
50
60
2
4
6
8
10
12
14
Normal
MK50
MK75
MK88
MK94
MK97
MK data
Normal data

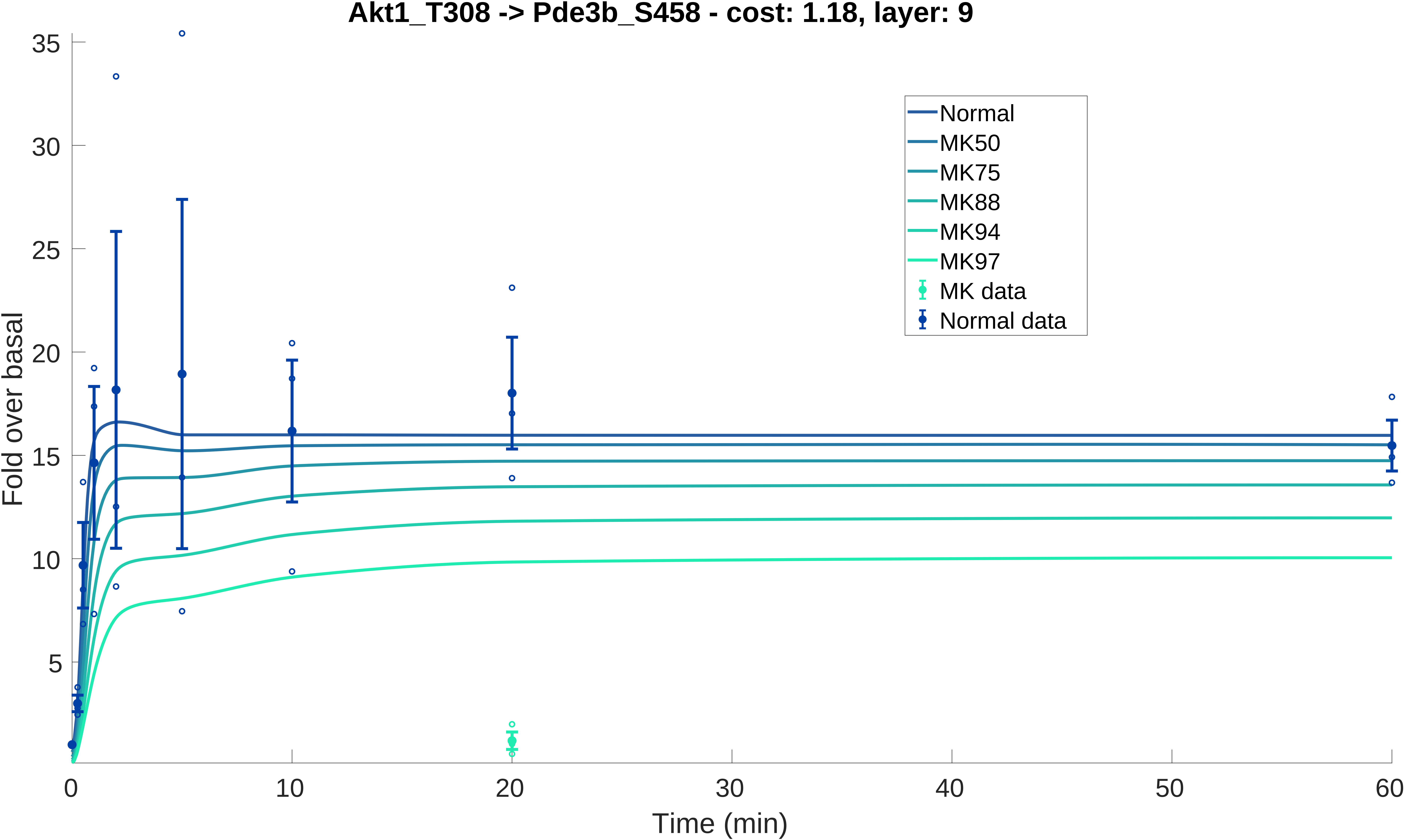

Akt1_T308 -> Pde3b_S458 - cost: 1.18, layer: 9
Fold over basal
35
30
25
20
15
10
5
0
10
20
30
40
50
60
Time (min)
Normal
MK50
MK75
MK88
MK94
MK97
MK data
Normal data

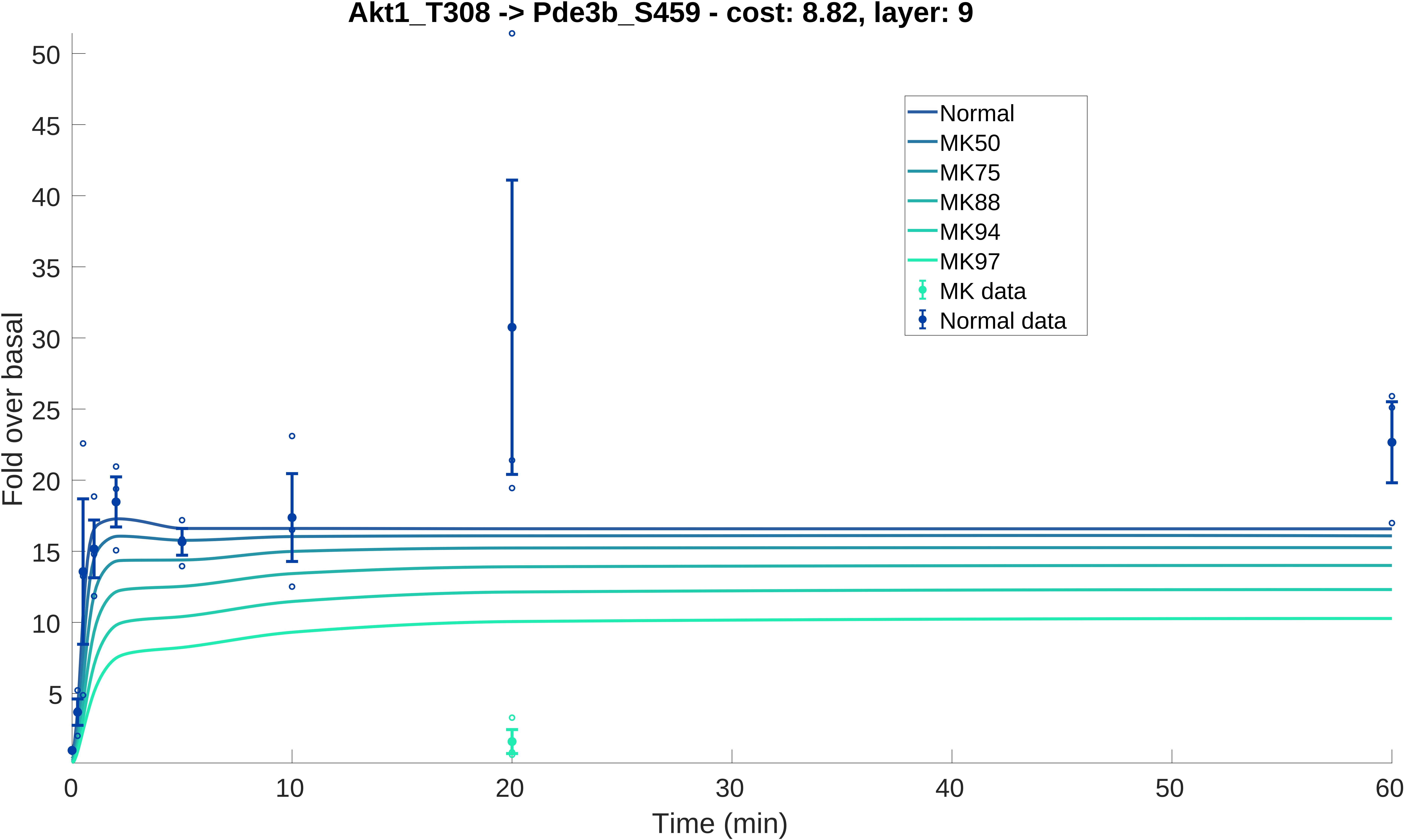

Akt1_T308 -> Pde3b_S459 - cost: 8.82, layer: 9
Fold over basal
Time (min)
0
10
20
30
40
50
60
5
10
15
20
25
30
35
40
45
50
Normal
MK50
MK75
MK88
MK94
MK97
MK data
Normal data

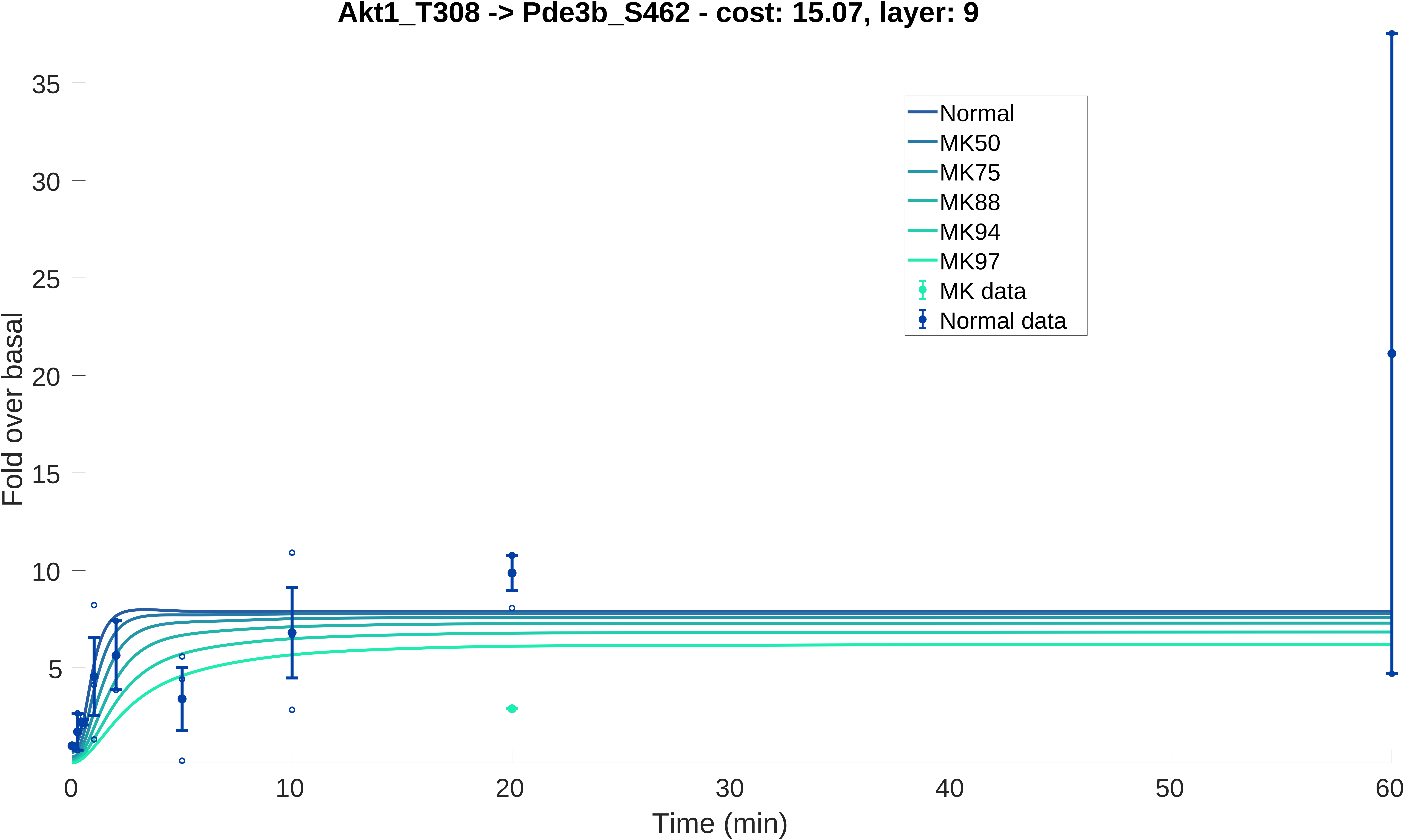

Akt1_T308 -> Pde3b_S462 - cost: 15.07, layer: 9
Fold over basal
Time (min)
0
10
20
30
40
50
60
5
10
15
20
25
30
35
Normal
MK50
MK75
MK88
MK94
MK97
MK data
Normal data

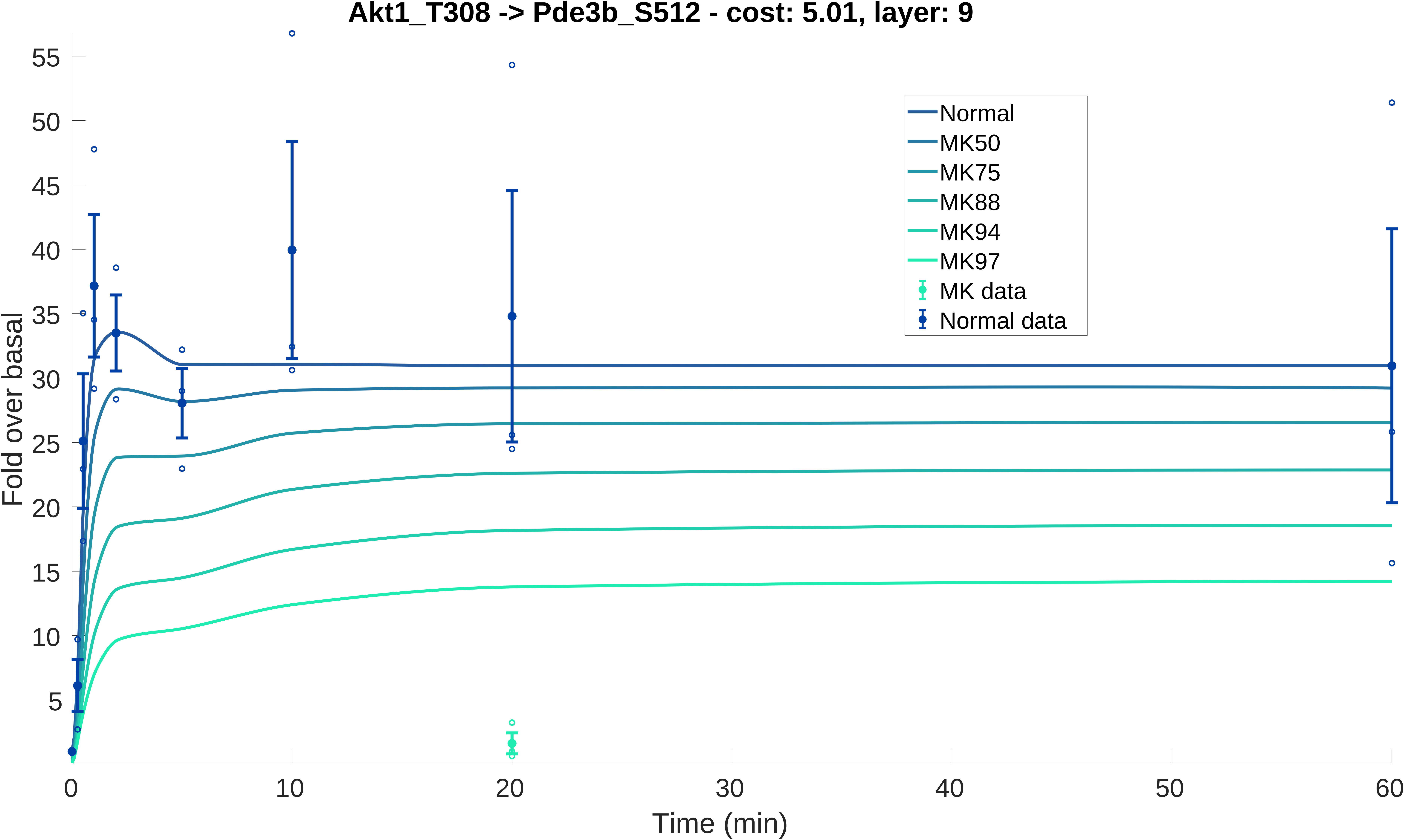

Akt1_T308 -> Pde3b_S512 - cost: 5.01, layer: 9
Fold over basal
Time (min)
0
10
20
30
40
50
60
5
10
15
20
25
30
35
40
45
50
55
Normal
MK50
MK75
MK88
MK94
MK97
MK data
Normal data

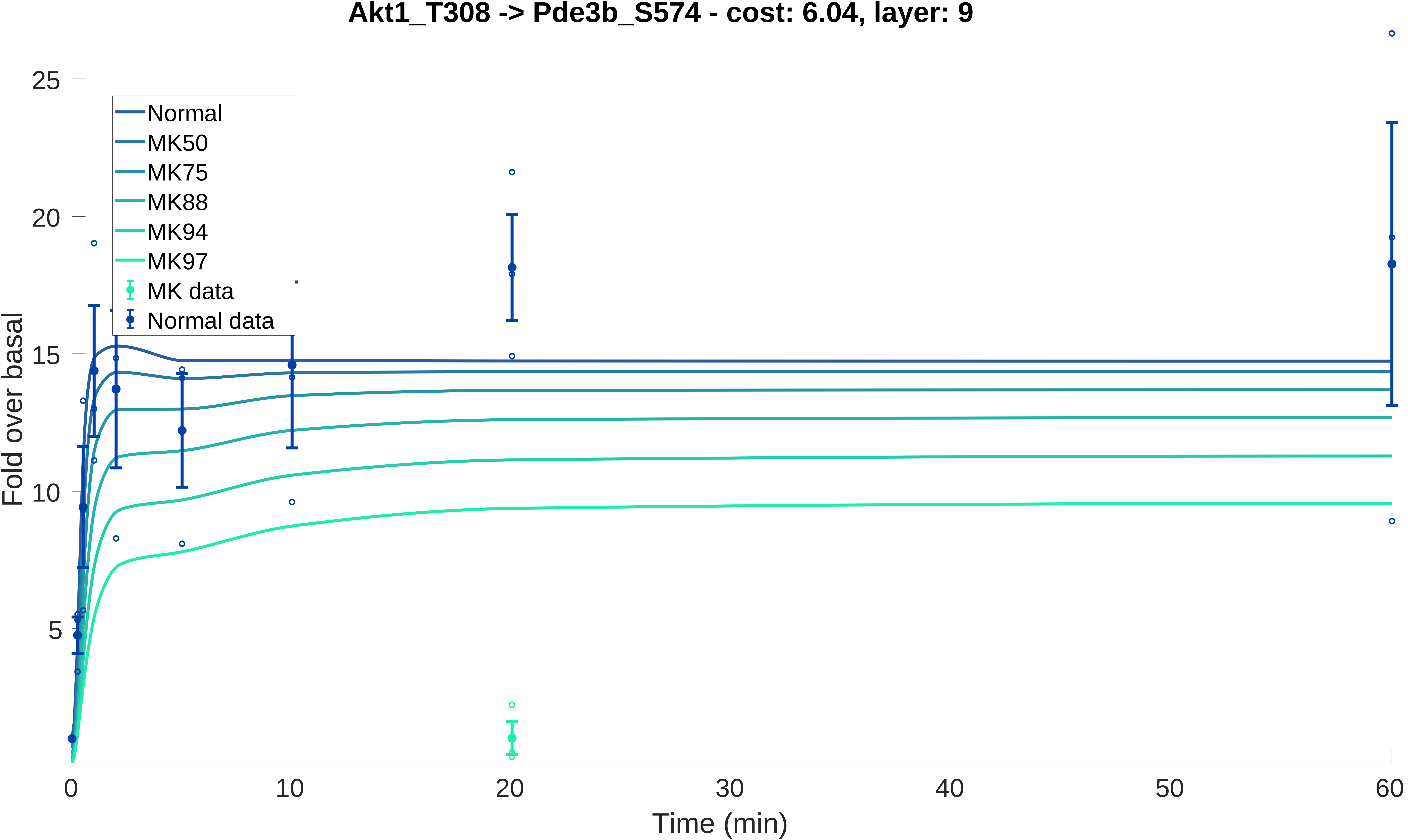

Akt1_T308 -> Pde3b_S574 - cost: 6.04, layer: 9
Fold over basal
Time (min)
0
10
20
30
40
50
60
5
10
15
20
25
Normal
MK50
MK75
MK88
MK94
MK97
MK data
Normal data

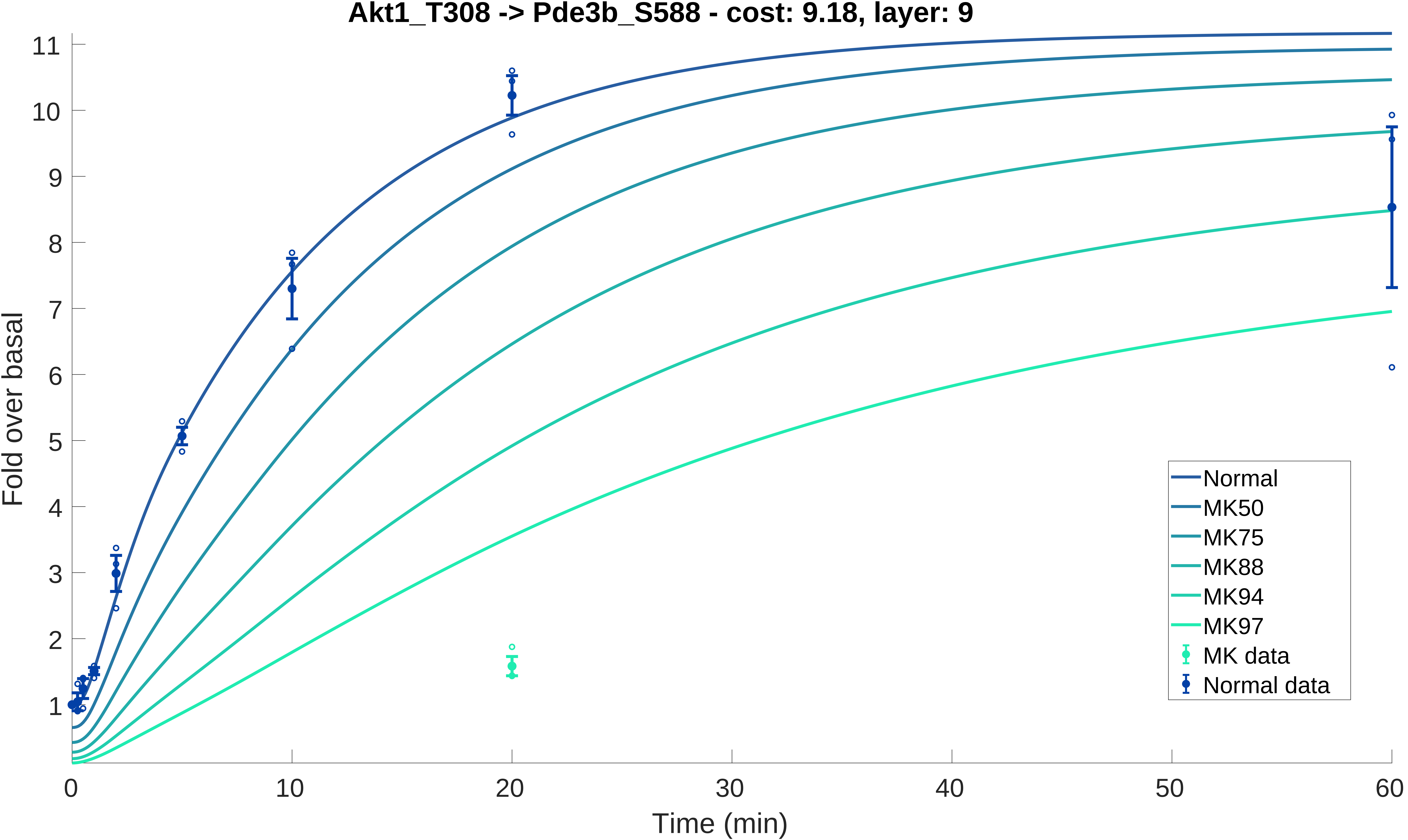

Akt1_T308 -> Pde3b_S588 - cost: 9.18, layer: 9
Fold over basal
Time (min)
Normal
MK50
MK75
MK88
MK94
MK97
MK data
Normal data

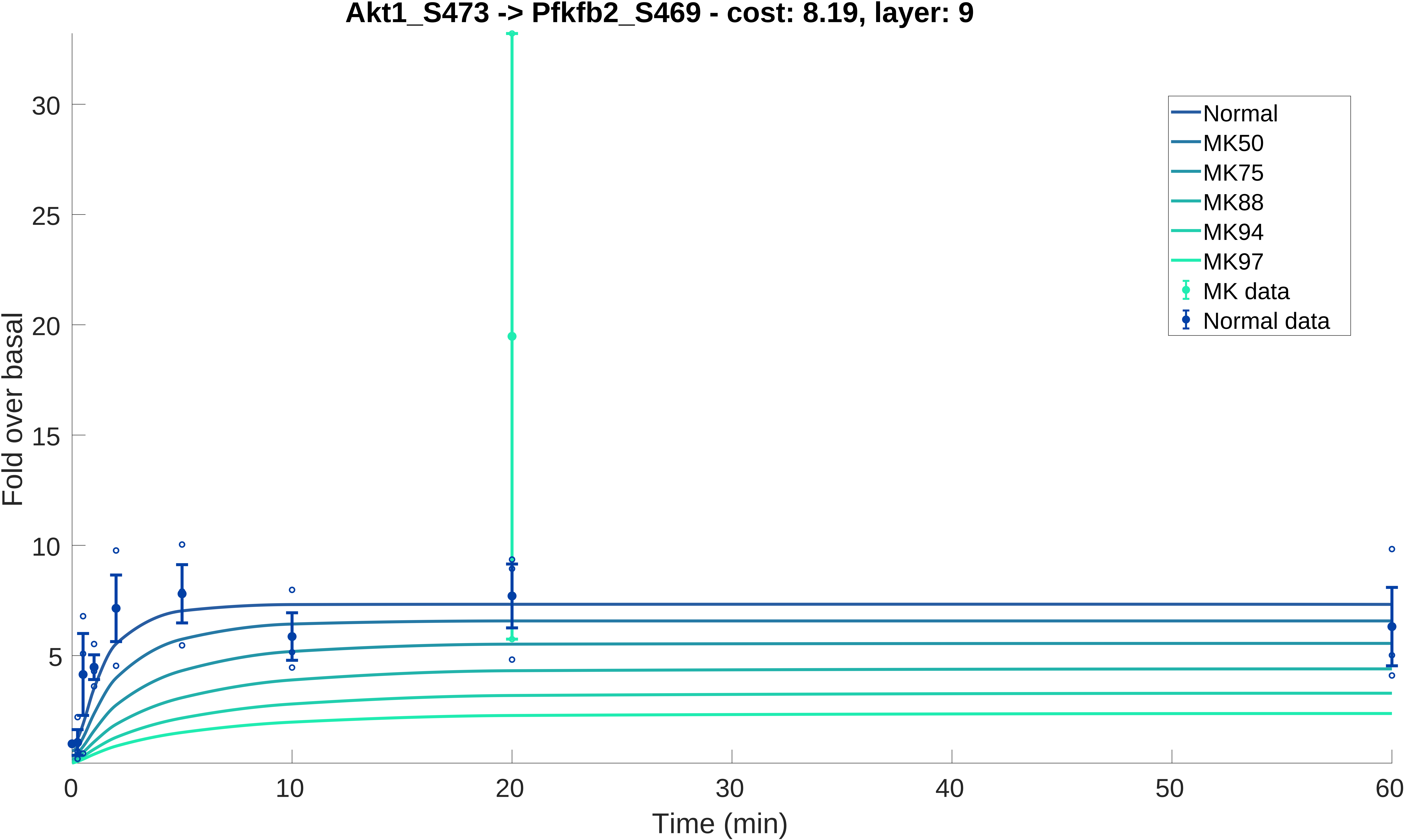

Akt1_S473 -> Pfkfb2_S469 - cost: 8.19, layer: 9
Fold over basal
Time (min)
Normal
MK50
MK75
MK88
MK94
MK97
MK data
Normal data

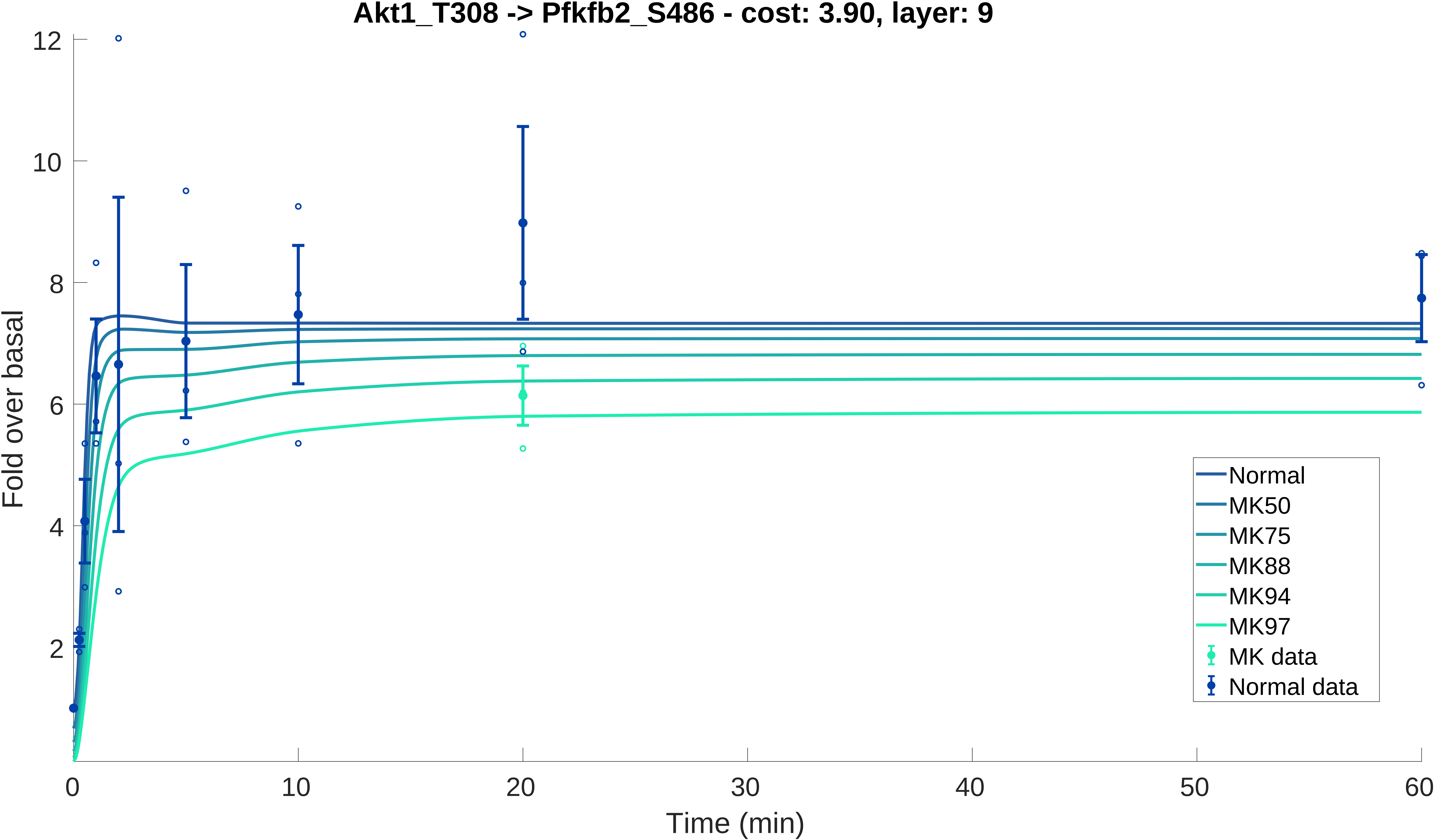

Akt1_T308 -> Pfkfb2_S486 - cost: 3.90, layer: 9
Fold over basal
Time (min)
0
10
20
30
40
50
60
2
4
6
8
10
12
Normal
MK50
MK75
MK88
MK94
MK97
MK data
Normal data

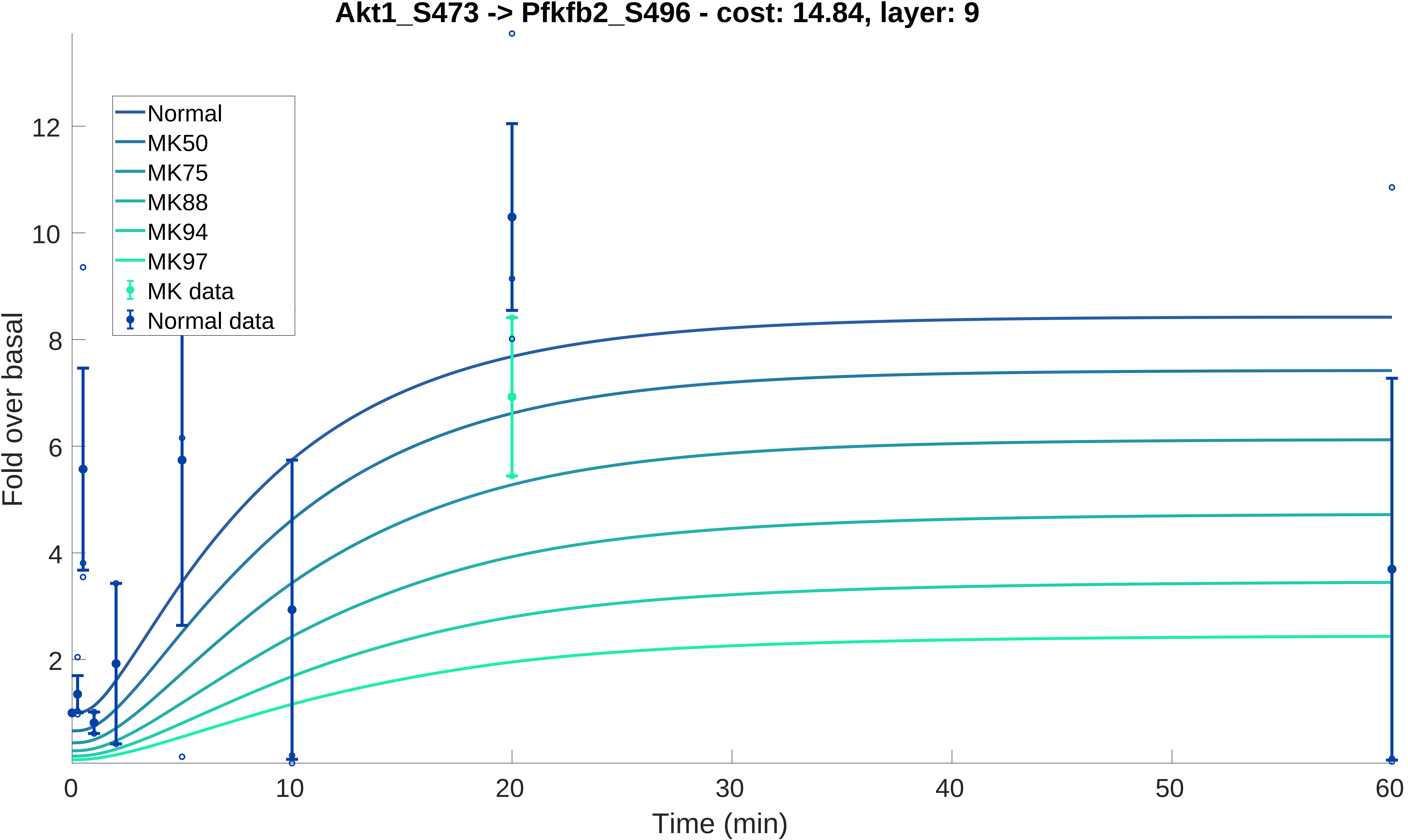

Akt1_S473 -> Pfkfb2_S496 - cost: 14.84, layer: 9
Normal
MK50
MK75
MK88
MK94
MK97
MK data
Normal data
Fold over basal
12
10
8
6
4
2
0
10
20
30
40
50
60
Time (min)

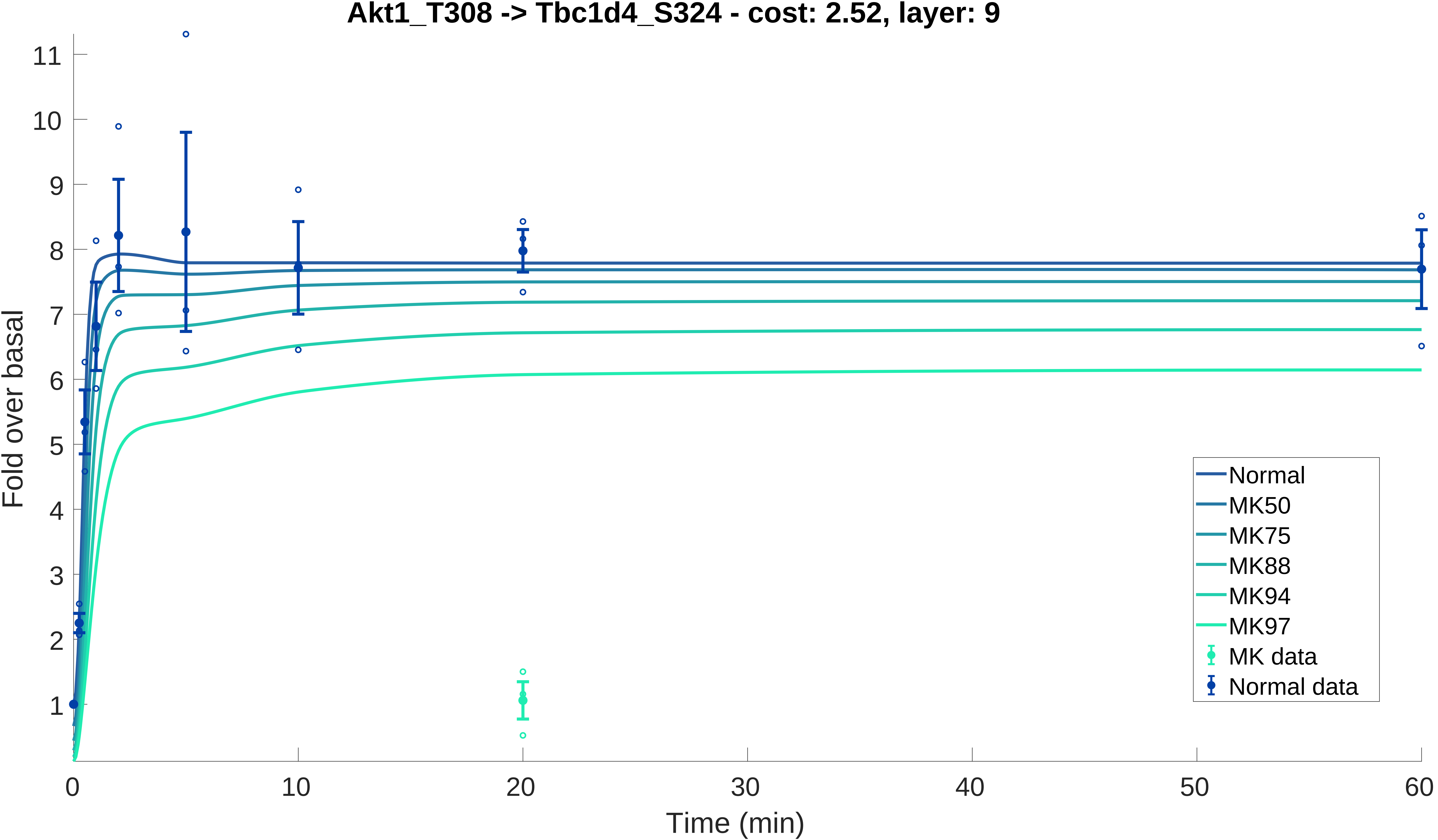
Akt1_T308 -> Tbc1d4_S324 - cost: 2.52, layer: 9
Fold over basal
Time (min)
1
2
3
4
5
6
7
8
9
10
11
0
10
20
30
40
50
60
Normal
MK50
MK75
MK88
MK94
MK97
MK data
Normal data

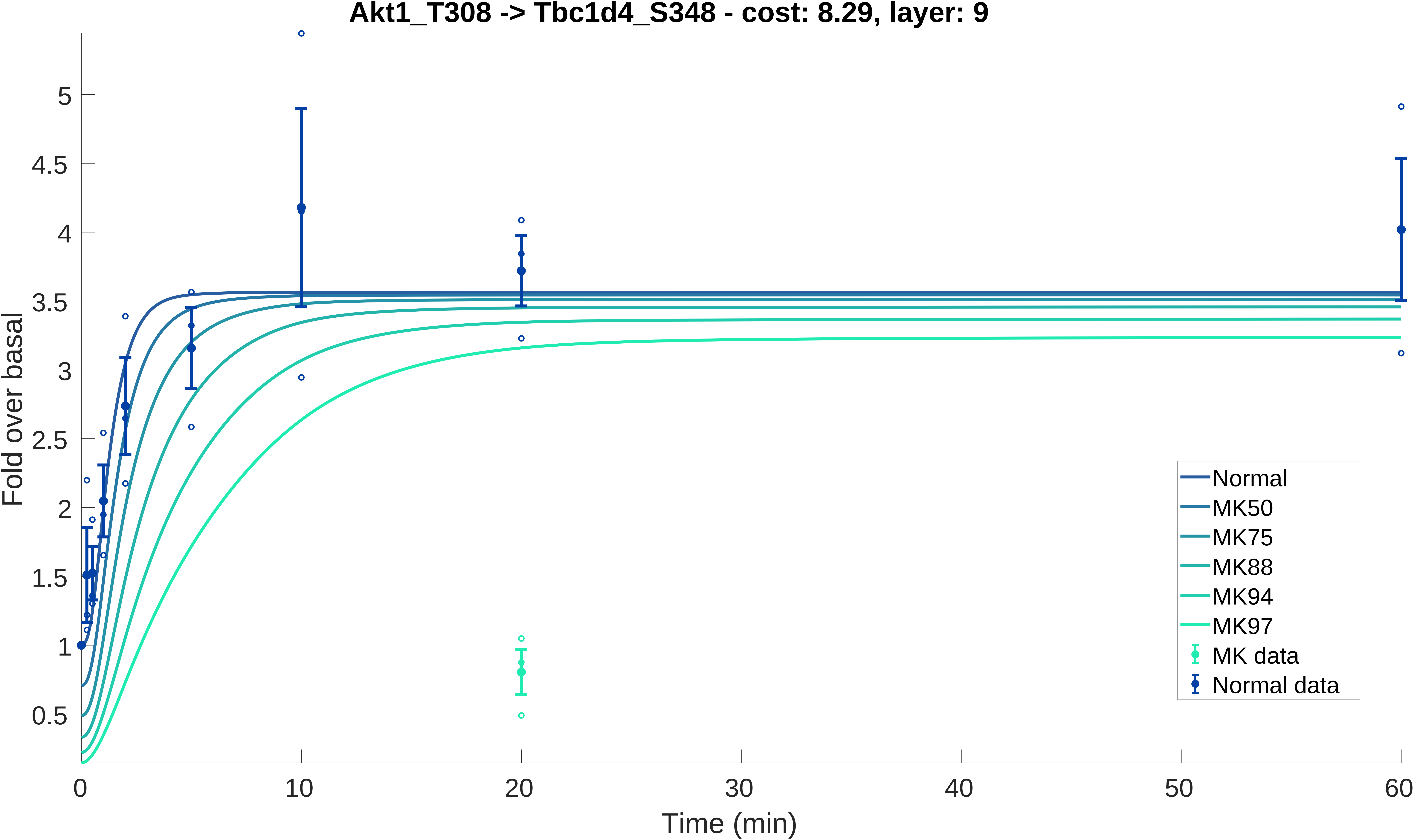
Akt1_T308 -> Tbc1d4_S348 - cost: 8.29, layer: 9
Fold over basal
Time (min)
0
10
20
30
40
50
60
0.5
1
1.5
2
2.5
3
3.5
4
4.5
5
Normal
MK50
MK75
MK88
MK94
MK97
MK data
Normal data

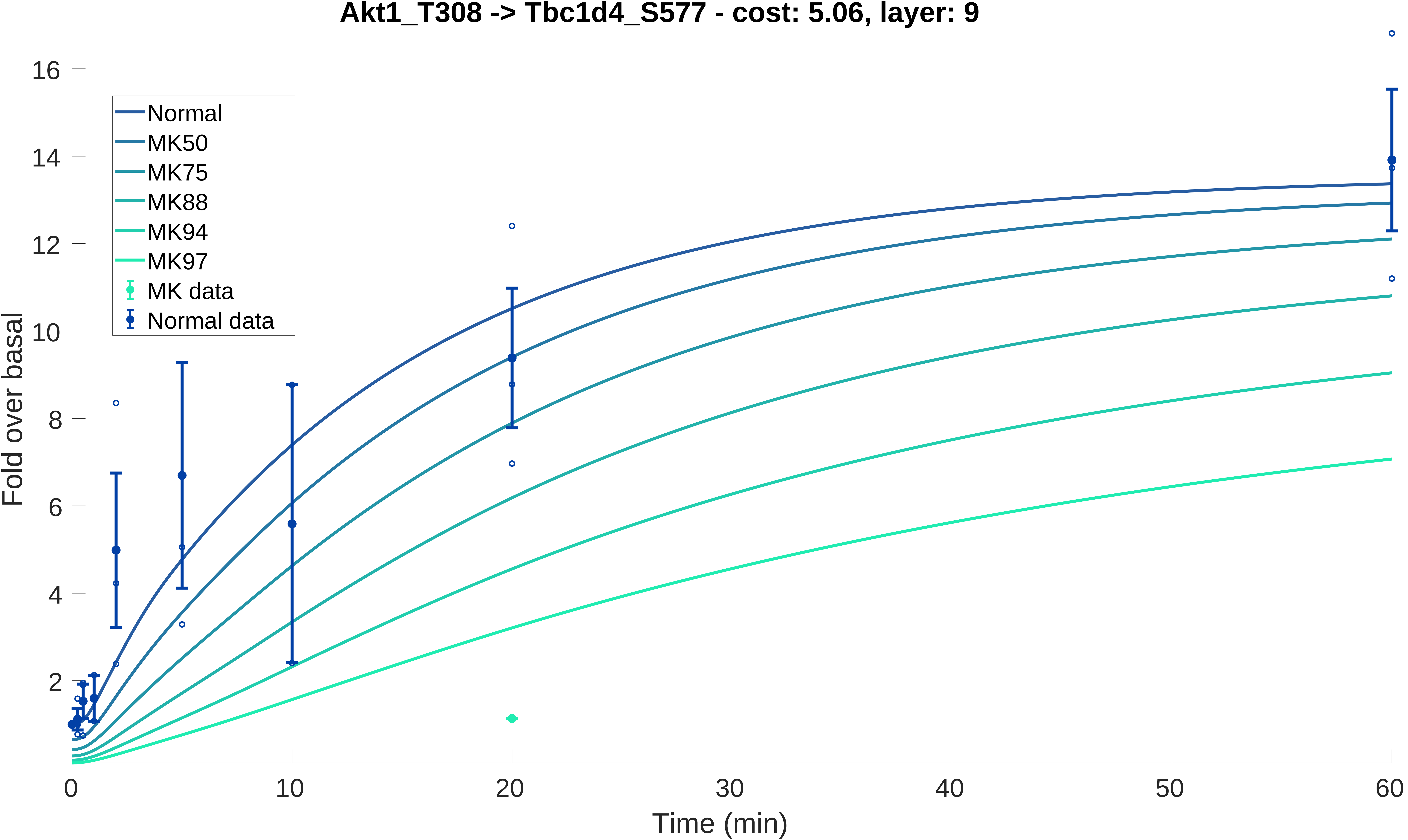
Akt1_T308 -> Tbc1d4_S577 - cost: 5.06, layer: 9
Fold over basal
Time (min)
Normal
MK50
MK75
MK88
MK94
MK97
MK data
Normal data
0
10
20
30
40
50
60
2
4
6
8
10
12
14
16

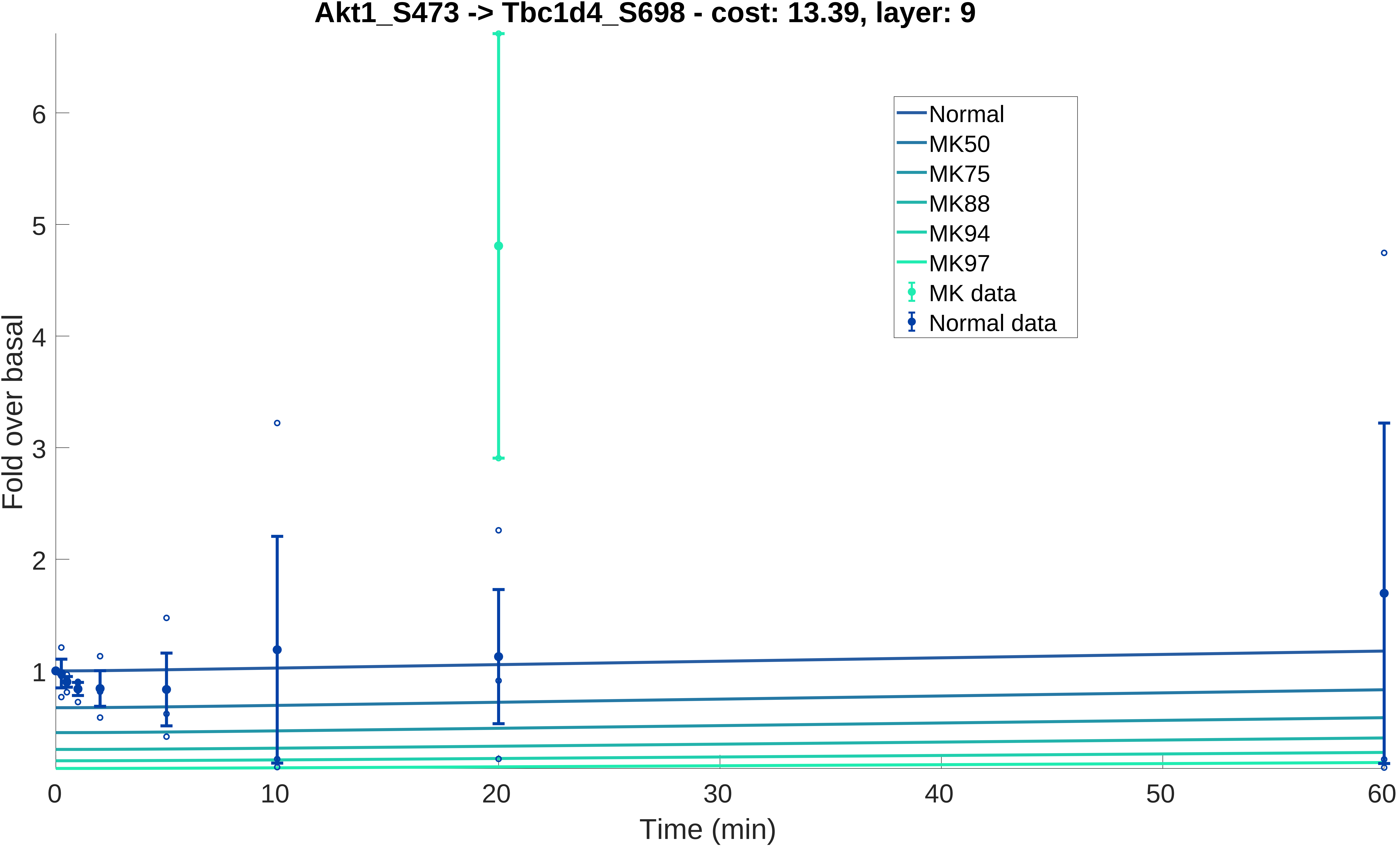

Akt1_S473 -> Tbc1d4_S698 - cost: 13.39, layer: 9
Fold over basal
Time (min)
0
10
20
30
40
50
60
1
2
3
4
5
6
Normal
MK50
MK75
MK88
MK94
MK97
MK data
Normal data

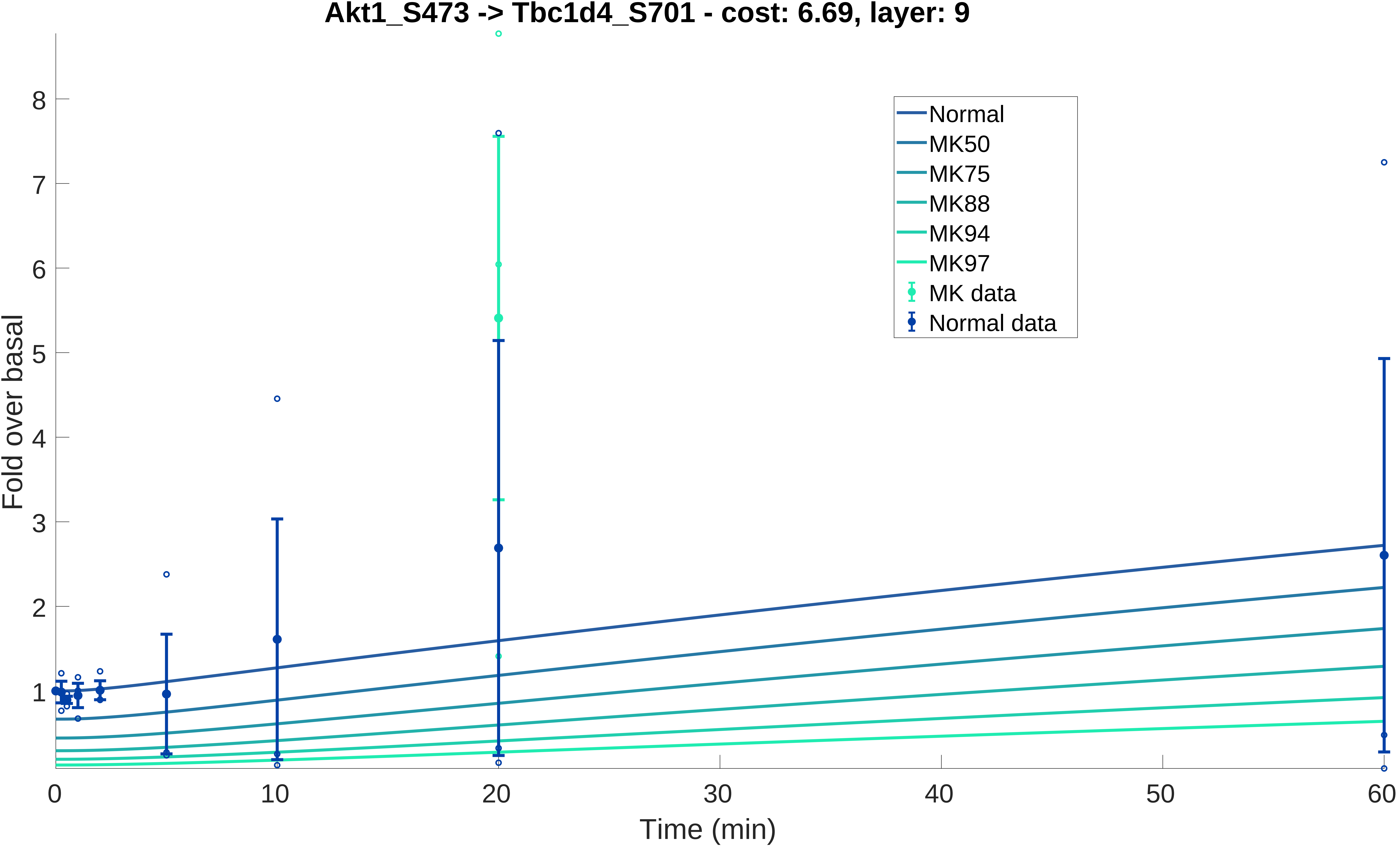

Akt1_S473 -> Tbc1d4_S701 - cost: 6.69, layer: 9
Fold over basal
Time (min)
0
10
20
30
40
50
60
1
2
3
4
5
6
7
8
Normal
MK50
MK75
MK88
MK94
MK97
MK data
Normal data

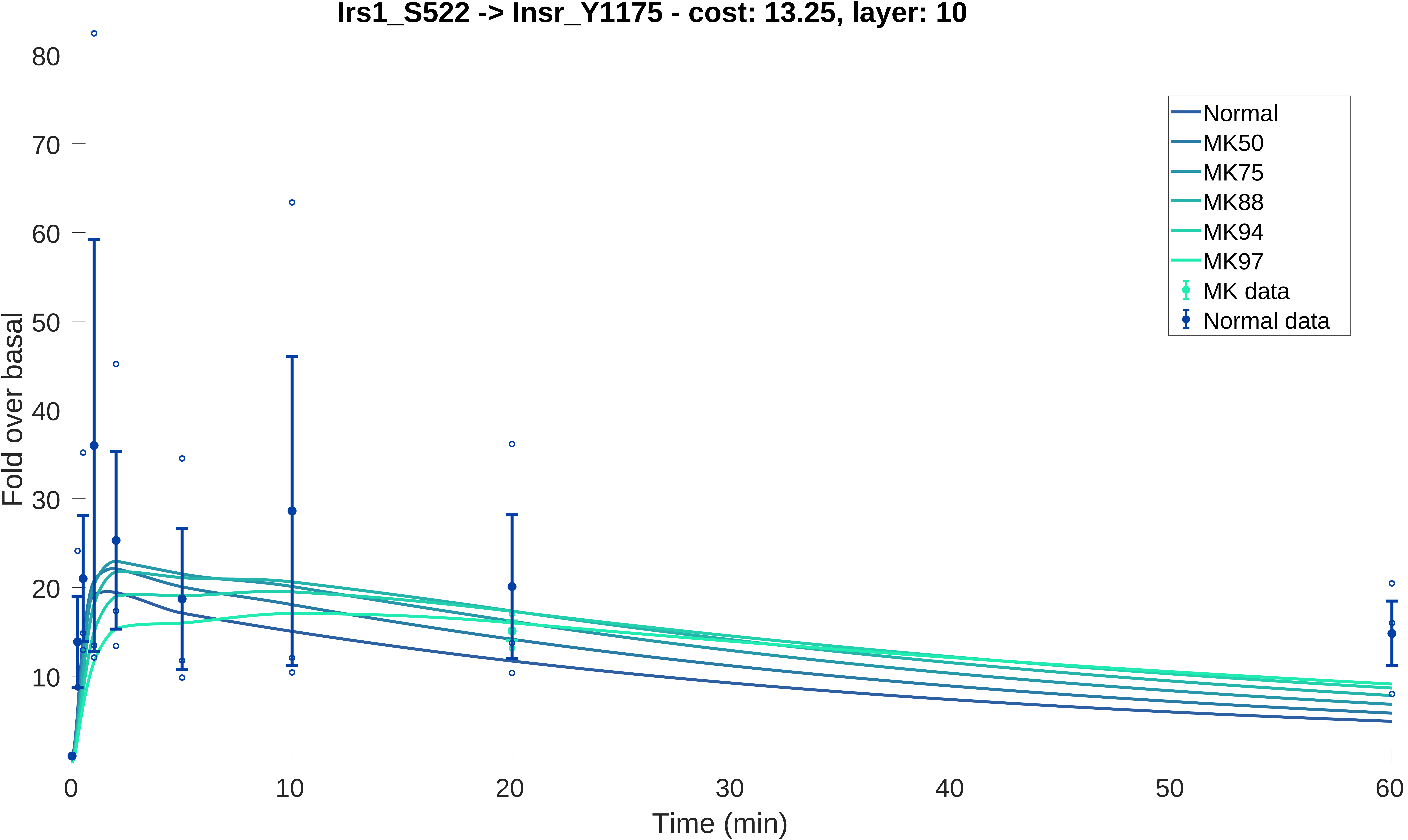

Irs1_S522 -> Insr_Y1175 - cost: 13.25, layer: 10
Fold over basal
Time (min)
0
10
20
30
40
50
60
70
80
Normal
MK50
MK75
MK88
MK94
MK97
MK data
Normal data

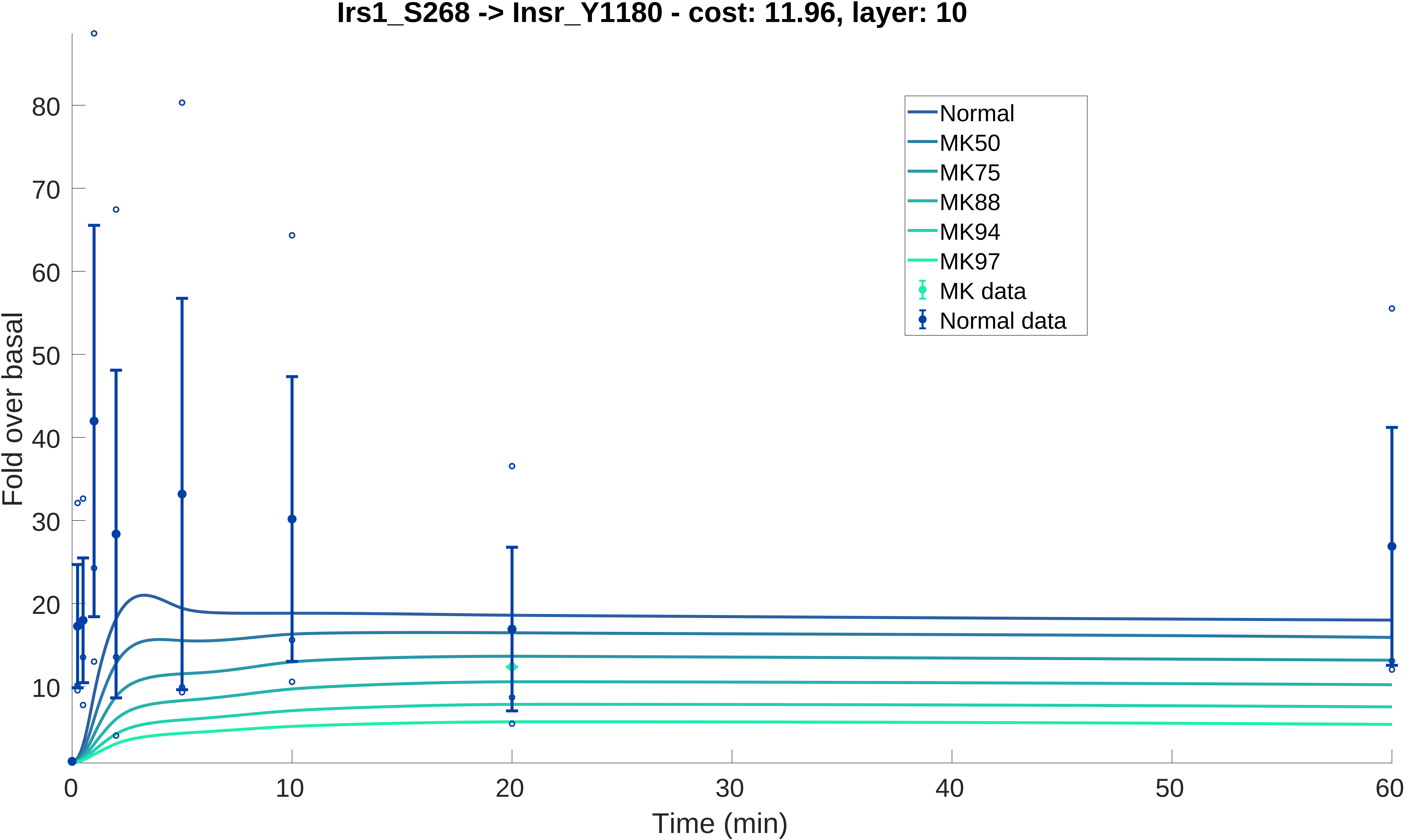

Irs1_S268 -> Insr_Y1180 - cost: 11.96, layer: 10
Fold over basal
Time (min)
80
70
60
50
40
30
20
10
0
10
20
30
40
50
60
Normal
MK50
MK75
MK88
MK94
MK97
MK data
Normal data

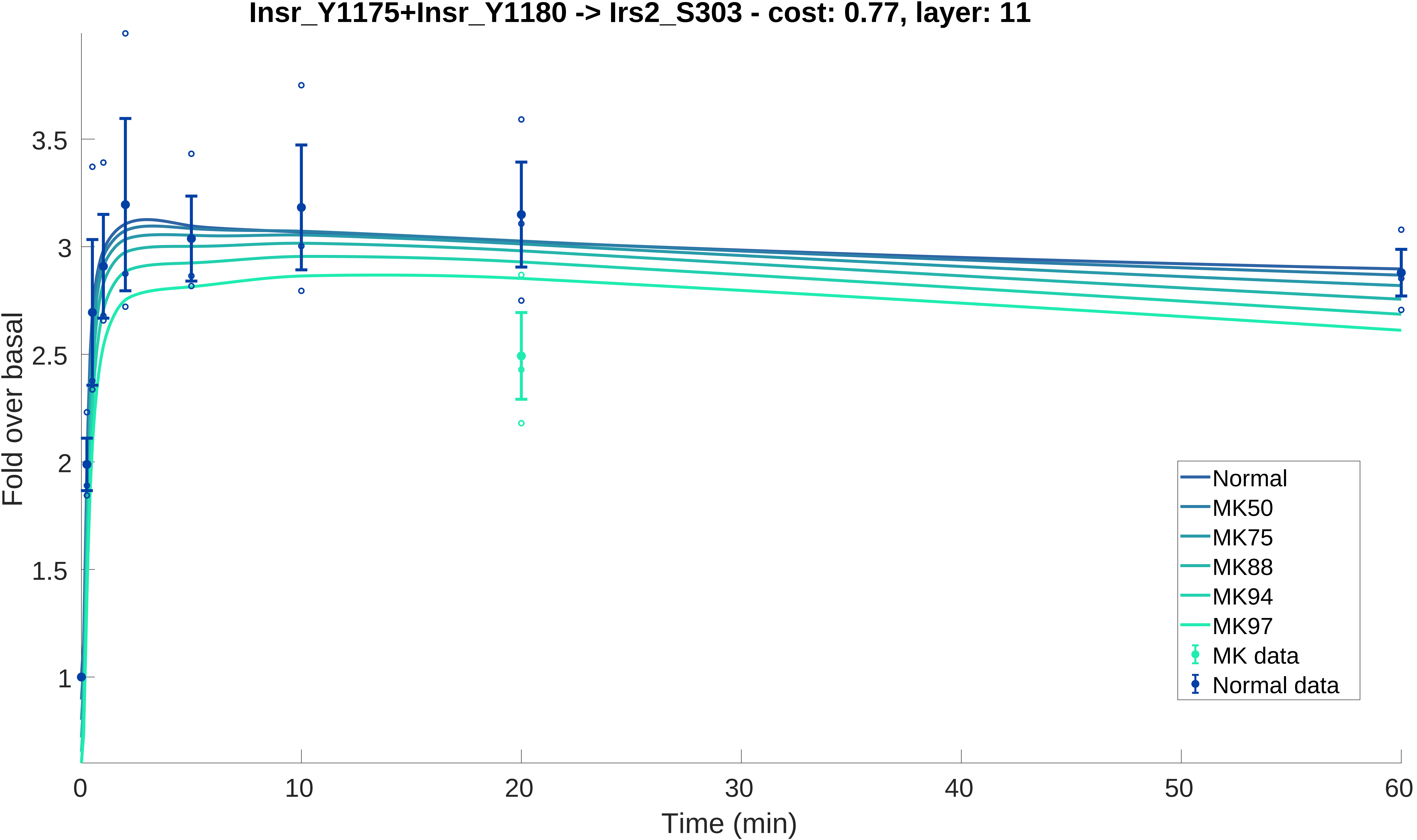
Insr_Y1175+Insr_Y1180 -> Irs2_S303 - cost: 0.77, layer: 11
Fold over basal
3.5
3
2.5
2
1.5
1
0
10
20
30
40
50
60
Time (min)
Normal
MK50
MK75
MK88
MK94
MK97
MK data
Normal data

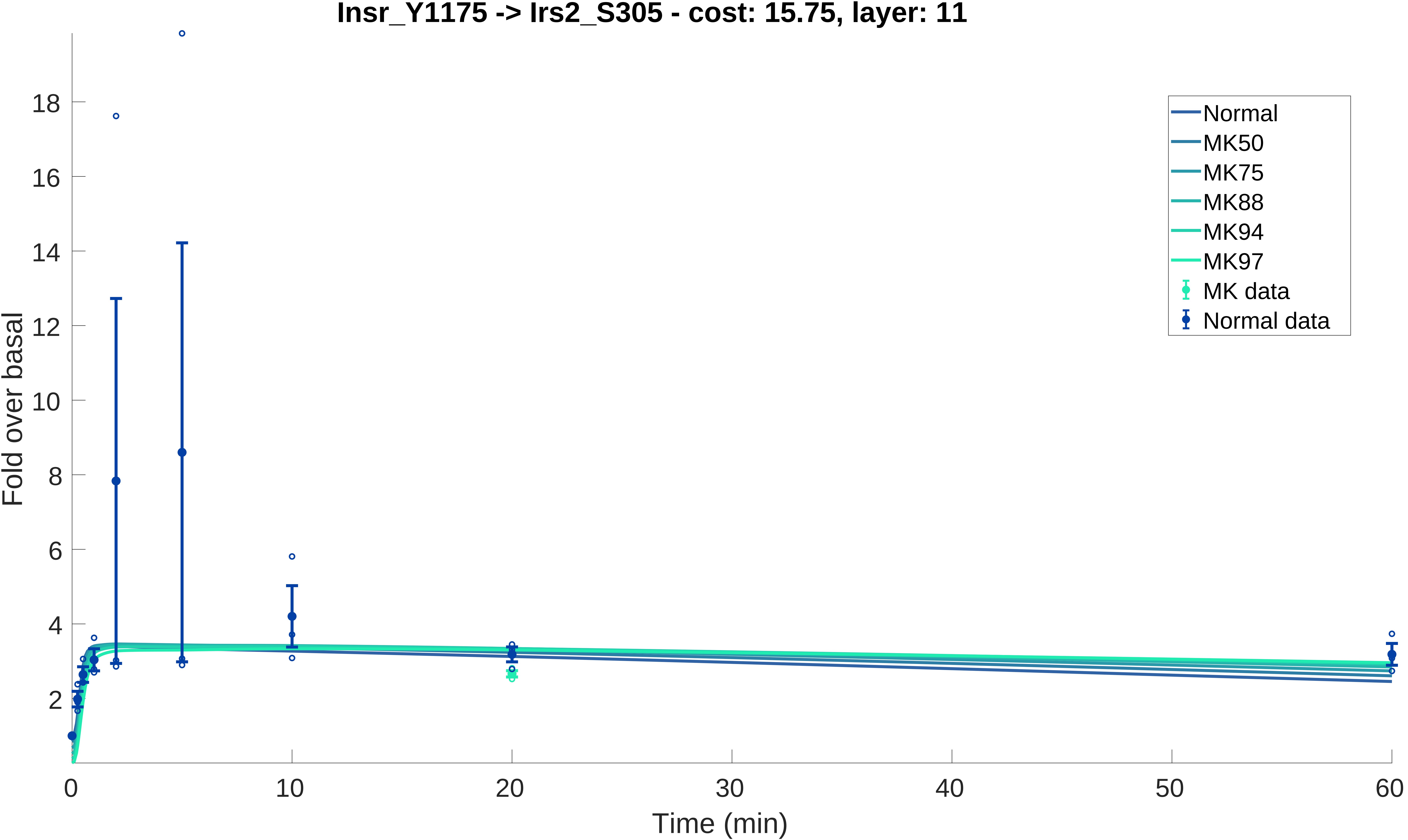

Insr_Y1175 -> Irs2_S305 - cost: 15.75, layer: 11
Fold over basal
Time (min)
0
10
20
30
40
50
60
2
4
6
8
10
12
14
16
18
Normal
MK50
MK75
MK88
MK94
MK97
MK data
Normal data

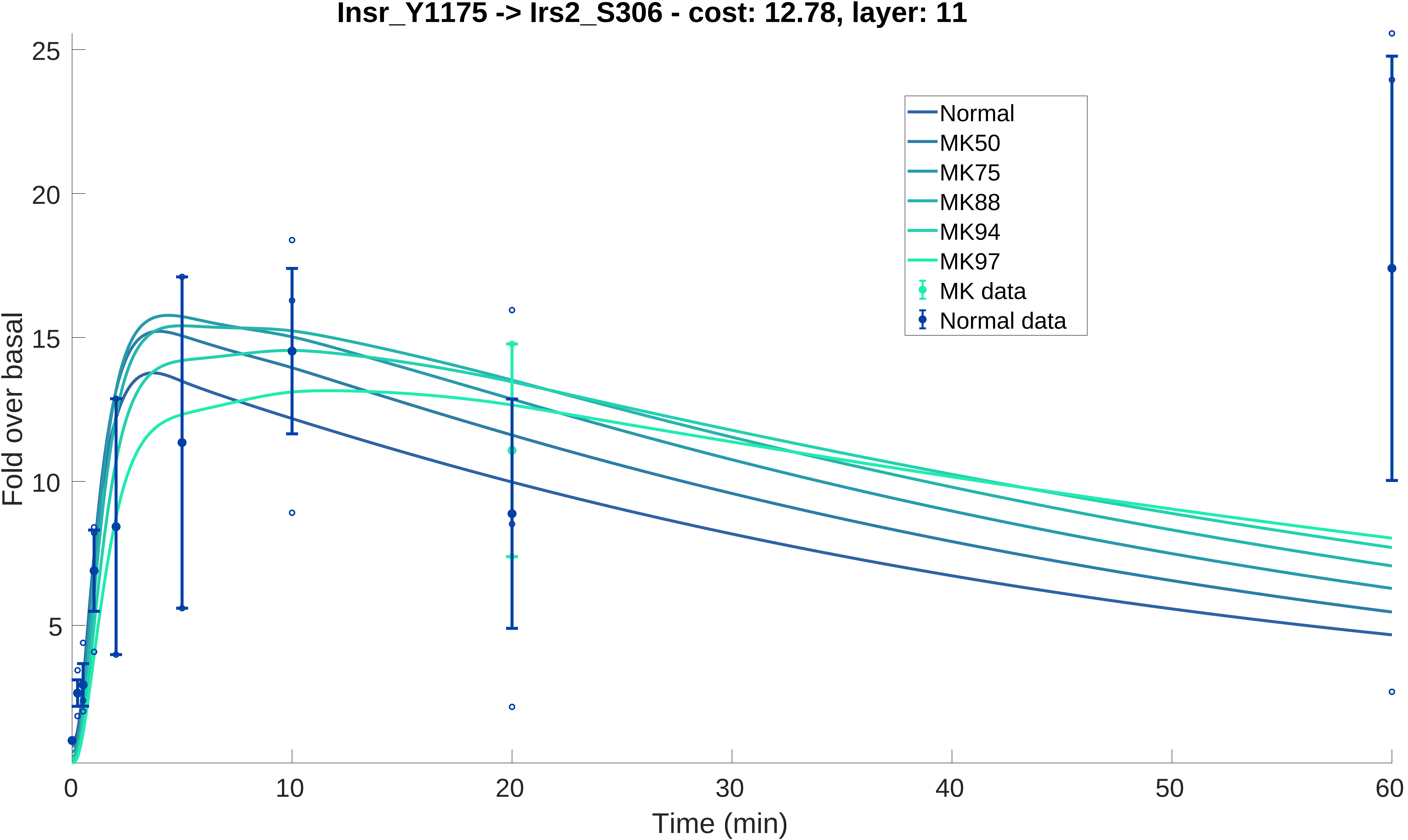
Insr_Y1175 -> Irs2_S306 - cost: 12.78, layer: 11
Fold over basal
Time (min)
Normal
MK50
MK75
MK88
MK94
MK97
MK data
Normal data
0
10
20
30
40
50
60
5
10
15
20
25

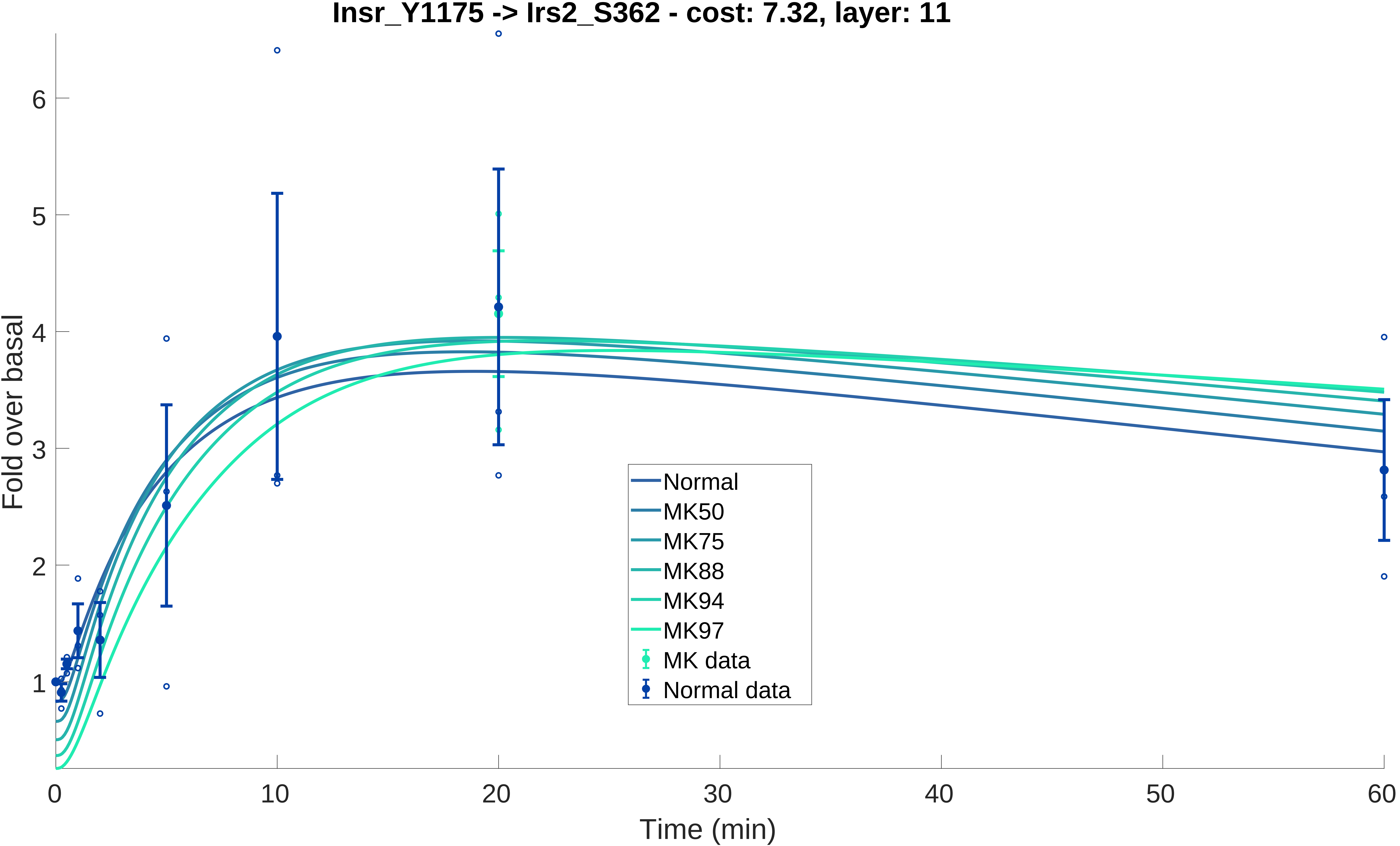

Insr_Y1175 -> Irs2_S362 - cost: 7.32, layer: 11
Fold over basal
Time (min)
0
10
20
30
40
50
60
1
2
3
4
5
6
Normal
MK50
MK75
MK88
MK94
MK97
MK data
Normal data

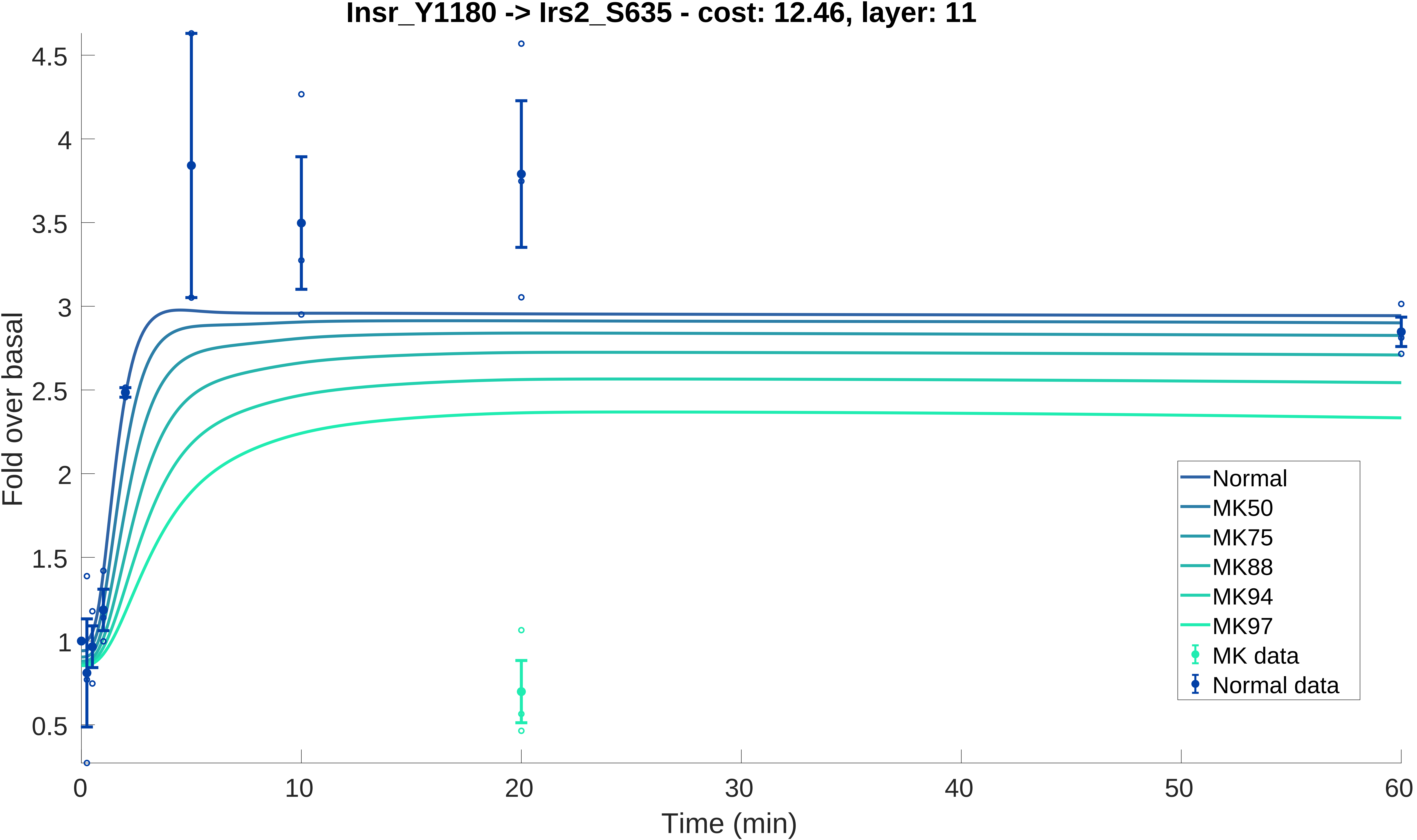

Insr_Y1180 -> Irs2_S635 - cost: 12.46, layer: 11
Fold over basal
Time (min)
0.5
1
1.5
2
2.5
3
3.5
4
4.5
0
10
20
30
40
50
60
Normal
MK50
MK75
MK88
MK94
MK97
MK data
Normal data

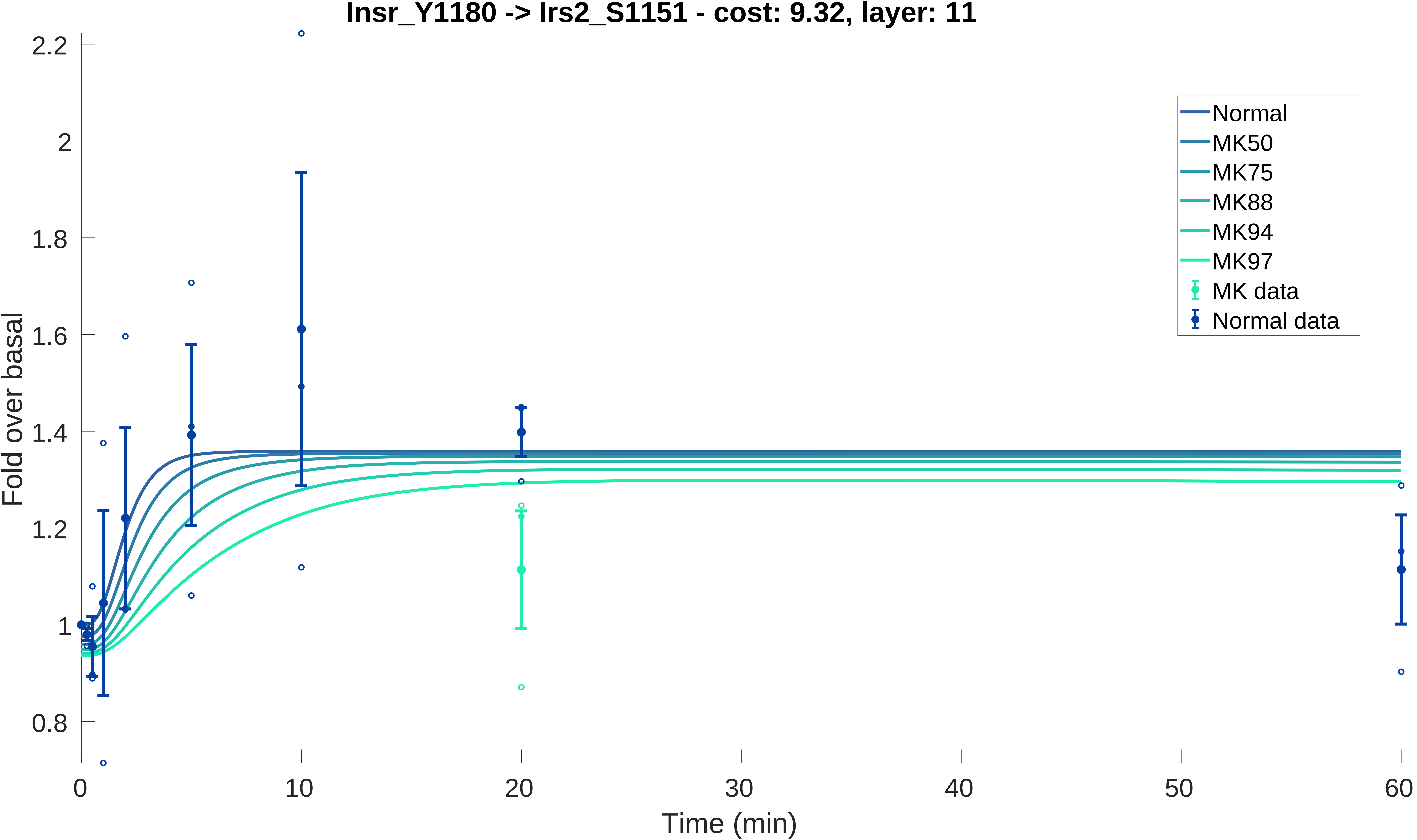

Insr_Y1180 -> Irs2_S1151 - cost: 9.32, layer: 11
Fold over basal
Time (min)
0.8
1
1.2
1.4
1.6
1.8
2
2.2
0
10
20
30
40
50
60
Normal
MK50
MK75
MK88
MK94
MK97
MK data
Normal data

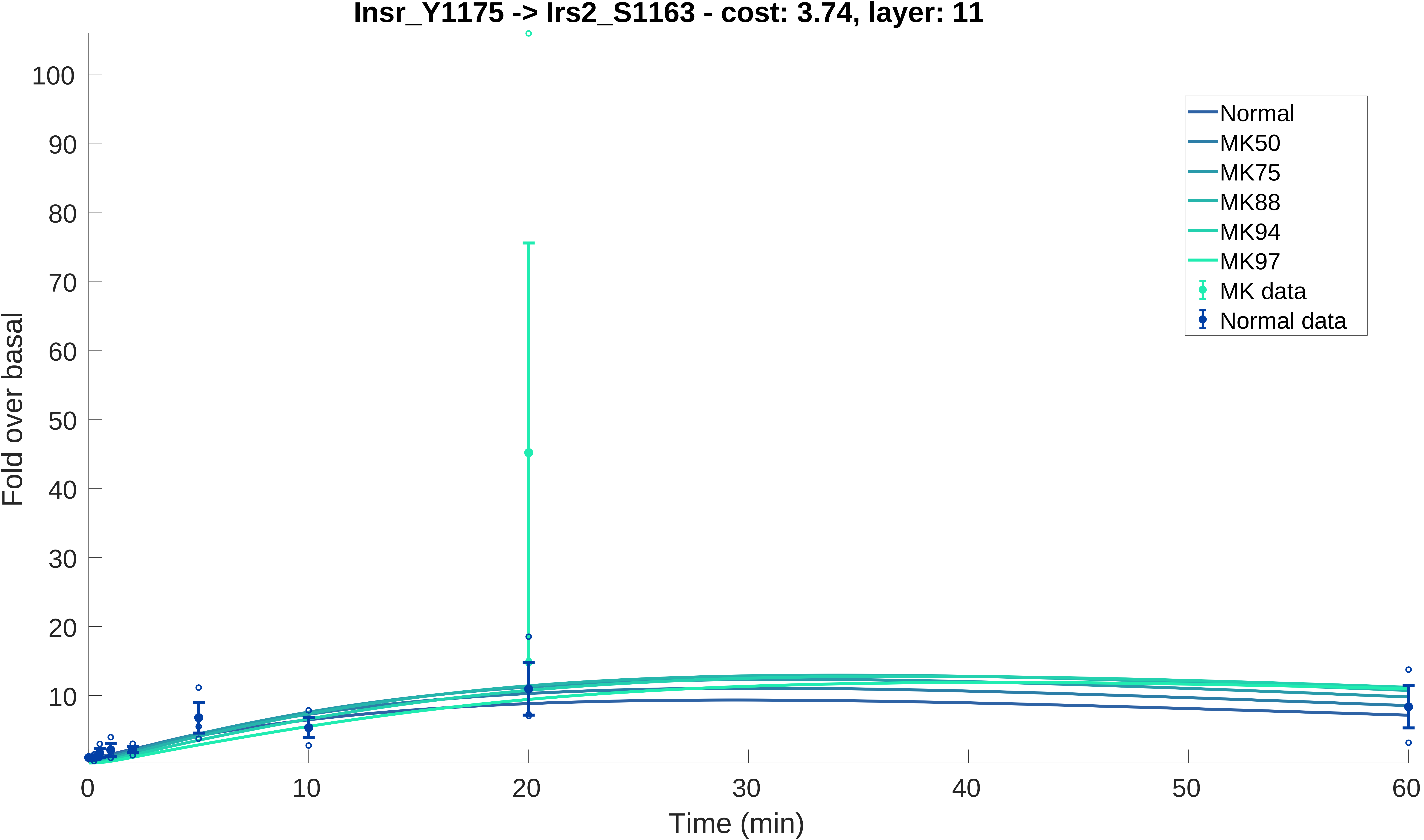
Insr_Y1175 -> Irs2_S1163 - cost: 3.74, layer: 11
Fold over basal
Time (min)
Normal
MK50
MK75
MK88
MK94
MK97
MK data
Normal data
0
10
20
30
40
50
60
70
80
90
100

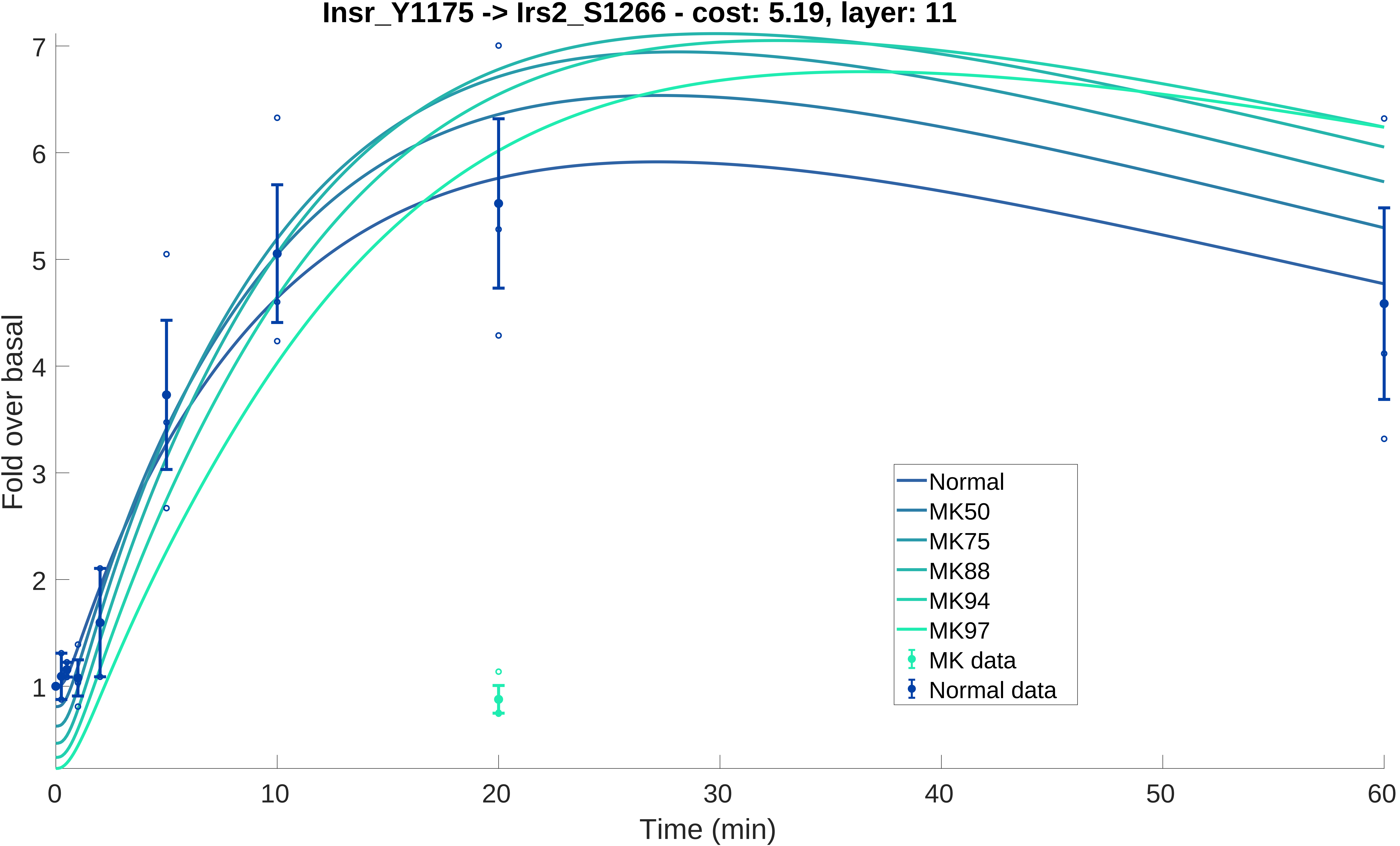

Insr_Y1175 -> Irs2_S1266 - cost: 5.19, layer: 11
Fold over basal
Time (min)
0
10
20
30
40
50
60
1
2
3
4
5
6
7
Normal
MK50
MK75
MK88
MK94
MK97
MK data
Normal data

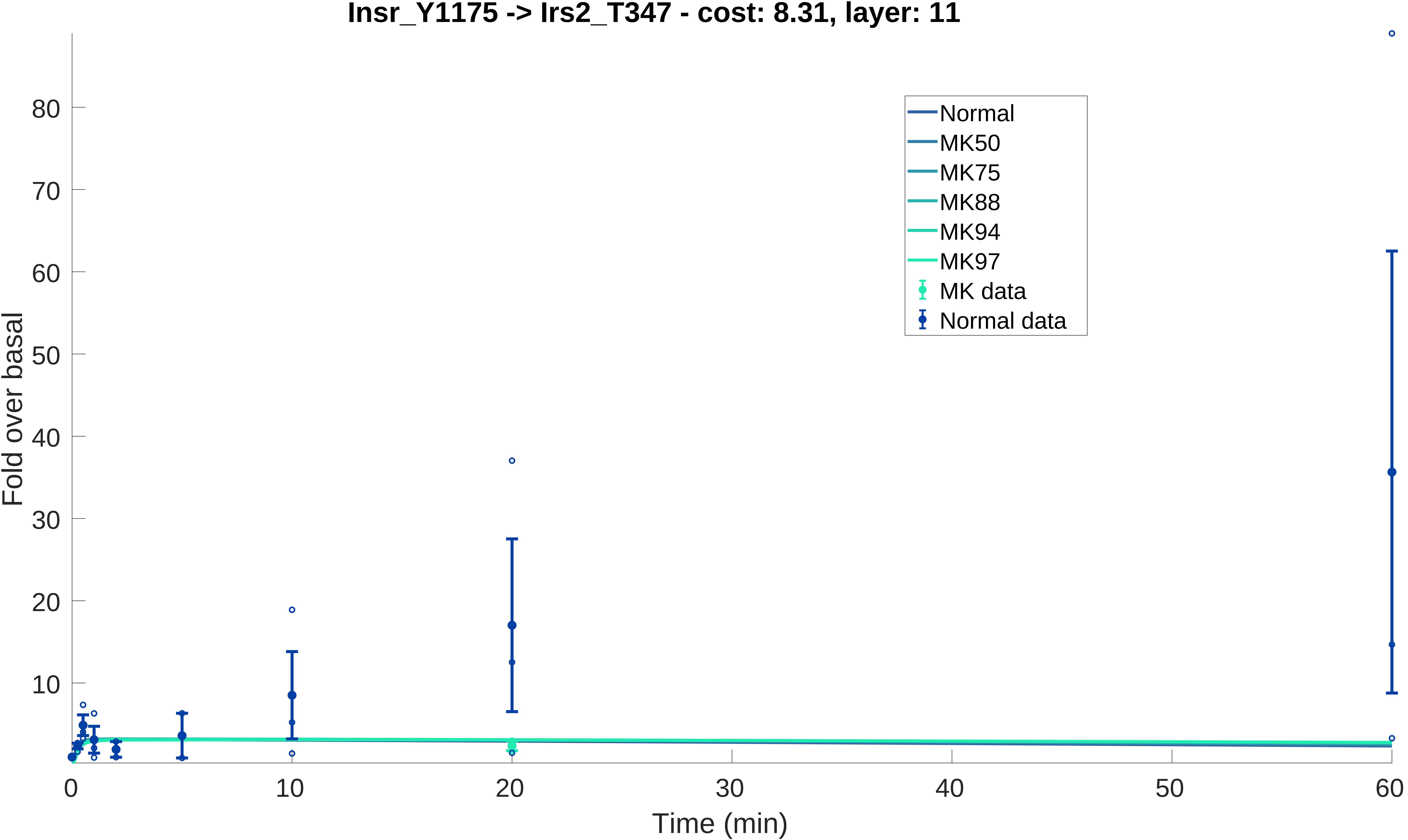

Insr_Y1175 -> Irs2_T347 - cost: 8.31, layer: 11
Fold over basal
Time (min)
0
10
20
30
40
50
60
80
70
60
50
40
30
20
10
Normal
MK50
MK75
MK88
MK94
MK97
MK data
Normal data

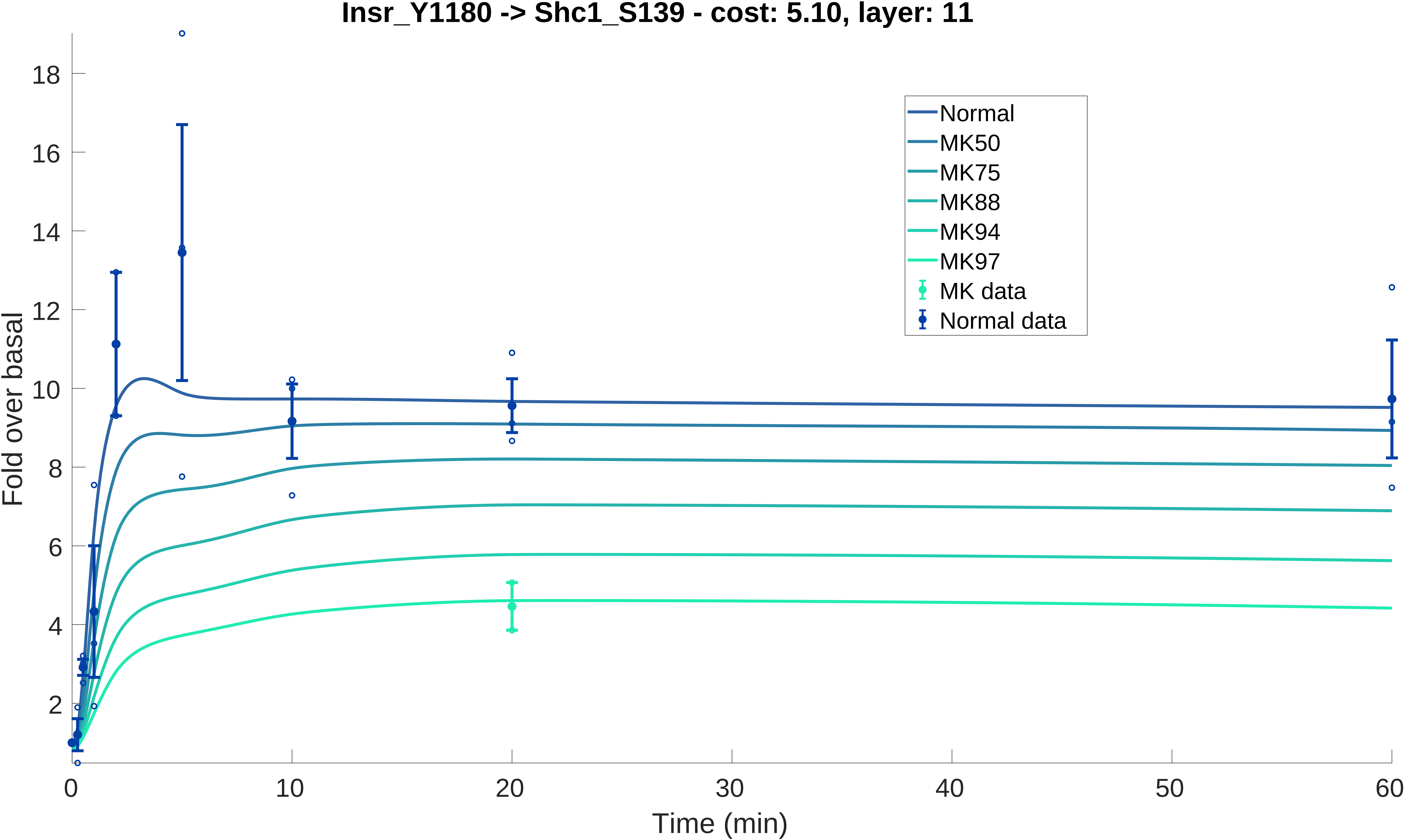

Insr_Y1180 -> Shc1_S139 - cost: 5.10, layer: 11
Fold over basal
Time (min)
Normal
MK50
MK75
MK88
MK94
MK97
MK data
Normal data
0
10
20
30
40
50
60
2
4
6
8
10
12
14
16
18

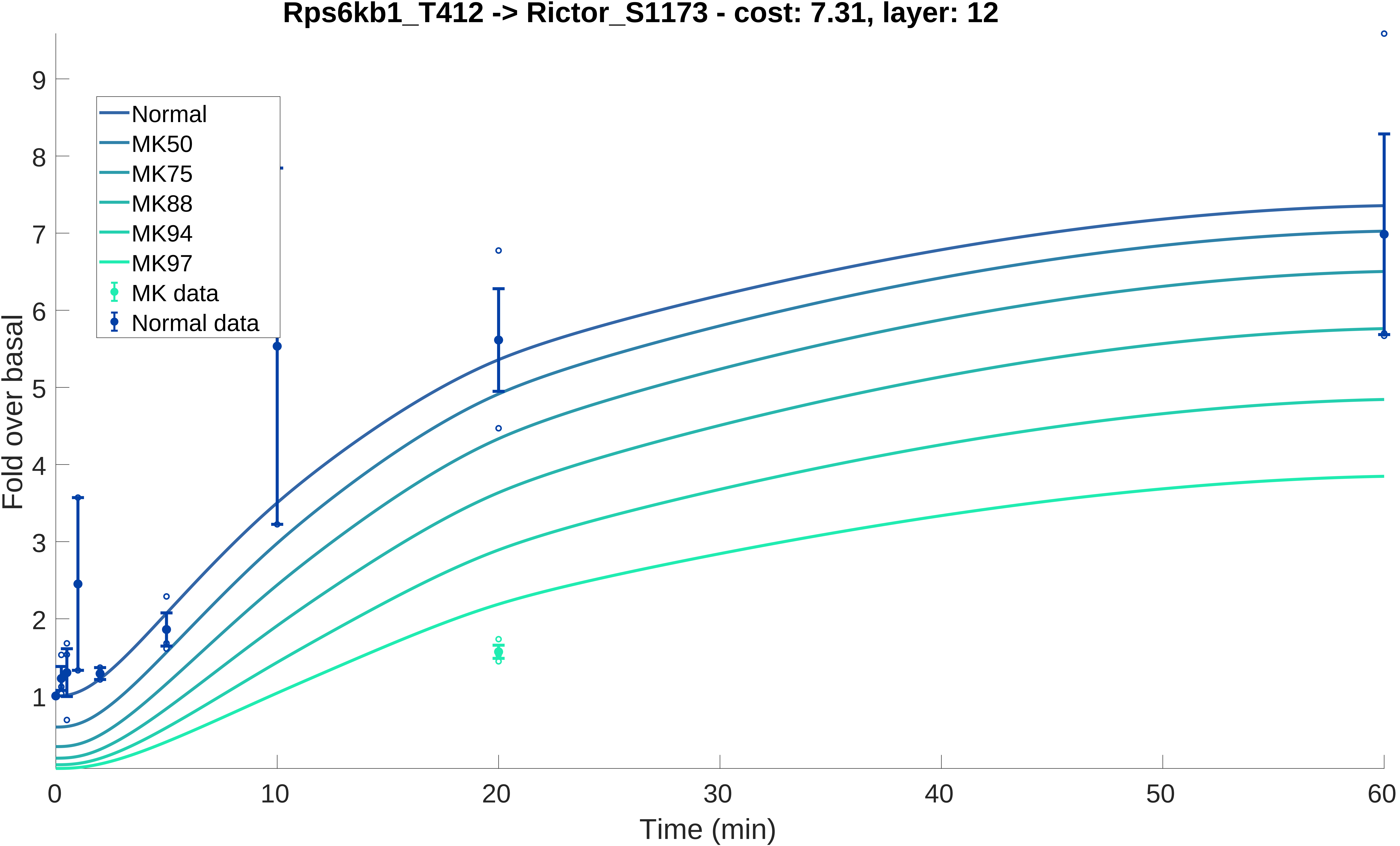
Rps6kb1_T412 -> Rictor_S1173 - cost: 7.31, layer: 12
Fold over basal
Time (min)
Normal
MK50
MK75
MK88
MK94
MK97
MK data
Normal data
0
10
20
30
40
50
60
1
2
3
4
5
6
7
8
9

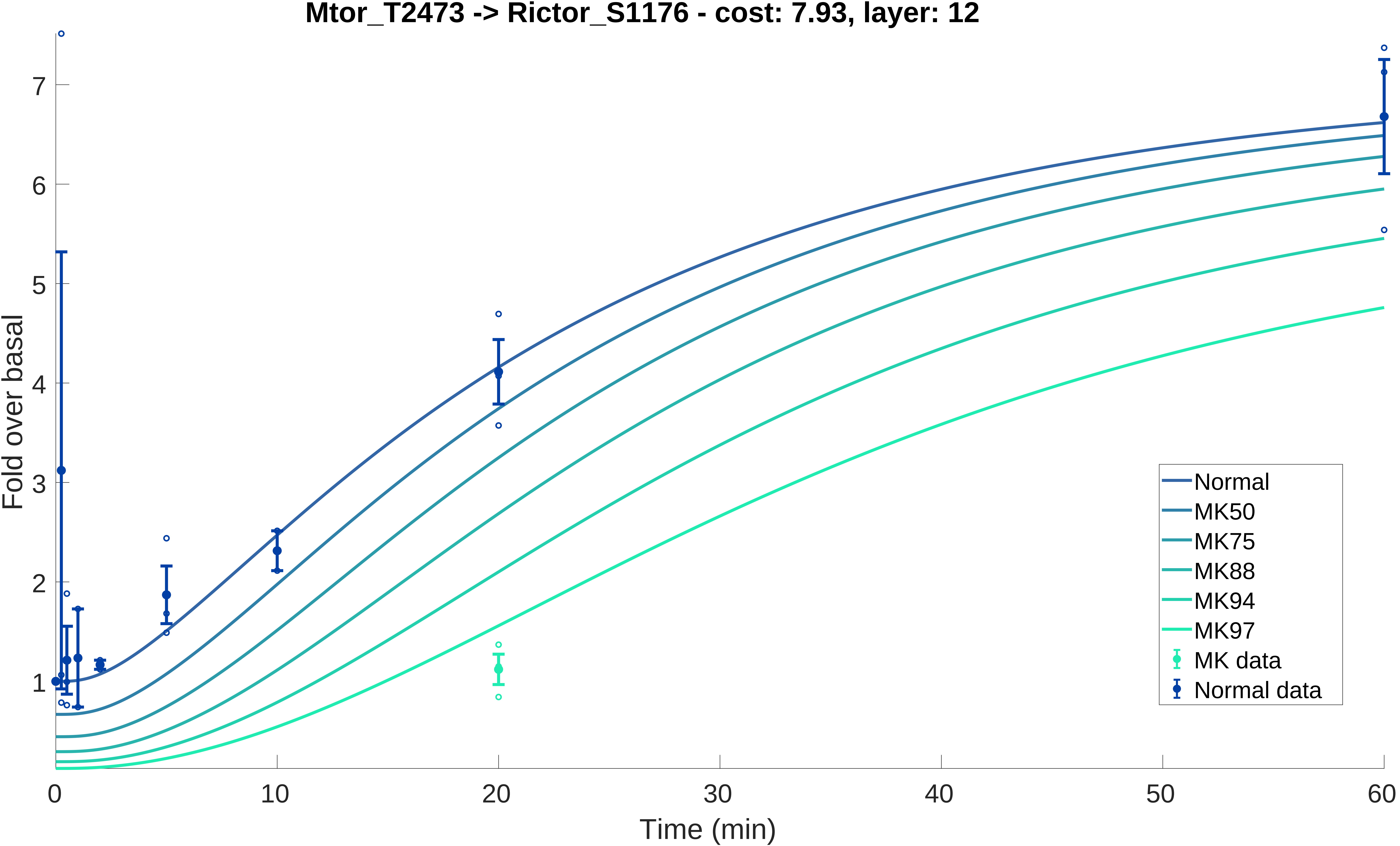

Mtor_T2473 -> Rictor_S1176 - cost: 7.93, layer: 12
Fold over basal
Time (min)
0
10
20
30
40
50
60
1
2
3
4
5
6
7
Normal
MK50
MK75
MK88
MK94
MK97
MK data
Normal data

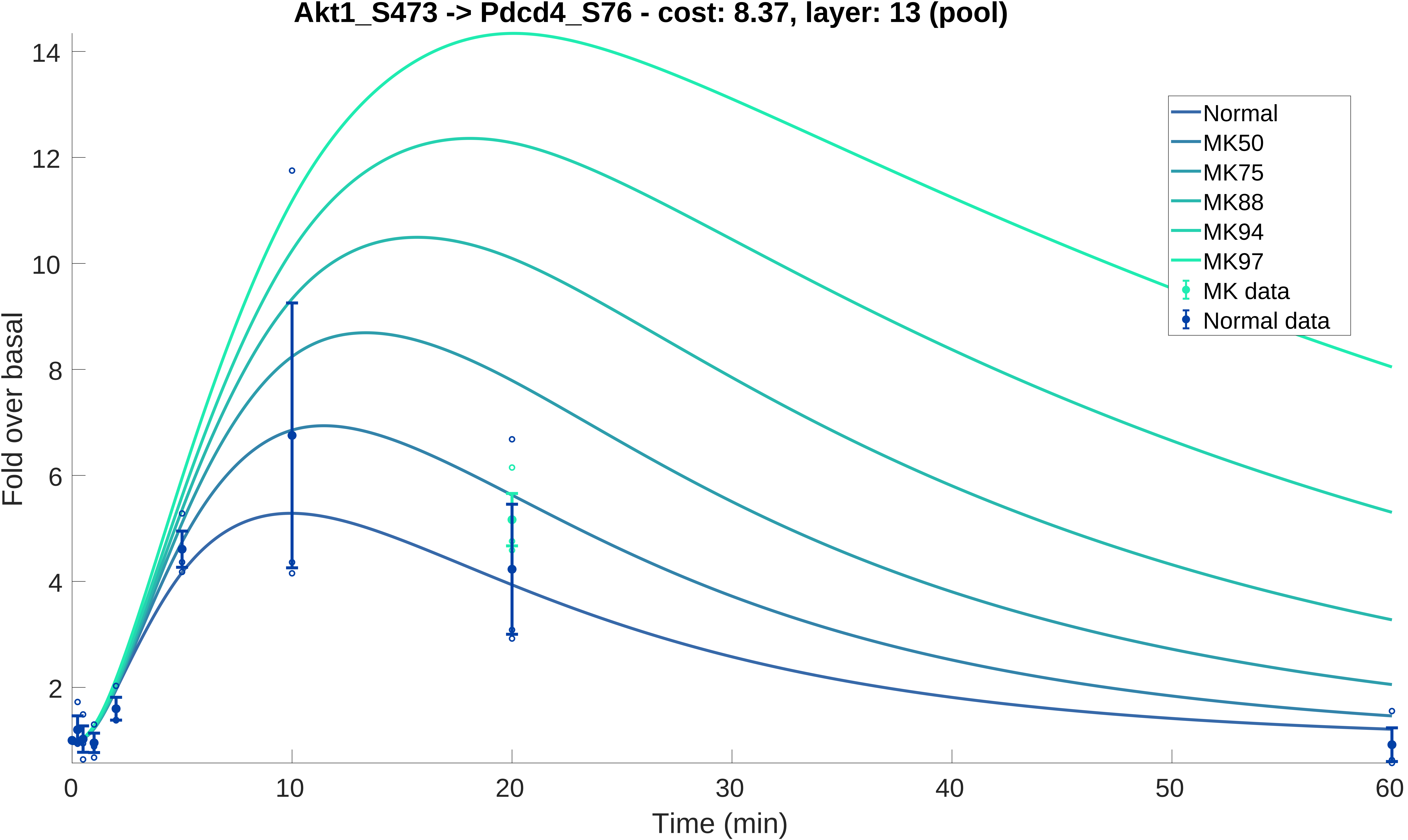
Akt1_S473 -> Pdcd4_S76 - cost: 8.37, layer: 13 (pool)
Fold over basal
Time (min)
Normal
MK50
MK75
MK88
MK94
MK97
MK data
Normal data
0
10
20
30
40
50
60
2
4
6
8
10
12
14

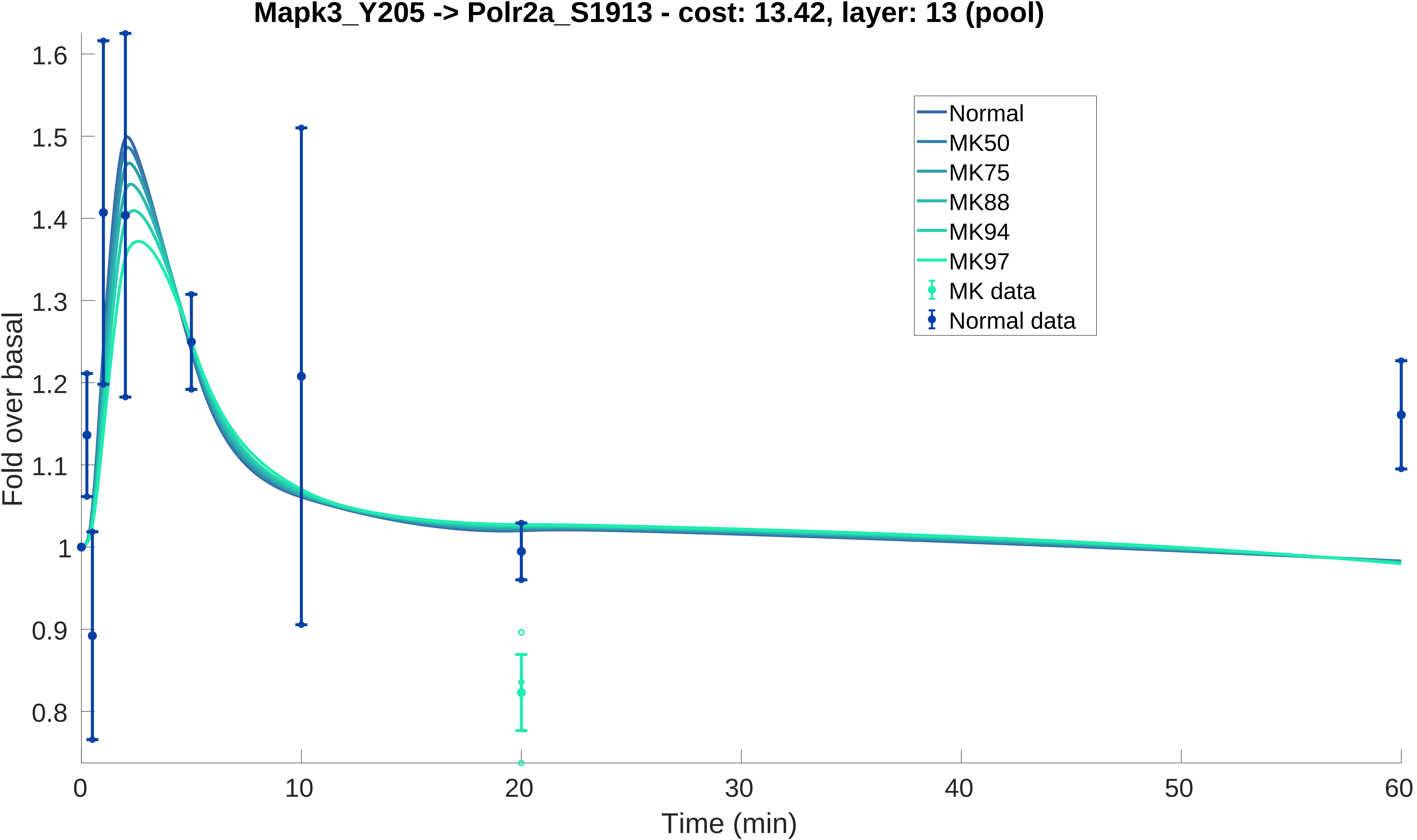
Mapk3_Y205 -> Polr2a_S1913 - cost: 13.42, layer: 13 (pool)
Fold over basal
Time (min)
Normal
MK50
MK75
MK88
MK94
MK97
MK data
Normal data
1.6
1.5
1.4
1.3
1.2
1.1
1
0.9
0.8
0
10
20
30
40
50
60

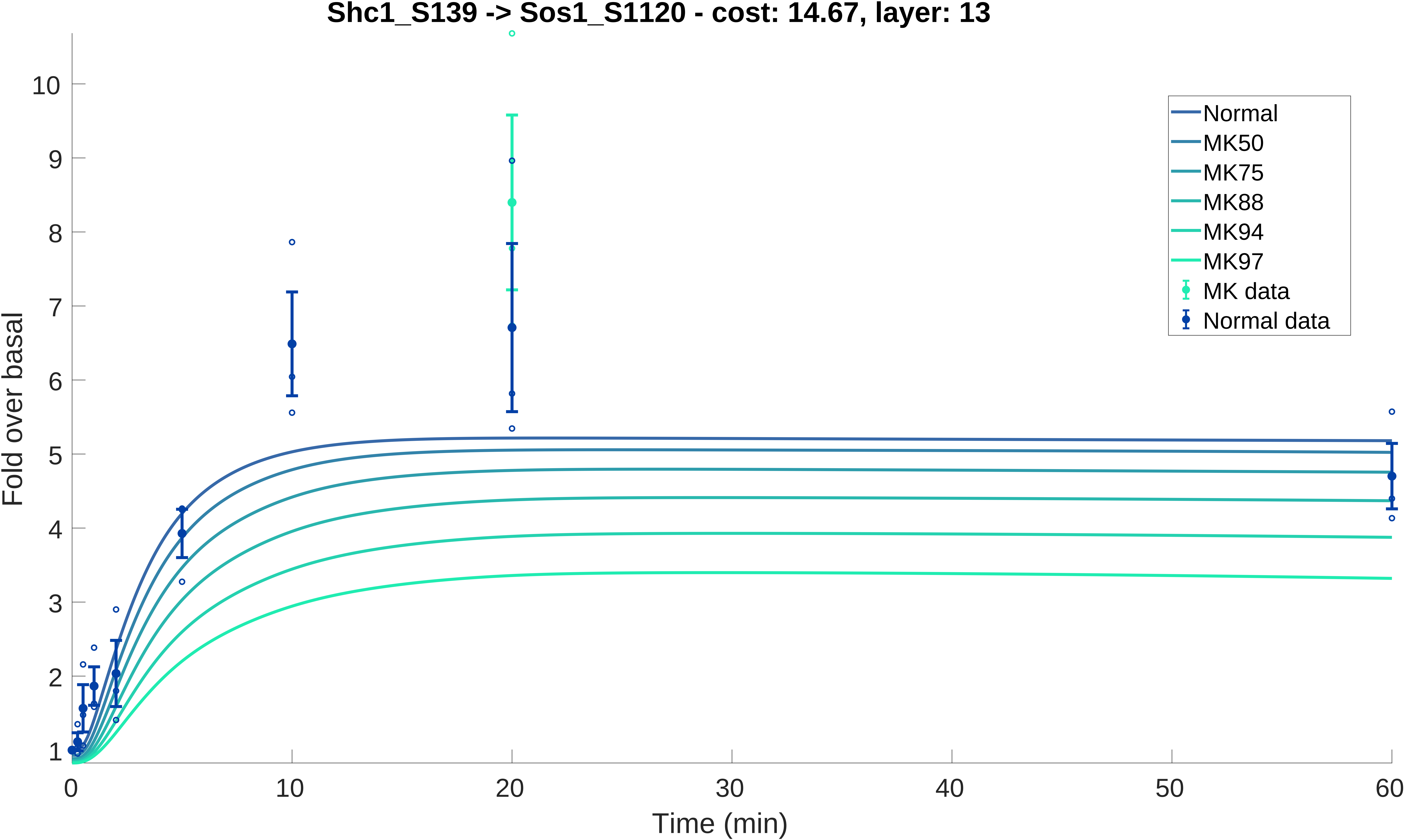

Shc1_S139 -> Sos1_S1120 - cost: 14.67, layer: 13
Normal
MK50
MK75
MK88
MK94
MK97
MK data
Normal data
Fold over basal
Time (min)
1
2
3
4
5
6
7
8
9
10
0
10
20
30
40
50
60

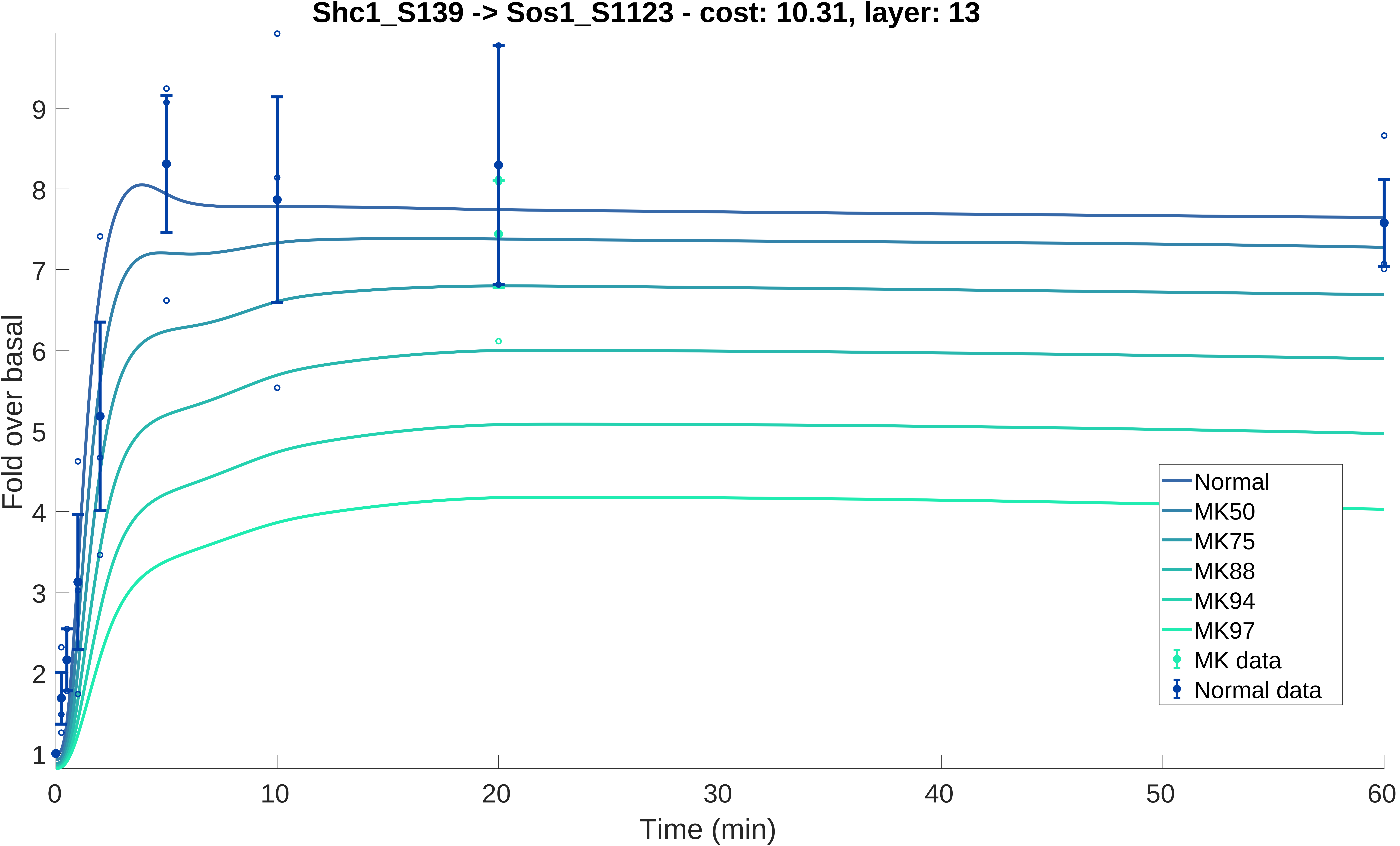

Shc1_S139 -> Sos1_S1123 - cost: 10.31, layer: 13
Fold over basal
Time (min)
1
2
3
4
5
6
7
8
9
0
10
20
30
40
50
60
Normal
MK50
MK75
MK88
MK94
MK97
MK data
Normal data

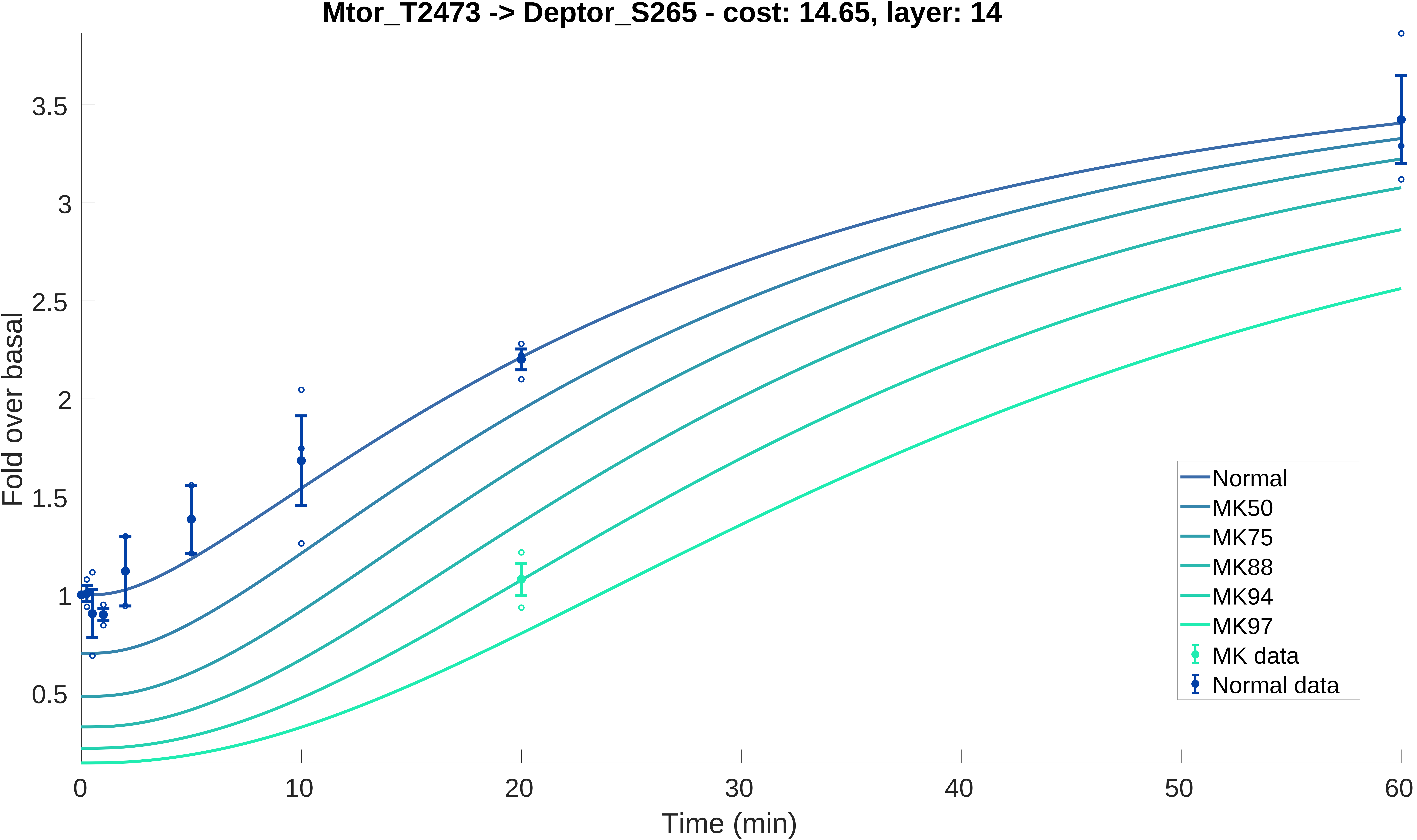

Mtor_T2473 -> Deptor_S265 - cost: 14.65, layer: 14
Fold over basal
3.5
3
2.5
2
1.5
1
0.5
0
10
20
30
40
50
60
Time (min)
Normal
MK50
MK75
MK88
MK94
MK97
MK data
Normal data

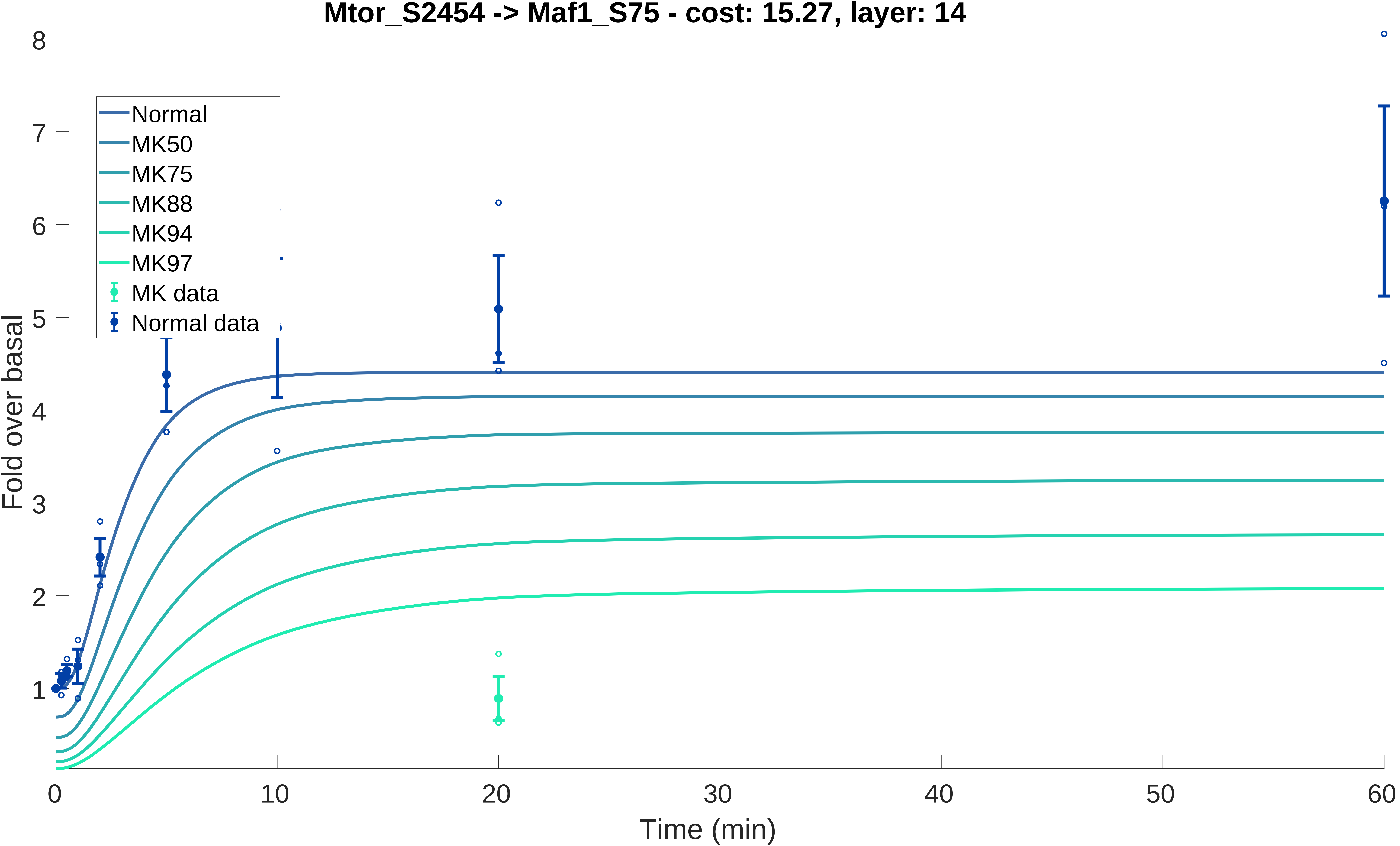

Mtor_S2454 -> Maf1_S75 - cost: 15.27, layer: 14
Normal
MK50
MK75
MK88
MK94
MK97
MK data
Normal data
Fold over basal
Time (min)
0
10
20
30
40
50
60
1
2
3
4
5
6
7
8

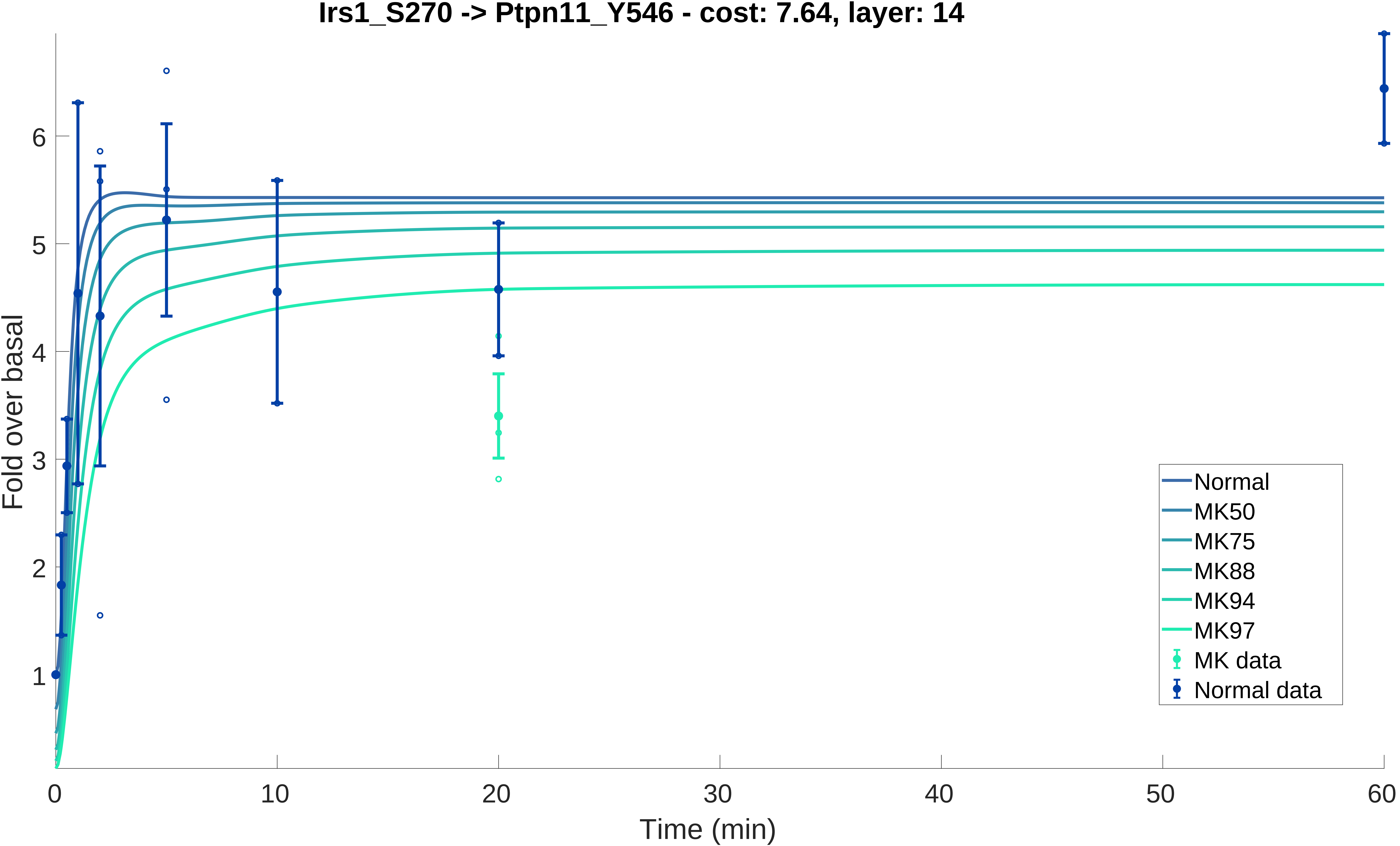
Irs1_S270 -> Ptpn11_Y546 - cost: 7.64, layer: 14
Fold over basal
Time (min)
0
10
20
30
40
50
60
1
2
3
4
5
6
Normal
MK50
MK75
MK88
MK94
MK97
MK data
Normal data

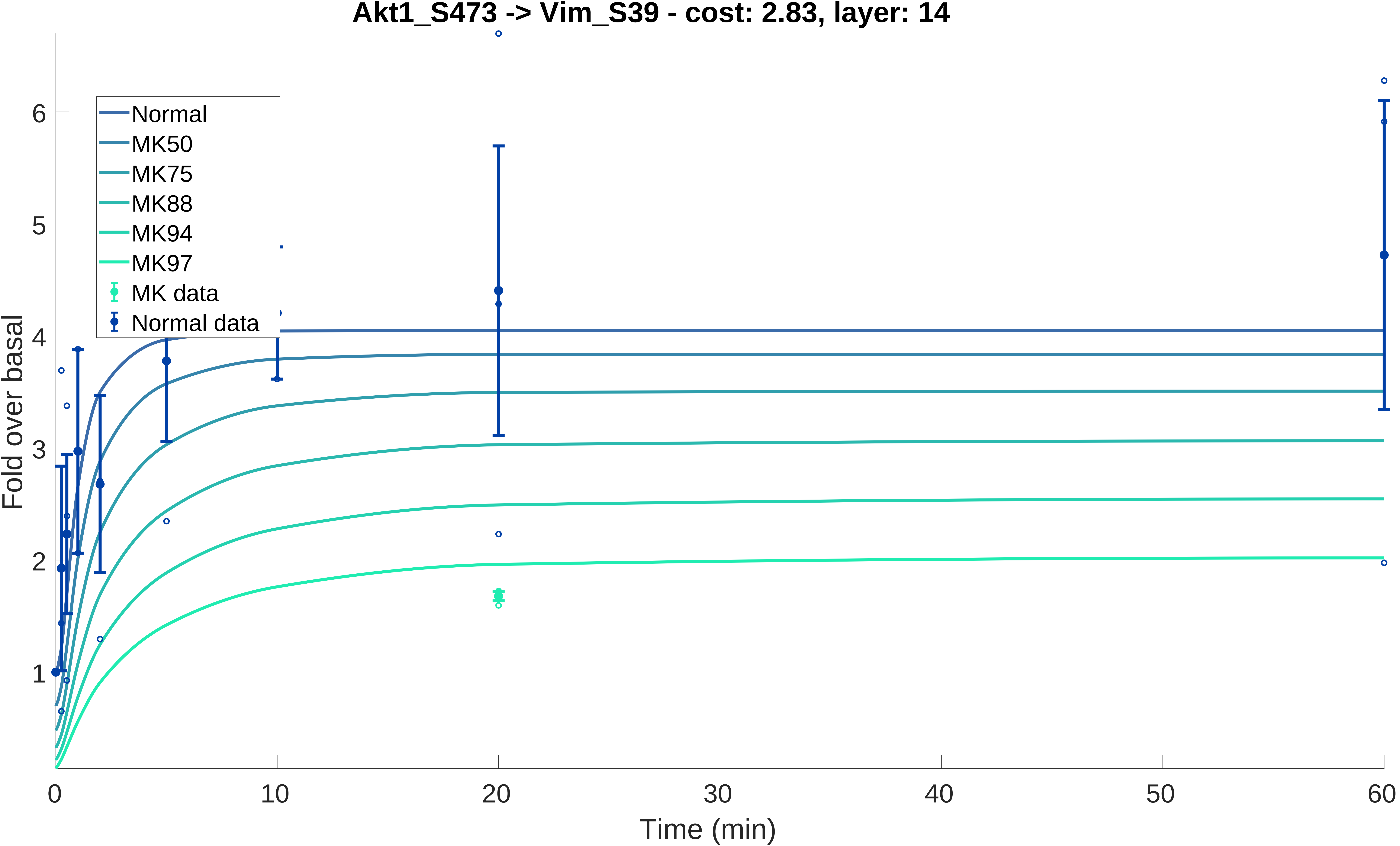

Akt1_S473 -> Vim_S39 - cost: 2.83, layer: 14
Fold over basal
Time (min)
Normal
MK50
MK75
MK88
MK94
MK97
MK data
Normal data
1
2
3
4
5
6
0
10
20
30
40
50
60

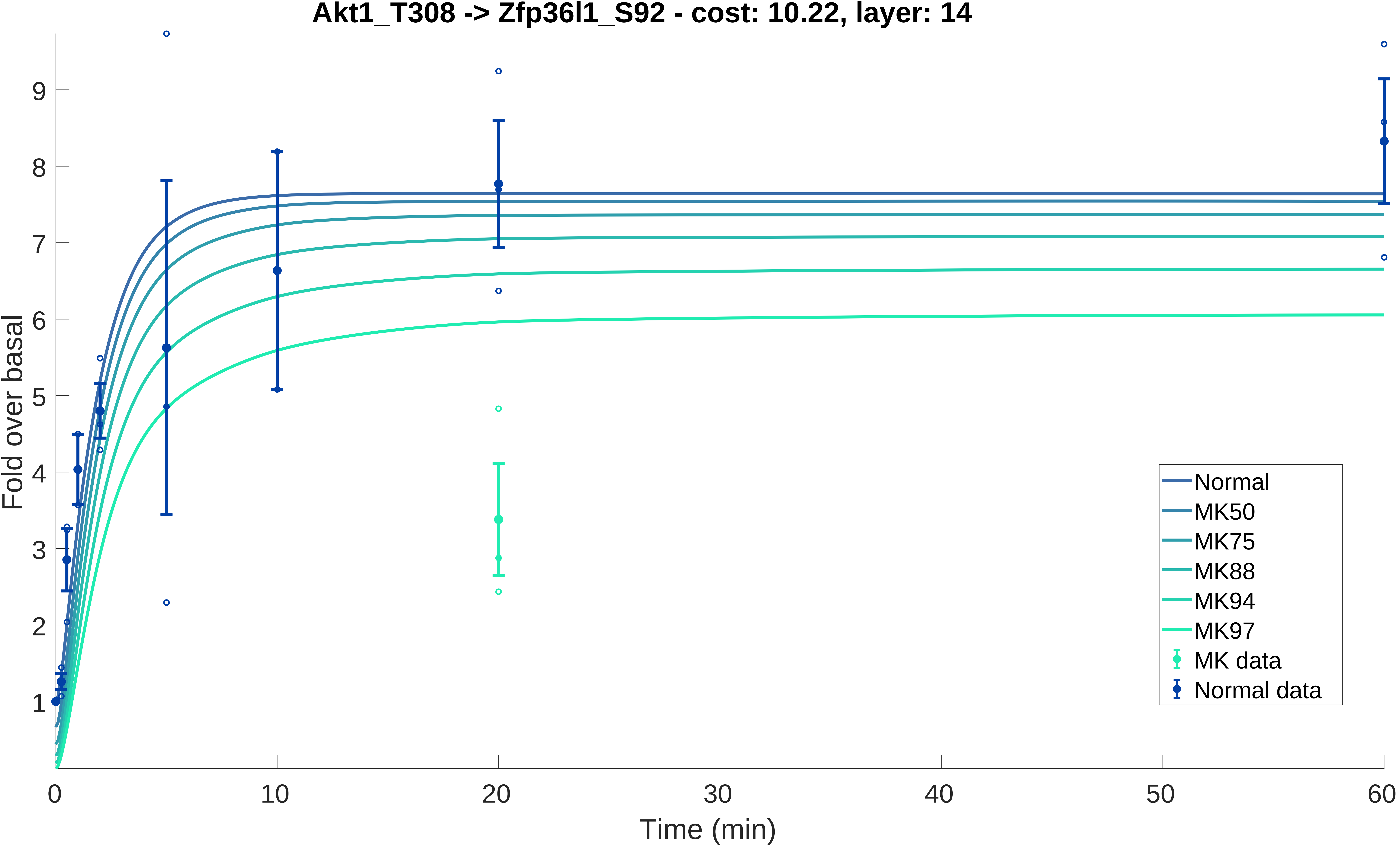

Akt1_T308 -> Zfp36l1_S92 - cost: 10.22, layer: 14
Fold over basal
Time (min)
0
10
20
30
40
50
60
1
2
3
4
5
6
7
8
9
Normal
MK50
MK75
MK88
MK94
MK97
MK data
Normal data

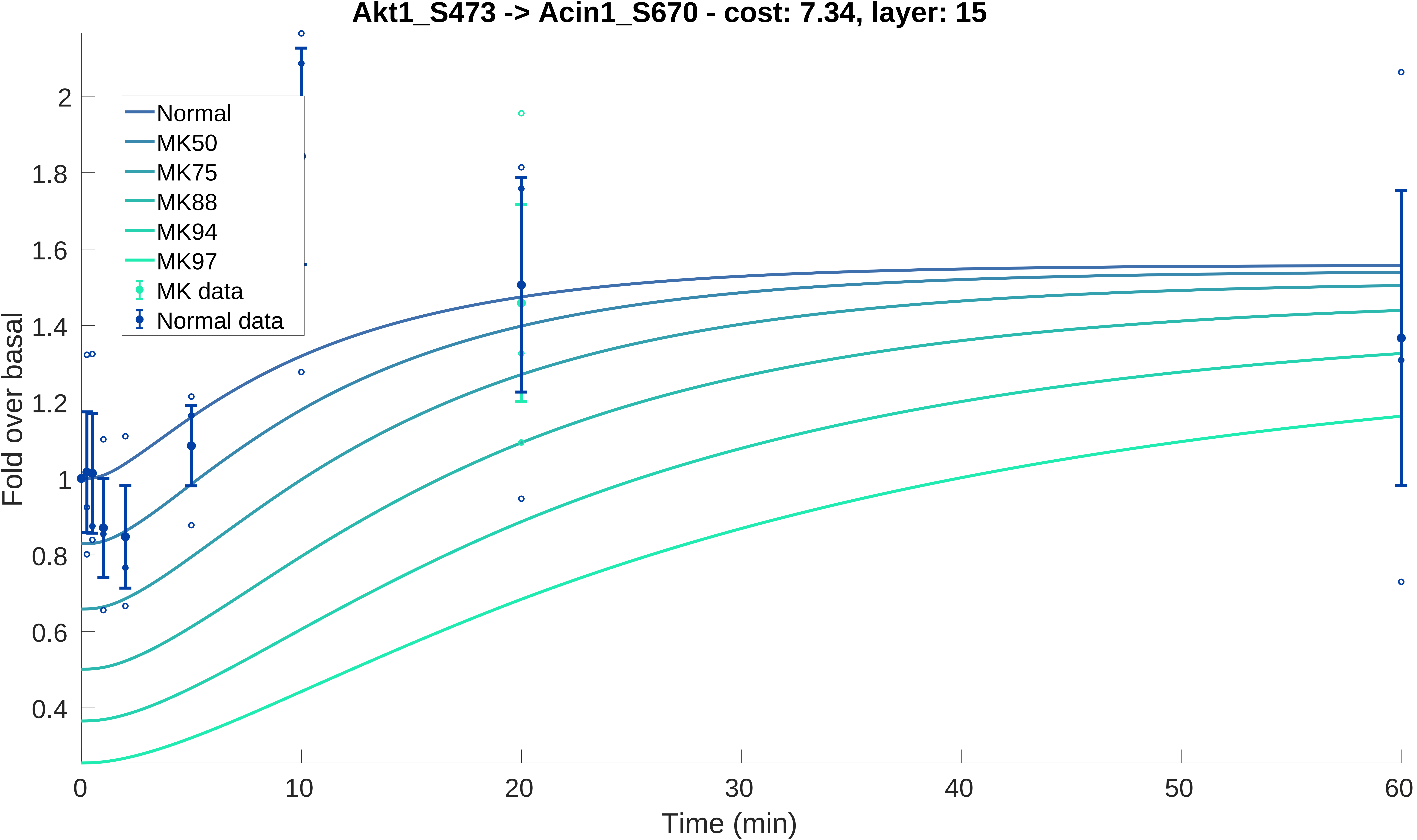

Akt1_S473 -> Acin1_S670 - cost: 7.34, layer: 15
Normal
MK50
MK75
MK88
MK94
MK97
MK data
Normal data
Fold over basal
Time (min)
0.4
0.6
0.8
1
1.2
1.4
1.6
1.8
2
0
10
20
30
40
50
60

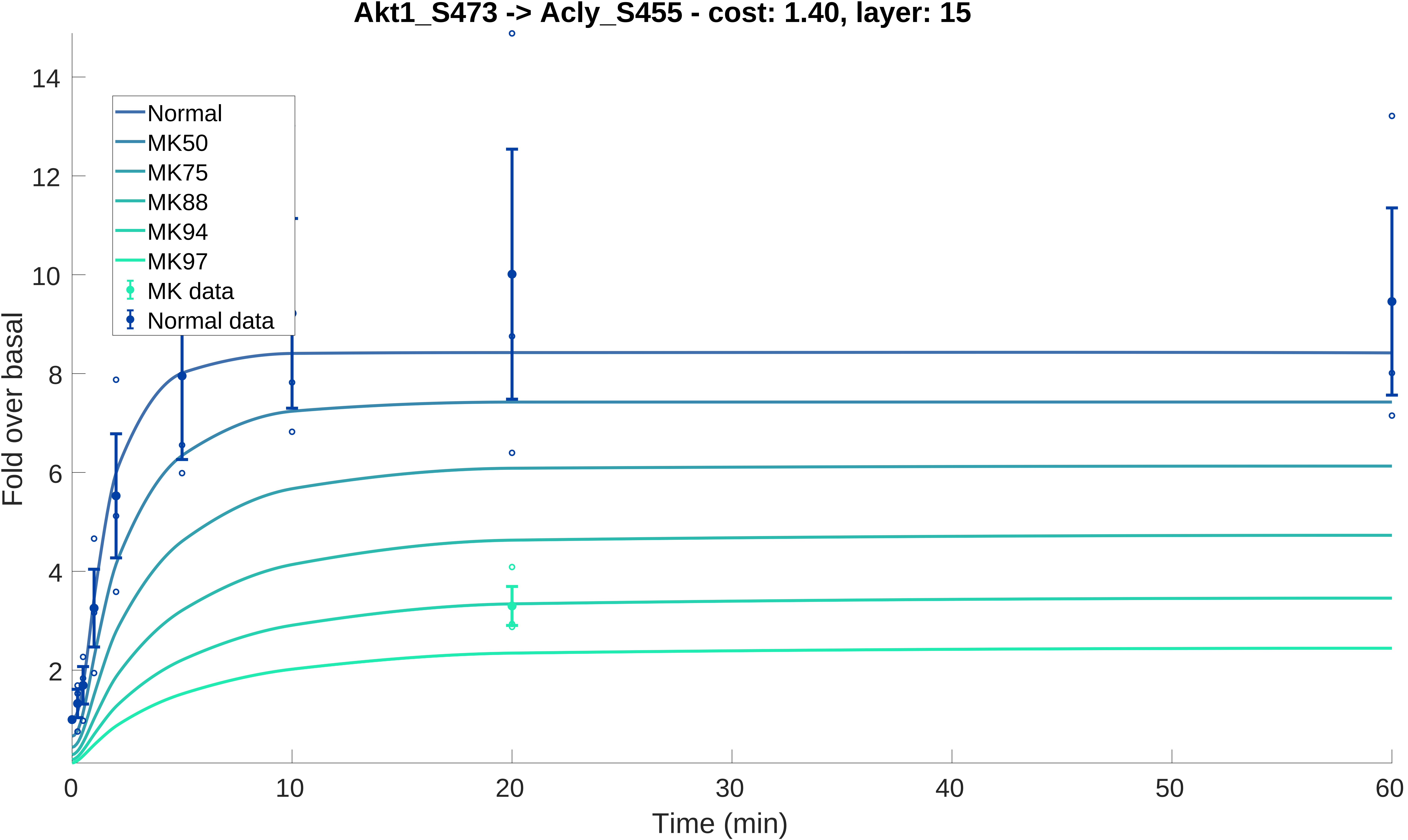

Akt1_S473 -> Acly_S455 - cost: 1.40, layer: 15
Normal
MK50
MK75
MK88
MK94
MK97
MK data
Normal data
Fold over basal
Time (min)
0
10
20
30
40
50
60
2
4
6
8
10
12
14

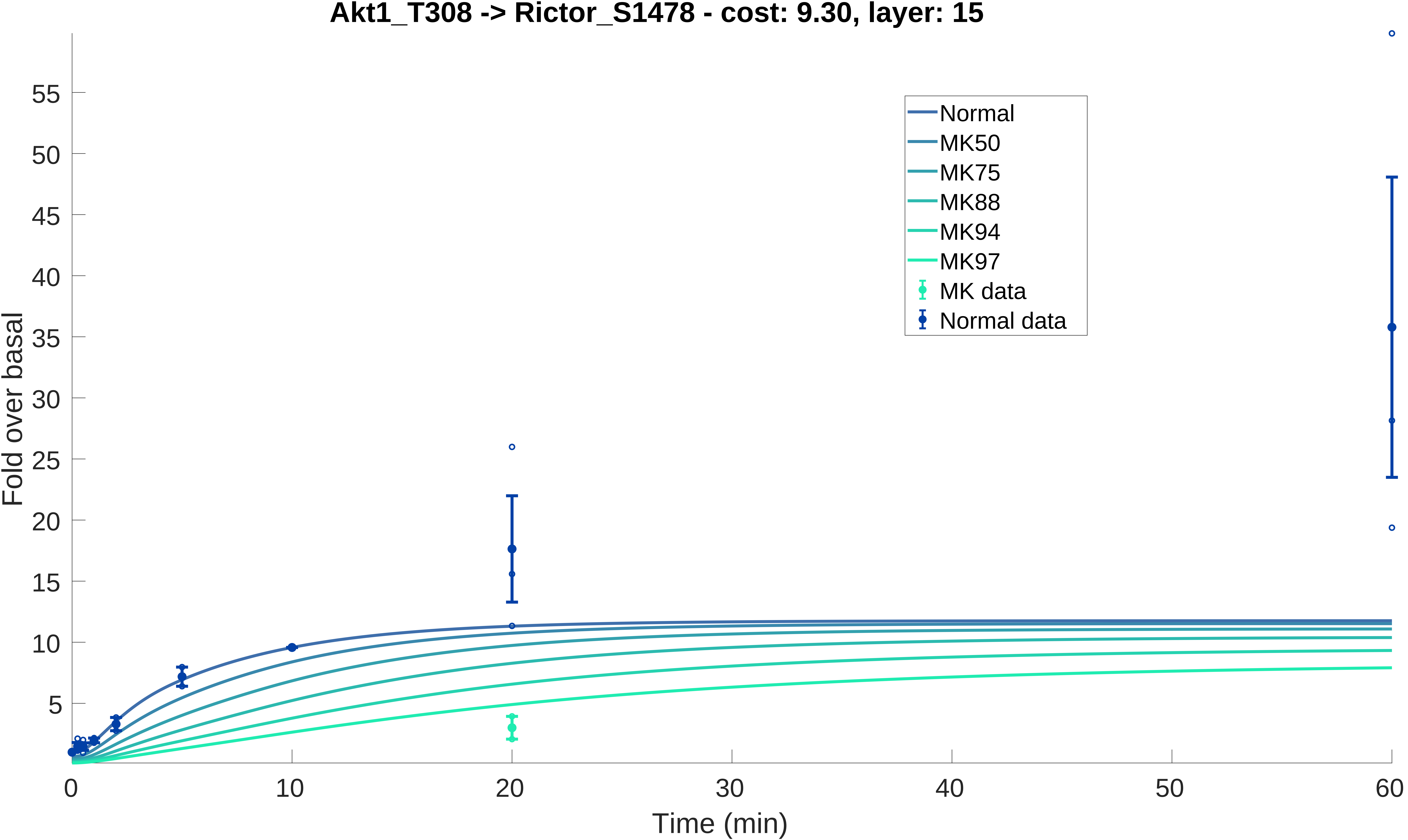
Akt1_T308 -> Rictor_S1478 - cost: 9.30, layer: 15
Normal
MK50
MK75
MK88
MK94
MK97
MK data
Normal data
Fold over basal
Time (min)
5
10
15
20
25
30
35
40
45
50
55
0
10
20
30
40
50
60

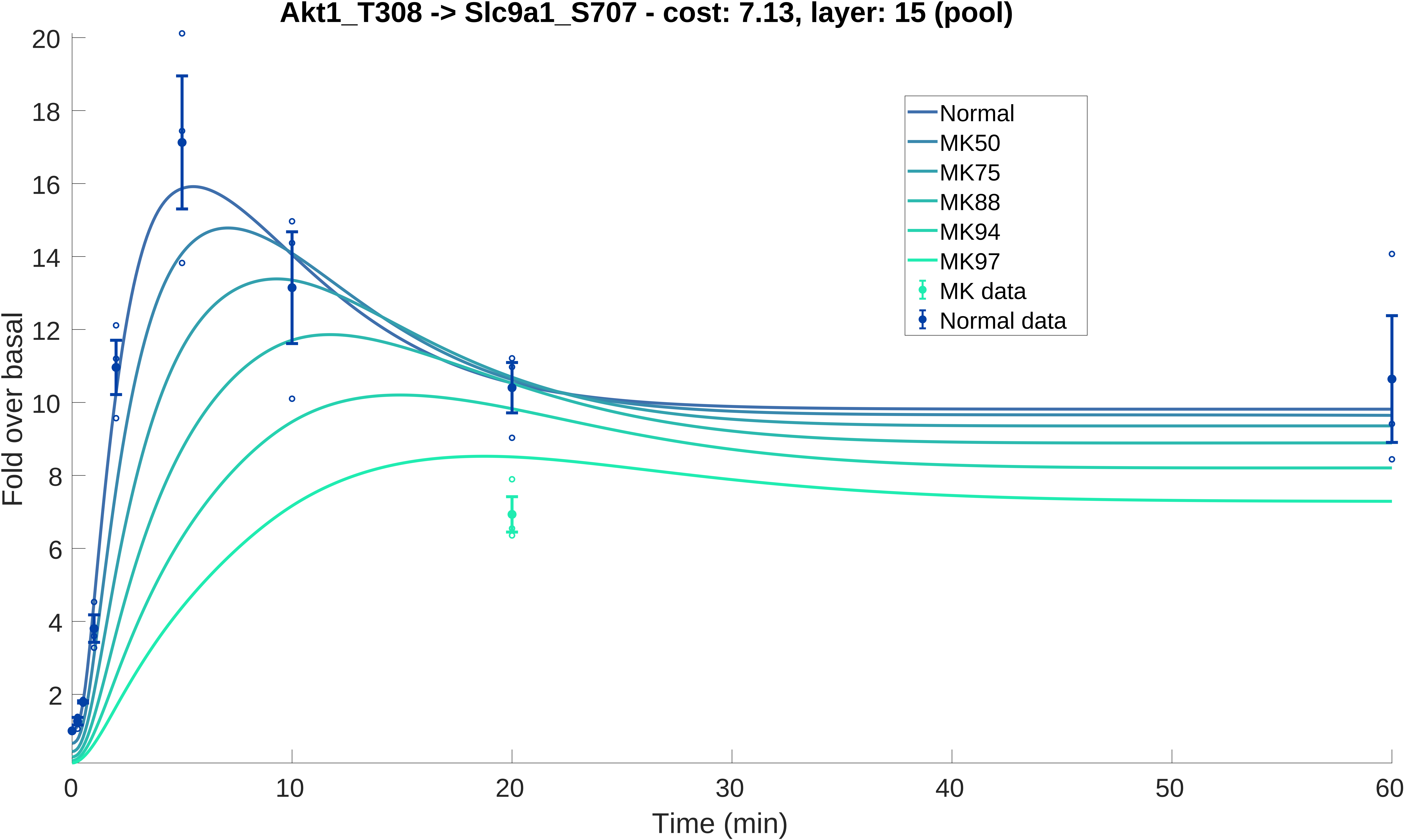

Akt1_T308 -> Slc9a1_S707 - cost: 7.13, layer: 15 (pool)
Fold over basal
Time (min)
0
10
20
30
40
50
60
2
4
6
8
10
12
14
16
18
20
Normal
MK50
MK75
MK88
MK94
MK97
MK data
Normal data

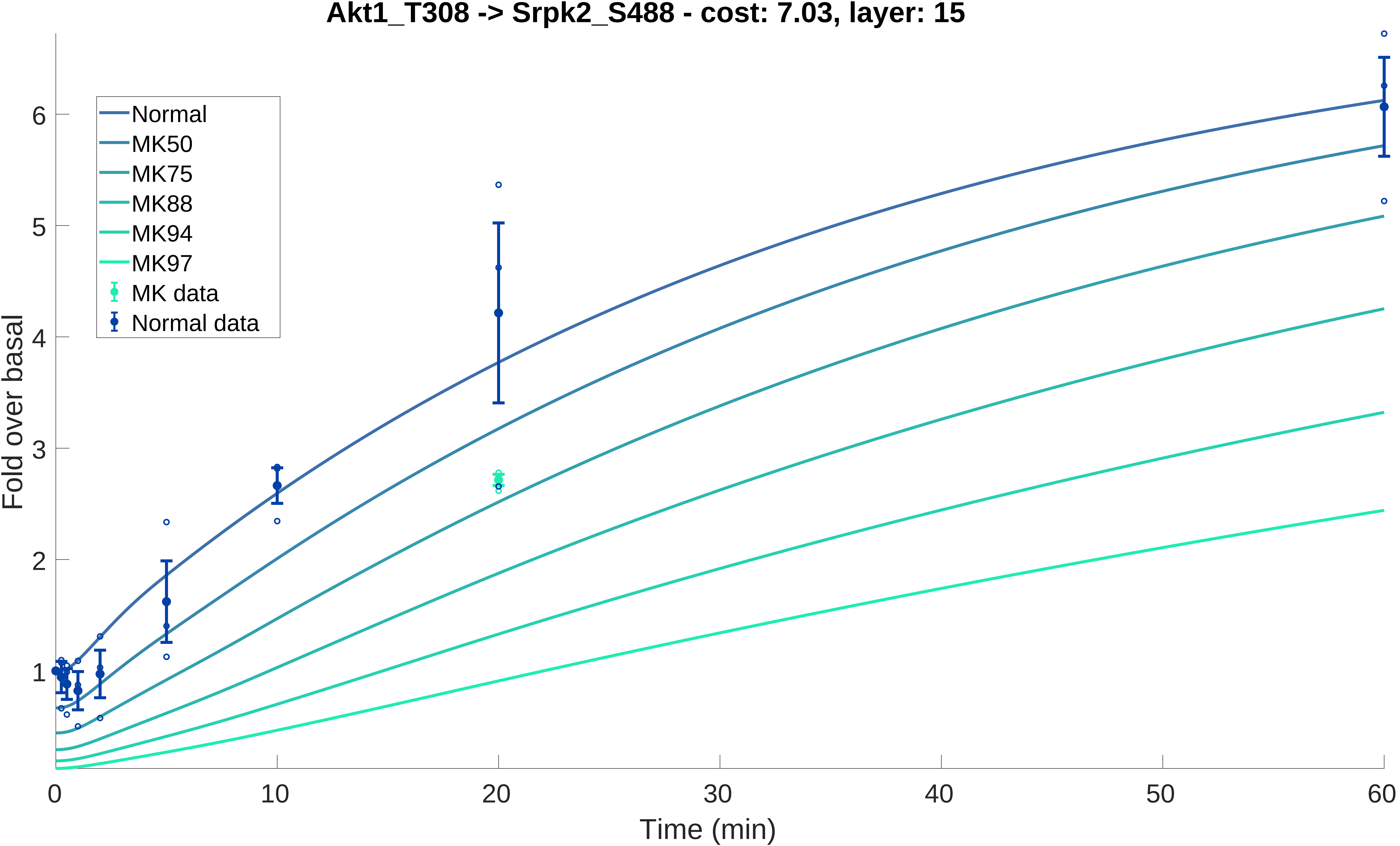

Akt1_T308 -> Srpk2_S488 - cost: 7.03, layer: 15
Normal
MK50
MK75
MK88
MK94
MK97
MK data
Normal data
Fold over basal
1
2
3
4
5
6
0
10
20
30
40
50
60
Time (min)

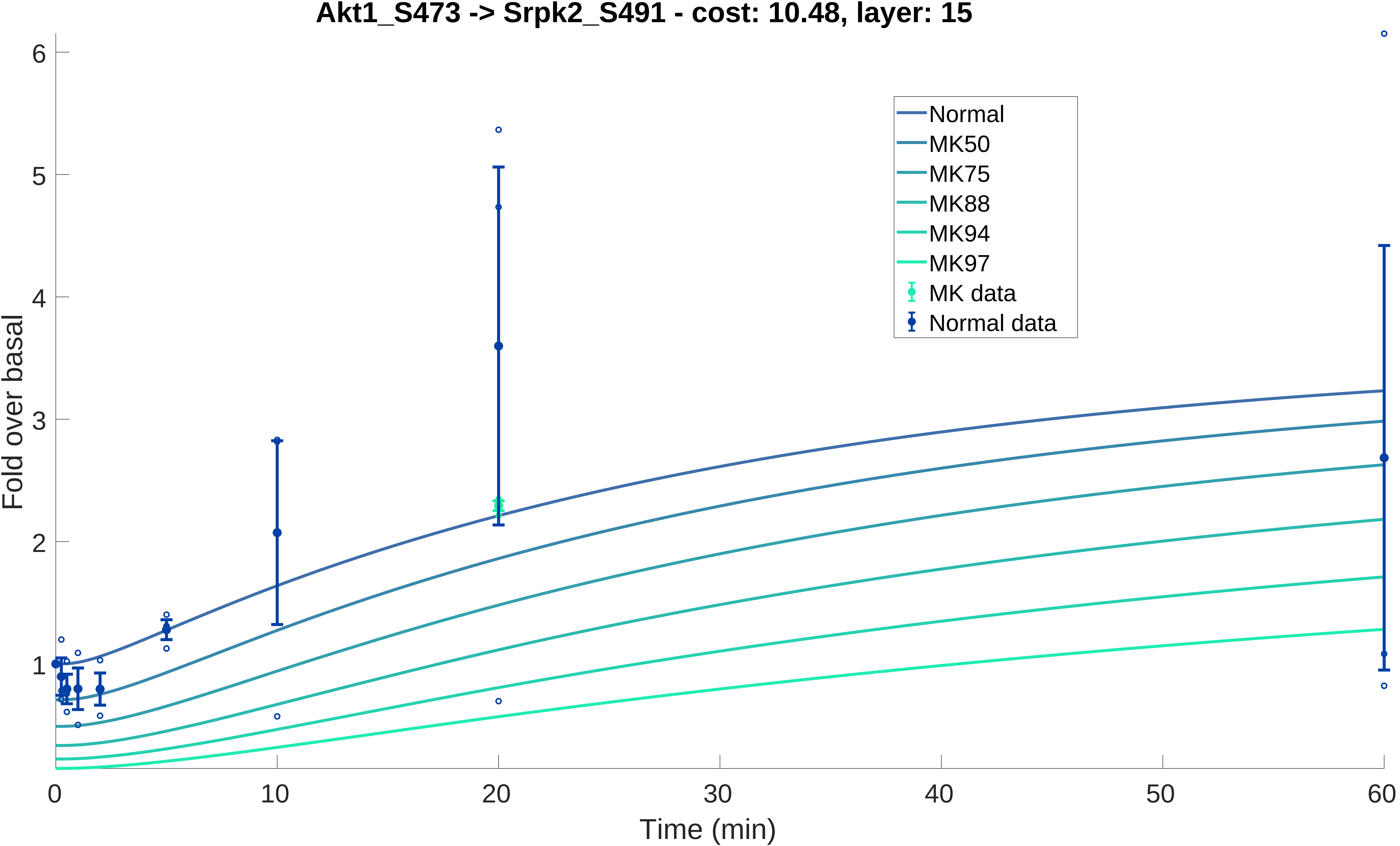

Akt1_S473 -> Srpk2_S491 - cost: 10.48, layer: 15
Fold over basal
Time (min)
Normal
MK50
MK75
MK88
MK94
MK97
MK data
Normal data
0
10
20
30
40
50
60
1
2
3
4
5
6

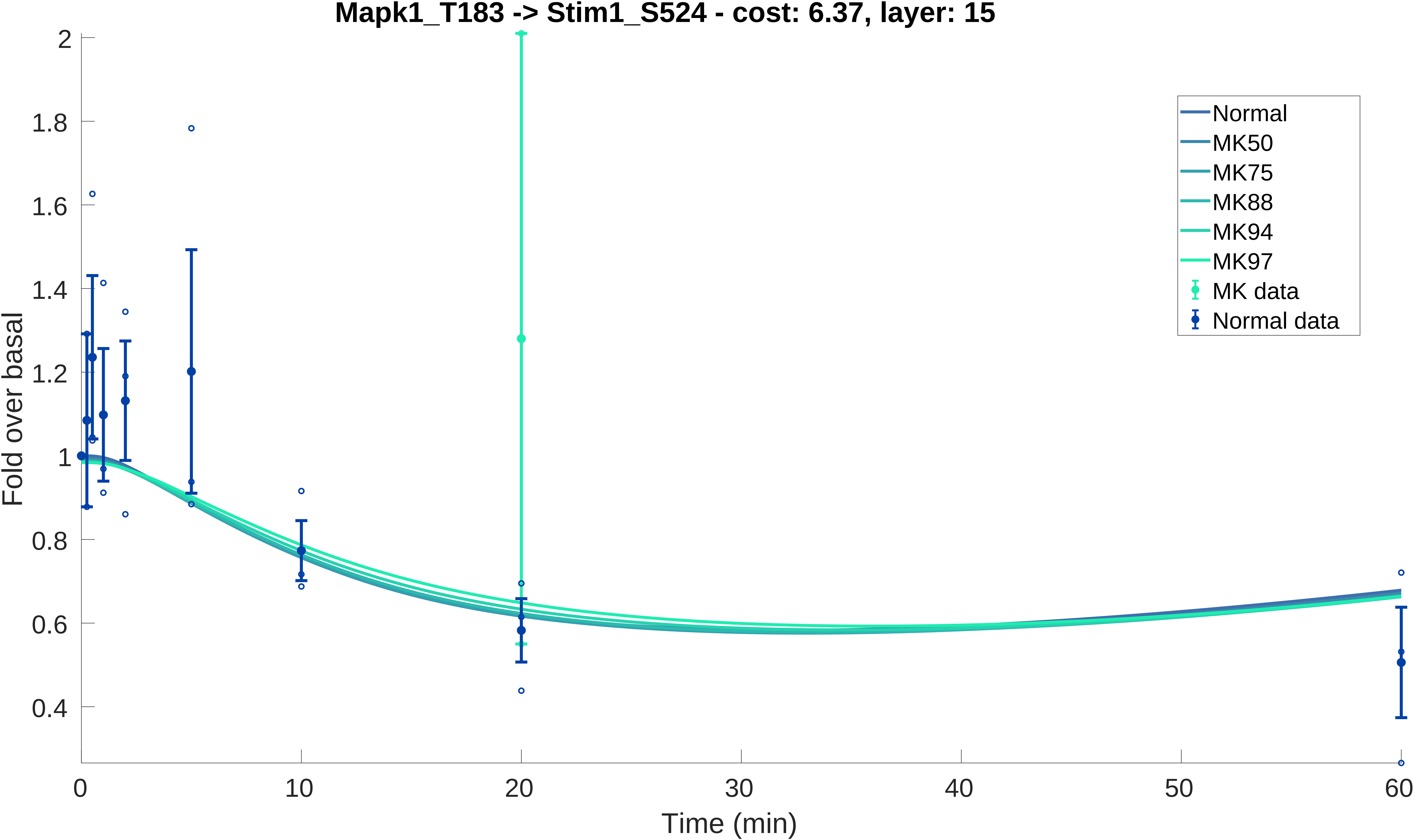
Mapk1_T183 -> Stim1_S524 - cost: 6.37, layer: 15
Fold over basal
Time (min)
2
1.8
1.6
1.4
1.2
1
0.8
0.6
0.4
0
10
20
30
40
50
60
Normal
MK50
MK75
MK88
MK94
MK97
MK data
Normal data

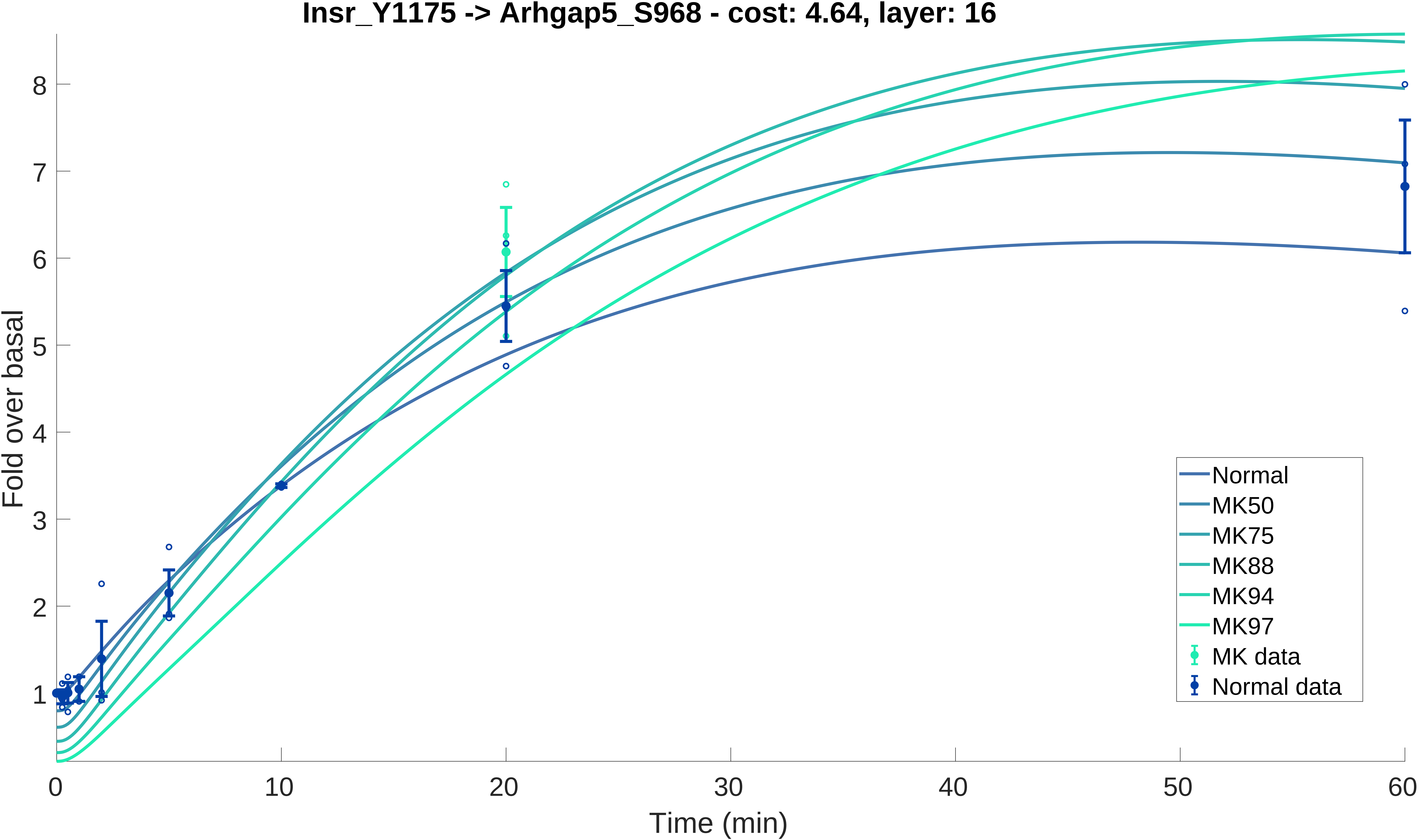

Insr_Y1175 -> Arhgap5_S968 - cost: 4.64, layer: 16
Fold over basal
Time (min)
0
10
20
30
40
50
60
1
2
3
4
5
6
7
8
Normal
MK50
MK75
MK88
MK94
MK97
MK data
Normal data

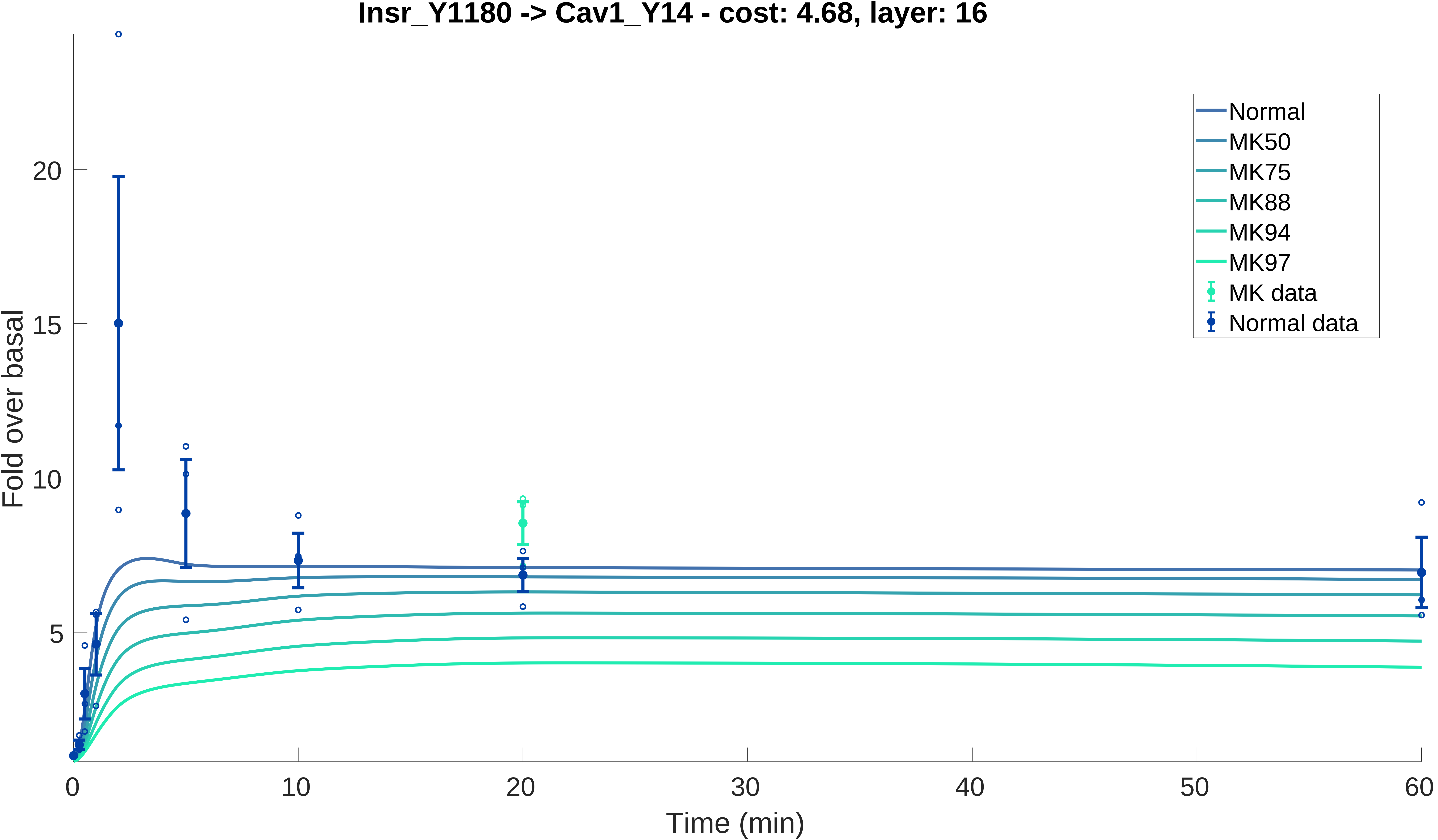
Insr_Y1180 -> Cav1_Y14 - cost: 4.68, layer: 16
Fold over basal
Time (min)
0
10
20
30
40
50
60
5
10
15
20
Normal
MK50
MK75
MK88
MK94
MK97
MK data
Normal data

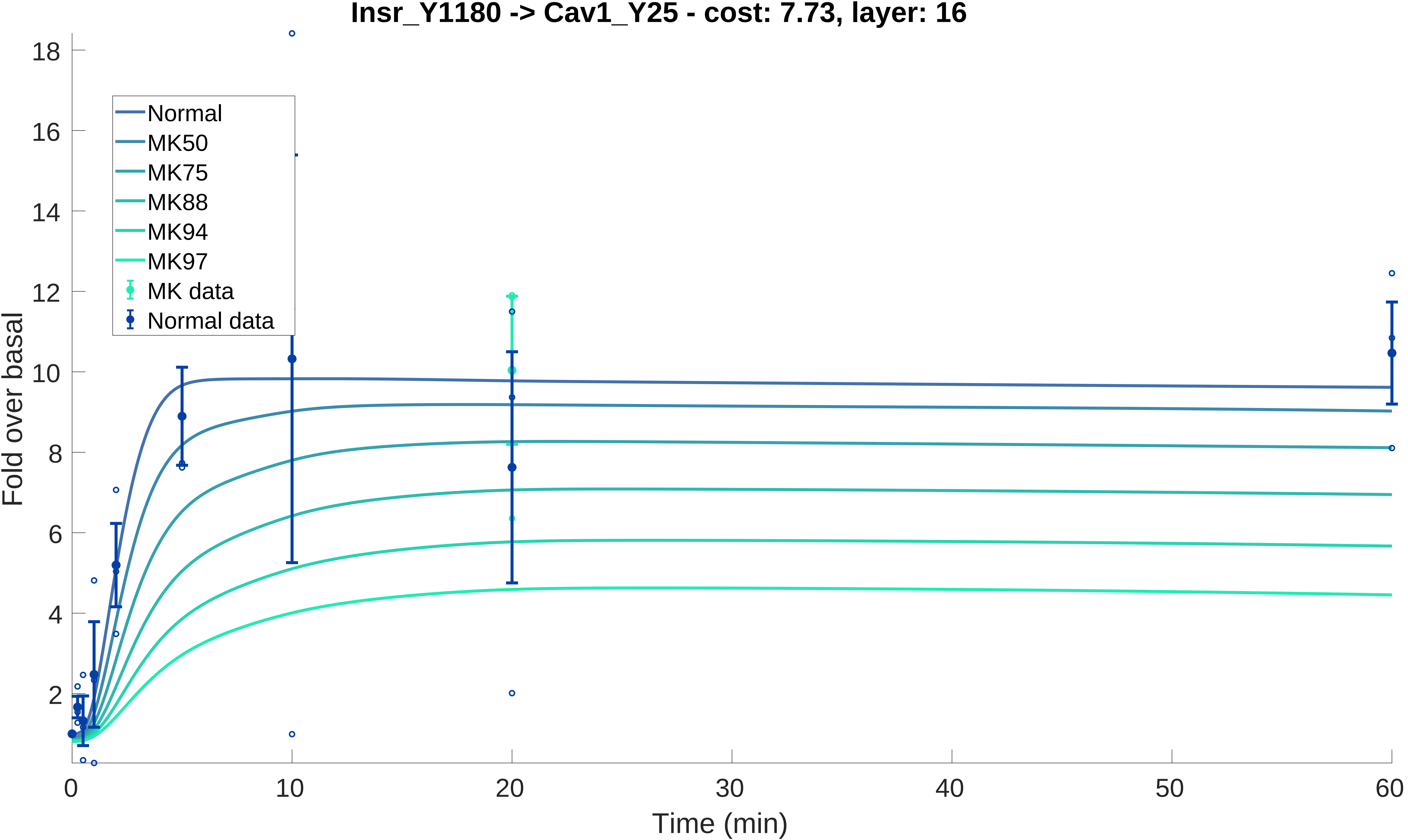

Insr_Y1180 -> Cav1_Y25 - cost: 7.73, layer: 16
Normal
MK50
MK75
MK88
MK94
MK97
MK data
Normal data
Fold over basal
Time (min)
0
10
20
30
40
50
60
2
4
6
8
10
12
14
16
18

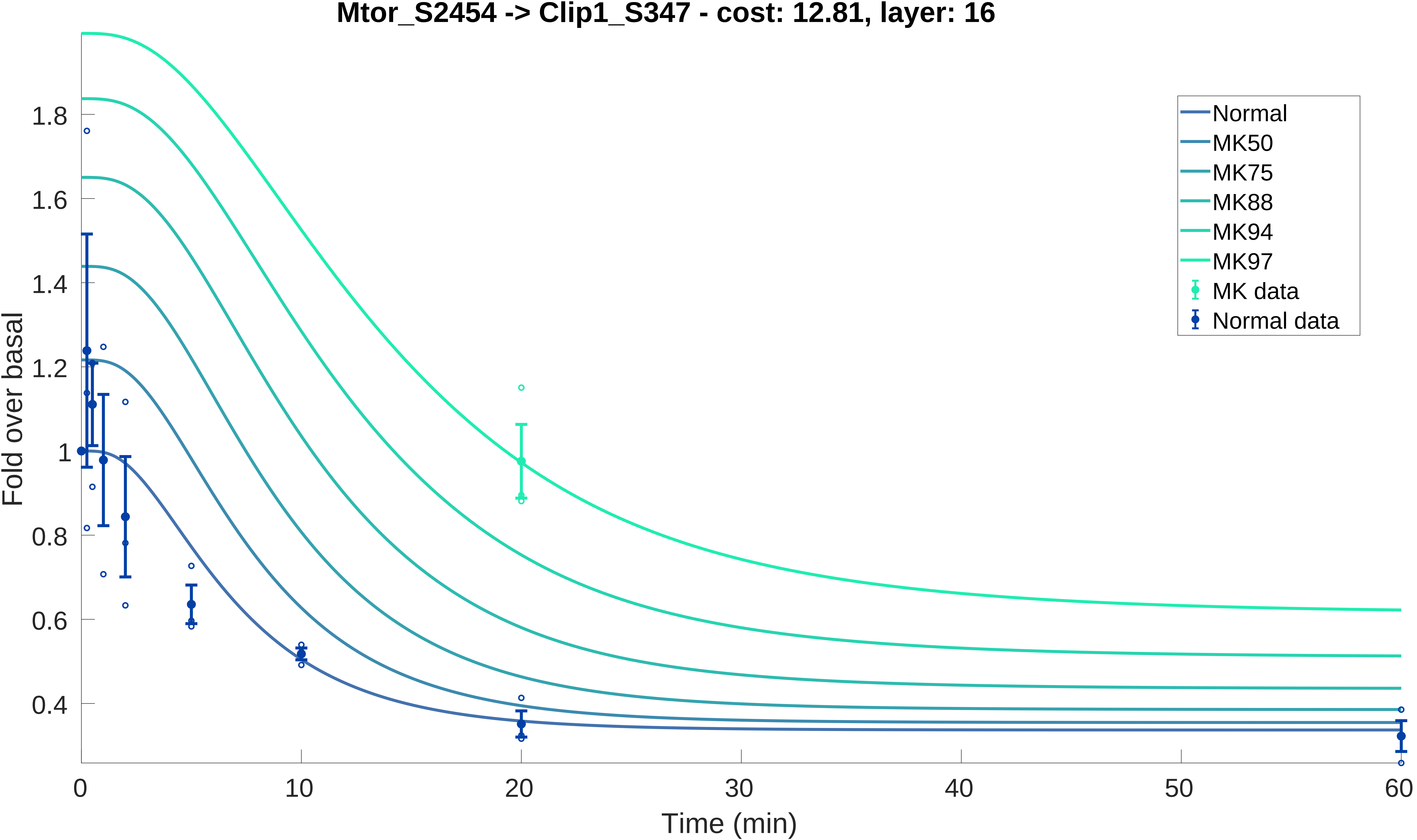

Mtor_S2454 -> Clip1_S347 - cost: 12.81, layer: 16
Fold over basal
Time (min)
Normal
MK50
MK75
MK88
MK94
MK97
MK data
Normal data

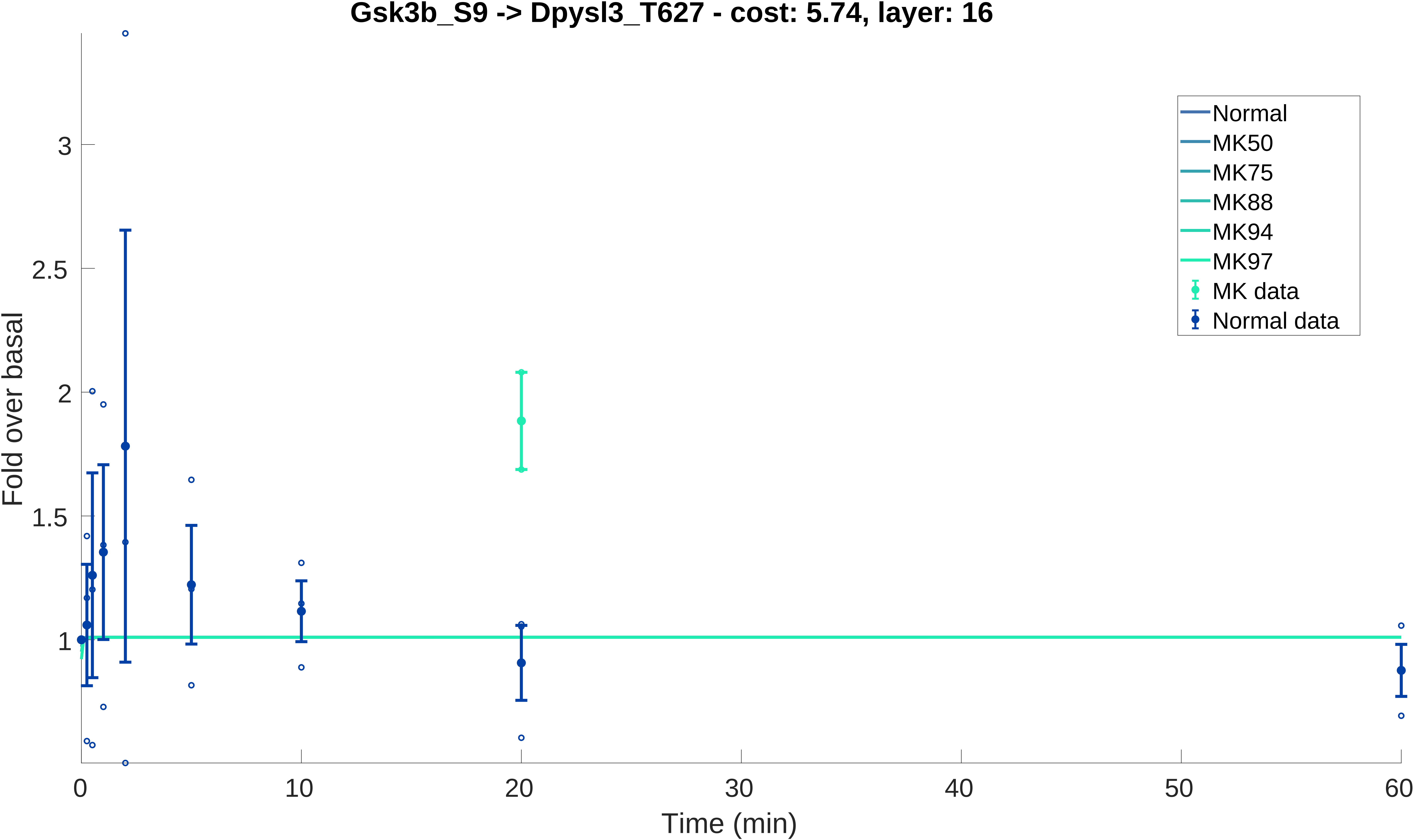
Gsk3b_S9 -> Dpysl3_T627 - cost: 5.74, layer: 16
Fold over basal
Time (min)
Normal
MK50
MK75
MK88
MK94
MK97
MK data
Normal data
3
2.5
2
1.5
1
0
10
20
30
40
50
60

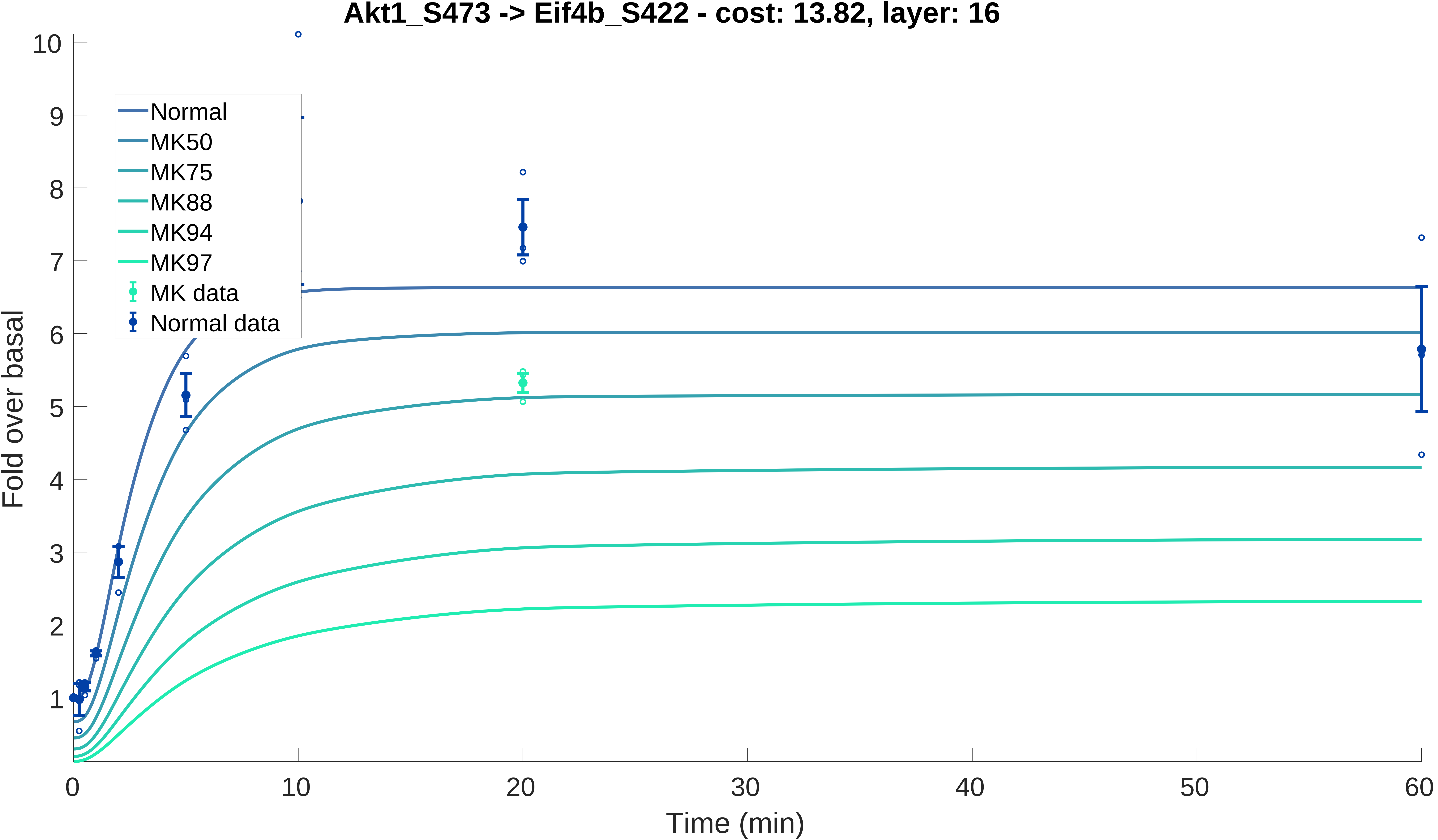

Akt1_S473 -> Eif4b_S422 - cost: 13.82, layer: 16
Normal
MK50
MK75
MK88
MK94
MK97
MK data
Normal data
Fold over basal
Time (min)
0
10
20
30
40
50
60
1
2
3
4
5
6
7
8
9
10

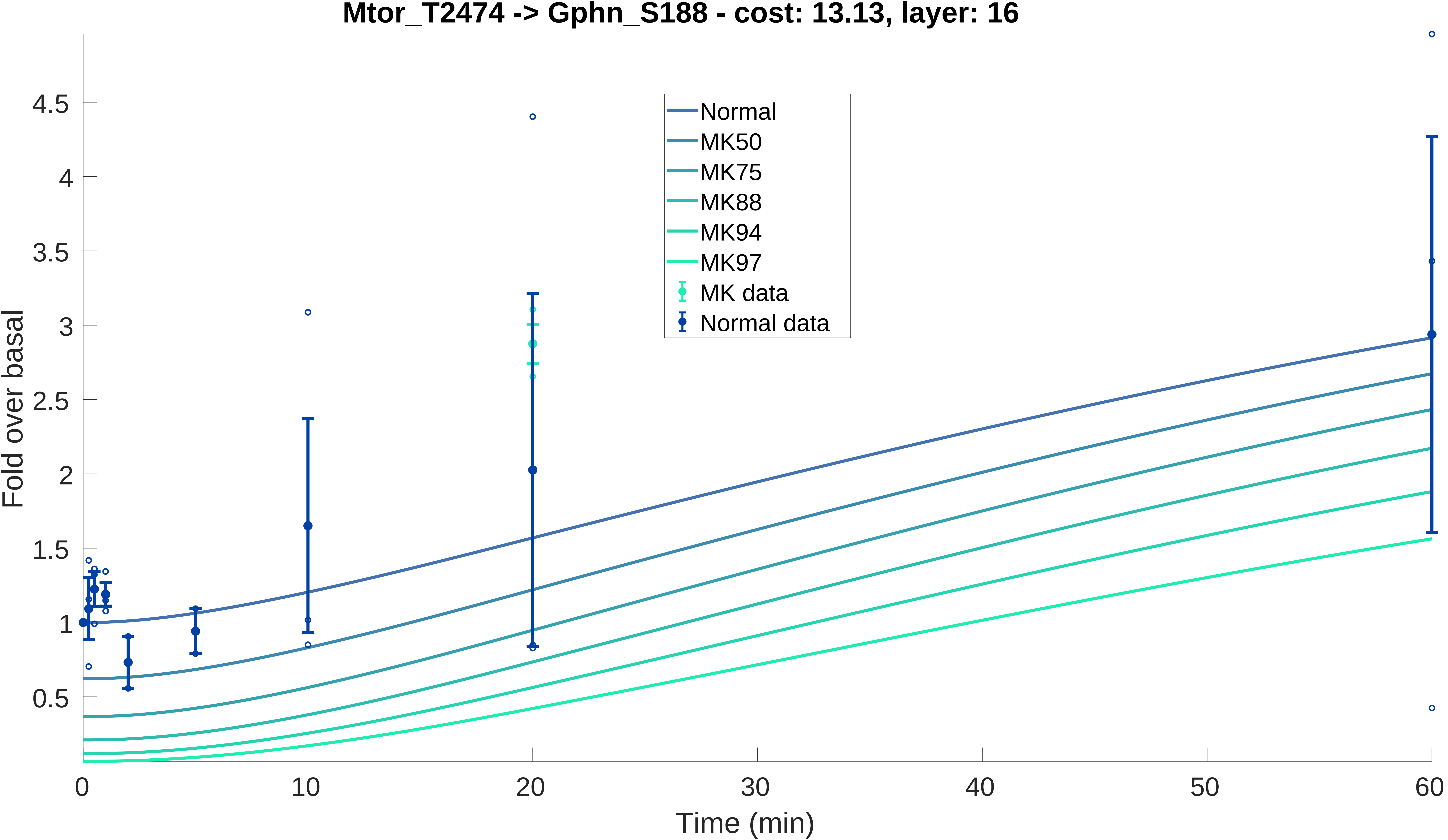
Mtor_T2474 -> Gphn_S188 - cost: 13.13, layer: 16
Normal
MK50
MK75
MK88
MK94
MK97
MK data
Normal data
Fold over basal
Time (min)
0.5
1
1.5
2
2.5
3
3.5
4
4.5
0
10
20
30
40
50
60

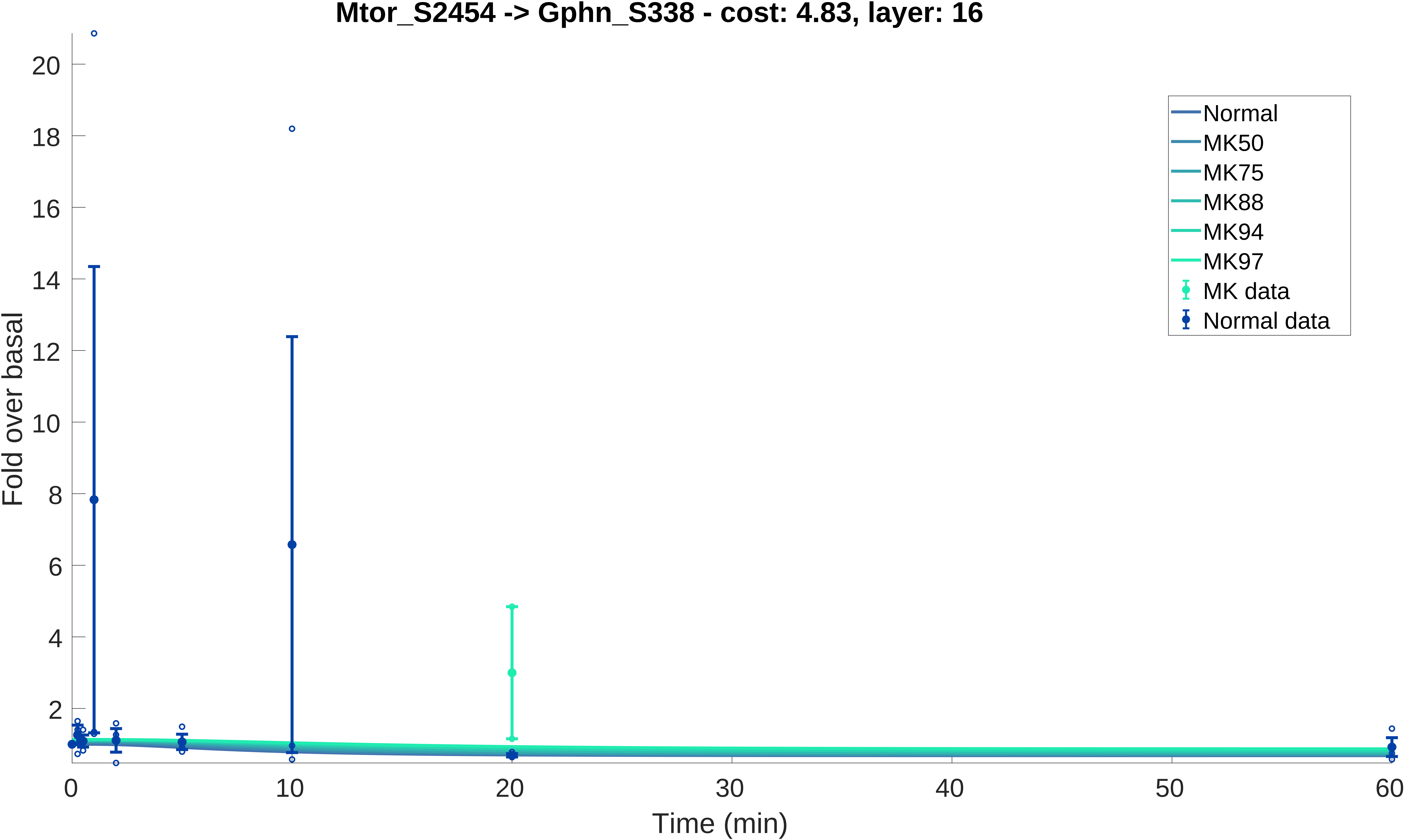

Mtor_S2454 -> Gphn_S338 - cost: 4.83, layer: 16
Fold over basal
Time (min)
0
10
20
30
40
50
60
2
4
6
8
10
12
14
16
18
20
Normal
MK50
MK75
MK88
MK94
MK97
MK data
Normal data

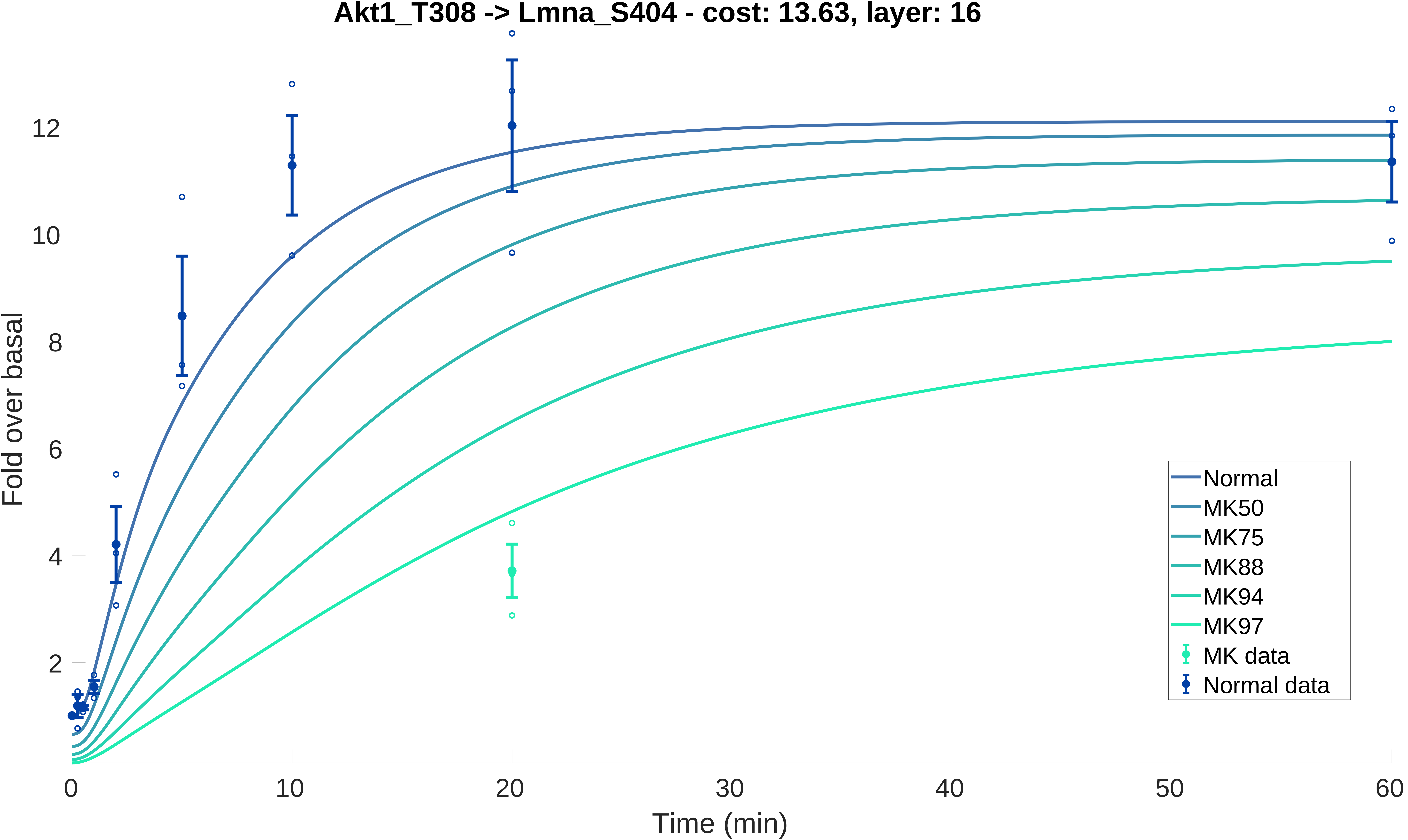
Akt1_T308 -> Lmna_S404 - cost: 13.63, layer: 16
Fold over basal
Time (min)
0
10
20
30
40
50
60
2
4
6
8
10
12
Normal
MK50
MK75
MK88
MK94
MK97
MK data
Normal data

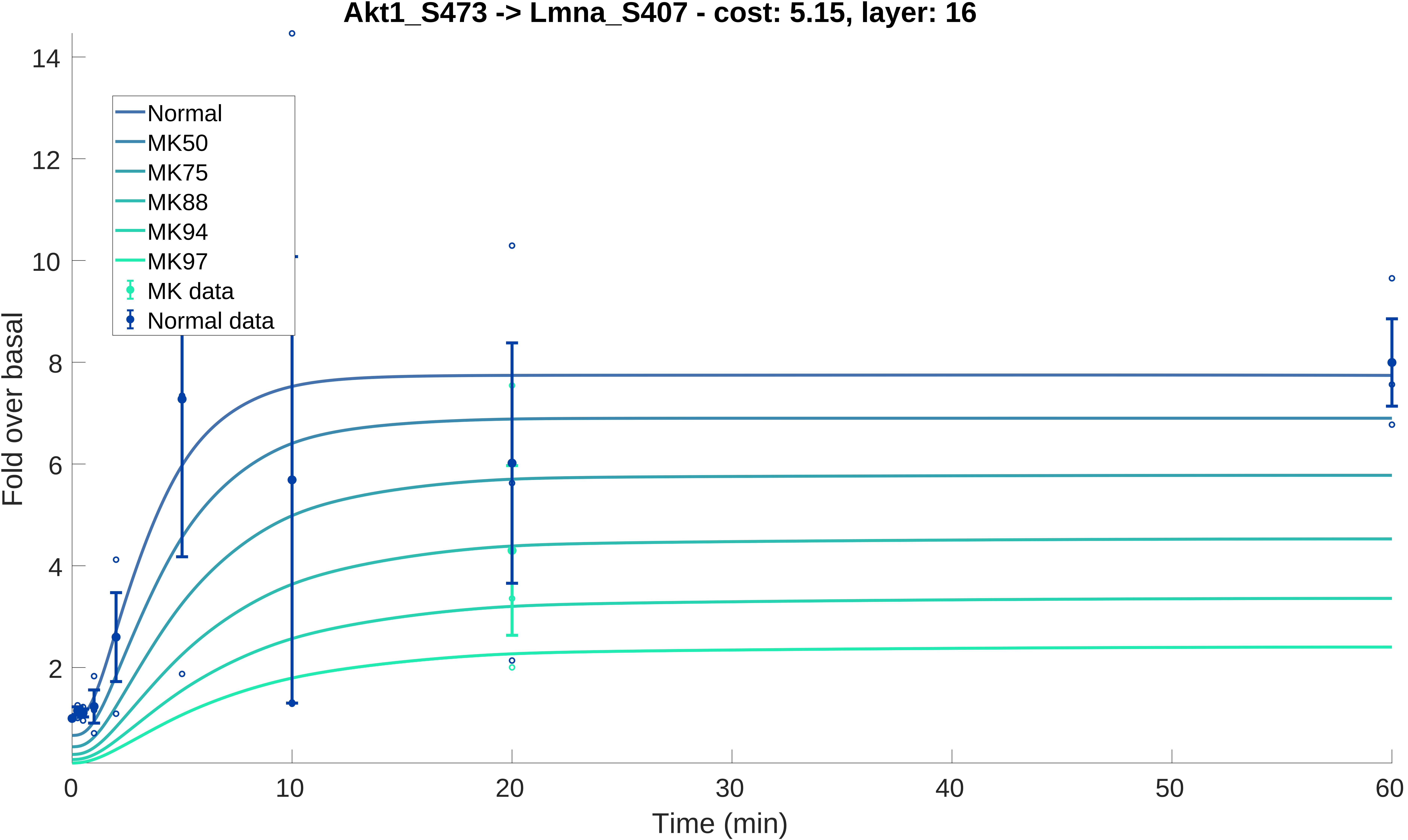
Akt1_S473 -> Lmna_S407 - cost: 5.15, layer: 16
Fold over basal
Time (min)
Normal
MK50
MK75
MK88
MK94
MK97
MK data
Normal data
0
10
20
30
40
50
60
2
4
6
8
10
12
14

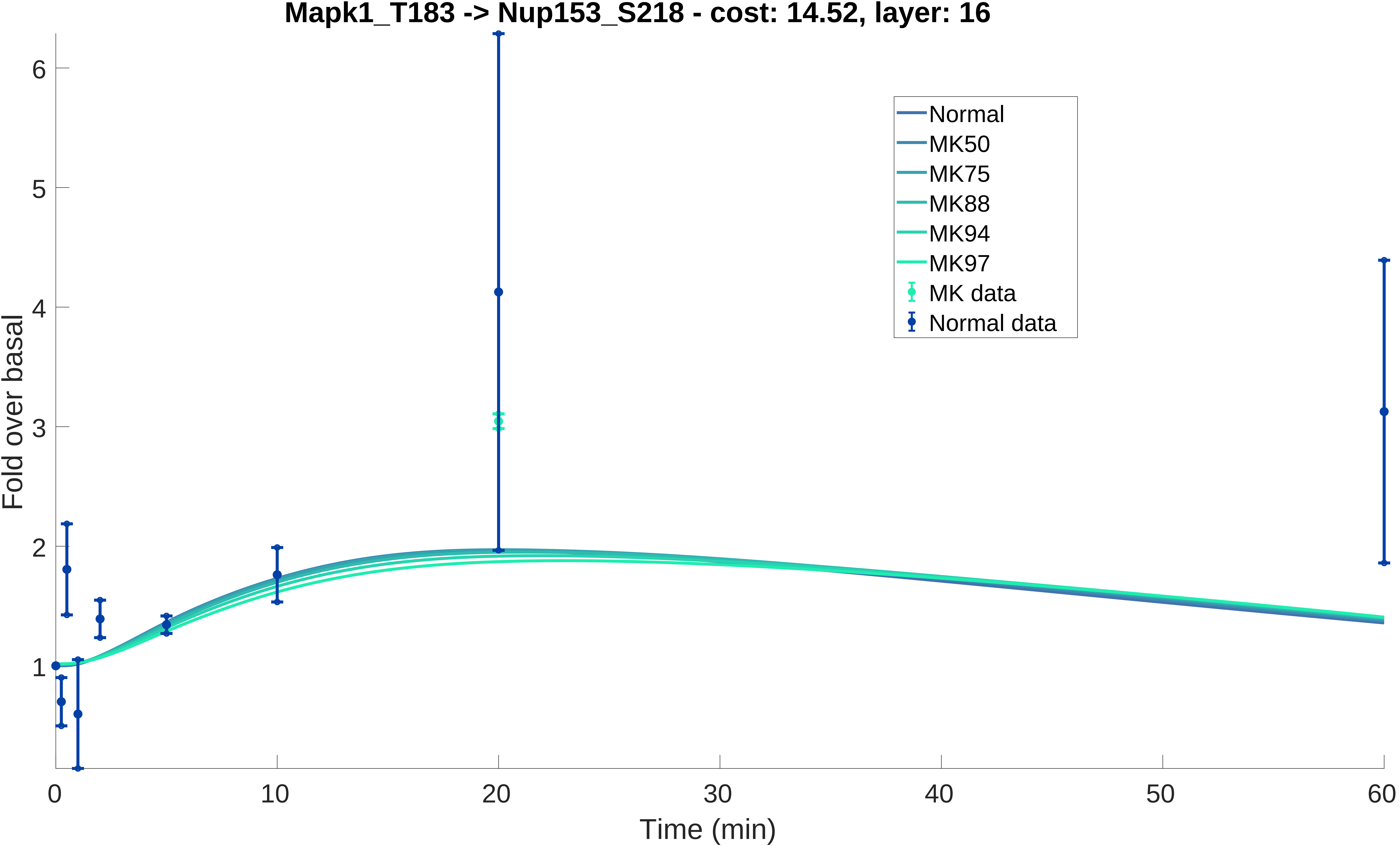

Mapk1_T183 -> Nup153_S218 - cost: 14.52, layer: 16
Fold over basal
Time (min)
0
10
20
30
40
50
60
1
2
3
4
5
6
Normal
MK50
MK75
MK88
MK94
MK97
MK data
Normal data

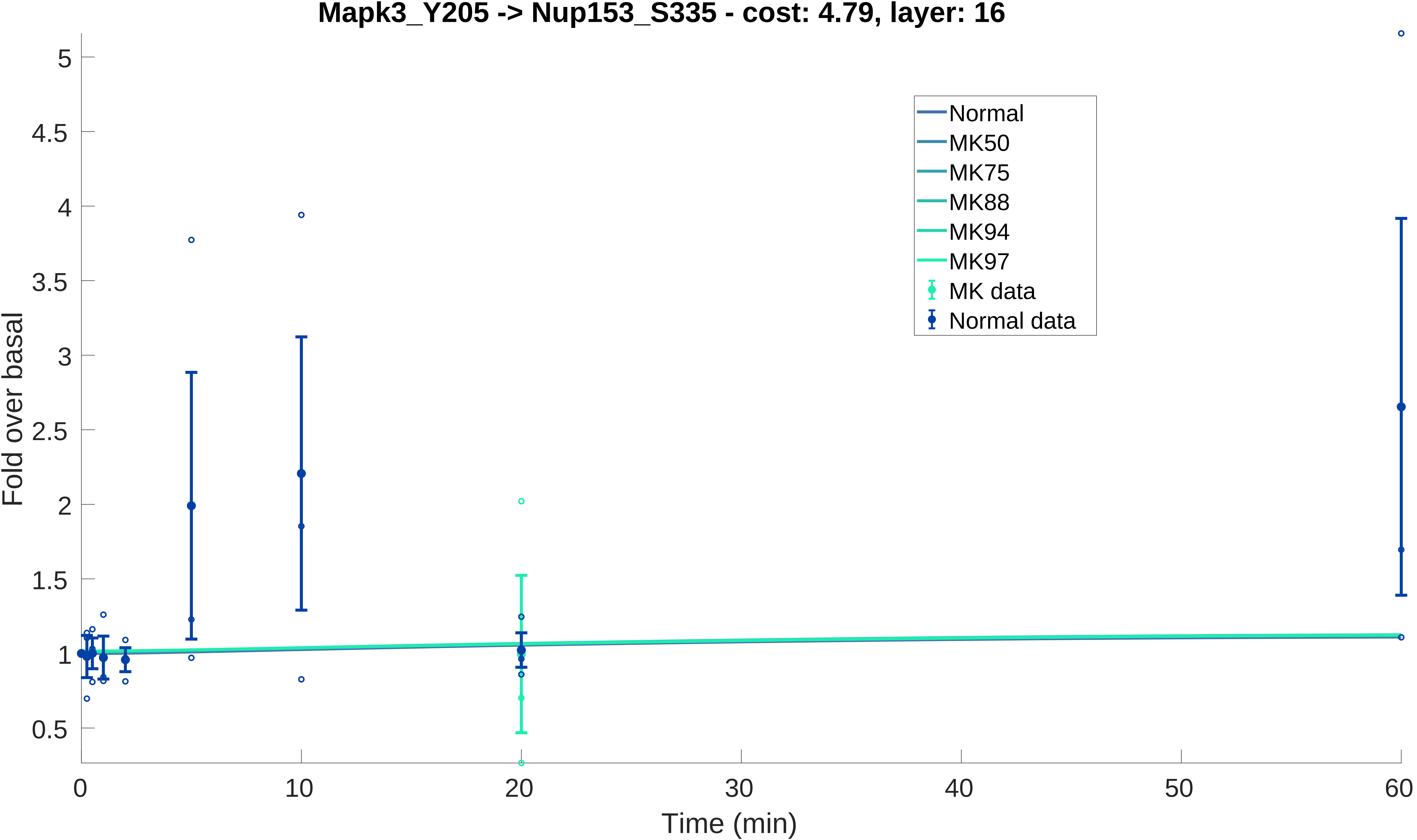

Mapk3_Y205 -> Nup153_S335 - cost: 4.79, layer: 16
Fold over basal
Time (min)
Normal
MK50
MK75
MK88
MK94
MK97
MK data
Normal data
0.5
1
1.5
2
2.5
3
3.5
4
4.5
5
0
10
20
30
40
50
60

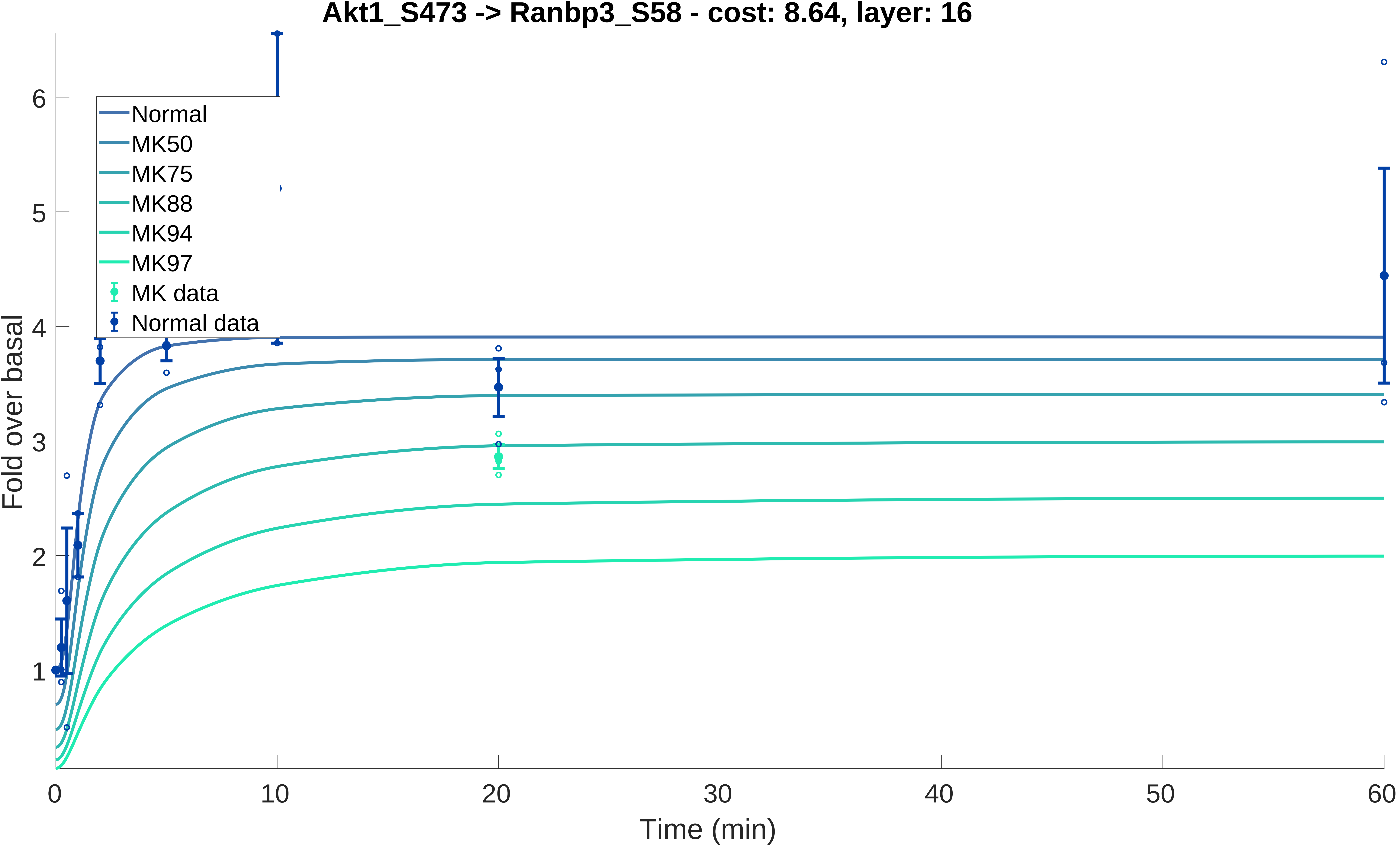

Akt1_S473 -> Ranbp3_S58 - cost: 8.64, layer: 16
Fold over basal
Time (min)
Normal
MK50
MK75
MK88
MK94
MK97
MK data
Normal data
1
2
3
4
5
6
0
10
20
30
40
50
60

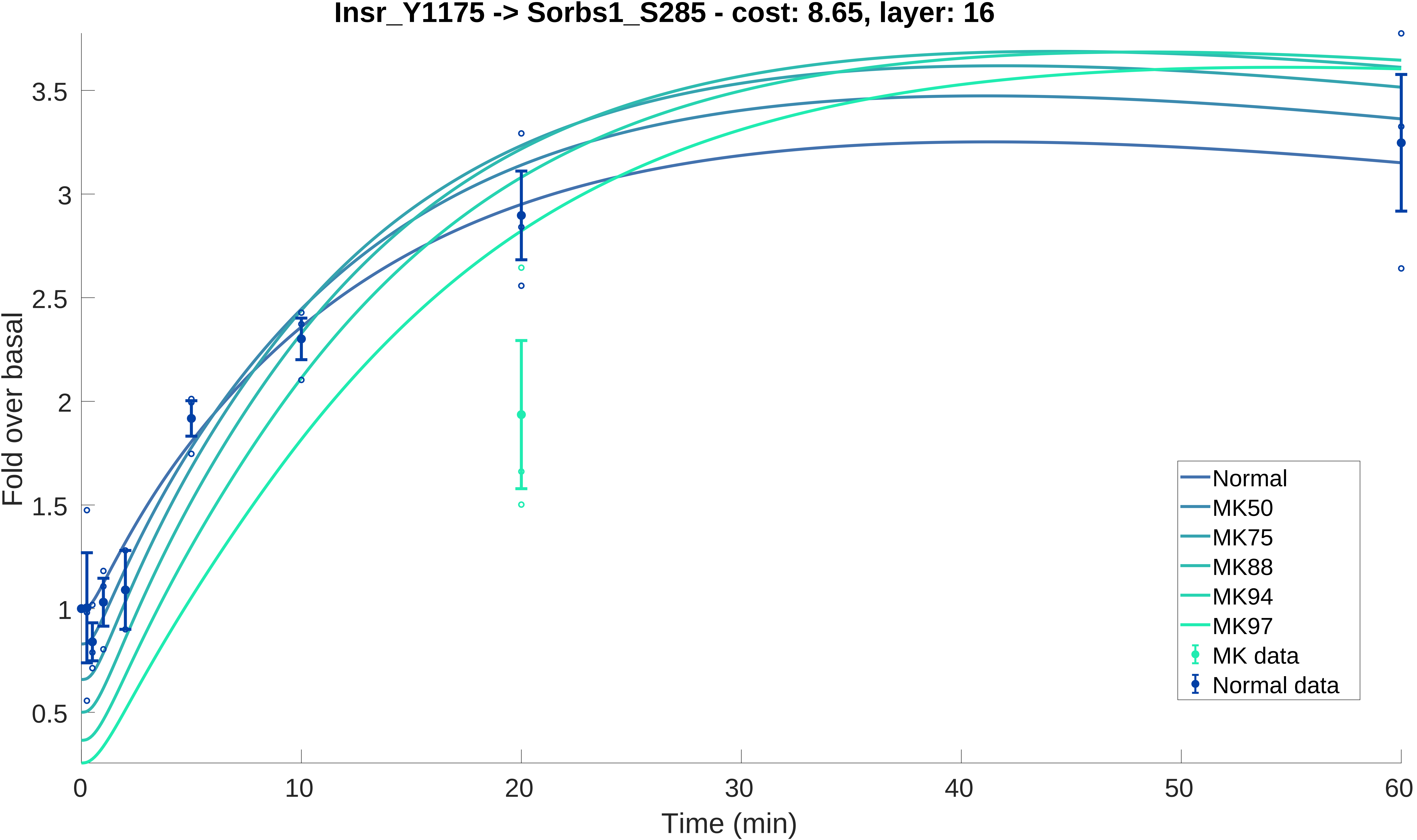

Insr_Y1175 -> Sorbs1_S285 - cost: 8.65, layer: 16
Fold over basal
Time (min)
0
10
20
30
40
50
60
0.5
1
1.5
2
2.5
3
3.5
Normal
MK50
MK75
MK88
MK94
MK97
MK data
Normal data

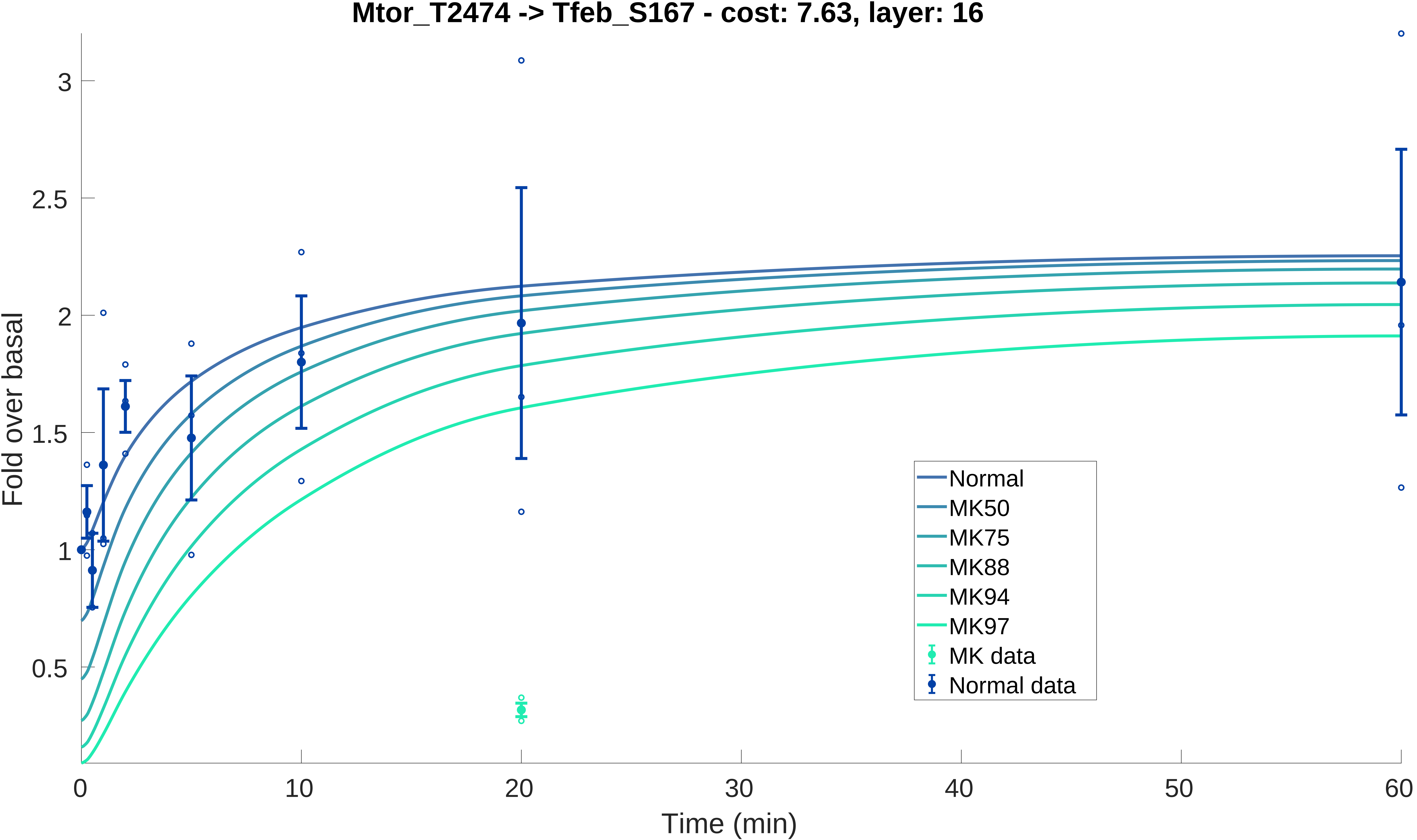
Mtor_T2474 -> Tfeb_S167 - cost: 7.63, layer: 16
Fold over basal
Time (min)
0.5
1
1.5
2
2.5
3
0
10
20
30
40
50
60
Normal
MK50
MK75
MK88
MK94
MK97
MK data
Normal data

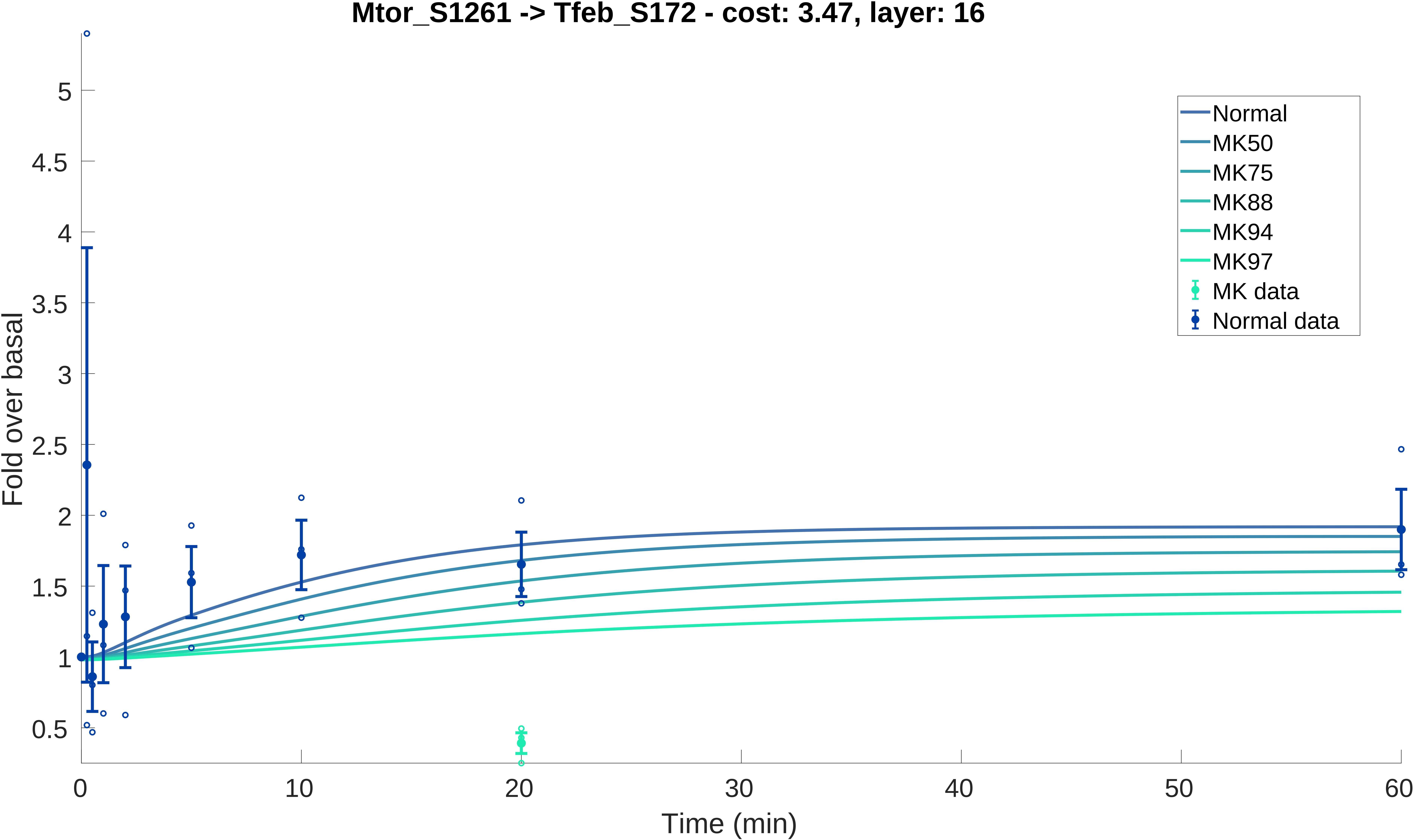

Mtor_S1261 -> Tfeb_S172 - cost: 3.47, layer: 16
Fold over basal
Time (min)
0.5
1
1.5
2
2.5
3
3.5
4
4.5
5
0
10
20
30
40
50
60
Normal
MK50
MK75
MK88
MK94
MK97
MK data
Normal data

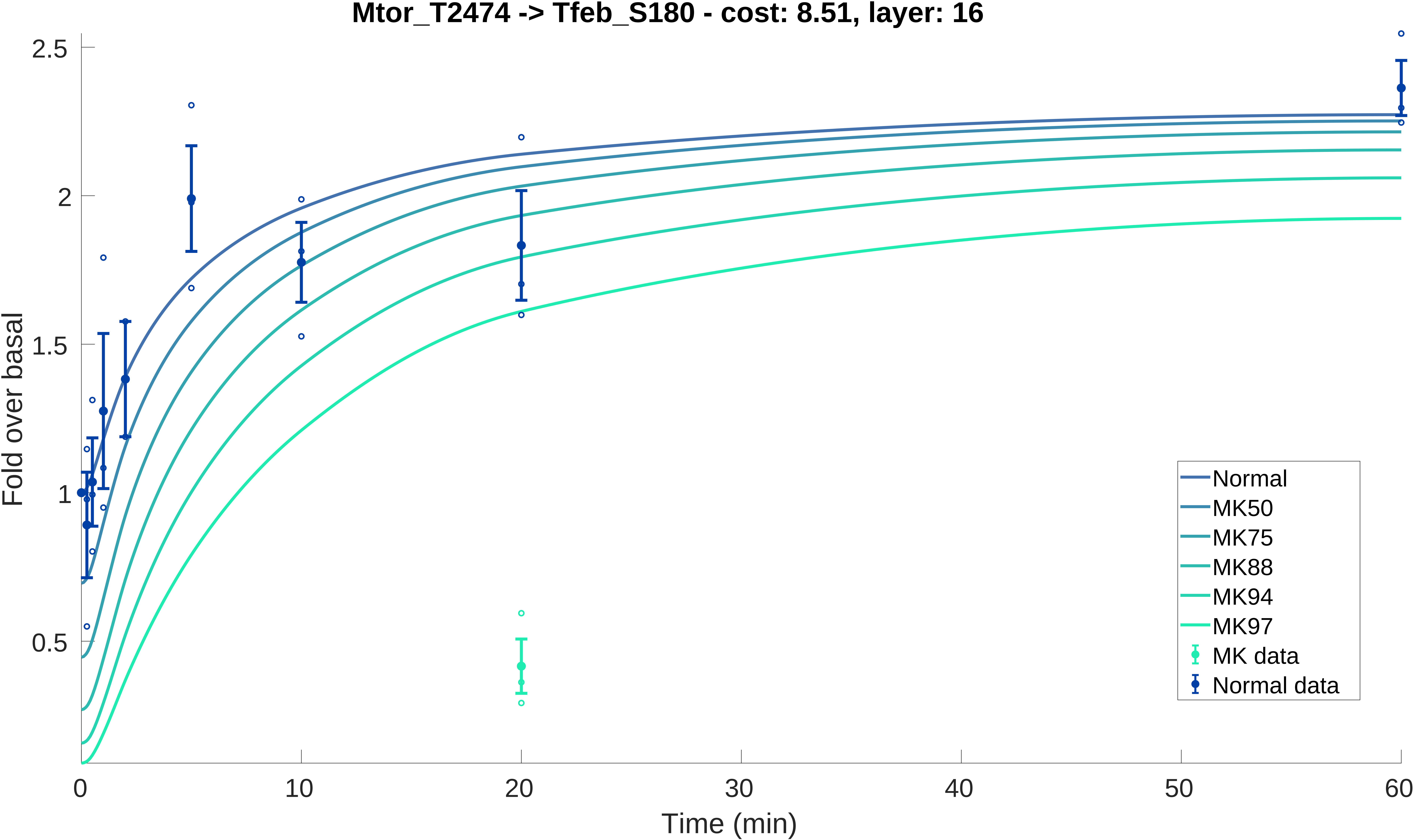

Mtor_T2474 -> Tfeb_S180 - cost: 8.51, layer: 16
Fold over basal
Time (min)
2.5
2
1.5
1
0.5
0
10
20
30
40
50
60
Normal
MK50
MK75
MK88
MK94
MK97
MK data
Normal data

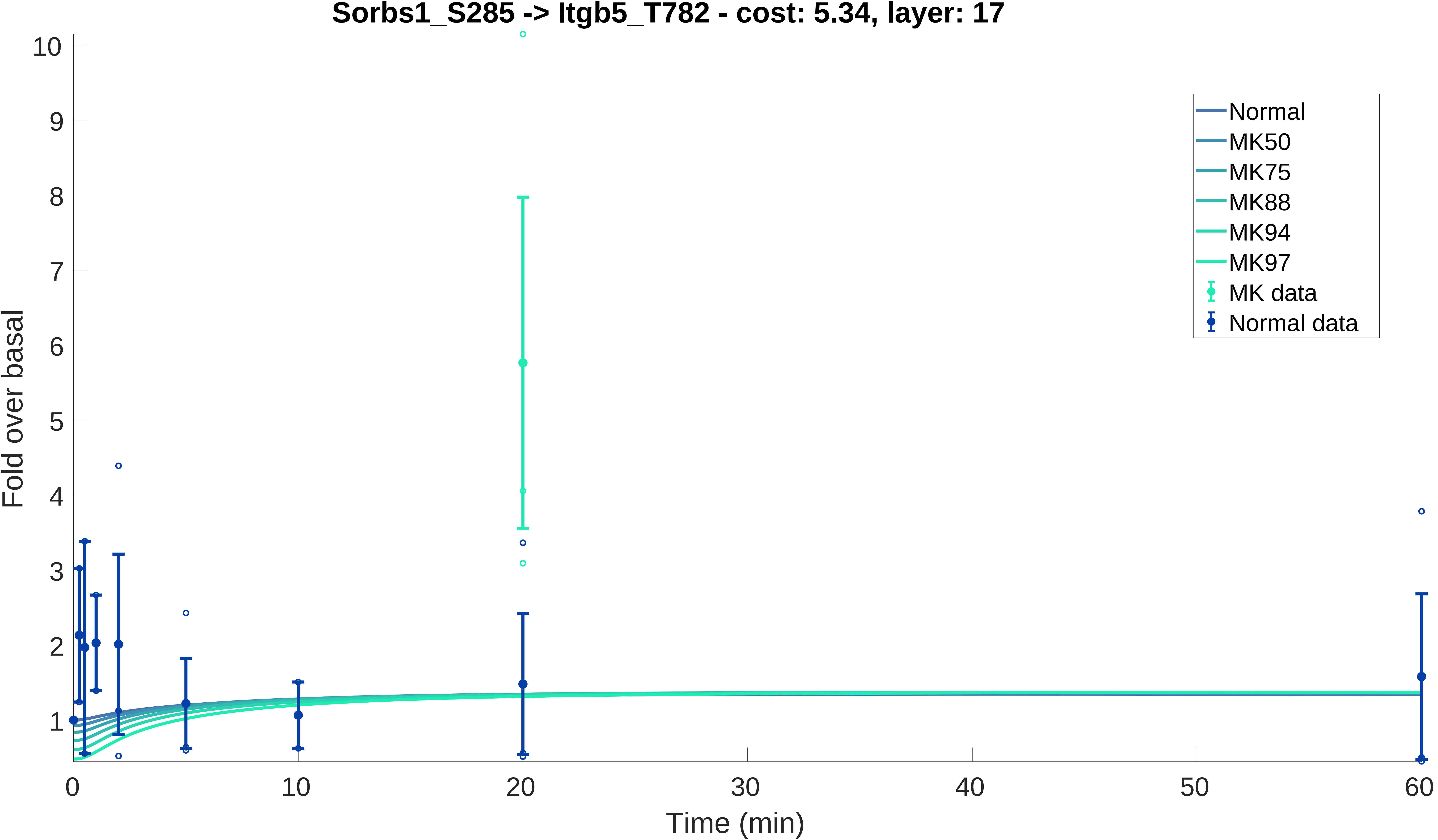
Sorbs1_S285 -> Itgb5_T782 - cost: 5.34, layer: 17
Fold over basal
Time (min)
Normal
MK50
MK75
MK88
MK94
MK97
MK data
Normal data
10
9
8
7
6
5
4
3
2
1
0
10
20
30
40
50
60

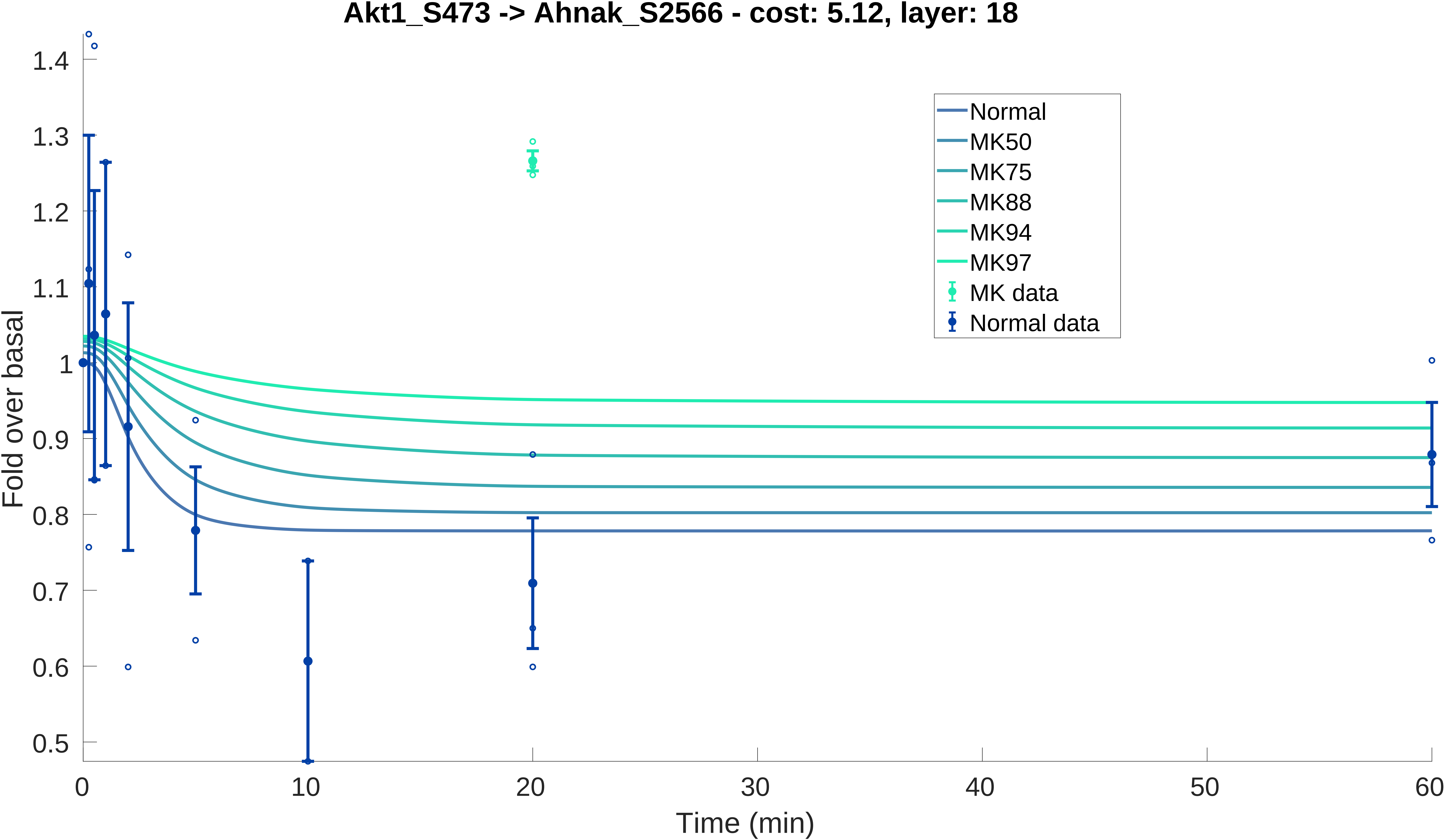

Akt1_S473 -> Ahnak_S2566 - cost: 5.12, layer: 18
Fold over basal
Time (min)
0
10
20
30
40
50
60
0.5
0.6
0.7
0.8
0.9
1
1.1
1.2
1.3
1.4
Normal
MK50
MK75
MK88
MK94
MK97
MK data
Normal data

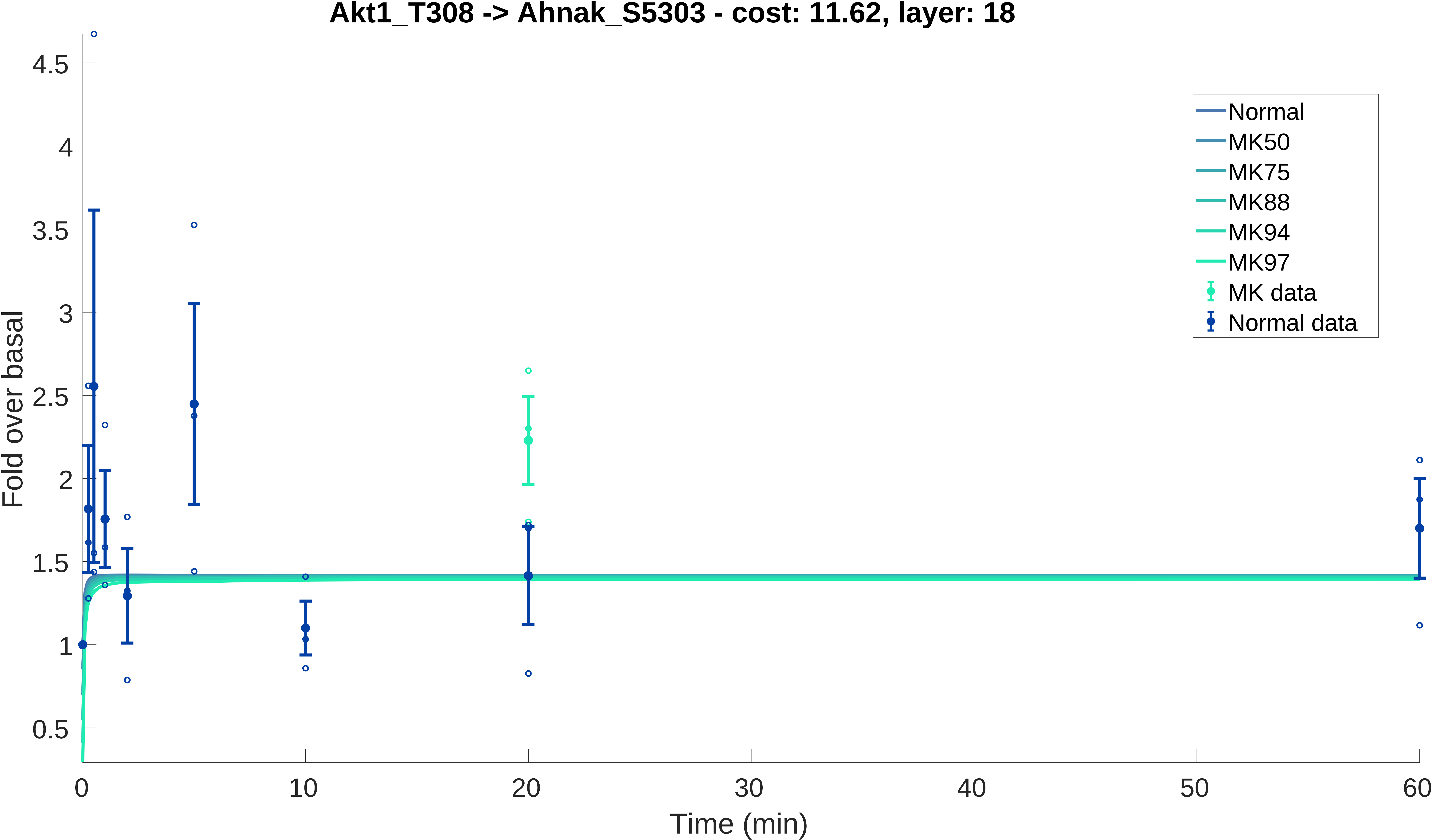
Akt1_T308 -> Ahnak_S5303 - cost: 11.62, layer: 18
Fold over basal
Time (min)
0.5
1
1.5
2
2.5
3
3.5
4
4.5
0
10
20
30
40
50
60
Normal
MK50
MK75
MK88
MK94
MK97
MK data
Normal data

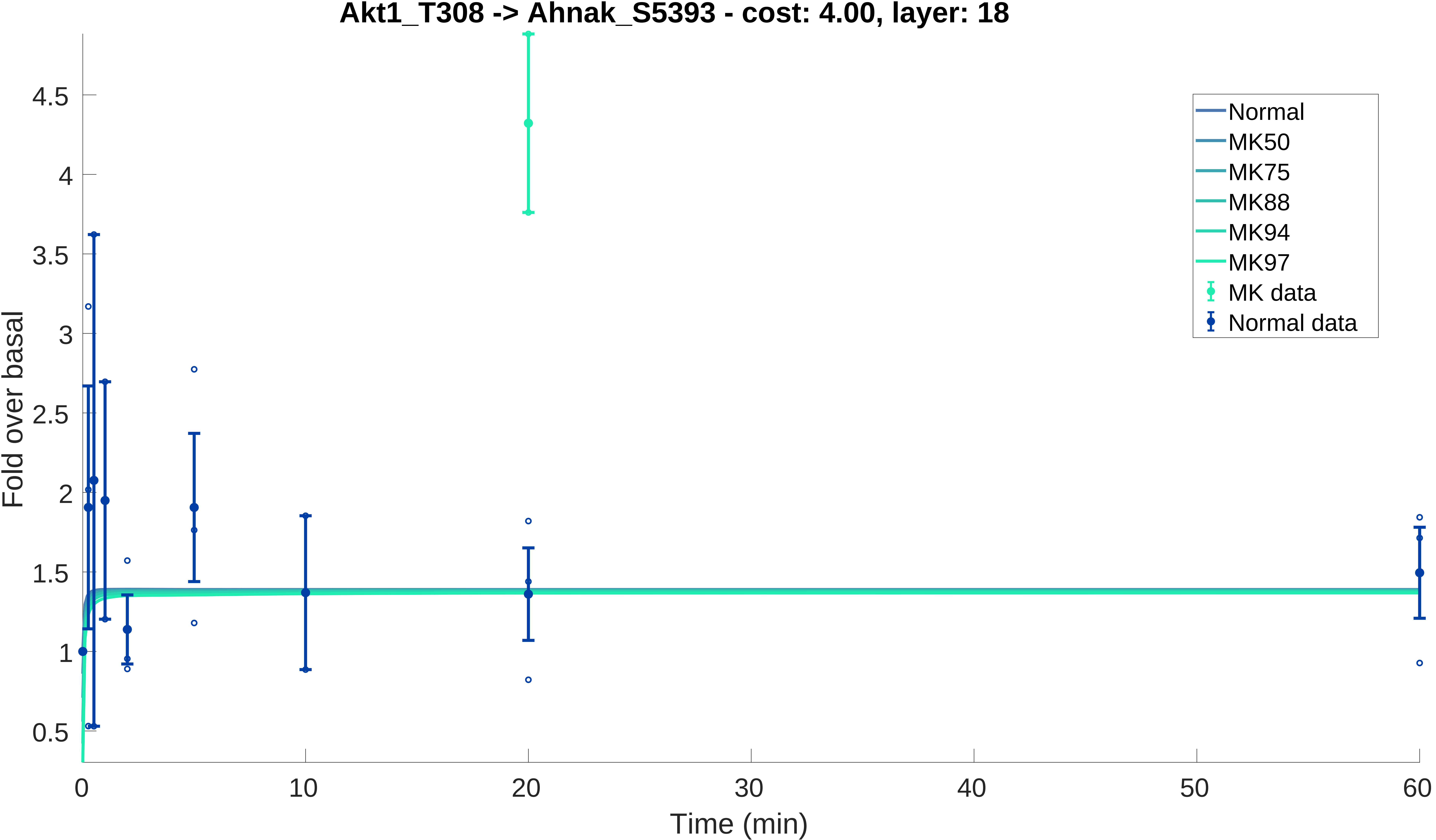
Akt1_T308 -> Ahnak_S5393 - cost: 4.00, layer: 18
Fold over basal
Time (min)
0
10
20
30
40
50
60
0.5
1
1.5
2
2.5
3
3.5
4
4.5
Normal
MK50
MK75
MK88
MK94
MK97
MK data
Normal data

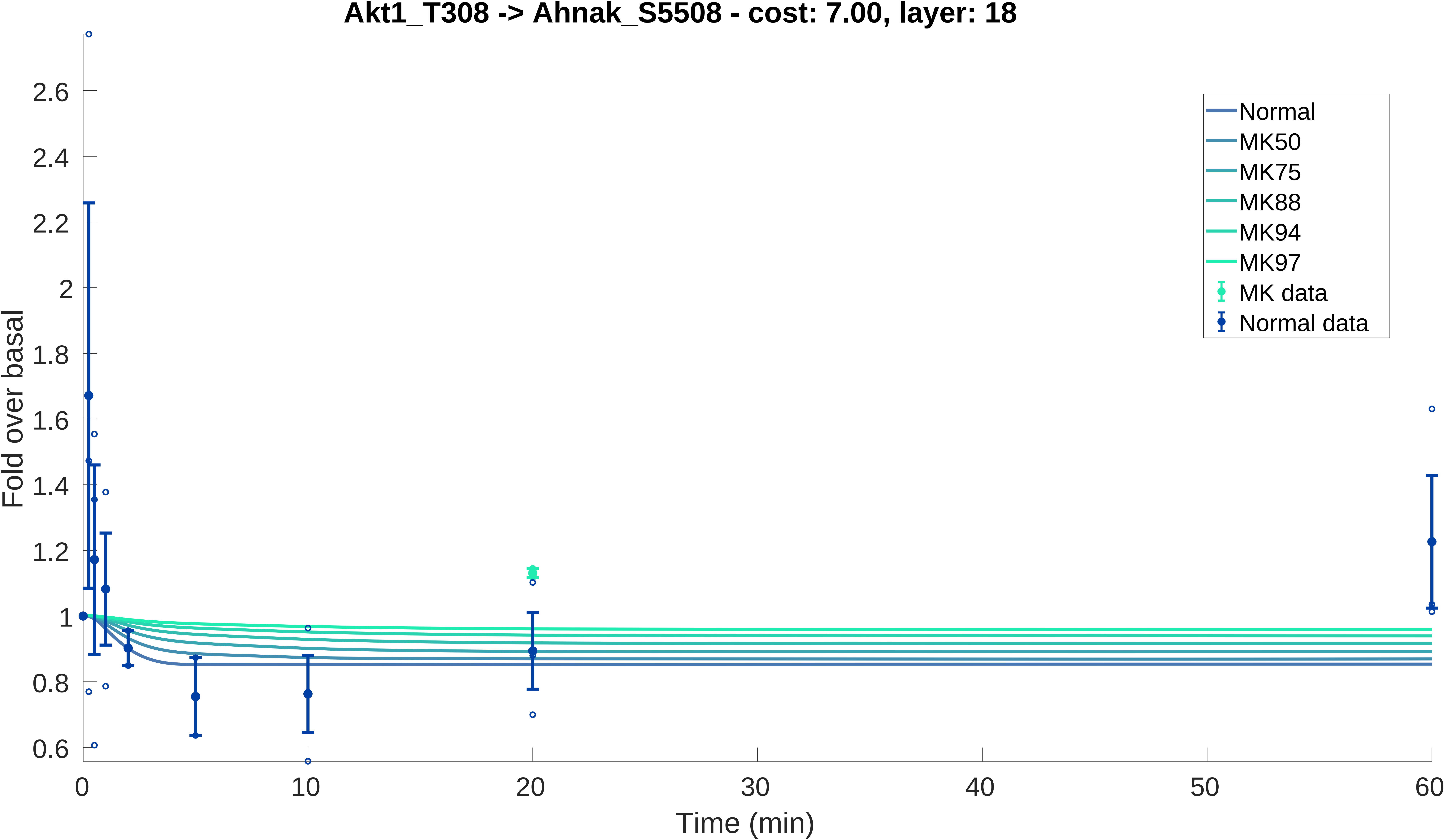
Akt1_T308 -> Ahnak_S5508 - cost: 7.00, layer: 18
Fold over basal
Time (min)
Normal
MK50
MK75
MK88
MK94
MK97
MK data
Normal data
2.6
2.4
2.2
2
1.8
1.6
1.4
1.2
1
0.8
0.6
0
10
20
30
40
50
60

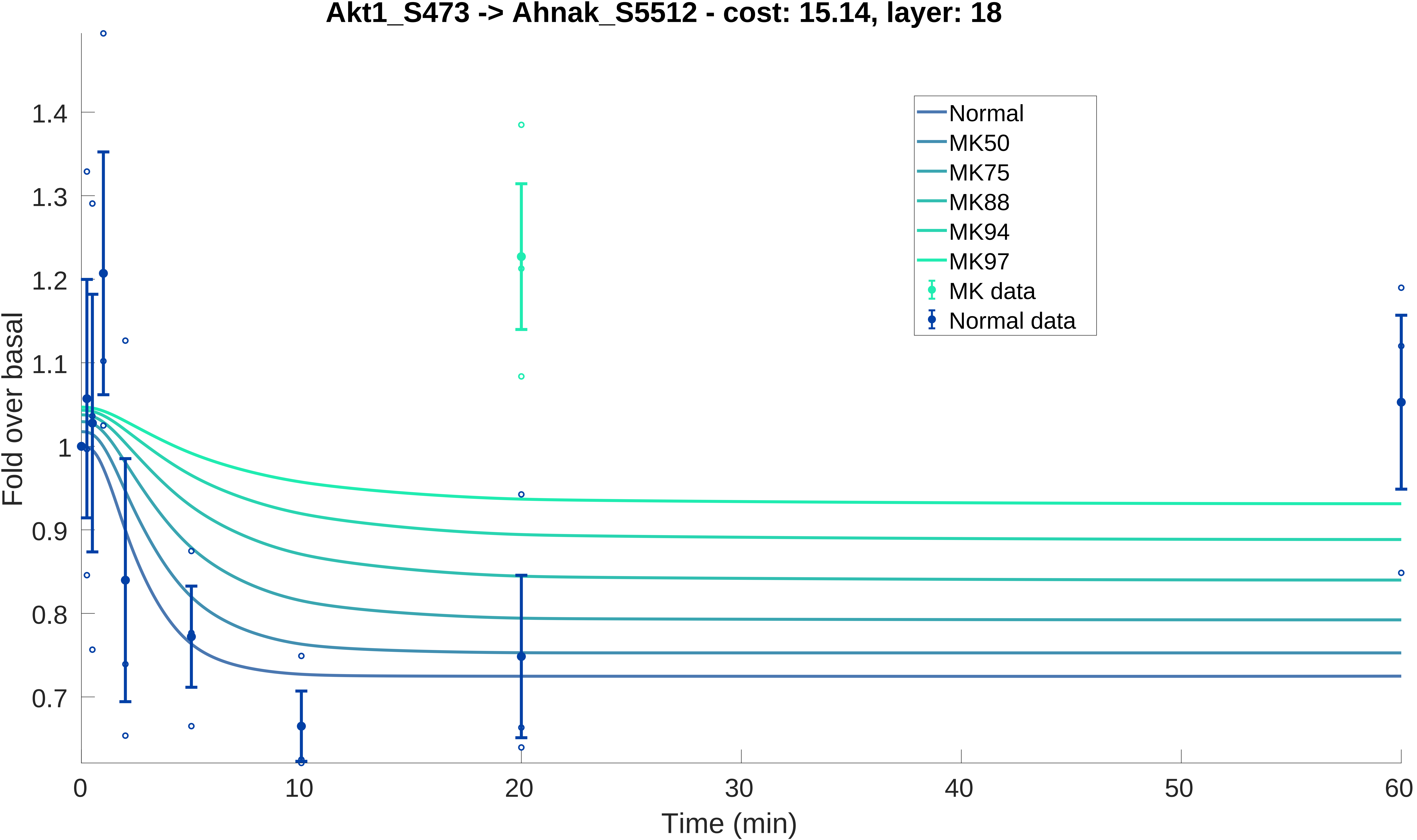

Akt1_S473 -> Ahnak_S5512 - cost: 15.14, layer: 18
Normal
MK50
MK75
MK88
MK94
MK97
MK data
Normal data
Fold over basal
1.4
1.3
1.2
1.1
1
0.9
0.8
0.7
Time (min)
0
10
20
30
40
50
60

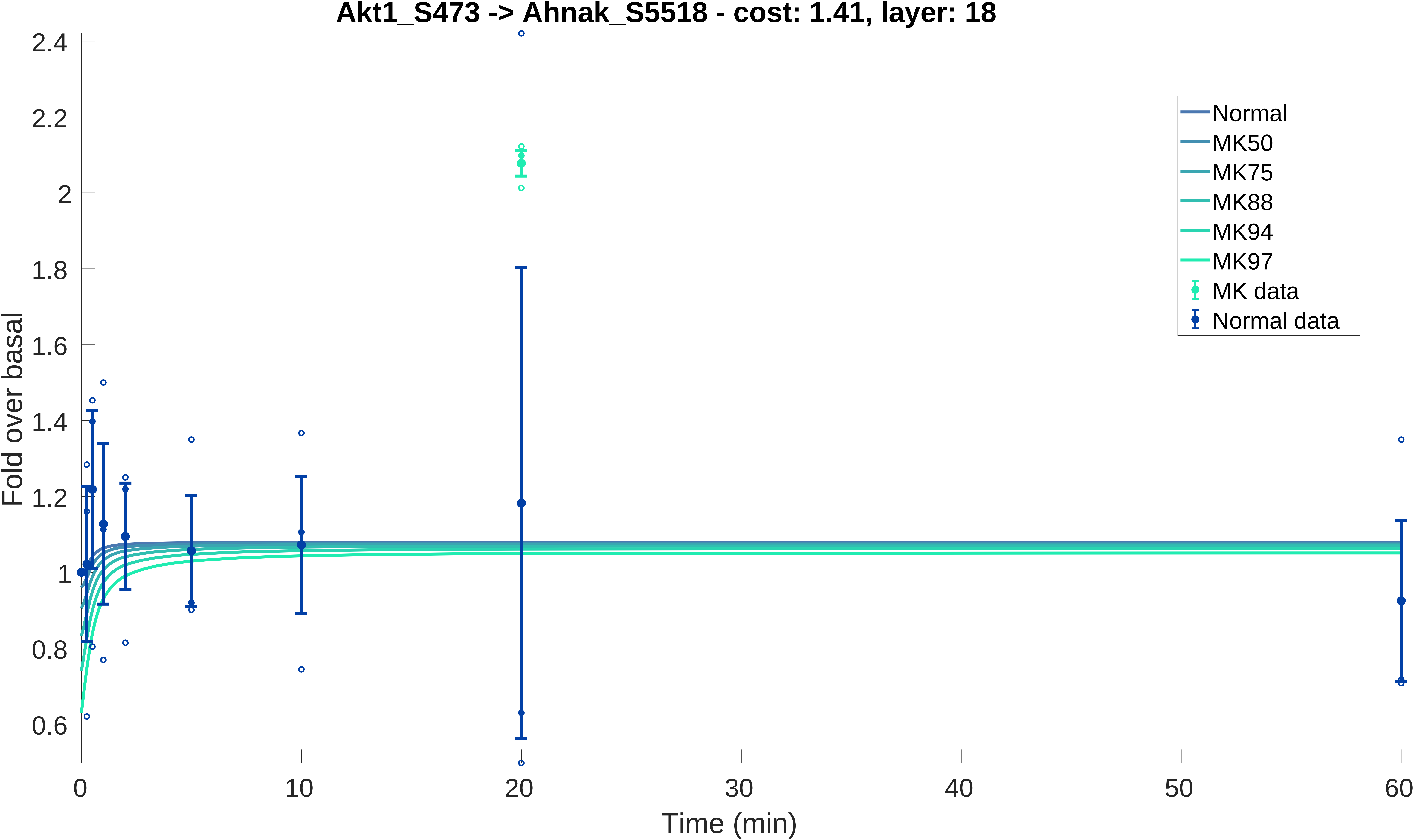
Akt1_S473 -> Ahnak_S5518 - cost: 1.41, layer: 18
Fold over basal
Time (min)
0
10
20
30
40
50
60
0.6
0.8
1
1.2
1.4
1.6
1.8
2
2.2
2.4
Normal
MK50
MK75
MK88
MK94
MK97
MK data
Normal data

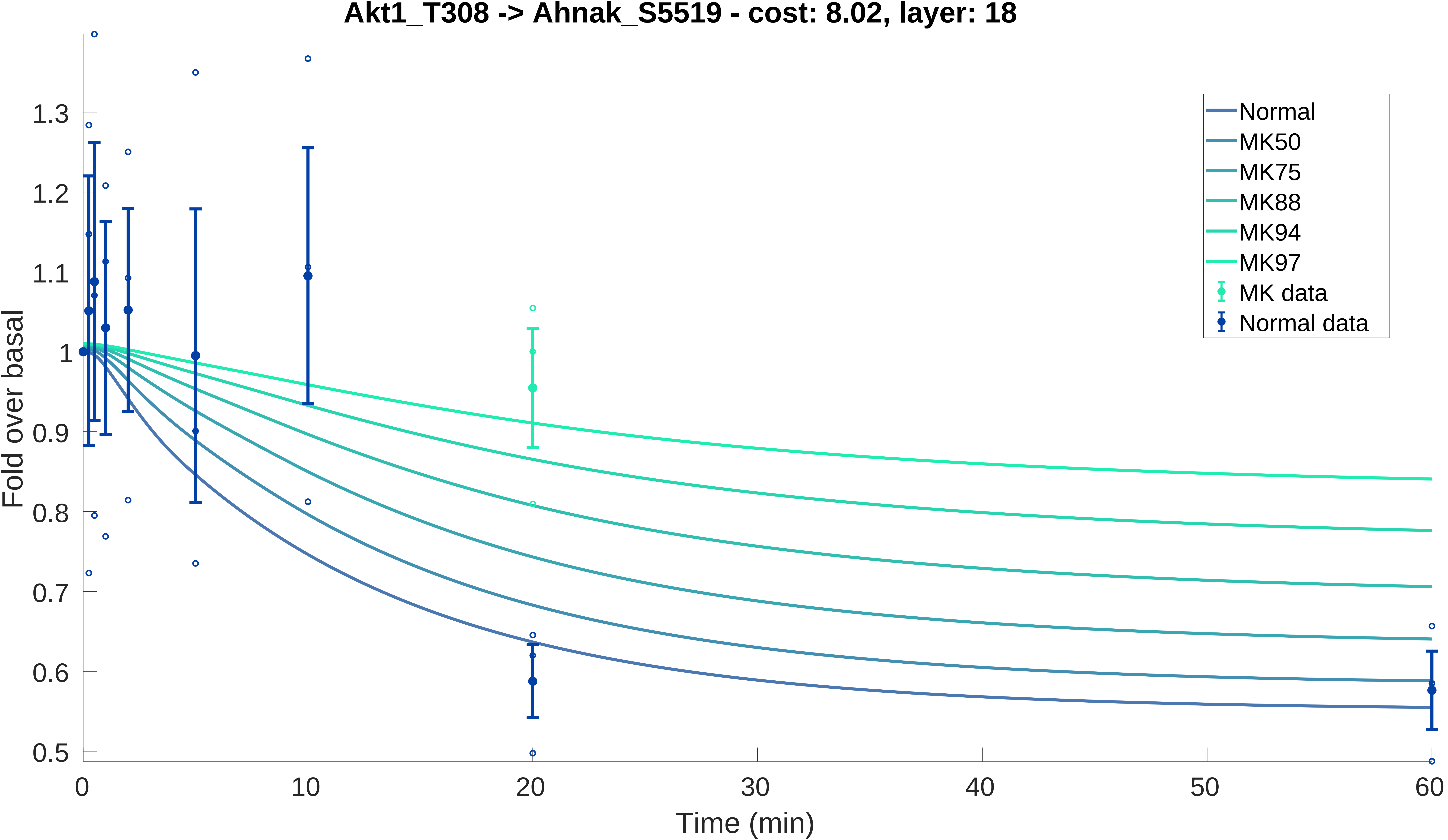

Akt1_T308 -> Ahnak_S5519 - cost: 8.02, layer: 18
Fold over basal
Time (min)
0.5
0.6
0.7
0.8
0.9
1
1.1
1.2
1.3
0
10
20
30
40
50
60
Normal
MK50
MK75
MK88
MK94
MK97
MK data
Normal data

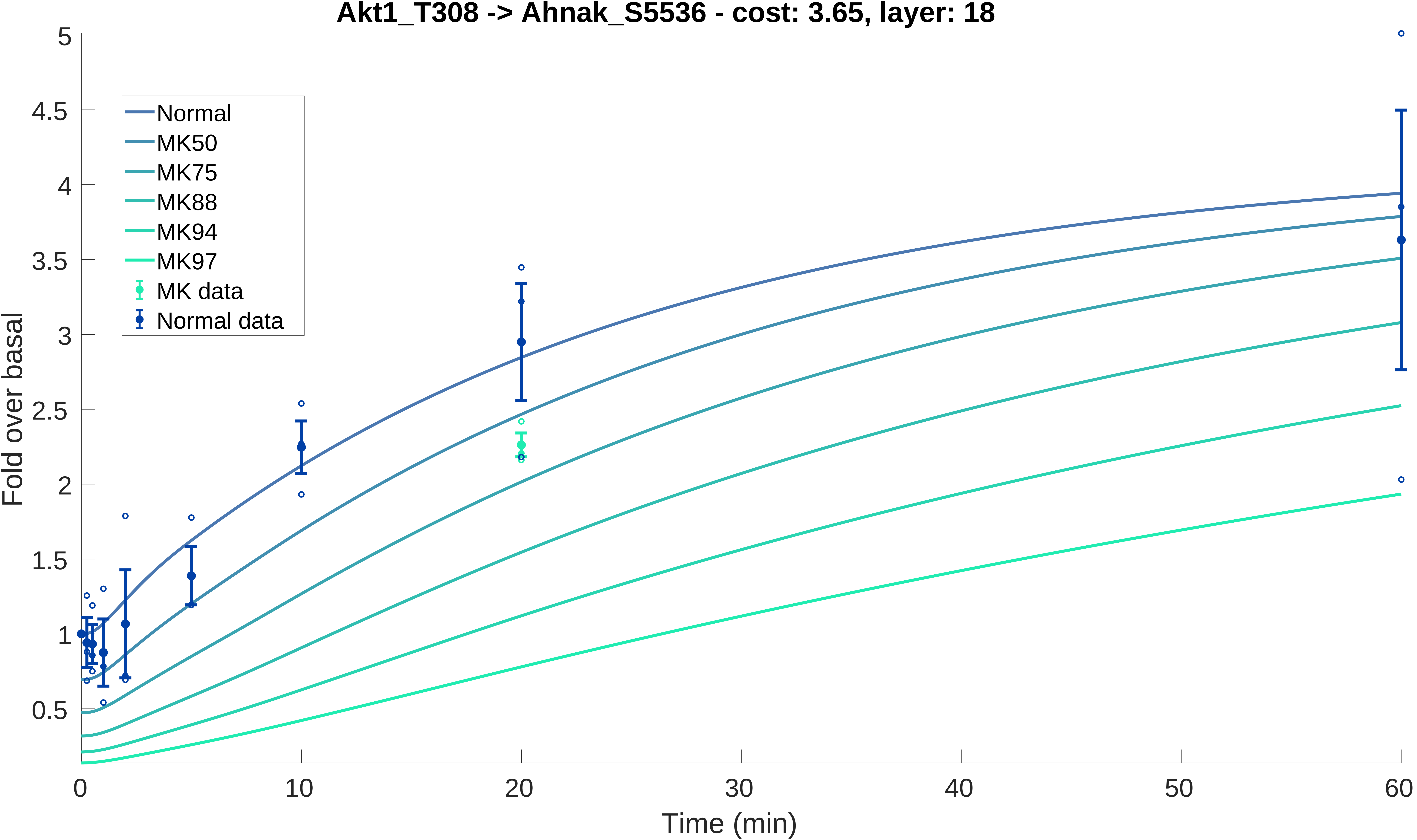

Akt1_T308 -> Ahnak_S5536 - cost: 3.65, layer: 18
Fold over basal
Time (min)
Normal
MK50
MK75
MK88
MK94
MK97
MK data
Normal data
0
10
20
30
40
50
60
0.5
1
1.5
2
2.5
3
3.5
4
4.5
5

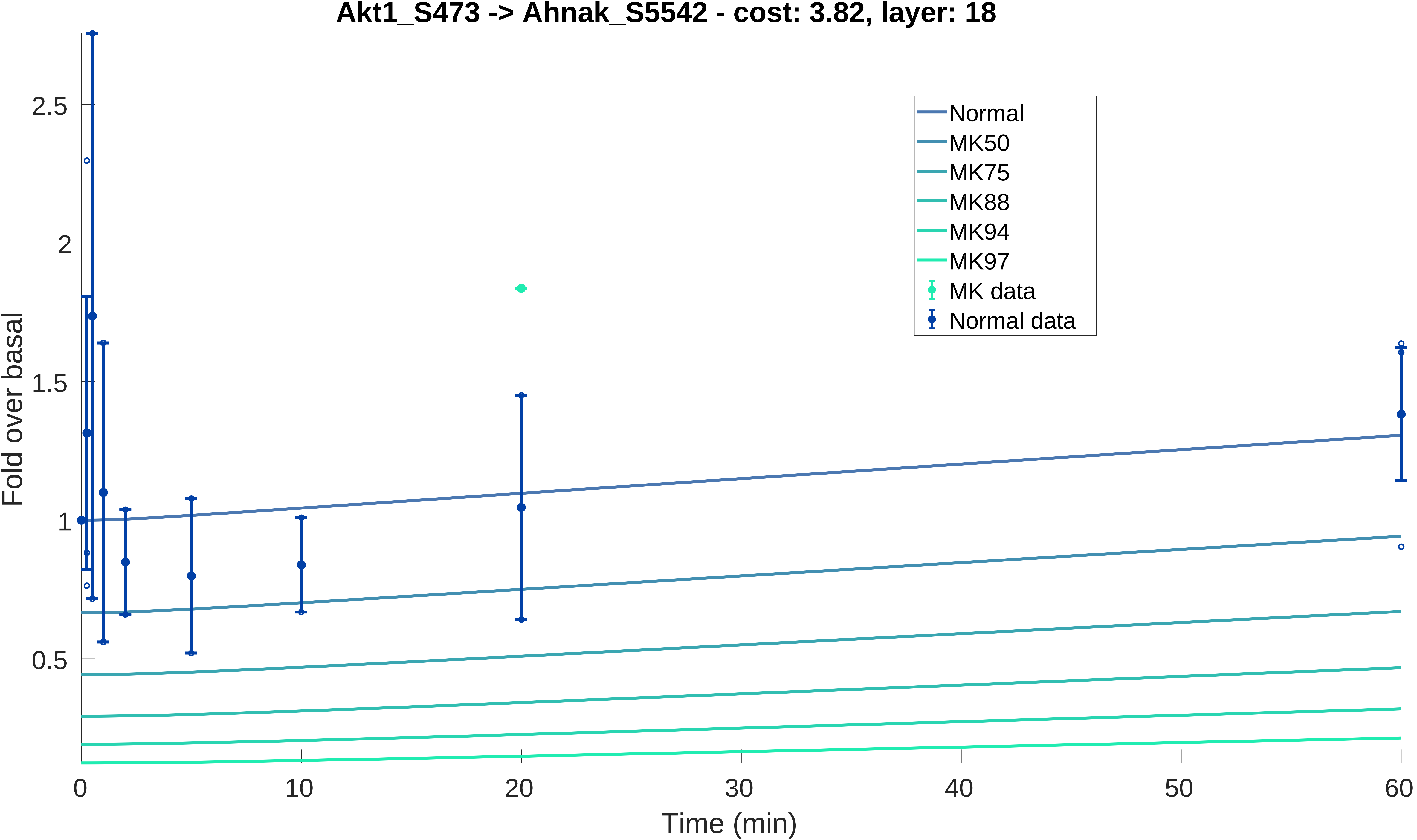
Akt1_S473 -> Ahnak_S5542 - cost: 3.82, layer: 18
Fold over basal
Time (min)
0.5
1
1.5
2
2.5
0
10
20
30
40
50
60
Normal
MK50
MK75
MK88
MK94
MK97
MK data
Normal data

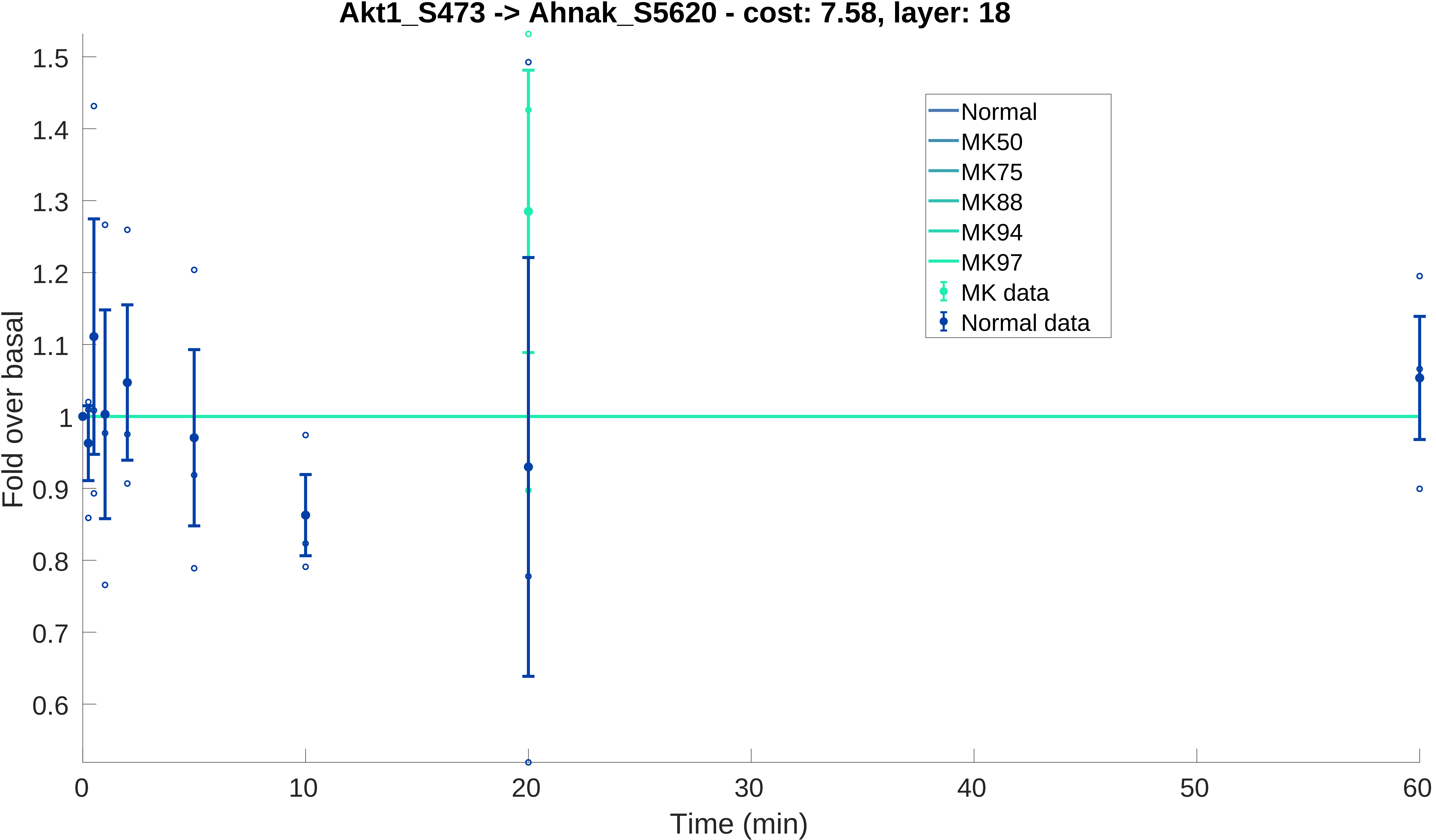

Akt1_S473 -> Ahnak_S5620 - cost: 7.58, layer: 18
Fold over basal
Time (min)
1.5
1.4
1.3
1.2
1.1
1
0.9
0.8
0.7
0.6
0
10
20
30
40
50
60
Normal
MK50
MK75
MK88
MK94
MK97
MK data
Normal data

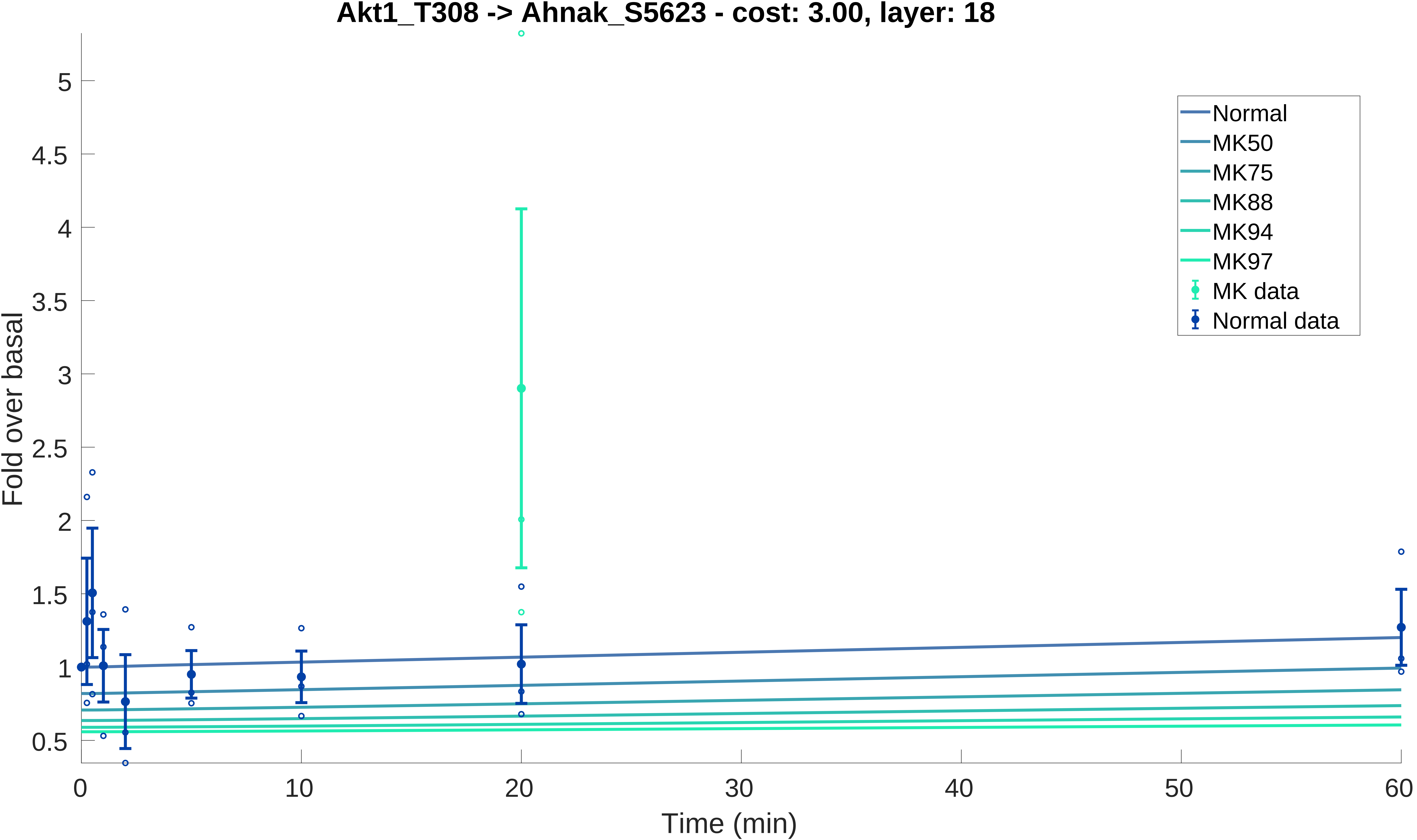
Akt1_T308 -> Ahnak_S5623 - cost: 3.00, layer: 18
Fold over basal
Time (min)
0.5
1
1.5
2
2.5
3
3.5
4
4.5
5
0
10
20
30
40
50
60
Normal
MK50
MK75
MK88
MK94
MK97
MK data
Normal data

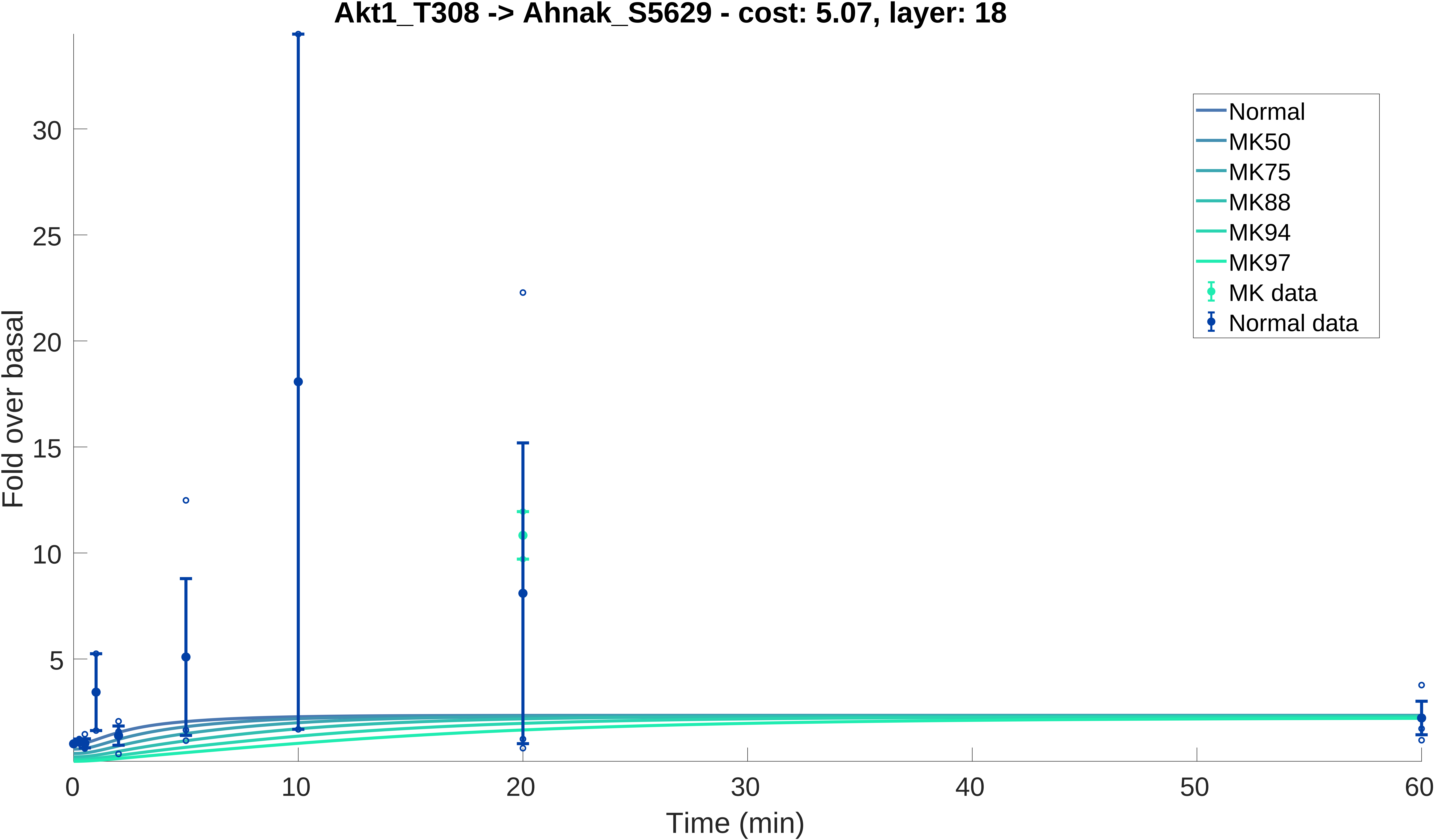

Akt1_T308 -> Ahnak_S5629 - cost: 5.07, layer: 18
Fold over basal
Time (min)
0
10
20
30
40
50
60
5
10
15
20
25
30
Normal
MK50
MK75
MK88
MK94
MK97
MK data
Normal data

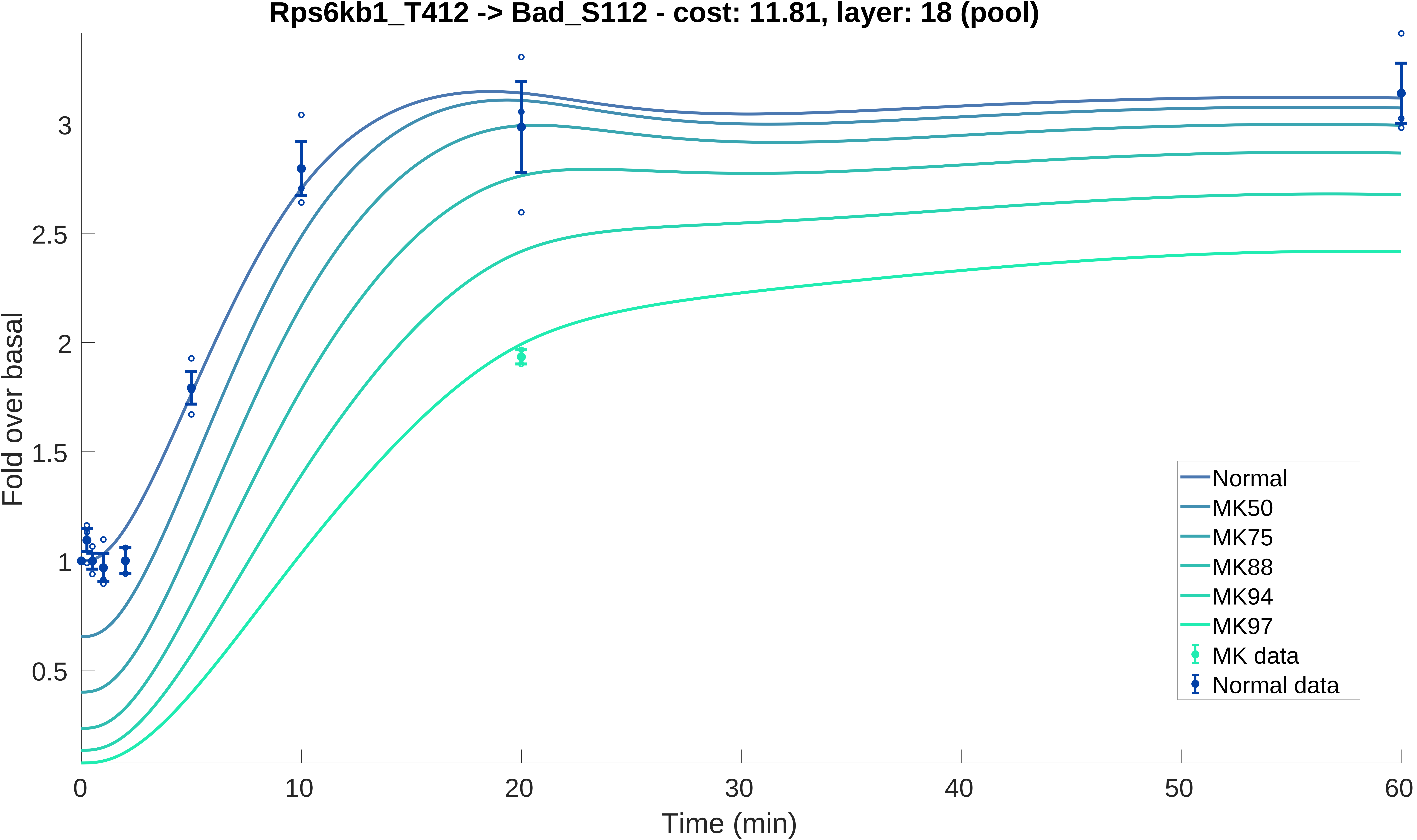

Rps6kb1_T412 -> Bad_S112 - cost: 11.81, layer: 18 (pool)
Fold over basal
3
2.5
2
1.5
1
0.5
0
10
20
30
40
50
60
Time (min)
Normal
MK50
MK75
MK88
MK94
MK97
MK data
Normal data

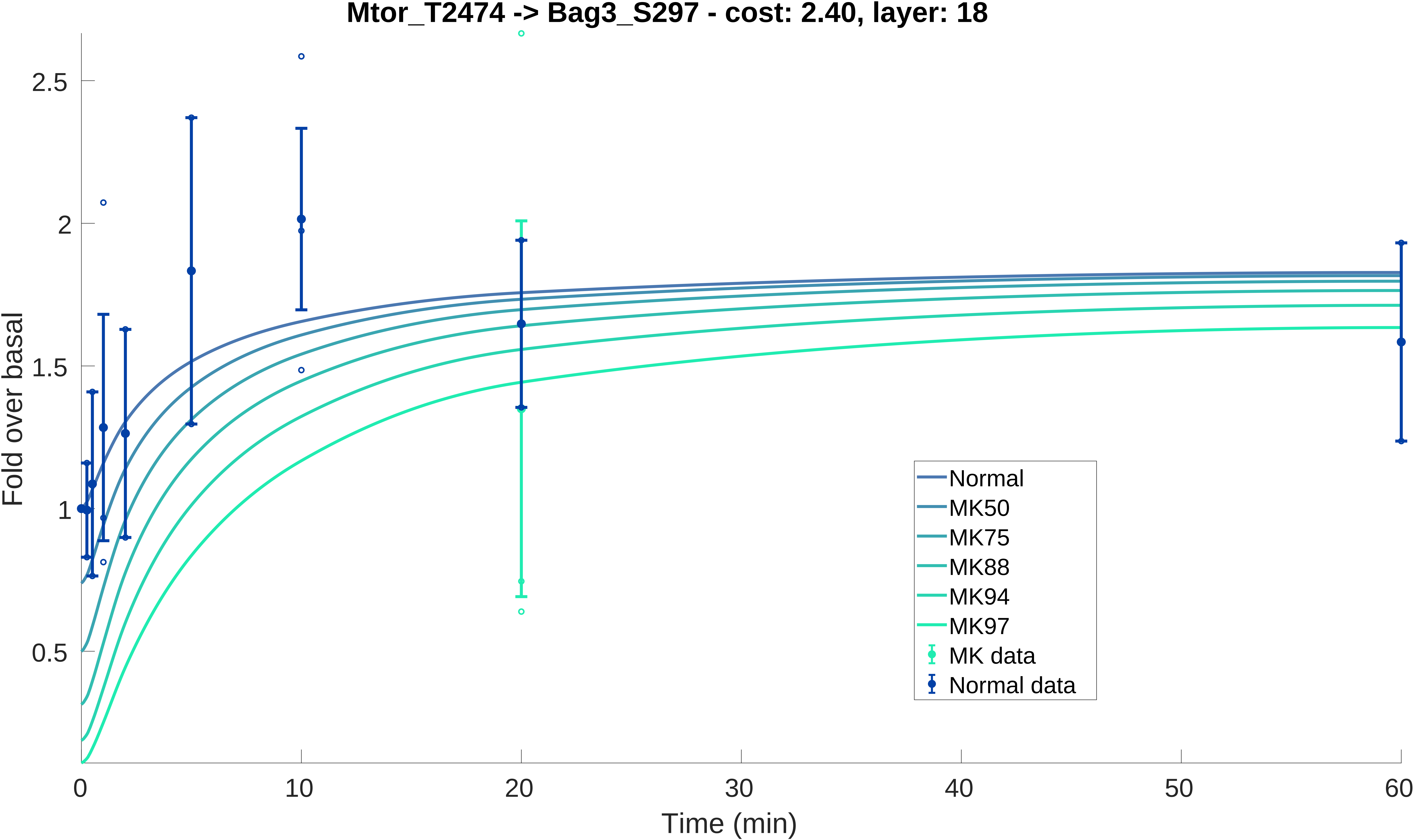
Mtor_T2474 -> Bag3_S297 - cost: 2.40, layer: 18
Fold over basal
Time (min)
0.5
1
1.5
2
2.5
0
10
20
30
40
50
60
Normal
MK50
MK75
MK88
MK94
MK97
MK data
Normal data

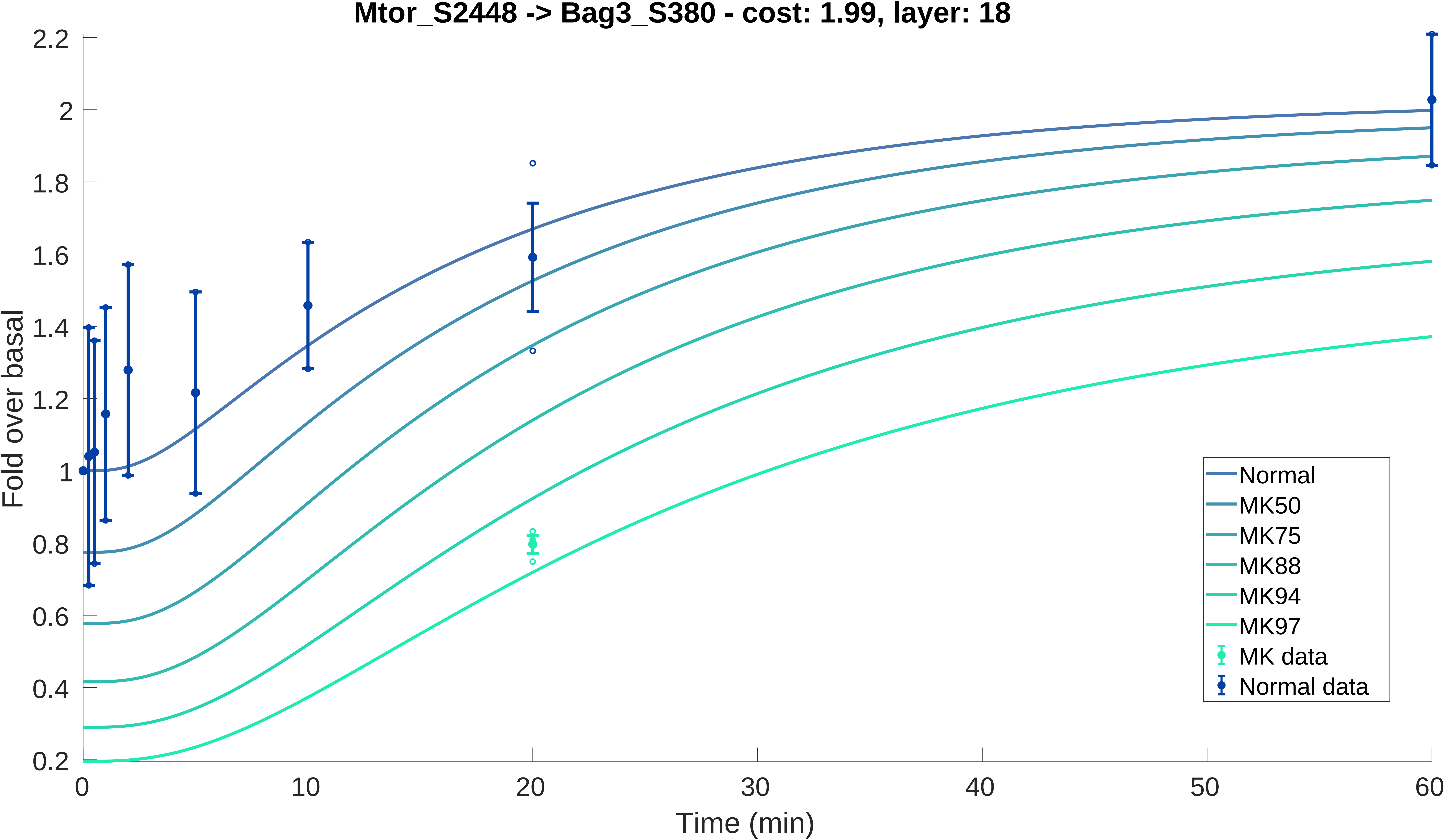

Mtor_S2448 -> Bag3_S380 - cost: 1.99, layer: 18
Fold over basal
2.2
2
1.8
1.6
1.4
1.2
1
0.8
0.6
0.4
0.2
0
10
20
30
40
50
60
Time (min)
Normal
MK50
MK75
MK88
MK94
MK97
MK data
Normal data

Akt1_T308 -> Flnc_S2234 - cost: 2.43, layer: 18

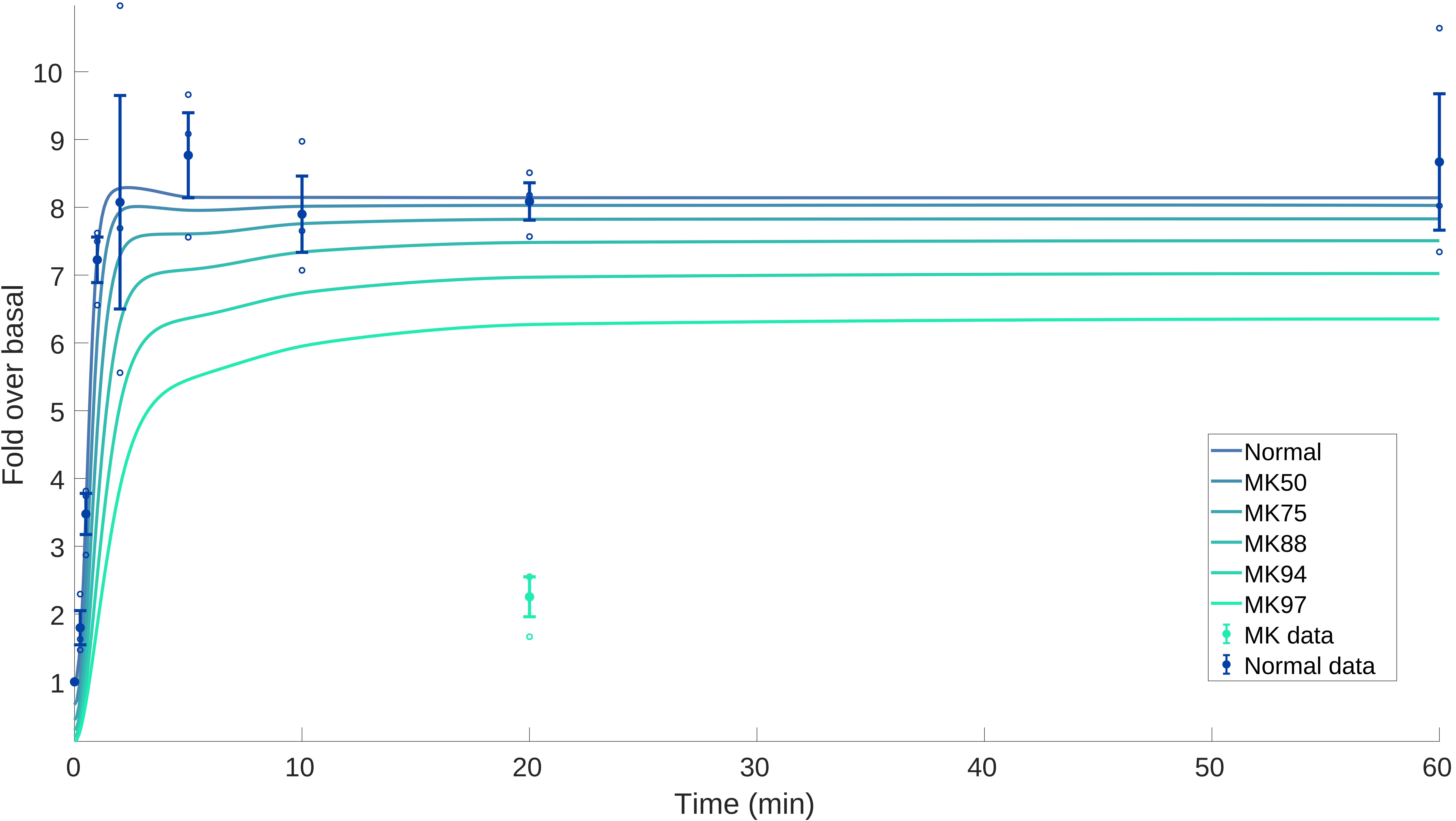

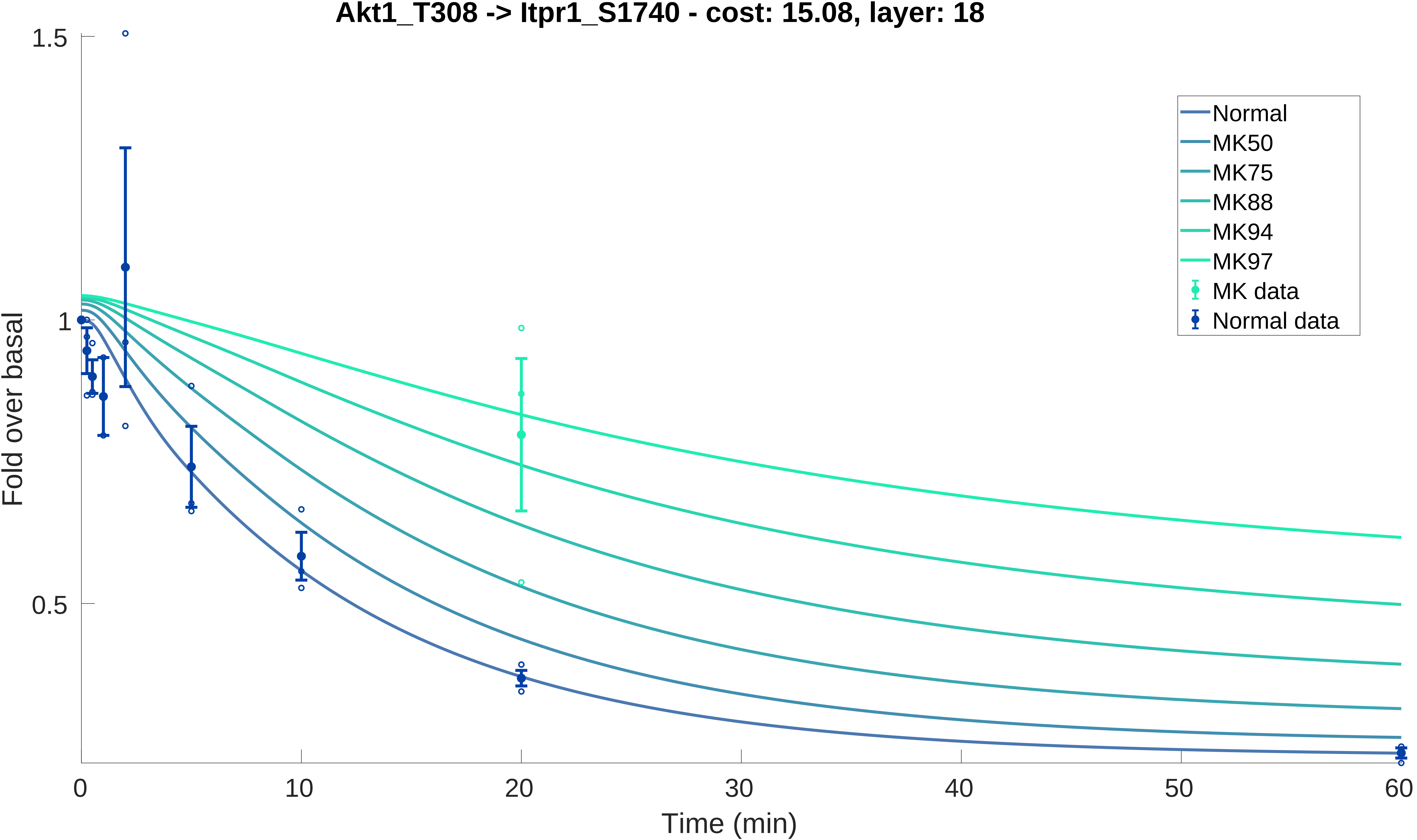

Akt1_T308 -> Itpr1_S1740 - cost: 15.08, layer: 18
1.5
1
0.5
Fold over basal
0
10
20
30
40
50
60
Time (min)
Normal
MK50
MK75
MK88
MK94
MK97
MK data
Normal data

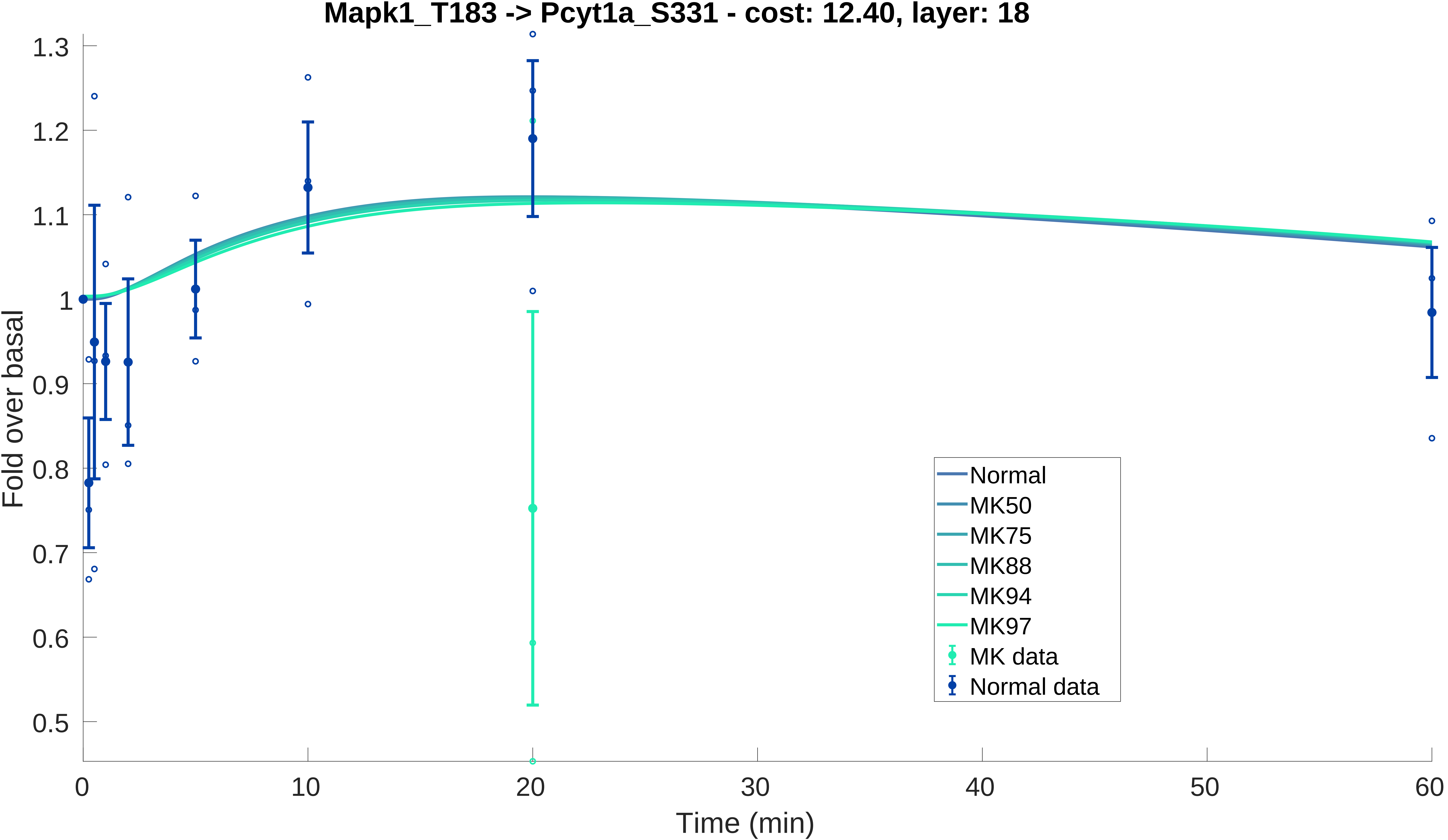

Mapk1_T183 -> Pcyt1a_S331 - cost: 12.40, layer: 18
Fold over basal
Time (min)
1.3
1.2
1.1
1
0.9
0.8
0.7
0.6
0.5
0
10
20
30
40
50
60
Normal
MK50
MK75
MK88
MK94
MK97
MK data
Normal data

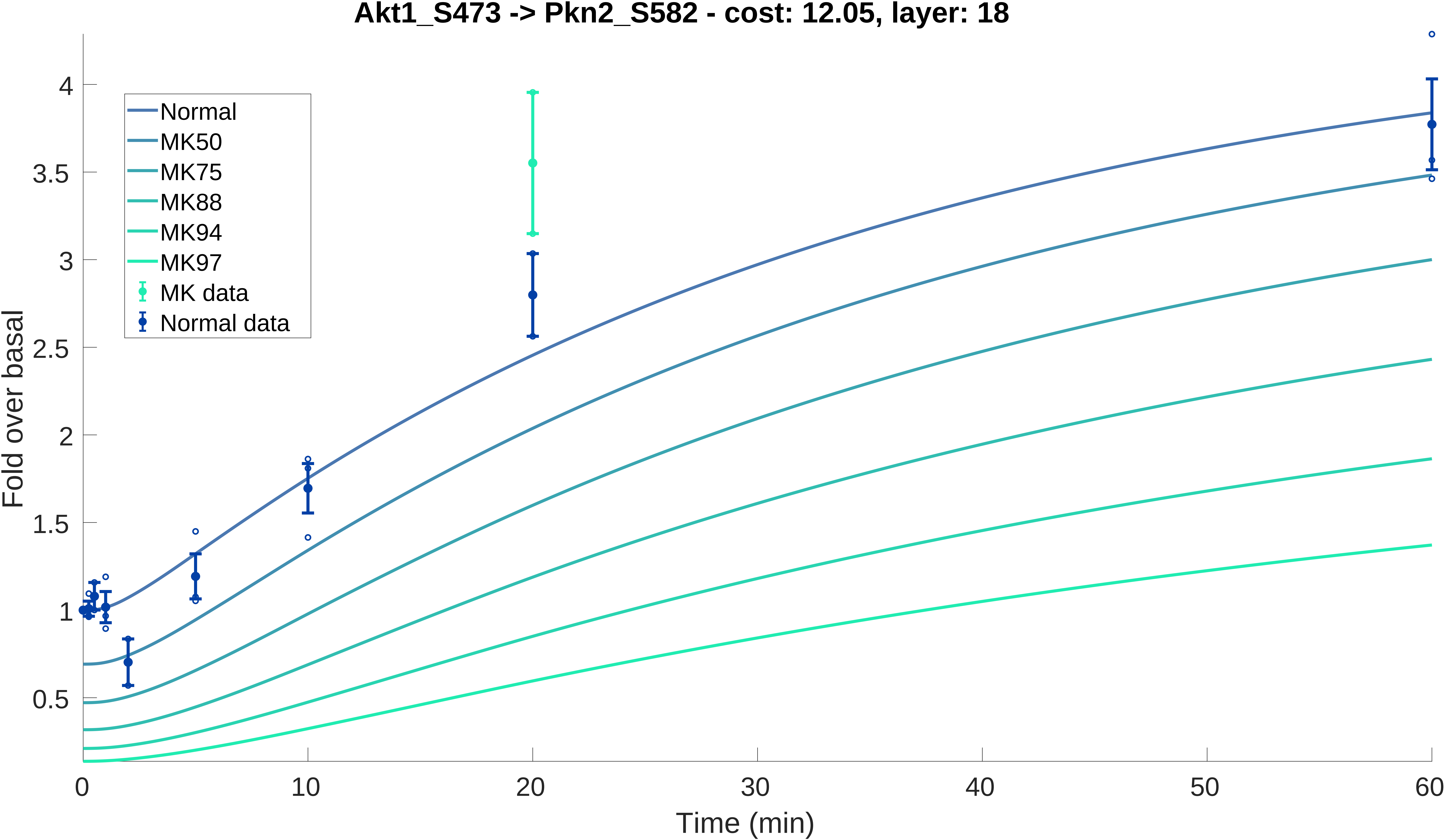

Akt1_S473 -> Pkn2_S582 - cost: 12.05, layer: 18
Normal
MK50
MK75
MK88
MK94
MK97
MK data
Normal data
Fold over basal
0.5
1
1.5
2
2.5
3
3.5
4
0
10
20
30
40
50
60
Time (min)

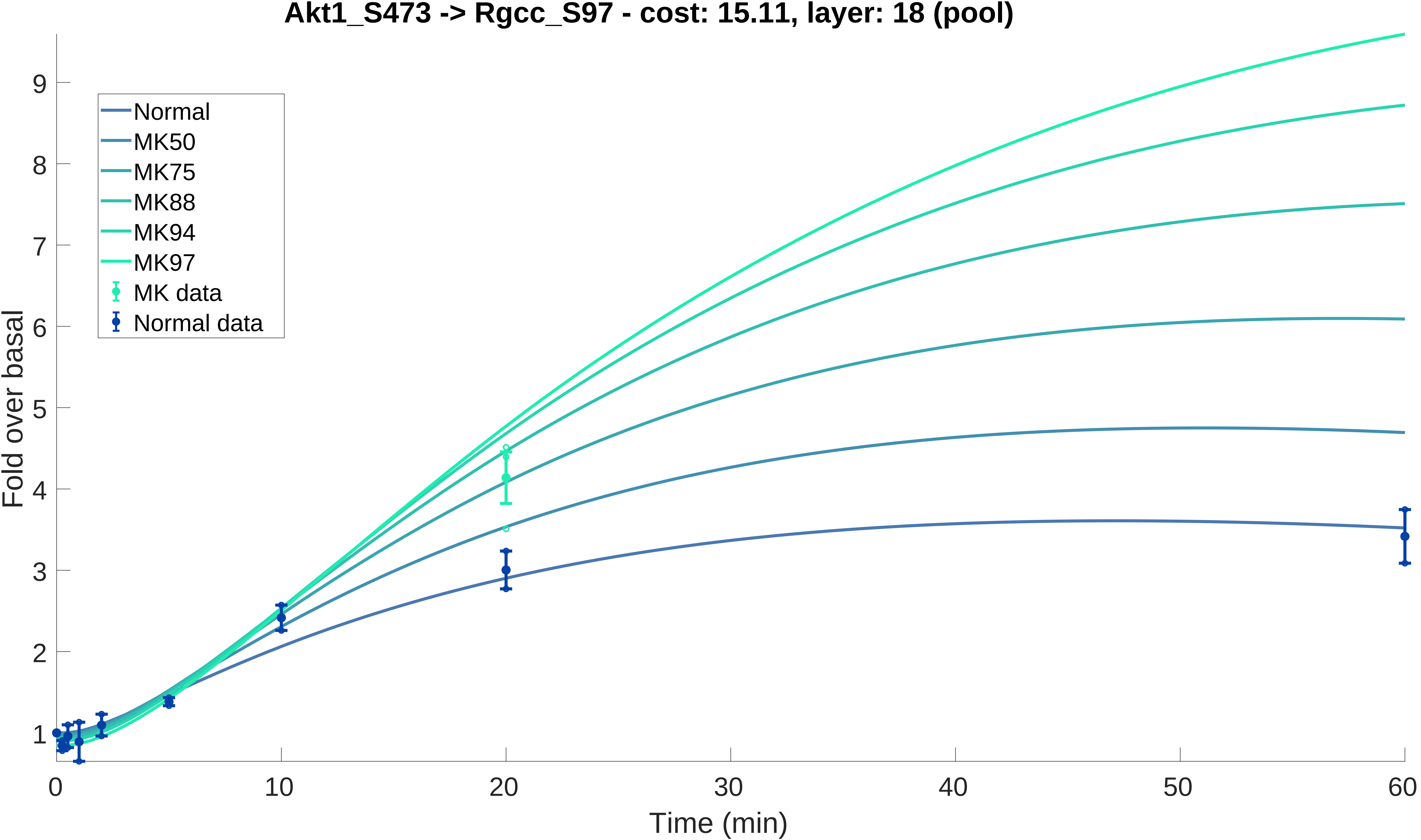

Akt1_S473 -> Rgcc_S97 - cost: 15.11, layer: 18 (pool)
Fold over basal
Time (min)
Normal
MK50
MK75
MK88
MK94
MK97
MK data
Normal data
1
2
3
4
5
6
7
8
9
0
10
20
30
40
50
60

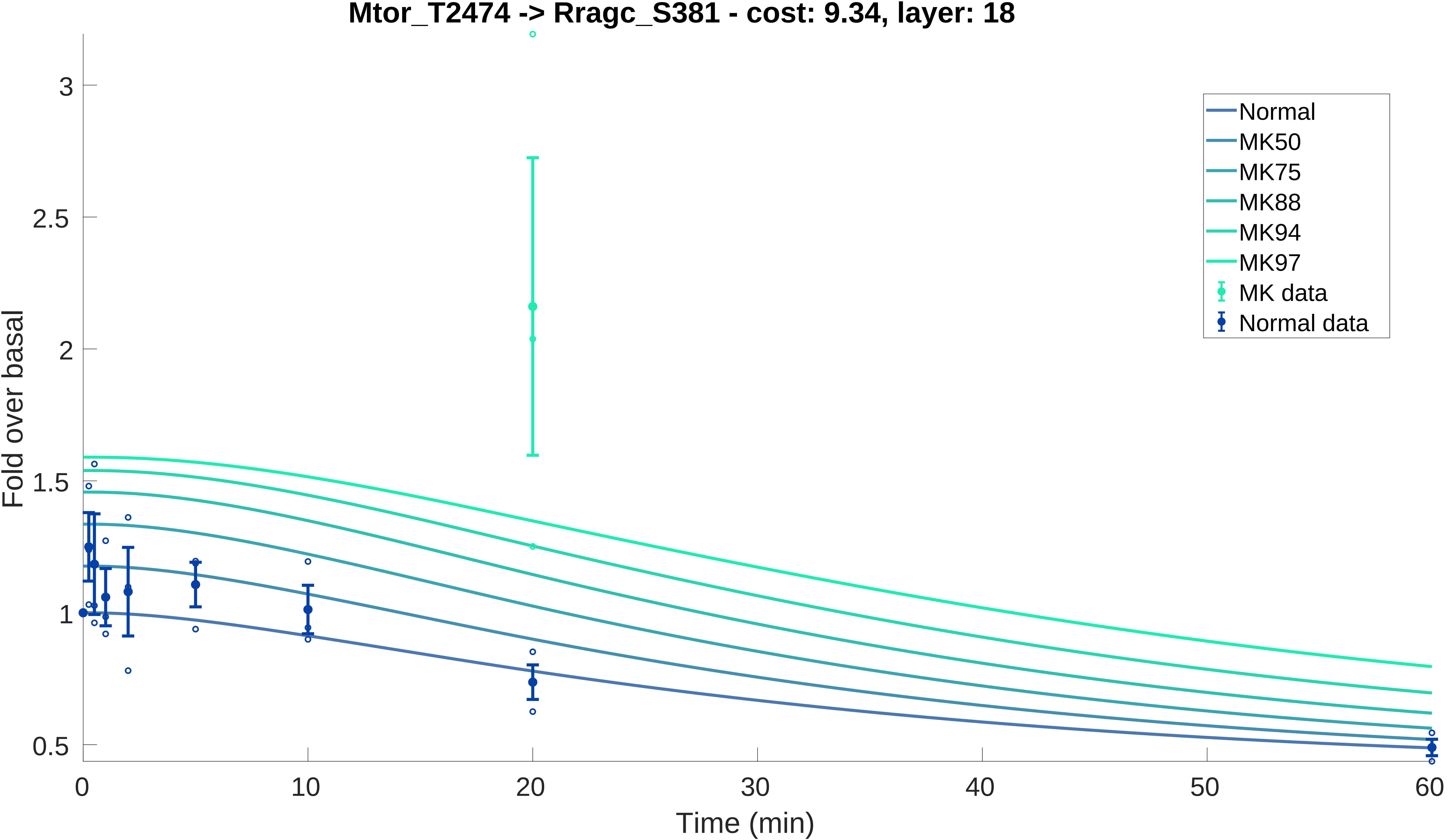
Mtor_T2474 -> Rragc_S381 - cost: 9.34, layer: 18
Fold over basal
Time (min)
Normal
MK50
MK75
MK88
MK94
MK97
MK data
Normal data
0.5
1
1.5
2
2.5
3
0
10
20
30
40
50
60

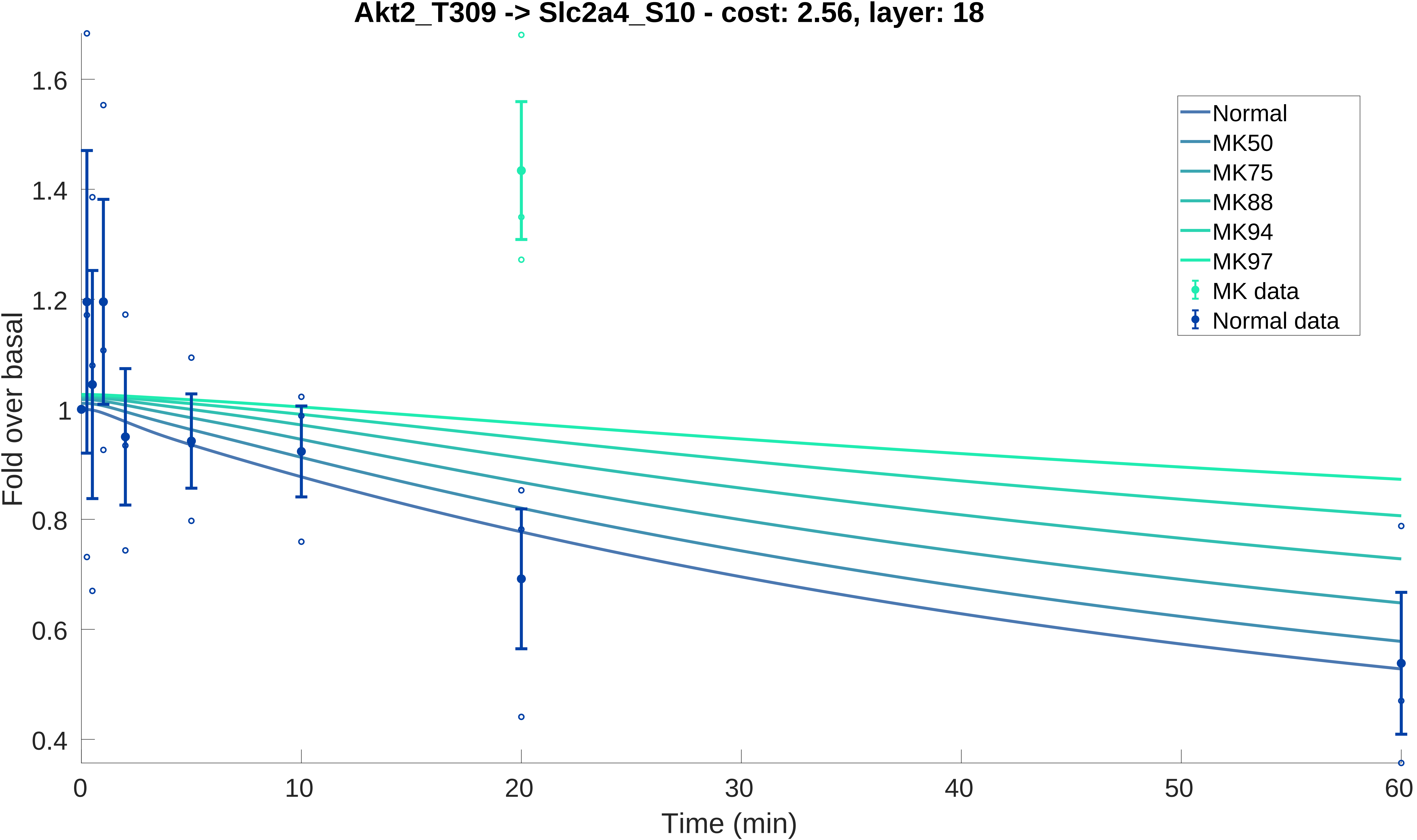
Akt2_T309 -> Slc2a4_S10 - cost: 2.56, layer: 18
Fold over basal
1.6
1.4
1.2
1
0.8
0.6
0.4
0
10
20
30
40
50
60
Time (min)
Normal
MK50
MK75
MK88
MK94
MK97
MK data
Normal data

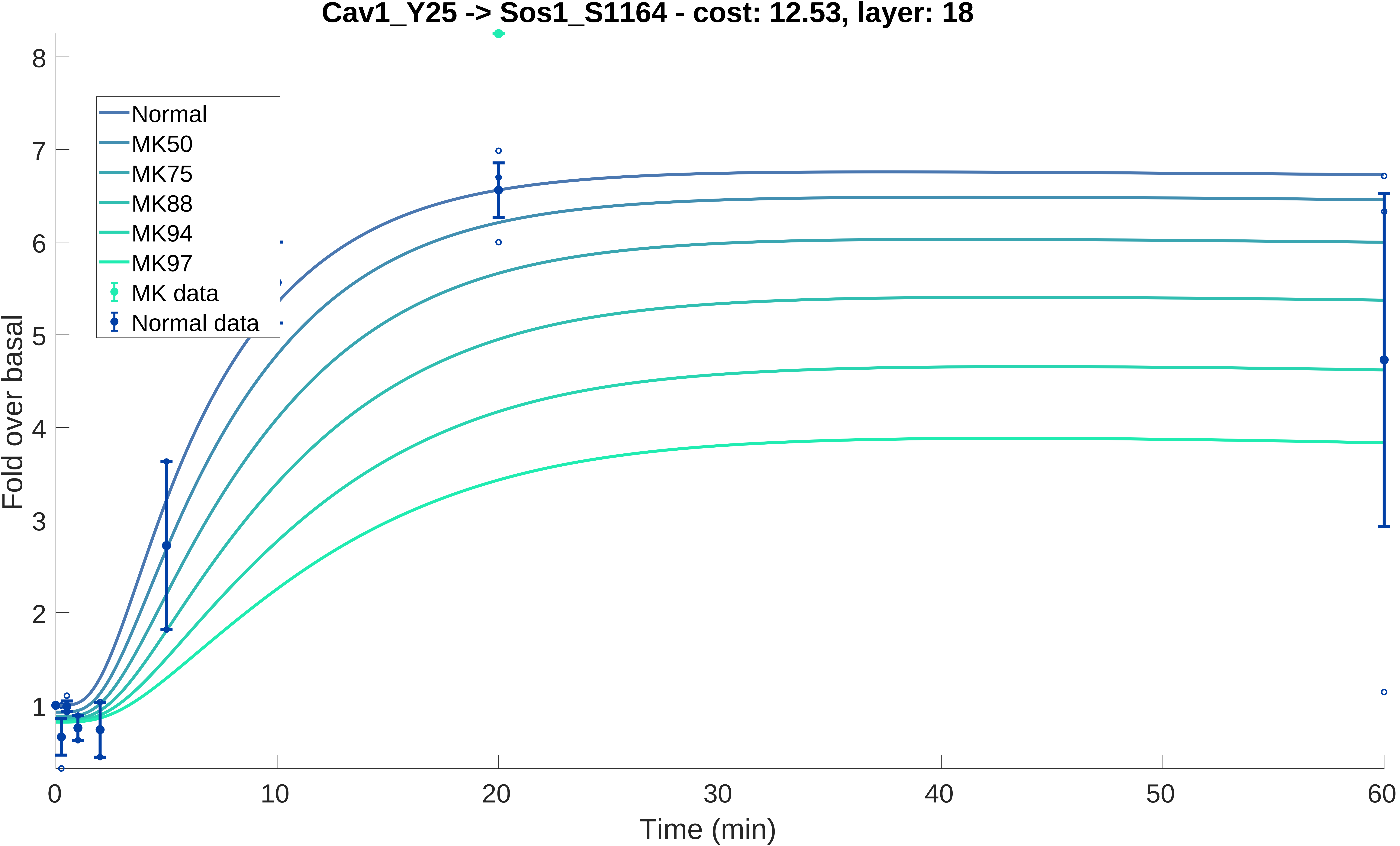

Cav1_Y25 -> Sos1_S1164 - cost: 12.53, layer: 18
Normal
MK50
MK75
MK88
MK94
MK97
MK data
Normal data
Fold over basal
Time (min)
0
10
20
30
40
50
60
1
2
3
4
5
6
7
8

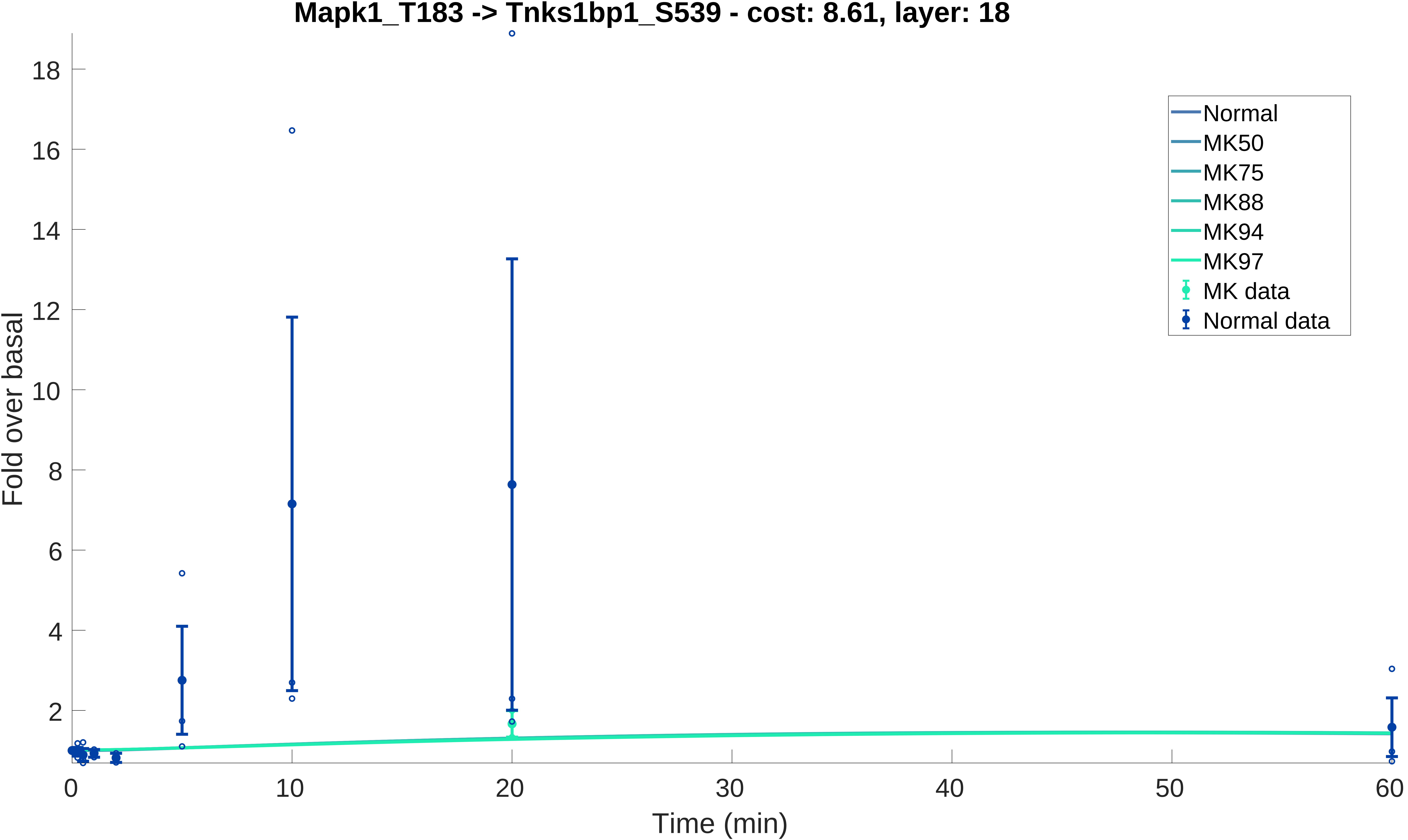

Mapk1_T183 -> Tnks1bp1_S539 - cost: 8.61, layer: 18
Fold over basal
Time (min)
0
2
4
6
8
10
12
14
16
18
0
10
20
30
40
50
60
Normal
MK50
MK75
MK88
MK94
MK97
MK data
Normal data

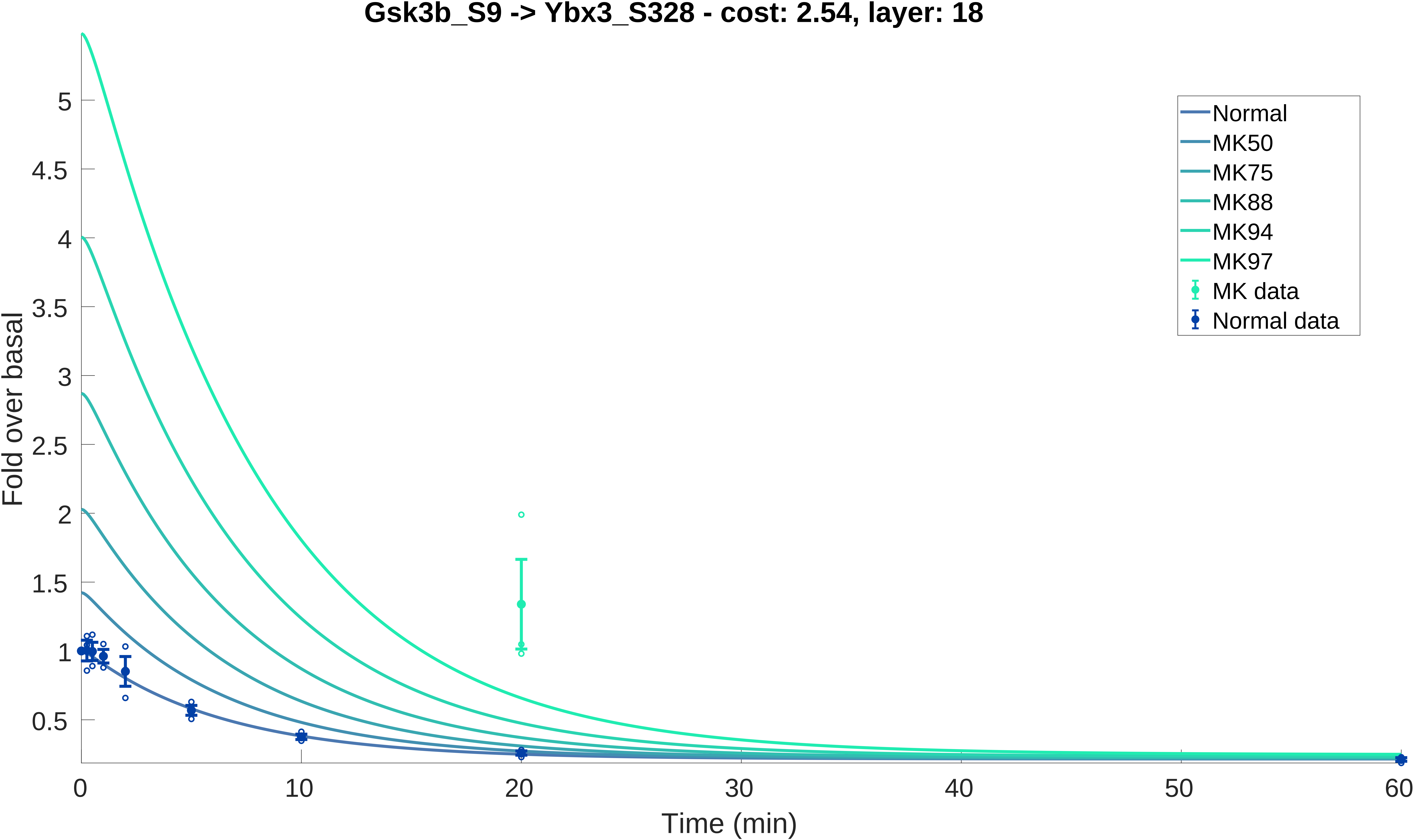

Gsk3b_S9 -> Ybx3_S328 - cost: 2.54, layer: 18
Fold over basal
Time (min)
0
10
20
30
40
50
60
0.5
1
1.5
2
2.5
3
3.5
4
4.5
5
Normal
MK50
MK75
MK88
MK94
MK97
MK data
Normal data

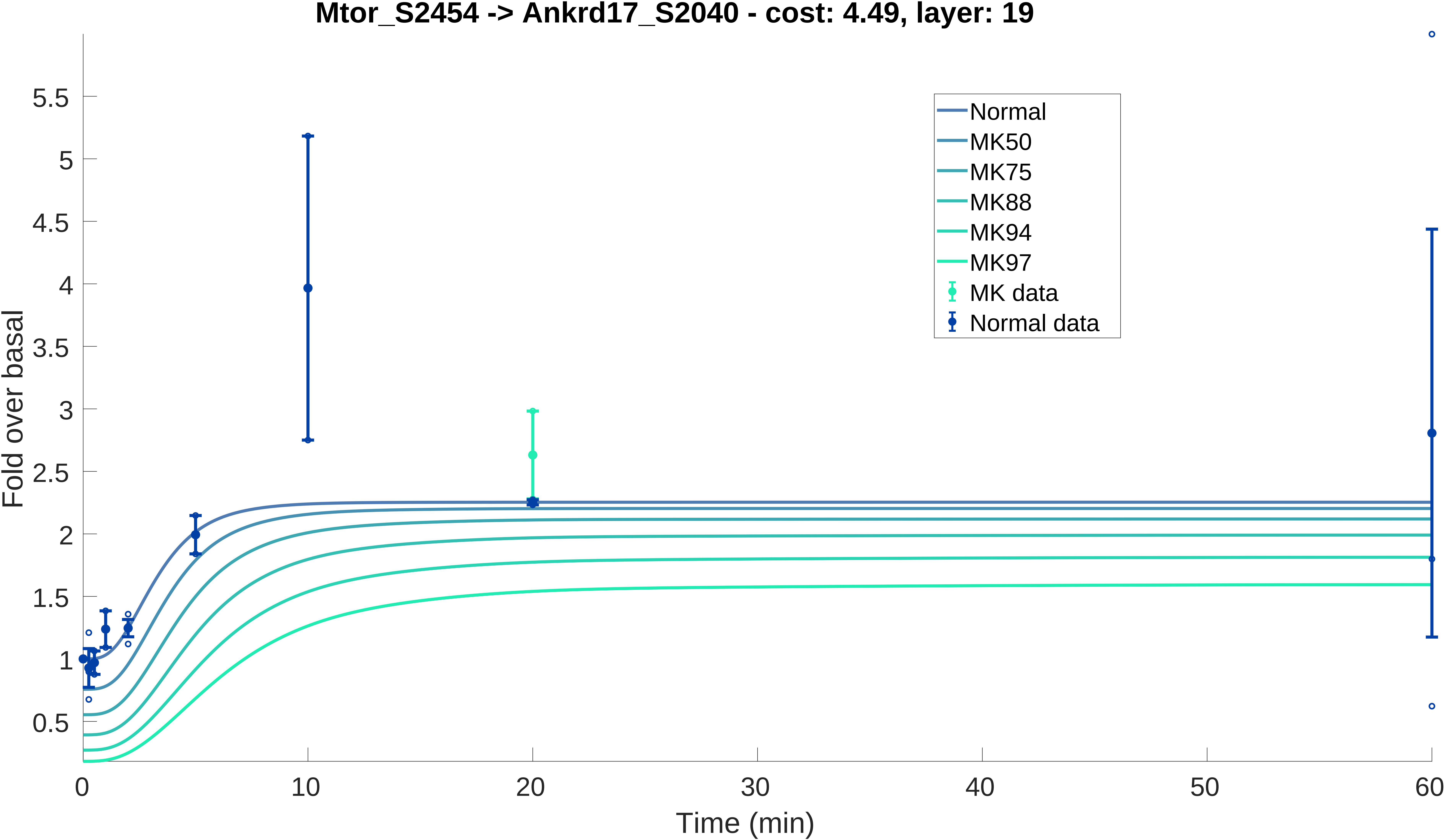

Mtor_S2454 -> Ankrd17_S2040 - cost: 4.49, layer: 19
Fold over basal
Time (min)
Normal
MK50
MK75
MK88
MK94
MK97
MK data
Normal data
0.5
1
1.5
2
2.5
3
3.5
4
4.5
5
5.5
0
10
20
30
40
50
60

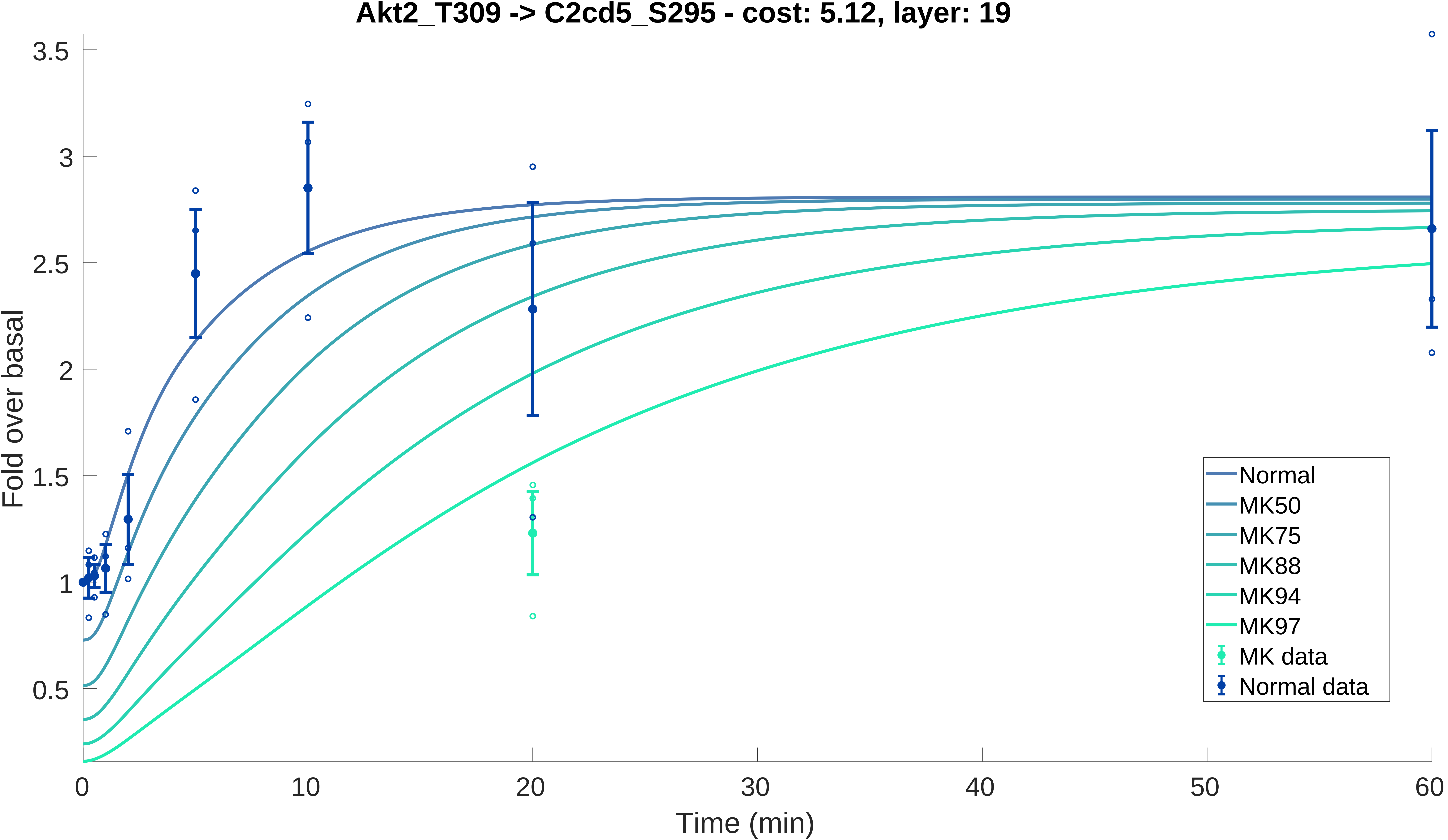

Akt2_T309 -> C2cd5_S295 - cost: 5.12, layer: 19
Fold over basal
Time (min)
0.5
1
1.5
2
2.5
3
3.5
0
10
20
30
40
50
60
Normal
MK50
MK75
MK88
MK94
MK97
MK data
Normal data

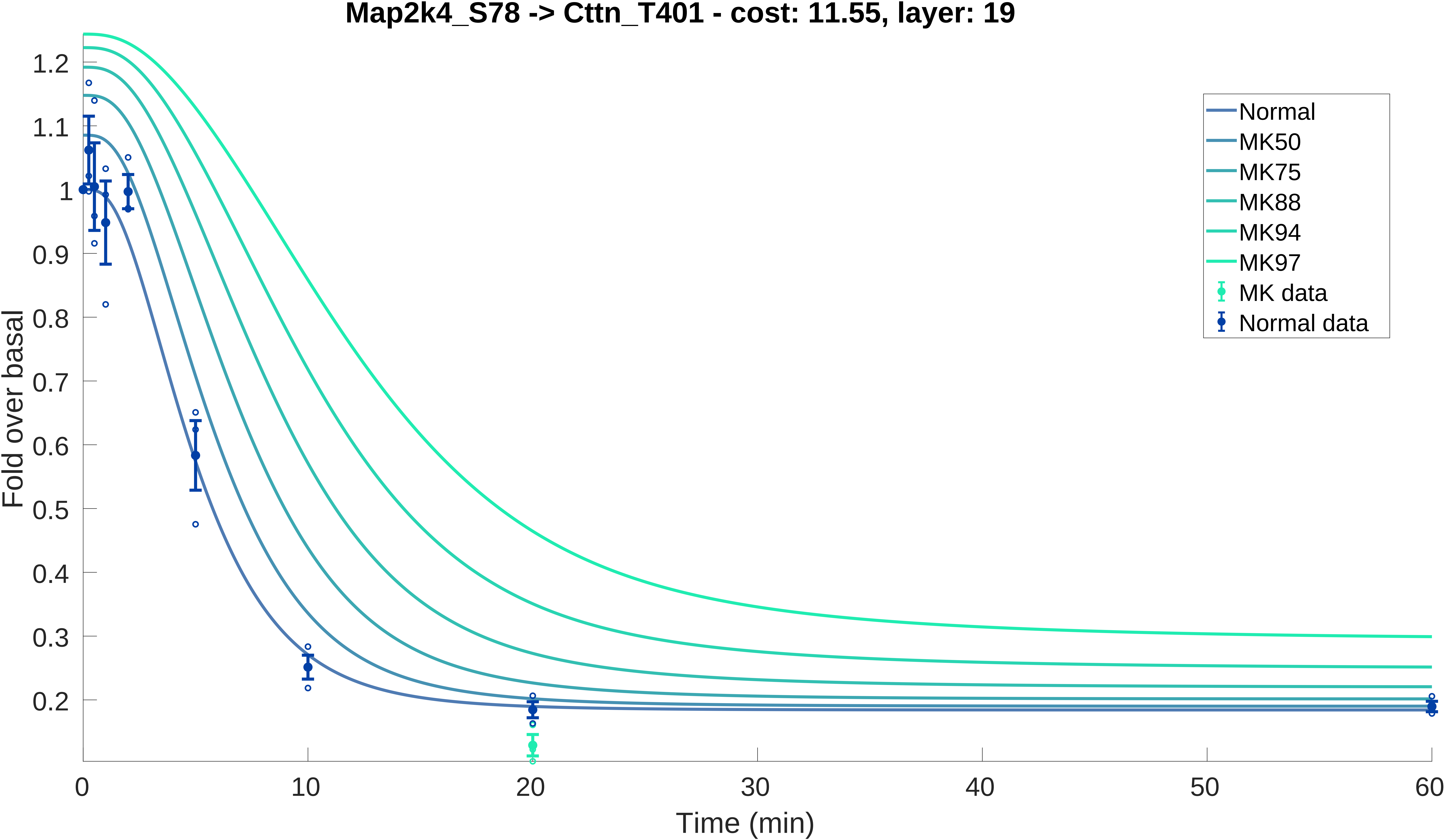
Map2k4_S78 -> Cttn_T401 - cost: 11.55, layer: 19
Fold over basal
Time (min)
0
10
20
30
40
50
60
0.2
0.3
0.4
0.5
0.6
0.7
0.8
0.9
1
1.1
1.2
Normal
MK50
MK75
MK88
MK94
MK97
MK data
Normal data

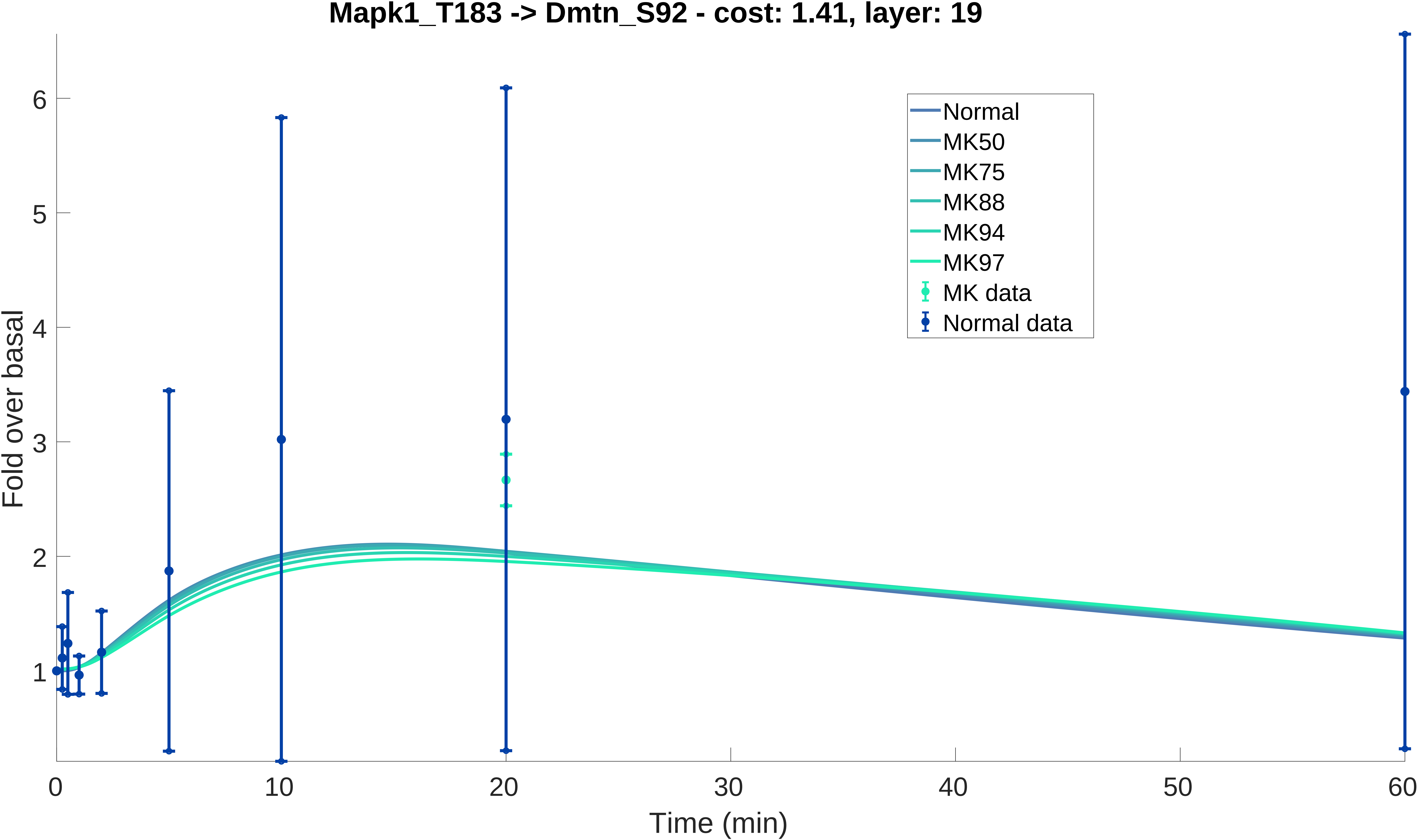

Mapk1_T183 -> Dmtn_S92 - cost: 1.41, layer: 19
Fold over basal
Time (min)
Normal
MK50
MK75
MK88
MK94
MK97
MK data
Normal data
1
2
3
4
5
6
0
10
20
30
40
50
60

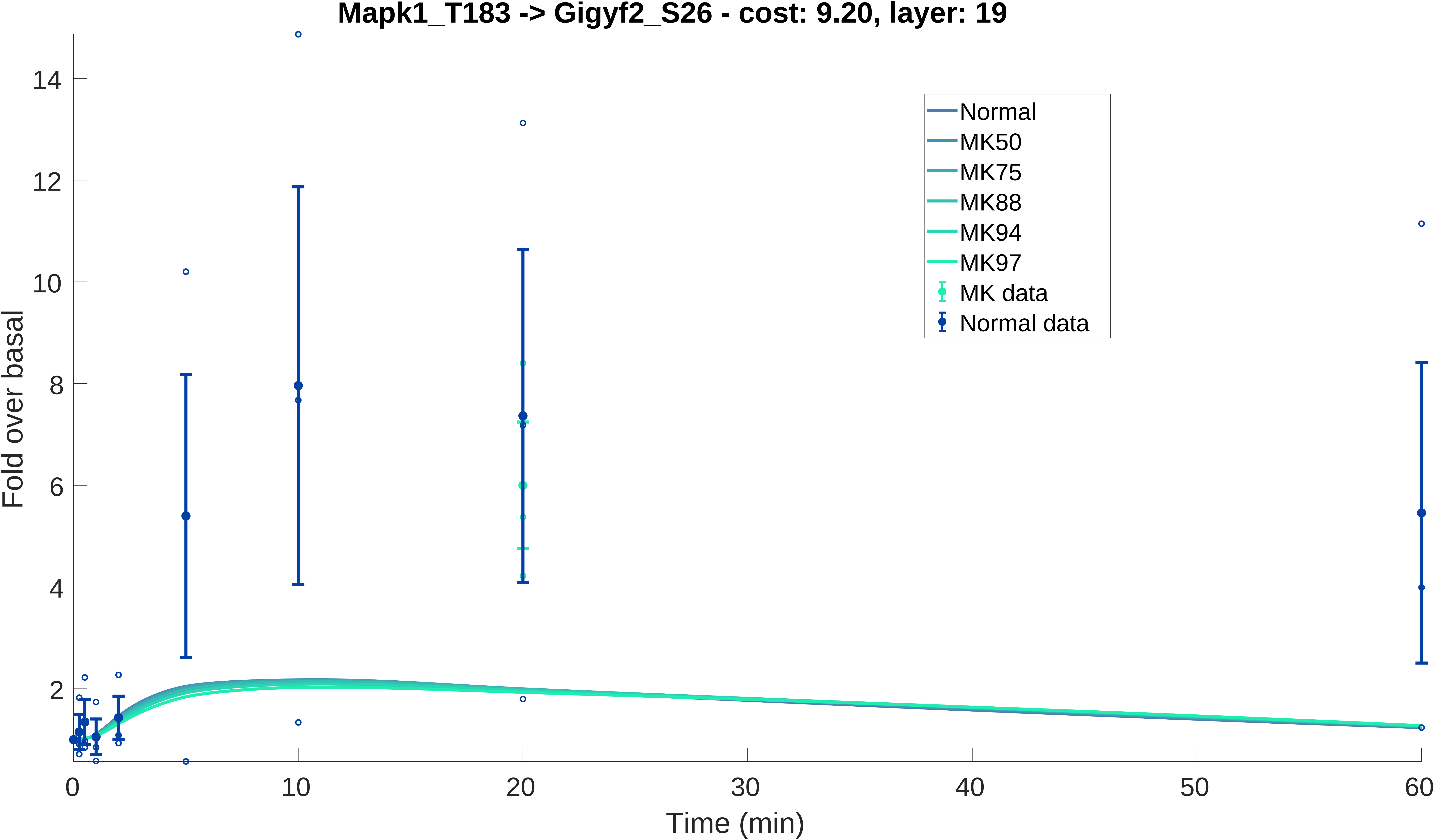

Mapk1_T183 -> Gigyf2_S26 - cost: 9.20, layer: 19
Fold over basal
Time (min)
0
10
20
30
40
50
60
2
4
6
8
10
12
14
Normal
MK50
MK75
MK88
MK94
MK97
MK data
Normal data

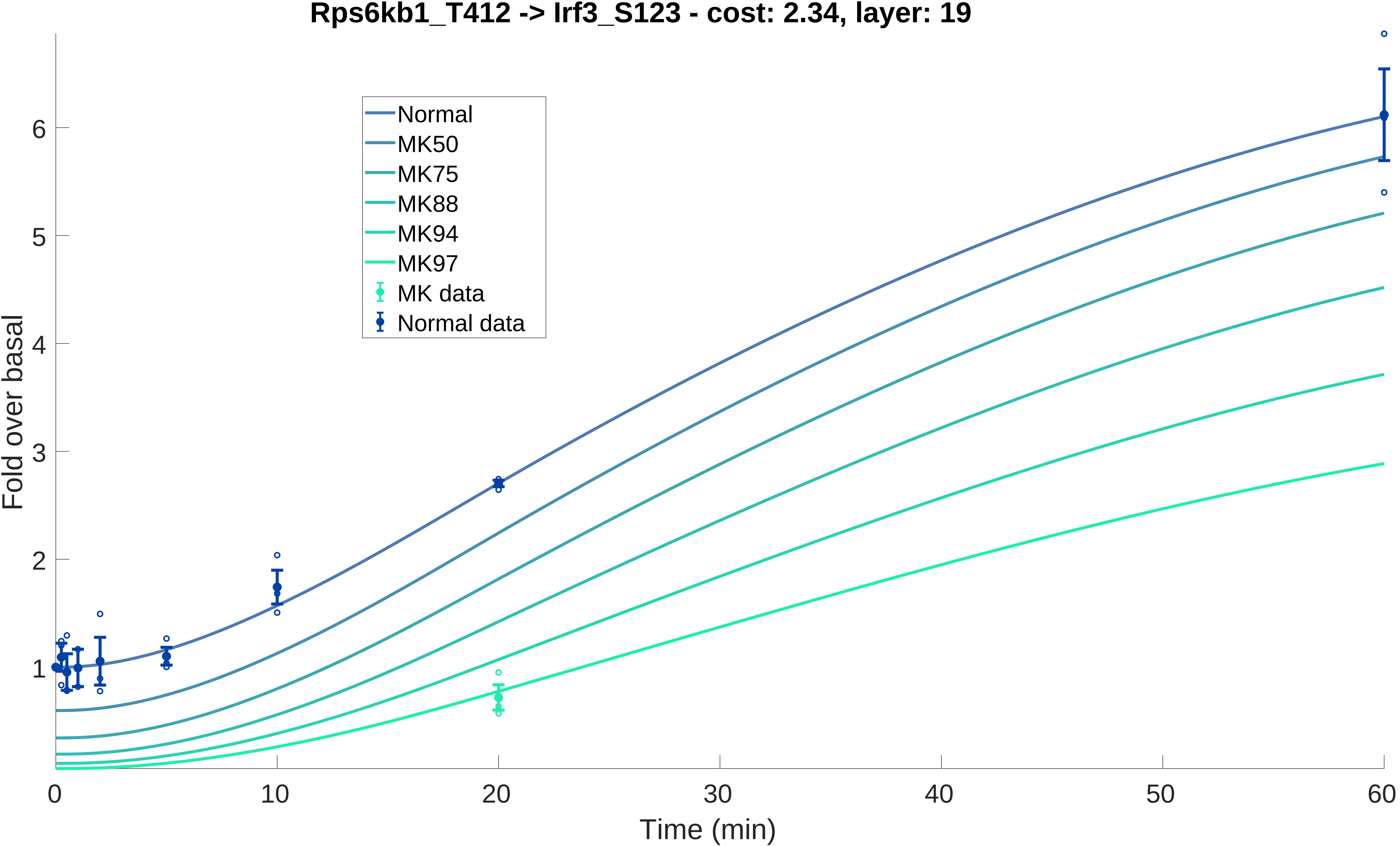
Rps6kb1_T412 -> Irf3_S123 - cost: 2.34, layer: 19
Normal
MK50
MK75
MK88
MK94
MK97
MK data
Normal data
Fold over basal
1
2
3
4
5
6
0
10
20
30
40
50
60
Time (min)

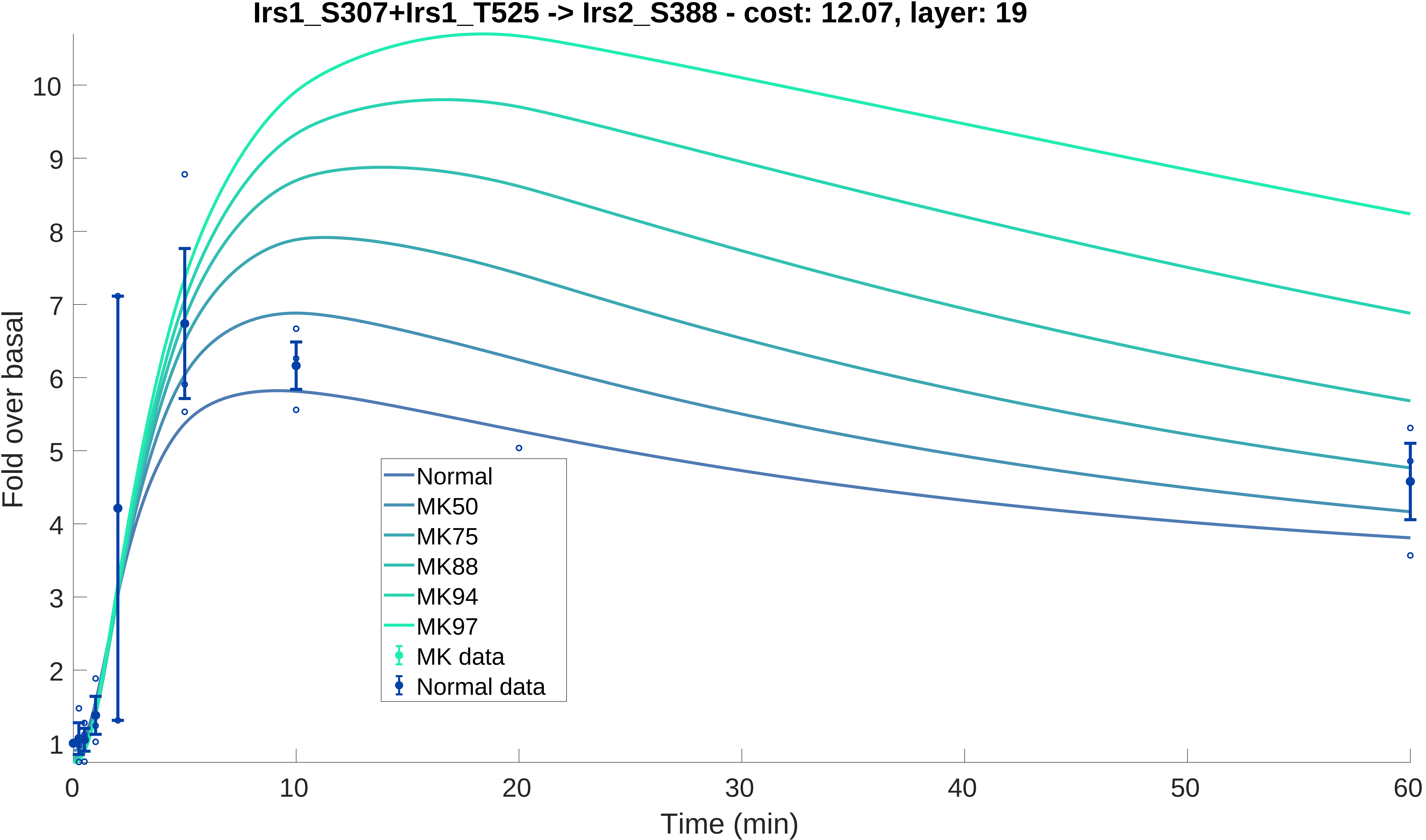

Irs1_S307+Irs1_T525 -> Irs2_S388 - cost: 12.07, layer: 19
Fold over basal
Time (min)
0
10
20
30
40
50
60
1
2
3
4
5
6
7
8
9
10
Normal
MK50
MK75
MK88
MK94
MK97
MK data
Normal data

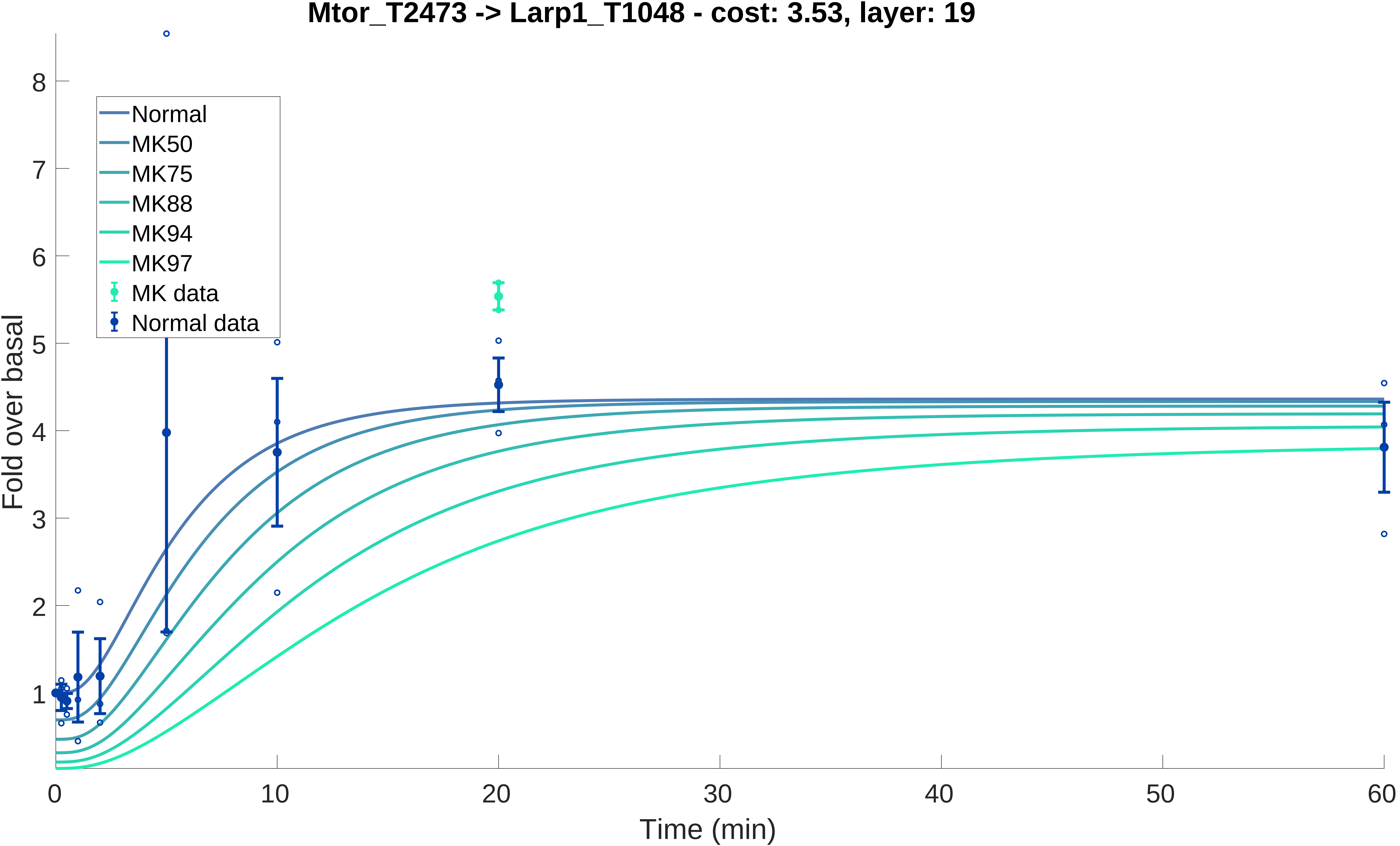

Mtor_T2473 -> Larp1_T1048 - cost: 3.53, layer: 19
Normal
MK50
MK75
MK88
MK94
MK97
MK data
Normal data
Fold over basal
Time (min)
0
10
20
30
40
50
60
1
2
3
4
5
6
7
8

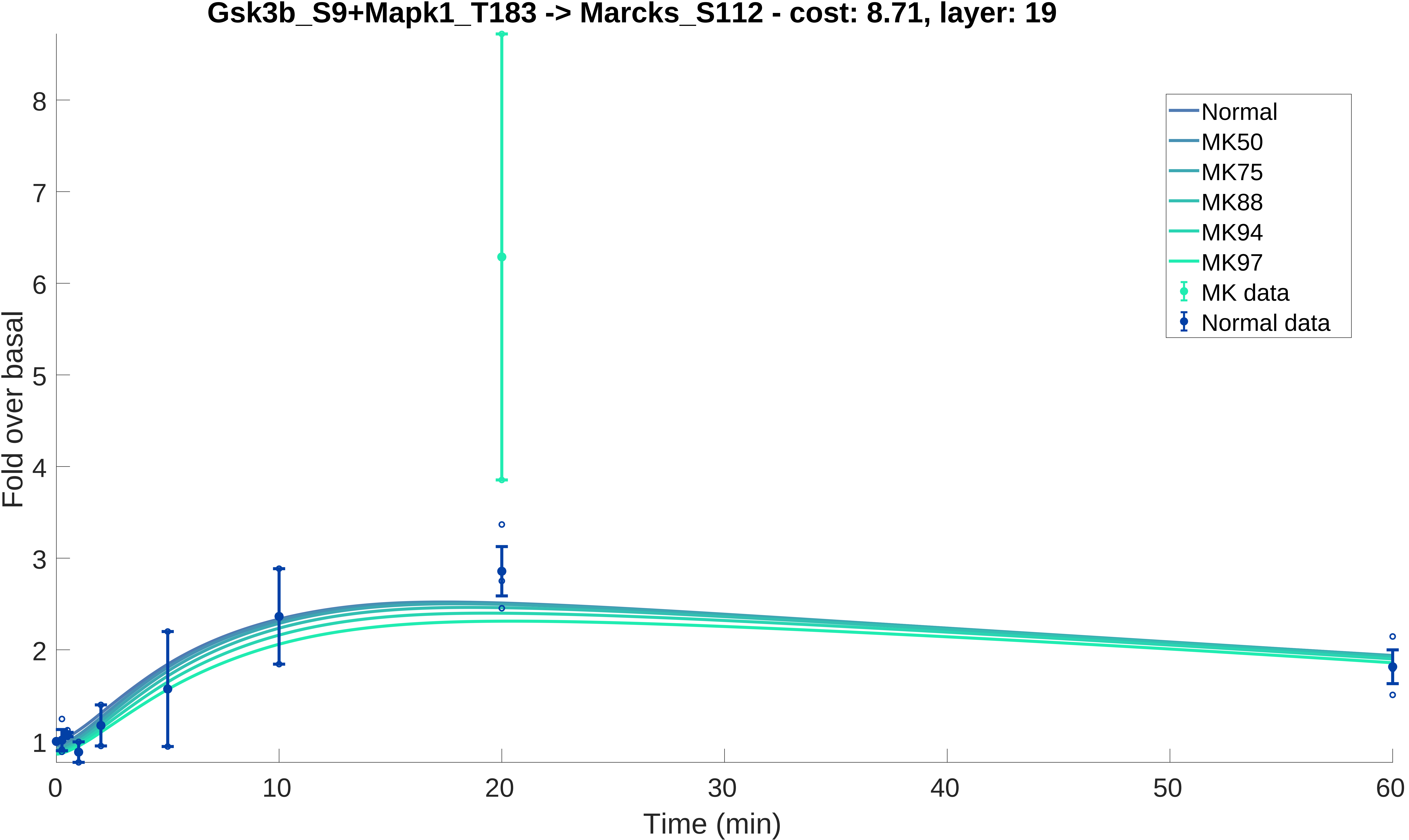

Gsk3b_S9+Mapk1_T183 -> Marcks_S112 - cost: 8.71, layer: 19
Fold over basal
Time (min)
0
10
20
30
40
50
60
1
2
3
4
5
6
7
8
Normal
MK50
MK75
MK88
MK94
MK97
MK data
Normal data

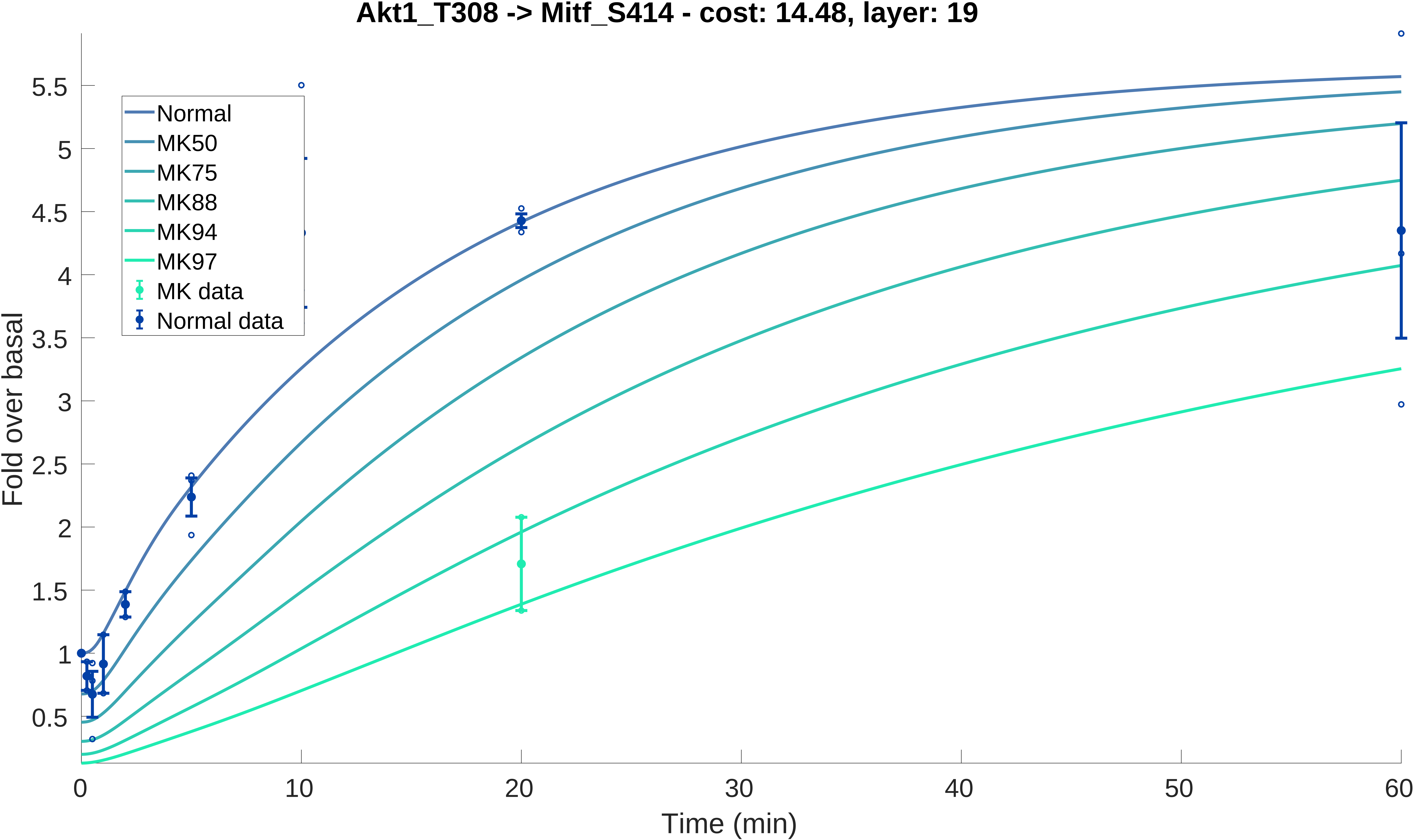

Akt1_T308 -> Mitf_S414 - cost: 14.48, layer: 19
Normal
MK50
MK75
MK88
MK94
MK97
MK data
Normal data
Fold over basal
Time (min)
0
10
20
30
40
50
60
0.5
1
1.5
2
2.5
3
3.5
4
4.5
5
5.5

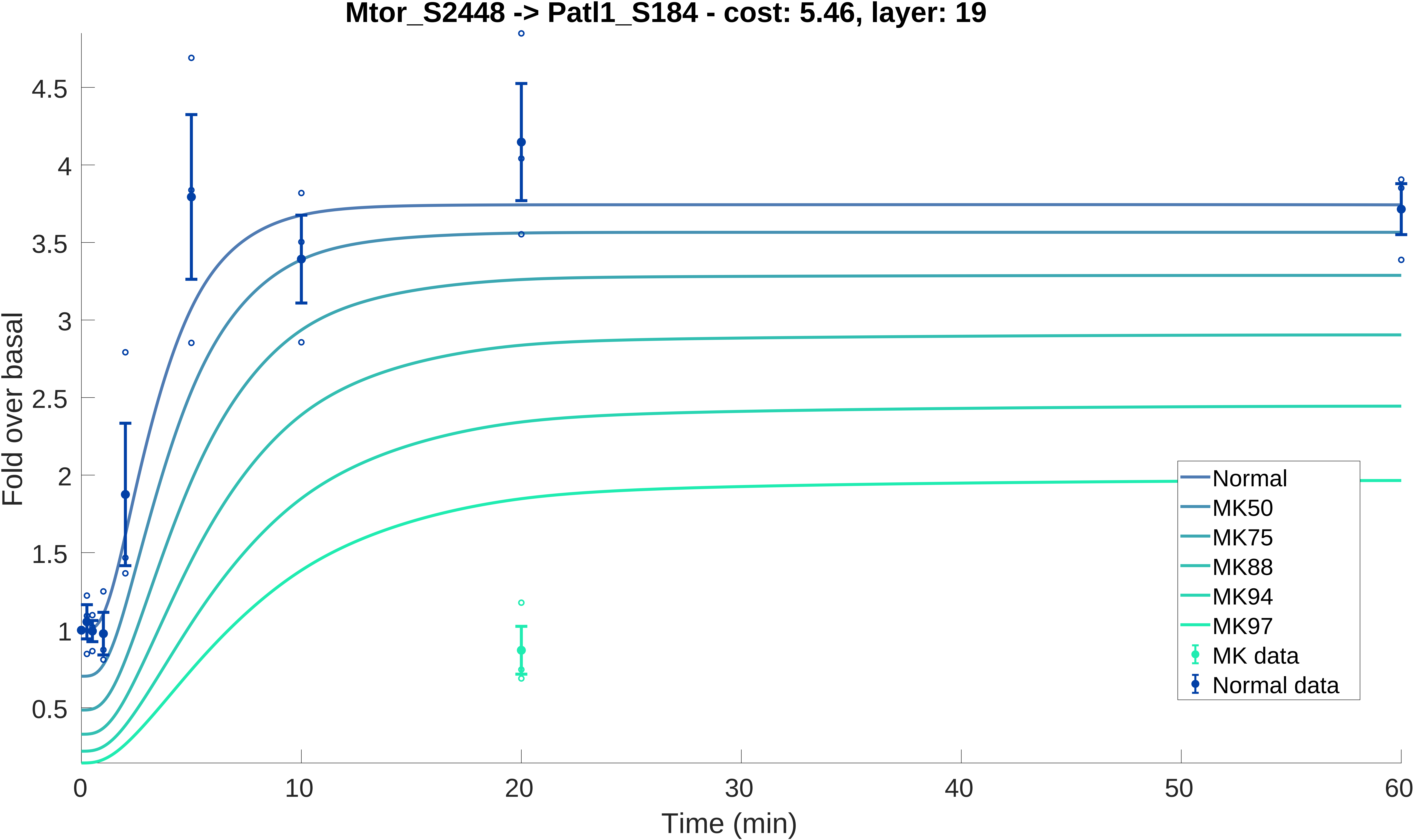

Mtor_S2448 -> Patl1_S184 - cost: 5.46, layer: 19
Fold over basal
Time (min)
0
10
20
30
40
50
60
0.5
1
1.5
2
2.5
3
3.5
4
4.5
Normal
MK50
MK75
MK88
MK94
MK97
MK data
Normal data

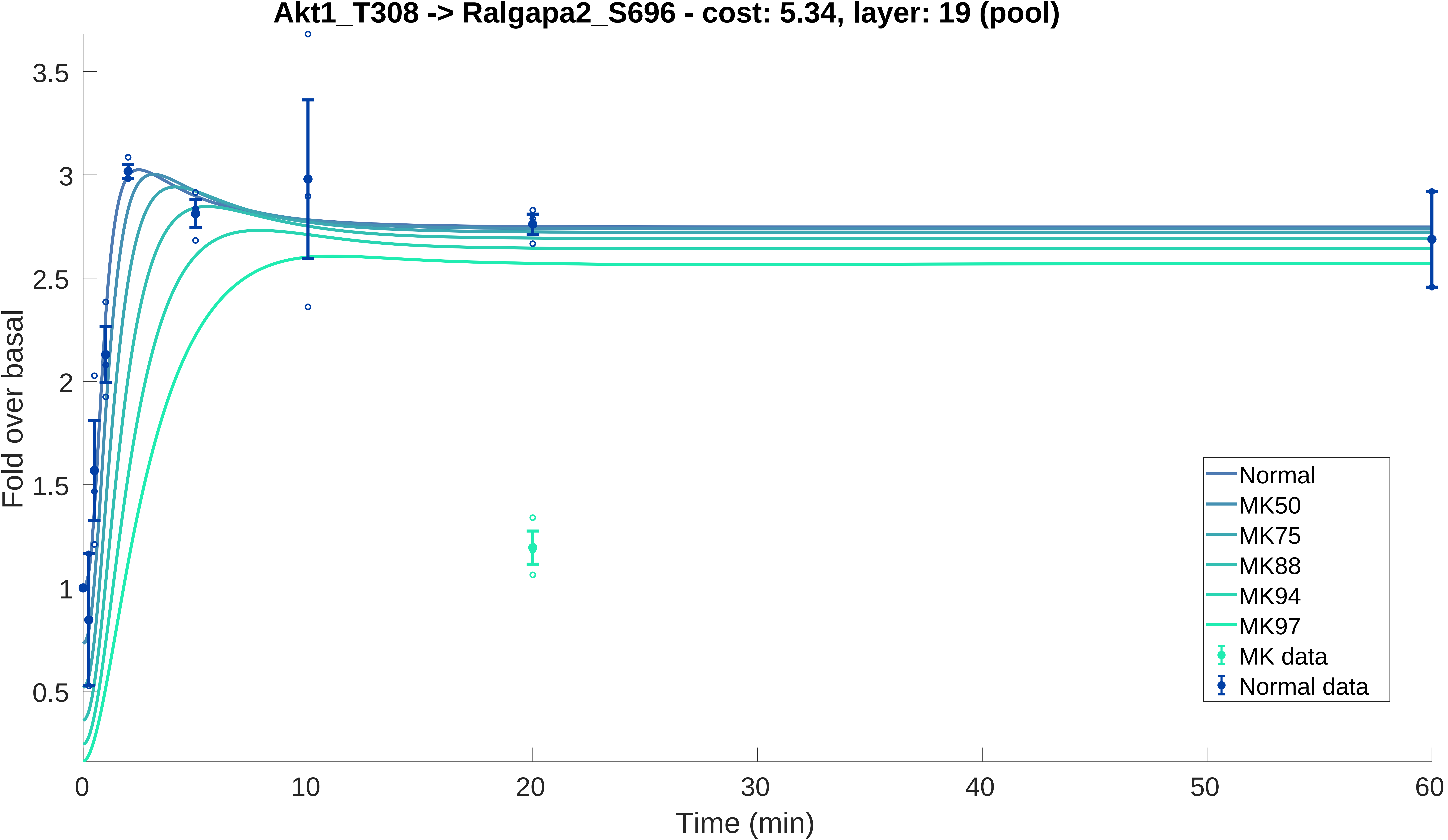
Akt1_T308 -> Ralgapa2_S696 - cost: 5.34, layer: 19 (pool)
Fold over basal
Time (min)
0.5
1
1.5
2
2.5
3
3.5
0
10
20
30
40
50
60
Normal
MK50
MK75
MK88
MK94
MK97
MK data
Normal data

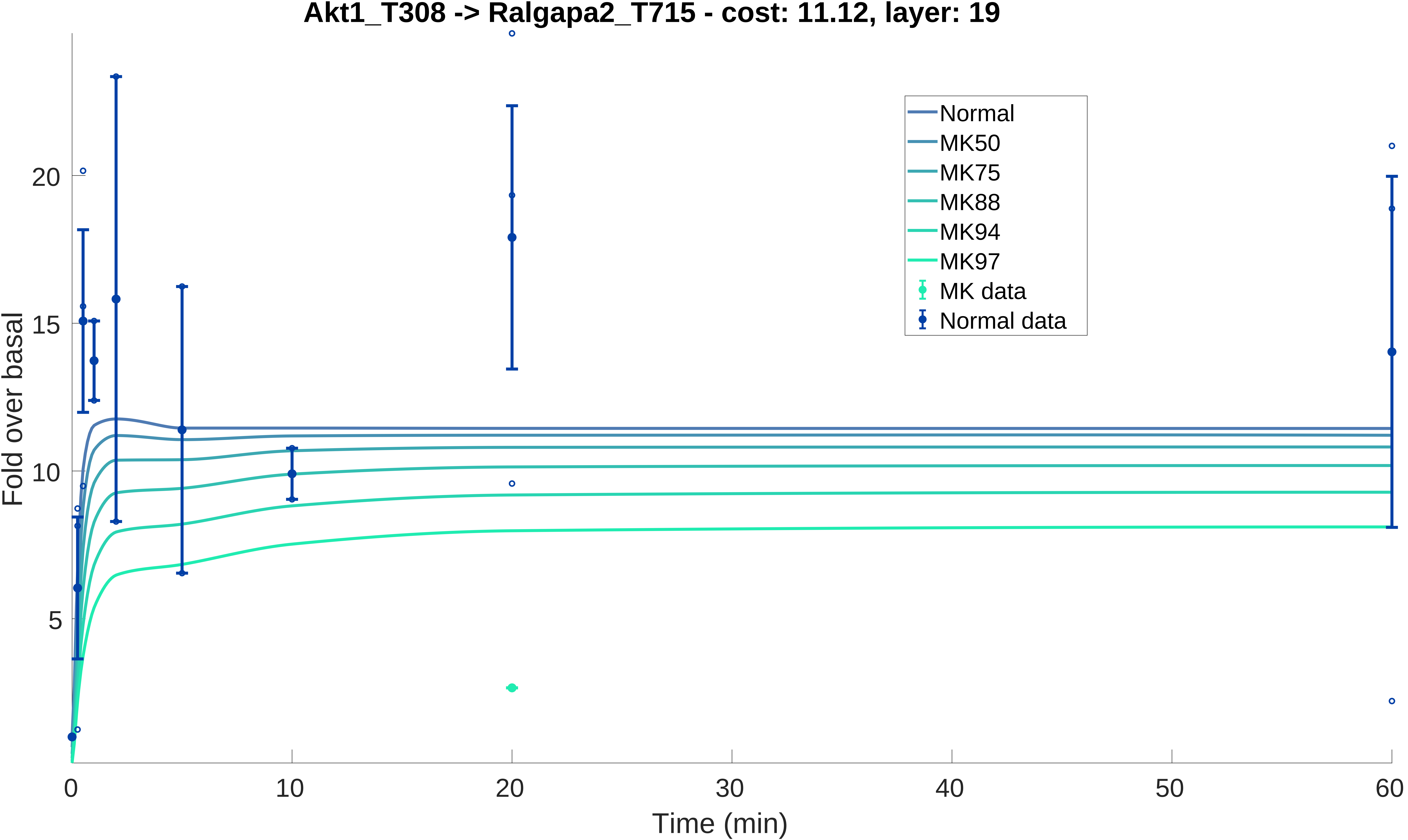
Akt1_T308 -> Ralgapa2_T715 - cost: 11.12, layer: 19
Fold over basal
Time (min)
0
10
20
30
40
50
60
5
10
15
20
Normal
MK50
MK75
MK88
MK94
MK97
MK data
Normal data

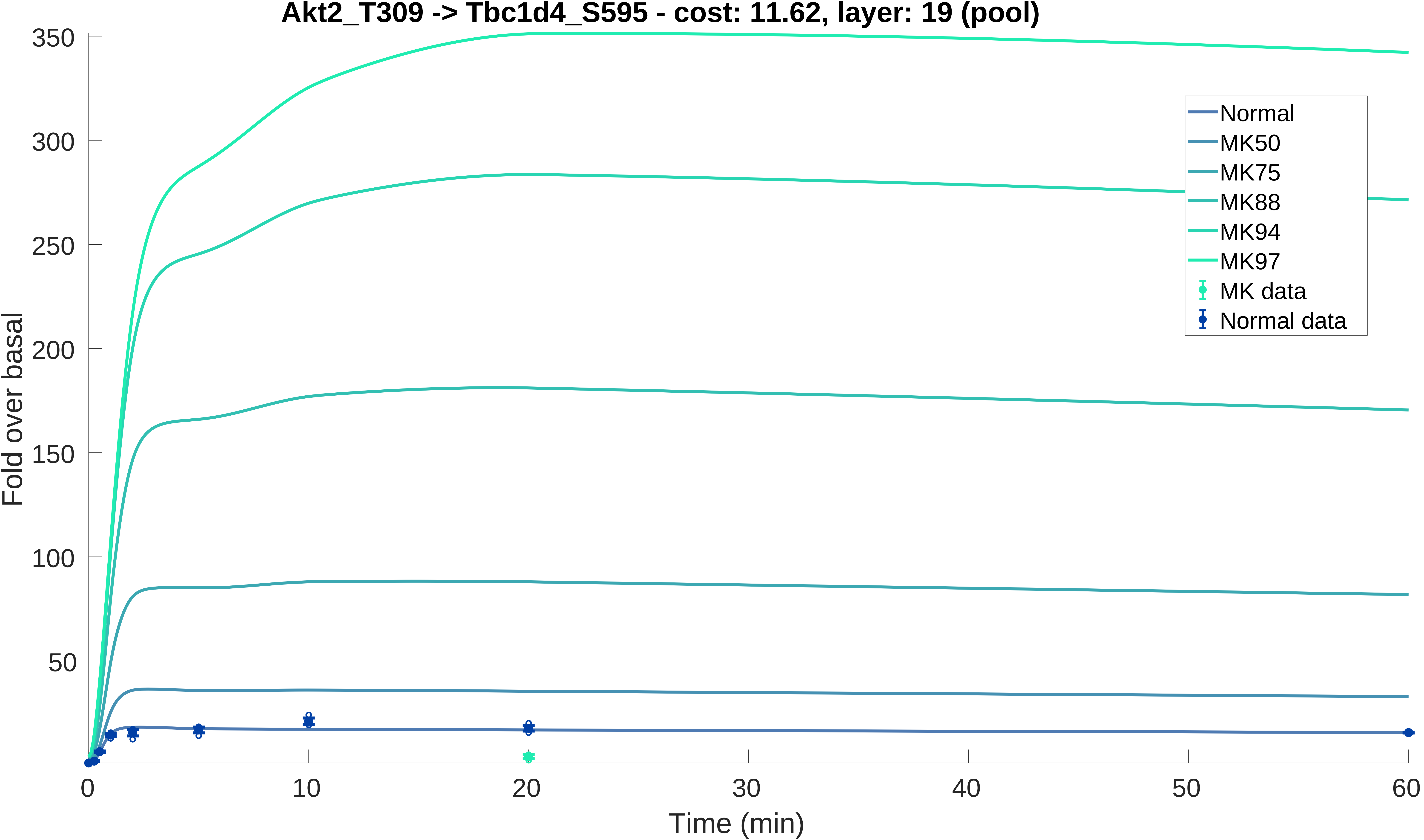

Akt2_T309 -> Tbc1d4_S595 - cost: 11.62, layer: 19 (pool)
Fold over basal
350
300
250
200
150
100
50
0
10
20
30
40
50
60
Time (min)
Normal
MK50
MK75
MK88
MK94
MK97
MK data
Normal data

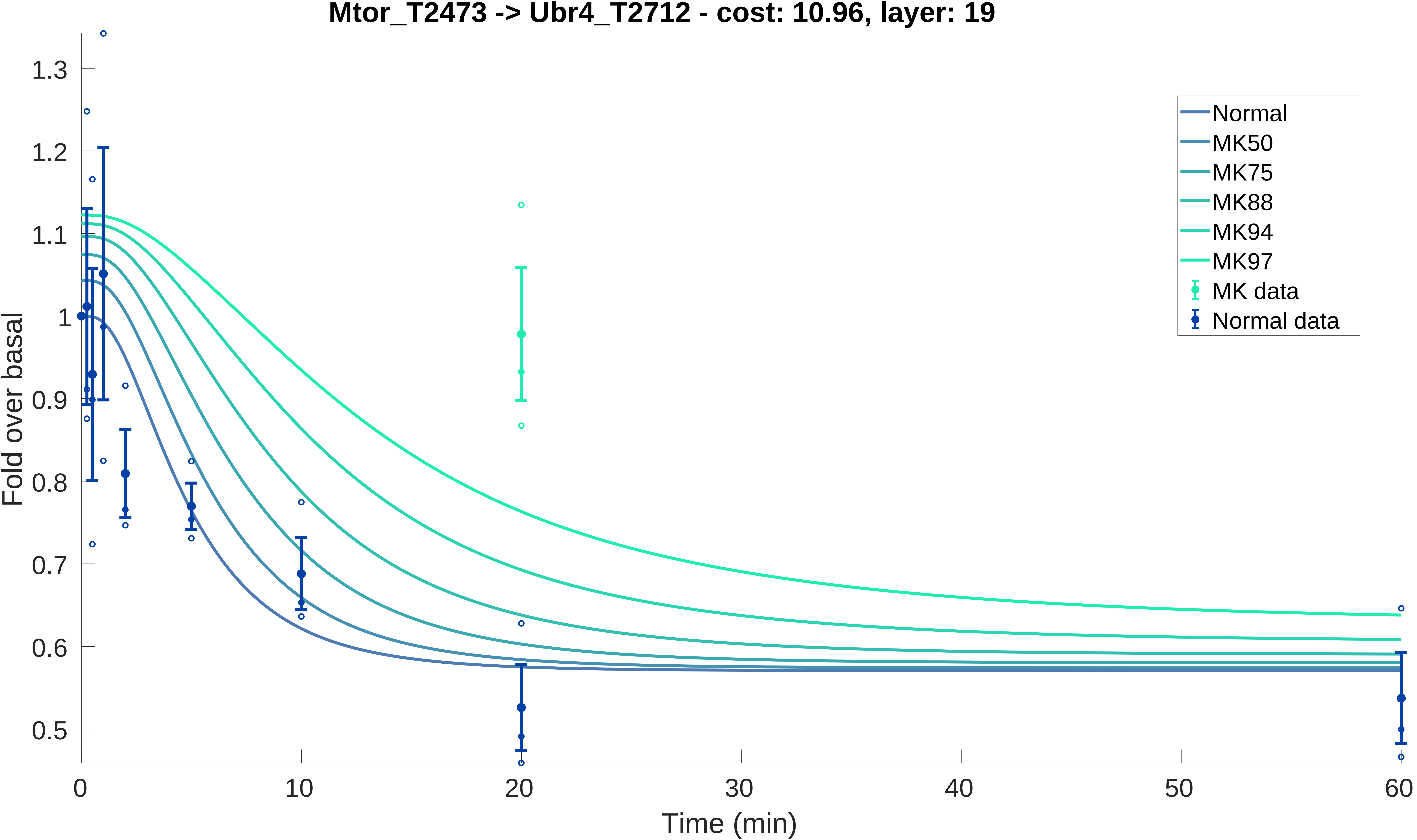

Mtor_T2473 -> Ubr4_T2712 - cost: 10.96, layer: 19
Fold over basal
Time (min)
0
10
20
30
40
50
60
0.5
0.6
0.7
0.8
0.9
1
1.1
1.2
1.3
Normal
MK50
MK75
MK88
MK94
MK97
MK data
Normal data

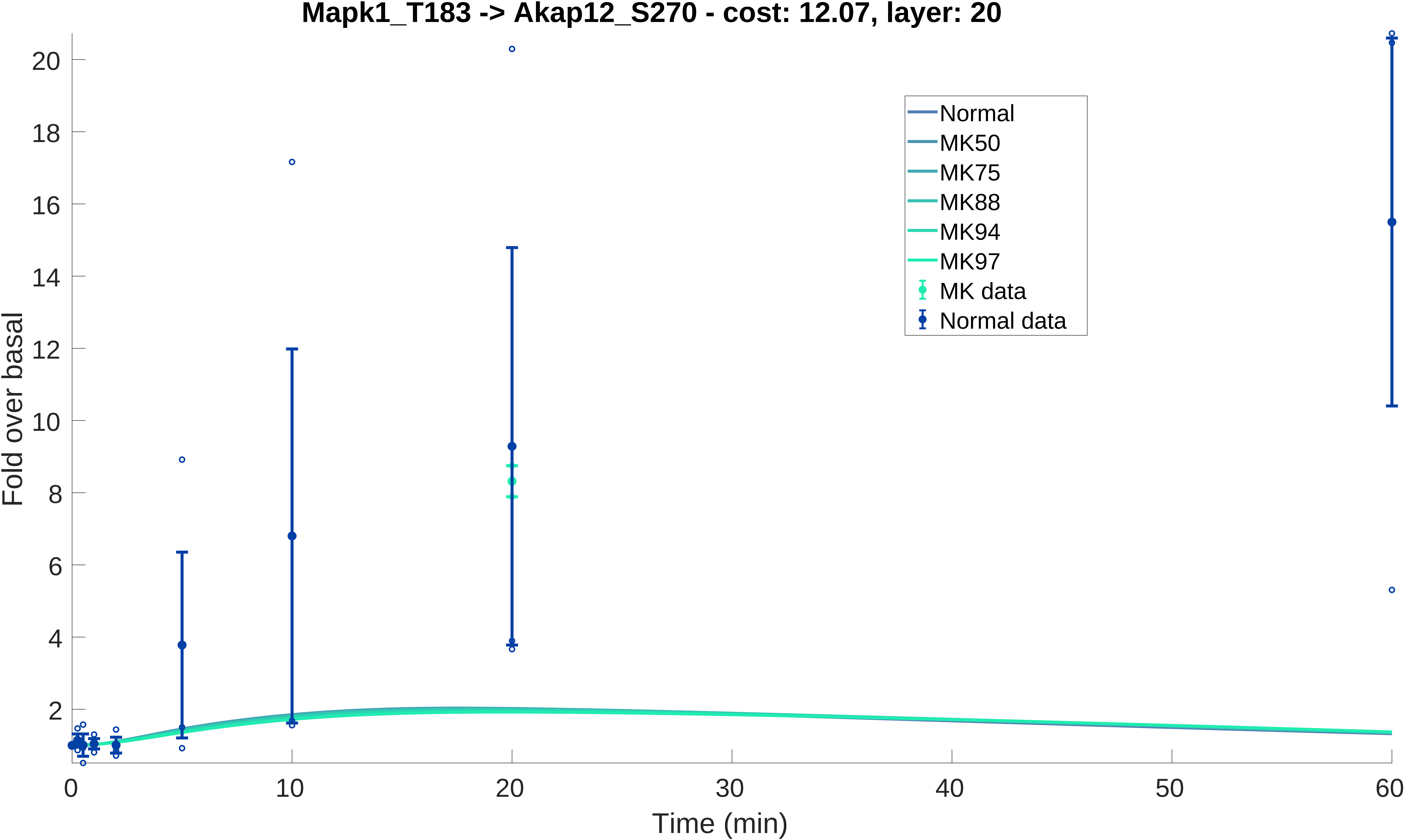

Mapk1_T183 -> Akap12_S270 - cost: 12.07, layer: 20
Fold over basal
Time (min)
0
10
20
30
40
50
60
2
4
6
8
10
12
14
16
18
20
Normal
MK50
MK75
MK88
MK94
MK97
MK data
Normal data

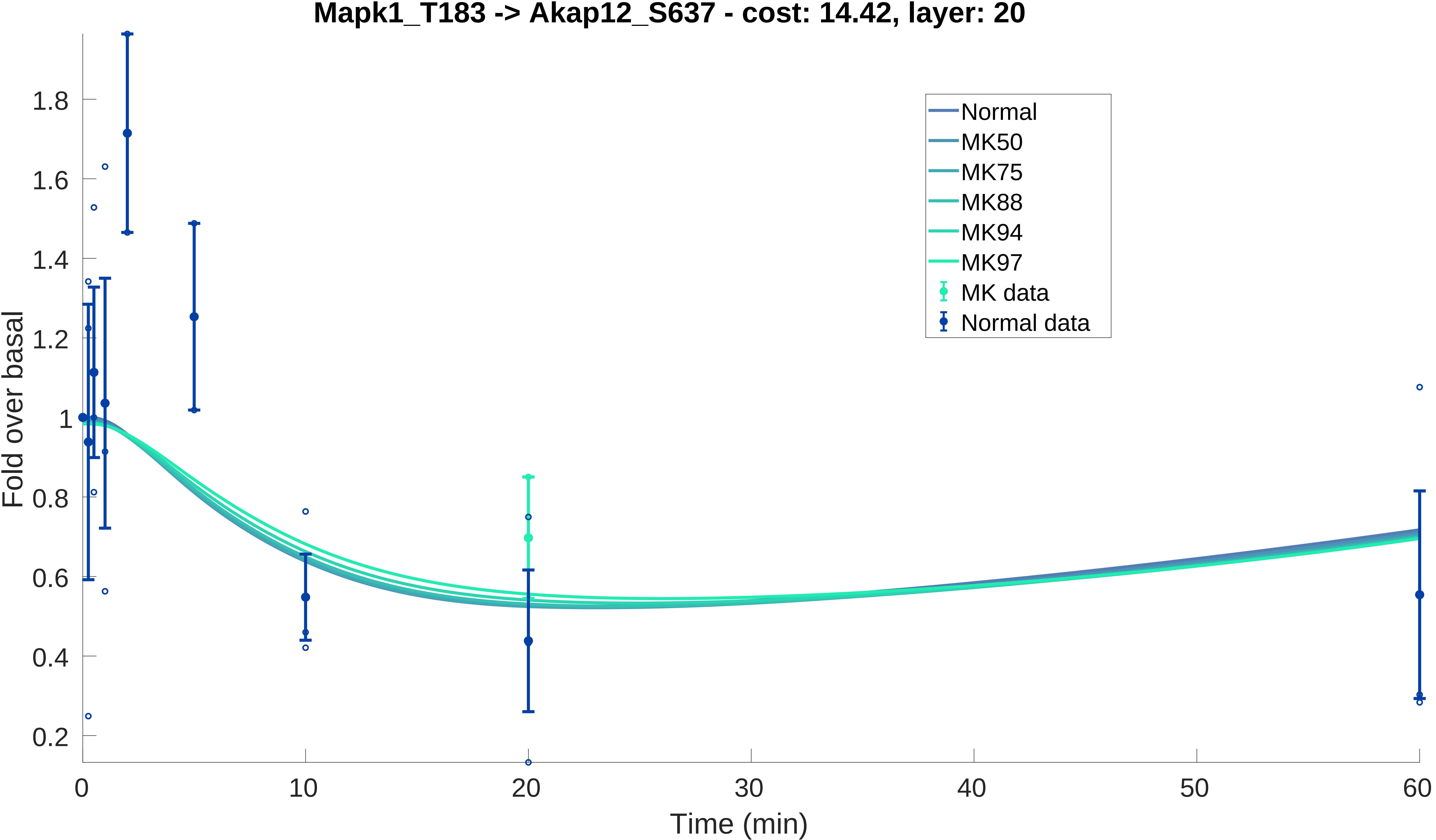
Mapk1_T183 -> Akap12_S637 - cost: 14.42, layer: 20
Fold over basal
Time (min)
Normal
MK50
MK75
MK88
MK94
MK97
MK data
Normal data
0
10
20
30
40
50
60
0.2
0.4
0.6
0.8
1
1.2
1.4
1.6
1.8

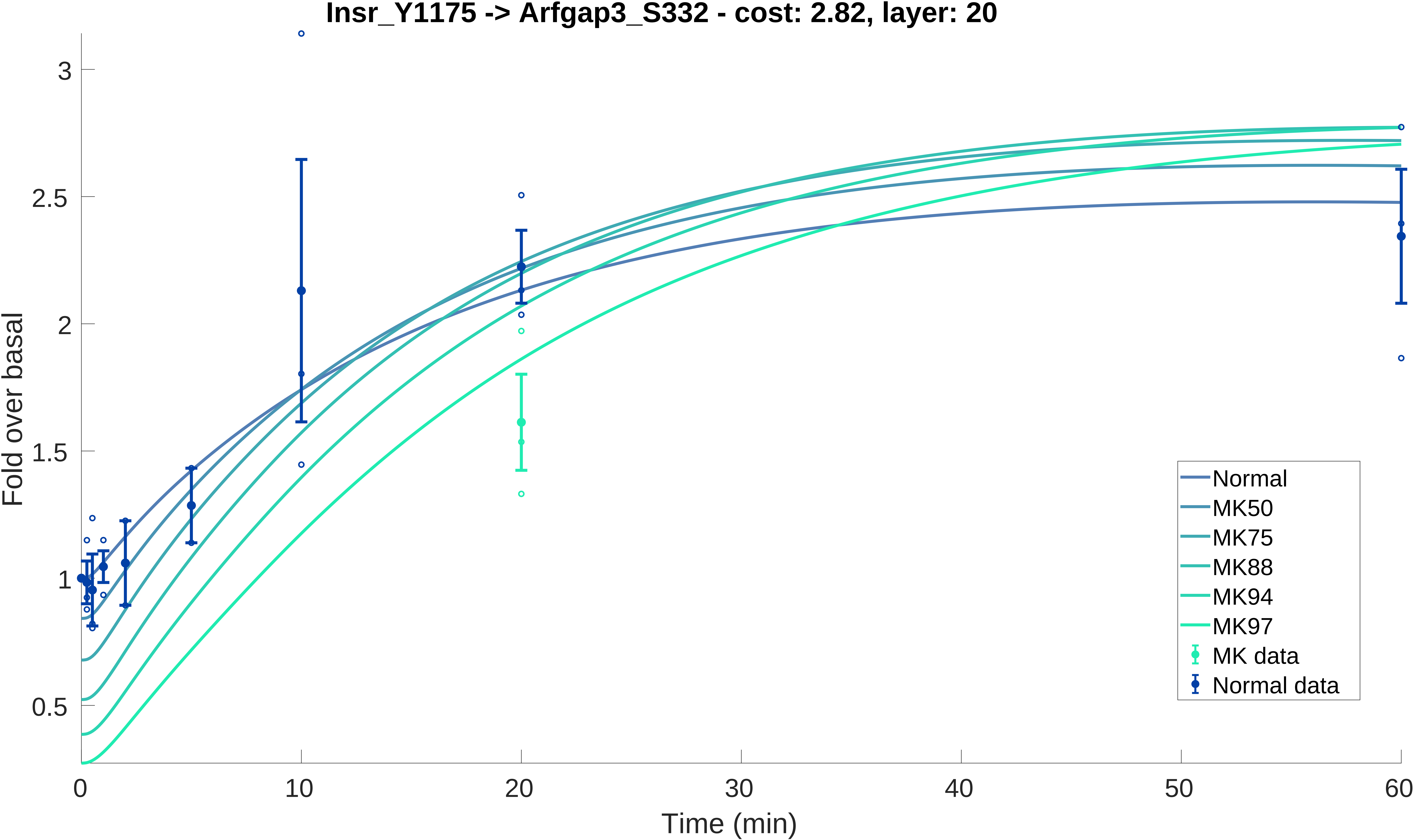

Insr_Y1175 -> Arfgap3_S332 - cost: 2.82, layer: 20
Fold over basal
3
2.5
2
1.5
1
0.5
0
10
20
30
40
50
60
Time (min)
Normal
MK50
MK75
MK88
MK94
MK97
MK data
Normal data

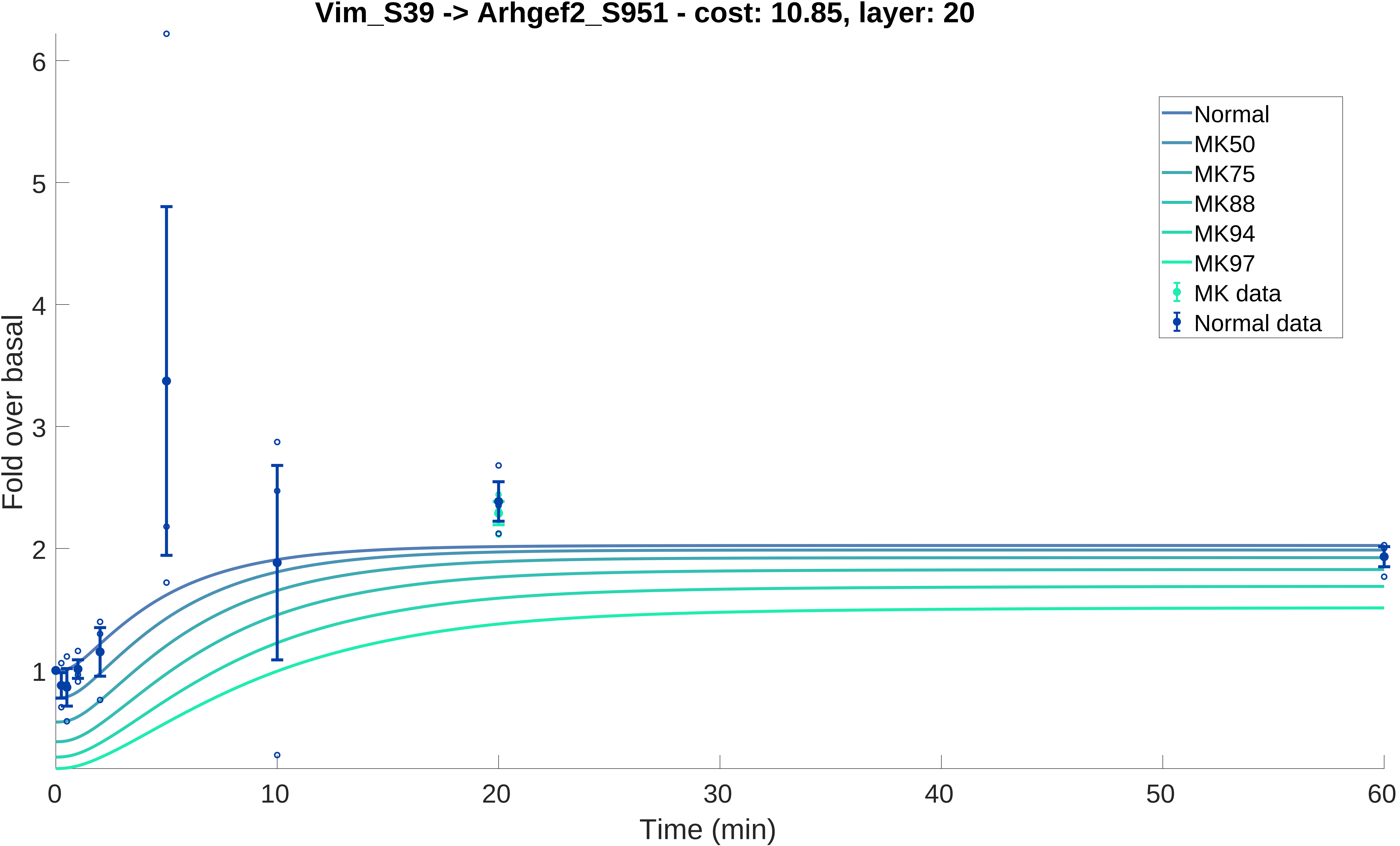

Vim_S39 -> Arhgef2_S951 - cost: 10.85, layer: 20
Fold over basal
Time (min)
0
10
20
30
40
50
60
1
2
3
4
5
6
Normal
MK50
MK75
MK88
MK94
MK97
MK data
Normal data

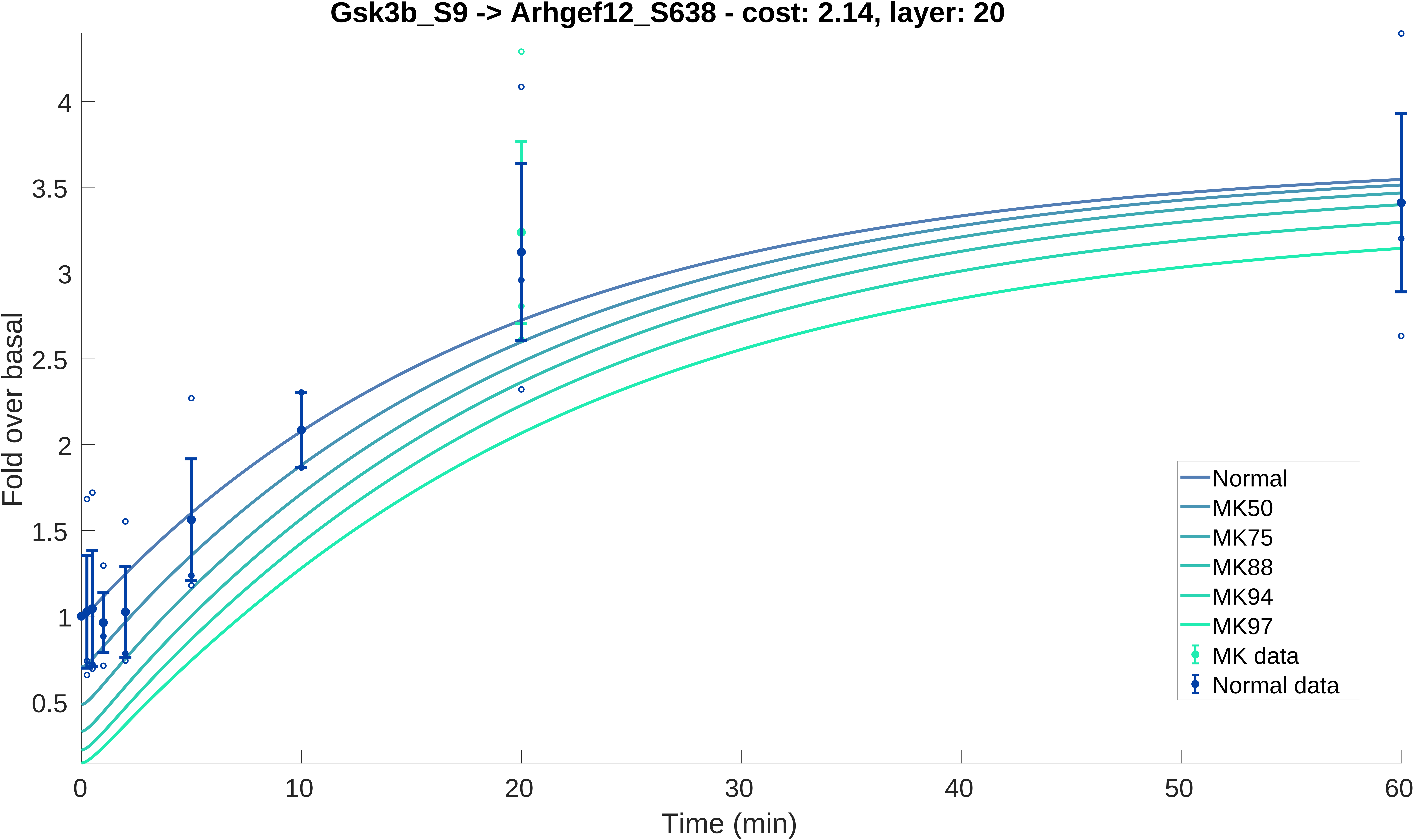
Gsk3b_S9 -> Arhgef12_S638 - cost: 2.14, layer: 20
Fold over basal
Time (min)
0.5
1
1.5
2
2.5
3
3.5
4
0
10
20
30
40
50
60
Normal
MK50
MK75
MK88
MK94
MK97
MK data
Normal data

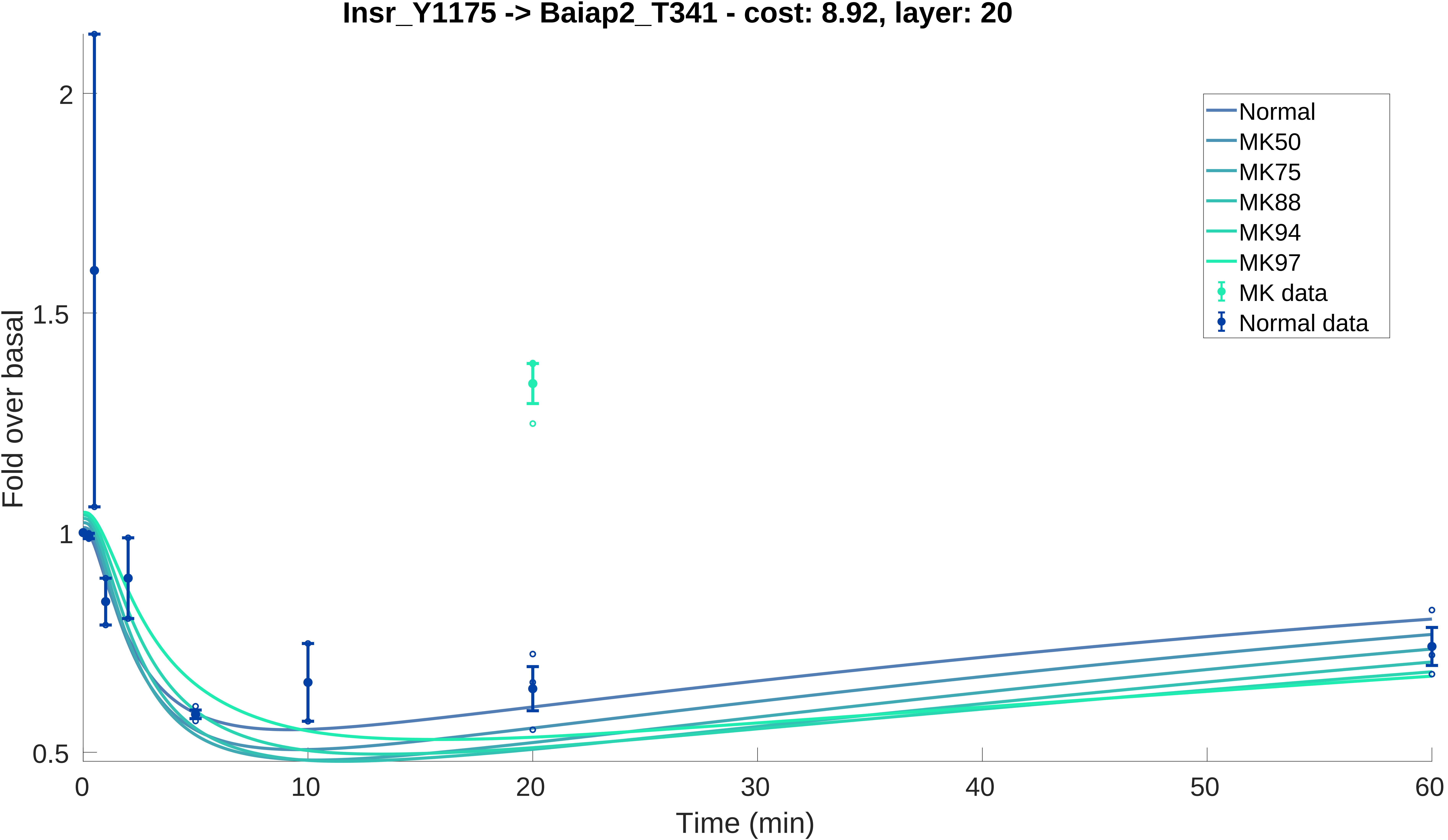

Insr_Y1175 -> Baiap2_T341 - cost: 8.92, layer: 20
Fold over basal
Time (min)
0.5
1
1.5
2
0
10
20
30
40
50
60
Normal
MK50
MK75
MK88
MK94
MK97
MK data
Normal data

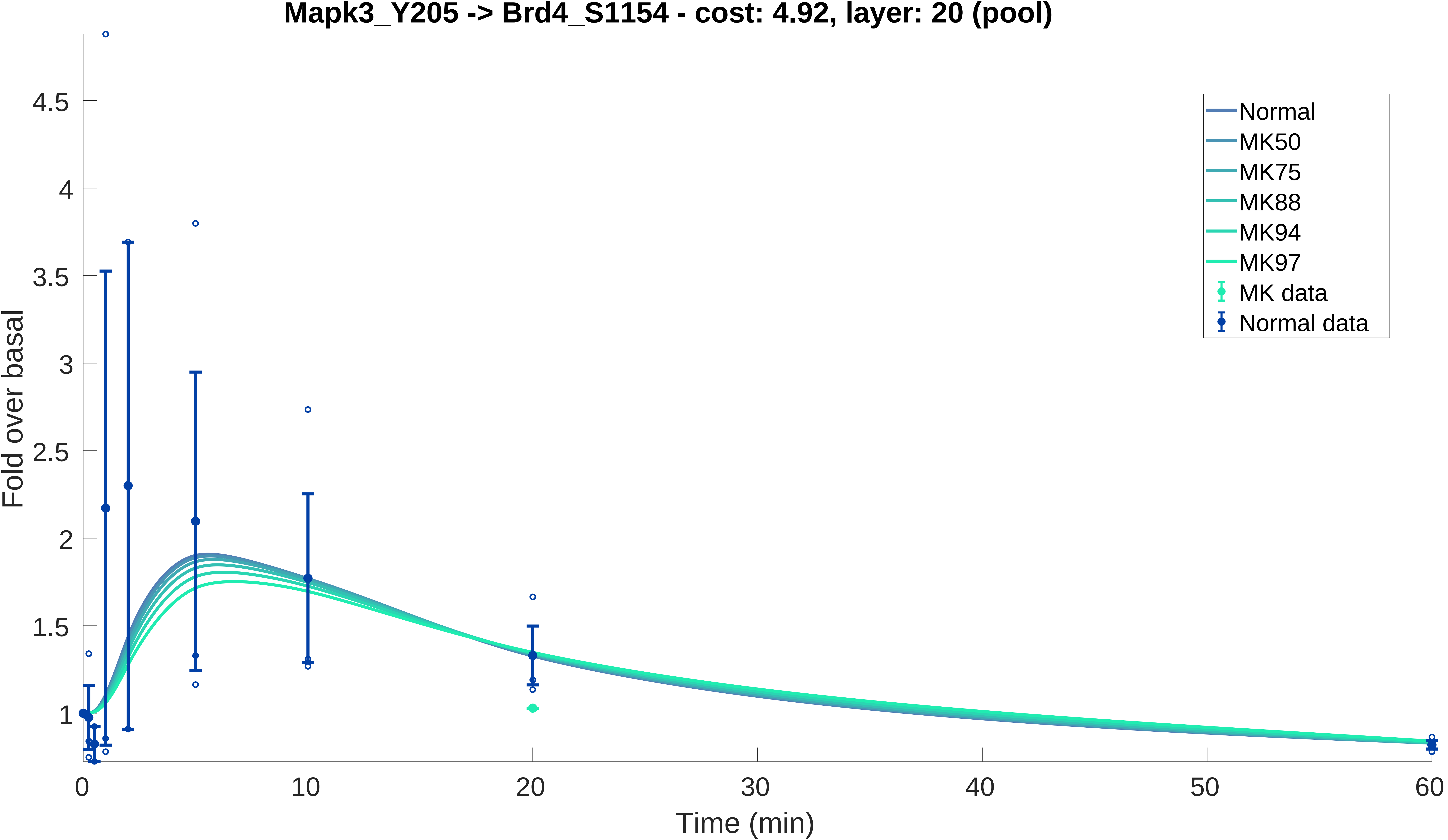

Mapk3_Y205 -> Brd4_S1154 - cost: 4.92, layer: 20 (pool)
Fold over basal
4.5
4
3.5
3
2.5
2
1.5
1
0
10
20
30
40
50
60
Time (min)
Normal
MK50
MK75
MK88
MK94
MK97
MK data
Normal data

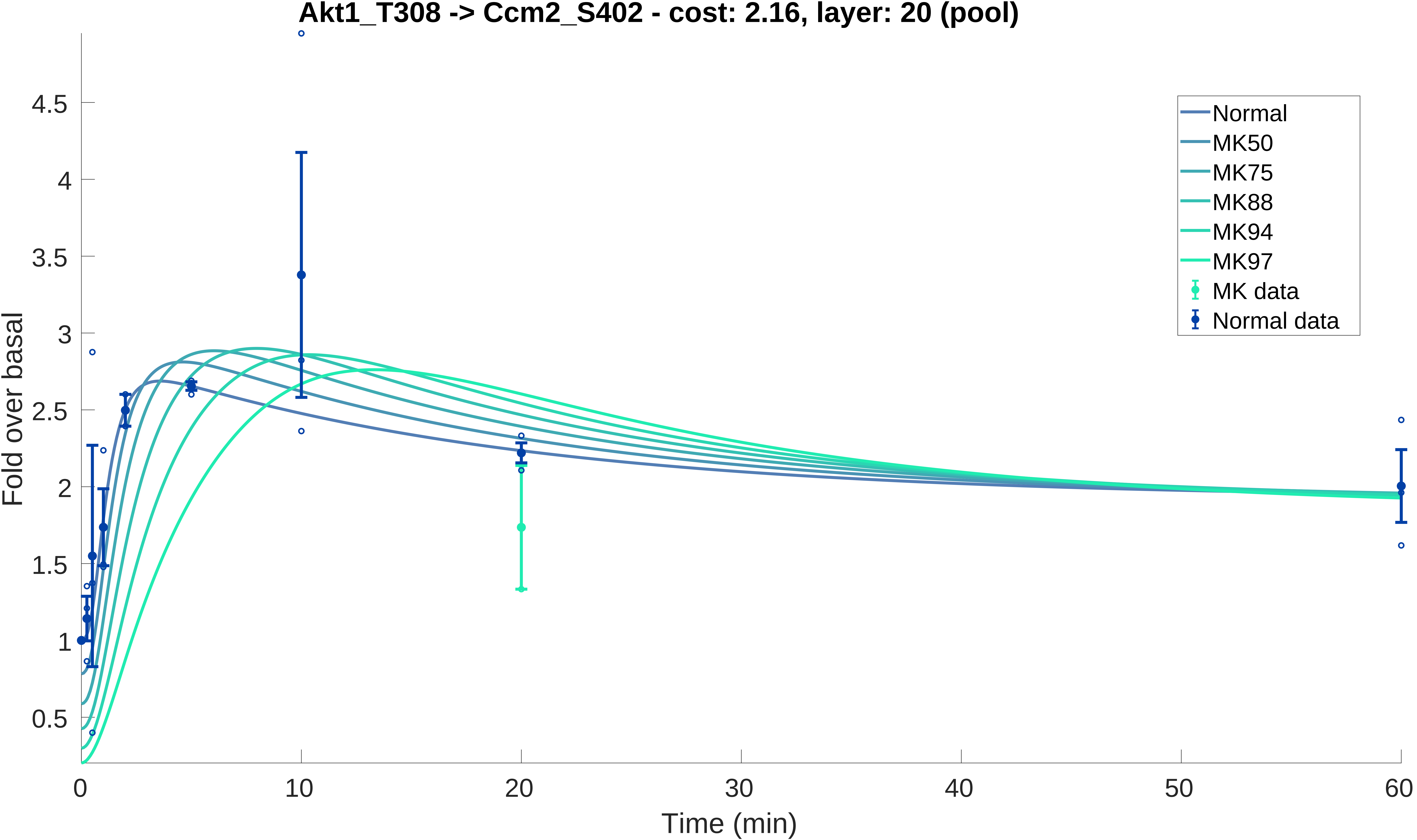

Akt1_T308 -> Ccm2_S402 - cost: 2.16, layer: 20 (pool)
Fold over basal
Time (min)
0.5
1
1.5
2
2.5
3
3.5
4
4.5
0
10
20
30
40
50
60
Normal
MK50
MK75
MK88
MK94
MK97
MK data
Normal data

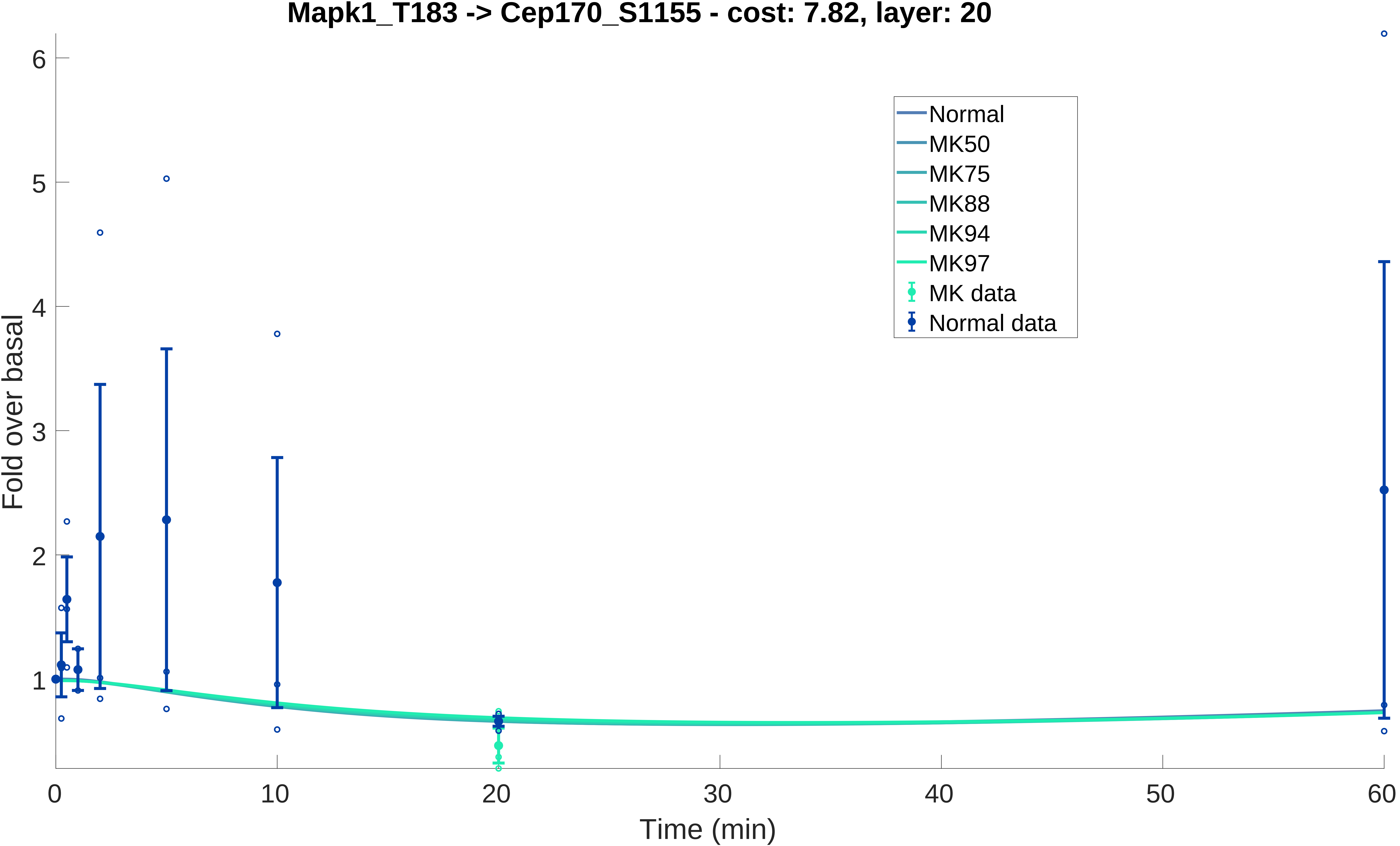

Mapk1_T183 -> Cep170_S1155 - cost: 7.82, layer: 20
Fold over basal
Time (min)
6
5
4
3
2
1
0
10
20
30
40
50
60
Normal
MK50
MK75
MK88
MK94
MK97
MK data
Normal data

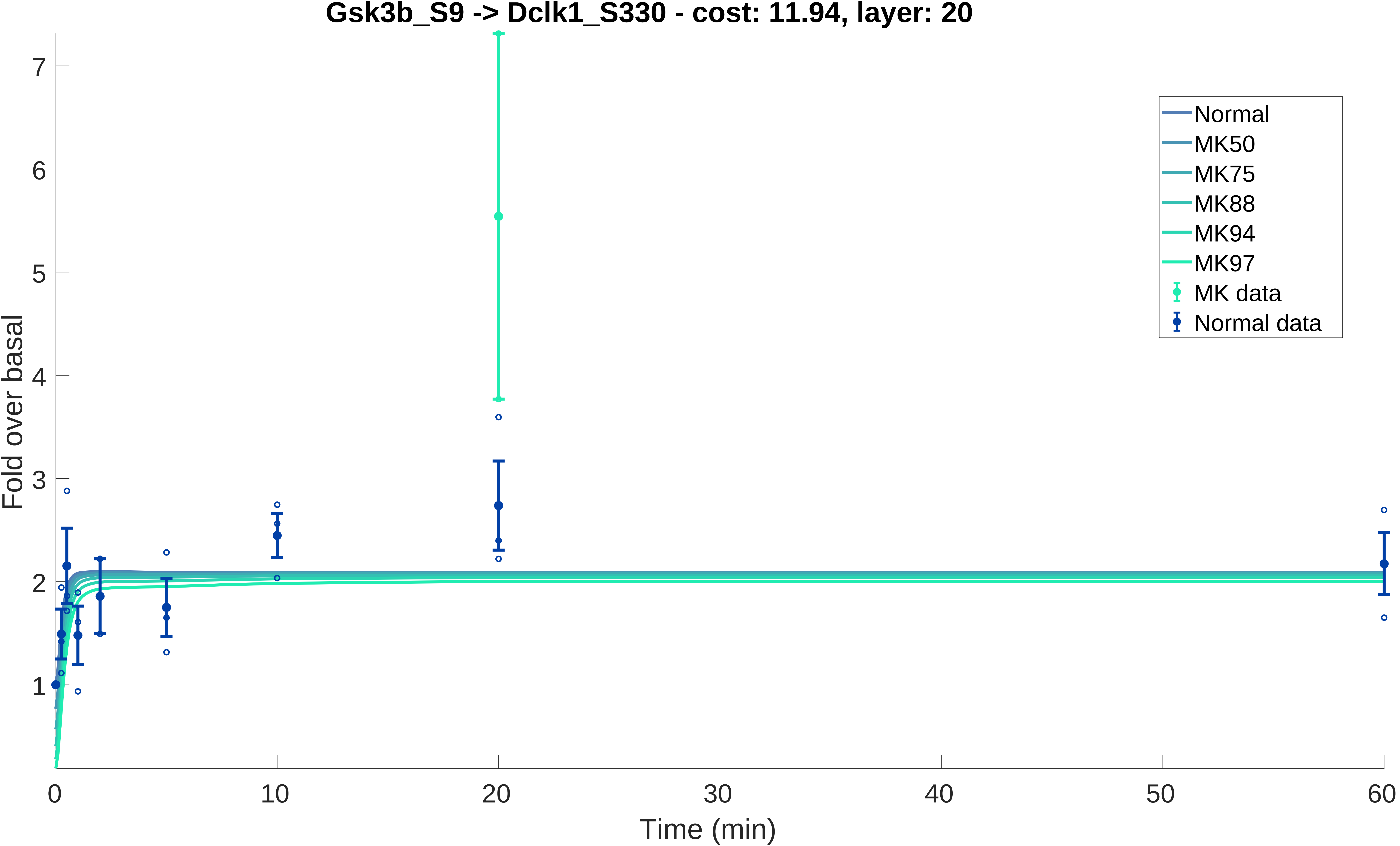

Gsk3b_S9 -> Dclk1_S330 - cost: 11.94, layer: 20
Fold over basal
Time (min)
0
10
20
30
40
50
60
1
2
3
4
5
6
7
Normal
MK50
MK75
MK88
MK94
MK97
MK data
Normal data

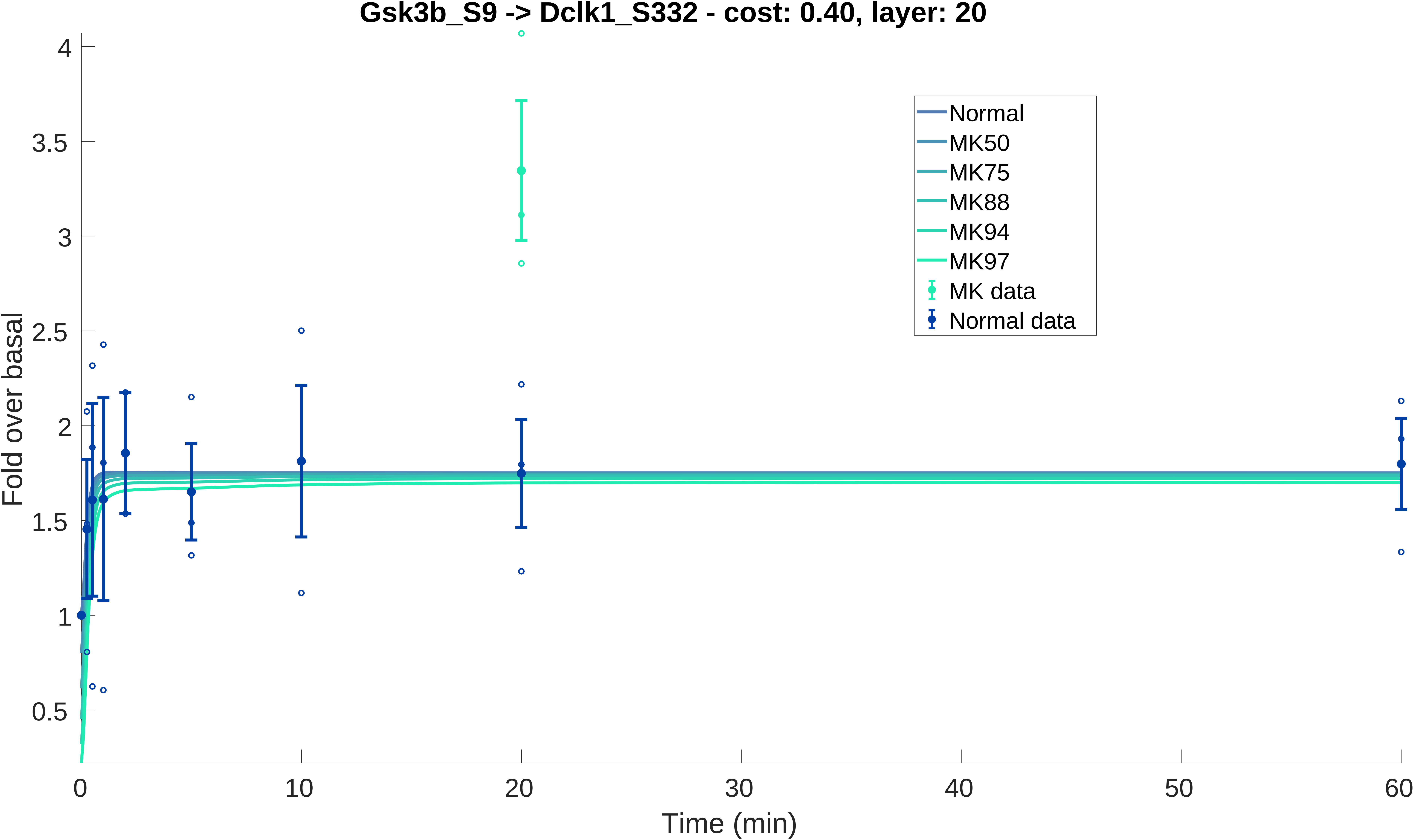

Gsk3b_S9 -> Dclk1_S332 - cost: 0.40, layer: 20
Fold over basal
Time (min)
0.5
1
1.5
2
2.5
3
3.5
4
0
10
20
30
40
50
60
Normal
MK50
MK75
MK88
MK94
MK97
MK data
Normal data

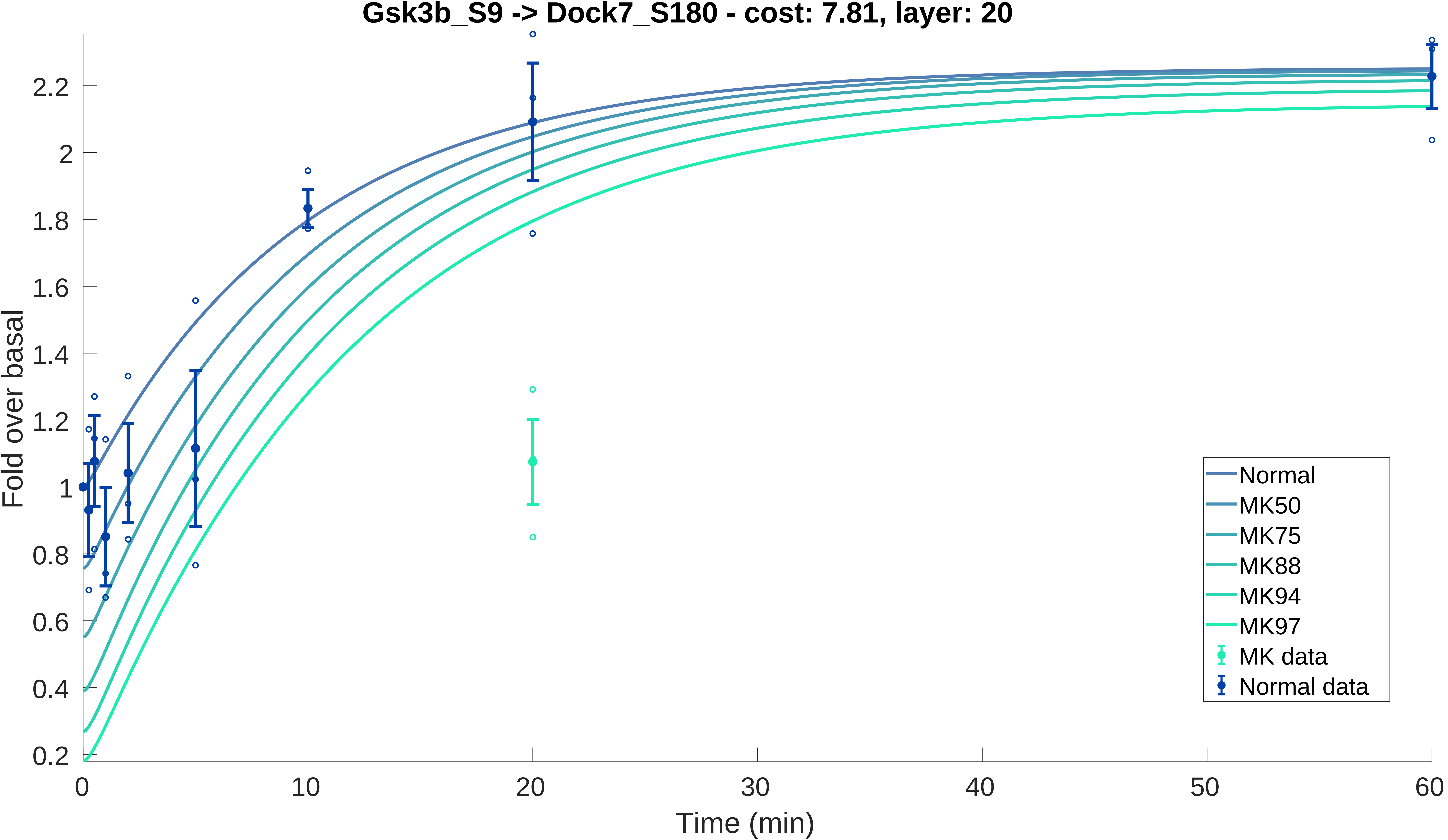

Gsk3b_S9 -> Dock7_S180 - cost: 7.81, layer: 20
Fold over basal
Time (min)
0.2
0.4
0.6
0.8
1
1.2
1.4
1.6
1.8
2
2.2
0
10
20
30
40
50
60
Normal
MK50
MK75
MK88
MK94
MK97
MK data
Normal data

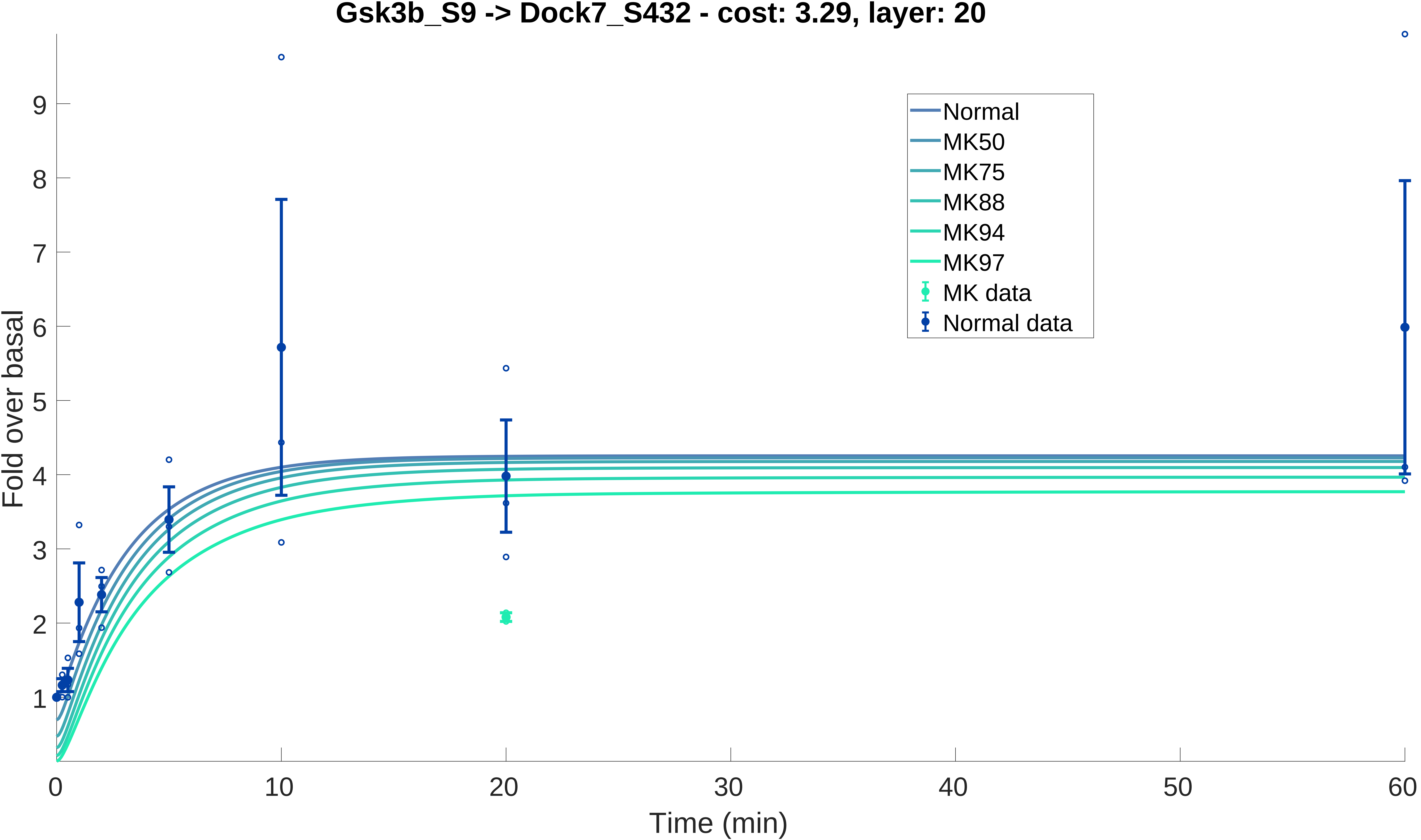

Gsk3b_S9 -> Dock7_S432 - cost: 3.29, layer: 20
Fold over basal
Time (min)
0
10
20
30
40
50
60
1
2
3
4
5
6
7
8
9
Normal
MK50
MK75
MK88
MK94
MK97
MK data
Normal data

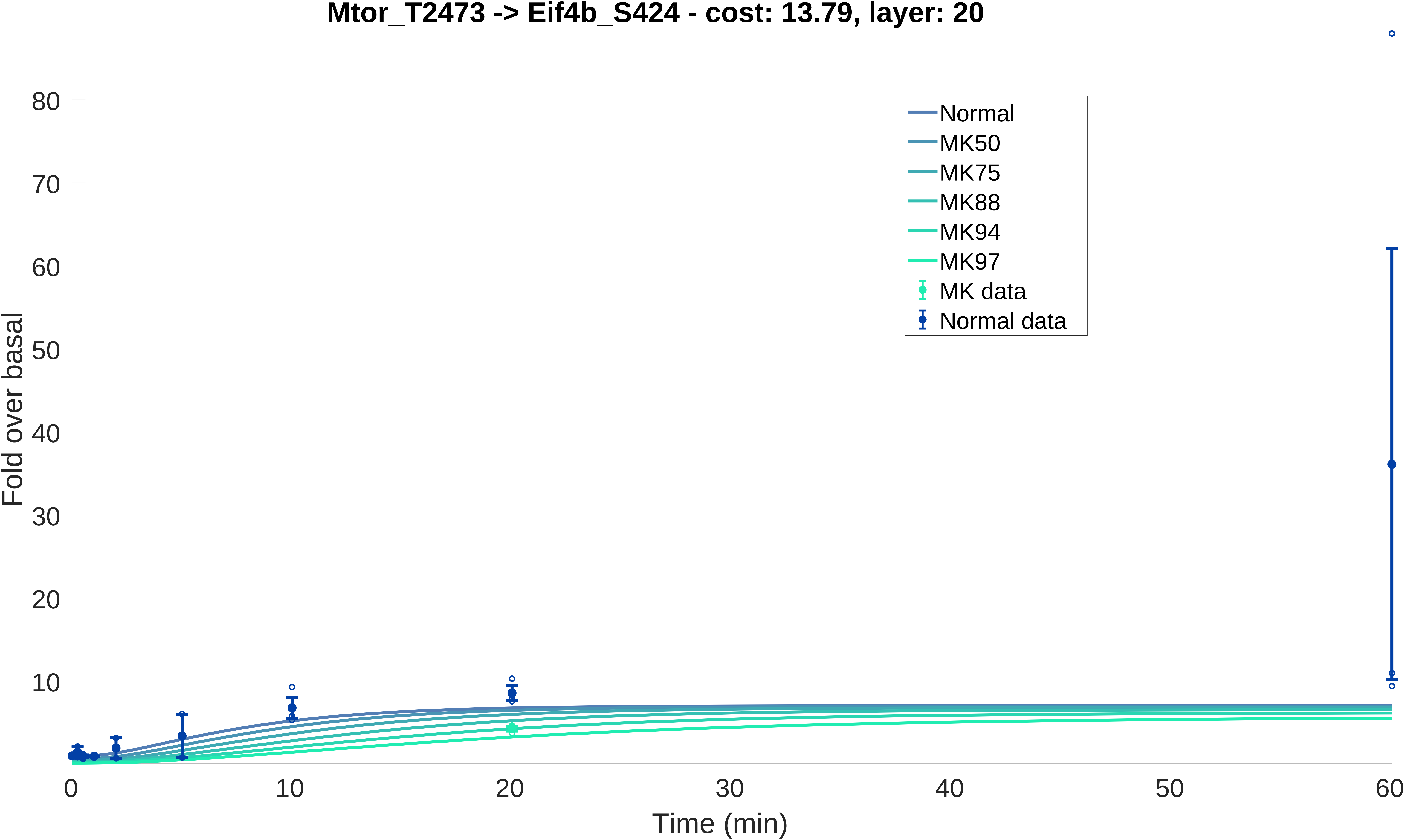
Mtor_T2473 -> Eif4b_S424 - cost: 13.79, layer: 20
Fold over basal
Time (min)
0
10
20
30
40
50
60
70
80
Normal
MK50
MK75
MK88
MK94
MK97
MK data
Normal data

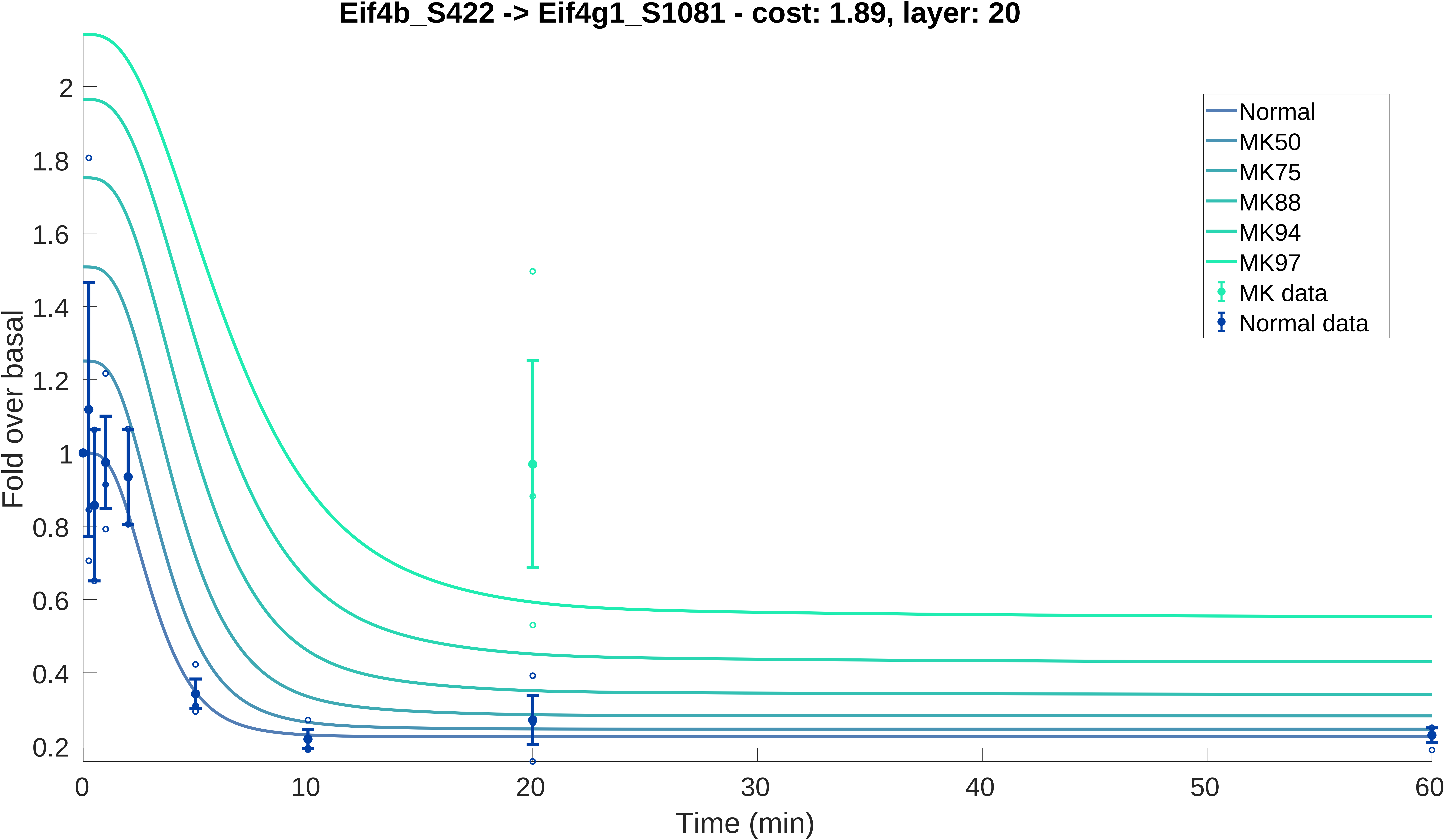
Eif4b_S422 -> Eif4g1_S1081 - cost: 1.89, layer: 20
Fold over basal
Time (min)
0
10
20
30
40
50
60
0.2
0.4
0.6
0.8
1
1.2
1.4
1.6
1.8
2
Normal
MK50
MK75
MK88
MK94
MK97
MK data
Normal data

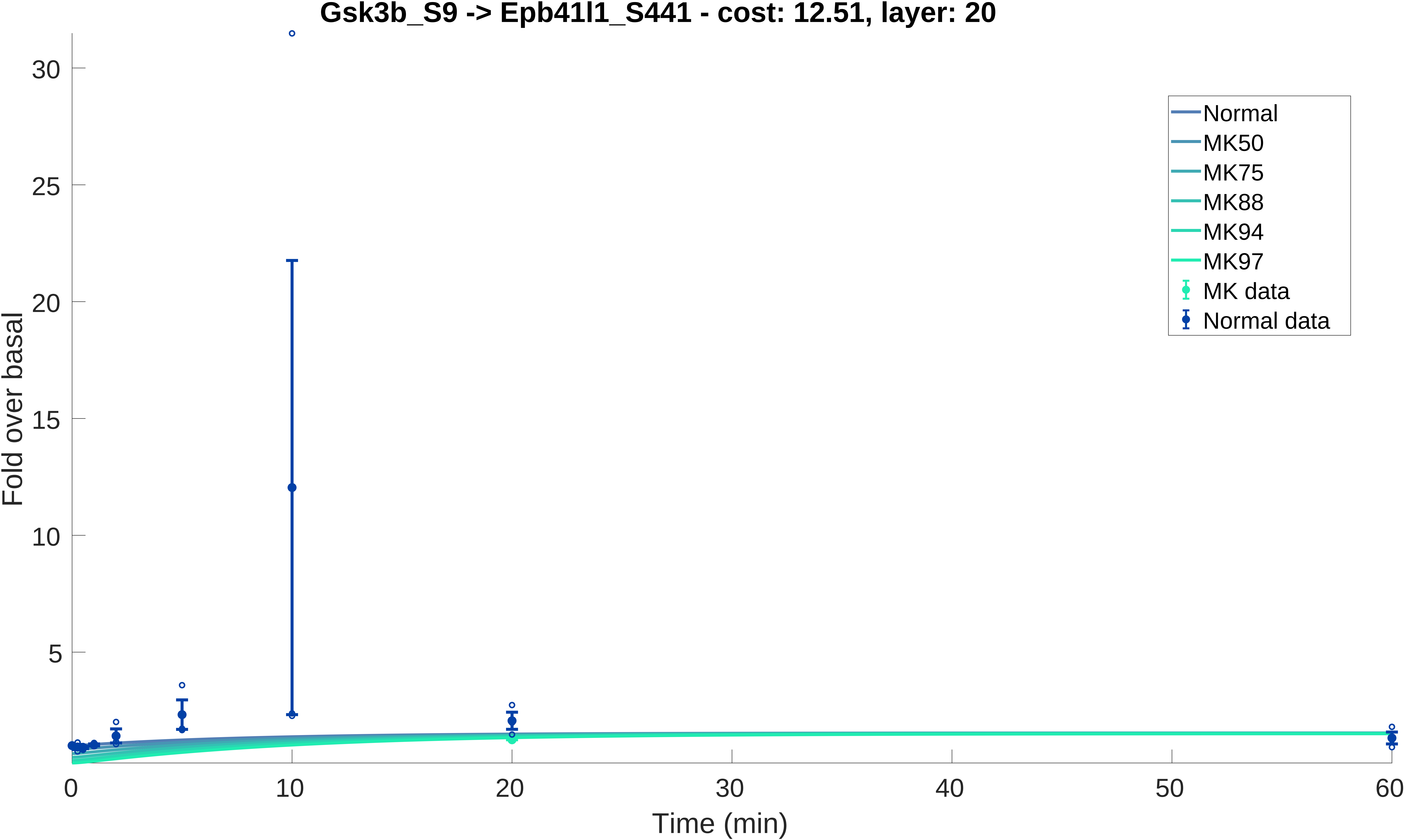
Gsk3b_S9 -> Epb41l1_S441 - cost: 12.51, layer: 20
Fold over basal
Time (min)
0
5
10
15
20
25
30
40
50
60
Normal
MK50
MK75
MK88
MK94
MK97
MK data
Normal data

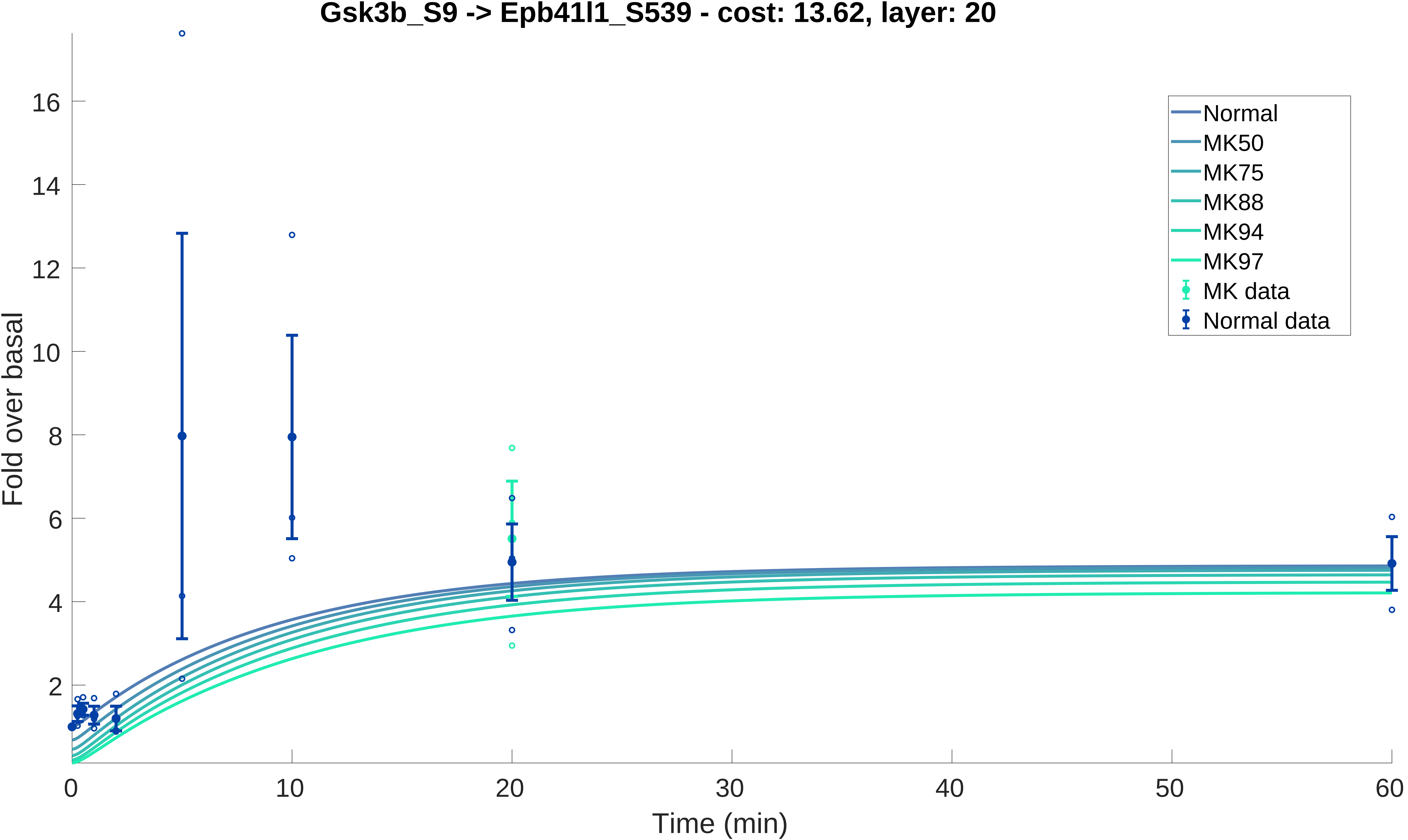
Gsk3b_S9 -> Epb41l1_S539 - cost: 13.62, layer: 20
Fold over basal
Time (min)
Normal
MK50
MK75
MK88
MK94
MK97
MK data
Normal data
0
10
20
30
40
50
60
2
4
6
8
10
12
14
16

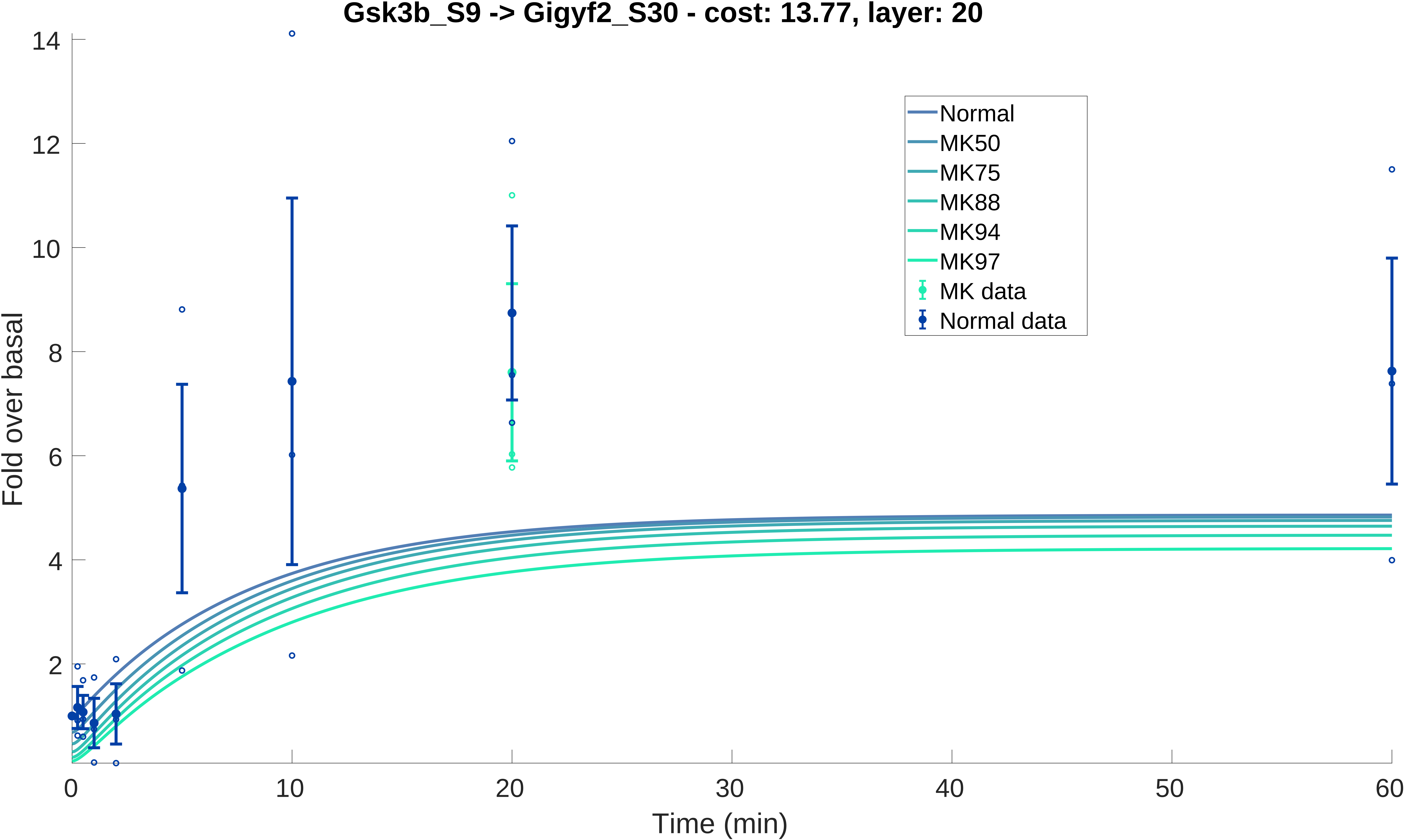

Gsk3b_S9 -> Gigyf2_S30 - cost: 13.77, layer: 20
Fold over basal
Time (min)
0
10
20
30
40
50
60
2
4
6
8
10
12
14
Normal
MK50
MK75
MK88
MK94
MK97
MK data
Normal data

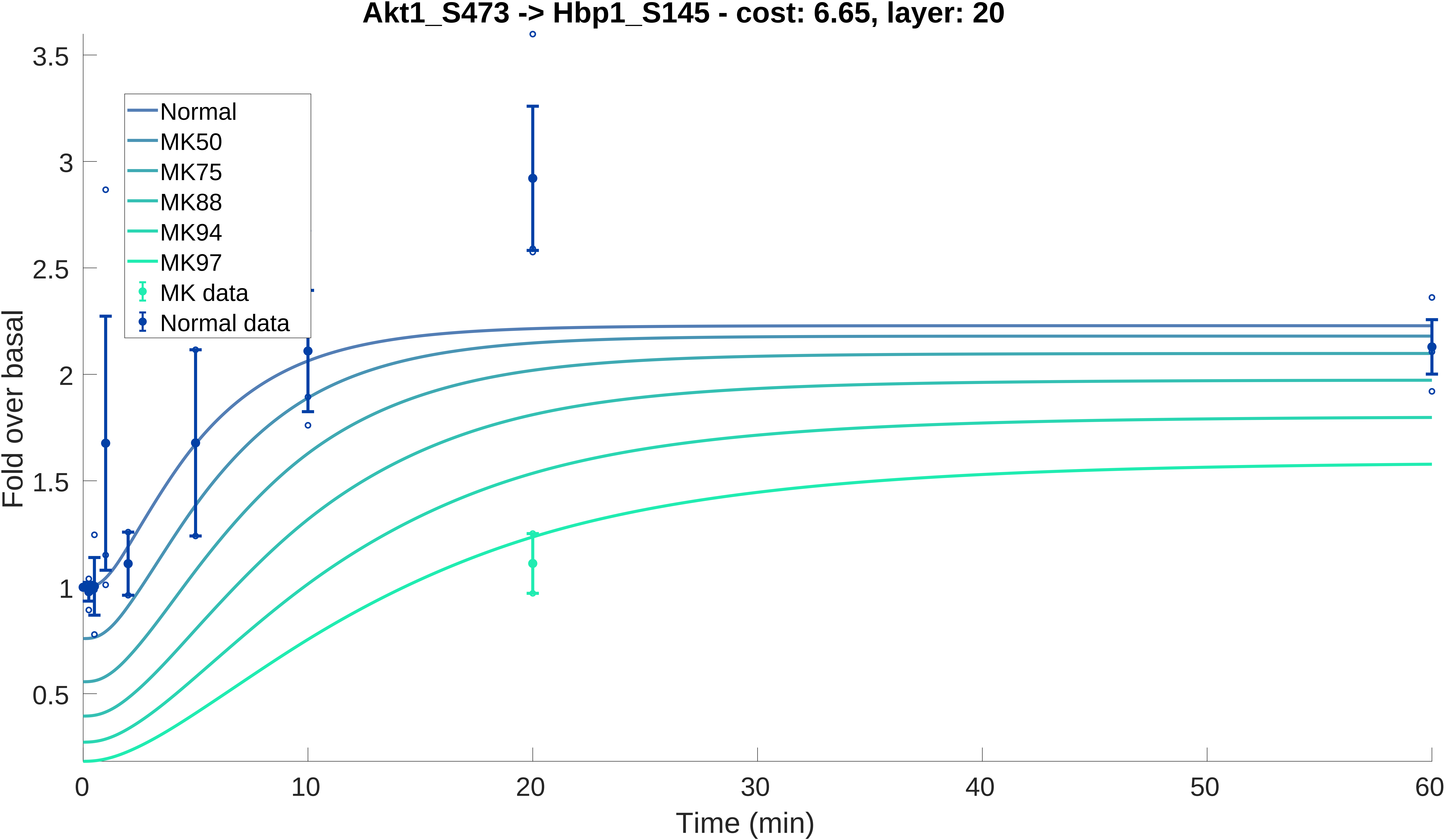

Akt1_S473 -> Hbp1_S145 - cost: 6.65, layer: 20
Normal
MK50
MK75
MK88
MK94
MK97
MK data
Normal data
Fold over basal
Time (min)
0.5
1
1.5
2
2.5
3
3.5
0
10
20
30
40
50
60

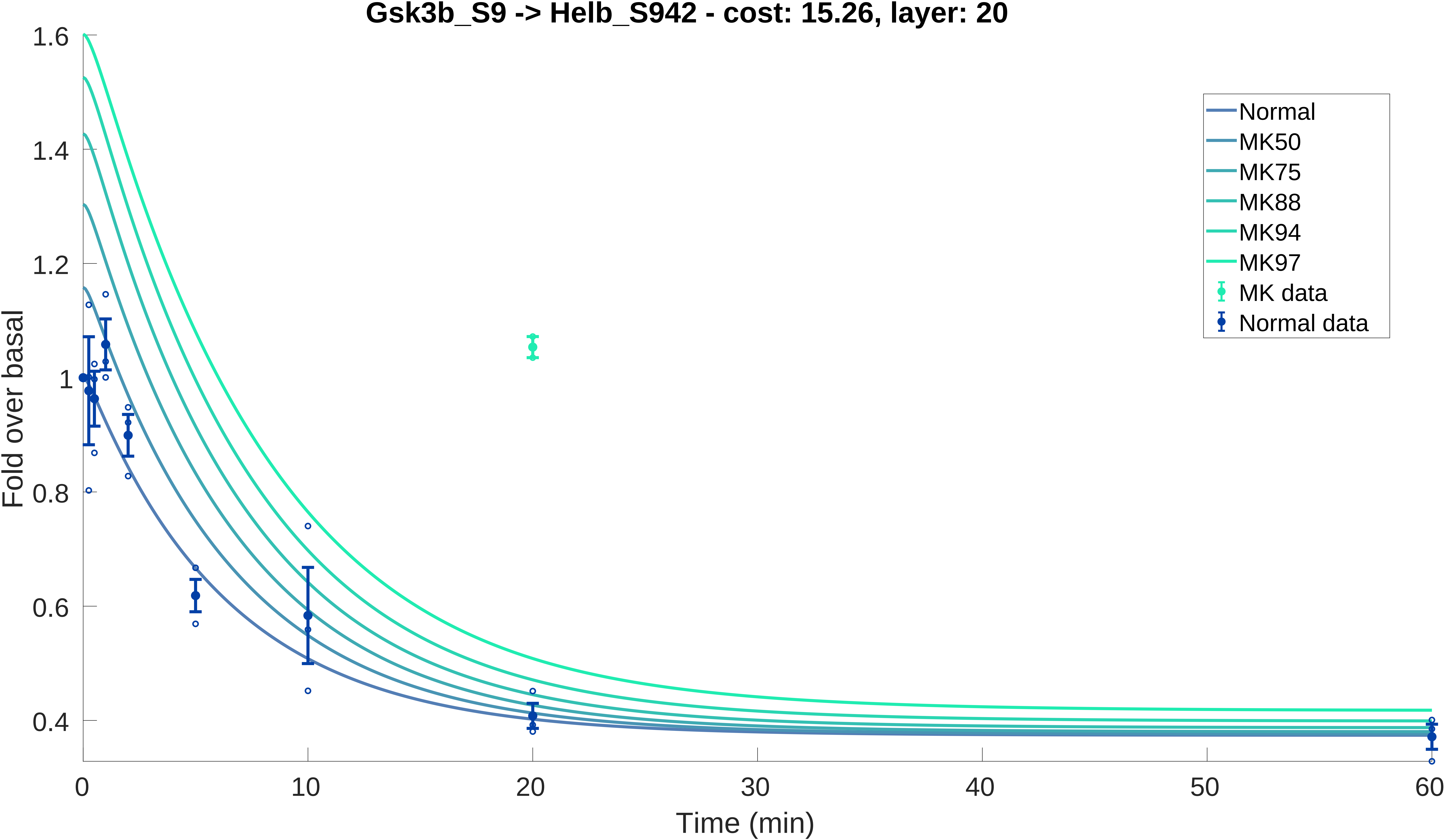
Gsk3b_S9 -> Helb_S942 - cost: 15.26, layer: 20
Fold over basal
Time (min)
0
10
20
30
40
50
60
0.4
0.6
0.8
1
1.2
1.4
1.6
Normal
MK50
MK75
MK88
MK94
MK97
MK data
Normal data

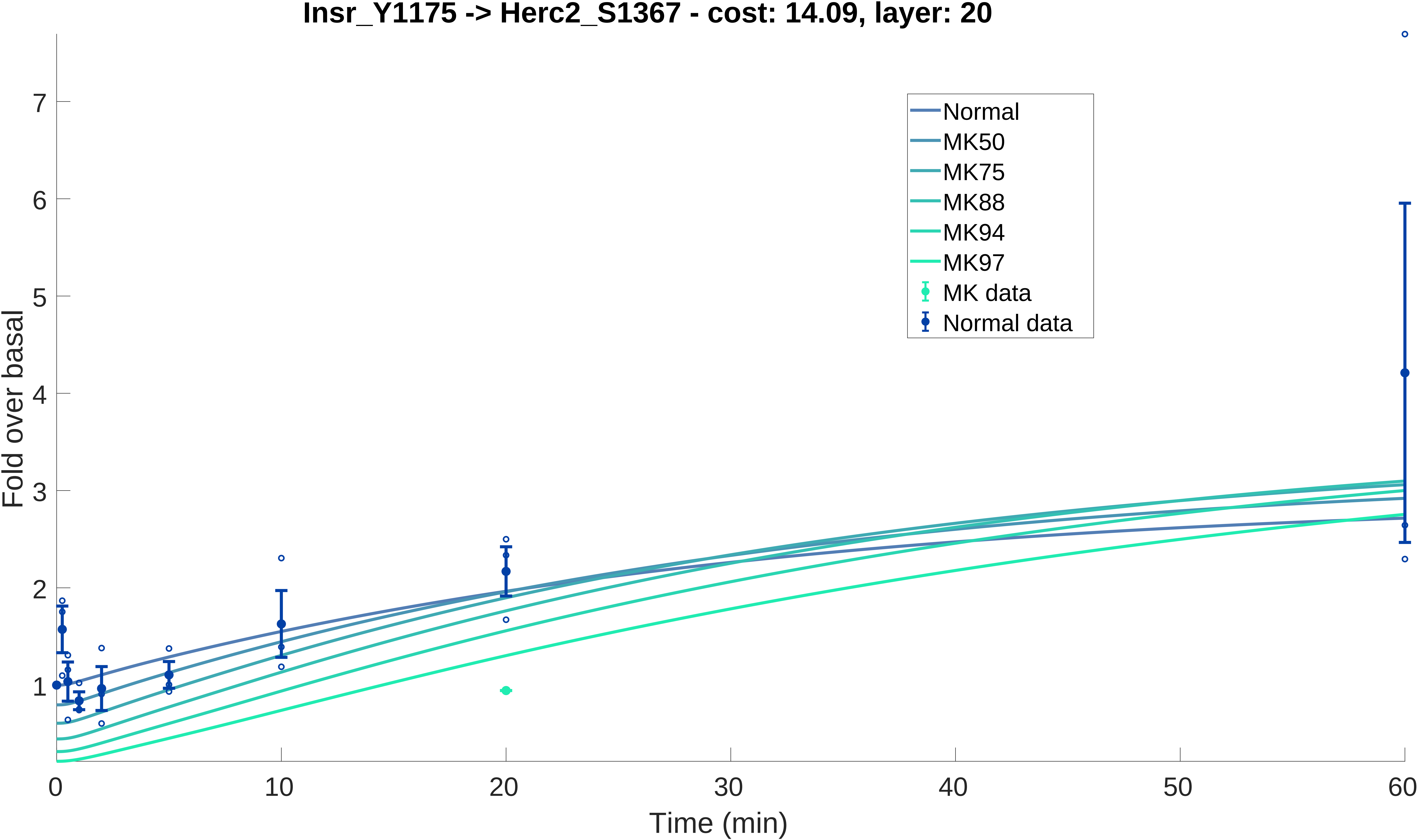
Insr_Y1175 -> Herc2_S1367 - cost: 14.09, layer: 20
Fold over basal
Time (min)
0
10
20
30
40
50
60
1
2
3
4
5
6
7
Normal
MK50
MK75
MK88
MK94
MK97
MK data
Normal data

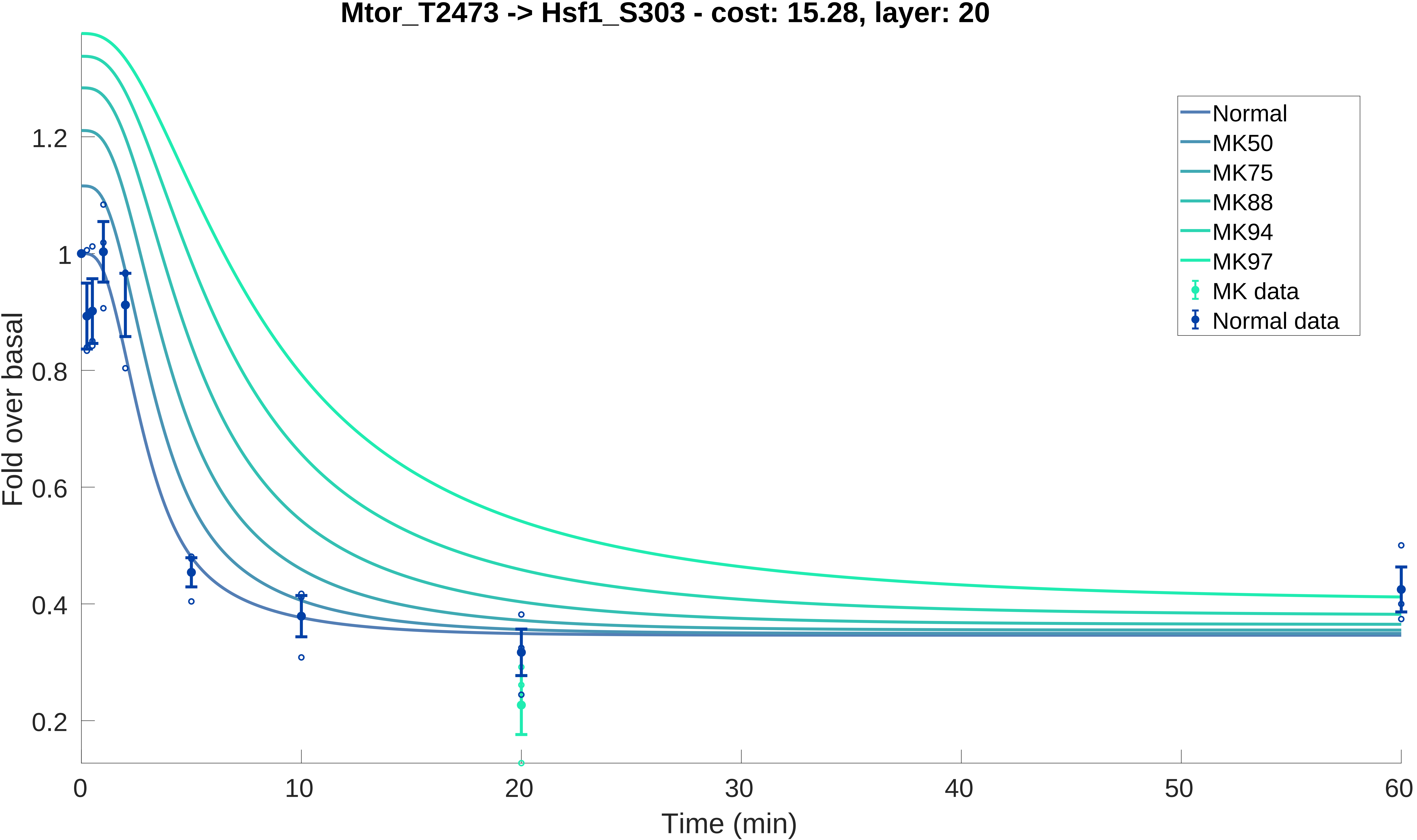
Mtor_T2473 -> Hsf1_S303 - cost: 15.28, layer: 20
Fold over basal
Time (min)
0.2
0.4
0.6
0.8
1
1.2
0
10
20
30
40
50
60
Normal
MK50
MK75
MK88
MK94
MK97
MK data
Normal data

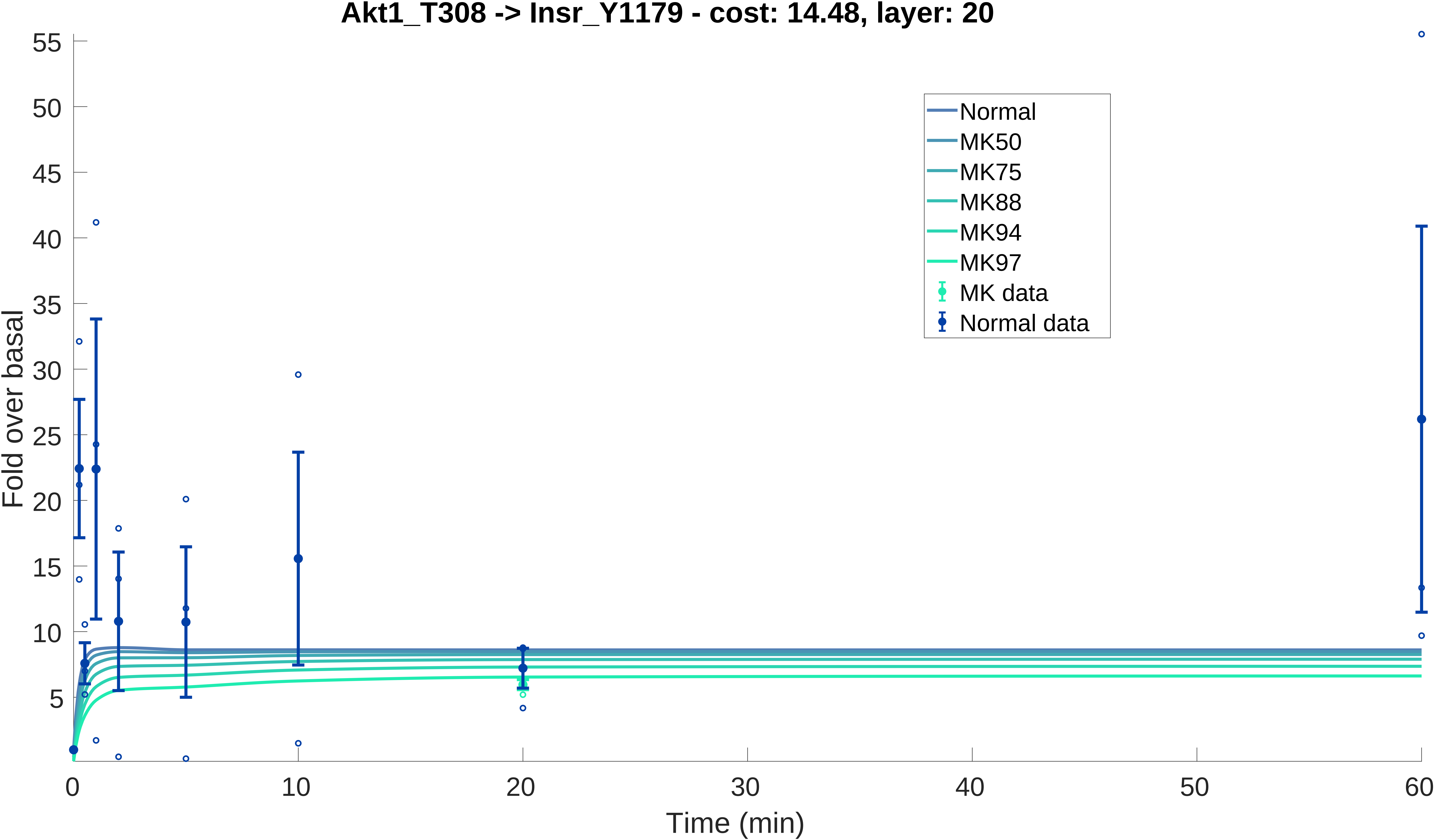
Akt1_T308 -> Insr_Y1179 - cost: 14.48, layer: 20
Fold over basal
Time (min)
0
10
20
30
40
50
60
5
10
15
20
25
30
35
40
45
50
55
Normal
MK50
MK75
MK88
MK94
MK97
MK data
Normal data

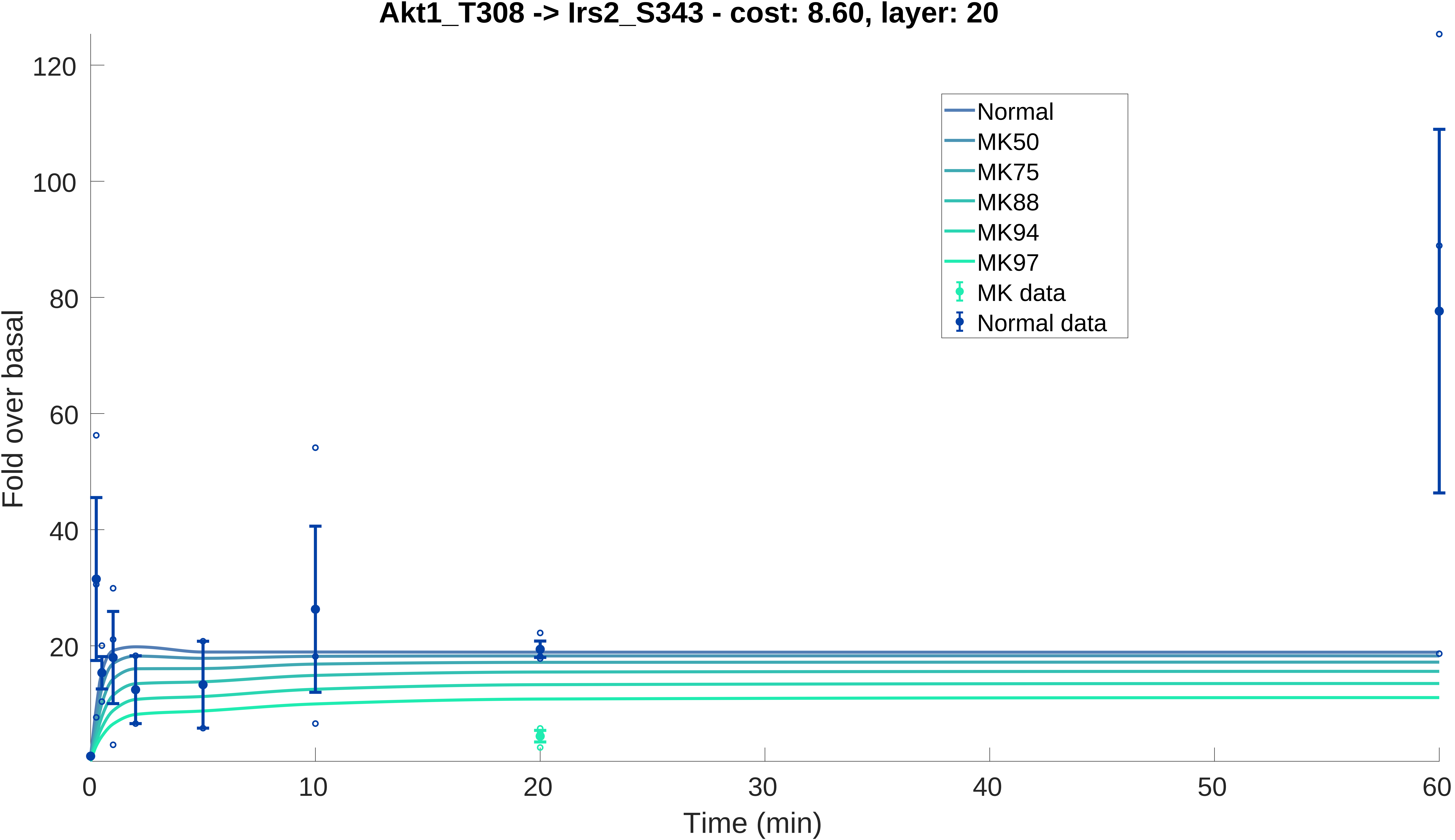

Akt1_T308 -> Irs2_S343 - cost: 8.60, layer: 20
Fold over basal
Time (min)
0
20
40
60
80
100
120
0
10
20
30
40
50
60
Normal
MK50
MK75
MK88
MK94
MK97
MK data
Normal data

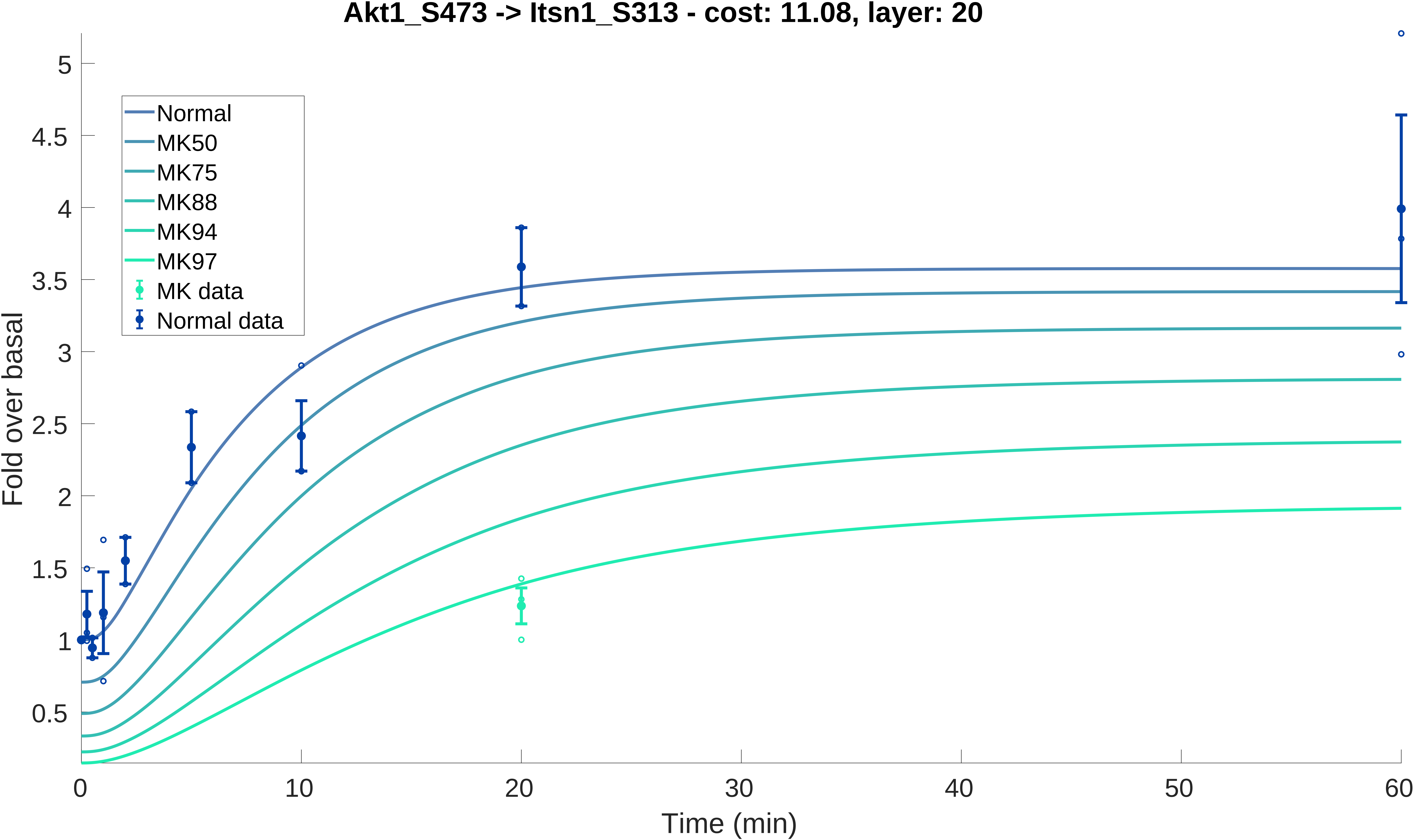
Akt1_S473 -> Itsn1_S313 - cost: 11.08, layer: 20
Normal
MK50
MK75
MK88
MK94
MK97
MK data
Normal data
Fold over basal
Time (min)
0.5
1
1.5
2
2.5
3
3.5
4
4.5
5
0
10
20
30
40
50
60

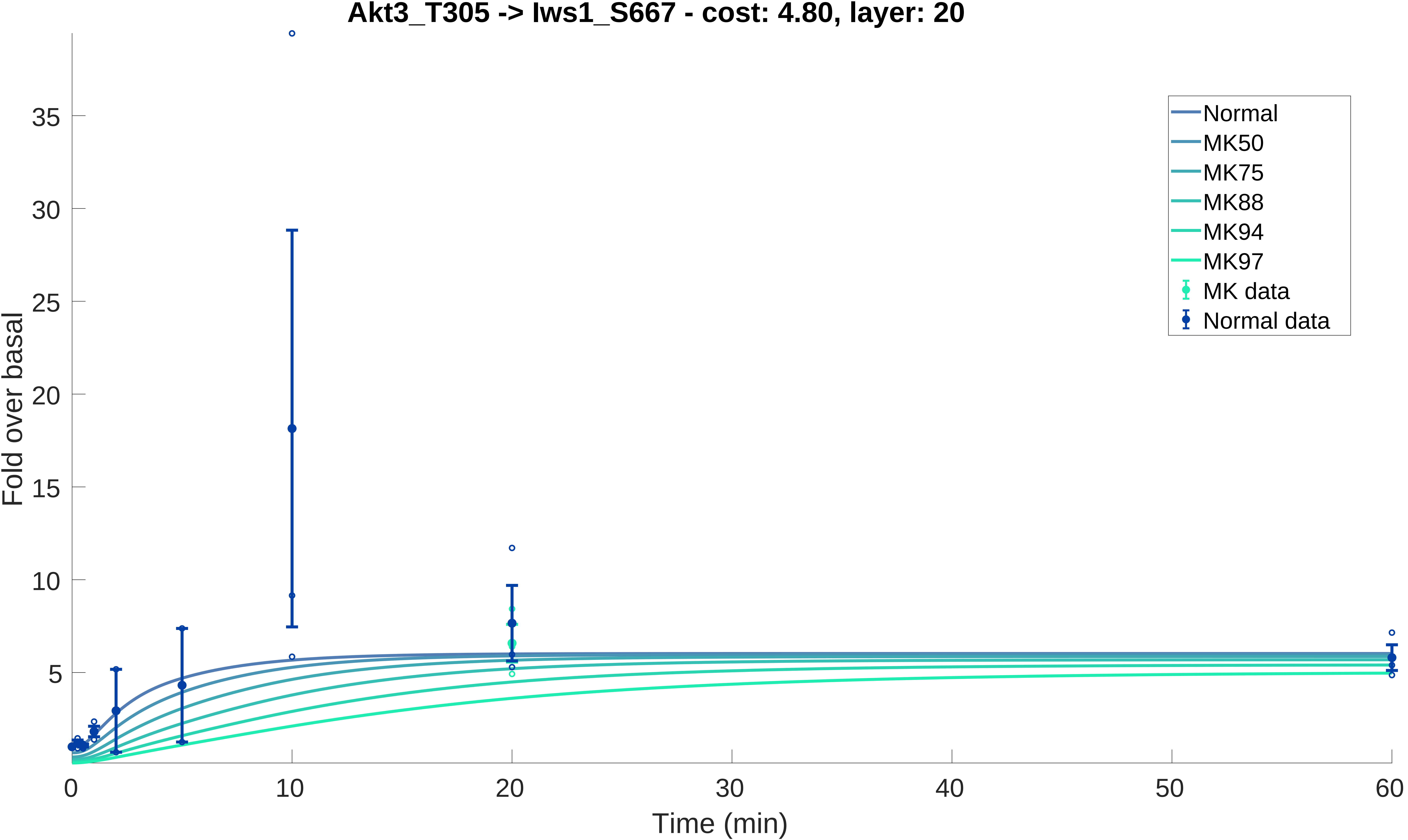

Akt3_T305 -> Iws1_S667 - cost: 4.80, layer: 20
Fold over basal
Time (min)
0
10
20
30
40
50
60
5
10
15
20
25
30
35
Normal
MK50
MK75
MK88
MK94
MK97
MK data
Normal data

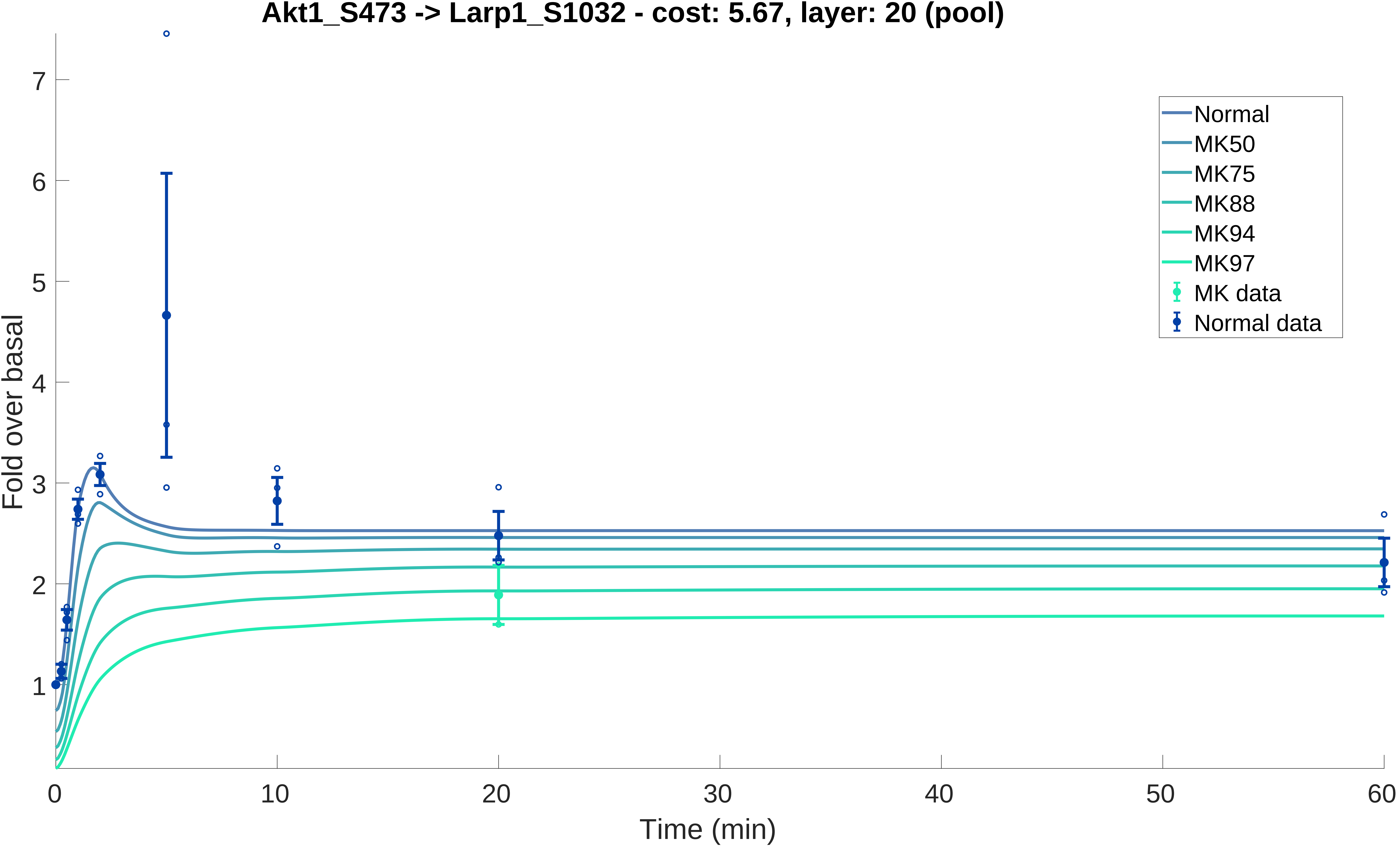
Akt1_S473 -> Larp1_S1032 - cost: 5.67, layer: 20 (pool)
Fold over basal
Time (min)
0
10
20
30
40
50
60
1
2
3
4
5
6
7
Normal
MK50
MK75
MK88
MK94
MK97
MK data
Normal data

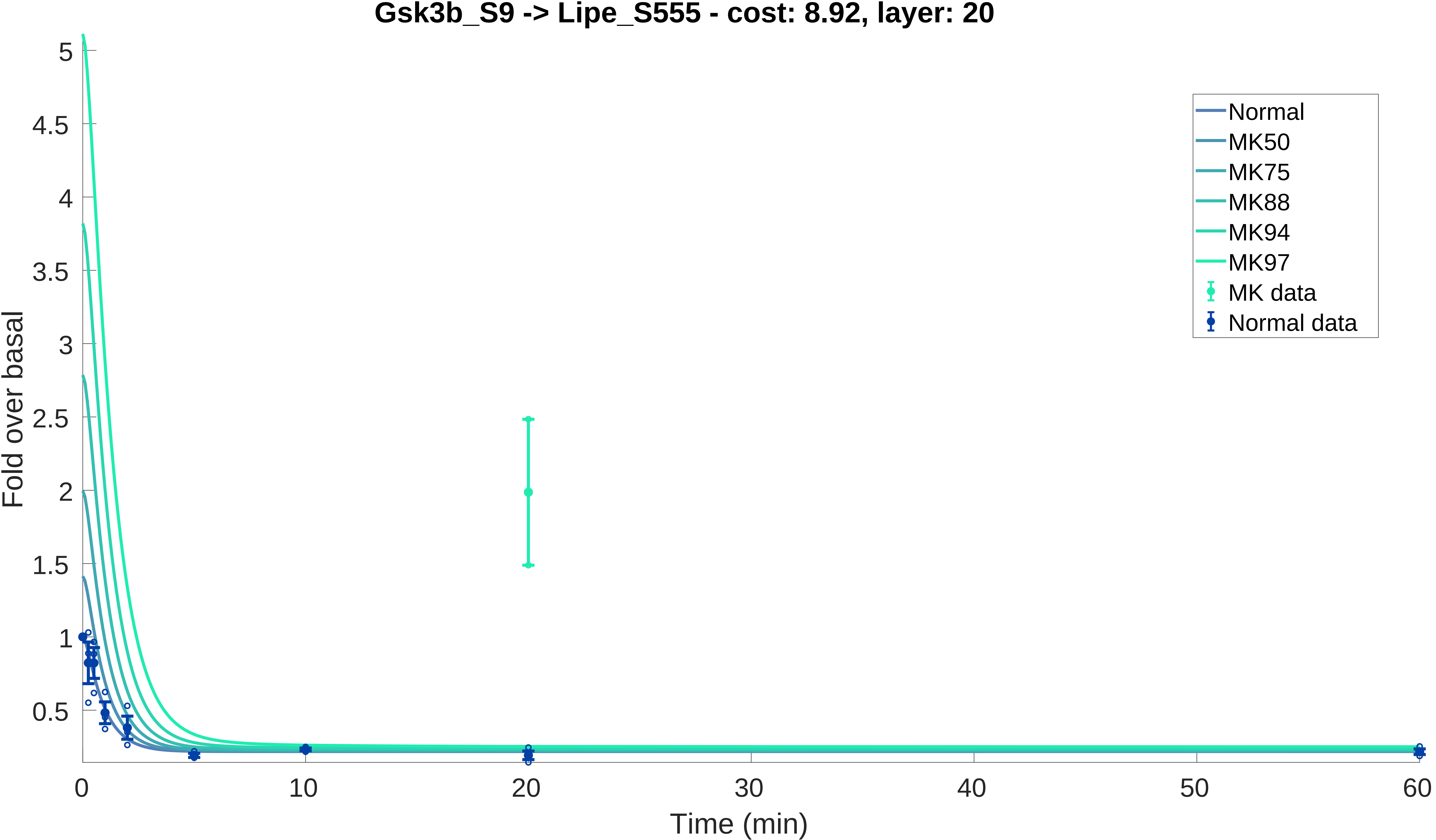
Gsk3b_S9 -> Lipe_S555 - cost: 8.92, layer: 20
Fold over basal
Time (min)
Normal
MK50
MK75
MK88
MK94
MK97
MK data
Normal data
5
4.5
4
3.5
3
2.5
2
1.5
1
0.5
0
10
20
30
40
50
60

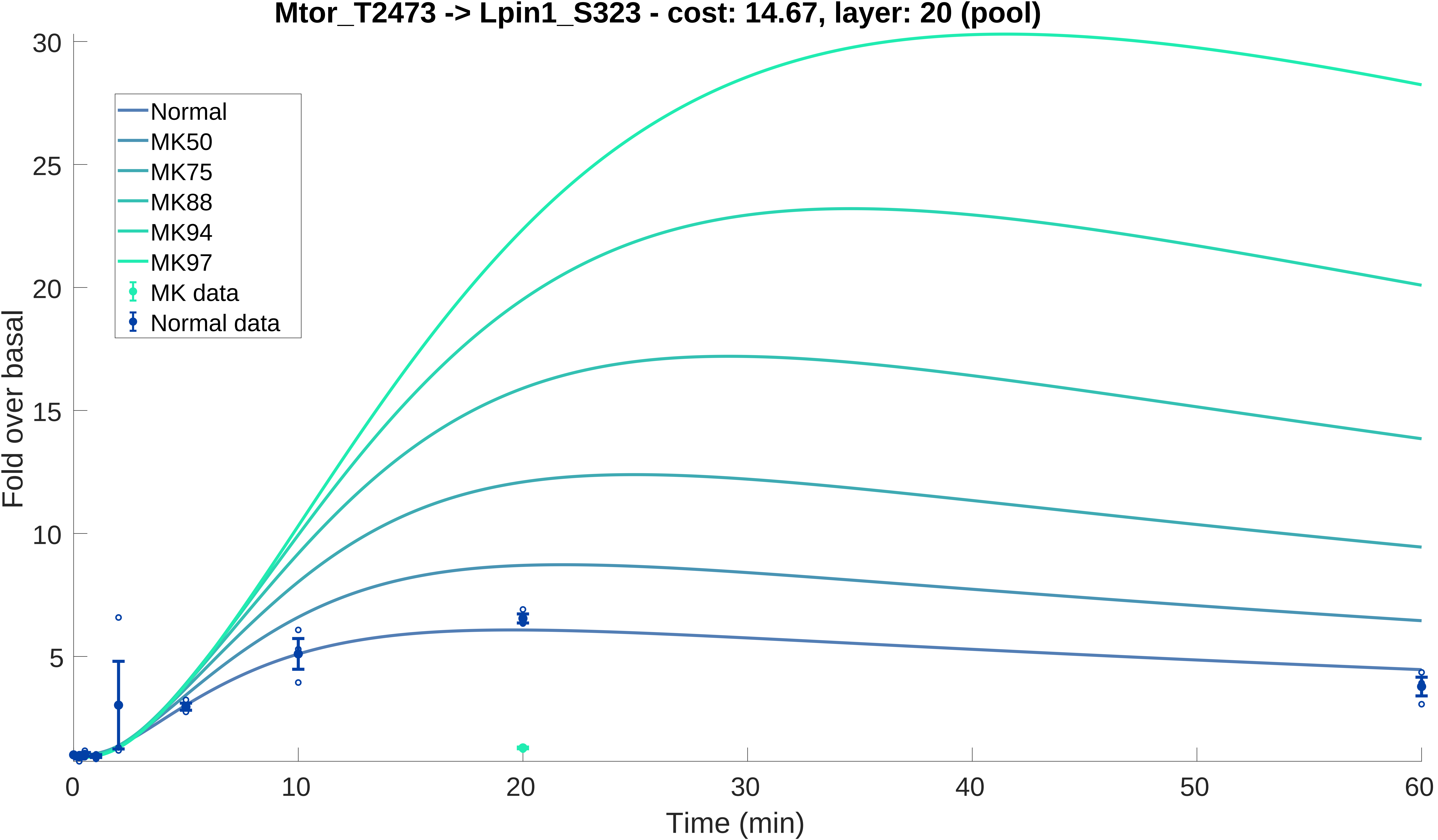
Mtor_T2473 -> Lpin1_S323 - cost: 14.67, layer: 20 (pool)
Fold over basal
Time (min)
0
10
20
30
40
50
60
5
10
15
20
25
30
Normal
MK50
MK75
MK88
MK94
MK97
MK data
Normal data

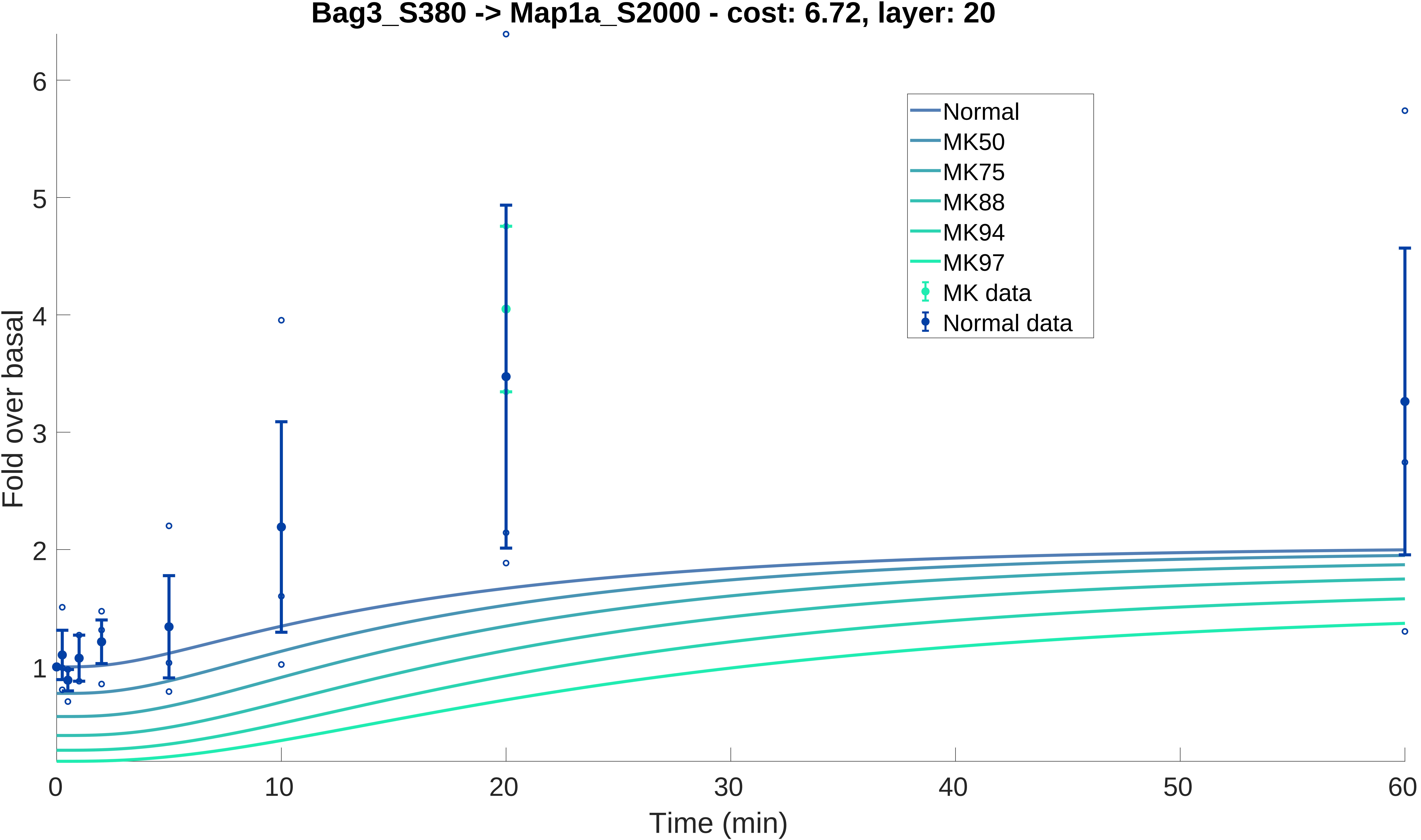
Bag3_S380 -> Map1a_S2000 - cost: 6.72, layer: 20
Fold over basal
Time (min)
0
10
20
30
40
50
60
1
2
3
4
5
6
Normal
MK50
MK75
MK88
MK94
MK97
MK data
Normal data

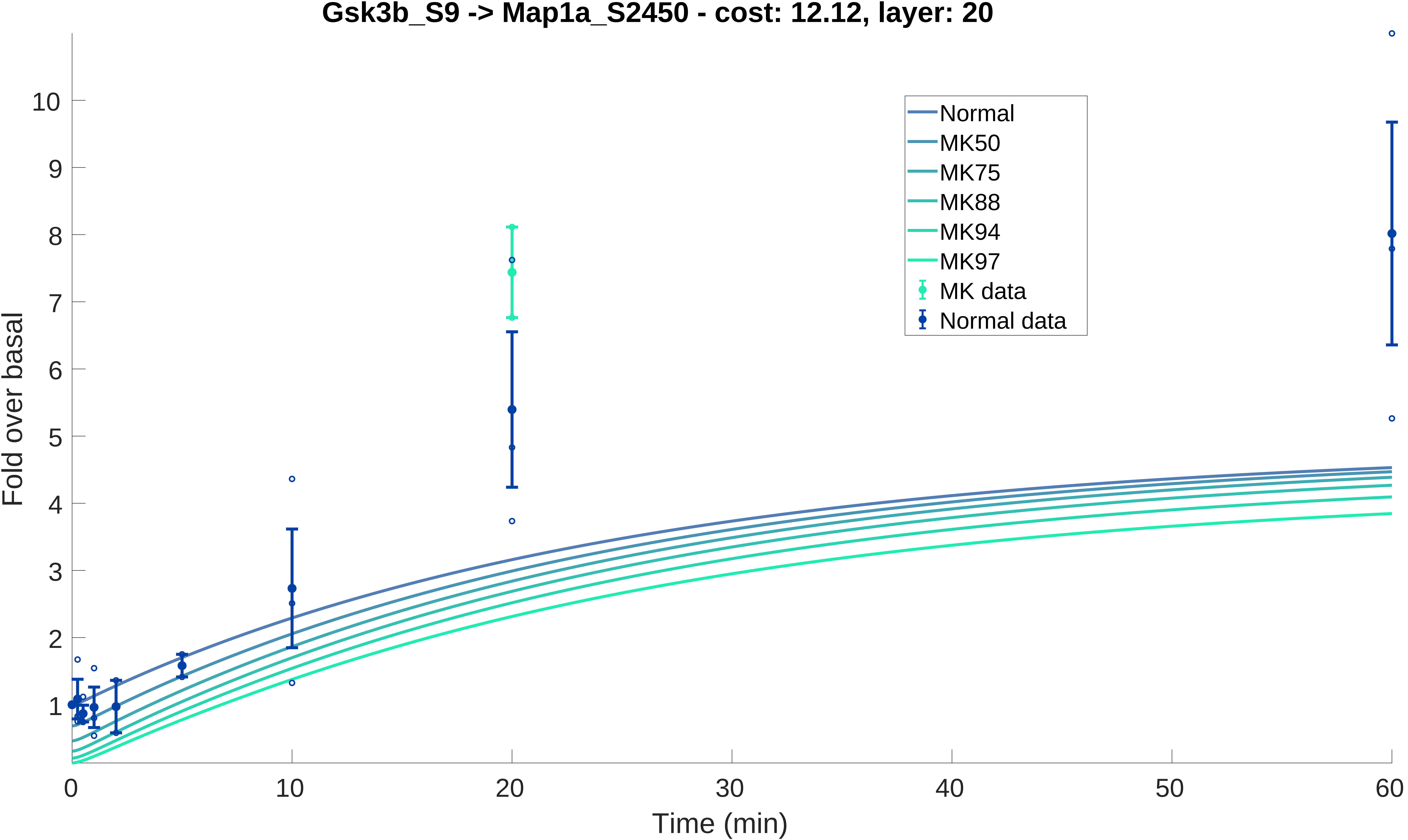
Gsk3b_S9 -> Map1a_S2450 - cost: 12.12, layer: 20
Fold over basal
Time (min)
Normal
MK50
MK75
MK88
MK94
MK97
MK data
Normal data
0
10
20
30
40
50
60
1
2
3
4
5
6
7
8
9
10

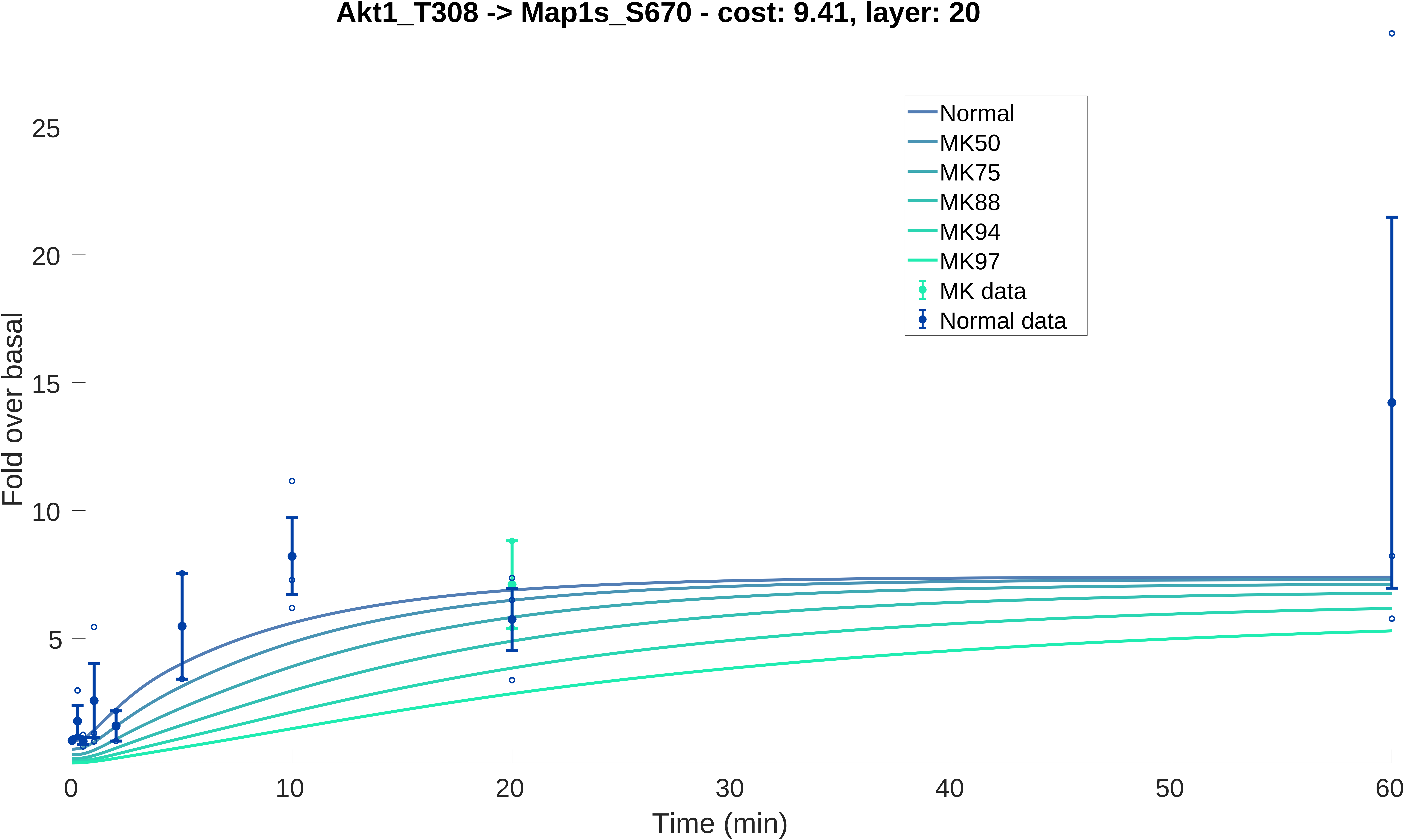
Akt1_T308 -> Map1s_S670 - cost: 9.41, layer: 20
Normal
MK50
MK75
MK88
MK94
MK97
MK data
Normal data
Fold over basal
Time (min)
0
10
20
30
40
50
60
5
10
15
20
25

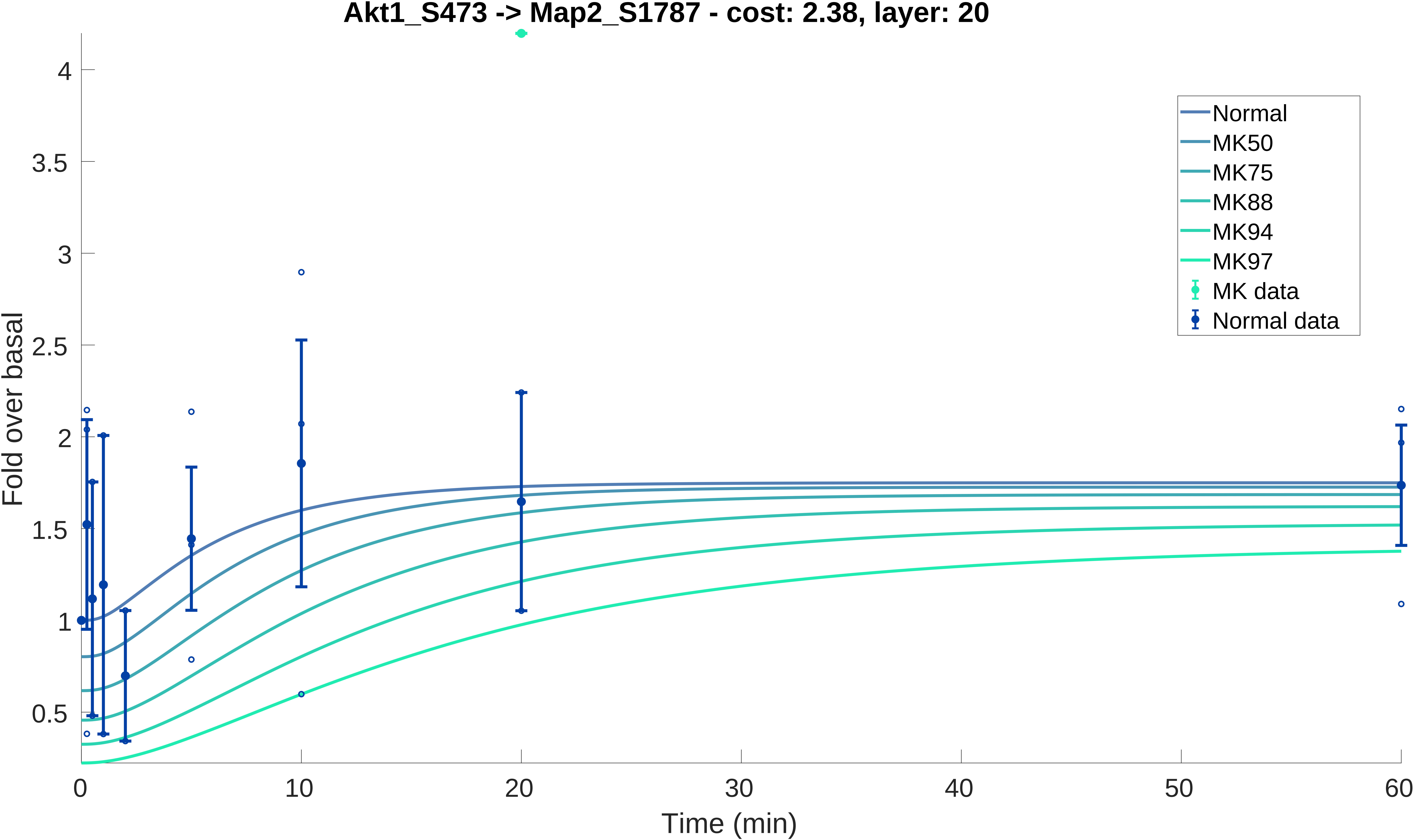

Akt1_S473 -> Map2_S1787 - cost: 2.38, layer: 20
Fold over basal
Time (min)
0.5
1
1.5
2
2.5
3
3.5
4
0
10
20
30
40
50
60
Normal
MK50
MK75
MK88
MK94
MK97
MK data
Normal data

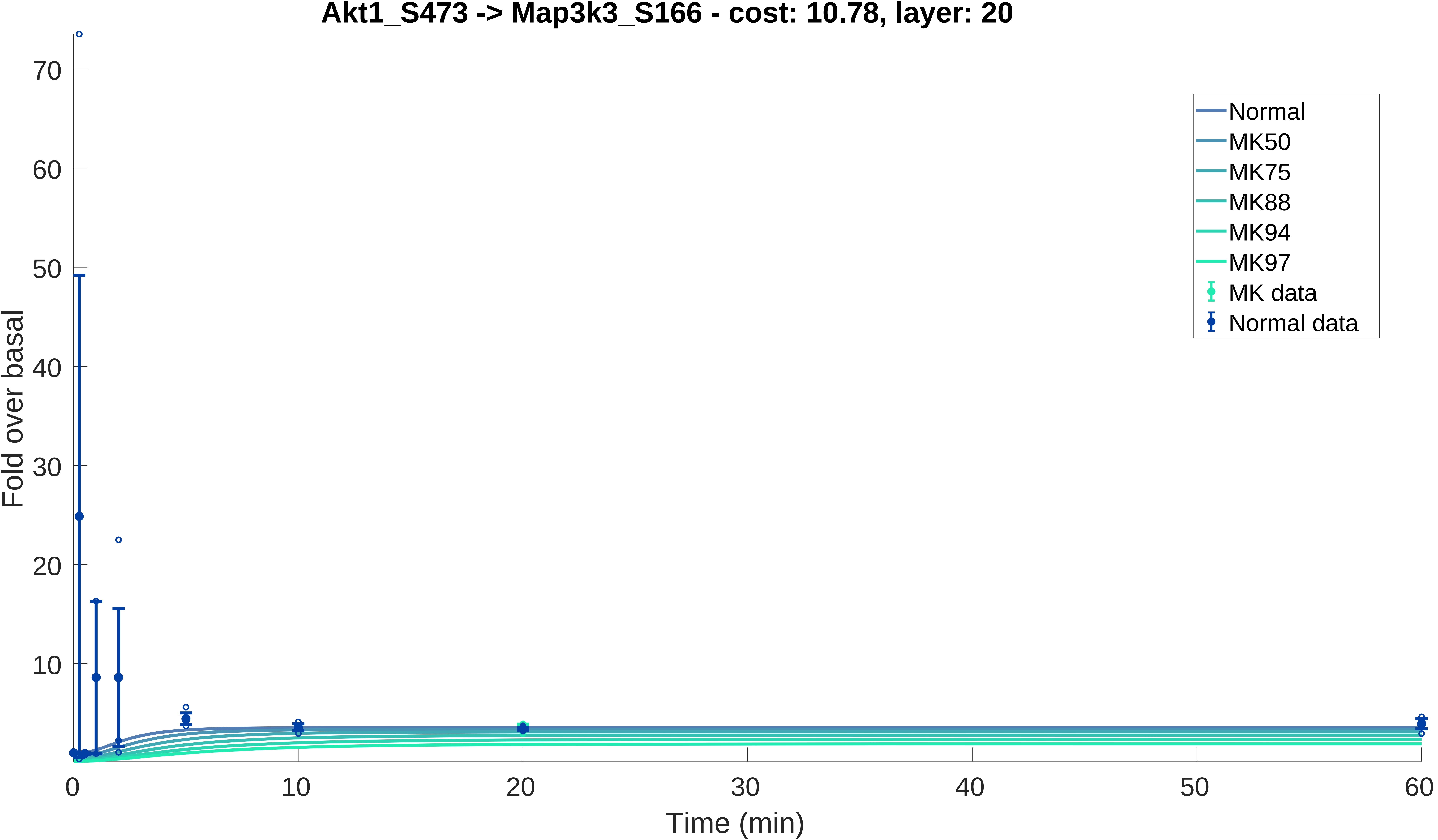

Akt1_S473 -> Map3k3_S166 - cost: 10.78, layer: 20
Fold over basal
Time (min)
0
10
20
30
40
50
60
70
Normal
MK50
MK75
MK88
MK94
MK97
MK data
Normal data

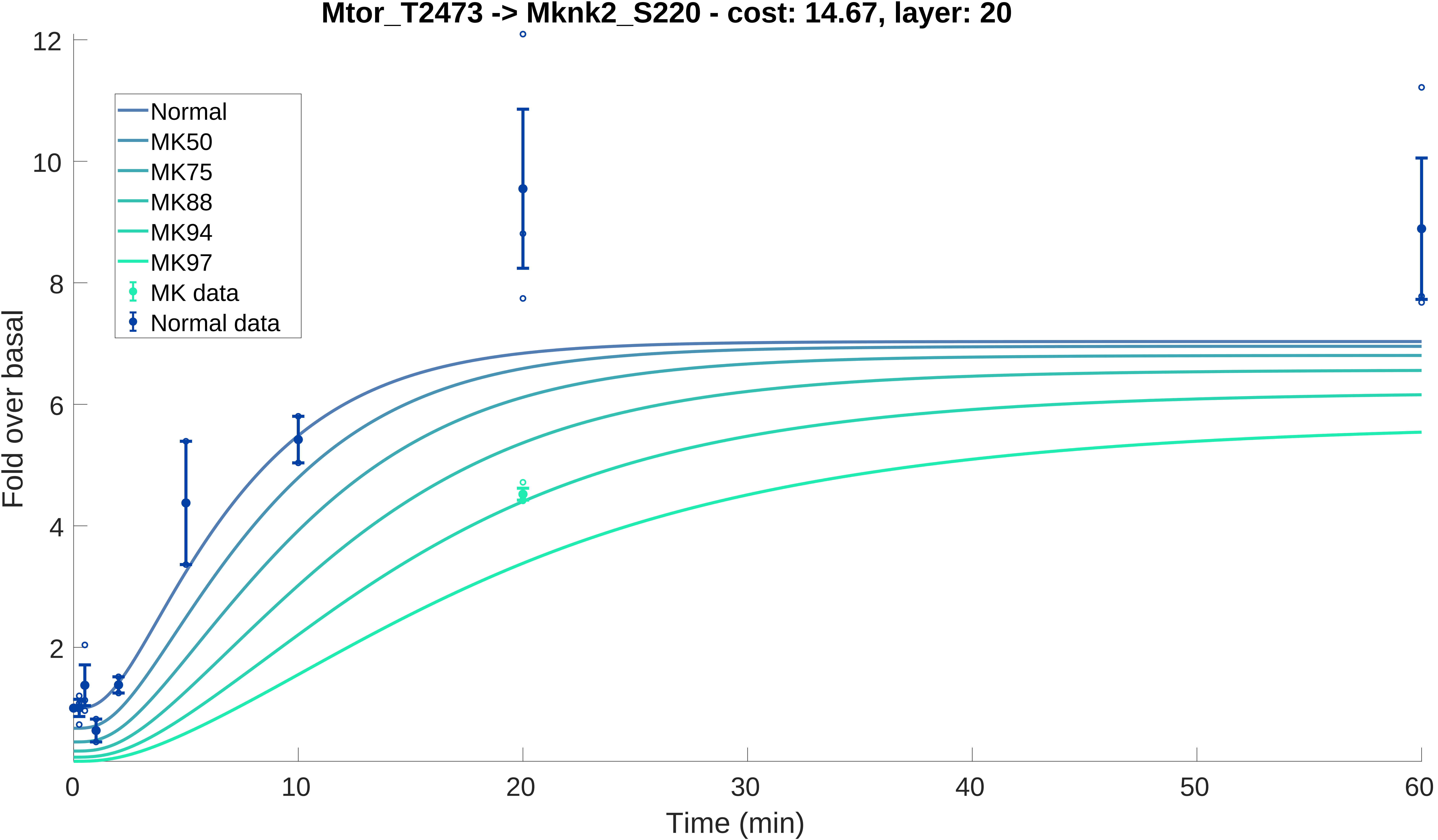
Mtor_T2473 -> Mknk2_S220 - cost: 14.67, layer: 20
Normal
MK50
MK75
MK88
MK94
MK97
MK data
Normal data
Fold over basal
Time (min)
0
10
20
30
40
50
60
2
4
6
8
10
12

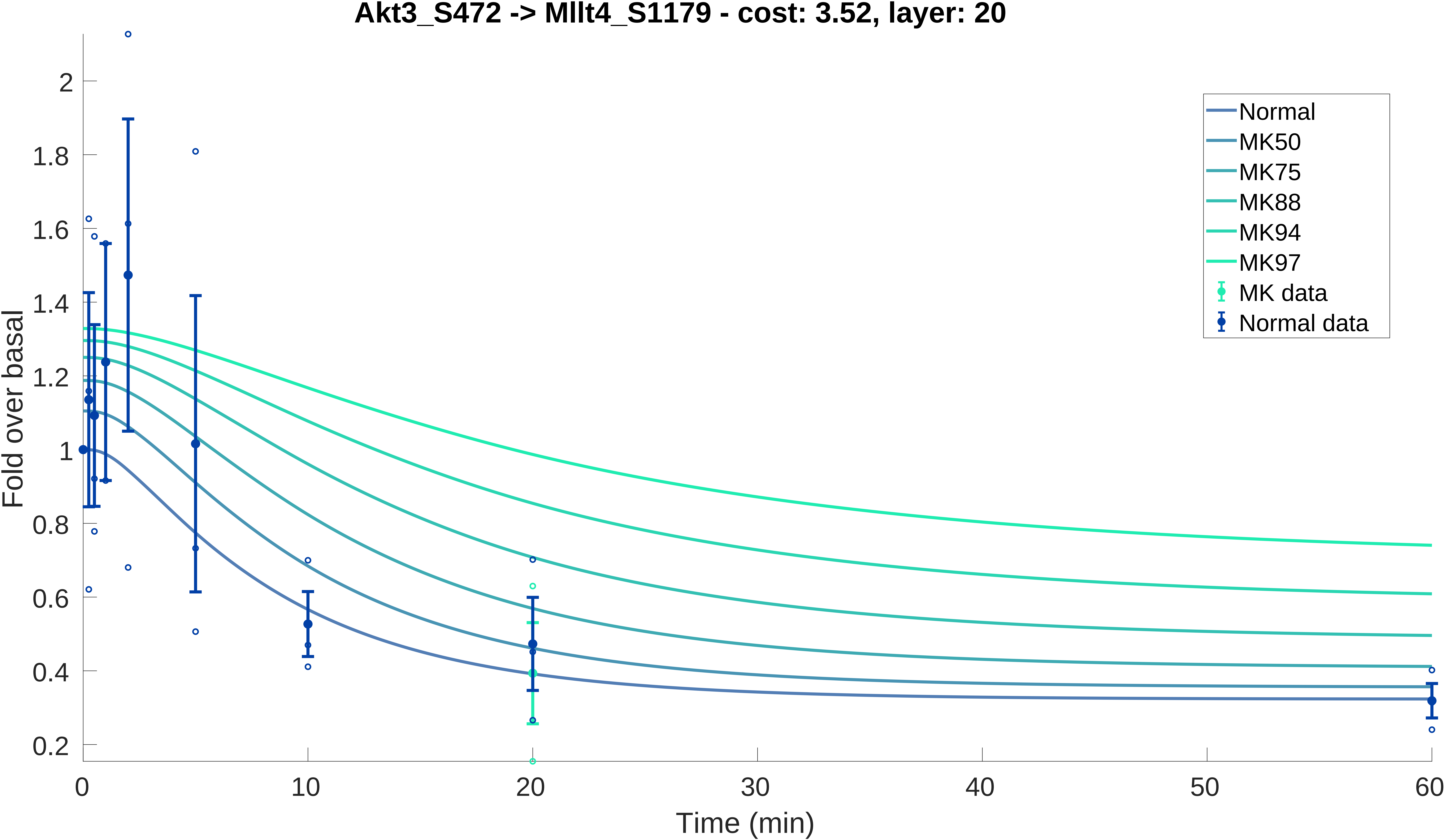
Akt3_S472 -> Mllt4_S1179 - cost: 3.52, layer: 20
Fold over basal
Time (min)
Normal
MK50
MK75
MK88
MK94
MK97
MK data
Normal data
0.2
0.4
0.6
0.8
1
1.2
1.4
1.6
1.8
2
0
10
20
30
40
50
60

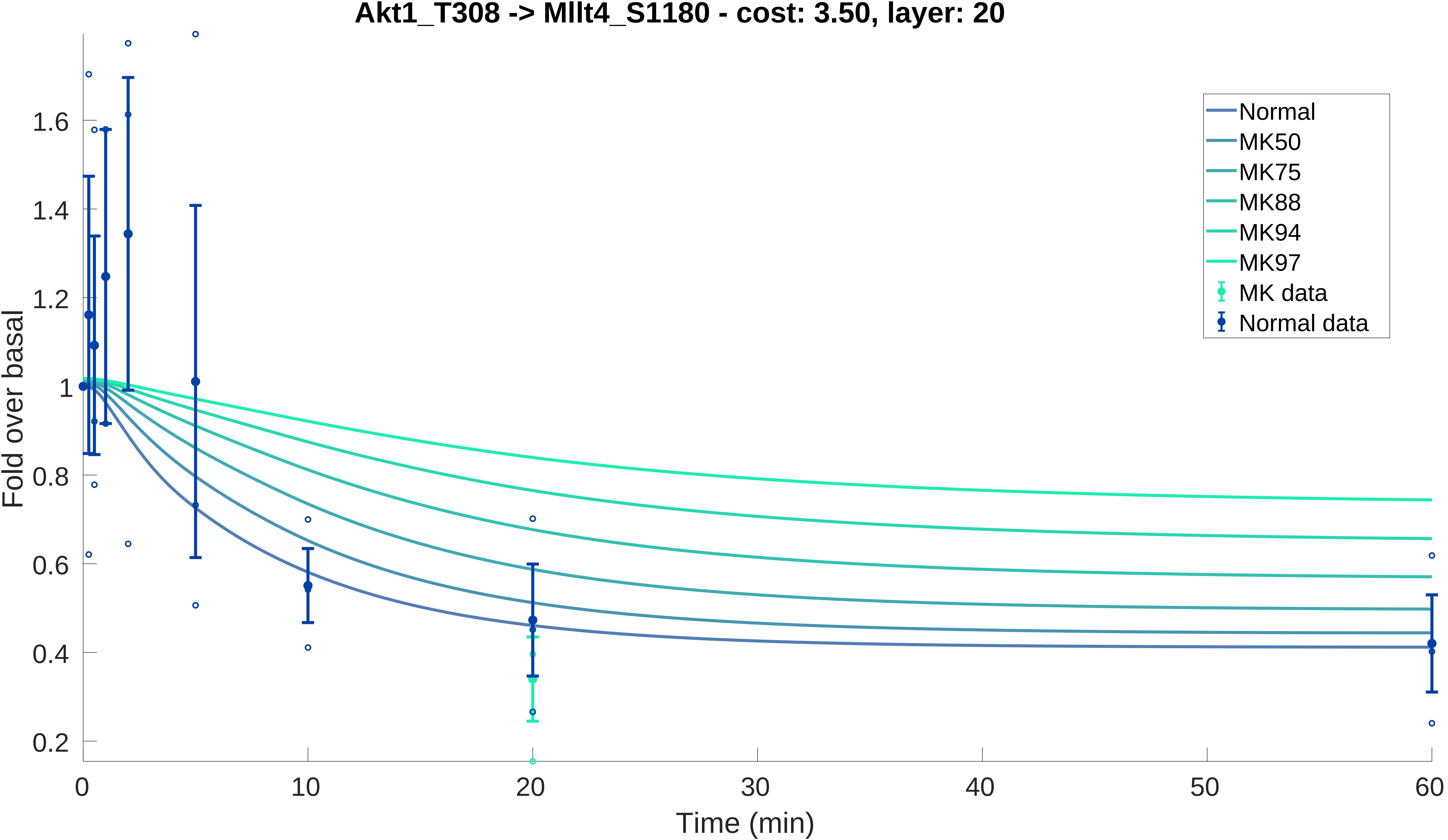

Akt1_T308 -> Mllt4_S1180 - cost: 3.50, layer: 20
Fold over basal
Time (min)
0.2
0.4
0.6
0.8
1
1.2
1.4
1.6
0
10
20
30
40
50
60
Normal
MK50
MK75
MK88
MK94
MK97
MK data
Normal data

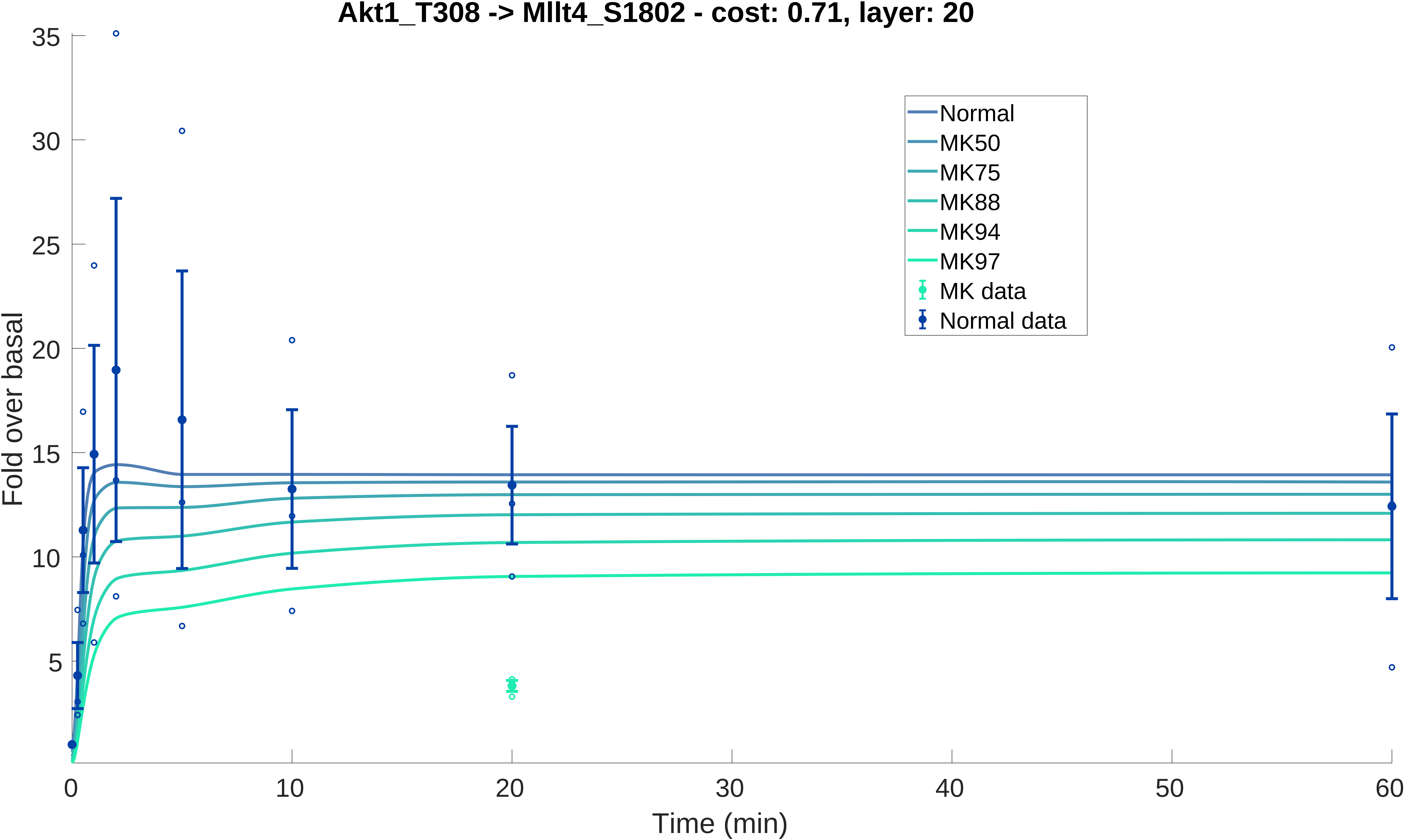

Akt1_T308 -> Mllt4_S1802 - cost: 0.71, layer: 20
Fold over basal
Time (min)
0
5
10
15
20
25
30
35
40
50
60
Normal
MK50
MK75
MK88
MK94
MK97
MK data
Normal data

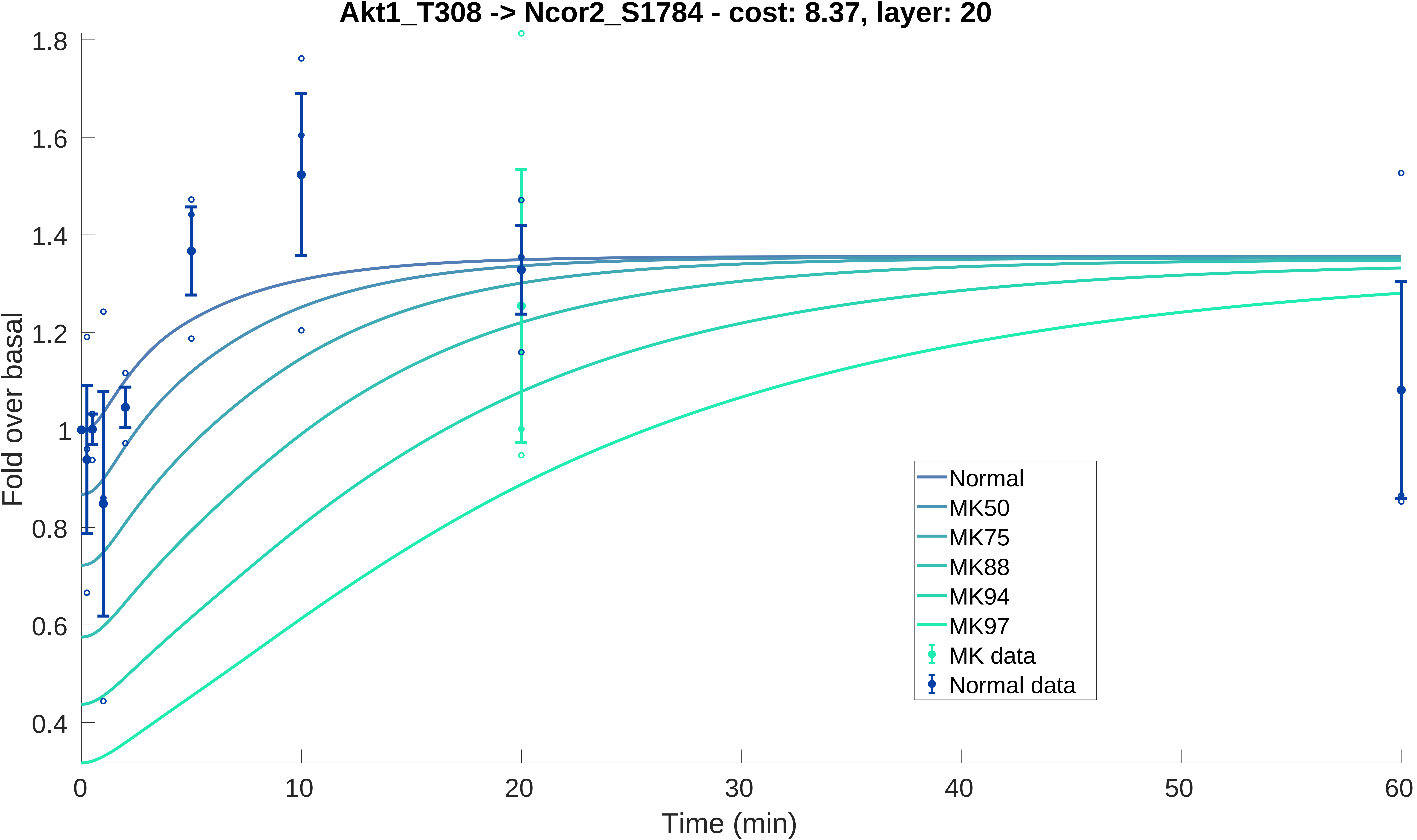
Akt1_T308 -> Ncor2_S1784 - cost: 8.37, layer: 20
Fold over basal
Time (min)
0
10
20
30
40
50
60
0.4
0.6
0.8
1
1.2
1.4
1.6
1.8
Normal
MK50
MK75
MK88
MK94
MK97
MK data
Normal data

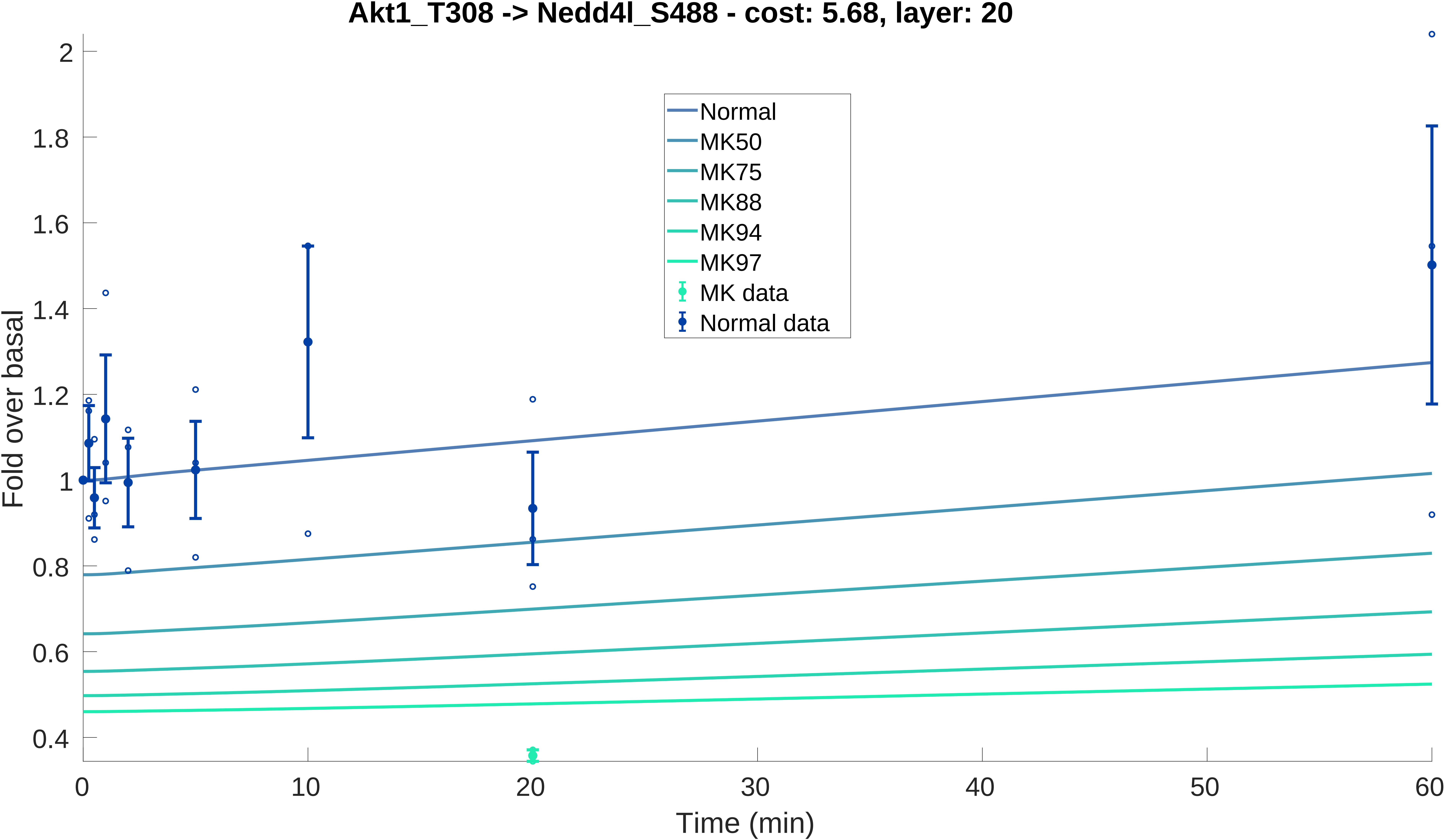

Akt1_T308 -> Nedd4l_S488 - cost: 5.68, layer: 20
Normal
MK50
MK75
MK88
MK94
MK97
MK data
Normal data
Fold over basal
Time (min)
0.4
0.6
0.8
1
1.2
1.4
1.6
1.8
2
0
10
20
30
40
50
60

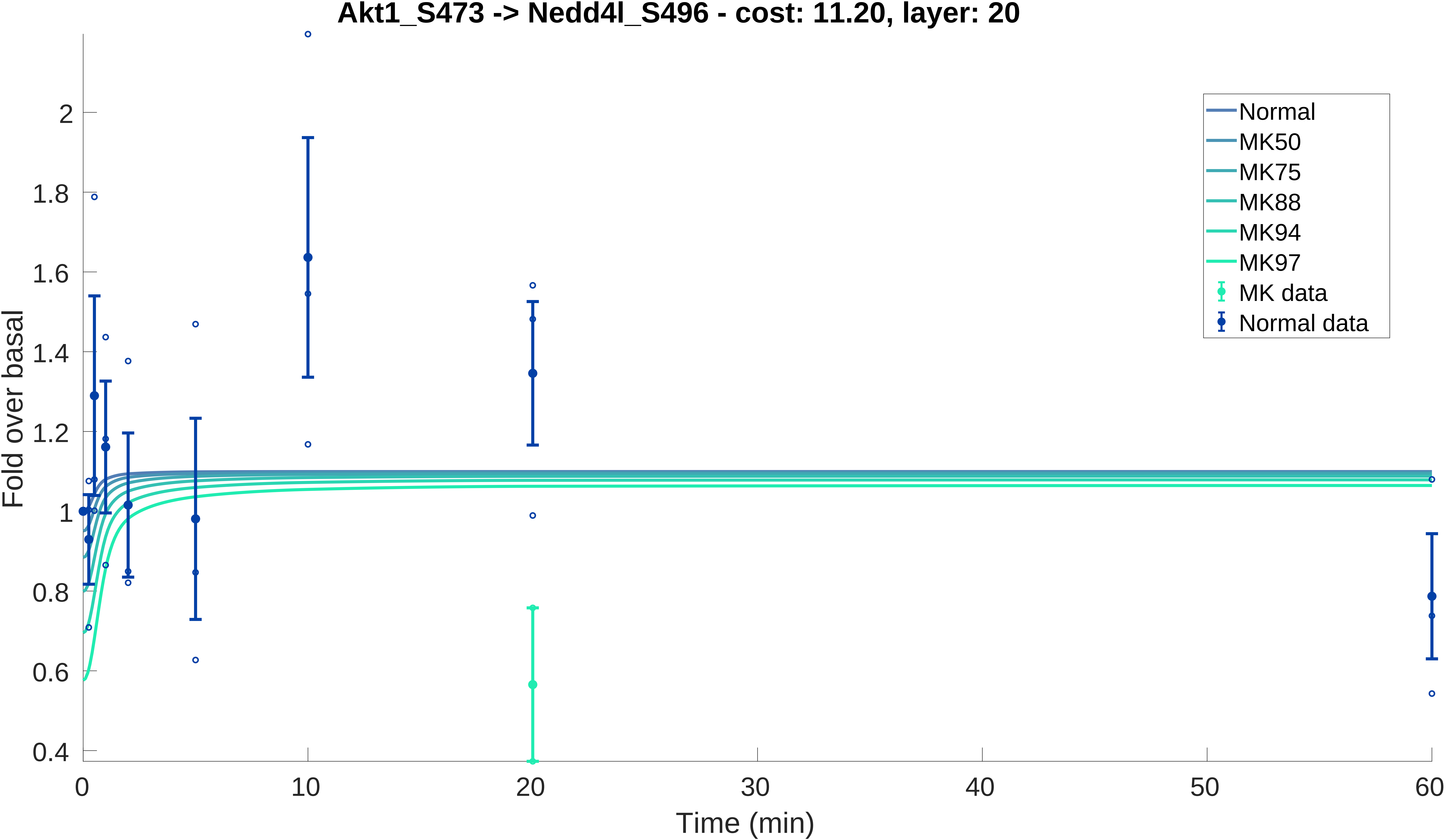
Akt1_S473 -> Nedd4l_S496 - cost: 11.20, layer: 20
Fold over basal
Time (min)
0.4
0.6
0.8
1
1.2
1.4
1.6
1.8
2
0
10
20
30
40
50
60
Normal
MK50
MK75
MK88
MK94
MK97
MK data
Normal data

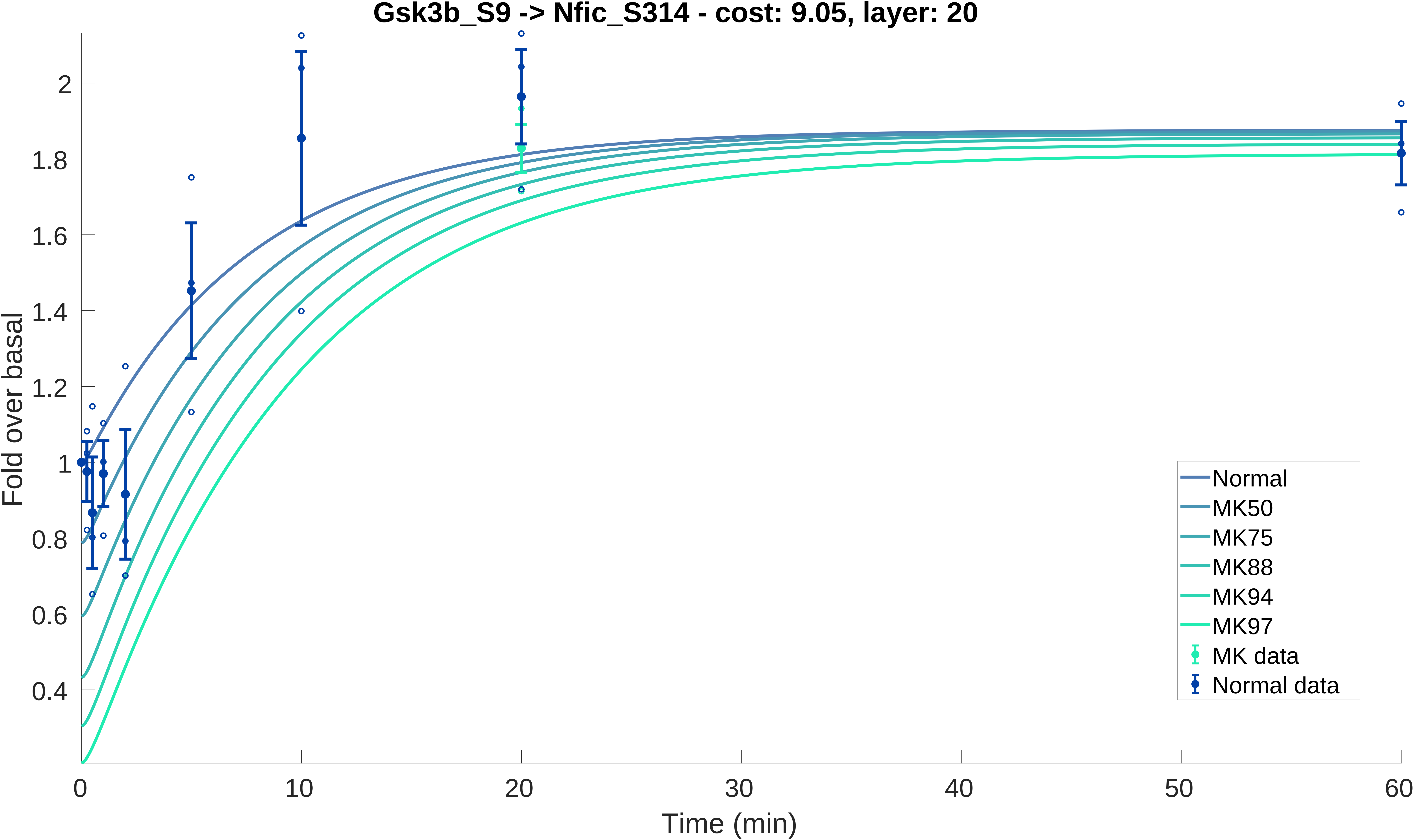

Gsk3b_S9 -> Nfic_S314 - cost: 9.05, layer: 20
Fold over basal
Time (min)
0
10
20
30
40
50
60
0.4
0.6
0.8
1
1.2
1.4
1.6
1.8
2
Normal
MK50
MK75
MK88
MK94
MK97
MK data
Normal data

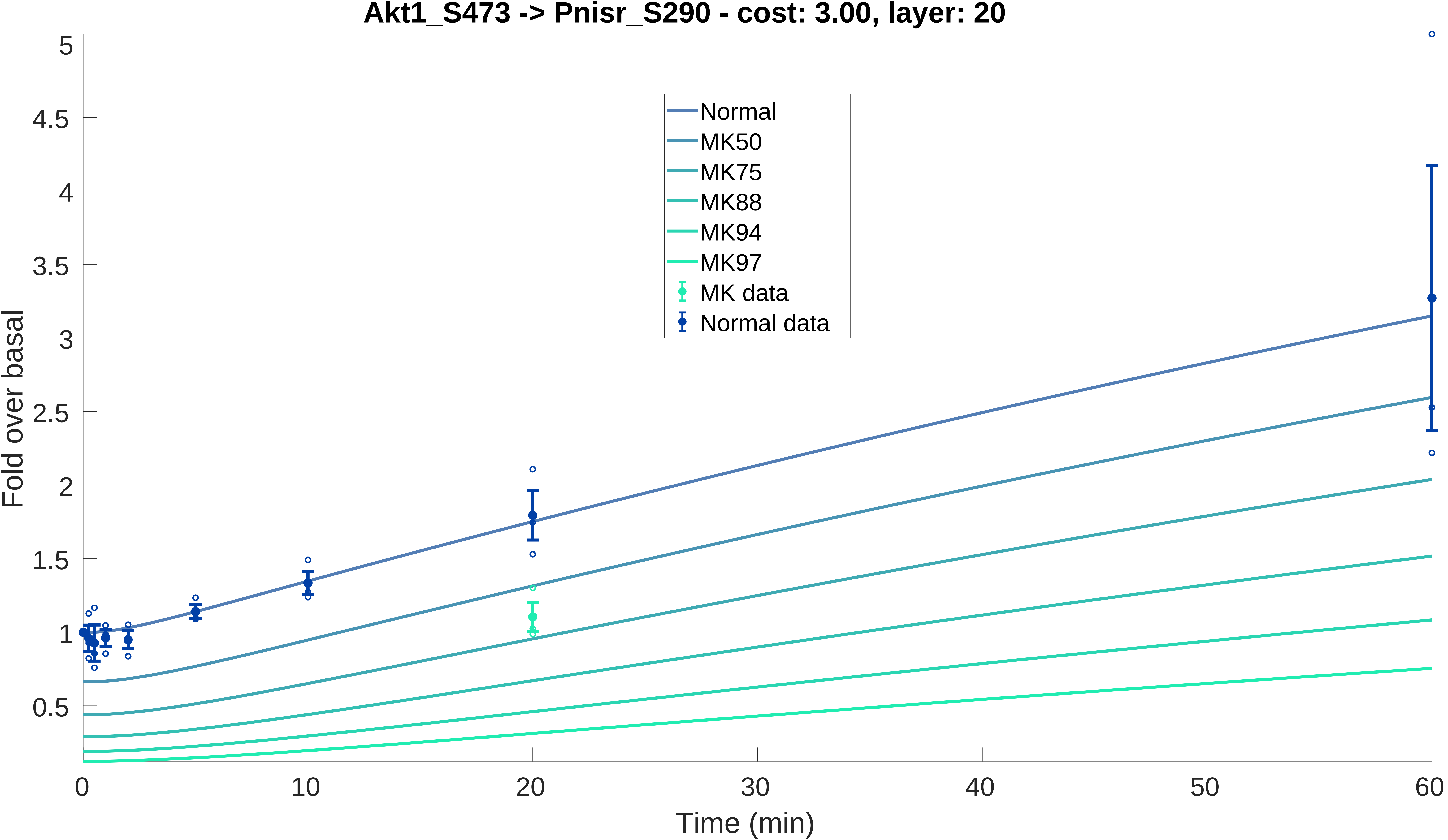

Akt1_S473 -> Pnisr_S290 - cost: 3.00, layer: 20
Fold over basal
Time (min)
Normal
MK50
MK75
MK88
MK94
MK97
MK data
Normal data
0
10
20
30
40
50
60
0.5
1
1.5
2
2.5
3
3.5
4
4.5
5

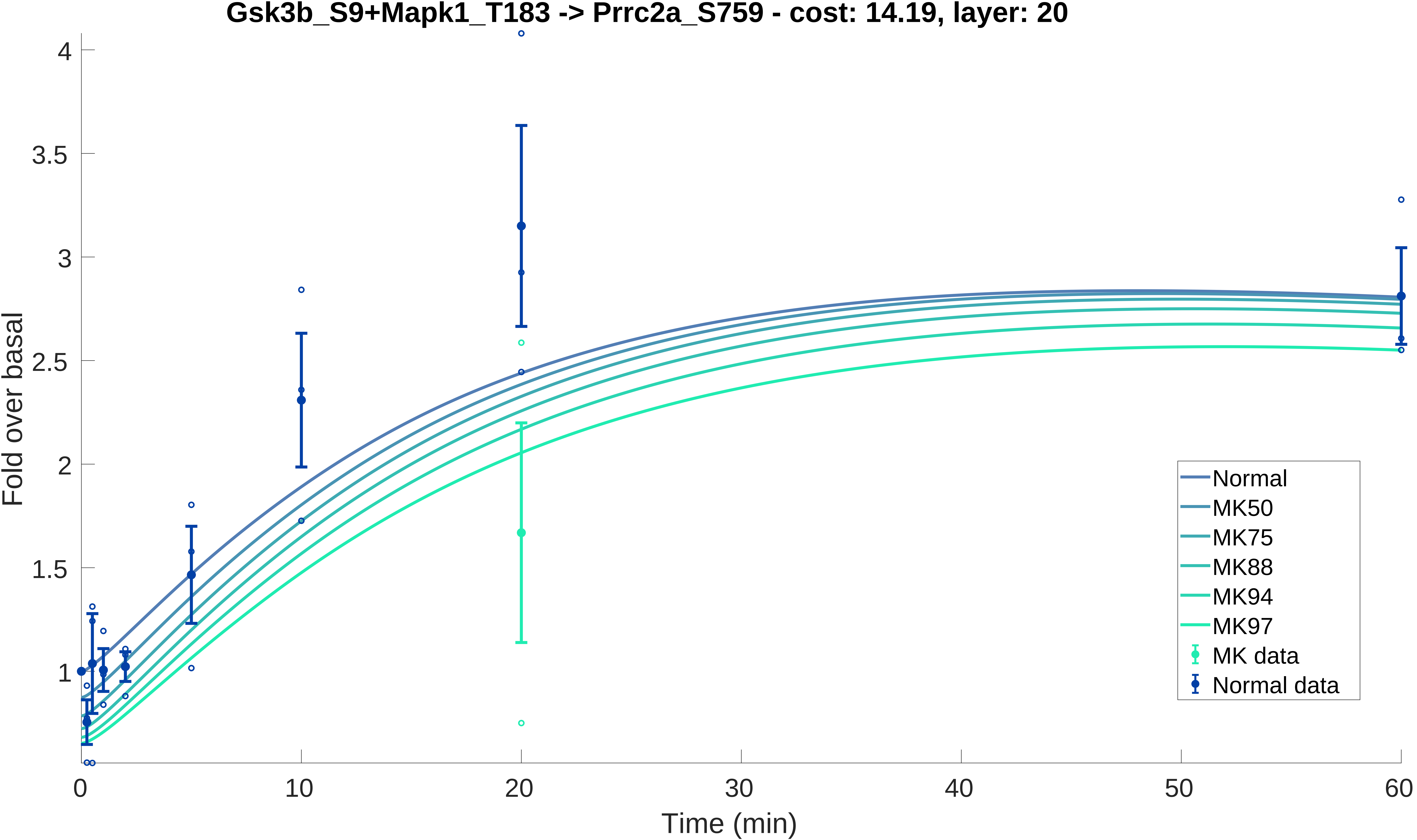

Gsk3b_S9+Mapk1_T183 -> Prrc2a_S759 - cost: 14.19, layer: 20
Fold over basal
Time (min)
0
10
20
30
40
50
60
1
1.5
2
2.5
3
3.5
4
Normal
MK50
MK75
MK88
MK94
MK97
MK data
Normal data

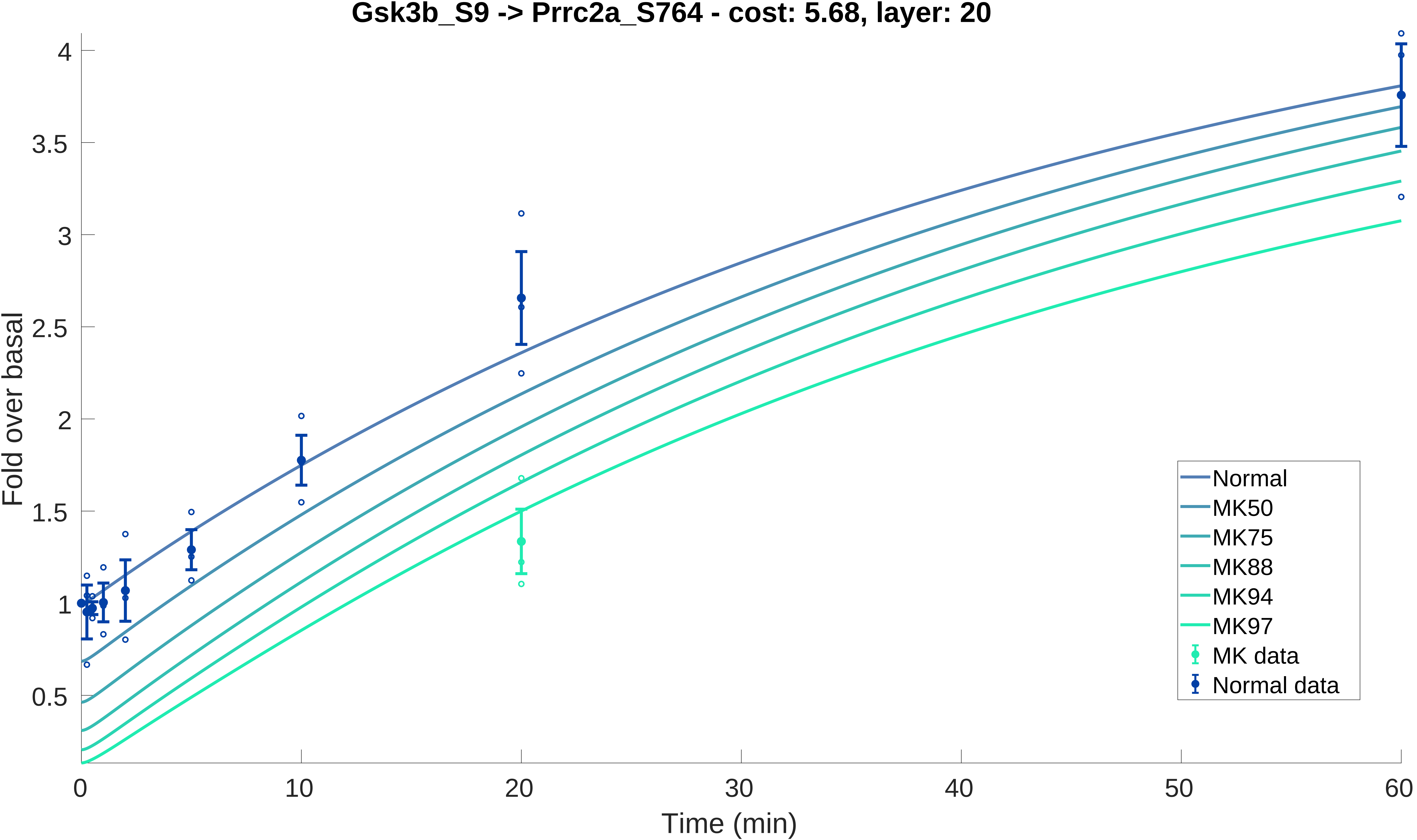

Gsk3b_S9 -> Prrc2a_S764 - cost: 5.68, layer: 20
Fold over basal
Time (min)
0.5
1
1.5
2
2.5
3
3.5
4
0
10
20
30
40
50
60
Normal
MK50
MK75
MK88
MK94
MK97
MK data
Normal data

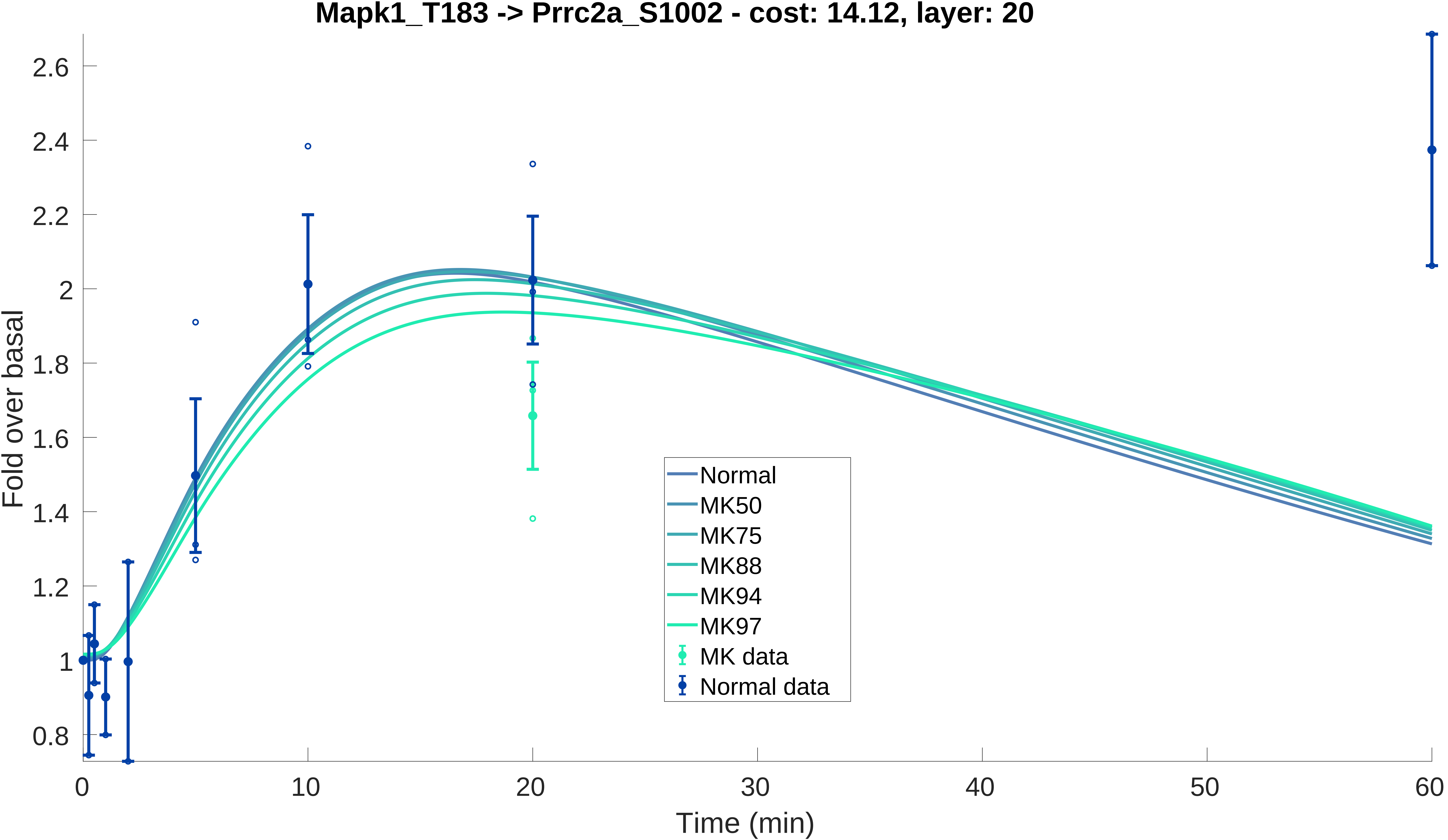

Mapk1_T183 -> Prrc2a_S1002 - cost: 14.12, layer: 20
Fold over basal
Time (min)
0.8
1
1.2
1.4
1.6
1.8
2
2.2
2.4
2.6
0
10
20
30
40
50
60
Normal
MK50
MK75
MK88
MK94
MK97
MK data
Normal data

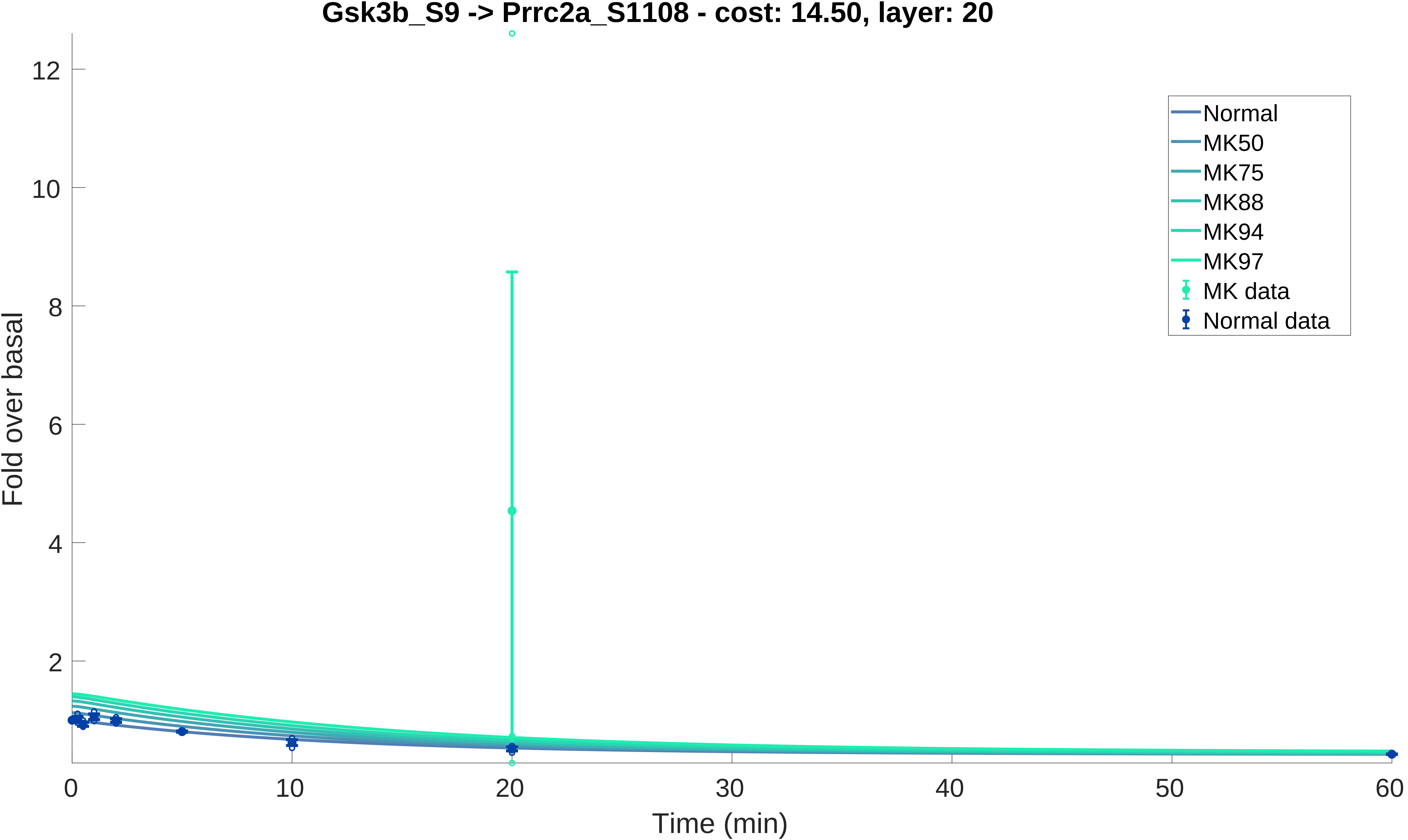

Gsk3b_S9 -> Prrc2a_S1108 - cost: 14.50, layer: 20
Fold over basal
Time (min)
0
10
20
30
40
50
60
2
4
6
8
10
12
Normal
MK50
MK75
MK88
MK94
MK97
MK data
Normal data

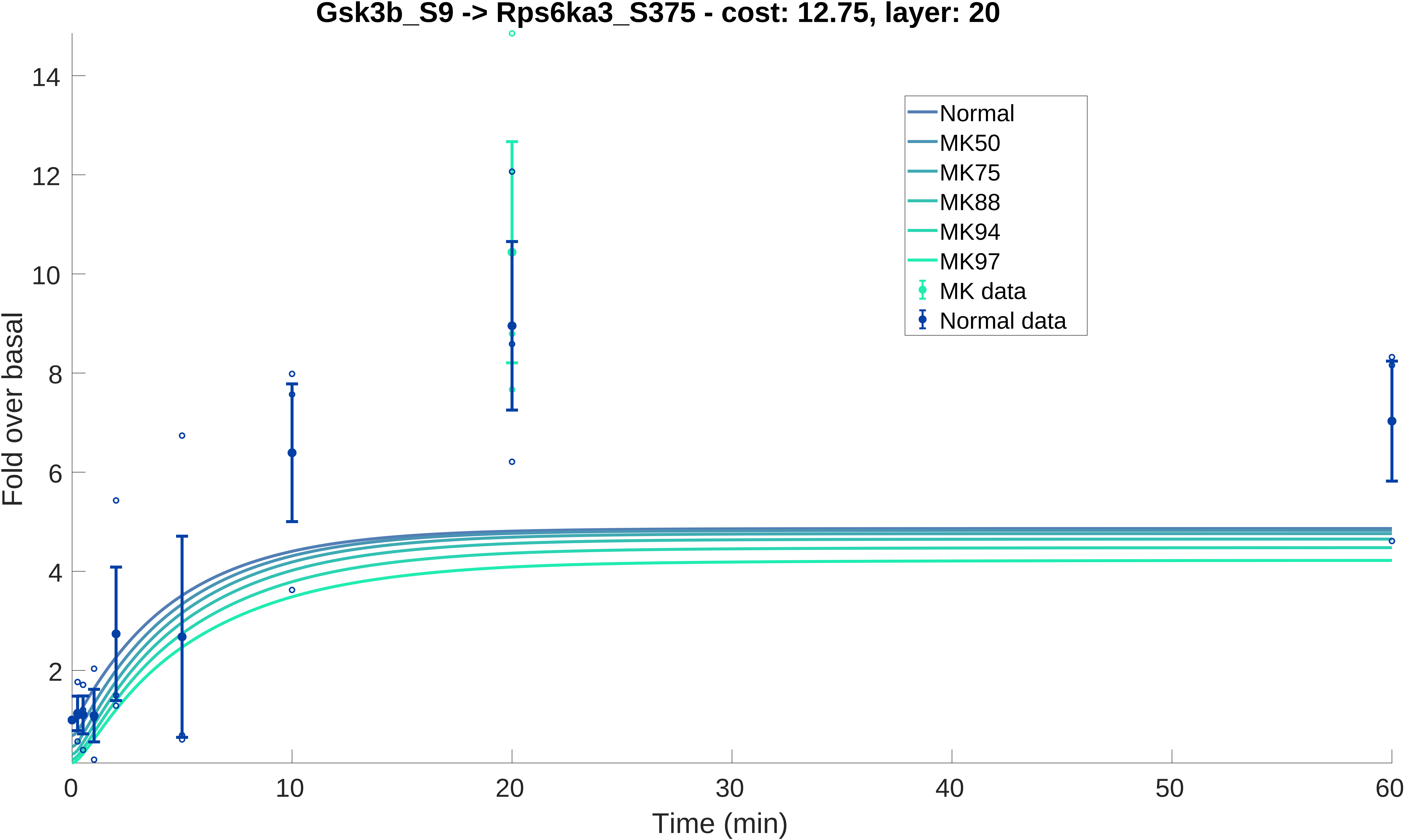

Gsk3b_S9 -> Rps6ka3_S375 - cost: 12.75, layer: 20
Fold over basal
Time (min)
0
2
4
6
8
10
12
14
0
10
20
30
40
50
60
Normal
MK50
MK75
MK88
MK94
MK97
MK data
Normal data

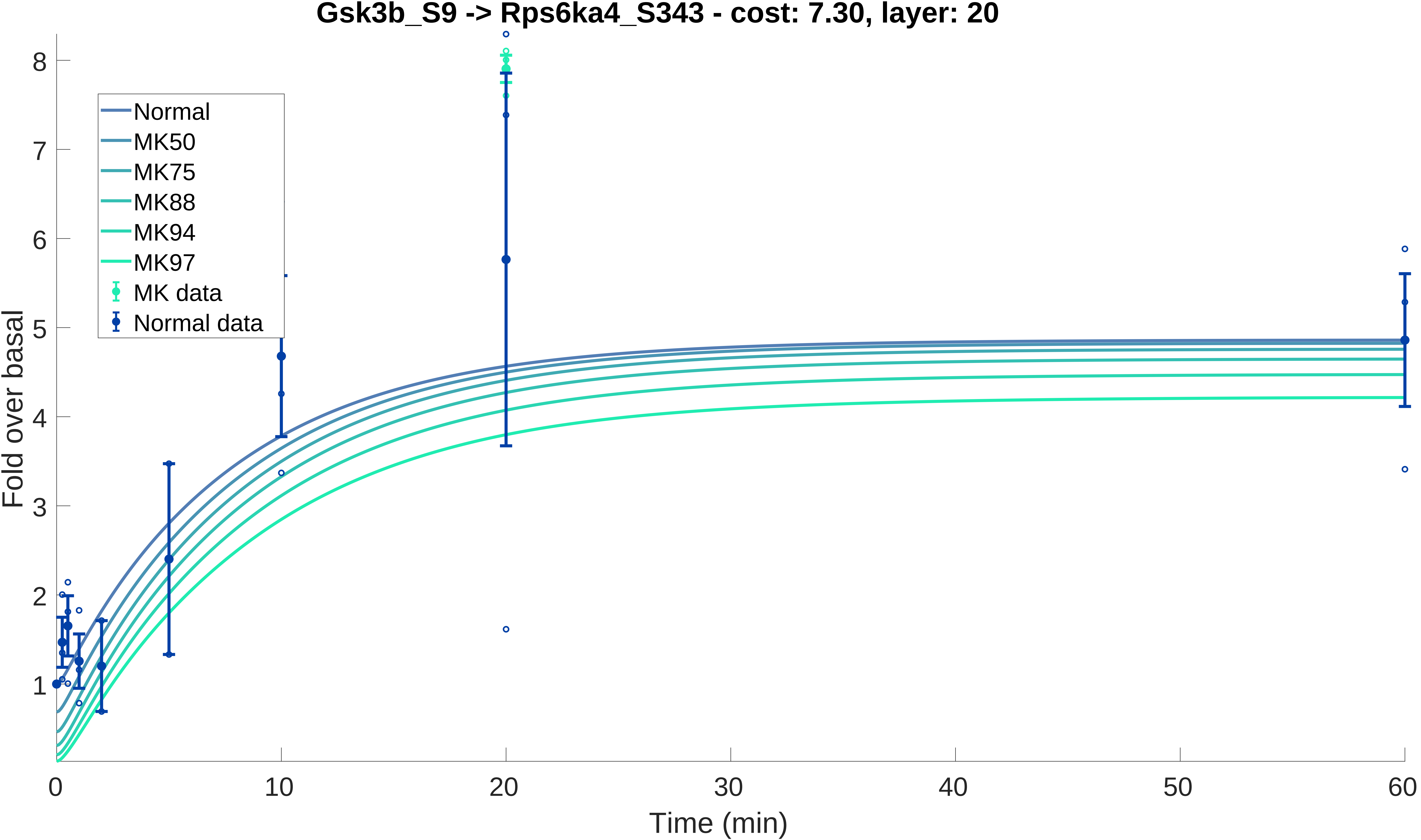

Gsk3b_S9 -> Rps6ka4_S343 - cost: 7.30, layer: 20
Normal
MK50
MK75
MK88
MK94
MK97
MK data
Normal data
Fold over basal
Time (min)
0
10
20
30
40
50
60
1
2
3
4
5
6
7
8

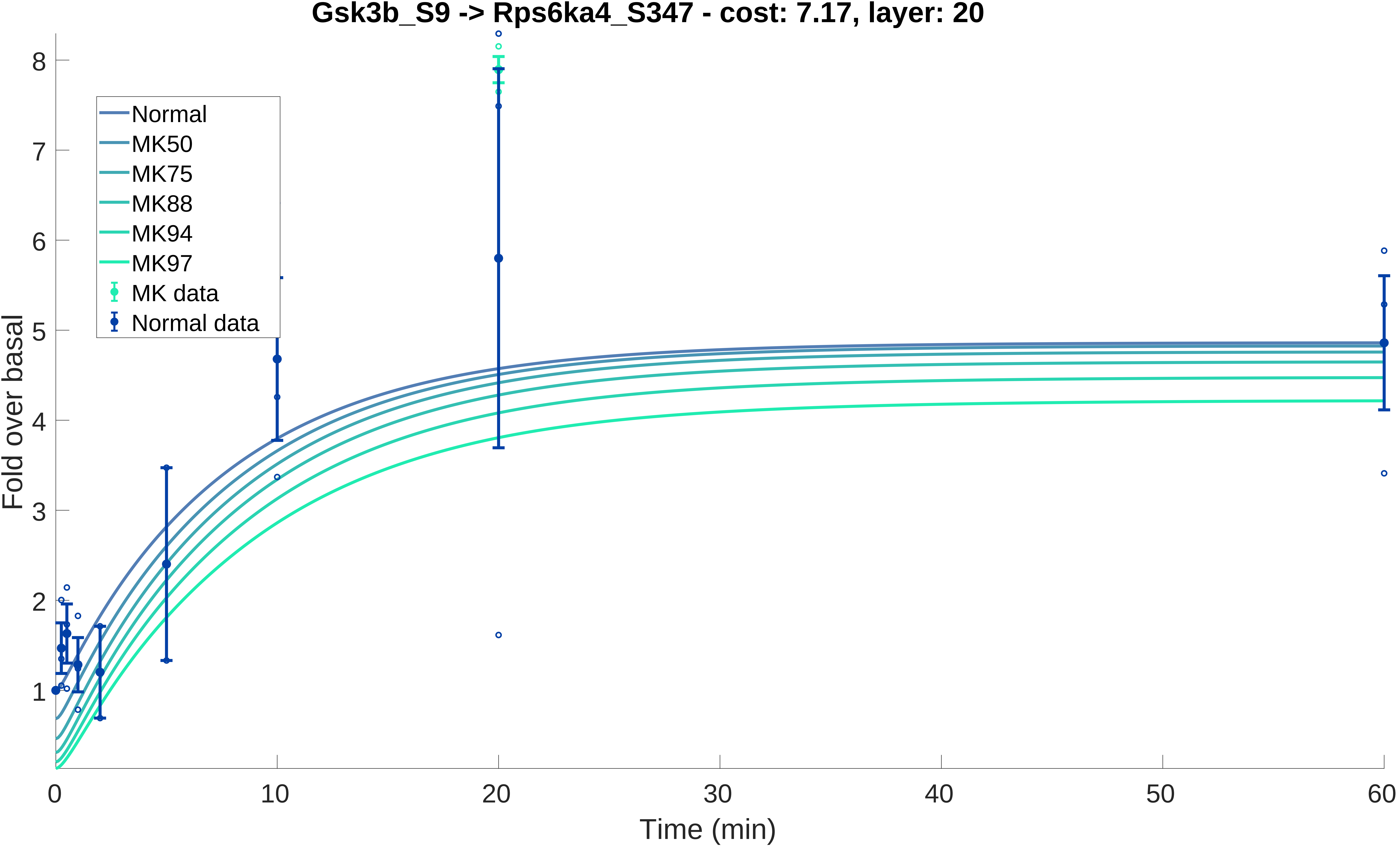

Gsk3b_S9 -> Rps6ka4_S347 - cost: 7.17, layer: 20
Normal
MK50
MK75
MK88
MK94
MK97
MK data
Normal data
Fold over basal
Time (min)
0
10
20
30
40
50
60
1
2
3
4
5
6
7
8

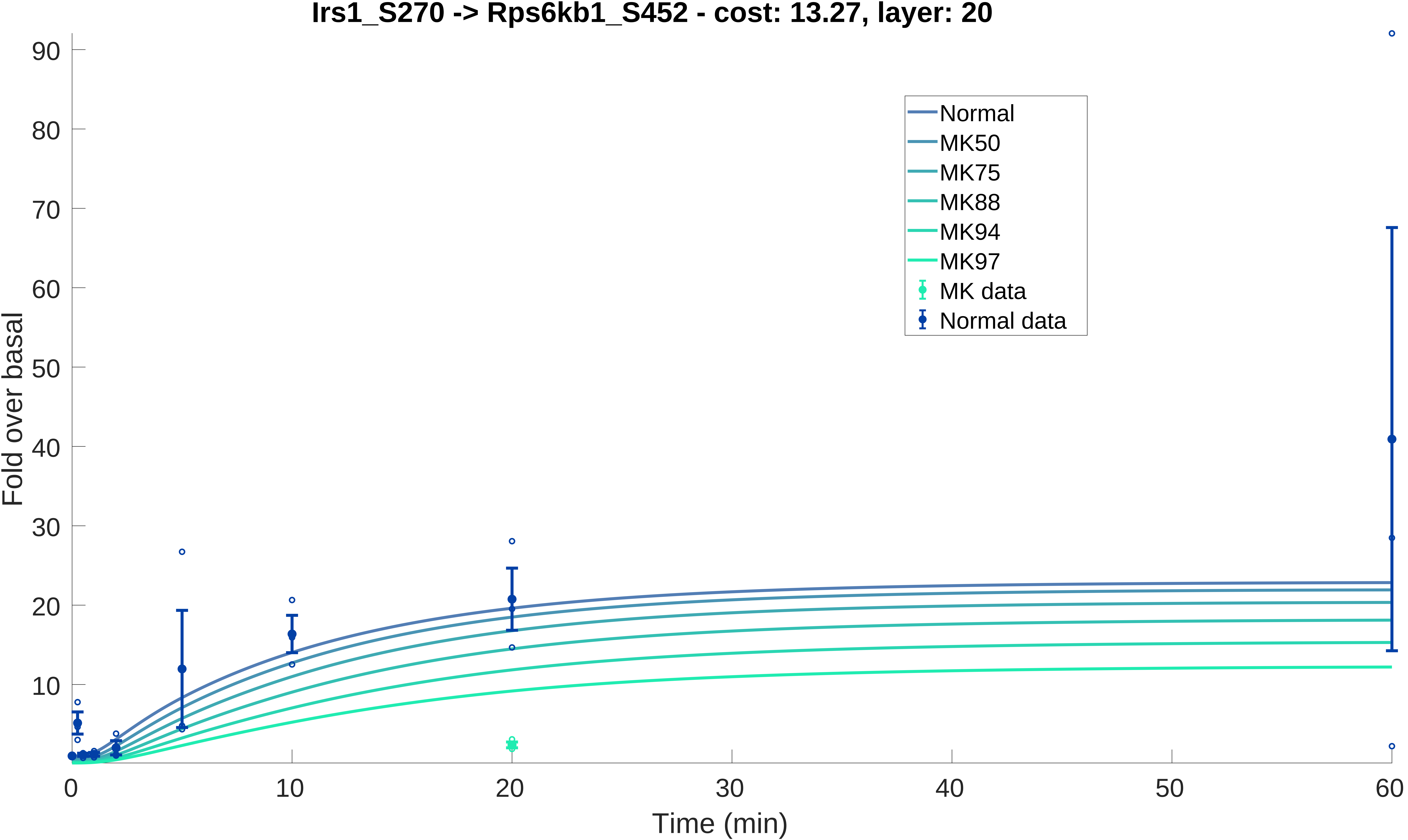

Irs1_S270 -> Rps6kb1_S452 - cost: 13.27, layer: 20
Fold over basal
Time (min)
0
10
20
30
40
50
60
70
80
90
Normal
MK50
MK75
MK88
MK94
MK97
MK data
Normal data

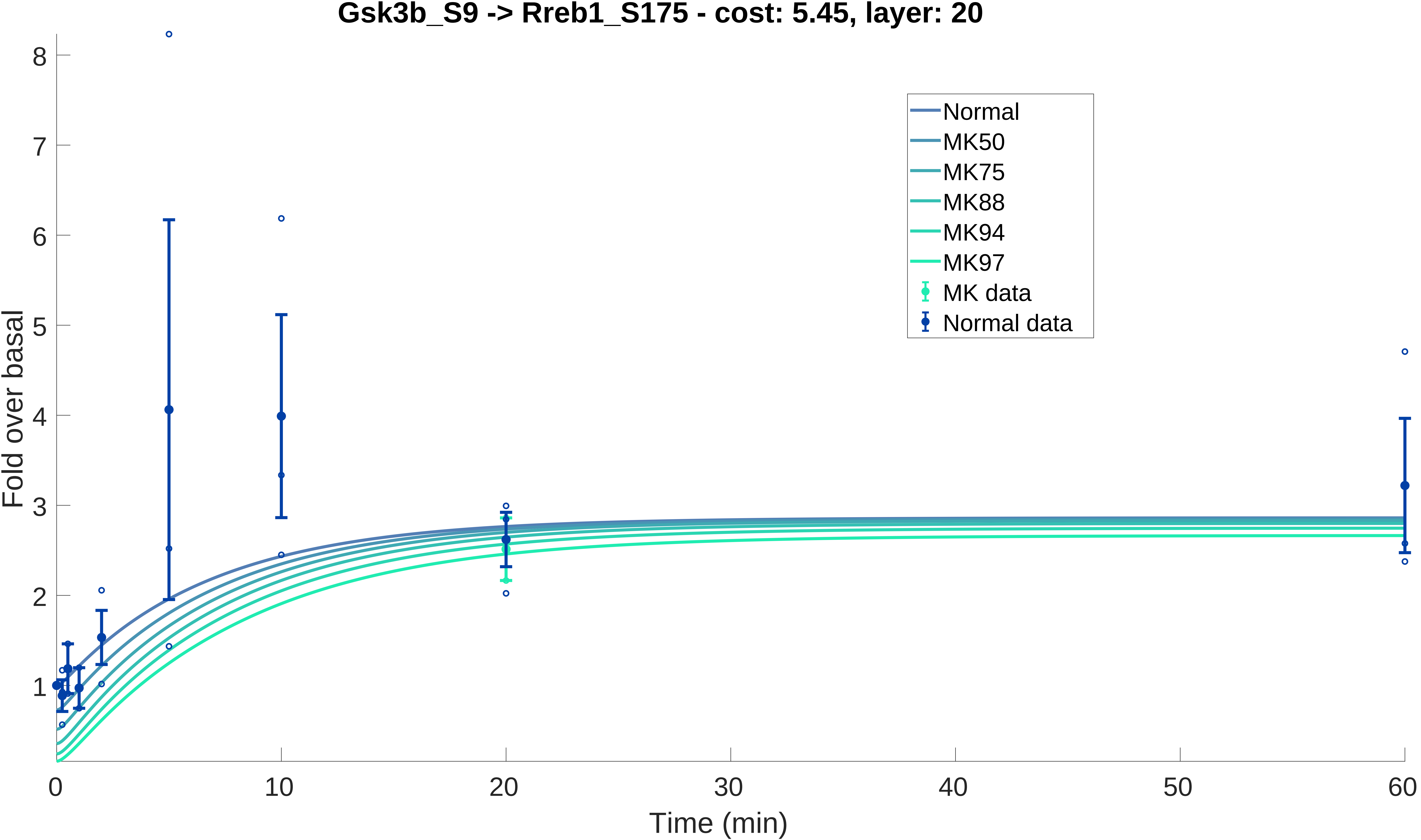

Gsk3b_S9 -> Rreb1_S175 - cost: 5.45, layer: 20
Fold over basal
Time (min)
0
10
20
30
40
50
60
1
2
3
4
5
6
7
8
Normal
MK50
MK75
MK88
MK94
MK97
MK data
Normal data

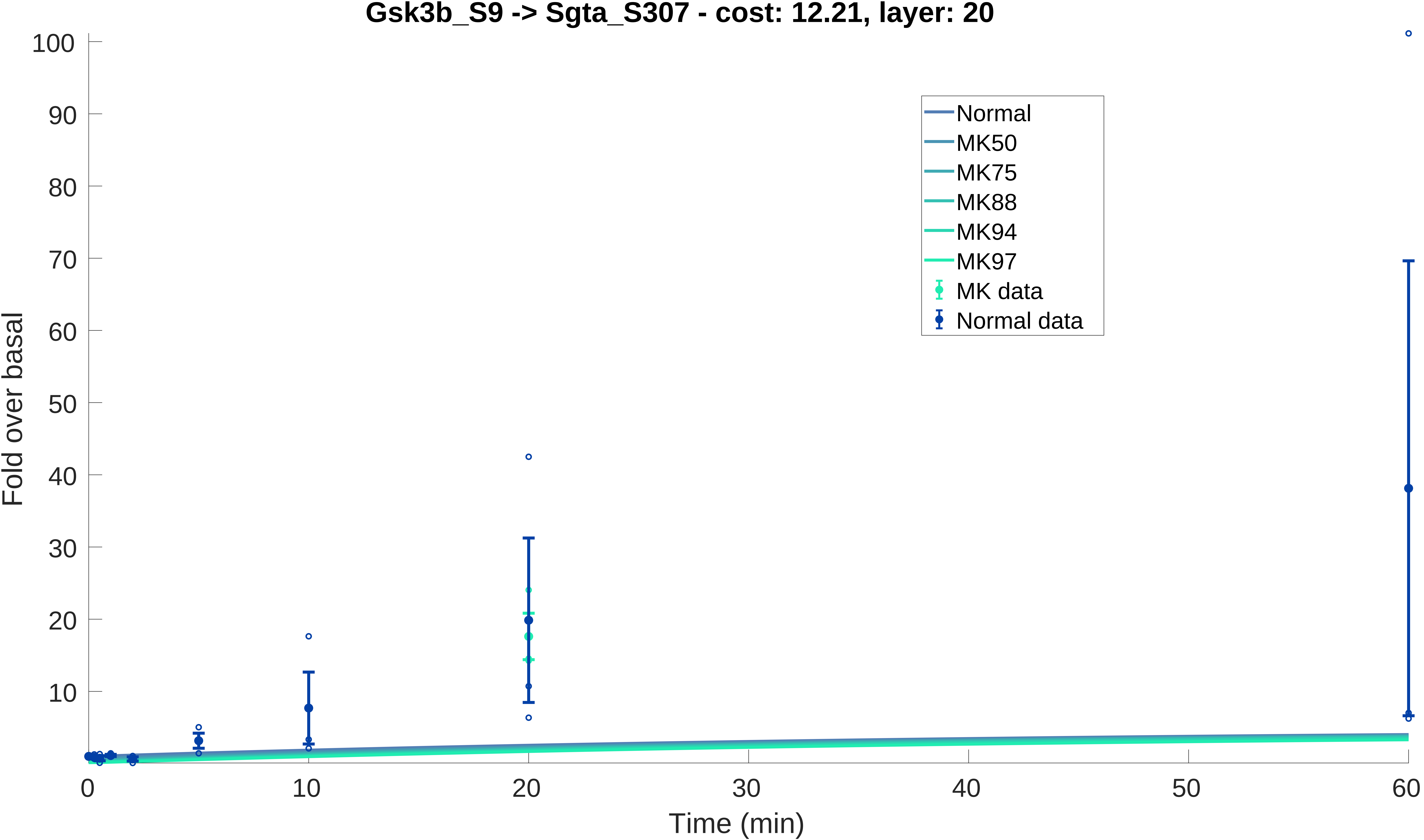

Gsk3b_S9 -> Sgta_S307 - cost: 12.21, layer: 20
Fold over basal
Time (min)
Normal
MK50
MK75
MK88
MK94
MK97
MK data
Normal data
100
90
80
70
60
50
40
30
20
10
0
10
20
30
40
50
60

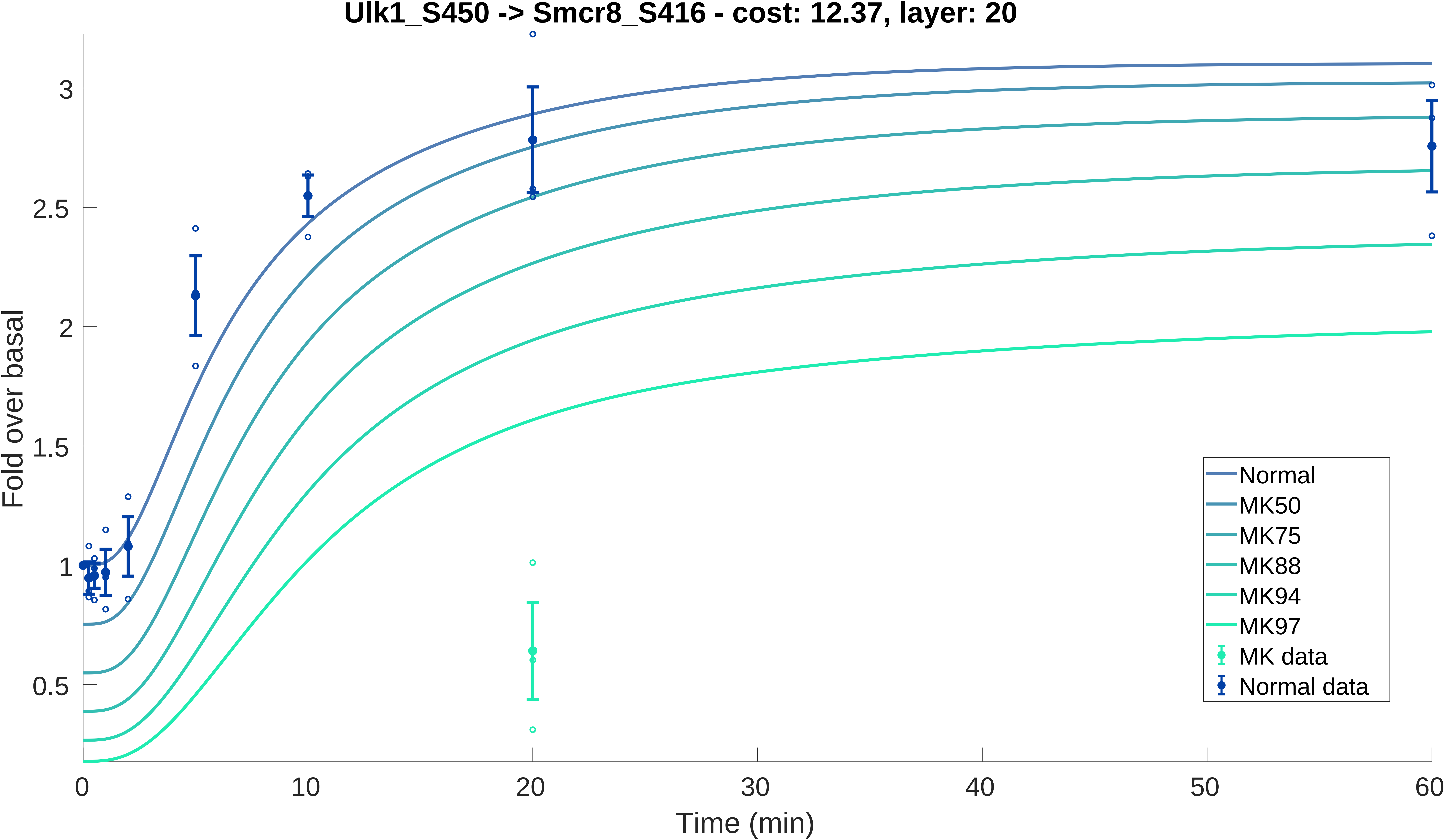

Ulk1_S450 -> Smcr8_S416 - cost: 12.37, layer: 20
Fold over basal
Time (min)
0
10
20
30
40
50
60
0.5
1
1.5
2
2.5
3
Normal
MK50
MK75
MK88
MK94
MK97
MK data
Normal data

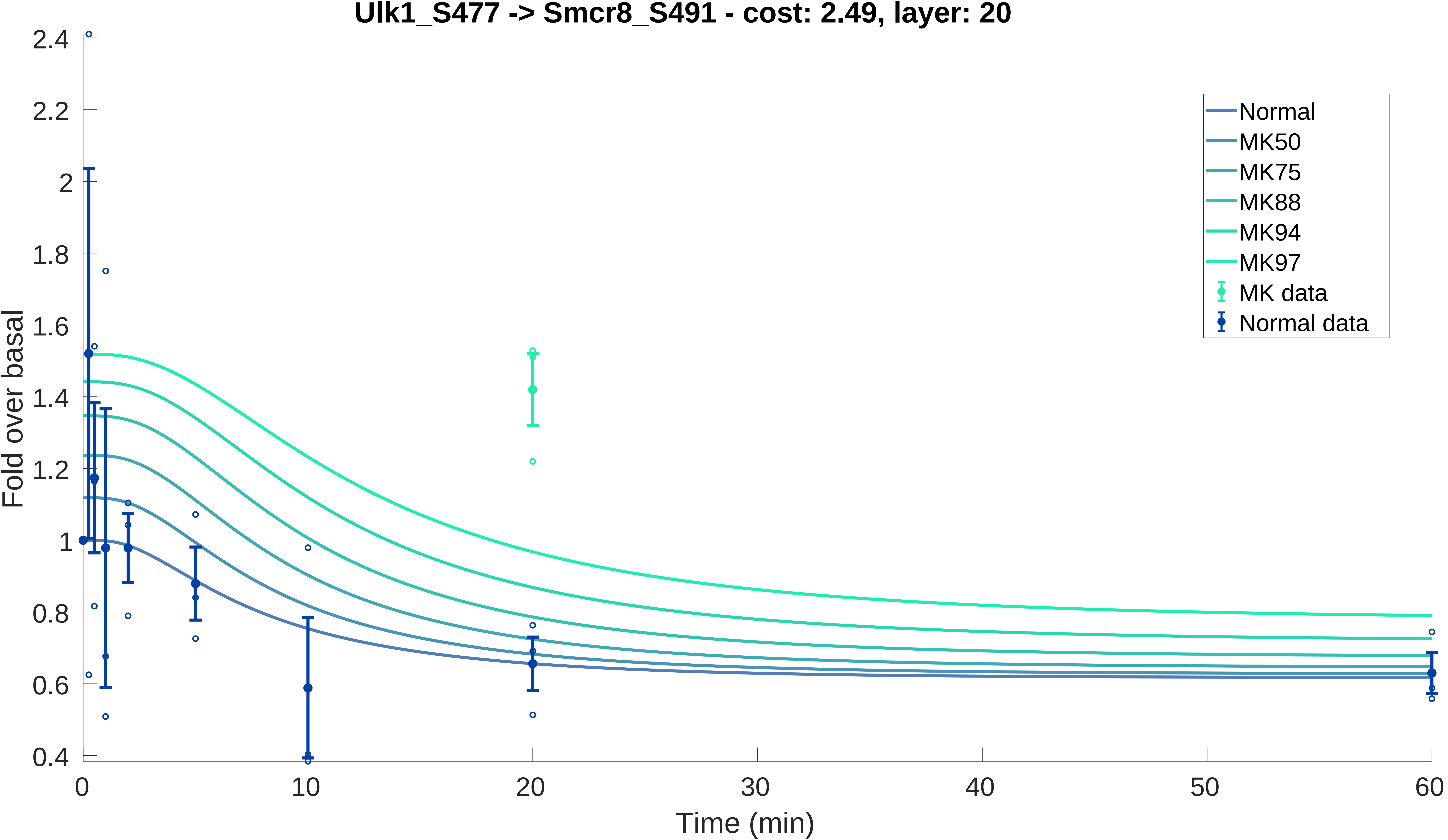

Ulk1_S477 -> Smcr8_S491 - cost: 2.49, layer: 20
Fold over basal
Time (min)
2.4
2.2
2
1.8
1.6
1.4
1.2
1
0.8
0.6
0.4
0
10
20
30
40
50
60
Normal
MK50
MK75
MK88
MK94
MK97
MK data
Normal data

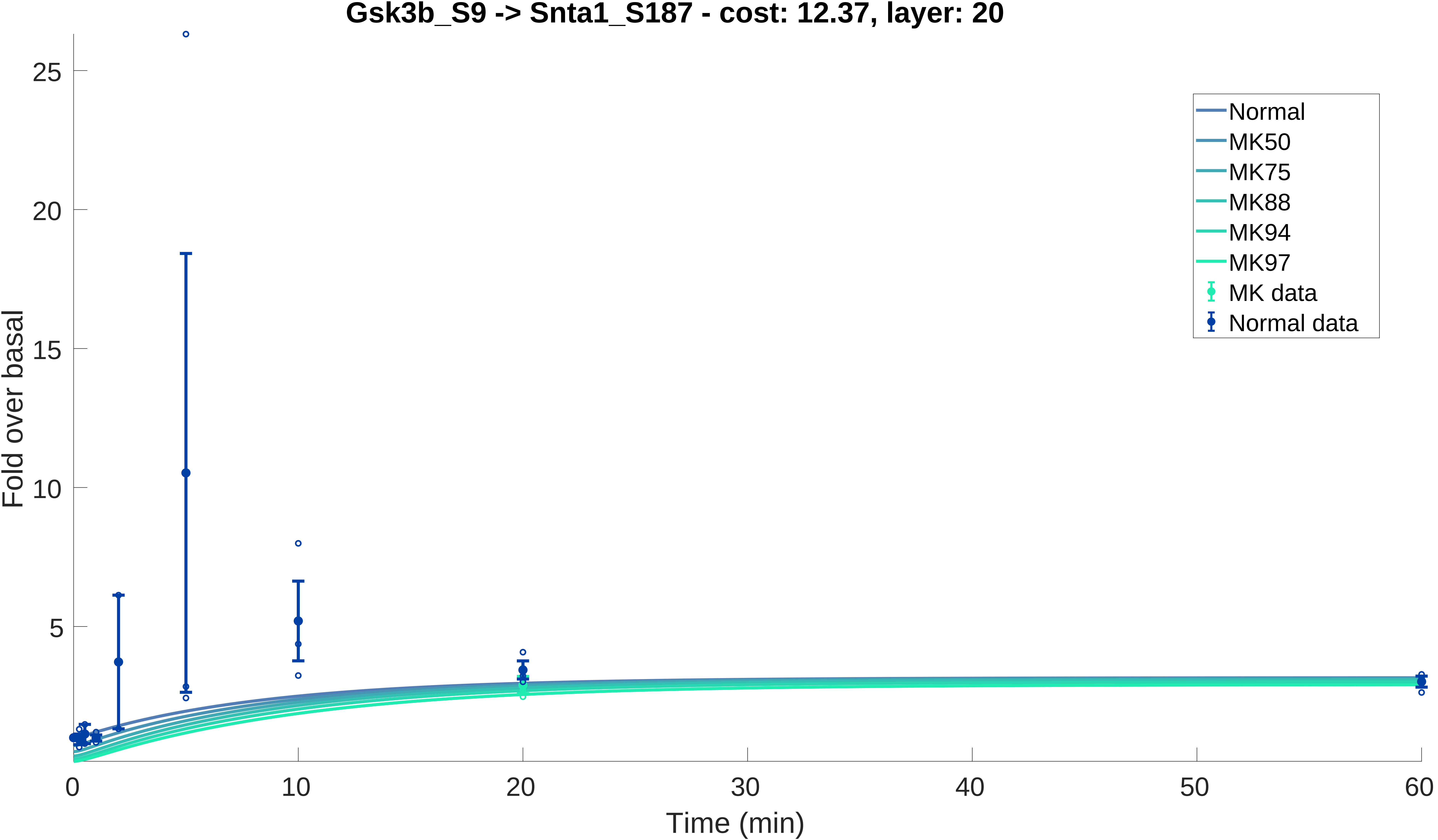

Gsk3b_S9 -> Snta1_S187 - cost: 12.37, layer: 20
Fold over basal
Time (min)
0
5
10
15
20
25
0
10
20
30
40
50
60
Normal
MK50
MK75
MK88
MK94
MK97
MK data
Normal data

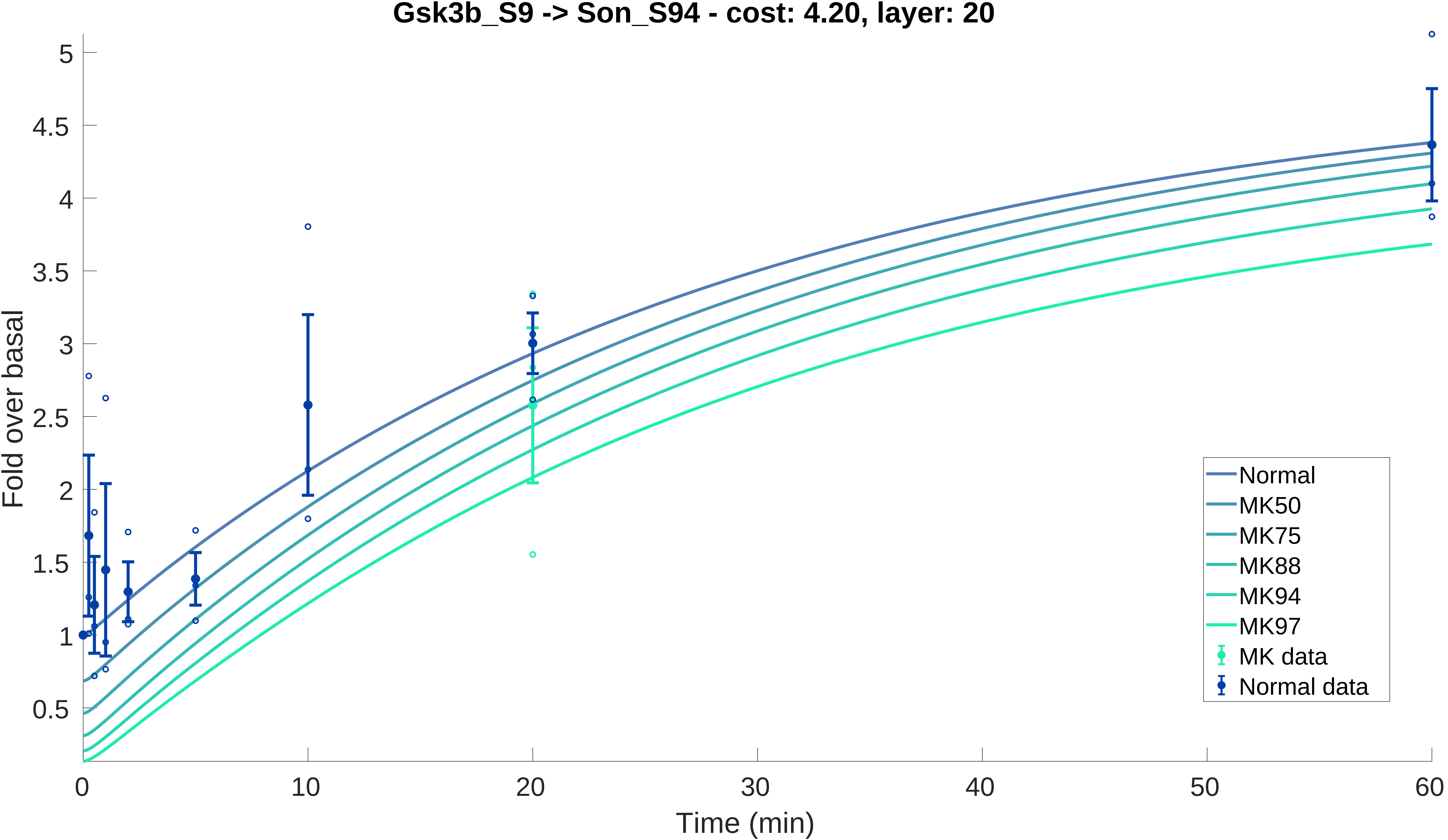

Gsk3b_S9 -> Son_S94 - cost: 4.20, layer: 20
Fold over basal
Time (min)
0
10
20
30
40
50
60
0.5
1
1.5
2
2.5
3
3.5
4
4.5
5
Normal
MK50
MK75
MK88
MK94
MK97
MK data
Normal data

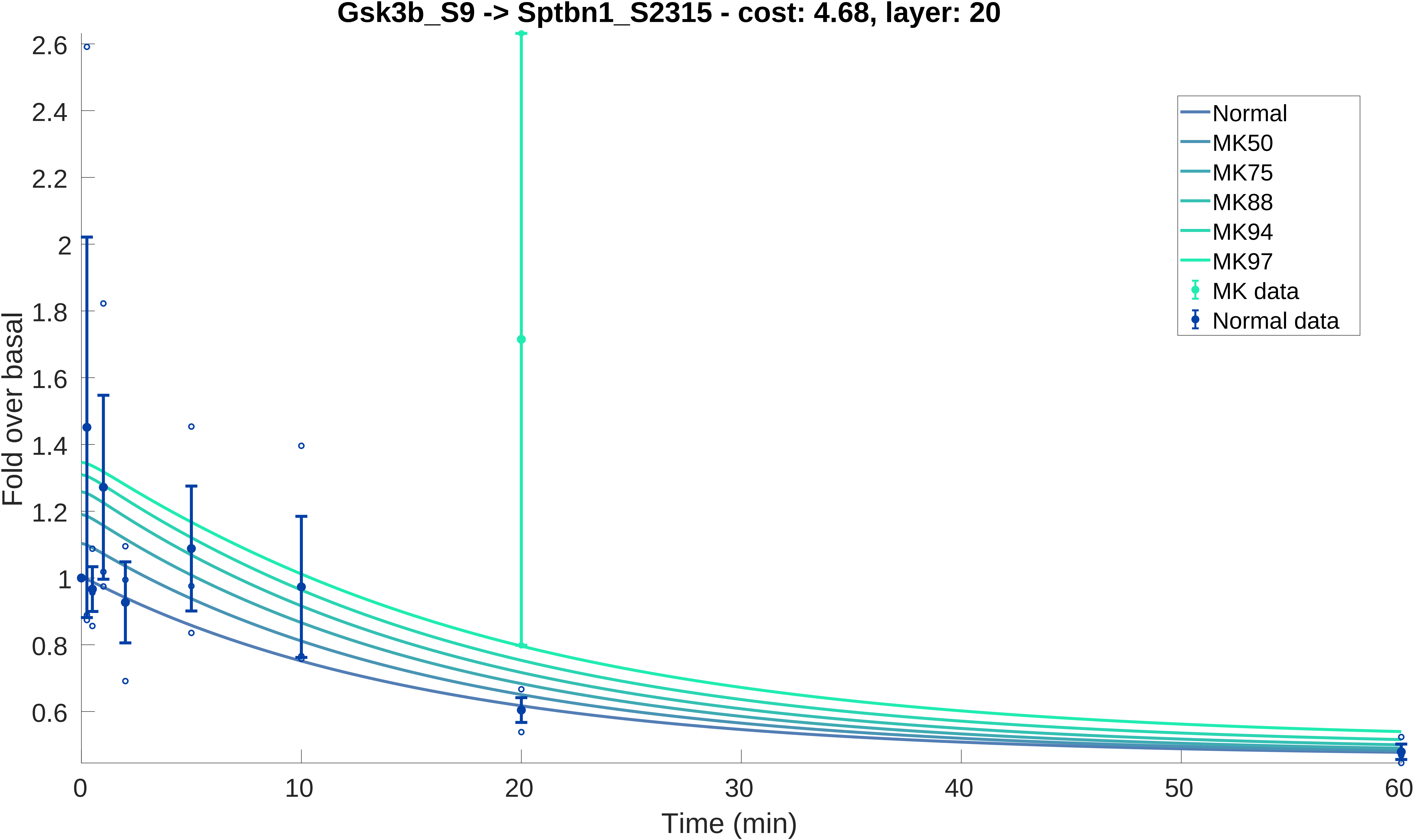

Gsk3b_S9 -> Sptbn1_S2315 - cost: 4.68, layer: 20
Fold over basal
Time (min)
Normal
MK50
MK75
MK88
MK94
MK97
MK data
Normal data
0
10
20
30
40
50
60
0.6
0.8
1
1.2
1.4
1.6
1.8
2
2.2
2.4
2.6

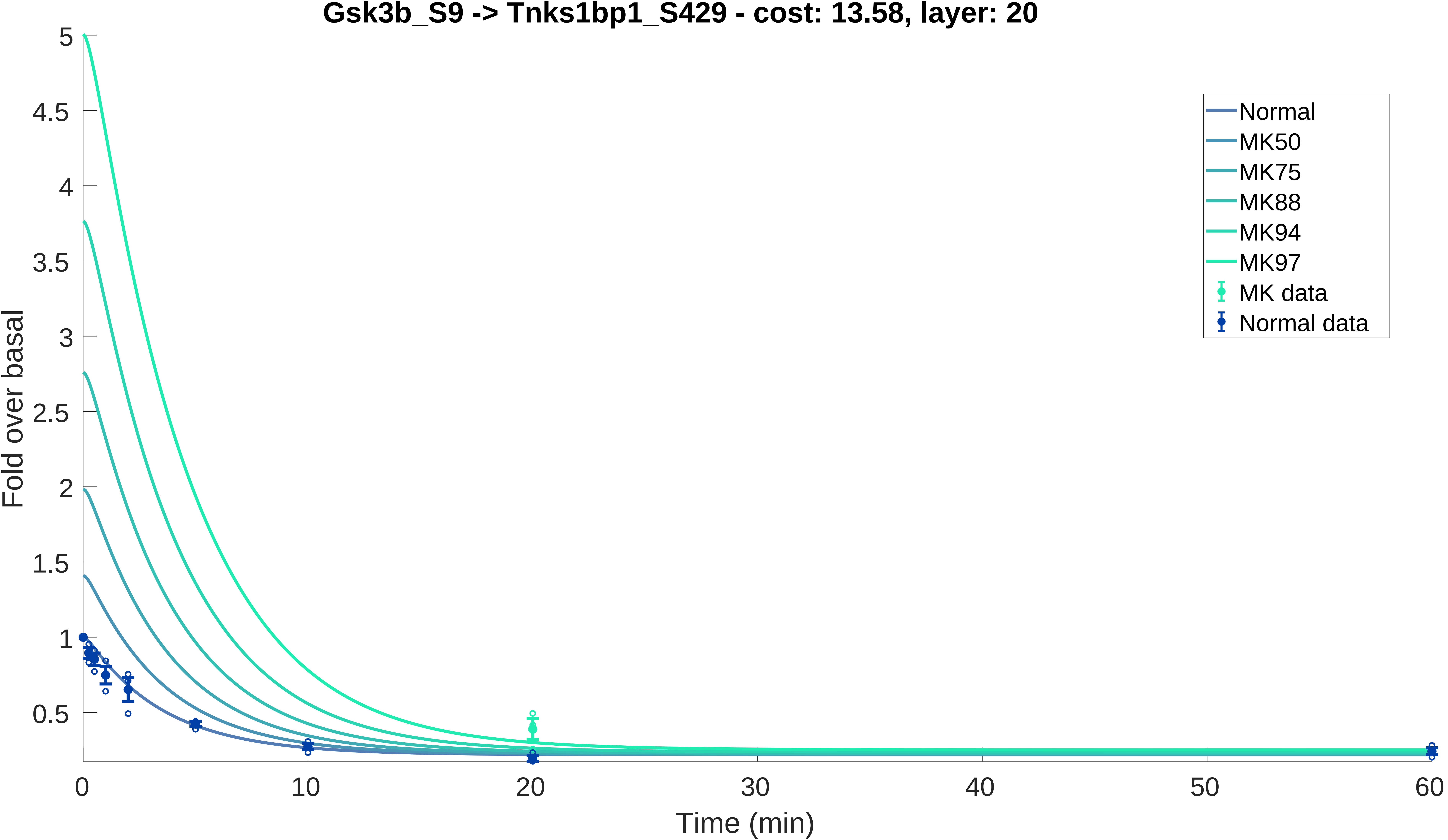

Gsk3b_S9 -> Tnks1bp1_S429 - cost: 13.58, layer: 20
Fold over basal
Time (min)
0
0.5
1
1.5
2
2.5
3
3.5
4
4.5
5
10
20
30
40
50
60
Normal
MK50
MK75
MK88
MK94
MK97
MK data
Normal data

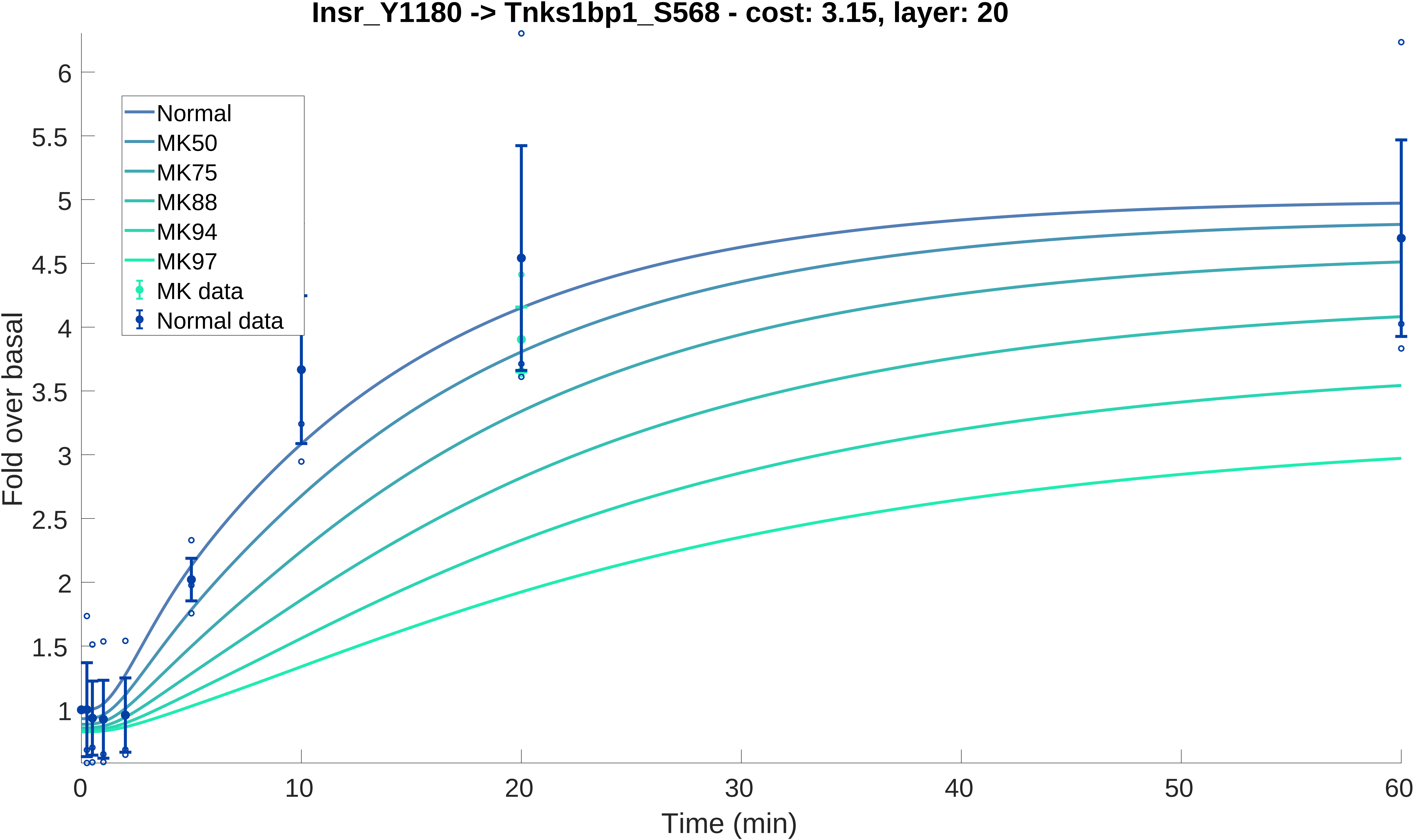

Insr_Y1180 -> Tnks1bp1_S568 - cost: 3.15, layer: 20
Normal
MK50
MK75
MK88
MK94
MK97
MK data
Normal data
Fold over basal
Time (min)
0
10
20
30
40
50
60
1
1.5
2
2.5
3
3.5
4
4.5
5
5.5
6

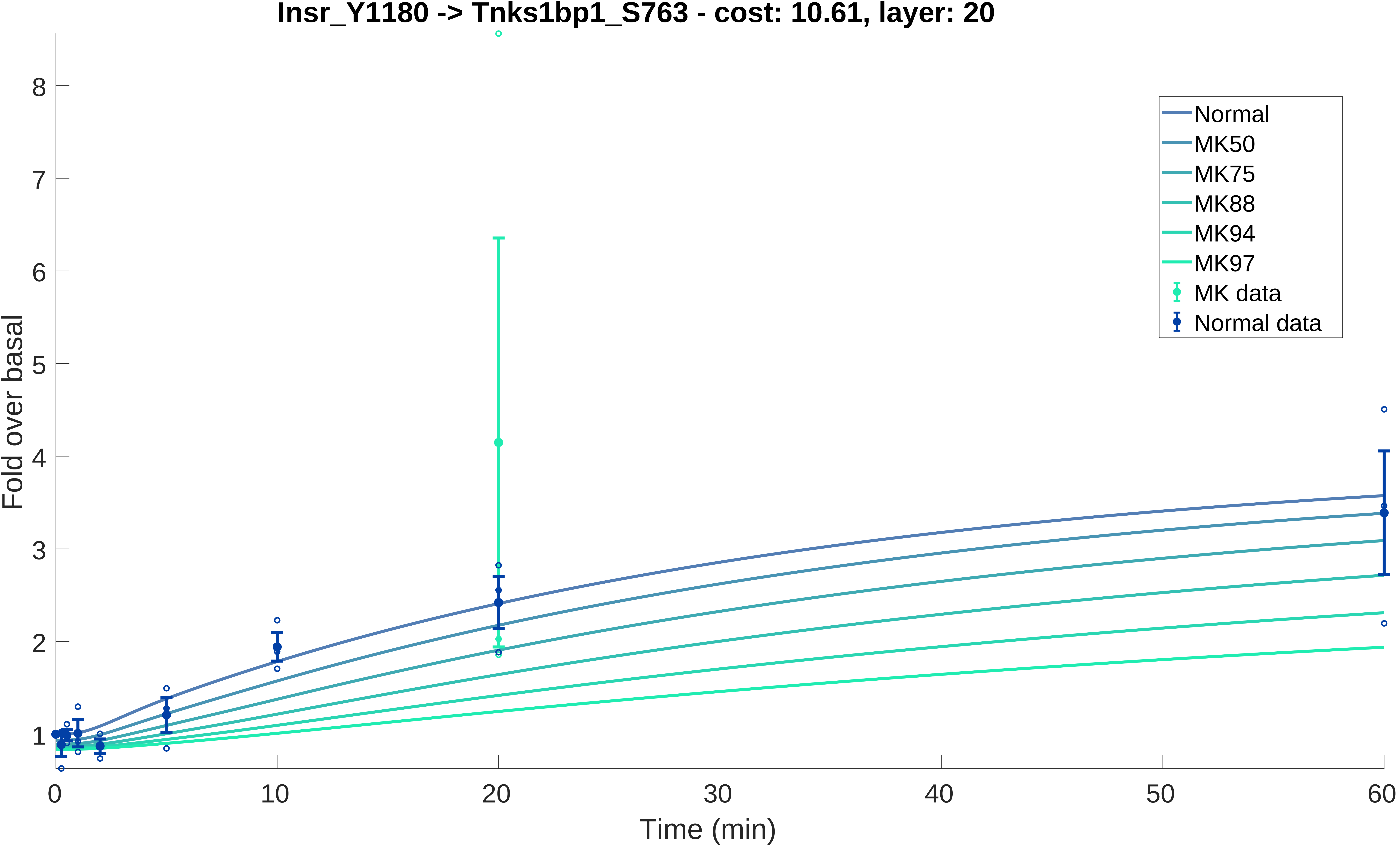

Insr_Y1180 -> Tnks1bp1_S763 - cost: 10.61, layer: 20
Fold over basal
Time (min)
Normal
MK50
MK75
MK88
MK94
MK97
MK data
Normal data
0
10
20
30
40
50
60
1
2
3
4
5
6
7
8

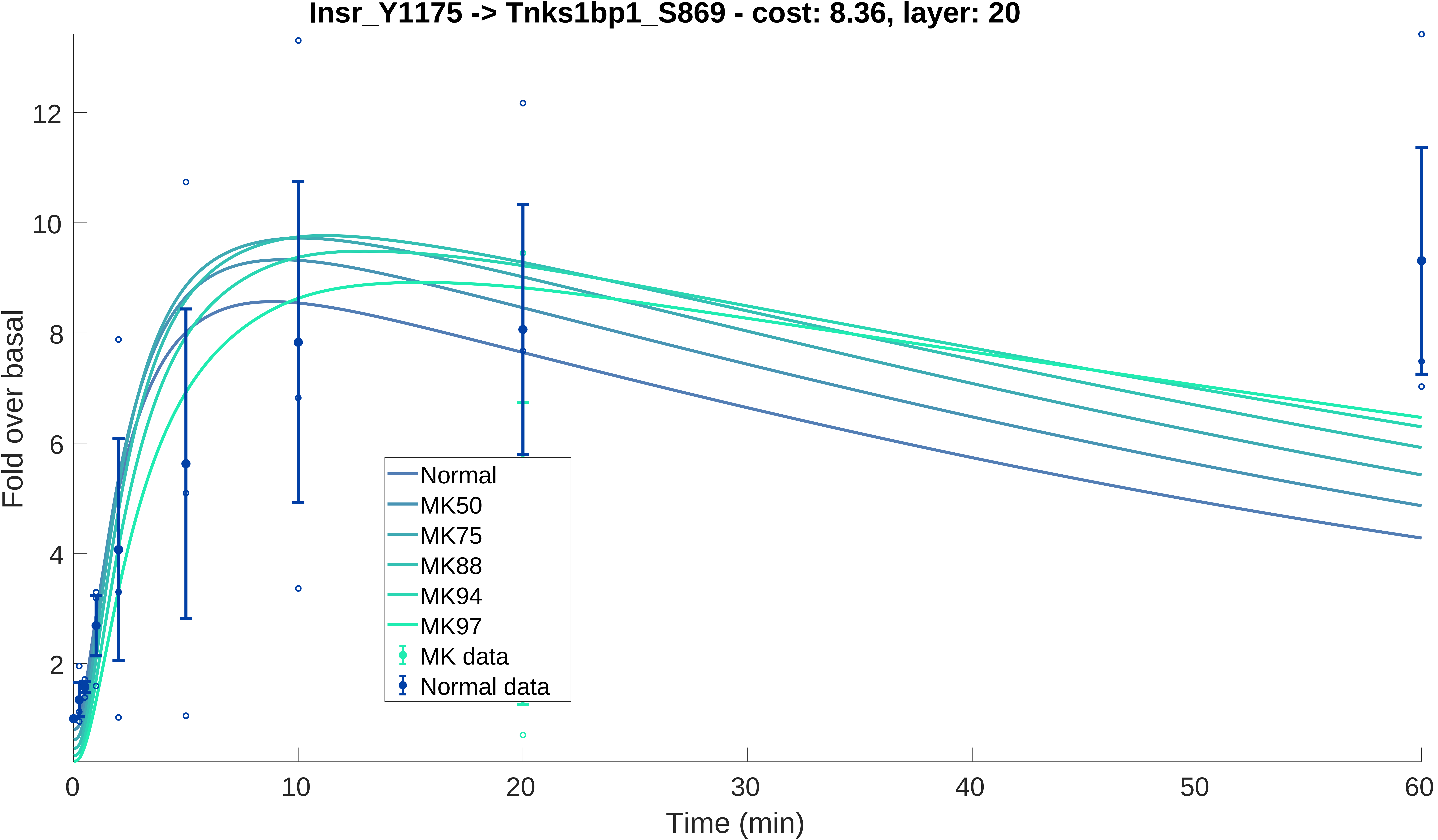

Insr_Y1175 -> Tnks1bp1_S869 - cost: 8.36, layer: 20
Fold over basal
Time (min)
0
10
20
30
40
50
60
2
4
6
8
10
12
Normal
MK50
MK75
MK88
MK94
MK97
MK data
Normal data

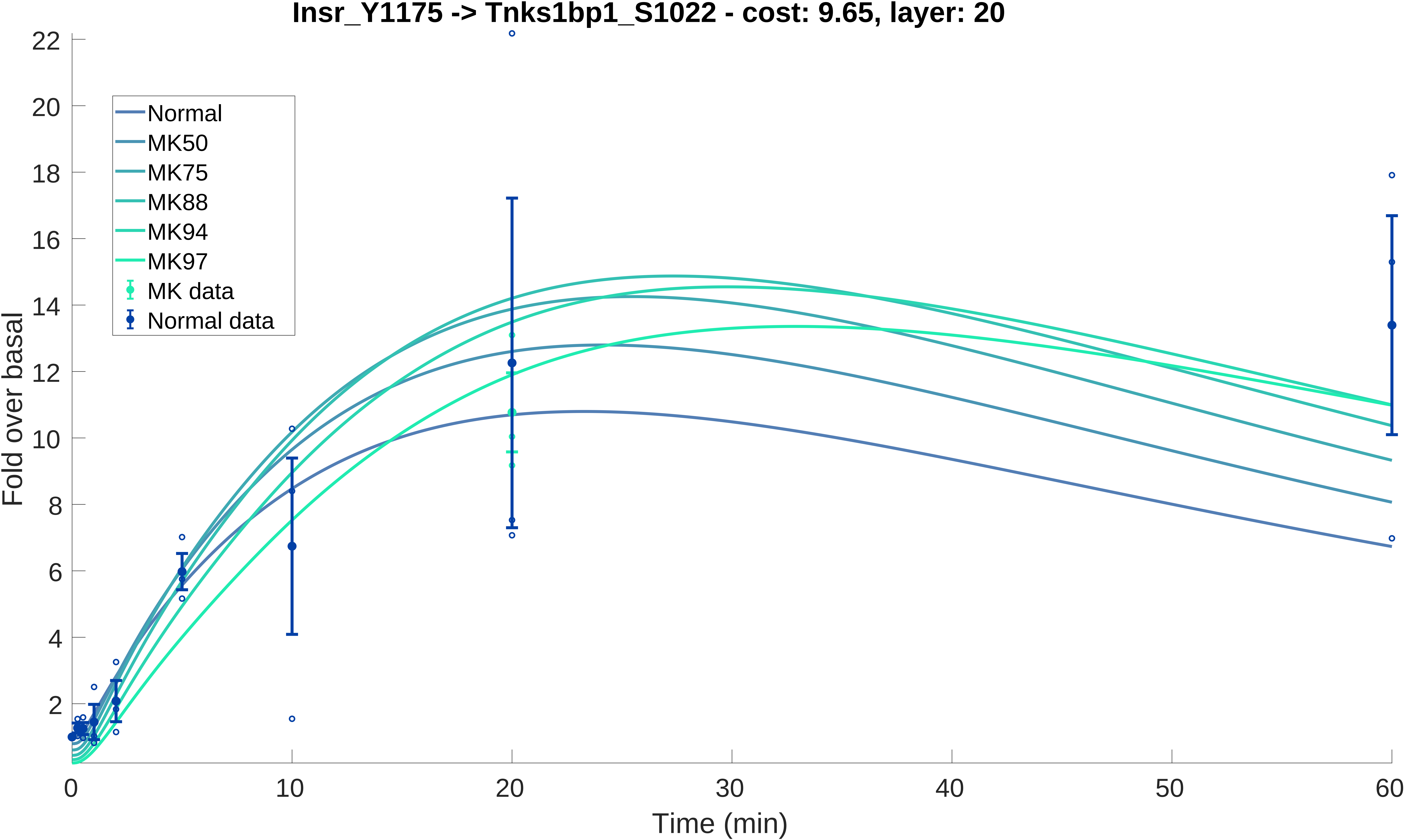

Insr_Y1175 -> Tnks1bp1_S1022 - cost: 9.65, layer: 20
Normal
MK50
MK75
MK88
MK94
MK97
MK data
Normal data
Fold over basal
Time (min)
0
10
20
30
40
50
60
2
4
6
8
10
12
14
16
18
20
22

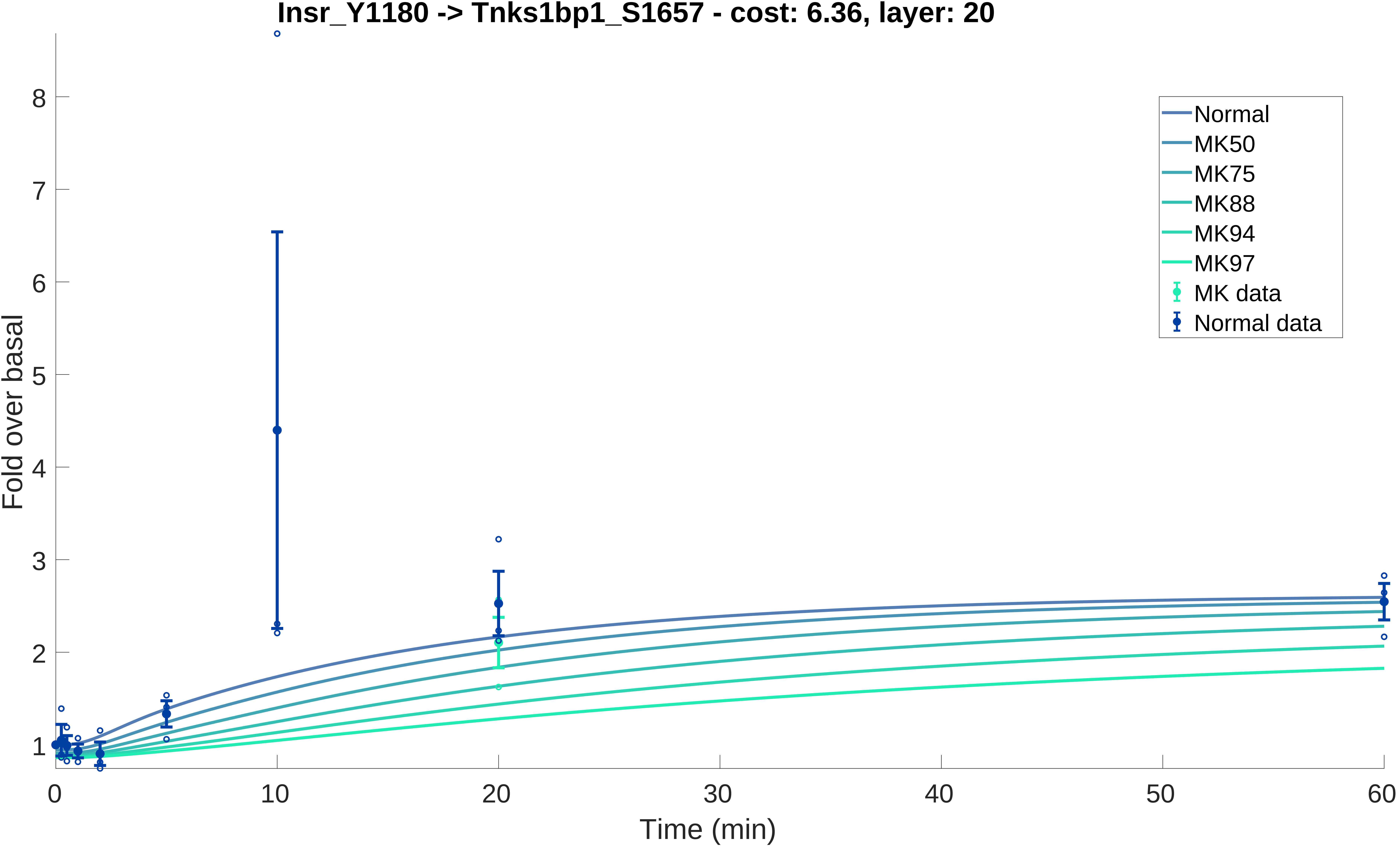

Insr_Y1180 -> Tnks1bp1_S1657 - cost: 6.36, layer: 20
Fold over basal
Time (min)
0
10
20
30
40
50
60
1
2
3
4
5
6
7
8
Normal
MK50
MK75
MK88
MK94
MK97
MK data
Normal data

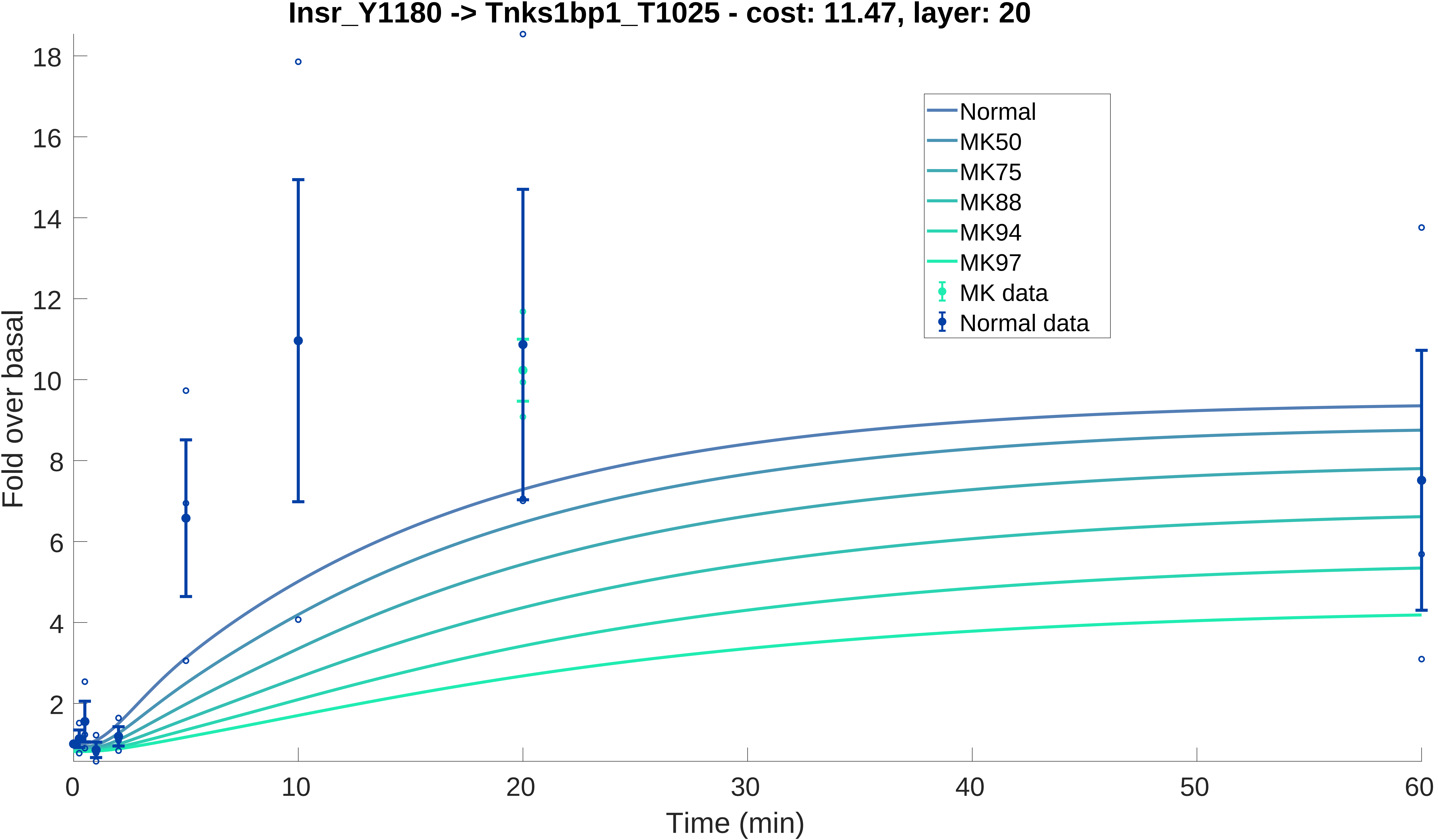
Insr_Y1180 -> Tnks1bp1_T1025 - cost: 11.47, layer: 20
Fold over basal
Time (min)
Normal
MK50
MK75
MK88
MK94
MK97
MK data
Normal data
0
10
20
30
40
50
60
2
4
6
8
10
12
14
16
18

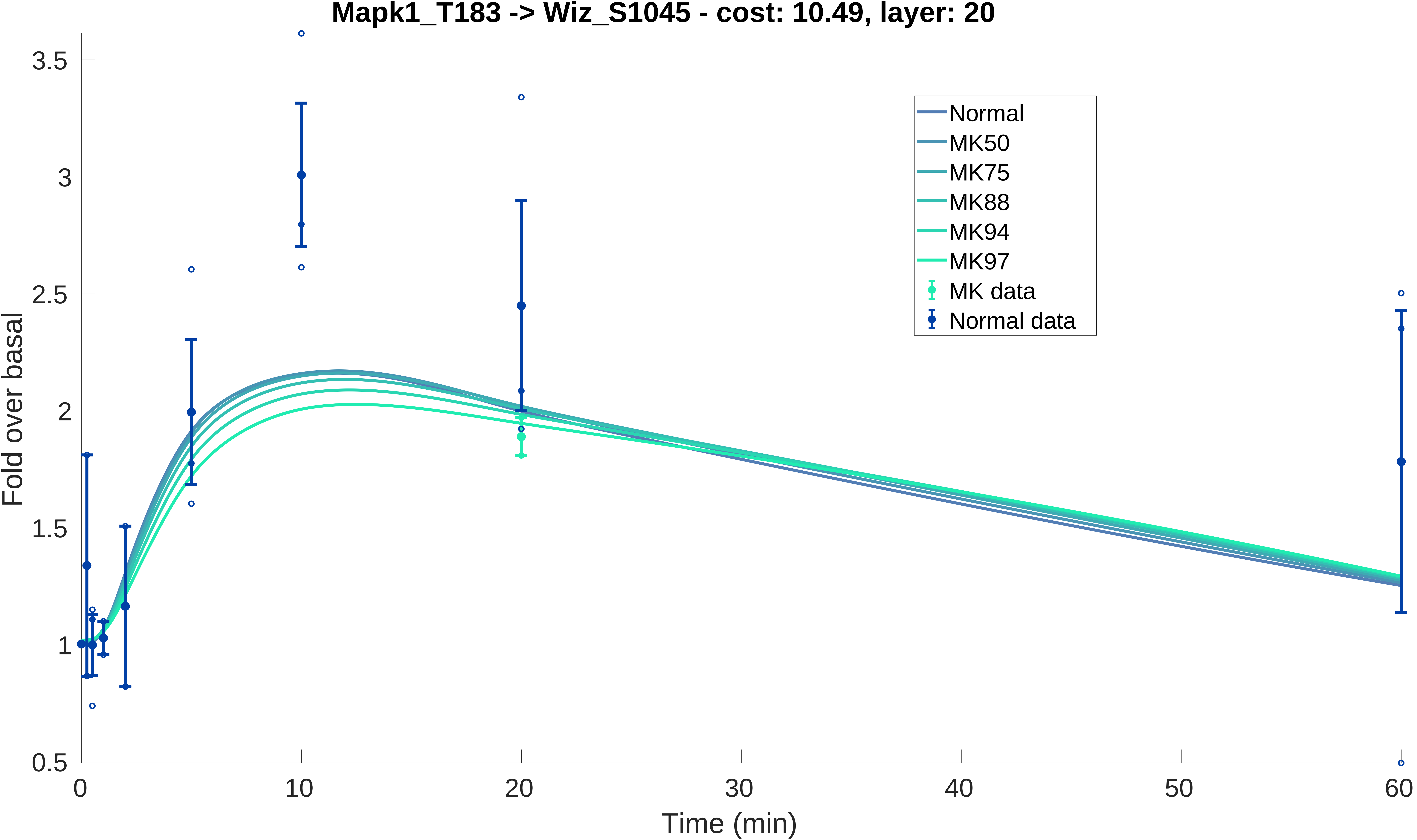

Mapk1_T183 -> Wiz_S1045 - cost: 10.49, layer: 20
Normal
MK50
MK75
MK88
MK94
MK97
MK data
Normal data
Fold over basal
3.5
3
2.5
2
1.5
1
0.5
0
10
20
30
40
50
60
Time (min)

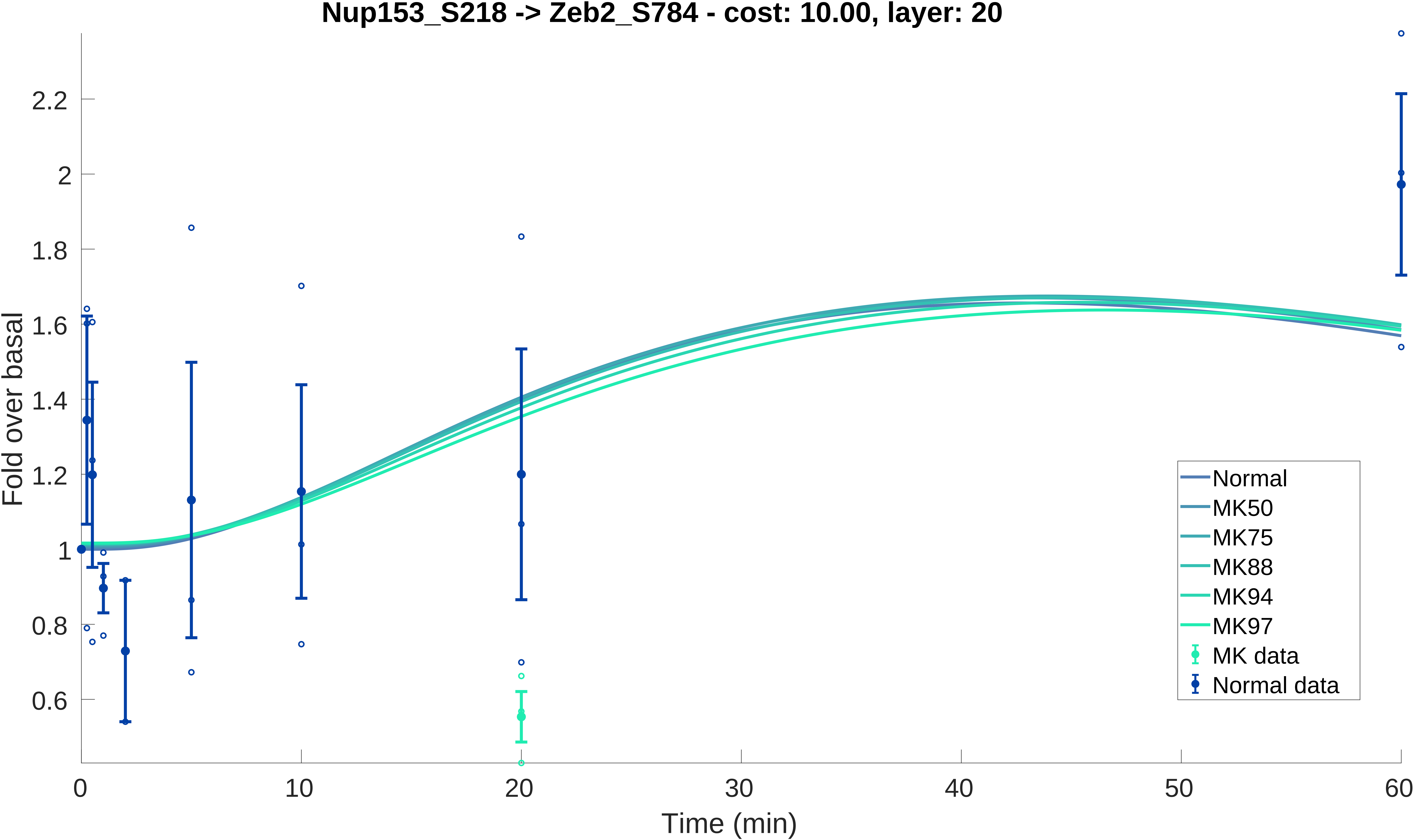

Nup153_S218 -> Zeb2_S784 - cost: 10.00, layer: 20
Fold over basal
Time (min)
0
10
20
30
40
50
60
0.6
0.8
1
1.2
1.4
1.6
1.8
2
2.2
Normal
MK50
MK75
MK88
MK94
MK97
MK data
Normal data

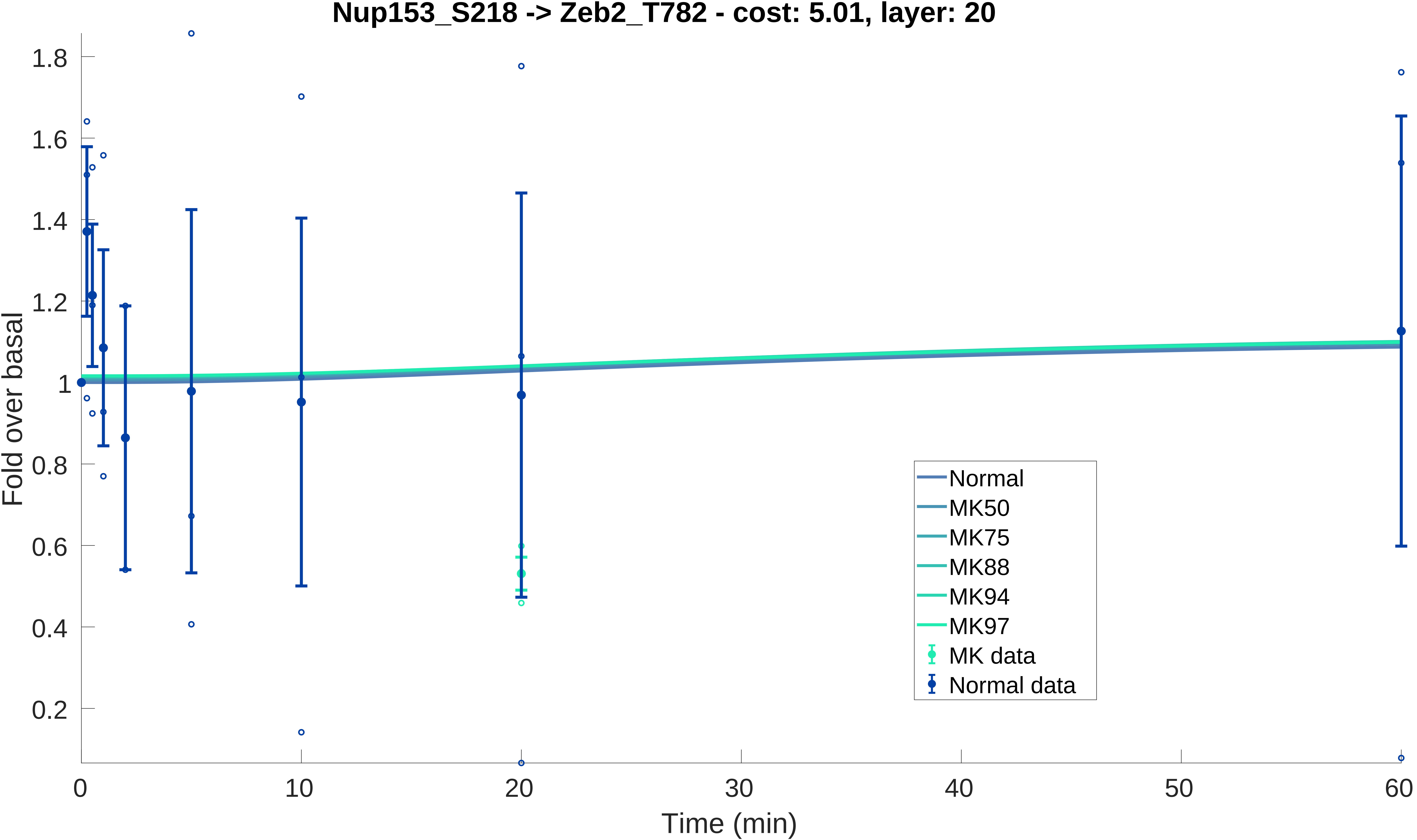
Nup153_S218 -> Zeb2_T782 - cost: 5.01, layer: 20
Fold over basal
Time (min)
1.8
1.6
1.4
1.2
1
0.8
0.6
0.4
0.2
0
10
20
30
40
50
60
Normal
MK50
MK75
MK88
MK94
MK97
MK data
Normal data

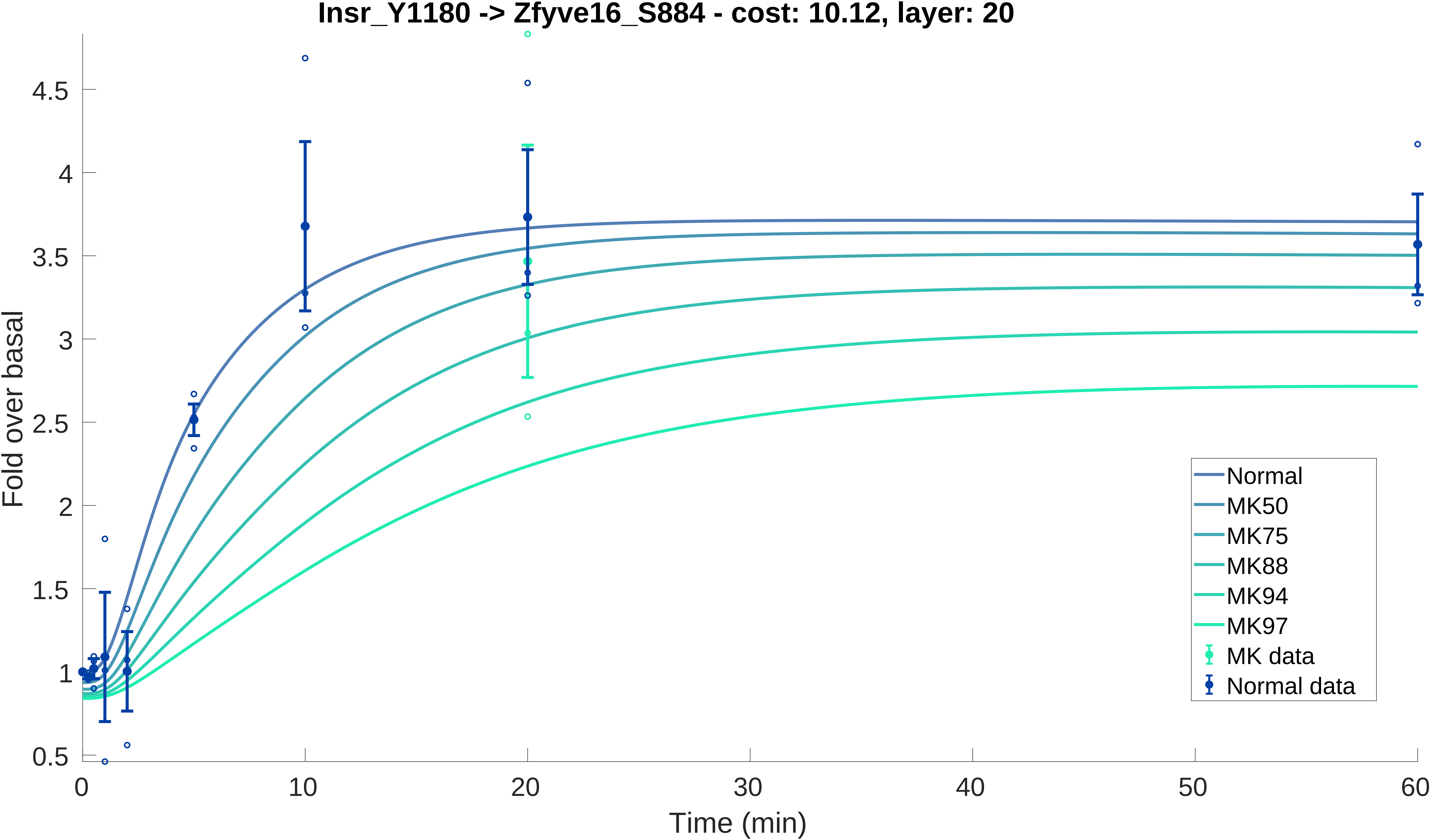

Insr_Y1180 -> Zfyve16_S884 - cost: 10.12, layer: 20
Fold over basal
4.5
4
3.5
3
2.5
2
1.5
1
0.5
0
10
20
30
40
50
60
Time (min)
Normal
MK50
MK75
MK88
MK94
MK97
MK data
Normal data

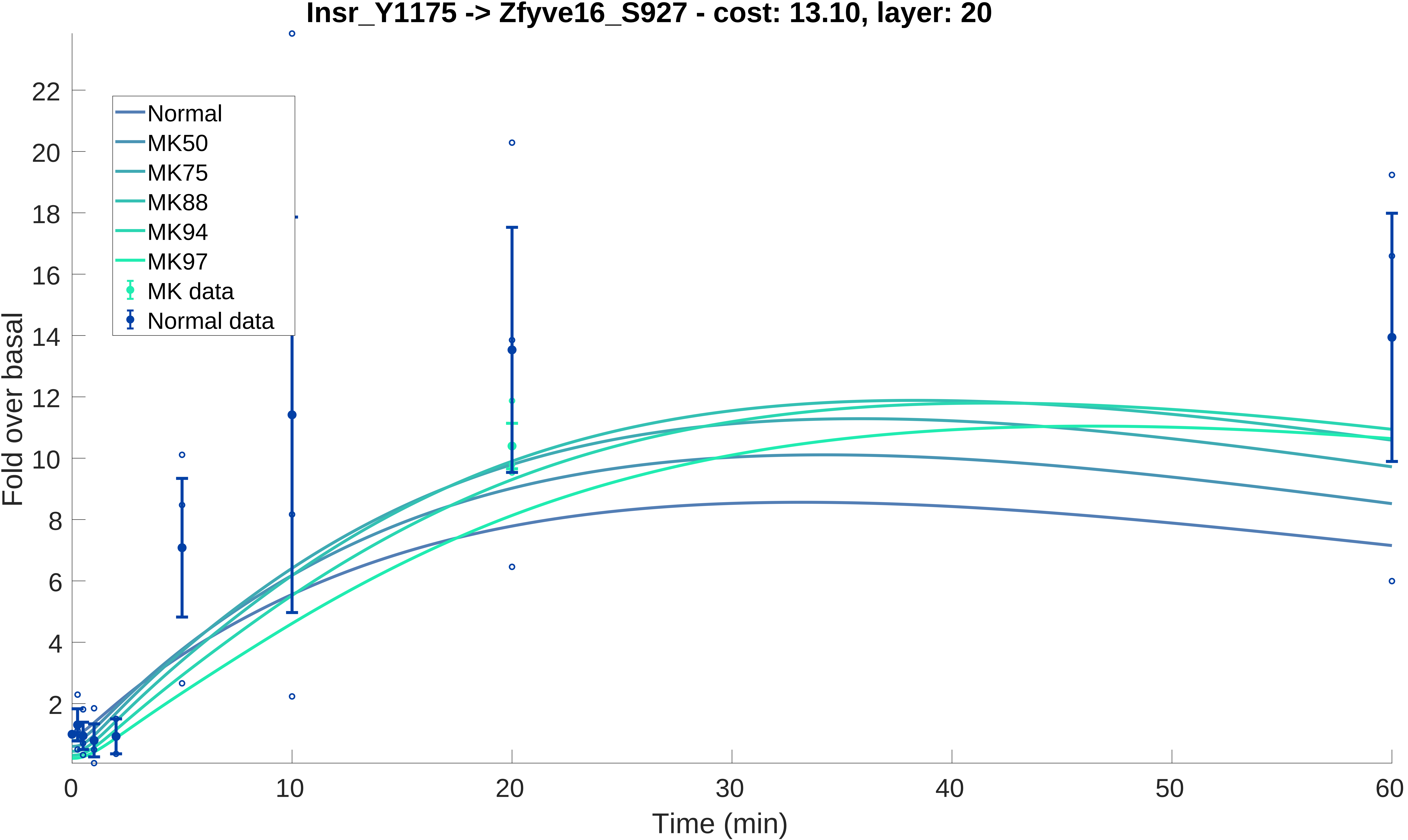

Insr_Y1175 -> Zfyve16_S927 - cost: 13.10, layer: 20
Normal
MK50
MK75
MK88
MK94
MK97
MK data
Normal data
Fold over basal
Time (min)
0
10
20
30
40
50
60
2
4
6
8
10
12
14
16
18
20
22

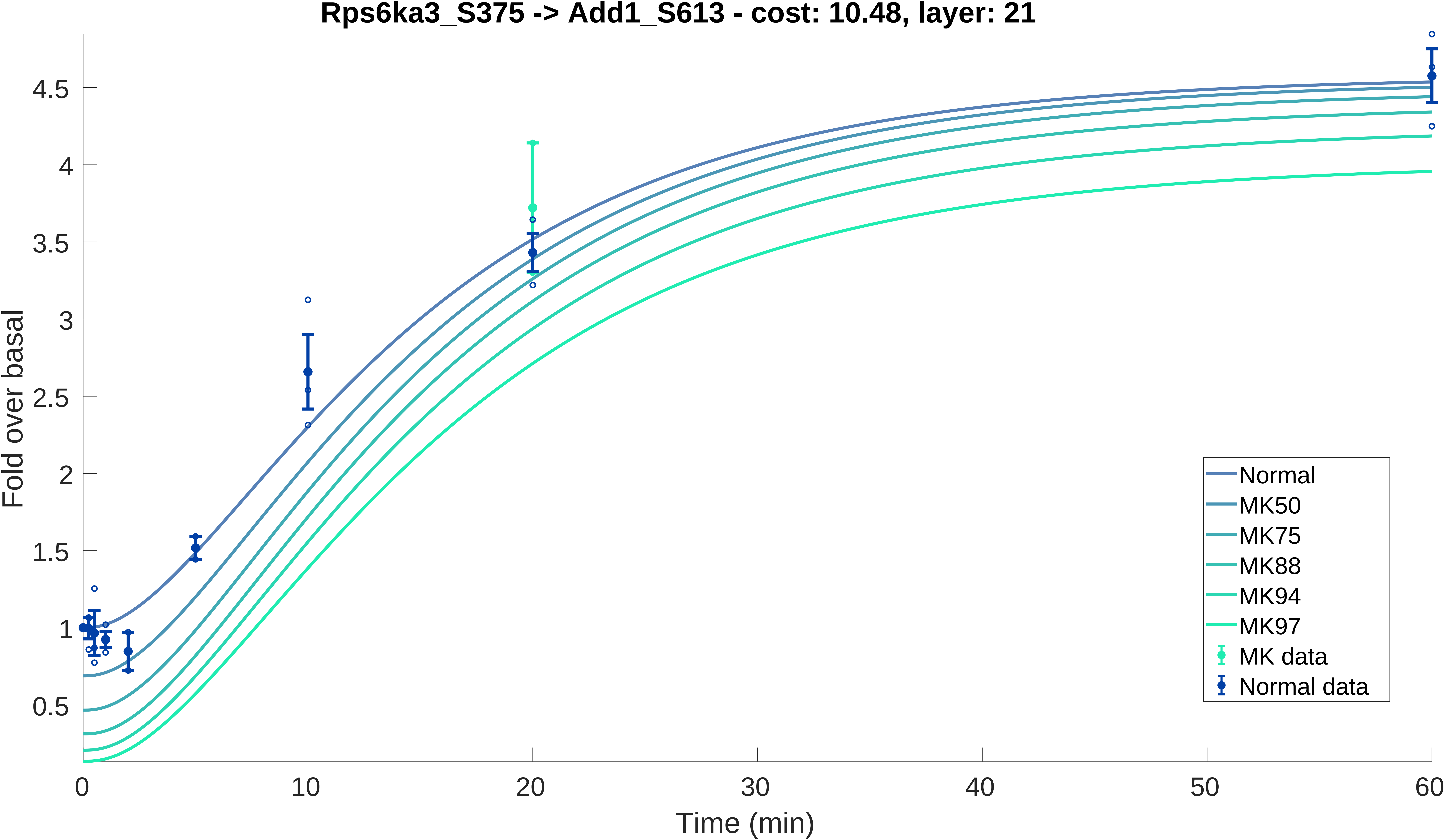
Rps6ka3_S375 -> Add1_S613 - cost: 10.48, layer: 21
Fold over basal
Time (min)
0.5
1
1.5
2
2.5
3
3.5
4
4.5
0
10
20
30
40
50
60
Normal
MK50
MK75
MK88
MK94
MK97
MK data
Normal data

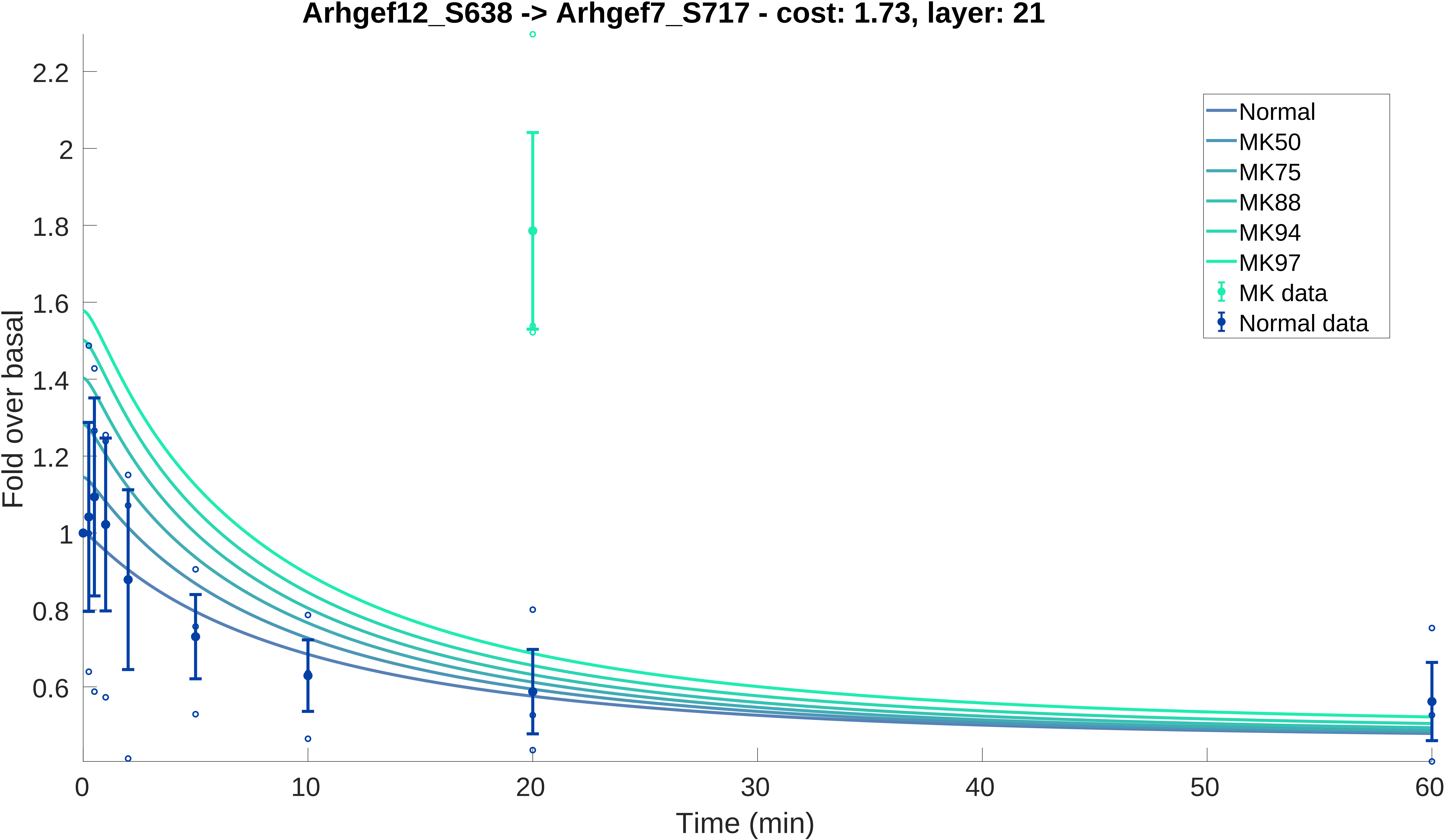
Arhgef12_S638 -> Arhgef7_S717 - cost: 1.73, layer: 21
Fold over basal
2.2
2
1.8
1.6
1.4
1.2
1
0.8
0.6
Time (min)
0
10
20
30
40
50
60
Normal
MK50
MK75
MK88
MK94
MK97
MK data
Normal data

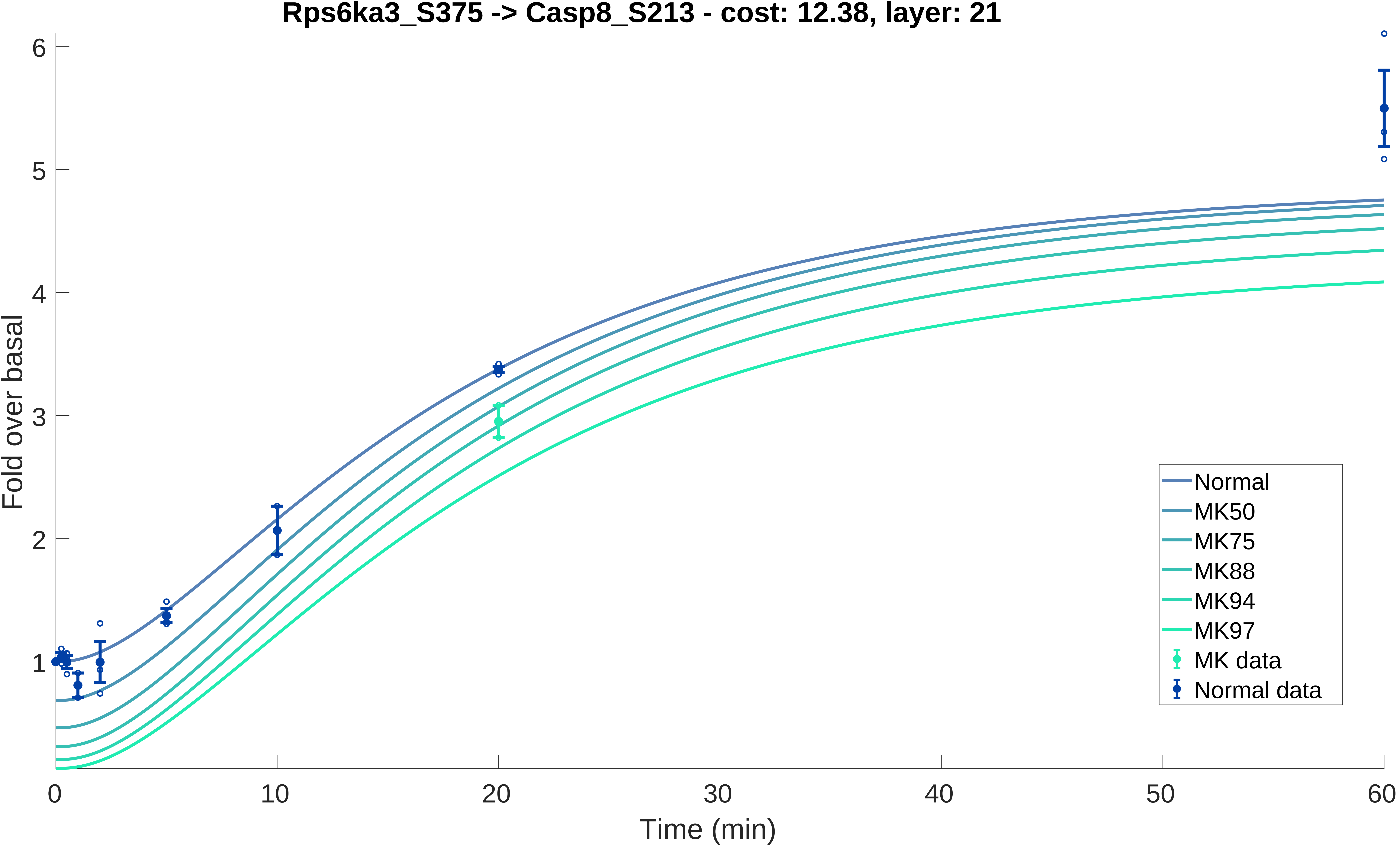

Rps6ka3_S375 -> Casp8_S213 - cost: 12.38, layer: 21
Fold over basal
Time (min)
0
10
20
30
40
50
60
1
2
3
4
5
6
Normal
MK50
MK75
MK88
MK94
MK97
MK data
Normal data

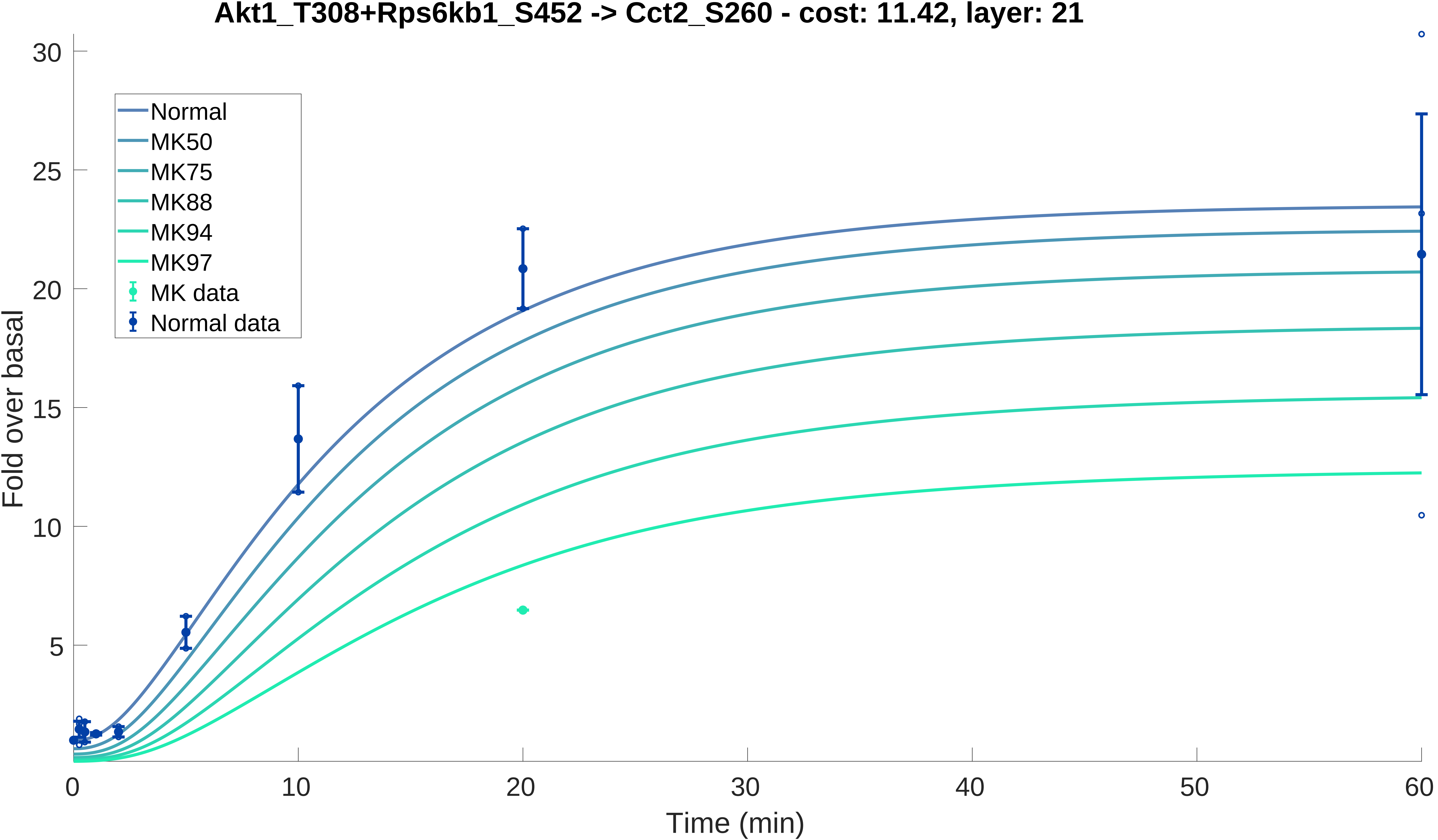

Akt1_T308+Rps6kb1_S452 -> Cct2_S260 - cost: 11.42, layer: 21
Fold over basal
Time (min)
0
10
20
30
40
50
60
5
10
15
20
25
30
Normal
MK50
MK75
MK88
MK94
MK97
MK data
Normal data

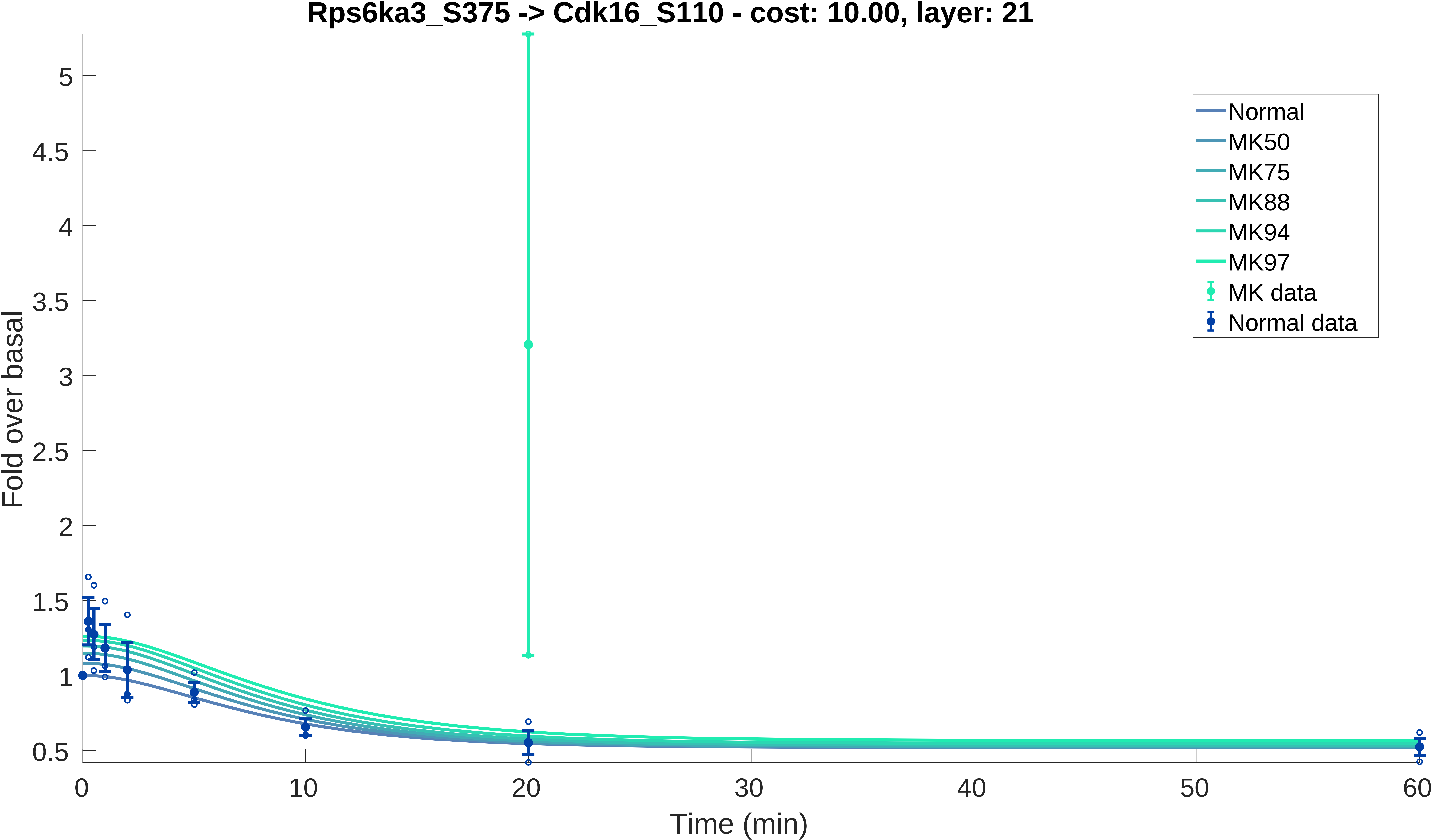
Rps6ka3_S375 -> Cdk16_S110 - cost: 10.00, layer: 21
Fold over basal
Time (min)
0
10
20
30
40
50
60
0.5
1
1.5
2
2.5
3
3.5
4
4.5
5
Normal
MK50
MK75
MK88
MK94
MK97
MK data
Normal data

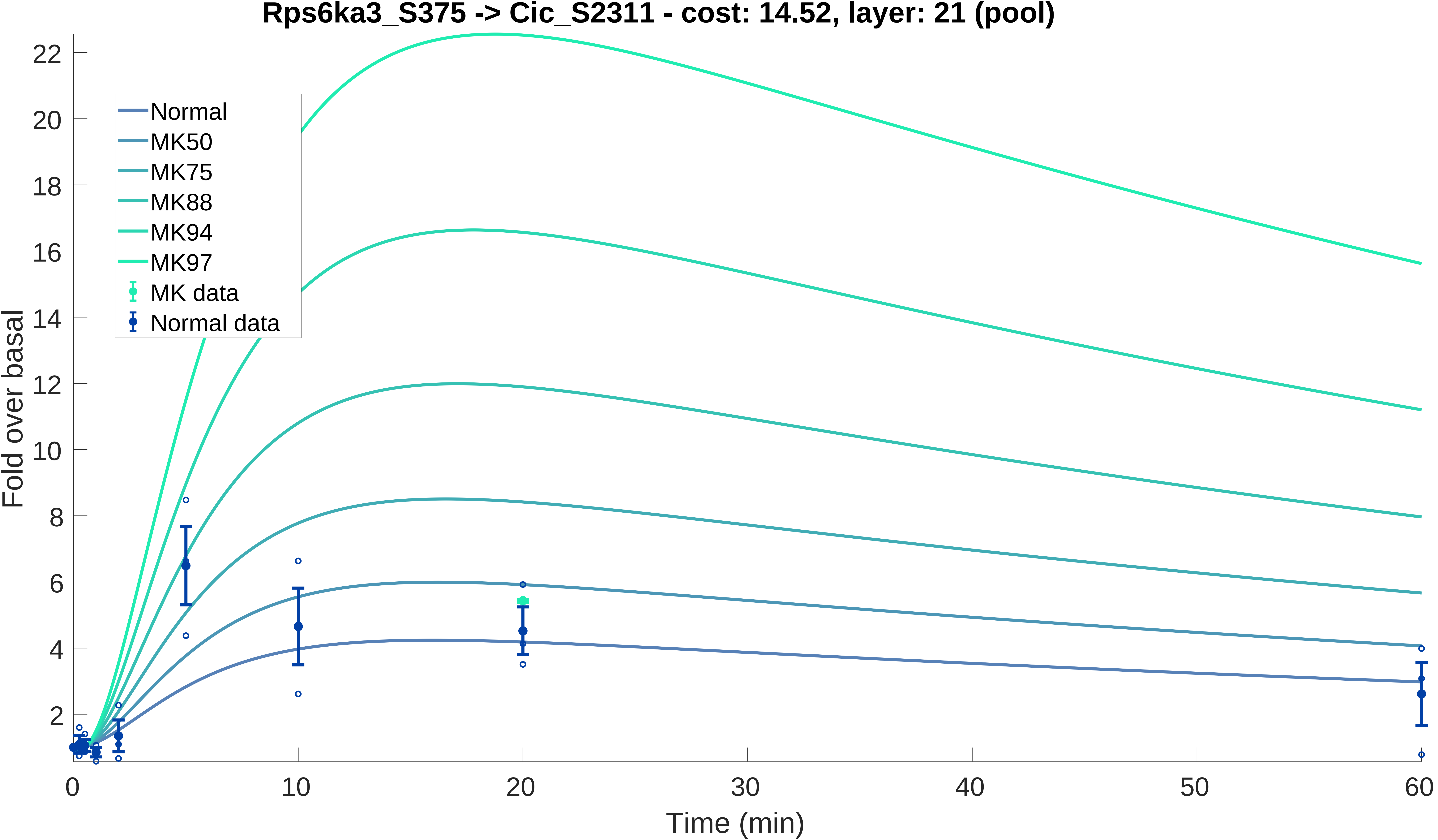

Rps6ka3_S375 -> Cic_S2311 - cost: 14.52, layer: 21 (pool)
Normal
MK50
MK75
MK88
MK94
MK97
MK data
Normal data
Fold over basal
Time (min)
0
10
20
30
40
50
60
2
4
6
8
10
12
14
16
18
20
22

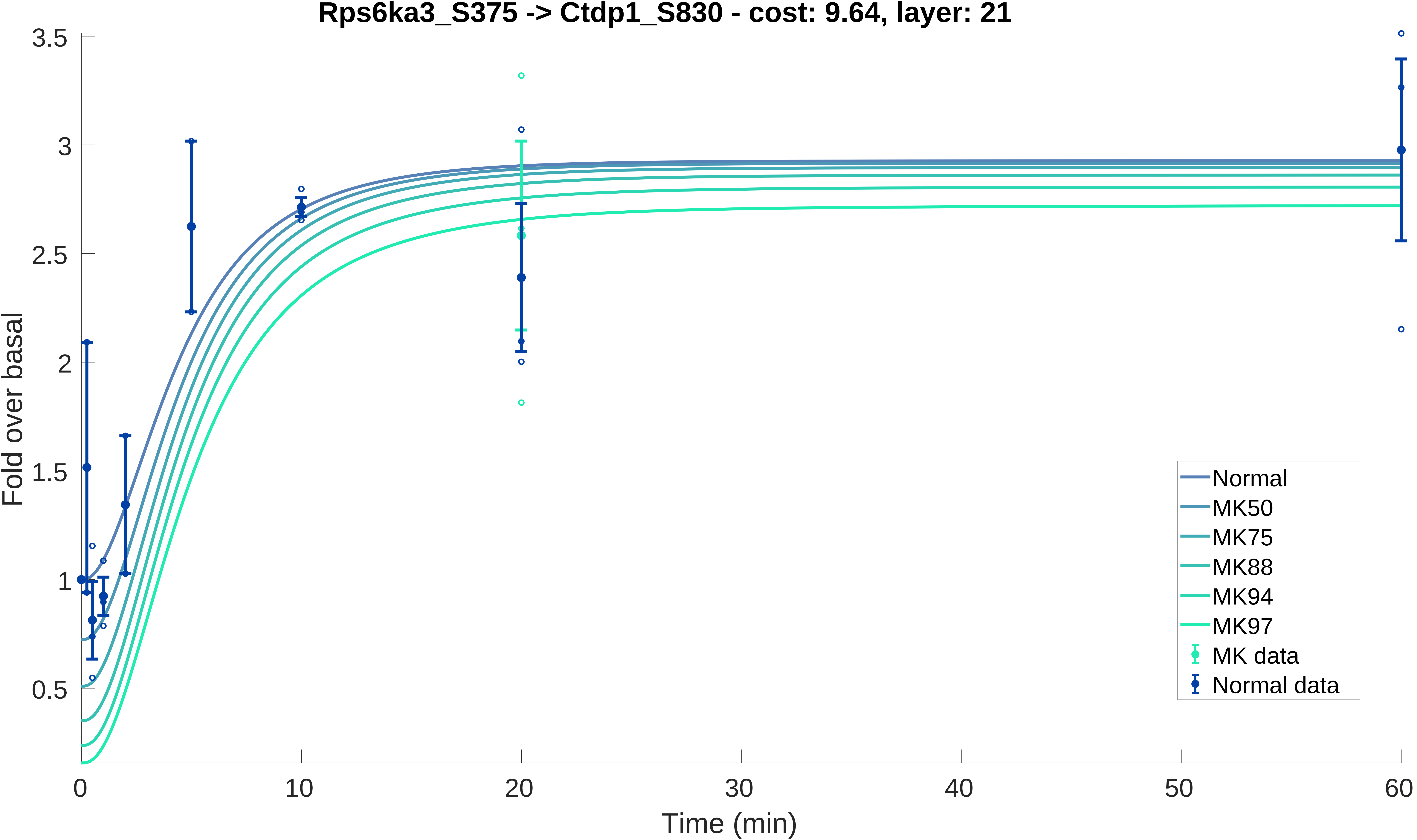

Rps6ka3_S375 -> Ctdp1_S830 - cost: 9.64, layer: 21
Fold over basal
Time (min)
0.5
1
1.5
2
2.5
3
3.5
0
10
20
30
40
50
60
Normal
MK50
MK75
MK88
MK94
MK97
MK data
Normal data

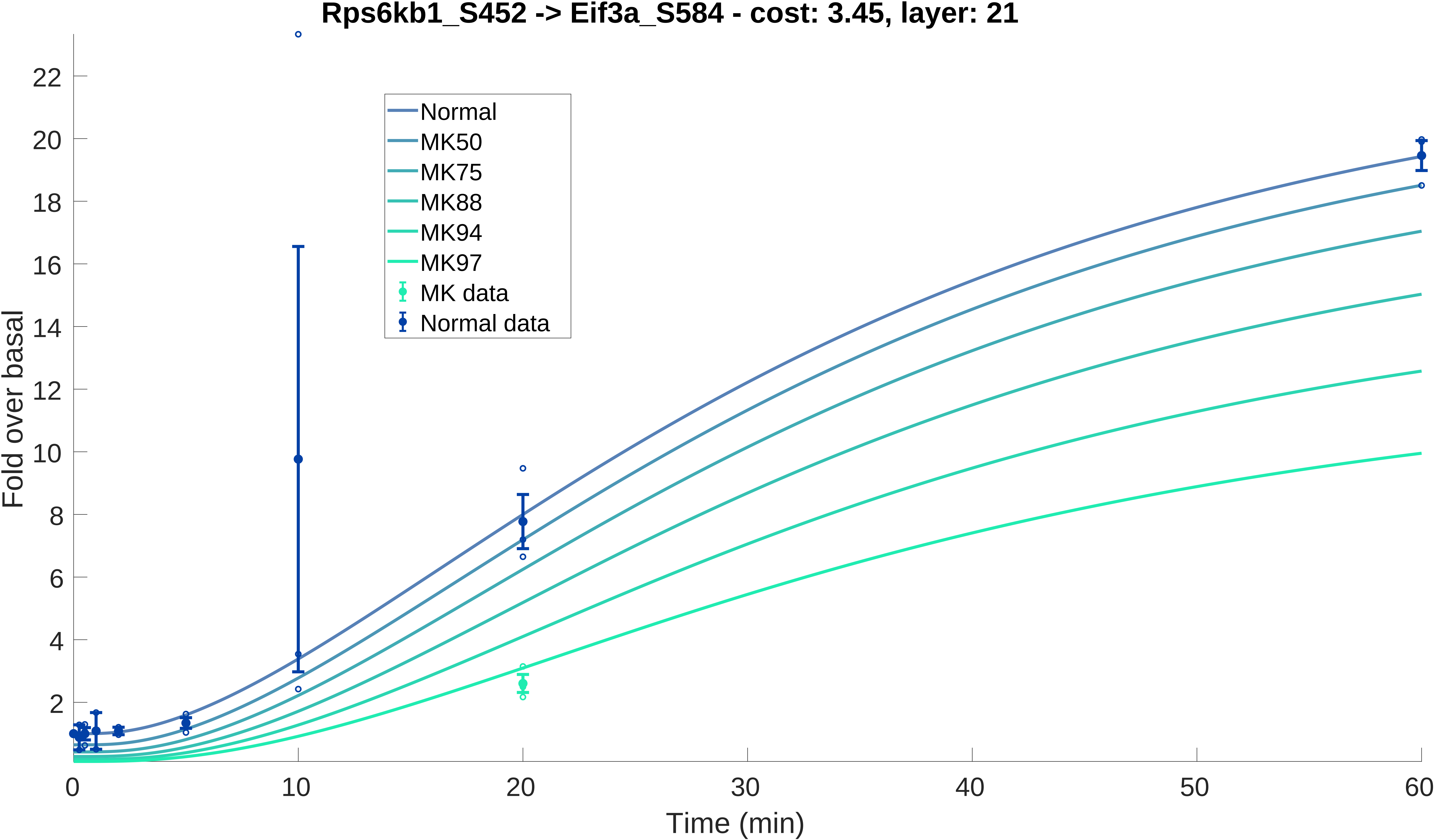

Rps6kb1_S452 -> Eif3a_S584 - cost: 3.45, layer: 21
Normal
MK50
MK75
MK88
MK94
MK97
MK data
Normal data
Fold over basal
Time (min)
0
10
20
30
40
50
60
2
4
6
8
10
12
14
16
18
20
22

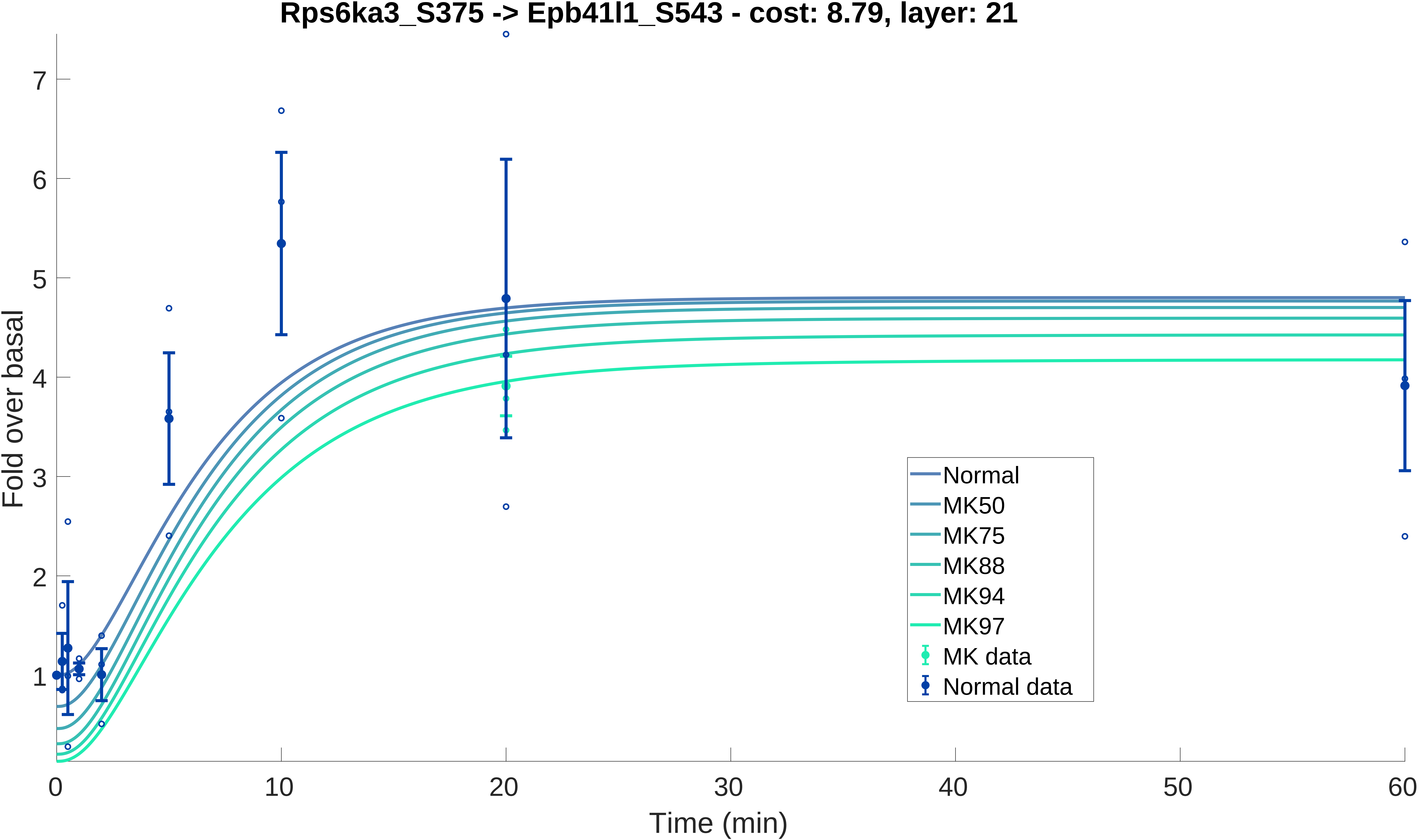

Rps6ka3_S375 -> Epb41l1_S543 - cost: 8.79, layer: 21
Fold over basal
Time (min)
0
10
20
30
40
50
60
1
2
3
4
5
6
7
Normal
MK50
MK75
MK88
MK94
MK97
MK data
Normal data

Gsk3b_S9+Rps6ka3_S375 -> Gys1_S711 - cost: 8.22, layer: 21

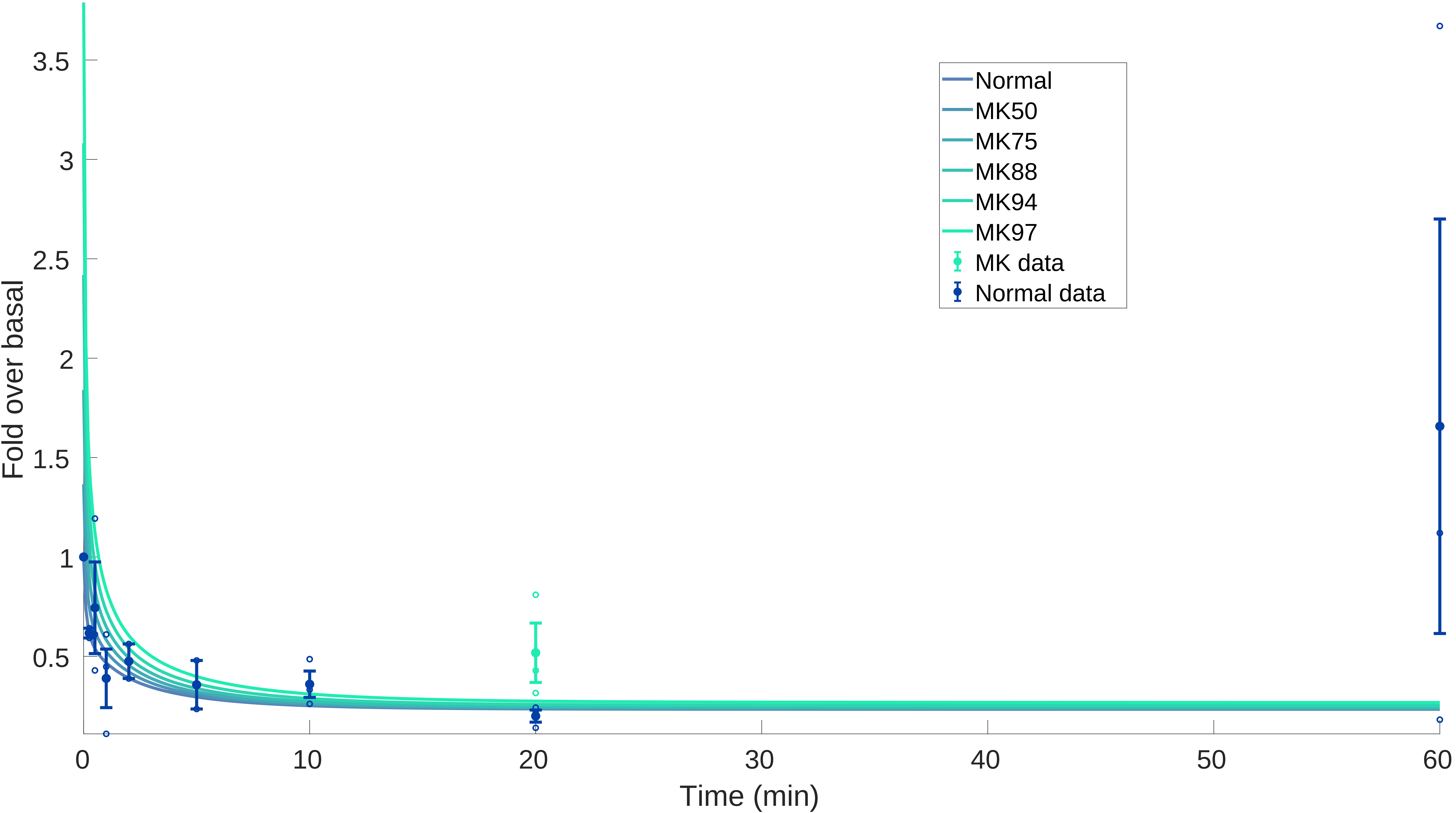

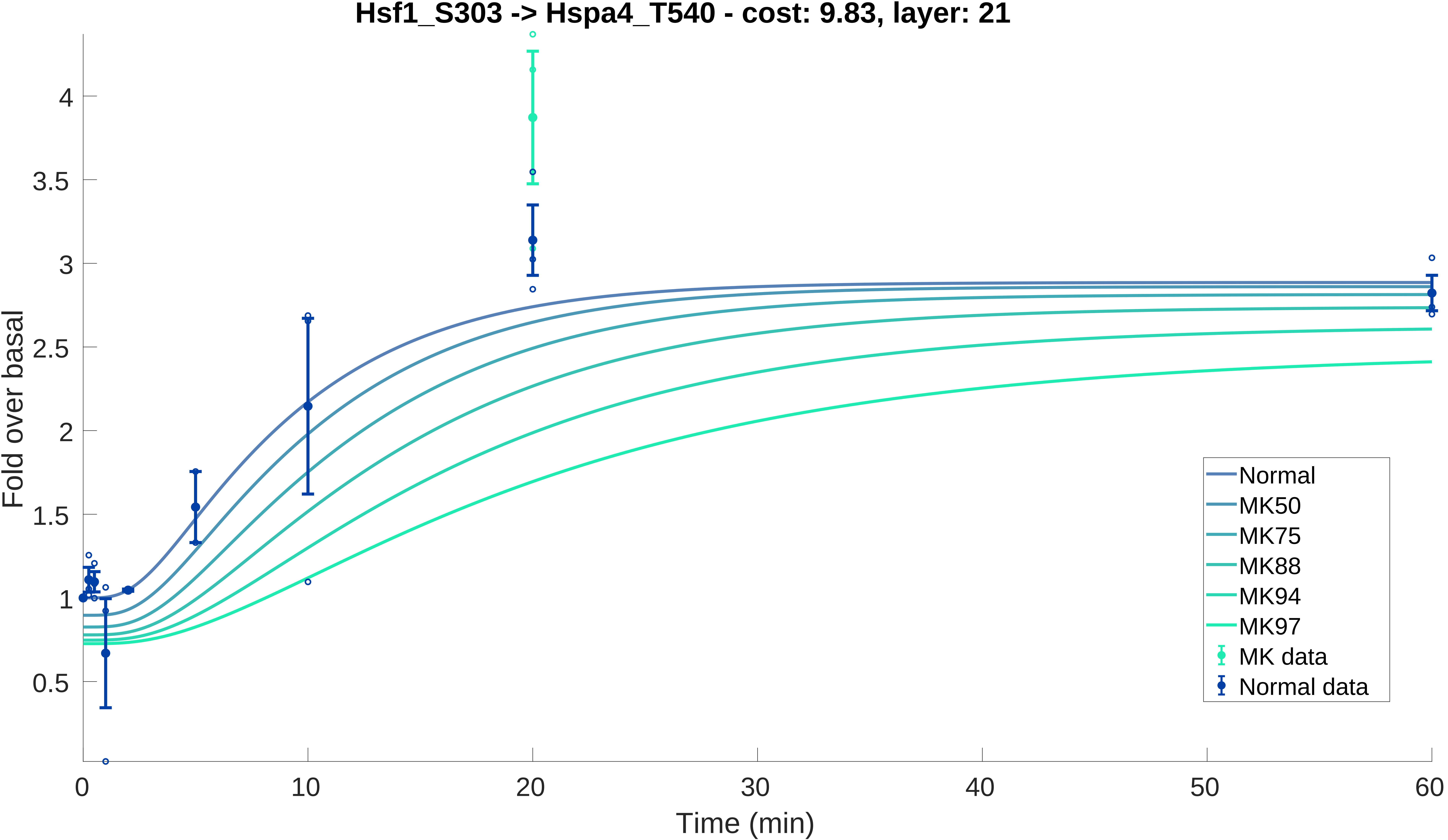
Hsf1_S303 -> Hspa4_T540 - cost: 9.83, layer: 21
Fold over basal
Time (min)
0.5
1
1.5
2
2.5
3
3.5
4
0
10
20
30
40
50
60
Normal
MK50
MK75
MK88
MK94
MK97
MK data
Normal data

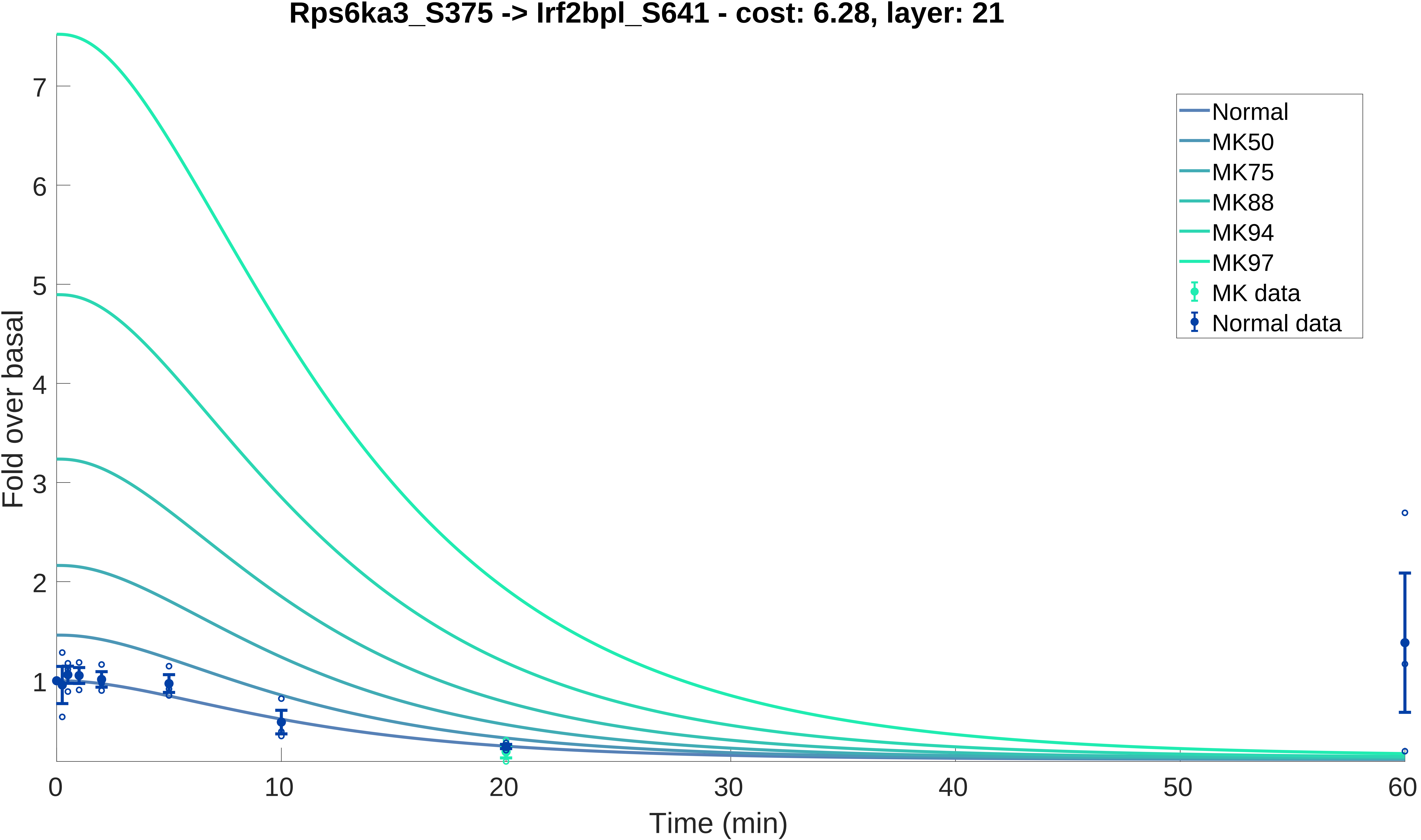
Rps6ka3_S375 -> Irf2bpl_S641 - cost: 6.28, layer: 21
Fold over basal
Time (min)
0
10
20
30
40
50
60
1
2
3
4
5
6
7
Normal
MK50
MK75
MK88
MK94
MK97
MK data
Normal data

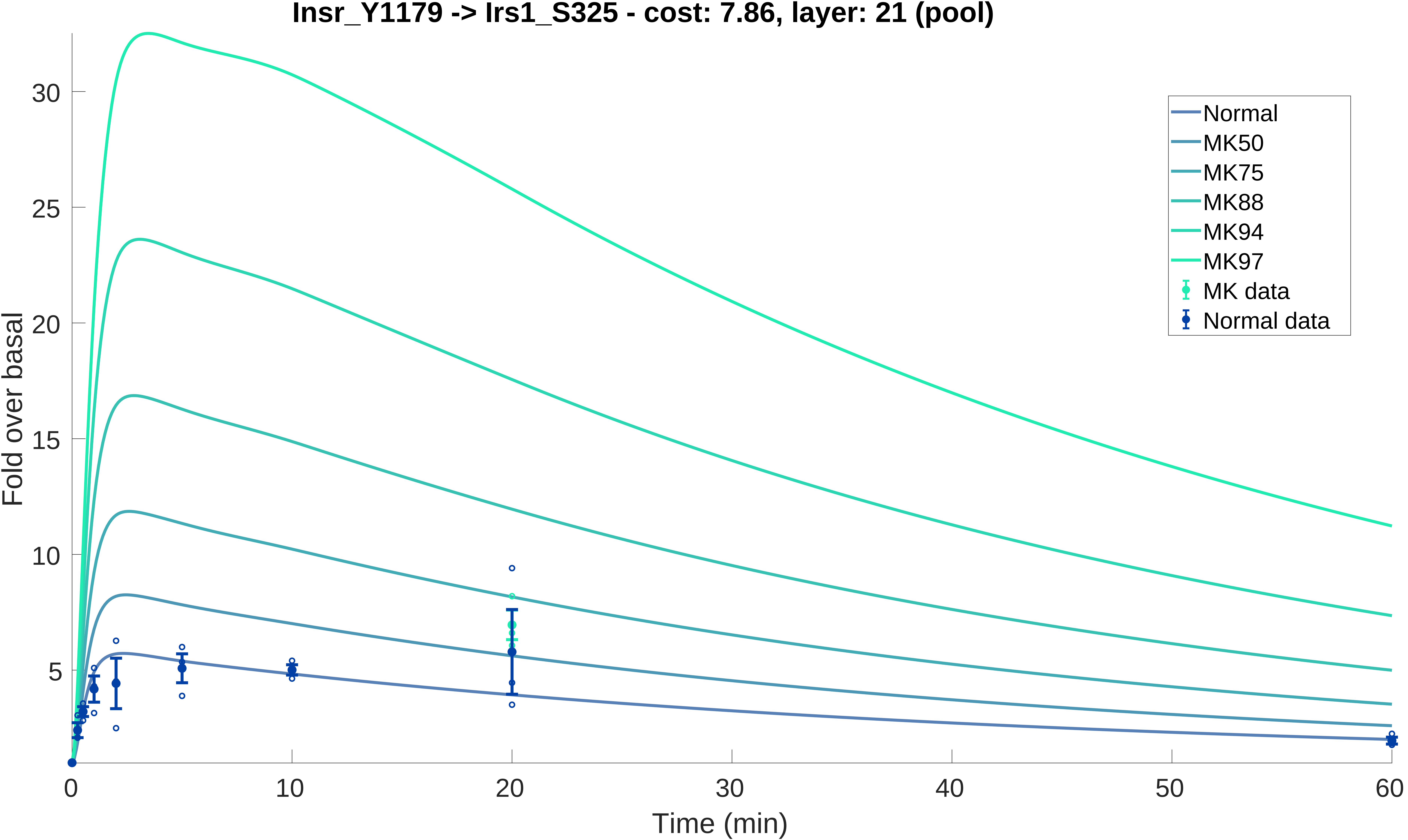
Insr_Y1179 -> Irs1_S325 - cost: 7.86, layer: 21 (pool)
Fold over basal
Time (min)
0
10
20
30
40
50
60
5
10
15
20
25
30
Normal
MK50
MK75
MK88
MK94
MK97
MK data
Normal data

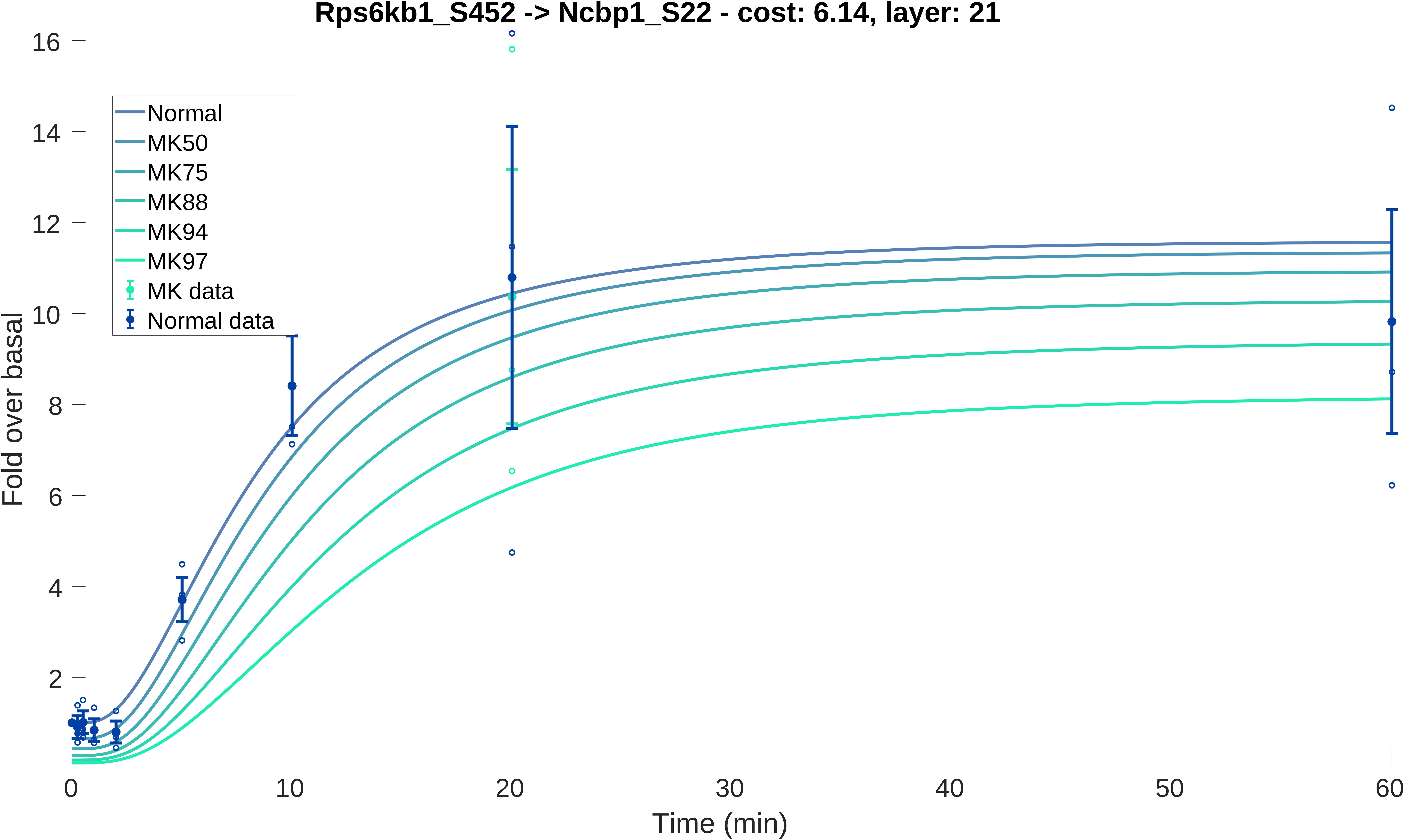
Rps6kb1_S452 -> Ncbp1_S22 - cost: 6.14, layer: 21
Normal
MK50
MK75
MK88
MK94
MK97
MK data
Normal data
Fold over basal
Time (min)
0
10
20
30
40
50
60
2
4
6
8
10
12
14
16

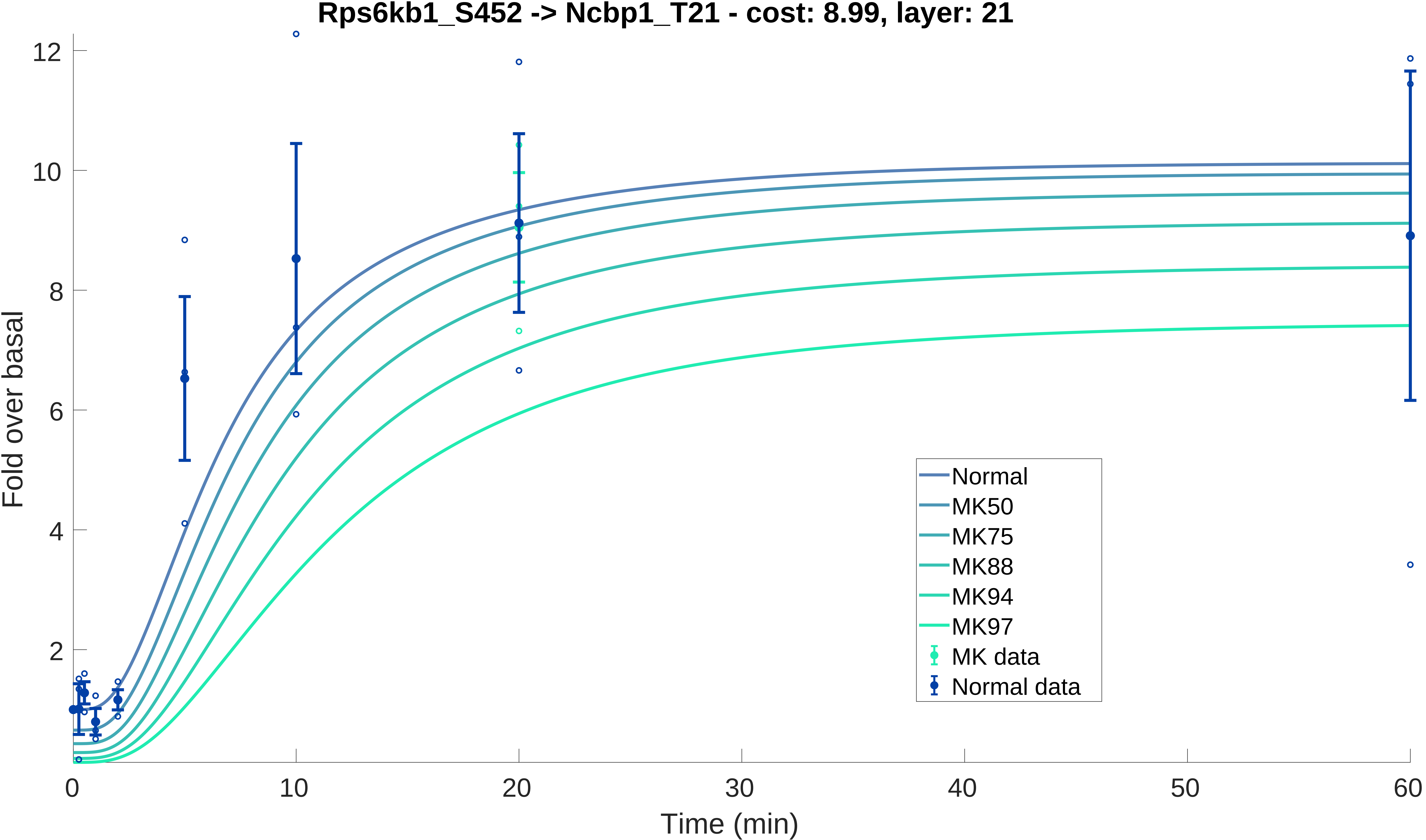

Rps6kb1_S452 -> Ncbp1_T21 - cost: 8.99, layer: 21
Fold over basal
Time (min)
0
10
20
30
40
50
60
2
4
6
8
10
12
Normal
MK50
MK75
MK88
MK94
MK97
MK data
Normal data

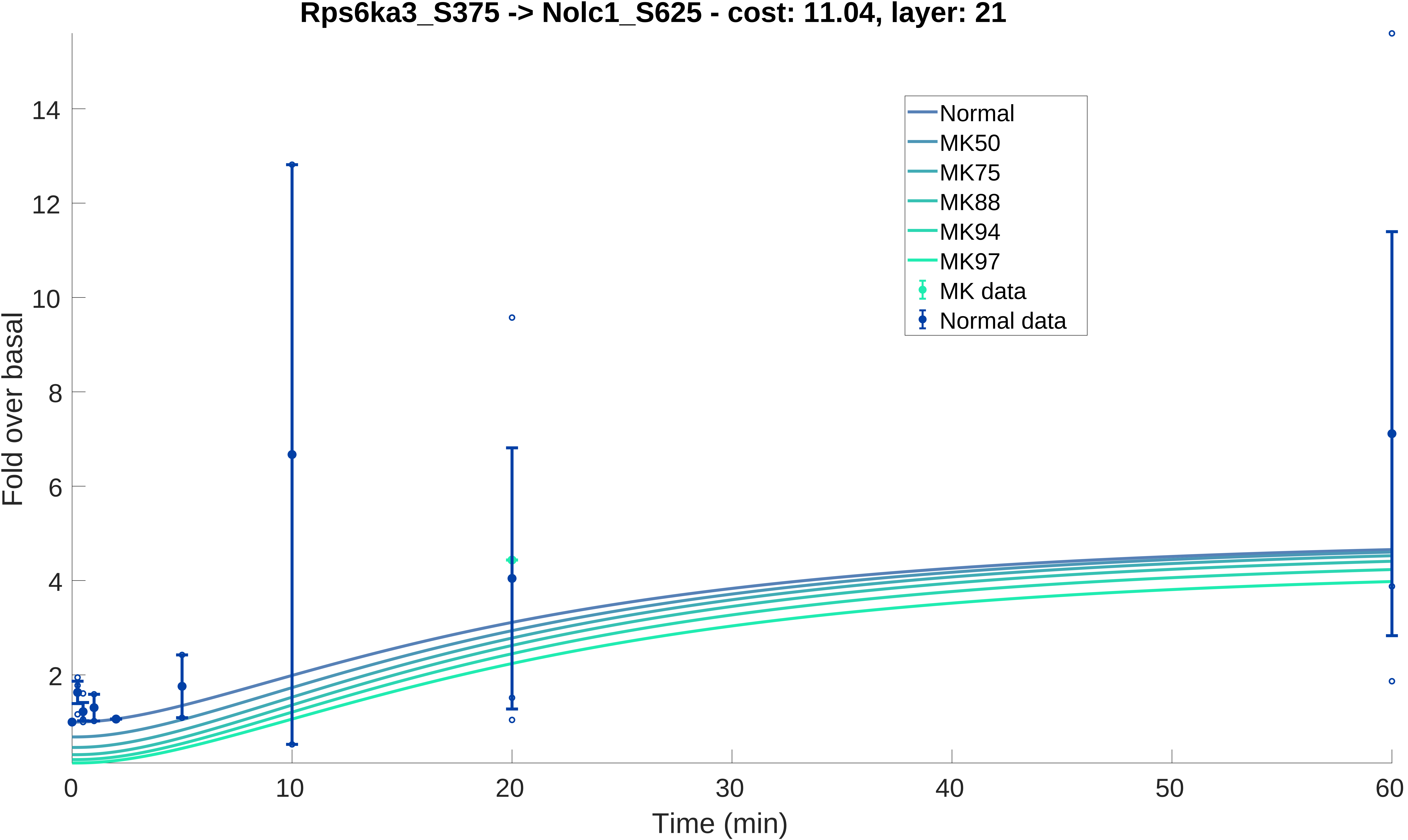

Rps6ka3_S375 -> Nolc1_S625 - cost: 11.04, layer: 21
Fold over basal
Time (min)
Normal
MK50
MK75
MK88
MK94
MK97
MK data
Normal data
0
10
20
30
40
50
60
2
4
6
8
10
12
14

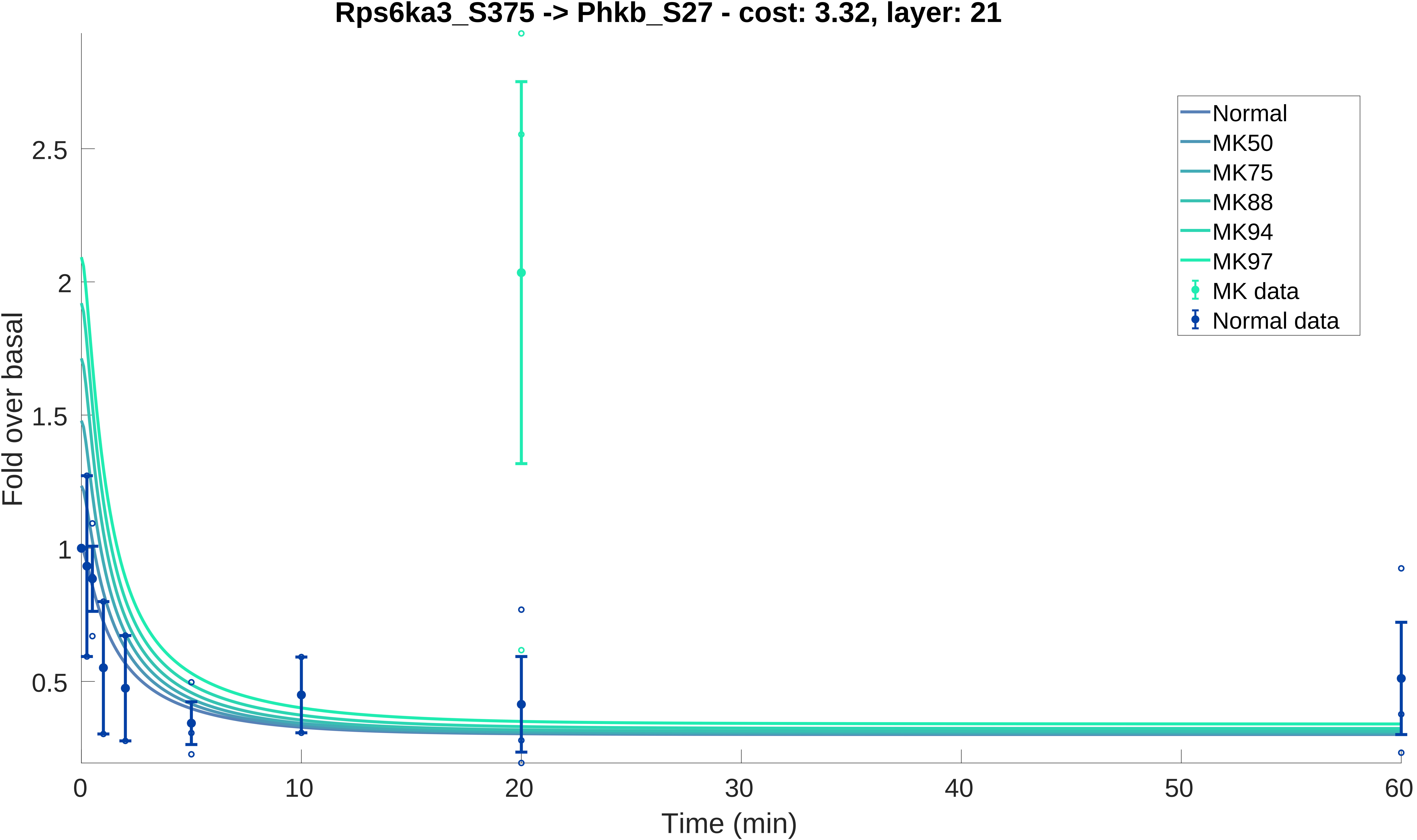

Rps6ka3_S375 -> Phkb_S27 - cost: 3.32, layer: 21
Fold over basal
Time (min)
0.5
1
1.5
2
2.5
0
10
20
30
40
50
60
Normal
MK50
MK75
MK88
MK94
MK97
MK data
Normal data

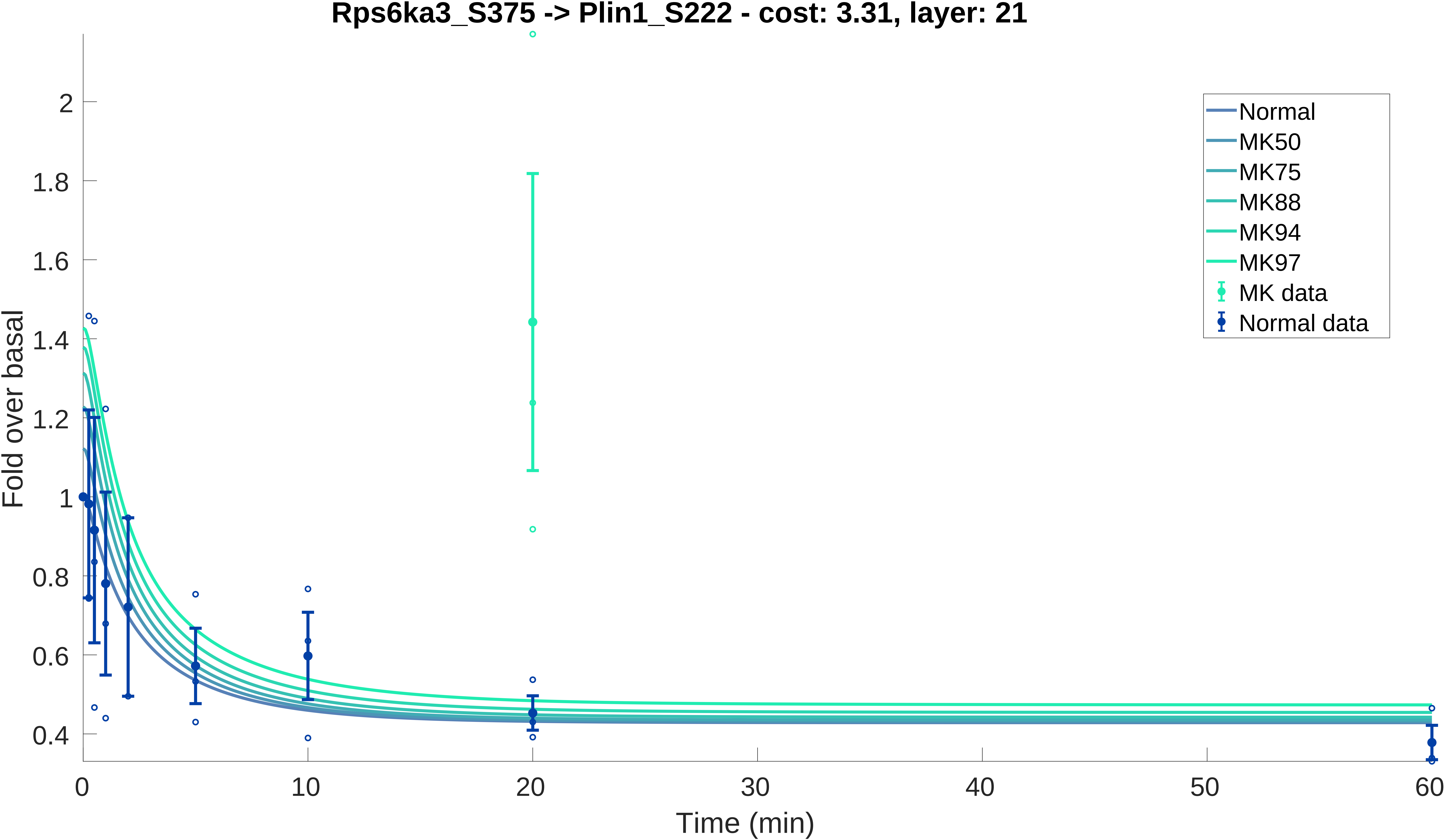

Rps6ka3_S375 -> Plin1_S222 - cost: 3.31, layer: 21
Fold over basal
Time (min)
0
10
20
30
40
50
60
0.4
0.6
0.8
1
1.2
1.4
1.6
1.8
2
Normal
MK50
MK75
MK88
MK94
MK97
MK data
Normal data

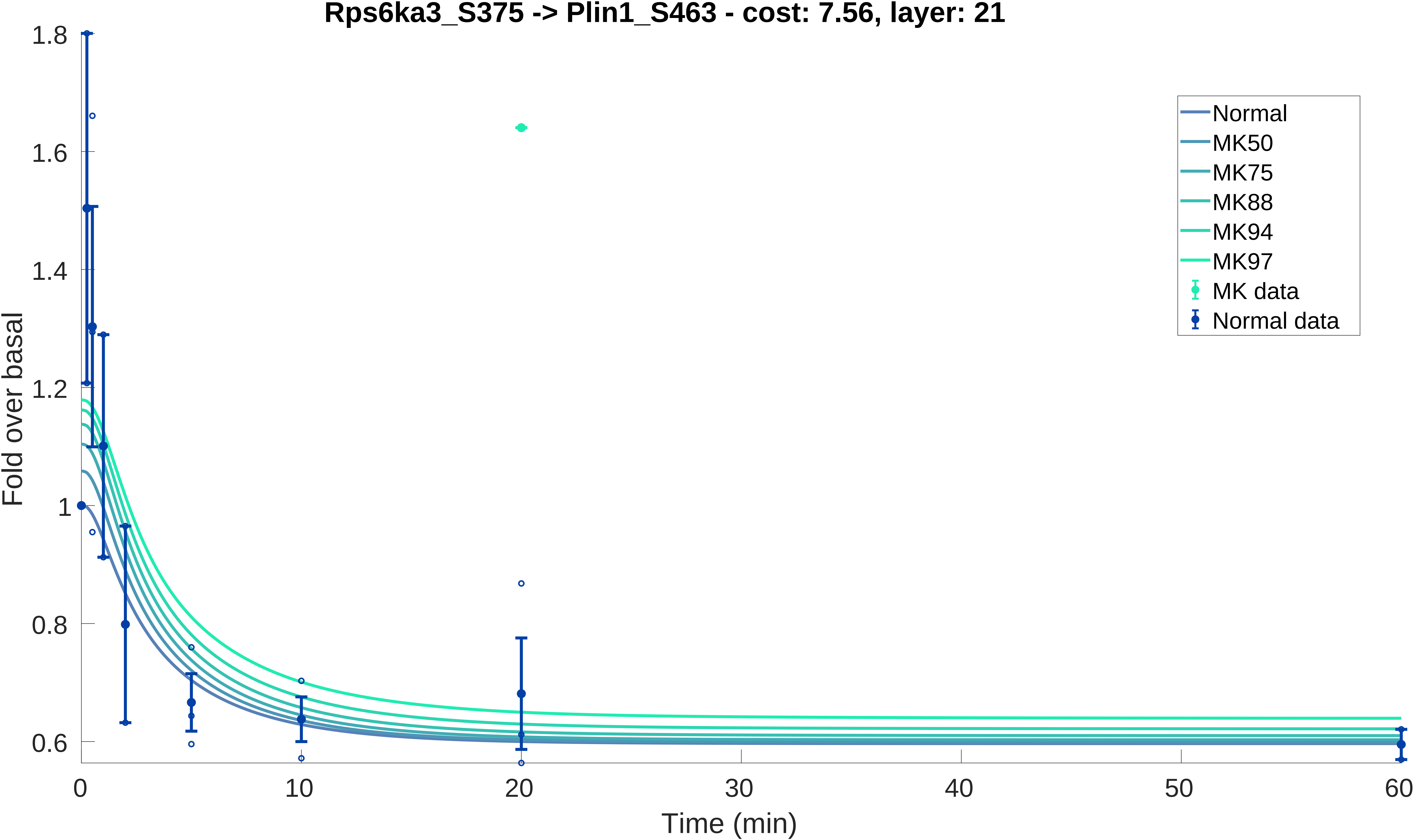

Rps6ka3_S375 -> Plin1_S463 - cost: 7.56, layer: 21
Fold over basal
Time (min)
1.8
1.6
1.4
1.2
1
0.8
0.6
0
10
20
30
40
50
60
Normal
MK50
MK75
MK88
MK94
MK97
MK data
Normal data

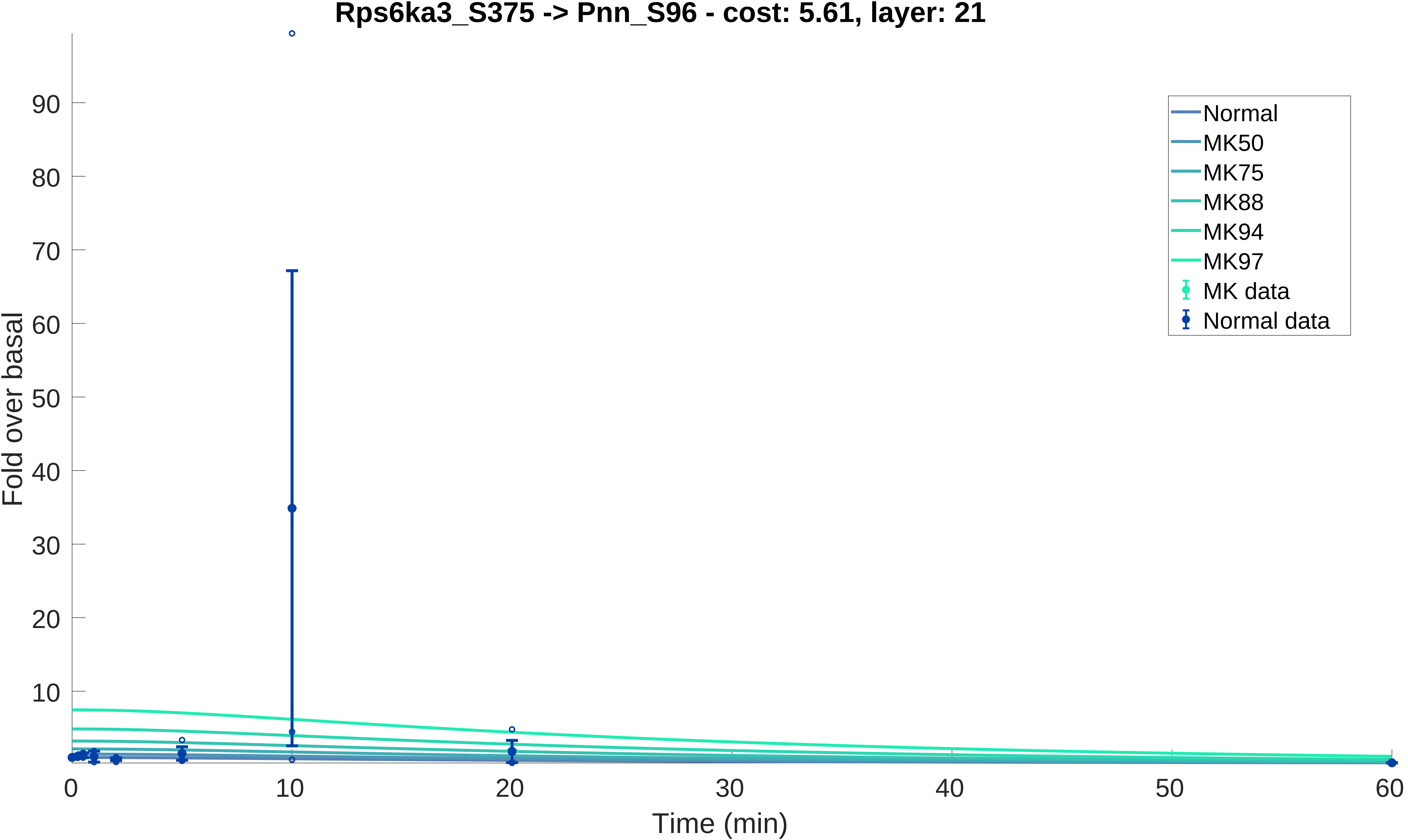
Rps6ka3_S375 -> Pnn_S96 - cost: 5.61, layer: 21
Fold over basal
Time (min)
0
10
20
30
40
50
60
70
80
90
Normal
MK50
MK75
MK88
MK94
MK97
MK data
Normal data

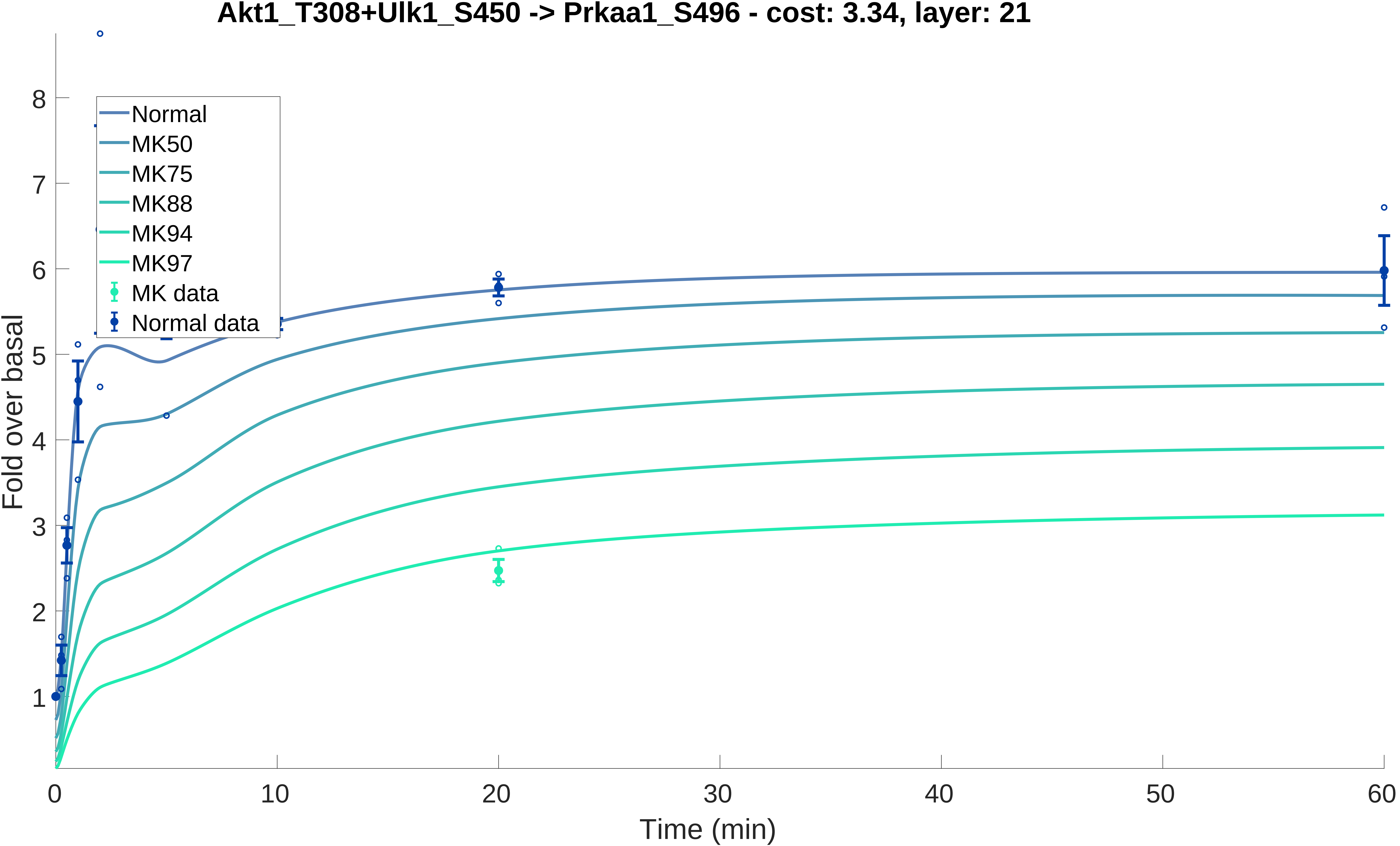
Akt1_T308+Ulk1_S450 -> Prkaa1_S496 - cost: 3.34, layer: 21
Normal
MK50
MK75
MK88
MK94
MK97
MK data
Normal data
Fold over basal
Time (min)
0
10
20
30
40
50
60
1
2
3
4
5
6
7
8

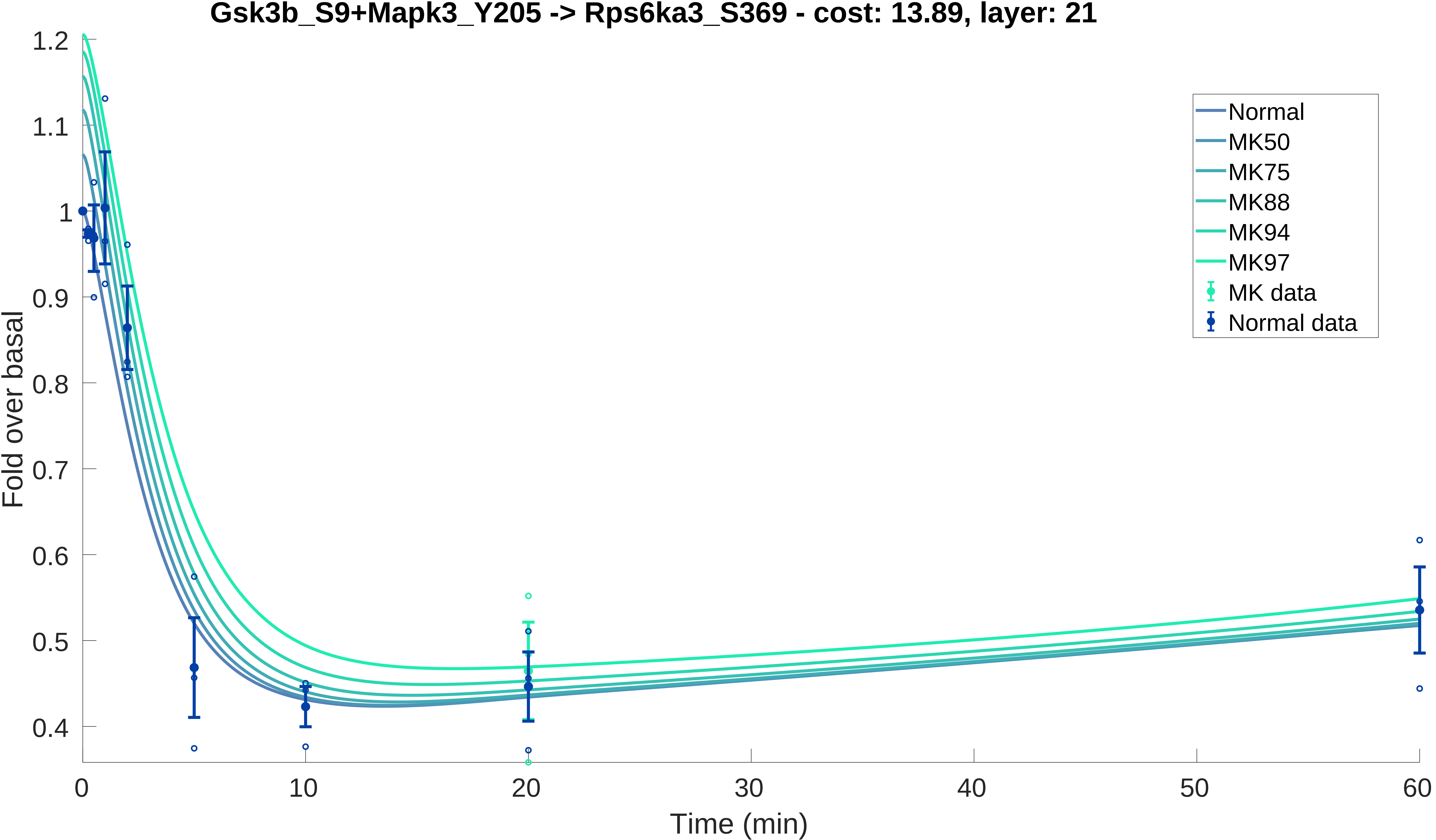
Gsk3b_S9+Mapk3_Y205 -> Rps6ka3_S369 - cost: 13.89, layer: 21
Fold over basal
Time (min)
1.2
1.1
1
0.9
0.8
0.7
0.6
0.5
0.4
0
10
20
30
40
50
60
Normal
MK50
MK75
MK88
MK94
MK97
MK data
Normal data

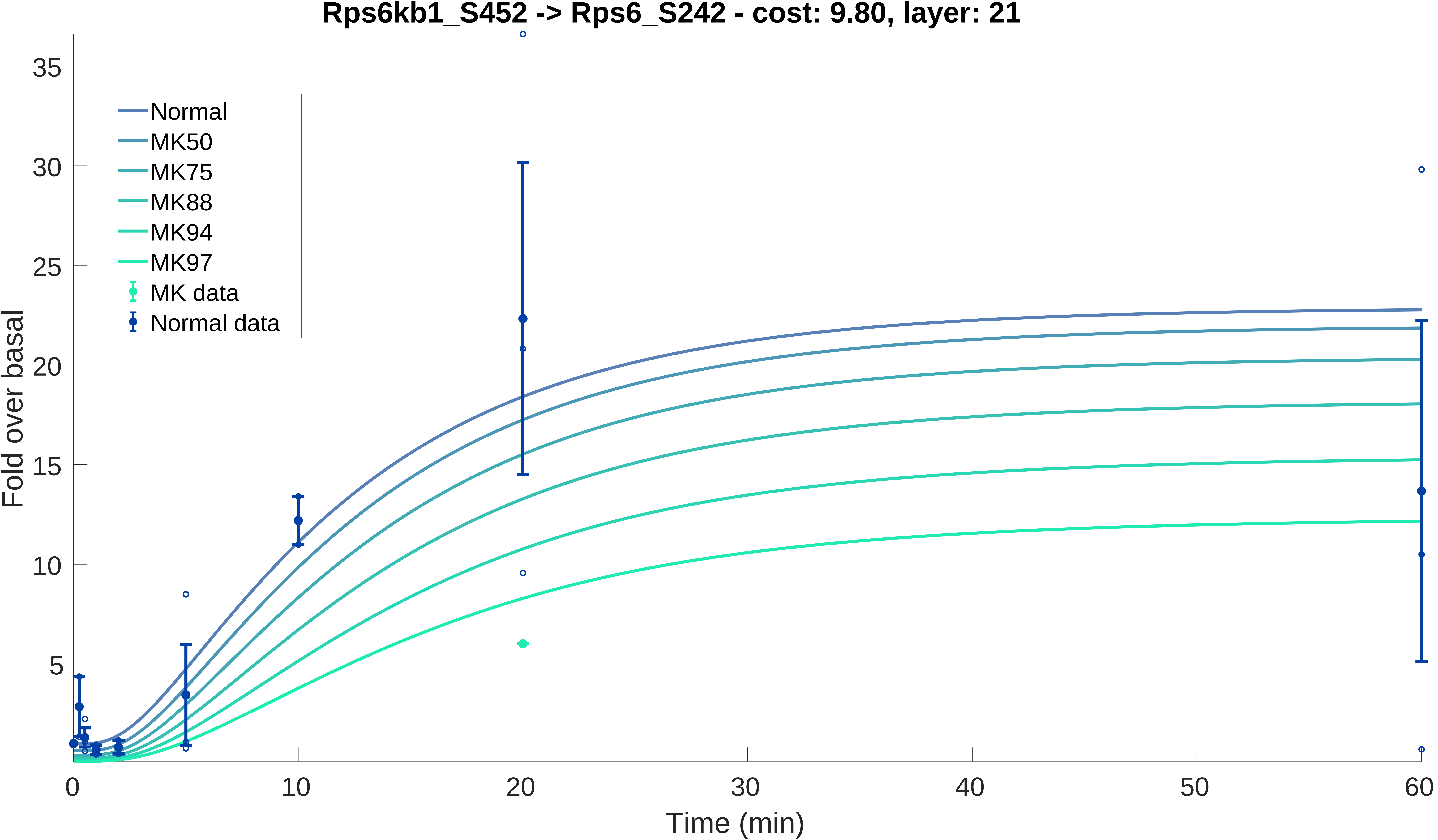

Rps6kb1_S452 -> Rps6_S242 - cost: 9.80, layer: 21
Normal
MK50
MK75
MK88
MK94
MK97
MK data
Normal data
Fold over basal
Time (min)
0
10
20
30
40
50
60
5
10
15
20
25
30
35

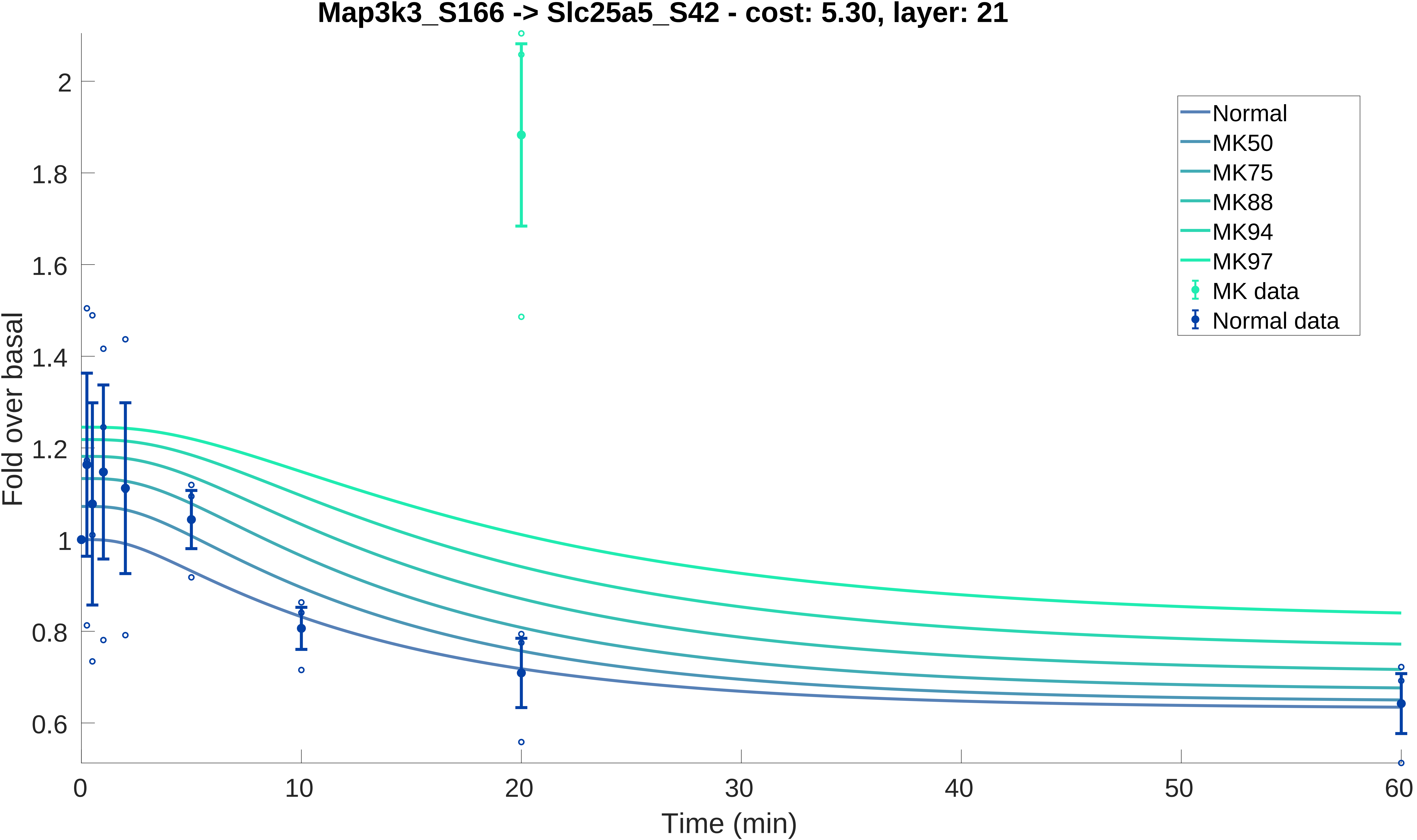

Map3k3_S166 -> Slc25a5_S42 - cost: 5.30, layer: 21
Fold over basal
Time (min)
0
10
20
30
40
50
60
0.6
0.8
1
1.2
1.4
1.6
1.8
2
Normal
MK50
MK75
MK88
MK94
MK97
MK data
Normal data

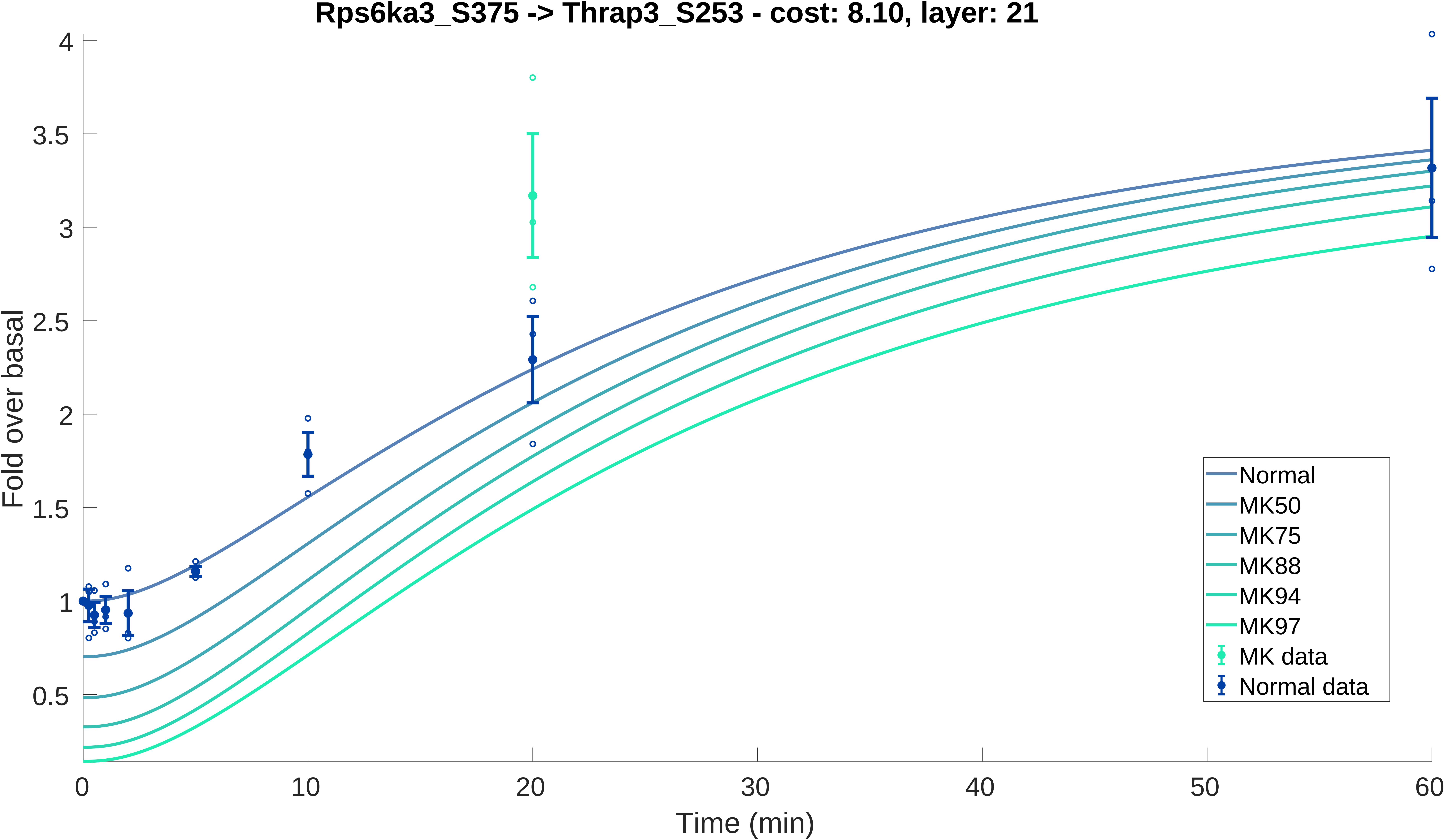
Rps6ka3_S375 -> Thrap3_S253 - cost: 8.10, layer: 21
Fold over basal
Time (min)
0
10
20
30
40
50
60
0.5
1
1.5
2
2.5
3
3.5
4
Normal
MK50
MK75
MK88
MK94
MK97
MK data
Normal data

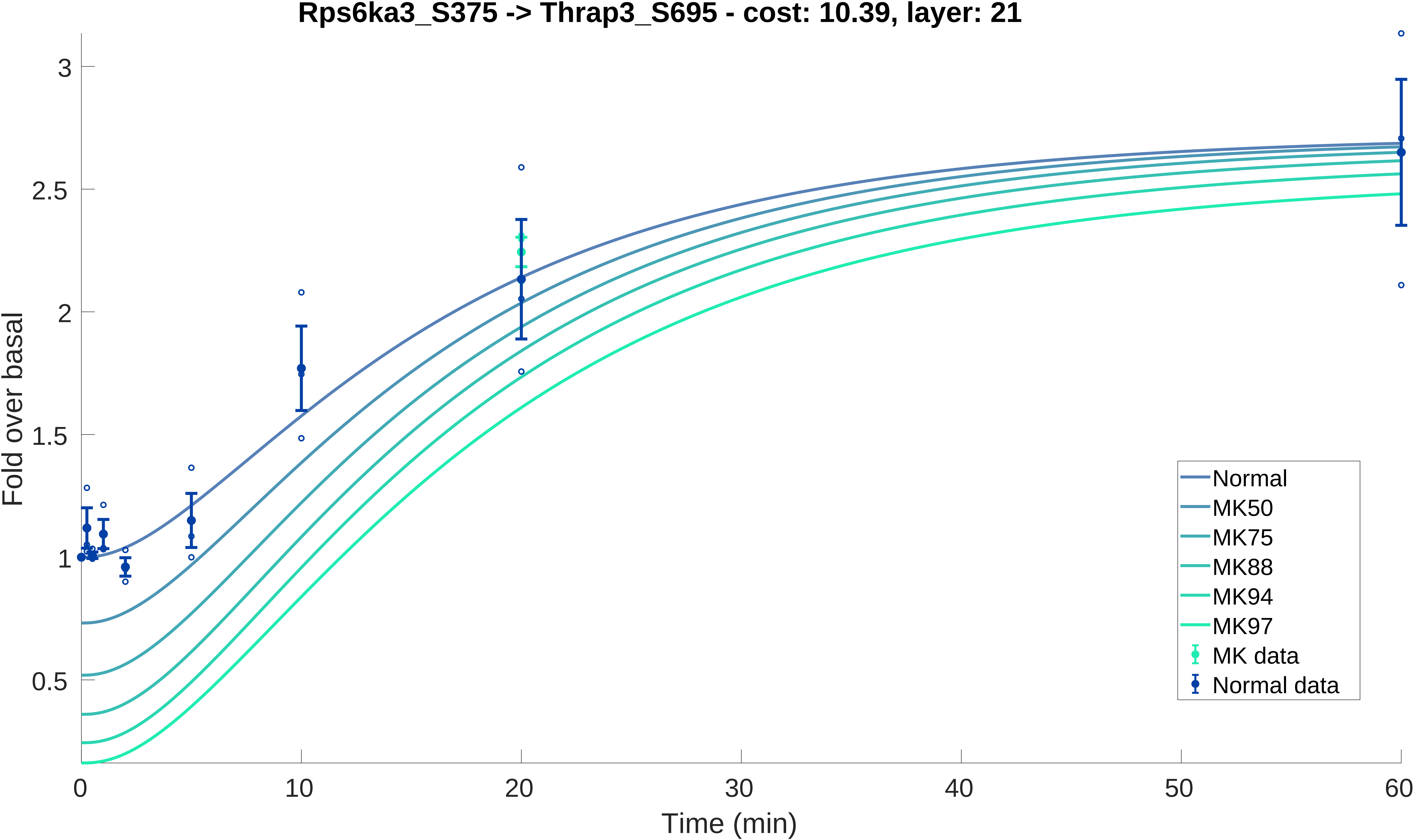

Rps6ka3_S375 -> Thrap3_S695 - cost: 10.39, layer: 21
Fold over basal
Time (min)
0.5
1
1.5
2
2.5
3
0
10
20
30
40
50
60
Normal
MK50
MK75
MK88
MK94
MK97
MK data
Normal data

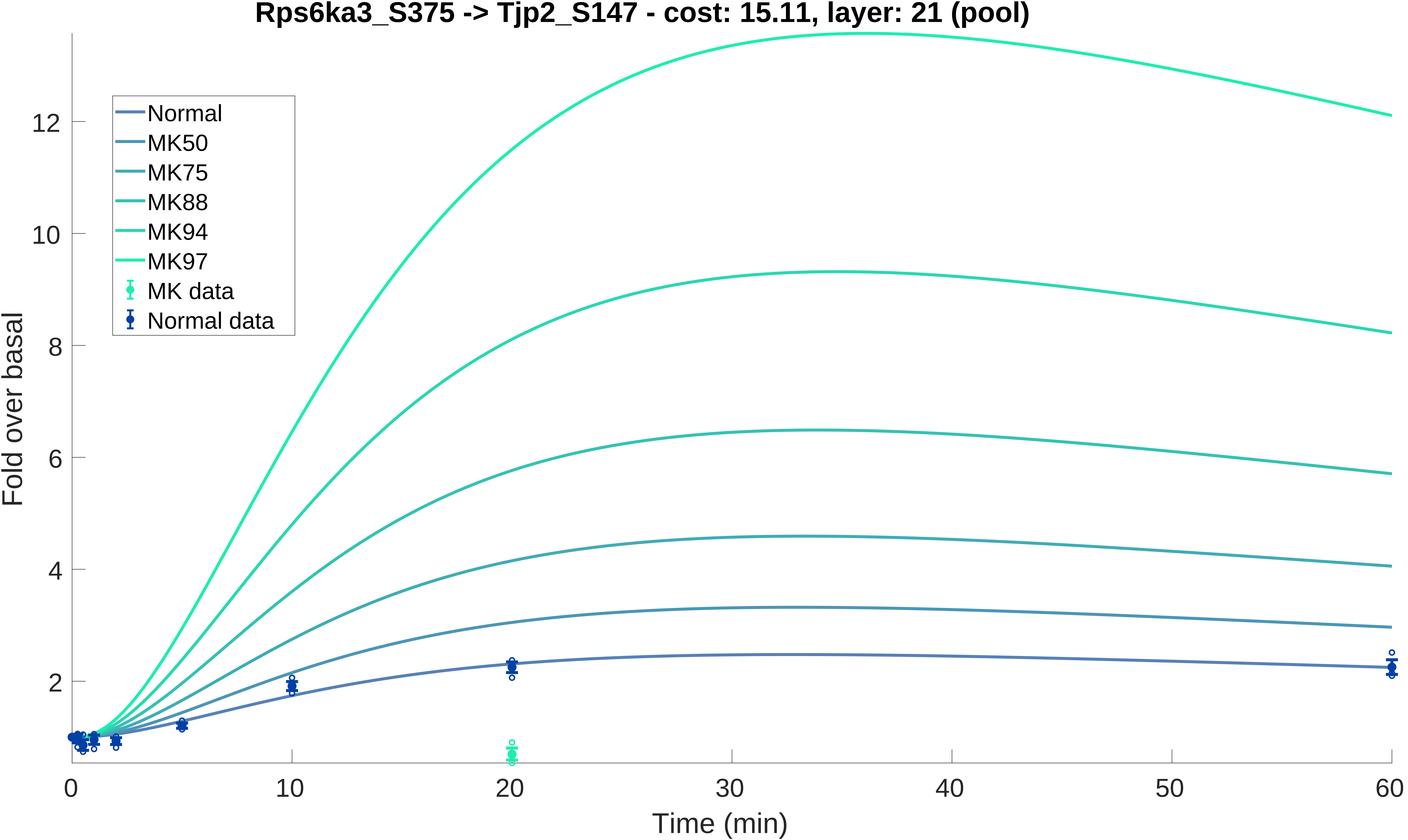

Rps6ka3_S375 -> Tjp2_S147 - cost: 15.11, layer: 21 (pool)
Normal
MK50
MK75
MK88
MK94
MK97
MK data
Normal data
Fold over basal
Time (min)
0
10
20
30
40
50
60
2
4
6
8
10
12

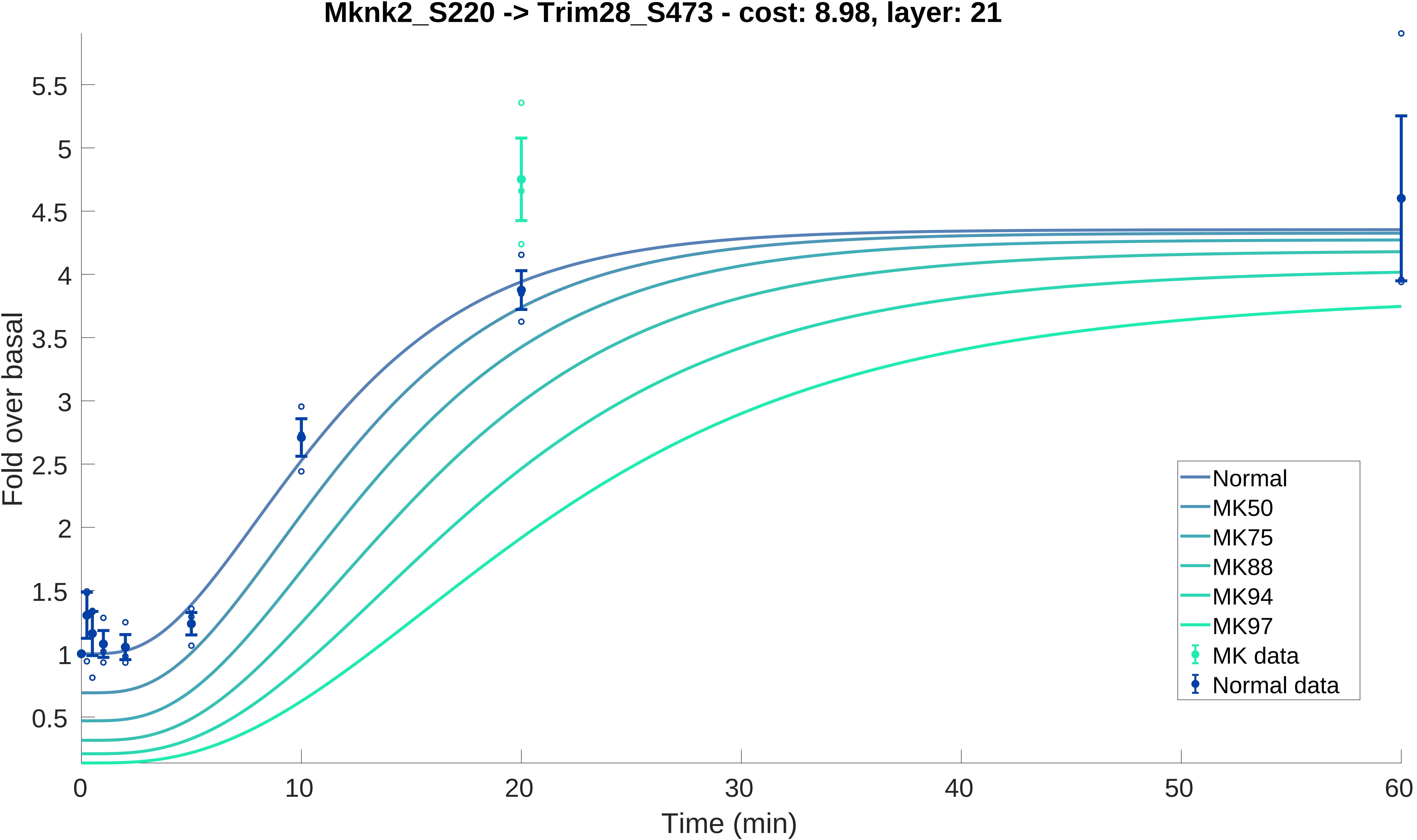

Mknk2_S220 -> Trim28_S473 - cost: 8.98, layer: 21
Fold over basal
Time (min)
0
10
20
30
40
50
60
0.5
1
1.5
2
2.5
3
3.5
4
4.5
5
5.5
Normal
MK50
MK75
MK88
MK94
MK97
MK data
Normal data

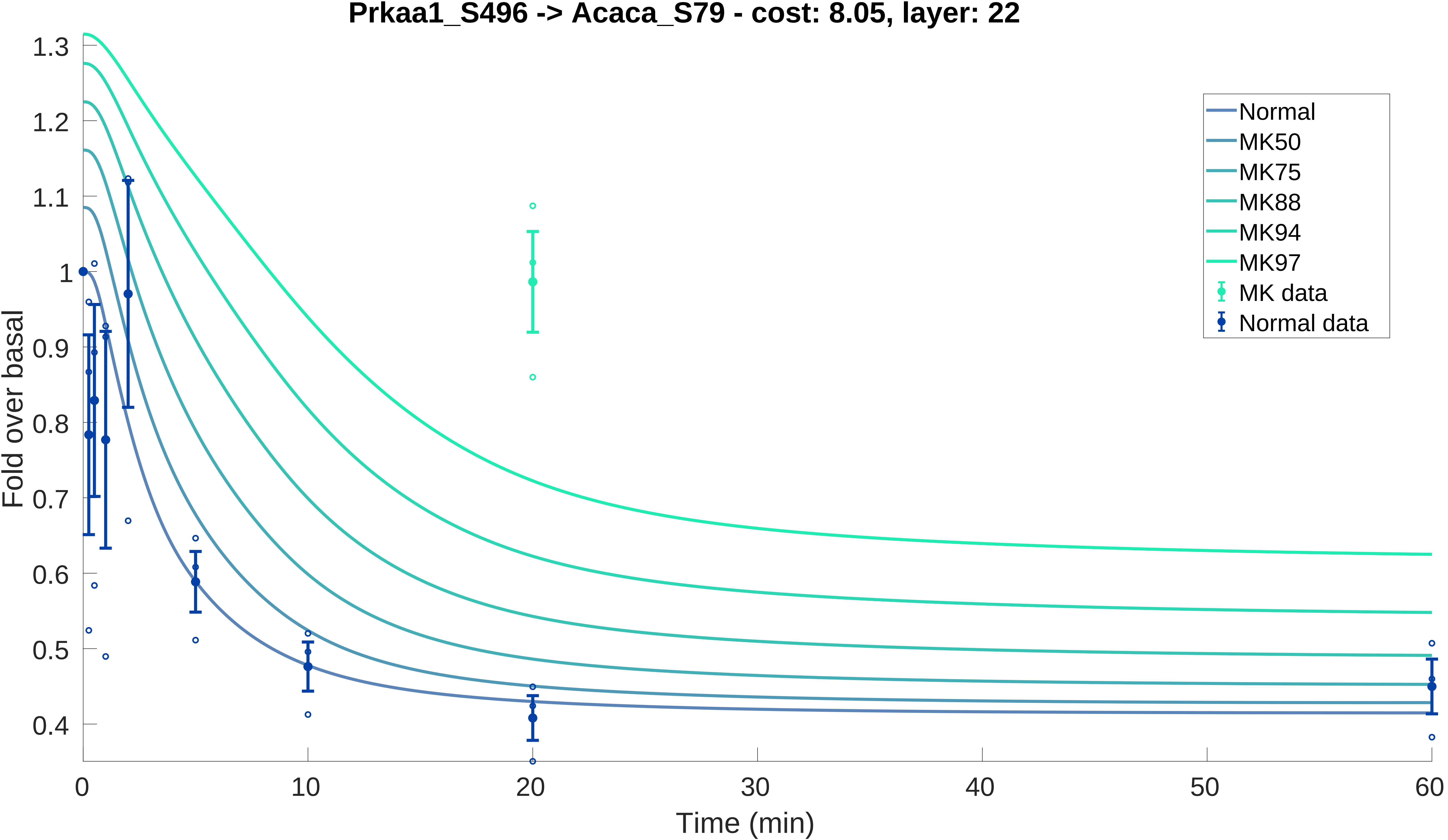

Prkaa1_S496 -> Acaca_S79 - cost: 8.05, layer: 22
Fold over basal
Time (min)
1.3
1.2
1.1
1
0.9
0.8
0.7
0.6
0.5
0.4
0
10
20
30
40
50
60
Normal
MK50
MK75
MK88
MK94
MK97
MK data
Normal data

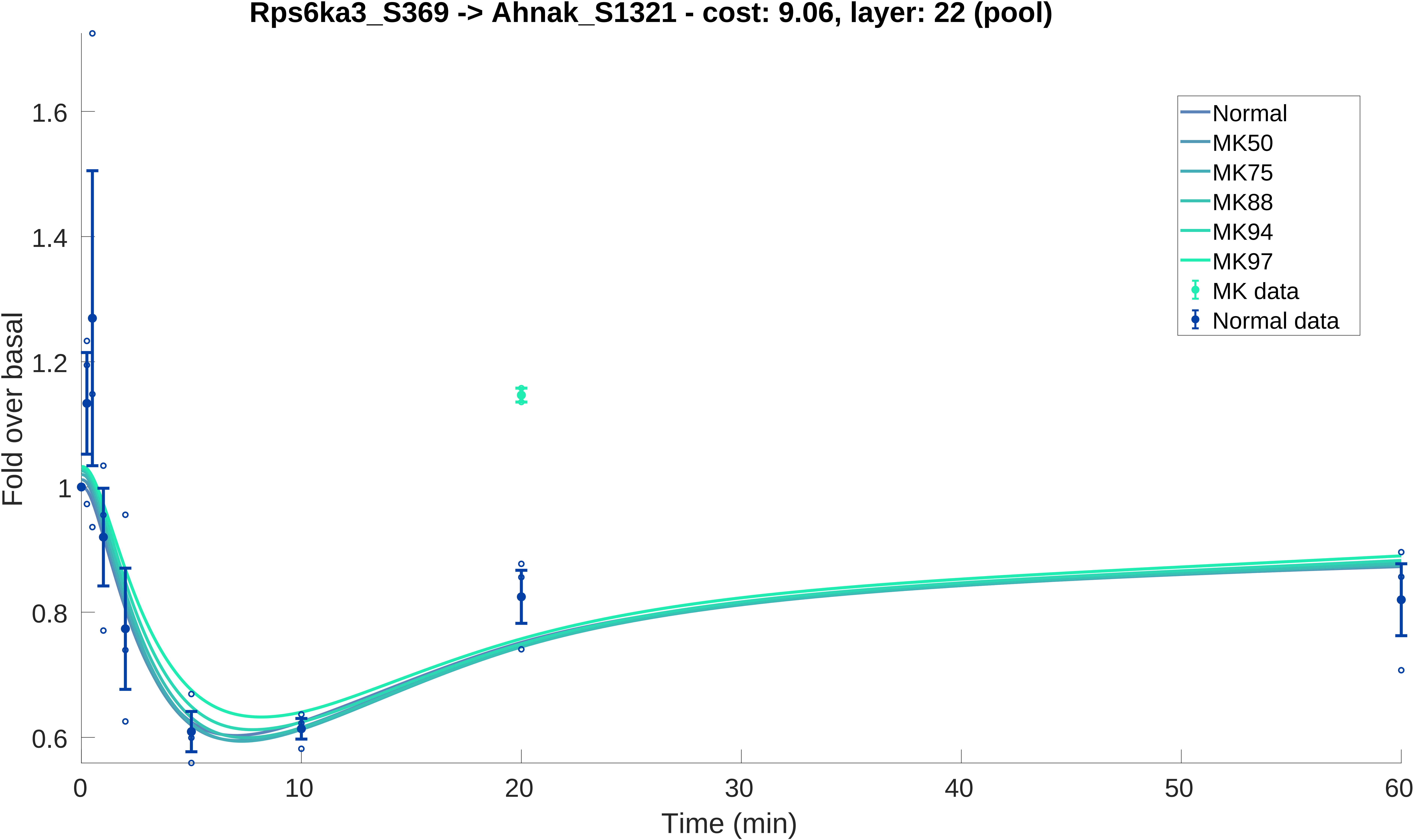
Rps6ka3_S369 -> Ahnak_S1321 - cost: 9.06, layer: 22 (pool)
Fold over basal
1.6
1.4
1.2
1
0.8
0.6
0
10
20
30
40
50
60
Time (min)
Normal
MK50
MK75
MK88
MK94
MK97
MK data
Normal data

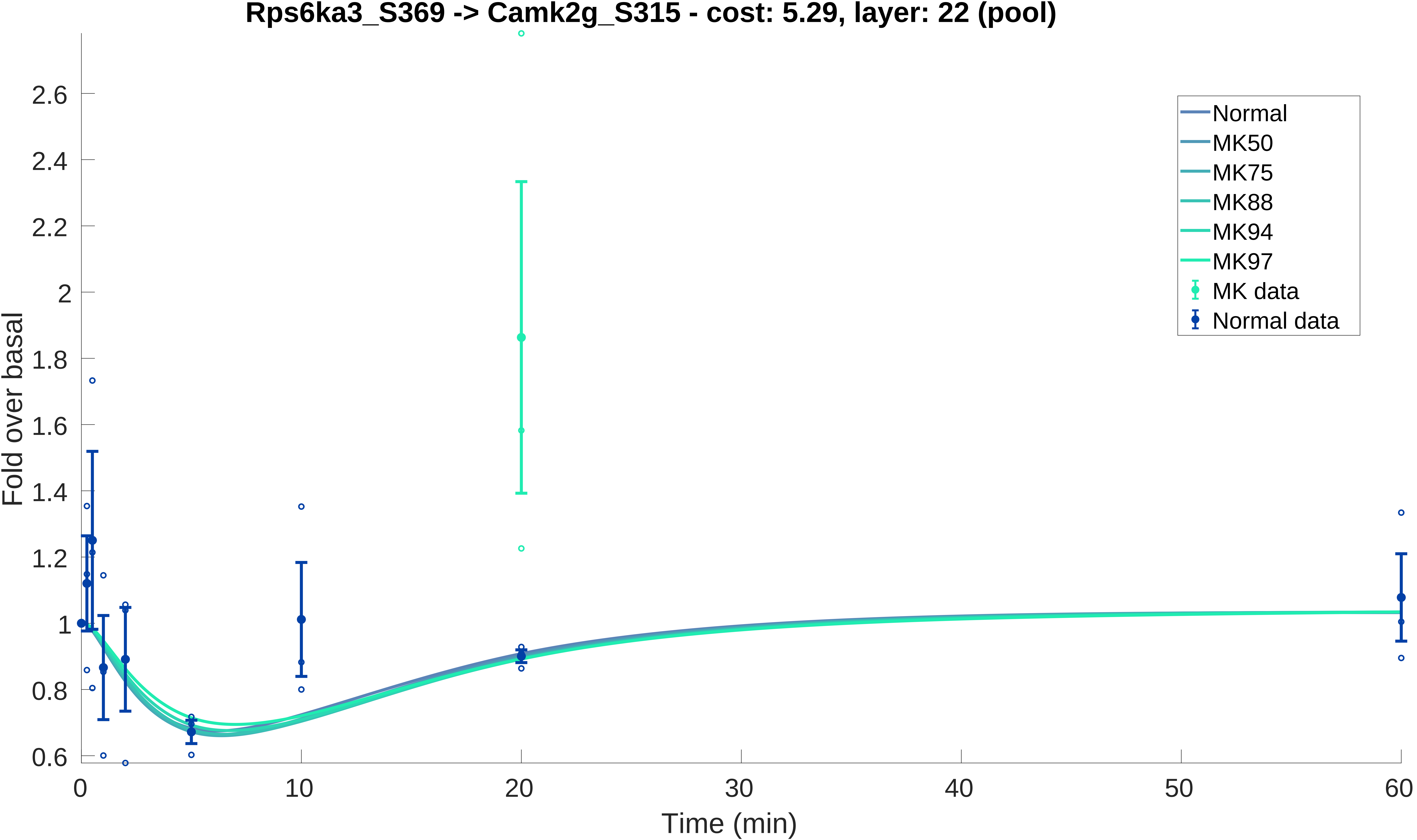

Rps6ka3_S369 -> Camk2g_S315 - cost: 5.29, layer: 22 (pool)
Fold over basal
Time (min)
0.6
0.8
1
1.2
1.4
1.6
1.8
2
2.2
2.4
2.6
0
10
20
30
40
50
60
Normal
MK50
MK75
MK88
MK94
MK97
MK data
Normal data

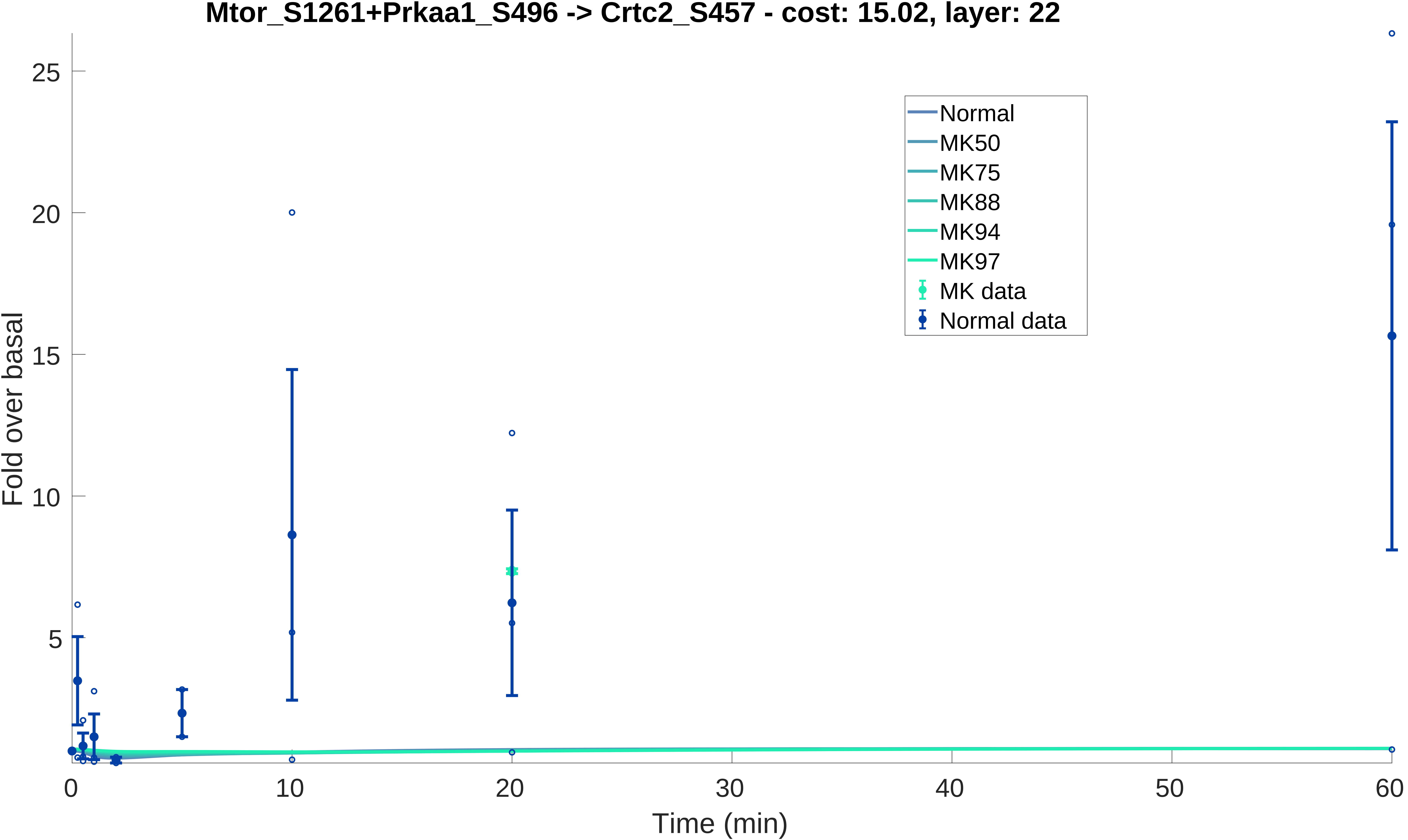

Mtor_S1261+Prkaa1_S496 -> Crtc2_S457 - cost: 15.02, layer: 22
Fold over basal
Time (min)
Normal
MK50
MK75
MK88
MK94
MK97
MK data
Normal data
0
10
20
30
40
50
60
5
10
15
20
25

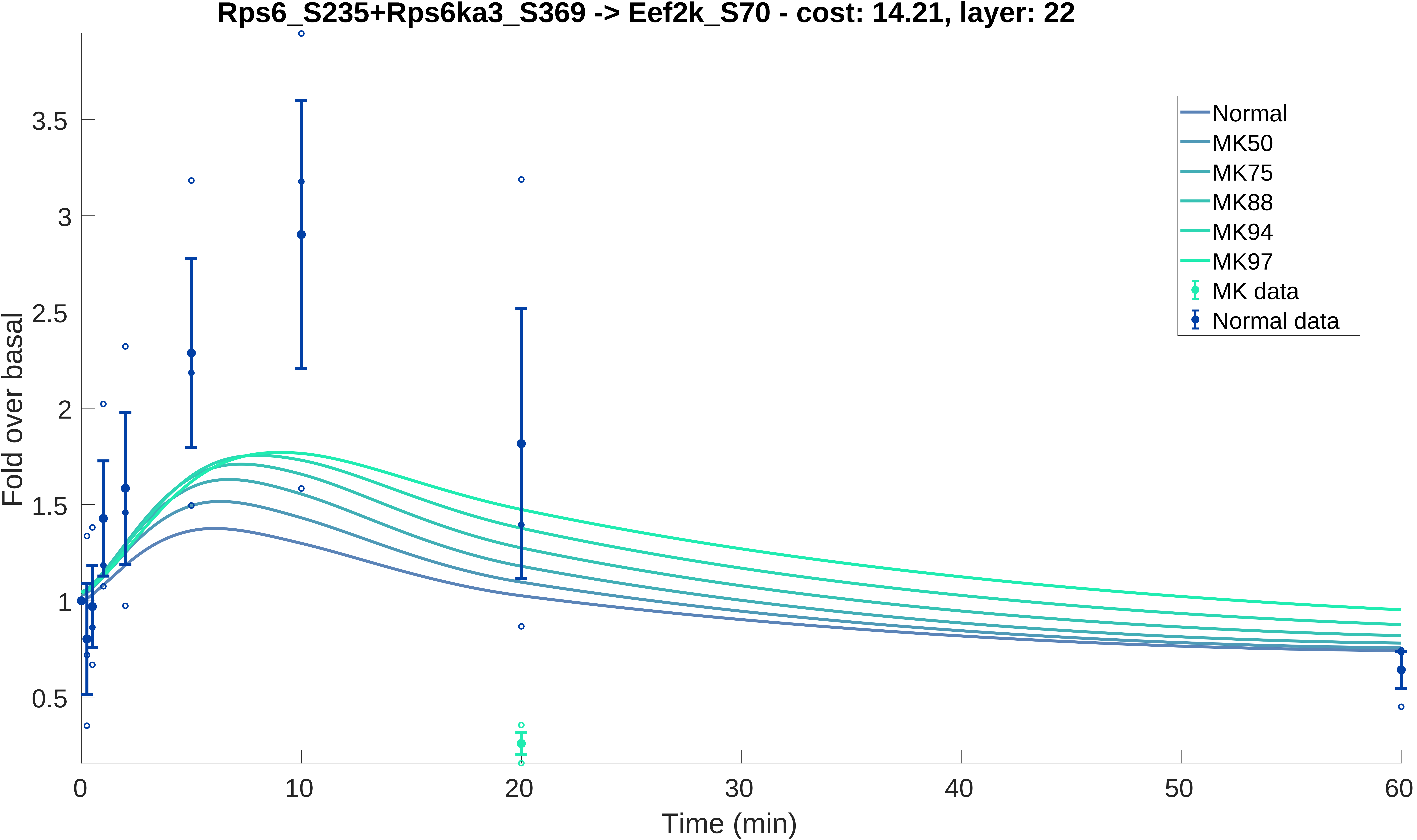
Rps6_S235+Rps6ka3_S369 -> Eef2k_S70 - cost: 14.21, layer: 22
Fold over basal
Time (min)
0.5
1
1.5
2
2.5
3
3.5
0
10
20
30
40
50
60
Normal
MK50
MK75
MK88
MK94
MK97
MK data
Normal data

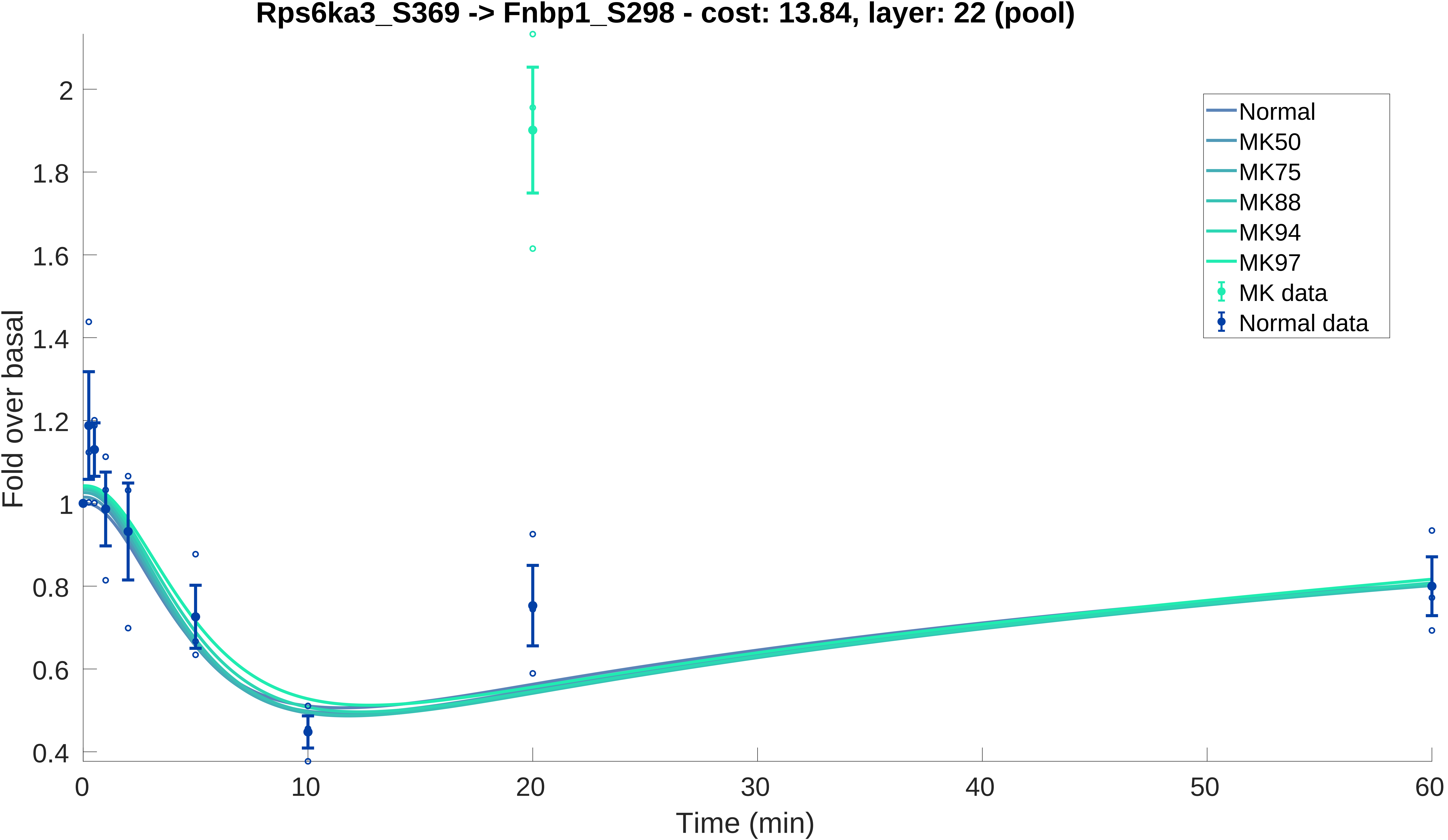
Rps6ka3_S369 -> Fnbp1_S298 - cost: 13.84, layer: 22 (pool)
Fold over basal
Time (min)
0.4
0.6
0.8
1
1.2
1.4
1.6
1.8
2
0
10
20
30
40
50
60
Normal
MK50
MK75
MK88
MK94
MK97
MK data
Normal data

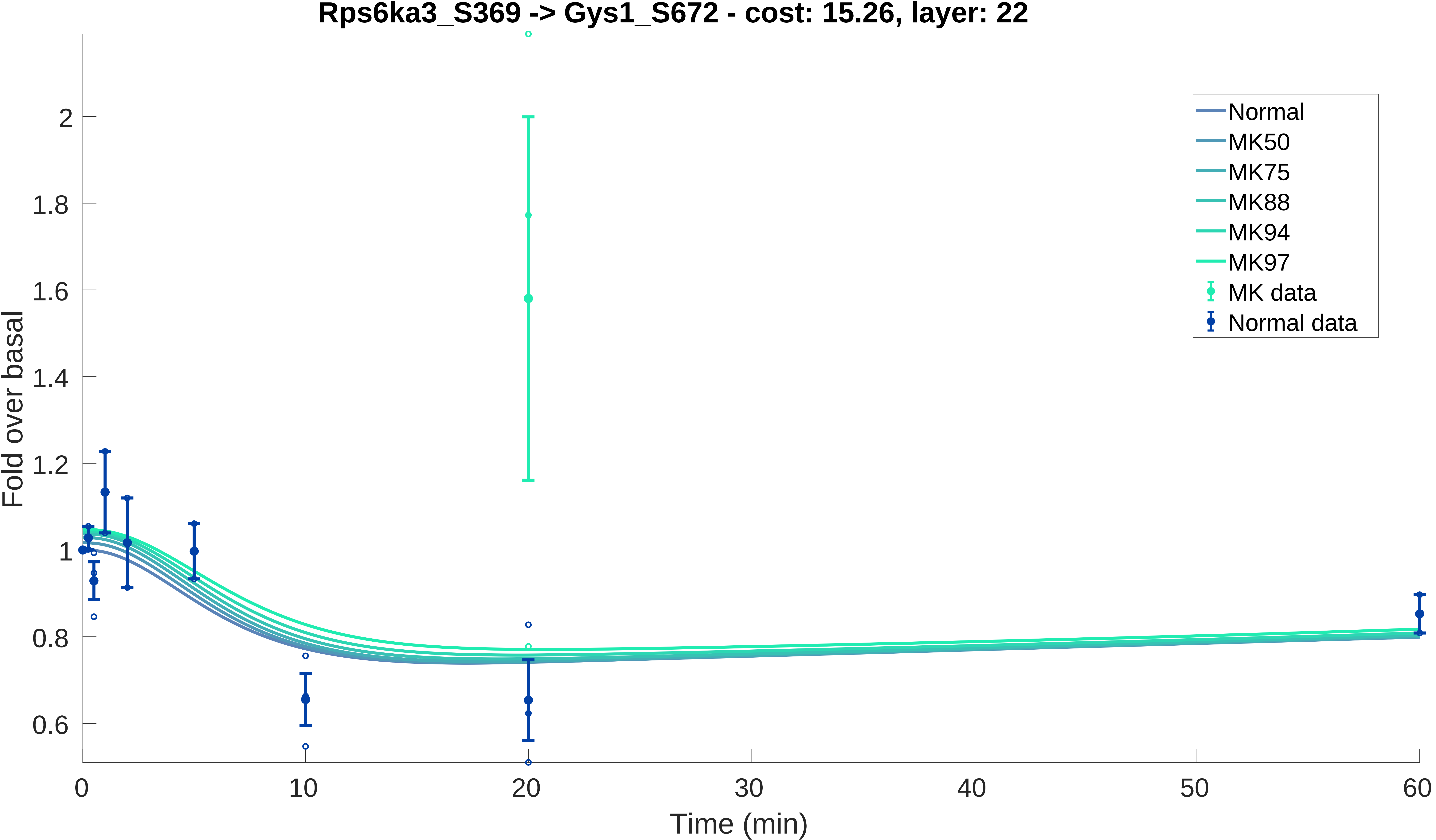

Rps6ka3_S369 -> Gys1_S672 - cost: 15.26, layer: 22
Fold over basal
Time (min)
0
10
20
30
40
50
60
0.6
0.8
1
1.2
1.4
1.6
1.8
2
Normal
MK50
MK75
MK88
MK94
MK97
MK data
Normal data

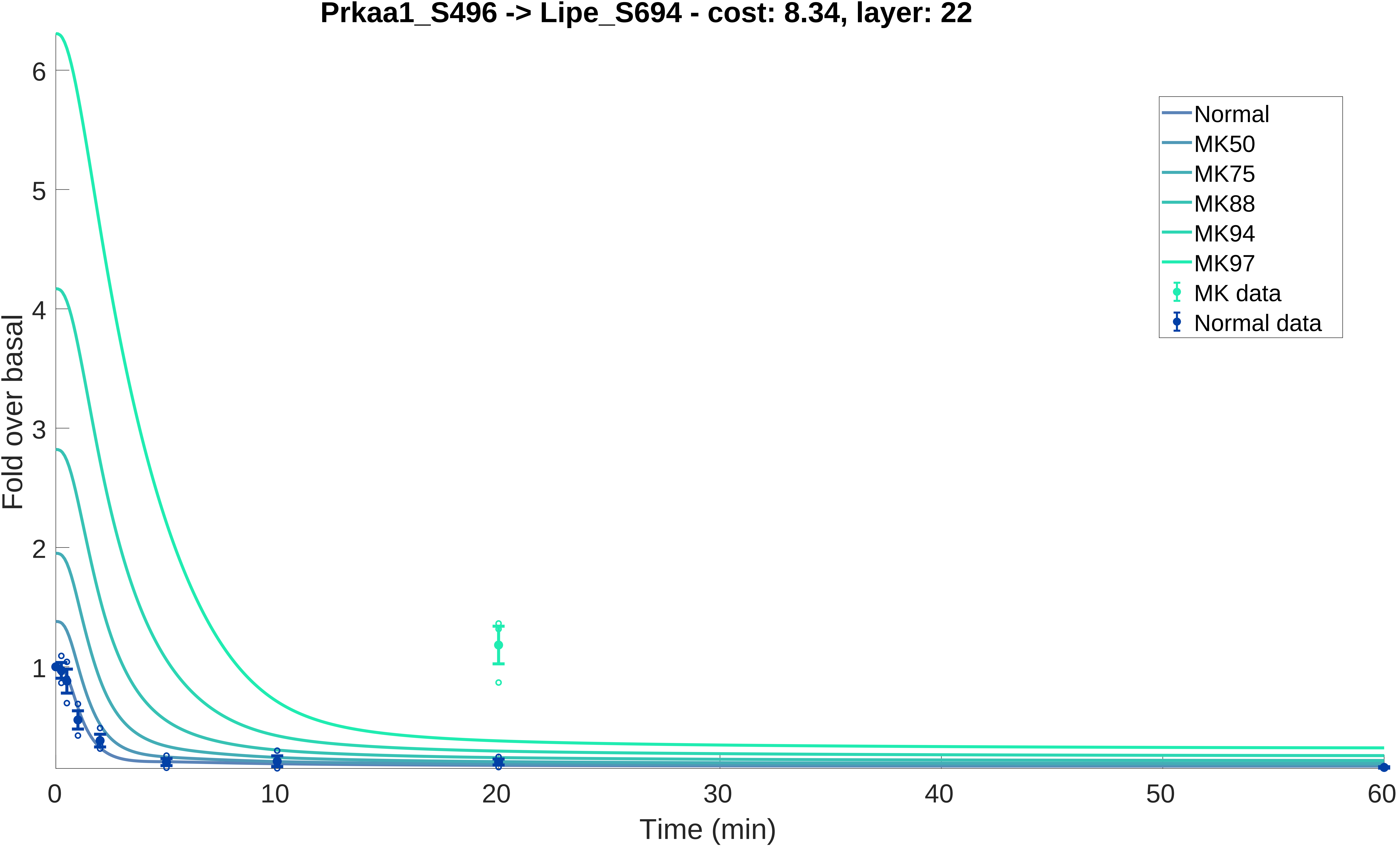
Prkaa1_S496 -> Lipe_S694 - cost: 8.34, layer: 22
Fold over basal
Time (min)
0
10
20
30
40
50
60
1
2
3
4
5
6
Normal
MK50
MK75
MK88
MK94
MK97
MK data
Normal data

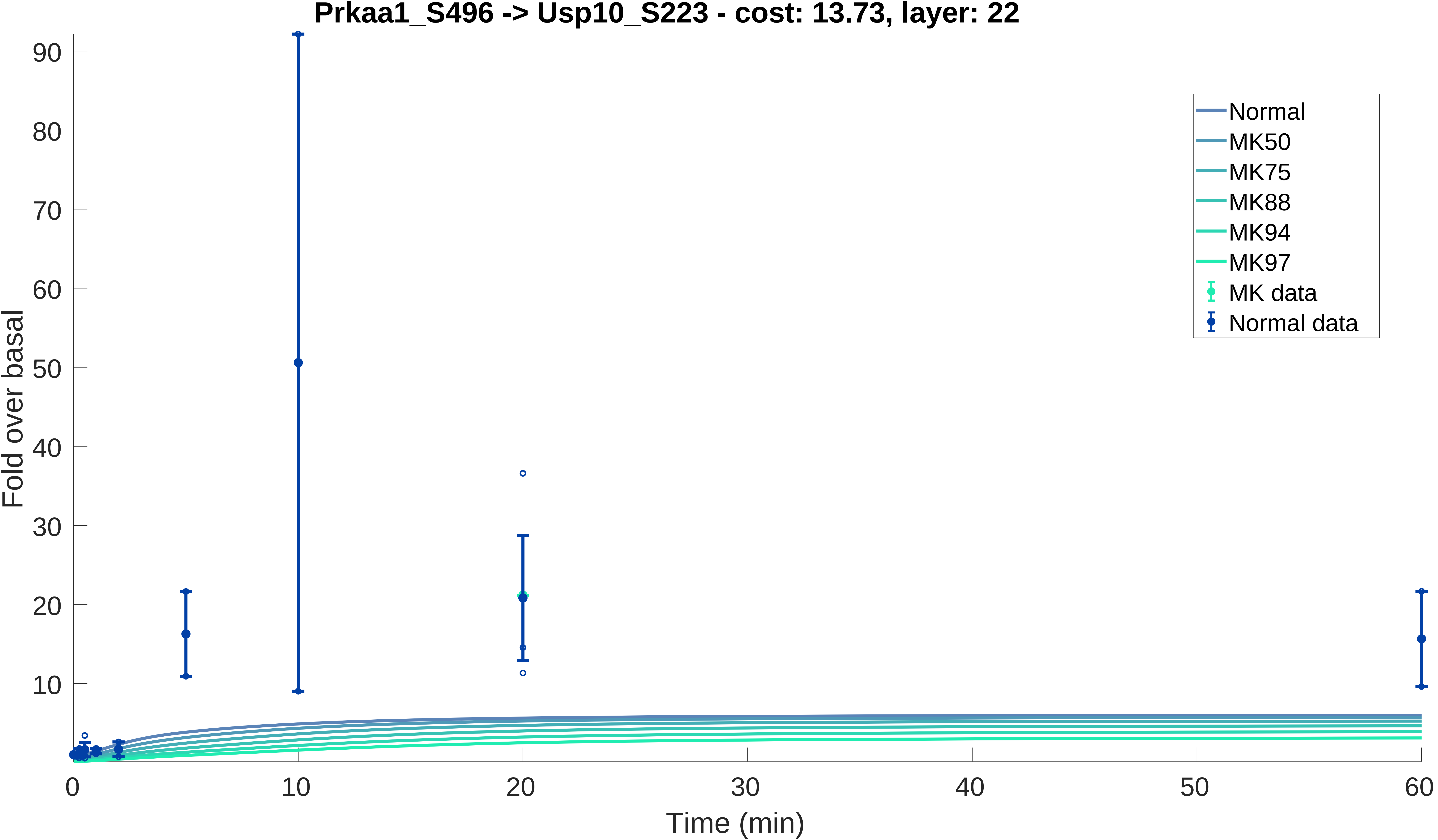

Prkaa1_S496 -> Usp10_S223 - cost: 13.73, layer: 22
Fold over basal
Time (min)
0
10
20
30
40
50
60
70
80
90
Normal
MK50
MK75
MK88
MK94
MK97
MK data
Normal data

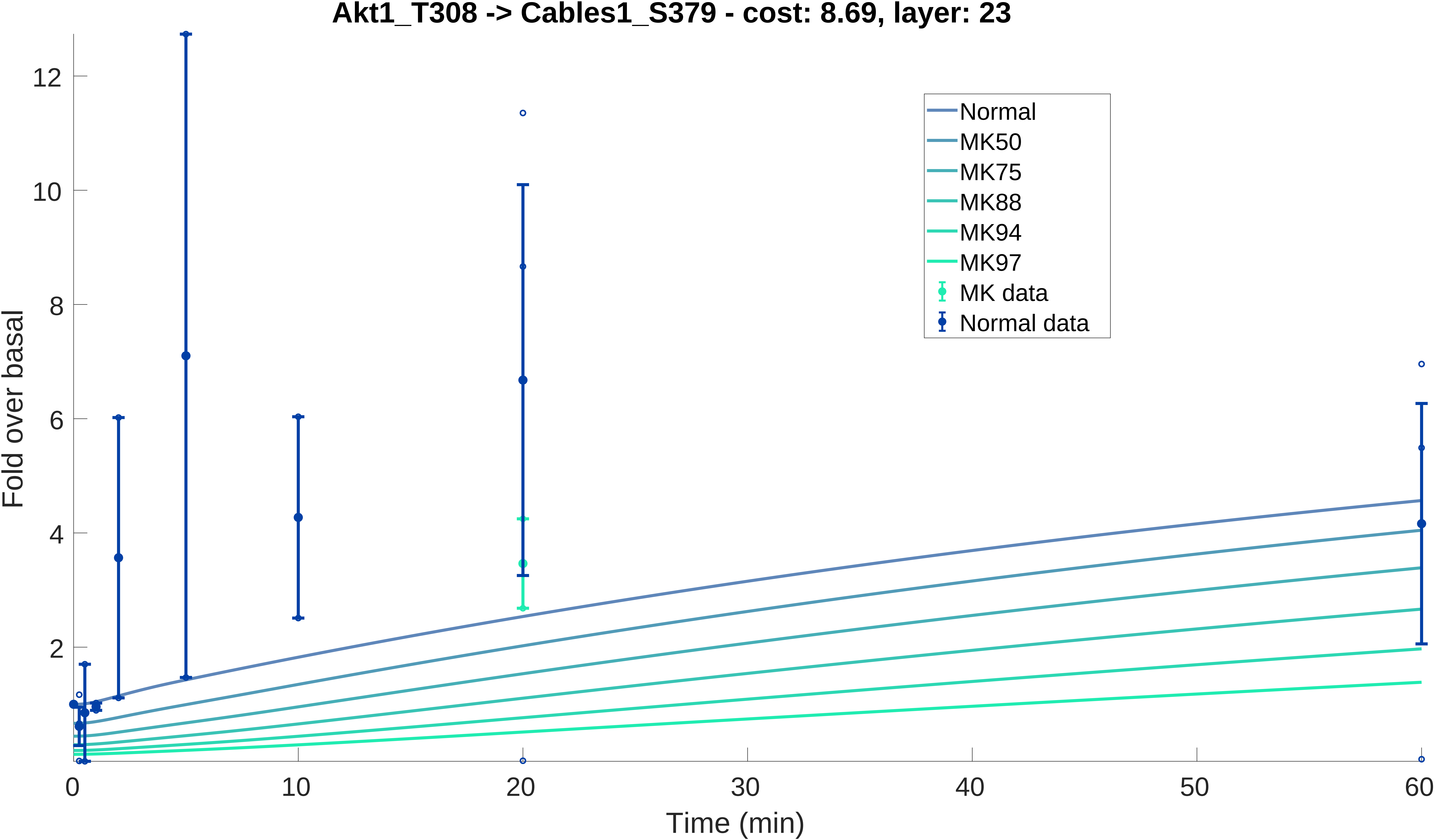
Akt1_T308 -> Cables1_S379 - cost: 8.69, layer: 23
Fold over basal
Time (min)
0
10
20
30
40
50
60
2
4
6
8
10
12
Normal
MK50
MK75
MK88
MK94
MK97
MK data
Normal data

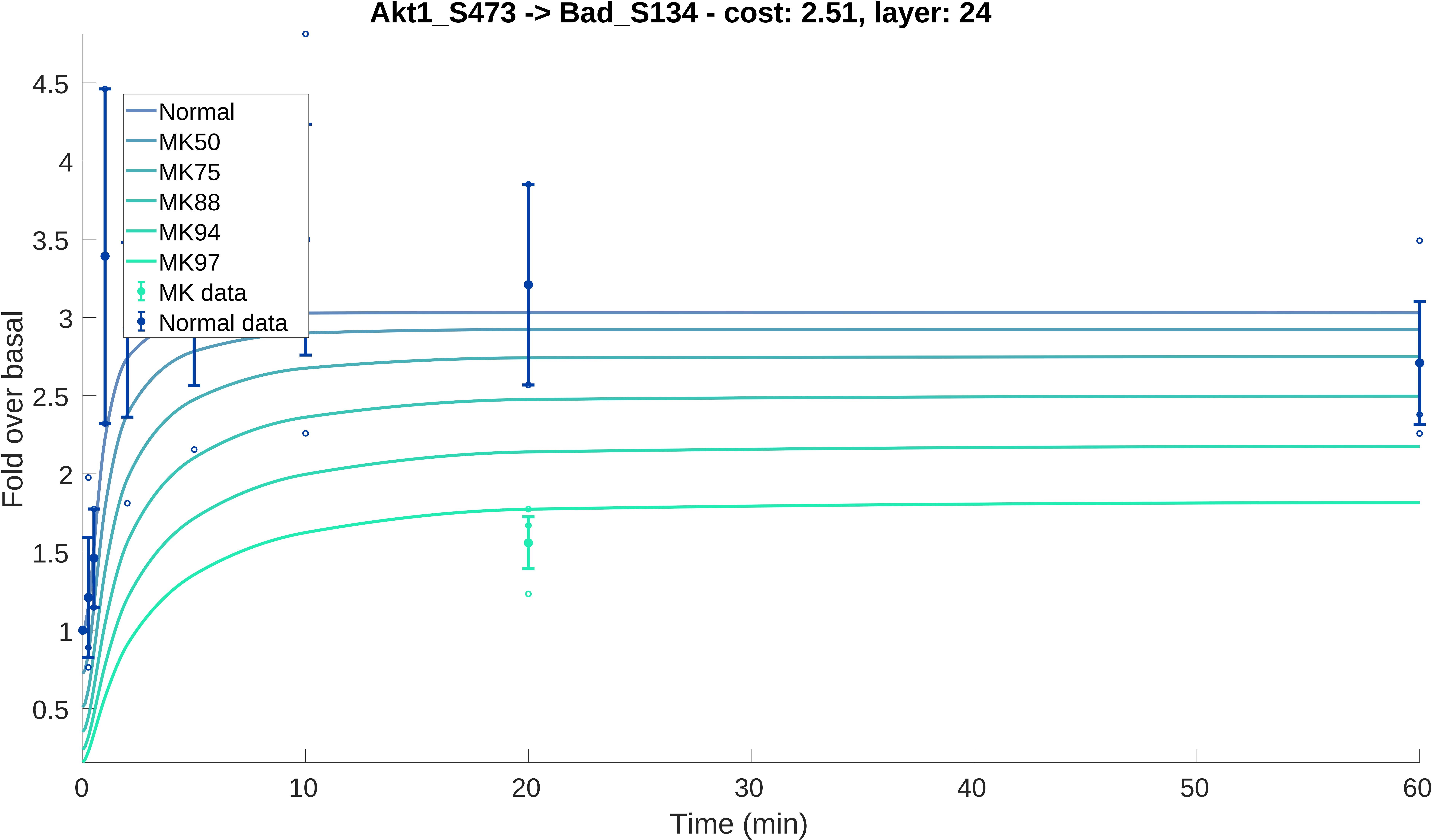

Akt1_S473 -> Bad_S134 - cost: 2.51, layer: 24
Normal
MK50
MK75
MK88
MK94
MK97
MK data
Normal data
Fold over basal
Time (min)
0.5
1
1.5
2
2.5
3
3.5
4
4.5
0
10
20
30
40
50
60

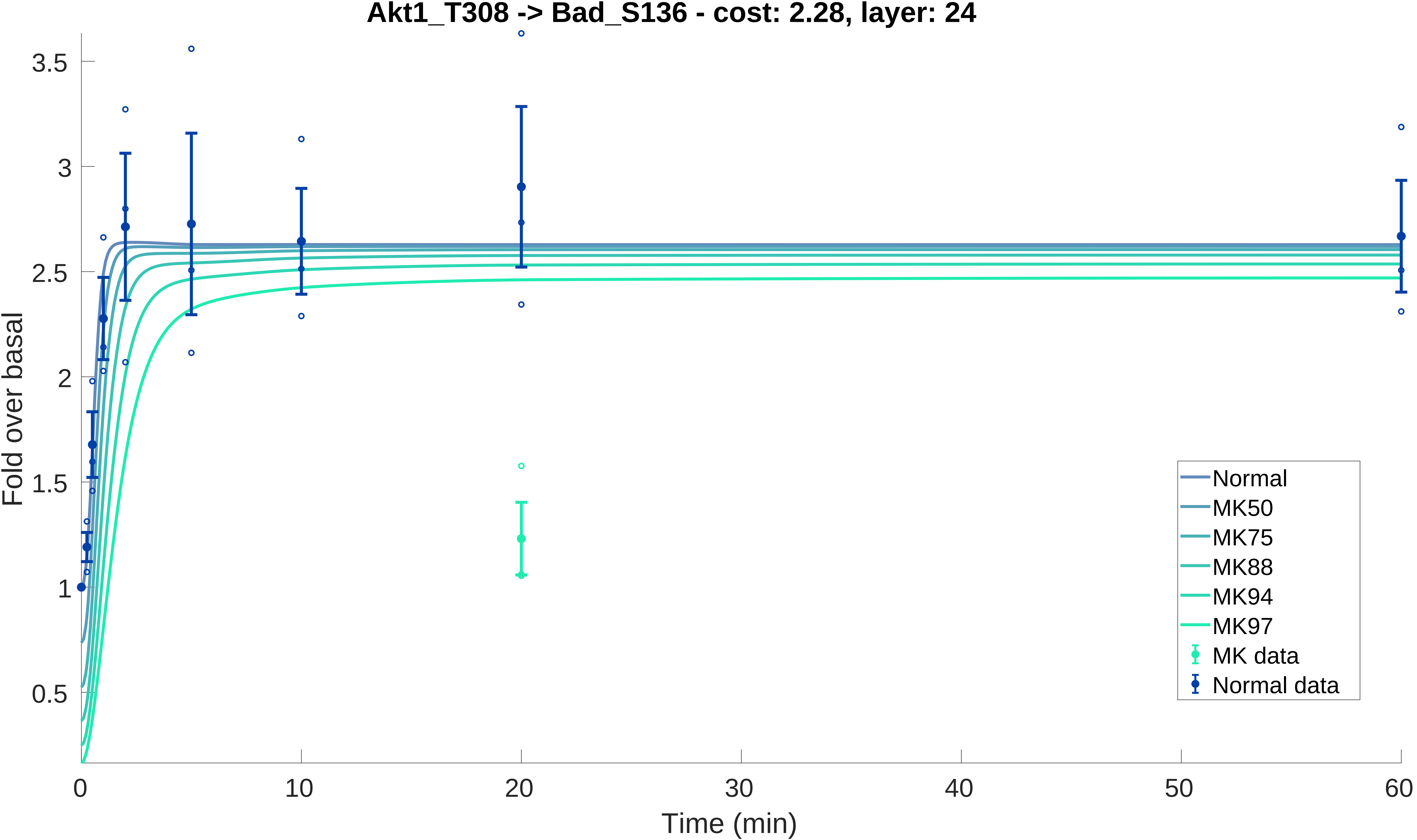

Akt1_T308 -> Bad_S136 - cost: 2.28, layer: 24
Fold over basal
Time (min)
0
10
20
30
40
50
60
0.5
1
1.5
2
2.5
3
3.5
Normal
MK50
MK75
MK88
MK94
MK97
MK data
Normal data

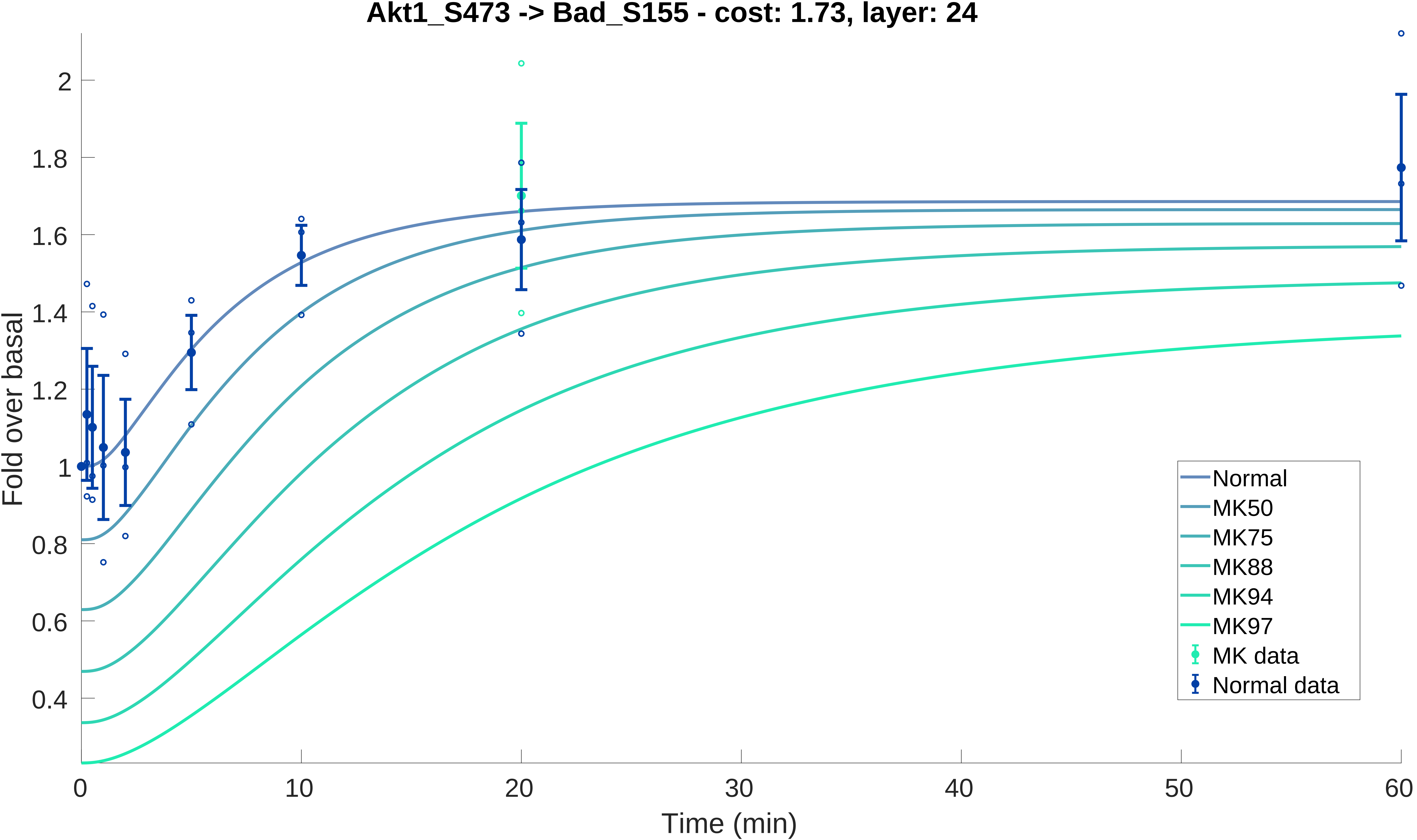

Akt1_S473 -> Bad_S155 - cost: 1.73, layer: 24
Fold over basal
Time (min)
0
10
20
30
40
50
60
0.4
0.6
0.8
1
1.2
1.4
1.6
1.8
2
Normal
MK50
MK75
MK88
MK94
MK97
MK data
Normal data

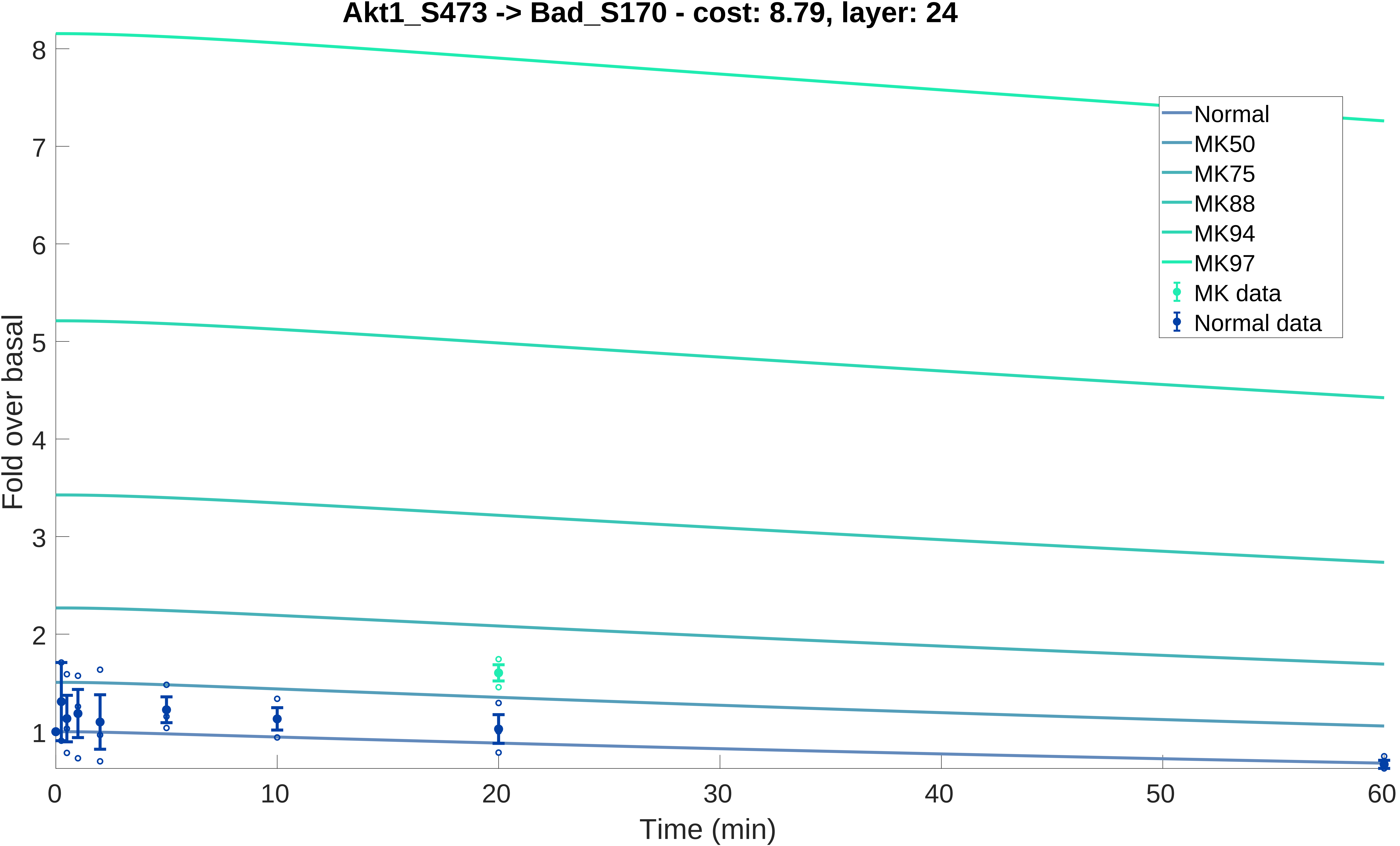

Akt1_S473 -> Bad_S170 - cost: 8.79, layer: 24
Fold over basal
Time (min)
0
10
20
30
40
50
60
1
2
3
4
5
6
7
8
Normal
MK50
MK75
MK88
MK94
MK97
MK data
Normal data

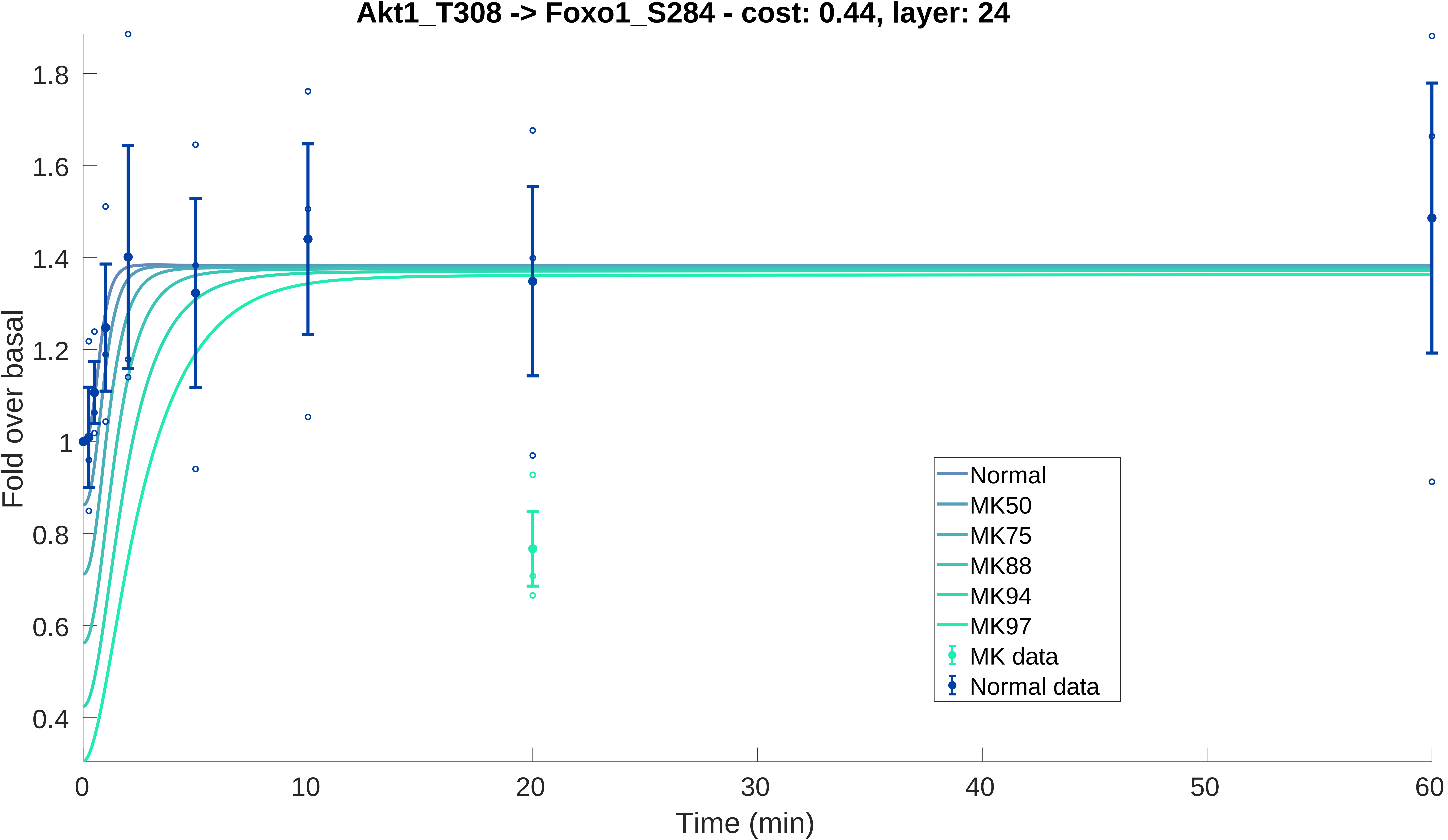

Akt1_T308 -> Foxo1_S284 - cost: 0.44, layer: 24
Fold over basal
1.8
1.6
1.4
1.2
1
0.8
0.6
0.4
0
10
20
30
40
50
60
Time (min)
Normal
MK50
MK75
MK88
MK94
MK97
MK data
Normal data

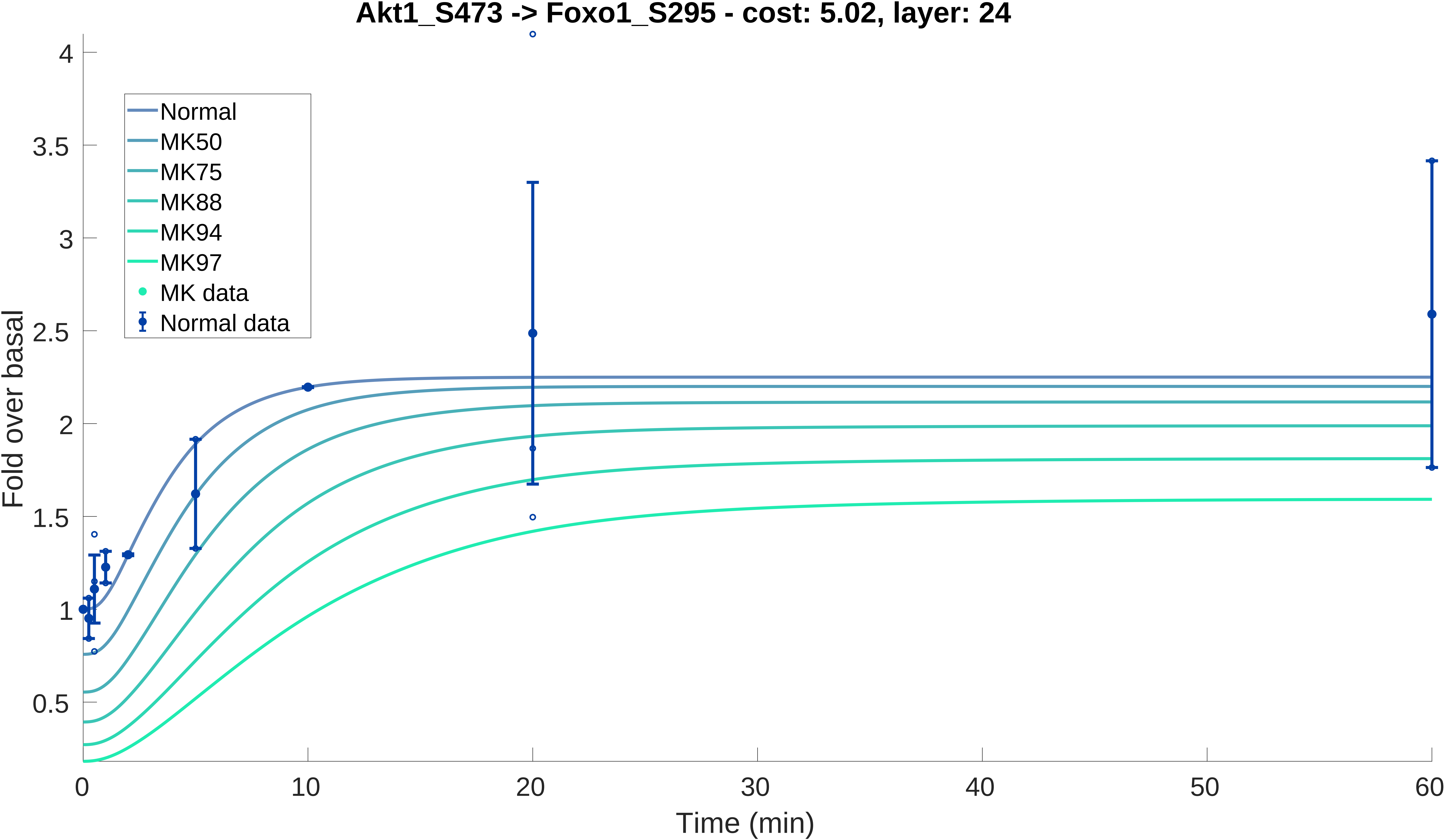

Akt1_S473 -> Foxo1_S295 - cost: 5.02, layer: 24
Fold over basal
Time (min)
Normal
MK50
MK75
MK88
MK94
MK97
MK data
Normal data
0.5
1
1.5
2
2.5
3
3.5
4
0
10
20
30
40
50
60

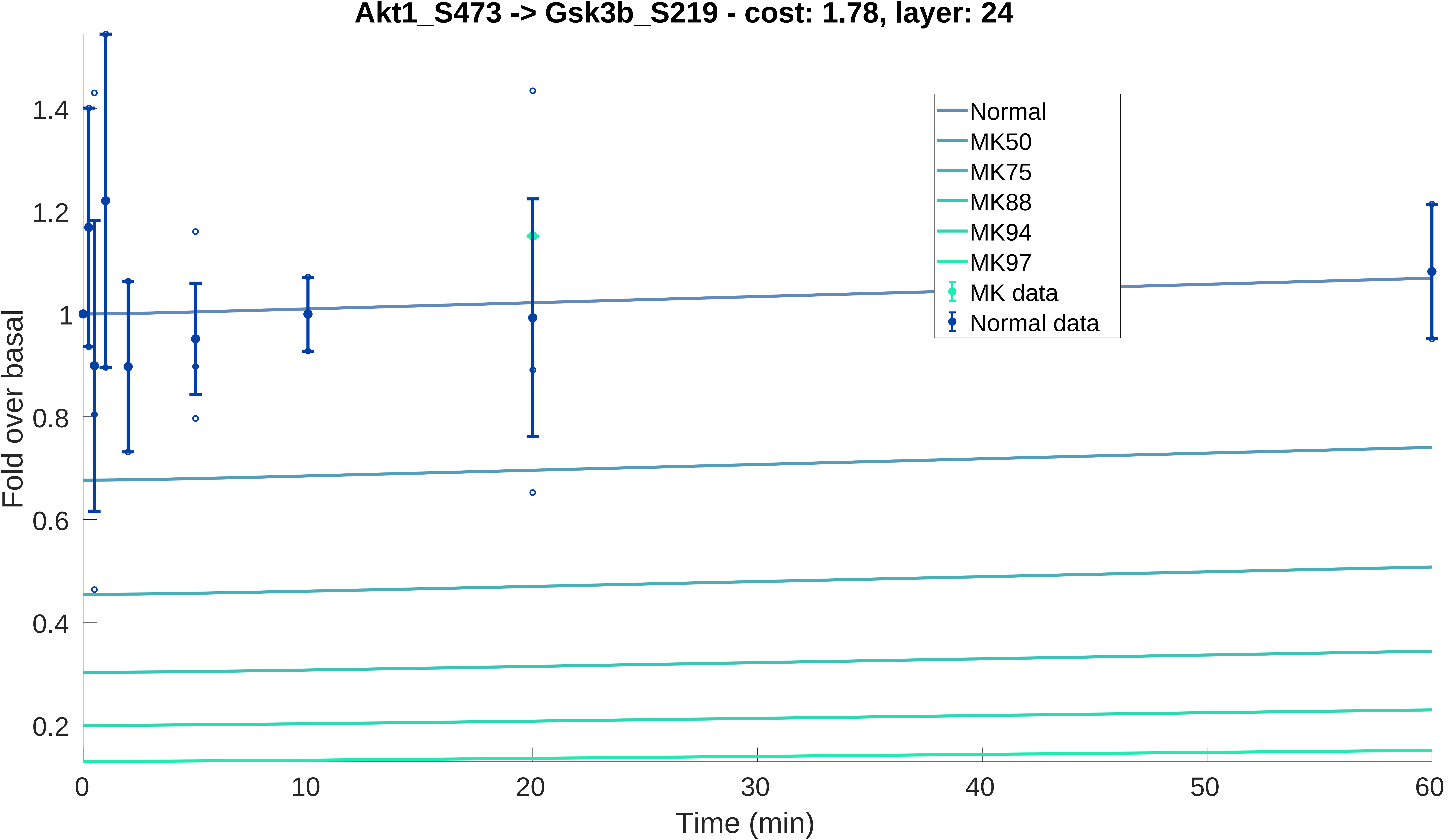
Akt1_S473 -> Gsk3b_S219 - cost: 1.78, layer: 24
Fold over basal
0.2
0.4
0.6
0.8
1
1.2
1.4
0
10
20
30
40
50
60
Time (min)
Normal
MK50
MK75
MK88
MK94
MK97
MK data
Normal data

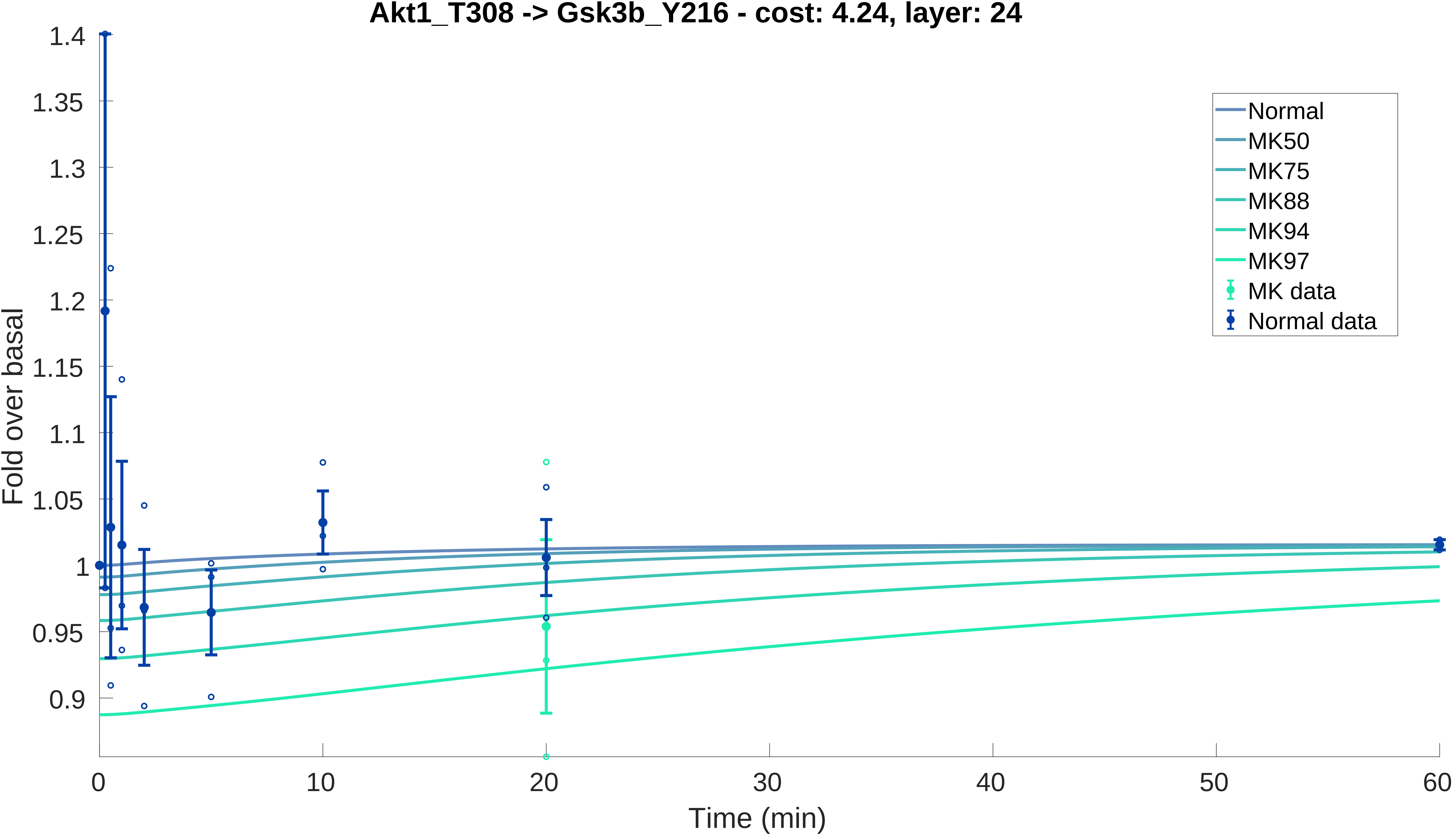

Akt1_T308 -> Gsk3b_Y216 - cost: 4.24, layer: 24
Fold over basal
Time (min)
1.4
1.35
1.3
1.25
1.2
1.15
1.1
1.05
1
0.95
0.9
0
10
20
30
40
50
60
Normal
MK50
MK75
MK88
MK94
MK97
MK data
Normal data

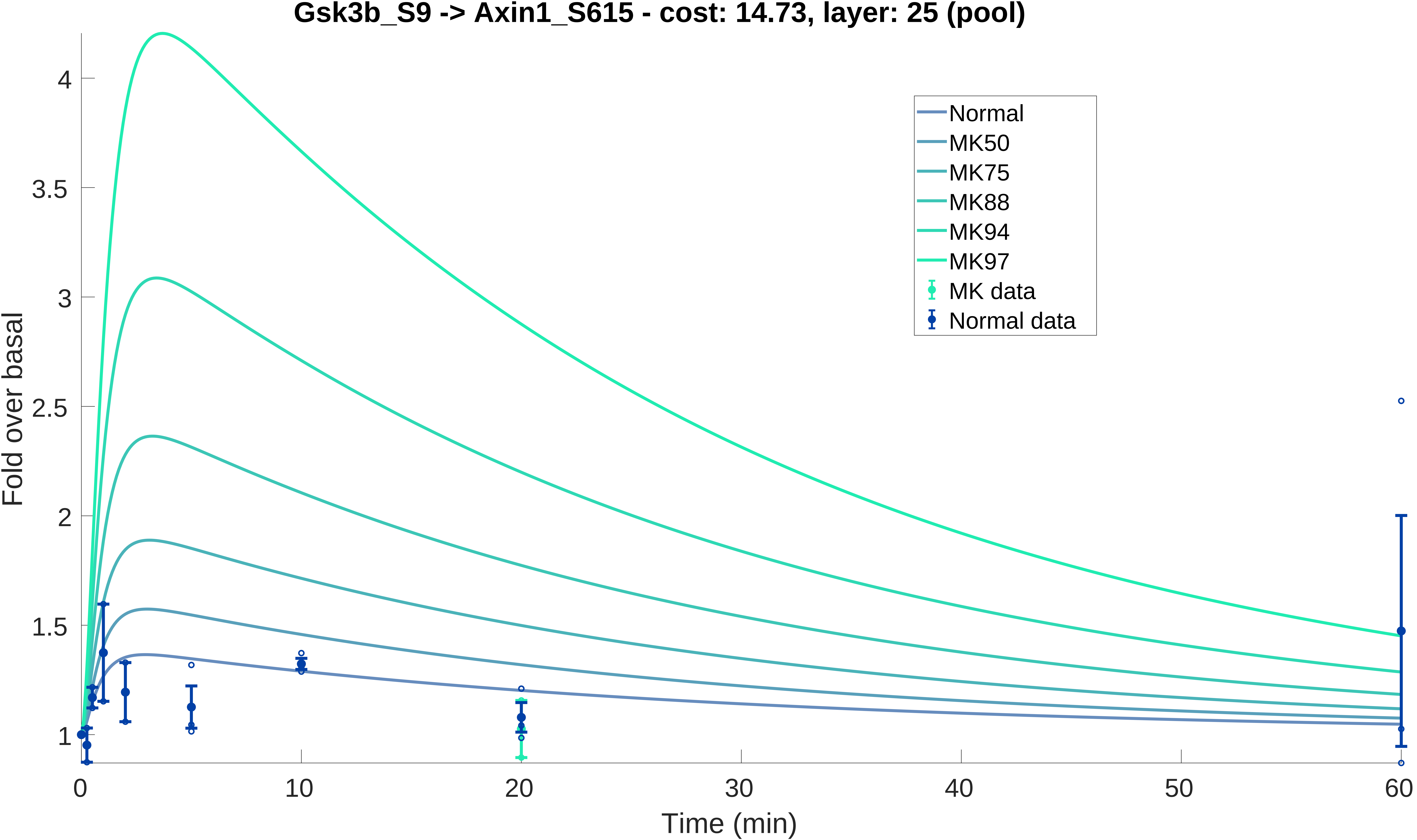

Gsk3b_S9 -> Axin1_S615 - cost: 14.73, layer: 25 (pool)
Normal
MK50
MK75
MK88
MK94
MK97
MK data
Normal data
Fold over basal
Time (min)
4
3.5
3
2.5
2
1.5
1
0
10
20
30
40
50
60

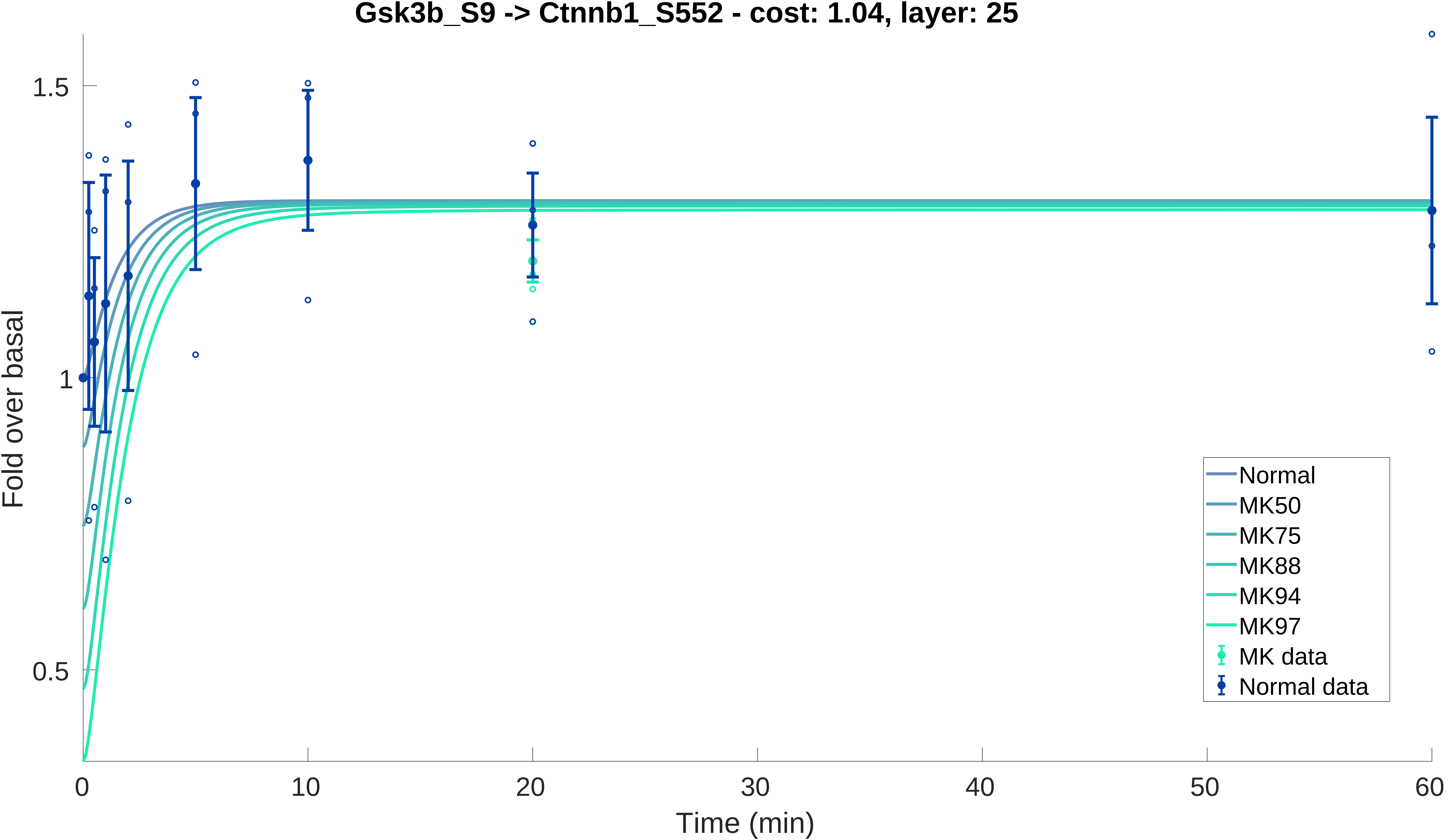
Gsk3b_S9 -> Ctnnb1_S552 - cost: 1.04, layer: 25
Fold over basal
1.5
1
0.5
0
10
20
30
40
50
60
Time (min)
Normal
MK50
MK75
MK88
MK94
MK97
MK data
Normal data

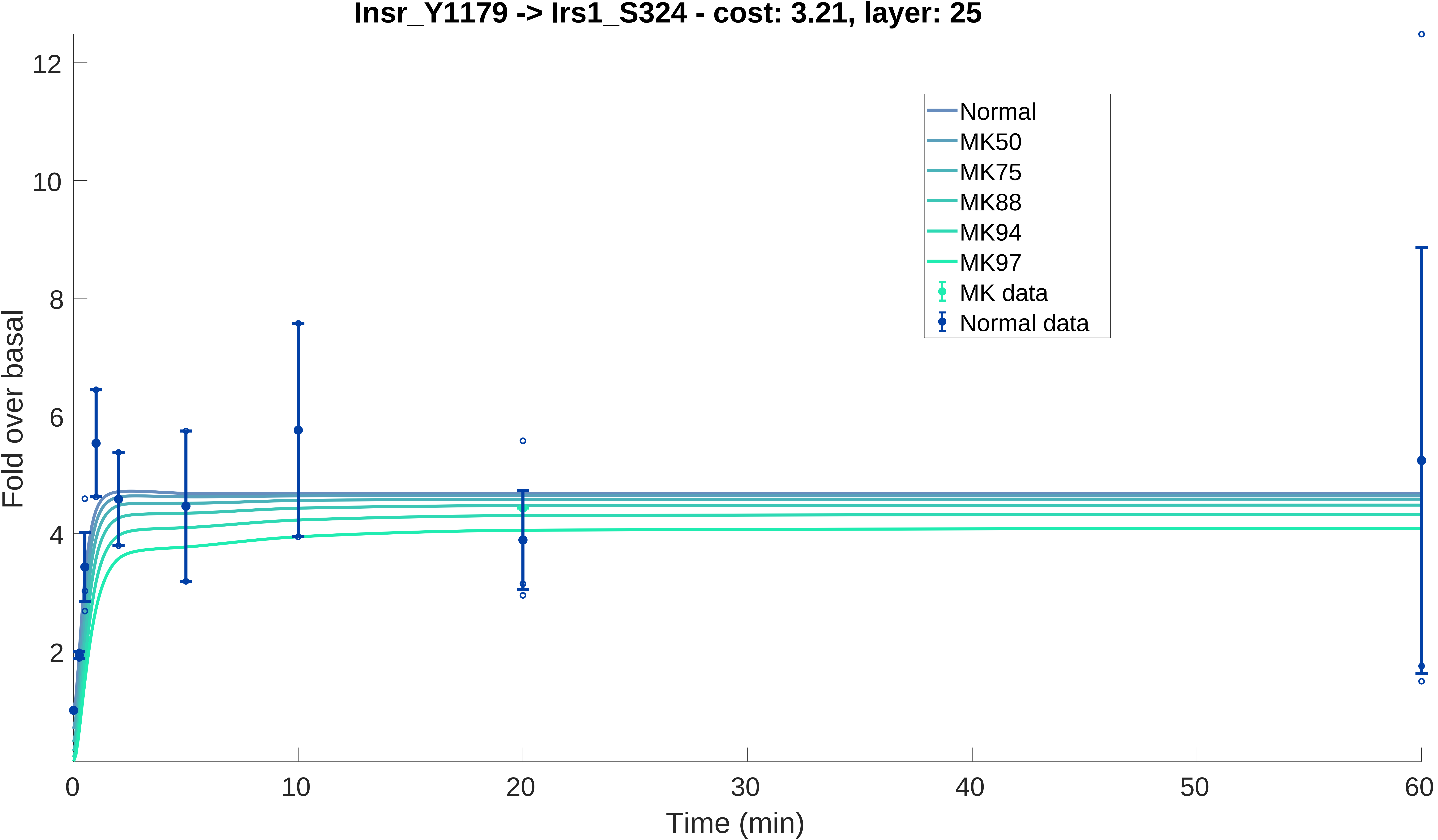

Insr_Y1179 -> Irs1_S324 - cost: 3.21, layer: 25
Fold over basal
Time (min)
0
10
20
30
40
50
60
2
4
6
8
10
12
Normal
MK50
MK75
MK88
MK94
MK97
MK data
Normal data

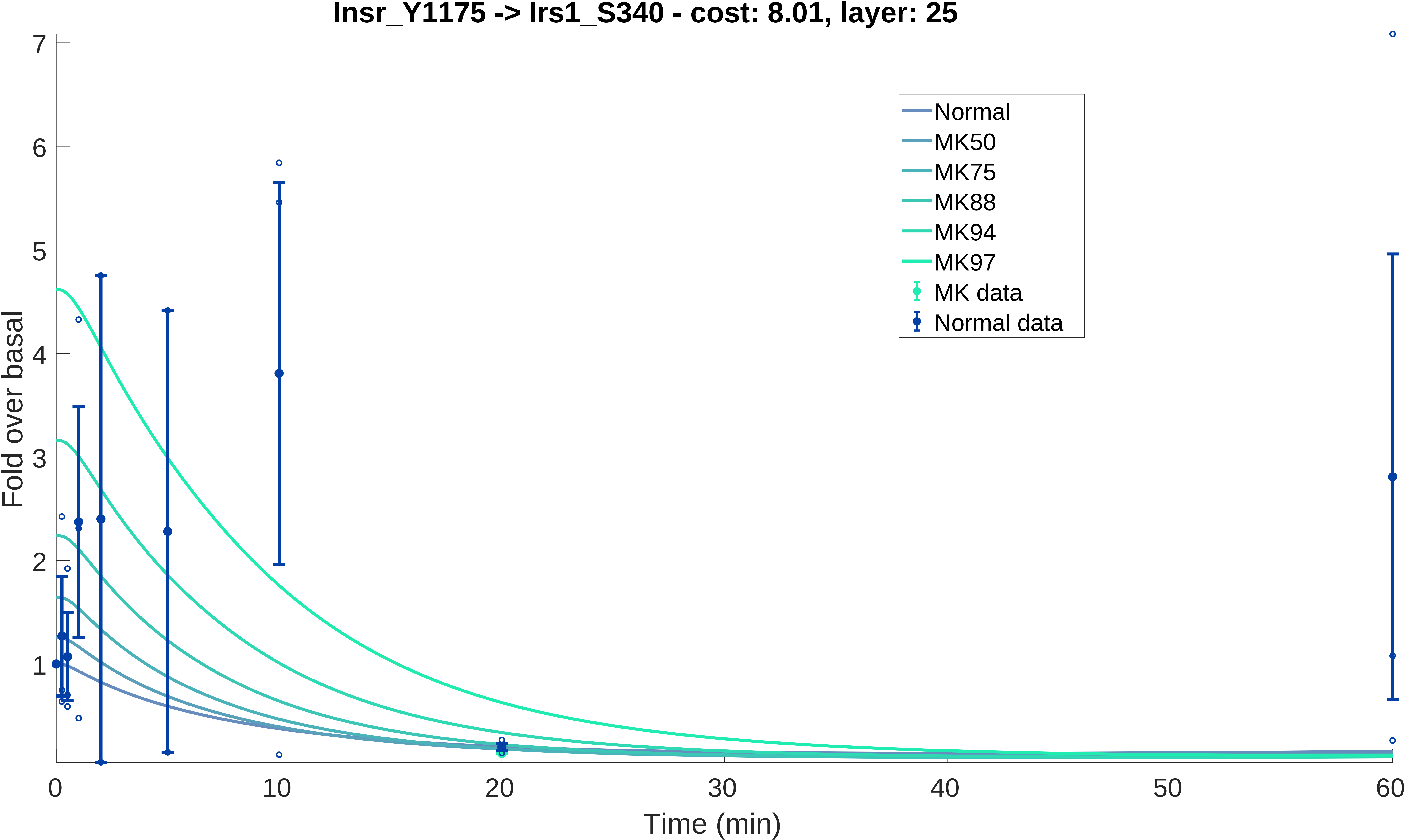

Insr_Y1175 -> Irs1_S340 - cost: 8.01, layer: 25
Fold over basal
Time (min)
Normal
MK50
MK75
MK88
MK94
MK97
MK data
Normal data
0
10
20
30
40
50
60
1
2
3
4
5
6
7

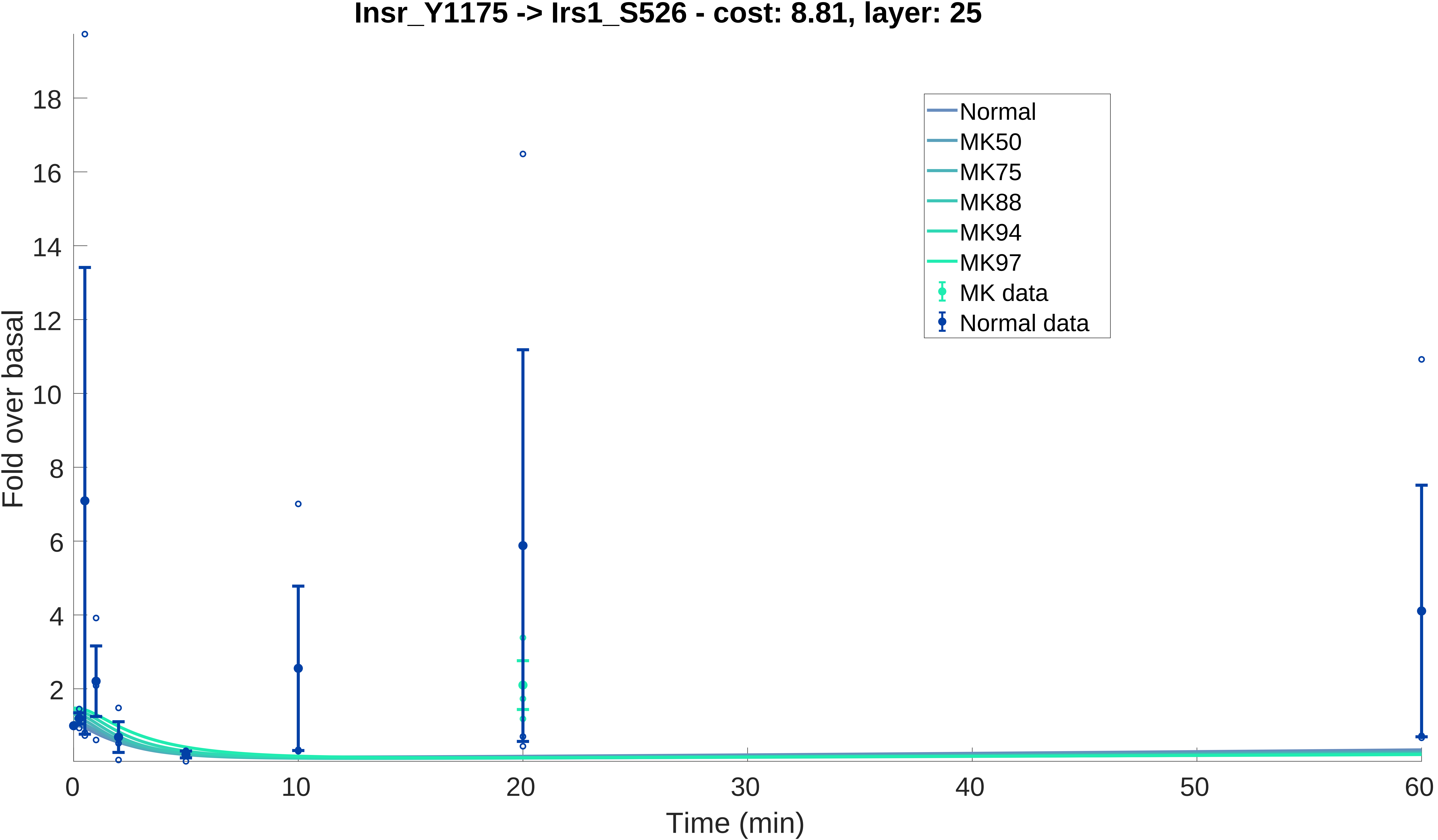

Insr_Y1175 -> Irs1_S526 - cost: 8.81, layer: 25
Fold over basal
Time (min)
0
10
20
30
40
50
60
2
4
6
8
10
12
14
16
18
Normal
MK50
MK75
MK88
MK94
MK97
MK data
Normal data

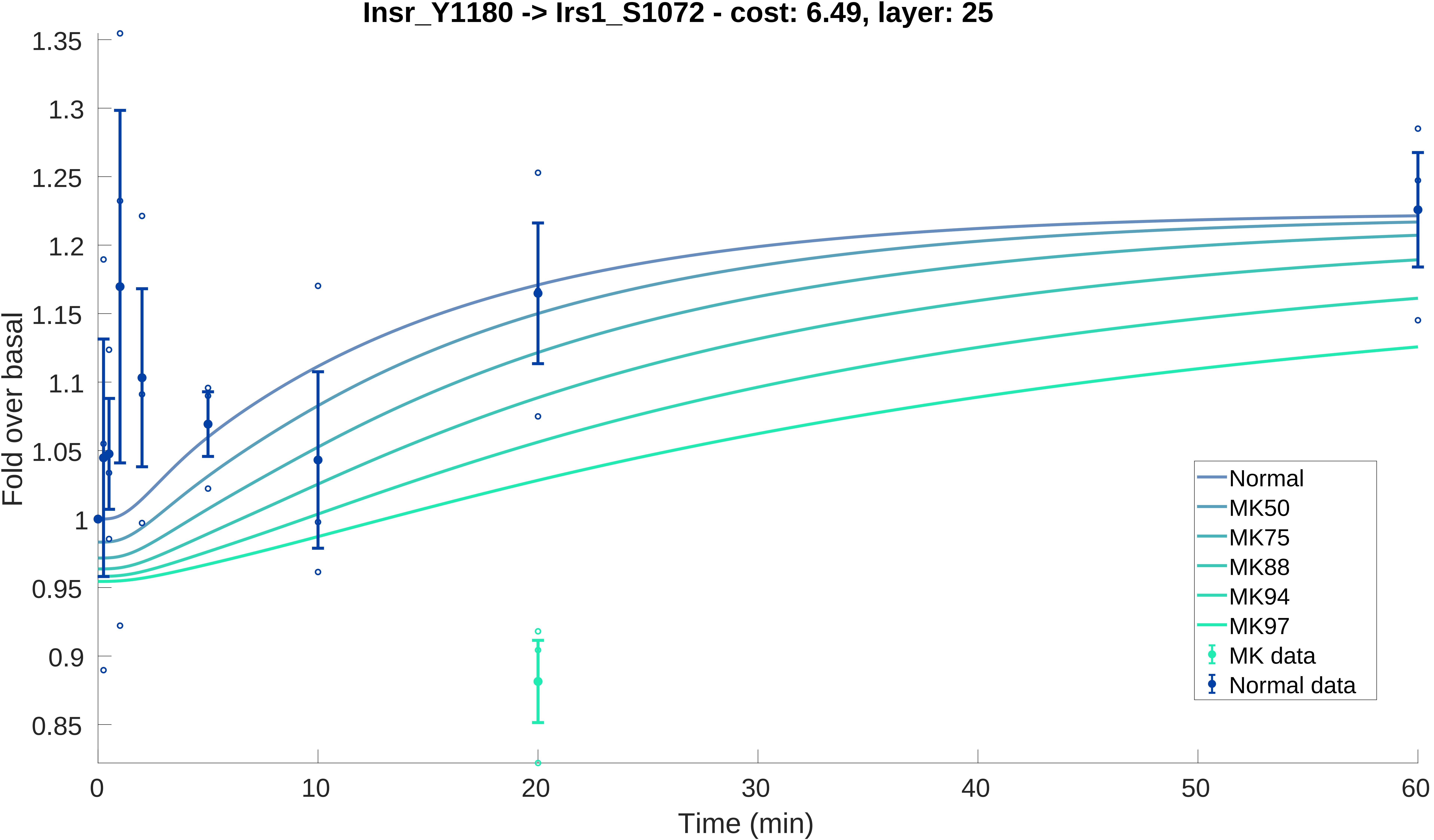

Insr_Y1180 -> Irs1_S1072 - cost: 6.49, layer: 25
1.35
1.3
1.25
1.2
1.15
1.1
1.05
1
0.95
0.9
0.85
Fold over basal
0
10
20
30
40
50
60
Time (min)
Normal
MK50
MK75
MK88
MK94
MK97
MK data
Normal data

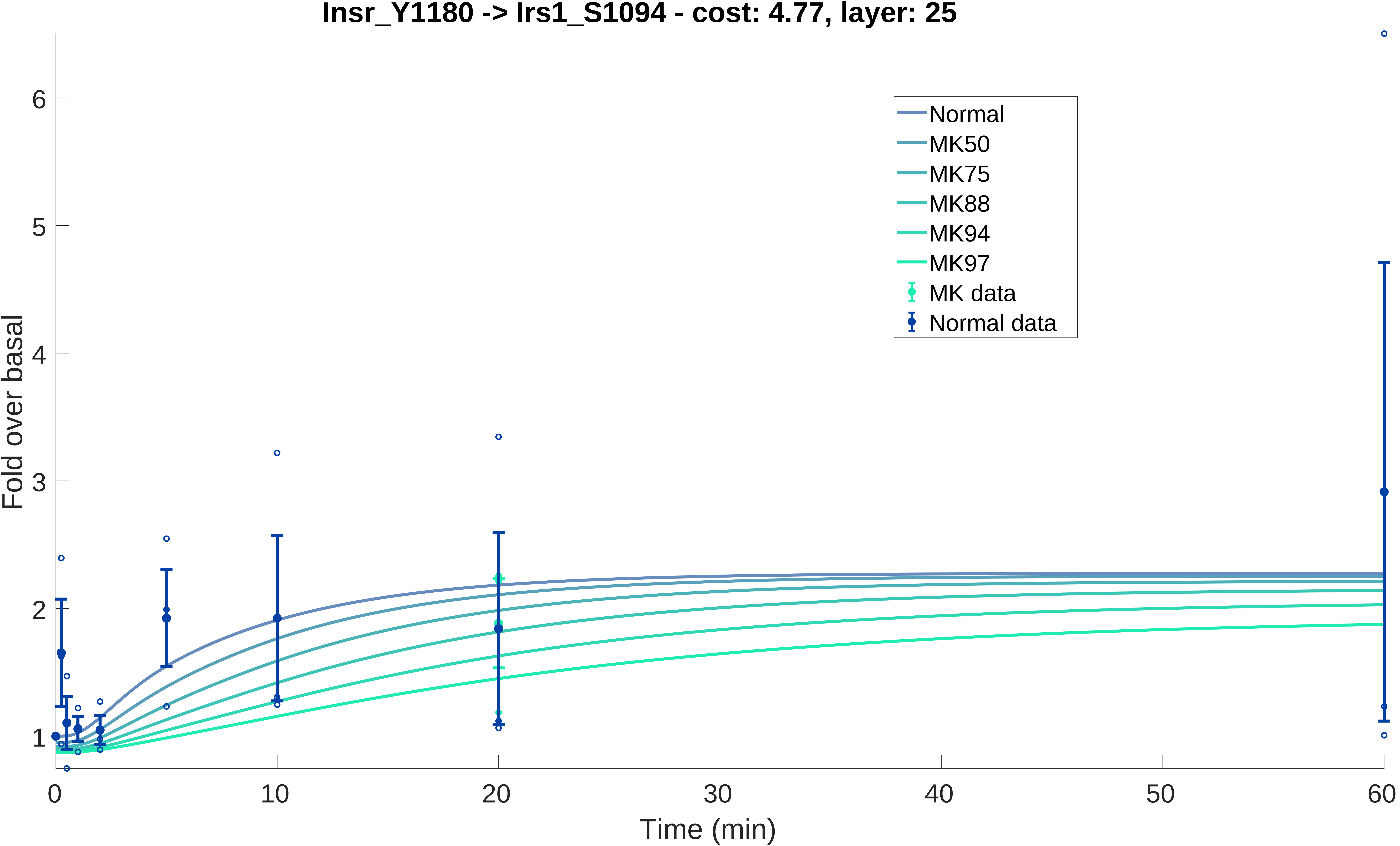

Insr_Y1180 -> Irs1_S1094 - cost: 4.77, layer: 25
Normal
MK50
MK75
MK88
MK94
MK97
MK data
Normal data
Fold over basal
Time (min)
1
2
3
4
5
6
0
10
20
30
40
50
60

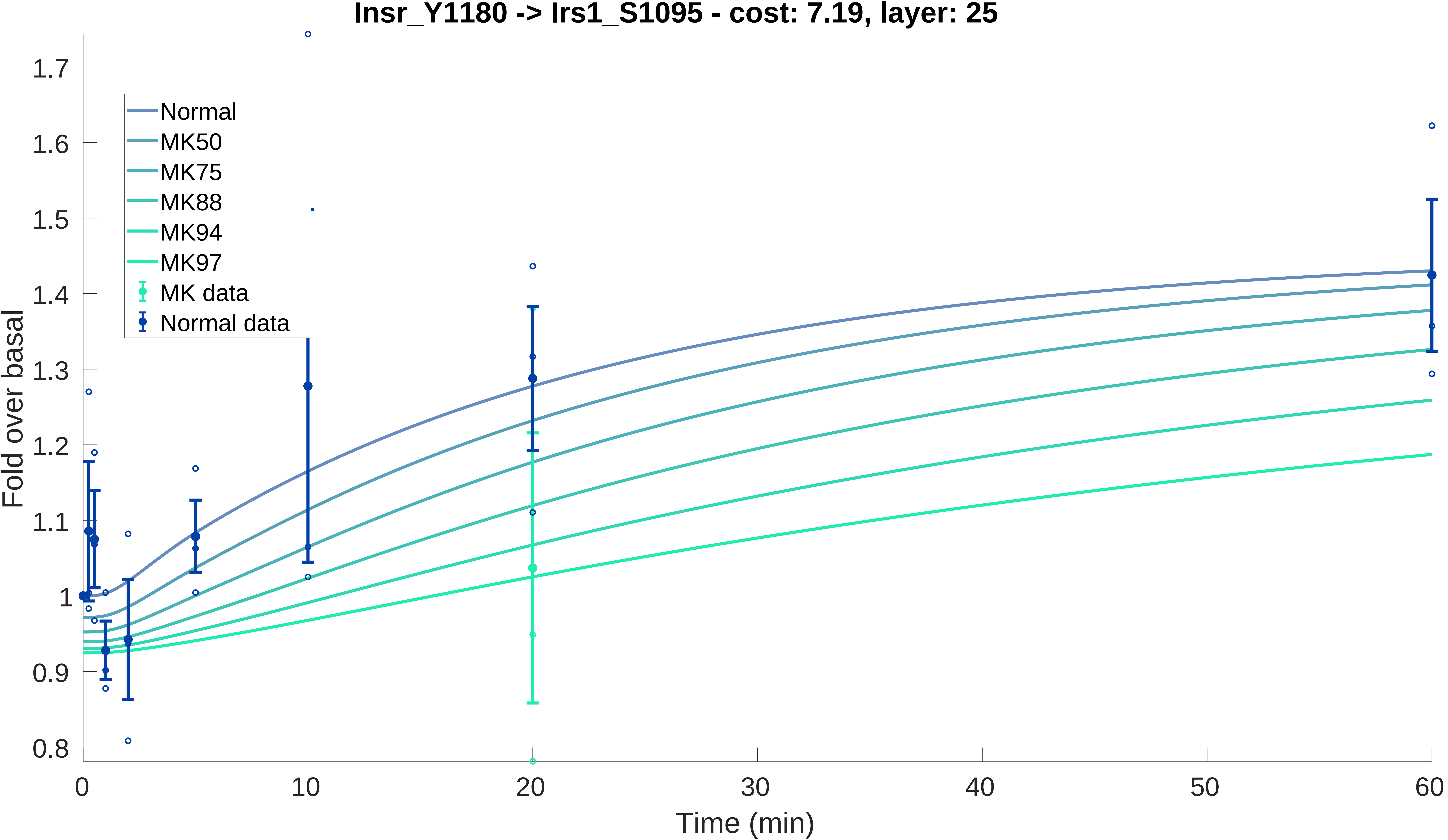
Insr_Y1180 -> Irs1_S1095 - cost: 7.19, layer: 25
Fold over basal
Time (min)
0.8
0.9
1
1.1
1.2
1.3
1.4
1.5
1.6
1.7
0
10
20
30
40
50
60
Normal
MK50
MK75
MK88
MK94
MK97
MK data
Normal data

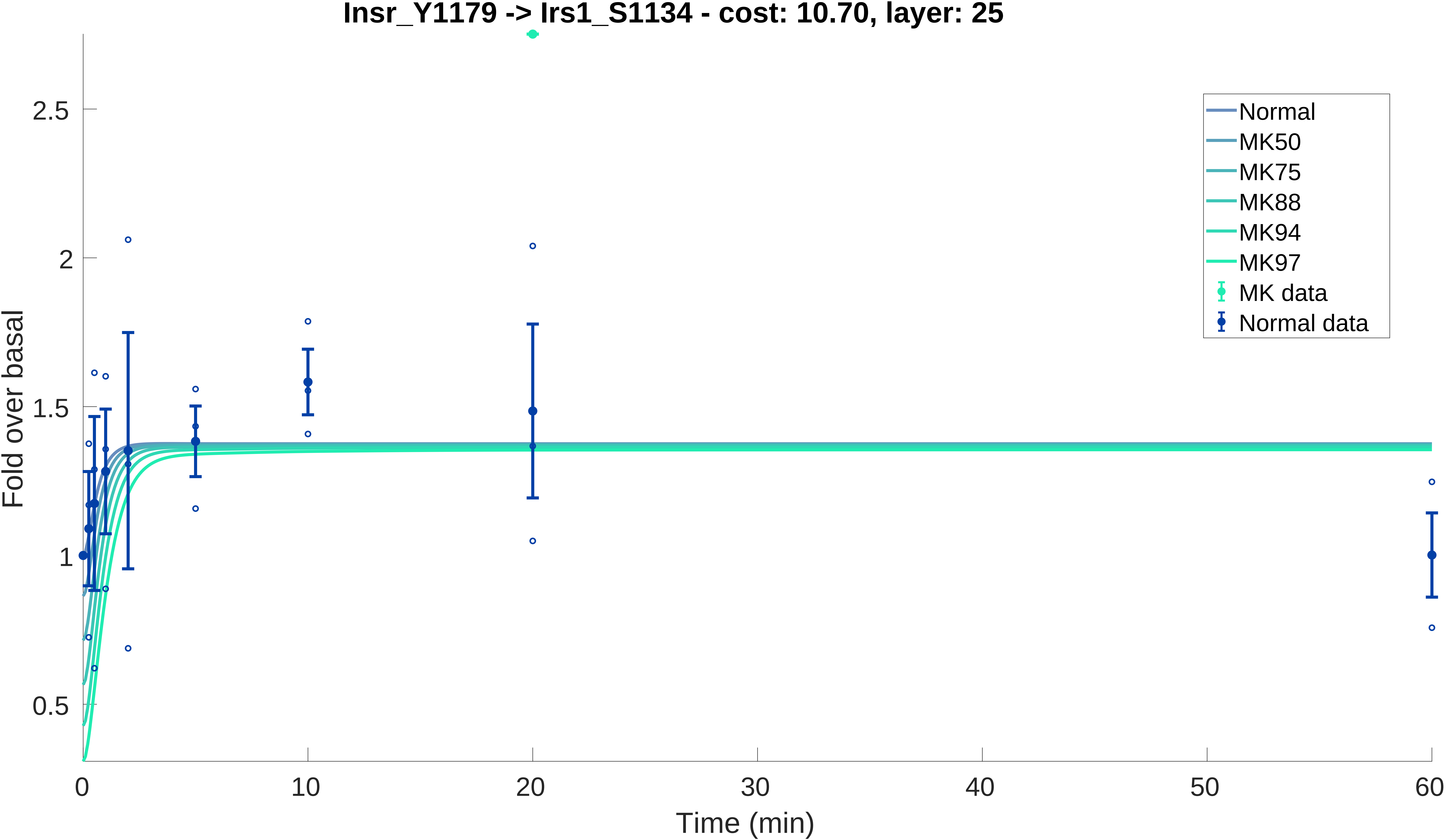

Insr_Y1179 -> Irs1_S1134 - cost: 10.70, layer: 25
Fold over basal
Time (min)
0.5
1
1.5
2
2.5
0
10
20
30
40
50
60
Normal
MK50
MK75
MK88
MK94
MK97
MK data
Normal data

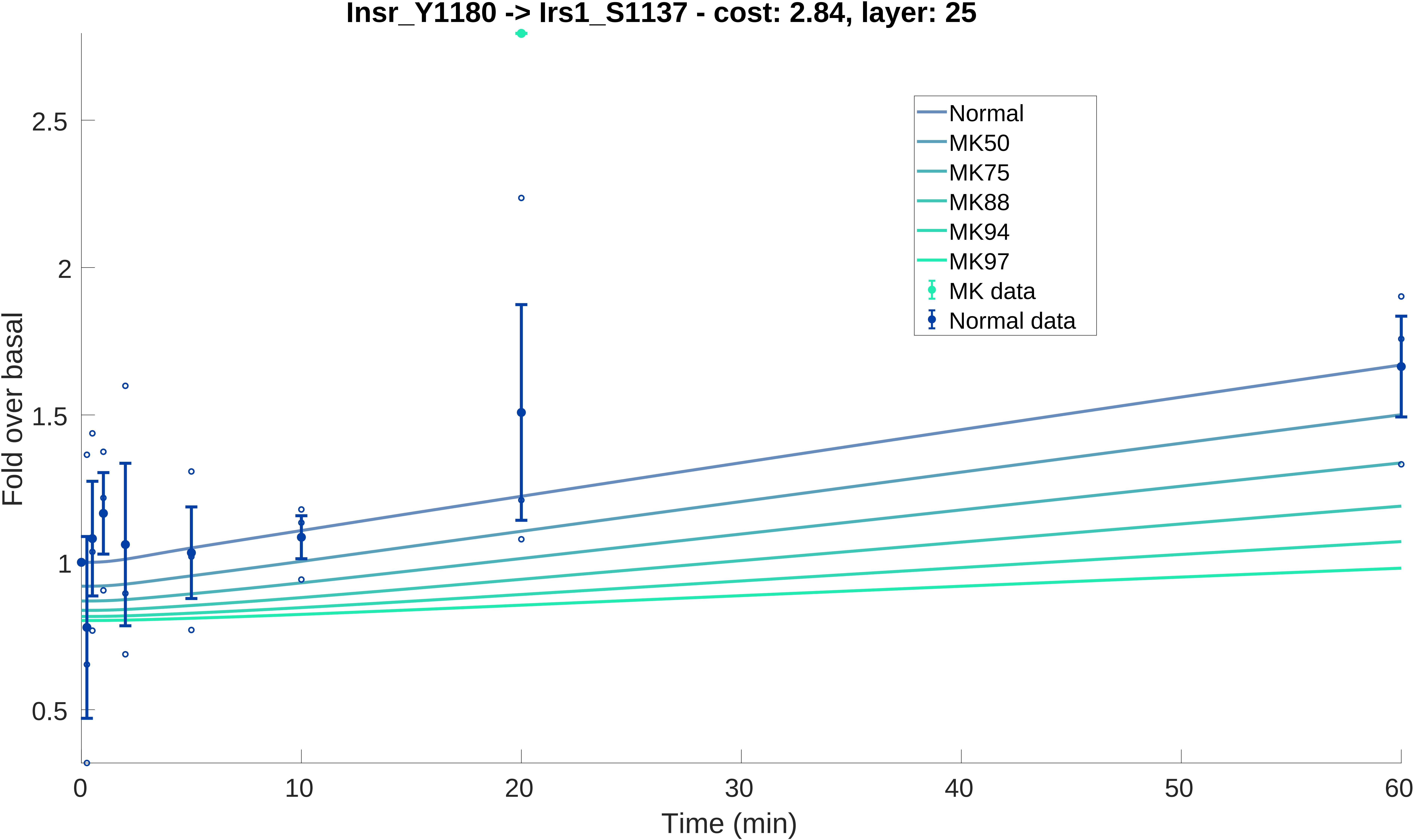

Insr_Y1180 -> Irs1_S1137 - cost: 2.84, layer: 25
Fold over basal
Time (min)
0
10
20
30
40
50
60
0.5
1
1.5
2
2.5
Normal
MK50
MK75
MK88
MK94
MK97
MK data
Normal data

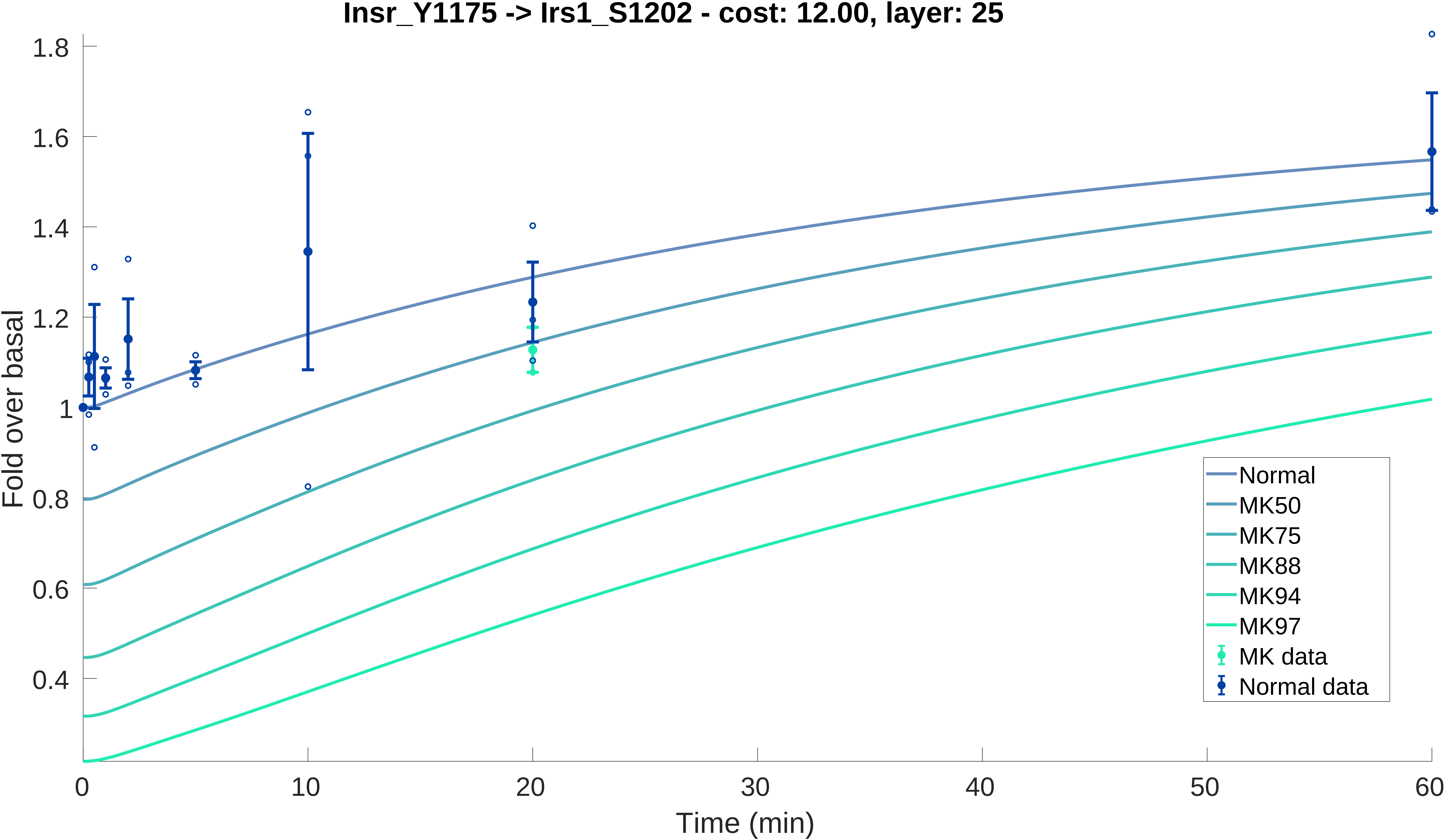
Insr_Y1175 -> Irs1_S1202 - cost: 12.00, layer: 25
Fold over basal
1.8
1.6
1.4
1.2
1
0.8
0.6
0.4
0
10
20
30
40
50
60
Time (min)
Normal
MK50
MK75
MK88
MK94
MK97
MK data
Normal data

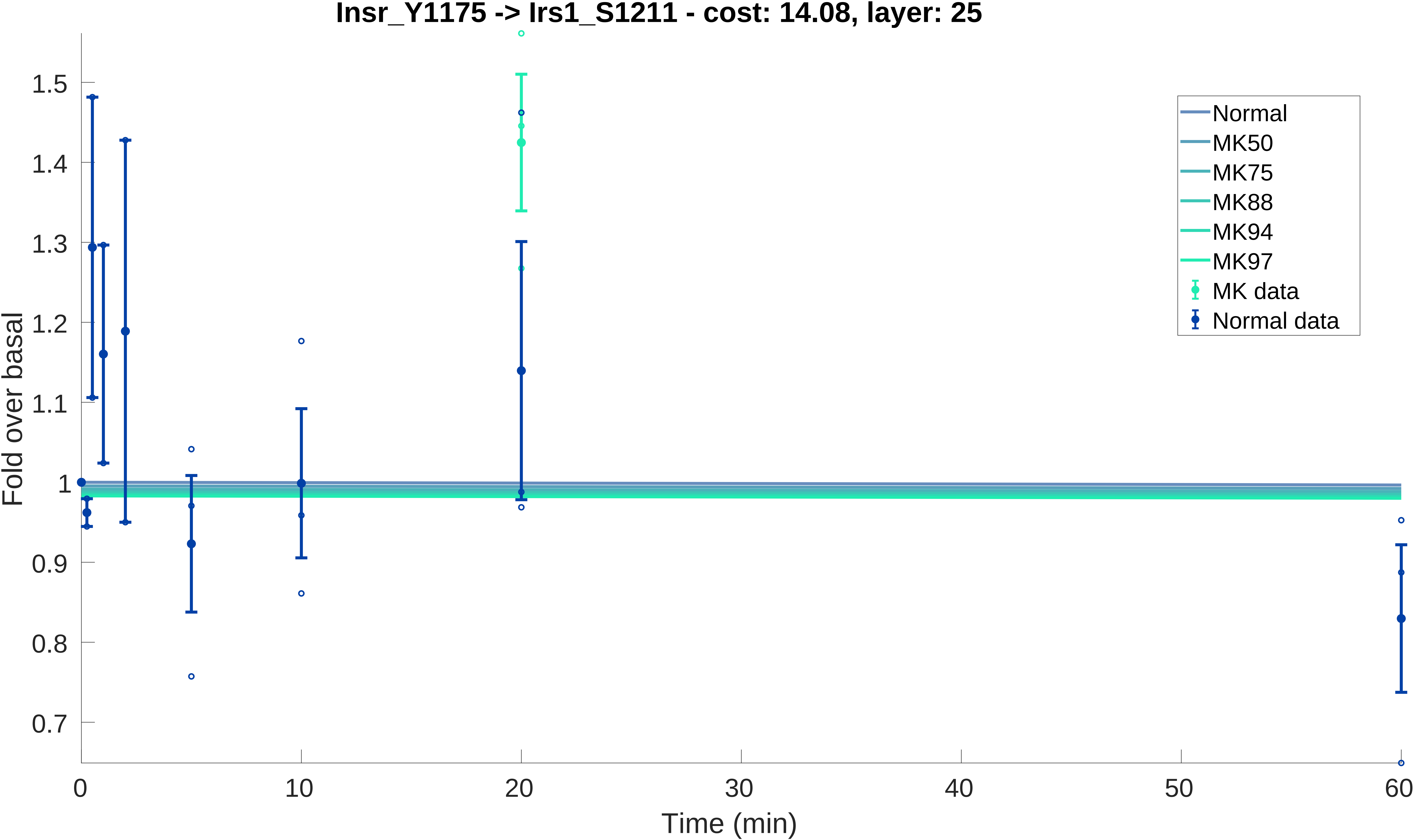

Insr_Y1175 -> Irs1_S1211 - cost: 14.08, layer: 25
Fold over basal
0.7
0.8
0.9
1
1.1
1.2
1.3
1.4
1.5
Time (min)
0
10
20
30
40
50
60
Normal
MK50
MK75
MK88
MK94
MK97
MK data
Normal data

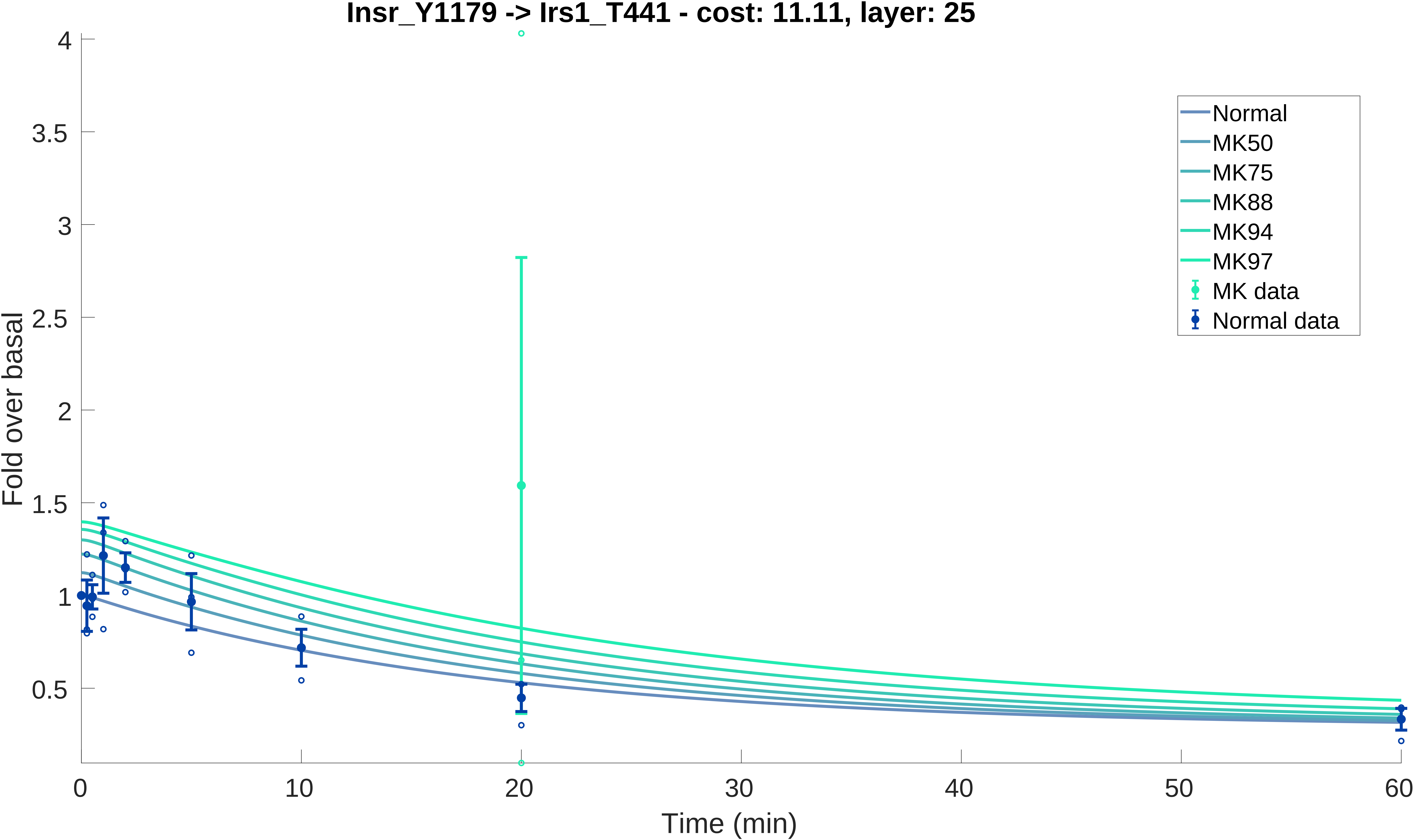

Insr_Y1179 -> Irs1_T441 - cost: 11.11, layer: 25
Fold over basal
Time (min)
0
10
20
30
40
50
60
0.5
1
1.5
2
2.5
3
3.5
4
Normal
MK50
MK75
MK88
MK94
MK97
MK data
Normal data

Mtor_T2473 -> Eif4ebp1_S64 - cost: 1.42, layer: 26

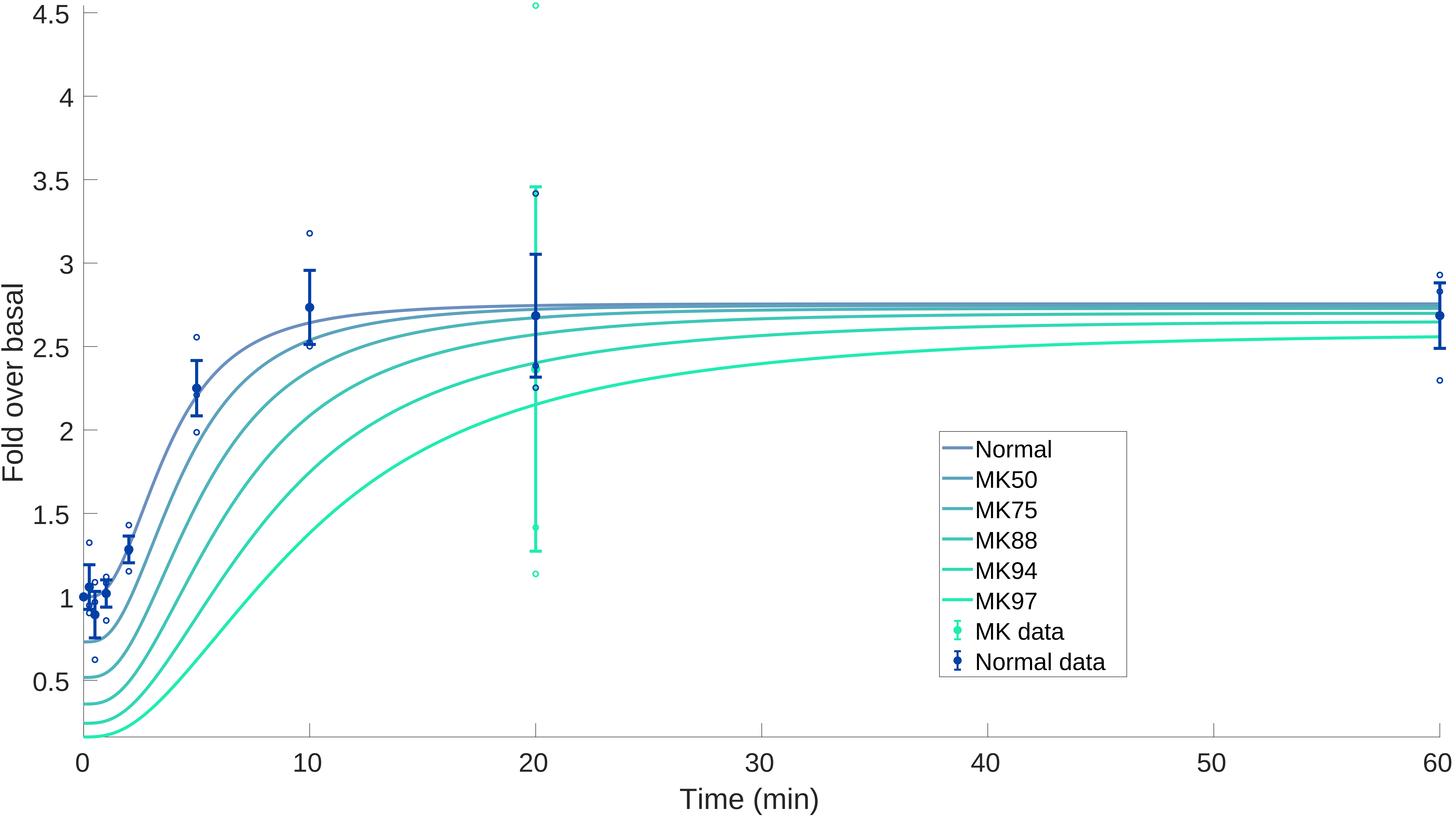

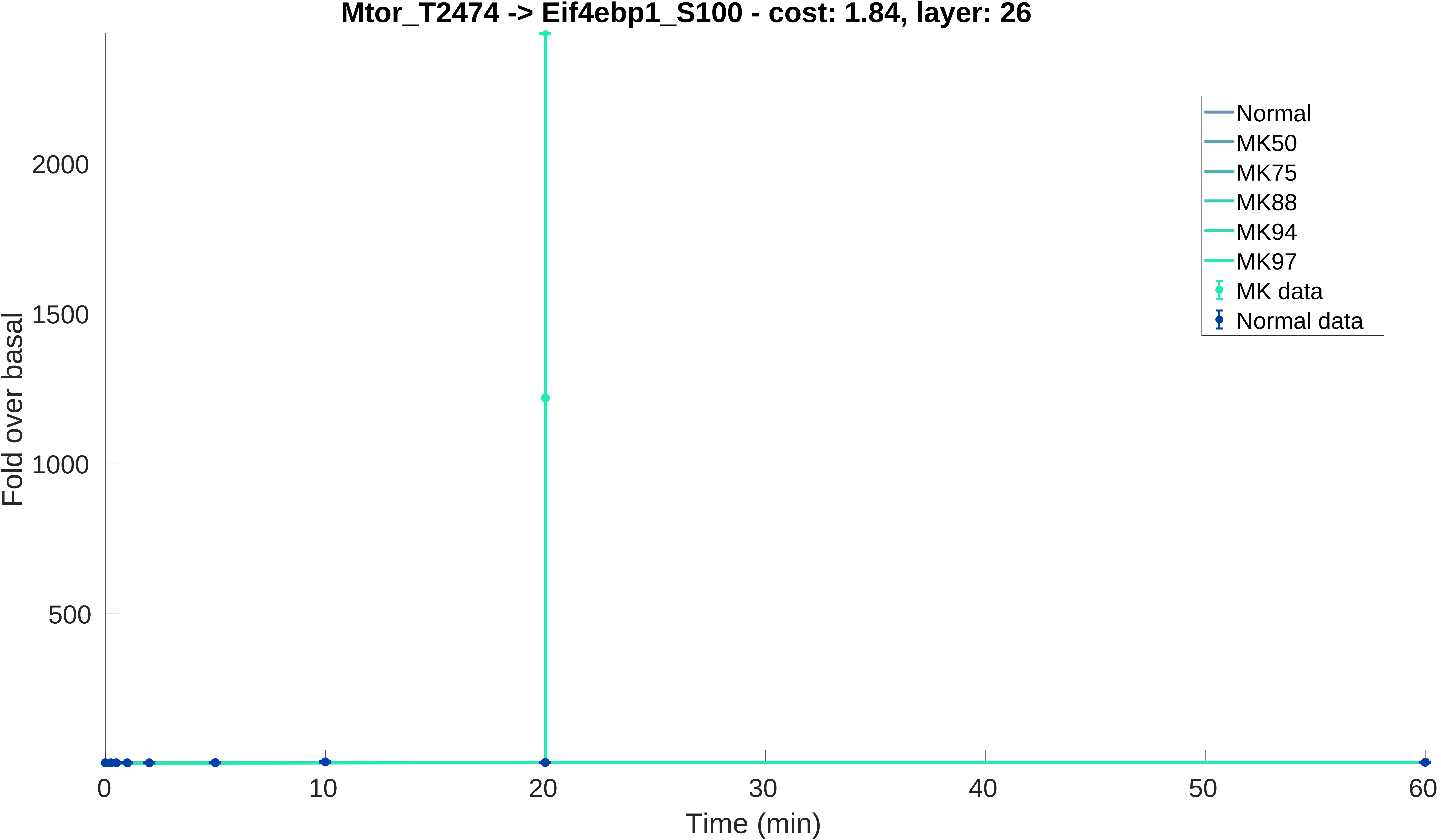
Mtor_T2474 -> Eif4ebp1_S100 - cost: 1.84, layer: 26
Fold over basal
2000
1500
1000
500
0
10
20
30
40
50
60
Time (min)
Normal
MK50
MK75
MK88
MK94
MK97
MK data
Normal data

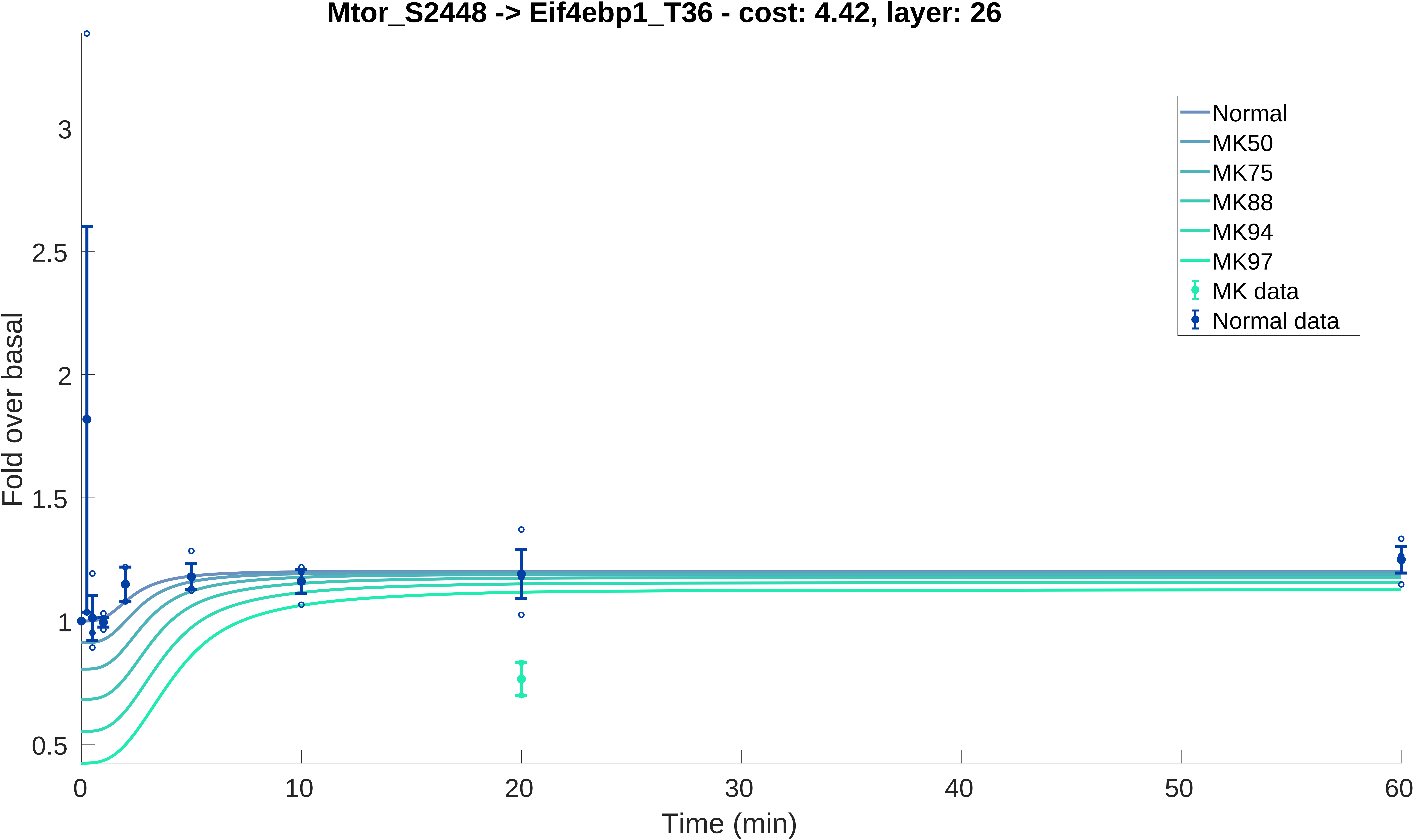

Mtor_S2448 -> Eif4ebp1_T36 - cost: 4.42, layer: 26
Fold over basal
Time (min)
0.5
1
1.5
2
2.5
3
0
10
20
30
40
50
60
Normal
MK50
MK75
MK88
MK94
MK97
MK data
Normal data

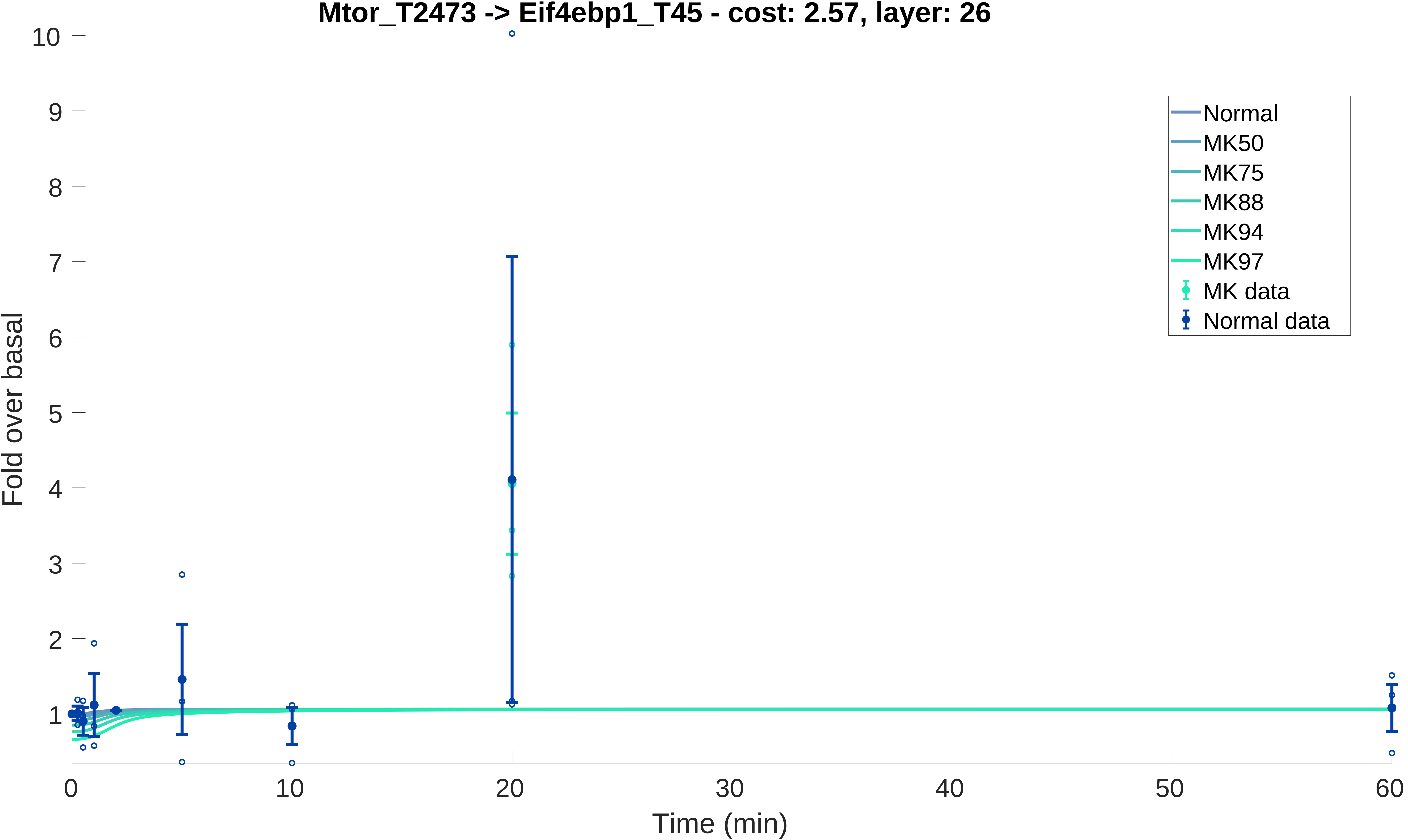

Mtor_T2473 -> Eif4ebp1_T45 - cost: 2.57, layer: 26
Fold over basal
Time (min)
10
9
8
7
6
5
4
3
2
1
0
10
20
30
40
50
60
Normal
MK50
MK75
MK88
MK94
MK97
MK data
Normal data

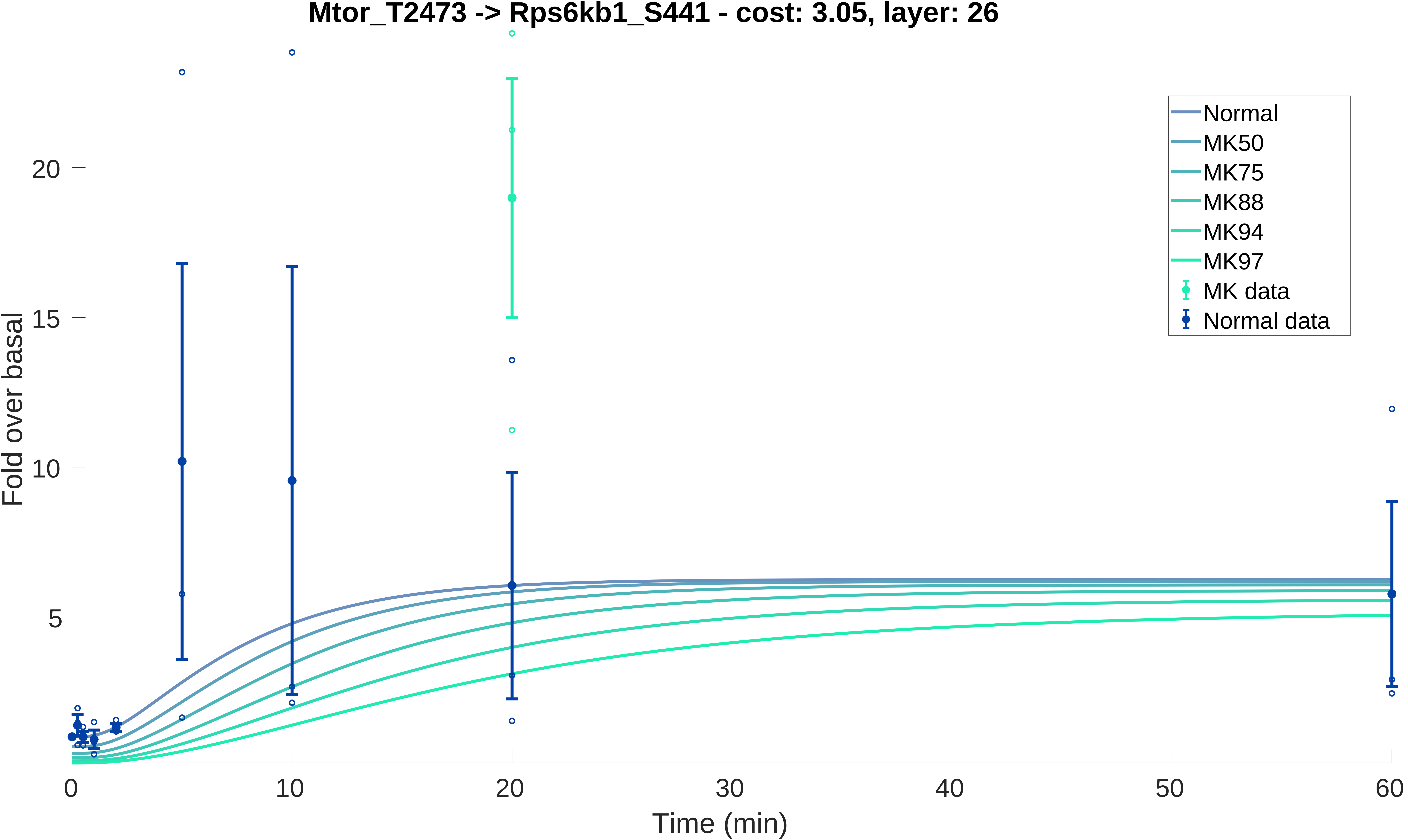

Mtor_T2473 -> Rps6kb1_S441 - cost: 3.05, layer: 26
Fold over basal
Time (min)
0
10
20
30
40
50
60
5
10
15
20
Normal
MK50
MK75
MK88
MK94
MK97
MK data
Normal data

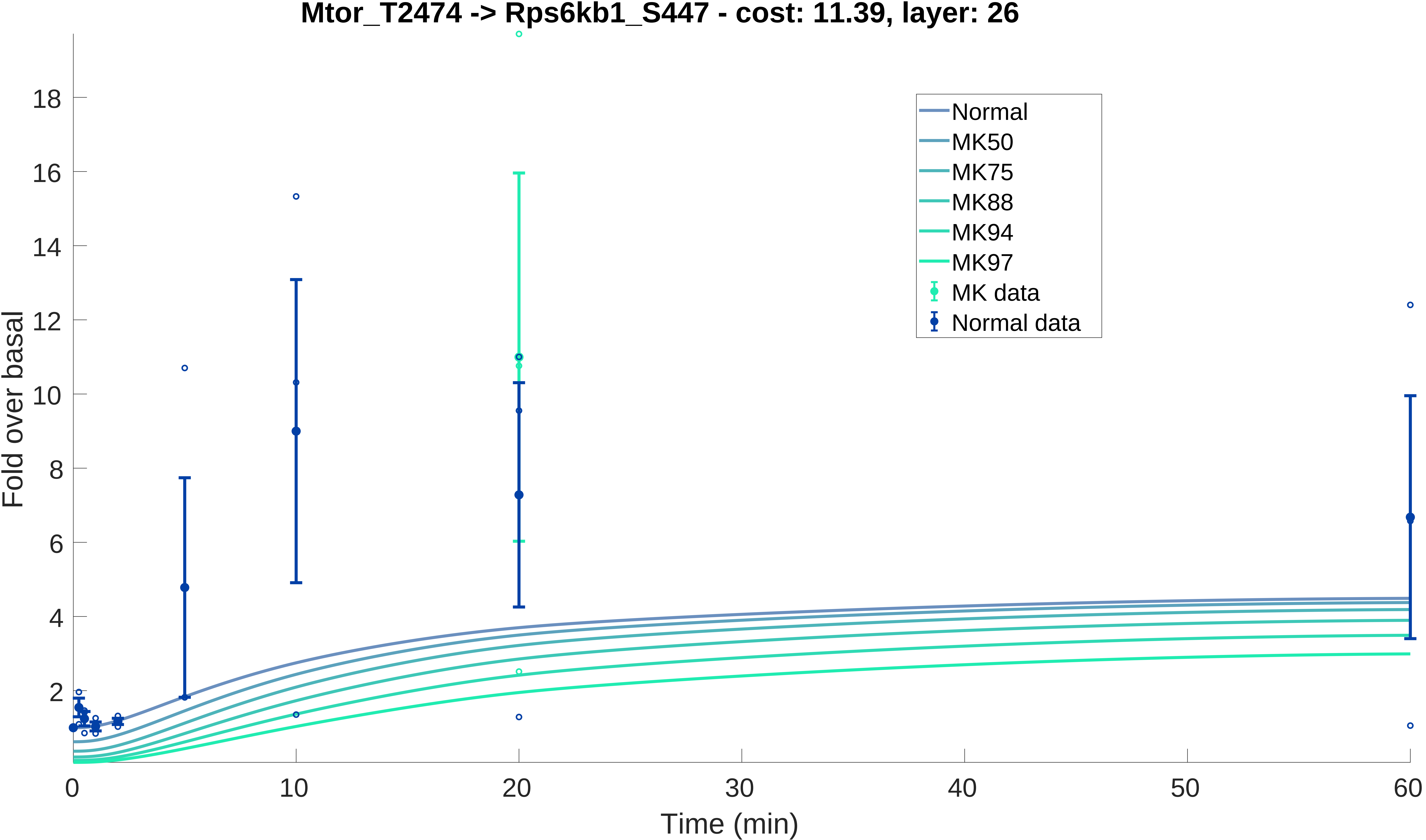

Mtor_T2474 -> Rps6kb1_S447 - cost: 11.39, layer: 26
Fold over basal
Time (min)
0
10
20
30
40
50
60
2
4
6
8
10
12
14
16
18
Normal
MK50
MK75
MK88
MK94
MK97
MK data
Normal data

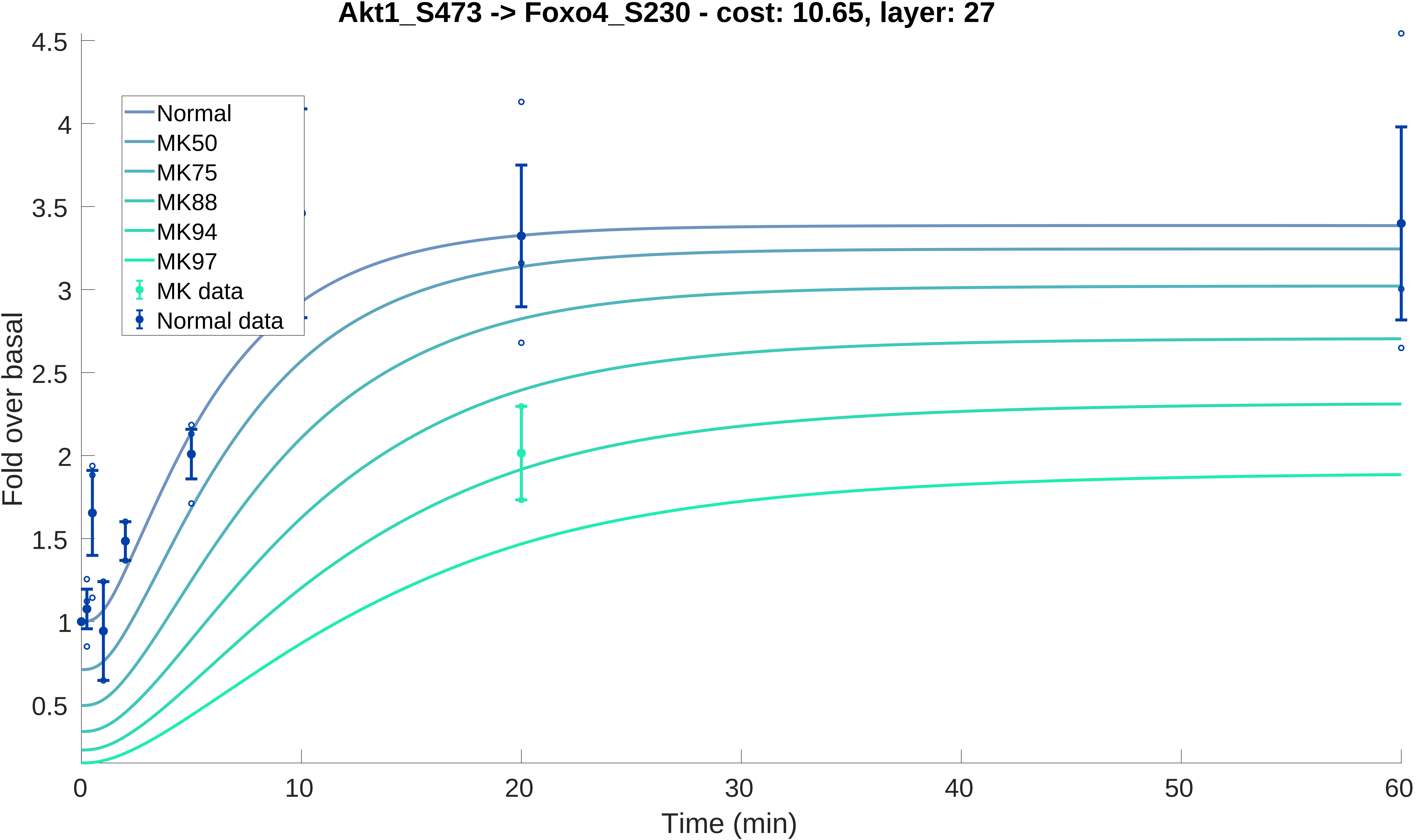

Akt1_S473 -> Foxo4_S230 - cost: 10.65, layer: 27
Normal
MK50
MK75
MK88
MK94
MK97
MK data
Normal data
Fold over basal
Time (min)
4.5
4
3.5
3
2.5
2
1.5
1
0.5
0
10
20
30
40
50
60

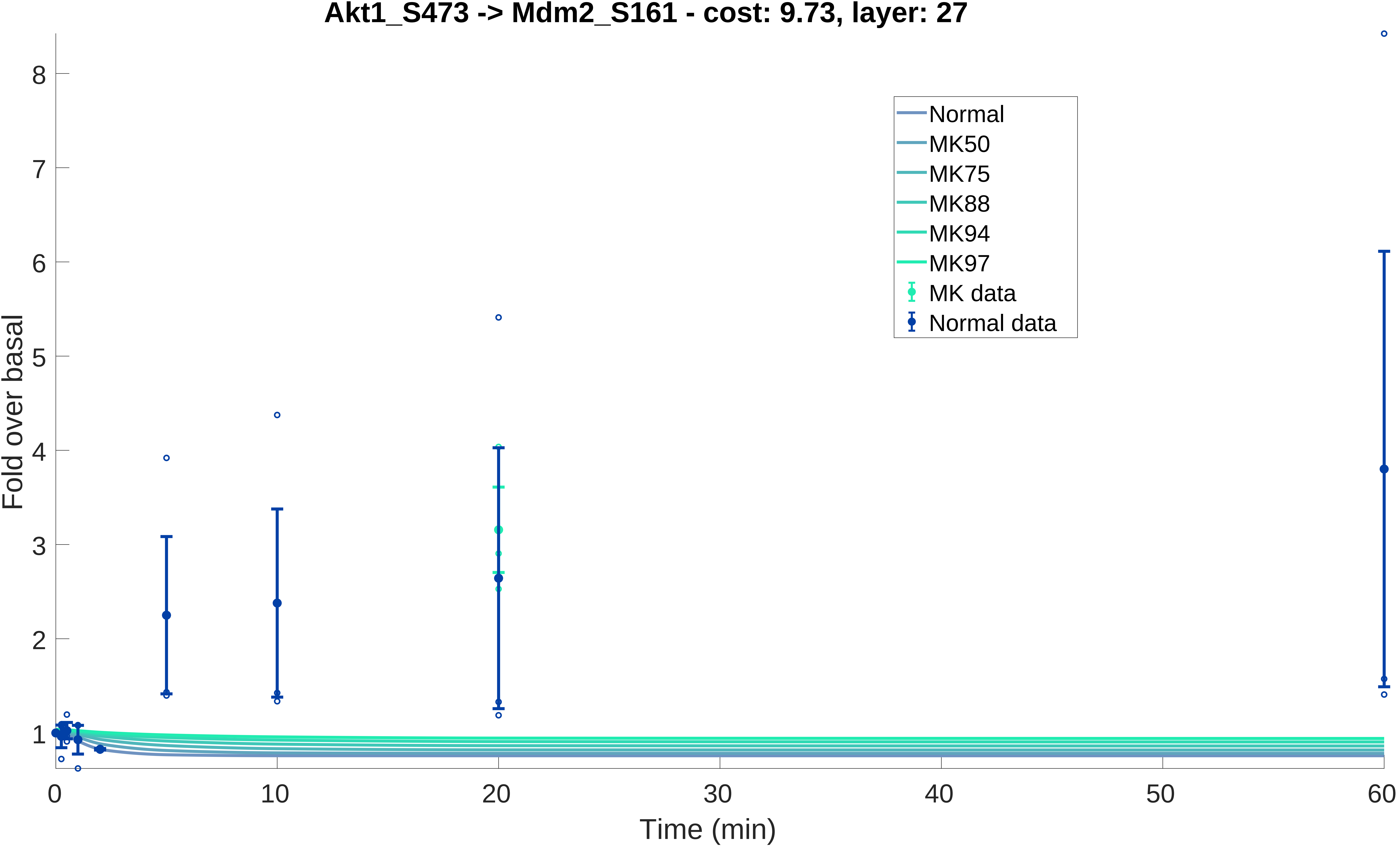

Akt1_S473 -> Mdm2_S161 - cost: 9.73, layer: 27
Fold over basal
Time (min)
Normal
MK50
MK75
MK88
MK94
MK97
MK data
Normal data
0
10
20
30
40
50
60
1
2
3
4
5
6
7
8

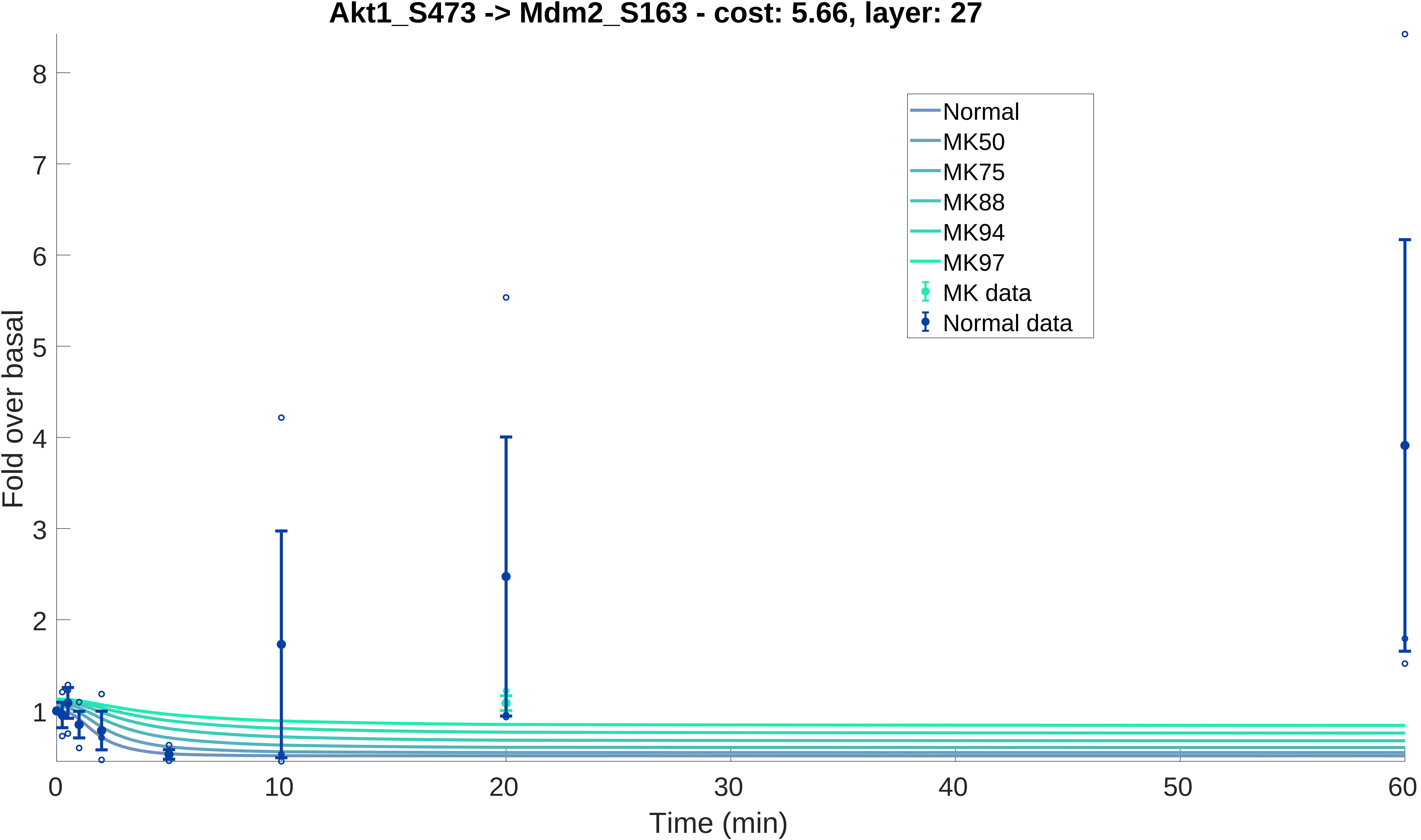

Akt1_S473 -> Mdm2_S163 - cost: 5.66, layer: 27
Fold over basal
Time (min)
0
10
20
30
40
50
60
1
2
3
4
5
6
7
8
Normal
MK50
MK75
MK88
MK94
MK97
MK data
Normal data

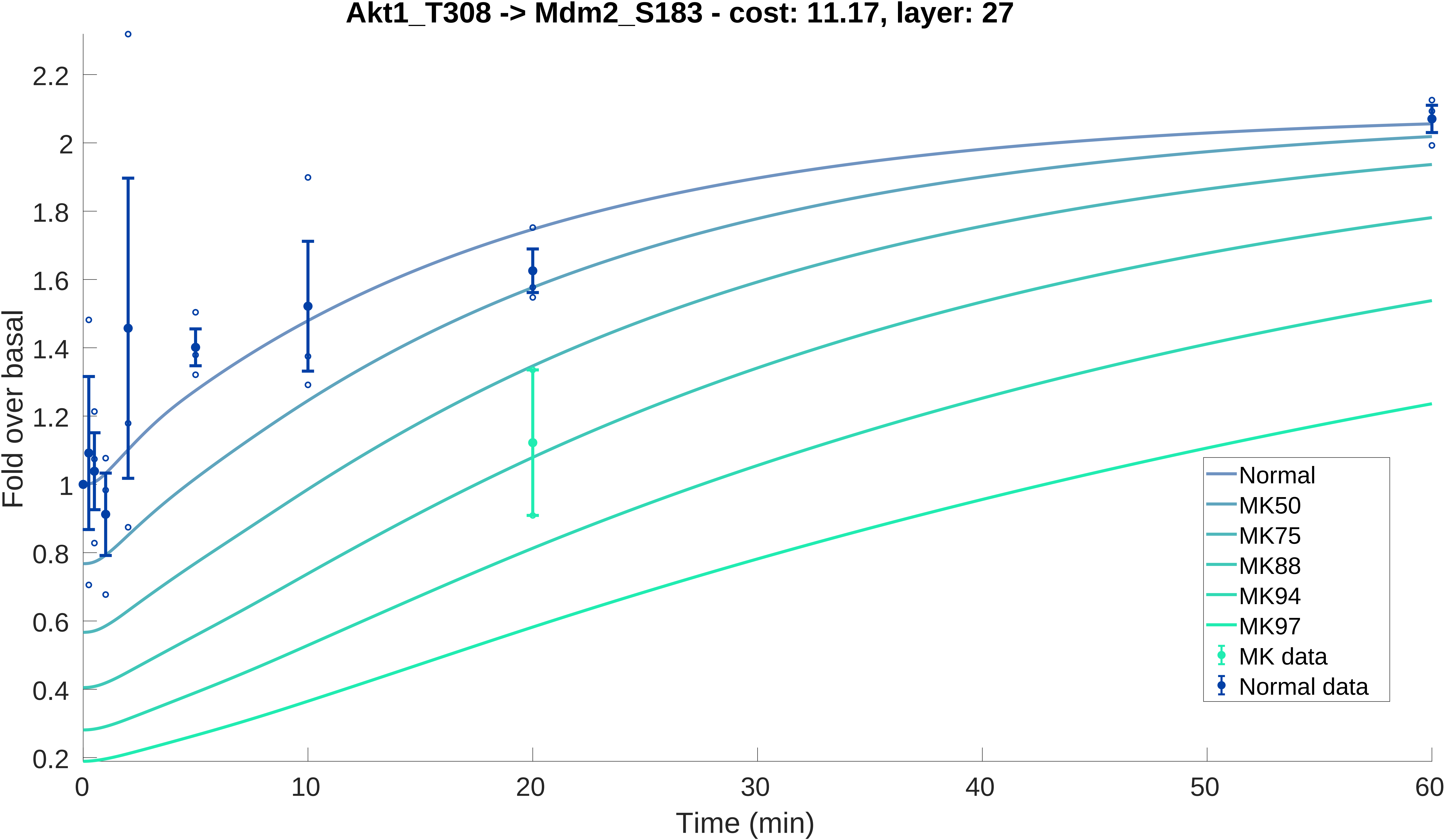

Akt1_T308 -> Mdm2_S183 - cost: 11.17, layer: 27
Fold over basal
2.2
2
1.8
1.6
1.4
1.2
1
0.8
0.6
0.4
0.2
0
10
20
30
40
50
60
Time (min)
Normal
MK50
MK75
MK88
MK94
MK97
MK data
Normal data

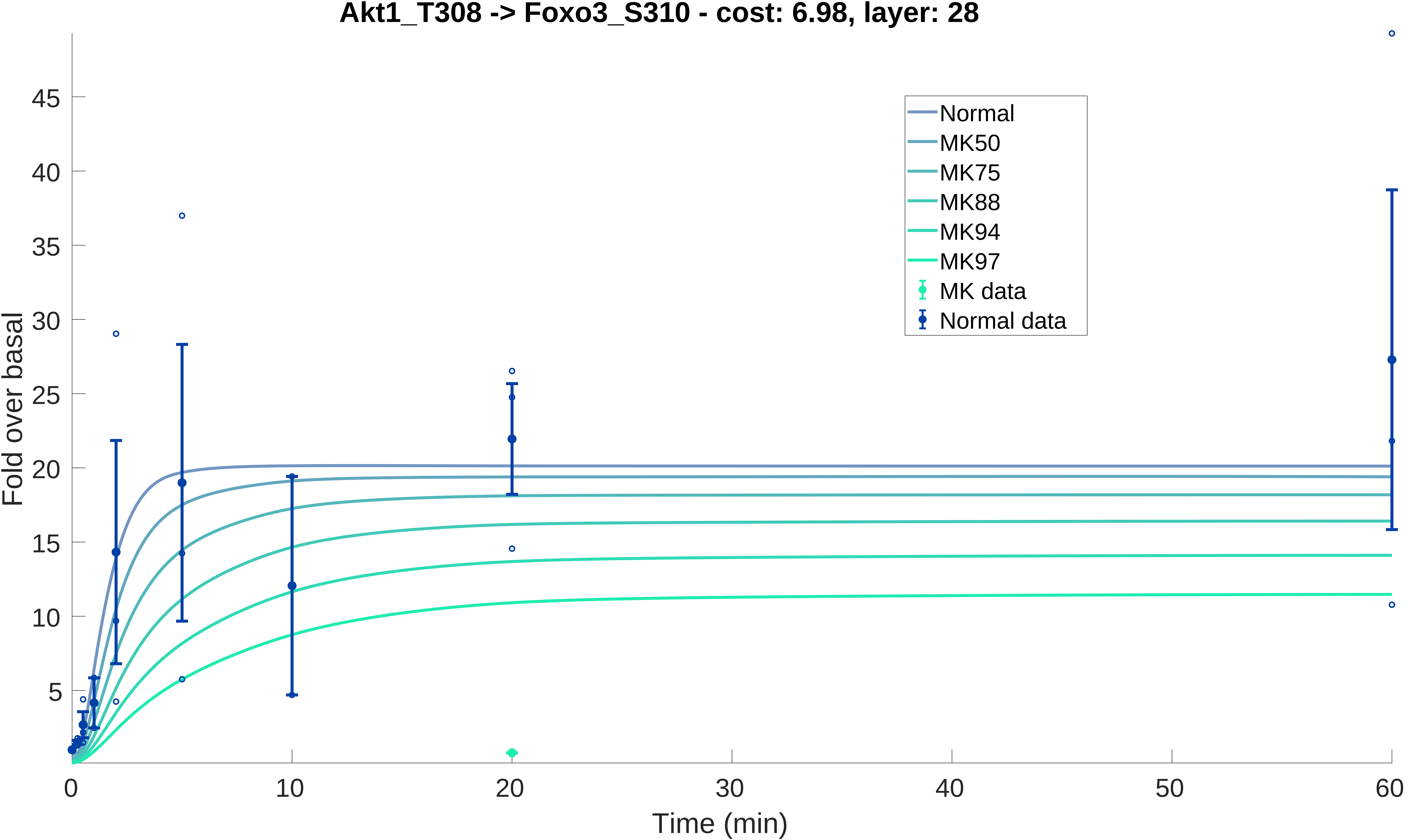
Akt1_T308 -> Foxo3_S310 - cost: 6.98, layer: 28
Fold over basal
Time (min)
0
10
20
30
40
50
60
5
10
15
20
25
30
35
40
45
Normal
MK50
MK75
MK88
MK94
MK97
MK data
Normal data

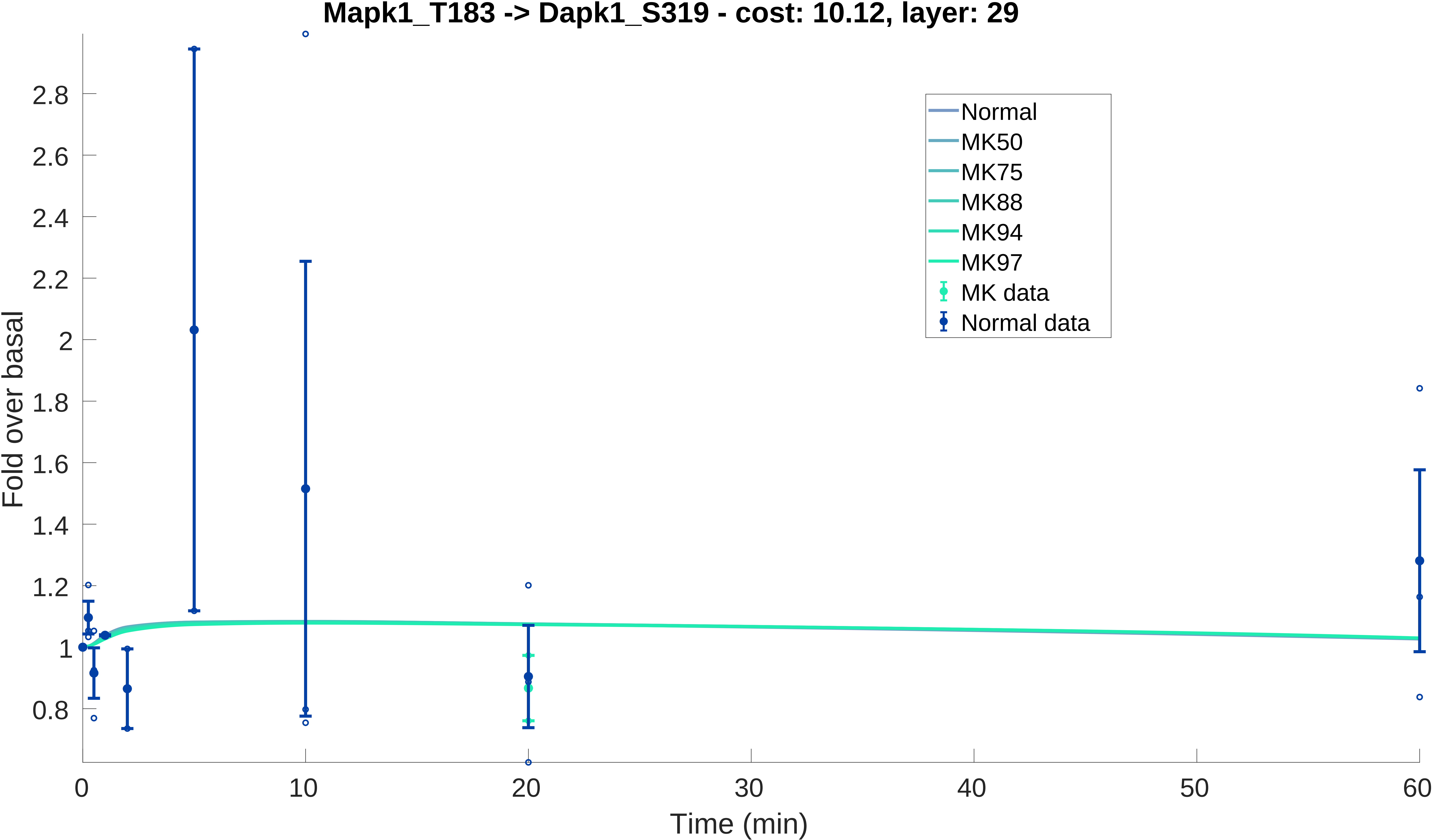
Mapk1_T183 -> Dapk1_S319 - cost: 10.12, layer: 29
Fold over basal
Time (min)
0
10
20
30
40
50
60
0.8
1
1.2
1.4
1.6
1.8
2
2.2
2.4
2.6
2.8
Normal
MK50
MK75
MK88
MK94
MK97
MK data
Normal data

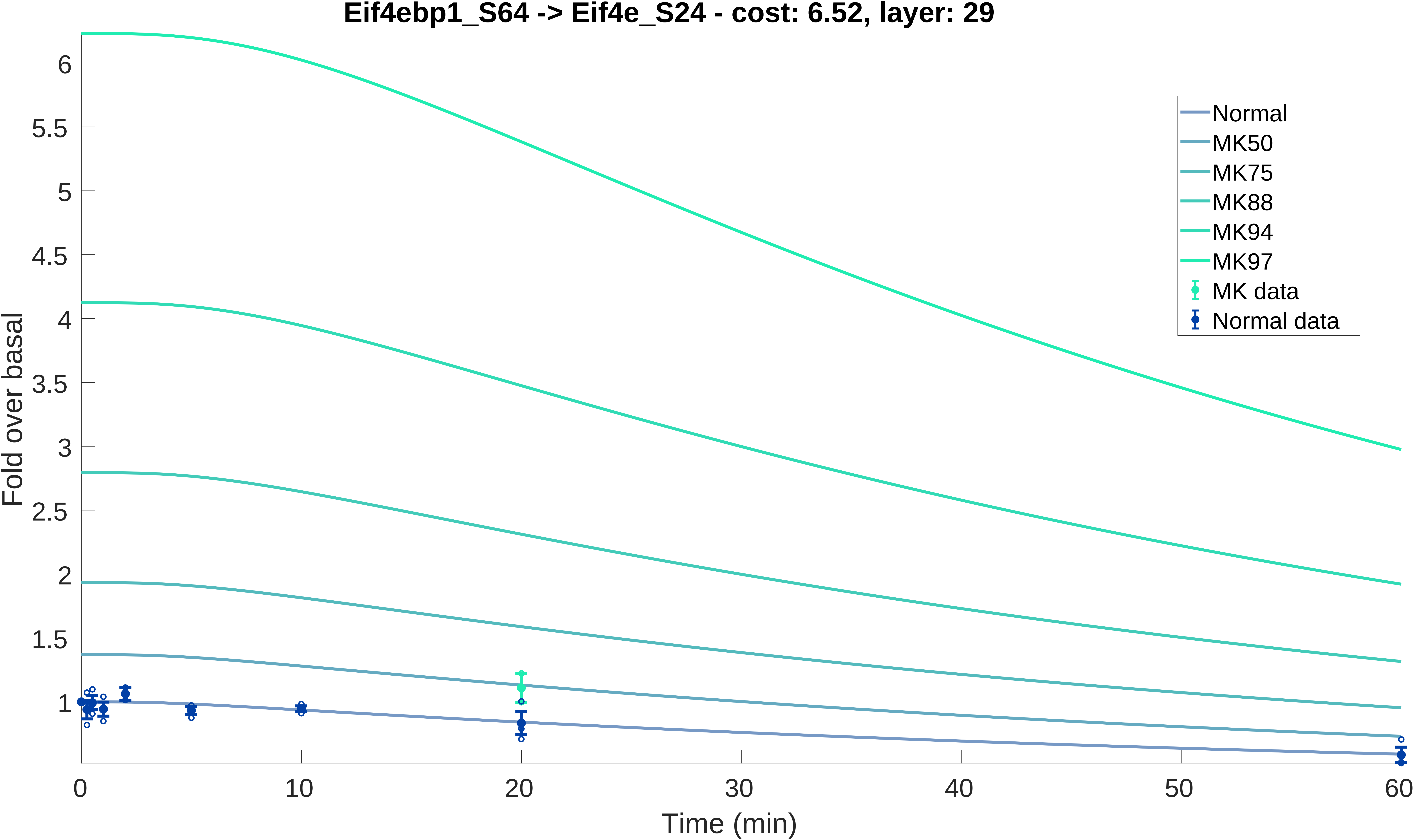

Eif4ebp1_S64 -> Eif4e_S24 - cost: 6.52, layer: 29
Fold over basal
Time (min)
Normal
MK50
MK75
MK88
MK94
MK97
MK data
Normal data
0
10
20
30
40
50
60
1
1.5
2
2.5
3
3.5
4
4.5
5
5.5
6

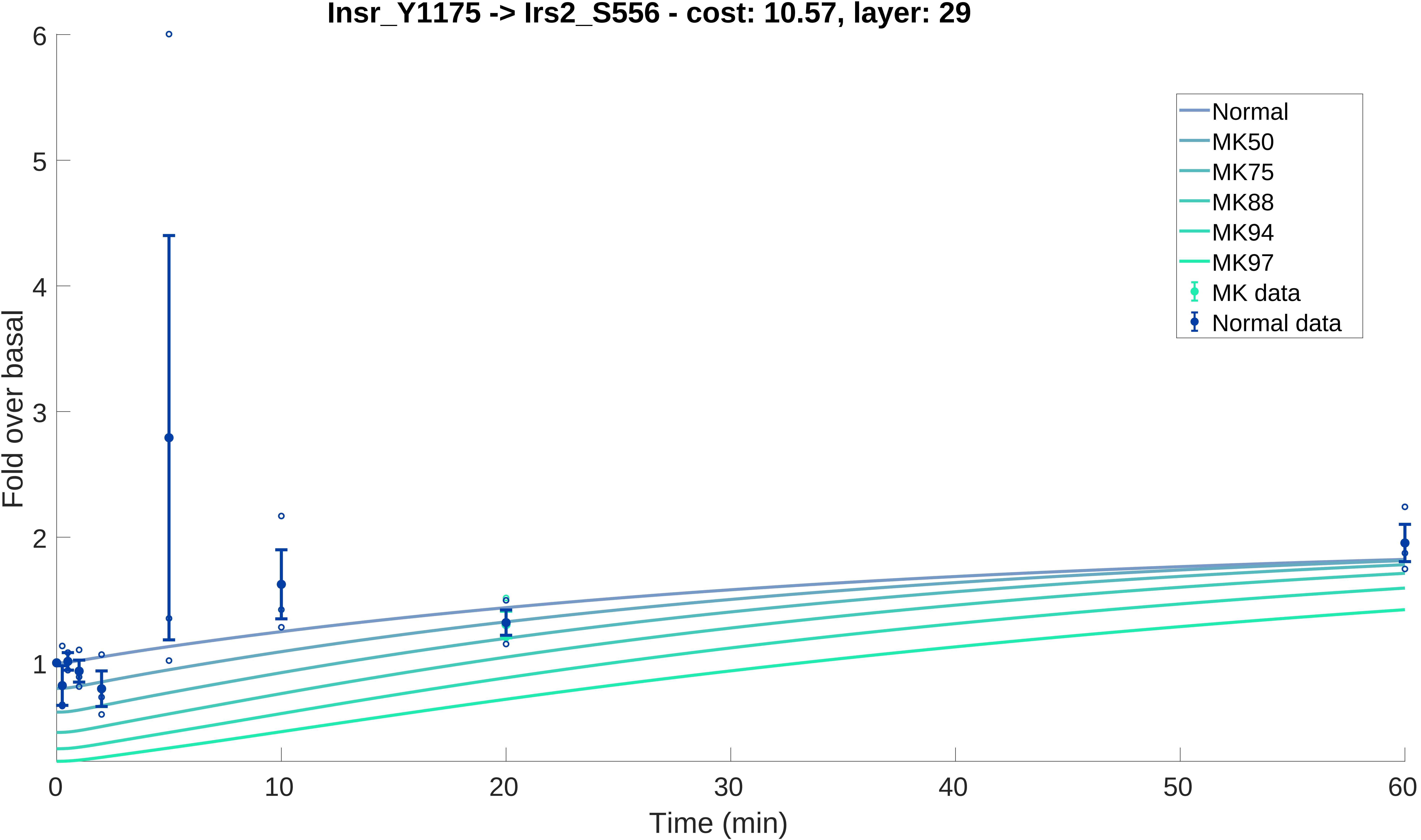

Insr_Y1175 -> Irs2_S556 - cost: 10.57, layer: 29
Fold over basal
Time (min)
0
10
20
30
40
50
60
1
2
3
4
5
6
Normal
MK50
MK75
MK88
MK94
MK97
MK data
Normal data

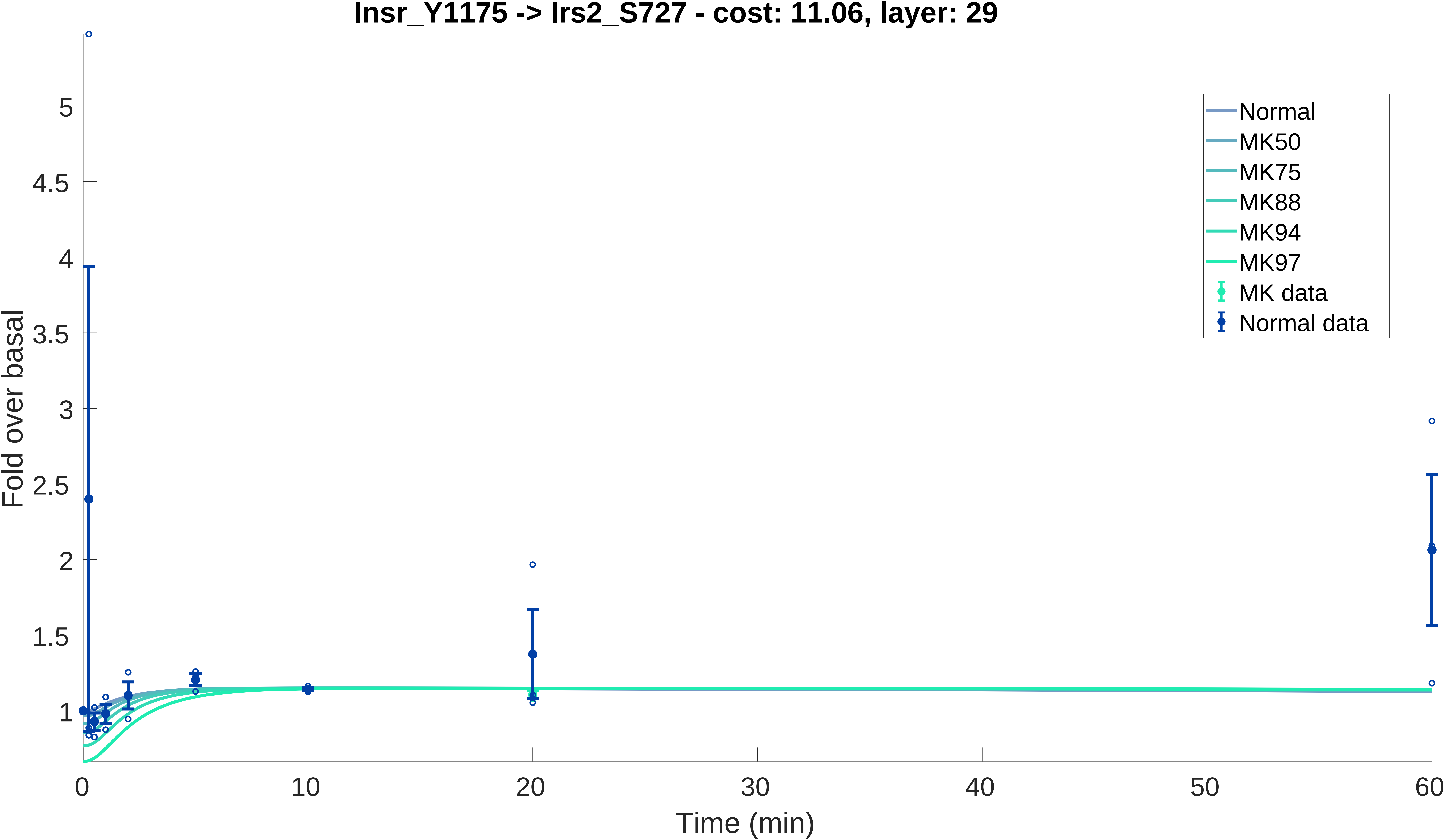
Insr_Y1175 -> Irs2_S727 - cost: 11.06, layer: 29
Fold over basal
Time (min)
Normal
MK50
MK75
MK88
MK94
MK97
MK data
Normal data
0
10
20
30
40
50
60
1
1.5
2
2.5
3
3.5
4
4.5
5

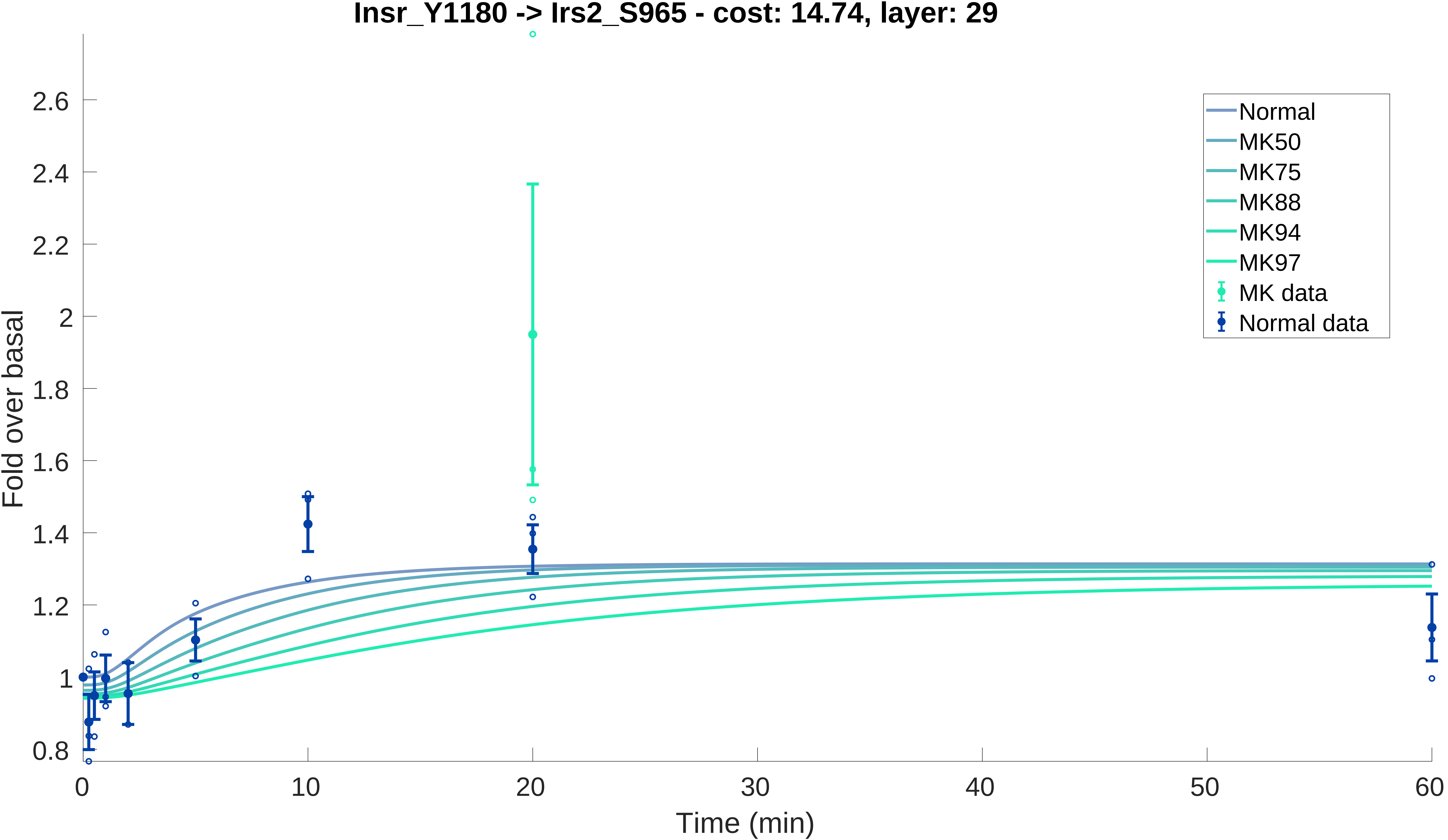

Insr_Y1180 -> Irs2_S965 - cost: 14.74, layer: 29
Fold over basal
2.6
2.4
2.2
2
1.8
1.6
1.4
1.2
1
0.8
0
10
20
30
40
50
60
Time (min)
Normal
MK50
MK75
MK88
MK94
MK97
MK data
Normal data

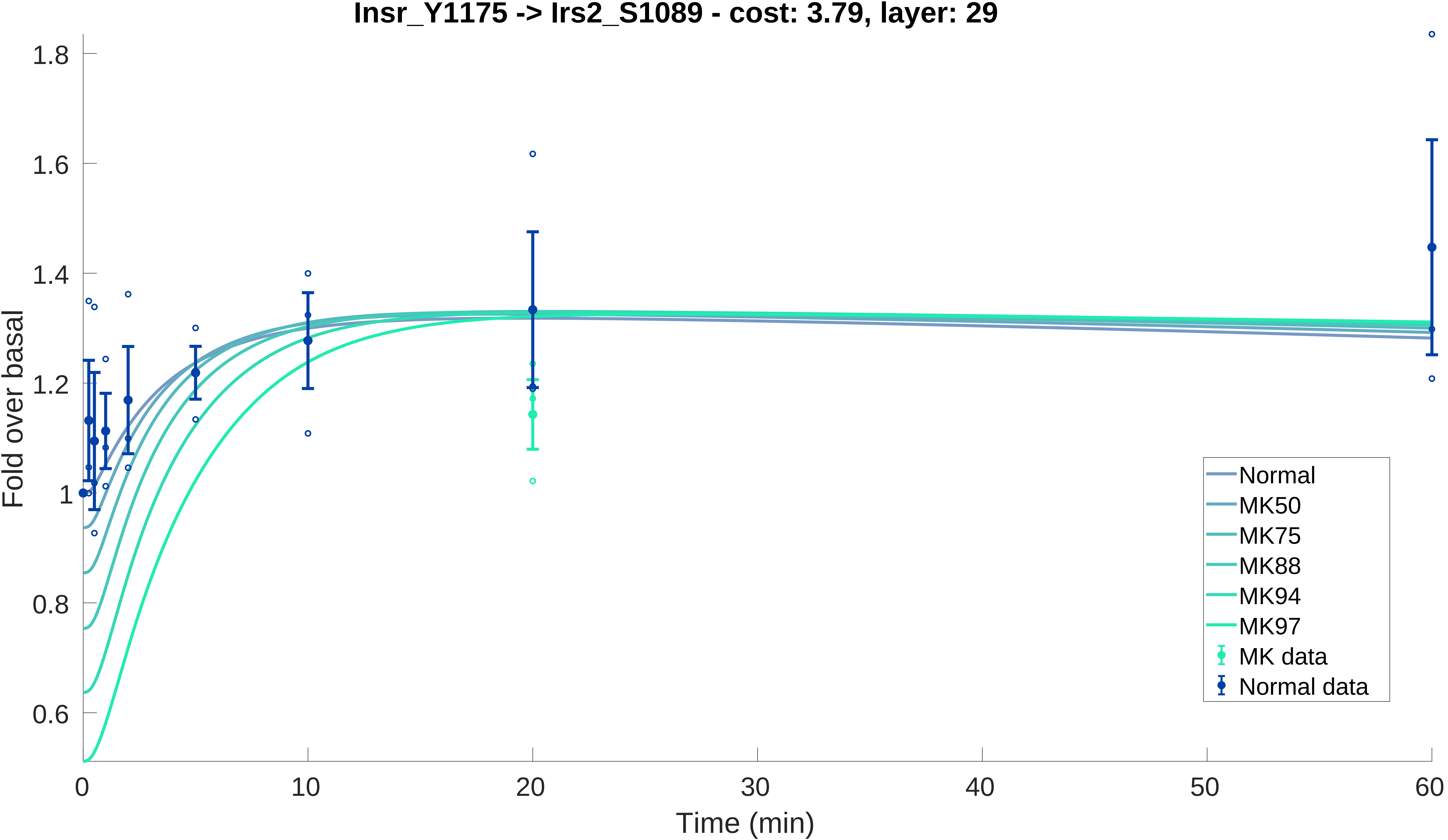
Insr_Y1175 -> Irs2_S1089 - cost: 3.79, layer: 29
Fold over basal
1.8
1.6
1.4
1.2
1
0.8
0.6
0
10
20
30
40
50
60
Time (min)
Normal
MK50
MK75
MK88
MK94
MK97
MK data
Normal data

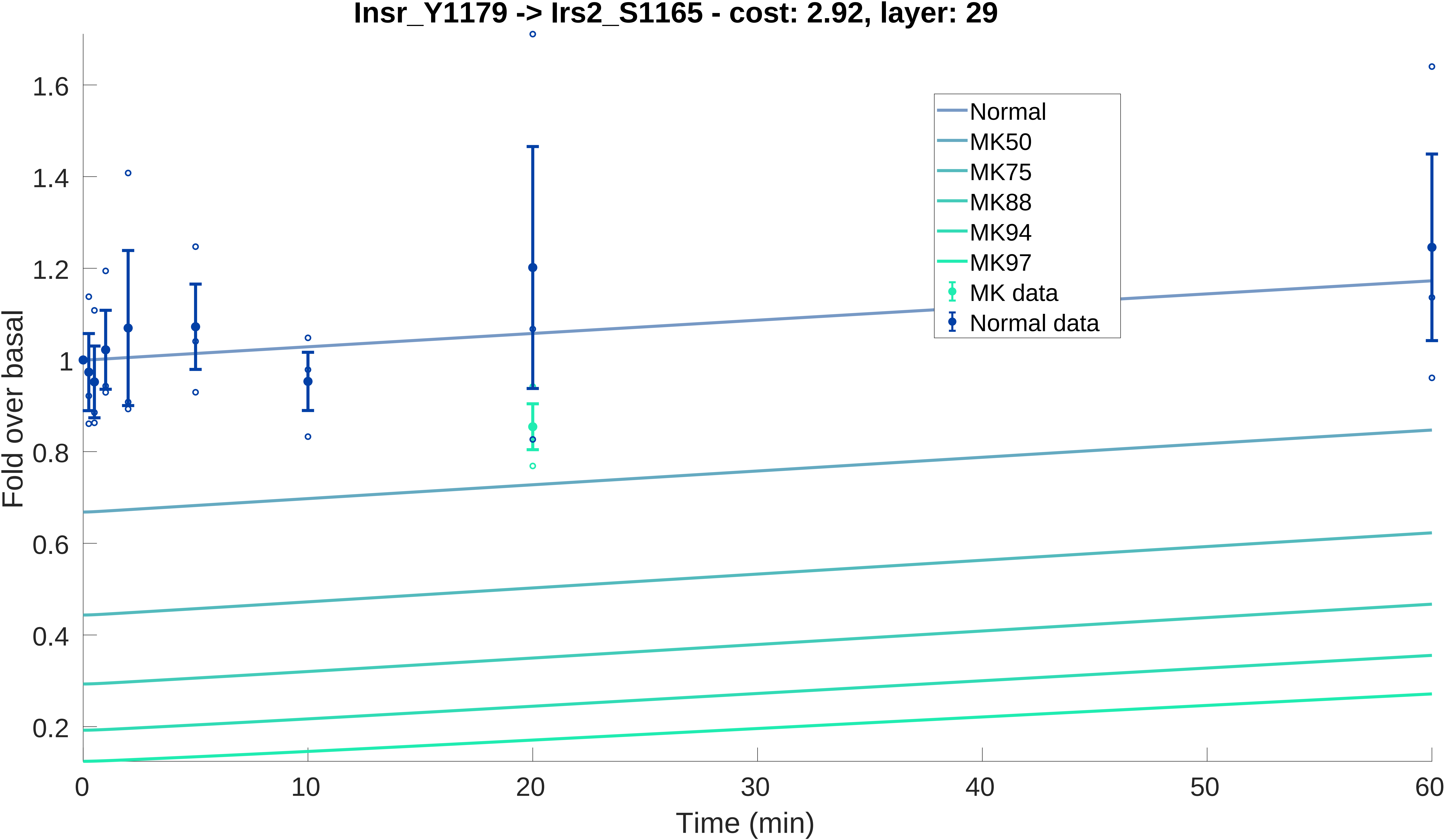
Insr_Y1179 -> Irs2_S1165 - cost: 2.92, layer: 29
Fold over basal
Time (min)
0
10
20
30
40
50
60
0.2
0.4
0.6
0.8
1
1.2
1.4
1.6
Normal
MK50
MK75
MK88
MK94
MK97
MK data
Normal data

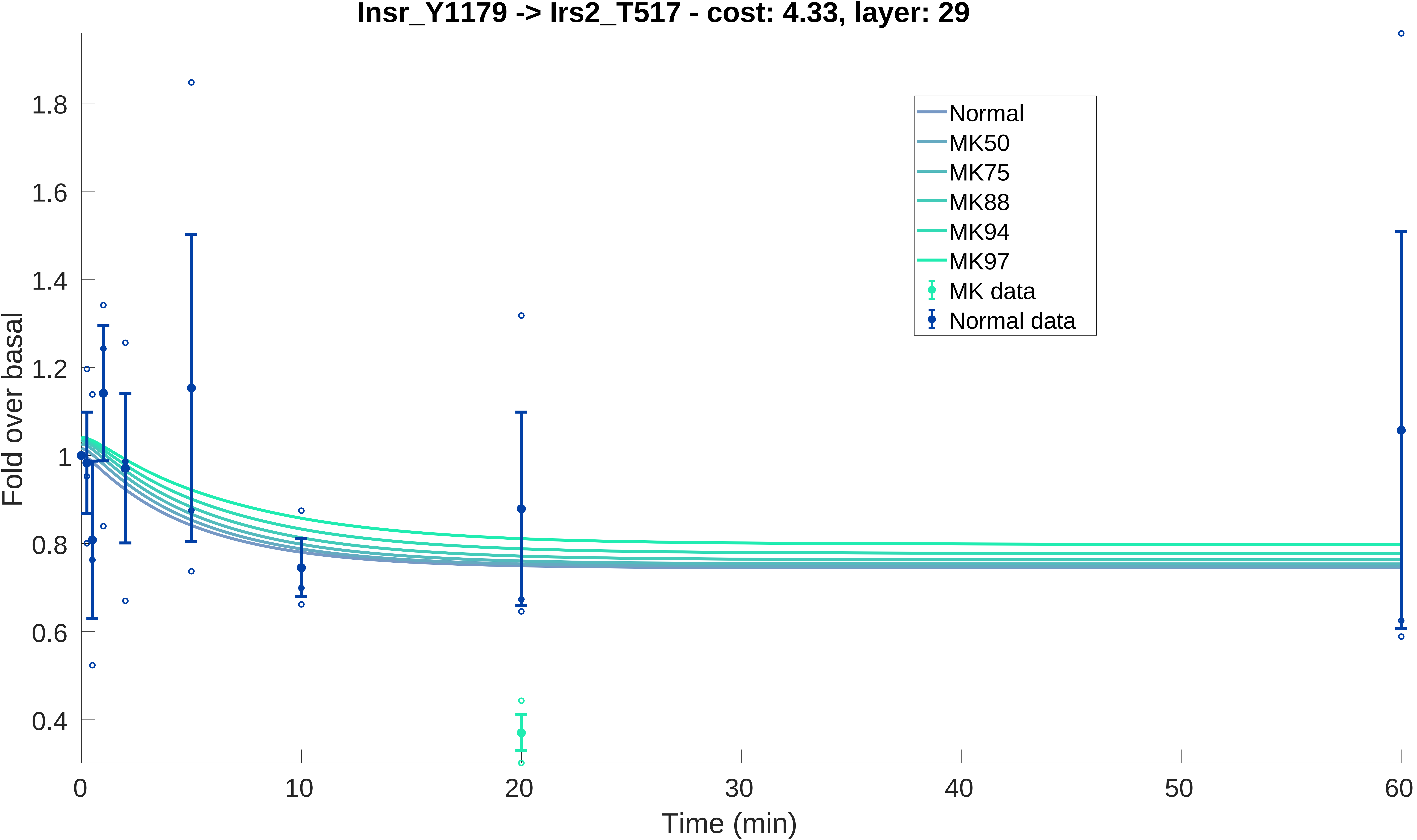
Insr_Y1179 -> Irs2_T517 - cost: 4.33, layer: 29
Fold over basal
Time (min)
1.8
1.6
1.4
1.2
1
0.8
0.6
0.4
0
10
20
30
40
50
60
Normal
MK50
MK75
MK88
MK94
MK97
MK data
Normal data

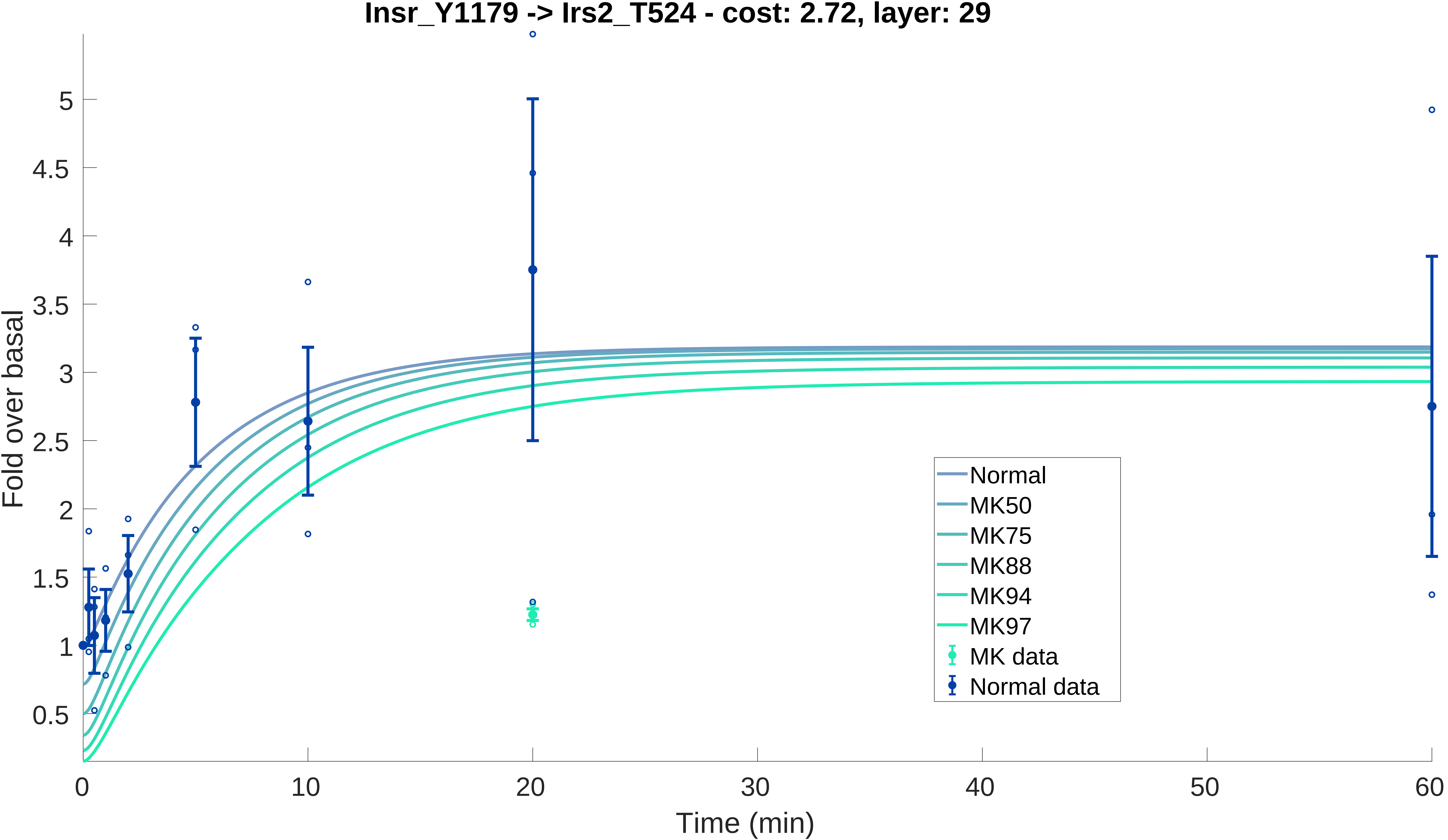

Insr_Y1179 -> Irs2_T524 - cost: 2.72, layer: 29
Fold over basal
Time (min)
0
10
20
30
40
50
60
0.5
1
1.5
2
2.5
3
3.5
4
4.5
5
Normal
MK50
MK75
MK88
MK94
MK97
MK data
Normal data

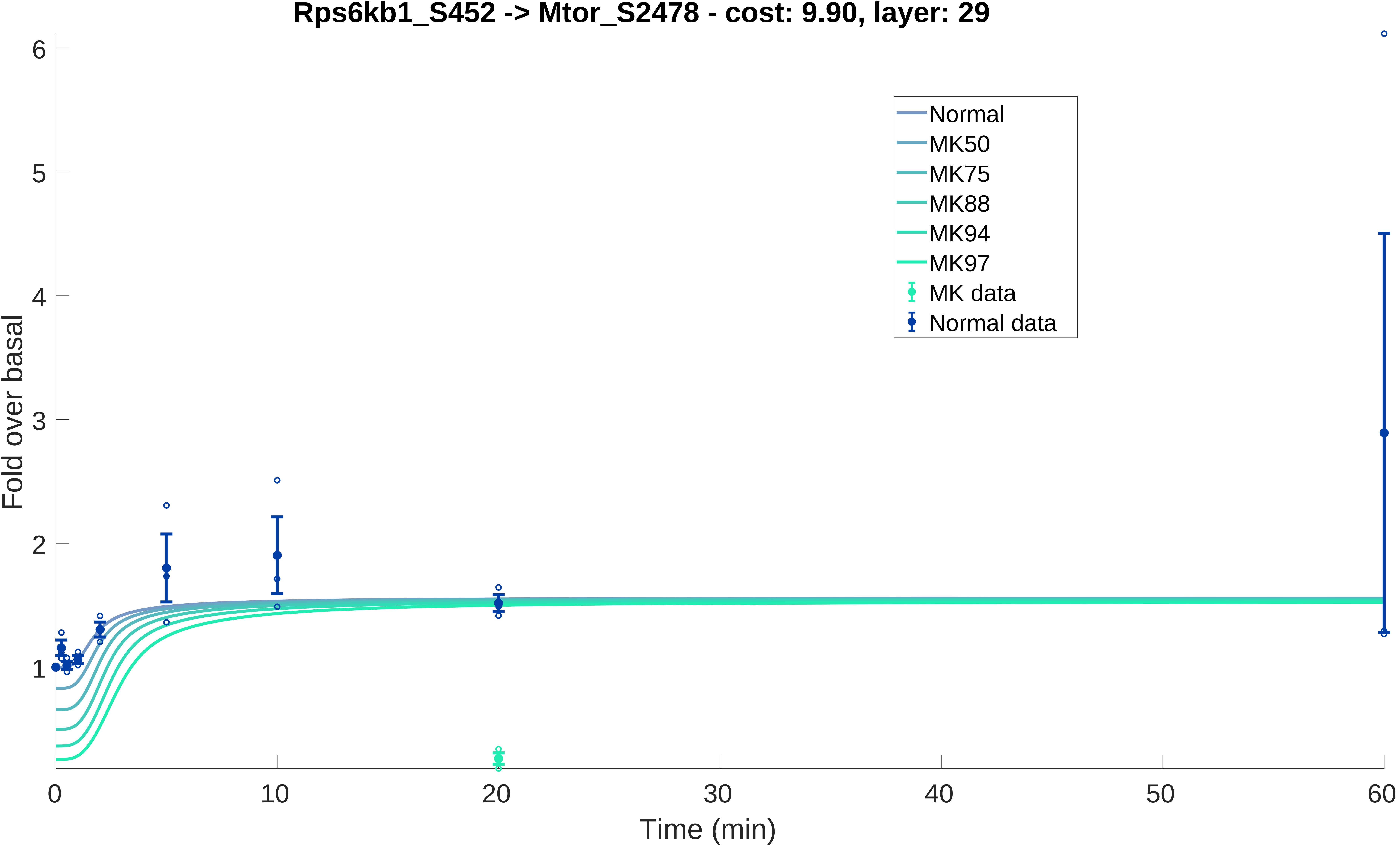
Rps6kb1_S452 -> Mtor_S2478 - cost: 9.90, layer: 29
Fold over basal
Time (min)
Normal
MK50
MK75
MK88
MK94
MK97
MK data
Normal data
0
10
20
30
40
50
60
1
2
3
4
5
6

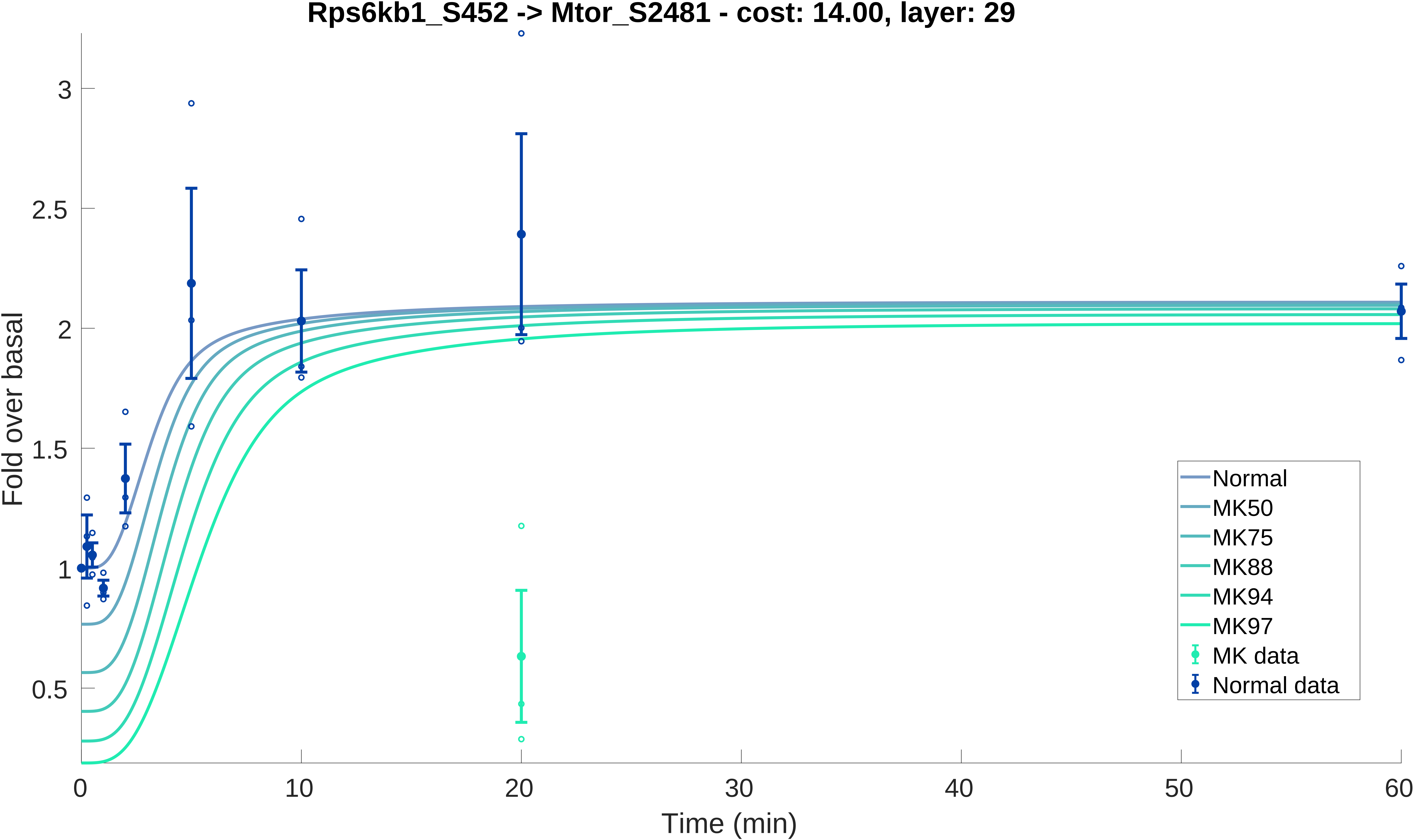
Rps6kb1_S452 -> Mtor_S2481 - cost: 14.00, layer: 29
Fold over basal
Time (min)
0
10
20
30
40
50
60
0.5
1
1.5
2
2.5
3
Normal
MK50
MK75
MK88
MK94
MK97
MK data
Normal data

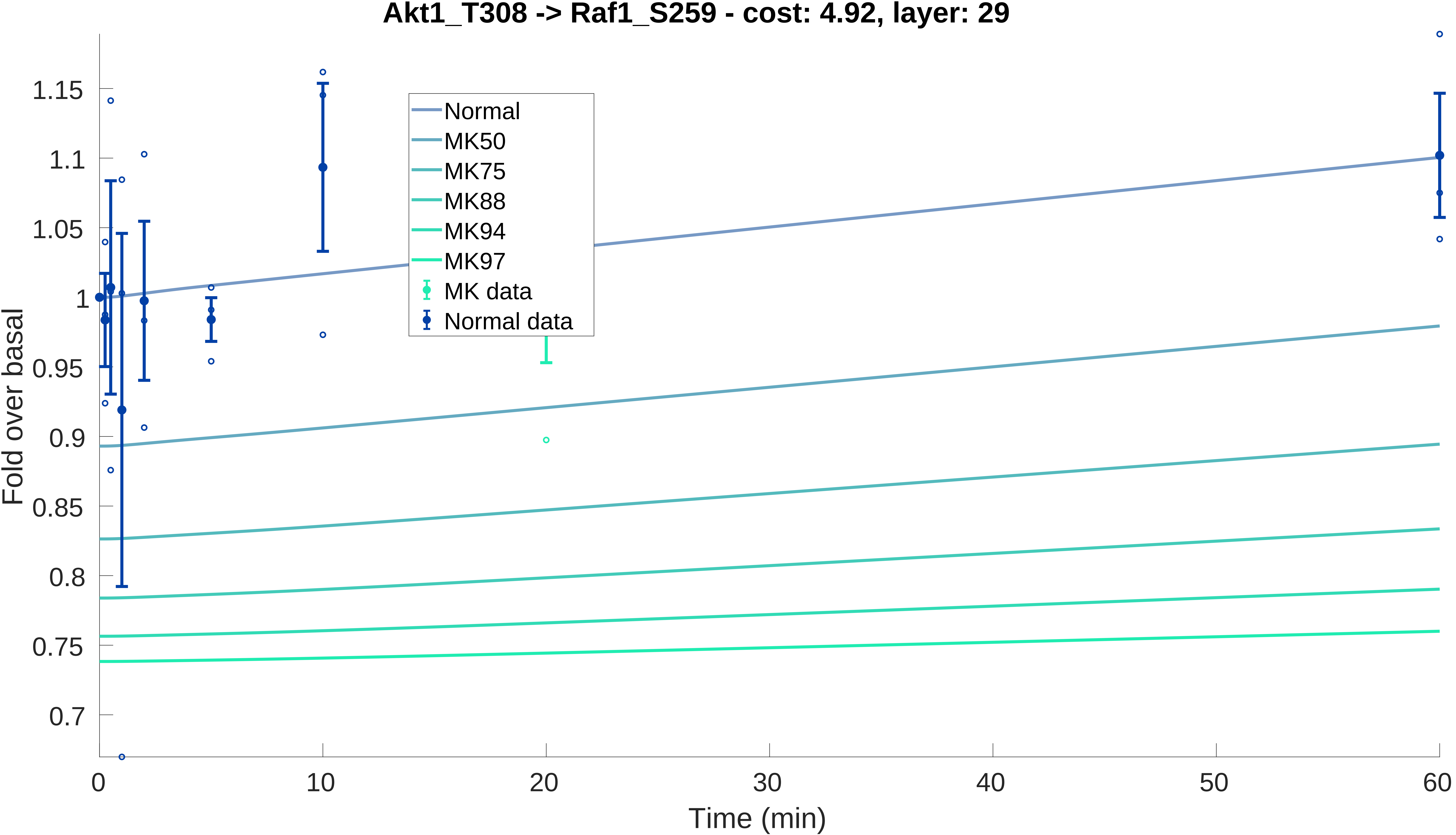
Akt1_T308 -> Raf1_S259 - cost: 4.92, layer: 29
Fold over basal
Time (min)
Normal
MK50
MK75
MK88
MK94
MK97
MK data
Normal data
1.15
1.1
1.05
1
0.95
0.9
0.85
0.8
0.75
0.7
0
10
20
30
40
50
60

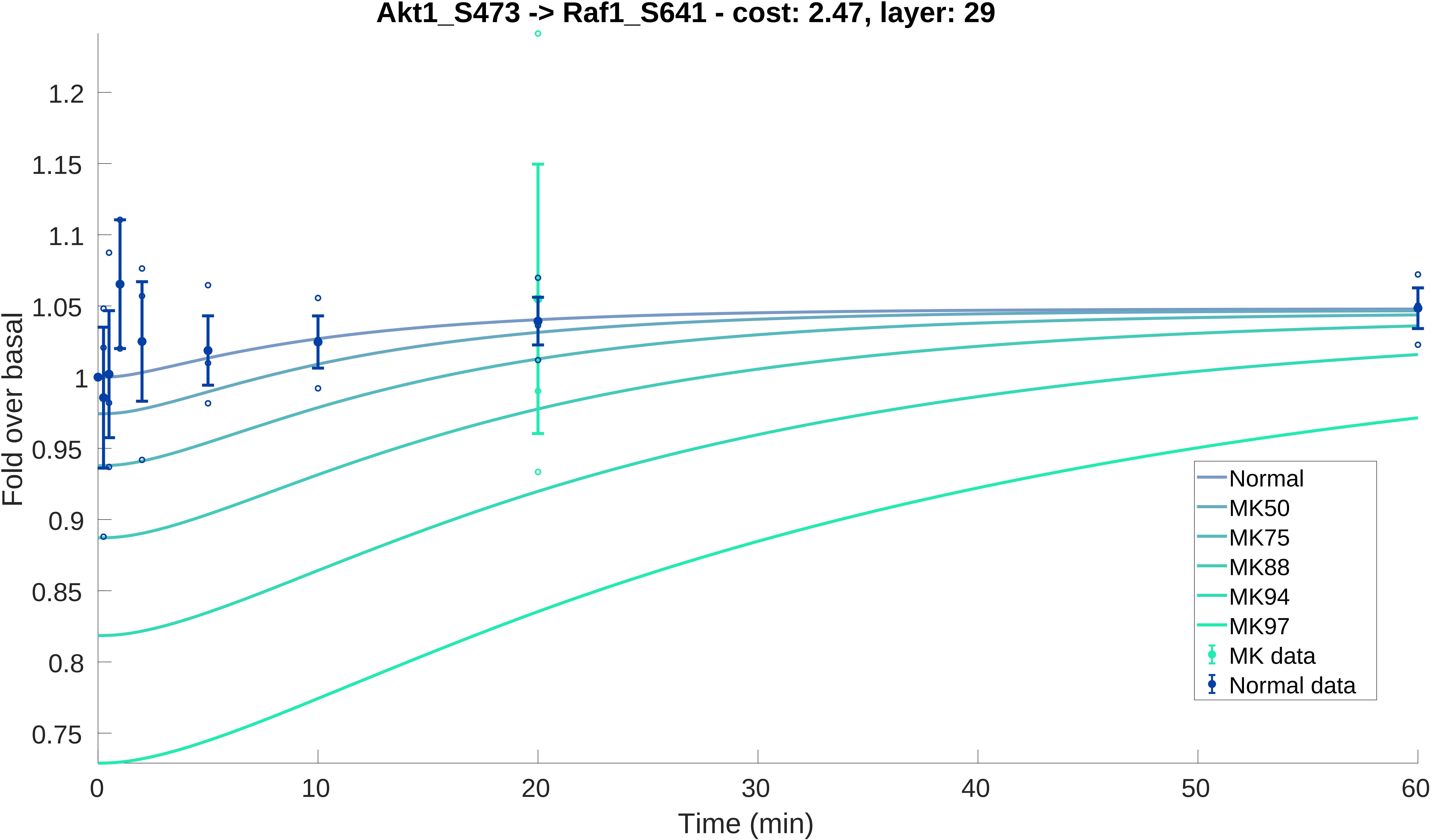

Akt1_S473 -> Raf1_S641 - cost: 2.47, layer: 29
Fold over basal
Time (min)
1.2
1.15
1.1
1.05
1
0.95
0.9
0.85
0.8
0.75
0
10
20
30
40
50
60
Normal
MK50
MK75
MK88
MK94
MK97
MK data
Normal data

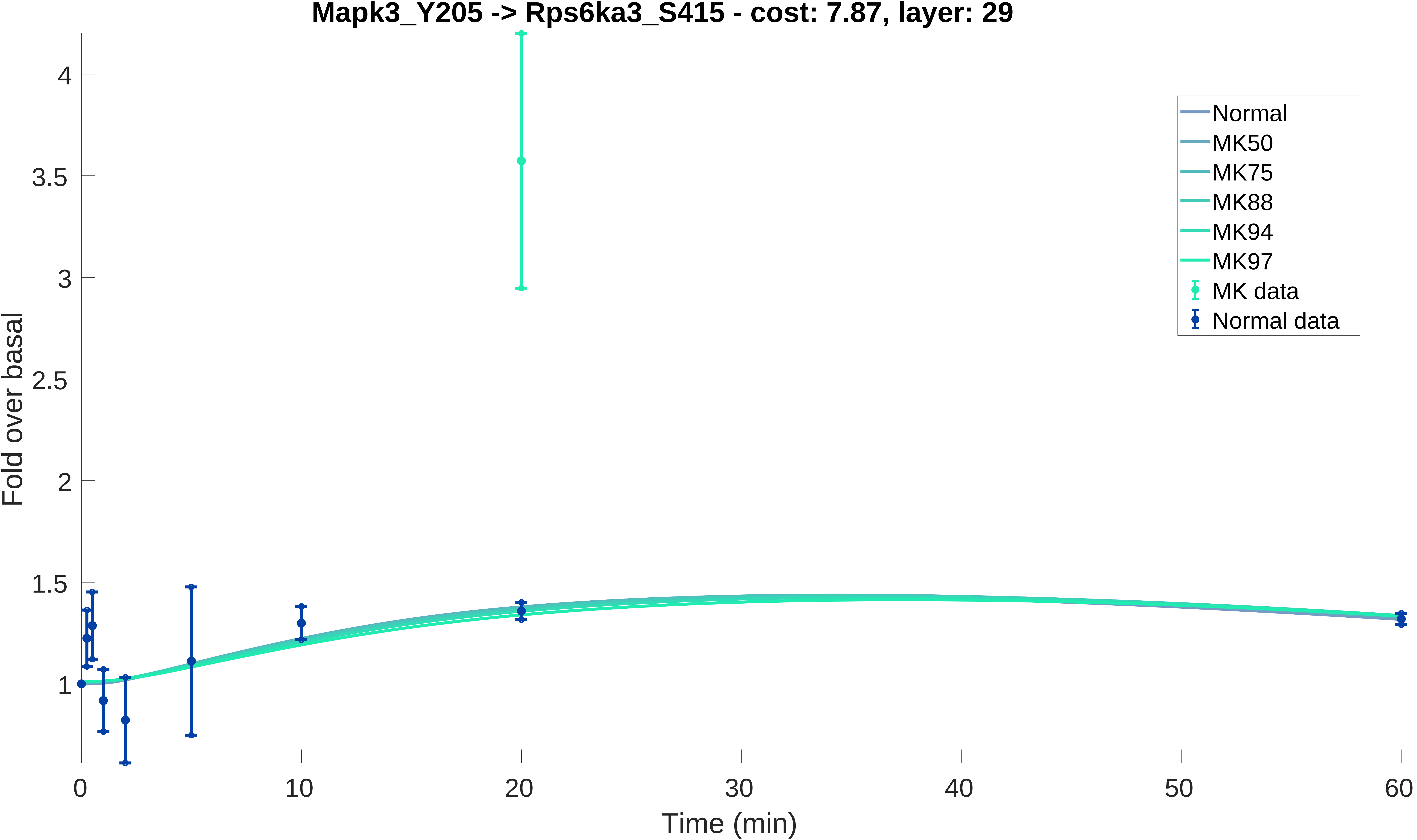
Mapk3_Y205 -> Rps6ka3_S415 - cost: 7.87, layer: 29
Fold over basal
Time (min)
0
10
20
30
40
50
60
1
1.5
2
2.5
3
3.5
4
Normal
MK50
MK75
MK88
MK94
MK97
MK data
Normal data

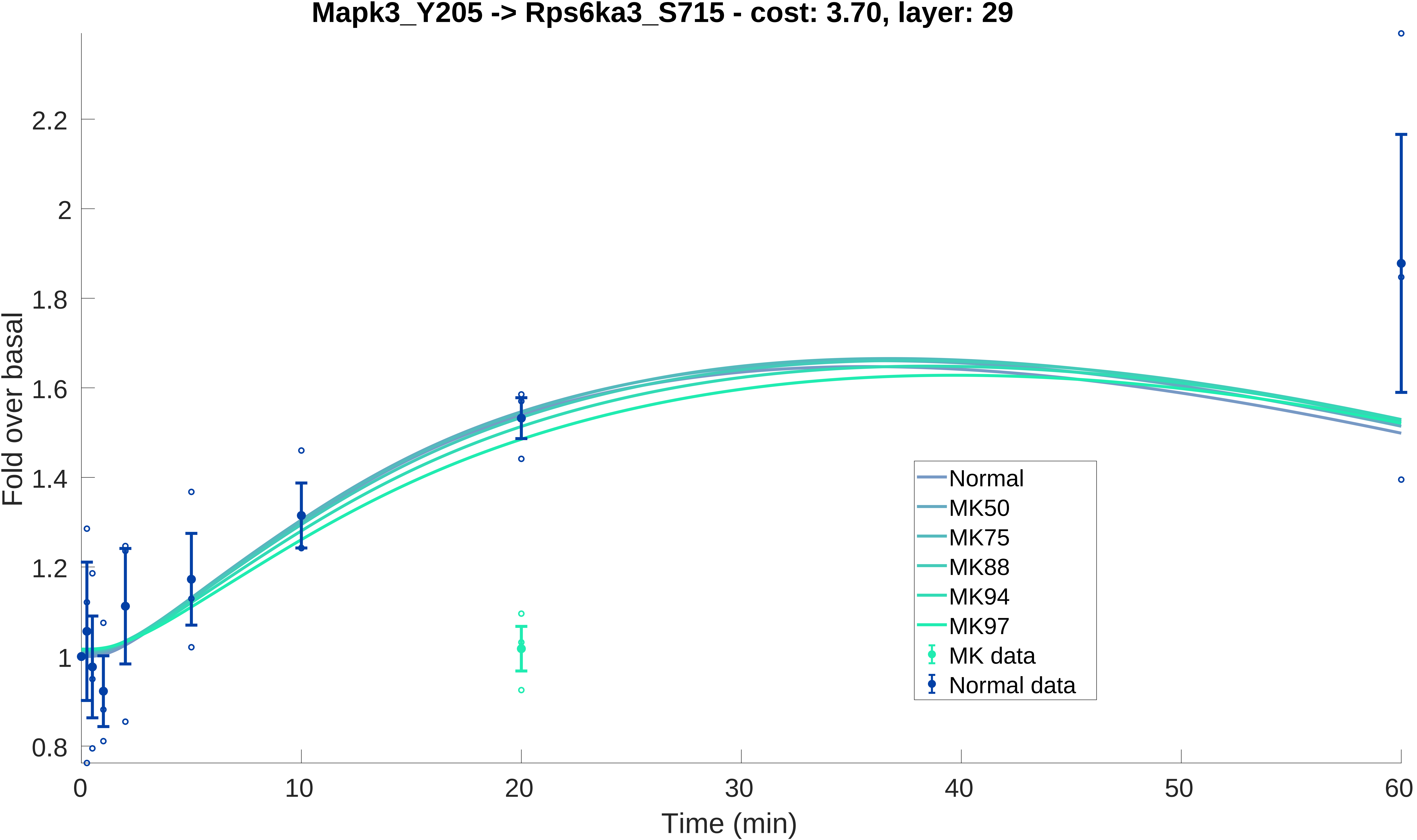
Mapk3_Y205 -> Rps6ka3_S715 - cost: 3.70, layer: 29
Fold over basal
Time (min)
0.8
1
1.2
1.4
1.6
1.8
2
2.2
0
10
20
30
40
50
60
Normal
MK50
MK75
MK88
MK94
MK97
MK data
Normal data

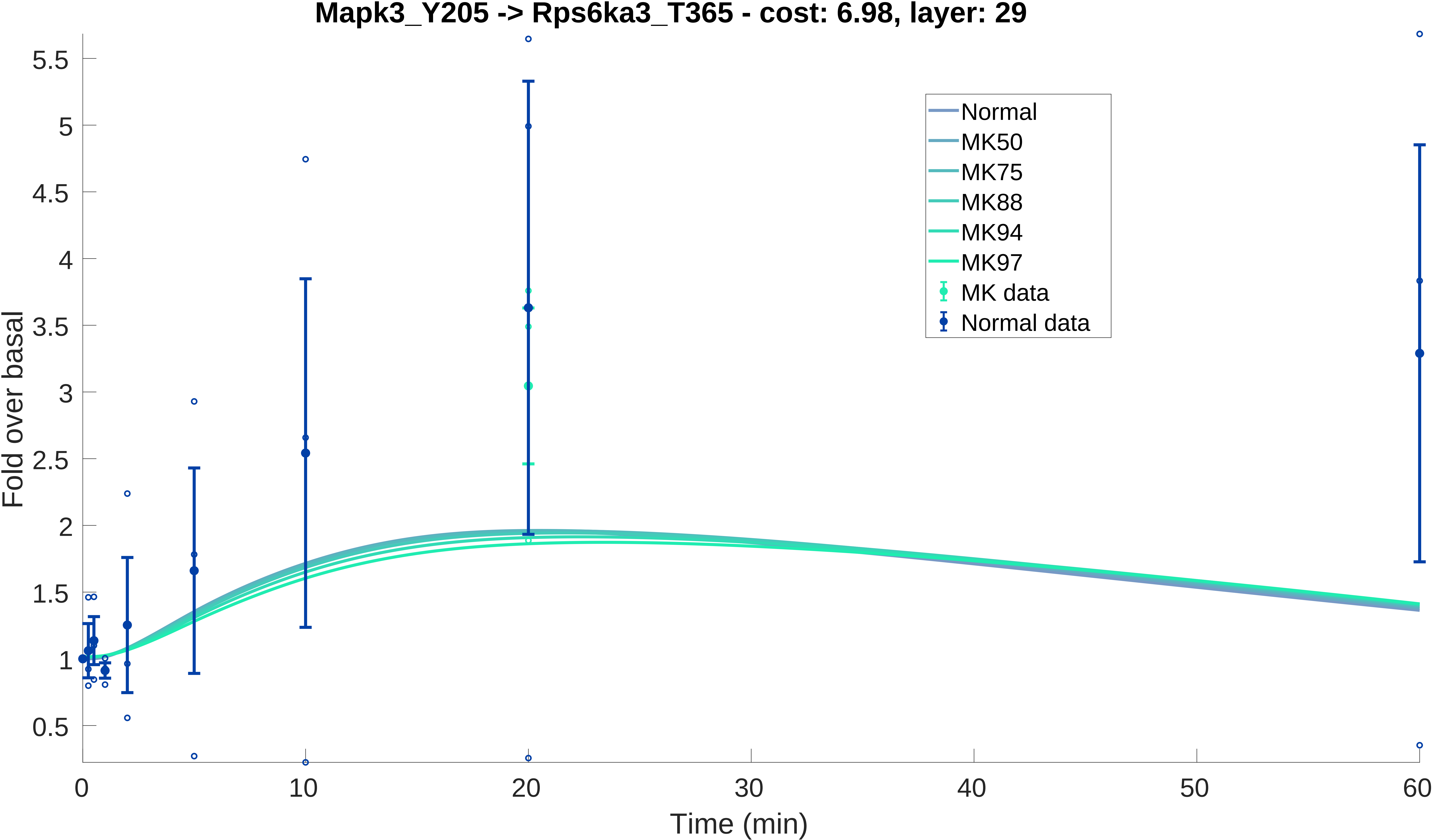
Mapk3_Y205 -> Rps6ka3_T365 - cost: 6.98, layer: 29
Normal
MK50
MK75
MK88
MK94
MK97
MK data
Normal data
Fold over basal
Time (min)
0
10
20
30
40
50
60
0.5
1
1.5
2
2.5
3
3.5
4
4.5
5
5.5

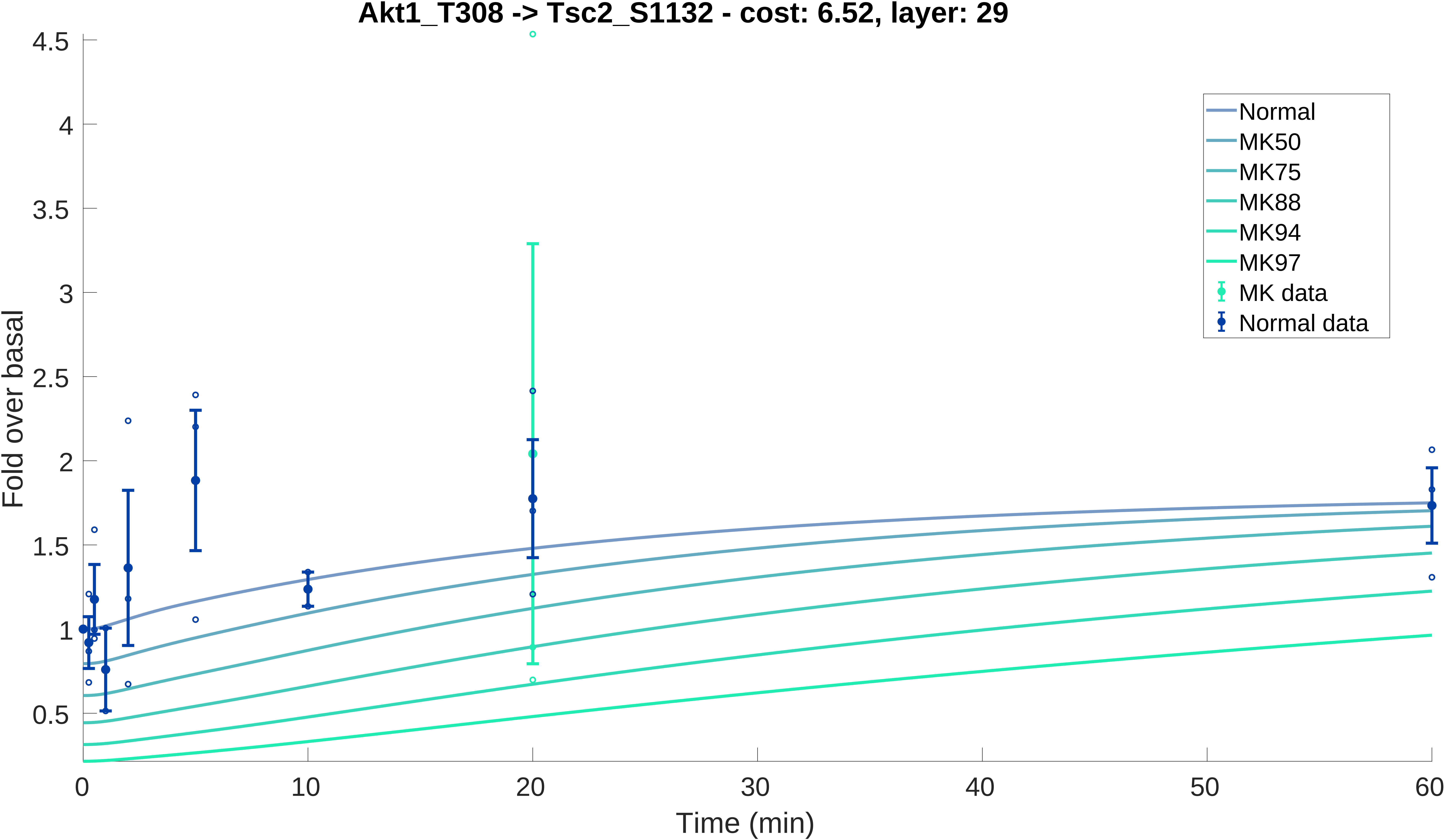

Akt1_T308 -> Tsc2_S1132 - cost: 6.52, layer: 29
Fold over basal
Time (min)
0.5
1
1.5
2
2.5
3
3.5
4
4.5
0
10
20
30
40
50
60
Normal
MK50
MK75
MK88
MK94
MK97
MK data
Normal data

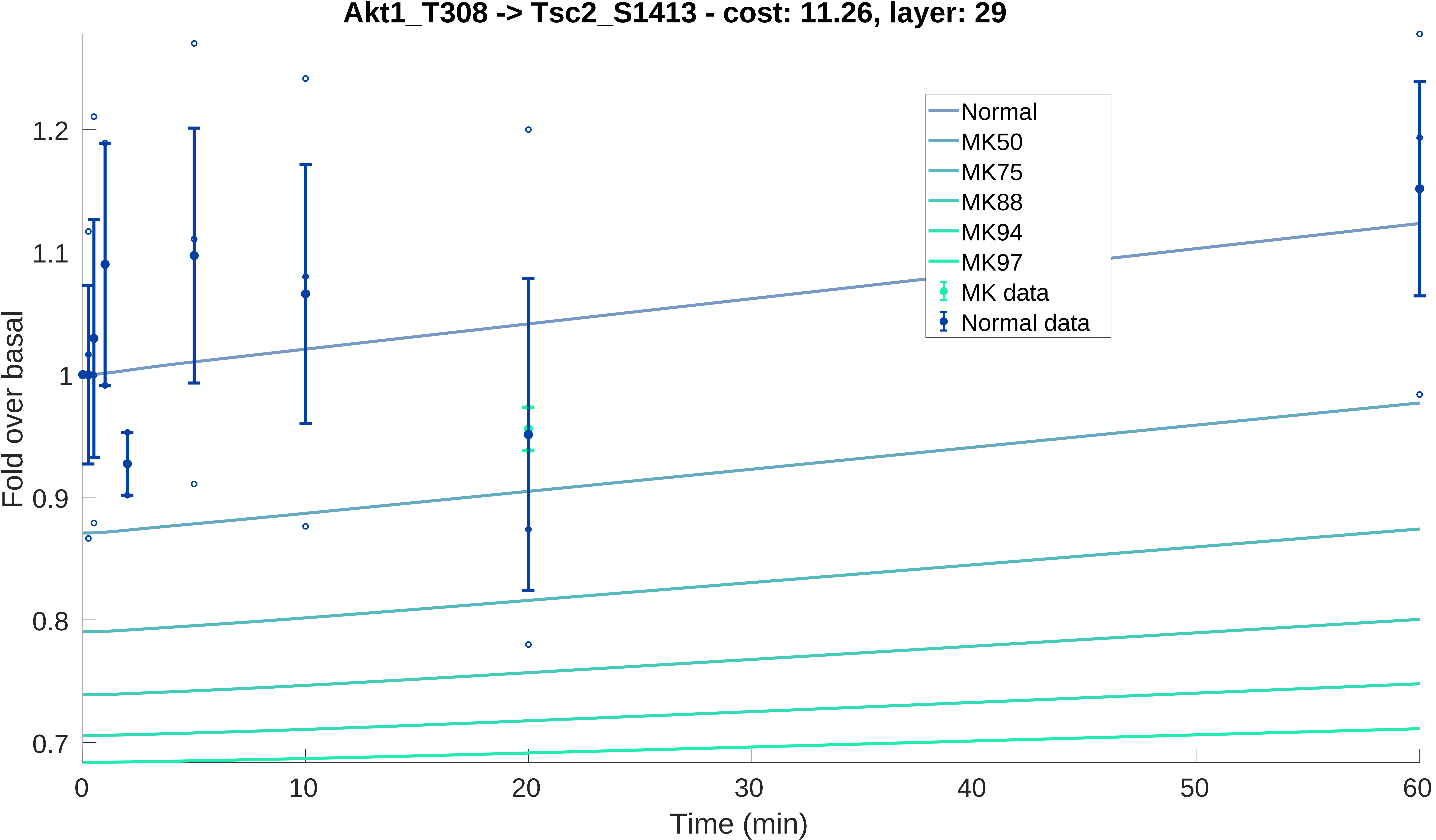
Akt1_T308 -> Tsc2_S1413 - cost: 11.26, layer: 29
Fold over basal
1.2
1.1
1
0.9
0.8
0.7
0
10
20
30
40
50
60
Time (min)
Normal
MK50
MK75
MK88
MK94
MK97
MK data
Normal data

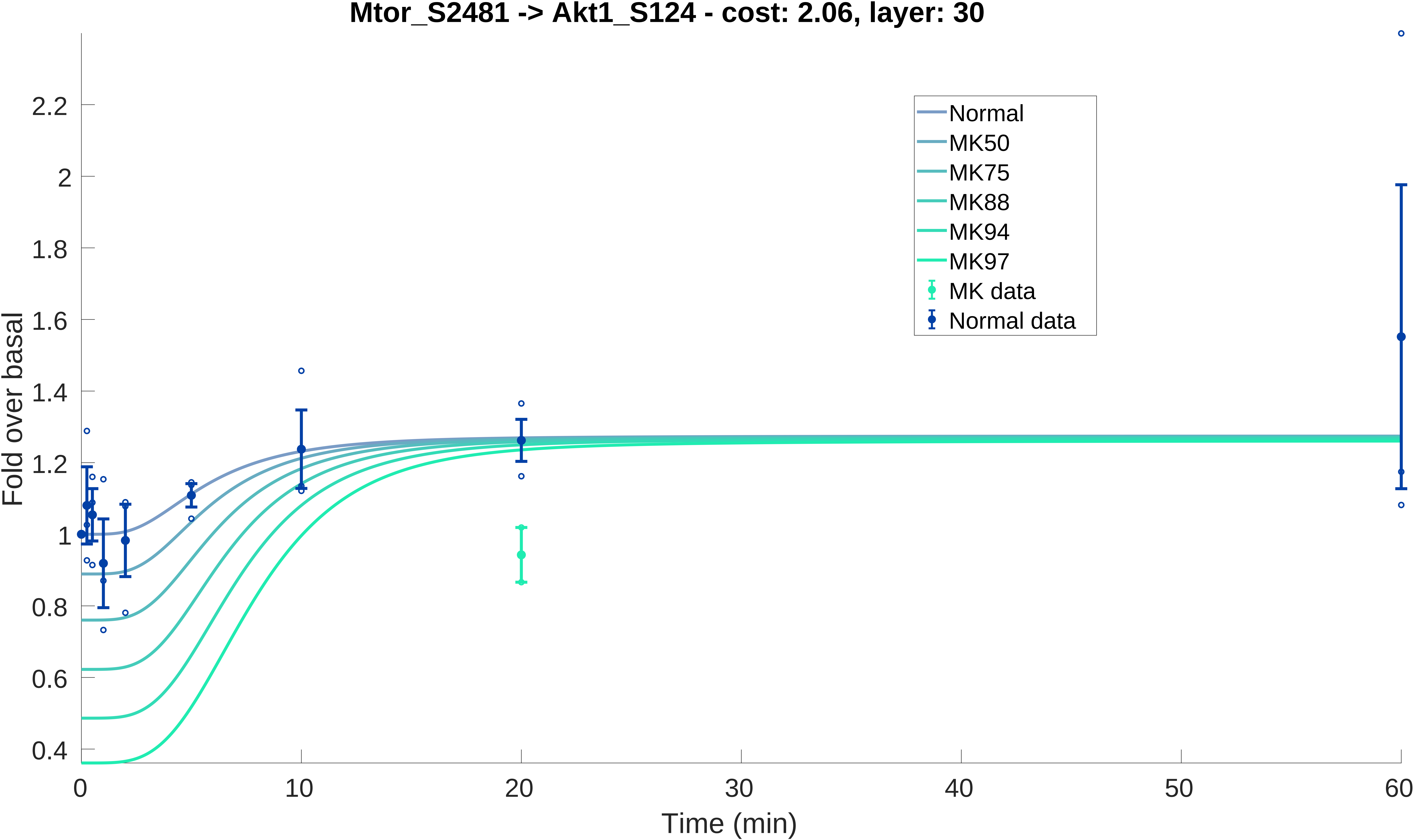

Mtor_S2481 -> Akt1_S124 - cost: 2.06, layer: 30
Fold over basal
Time (min)
0.4
0.6
0.8
1
1.2
1.4
1.6
1.8
2
2.2
0
10
20
30
40
50
60
Normal
MK50
MK75
MK88
MK94
MK97
MK data
Normal data

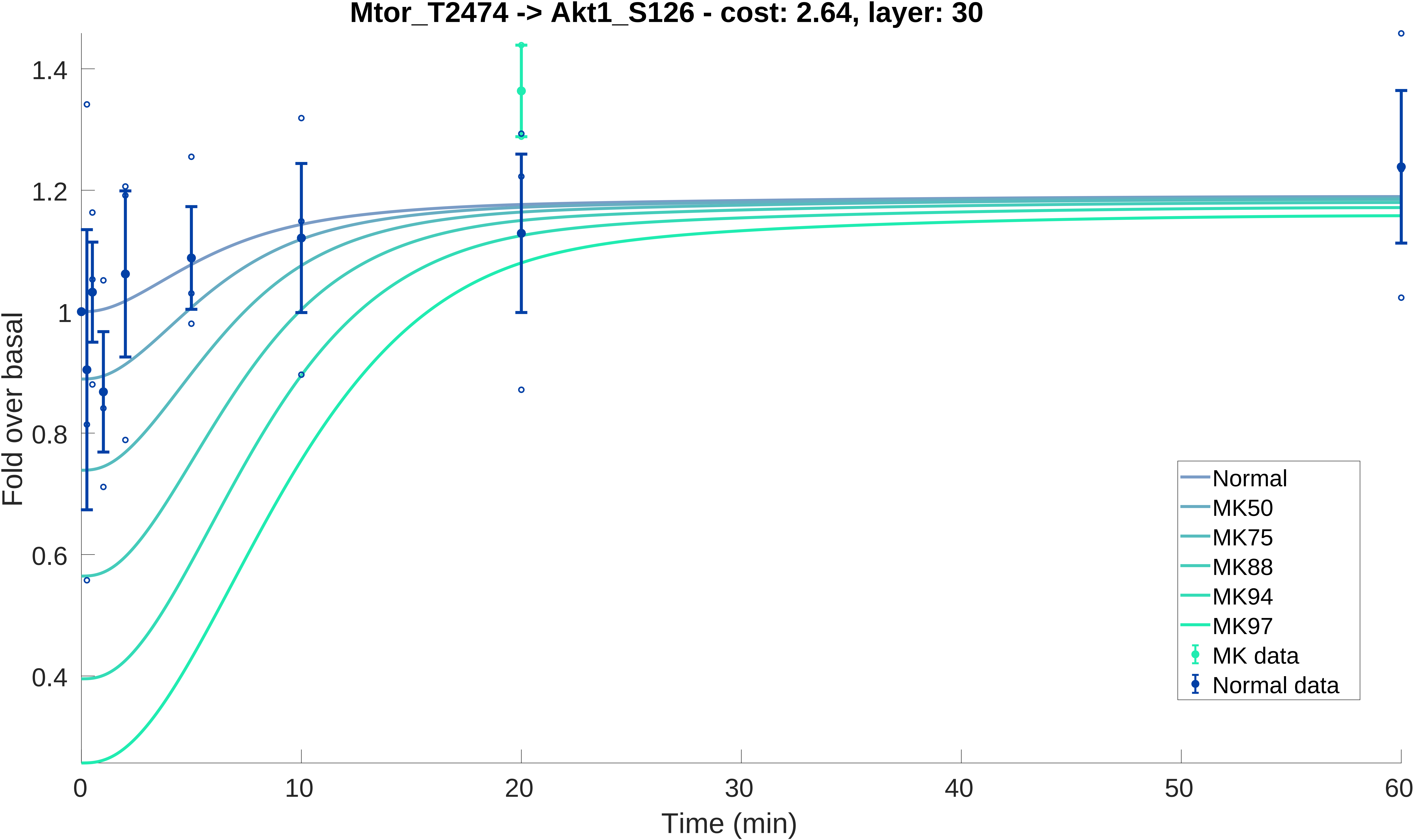
Mtor_T2474 -> Akt1_S126 - cost: 2.64, layer: 30
Fold over basal
1.4
1.2
1
0.8
0.6
0.4
0
10
20
30
40
50
60
Time (min)
Normal
MK50
MK75
MK88
MK94
MK97
MK data
Normal data

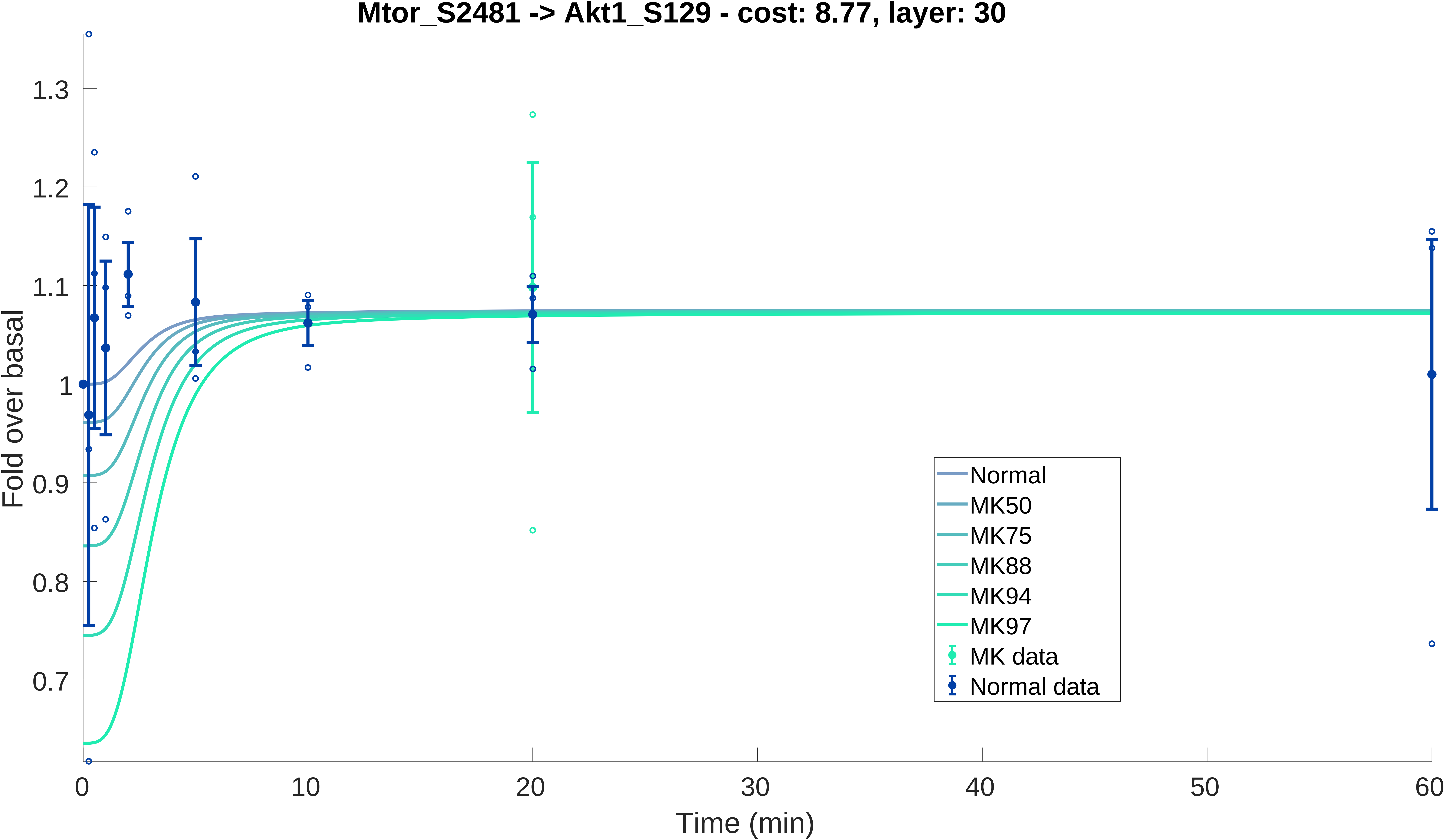

Mtor_S2481 -> Akt1_S129 - cost: 8.77, layer: 30
Fold over basal
Time (min)
1.3
1.2
1.1
1
0.9
0.8
0.7
0
10
20
30
40
50
60
Normal
MK50
MK75
MK88
MK94
MK97
MK data
Normal data

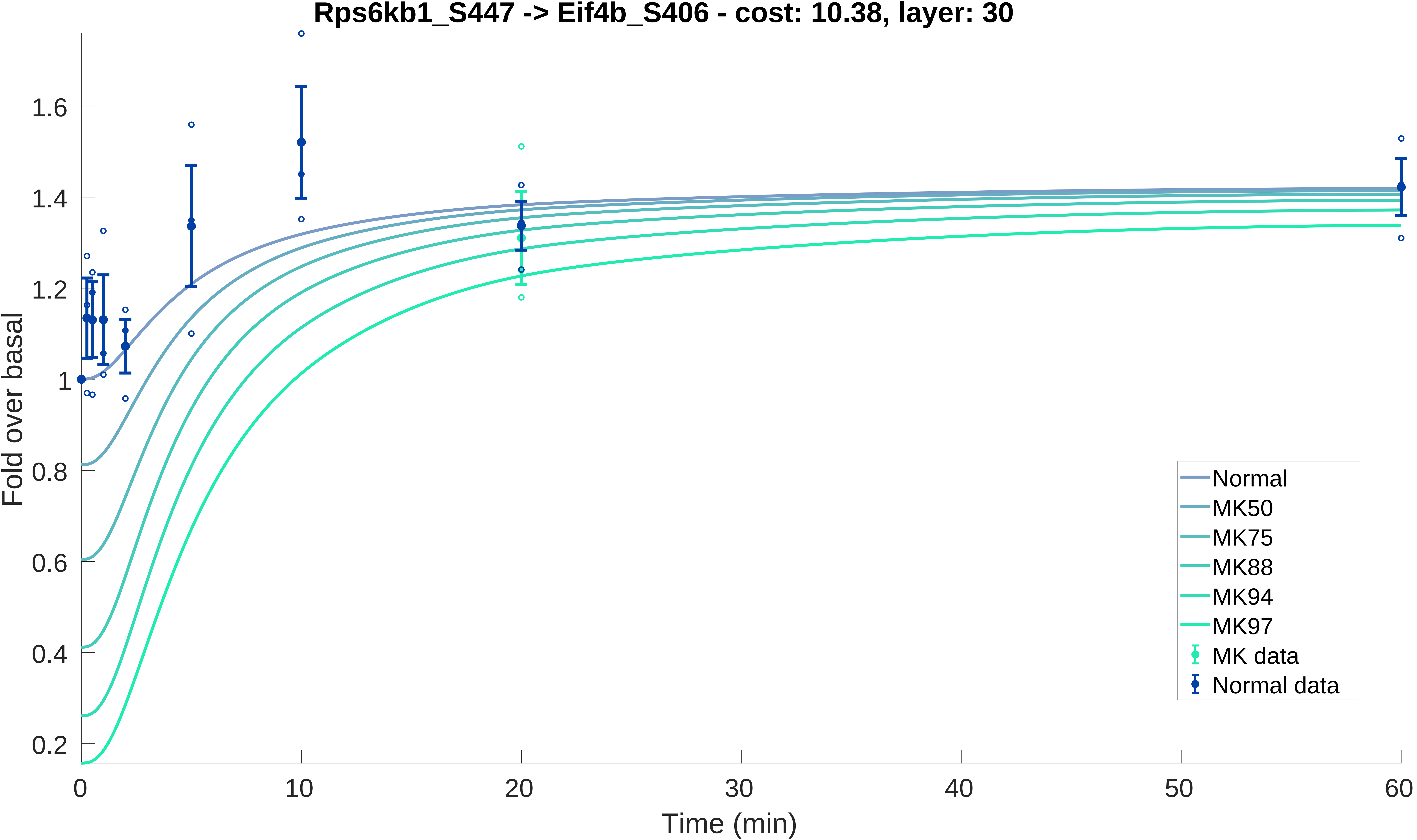

Rps6kb1_S447 -> Eif4b_S406 - cost: 10.38, layer: 30
Fold over basal
Time (min)
0.2
0.4
0.6
0.8
1
1.2
1.4
1.6
0
10
20
30
40
50
60
Normal
MK50
MK75
MK88
MK94
MK97
MK data
Normal data

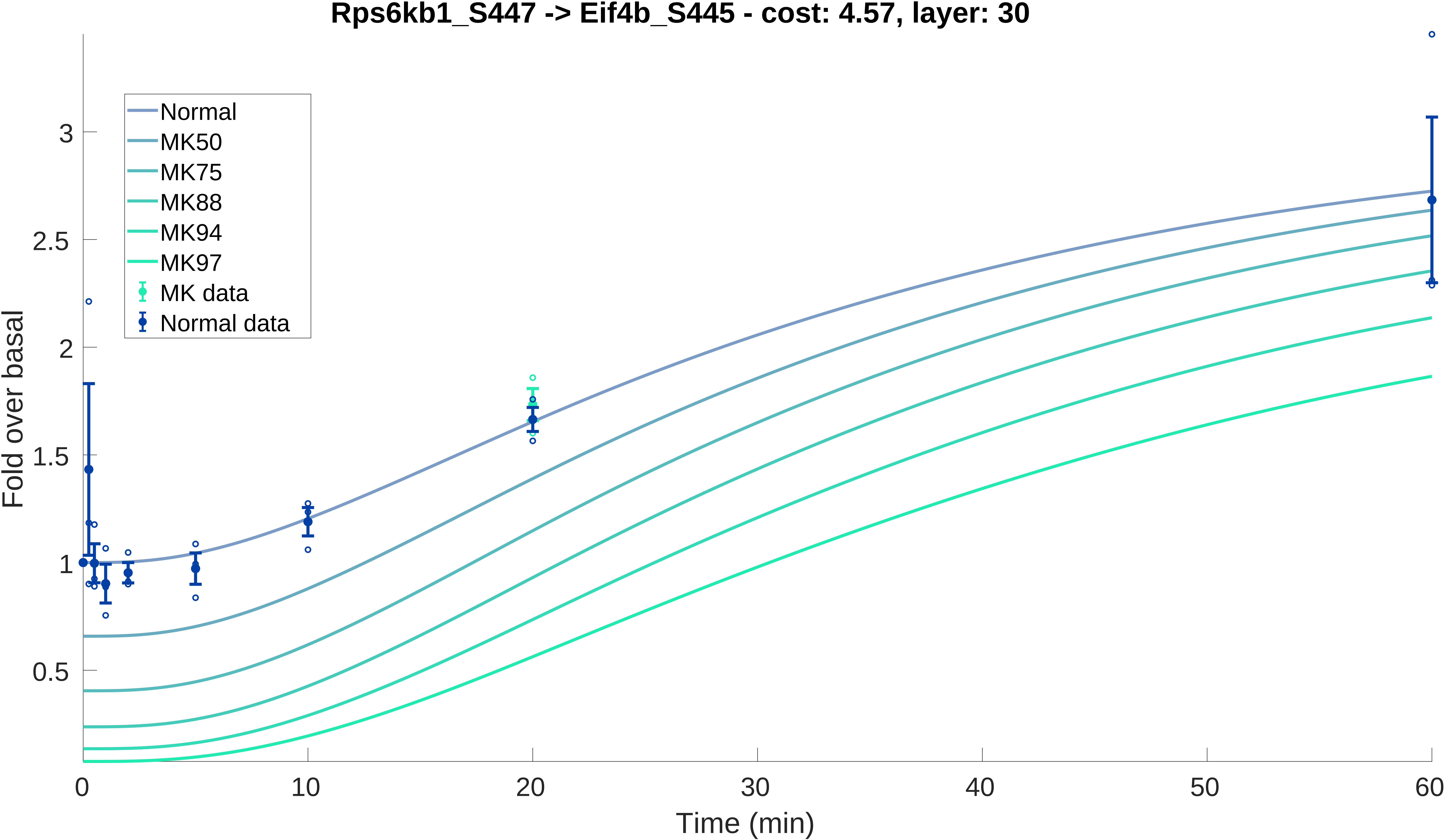
Rps6kb1_S447 -> Eif4b_S445 - cost: 4.57, layer: 30
Fold over basal
Time (min)
0.5
1
1.5
2
2.5
3
0
10
20
30
40
50
60
Normal
MK50
MK75
MK88
MK94
MK97
MK data
Normal data

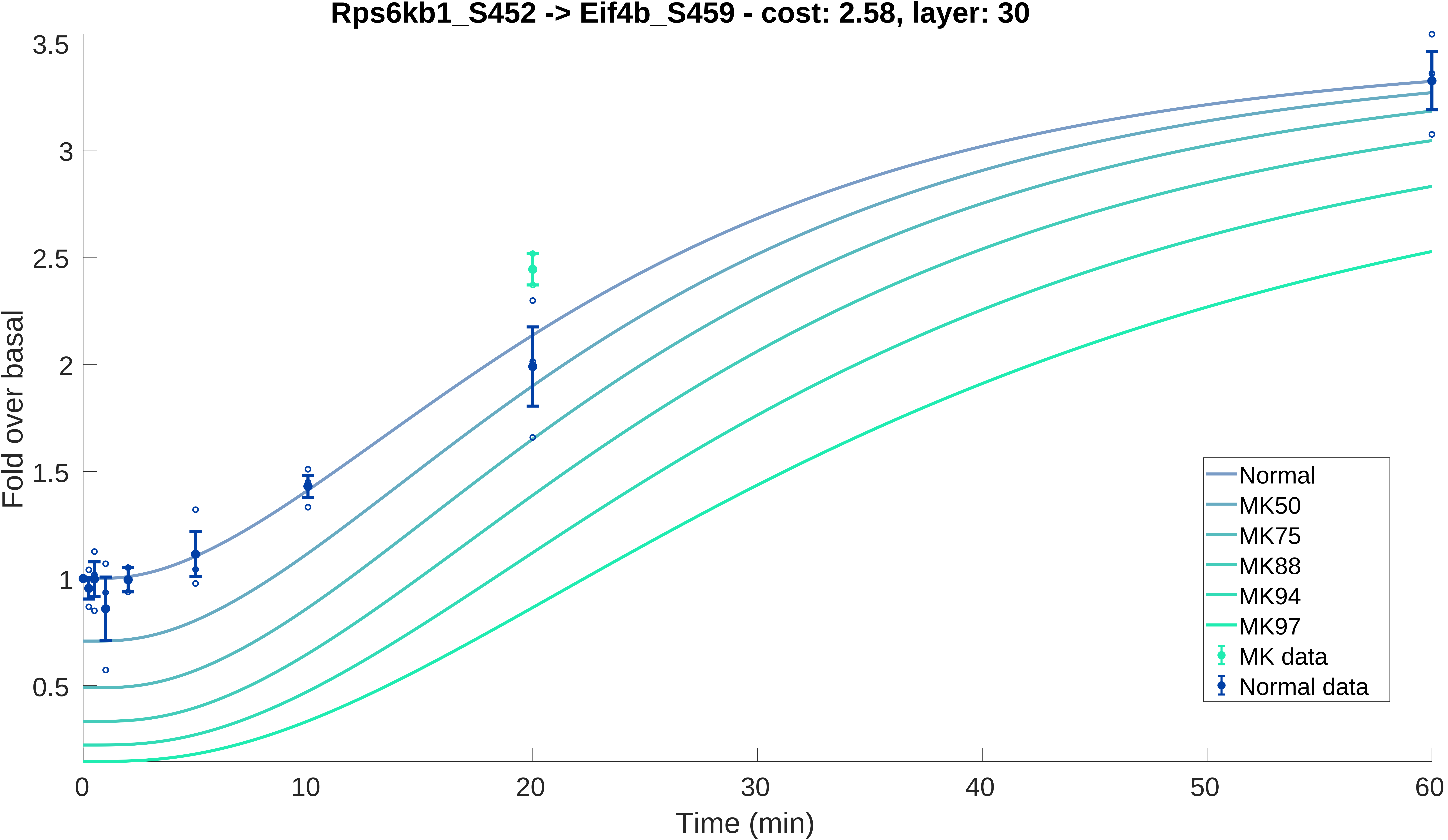
Rps6kb1_S452 -> Eif4b_S459 - cost: 2.58, layer: 30
Fold over basal
3.5
3
2.5
2
1.5
1
0.5
0
10
20
30
40
50
60
Time (min)
Normal
MK50
MK75
MK88
MK94
MK97
MK data
Normal data

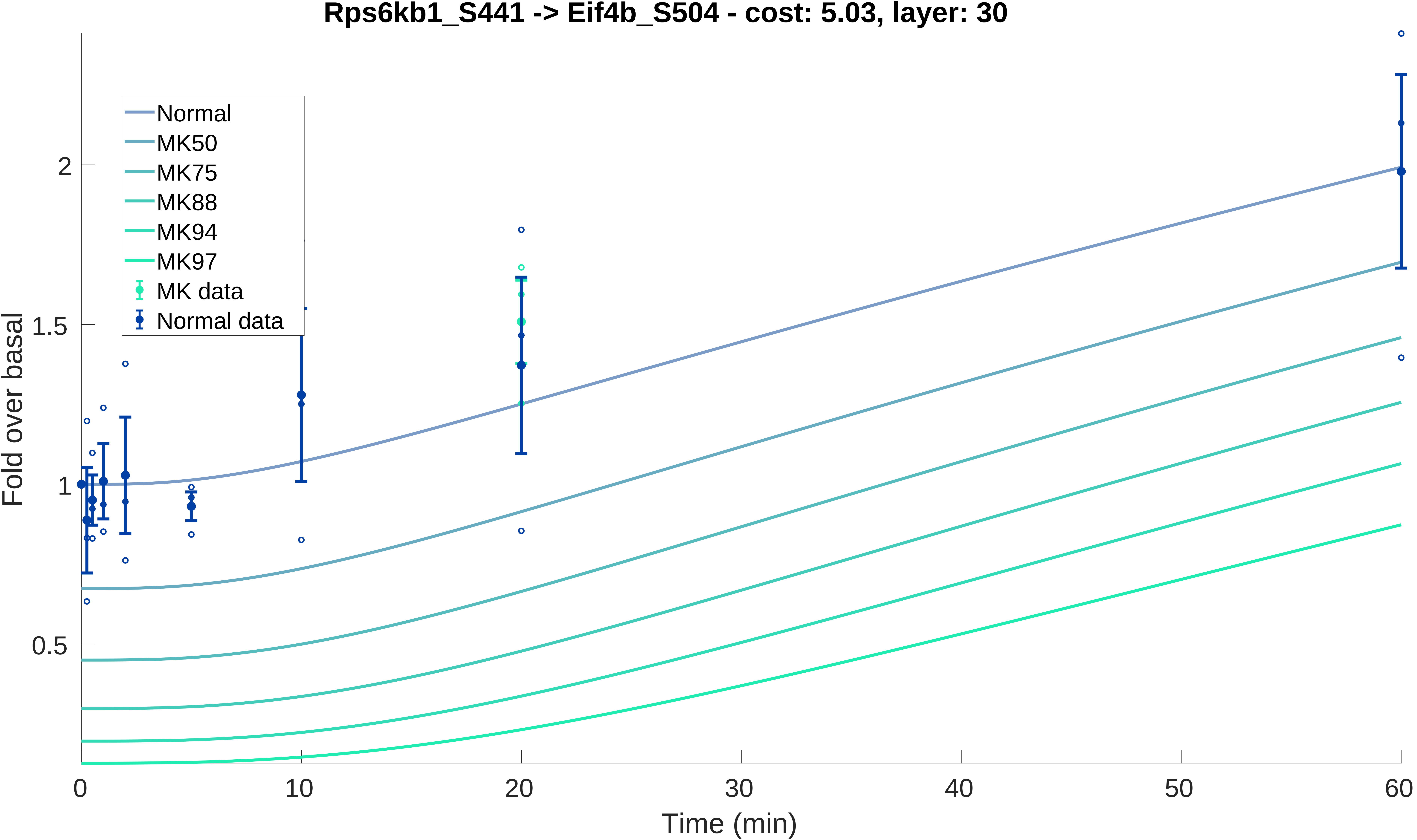

Rps6kb1_S441 -> Eif4b_S504 - cost: 5.03, layer: 30
Normal
MK50
MK75
MK88
MK94
MK97
MK data
Normal data
Fold over basal
Time (min)
0.5
1
1.5
2
0
10
20
30
40
50
60

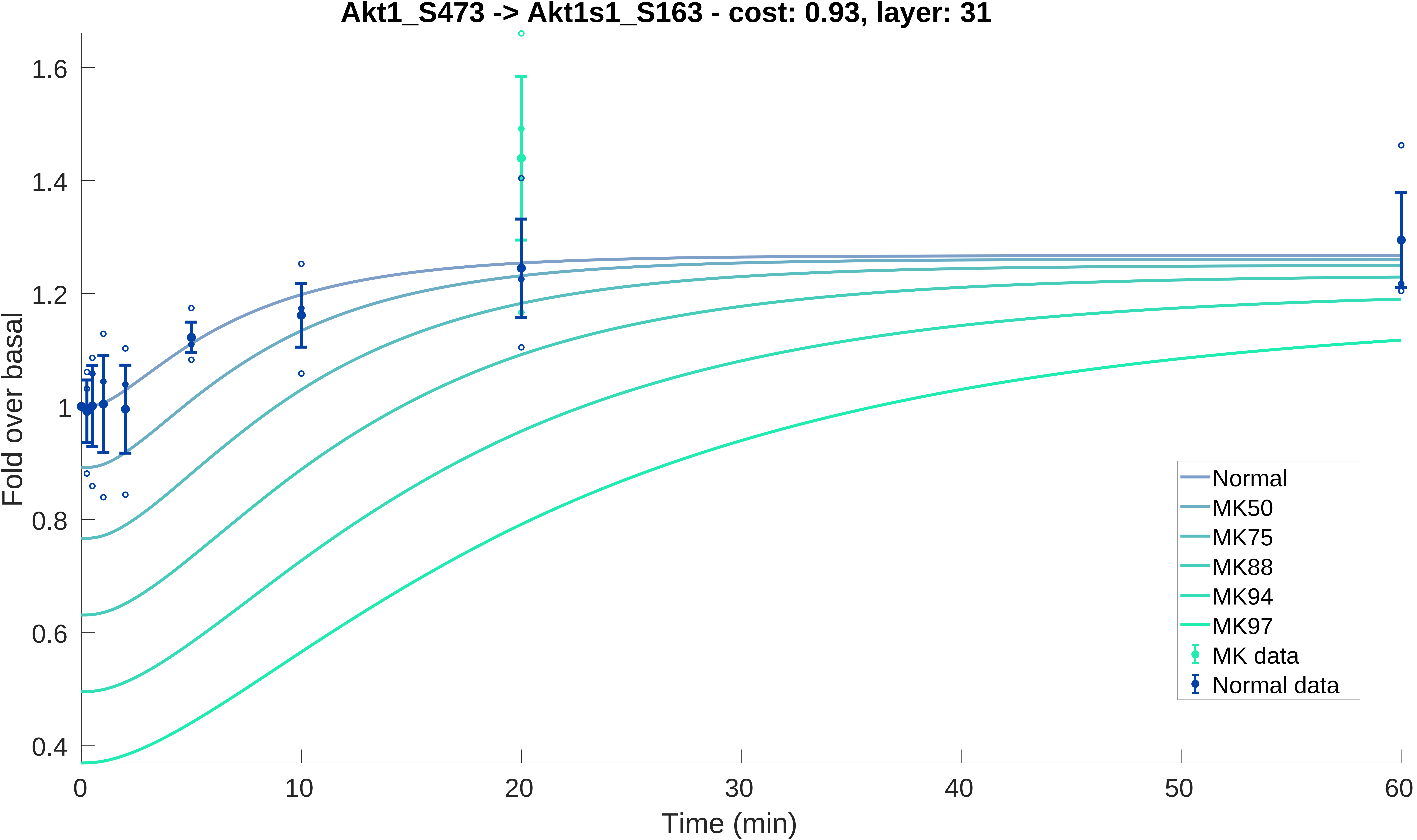
Akt1_S473 -> Akt1s1_S163 - cost: 0.93, layer: 31
Fold over basal
Time (min)
1.6
1.4
1.2
1
0.8
0.6
0.4
0
10
20
30
40
50
60
Normal
MK50
MK75
MK88
MK94
MK97
MK data
Normal data

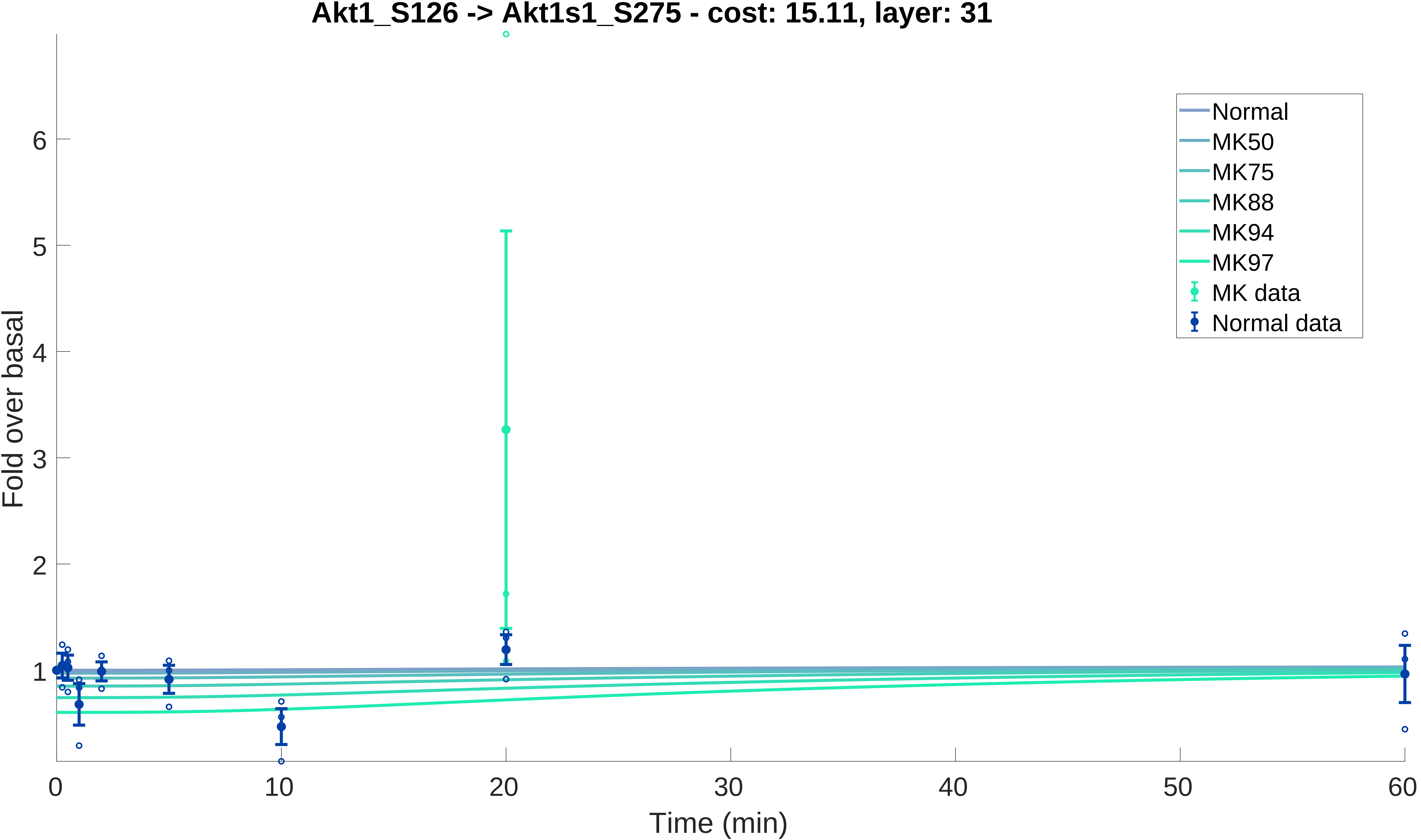

Akt1_S126 -> Akt1s1_S275 - cost: 15.11, layer: 31
Fold over basal
1
2
3
4
5
6
Time (min)
0
10
20
30
40
50
60
Normal
MK50
MK75
MK88
MK94
MK97
MK data
Normal data

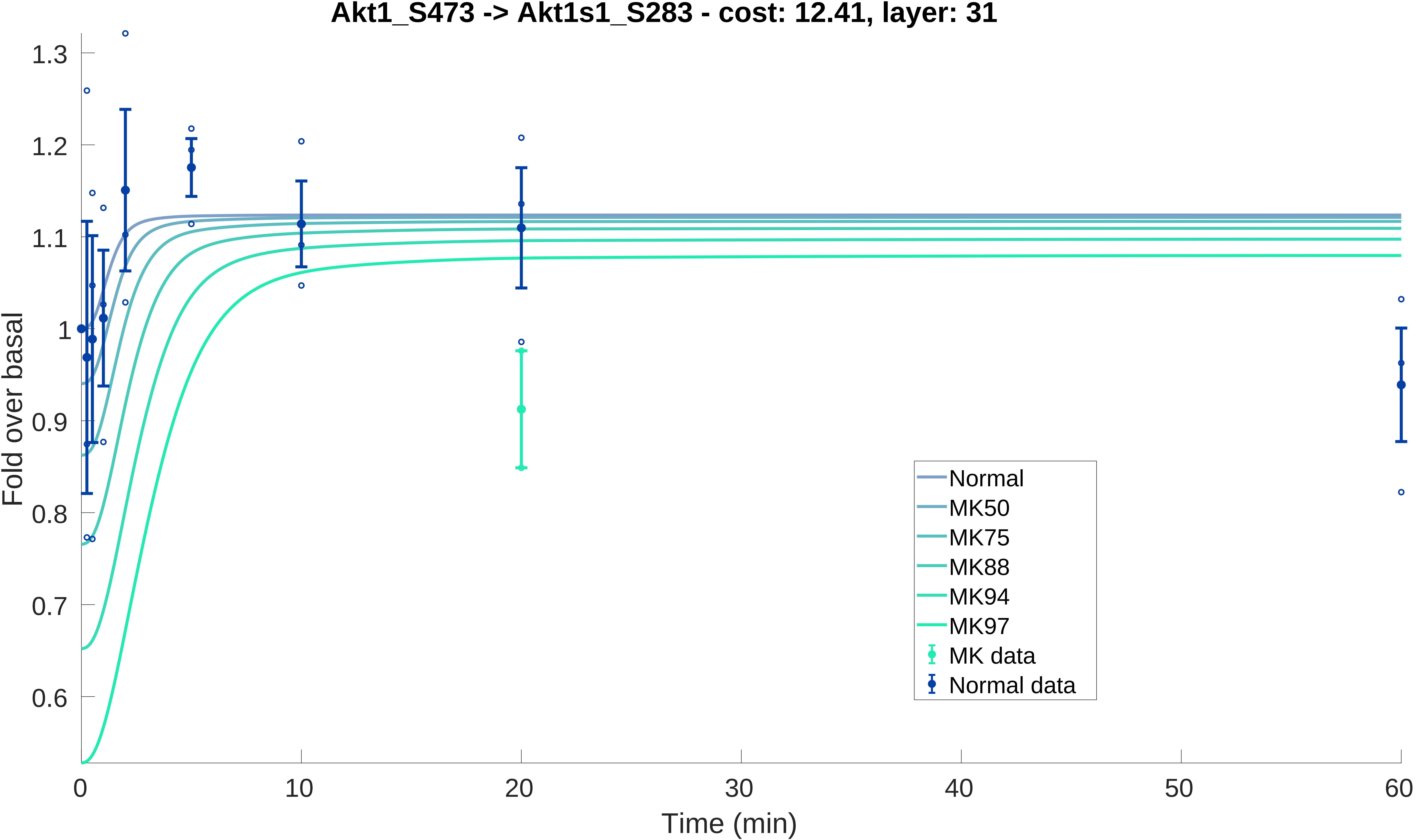

Akt1_S473 -> Akt1s1_S283 - cost: 12.41, layer: 31
Fold over basal
1.3
1.2
1.1
1
0.9
0.8
0.7
0.6
0
10
20
30
40
50
60
Time (min)
Normal
MK50
MK75
MK88
MK94
MK97
MK data
Normal data

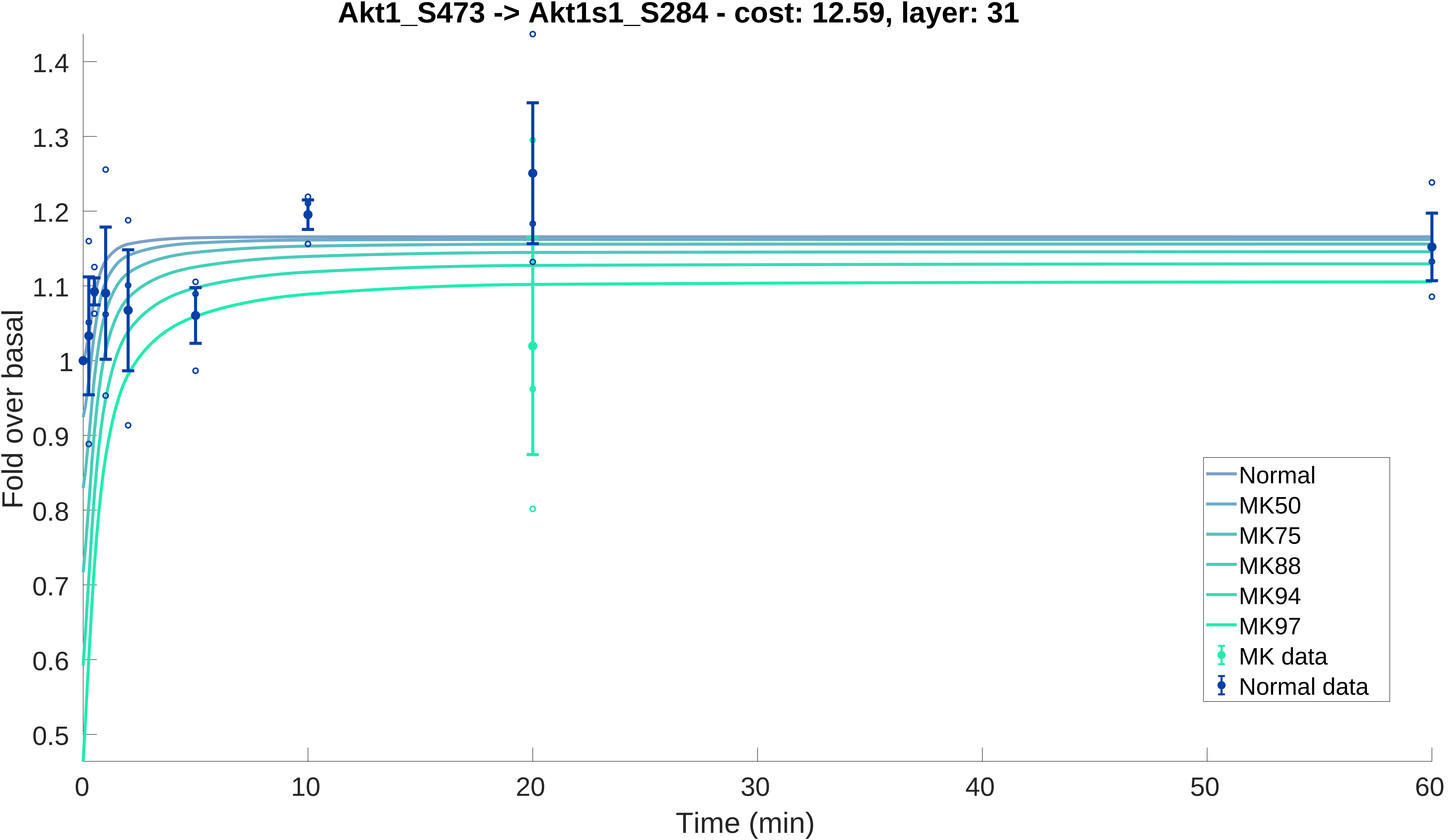
Akt1_S473 -> Akt1s1_S284 - cost: 12.59, layer: 31
Fold over basal
1.4
1.3
1.2
1.1
1
0.9
0.8
0.7
0.6
0.5
0
10
20
30
40
50
60
Time (min)
Normal
MK50
MK75
MK88
MK94
MK97
MK data
Normal data

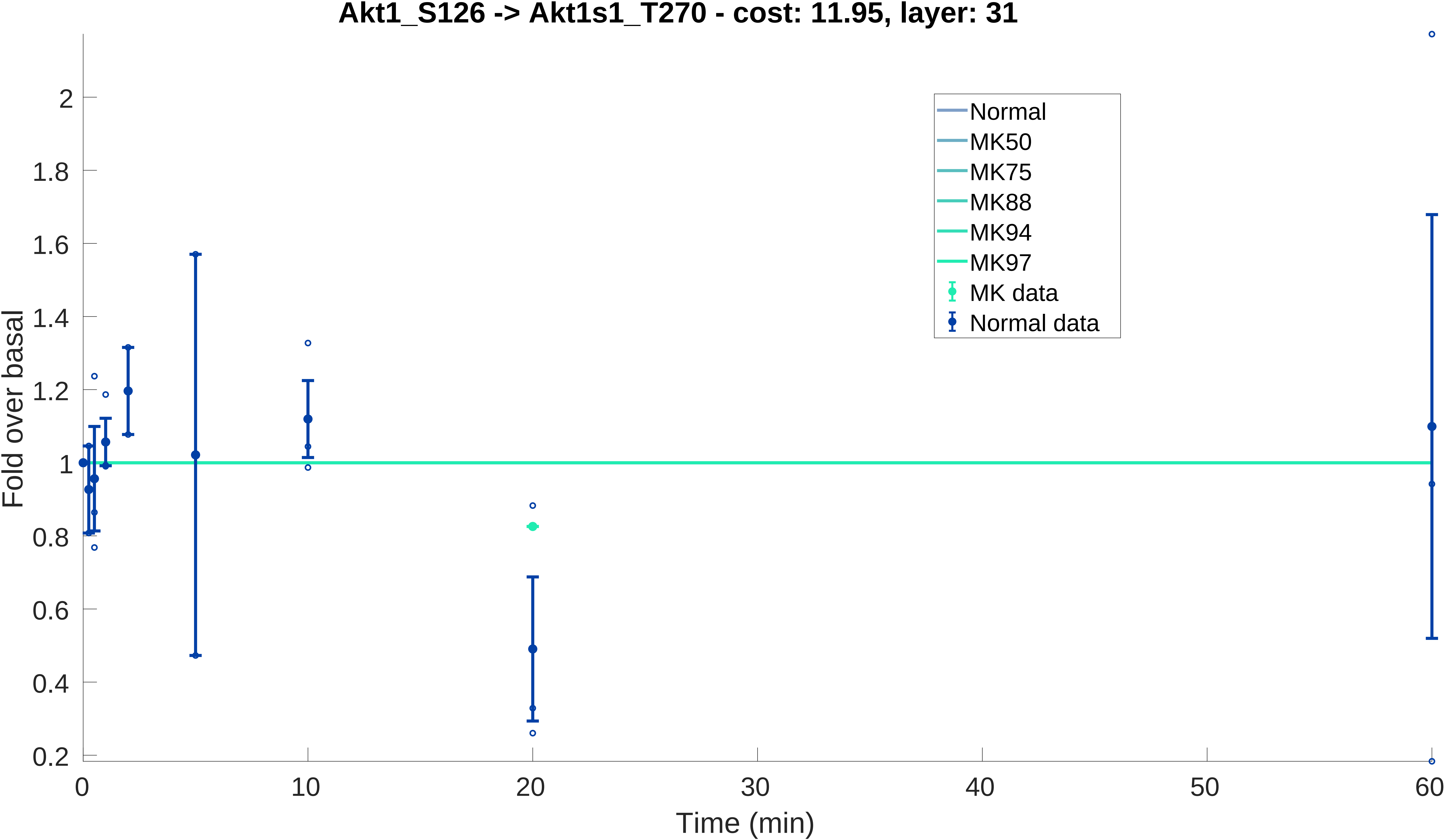

Akt1_S126 -> Akt1s1_T270 - cost: 11.95, layer: 31
Fold over basal
Time (min)
Normal
MK50
MK75
MK88
MK94
MK97
MK data
Normal data
2
1.8
1.6
1.4
1.2
1
0.8
0.6
0.4
0.2
0
10
20
30
40
50
60

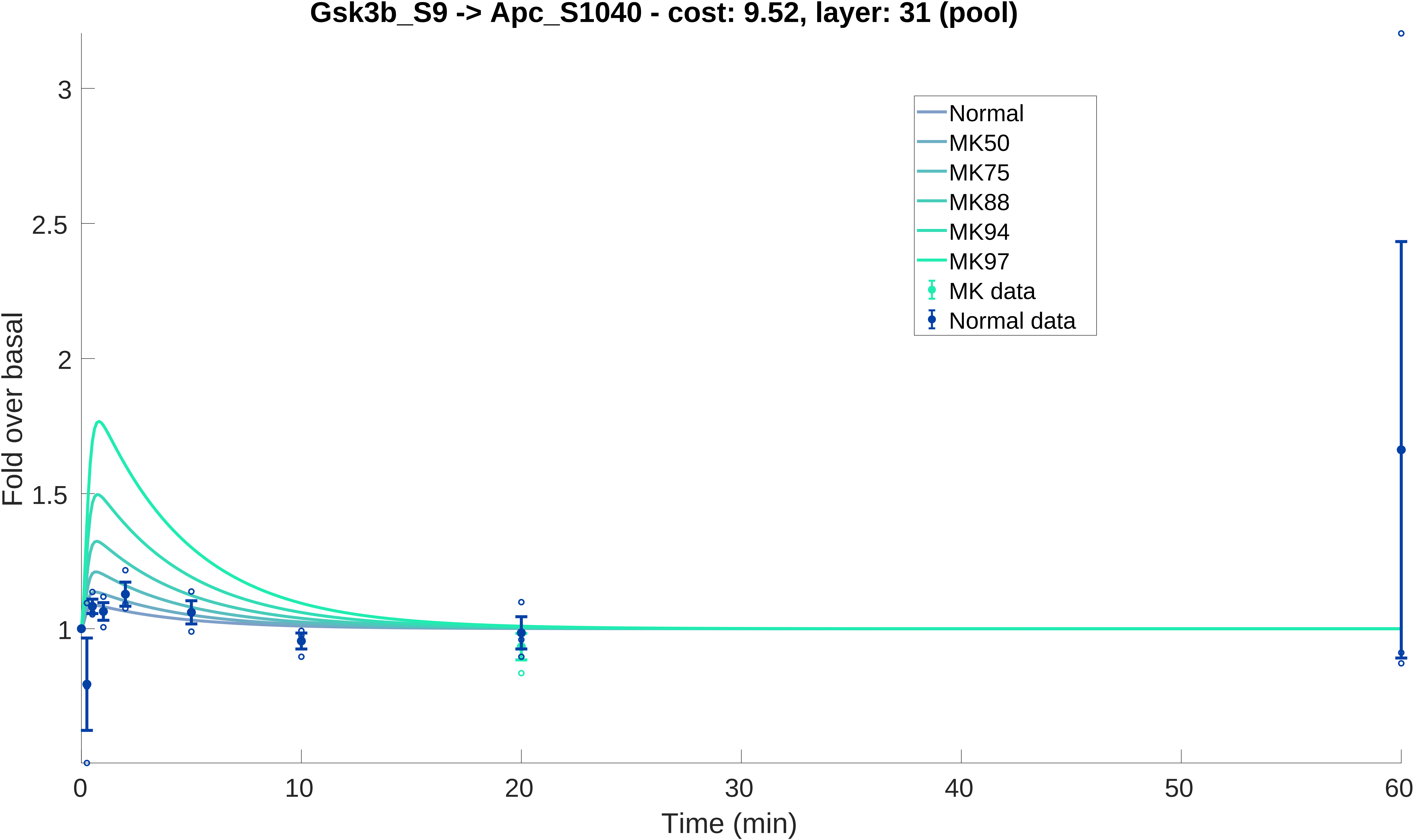
Gsk3b_S9 -> Apc_S1040 - cost: 9.52, layer: 31 (pool)
Normal
MK50
MK75
MK88
MK94
MK97
MK data
Normal data
Fold over basal
Time (min)
0
10
20
30
40
50
60
1
1.5
2
2.5
3

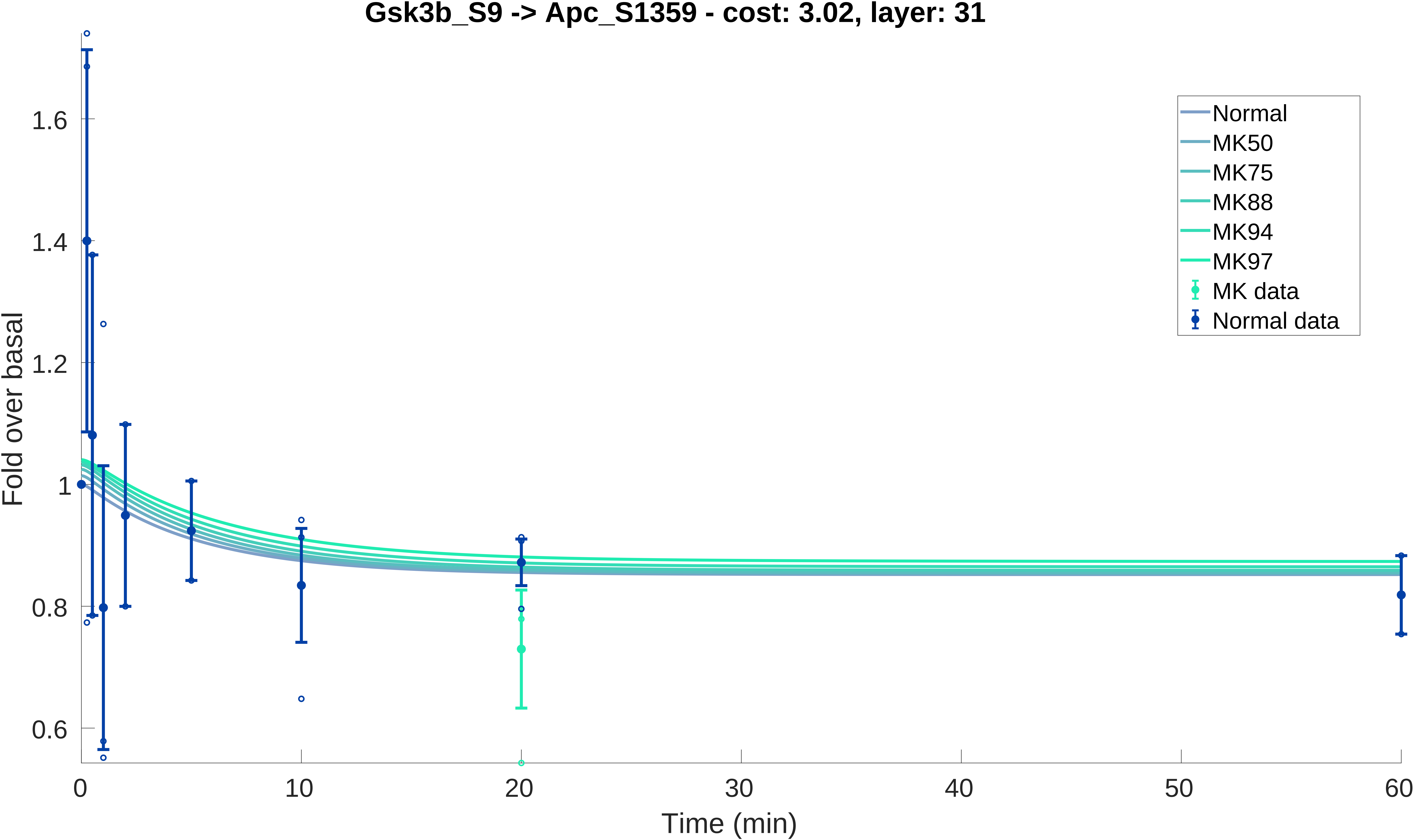

Gsk3b_S9 -> Apc_S1359 - cost: 3.02, layer: 31
Fold over basal
Time (min)
Normal
MK50
MK75
MK88
MK94
MK97
MK data
Normal data
0.6
0.8
1
1.2
1.4
1.6
0
10
20
30
40
50
60

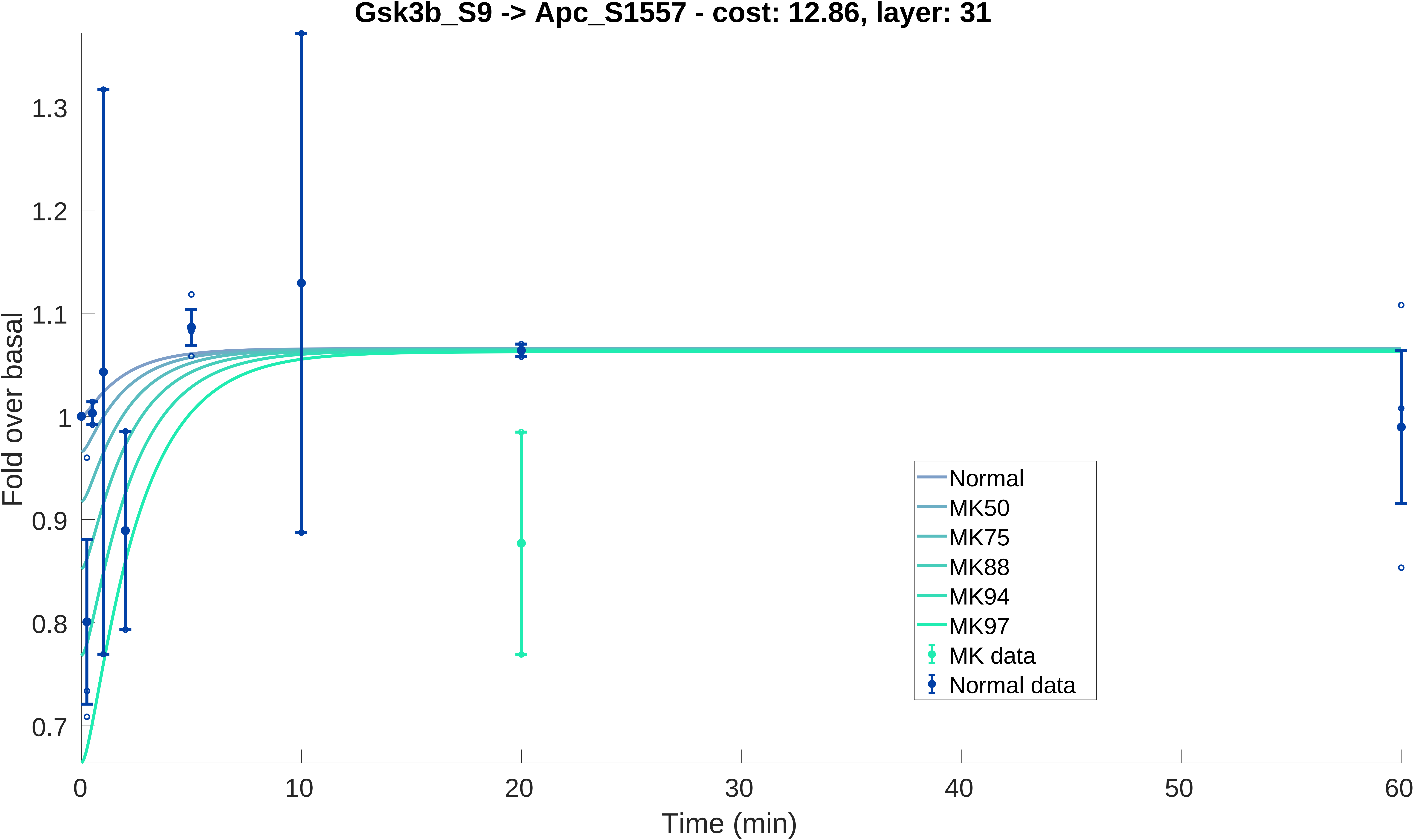

Gsk3b_S9 -> Apc_S1557 - cost: 12.86, layer: 31
Fold over basal
1.3
1.2
1.1
1
0.9
0.8
0.7
0
10
20
30
40
50
60
Time (min)
Normal
MK50
MK75
MK88
MK94
MK97
MK data
Normal data

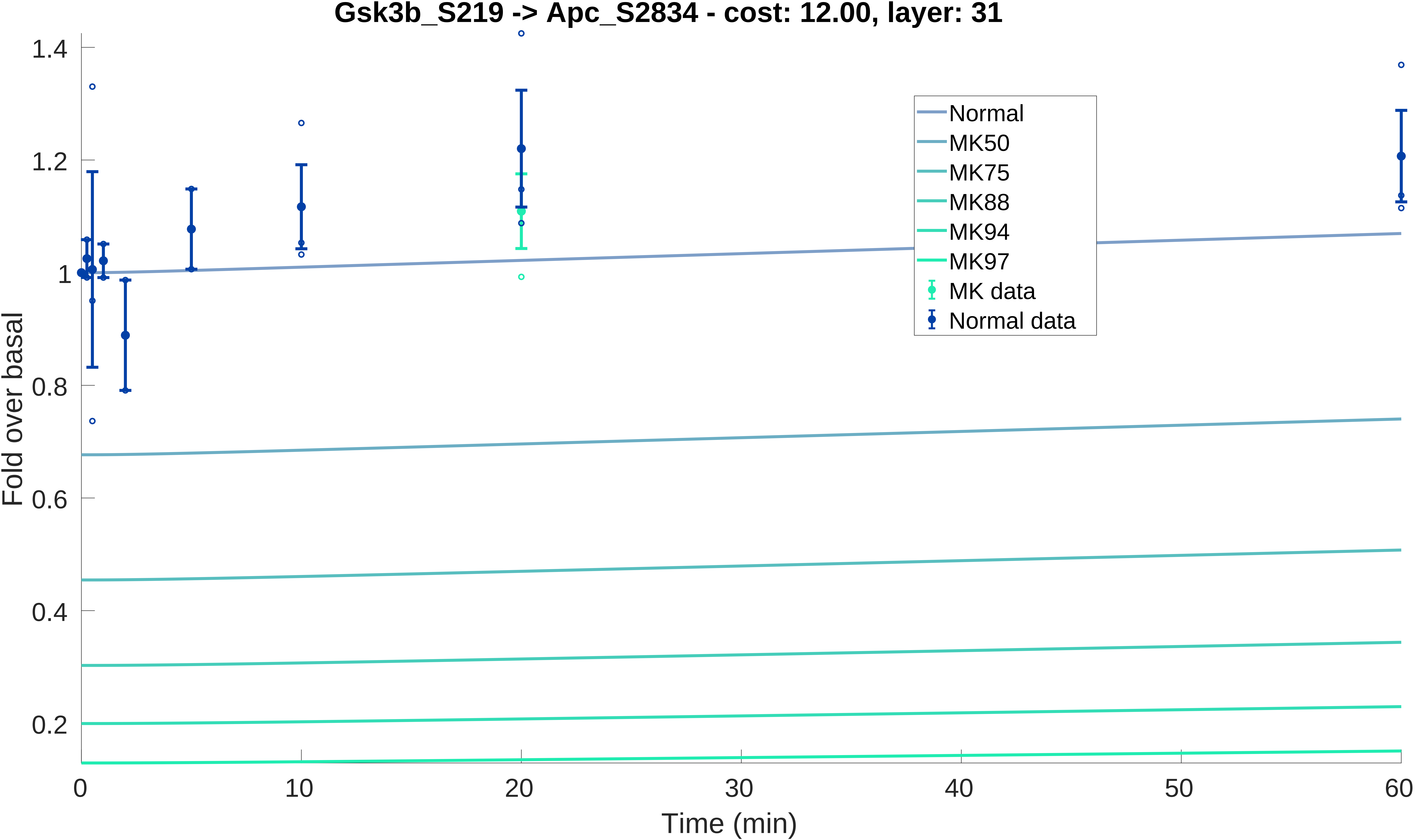

Gsk3b_S219 -> Apc_S2834 - cost: 12.00, layer: 31
Fold over basal
Time (min)
1.4
1.2
1
0.8
0.6
0.4
0.2
0
10
20
30
40
50
60
Normal
MK50
MK75
MK88
MK94
MK97
MK data
Normal data

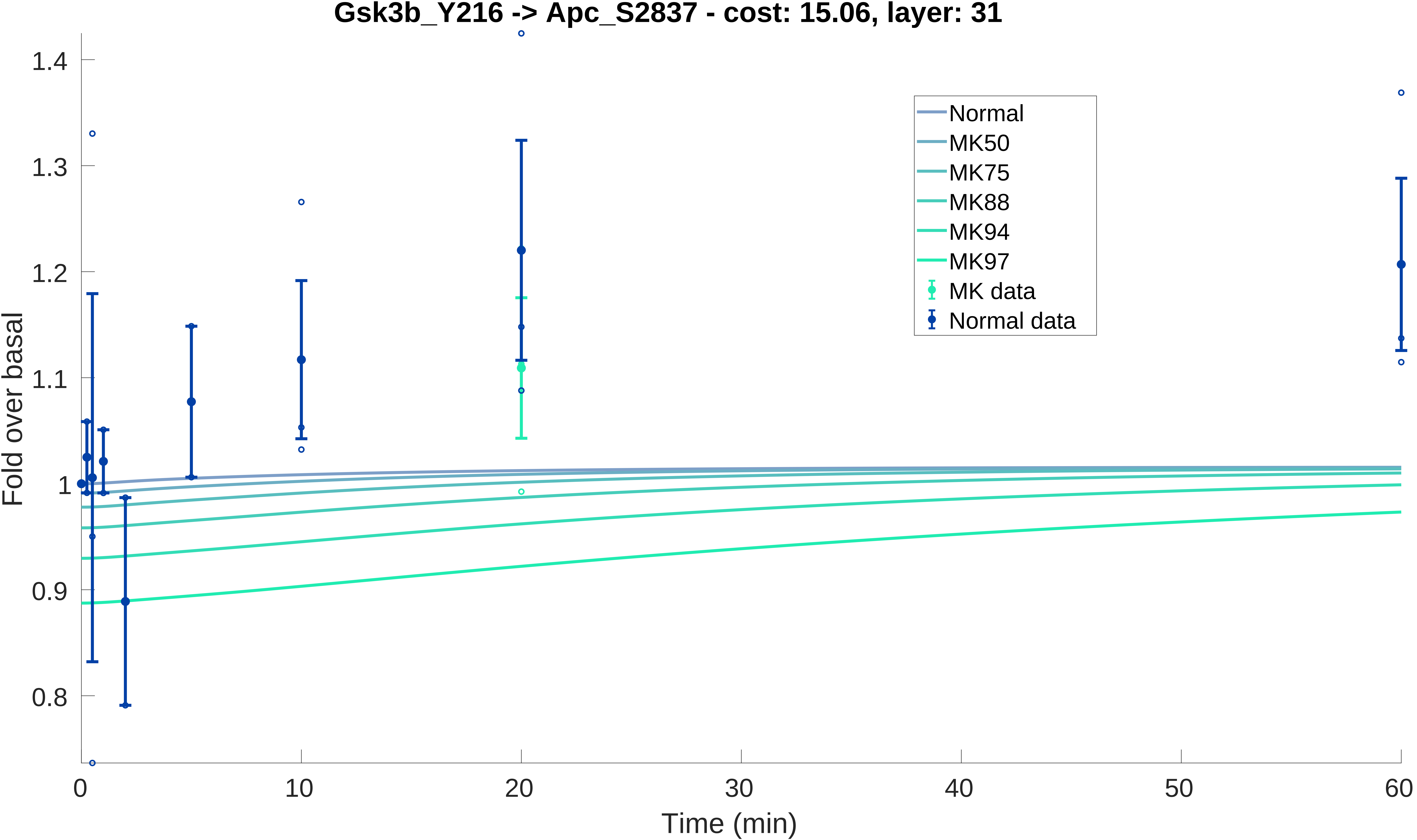
Gsk3b_Y216 -> Apc_S2837 - cost: 15.06, layer: 31
Fold over basal
1.4
1.3
1.2
1.1
1
0.9
0.8
Time (min)
0
10
20
30
40
50
60
Normal
MK50
MK75
MK88
MK94
MK97
MK data
Normal data

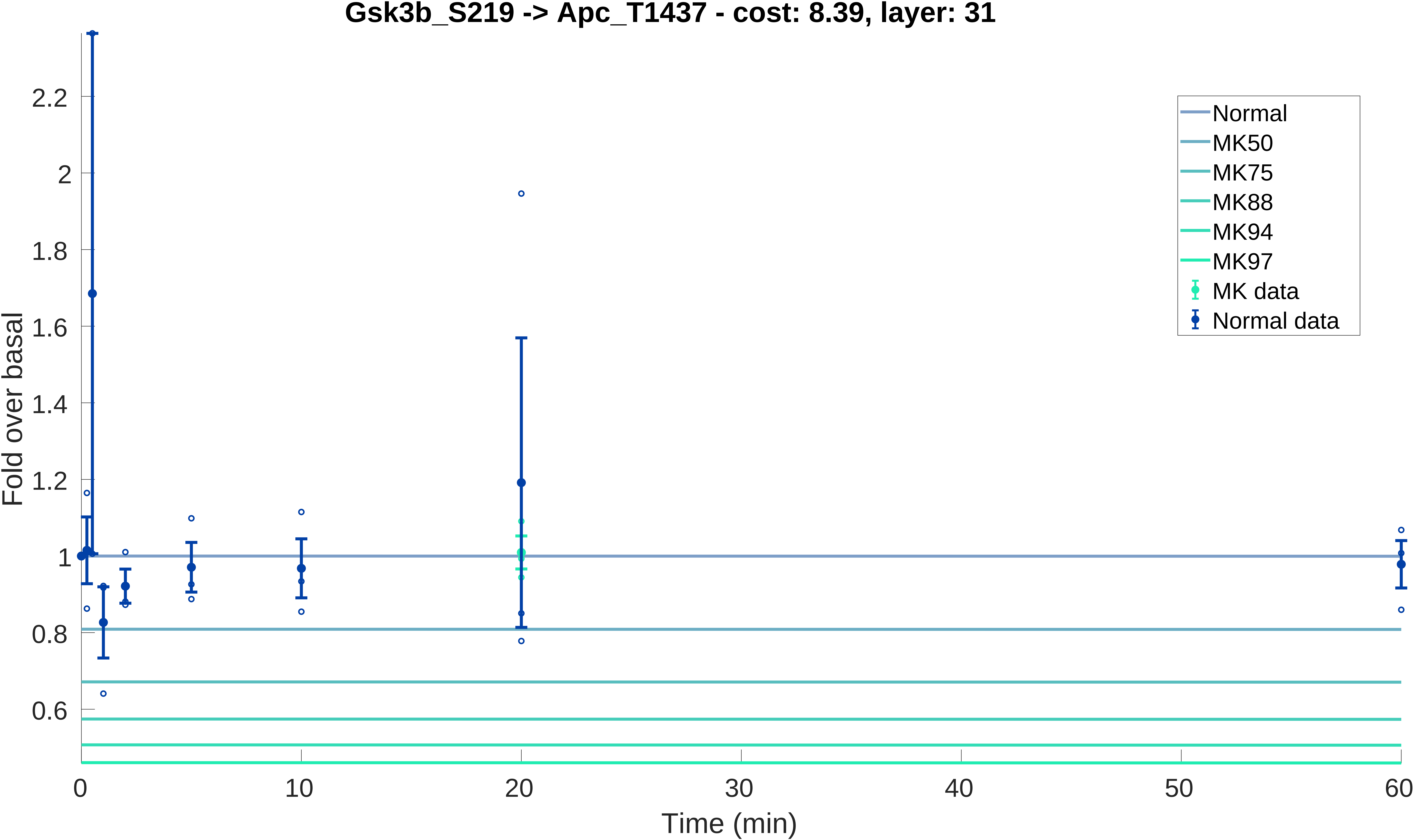

Gsk3b_S219 -> Apc_T1437 - cost: 8.39, layer: 31
Fold over basal
Time (min)
0
10
20
30
40
50
60
0.6
0.8
1
1.2
1.4
1.6
1.8
2
2.2
Normal
MK50
MK75
MK88
MK94
MK97
MK data
Normal data

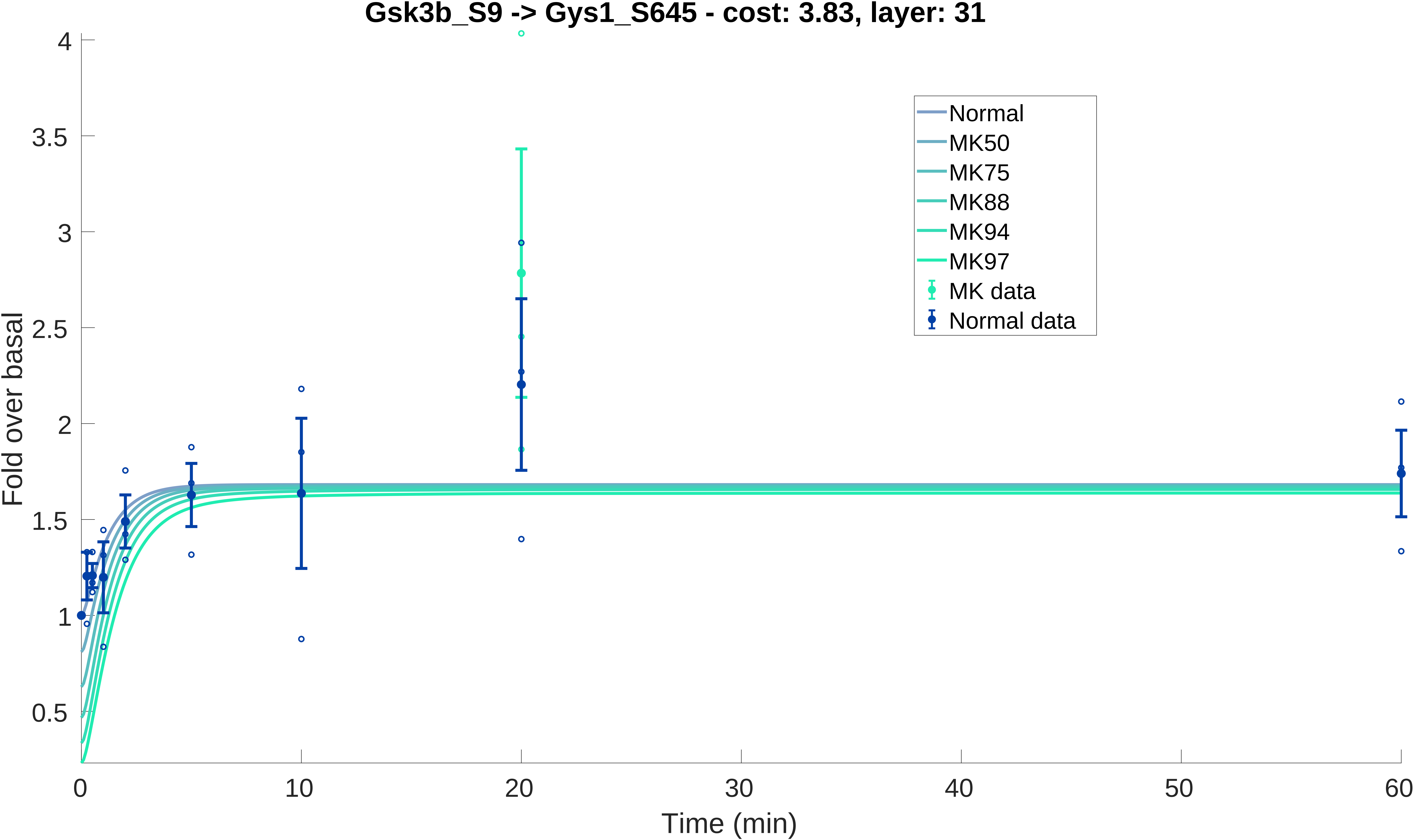
Gsk3b_S9 -> Gys1_S645 - cost: 3.83, layer: 31
Fold over basal
Time (min)
Normal
MK50
MK75
MK88
MK94
MK97
MK data
Normal data
0
10
20
30
40
50
60
0.5
1
1.5
2
2.5
3
3.5
4

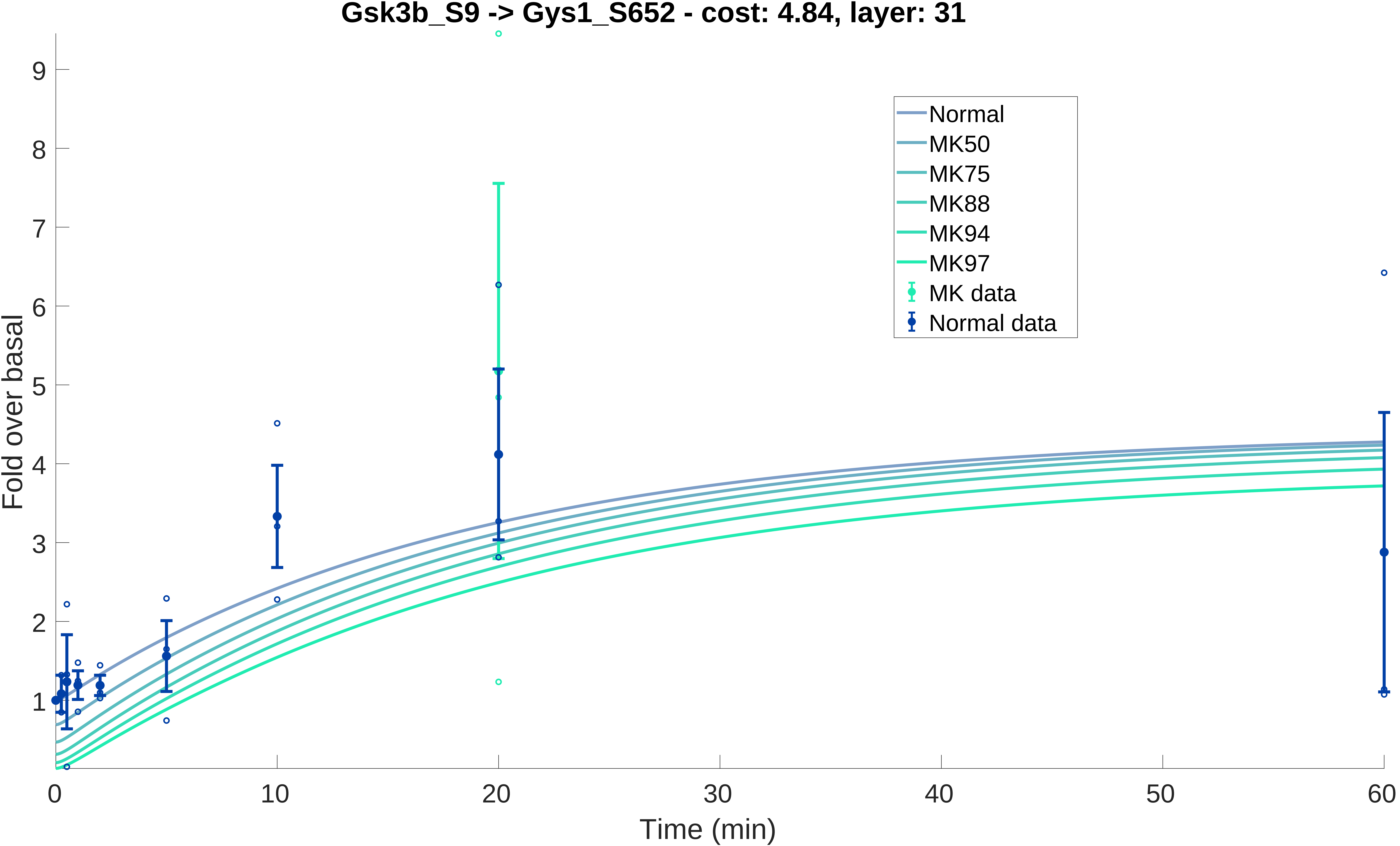

Gsk3b_S9 -> Gys1_S652 - cost: 4.84, layer: 31
Fold over basal
Time (min)
0
10
20
30
40
50
60
1
2
3
4
5
6
7
8
9
Normal
MK50
MK75
MK88
MK94
MK97
MK data
Normal data

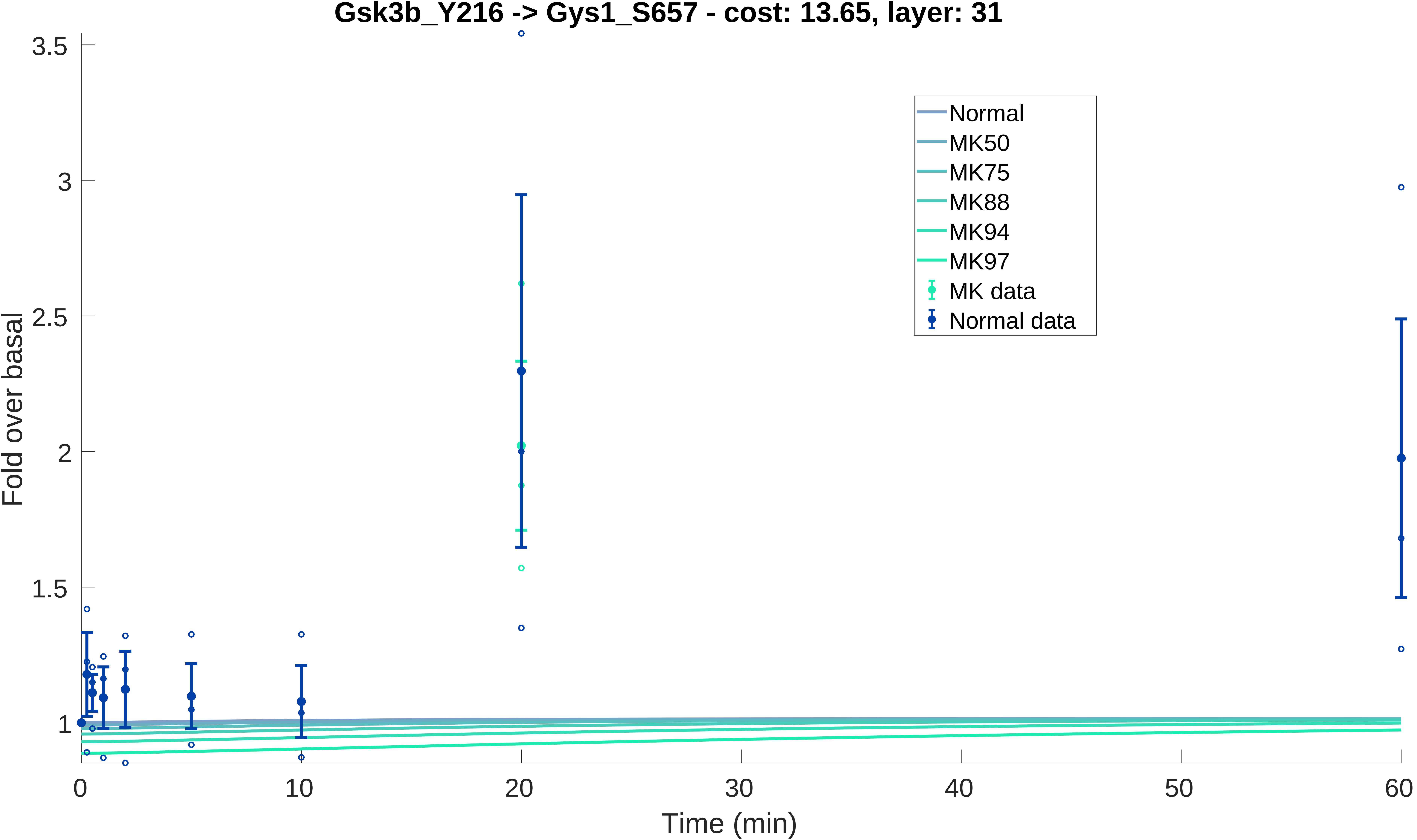
Gsk3b_Y216 -> Gys1_S657 - cost: 13.65, layer: 31
Fold over basal
Time (min)
3.5
3
2.5
2
1.5
1
0
10
20
30
40
50
60
Normal
MK50
MK75
MK88
MK94
MK97
MK data
Normal data

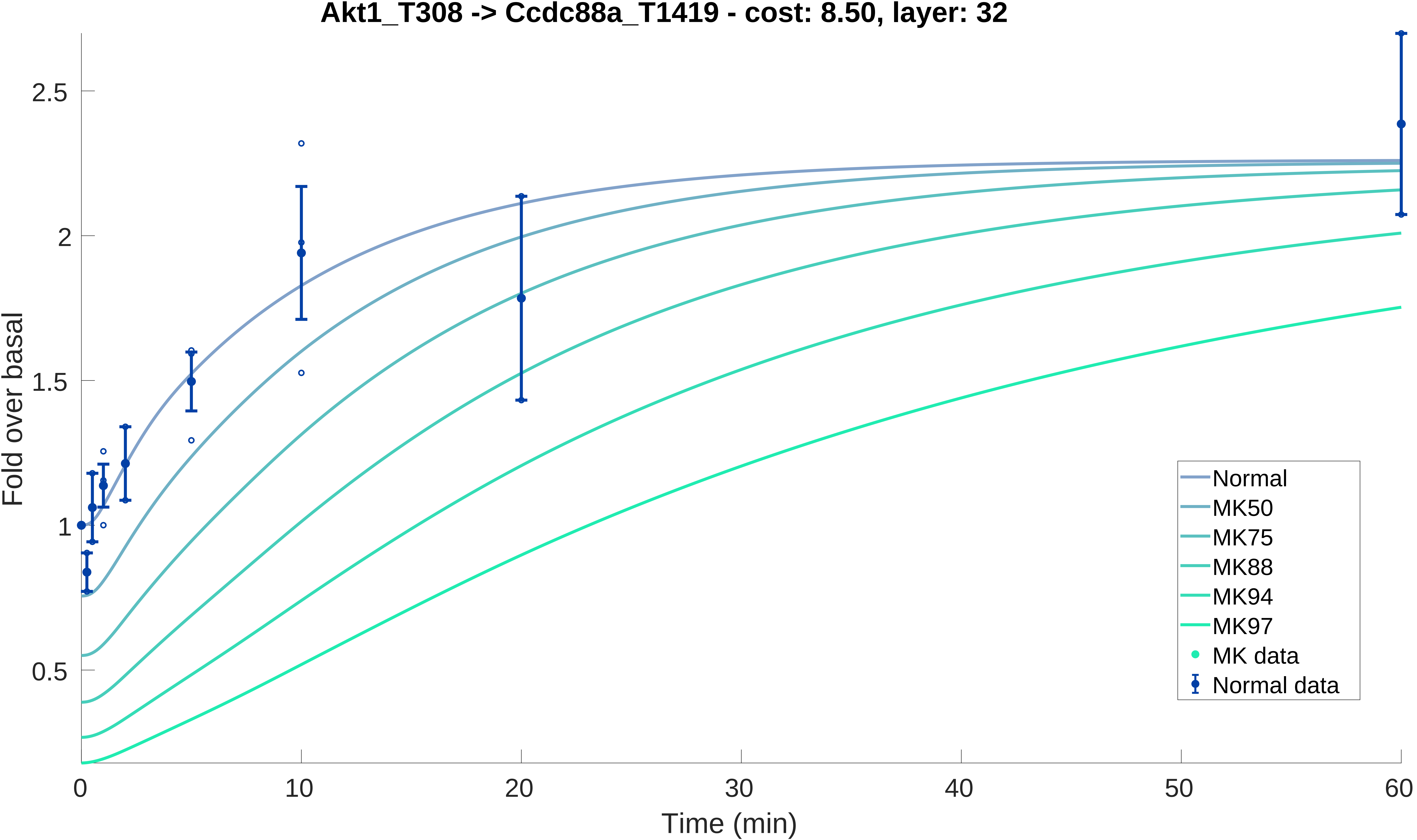

Akt1_T308 -> Ccdc88a_T1419 - cost: 8.50, layer: 32
Fold over basal
Time (min)
0
10
20
30
40
50
60
0.5
1
1.5
2
2.5
Normal
MK50
MK75
MK88
MK94
MK97
MK data
Normal data

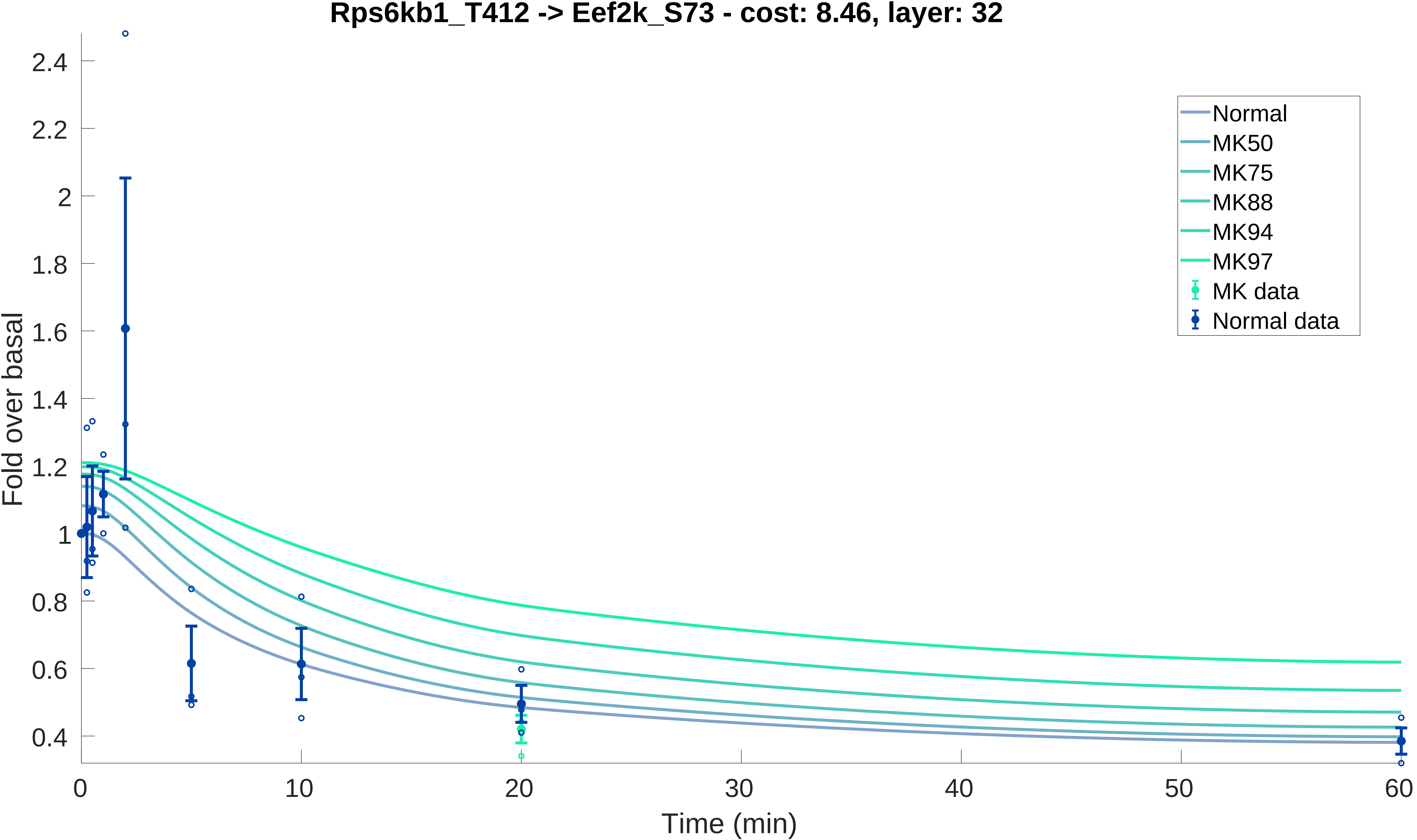
Rps6kb1_T412 -> Eef2k_S73 - cost: 8.46, layer: 32
Fold over basal
Time (min)
Normal
MK50
MK75
MK88
MK94
MK97
MK data
Normal data
0
10
20
30
40
50
60
0.4
0.6
0.8
1
1.2
1.4
1.6
1.8
2
2.2
2.4

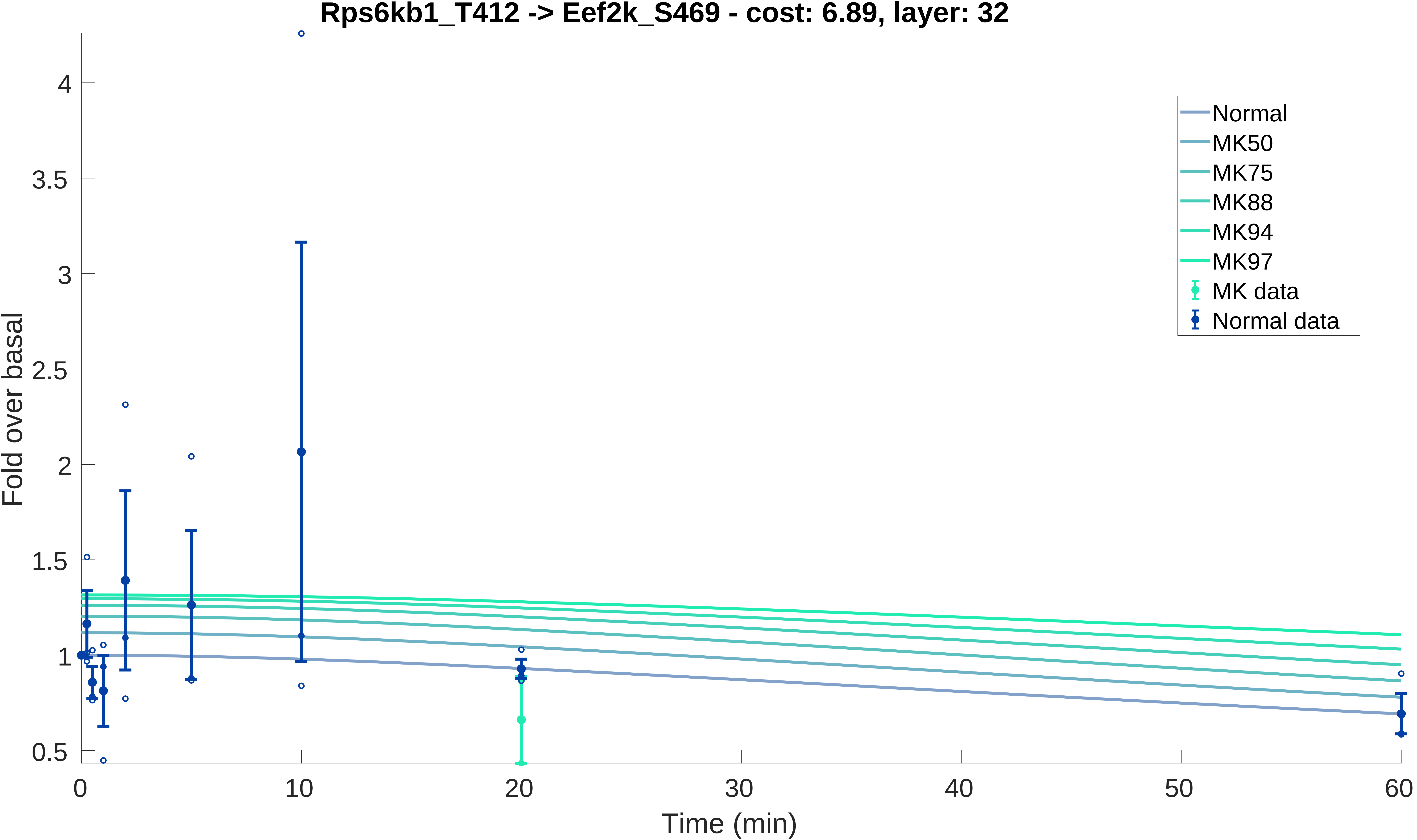
Rps6kb1_T412 -> Eef2k_S469 - cost: 6.89, layer: 32
Fold over basal
Time (min)
4
3.5
3
2.5
2
1.5
1
0.5
0
10
20
30
40
50
60
Normal
MK50
MK75
MK88
MK94
MK97
MK data
Normal data

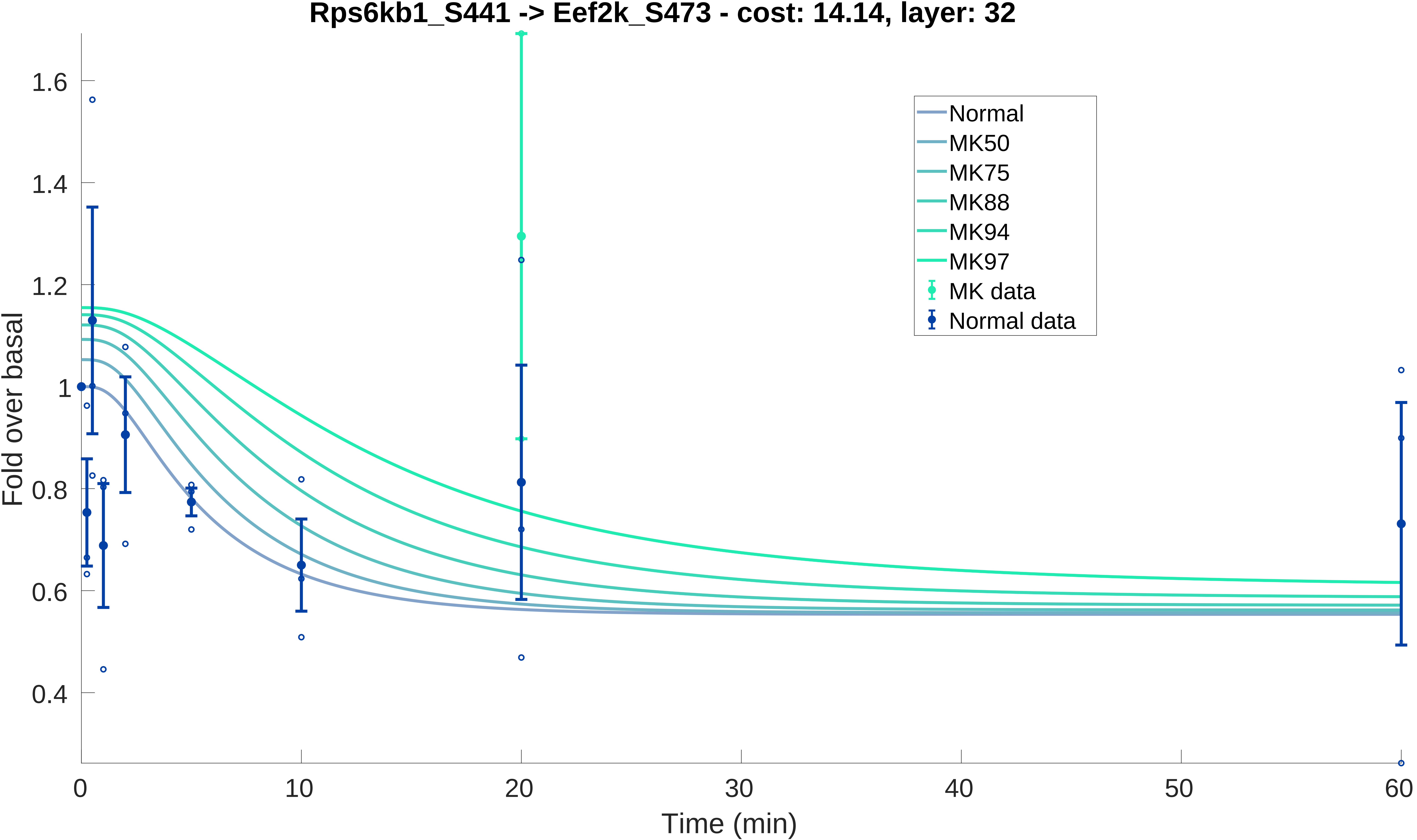

Rps6kb1_S441 -> Eef2k_S473 - cost: 14.14, layer: 32
Fold over basal
Time (min)
0.4
0.6
0.8
1
1.2
1.4
1.6
0
10
20
30
40
50
60
Normal
MK50
MK75
MK88
MK94
MK97
MK data
Normal data

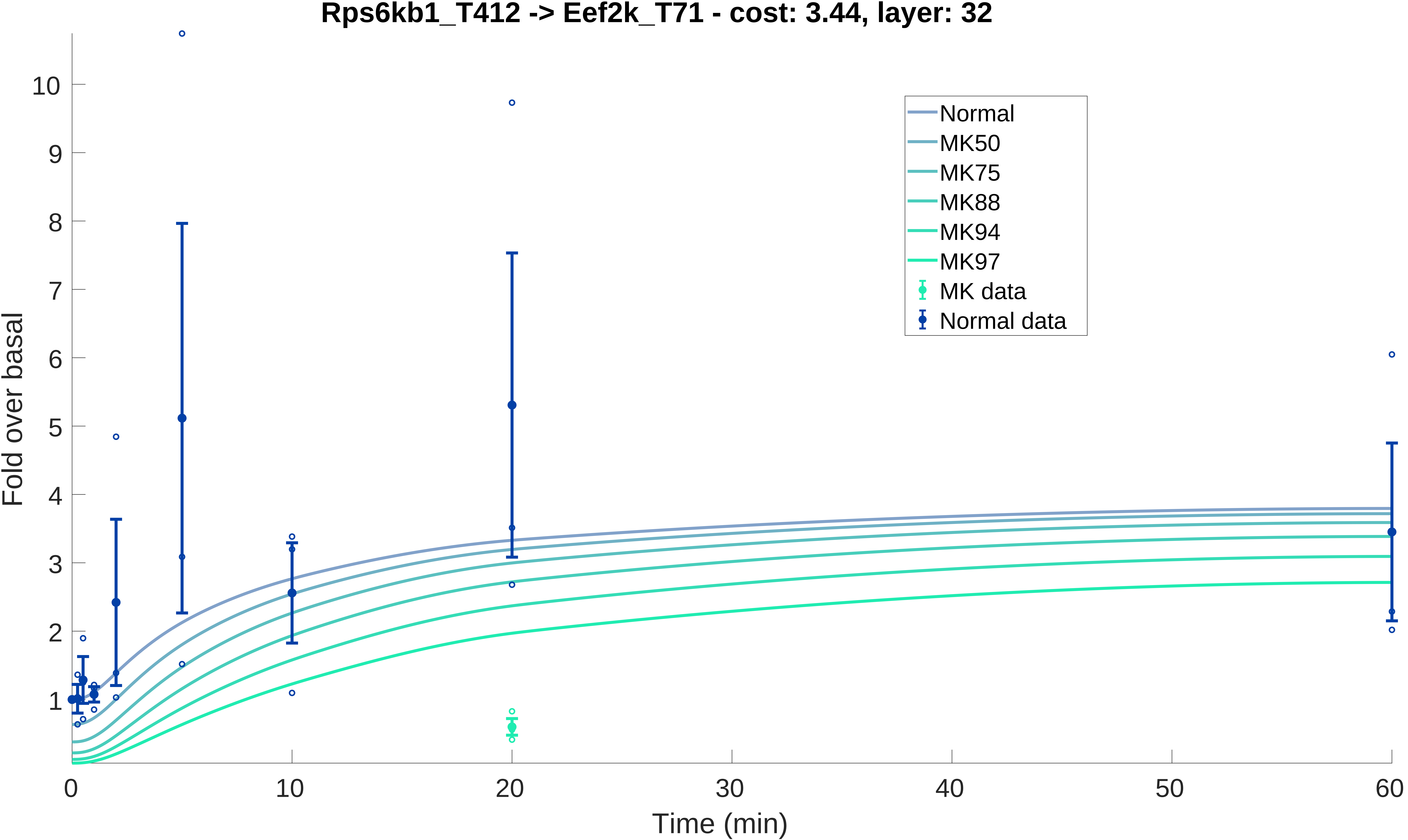

Rps6kb1_T412 -> Eef2k_T71 - cost: 3.44, layer: 32
Normal
MK50
MK75
MK88
MK94
MK97
MK data
Normal data
Fold over basal
Time (min)
1
2
3
4
5
6
7
8
9
10
0
10
20
30
40
50
60

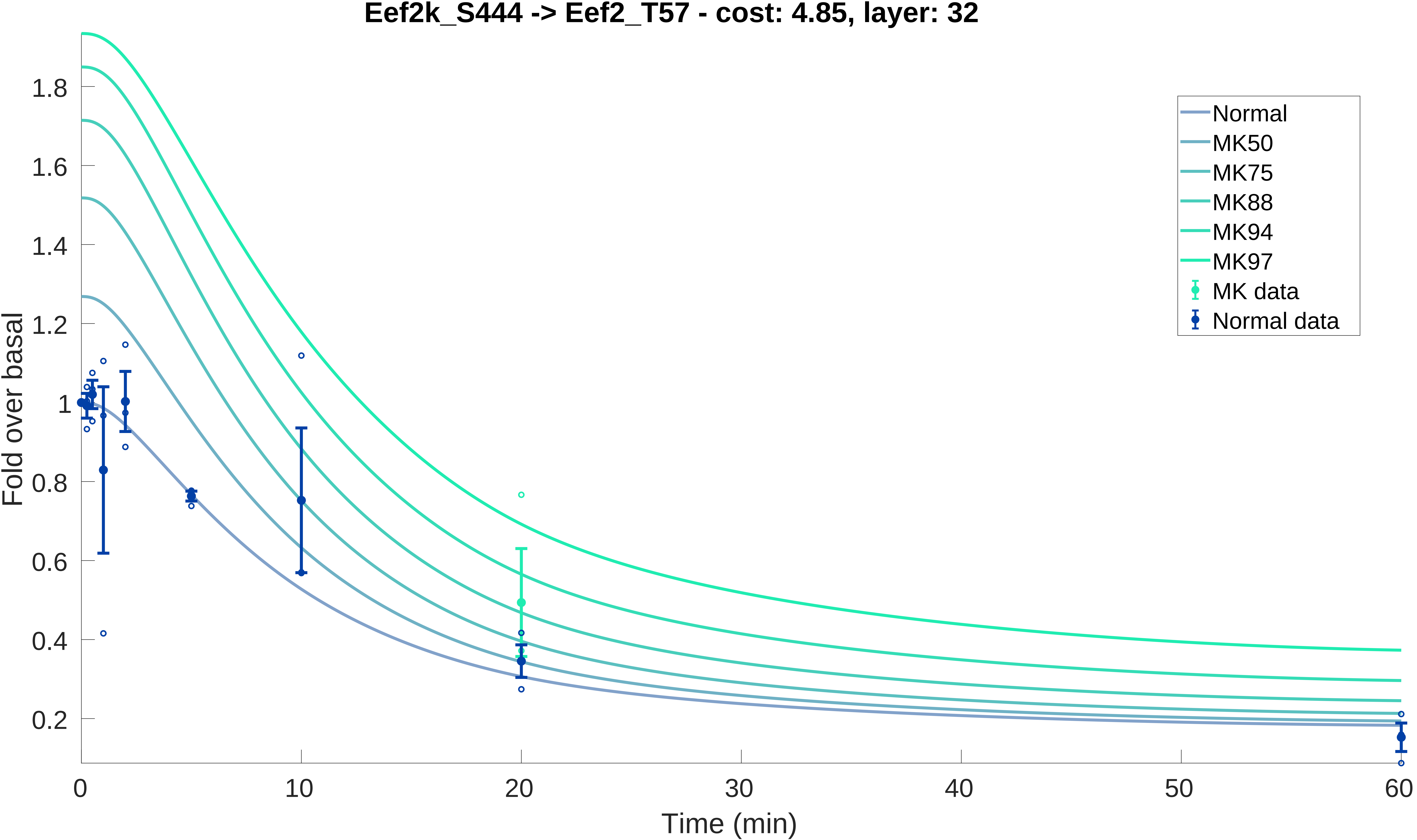

Eef2k_S444 -> Eef2_T57 - cost: 4.85, layer: 32
Fold over basal
Time (min)
0
10
20
30
40
50
60
0.2
0.4
0.6
0.8
1
1.2
1.4
1.6
1.8
Normal
MK50
MK75
MK88
MK94
MK97
MK data
Normal data

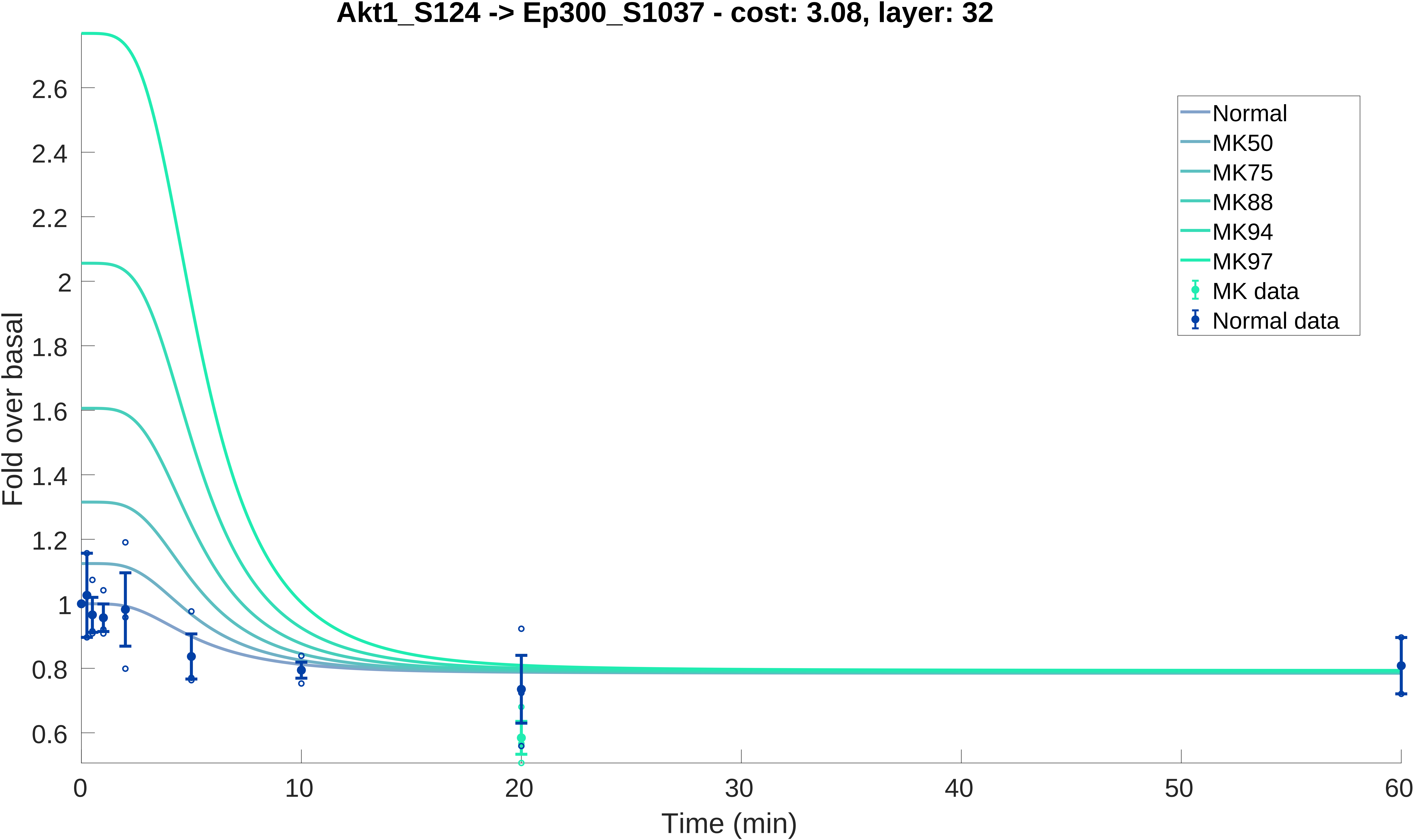

Akt1_S124 -> Ep300_S1037 - cost: 3.08, layer: 32
Fold over basal
Time (min)
Normal
MK50
MK75
MK88
MK94
MK97
MK data
Normal data
0
10
20
30
40
50
60
0.6
0.8
1
1.2
1.4
1.6
1.8
2
2.2
2.4
2.6

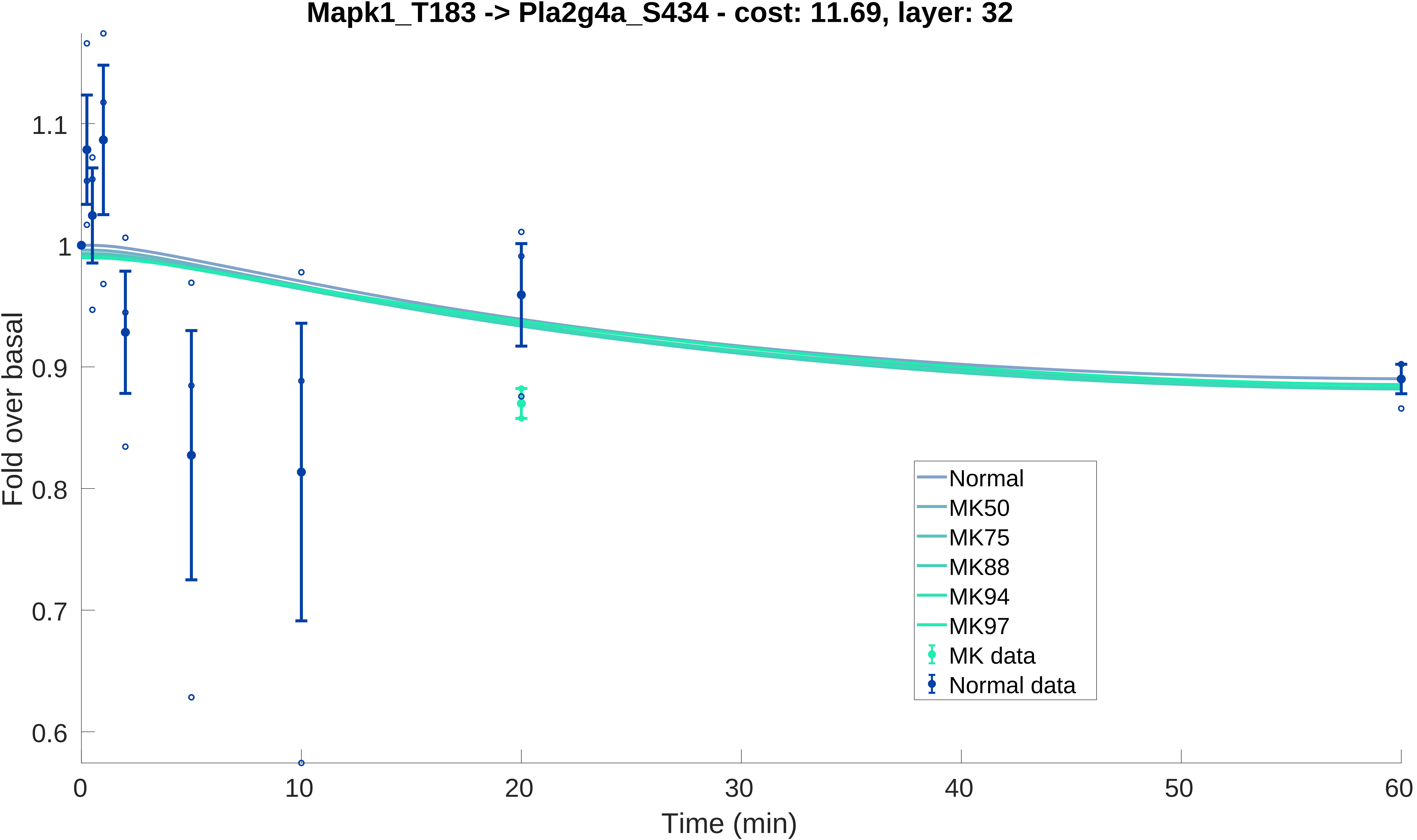

Mapk1_T183 -> Pla2g4a_S434 - cost: 11.69, layer: 32
Fold over basal
1.1
1
0.9
0.8
0.7
0.6
0
10
20
30
40
50
60
Time (min)
Normal
MK50
MK75
MK88
MK94
MK97
MK data
Normal data

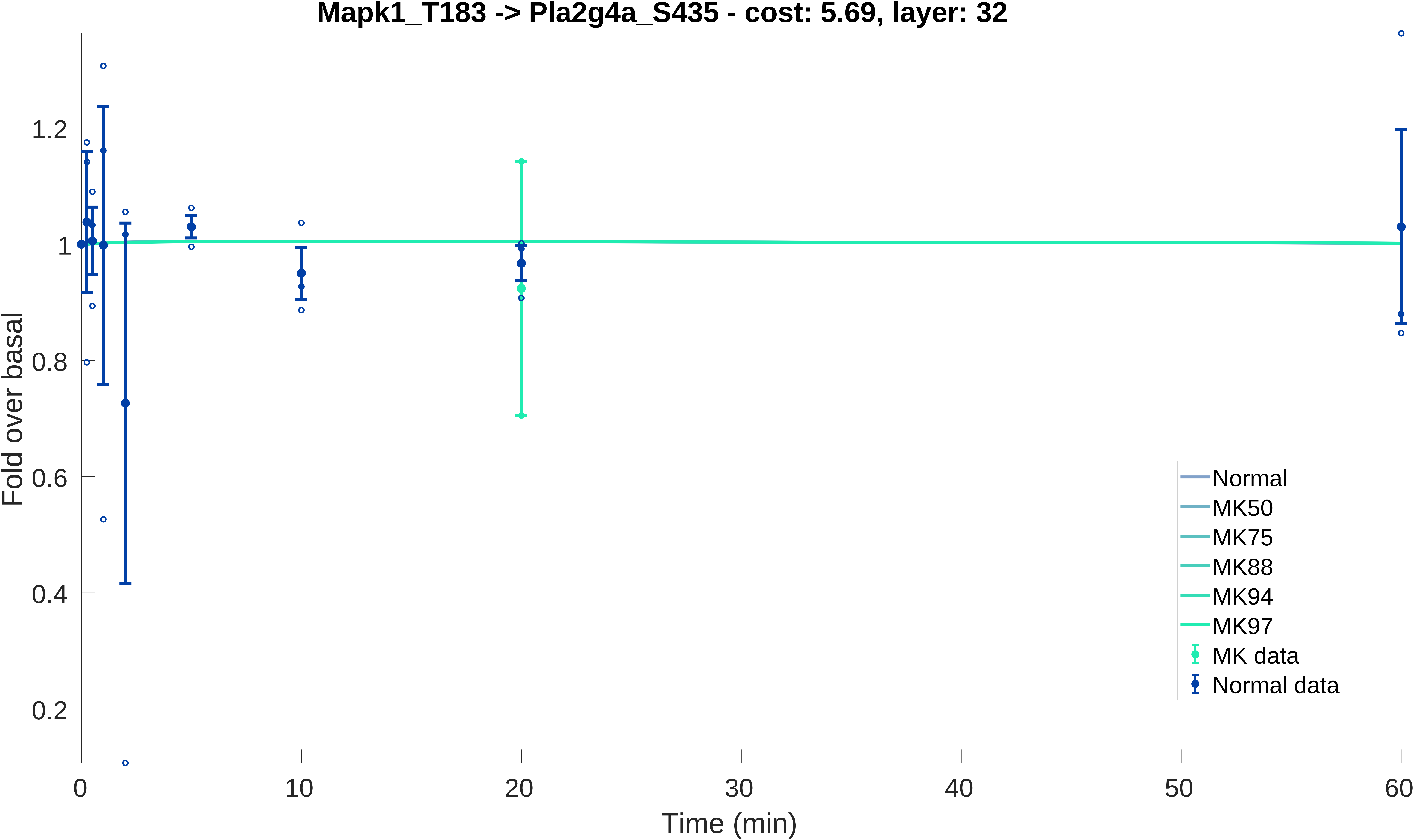

Mapk1_T183 -> Pla2g4a_S435 - cost: 5.69, layer: 32
Fold over basal
1.2
1
0.8
0.6
0.4
0.2
0
10
20
30
40
50
60
Time (min)
Normal
MK50
MK75
MK88
MK94
MK97
MK data
Normal data

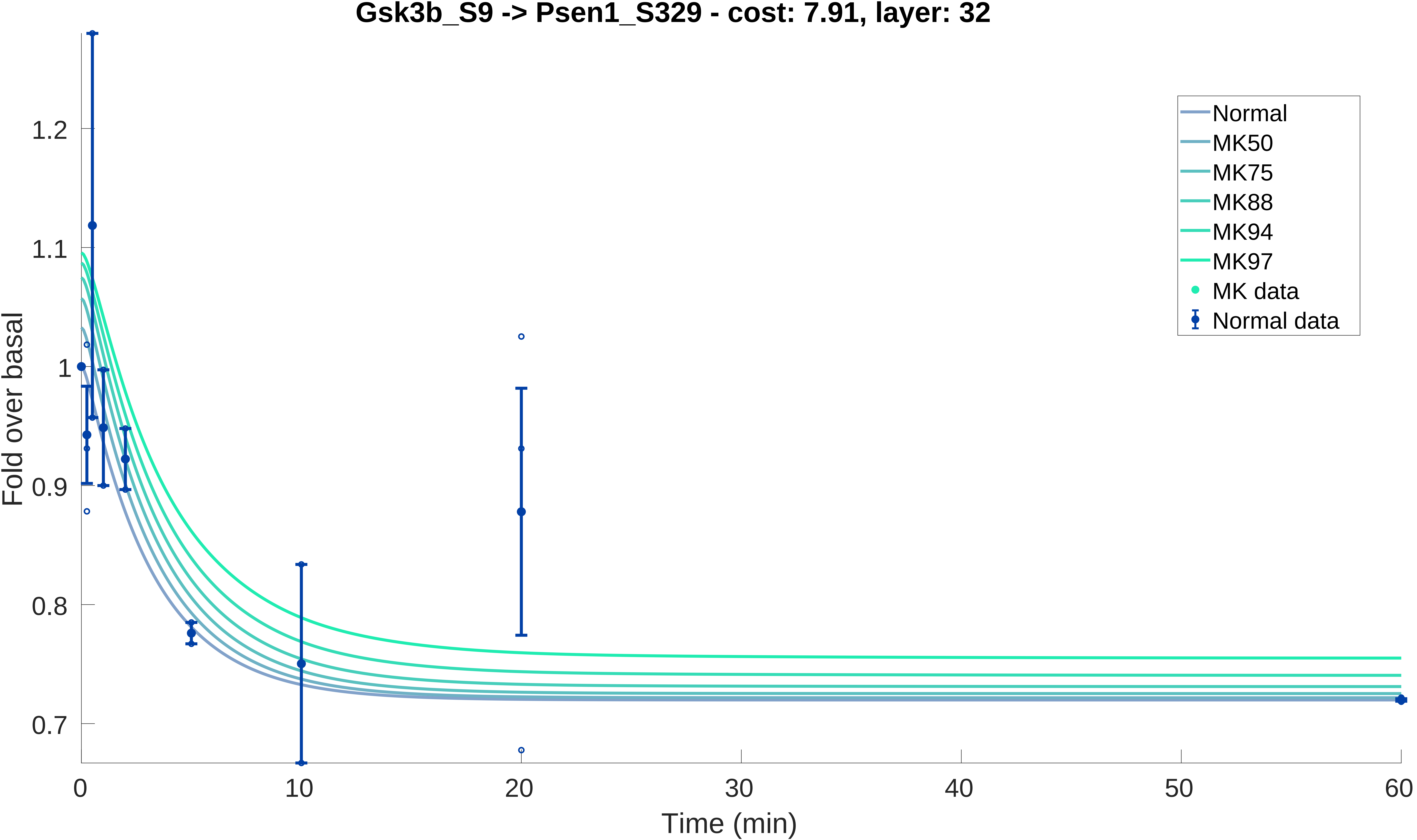

Gsk3b_S9 -> Psen1_S329 - cost: 7.91, layer: 32
Fold over basal
Time (min)
1.2
1.1
1
0.9
0.8
0.7
0
10
20
30
40
50
60
Normal
MK50
MK75
MK88
MK94
MK97
MK data
Normal data

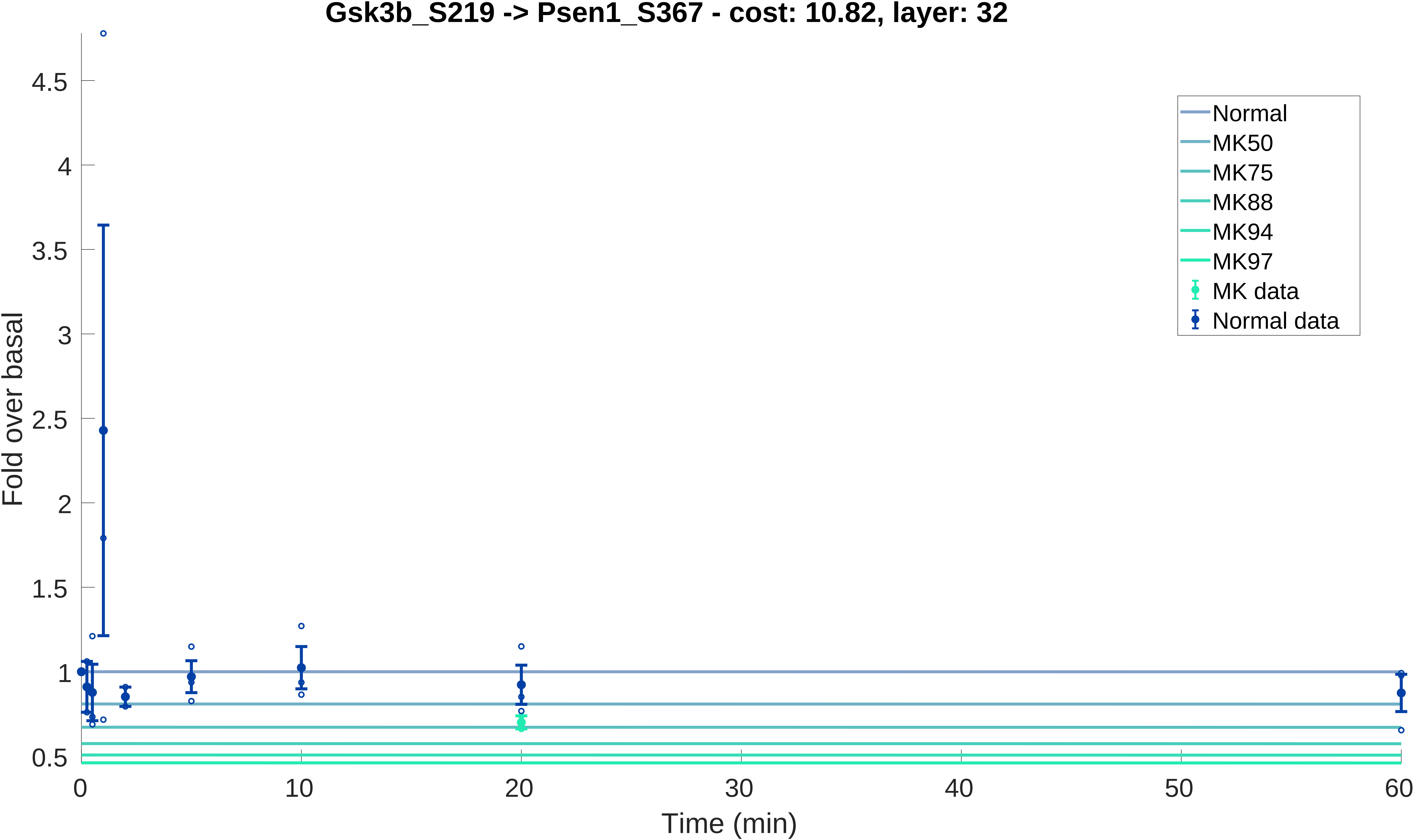

Gsk3b_S219 -> Psen1_S367 - cost: 10.82, layer: 32
Fold over basal
Time (min)
0.5
1
1.5
2
2.5
3
3.5
4
4.5
0
10
20
30
40
50
60
Normal
MK50
MK75
MK88
MK94
MK97
MK data
Normal data

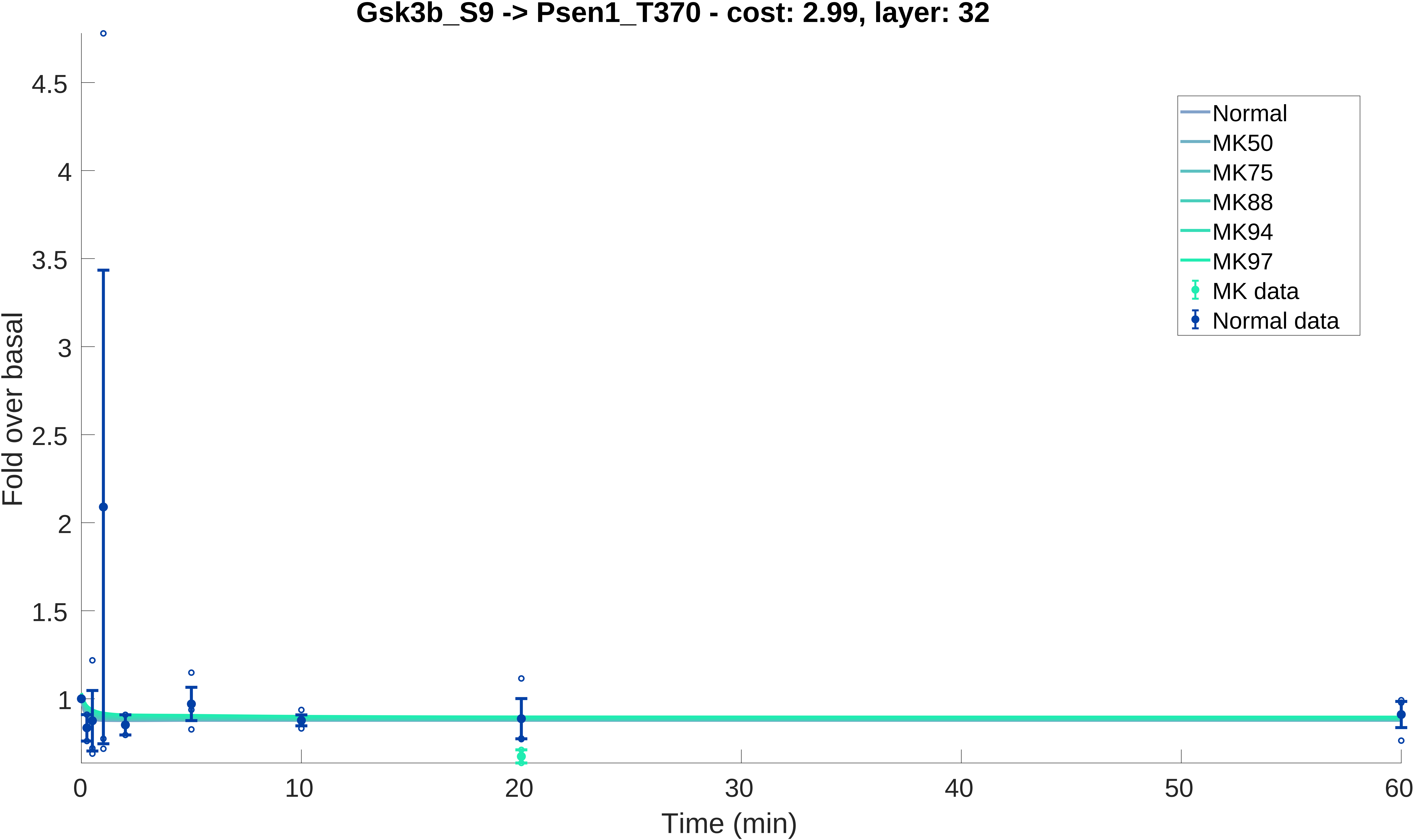
Gsk3b_S9 -> Psen1_T370 - cost: 2.99, layer: 32
Fold over basal
Time (min)
4.5
4
3.5
3
2.5
2
1.5
1
0
10
20
30
40
50
60
Normal
MK50
MK75
MK88
MK94
MK97
MK data
Normal data

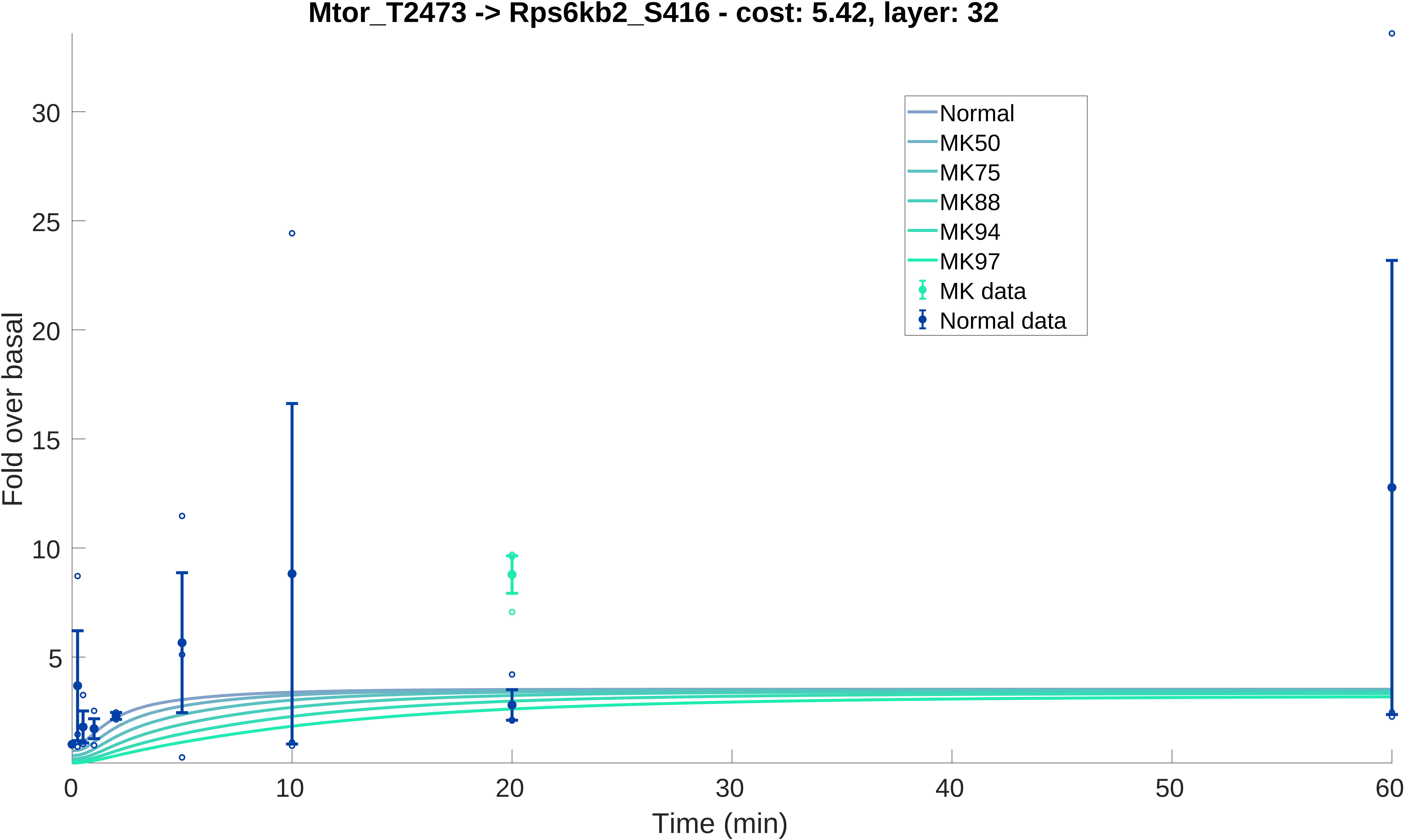

Mtor_T2473 -> Rps6kb2_S416 - cost: 5.42, layer: 32
Fold over basal
Time (min)
0
10
20
30
40
50
60
5
10
15
20
25
30
Normal
MK50
MK75
MK88
MK94
MK97
MK data
Normal data

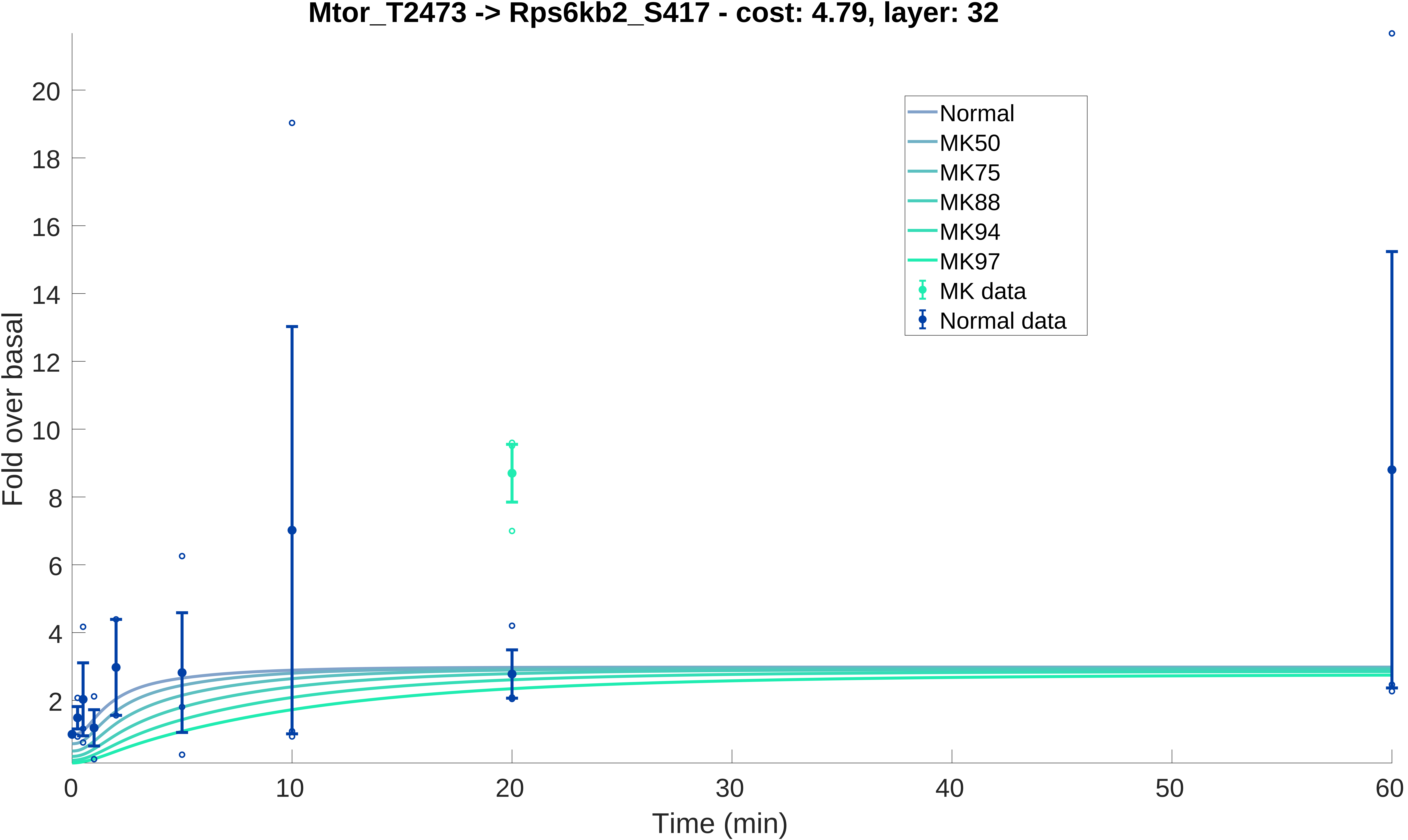
Mtor_T2473 -> Rps6kb2_S417 - cost: 4.79, layer: 32
Fold over basal
Time (min)
0
10
20
30
40
50
60
2
4
6
8
10
12
14
16
18
20
Normal
MK50
MK75
MK88
MK94
MK97
MK data
Normal data

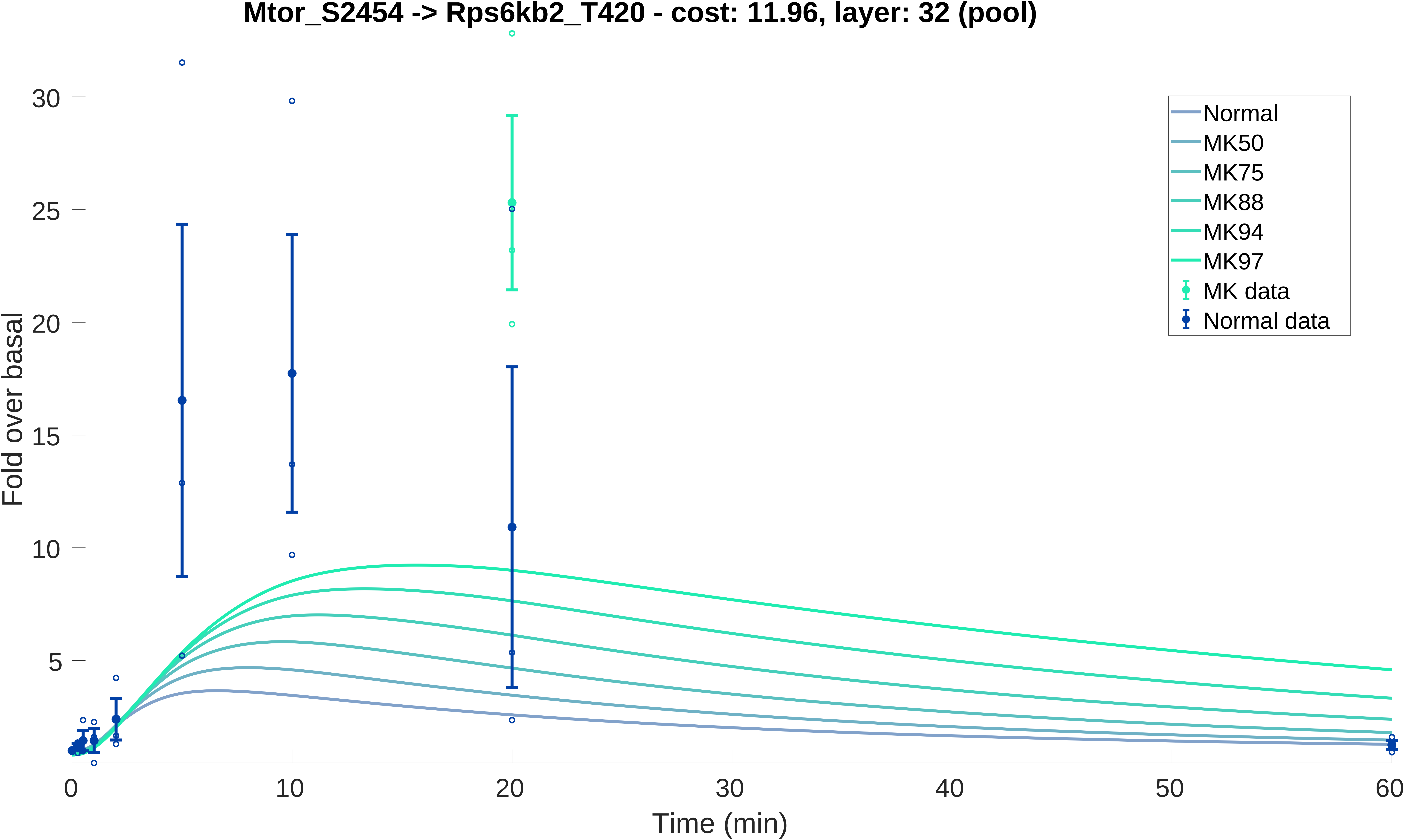

Mtor_S2454 -> Rps6kb2_T420 - cost: 11.96, layer: 32 (pool)
Fold over basal
Time (min)
0
10
20
30
40
50
60
5
10
15
20
25
30
Normal
MK50
MK75
MK88
MK94
MK97
MK data
Normal data

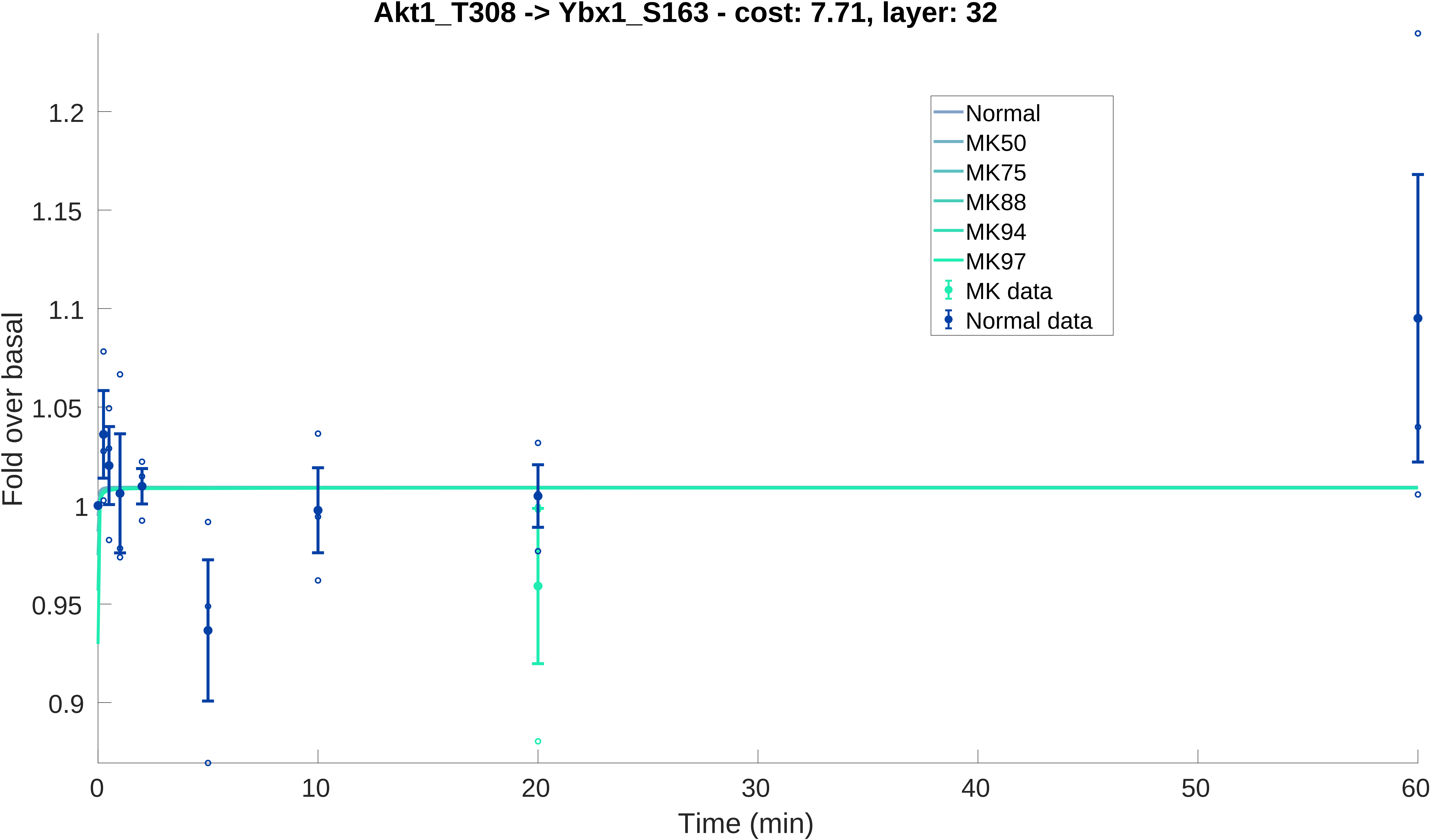

Akt1_T308 -> Ybx1_S163 - cost: 7.71, layer: 32
Fold over basal
Time (min)
1.2
1.15
1.1
1.05
1
0.95
0.9
0
10
20
30
40
50
60
Normal
MK50
MK75
MK88
MK94
MK97
MK data
Normal data

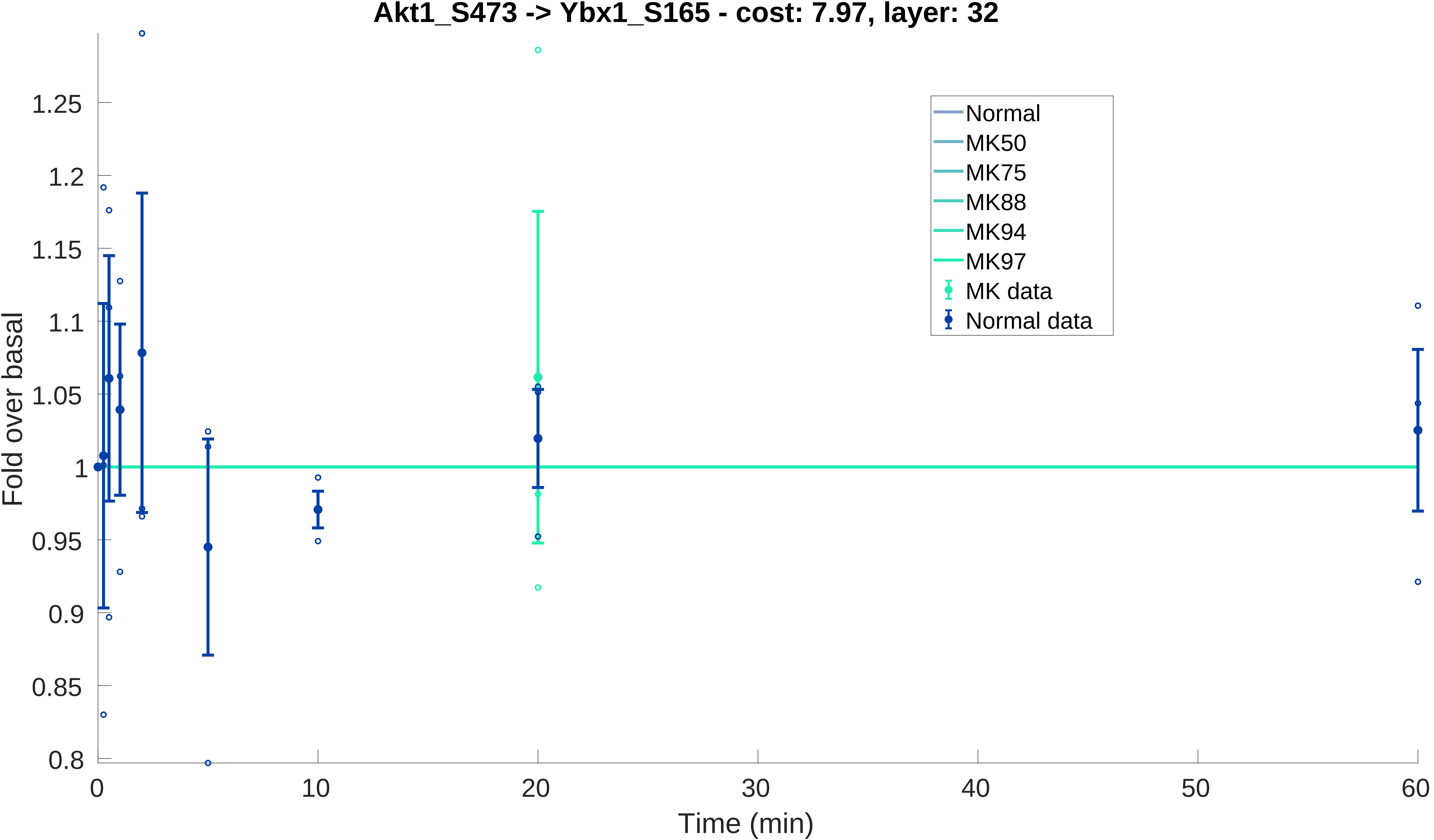
Akt1_S473 -> Ybx1_S165 - cost: 7.97, layer: 32
Fold over basal
Time (min)
Normal
MK50
MK75
MK88
MK94
MK97
MK data
Normal data
1.25
1.2
1.15
1.1
1.05
1
0.95
0.9
0.85
0.8
0
10
20
30
40
50
60

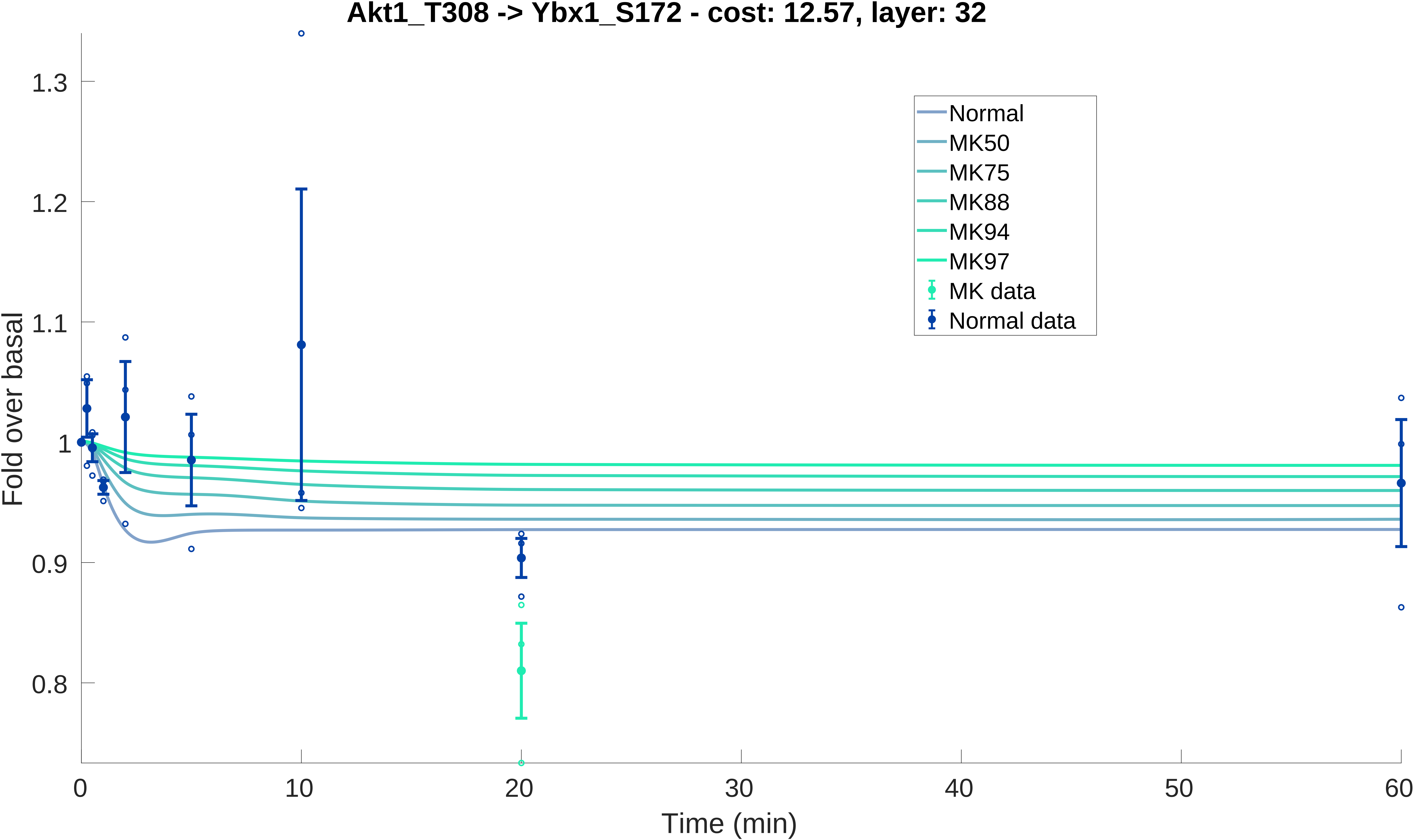
Akt1_T308 -> Ybx1_S172 - cost: 12.57, layer: 32
Fold over basal
1.3
1.2
1.1
1
0.9
0.8
0
10
20
30
40
50
60
Time (min)
Normal
MK50
MK75
MK88
MK94
MK97
MK data
Normal data

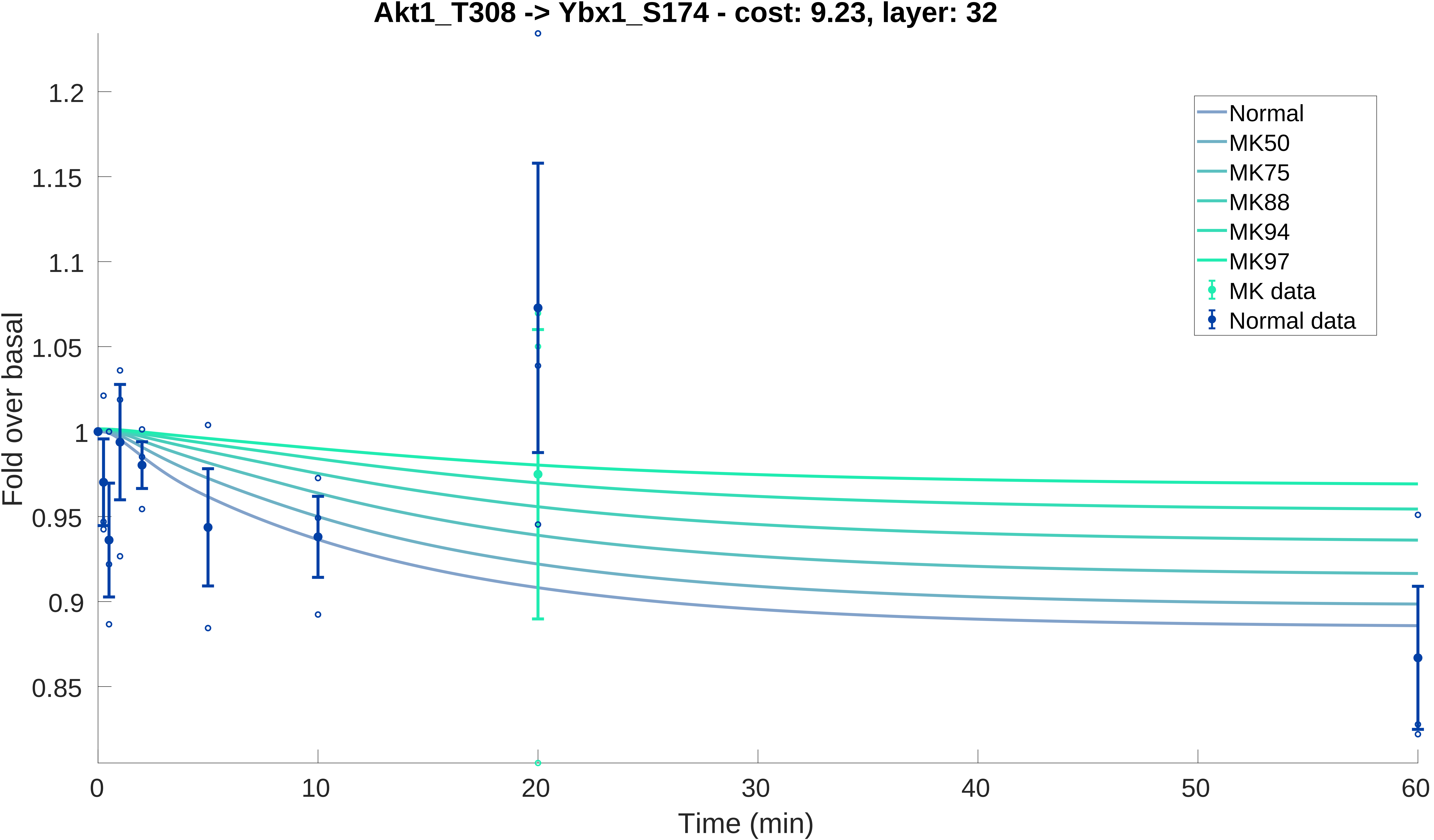
Akt1_T308 -> Ybx1_S174 - cost: 9.23, layer: 32
Fold over basal
1.2
1.15
1.1
1.05
1
0.95
0.9
0.85
0
10
20
30
40
50
60
Time (min)
Normal
MK50
MK75
MK88
MK94
MK97
MK data
Normal data

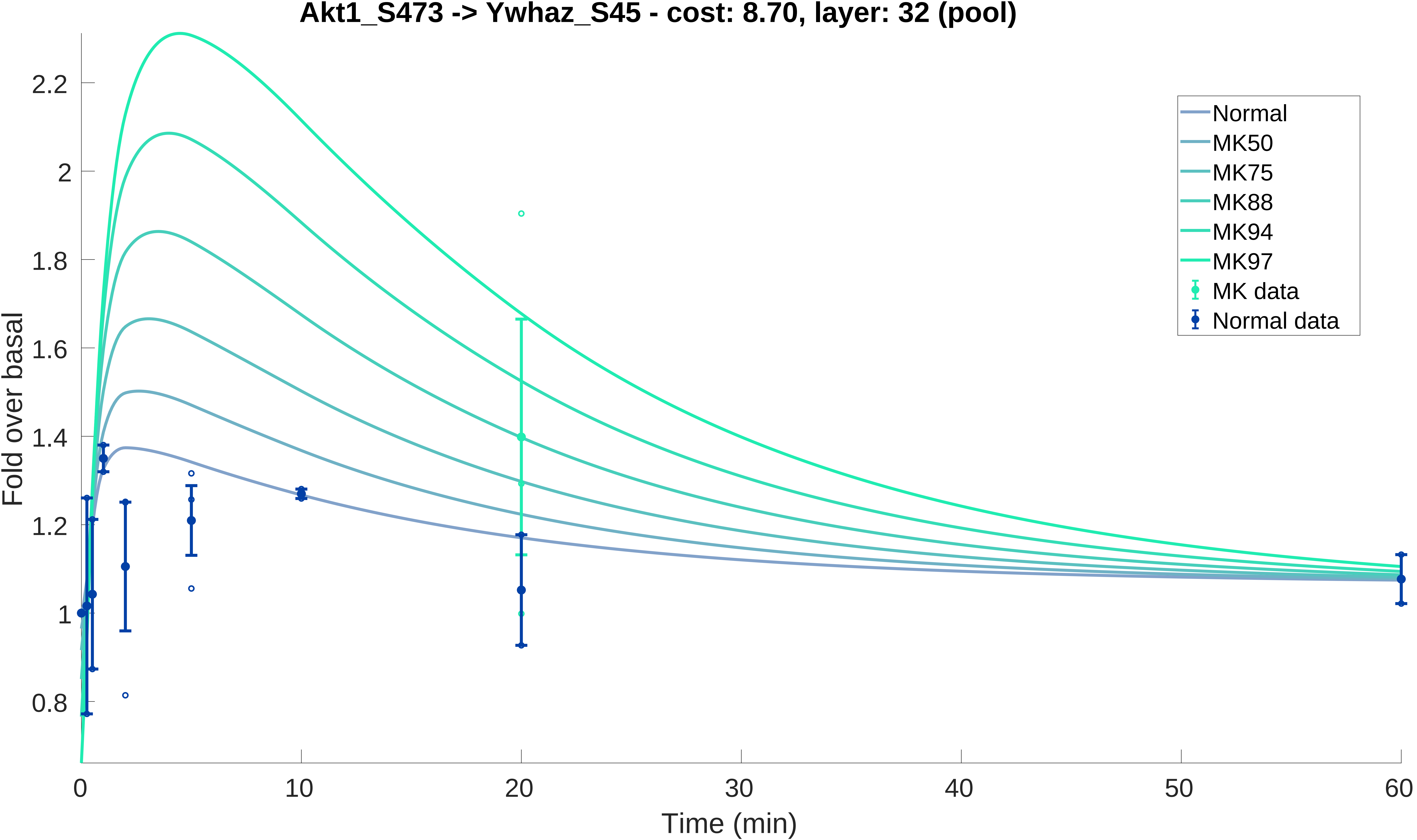
Akt1_S473 -> Ywhaz_S45 - cost: 8.70, layer: 32 (pool)
Fold over basal
Time (min)
0.8
1
1.2
1.4
1.6
1.8
2
2.2
0
10
20
30
40
50
60
Normal
MK50
MK75
MK88
MK94
MK97
MK data
Normal data

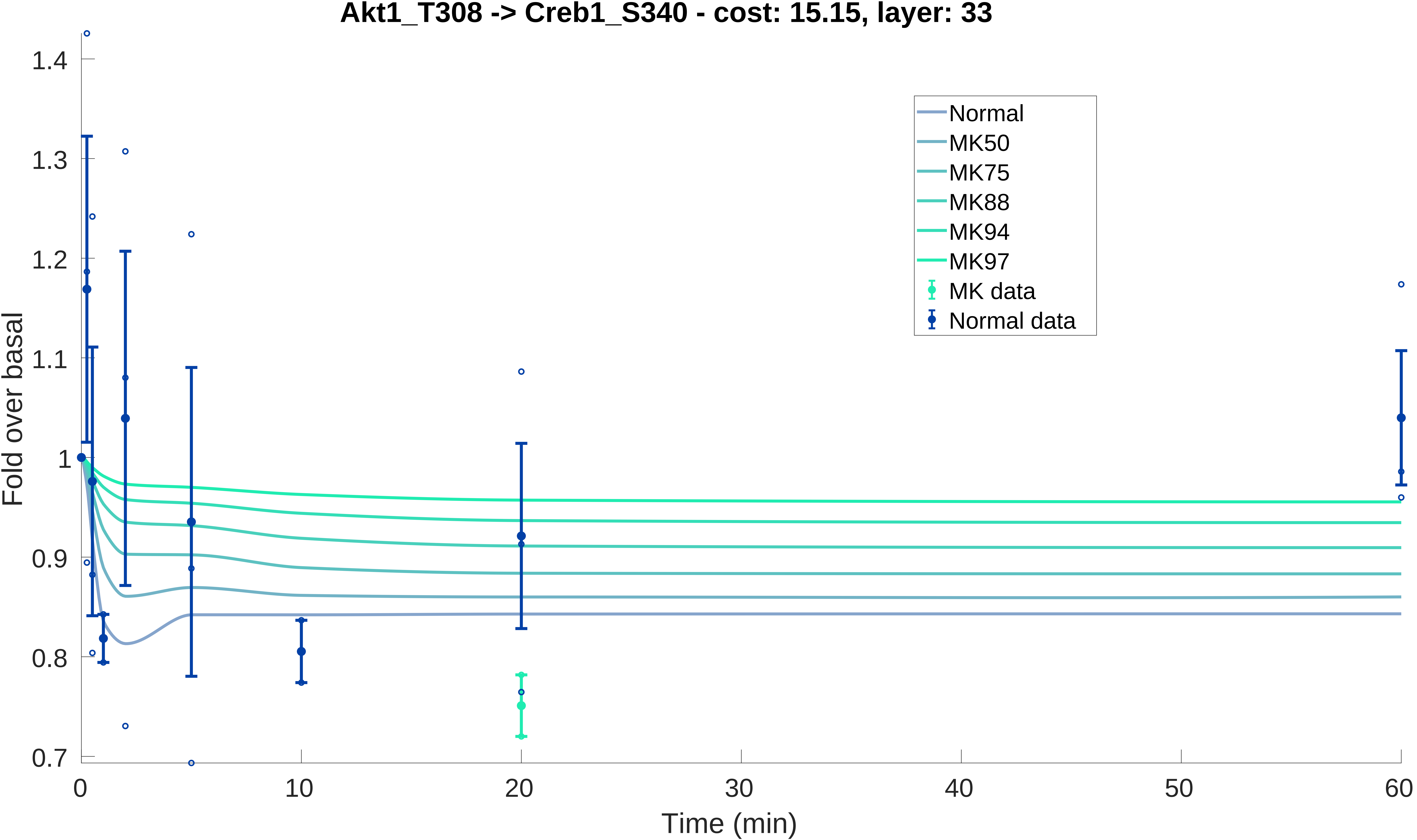
Akt1_T308 -> Creb1_S340 - cost: 15.15, layer: 33
Fold over basal
Time (min)
Normal
MK50
MK75
MK88
MK94
MK97
MK data
Normal data
1.4
1.3
1.2
1.1
1
0.9
0.8
0.7
0
10
20
30
40
50
60

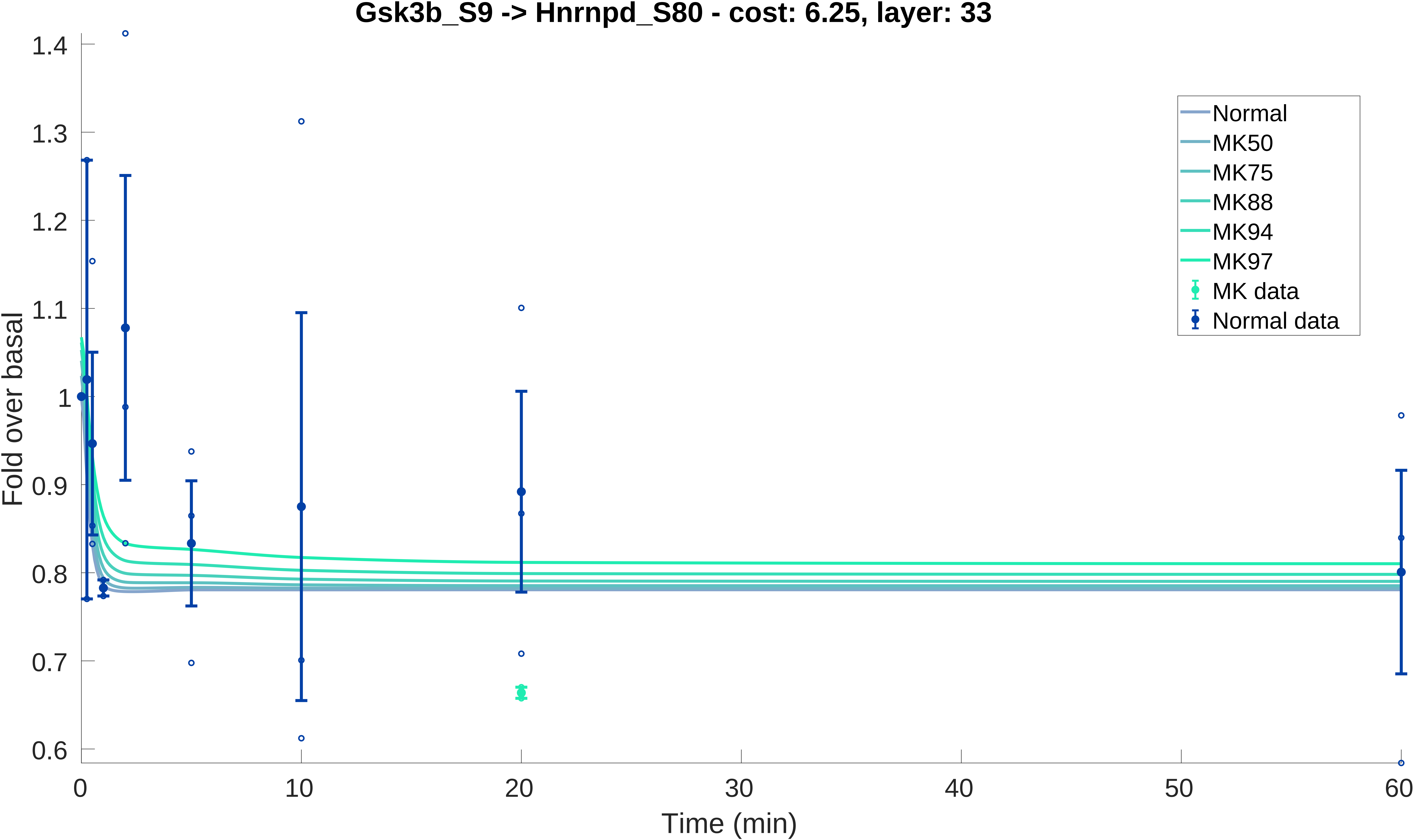

Gsk3b_S9 -> Hnrnpd_S80 - cost: 6.25, layer: 33
Fold over basal
Time (min)
0
10
20
30
40
50
60
0.6
0.7
0.8
0.9
1
1.1
1.2
1.3
1.4
Normal
MK50
MK75
MK88
MK94
MK97
MK data
Normal data

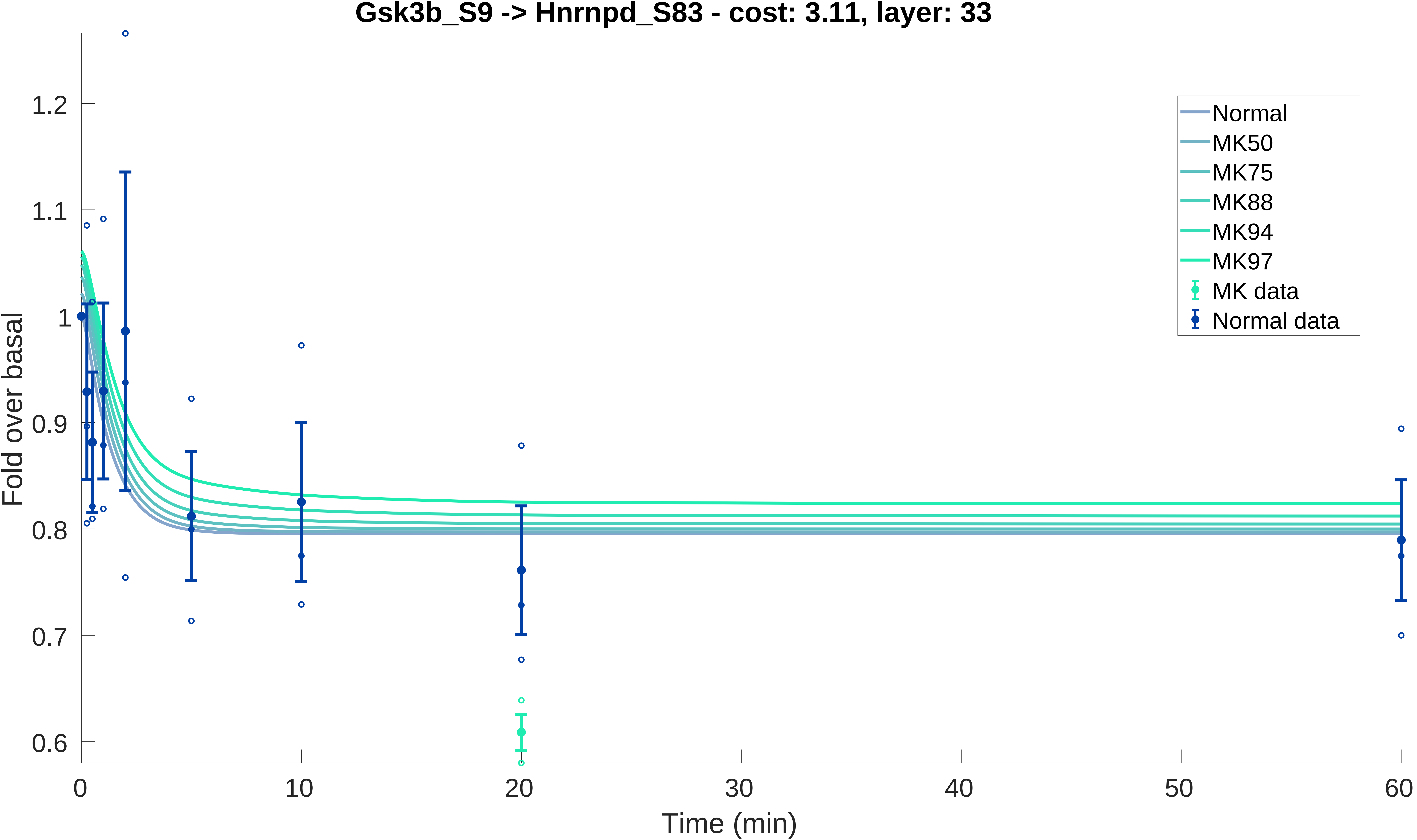

Gsk3b_S9 -> Hnrnpd_S83 - cost: 3.11, layer: 33
Fold over basal
Time (min)
1.2
1.1
1
0.9
0.8
0.7
0.6
0
10
20
30
40
50
60
Normal
MK50
MK75
MK88
MK94
MK97
MK data
Normal data

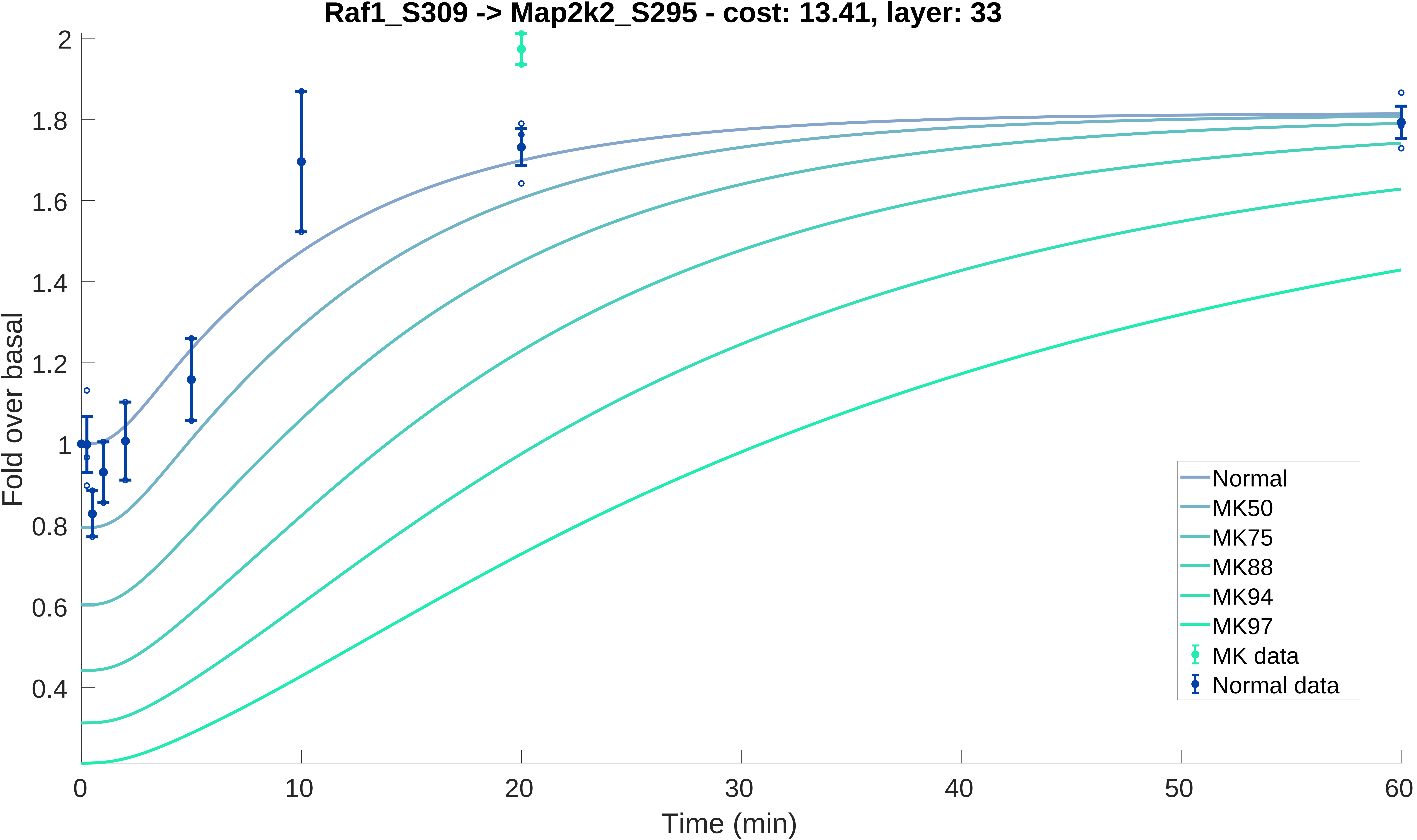

Raf1_S309 -> Map2k2_S295 - cost: 13.41, layer: 33
Fold over basal
Time (min)
2
1.8
1.6
1.4
1.2
1
0.8
0.6
0.4
0
10
20
30
40
50
60
Normal
MK50
MK75
MK88
MK94
MK97
MK data
Normal data

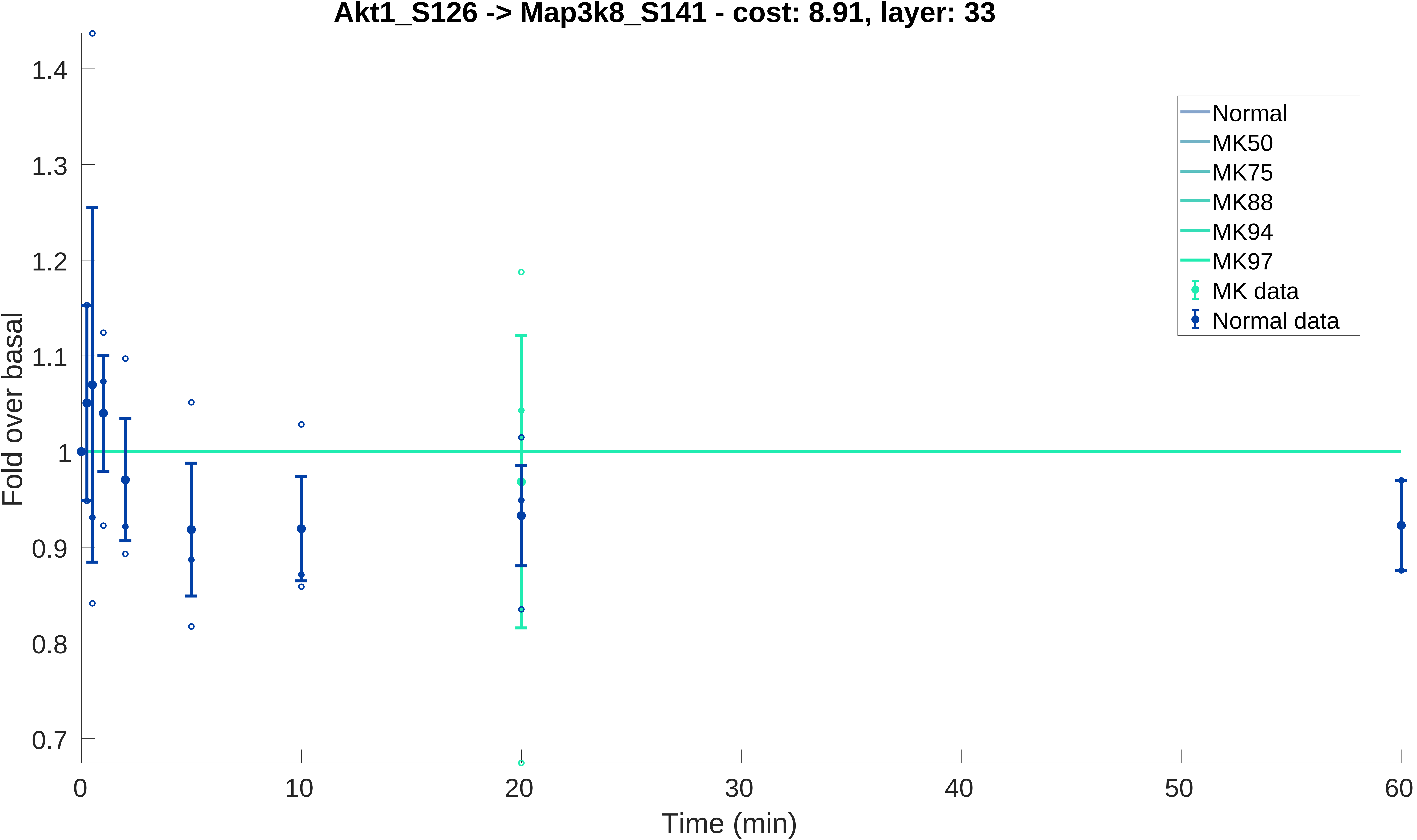

Akt1_S126 -> Map3k8_S141 - cost: 8.91, layer: 33
Fold over basal
Time (min)
1.4
1.3
1.2
1.1
1
0.9
0.8
0.7
0
10
20
30
40
50
60
Normal
MK50
MK75
MK88
MK94
MK97
MK data
Normal data

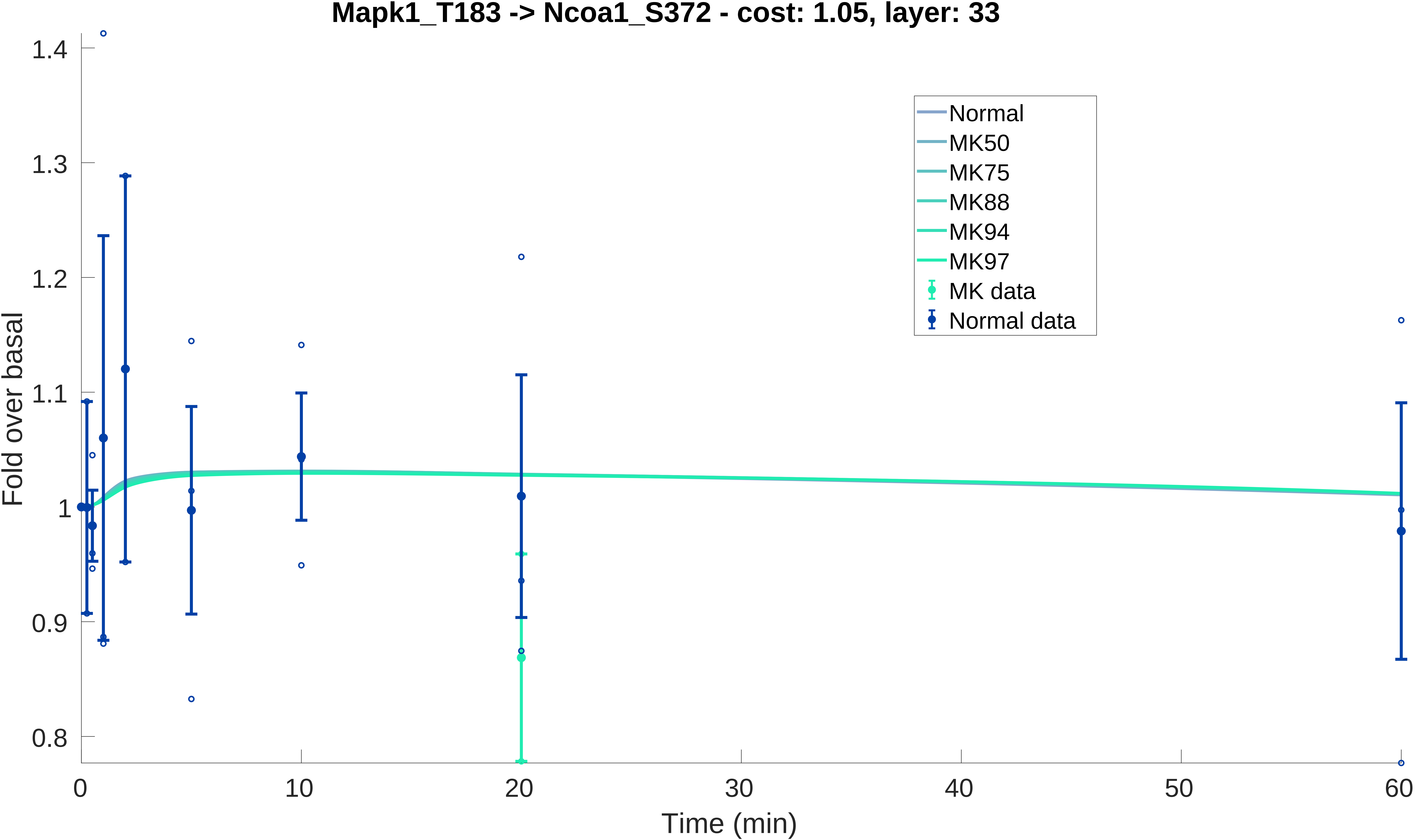

Mapk1_T183 -> Ncoa1_S372 - cost: 1.05, layer: 33
Fold over basal
Time (min)
Normal
MK50
MK75
MK88
MK94
MK97
MK data
Normal data
1.4
1.3
1.2
1.1
1
0.9
0.8
0
10
20
30
40
50
60

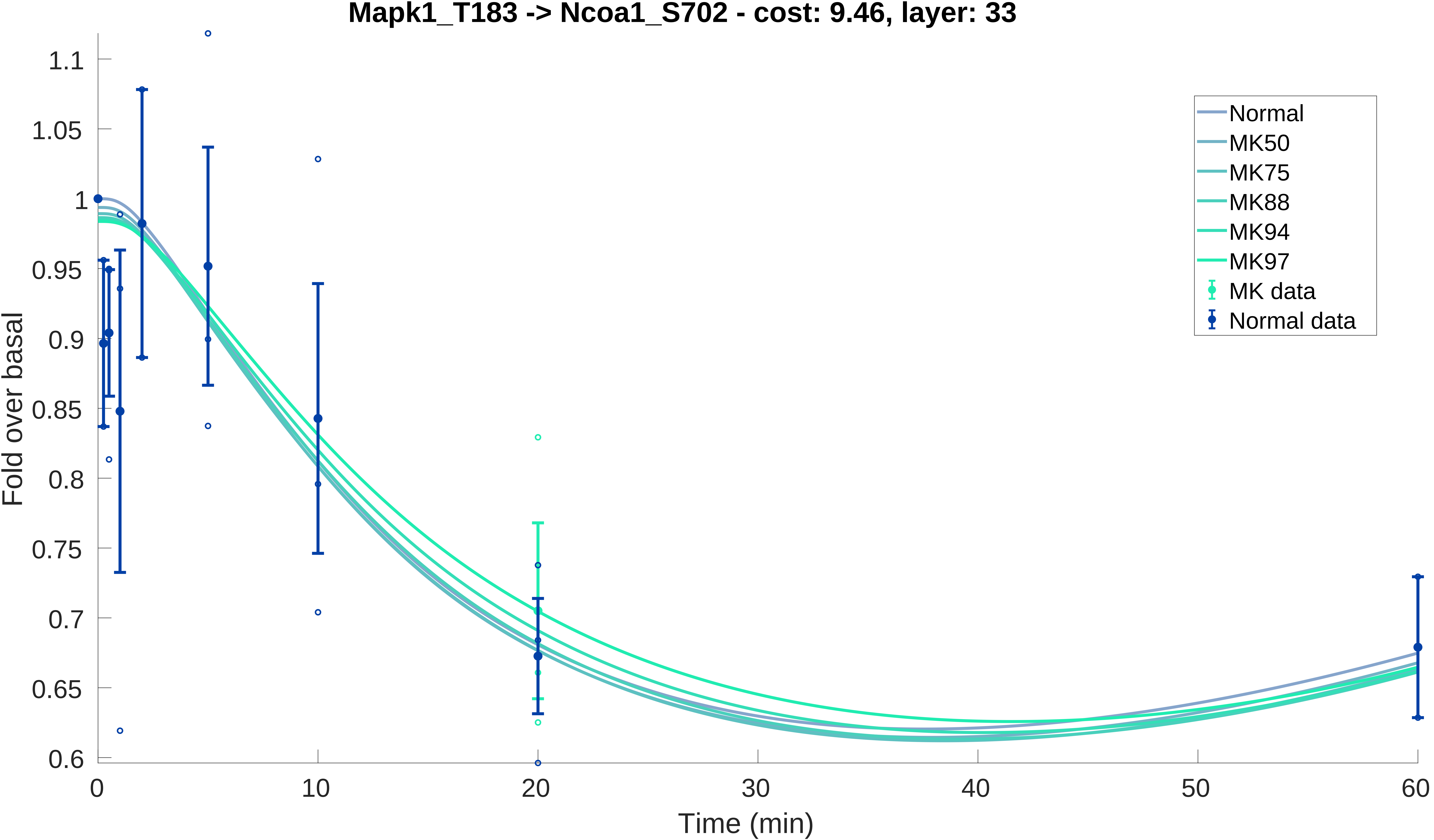

Mapk1_T183 -> Ncoa1_S702 - cost: 9.46, layer: 33
Fold over basal
Time (min)
0.6
0.65
0.7
0.75
0.8
0.85
0.9
0.95
1
1.05
1.1
0
10
20
30
40
50
60
Normal
MK50
MK75
MK88
MK94
MK97
MK data
Normal data

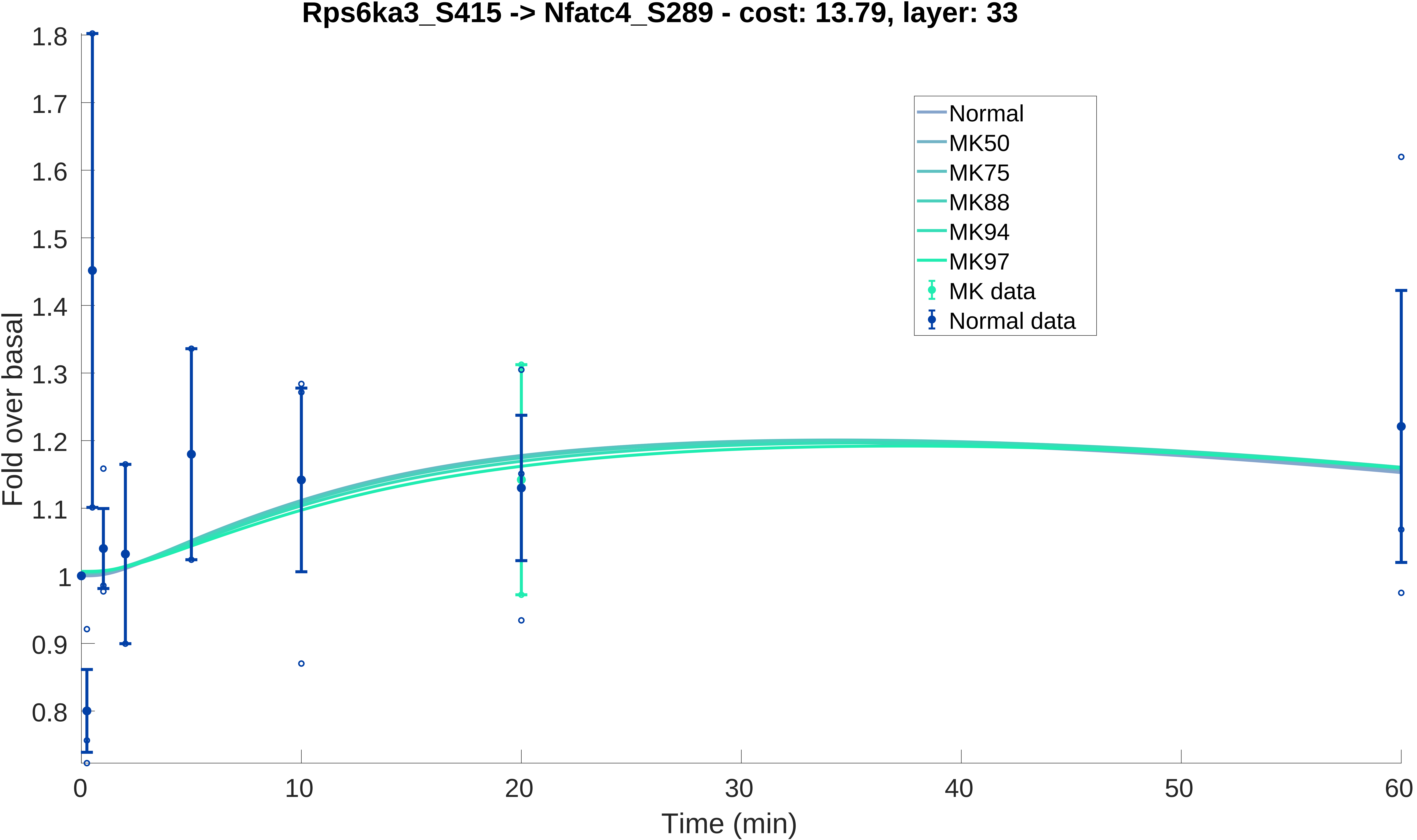
Rps6ka3_S415 -> Nfatc4_S289 - cost: 13.79, layer: 33
Fold over basal
Time (min)
Normal
MK50
MK75
MK88
MK94
MK97
MK data
Normal data
0.8
0.9
1
1.1
1.2
1.3
1.4
1.5
1.6
1.7
1.8
0
10
20
30
40
50
60

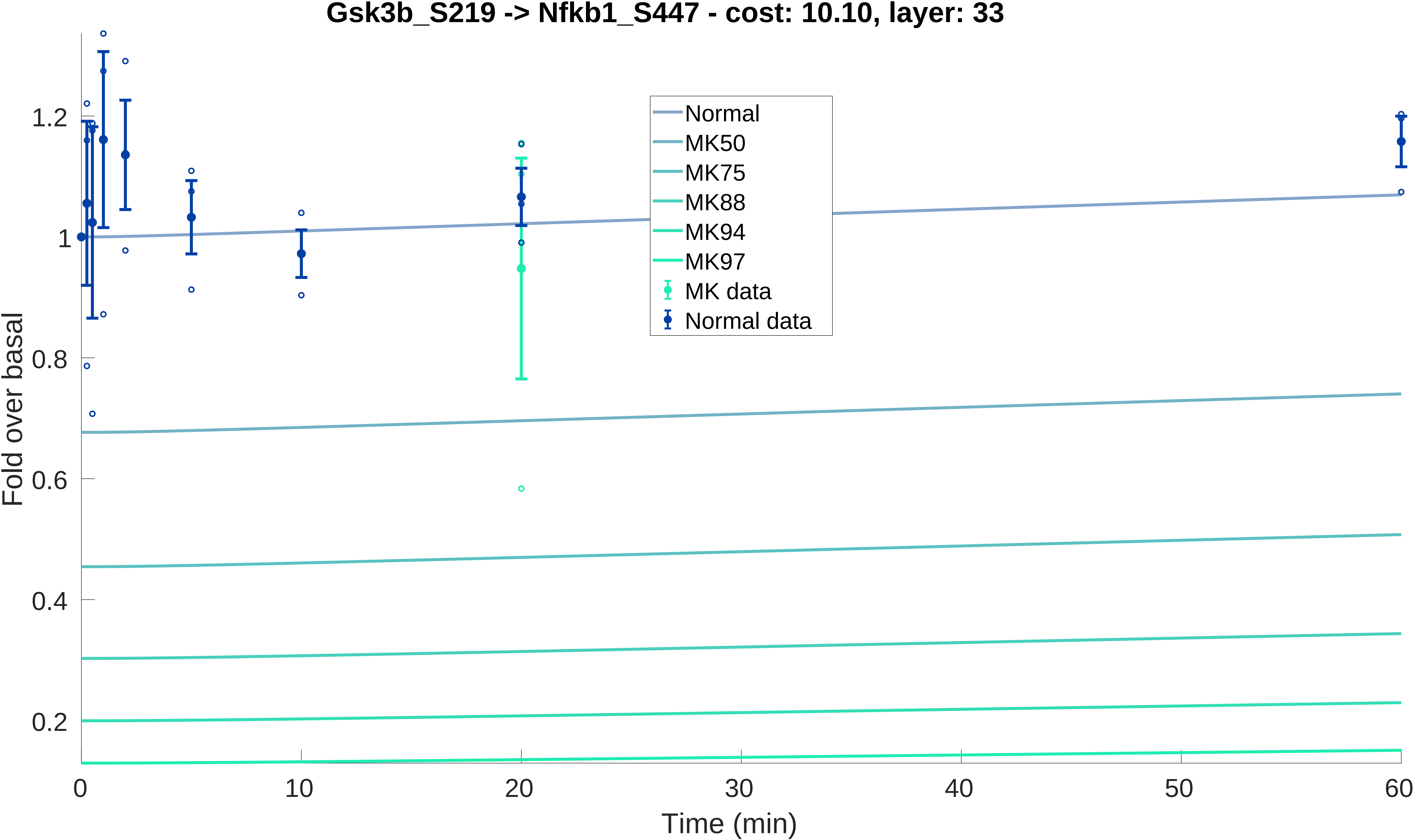
Gsk3b_S219 -> Nfkb1_S447 - cost: 10.10, layer: 33
Fold over basal
Time (min)
Normal
MK50
MK75
MK88
MK94
MK97
MK data
Normal data
0
10
20
30
40
50
60
0.2
0.4
0.6
0.8
1
1.2

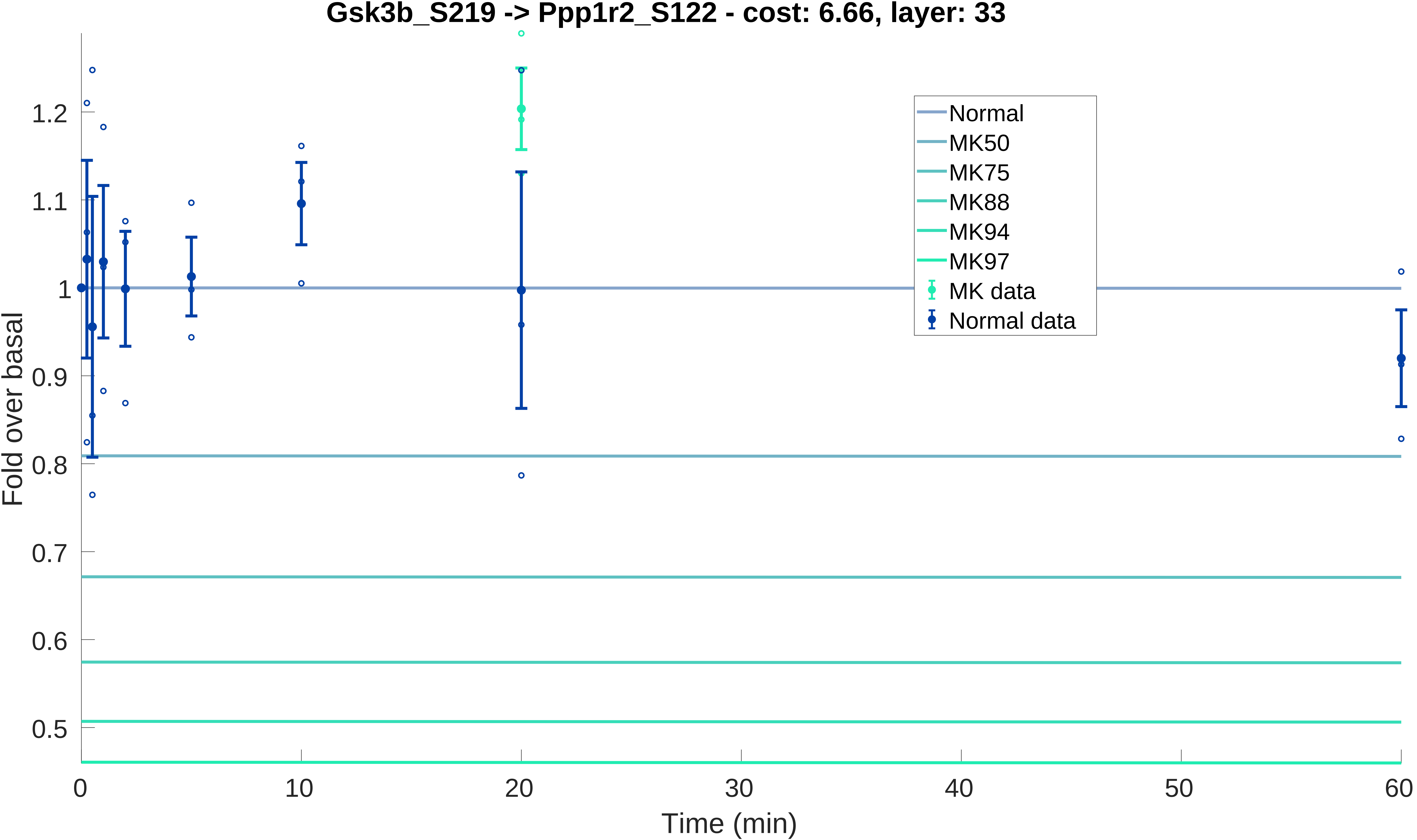
Gsk3b_S219 -> Ppp1r2_S122 - cost: 6.66, layer: 33
Fold over basal
Time (min)
1.2
1.1
1
0.9
0.8
0.7
0.6
0.5
0
10
20
30
40
50
60
Normal
MK50
MK75
MK88
MK94
MK97
MK data
Normal data

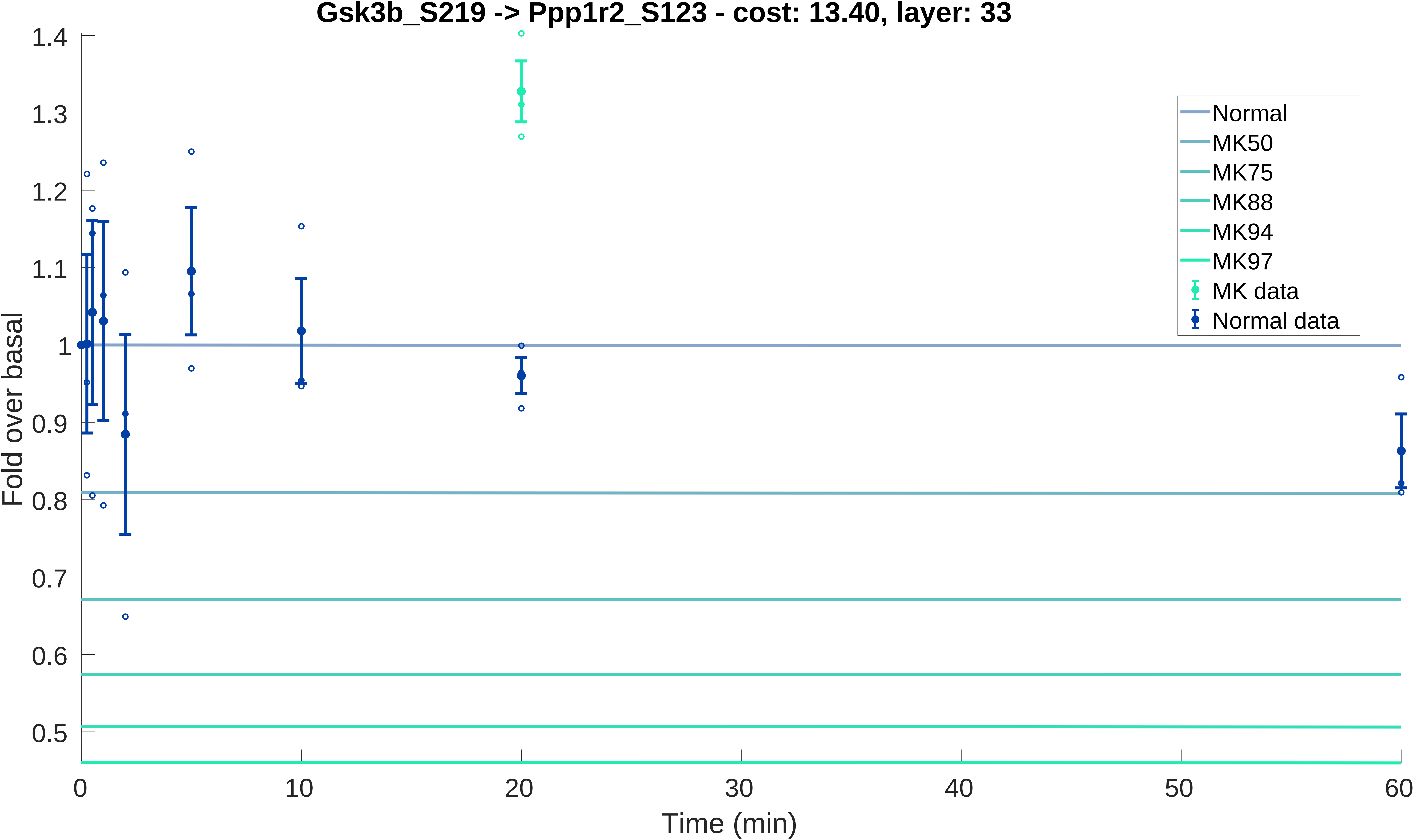

Gsk3b_S219 -> Ppp1r2_S123 - cost: 13.40, layer: 33
Fold over basal
Time (min)
1.4
1.3
1.2
1.1
1
0.9
0.8
0.7
0.6
0.5
0
10
20
30
40
50
60
Normal
MK50
MK75
MK88
MK94
MK97
MK data
Normal data

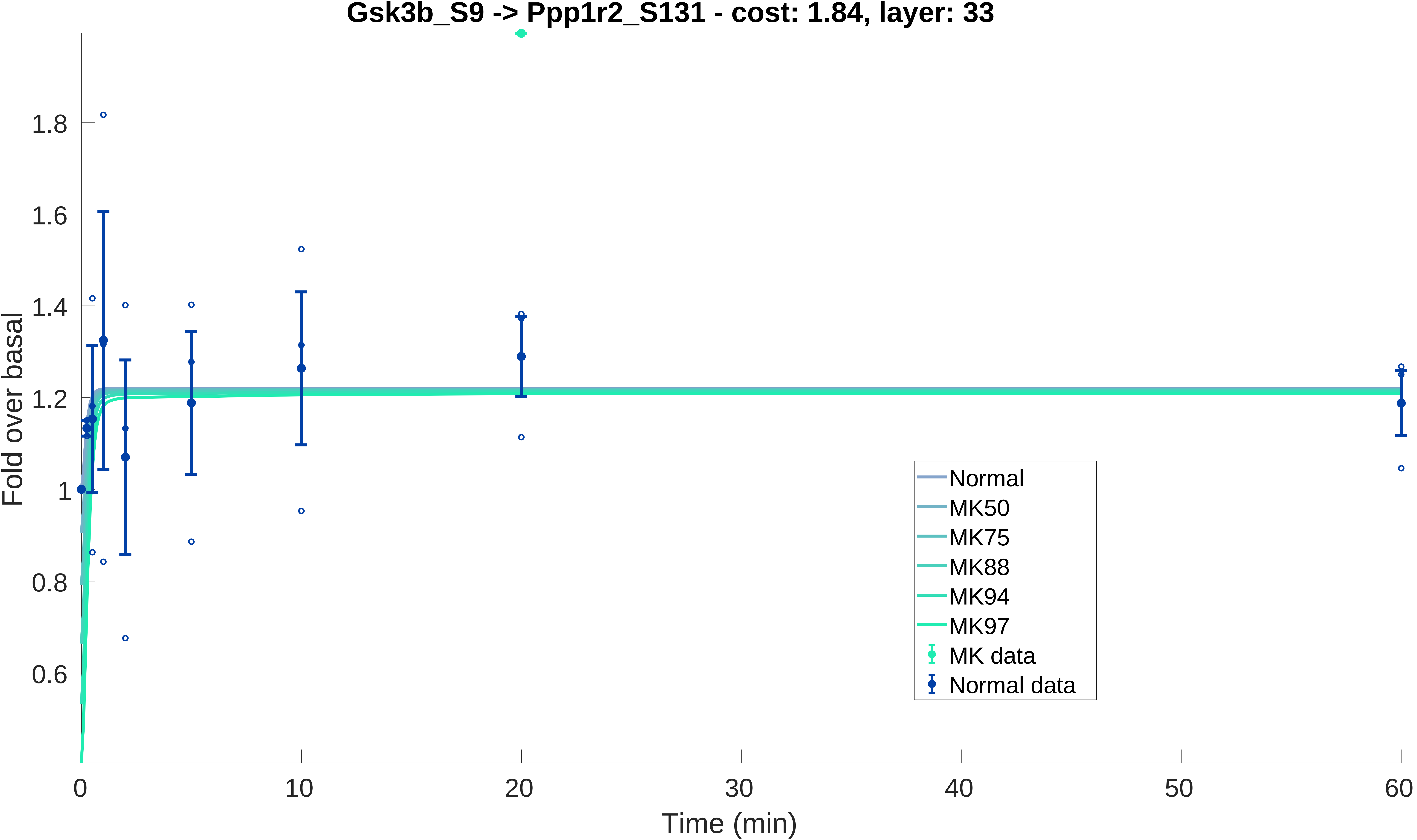
Gsk3b_S9 -> Ppp1r2_S131 - cost: 1.84, layer: 33
Fold over basal
1.8
1.6
1.4
1.2
1
0.8
0.6
0
10
20
30
40
50
60
Time (min)
Normal
MK50
MK75
MK88
MK94
MK97
MK data
Normal data

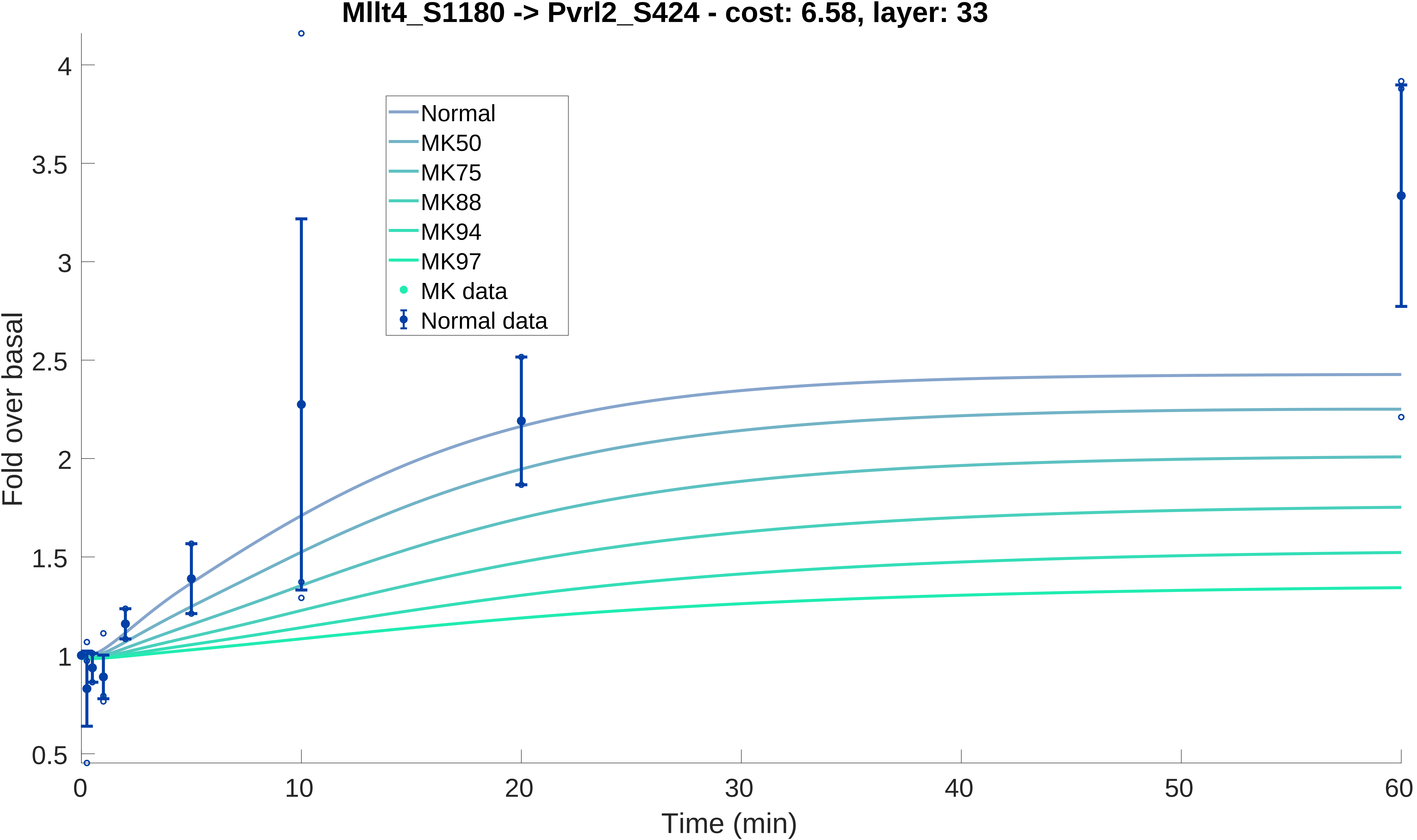
Mllt4_S1180 -> Pvrl2_S424 - cost: 6.58, layer: 33
Fold over basal
Time (min)
0.5
1
1.5
2
2.5
3
3.5
4
0
10
20
30
40
50
60
Normal
MK50
MK75
MK88
MK94
MK97
MK data
Normal data

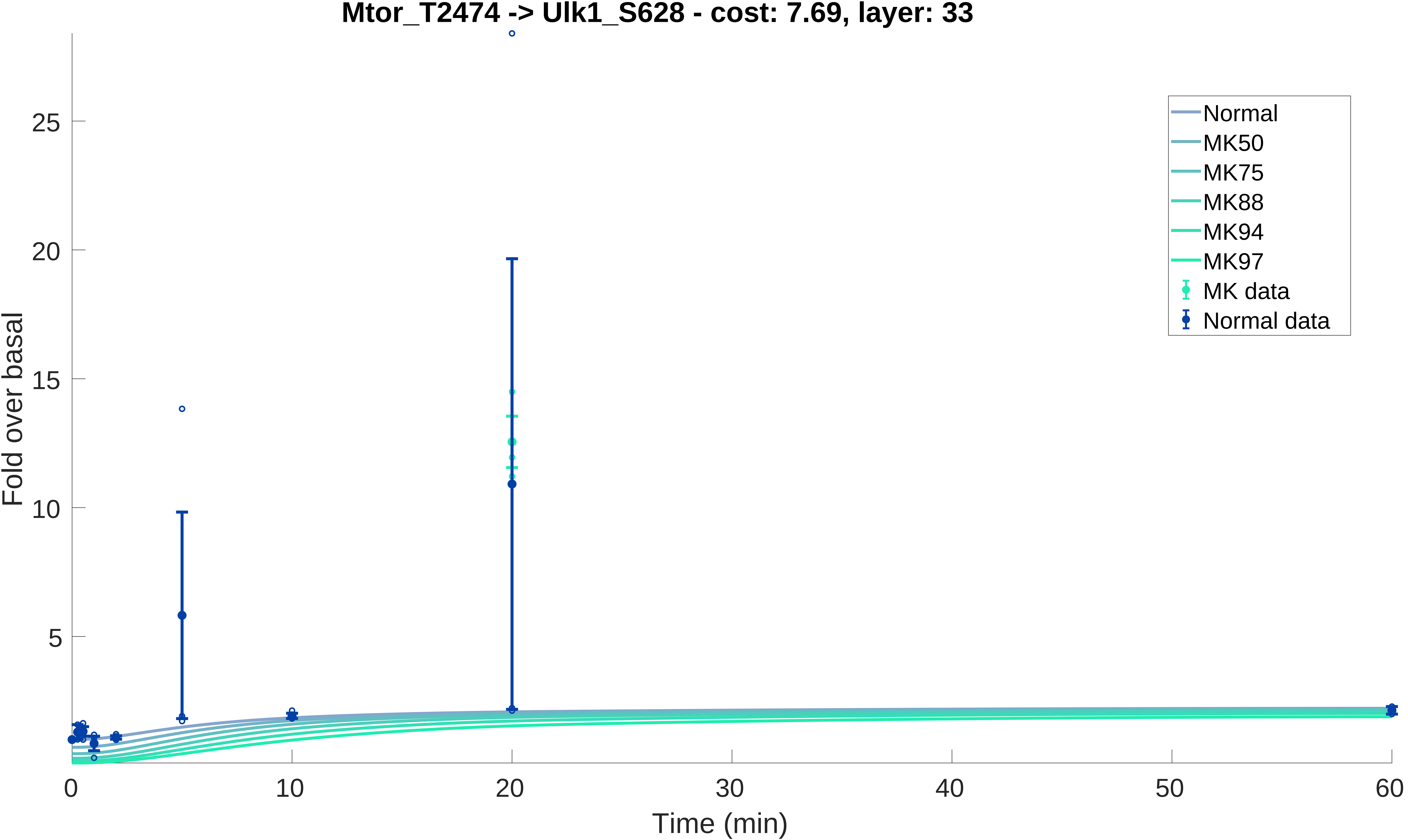
Mtor_T2474 -> Ulk1_S628 - cost: 7.69, layer: 33
Fold over basal
Time (min)
Normal
MK50
MK75
MK88
MK94
MK97
MK data
Normal data
0
10
20
30
40
50
60
5
10
15
20
25

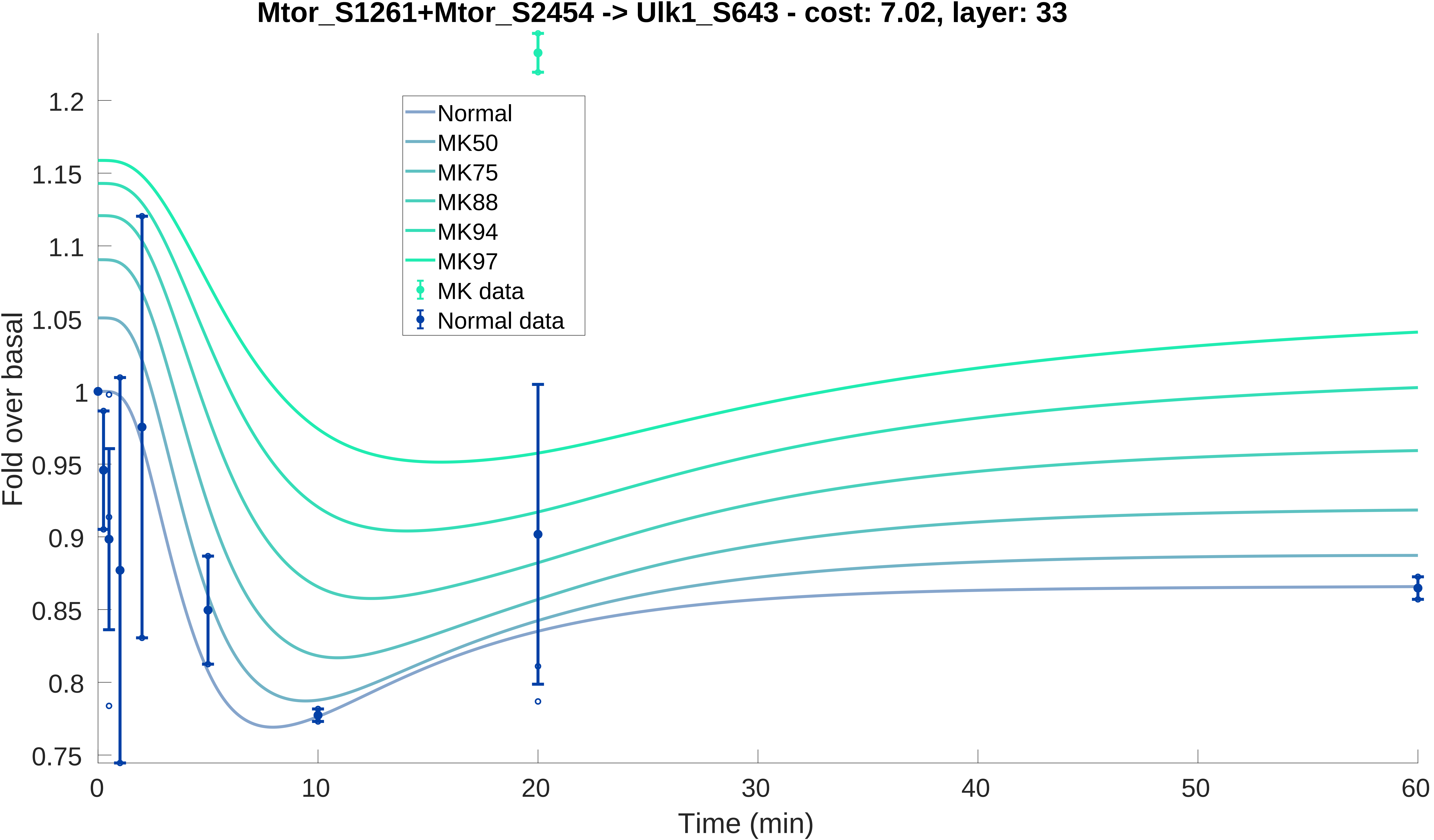

Mtor_S1261+Mtor_S2454 -> Ulk1_S643 - cost: 7.02, layer: 33
Normal
MK50
MK75
MK88
MK94
MK97
MK data
Normal data
Fold over basal
Time (min)
0.75
0.8
0.85
0.9
0.95
1
1.05
1.1
1.15
1.2
0
10
20
30
40
50
60

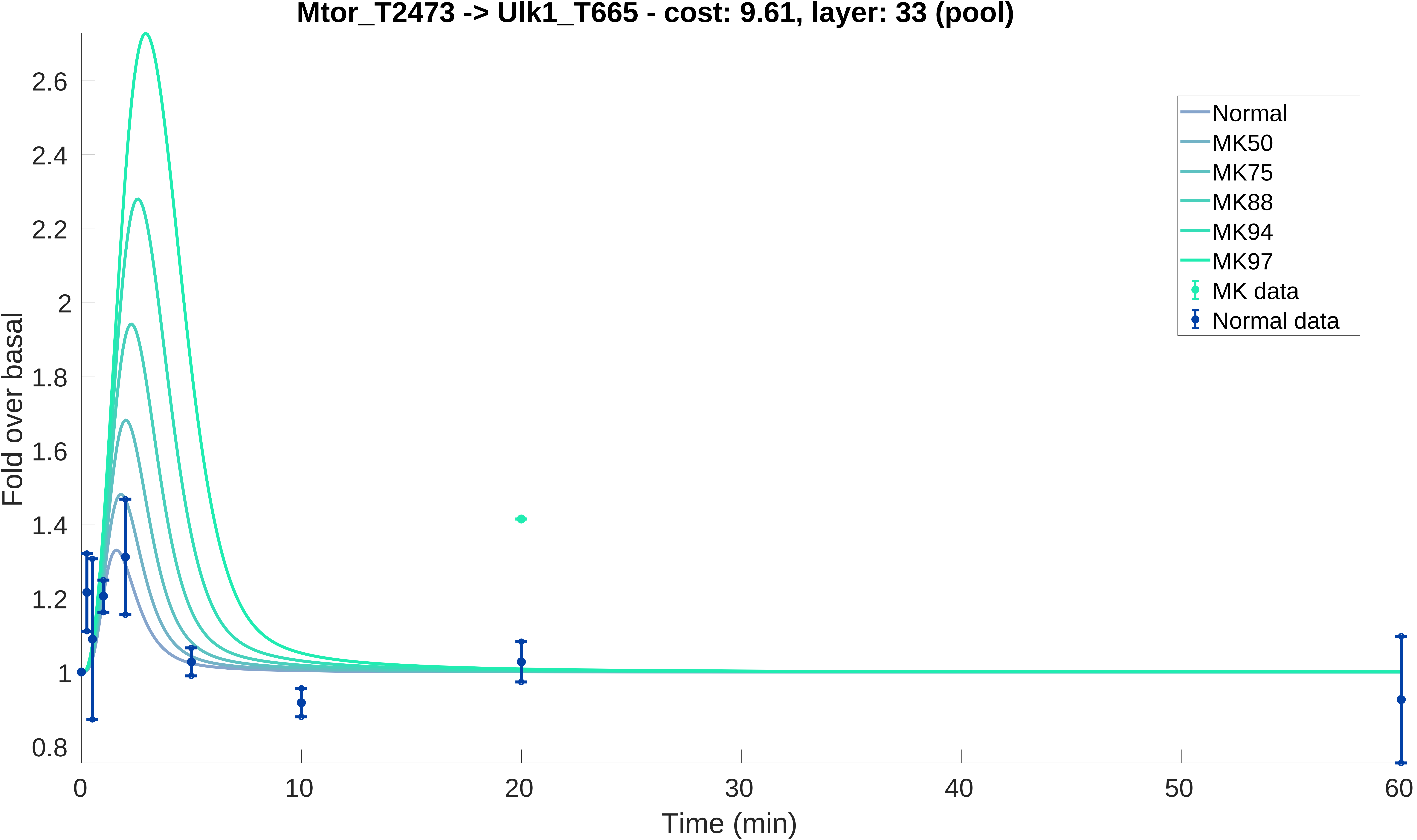

Mtor_T2473 -> Ulk1_T665 - cost: 9.61, layer: 33 (pool)
Fold over basal
Time (min)
0
10
20
30
40
50
60
0.8
1
1.2
1.4
1.6
1.8
2
2.2
2.4
2.6
Normal
MK50
MK75
MK88
MK94
MK97
MK data
Normal data

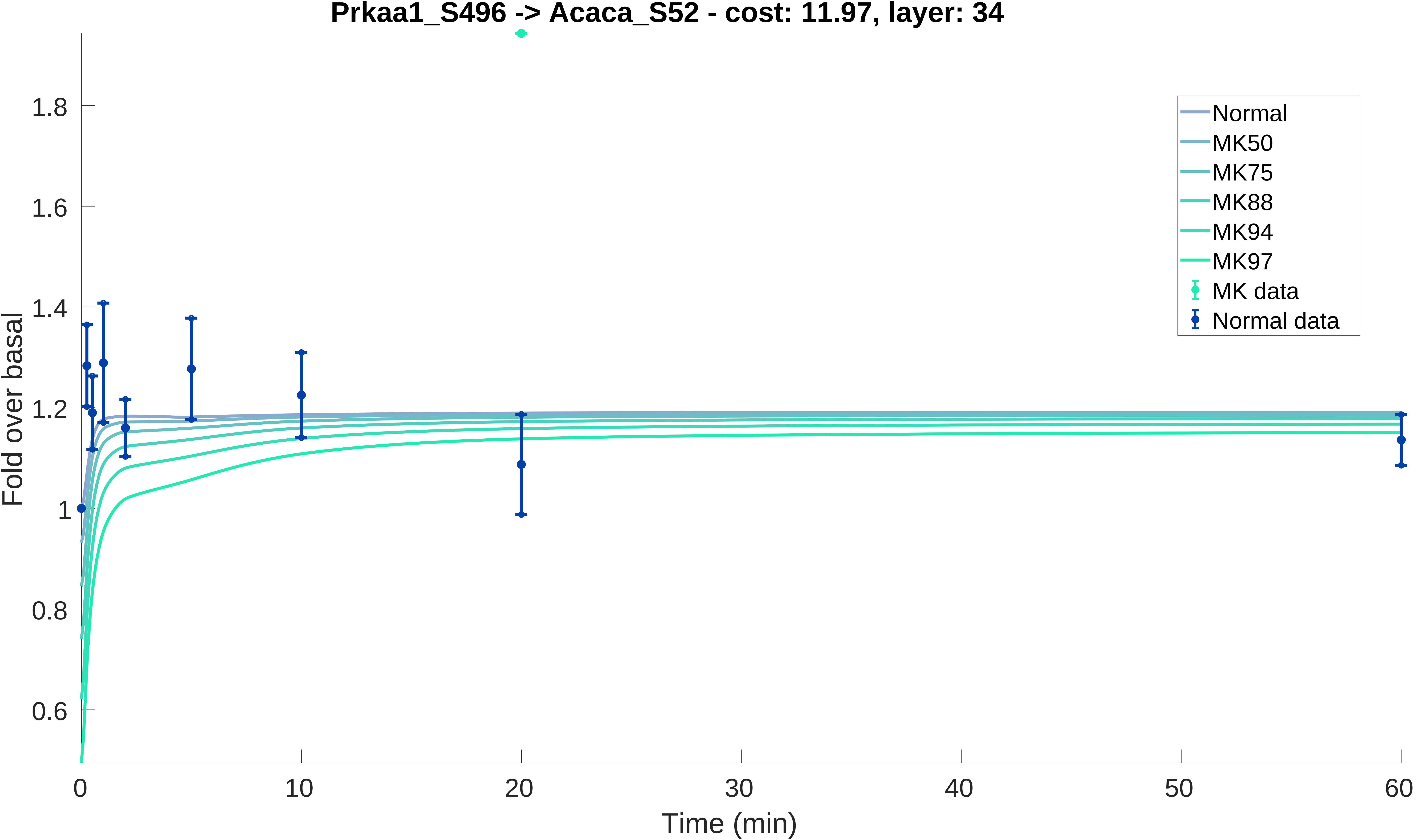

Prkaa1_S496 -> Acaca_S52 - cost: 11.97, layer: 34
Fold over basal
Time (min)
0.6
0.8
1
1.2
1.4
1.6
1.8
0
10
20
30
40
50
60
Normal
MK50
MK75
MK88
MK94
MK97
MK data
Normal data

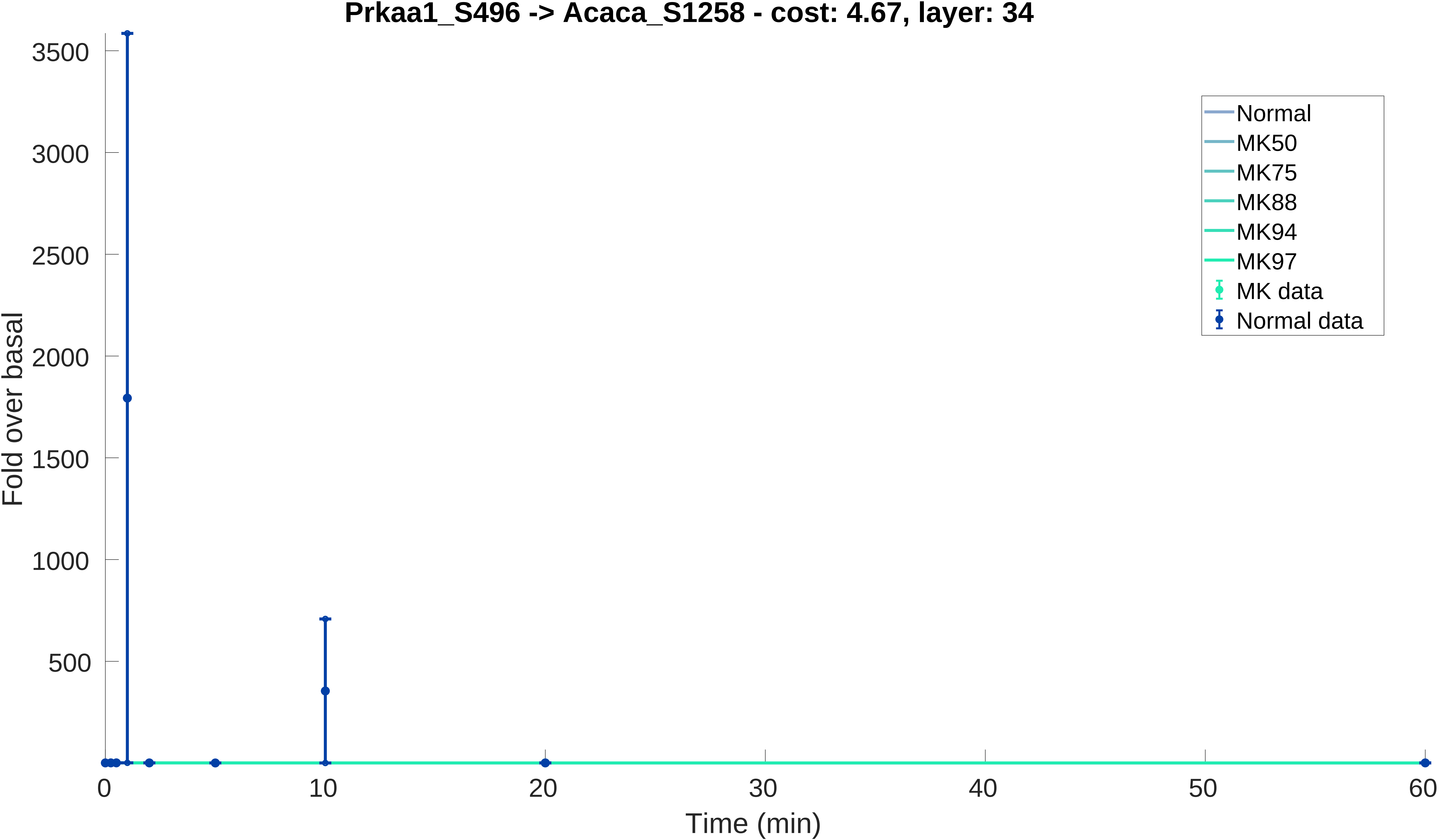
Prrkaa1_S496 -> Acaca_S1258 - cost: 4.67, layer: 34
Fold over basal
Time (min)
0
10
20
30
40
50
60
500
1000
1500
2000
2500
3000
3500
Normal
MK50
MK75
MK88
MK94
MK97
MK data
Normal data

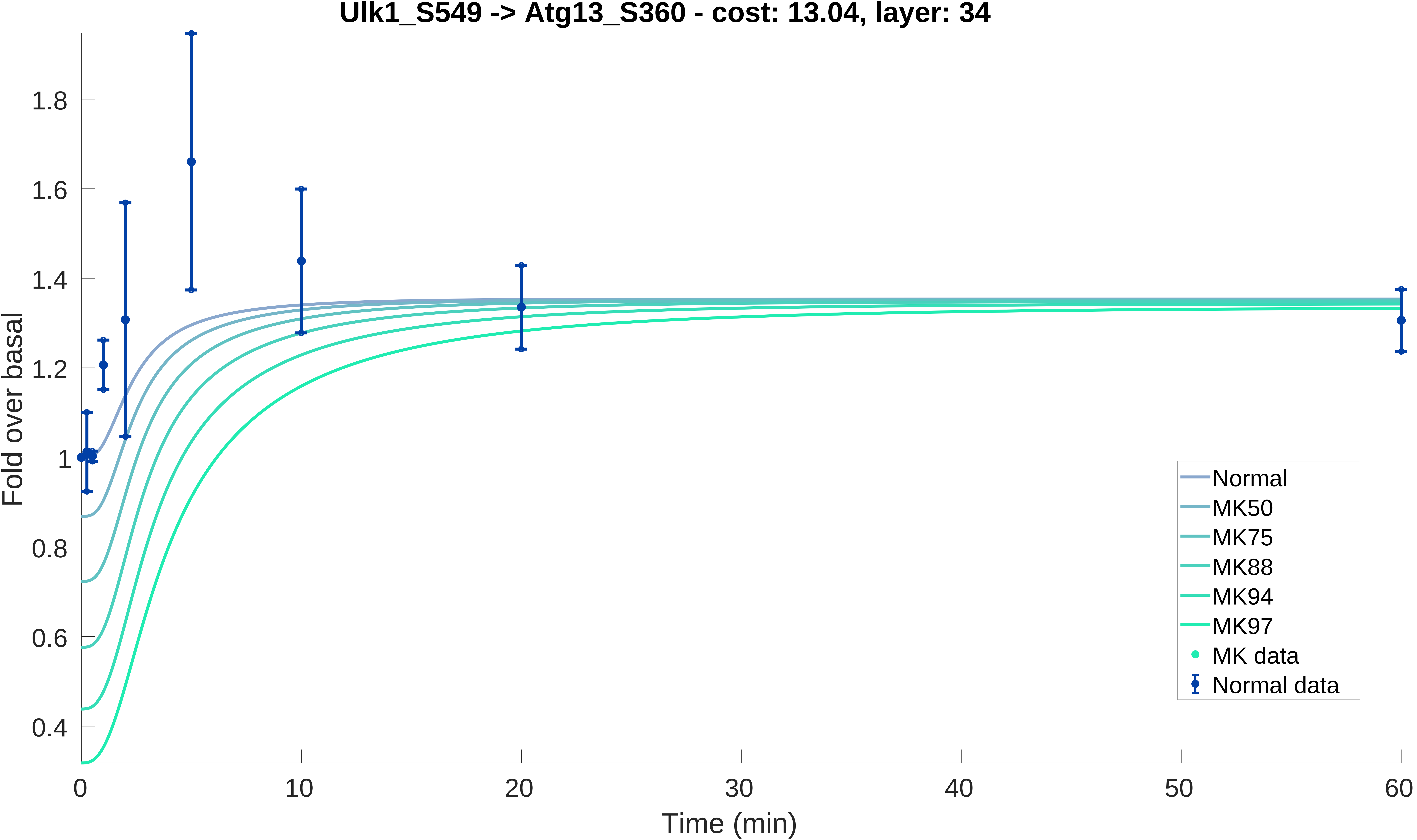

Ulk1_S549 -> Atg13_S360 - cost: 13.04, layer: 34
Fold over basal
1.8
1.6
1.4
1.2
1
0.8
0.6
0.4
0
10
20
30
40
50
60
Time (min)
Normal
MK50
MK75
MK88
MK94
MK97
MK data
Normal data

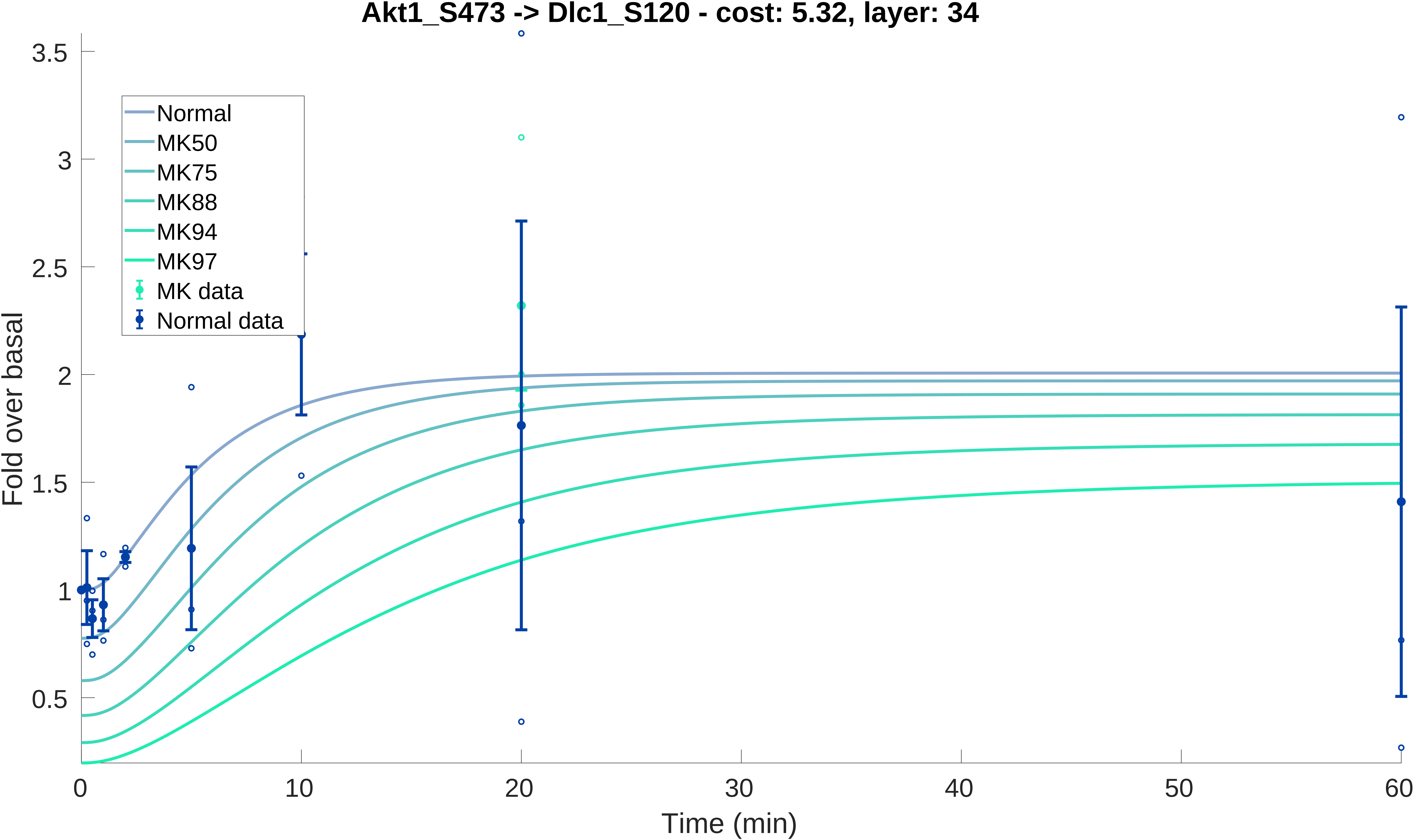

Akt1_S473 -> Dlc1_S120 - cost: 5.32, layer: 34
Normal
MK50
MK75
MK88
MK94
MK97
MK data
Normal data
Fold over basal
Time (min)
3.5
3
2.5
2
1.5
1
0.5
0
10
20
30
40
50
60

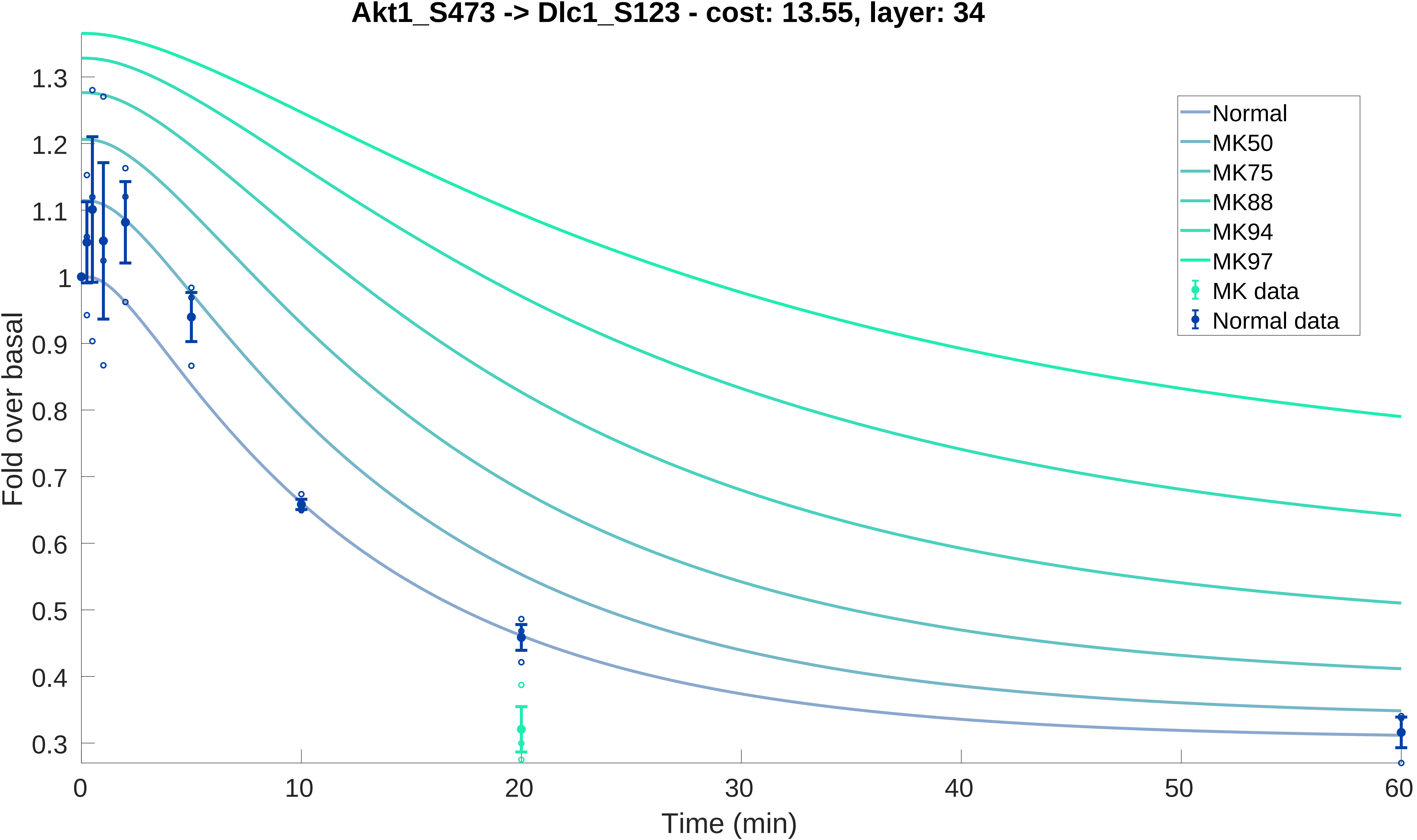

Akt1_S473 -> Dlc1_S123 - cost: 13.55, layer: 34
Fold over basal
Time (min)
1.3
1.2
1.1
1
0.9
0.8
0.7
0.6
0.5
0.4
0.3
0
10
20
30
40
50
60
Normal
MK50
MK75
MK88
MK94
MK97
MK data
Normal data

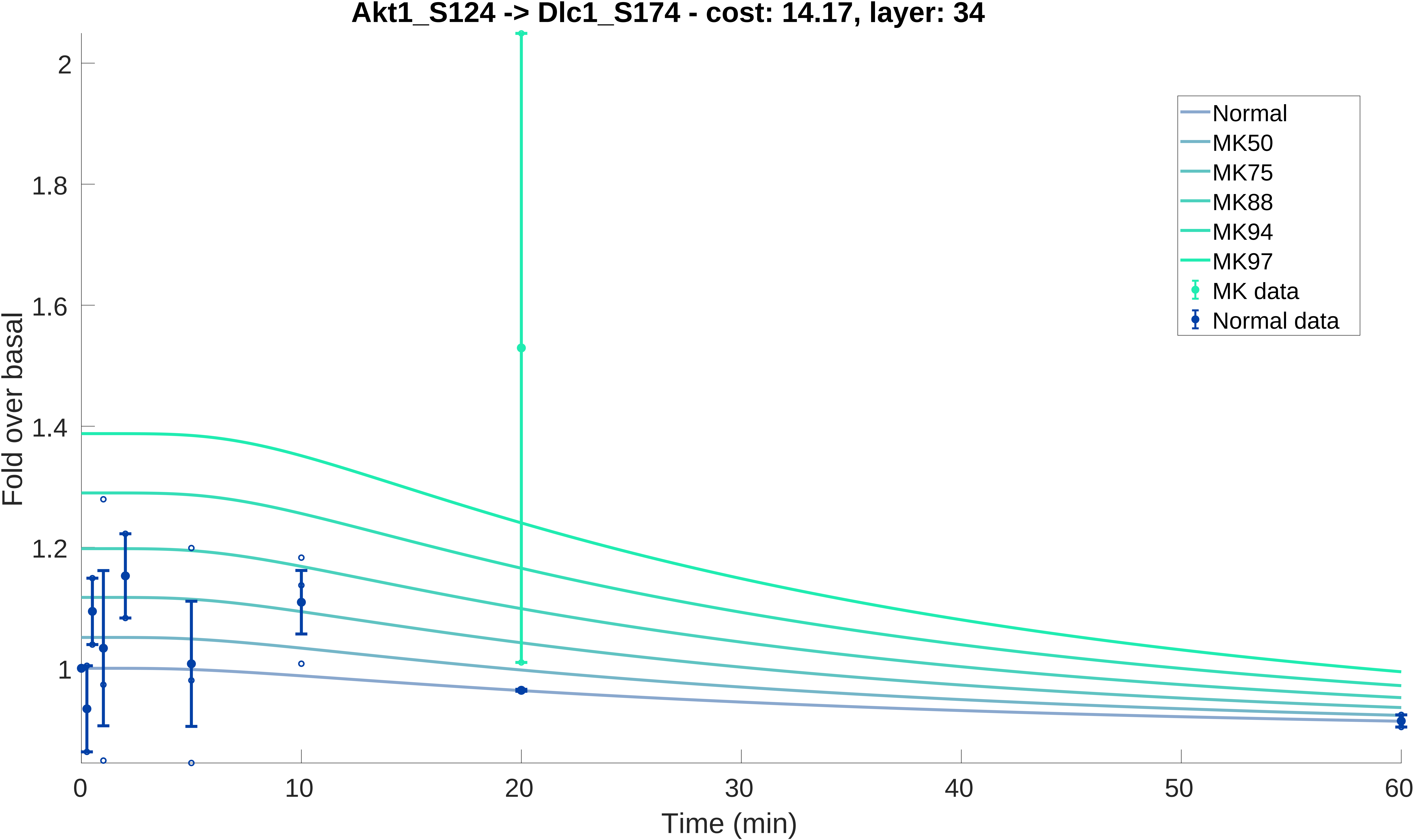

Akt1_S124 -> Dlc1_S174 - cost: 14.17, layer: 34
Fold over basal
Time (min)
2
1.8
1.6
1.4
1.2
1
0
10
20
30
40
50
60
Normal
MK50
MK75
MK88
MK94
MK97
MK data
Normal data

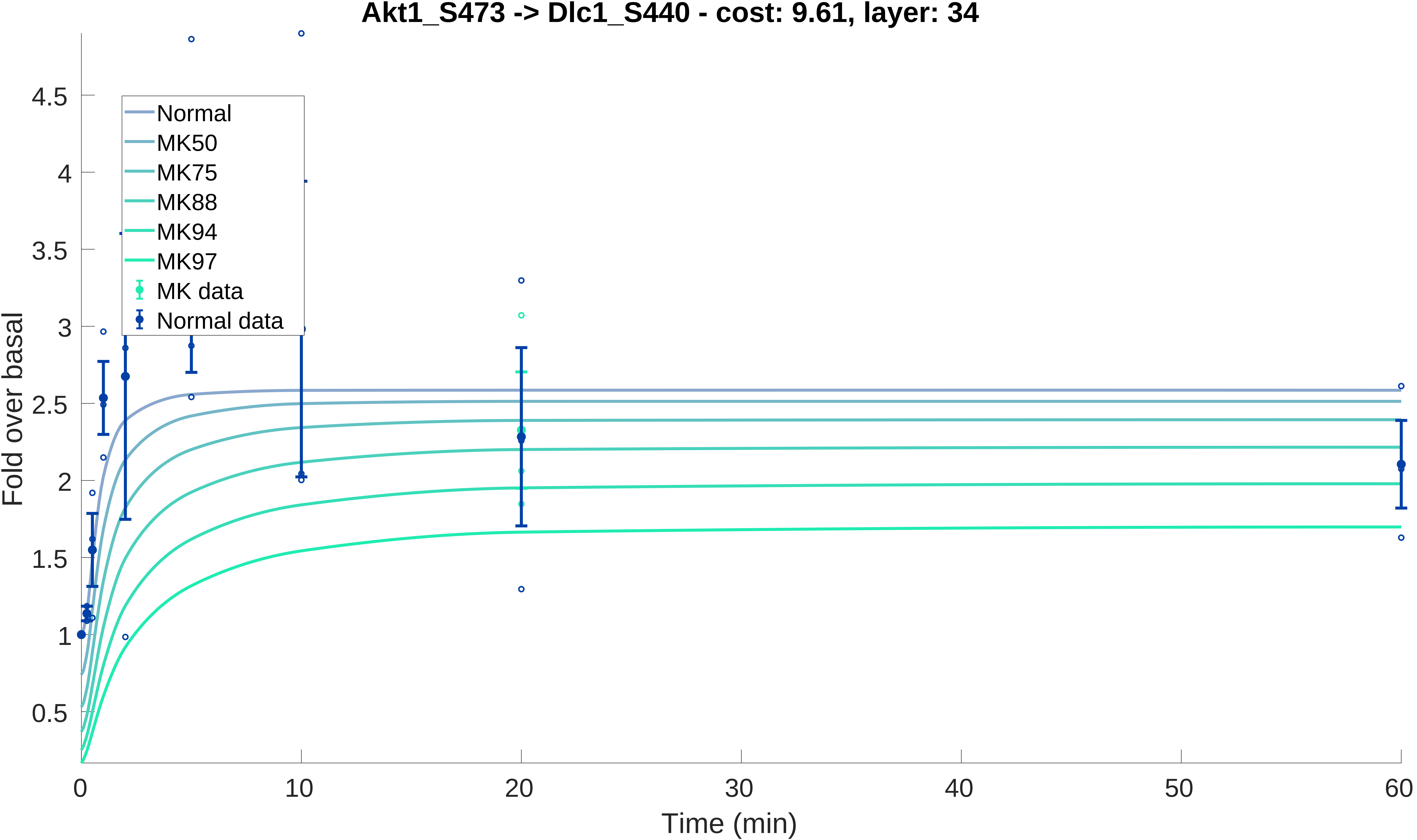

Akt1_S473 -> Dlc1_S440 - cost: 9.61, layer: 34
Normal
MK50
MK75
MK88
MK94
MK97
MK data
Normal data
Fold over basal
Time (min)
0.5
1
1.5
2
2.5
3
3.5
4
4.5
0
10
20
30
40
50
60

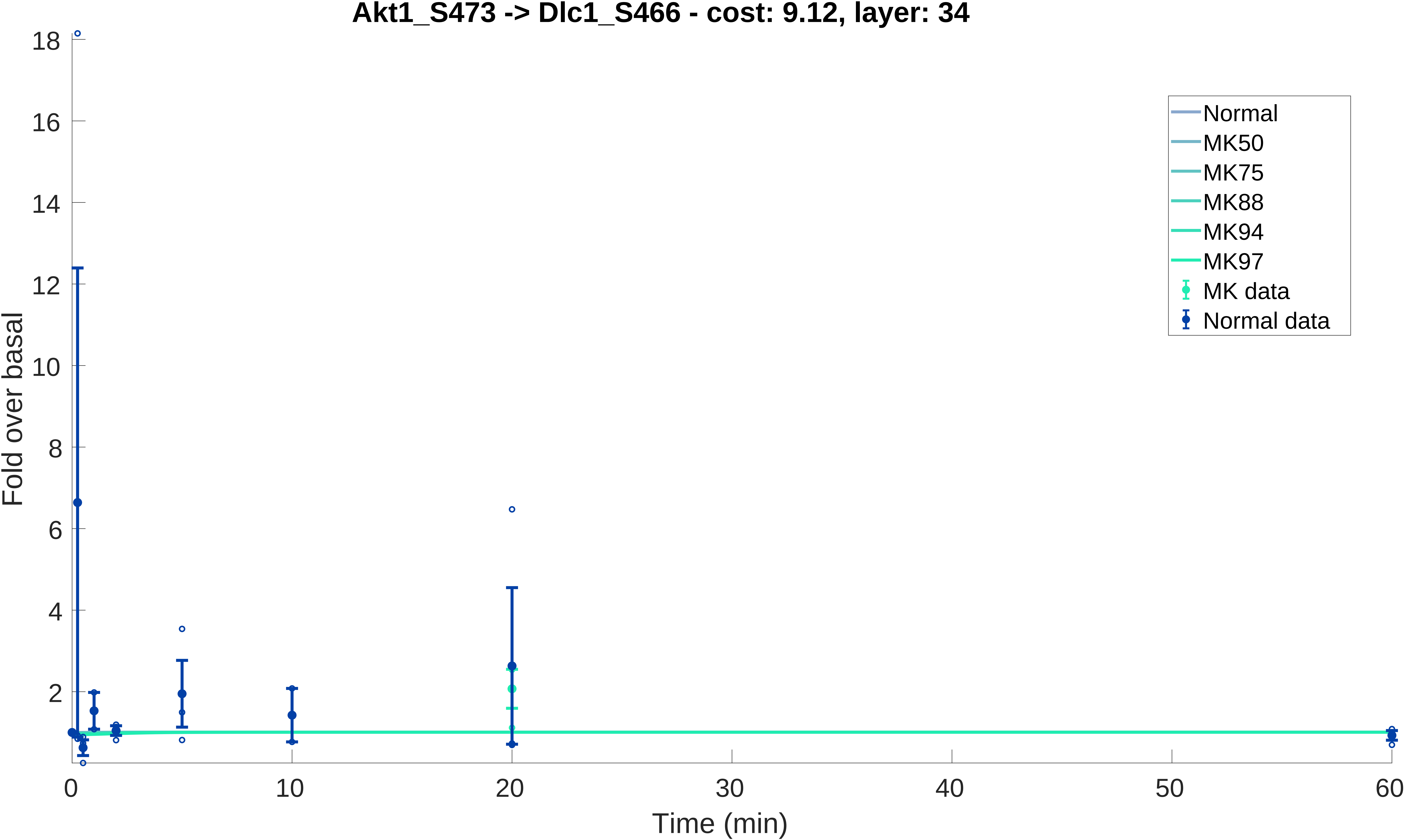

Akt1_S473 -> Dlc1_S466 - cost: 9.12, layer: 34
Fold over basal
Time (min)
Normal
MK50
MK75
MK88
MK94
MK97
MK data
Normal data
0
10
20
30
40
50
60
2
4
6
8
10
12
14
16
18

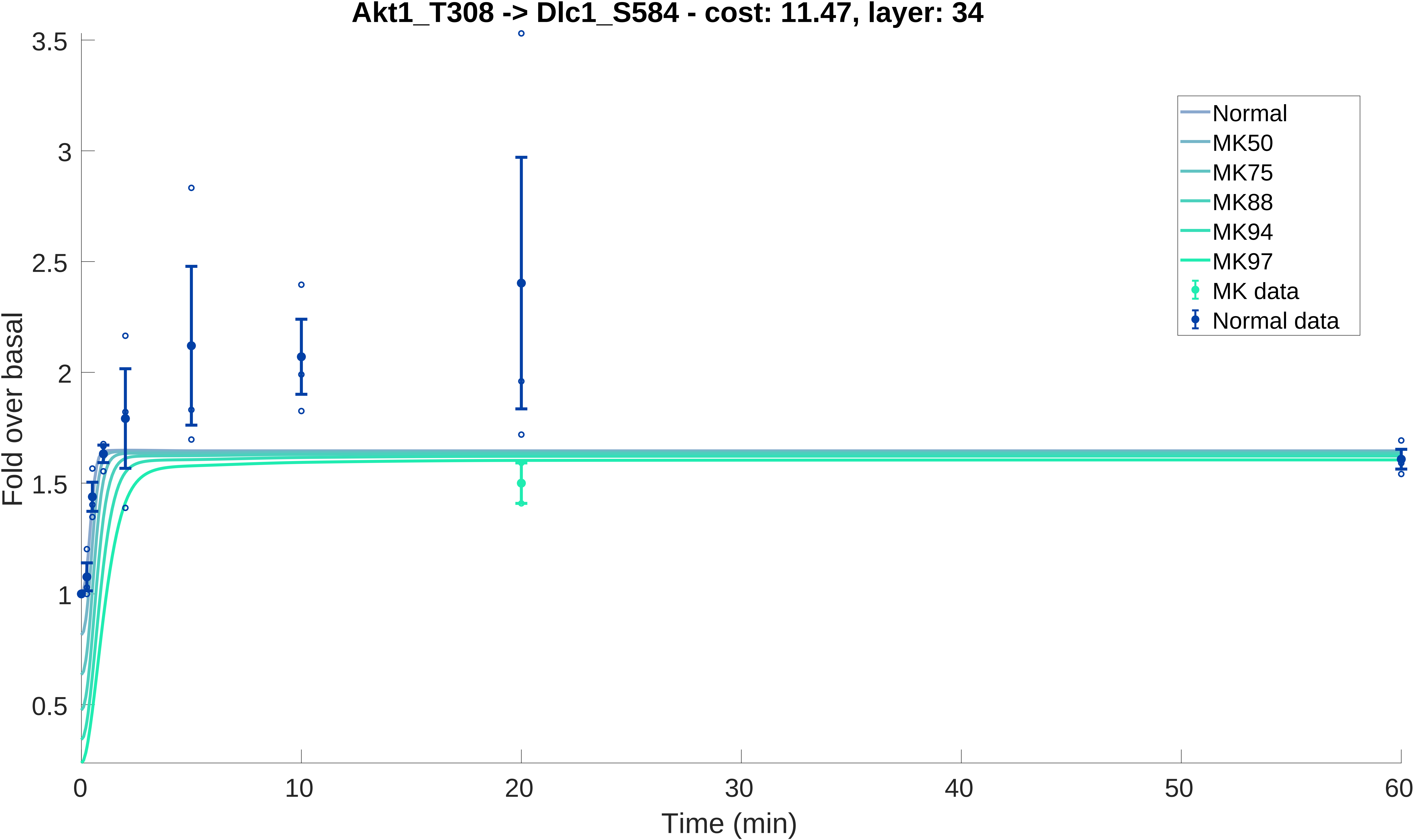

Akt1_T308 -> Dlc1_S584 - cost: 11.47, layer: 34
Fold over basal
Time (min)
3.5
3
2.5
2
1.5
1
0.5
0
10
20
30
40
50
60
Normal
MK50
MK75
MK88
MK94
MK97
MK data
Normal data

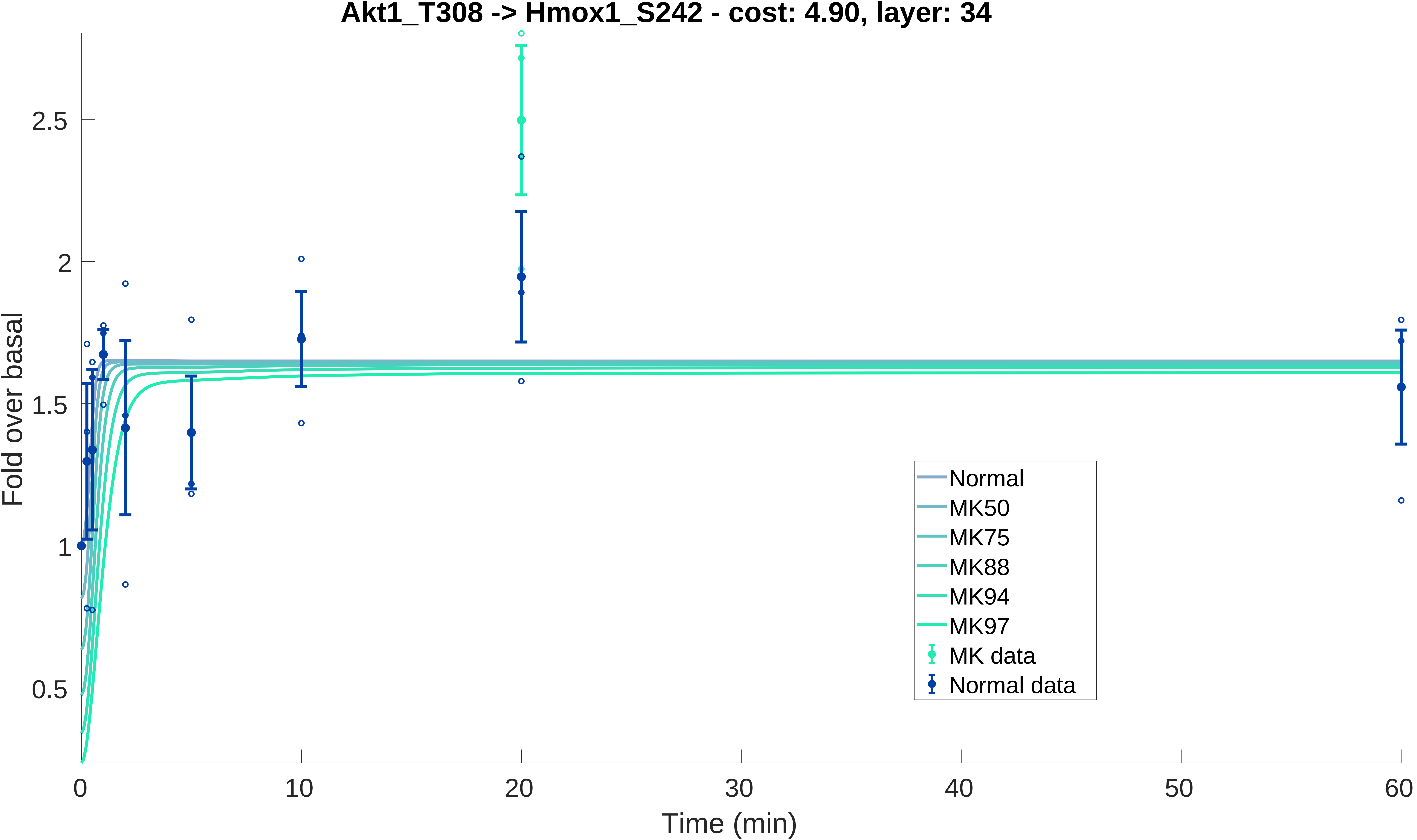
Akt1_T308 -> Hmox1_S242 - cost: 4.90, layer: 34
Fold over basal
Time (min)
0
10
20
30
40
50
60
0.5
1
1.5
2
2.5
Normal
MK50
MK75
MK88
MK94
MK97
MK data
Normal data

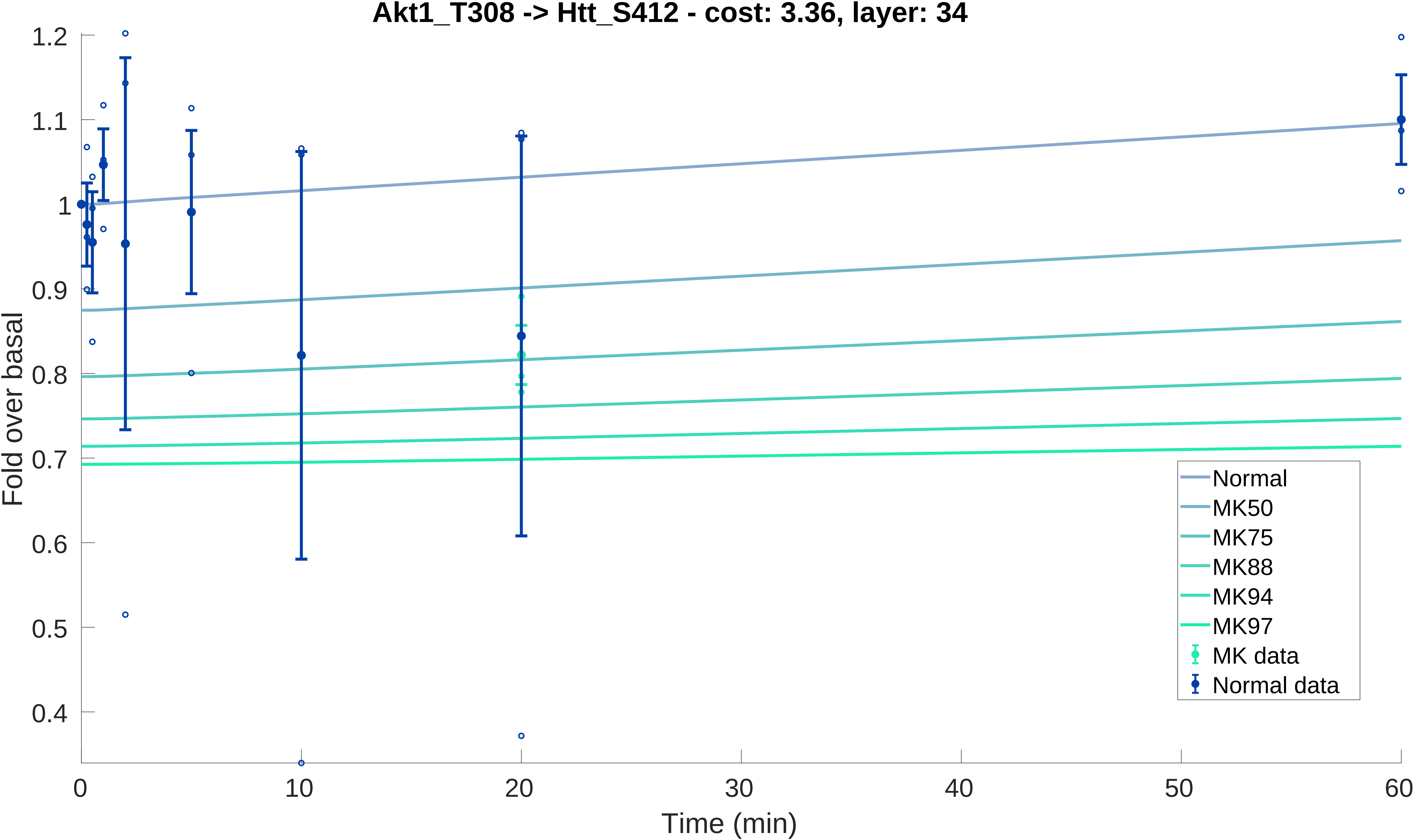

Akt1_T308 -> Htt_S412 - cost: 3.36, layer: 34
Fold over basal
Time (min)
0
10
20
30
40
50
60
0.4
0.5
0.6
0.7
0.8
0.9
1
1.1
1.2
Normal
MK50
MK75
MK88
MK94
MK97
MK data
Normal data

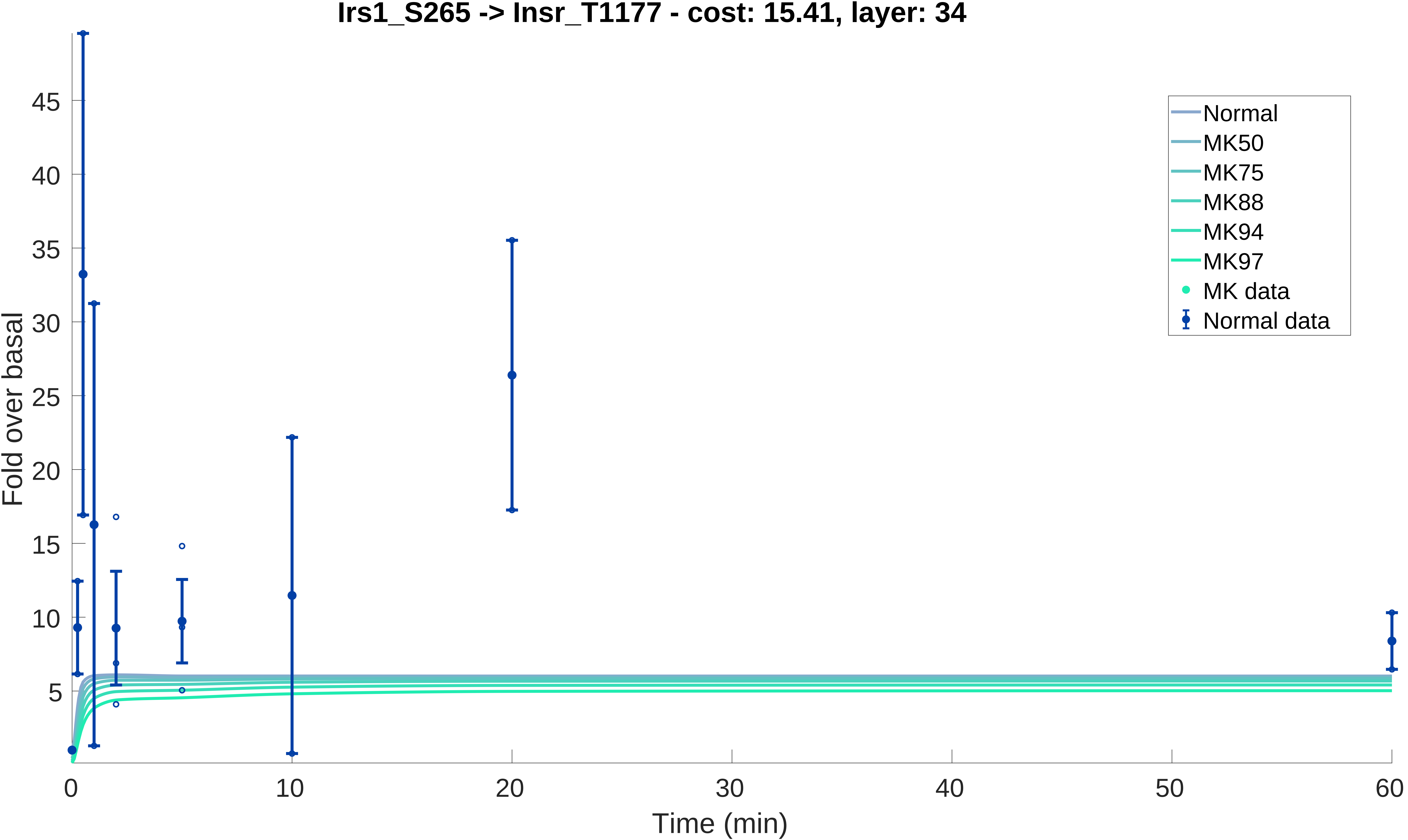

Irs1_S265 -> Insr_T1177 - cost: 15.41, layer: 34
Fold over basal
Time (min)
0
10
20
30
40
50
60
5
10
15
20
25
30
35
40
45
Normal
MK50
MK75
MK88
MK94
MK97
MK data
Normal data

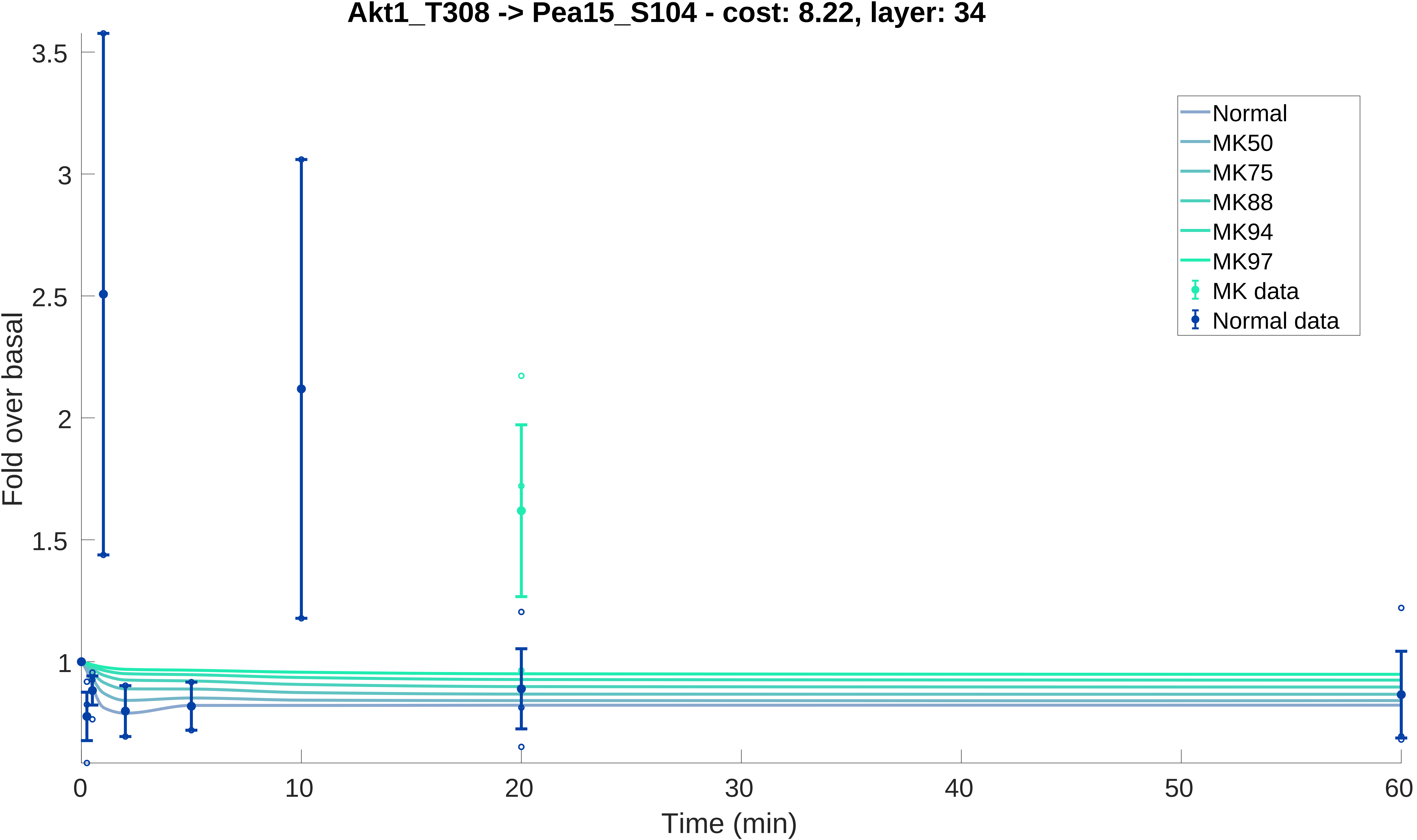

Akt1_T308 -> Pea15_S104 - cost: 8.22, layer: 34
Fold over basal
3.5
3
2.5
2
1.5
1
0
10
20
30
40
50
60
Time (min)
Normal
MK50
MK75
MK88
MK94
MK97
MK data
Normal data

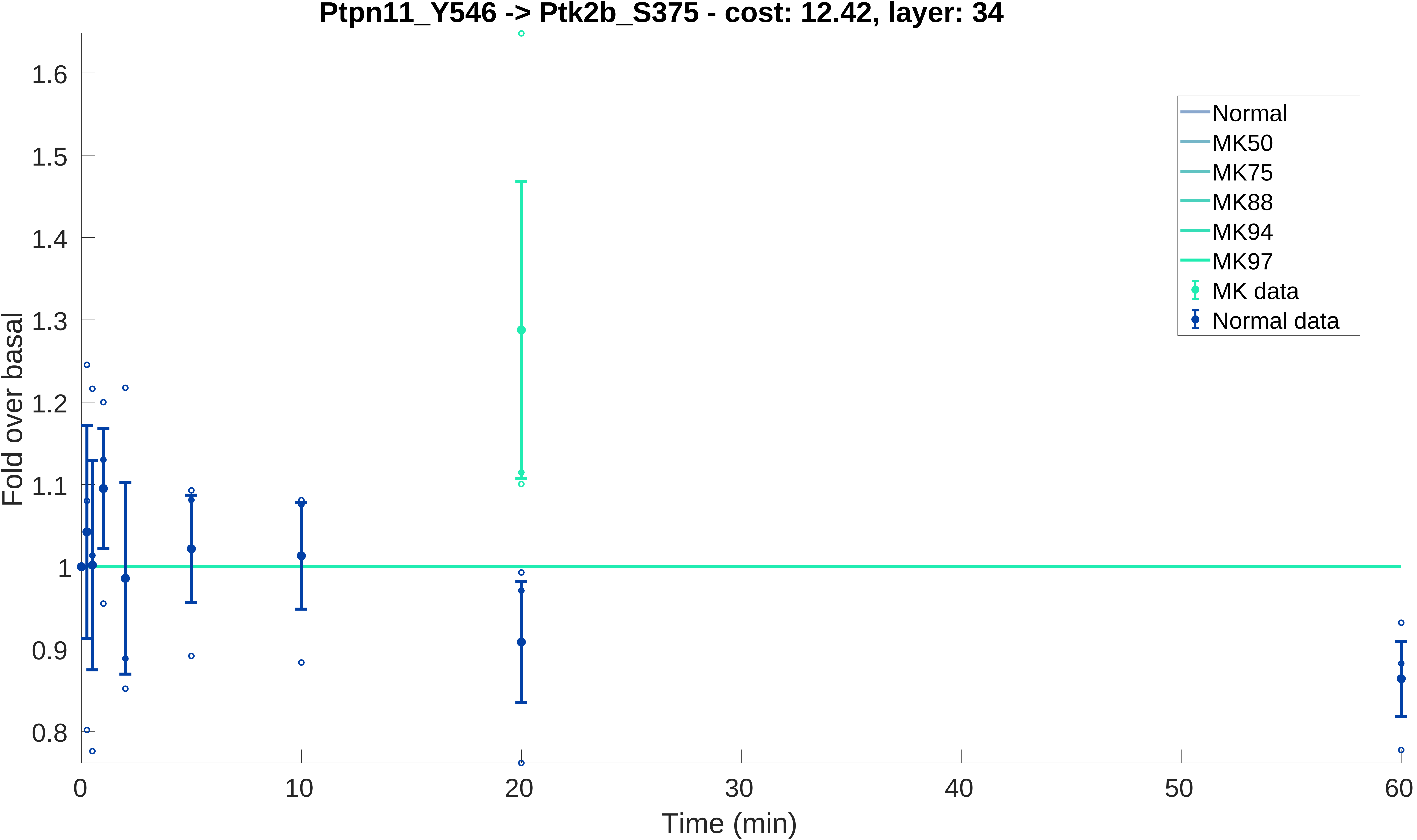

Ptpn11_Y546 -> Ptk2b_S375 - cost: 12.42, layer: 34
Fold over basal
1.6
1.5
1.4
1.3
1.2
1.1
1
0.9
0.8
0
10
20
30
40
50
60
Time (min)
Normal
MK50
MK75
MK88
MK94
MK97
MK data
Normal data

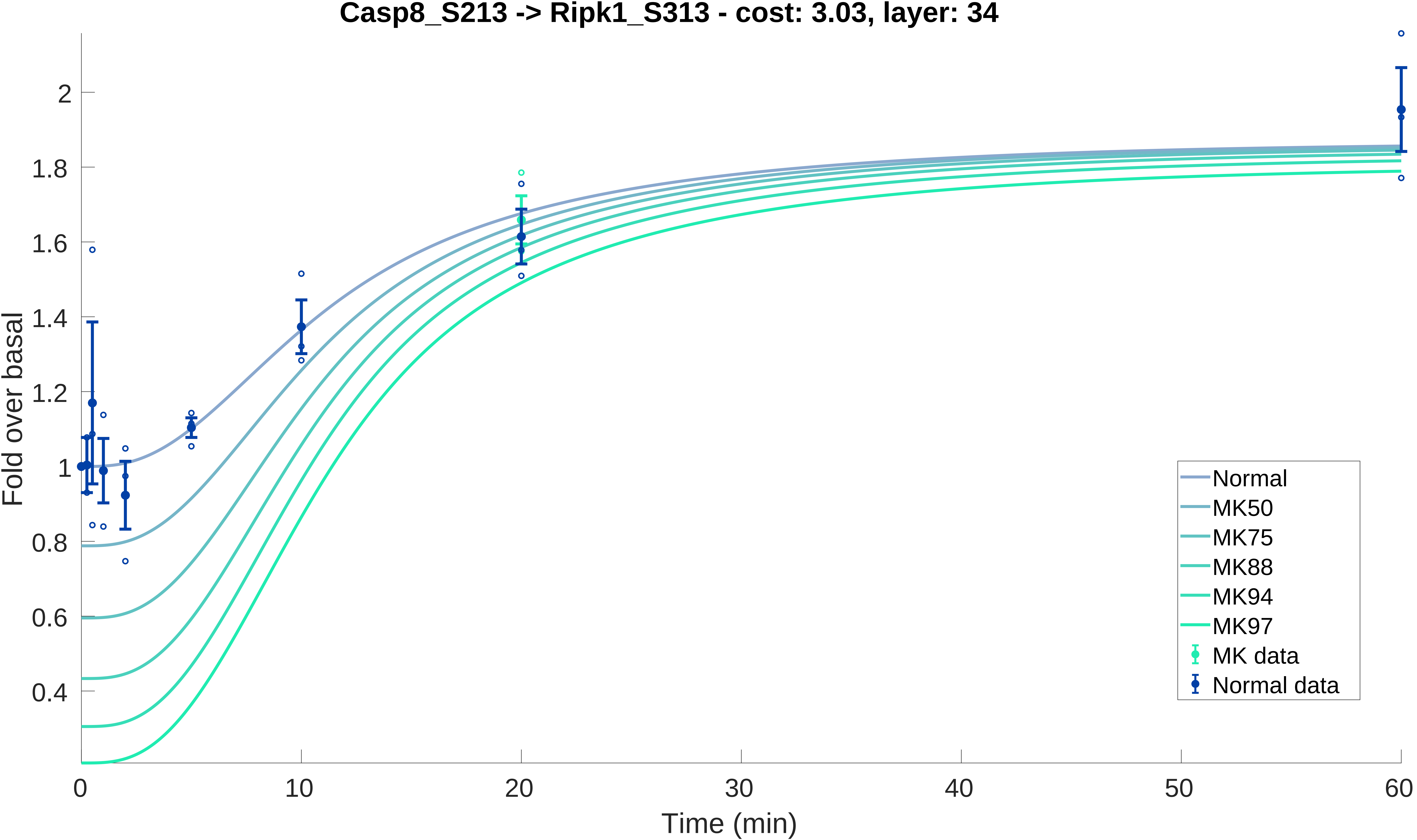

Casp8_S213 -> Ripk1_S313 - cost: 3.03, layer: 34
Fold over basal
2
1.8
1.6
1.4
1.2
1
0.8
0.6
0.4
0
10
20
30
40
50
60
Time (min)
Normal
MK50
MK75
MK88
MK94
MK97
MK data
Normal data

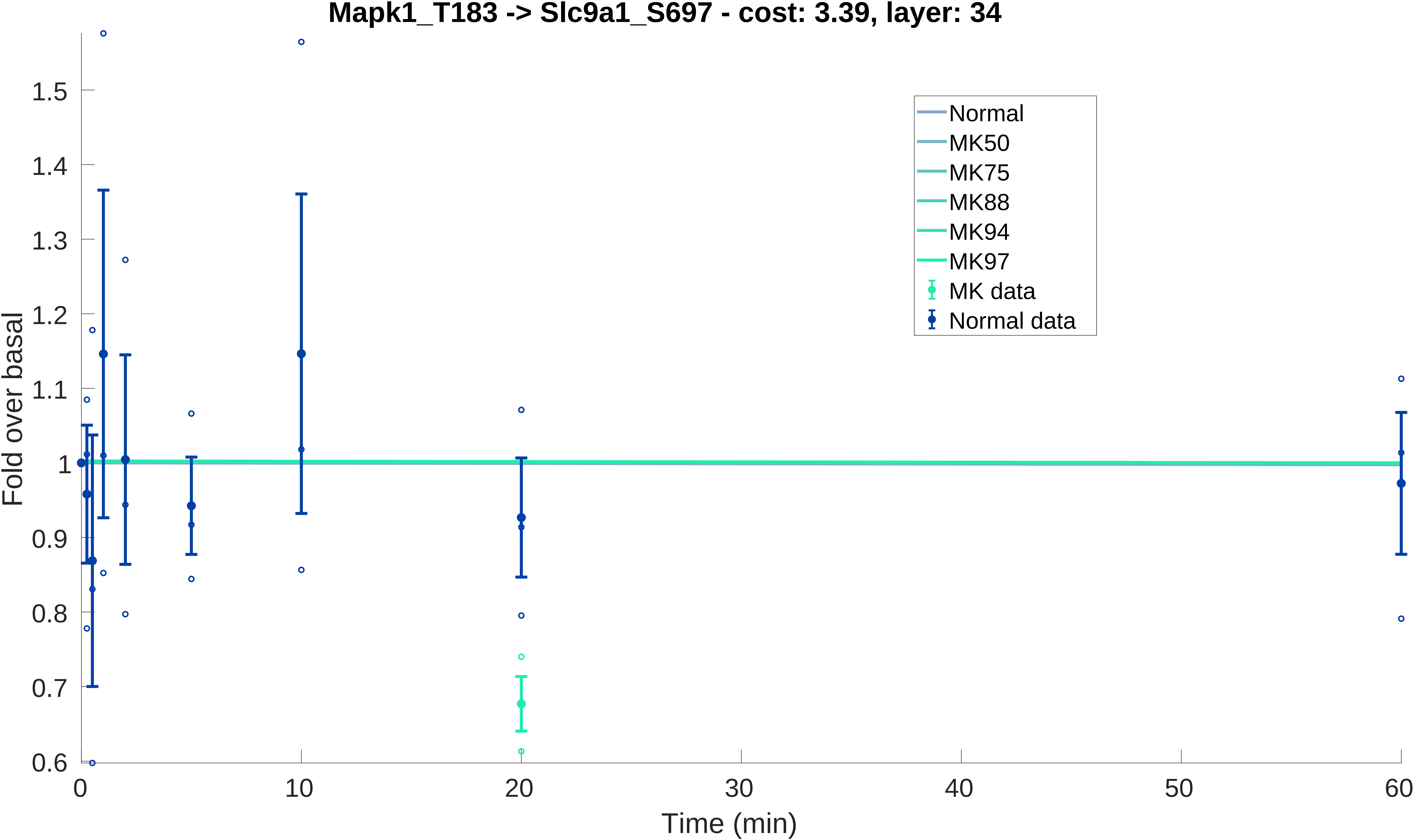
Mapk1_T183 -> Slc9a1_S697 - cost: 3.39, layer: 34
Fold over basal
Time (min)
Normal
MK50
MK75
MK88
MK94
MK97
MK data
Normal data
1.5
1.4
1.3
1.2
1.1
1
0.9
0.8
0.7
0.6
0
10
20
30
40
50
60

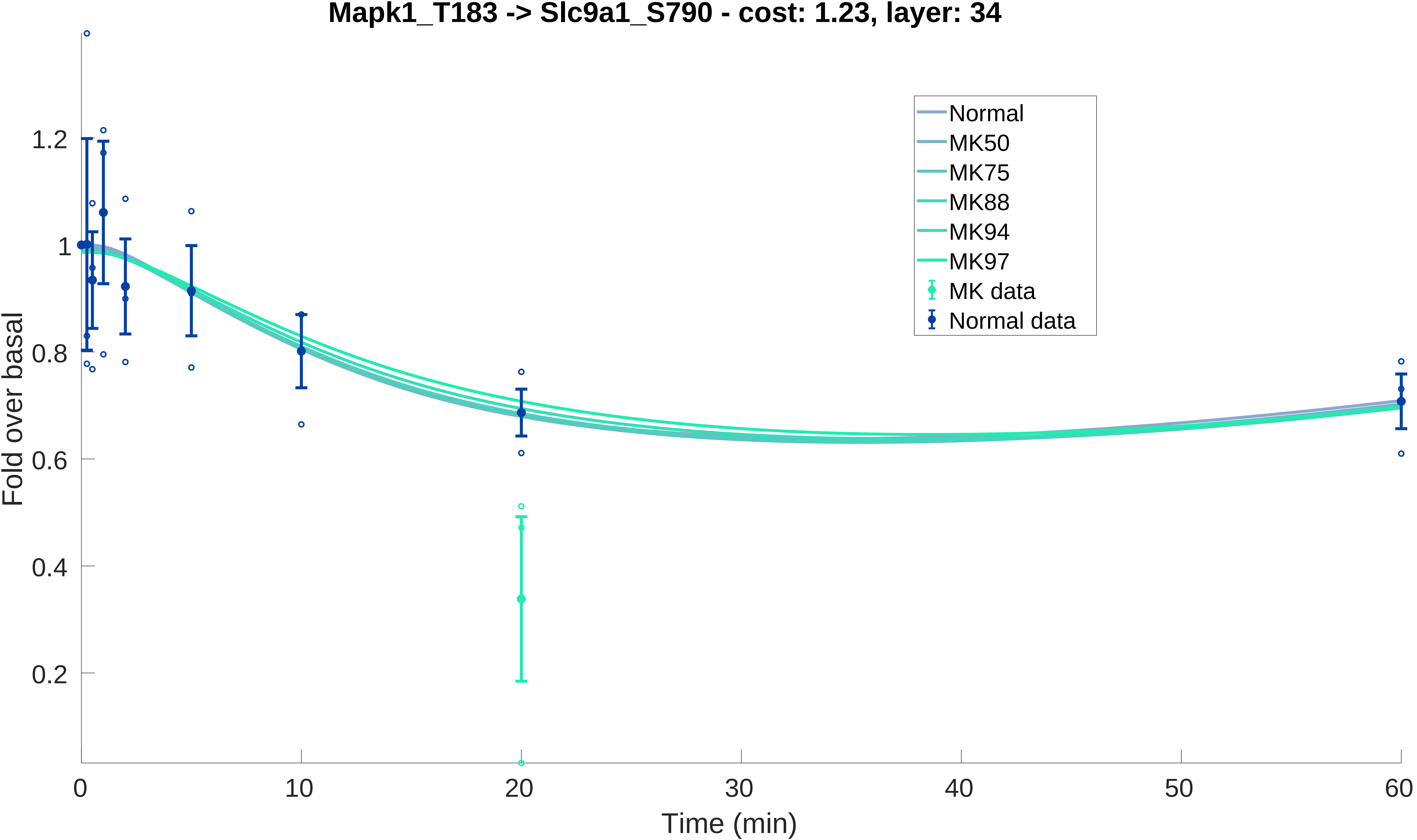

Mapk1_T183 -> Slc9a1_S790 - cost: 1.23, layer: 34
Normal
MK50
MK75
MK88
MK94
MK97
MK data
Normal data
Fold over basal
Time (min)
0.2
0.4
0.6
0.8
1
1.2
0
10
20
30
40
50
60

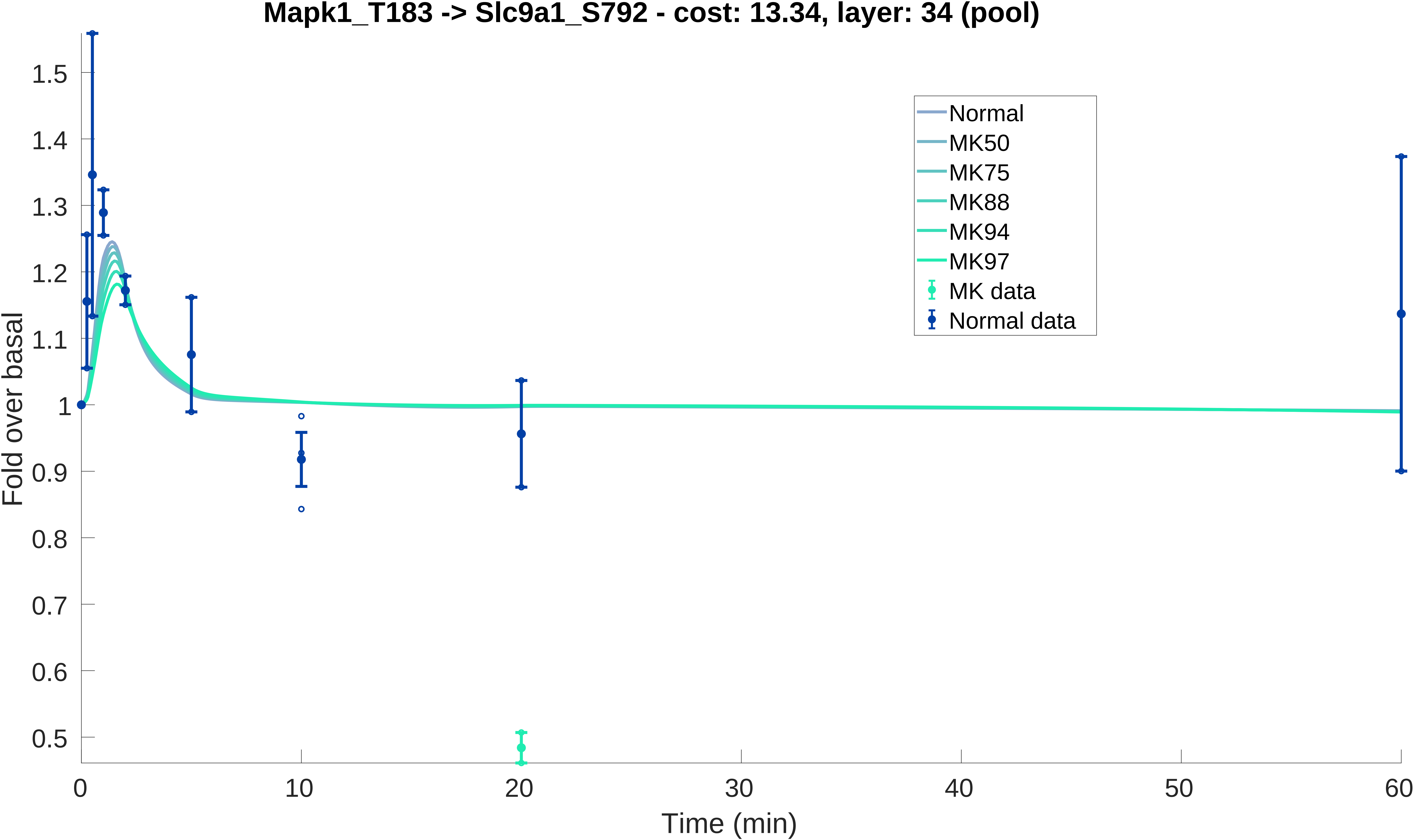

Mapk1_T183 -> Slc9a1_S792 - cost: 13.34, layer: 34 (pool)
Fold over basal
Time (min)
0
10
20
30
40
50
60
0.5
0.6
0.7
0.8
0.9
1
1.1
1.2
1.3
1.4
1.5
Normal
MK50
MK75
MK88
MK94
MK97
MK data
Normal data

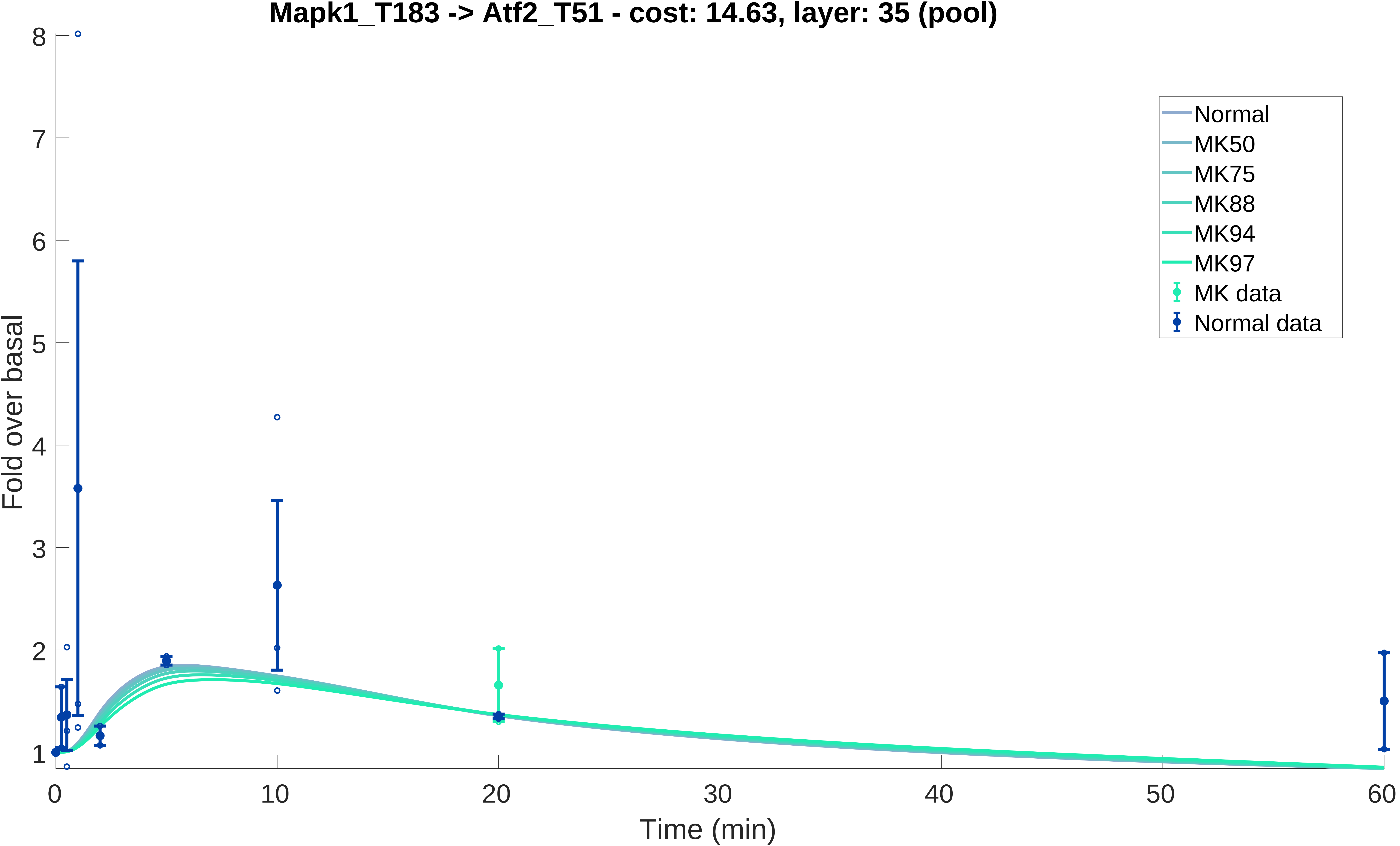

Mapk1_T183 -> Atf2_T51 - cost: 14.63, layer: 35 (pool)
Fold over basal
Time (min)
0
10
20
30
40
50
60
1
2
3
4
5
6
7
8
Normal
MK50
MK75
MK88
MK94
MK97
MK data
Normal data

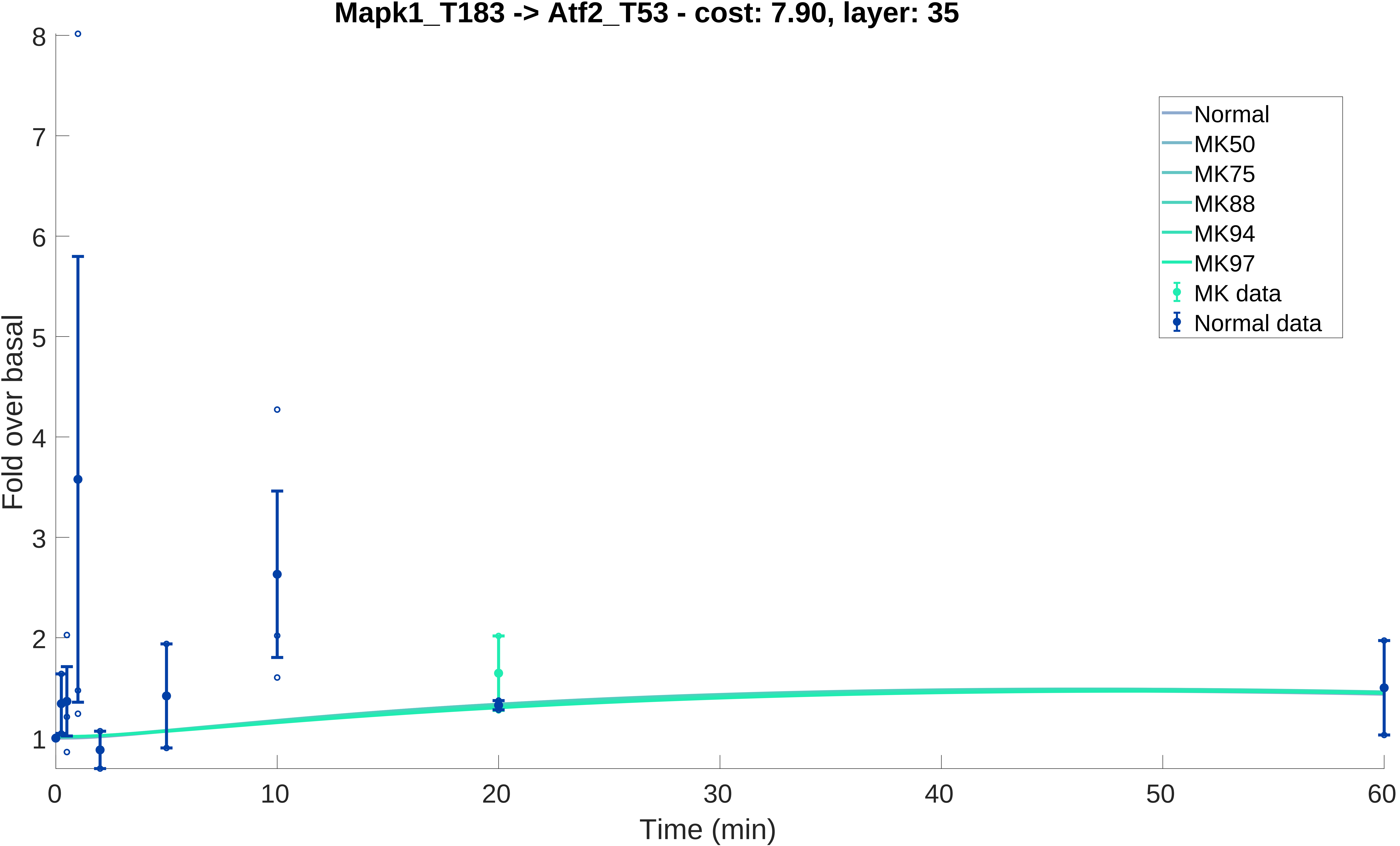
Mapk1_T183 -> Atf2_T53 - cost: 7.90, layer: 35
Fold over basal
Time (min)
Normal
MK50
MK75
MK88
MK94
MK97
MK data
Normal data
0
10
20
30
40
50
60
1
2
3
4
5
6
7
8

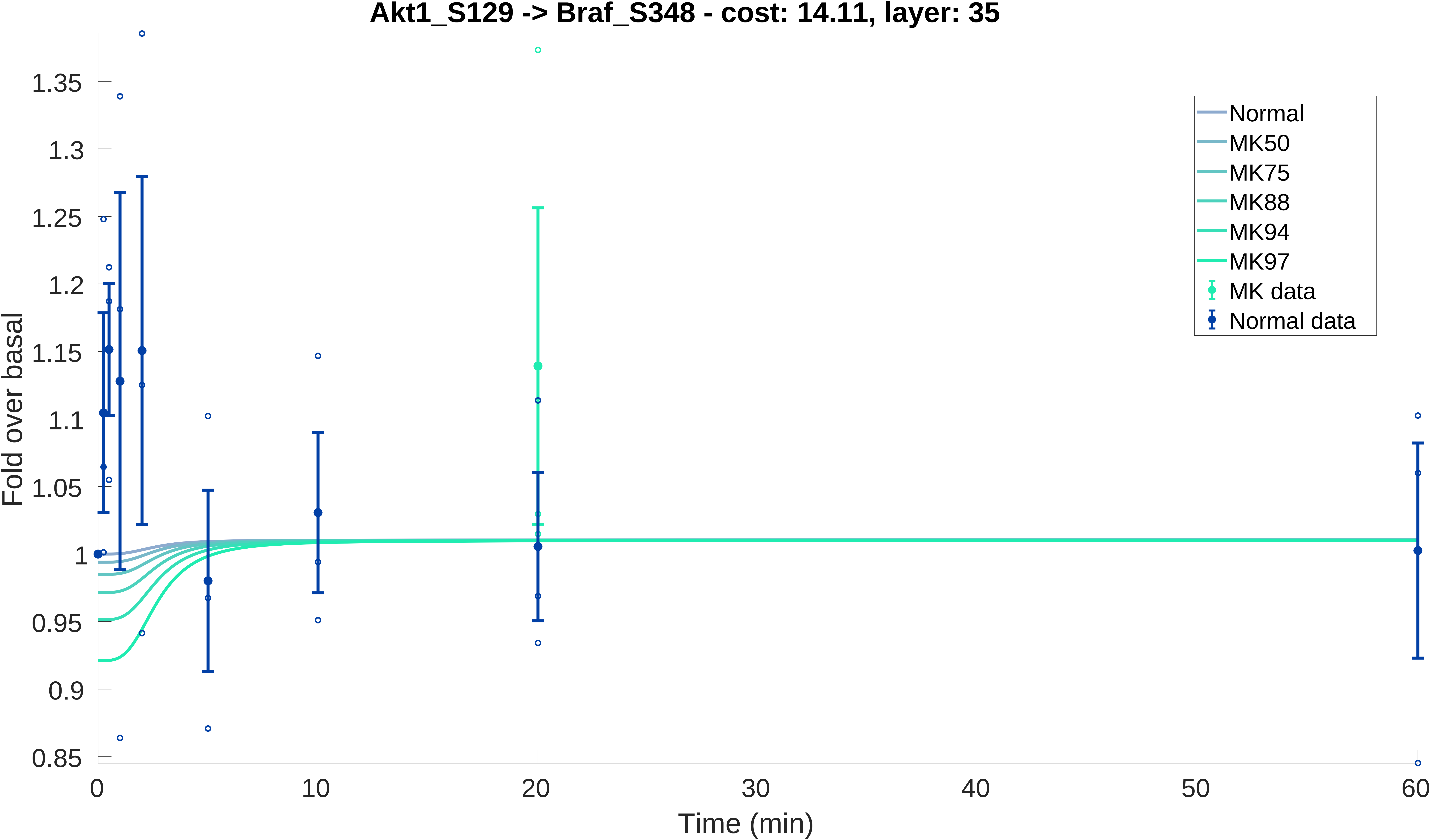
Akt1_S129 -> Braf_S348 - cost: 14.11, layer: 35
Fold over basal
Time (min)
1.35
1.3
1.25
1.2
1.15
1.1
1.05
1
0.95
0.9
0.85
0
10
20
30
40
50
60
Normal
MK50
MK75
MK88
MK94
MK97
MK data
Normal data

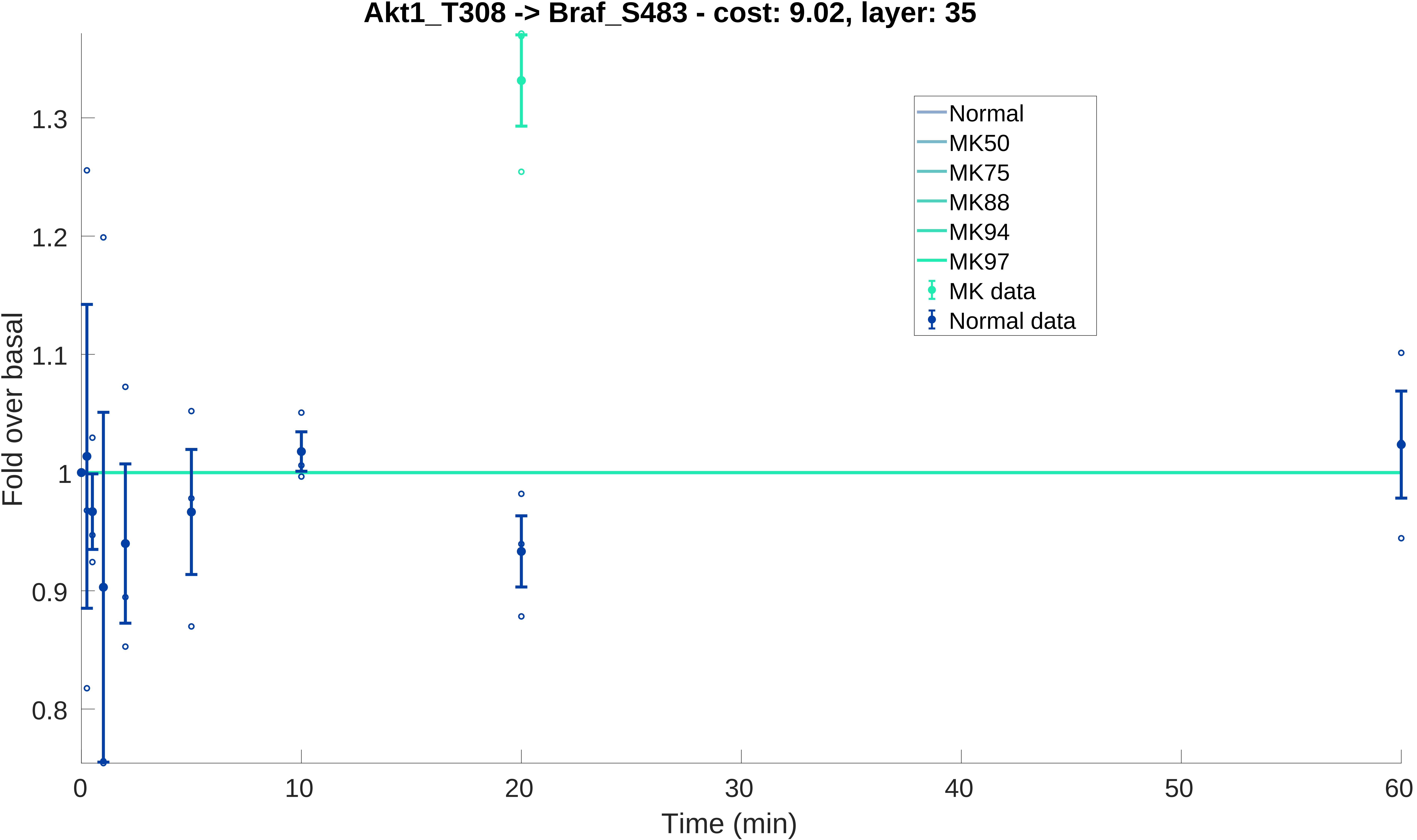

Akt1_T308 -> Braf_S483 - cost: 9.02, layer: 35
Fold over basal
Time (min)
1.3
1.2
1.1
1
0.9
0.8
0
10
20
30
40
50
60
Normal
MK50
MK75
MK88
MK94
MK97
MK data
Normal data

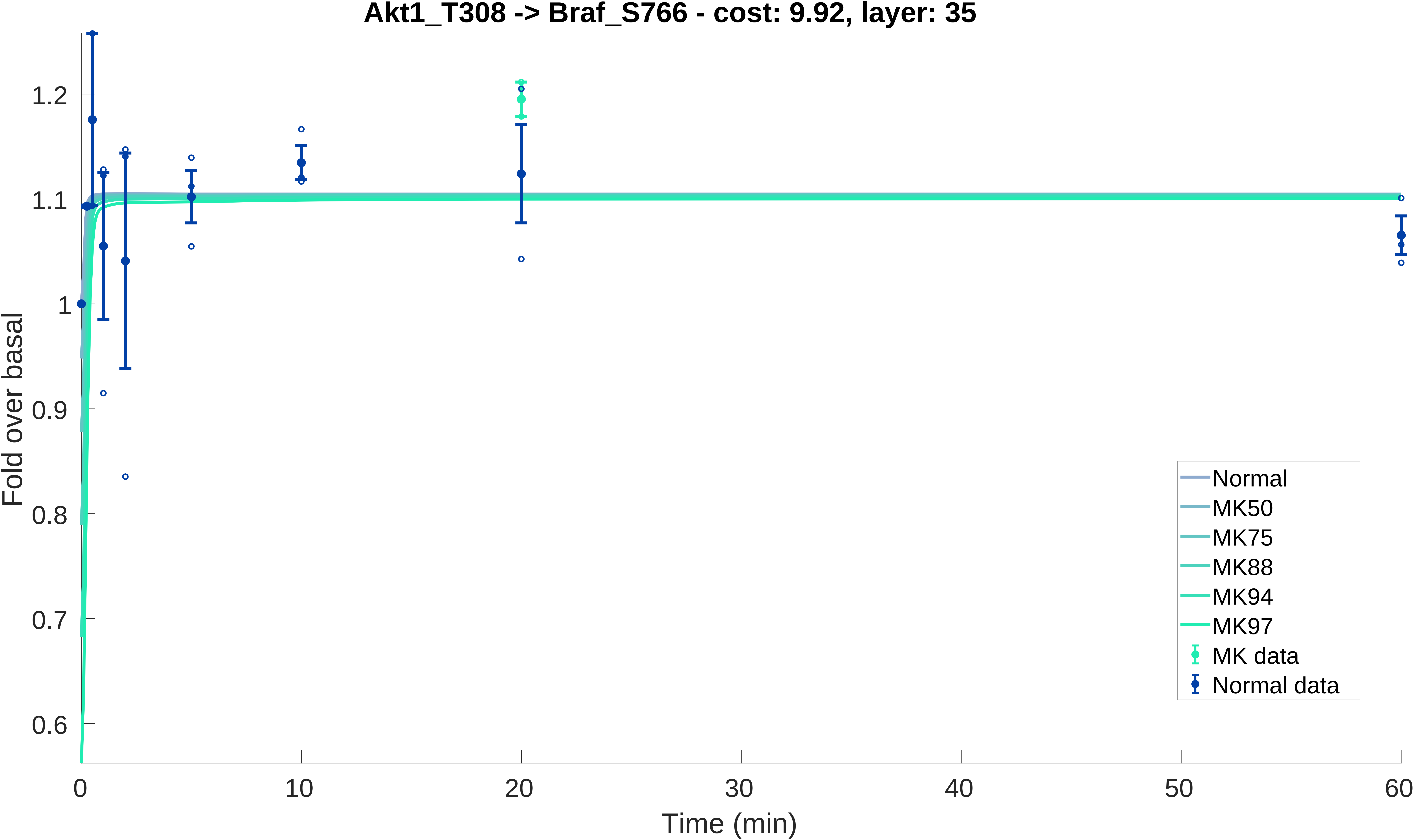

Akt1_T308 -> Braf_S766 - cost: 9.92, layer: 35
Fold over basal
1.2
1.1
1
0.9
0.8
0.7
0.6
0
10
20
30
40
50
60
Time (min)
Normal
MK50
MK75
MK88
MK94
MK97
MK data
Normal data

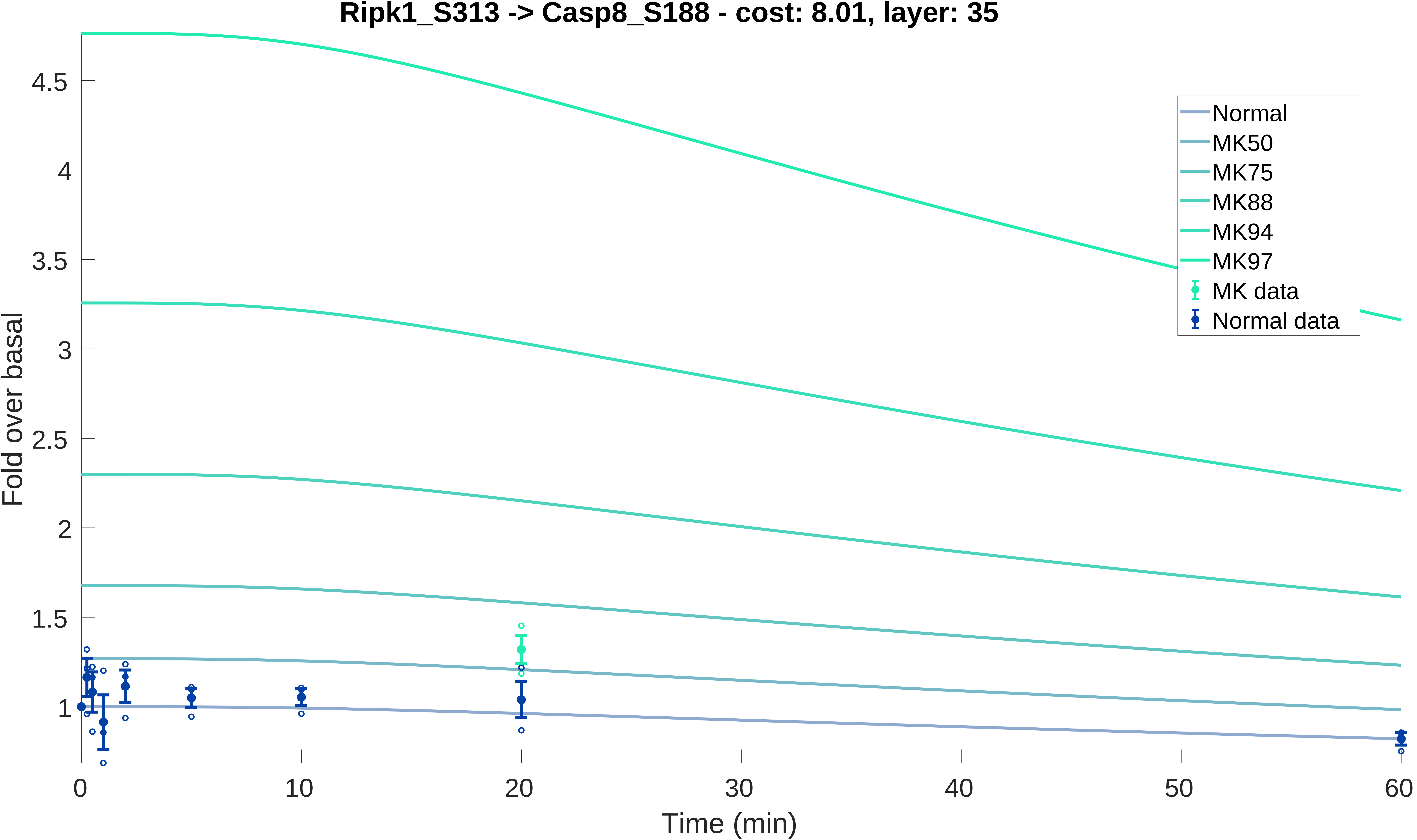
Ripk1_S313 -> Casp8_S188 - cost: 8.01, layer: 35
Fold over basal
Time (min)
Normal
MK50
MK75
MK88
MK94
MK97
MK data
Normal data
4.5
4
3.5
3
2.5
2
1.5
1
0
10
20
30
40
50
60

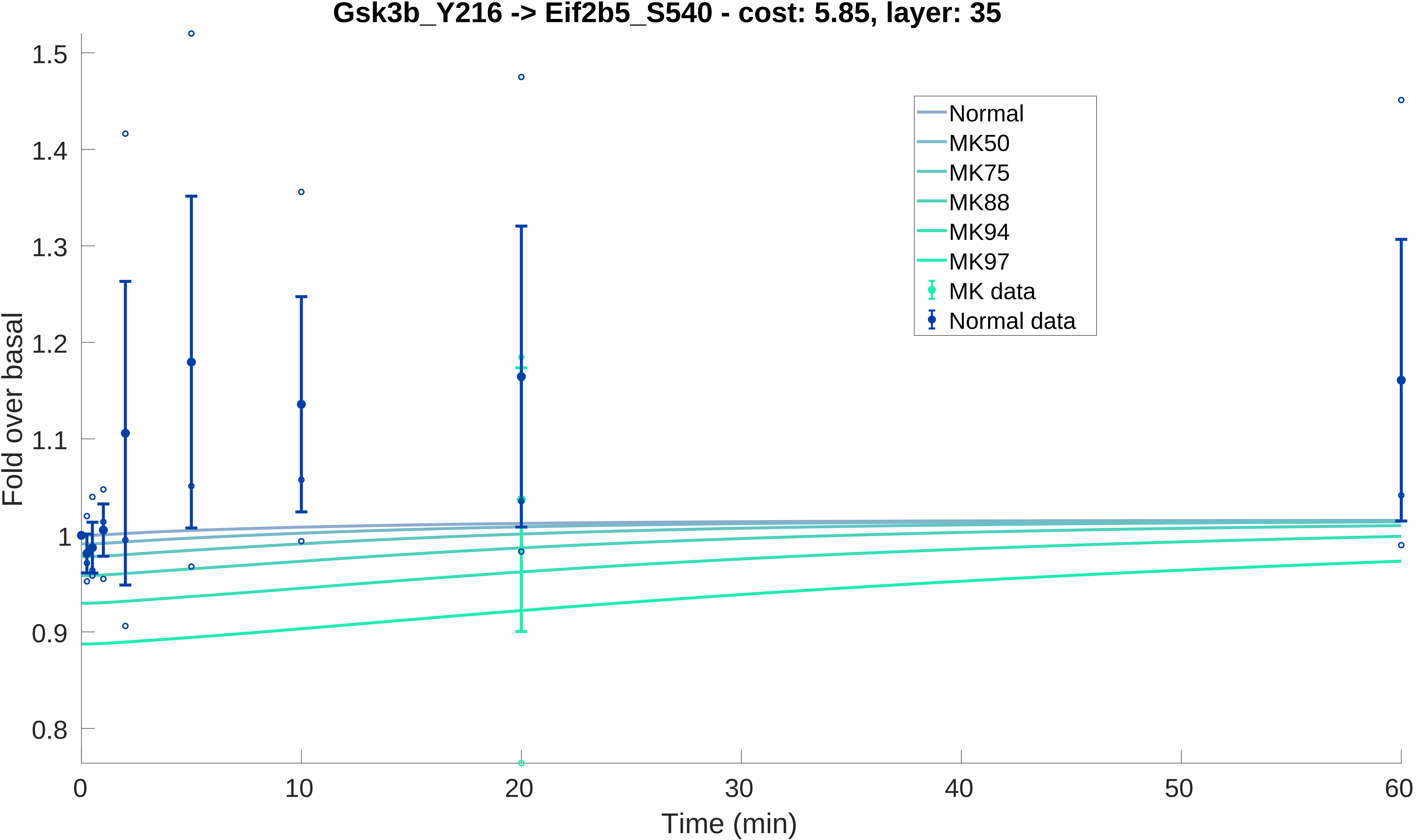

Gsk3b_Y216 -> Eif2b5_S540 - cost: 5.85, layer: 35
Fold over basal
Time (min)
0.8
0.9
1
1.1
1.2
1.3
1.4
1.5
0
10
20
30
40
50
60
Normal
MK50
MK75
MK88
MK94
MK97
MK data
Normal data

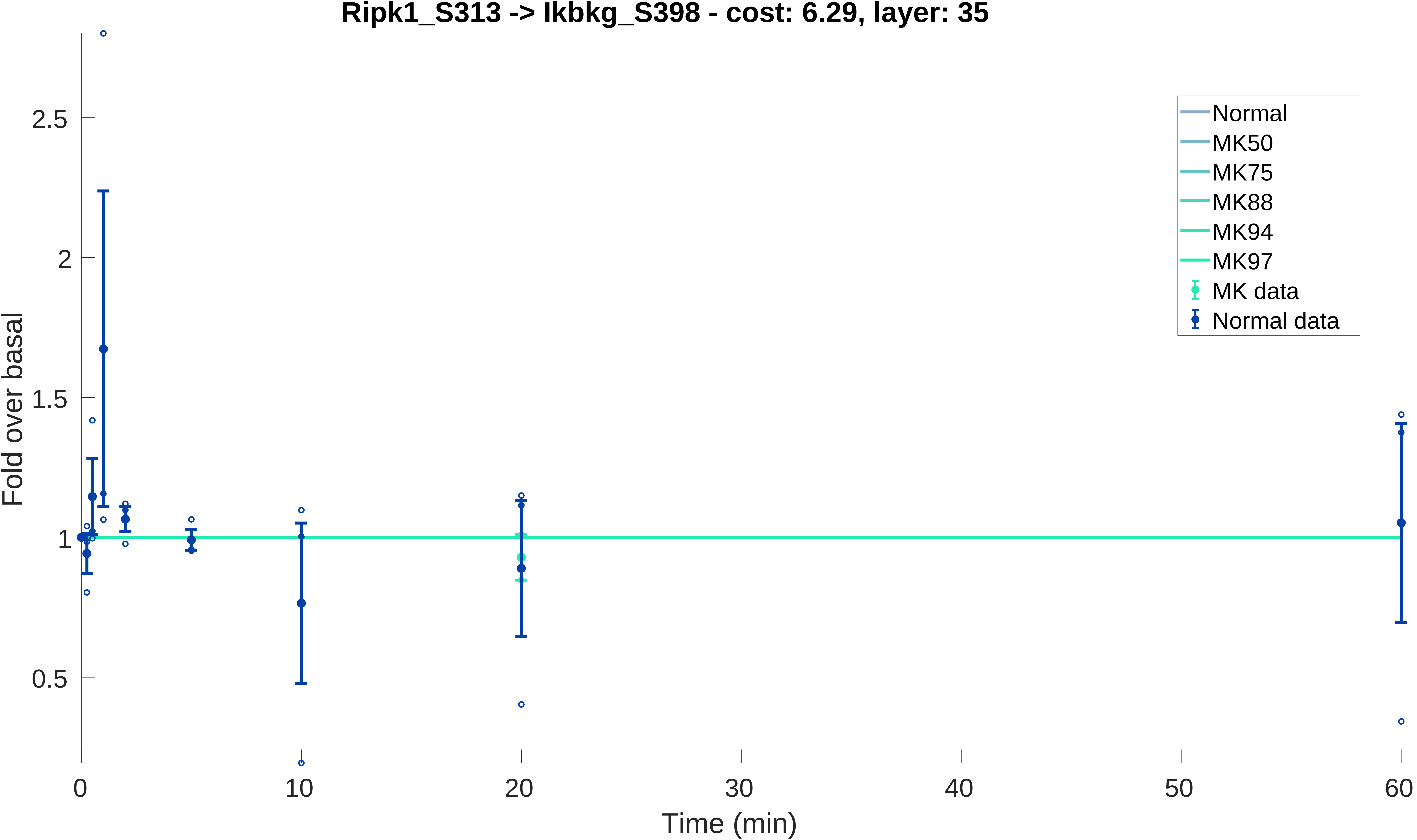

Ripk1_S313 -> Ikbkg_S398 - cost: 6.29, layer: 35
Fold over basal
Time (min)
0
10
20
30
40
50
60
0.5
1
1.5
2
2.5
Normal
MK50
MK75
MK88
MK94
MK97
MK data
Normal data

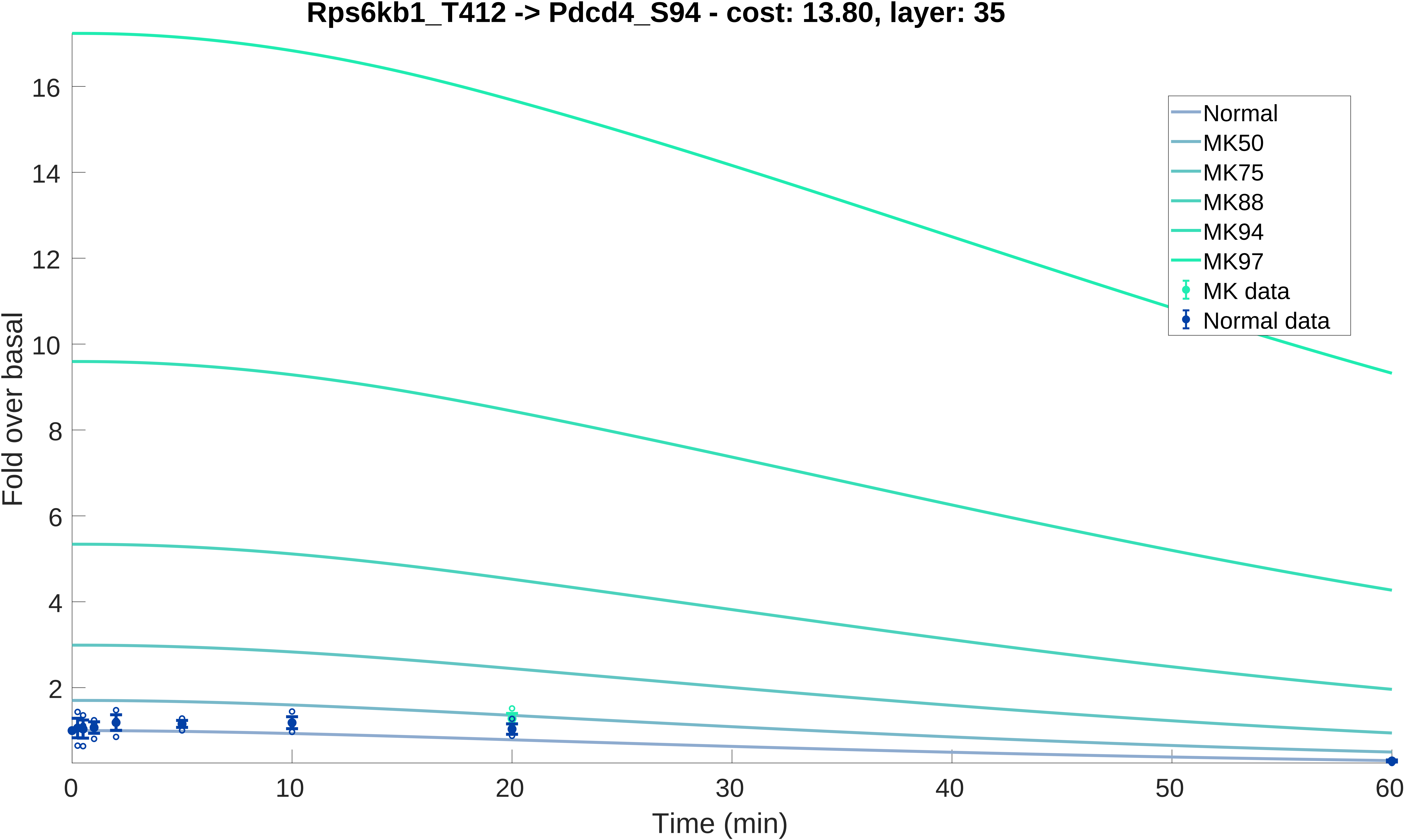

Rps6kb1_T412 -> Pdcd4_S94 - cost: 13.80, layer: 35
Fold over basal
Time (min)
0
10
20
30
40
50
60
2
4
6
8
10
12
14
16
Normal
MK50
MK75
MK88
MK94
MK97
MK data
Normal data

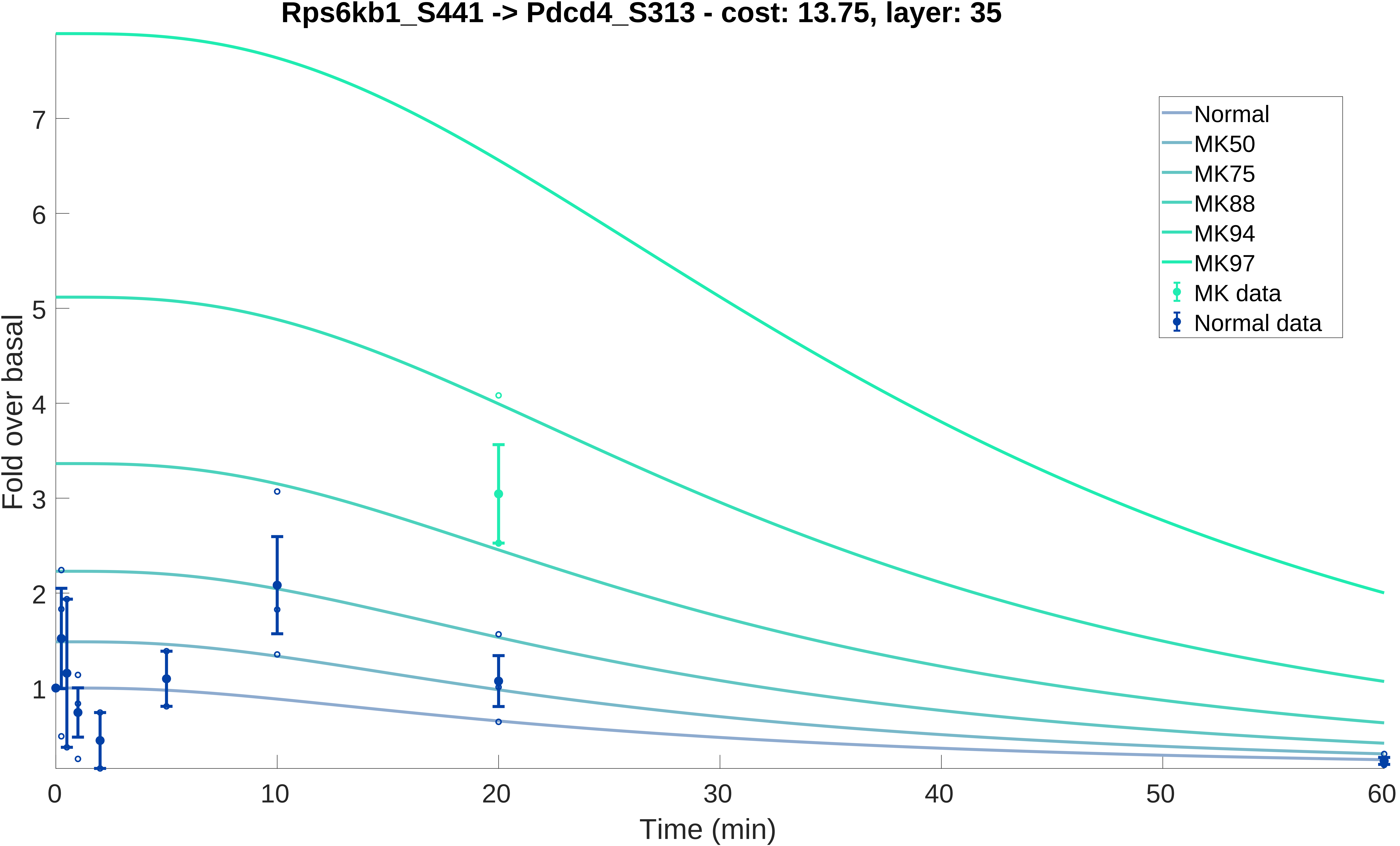

Rps6kb1_S441 -> Pdcd4_S313 - cost: 13.75, layer: 35
Fold over basal
Time (min)
0
10
20
30
40
50
60
1
2
3
4
5
6
7
Normal
MK50
MK75
MK88
MK94
MK97
MK data
Normal data

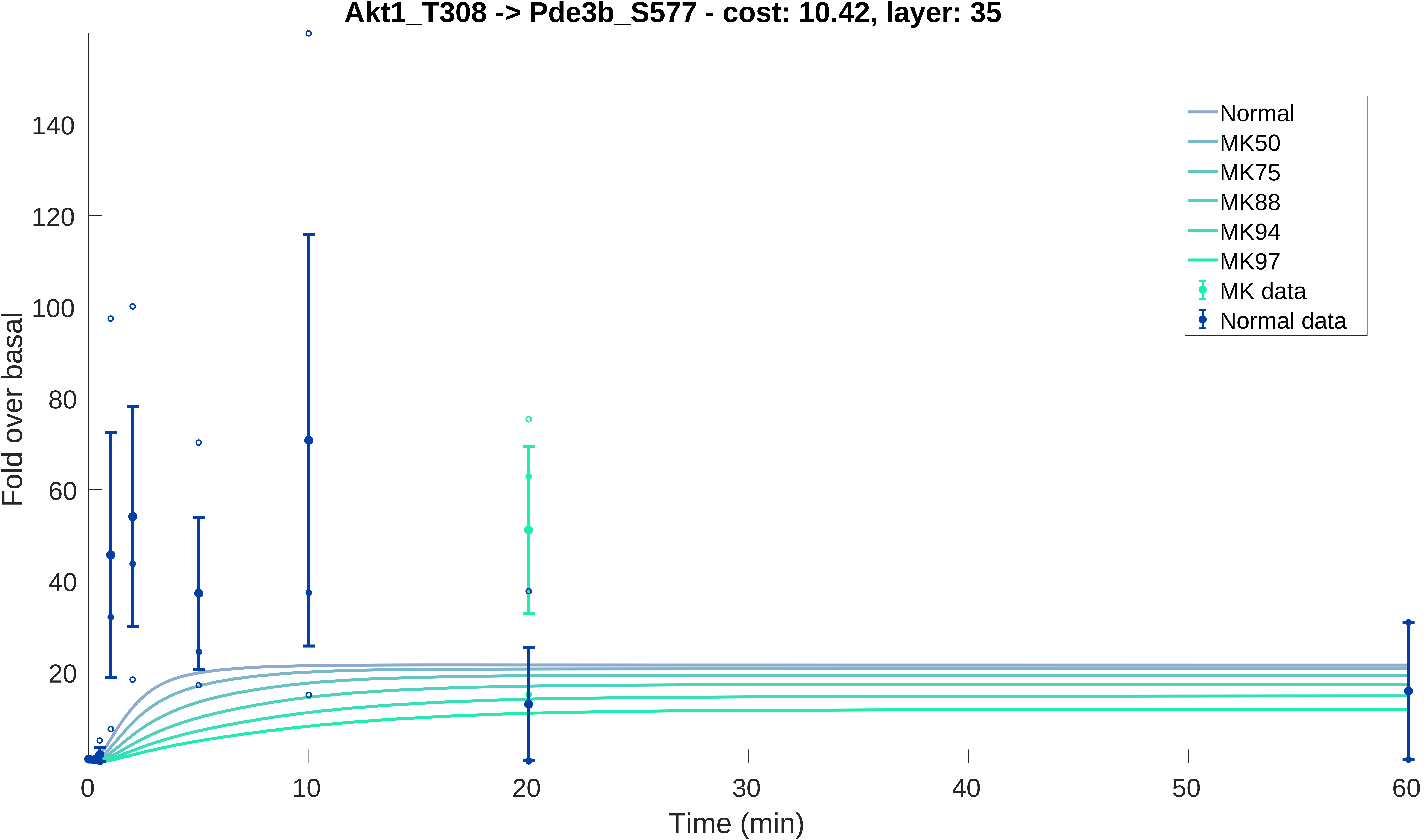

Akt1_T308 -> Pde3b_S577 - cost: 10.42, layer: 35
Fold over basal
Time (min)
0
10
20
30
40
50
60
20
40
60
80
100
120
140
Normal
MK50
MK75
MK88
MK94
MK97
MK data
Normal data

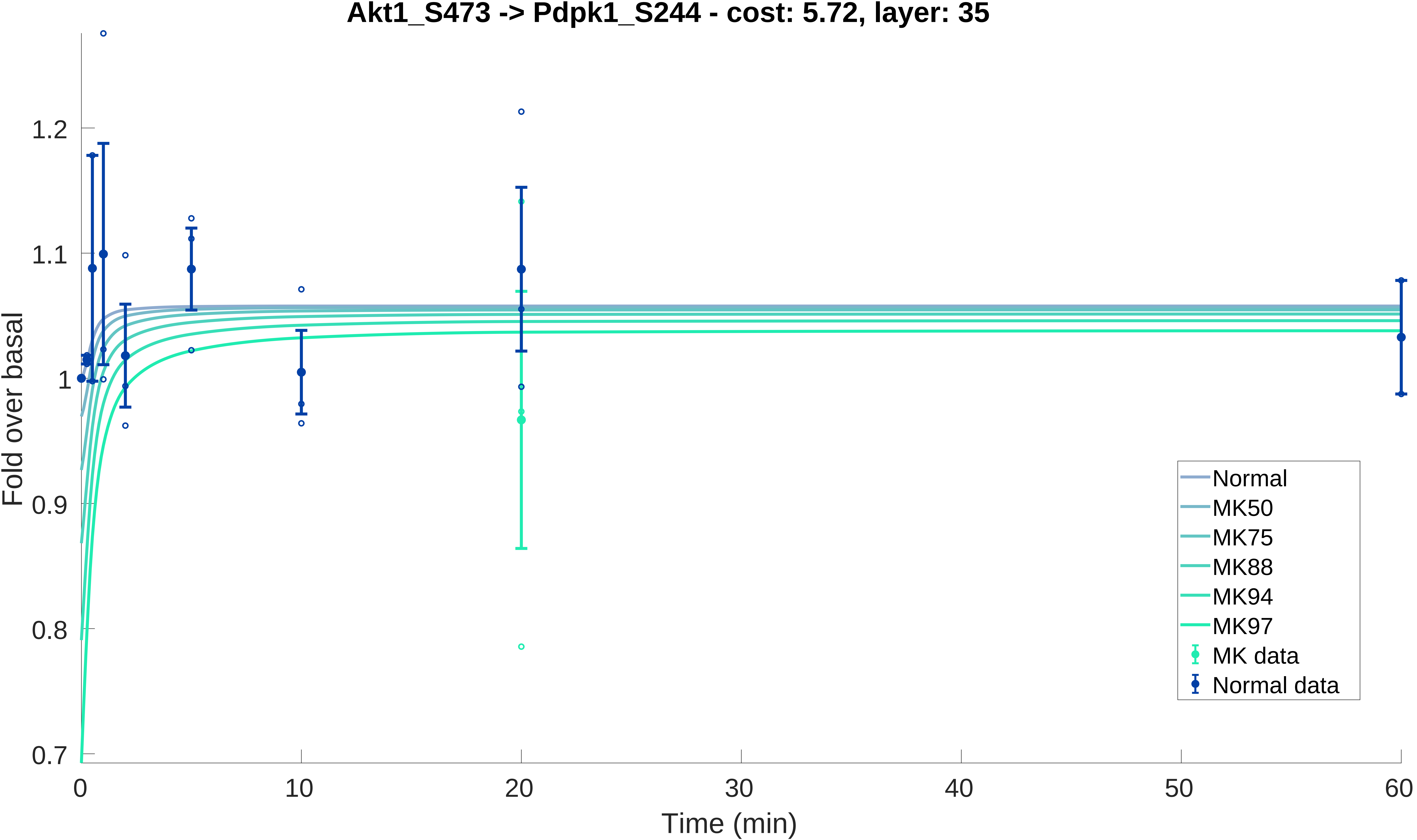

Akt1_S473 -> Pdpk1_S244 - cost: 5.72, layer: 35
Fold over basal
Time (min)
0.7
0.8
0.9
1
1.1
1.2
0
10
20
30
40
50
60
Normal
MK50
MK75
MK88
MK94
MK97
MK data
Normal data

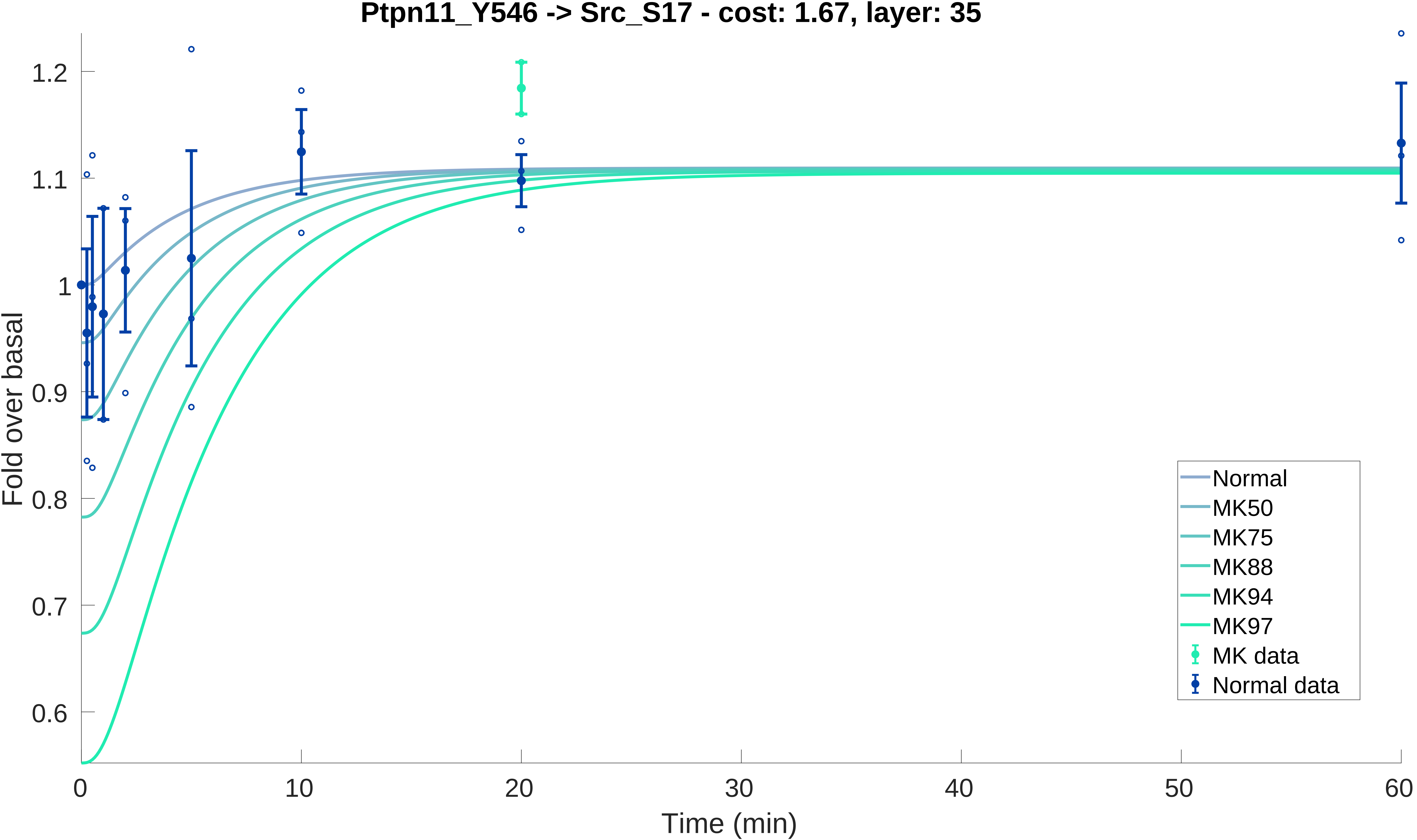

Ptpn11_Y546 -> Src_S17 - cost: 1.67, layer: 35
Fold over basal
1.2
1.1
1
0.9
0.8
0.7
0.6
0
10
20
30
40
50
60
Time (min)
Normal
MK50
MK75
MK88
MK94
MK97
MK data
Normal data

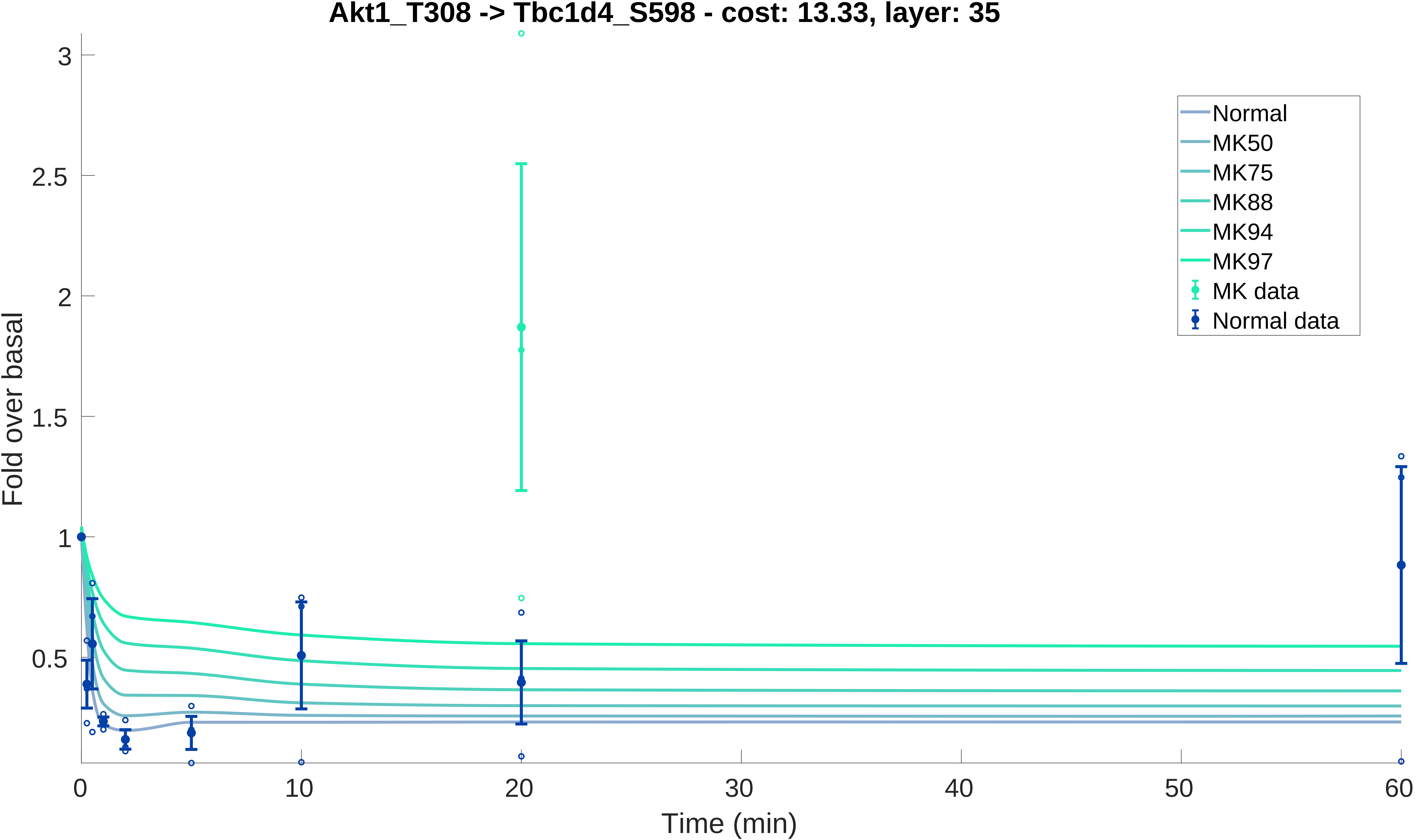
Akt1_T308 -> Tbc1d4_S598 - cost: 13.33, layer: 35
Fold over basal
3
2.5
2
1.5
1
0.5
0
10
20
30
40
50
60
Time (min)
Normal
MK50
MK75
MK88
MK94
MK97
MK data
Normal data

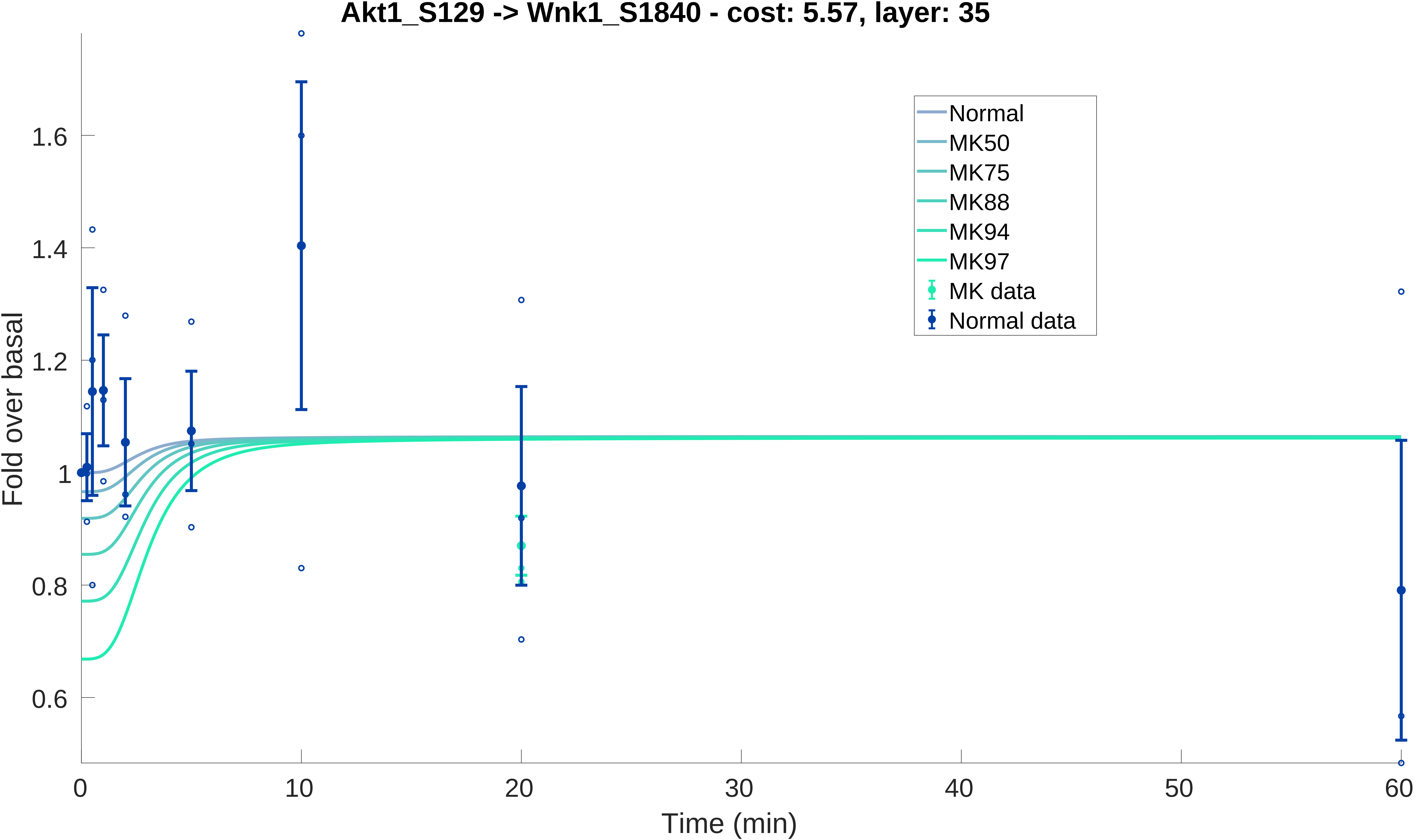

Akt1_S129 -> Wnk1_S1840 - cost: 5.57, layer: 35
Fold over basal
Time (min)
0.6
0.8
1
1.2
1.4
1.6
0
10
20
30
40
50
60
Normal
MK50
MK75
MK88
MK94
MK97
MK data
Normal data

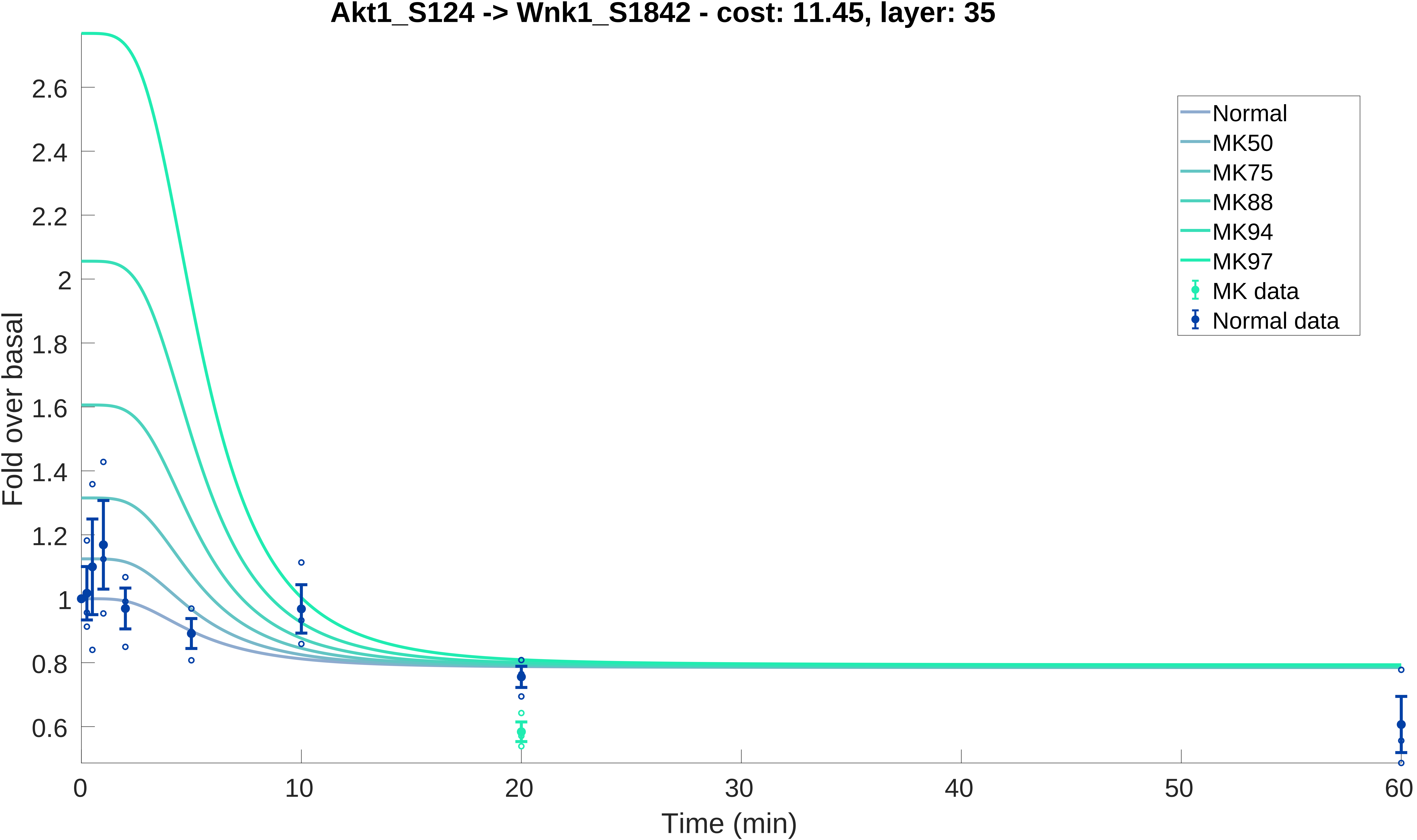

Akt1_S124 -> Wnk1_S1842 - cost: 11.45, layer: 35
Fold over basal
Time (min)
0.6
0.8
1
1.2
1.4
1.6
1.8
2
2.2
2.4
2.6
0
10
20
30
40
50
60
Normal
MK50
MK75
MK88
MK94
MK97
MK data
Normal data

Akt1_T308 -> Wnk1_S1845 - cost: 6.77, layer: 35

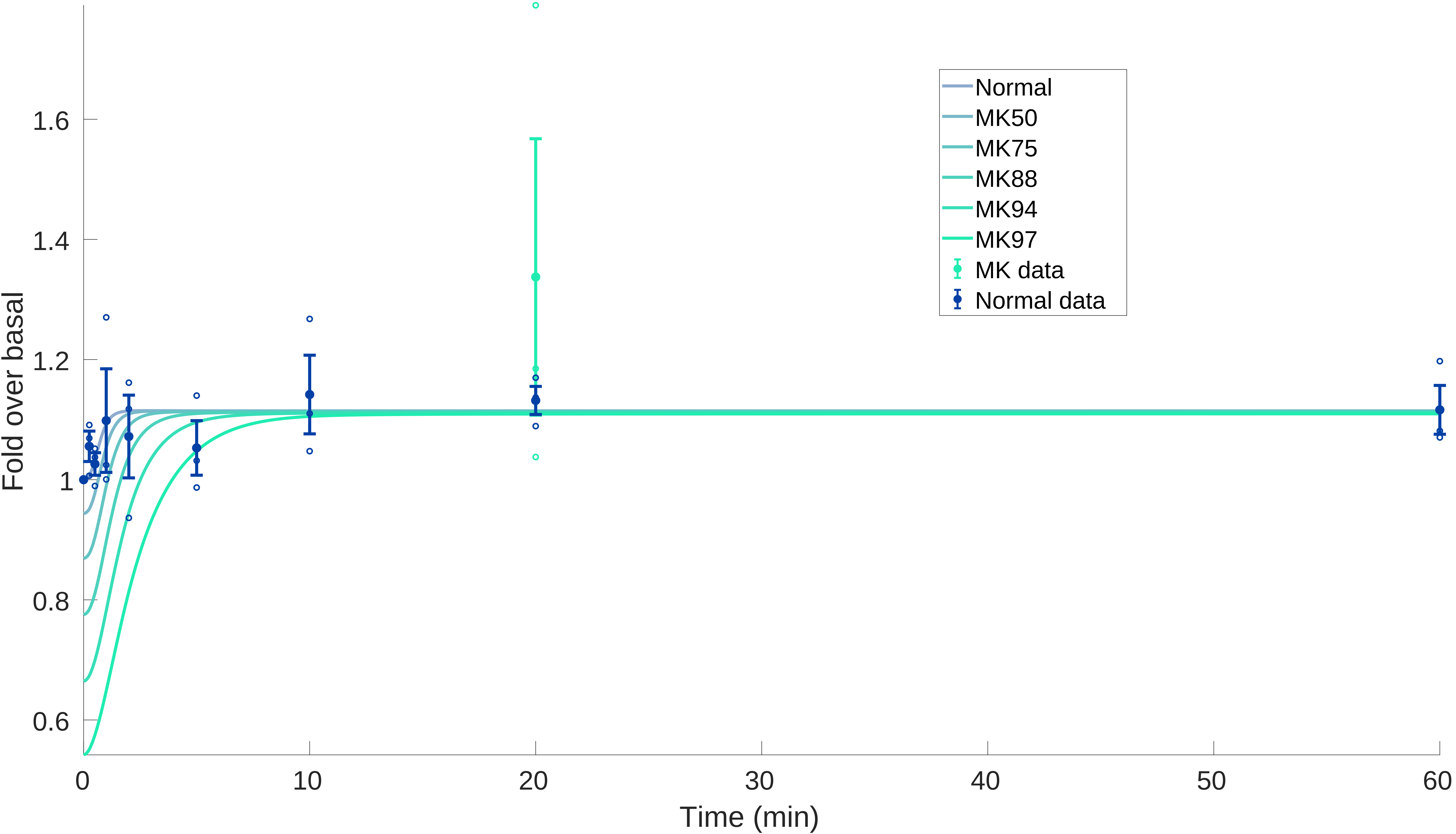

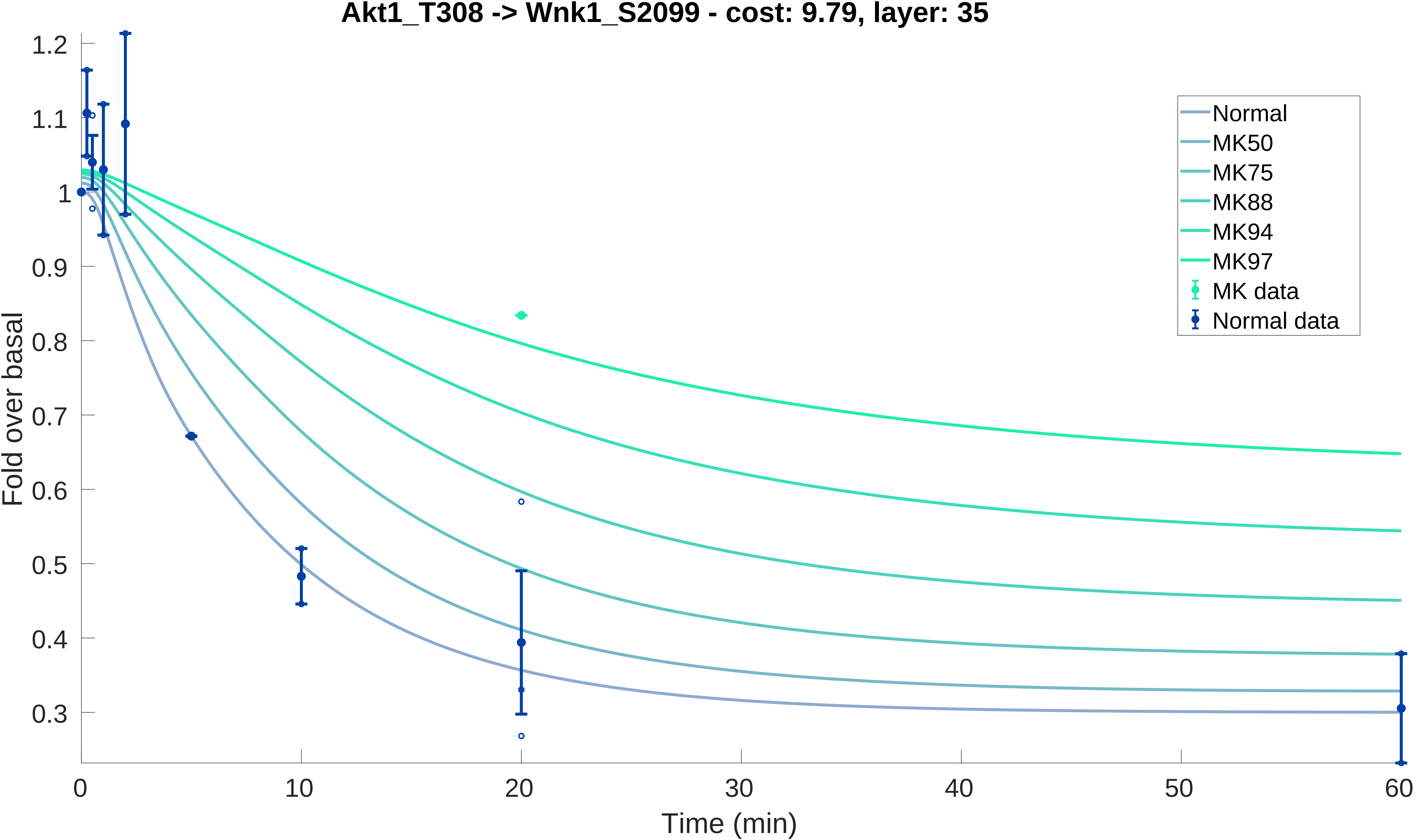

Akt1_T308 -> Wnk1_S2099 - cost: 9.79, layer: 35
Fold over basal
Time (min)
1.2
1.1
1
0.9
0.8
0.7
0.6
0.5
0.4
0.3
0
10
20
30
40
50
60
Normal
MK50
MK75
MK88
MK94
MK97
MK data
Normal data

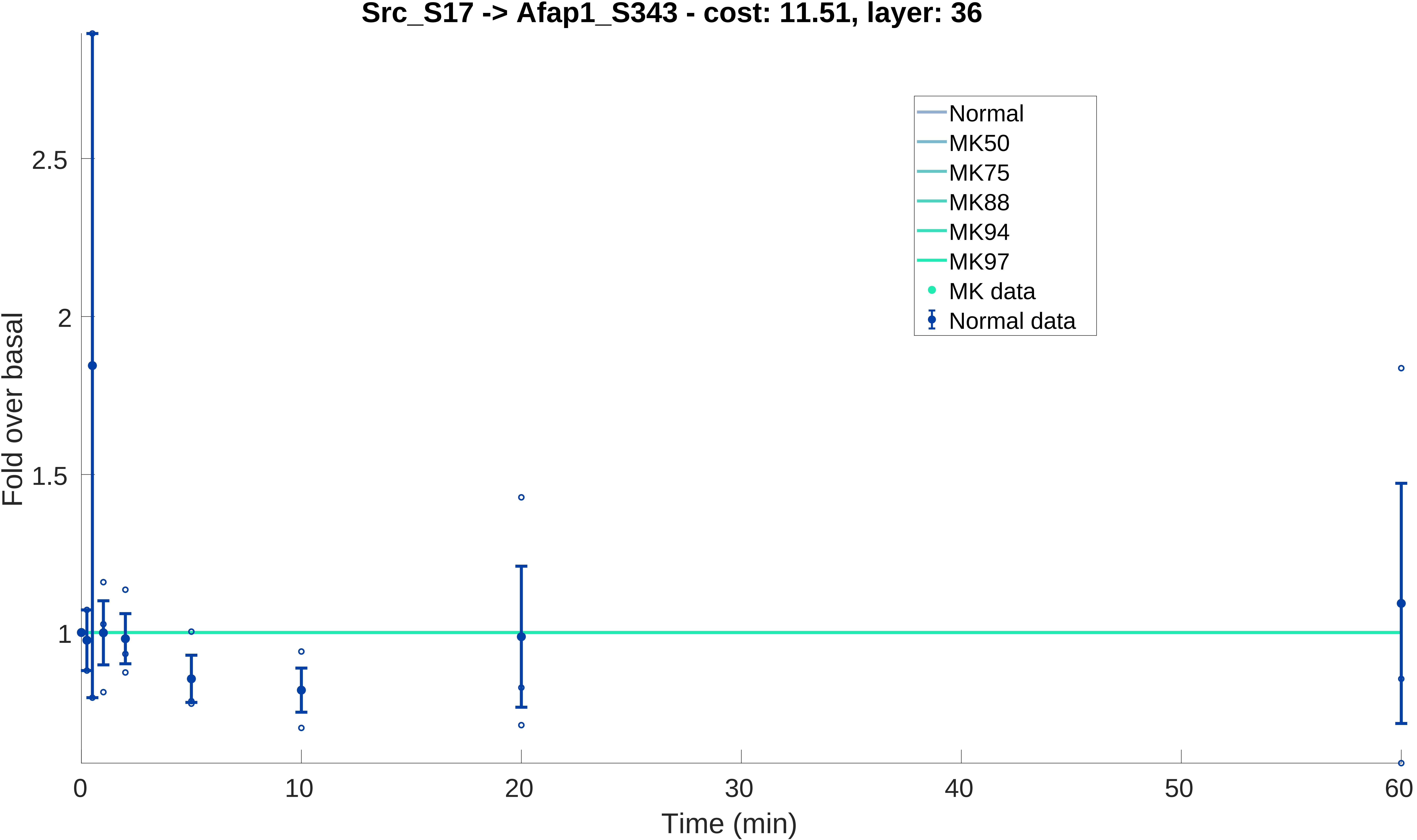

Src_S17 -> Afap1_S343 - cost: 11.51, layer: 36
Fold over basal
2.5
2
1.5
1
0
10
20
30
40
50
60
Time (min)
Normal
MK50
MK75
MK88
MK94
MK97
MK data
Normal data

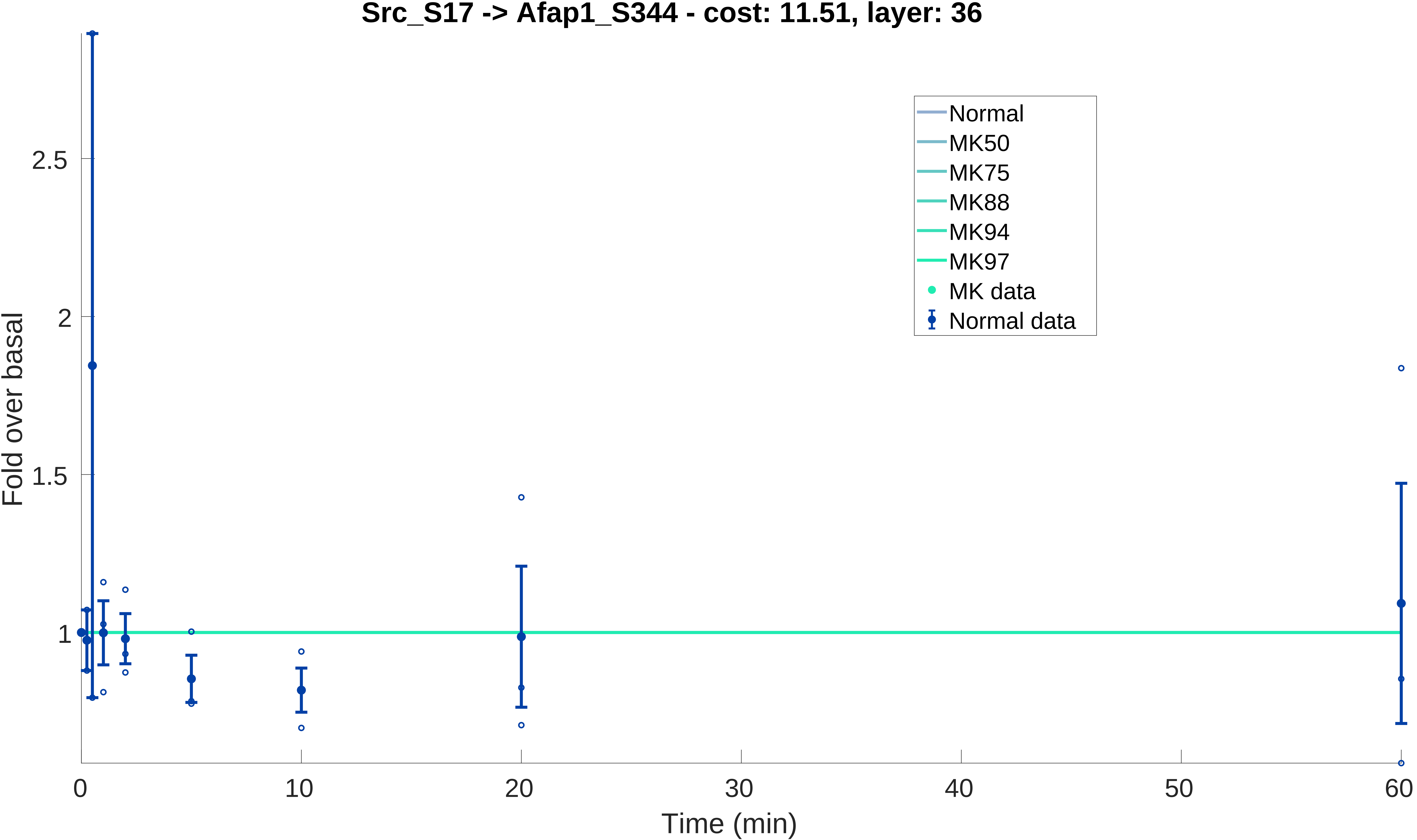
Src_S17 -> Afap1_S344 - cost: 11.51, layer: 36
Fold over basal
2.5
2
1.5
1
0
10
20
30
40
50
60
Time (min)
Normal
MK50
MK75
MK88
MK94
MK97
MK data
Normal data

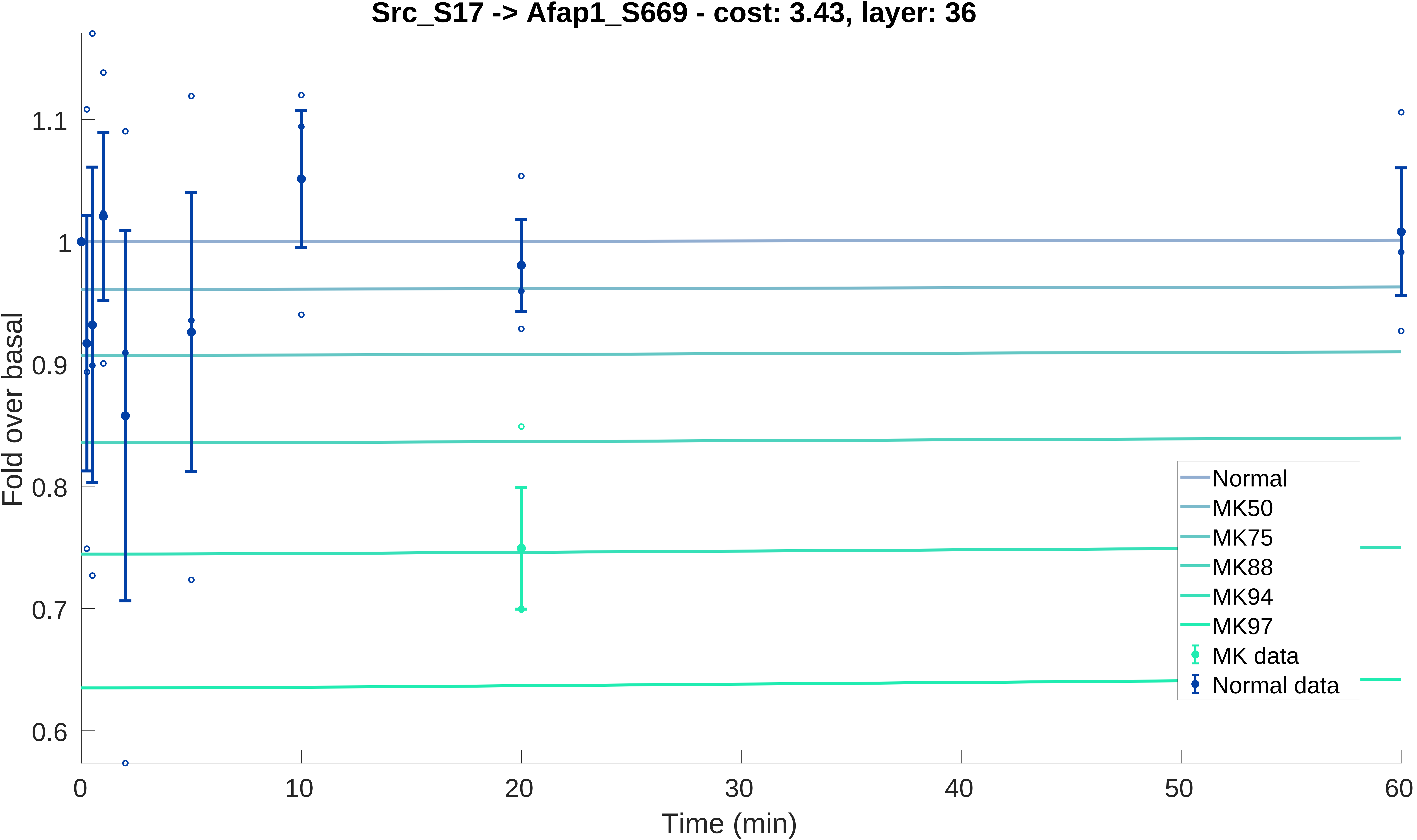

Src_S17 -> Afap1_S669 - cost: 3.43, layer: 36
Fold over basal
1.1
1
0.9
0.8
0.7
0.6
0
10
20
30
40
50
60
Time (min)
Normal
MK50
MK75
MK88
MK94
MK97
MK data
Normal data

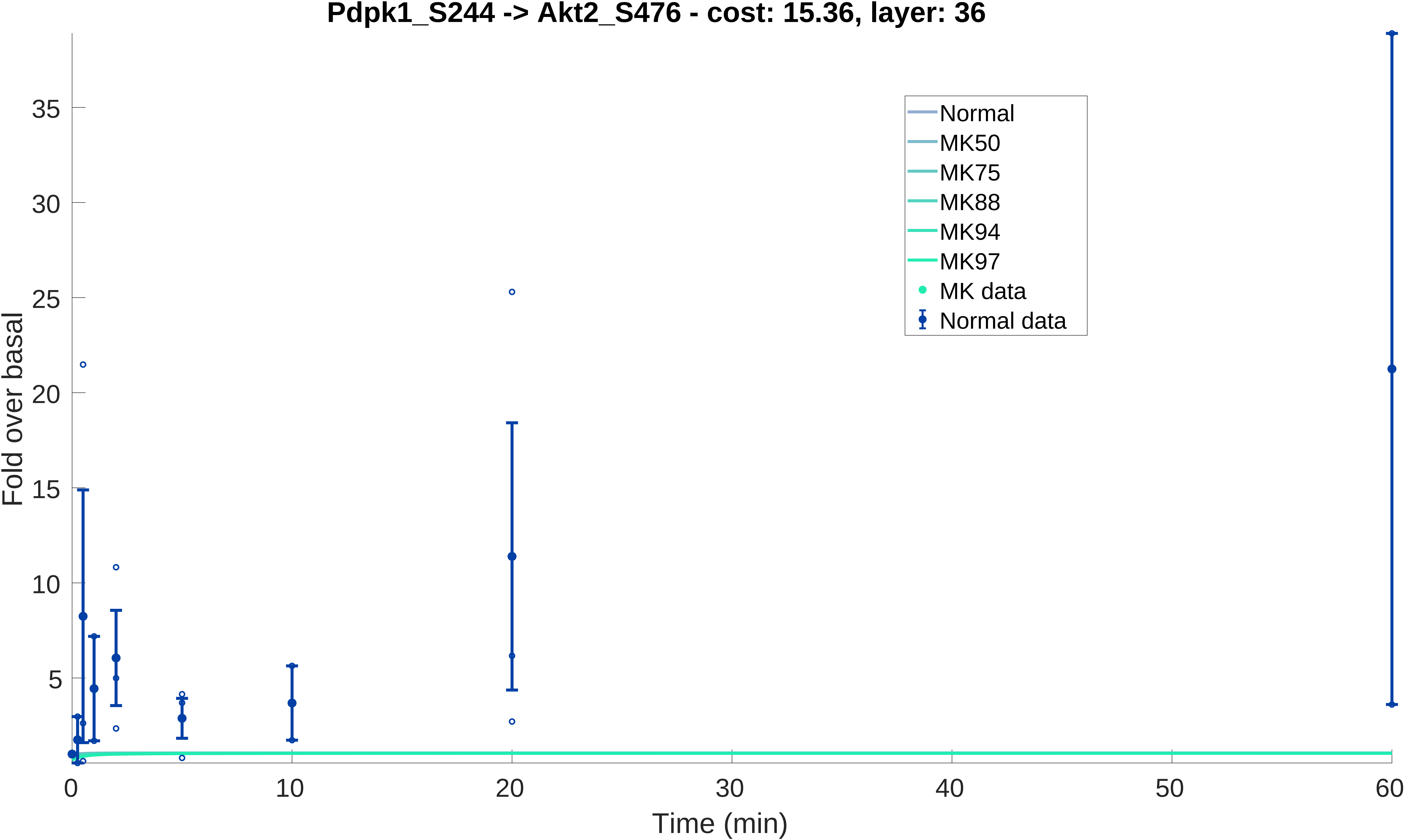
Pdpk1_S244 -> Akt2_S476 - cost: 15.36, layer: 36
Fold over basal
Time (min)
0
10
20
30
40
50
60
5
10
15
20
25
30
35
Normal
MK50
MK75
MK88
MK94
MK97
MK data
Normal data

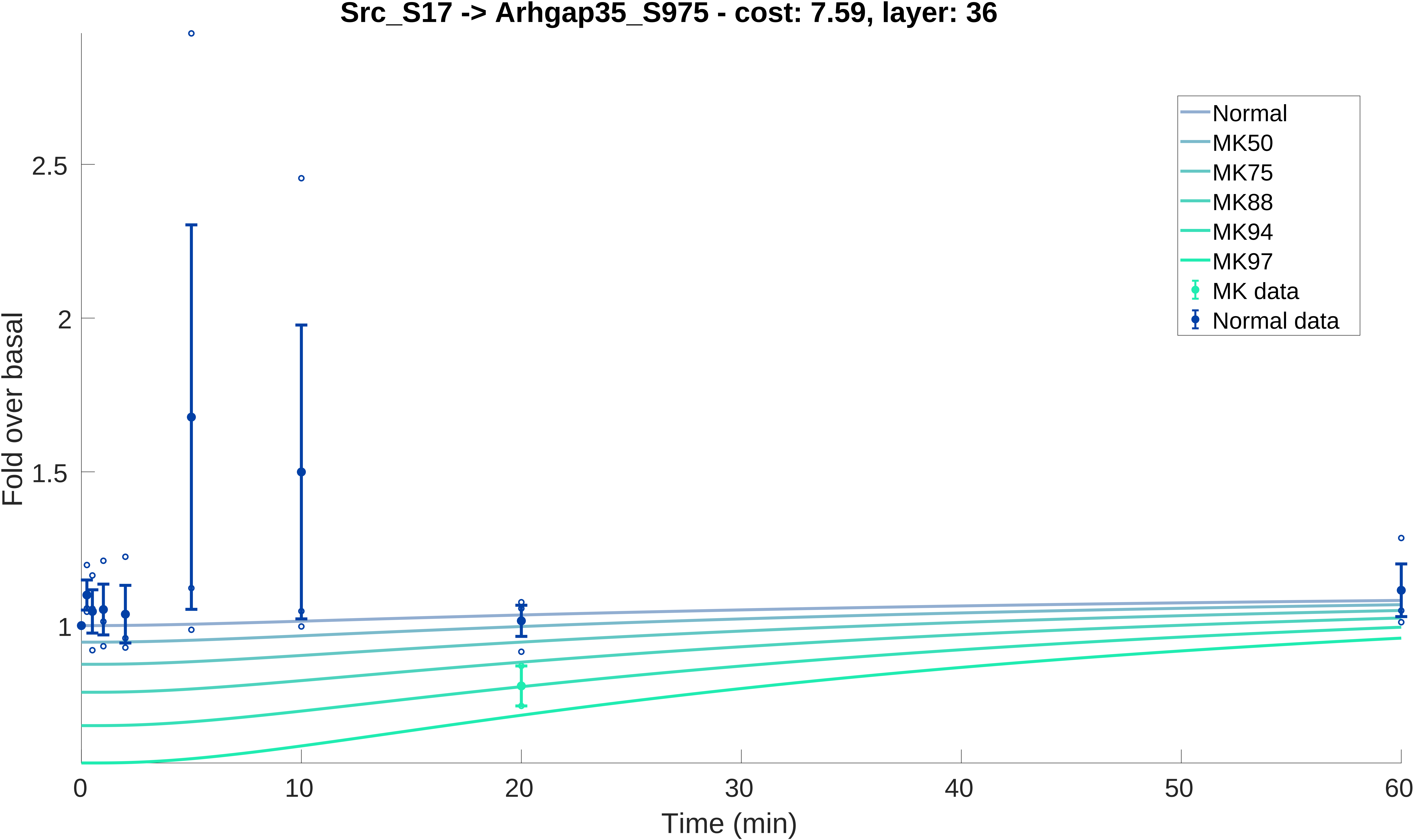

Src_S17 -> Arhgap35_S975 - cost: 7.59, layer: 36
Fold over basal
2.5
2
1.5
1
0
10
20
30
40
50
60
Time (min)
Normal
MK50
MK75
MK88
MK94
MK97
MK data
Normal data

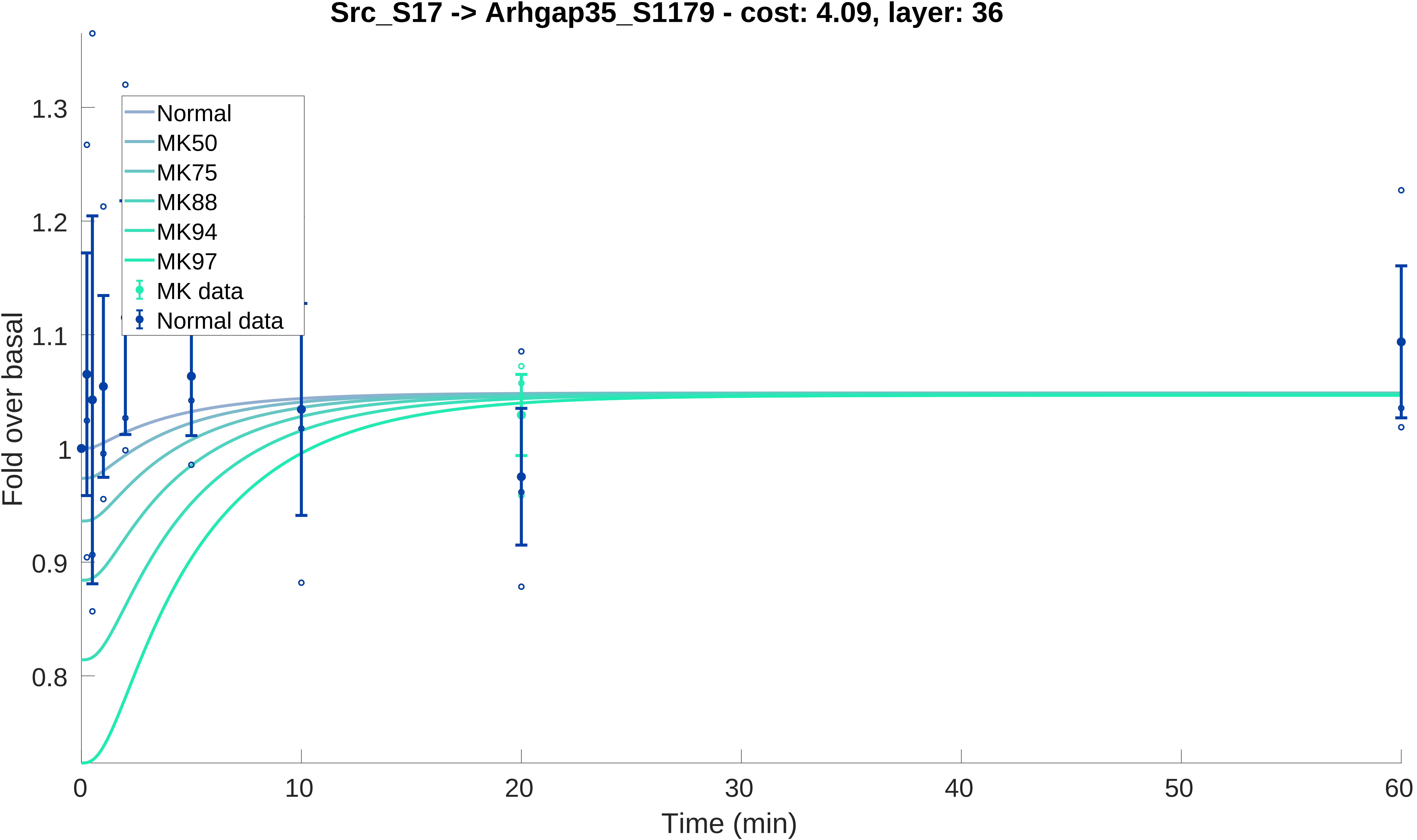

Src_S17 -> Arhgap35_S1179 - cost: 4.09, layer: 36
Normal
MK50
MK75
MK88
MK94
MK97
MK data
Normal data
Fold over basal
1.3
1.2
1.1
1
0.9
0.8
0
10
20
30
40
50
60
Time (min)

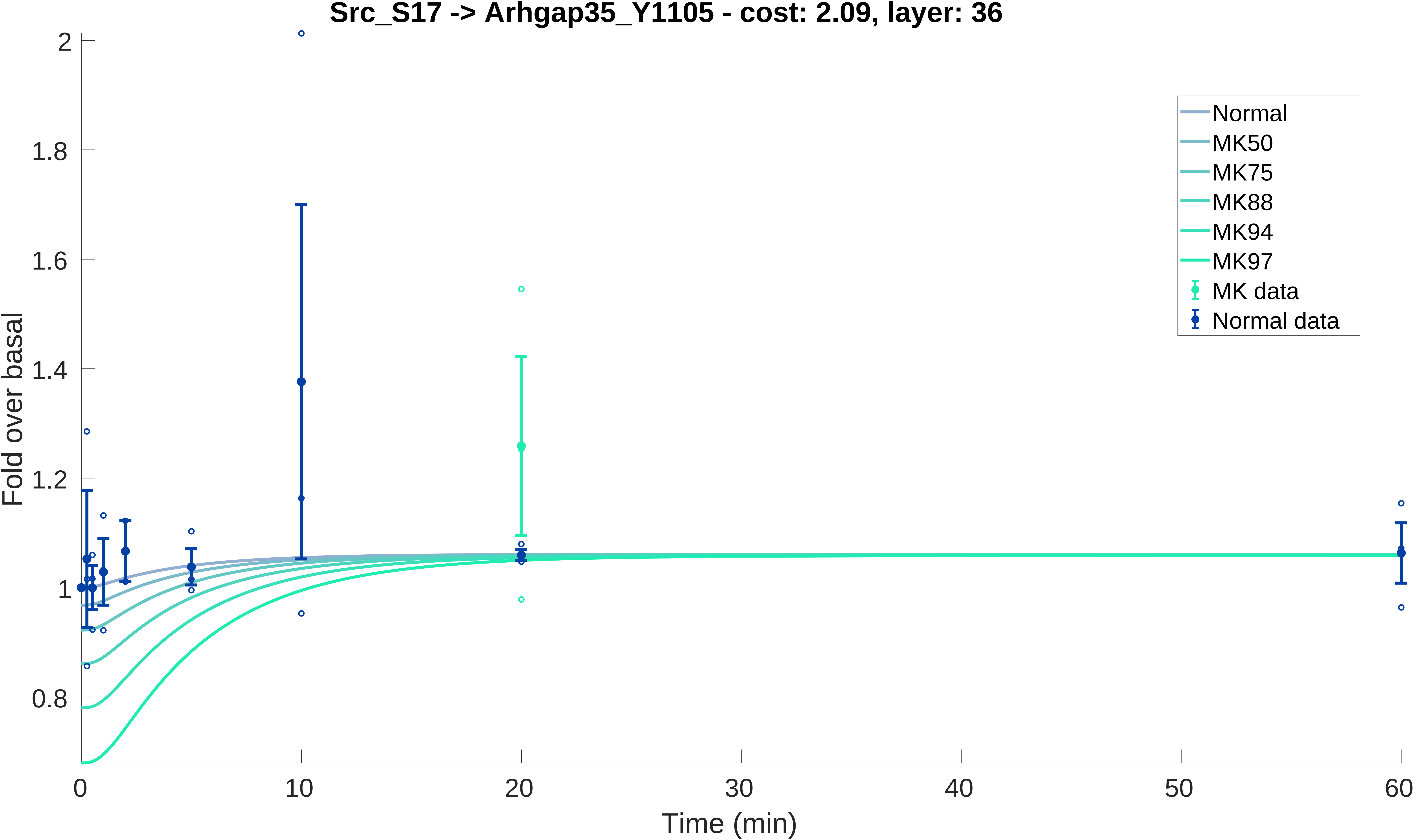

Src_S17 -> Arhgap35_Y1105 - cost: 2.09, layer: 36
Fold over basal
2
1.8
1.6
1.4
1.2
1
0.8
0
10
20
30
40
50
60
Time (min)
Normal
MK50
MK75
MK88
MK94
MK97
MK data
Normal data

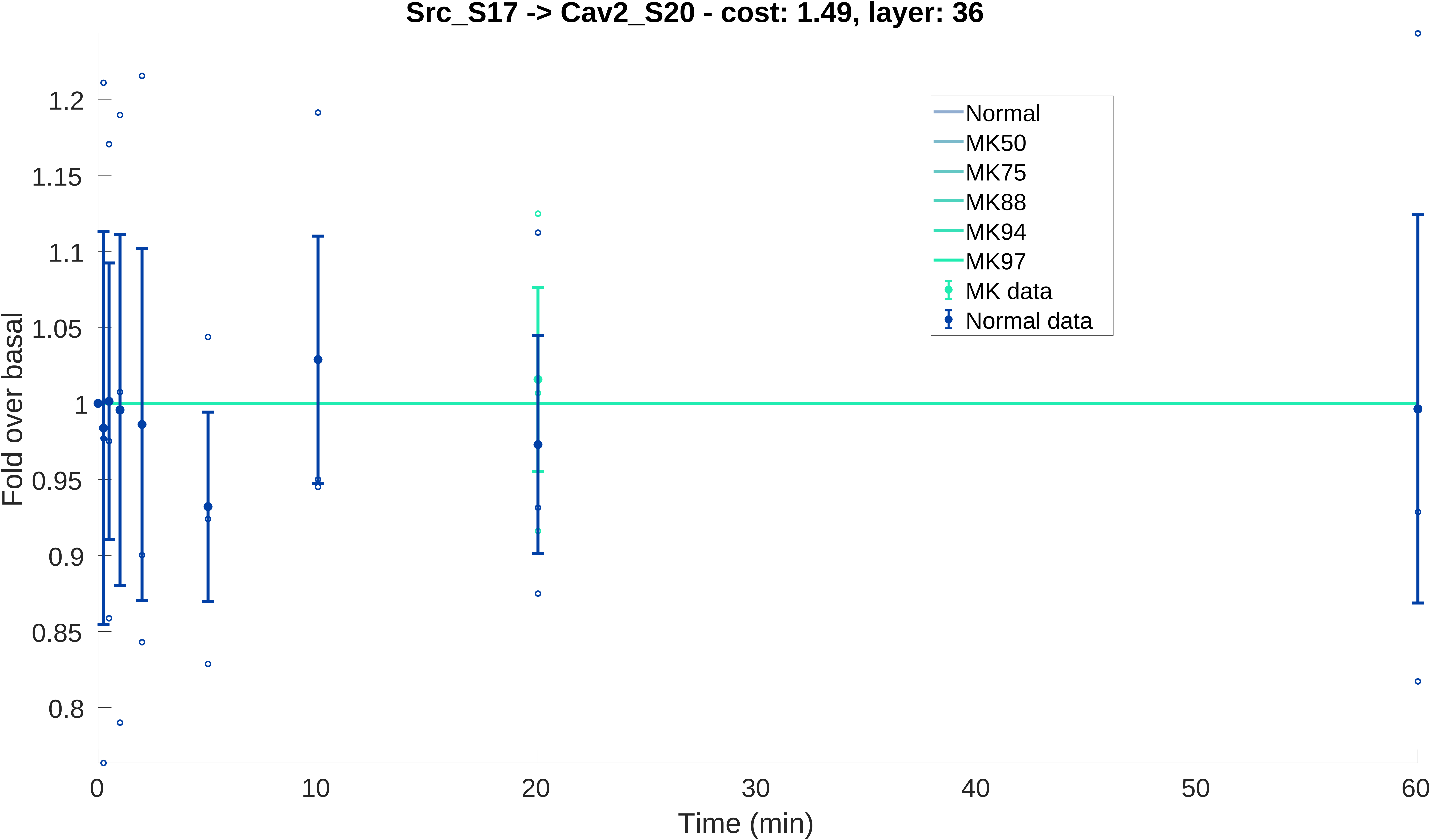

Src_S17 -> Cav2_S20 - cost: 1.49, layer: 36
Fold over basal
1.2
1.15
1.1
1.05
1
0.95
0.9
0.85
0.8
0
10
20
30
40
50
60
Time (min)
Normal
MK50
MK75
MK88
MK94
MK97
MK data
Normal data

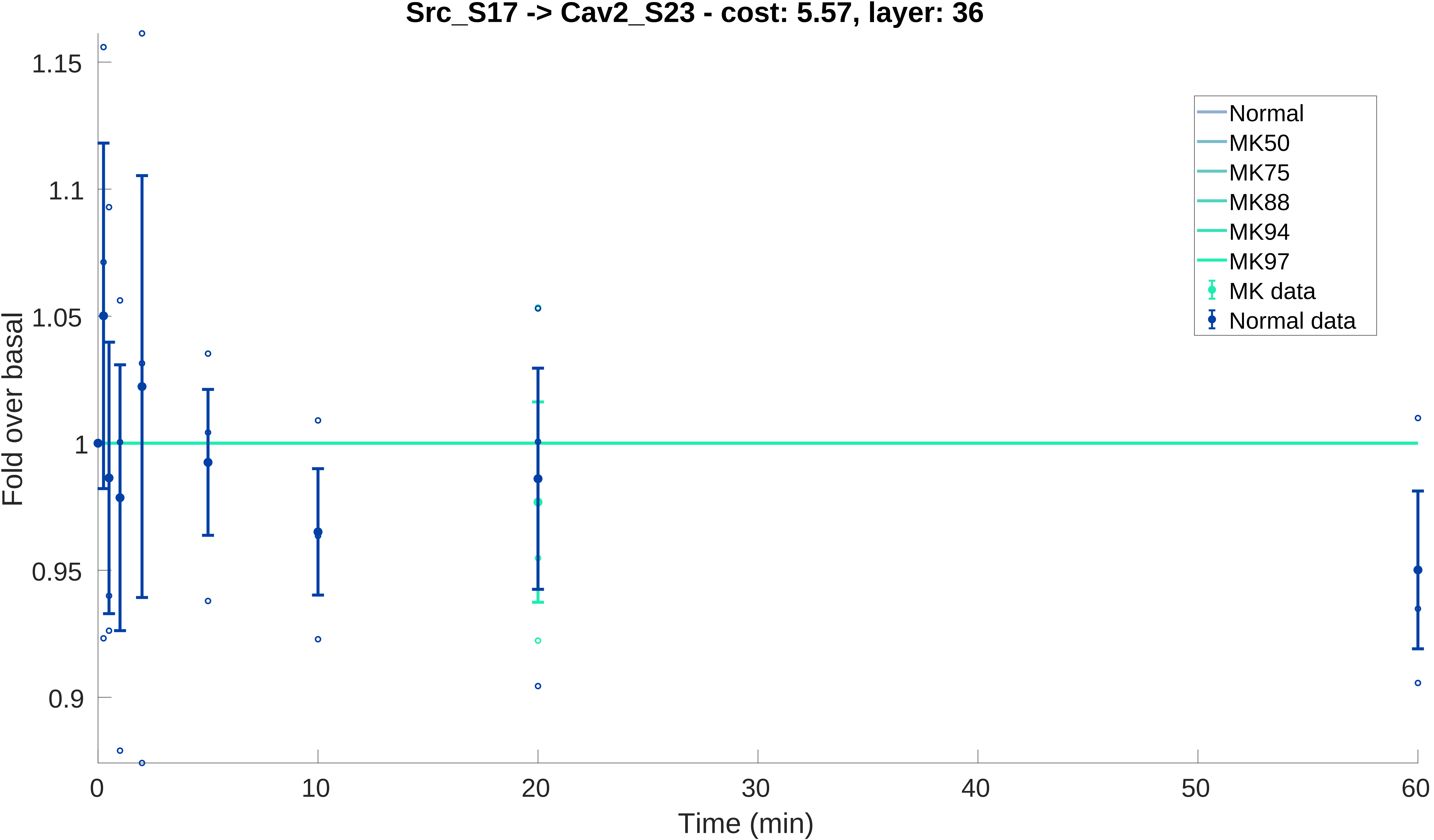
Src_S17 -> Cav2_S23 - cost: 5.57, layer: 36
Fold over basal
1.15
1.1
1.05
1
0.95
0.9
0
10
20
30
40
50
60
Time (min)
Normal
MK50
MK75
MK88
MK94
MK97
MK data
Normal data

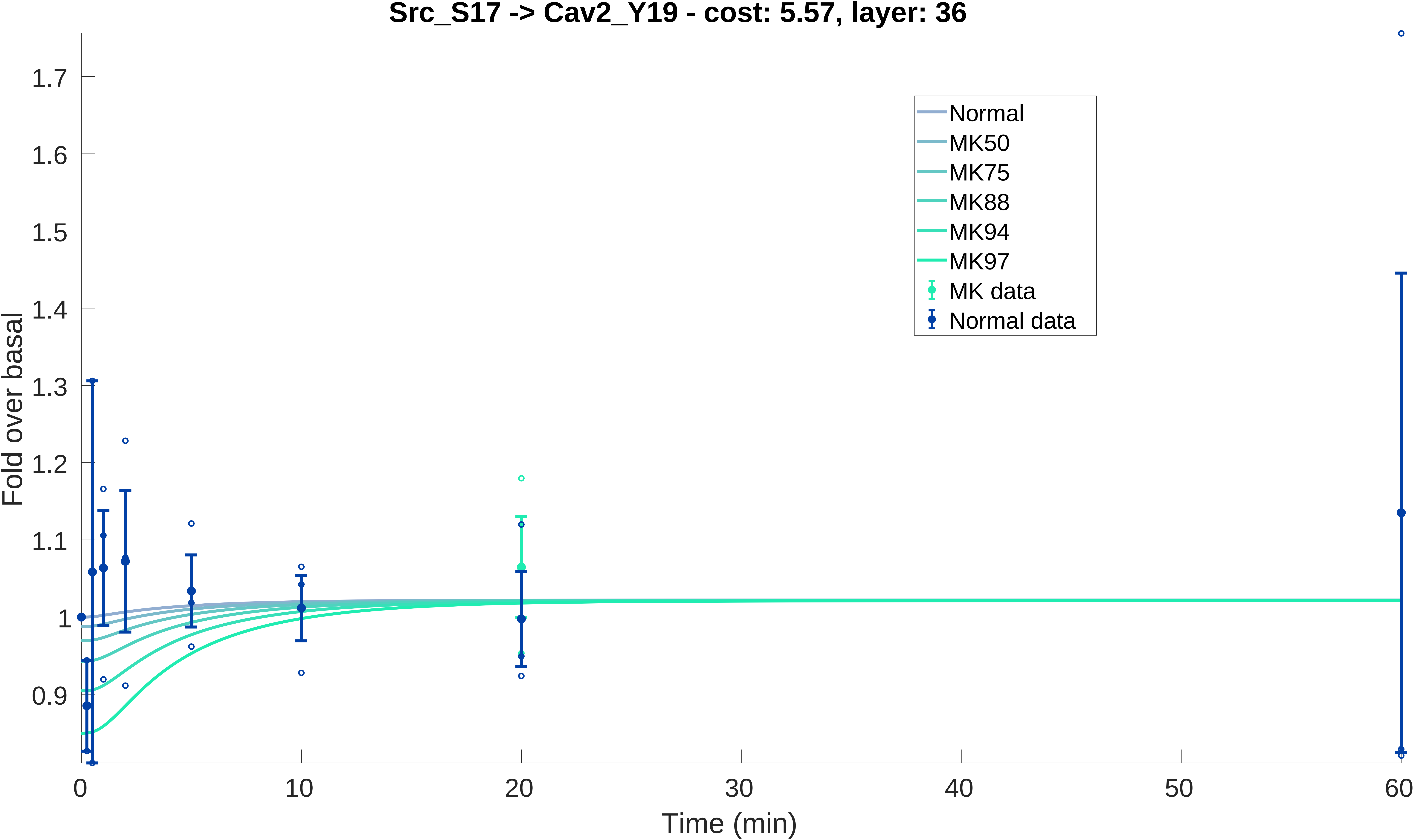
Src_S17 -> Cav2_Y19 - cost: 5.57, layer: 36
Fold over basal
Time (min)
1.7
1.6
1.5
1.4
1.3
1.2
1.1
1
0.9
0
10
20
30
40
50
60
Normal
MK50
MK75
MK88
MK94
MK97
MK data
Normal data

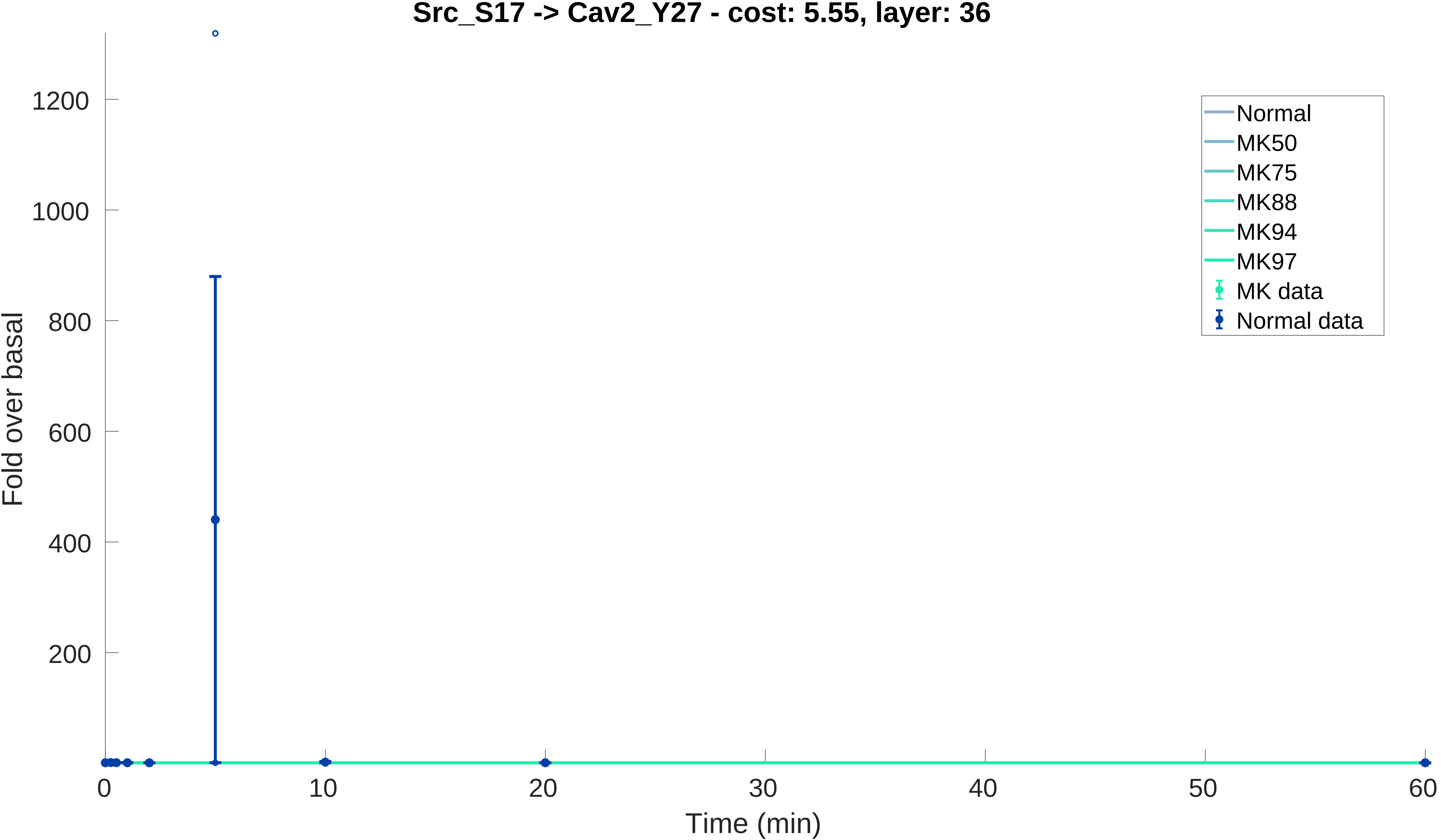

Src_S17 -> Cav2_Y27 - cost: 5.55, layer: 36
Fold over basal
1200
1000
800
600
400
200
0
10
20
30
40
50
60
Time (min)
Normal
MK50
MK75
MK88
MK94
MK97
MK data
Normal data

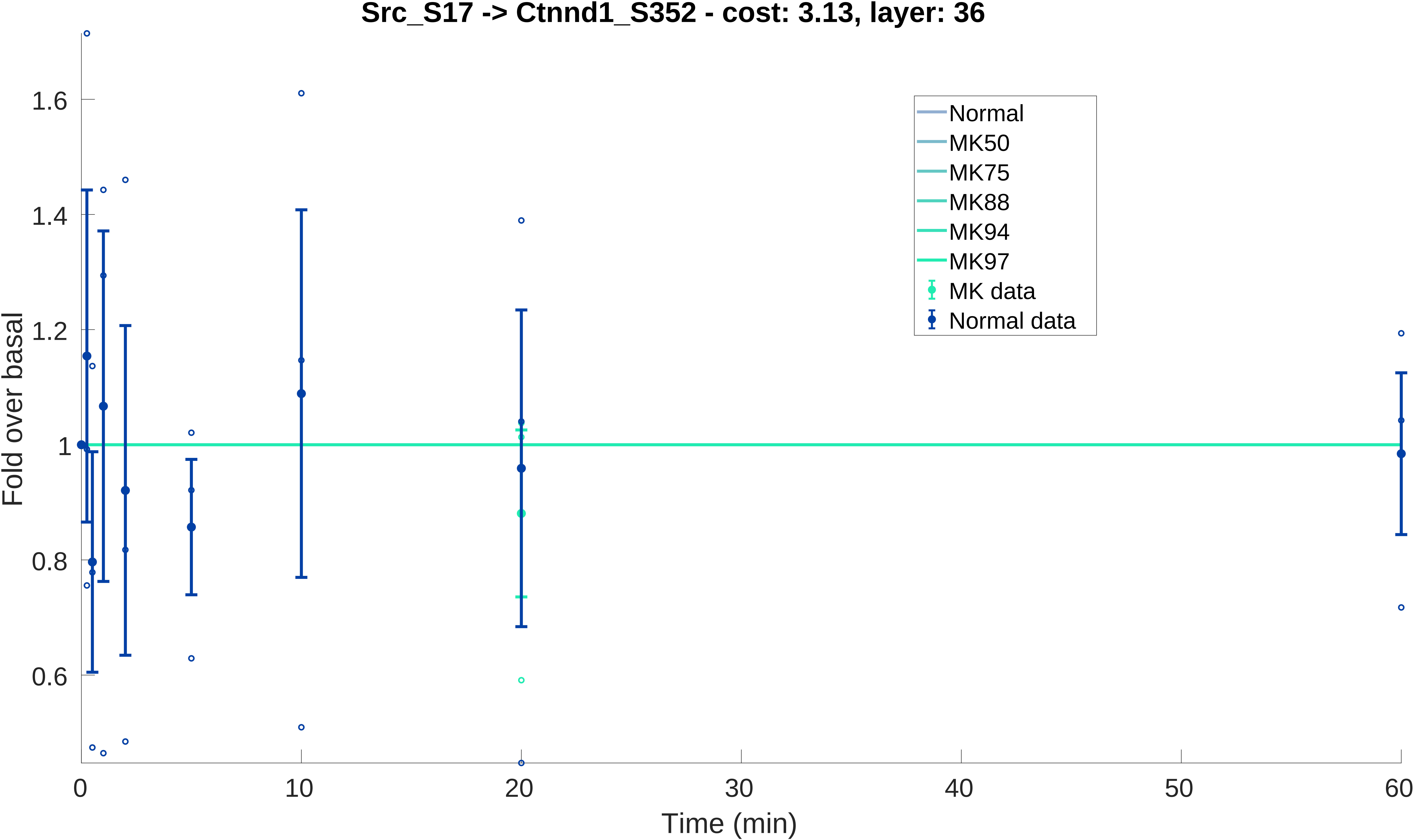

Src_S17 -> Ctnnd1_S352 - cost: 3.13, layer: 36
Fold over basal
Time (min)
0
10
20
30
40
50
60
0.6
0.8
1
1.2
1.4
1.6
Normal
MK50
MK75
MK88
MK94
MK97
MK data
Normal data

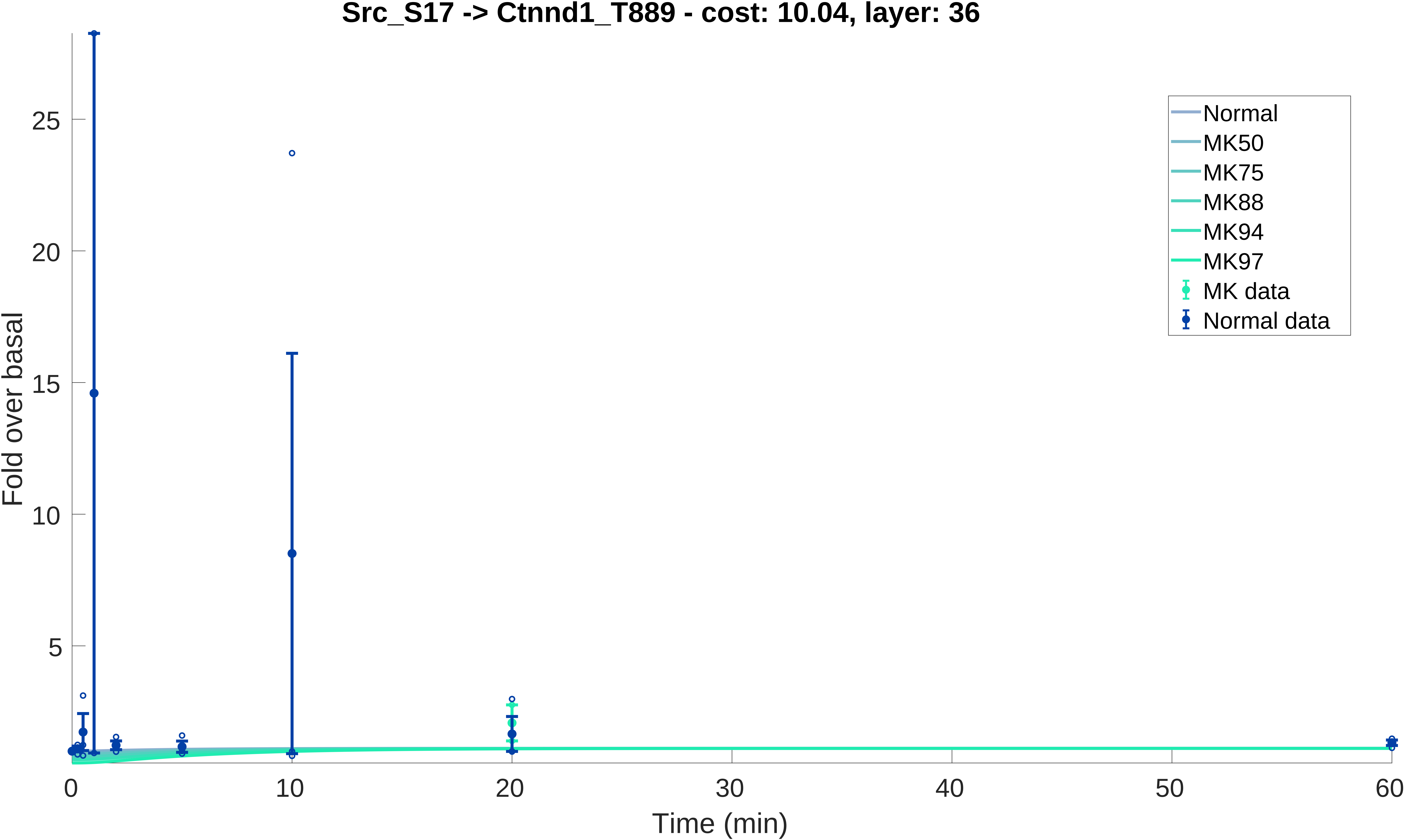

Src_S17 -> Ctnnd1_T889 - cost: 10.04, layer: 36
Fold over basal
Time (min)
0
10
20
30
40
50
60
5
10
15
20
25
Normal
MK50
MK75
MK88
MK94
MK97
MK data
Normal data

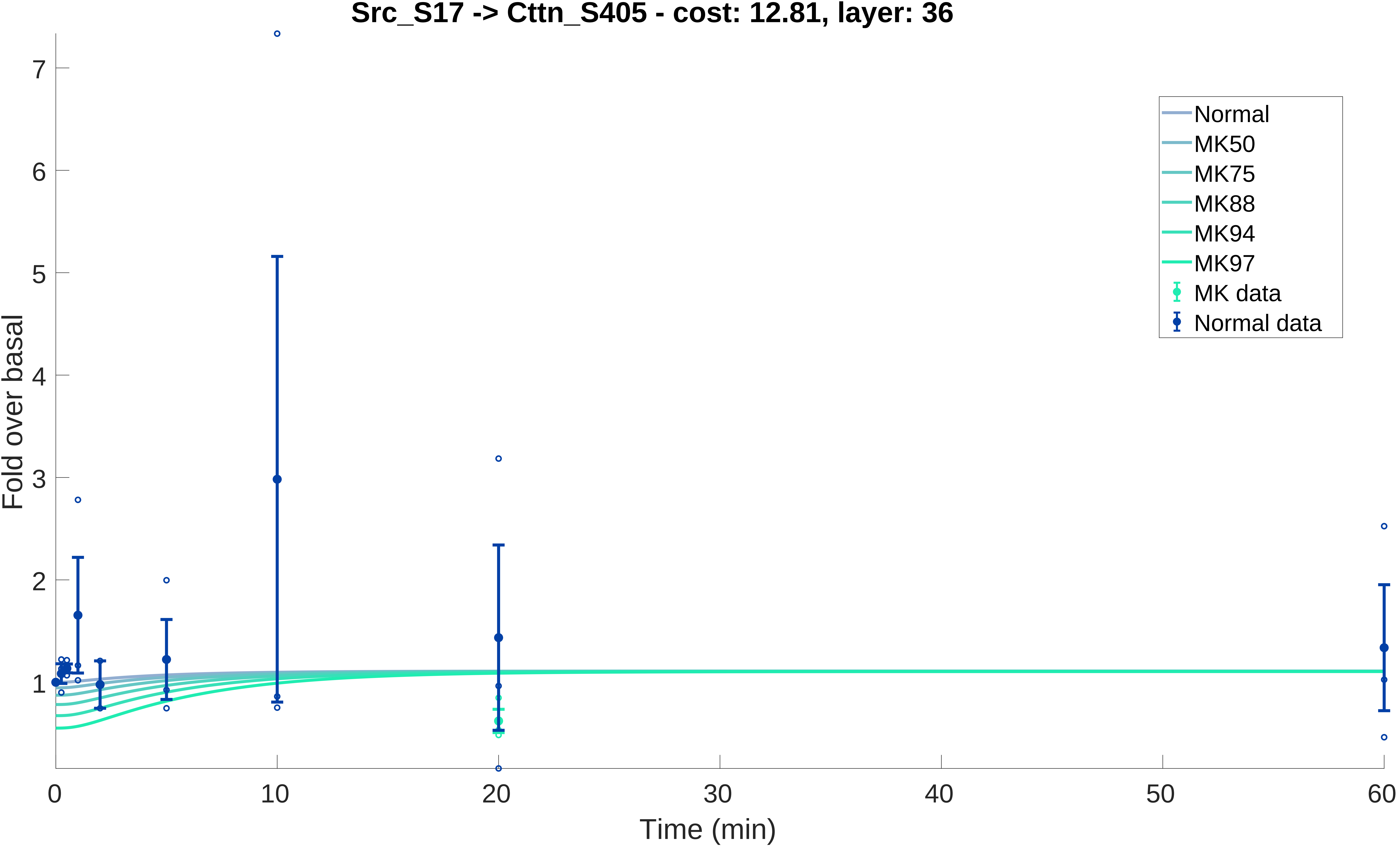

Src_S17 -> Cttn_S405 - cost: 12.81, layer: 36
Fold over basal
Time (min)
0
10
20
30
40
50
60
1
2
3
4
5
6
7
Normal
MK50
MK75
MK88
MK94
MK97
MK data
Normal data

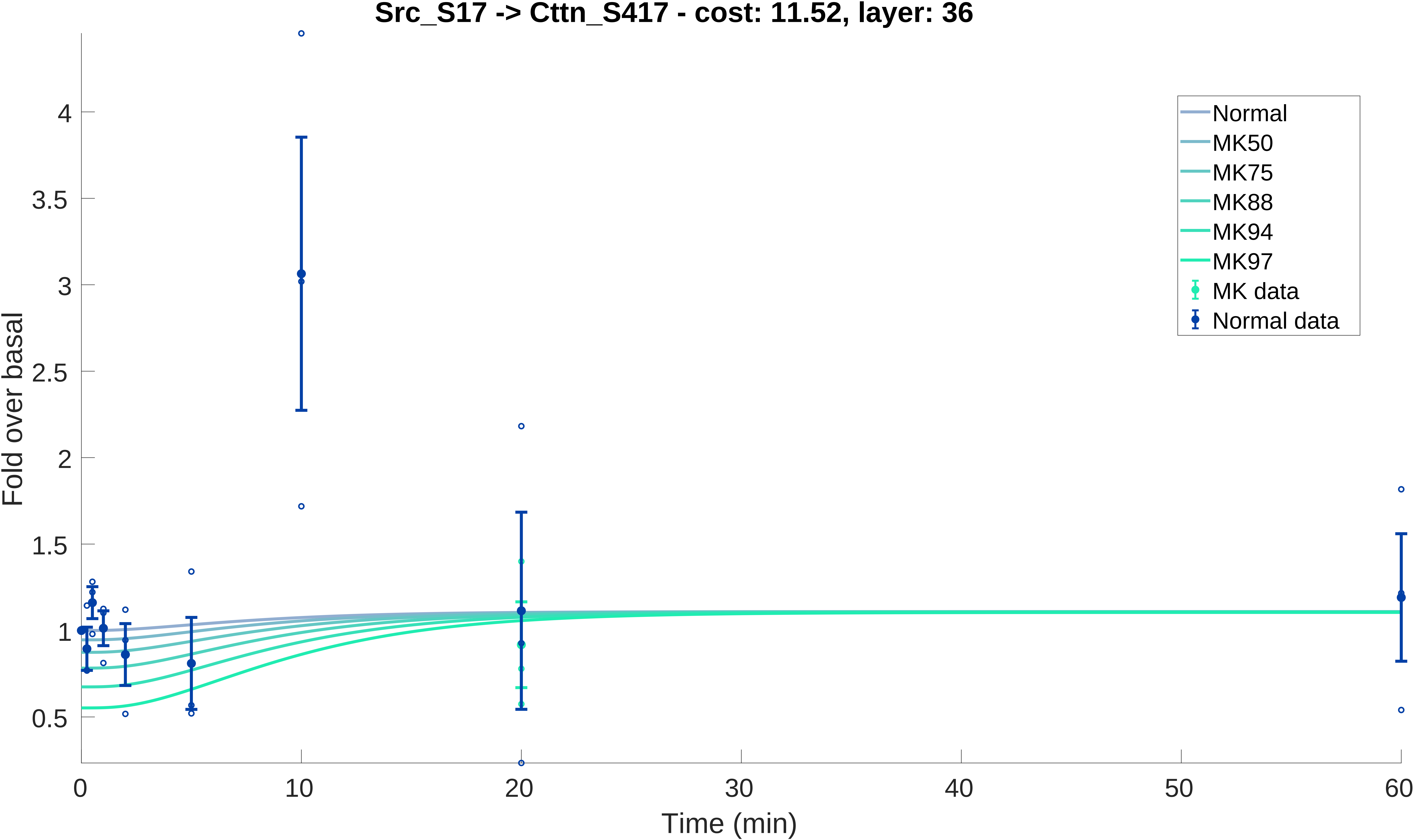

Src_S17 -> Cttn_S417 - cost: 11.52, layer: 36
Fold over basal
Time (min)
0.5
1
1.5
2
2.5
3
3.5
4
0
10
20
30
40
50
60
Normal
MK50
MK75
MK88
MK94
MK97
MK data
Normal data

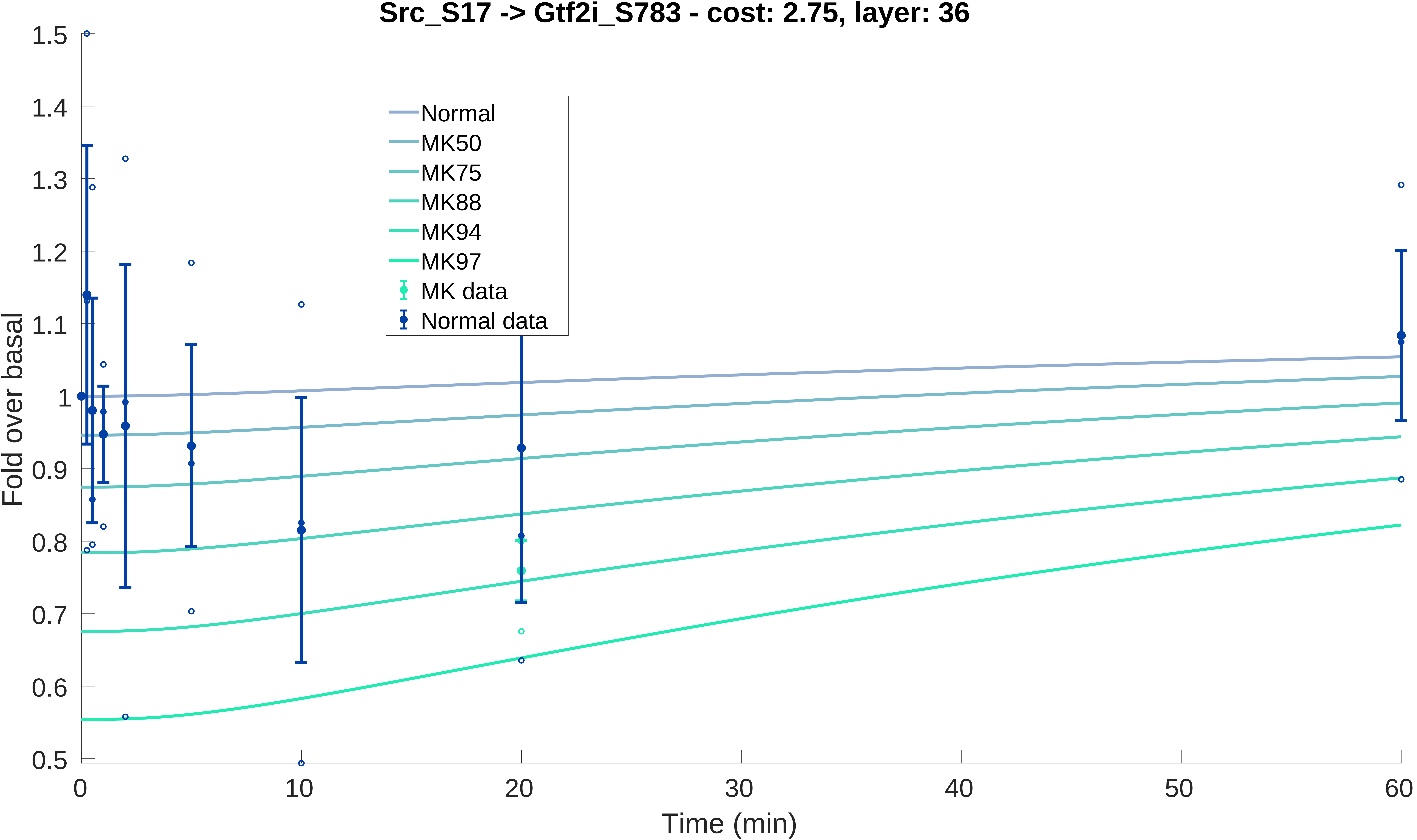

Src_S17 -> Gtf2i_S783 - cost: 2.75, layer: 36
Fold over basal
Time (min)
Normal
MK50
MK75
MK88
MK94
MK97
MK data
Normal data
1.5
1.4
1.3
1.2
1.1
1
0.9
0.8
0.7
0.6
0.5
0
10
20
30
40
50
60

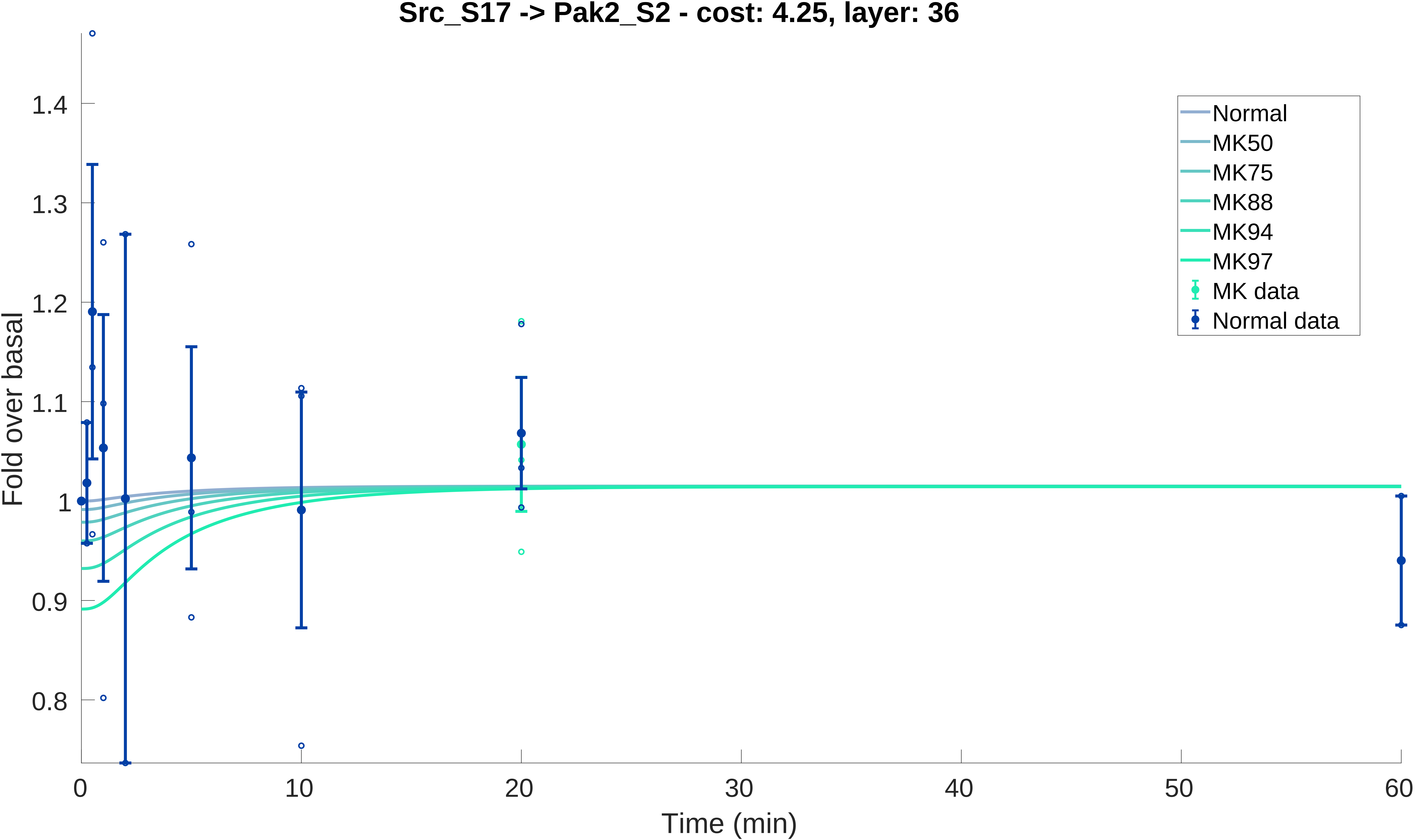
Src_S17 -> Pak2_S2 - cost: 4.25, layer: 36
Fold over basal
Time (min)
0
10
20
30
40
50
60
0.8
0.9
1
1.1
1.2
1.3
1.4
Normal
MK50
MK75
MK88
MK94
MK97
MK data
Normal data

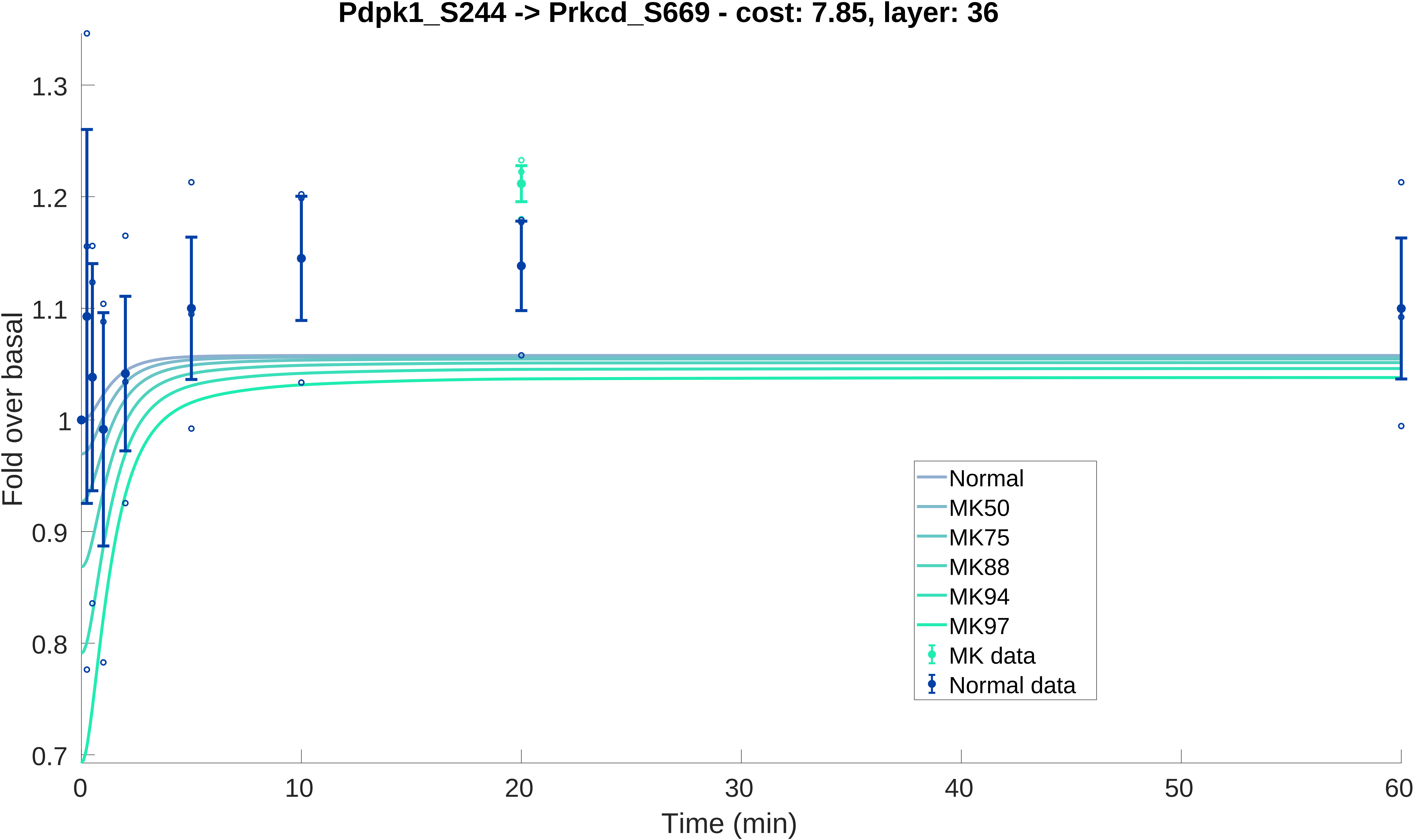

Pdpk1_S244 -> Prkcd_S669 - cost: 7.85, layer: 36
Fold over basal
Time (min)
0.7
0.8
0.9
1
1.1
1.2
1.3
0
10
20
30
40
50
60
Normal
MK50
MK75
MK88
MK94
MK97
MK data
Normal data

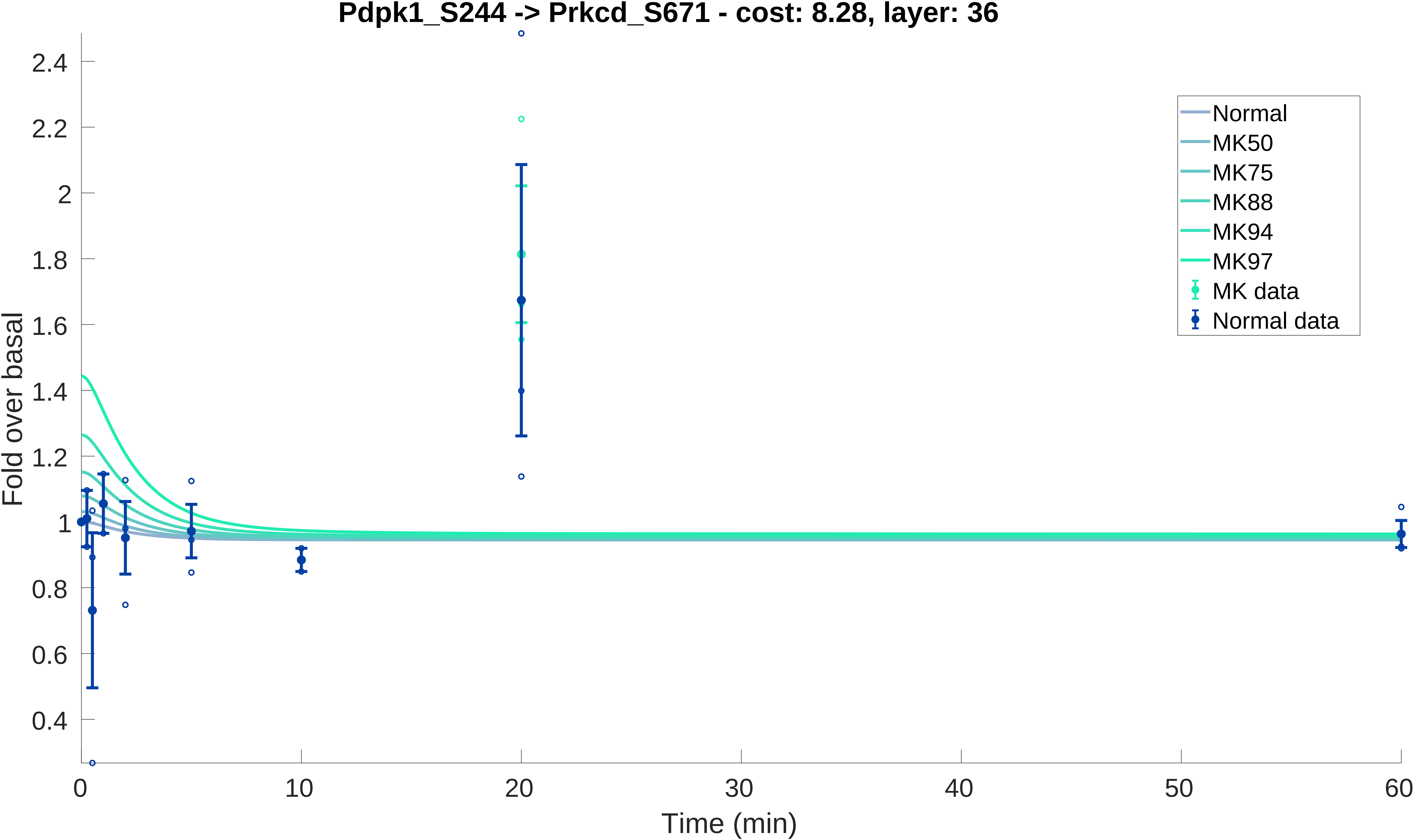

Pdpk1_S244 -> Prkcd_S671 - cost: 8.28, layer: 36
Fold over basal
Time (min)
2.4
2.2
2
1.8
1.6
1.4
1.2
1
0.8
0.6
0.4
0
10
20
30
40
50
60
Normal
MK50
MK75
MK88
MK94
MK97
MK data
Normal data

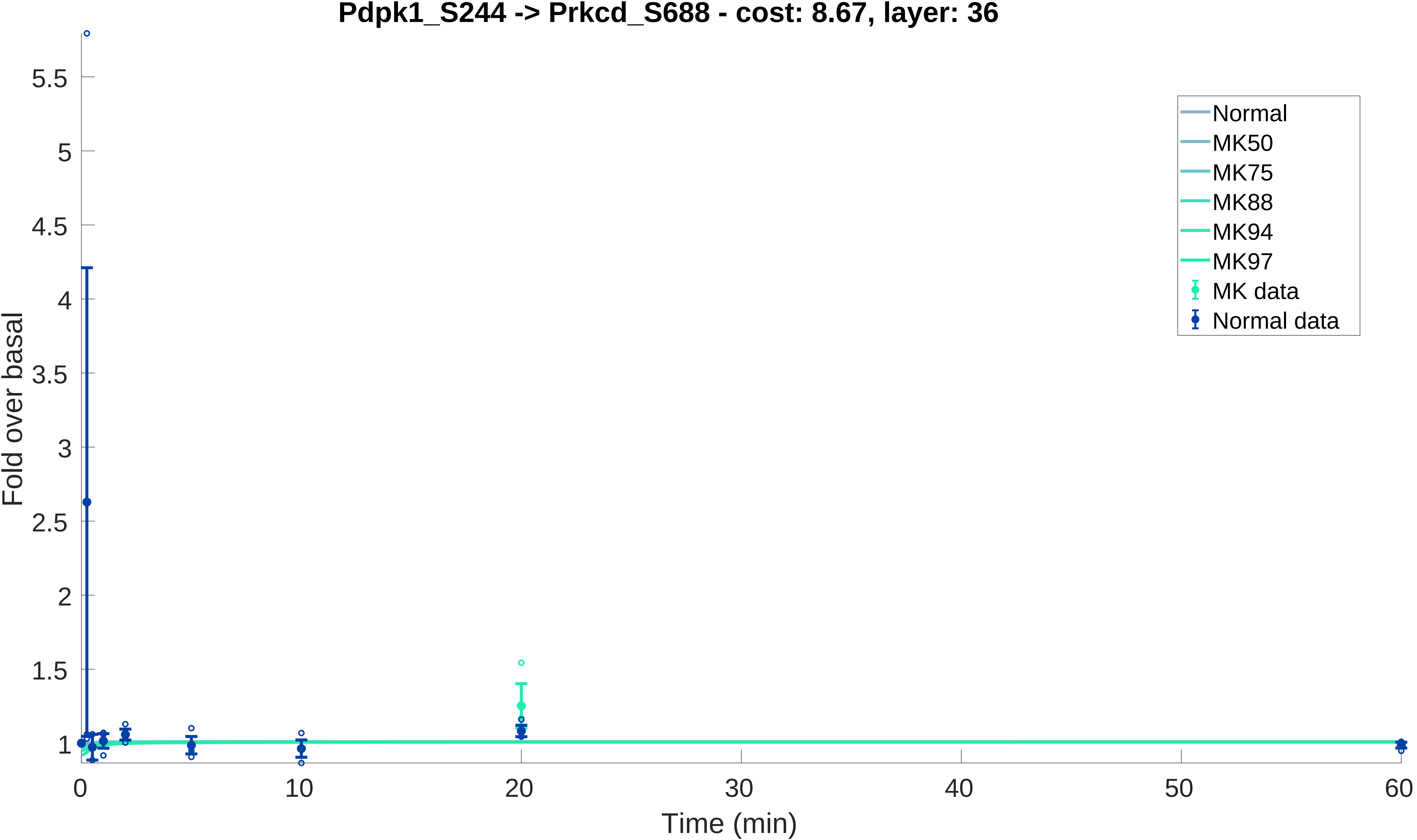
Pdpk1_S244 -> Prkcd_S688 - cost: 8.67, layer: 36
Fold over basal
Time (min)
5.5
5
4.5
4
3.5
3
2.5
2
1.5
1
0
10
20
30
40
50
60
Normal
MK50
MK75
MK88
MK94
MK97
MK data
Normal data

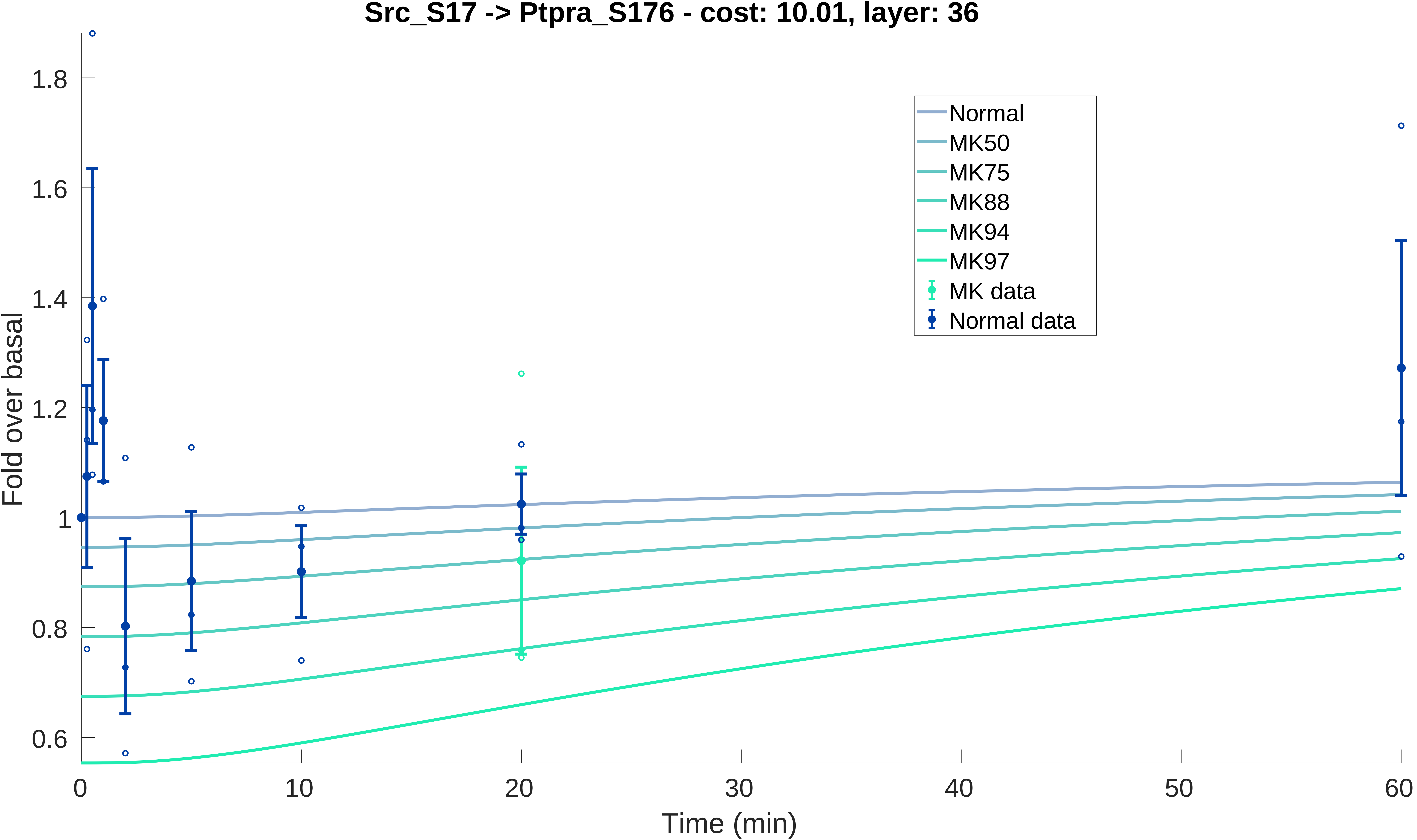

Src_S17 -> Ptpra_S176 - cost: 10.01, layer: 36
Fold over basal
Time (min)
0.6
0.8
1
1.2
1.4
1.6
1.8
0
10
20
30
40
50
60
Normal
MK50
MK75
MK88
MK94
MK97
MK data
Normal data

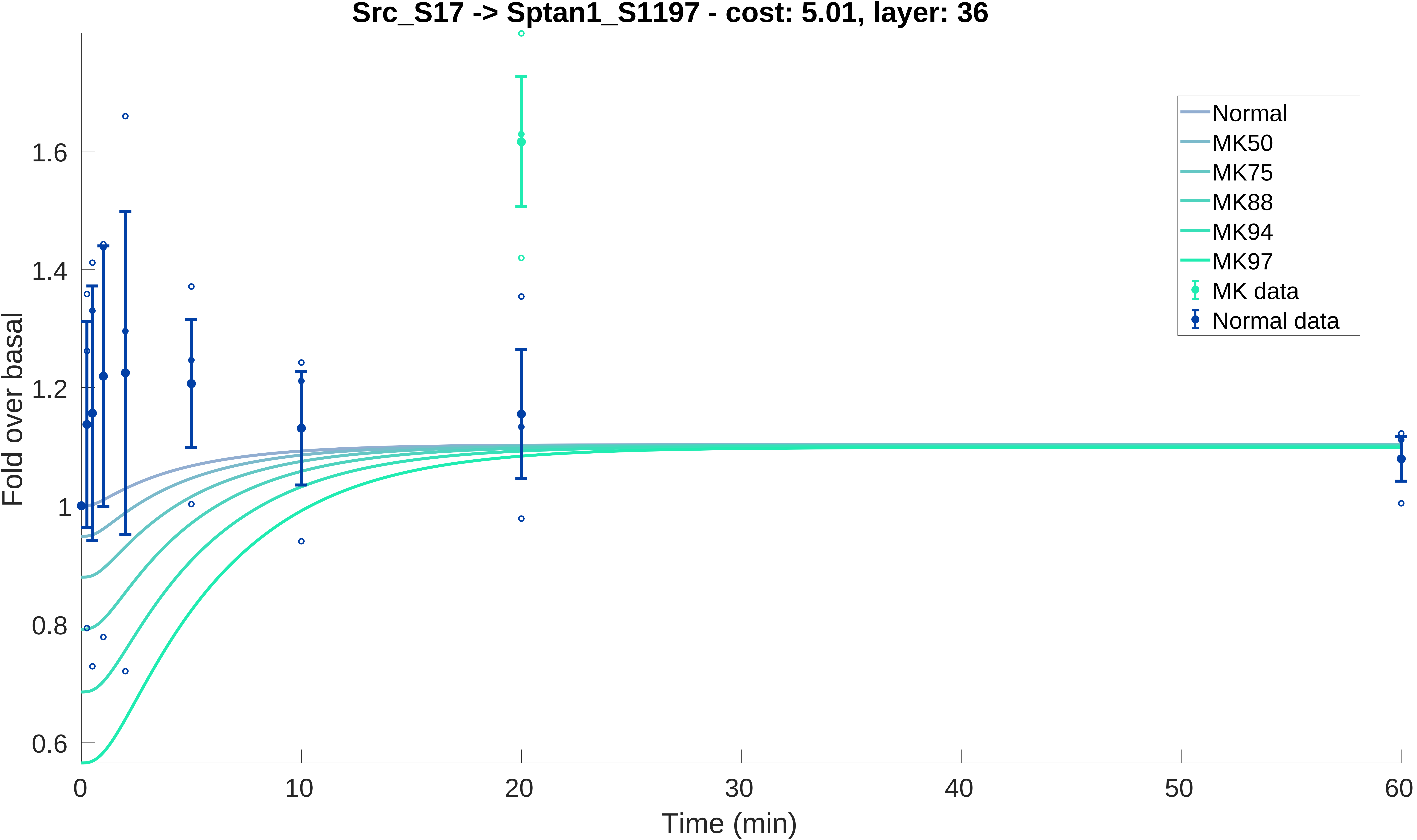

Src_S17 -> Sptan1_S1197 - cost: 5.01, layer: 36
Normal
MK50
MK75
MK88
MK94
MK97
MK data
Normal data
Fold over basal
Time (min)
0.6
0.8
1
1.2
1.4
1.6
0
10
20
30
40
50
60

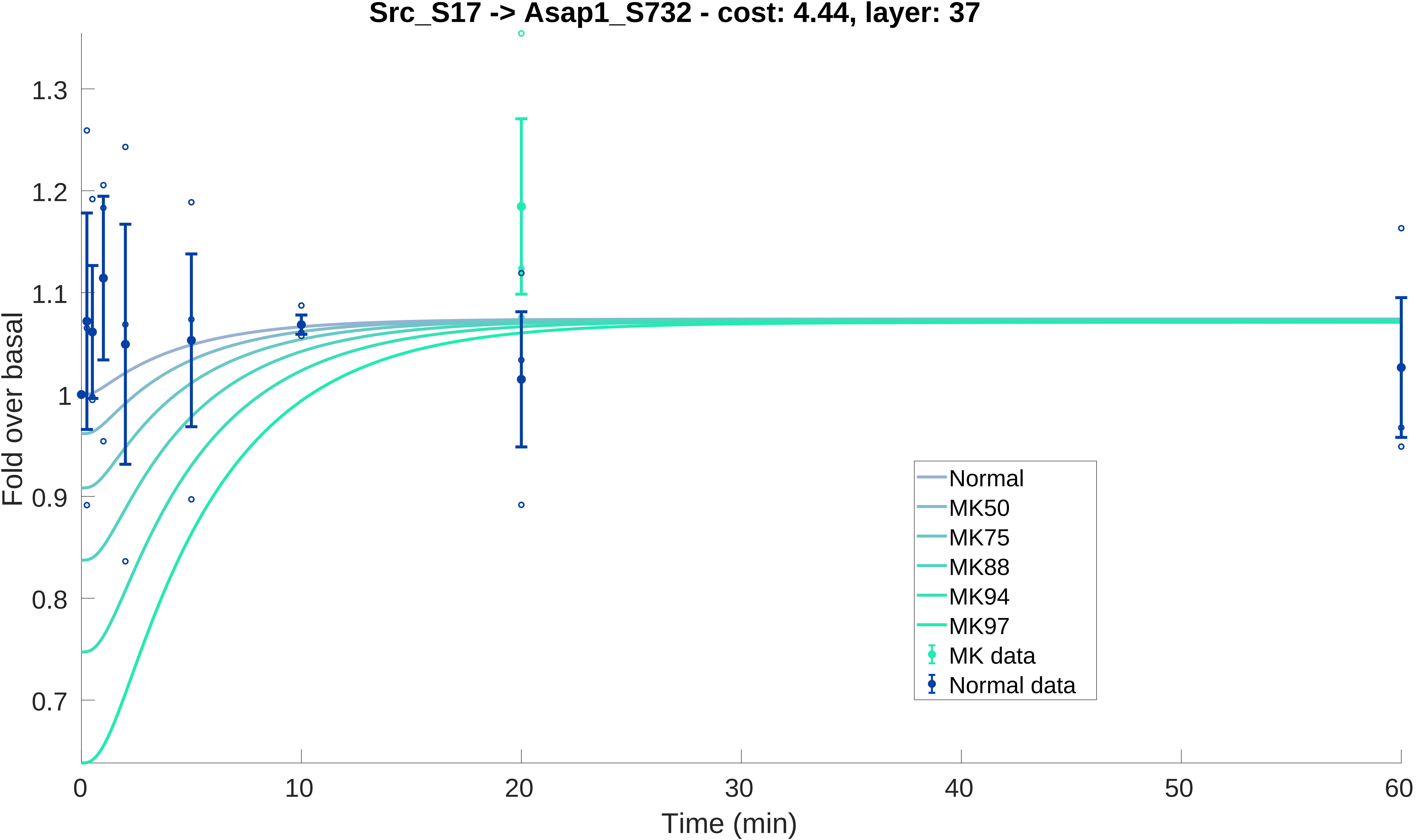
Src_S17 -> Asap1_S732 - cost: 4.44, layer: 37
Fold over basal
Time (min)
1.3
1.2
1.1
1
0.9
0.8
0.7
0
10
20
30
40
50
60
Normal
MK50
MK75
MK88
MK94
MK97
MK data
Normal data

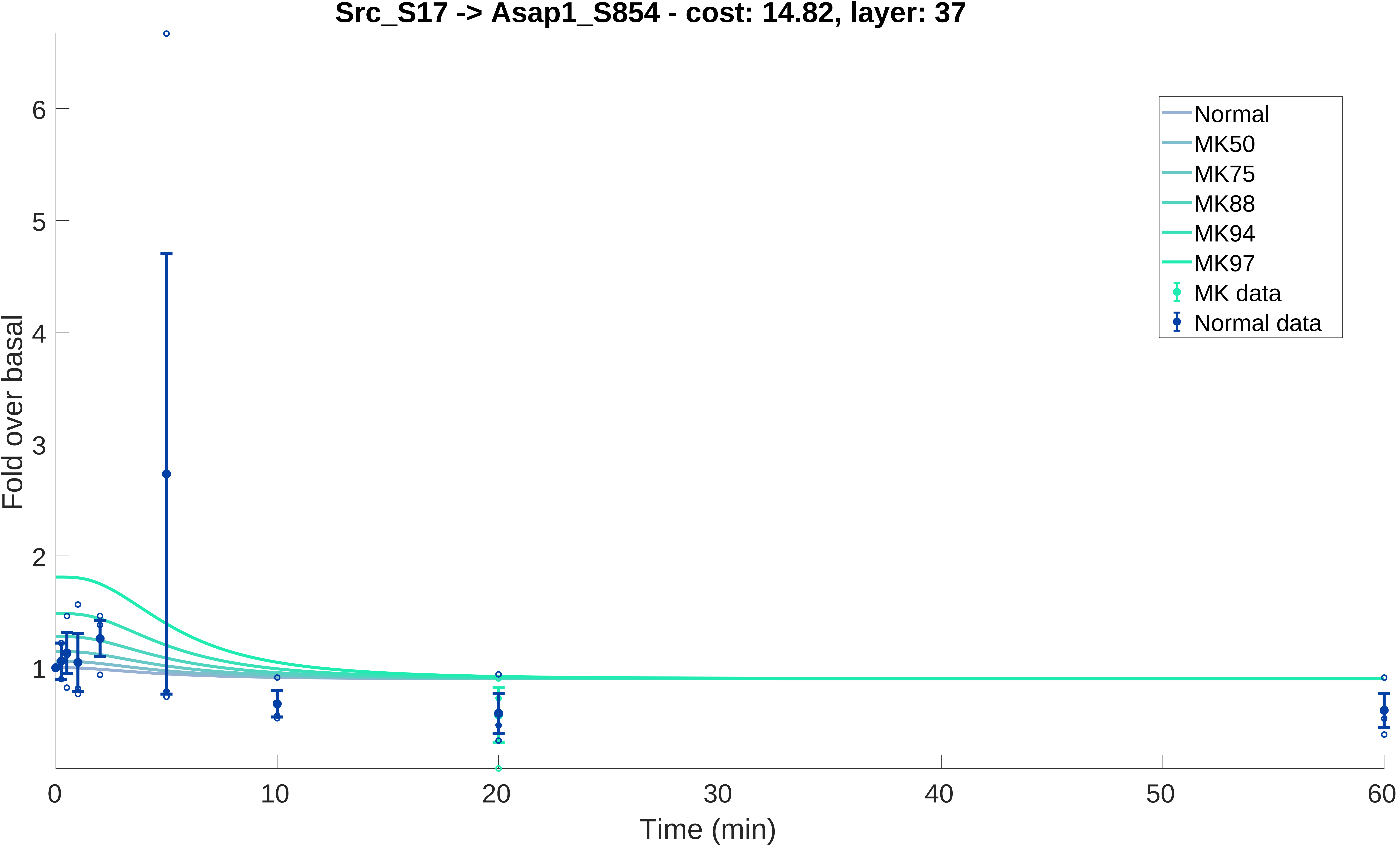

Src_S17 -> Asap1_S854 - cost: 14.82, layer: 37
Fold over basal
Time (min)
0
10
20
30
40
50
60
1
2
3
4
5
6
Normal
MK50
MK75
MK88
MK94
MK97
MK data
Normal data

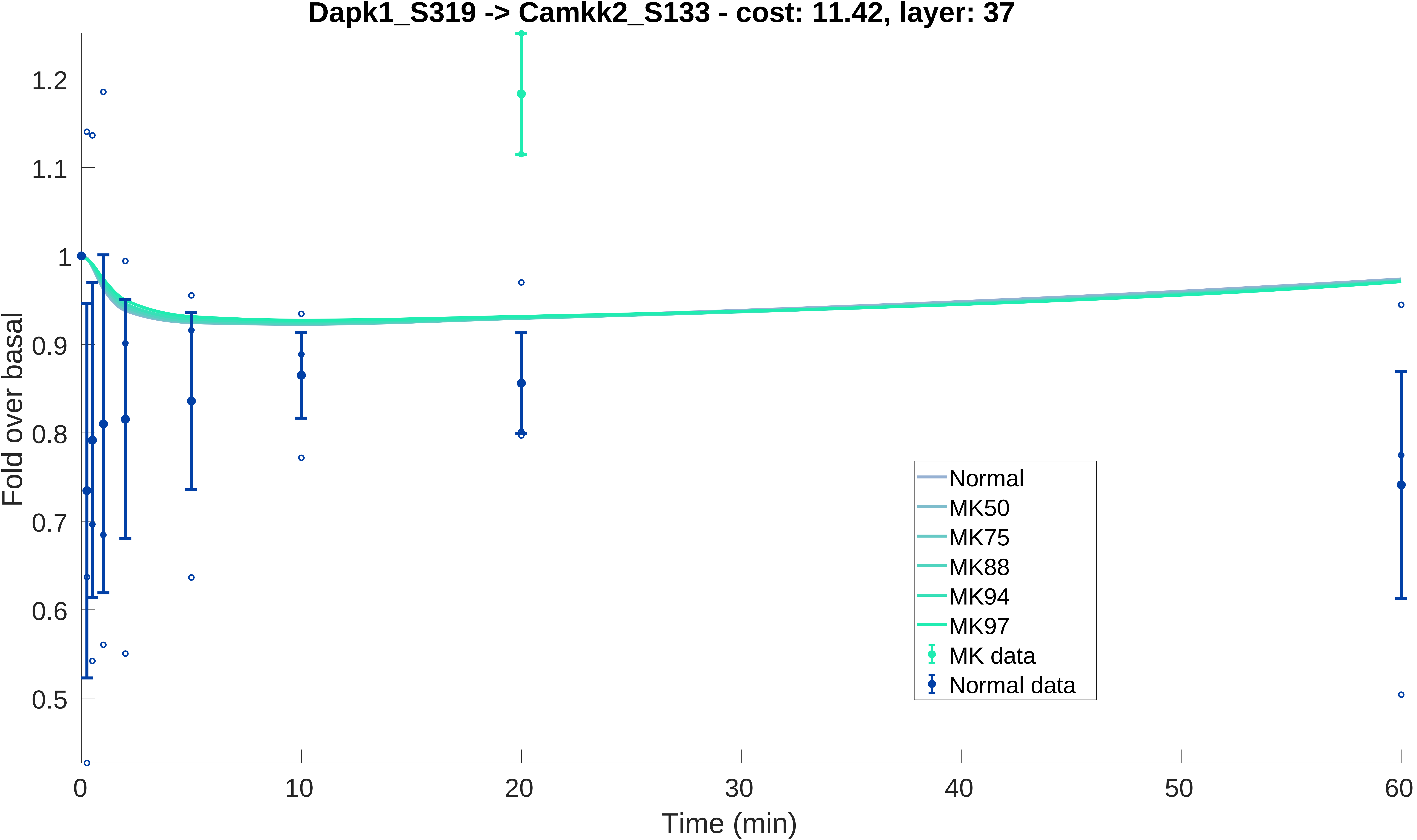
Dapk1_S319 -> Camkk2_S133 - cost: 11.42, layer: 37
Fold over basal
1.2
1.1
1
0.9
0.8
0.7
0.6
0.5
0
10
20
30
40
50
60
Time (min)
Normal
MK50
MK75
MK88
MK94
MK97
MK data
Normal data

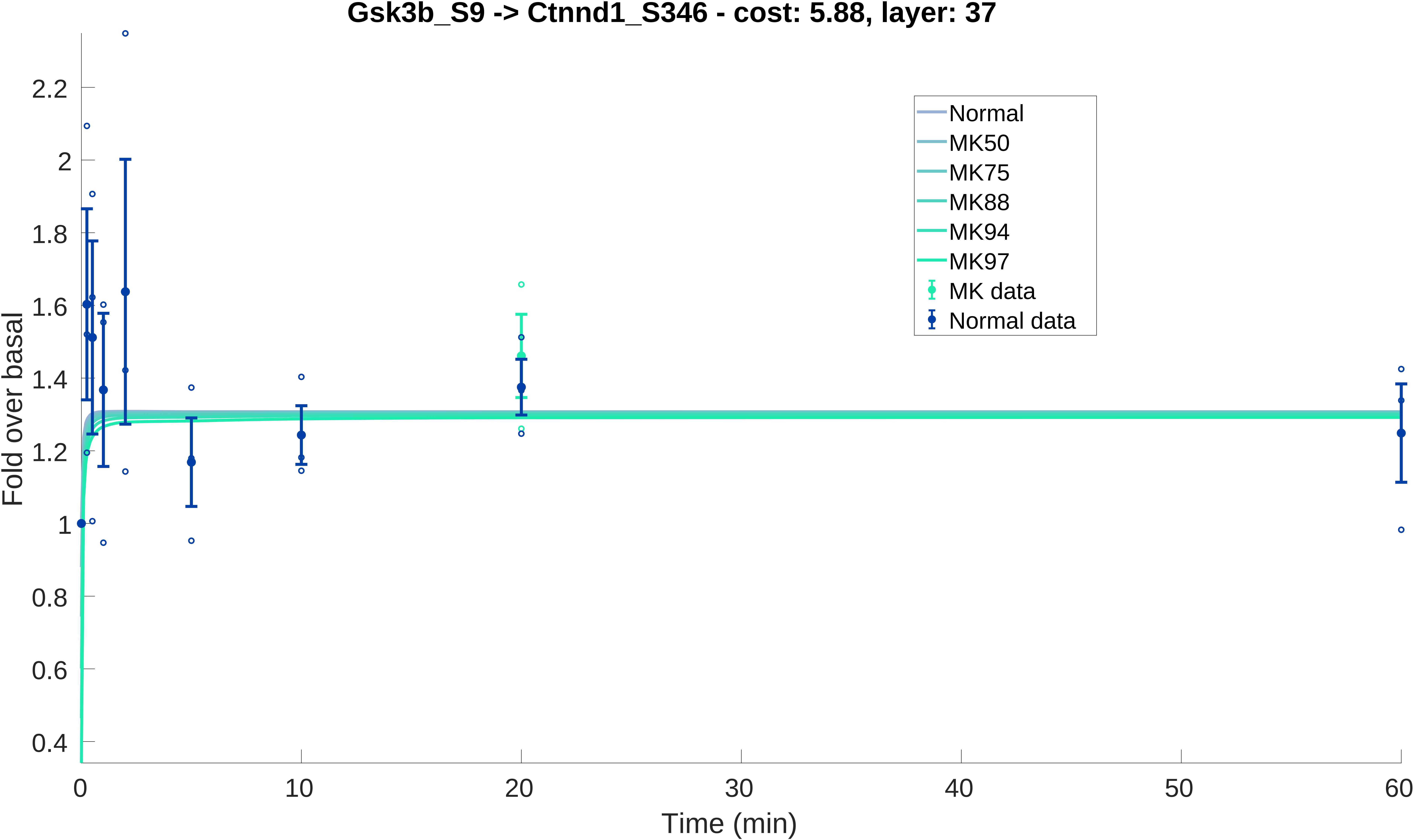
Gsk3b_S9 -> Ctnnd1_S346 - cost: 5.88, layer: 37
Fold over basal
Time (min)
0.4
0.6
0.8
1
1.2
1.4
1.6
1.8
2
2.2
0
10
20
30
40
50
60
Normal
MK50
MK75
MK88
MK94
MK97
MK data
Normal data

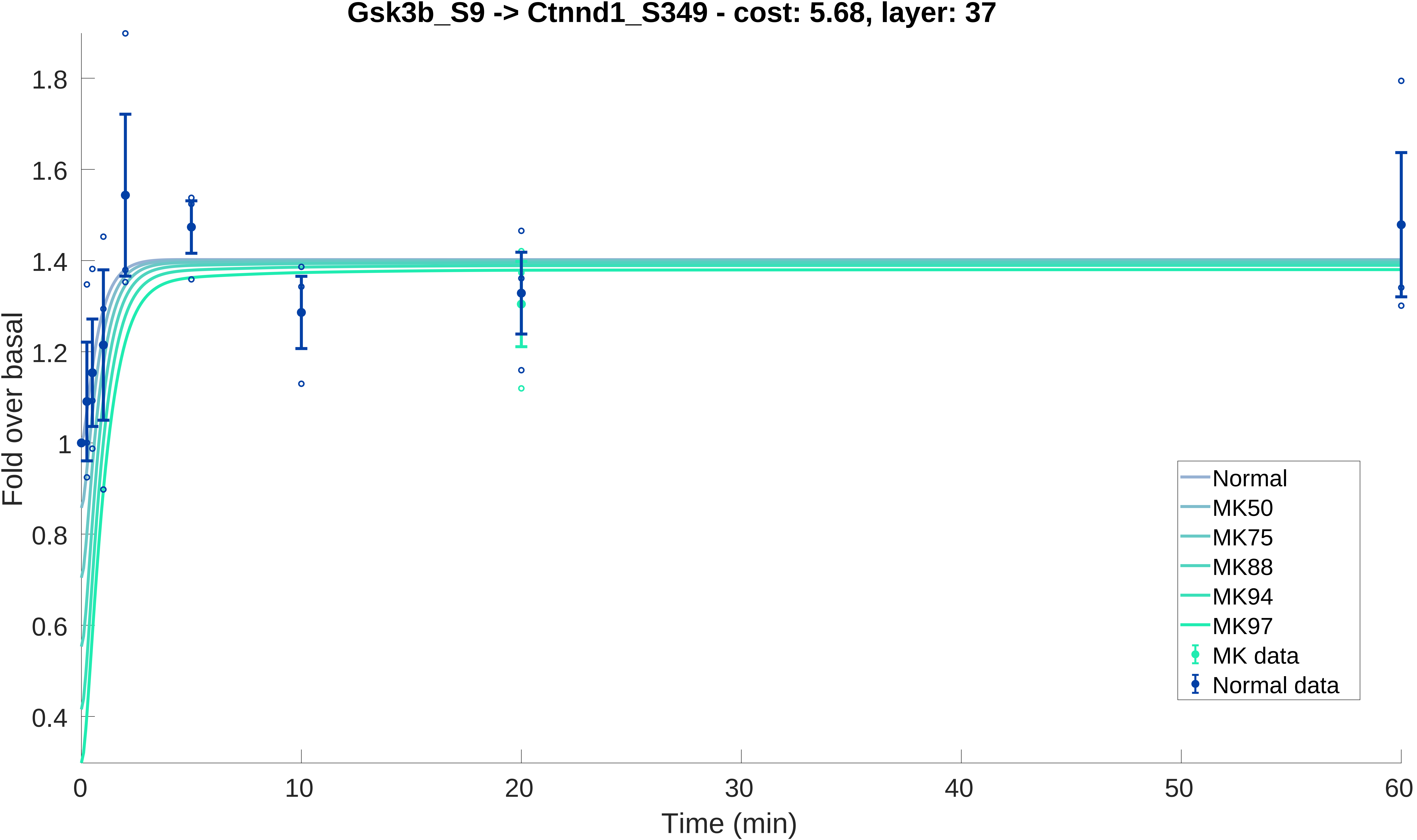

Gsk3b_S9 -> Ctnnd1_S349 - cost: 5.68, layer: 37
Fold over basal
Time (min)
0.4
0.6
0.8
1
1.2
1.4
1.6
1.8
0
10
20
30
40
50
60
Normal
MK50
MK75
MK88
MK94
MK97
MK data
Normal data

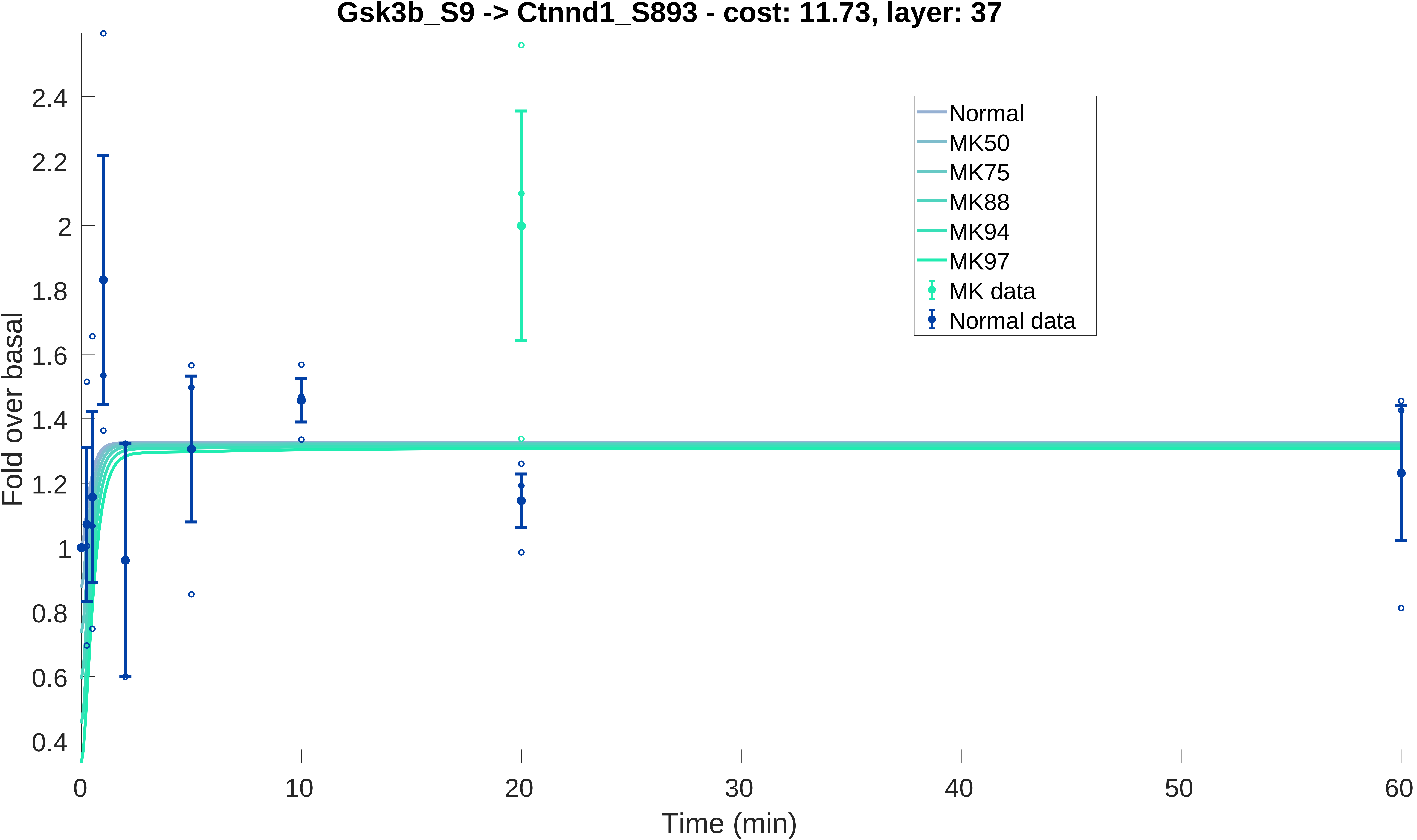
Gsk3b_S9 -> Ctnnd1_S893 - cost: 11.73, layer: 37
Fold over basal
Time (min)
0
10
20
30
40
50
60
0.4
0.6
0.8
1
1.2
1.4
1.6
1.8
2
2.2
2.4
Normal
MK50
MK75
MK88
MK94
MK97
MK data
Normal data

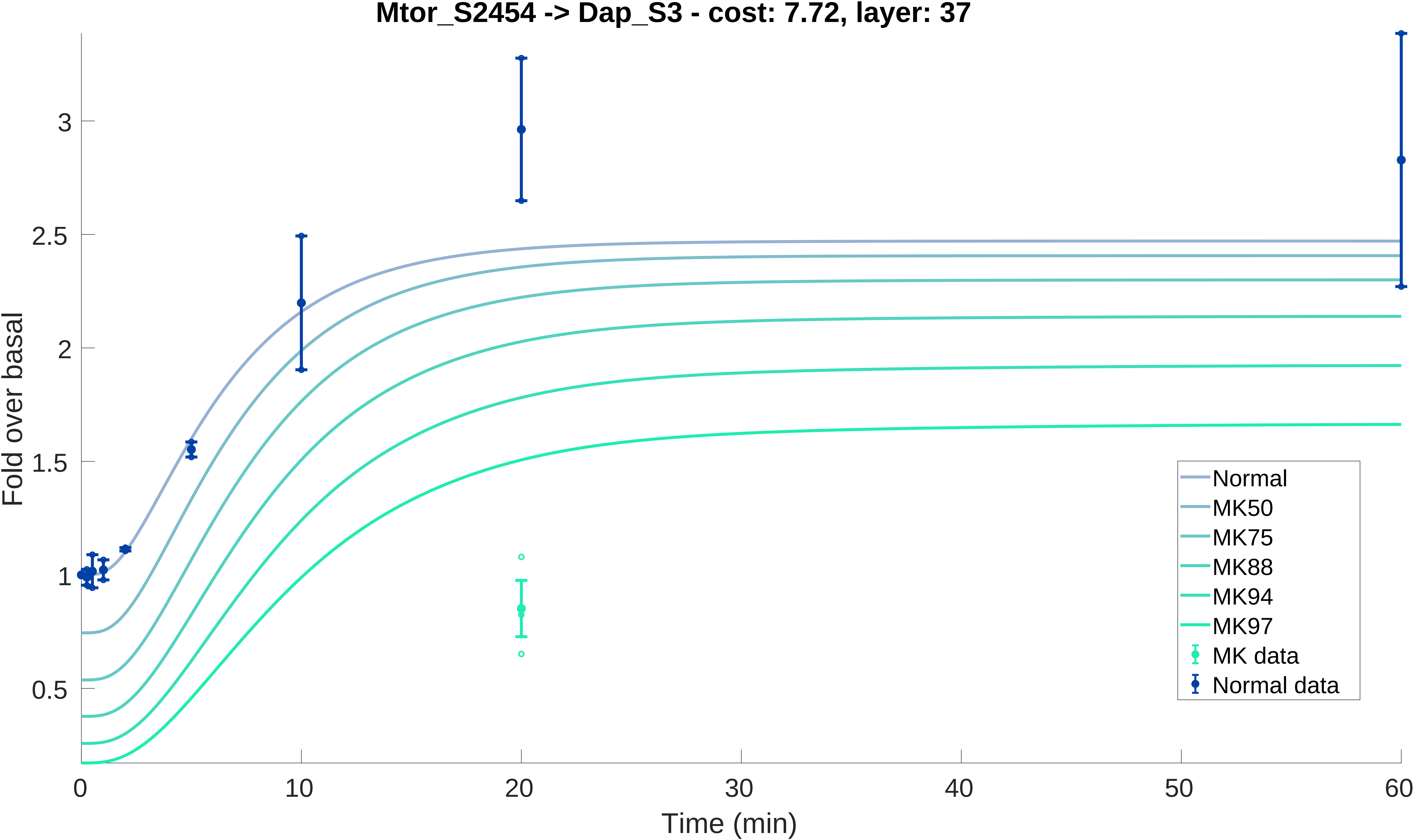
Mtor_S2454 -> Dap_S3 - cost: 7.72, layer: 37
Fold over basal
Time (min)
0.5
1
1.5
2
2.5
3
0
10
20
30
40
50
60
Normal
MK50
MK75
MK88
MK94
MK97
MK data
Normal data

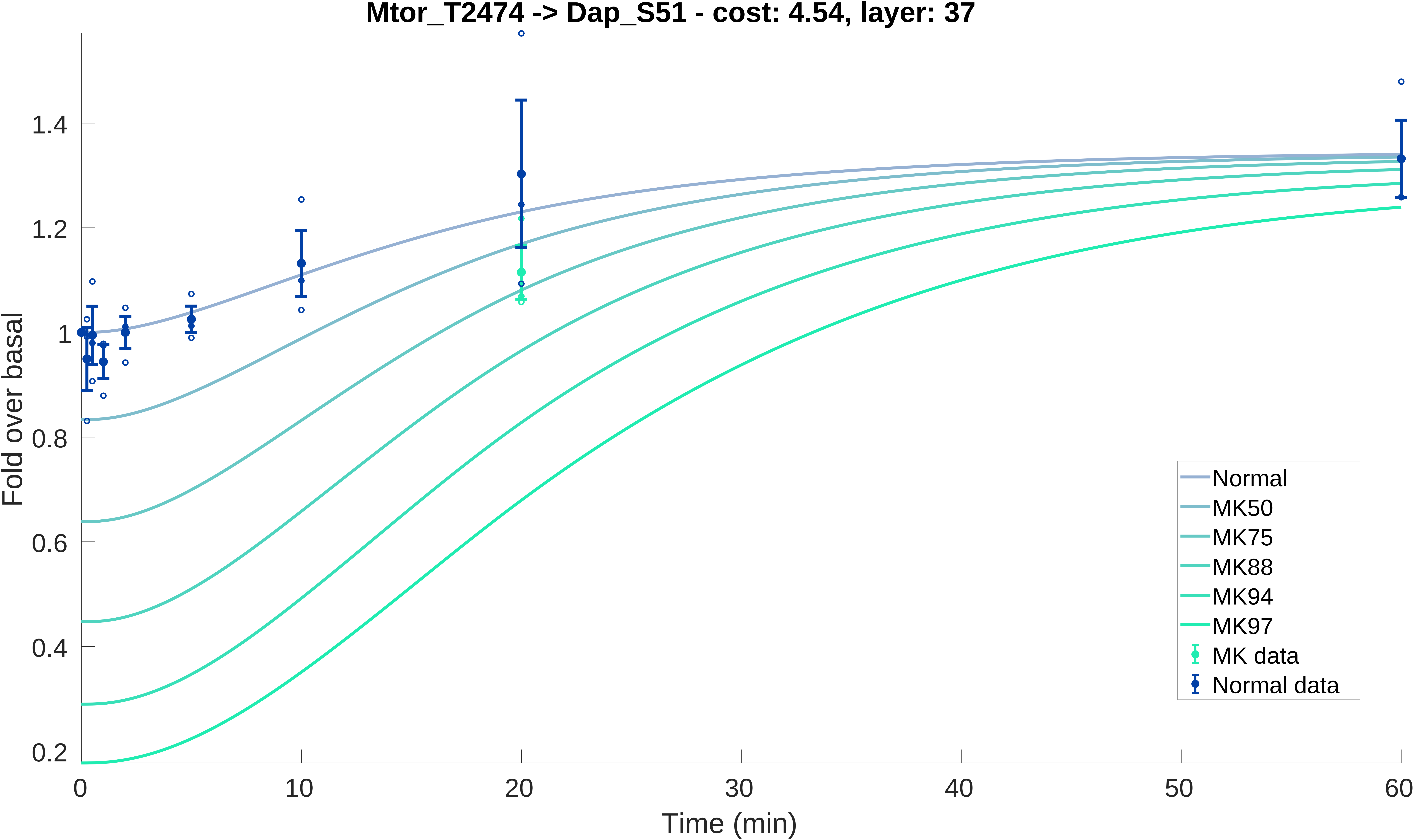
Mtor_T2474 -> Dap_S51 - cost: 4.54, layer: 37
Fold over basal
1.4
1.2
1
0.8
0.6
0.4
0.2
0
10
20
30
40
50
60
Time (min)
Normal
MK50
MK75
MK88
MK94
MK97
MK data
Normal data

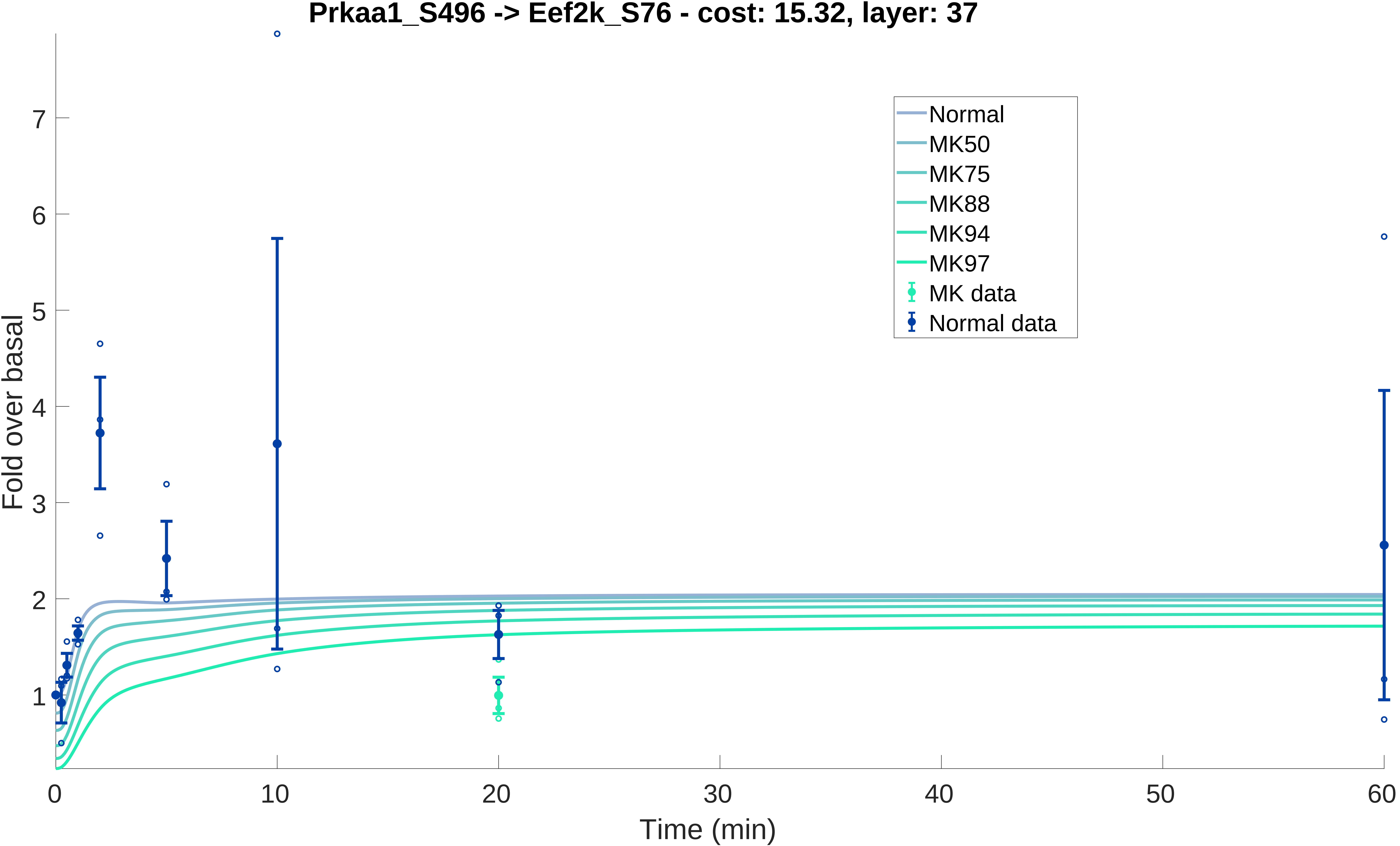

Prkaa1_S496 -> Eef2k_S76 - cost: 15.32, layer: 37
Fold over basal
Time (min)
Normal
MK50
MK75
MK88
MK94
MK97
MK data
Normal data
1
2
3
4
5
6
7
0
10
20
30
40
50
60

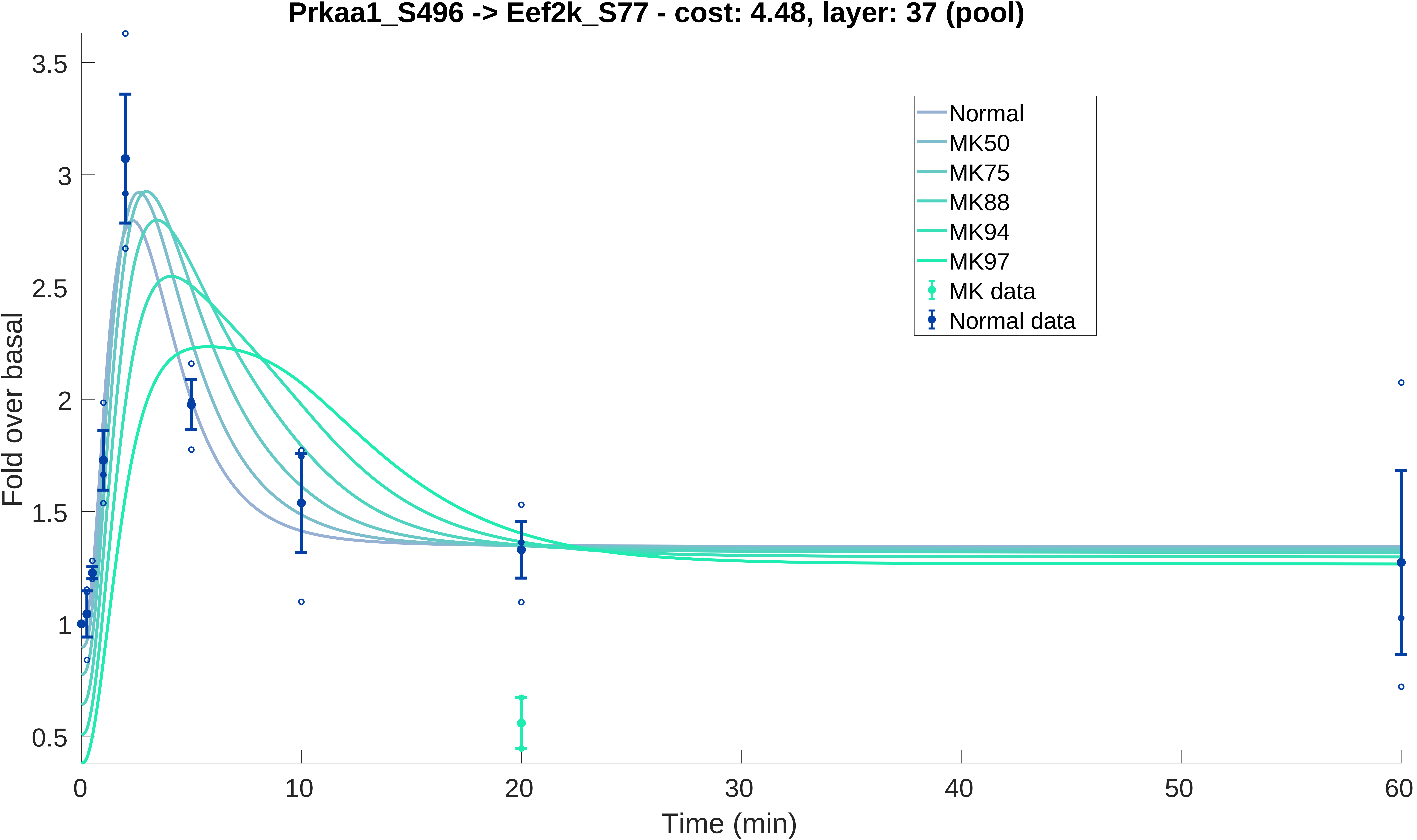

Prkaa1_S496 -> Eef2k_S77 - cost: 4.48, layer: 37 (pool)
Fold over basal
Time (min)
0.5
1
1.5
2
2.5
3
3.5
0
10
20
30
40
50
60
Normal
MK50
MK75
MK88
MK94
MK97
MK data
Normal data

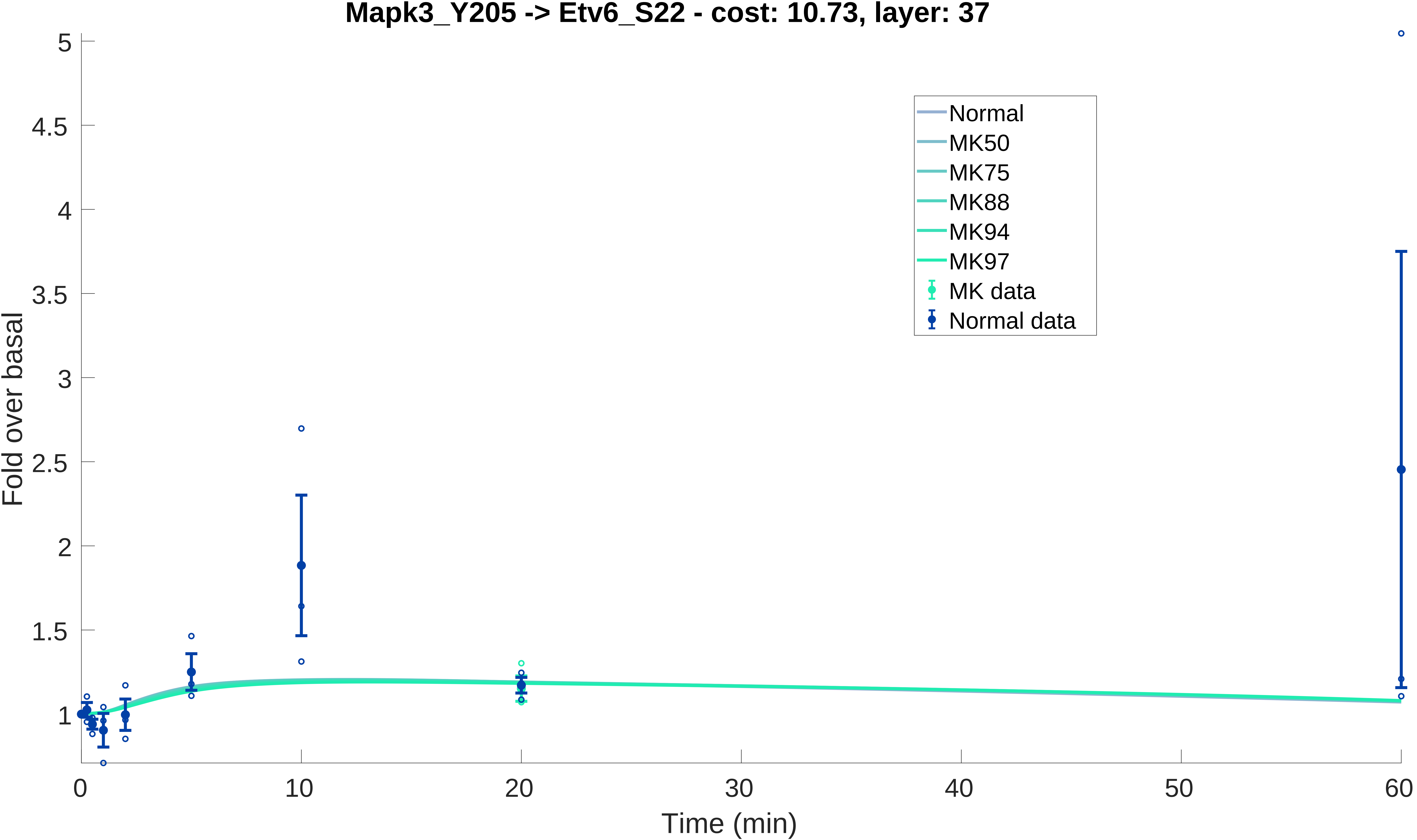

Mapk3_Y205 -> Etv6_S22 - cost: 10.73, layer: 37
Fold over basal
Time (min)
5
4.5
4
3.5
3
2.5
2
1.5
1
0
10
20
30
40
50
60
Normal
MK50
MK75
MK88
MK94
MK97
MK data
Normal data

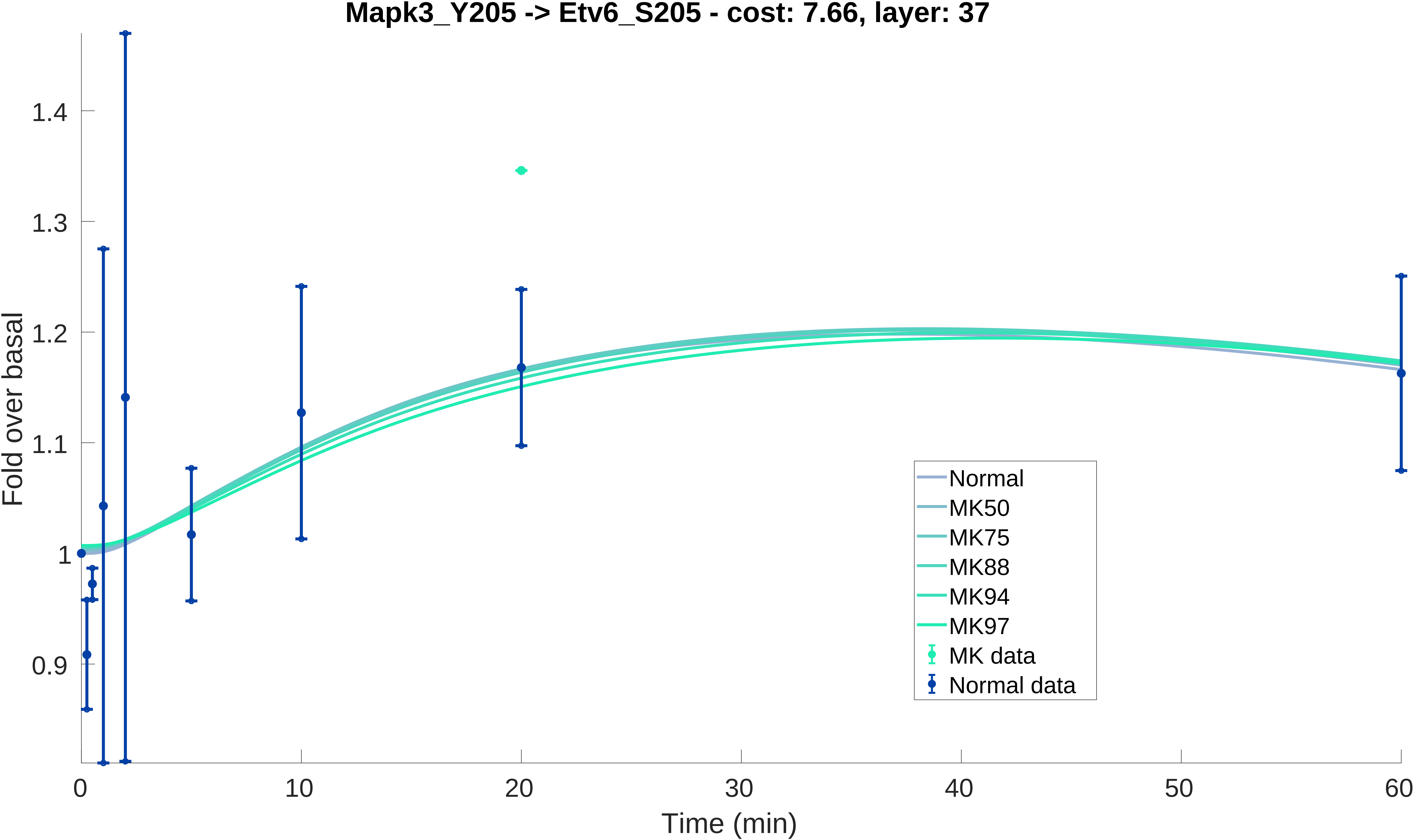

Mapk3_Y205 -> Etv6_S205 - cost: 7.66, layer: 37
Fold over basal
1.4
1.3
1.2
1.1
1
0.9
Time (min)
0
10
20
30
40
50
60
Normal
MK50
MK75
MK88
MK94
MK97
MK data
Normal data

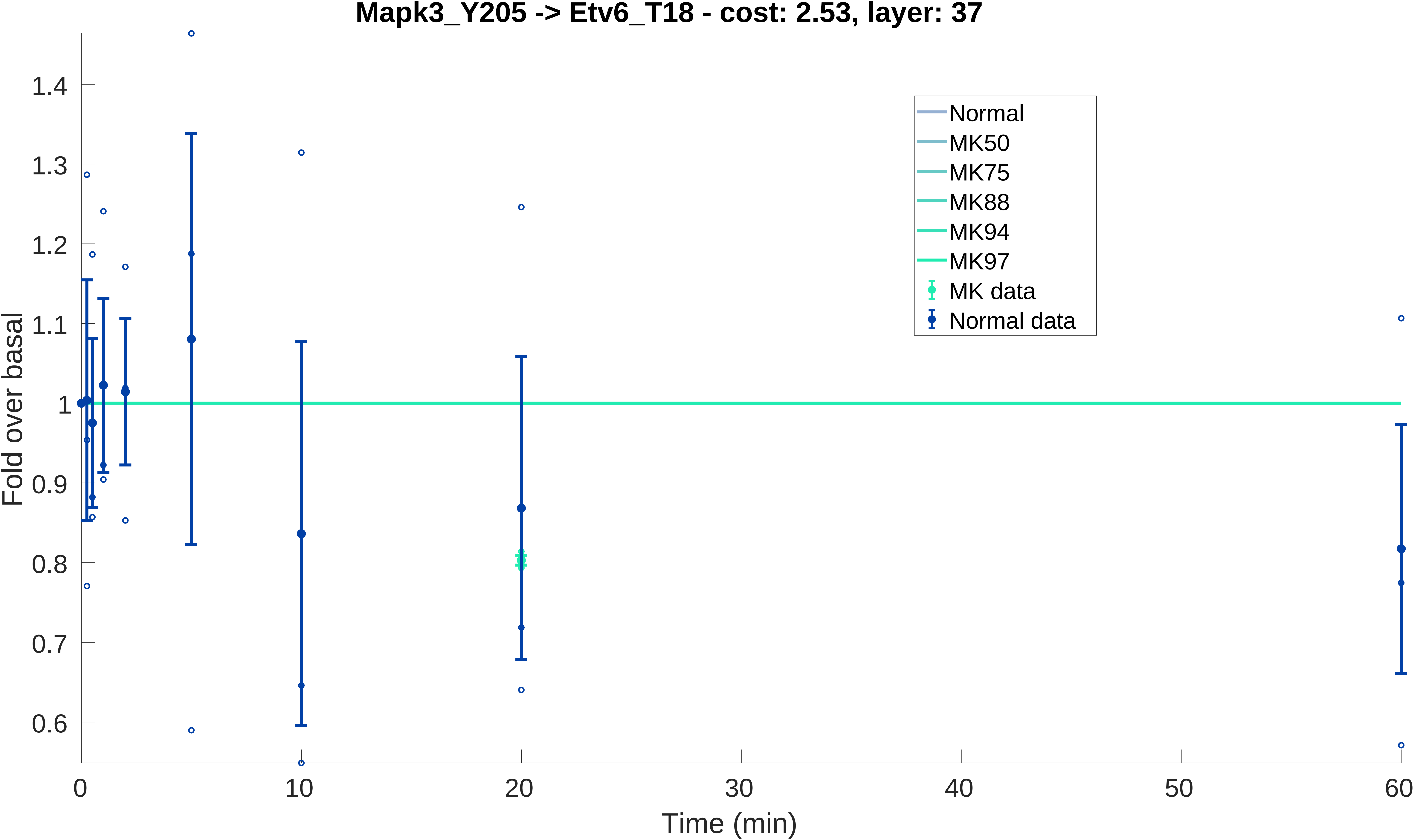

Mapk3_Y205 -> Etv6_T18 - cost: 2.53, layer: 37
Fold over basal
Time (min)
Normal
MK50
MK75
MK88
MK94
MK97
MK data
Normal data
1.4
1.3
1.2
1.1
1
0.9
0.8
0.7
0.6
0
10
20
30
40
50
60

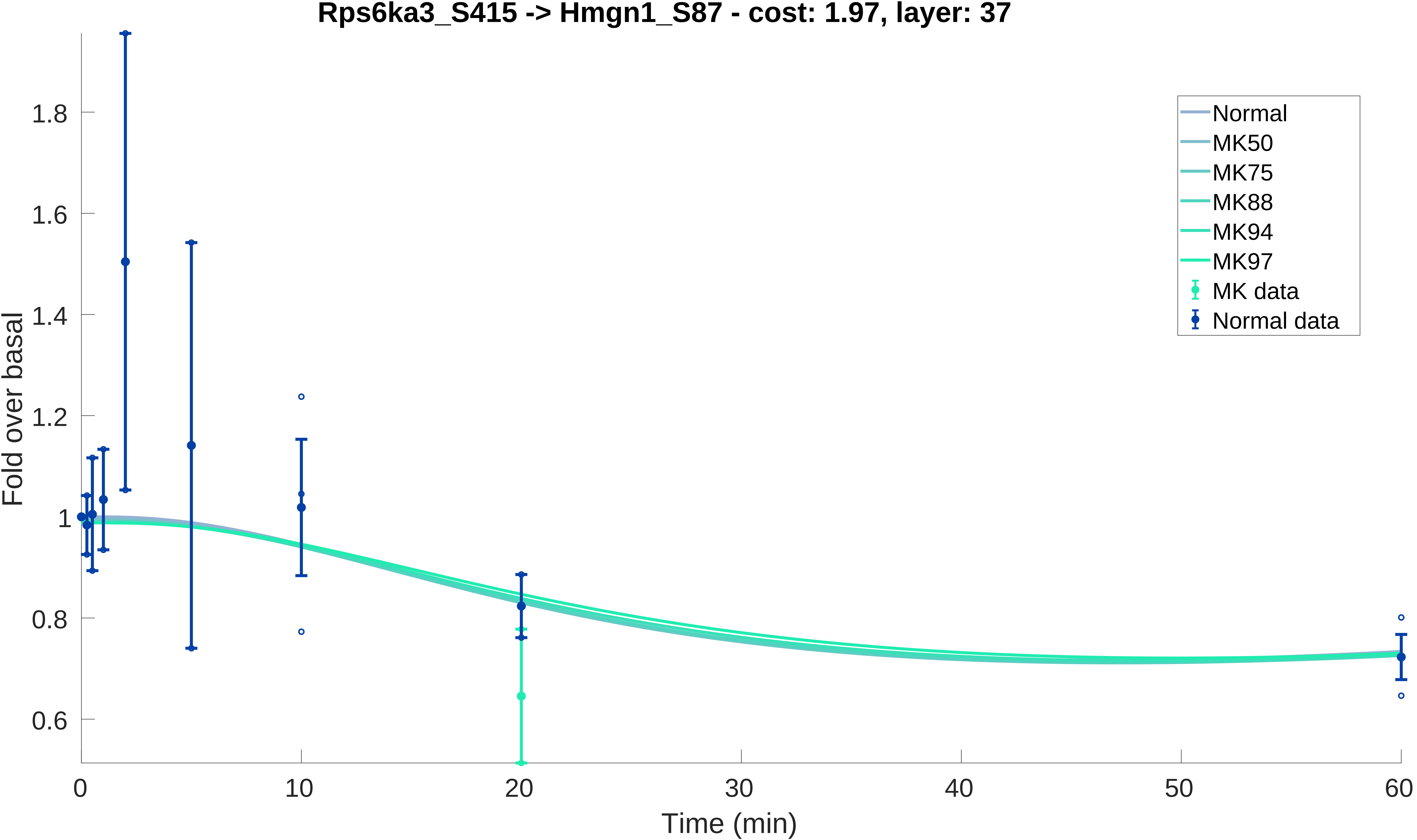

Rps6ka3_S415 -> Hmgn1_S87 - cost: 1.97, layer: 37
Fold over basal
Time (min)
0.6
0.8
1
1.2
1.4
1.6
1.8
0
10
20
30
40
50
60
Normal
MK50
MK75
MK88
MK94
MK97
MK data
Normal data

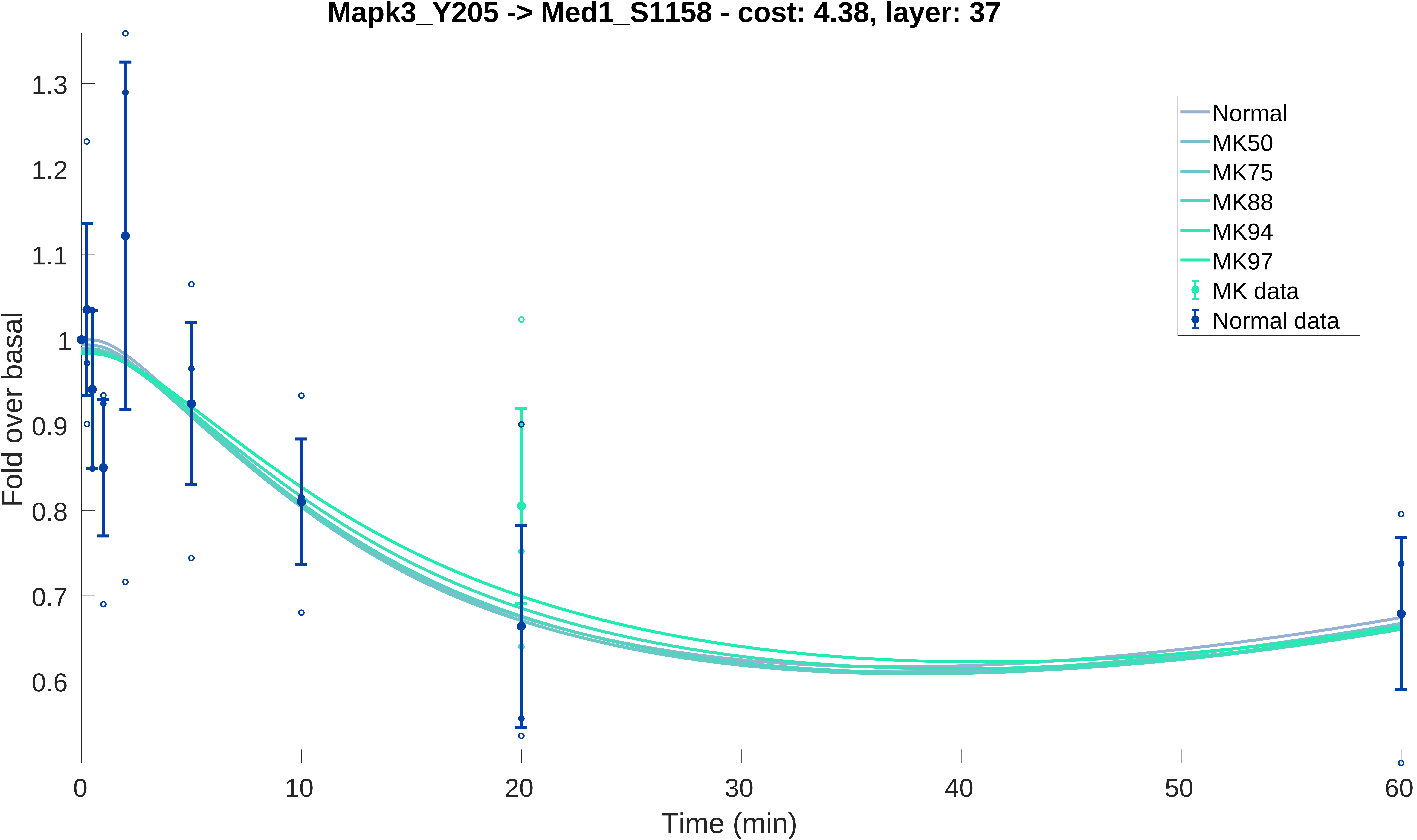

Mapk3_Y205 -> Med1_S1158 - cost: 4.38, layer: 37
Fold over basal
Time (min)
1.3
1.2
1.1
1
0.9
0.8
0.7
0.6
0
10
20
30
40
50
60
Normal
MK50
MK75
MK88
MK94
MK97
MK data
Normal data

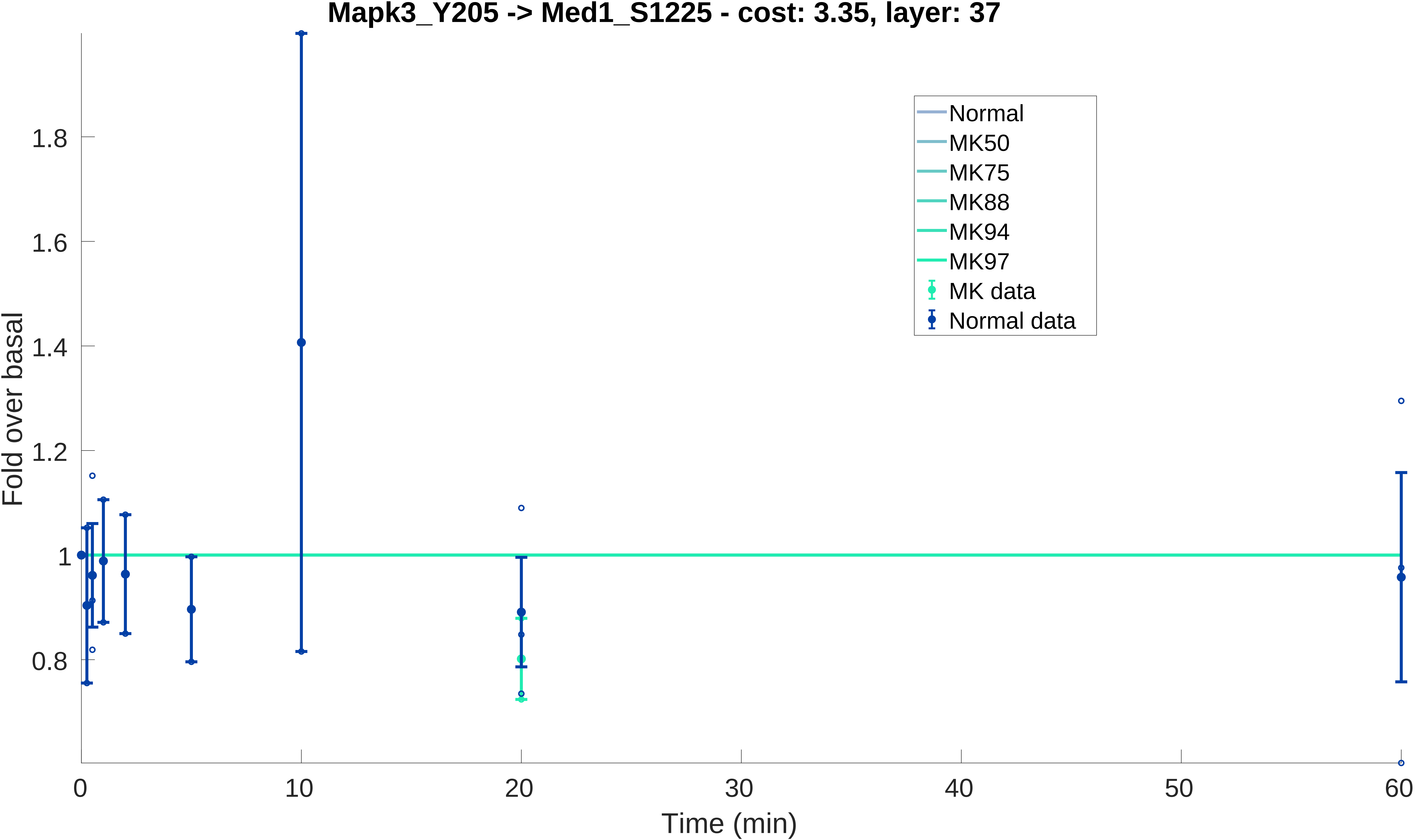

Mapk3_Y205 -> Med1_S1225 - cost: 3.35, layer: 37
Fold over basal
Time (min)
0.8
1
1.2
1.4
1.6
1.8
0
10
20
30
40
50
60
Normal
MK50
MK75
MK88
MK94
MK97
MK data
Normal data

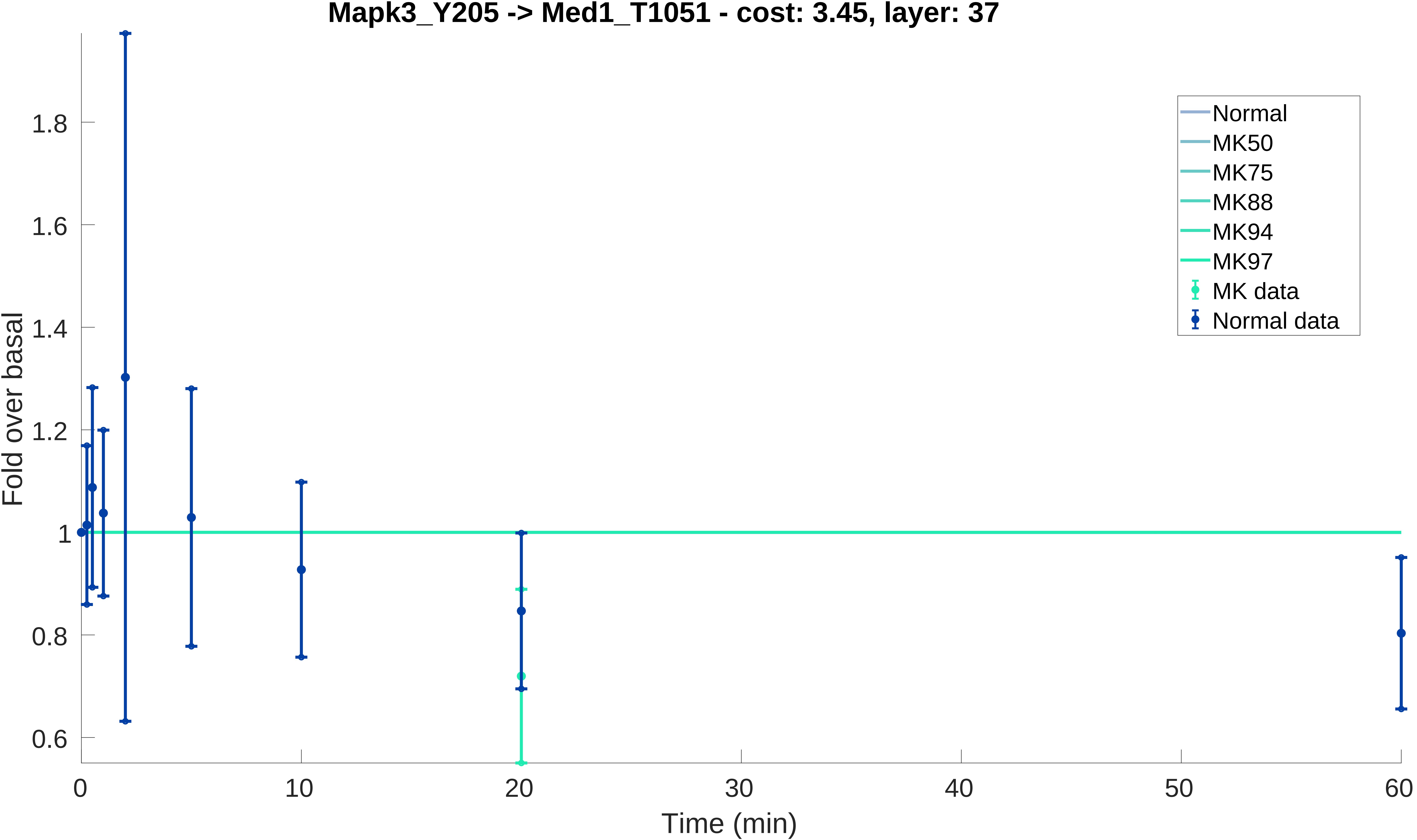

Mapk3_Y205 -> Med1_T1051 - cost: 3.45, layer: 37
Fold over basal
1.8
1.6
1.4
1.2
1
0.8
0.6
0
10
20
30
40
50
60
Time (min)
Normal
MK50
MK75
MK88
MK94
MK97
MK data
Normal data

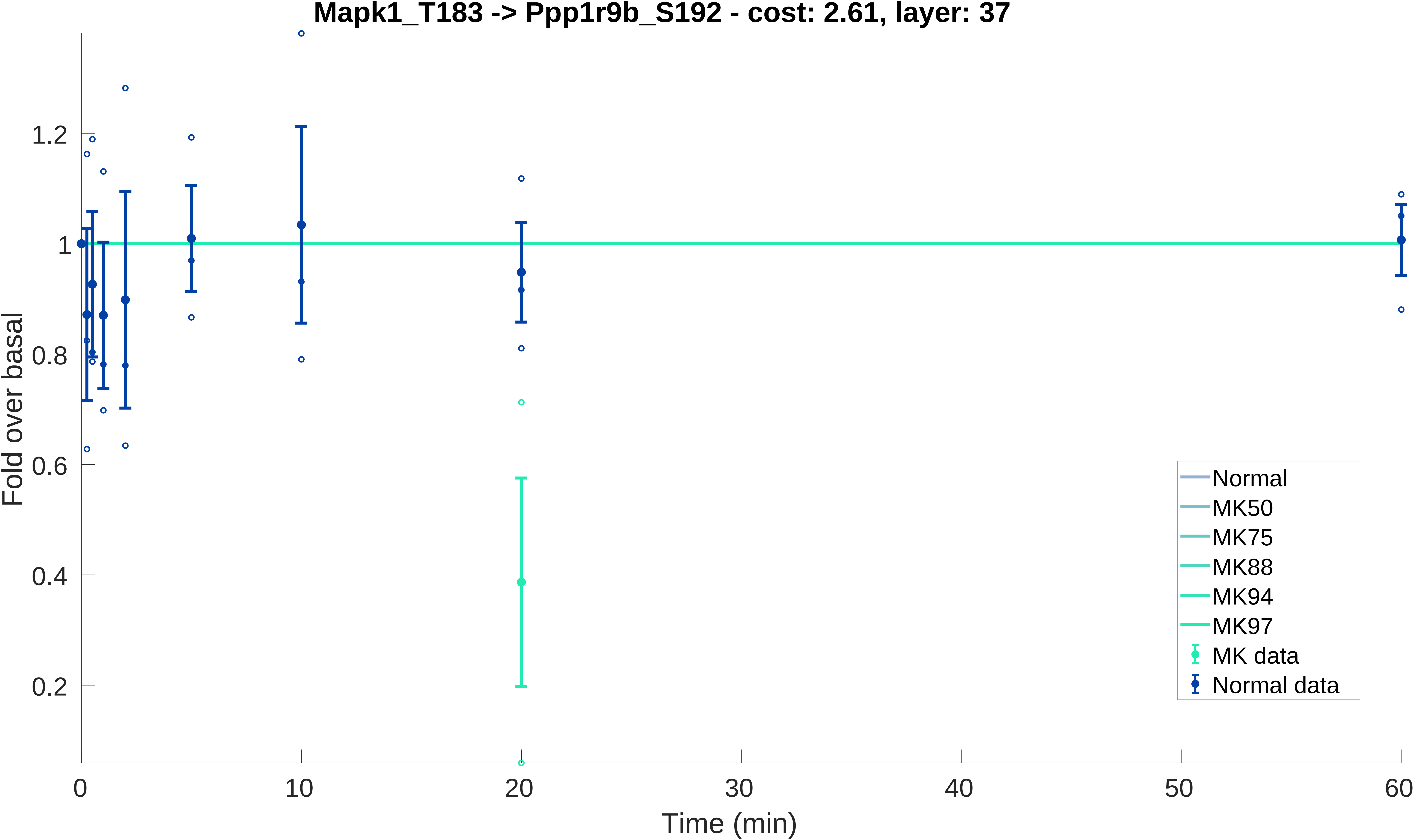

Mapk1_T183 -> Ppp1r9b_S192 - cost: 2.61, layer: 37
Fold over basal
1.2
1
0.8
0.6
0.4
0.2
0
10
20
30
40
50
60
Time (min)
Normal
MK50
MK75
MK88
MK94
MK97
MK data
Normal data

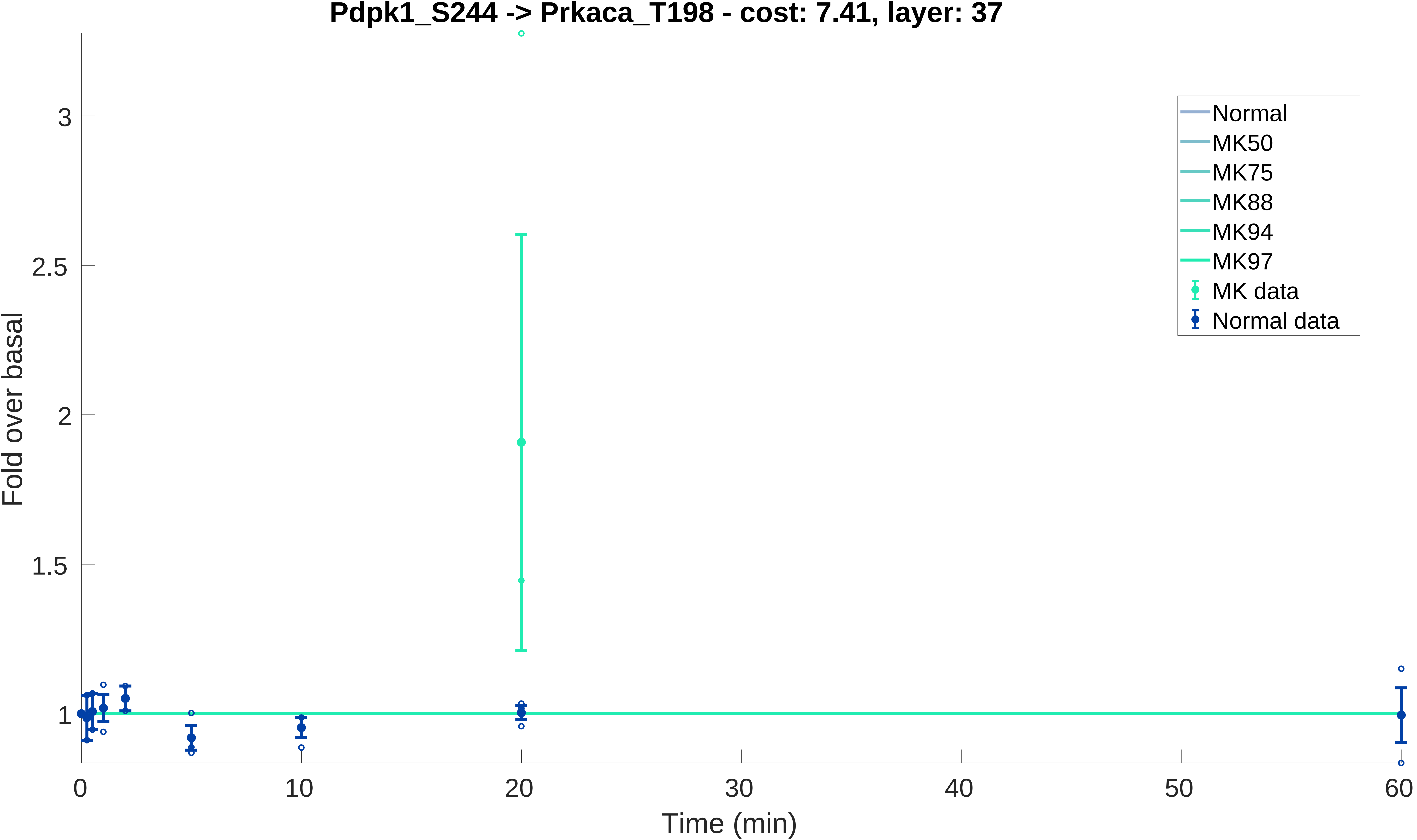
Pdpk1_S244 -> Prkaca_T198 - cost: 7.41, layer: 37
Fold over basal
3
2.5
2
1.5
1
Time (min)
0
10
20
30
40
50
60
Normal
MK50
MK75
MK88
MK94
MK97
MK data
Normal data

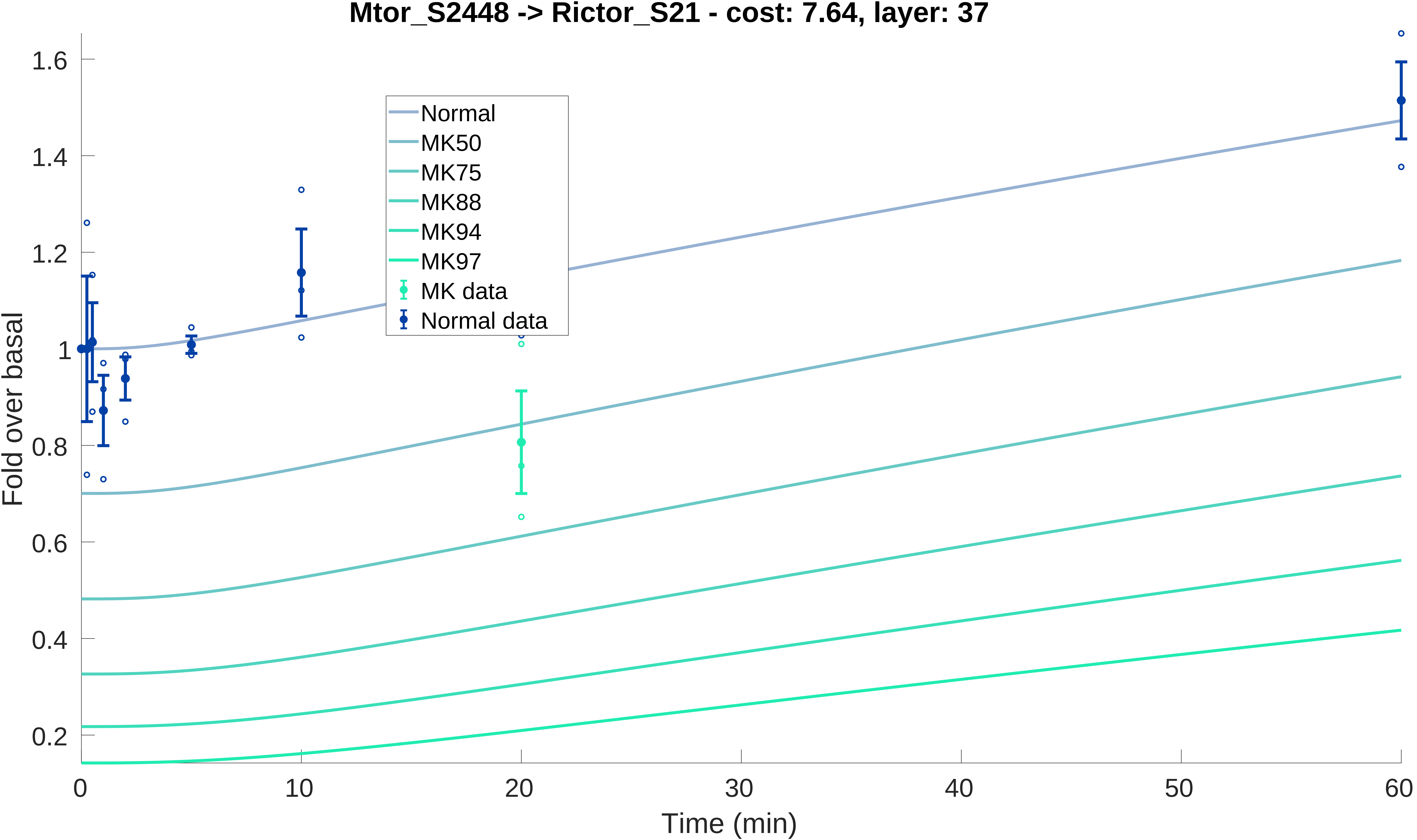
Mtor_S2448 -> Rictor_S21 - cost: 7.64, layer: 37
Normal
MK50
MK75
MK88
MK94
MK97
MK data
Normal data
Fold over basal
1.6
1.4
1.2
1
0.8
0.6
0.4
0.2
0
10
20
30
40
50
60
Time (min)

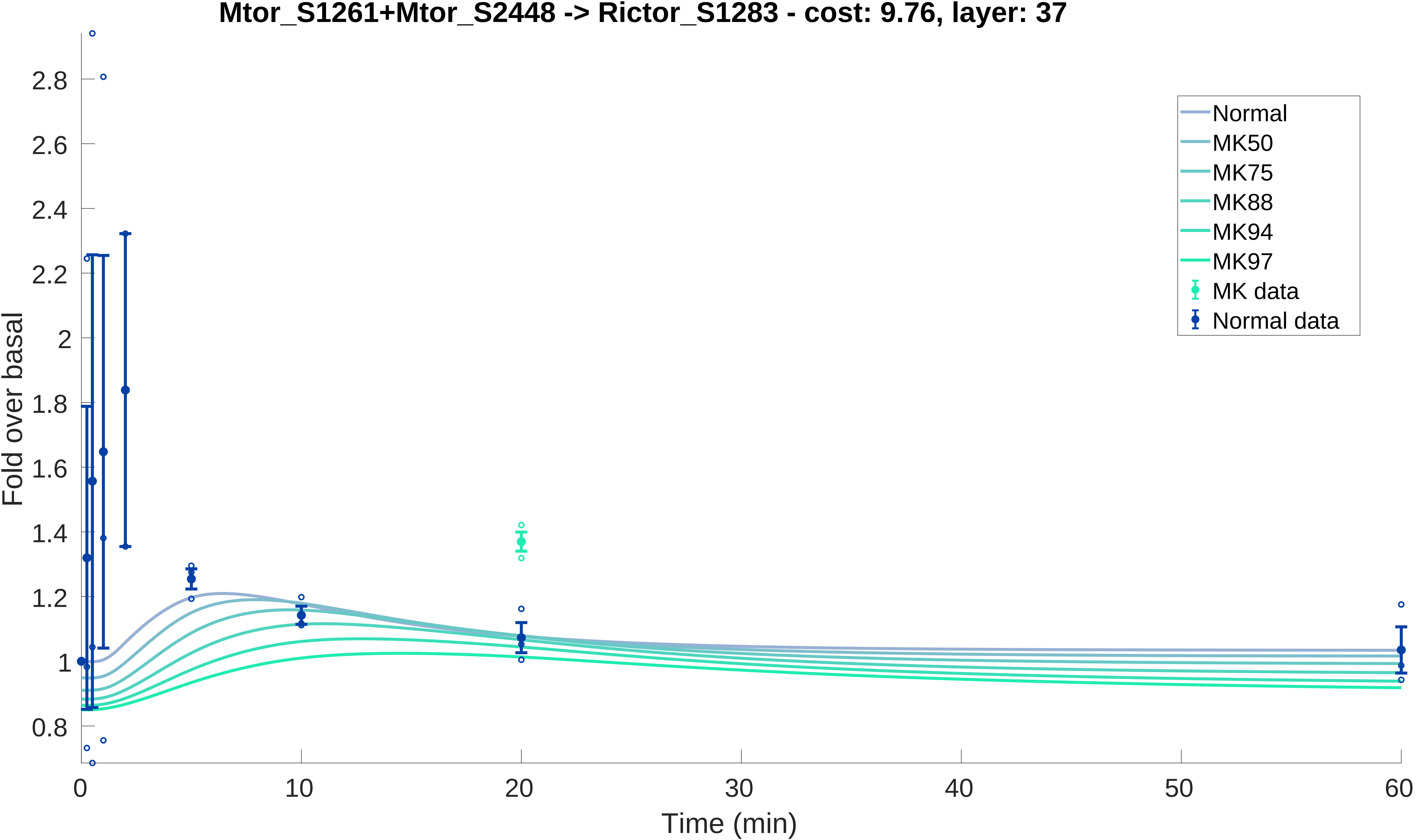

Mtor_S1261+Mtor_S2448 -> Rictor_S1283 - cost: 9.76, layer: 37
Fold over basal
Time (min)
0.8
1
1.2
1.4
1.6
1.8
2
2.2
2.4
2.6
2.8
0
10
20
30
40
50
60
Normal
MK50
MK75
MK88
MK94
MK97
MK data
Normal data

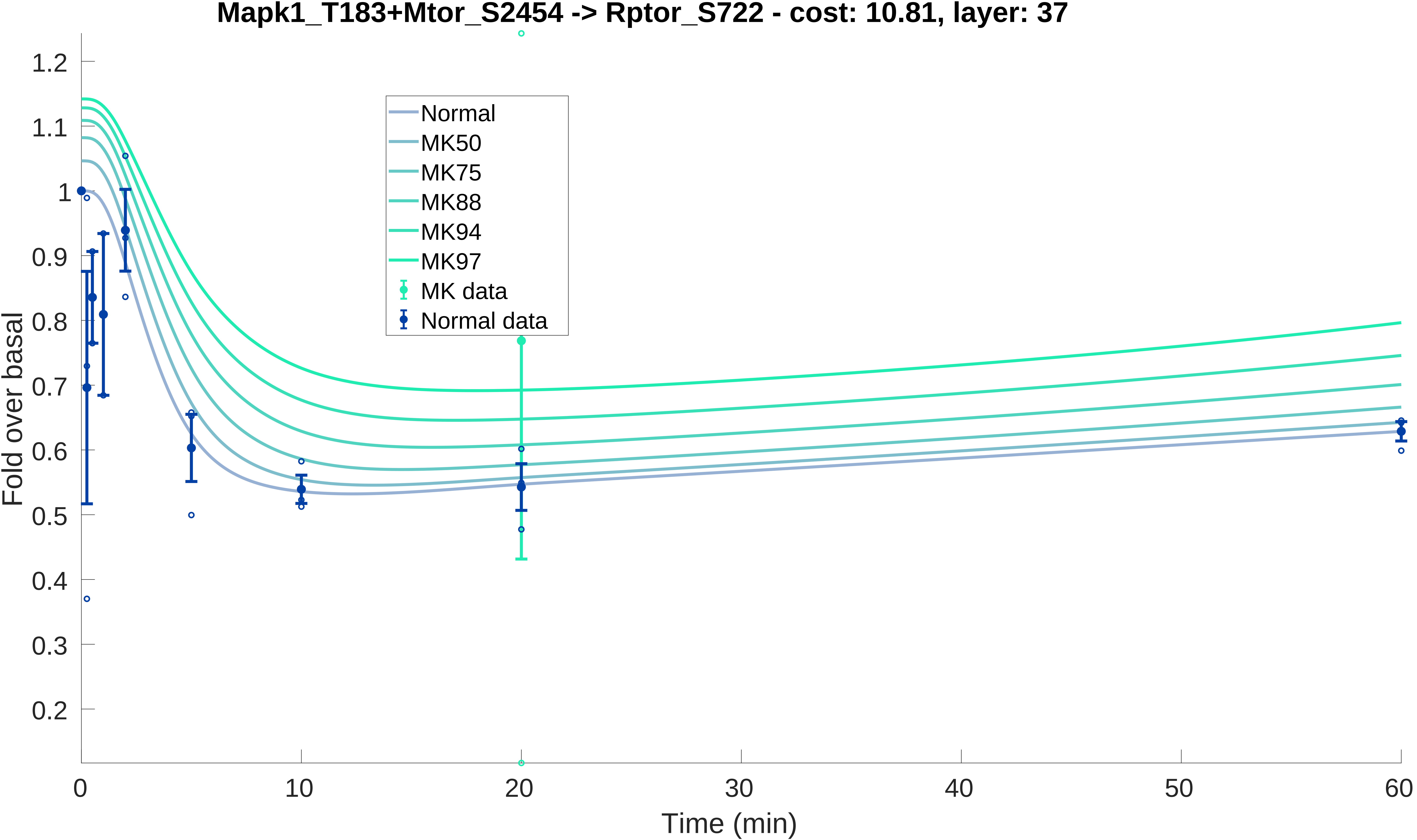

Mapk1_T183+Mtor_S2454 -> Rptor_S722 - cost: 10.81, layer: 37
Fold over basal
Time (min)
1.2
1.1
1
0.9
0.8
0.7
0.6
0.5
0.4
0.3
0.2
0
10
20
30
40
50
60
Normal
MK50
MK75
MK88
MK94
MK97
MK data
Normal data

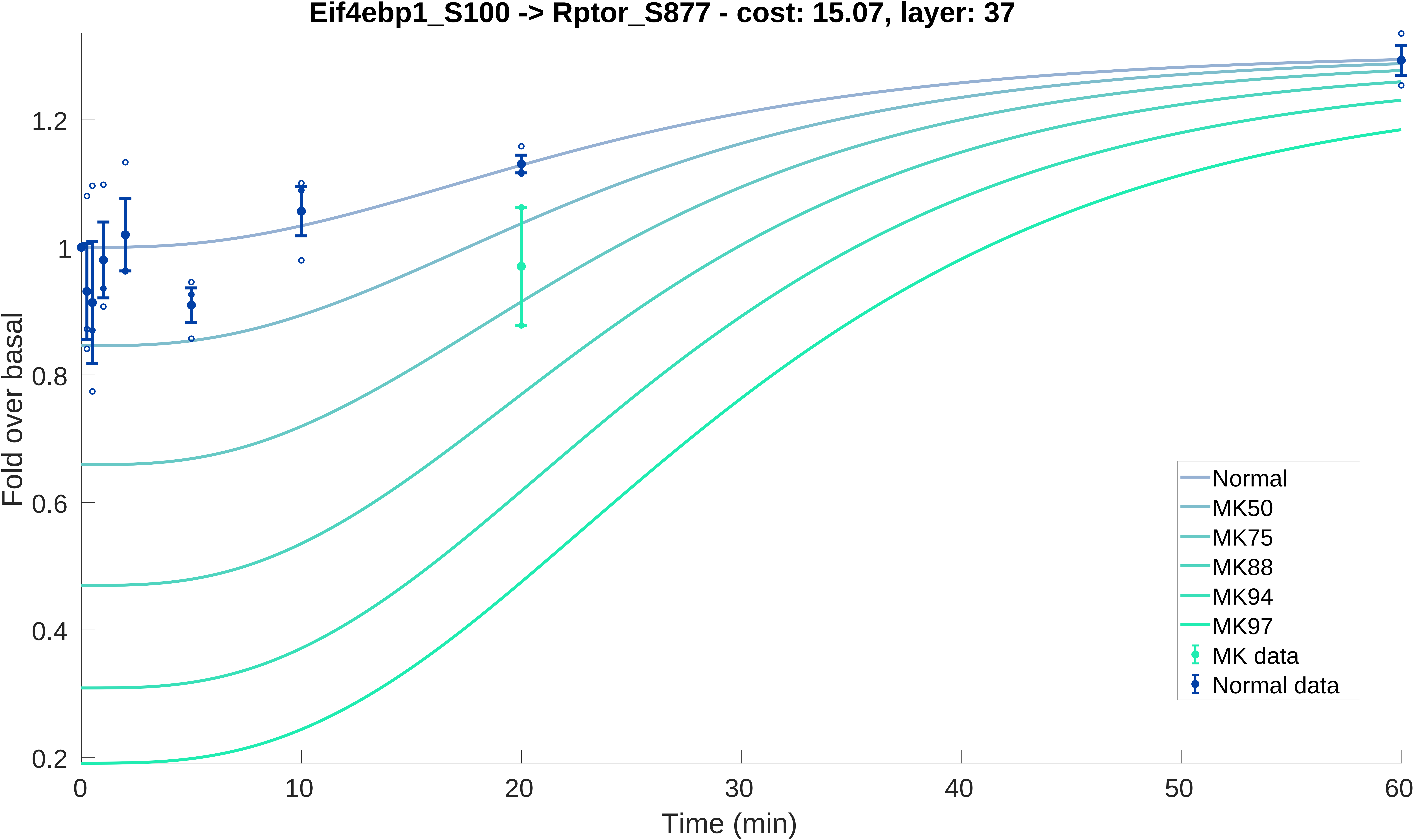
Eif4ebp1_S100 -> Rptor_S877 - cost: 15.07, layer: 37
Fold over basal
1.2
1
0.8
0.6
0.4
0.2
0
10
20
30
40
50
60
Time (min)
Normal
MK50
MK75
MK88
MK94
MK97
MK data
Normal data

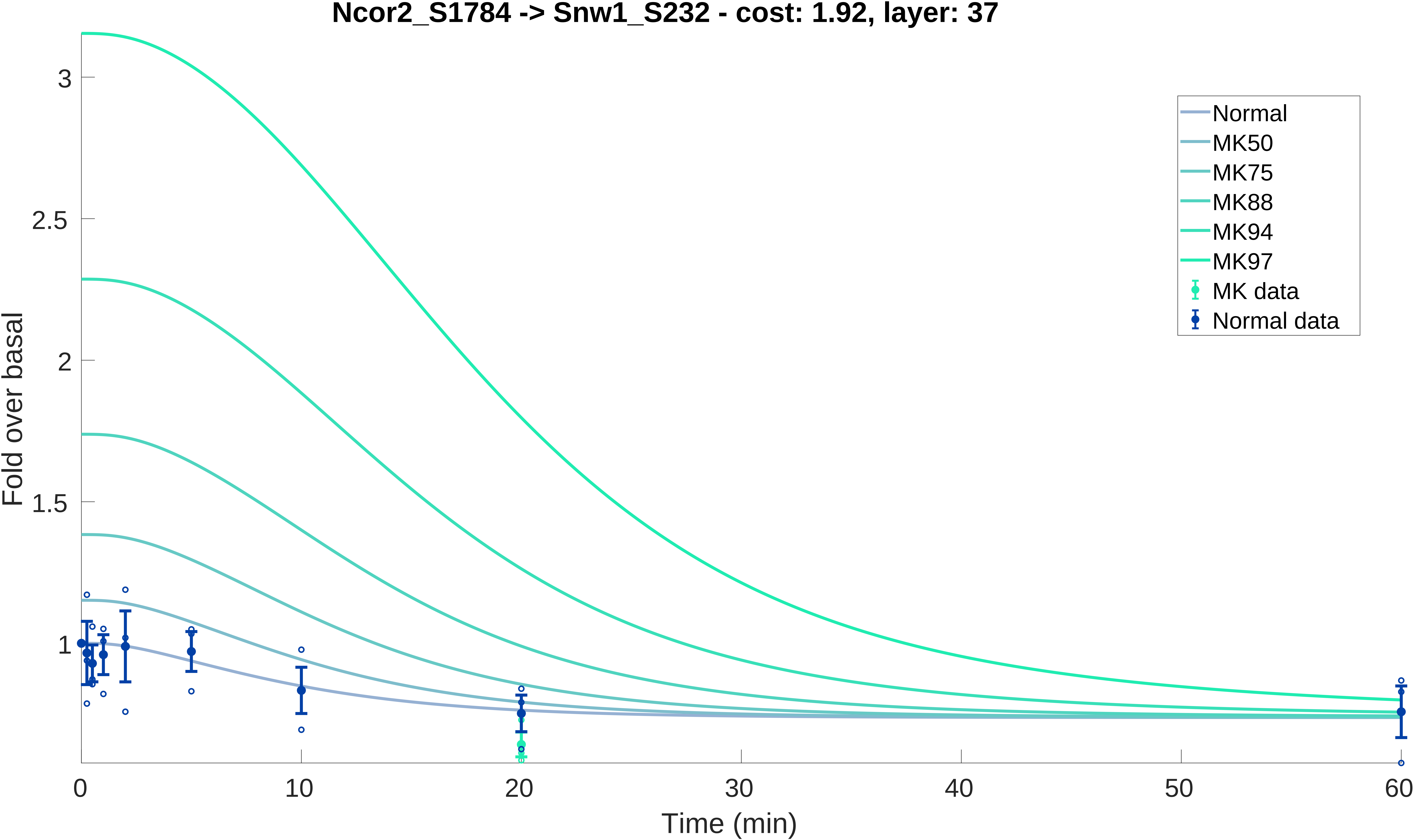
Ncor2_S1784 -> Snw1_S232 - cost: 1.92, layer: 37
Fold over basal
3
2.5
2
1.5
1
Time (min)
0
10
20
30
40
50
60
Normal
MK50
MK75
MK88
MK94
MK97
MK data
Normal data

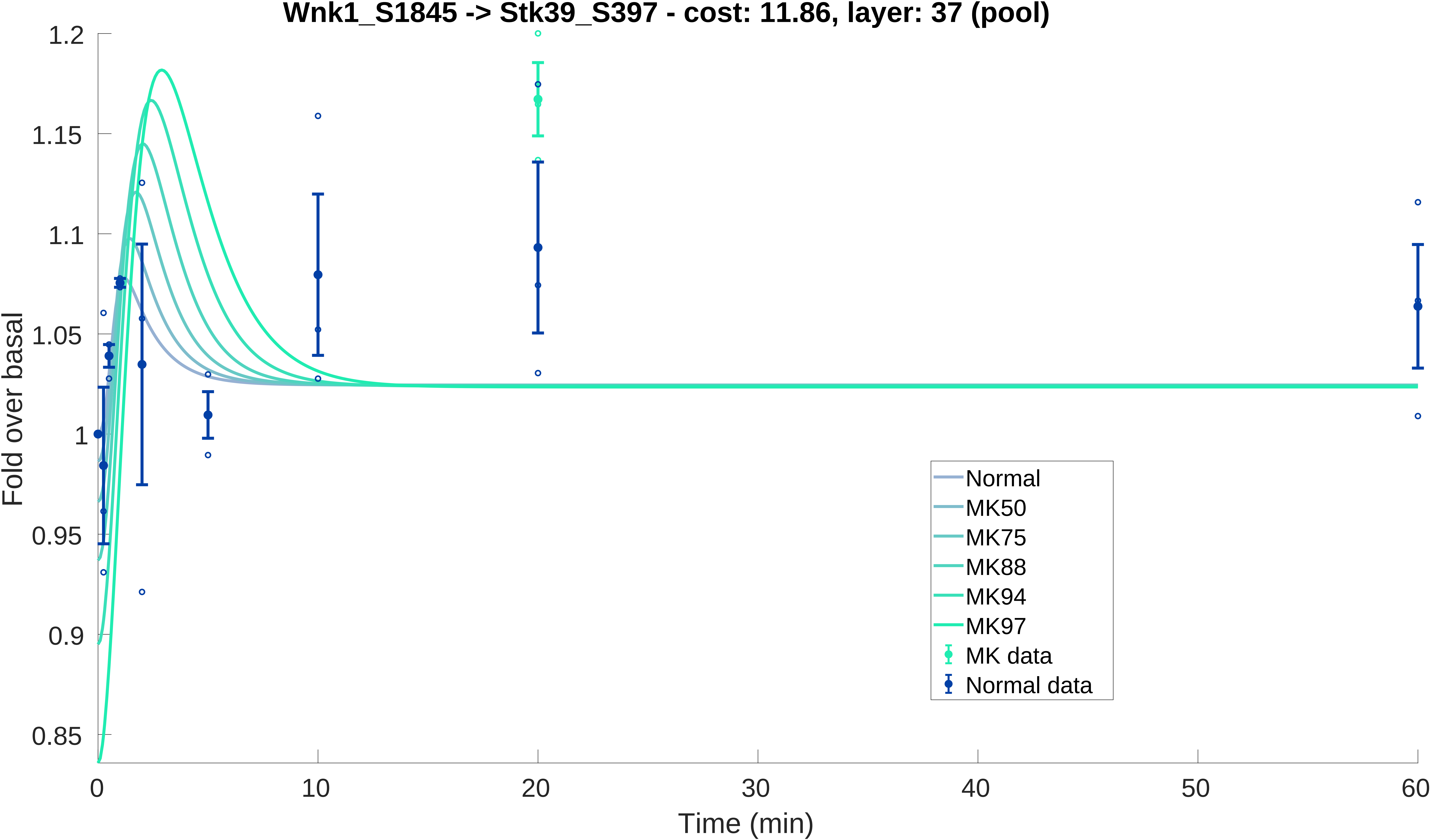

Wnk1_S1845 -> Stk39_S397 - cost: 11.86, layer: 37 (pool)
Fold over basal
Time (min)
1.2
1.15
1.1
1.05
1
0.95
0.9
0.85
0
10
20
30
40
50
60
Normal
MK50
MK75
MK88
MK94
MK97
MK data
Normal data

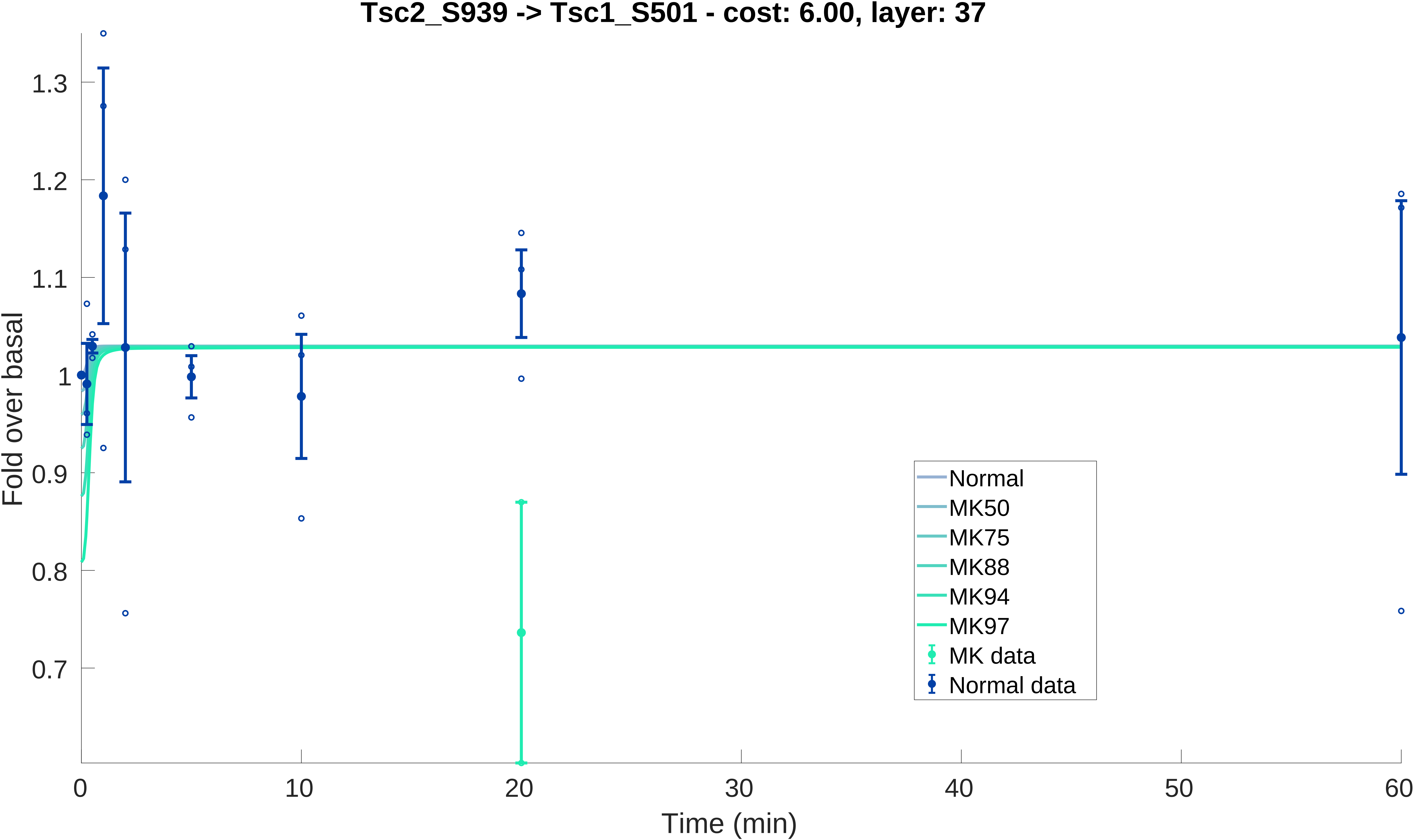

Tsc2_S939 -> Tsc1_S501 - cost: 6.00, layer: 37
Fold over basal
Time (min)
1.3
1.2
1.1
1
0.9
0.8
0.7
0
10
20
30
40
50
60
Normal
MK50
MK75
MK88
MK94
MK97
MK data
Normal data

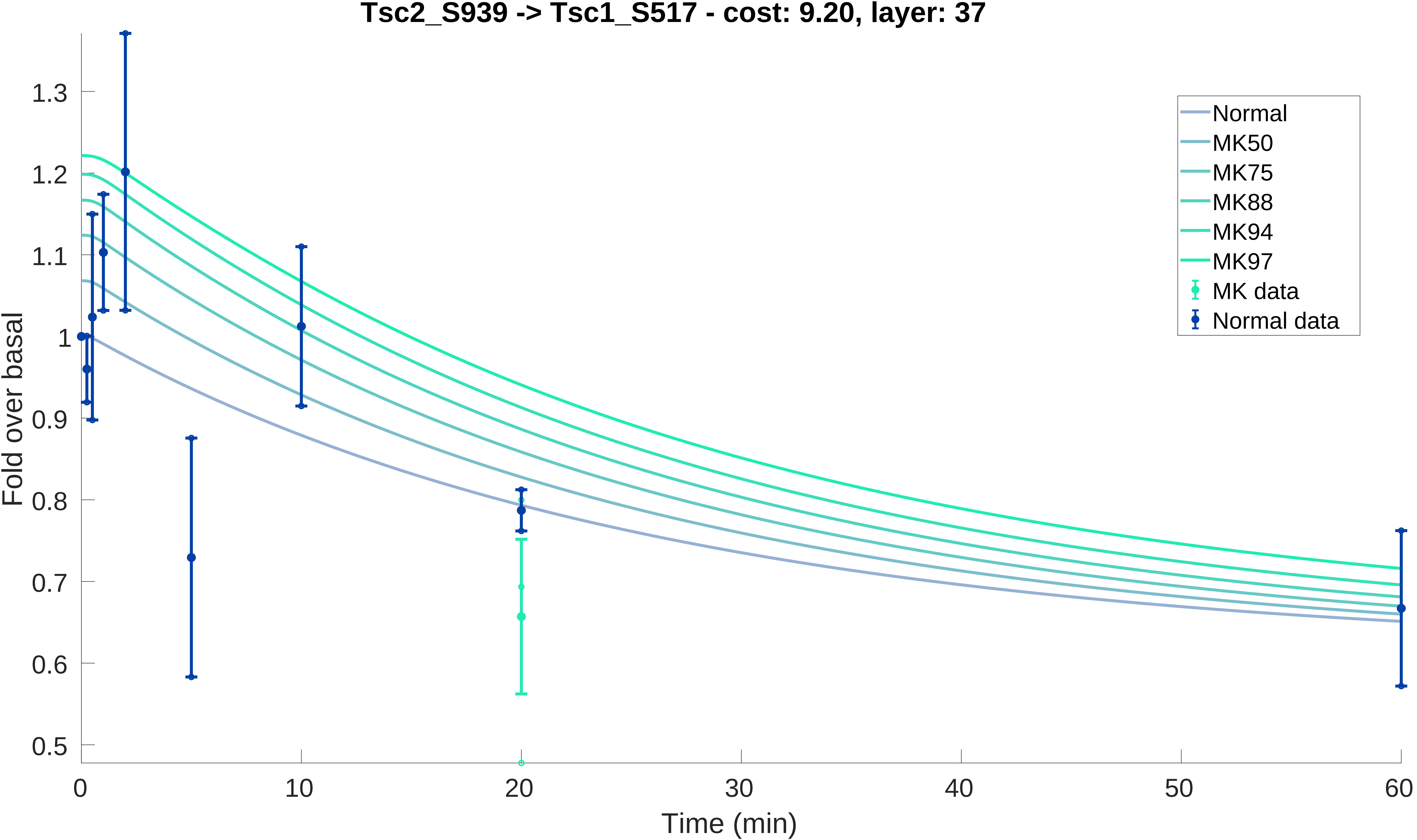

Tsc2_S939 -> Tsc1_S517 - cost: 9.20, layer: 37
Fold over basal
Time (min)
0.5
0.6
0.7
0.8
0.9
1
1.1
1.2
1.3
0
10
20
30
40
50
60
Normal
MK50
MK75
MK88
MK94
MK97
MK data
Normal data

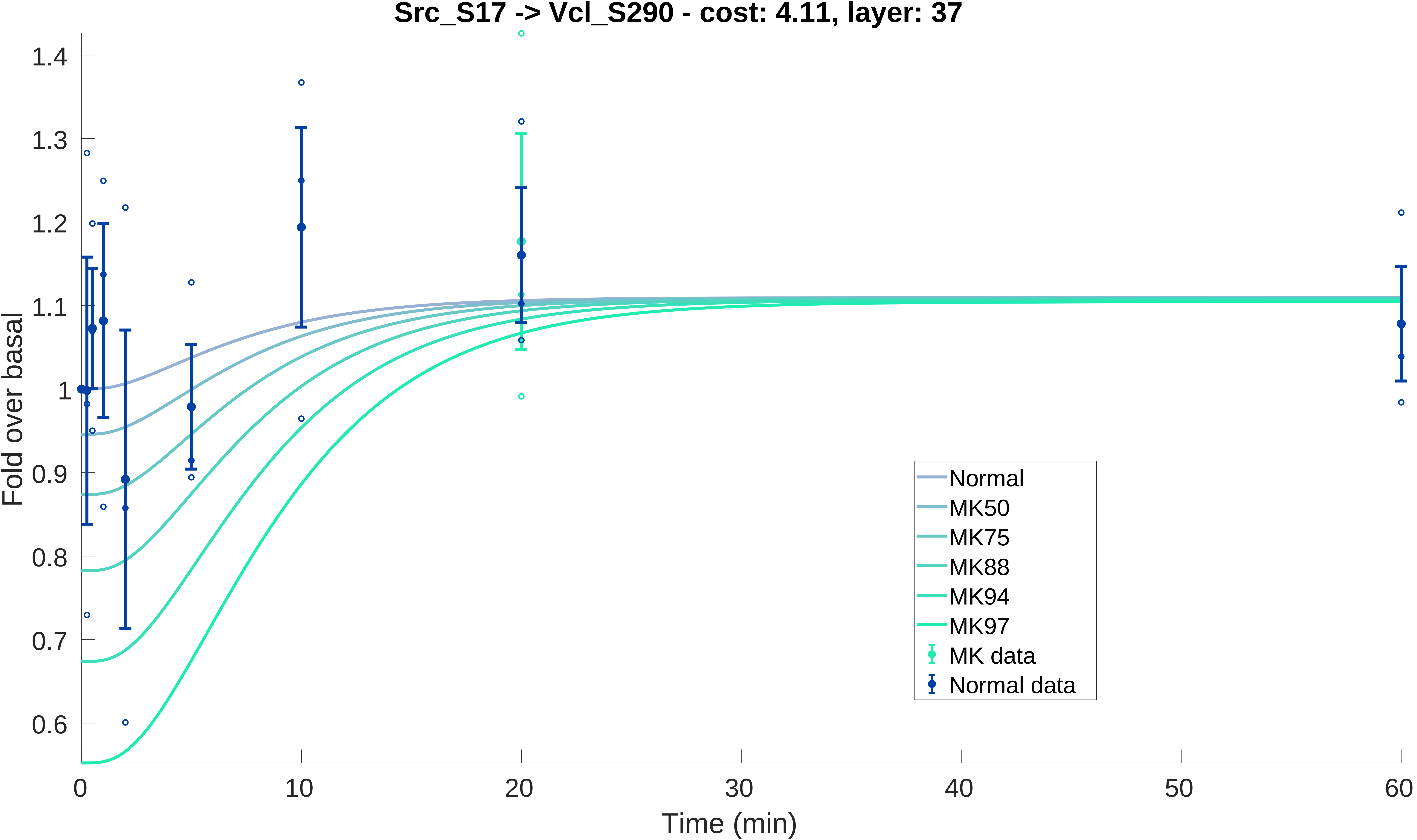

Src_S17 -> Vcl_S290 - cost: 4.11, layer: 37
Fold over basal
Time (min)
1.4
1.3
1.2
1.1
1
0.9
0.8
0.7
0.6
0
10
20
30
40
50
60
Normal
MK50
MK75
MK88
MK94
MK97
MK data
Normal data

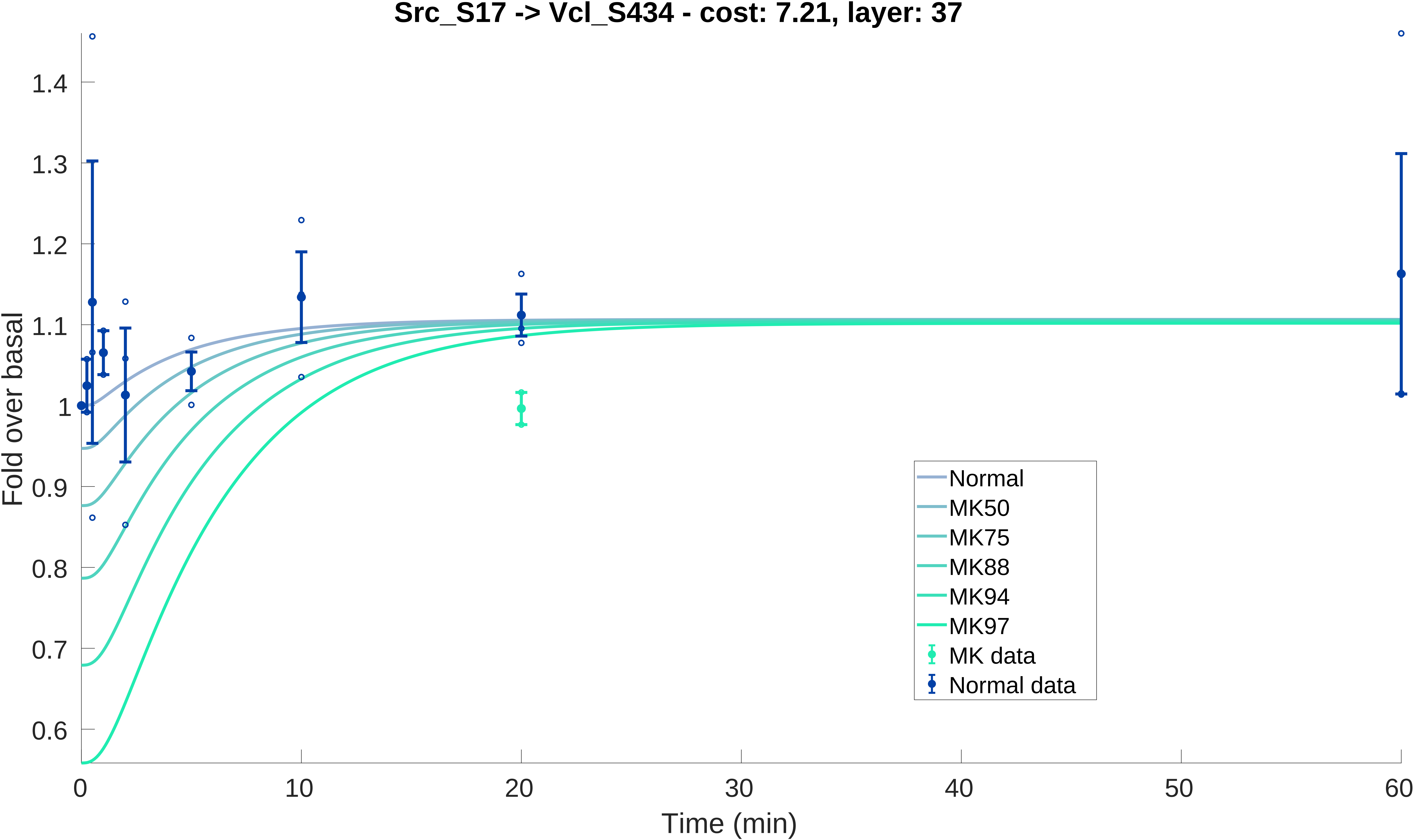

Src_S17 -> Vcl_S434 - cost: 7.21, layer: 37
Fold over basal
1.4
1.3
1.2
1.1
1
0.9
0.8
0.7
0.6
0
10
20
30
40
50
60
Time (min)
Normal
MK50
MK75
MK88
MK94
MK97
MK data
Normal data

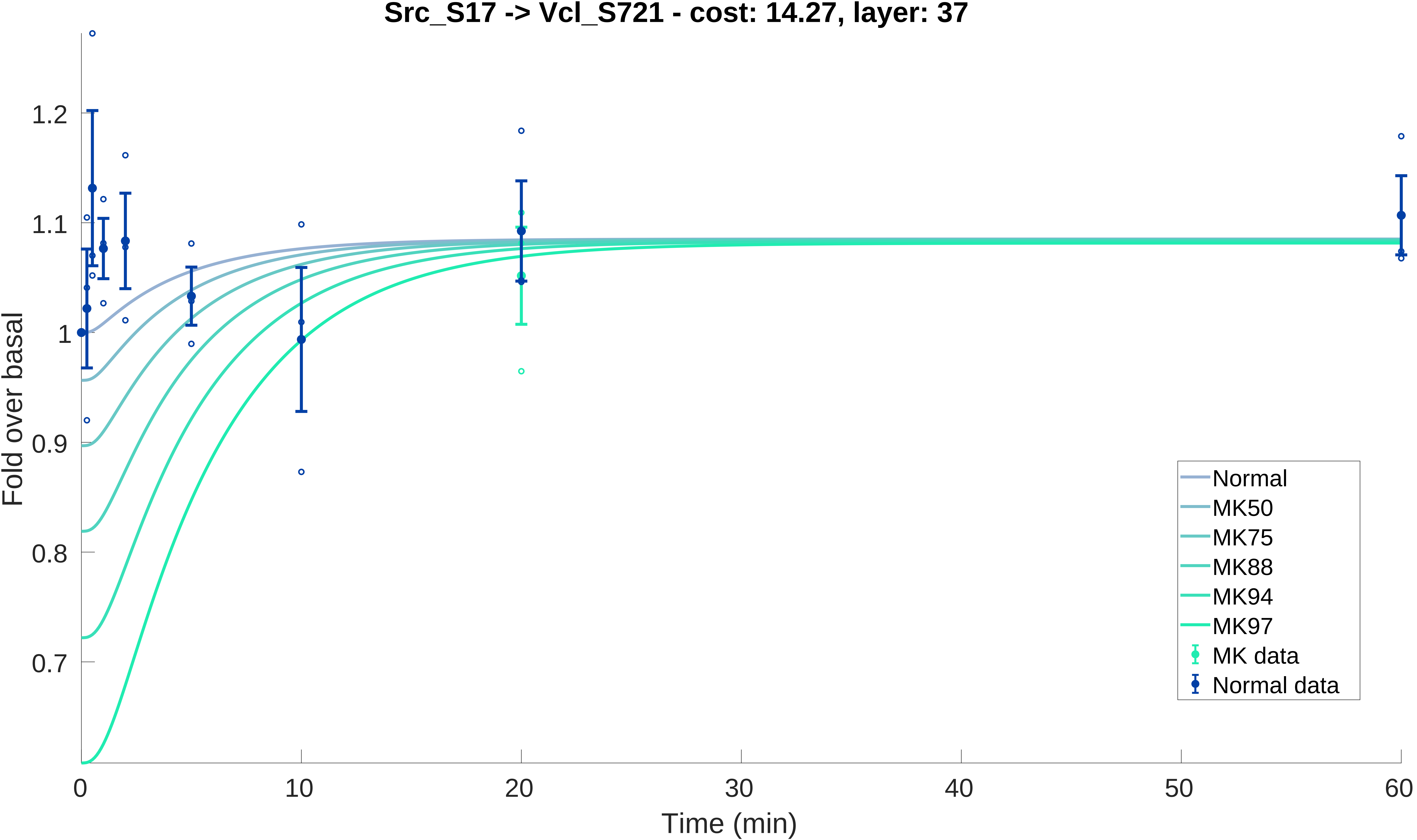
Src_S17 -> Vcl_S721 - cost: 14.27, layer: 37
Fold over basal
Time (min)
1.2
1.1
1
0.9
0.8
0.7
0
10
20
30
40
50
60
Normal
MK50
MK75
MK88
MK94
MK97
MK data
Normal data

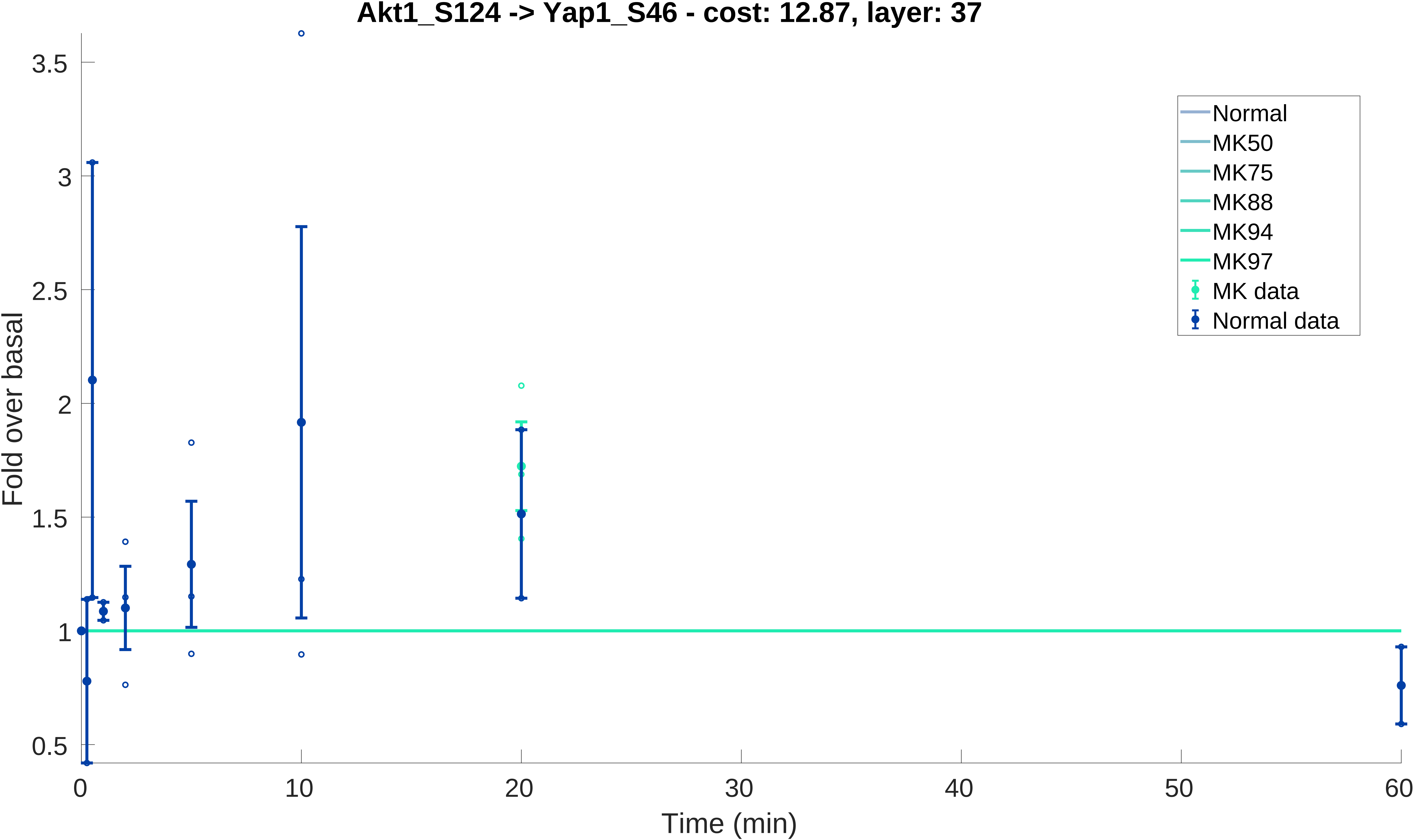

Akt1_S124 -> Yap1_S46 - cost: 12.87, layer: 37
Fold over basal
Time (min)
0.5
1
1.5
2
2.5
3
3.5
0
10
20
30
40
50
60
Normal
MK50
MK75
MK88
MK94
MK97
MK data
Normal data

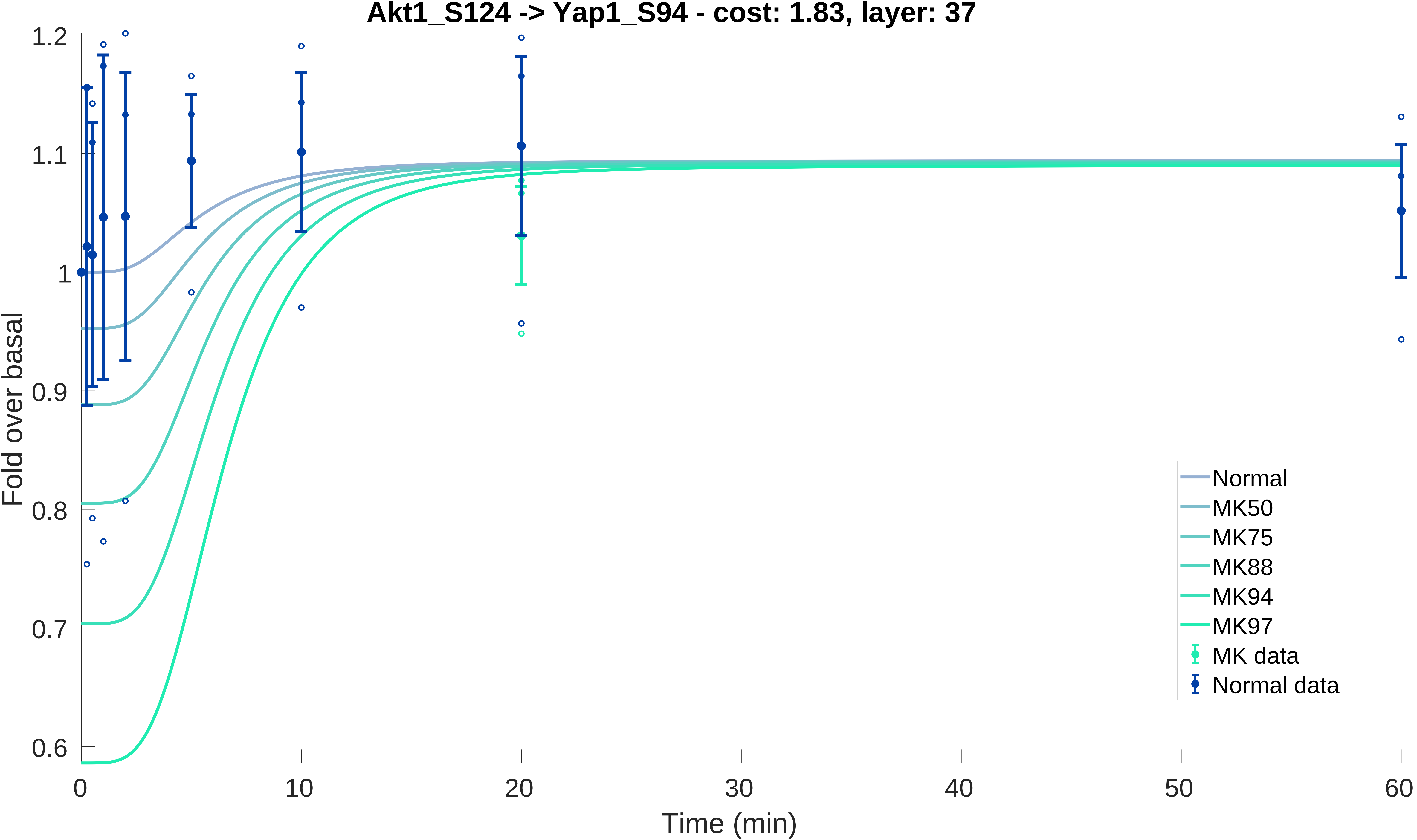

Akt1_S124 -> Yap1_S94 - cost: 1.83, layer: 37
Fold over basal
1.2
1.1
1
0.9
0.8
0.7
0.6
0
10
20
30
40
50
60
Time (min)
Normal
MK50
MK75
MK88
MK94
MK97
MK data
Normal data

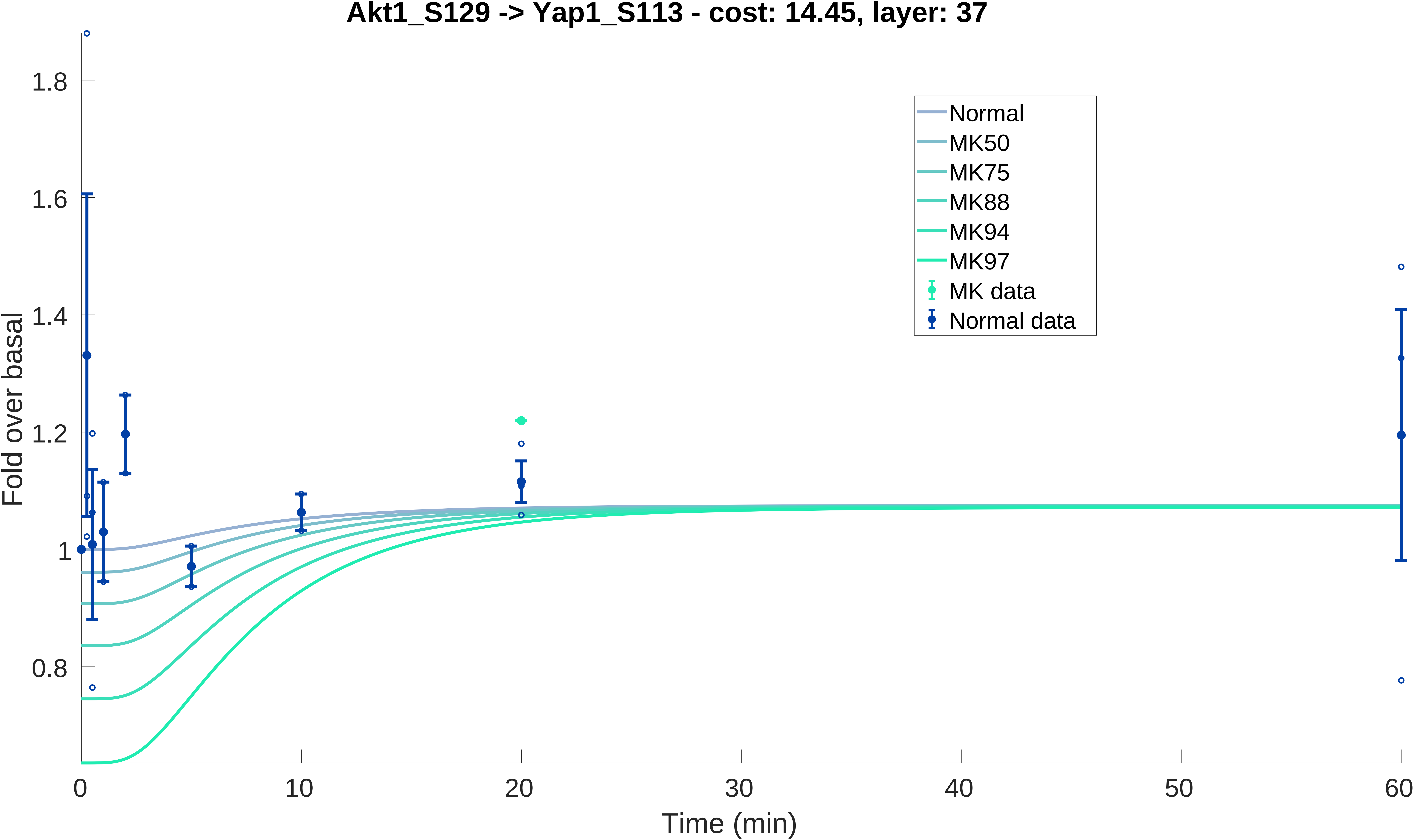

Akt1_S129 -> Yap1_S113 - cost: 14.45, layer: 37
Fold over basal
1.8
1.6
1.4
1.2
1
0.8
0
10
20
30
40
50
60
Time (min)
Normal
MK50
MK75
MK88
MK94
MK97
MK data
Normal data

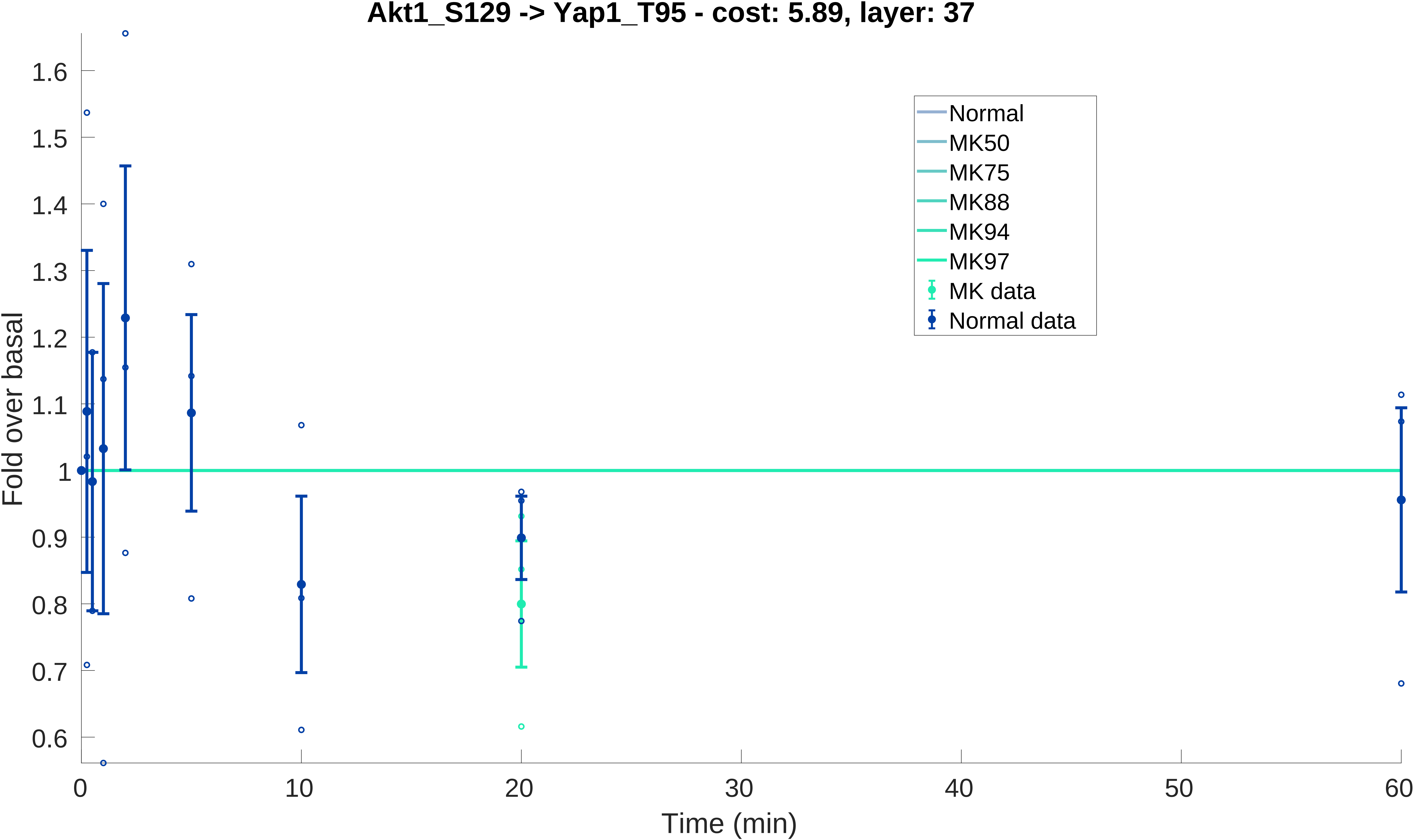

Akt1_S129 -> Yap1_T95 - cost: 5.89, layer: 37
Fold over basal
Time (min)
Normal
MK50
MK75
MK88
MK94
MK97
MK data
Normal data
0.6
0.7
0.8
0.9
1
1.1
1.2
1.3
1.4
1.5
1.6
0
10
20
30
40
50
60

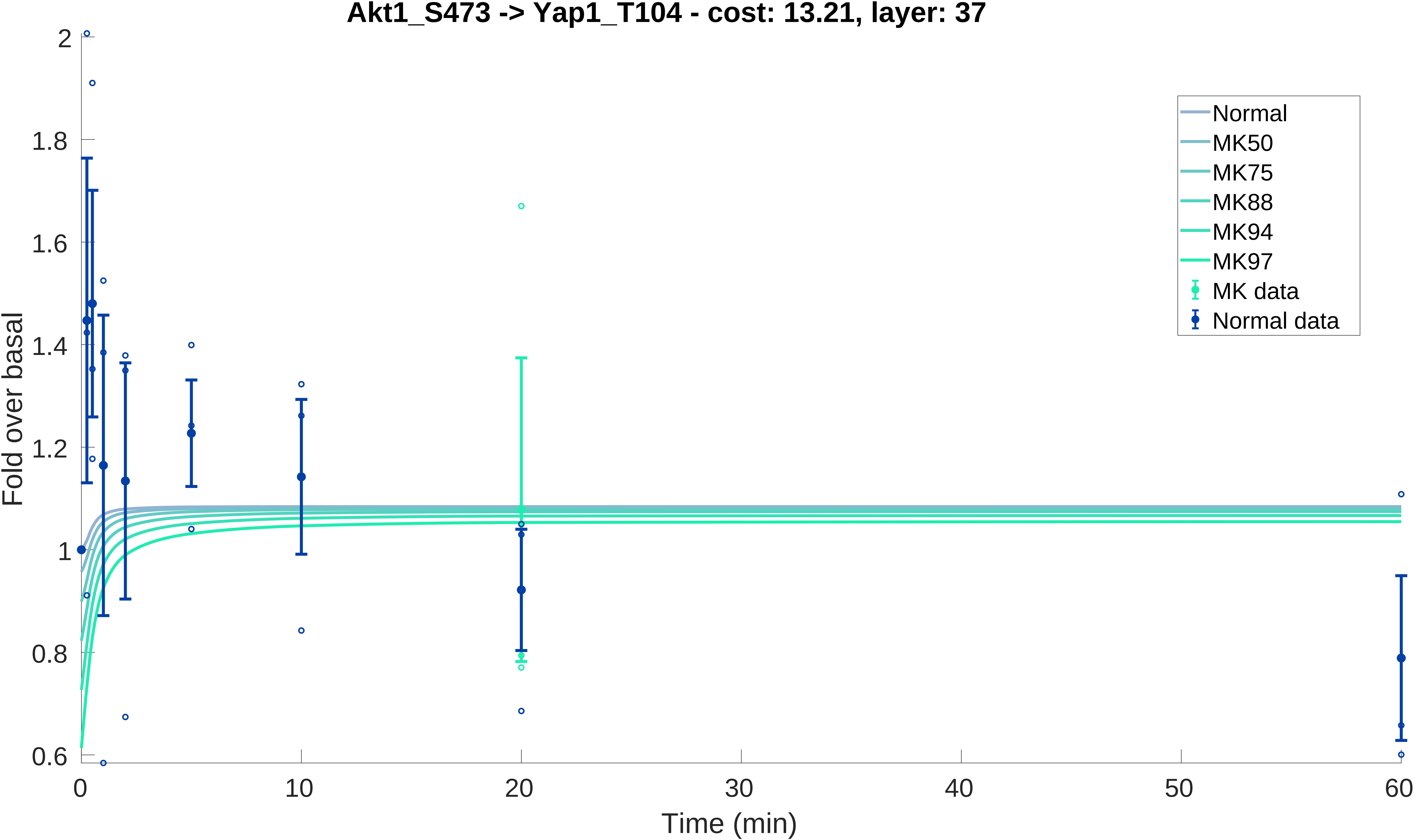

Akt1_S473 -> Yap1_T104 - cost: 13.21, layer: 37
Fold over basal
Time (min)
2
1.8
1.6
1.4
1.2
1
0.8
0.6
0
10
20
30
40
50
60
Normal
MK50
MK75
MK88
MK94
MK97
MK data
Normal data

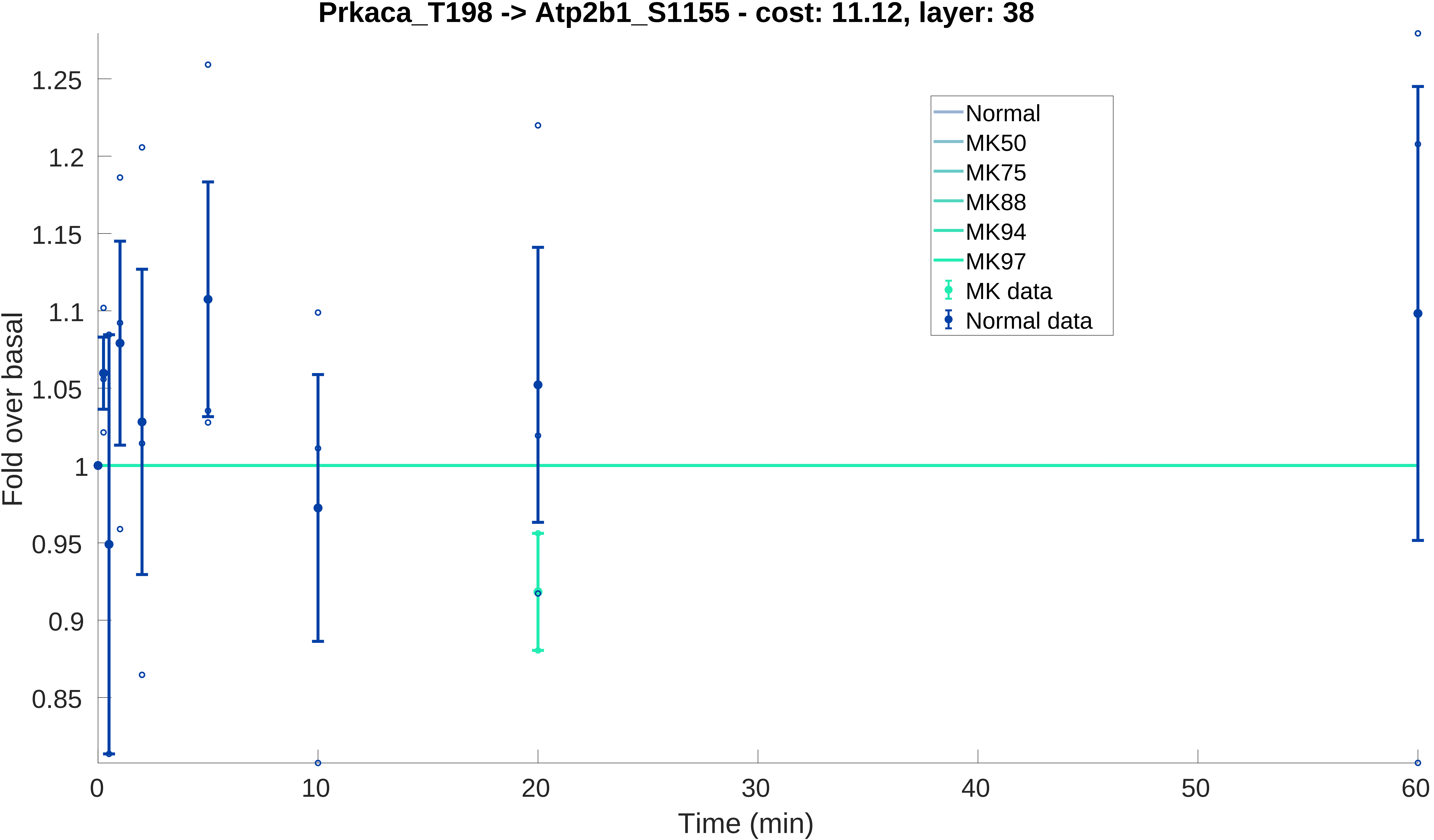

Prkaca_T198 -> Atp2b1_S1155 - cost: 11.12, layer: 38
Fold over basal
Time (min)
1.25
1.2
1.15
1.1
1.05
1
0.95
0.9
0.85
0
10
20
30
40
50
60
Normal
MK50
MK75
MK88
MK94
MK97
MK data
Normal data

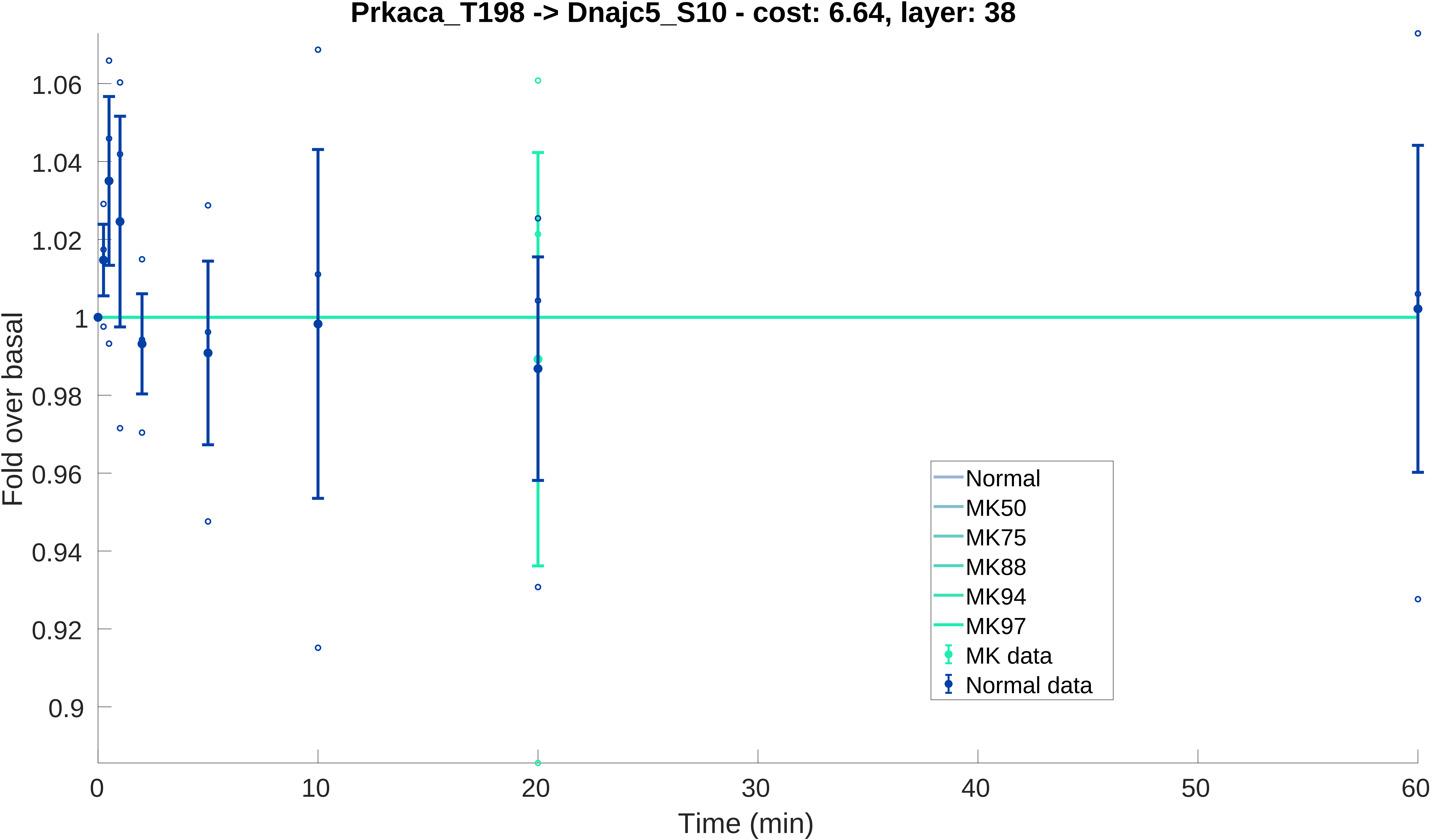

Prkaca_T198 -> Dnajc5_S10 - cost: 6.64, layer: 38
Fold over basal
1.06
1.04
1.02
1
0.98
0.96
0.94
0.92
0.9
0
10
20
30
40
50
60
Time (min)
Normal
MK50
MK75
MK88
MK94
MK97
MK data
Normal data

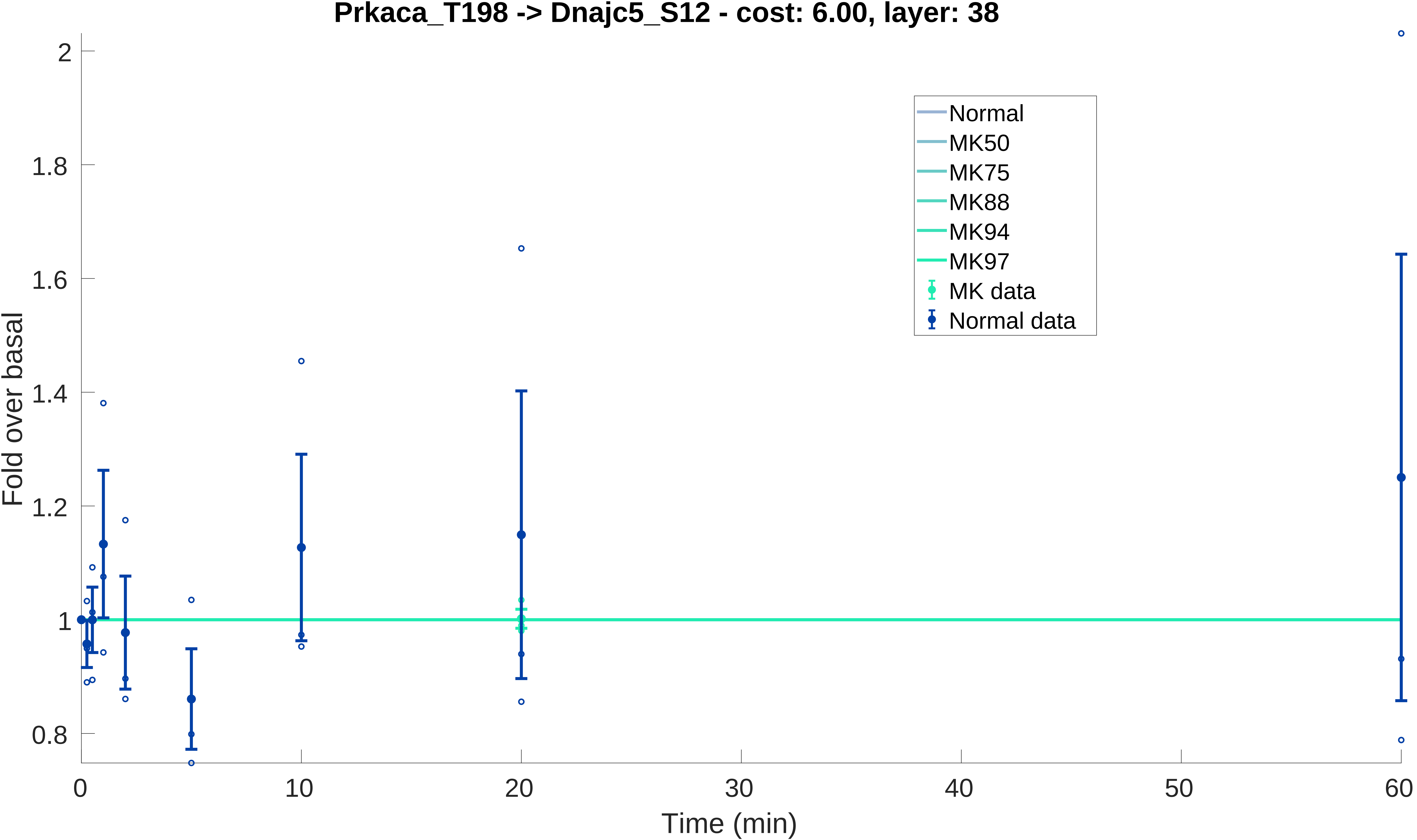

Prkaca_T198 -> Dnajc5_S12 - cost: 6.00, layer: 38
Fold over basal
Time (min)
0.8
1
1.2
1.4
1.6
1.8
2
0
10
20
30
40
50
60
Normal
MK50
MK75
MK88
MK94
MK97
MK data
Normal data

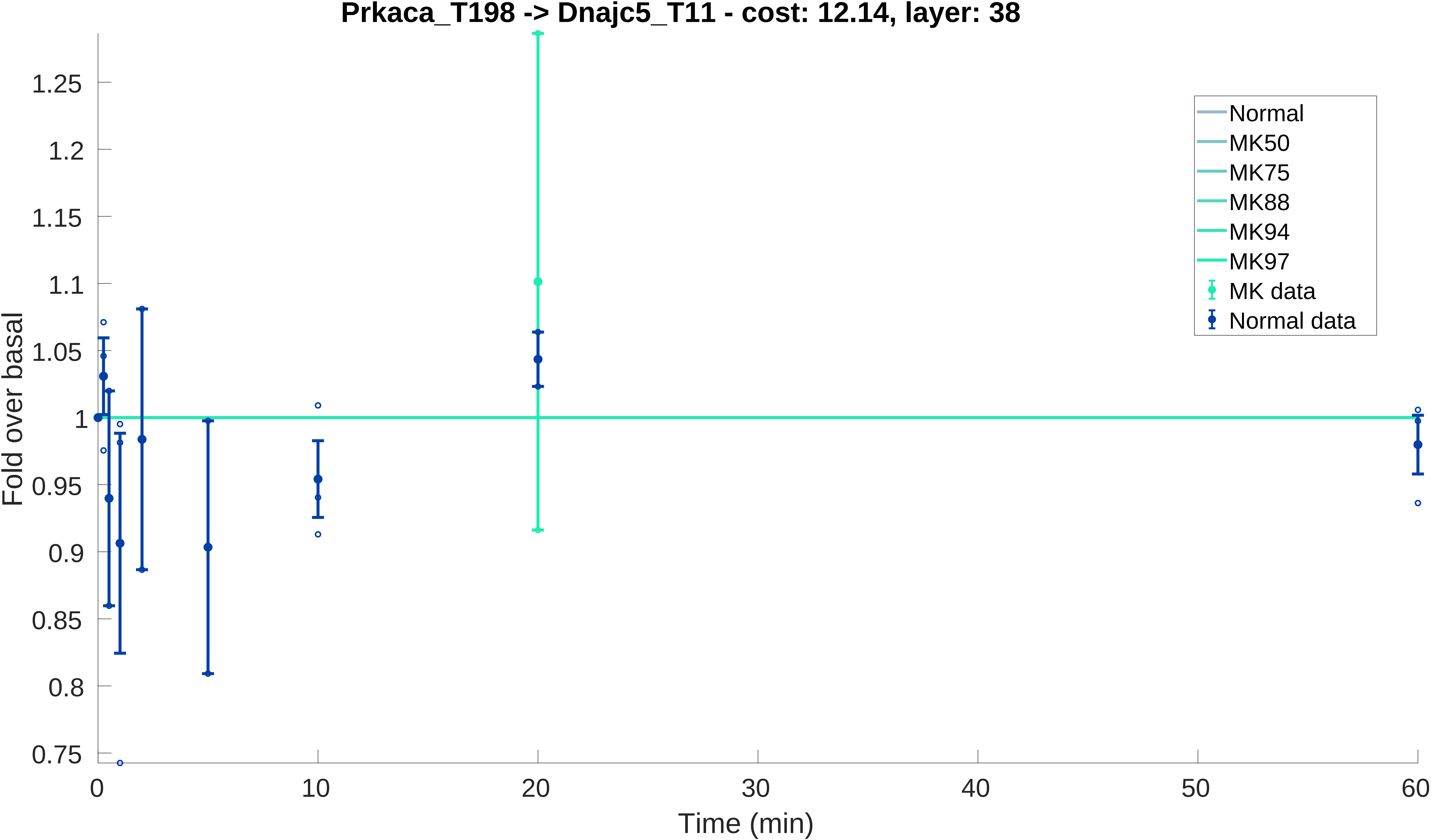

Prkaca_T198 -> Dnajc5_T11 - cost: 12.14, layer: 38
Fold over basal
Time (min)
0.75
0.8
0.85
0.9
0.95
1
1.05
1.1
1.15
1.2
1.25
0
10
20
30
40
50
60
Normal
MK50
MK75
MK88
MK94
MK97
MK data
Normal data

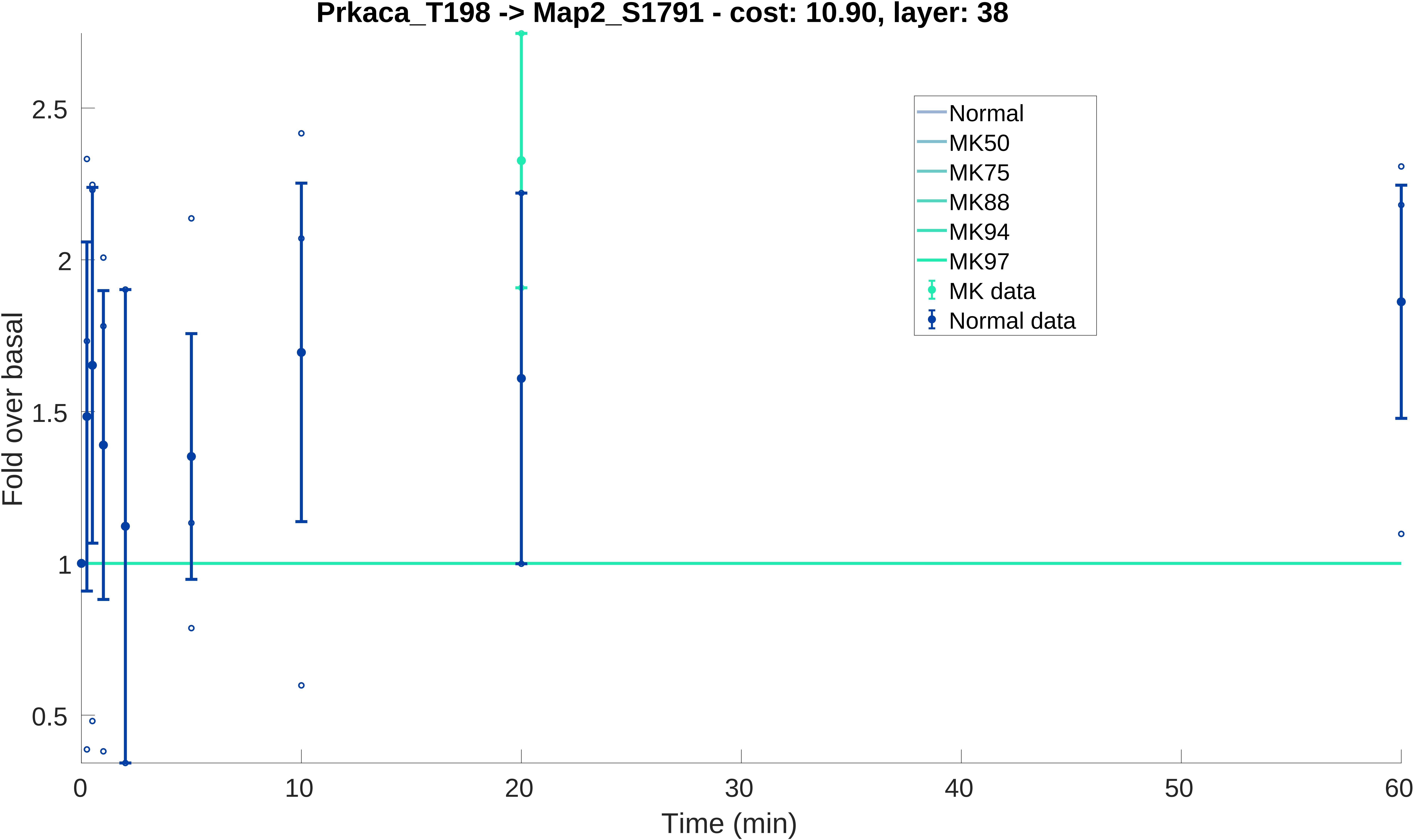

Prrkaca_T198 -> Map2_S1791 - cost: 10.90, layer: 38
Fold over basal
Time (min)
0.5
1
1.5
2
2.5
0
10
20
30
40
50
60
Normal
MK50
MK75
MK88
MK94
MK97
MK data
Normal data

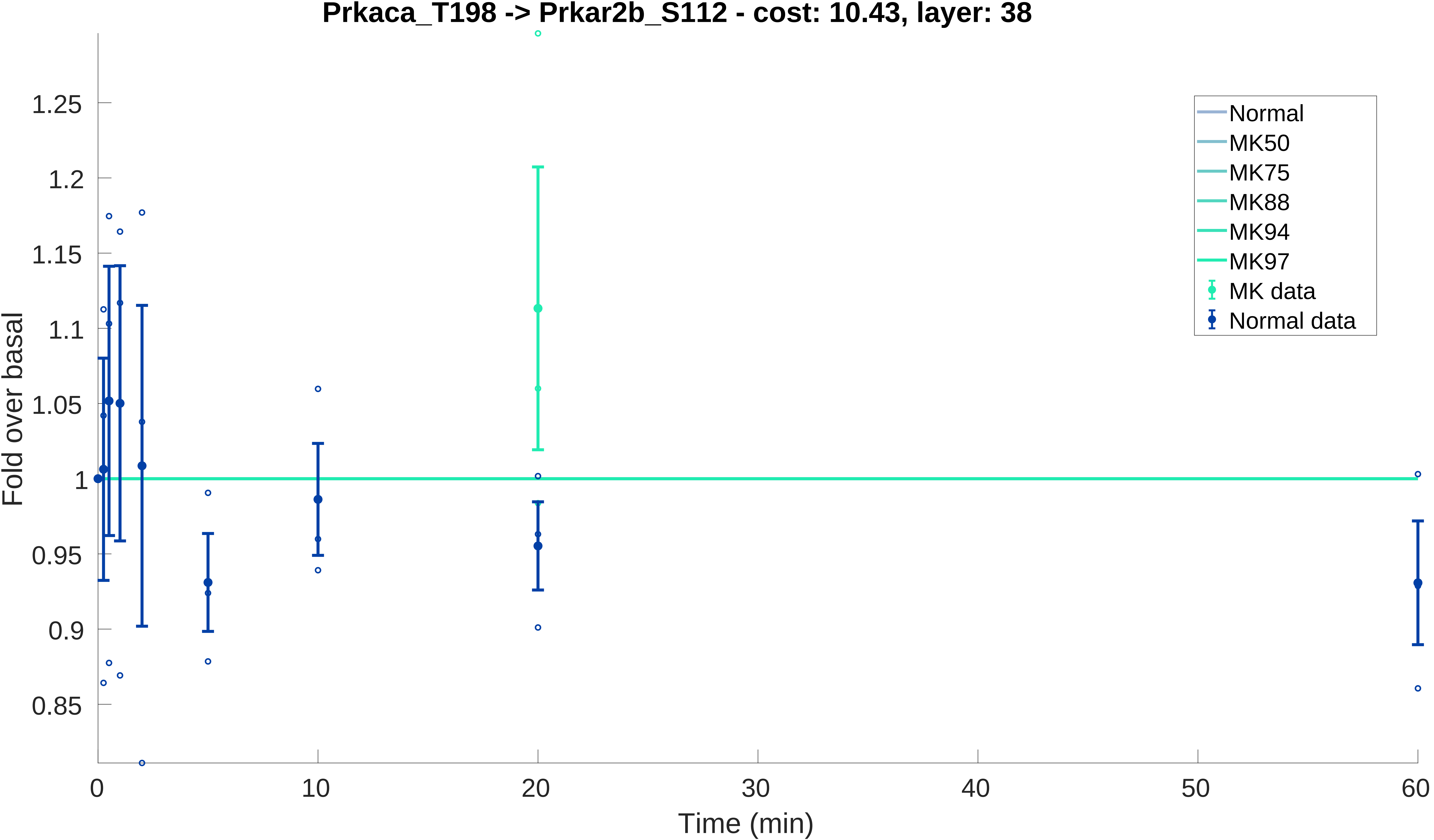

Prkaca_T198 -> Prkar2b_S112 - cost: 10.43, layer: 38
Fold over basal
Time (min)
1.25
1.2
1.15
1.1
1.05
1
0.95
0.9
0.85
0
10
20
30
40
50
60
Normal
MK50
MK75
MK88
MK94
MK97
MK data
Normal data

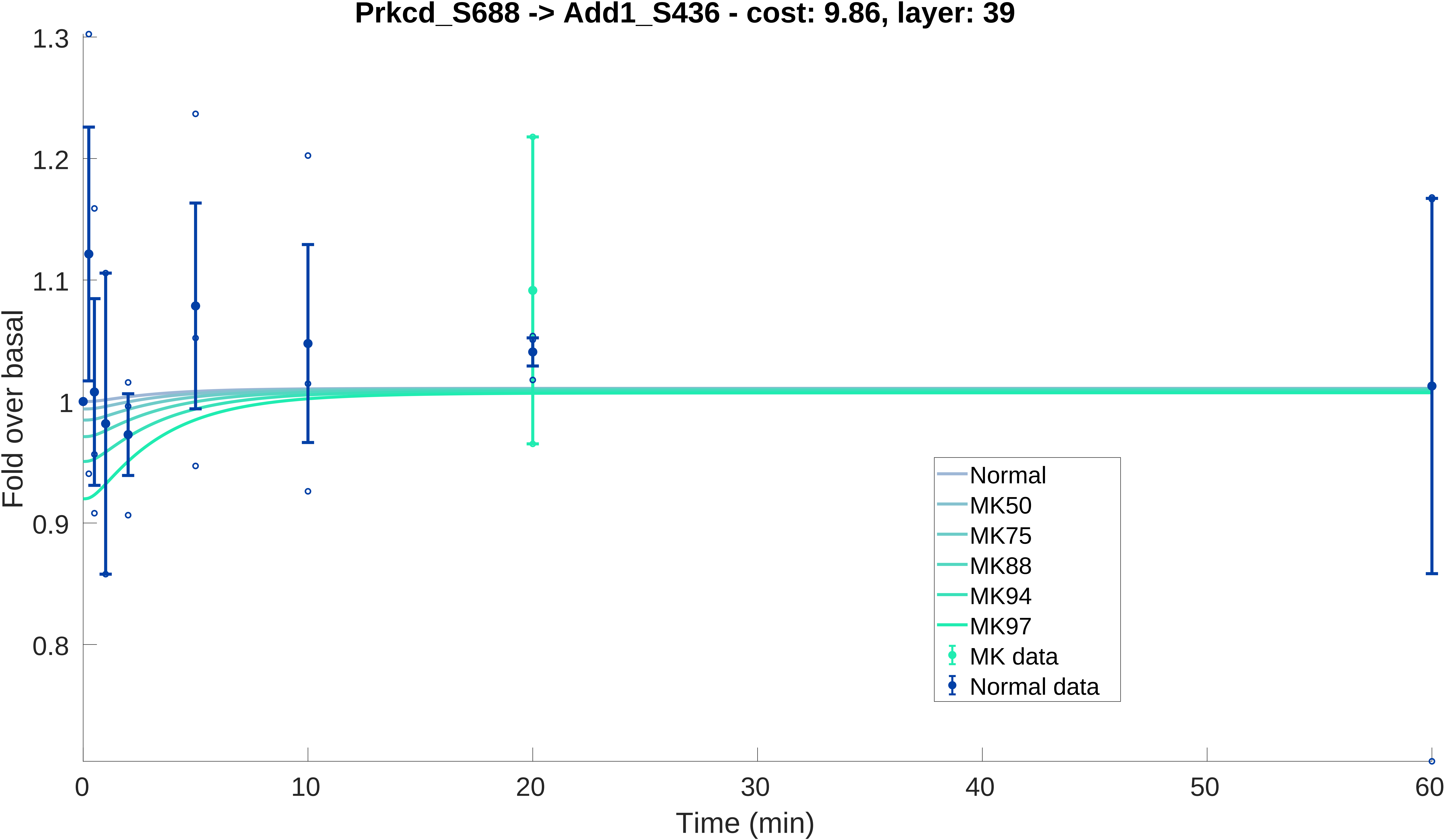

Prkcd_S688 -> Add1_S436 - cost: 9.86, layer: 39
Fold over basal
Time (min)
1.3
1.2
1.1
1
0.9
0.8
0
10
20
30
40
50
60
Normal
MK50
MK75
MK88
MK94
MK97
MK data
Normal data

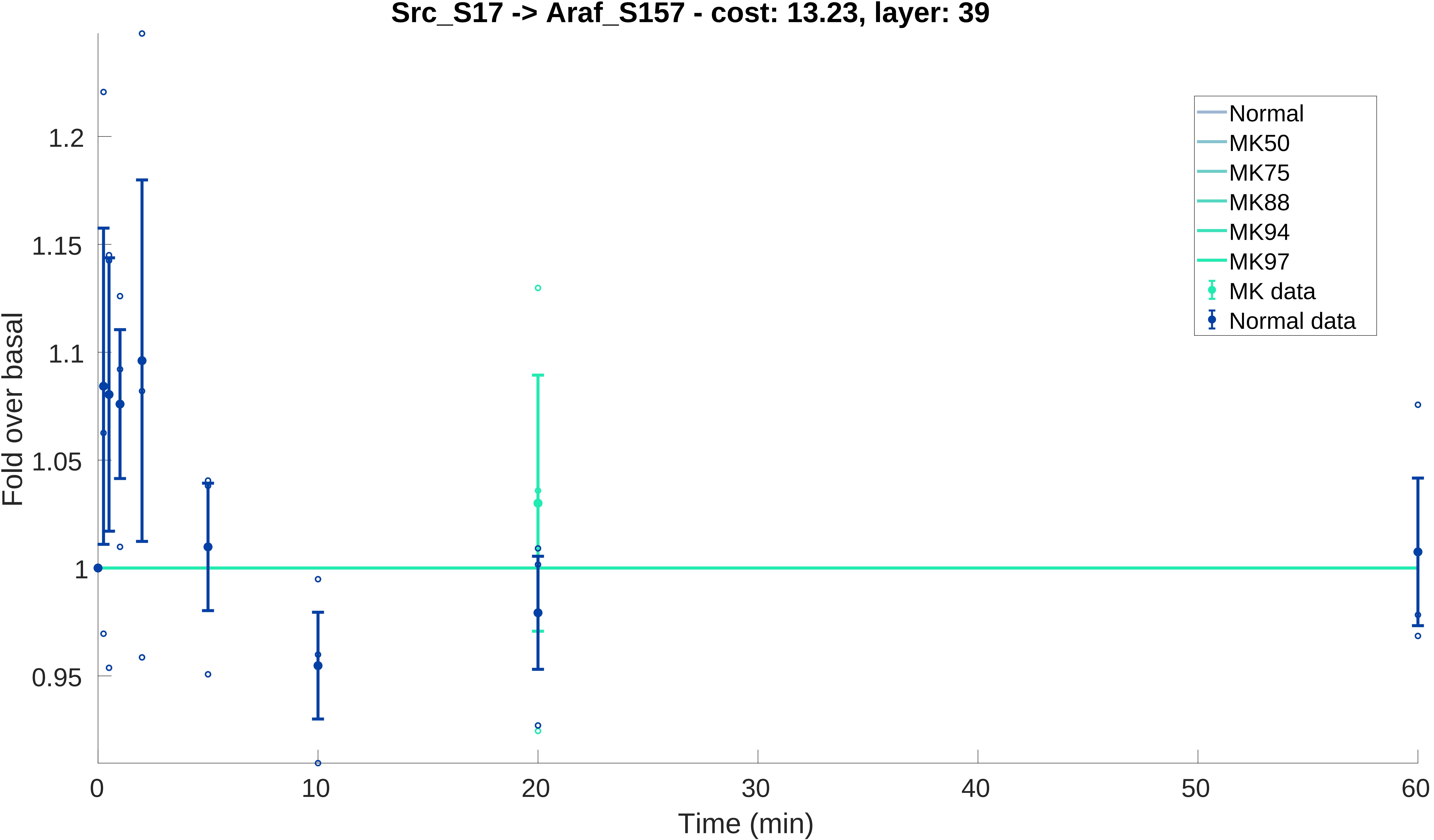
Src_S17 -> Araf_S157 - cost: 13.23, layer: 39
Fold over basal
1.2
1.15
1.1
1.05
1
0.95
0
10
20
30
40
50
60
Time (min)
Normal
MK50
MK75
MK88
MK94
MK97
MK data
Normal data

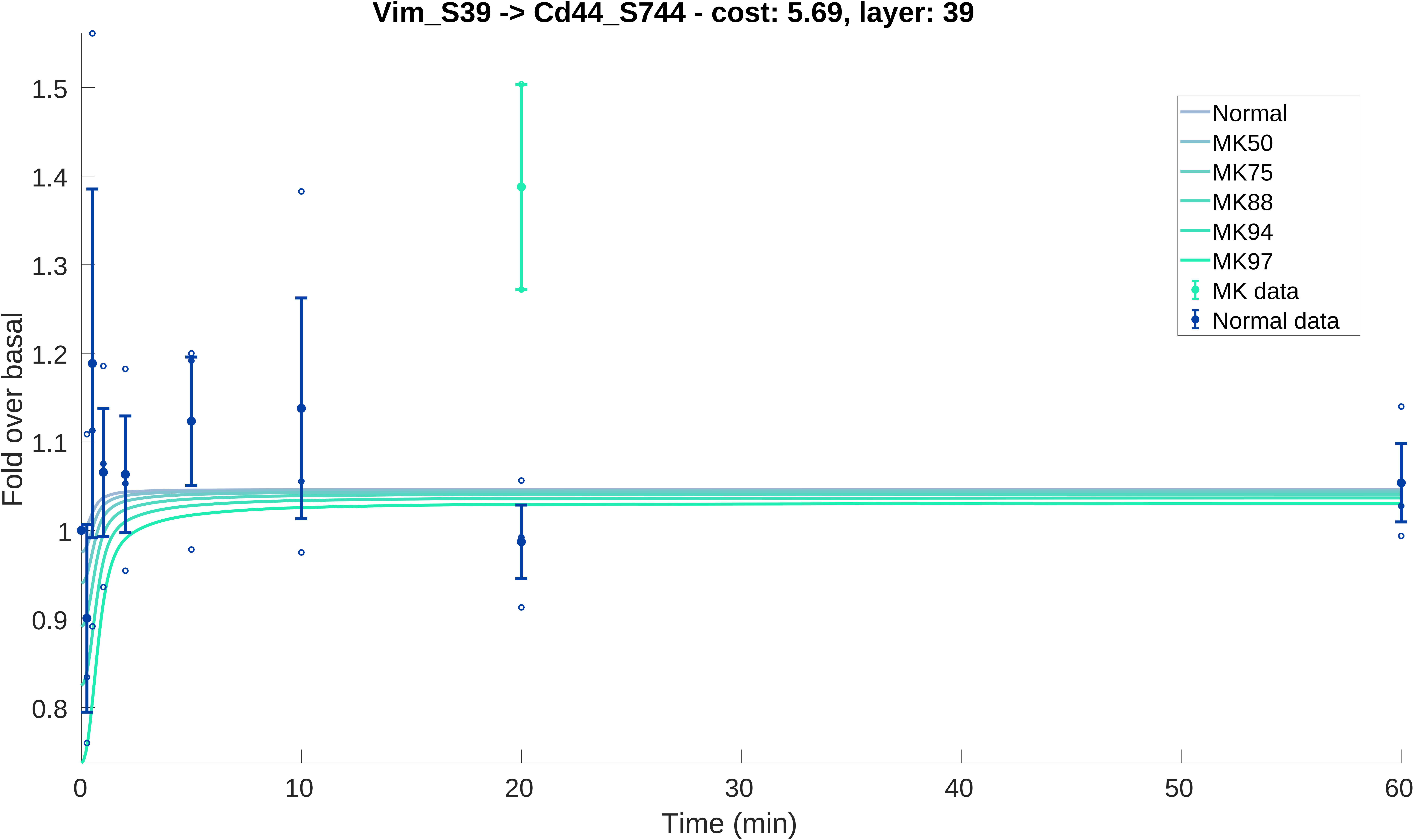

Vim_S39 -> Cd44_S744 - cost: 5.69, layer: 39
Fold over basal
Time (min)
0.8
0.9
1
1.1
1.2
1.3
1.4
1.5
0
10
20
30
40
50
60
Normal
MK50
MK75
MK88
MK94
MK97
MK data
Normal data

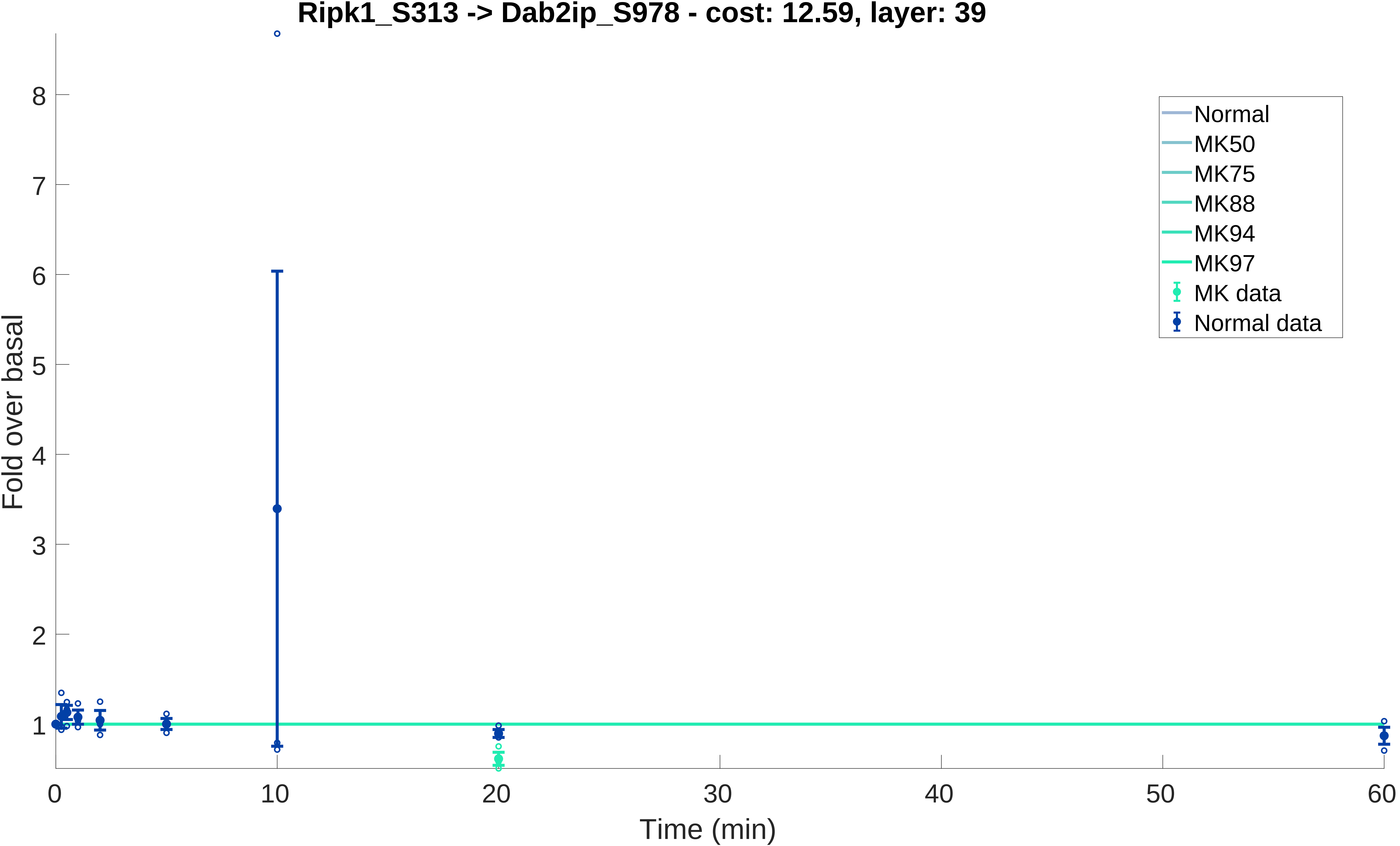

Ripk1_S313 -> Dab2ip_S978 - cost: 12.59, layer: 39
Fold over basal
Time (min)
0
10
20
30
40
50
60
1
2
3
4
5
6
7
8
Normal
MK50
MK75
MK88
MK94
MK97
MK data
Normal data

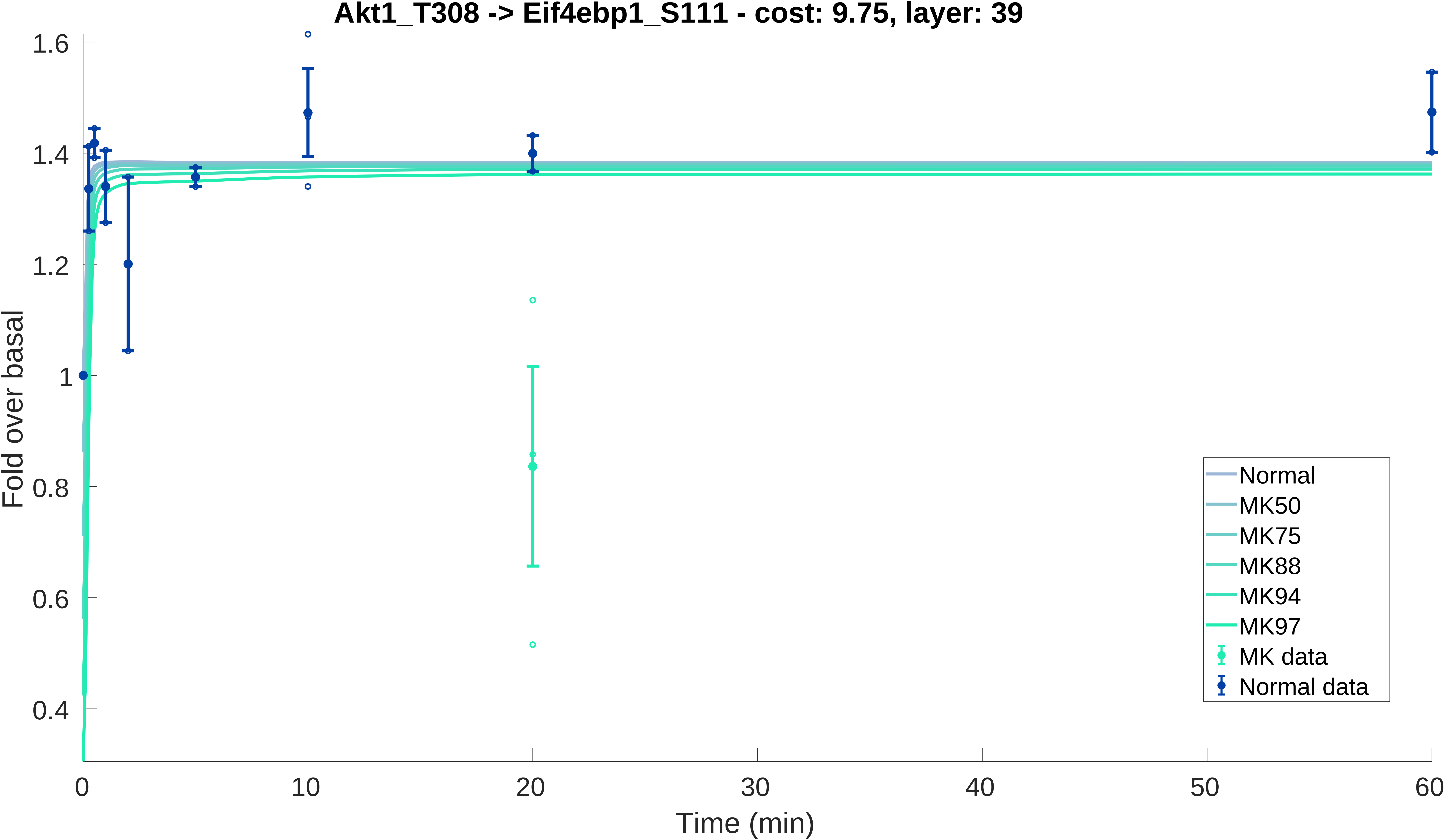

Akt1_T308 -> Eif4ebp1_S111 - cost: 9.75, layer: 39
Fold over basal
Time (min)
1.6
1.4
1.2
1
0.8
0.6
0.4
0
10
20
30
40
50
60
Normal
MK50
MK75
MK88
MK94
MK97
MK data
Normal data

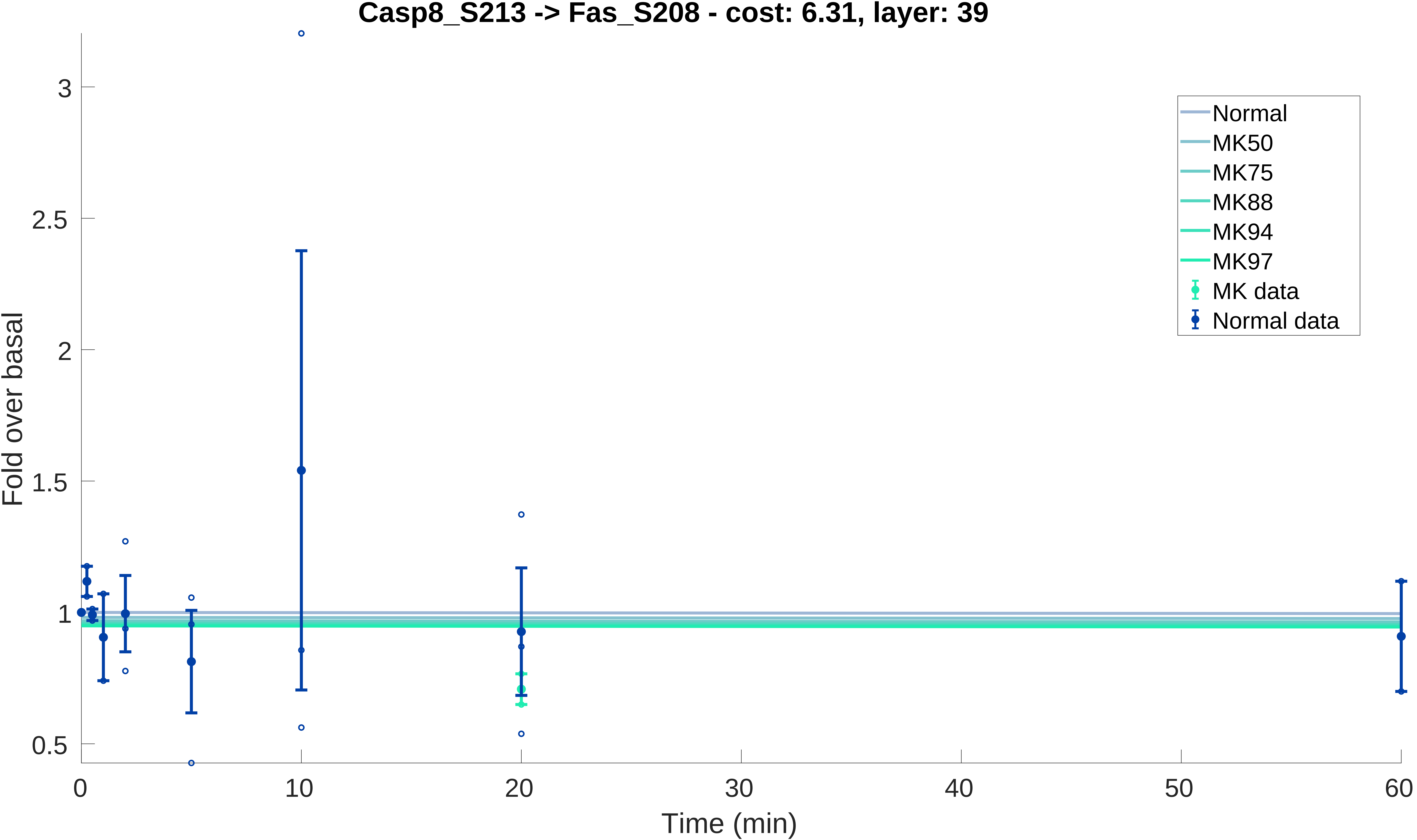

Casp8_S213 -> Fas_S208 - cost: 6.31, layer: 39
Fold over basal
Time (min)
0.5
1
1.5
2
2.5
3
0
10
20
30
40
50
60
Normal
MK50
MK75
MK88
MK94
MK97
MK data
Normal data

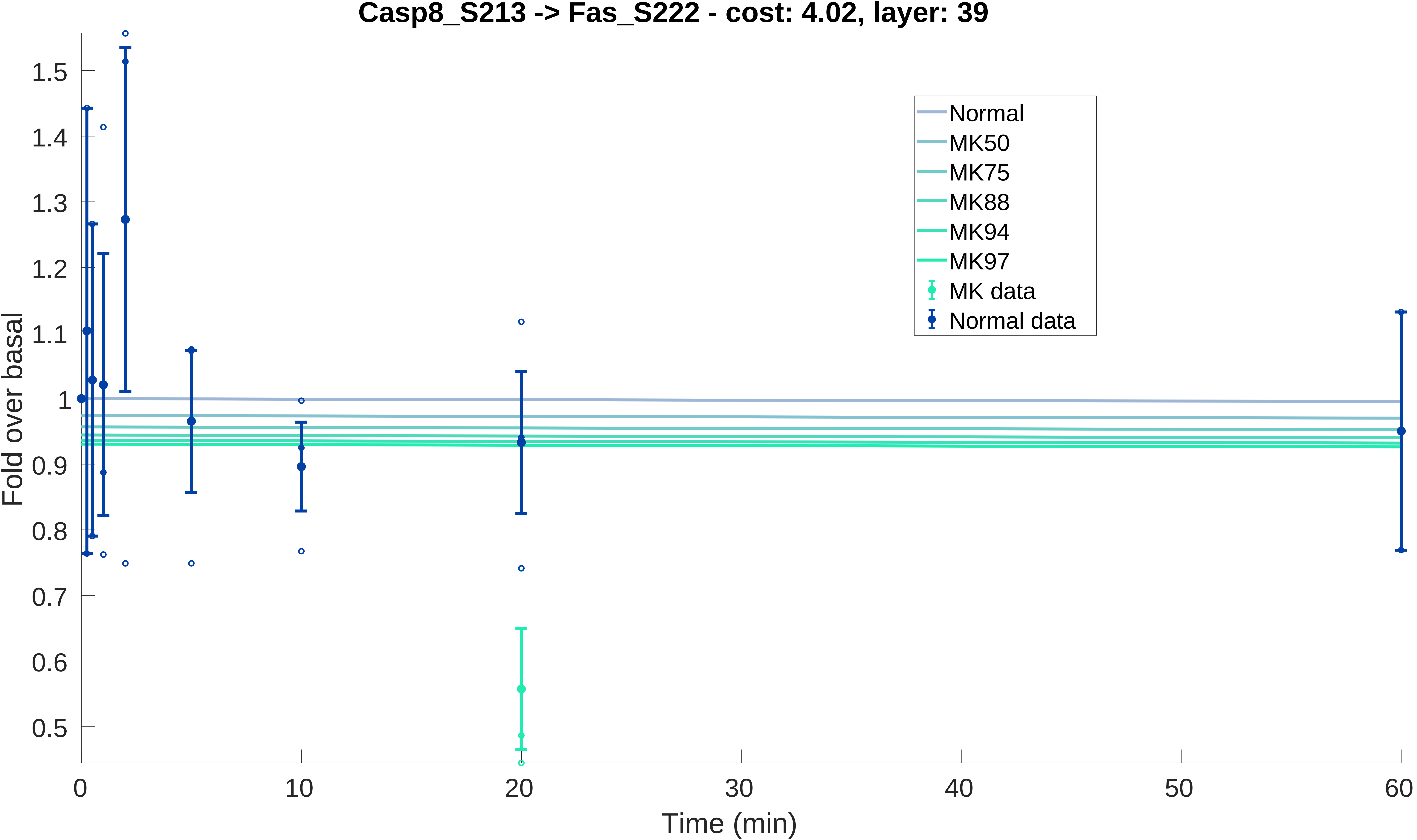
Casp8_S213 -> Fas_S222 - cost: 4.02, layer: 39
Fold over basal
Time (min)
Normal
MK50
MK75
MK88
MK94
MK97
MK data
Normal data
1.5
1.4
1.3
1.2
1.1
1
0.9
0.8
0.7
0.6
0.5
0
10
20
30
40
50
60

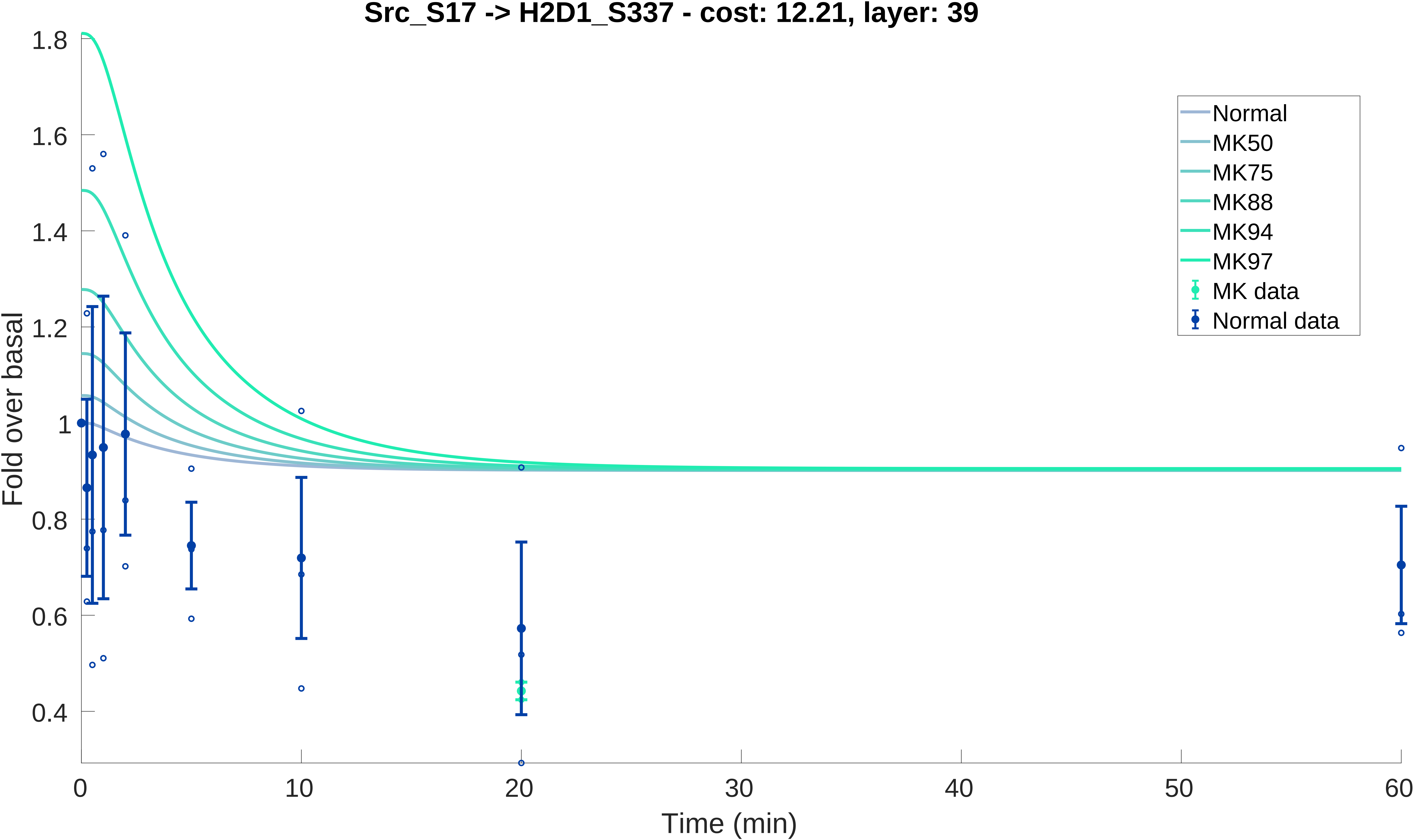
Src_S17 -> H2D1_S337 - cost: 12.21, layer: 39
Fold over basal
Time (min)
Normal
MK50
MK75
MK88
MK94
MK97
MK data
Normal data
1.8
1.6
1.4
1.2
1
0.8
0.6
0.4
0
10
20
30
40
50
60

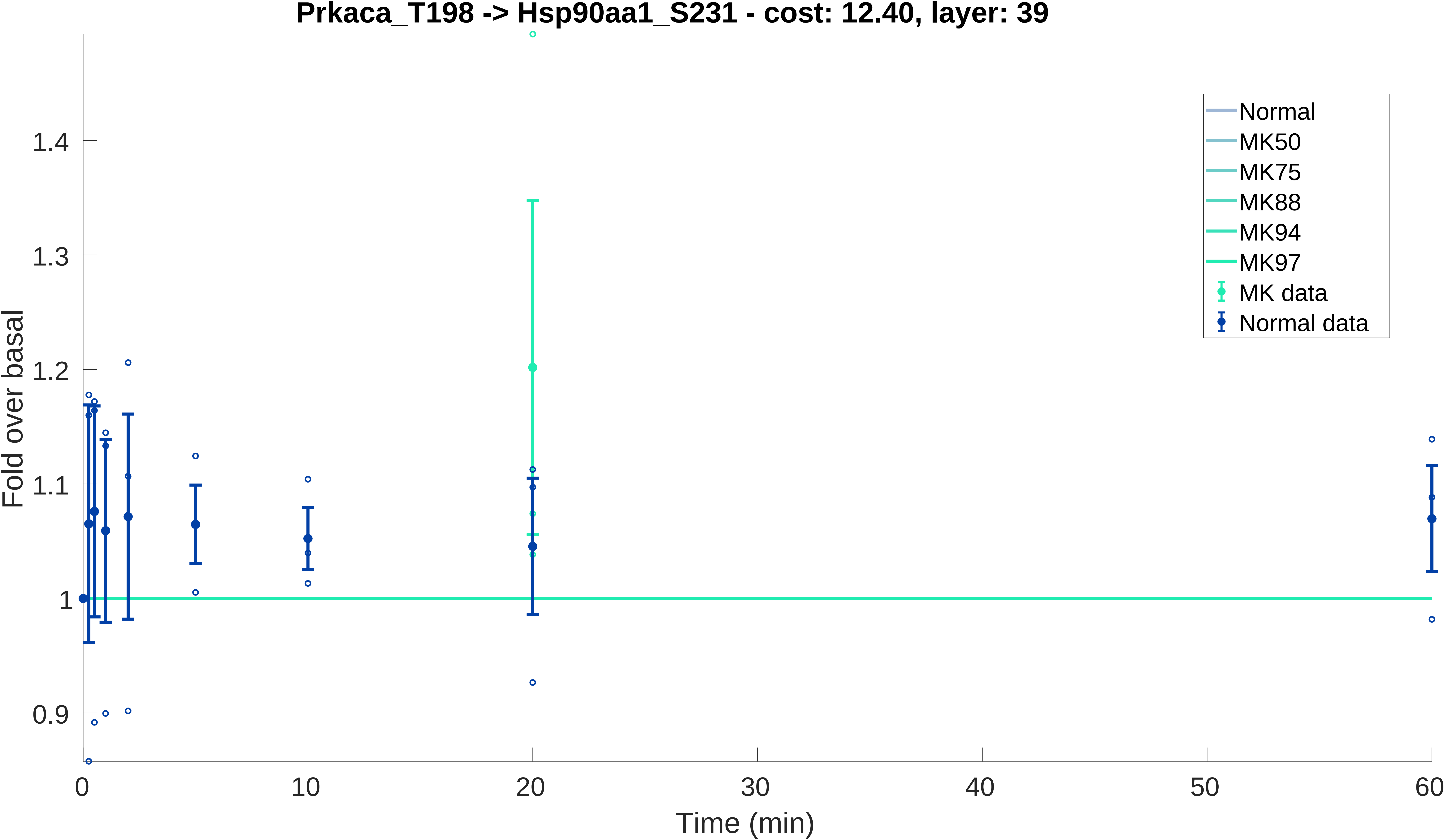
Prkaca_T198 -> Hsp90aa1_S231 - cost: 12.40, layer: 39
Fold over basal
1.4
1.3
1.2
1.1
1
0.9
0
10
20
30
40
50
60
Time (min)
Normal
MK50
MK75
MK88
MK94
MK97
MK data
Normal data

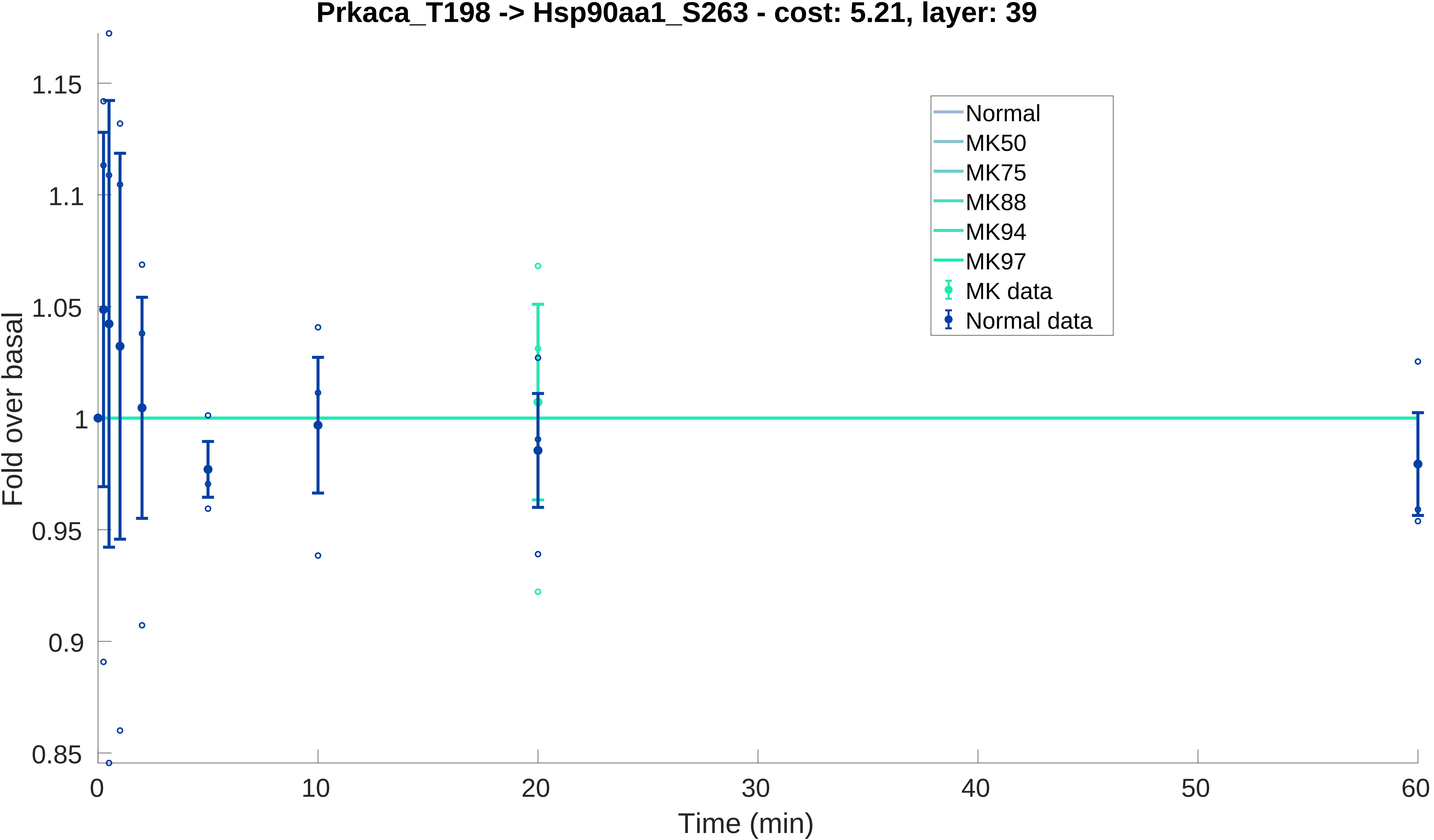

Prrkaca_T198 -> Hsp90aa1_S263 - cost: 5.21, layer: 39
Fold over basal
1.15
1.1
1.05
1
0.95
0.9
0.85
0
10
20
30
40
50
60
Time (min)
Normal
MK50
MK75
MK88
MK94
MK97
MK data
Normal data

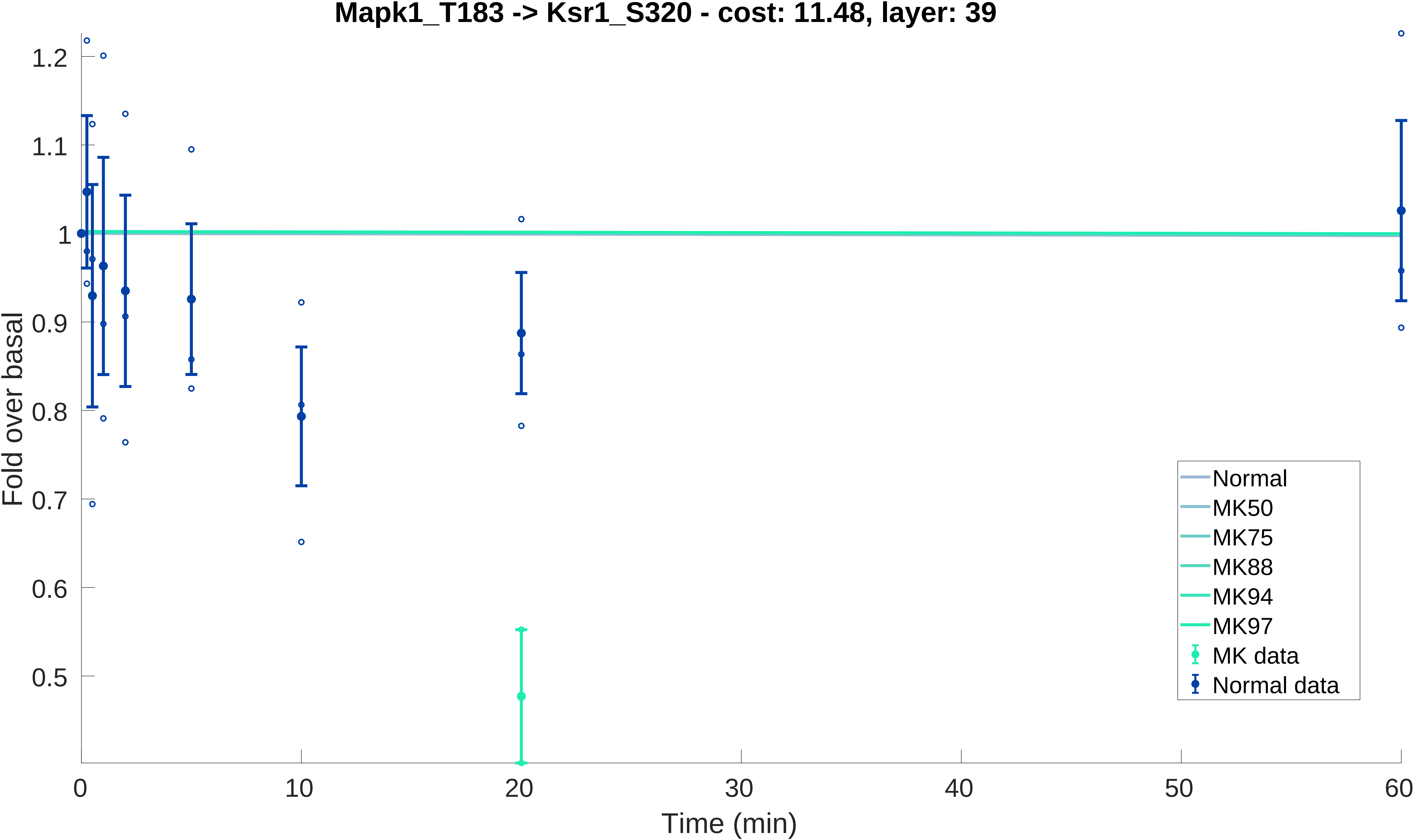

Mapk1_T183 -> Ksr1_S320 - cost: 11.48, layer: 39
Fold over basal
Time (min)
1.2
1.1
1
0.9
0.8
0.7
0.6
0.5
0
10
20
30
40
50
60
Normal
MK50
MK75
MK88
MK94
MK97
MK data
Normal data

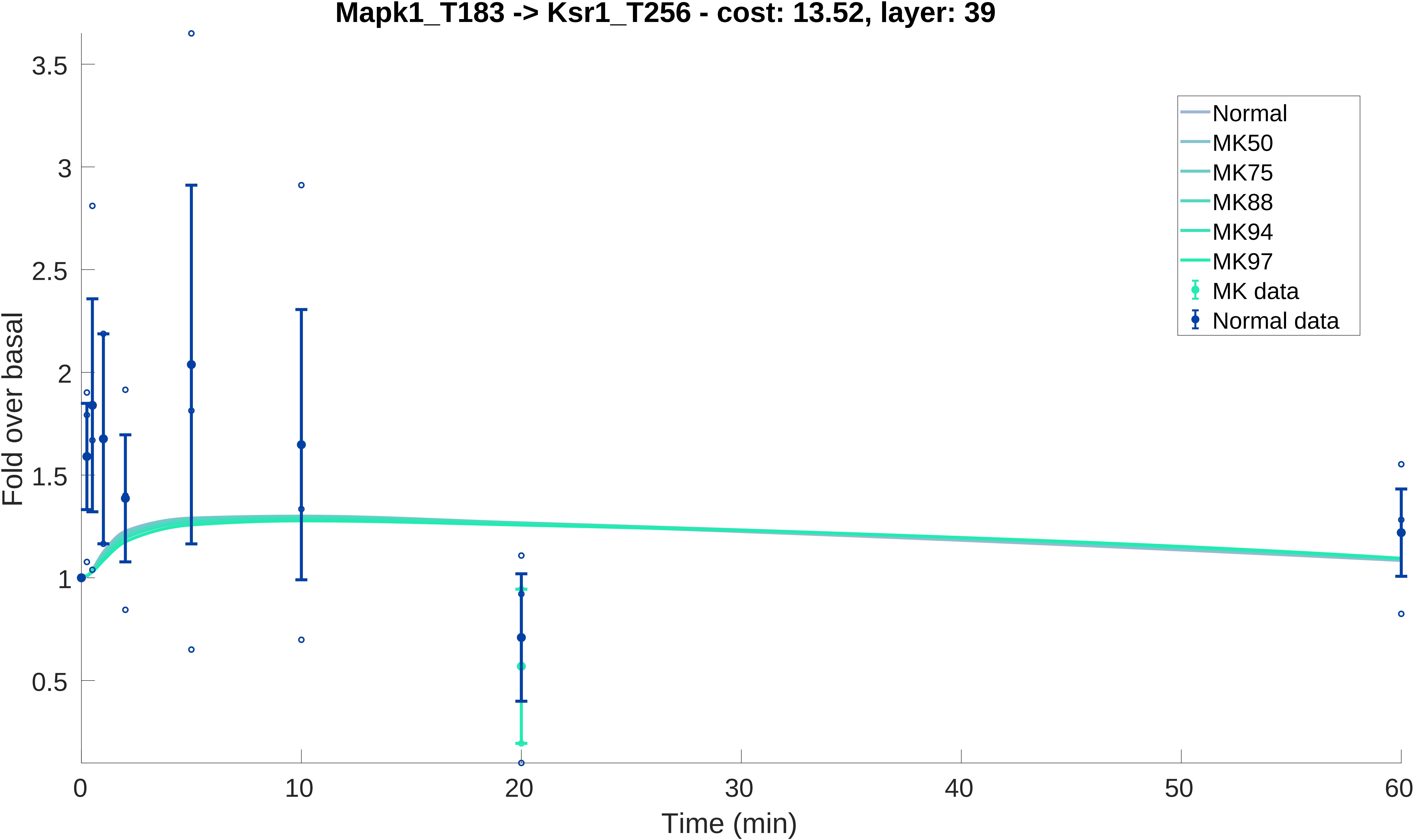

Mapk1_T183 -> Ksr1_T256 - cost: 13.52, layer: 39
Fold over basal
Time (min)
0.5
1
1.5
2
2.5
3
3.5
0
10
20
30
40
50
60
Normal
MK50
MK75
MK88
MK94
MK97
MK data
Normal data

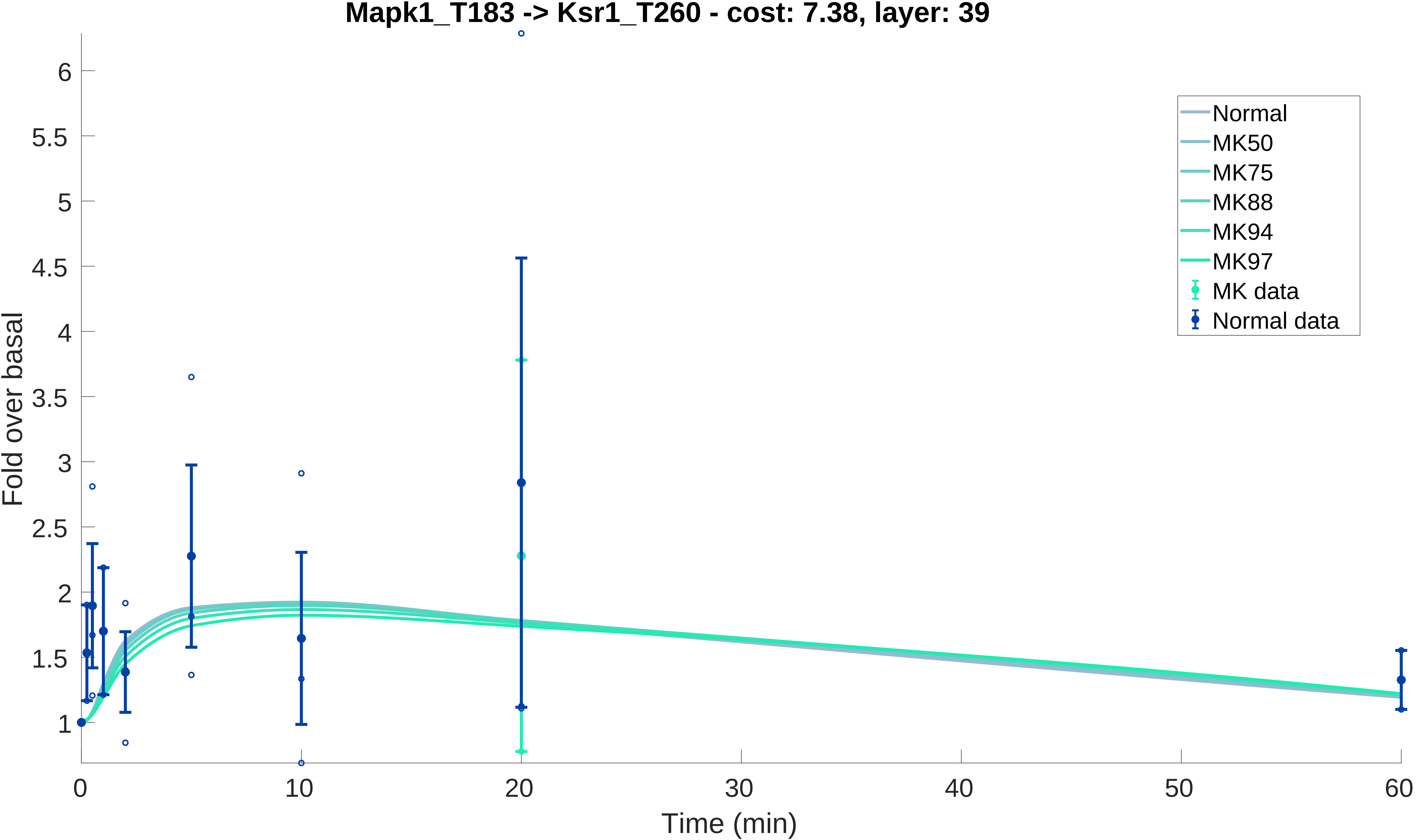
Mapk1_T183 -> Ksr1_T260 - cost: 7.38, layer: 39
Fold over basal
Time (min)
Normal
MK50
MK75
MK88
MK94
MK97
MK data
Normal data
1
1.5
2
2.5
3
3.5
4
4.5
5
5.5
6
0
10
20
30
40
50
60

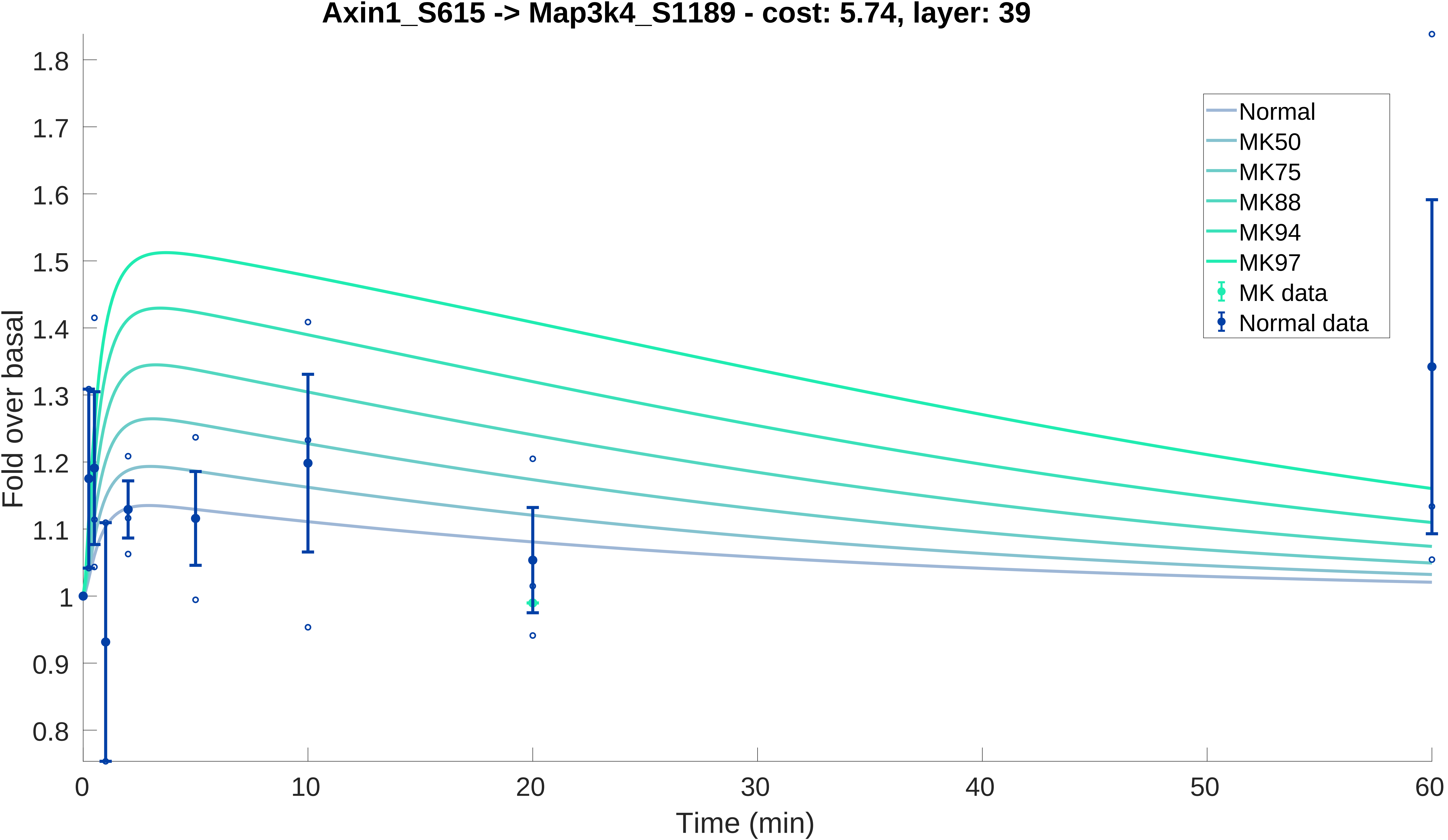
Axin1_S615 -> Map3k4_S1189 - cost: 5.74, layer: 39
Fold over basal
Time (min)
1.8
1.7
1.6
1.5
1.4
1.3
1.2
1.1
1
0.9
0.8
0
10
20
30
40
50
60
Normal
MK50
MK75
MK88
MK94
MK97
MK data
Normal data

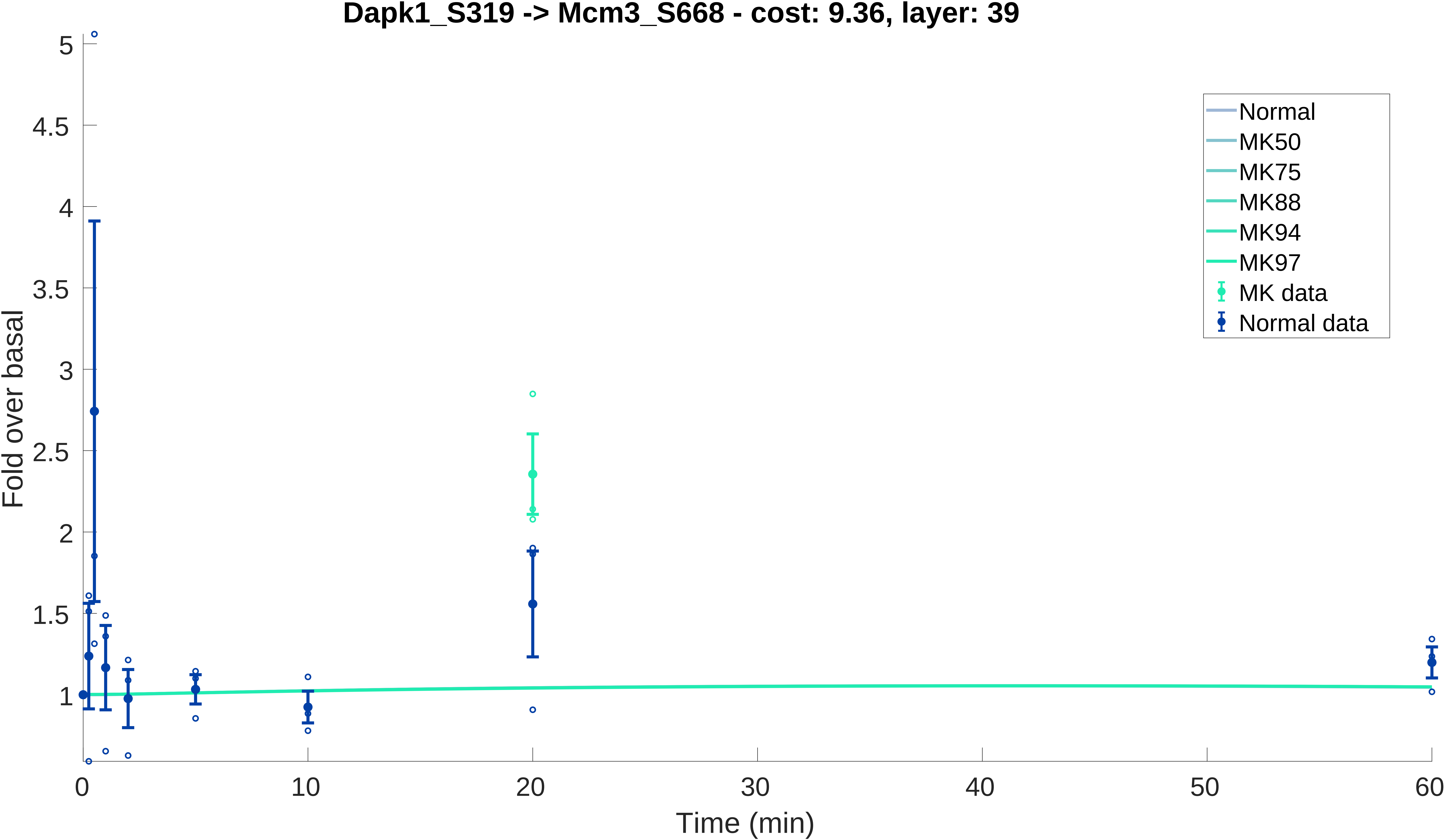
Dapk1_S319 -> Mcm3_S668 - cost: 9.36, layer: 39
Fold over basal
Time (min)
Normal
MK50
MK75
MK88
MK94
MK97
MK data
Normal data
0
10
20
30
40
50
60
1
1.5
2
2.5
3
3.5
4
4.5
5

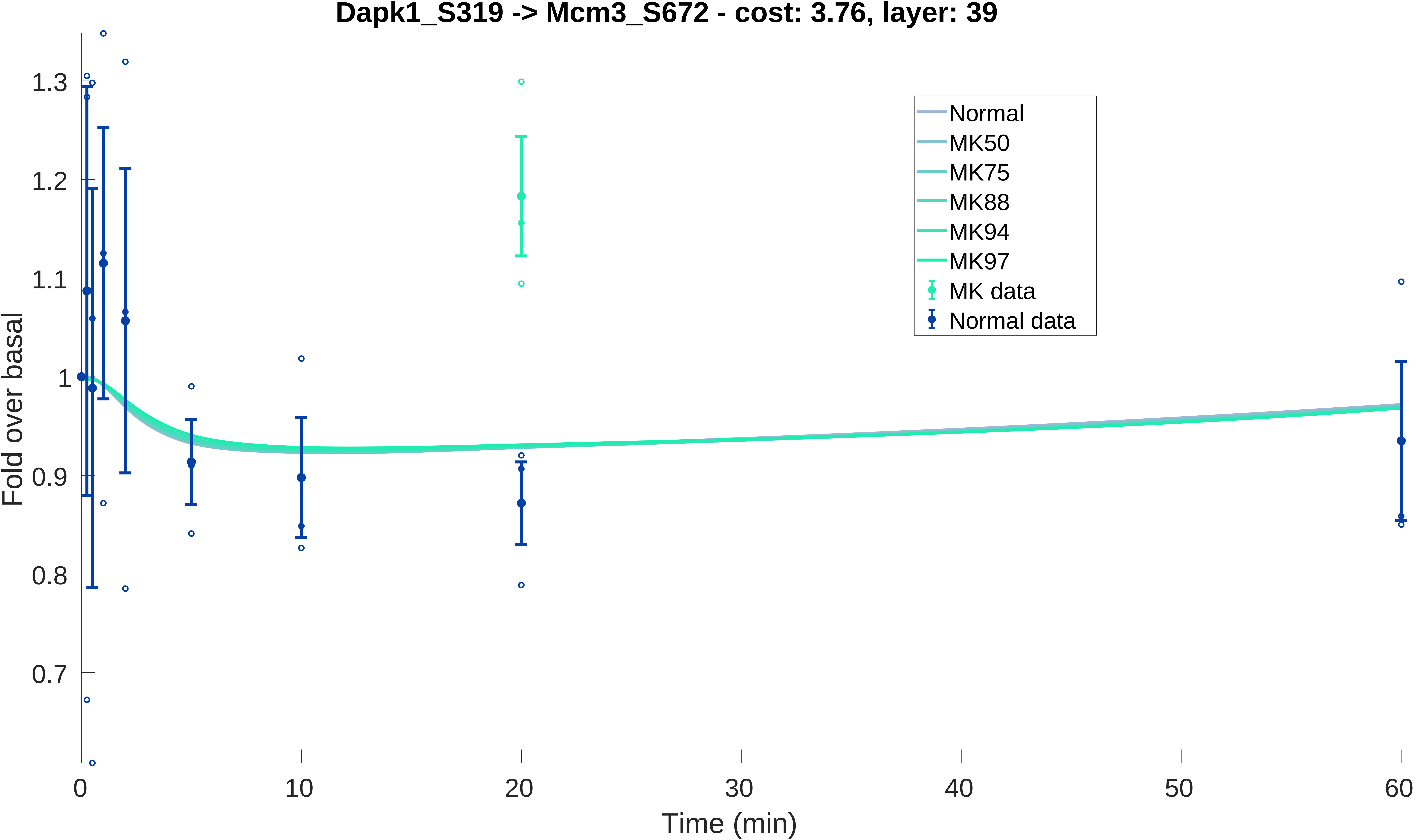

Dapk1_S319 -> Mcm3_S672 - cost: 3.76, layer: 39
Fold over basal
Time (min)
0.7
0.8
0.9
1
1.1
1.2
1.3
0
10
20
30
40
50
60
Normal
MK50
MK75
MK88
MK94
MK97
MK data
Normal data

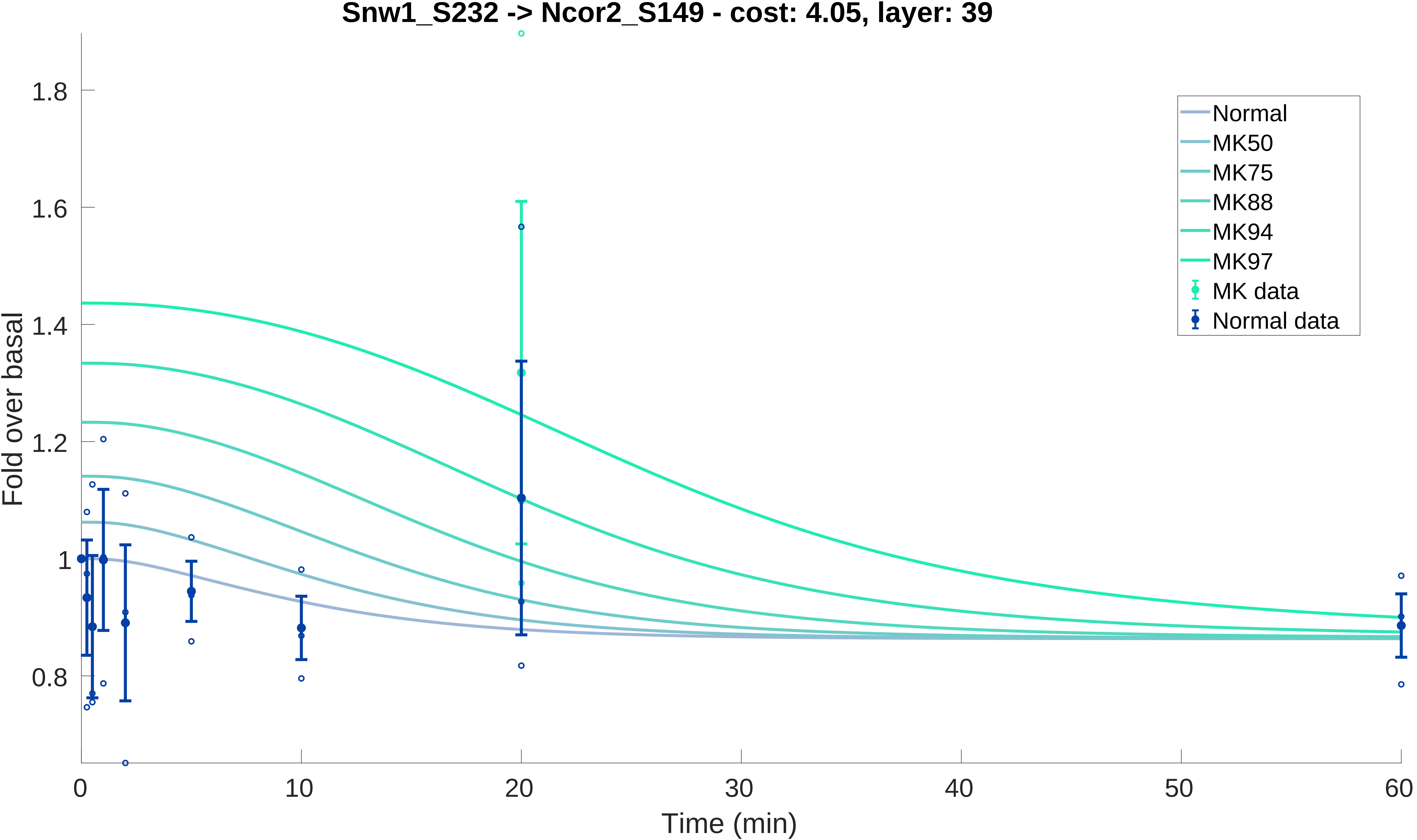
Snw1_S232 -> Ncor2_S149 - cost: 4.05, layer: 39
Fold over basal
Time (min)
0
10
20
30
40
50
60
0.8
1
1.2
1.4
1.6
1.8
Normal
MK50
MK75
MK88
MK94
MK97
MK data
Normal data

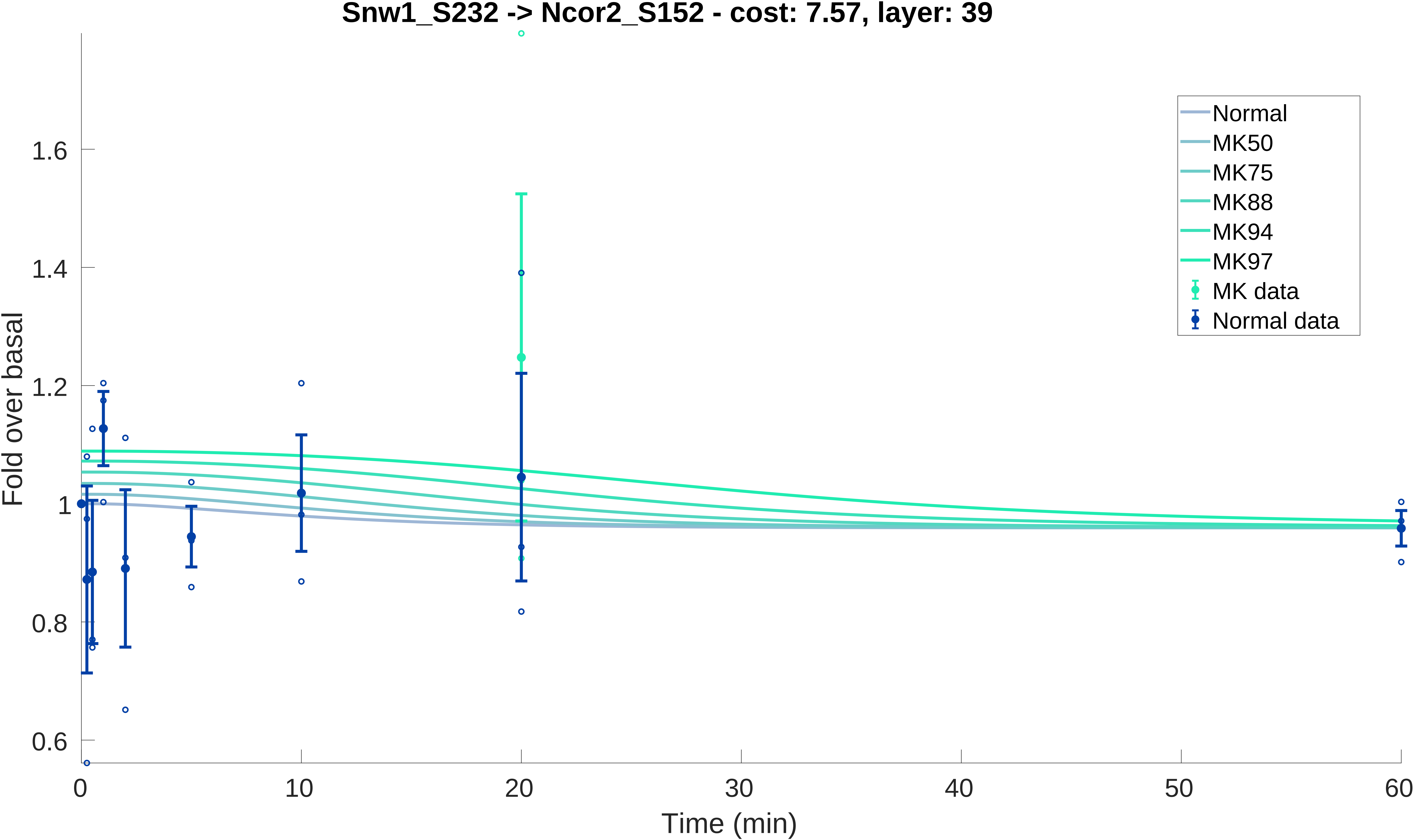

Snw1_S232 -> Ncor2_S152 - cost: 7.57, layer: 39
Fold over basal
1.6
1.4
1.2
1
0.8
0.6
0
10
20
30
40
50
60
Time (min)
Normal
MK50
MK75
MK88
MK94
MK97
MK data
Normal data

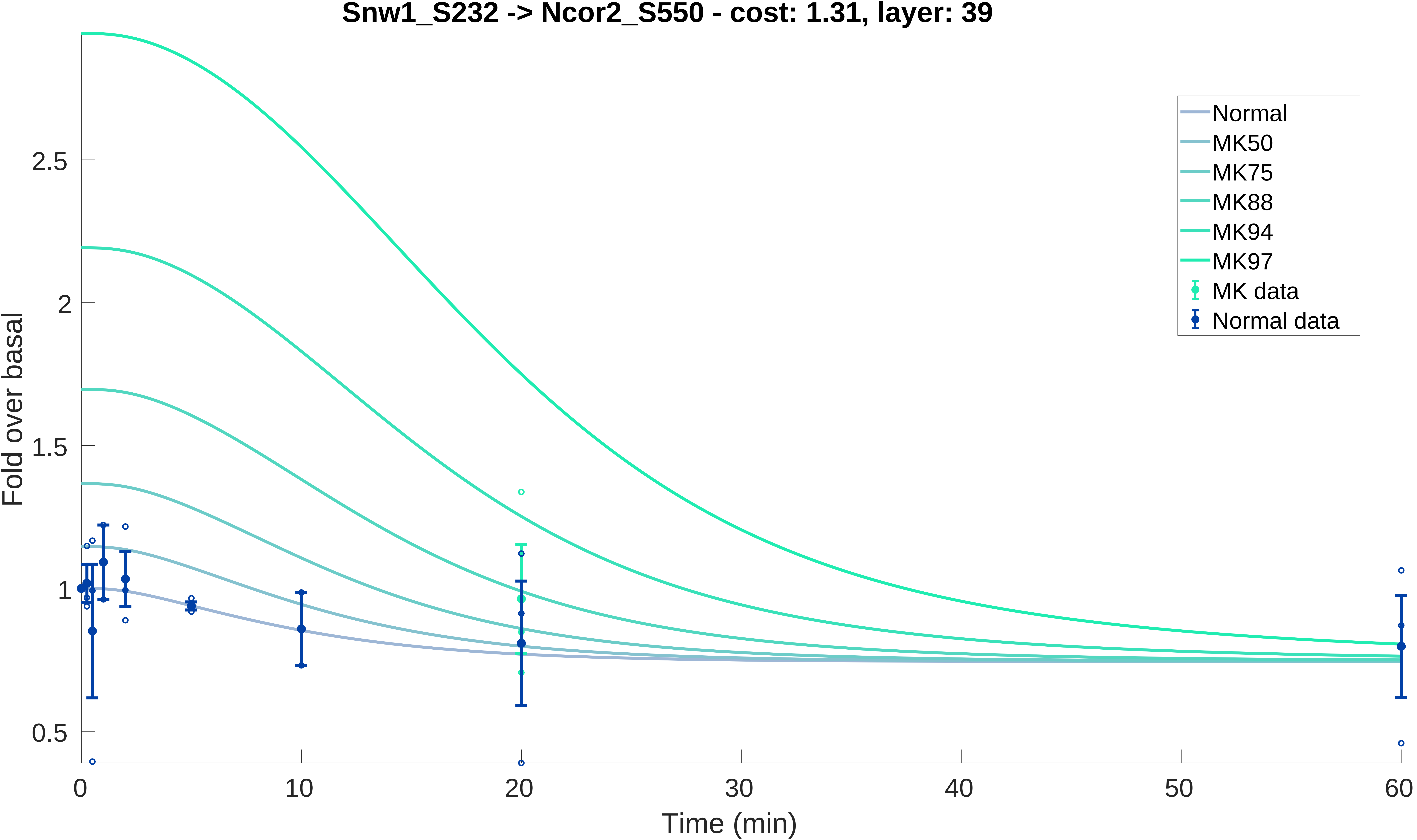

Snw1_S232 -> Ncor2_S550 - cost: 1.31, layer: 39
Fold over basal
Time (min)
0.5
1
1.5
2
2.5
0
10
20
30
40
50
60
Normal
MK50
MK75
MK88
MK94
MK97
MK data
Normal data

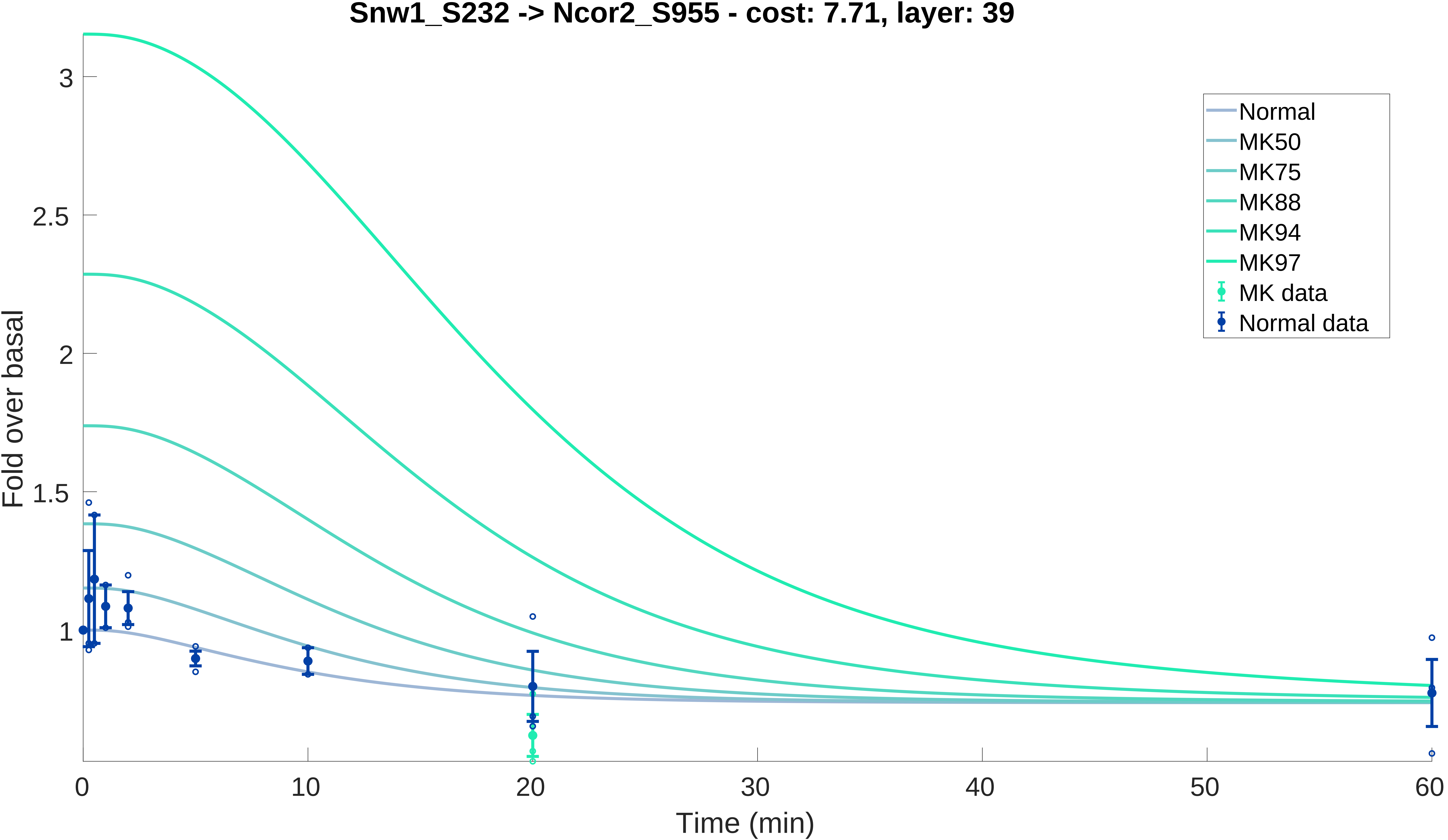

Snw1_S232 -> Ncor2_S955 - cost: 7.71, layer: 39
Normal
MK50
MK75
MK88
MK94
MK97
MK data
Normal data
Fold over basal
3
2.5
2
1.5
1
0
10
20
30
40
50
60
Time (min)

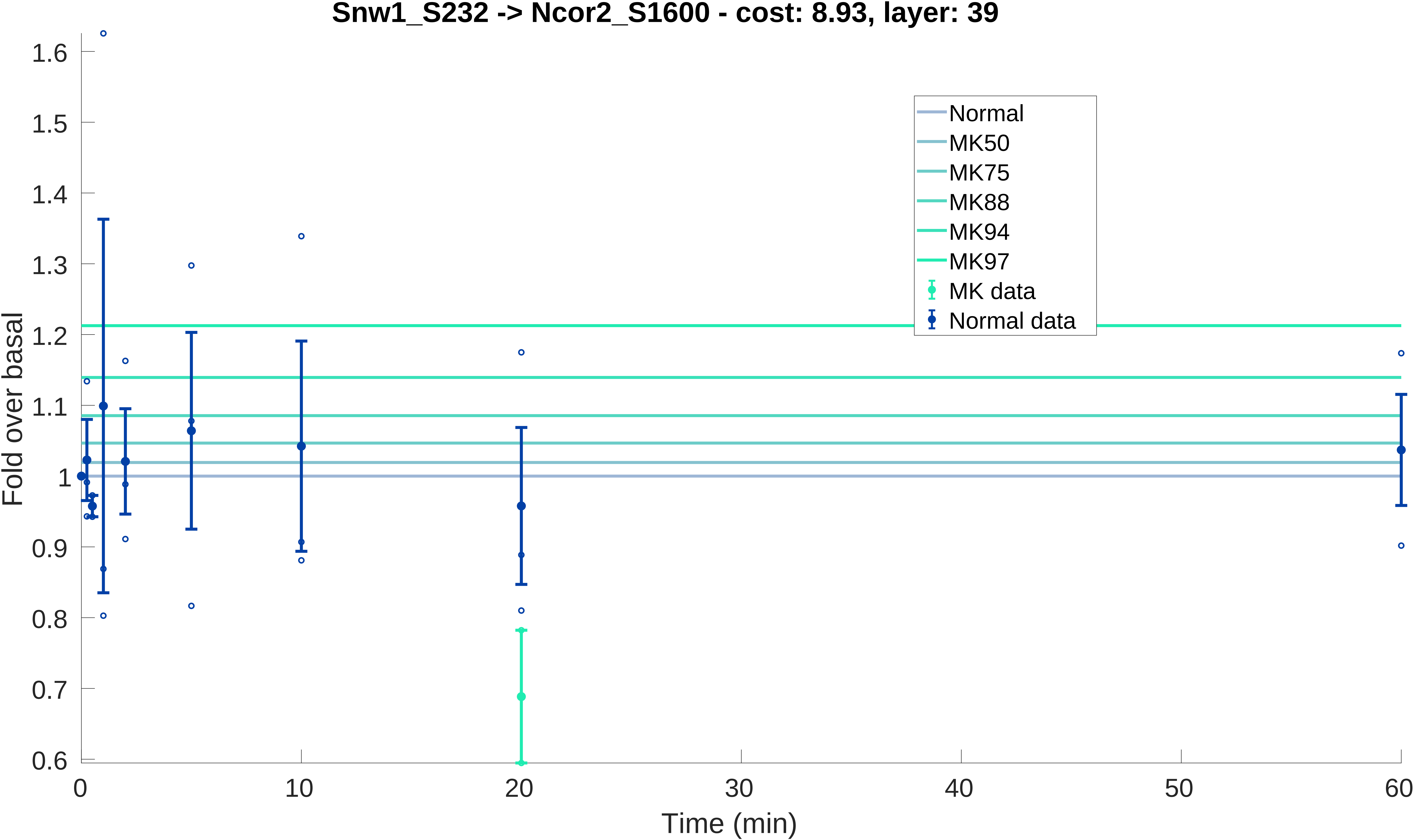

Snw1_S232 -> Ncor2_S1600 - cost: 8.93, layer: 39
Fold over basal
Time (min)
0.6
0.7
0.8
0.9
1
1.1
1.2
1.3
1.4
1.5
1.6
0
10
20
30
40
50
60
Normal
MK50
MK75
MK88
MK94
MK97
MK data
Normal data

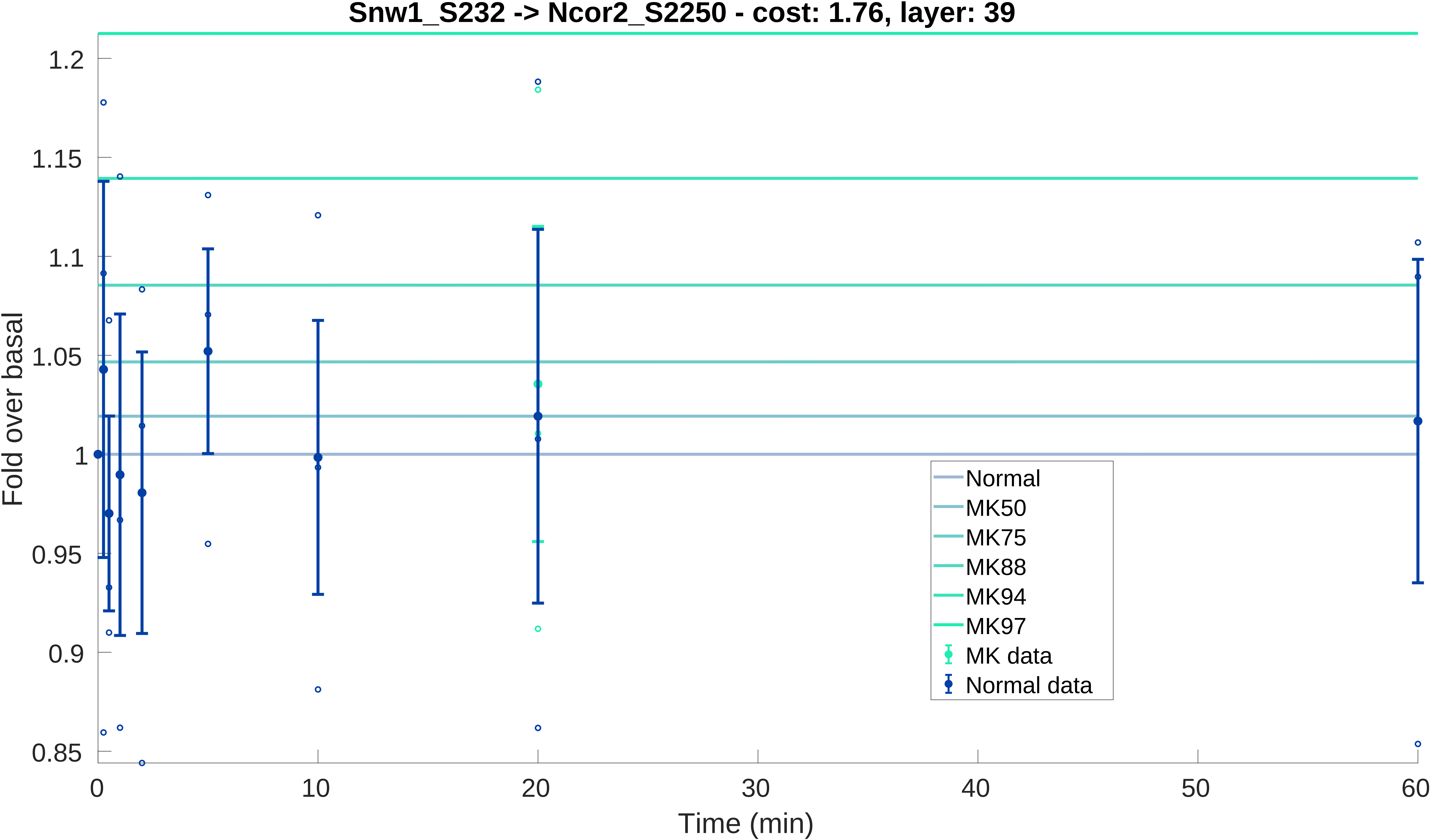
Snw1_S232 -> Ncor2_S2250 - cost: 1.76, layer: 39
Fold over basal
Time (min)
1.2
1.15
1.1
1.05
1
0.95
0.9
0.85
0
10
20
30
40
50
60
Normal
MK50
MK75
MK88
MK94
MK97
MK data
Normal data

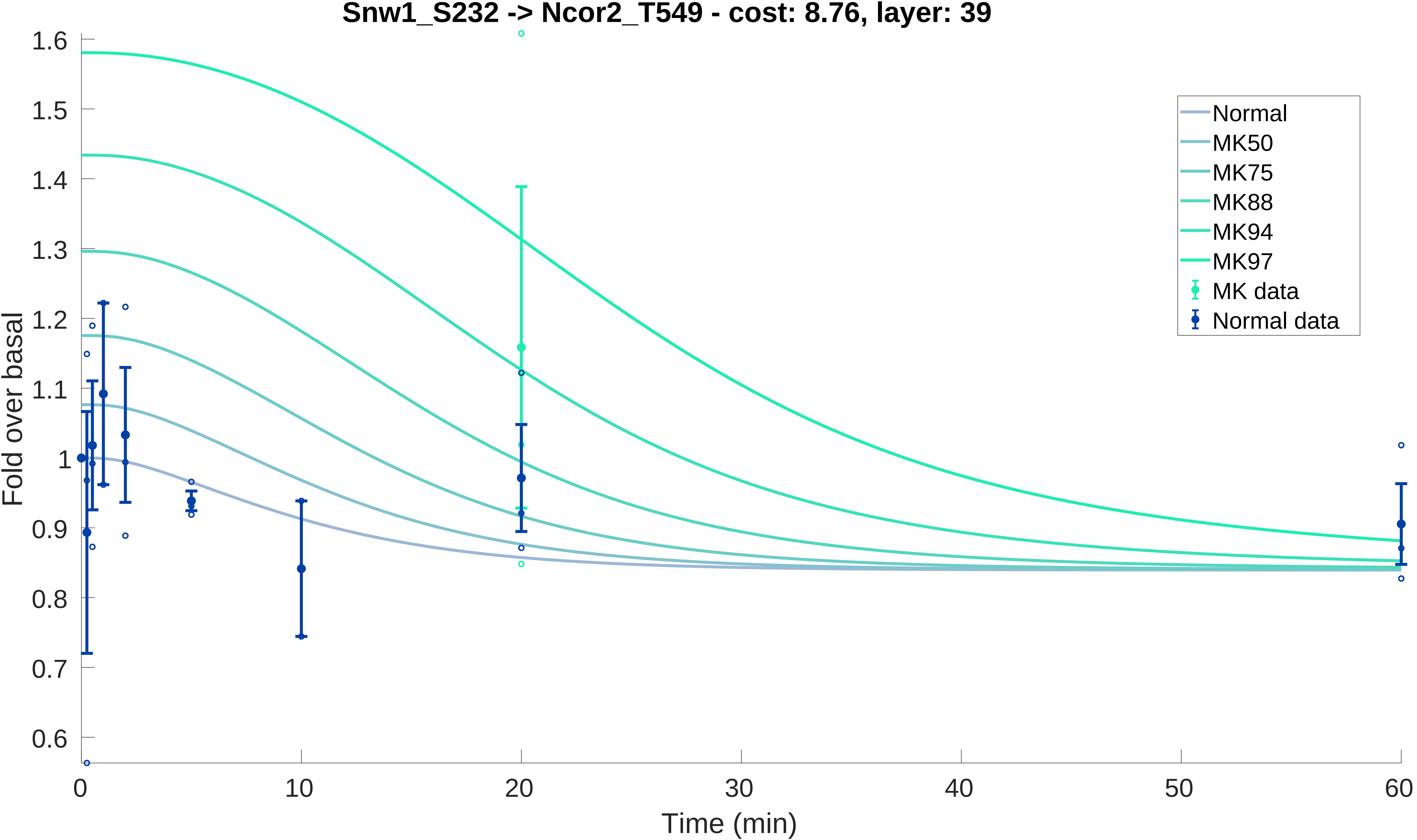

Snw1_S232 -> Ncor2_T549 - cost: 8.76, layer: 39
Fold over basal
Time (min)
1.6
1.5
1.4
1.3
1.2
1.1
1
0.9
0.8
0.7
0.6
0
10
20
30
40
50
60
Normal
MK50
MK75
MK88
MK94
MK97
MK data
Normal data

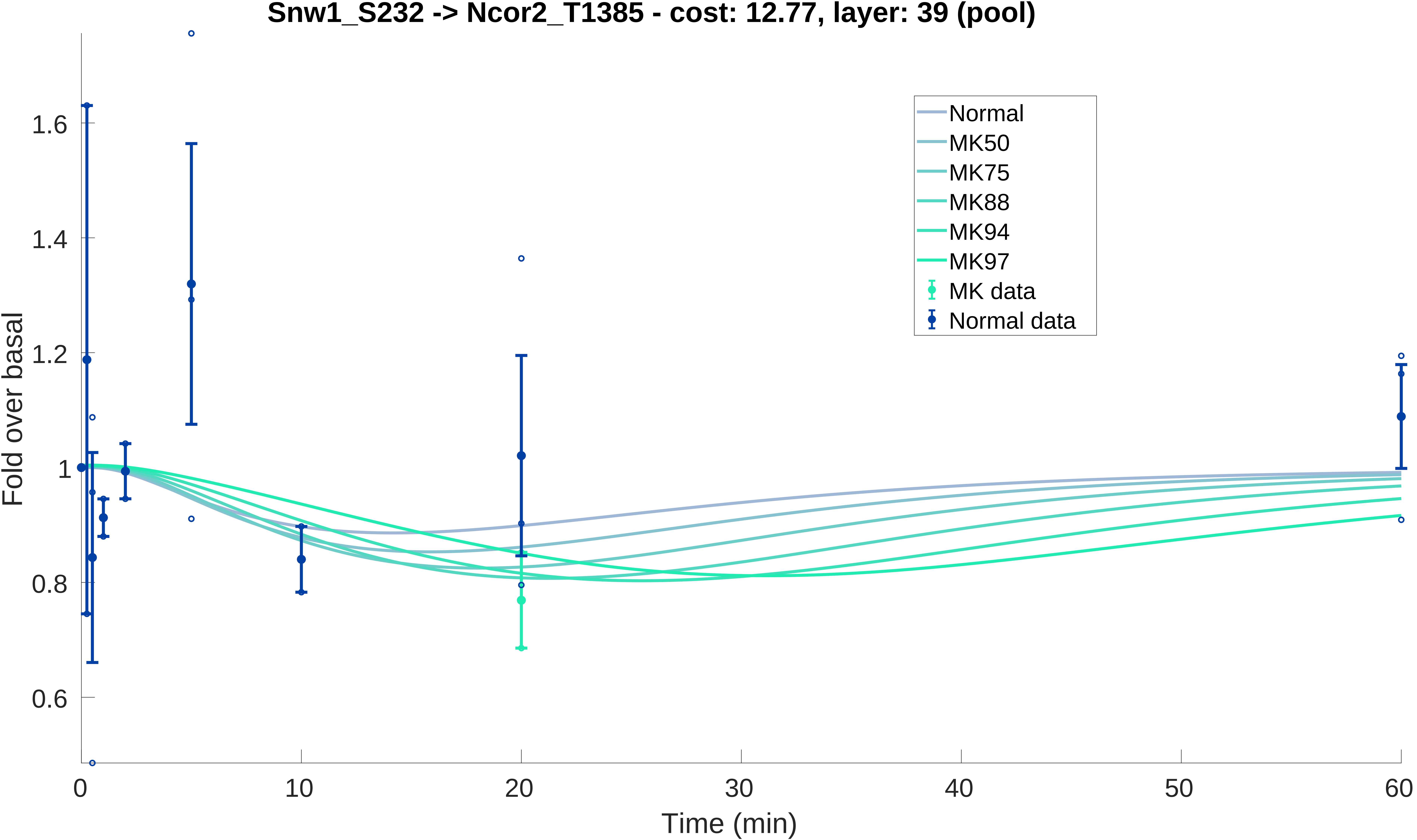
Snw1_S232 -> Ncor2_T1385 - cost: 12.77, layer: 39 (pool)
Fold over basal
Time (min)
1.6
1.4
1.2
1
0.8
0.6
0
10
20
30
40
50
60
Normal
MK50
MK75
MK88
MK94
MK97
MK data
Normal data

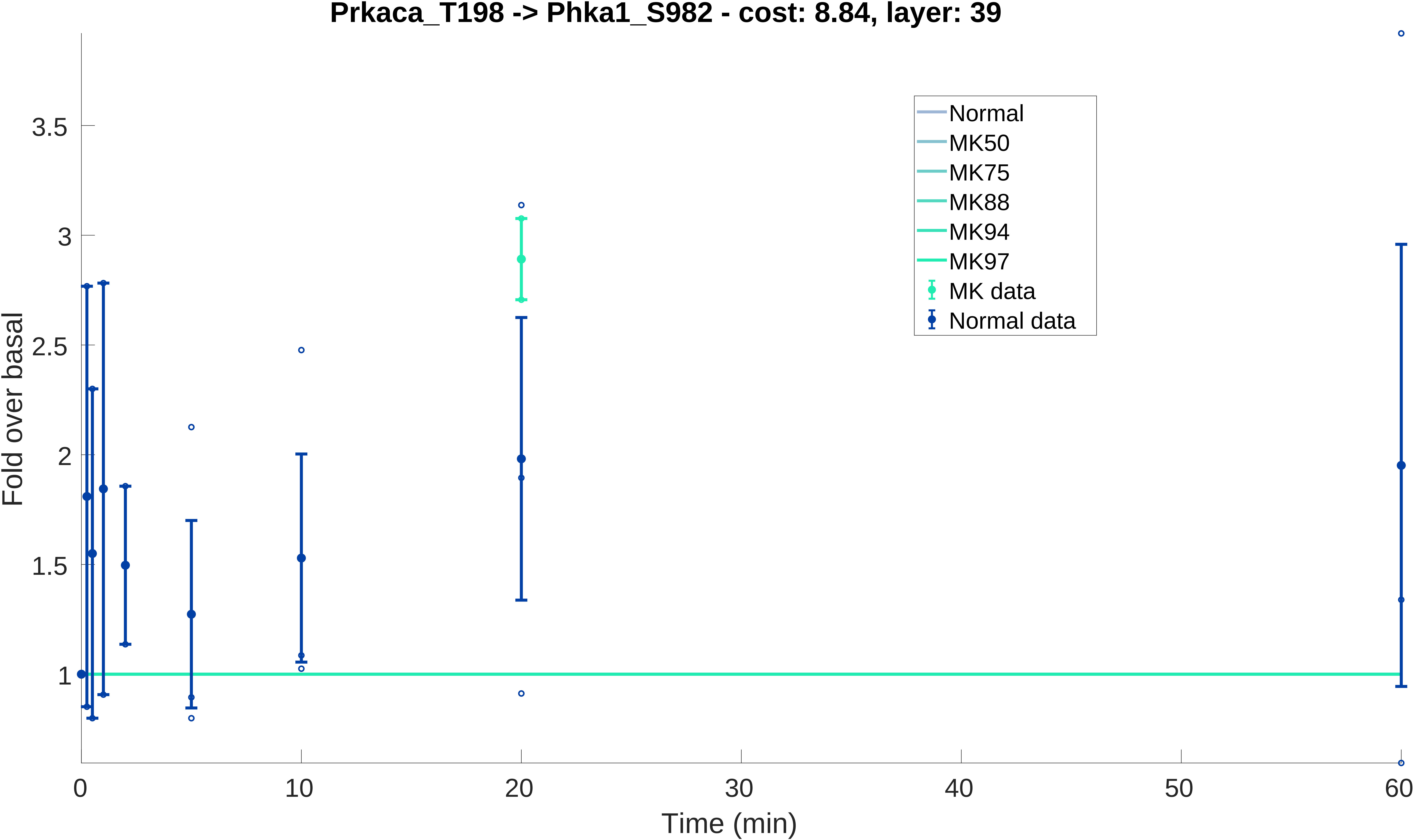

Prkaca_T198 -> Phka1_S982 - cost: 8.84, layer: 39
Fold over basal
Time (min)
3.5
3
2.5
2
1.5
1
0
10
20
30
40
50
60
Normal
MK50
MK75
MK88
MK94
MK97
MK data
Normal data

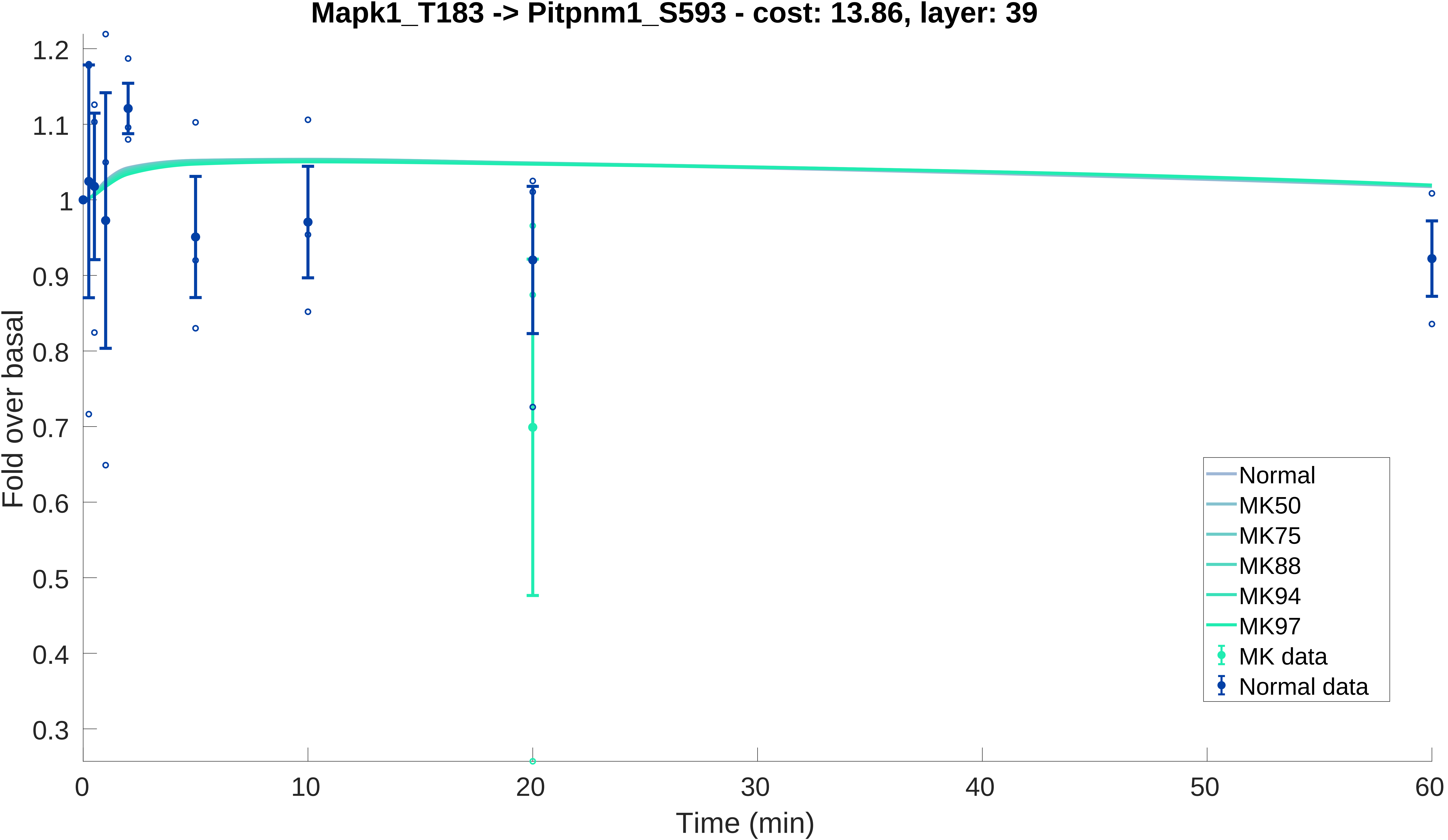

Mapk1_T183 -> Pitpnm1_S593 - cost: 13.86, layer: 39
Fold over basal
Time (min)
0
10
20
30
40
50
60
0.3
0.4
0.5
0.6
0.7
0.8
0.9
1
1.1
1.2
Normal
MK50
MK75
MK88
MK94
MK97
MK data
Normal data

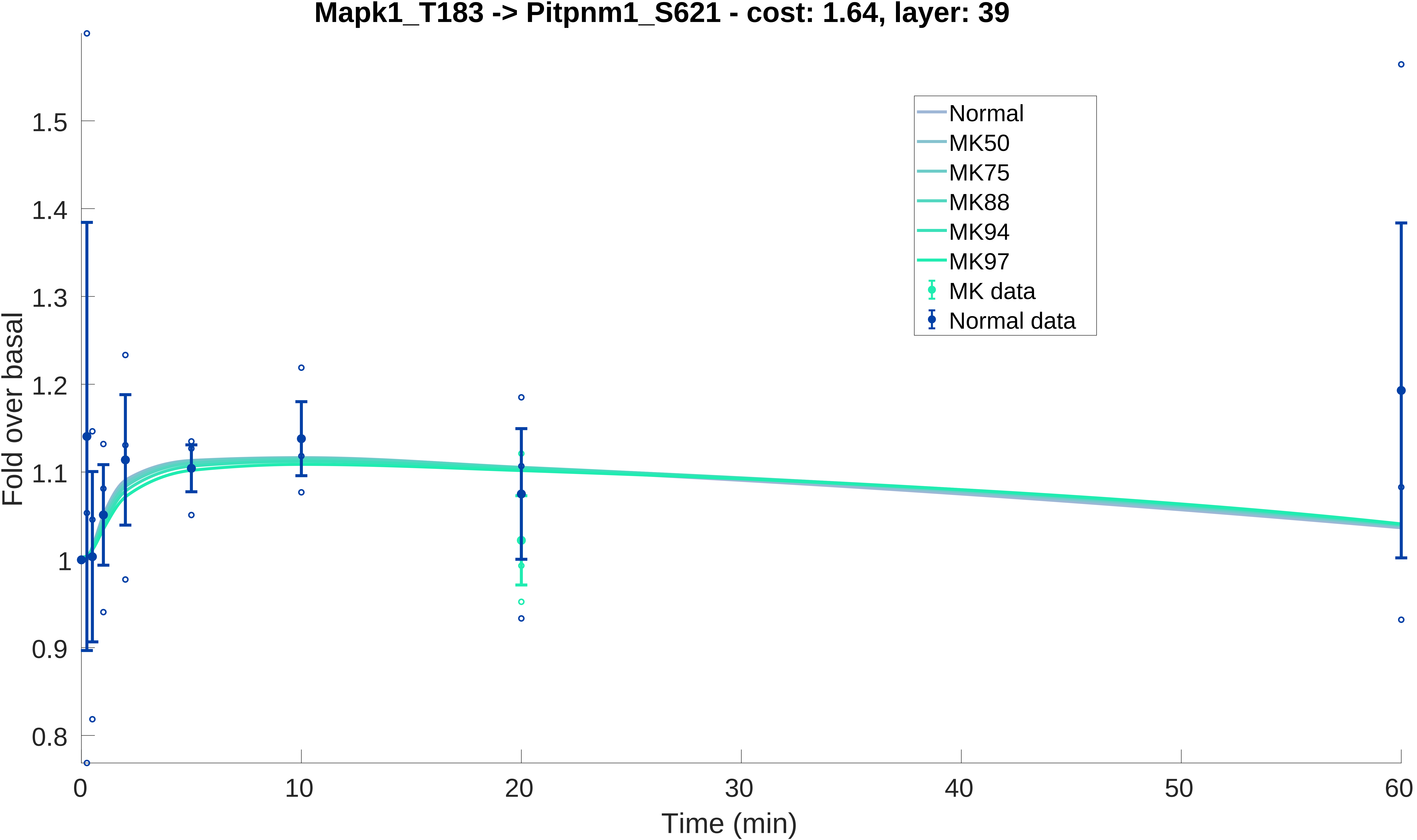

Mapk1_T183 -> Pitpnm1_S621 - cost: 1.64, layer: 39
Normal
MK50
MK75
MK88
MK94
MK97
MK data
Normal data
Fold over basal
1.5
1.4
1.3
1.2
1.1
1
0.9
0.8
0
10
20
30
40
50
60
Time (min)

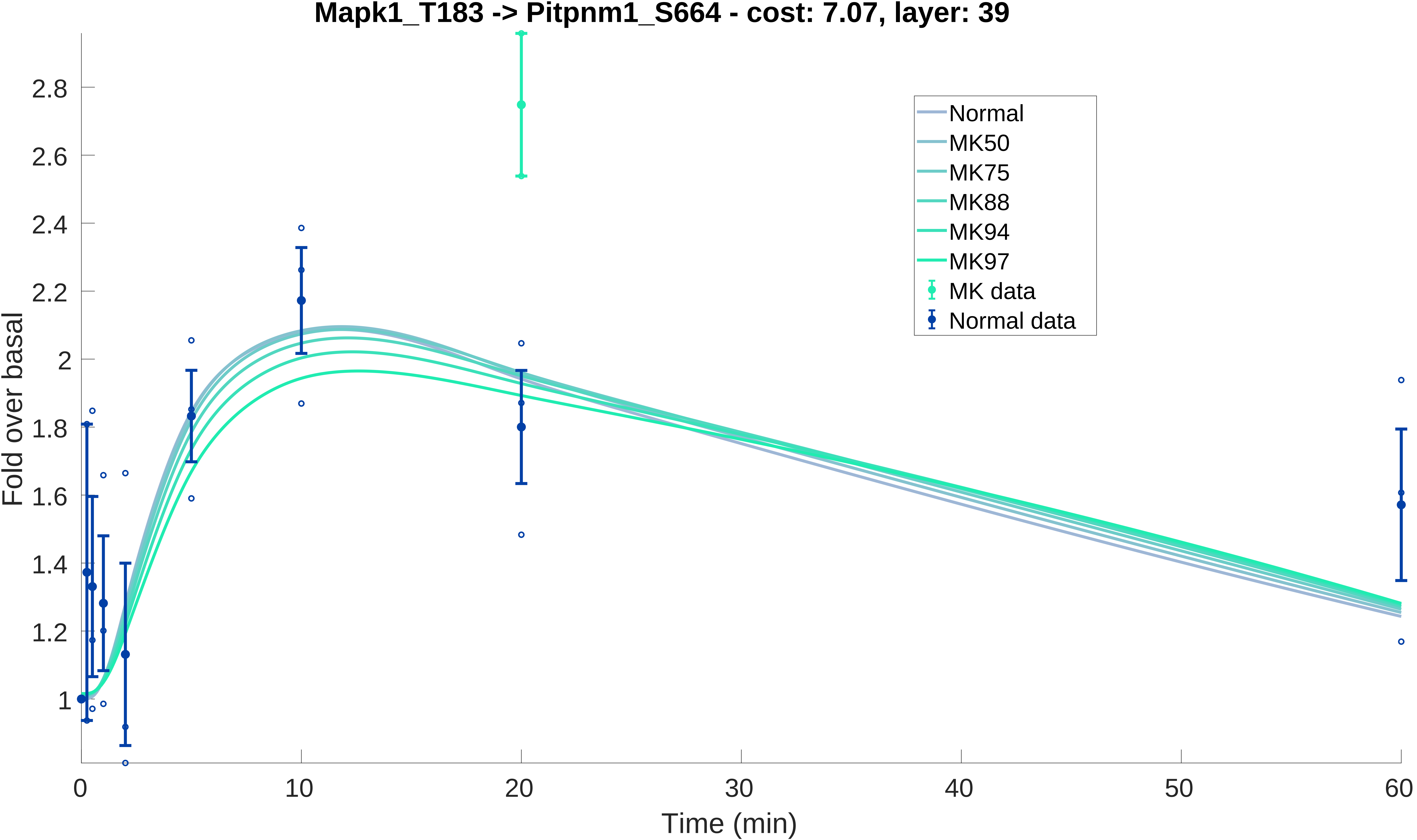

Mapk1_T183 -> Pitpnm1_S664 - cost: 7.07, layer: 39
Fold over basal
Time (min)
0
10
20
30
40
50
60
1
1.2
1.4
1.6
1.8
2
2.2
2.4
2.6
2.8
Normal
MK50
MK75
MK88
MK94
MK97
MK data
Normal data

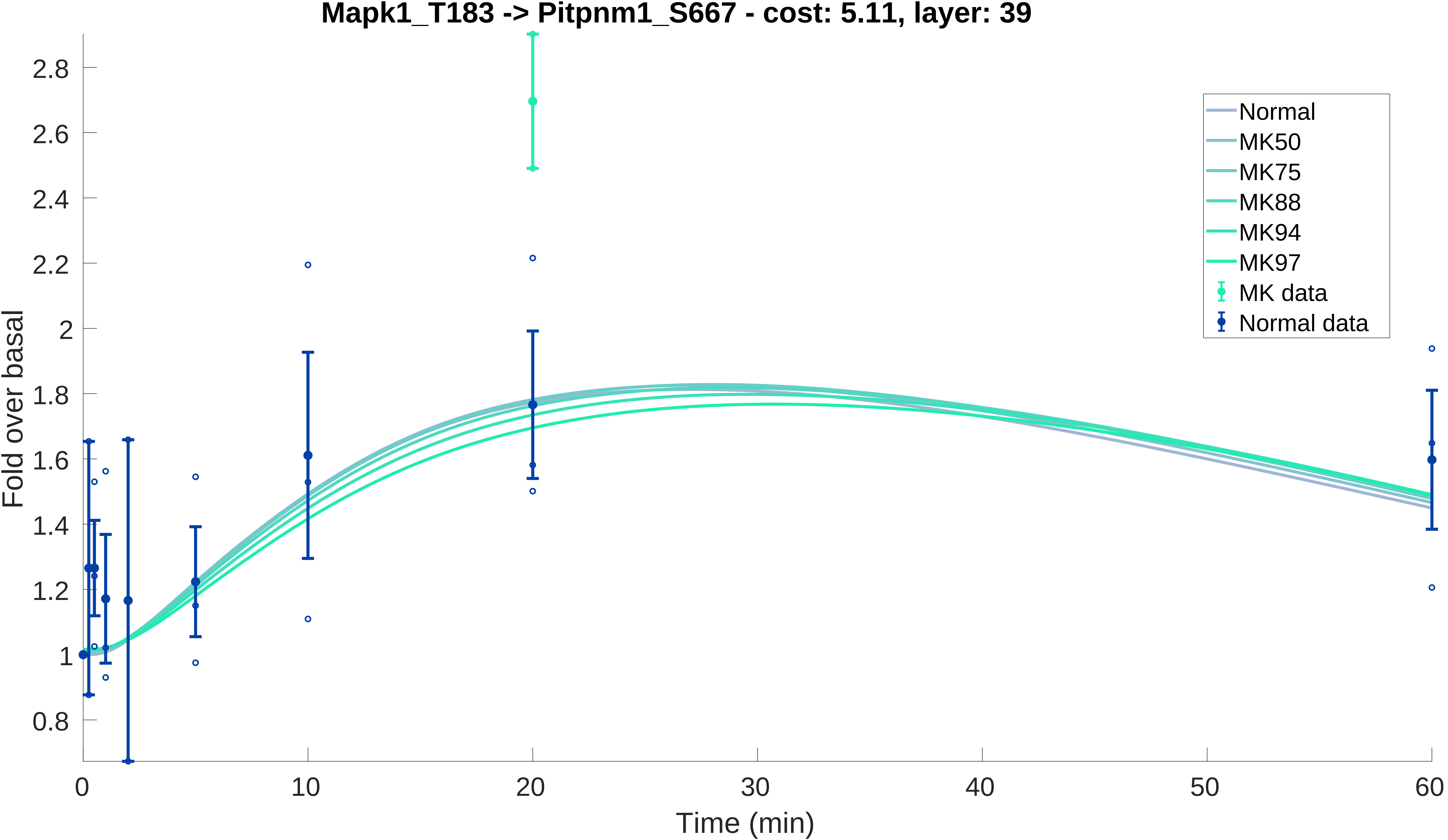

Mapk1_T183 -> Pitpnm1_S667 - cost: 5.11, layer: 39
Fold over basal
Time (min)
0.8
1
1.2
1.4
1.6
1.8
2
2.2
2.4
2.6
2.8
0
10
20
30
40
50
60
Normal
MK50
MK75
MK88
MK94
MK97
MK data
Normal data

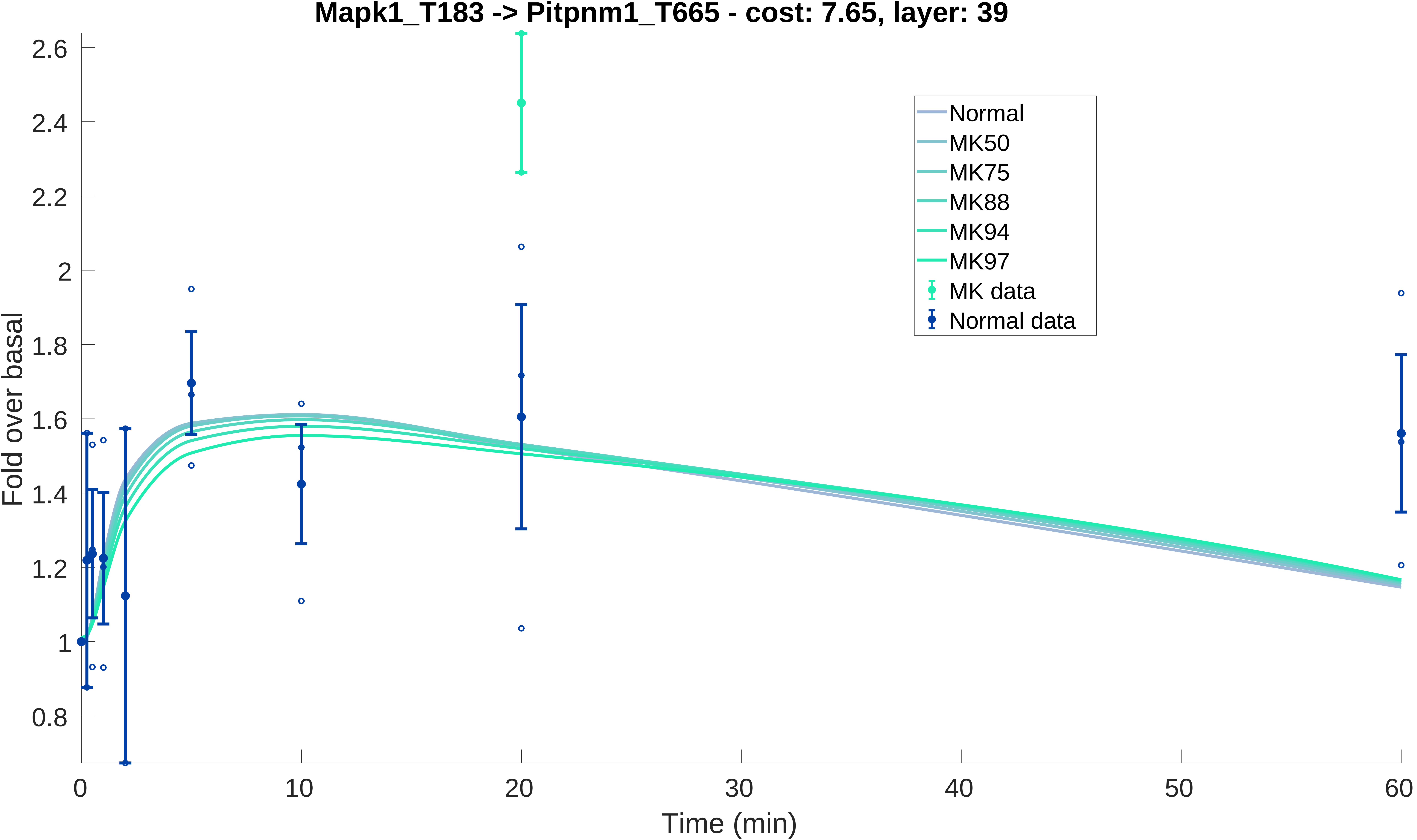
Mapk1_T183 -> Pitpnm1_T665 - cost: 7.65, layer: 39
Fold over basal
Time (min)
0.8
1
1.2
1.4
1.6
1.8
2
2.2
2.4
2.6
0
10
20
30
40
50
60
Normal
MK50
MK75
MK88
MK94
MK97
MK data
Normal data

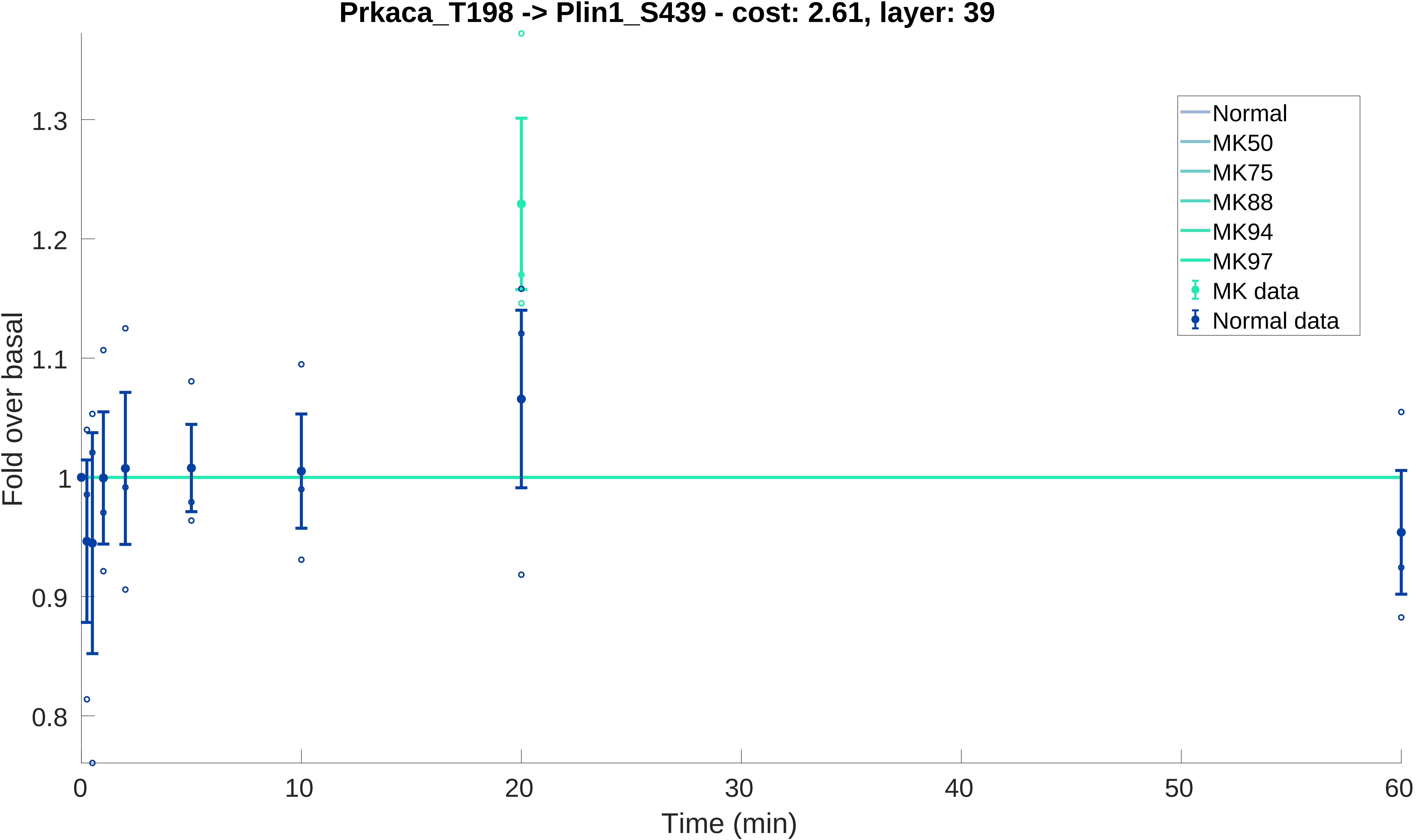

Prkaca_T198 -> Plin1_S439 - cost: 2.61, layer: 39
Fold over basal
Time (min)
1.3
1.2
1.1
1
0.9
0.8
0
10
20
30
40
50
60
Normal
MK50
MK75
MK88
MK94
MK97
MK data
Normal data

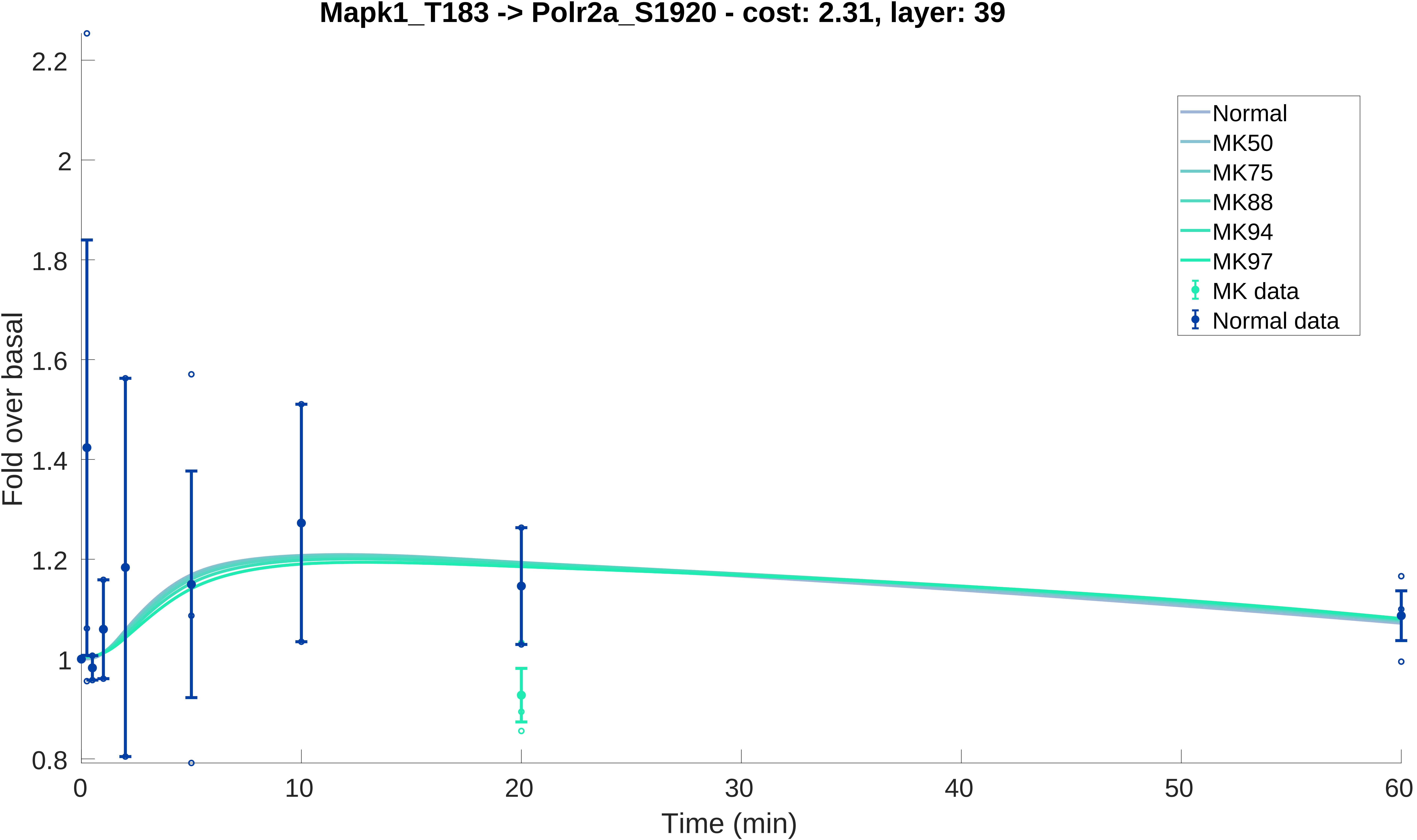

Mapk1_T183 -> Polr2a_S1920 - cost: 2.31, layer: 39
Fold over basal
Time (min)
2.2
2
1.8
1.6
1.4
1.2
1
0.8
0
10
20
30
40
50
60
Normal
MK50
MK75
MK88
MK94
MK97
MK data
Normal data

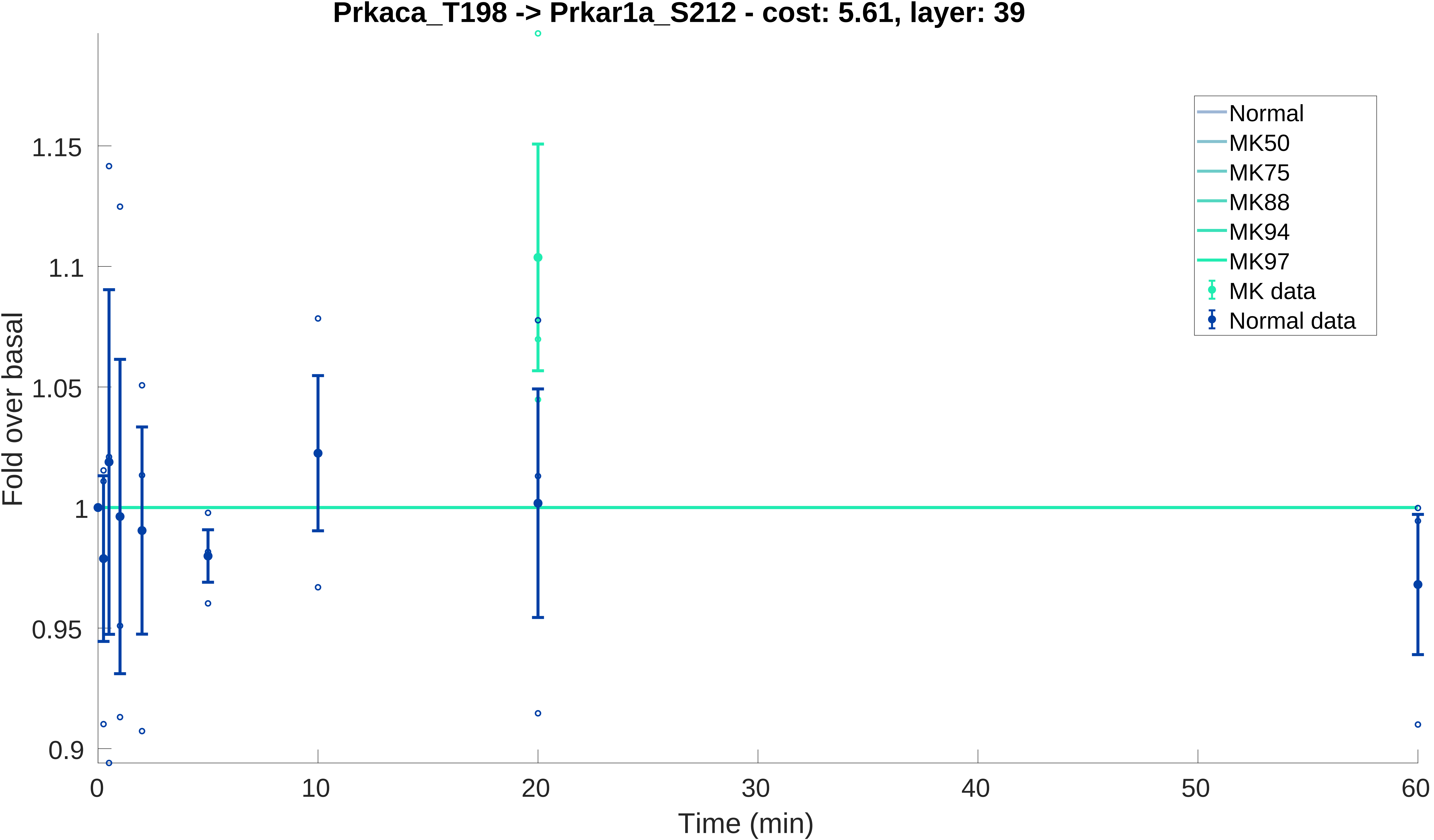

Prrkaca_T198 -> Prkar1a_S212 - cost: 5.61, layer: 39
Fold over basal
1.15
1.1
1.05
1
0.95
0.9
0
10
20
30
40
50
60
Time (min)
Normal
MK50
MK75
MK88
MK94
MK97
MK data
Normal data

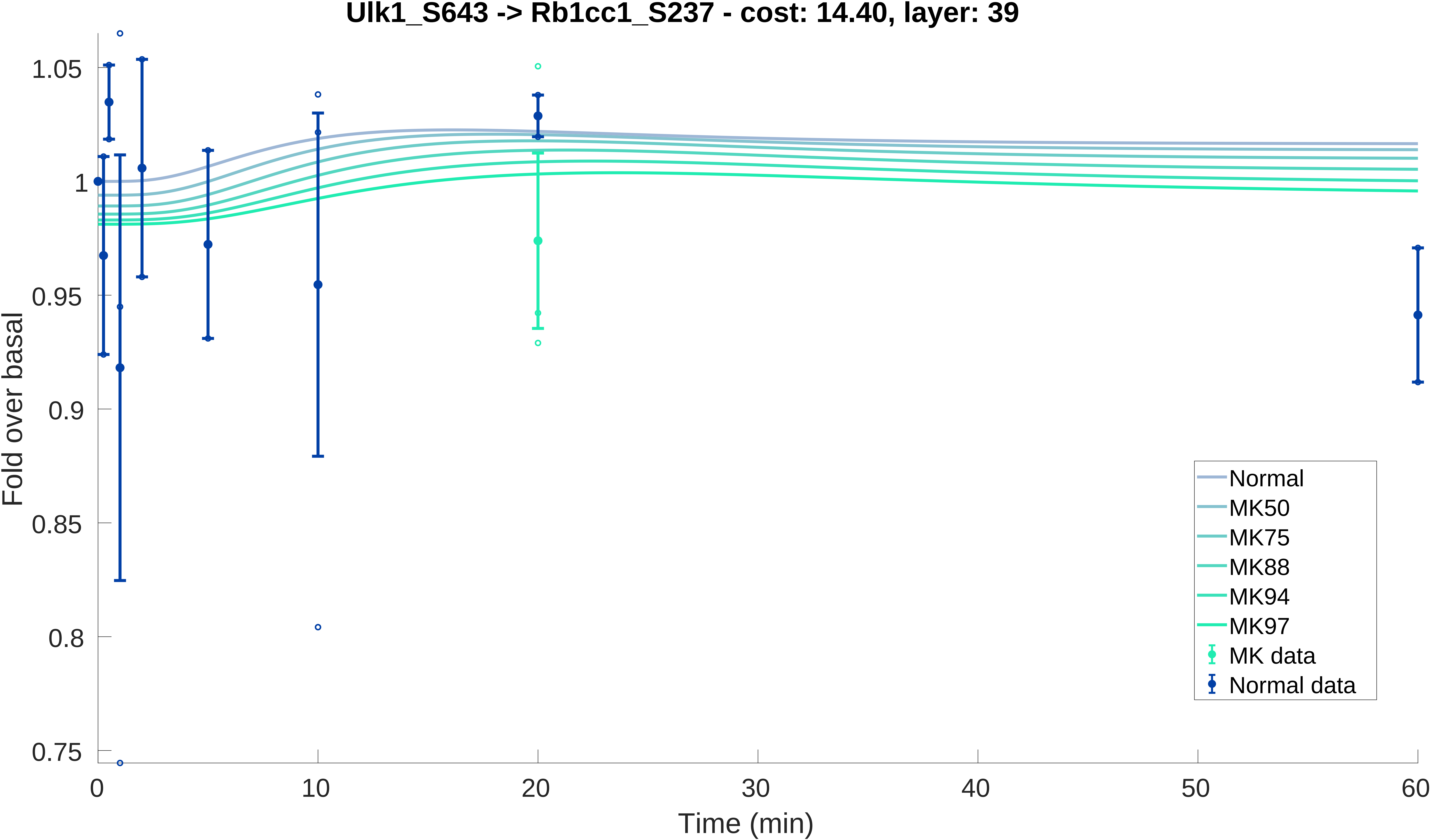
Ulk1_S643 -> Rb1cc1_S237 - cost: 14.40, layer: 39
Fold over basal
1.05
1
0.95
0.9
0.85
0.8
0.75
0
10
20
30
40
50
60
Time (min)
Normal
MK50
MK75
MK88
MK94
MK97
MK data
Normal data

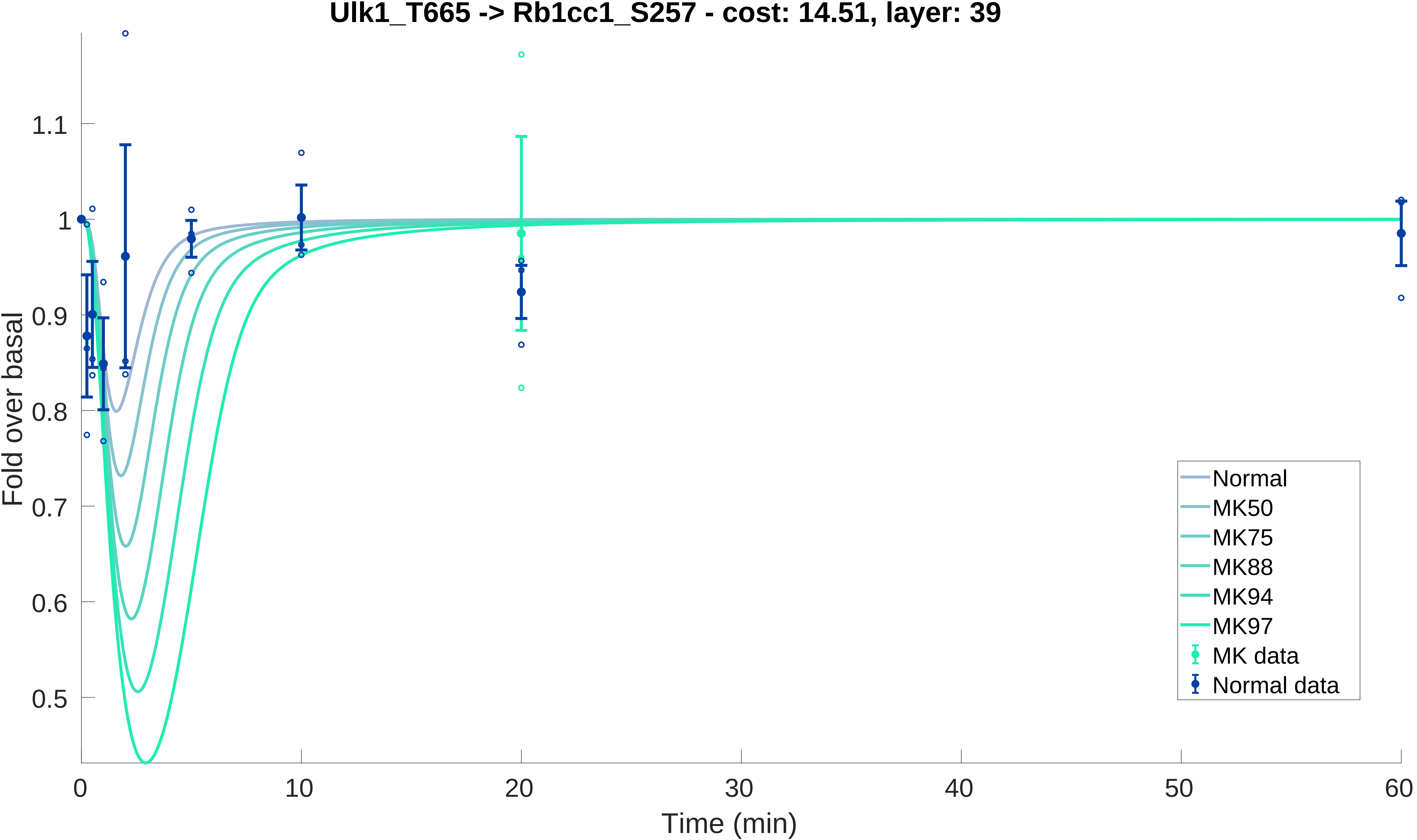
Ulk1_T665 -> Rb1cc1_S257 - cost: 14.51, layer: 39
Fold over basal
1.1
1
0.9
0.8
0.7
0.6
0.5
0
10
20
30
40
50
60
Time (min)
Normal
MK50
MK75
MK88
MK94
MK97
MK data
Normal data

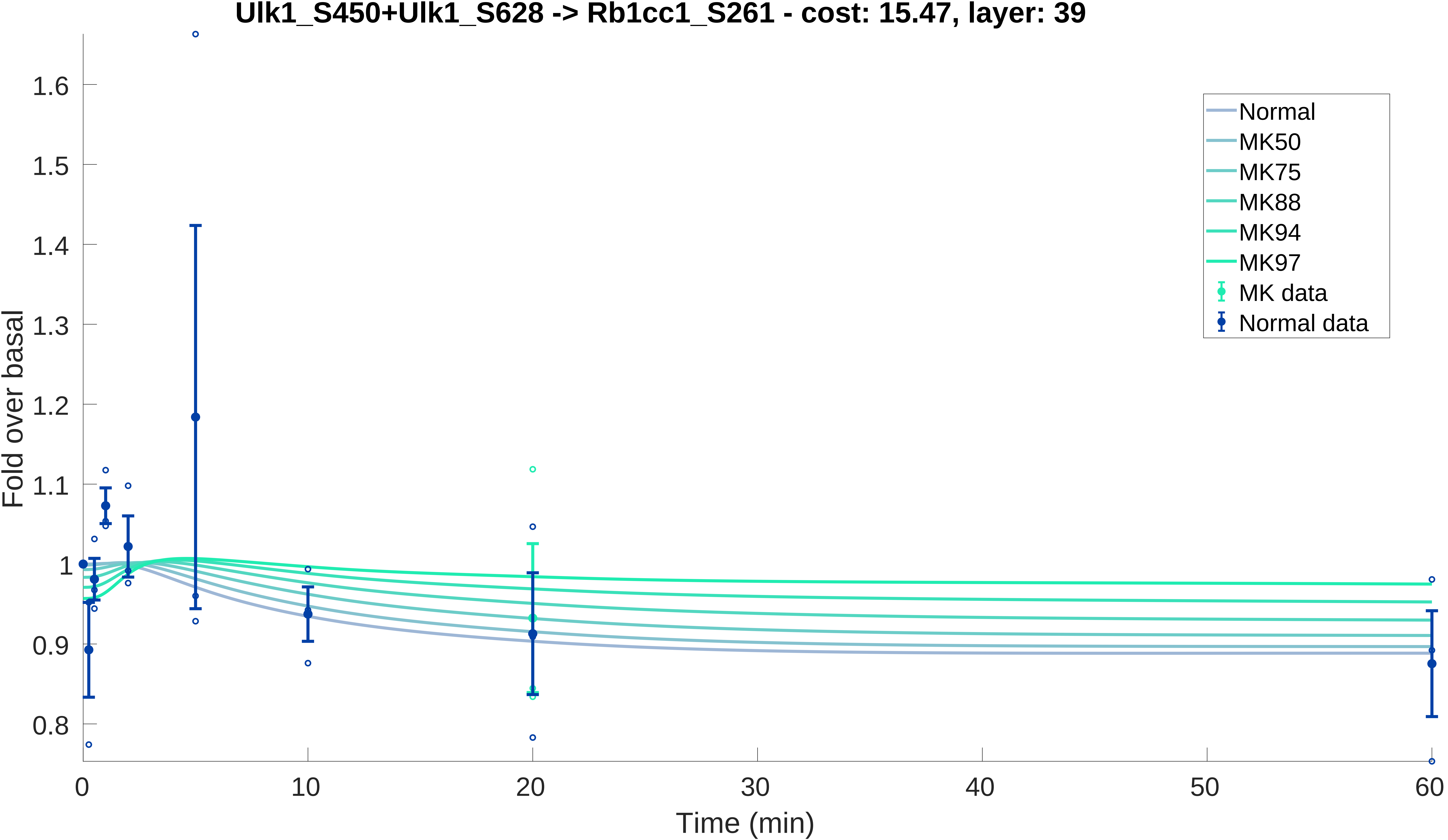
Ulk1_S450+Ulk1_S628 -> Rb1cc1_S261 - cost: 15.47, layer: 39
Fold over basal
1.6
1.5
1.4
1.3
1.2
1.1
1
0.9
0.8
0
10
20
30
40
50
60
Time (min)
Normal
MK50
MK75
MK88
MK94
MK97
MK data
Normal data

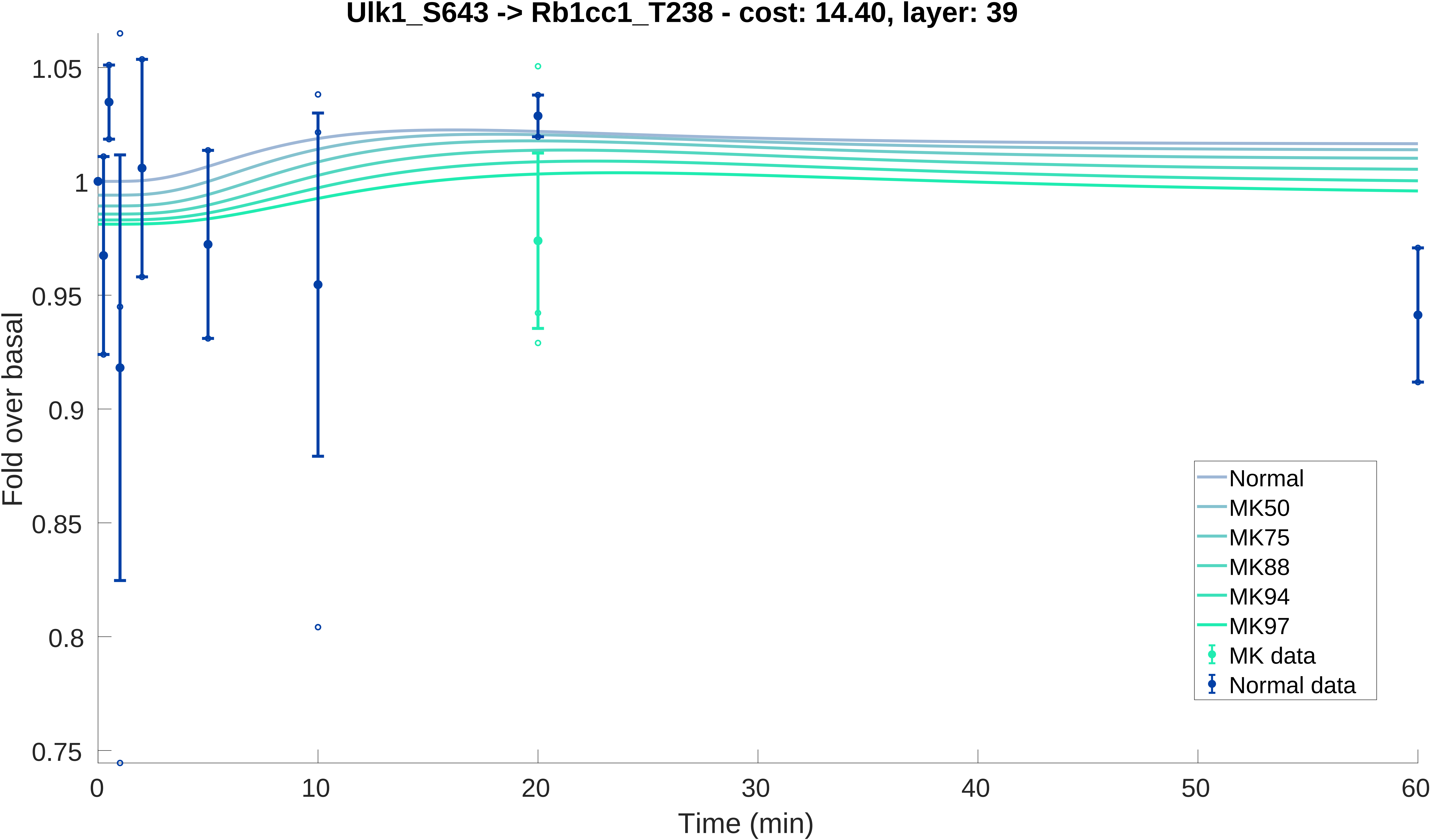

Ulk1_S643 -> Rb1cc1_T238 - cost: 14.40, layer: 39
Fold over basal
1.05
1
0.95
0.9
0.85
0.8
0.75
0
10
20
30
40
50
60
Time (min)
Normal
MK50
MK75
MK88
MK94
MK97
MK data
Normal data

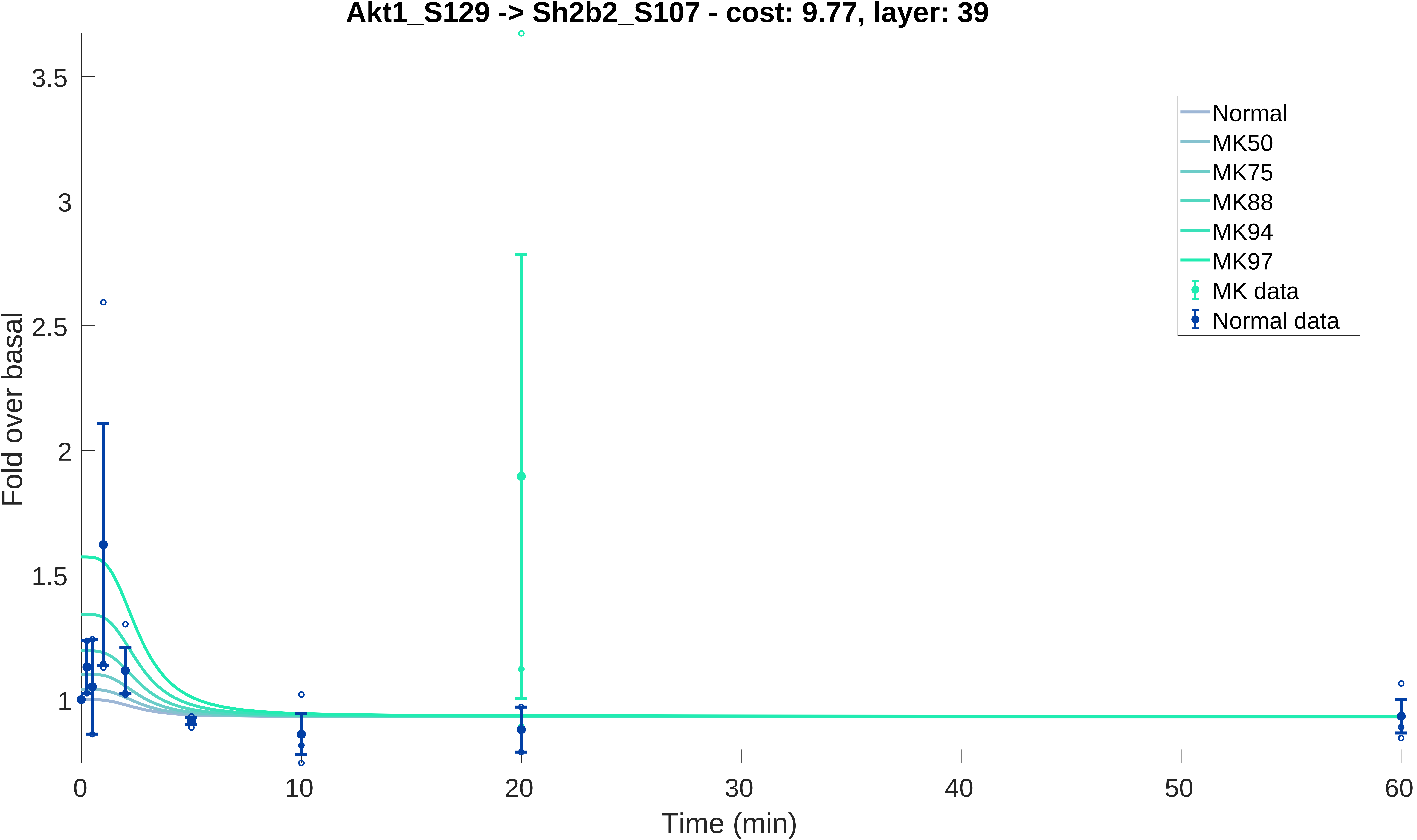

Akt1_S129 -> Sh2b2_S107 - cost: 9.77, layer: 39
Fold over basal
Time (min)
Normal
MK50
MK75
MK88
MK94
MK97
MK data
Normal data

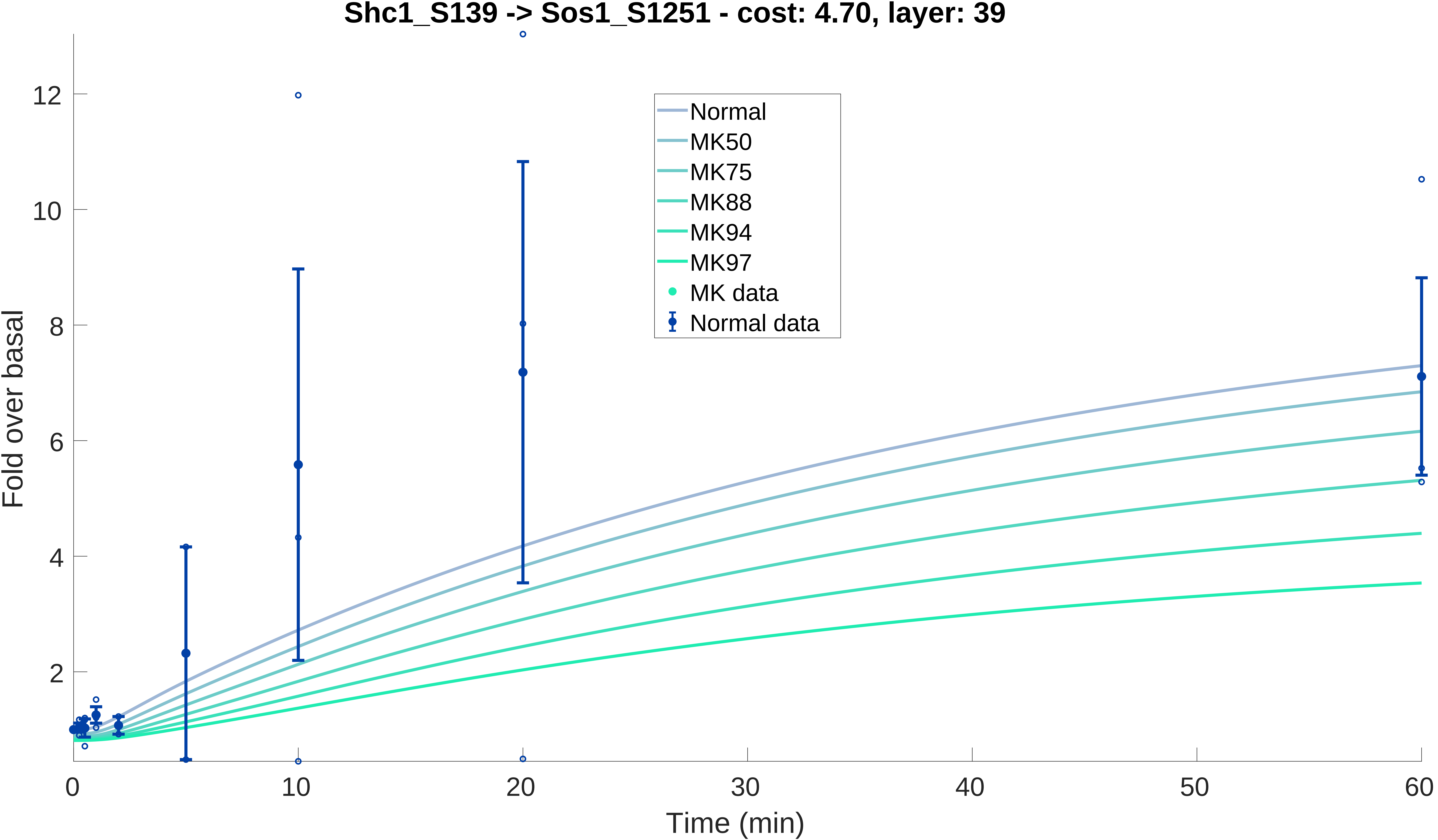

Shc1_S139 -> Sos1_S1251 - cost: 4.70, layer: 39
Normal
MK50
MK75
MK88
MK94
MK97
MK data
Normal data
Fold over basal
Time (min)
0
10
20
30
40
50
60
2
4
6
8
10
12

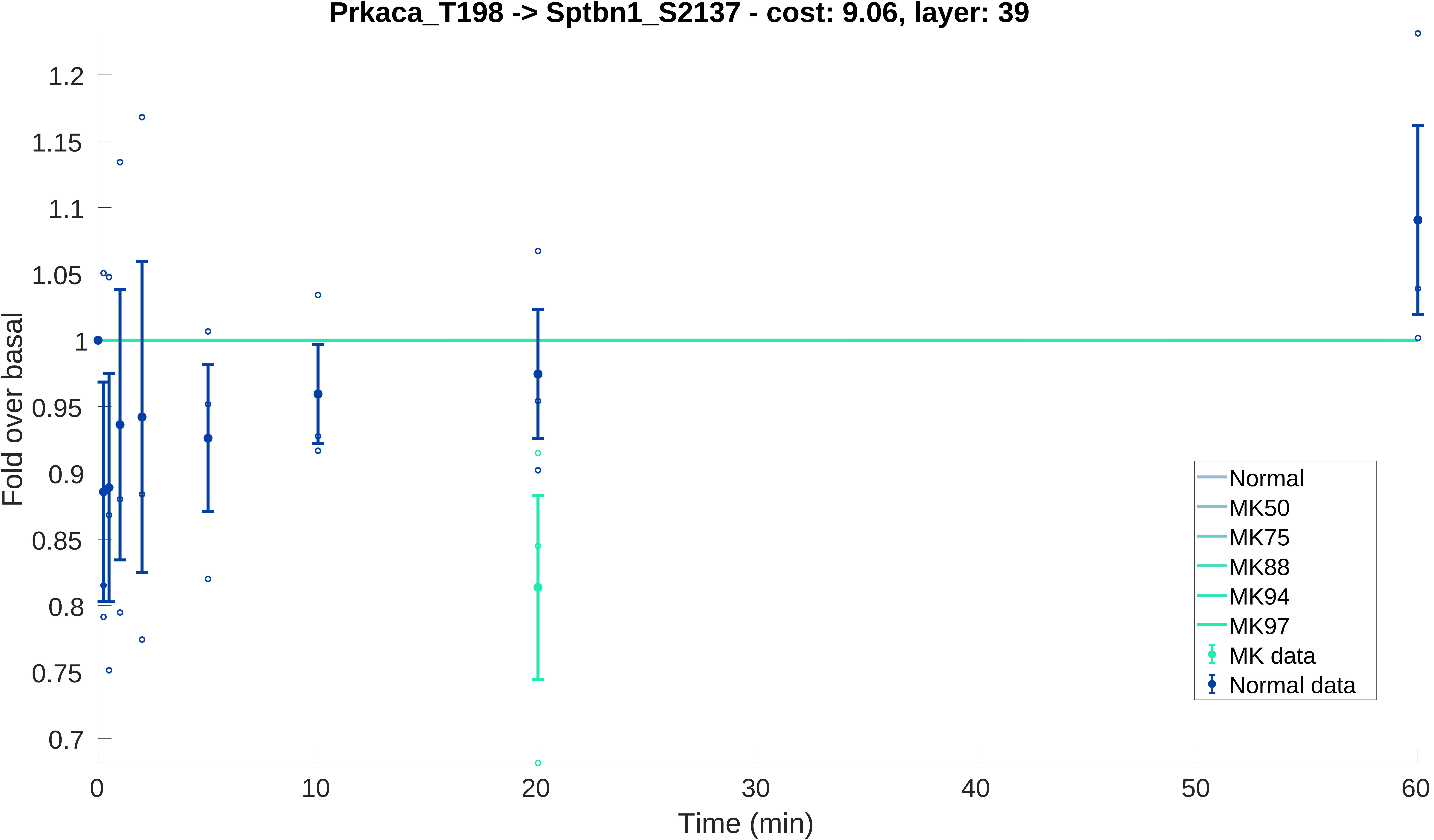
Prkaca_T198 -> Sptbn1_S2137 - cost: 9.06, layer: 39
Fold over basal
Time (min)
1.2
1.15
1.1
1.05
1
0.95
0.9
0.85
0.8
0.75
0.7
0
10
20
30
40
50
60
Normal
MK50
MK75
MK88
MK94
MK97
MK data
Normal data

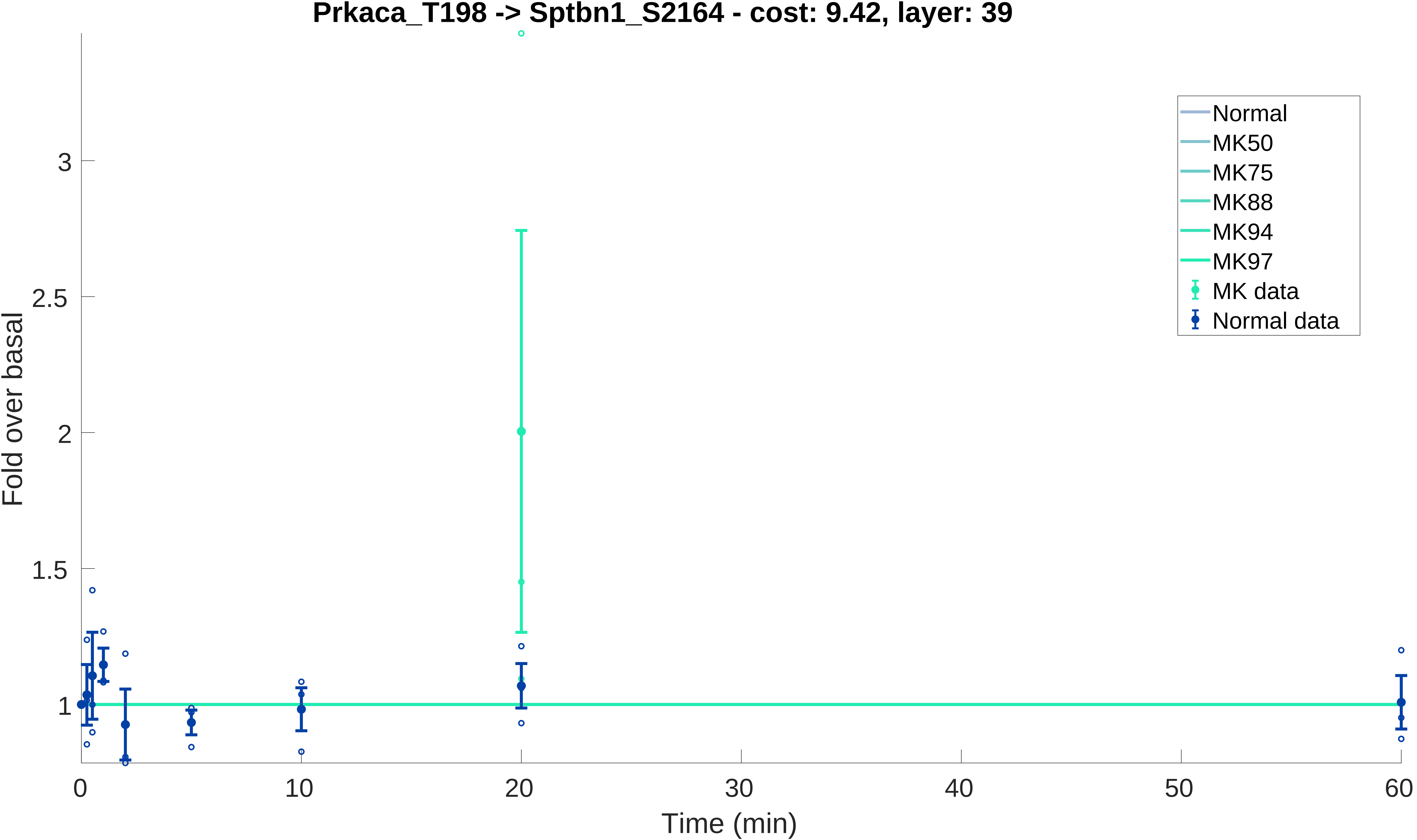

Prkaca_T198 -> Sptbn1_S2164 - cost: 9.42, layer: 39
Normal
MK50
MK75
MK88
MK94
MK97
MK data
Normal data
Fold over basal
Time (min)
0
10
20
30
40
50
60
1
1.5
2
2.5
3

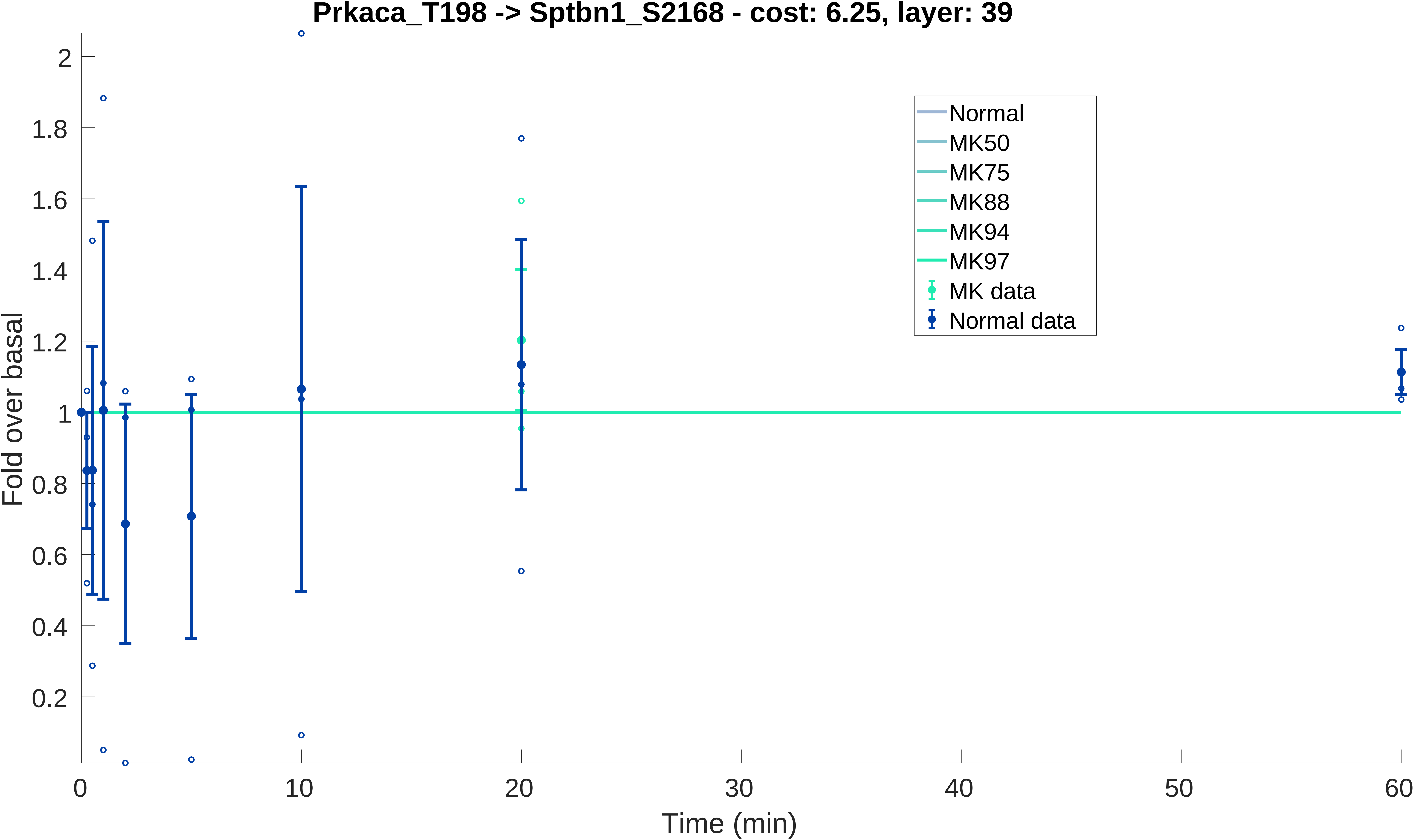

Prkaca_T198 -> Sptbn1_S2168 - cost: 6.25, layer: 39
Fold over basal
Time (min)
Normal
MK50
MK75
MK88
MK94
MK97
MK data
Normal data
0
10
20
30
40
50
60
0.2
0.4
0.6
0.8
1
1.2
1.4
1.6
1.8
2

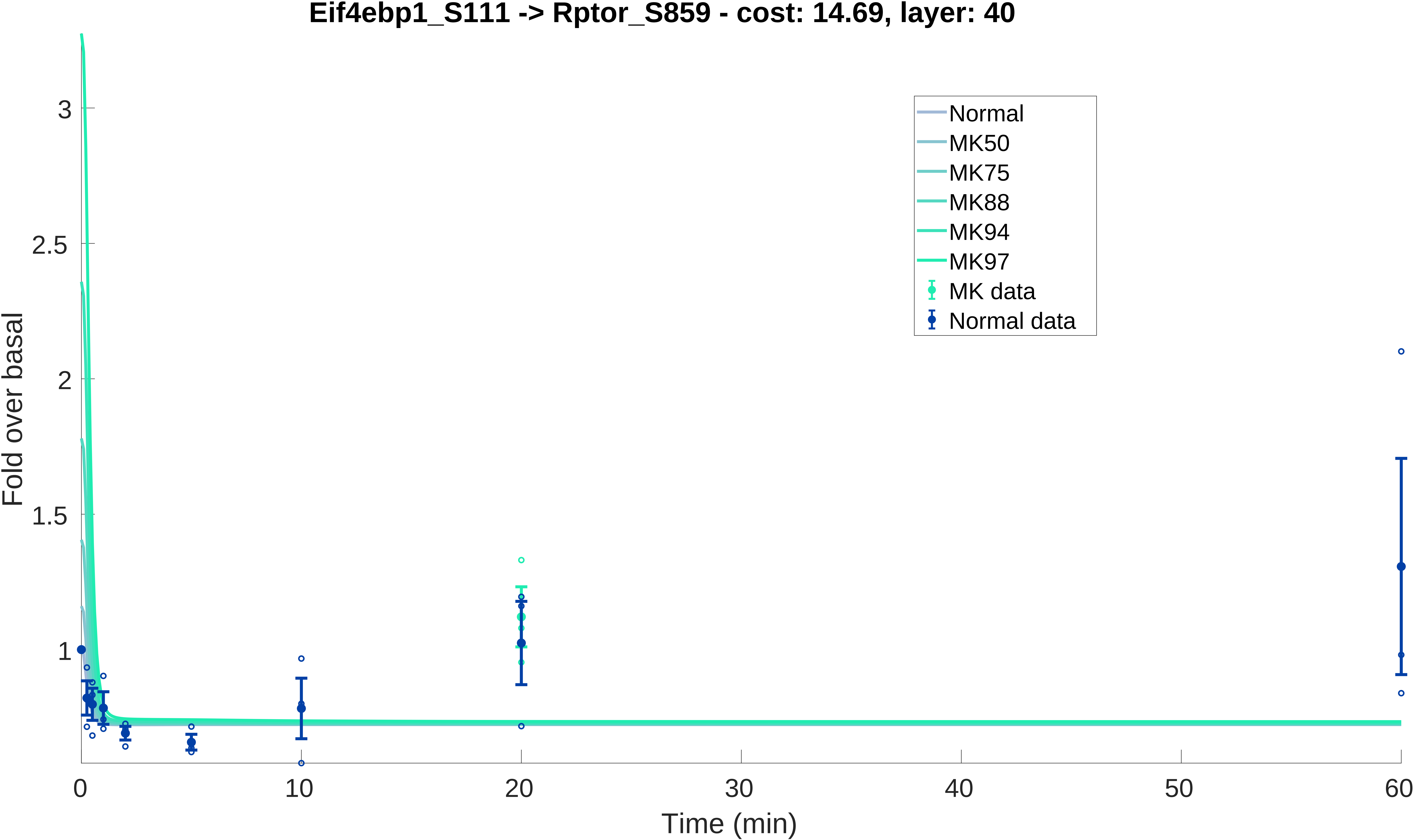
Eif4ebp1_S111 -> Rptor_S859 - cost: 14.69, layer: 40
Fold over basal
3
2.5
2
1.5
1
0
10
20
30
40
50
60
Time (min)
Normal
MK50
MK75
MK88
MK94
MK97
MK data
Normal data

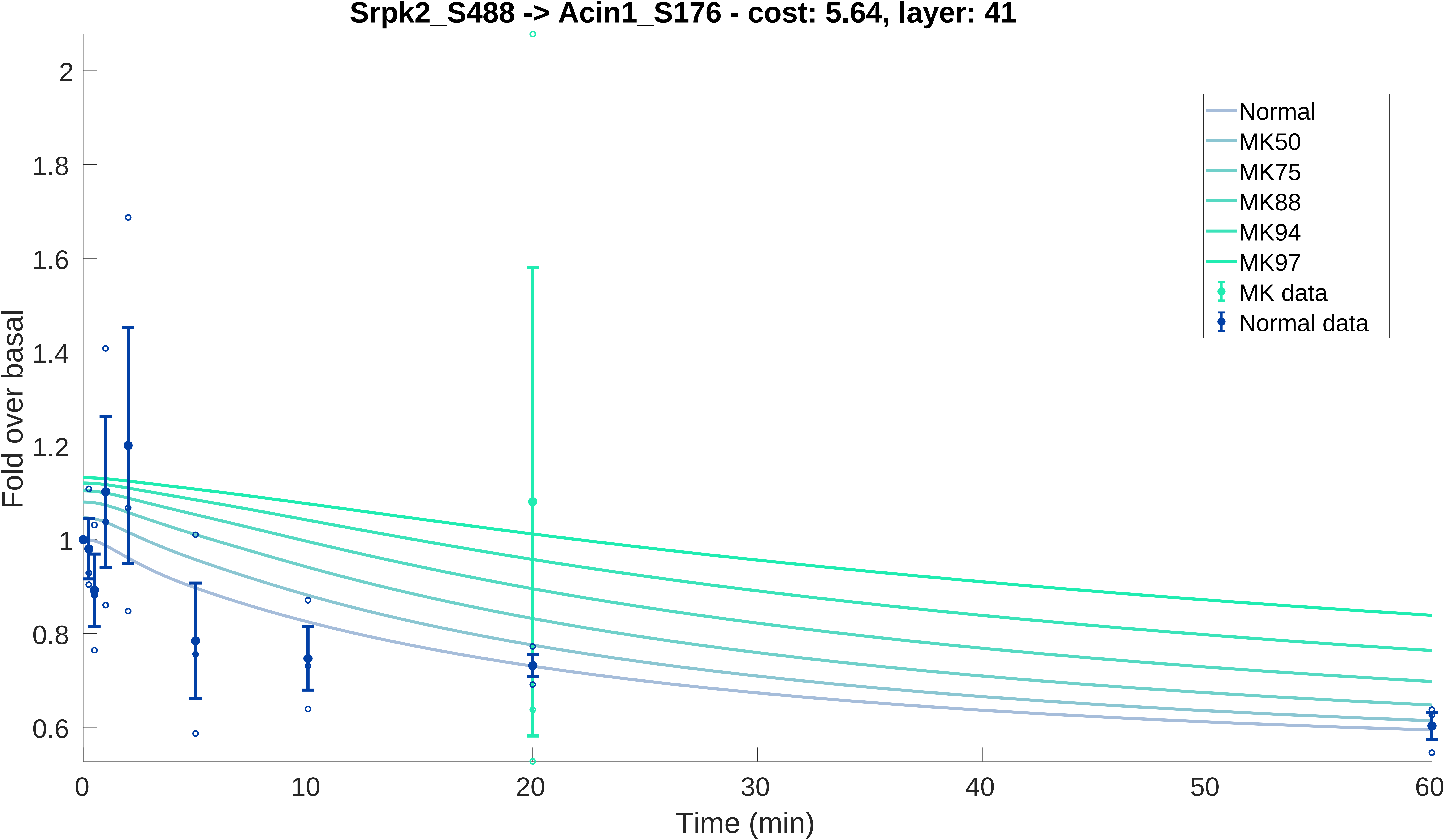
Srpk2_S488 -> Acin1_S176 - cost: 5.64, layer: 41
Fold over basal
Time (min)
0
10
20
30
40
50
60
0.6
0.8
1
1.2
1.4
1.6
1.8
2
Normal
MK50
MK75
MK88
MK94
MK97
MK data
Normal data

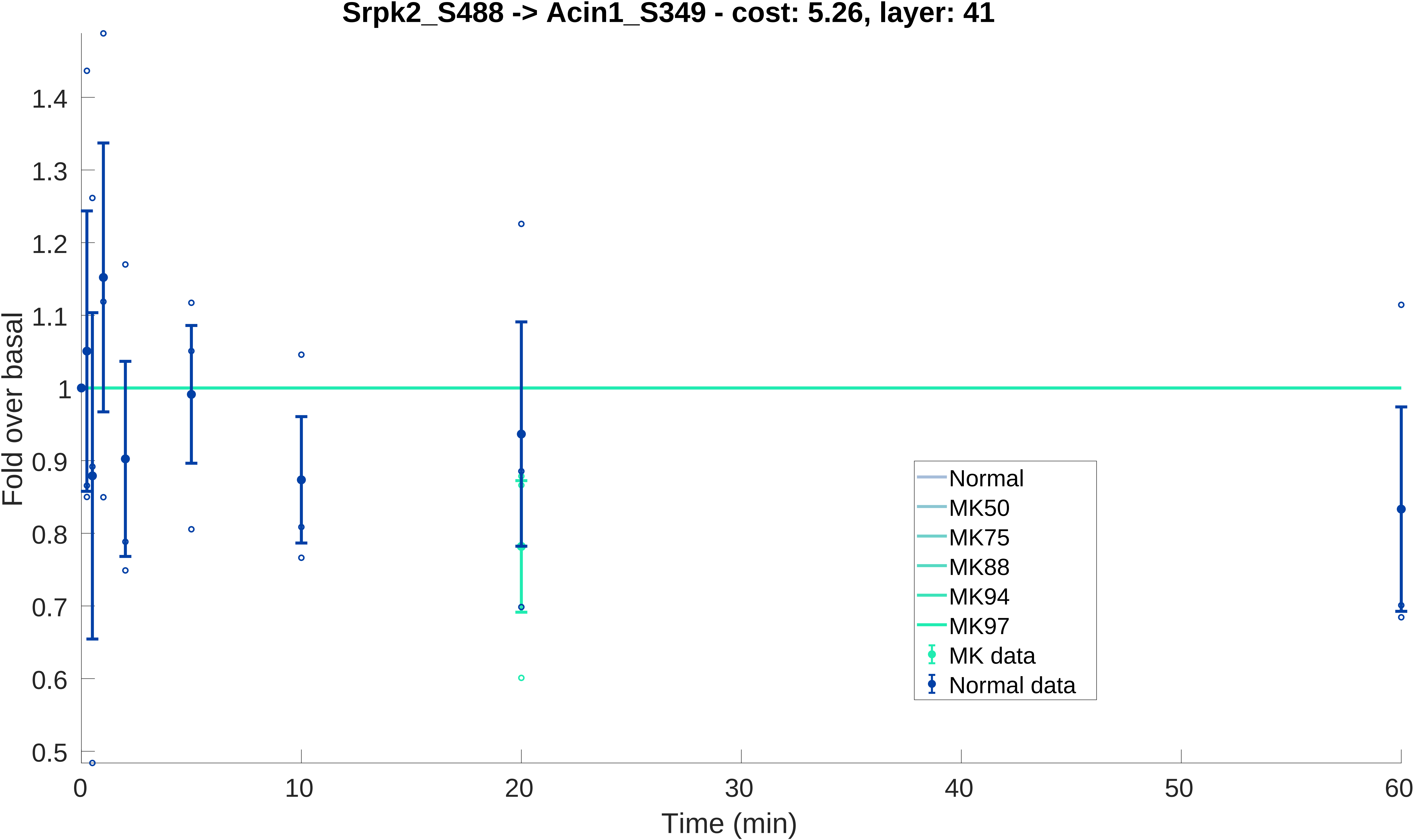
Srpk2_S488 -> Acin1_S349 - cost: 5.26, layer: 41
Fold over basal
Time (min)
0
10
20
30
40
50
60
0.5
0.6
0.7
0.8
0.9
1
1.1
1.2
1.3
1.4
Normal
MK50
MK75
MK88
MK94
MK97
MK data
Normal data

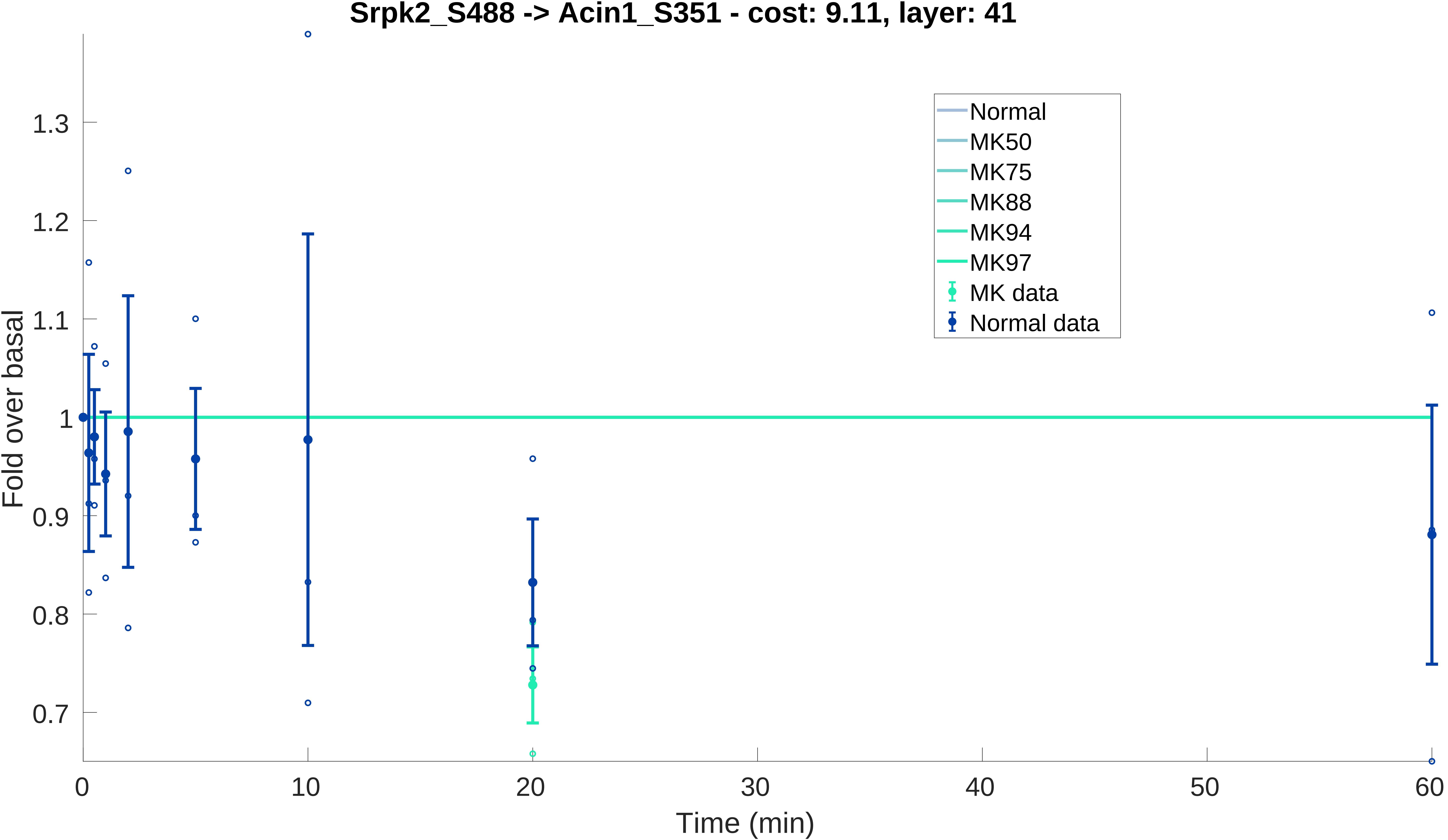

Srpk2_S488 -> Acin1_S351 - cost: 9.11, layer: 41
Fold over basal
Time (min)
Normal
MK50
MK75
MK88
MK94
MK97
MK data
Normal data
1.3
1.2
1.1
1
0.9
0.8
0.7
0
10
20
30
40
50
60

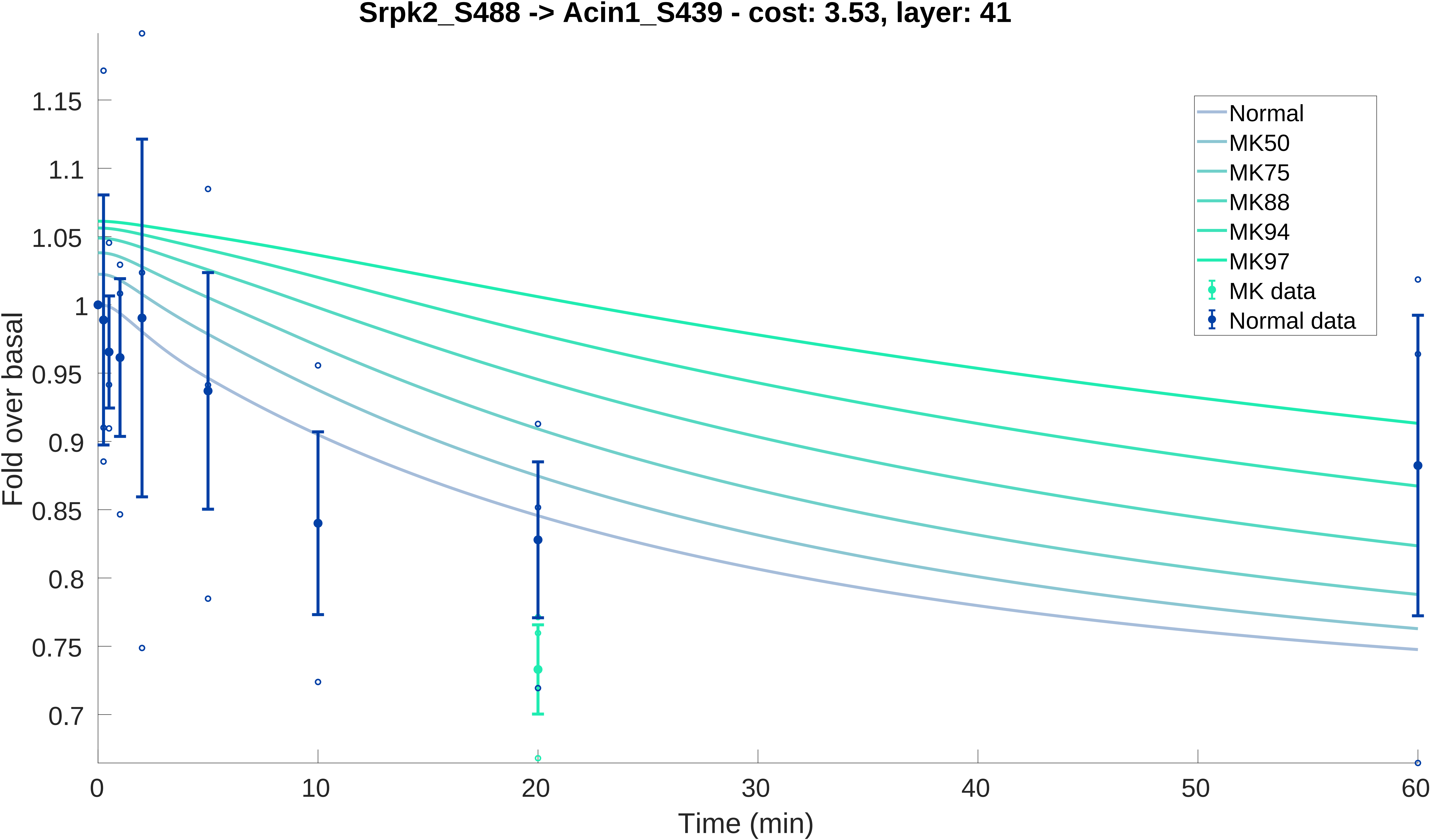
Srpk2_S488 -> Acin1_S439 - cost: 3.53, layer: 41
Fold over basal
Time (min)
Normal
MK50
MK75
MK88
MK94
MK97
MK data
Normal data
1.15
1.1
1.05
1
0.95
0.9
0.85
0.8
0.75
0.7
0
10
20
30
40
50
60

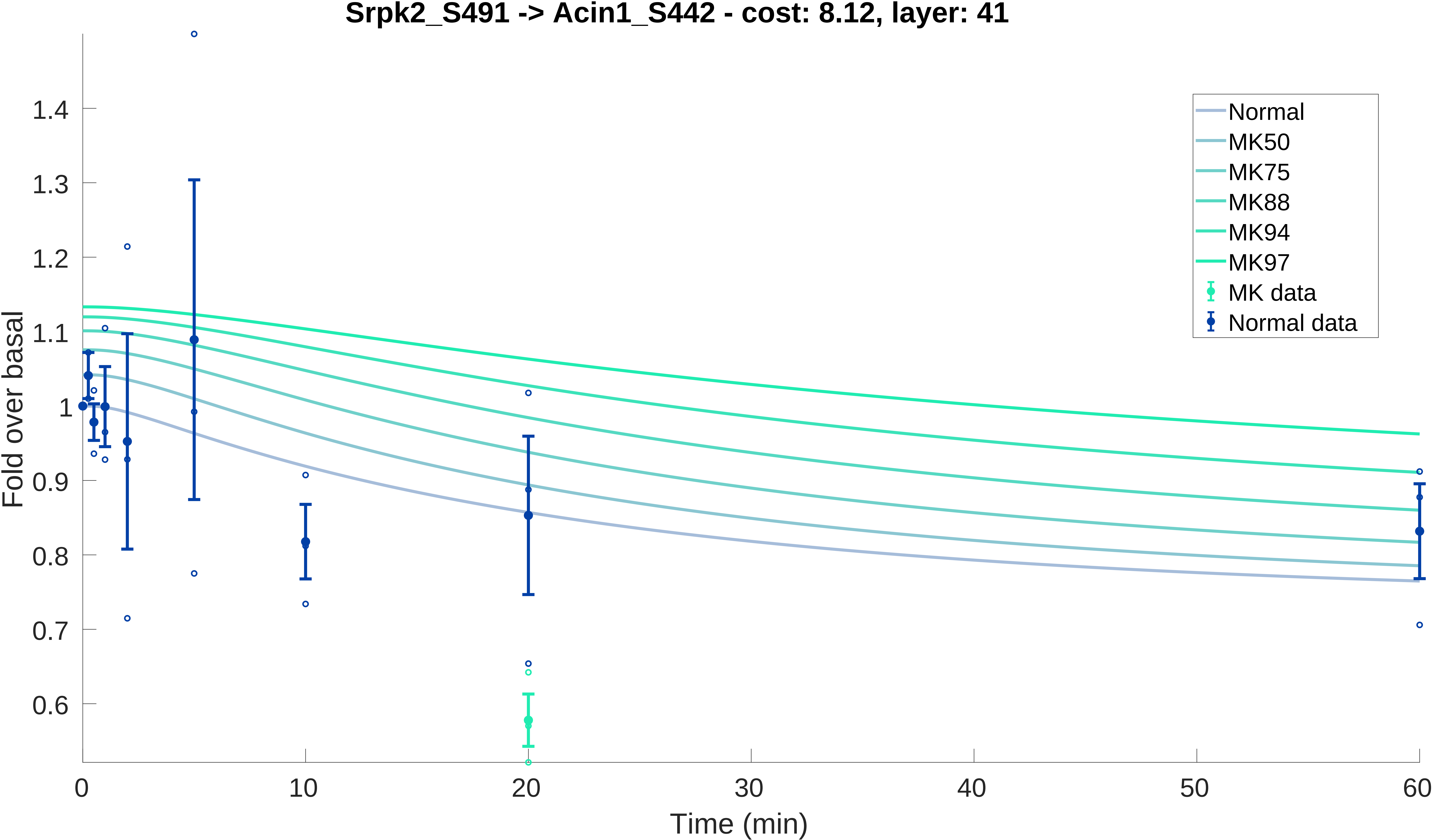

Srpk2_S491 -> Acin1_S442 - cost: 8.12, layer: 41
Fold over basal
Time (min)
1.4
1.3
1.2
1.1
1
0.9
0.8
0.7
0.6
0
10
20
30
40
50
60
Normal
MK50
MK75
MK88
MK94
MK97
MK data
Normal data

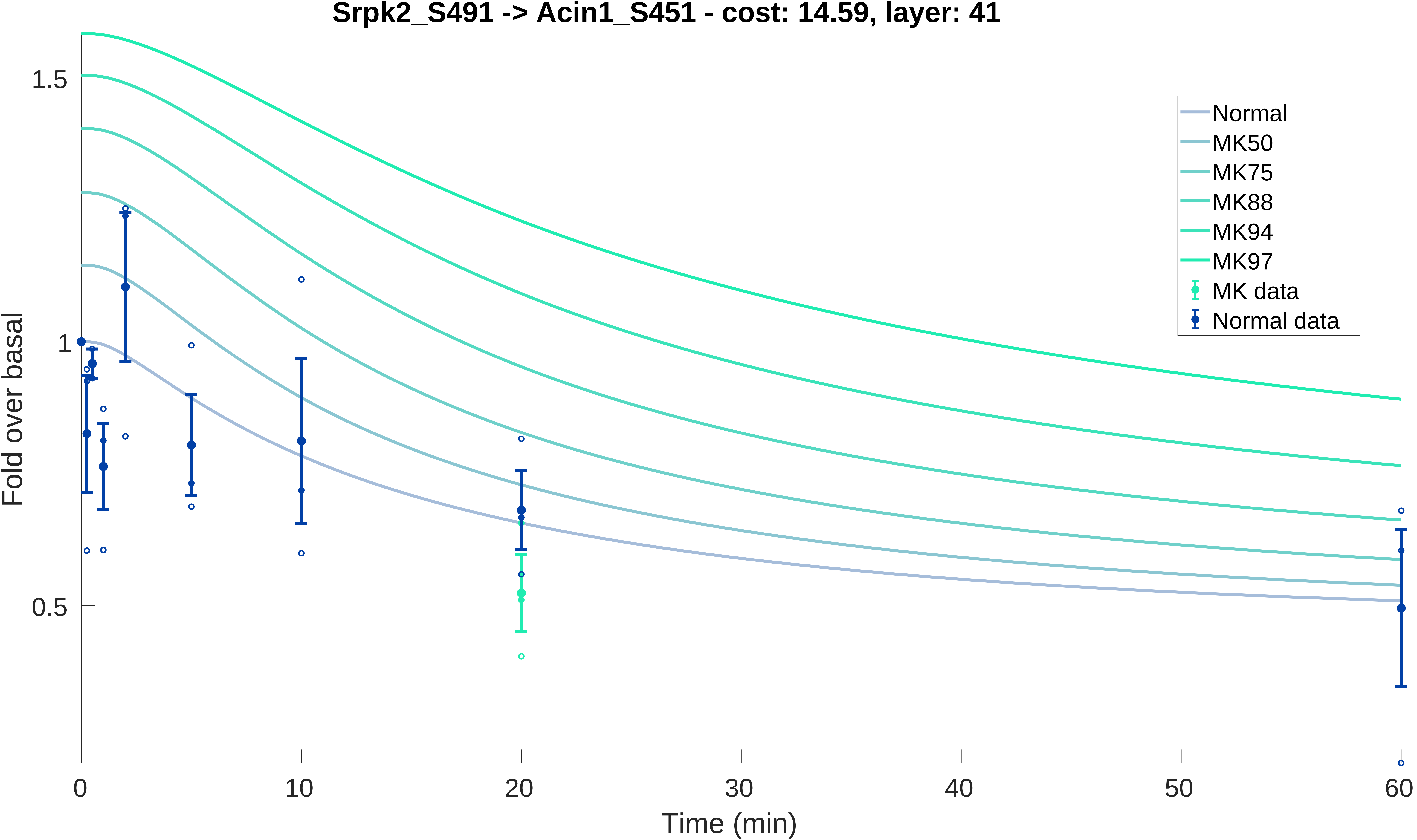
Srpk2_S491 -> Acin1_S451 - cost: 14.59, layer: 41
Fold over basal
1.5
1
0.5
Time (min)
0
10
20
30
40
50
60
Normal
MK50
MK75
MK88
MK94
MK97
MK data
Normal data

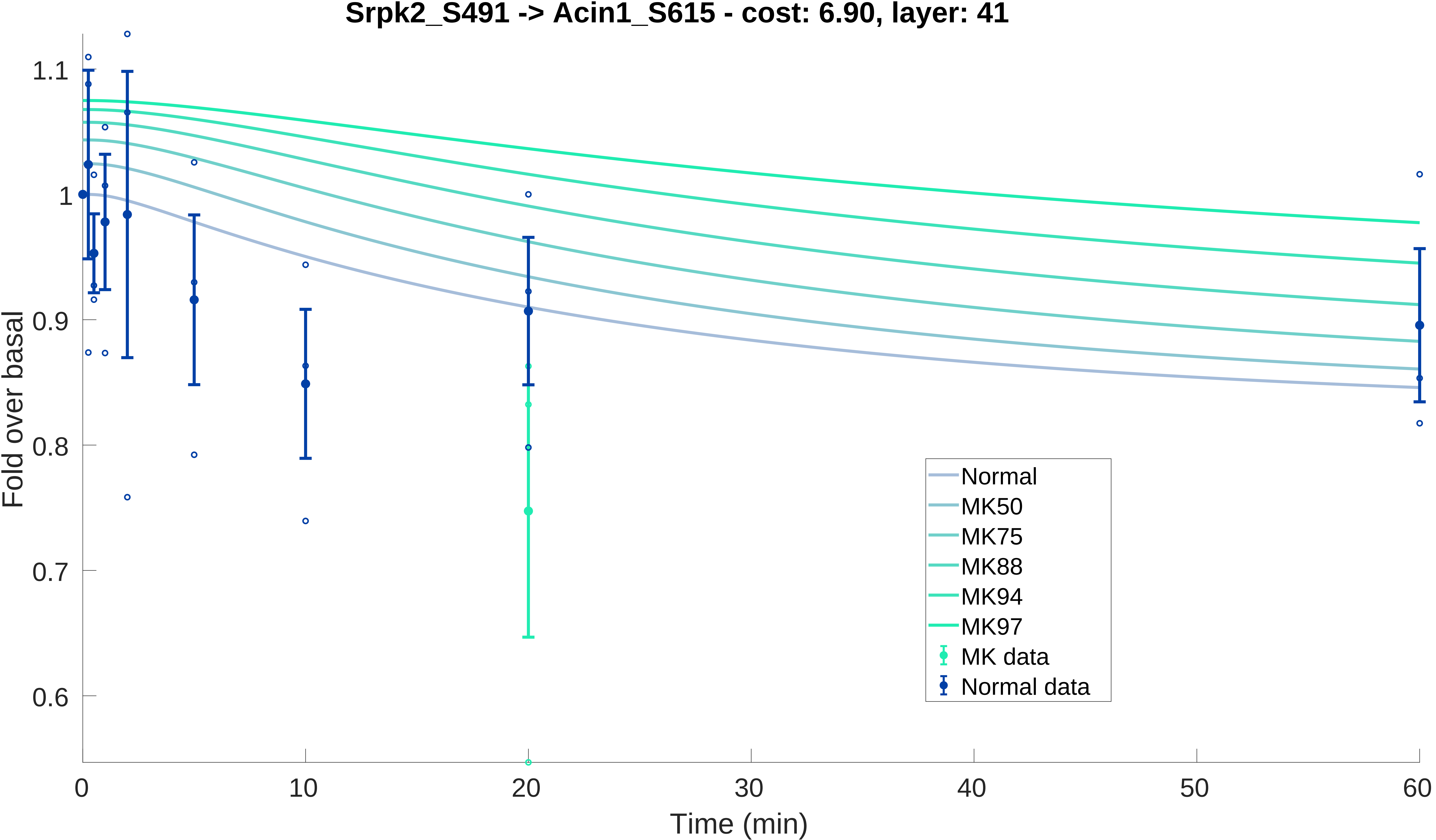

Srpk2_S491 -> Acin1_S615 - cost: 6.90, layer: 41
Fold over basal
Time (min)
Normal
MK50
MK75
MK88
MK94
MK97
MK data
Normal data

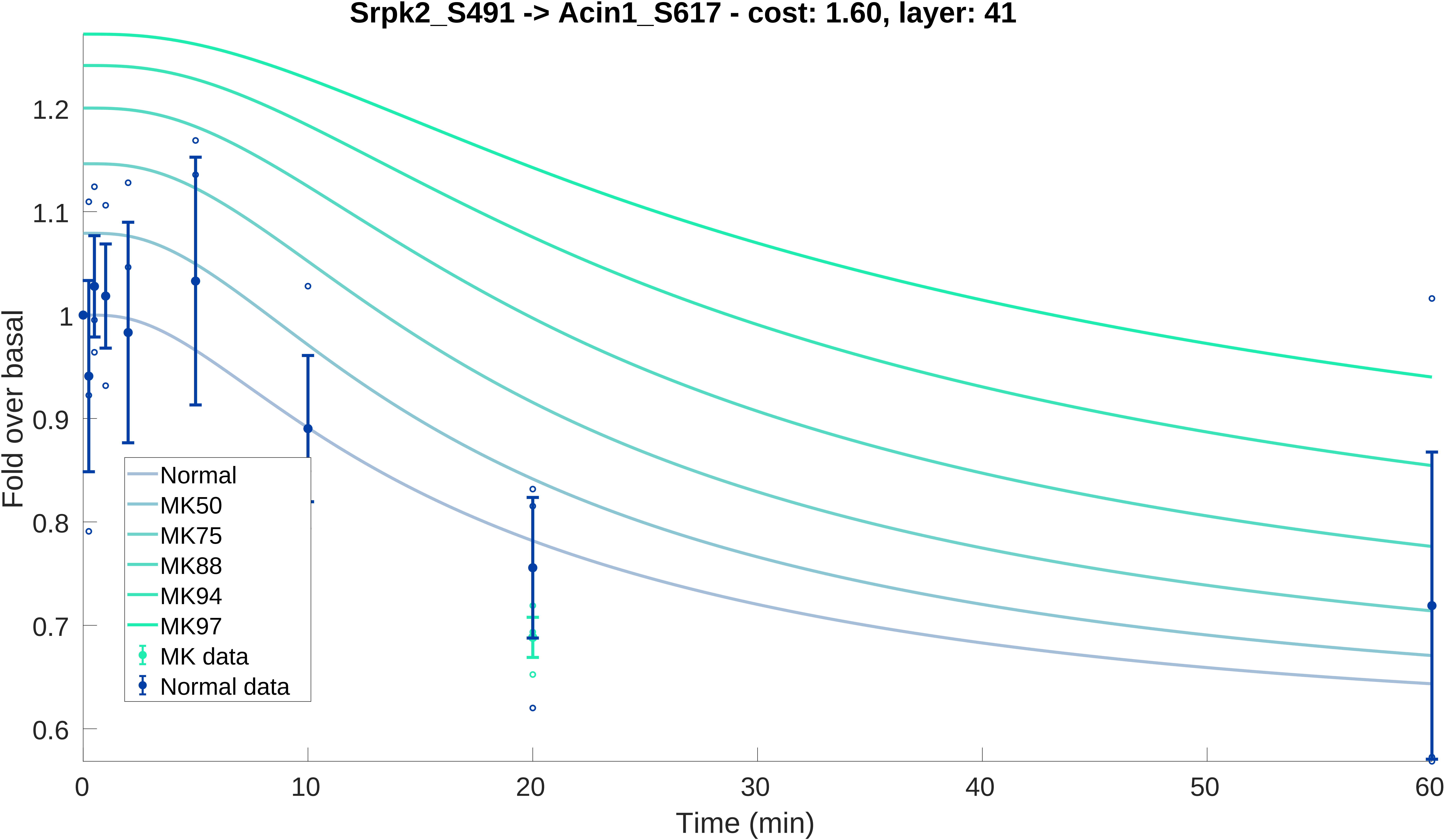
Srpk2_S491 -> Acin1_S617 - cost: 1.60, layer: 41
Fold over basal
Time (min)
1.2
1.1
1
0.9
0.8
0.7
0.6
0
10
20
30
40
50
60
Normal
MK50
MK75
MK88
MK94
MK97
MK data
Normal data

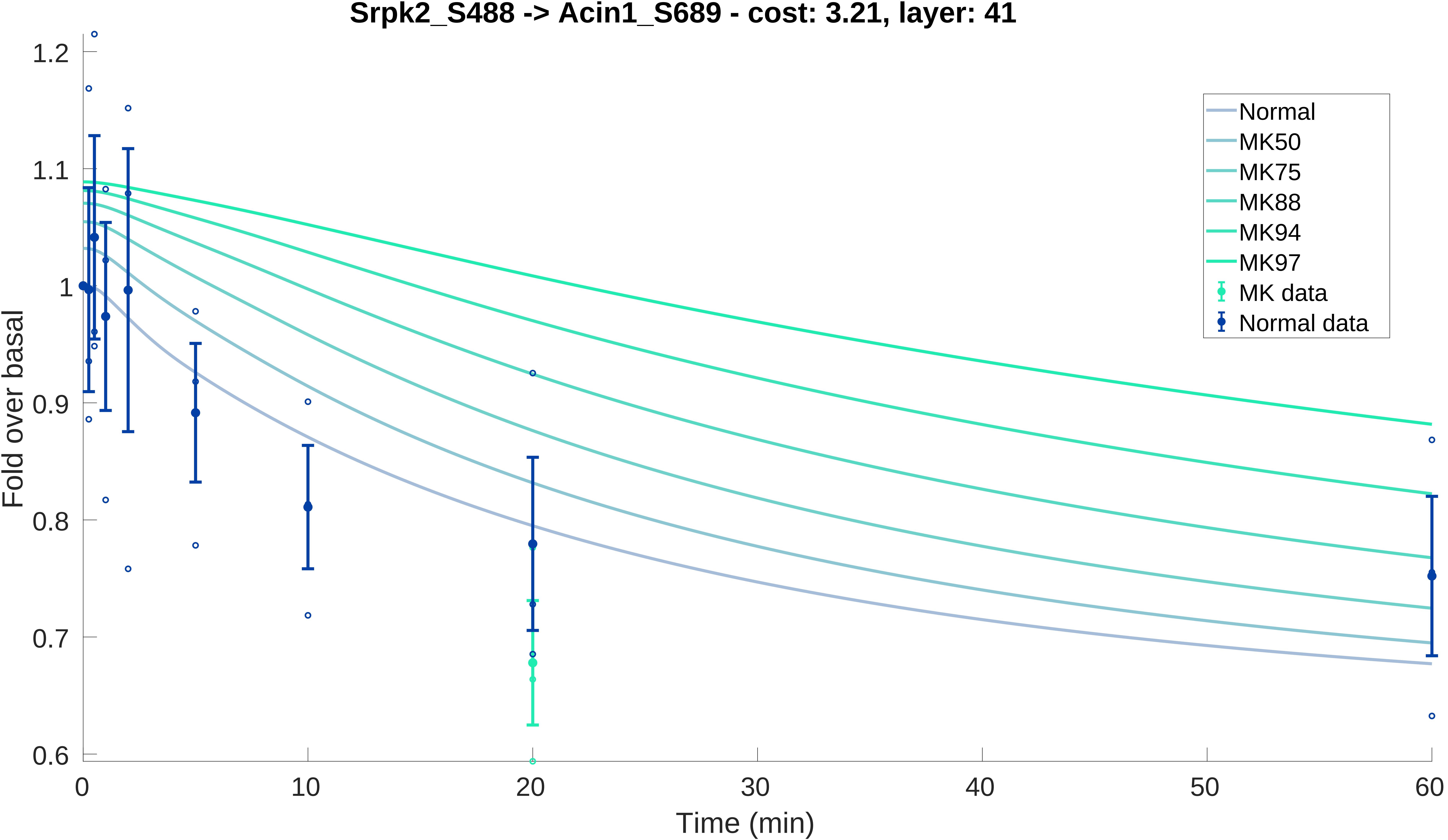

Srpk2_S488 -> Acin1_S689 - cost: 3.21, layer: 41
Fold over basal
Time (min)
1.2
1.1
1
0.9
0.8
0.7
0.6
0
10
20
30
40
50
60
Normal
MK50
MK75
MK88
MK94
MK97
MK data
Normal data

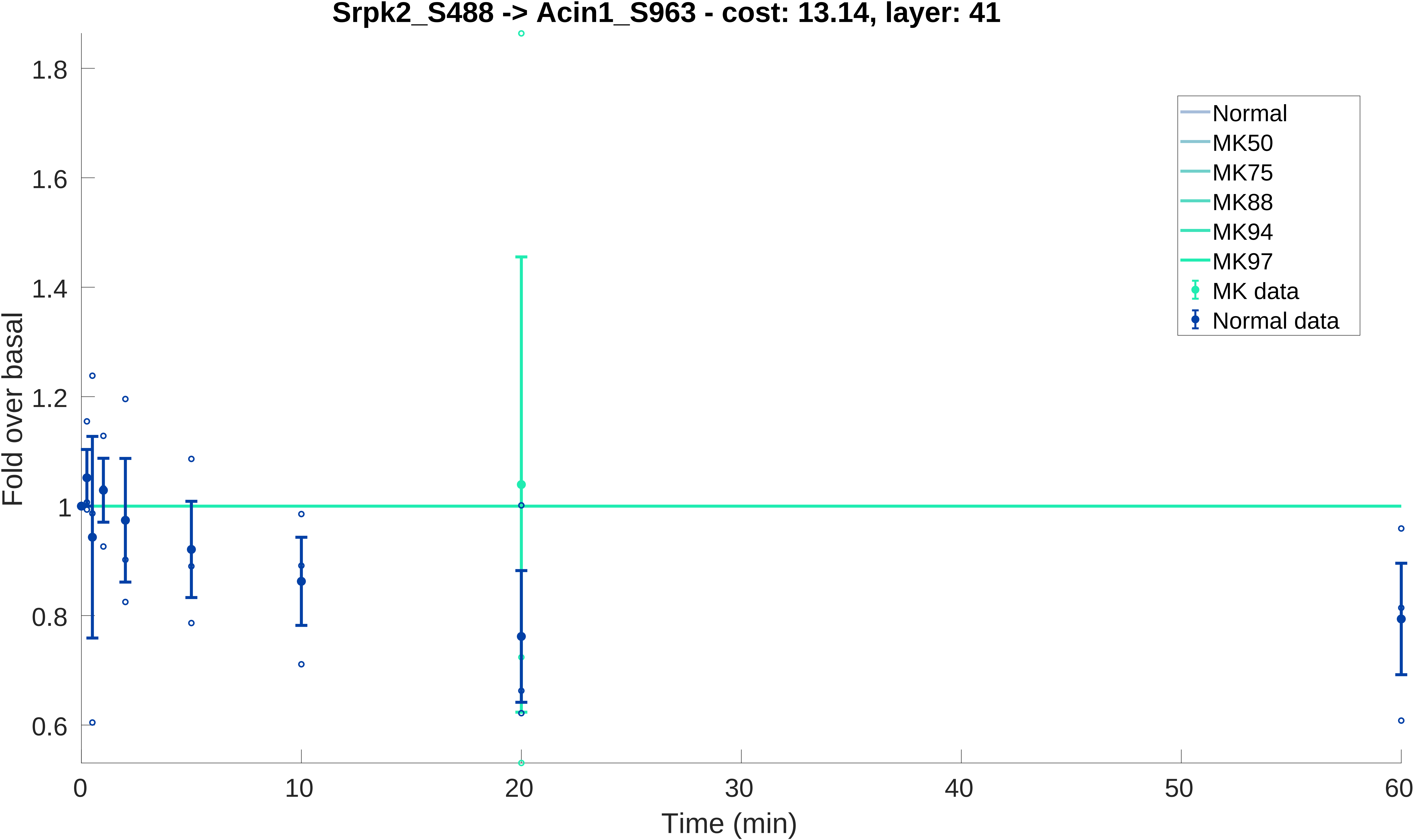
Srpk2_S488 -> Acin1_S963 - cost: 13.14, layer: 41
Fold over basal
Time (min)
0.6
0.8
1
1.2
1.4
1.6
1.8
0
10
20
30
40
50
60
Normal
MK50
MK75
MK88
MK94
MK97
MK data
Normal data

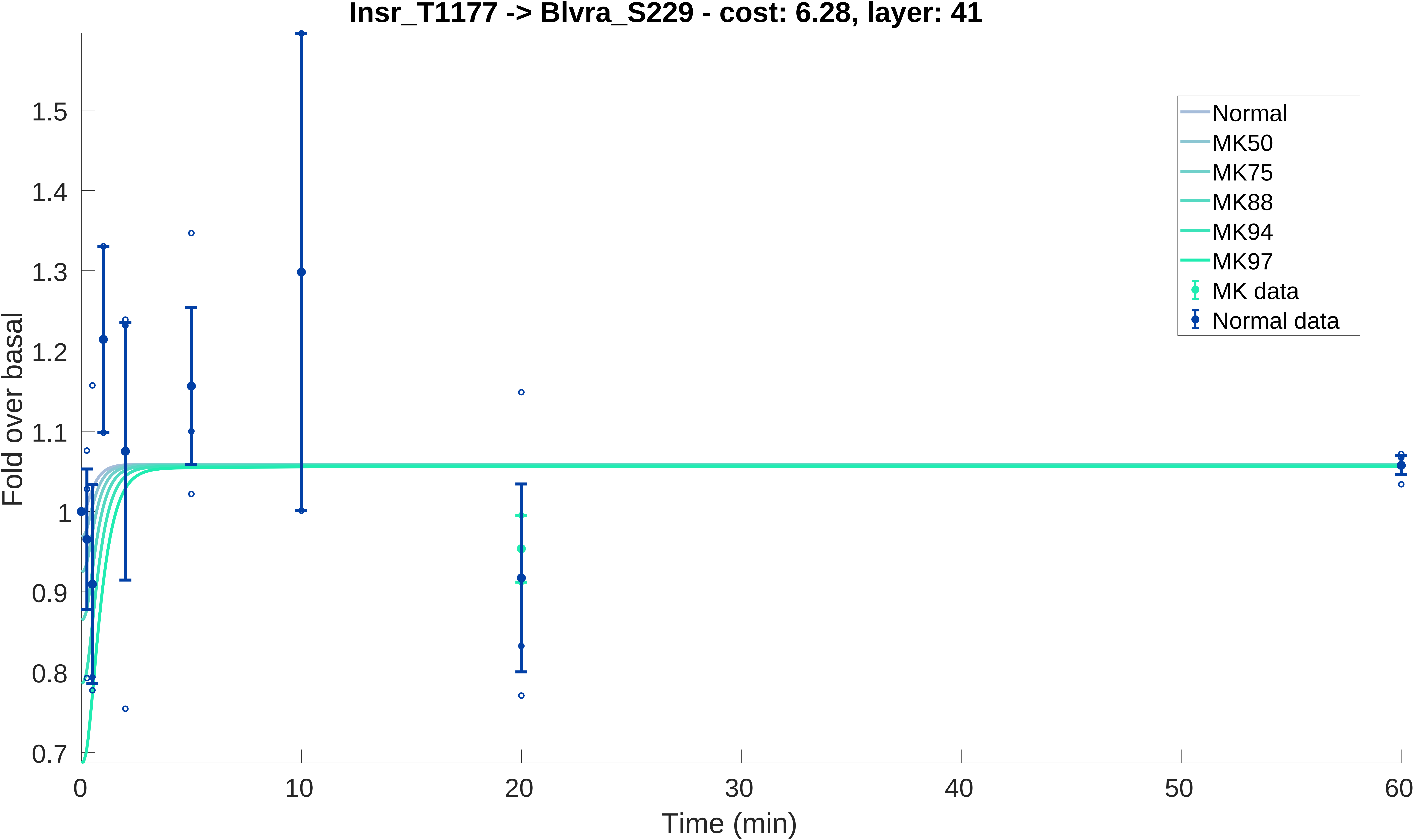
Insr_T1177 -> Blvra_S229 - cost: 6.28, layer: 41
Fold over basal
Time (min)
0.7
0.8
0.9
1
1.1
1.2
1.3
1.4
1.5
0
10
20
30
40
50
60
Normal
MK50
MK75
MK88
MK94
MK97
MK data
Normal data

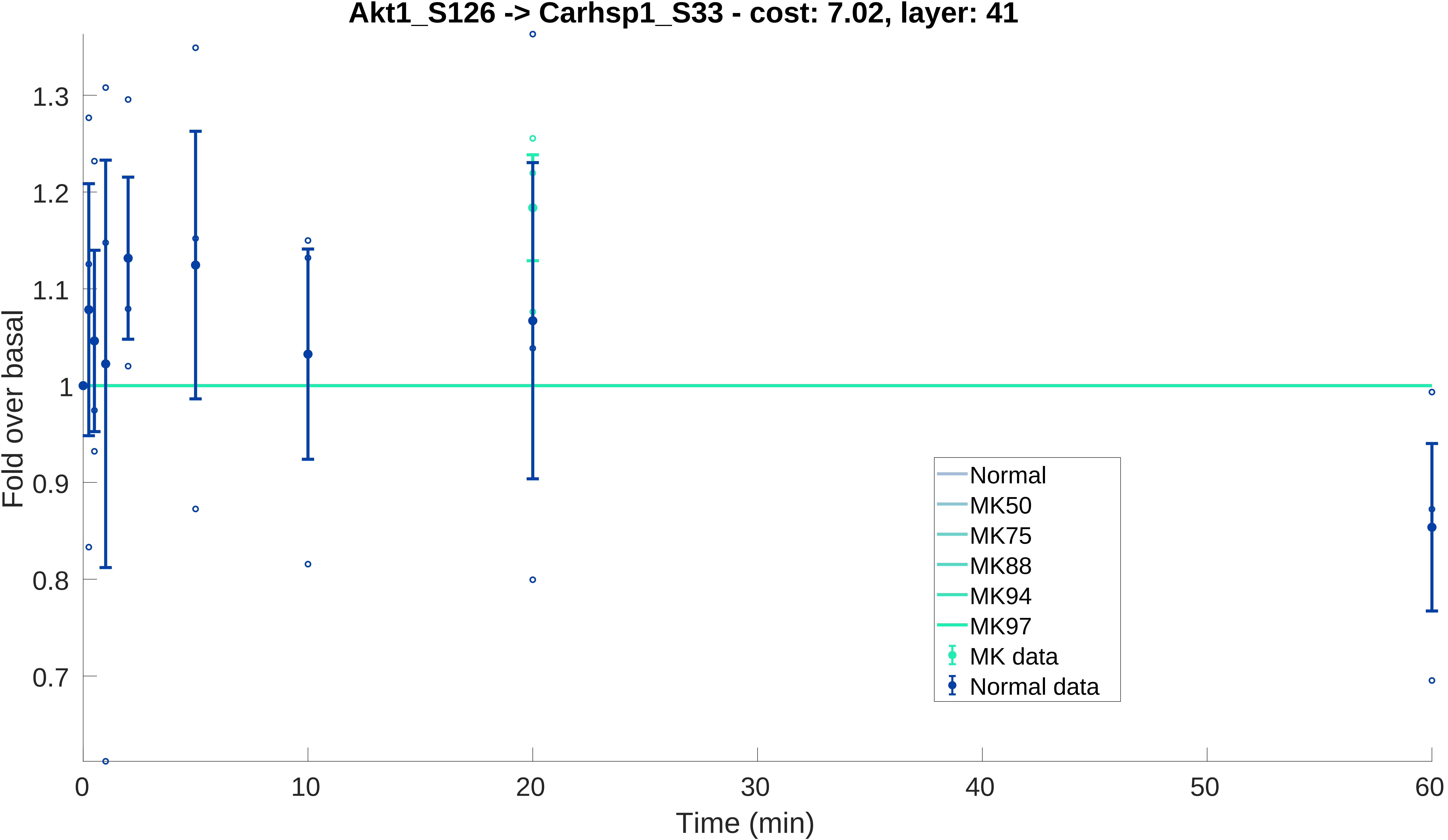

Akt1_S126 -> Carhsp1_S33 - cost: 7.02, layer: 41
Fold over basal
1.3
1.2
1.1
1
0.9
0.8
0.7
Time (min)
0
10
20
30
40
50
60
Normal
MK50
MK75
MK88
MK94
MK97
MK data
Normal data

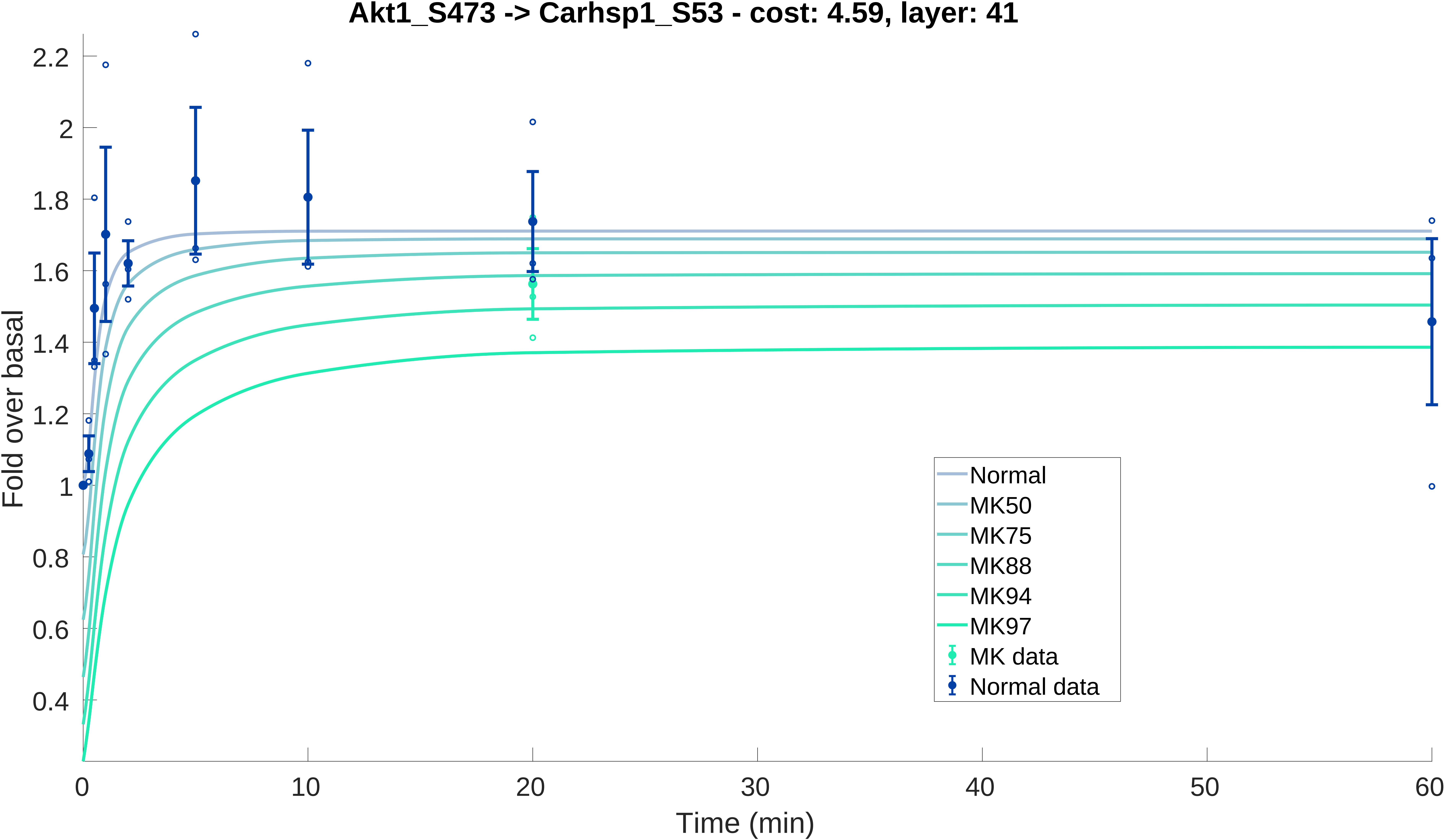
Akt1_S473 -> Carhsp1_S53 - cost: 4.59, layer: 41
Fold over basal
Time (min)
0
10
20
30
40
50
60
0.4
0.6
0.8
1
1.2
1.4
1.6
1.8
2
2.2
Normal
MK50
MK75
MK88
MK94
MK97
MK data
Normal data

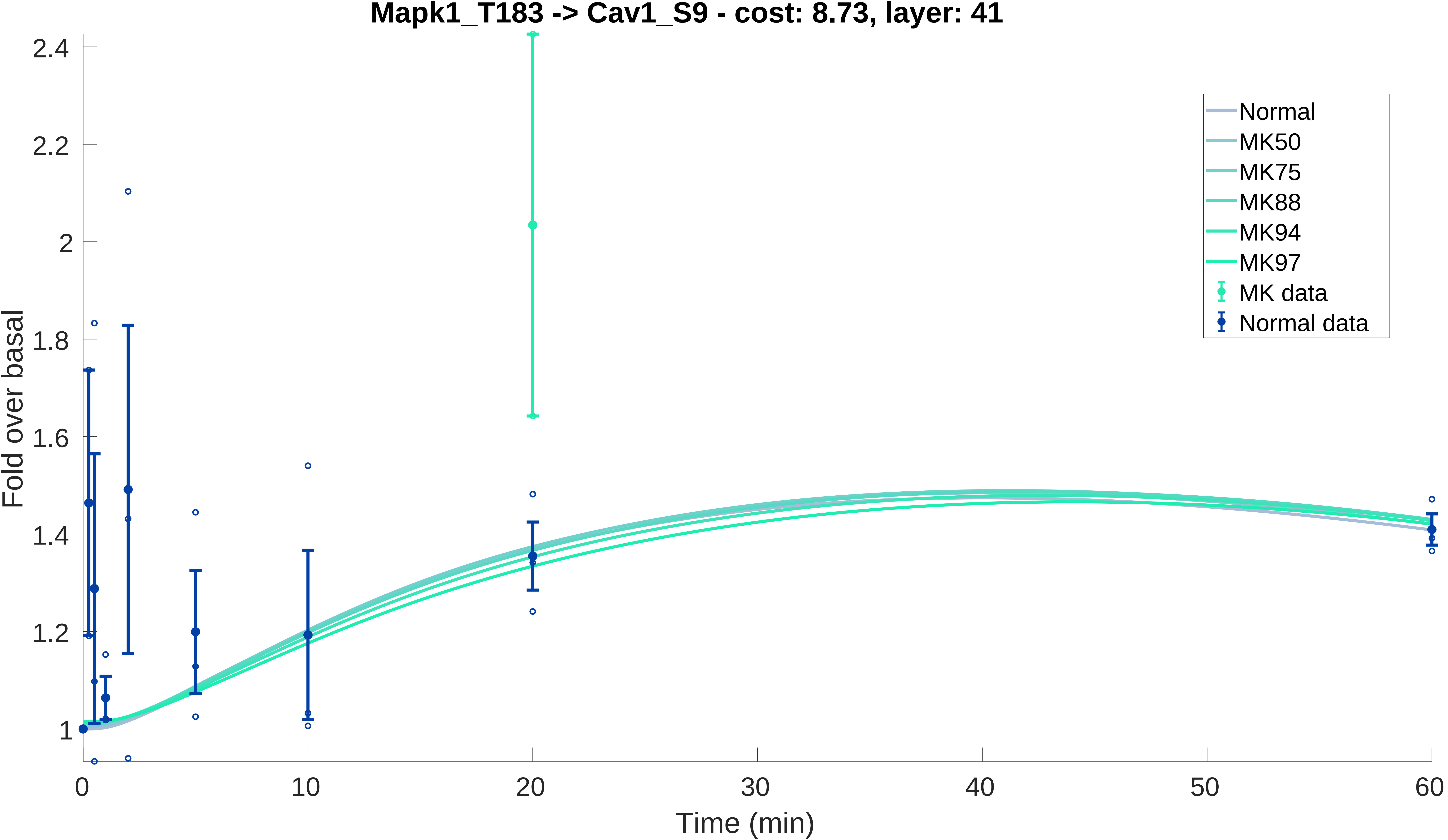

Mapk1_T183 -> Cav1_S9 - cost: 8.73, layer: 41
Fold over basal
Time (min)
2.4
2.2
2
1.8
1.6
1.4
1.2
1
0
10
20
30
40
50
60
Normal
MK50
MK75
MK88
MK94
MK97
MK data
Normal data

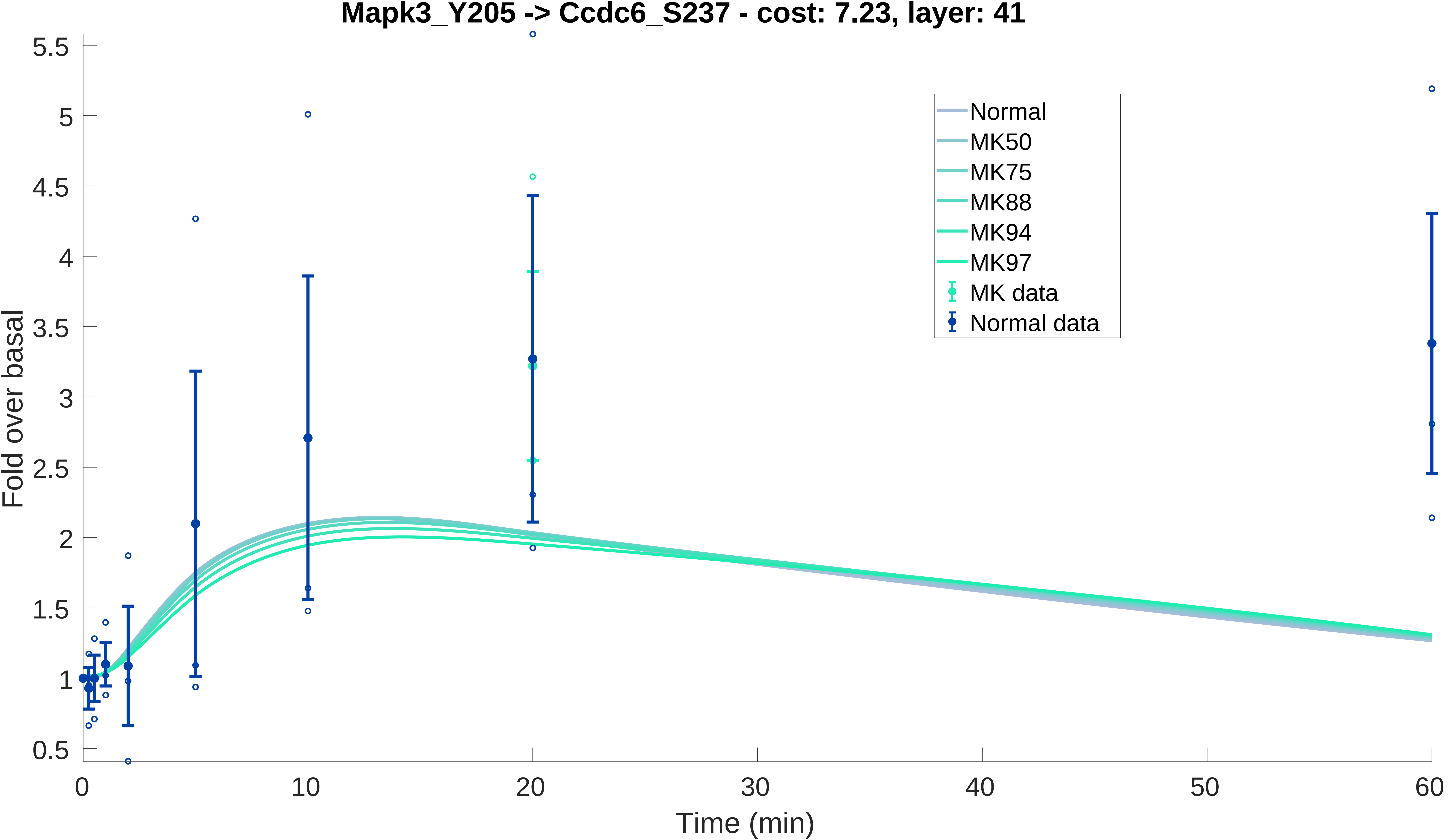

Mapk3_Y205 -> Ccdc6_S237 - cost: 7.23, layer: 41
Fold over basal
Time (min)
Normal
MK50
MK75
MK88
MK94
MK97
MK data
Normal data
0.5
1
1.5
2
2.5
3
3.5
4
4.5
5
5.5
0
10
20
30
40
50
60

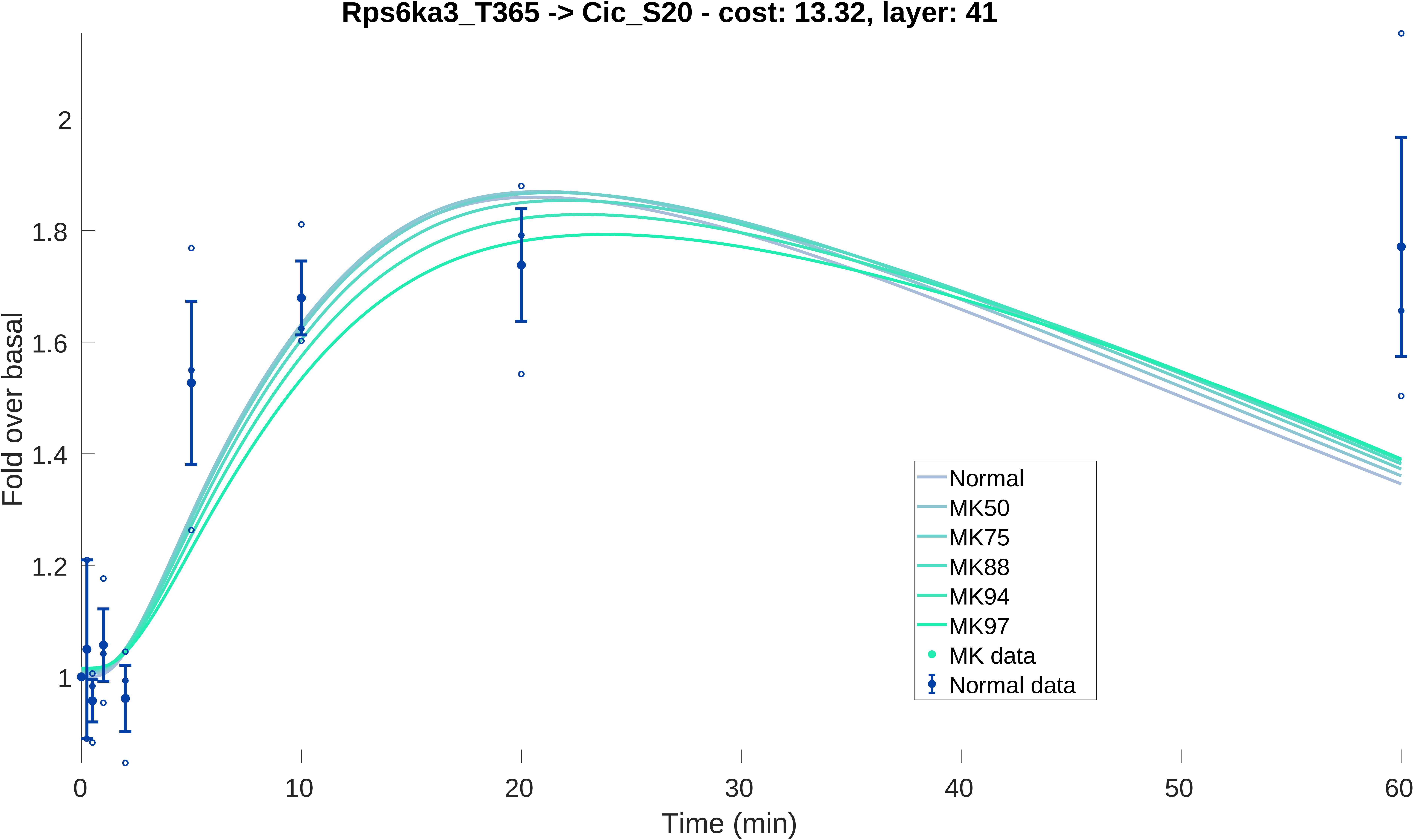

Rps6ka3_T365 -> Cic_S20 - cost: 13.32, layer: 41
Fold over basal
Time (min)
0
10
20
30
40
50
60
1
1.2
1.4
1.6
1.8
2
Normal
MK50
MK75
MK88
MK94
MK97
MK data
Normal data

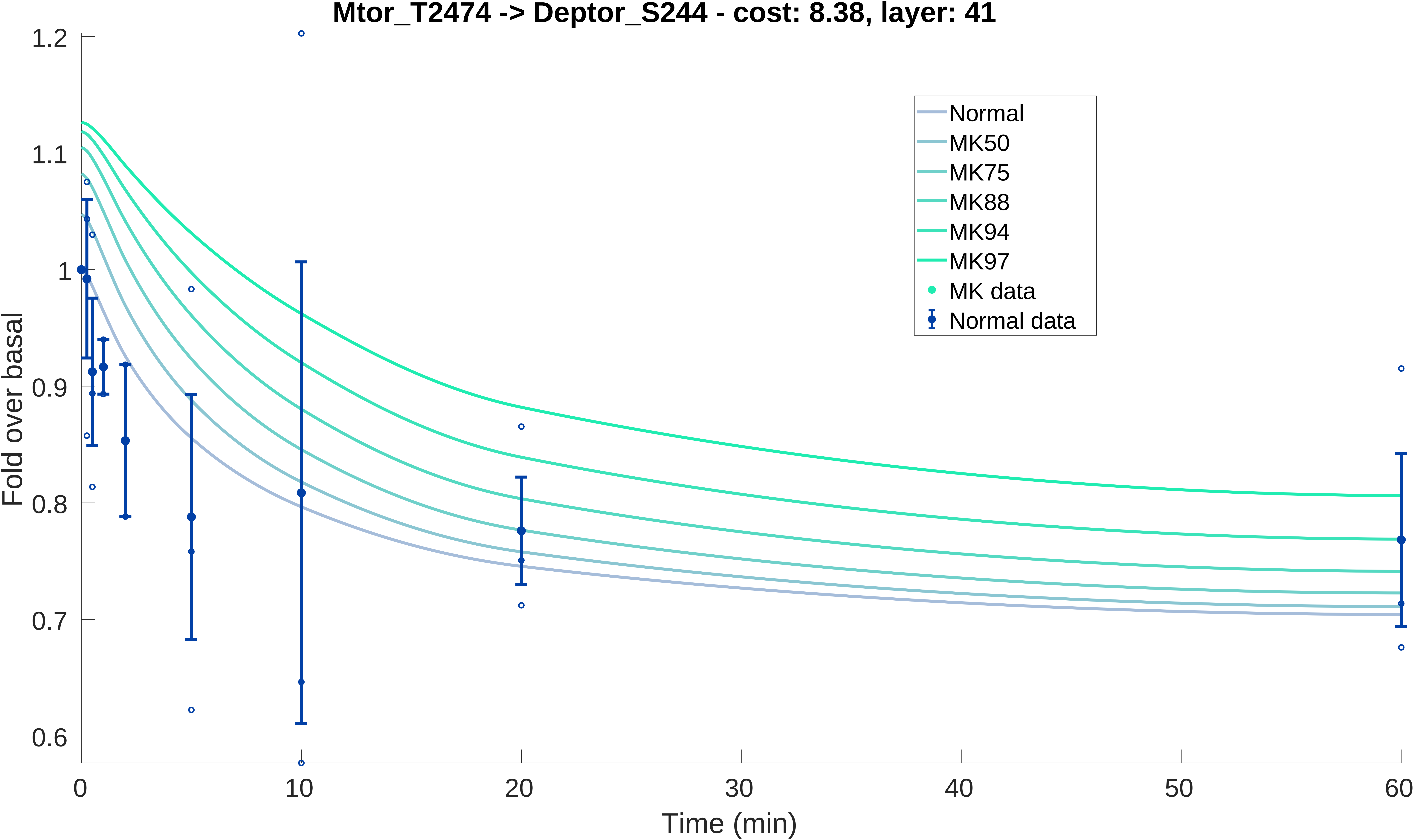

Mtor_T2474 -> Deptor_S244 - cost: 8.38, layer: 41
Fold over basal
Time (min)
1.2
1.1
1
0.9
0.8
0.7
0.6
0
10
20
30
40
50
60
Normal
MK50
MK75
MK88
MK94
MK97
MK data
Normal data

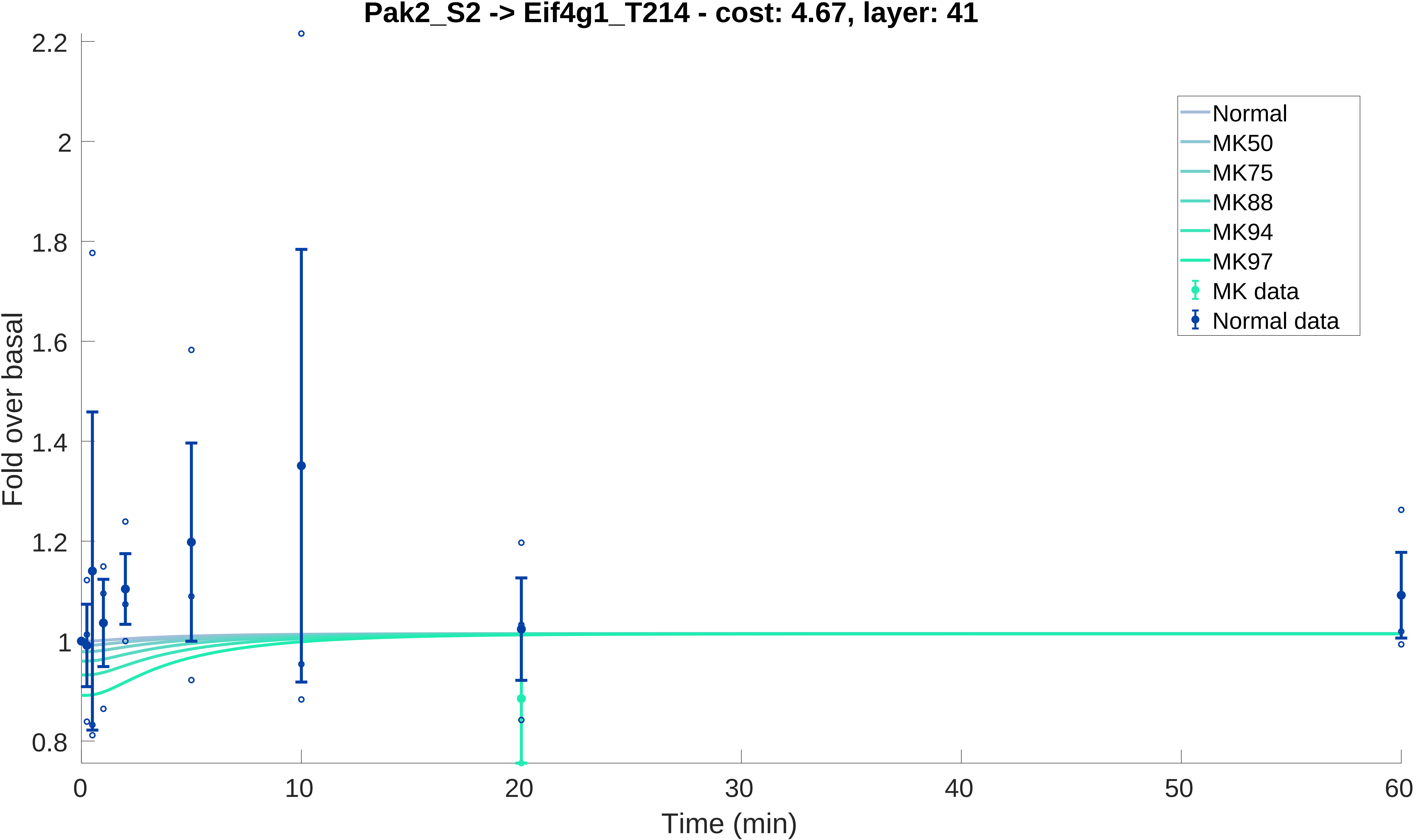

Pak2_S2 -> Eif4g1_T214 - cost: 4.67, layer: 41
Fold over basal
0.8
1
1.2
1.4
1.6
1.8
2
2.2
Time (min)
0
10
20
30
40
50
60
Normal
MK50
MK75
MK88
MK94
MK97
MK data
Normal data

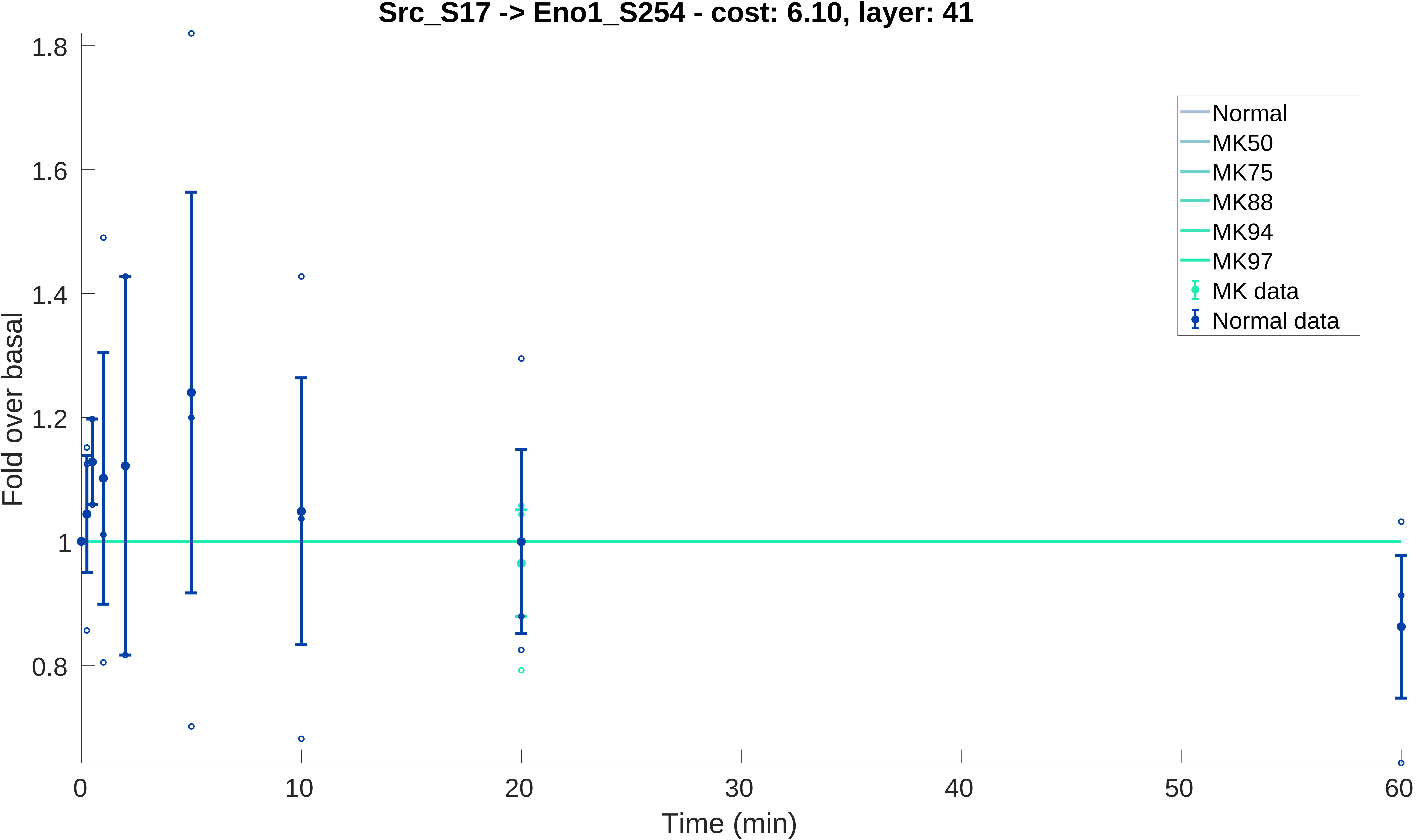

Src_S17 -> Eno1_S254 - cost: 6.10, layer: 41
Fold over basal
Time (min)
0.8
1
1.2
1.4
1.6
1.8
0
10
20
30
40
50
60
Normal
MK50
MK75
MK88
MK94
MK97
MK data
Normal data

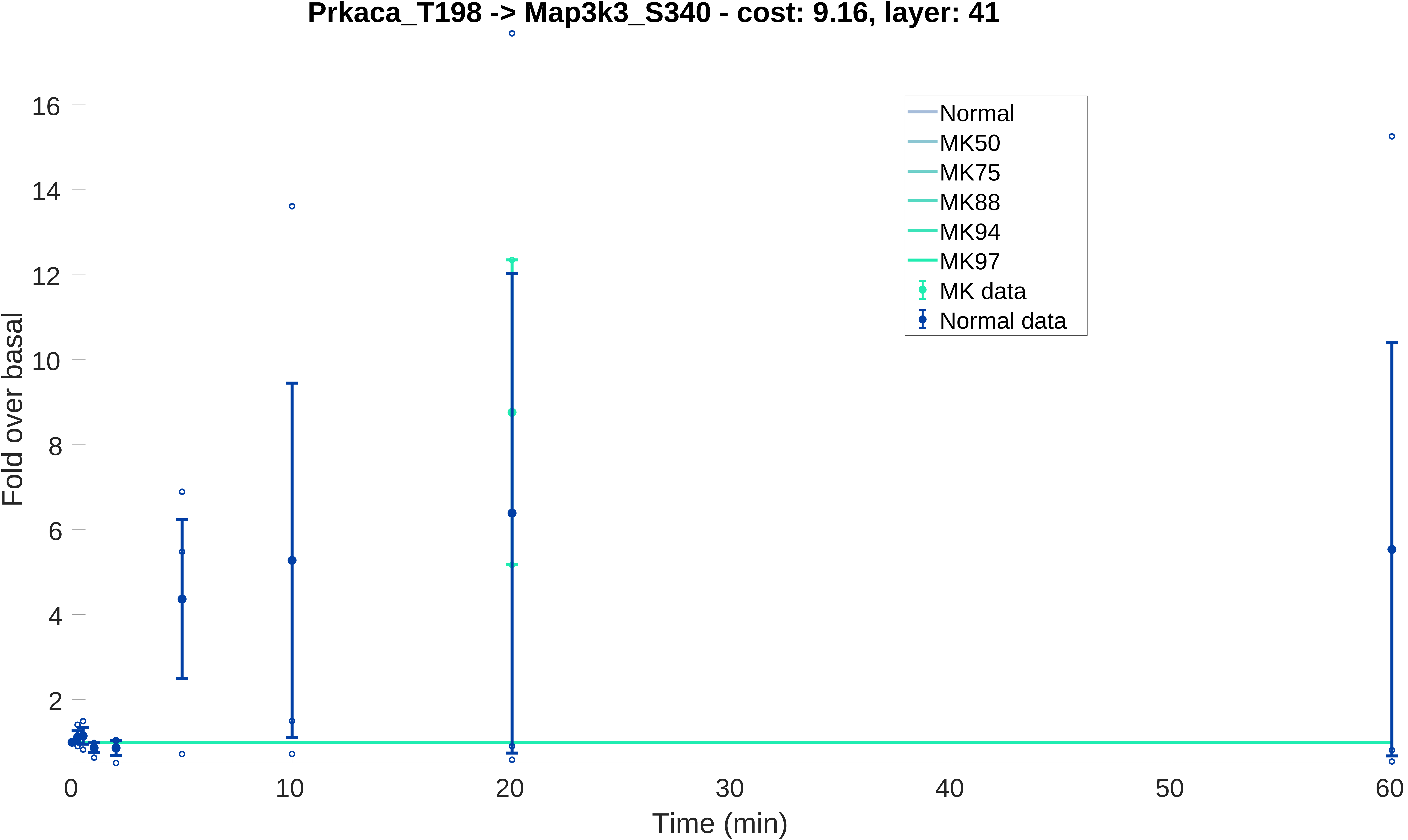
Prrkaca_T198 -> Map3k3_S340 - cost: 9.16, layer: 41
Fold over basal
Time (min)
0
10
20
30
40
50
60
2
4
6
8
10
12
14
16
Normal
MK50
MK75
MK88
MK94
MK97
MK data
Normal data

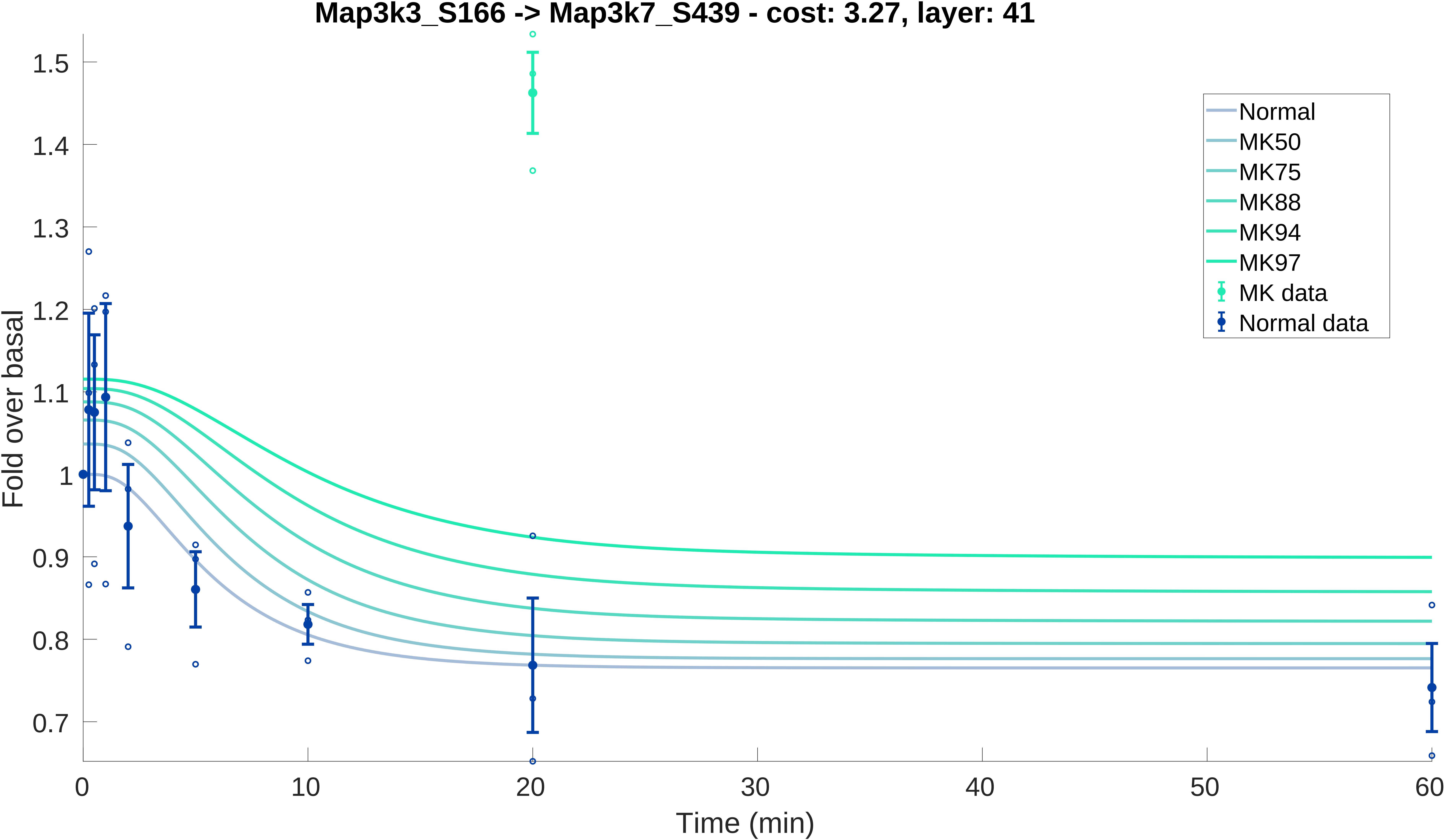

Map3k3_S166 -> Map3k7_S439 - cost: 3.27, layer: 41
Fold over basal
Time (min)
0
10
20
30
40
50
60
0.7
0.8
0.9
1
1.1
1.2
1.3
1.4
1.5
Normal
MK50
MK75
MK88
MK94
MK97
MK data
Normal data

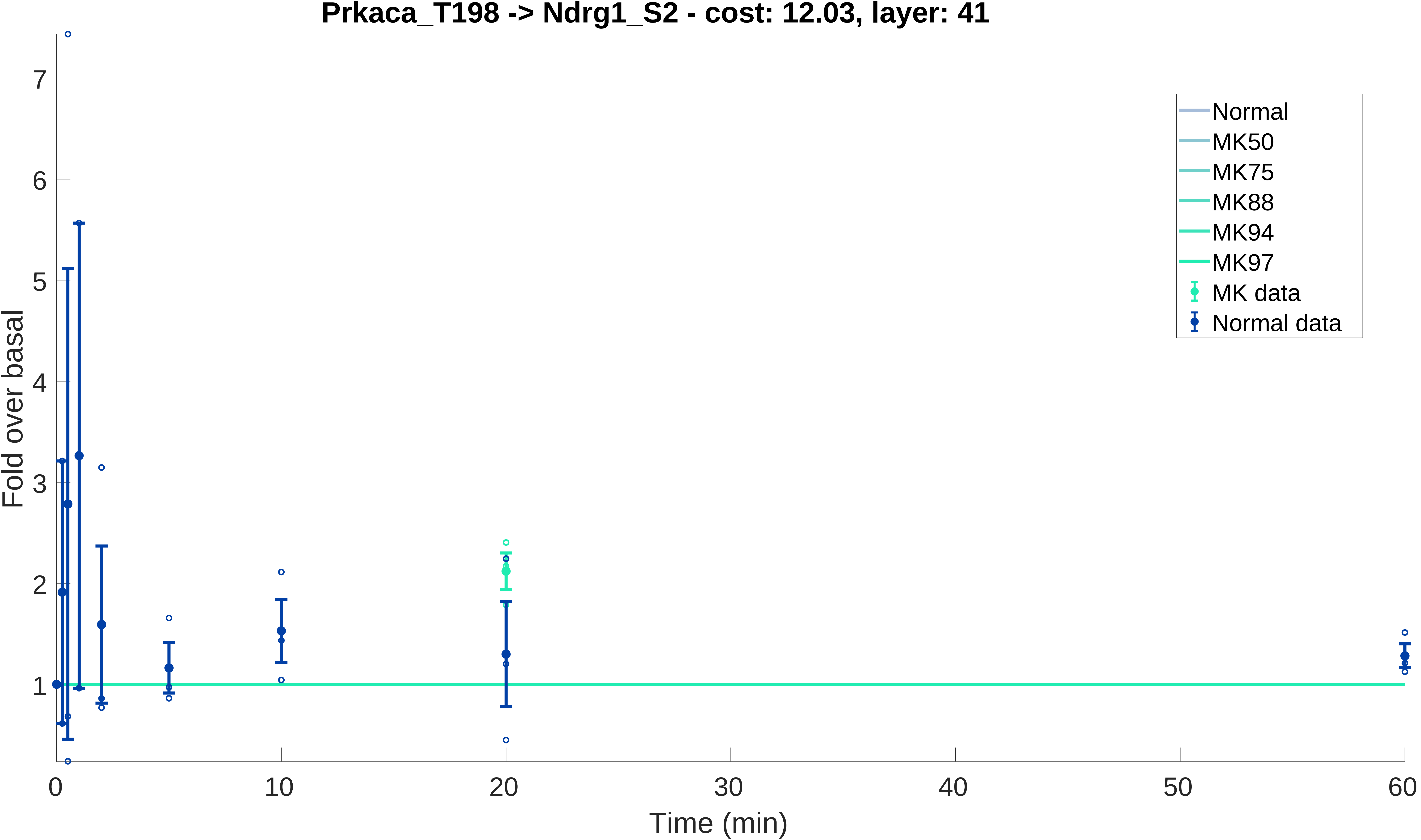
Prkaca_T198 -> Ndrg1_S2 - cost: 12.03, layer: 41
Fold over basal
Time (min)
0
10
20
30
40
50
60
1
2
3
4
5
6
7
Normal
MK50
MK75
MK88
MK94
MK97
MK data
Normal data

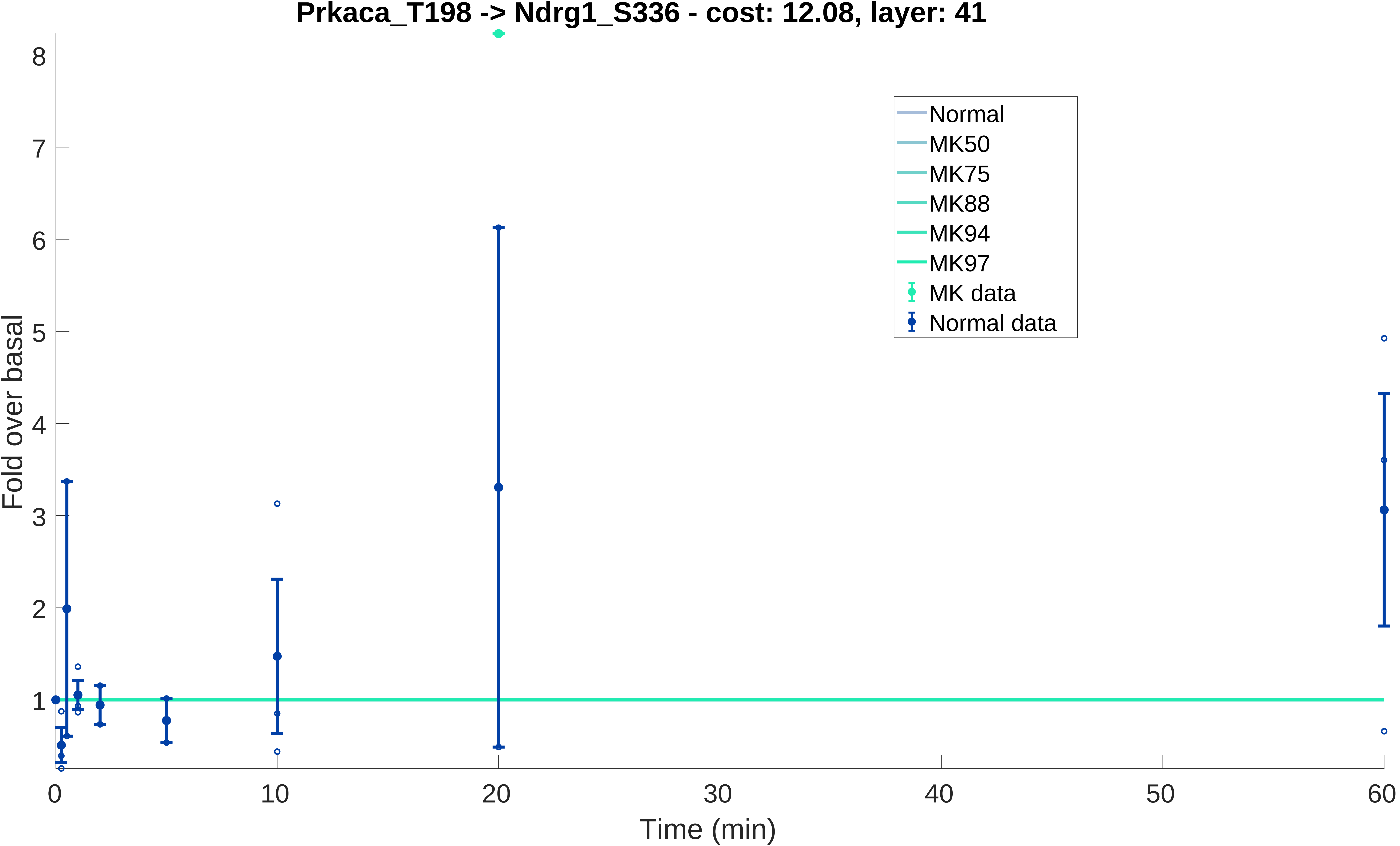

Prkaca_T198 -> Ndrg1_S336 - cost: 12.08, layer: 41
Fold over basal
Time (min)
Normal
MK50
MK75
MK88
MK94
MK97
MK data
Normal data
0
10
20
30
40
50
60
1
2
3
4
5
6
7
8

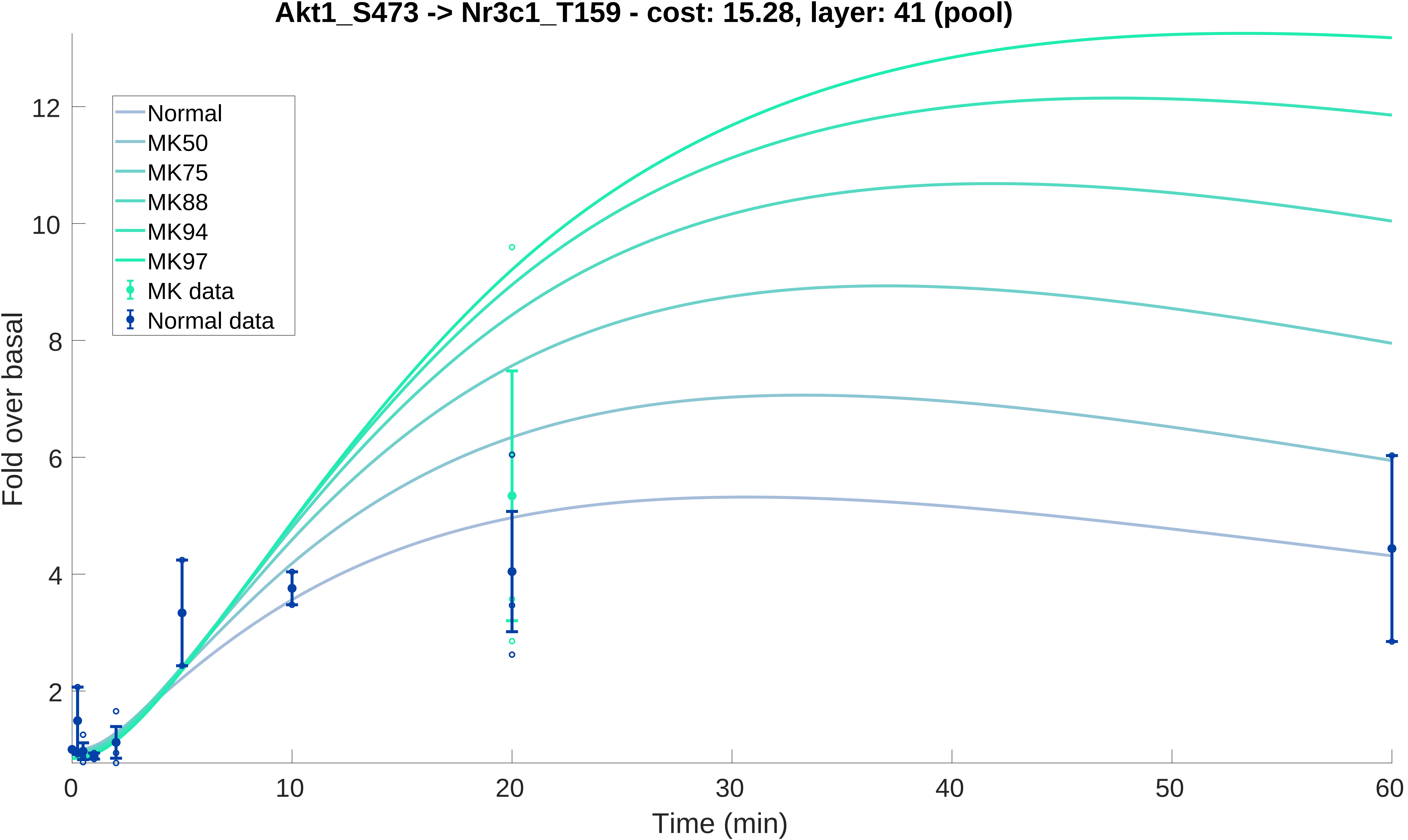

Akt1_S473 -> Nr3c1_T159 - cost: 15.28, layer: 41 (pool)
Normal
MK50
MK75
MK88
MK94
MK97
MK data
Normal data
Fold over basal
Time (min)
0
10
20
30
40
50
60
2
4
6
8
10
12

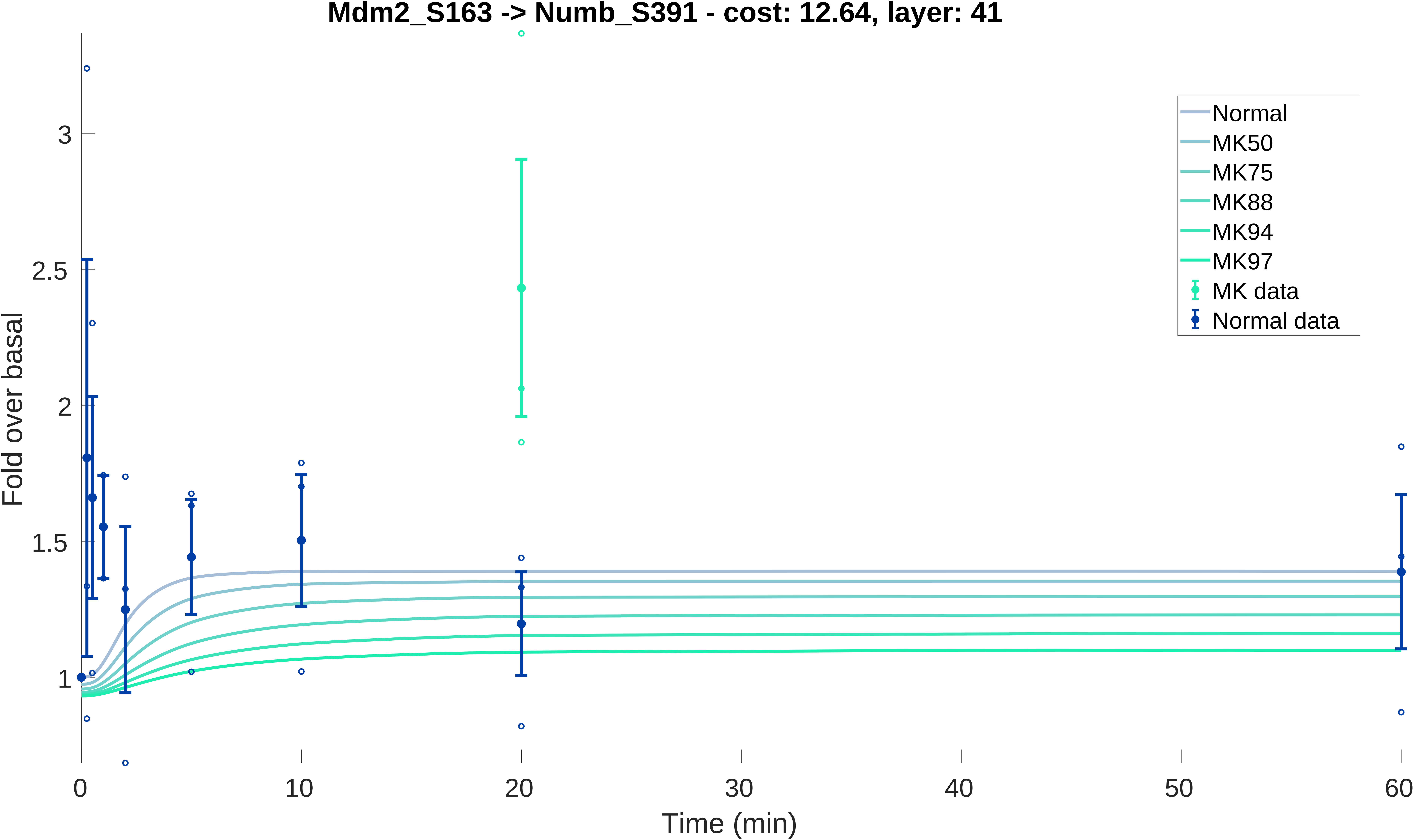
Mdm2_S163 -> Numb_S391 - cost: 12.64, layer: 41
Fold over basal
Time (min)
Normal
MK50
MK75
MK88
MK94
MK97
MK data
Normal data
0
10
20
30
40
50
60
1
1.5
2
2.5
3

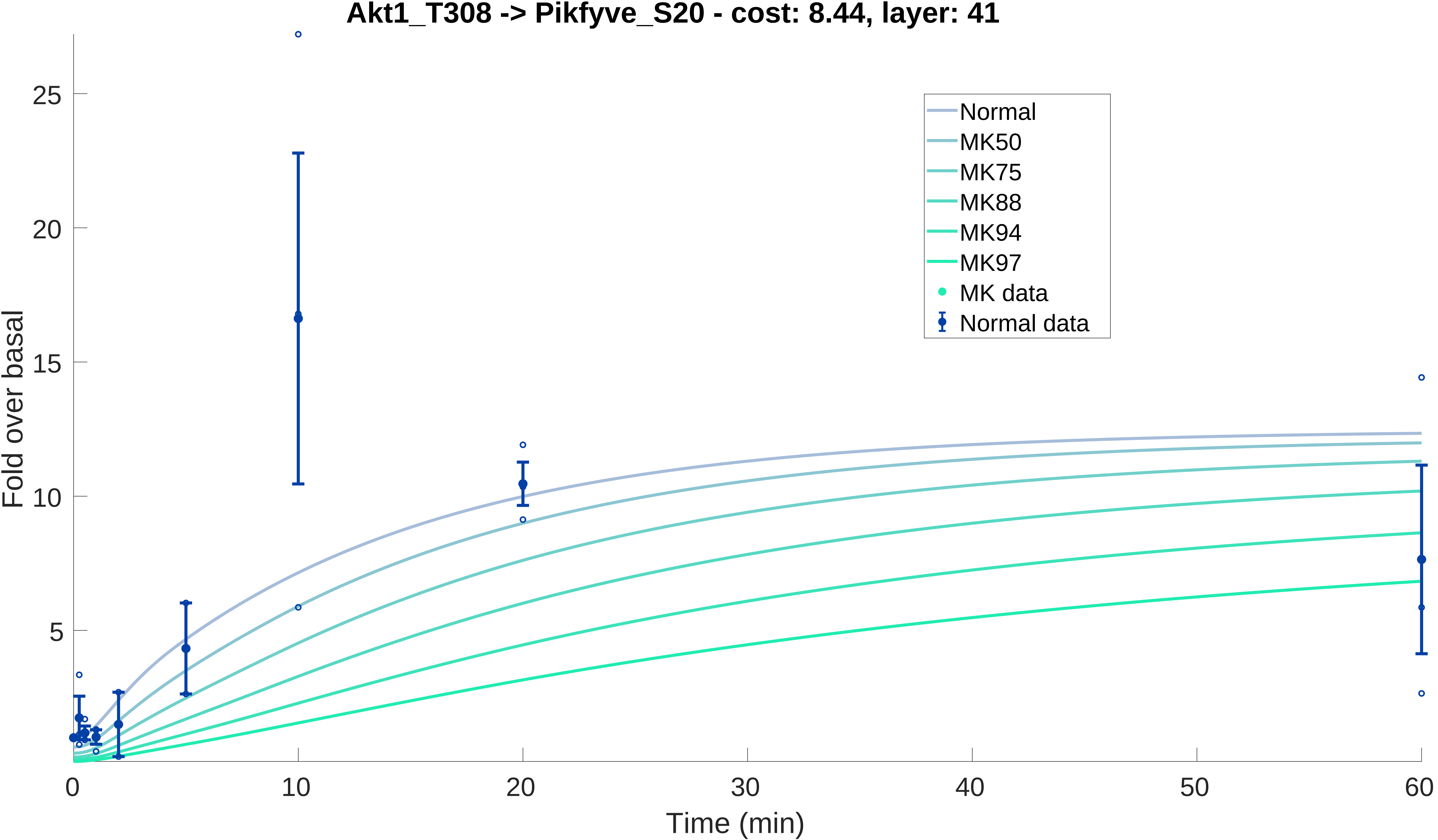

Akt1_T308 -> Pikfyve_S20 - cost: 8.44, layer: 41
Fold over basal
Time (min)
0
10
20
30
40
50
60
5
10
15
20
25
Normal
MK50
MK75
MK88
MK94
MK97
MK data
Normal data

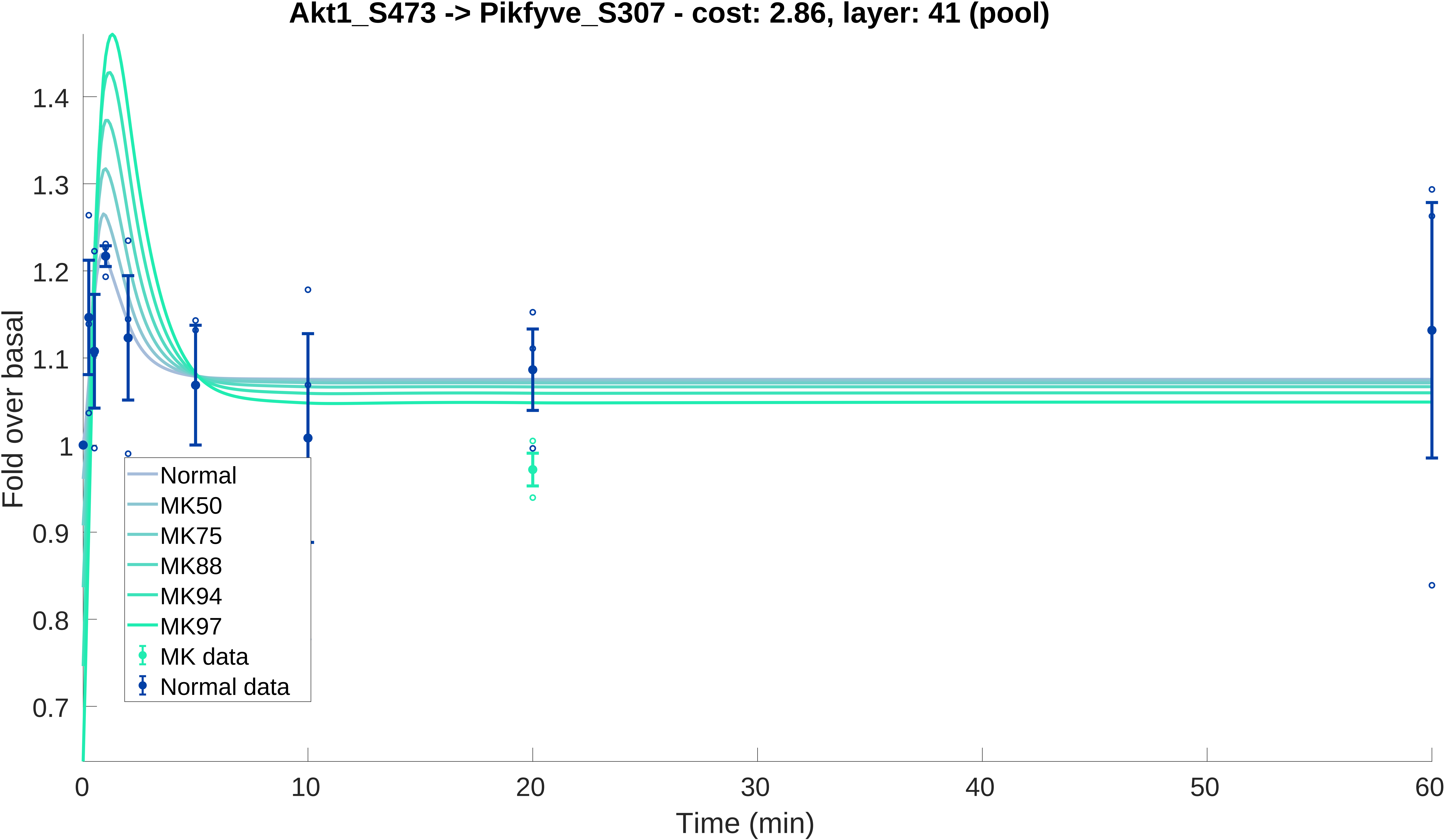

Akt1_S473 -> Pikfyve_S307 - cost: 2.86, layer: 41 (pool)
Fold over basal
1.4
1.3
1.2
1.1
1
0.9
0.8
0.7
0
10
20
30
40
50
60
Time (min)
Normal
MK50
MK75
MK88
MK94
MK97
MK data
Normal data

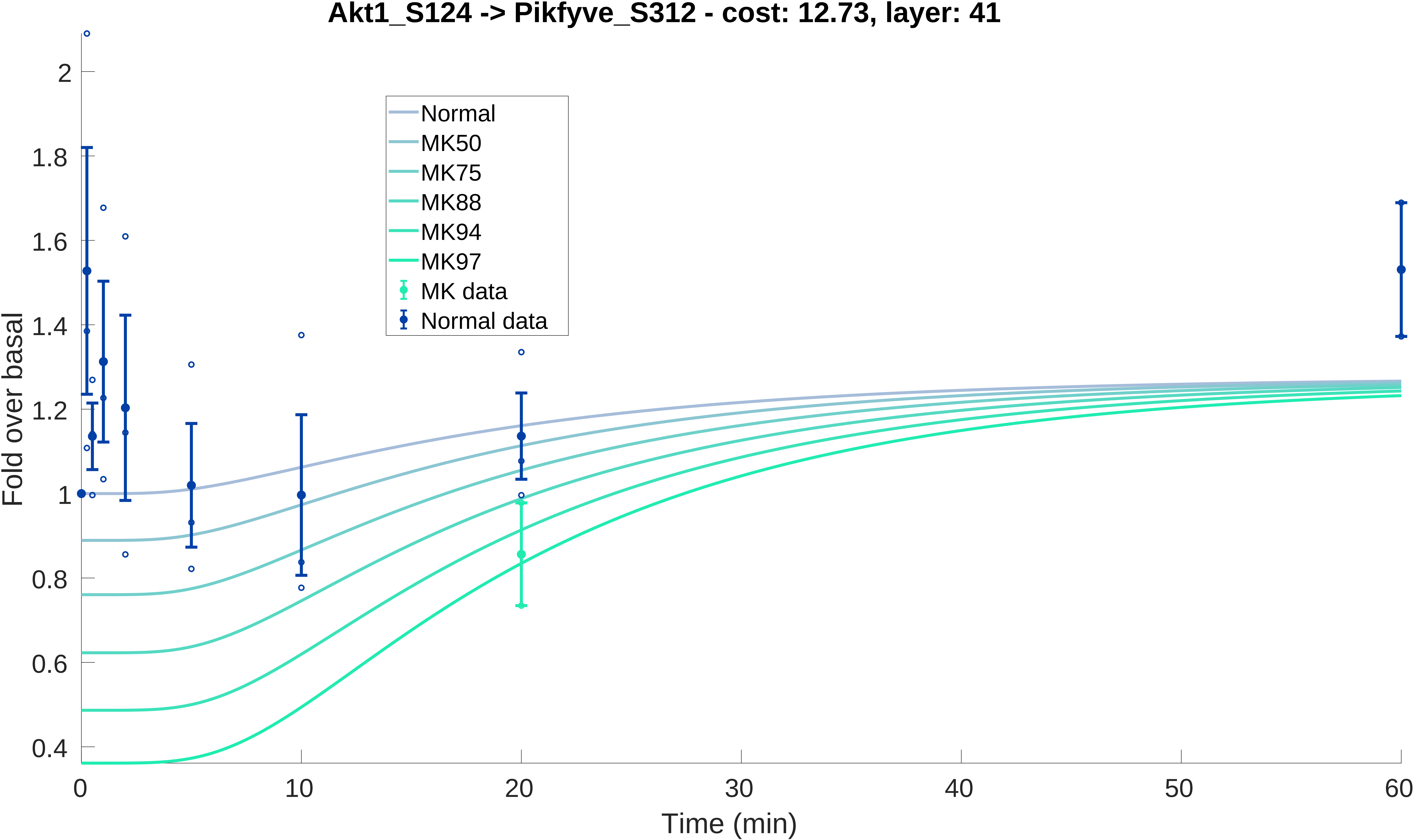

Akt1_S124 -> Pikfyve_S312 - cost: 12.73, layer: 41
Normal
MK50
MK75
MK88
MK94
MK97
MK data
Normal data
Fold over basal
Time (min)
0.4
0.6
0.8
1
1.2
1.4
1.6
1.8
2
0
10
20
30
40
50
60

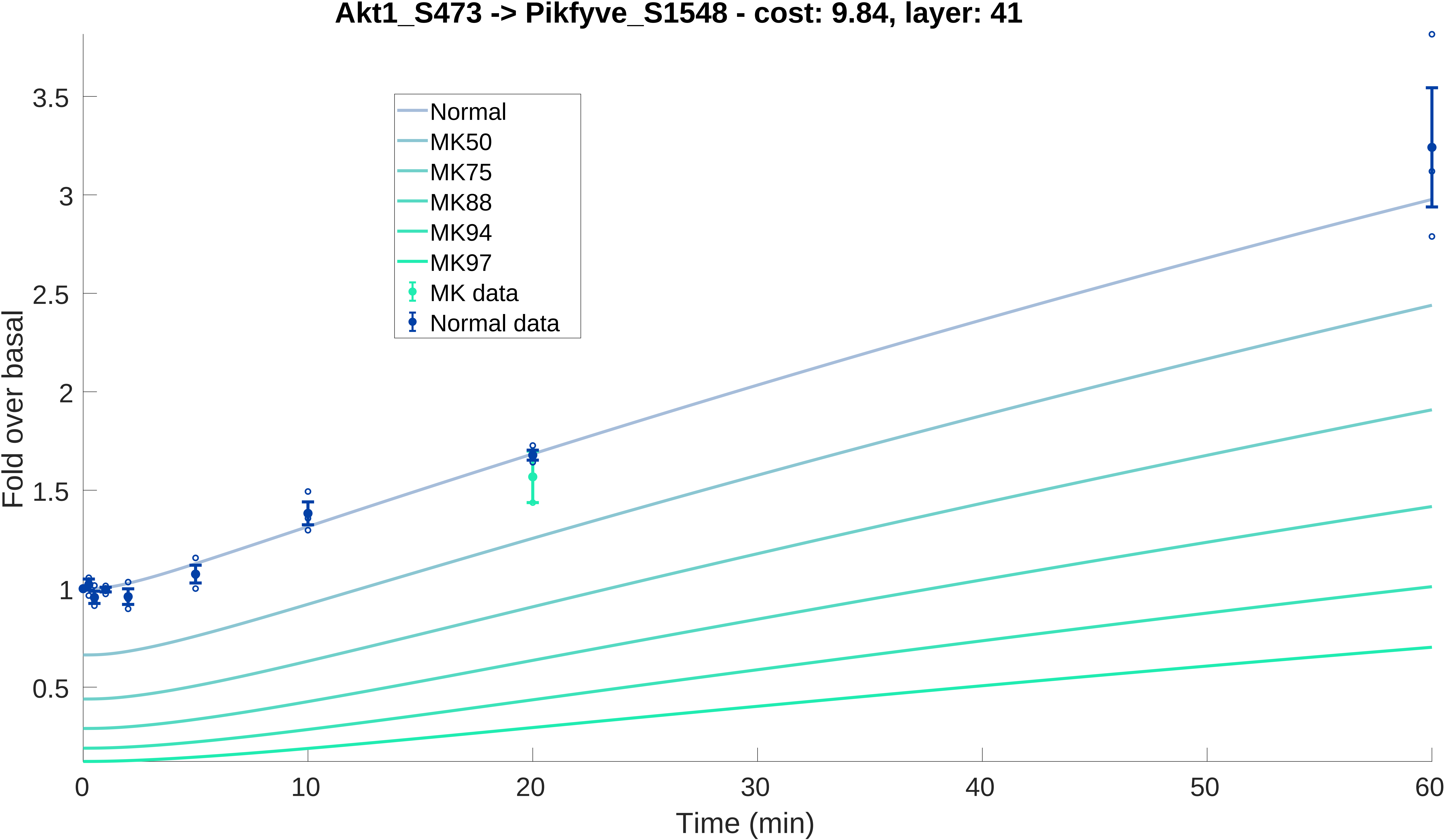

Akt1_S473 -> Pikfyve_S1548 - cost: 9.84, layer: 41
Normal
MK50
MK75
MK88
MK94
MK97
MK data
Normal data
Fold over basal
0.5
1
1.5
2
2.5
3
3.5
0
10
20
30
40
50
60
Time (min)

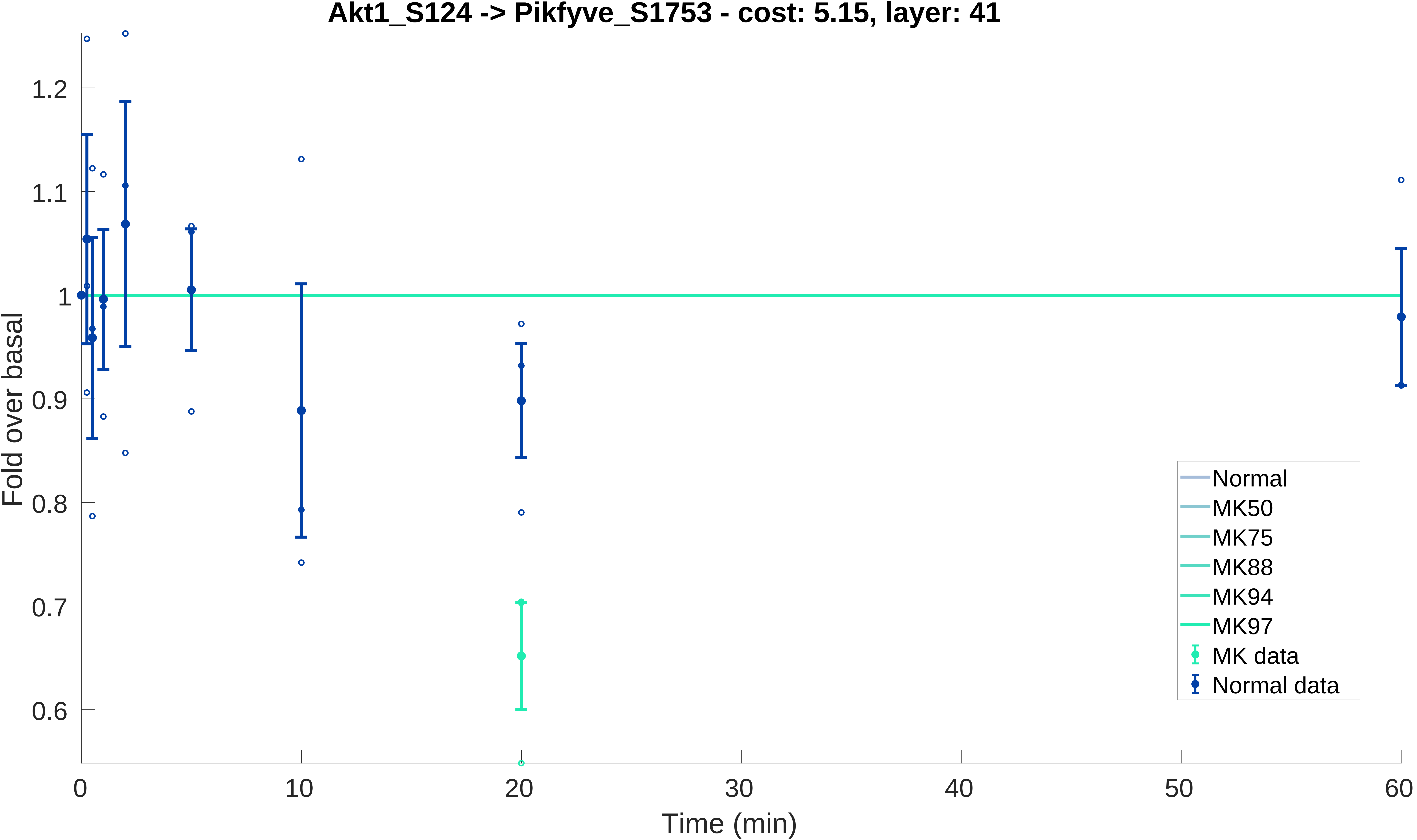

Akt1_S124 -> Pikfyve_S1753 - cost: 5.15, layer: 41
Fold over basal
Time (min)
0.6
0.7
0.8
0.9
1
1.1
1.2
0
10
20
30
40
50
60
Normal
MK50
MK75
MK88
MK94
MK97
MK data
Normal data

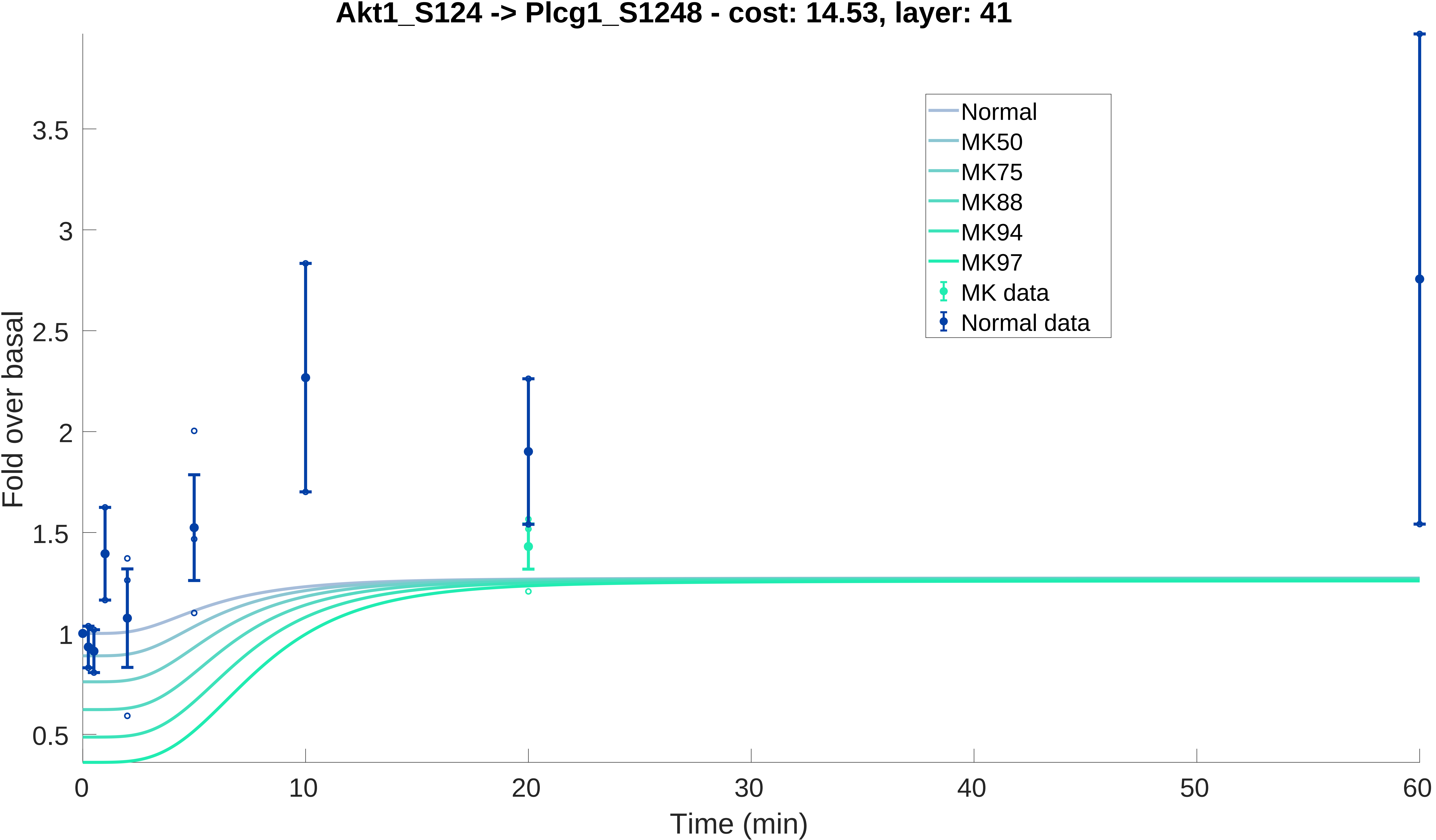

Akt1_S124 -> Plcg1_S1248 - cost: 14.53, layer: 41
Fold over basal
Time (min)
0.5
1
1.5
2
2.5
3
3.5
0
10
20
30
40
50
60
Normal
MK50
MK75
MK88
MK94
MK97
MK data
Normal data

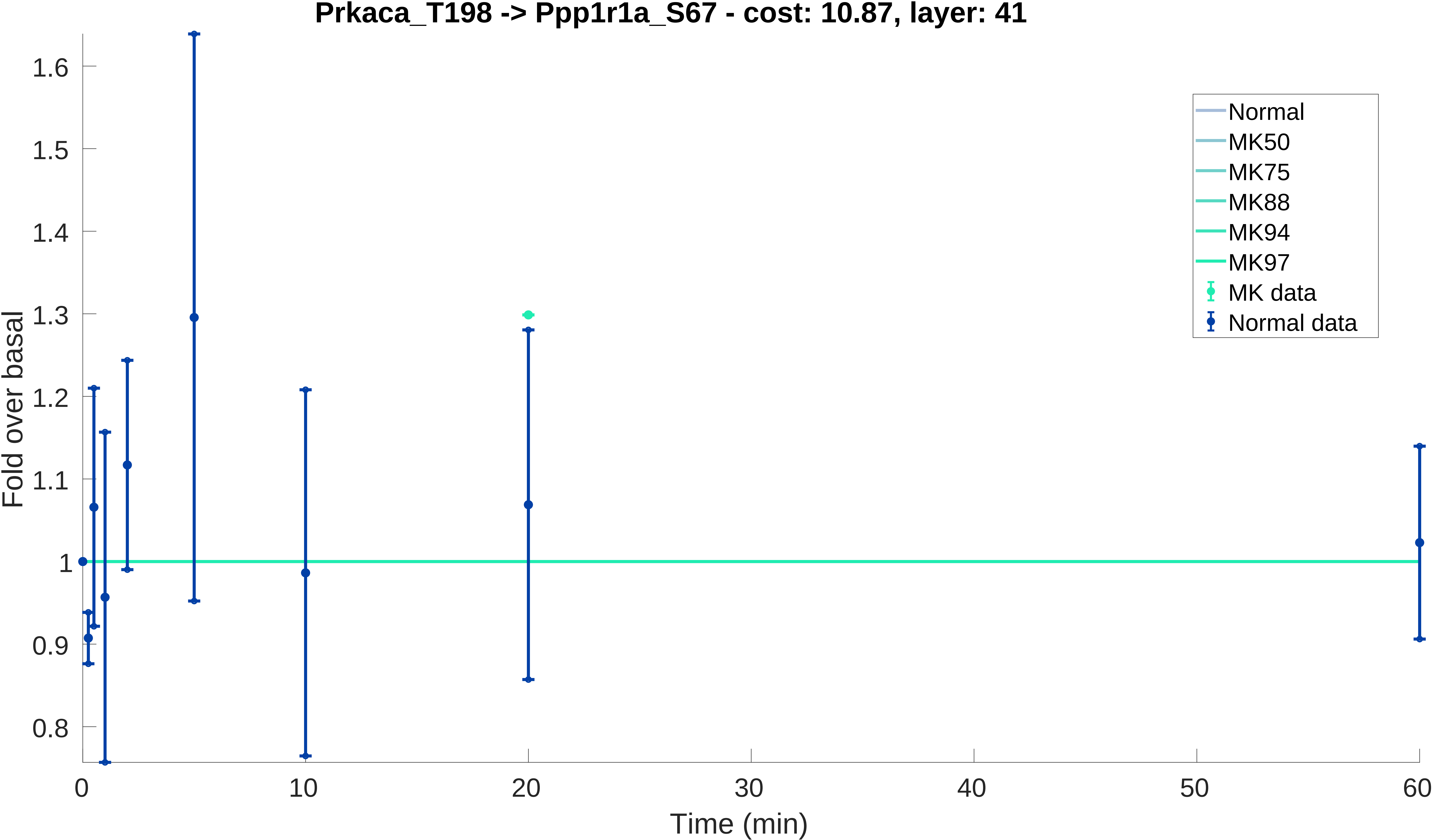

Prkaca_T198 -> Ppp1r1a_S67 - cost: 10.87, layer: 41
Fold over basal
Time (min)
1.6
1.5
1.4
1.3
1.2
1.1
1
0.9
0.8
0
10
20
30
40
50
60
Normal
MK50
MK75
MK88
MK94
MK97
MK data
Normal data

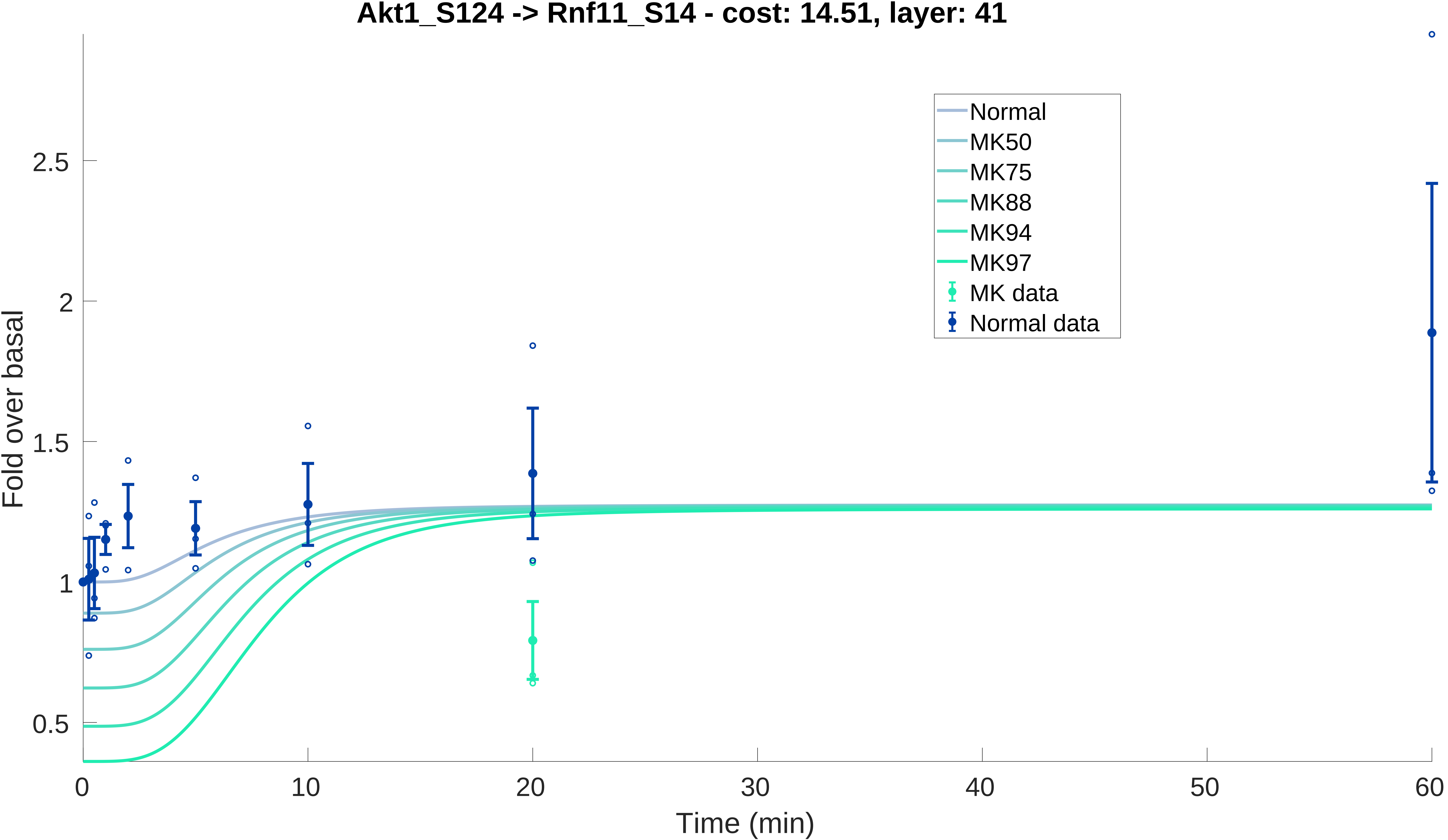

Akt1_S124 -> Rnf11_S14 - cost: 14.51, layer: 41
Fold over basal
0.5
1
1.5
2
2.5
0
10
20
30
40
50
60
Time (min)
Normal
MK50
MK75
MK88
MK94
MK97
MK data
Normal data

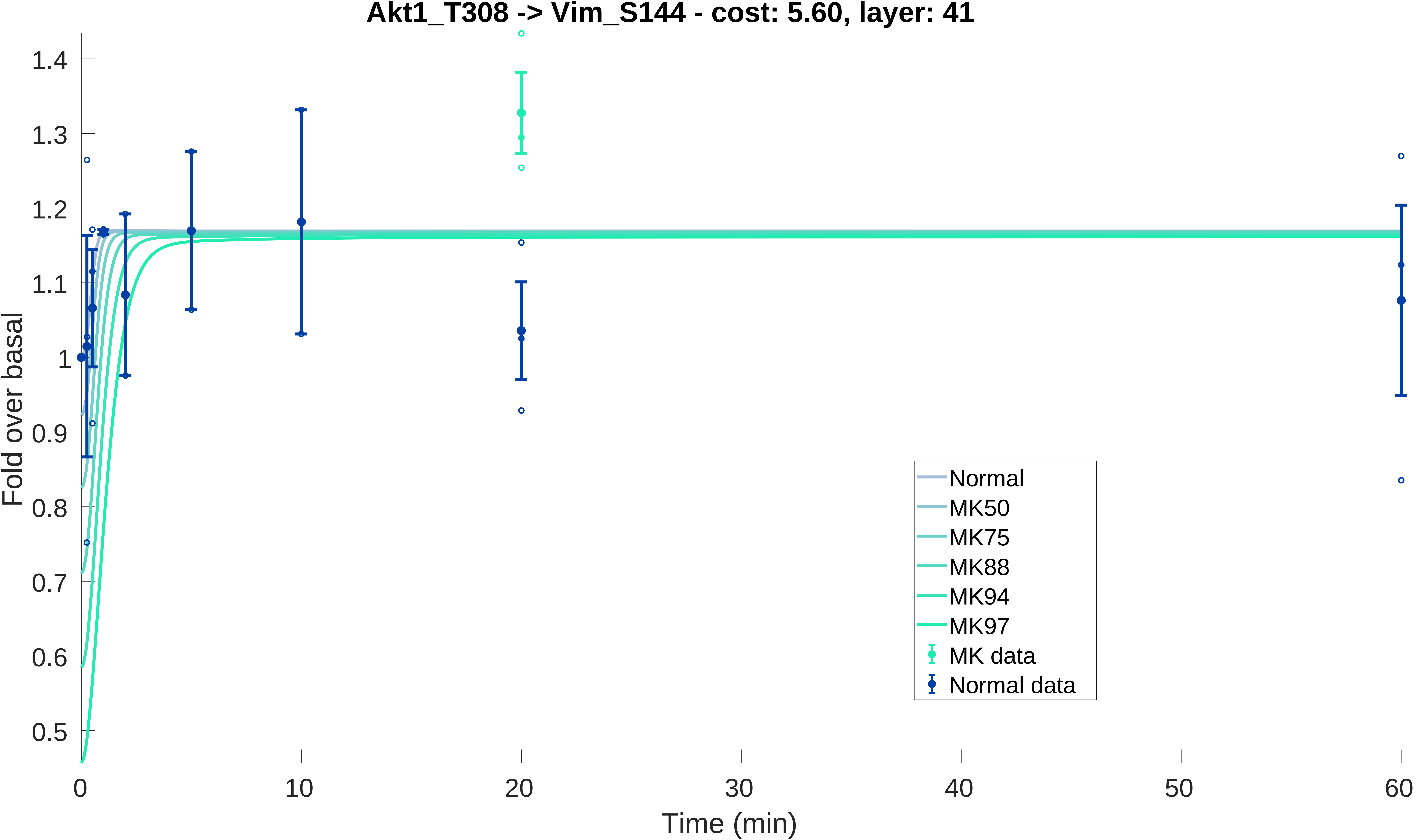
Akt1_T308 -> Vim_S144 - cost: 5.60, layer: 41
Fold over basal
Time (min)
1.4
1.3
1.2
1.1
1
0.9
0.8
0.7
0.6
0.5
0
10
20
30
40
50
60
Normal
MK50
MK75
MK88
MK94
MK97
MK data
Normal data

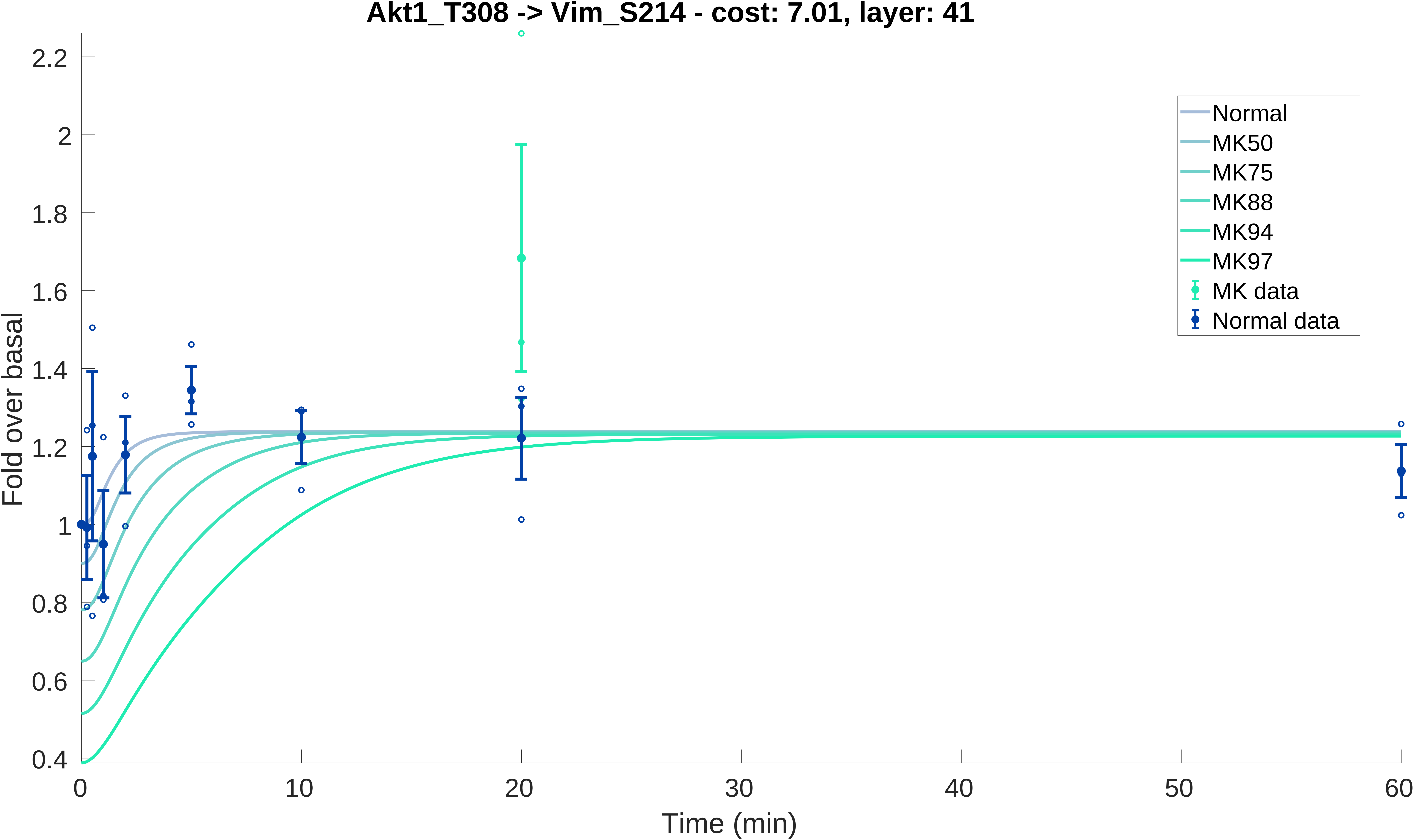

Akt1_T308 -> Vim_S214 - cost: 7.01, layer: 41
Fold over basal
Time (min)
0.4
0.6
0.8
1
1.2
1.4
1.6
1.8
2
2.2
0
10
20
30
40
50
60
Normal
MK50
MK75
MK88
MK94
MK97
MK data
Normal data

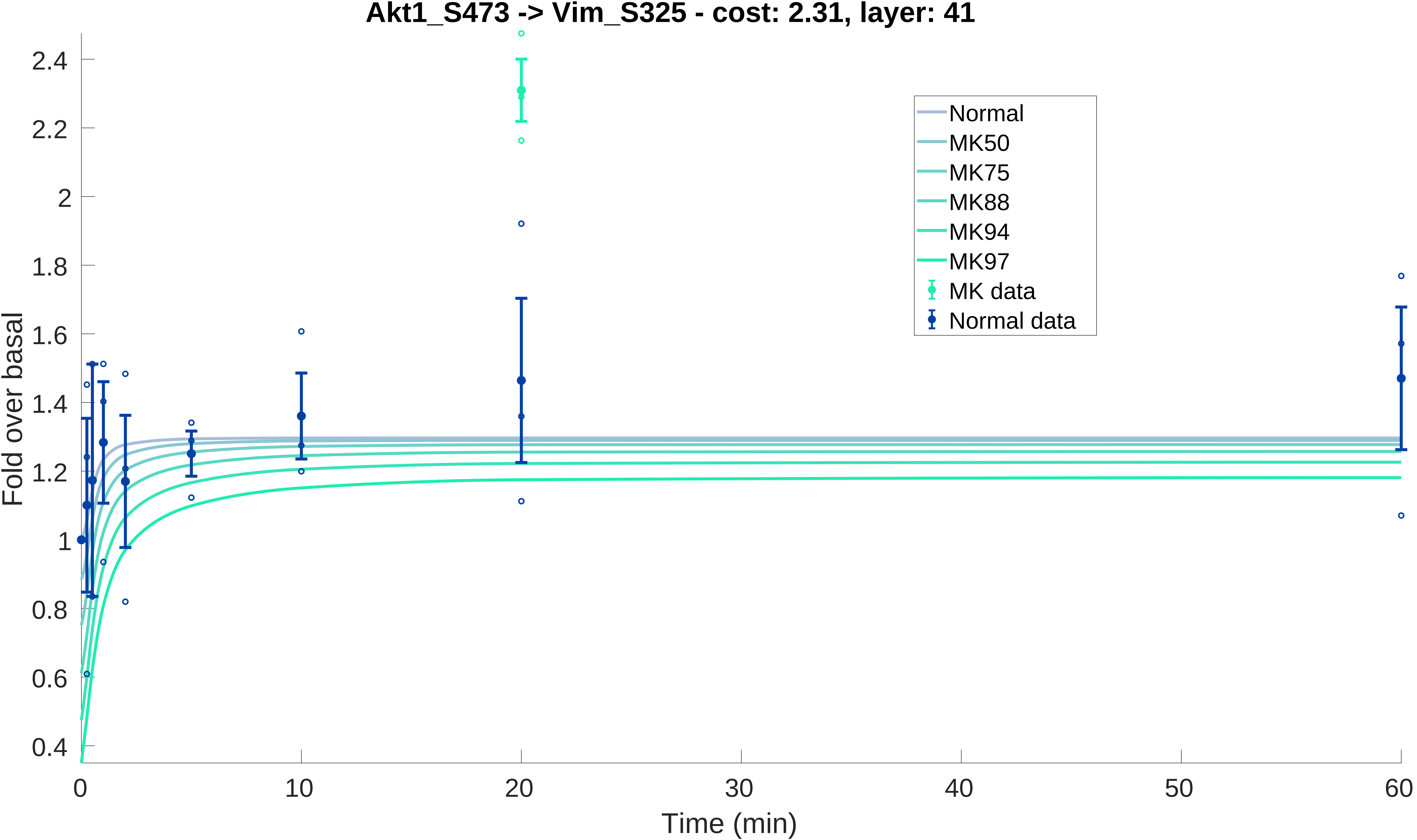

Akt1_S473 -> Vim_S325 - cost: 2.31, layer: 41
Fold over basal
Time (min)
Normal
MK50
MK75
MK88
MK94
MK97
MK data
Normal data
2.4
2.2
2
1.8
1.6
1.4
1.2
1
0.8
0.6
0.4
0
10
20
30
40
50
60

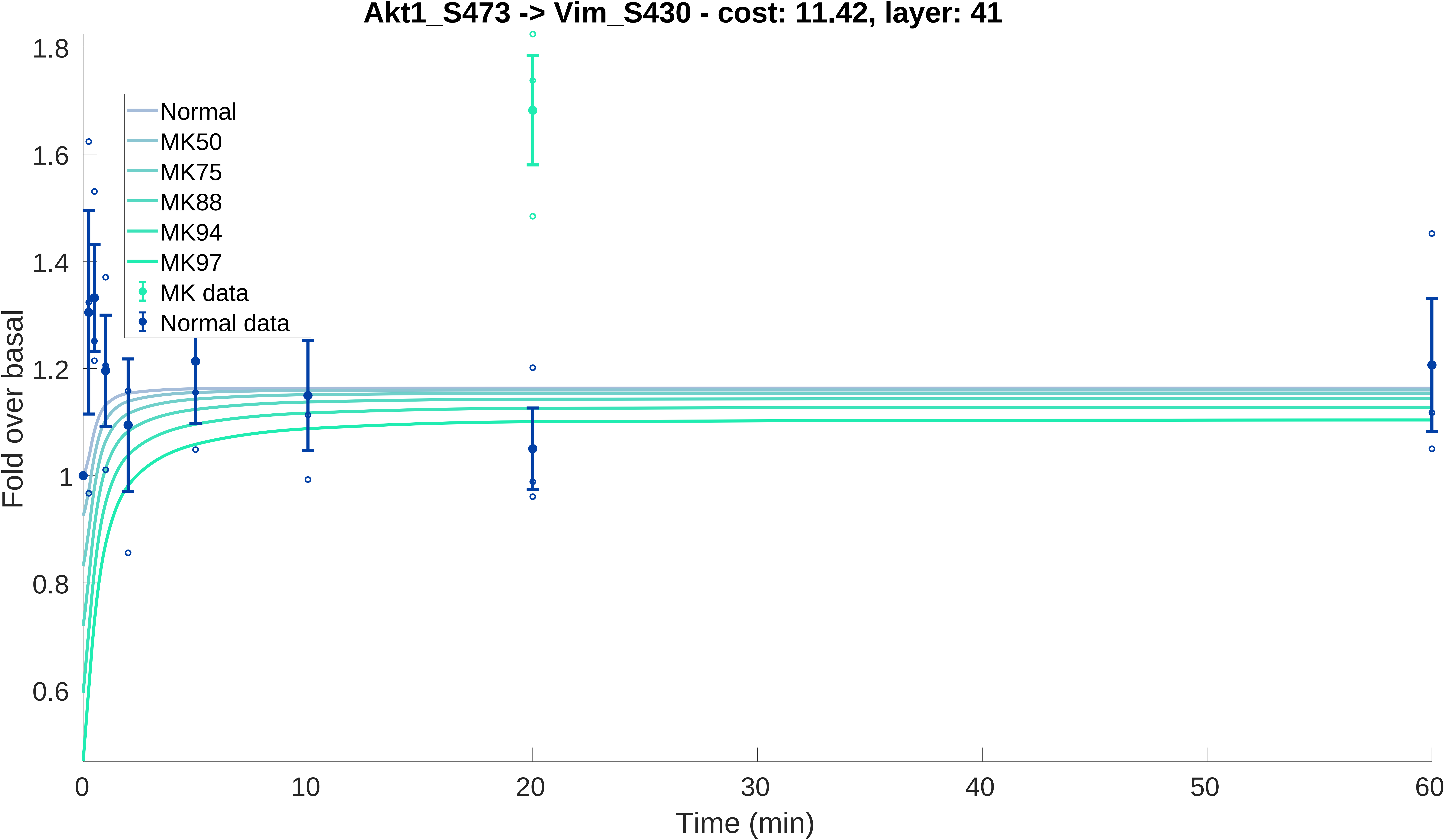

Akt1_S473 -> Vim_S430 - cost: 11.42, layer: 41
Fold over basal
Time (min)
Normal
MK50
MK75
MK88
MK94
MK97
MK data
Normal data
1.8
1.6
1.4
1.2
1
0.8
0.6
0
10
20
30
40
50
60

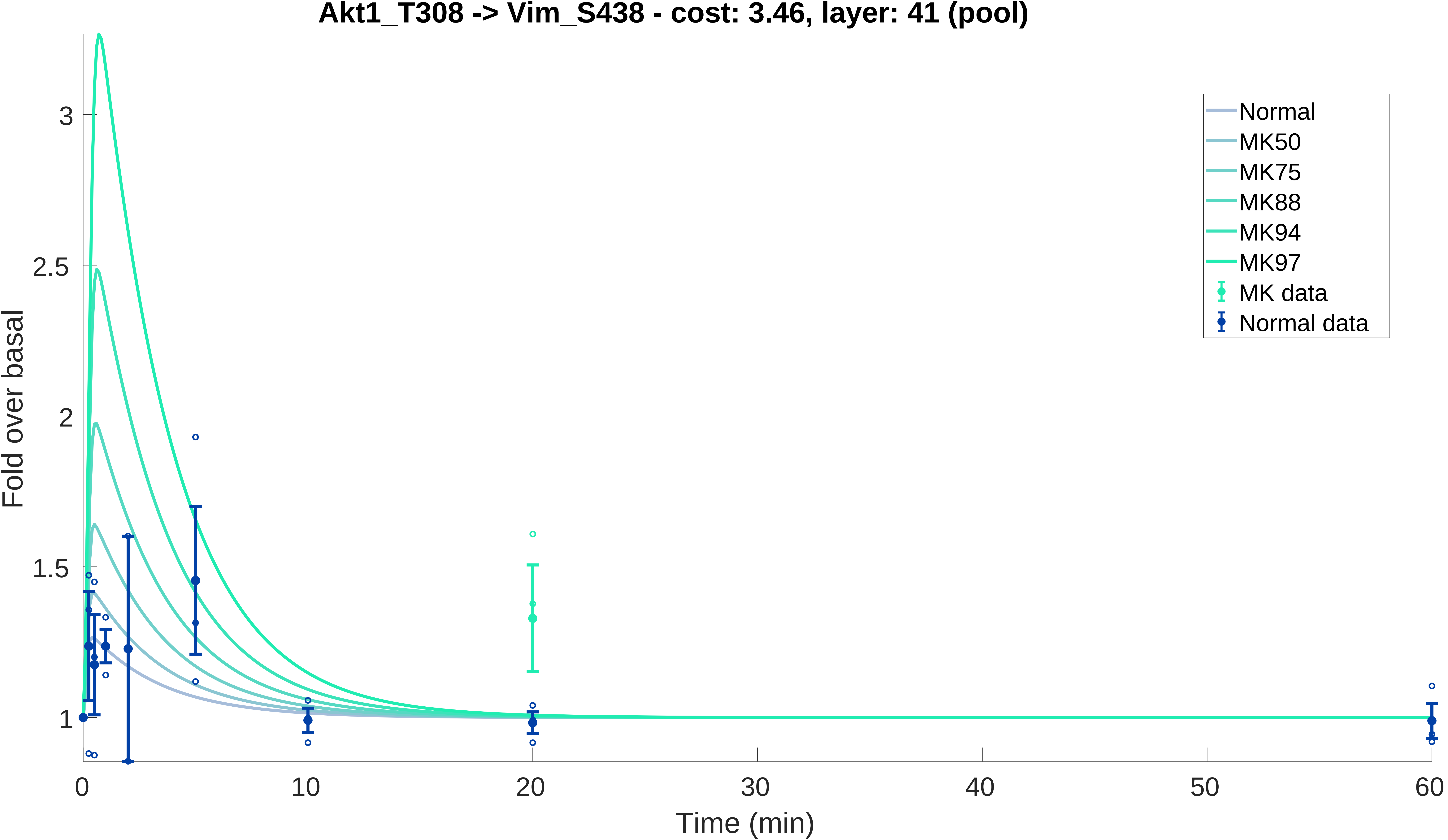
Akt1_T308 -> Vim_S438 - cost: 3.46, layer: 41 (pool)
Fold over basal
Time (min)
Normal
MK50
MK75
MK88
MK94
MK97
MK data
Normal data
1
1.5
2
2.5
3
0
10
20
30
40
50
60

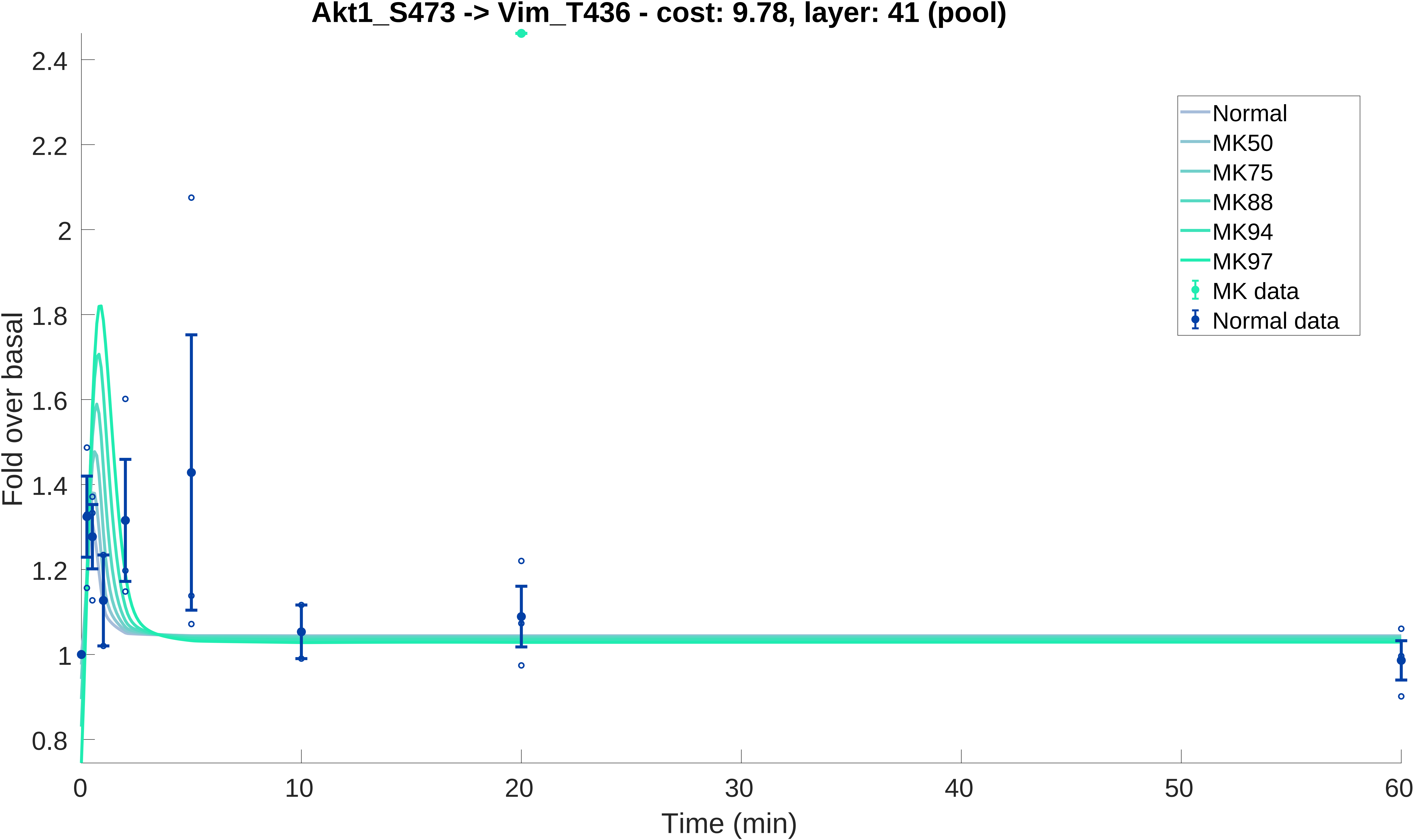
Akt1_S473 -> Vim_T436 - cost: 9.78, layer: 41 (pool)
Fold over basal
Time (min)
0.8
1
1.2
1.4
1.6
1.8
2
2.2
2.4
0
10
20
30
40
50
60
Normal
MK50
MK75
MK88
MK94
MK97
MK data
Normal data

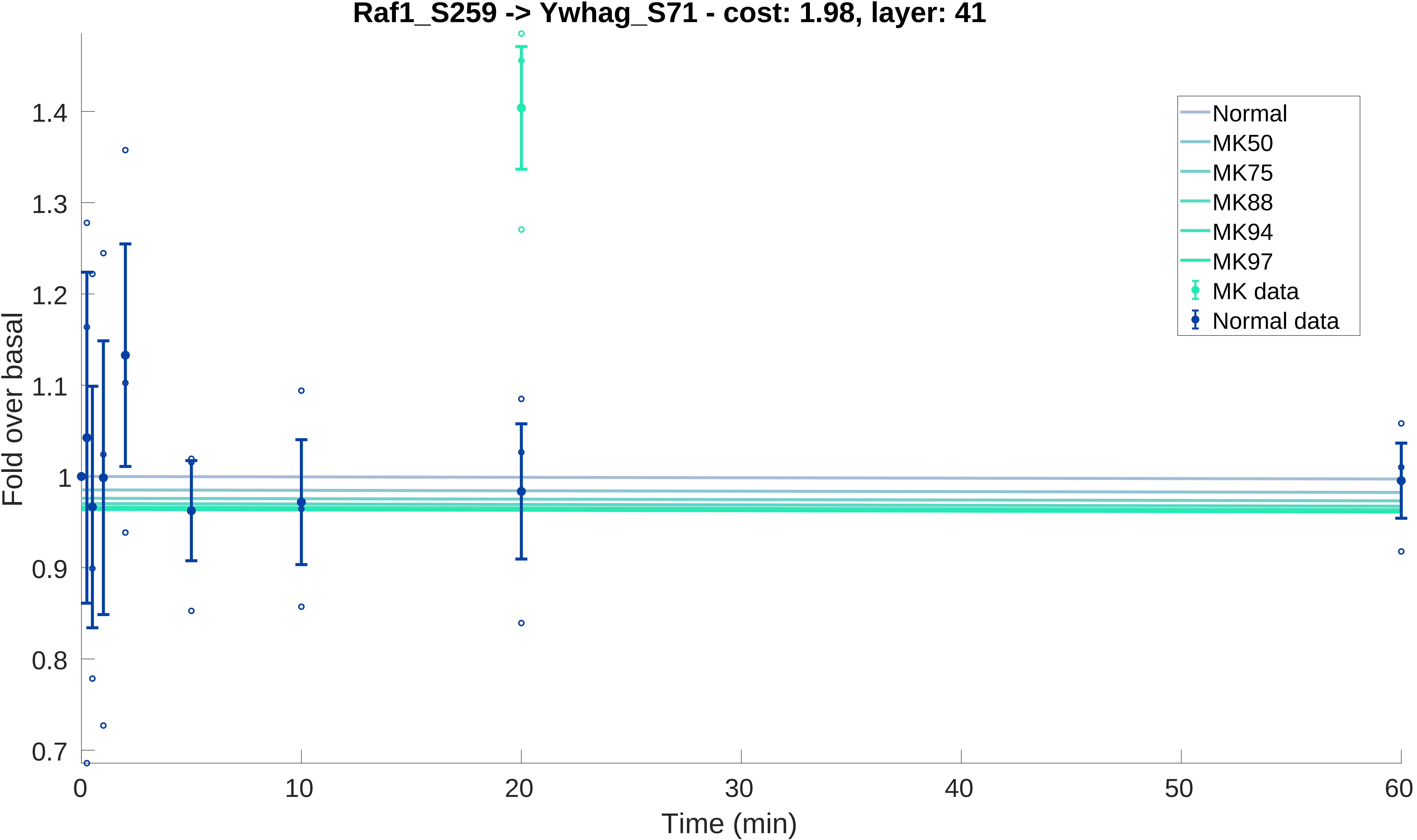

Raf1_S259 -> Ywhag_S71 - cost: 1.98, layer: 41
Fold over basal
Time (min)
0.7
0.8
0.9
1
1.1
1.2
1.3
1.4
0
10
20
30
40
50
60
Normal
MK50
MK75
MK88
MK94
MK97
MK data
Normal data

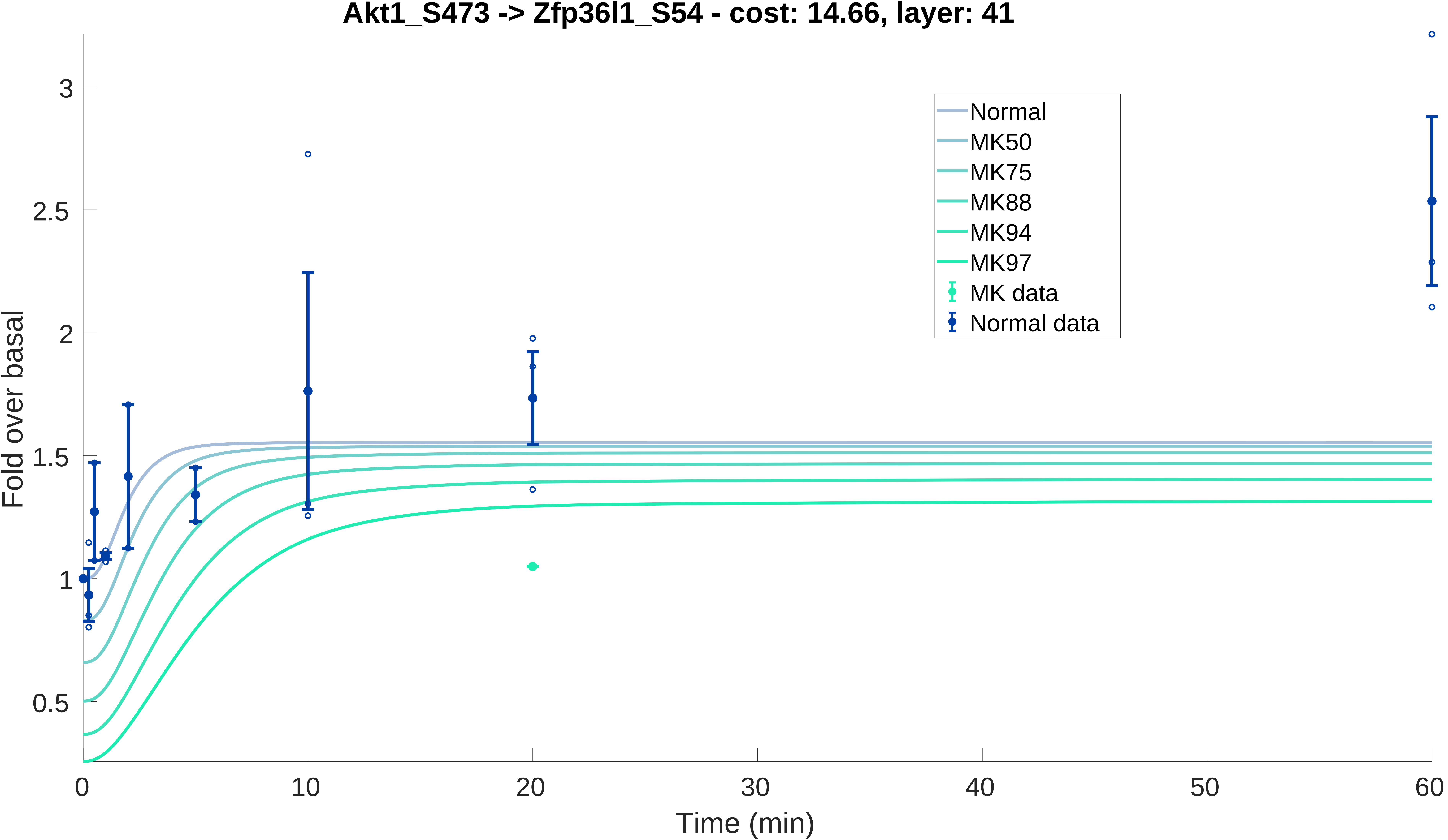

Akt1_S473 -> Zfp36l1_S54 - cost: 14.66, layer: 41
Fold over basal
Time (min)
0
10
20
30
40
50
60
0.5
1
1.5
2
2.5
3
Normal
MK50
MK75
MK88
MK94
MK97
MK data
Normal data

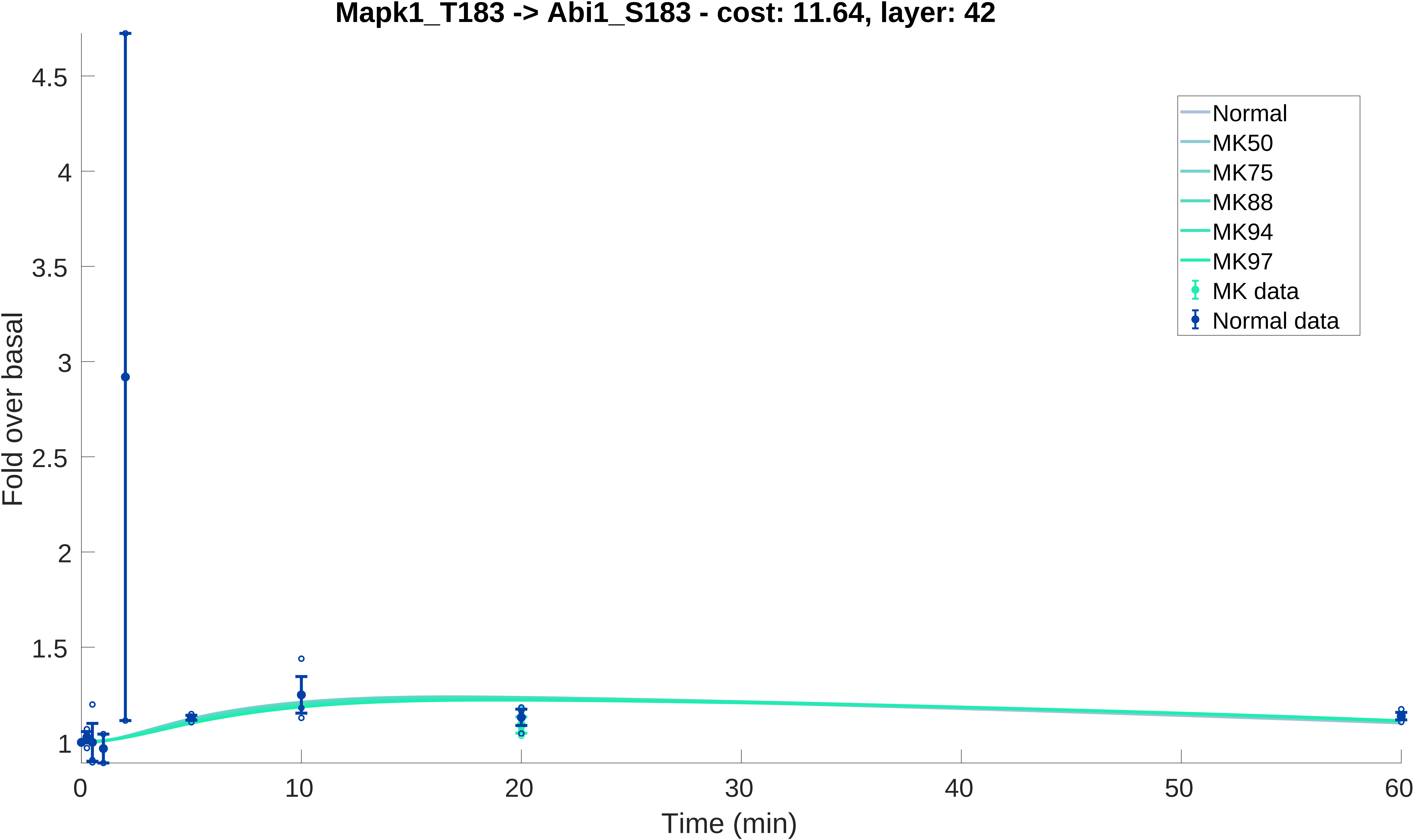

Mapk1_T183 -> Abi1_S183 - cost: 11.64, layer: 42
Fold over basal
Time (min)
1
1.5
2
2.5
3
3.5
4
4.5
0
10
20
30
40
50
60
Normal
MK50
MK75
MK88
MK94
MK97
MK data
Normal data

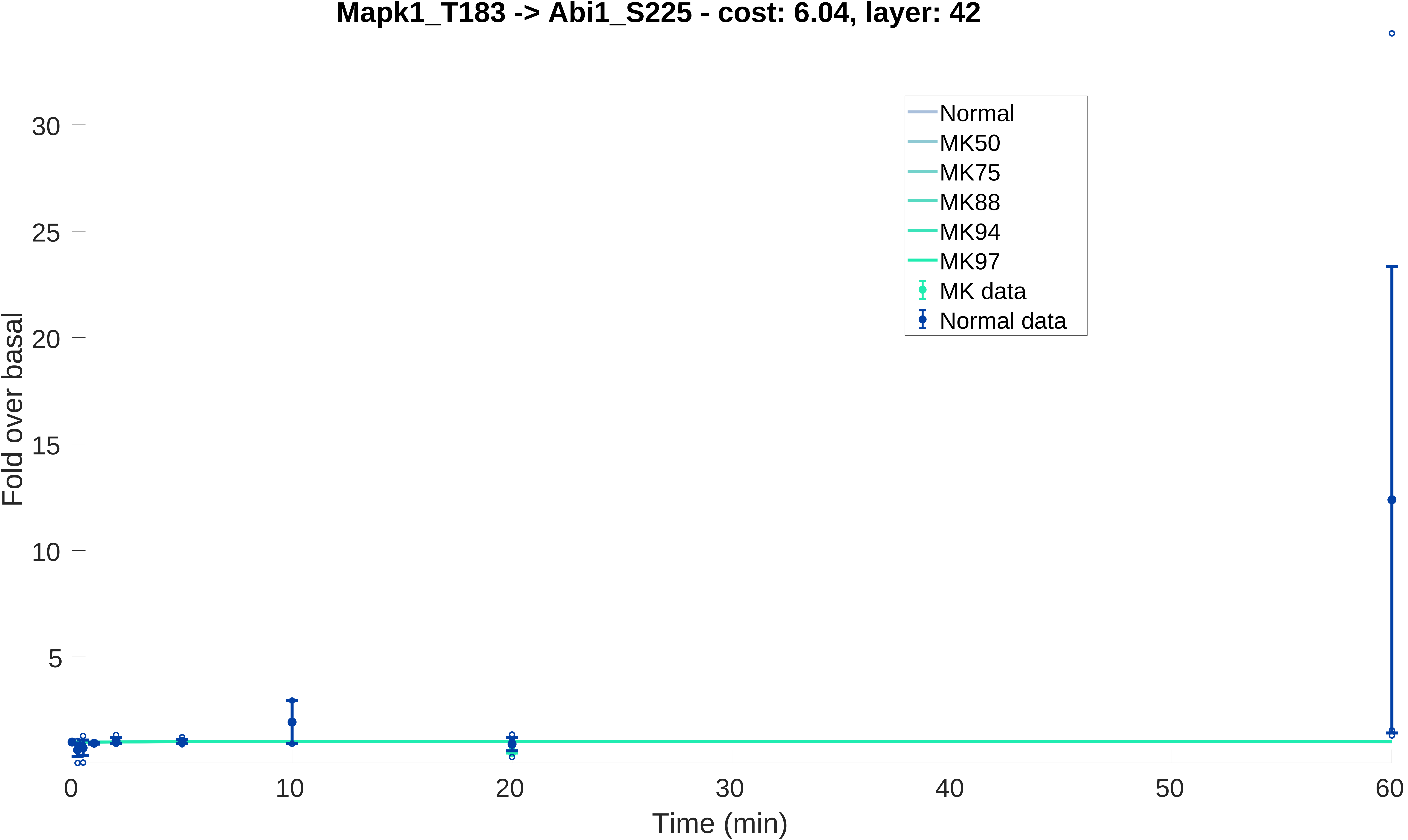

Mapk1_T183 -> Abi1_S225 - cost: 6.04, layer: 42
Fold over basal
Time (min)
0
10
20
30
40
50
60
5
10
15
20
25
30
Normal
MK50
MK75
MK88
MK94
MK97
MK data
Normal data

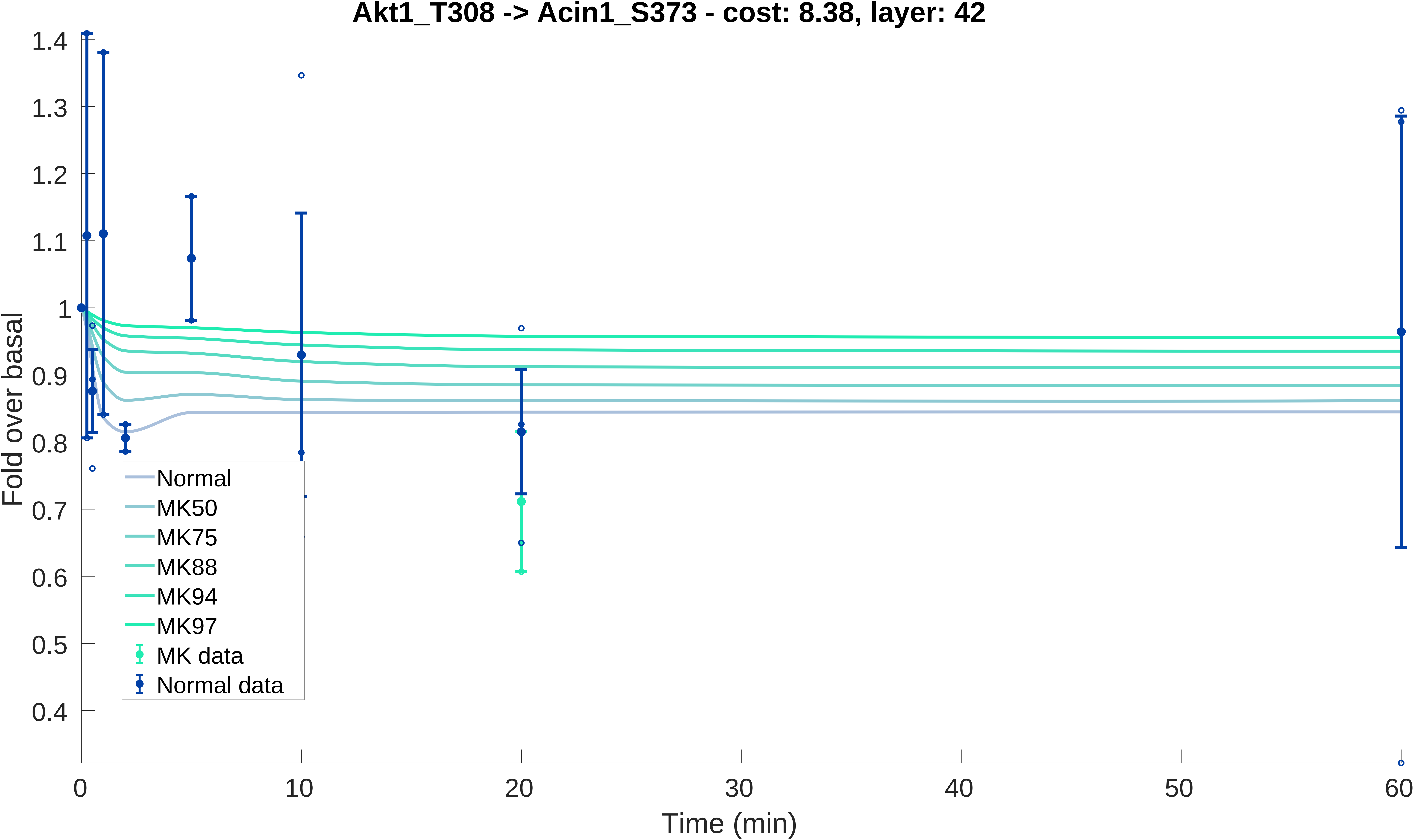

Akt1_T308 -> Acin1_S373 - cost: 8.38, layer: 42
Fold over basal
Time (min)
1.4
1.3
1.2
1.1
1
0.9
0.8
0.7
0.6
0.5
0.4
0
10
20
30
40
50
60
Normal
MK50
MK75
MK88
MK94
MK97
MK data
Normal data

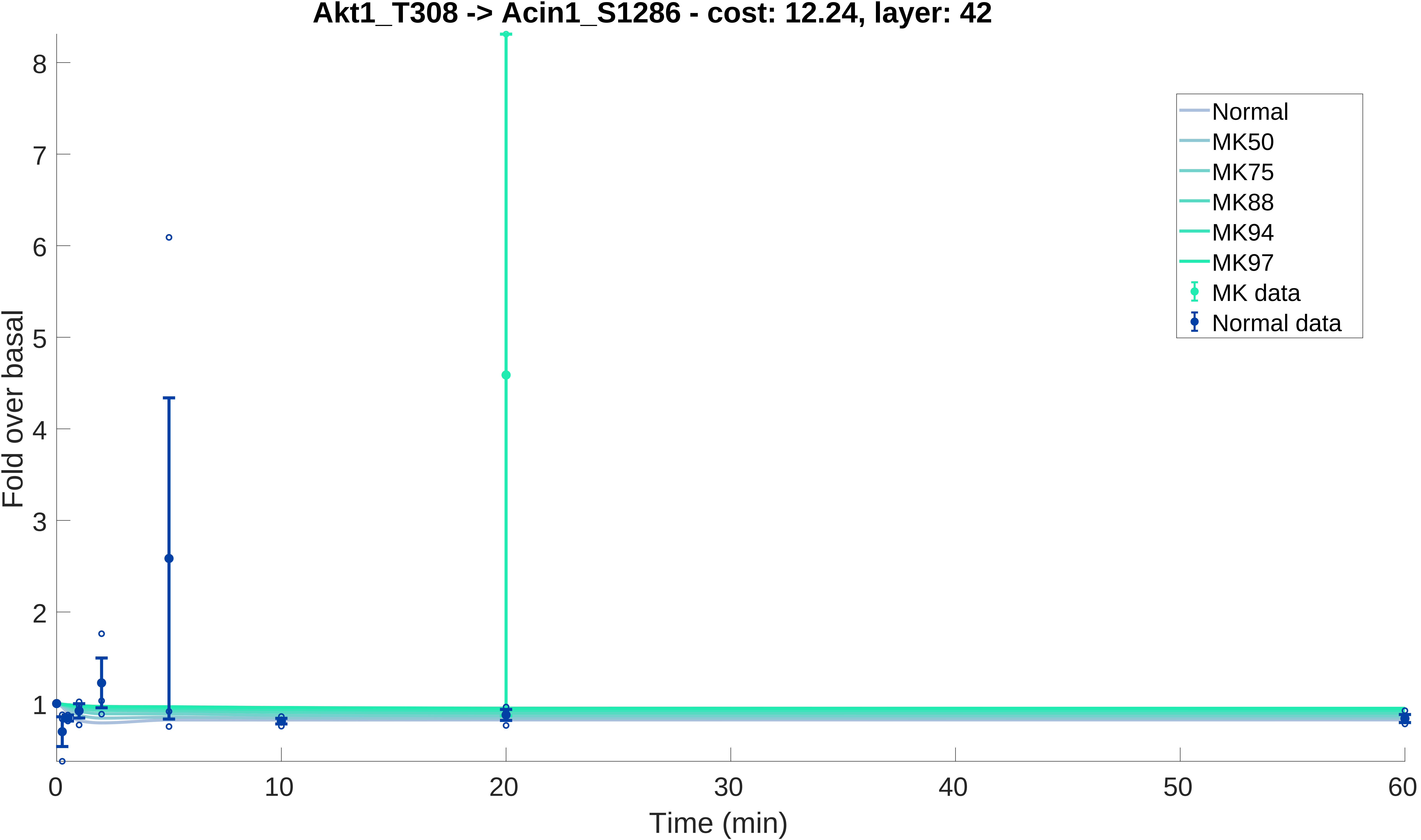

Akt1_T308 -> Acin1_S1286 - cost: 12.24, layer: 42
Fold over basal
Time (min)
Normal
MK50
MK75
MK88
MK94
MK97
MK data
Normal data
0
10
20
30
40
50
60
1
2
3
4
5
6
7
8

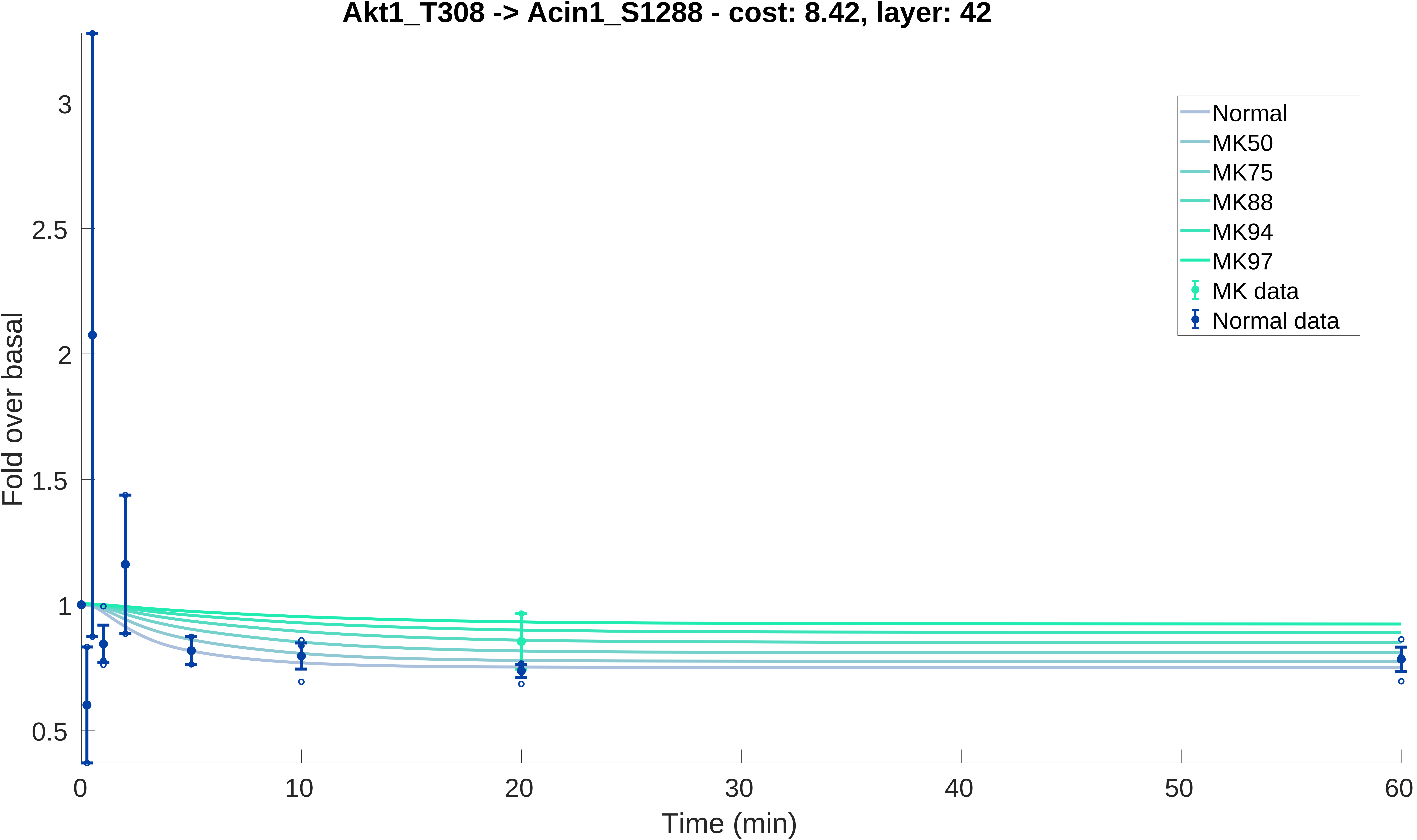

Akt1_T308 -> Acin1_S1288 - cost: 8.42, layer: 42
Fold over basal
Time (min)
0.5
1
1.5
2
2.5
3
0
10
20
30
40
50
60
Normal
MK50
MK75
MK88
MK94
MK97
MK data
Normal data

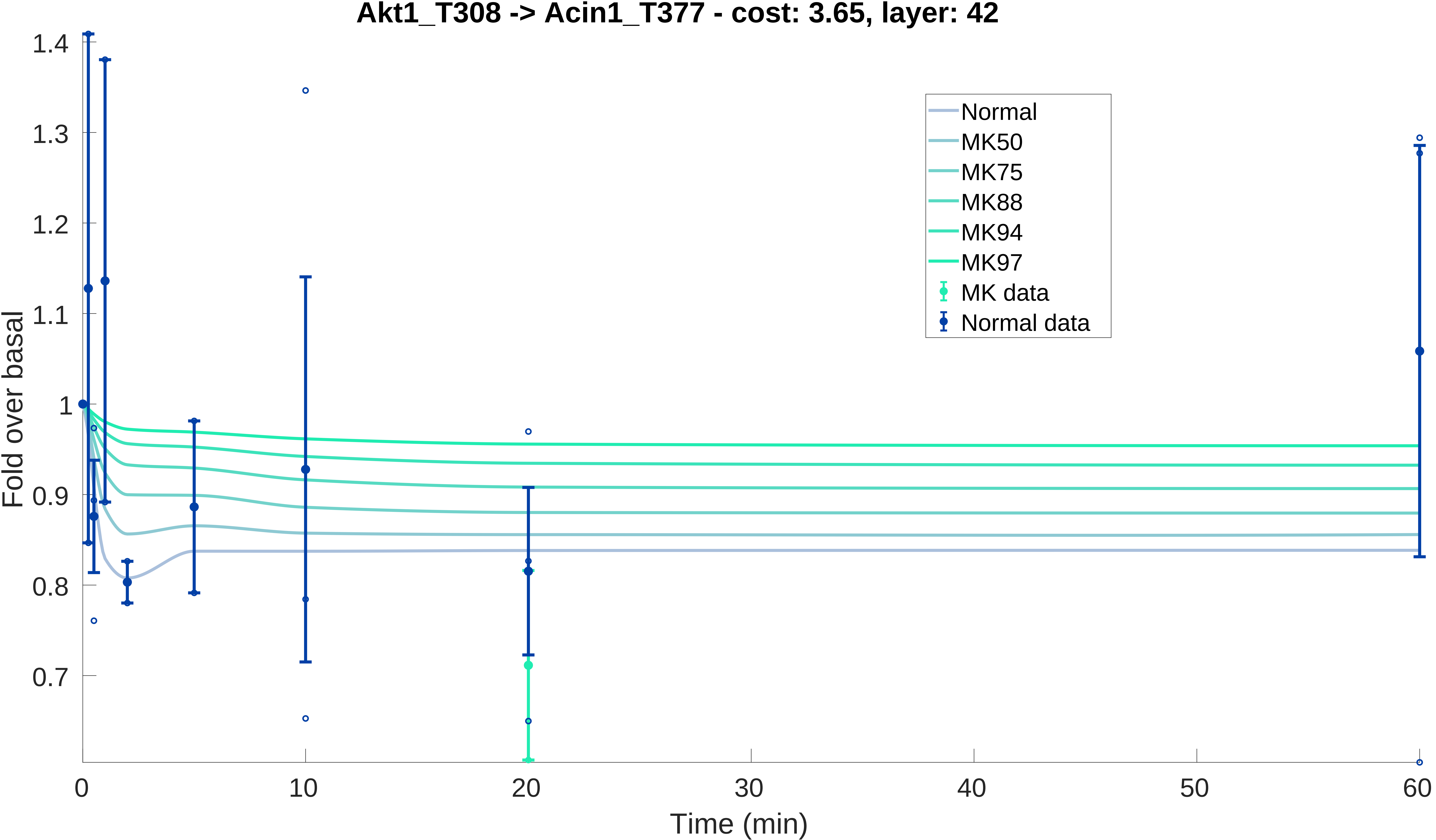

Akt1_T308 -> Acin1_T377 - cost: 3.65, layer: 42
Fold over basal
Time (min)
Normal
MK50
MK75
MK88
MK94
MK97
MK data
Normal data
1.4
1.3
1.2
1.1
1
0.9
0.8
0.7
0
10
20
30
40
50
60

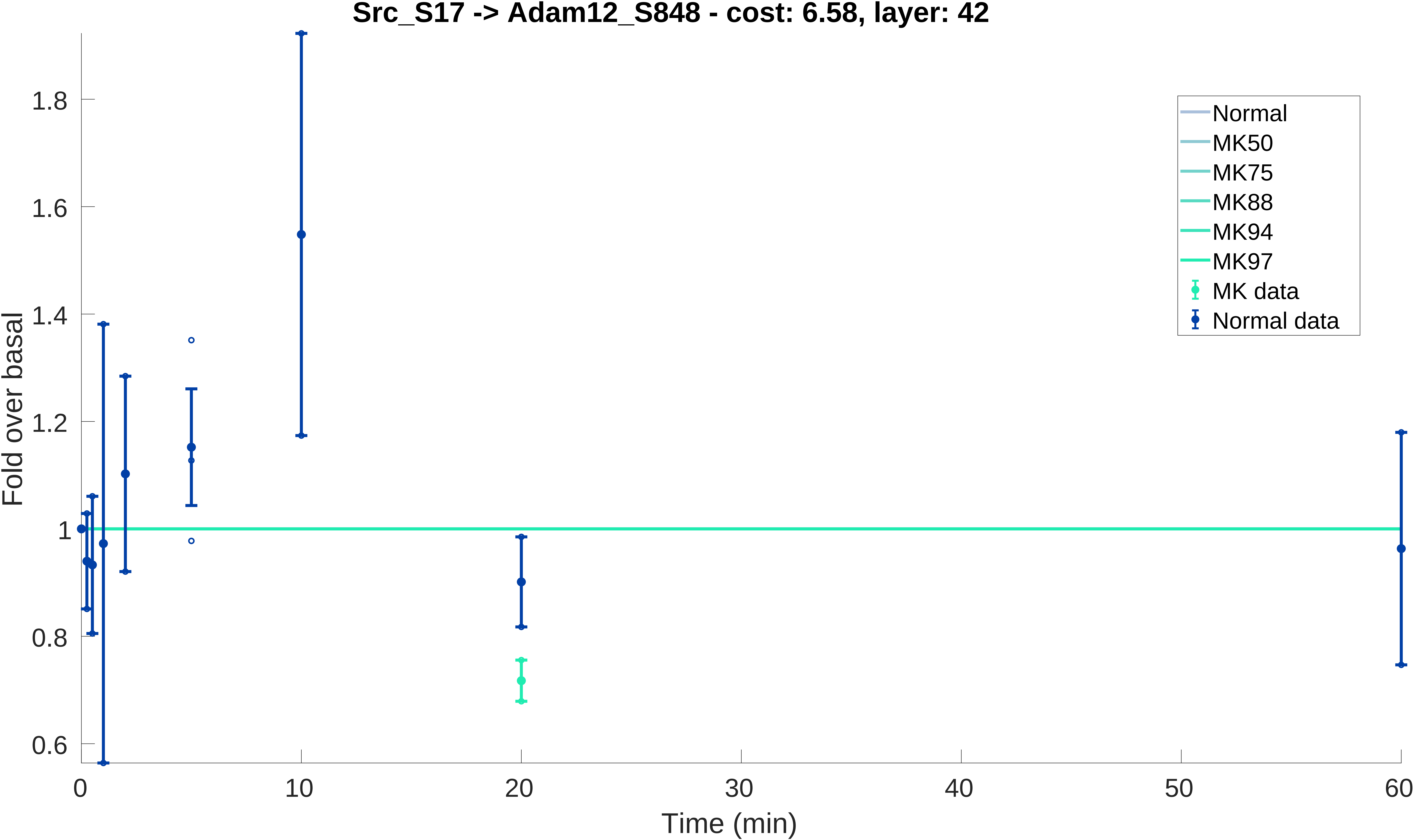

Src_S17 -> Adam12_S848 - cost: 6.58, layer: 42
Fold over basal
Time (min)
0.6
0.8
1
1.2
1.4
1.6
1.8
0
10
20
30
40
50
60
Normal
MK50
MK75
MK88
MK94
MK97
MK data
Normal data

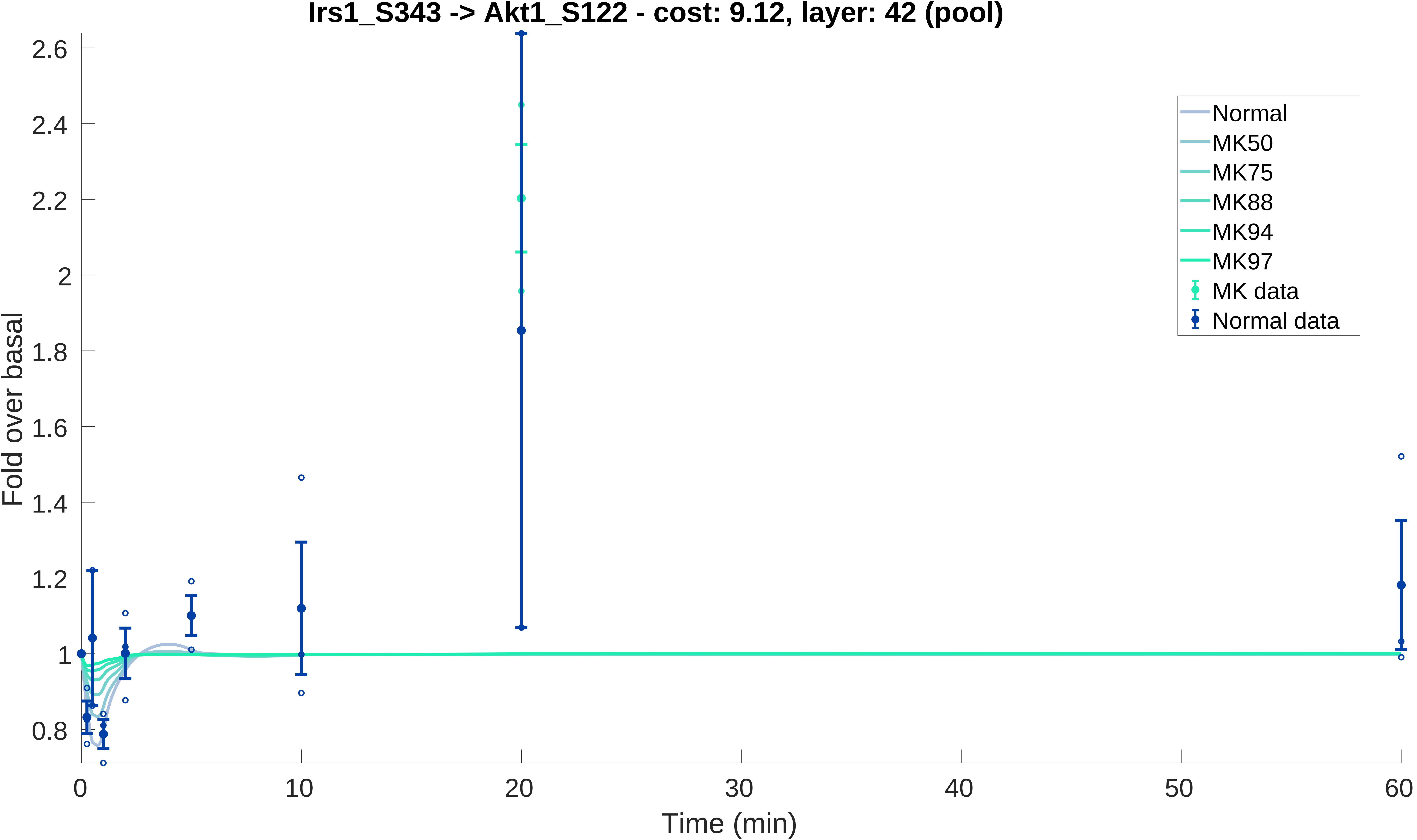
Irs1_S343 -> Akt1_S122 - cost: 9.12, layer: 42 (pool)
Fold over basal
Time (min)
Normal
MK50
MK75
MK88
MK94
MK97
MK data
Normal data
0.8
1
1.2
1.4
1.6
1.8
2
2.2
2.4
2.6
0
10
20
30
40
50
60

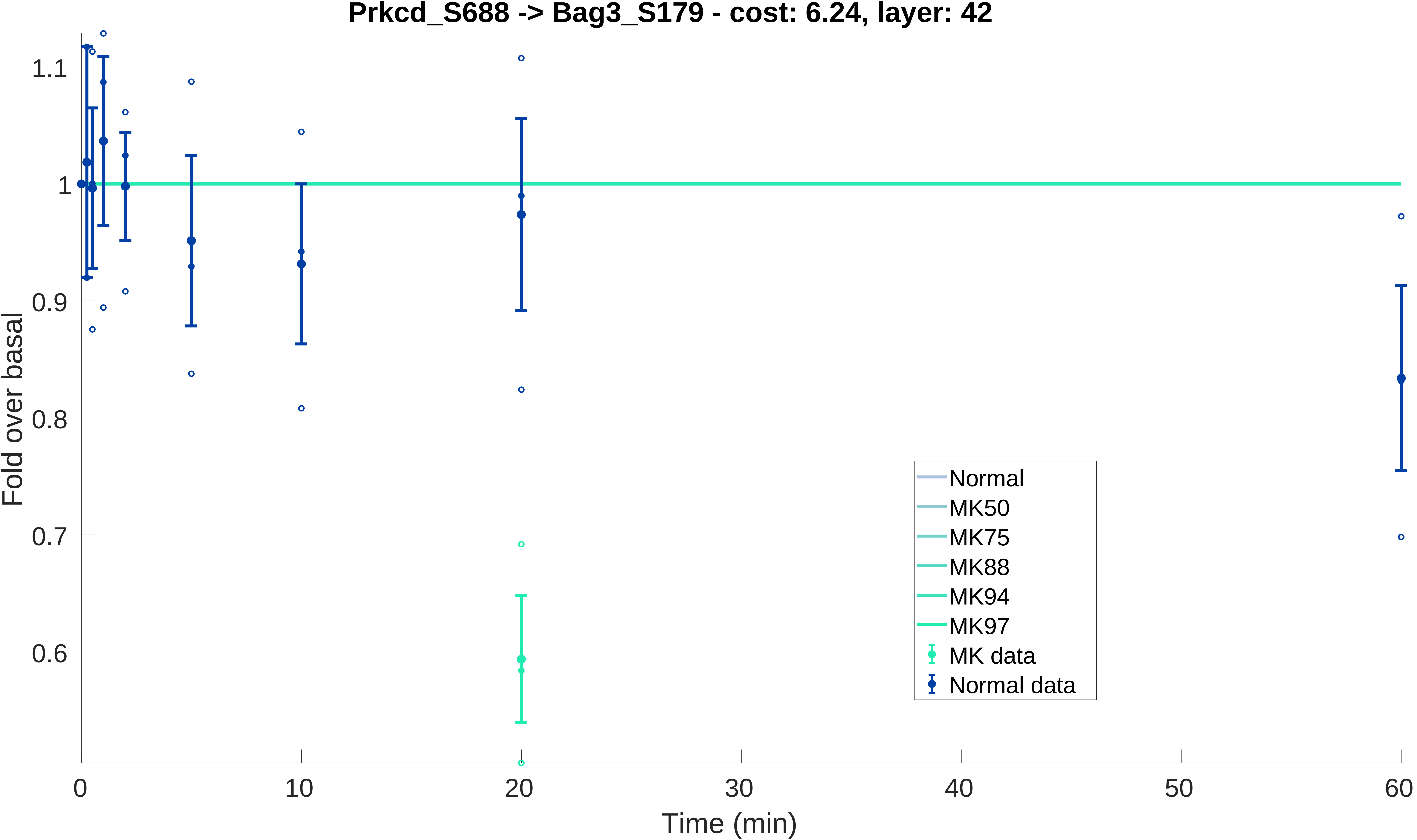
Prkcd_S688 -> Bag3_S179 - cost: 6.24, layer: 42
Fold over basal
Time (min)
1.1
1
0.9
0.8
0.7
0.6
0
10
20
30
40
50
60
Normal
MK50
MK75
MK88
MK94
MK97
MK data
Normal data

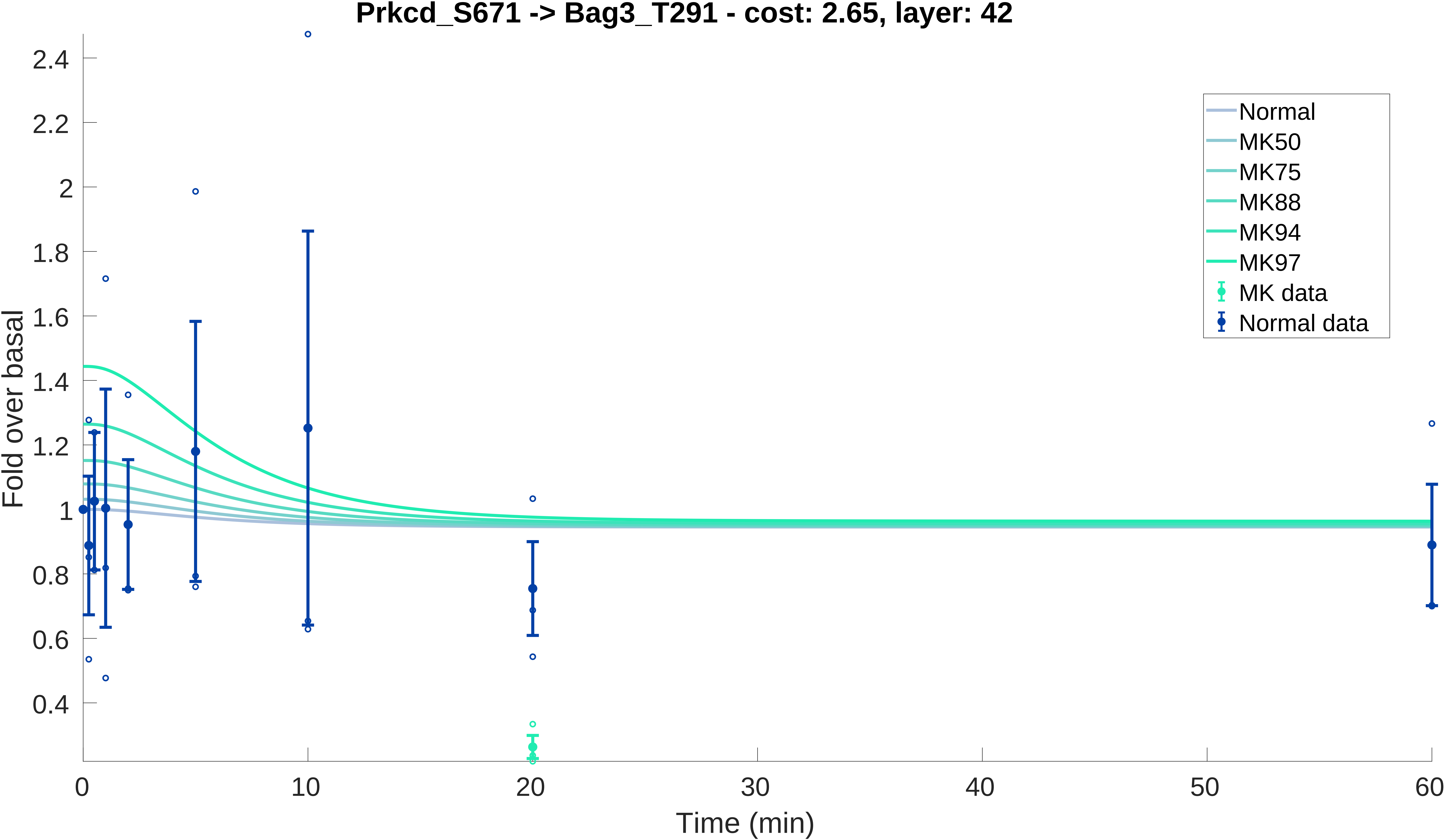

Prkcd_S671 -> Bag3_T291 - cost: 2.65, layer: 42
Fold over basal
Time (min)
0
10
20
30
40
50
60
0.4
0.6
0.8
1
1.2
1.4
1.6
1.8
2
2.2
2.4
Normal
MK50
MK75
MK88
MK94
MK97
MK data
Normal data

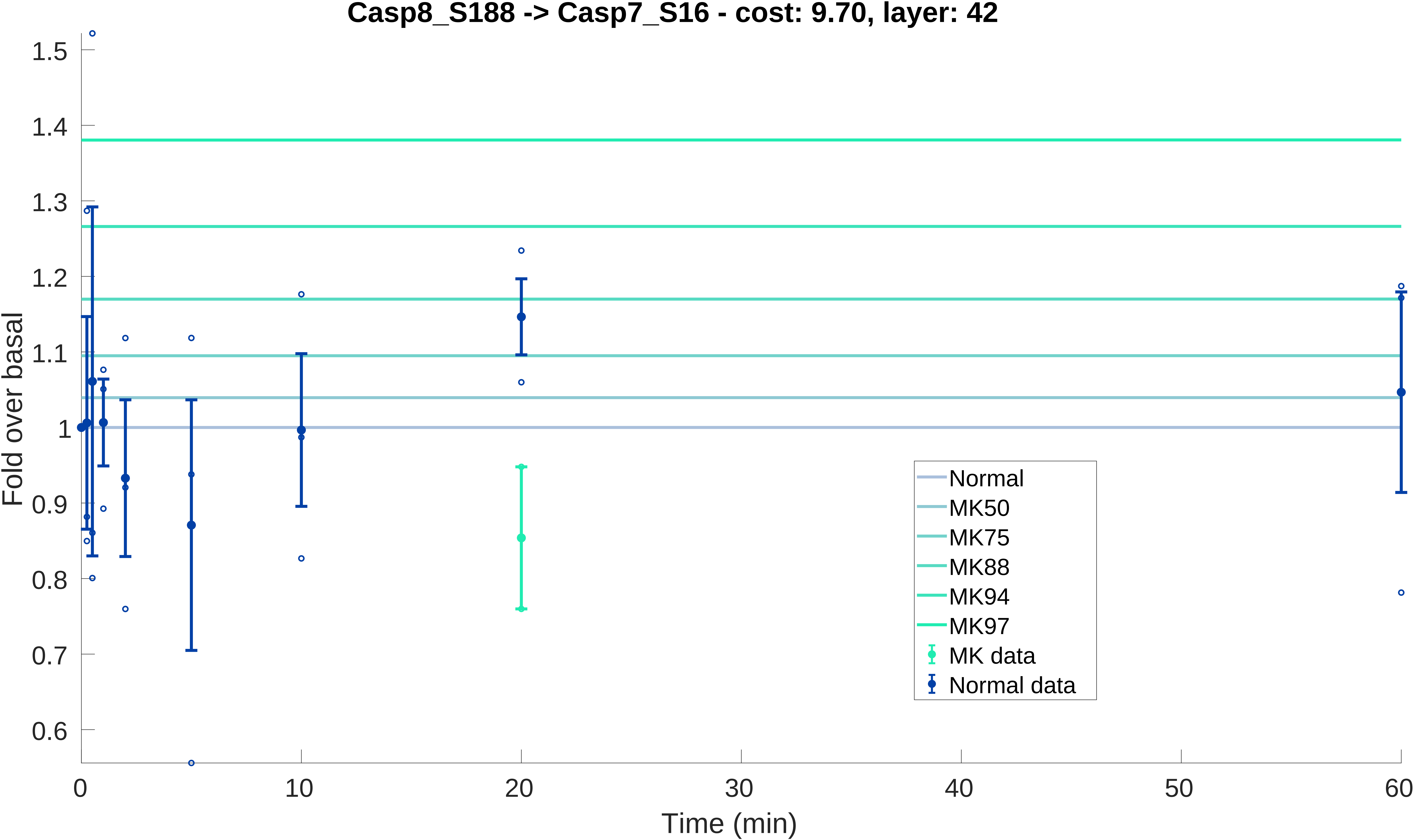

Casp8_S188 -> Casp7_S16 - cost: 9.70, layer: 42
Fold over basal
Time (min)
0.6
0.7
0.8
0.9
1
1.1
1.2
1.3
1.4
1.5
0
10
20
30
40
50
60
Normal
MK50
MK75
MK88
MK94
MK97
MK data
Normal data

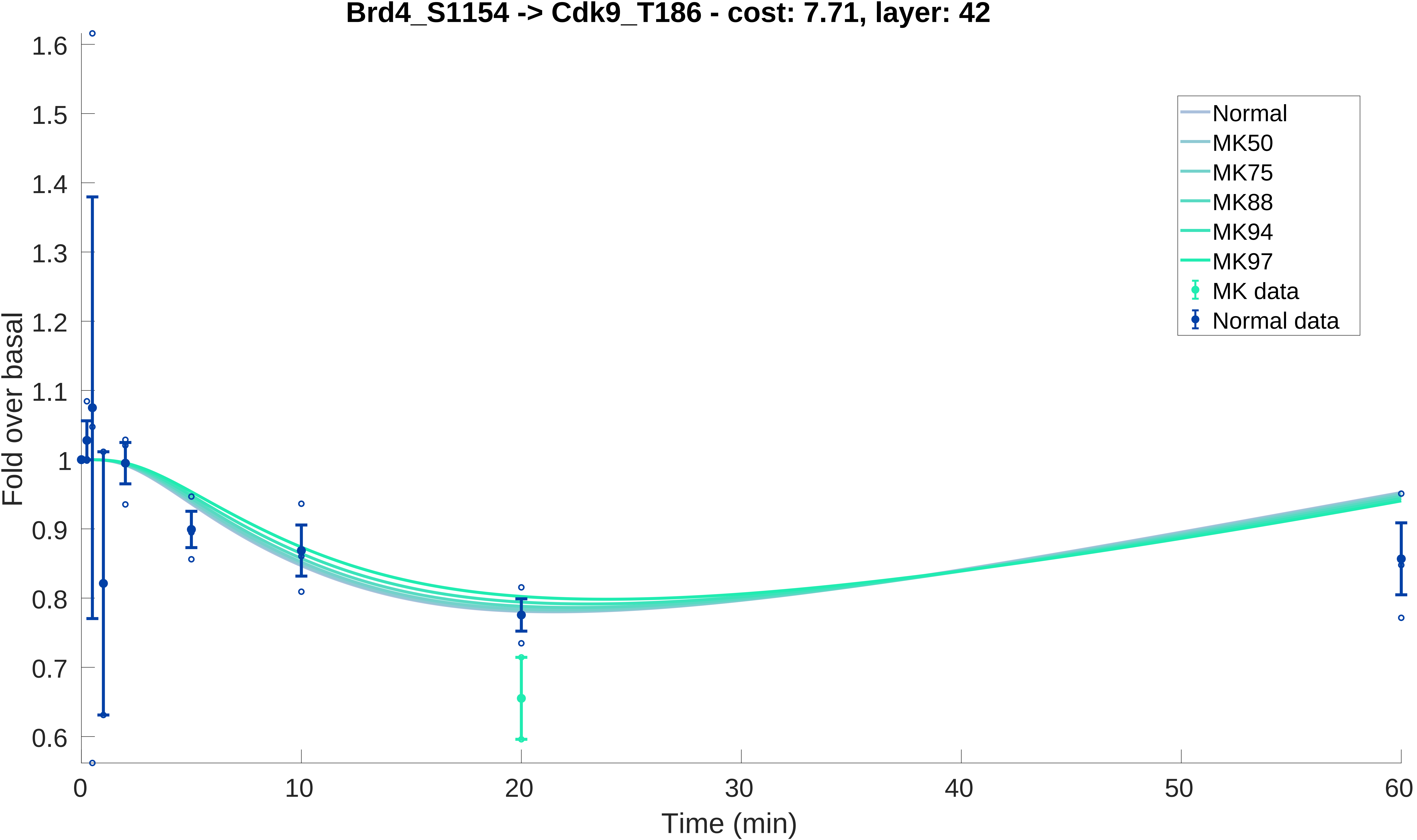

Brd4_S1154 -> Cdk9_T186 - cost: 7.71, layer: 42
Fold over basal
Time (min)
1.6
1.5
1.4
1.3
1.2
1.1
1
0.9
0.8
0.7
0.6
0
10
20
30
40
50
60
Normal
MK50
MK75
MK88
MK94
MK97
MK data
Normal data

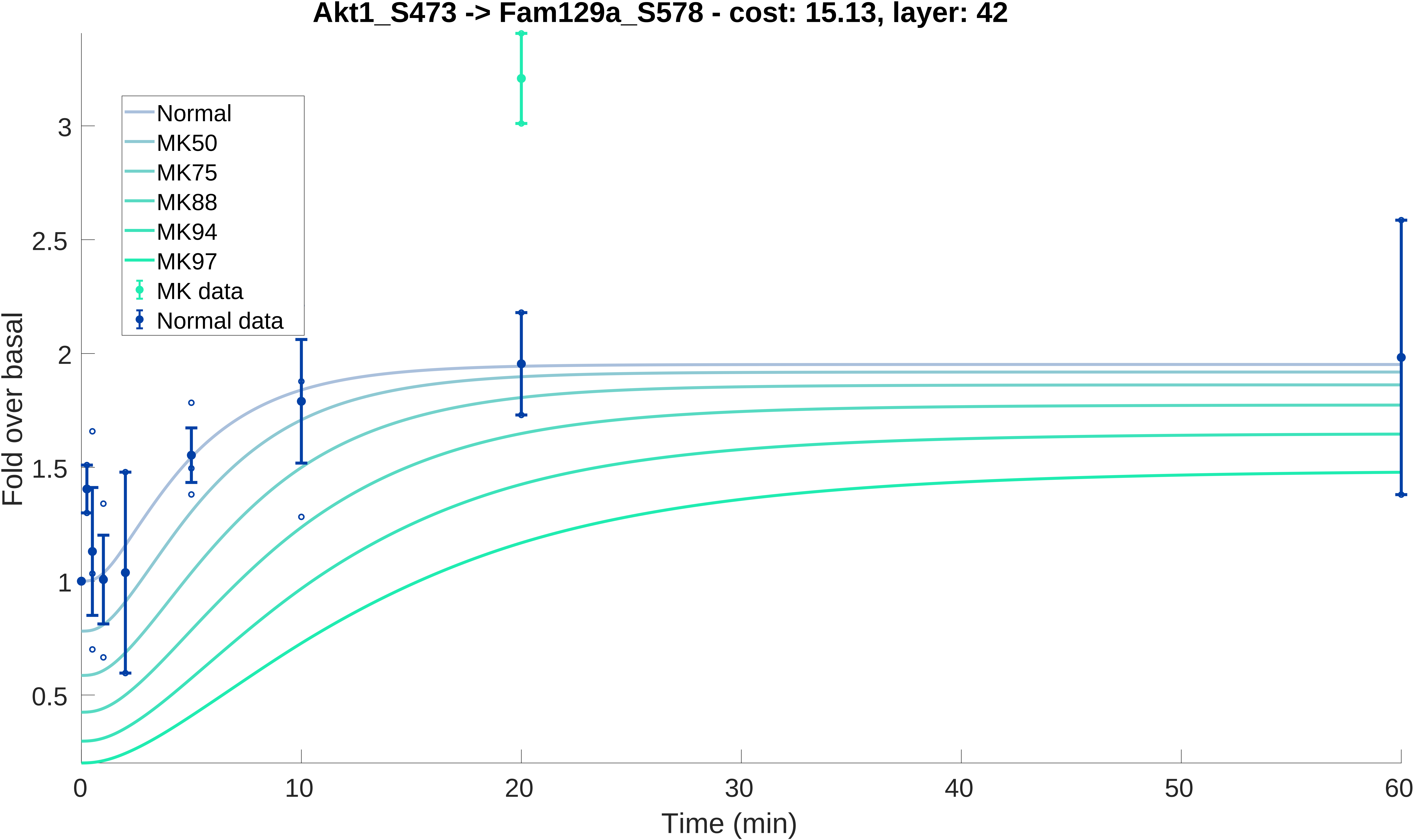

Akt1_S473 -> Fam129a_S578 - cost: 15.13, layer: 42
Normal
MK50
MK75
MK88
MK94
MK97
MK data
Normal data
Fold over basal
3
2.5
2
1.5
1
0.5
0
10
20
30
40
50
60
Time (min)

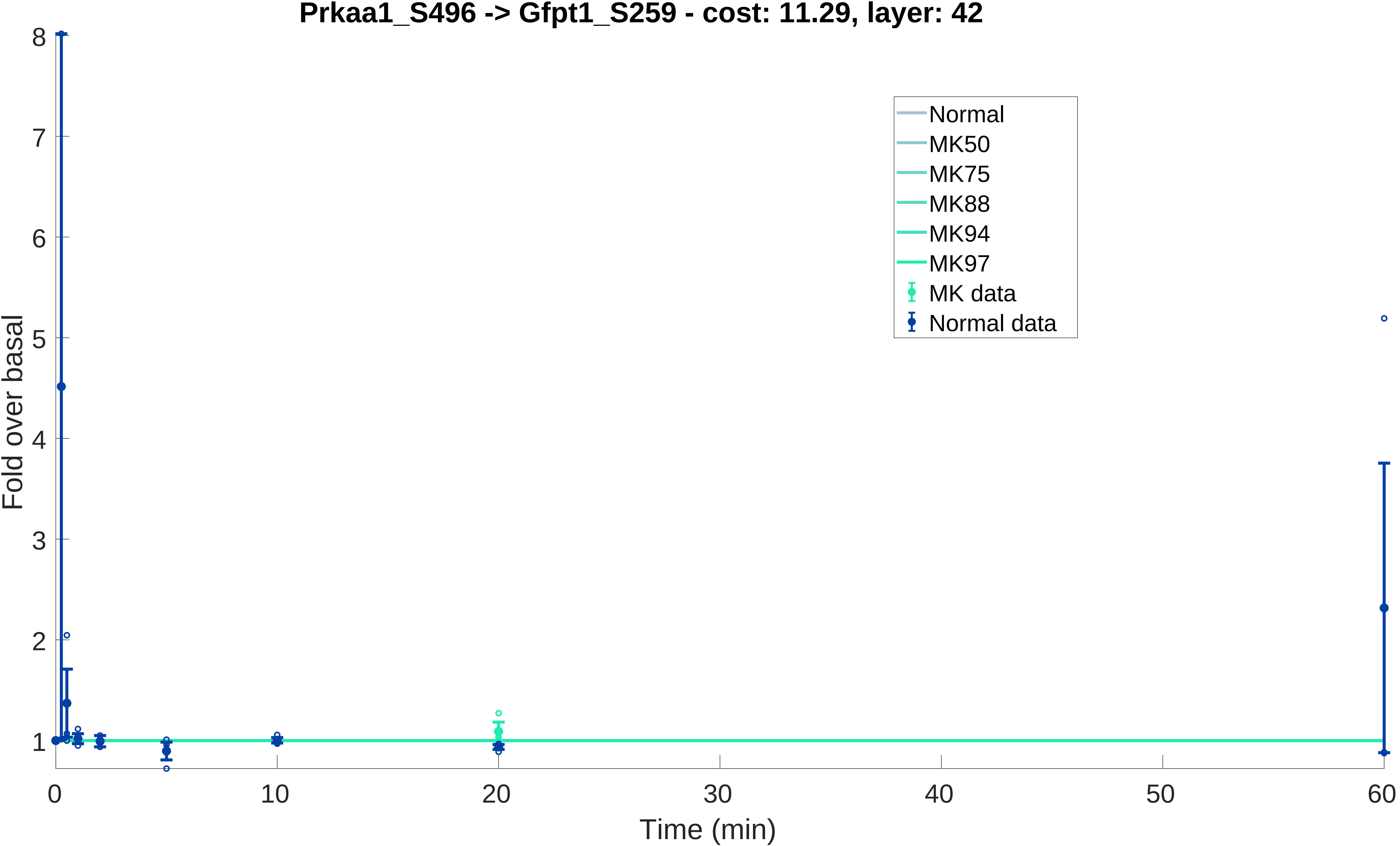

Prkaa1_S496 -> Gfpt1_S259 - cost: 11.29, layer: 42
Fold over basal
Time (min)
8
7
6
5
4
3
2
1
0
10
20
30
40
50
60
Normal
MK50
MK75
MK88
MK94
MK97
MK data
Normal data

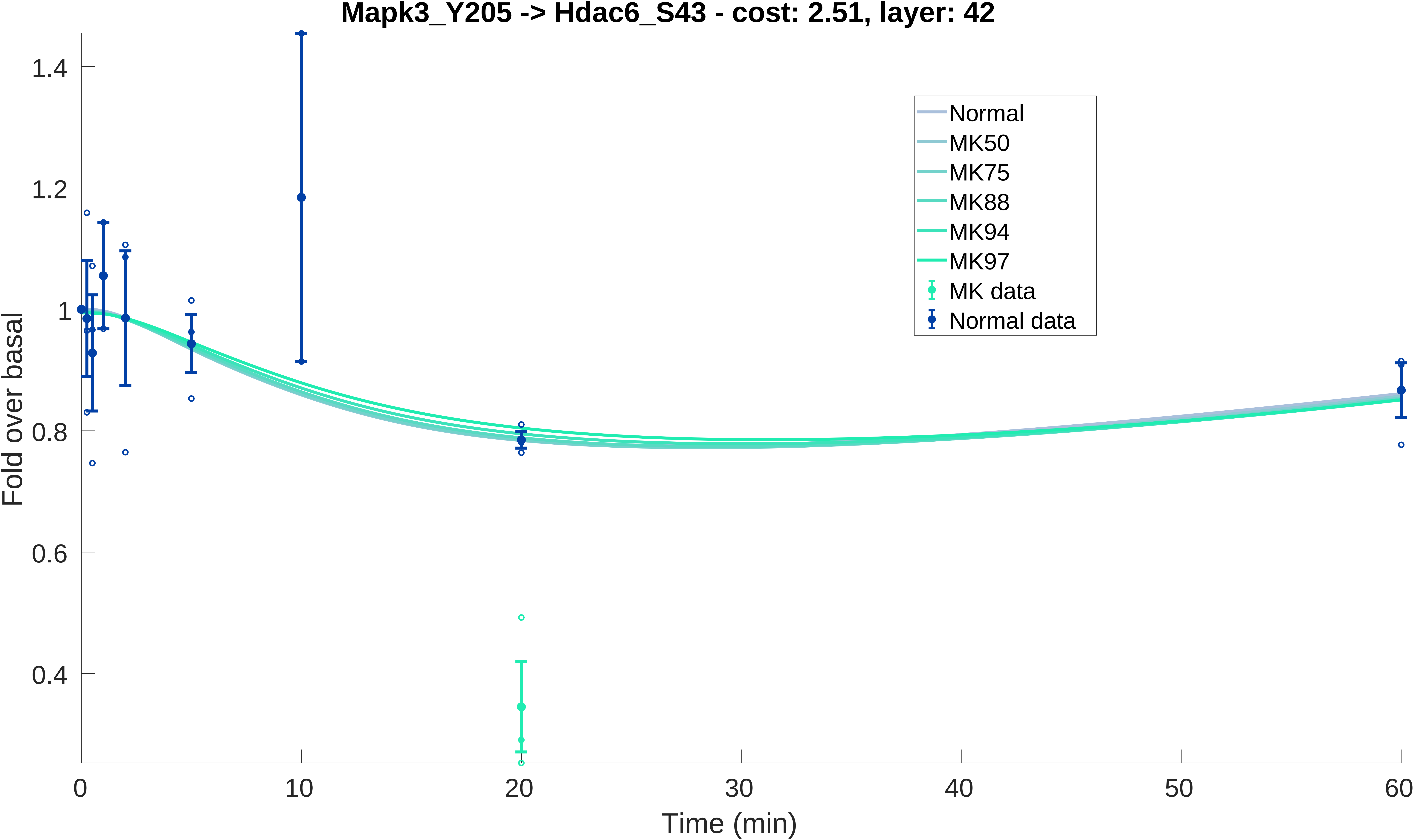
Mapk3_Y205 -> Hdac6_S43 - cost: 2.51, layer: 42
Fold over basal
1.4
1.2
1
0.8
0.6
0.4
Time (min)
0
10
20
30
40
50
60
Normal
MK50
MK75
MK88
MK94
MK97
MK data
Normal data

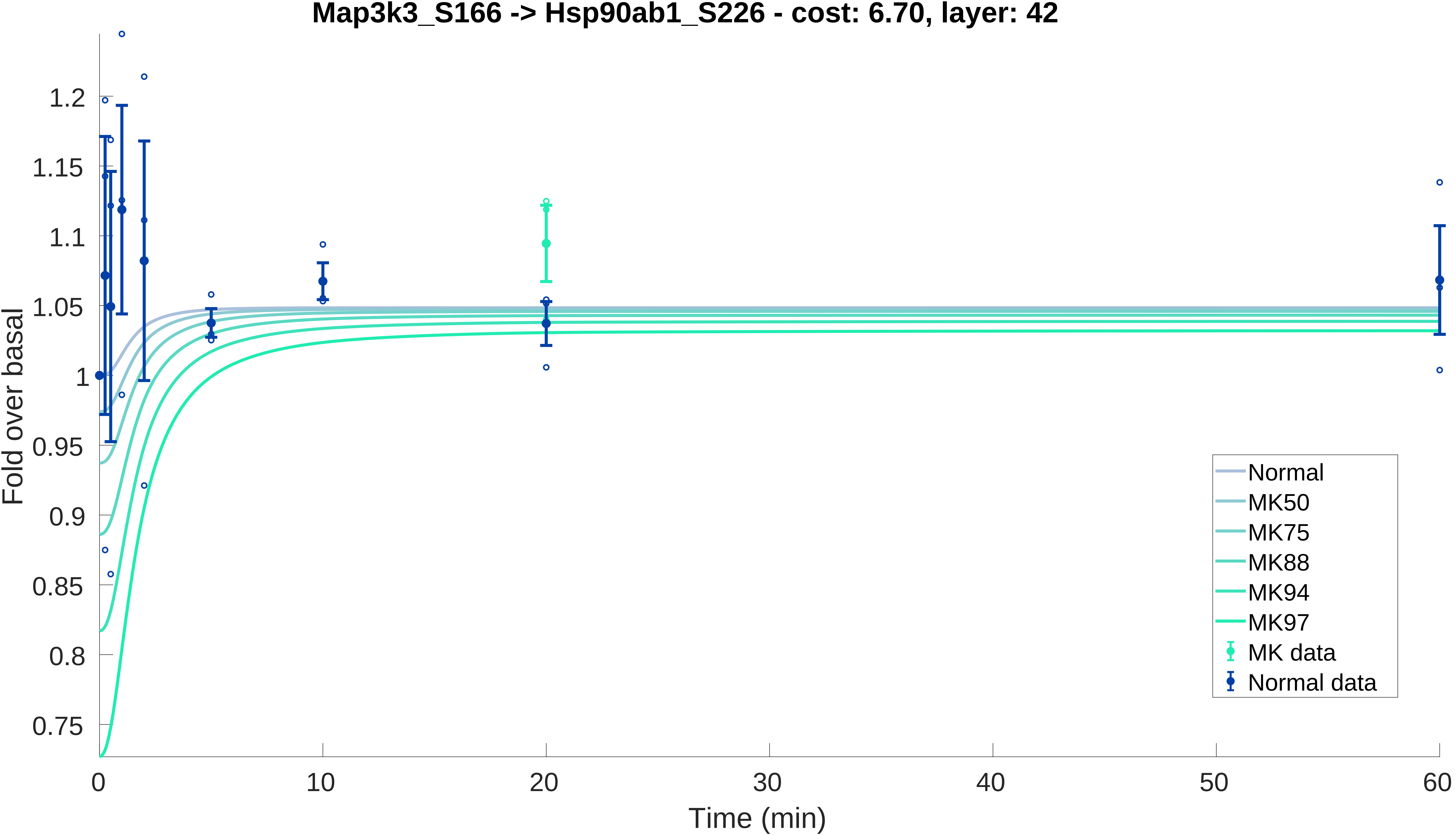

Map3k3_S166 -> Hsp90ab1_S226 - cost: 6.70, layer: 42
Fold over basal
Time (min)
0.75
0.8
0.85
0.9
0.95
1
1.05
1.1
1.15
1.2
0
10
20
30
40
50
60
Normal
MK50
MK75
MK88
MK94
MK97
MK data
Normal data

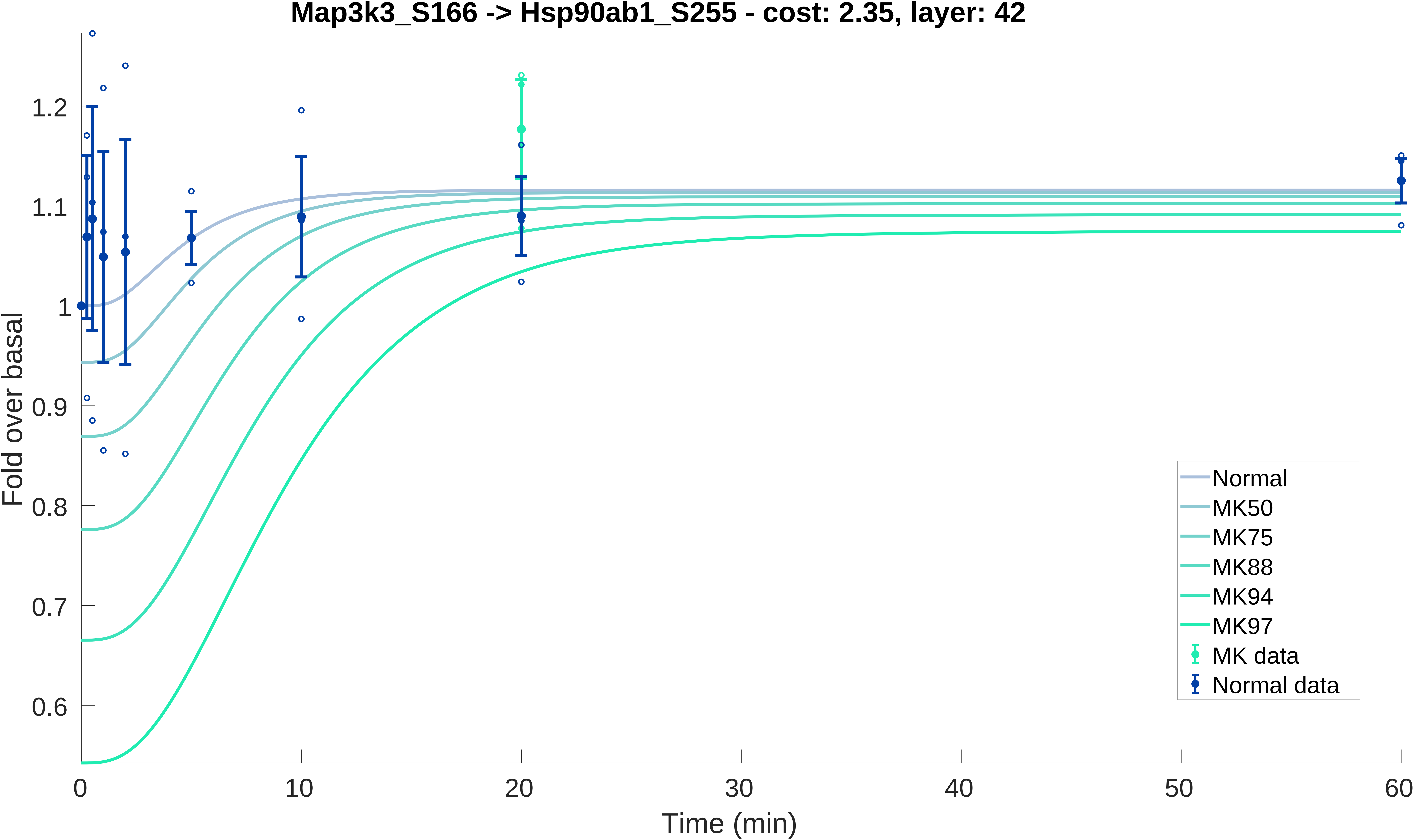

Map3k3_S166 -> Hsp90ab1_S255 - cost: 2.35, layer: 42
Fold over basal
1.2
1.1
1
0.9
0.8
0.7
0.6
0
10
20
30
40
50
60
Time (min)
Normal
MK50
MK75
MK88
MK94
MK97
MK data
Normal data

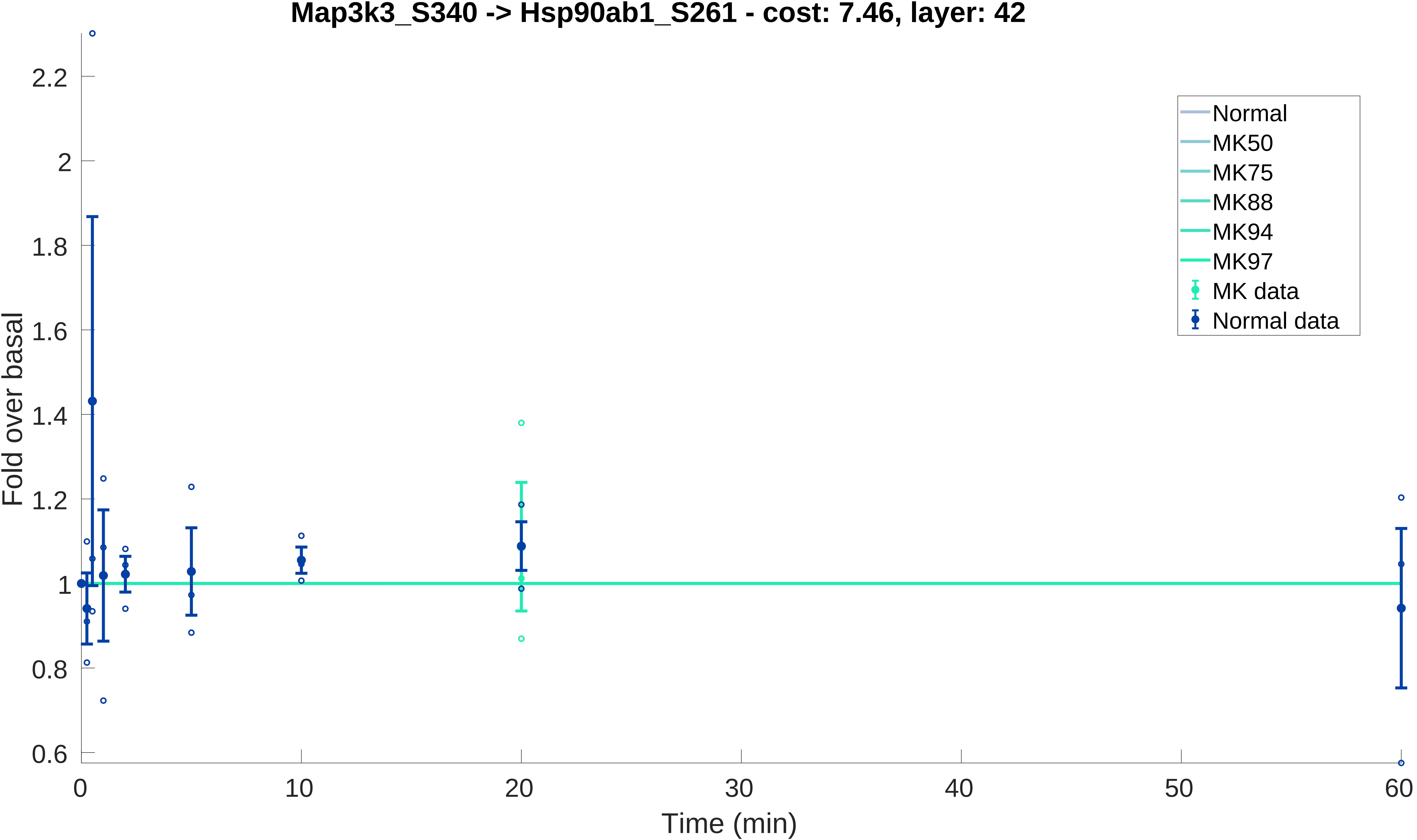

Map3k3_S340 -> Hsp90ab1_S261 - cost: 7.46, layer: 42
Fold over basal
Time (min)
Normal
MK50
MK75
MK88
MK94
MK97
MK data
Normal data
2.2
2
1.8
1.6
1.4
1.2
1
0.8
0.6
0
10
20
30
40
50
60

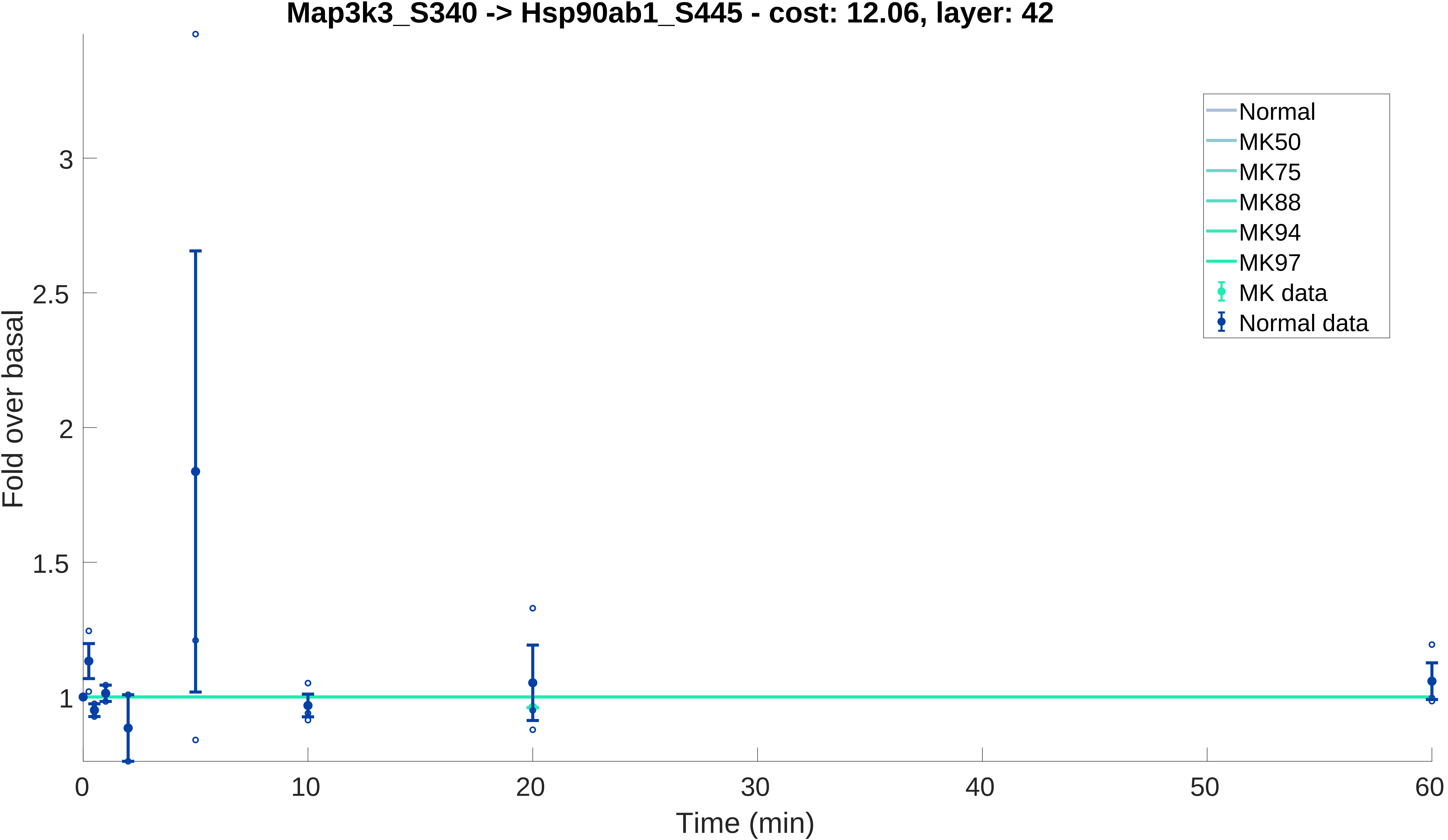

Map3k3_S340 -> Hsp90ab1_S445 - cost: 12.06, layer: 42
Fold over basal
Time (min)
Normal
MK50
MK75
MK88
MK94
MK97
MK data
Normal data
3
2.5
2
1.5
1
0
10
20
30
40
50
60

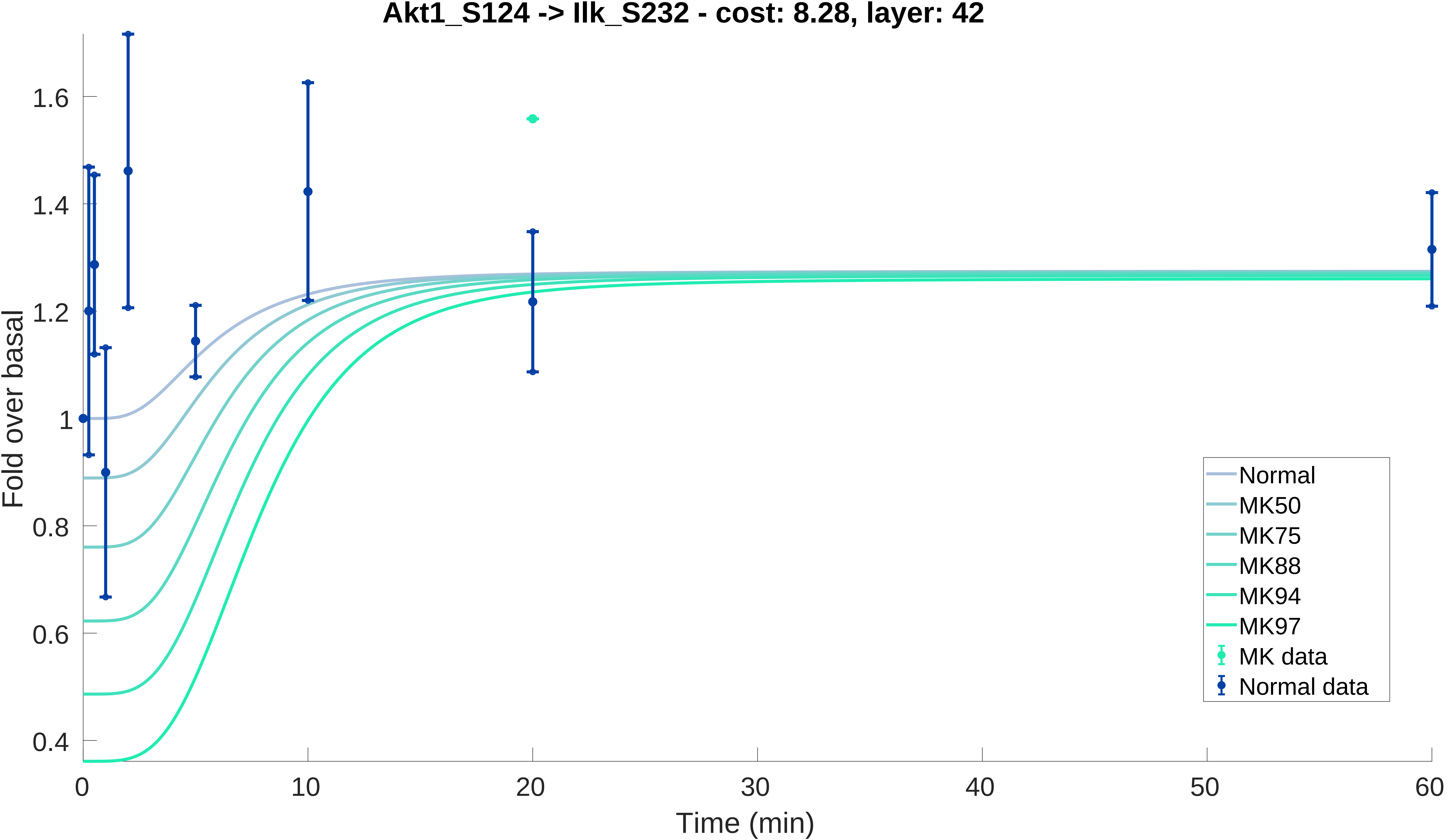

Akt1_S124 -> Ilk_S232 - cost: 8.28, layer: 42
Fold over basal
1.6
1.4
1.2
1
0.8
0.6
0.4
0
10
20
30
40
50
60
Time (min)
Normal
MK50
MK75
MK88
MK94
MK97
MK data
Normal data

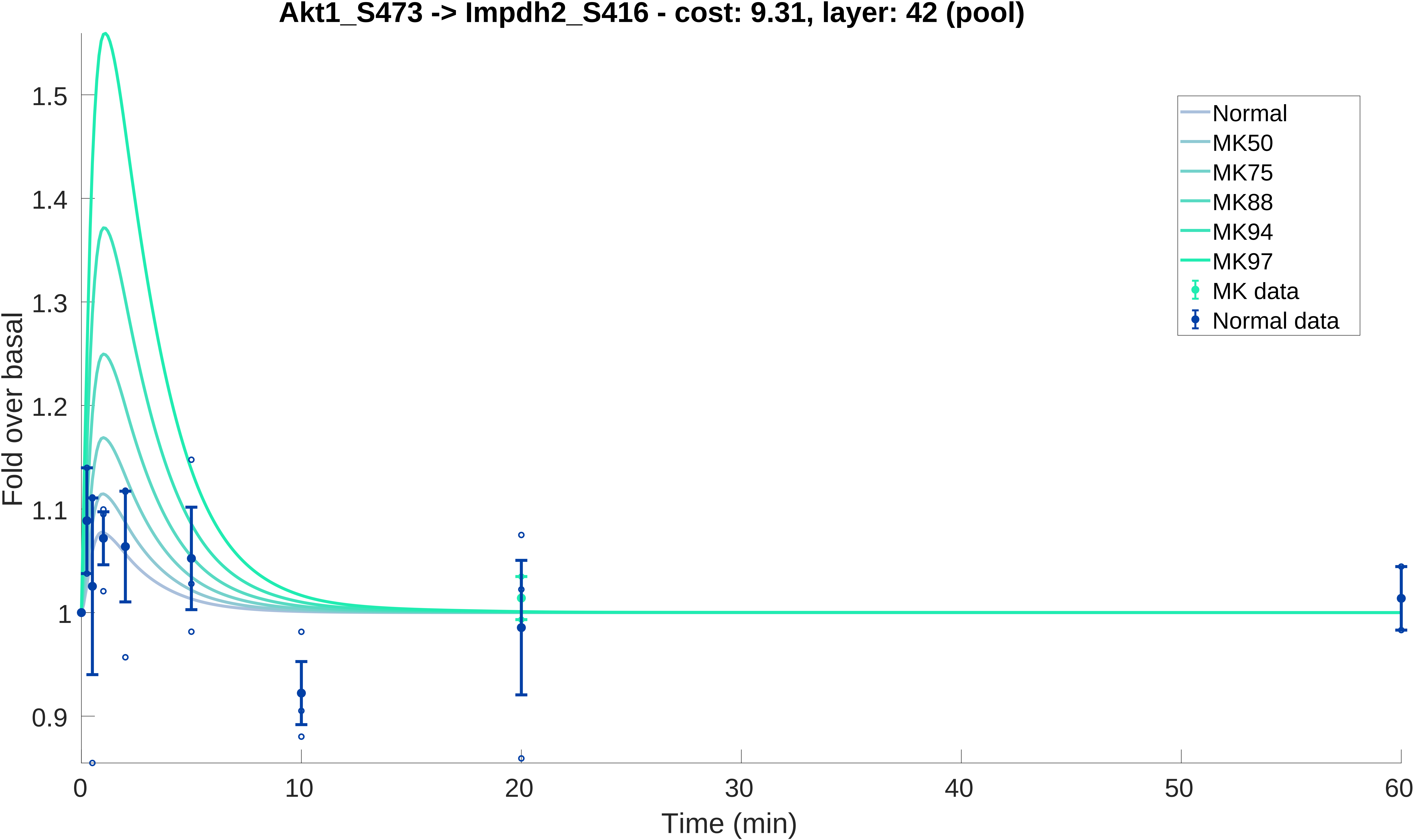

Akt1_S473 -> Impdh2_S416 - cost: 9.31, layer: 42 (pool)
Fold over basal
1.5
1.4
1.3
1.2
1.1
1
0.9
0
10
20
30
40
50
60
Time (min)
Normal
MK50
MK75
MK88
MK94
MK97
MK data
Normal data

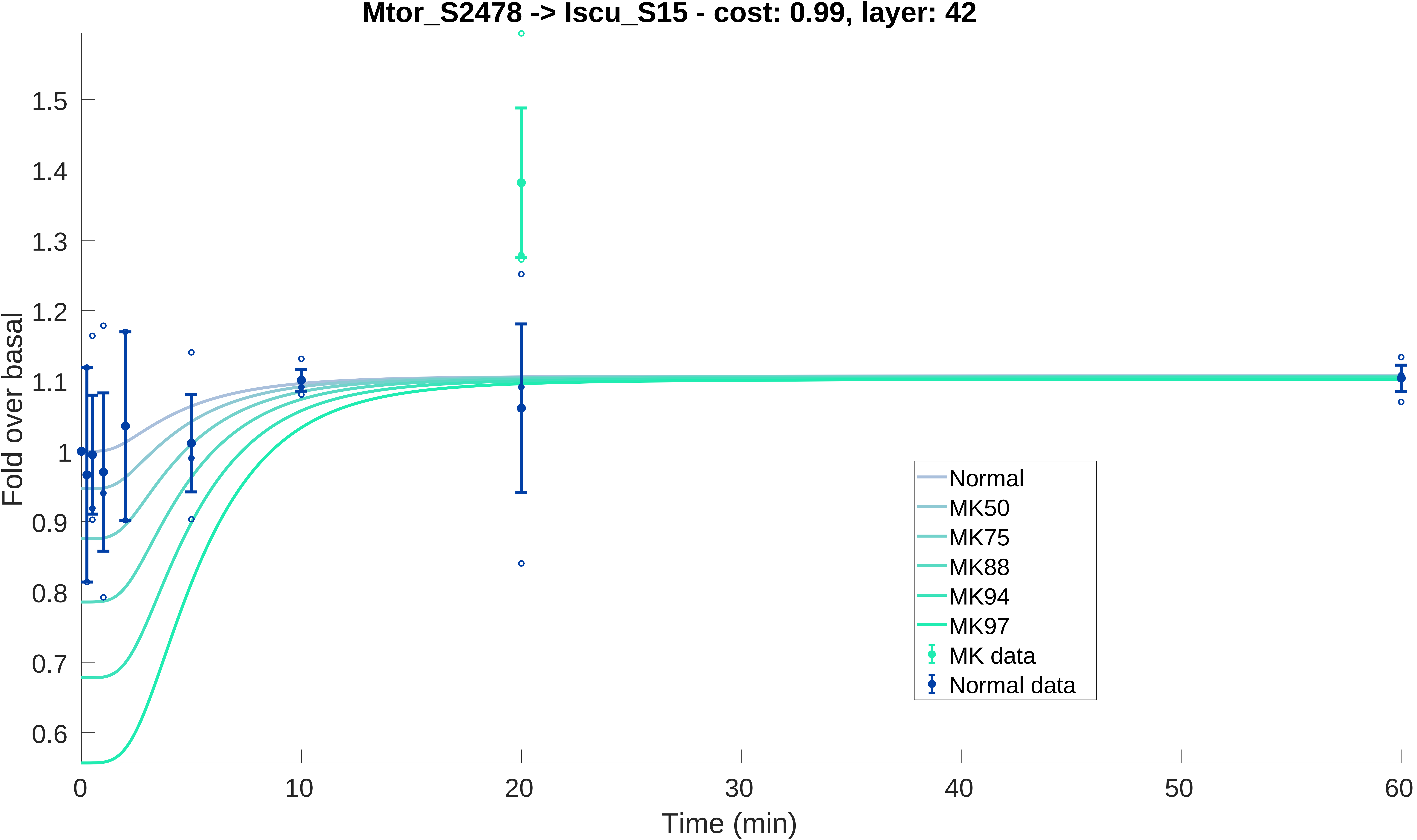
Mtor_S2478 -> Iscu_S15 - cost: 0.99, layer: 42
Fold over basal
Time (min)
1.5
1.4
1.3
1.2
1.1
1
0.9
0.8
0.7
0.6
0
10
20
30
40
50
60
Normal
MK50
MK75
MK88
MK94
MK97
MK data
Normal data

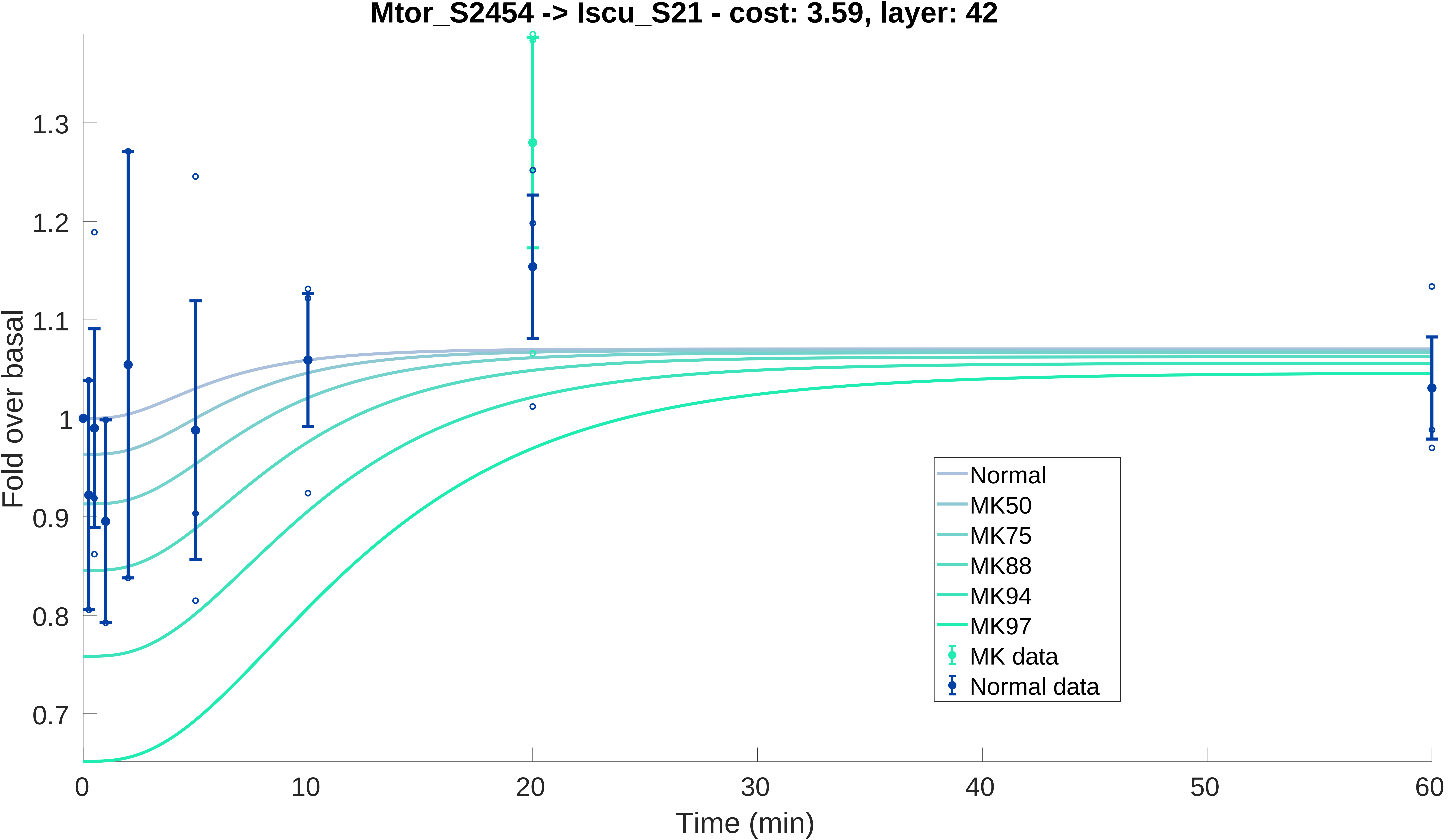

Mtor_S2454 -> Iscu_S21 - cost: 3.59, layer: 42
Fold over basal
Time (min)
0.7
0.8
0.9
1
1.1
1.2
1.3
0
10
20
30
40
50
60
Normal
MK50
MK75
MK88
MK94
MK97
MK data
Normal data

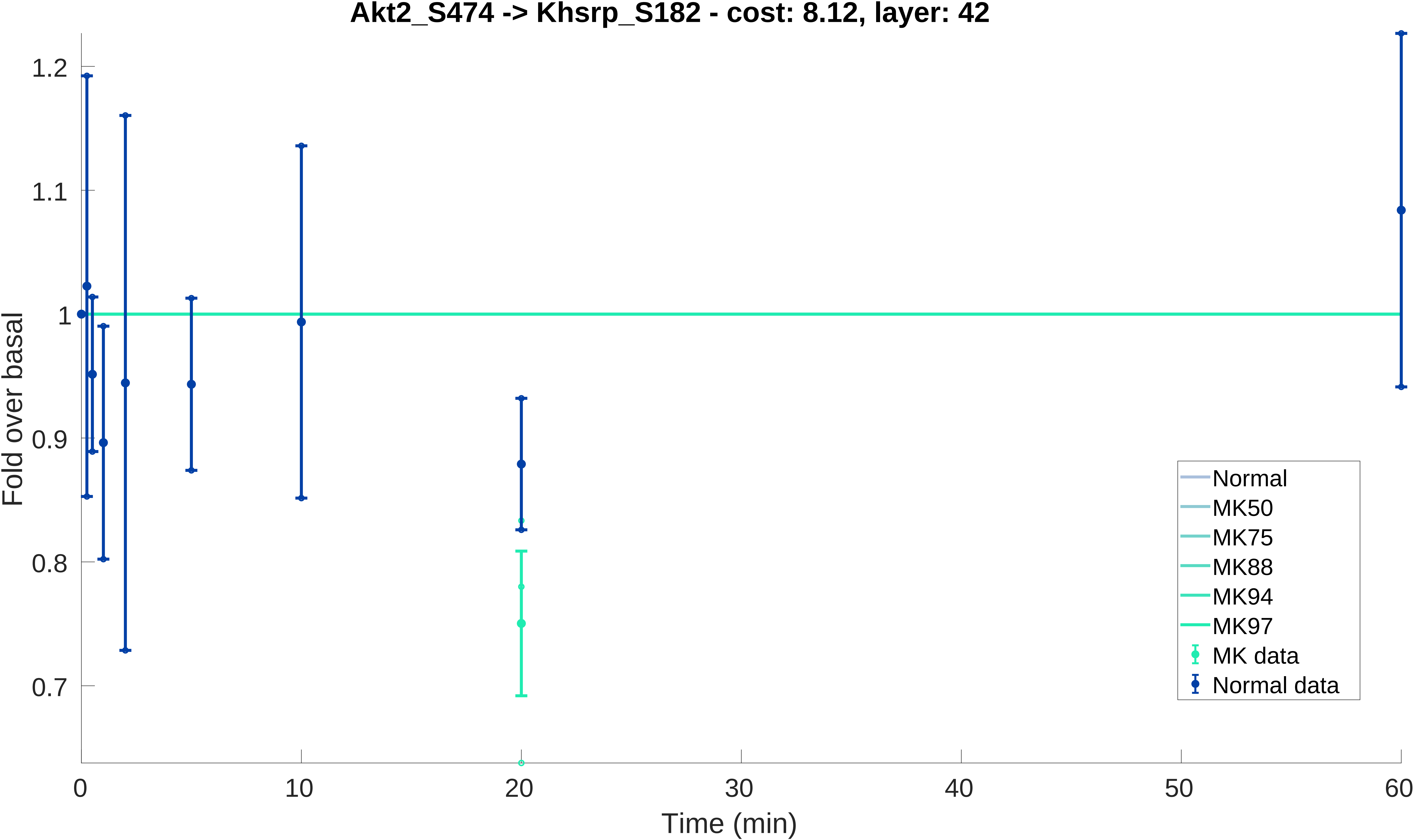

Akt2_S474 -> Khsrp_S182 - cost: 8.12, layer: 42
Fold over basal
Time (min)
1.2
1.1
1
0.9
0.8
0.7
0
10
20
30
40
50
60
Normal
MK50
MK75
MK88
MK94
MK97
MK data
Normal data

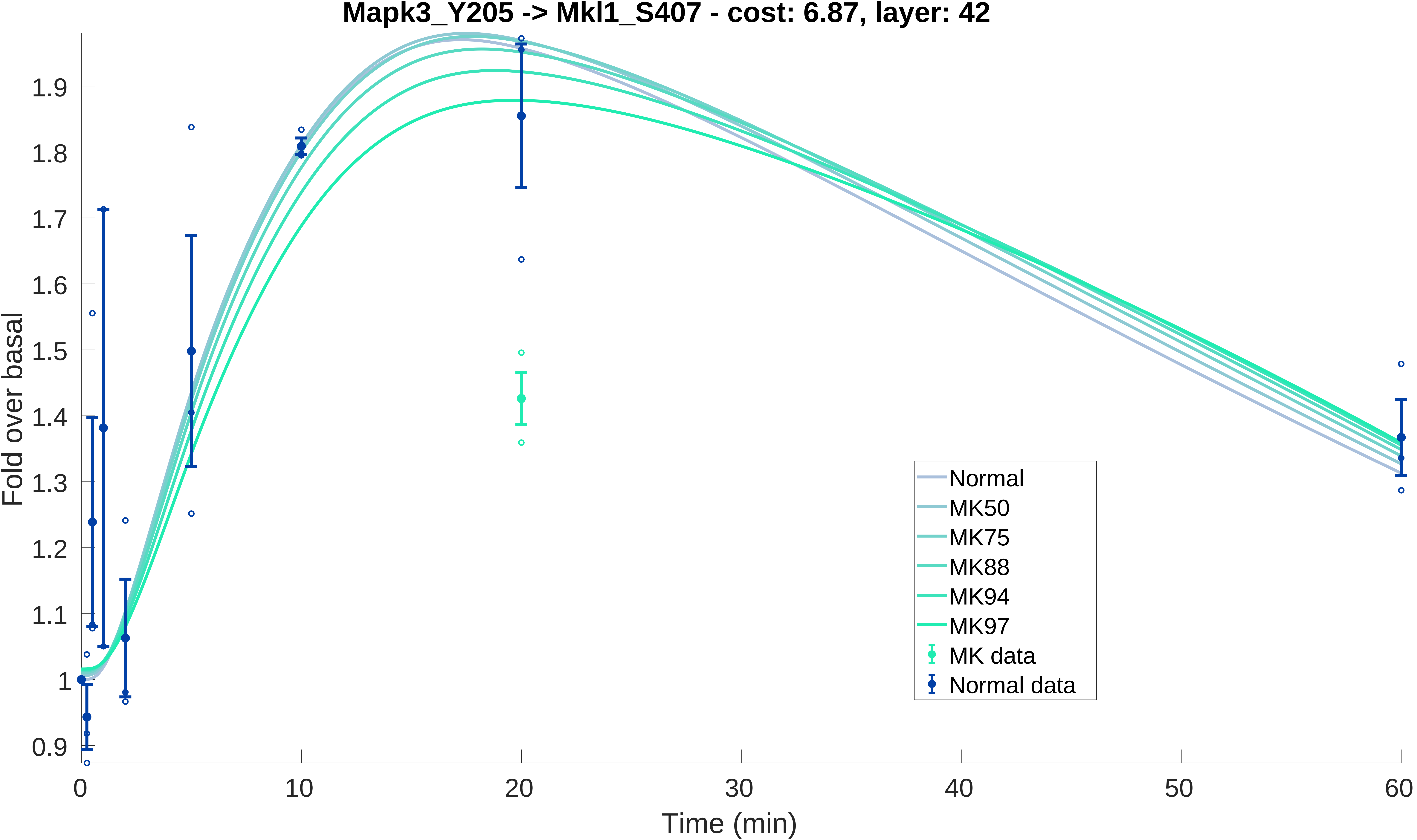

Mapk3_Y205 -> Mkl1_S407 - cost: 6.87, layer: 42
Fold over basal
Time (min)
0.9
1
1.1
1.2
1.3
1.4
1.5
1.6
1.7
1.8
1.9
0
10
20
30
40
50
60
Normal
MK50
MK75
MK88
MK94
MK97
MK data
Normal data

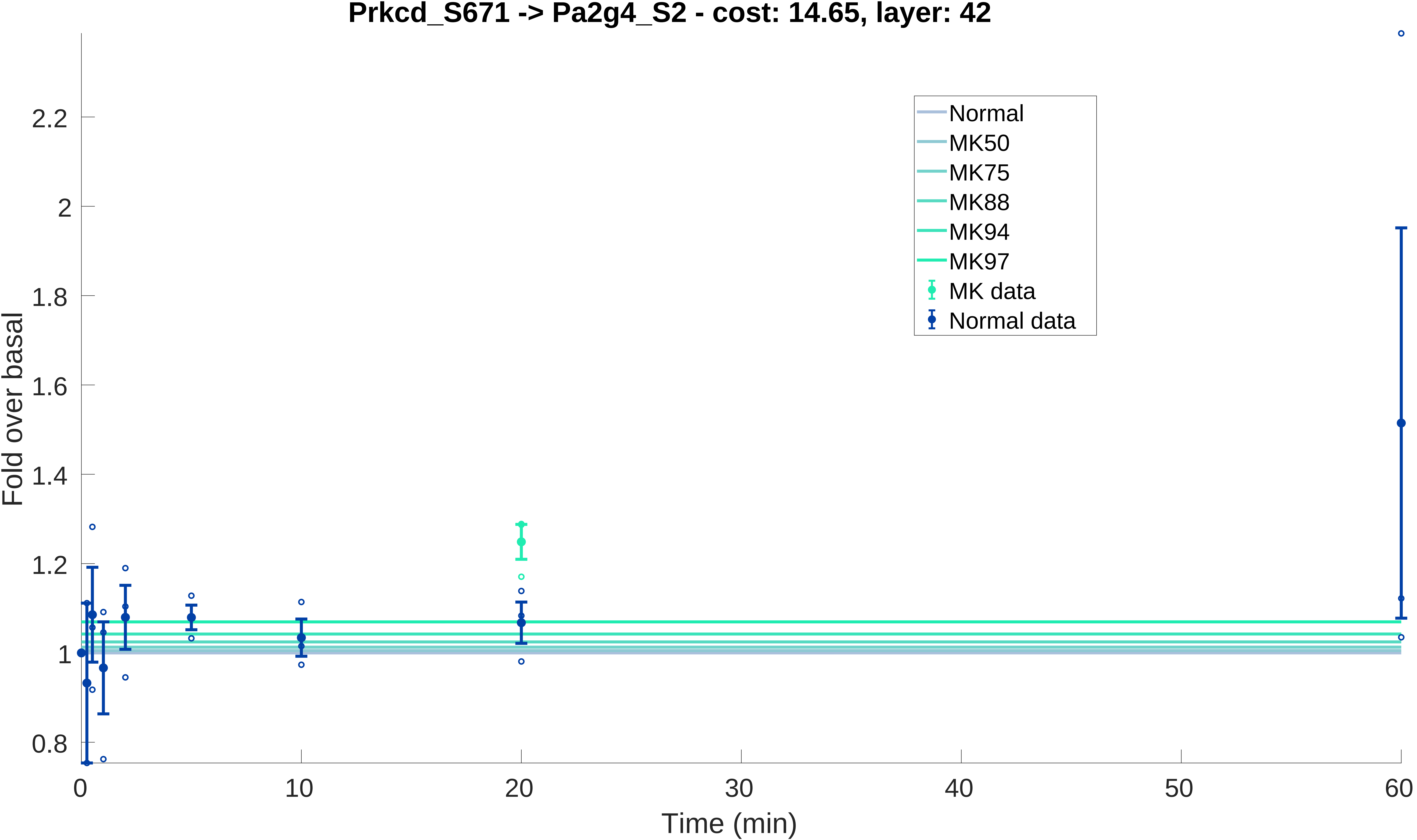

Prkcd_S671 -> Pa2g4_S2 - cost: 14.65, layer: 42
Fold over basal
2.2
2
1.8
1.6
1.4
1.2
1
0.8
0
10
20
30
40
50
60
Time (min)
Normal
MK50
MK75
MK88
MK94
MK97
MK data
Normal data

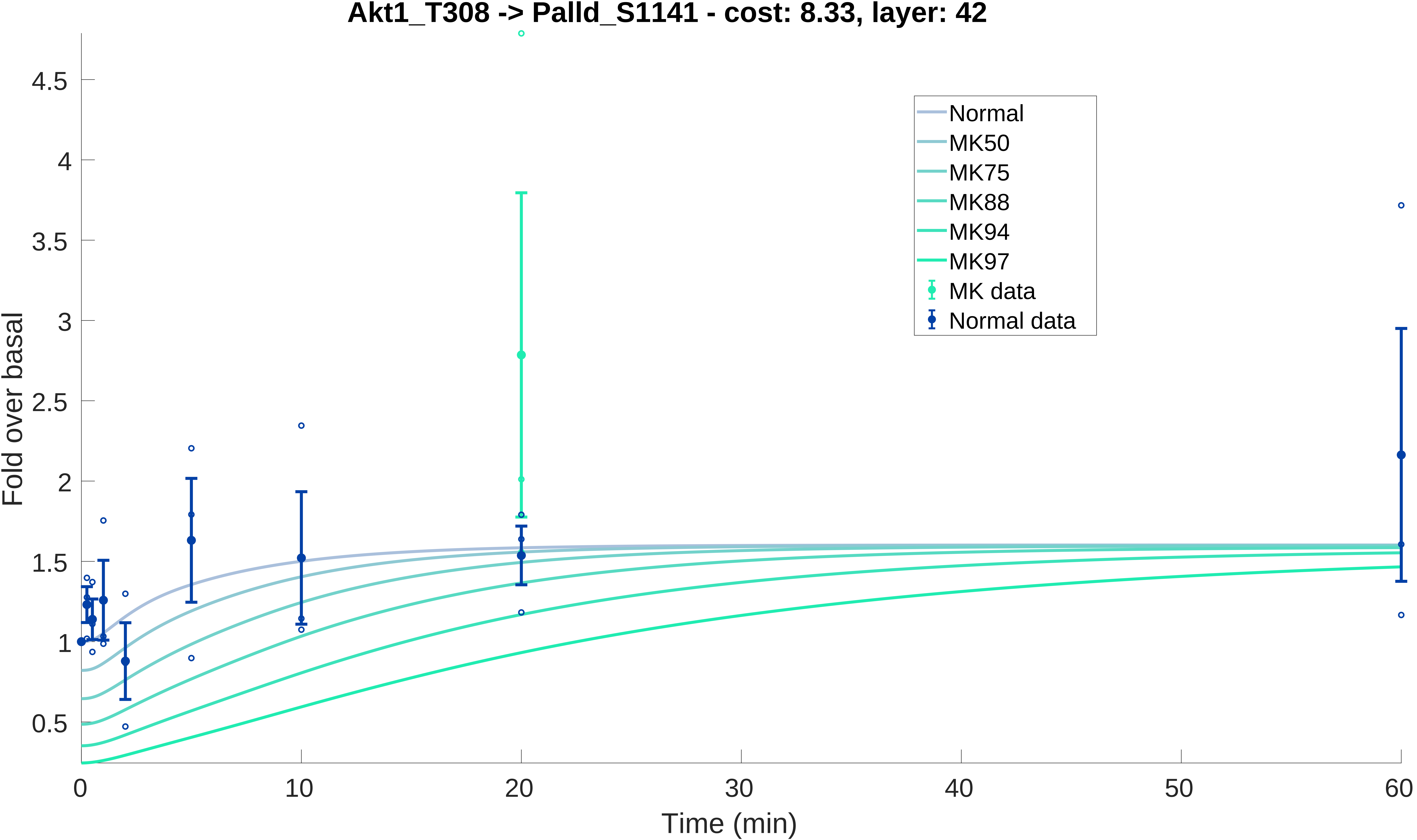

Akt1_T308 -> Palld_S1141 - cost: 8.33, layer: 42
Normal
MK50
MK75
MK88
MK94
MK97
MK data
Normal data
Fold over basal
Time (min)
0
10
20
30
40
50
60
0.5
1
1.5
2
2.5
3
3.5
4
4.5

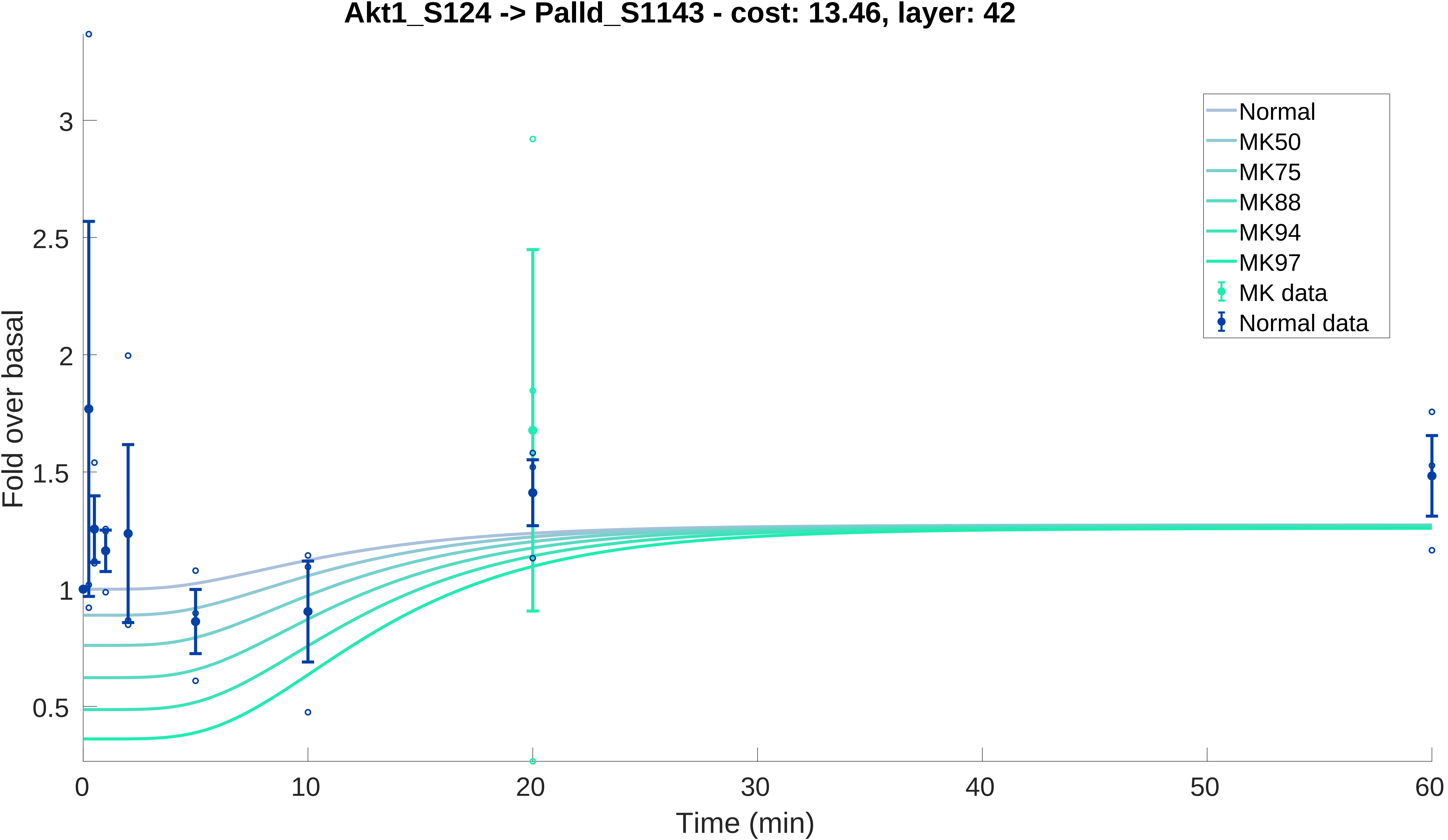

Akt1_S124 -> Palld_S1143 - cost: 13.46, layer: 42
Fold over basal
3
2.5
2
1.5
1
0.5
0
10
20
30
40
50
60
Time (min)
Normal
MK50
MK75
MK88
MK94
MK97
MK data
Normal data

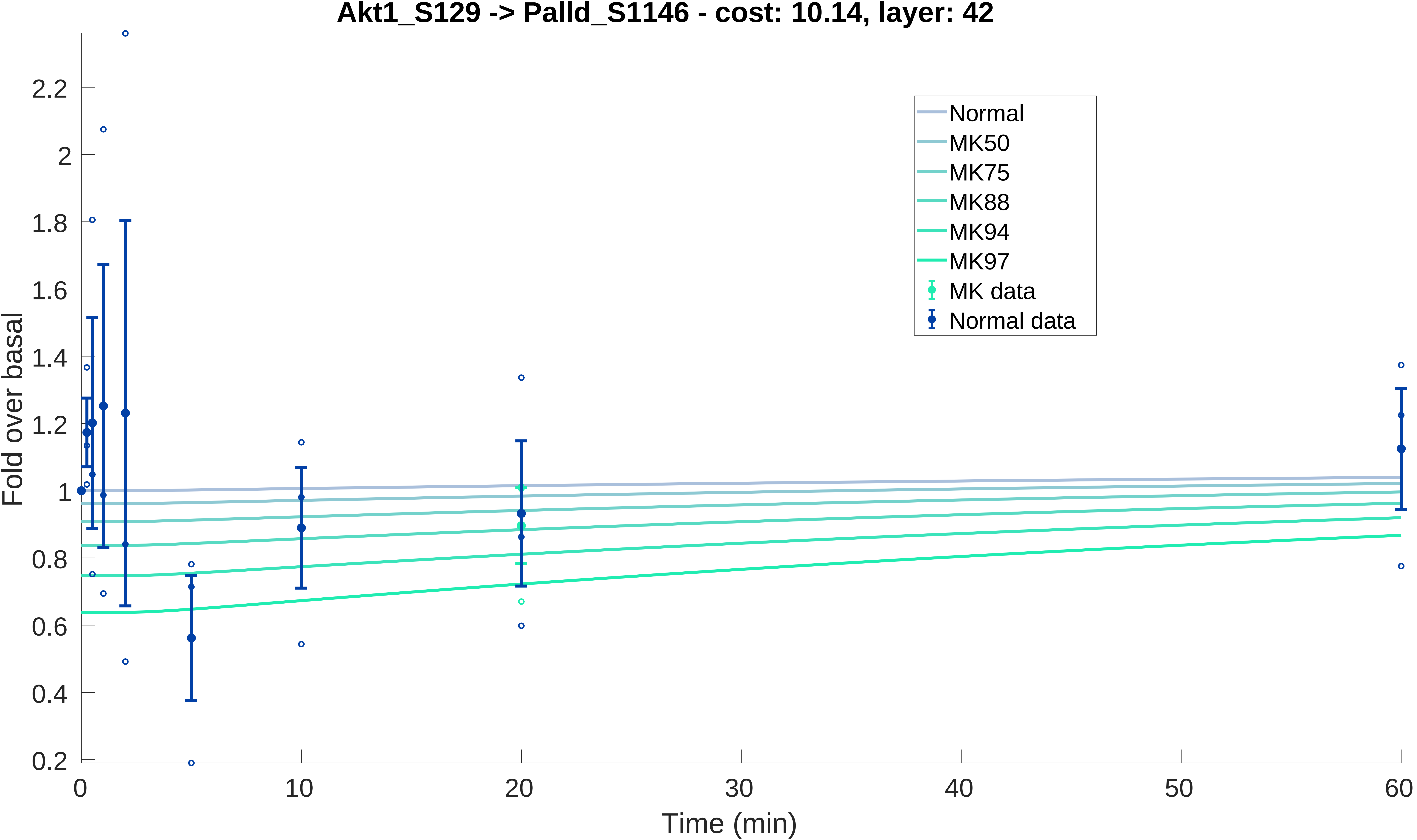
Akt1_S129 -> Palld_S1146 - cost: 10.14, layer: 42
Fold over basal
Time (min)
0
10
20
30
40
50
60
0.2
0.4
0.6
0.8
1
1.2
1.4
1.6
1.8
2
2.2
Normal
MK50
MK75
MK88
MK94
MK97
MK data
Normal data

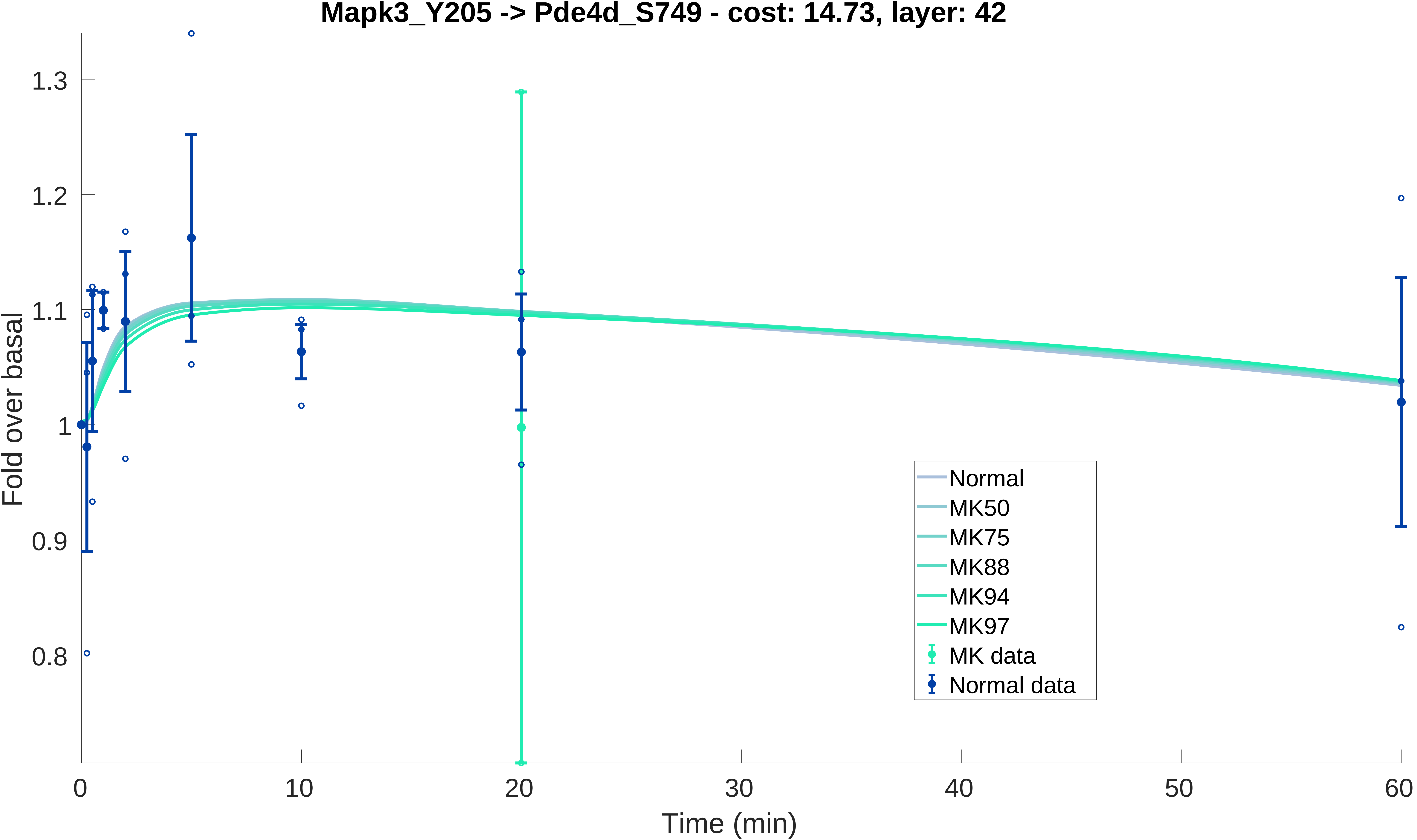

Mapk3_Y205 -> Pde4d_S749 - cost: 14.73, layer: 42
Fold over basal
Time (min)
1.3
1.2
1.1
1
0.9
0.8
0
10
20
30
40
50
60
Normal
MK50
MK75
MK88
MK94
MK97
MK data
Normal data

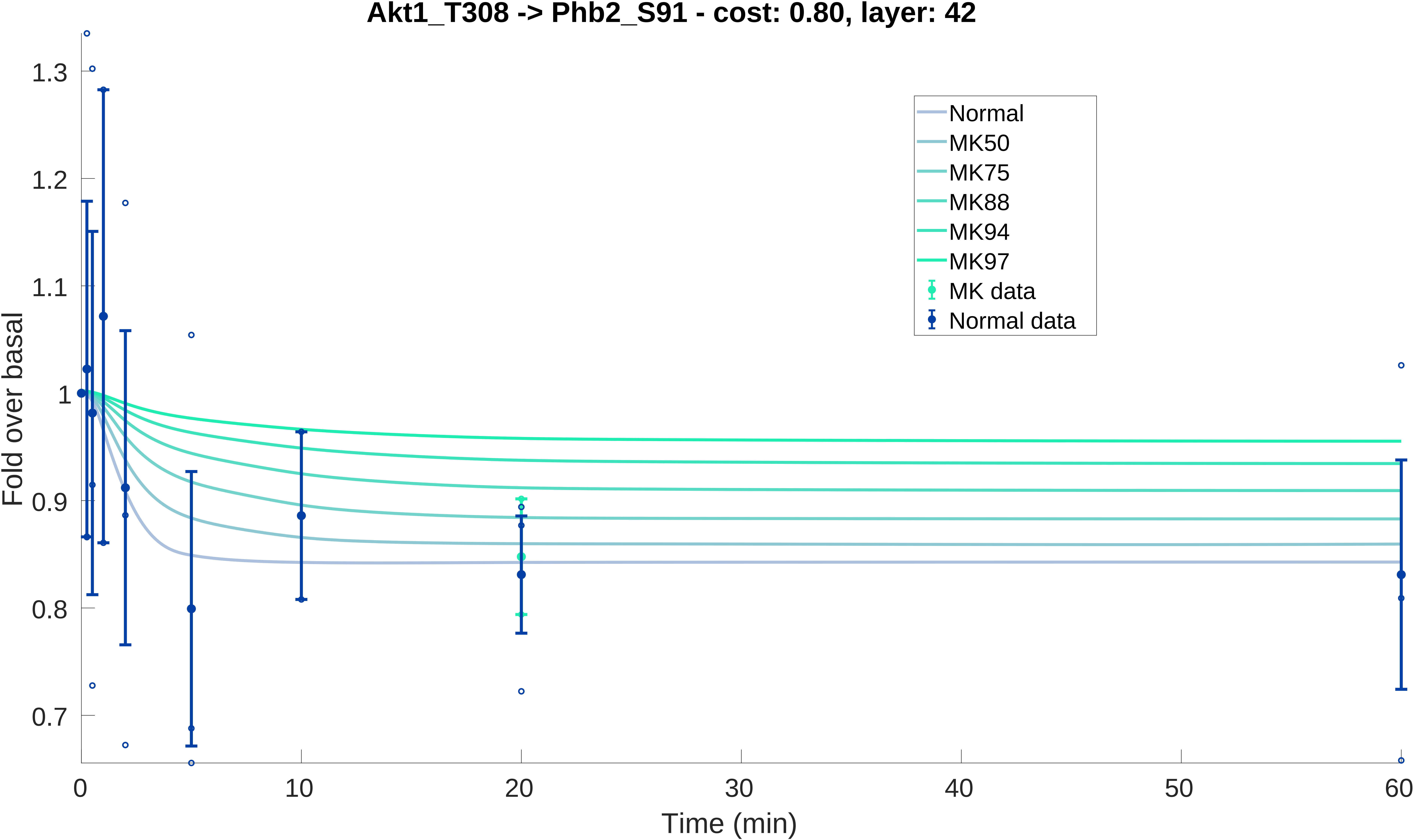

Akt1_T308 -> Phb2_S91 - cost: 0.80, layer: 42
Fold over basal
Time (min)
0.7
0.8
0.9
1
1.1
1.2
1.3
0
10
20
30
40
50
60
Normal
MK50
MK75
MK88
MK94
MK97
MK data
Normal data

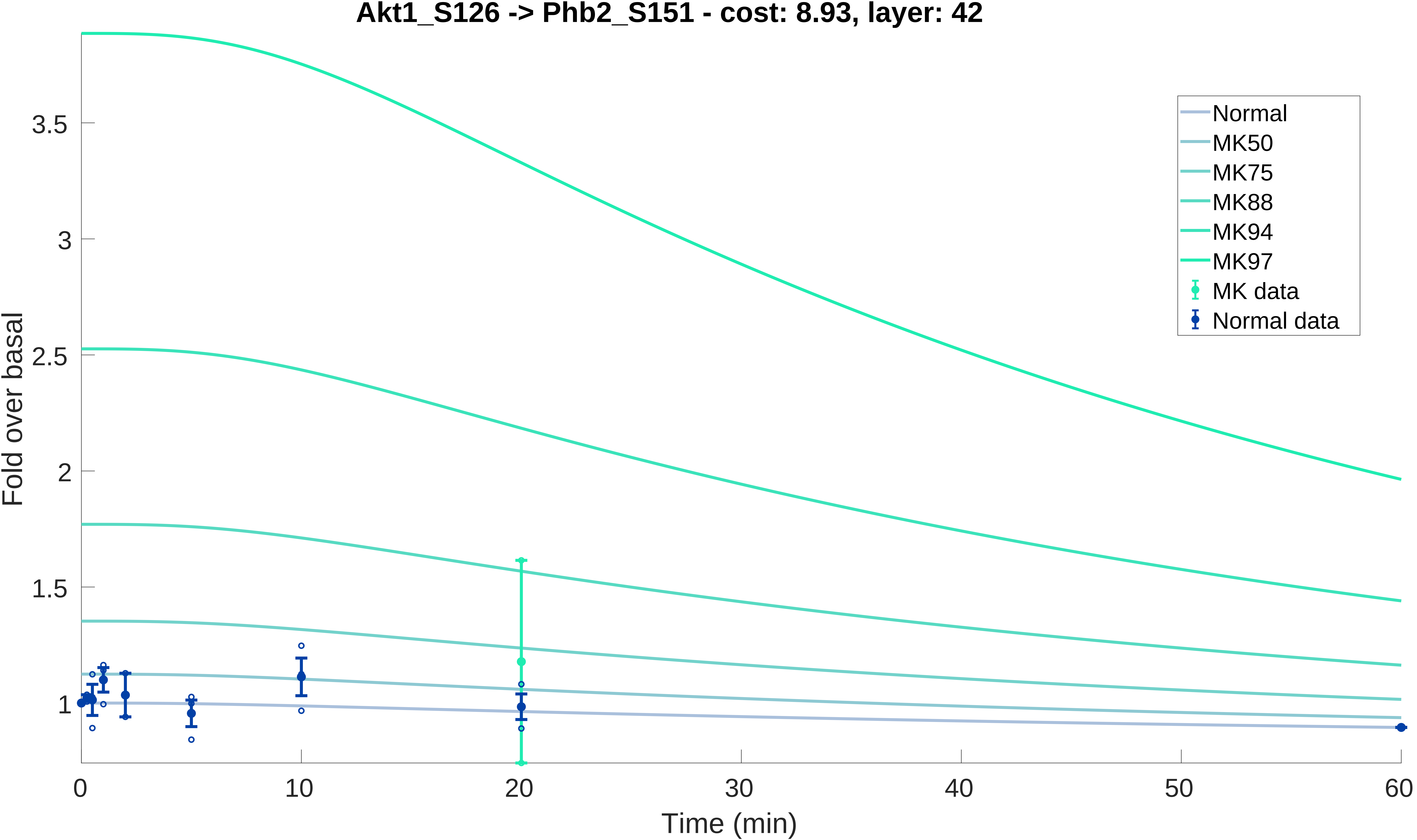
Akt1_S126 -> Phb2_S151 - cost: 8.93, layer: 42
Fold over basal
Time (min)
3.5
3
2.5
2
1.5
1
0
10
20
30
40
50
60
Normal
MK50
MK75
MK88
MK94
MK97
MK data
Normal data

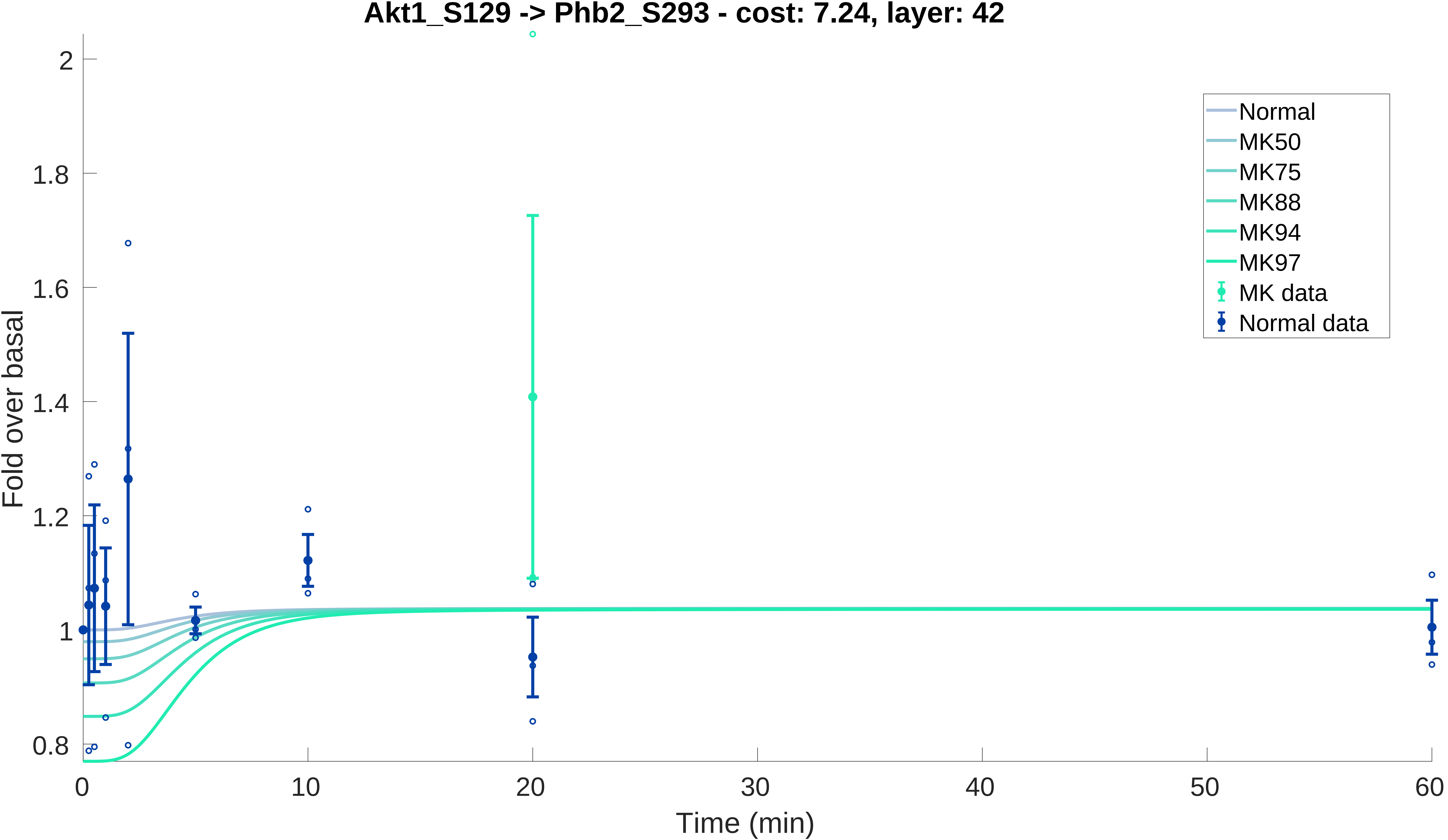
Akt1_S129 -> Phb2_S293 - cost: 7.24, layer: 42
Fold over basal
Time (min)
Normal
MK50
MK75
MK88
MK94
MK97
MK data
Normal data
0
10
20
30
40
50
60
0.8
1
1.2
1.4
1.6
1.8
2

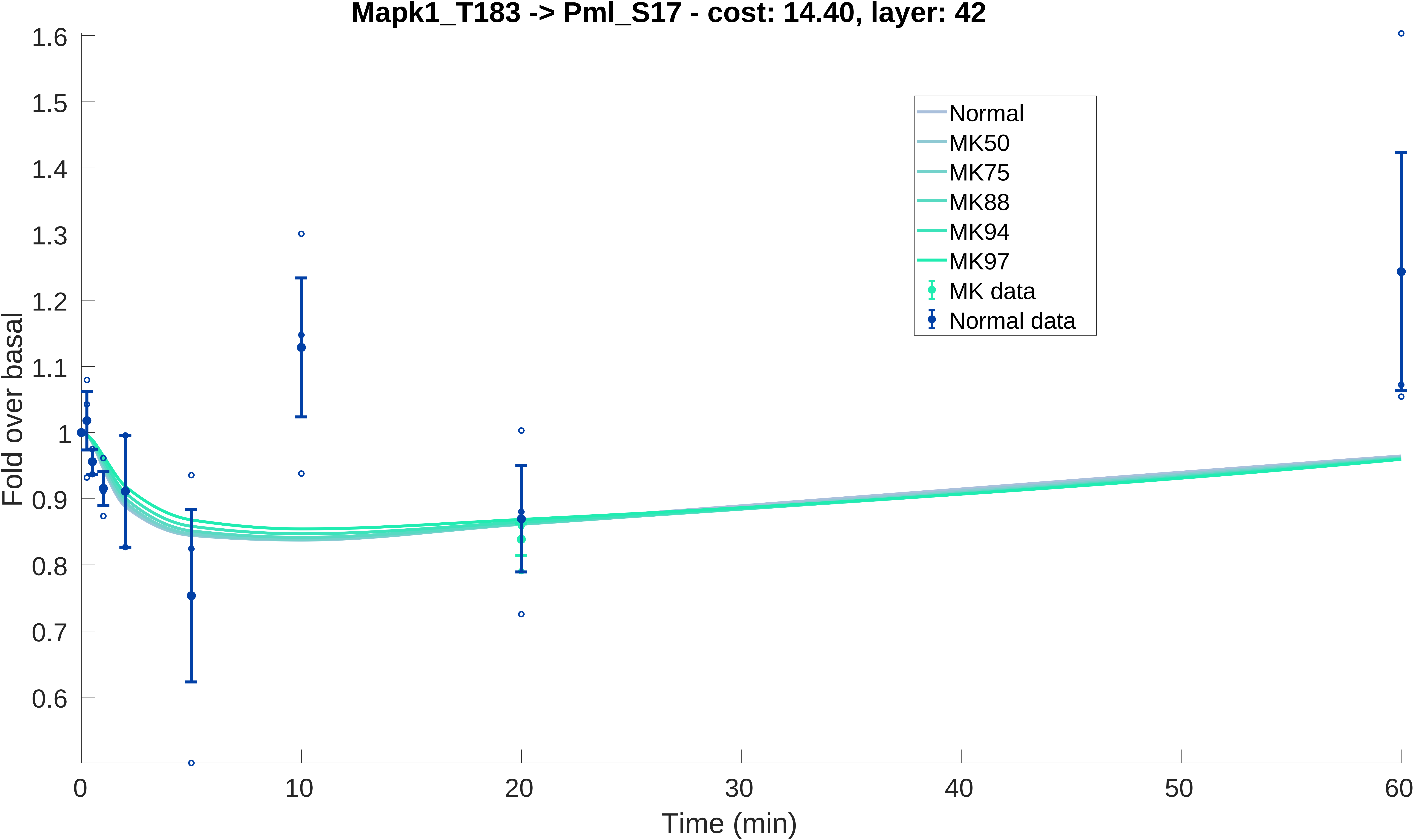
Mapk1_T183 -> Pml_S17 - cost: 14.40, layer: 42
Fold over basal
1.6
1.5
1.4
1.3
1.2
1.1
1
0.9
0.8
0.7
0.6
0
10
20
30
40
50
60
Time (min)
Normal
MK50
MK75
MK88
MK94
MK97
MK data
Normal data

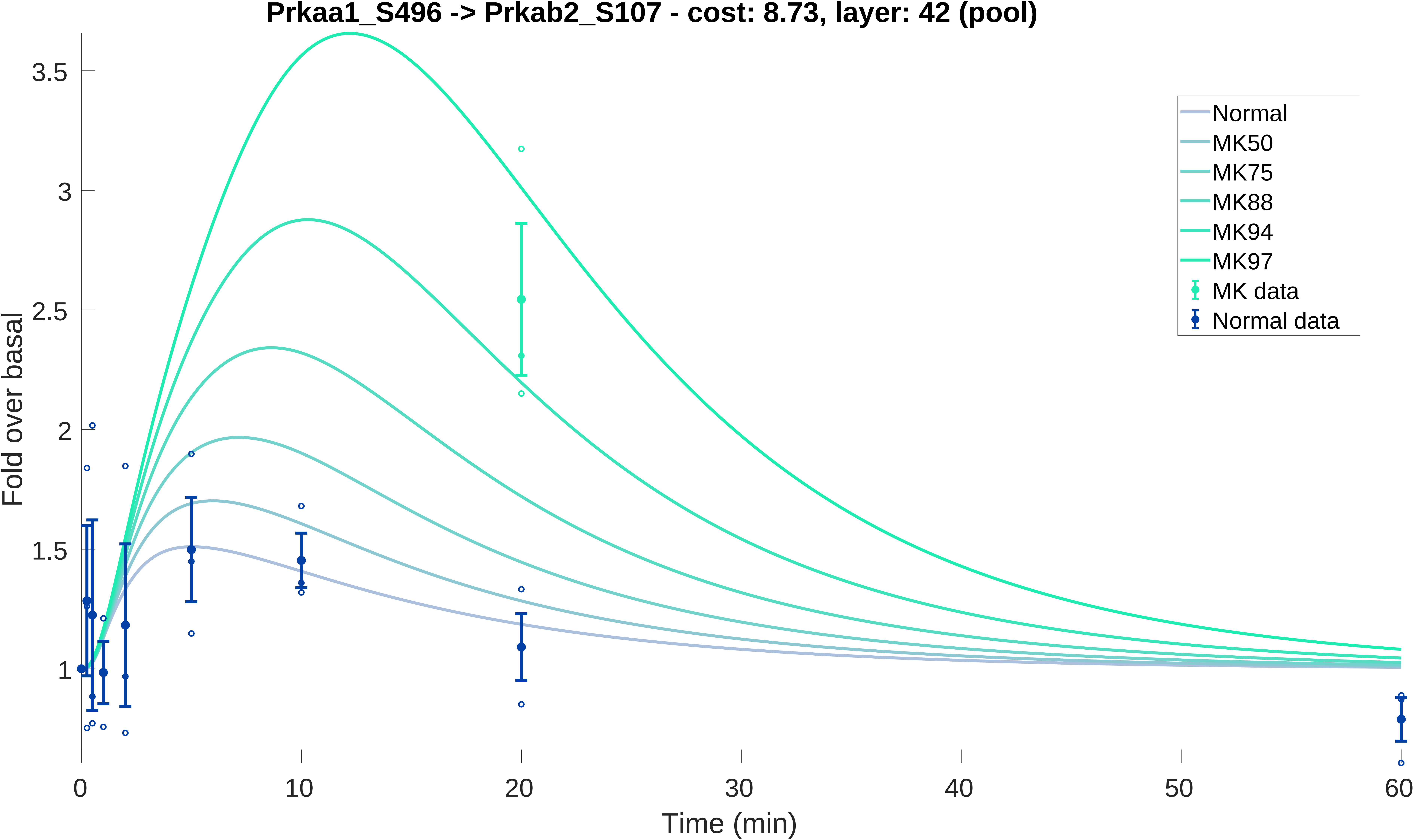

Prkaa1_S496 -> Prkab2_S107 - cost: 8.73, layer: 42 (pool)
Fold over basal
Time (min)
3.5
3
2.5
2
1.5
1
0
10
20
30
40
50
60
Normal
MK50
MK75
MK88
MK94
MK97
MK data
Normal data

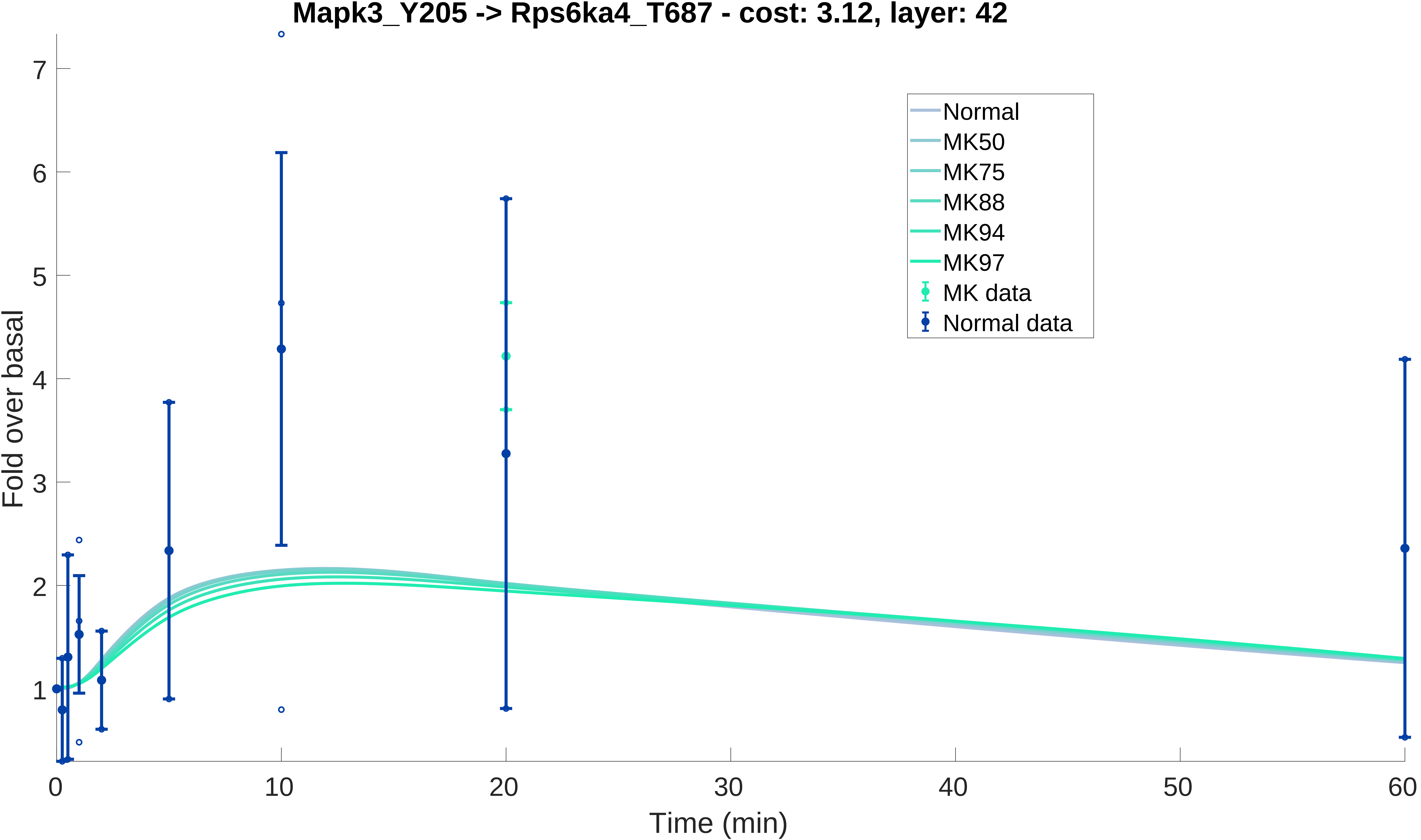

Mapk3_Y205 -> Rps6ka4_T687 - cost: 3.12, layer: 42
Fold over basal
Time (min)
0
10
20
30
40
50
60
1
2
3
4
5
6
7
Normal
MK50
MK75
MK88
MK94
MK97
MK data
Normal data

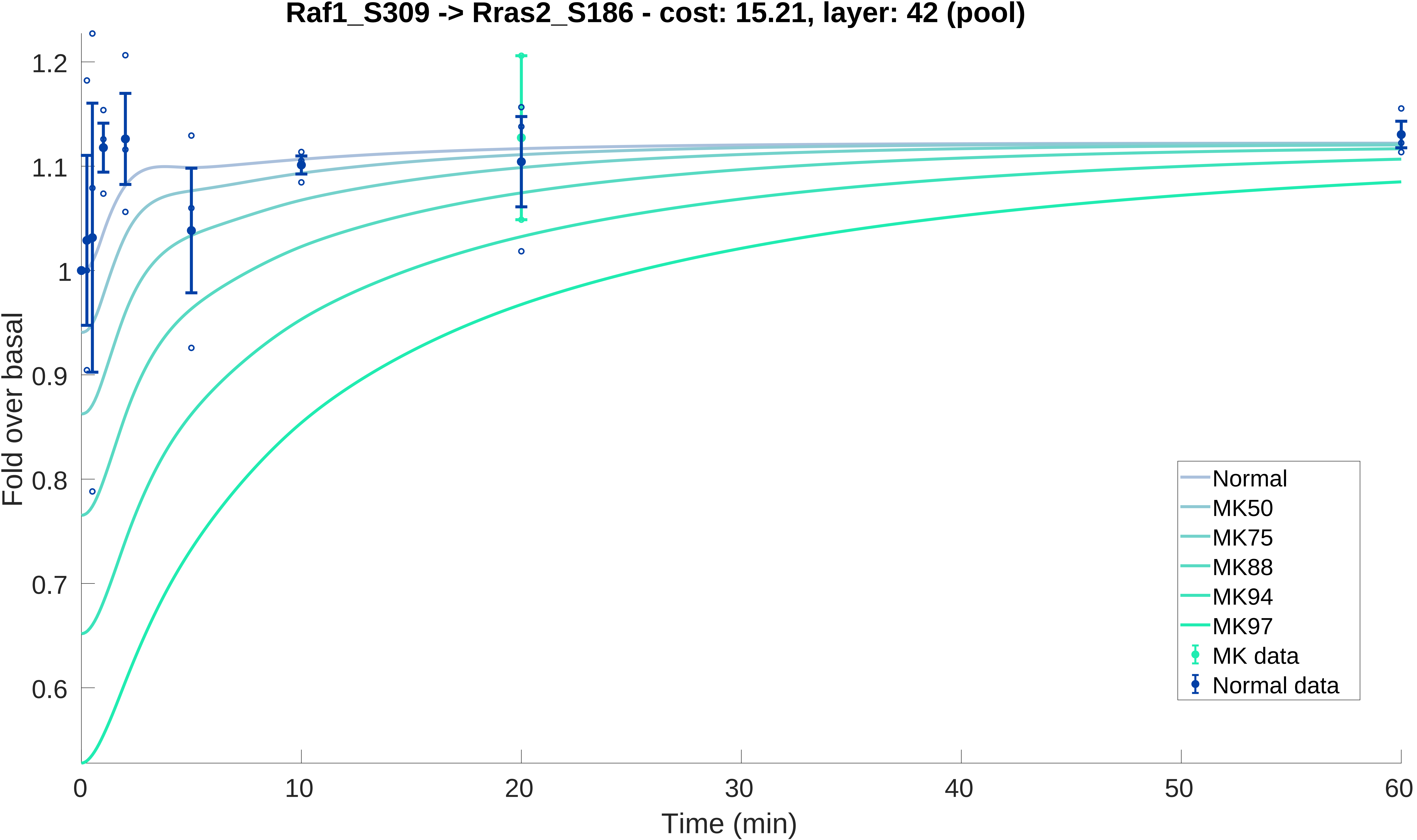

Raf1_S309 -> Rras2_S186 - cost: 15.21, layer: 42 (pool)
Fold over basal
Time (min)
1.2
1.1
1
0.9
0.8
0.7
0.6
0
10
20
30
40
50
60
Normal
MK50
MK75
MK88
MK94
MK97
MK data
Normal data

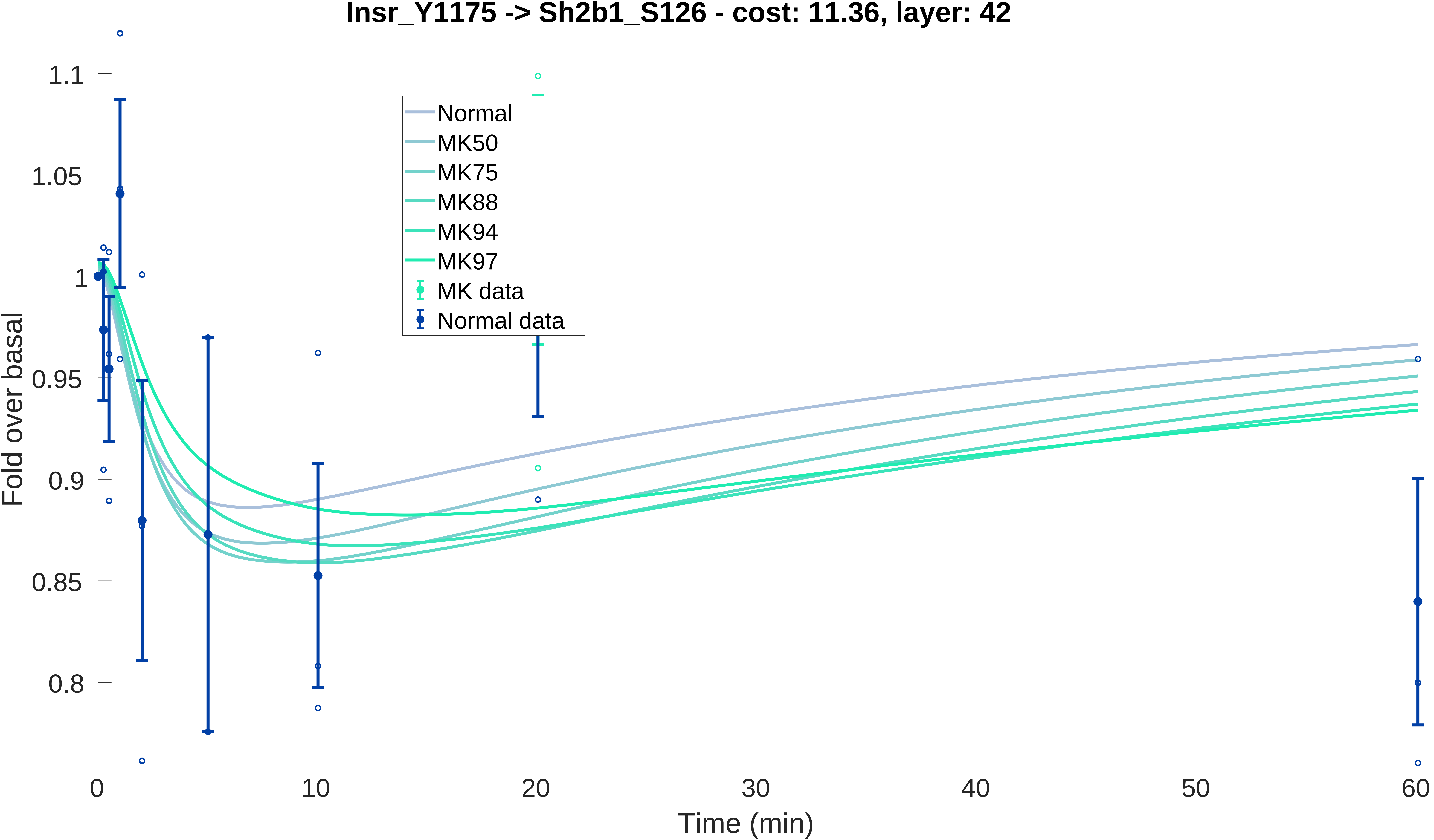
Insr_Y1175 -> Sh2b1_S126 - cost: 11.36, layer: 42
Normal
MK50
MK75
MK88
MK94
MK97
MK data
Normal data
Fold over basal
1.1
1.05
1
0.95
0.9
0.85
0.8
0
10
20
30
40
50
60
Time (min)

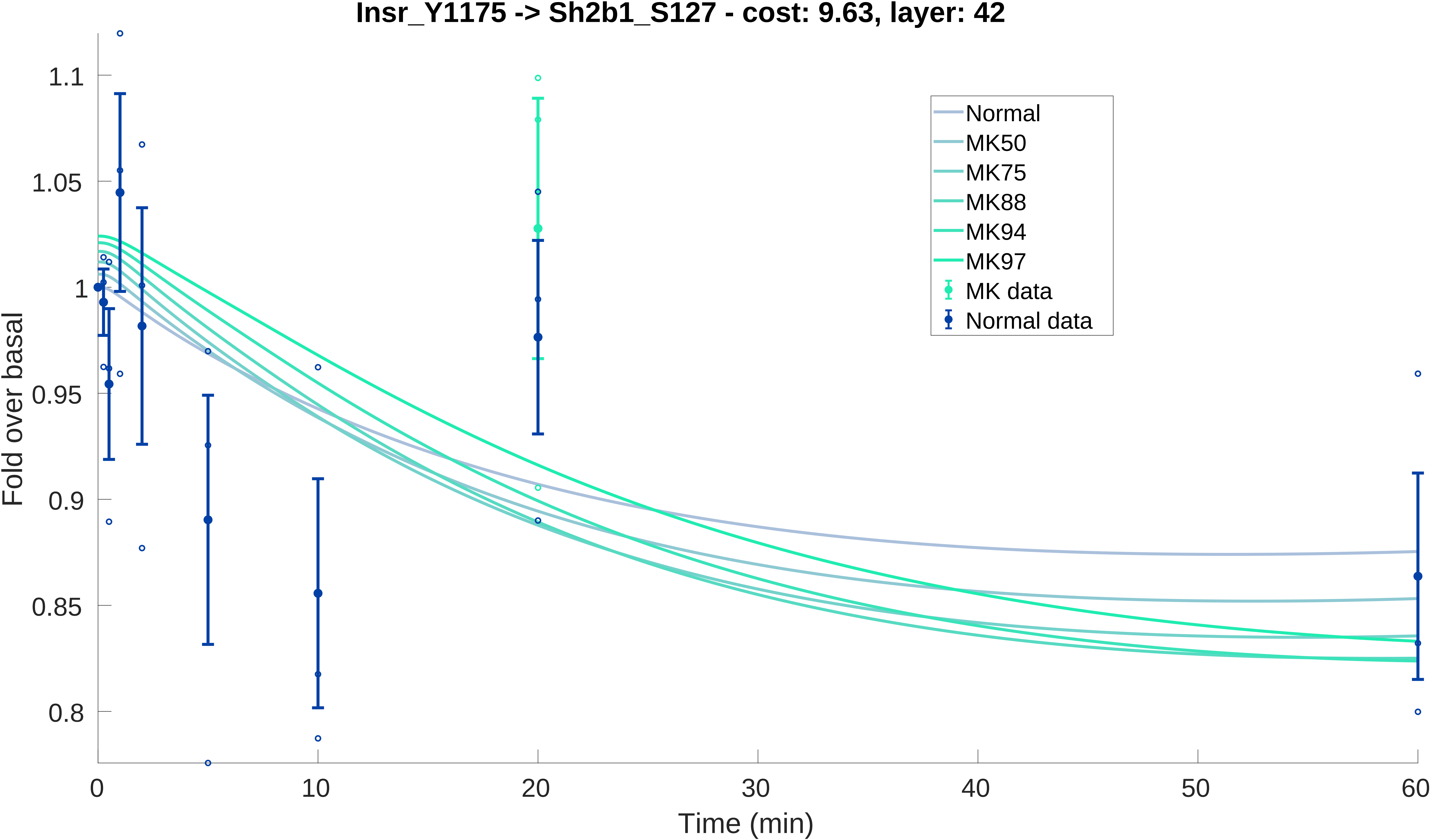

Insr_Y1175 -> Sh2b1_S127 - cost: 9.63, layer: 42
Fold over basal
1.1
1.05
1
0.95
0.9
0.85
0.8
Normal
MK50
MK75
MK88
MK94
MK97
MK data
Normal data
0
10
20
30
40
50
60
Time (min)

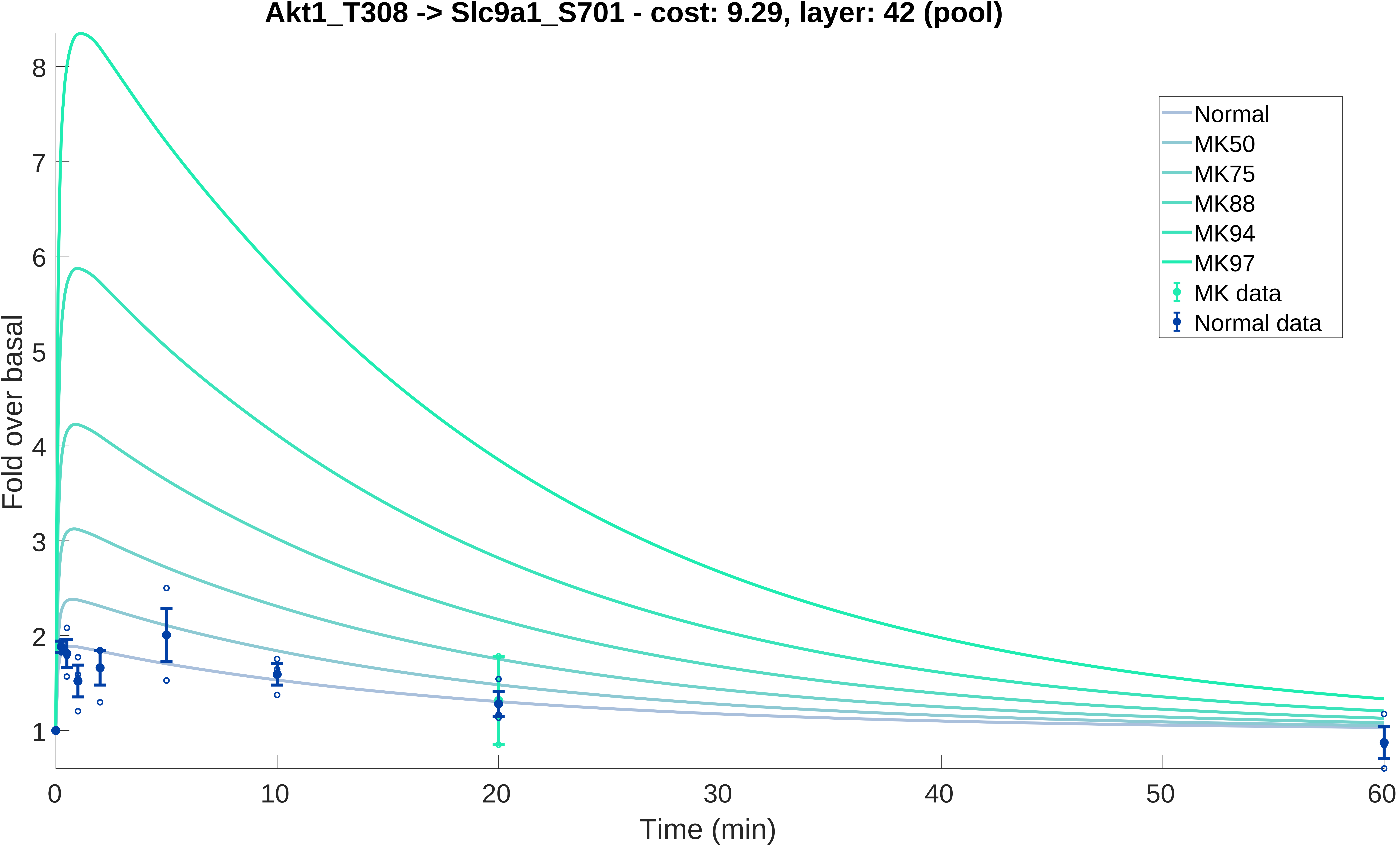
Akt1_T308 -> Slc9a1_S701 - cost: 9.29, layer: 42 (pool)
Fold over basal
Time (min)
Normal
MK50
MK75
MK88
MK94
MK97
MK data
Normal data
1
2
3
4
5
6
7
8
0
10
20
30
40
50
60

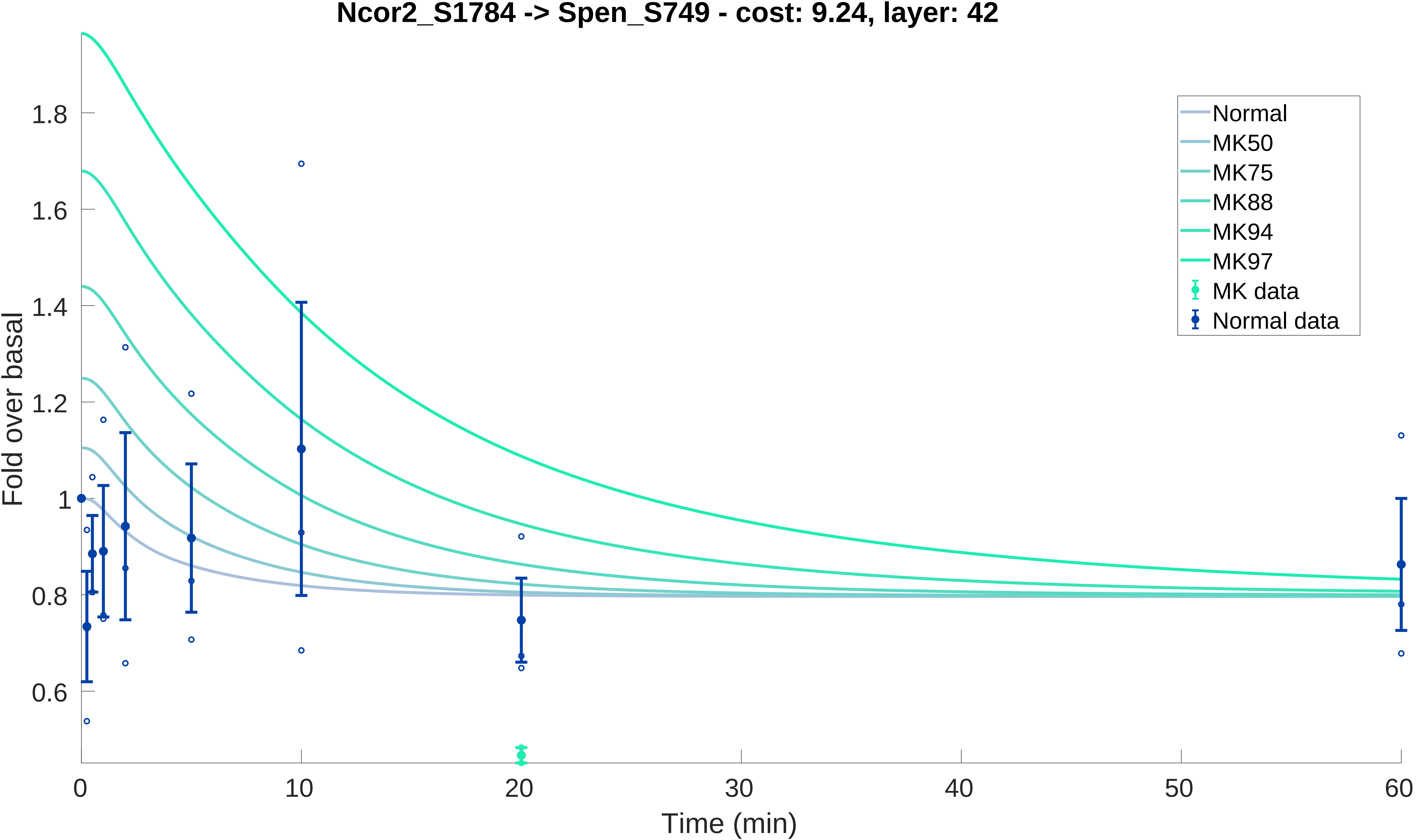

Ncor2_S1784 -> Spen_S749 - cost: 9.24, layer: 42
Fold over basal
Time (min)
0.6
0.8
1
1.2
1.4
1.6
1.8
0
10
20
30
40
50
60
Normal
MK50
MK75
MK88
MK94
MK97
MK data
Normal data

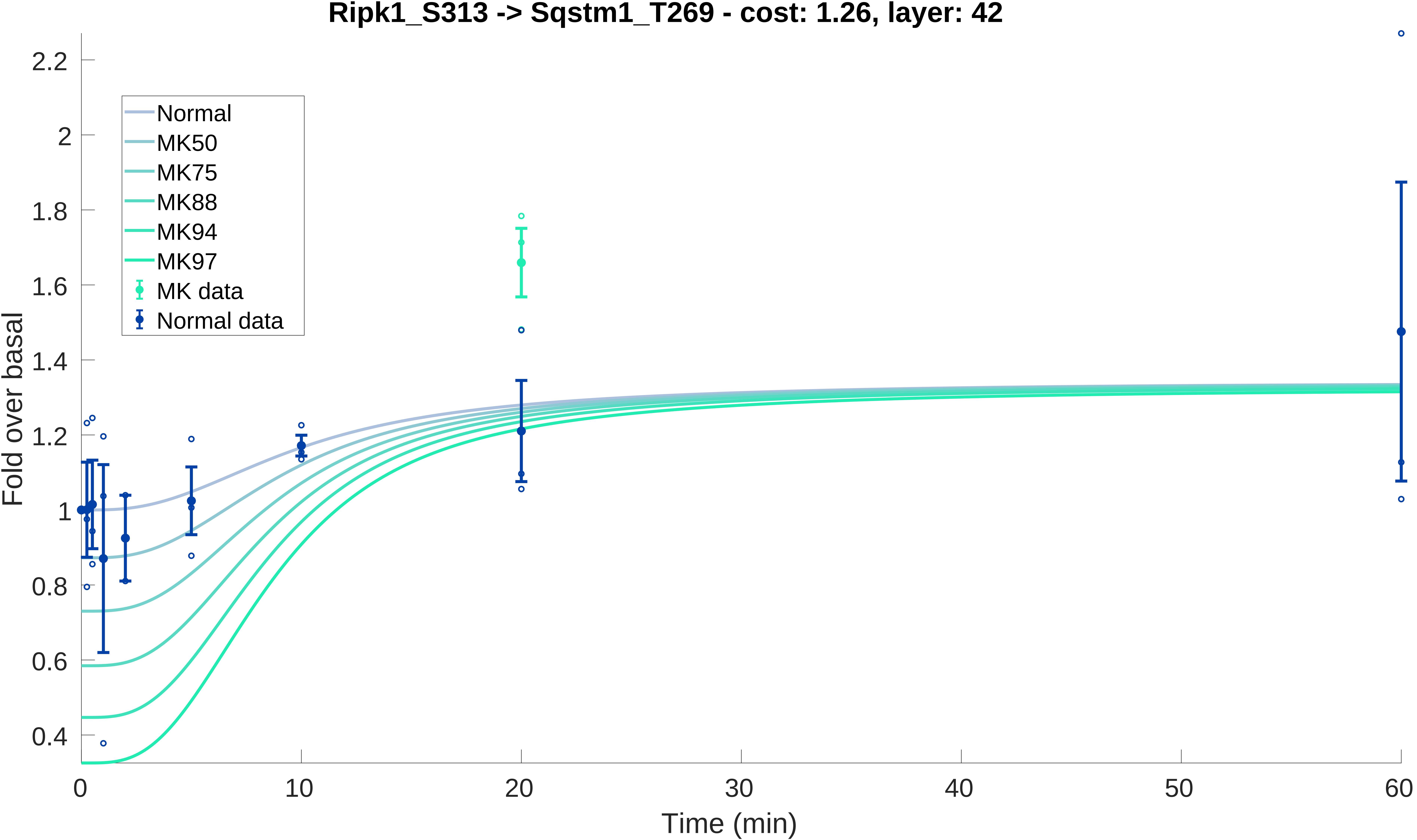
Ripk1_S313 -> Sqstm1_T269 - cost: 1.26, layer: 42
Normal
MK50
MK75
MK88
MK94
MK97
MK data
Normal data
Fold over basal
Time (min)
0.4
0.6
0.8
1
1.2
1.4
1.6
1.8
2
2.2
0
10
20
30
40
50
60

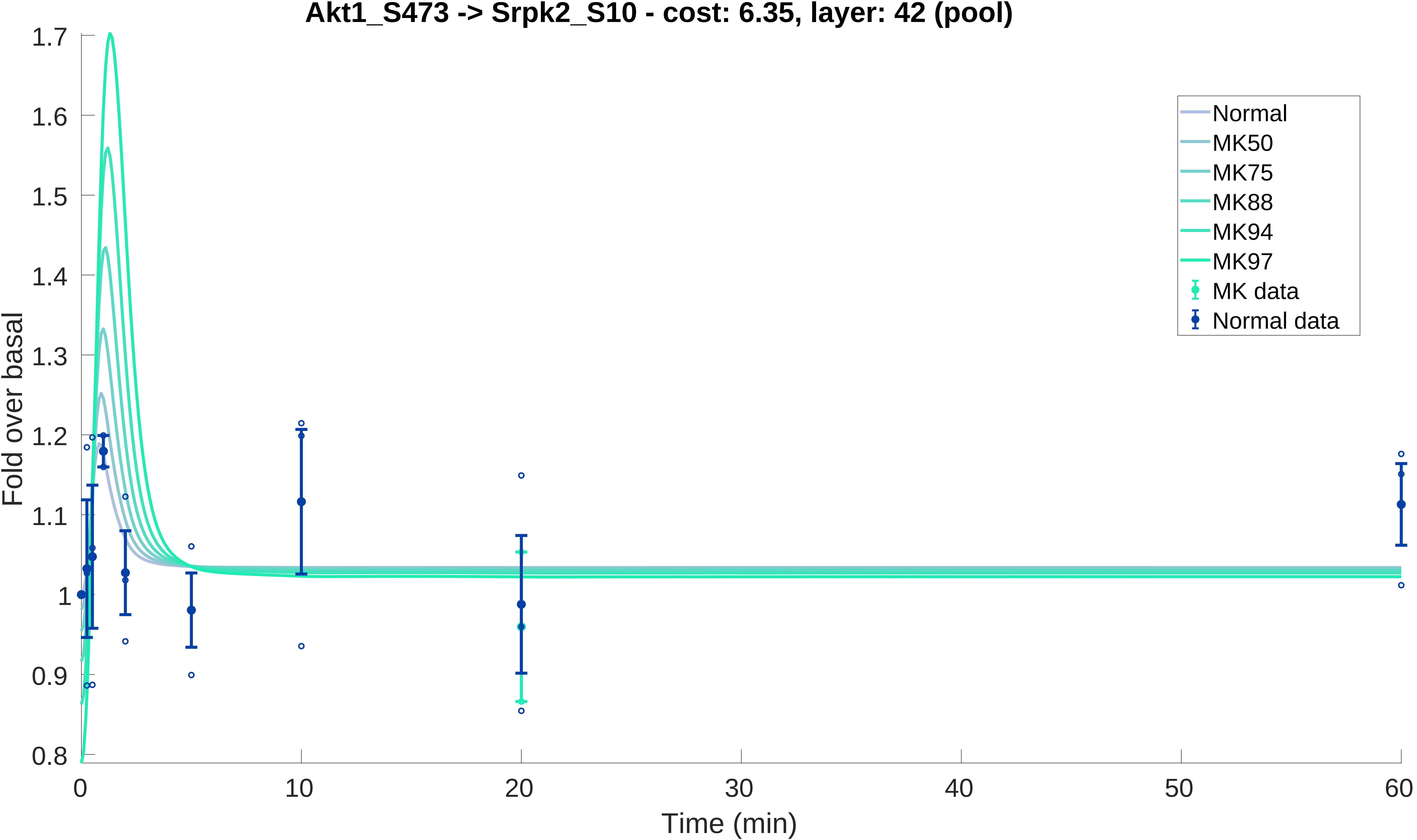

Akt1_S473 -> Srpk2_S10 - cost: 6.35, layer: 42 (pool)
Fold over basal
Time (min)
Normal
MK50
MK75
MK88
MK94
MK97
MK data
Normal data
0.8
0.9
1
1.1
1.2
1.3
1.4
1.5
1.6
1.7
0
10
20
30
40
50
60

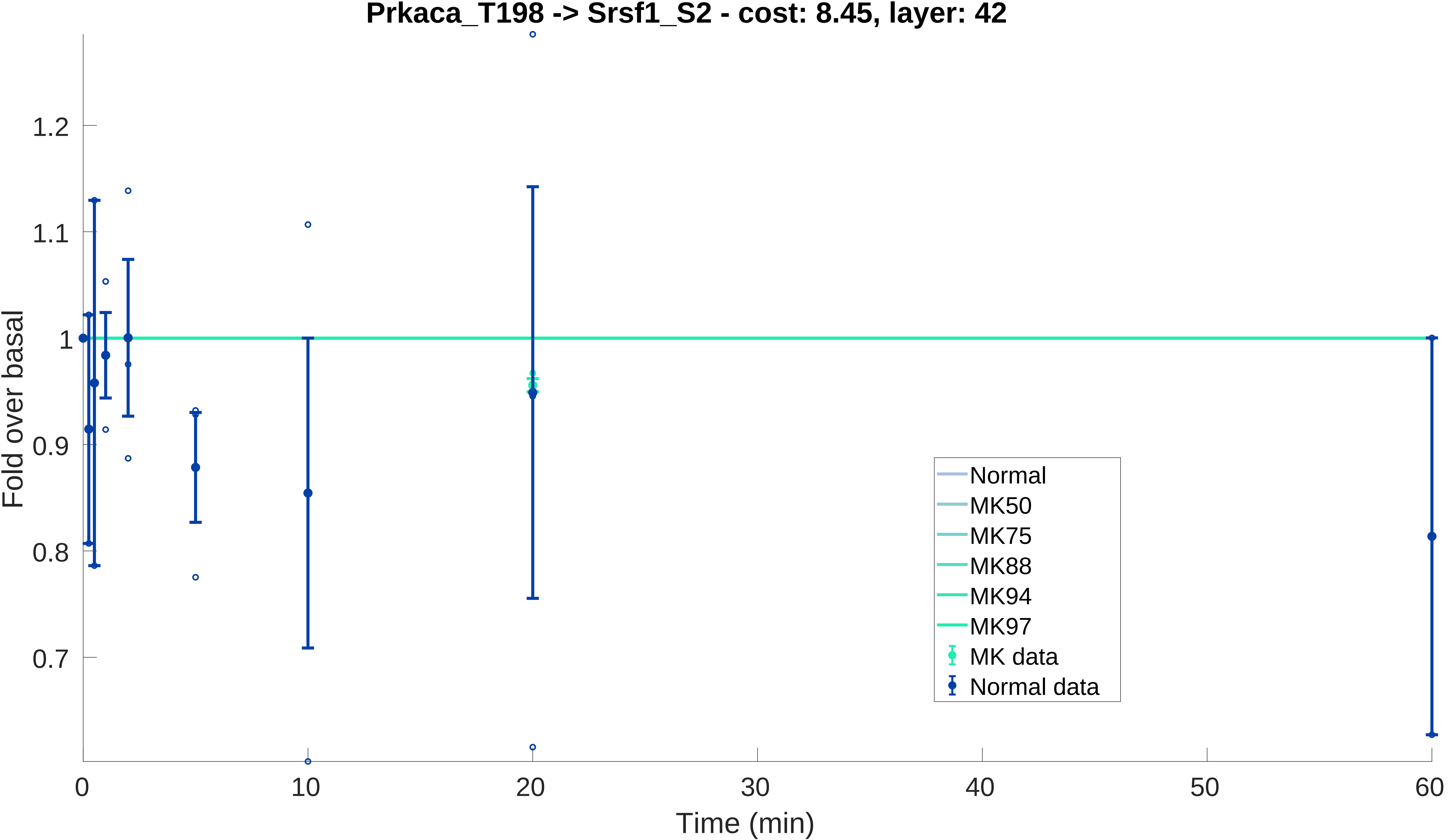

Prkaca_T198 -> Srsf1_S2 - cost: 8.45, layer: 42
Fold over basal
1.2
1.1
1
0.9
0.8
0.7
0
10
20
30
40
50
60
Time (min)
Normal
MK50
MK75
MK88
MK94
MK97
MK data
Normal data

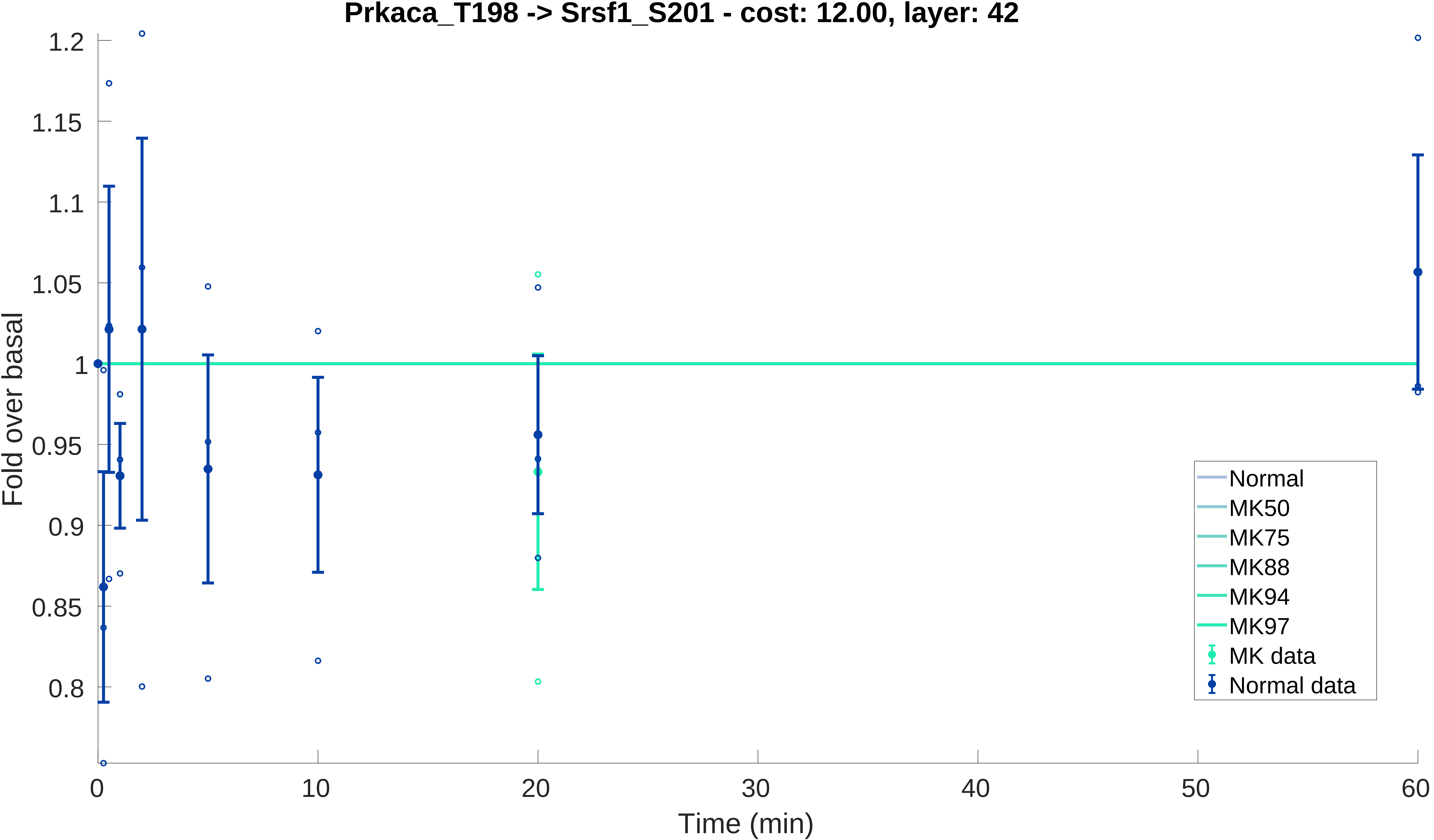

Prrkaca_T198 -> Srsf1_S201 - cost: 12.00, layer: 42
Fold over basal
1.2
1.15
1.1
1.05
1
0.95
0.9
0.85
0.8
0
10
20
30
40
50
60
Time (min)
Normal
MK50
MK75
MK88
MK94
MK97
MK data
Normal data

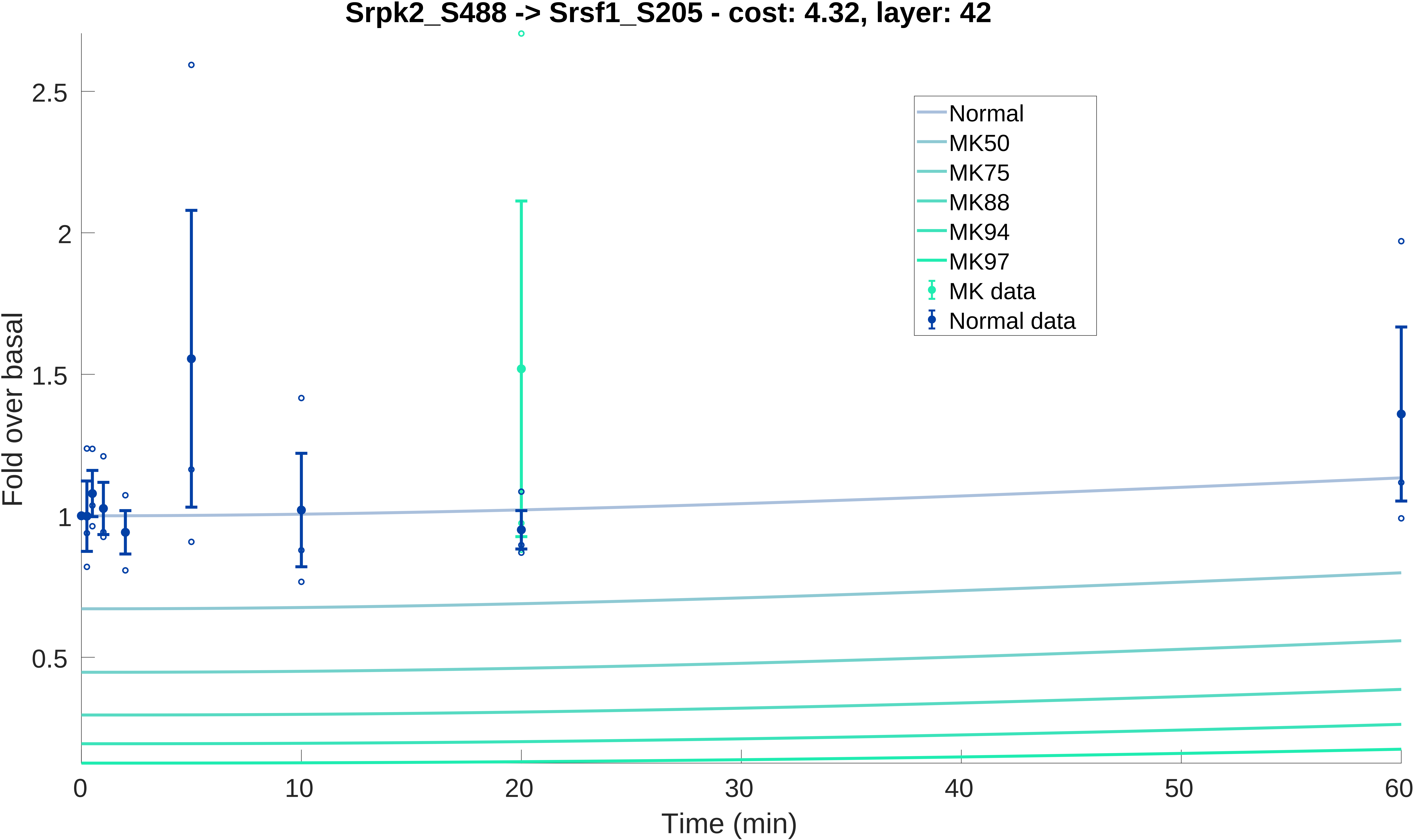

Srpk2_S488 -> Srsf1_S205 - cost: 4.32, layer: 42
Fold over basal
Time (min)
0
10
20
30
40
50
60
0.5
1
1.5
2
2.5
Normal
MK50
MK75
MK88
MK94
MK97
MK data
Normal data

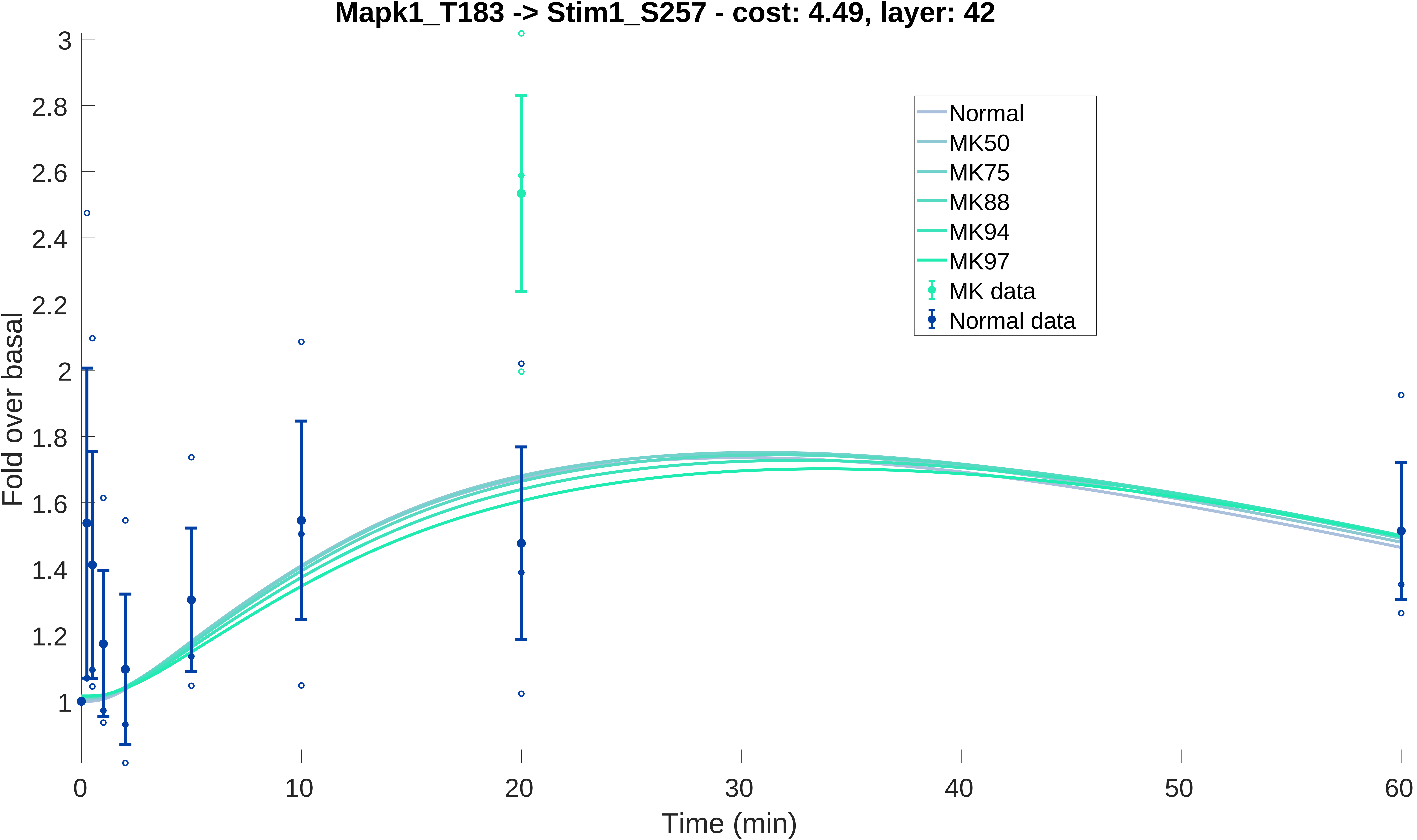
Mapk1_T183 -> Stim1_S257 - cost: 4.49, layer: 42
Fold over basal
Time (min)
Normal
MK50
MK75
MK88
MK94
MK97
MK data
Normal data
3
2.8
2.6
2.4
2.2
2
1.8
1.6
1.4
1.2
1
0
10
20
30
40
50
60

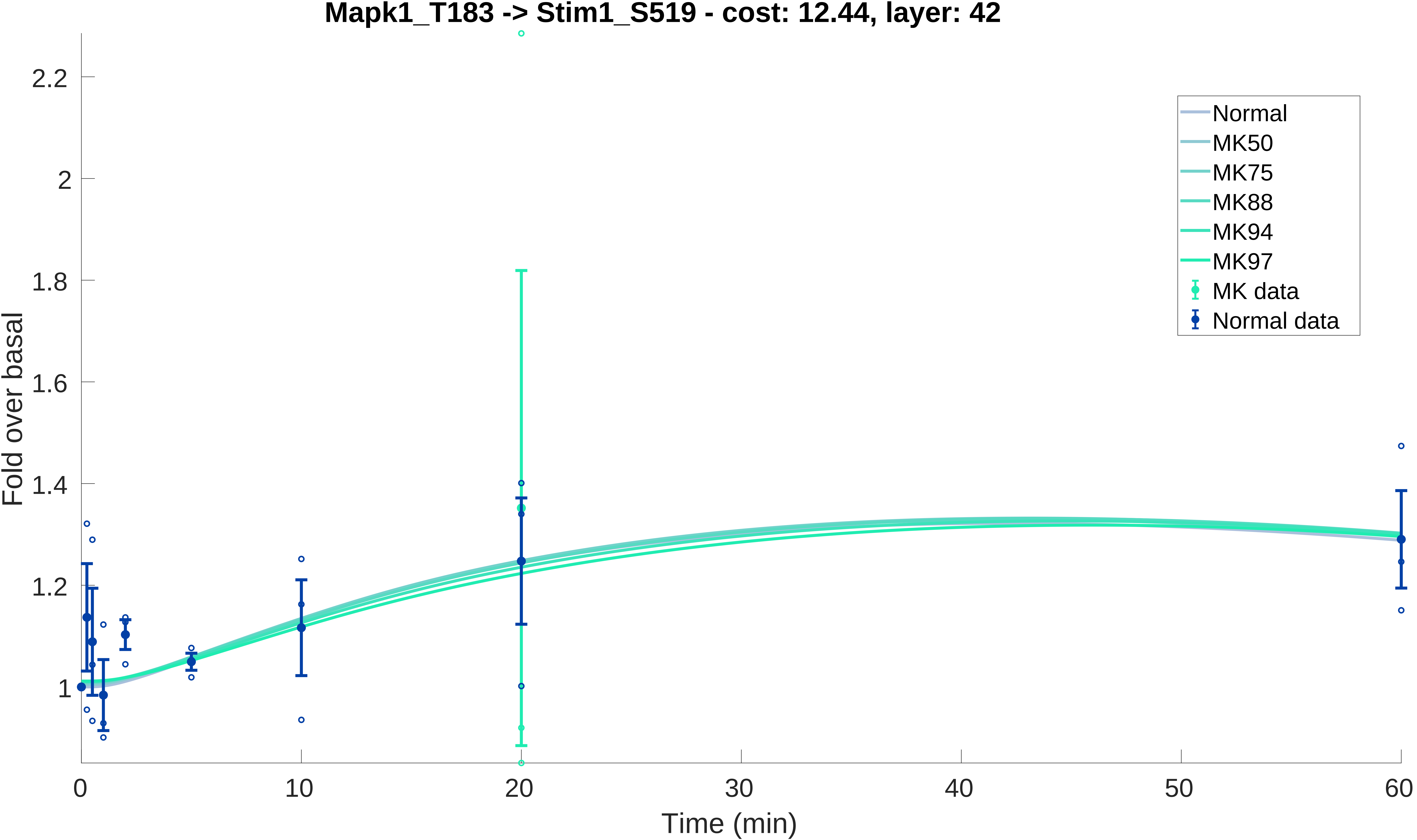

Mapk1_T183 -> Stim1_S519 - cost: 12.44, layer: 42
Fold over basal
2.2
2
1.8
1.6
1.4
1.2
1
0
10
20
30
40
50
60
Time (min)
Normal
MK50
MK75
MK88
MK94
MK97
MK data
Normal data

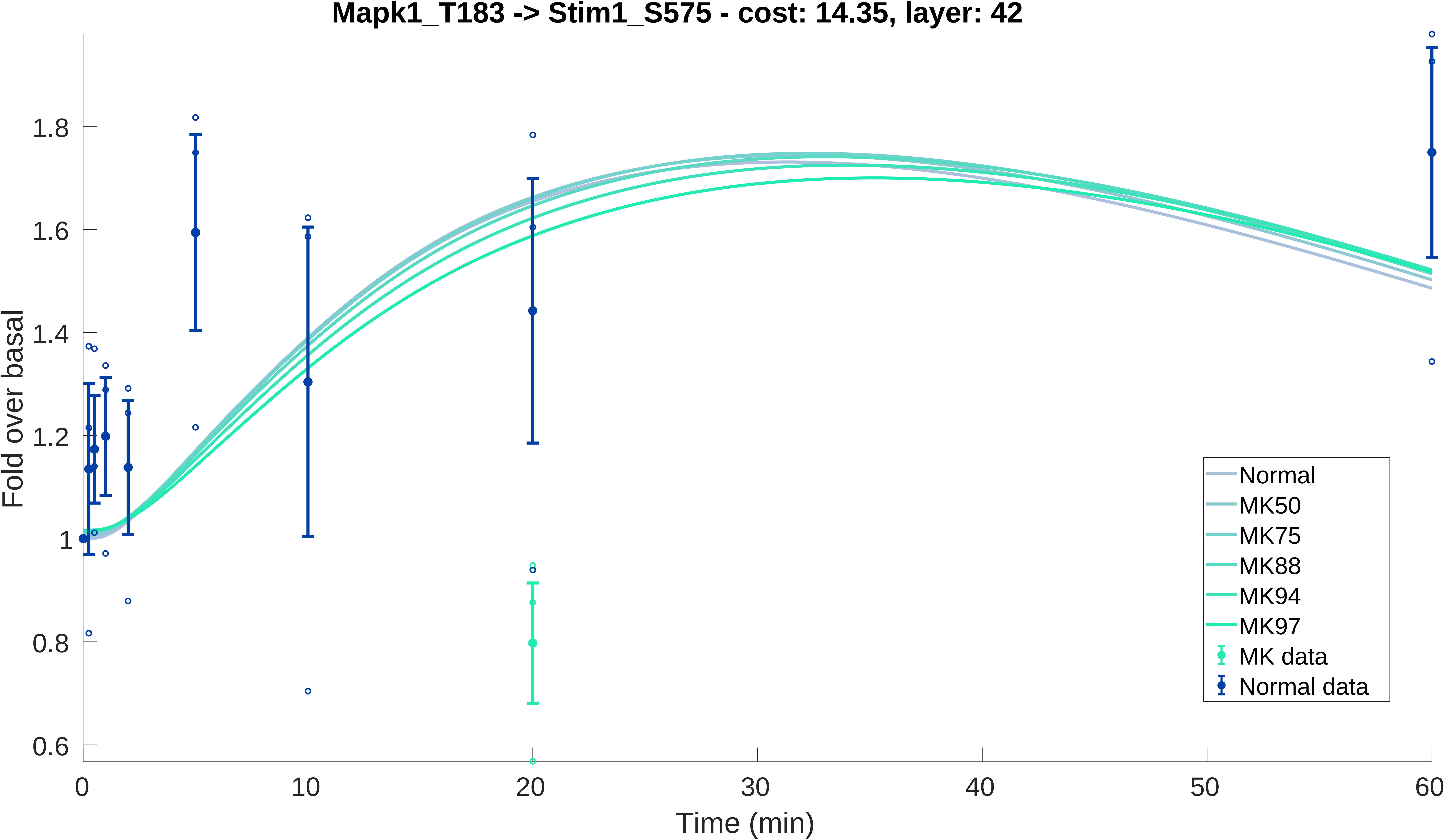
Mapk1_T183 -> Stim1_S575 - cost: 14.35, layer: 42
Fold over basal
1.8
1.6
1.4
1.2
1
0.8
0.6
0
10
20
30
40
50
60
Time (min)
Normal
MK50
MK75
MK88
MK94
MK97
MK data
Normal data

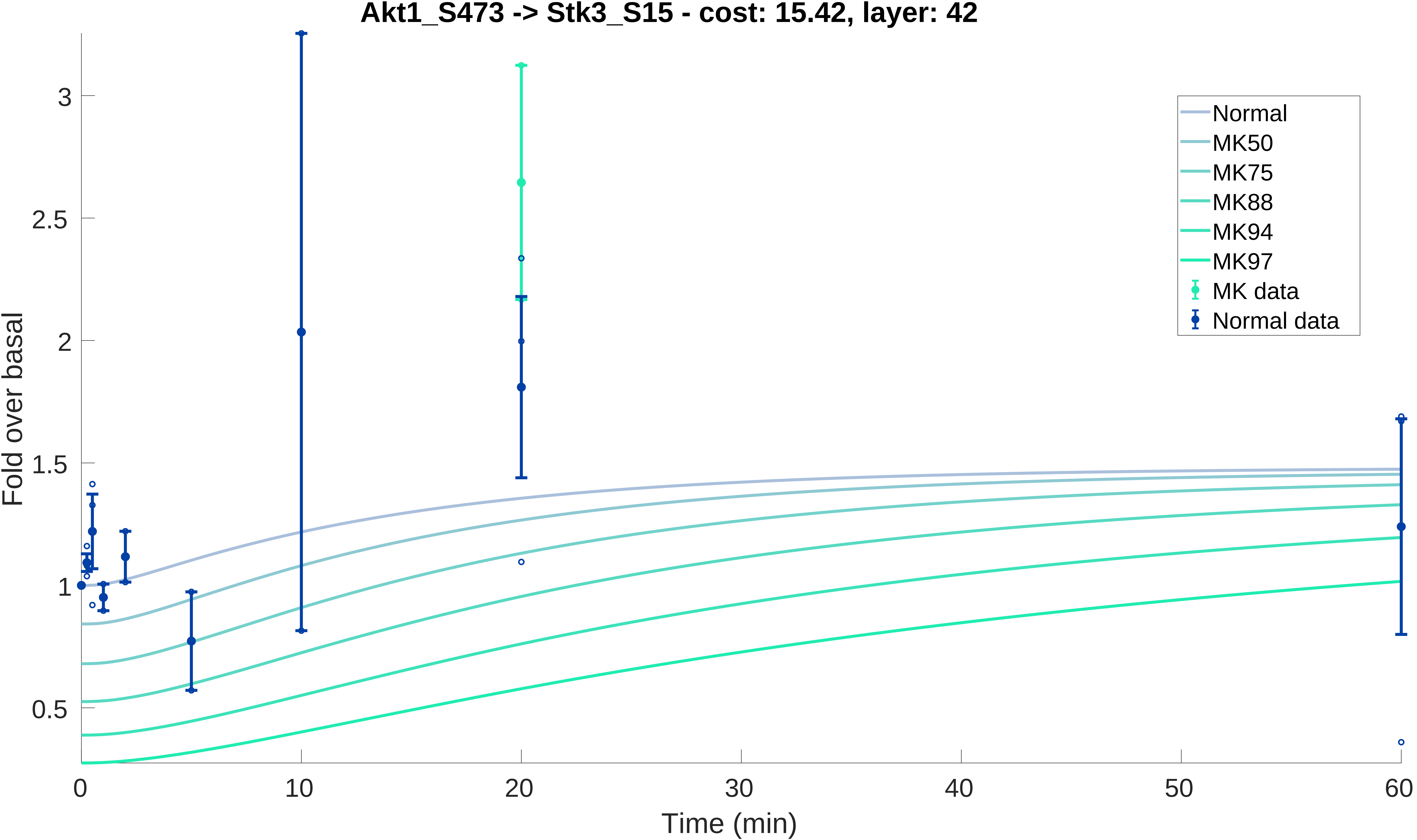

Akt1_S473 -> Stk3_S15 - cost: 15.42, layer: 42
Fold over basal
Time (min)
0
10
20
30
40
50
60
0.5
1
1.5
2
2.5
3
Normal
MK50
MK75
MK88
MK94
MK97
MK data
Normal data

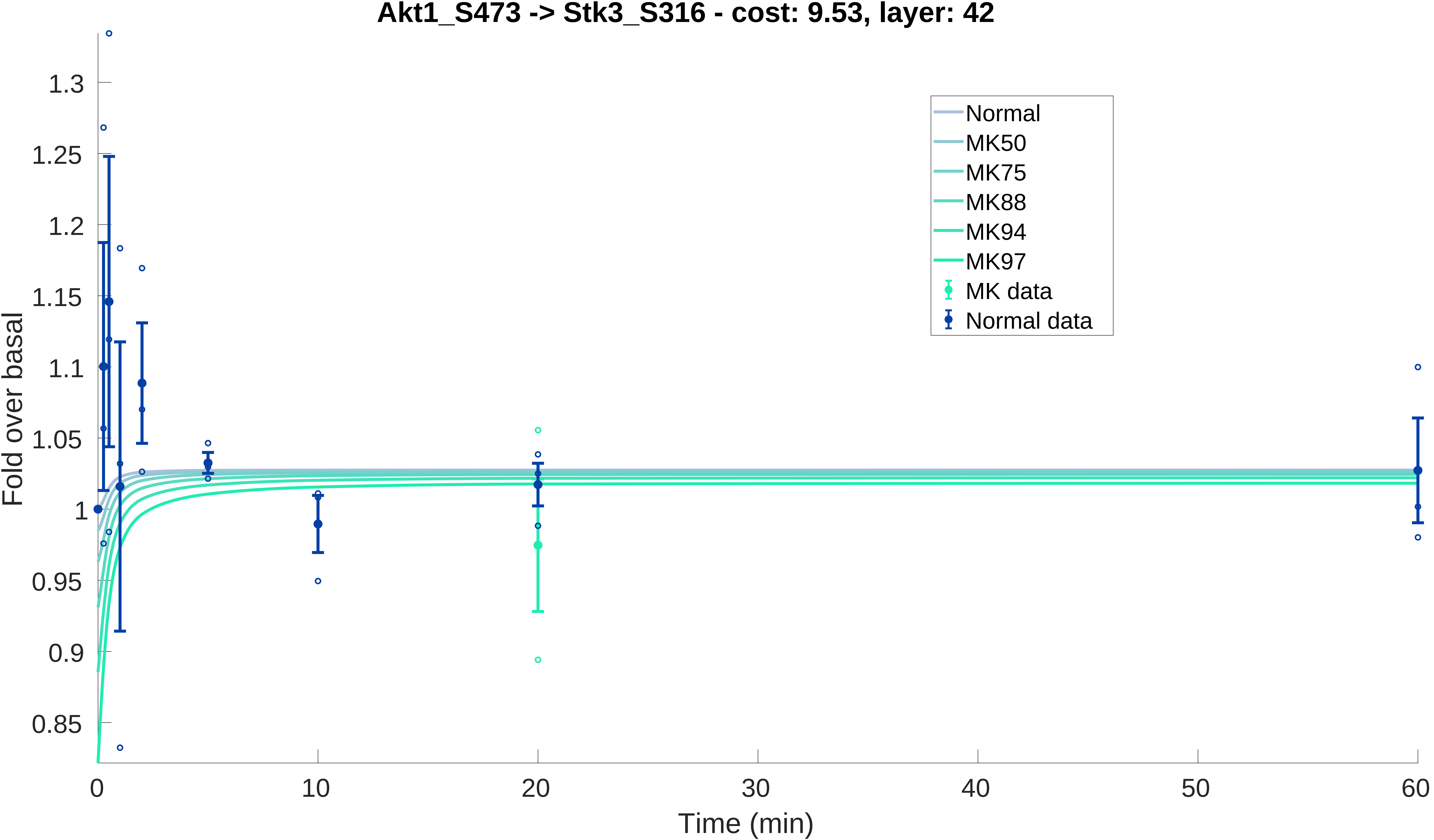

Akt1_S473 -> Stk3_S316 - cost: 9.53, layer: 42
Fold over basal
Time (min)
1.3
1.25
1.2
1.15
1.1
1.05
1
0.95
0.9
0.85
0
10
20
30
40
50
60
Normal
MK50
MK75
MK88
MK94
MK97
MK data
Normal data

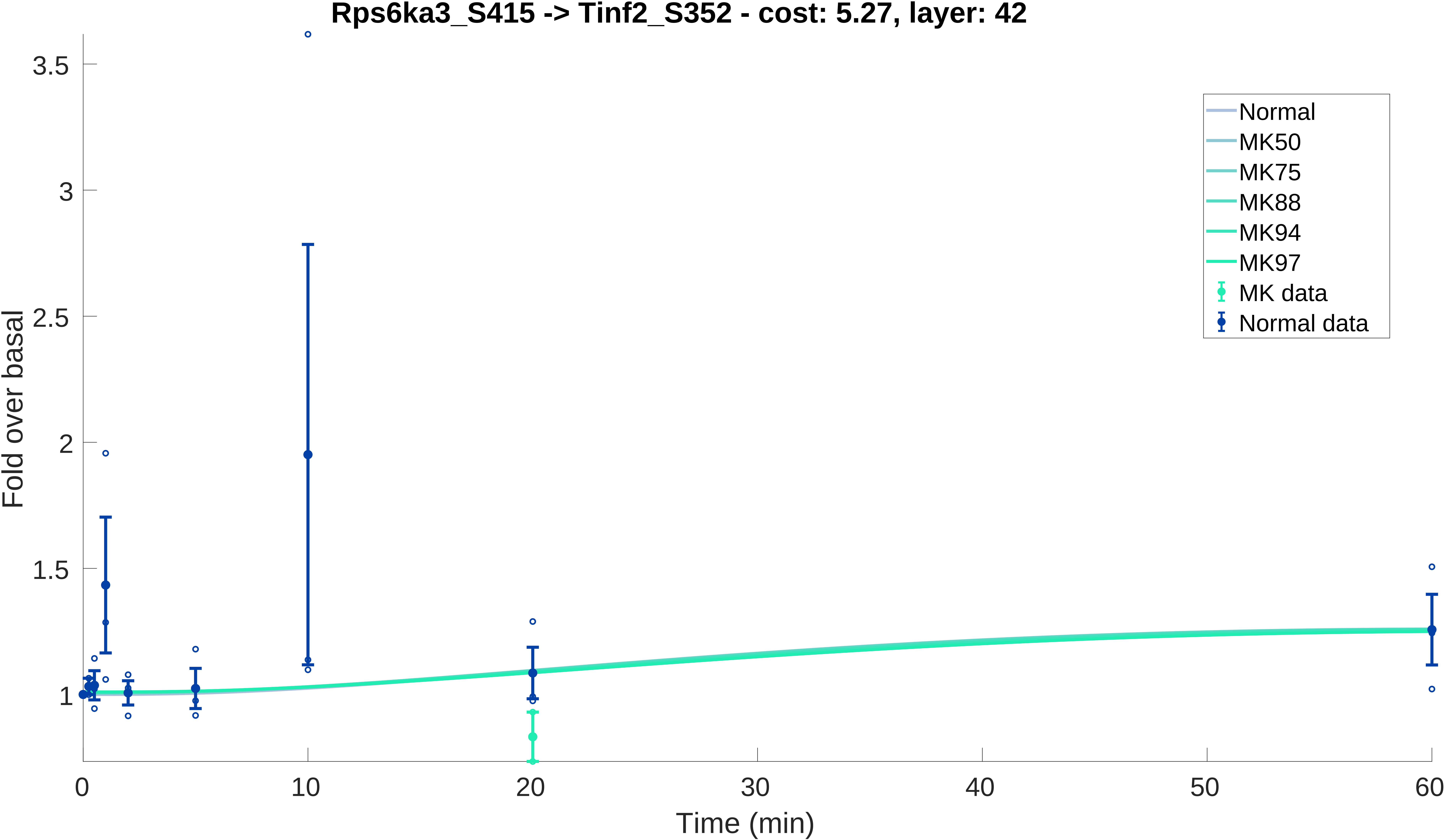
Rps6ka3_S415 -> Tinf2_S352 - cost: 5.27, layer: 42
Fold over basal
Time (min)
3.5
3
2.5
2
1.5
1
0
10
20
30
40
50
60
Normal
MK50
MK75
MK88
MK94
MK97
MK data
Normal data

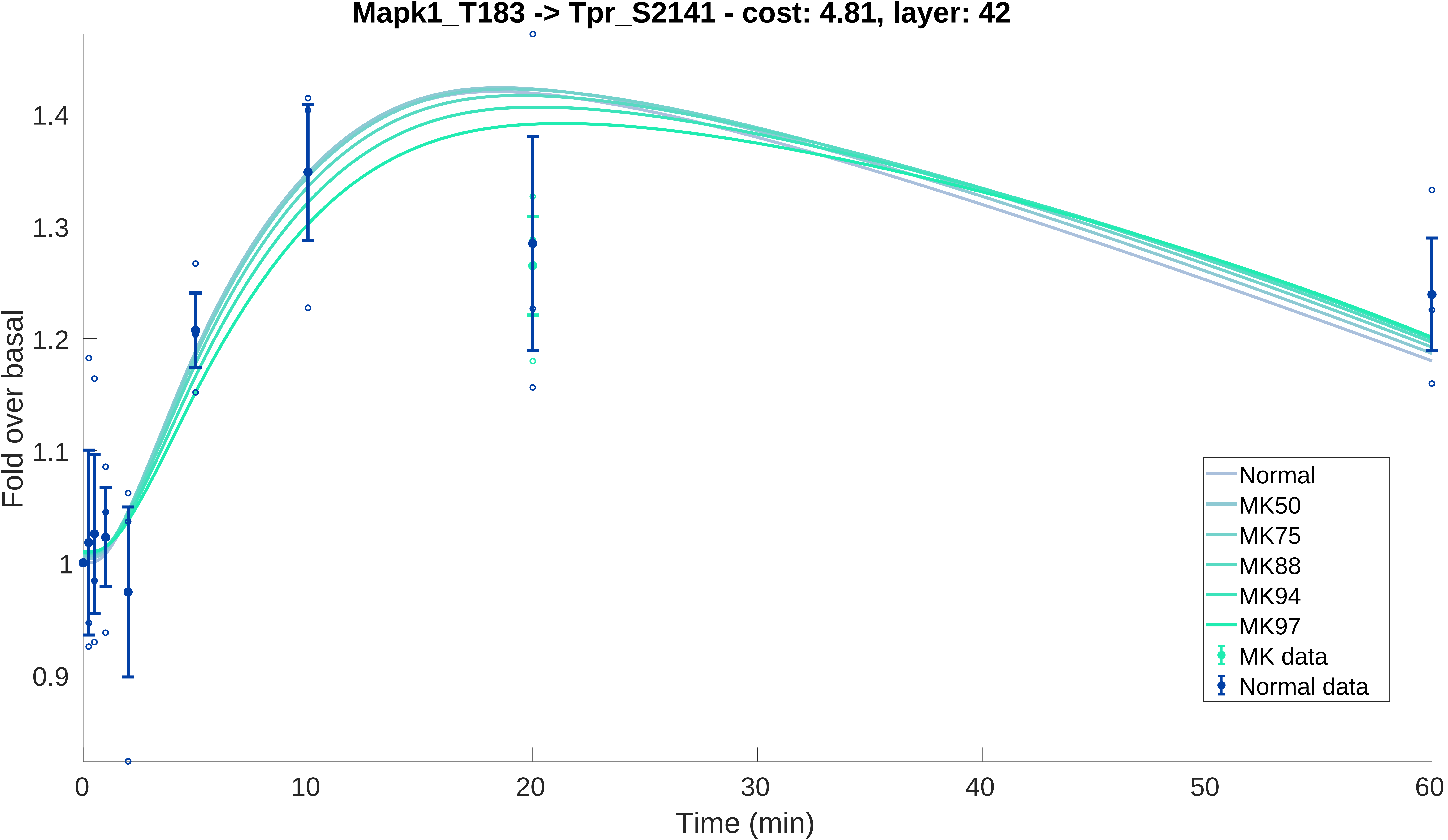
Mapk1_T183 -> Tpr_S2141 - cost: 4.81, layer: 42
Fold over basal
1.4
1.3
1.2
1.1
1
0.9
0
10
20
30
40
50
60
Time (min)
Normal
MK50
MK75
MK88
MK94
MK97
MK data
Normal data

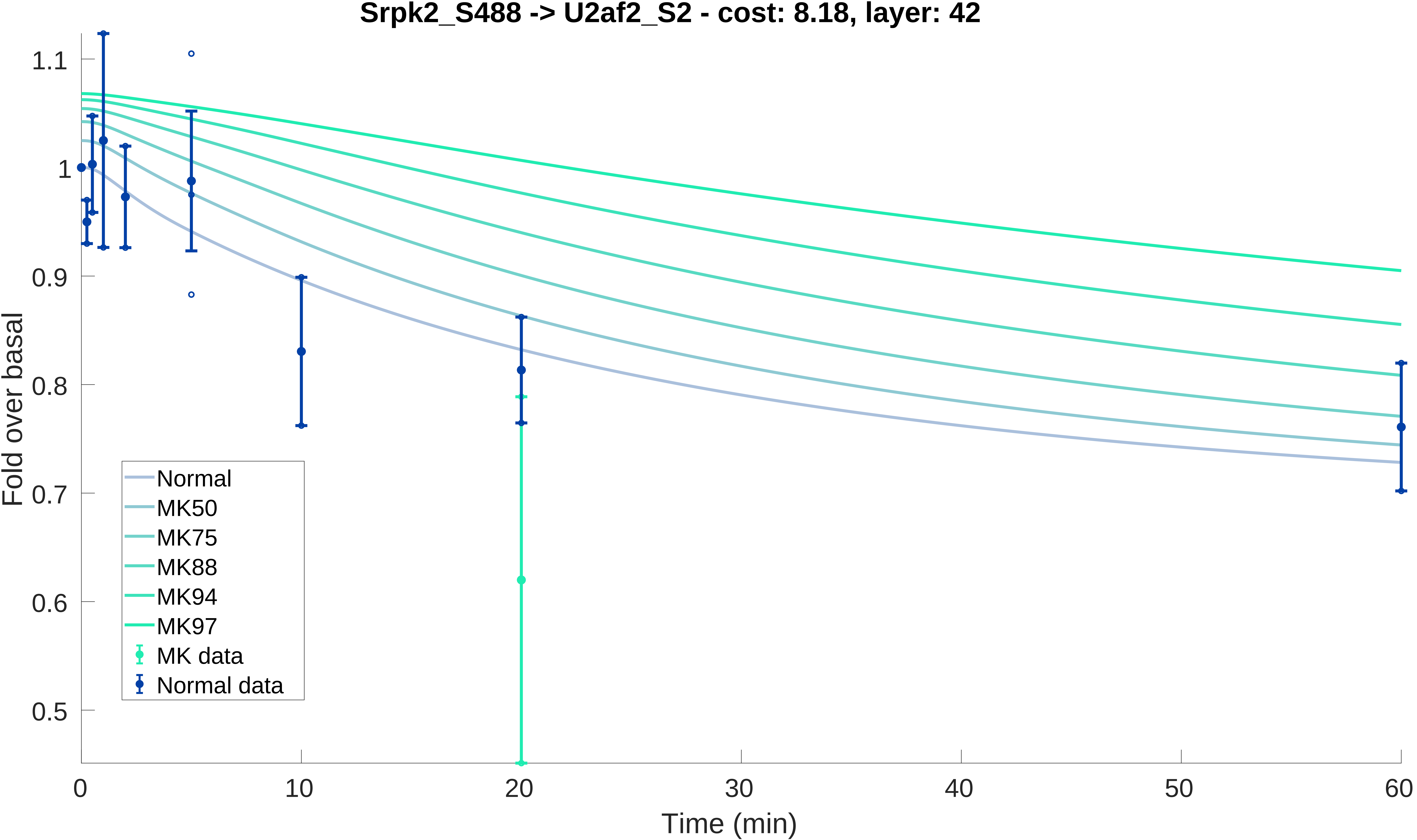

Srpk2_S488 -> U2af2_S2 - cost: 8.18, layer: 42
Fold over basal
1.1
1
0.9
0.8
0.7
0.6
0.5
0
10
20
30
40
50
60
Time (min)
Normal
MK50
MK75
MK88
MK94
MK97
MK data
Normal data

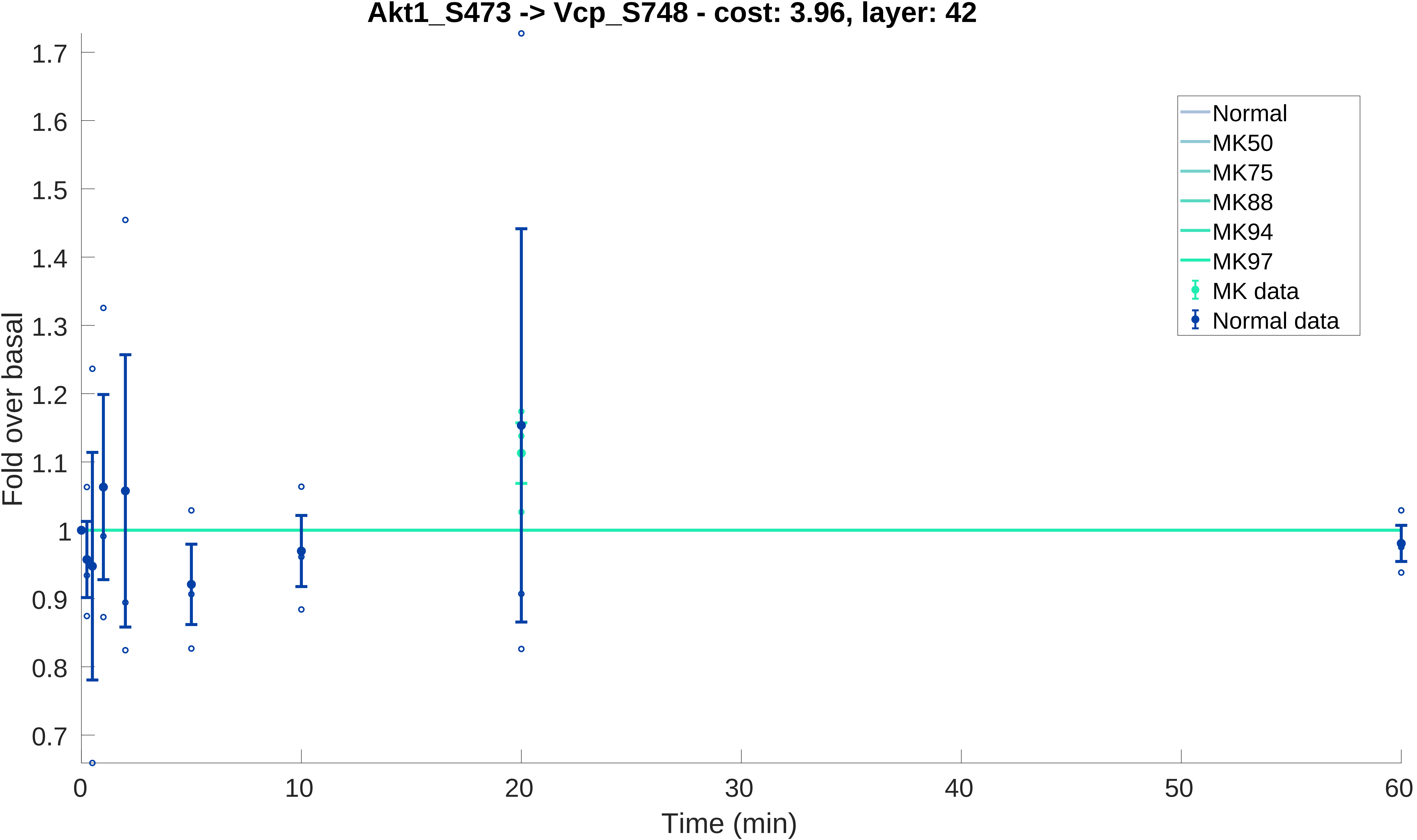
Akt1_S473 -> Vcp_S748 - cost: 3.96, layer: 42
Fold over basal
Time (min)
0
10
20
30
40
50
60
0.7
0.8
0.9
1
1.1
1.2
1.3
1.4
1.5
1.6
1.7
Normal
MK50
MK75
MK88
MK94
MK97
MK data
Normal data

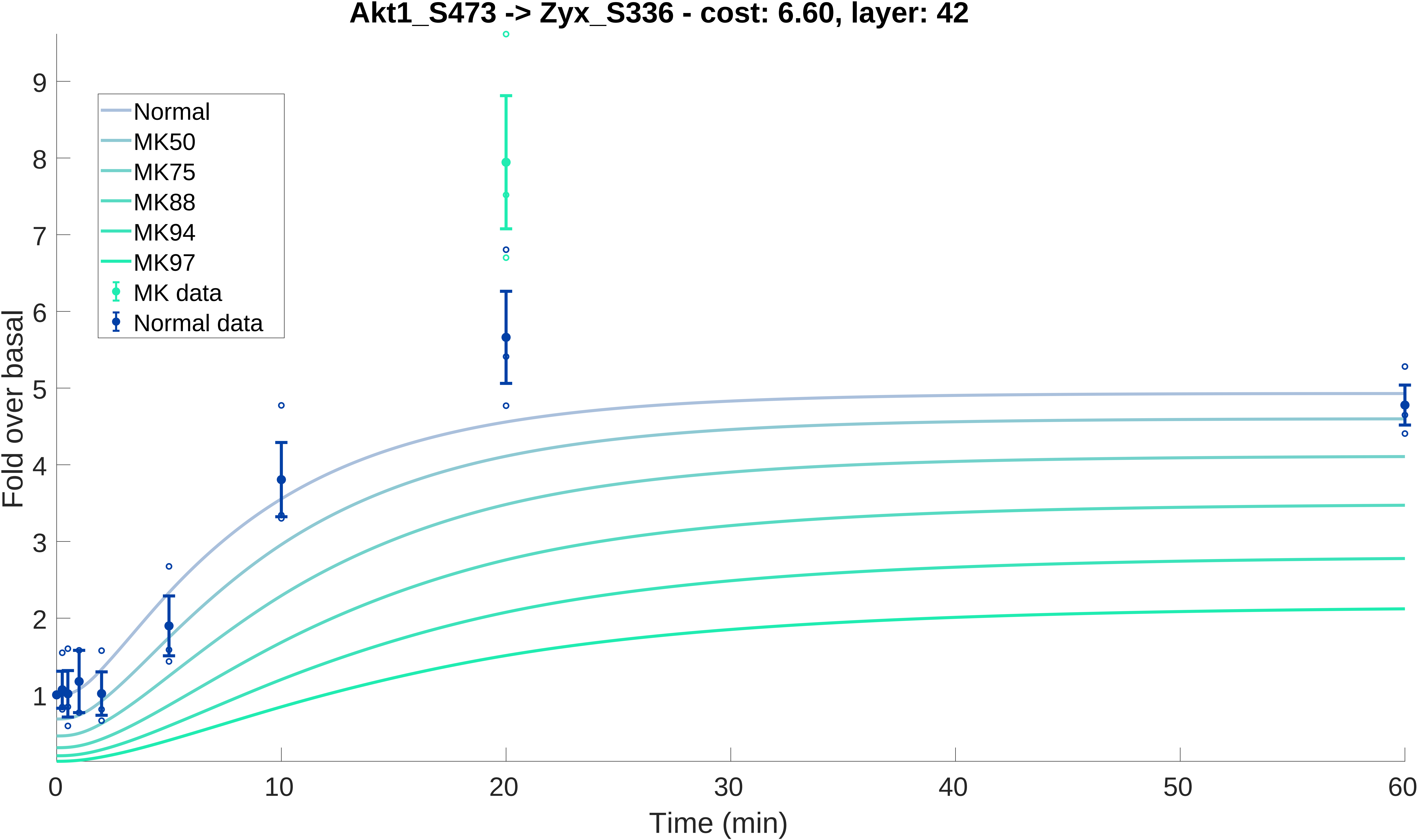

Akt1_S473 -> Zyx_S336 - cost: 6.60, layer: 42
Normal
MK50
MK75
MK88
MK94
MK97
MK data
Normal data
Fold over basal
Time (min)
0
10
20
30
40
50
60
1
2
3
4
5
6
7
8
9

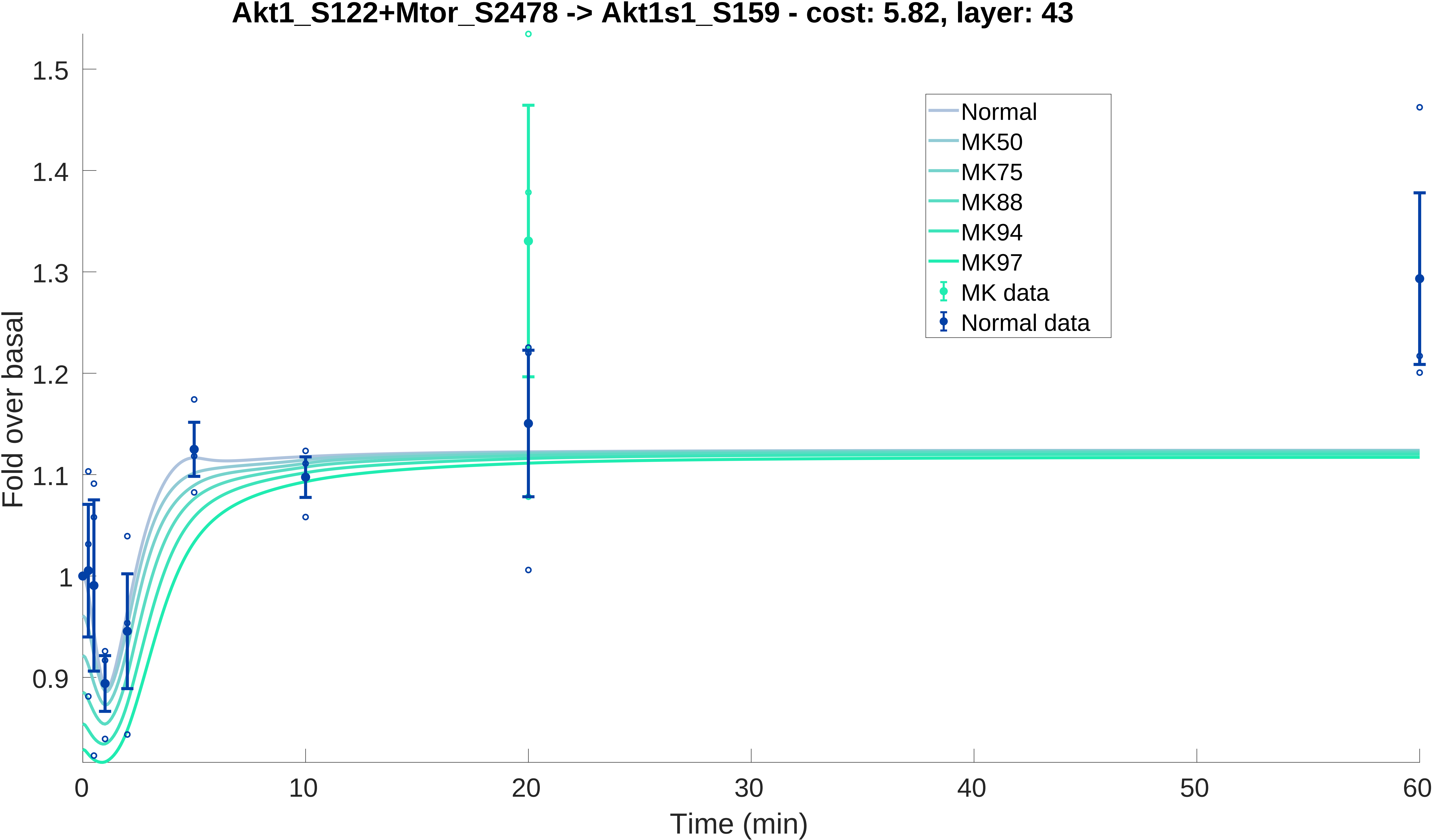
Akt1_S122+Mtor_S2478 -> Akt1s1_S159 - cost: 5.82, layer: 43
Fold over basal
1.5
1.4
1.3
1.2
1.1
1
0.9
0
10
20
30
40
50
60
Time (min)
Normal
MK50
MK75
MK88
MK94
MK97
MK data
Normal data

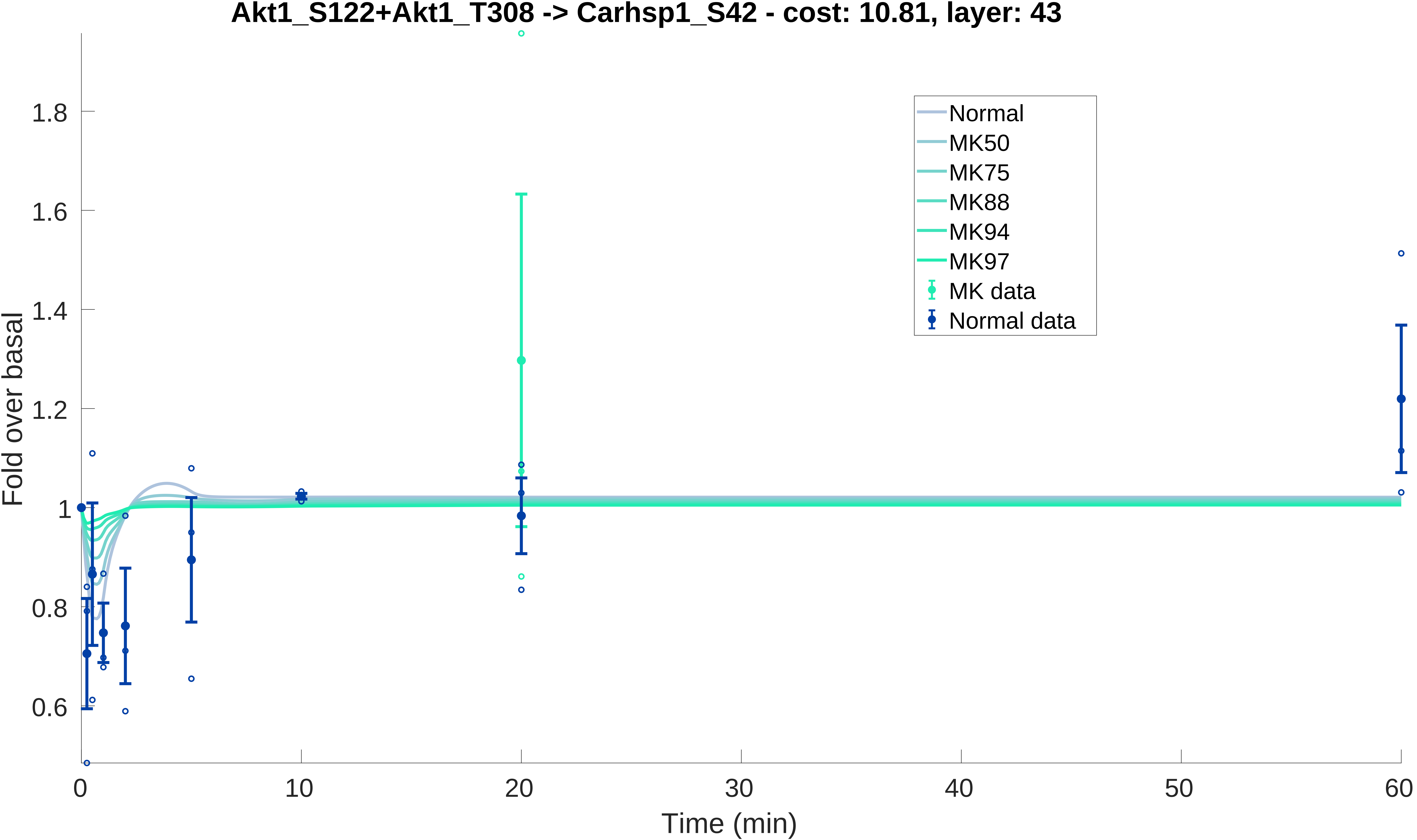

Akt1_S122+Akt1_T308 -> Carhsp1_S42 - cost: 10.81, layer: 43
Normal
MK50
MK75
MK88
MK94
MK97
MK data
Normal data
Fold over basal
Time (min)
0.6
0.8
1
1.2
1.4
1.6
1.8
0
10
20
30
40
50
60

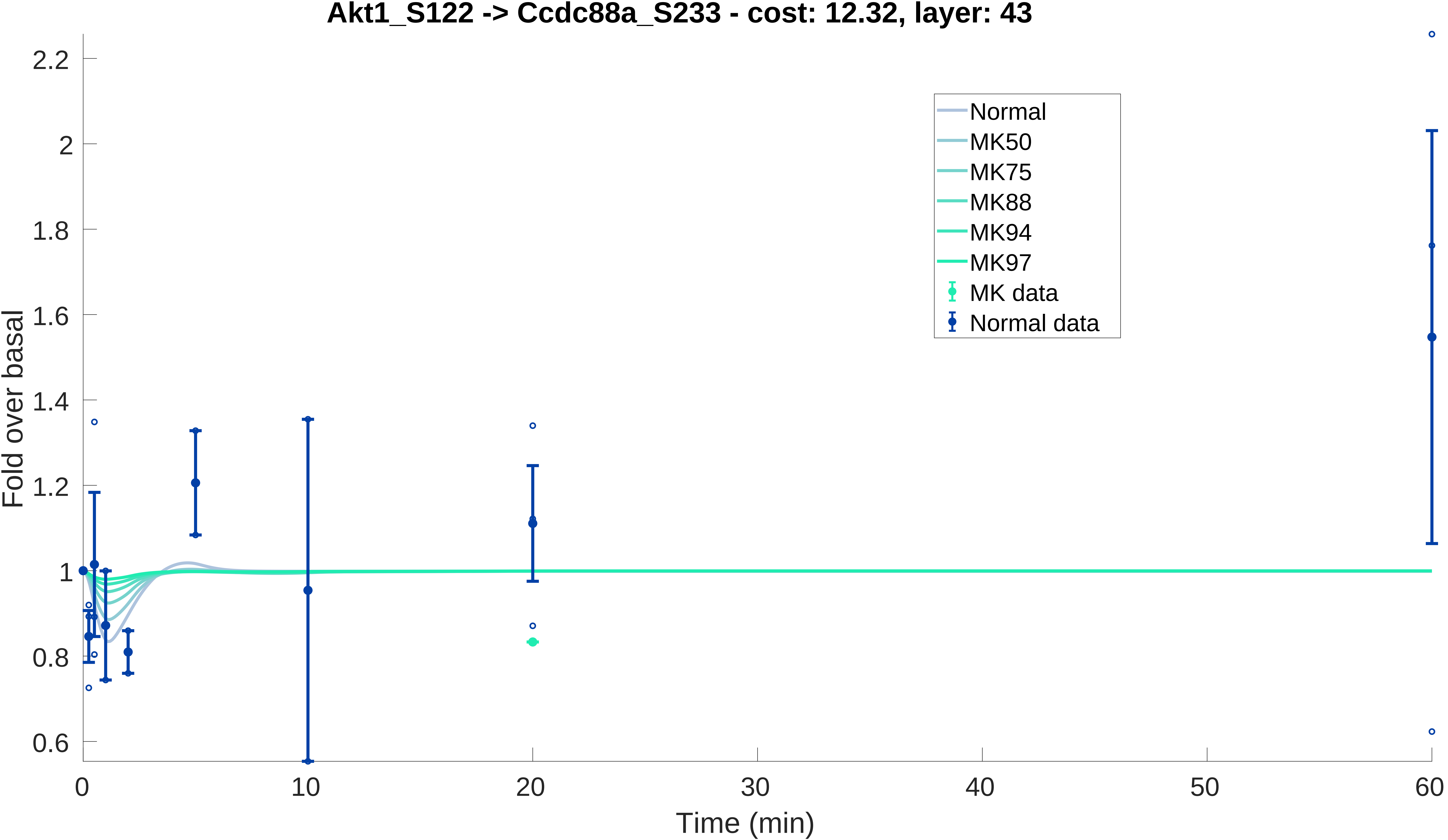
Akt1_S122 -> Ccdc88a_S233 - cost: 12.32, layer: 43
Fold over basal
Time (min)
0.6
0.8
1
1.2
1.4
1.6
1.8
2
2.2
0
10
20
30
40
50
60
Normal
MK50
MK75
MK88
MK94
MK97
MK data
Normal data

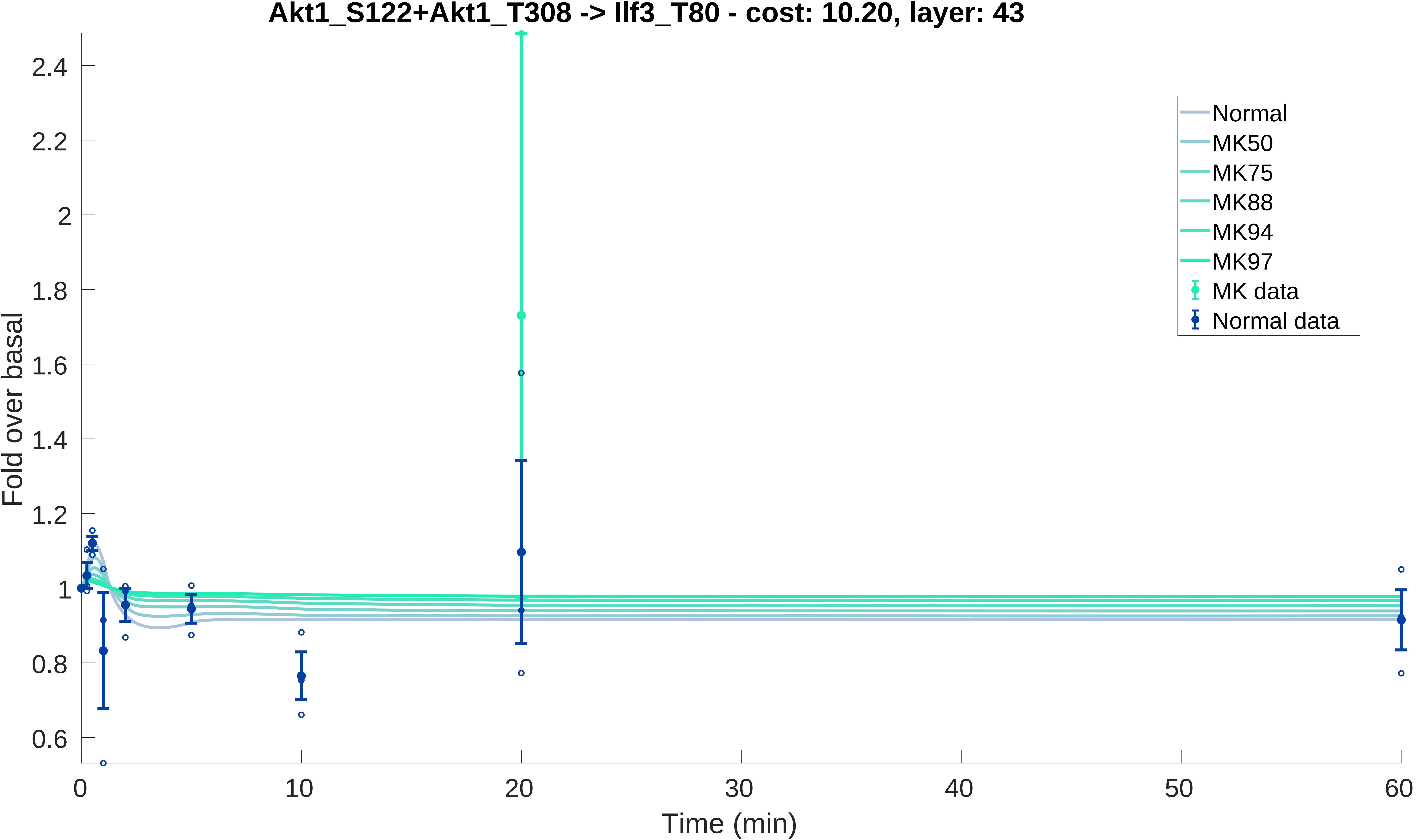

Akt1_S122+Akt1_T308 -> Ilf3_T80 - cost: 10.20, layer: 43
Fold over basal
Time (min)
2.4
2.2
2
1.8
1.6
1.4
1.2
1
0.8
0.6
0
10
20
30
40
50
60
Normal
MK50
MK75
MK88
MK94
MK97
MK data
Normal data

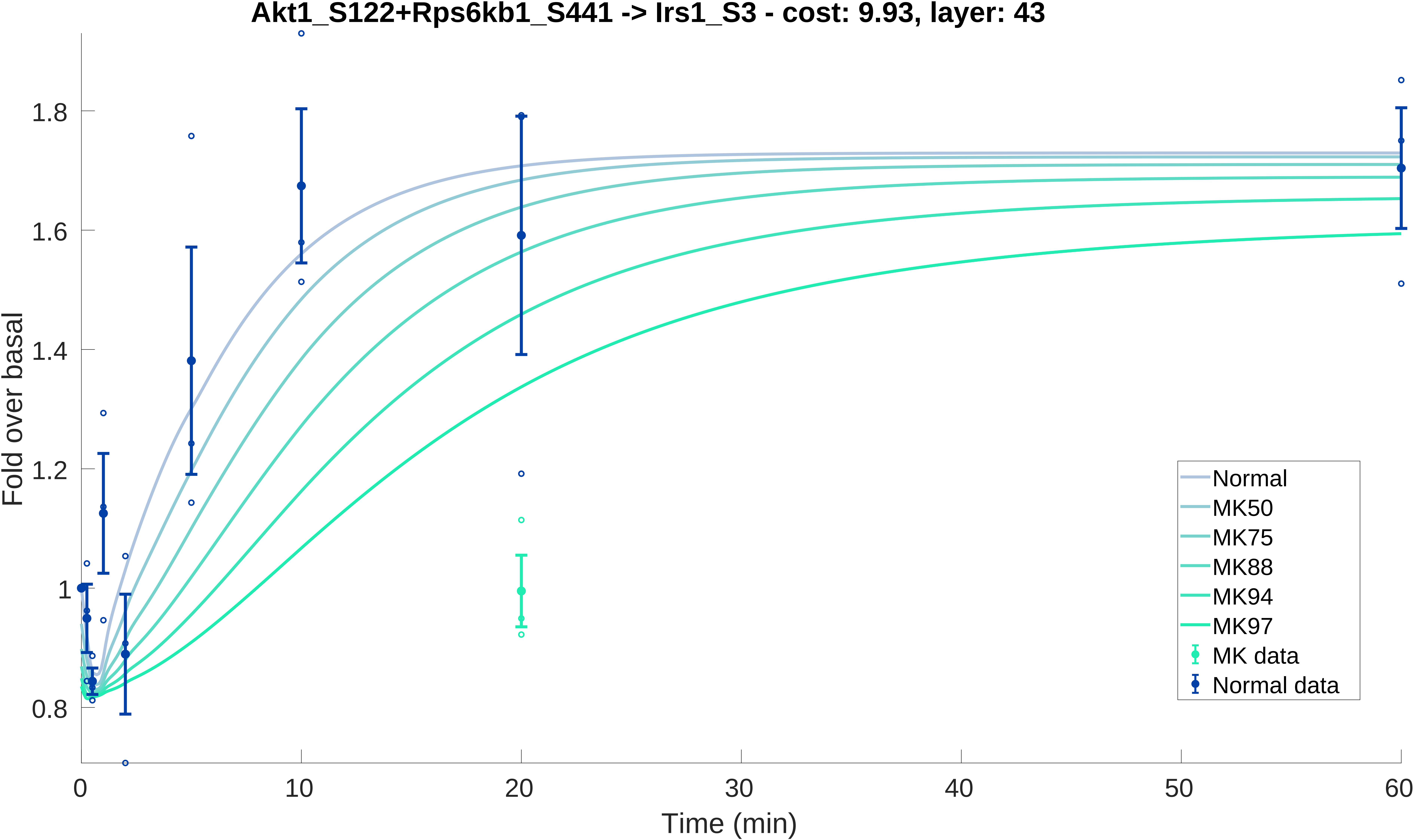

Akt1_S122+Rps6kb1_S441 -> Irs1_S3 - cost: 9.93, layer: 43
Fold over basal
Time (min)
0.8
1
1.2
1.4
1.6
1.8
0
10
20
30
40
50
60
Normal
MK50
MK75
MK88
MK94
MK97
MK data
Normal data

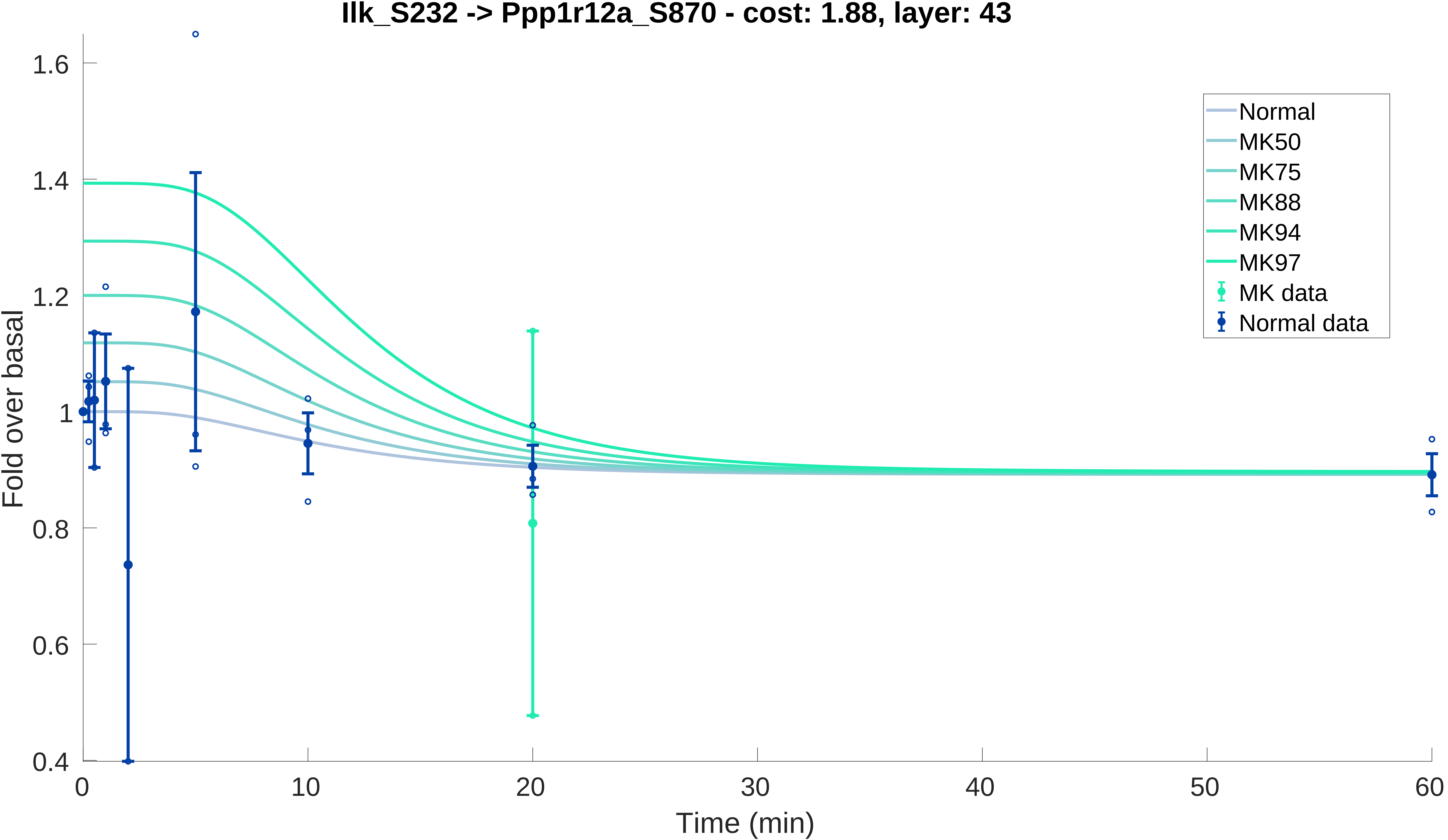

Ilk_S232 -> Ppp1r12a_S870 - cost: 1.88, layer: 43
Fold over basal
Time (min)
0.4
0.6
0.8
1
1.2
1.4
1.6
0
10
20
30
40
50
60
Normal
MK50
MK75
MK88
MK94
MK97
MK data
Normal data

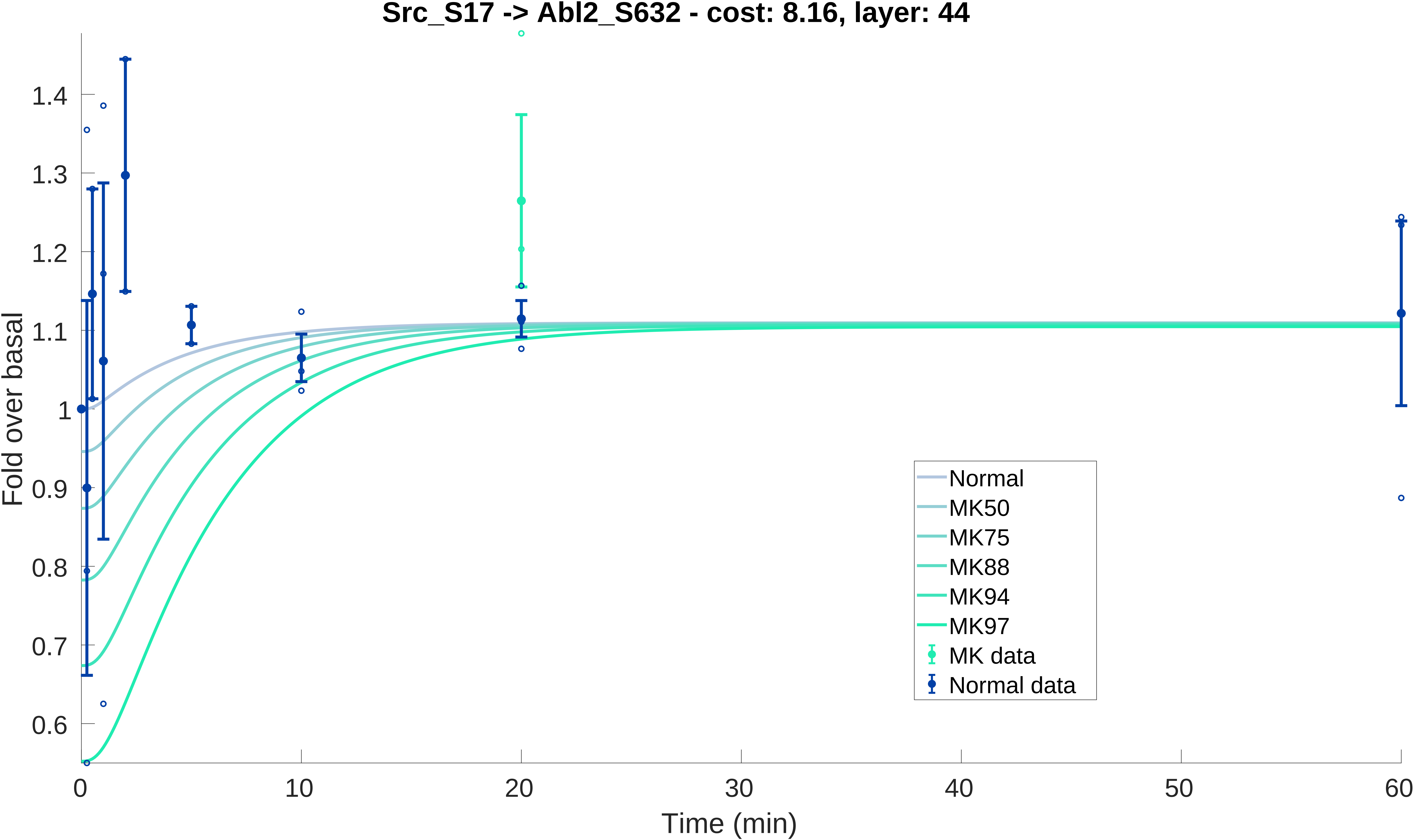

Src_S17 -> Abl2_S632 - cost: 8.16, layer: 44
Fold over basal
Time (min)
0.6
0.7
0.8
0.9
1
1.1
1.2
1.3
1.4
0
10
20
30
40
50
60
Normal
MK50
MK75
MK88
MK94
MK97
MK data
Normal data

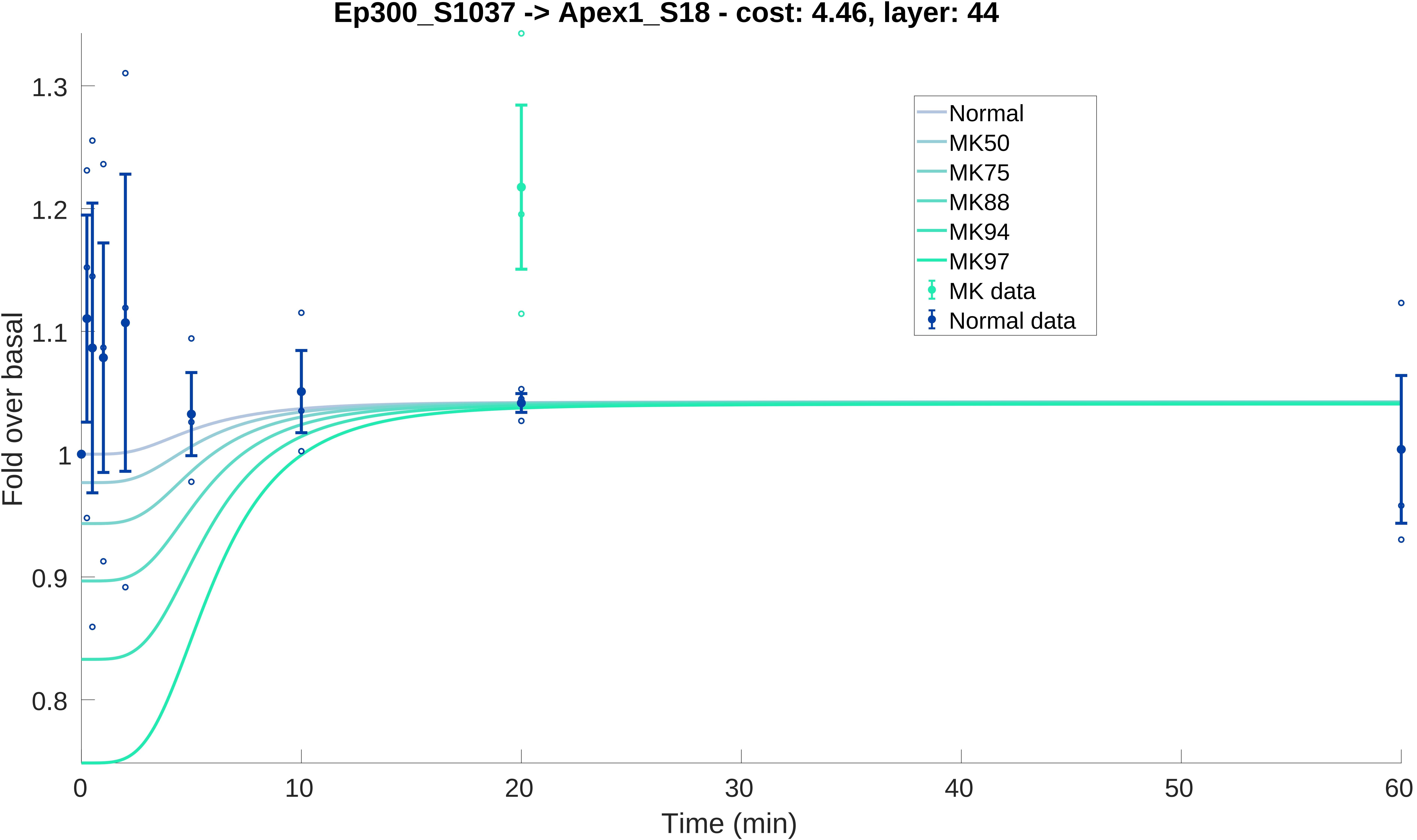

Ep300_S1037 -> Apex1_S18 - cost: 4.46, layer: 44
Fold over basal
Time (min)
1.3
1.2
1.1
1
0.9
0.8
0
10
20
30
40
50
60
Normal
MK50
MK75
MK88
MK94
MK97
MK data
Normal data

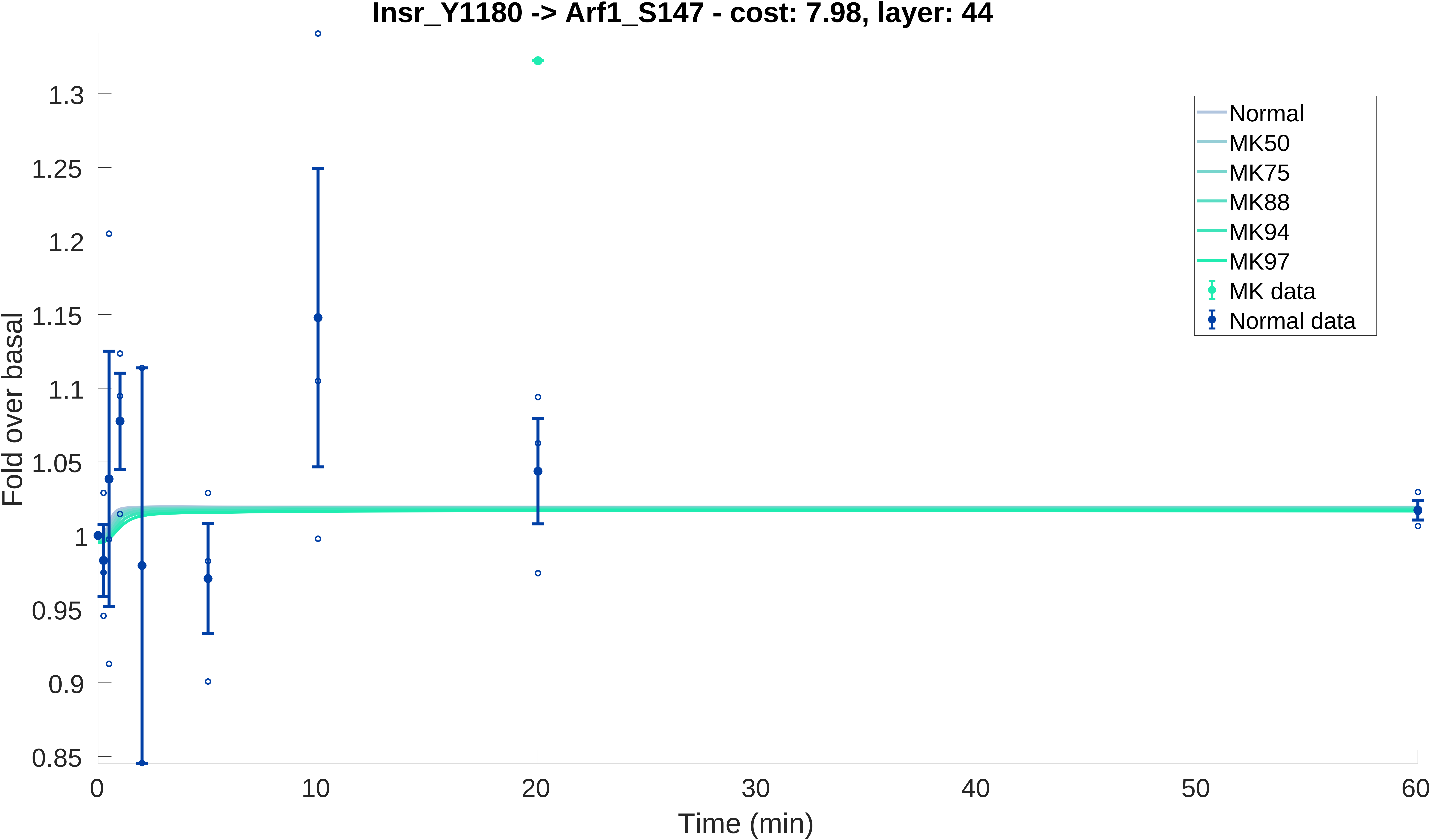
Insr_Y1180 -> Arf1_S147 - cost: 7.98, layer: 44
Fold over basal
Time (min)
Normal
MK50
MK75
MK88
MK94
MK97
MK data
Normal data
0
10
20
30
40
50
60
0.85
0.9
0.95
1
1.05
1.1
1.15
1.2
1.25
1.3

Insr_Y1180 -> Arhgap5_S1125 - cost: 11.37, layer: 44 (pool)

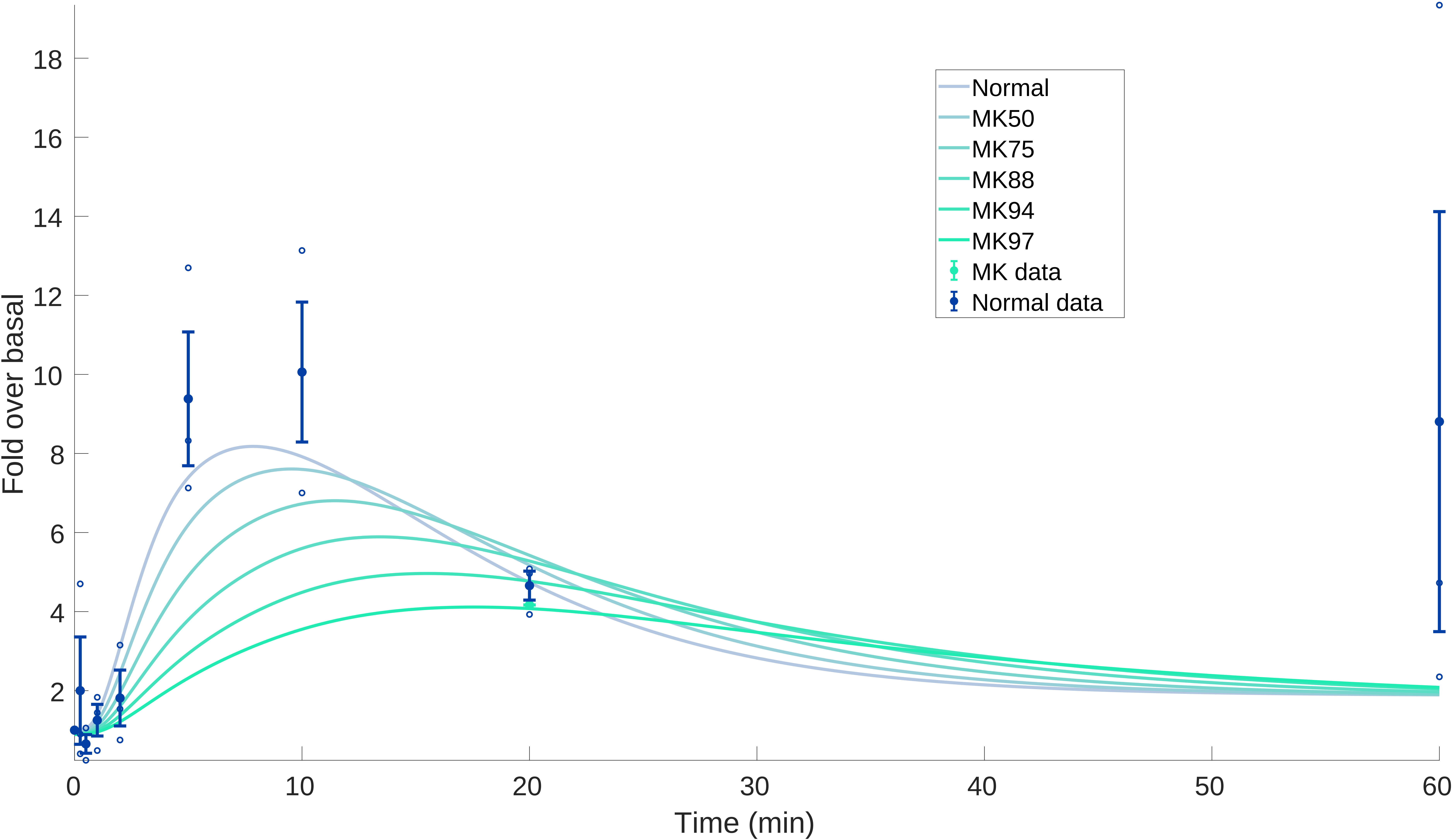

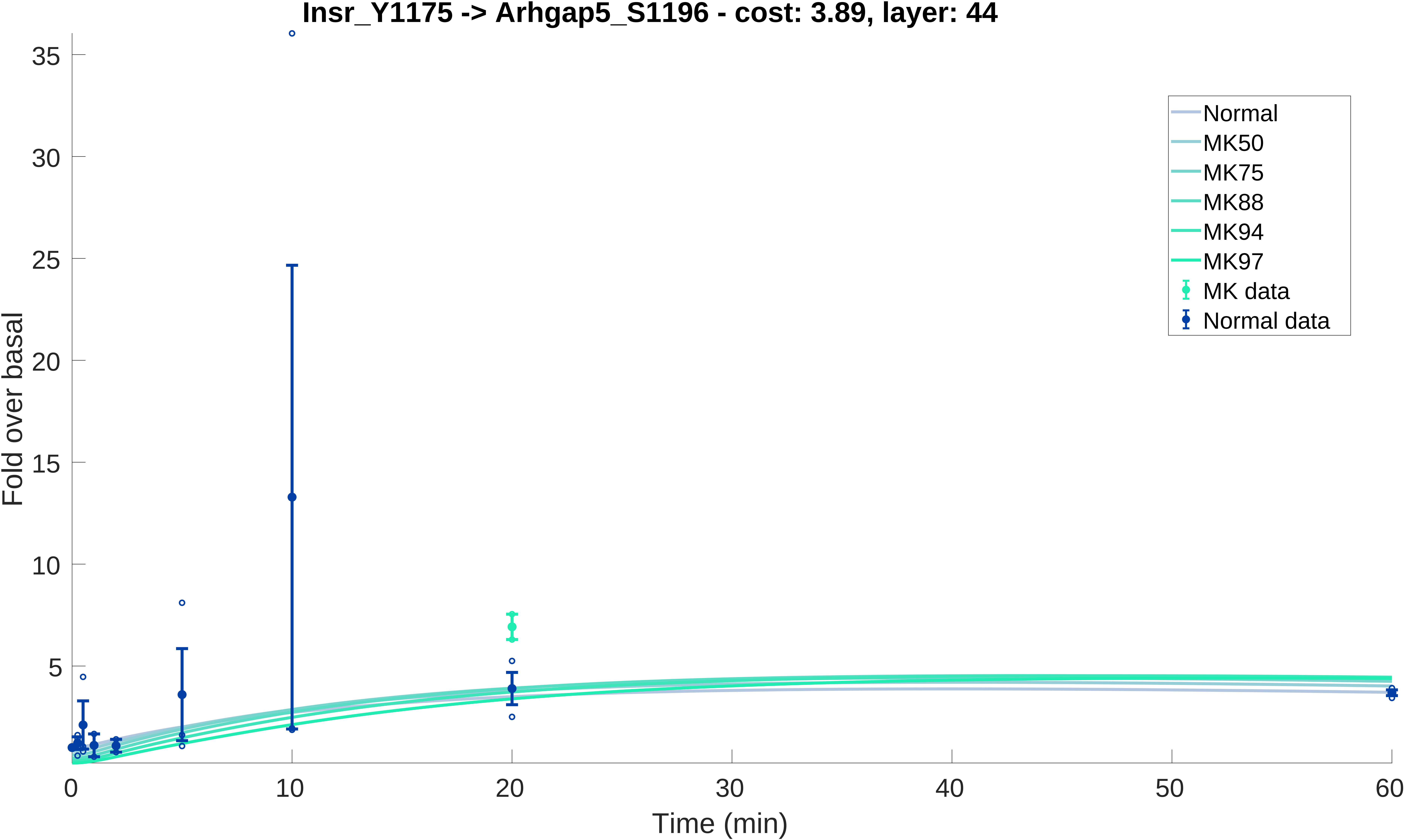

Insr_Y1175 -> Arhgap5_S1196 - cost: 3.89, layer: 44
Fold over basal
Time (min)
0
10
20
30
40
50
60
5
10
15
20
25
30
35
Normal
MK50
MK75
MK88
MK94
MK97
MK data
Normal data

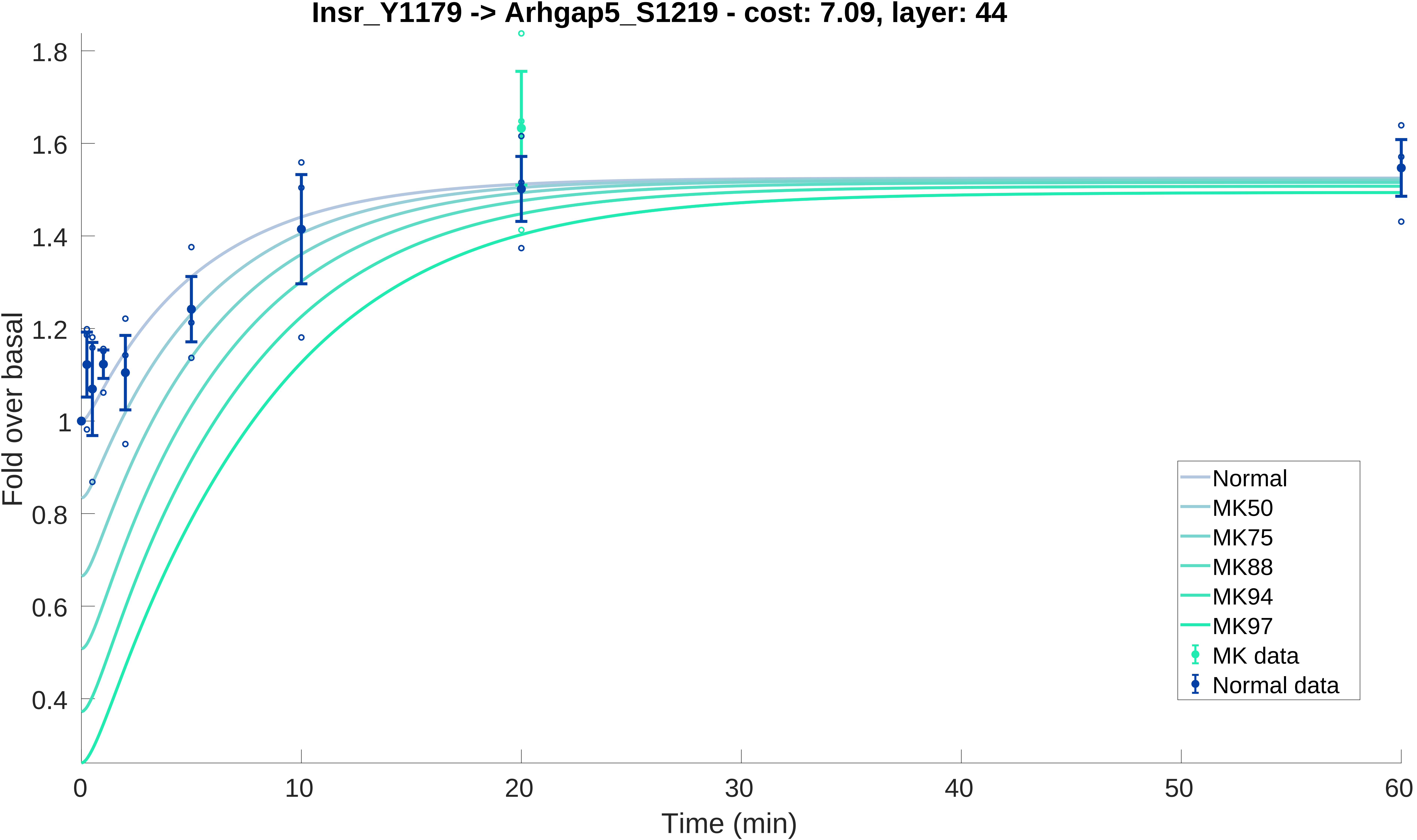

Insr_Y1179 -> Arhgap5_S1219 - cost: 7.09, layer: 44
Fold over basal
1.8
1.6
1.4
1.2
1
0.8
0.6
0.4
0
10
20
30
40
50
60
Time (min)
Normal
MK50
MK75
MK88
MK94
MK97
MK data
Normal data

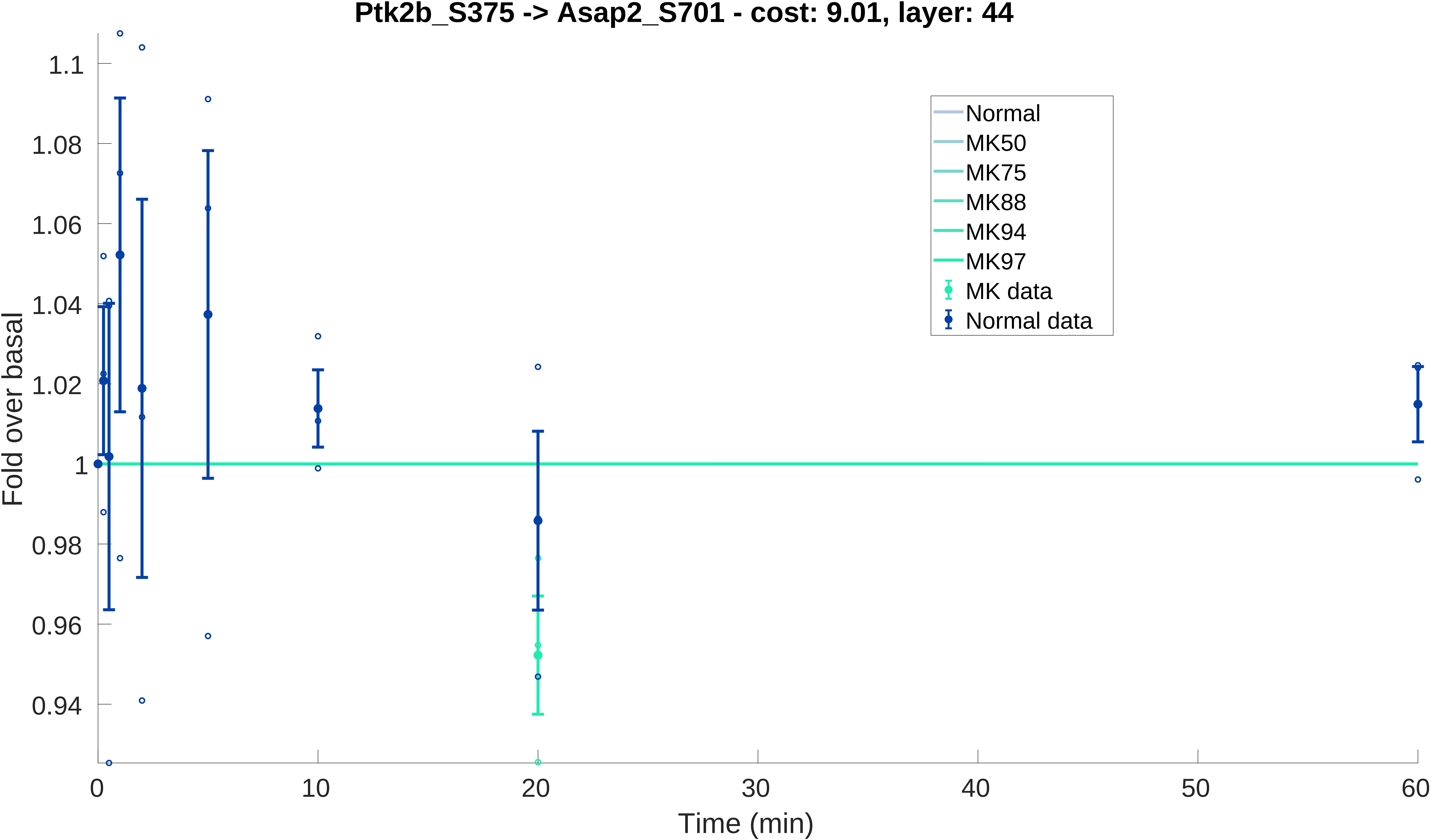

Ptk2b_S375 -> Asap2_S701 - cost: 9.01, layer: 44
Fold over basal
1.1
1.08
1.06
1.04
1.02
1
0.98
0.96
0.94
0
10
20
30
40
50
60
Time (min)
Normal
MK50
MK75
MK88
MK94
MK97
MK data
Normal data

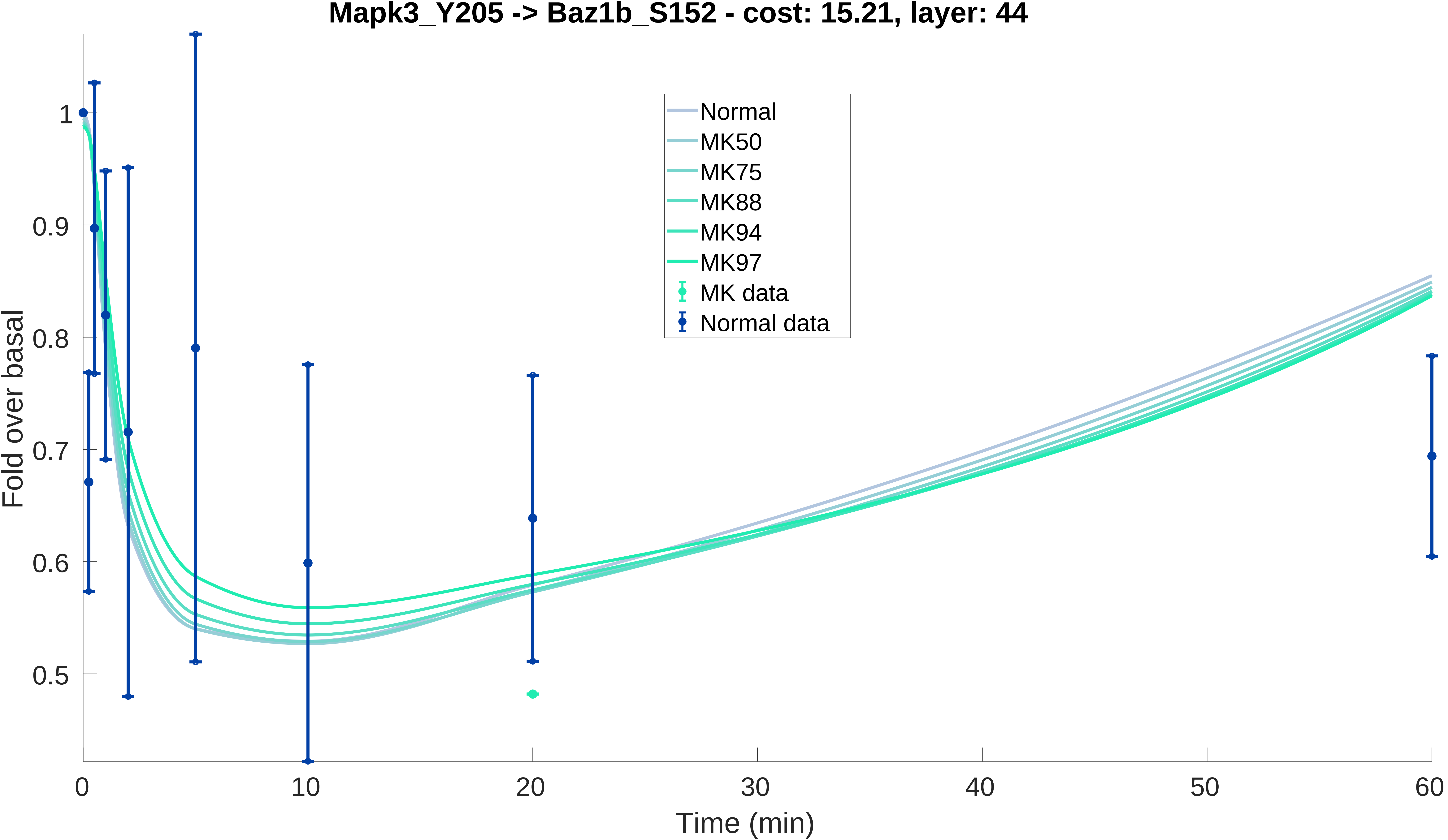
Mapk3_Y205 -> Baz1b_S152 - cost: 15.21, layer: 44
Fold over basal
1
0.9
0.8
0.7
0.6
0.5
0
10
20
30
40
50
60
Time (min)
Normal
MK50
MK75
MK88
MK94
MK97
MK data
Normal data

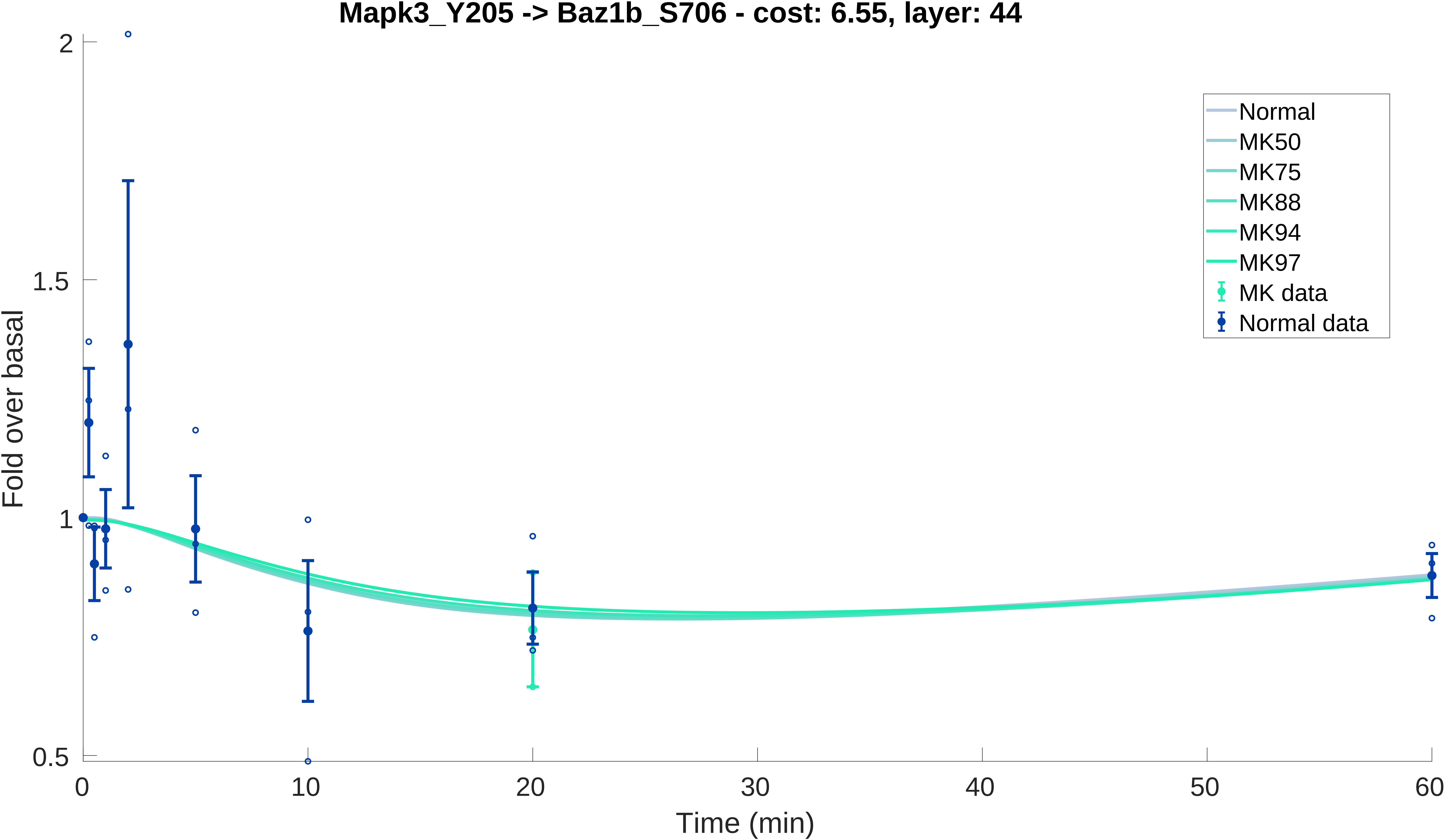

Mapk3_Y205 -> Baz1b_S706 - cost: 6.55, layer: 44
Fold over basal
Time (min)
2
1.5
1
0.5
0
10
20
30
40
50
60
Normal
MK50
MK75
MK88
MK94
MK97
MK data
Normal data

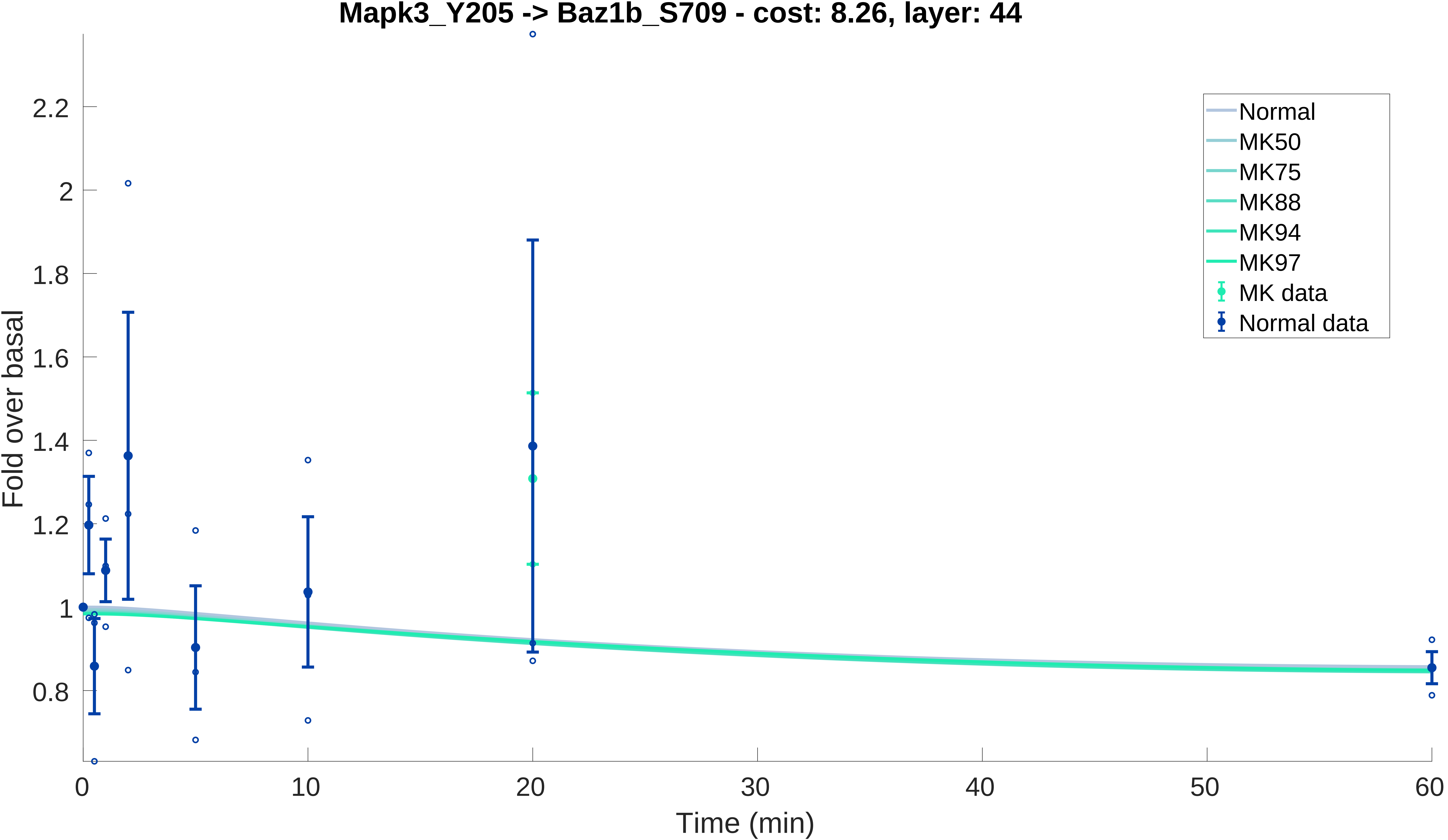
Mapk3_Y205 -> Baz1b_S709 - cost: 8.26, layer: 44
Fold over basal
Time (min)
0.8
1
1.2
1.4
1.6
1.8
2
2.2
0
10
20
30
40
50
60
Normal
MK50
MK75
MK88
MK94
MK97
MK data
Normal data

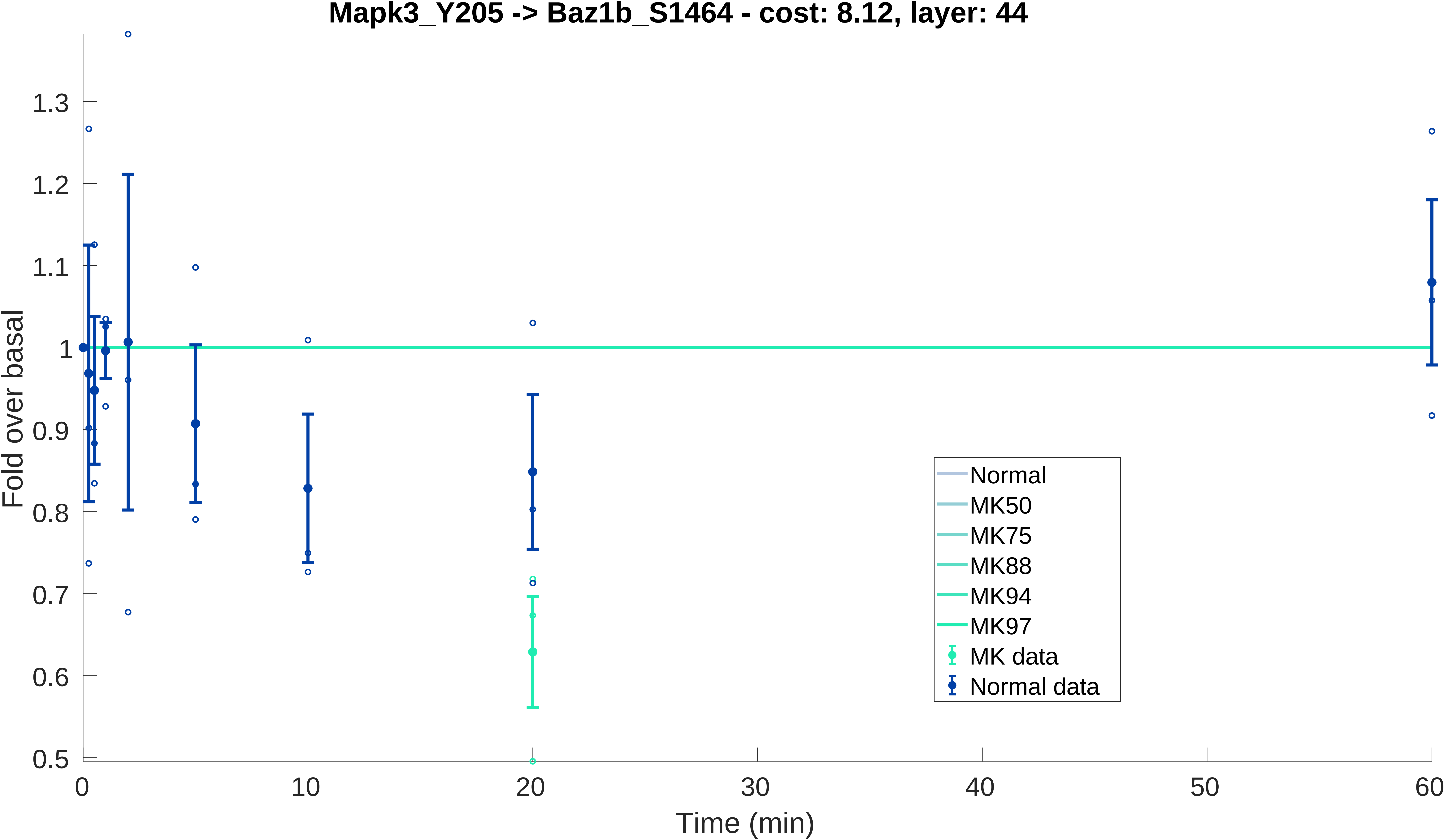

Mapk3_Y205 -> Baz1b_S1464 - cost: 8.12, layer: 44
Fold over basal
Time (min)
1.3
1.2
1.1
1
0.9
0.8
0.7
0.6
0.5
0
10
20
30
40
50
60
Normal
MK50
MK75
MK88
MK94
MK97
MK data
Normal data

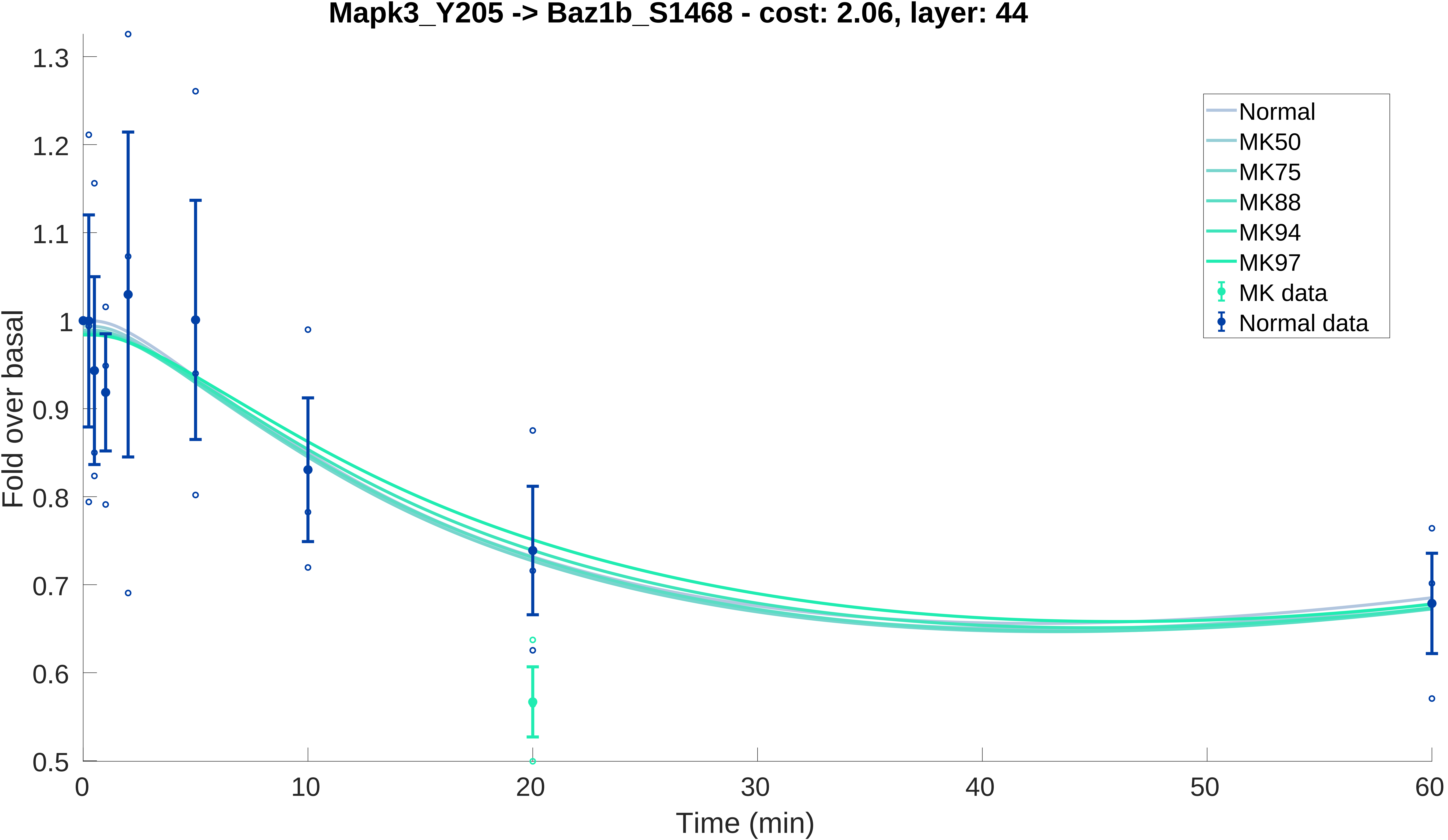

Mapk3_Y205 -> Baz1b_S1468 - cost: 2.06, layer: 44
Fold over basal
Time (min)
Normal
MK50
MK75
MK88
MK94
MK97
MK data
Normal data
1.3
1.2
1.1
1
0.9
0.8
0.7
0.6
0.5
0
10
20
30
40
50
60

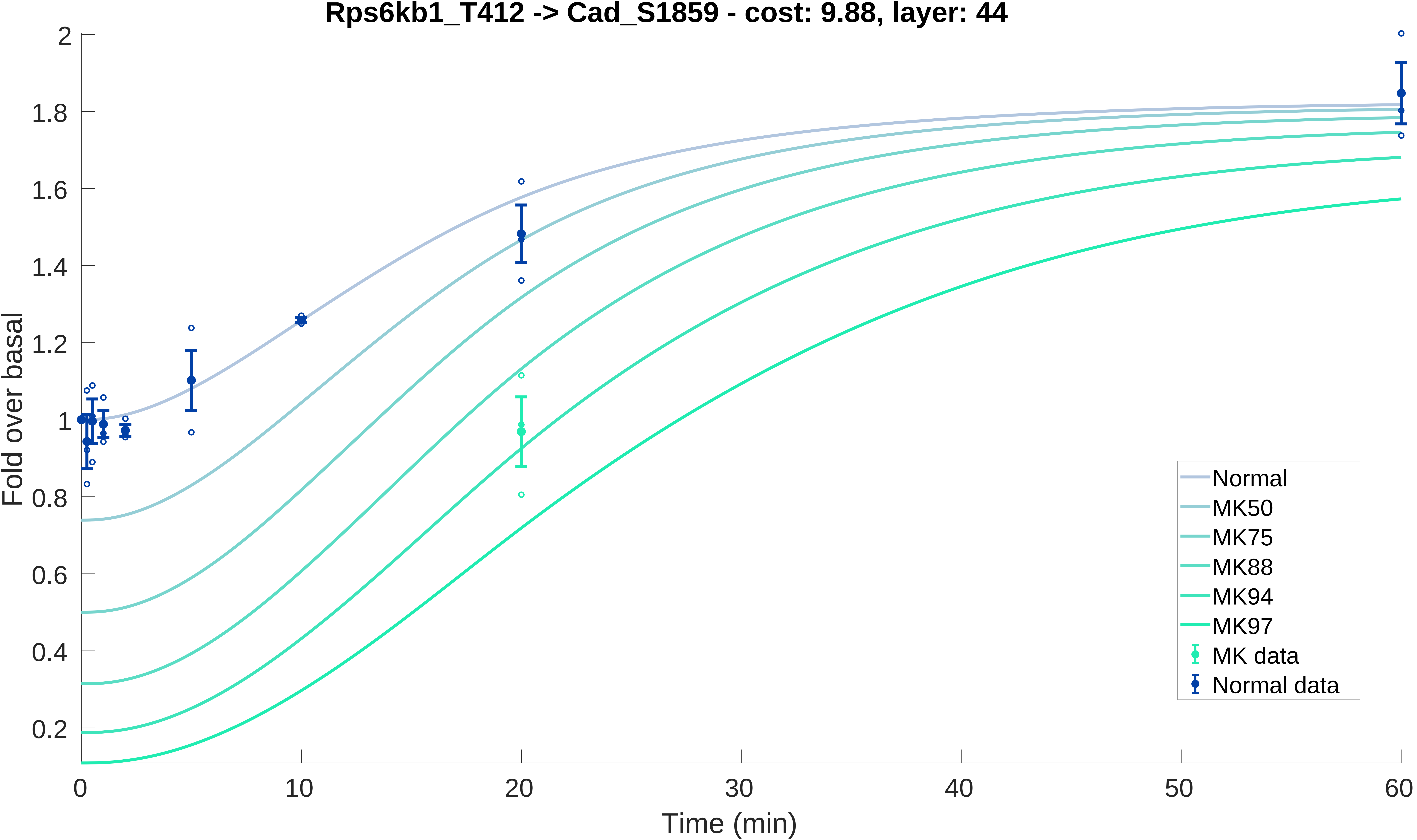

Rps6kb1_T412 -> Cad_S1859 - cost: 9.88, layer: 44
Fold over basal
Time (min)
2
1.8
1.6
1.4
1.2
1
0.8
0.6
0.4
0.2
0
10
20
30
40
50
60
Normal
MK50
MK75
MK88
MK94
MK97
MK data
Normal data

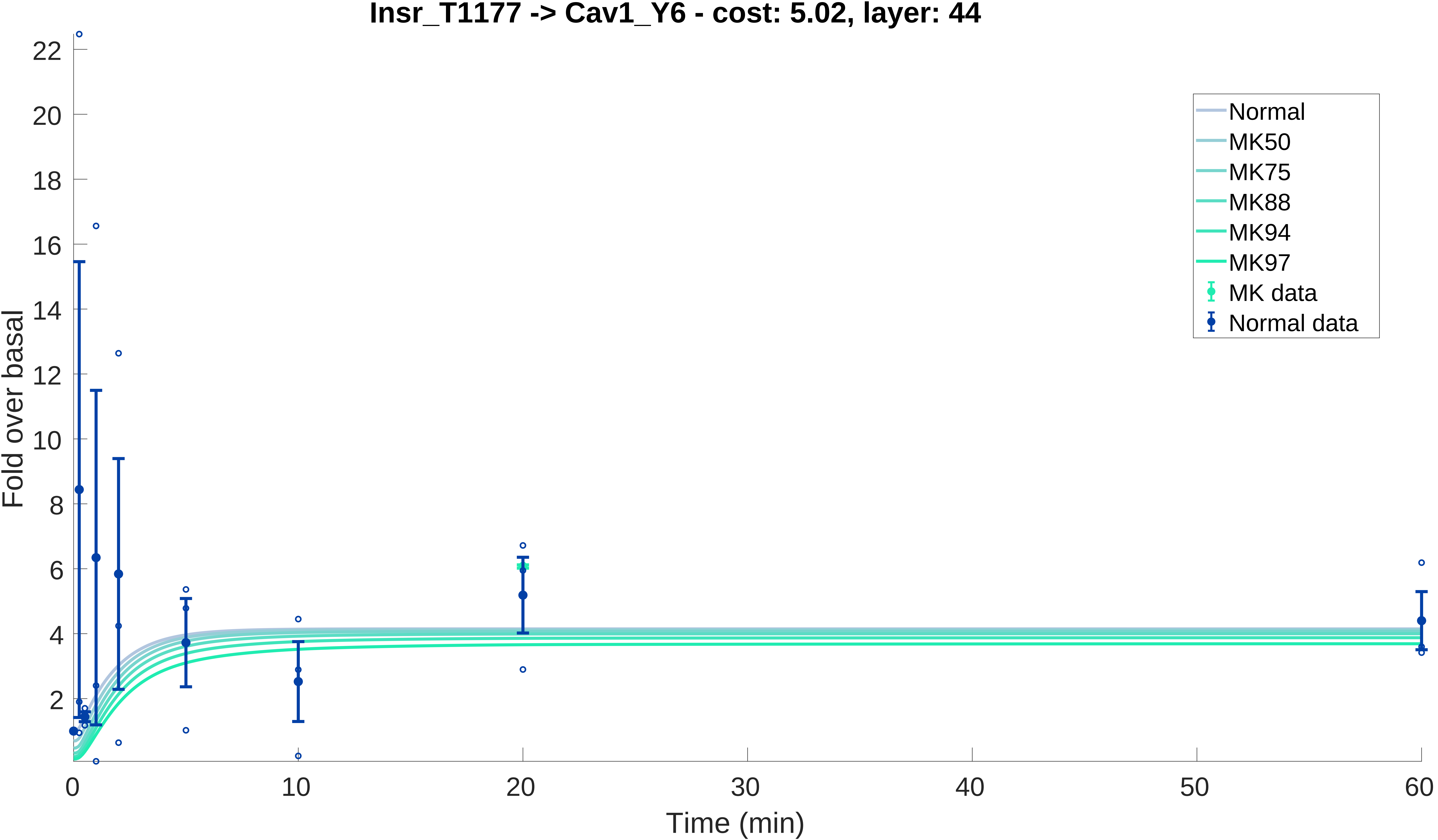

Insr_T1177 -> Cav1_Y6 - cost: 5.02, layer: 44
Fold over basal
Time (min)
0
10
20
30
40
50
60
2
4
6
8
10
12
14
16
18
20
22
Normal
MK50
MK75
MK88
MK94
MK97
MK data
Normal data

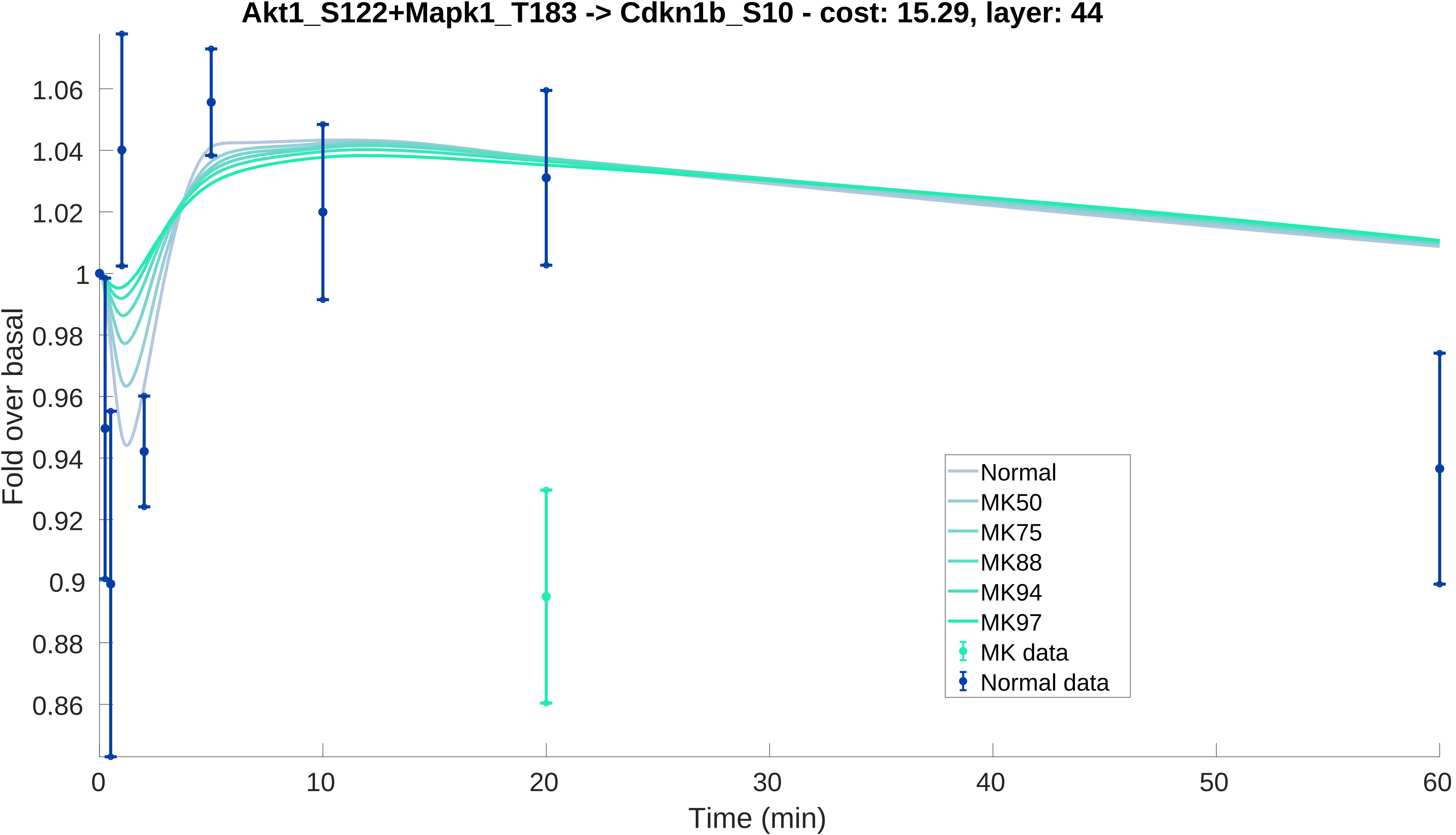

Akt1_S122+Mapk1_T183 -> Cdkn1b_S10 - cost: 15.29, layer: 44
Fold over basal
1.06
1.04
1.02
1
0.98
0.96
0.94
0.92
0.9
0.88
0.86
0
10
20
30
40
50
60
Time (min)
Normal
MK50
MK75
MK88
MK94
MK97
MK data
Normal data

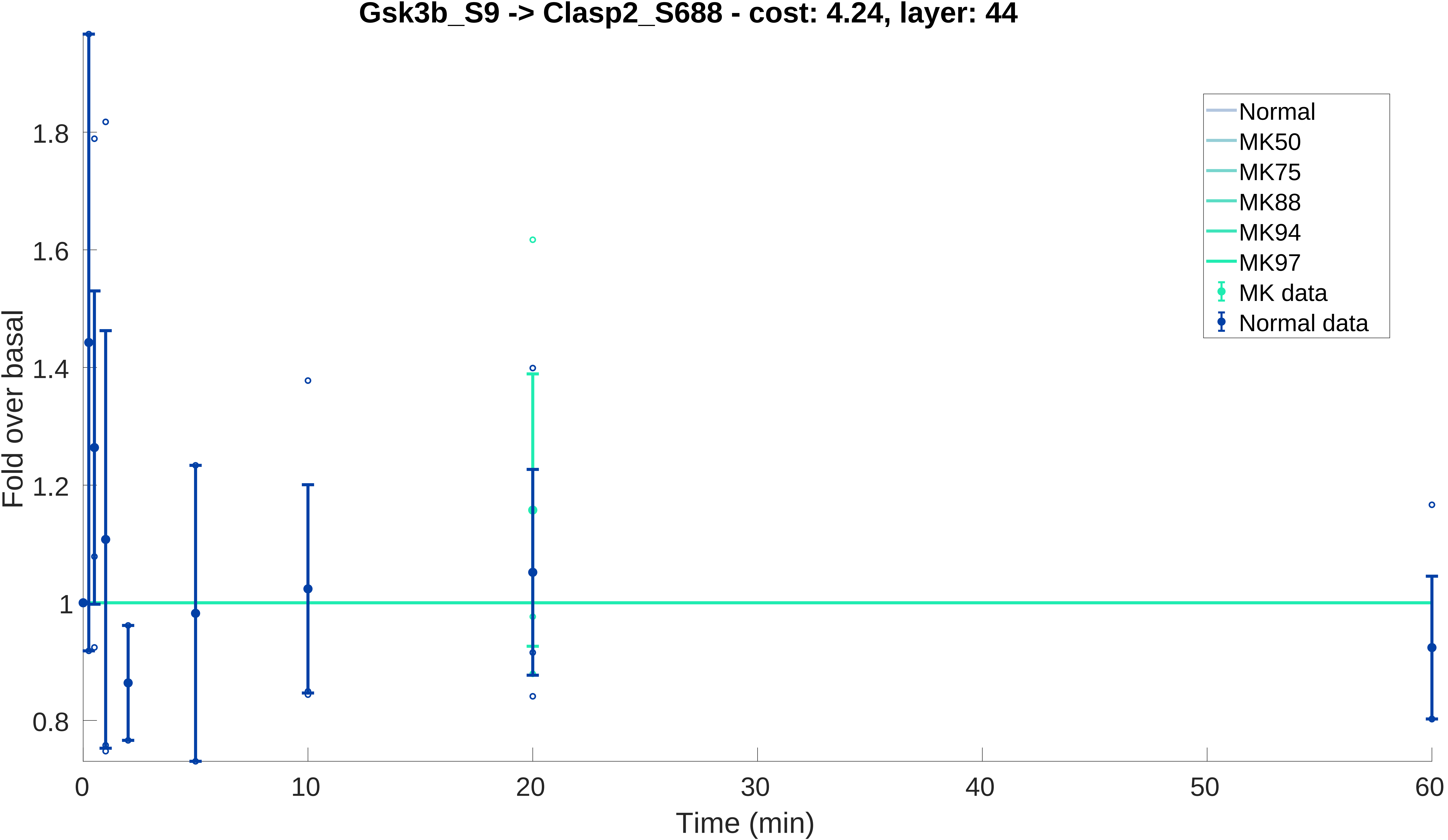
Gsk3b_S9 -> Clasp2_S688 - cost: 4.24, layer: 44
Fold over basal
Time (min)
0.8
1
1.2
1.4
1.6
1.8
0
10
20
30
40
50
60
Normal
MK50
MK75
MK88
MK94
MK97
MK data
Normal data

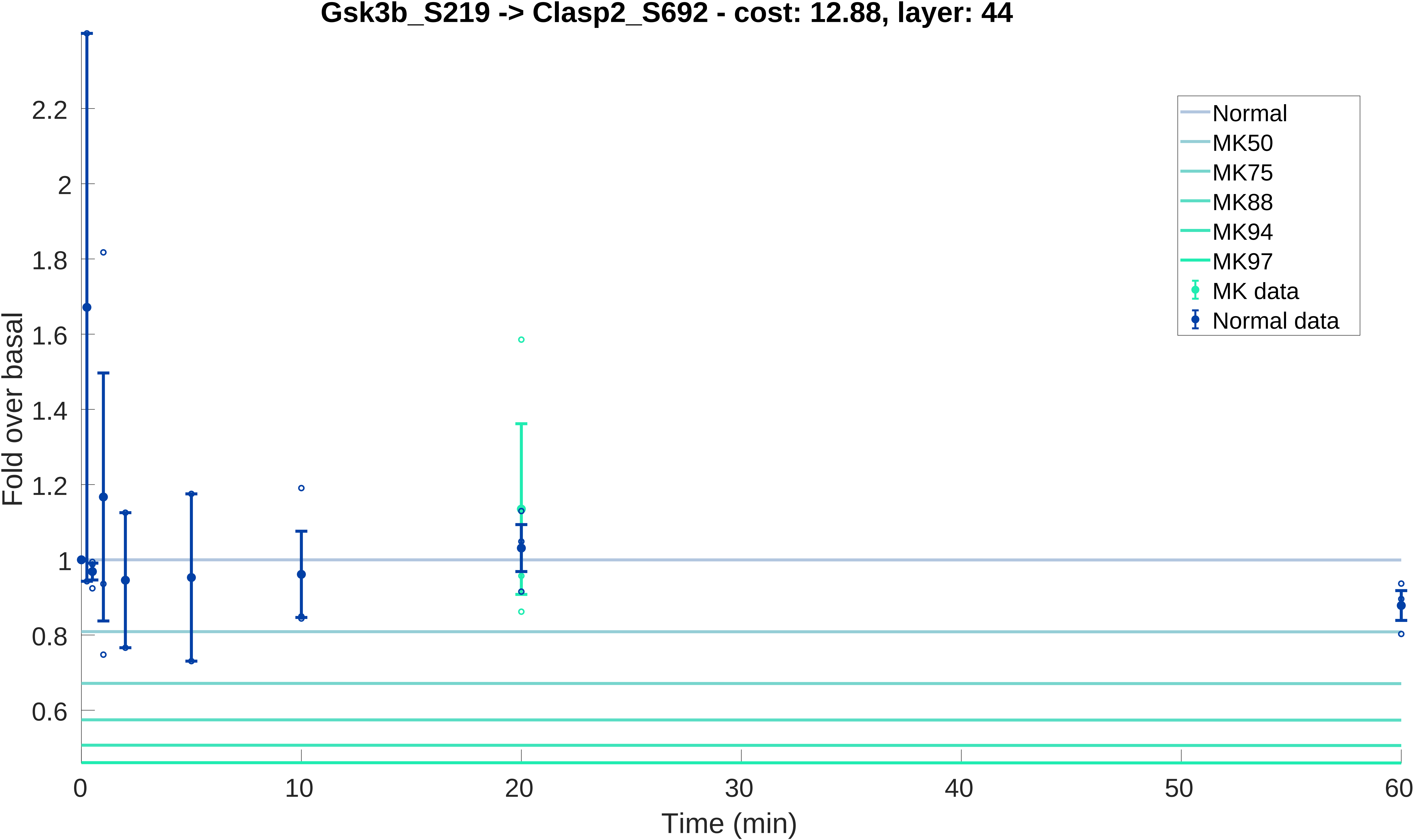
Gsk3b_S219 -> Clasp2_S692 - cost: 12.88, layer: 44
Fold over basal
Time (min)
0
10
20
30
40
50
60
0.6
0.8
1
1.2
1.4
1.6
1.8
2
2.2
Normal
MK50
MK75
MK88
MK94
MK97
MK data
Normal data

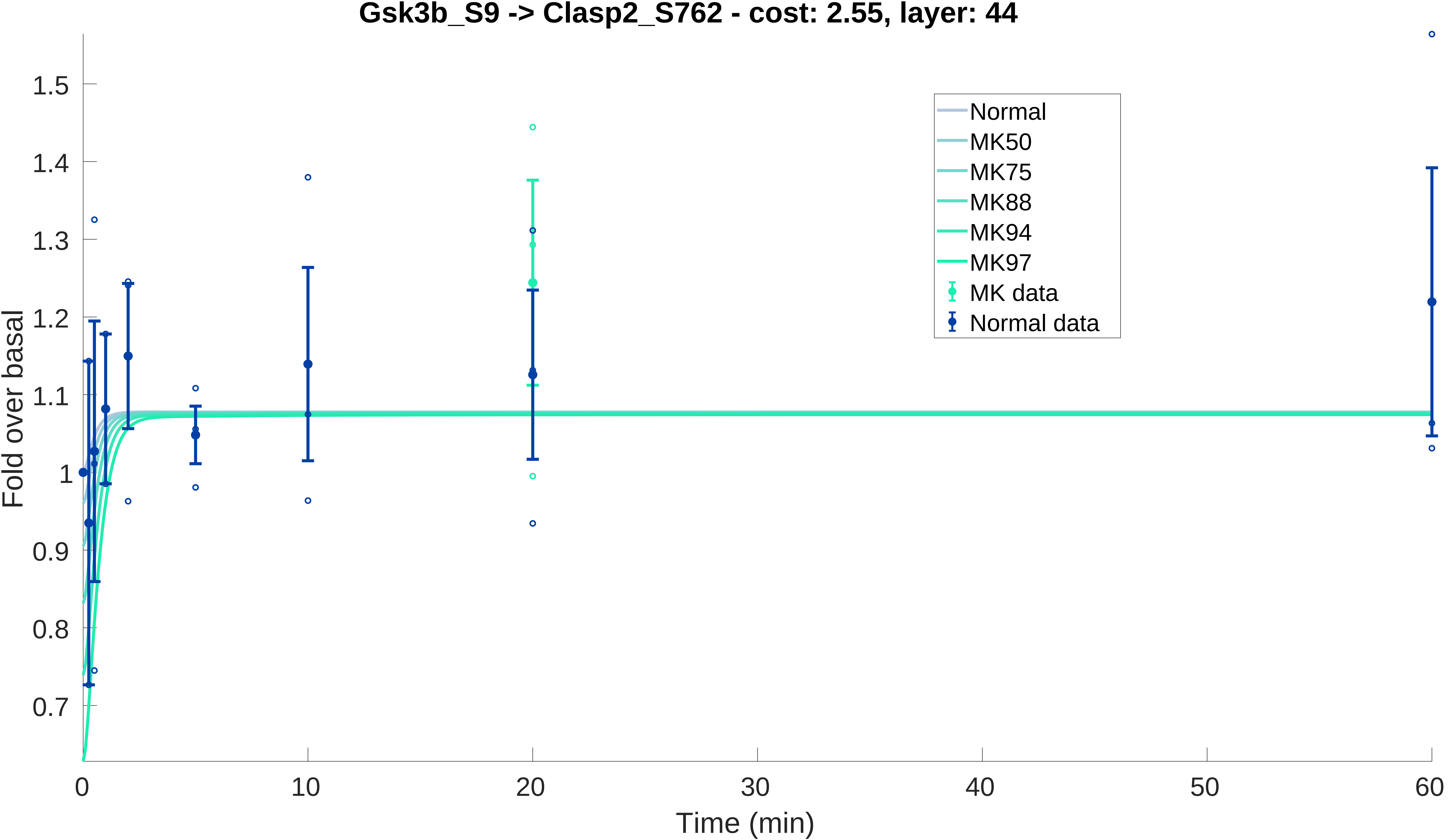
Gsk3b_S9 -> Clasp2_S762 - cost: 2.55, layer: 44
Fold over basal
Time (min)
0
10
20
30
40
50
60
0.7
0.8
0.9
1
1.1
1.2
1.3
1.4
1.5
Normal
MK50
MK75
MK88
MK94
MK97
MK data
Normal data

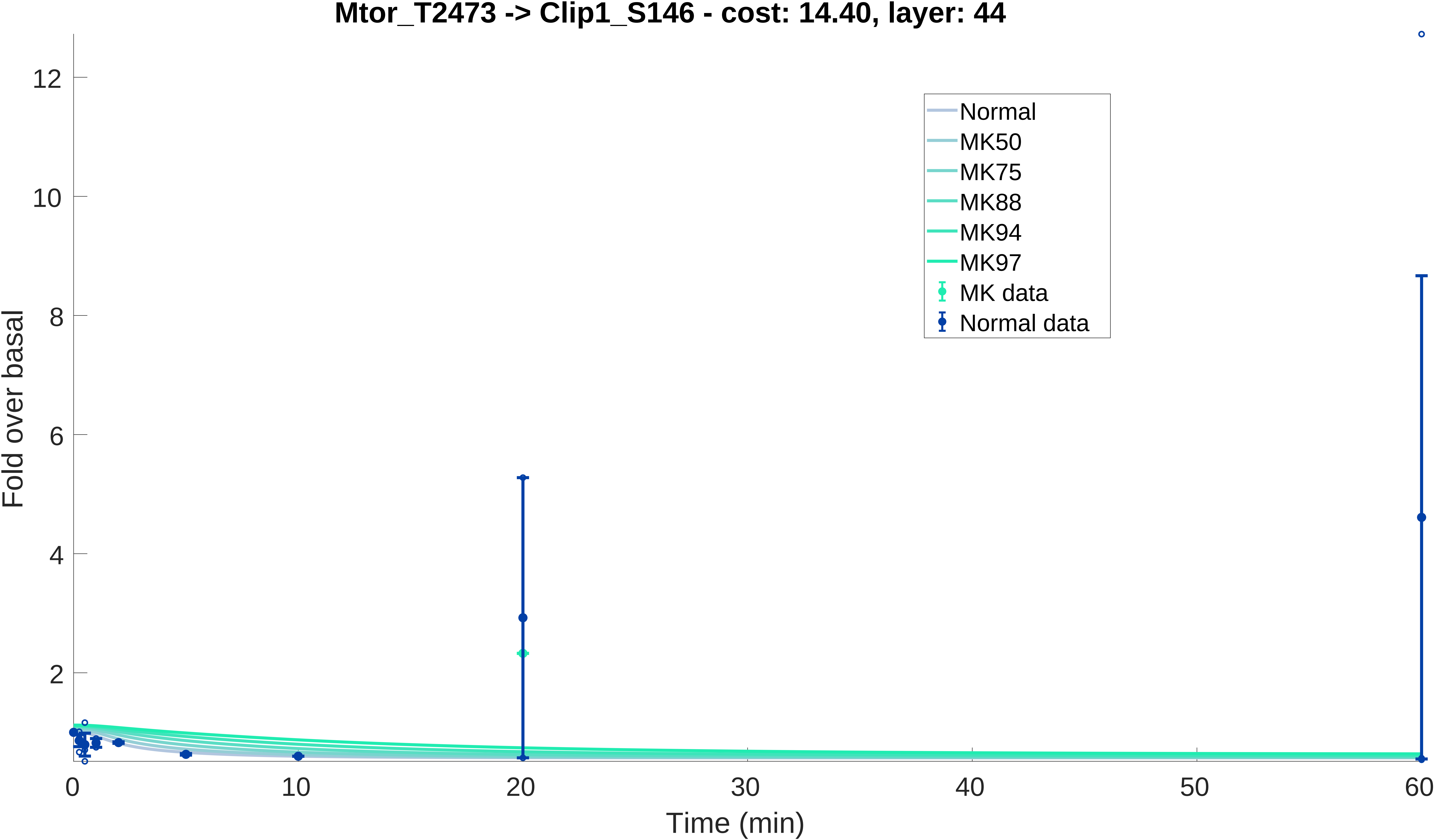

Mtor_T2473 -> Clip1_S146 - cost: 14.40, layer: 44
Fold over basal
Time (min)
Normal
MK50
MK75
MK88
MK94
MK97
MK data
Normal data
0
10
20
30
40
50
60
2
4
6
8
10
12

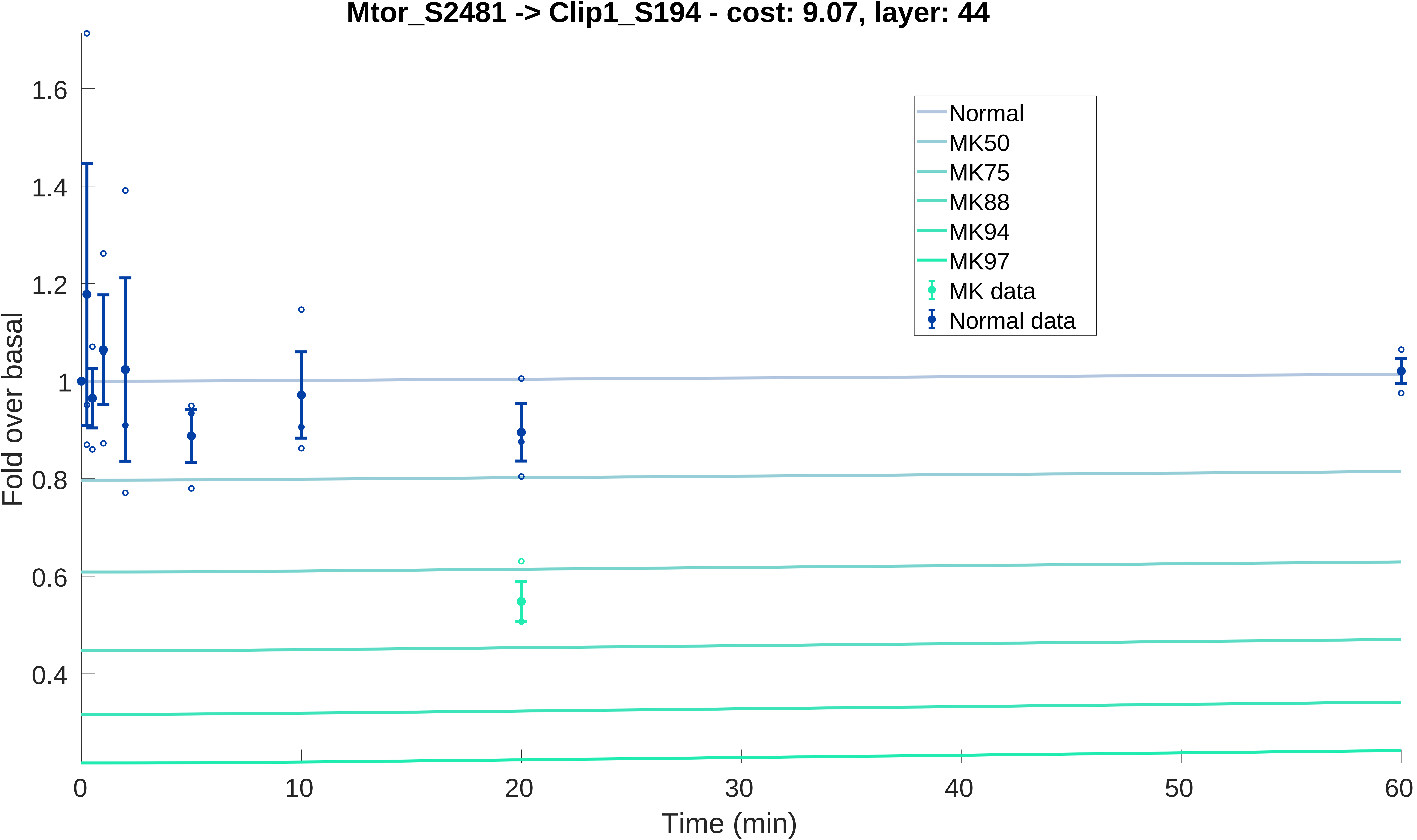
Mtor_S2481 -> Clip1_S194 - cost: 9.07, layer: 44
Fold over basal
Time (min)
1.6
1.4
1.2
1
0.8
0.6
0.4
0
10
20
30
40
50
60
Normal
MK50
MK75
MK88
MK94
MK97
MK data
Normal data

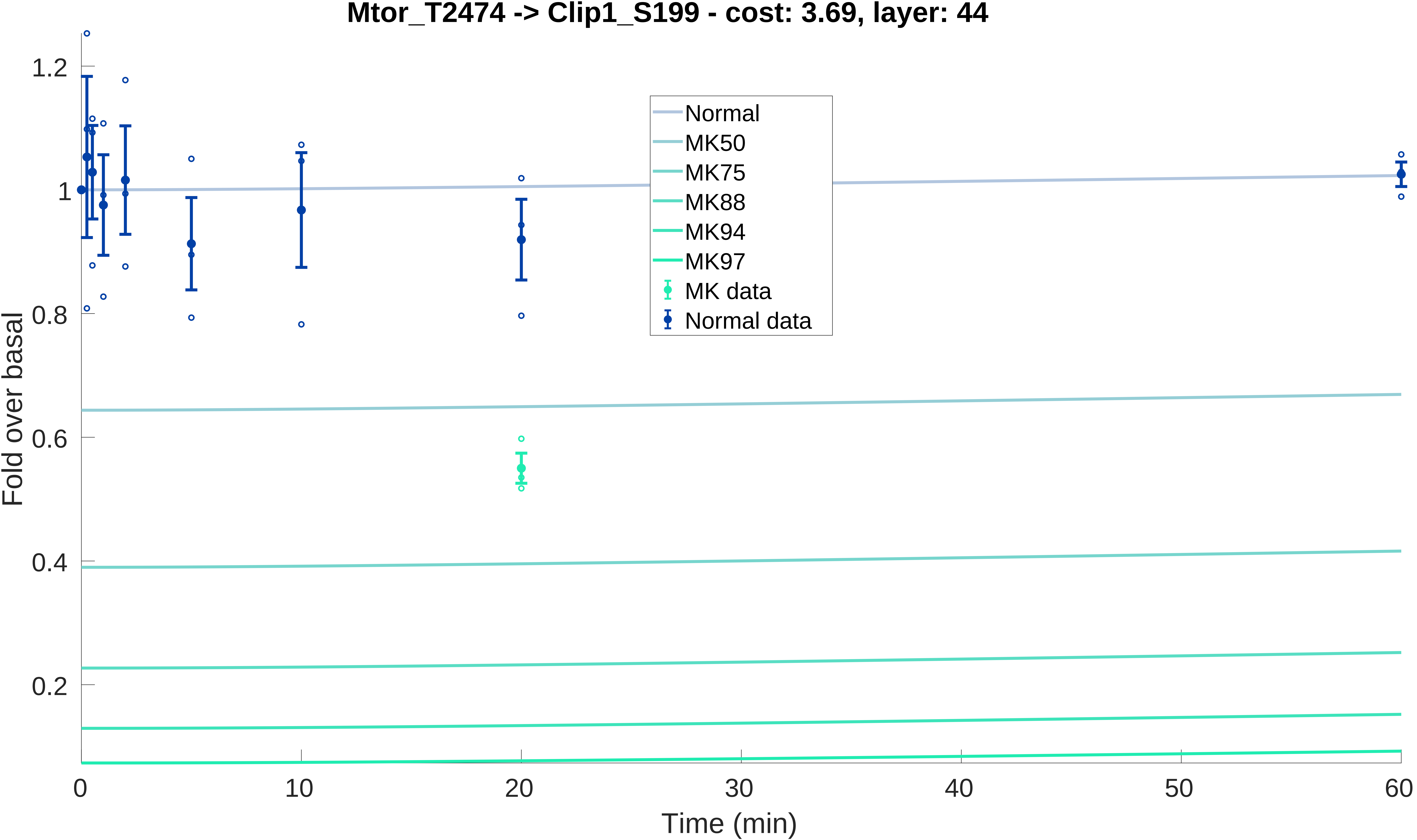
Mtor_T2474 -> Clip1_S199 - cost: 3.69, layer: 44
Fold over basal
Time (min)
0
10
20
30
40
50
60
0.2
0.4
0.6
0.8
1
1.2
Normal
MK50
MK75
MK88
MK94
MK97
MK data
Normal data

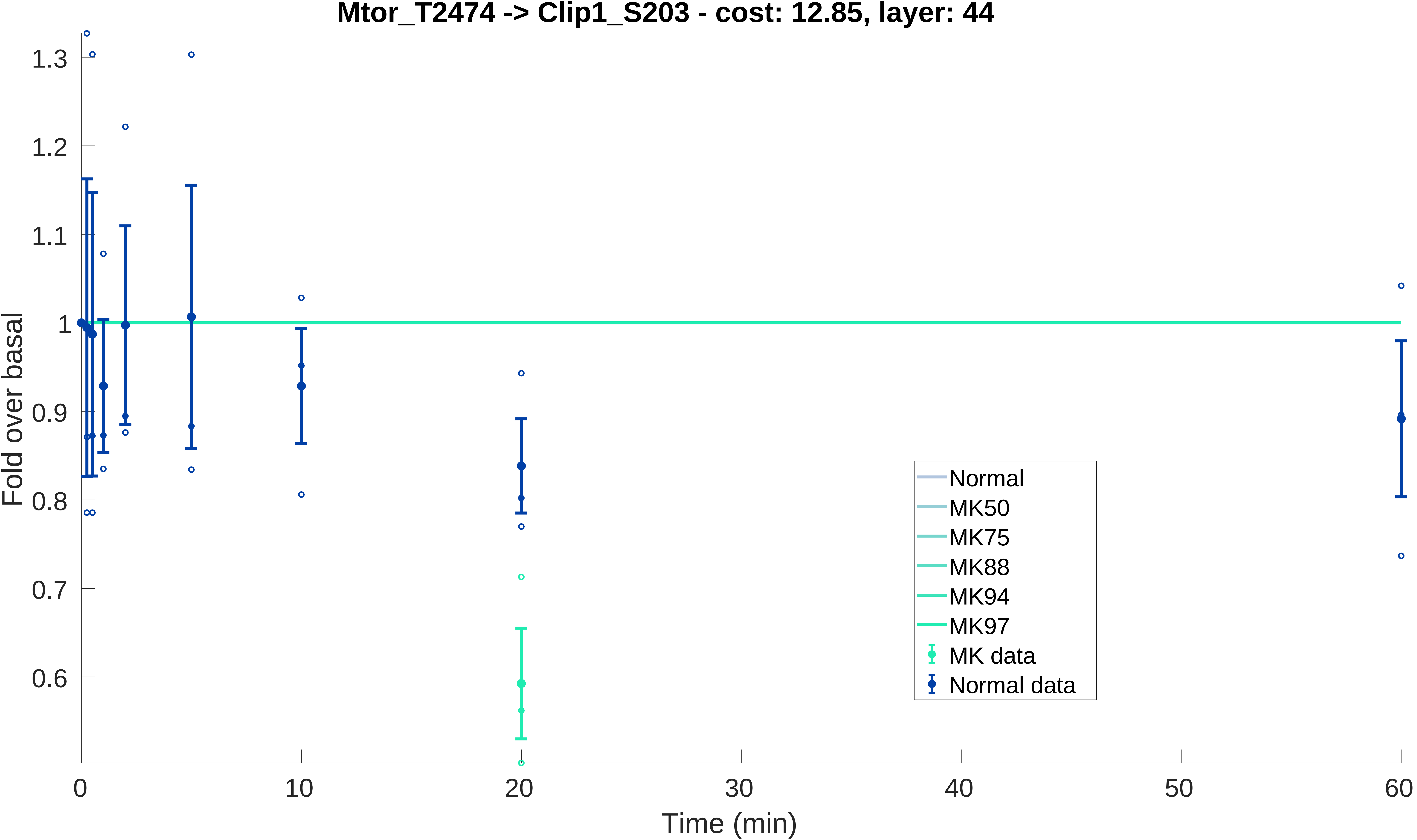

Mtor_T2474 -> Clip1_S203 - cost: 12.85, layer: 44
Fold over basal
Time (min)
1.3
1.2
1.1
1
0.9
0.8
0.7
0.6
0
10
20
30
40
50
60
Normal
MK50
MK75
MK88
MK94
MK97
MK data
Normal data

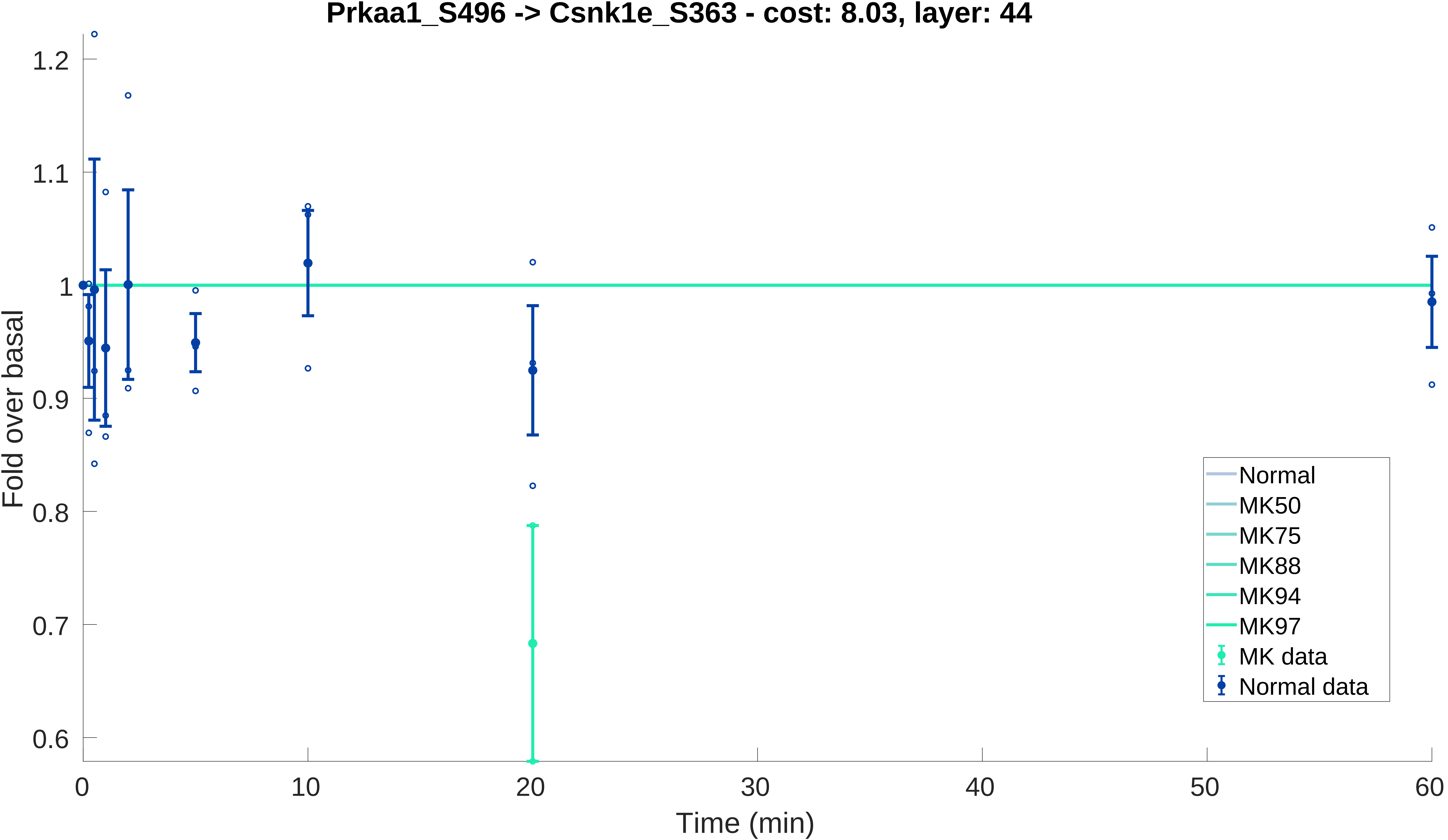

Prkaa1_S496 -> Csnk1e_S363 - cost: 8.03, layer: 44
Fold over basal
1.2
1.1
1
0.9
0.8
0.7
0.6
0
10
20
30
40
50
60
Time (min)
Normal
MK50
MK75
MK88
MK94
MK97
MK data
Normal data

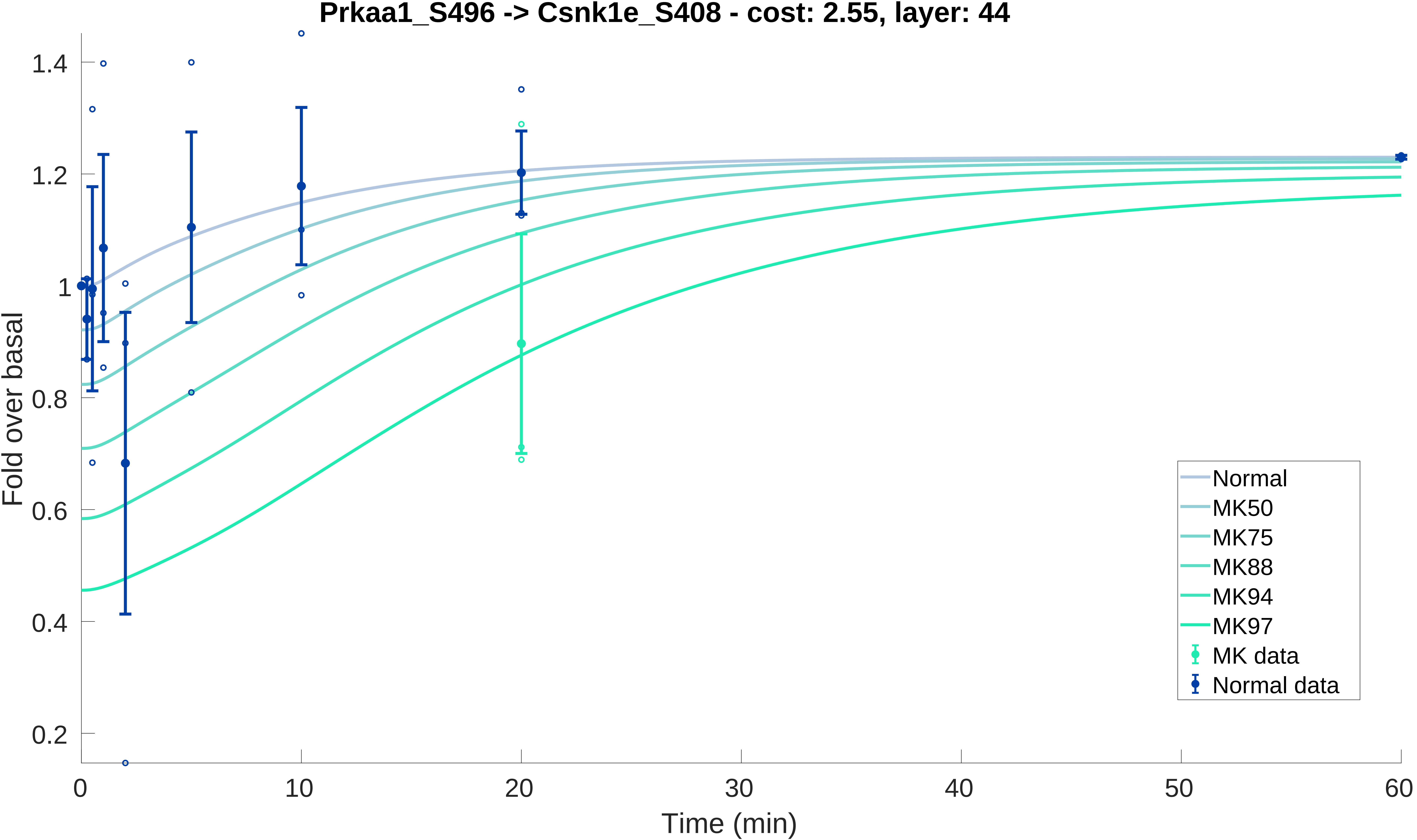
Prkaa1_S496 -> Csnk1e_S408 - cost: 2.55, layer: 44
Fold over basal
Time (min)
0.2
0.4
0.6
0.8
1
1.2
1.4
0
10
20
30
40
50
60
Normal
MK50
MK75
MK88
MK94
MK97
MK data
Normal data

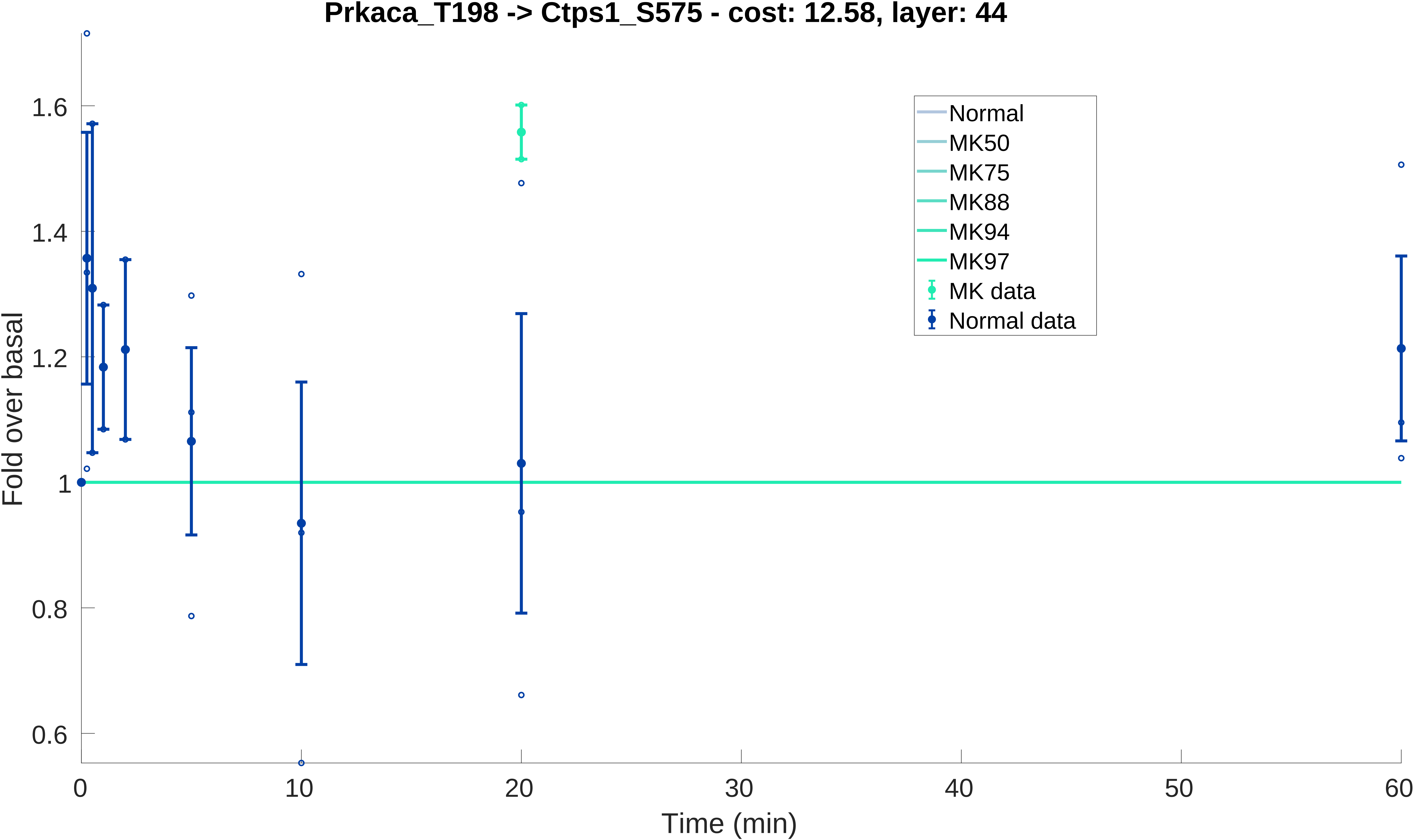

Prkaca_T198 -> Ctps1_S575 - cost: 12.58, layer: 44
Fold over basal
Time (min)
Normal
MK50
MK75
MK88
MK94
MK97
MK data
Normal data
0.6
0.8
1
1.2
1.4
1.6
0
10
20
30
40
50
60

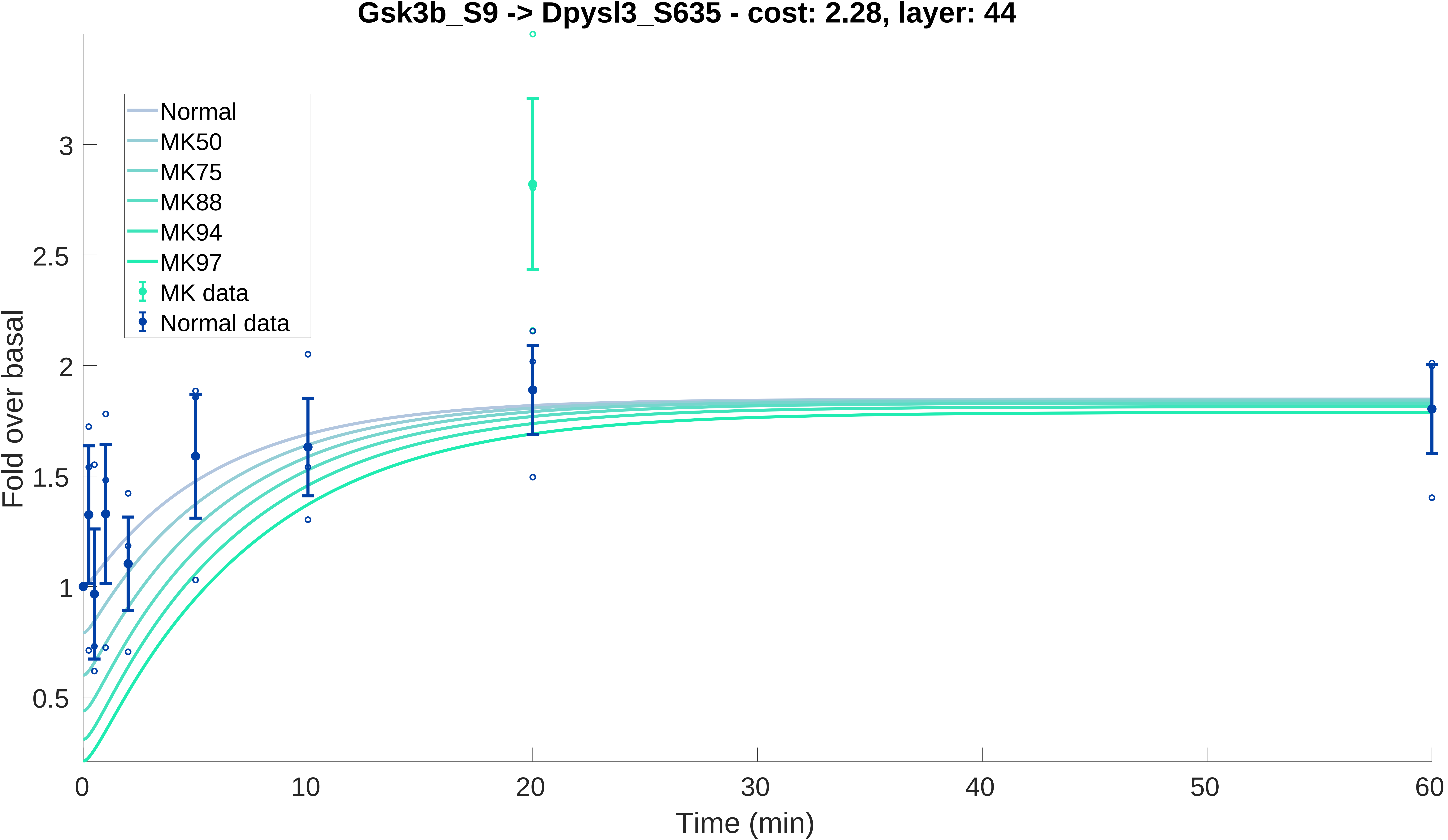

Gsk3b_S9 -> Dpysl3_S635 - cost: 2.28, layer: 44
Normal
MK50
MK75
MK88
MK94
MK97
MK data
Normal data
Fold over basal
Time (min)
0
10
20
30
40
50
60
0.5
1
1.5
2
2.5
3

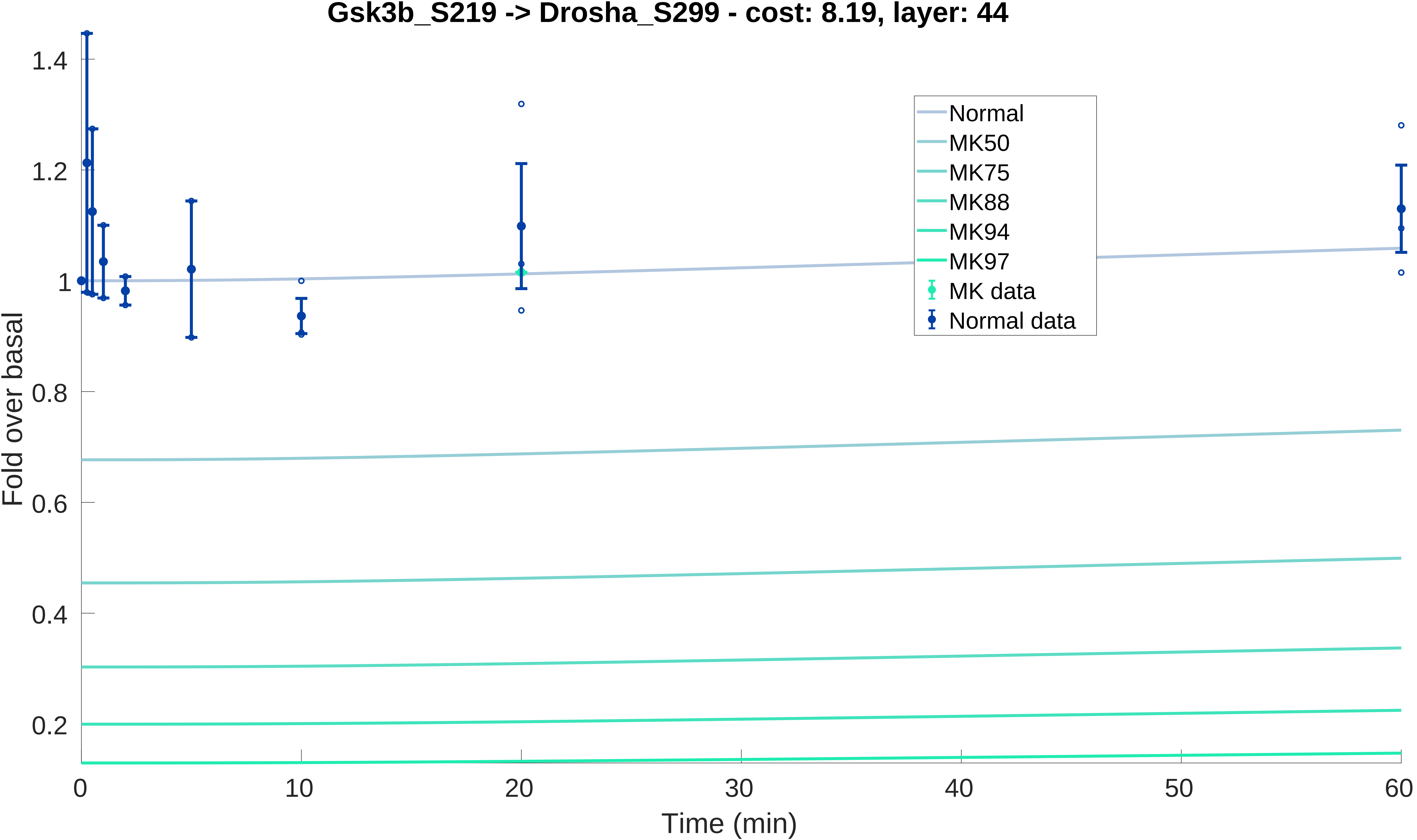
Gsk3b_S219 -> Drosha_S299 - cost: 8.19, layer: 44
Normal
MK50
MK75
MK88
MK94
MK97
MK data
Normal data
Fold over basal
Time (min)
0
10
20
30
40
50
60
0.2
0.4
0.6
0.8
1
1.2
1.4

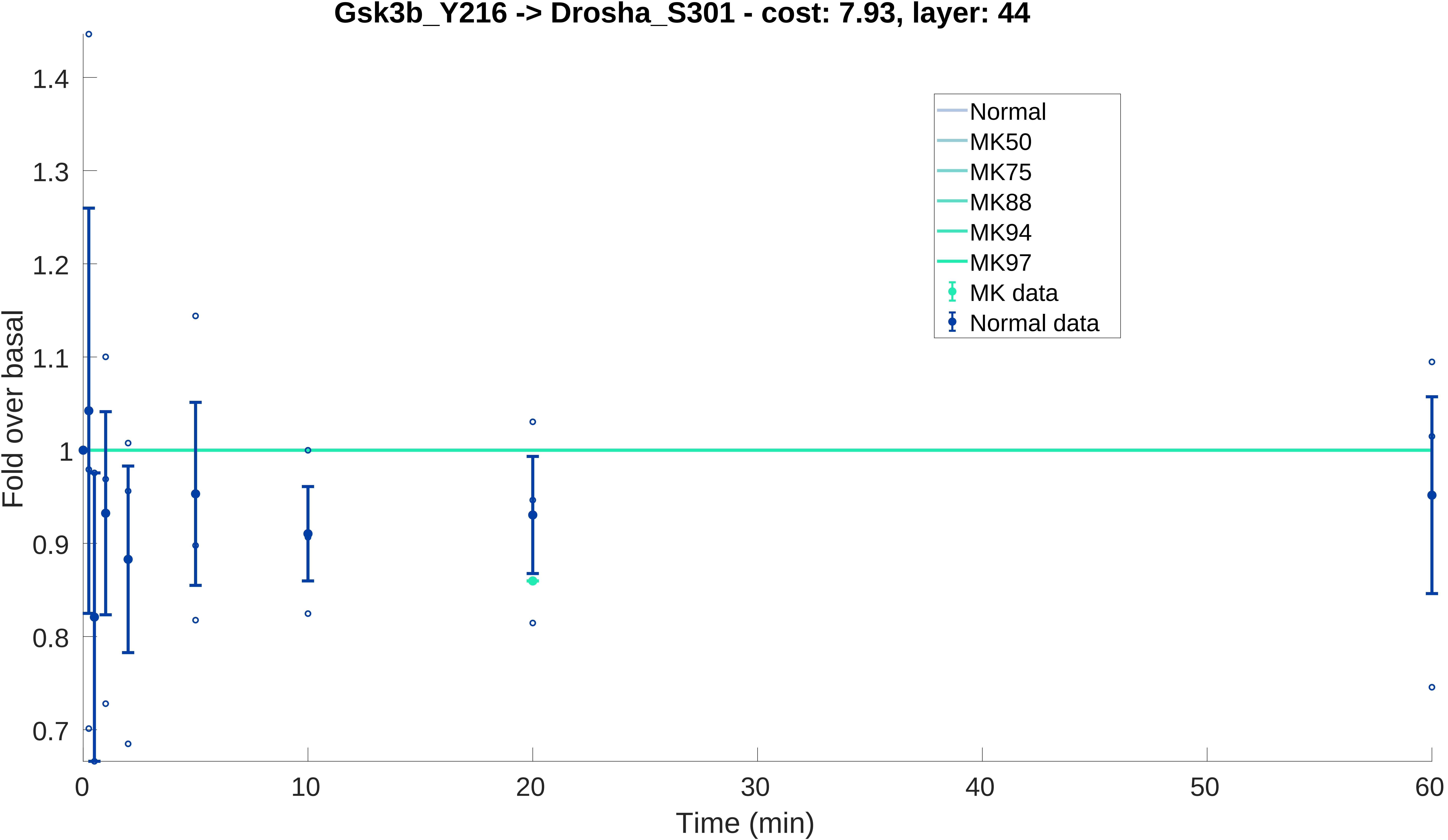

Gsk3b_Y216 -> Drosha_S301 - cost: 7.93, layer: 44
Fold over basal
Time (min)
1.4
1.3
1.2
1.1
1
0.9
0.8
0.7
0
10
20
30
40
50
60
Normal
MK50
MK75
MK88
MK94
MK97
MK data
Normal data

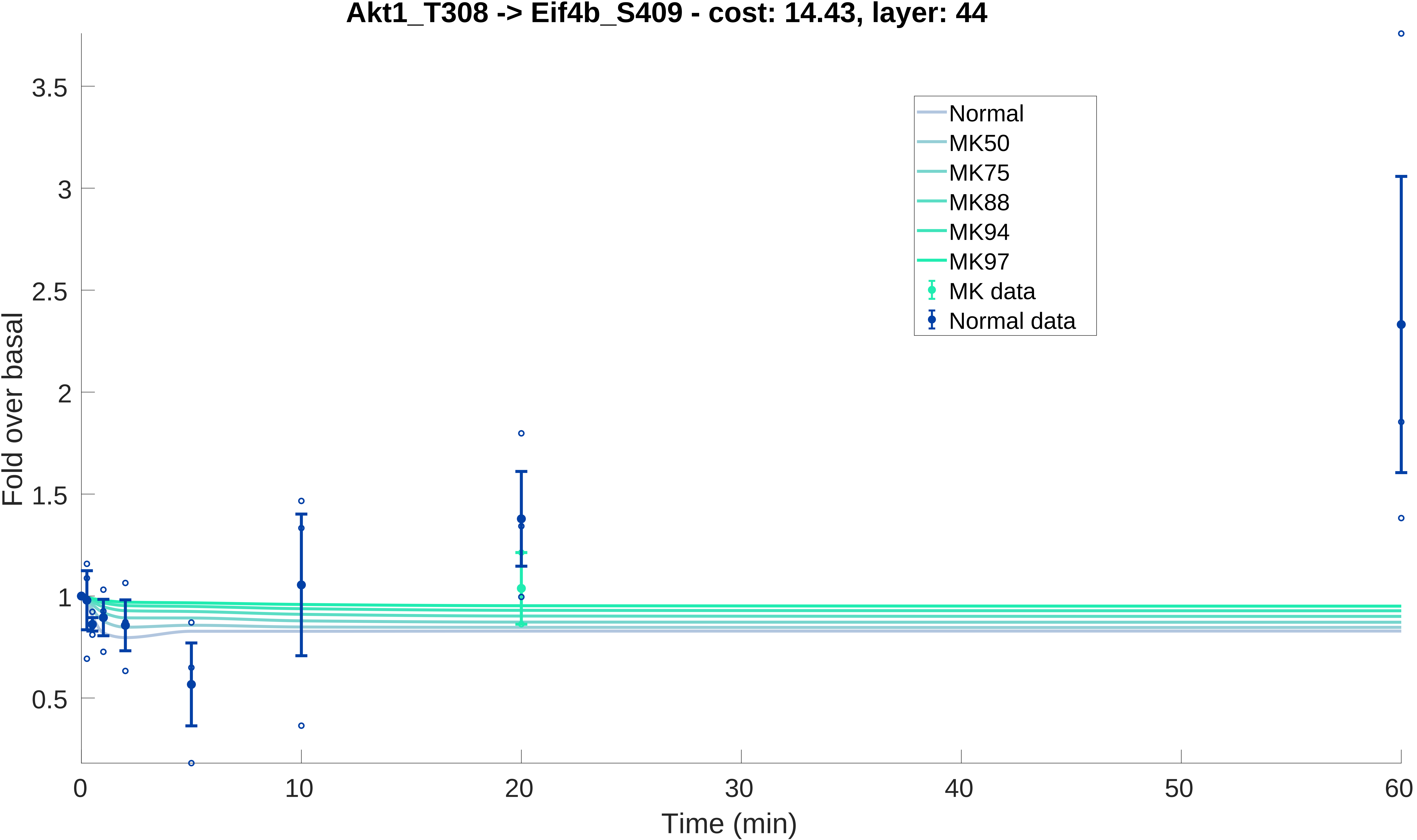

Akt1_T308 -> Eif4b_S409 - cost: 14.43, layer: 44
Normal
MK50
MK75
MK88
MK94
MK97
MK data
Normal data
Fold over basal
Time (min)
0.5
1
1.5
2
2.5
3
3.5
0
10
20
30
40
50
60

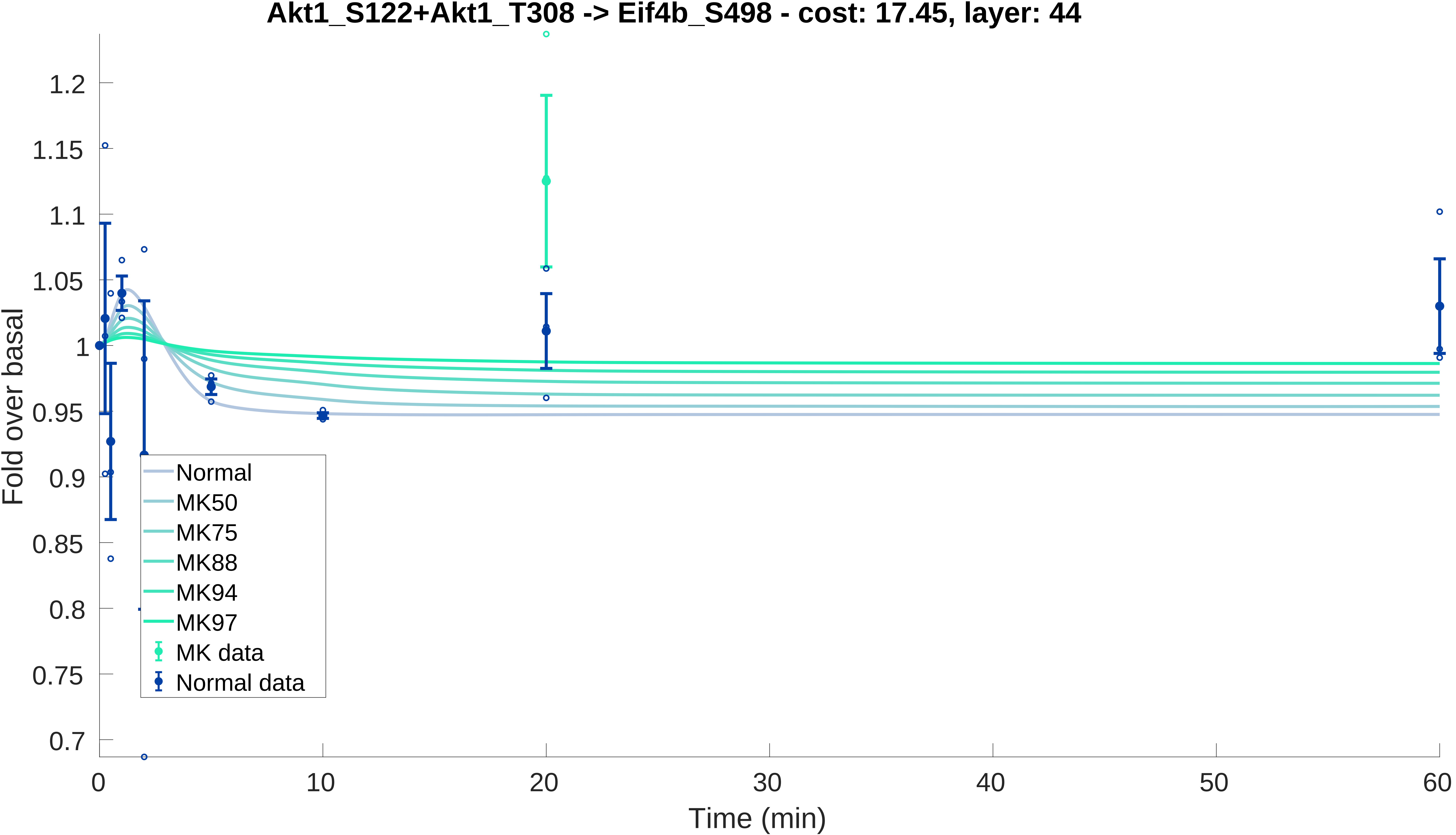
Akt1_S122+Akt1_T308 -> Eif4b_S498 - cost: 17.45, layer: 44
Fold over basal
Time (min)
1.2
1.15
1.1
1.05
1
0.95
0.9
0.85
0.8
0.75
0.7
0
10
20
30
40
50
60
Normal
MK50
MK75
MK88
MK94
MK97
MK data
Normal data

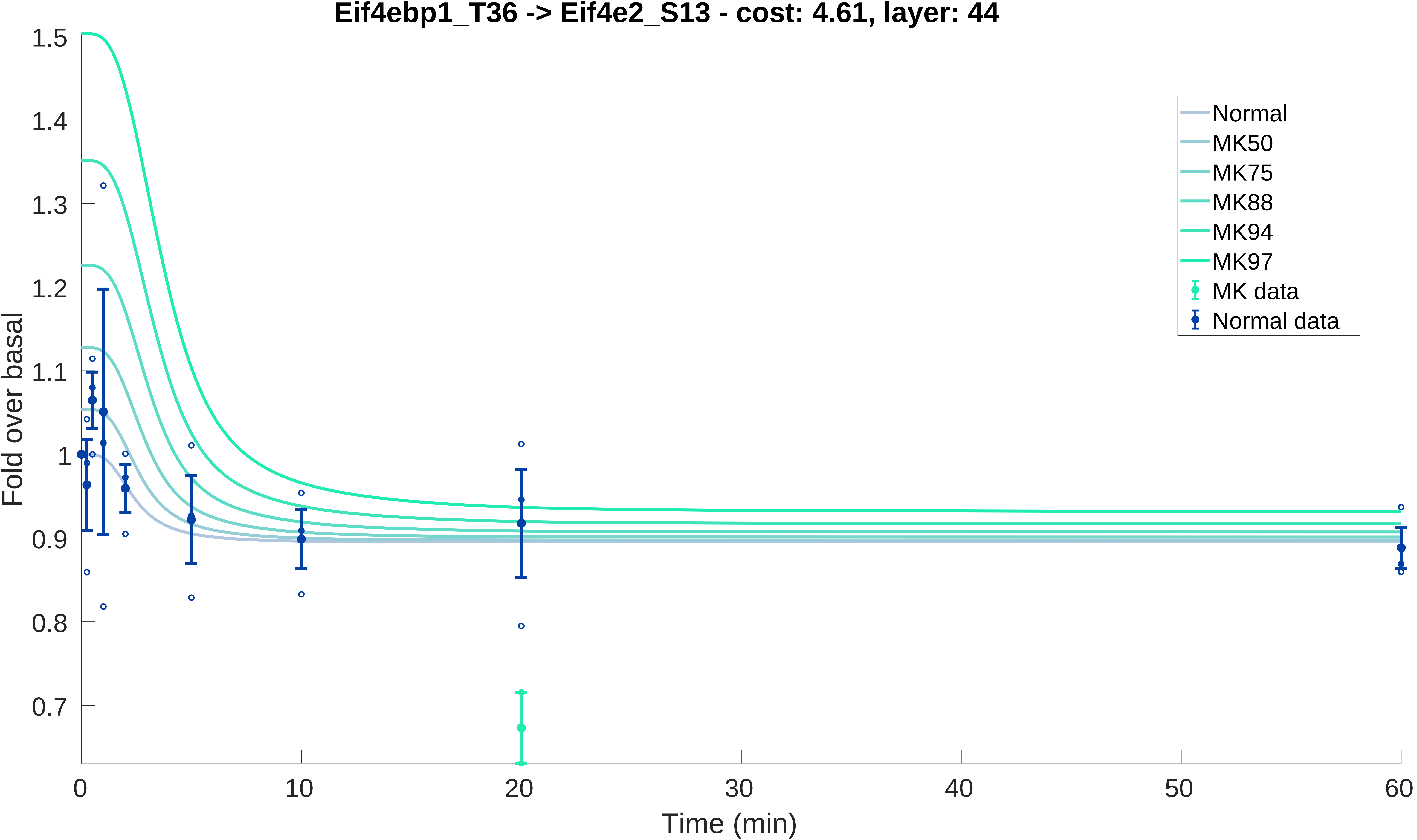
Eif4ebp1_T36 -> Eif4e2_S13 - cost: 4.61, layer: 44
Fold over basal
Time (min)
Normal
MK50
MK75
MK88
MK94
MK97
MK data
Normal data
1.5
1.4
1.3
1.2
1.1
1
0.9
0.8
0.7
0
10
20
30
40
50
60

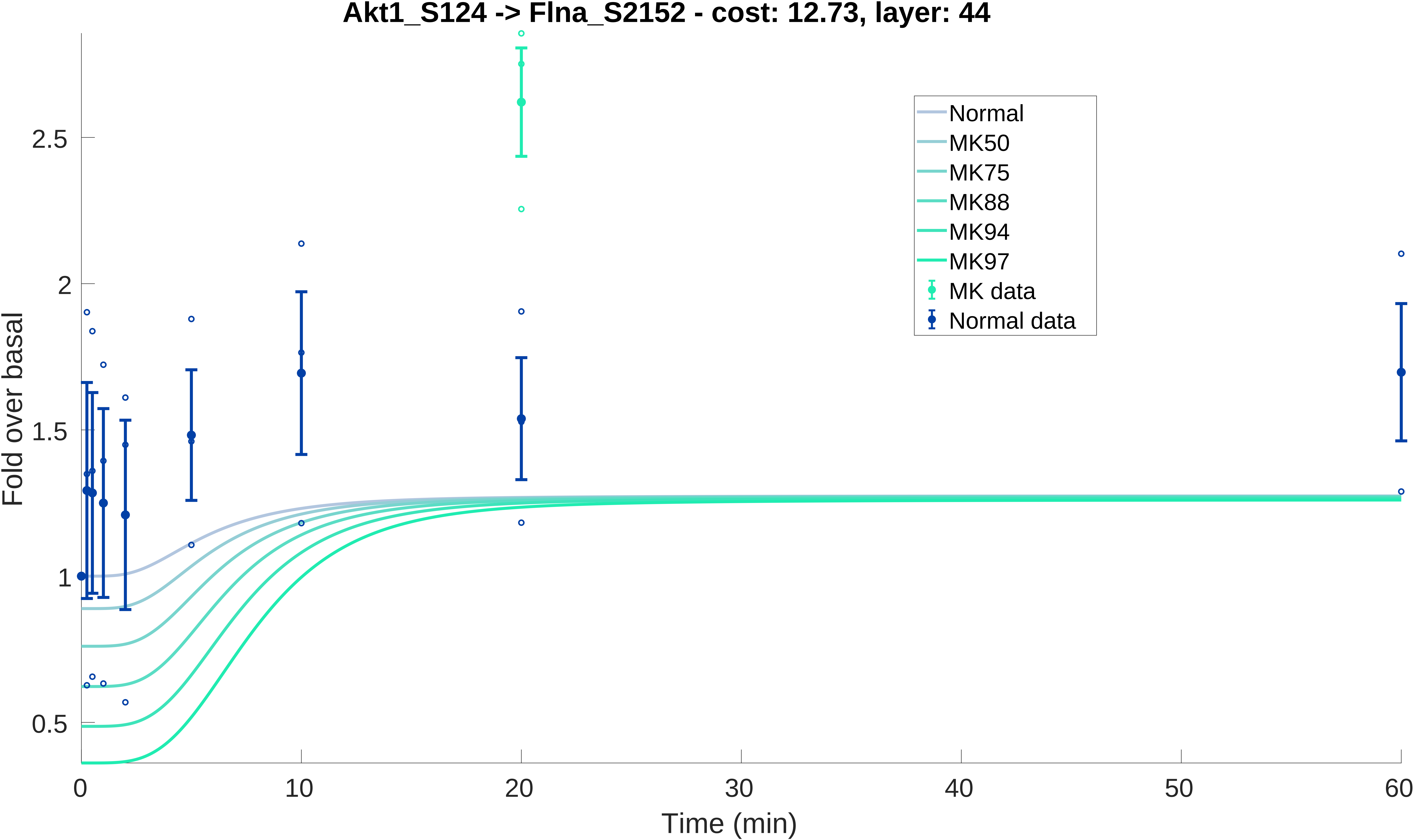

Akt1_S124 -> Flna_S2152 - cost: 12.73, layer: 44
Fold over basal
Time (min)
Normal
MK50
MK75
MK88
MK94
MK97
MK data
Normal data
0.5
1
1.5
2
2.5
0
10
20
30
40
50
60

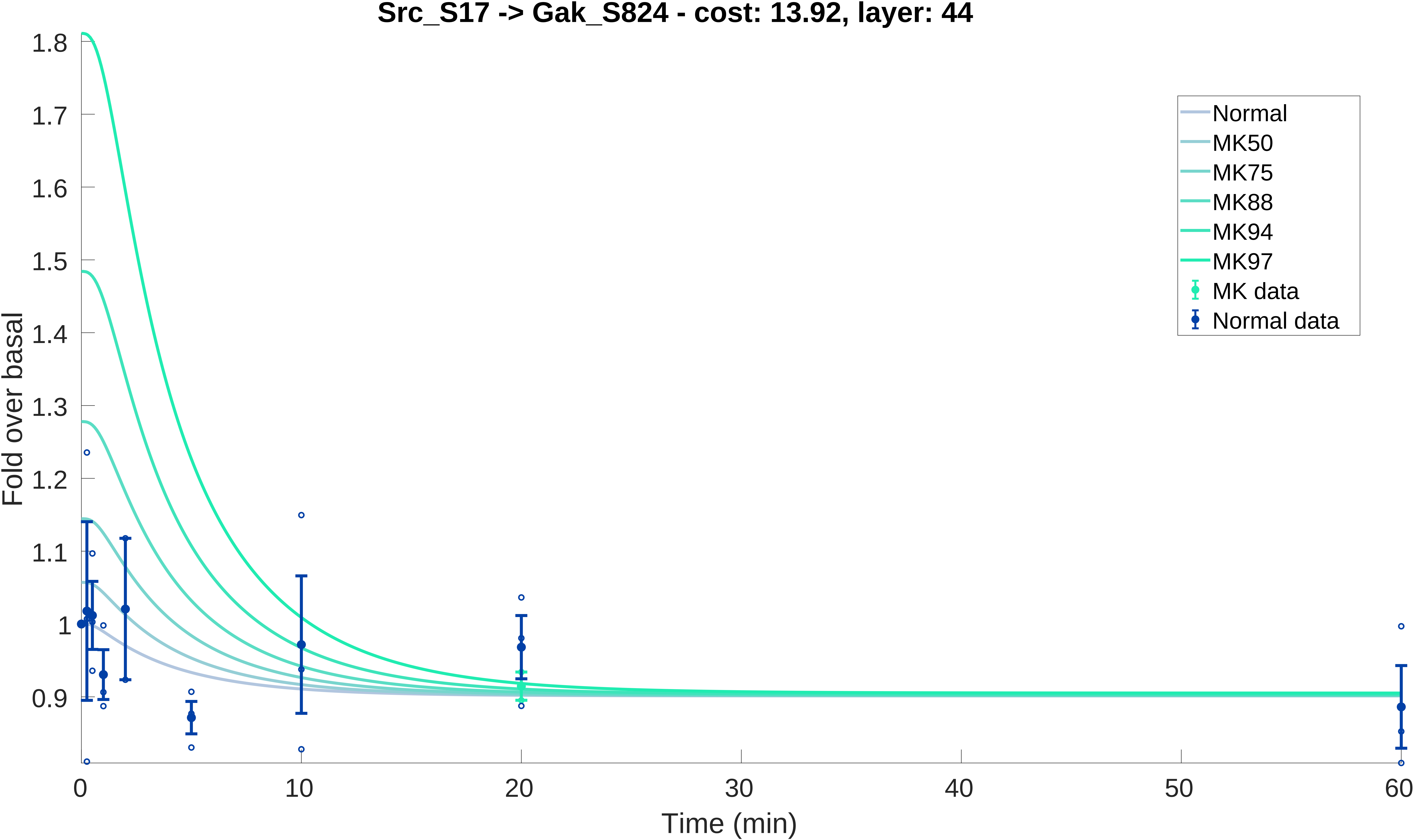

Src_S17 -> Gak_S824 - cost: 13.92, layer: 44
Fold over basal
1.8
1.7
1.6
1.5
1.4
1.3
1.2
1.1
1
0.9
0
10
20
30
40
50
60
Time (min)
Normal
MK50
MK75
MK88
MK94
MK97
MK data
Normal data

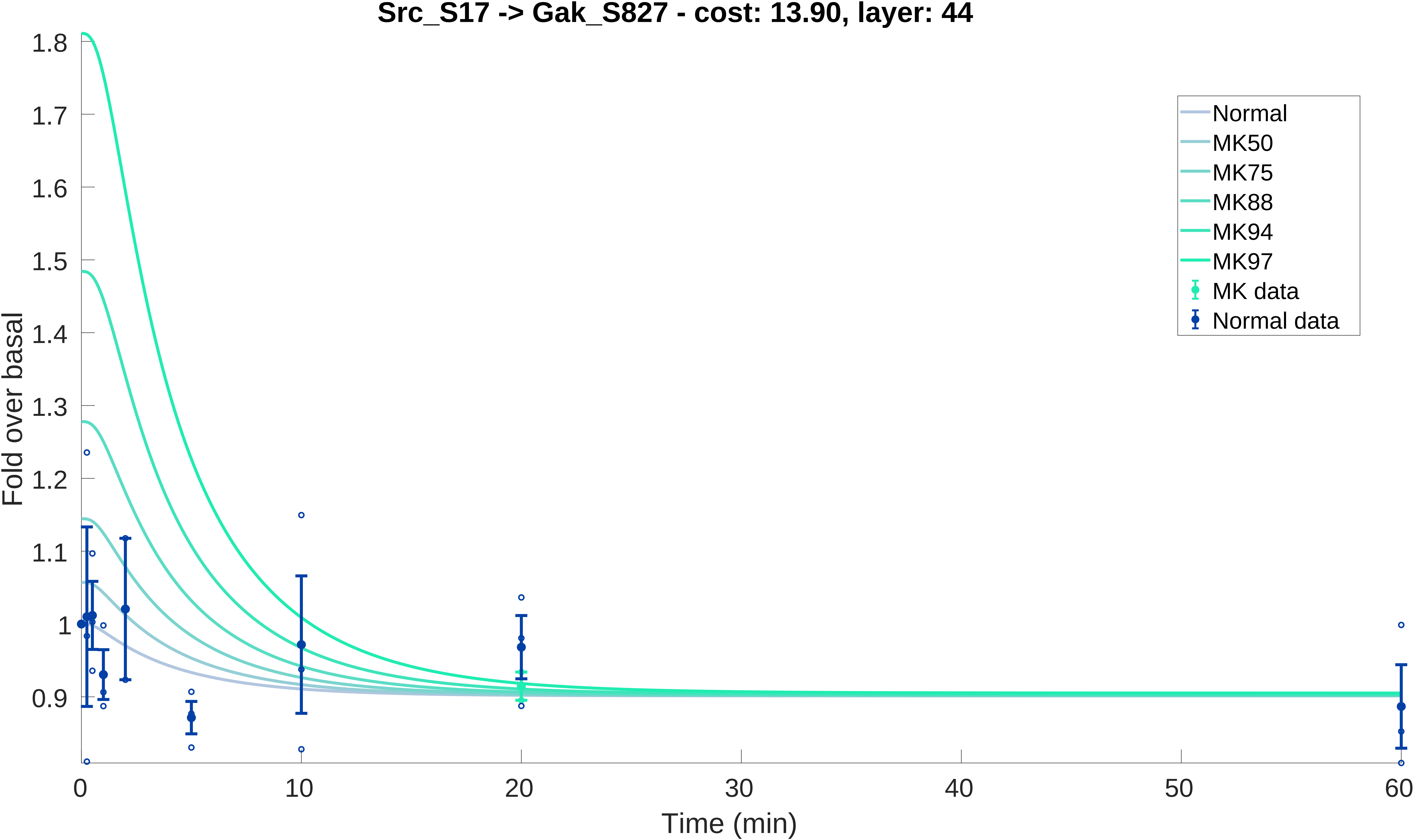

Src_S17 -> Gak_S827 - cost: 13.90, layer: 44
1.8
1.7
1.6
1.5
1.4
1.3
1.2
1.1
1
0.9
Fold over basal
0
10
20
30
40
50
60
Time (min)
Normal
MK50
MK75
MK88
MK94
MK97
MK data
Normal data

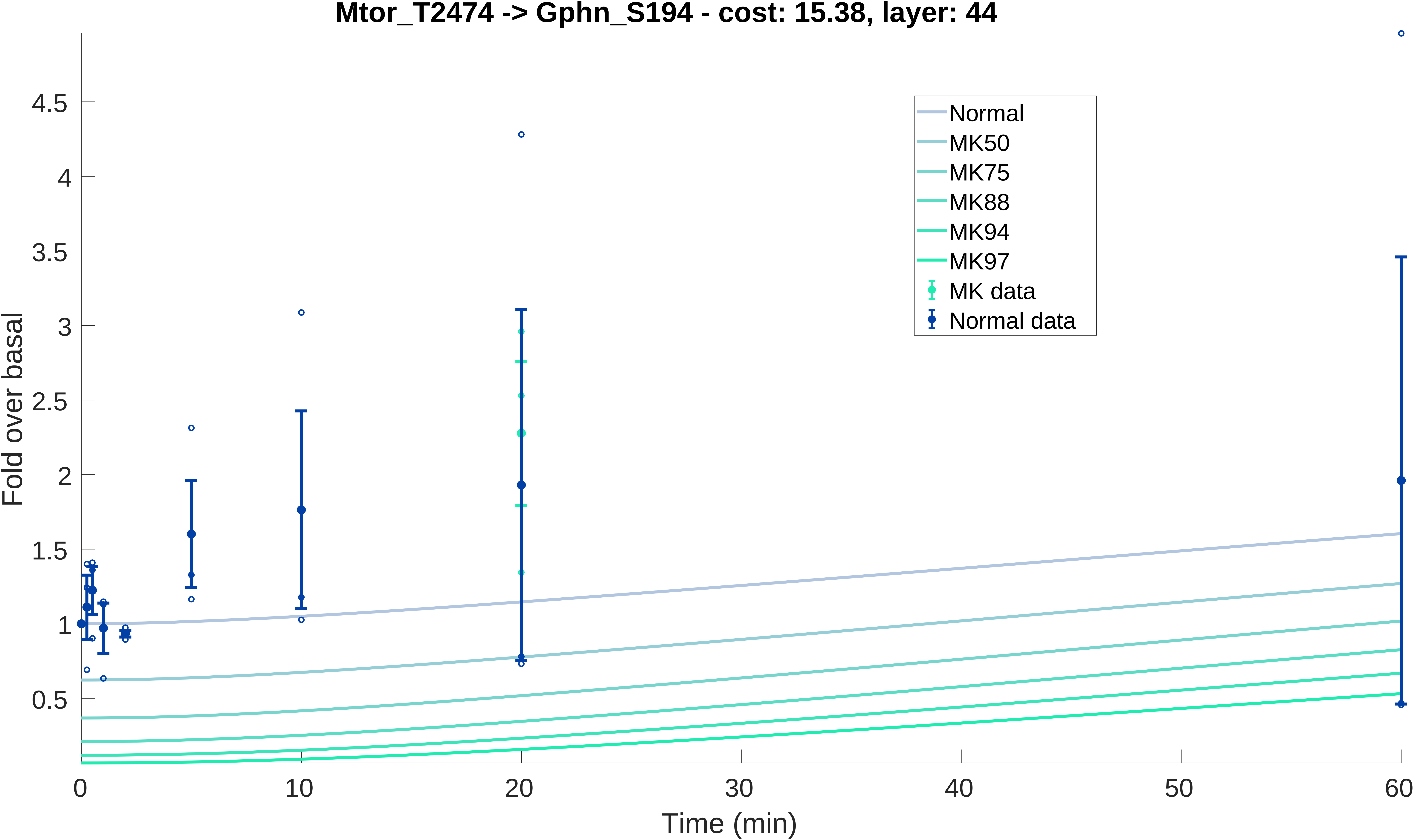

Mtor_T2474 -> Gphn_S194 - cost: 15.38, layer: 44
Normal
MK50
MK75
MK88
MK94
MK97
MK data
Normal data
Fold over basal
Time (min)
0.5
1
1.5
2
2.5
3
3.5
4
4.5
0
10
20
30
40
50
60

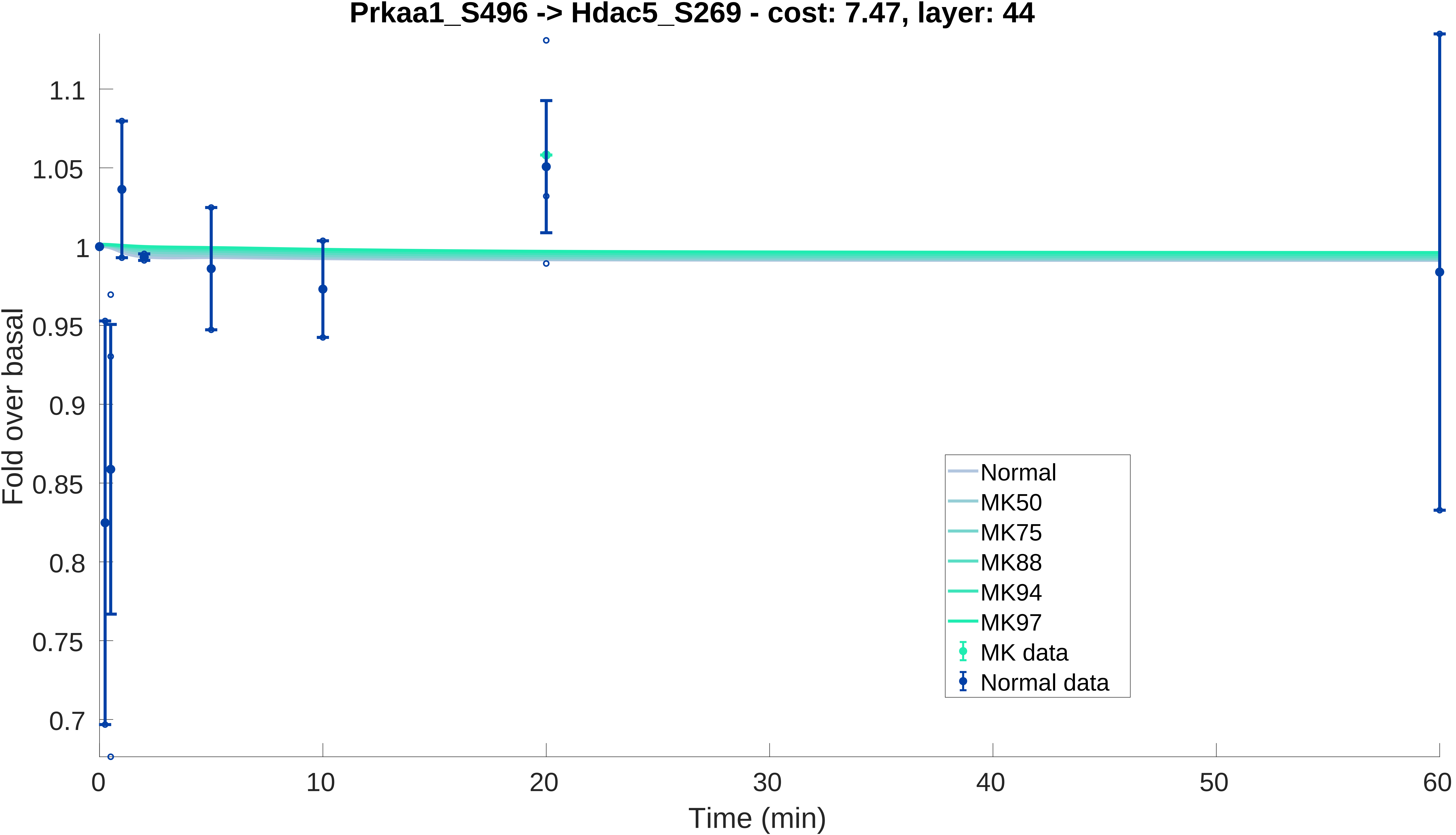

Prrkaa1_S496 -> Hdac5_S269 - cost: 7.47, layer: 44
Fold over basal
Time (min)
1.1
1.05
1
0.95
0.9
0.85
0.8
0.75
0.7
0
10
20
30
40
50
60
Normal
MK50
MK75
MK88
MK94
MK97
MK data
Normal data

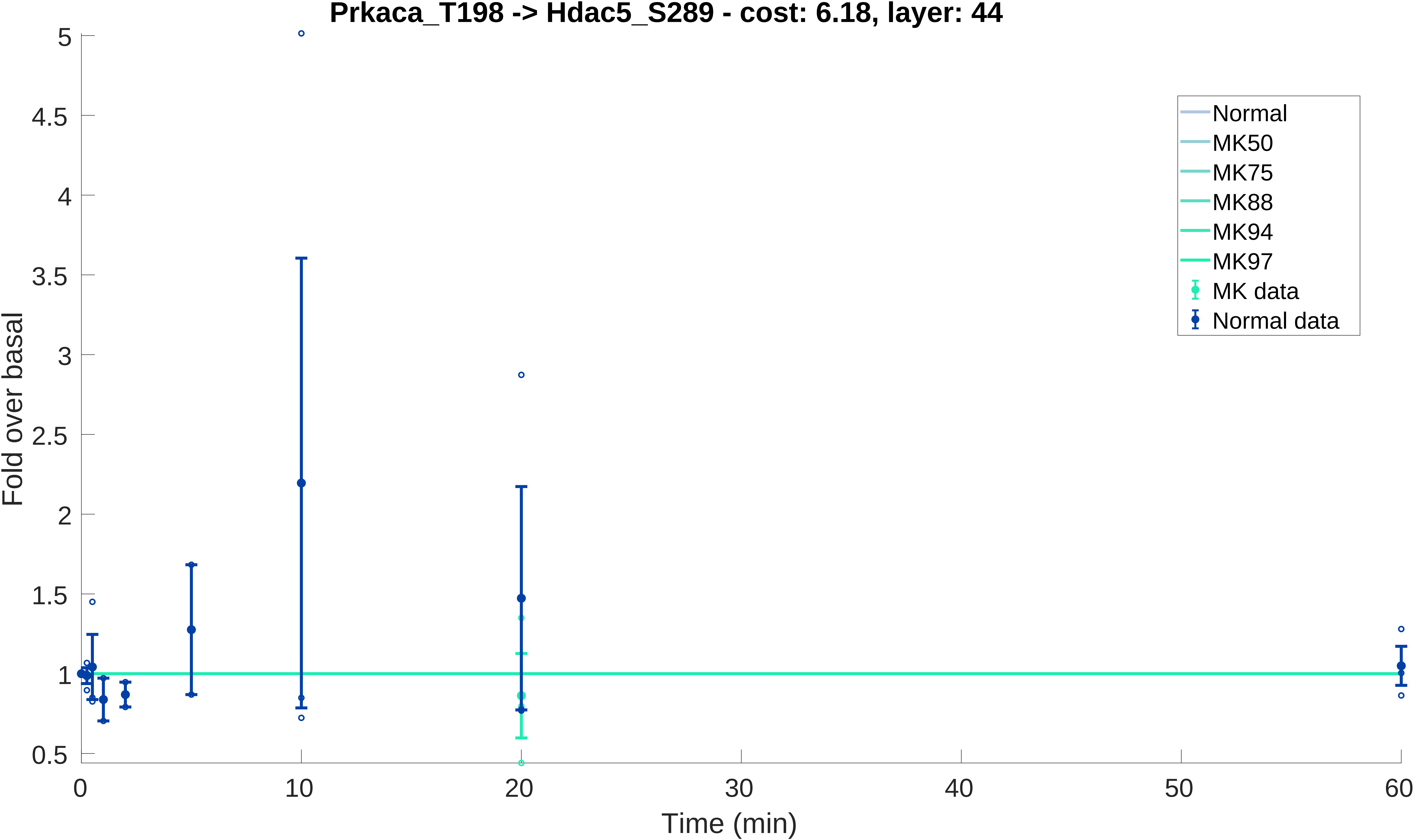

Prkaca_T198 -> Hdac5_S289 - cost: 6.18, layer: 44
Fold over basal
Time (min)
0.5
1
1.5
2
2.5
3
3.5
4
4.5
5
0
10
20
30
40
50
60
Normal
MK50
MK75
MK88
MK94
MK97
MK data
Normal data

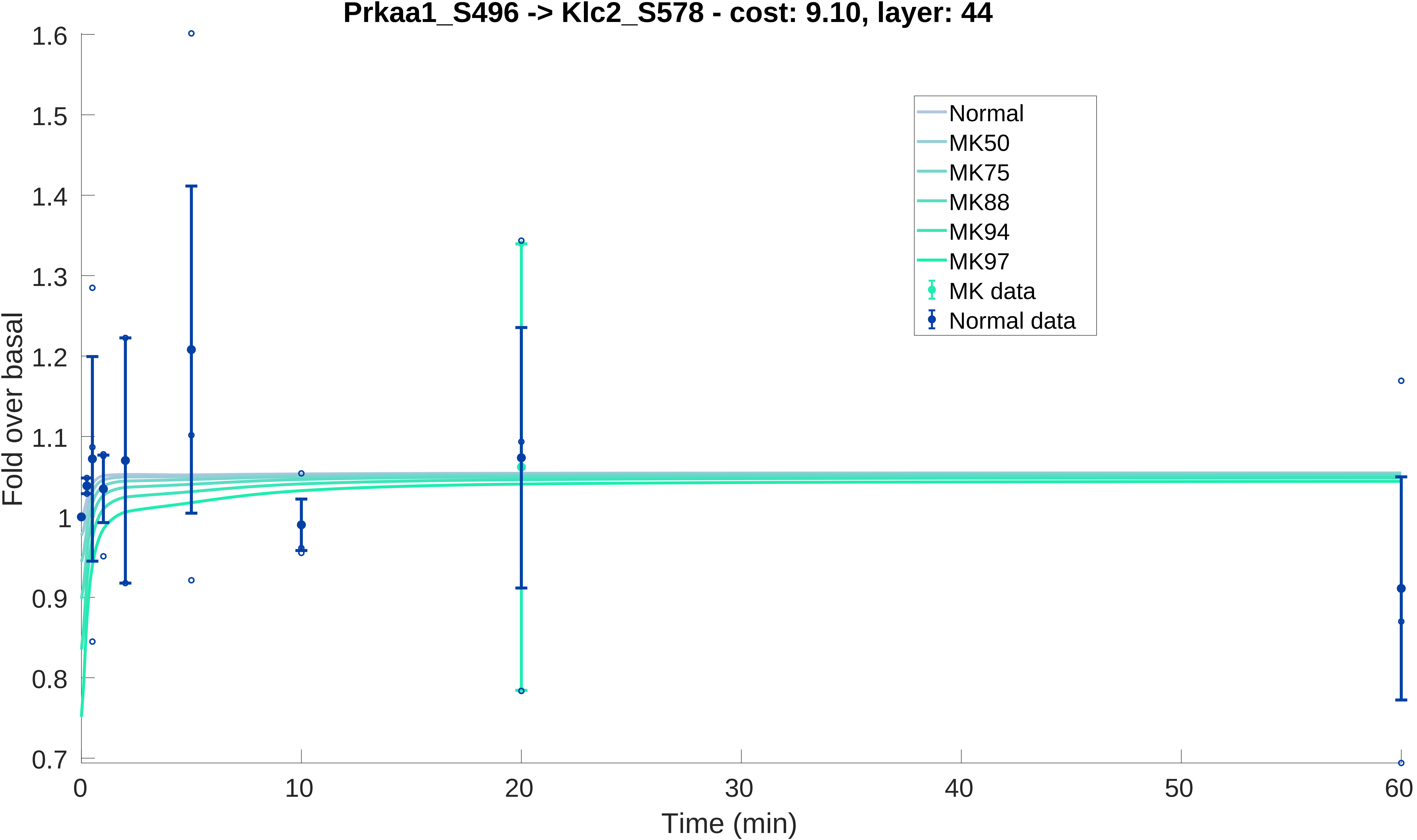
Prkaa1_S496 -> Klc2_S578 - cost: 9.10, layer: 44
Fold over basal
Time (min)
1.6
1.5
1.4
1.3
1.2
1.1
1
0.9
0.8
0.7
0
10
20
30
40
50
60
Normal
MK50
MK75
MK88
MK94
MK97
MK data
Normal data

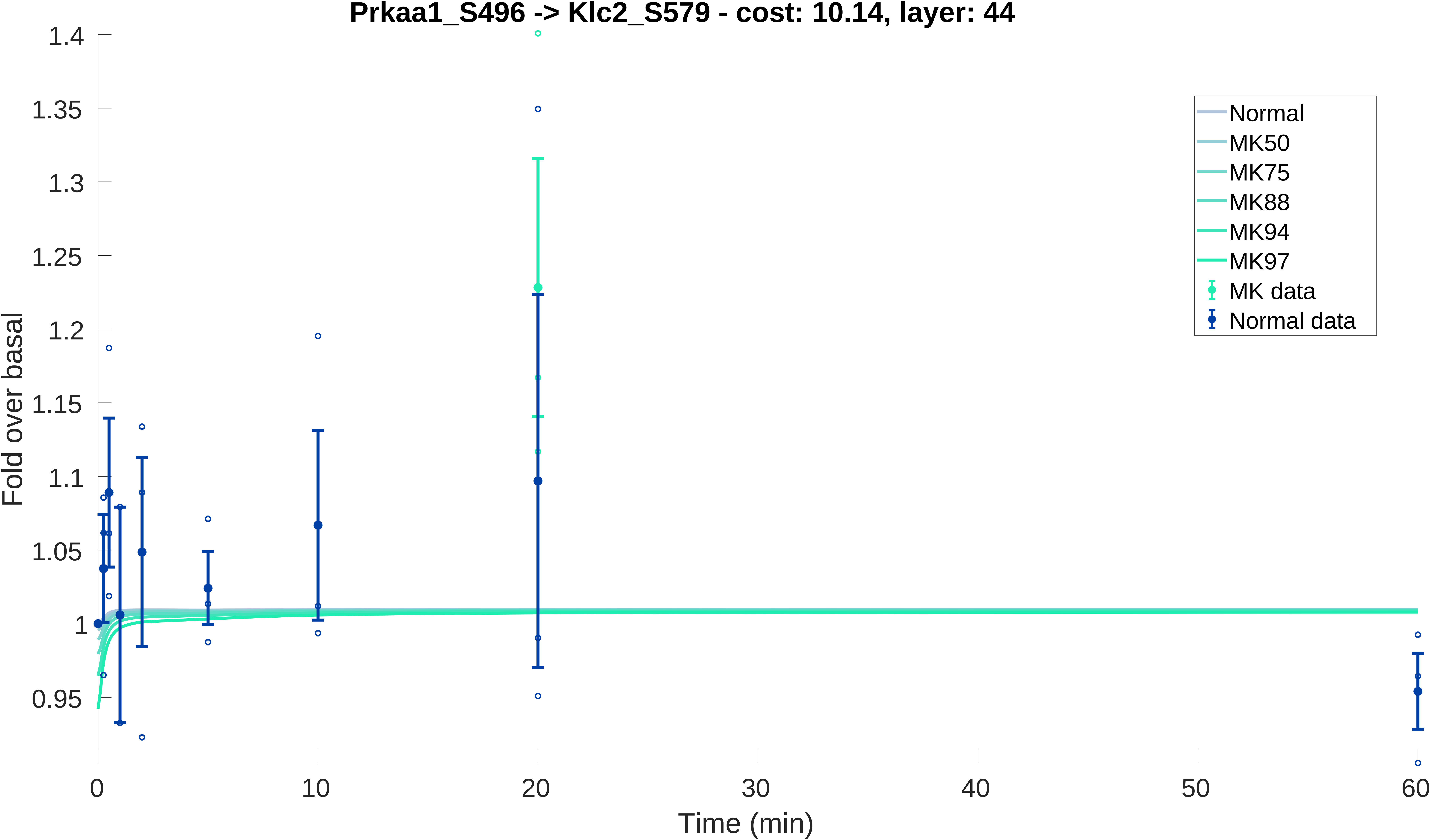

Prrkaa1_S496 -> Klc2_S579 - cost: 10.14, layer: 44
Fold over basal
Time (min)
Normal
MK50
MK75
MK88
MK94
MK97
MK data
Normal data
1.4
1.35
1.3
1.25
1.2
1.15
1.1
1.05
1
0.95
0
10
20
30
40
50
60

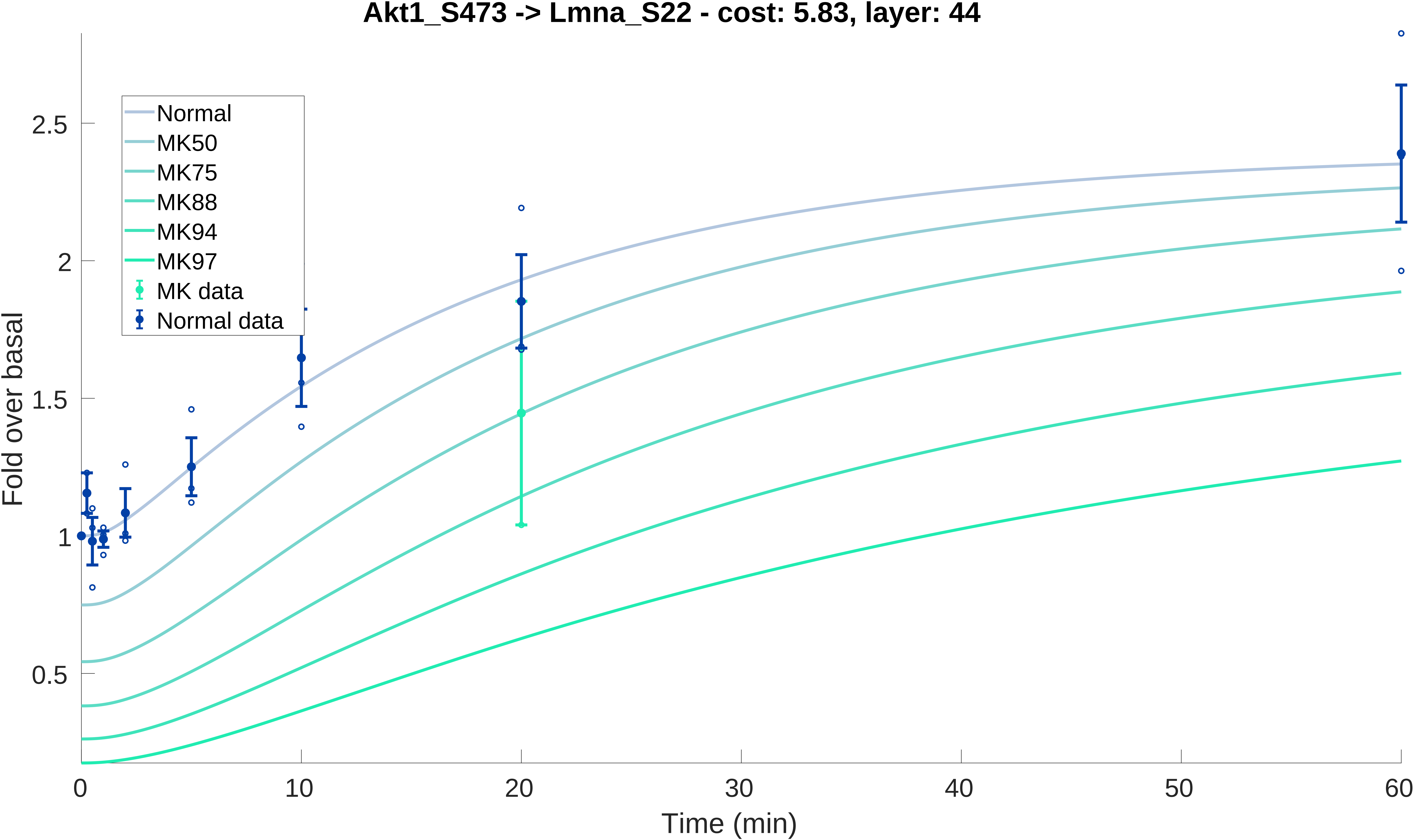
Akt1_S473 -> Lmna_S22 - cost: 5.83, layer: 44
Normal
MK50
MK75
MK88
MK94
MK97
MK data
Normal data
Fold over basal
Time (min)
0.5
1
1.5
2
2.5
0
10
20
30
40
50
60

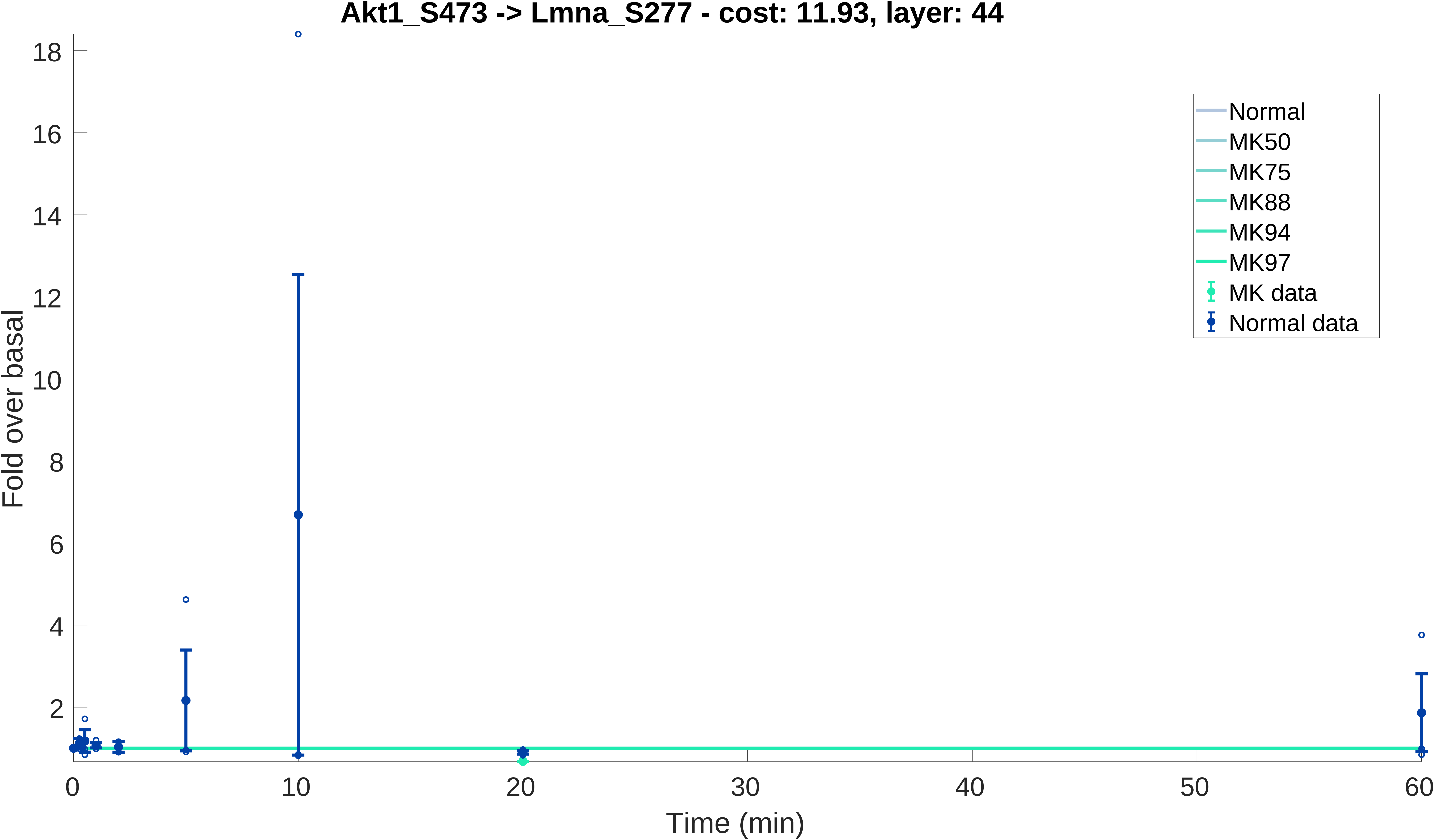
Akt1_S473 -> Lmna_S277 - cost: 11.93, layer: 44
Fold over basal
Time (min)
0
10
20
30
40
50
60
2
4
6
8
10
12
14
16
18
Normal
MK50
MK75
MK88
MK94
MK97
MK data
Normal data

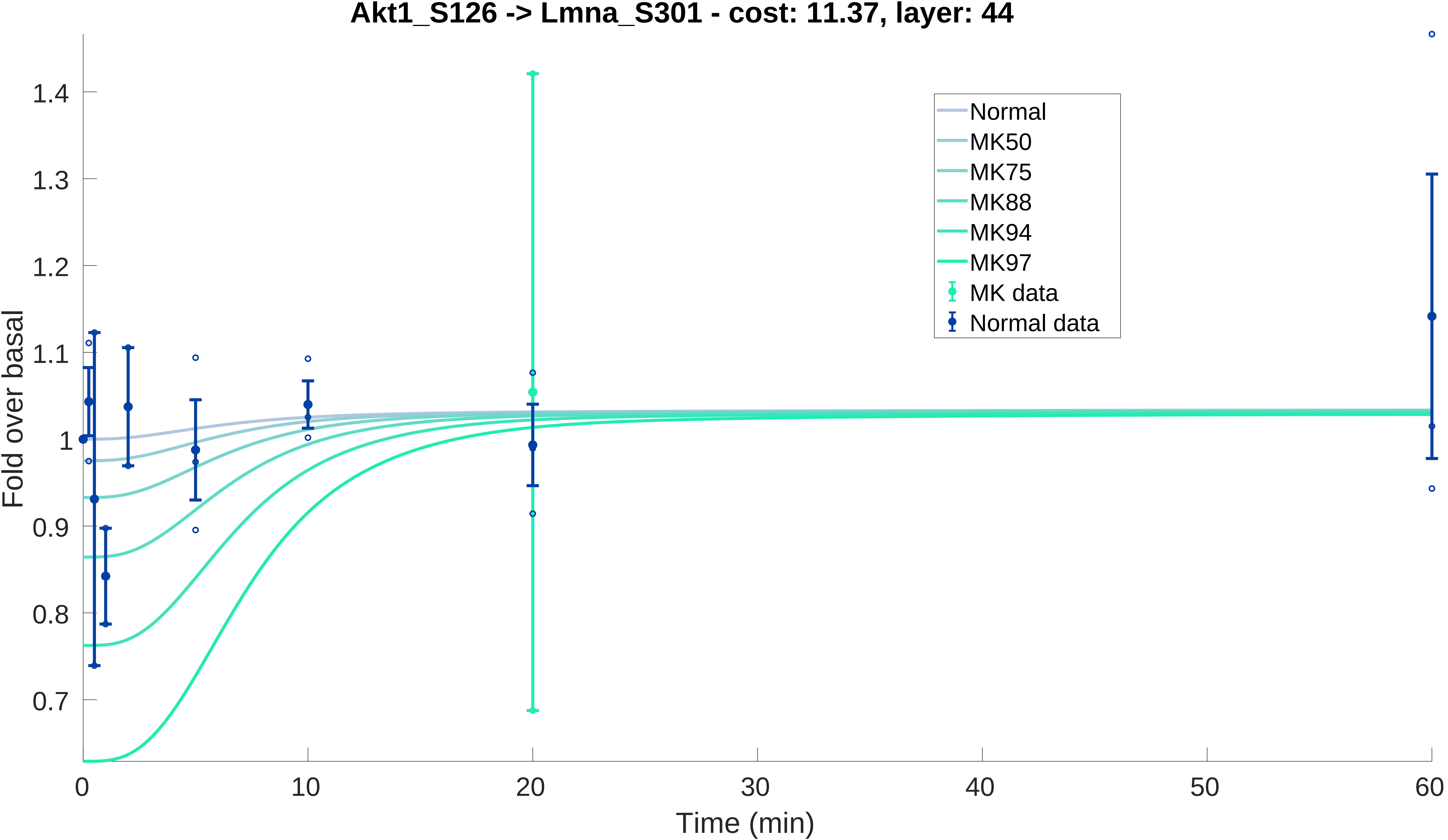

Akt1_S126 -> Lmna_S301 - cost: 11.37, layer: 44
Fold over basal
Time (min)
0
10
20
30
40
50
60
0.7
0.8
0.9
1
1.1
1.2
1.3
1.4
Normal
MK50
MK75
MK88
MK94
MK97
MK data
Normal data

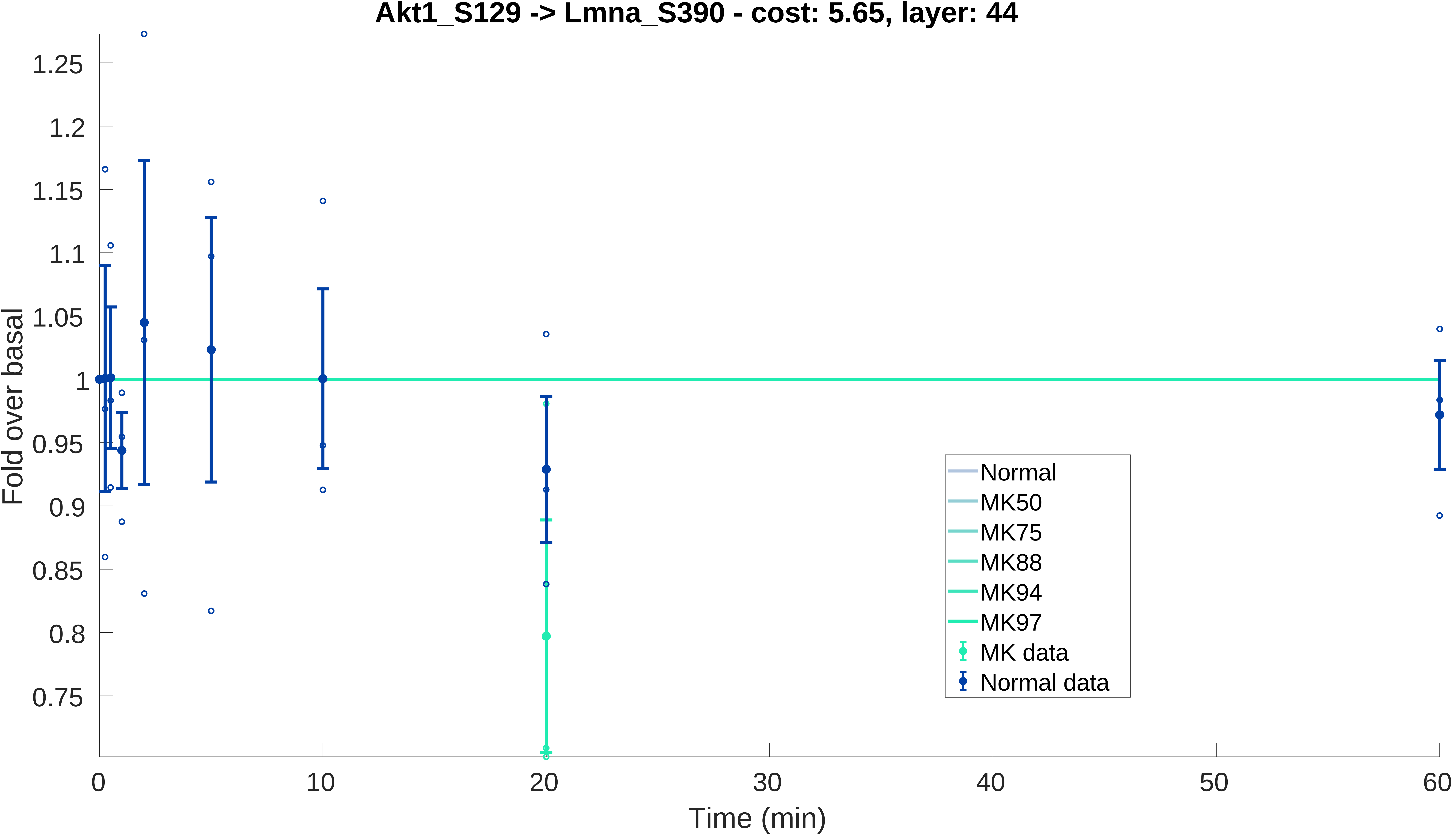

Akt1_S129 -> Lmna_S390 - cost: 5.65, layer: 44
Fold over basal
1.25
1.2
1.15
1.1
1.05
1
0.95
0.9
0.85
0.8
0.75
0
10
20
30
40
50
60
Time (min)
Normal
MK50
MK75
MK88
MK94
MK97
MK data
Normal data

Akt1_T308 -> Lmna_S392 - cost: 9.74, layer: 44

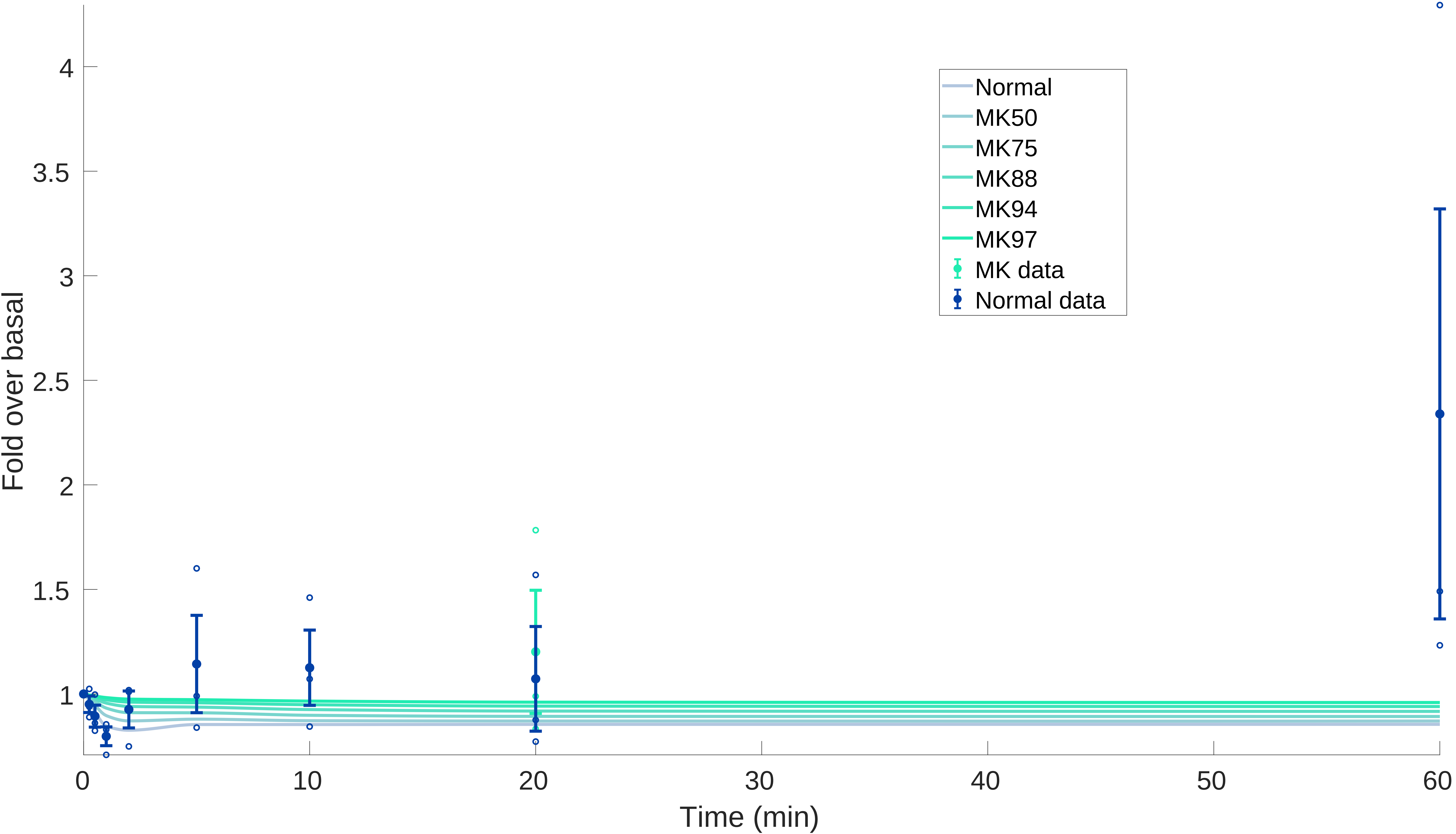

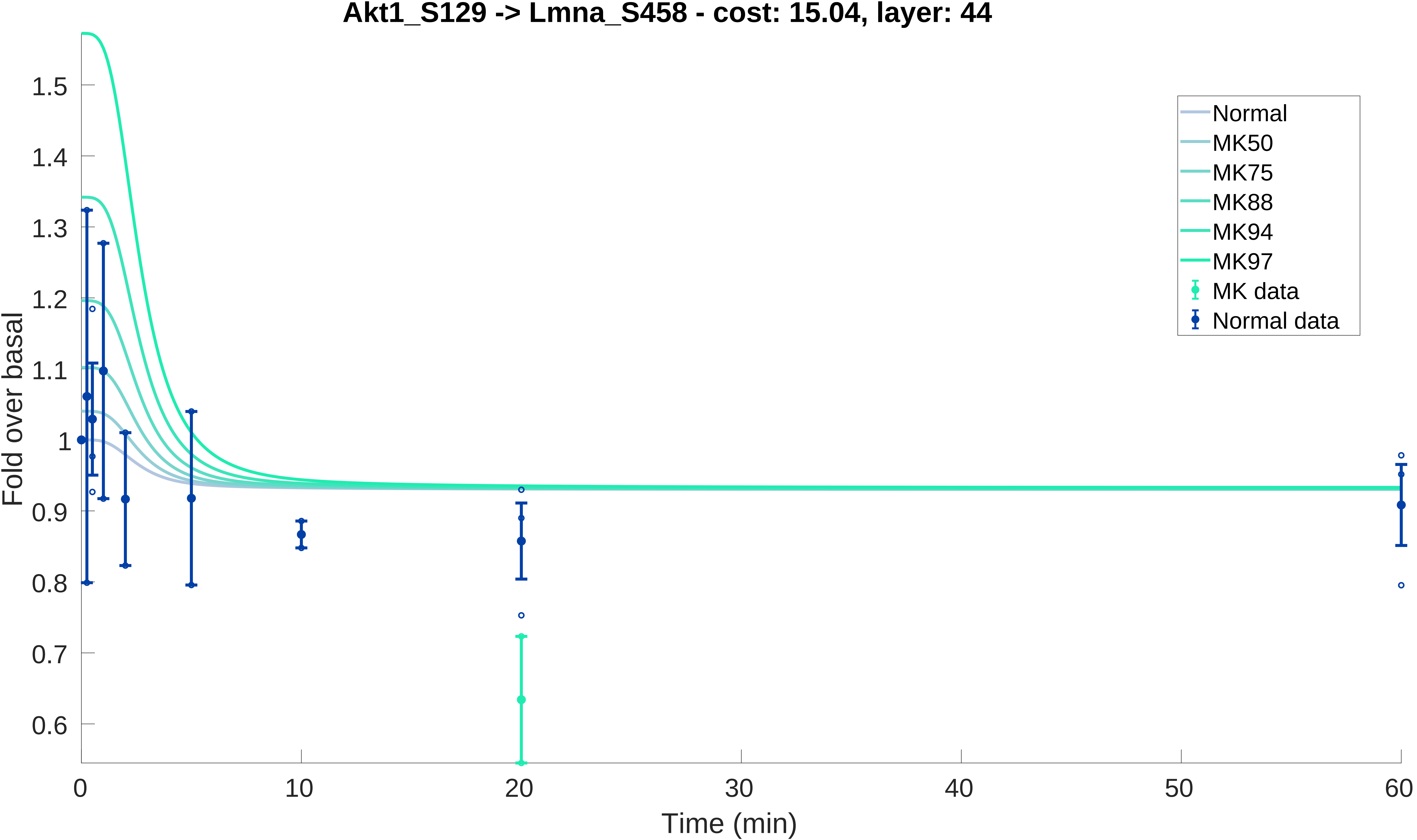
Akt1_S129 -> Lmna_S458 - cost: 15.04, layer: 44
Fold over basal
Time (min)
Normal
MK50
MK75
MK88
MK94
MK97
MK data
Normal data
1.5
1.4
1.3
1.2
1.1
1
0.9
0.8
0.7
0.6
0
10
20
30
40
50
60

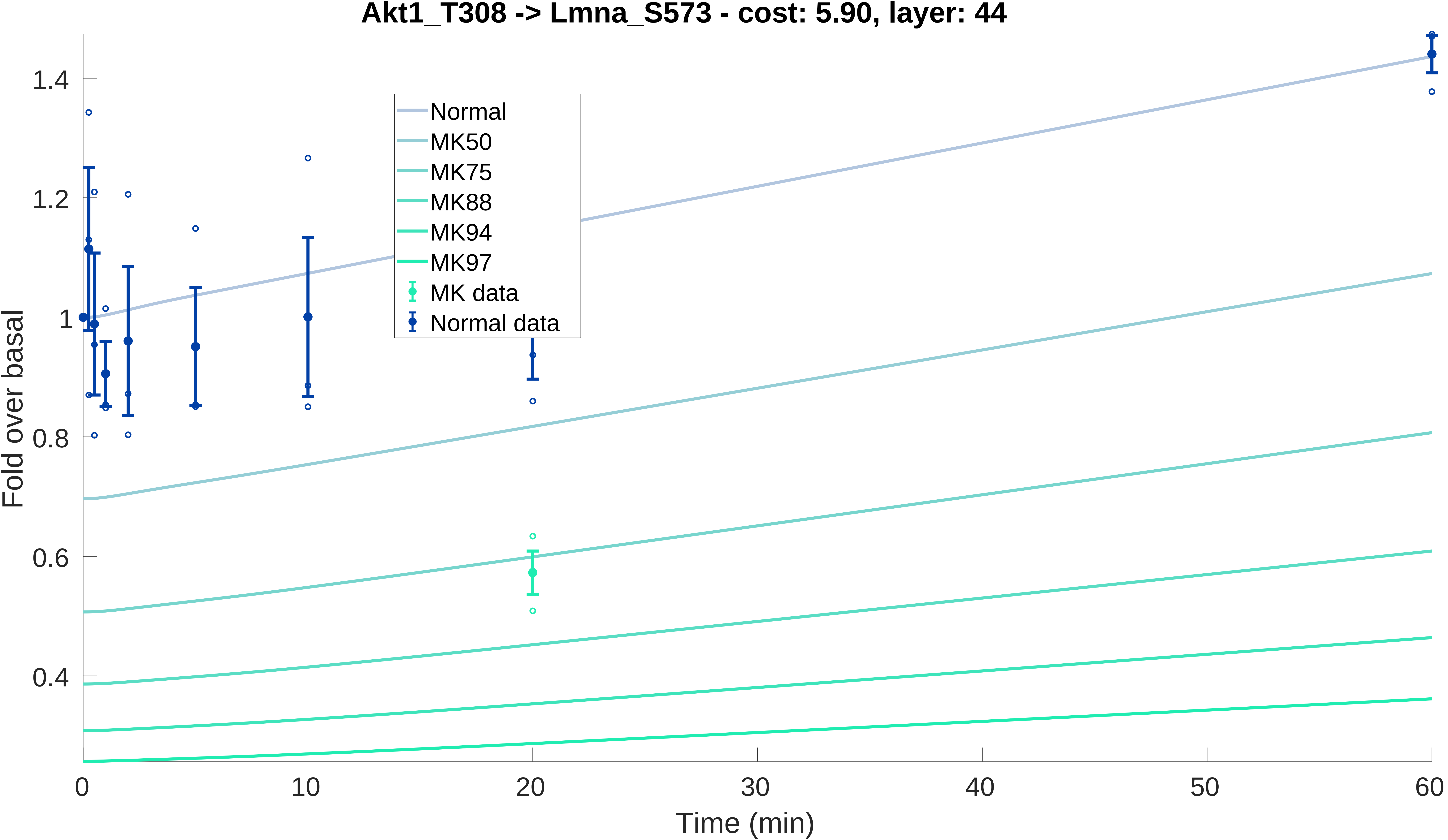

Akt1_T308 -> Lmna_S573 - cost: 5.90, layer: 44
Fold over basal
1.4
1.2
1
0.8
0.6
0.4
0
10
20
30
40
50
60
Time (min)
Normal
MK50
MK75
MK88
MK94
MK97
MK data
Normal data

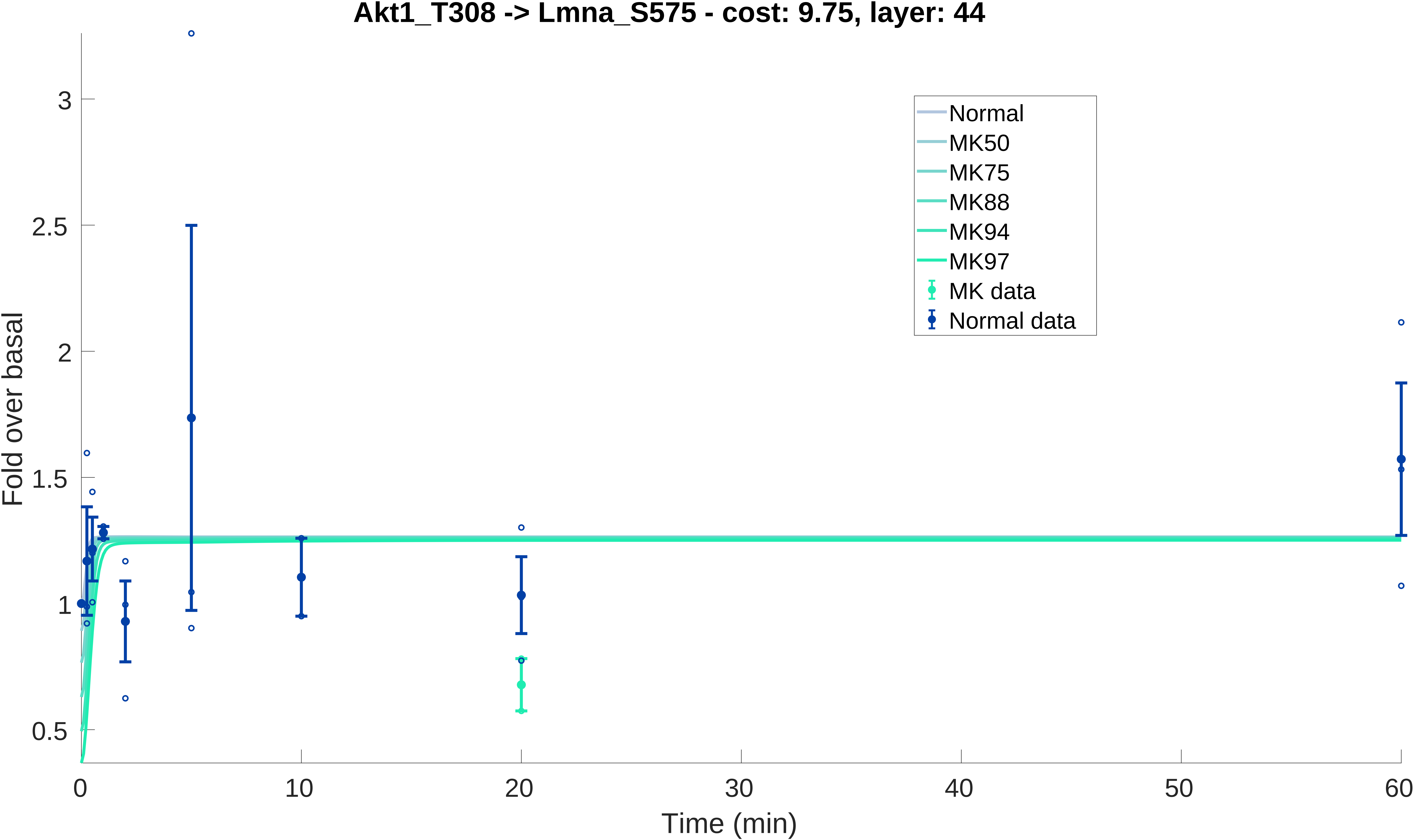

Akt1_T308 -> Lmna_S575 - cost: 9.75, layer: 44
Fold over basal
Time (min)
0.5
1
1.5
2
2.5
3
0
10
20
30
40
50
60
Normal
MK50
MK75
MK88
MK94
MK97
MK data
Normal data

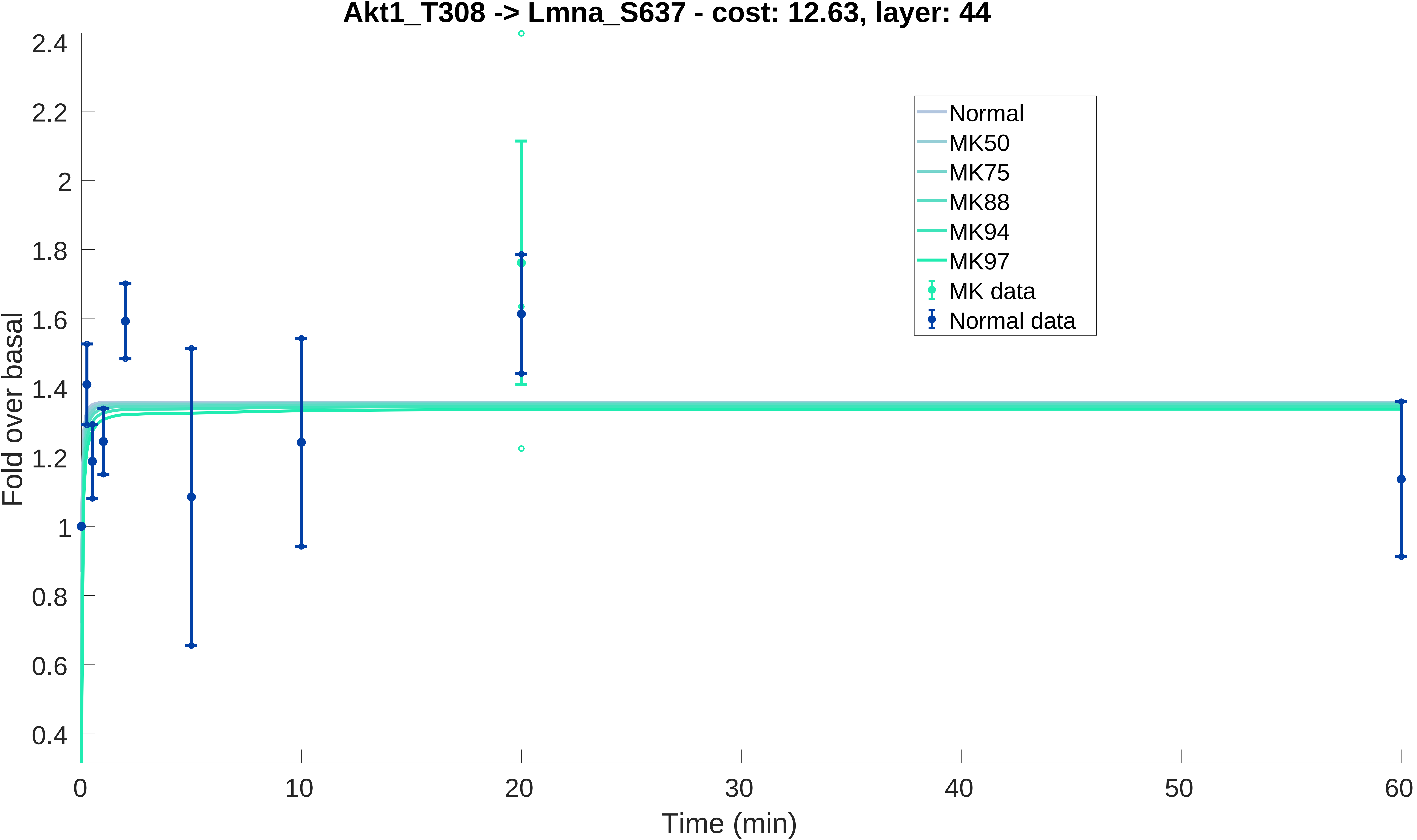

Akt1_T308 -> Lmna_S637 - cost: 12.63, layer: 44
Fold over basal
Time (min)
2.4
2.2
2
1.8
1.6
1.4
1.2
1
0.8
0.6
0.4
0
10
20
30
40
50
60
Normal
MK50
MK75
MK88
MK94
MK97
MK data
Normal data

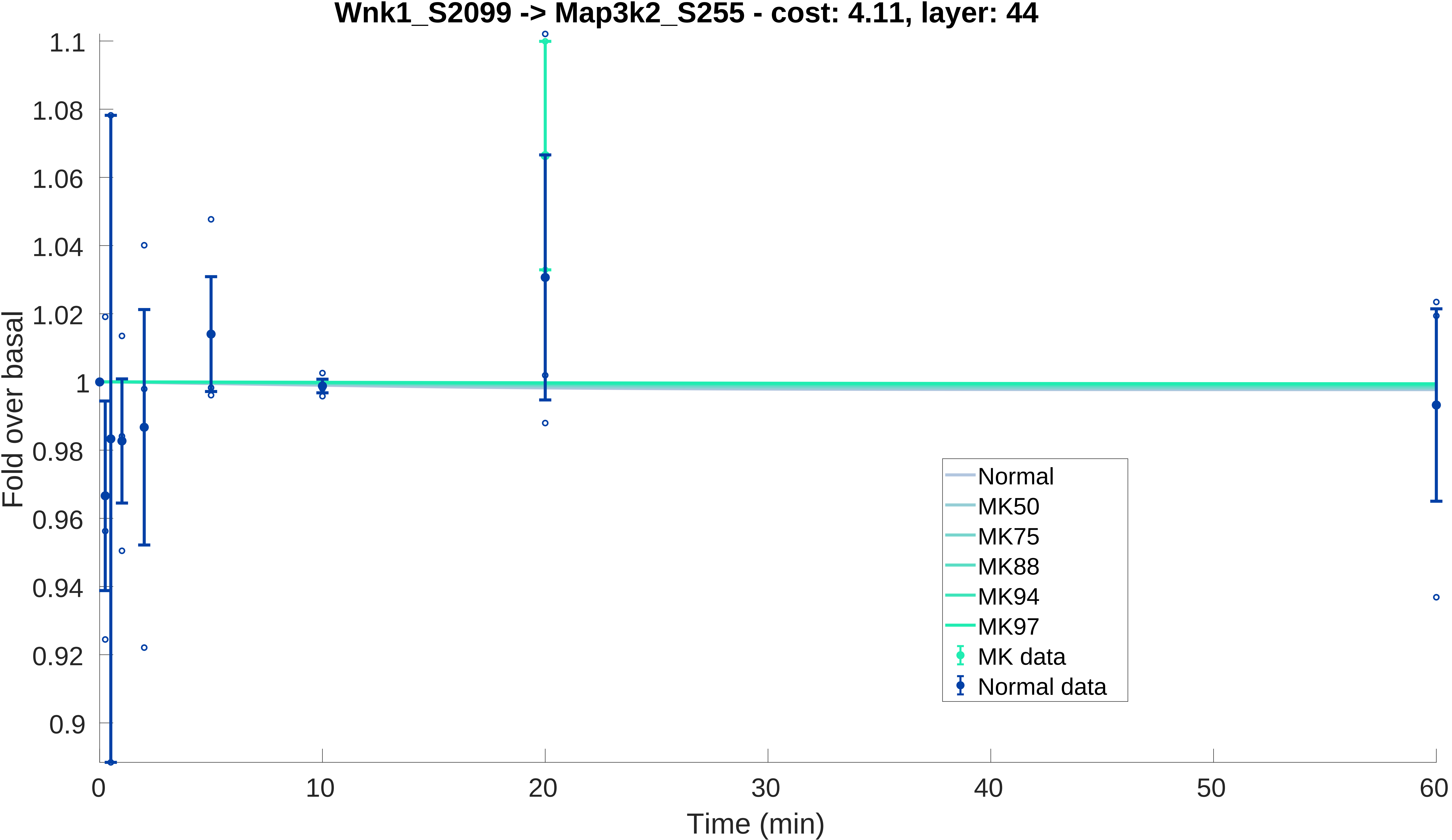
Wnk1_S2099 -> Map3k2_S255 - cost: 4.11, layer: 44
Fold over basal
Time (min)
1.1
1.08
1.06
1.04
1.02
1
0.98
0.96
0.94
0.92
0.9
0
10
20
30
40
50
60
Normal
MK50
MK75
MK88
MK94
MK97
MK data
Normal data

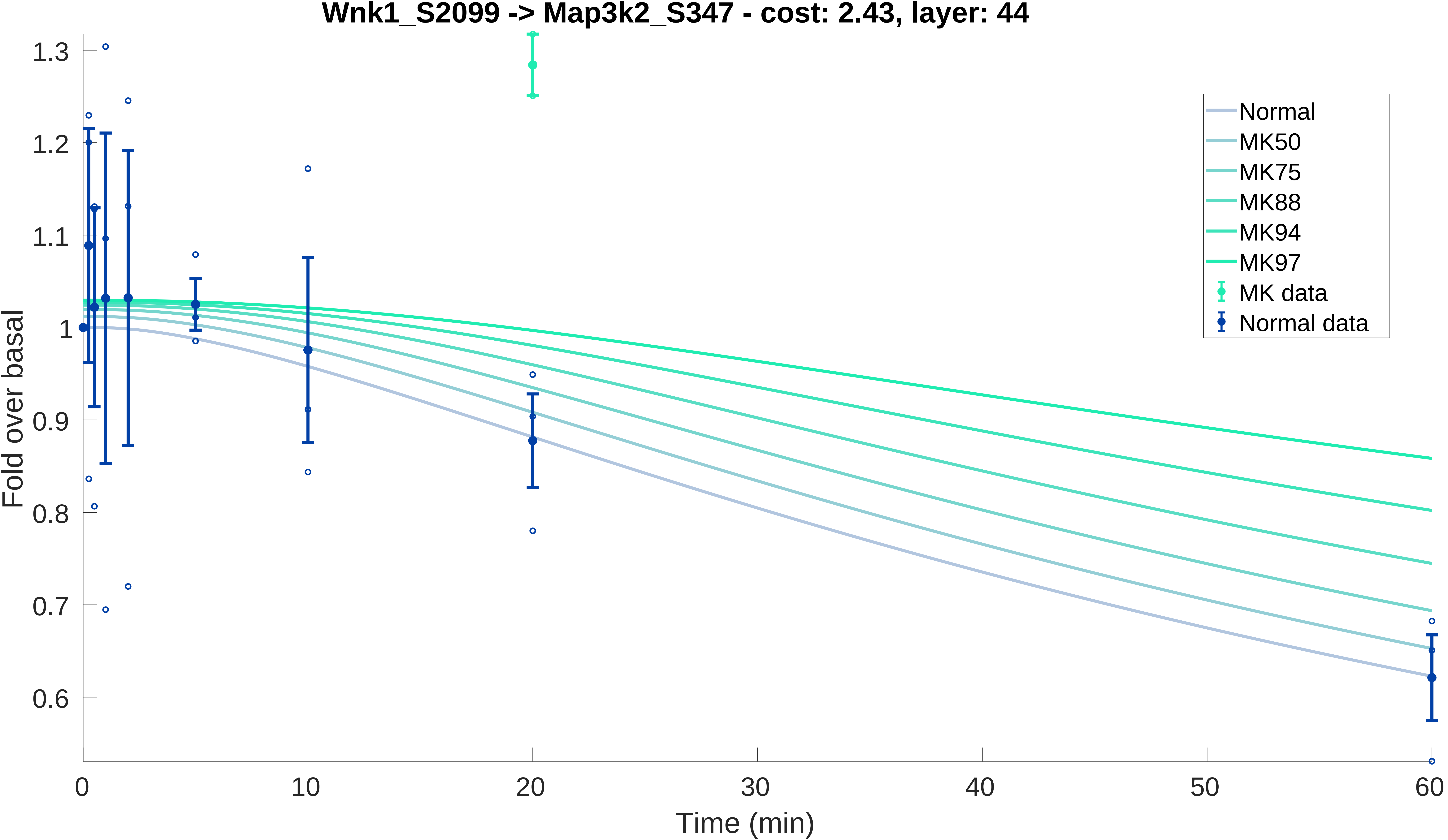

Wnk1_S2099 -> Map3k2_S347 - cost: 2.43, layer: 44
Fold over basal
Time (min)
1.3
1.2
1.1
1
0.9
0.8
0.7
0.6
0
10
20
30
40
50
60
Normal
MK50
MK75
MK88
MK94
MK97
MK data
Normal data

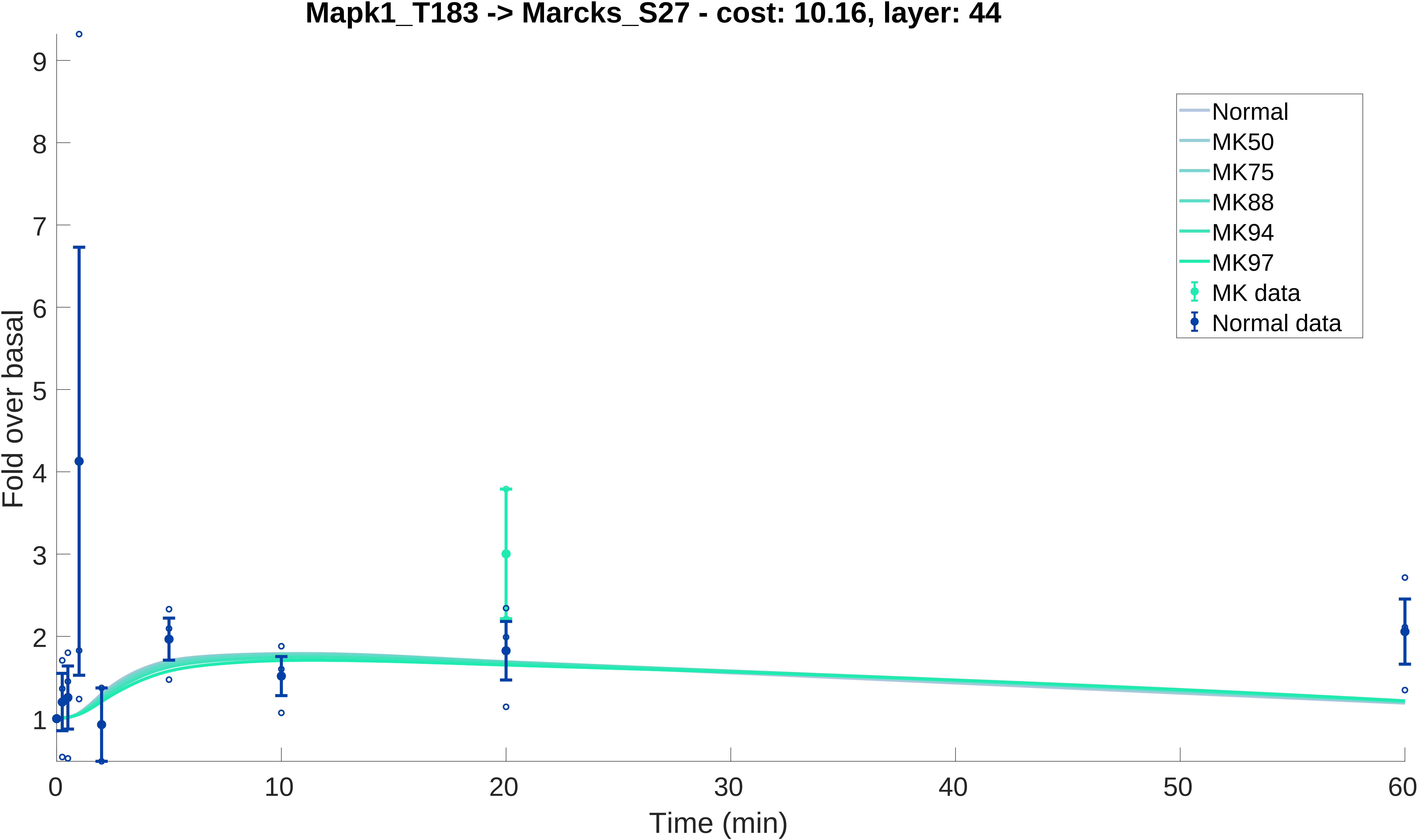

Mapk1_T183 -> Marcks_S27 - cost: 10.16, layer: 44
Fold over basal
Time (min)
0
10
20
30
40
50
60
1
2
3
4
5
6
7
8
9
Normal
MK50
MK75
MK88
MK94
MK97
MK data
Normal data

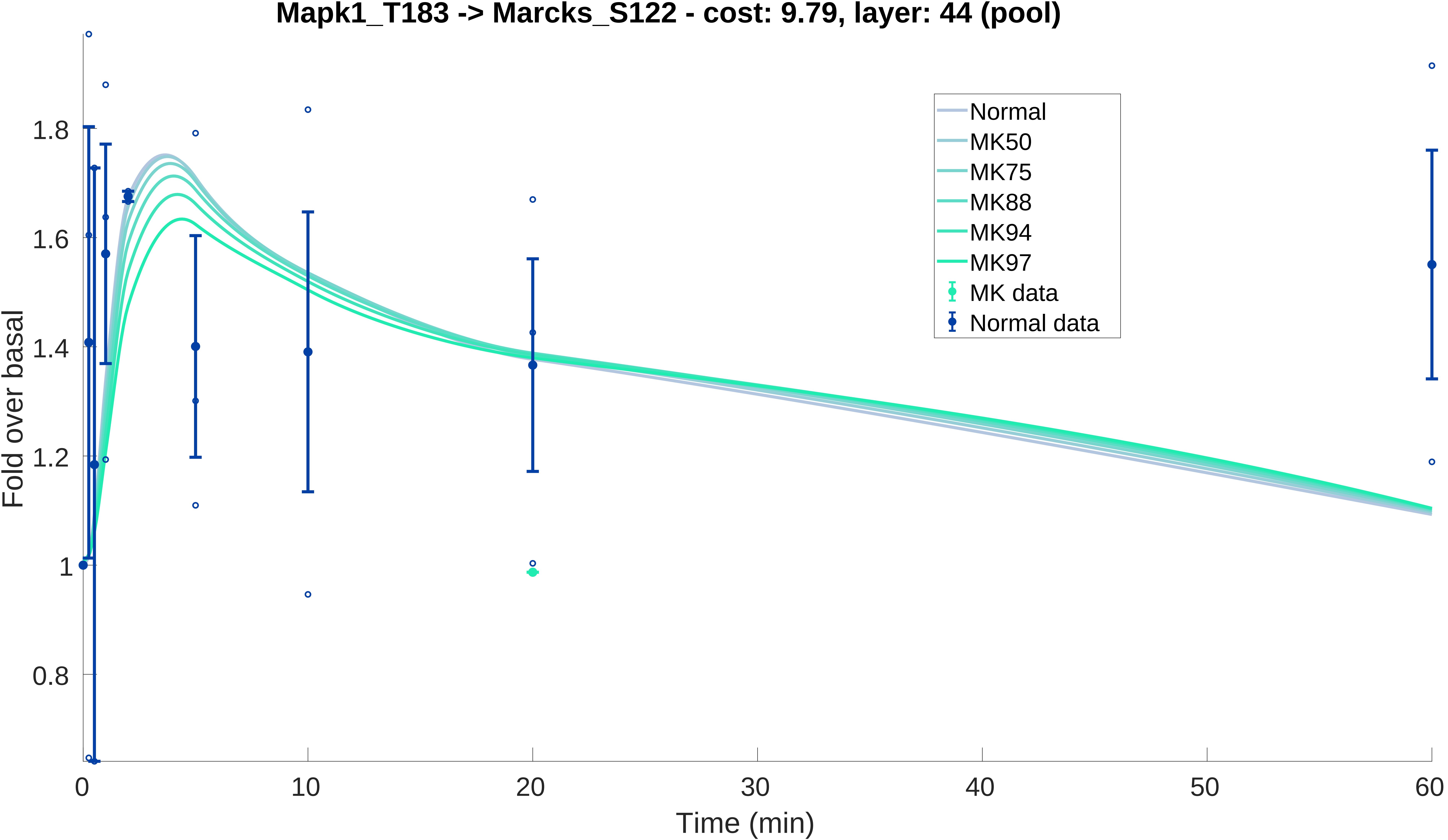

Mapk1_T183 -> Marcks_S122 - cost: 9.79, layer: 44 (pool)
Fold over basal
Time (min)
0.8
1
1.2
1.4
1.6
1.8
0
10
20
30
40
50
60
Normal
MK50
MK75
MK88
MK94
MK97
MK data
Normal data

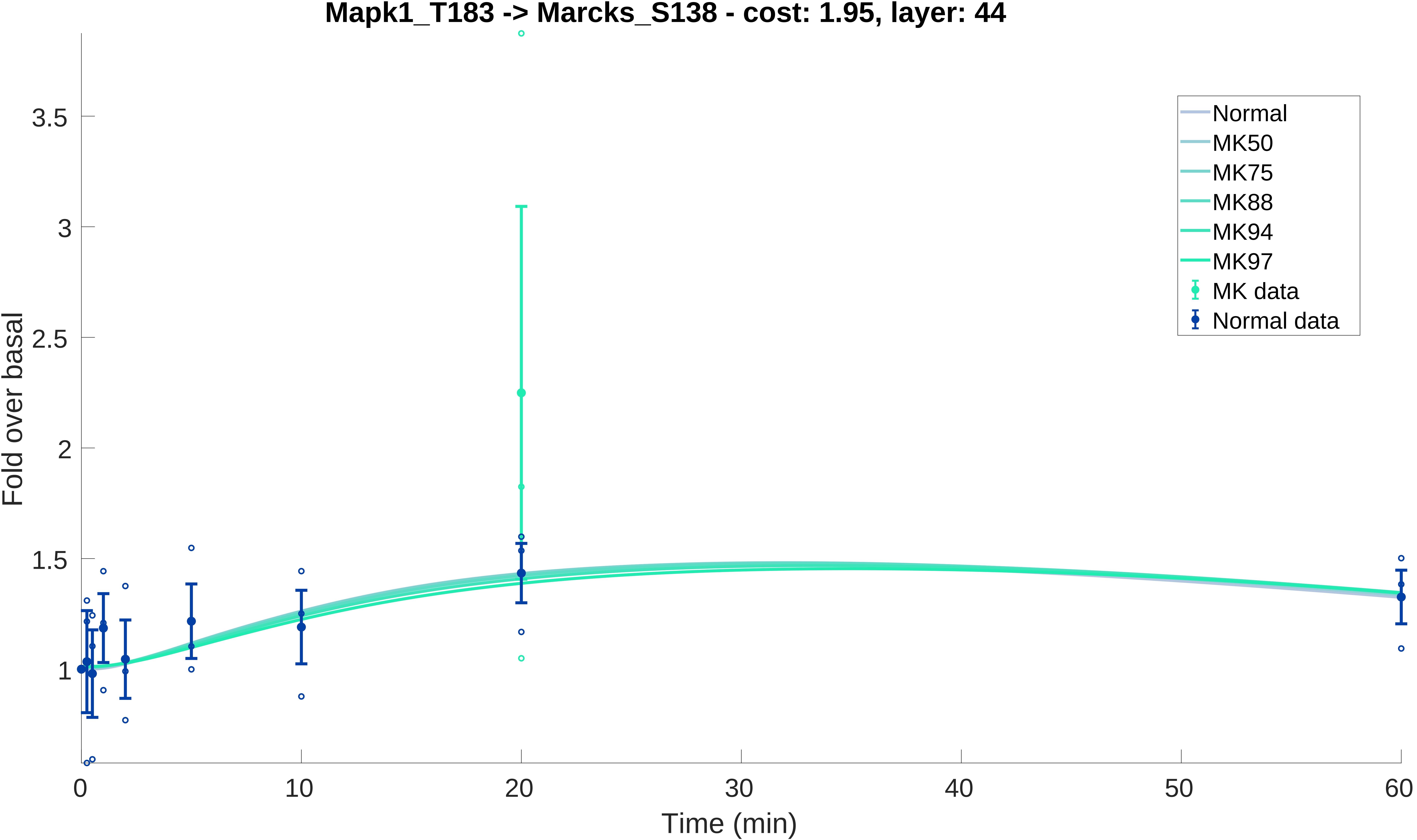
Mapk1_T183 -> Marcks_S138 - cost: 1.95, layer: 44
Fold over basal
Time (min)
0
10
20
30
40
50
60
1
1.5
2
2.5
3
3.5
Normal
MK50
MK75
MK88
MK94
MK97
MK data
Normal data

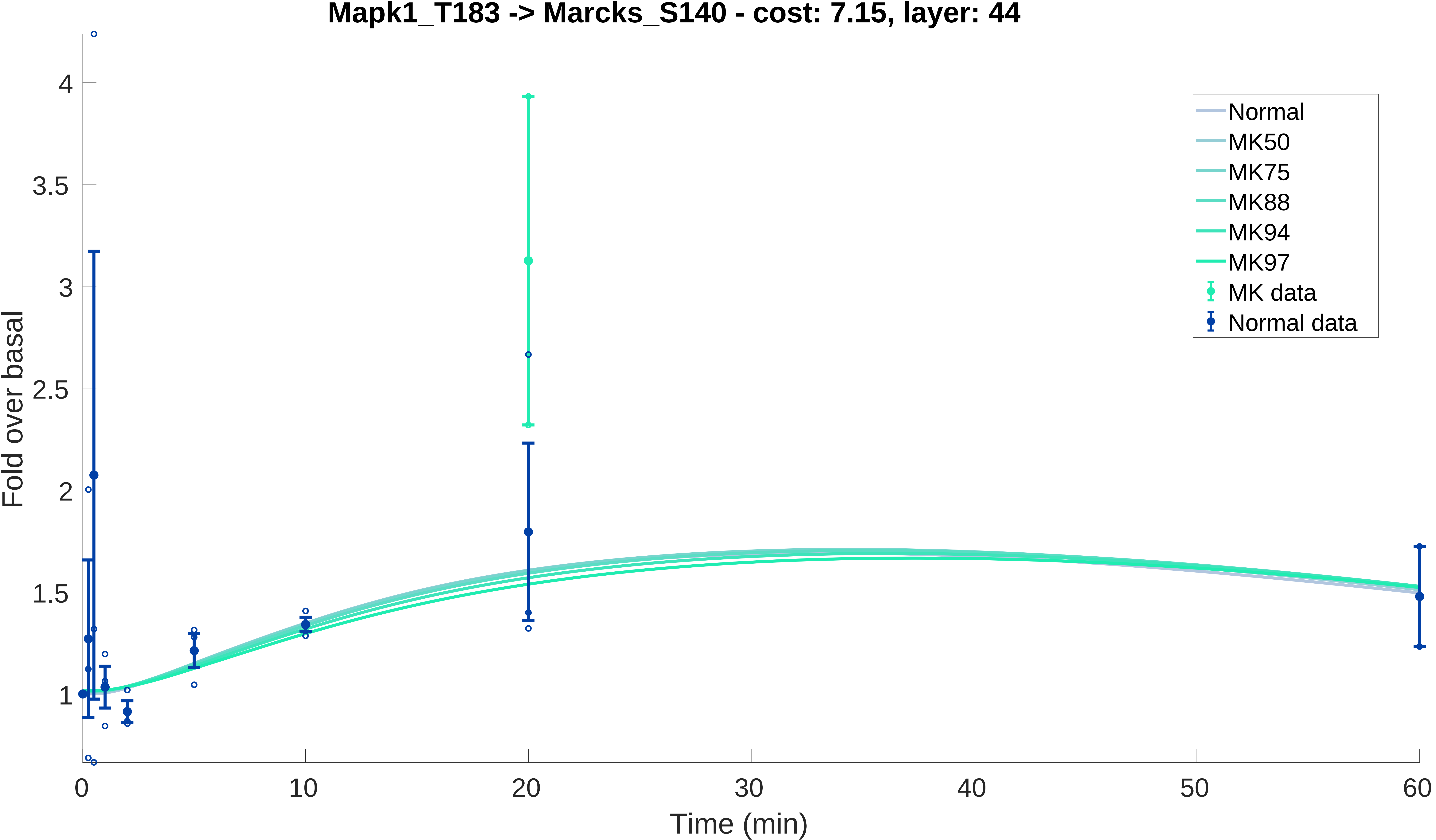

Mapk1_T183 -> Marcks_S140 - cost: 7.15, layer: 44
Fold over basal
Time (min)
0
10
20
30
40
50
60
1
1.5
2
2.5
3
3.5
4
Normal
MK50
MK75
MK88
MK94
MK97
MK data
Normal data

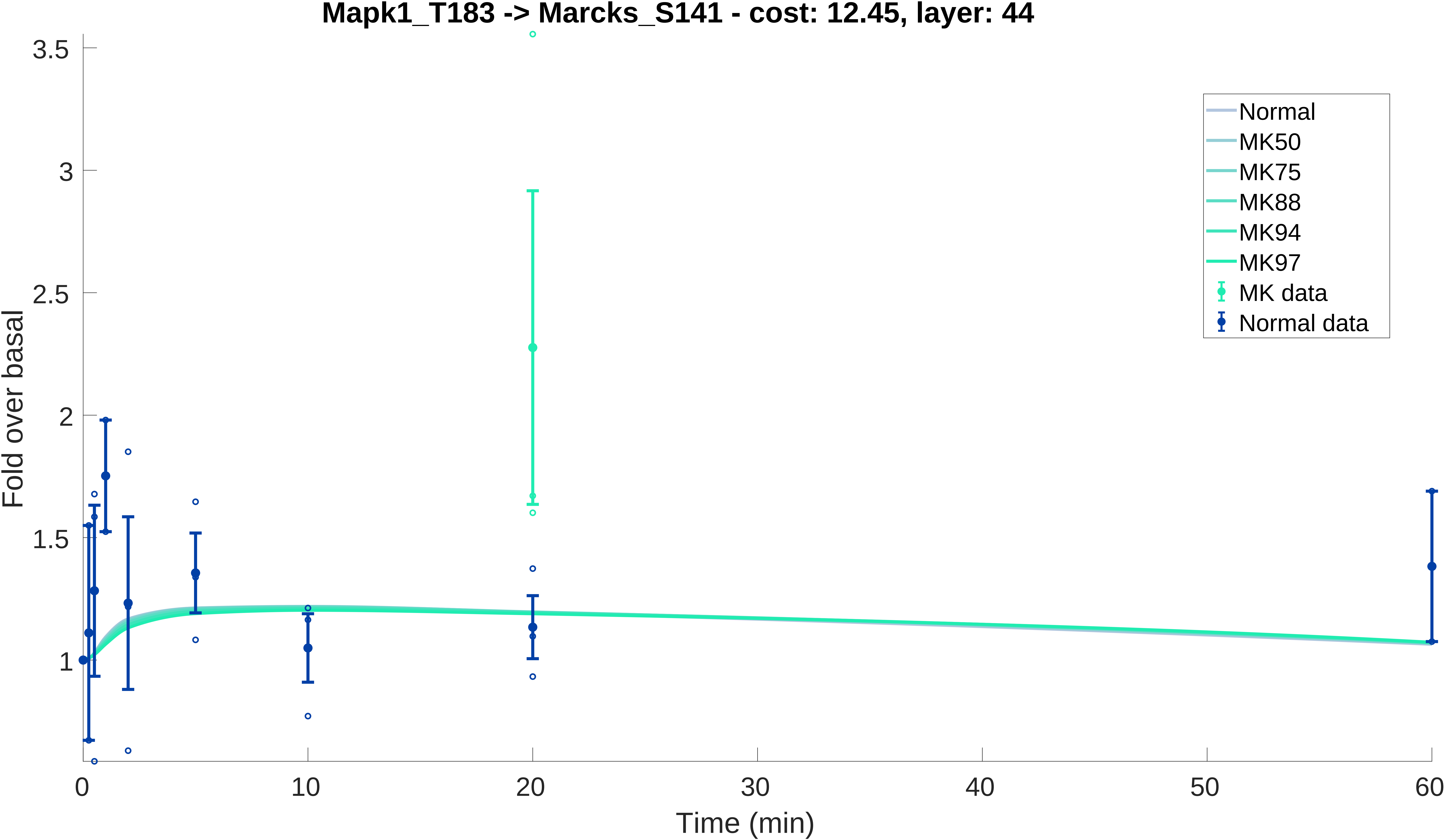
Mapk1_T183 -> Marcks_S141 - cost: 12.45, layer: 44
Fold over basal
3.5
3
2.5
2
1.5
1
Time (min)
0
10
20
30
40
50
60
Normal
MK50
MK75
MK88
MK94
MK97
MK data
Normal data

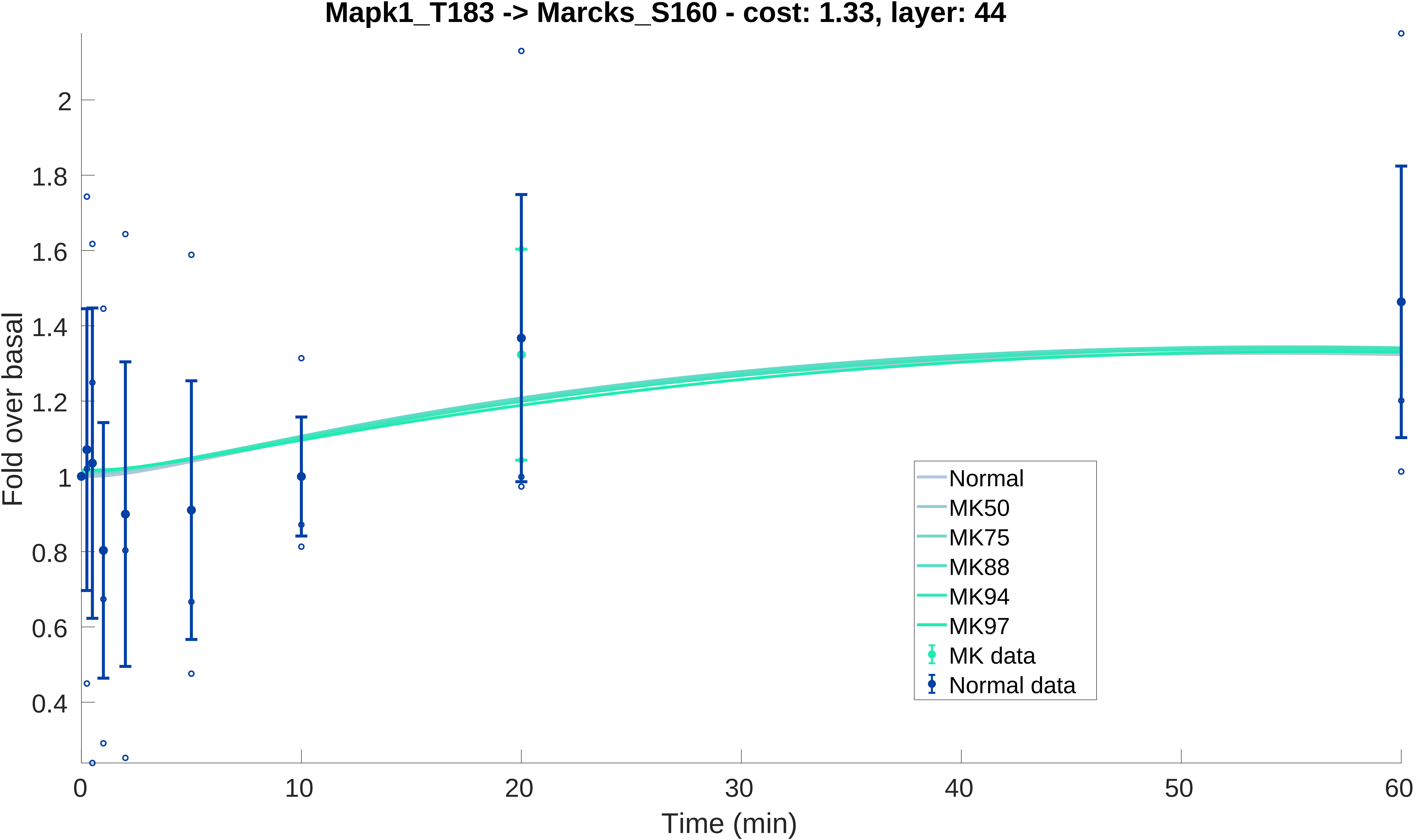

Mapk1_T183 -> Marcks_S160 - cost: 1.33, layer: 44
Fold over basal
Time (min)
0
10
20
30
40
50
60
0.4
0.6
0.8
1
1.2
1.4
1.6
1.8
2
Normal
MK50
MK75
MK88
MK94
MK97
MK data
Normal data

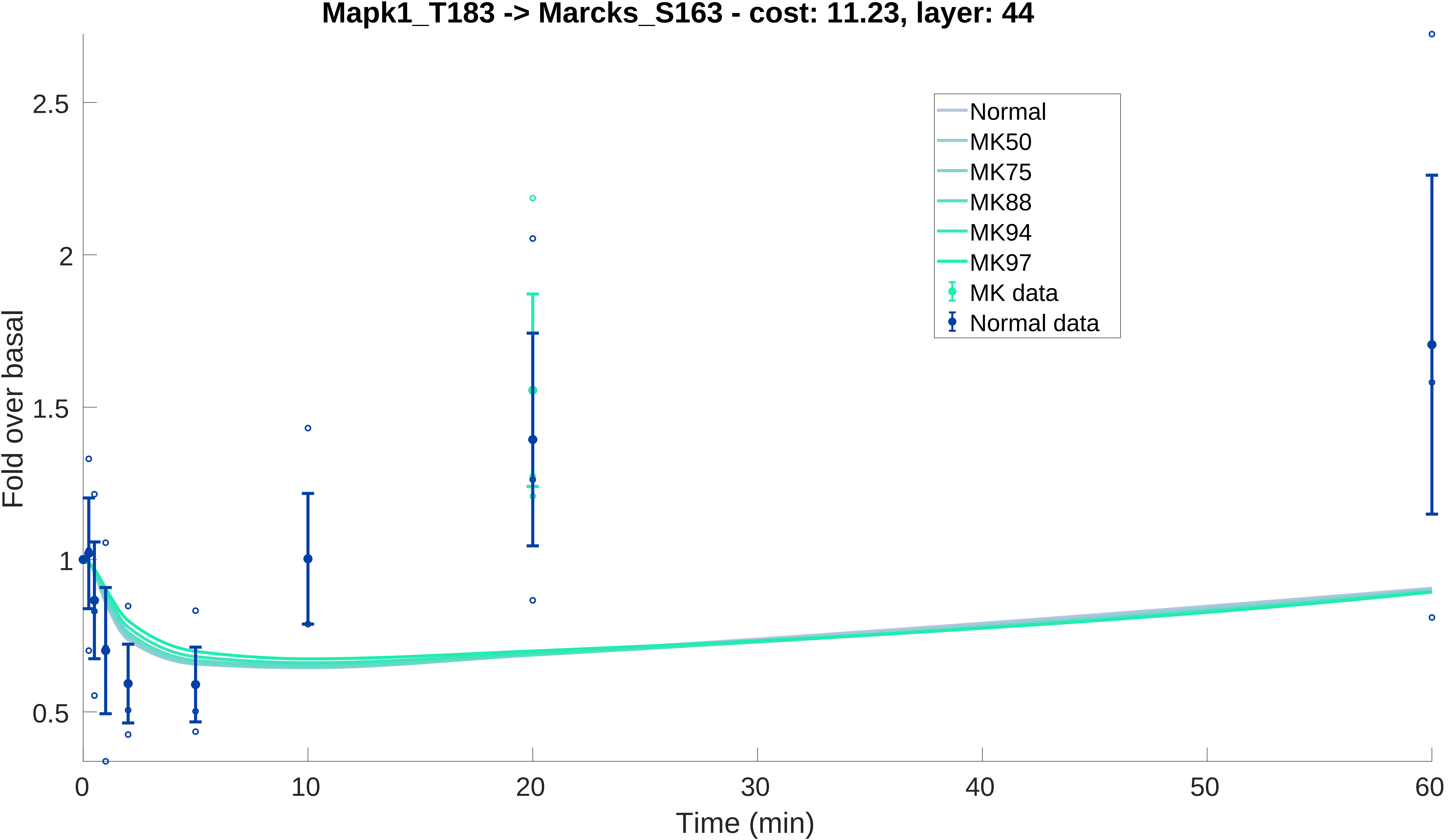

Mapk1_T183 -> Marcks_S163 - cost: 11.23, layer: 44
Fold over basal
Time (min)
Normal
MK50
MK75
MK88
MK94
MK97
MK data
Normal data
0.5
1
1.5
2
2.5
0
10
20
30
40
50
60

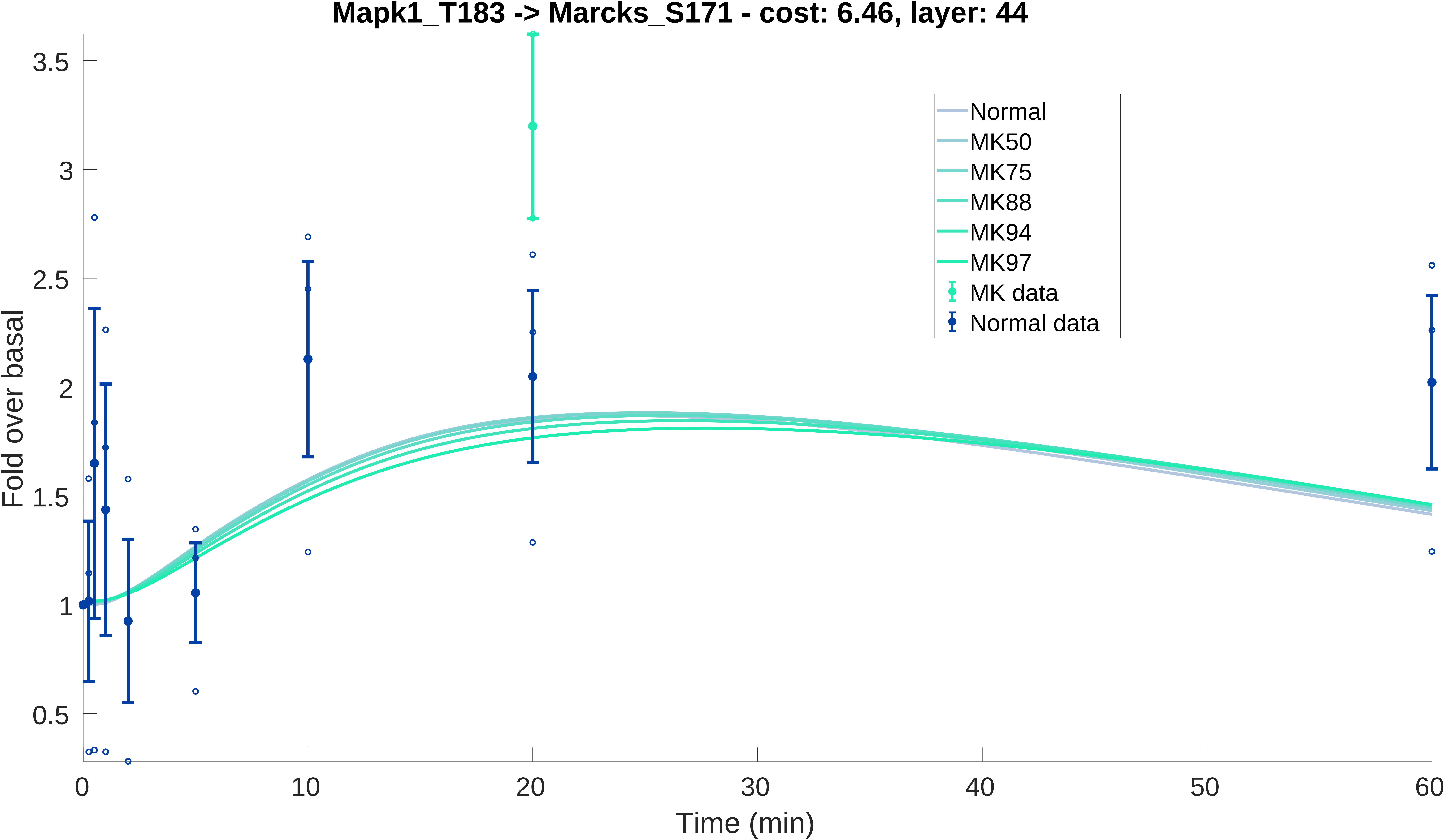

Mapk1_T183 -> Marcks_S171 - cost: 6.46, layer: 44
Fold over basal
Time (min)
0.5
1
1.5
2
2.5
3
3.5
0
10
20
30
40
50
60
Normal
MK50
MK75
MK88
MK94
MK97
MK data
Normal data

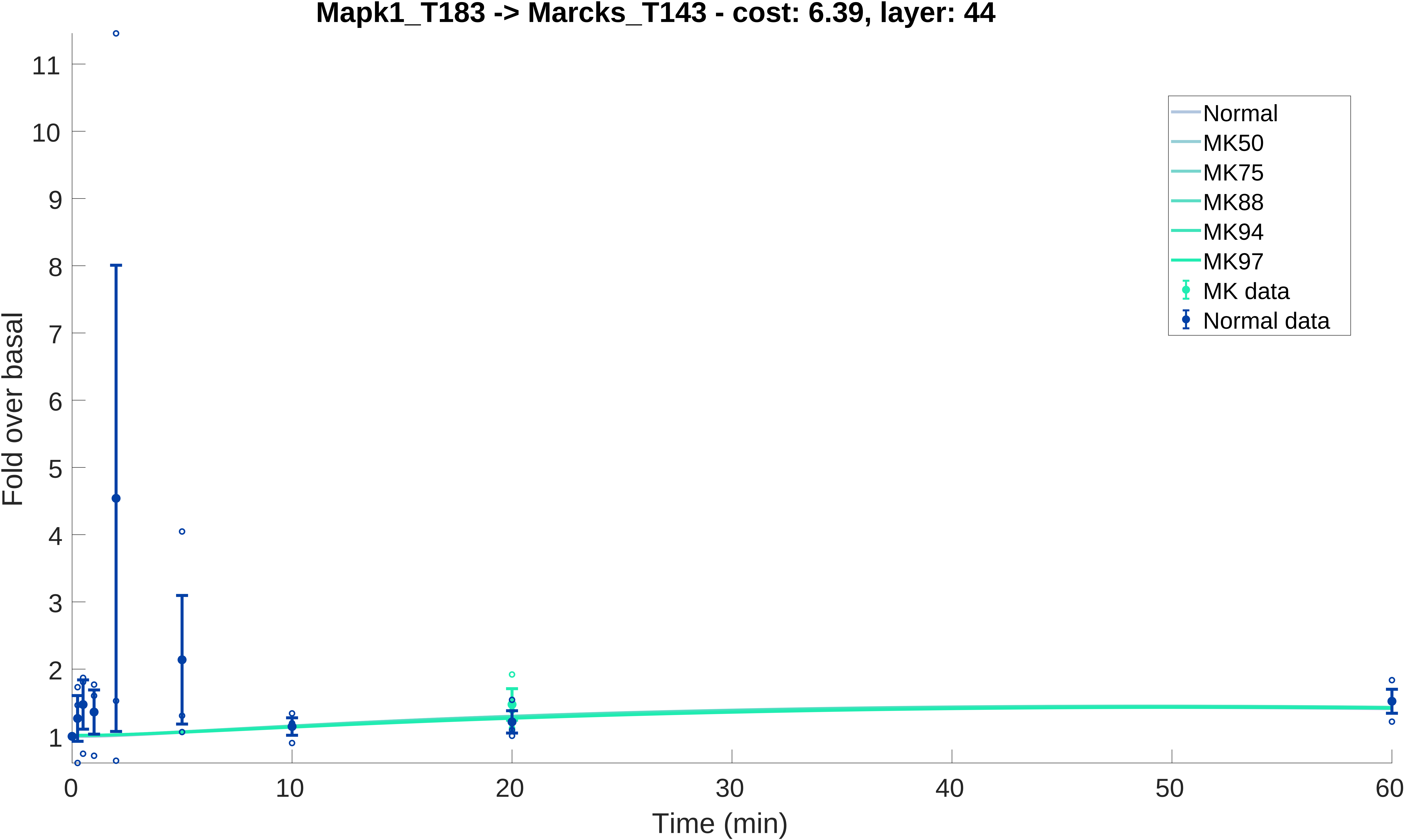

Mapk1_T183 -> Marcks_T143 - cost: 6.39, layer: 44
Fold over basal
Time (min)
0
10
20
30
40
50
60
1
2
3
4
5
6
7
8
9
10
11
Normal
MK50
MK75
MK88
MK94
MK97
MK data
Normal data

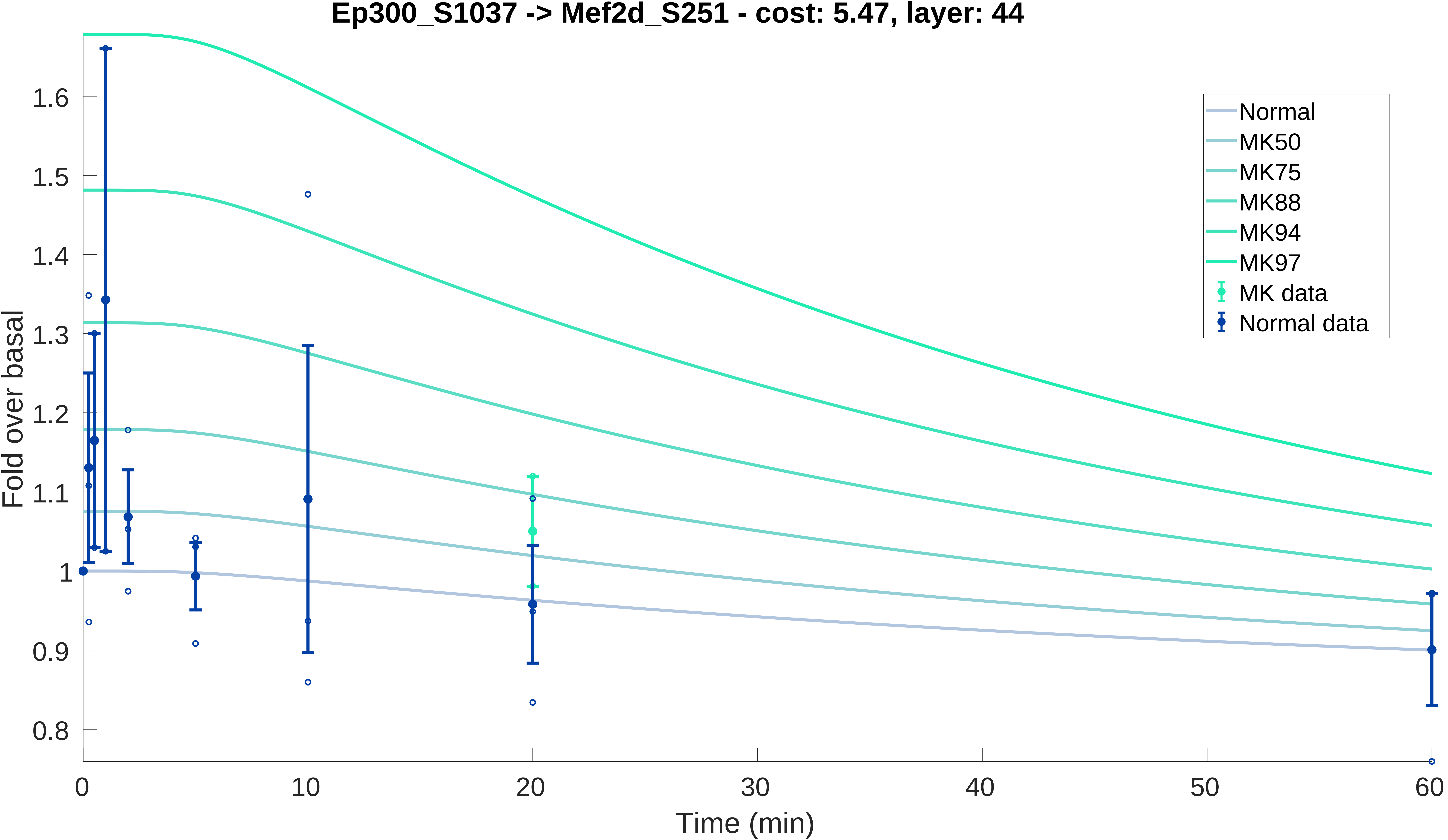

Ep300_S1037 -> Mef2d_S251 - cost: 5.47, layer: 44
Fold over basal
1.6
1.5
1.4
1.3
1.2
1.1
1
0.9
0.8
0
10
20
30
40
50
60
Time (min)
Normal
MK50
MK75
MK88
MK94
MK97
MK data
Normal data

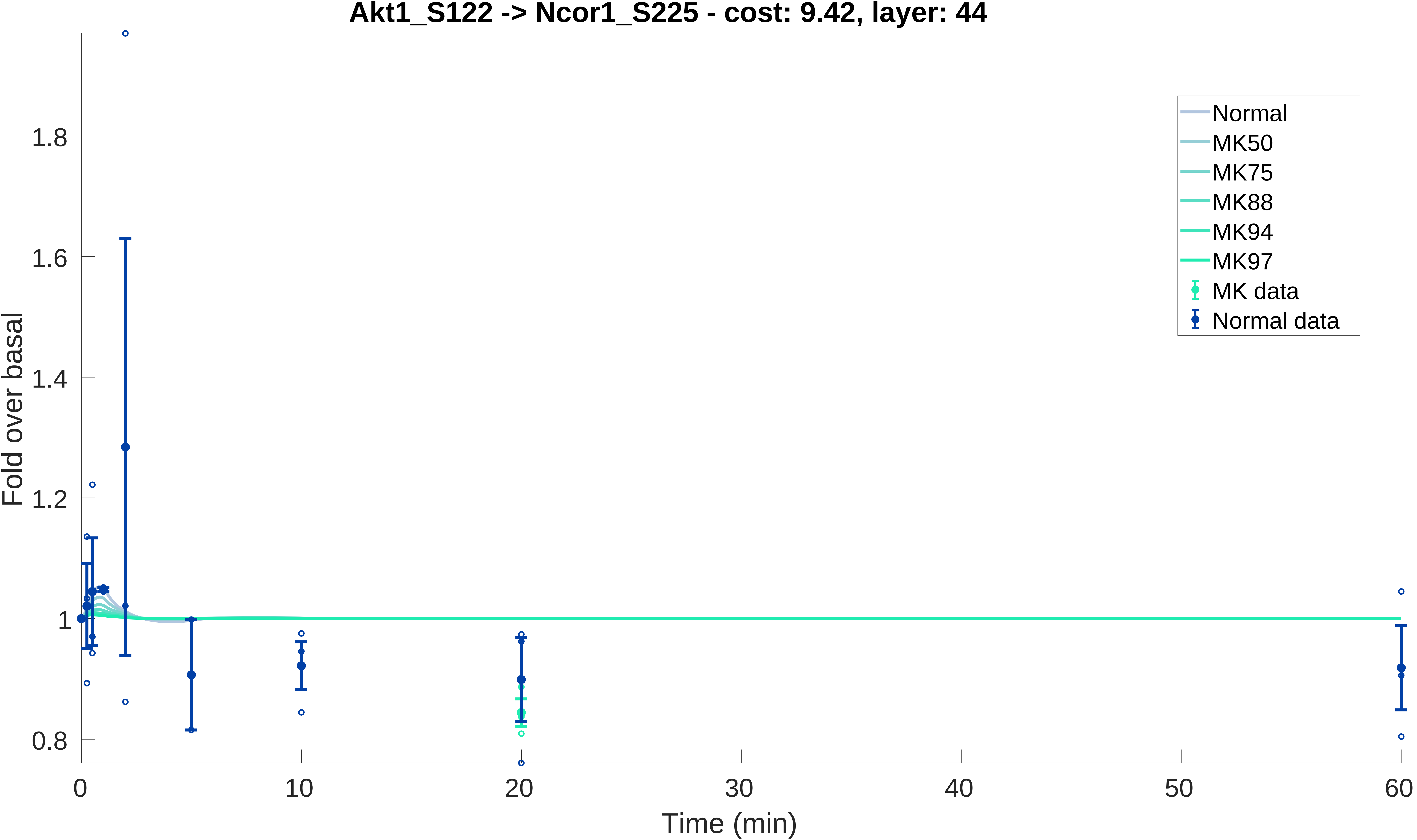

Akt1_S122 -> Ncor1_S225 - cost: 9.42, layer: 44
Fold over basal
Time (min)
0.8
1
1.2
1.4
1.6
1.8
0
10
20
30
40
50
60
Normal
MK50
MK75
MK88
MK94
MK97
MK data
Normal data

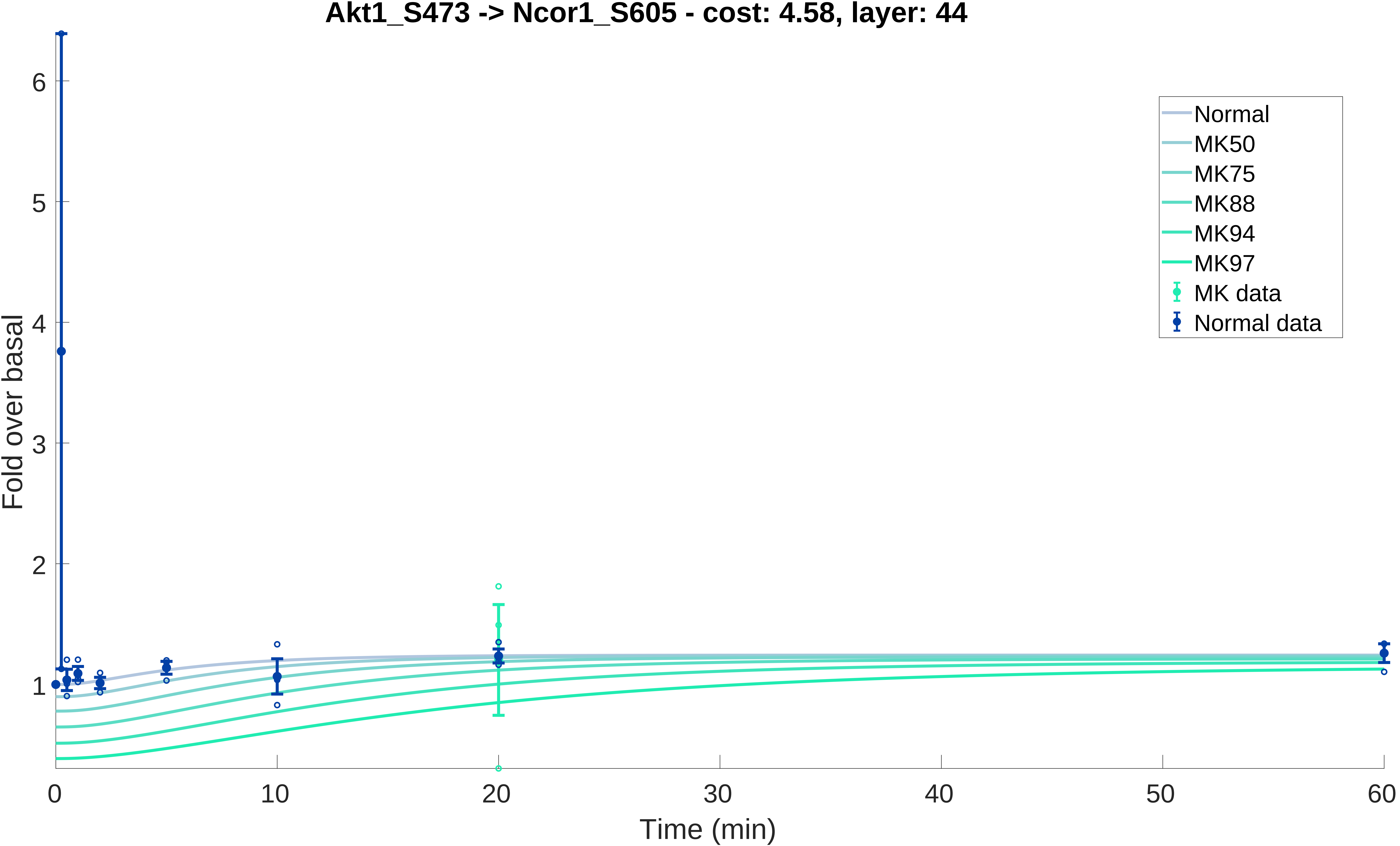

Akt1_S473 -> Ncor1_S605 - cost: 4.58, layer: 44
Fold over basal
Time (min)
Normal
MK50
MK75
MK88
MK94
MK97
MK data
Normal data
0
10
20
30
40
50
60
1
2
3
4
5
6

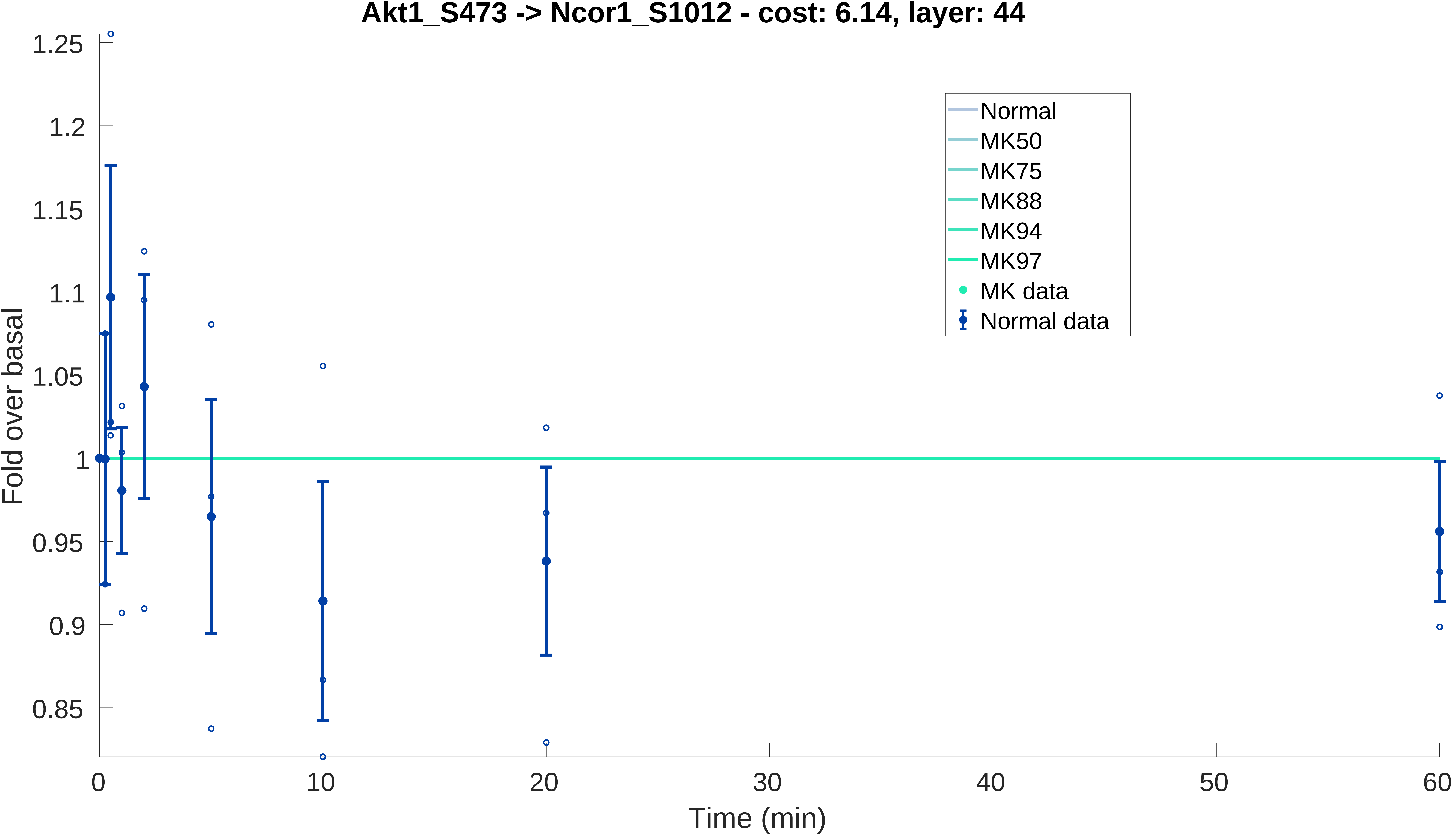

Akt1_S473 -> Ncor1_S1012 - cost: 6.14, layer: 44
Fold over basal
Time (min)
1.25
1.2
1.15
1.1
1.05
1
0.95
0.9
0.85
0
10
20
30
40
50
60
Normal
MK50
MK75
MK88
MK94
MK97
MK data
Normal data

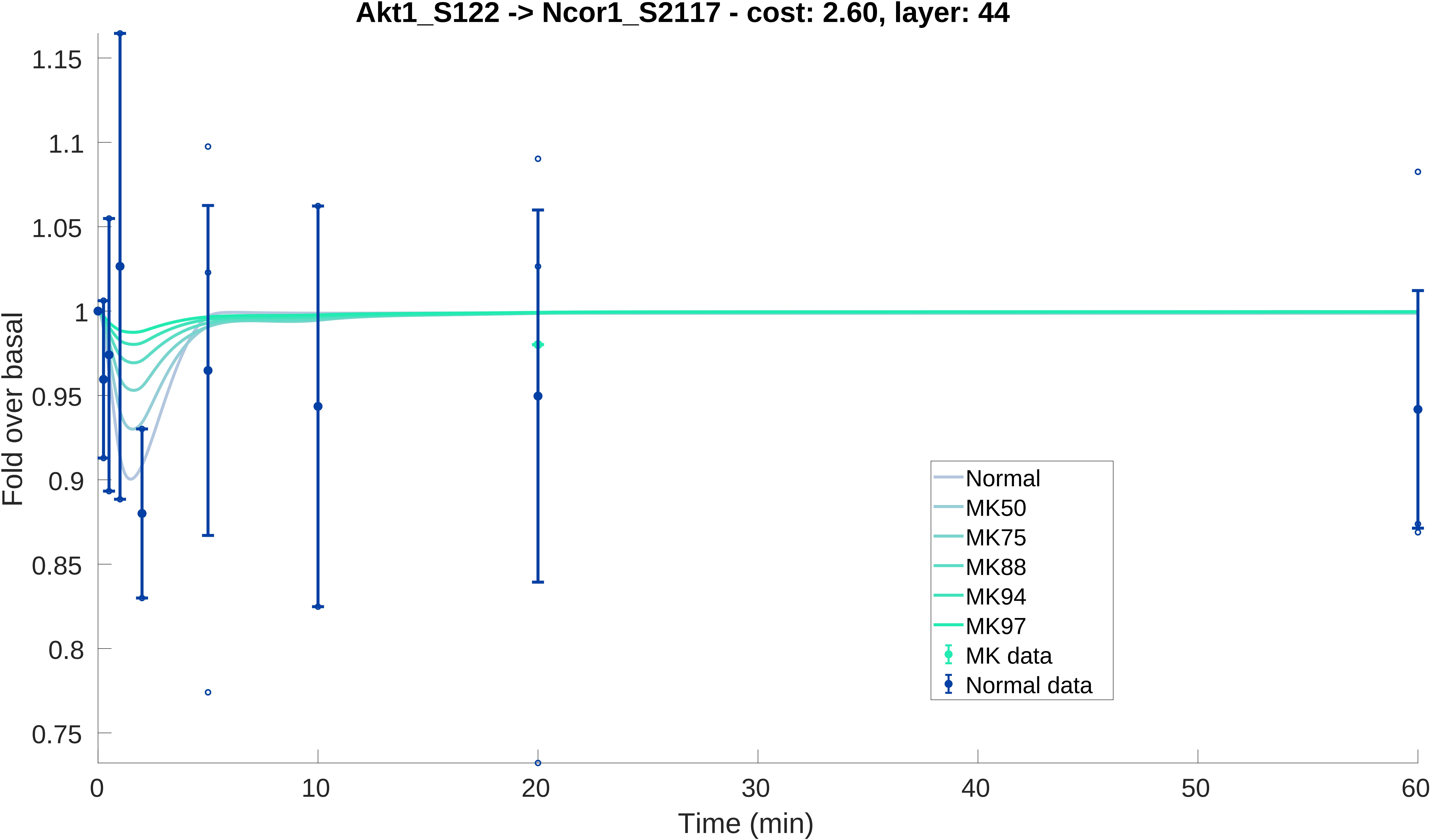

Akt1_S122 -> Ncor1_S2117 - cost: 2.60, layer: 44
Fold over basal
1.15
1.1
1.05
1
0.95
0.9
0.85
0.8
0.75
0
10
20
30
40
50
60
Time (min)
Normal
MK50
MK75
MK88
MK94
MK97
MK data
Normal data

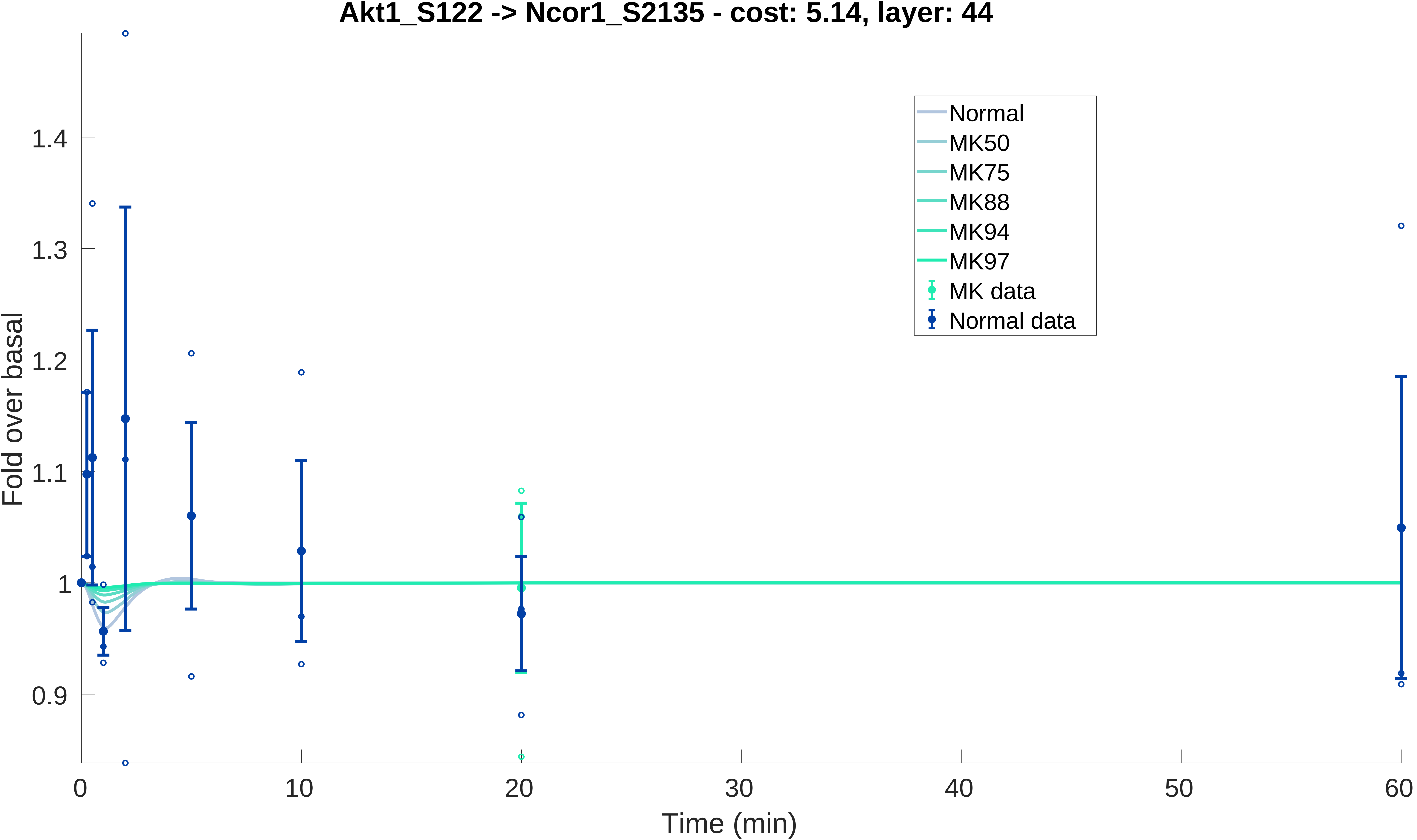

Akt1_S122 -> Ncor1_S2135 - cost: 5.14, layer: 44
Normal
MK50
MK75
MK88
MK94
MK97
MK data
Normal data
Fold over basal
1.4
1.3
1.2
1.1
1
0.9
0
10
20
30
40
50
60
Time (min)

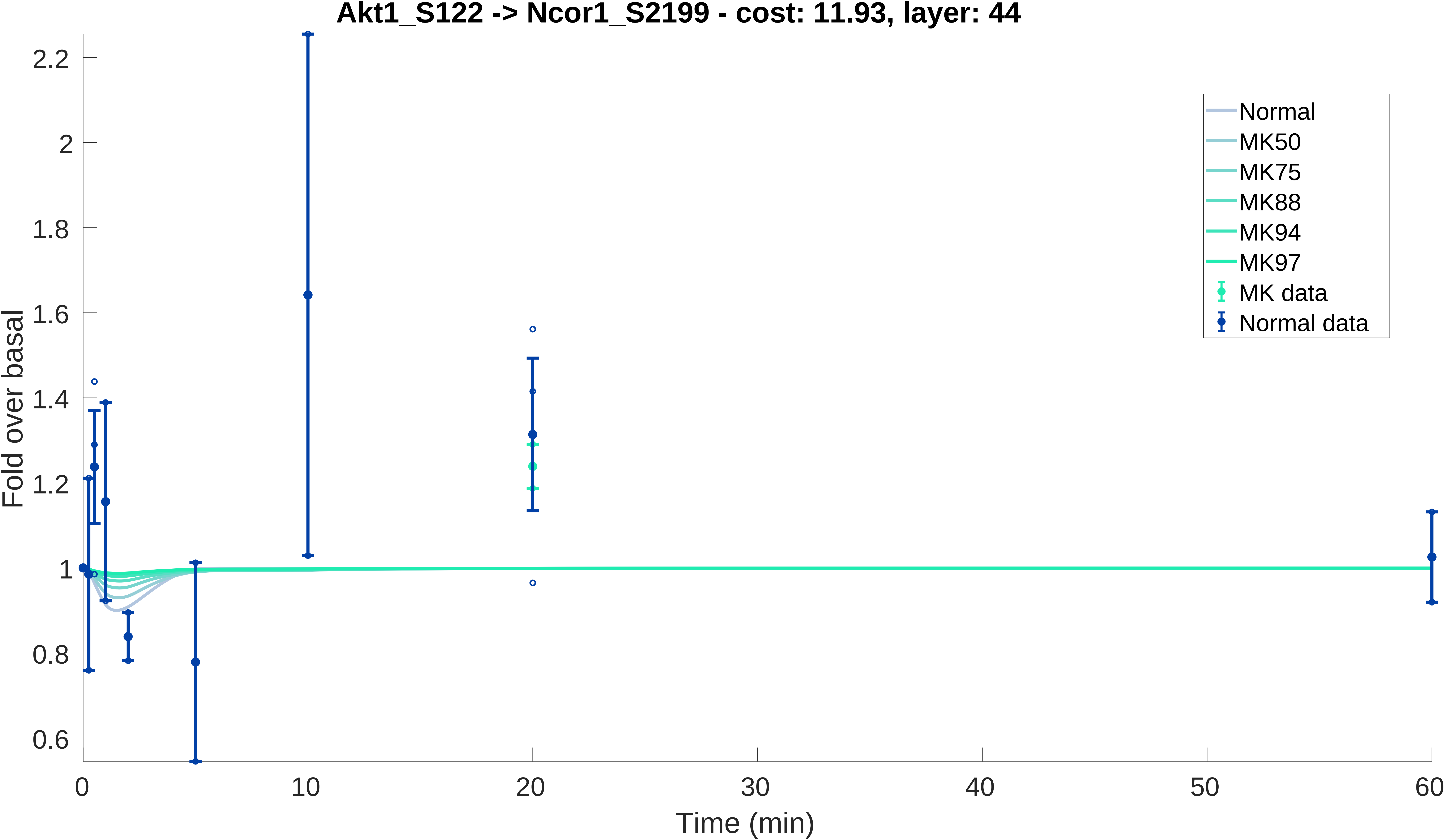

Akt1_S122 -> Ncor1_S2199 - cost: 11.93, layer: 44
Fold over basal
Time (min)
0
10
20
30
40
50
60
0.6
0.8
1
1.2
1.4
1.6
1.8
2
2.2
Normal
MK50
MK75
MK88
MK94
MK97
MK data
Normal data

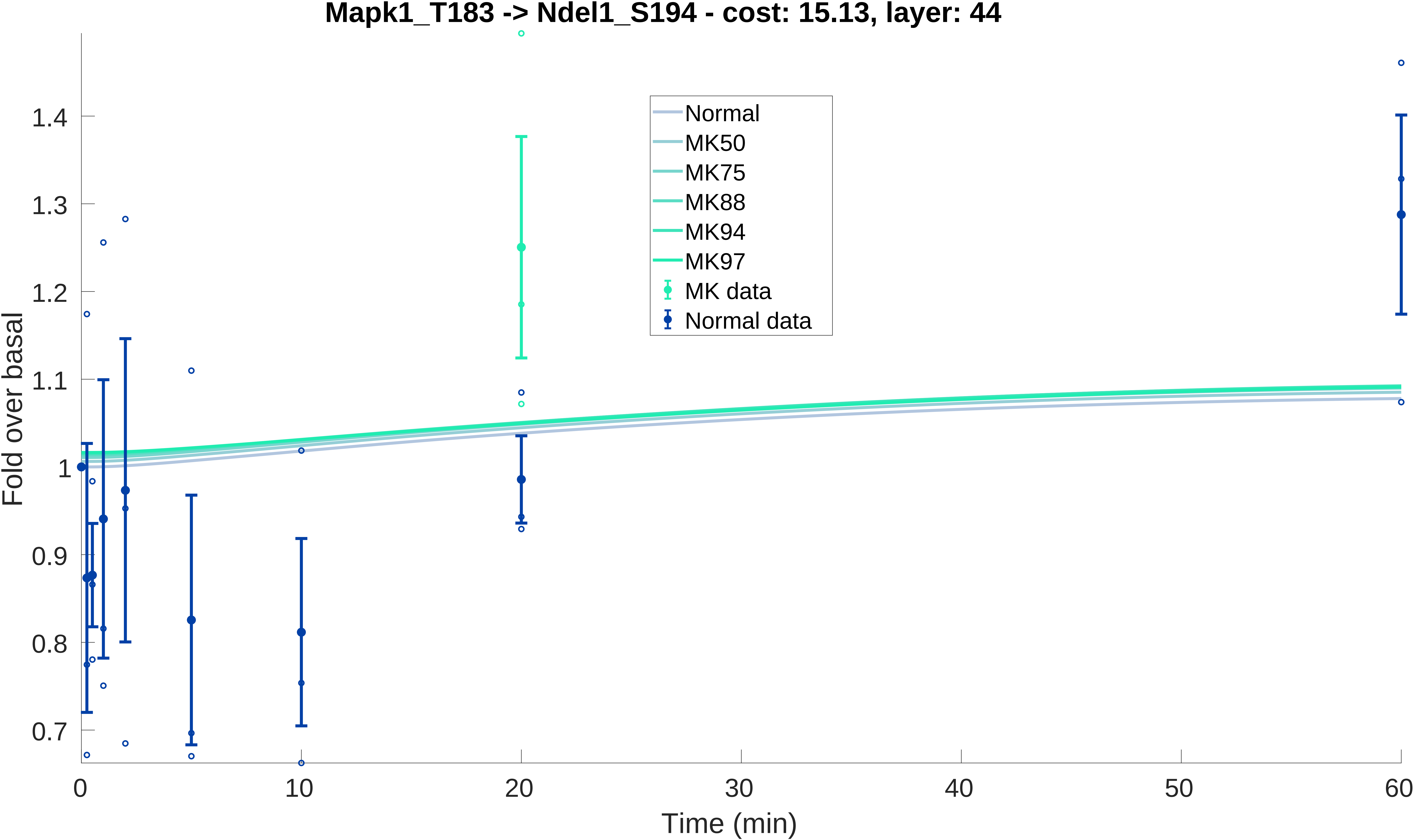
Mapk1_T183 -> Ndel1_S194 - cost: 15.13, layer: 44
Fold over basal
Time (min)
0.7
0.8
0.9
1
1.1
1.2
1.3
1.4
0
10
20
30
40
50
60
Normal
MK50
MK75
MK88
MK94
MK97
MK data
Normal data

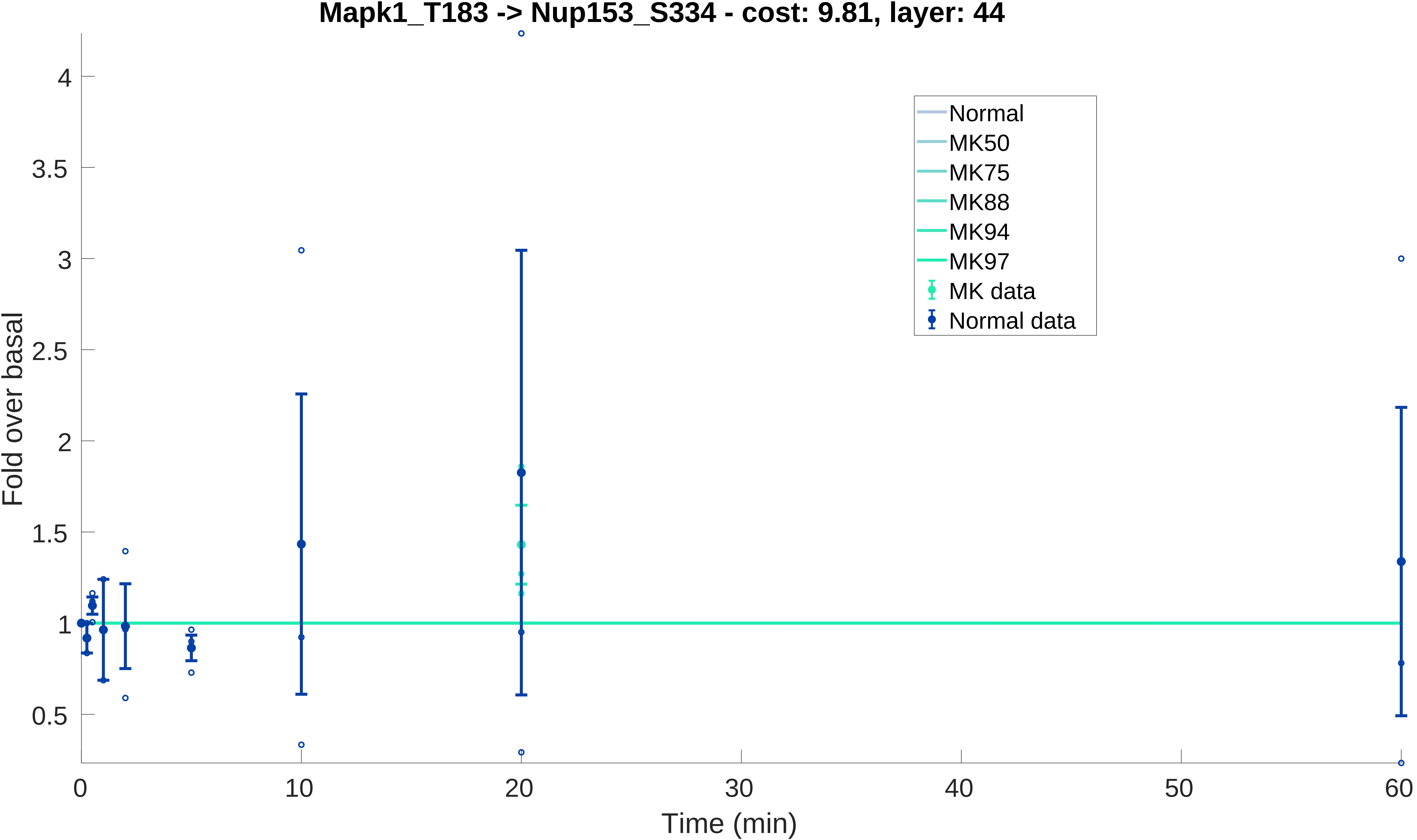
Mapk1_T183 -> Nup153_S334 - cost: 9.81, layer: 44
Fold over basal
Time (min)
4
3.5
3
2.5
2
1.5
1
0.5
0
10
20
30
40
50
60
Normal
MK50
MK75
MK88
MK94
MK97
MK data
Normal data

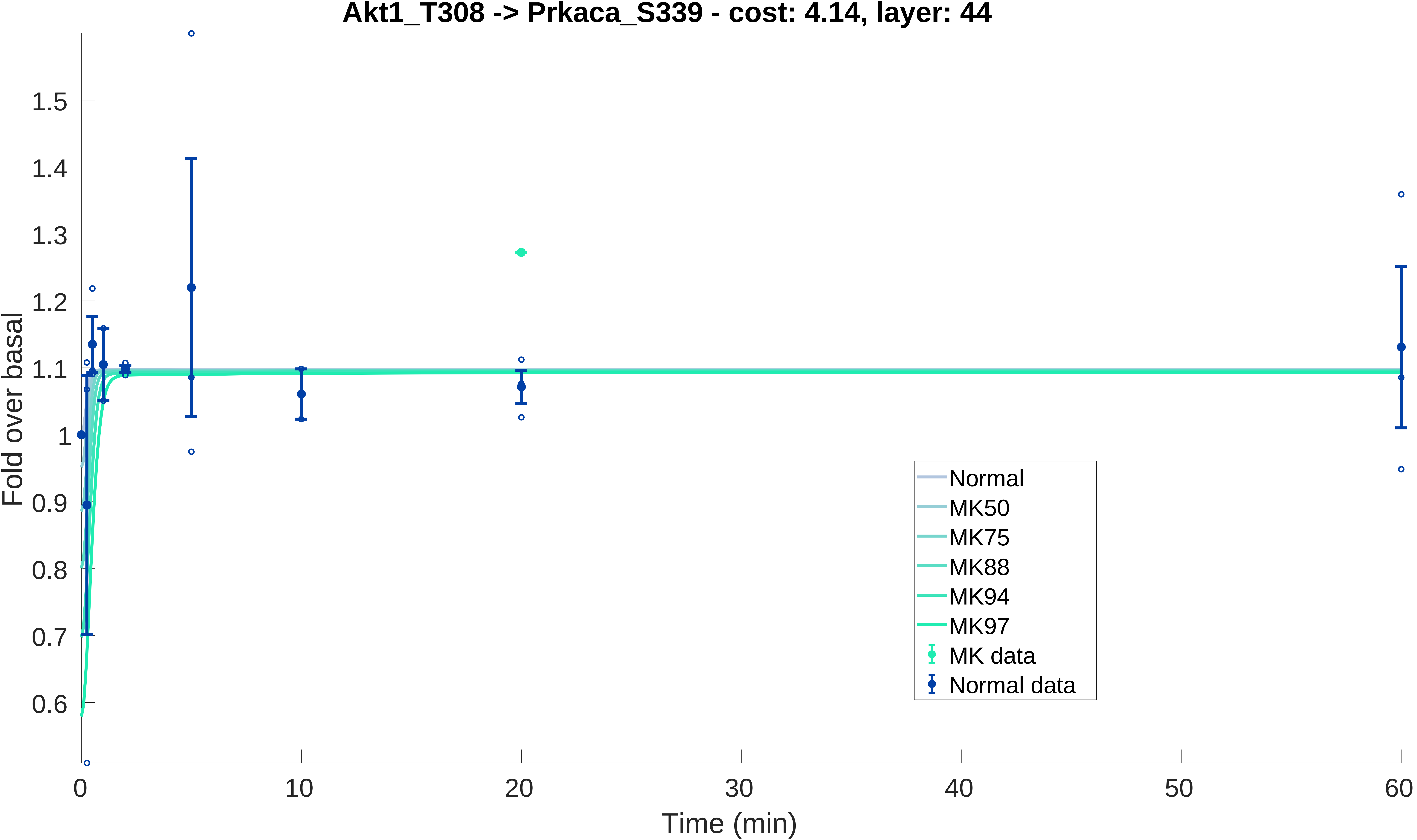
Akt1_T308 -> Prkaca_S339 - cost: 4.14, layer: 44
Fold over basal
Time (min)
0.6
0.7
0.8
0.9
1
1.1
1.2
1.3
1.4
1.5
0
10
20
30
40
50
60
Normal
MK50
MK75
MK88
MK94
MK97
MK data
Normal data

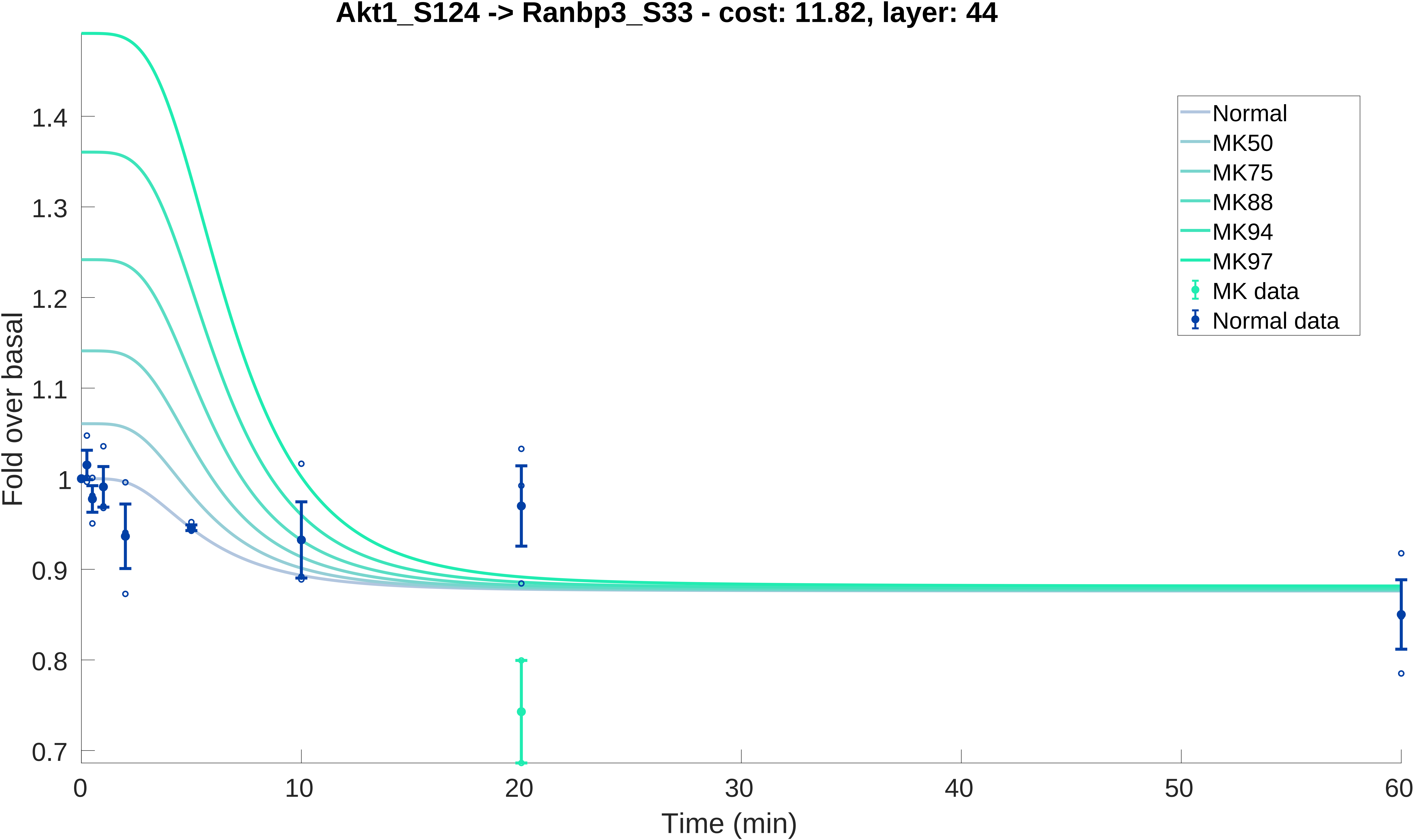

Akt1_S124 -> Ranbp3_S33 - cost: 11.82, layer: 44
Fold over basal
Time (min)
1.4
1.3
1.2
1.1
1
0.9
0.8
0.7
0
10
20
30
40
50
60
Normal
MK50
MK75
MK88
MK94
MK97
MK data
Normal data

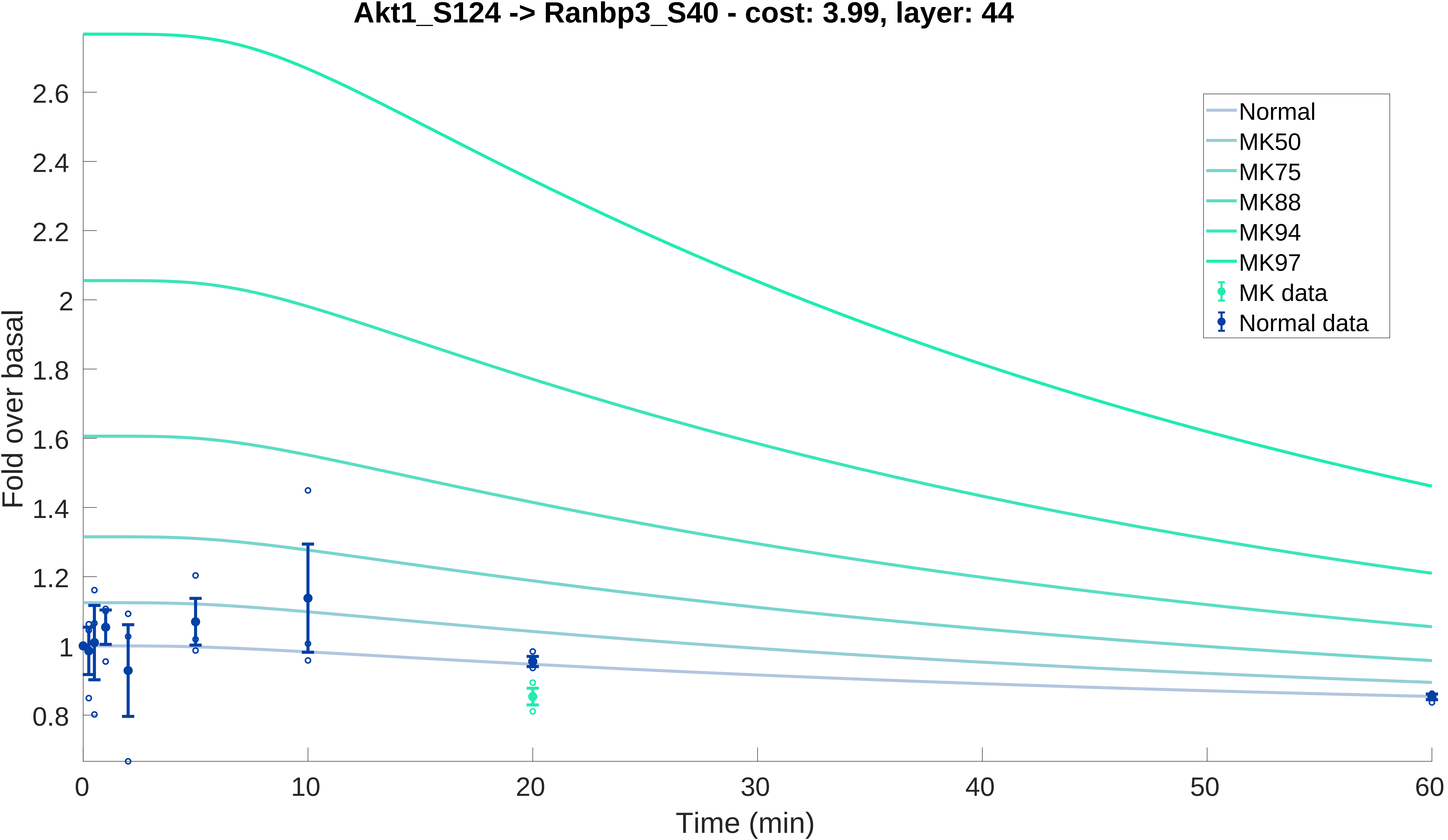

Akt1_S124 -> Ranbp3_S40 - cost: 3.99, layer: 44
Fold over basal
Time (min)
2.6
2.4
2.2
2
1.8
1.6
1.4
1.2
1
0.8
0
10
20
30
40
50
60
Normal
MK50
MK75
MK88
MK94
MK97
MK data
Normal data

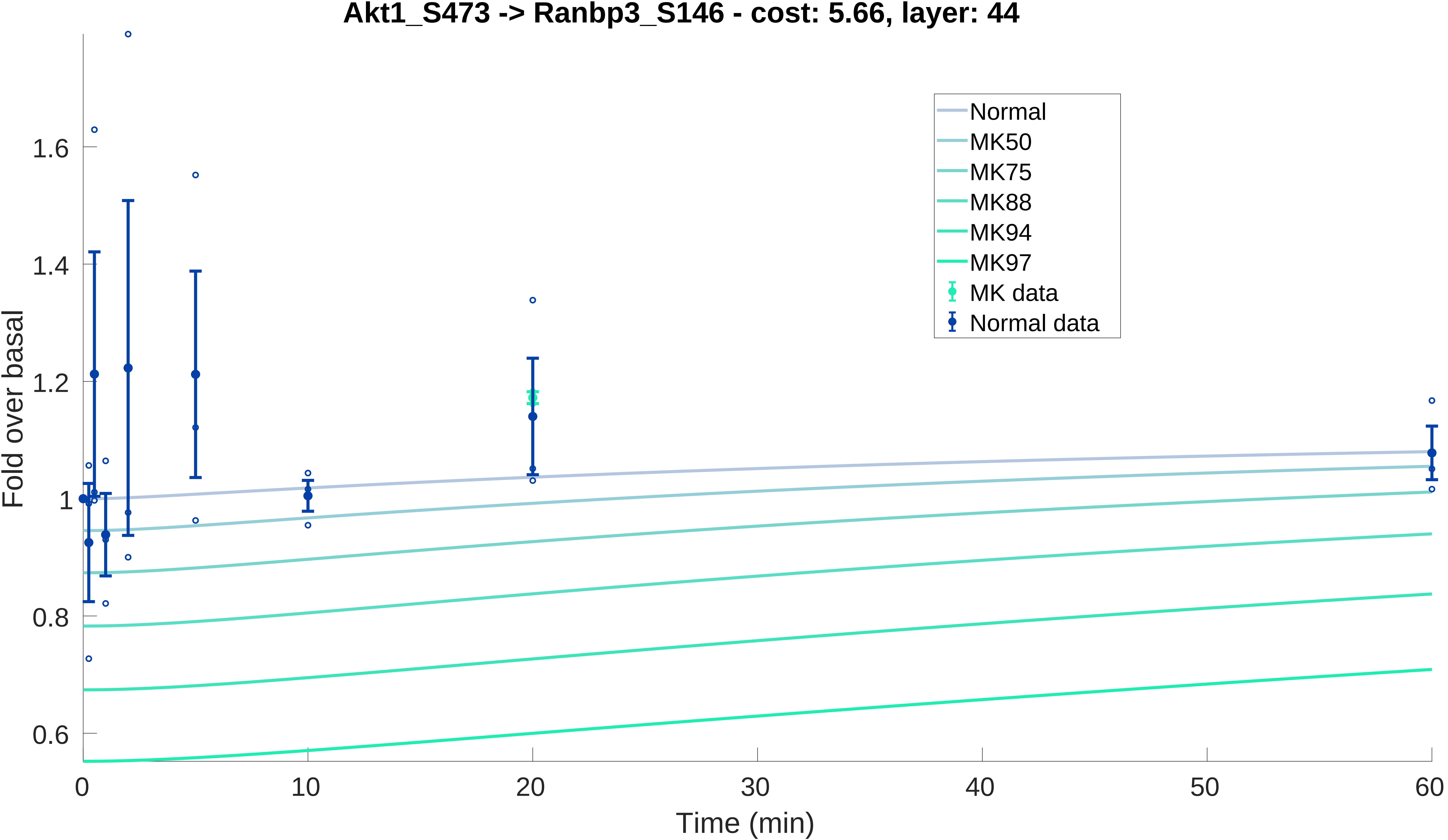
Akt1_S473 -> Ranbp3_S146 - cost: 5.66, layer: 44
Fold over basal
1.6
1.4
1.2
1
0.8
0.6
0
10
20
30
40
50
60
Time (min)
Normal
MK50
MK75
MK88
MK94
MK97
MK data
Normal data

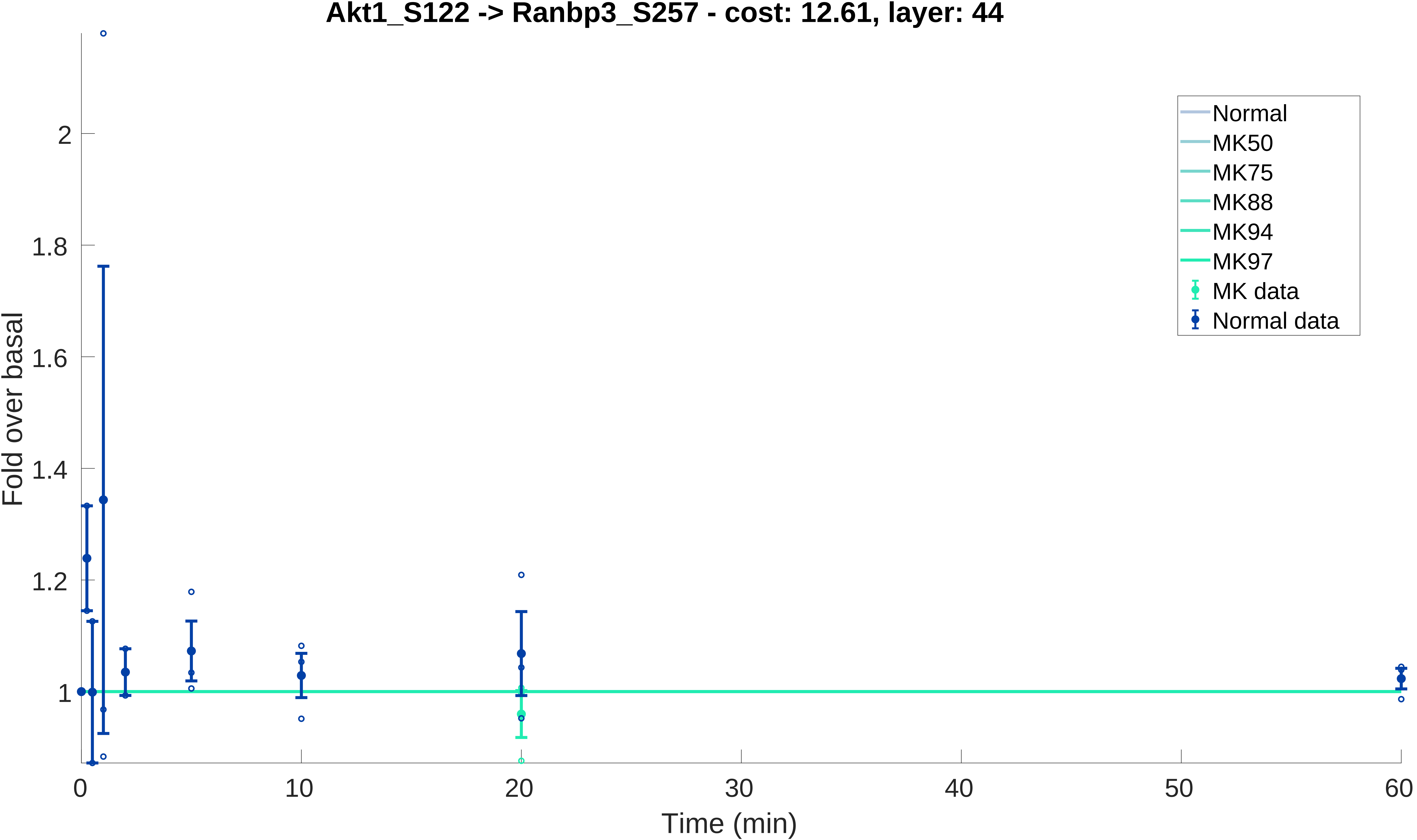

Akt1_S122 -> Ranbp3_S257 - cost: 12.61, layer: 44
Fold over basal
Time (min)
0
10
20
30
40
50
60
1
1.2
1.4
1.6
1.8
2
Normal
MK50
MK75
MK88
MK94
MK97
MK data
Normal data

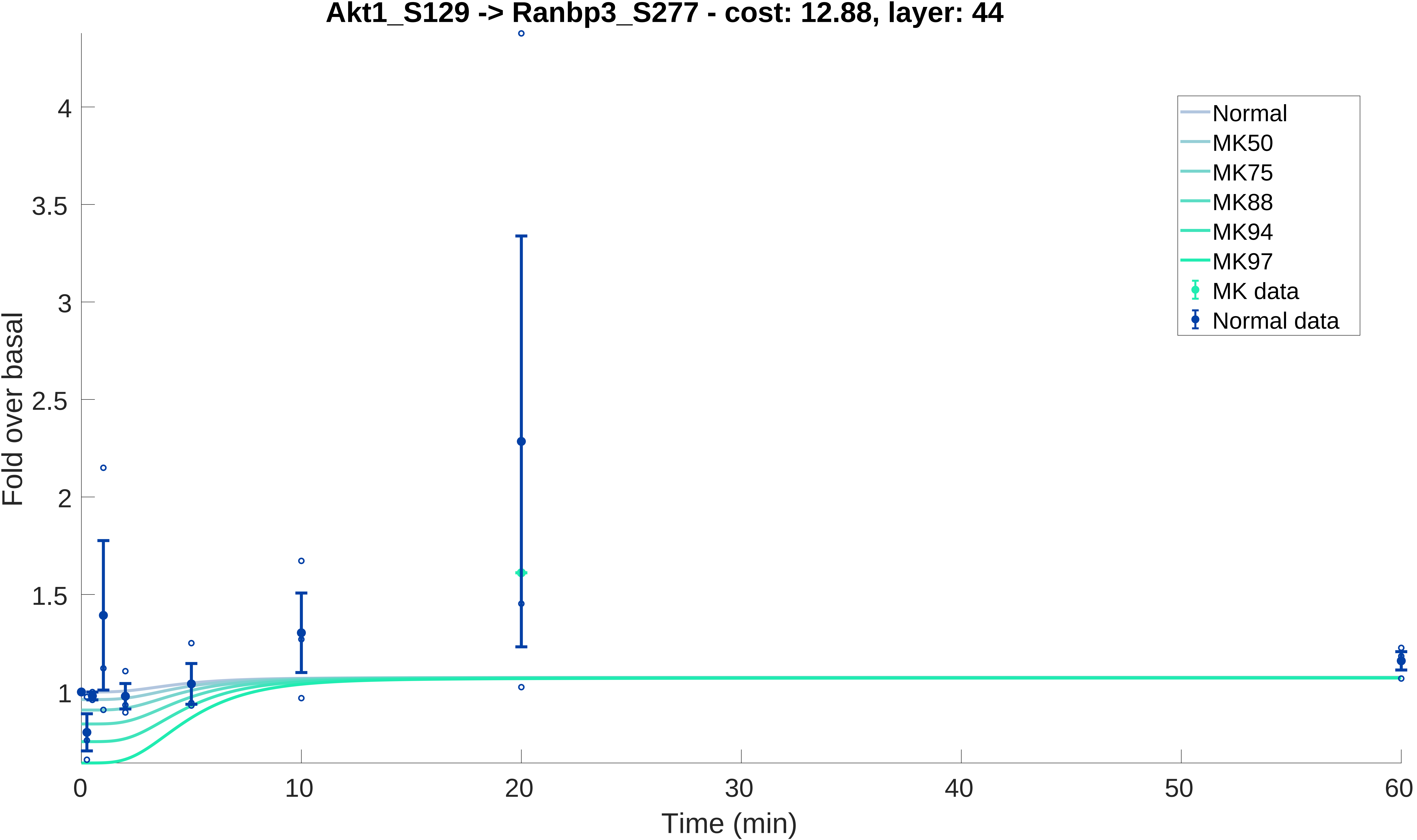
Akt1_S129 -> Ranbp3_S277 - cost: 12.88, layer: 44
Fold over basal
Time (min)
Normal
MK50
MK75
MK88
MK94
MK97
MK data
Normal data
1
1.5
2
2.5
3
3.5
4
0
10
20
30
40
50
60

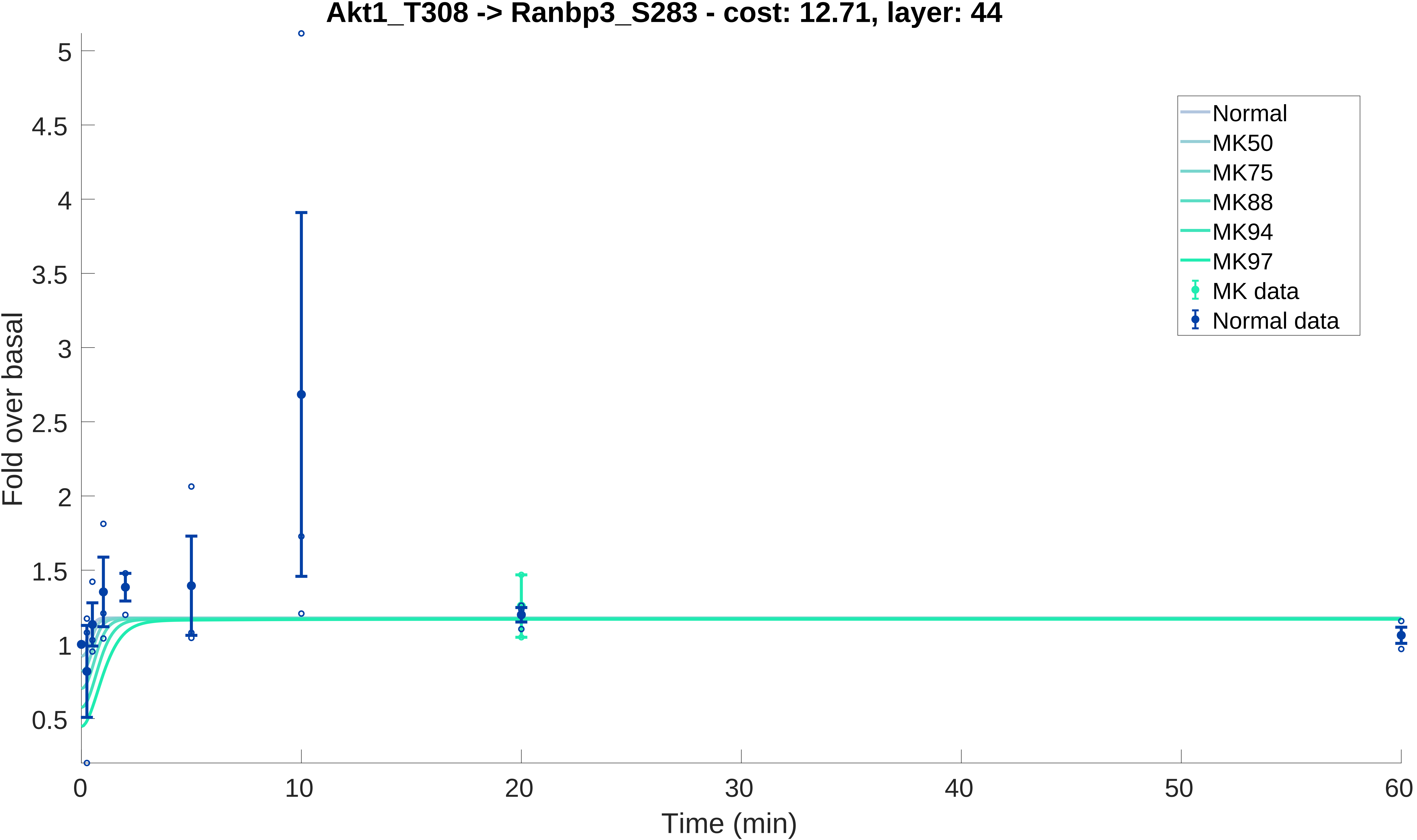

Akt1_T308 -> Ranbp3_S283 - cost: 12.71, layer: 44
Fold over basal
Time (min)
0.5
1
1.5
2
2.5
3
3.5
4
4.5
5
0
10
20
30
40
50
60
Normal
MK50
MK75
MK88
MK94
MK97
MK data
Normal data

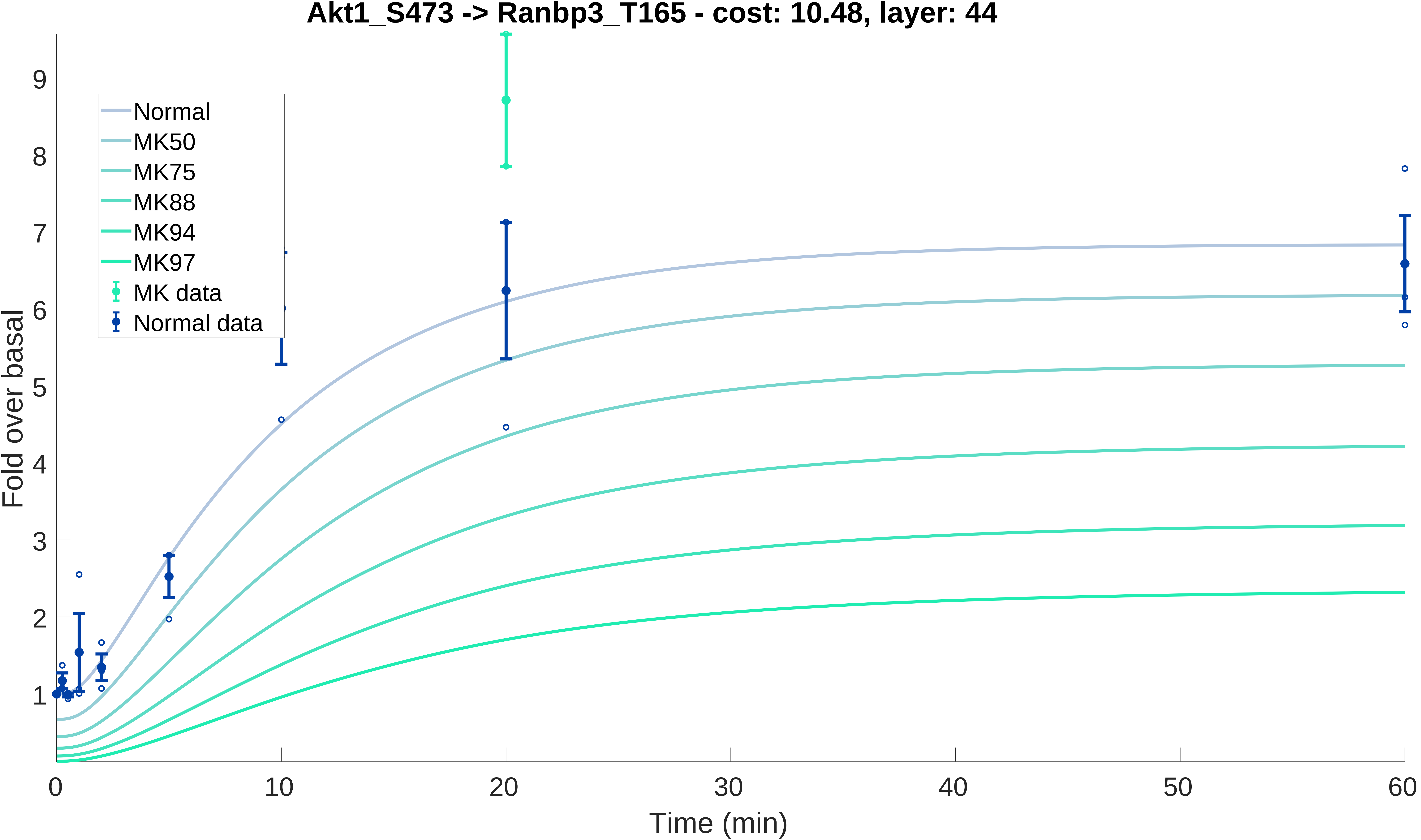

Akt1_S473 -> Ranbp3_T165 - cost: 10.48, layer: 44
Normal
MK50
MK75
MK88
MK94
MK97
MK data
Normal data
Fold over basal
Time (min)
0
10
20
30
40
50
60
1
2
3
4
5
6
7
8
9

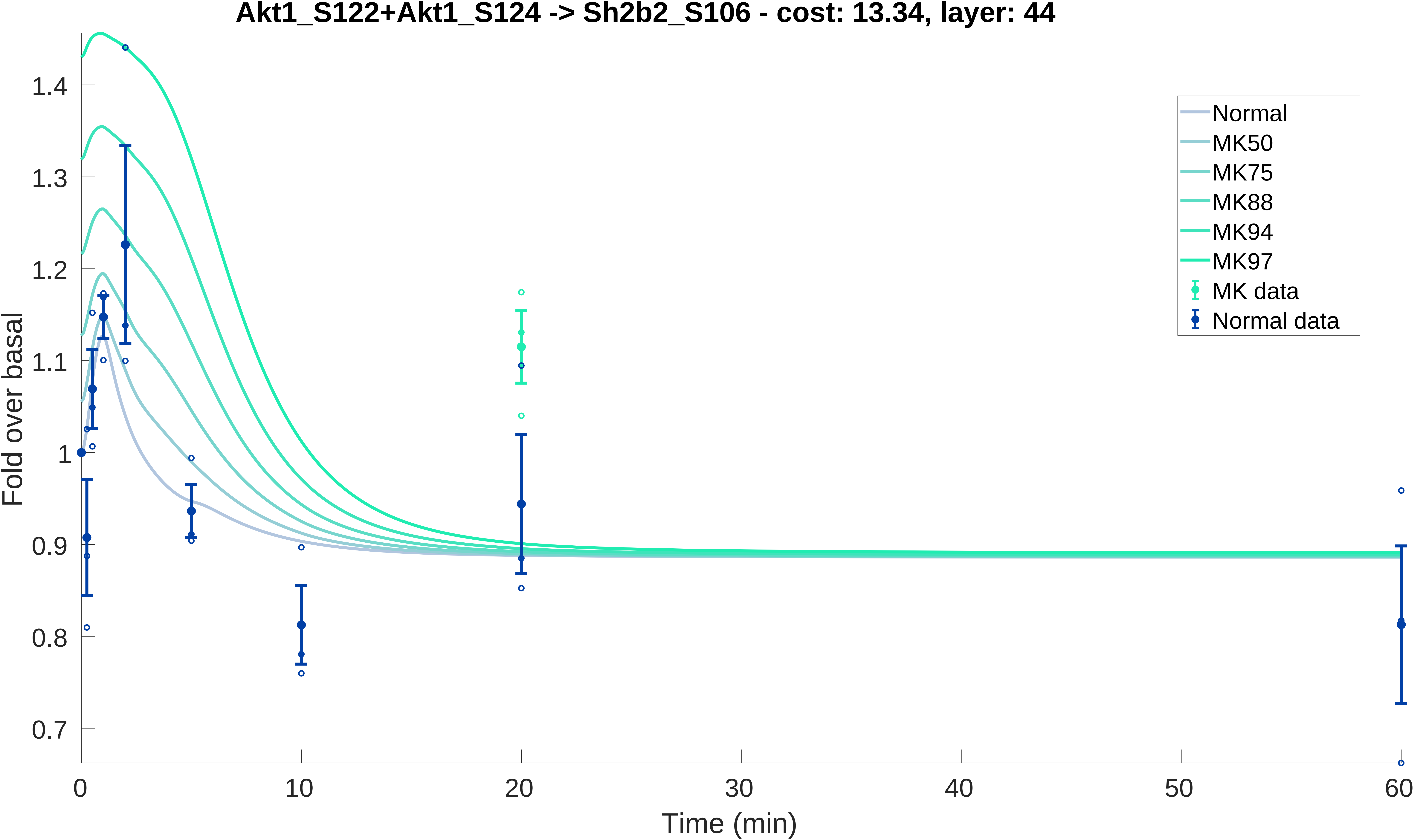

Akt1_S122+Akt1_S124 -> Sh2b2_S106 - cost: 13.34, layer: 44
Fold over basal
Time (min)
0
10
20
30
40
50
60
0.7
0.8
0.9
1
1.1
1.2
1.3
1.4
Normal
MK50
MK75
MK88
MK94
MK97
MK data
Normal data

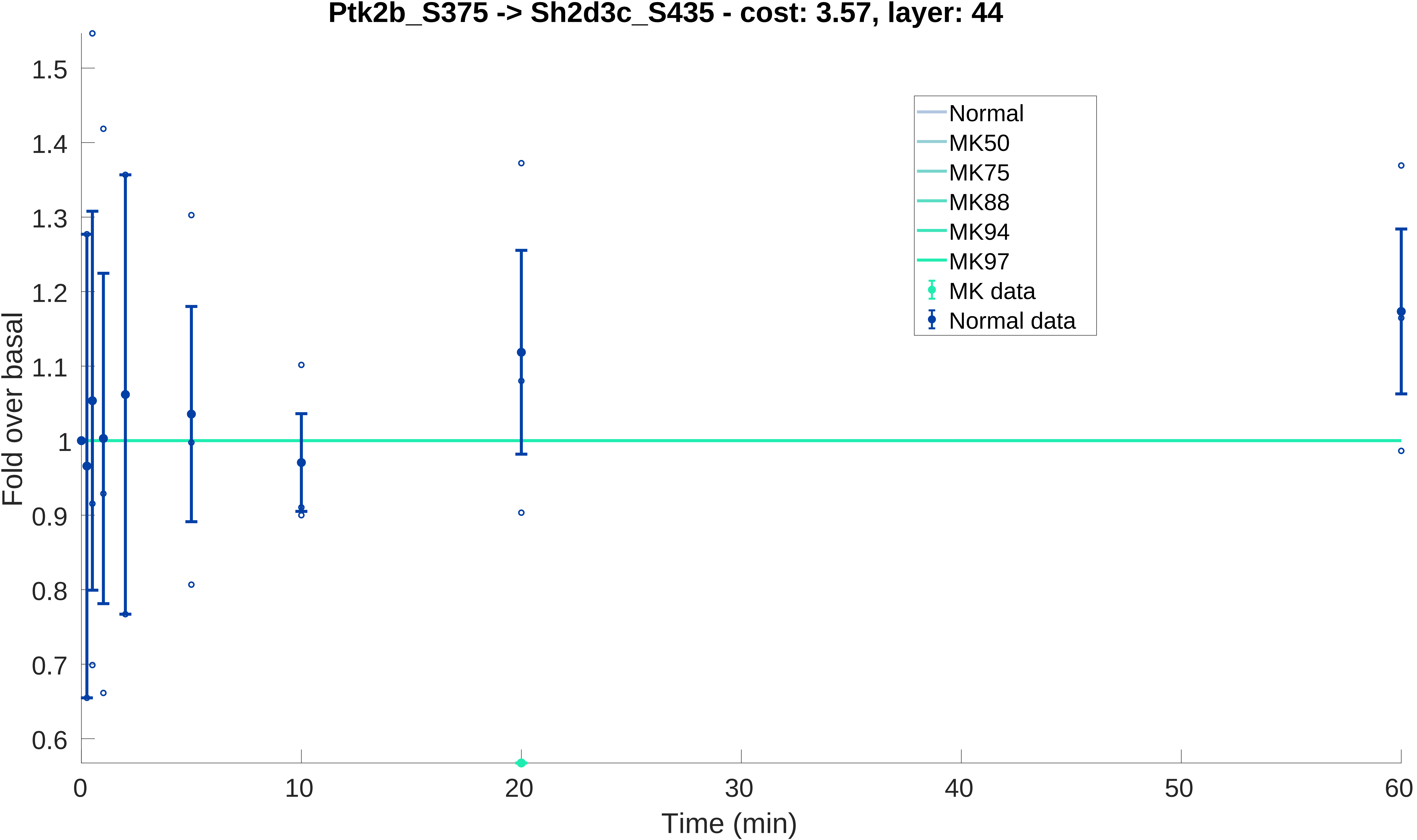
Ptk2b_S375 -> Sh2d3c_S435 - cost: 3.57, layer: 44
Normal
MK50
MK75
MK88
MK94
MK97
MK data
Normal data
Fold over basal
Time (min)
0.6
0.7
0.8
0.9
1
1.1
1.2
1.3
1.4
1.5
0
10
20
30
40
50
60

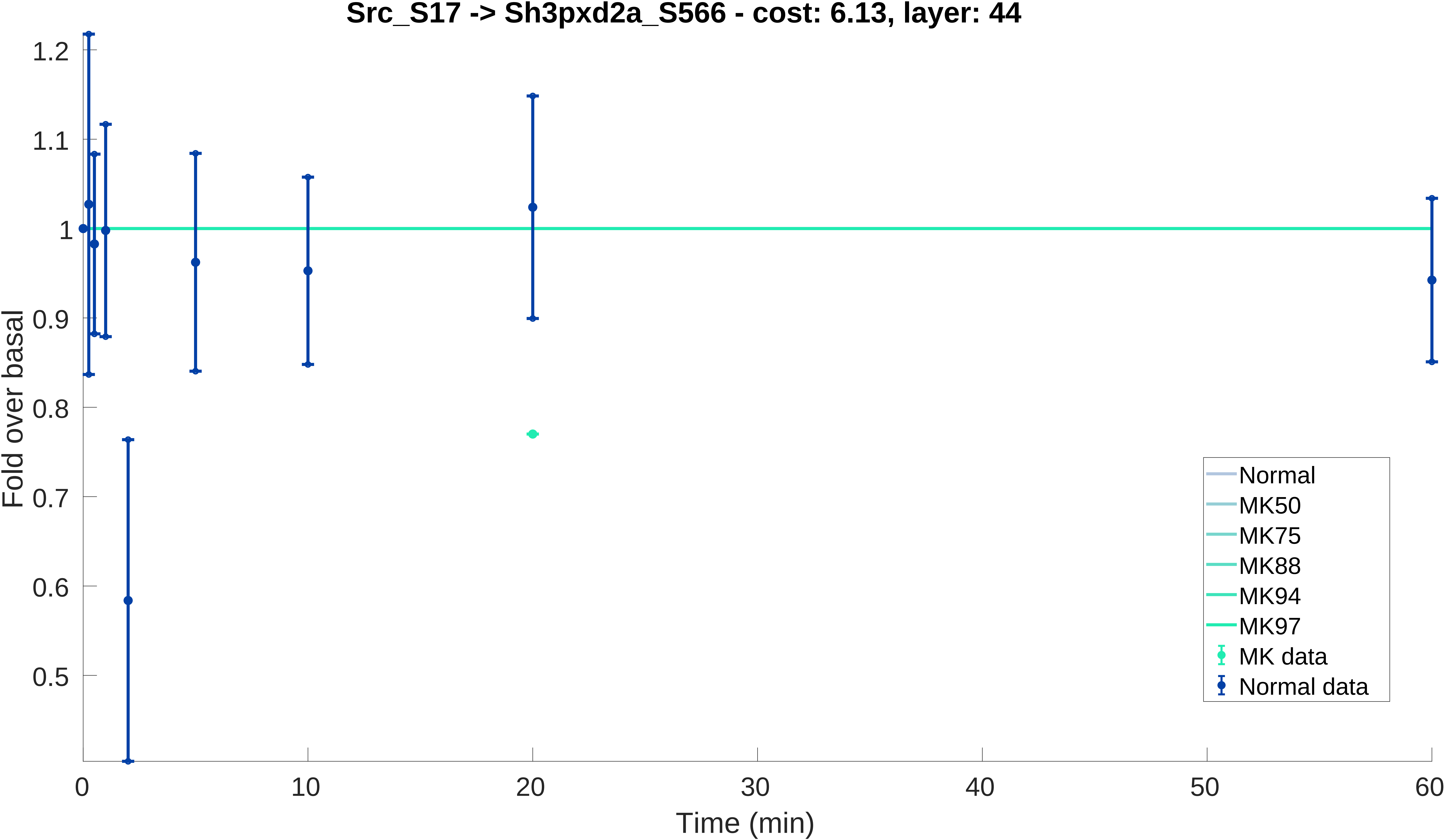

Src_S17 -> Sh3pxd2a_S566 - cost: 6.13, layer: 44
Fold over basal
1.2
1.1
1
0.9
0.8
0.7
0.6
0.5
0
10
20
30
40
50
60
Time (min)
Normal
MK50
MK75
MK88
MK94
MK97
MK data
Normal data

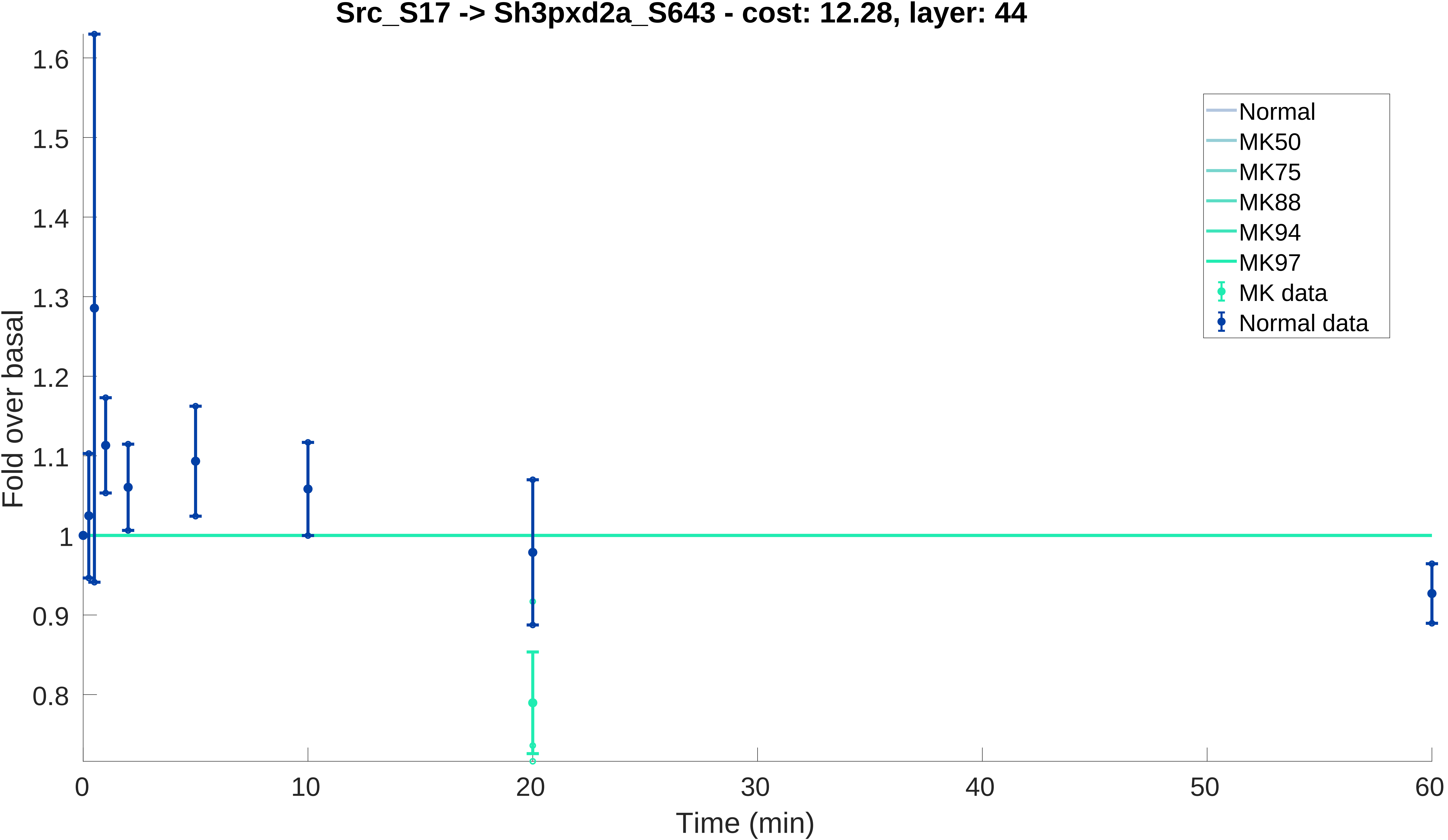

Src_S17 -> Sh3pxd2a_S643 - cost: 12.28, layer: 44
Fold over basal
1.6
1.5
1.4
1.3
1.2
1.1
1
0.9
0.8
0
10
20
30
40
50
60
Time (min)
Normal
MK50
MK75
MK88
MK94
MK97
MK data
Normal data

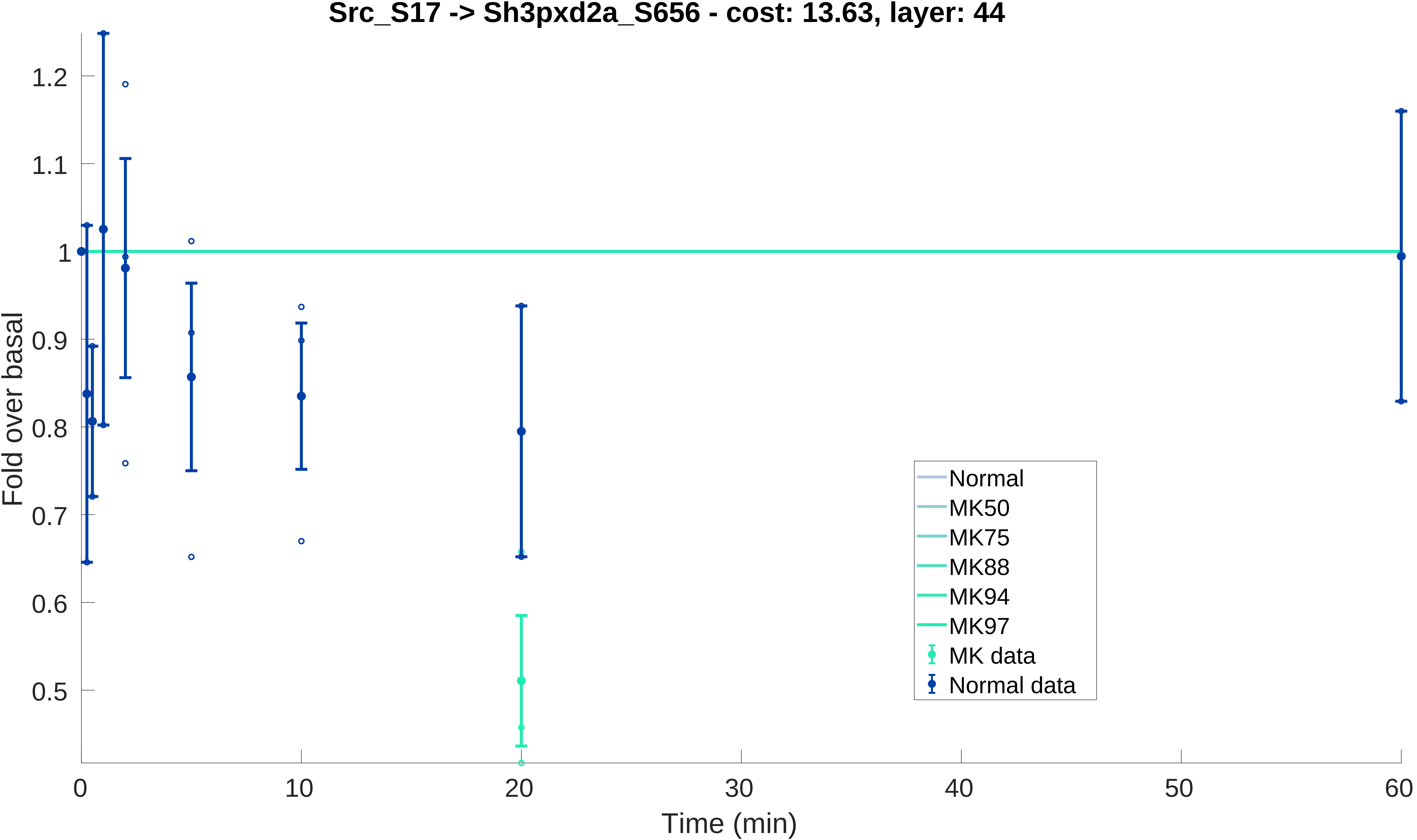
Src_S17 -> Sh3pxd2a_S656 - cost: 13.63, layer: 44
Fold over basal
1.2
1.1
1
0.9
0.8
0.7
0.6
0.5
0
10
20
30
40
50
60
Time (min)
Normal
MK50
MK75
MK88
MK94
MK97
MK data
Normal data

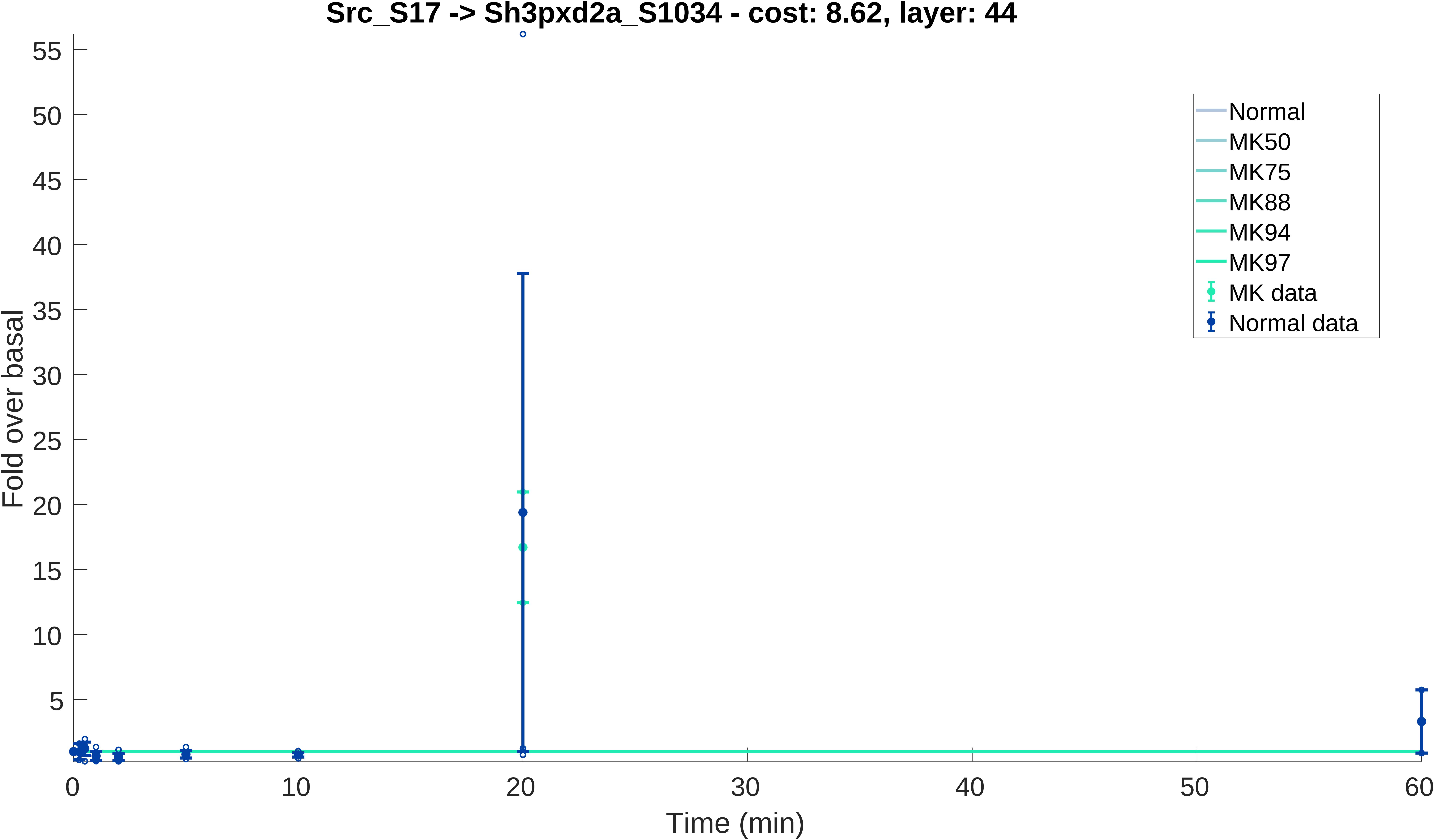

Src_S17 -> Sh3pxd2a_S1034 - cost: 8.62, layer: 44
Fold over basal
Time (min)
0
10
20
30
40
50
60
5
10
15
20
25
30
35
40
45
50
55
Normal
MK50
MK75
MK88
MK94
MK97
MK data
Normal data

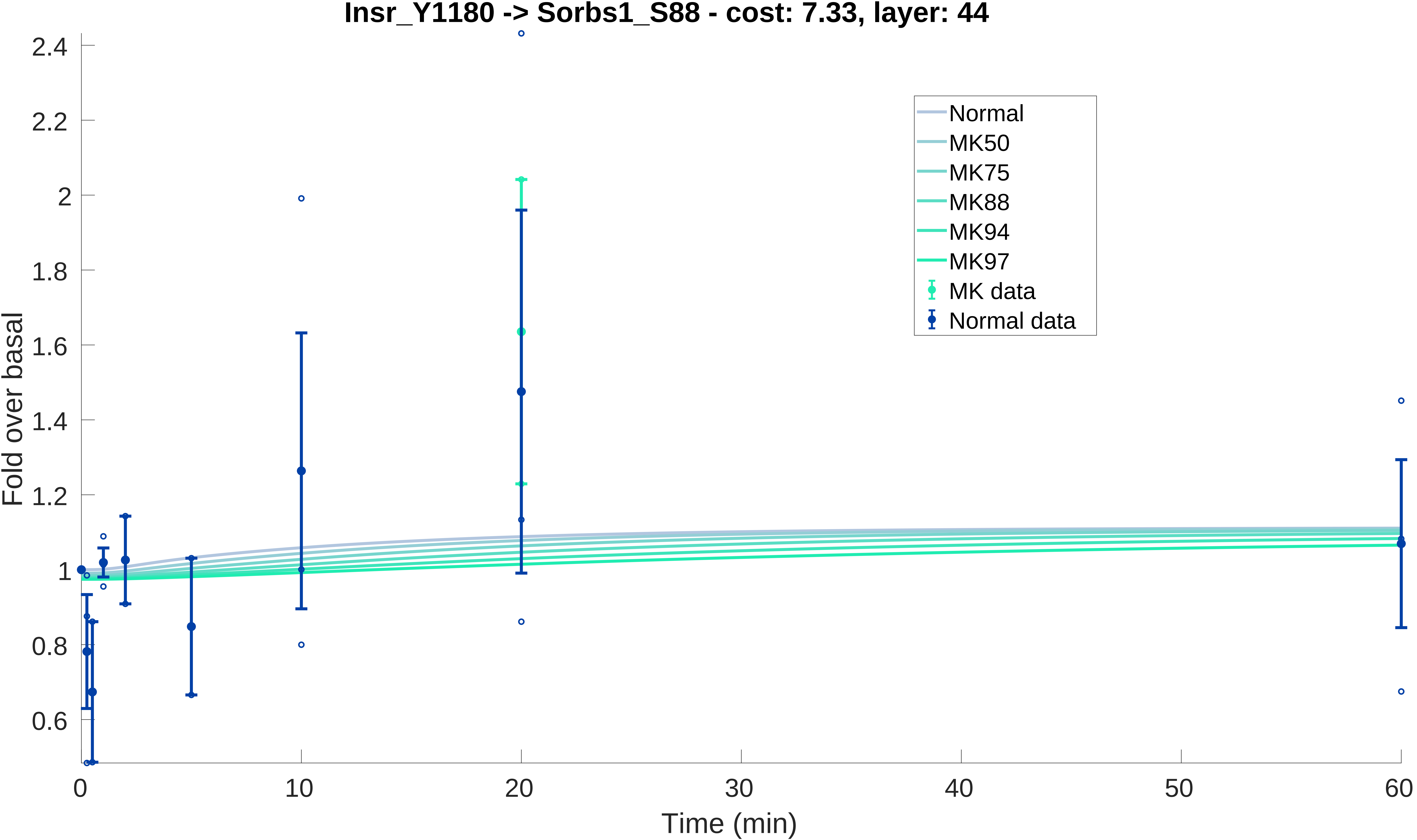

Insr_Y1180 -> Sorbs1_S88 - cost: 7.33, layer: 44
Normal
MK50
MK75
MK88
MK94
MK97
MK data
Normal data
Fold over basal
Time (min)
2.4
2.2
2
1.8
1.6
1.4
1.2
1
0.8
0.6
0
10
20
30
40
50
60

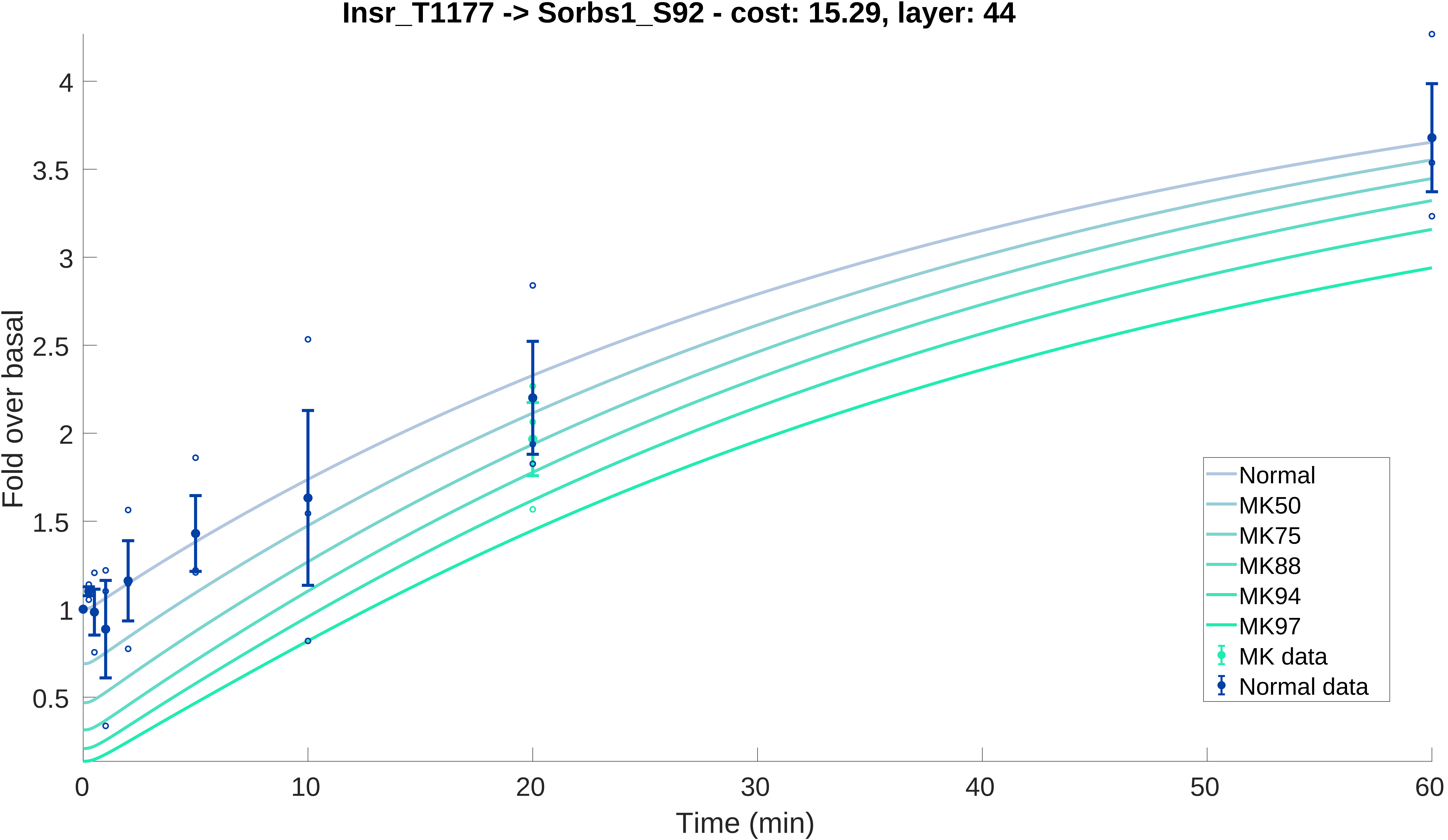

Insr_T1177 -> Sorbs1_S92 - cost: 15.29, layer: 44
Fold over basal
Time (min)
0
10
20
30
40
50
60
0.5
1
1.5
2
2.5
3
3.5
4
Normal
MK50
MK75
MK88
MK94
MK97
MK data
Normal data

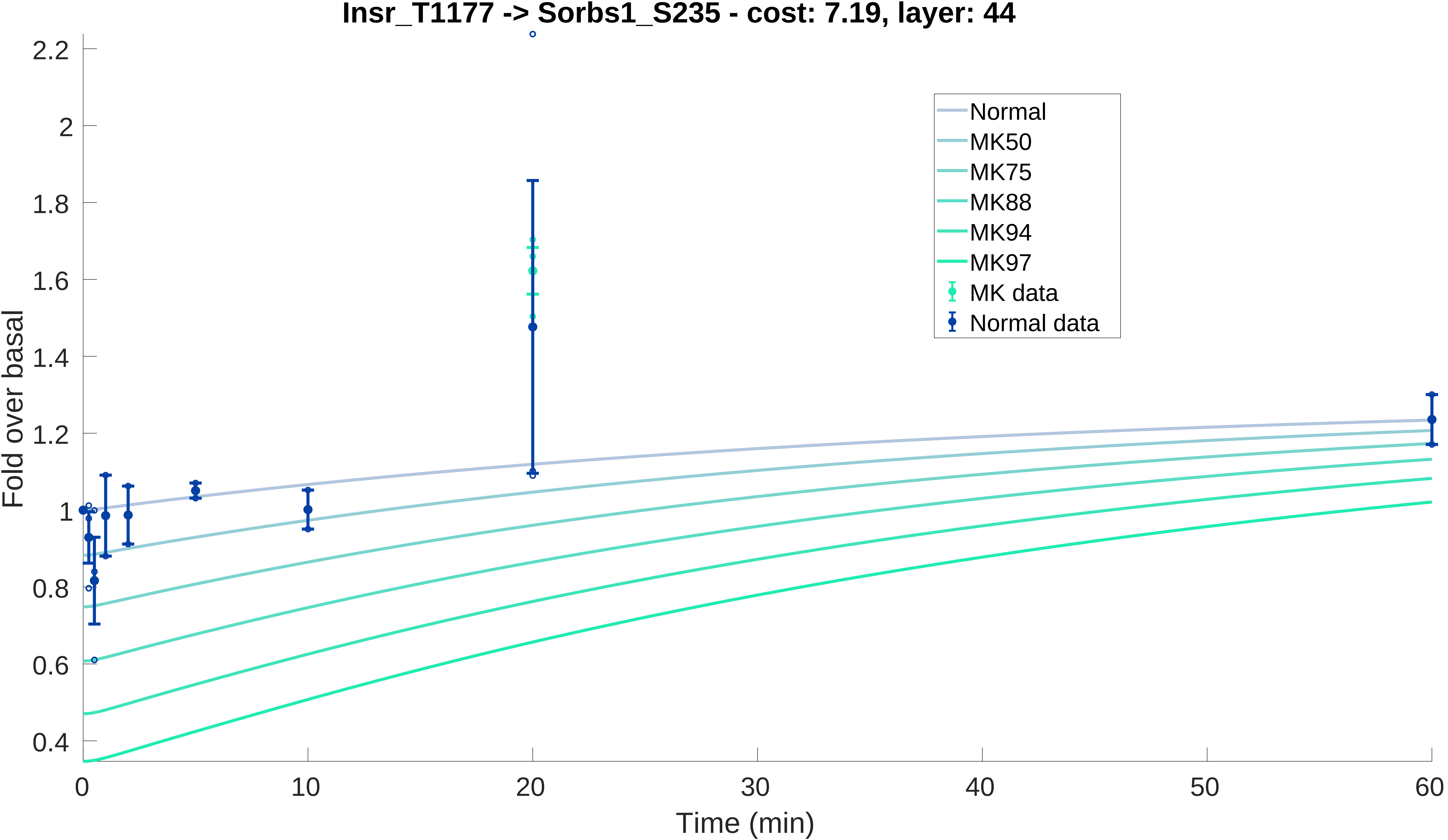

Insr_T1177 -> Sorbs1_S235 - cost: 7.19, layer: 44
Fold over basal
Time (min)
Normal
MK50
MK75
MK88
MK94
MK97
MK data
Normal data
2.2
2
1.8
1.6
1.4
1.2
1
0.8
0.6
0.4
0
10
20
30
40
50
60

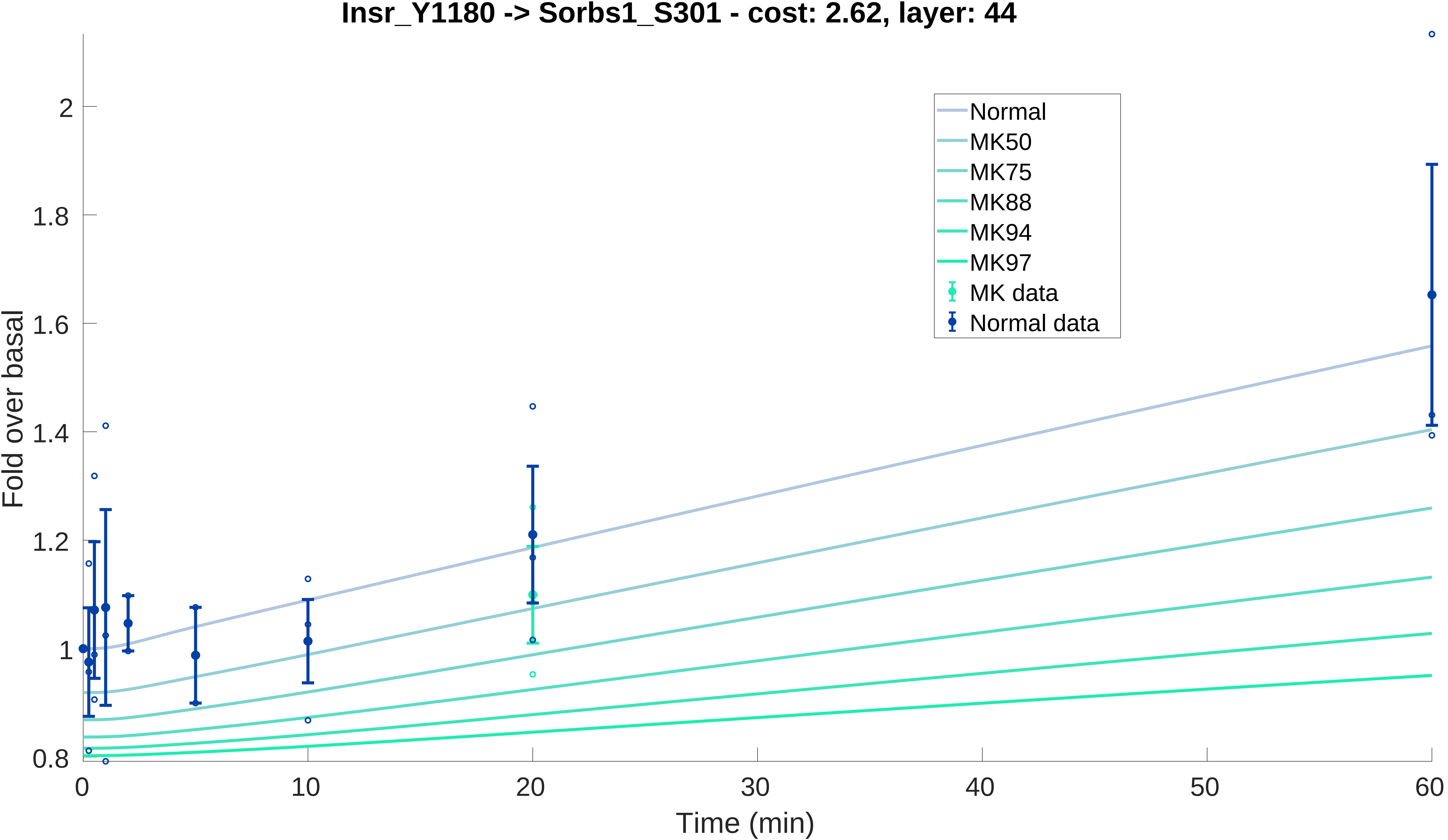

Insr_Y1180 -> Sorbs1_S301 - cost: 2.62, layer: 44
Fold over basal
Time (min)
0.8
1
1.2
1.4
1.6
1.8
2
0
10
20
30
40
50
60
Normal
MK50
MK75
MK88
MK94
MK97
MK data
Normal data

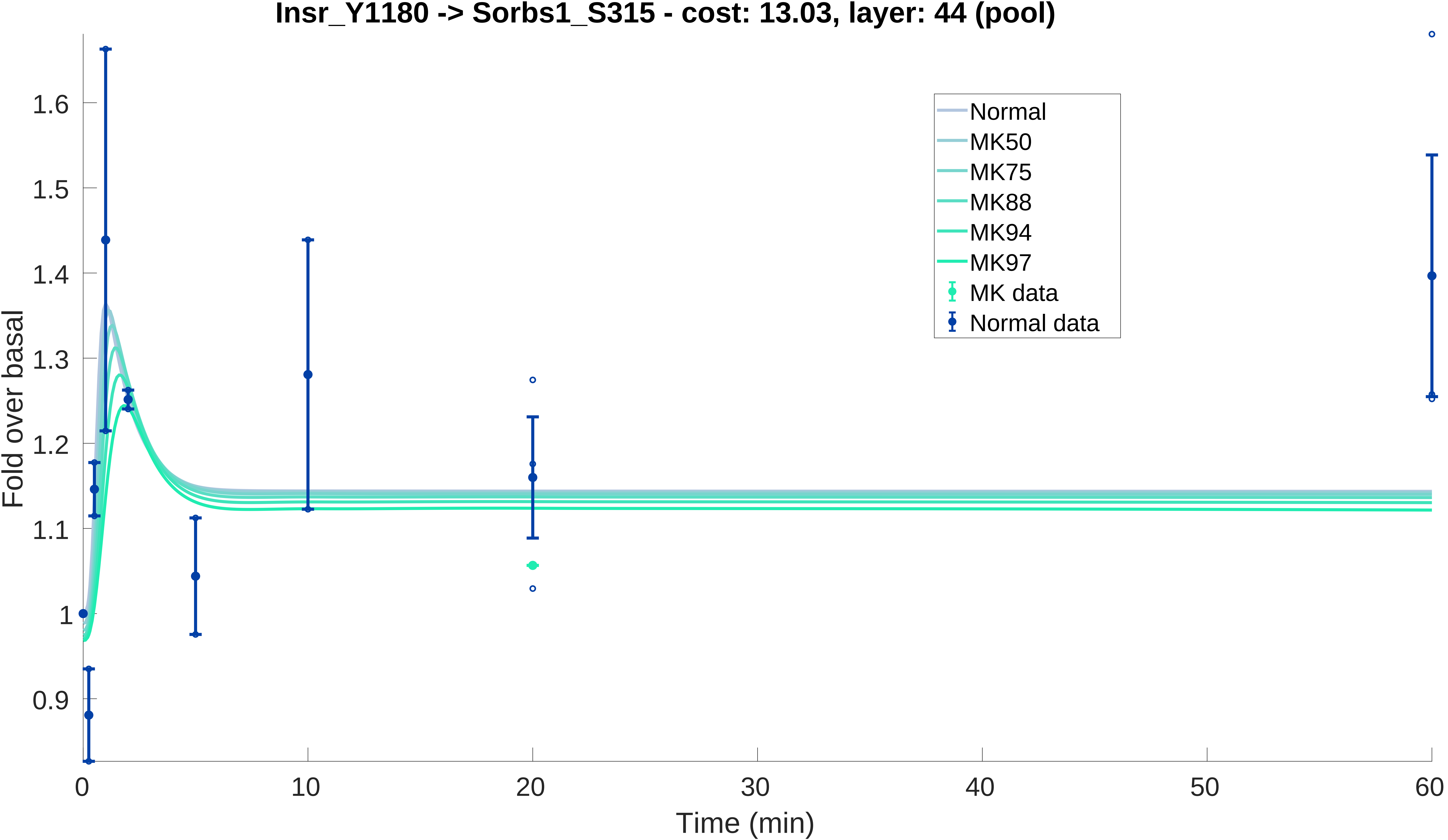
Insr_Y1180 -> Sorbs1_S315 - cost: 13.03, layer: 44 (pool)
Fold over basal
Time (min)
1.6
1.5
1.4
1.3
1.2
1.1
1
0.9
0
10
20
30
40
50
60
Normal
MK50
MK75
MK88
MK94
MK97
MK data
Normal data

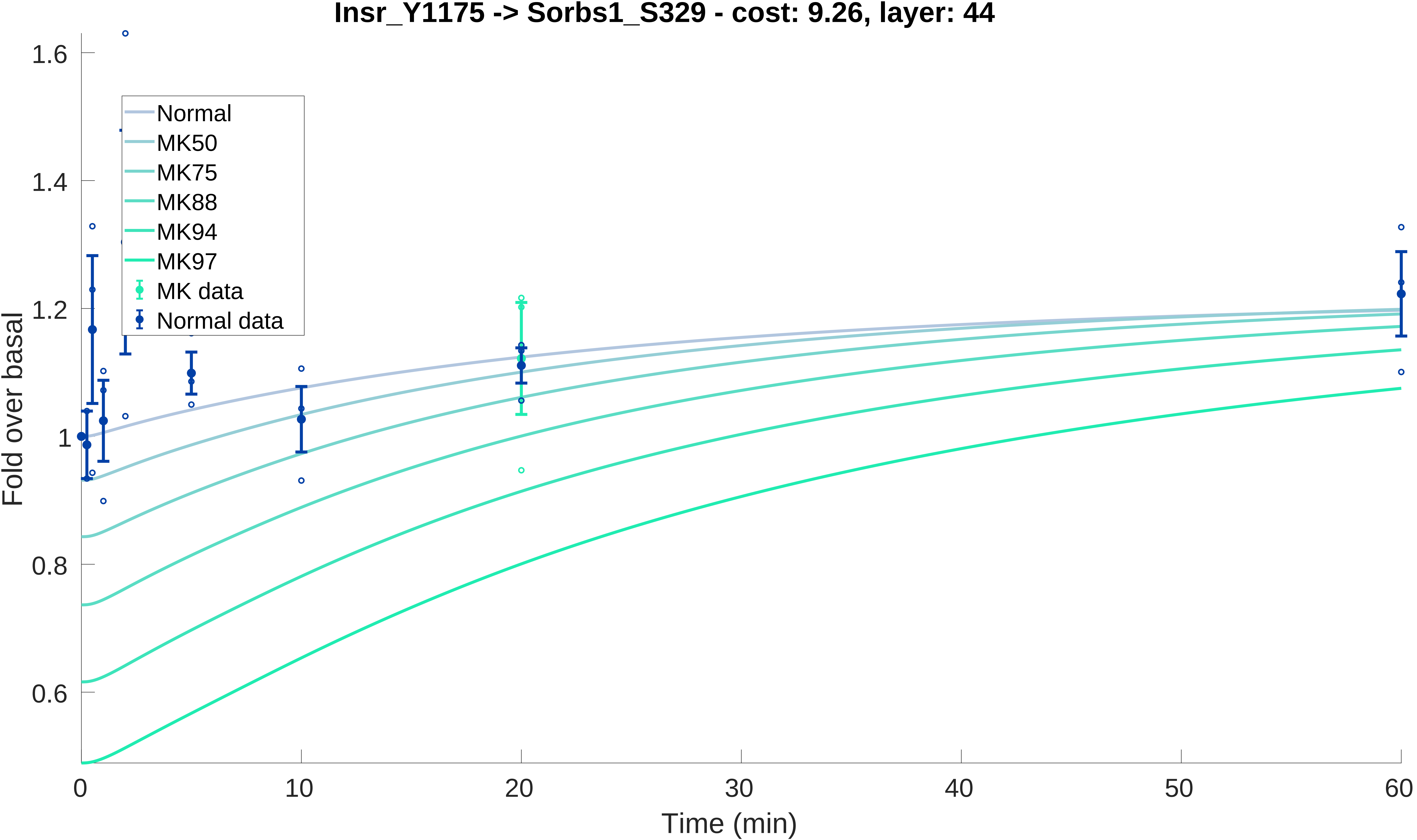

Insr_Y1175 -> Sorbs1_S329 - cost: 9.26, layer: 44
Normal
MK50
MK75
MK88
MK94
MK97
MK data
Normal data
Fold over basal
1.6
1.4
1.2
1
0.8
0.6
0
10
20
30
40
50
60
Time (min)

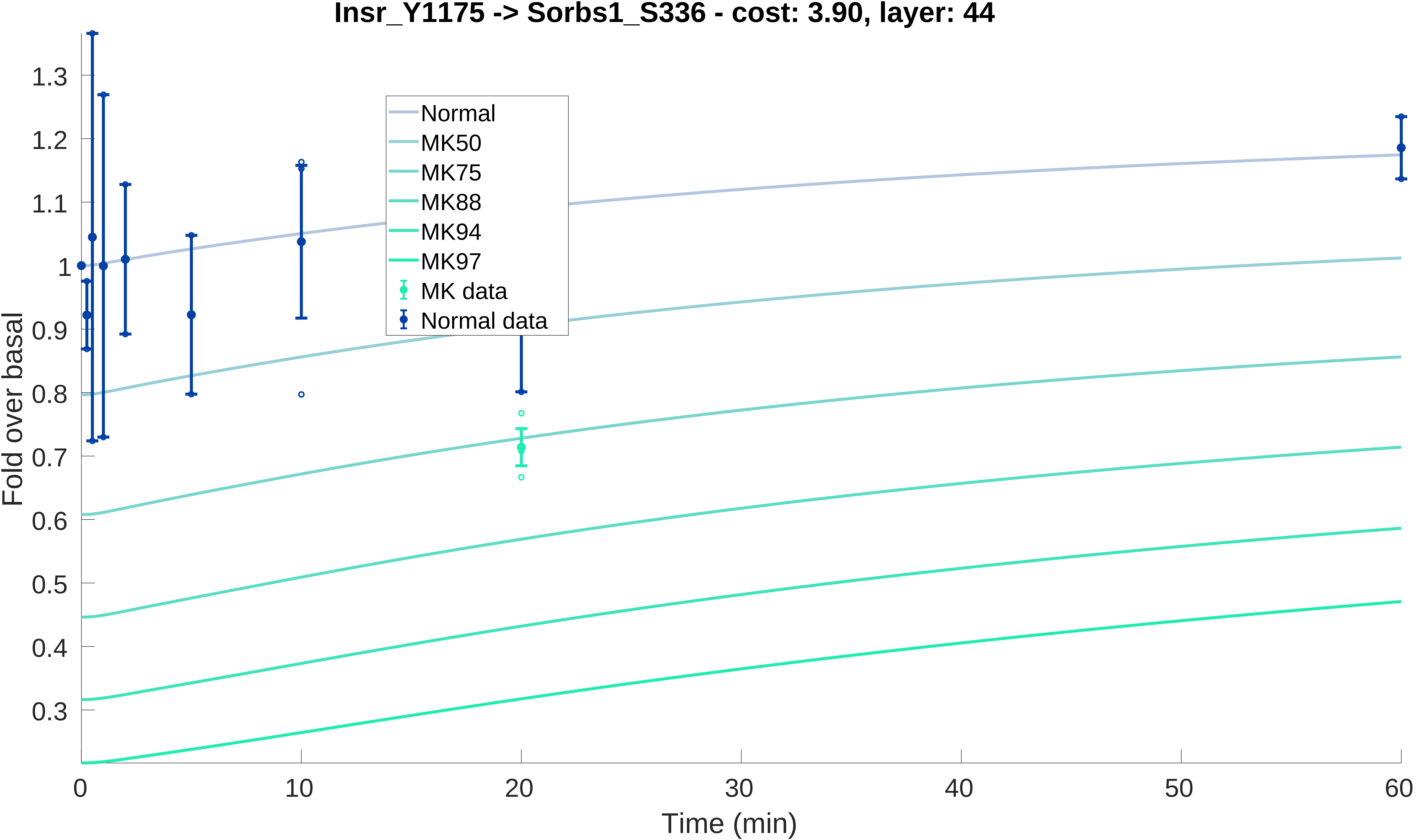

Insr_Y1175 -> Sorbs1_S336 - cost: 3.90, layer: 44
Fold over basal
Time (min)
1.3
1.2
1.1
1
0.9
0.8
0.7
0.6
0.5
0.4
0.3
0
10
20
30
40
50
60
Normal
MK50
MK75
MK88
MK94
MK97
MK data
Normal data

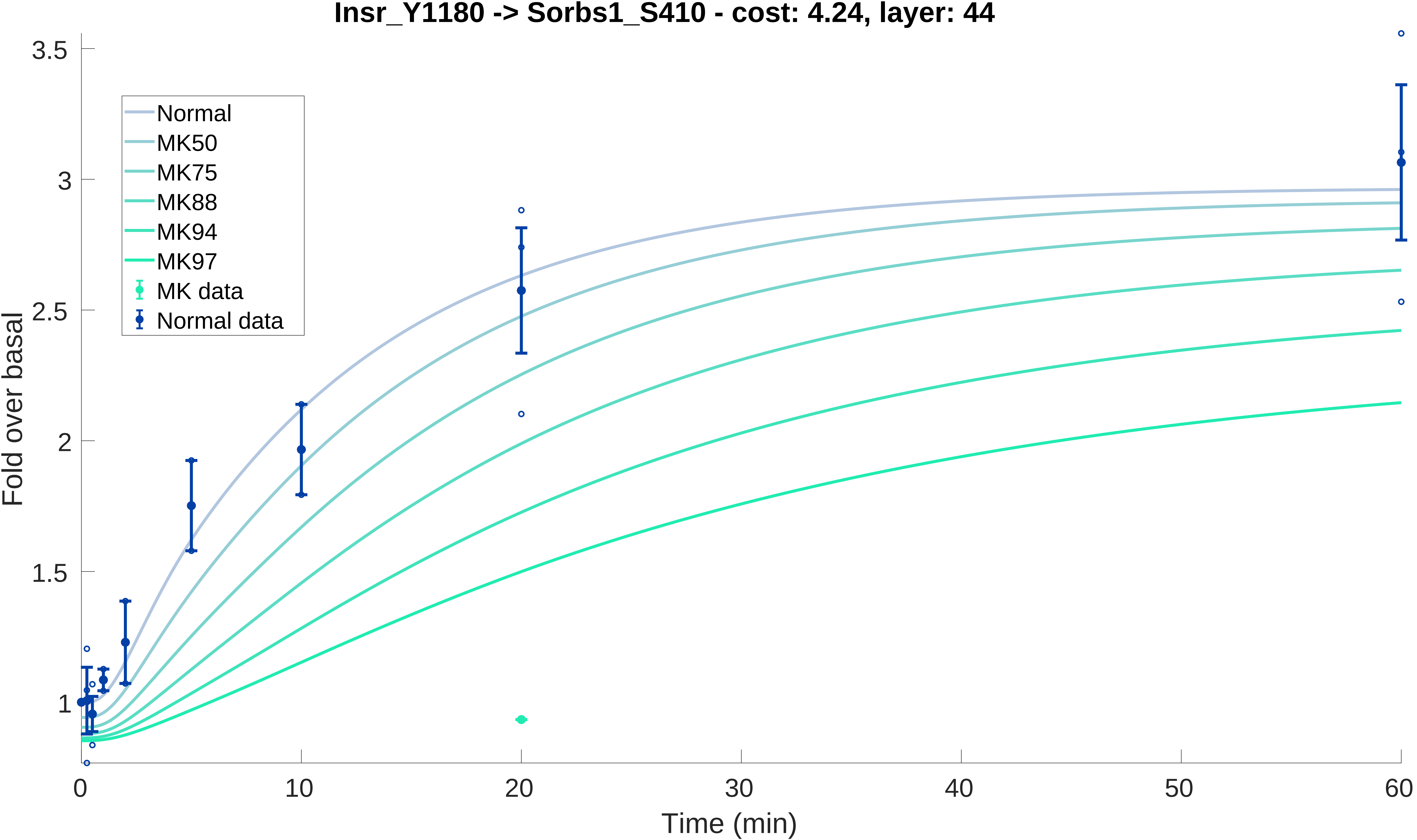

Insr_Y1180 -> Sorbs1_S410 - cost: 4.24, layer: 44
Fold over basal
Time (min)
Normal
MK50
MK75
MK88
MK94
MK97
MK data
Normal data
3.5
3
2.5
2
1.5
1
0
10
20
30
40
50
60

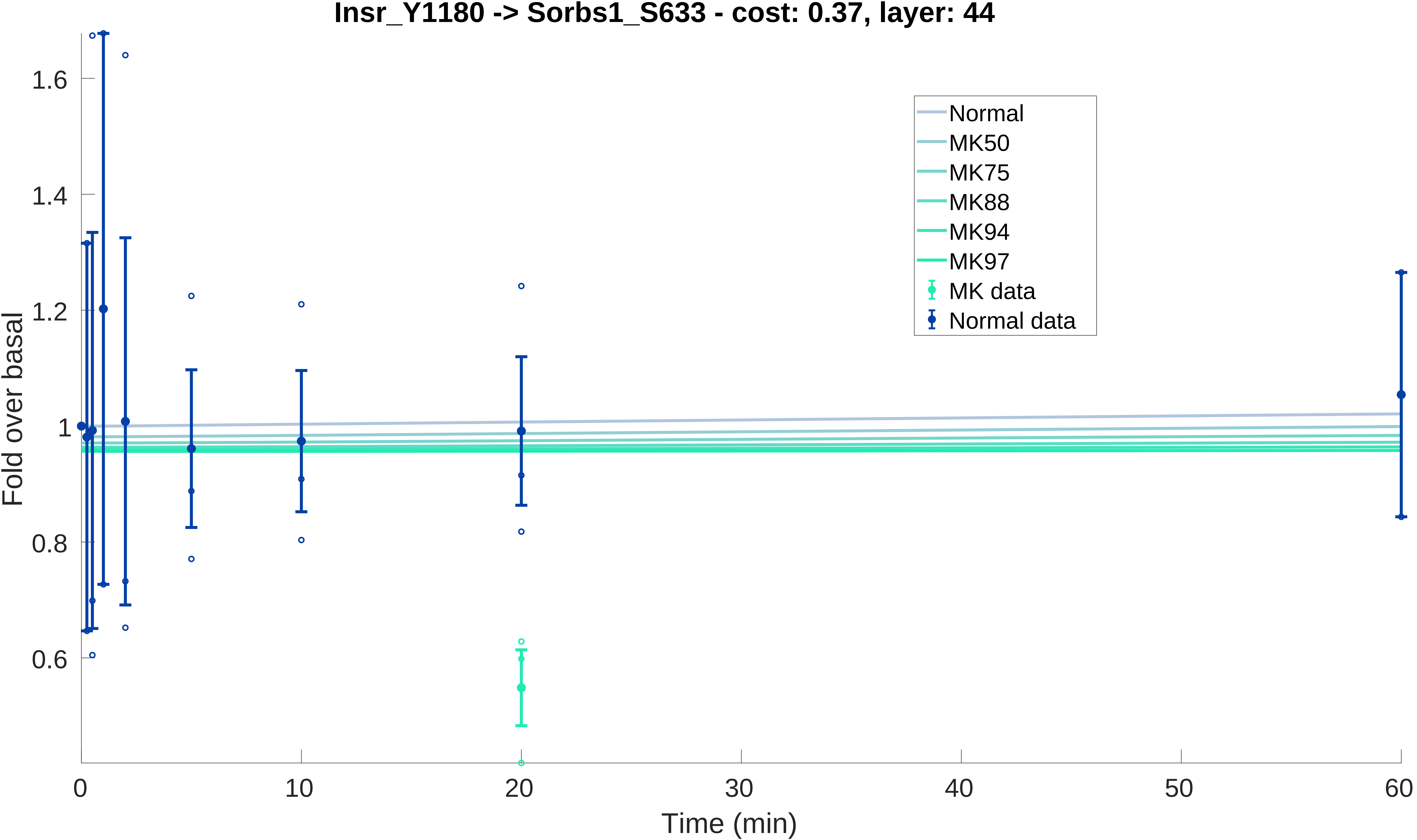

Insr_Y1180 -> Sorbs1_S633 - cost: 0.37, layer: 44
Fold over basal
Time (min)
1.6
1.4
1.2
1
0.8
0.6
0
10
20
30
40
50
60
Normal
MK50
MK75
MK88
MK94
MK97
MK data
Normal data

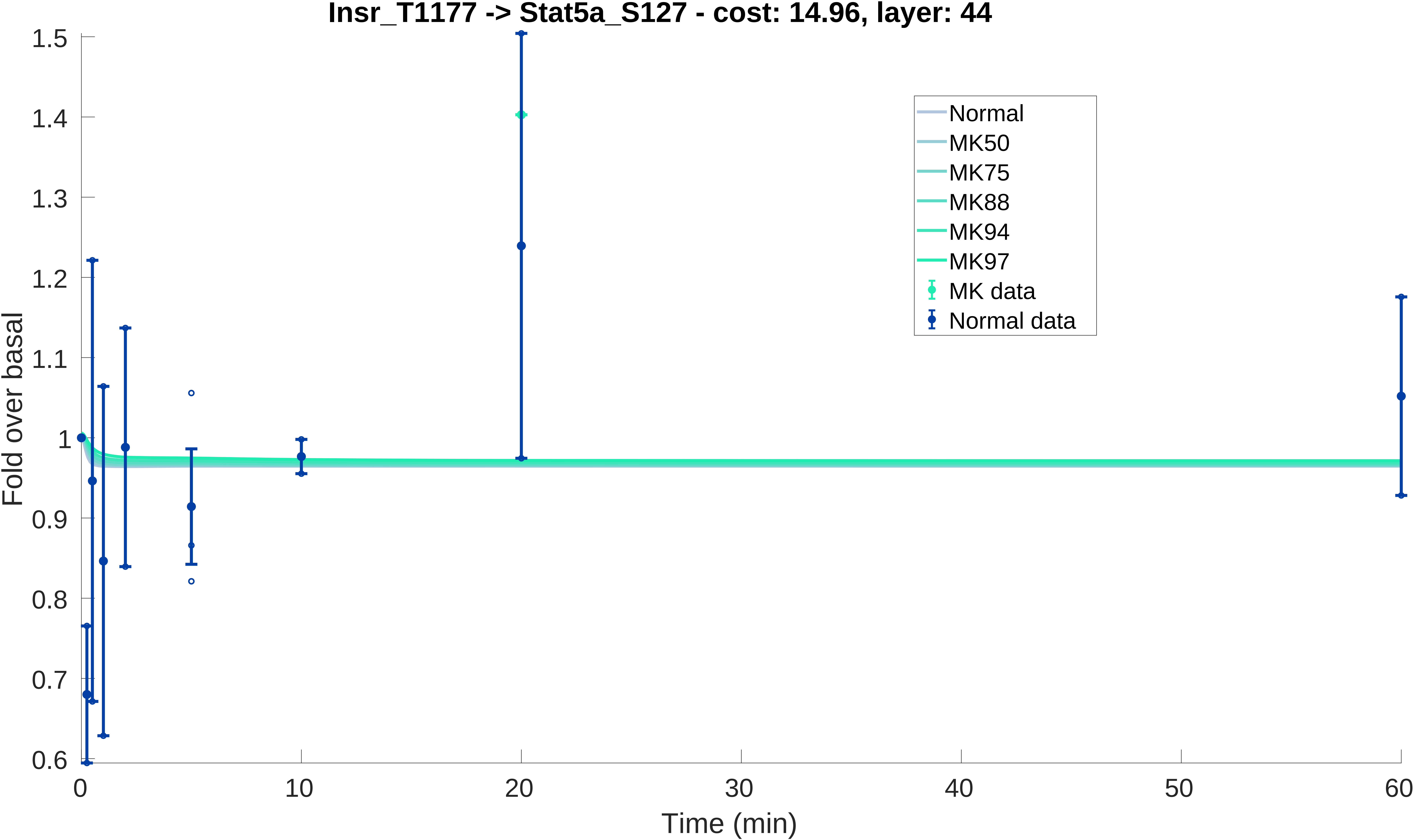

Insr_T1177 -> Stat5a_S127 - cost: 14.96, layer: 44
Fold over basal
Time (min)
1.5
1.4
1.3
1.2
1.1
1
0.9
0.8
0.7
0.6
0
10
20
30
40
50
60
Normal
MK50
MK75
MK88
MK94
MK97
MK data
Normal data

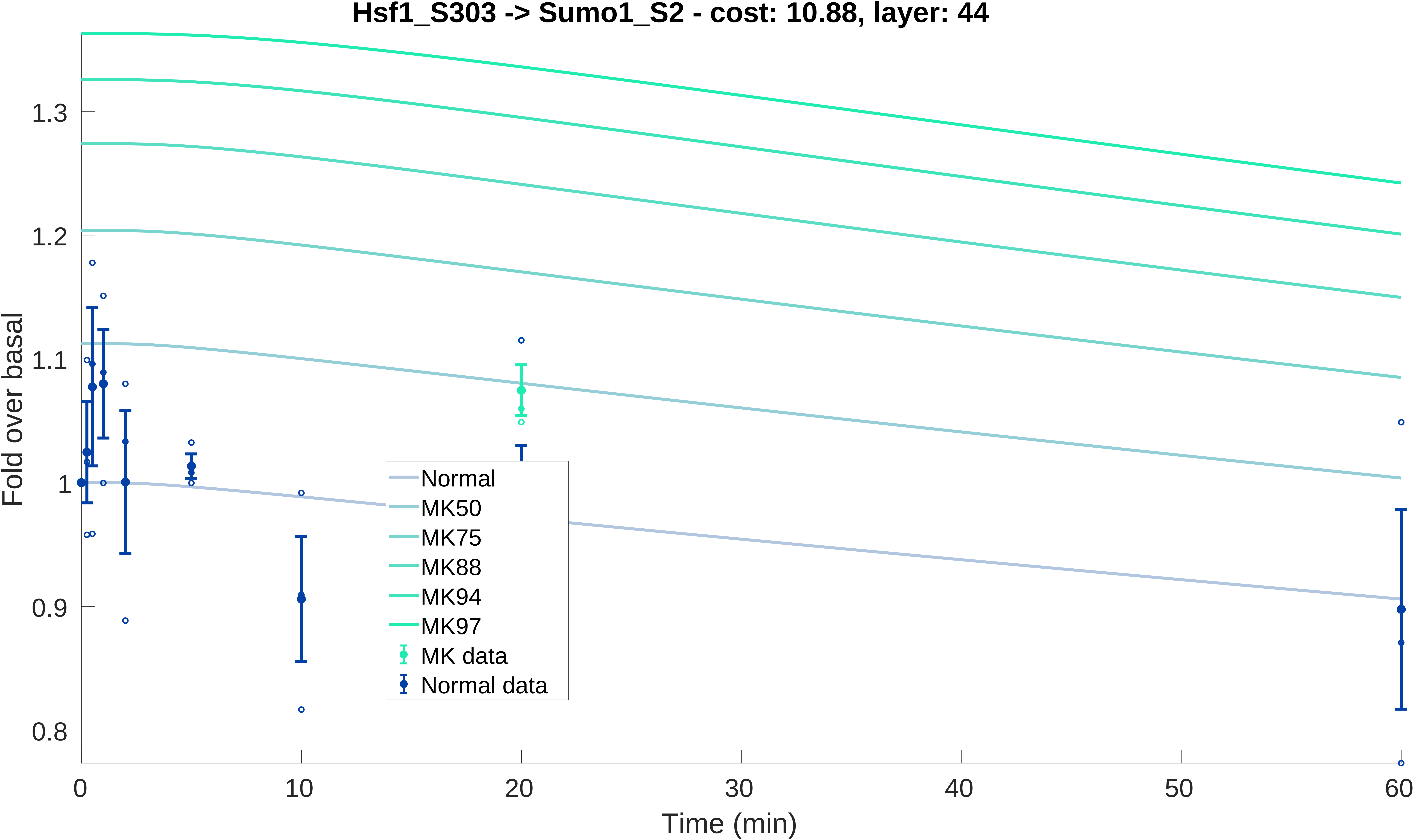

Hsf1_S303 -> Sumo1_S2 - cost: 10.88, layer: 44
Fold over basal
Time (min)
0.8
0.9
1
1.1
1.2
1.3
0
10
20
30
40
50
60
Normal
MK50
MK75
MK88
MK94
MK97
MK data
Normal data

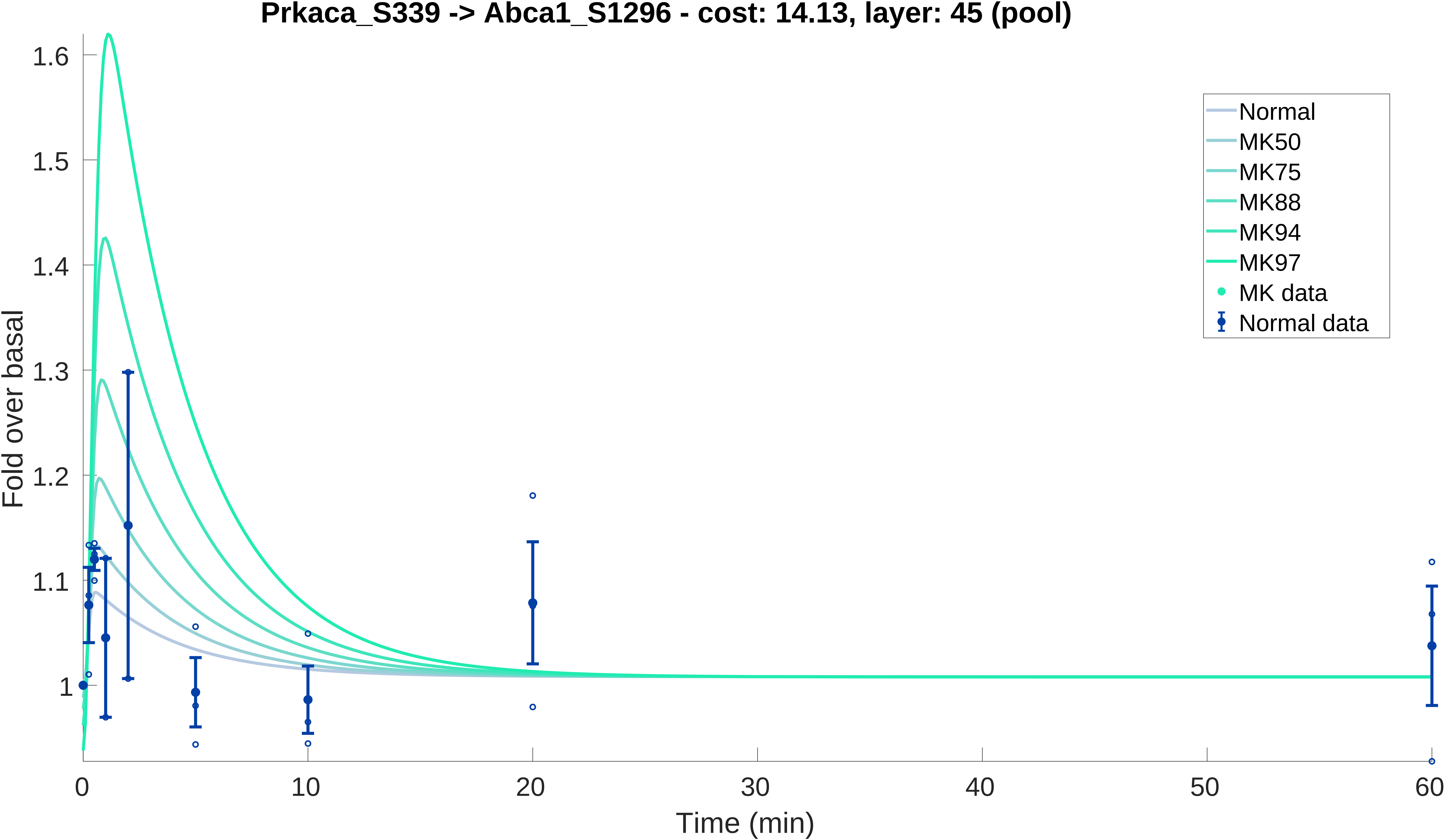

Prkaca_S339 -> Abca1_S1296 - cost: 14.13, layer: 45 (pool)
Fold over basal
1.6
1.5
1.4
1.3
1.2
1.1
1
0
10
20
30
40
50
60
Time (min)
Normal
MK50
MK75
MK88
MK94
MK97
MK data
Normal data

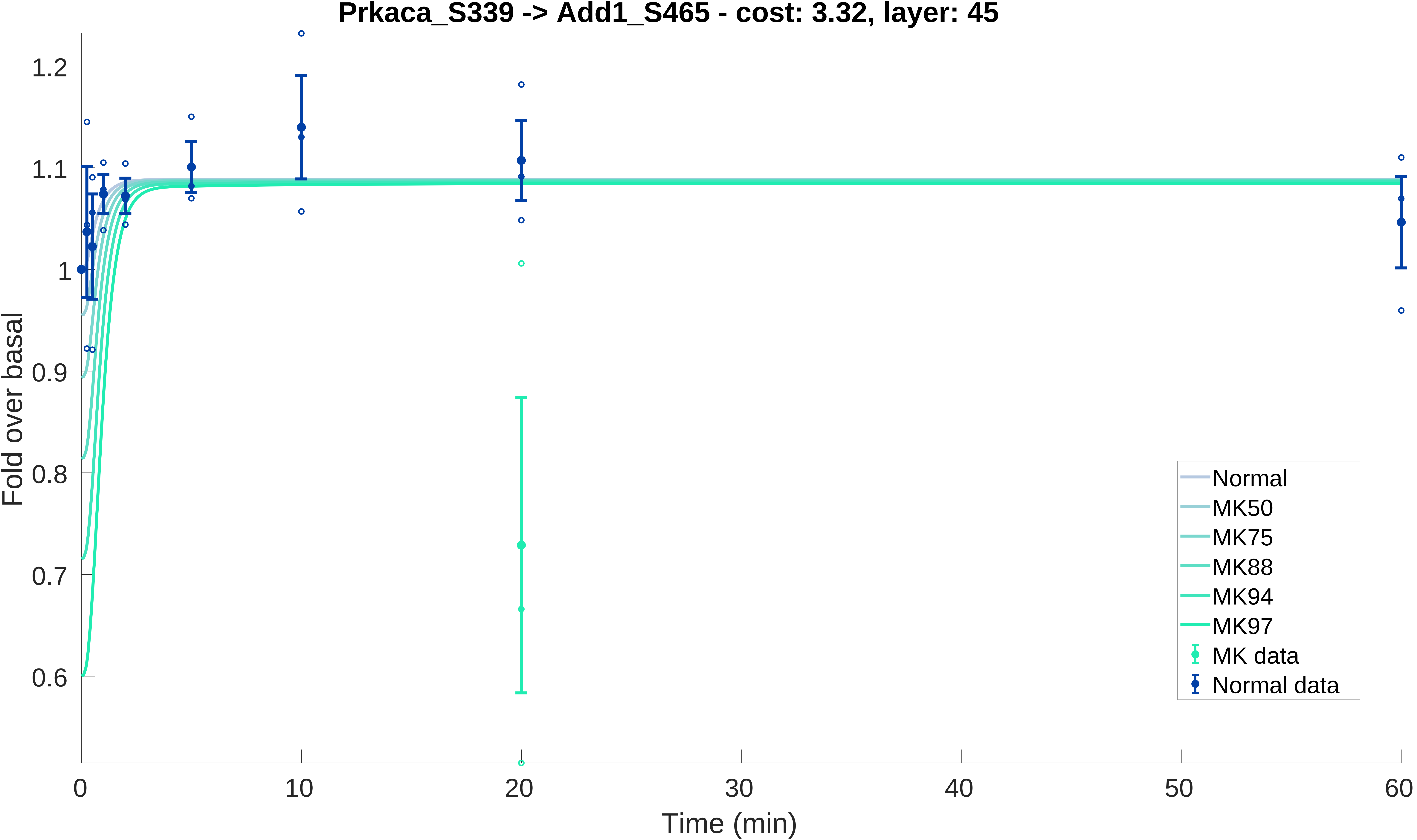

Prrkaca_S339 -> Add1_S465 - cost: 3.32, layer: 45
Fold over basal
Time (min)
0
10
20
30
40
50
60
0.6
0.7
0.8
0.9
1
1.1
1.2
Normal
MK50
MK75
MK88
MK94
MK97
MK data
Normal data

Prkaca_S339 -> Add1_T610 - cost: 13.15, layer: 45

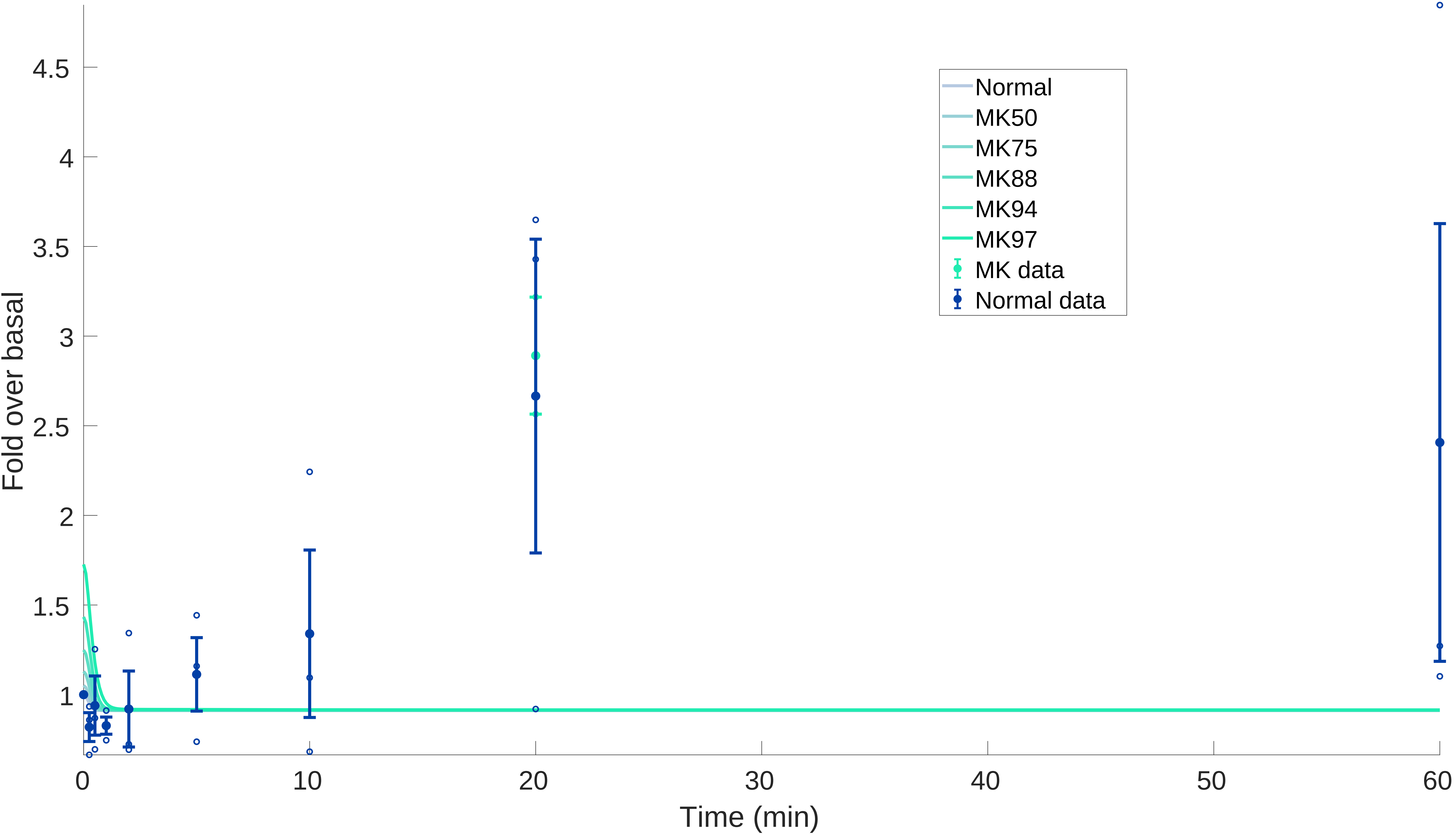

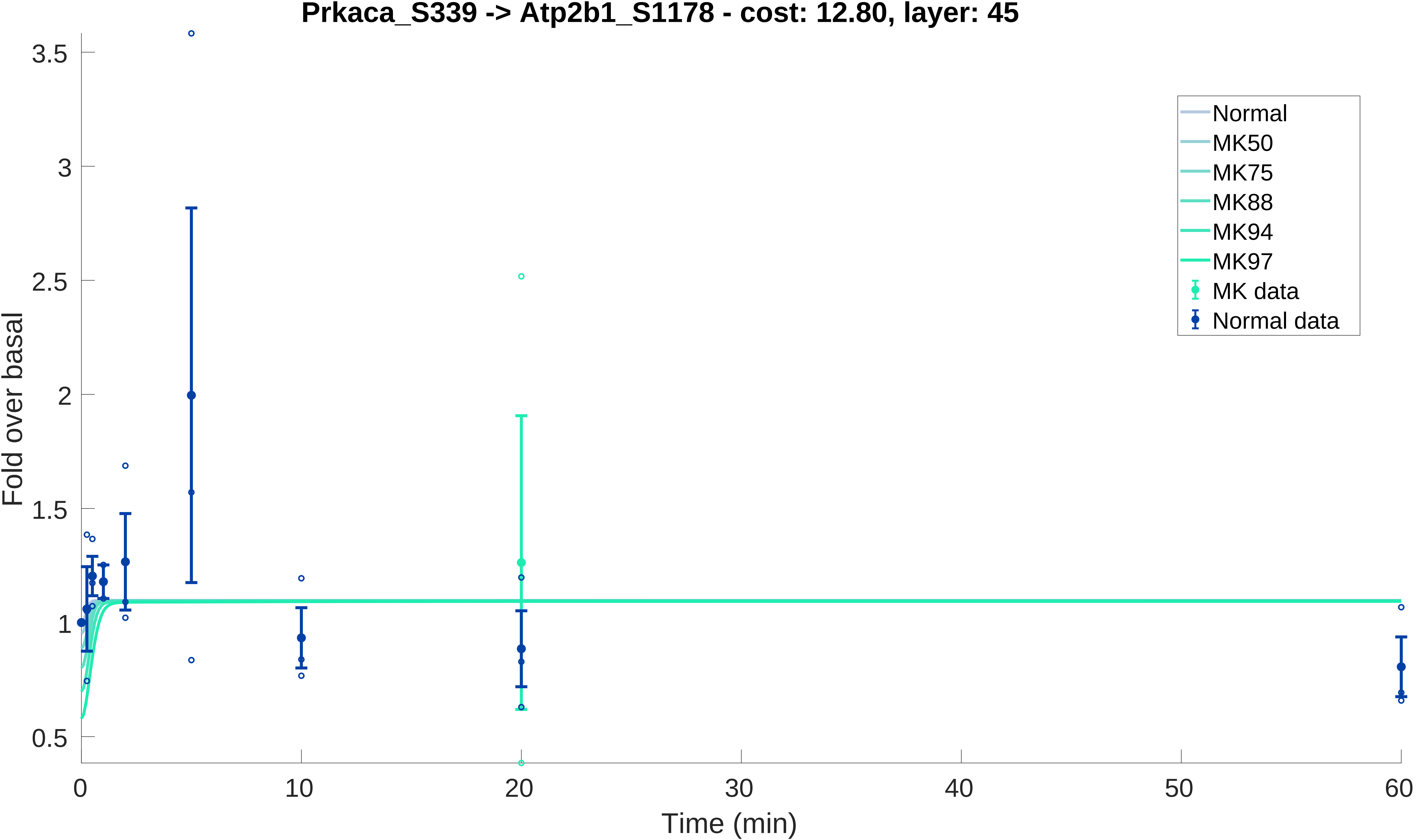

Prkaca_S339 -> Atp2b1_S1178 - cost: 12.80, layer: 45
Fold over basal
Time (min)
0.5
1
1.5
2
2.5
3
3.5
0
10
20
30
40
50
60
Normal
MK50
MK75
MK88
MK94
MK97
MK data
Normal data

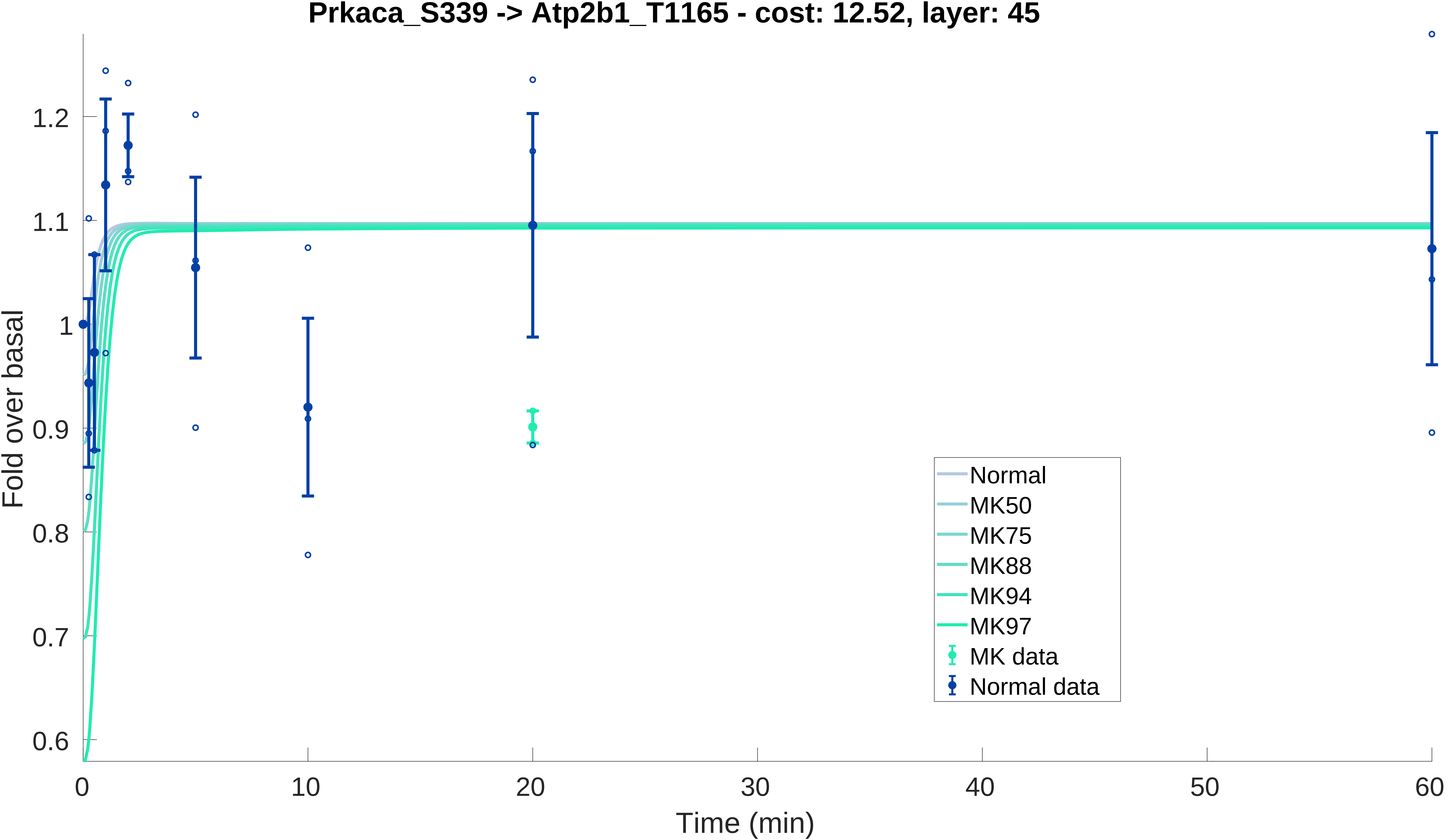

Prkaca_S339 -> Atp2b1_T1165 - cost: 12.52, layer: 45
Fold over basal
1.2
1.1
1
0.9
0.8
0.7
0.6
0
10
20
30
40
50
60
Time (min)
Normal
MK50
MK75
MK88
MK94
MK97
MK data
Normal data

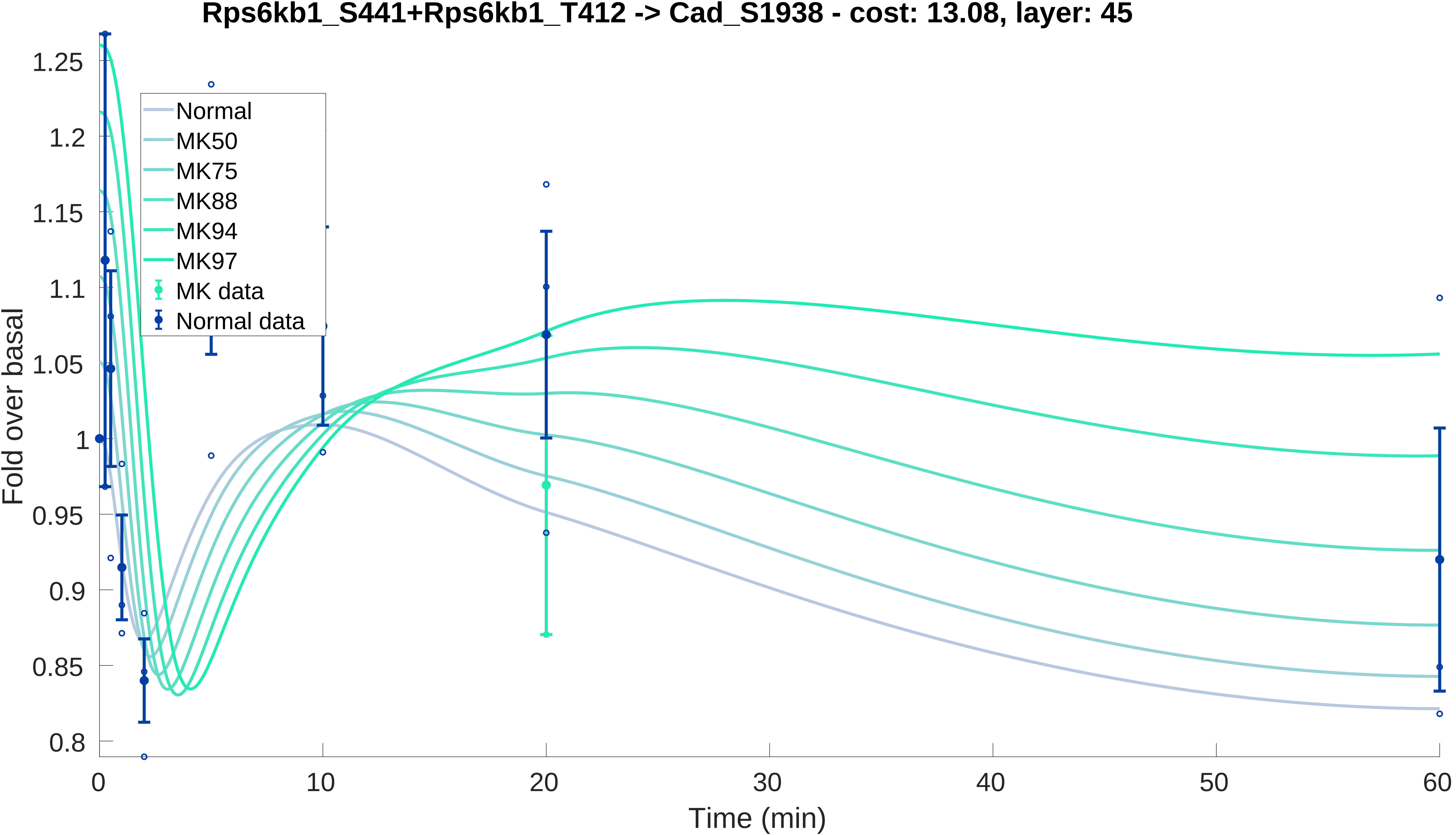

Rps6kb1_S441+Rps6kb1_T412 -> Cad_S1938 - cost: 13.08, layer: 45
Normal
MK50
MK75
MK88
MK94
MK97
MK data
Normal data
Fold over basal
Time (min)
1.25
1.2
1.15
1.1
1.05
1
0.95
0.9
0.85
0.8
0
10
20
30
40
50
60

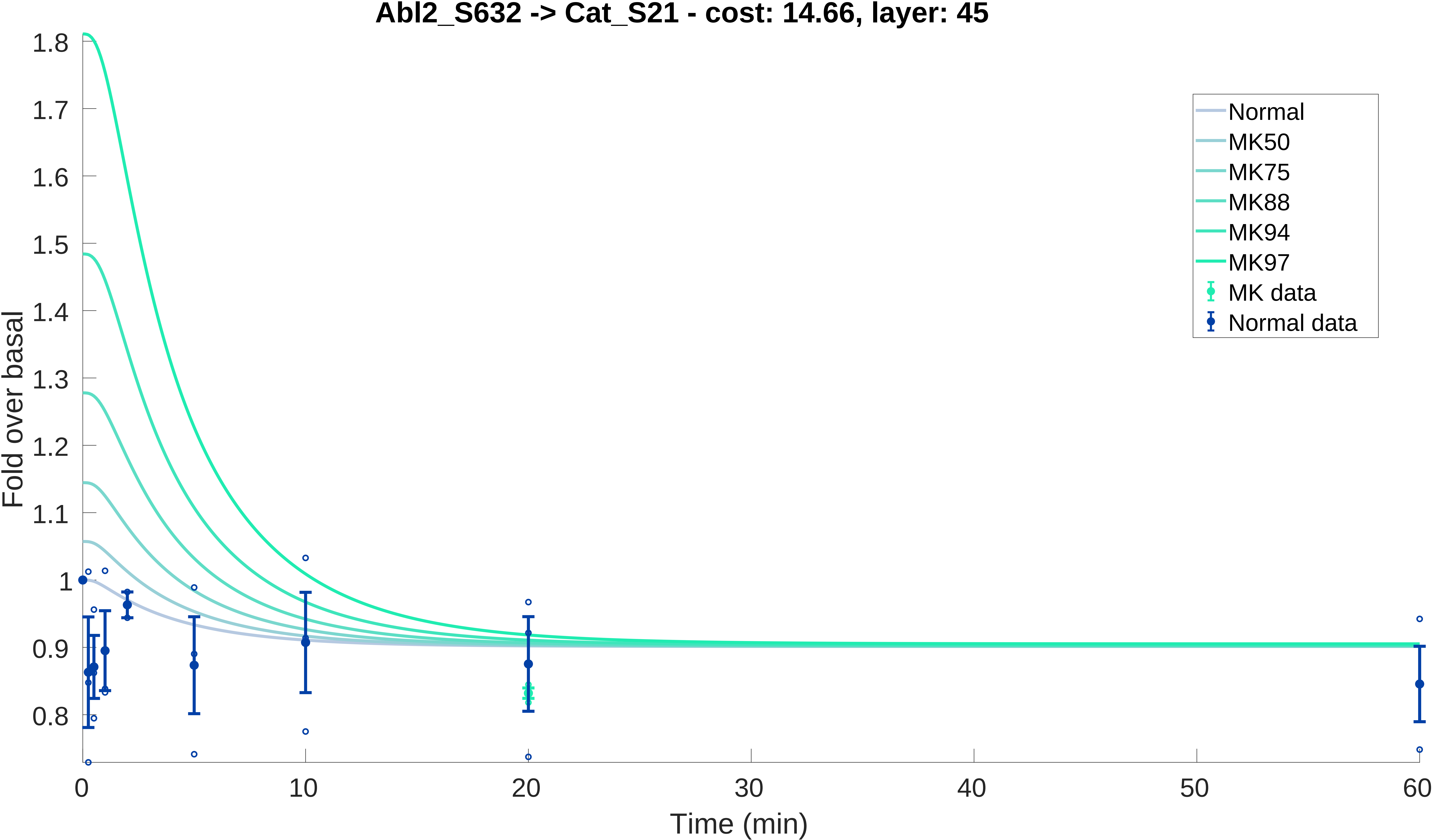
Abl2_S632 -> Cat_S21 - cost: 14.66, layer: 45
Fold over basal
1.8
1.7
1.6
1.5
1.4
1.3
1.2
1.1
1
0.9
0.8
0
10
20
30
40
50
60
Time (min)
Normal
MK50
MK75
MK88
MK94
MK97
MK data
Normal data

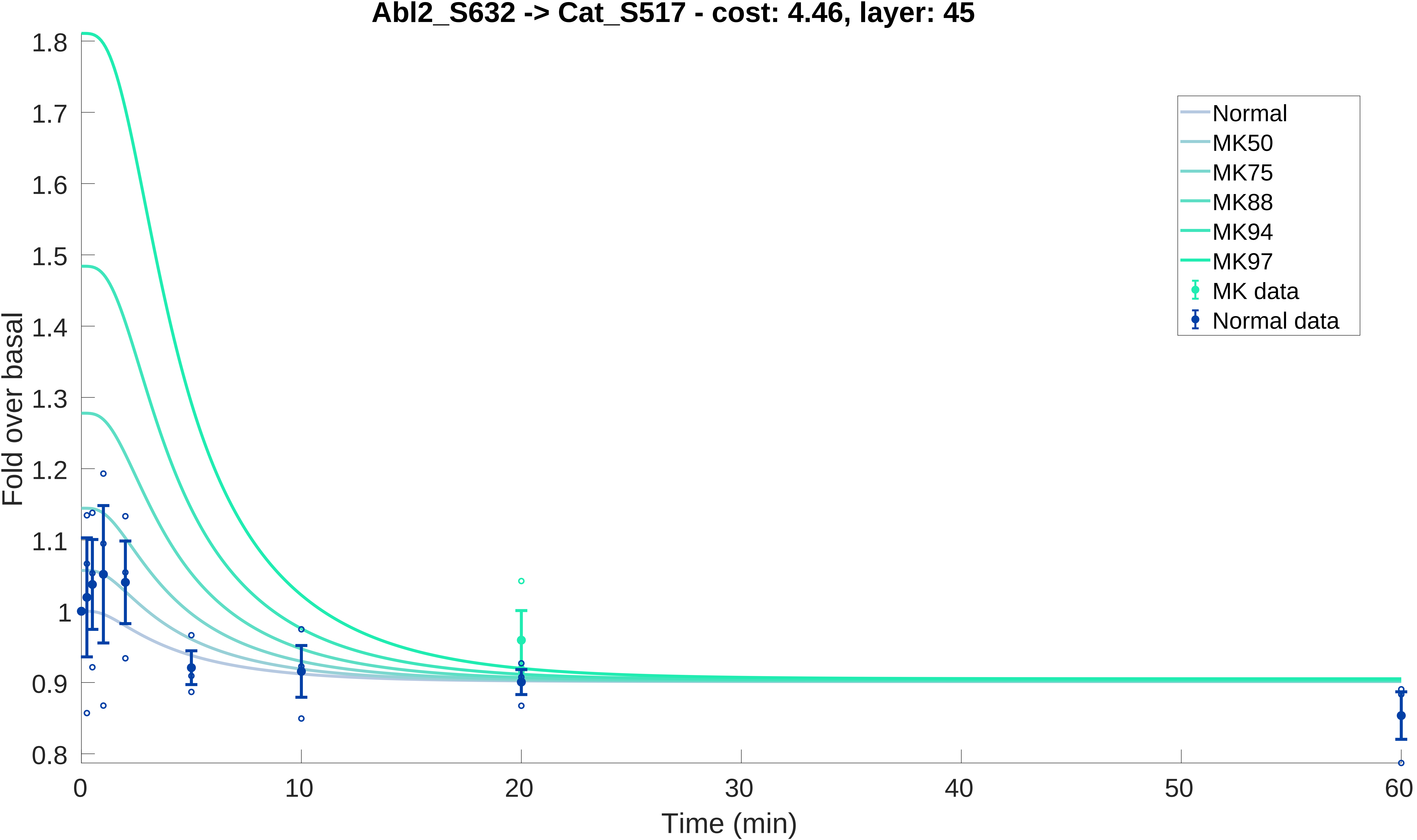

Abl2_S632 -> Cat_S517 - cost: 4.46, layer: 45
Fold over basal
Time (min)
Normal
MK50
MK75
MK88
MK94
MK97
MK data
Normal data
1.8
1.7
1.6
1.5
1.4
1.3
1.2
1.1
1
0.9
0.8
0
10
20
30
40
50
60

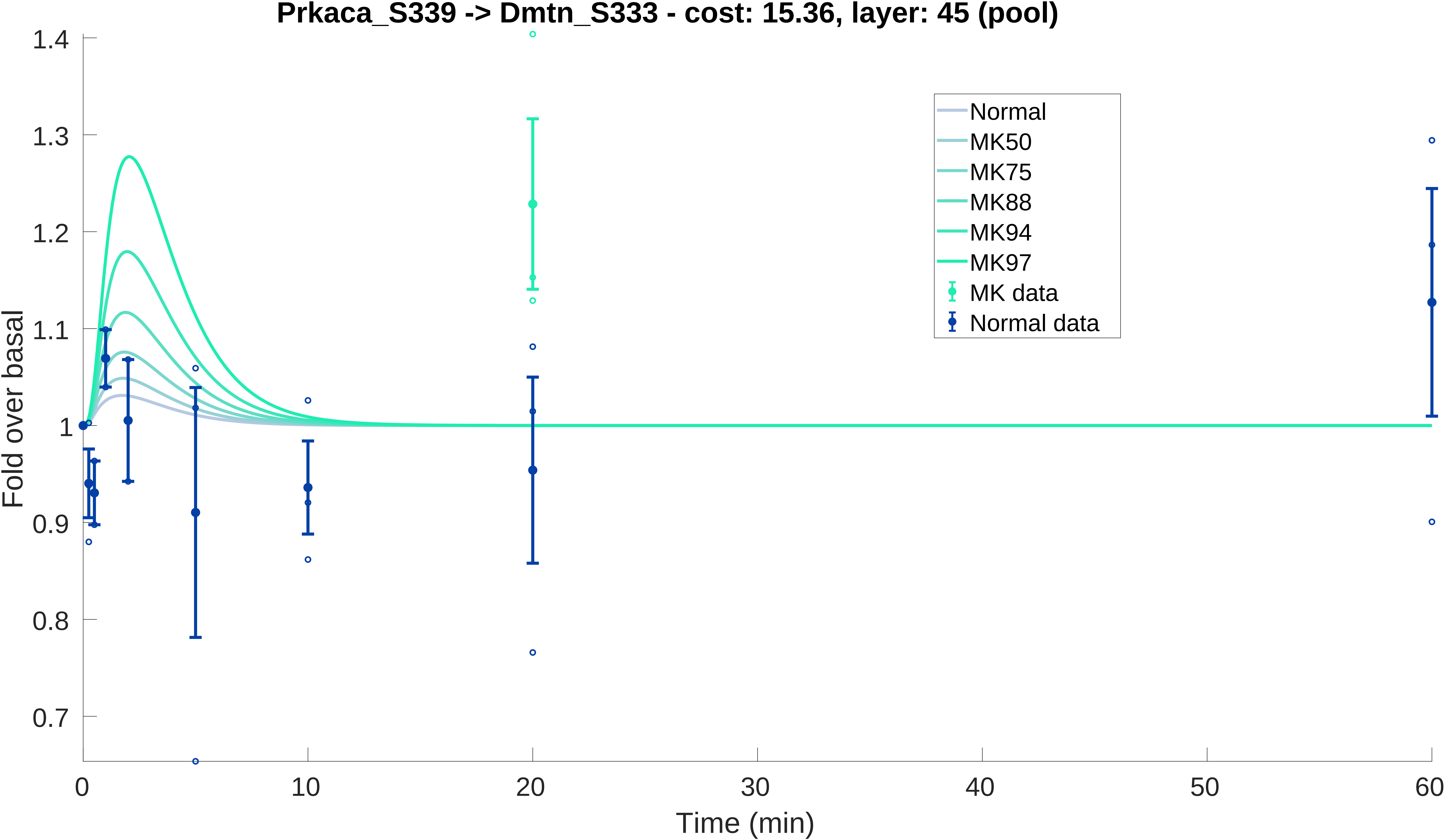
Prkaca_S339 -> Dmtn_S333 - cost: 15.36, layer: 45 (pool)
Fold over basal
Time (min)
1.4
1.3
1.2
1.1
1
0.9
0.8
0.7
0
10
20
30
40
50
60
Normal
MK50
MK75
MK88
MK94
MK97
MK data
Normal data

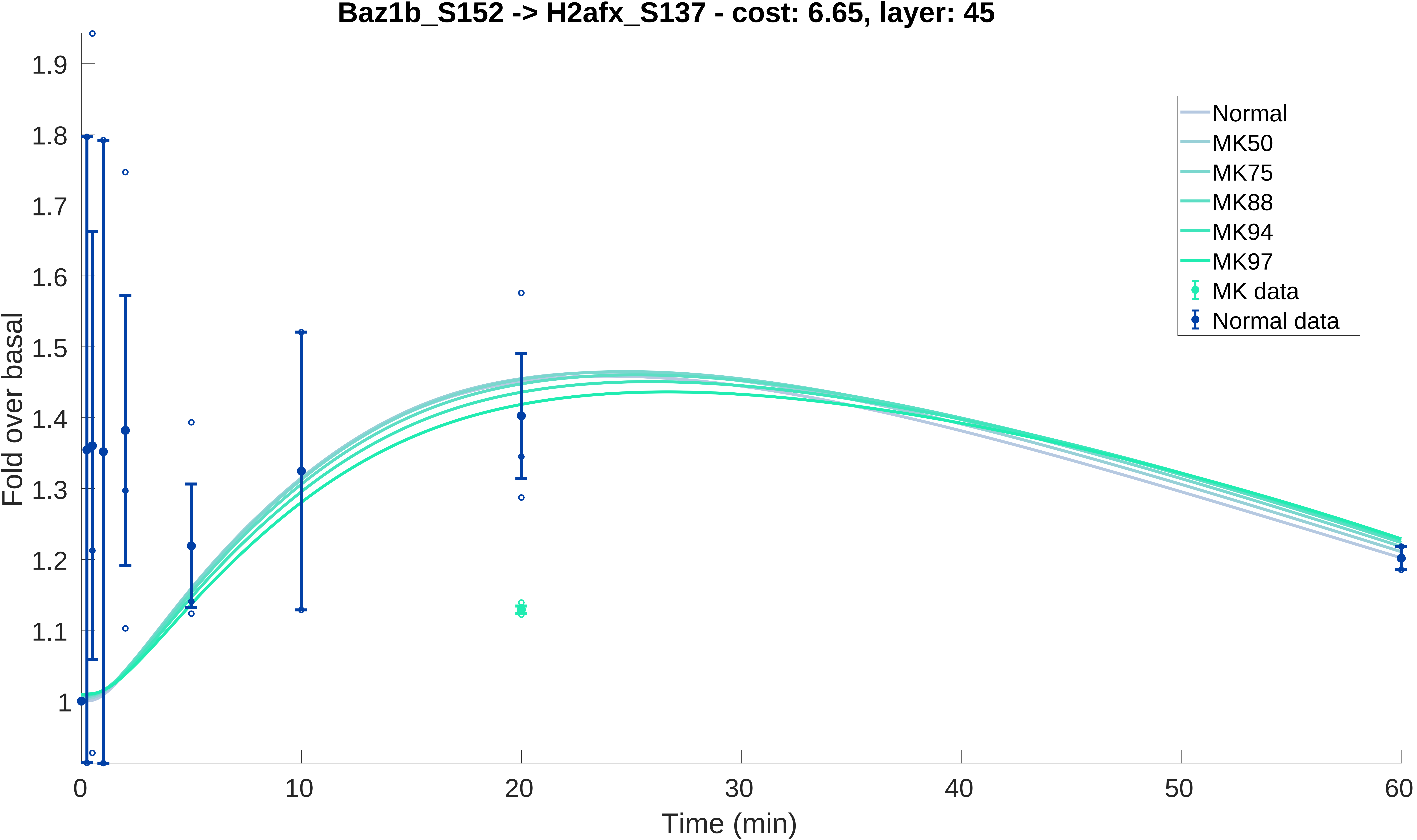

Baz1b_S152 -> H2afx_S137 - cost: 6.65, layer: 45
Fold over basal
Time (min)
1.9
1.8
1.7
1.6
1.5
1.4
1.3
1.2
1.1
1
0
10
20
30
40
50
60
Normal
MK50
MK75
MK88
MK94
MK97
MK data
Normal data

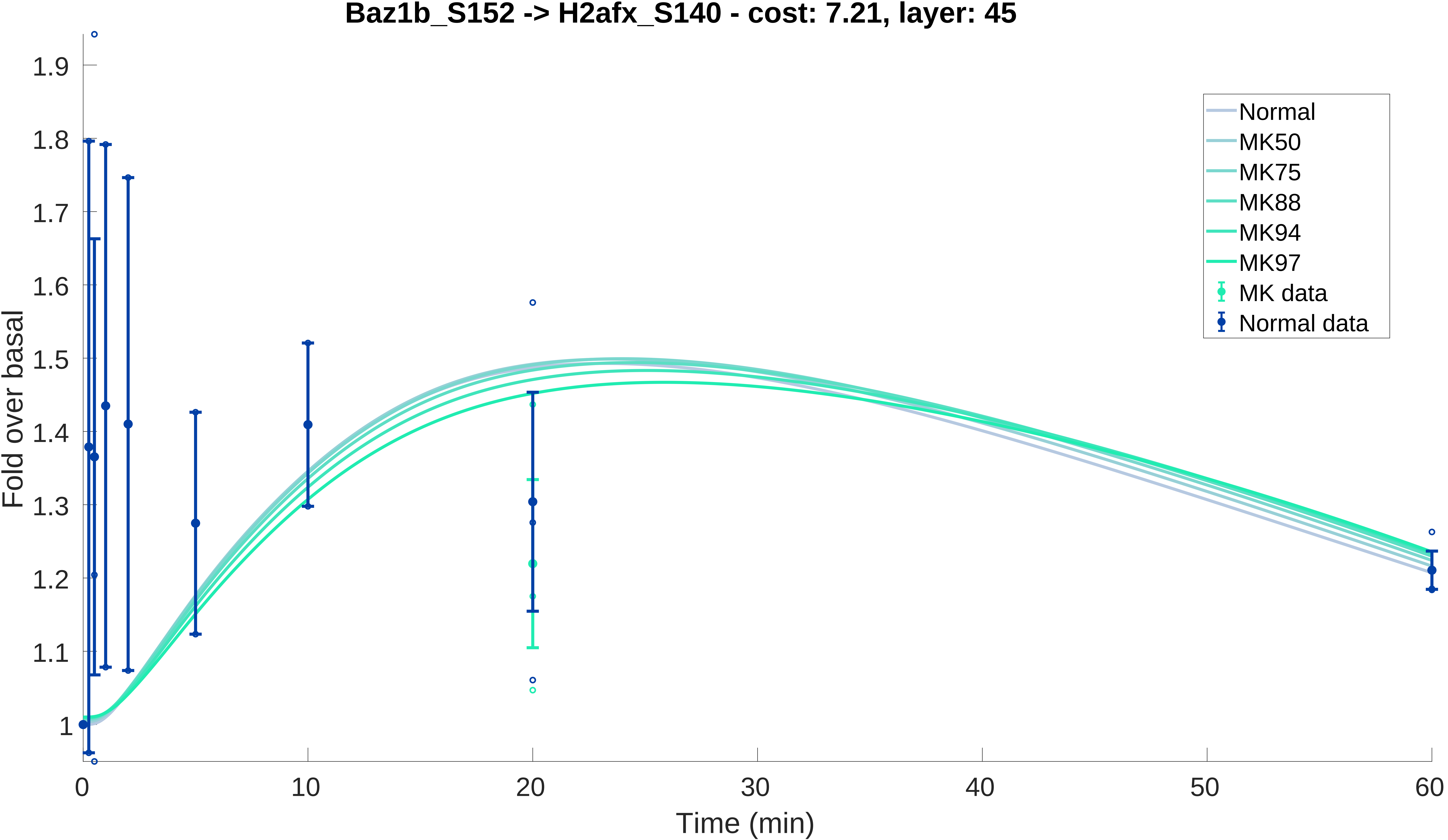

Baz1b_S152 -> H2afx_S140 - cost: 7.21, layer: 45
Fold over basal
Time (min)
1.9
1.8
1.7
1.6
1.5
1.4
1.3
1.2
1.1
1
0
10
20
30
40
50
60
Normal
MK50
MK75
MK88
MK94
MK97
MK data
Normal data

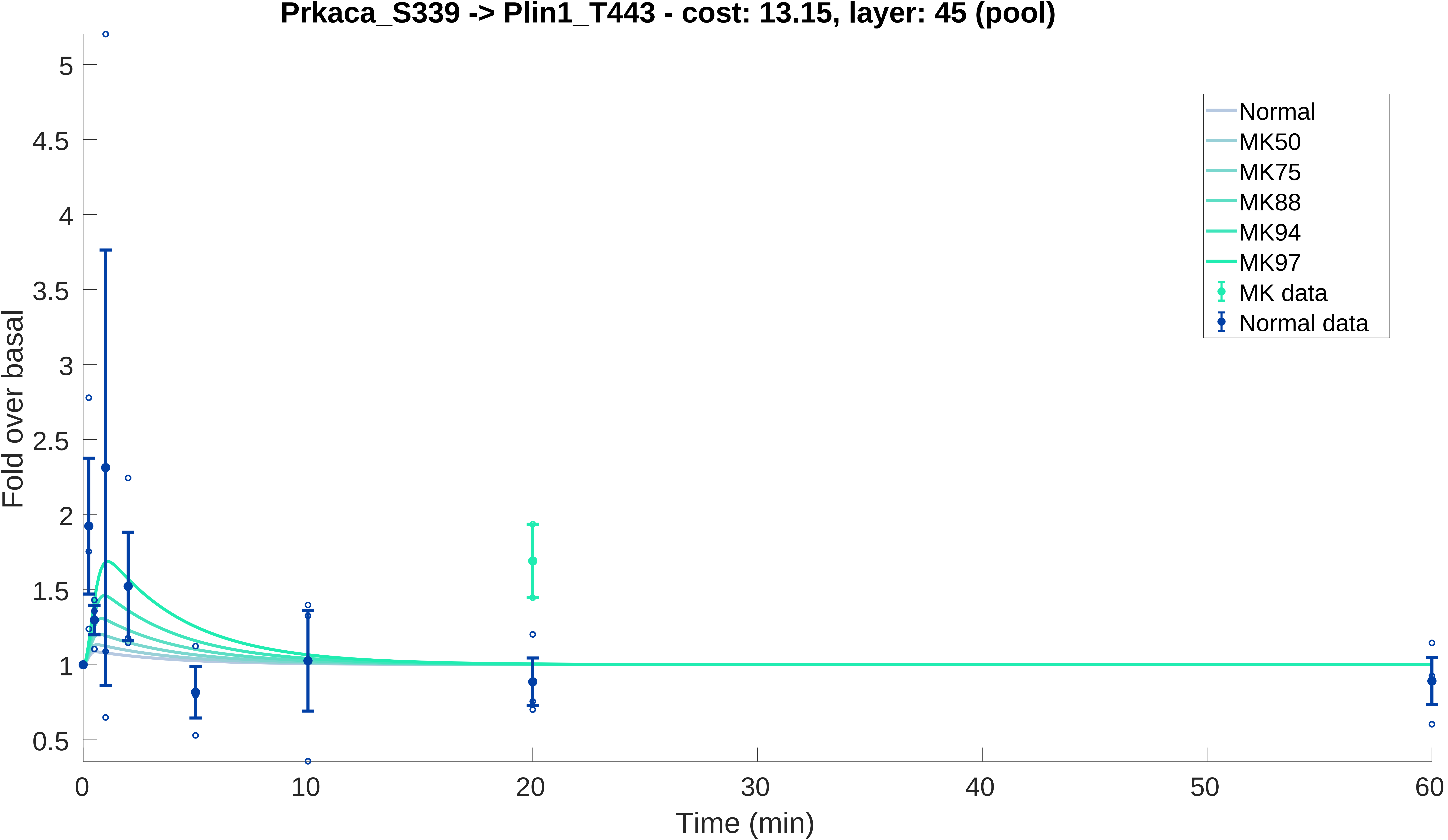

Prkaca_S339 -> Plin1_T443 - cost: 13.15, layer: 45 (pool)
Fold over basal
Time (min)
0.5
1
1.5
2
2.5
3
3.5
4
4.5
5
0
10
20
30
40
50
60
Normal
MK50
MK75
MK88
MK94
MK97
MK data
Normal data

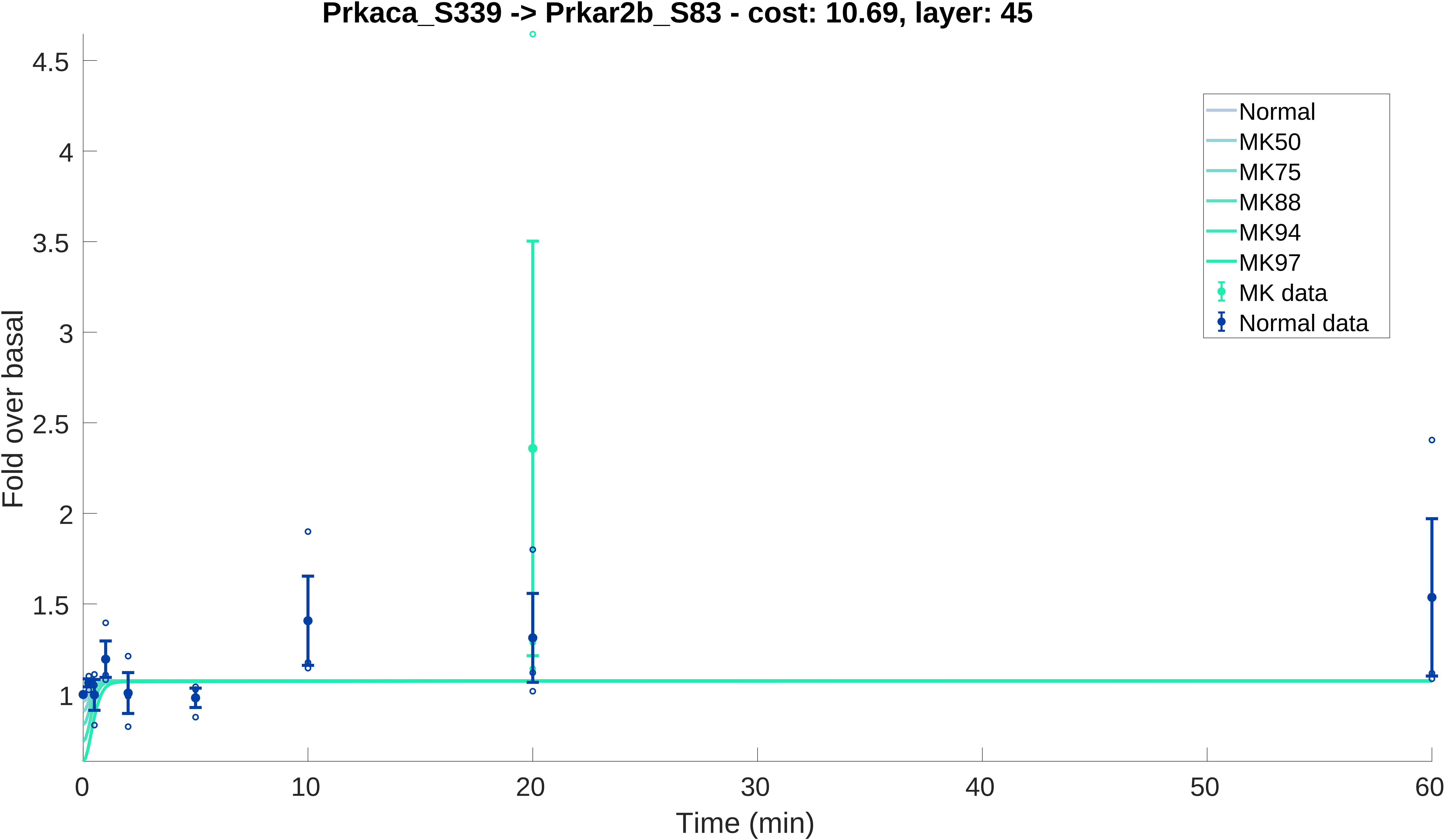
Prkaca_S339 -> Prkar2b_S83 - cost: 10.69, layer: 45
Fold over basal
Time (min)
4.5
4
3.5
3
2.5
2
1.5
1
0
10
20
30
40
50
60
Normal
MK50
MK75
MK88
MK94
MK97
MK data
Normal data

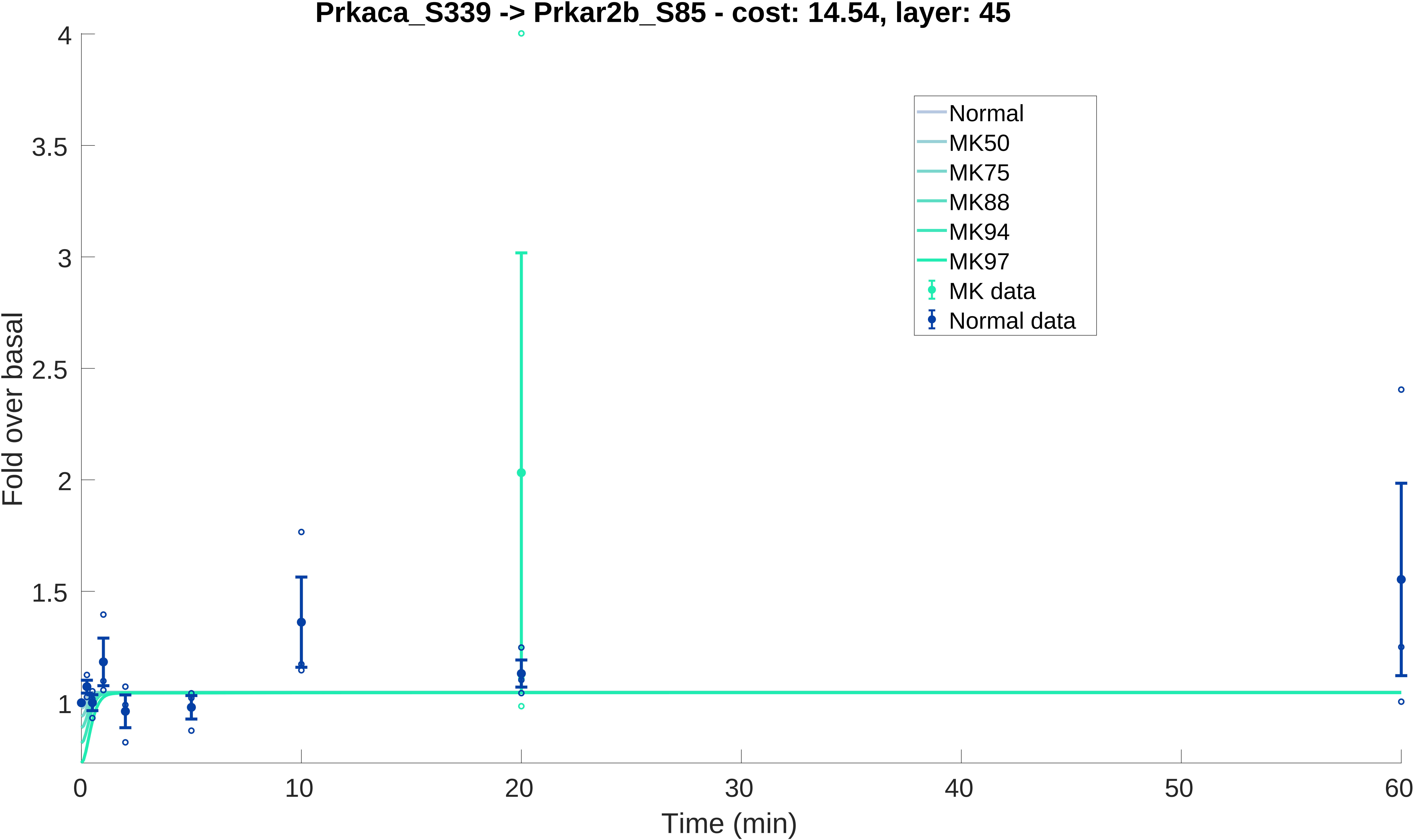

Prkaca_S339 -> Prkar2b_S85 - cost: 14.54, layer: 45
Fold over basal
Time (min)
4
3.5
3
2.5
2
1.5
1
0
10
20
30
40
50
60
Normal
MK50
MK75
MK88
MK94
MK97
MK data
Normal data

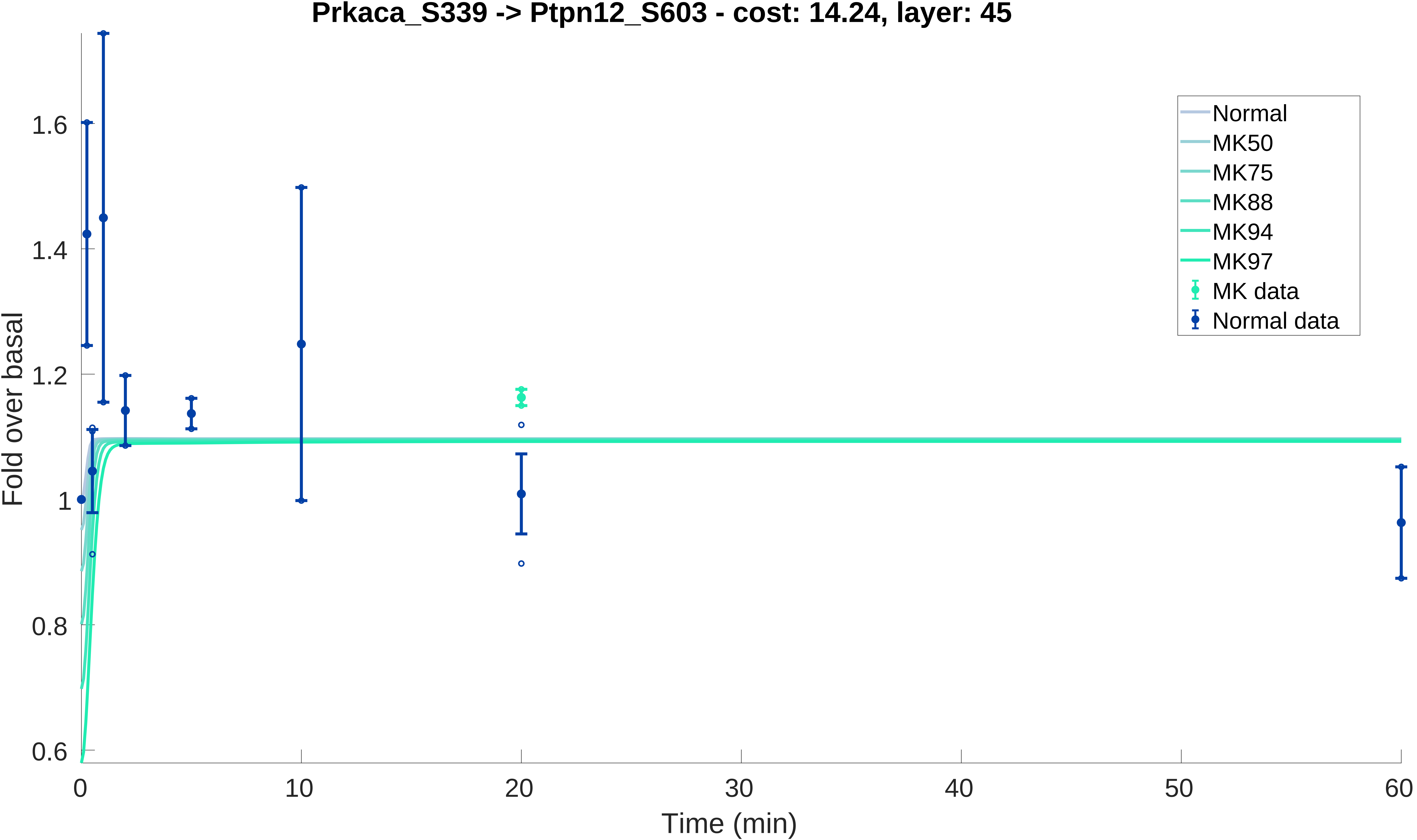

Prkaca_S339 -> Ptpn12_S603 - cost: 14.24, layer: 45
Fold over basal
Time (min)
0.6
0.8
1
1.2
1.4
1.6
0
10
20
30
40
50
60
Normal
MK50
MK75
MK88
MK94
MK97
MK data
Normal data

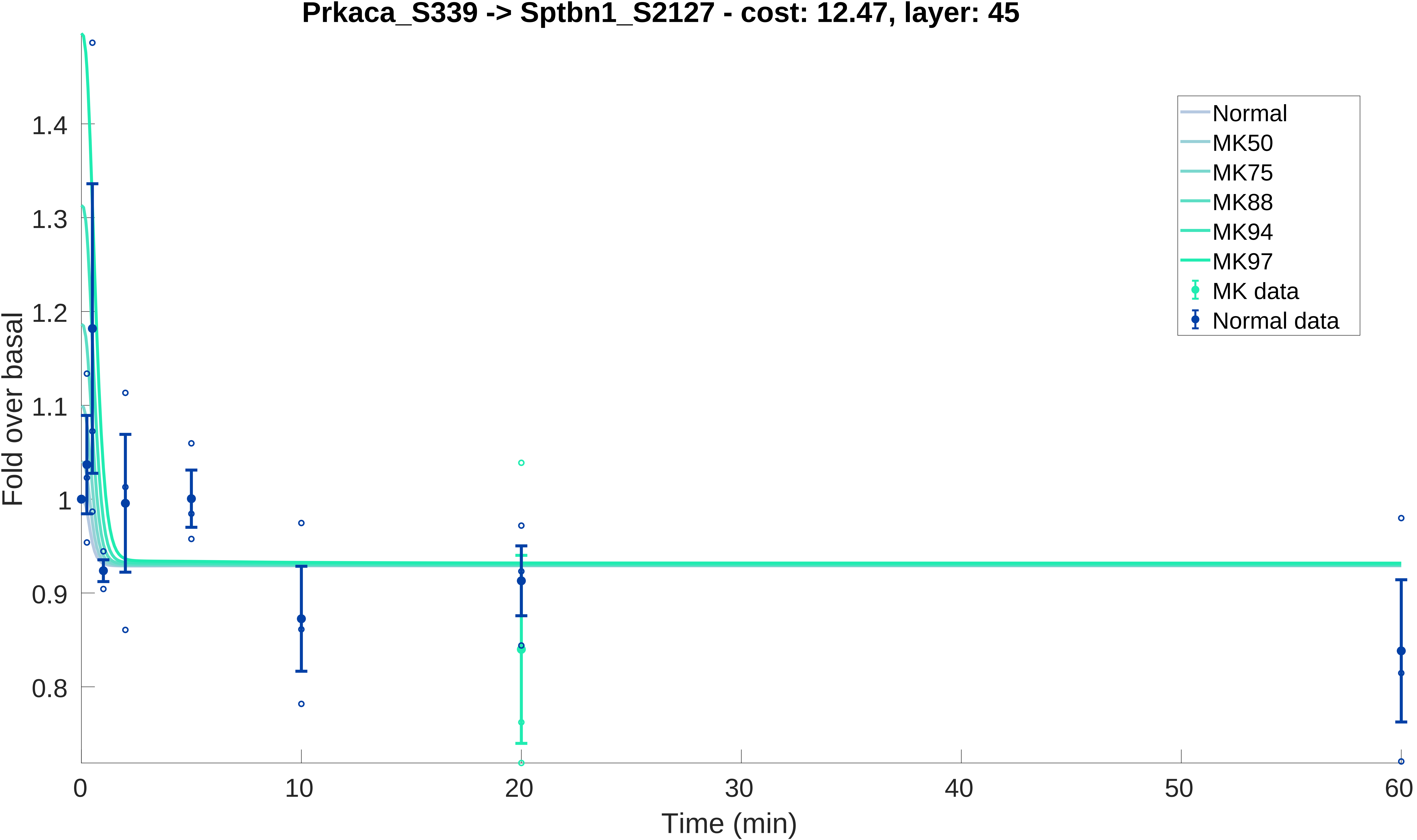

Prkaca_S339 -> Sptbn1_S2127 - cost: 12.47, layer: 45
Fold over basal
Time (min)
0.8
0.9
1
1.1
1.2
1.3
1.4
0
10
20
30
40
50
60
Normal
MK50
MK75
MK88
MK94
MK97
MK data
Normal data

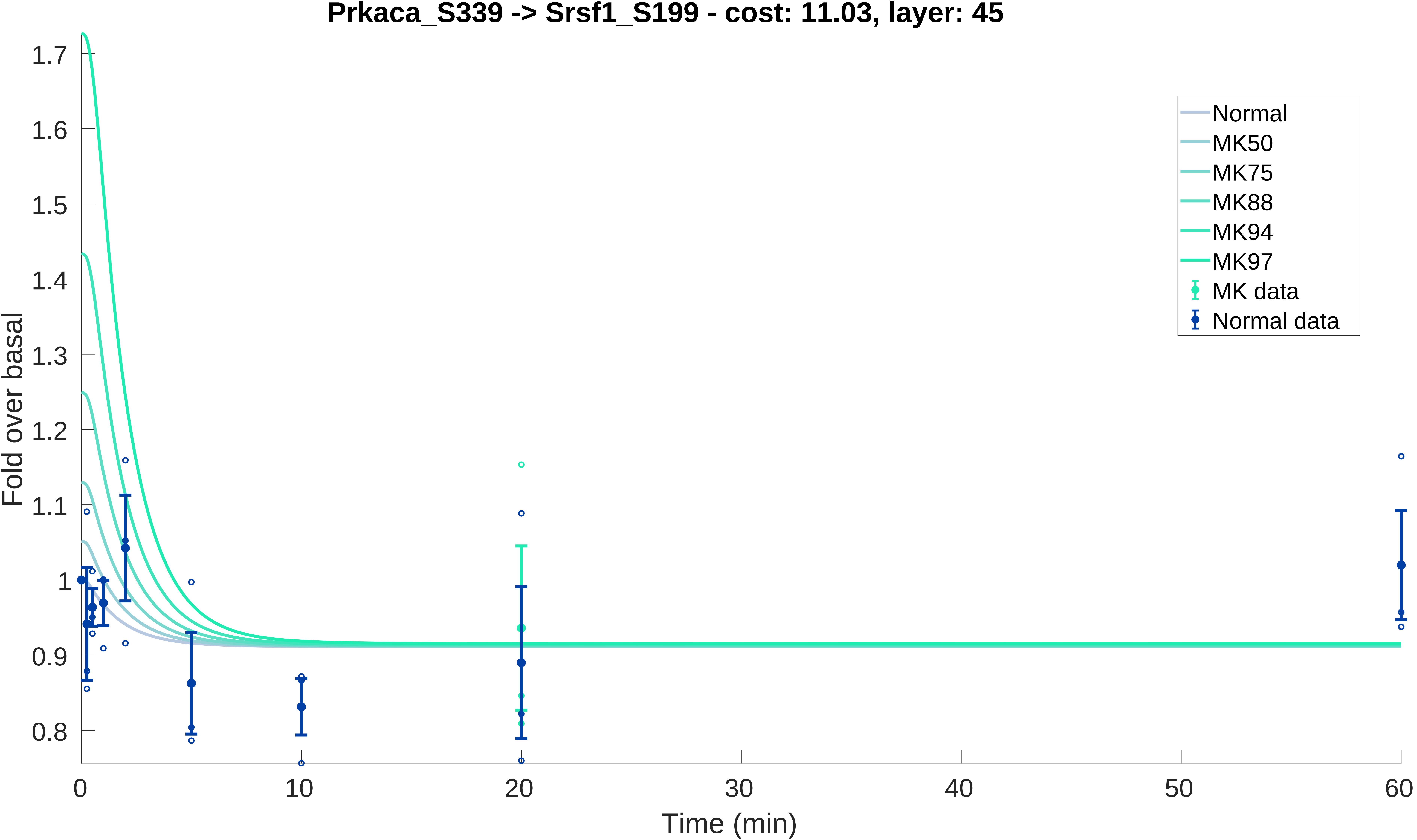

Prrkaca_S339 -> Srsf1_S199 - cost: 11.03, layer: 45
Fold over basal
Time (min)
Normal
MK50
MK75
MK88
MK94
MK97
MK data
Normal data

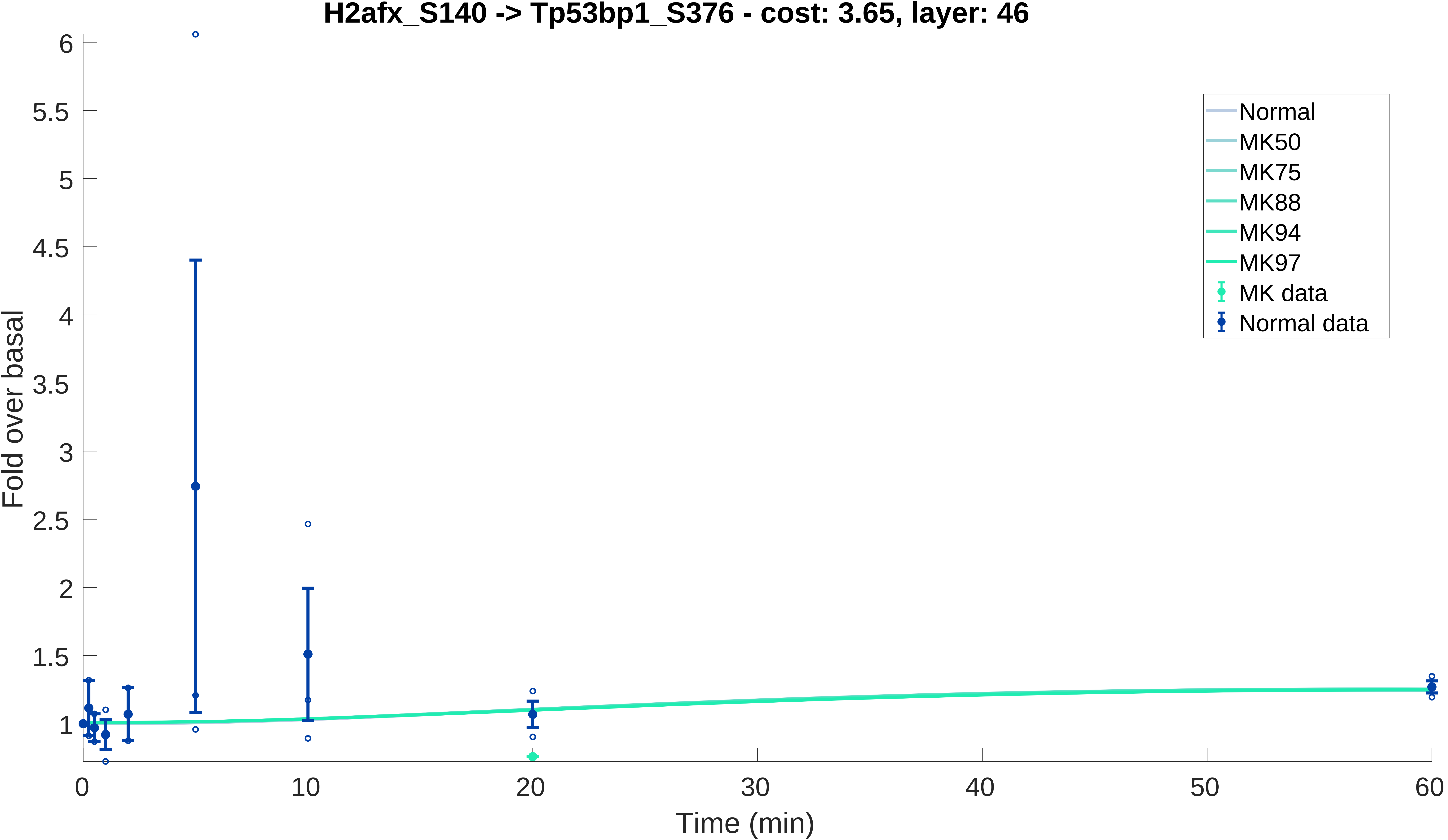

H2afx_S140 -> Tp53bp1_S376 - cost: 3.65, layer: 46
Fold over basal
Time (min)
0
10
20
30
40
50
60
1
1.5
2
2.5
3
3.5
4
4.5
5
5.5
6
Normal
MK50
MK75
MK88
MK94
MK97
MK data
Normal data

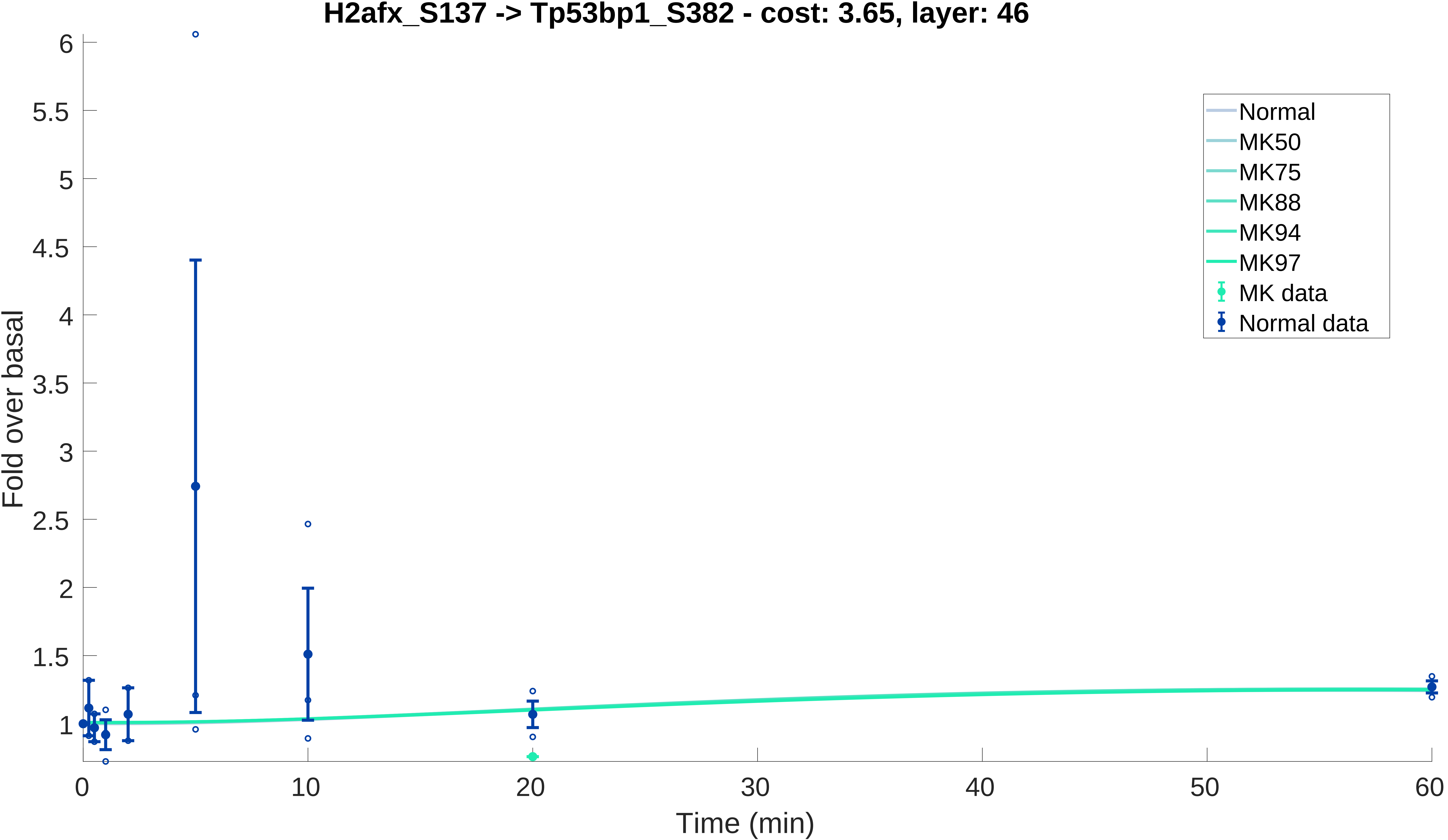
H2afx_S137 -> Tp53bp1_S382 - cost: 3.65, layer: 46
Fold over basal
Time (min)
0
10
20
30
40
50
60
1
1.5
2
2.5
3
3.5
4
4.5
5
5.5
6
Normal
MK50
MK75
MK88
MK94
MK97
MK data
Normal data

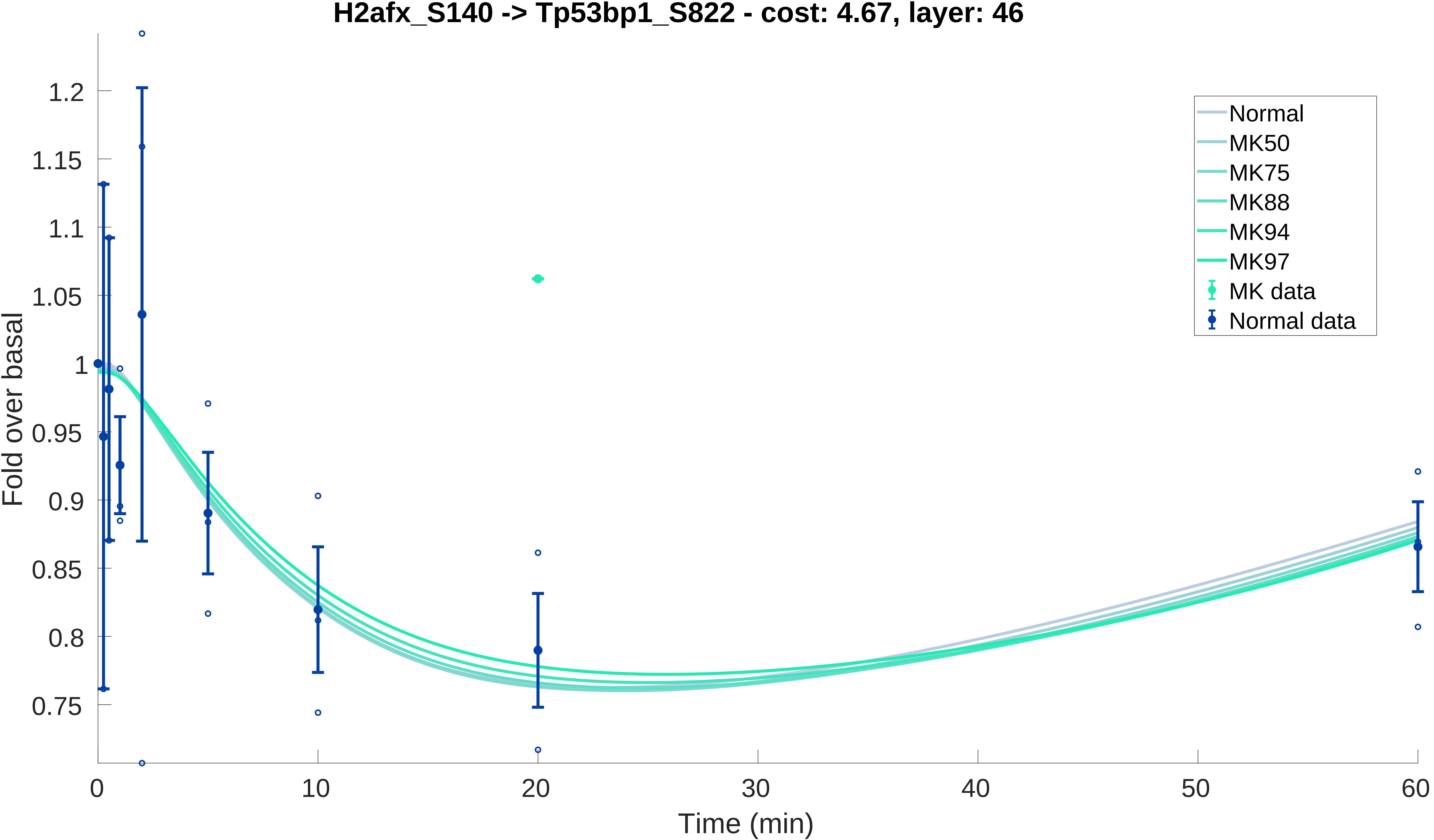

H2afx_S140 -> Tp53bp1_S822 - cost: 4.67, layer: 46
Fold over basal
1.2
1.15
1.1
1.05
1
0.95
0.9
0.85
0.8
0.75
0
10
20
30
40
50
60
Time (min)
Normal
MK50
MK75
MK88
MK94
MK97
MK data
Normal data

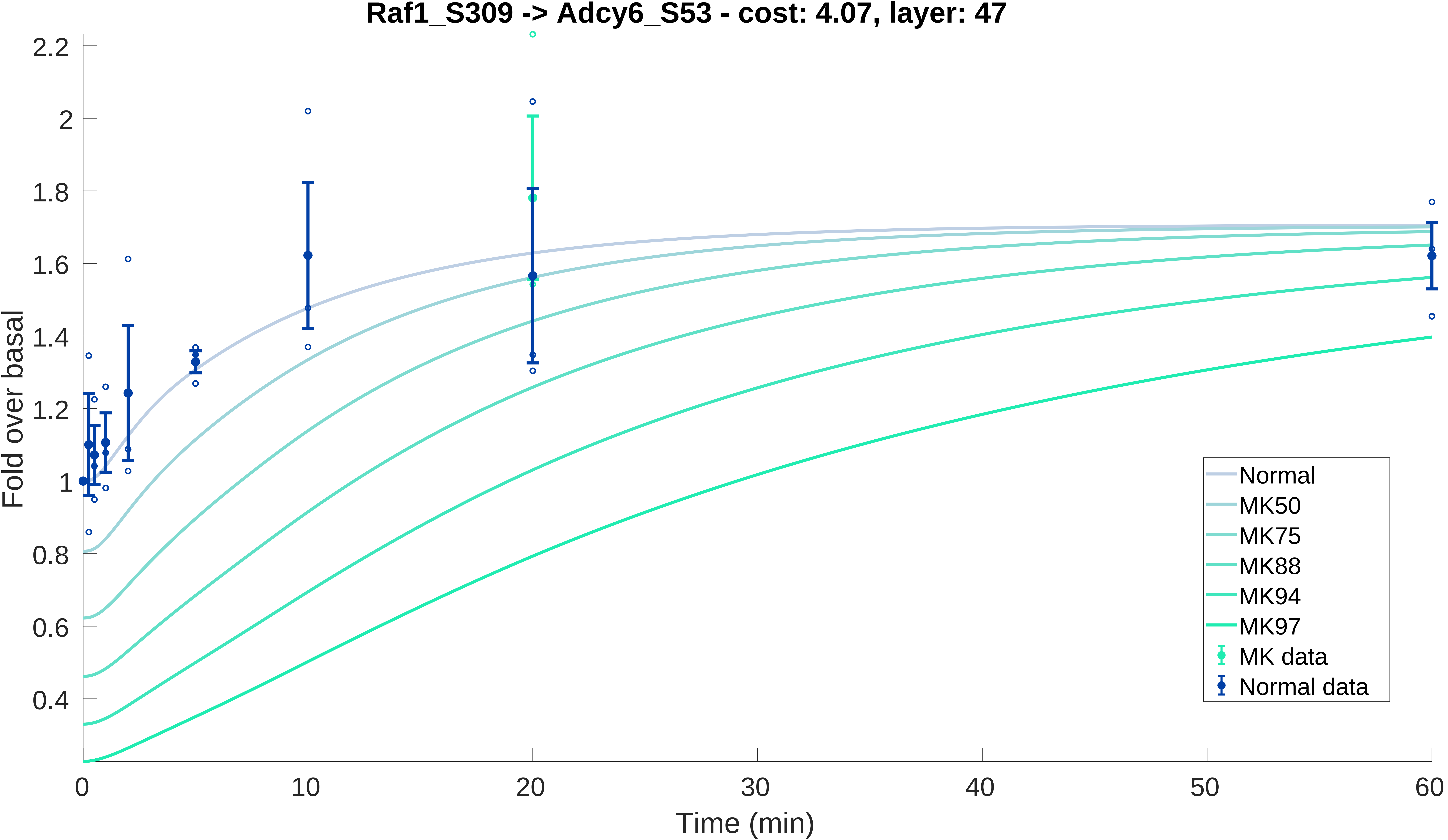

Raf1_S309 -> Adcy6_S53 - cost: 4.07, layer: 47
Fold over basal
Time (min)
0
10
20
30
40
50
60
0.4
0.6
0.8
1
1.2
1.4
1.6
1.8
2
2.2
Normal
MK50
MK75
MK88
MK94
MK97
MK data
Normal data

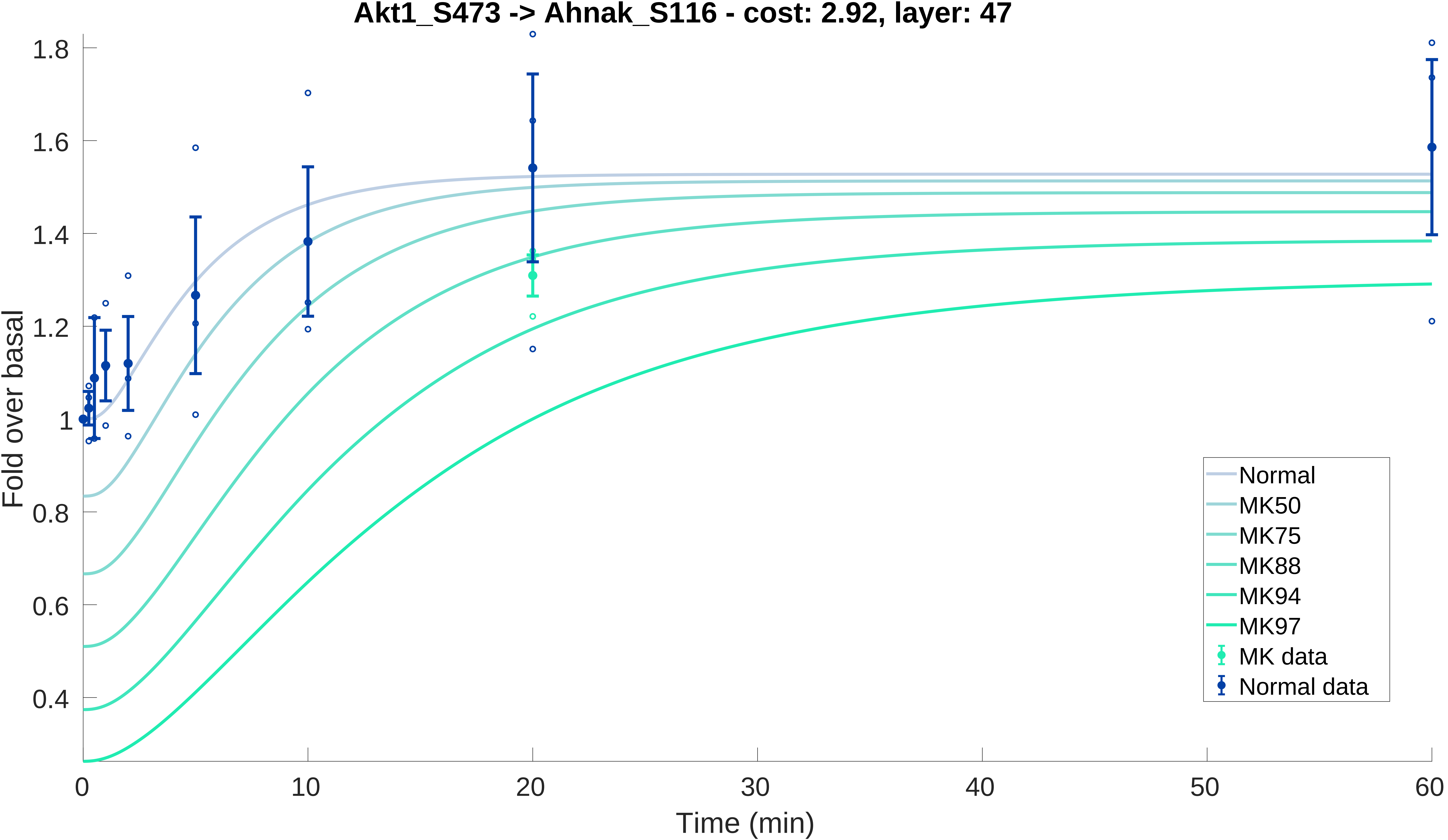
Akt1_S473 -> Ahnak_S116 - cost: 2.92, layer: 47
Fold over basal
Time (min)
1.8
1.6
1.4
1.2
1
0.8
0.6
0.4
0
10
20
30
40
50
60
Normal
MK50
MK75
MK88
MK94
MK97
MK data
Normal data

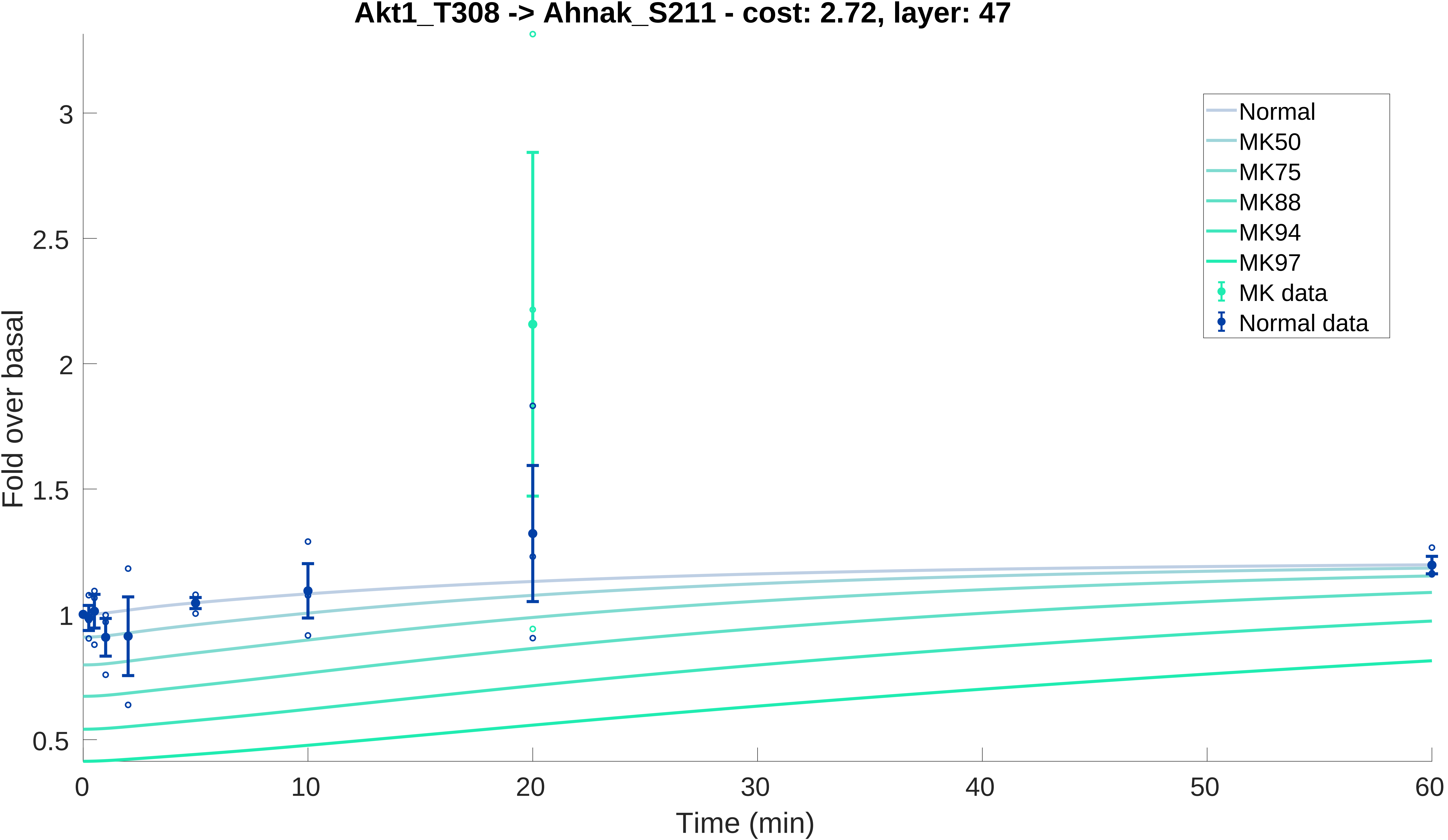
Akt1_T308 -> Ahnak_S211 - cost: 2.72, layer: 47
Fold over basal
Time (min)
0.5
1
1.5
2
2.5
3
0
10
20
30
40
50
60
Normal
MK50
MK75
MK88
MK94
MK97
MK data
Normal data

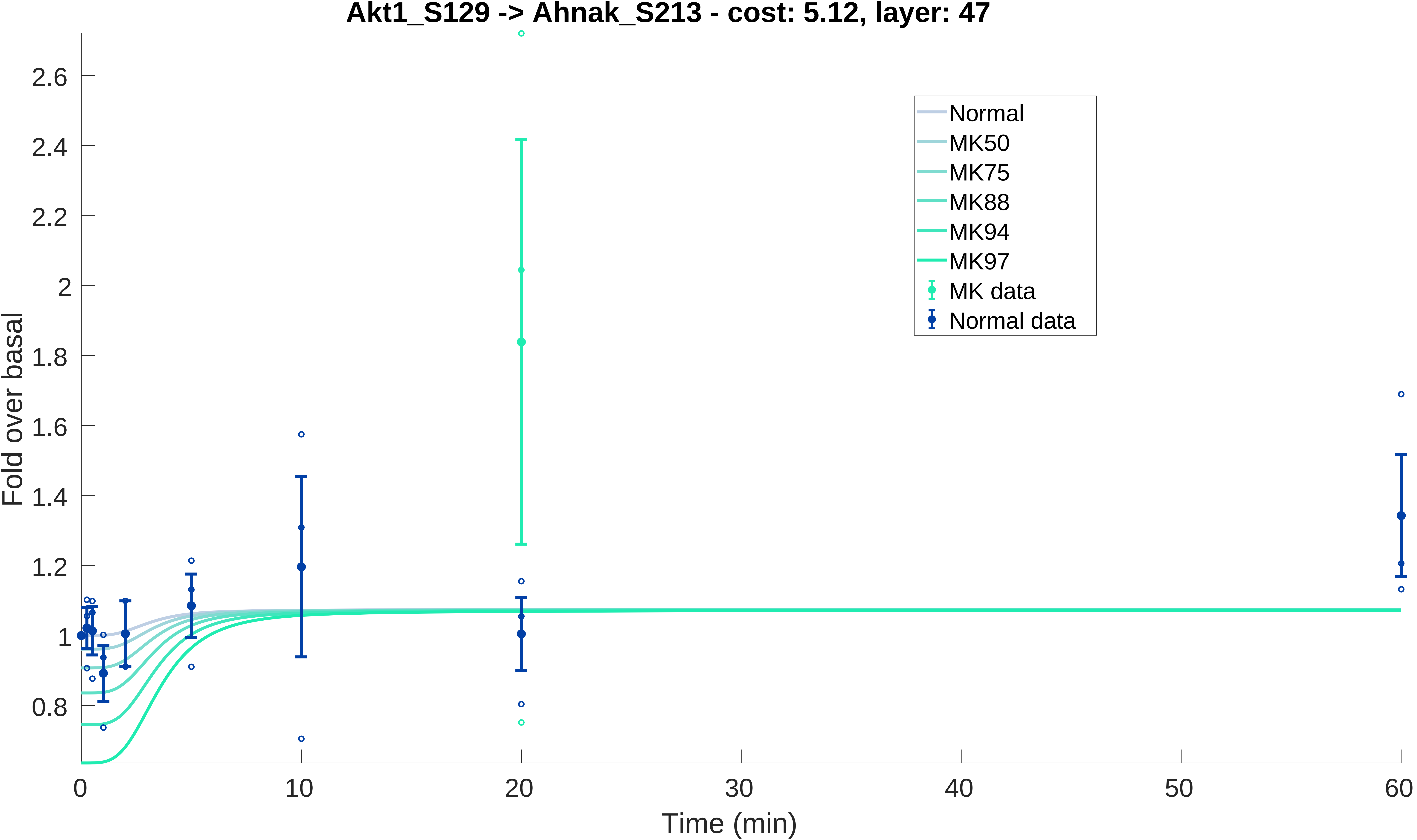

Akt1_S129 -> Ahnak_S213 - cost: 5.12, layer: 47
Fold over basal
2.6
2.4
2.2
2
1.8
1.6
1.4
1.2
1
0.8
0
10
20
30
40
50
60
Time (min)
Normal
MK50
MK75
MK88
MK94
MK97
MK data
Normal data

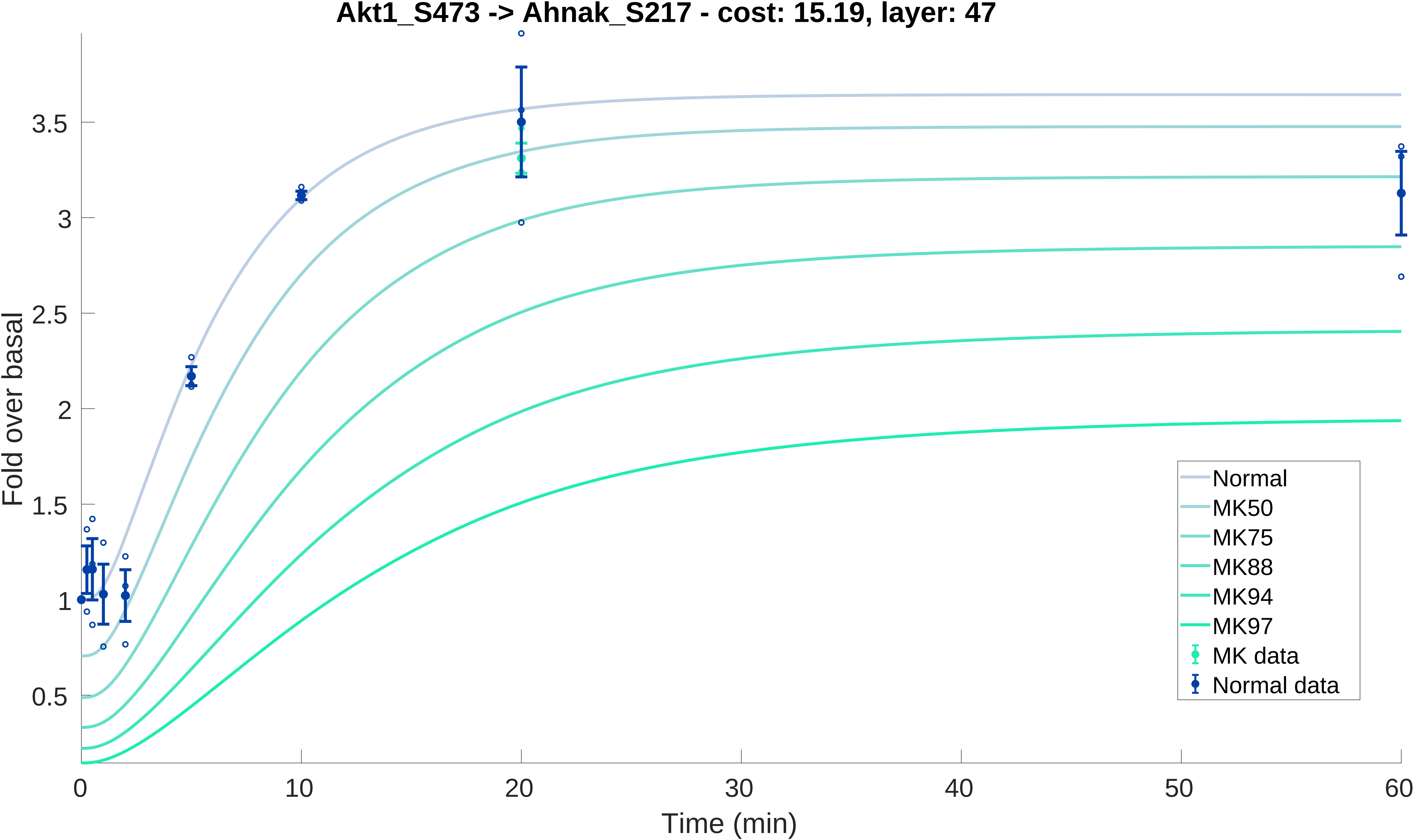

Akt1_S473 -> Ahnak_S217 - cost: 15.19, layer: 47
Fold over basal
0.5
1
1.5
2
2.5
3
3.5
0
10
20
30
40
50
60
Time (min)
Normal
MK50
MK75
MK88
MK94
MK97
MK data
Normal data

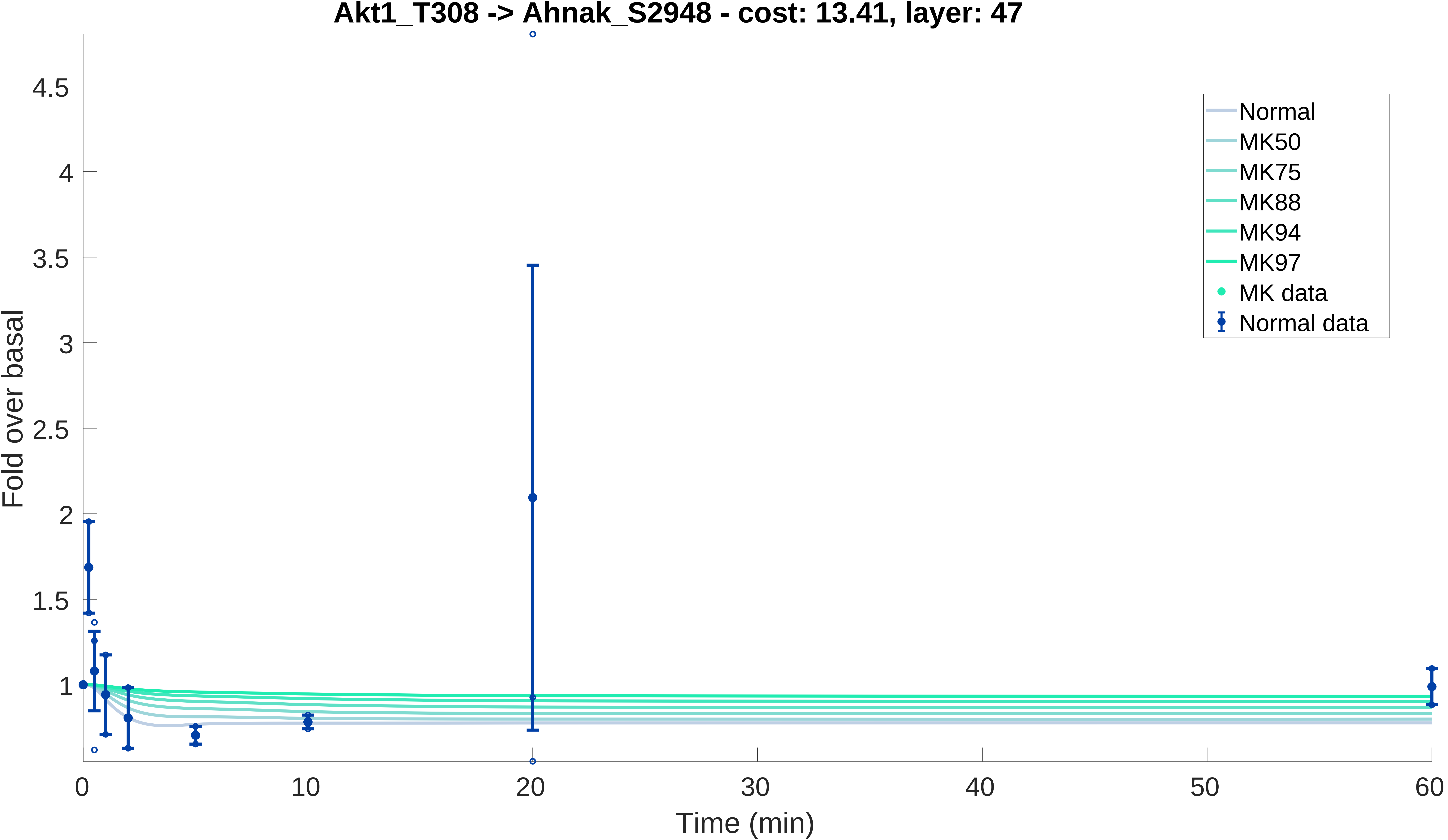

Akt1_T308 -> Ahnak_S2948 - cost: 13.41, layer: 47
Fold over basal
Time (min)
Normal
MK50
MK75
MK88
MK94
MK97
MK data
Normal data
4.5
4
3.5
3
2.5
2
1.5
1
0
10
20
30
40
50
60

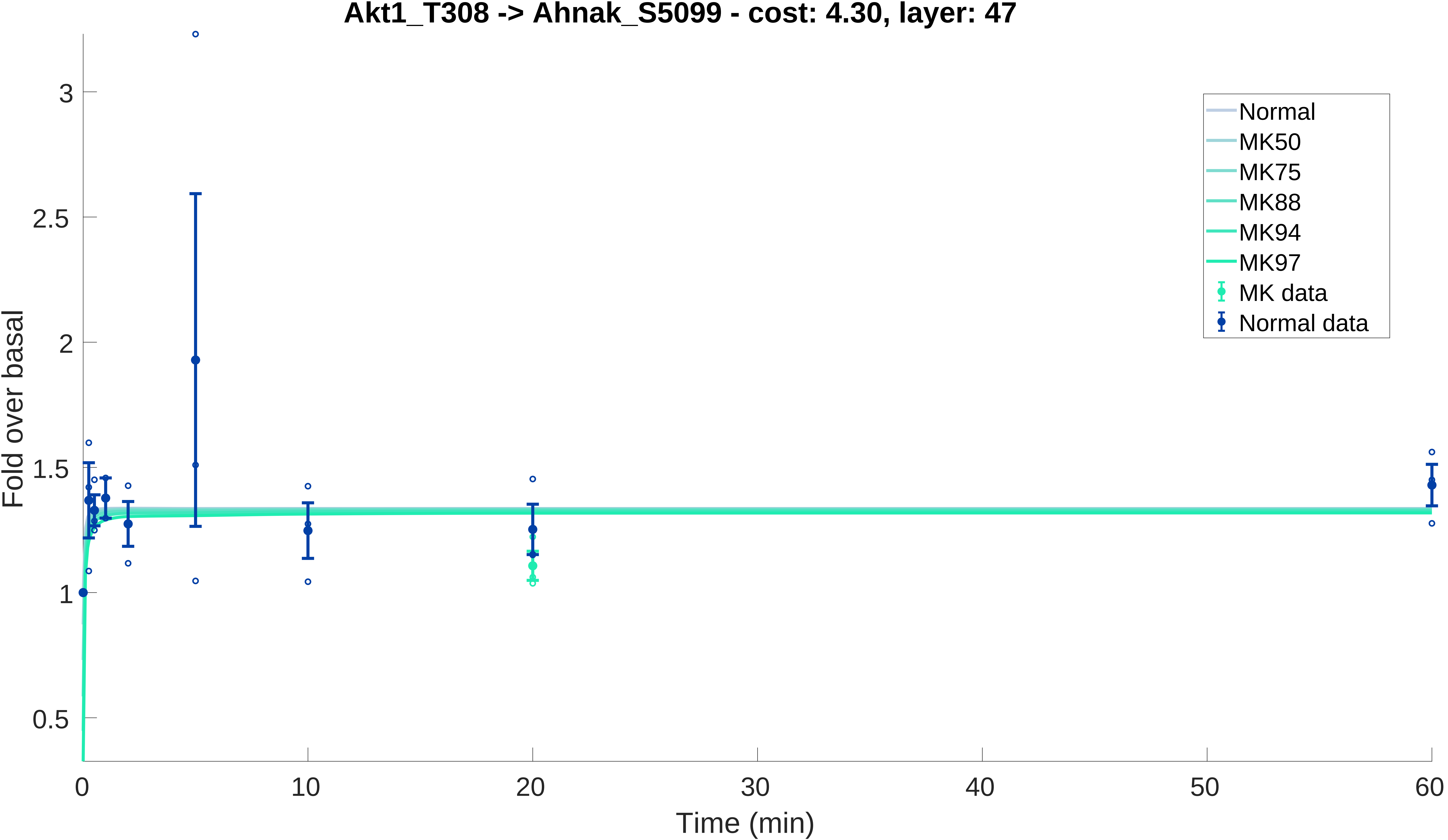
Akt1_T308 -> Ahnak_S5099 - cost: 4.30, layer: 47
Fold over basal
Time (min)
0.5
1
1.5
2
2.5
3
0
10
20
30
40
50
60
Normal
MK50
MK75
MK88
MK94
MK97
MK data
Normal data

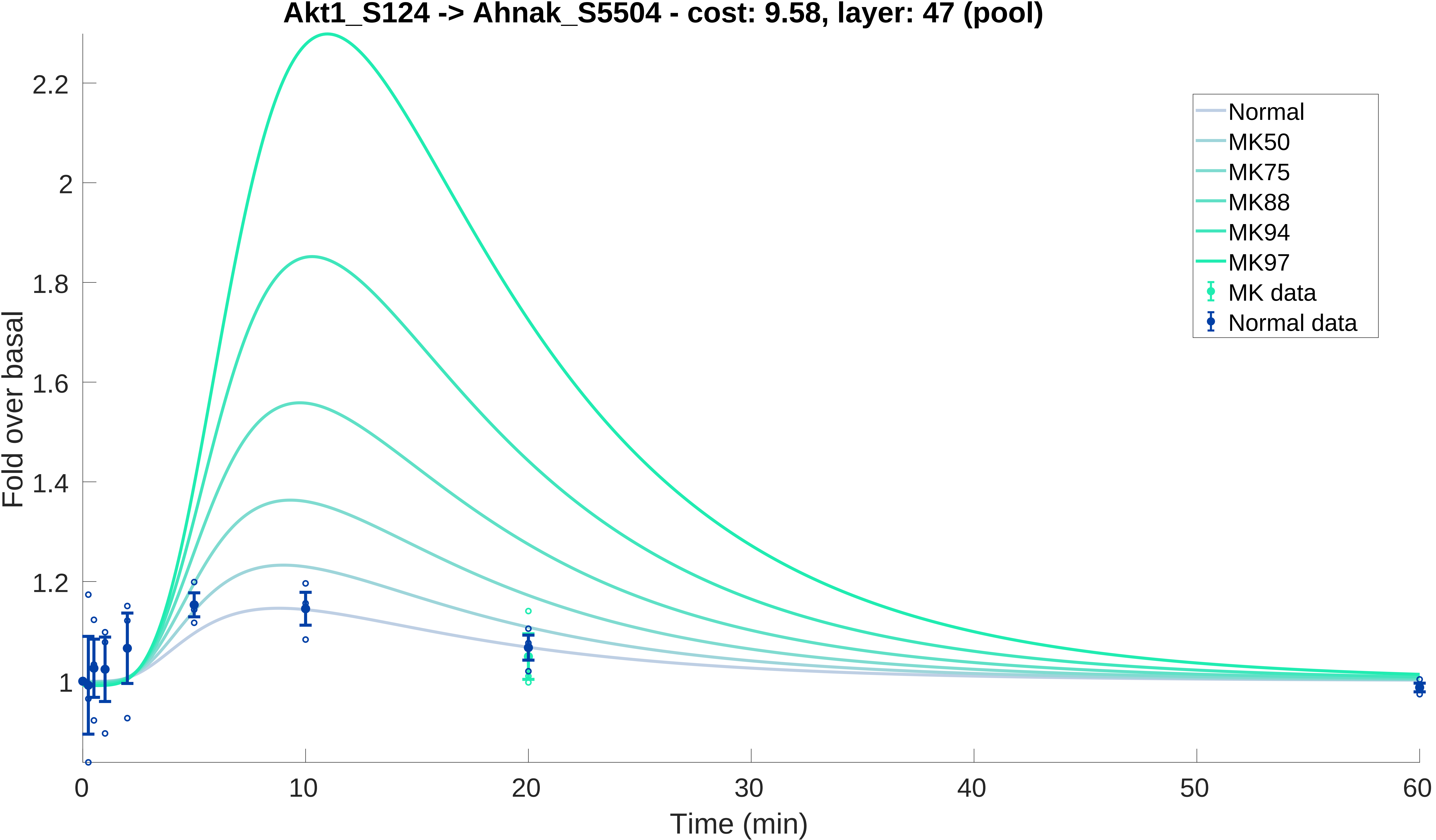

Akt1_S124 -> Ahnak_S5504 - cost: 9.58, layer: 47 (pool)
Fold over basal
Time (min)
2.2
2
1.8
1.6
1.4
1.2
1
0
10
20
30
40
50
60
Normal
MK50
MK75
MK88
MK94
MK97
MK data
Normal data

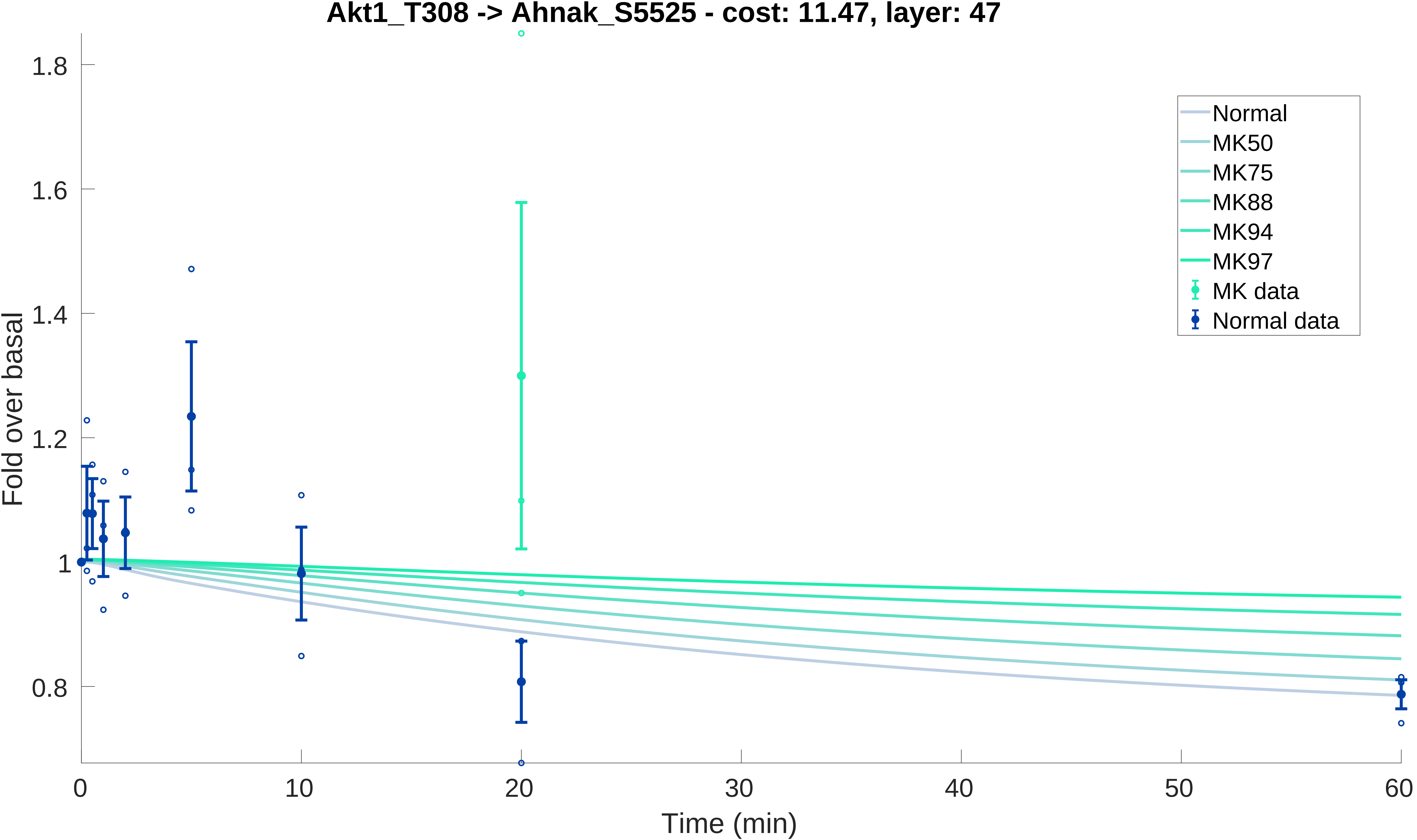

Akt1_T308 -> Ahnak_S5525 - cost: 11.47, layer: 47
Fold over basal
1.8
1.6
1.4
1.2
1
0.8
0
10
20
30
40
50
60
Time (min)
Normal
MK50
MK75
MK88
MK94
MK97
MK data
Normal data

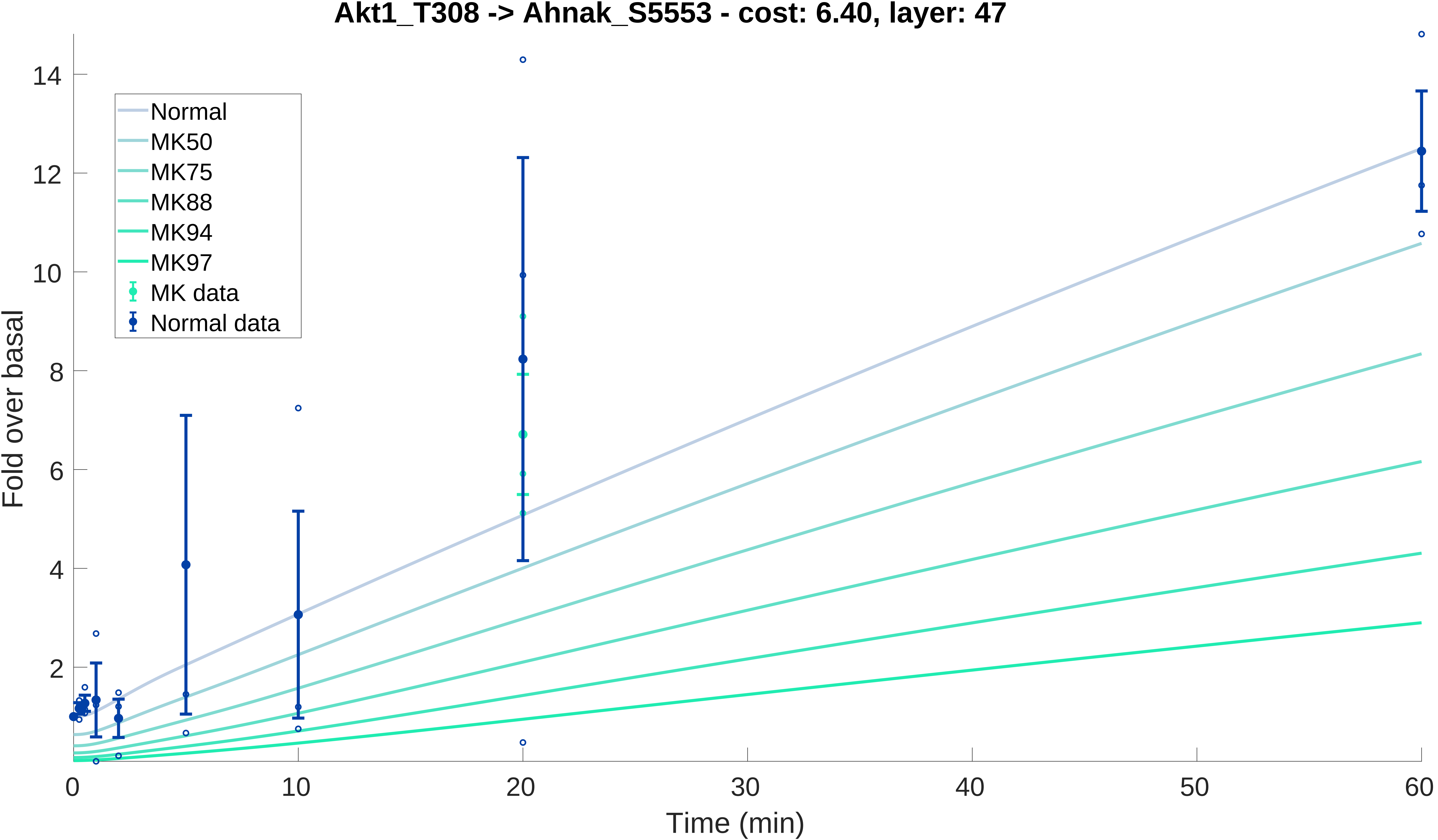

Akt1_T308 -> Ahnak_S5553 - cost: 6.40, layer: 47
Normal
MK50
MK75
MK88
MK94
MK97
MK data
Normal data
Fold over basal
Time (min)
0
10
20
30
40
50
60
2
4
6
8
10
12
14

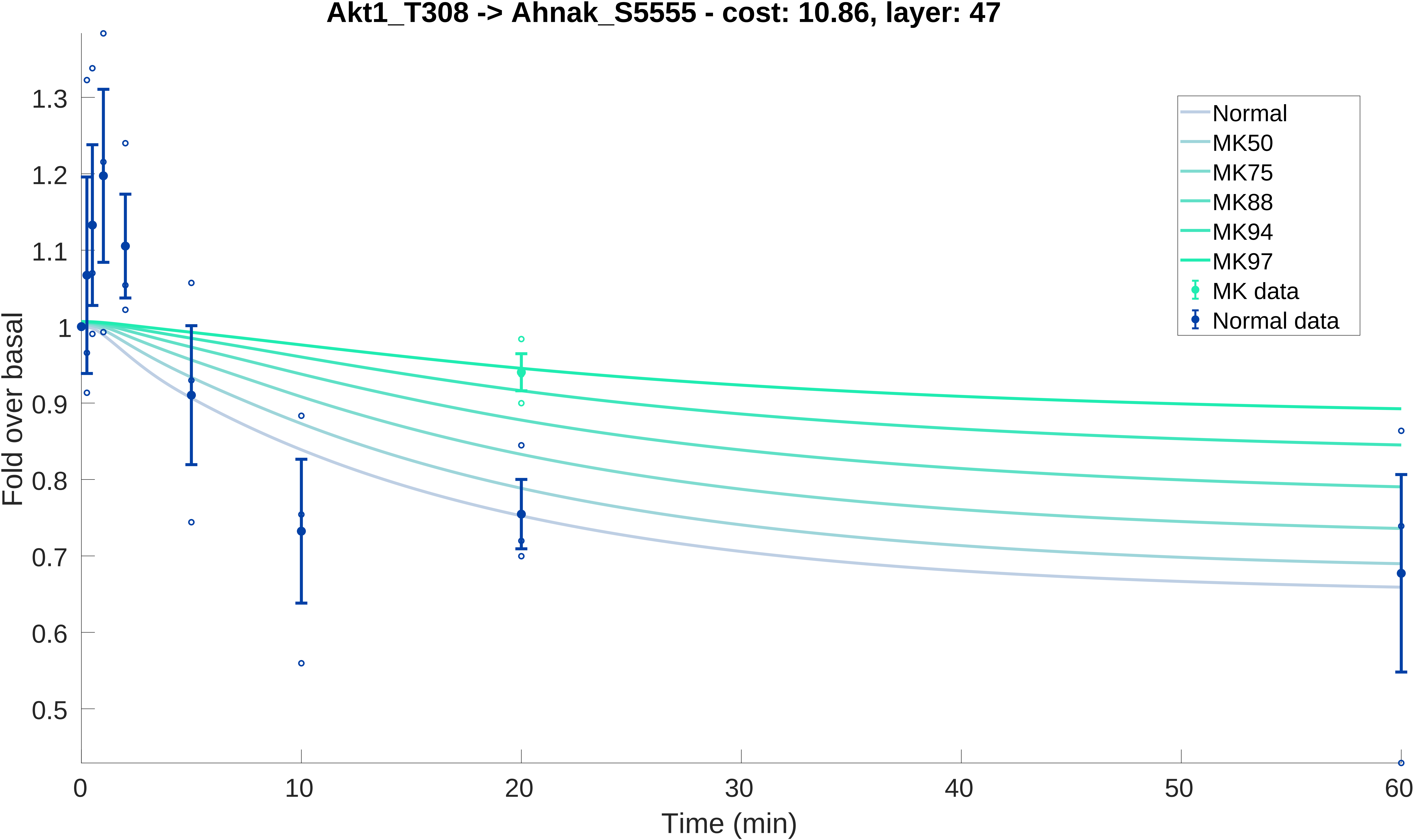

Akt1_T308 -> Ahnak_S5555 - cost: 10.86, layer: 47
Fold over basal
Time (min)
1.3
1.2
1.1
1
0.9
0.8
0.7
0.6
0.5
0
10
20
30
40
50
60
Normal
MK50
MK75
MK88
MK94
MK97
MK data
Normal data

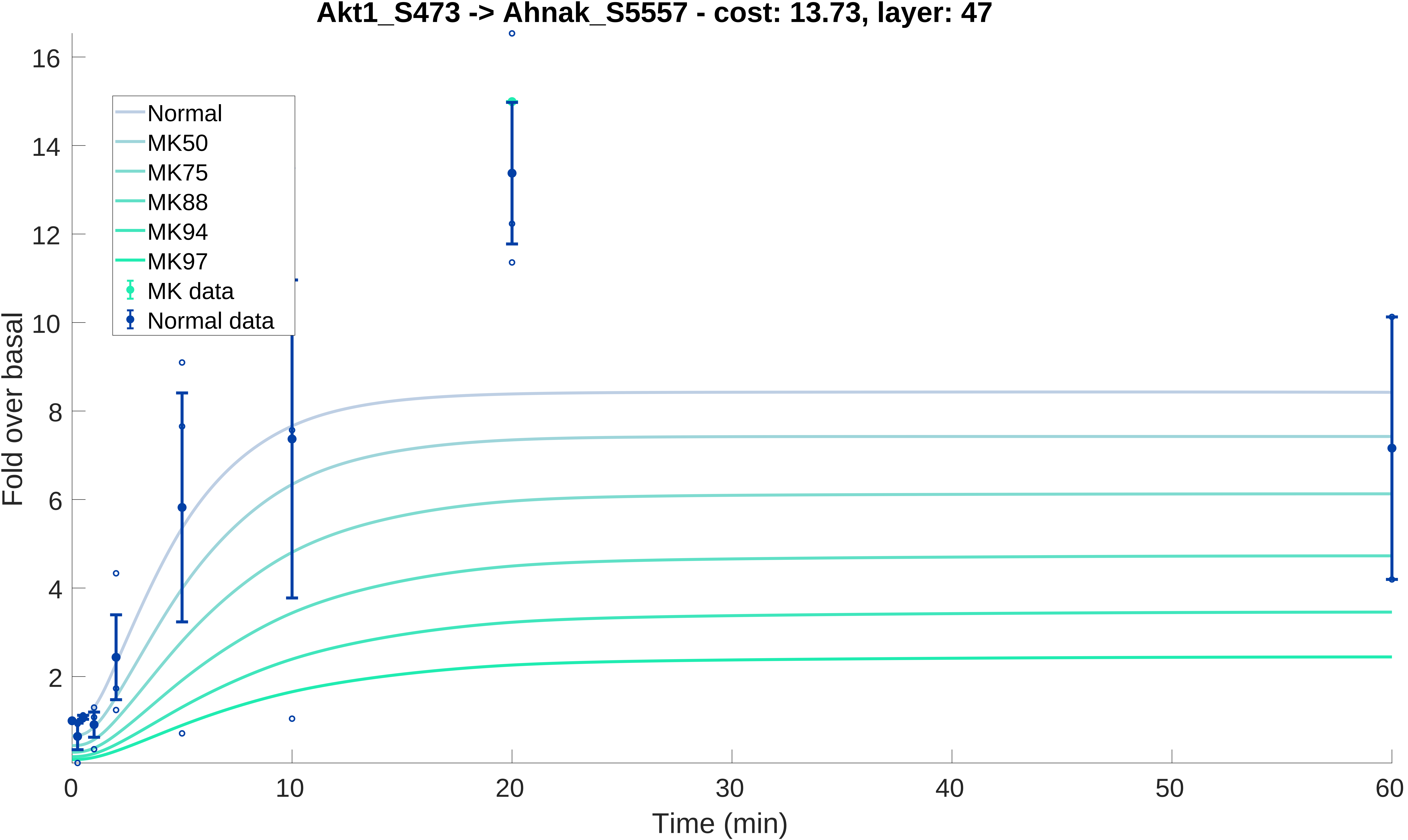

Akt1_S473 -> Ahnak_S5557 - cost: 13.73, layer: 47
Normal
MK50
MK75
MK88
MK94
MK97
MK data
Normal data
Fold over basal
Time (min)
0
10
20
30
40
50
60
2
4
6
8
10
12
14
16

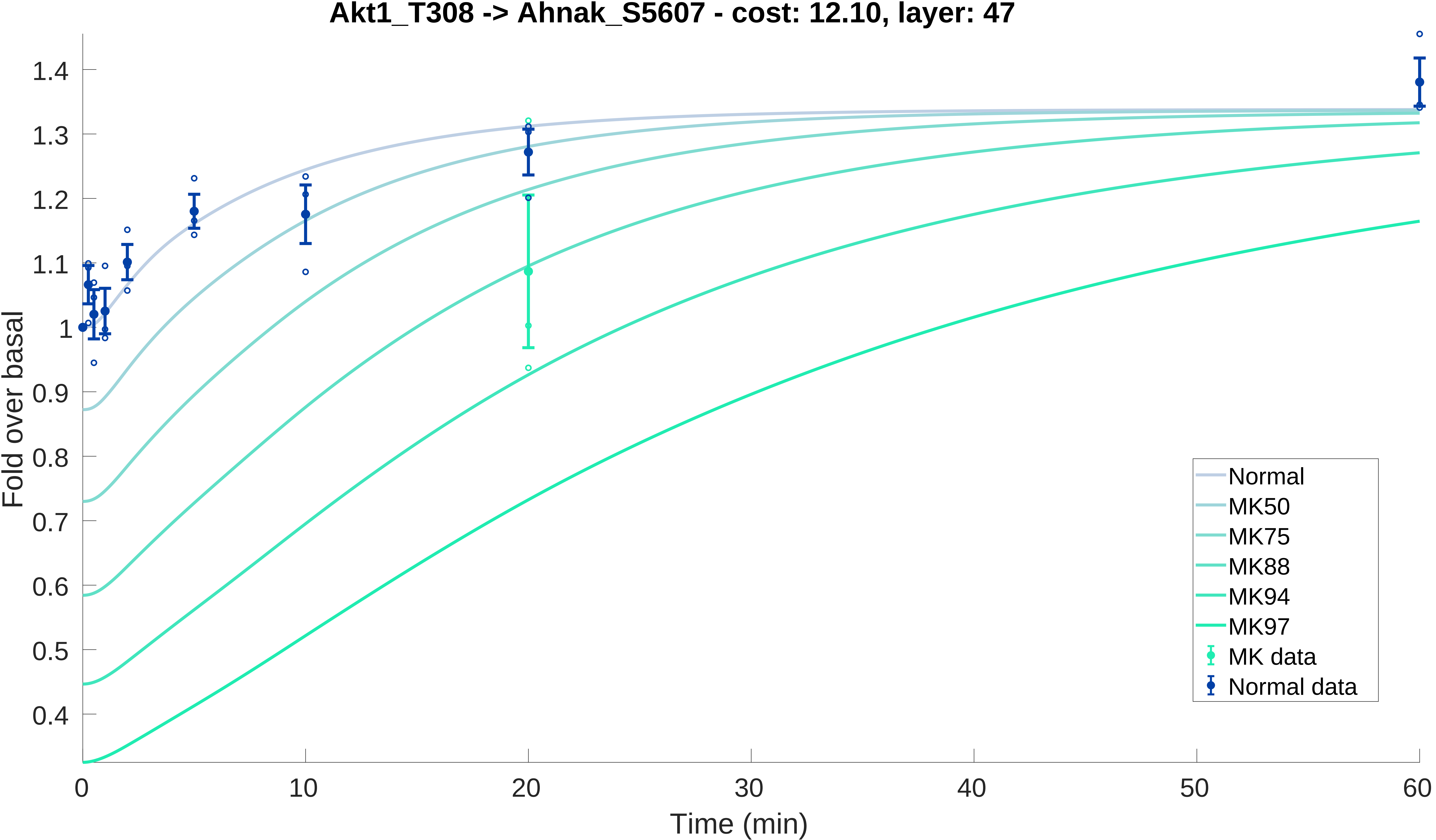

Akt1_T308 -> Ahnak_S5607 - cost: 12.10, layer: 47
Fold over basal
Time (min)
1.4
1.3
1.2
1.1
1
0.9
0.8
0.7
0.6
0.5
0.4
0
10
20
30
40
50
60
Normal
MK50
MK75
MK88
MK94
MK97
MK data
Normal data

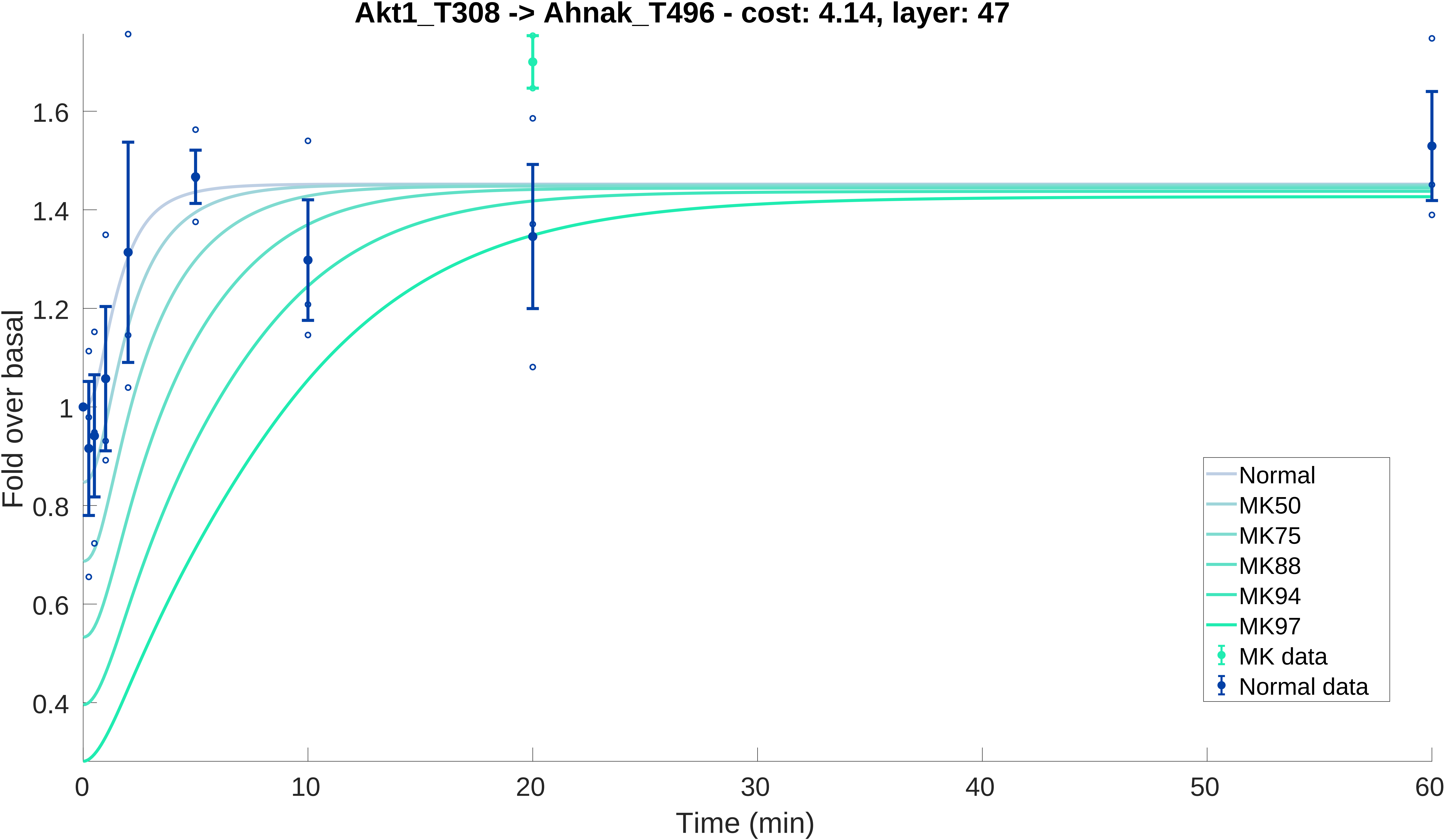
Akt1_T308 -> Ahnak_T496 - cost: 4.14, layer: 47
Fold over basal
Time (min)
0
10
20
30
40
50
60
0.4
0.6
0.8
1
1.2
1.4
1.6
Normal
MK50
MK75
MK88
MK94
MK97
MK data
Normal data

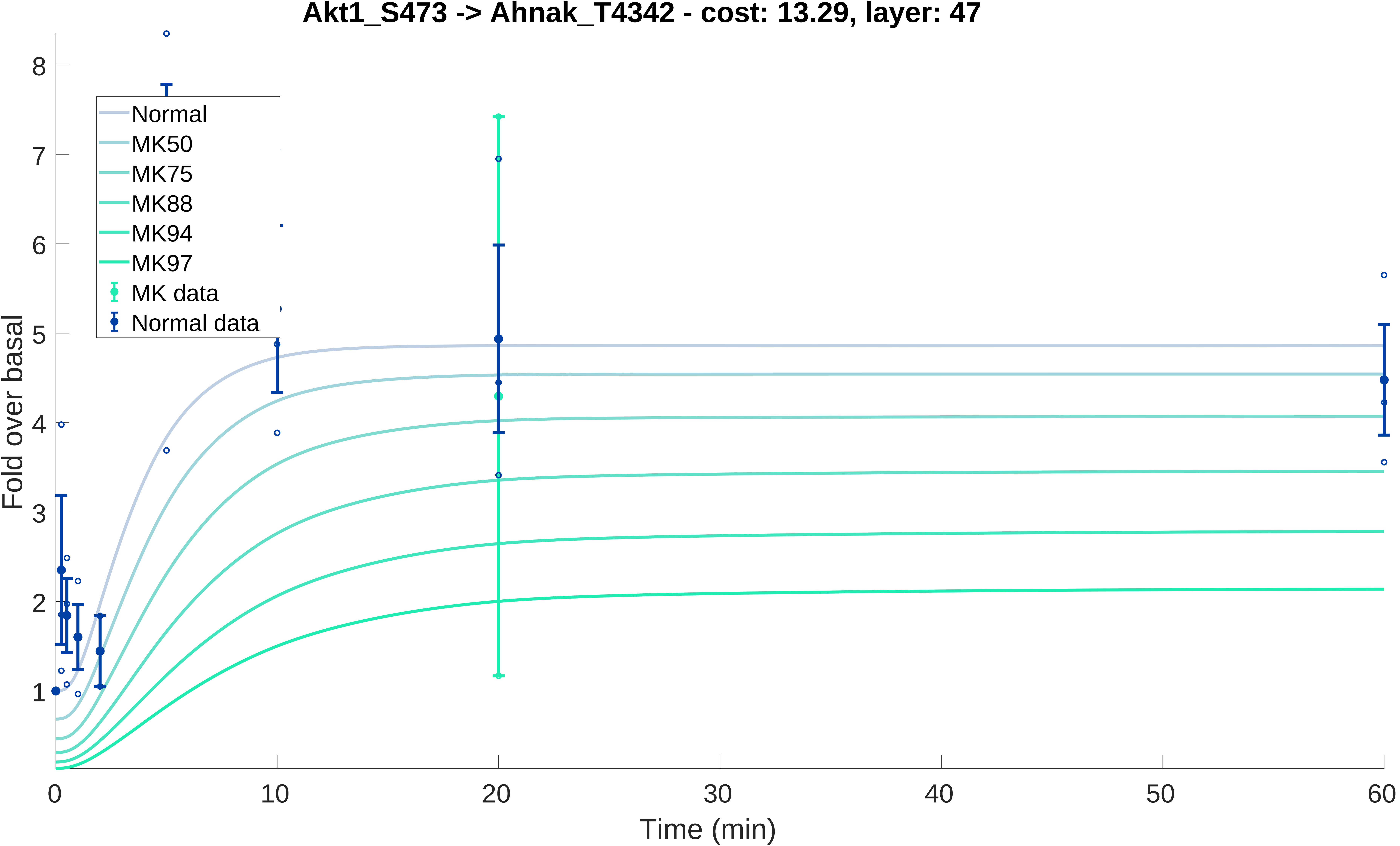
Akt1_S473 -> Ahnak_T4342 - cost: 13.29, layer: 47
Normal
MK50
MK75
MK88
MK94
MK97
MK data
Normal data
Fold over basal
Time (min)
1
2
3
4
5
6
7
8
0
10
20
30
40
50
60

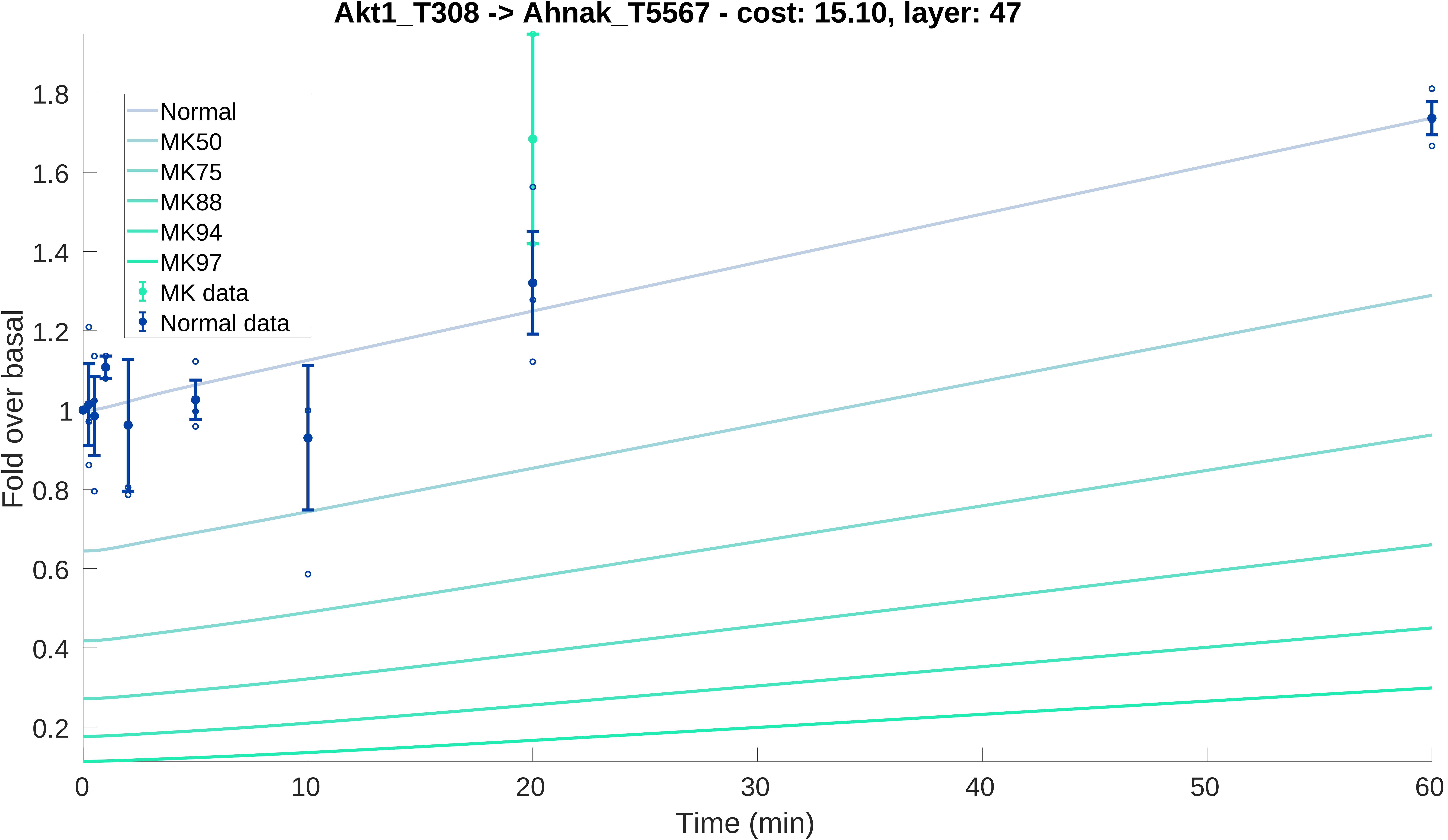

Akt1_T308 -> Ahnak_T5567 - cost: 15.10, layer: 47
Normal
MK50
MK75
MK88
MK94
MK97
MK data
Normal data
Fold over basal
Time (min)
0
10
20
30
40
50
60
0.2
0.4
0.6
0.8
1
1.2
1.4
1.6
1.8

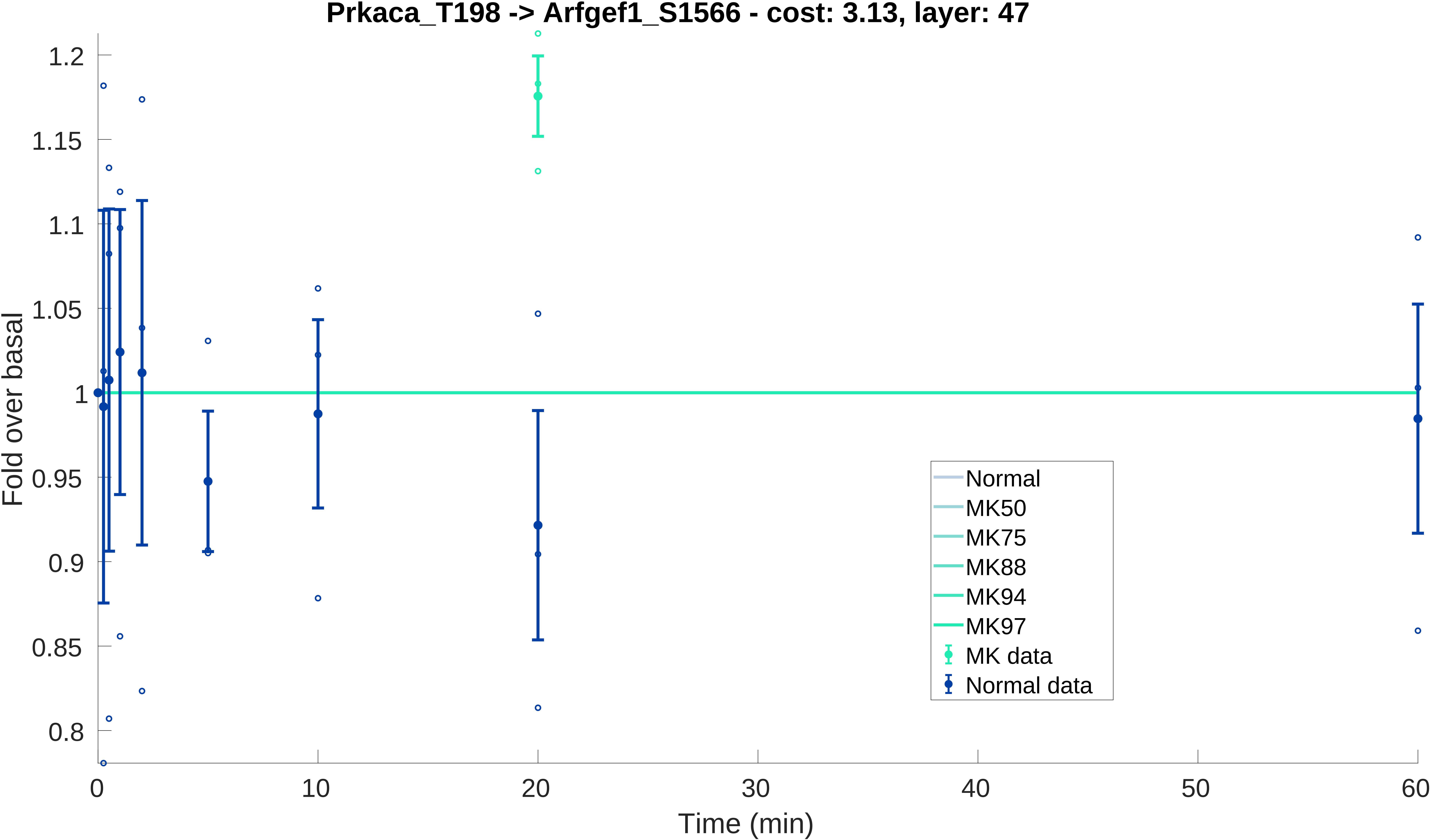

Prkaca_T198 -> Arfgef1_S1566 - cost: 3.13, layer: 47
Fold over basal
1.2
1.15
1.1
1.05
1
0.95
0.9
0.85
0.8
0
10
20
30
40
50
60
Time (min)
Normal
MK50
MK75
MK88
MK94
MK97
MK data
Normal data

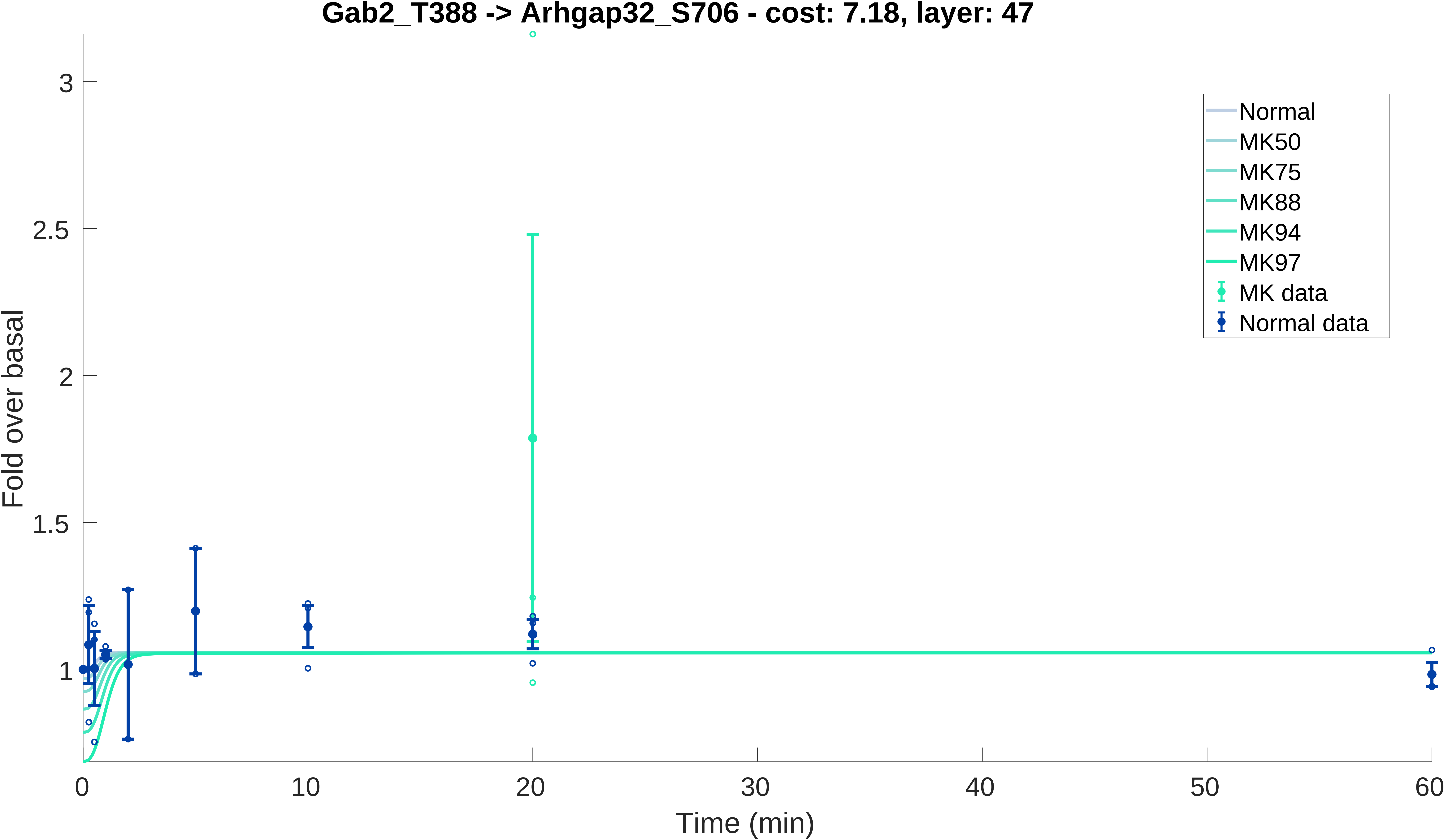
Gab2_T388 -> Arhgap32_S706 - cost: 7.18, layer: 47
Fold over basal
Time (min)
3
2.5
2
1.5
1
0
10
20
30
40
50
60
Normal
MK50
MK75
MK88
MK94
MK97
MK data
Normal data

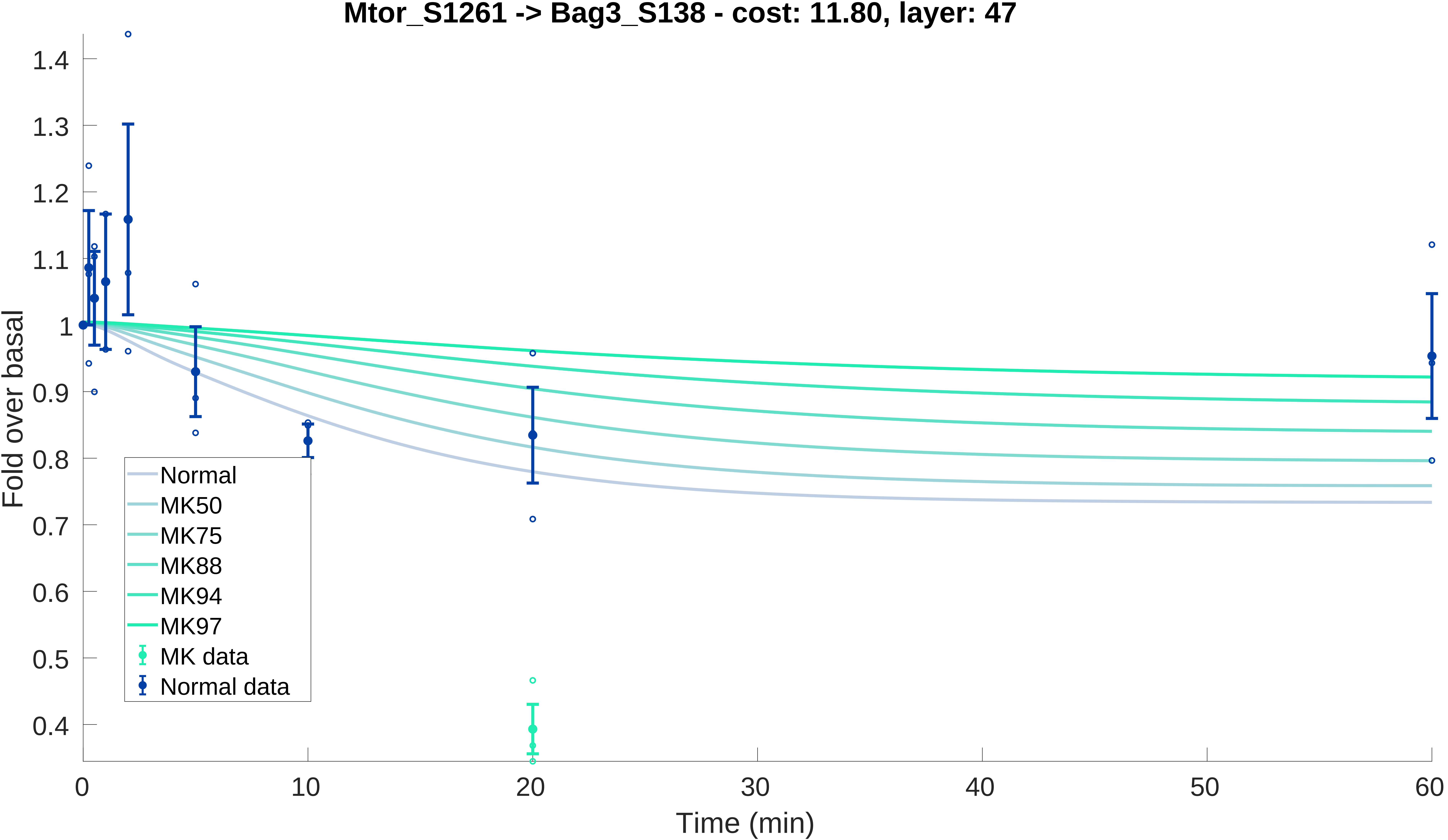

Mtor_S1261 -> Bag3_S138 - cost: 11.80, layer: 47
Fold over basal
Time (min)
1.4
1.3
1.2
1.1
1
0.9
0.8
0.7
0.6
0.5
0.4
0
10
20
30
40
50
60
Normal
MK50
MK75
MK88
MK94
MK97
MK data
Normal data

Mtor_S2478 -> Bag3_S270 - cost: 13.47, layer: 47 (pool)

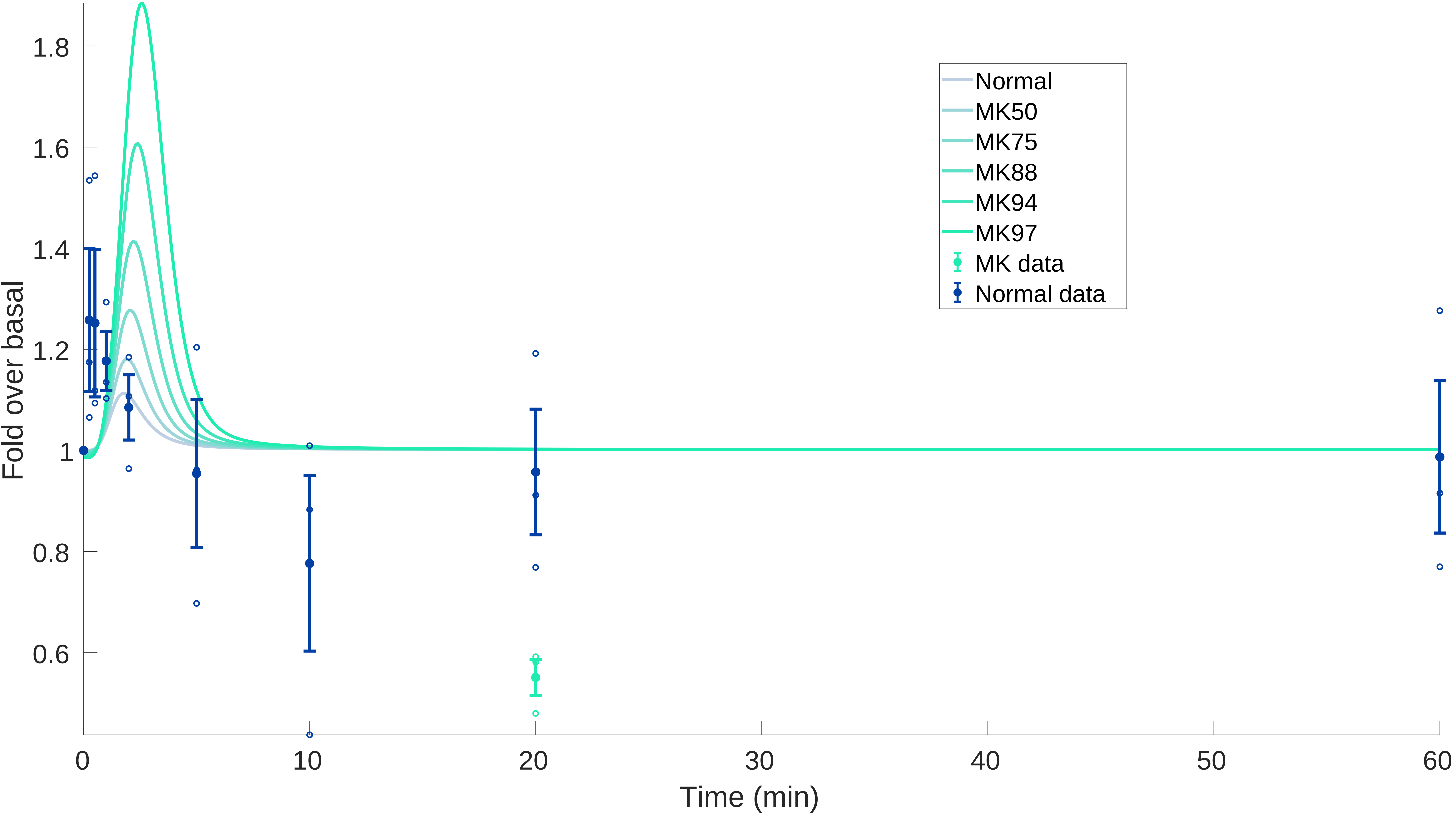

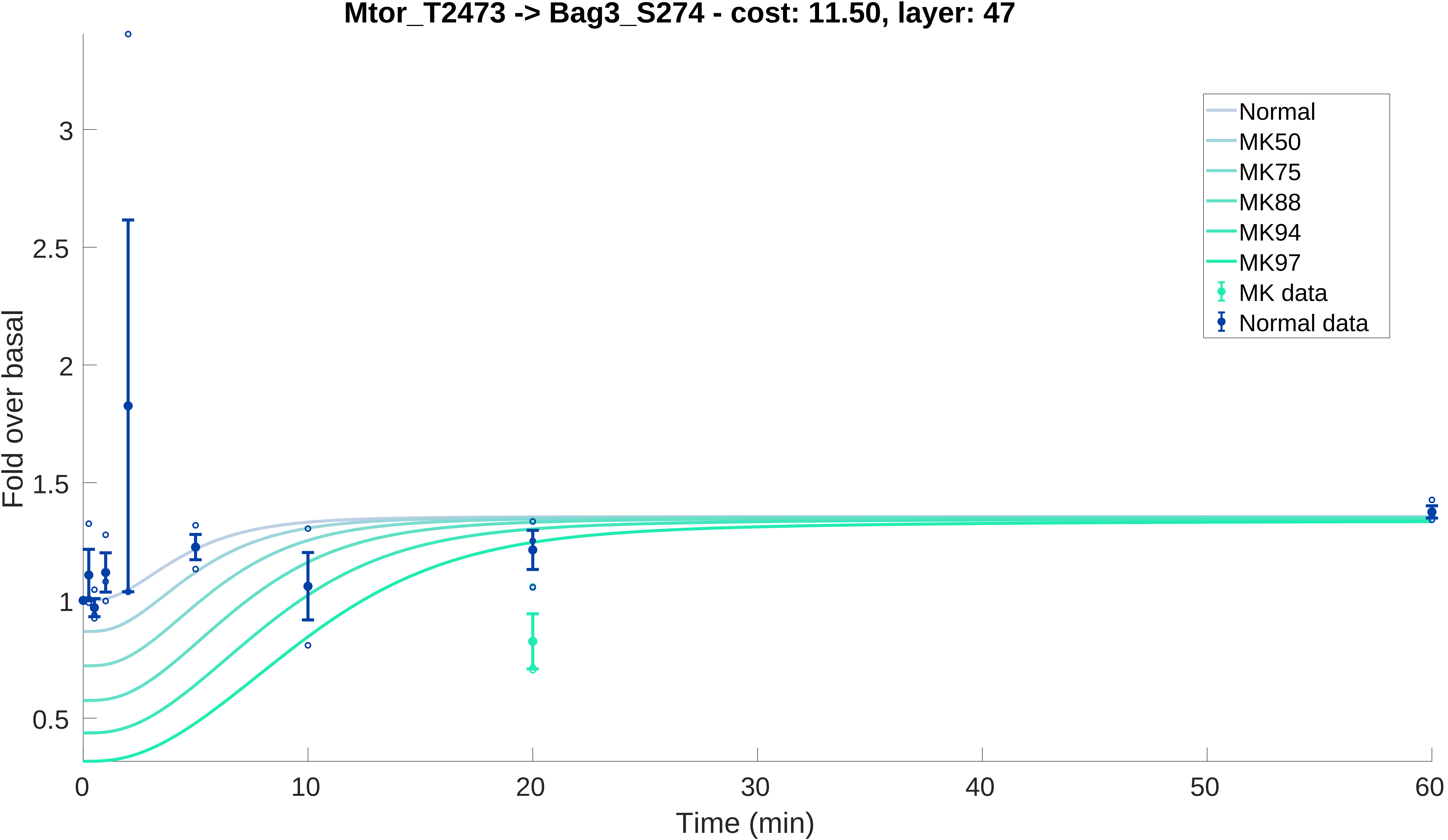

Mtor_T2473 -> Bag3_S274 - cost: 11.50, layer: 47
Fold over basal
Time (min)
Normal
MK50
MK75
MK88
MK94
MK97
MK data
Normal data
0.5
1
1.5
2
2.5
3
0
10
20
30
40
50
60

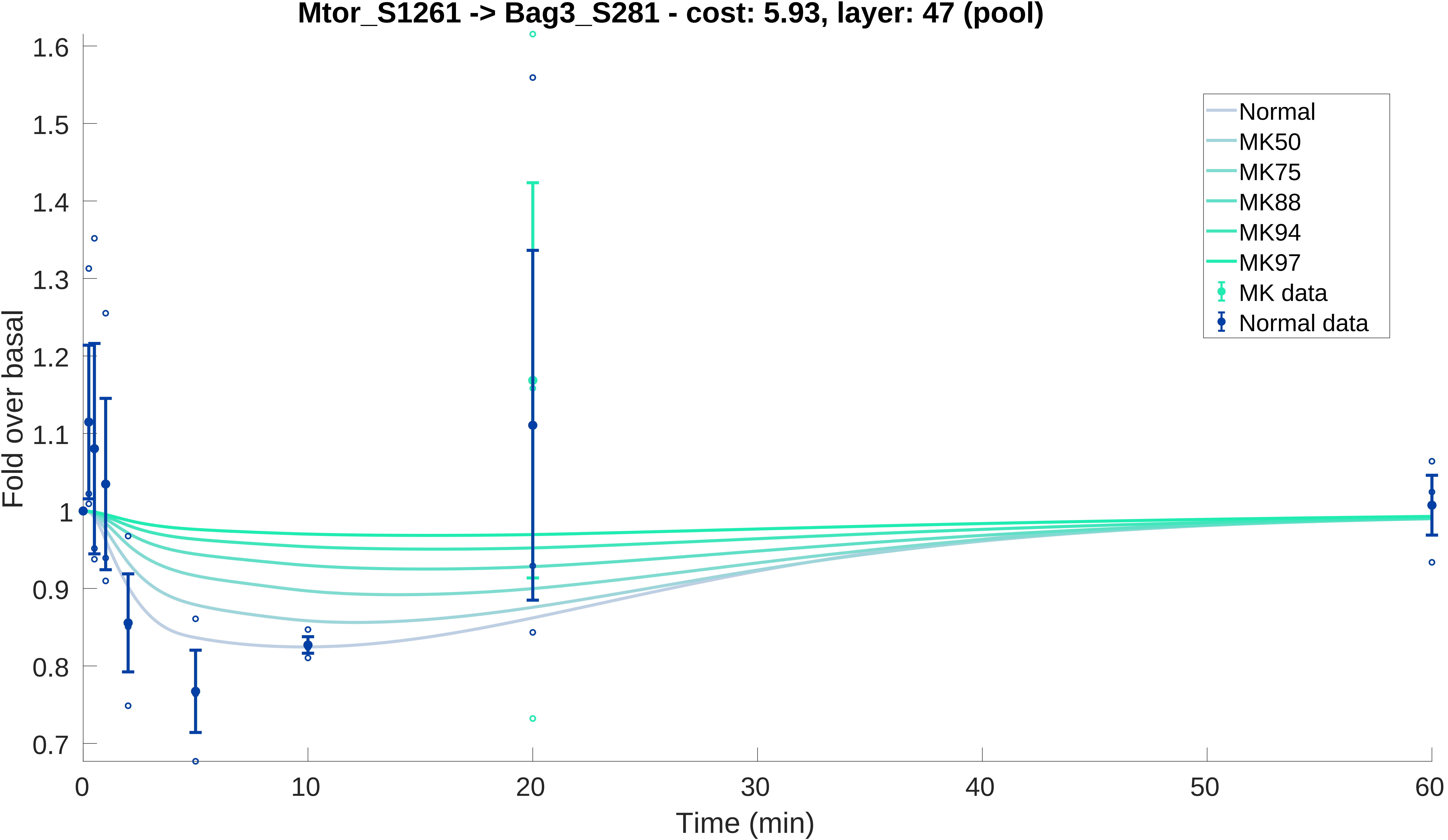

Mtor_S1261 -> Bag3_S281 - cost: 5.93, layer: 47 (pool)
Fold over basal
Time (min)
1.6
1.5
1.4
1.3
1.2
1.1
1
0.9
0.8
0.7
0
10
20
30
40
50
60
Normal
MK50
MK75
MK88
MK94
MK97
MK data
Normal data

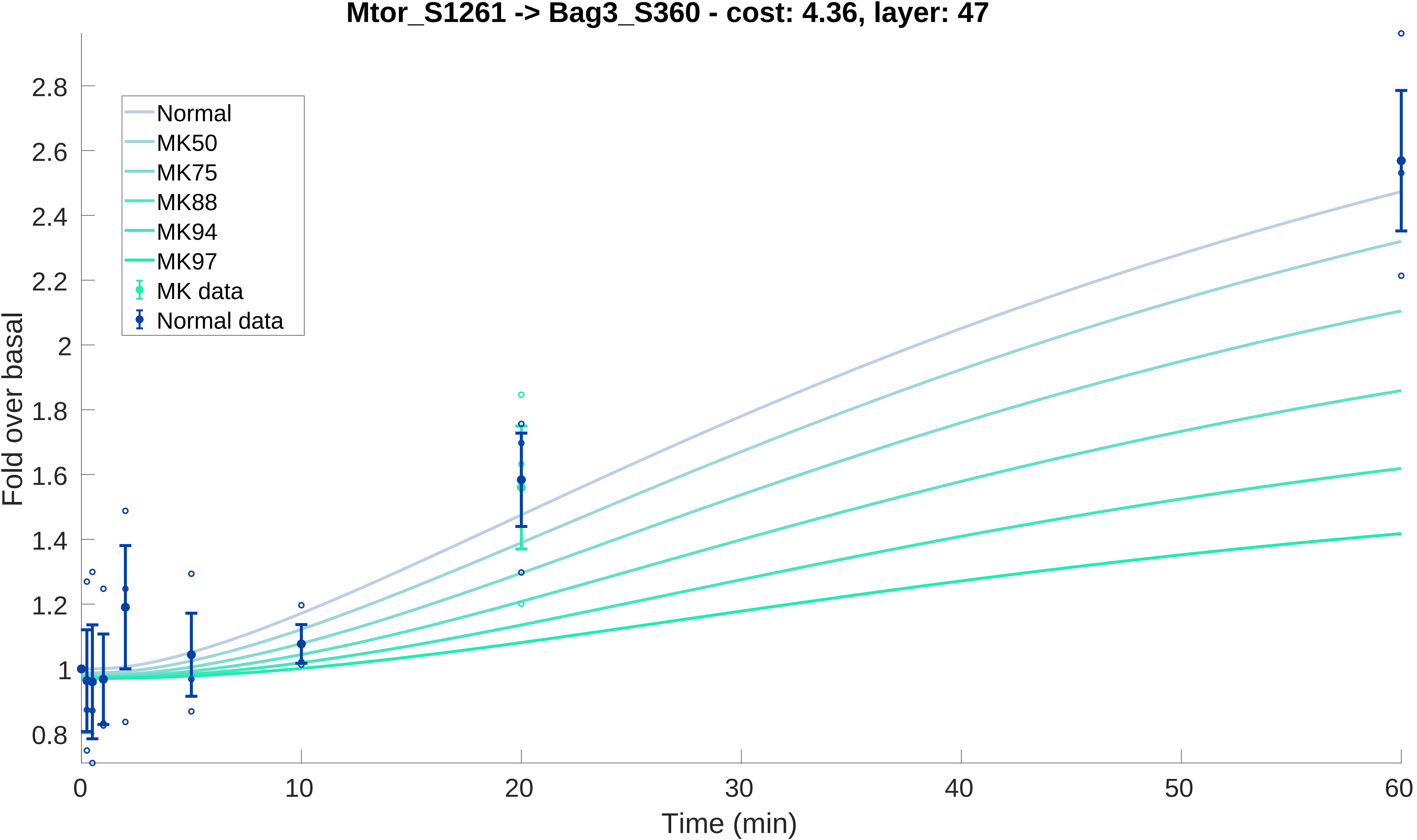

Mtor_S1261 -> Bag3_S360 - cost: 4.36, layer: 47
Normal
MK50
MK75
MK88
MK94
MK97
MK data
Normal data
Fold over basal
2.8
2.6
2.4
2.2
2
1.8
1.6
1.4
1.2
1
0.8
0
10
20
30
40
50
60
Time (min)

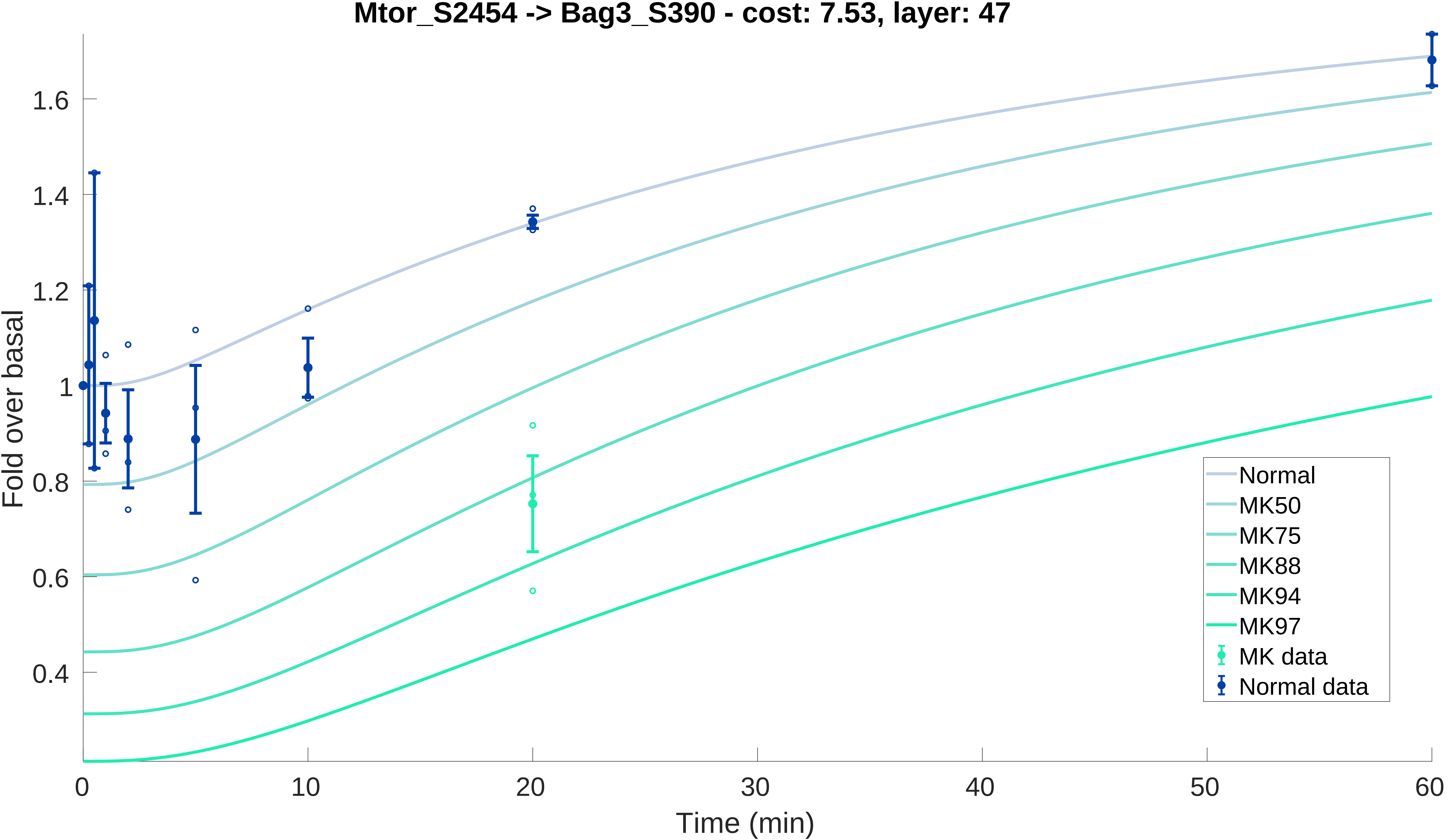

Mtor_S2454 -> Bag3_S390 - cost: 7.53, layer: 47
Fold over basal
1.6
1.4
1.2
1
0.8
0.6
0.4
0
10
20
30
40
50
60
Time (min)
Normal
MK50
MK75
MK88
MK94
MK97
MK data
Normal data

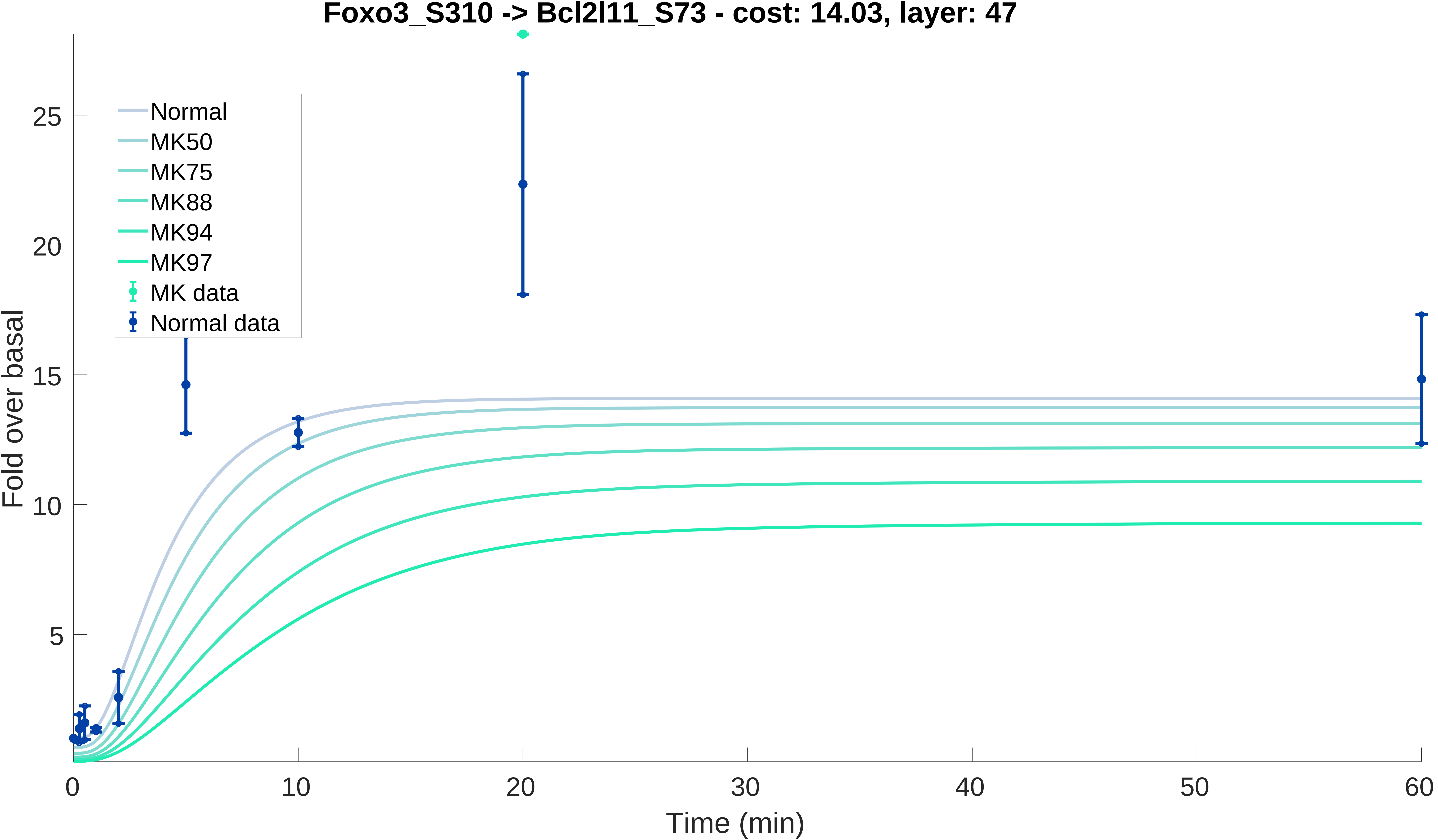

Foxo3_S310 -> Bcl2l11_S73 - cost: 14.03, layer: 47
Normal
MK50
MK75
MK88
MK94
MK97
MK data
Normal data
Fold over basal
Time (min)
0
10
20
30
40
50
60
5
10
15
20
25

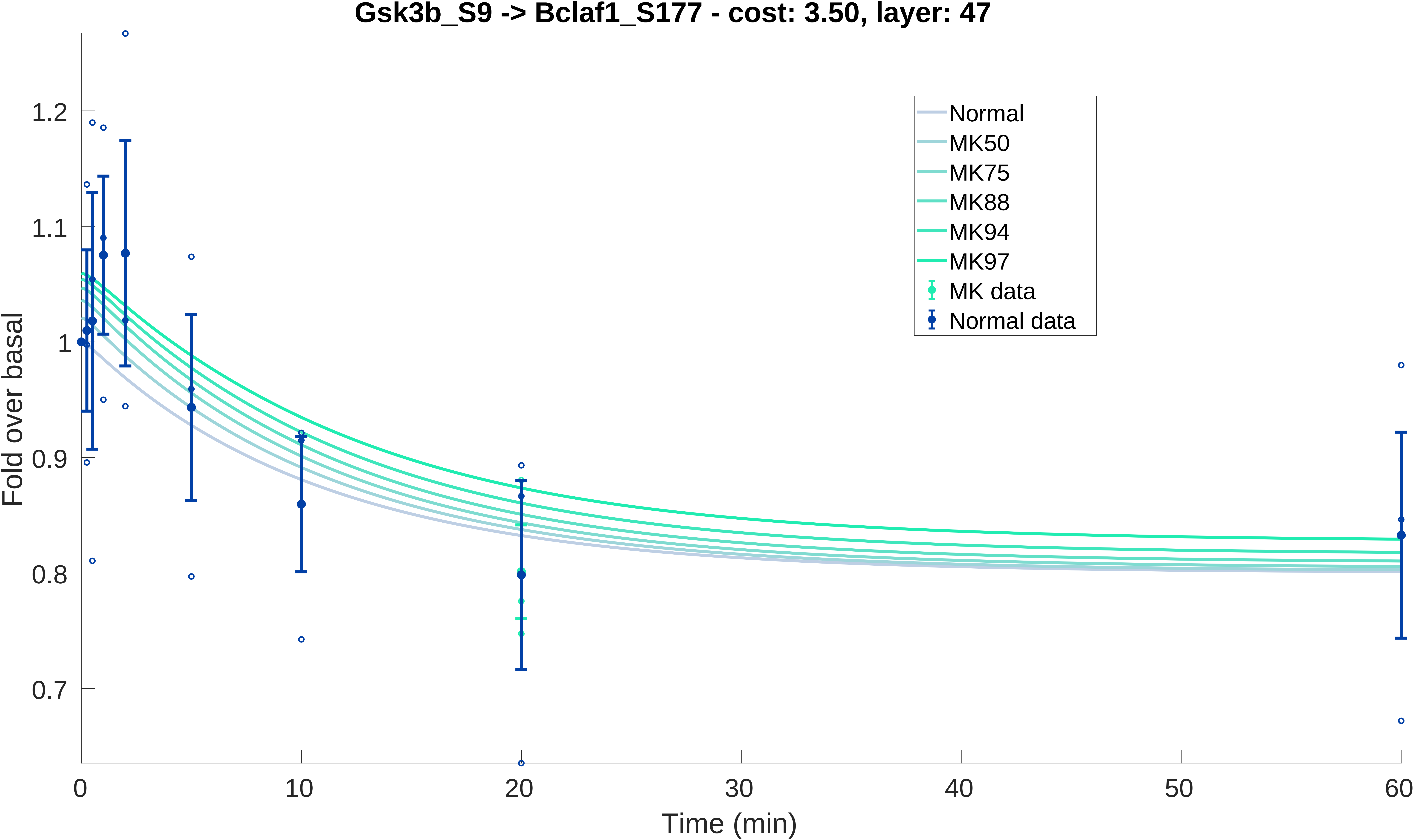

Gsk3b_S9 -> Bclaf1_S177 - cost: 3.50, layer: 47
Normal
MK50
MK75
MK88
MK94
MK97
MK data
Normal data
Fold over basal
Time (min)
0.7
0.8
0.9
1
1.1
1.2
0
10
20
30
40
50
60

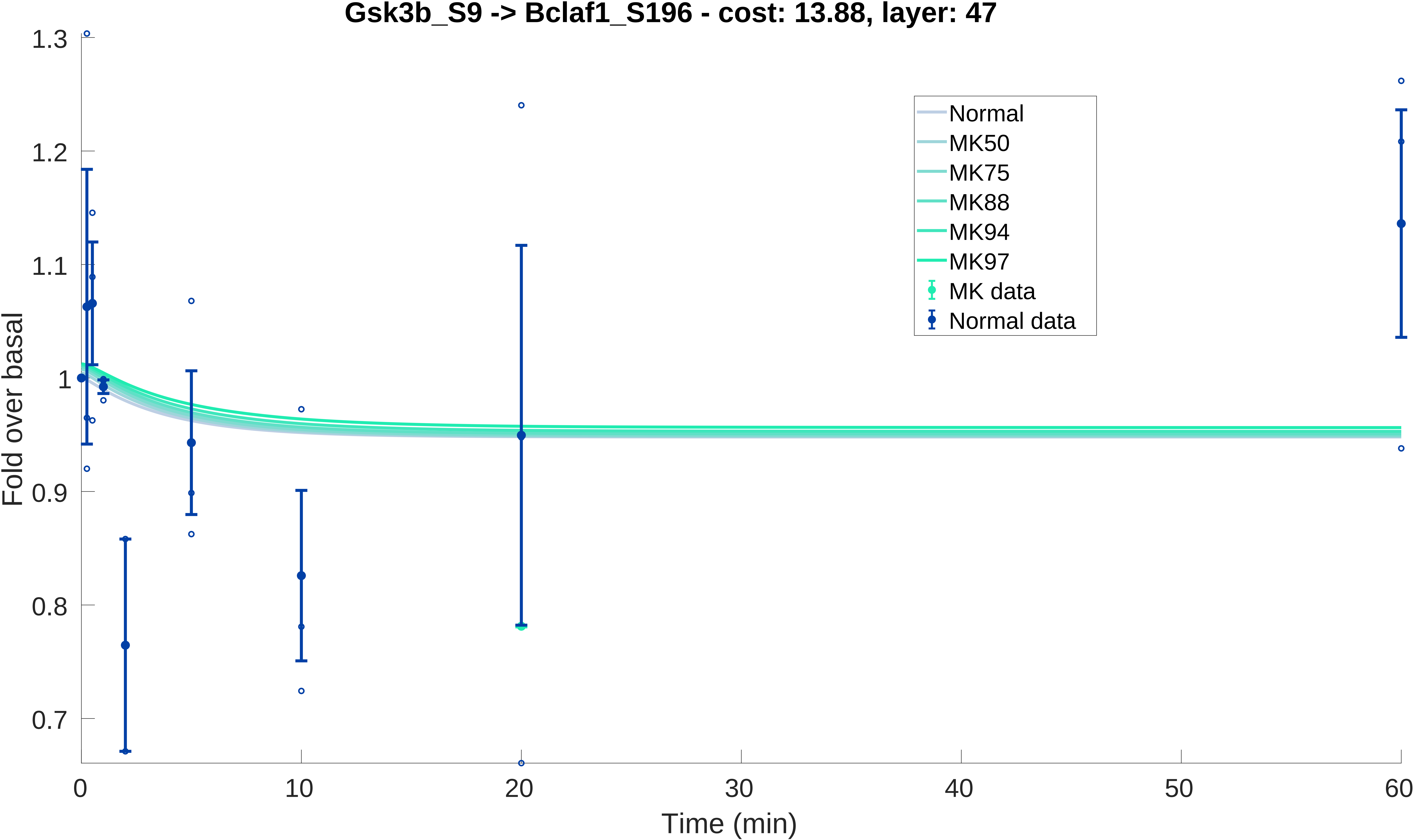
Gsk3b_S9 -> Bclaf1_S196 - cost: 13.88, layer: 47
Fold over basal
Time (min)
Normal
MK50
MK75
MK88
MK94
MK97
MK data
Normal data
1.3
1.2
1.1
1
0.9
0.8
0.7
0
10
20
30
40
50
60

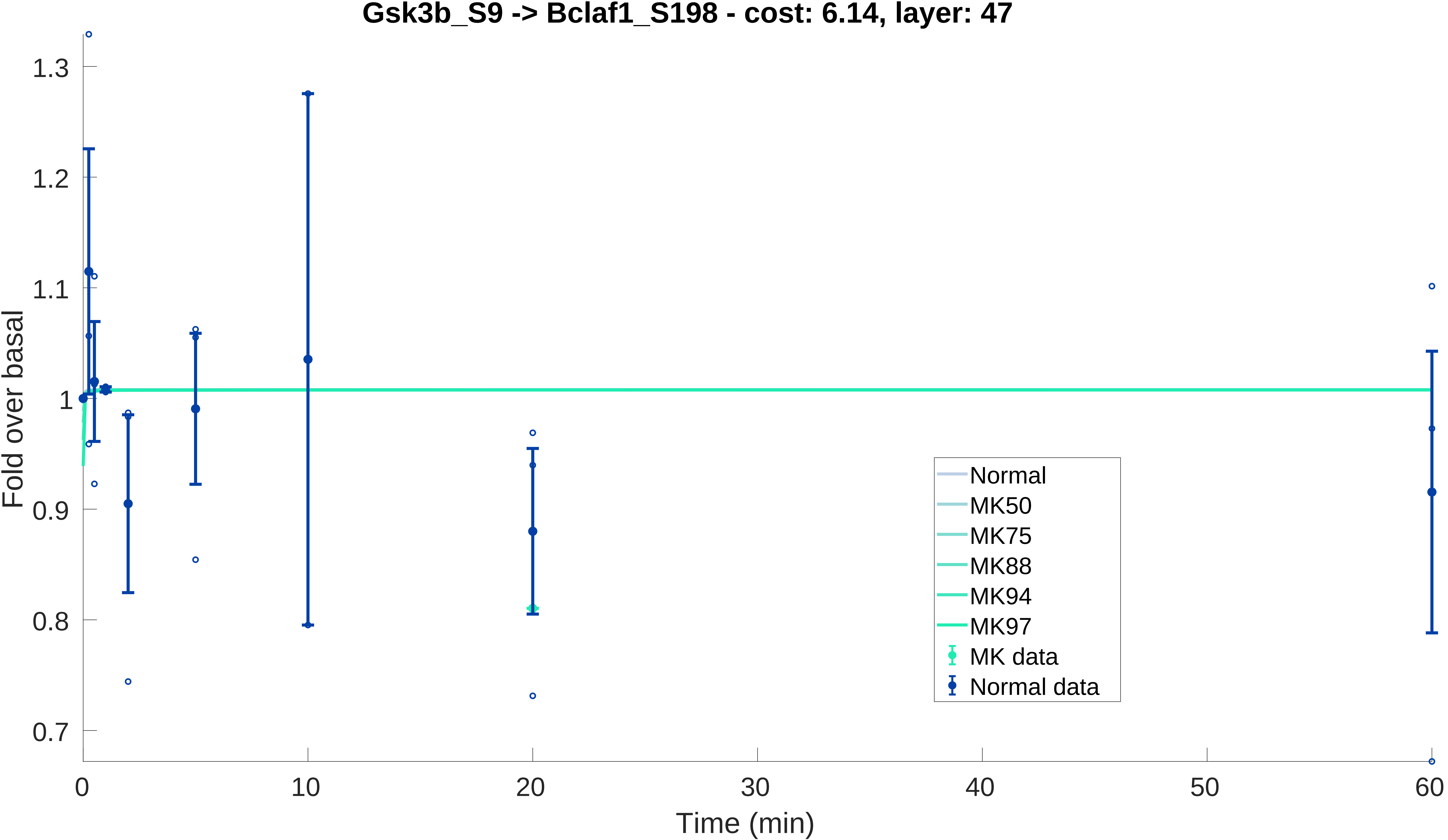
Gsk3b_S9 -> Bclaf1_S198 - cost: 6.14, layer: 47
Fold over basal
Time (min)
1.3
1.2
1.1
1
0.9
0.8
0.7
0
10
20
30
40
50
60
Normal
MK50
MK75
MK88
MK94
MK97
MK data
Normal data

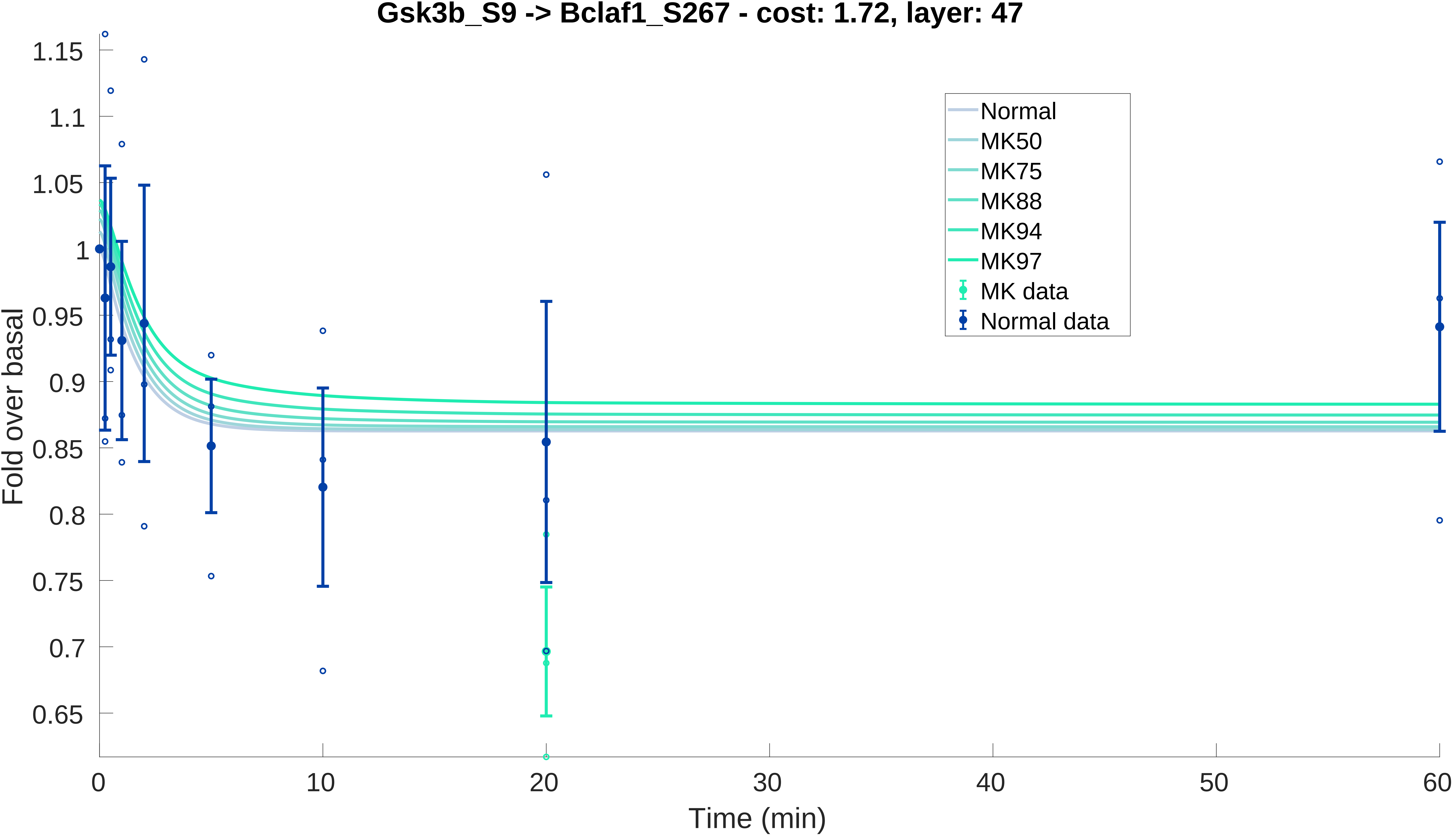

Gsk3b_S9 -> Bclaf1_S267 - cost: 1.72, layer: 47
Fold over basal
1.15
1.1
1.05
1
0.95
0.9
0.85
0.8
0.75
0.7
0.65
Time (min)
0
10
20
30
40
50
60
Normal
MK50
MK75
MK88
MK94
MK97
MK data
Normal data

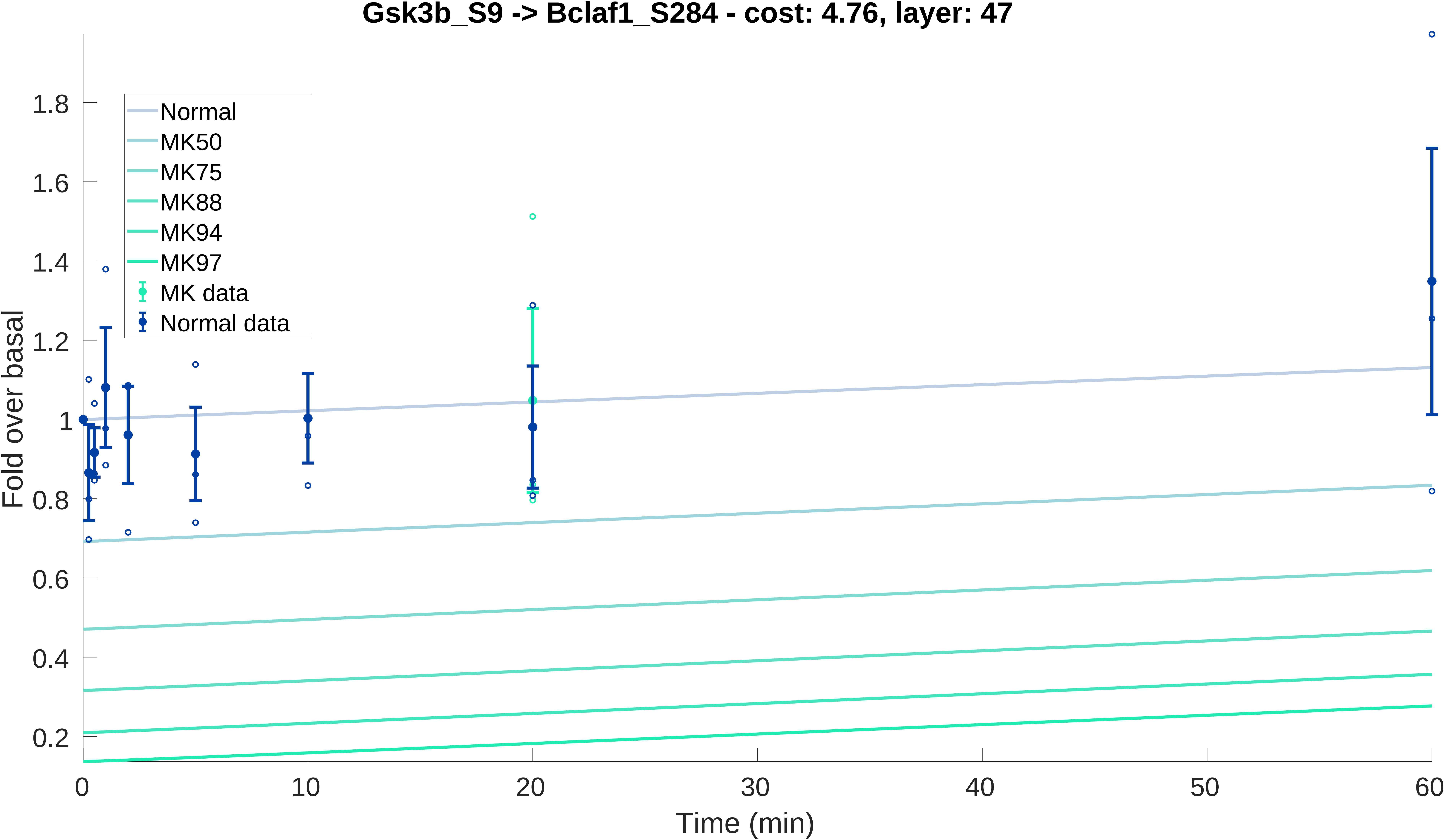

Gsk3b_S9 -> Bclaf1_S284 - cost: 4.76, layer: 47
Normal
MK50
MK75
MK88
MK94
MK97
MK data
Normal data
Fold over basal
0.2
0.4
0.6
0.8
1
1.2
1.4
1.6
1.8
0
10
20
30
40
50
60
Time (min)

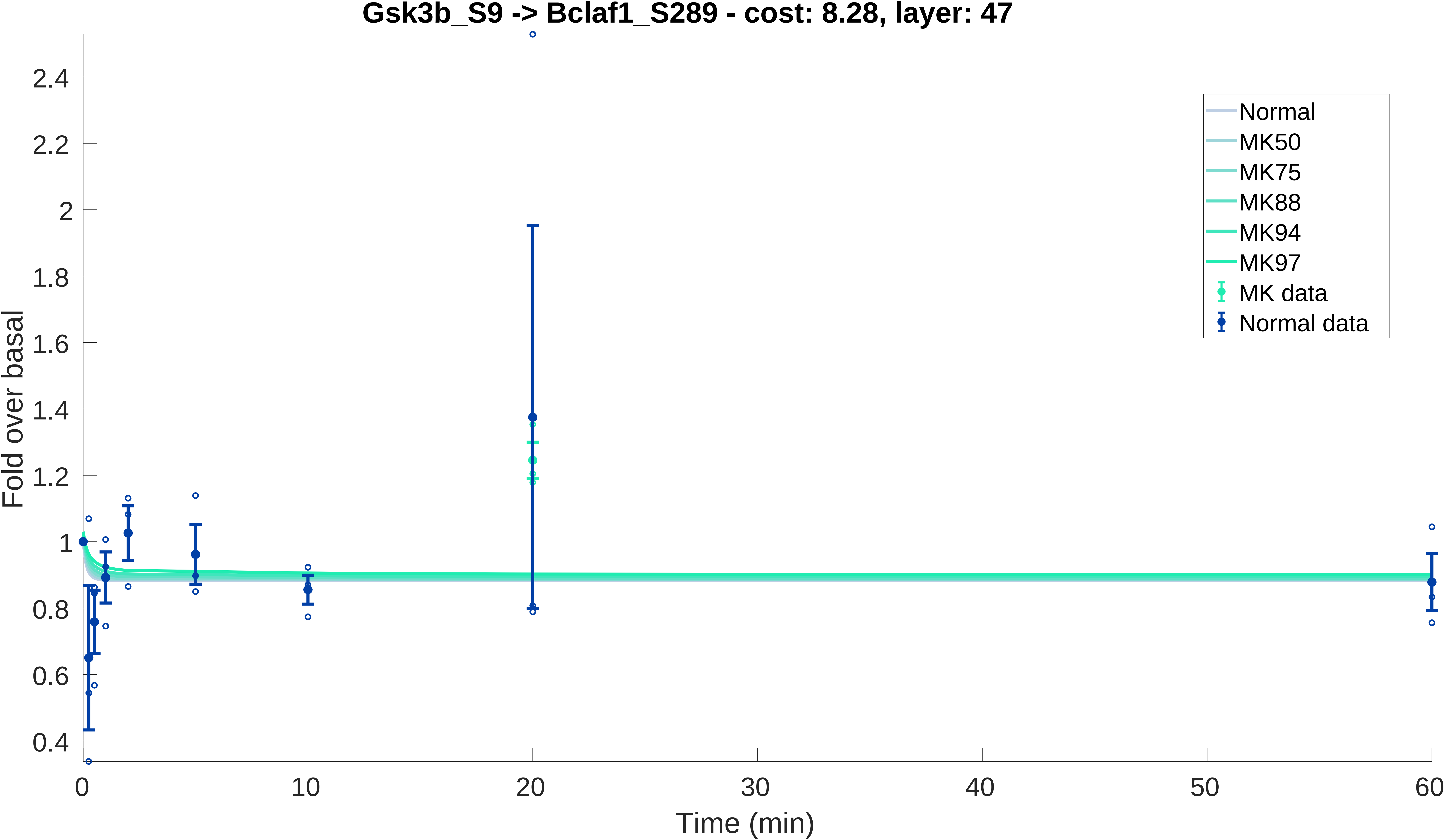

Gsk3b_S9 -> Bclaf1_S289 - cost: 8.28, layer: 47
Fold over basal
Time (min)
0
10
20
30
40
50
60
0.4
0.6
0.8
1
1.2
1.4
1.6
1.8
2
2.2
2.4
Normal
MK50
MK75
MK88
MK94
MK97
MK data
Normal data

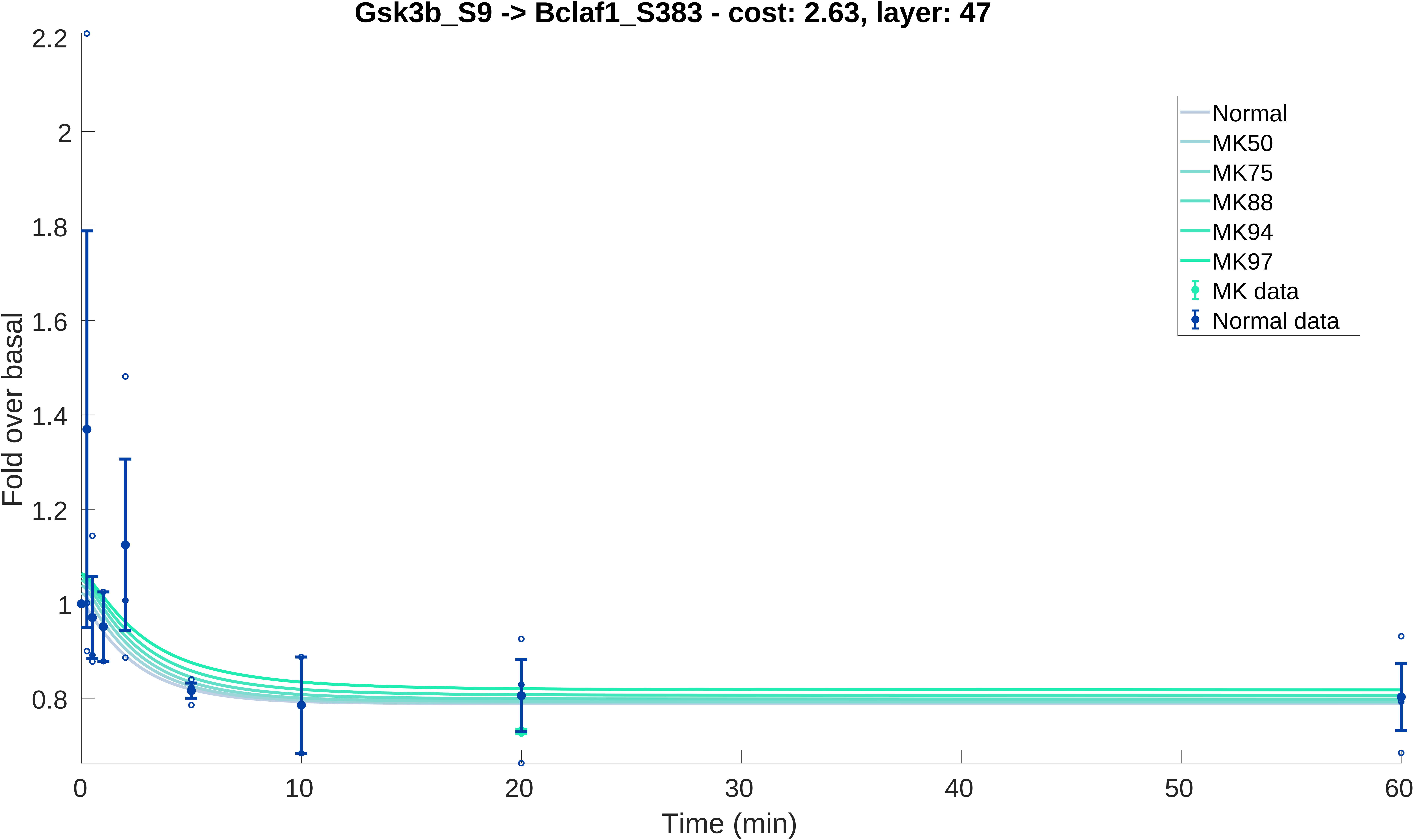

Gsk3b_S9 -> Bclaf1_S383 - cost: 2.63, layer: 47
Fold over basal
Time (min)
2.2
2
1.8
1.6
1.4
1.2
1
0.8
0
10
20
30
40
50
60
Normal
MK50
MK75
MK88
MK94
MK97
MK data
Normal data

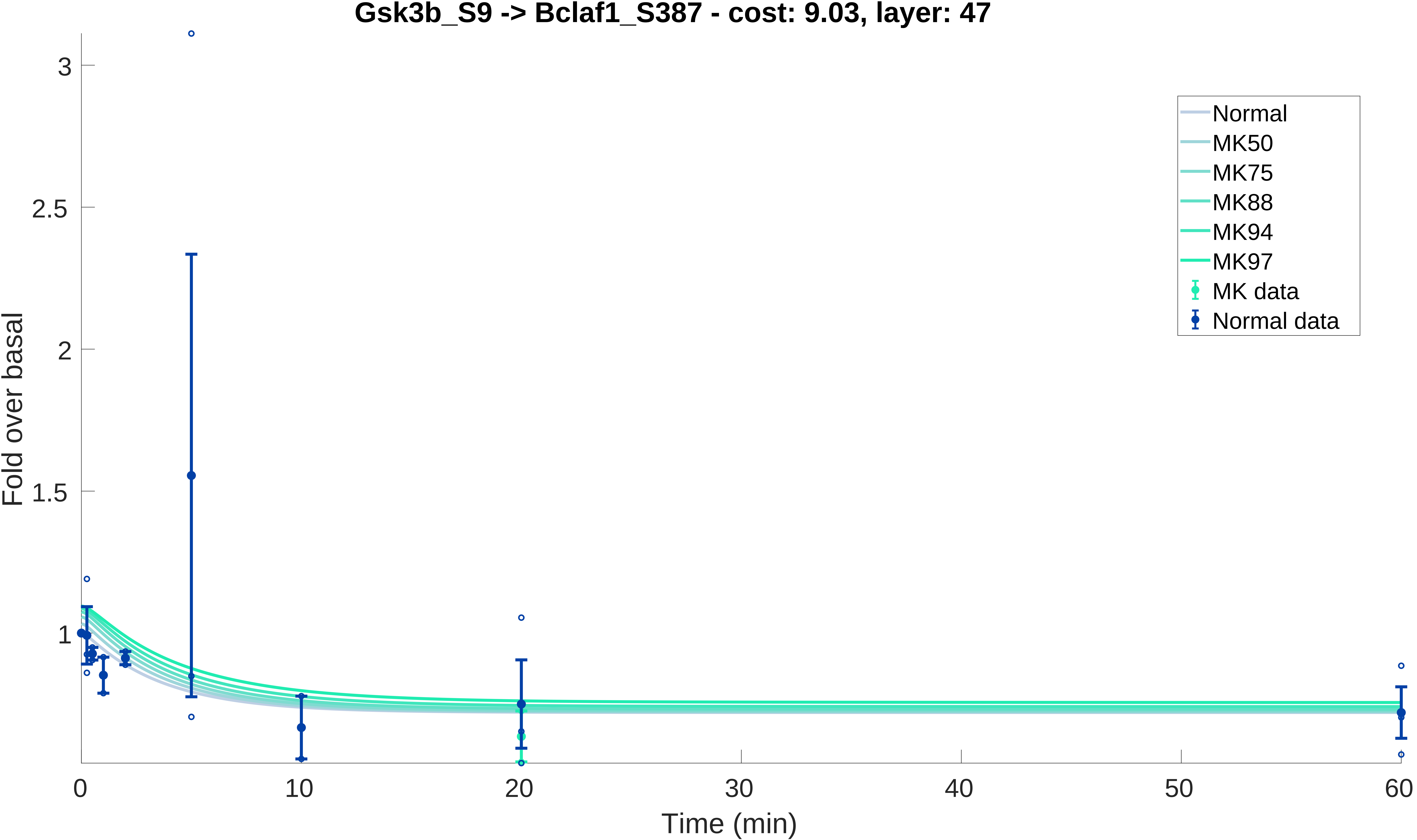

Gsk3b_S9 -> Bclaf1_S387 - cost: 9.03, layer: 47
Fold over basal
Time (min)
Normal
MK50
MK75
MK88
MK94
MK97
MK data
Normal data
3
2.5
2
1.5
1
0
10
20
30
40
50
60

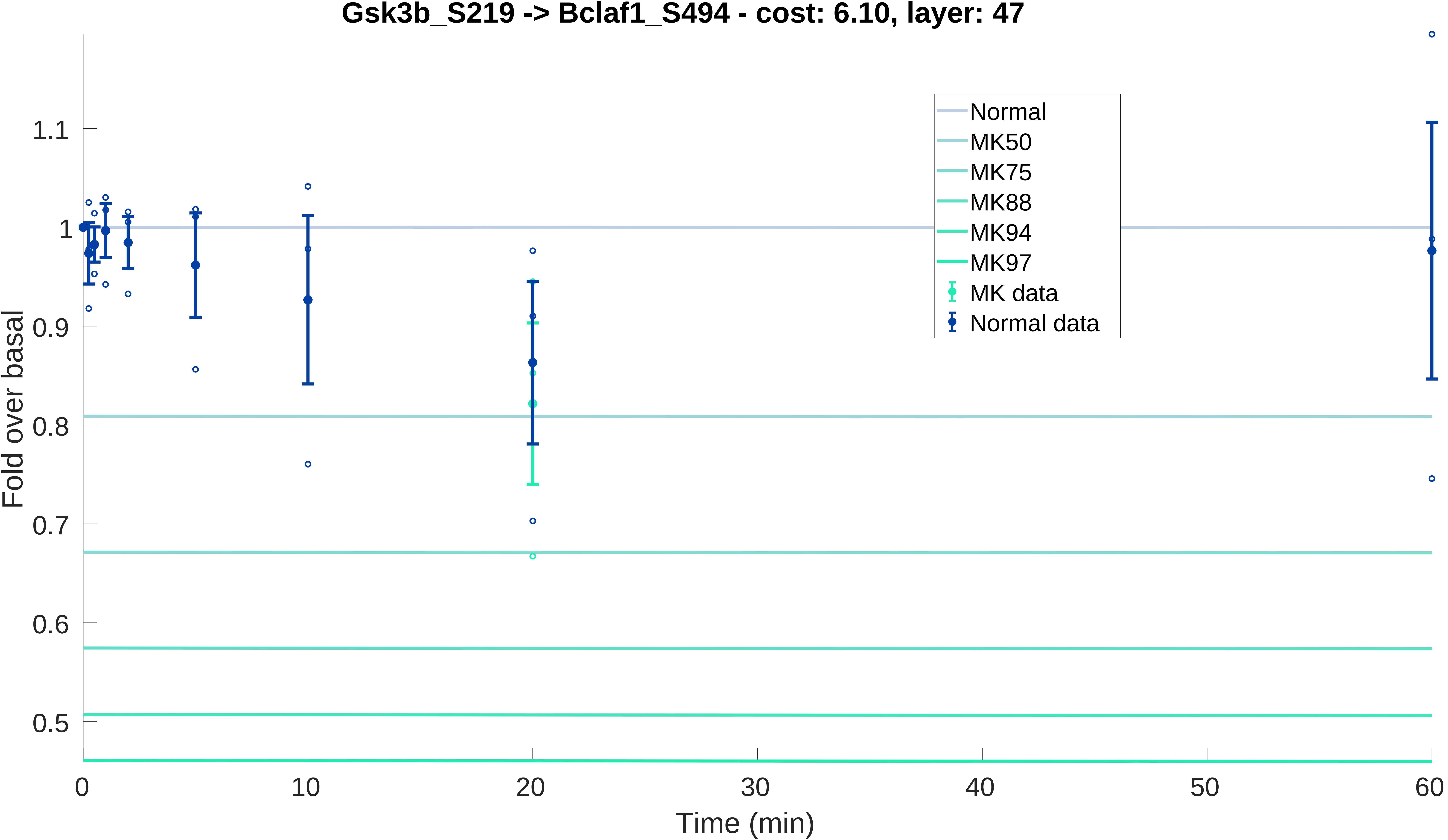
Gsk3b_S219 -> Bclaf1_S494 - cost: 6.10, layer: 47
Normal
MK50
MK75
MK88
MK94
MK97
MK data
Normal data
Fold over basal
1.1
1
0.9
0.8
0.7
0.6
0.5
0
10
20
30
40
50
60
Time (min)

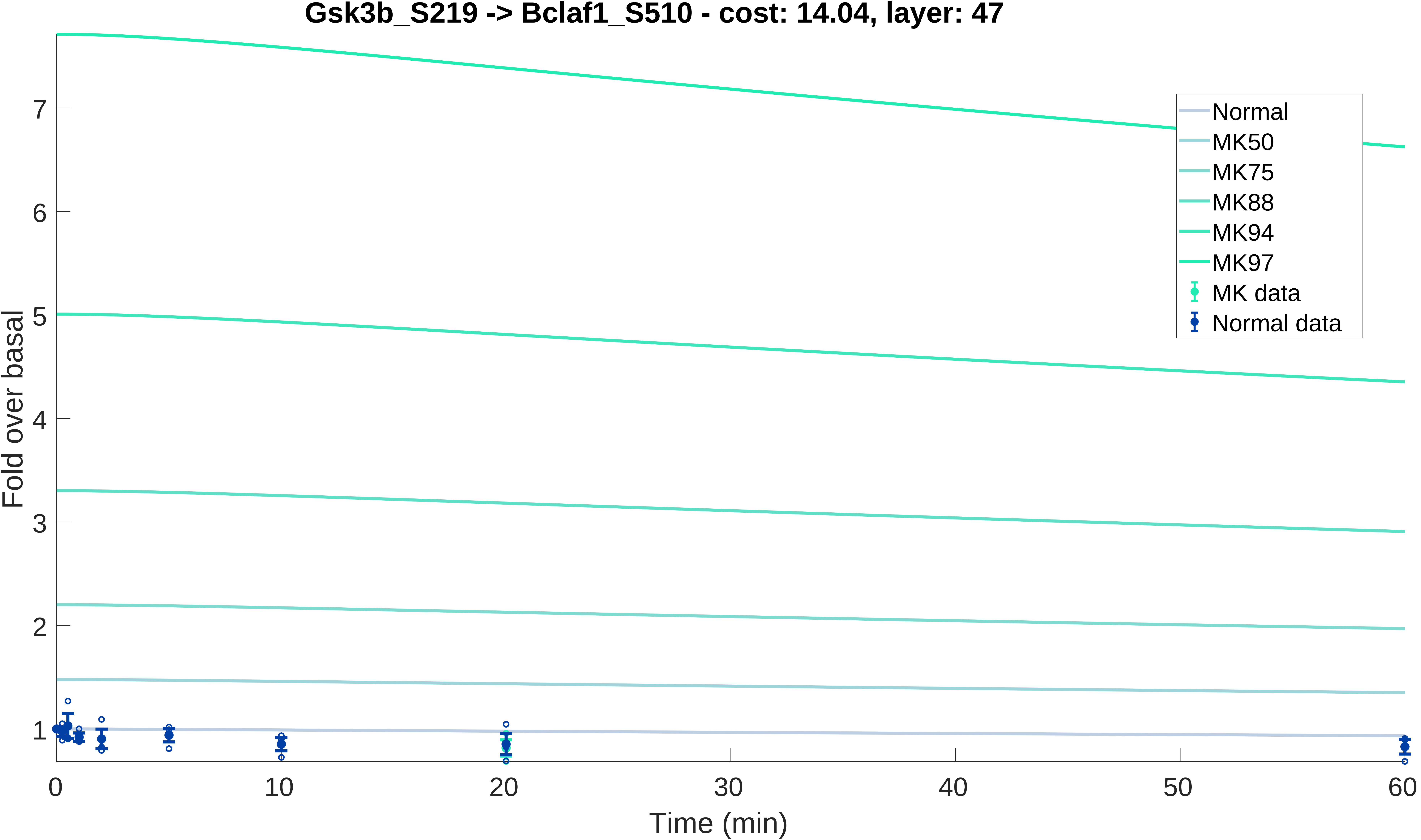

Gsk3b_S219 -> Bclaf1_S510 - cost: 14.04, layer: 47
Fold over basal
Time (min)
1
2
3
4
5
6
7
0
10
20
30
40
50
60
Normal
MK50
MK75
MK88
MK94
MK97
MK data
Normal data

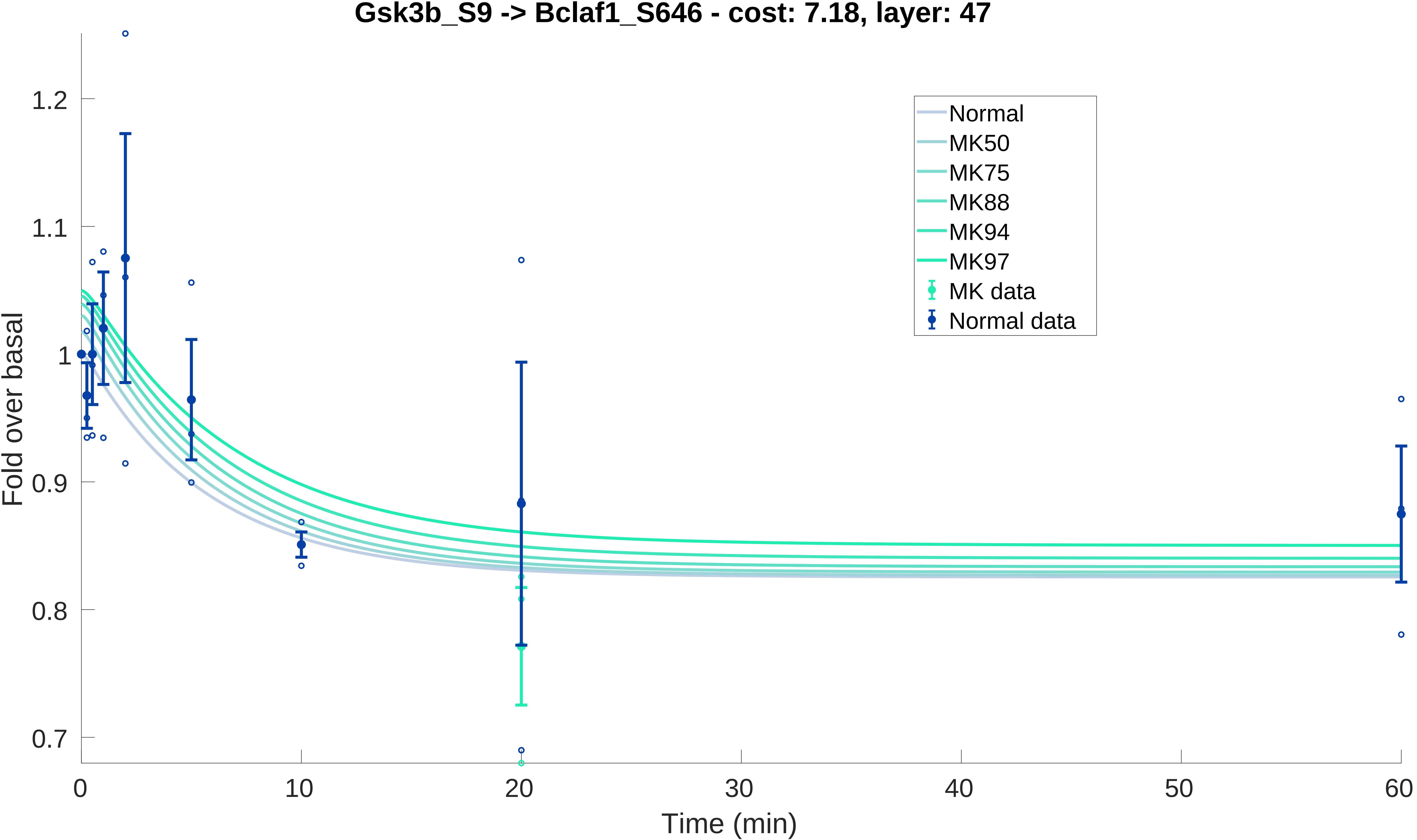
Gsk3b_S9 -> Bclaf1_S646 - cost: 7.18, layer: 47
Fold over basal
Time (min)
0.7
0.8
0.9
1
1.1
1.2
0
10
20
30
40
50
60
Normal
MK50
MK75
MK88
MK94
MK97
MK data
Normal data

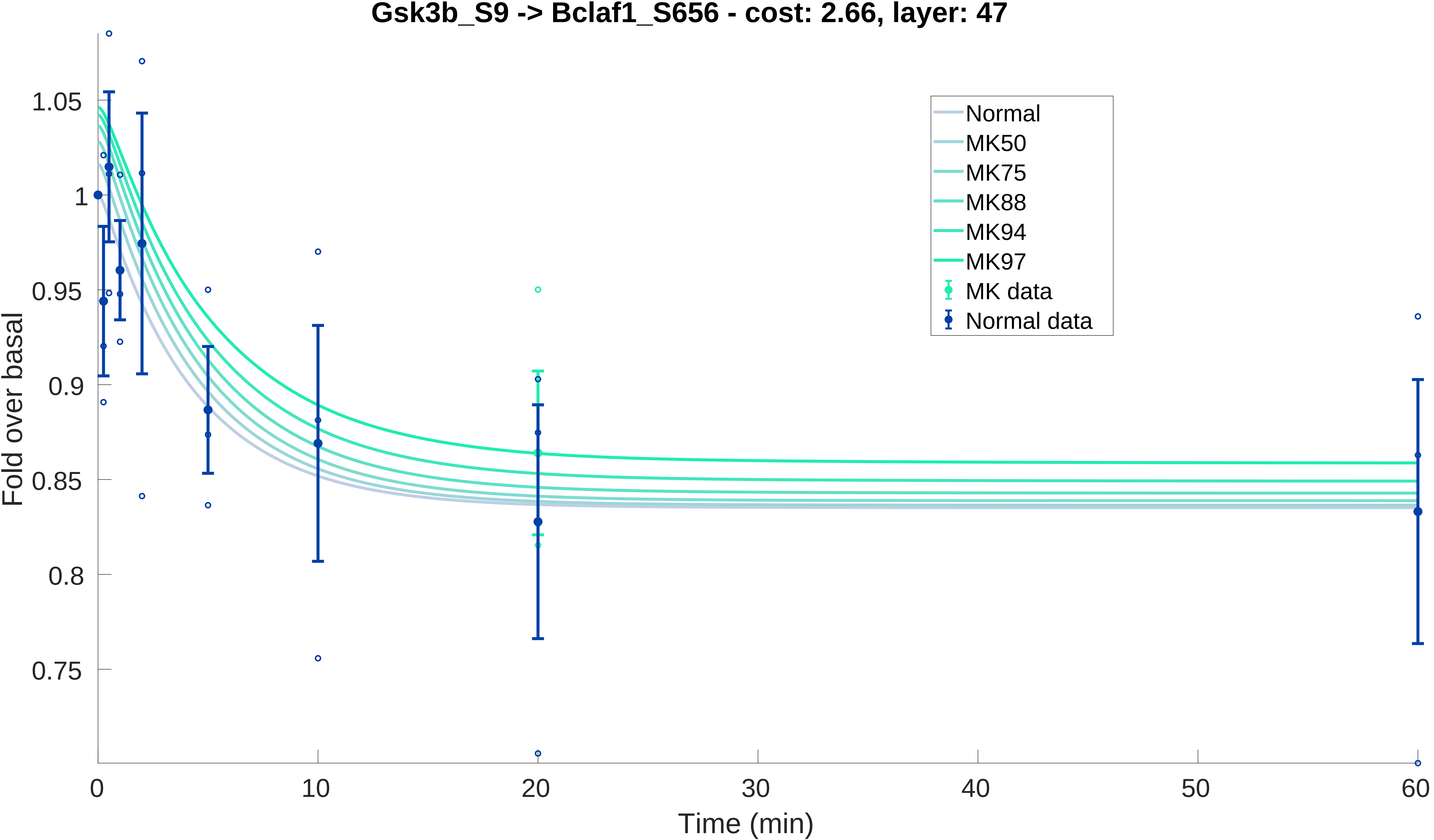
Gsk3b_S9 -> Bclaf1_S656 - cost: 2.66, layer: 47
Fold over basal
Time (min)
1.05
1
0.95
0.9
0.85
0.8
0.75
0
10
20
30
40
50
60
Normal
MK50
MK75
MK88
MK94
MK97
MK data
Normal data

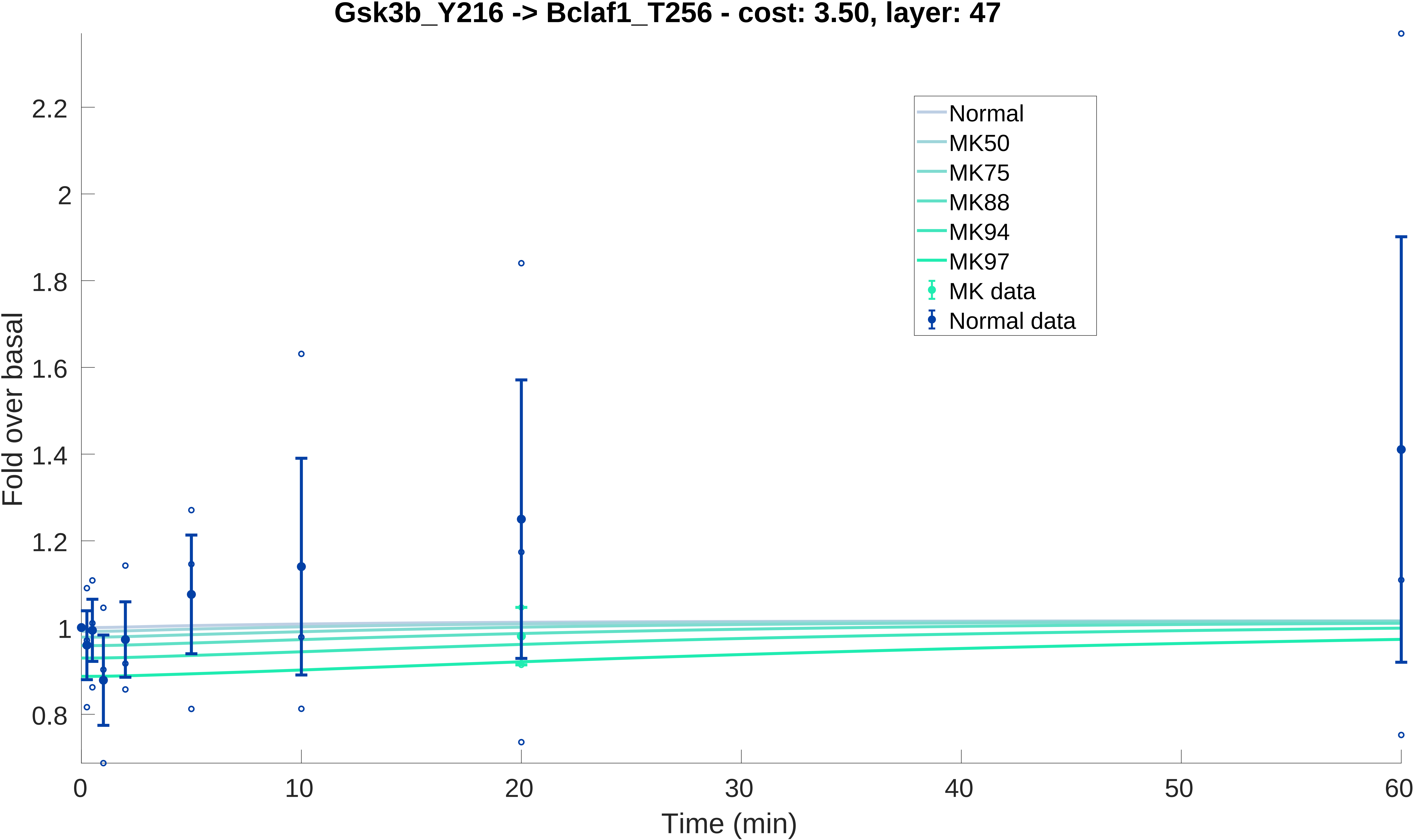

Gsk3b_Y216 -> Bclaf1_T256 - cost: 3.50, layer: 47
Fold over basal
Time (min)
0.8
1
1.2
1.4
1.6
1.8
2
2.2
0
10
20
30
40
50
60
Normal
MK50
MK75
MK88
MK94
MK97
MK data
Normal data

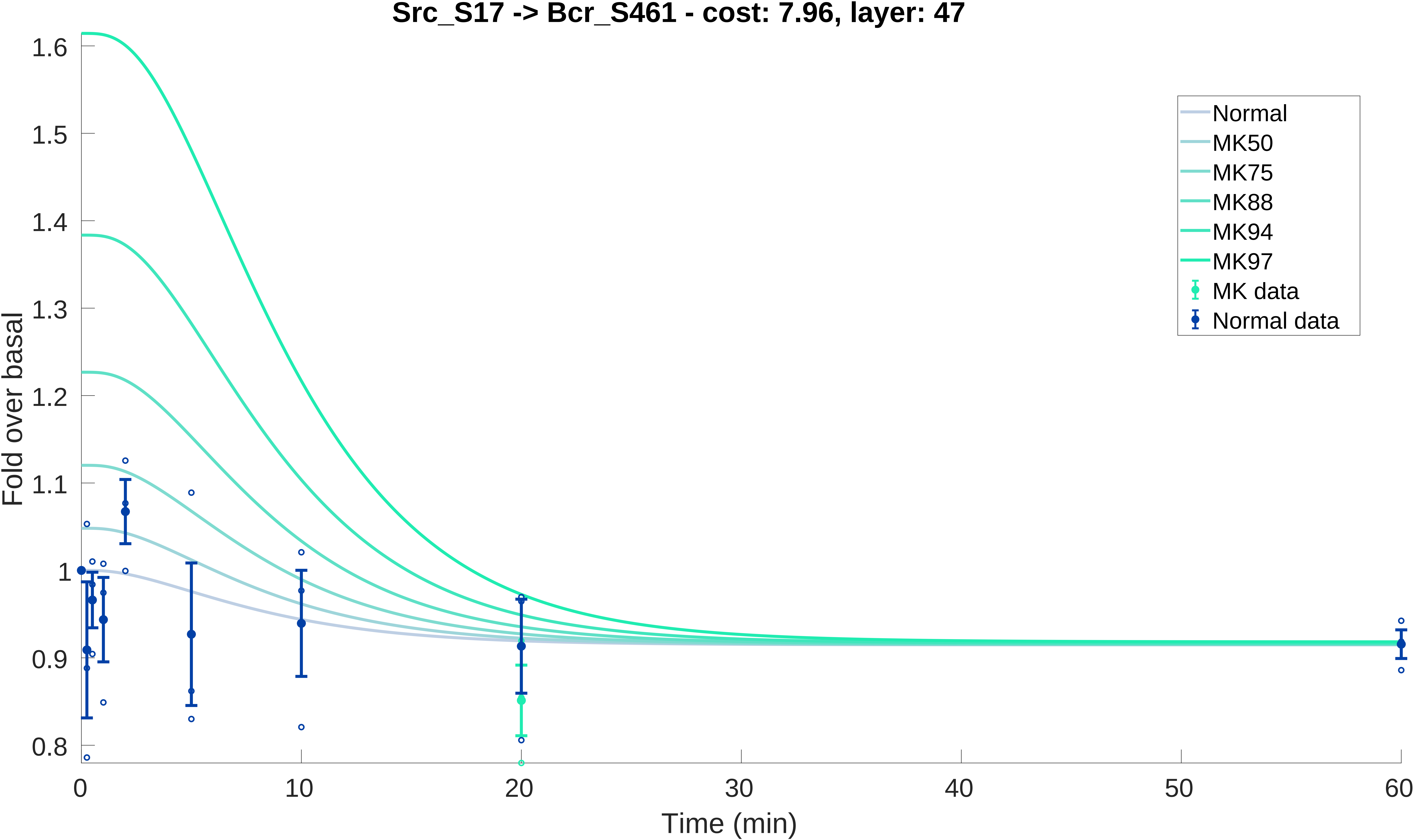

Src_S17 -> Bcr_S461 - cost: 7.96, layer: 47
Fold over basal
1.6
1.5
1.4
1.3
1.2
1.1
1
0.9
0.8
0
10
20
30
40
50
60
Time (min)
Normal
MK50
MK75
MK88
MK94
MK97
MK data
Normal data

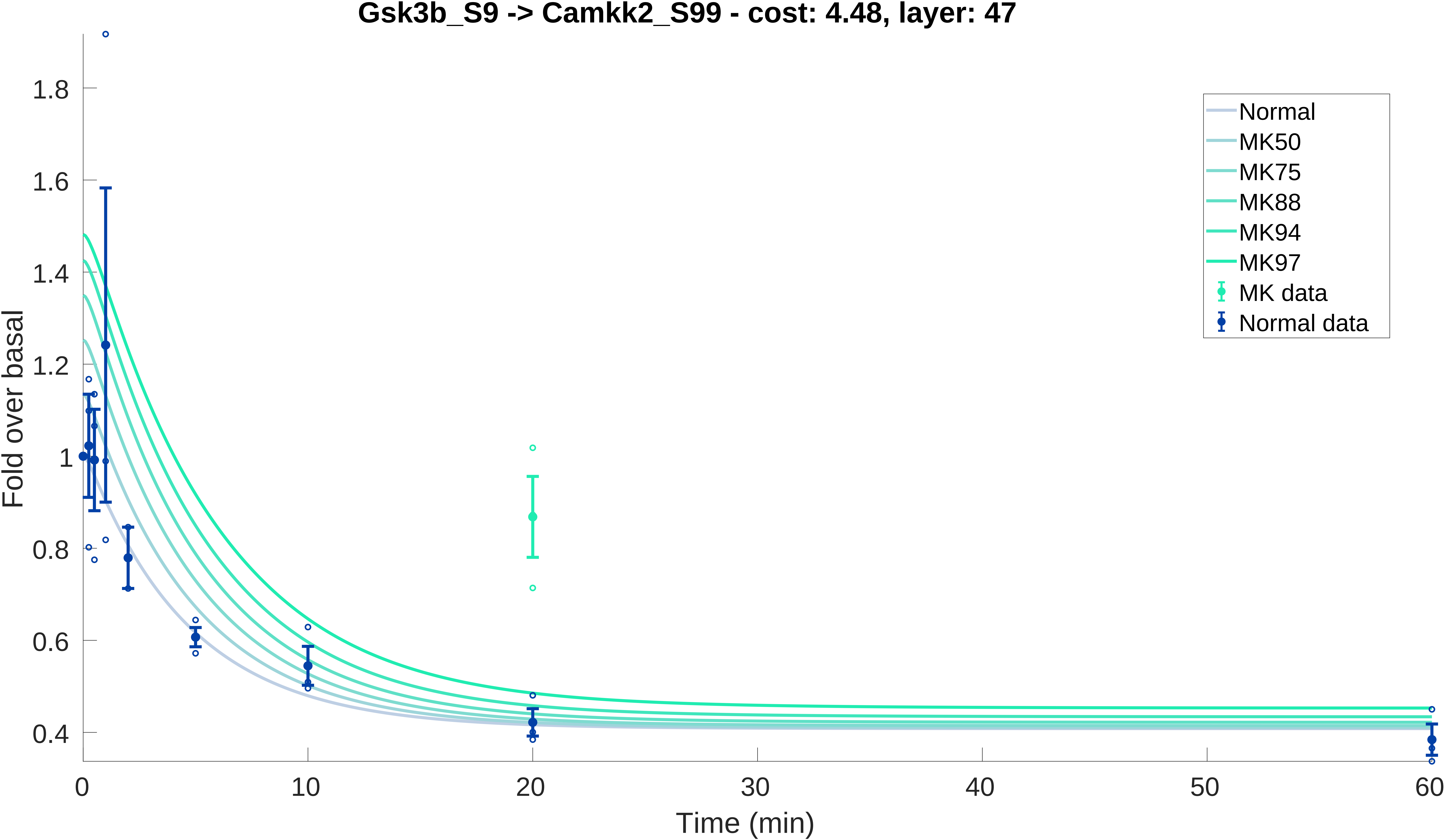
Gsk3b_S9 -> Camkk2_S99 - cost: 4.48, layer: 47
Fold over basal
Time (min)
0
10
20
30
40
50
60
0.4
0.6
0.8
1
1.2
1.4
1.6
1.8
Normal
MK50
MK75
MK88
MK94
MK97
MK data
Normal data

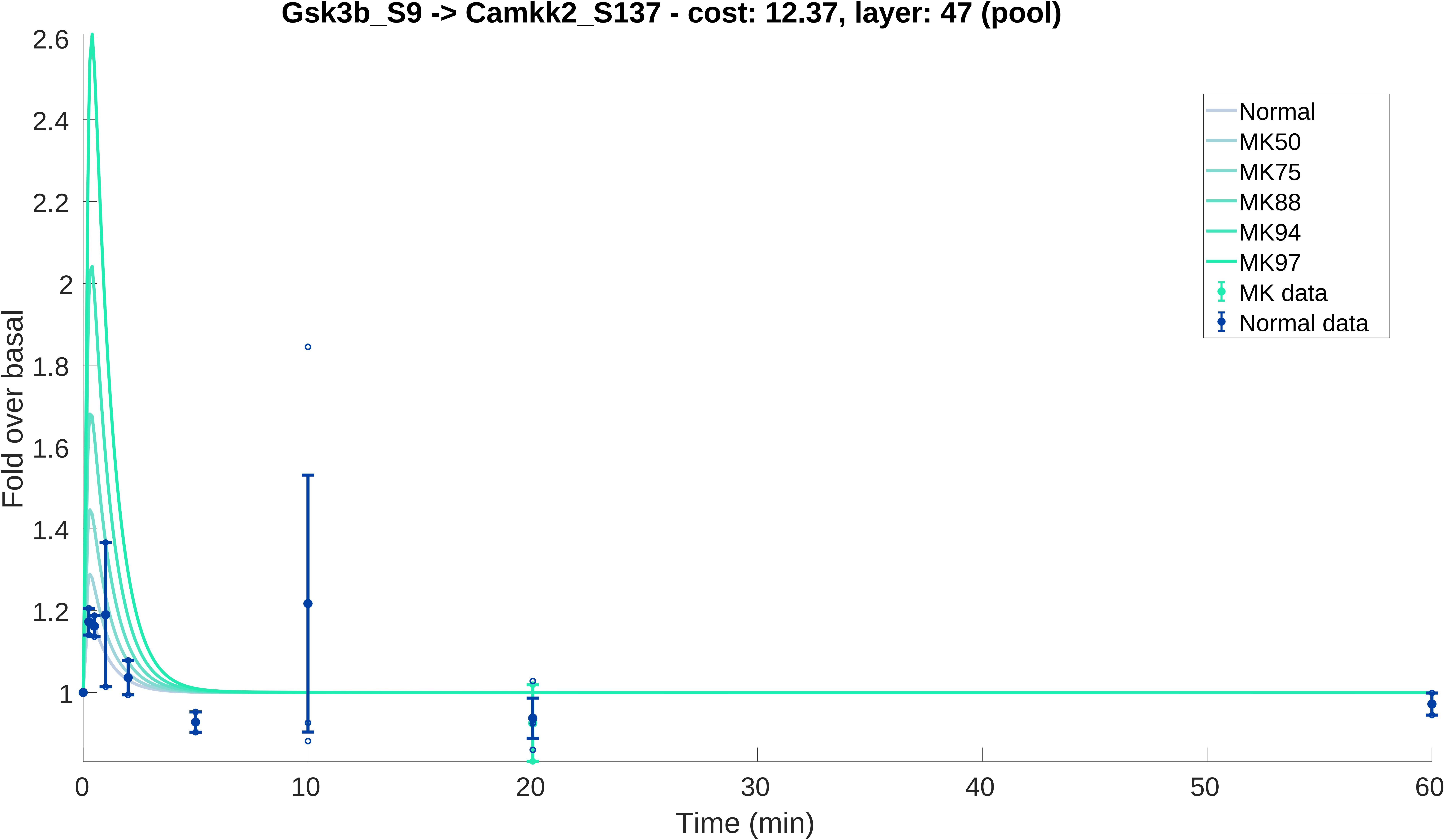

Gsk3b_S9 -> Camkk2_S137 - cost: 12.37, layer: 47 (pool)
Fold over basal
Time (min)
2.6
2.4
2.2
2
1.8
1.6
1.4
1.2
1
0
10
20
30
40
50
60
Normal
MK50
MK75
MK88
MK94
MK97
MK data
Normal data

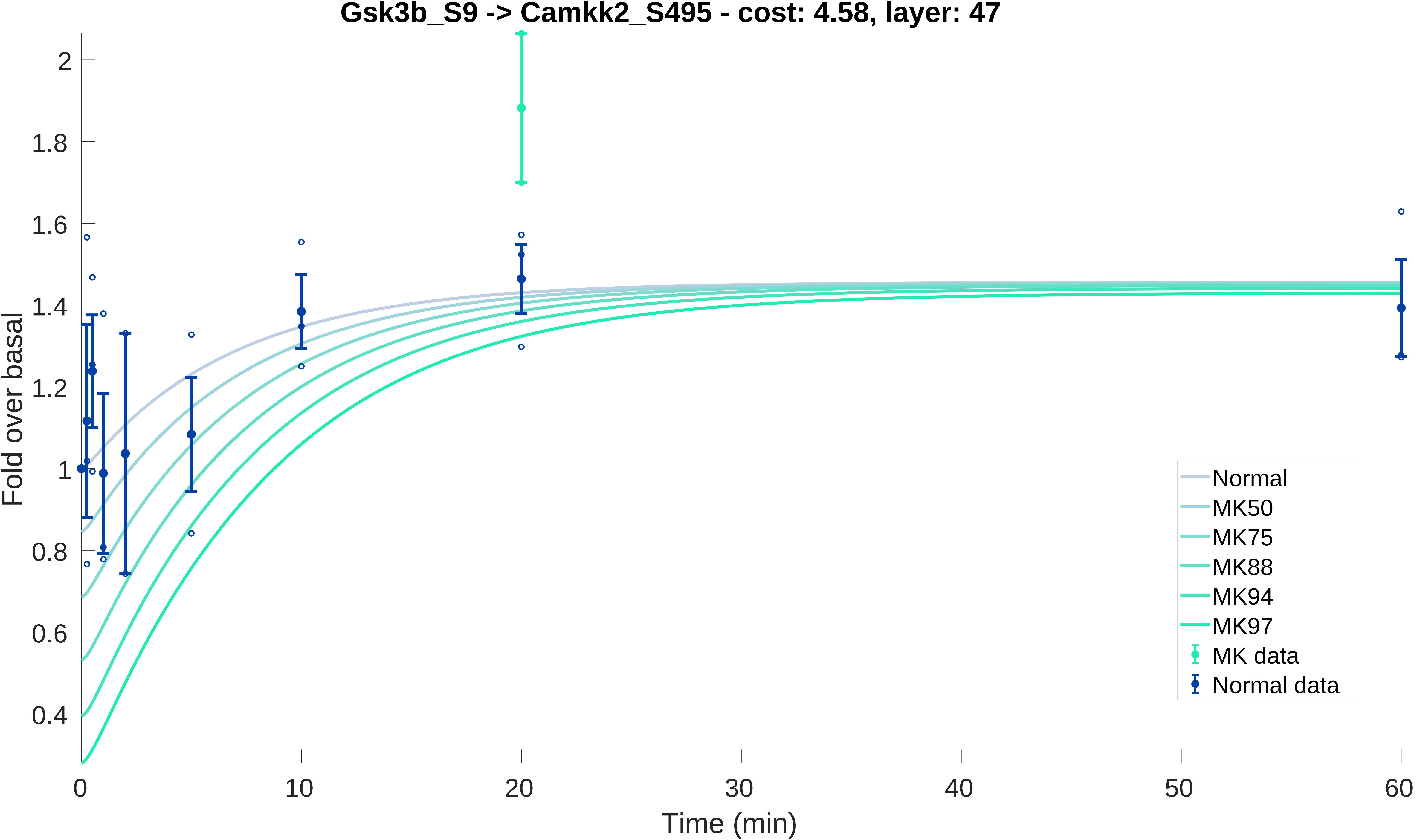

Gsk3b_S9 -> Camkk2_S495 - cost: 4.58, layer: 47
Fold over basal
Time (min)
0.4
0.6
0.8
1
1.2
1.4
1.6
1.8
2
0
10
20
30
40
50
60
Normal
MK50
MK75
MK88
MK94
MK97
MK data
Normal data

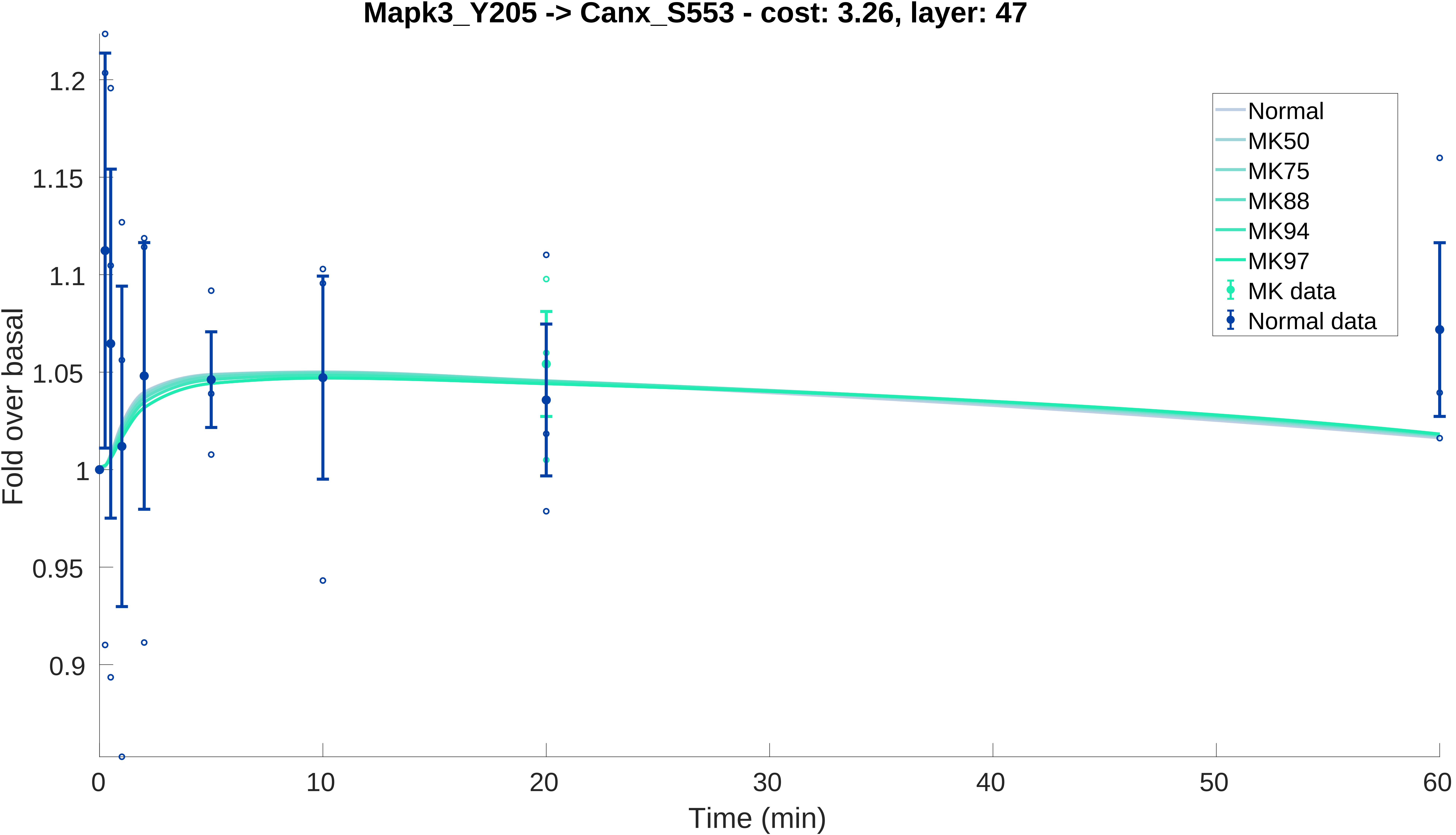
Mapk3_Y205 -> Canx_S553 - cost: 3.26, layer: 47
Fold over basal
Time (min)
0
10
20
30
40
50
60
0.9
0.95
1
1.05
1.1
1.15
1.2
Normal
MK50
MK75
MK88
MK94
MK97
MK data
Normal data

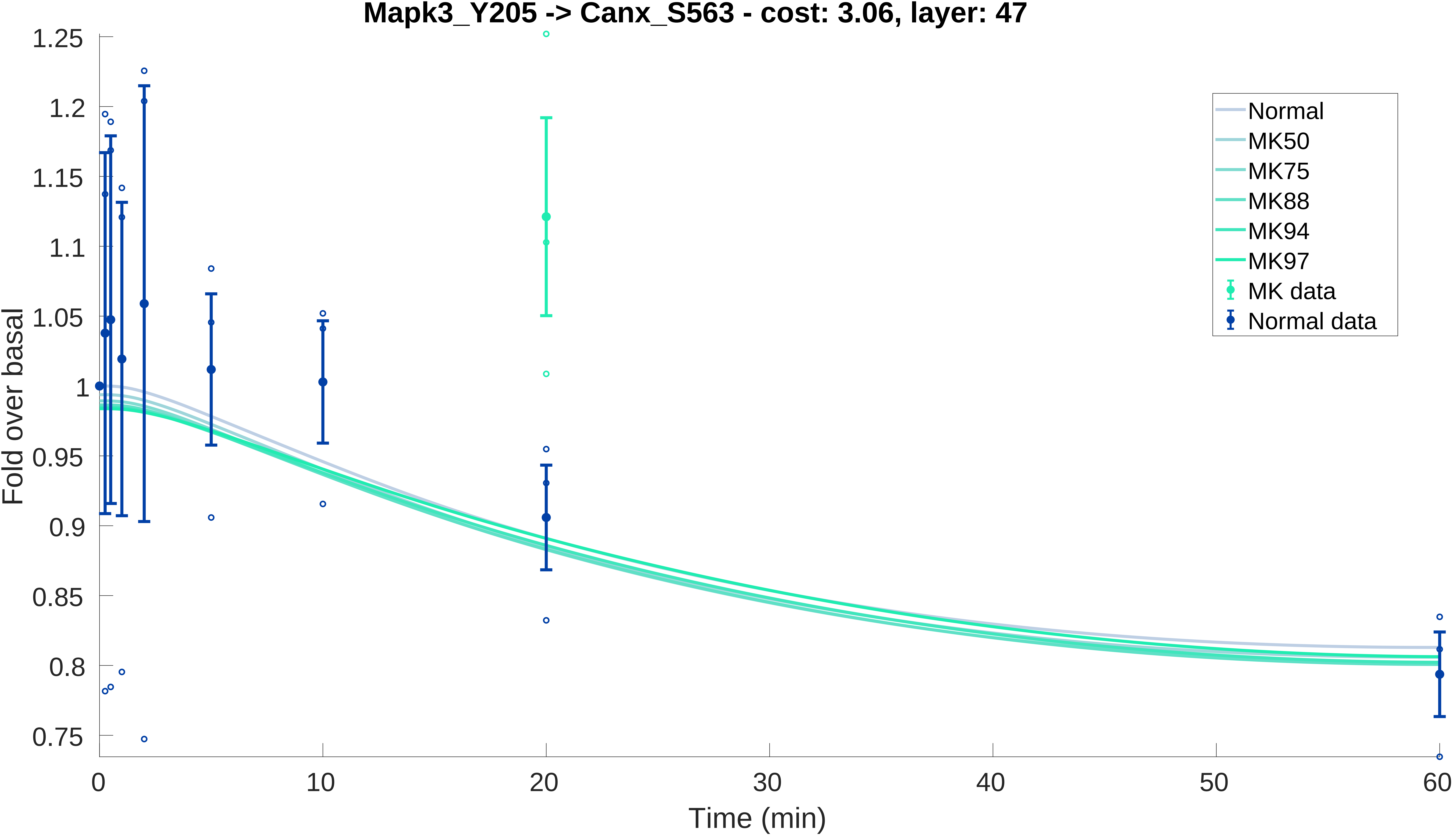

Mapk3_Y205 -> Canx_S563 - cost: 3.06, layer: 47
Fold over basal
1.25
1.2
1.15
1.1
1.05
1
0.95
0.9
0.85
0.8
0.75
0
10
20
30
40
50
60
Time (min)
Normal
MK50
MK75
MK88
MK94
MK97
MK data
Normal data

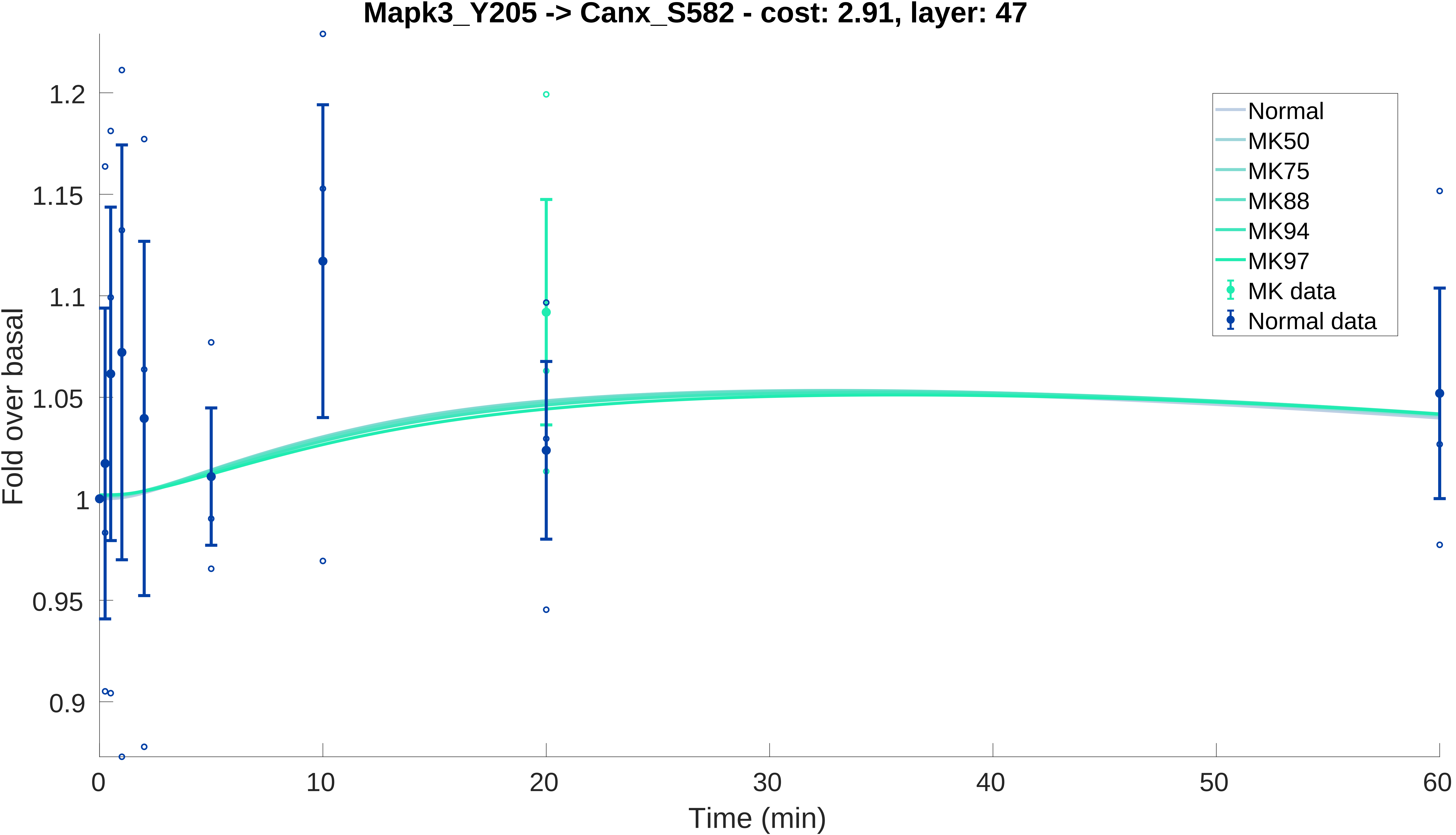

Mapk3_Y205 -> Canx_S582 - cost: 2.91, layer: 47
Fold over basal
Time (min)
0.9
0.95
1
1.05
1.1
1.15
1.2
0
10
20
30
40
50
60
Normal
MK50
MK75
MK88
MK94
MK97
MK data
Normal data

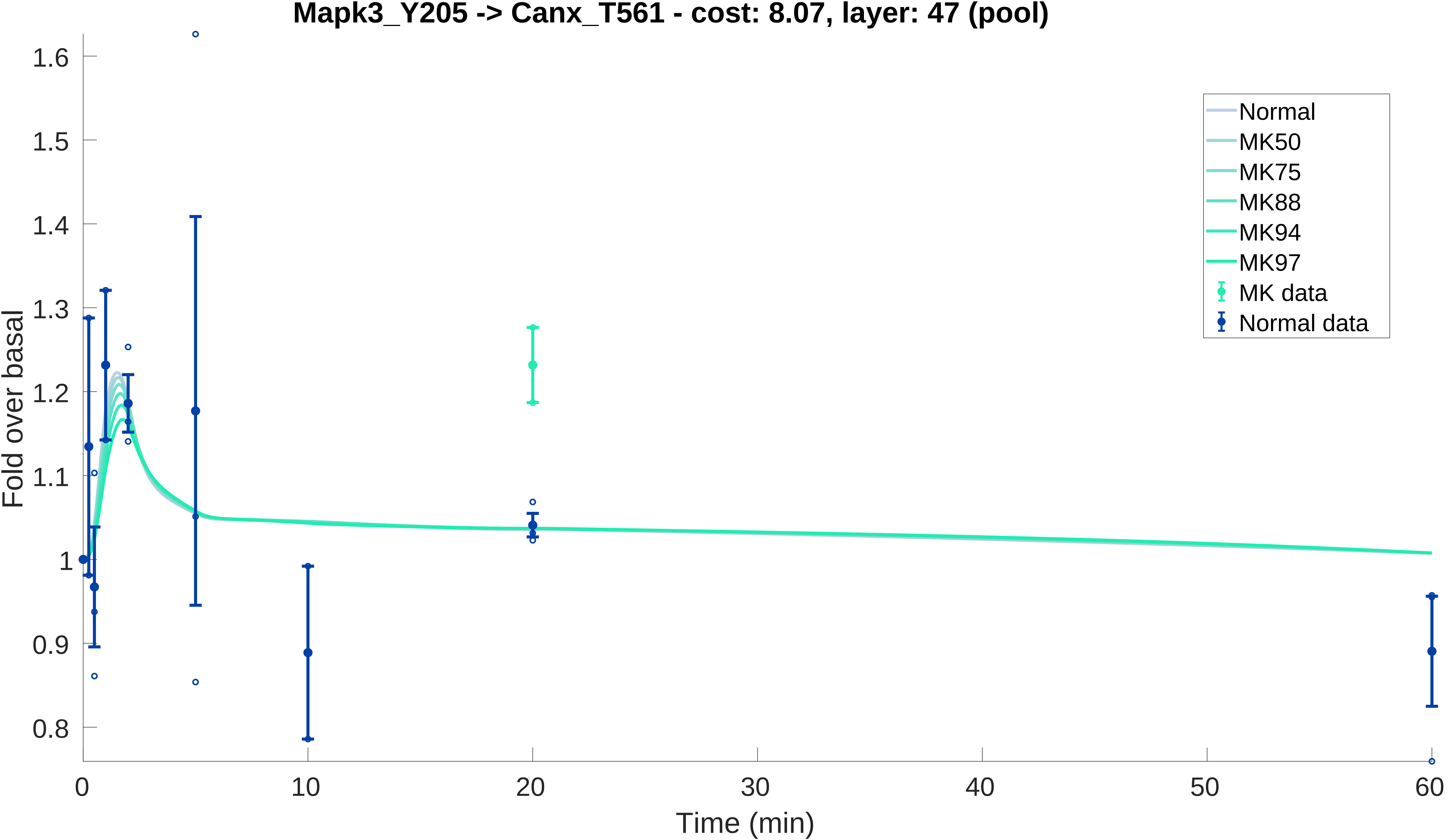

Mapk3_Y205 -> Canx_T561 - cost: 8.07, layer: 47 (pool)
Fold over basal
Time (min)
0
10
20
30
40
50
60
0.8
0.9
1
1.1
1.2
1.3
1.4
1.5
1.6
Normal
MK50
MK75
MK88
MK94
MK97
MK data
Normal data

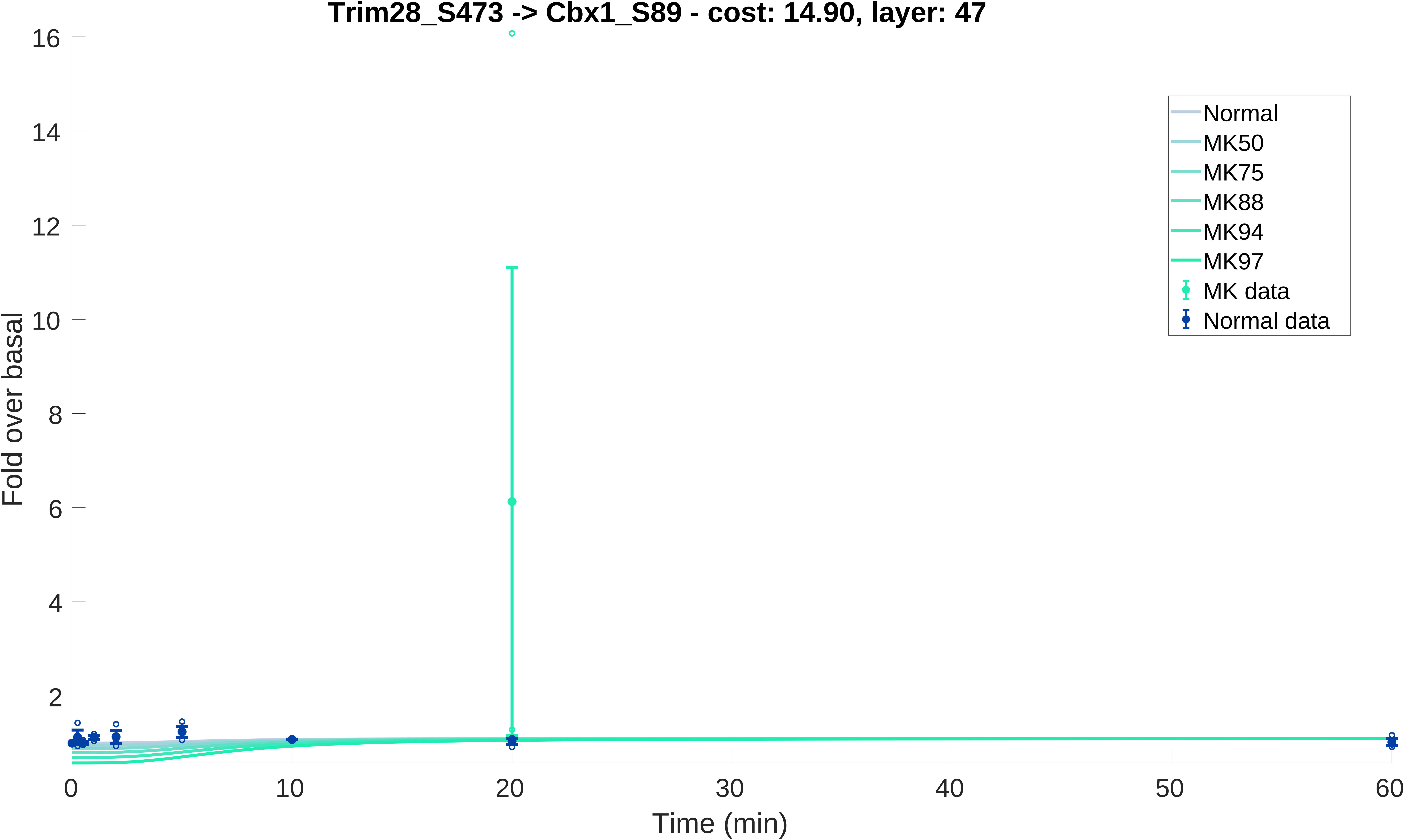
Trim28_S473 -> Cbx1_S89 - cost: 14.90, layer: 47
Fold over basal
Time (min)
0
10
20
30
40
50
60
2
4
6
8
10
12
14
16
Normal
MK50
MK75
MK88
MK94
MK97
MK data
Normal data

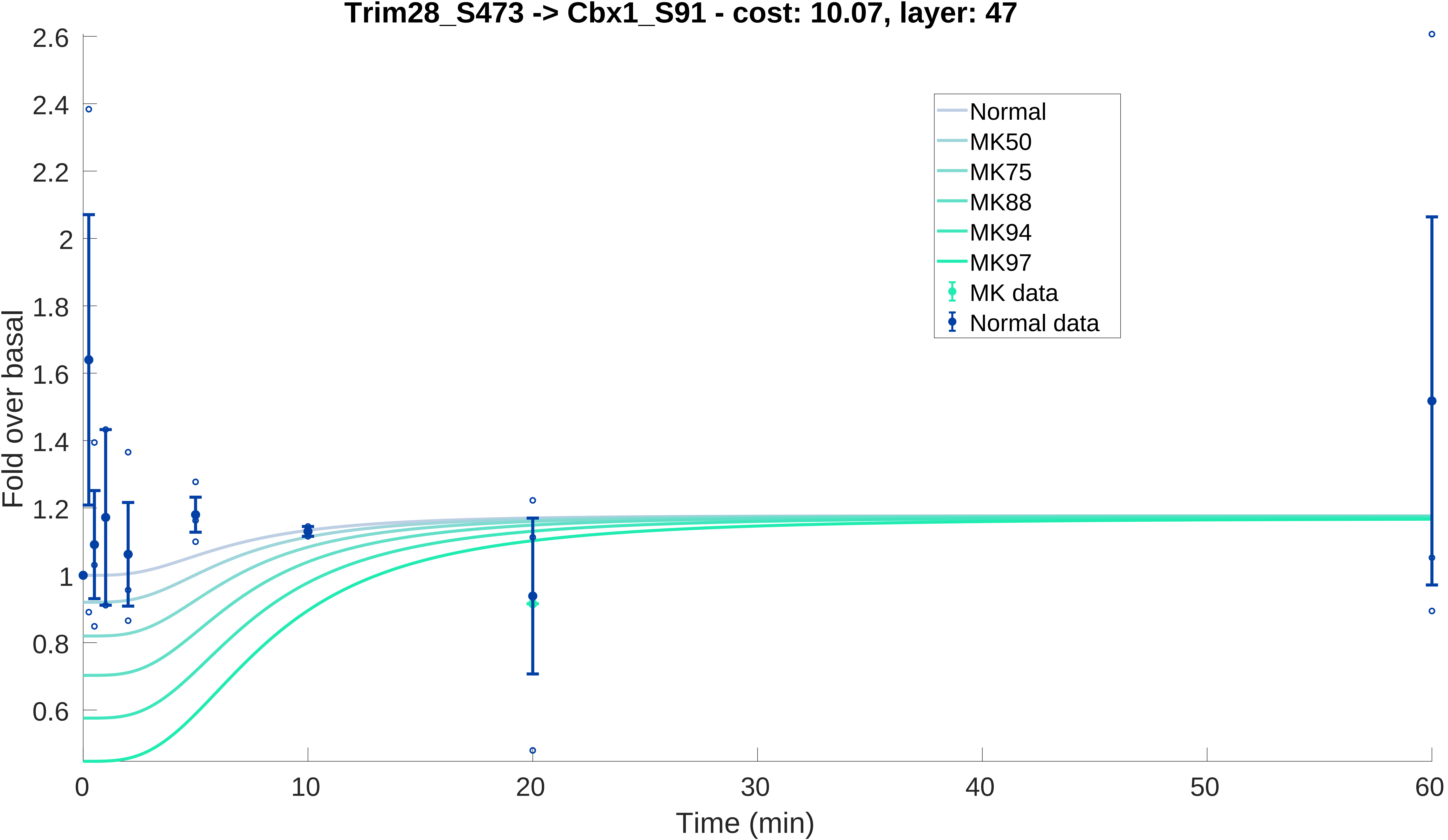
Trim28_S473 -> Cbx1_S91 - cost: 10.07, layer: 47
Fold over basal
Time (min)
Normal
MK50
MK75
MK88
MK94
MK97
MK data
Normal data
0
10
20
30
40
50
60
0.6
0.8
1
1.2
1.4
1.6
1.8
2
2.2
2.4
2.6

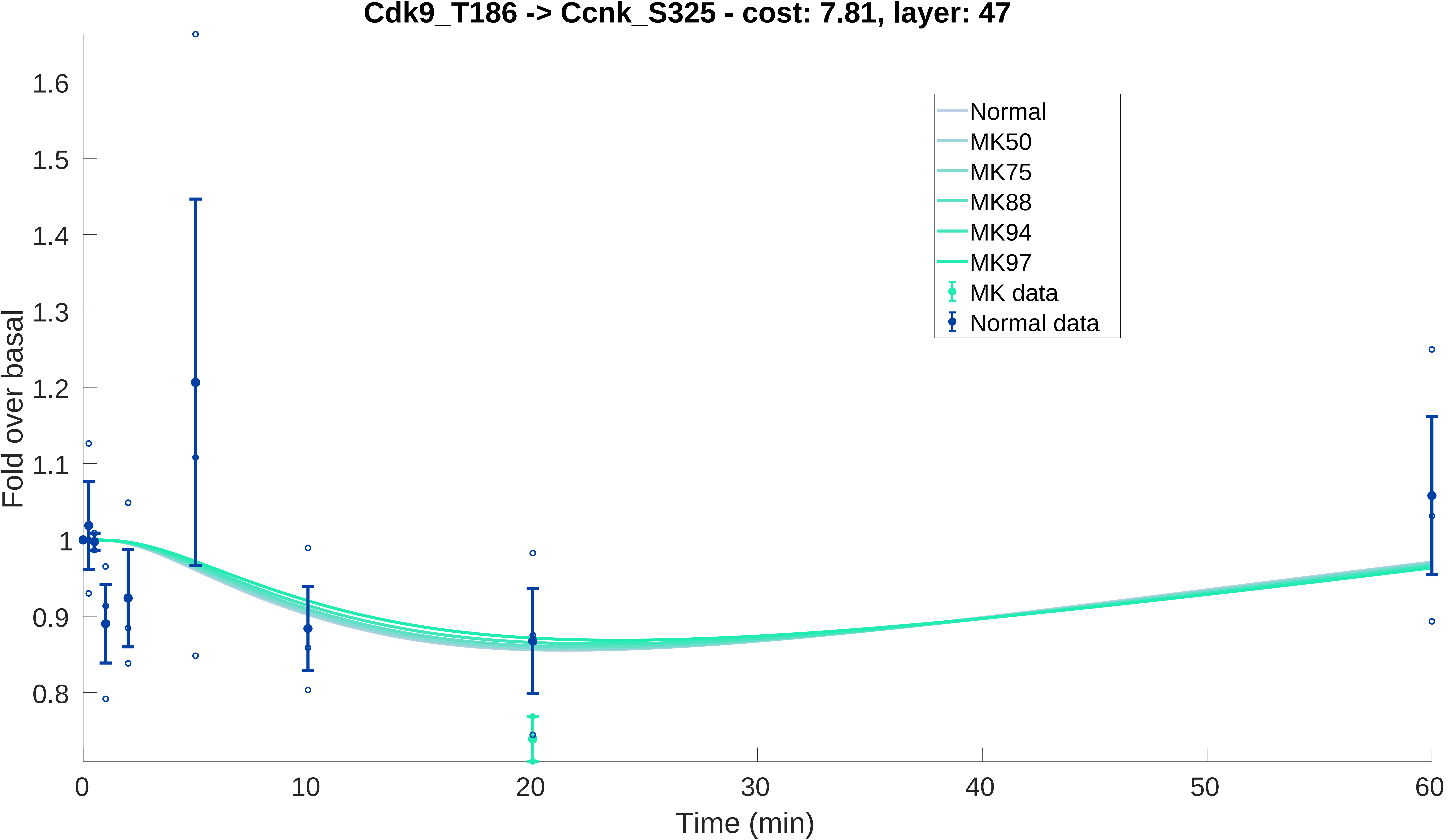

Cdk9_T186 -> Ccnk_S325 - cost: 7.81, layer: 47
Fold over basal
Time (min)
0
10
20
30
40
50
60
0.8
0.9
1
1.1
1.2
1.3
1.4
1.5
1.6
Normal
MK50
MK75
MK88
MK94
MK97
MK data
Normal data

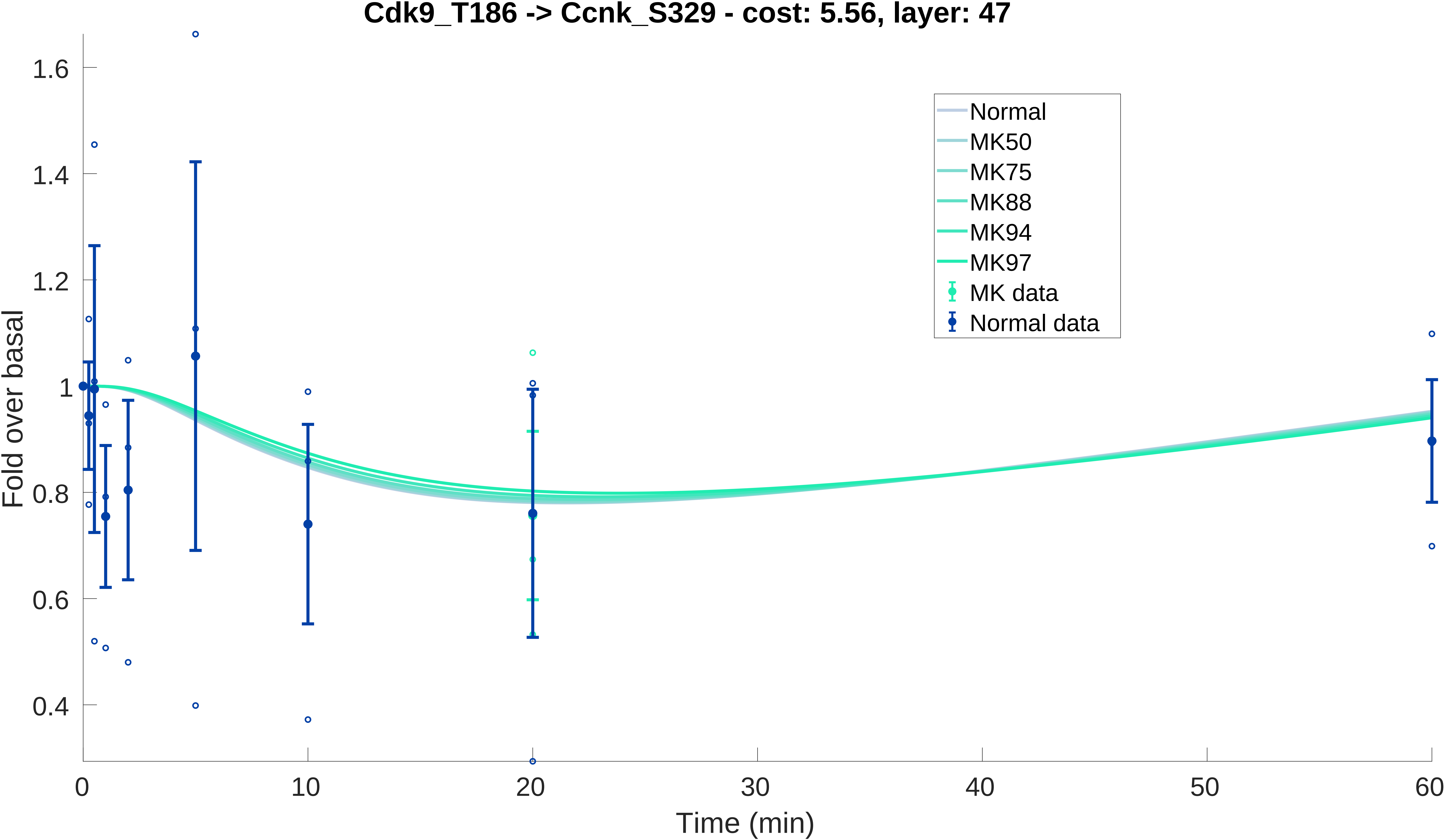

Cdk9_T186 -> Ccnk_S329 - cost: 5.56, layer: 47
Fold over basal
1.6
1.4
1.2
1
0.8
0.6
0.4
0
10
20
30
40
50
60
Time (min)
Normal
MK50
MK75
MK88
MK94
MK97
MK data
Normal data

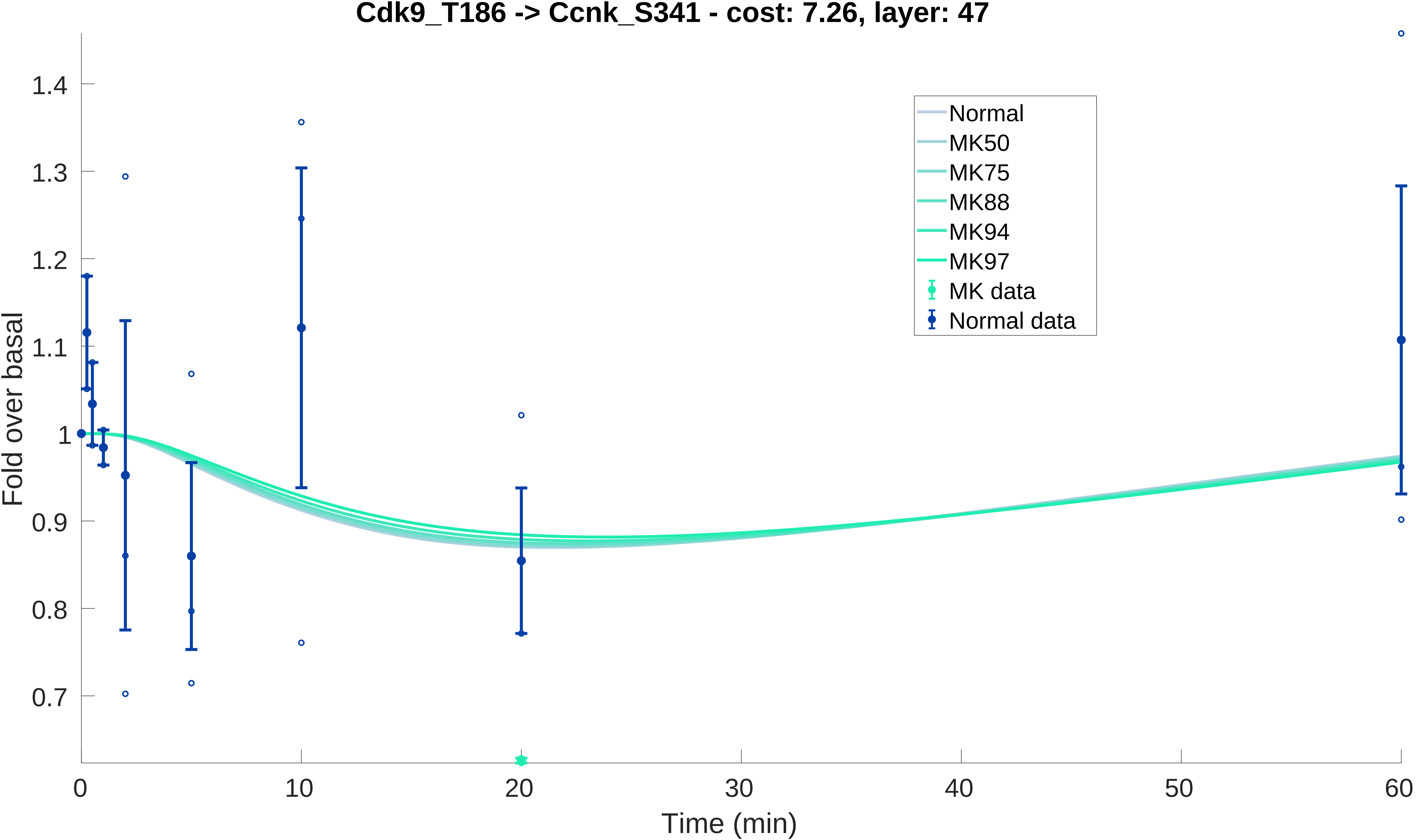

Cdk9_T186 -> Ccnk_S341 - cost: 7.26, layer: 47
Fold over basal
Time (min)
1.4
1.3
1.2
1.1
1
0.9
0.8
0.7
0
10
20
30
40
50
60
Normal
MK50
MK75
MK88
MK94
MK97
MK data
Normal data

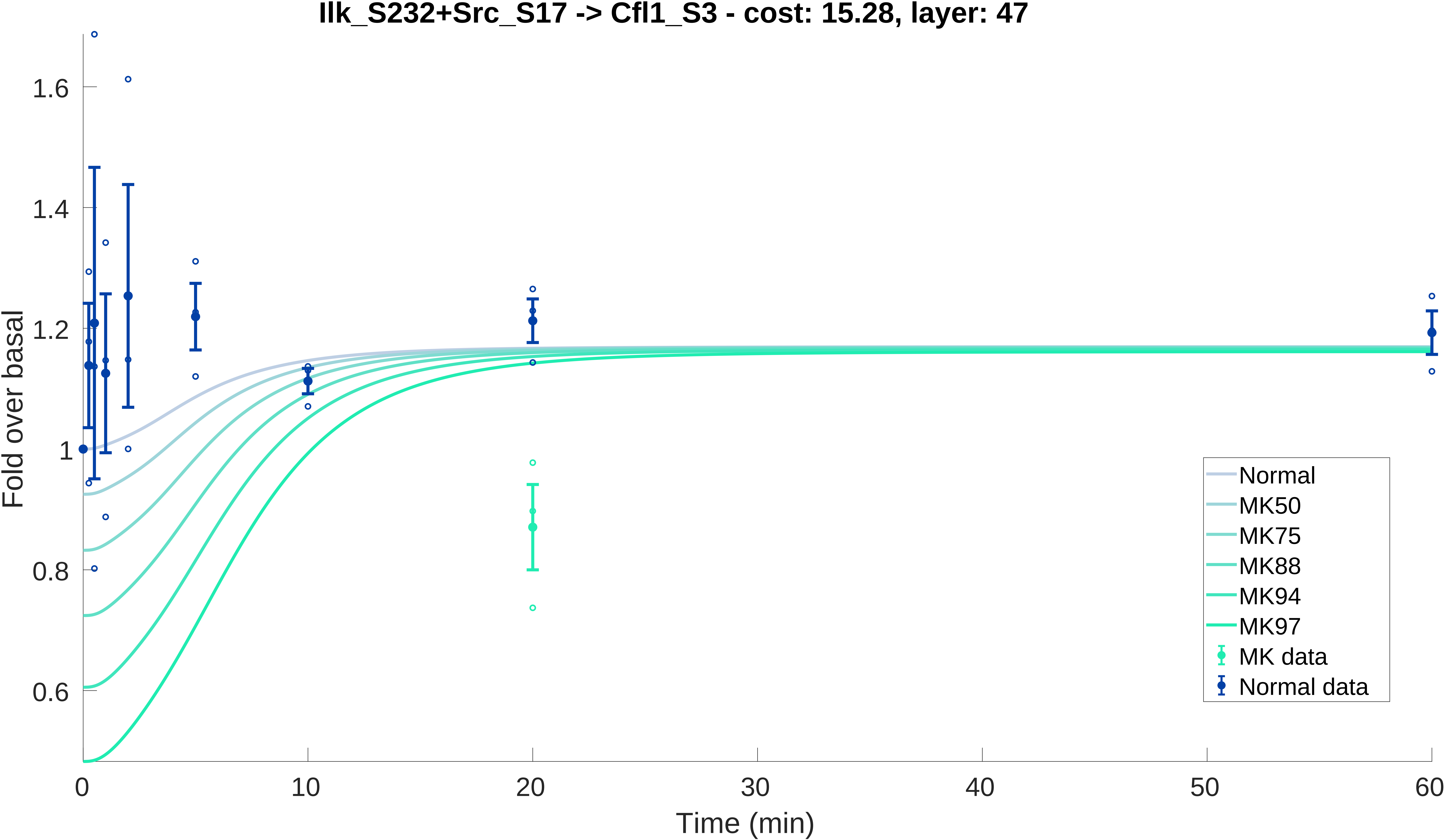

Ilk_S232+Src_S17 -> Cfl1_S3 - cost: 15.28, layer: 47
Fold over basal
1.6
1.4
1.2
1
0.8
0.6
0
10
20
30
40
50
60
Time (min)
Normal
MK50
MK75
MK88
MK94
MK97
MK data
Normal data

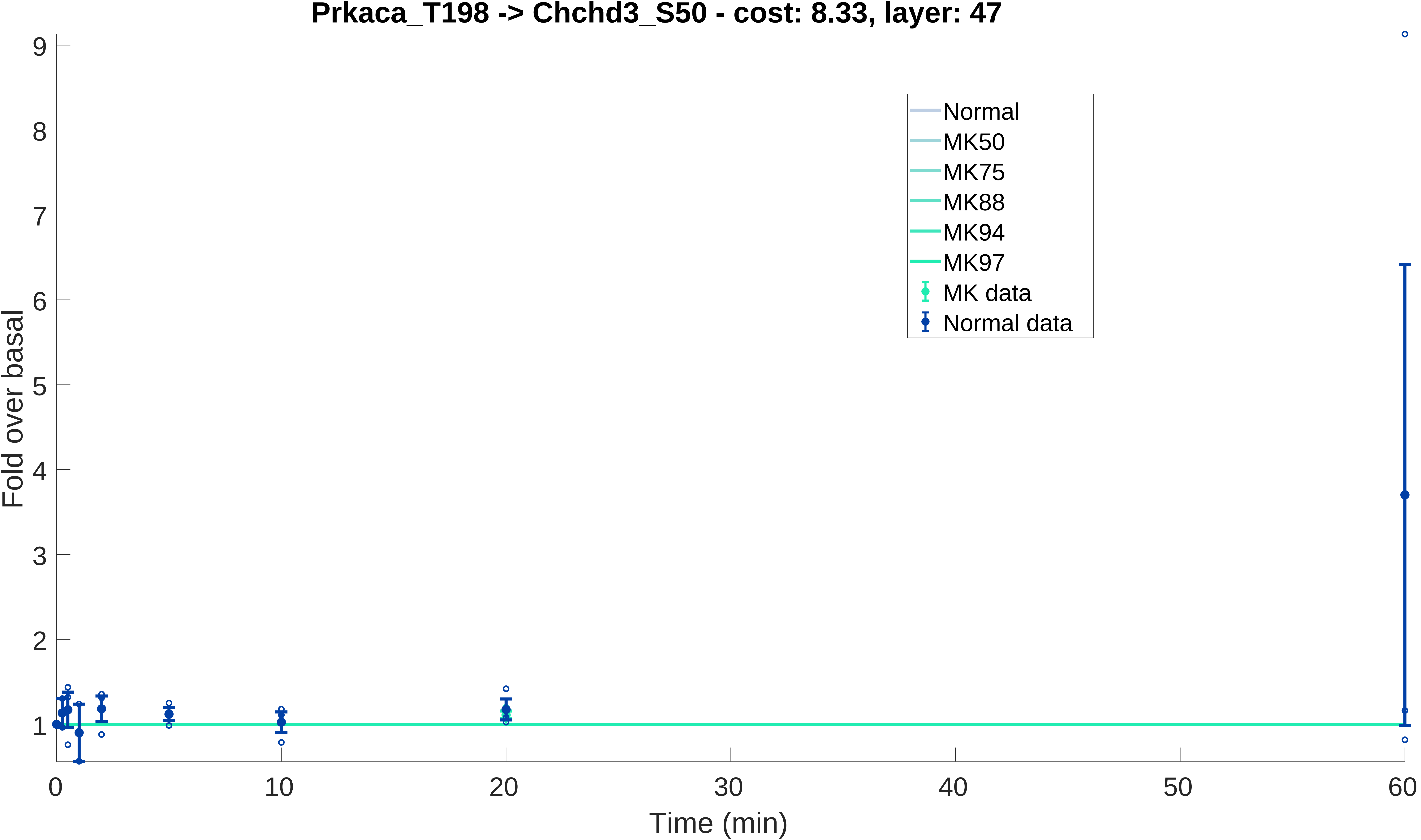

Prrkaca_T198 -> Chchd3_S50 - cost: 8.33, layer: 47
Fold over basal
Time (min)
0
10
20
30
40
50
60
1
2
3
4
5
6
7
8
9
Normal
MK50
MK75
MK88
MK94
MK97
MK data
Normal data

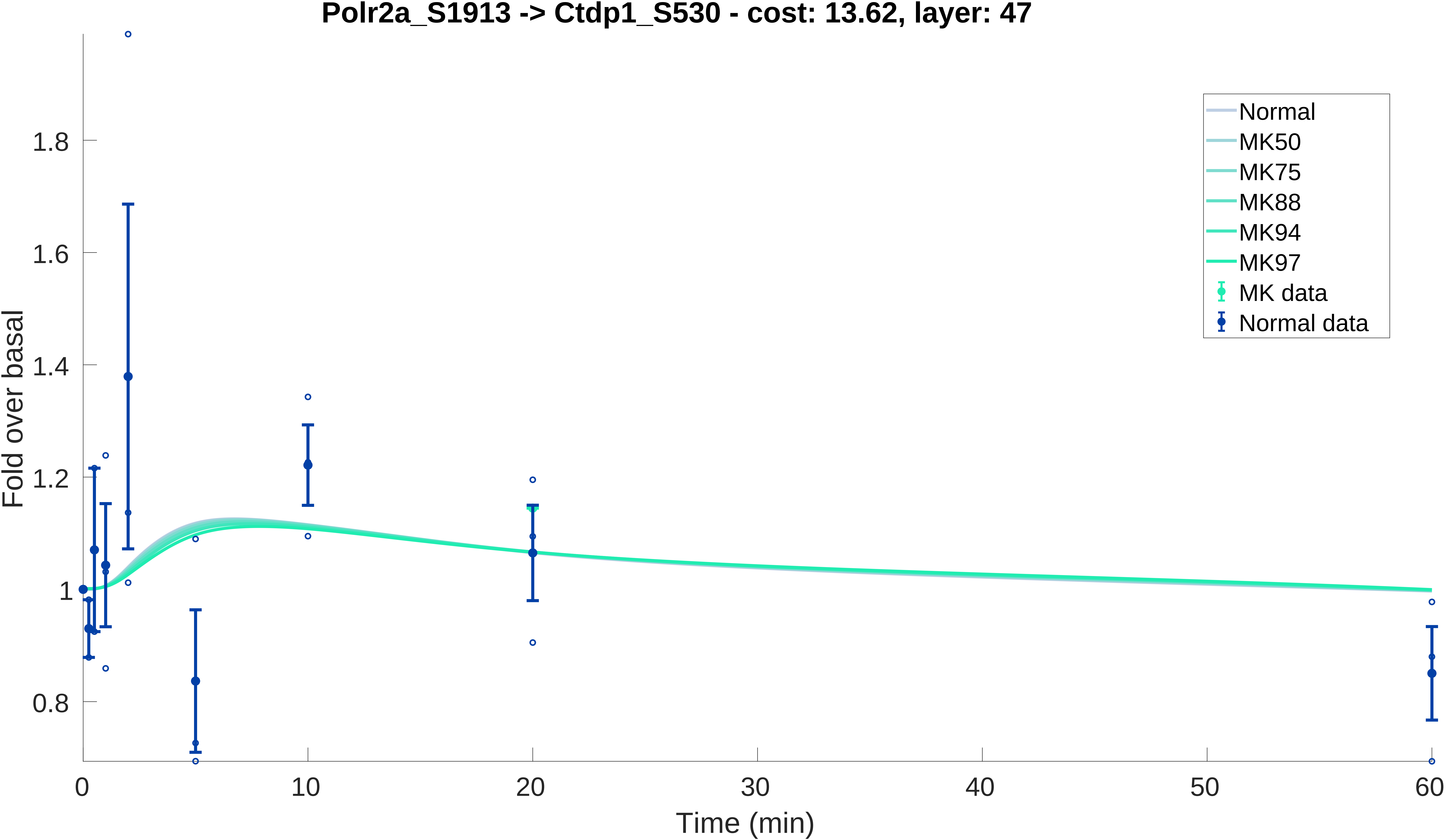

Polr2a_S1913 -> Ctdp1_S530 - cost: 13.62, layer: 47
Fold over basal
1.8
1.6
1.4
1.2
1
0.8
0
10
20
30
40
50
60
Time (min)
Normal
MK50
MK75
MK88
MK94
MK97
MK data
Normal data

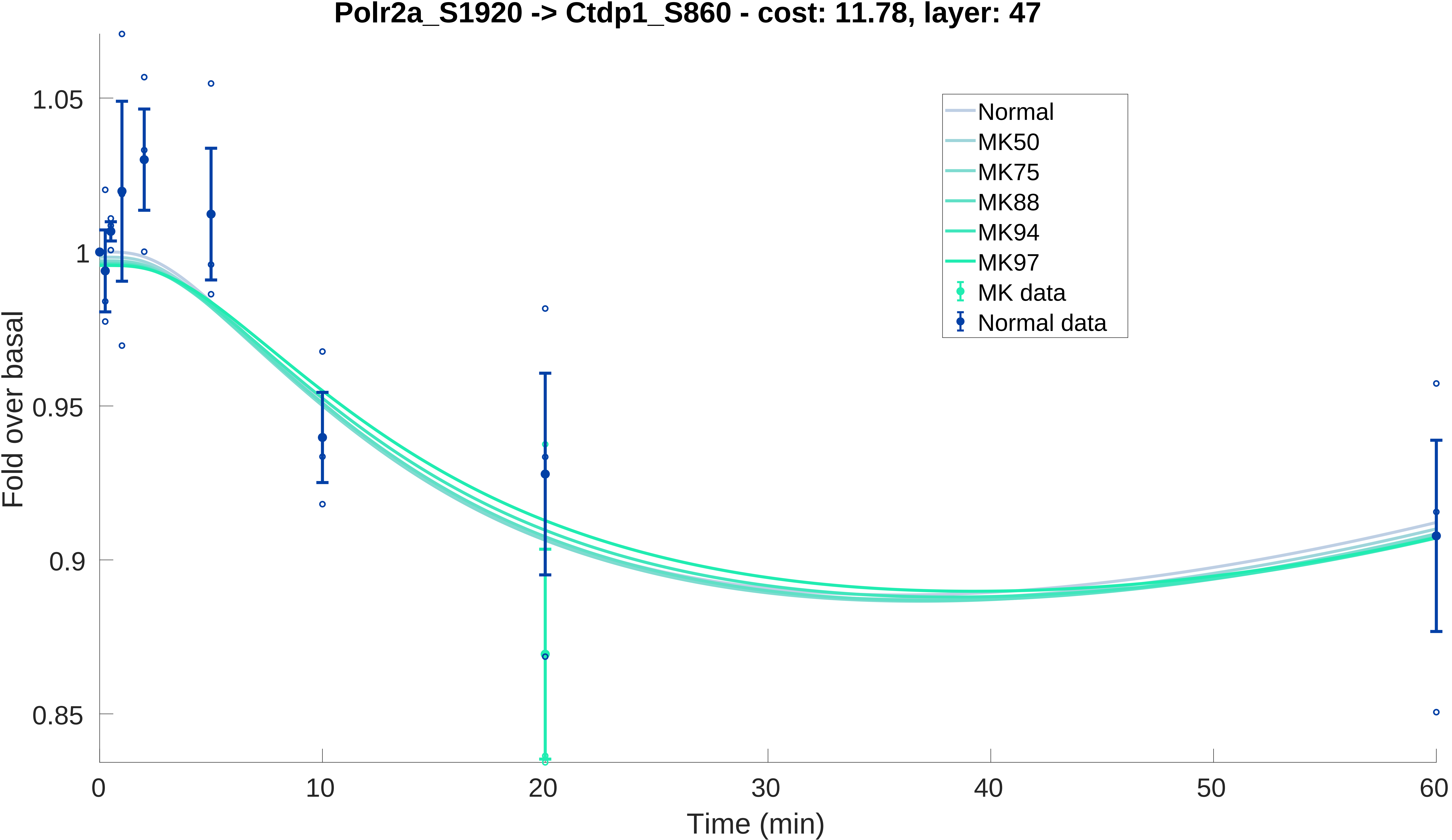

Polr2a_S1920 -> Ctdp1_S860 - cost: 11.78, layer: 47
Fold over basal
1.05
1
0.95
0.9
0.85
0
10
20
30
40
50
60
Time (min)
Normal
MK50
MK75
MK88
MK94
MK97
MK data
Normal data

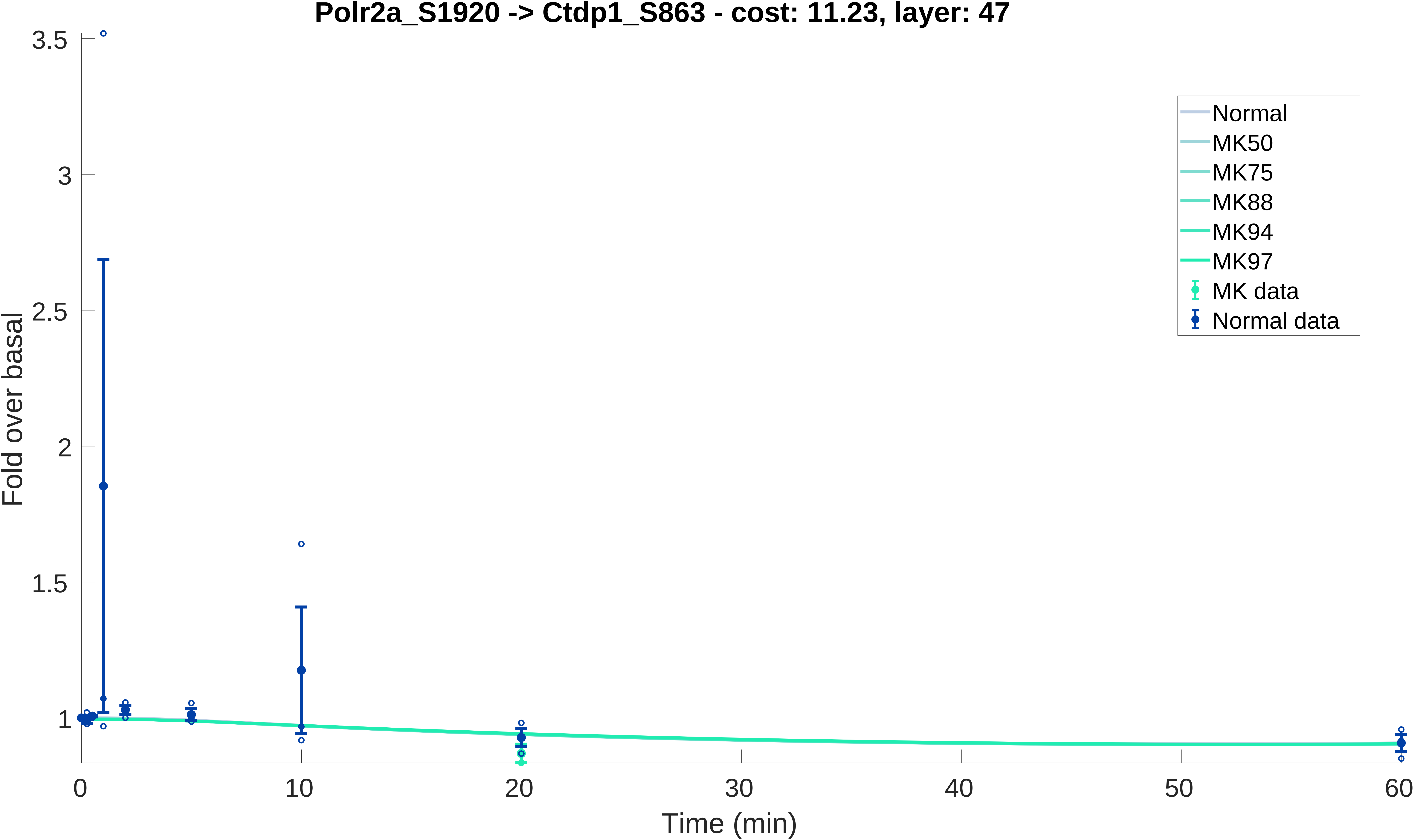
Polr2a_S1920 -> Ctdp1_S863 - cost: 11.23, layer: 47
Fold over basal
3.5
3
2.5
2
1.5
1
0
10
20
30
40
50
60
Time (min)
Normal
MK50
MK75
MK88
MK94
MK97
MK data
Normal data

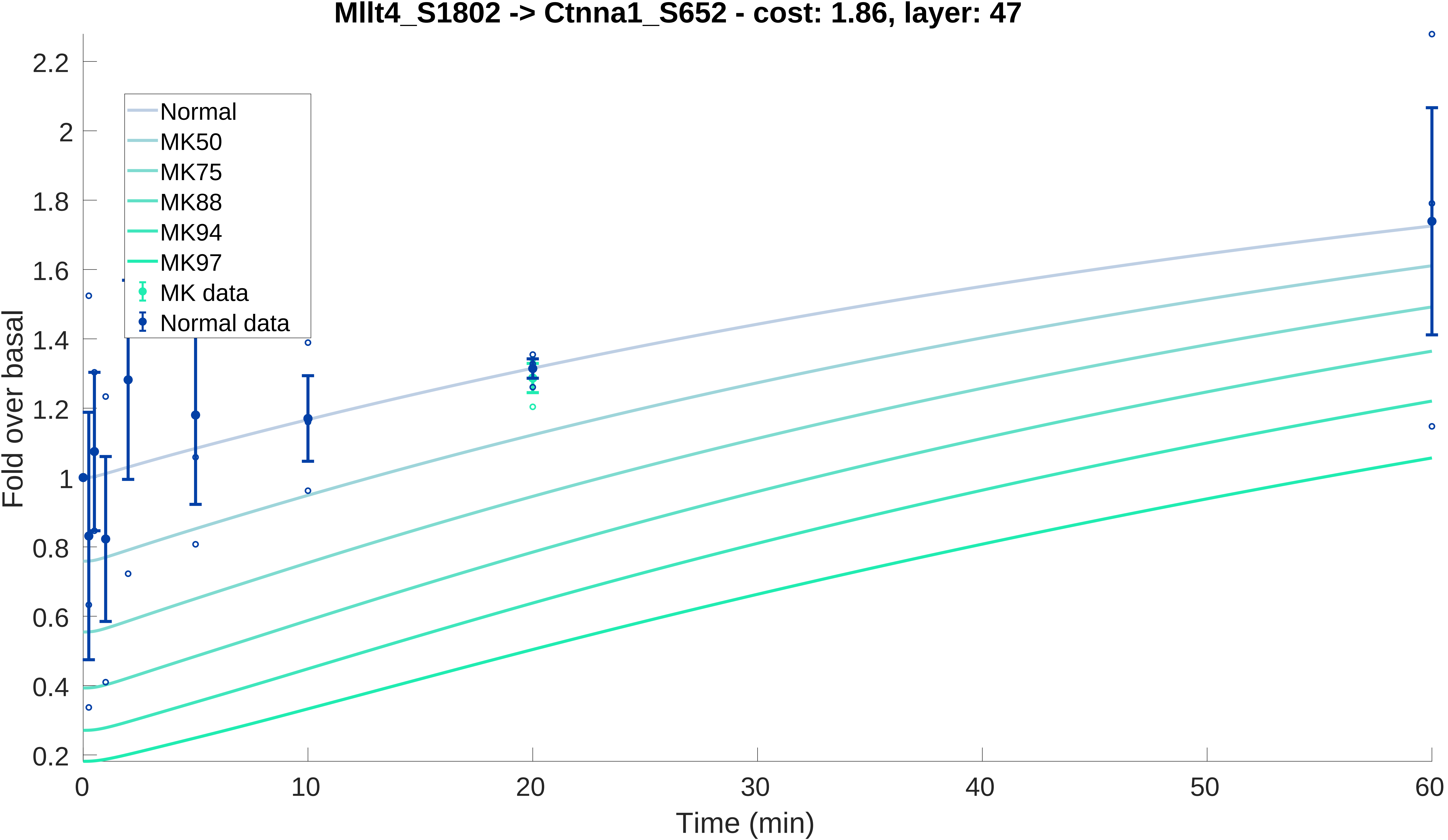

Mllt4_S1802 -> Ctnna1_S652 - cost: 1.86, layer: 47
Fold over basal
Time (min)
0
10
20
30
40
50
60
0.2
0.4
0.6
0.8
1
1.2
1.4
1.6
1.8
2
2.2
Normal
MK50
MK75
MK88
MK94
MK97
MK data
Normal data

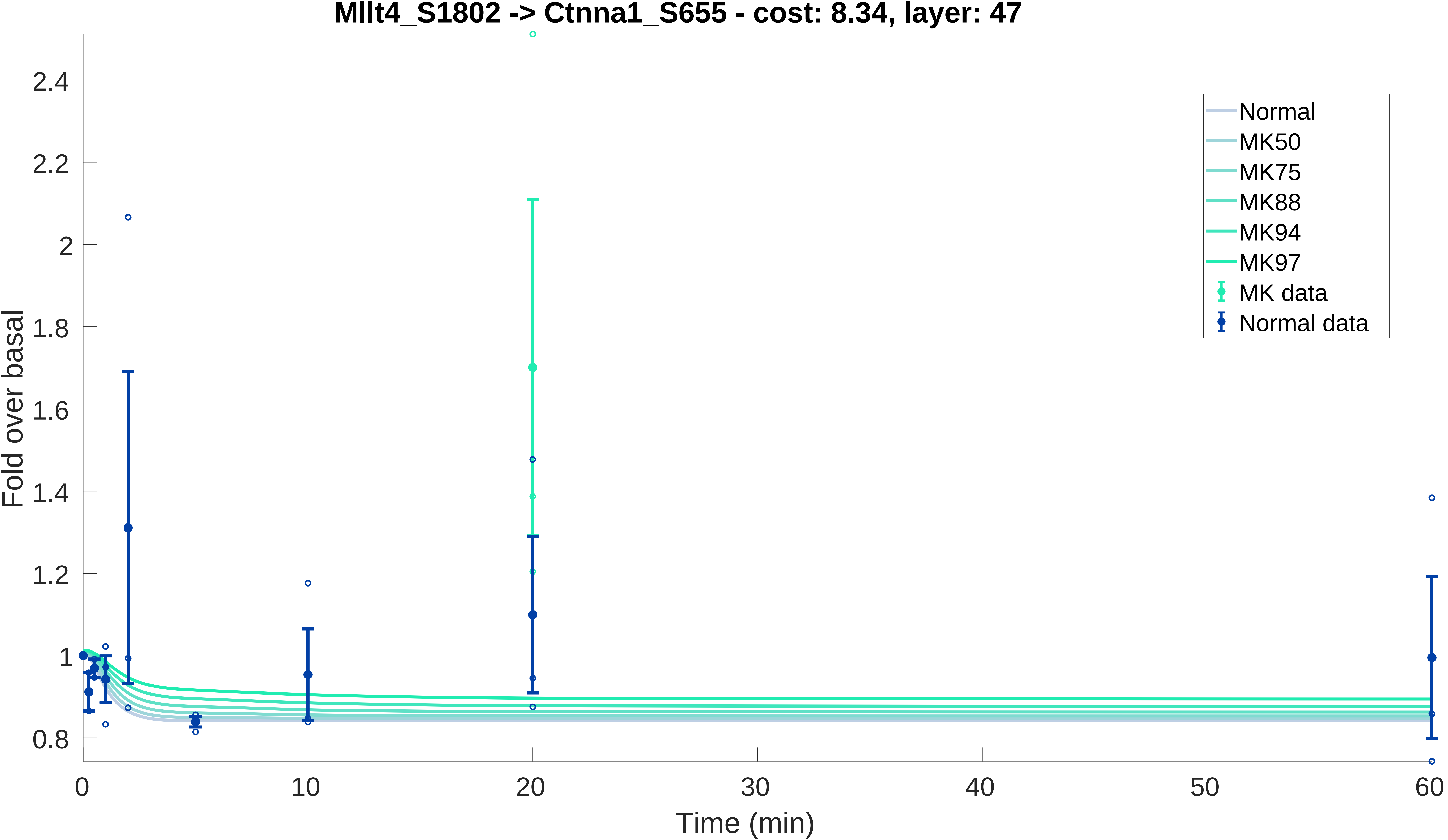

Mllt4_S1802 -> Ctnna1_S655 - cost: 8.34, layer: 47
Fold over basal
Time (min)
0.8
1
1.2
1.4
1.6
1.8
2
2.2
2.4
0
10
20
30
40
50
60
Normal
MK50
MK75
MK88
MK94
MK97
MK data
Normal data

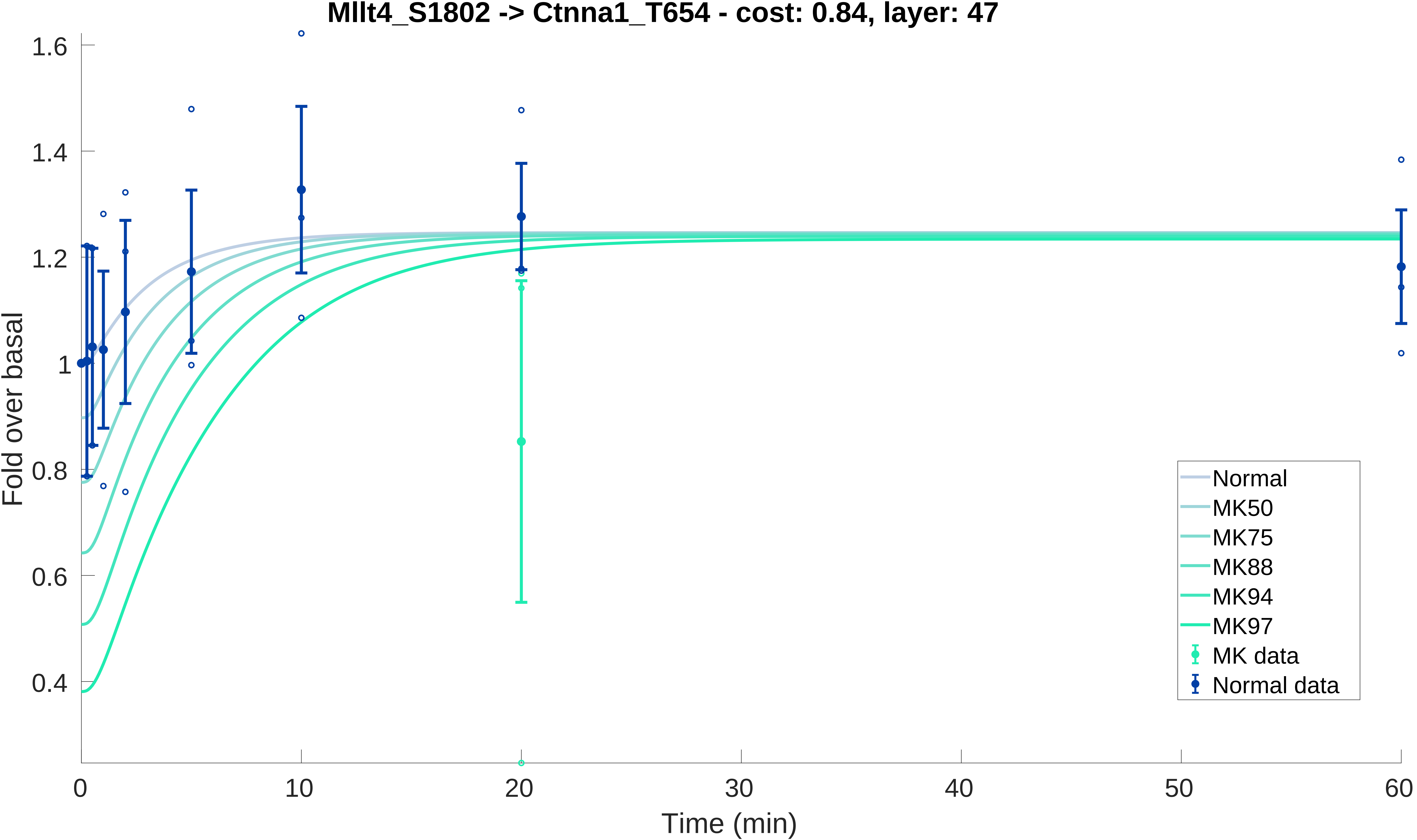
Mllt4_S1802 -> Ctnna1_T654 - cost: 0.84, layer: 47
Fold over basal
1.6
1.4
1.2
1
0.8
0.6
0.4
0
10
20
30
40
50
60
Time (min)
Normal
MK50
MK75
MK88
MK94
MK97
MK data
Normal data

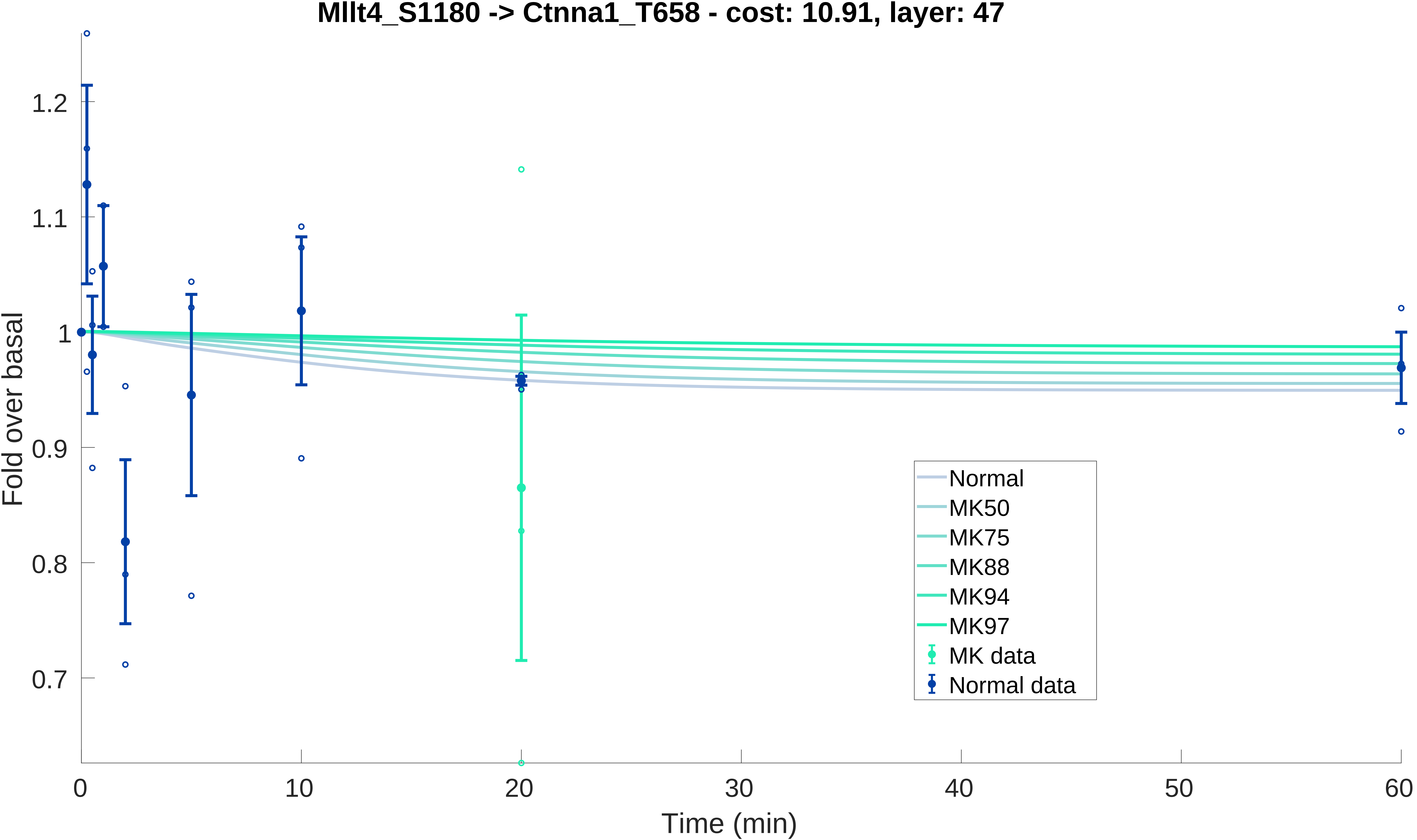

Mllt4_S1180 -> Ctnna1_T658 - cost: 10.91, layer: 47
Fold over basal
1.2
1.1
1
0.9
0.8
0.7
0
10
20
30
40
50
60
Time (min)
Normal
MK50
MK75
MK88
MK94
MK97
MK data
Normal data

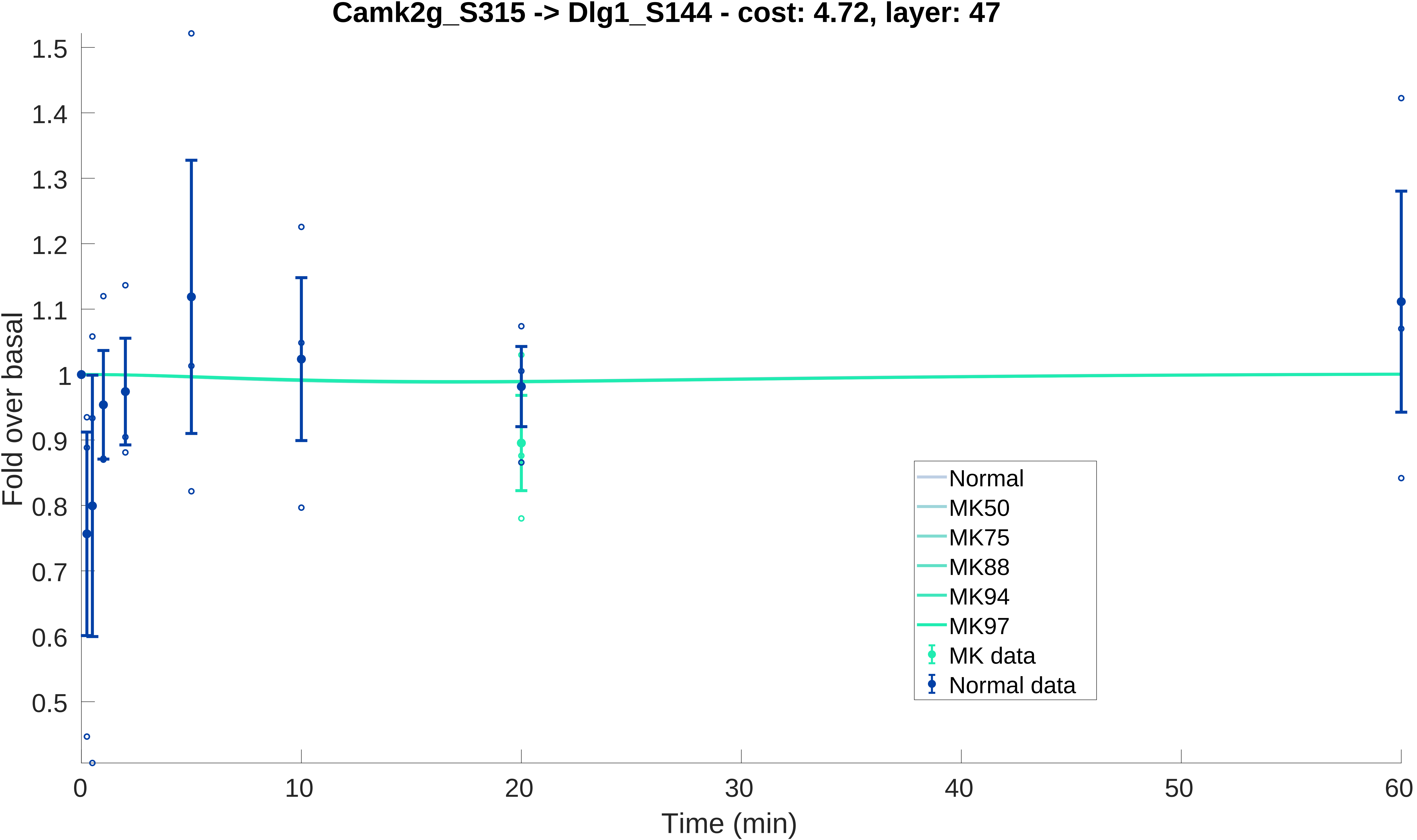
Camk2g_S315 -> Dlg1_S144 - cost: 4.72, layer: 47
Fold over basal
Time (min)
0
10
20
30
40
50
60
0.5
0.6
0.7
0.8
0.9
1
1.1
1.2
1.3
1.4
1.5
Normal
MK50
MK75
MK88
MK94
MK97
MK data
Normal data

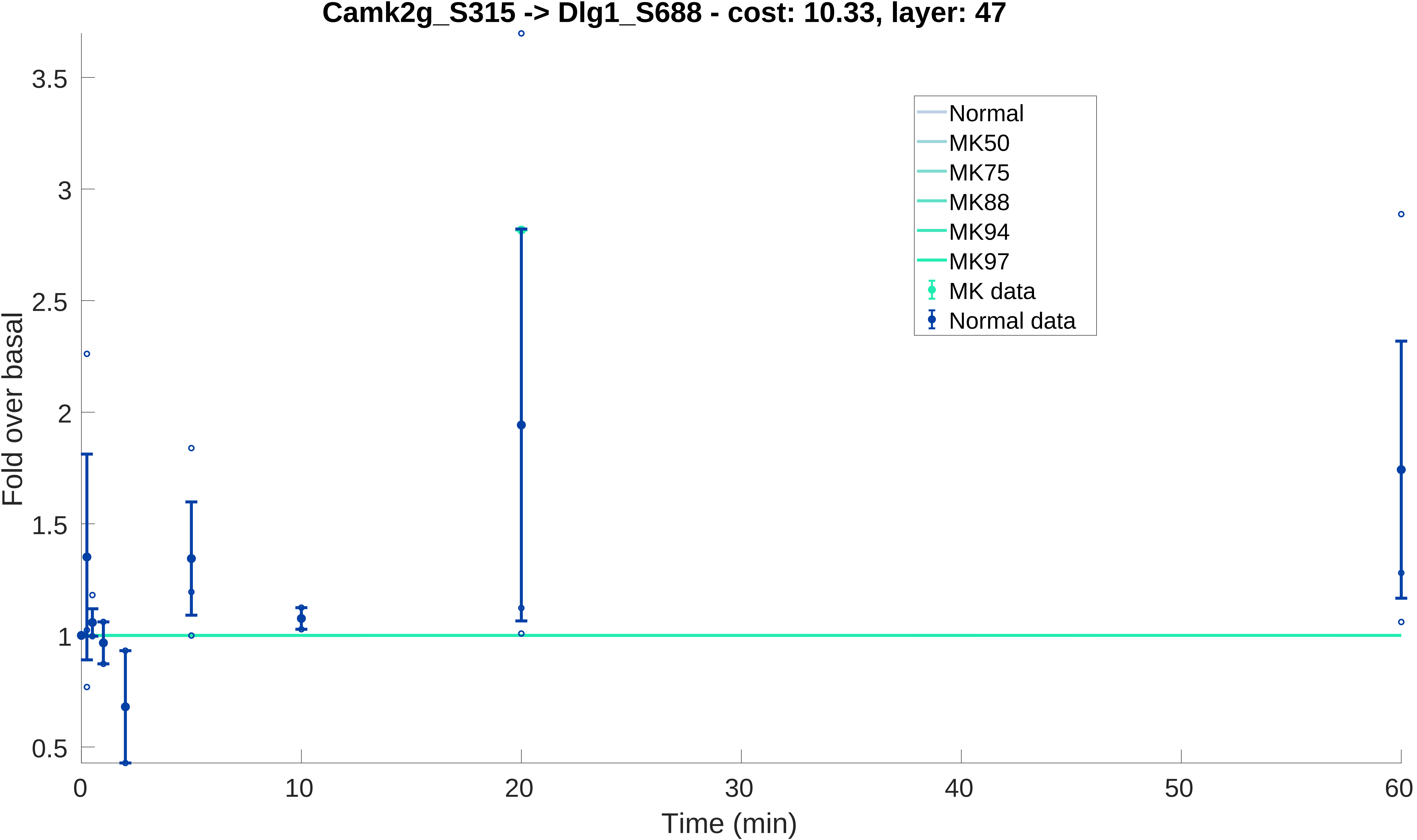
Camk2g_S315 -> Dlg1_S688 - cost: 10.33, layer: 47
Fold over basal
Time (min)
3.5
3
2.5
2
1.5
1
0.5
0
10
20
30
40
50
60
Normal
MK50
MK75
MK88
MK94
MK97
MK data
Normal data

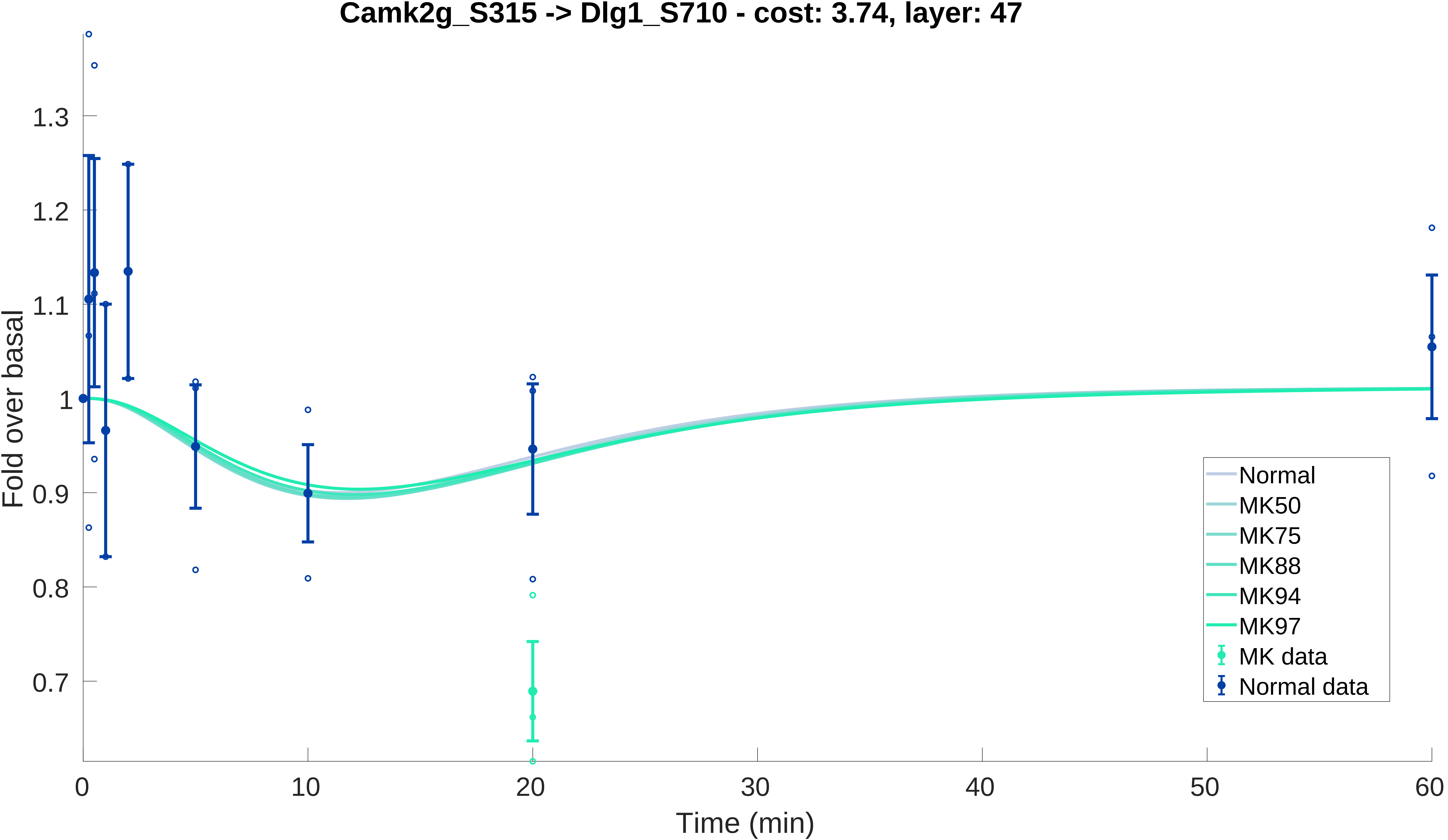

Camk2g_S315 -> Dlg1_S710 - cost: 3.74, layer: 47
Fold over basal
1.3
1.2
1.1
1
0.9
0.8
0.7
0
10
20
30
40
50
60
Time (min)
Normal
MK50
MK75
MK88
MK94
MK97
MK data
Normal data

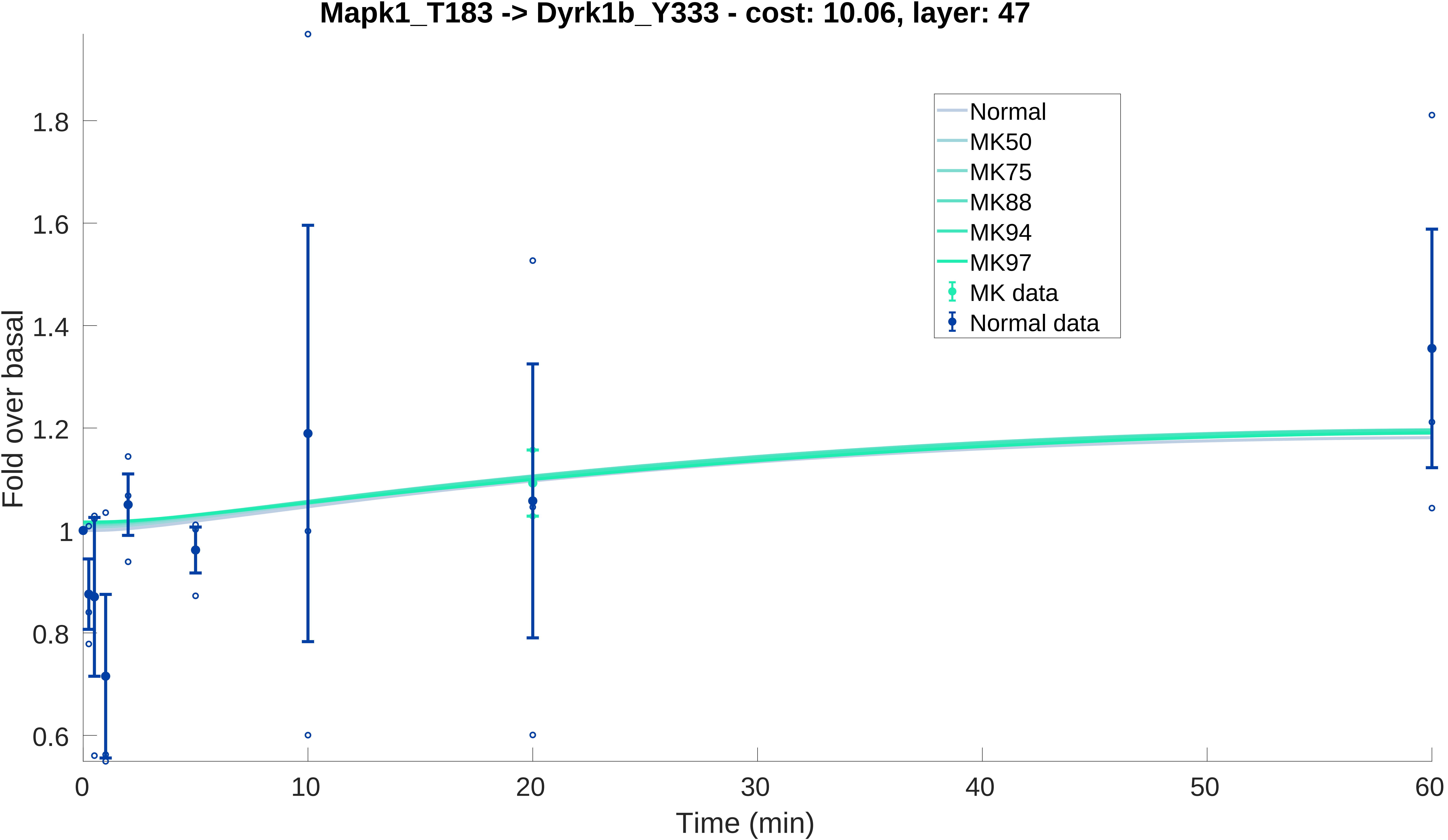

Mapk1_T183 -> Dyrk1b_Y333 - cost: 10.06, layer: 47
Normal
MK50
MK75
MK88
MK94
MK97
MK data
Normal data
Fold over basal
1.8
1.6
1.4
1.2
1
0.8
0.6
0
10
20
30
40
50
60
Time (min)

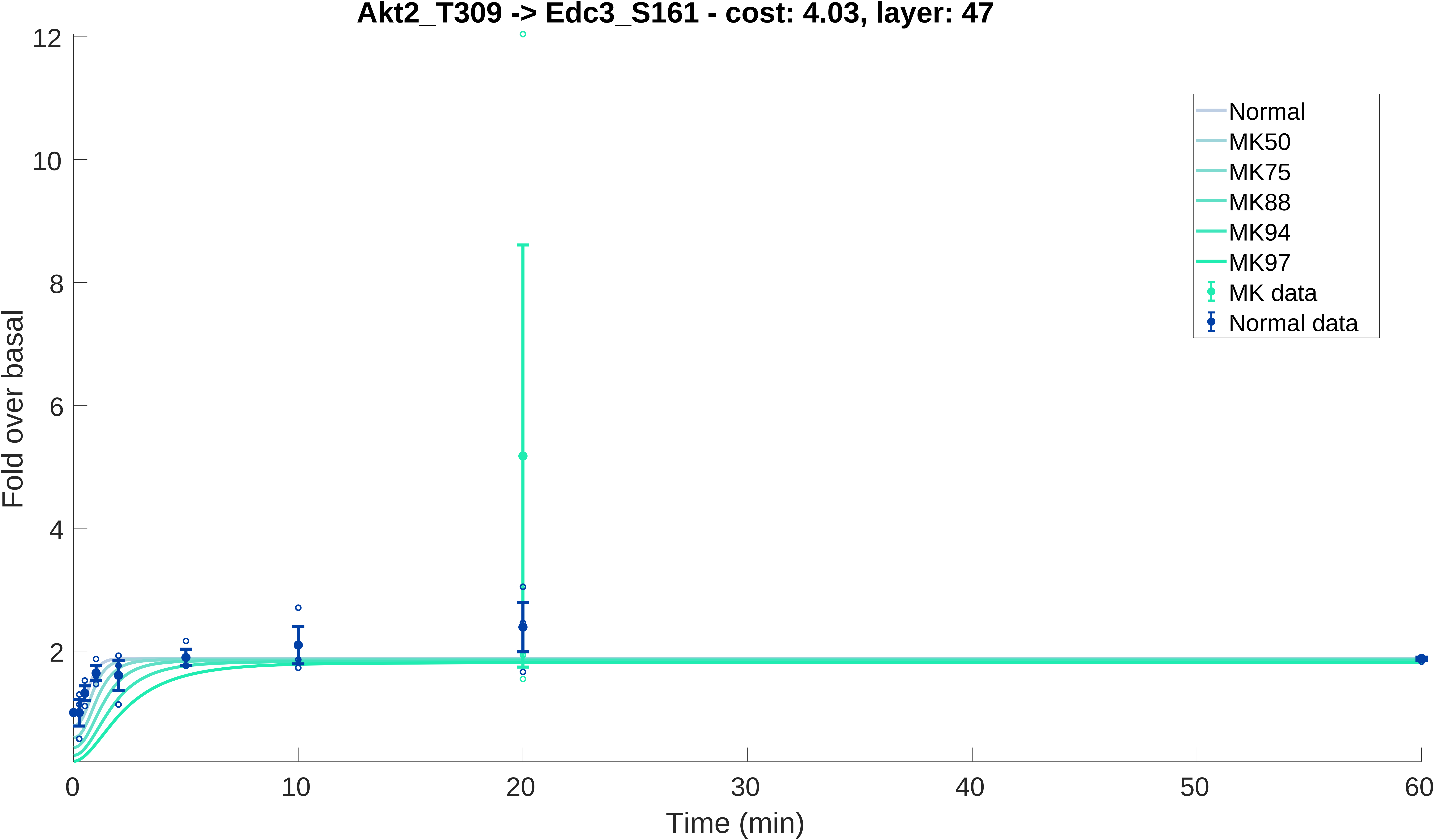

Akt2_T309 -> Edc3_S161 - cost: 4.03, layer: 47
Fold over basal
Time (min)
0
10
20
30
40
50
60
2
4
6
8
10
12
Normal
MK50
MK75
MK88
MK94
MK97
MK data
Normal data

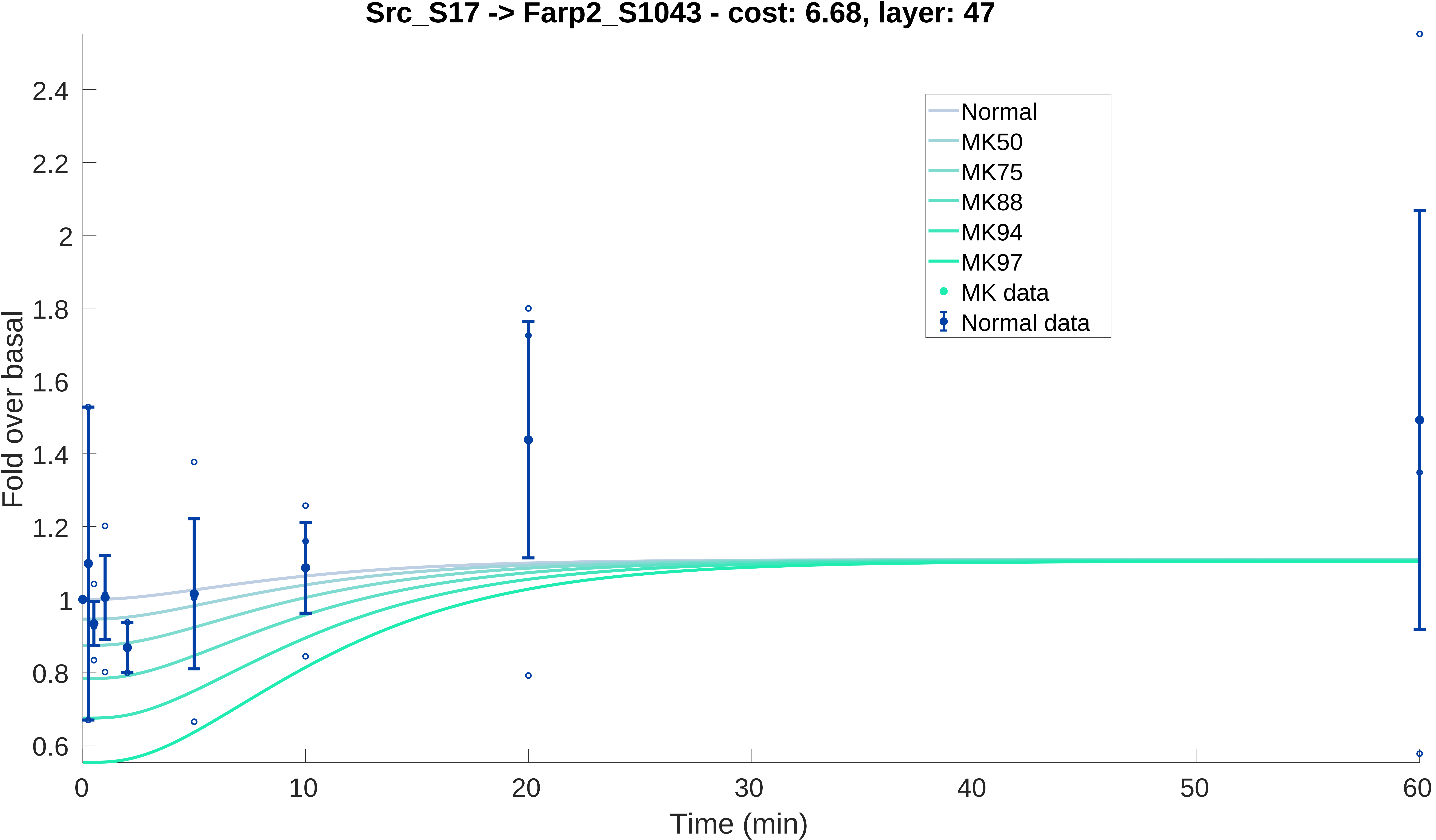

Src_S17 -> Farp2_S1043 - cost: 6.68, layer: 47
Fold over basal
2.4
2.2
2
1.8
1.6
1.4
1.2
1
0.8
0.6
0
10
20
30
40
50
60
Time (min)
Normal
MK50
MK75
MK88
MK94
MK97
MK data
Normal data

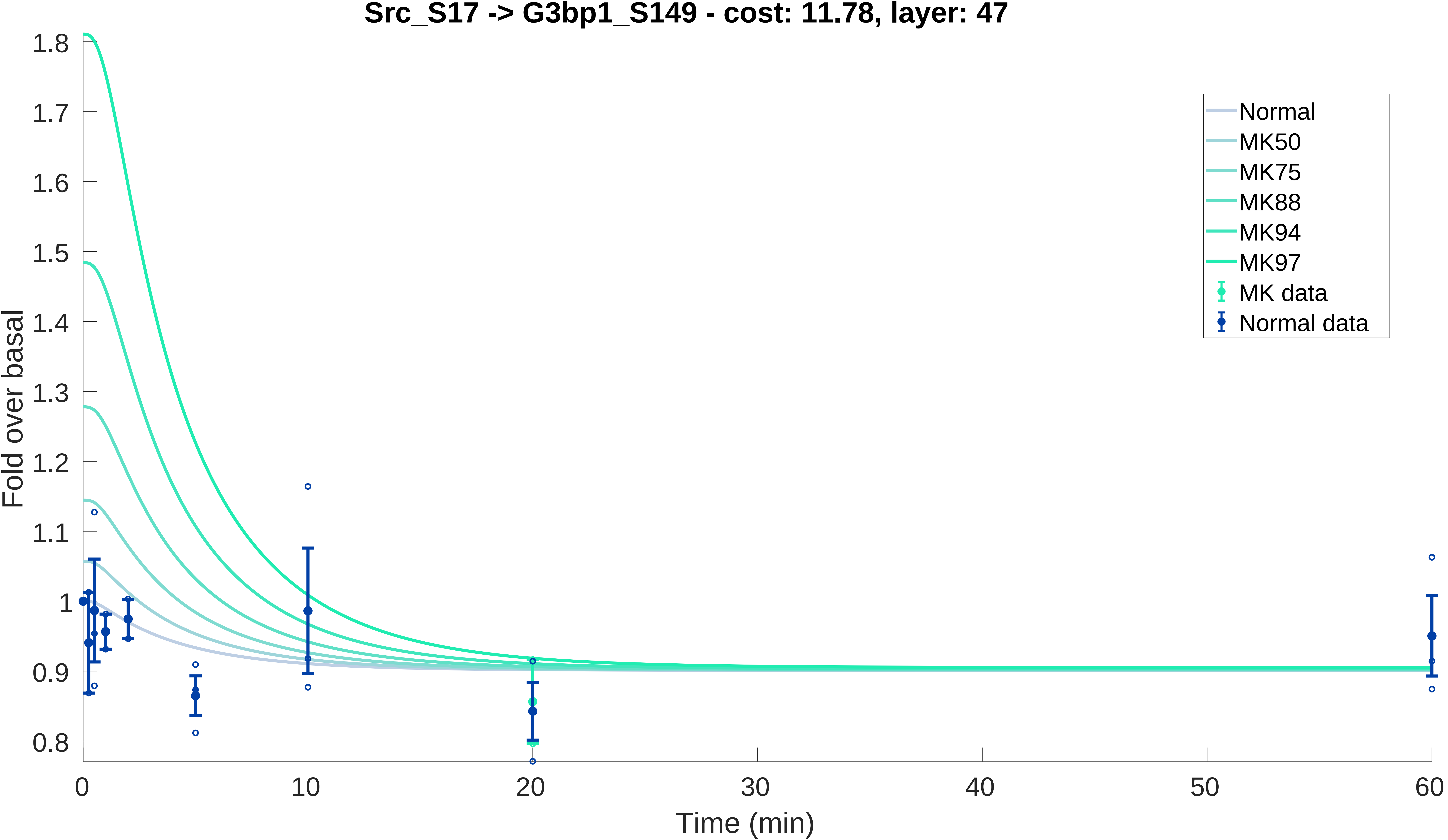
Src_S17 -> G3bp1_S149 - cost: 11.78, layer: 47
Fold over basal
Time (min)
Normal
MK50
MK75
MK88
MK94
MK97
MK data
Normal data
0.8
0.9
1
1.1
1.2
1.3
1.4
1.5
1.6
1.7
1.8
0
10
20
30
40
50
60

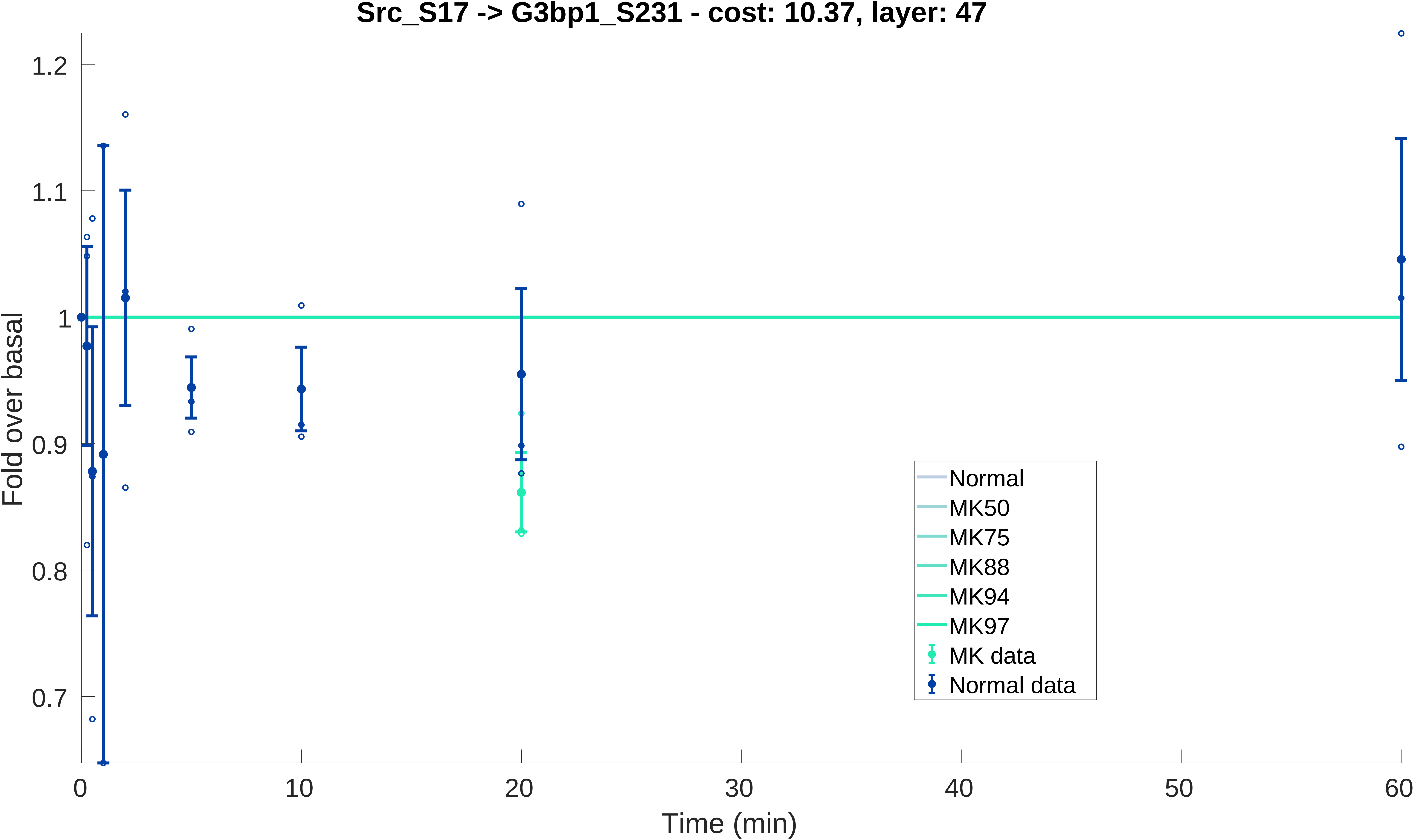

Src_S17 -> G3bp1_S231 - cost: 10.37, layer: 47
Fold over basal
Time (min)
1.2
1.1
1
0.9
0.8
0.7
0
10
20
30
40
50
60
Normal
MK50
MK75
MK88
MK94
MK97
MK data
Normal data

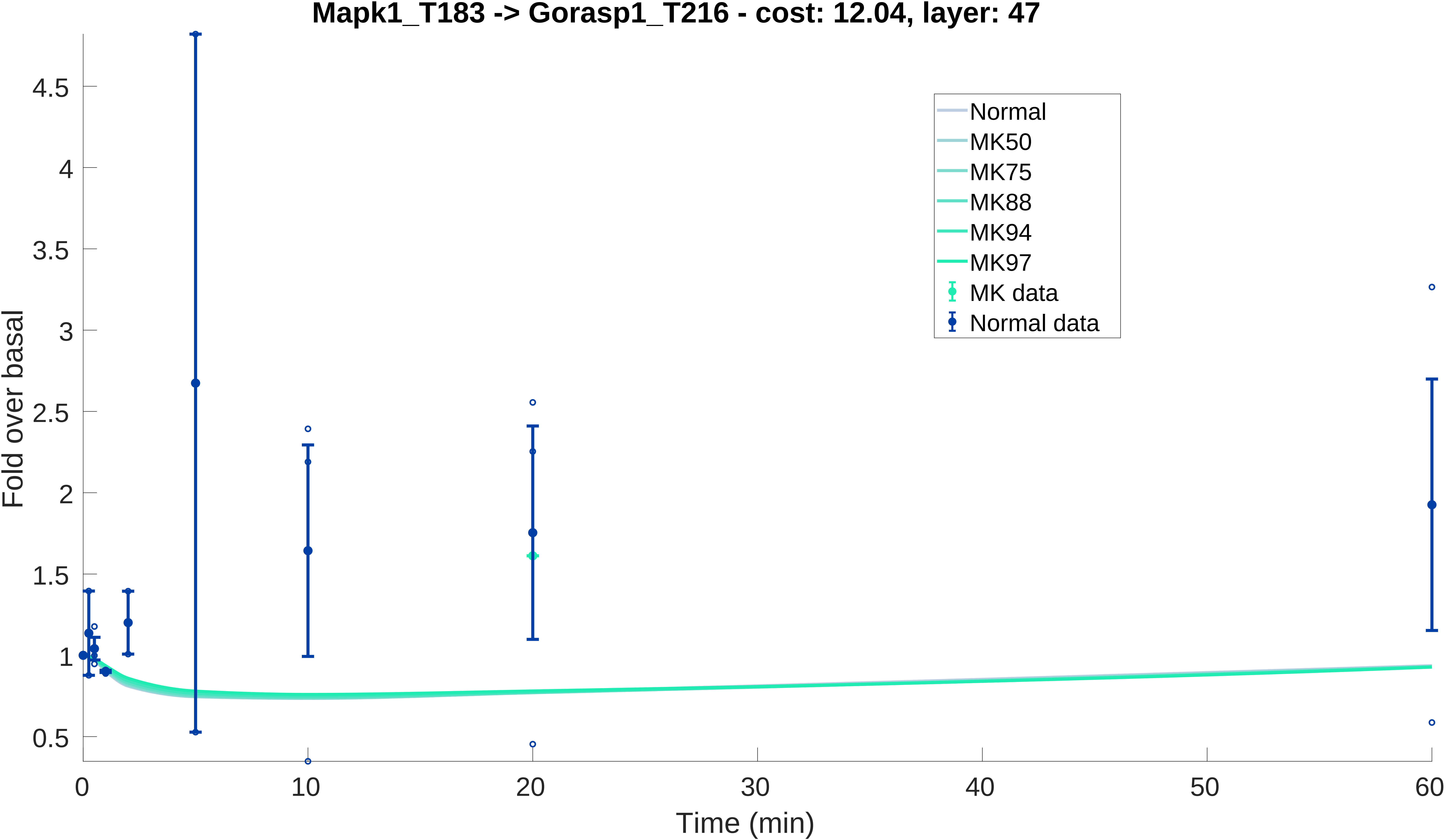

Mapk1_T183 -> Gorasp1_T216 - cost: 12.04, layer: 47
Fold over basal
Time (min)
0.5
1
1.5
2
2.5
3
3.5
4
4.5
0
10
20
30
40
50
60
Normal
MK50
MK75
MK88
MK94
MK97
MK data
Normal data

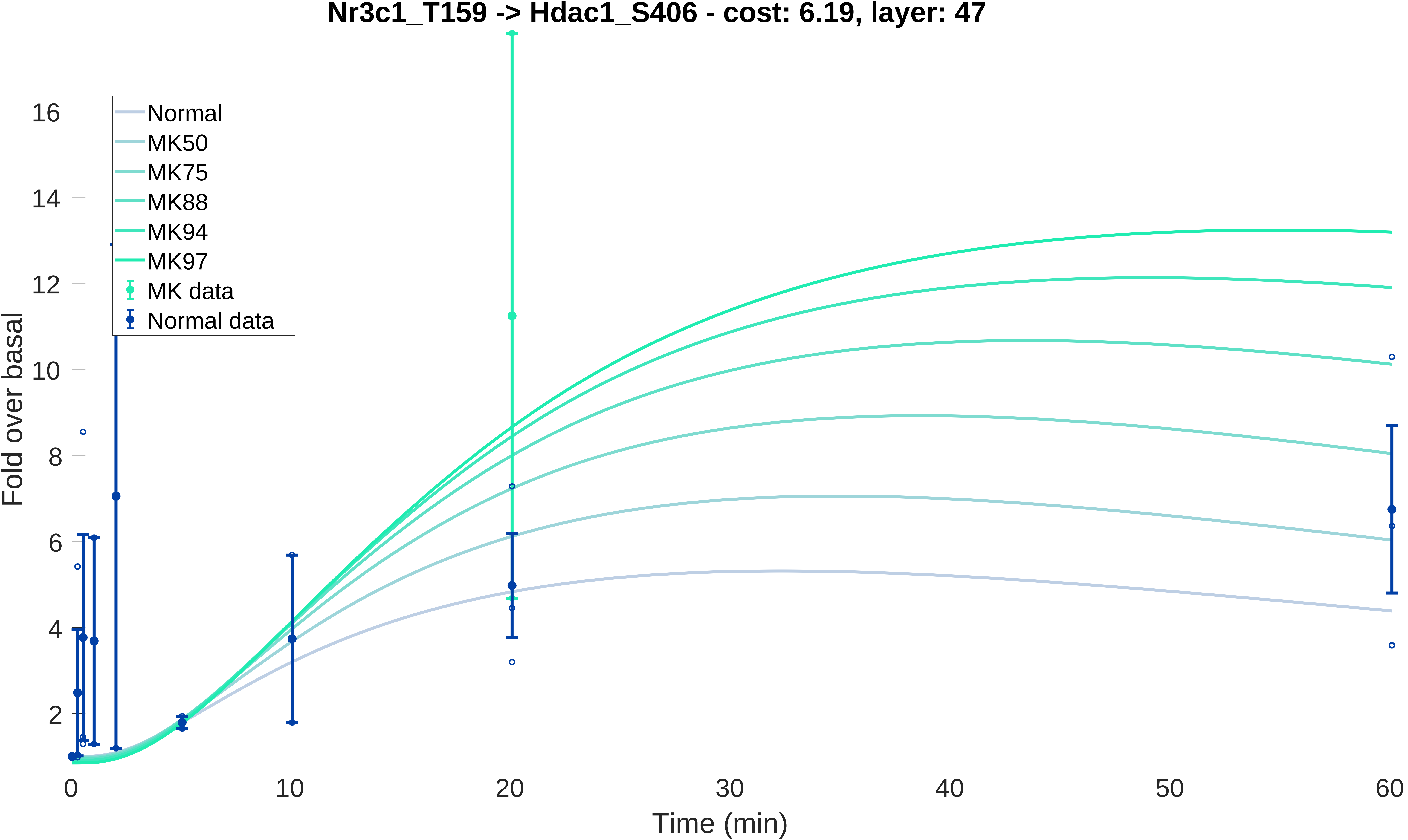

Nr3c1_T159 -> Hdac1_S406 - cost: 6.19, layer: 47
Fold over basal
Time (min)
Normal
MK50
MK75
MK88
MK94
MK97
MK data
Normal data
0
10
20
30
40
50
60
2
4
6
8
10
12
14
16

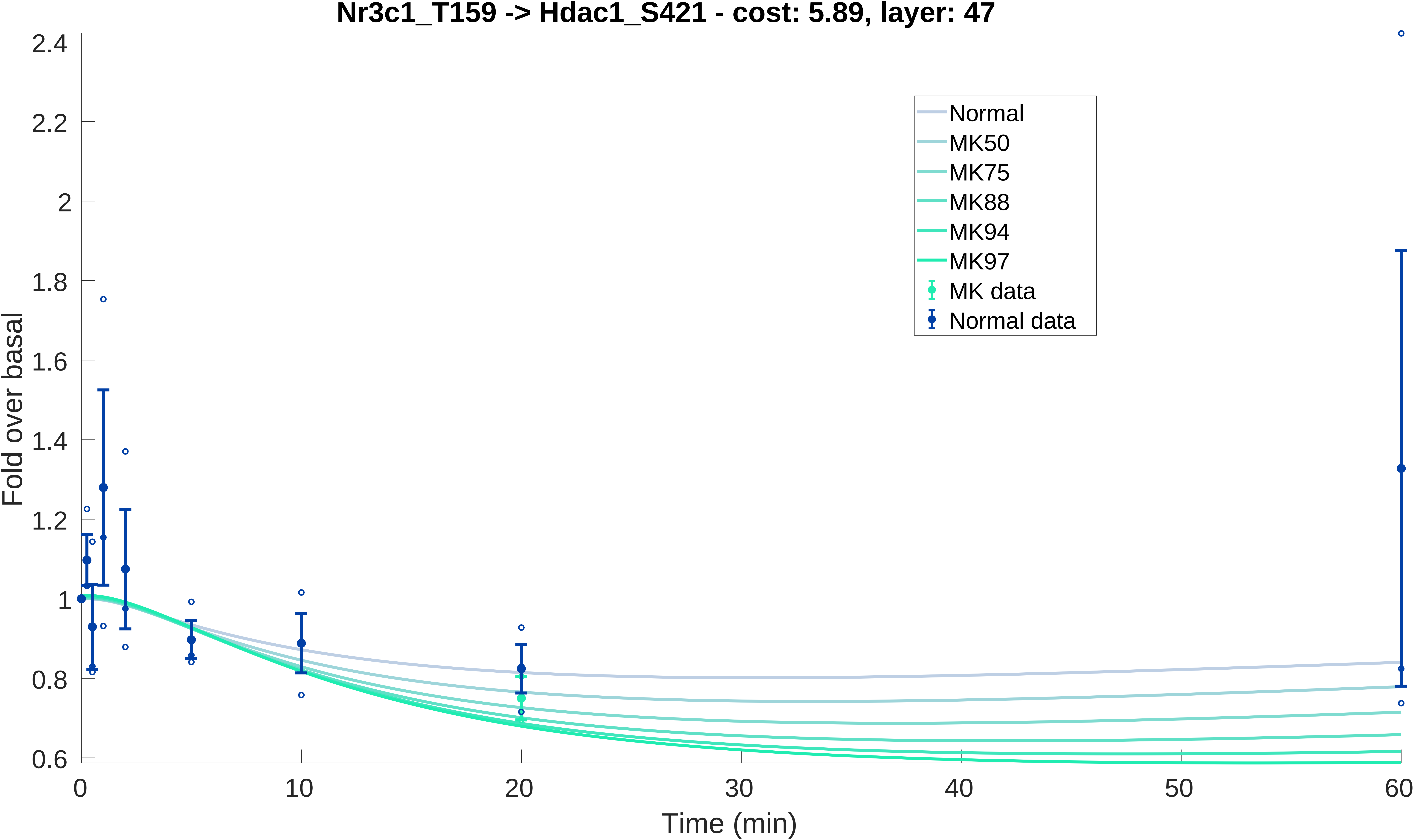

Nr3c1_T159 -> Hdac1_S421 - cost: 5.89, layer: 47
Fold over basal
Time (min)
Normal
MK50
MK75
MK88
MK94
MK97
MK data
Normal data
2.4
2.2
2
1.8
1.6
1.4
1.2
1
0.8
0.6
0
10
20
30
40
50
60

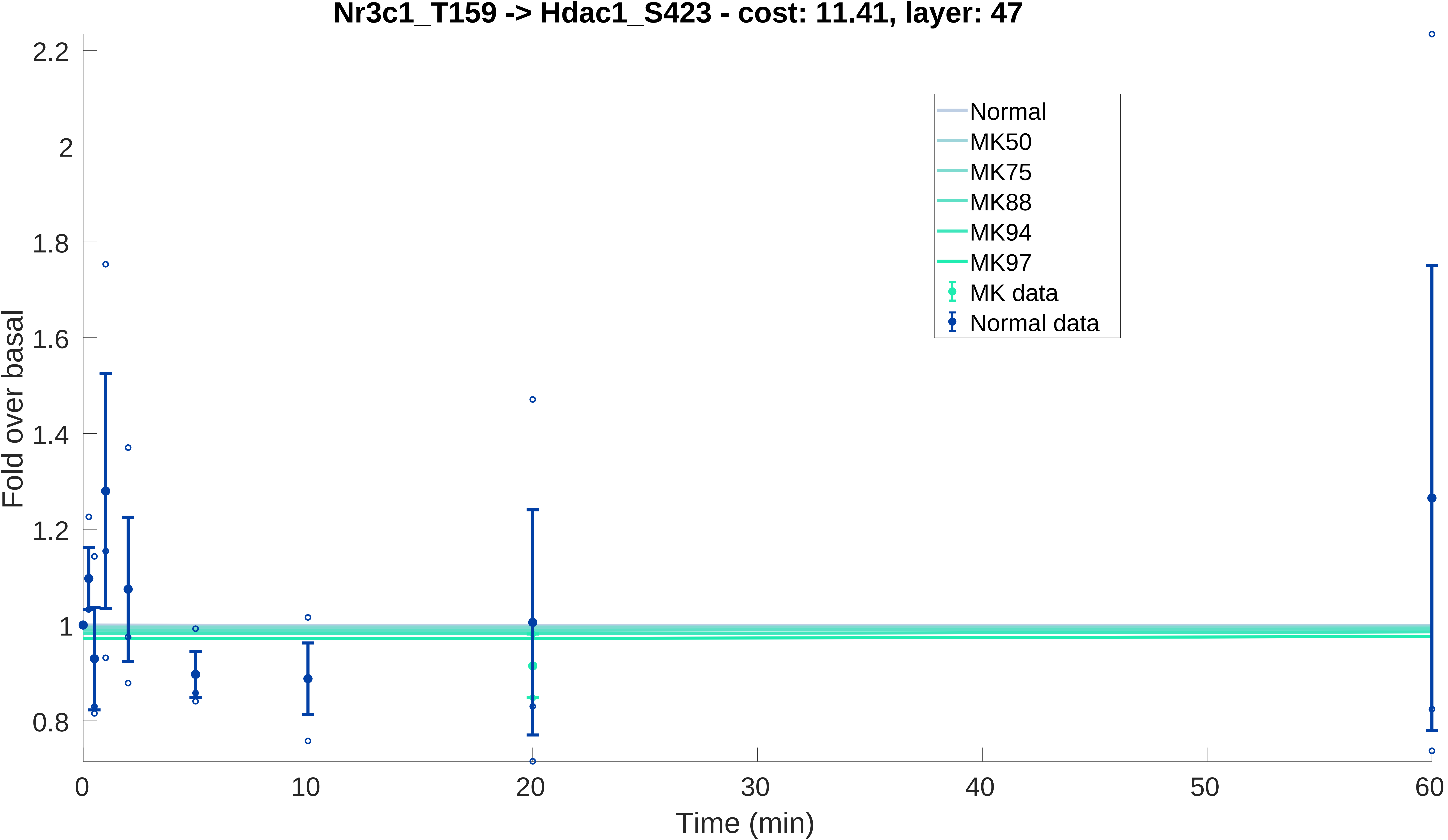

Nr3c1_T159 -> Hdac1_S423 - cost: 11.41, layer: 47
Fold over basal
Time (min)
0.8
1
1.2
1.4
1.6
1.8
2
2.2
0
10
20
30
40
50
60
Normal
MK50
MK75
MK88
MK94
MK97
MK data
Normal data

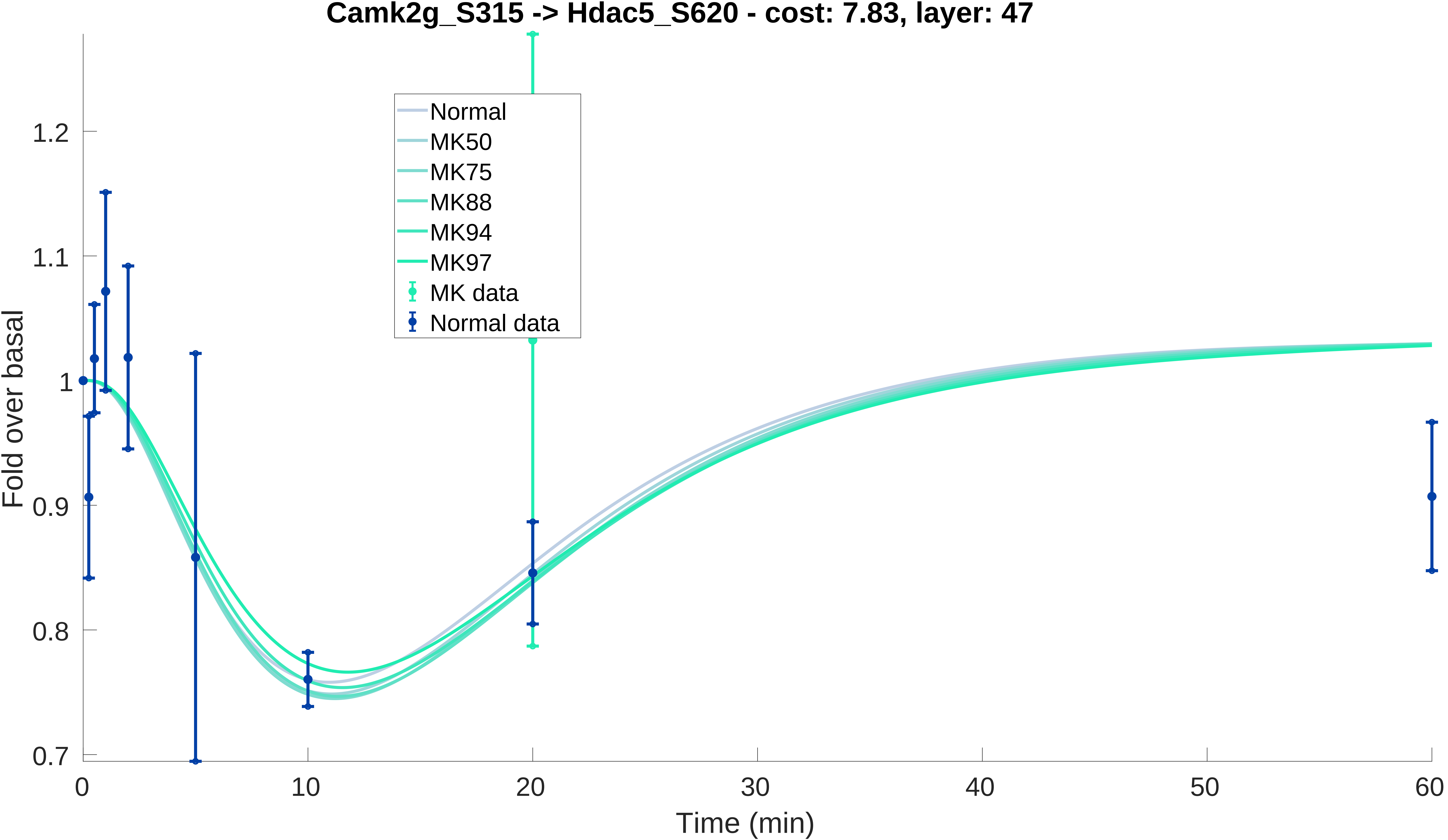
Camk2g_S315 -> Hdac5_S620 - cost: 7.83, layer: 47
Normal
MK50
MK75
MK88
MK94
MK97
MK data
Normal data
Fold over basal
1.2
1.1
1
0.9
0.8
0.7
0
10
20
30
40
50
60
Time (min)

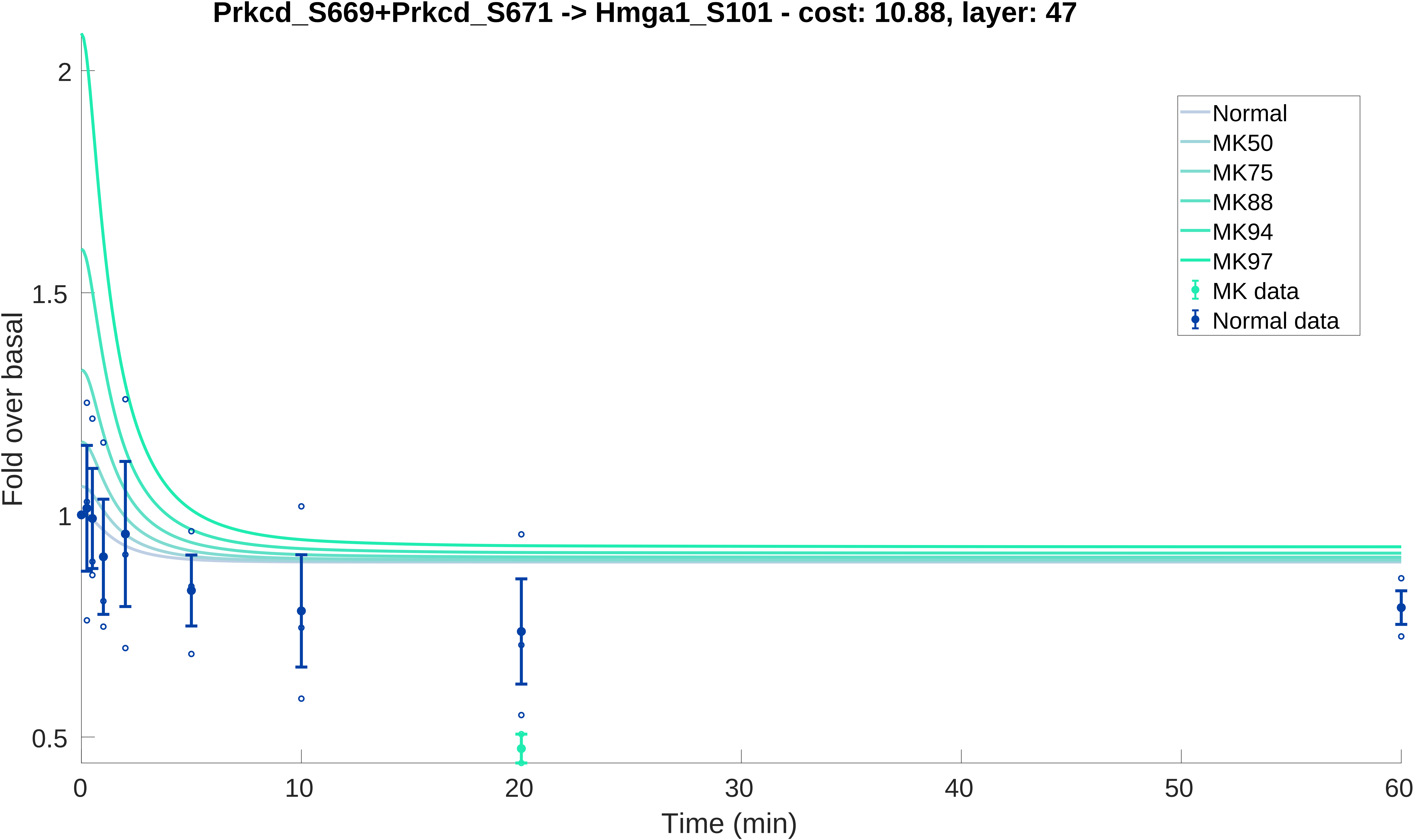

Prkcd_S669+Prkcd_S671 -> Hmga1_S101 - cost: 10.88, layer: 47
Fold over basal
Time (min)
0.5
1
1.5
2
0
10
20
30
40
50
60
Normal
MK50
MK75
MK88
MK94
MK97
MK data
Normal data

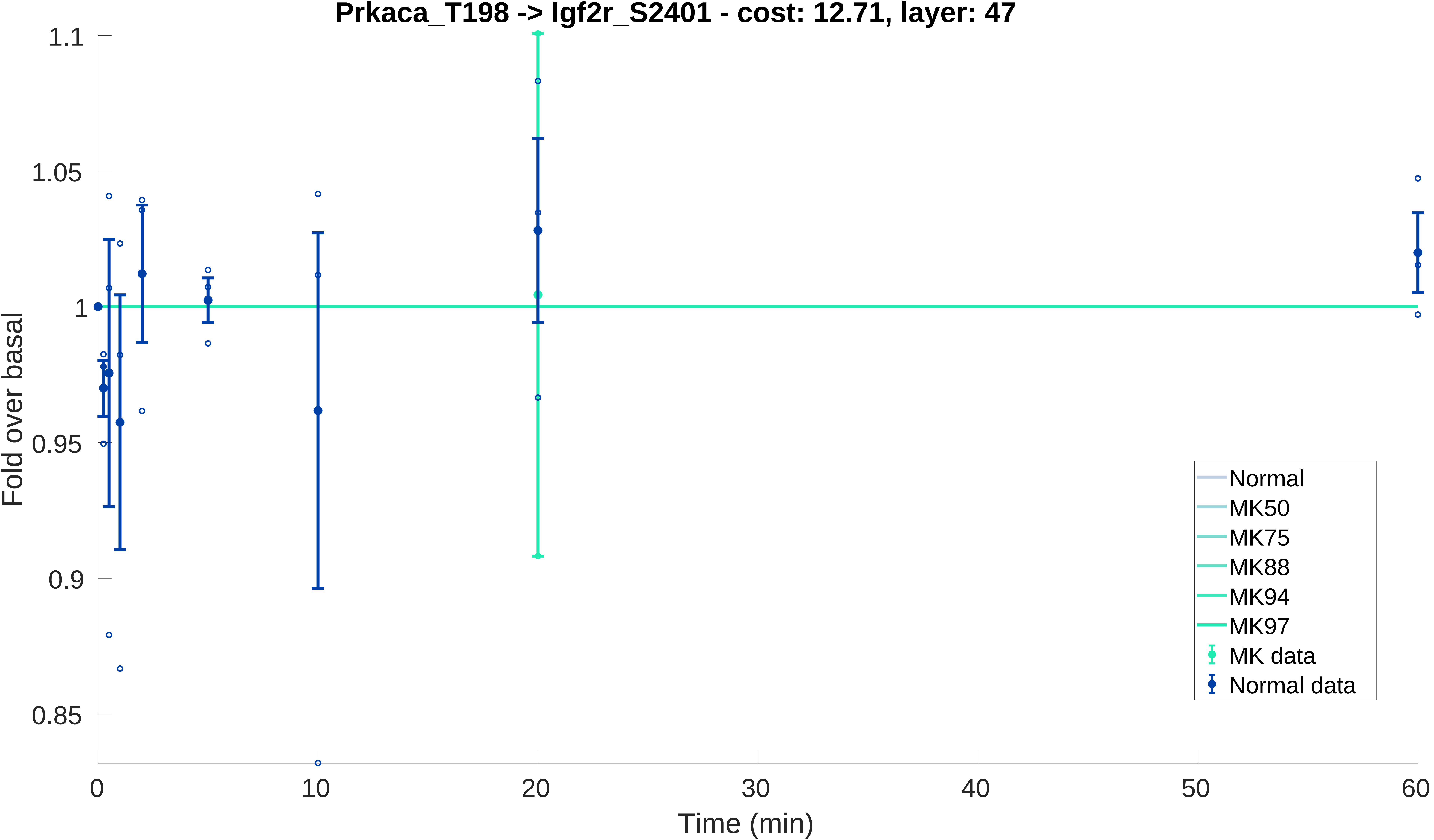

Prkaca_T198 -> Igf2r_S2401 - cost: 12.71, layer: 47
Fold over basal
1.1
1.05
1
0.95
0.9
0.85
0
10
20
30
40
50
60
Time (min)
Normal
MK50
MK75
MK88
MK94
MK97
MK data
Normal data

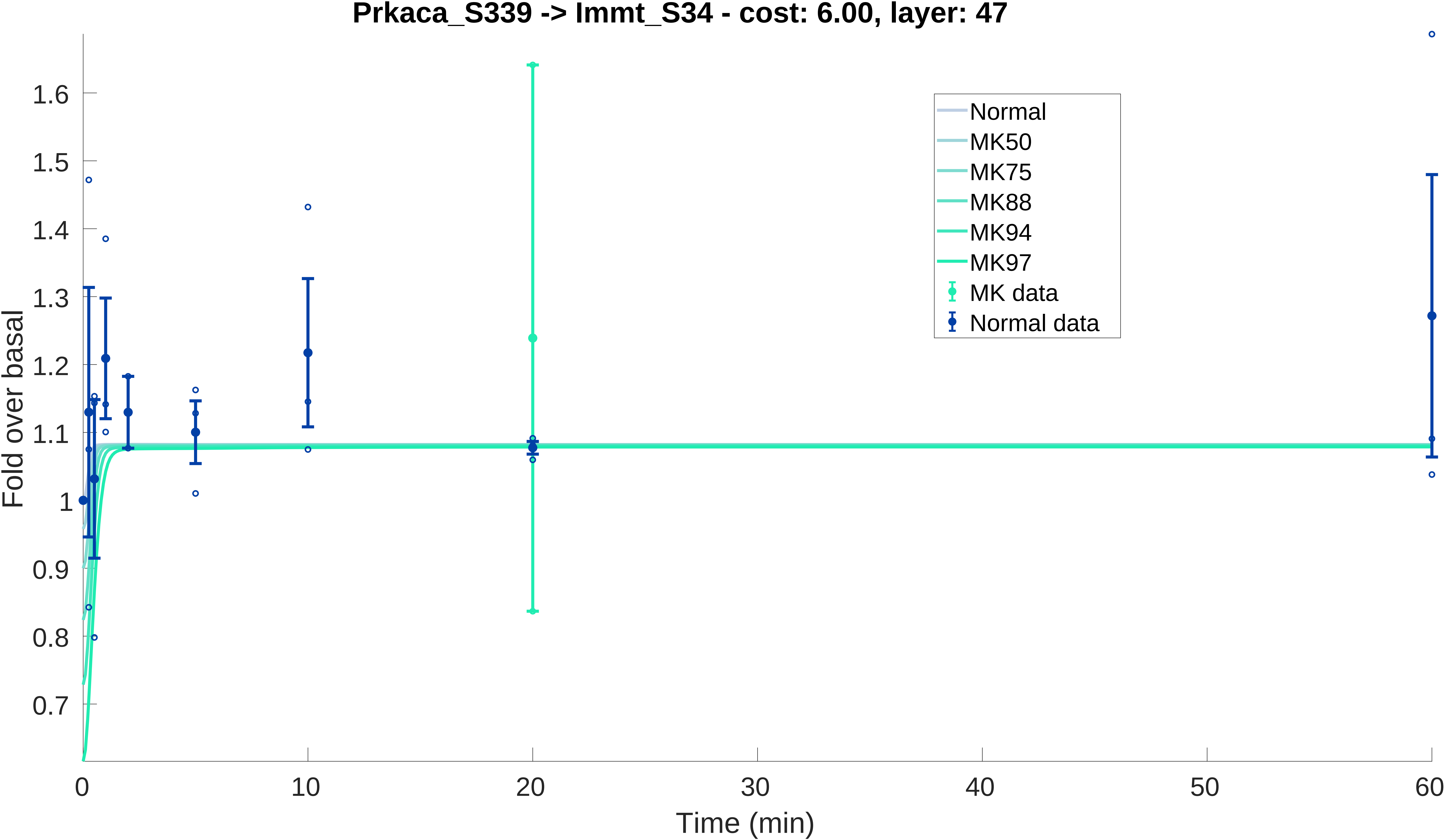
Prrkaca_S339 -> Immt_S34 - cost: 6.00, layer: 47
Fold over basal
Time (min)
Normal
MK50
MK75
MK88
MK94
MK97
MK data
Normal data
1.6
1.5
1.4
1.3
1.2
1.1
1
0.9
0.8
0.7
0
10
20
30
40
50
60

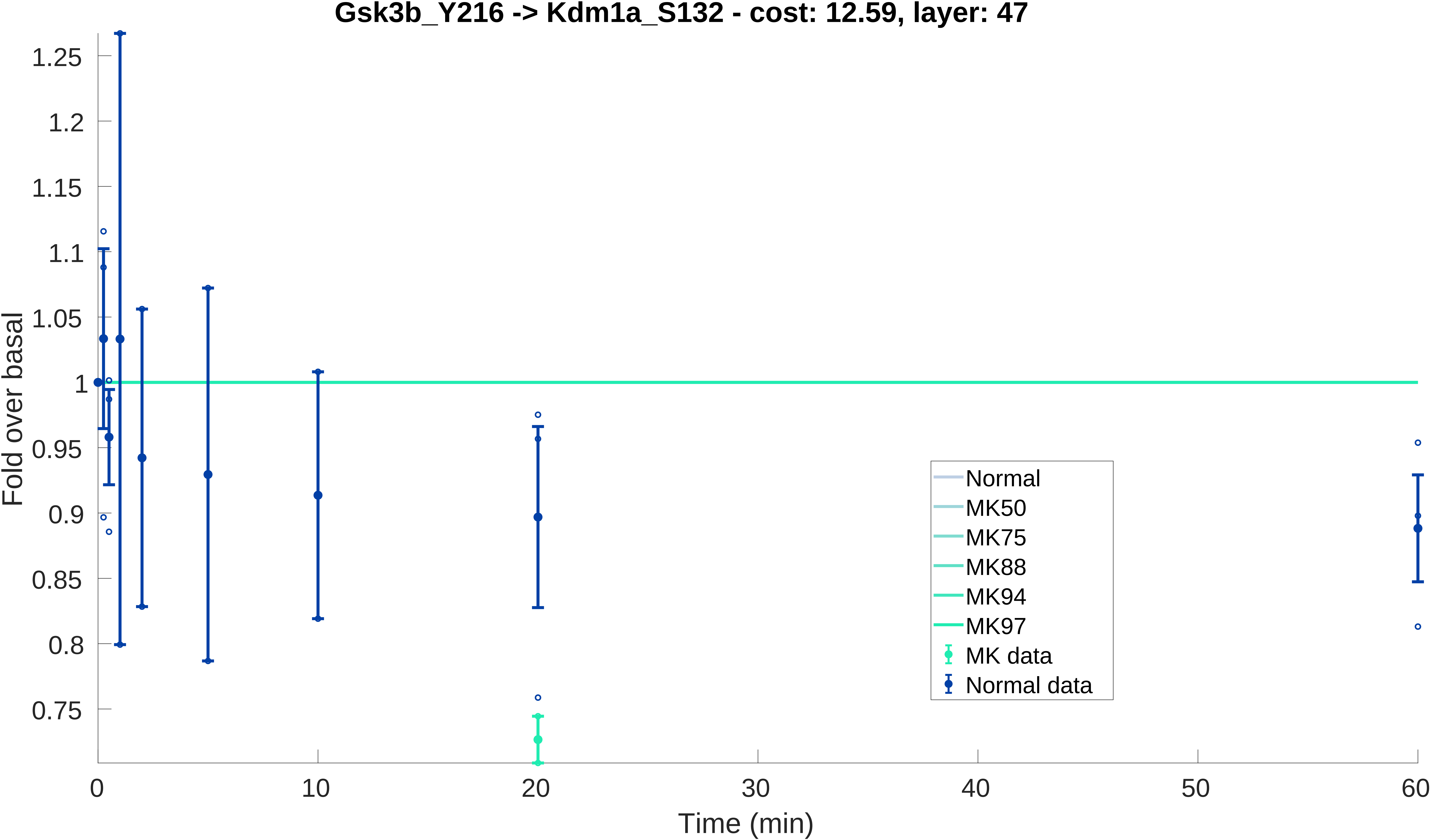
Gsk3b_Y216 -> Kdm1a_S132 - cost: 12.59, layer: 47
Fold over basal
Time (min)
1.25
1.2
1.15
1.1
1.05
1
0.95
0.9
0.85
0.8
0.75
0
10
20
30
40
50
60
Normal
MK50
MK75
MK88
MK94
MK97
MK data
Normal data

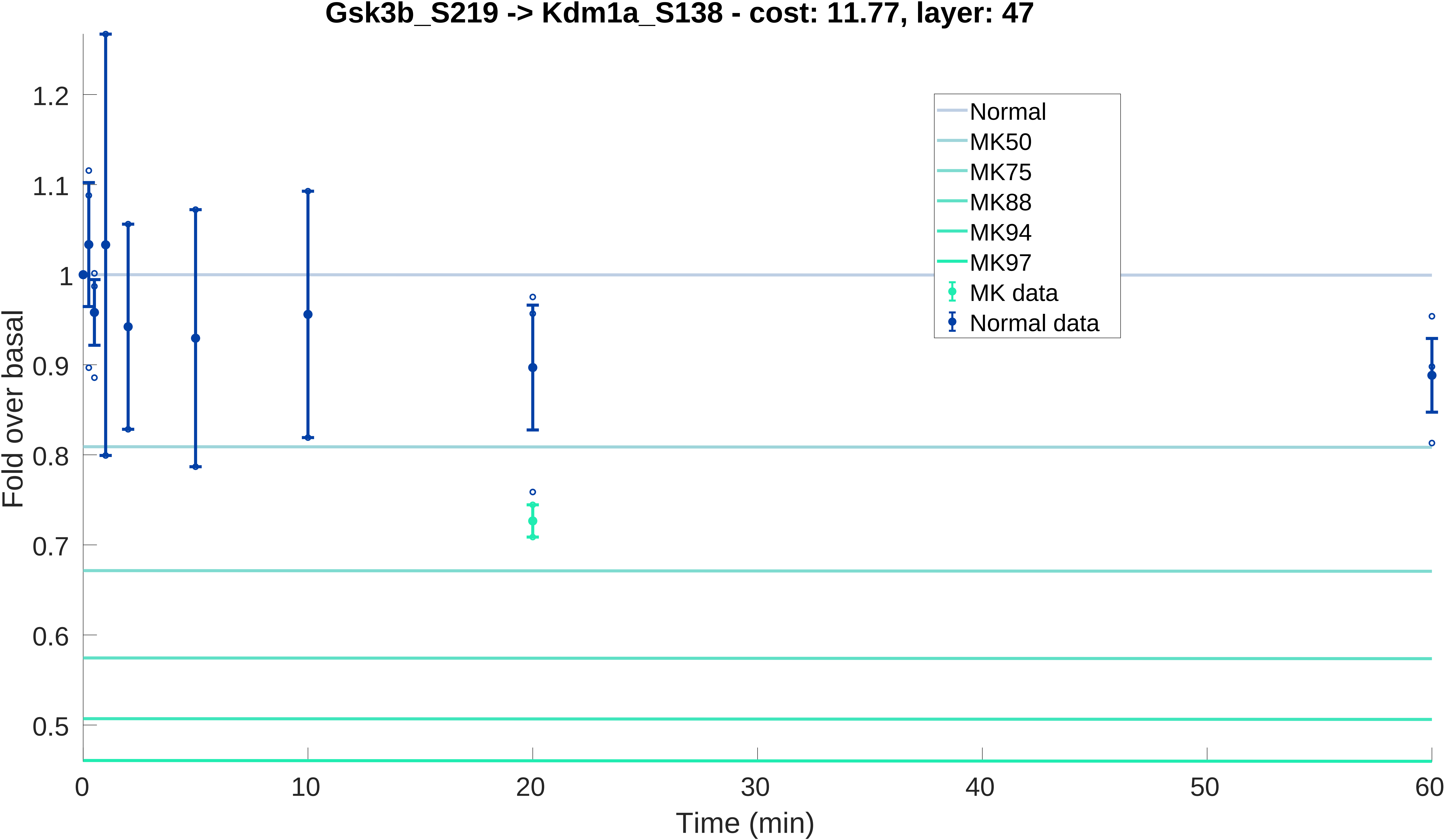

Gsk3b_S219 -> Kdm1a_S138 - cost: 11.77, layer: 47
Fold over basal
Time (min)
0
10
20
30
40
50
60
0.5
0.6
0.7
0.8
0.9
1
1.1
1.2
Normal
MK50
MK75
MK88
MK94
MK97
MK data
Normal data

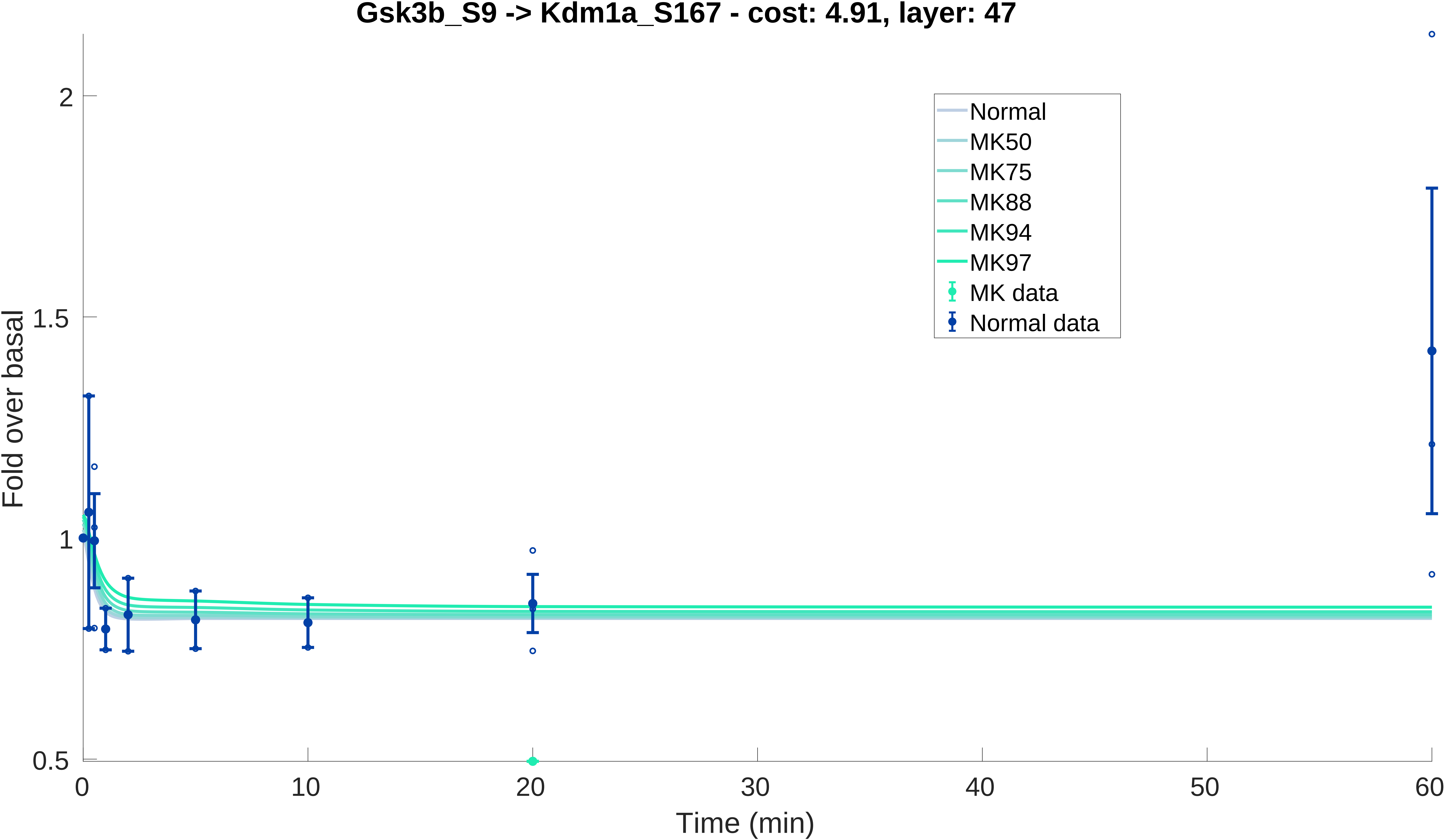
Gsk3b_S9 -> Kdm1a_S167 - cost: 4.91, layer: 47
Fold over basal
Time (min)
0.5
1
1.5
2
0
10
20
30
40
50
60
Normal
MK50
MK75
MK88
MK94
MK97
MK data
Normal data

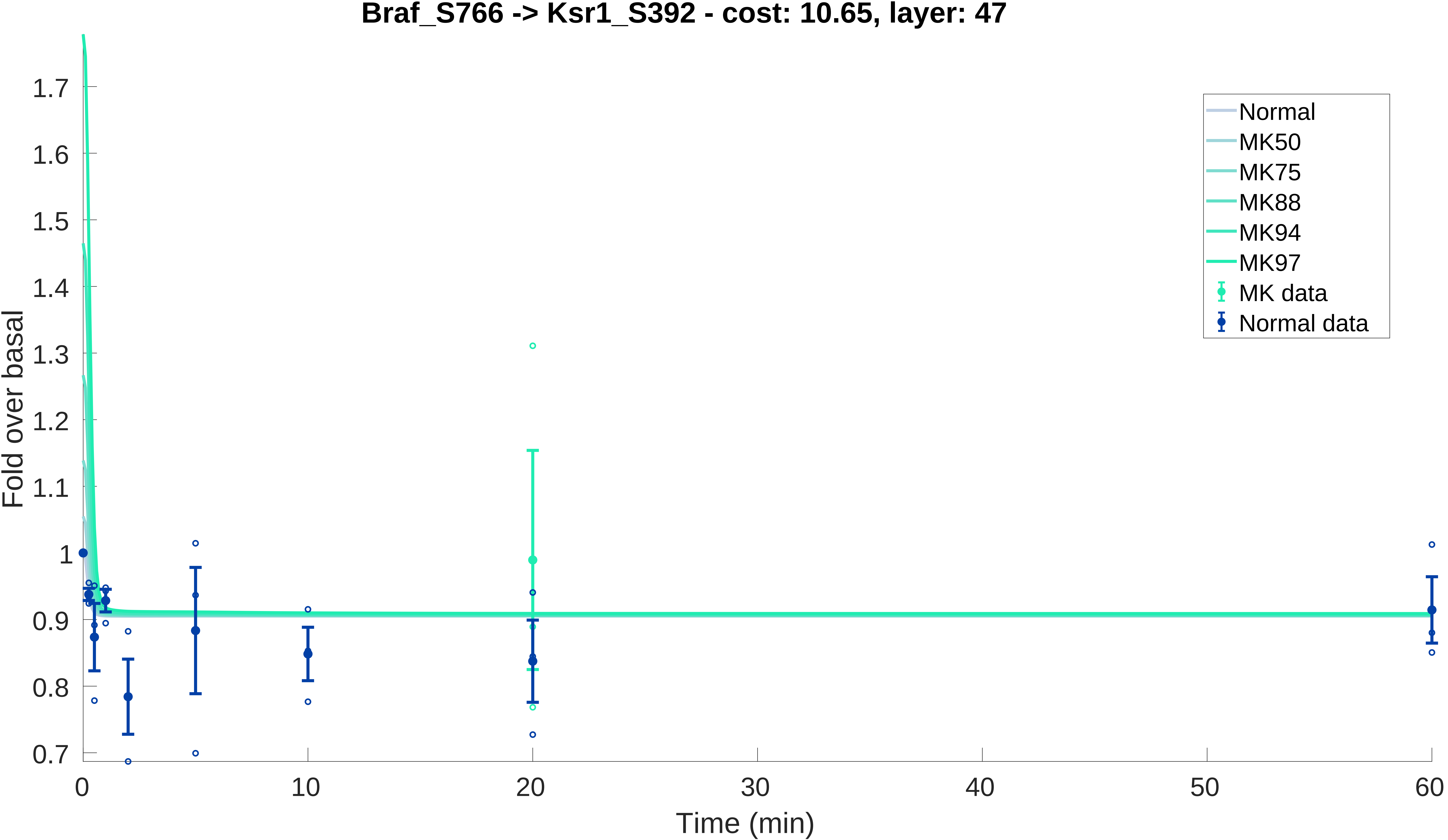

Braf_S766 -> Ksr1_S392 - cost: 10.65, layer: 47
Fold over basal
Time (min)
Normal
MK50
MK75
MK88
MK94
MK97
MK data
Normal data
0.7
0.8
0.9
1
1.1
1.2
1.3
1.4
1.5
1.6
1.7
0
10
20
30
40
50
60

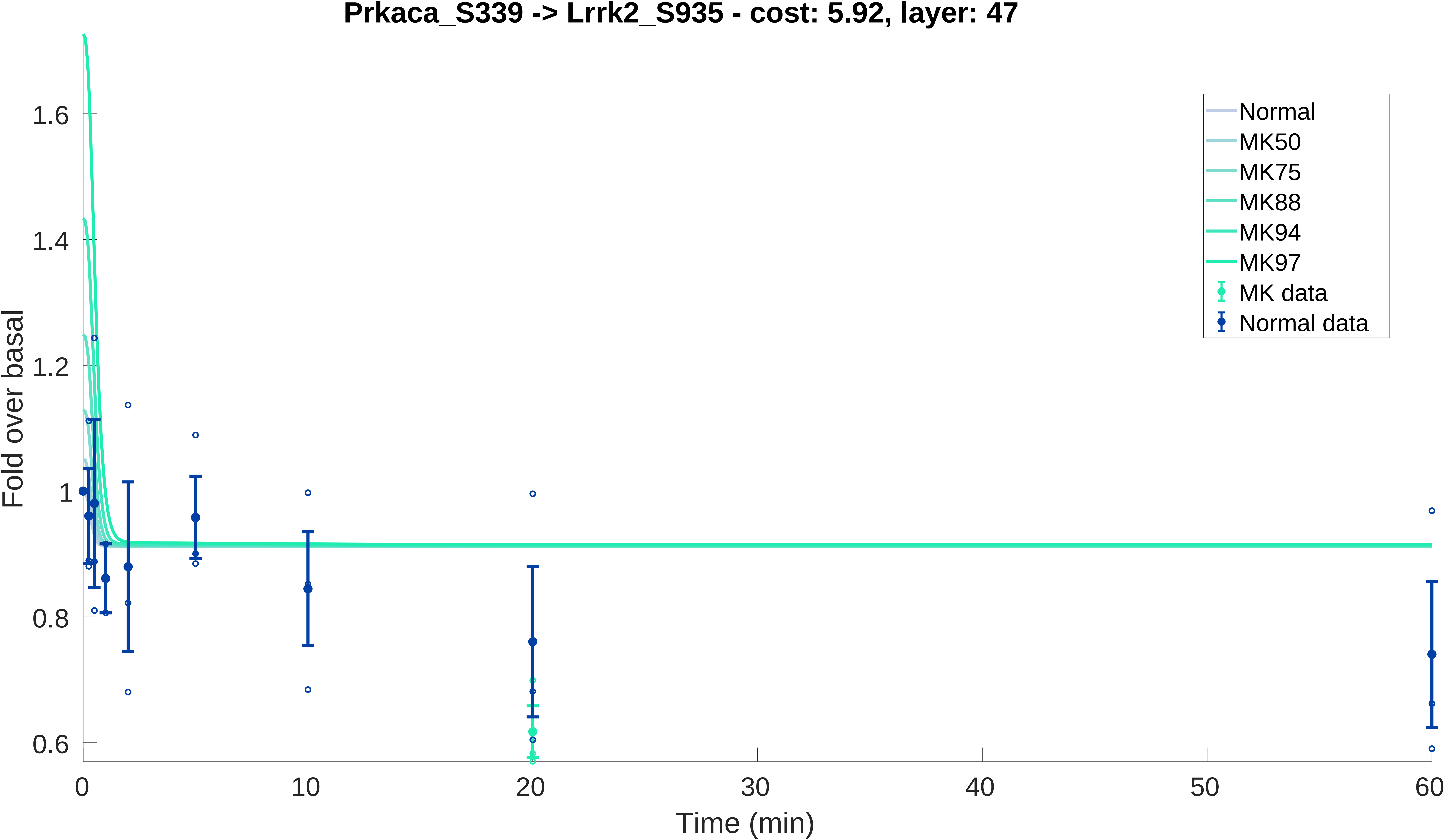

Prkaca_S339 -> Lrrk2_S935 - cost: 5.92, layer: 47
Fold over basal
Time (min)
1.6
1.4
1.2
1
0.8
0.6
0
10
20
30
40
50
60
Normal
MK50
MK75
MK88
MK94
MK97
MK data
Normal data

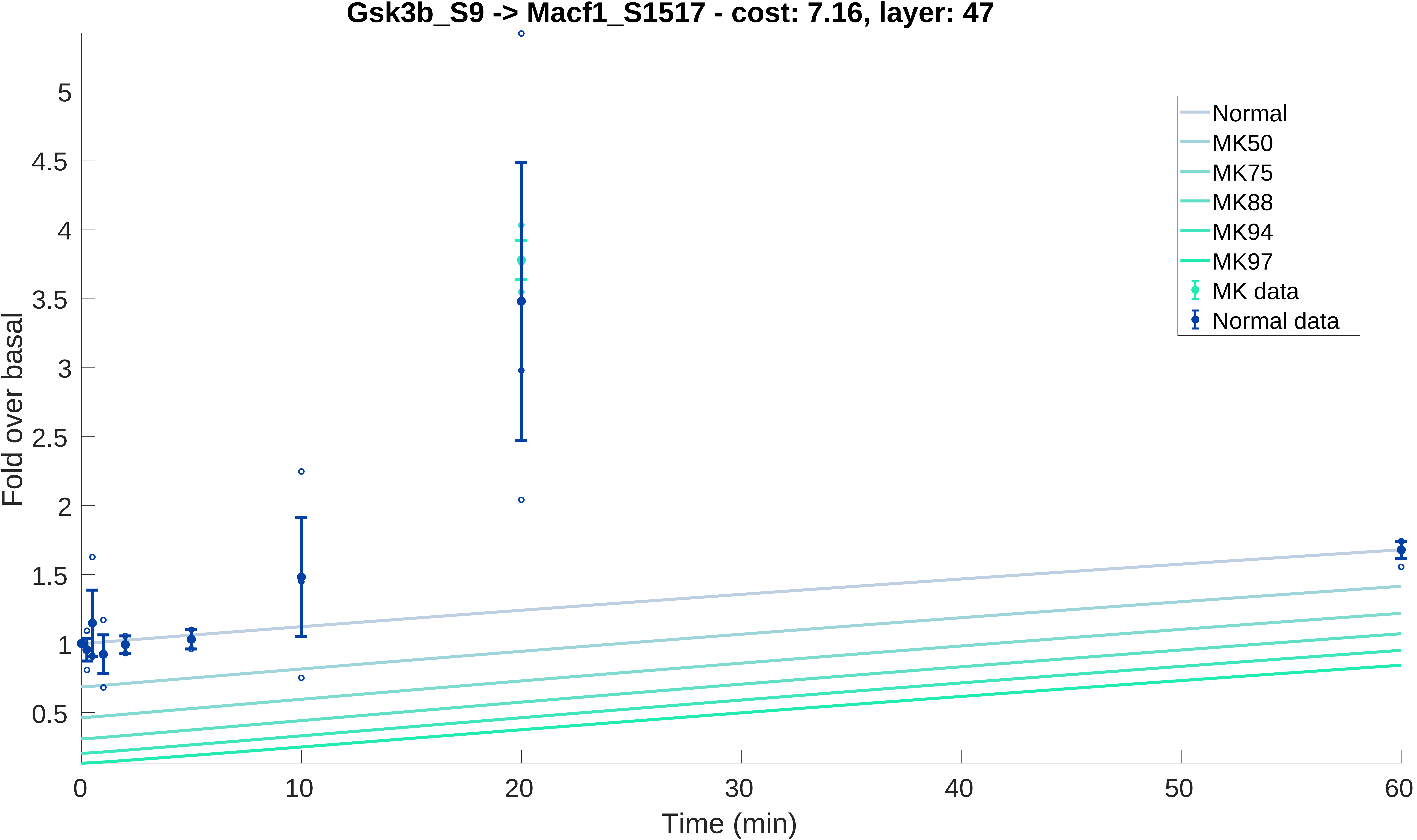

Gsk3b_S9 -> Macf1_S1517 - cost: 7.16, layer: 47
Fold over basal
Time (min)
0
10
20
30
40
50
60
0.5
1
1.5
2
2.5
3
3.5
4
4.5
5
Normal
MK50
MK75
MK88
MK94
MK97
MK data
Normal data

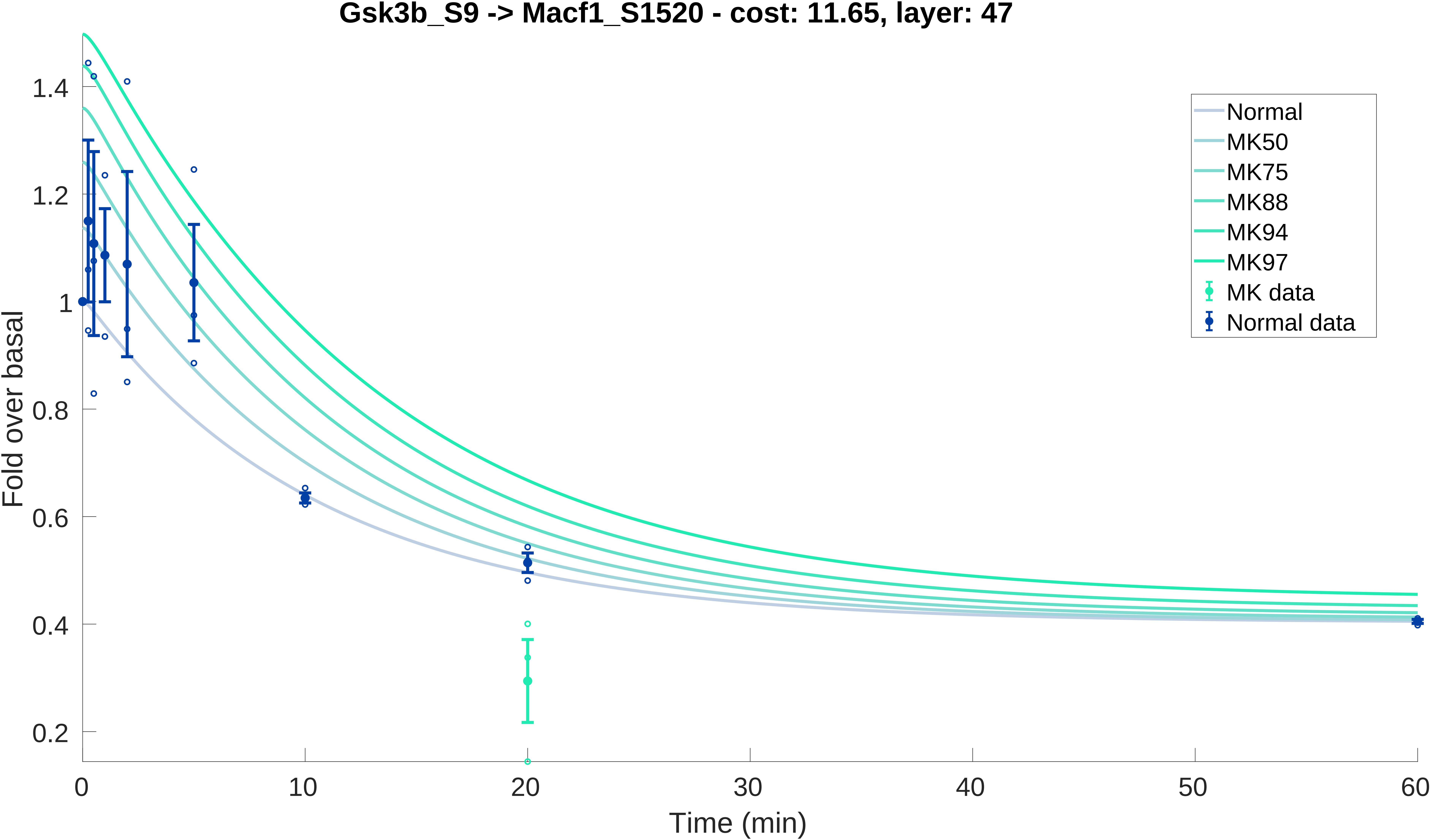
Gsk3b_S9 -> Macf1_S1520 - cost: 11.65, layer: 47
Fold over basal
Time (min)
Normal
MK50
MK75
MK88
MK94
MK97
MK data
Normal data
0.2
0.4
0.6
0.8
1
1.2
1.4
0
10
20
30
40
50
60

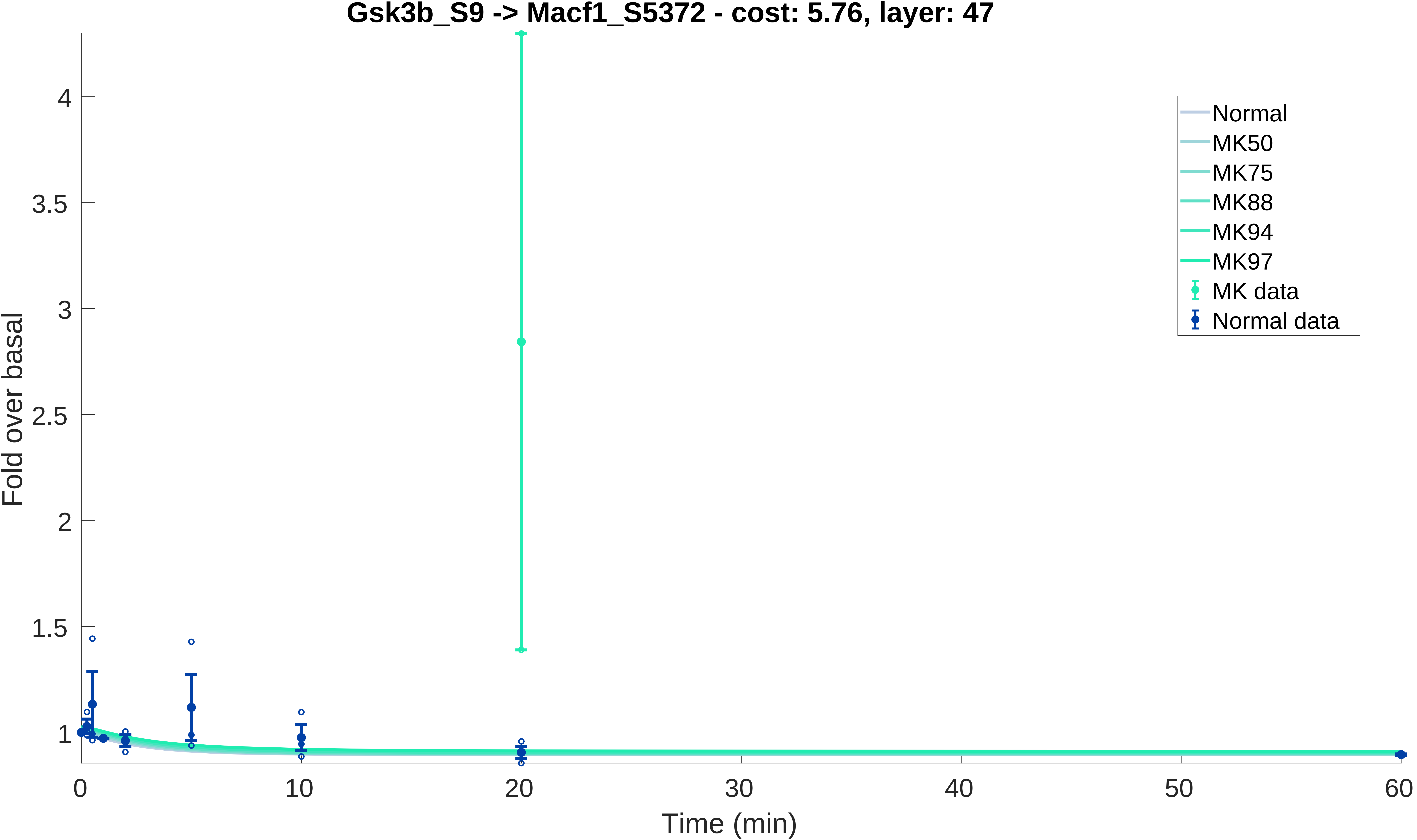

Gsk3b_S9 -> Macf1_S5372 - cost: 5.76, layer: 47
Fold over basal
Time (min)
4
3.5
3
2.5
2
1.5
1
0
10
20
30
40
50
60
Normal
MK50
MK75
MK88
MK94
MK97
MK data
Normal data

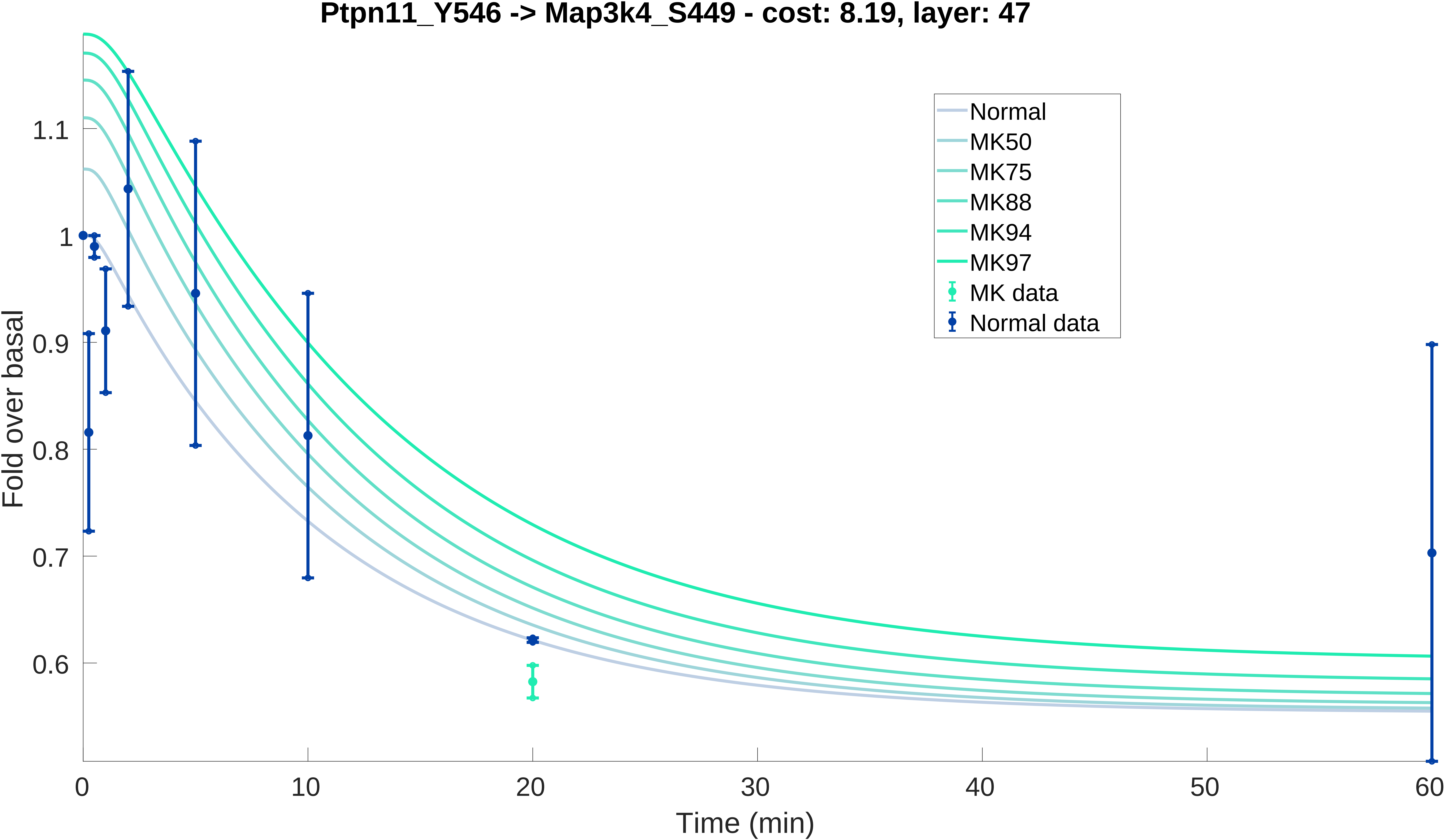
Ptpn11_Y546 -> Map3k4_S449 - cost: 8.19, layer: 47
Fold over basal
Time (min)
0
10
20
30
40
50
60
0.6
0.7
0.8
0.9
1
1.1
Normal
MK50
MK75
MK88
MK94
MK97
MK data
Normal data

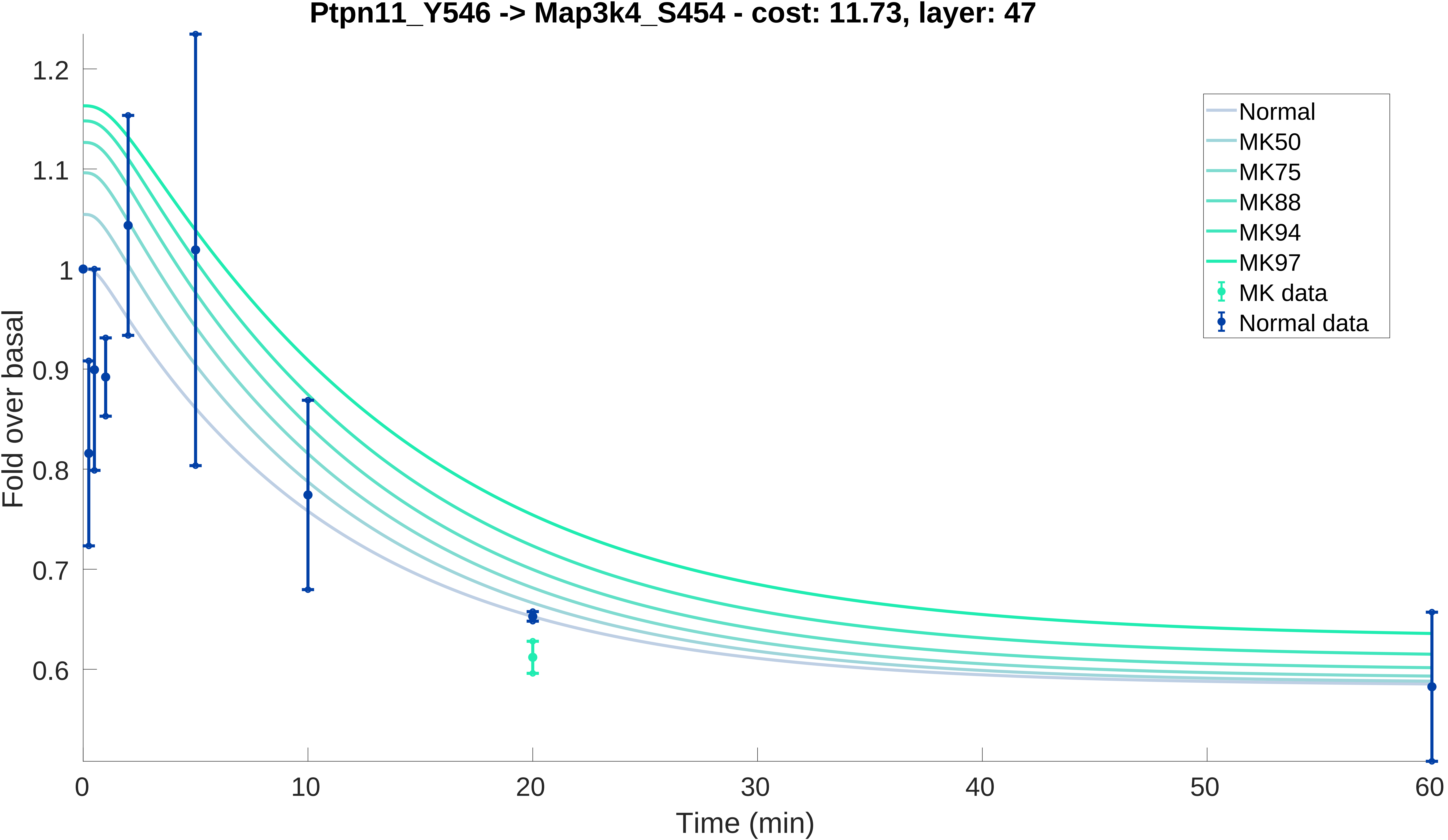
Ptpn11_Y546 -> Map3k4_S454 - cost: 11.73, layer: 47
Fold over basal
1.2
1.1
1
0.9
0.8
0.7
0.6
0
10
20
30
40
50
60
Time (min)
Normal
MK50
MK75
MK88
MK94
MK97
MK data
Normal data

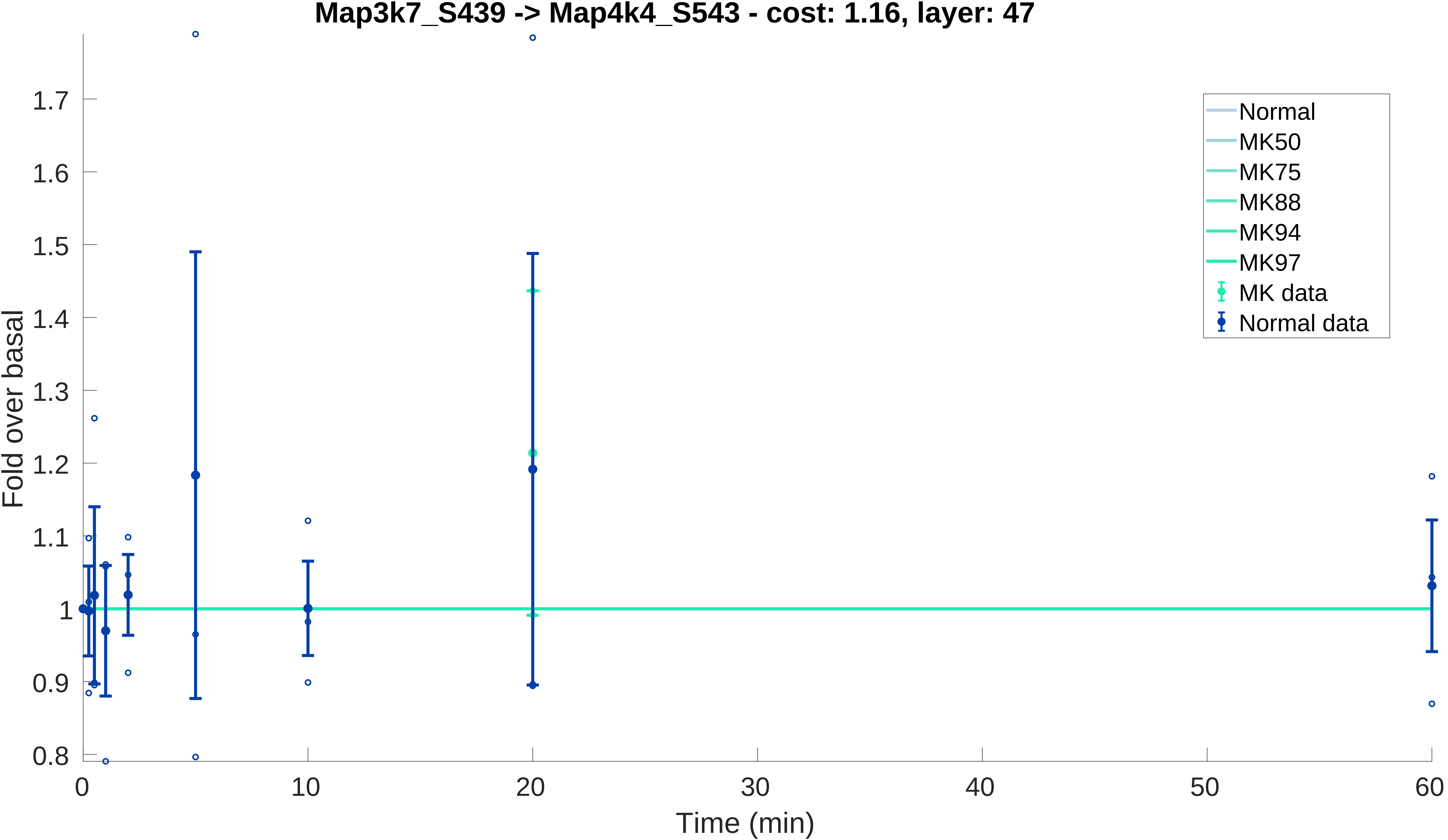

Map3k7_S439 -> Map4k4_S543 - cost: 1.16, layer: 47
Fold over basal
Time (min)
Normal
MK50
MK75
MK88
MK94
MK97
MK data
Normal data
0.8
0.9
1
1.1
1.2
1.3
1.4
1.5
1.6
1.7
0
10
20
30
40
50
60

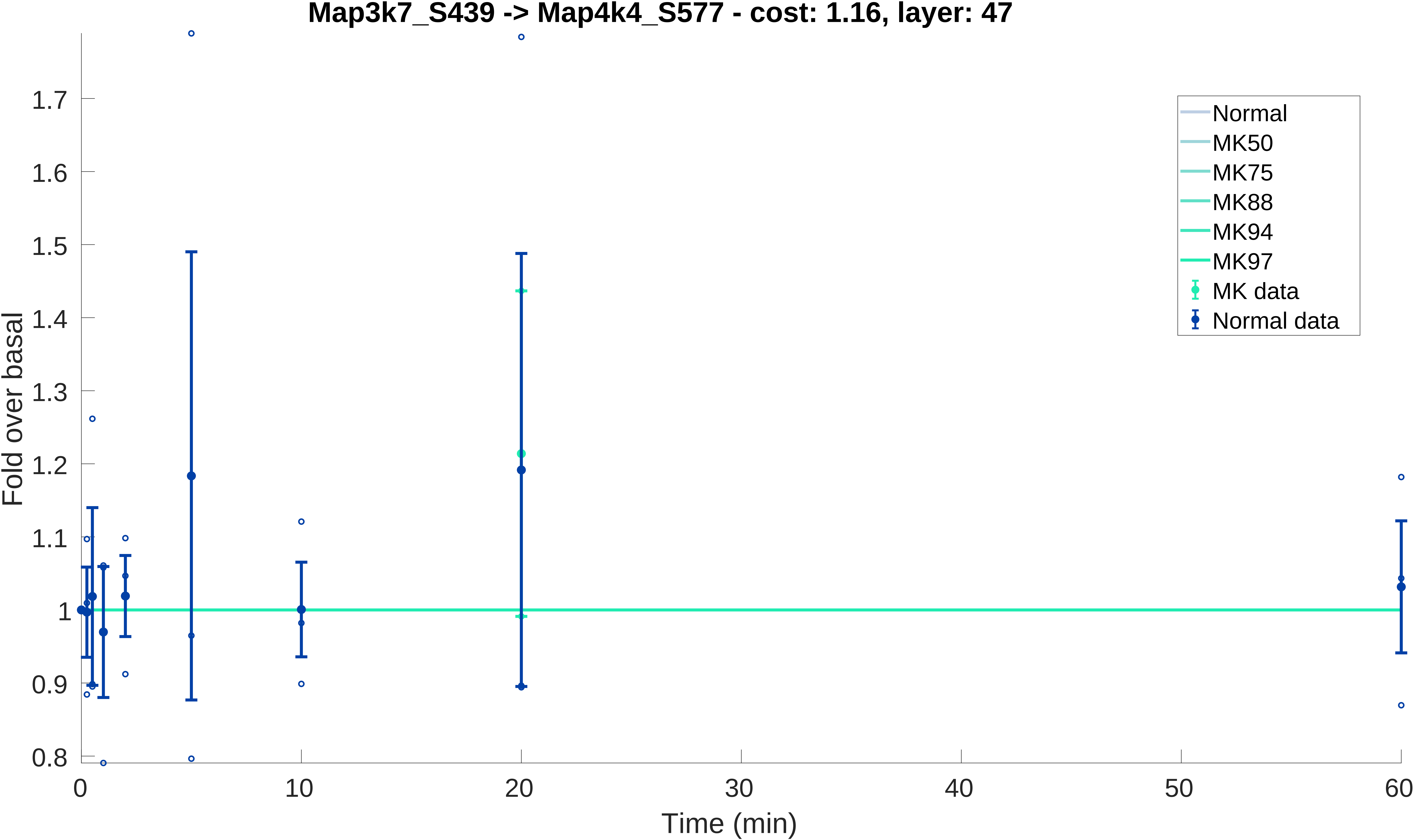
Map3k7_S439 -> Map4k4_S577 - cost: 1.16, layer: 47
Fold over basal
Time (min)
0.8
0.9
1
1.1
1.2
1.3
1.4
1.5
1.6
1.7
0
10
20
30
40
50
60
Normal
MK50
MK75
MK88
MK94
MK97
MK data
Normal data

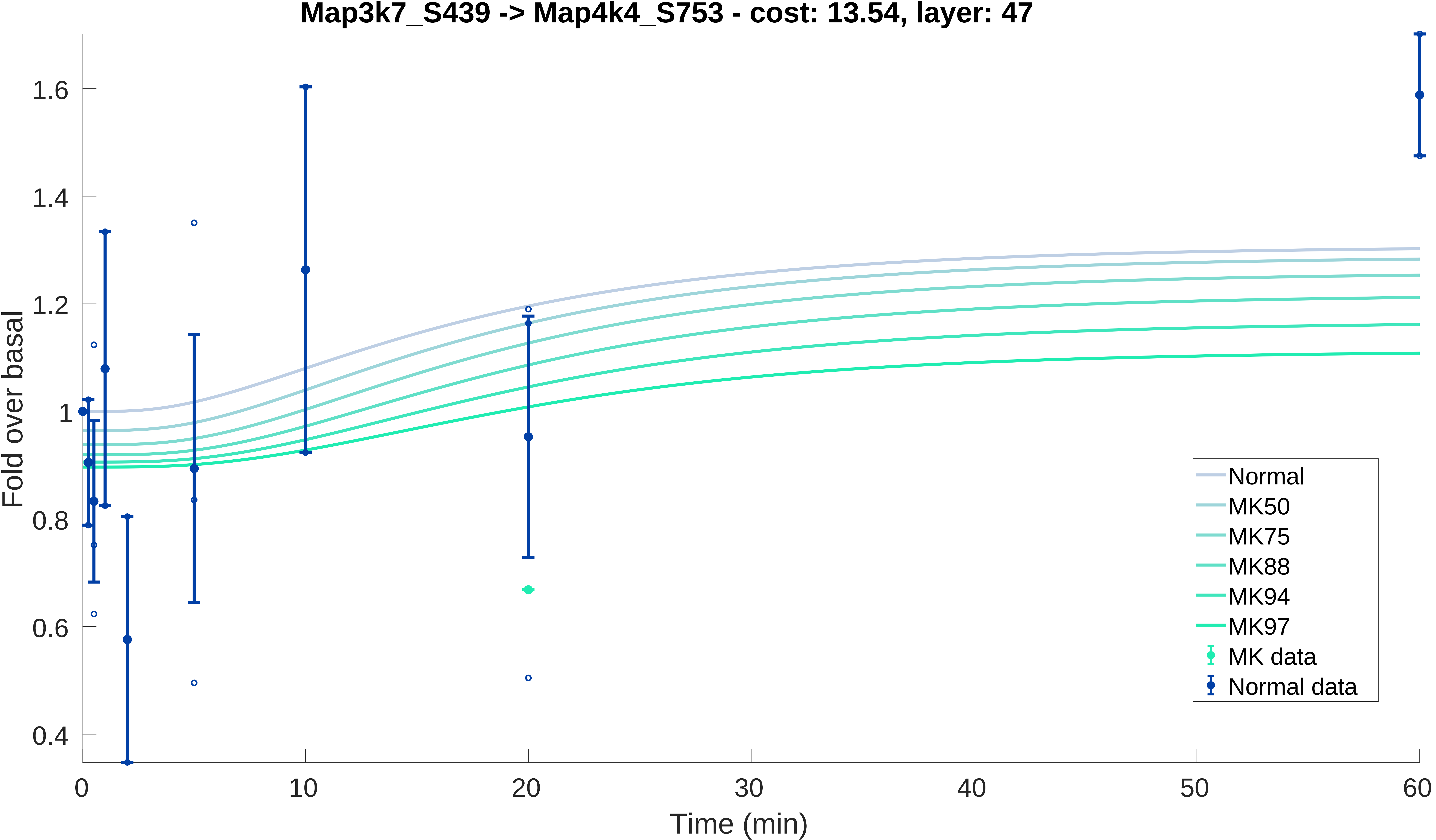
Map3k7_S439 -> Map4k4_S753 - cost: 13.54, layer: 47
Fold over basal
1.6
1.4
1.2
1
0.8
0.6
0.4
0
10
20
30
40
50
60
Time (min)
Normal
MK50
MK75
MK88
MK94
MK97
MK data
Normal data

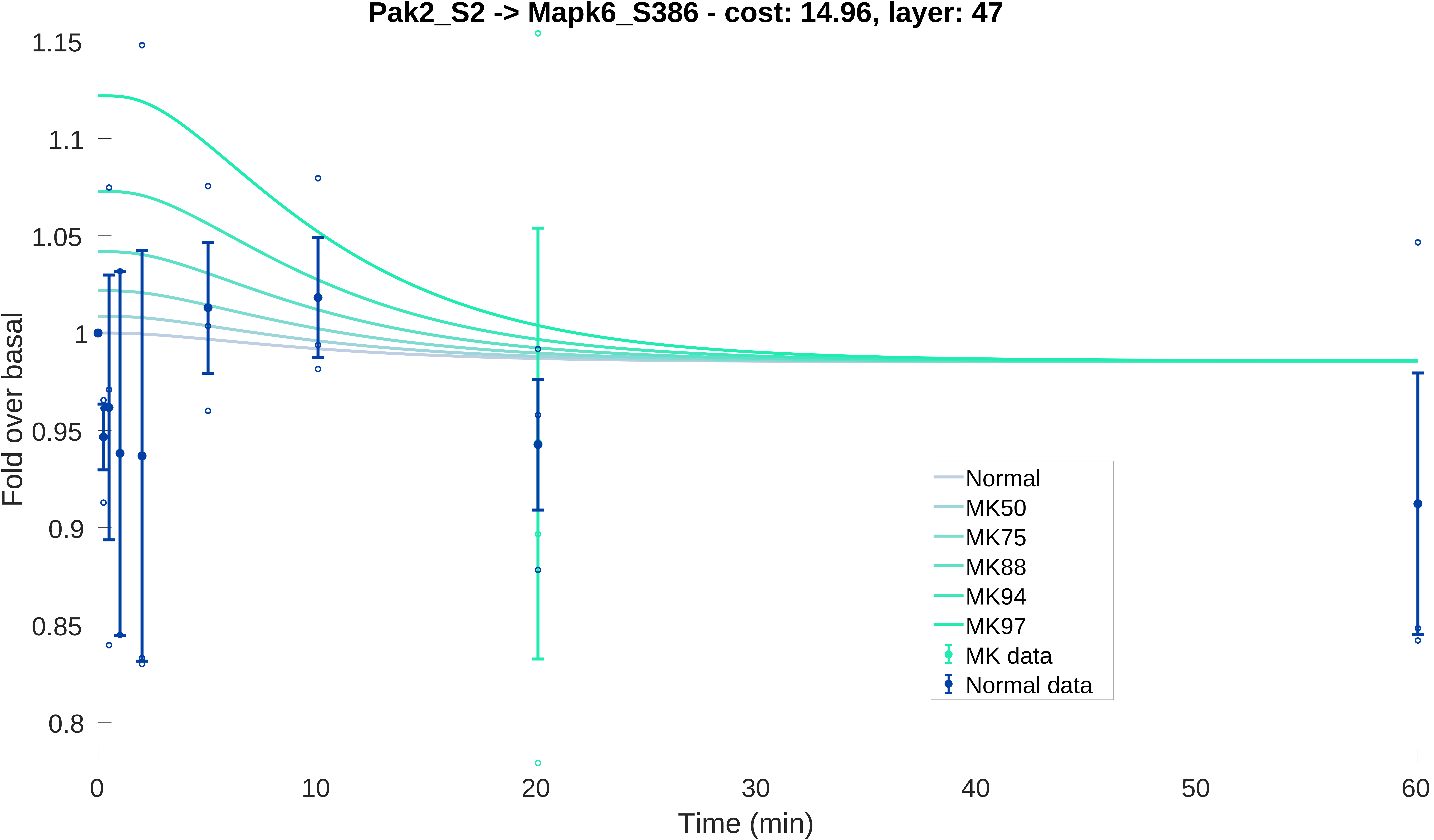

Pak2_S2 -> Mapk6_S386 - cost: 14.96, layer: 47
Fold over basal
Time (min)
1.15
1.1
1.05
1
0.95
0.9
0.85
0.8
0
10
20
30
40
50
60
Normal
MK50
MK75
MK88
MK94
MK97
MK data
Normal data

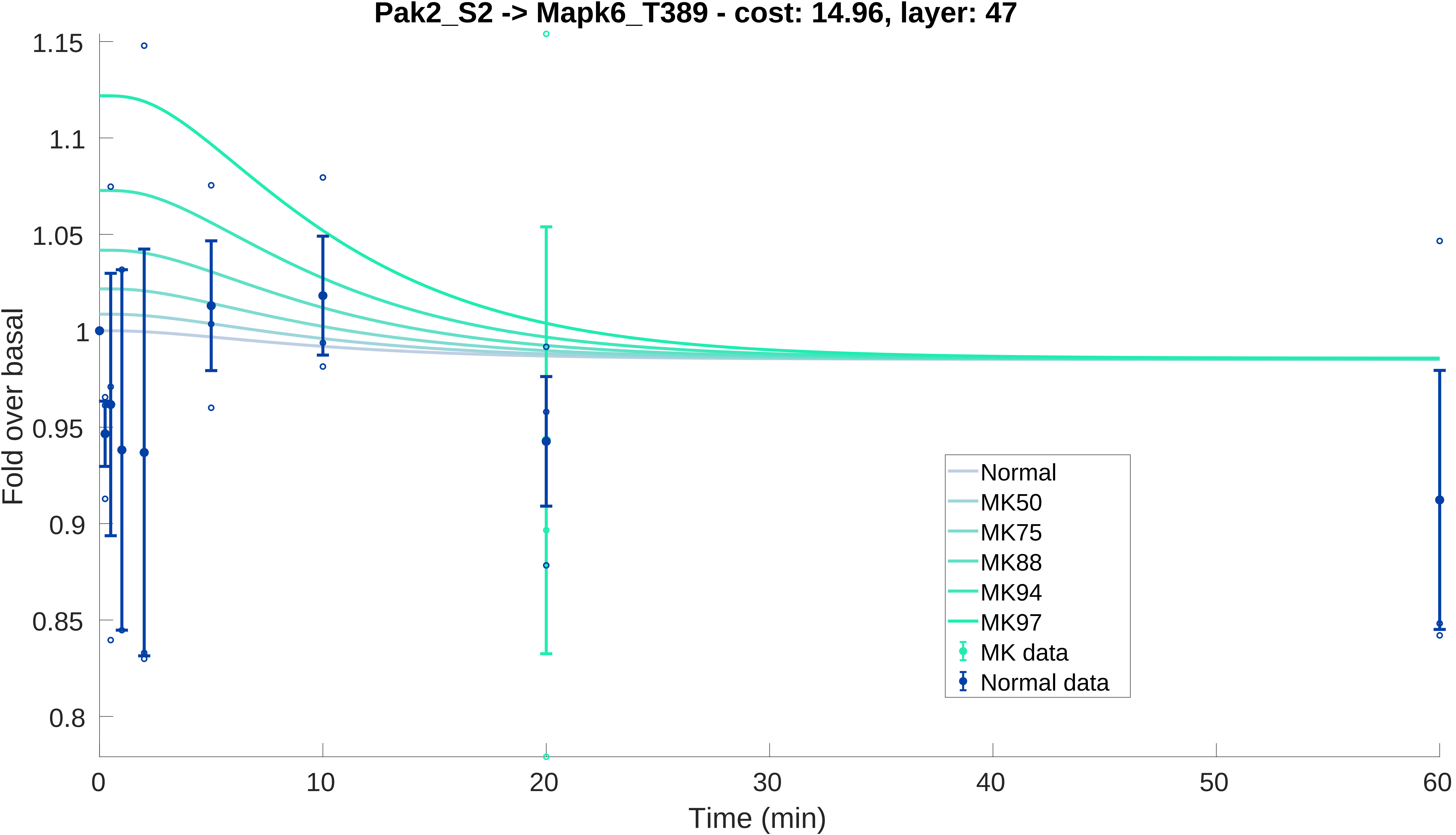

Pak2_S2 -> Mapk6_T389 - cost: 14.96, layer: 47
Fold over basal
Time (min)
1.15
1.1
1.05
1
0.95
0.9
0.85
0.8
0
10
20
30
40
50
60
Normal
MK50
MK75
MK88
MK94
MK97
MK data
Normal data

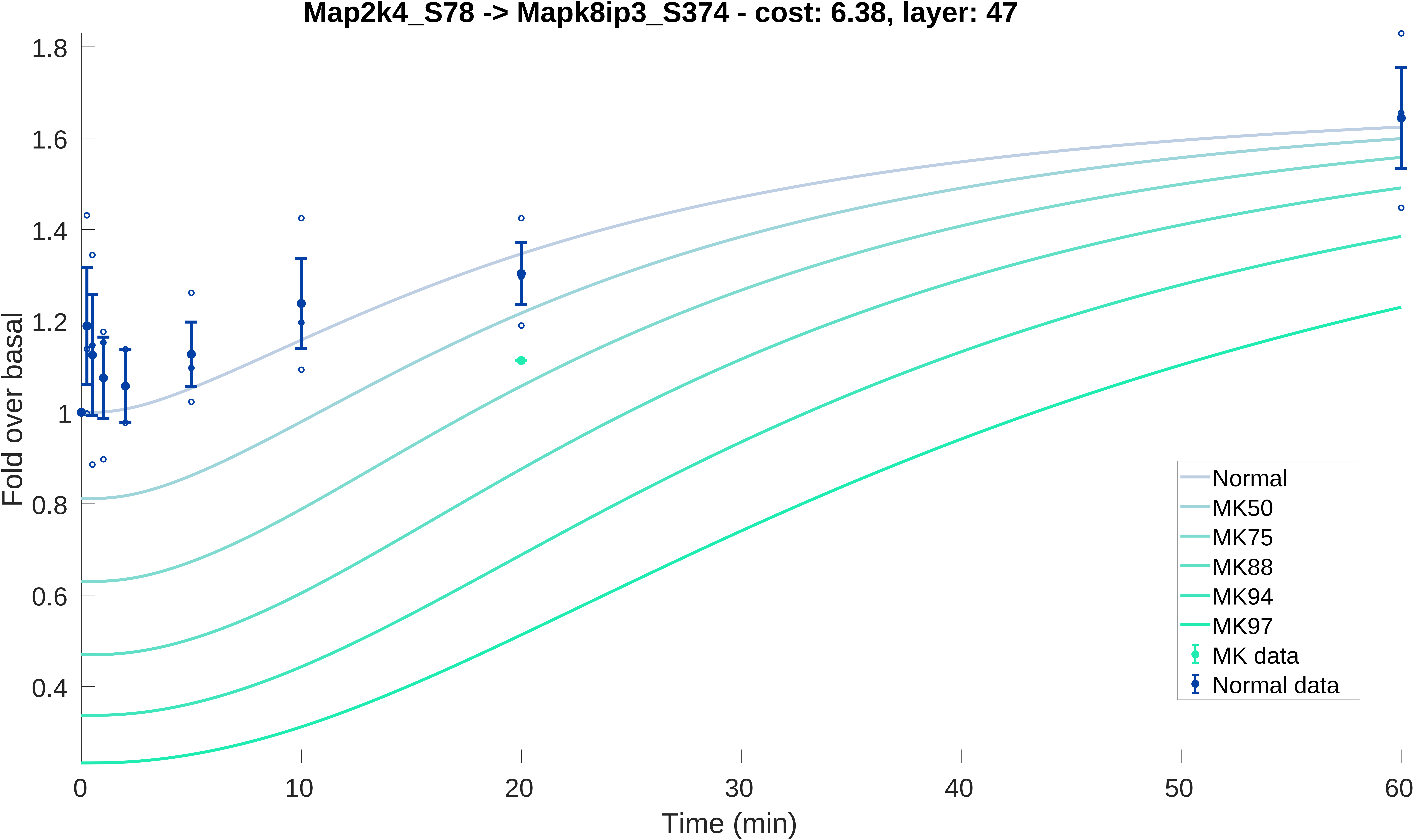

Map2k4_S78 -> Mapk8ip3_S374 - cost: 6.38, layer: 47
Fold over basal
Time (min)
0.4
0.6
0.8
1
1.2
1.4
1.6
1.8
0
10
20
30
40
50
60
Normal
MK50
MK75
MK88
MK94
MK97
MK data
Normal data

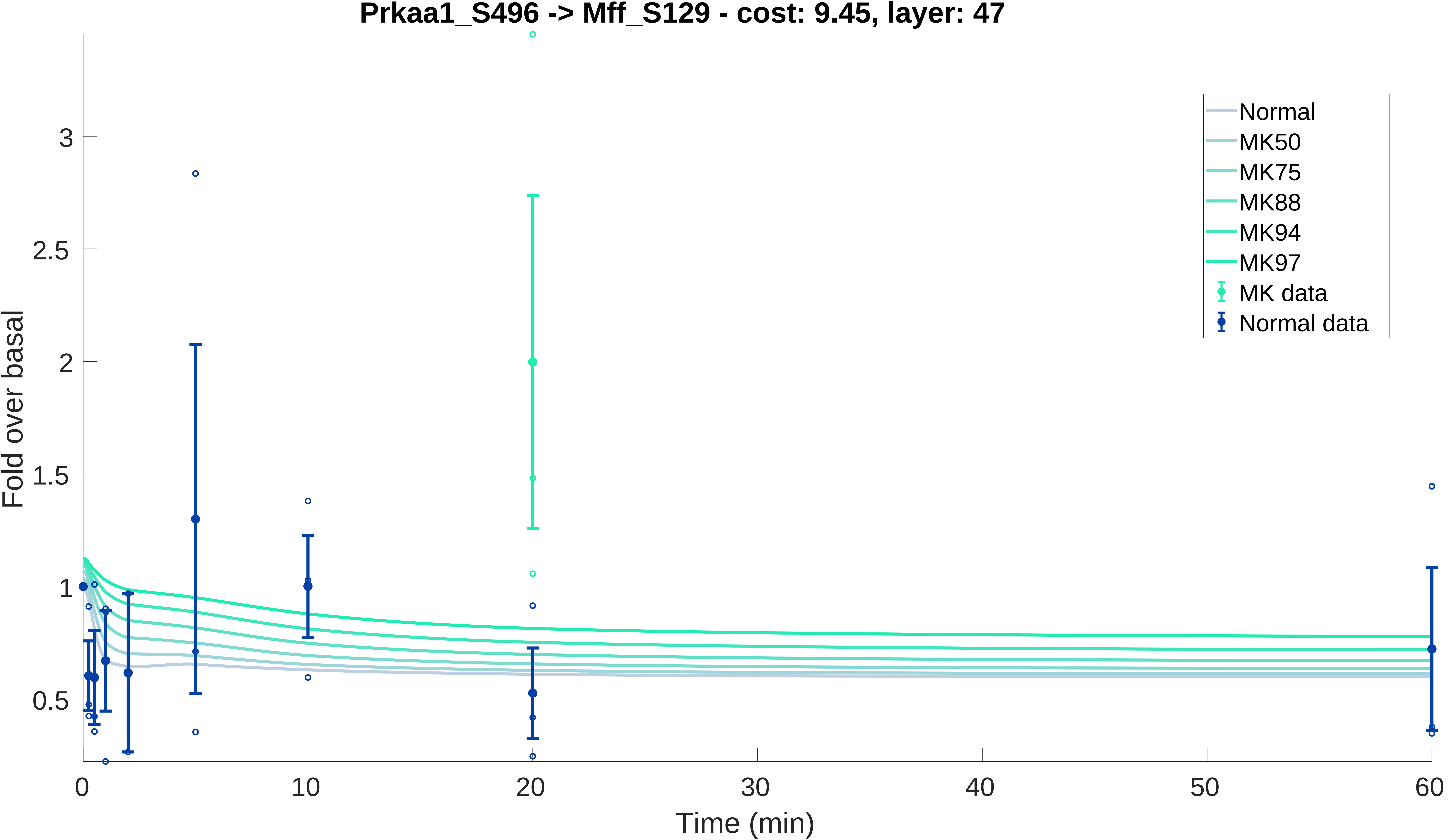

Prkaa1_S496 -> Mff_S129 - cost: 9.45, layer: 47
Fold over basal
Time (min)
0.5
1
1.5
2
2.5
3
0
10
20
30
40
50
60
Normal
MK50
MK75
MK88
MK94
MK97
MK data
Normal data

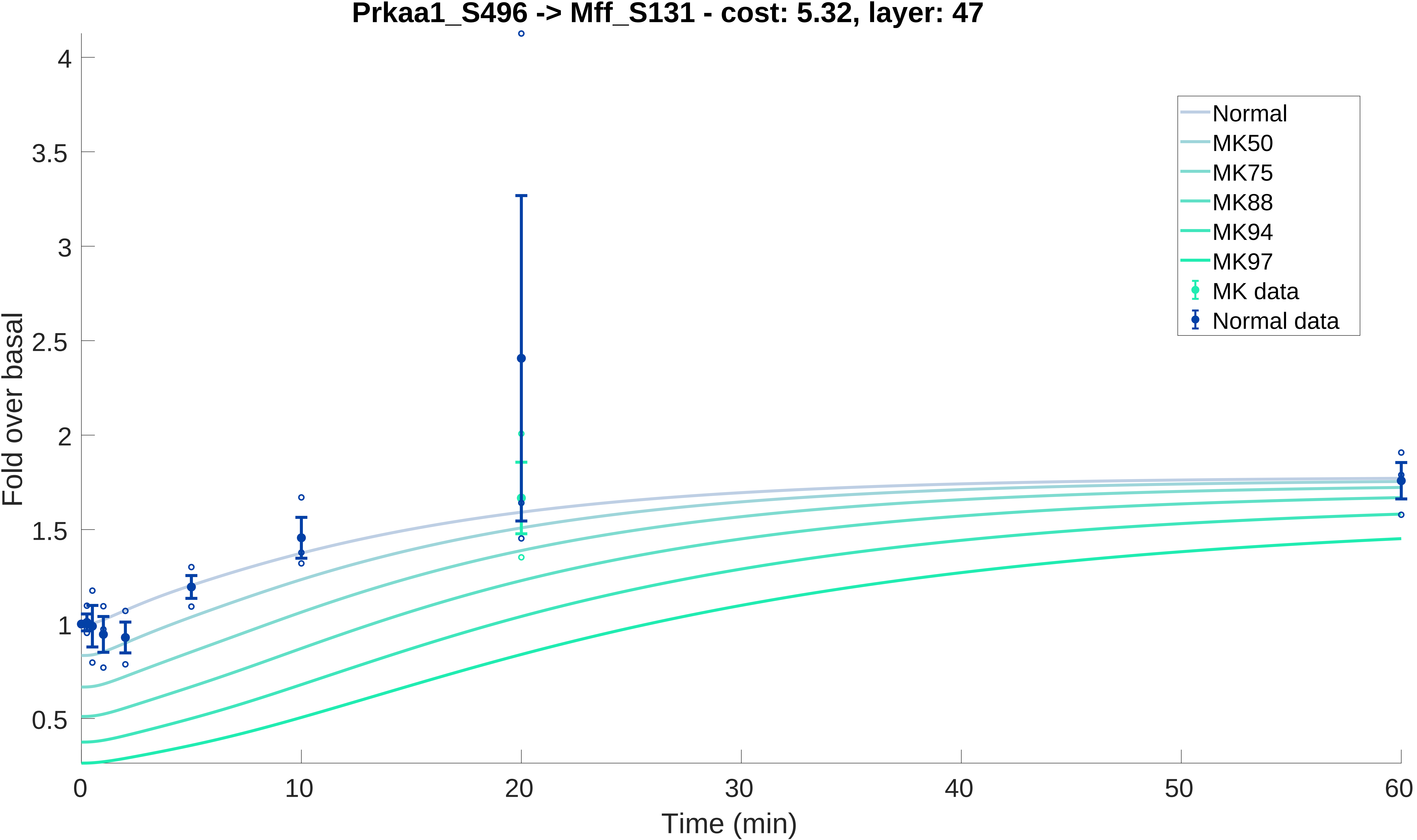

Prkaa1_S496 -> Mff_S131 - cost: 5.32, layer: 47
Fold over basal
Time (min)
0
10
20
30
40
50
60
0.5
1
1.5
2
2.5
3
3.5
4
Normal
MK50
MK75
MK88
MK94
MK97
MK data
Normal data

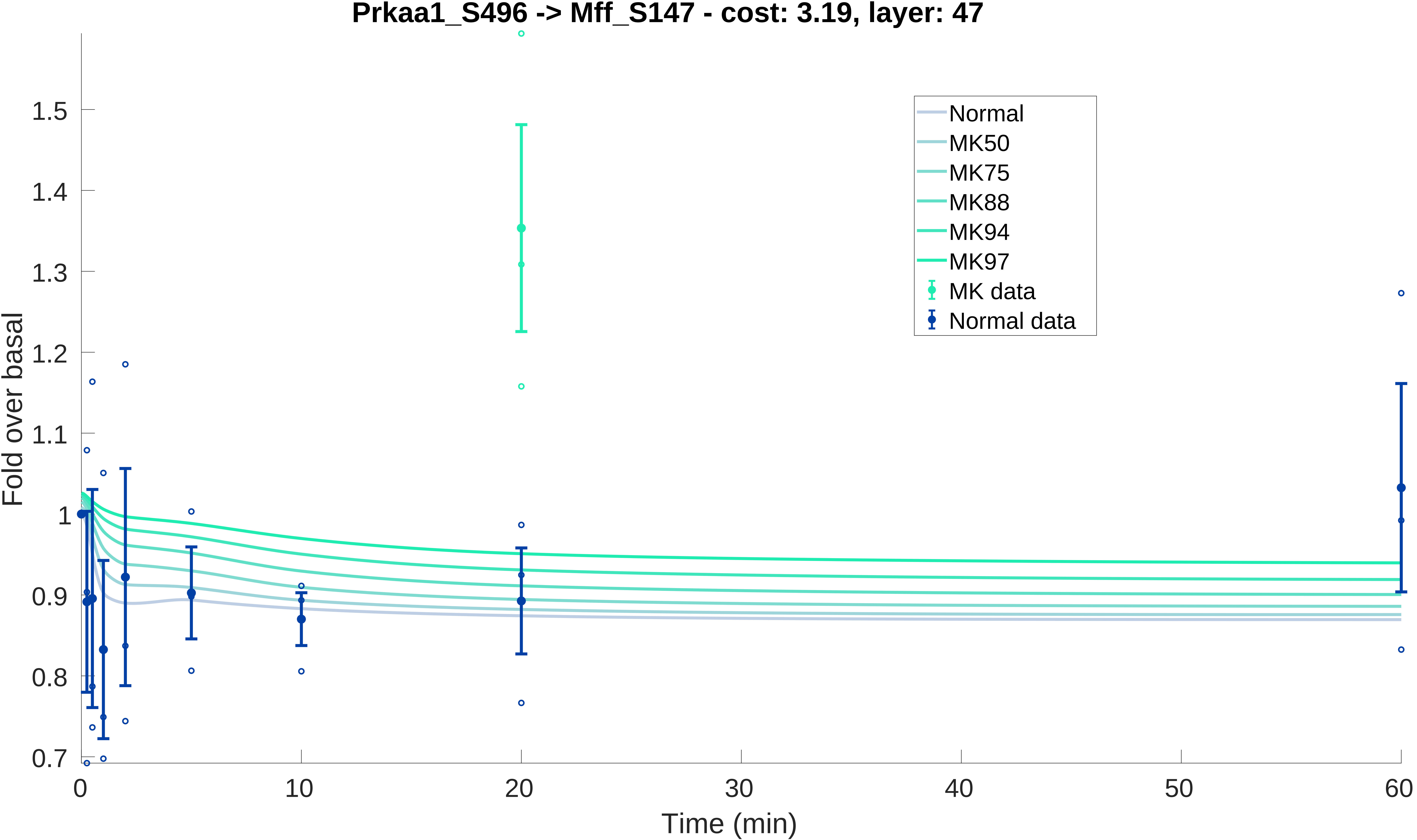

Prkaa1_S496 -> Mff_S147 - cost: 3.19, layer: 47
Fold over basal
Time (min)
0.7
0.8
0.9
1
1.1
1.2
1.3
1.4
1.5
0
10
20
30
40
50
60
Normal
MK50
MK75
MK88
MK94
MK97
MK data
Normal data

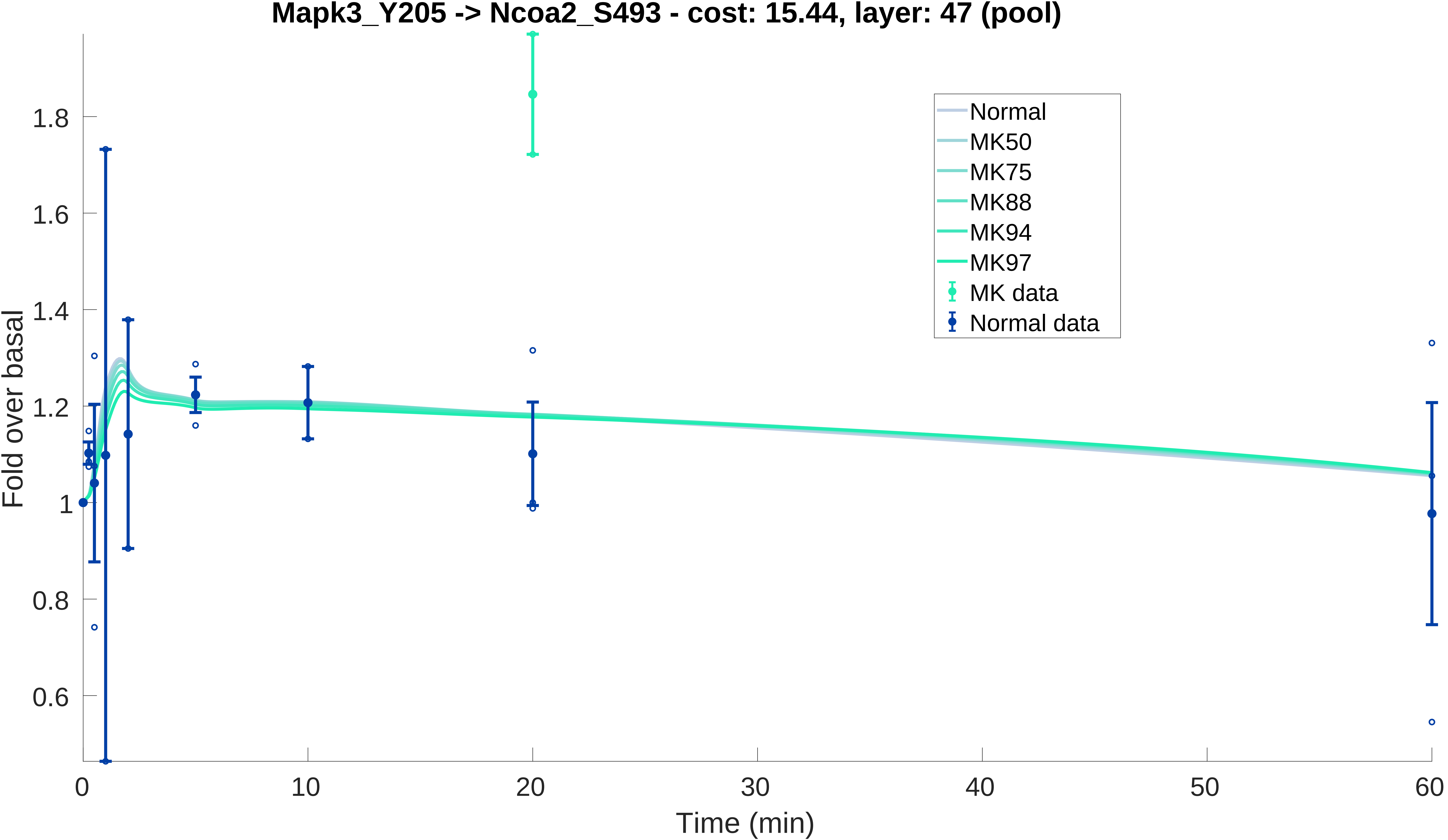

Mapk3_Y205 -> Ncoa2_S493 - cost: 15.44, layer: 47 (pool)
Fold over basal
Time (min)
0
10
20
30
40
50
60
0.6
0.8
1
1.2
1.4
1.6
1.8
Normal
MK50
MK75
MK88
MK94
MK97
MK data
Normal data

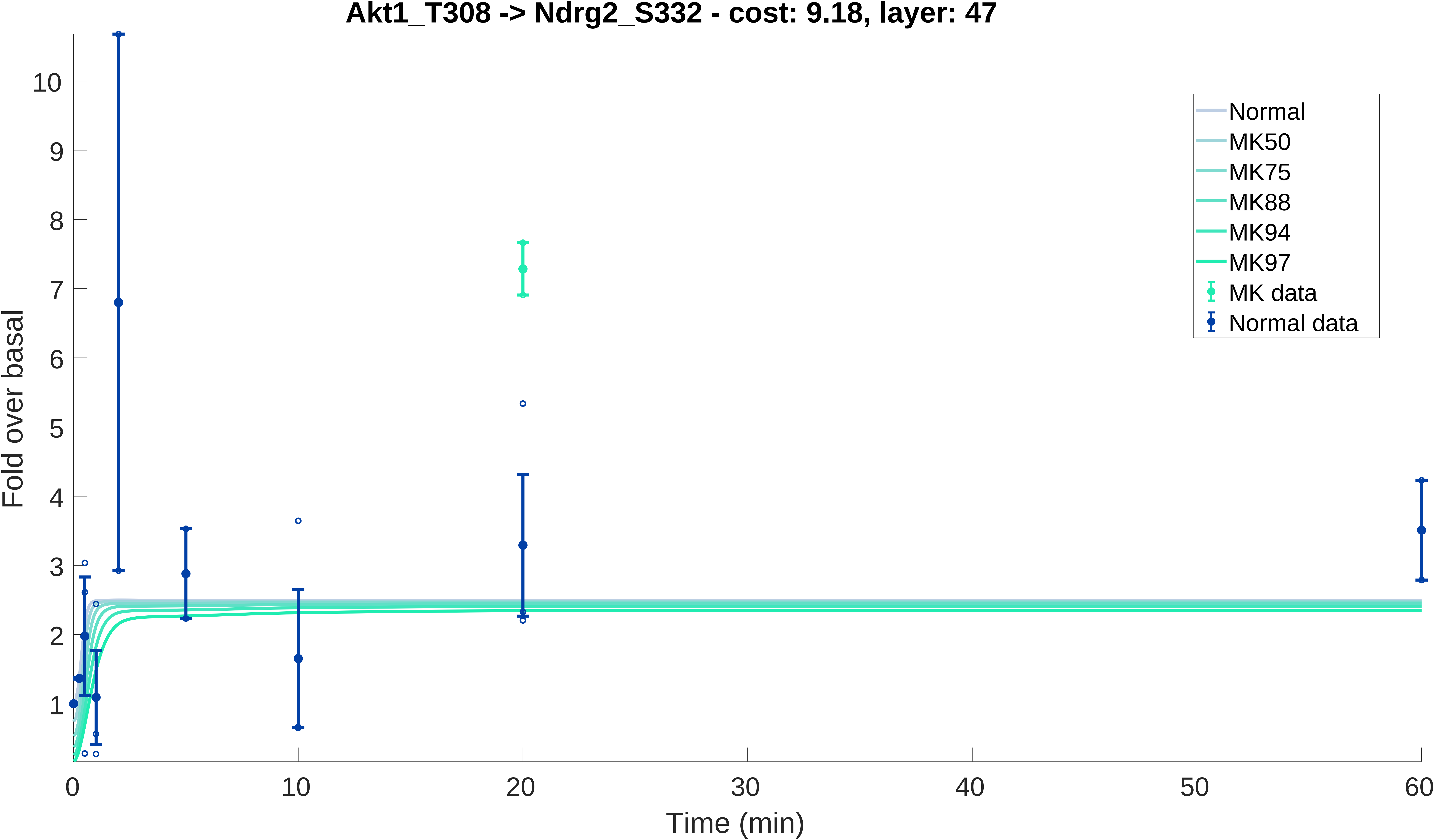
Akt1_T308 -> Ndrg2_S332 - cost: 9.18, layer: 47
Fold over basal
Time (min)
0
10
20
30
40
50
60
1
2
3
4
5
6
7
8
9
10
Normal
MK50
MK75
MK88
MK94
MK97
MK data
Normal data

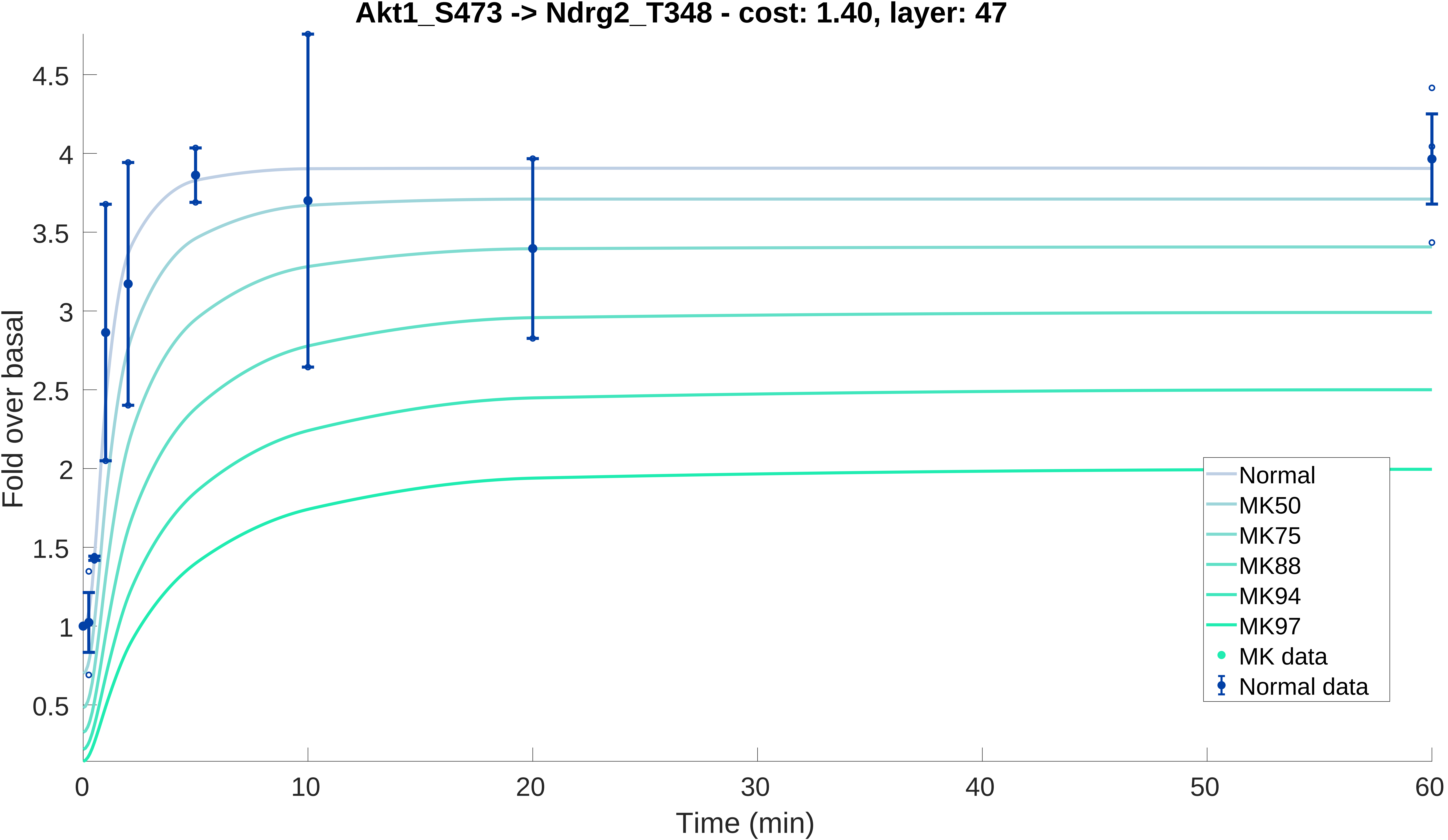
Akt1_S473 -> Ndrg2_T348 - cost: 1.40, layer: 47
Fold over basal
Time (min)
0
10
20
30
40
50
60
0.5
1
1.5
2
2.5
3
3.5
4
4.5
Normal
MK50
MK75
MK88
MK94
MK97
MK data
Normal data

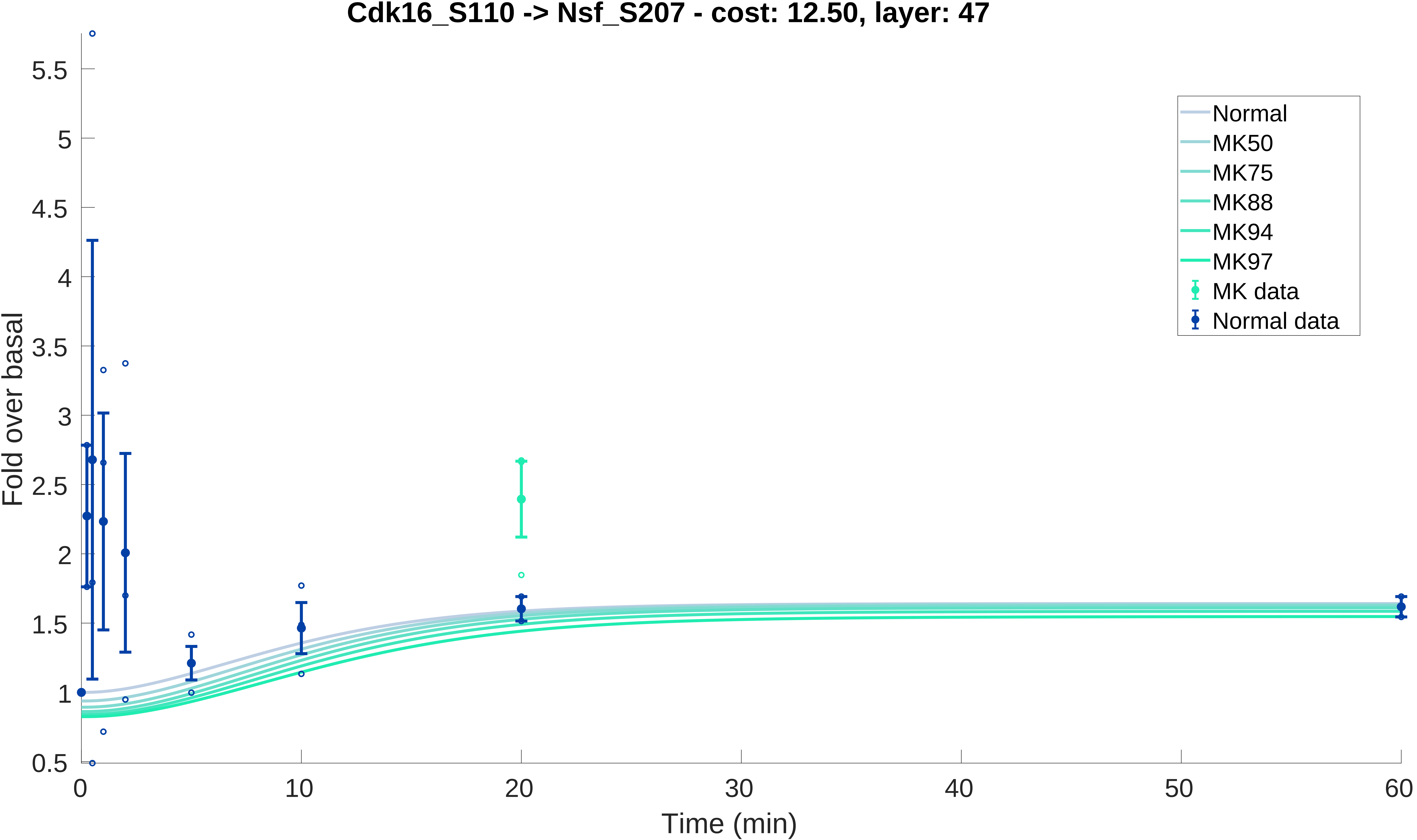

Cdk16_S110 -> Nsf_S207 - cost: 12.50, layer: 47
Fold over basal
Time (min)
0.5
1
1.5
2
2.5
3
3.5
4
4.5
5
5.5
0
10
20
30
40
50
60
Normal
MK50
MK75
MK88
MK94
MK97
MK data
Normal data

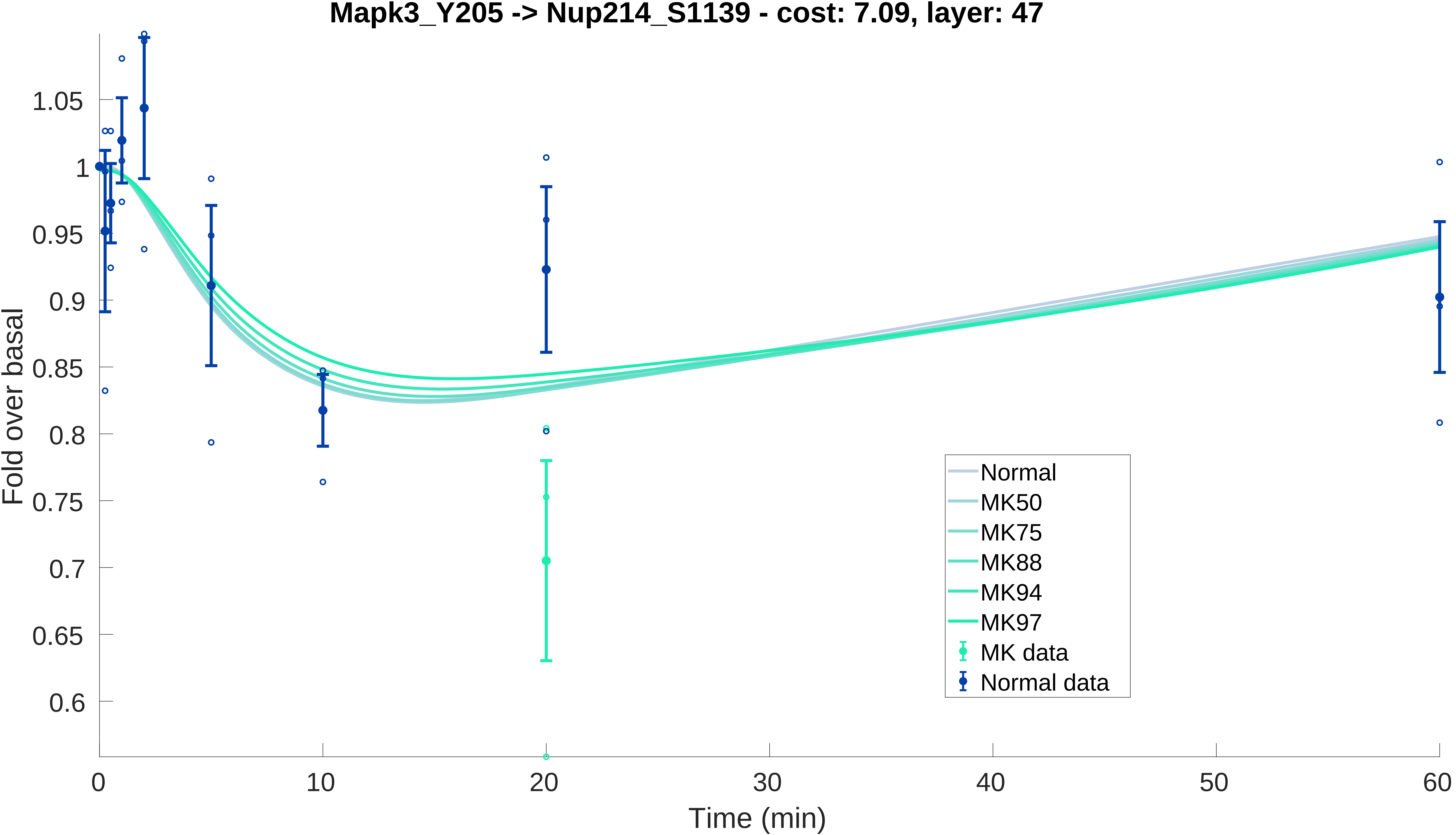
Mapk3_Y205 -> Nup214_S1139 - cost: 7.09, layer: 47
Fold over basal
Time (min)
1.05
1
0.95
0.9
0.85
0.8
0.75
0.7
0.65
0.6
0
10
20
30
40
50
60
Normal
MK50
MK75
MK88
MK94
MK97
MK data
Normal data

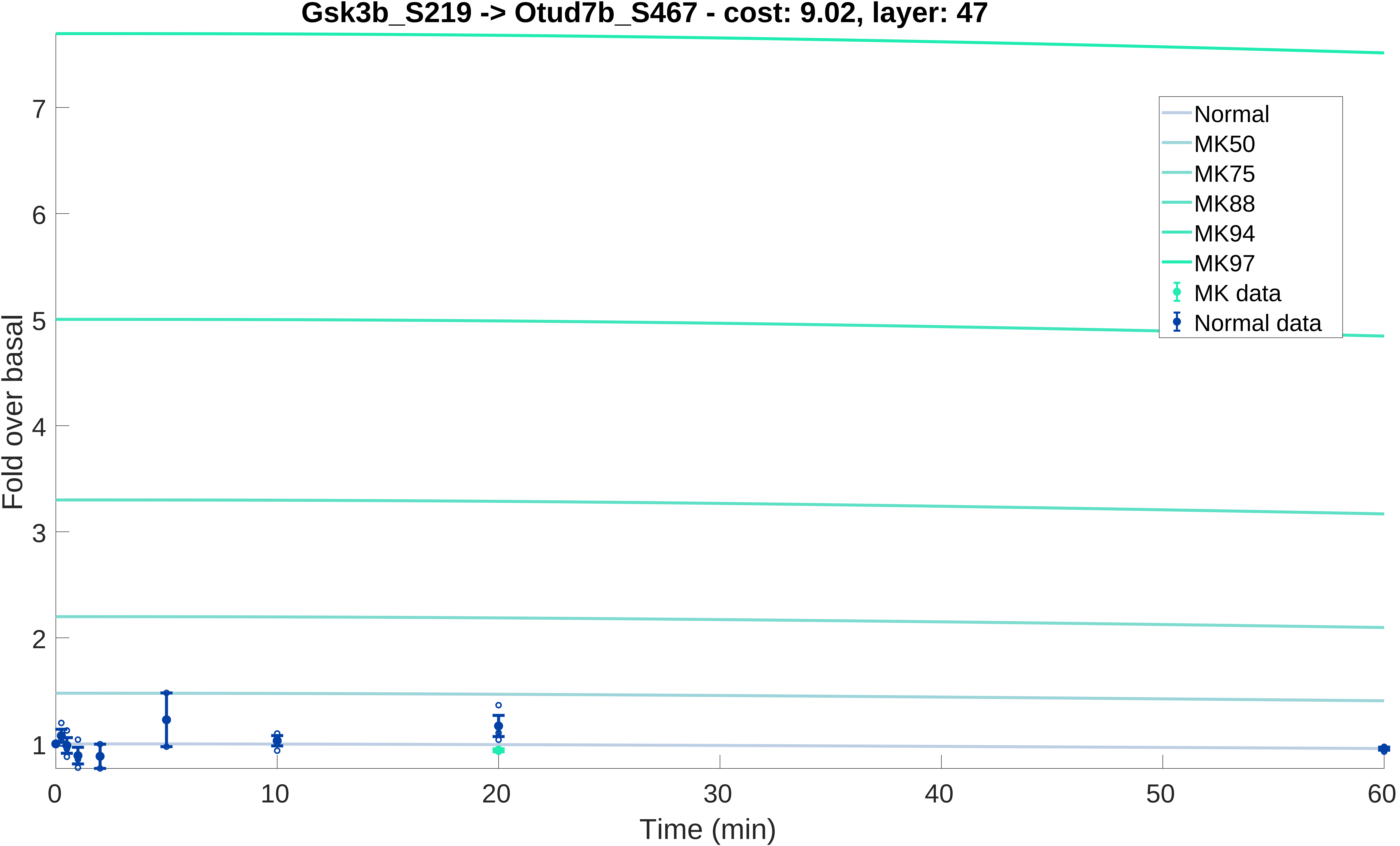

Gsk3b_S219 -> Otud7b_S467 - cost: 9.02, layer: 47
Fold over basal
Time (min)
Normal
MK50
MK75
MK88
MK94
MK97
MK data
Normal data
1
2
3
4
5
6
7
0
10
20
30
40
50
60

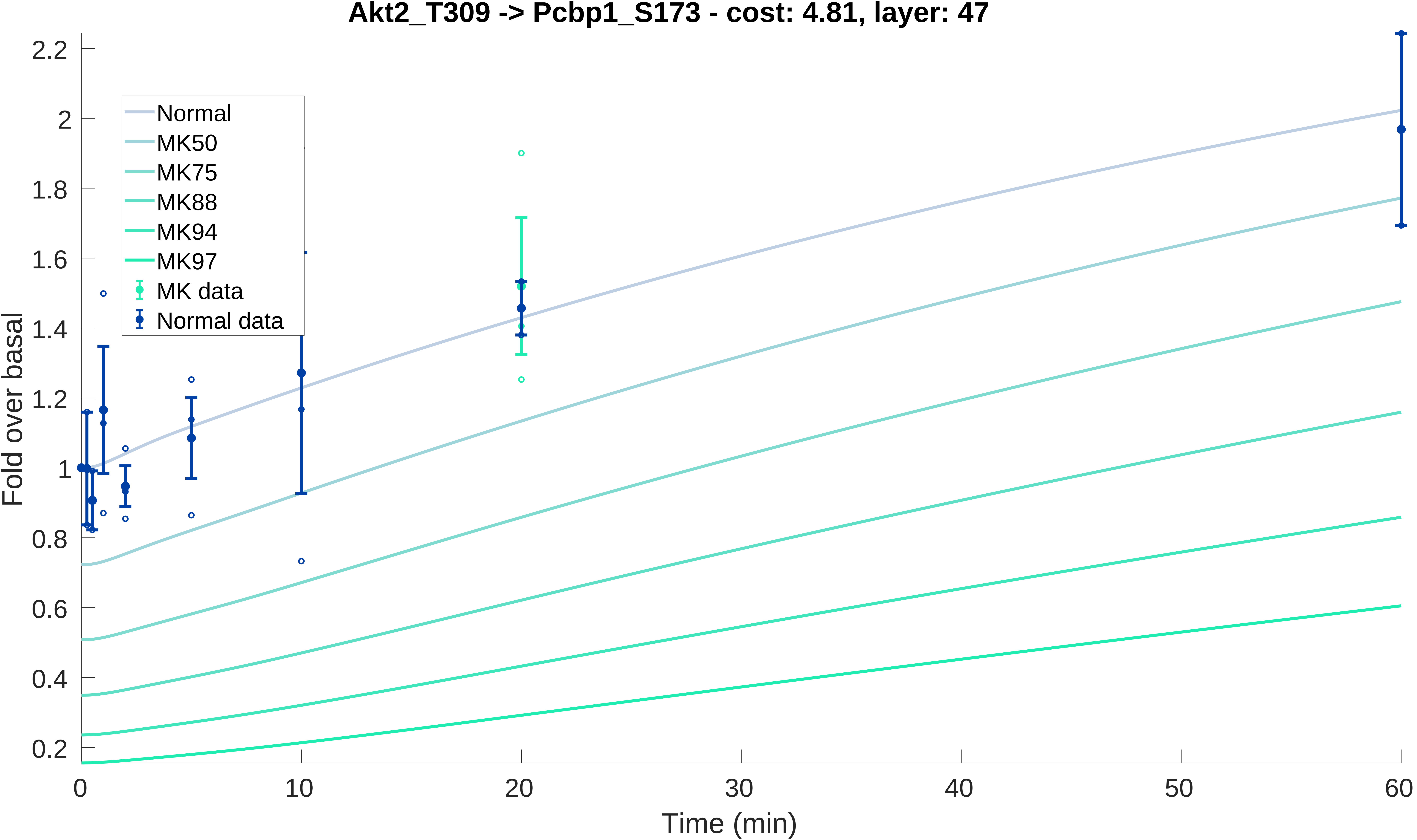

Akt2_T309 -> Pcbp1_S173 - cost: 4.81, layer: 47
Normal
MK50
MK75
MK88
MK94
MK97
MK data
Normal data
Fold over basal
Time (min)
0.2
0.4
0.6
0.8
1
1.2
1.4
1.6
1.8
2
2.2
0
10
20
30
40
50
60

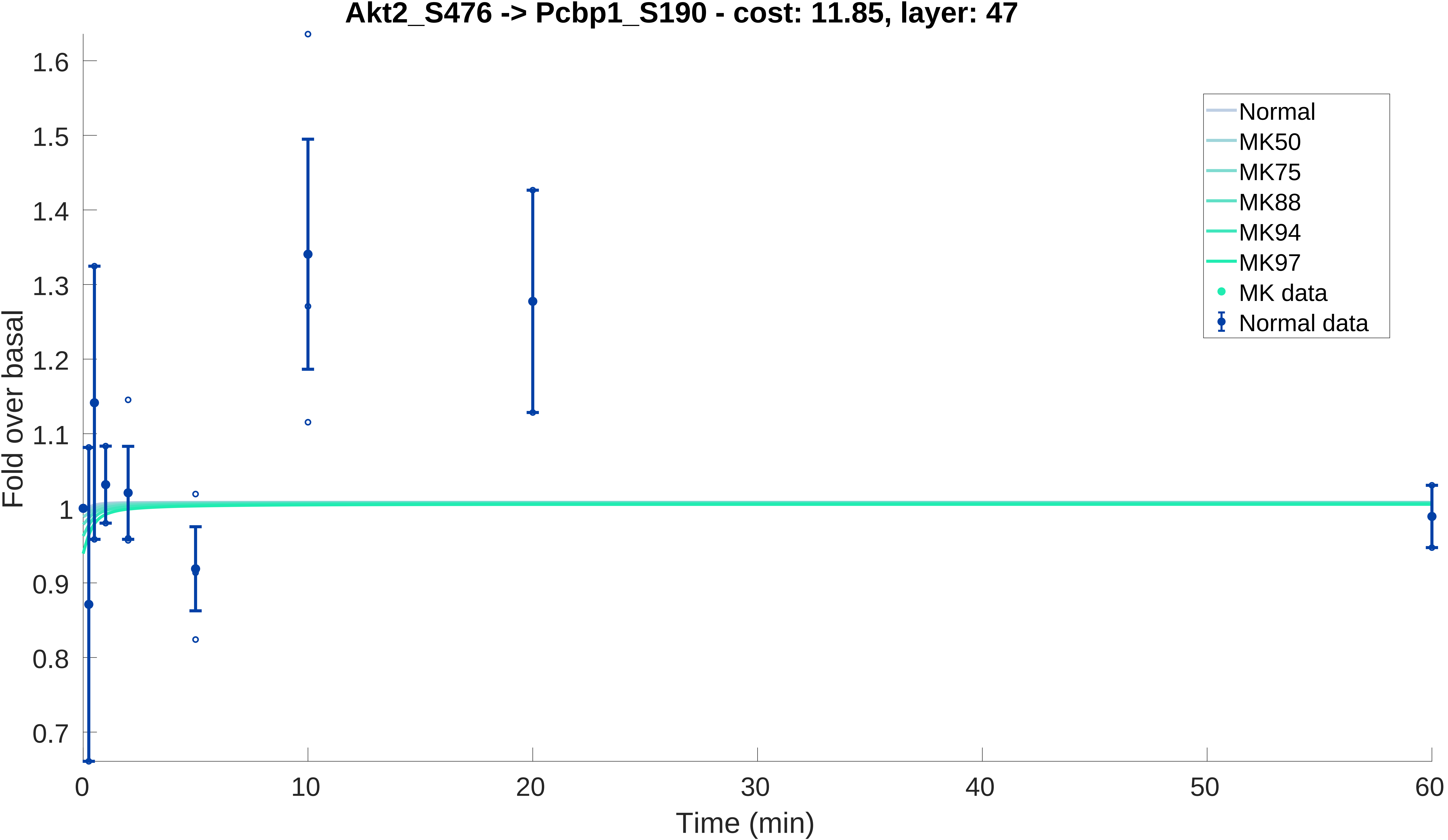
Akt2_S476 -> Pcbp1_S190 - cost: 11.85, layer: 47
Fold over basal
1.6
1.5
1.4
1.3
1.2
1.1
1
0.9
0.8
0.7
0
10
20
30
40
50
60
Time (min)
Normal
MK50
MK75
MK88
MK94
MK97
MK data
Normal data

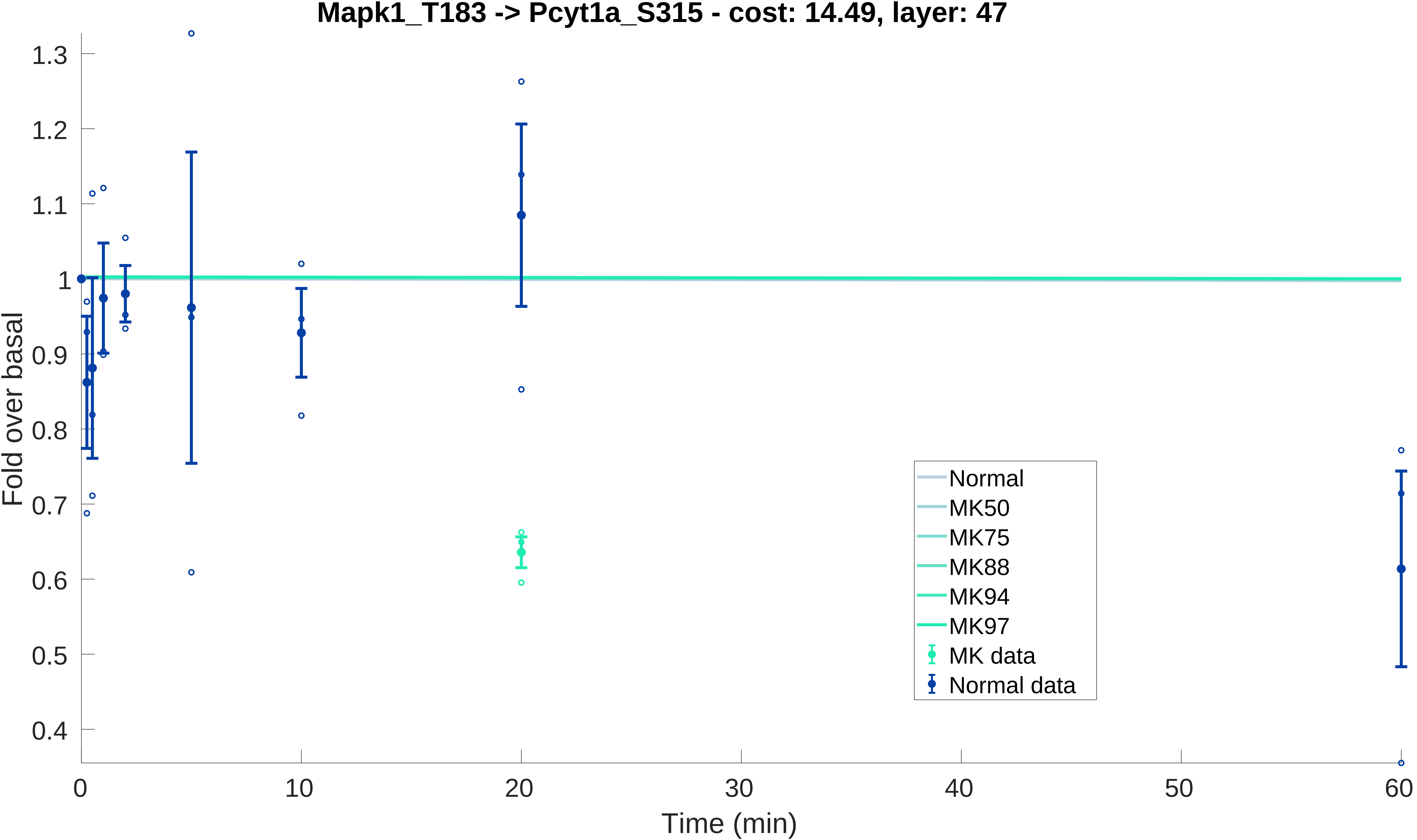

Mapk1_T183 -> Pcyt1a_S315 - cost: 14.49, layer: 47
Fold over basal
Time (min)
1.3
1.2
1.1
1
0.9
0.8
0.7
0.6
0.5
0.4
0
10
20
30
40
50
60
Normal
MK50
MK75
MK88
MK94
MK97
MK data
Normal data

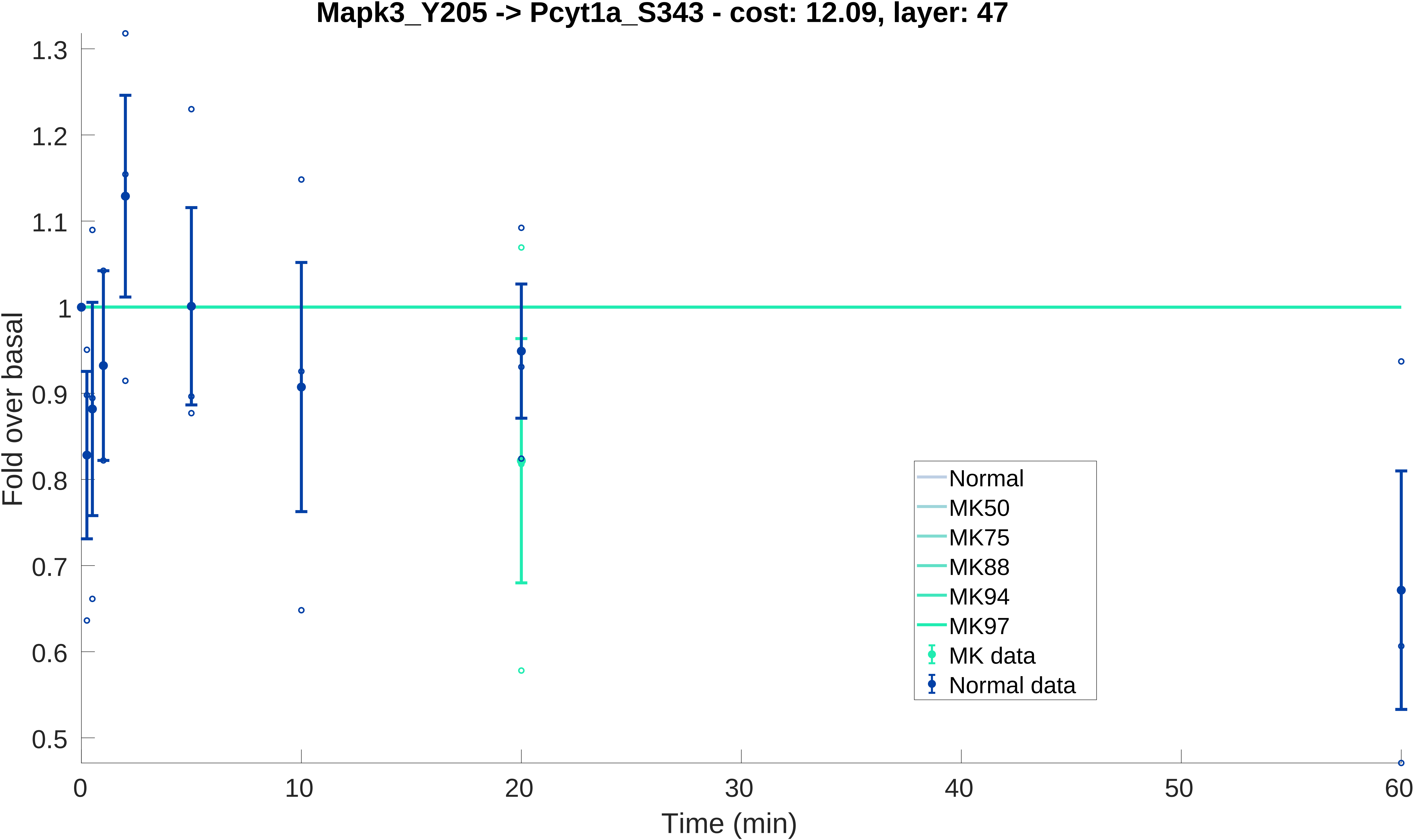

Mapk3_Y205 -> Pcyt1a_S343 - cost: 12.09, layer: 47
Fold over basal
Time (min)
1.3
1.2
1.1
1
0.9
0.8
0.7
0.6
0.5
0
10
20
30
40
50
60
Normal
MK50
MK75
MK88
MK94
MK97
MK data
Normal data

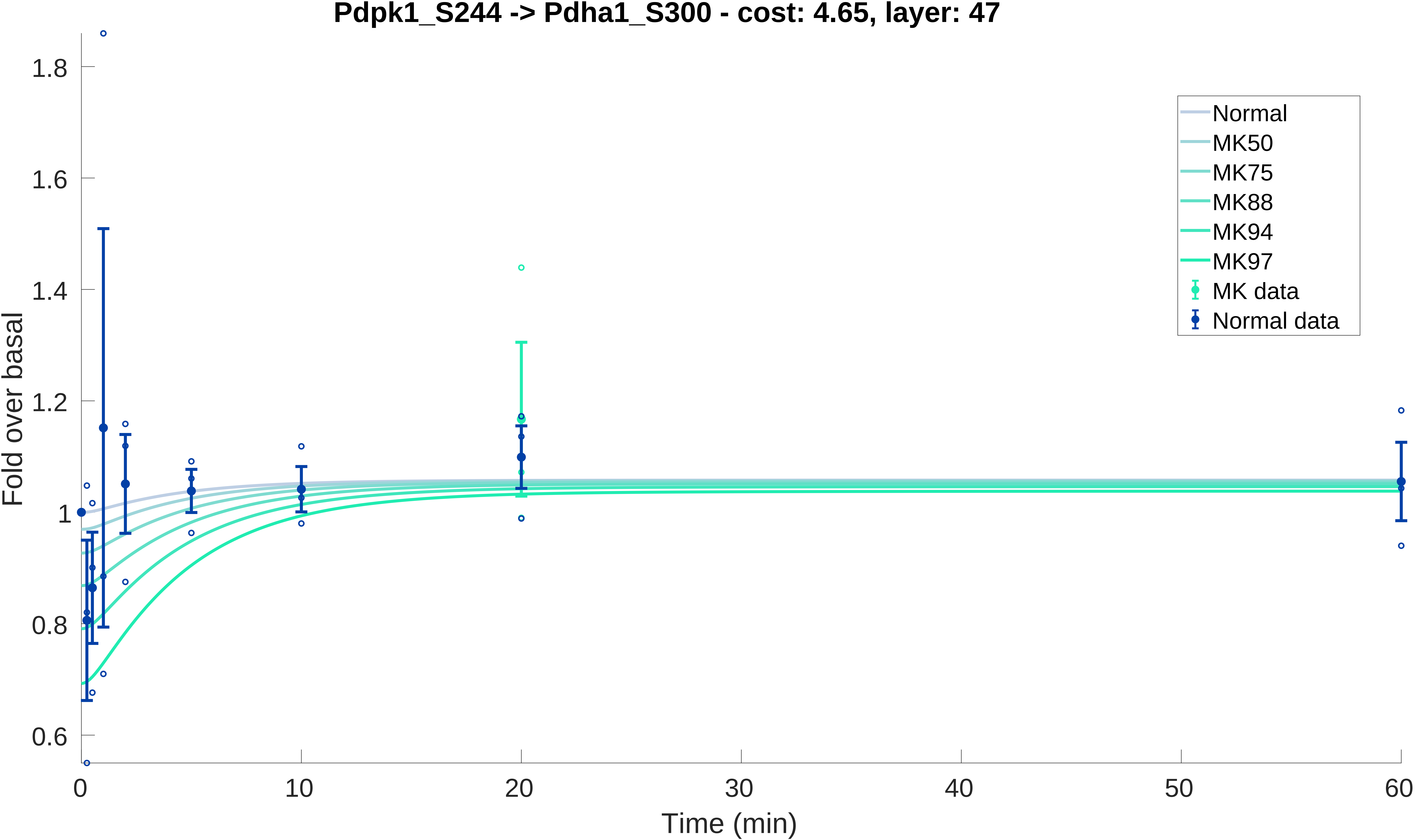

Pdpk1_S244 -> Pdha1_S300 - cost: 4.65, layer: 47
Fold over basal
Time (min)
0
10
20
30
40
50
60
0.6
0.8
1
1.2
1.4
1.6
1.8
Normal
MK50
MK75
MK88
MK94
MK97
MK data
Normal data

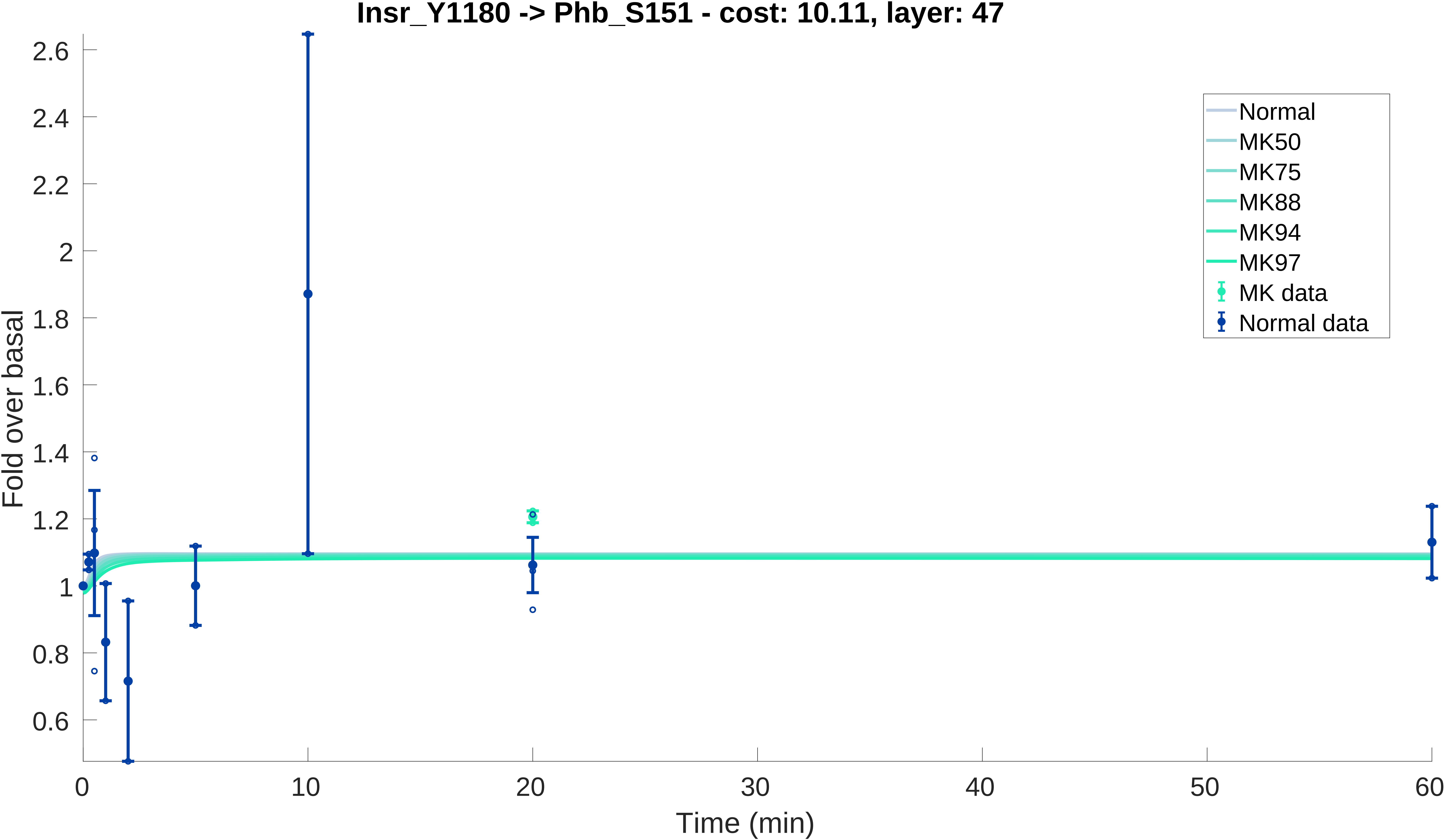
Insr_Y1180 -> Phb_S151 - cost: 10.11, layer: 47
Fold over basal
Time (min)
Normal
MK50
MK75
MK88
MK94
MK97
MK data
Normal data
2.6
2.4
2.2
2
1.8
1.6
1.4
1.2
1
0.8
0.6
0
10
20
30
40
50
60

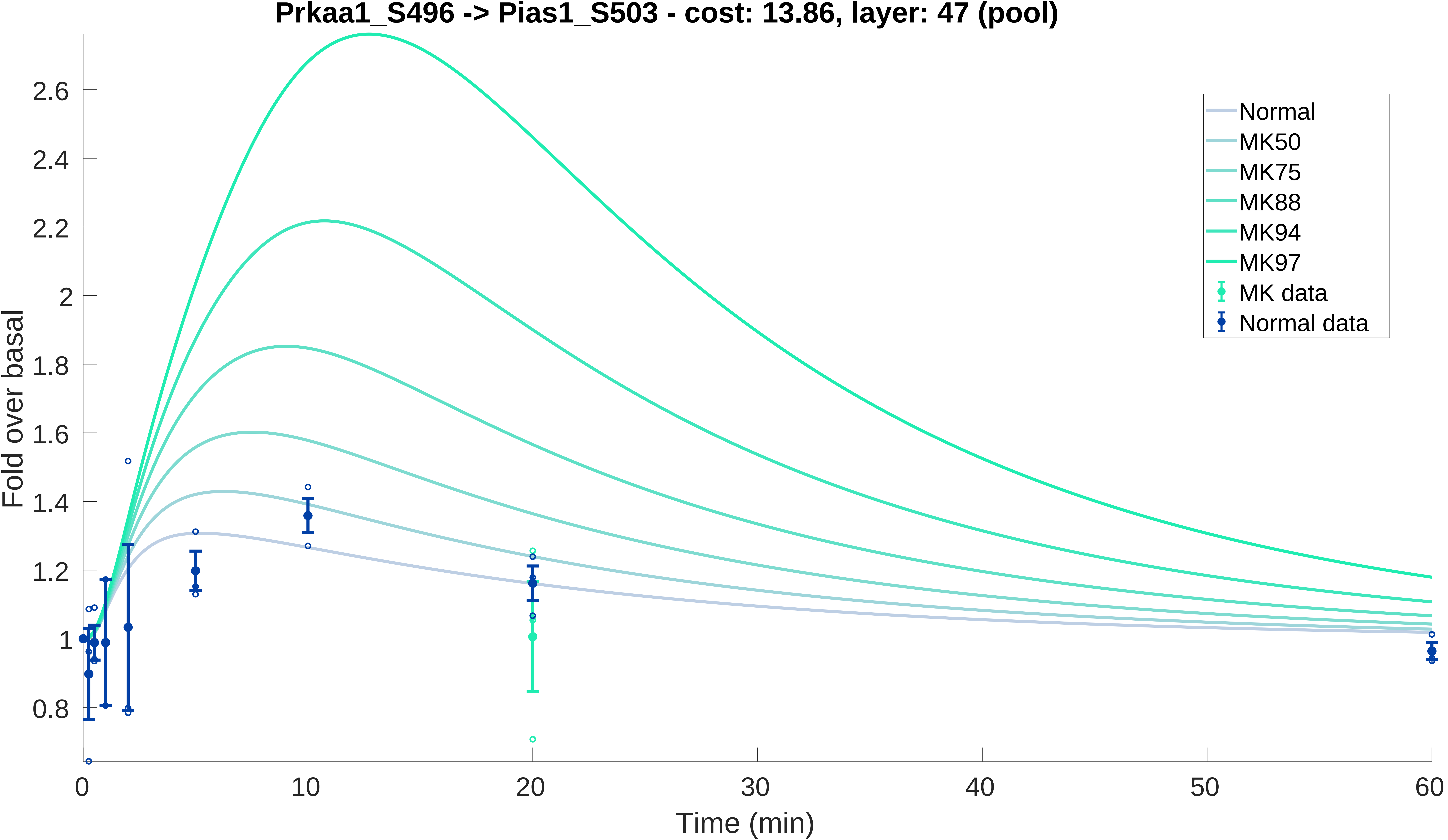

Prkaa1_S496 -> Pias1_S503 - cost: 13.86, layer: 47 (pool)
Fold over basal
Time (min)
0.8
1
1.2
1.4
1.6
1.8
2
2.2
2.4
2.6
0
10
20
30
40
50
60
Normal
MK50
MK75
MK88
MK94
MK97
MK data
Normal data

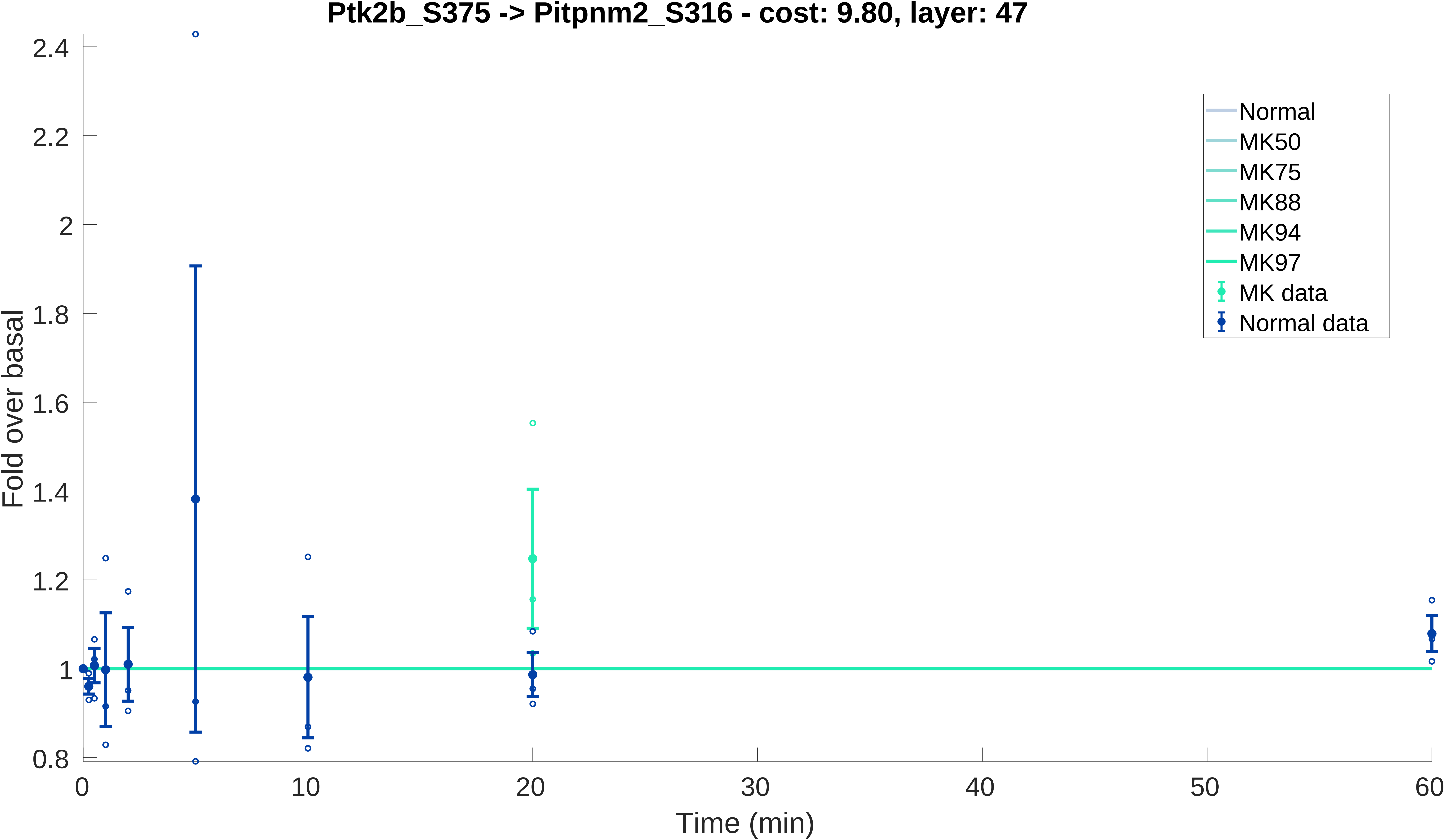

Ptk2b_S375 -> Pitpnm2_S316 - cost: 9.80, layer: 47
Fold over basal
Time (min)
2.4
2.2
2
1.8
1.6
1.4
1.2
1
0.8
0
10
20
30
40
50
60
Normal
MK50
MK75
MK88
MK94
MK97
MK data
Normal data

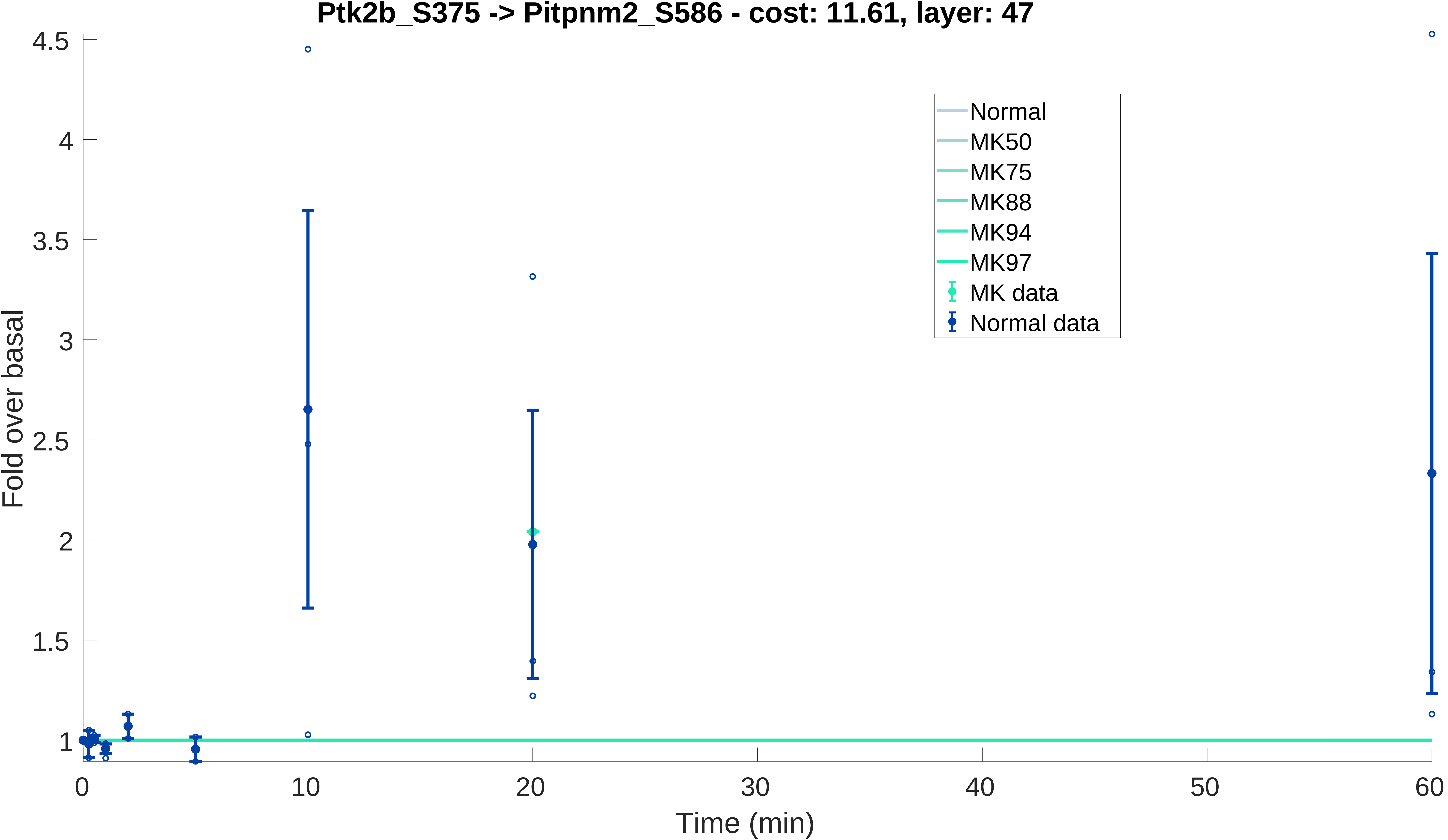

Ptk2b_S375 -> Pitpnm2_S586 - cost: 11.61, layer: 47
Fold over basal
Time (min)
4.5
4
3.5
3
2.5
2
1.5
1
0
10
20
30
40
50
60
Normal
MK50
MK75
MK88
MK94
MK97
MK data
Normal data

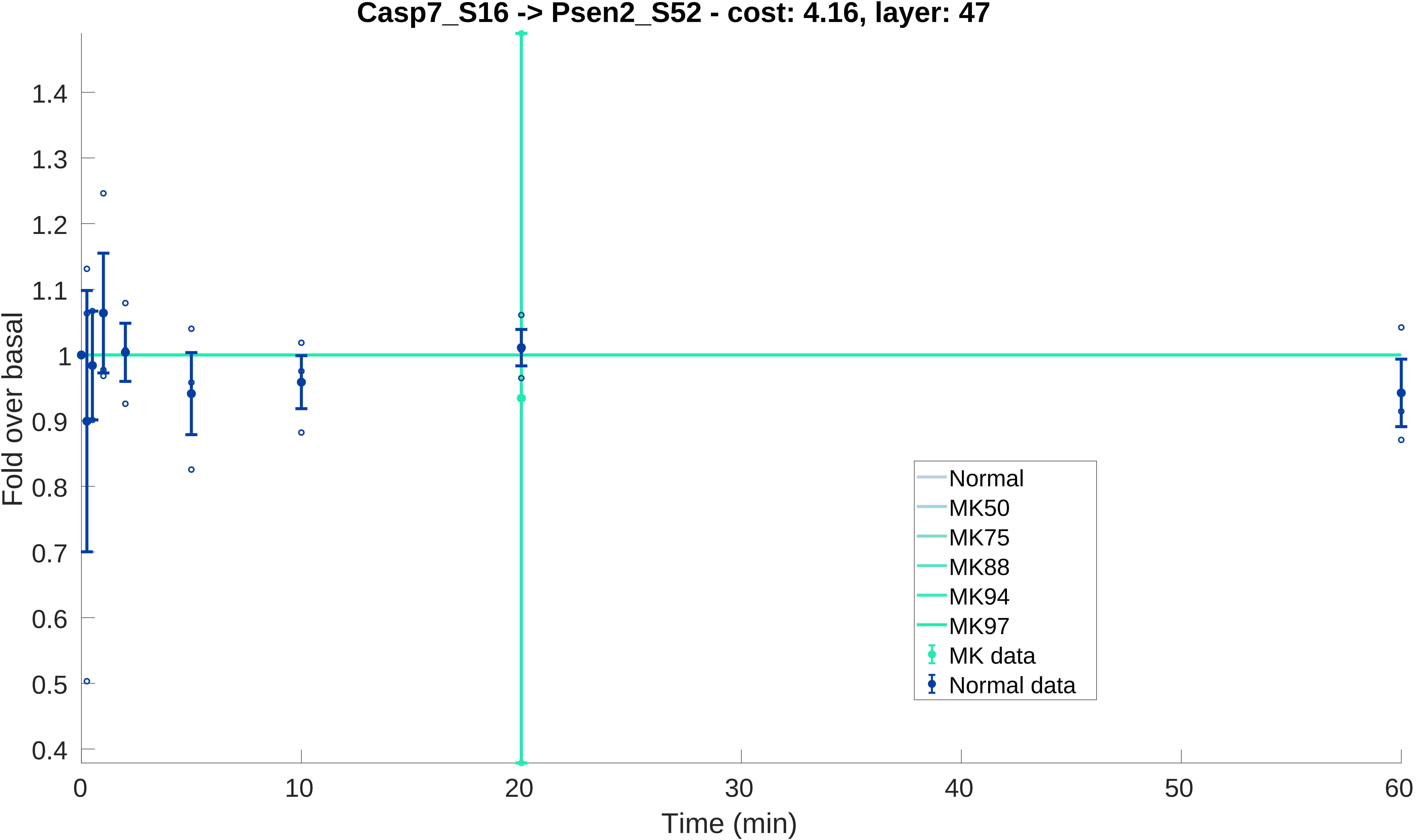

Casp7_S16 -> Psen2_S52 - cost: 4.16, layer: 47
Fold over basal
Time (min)
1.4
1.3
1.2
1.1
1
0.9
0.8
0.7
0.6
0.5
0.4
0
10
20
30
40
50
60
Normal
MK50
MK75
MK88
MK94
MK97
MK data
Normal data

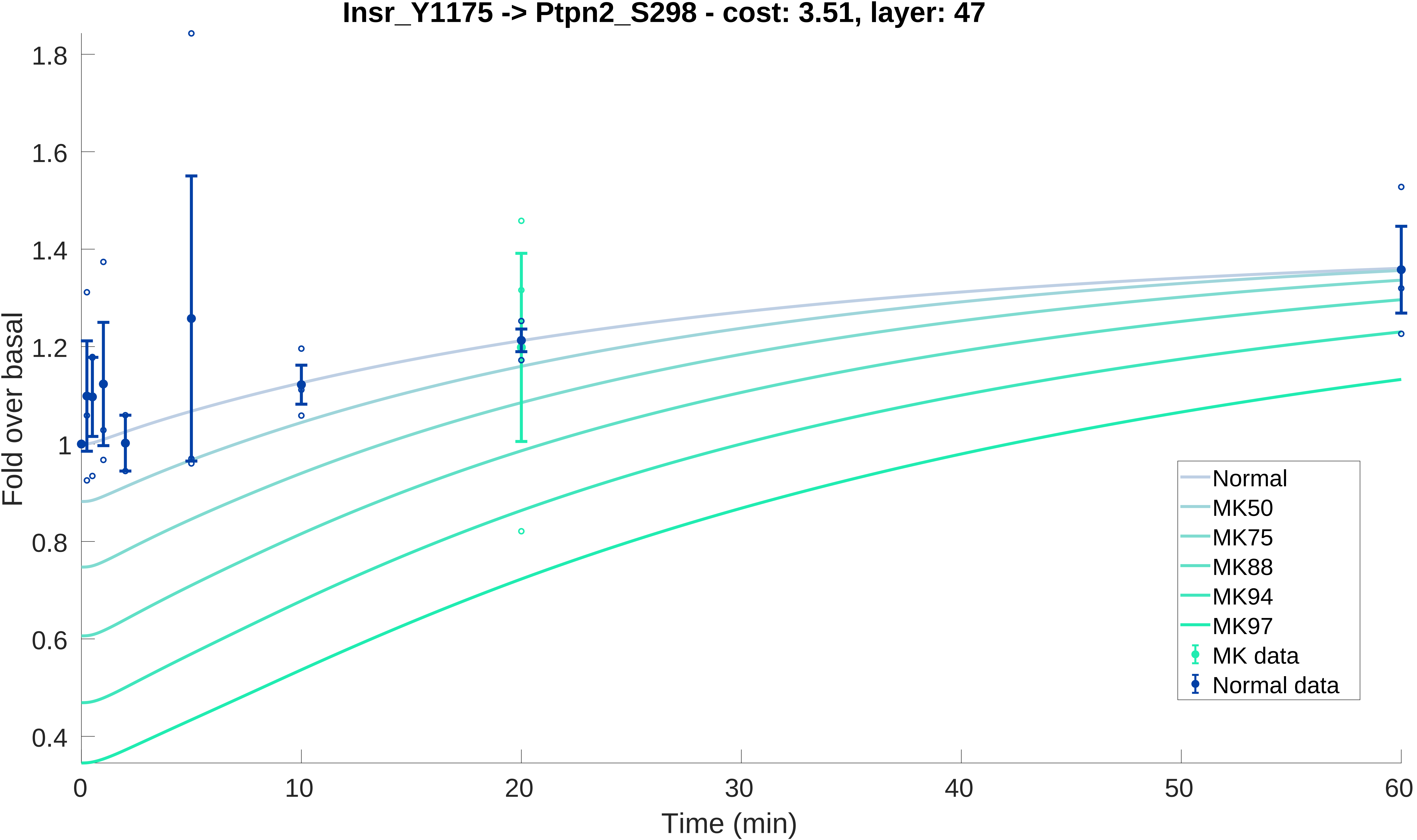
Insr_Y1175 -> Ptpn2_S298 - cost: 3.51, layer: 47
Fold over basal
1.8
1.6
1.4
1.2
1
0.8
0.6
0.4
0
10
20
30
40
50
60
Time (min)
Normal
MK50
MK75
MK88
MK94
MK97
MK data
Normal data

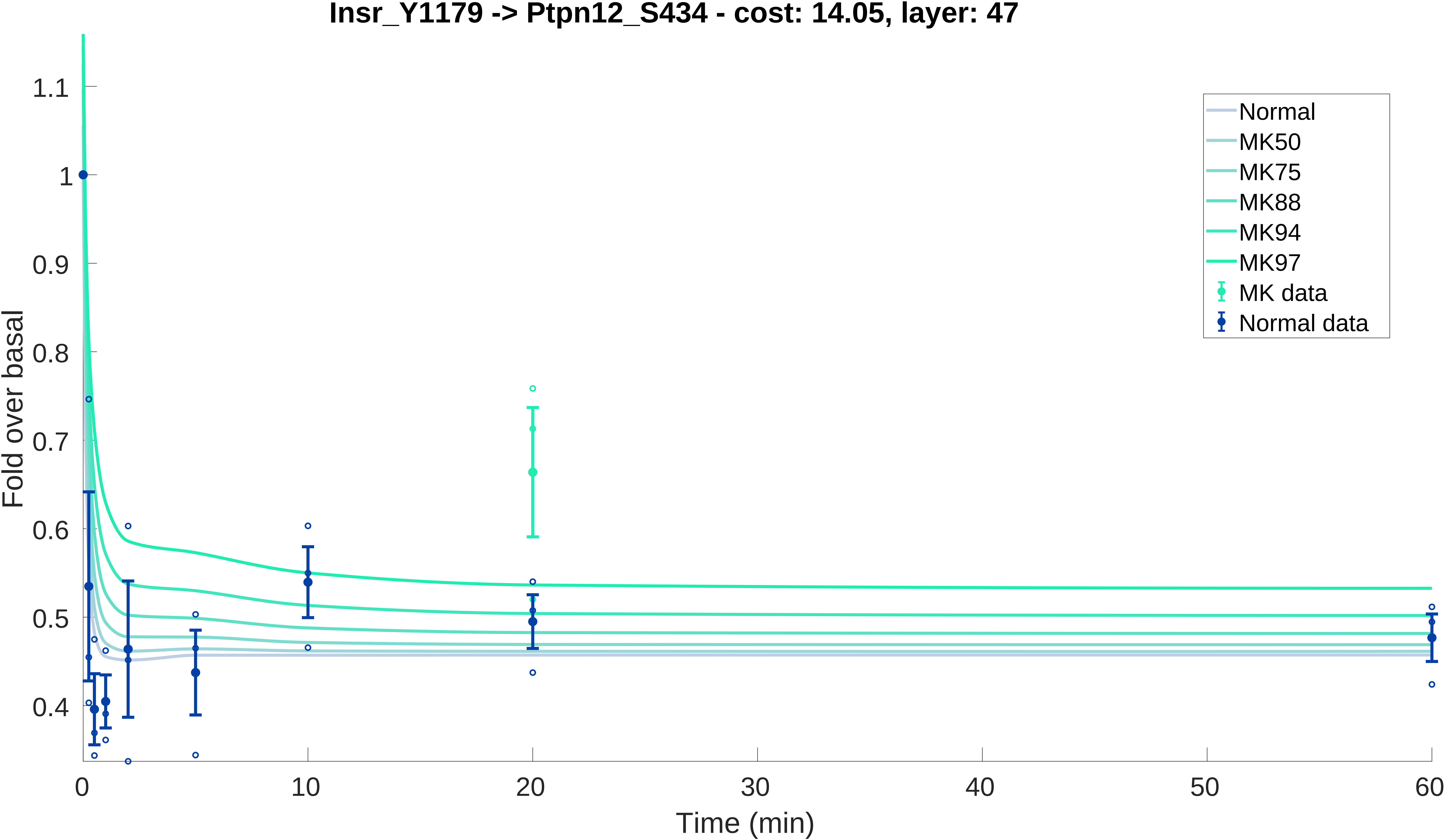
Insr_Y1179 -> Ptpn12_S434 - cost: 14.05, layer: 47
Fold over basal
Time (min)
Normal
MK50
MK75
MK88
MK94
MK97
MK data
Normal data
1.1
1
0.9
0.8
0.7
0.6
0.5
0.4
0
10
20
30
40
50
60

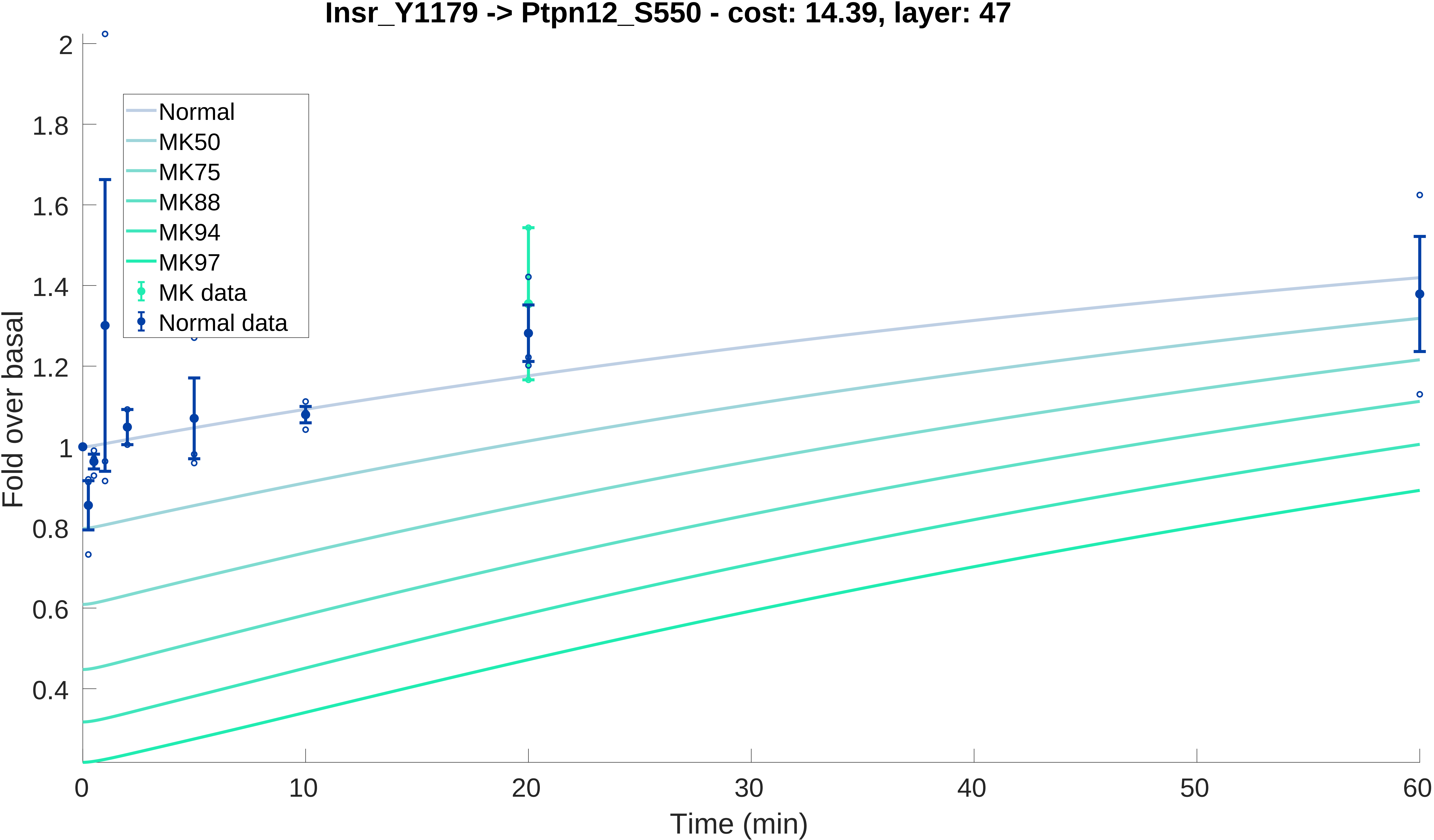

Insr_Y1179 -> Ptpn12_S550 - cost: 14.39, layer: 47
Normal
MK50
MK75
MK88
MK94
MK97
MK data
Normal data
Fold over basal
Time (min)
2
1.8
1.6
1.4
1.2
1
0.8
0.6
0.4
0
10
20
30
40
50
60

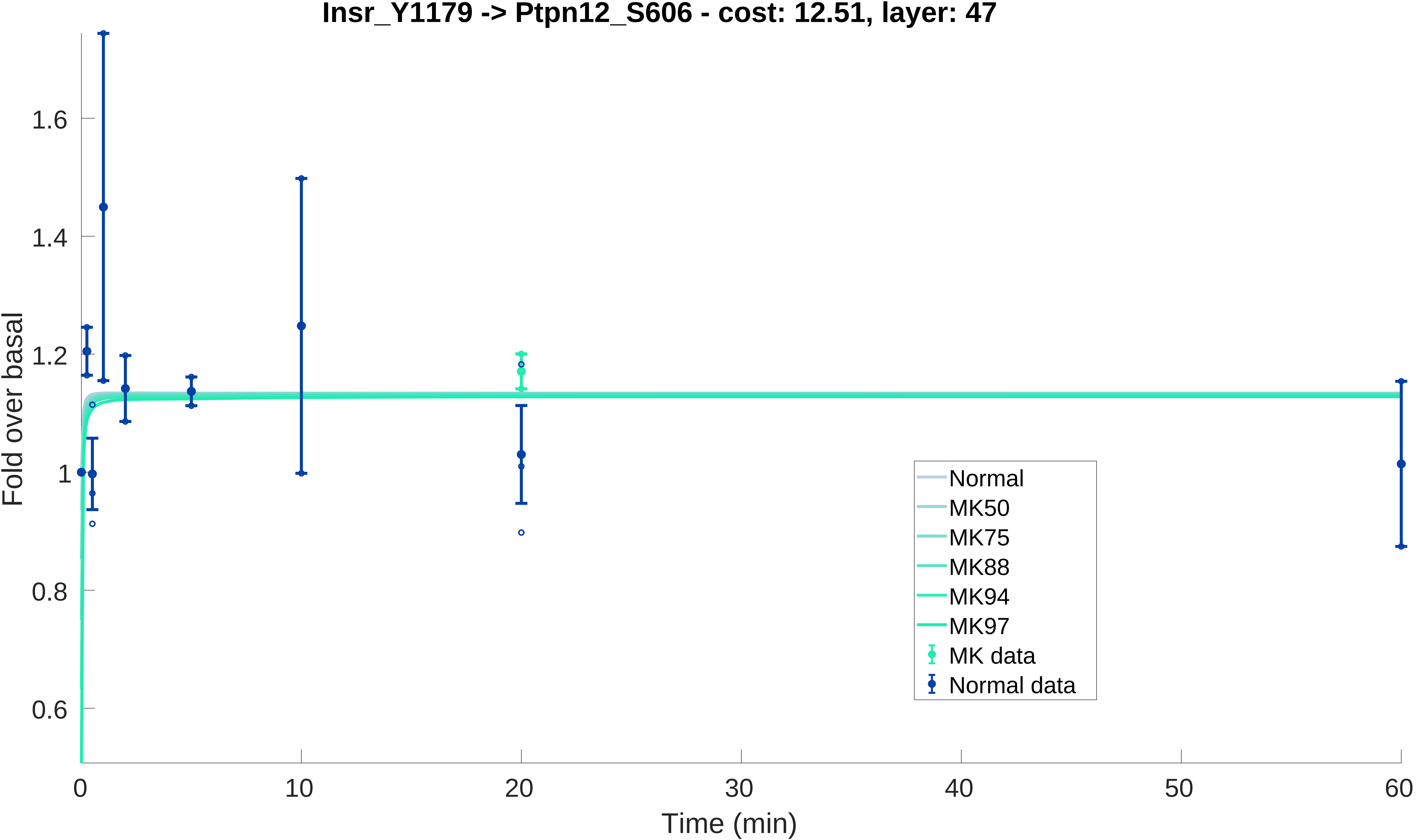
Insr_Y1179 -> Ptpn12_S606 - cost: 12.51, layer: 47
Fold over basal
1.6
1.4
1.2
1
0.8
0.6
0
10
20
30
40
50
60
Time (min)
Normal
MK50
MK75
MK88
MK94
MK97
MK data
Normal data

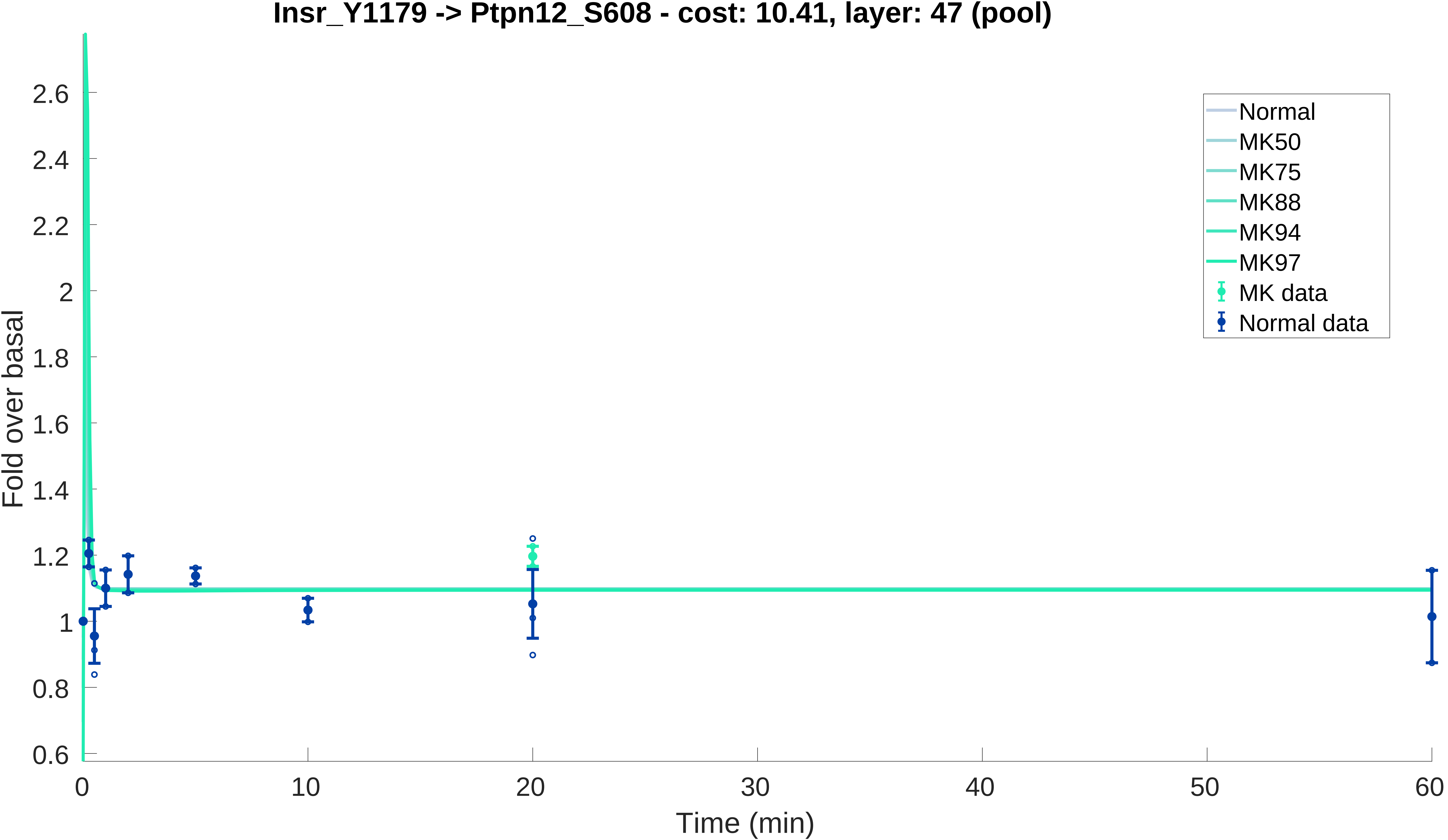

Insr_Y1179 -> Ptpn12_S608 - cost: 10.41, layer: 47 (pool)
Fold over basal
Time (min)
Normal
MK50
MK75
MK88
MK94
MK97
MK data
Normal data
0.6
0.8
1
1.2
1.4
1.6
1.8
2
2.2
2.4
2.6
0
10
20
30
40
50
60

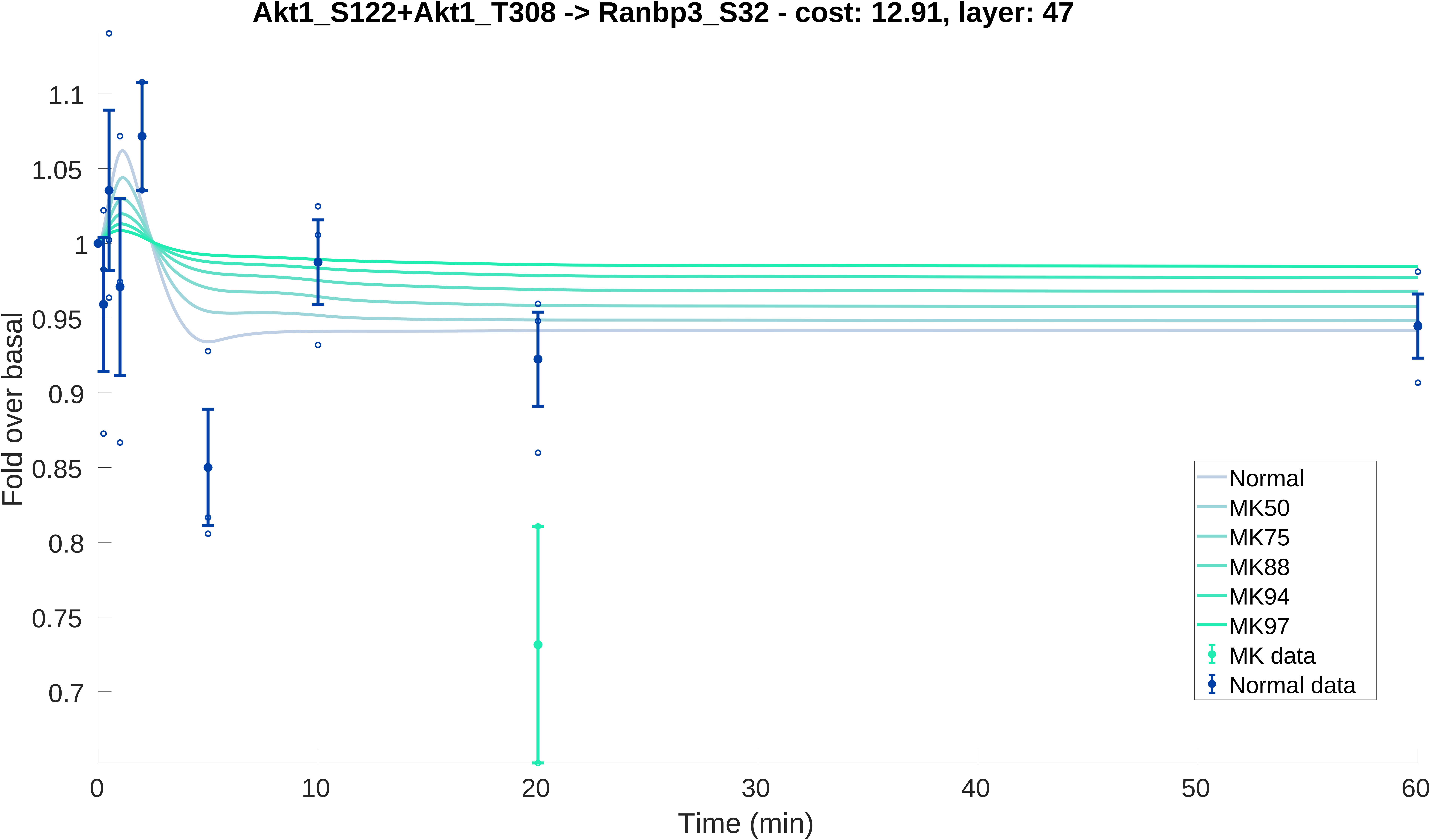

Akt1_S122+Akt1_T308 -> Ranbp3_S32 - cost: 12.91, layer: 47
Fold over basal
1.1
1.05
1
0.95
0.9
0.85
0.8
0.75
0.7
0
10
20
30
40
50
60
Time (min)
Normal
MK50
MK75
MK88
MK94
MK97
MK data
Normal data

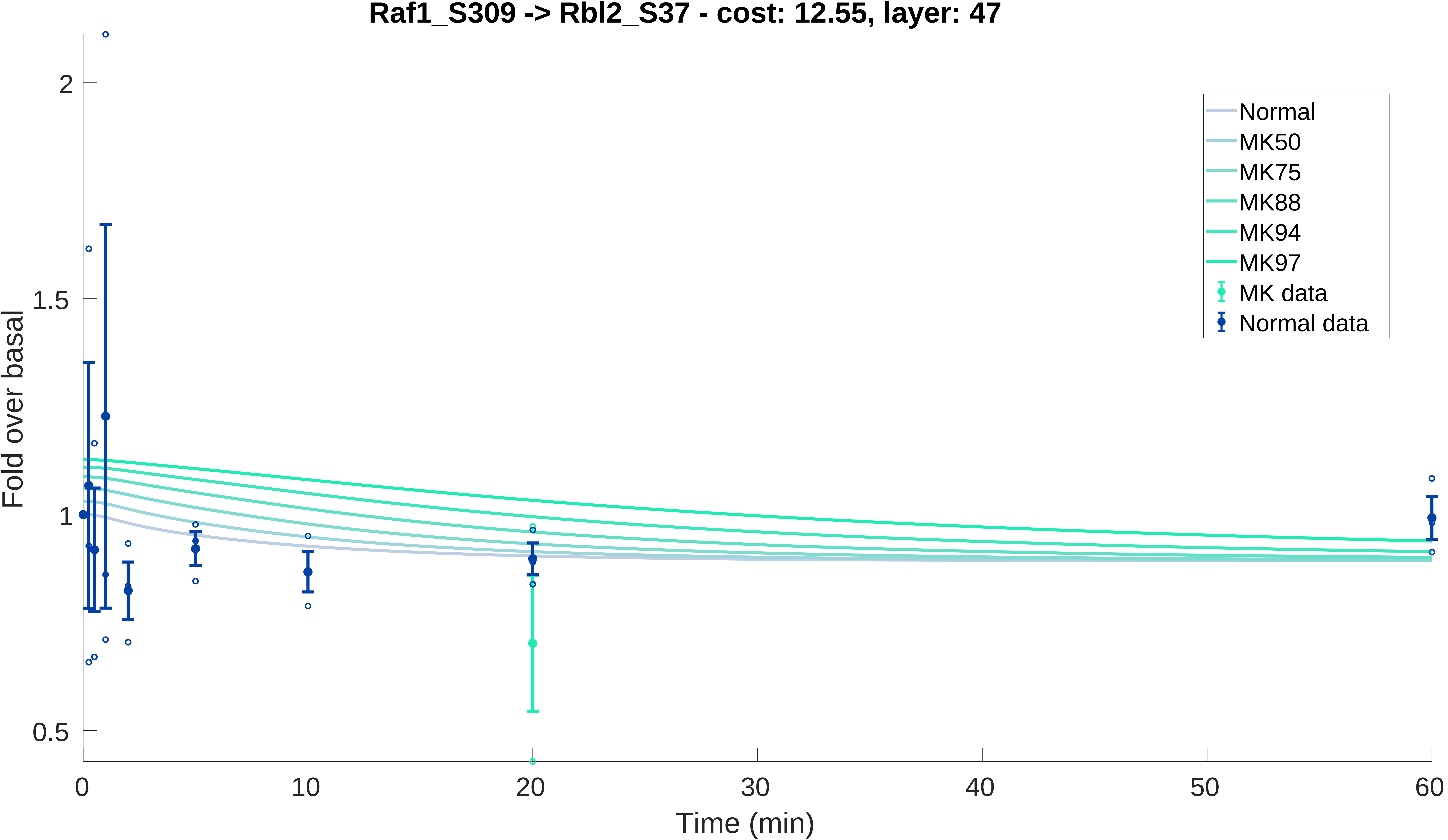

Raf1_S309 -> Rbl2_S37 - cost: 12.55, layer: 47
Fold over basal
Time (min)
0
10
20
30
40
50
60
0.5
1
1.5
2
Normal
MK50
MK75
MK88
MK94
MK97
MK data
Normal data

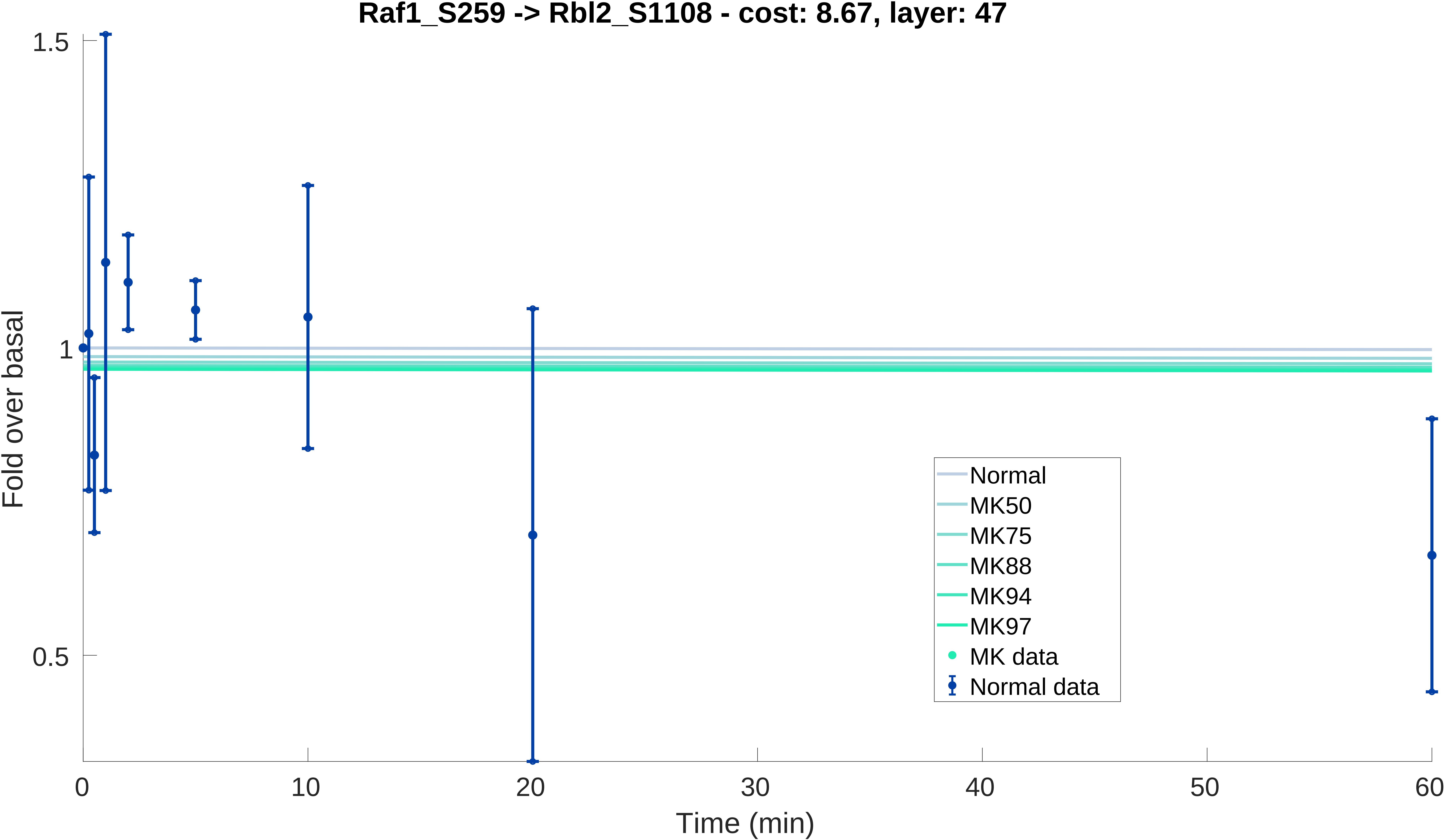

Raf1_S259 -> Rbl2_S1108 - cost: 8.67, layer: 47
Fold over basal
1.5
1
0.5
0
10
20
30
40
50
60
Time (min)
Normal
MK50
MK75
MK88
MK94
MK97
MK data
Normal data

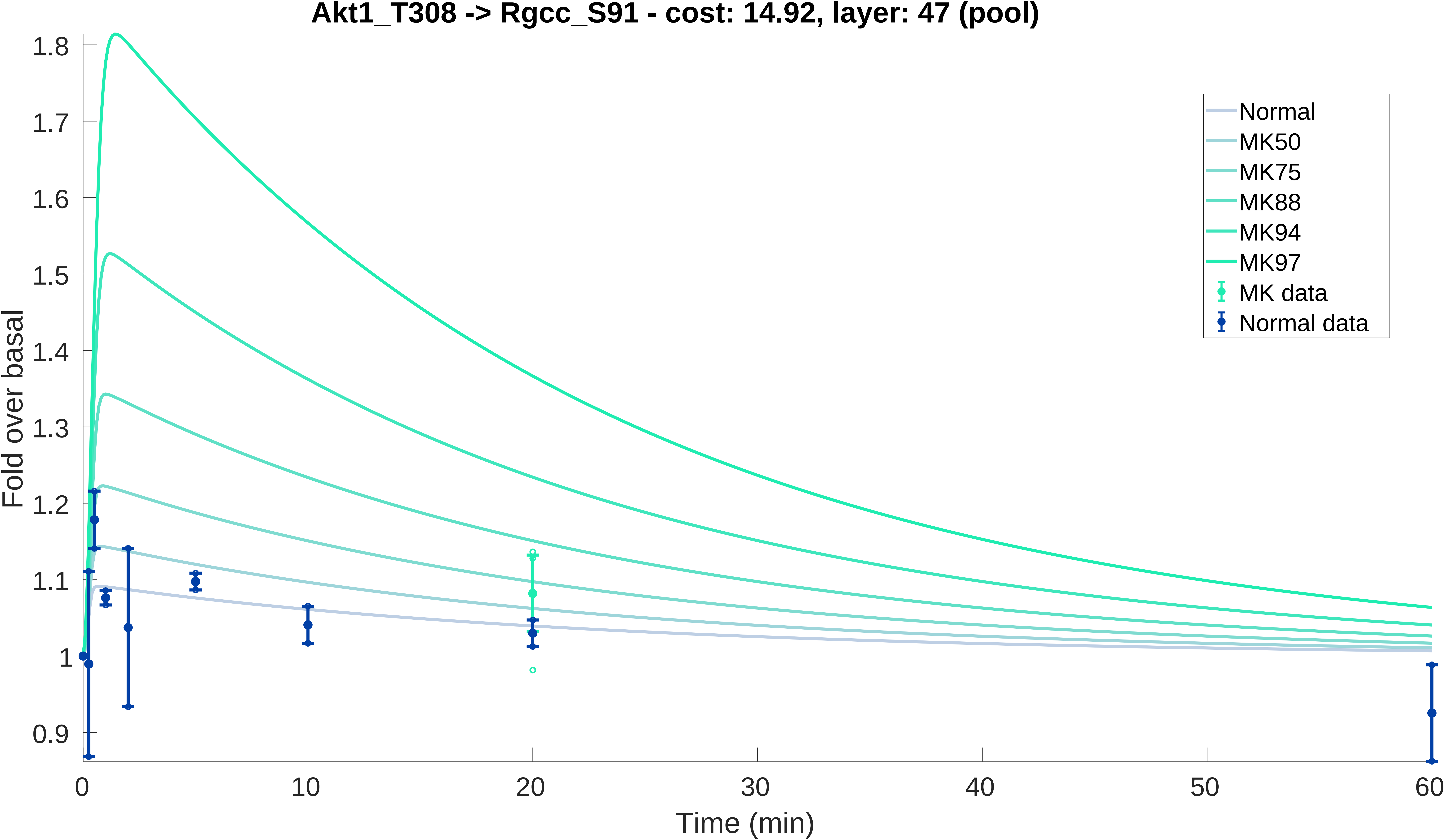

Akt1_T308 -> Rgcc_S91 - cost: 14.92, layer: 47 (pool)
Fold over basal
Time (min)
1.8
1.7
1.6
1.5
1.4
1.3
1.2
1.1
1
0.9
0
10
20
30
40
50
60
Normal
MK50
MK75
MK88
MK94
MK97
MK data
Normal data

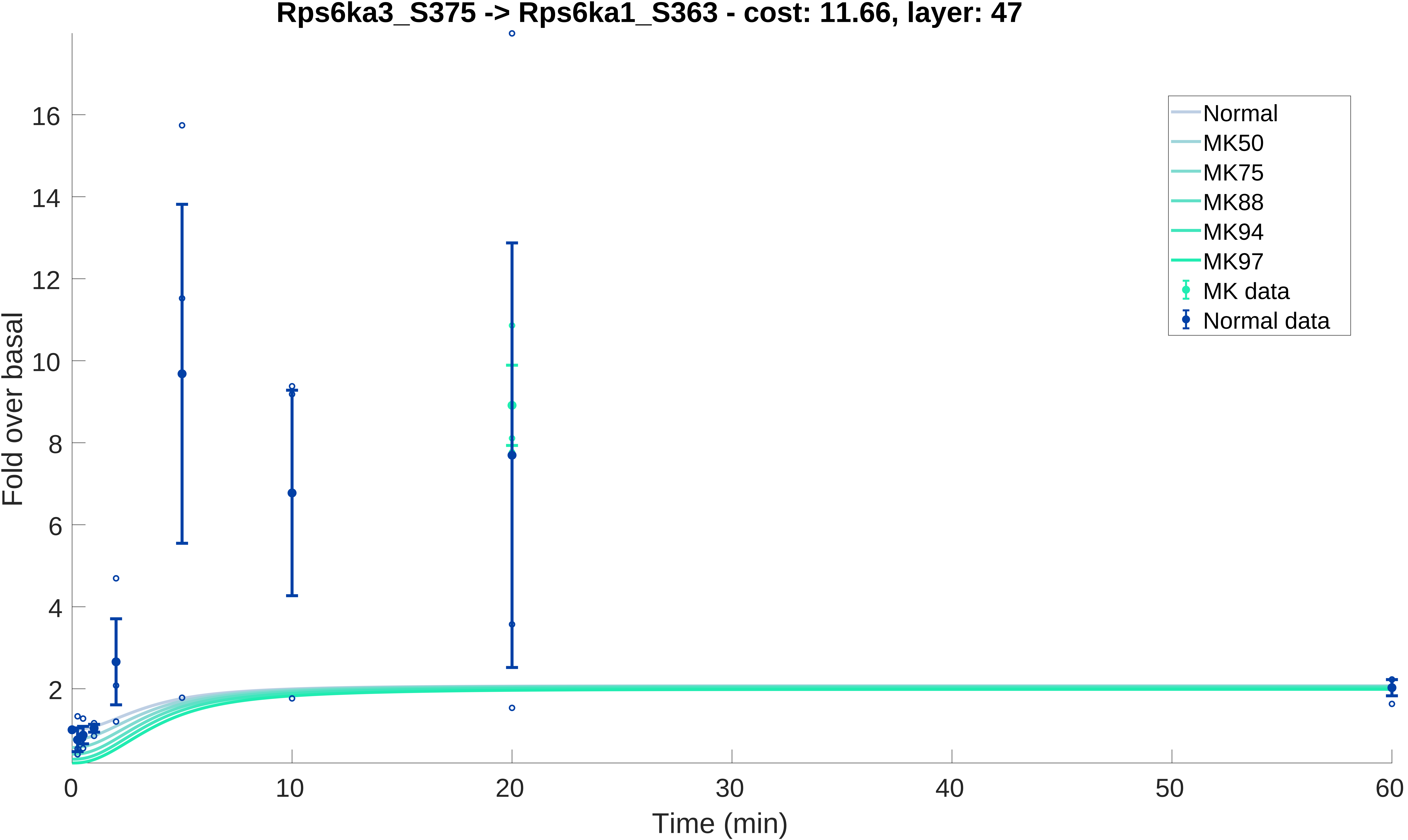
Rps6ka3_S375 -> Rps6ka1_S363 - cost: 11.66, layer: 47
Fold over basal
Time (min)
0
10
20
30
40
50
60
2
4
6
8
10
12
14
16
Normal
MK50
MK75
MK88
MK94
MK97
MK data
Normal data

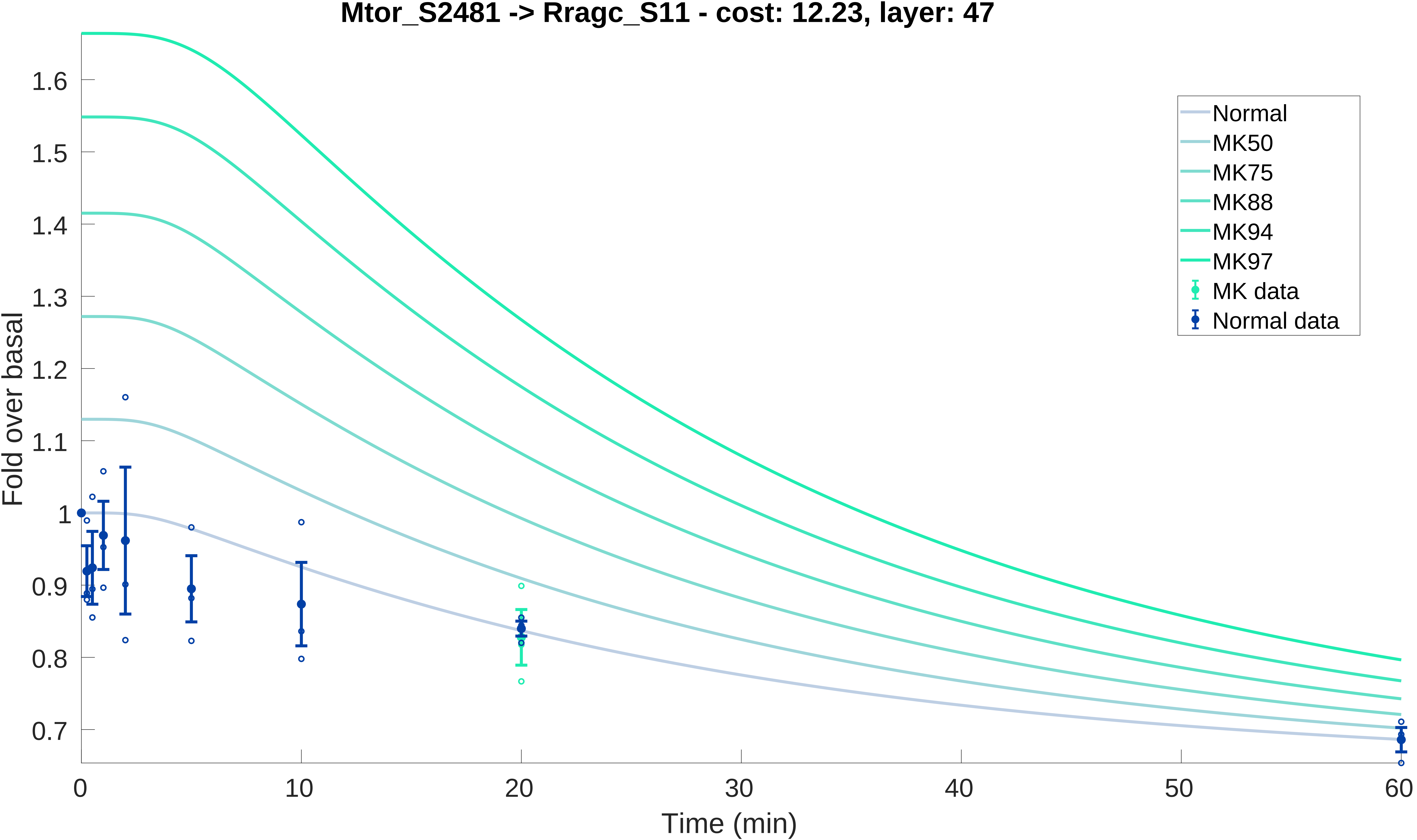

Mtor_S2481 -> Rragc_S11 - cost: 12.23, layer: 47
Fold over basal
Time (min)
1.6
1.5
1.4
1.3
1.2
1.1
1
0.9
0.8
0.7
0
10
20
30
40
50
60
Normal
MK50
MK75
MK88
MK94
MK97
MK data
Normal data

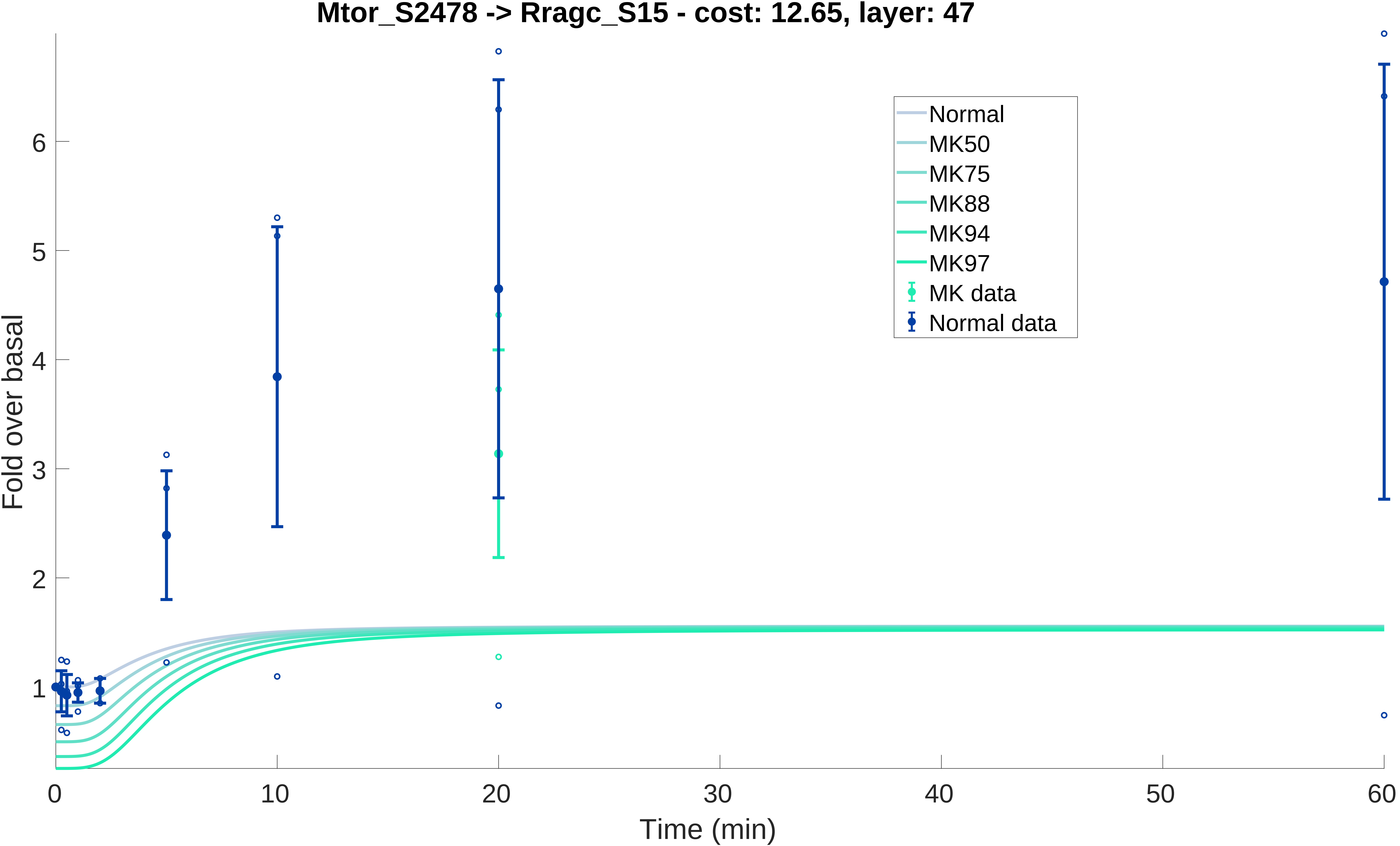

Mtor_S2478 -> Rragc_S15 - cost: 12.65, layer: 47
Fold over basal
Time (min)
Normal
MK50
MK75
MK88
MK94
MK97
MK data
Normal data
0
10
20
30
40
50
60
1
2
3
4
5
6

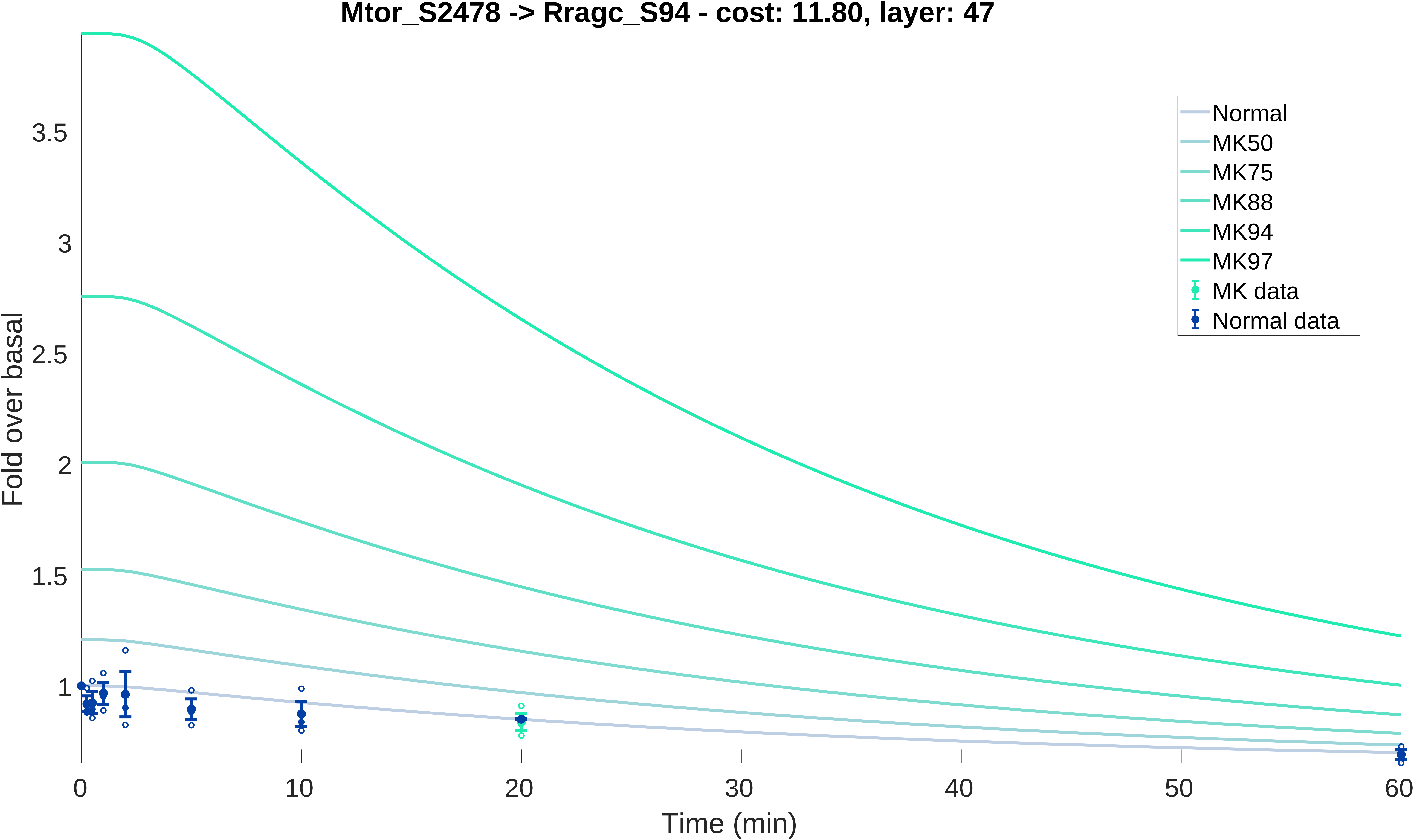
Mtor_S2478 -> Rragc_S94 - cost: 11.80, layer: 47
Fold over basal
Time (min)
3.5
3
2.5
2
1.5
1
0
10
20
30
40
50
60
Normal
MK50
MK75
MK88
MK94
MK97
MK data
Normal data

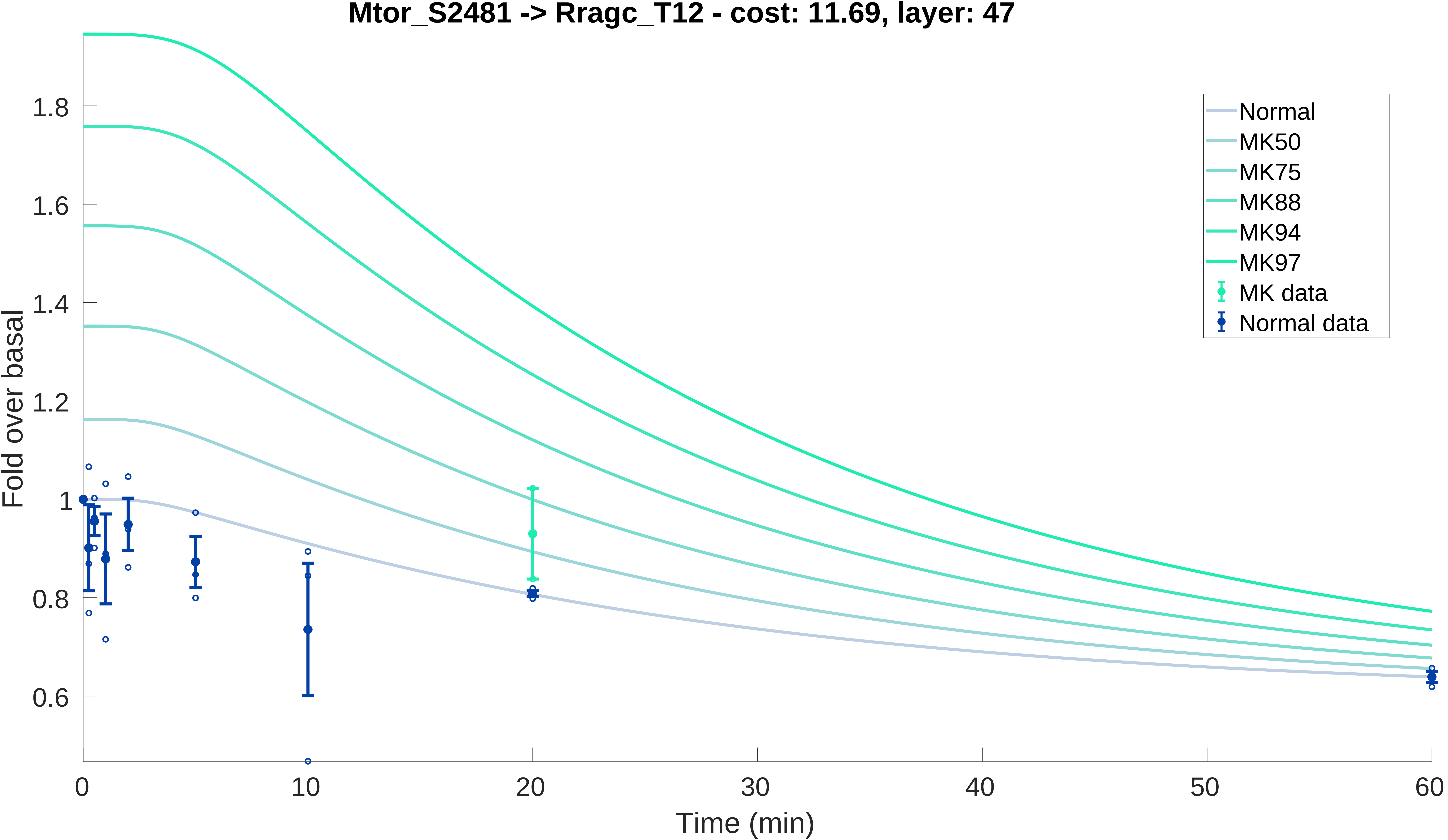

Mtor_S2481 -> Rragc_T12 - cost: 11.69, layer: 47
Fold over basal
Time (min)
1.8
1.6
1.4
1.2
1
0.8
0.6
0
10
20
30
40
50
60
Normal
MK50
MK75
MK88
MK94
MK97
MK data
Normal data

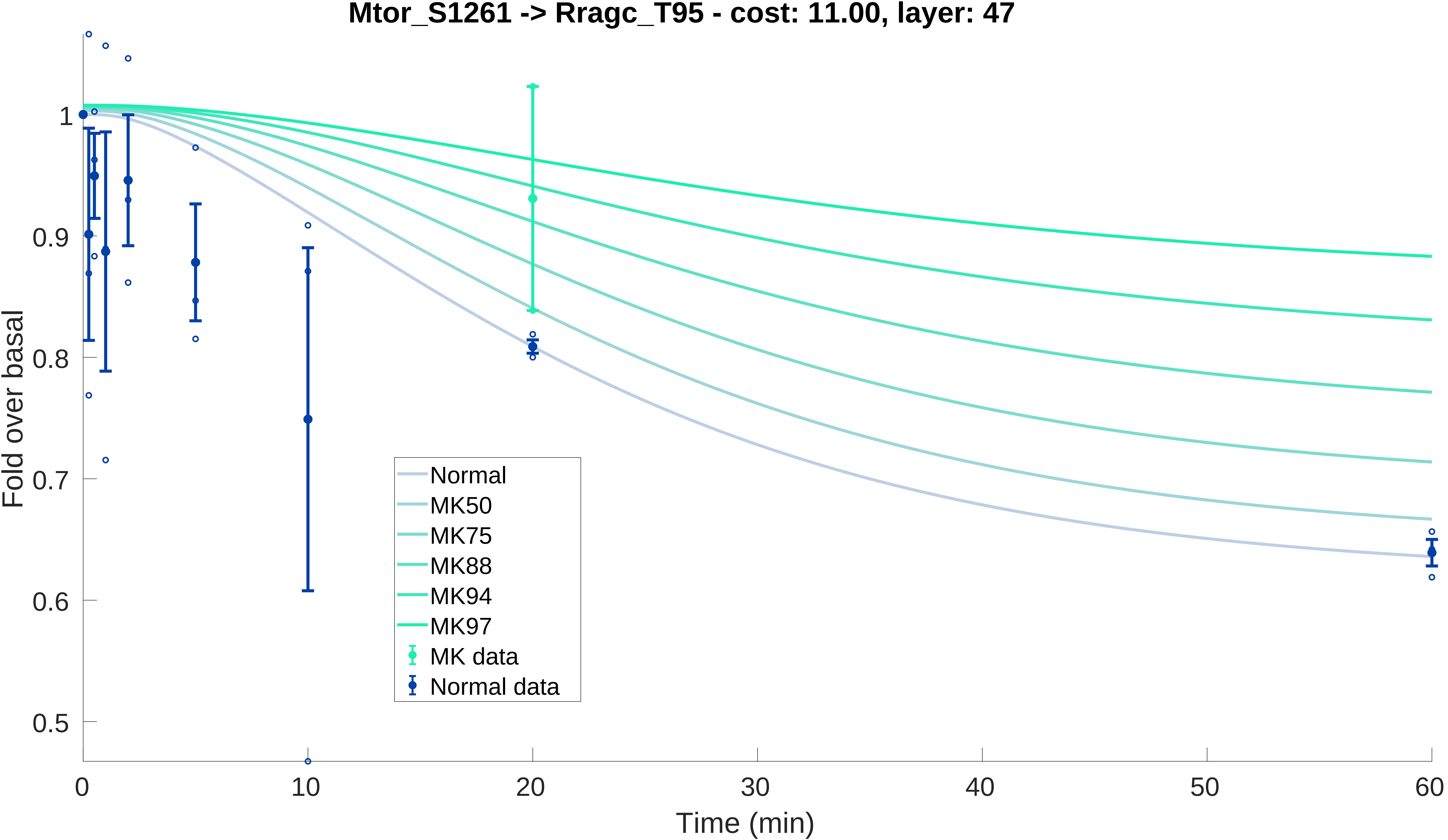

Mtor_S1261 -> Rragc_T95 - cost: 11.00, layer: 47
Fold over basal
Time (min)
1
0.9
0.8
0.7
0.6
0.5
0
10
20
30
40
50
60
Normal
MK50
MK75
MK88
MK94
MK97
MK data
Normal data

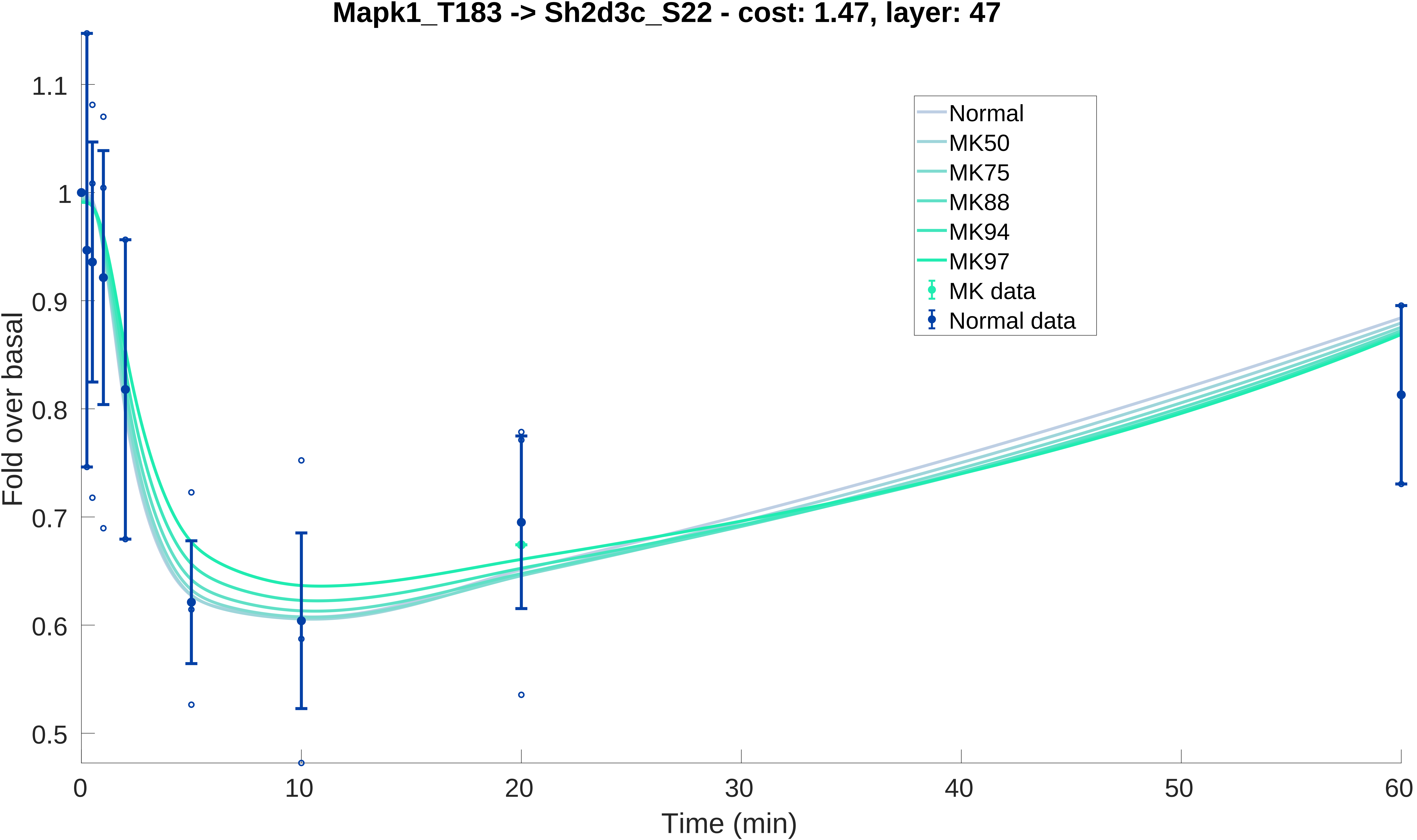

Mapk1_T183 -> Sh2d3c_S22 - cost: 1.47, layer: 47
Fold over basal
Time (min)
Normal
MK50
MK75
MK88
MK94
MK97
MK data
Normal data
1.1
1
0.9
0.8
0.7
0.6
0.5
0
10
20
30
40
50
60

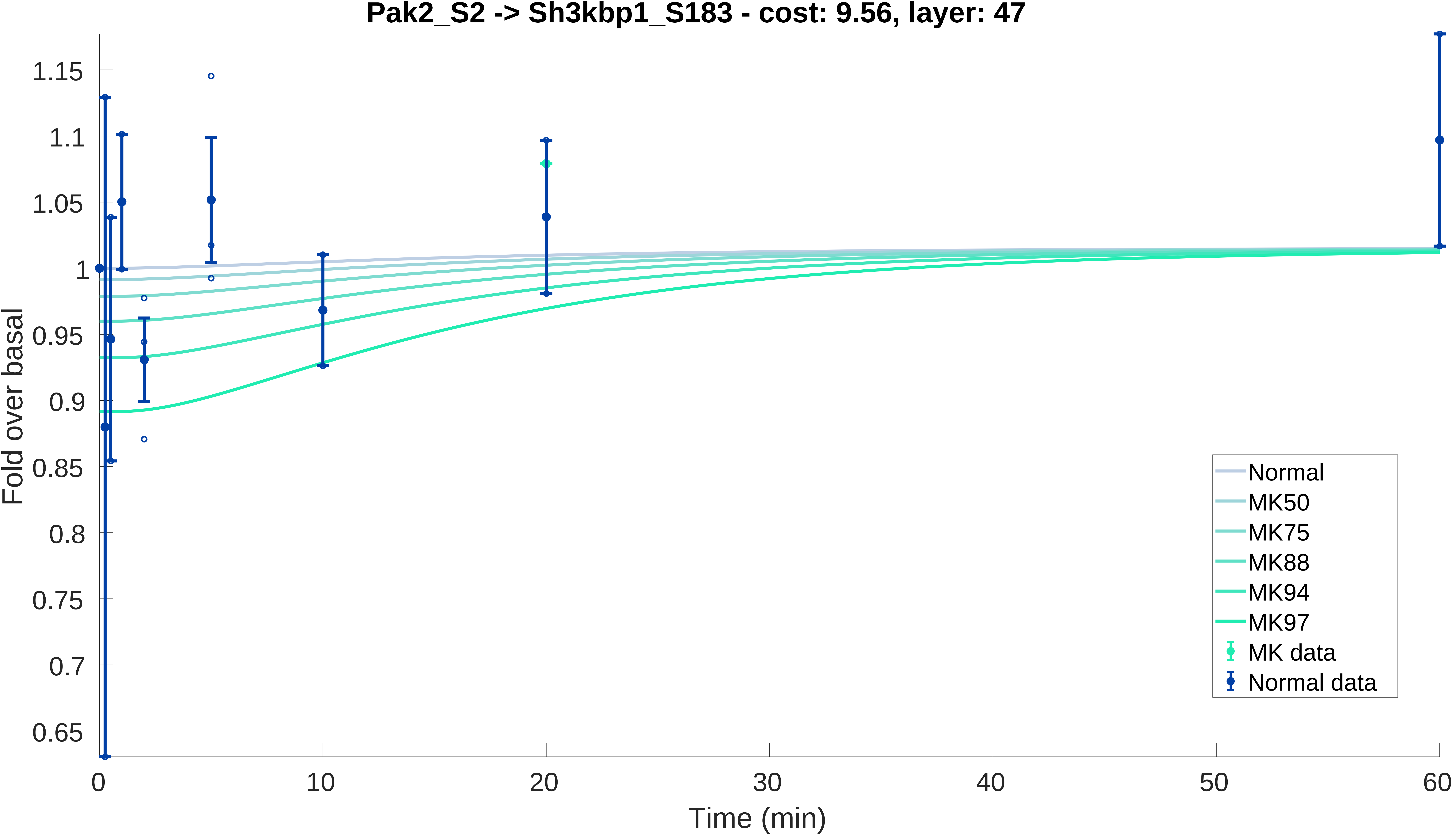

Pak2_S2 -> Sh3kbp1_S183 - cost: 9.56, layer: 47
Fold over basal
Time (min)
1.15
1.1
1.05
1
0.95
0.9
0.85
0.8
0.75
0.7
0.65
0
10
20
30
40
50
60
Normal
MK50
MK75
MK88
MK94
MK97
MK data
Normal data

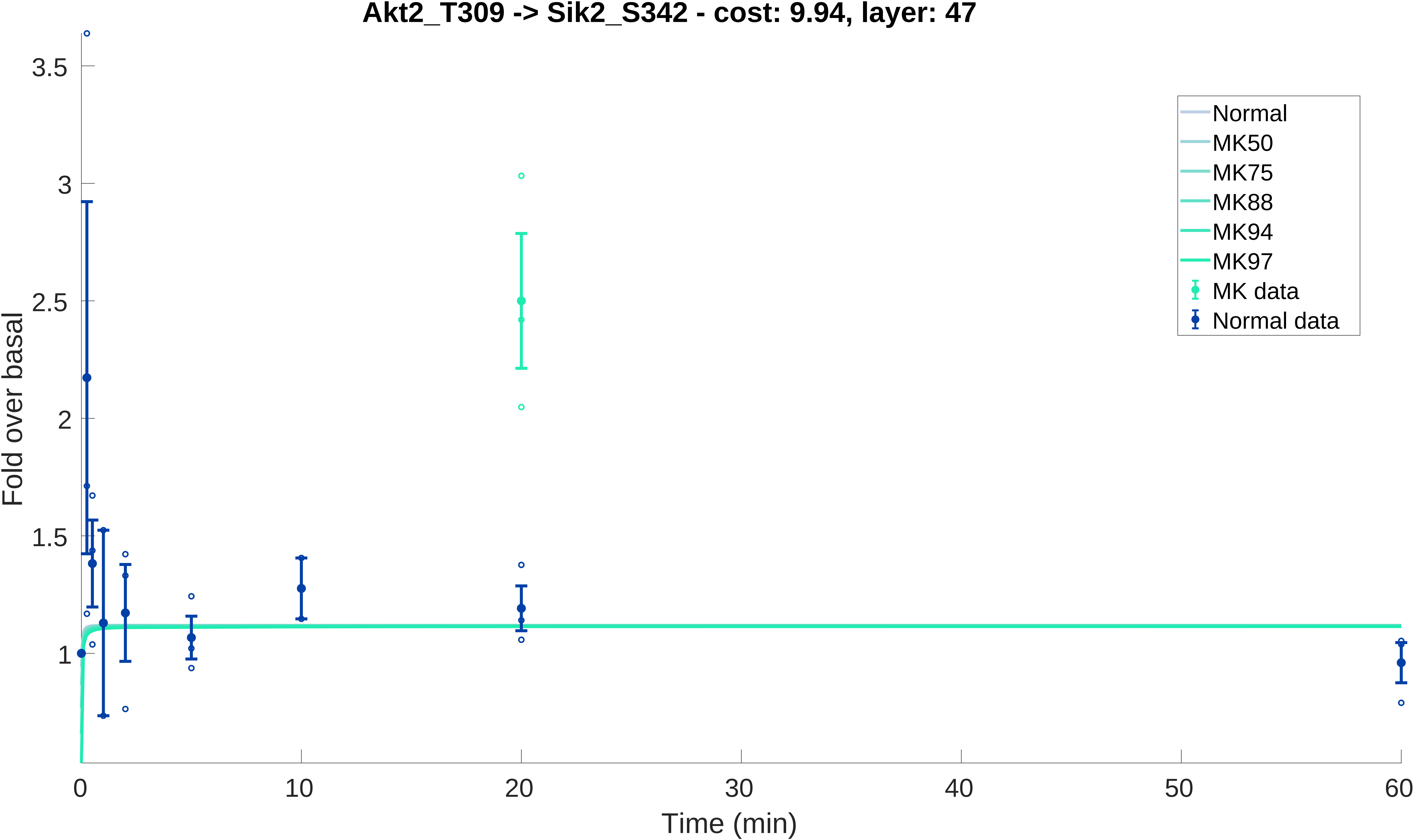

Akt2_T309 -> Sik2_S342 - cost: 9.94, layer: 47
Fold over basal
Time (min)
3.5
3
2.5
2
1.5
1
0
10
20
30
40
50
60
Normal
MK50
MK75
MK88
MK94
MK97
MK data
Normal data

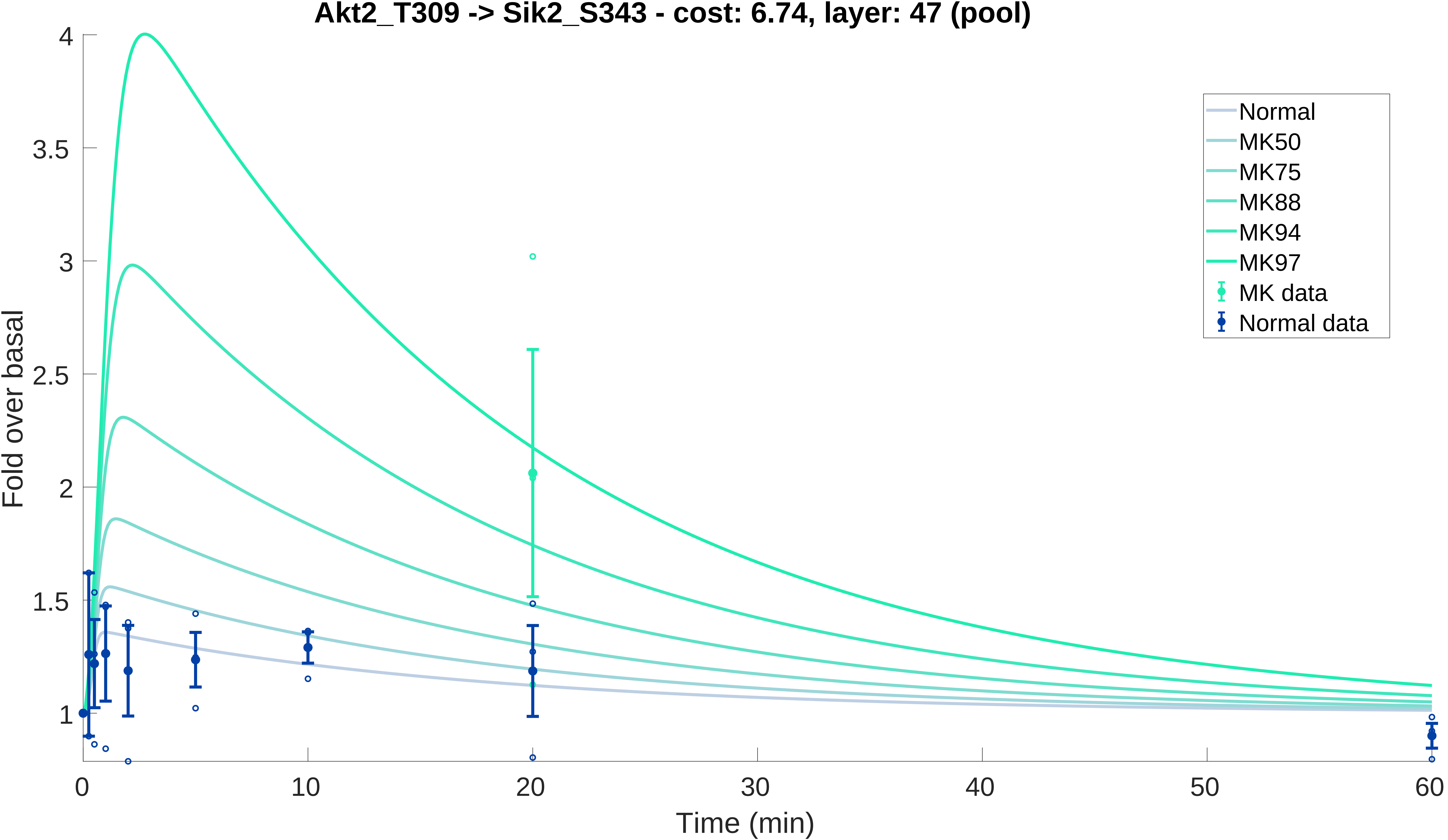
Akt2_T309 -> Sik2_S343 - cost: 6.74, layer: 47 (pool)
Fold over basal
Time (min)
4
3.5
3
2.5
2
1.5
1
0
10
20
30
40
50
60
Normal
MK50
MK75
MK88
MK94
MK97
MK data
Normal data

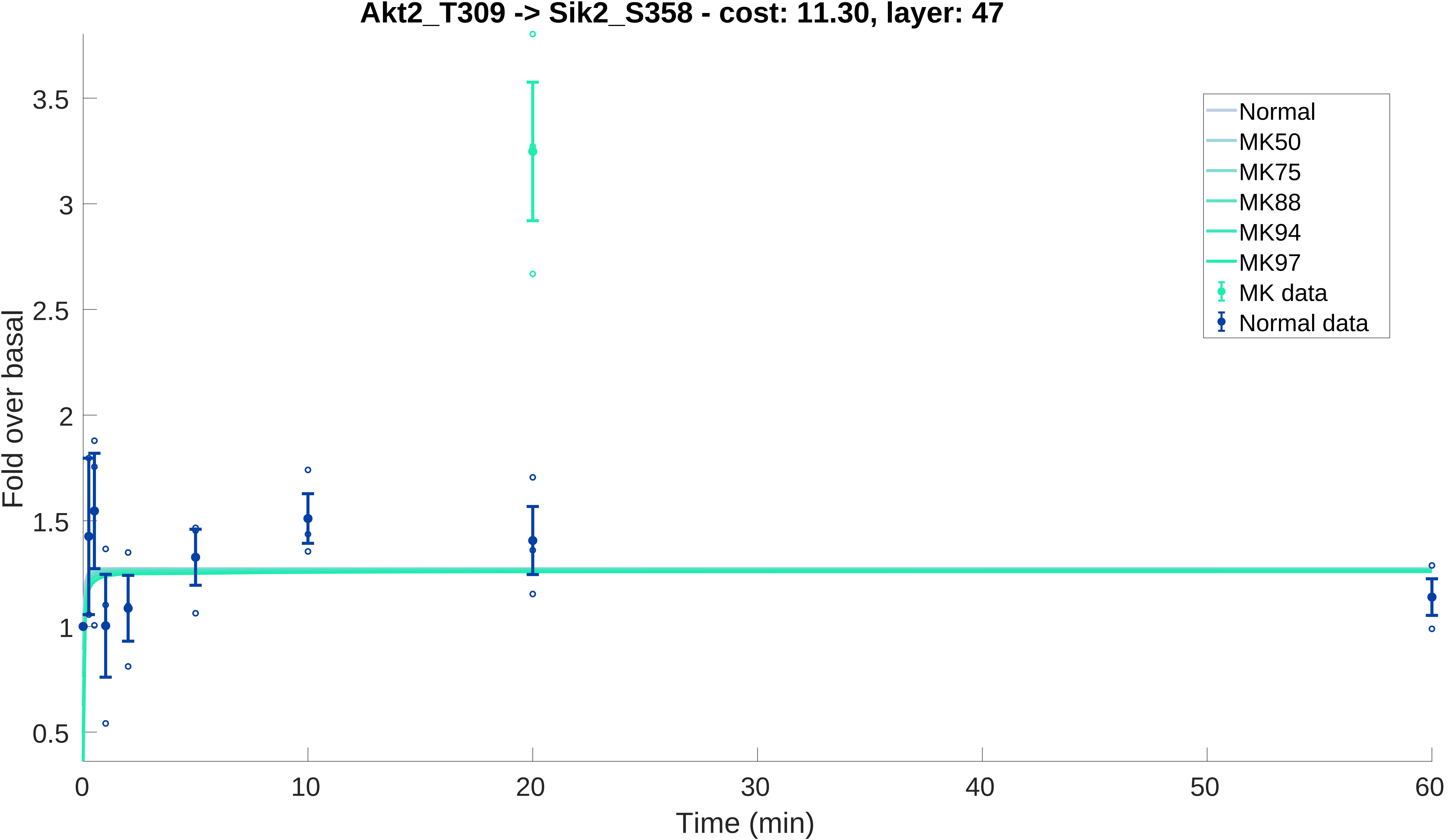

Akt2_T309 -> Sik2_S358 - cost: 11.30, layer: 47
Fold over basal
Time (min)
0.5
1
1.5
2
2.5
3
3.5
0
10
20
30
40
50
60
Normal
MK50
MK75
MK88
MK94
MK97
MK data
Normal data

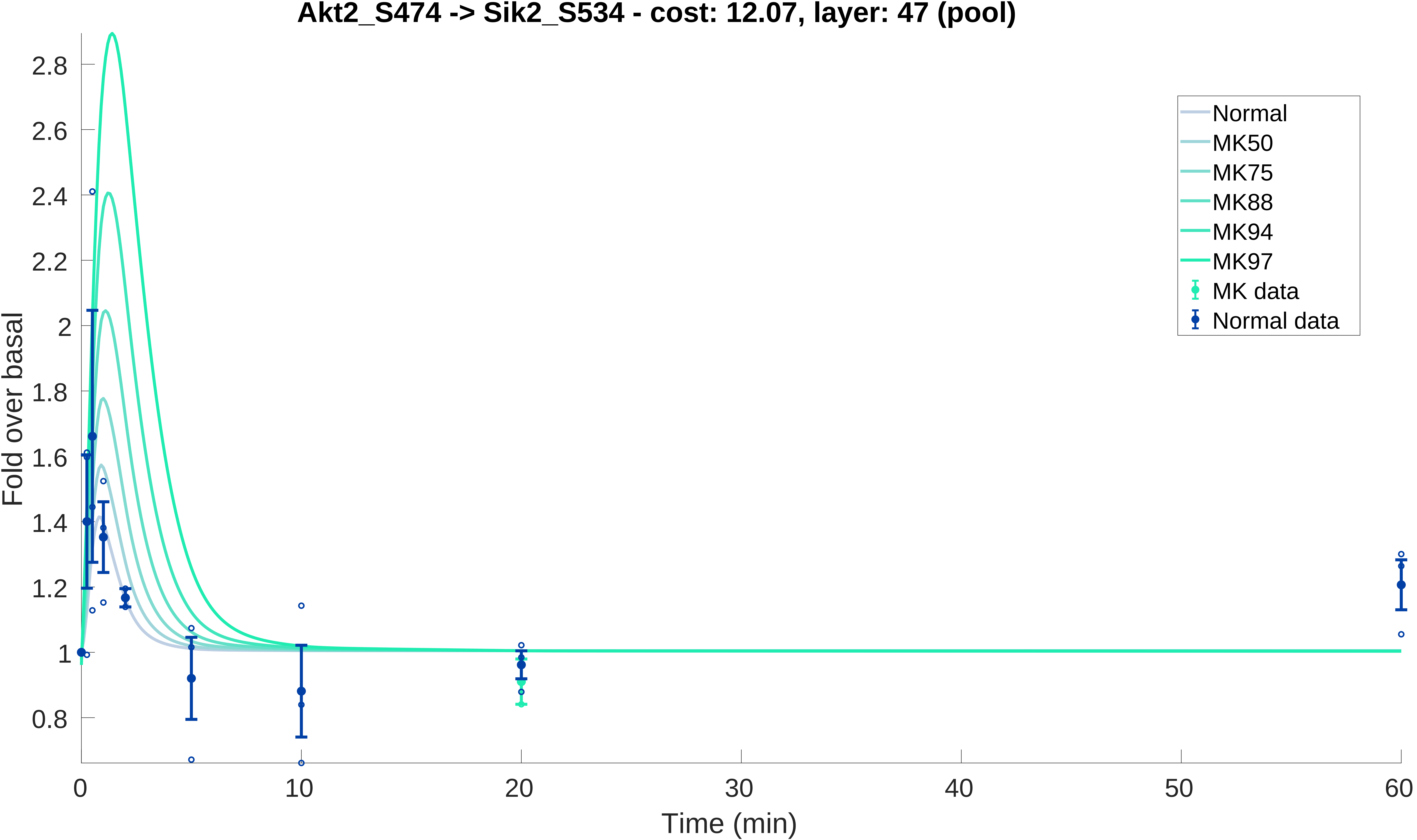

Akt2_S474 -> Sik2_S534 - cost: 12.07, layer: 47 (pool)
Fold over basal
Time (min)
0.8
1
1.2
1.4
1.6
1.8
2
2.2
2.4
2.6
2.8
0
10
20
30
40
50
60
Normal
MK50
MK75
MK88
MK94
MK97
MK data
Normal data

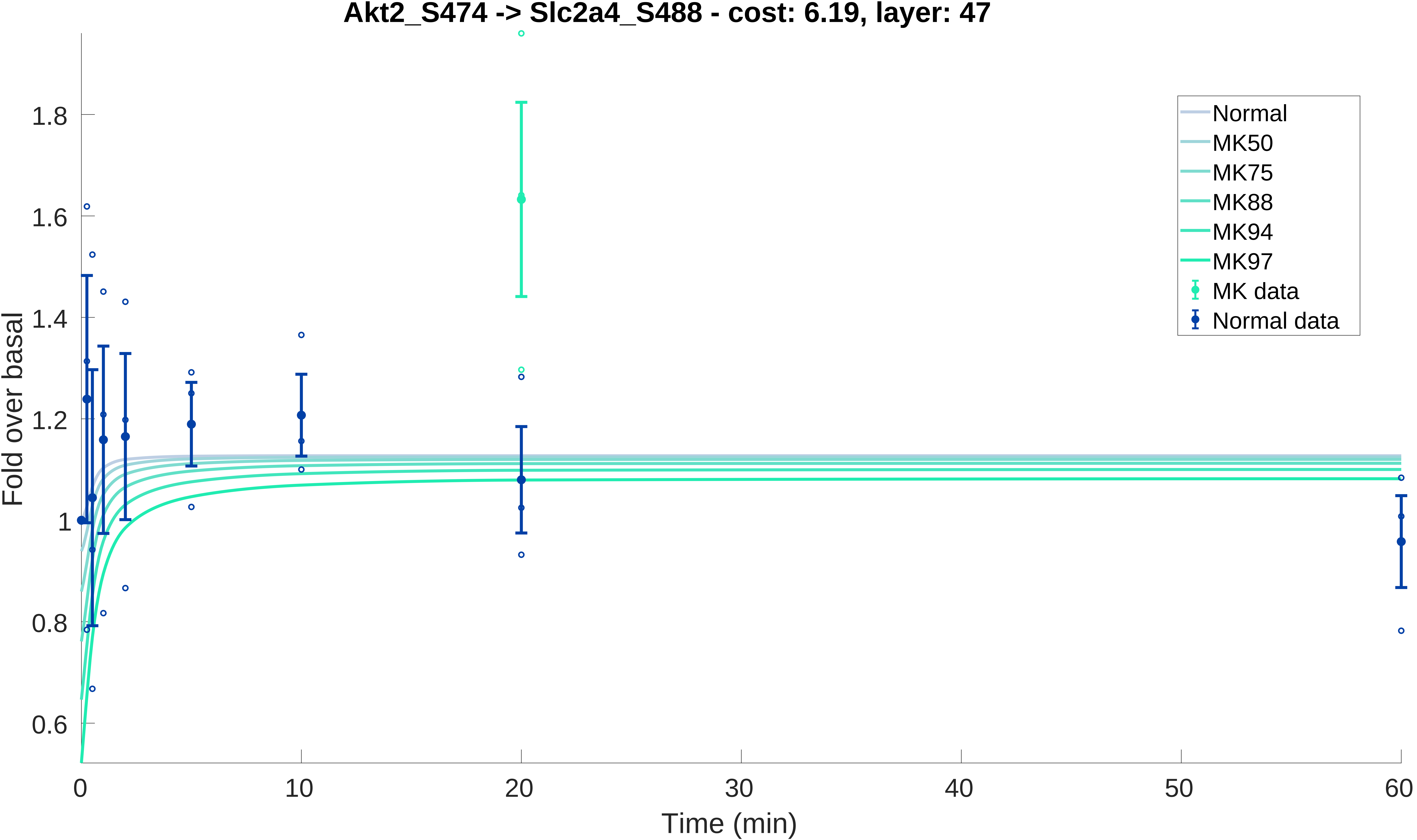
Akt2_S474 -> Slc2a4_S488 - cost: 6.19, layer: 47
Normal
MK50
MK75
MK88
MK94
MK97
MK data
Normal data
Fold over basal
Time (min)
0
10
20
30
40
50
60
0.6
0.8
1
1.2
1.4
1.6
1.8

Src_S17 -> Slc25a4_S149 - cost: 13.90, layer: 47

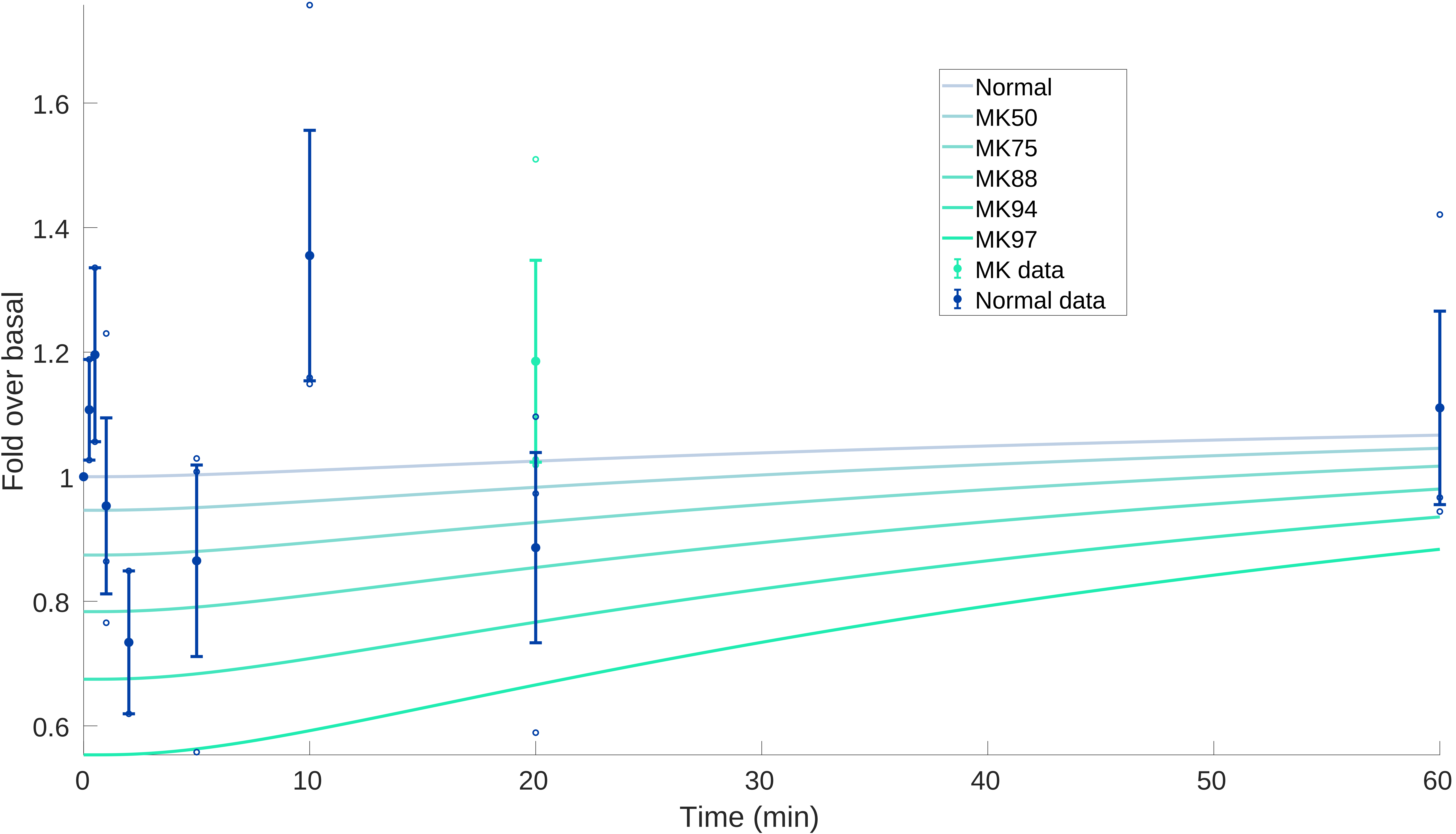

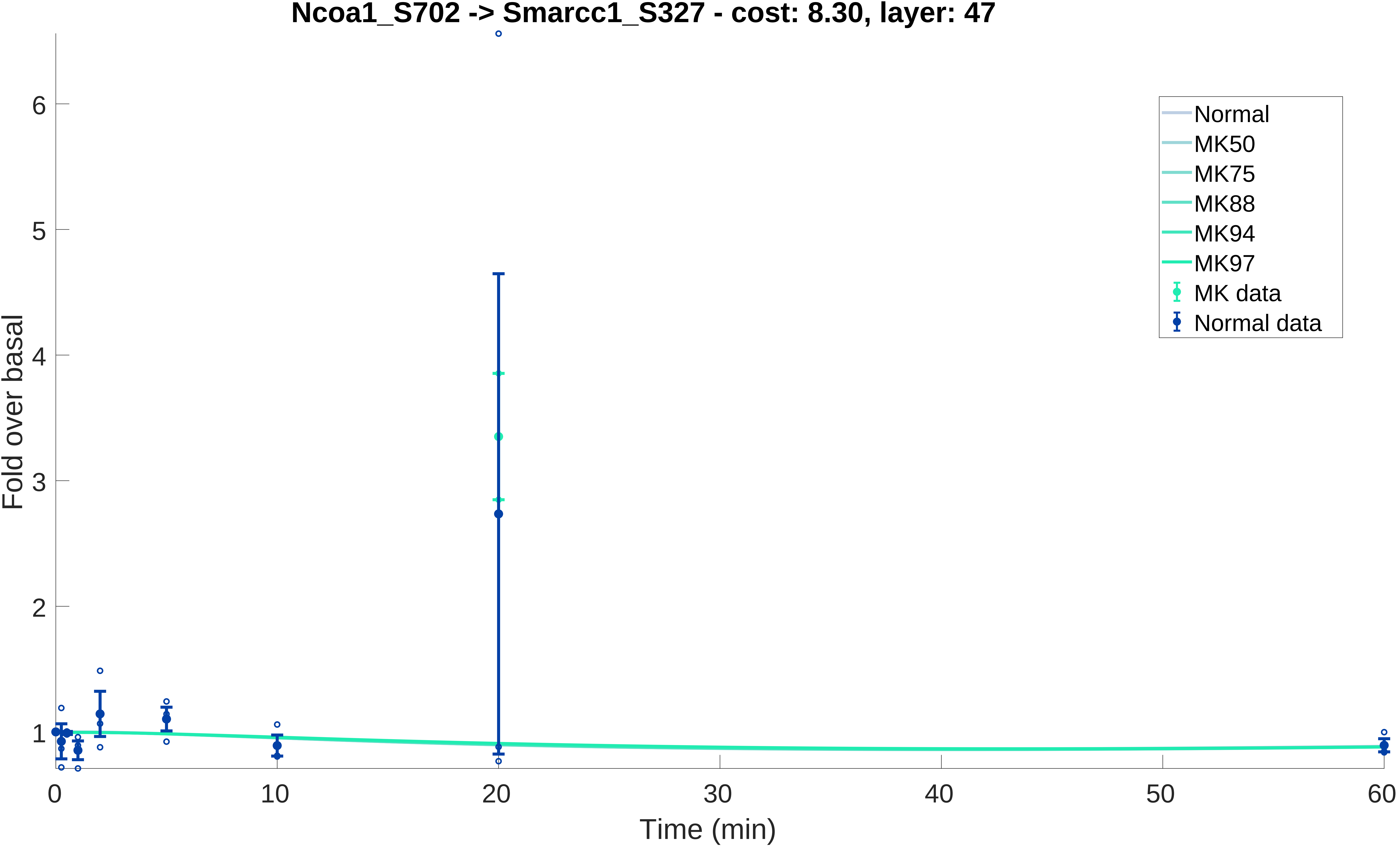
Ncoa1_S702 -> Smarcc1_S327 - cost: 8.30, layer: 47
Fold over basal
Time (min)
0
10
20
30
40
50
60
1
2
3
4
5
6
Normal
MK50
MK75
MK88
MK94
MK97
MK data
Normal data

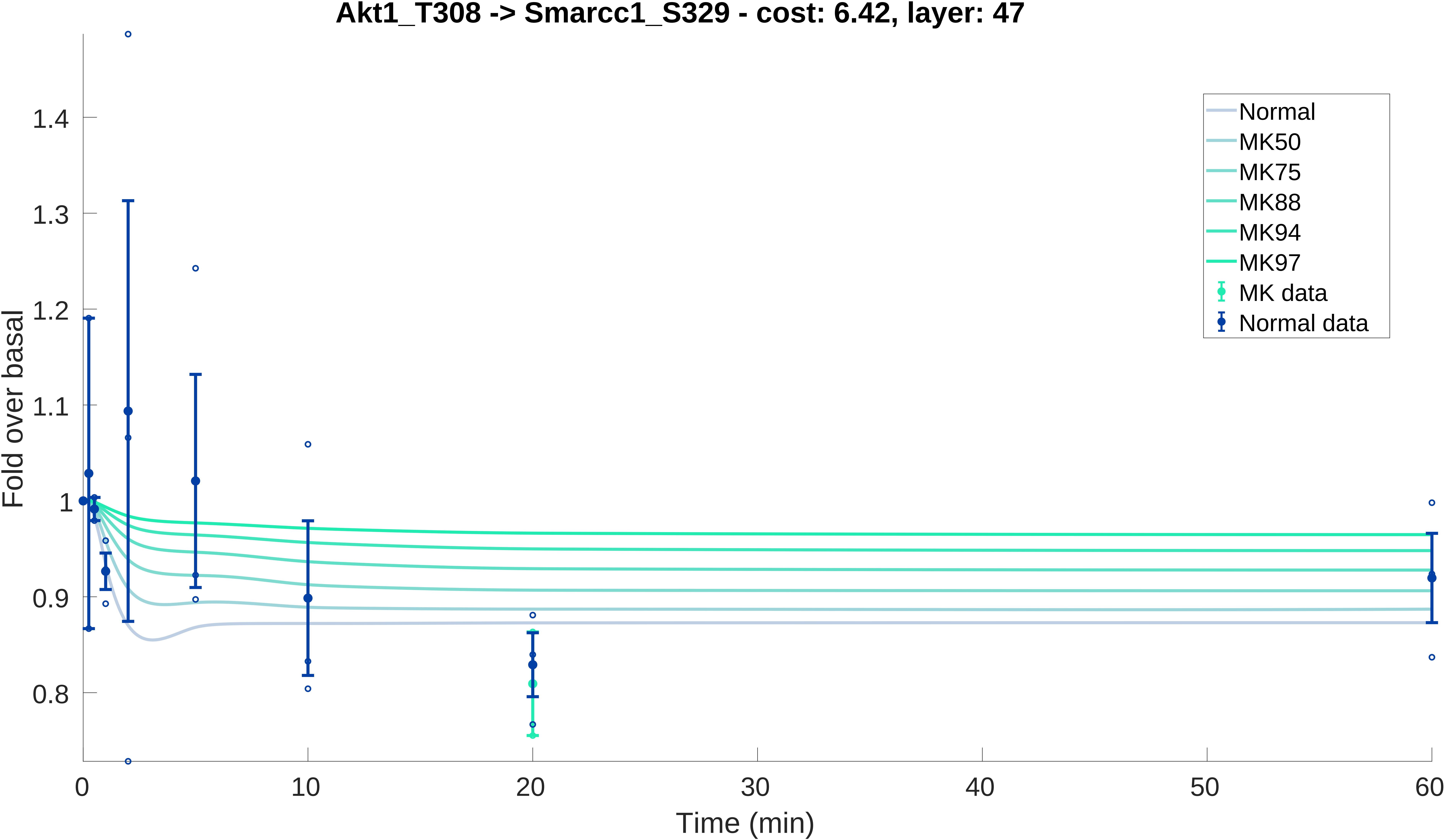

Akt1_T308 -> Smarcc1_S329 - cost: 6.42, layer: 47
Fold over basal
Time (min)
0.8
0.9
1
1.1
1.2
1.3
1.4
0
10
20
30
40
50
60
Normal
MK50
MK75
MK88
MK94
MK97
MK data
Normal data

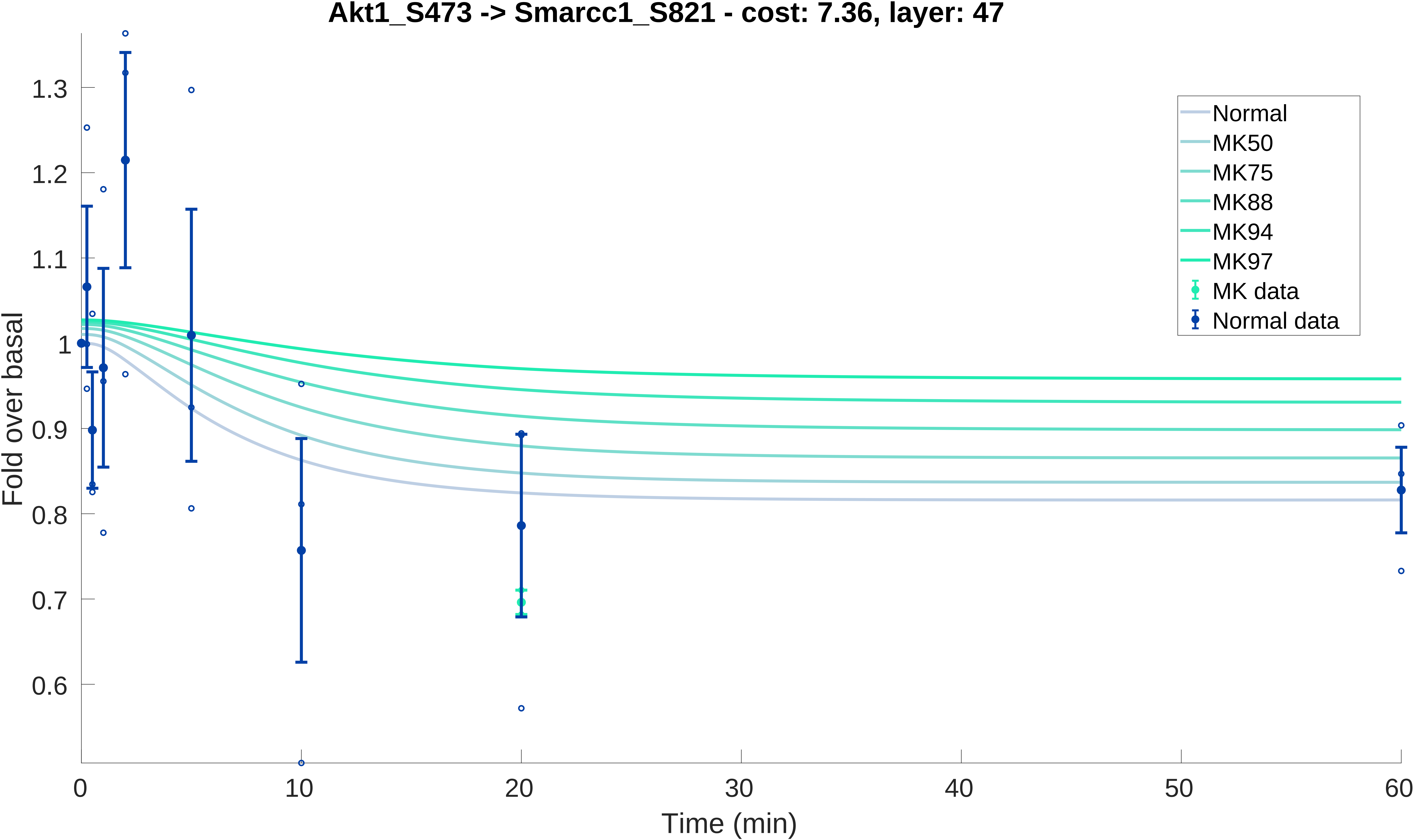

Akt1_S473 -> Smarcc1_S821 - cost: 7.36, layer: 47
Fold over basal
Time (min)
1.3
1.2
1.1
1
0.9
0.8
0.7
0.6
0
10
20
30
40
50
60
Normal
MK50
MK75
MK88
MK94
MK97
MK data
Normal data

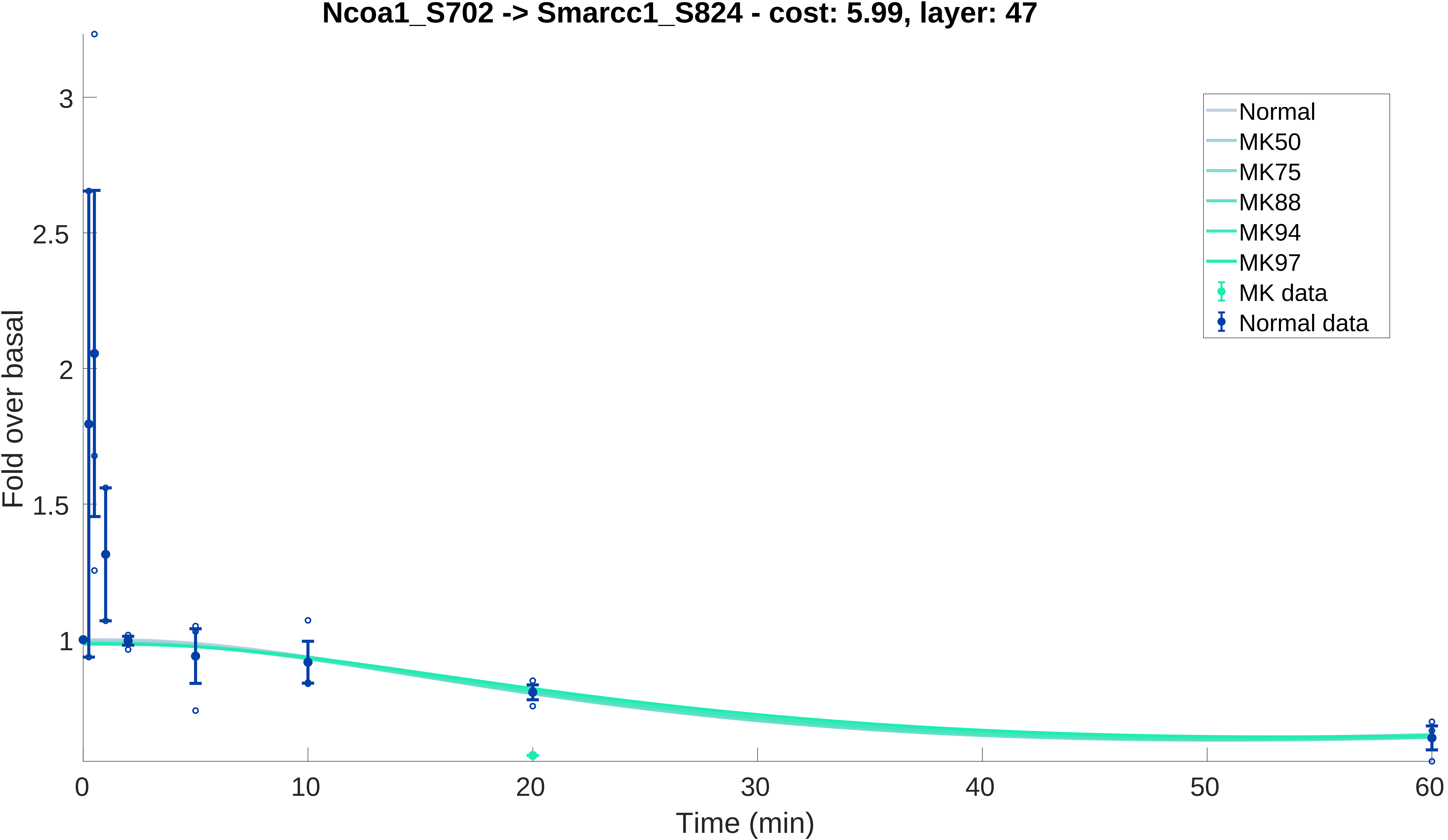
Ncoa1_S702 -> Smarcc1_S824 - cost: 5.99, layer: 47
Fold over basal
3
2.5
2
1.5
1
Time (min)
0
10
20
30
40
50
60
Normal
MK50
MK75
MK88
MK94
MK97
MK data
Normal data

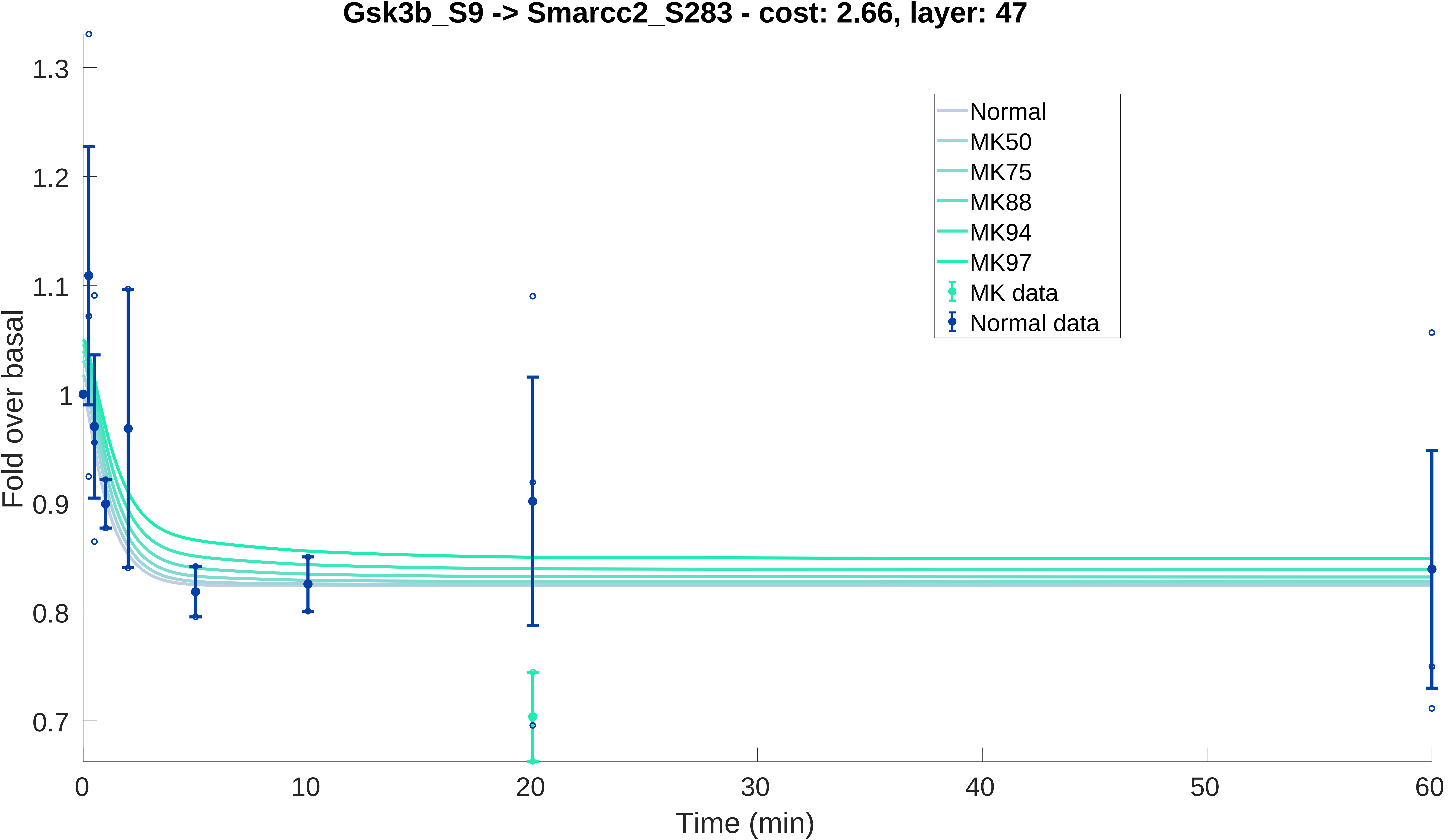
Gsk3b_S9 -> Smarcc2_S283 - cost: 2.66, layer: 47
Fold over basal
1.3
1.2
1.1
1
0.9
0.8
0.7
0
10
20
30
40
50
60
Time (min)
Normal
MK50
MK75
MK88
MK94
MK97
MK data
Normal data

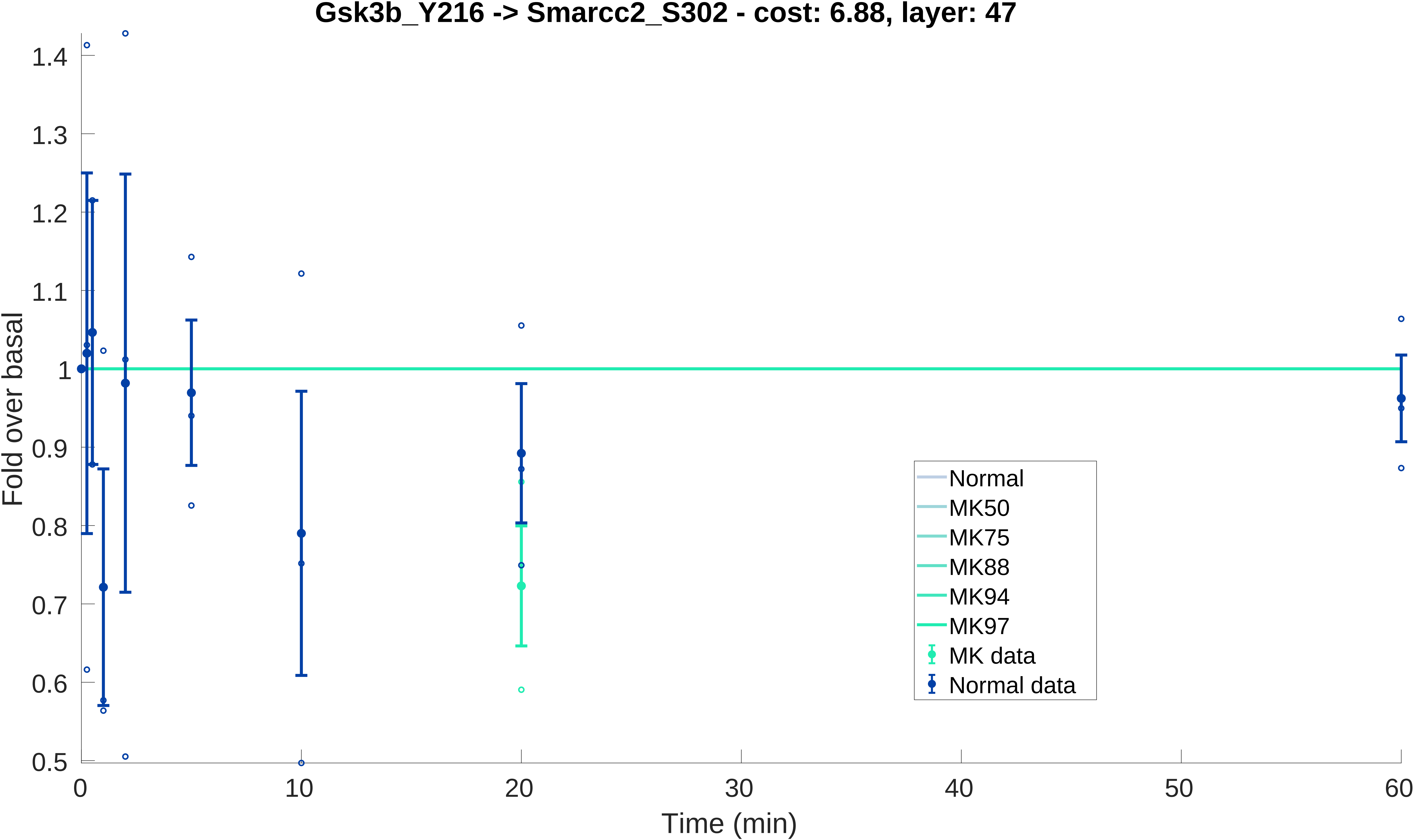

Gsk3b_Y216 -> Smarcc2_S302 - cost: 6.88, layer: 47
Fold over basal
1.4
1.3
1.2
1.1
1
0.9
0.8
0.7
0.6
0.5
0
10
20
30
40
50
60
Time (min)
Normal
MK50
MK75
MK88
MK94
MK97
MK data
Normal data

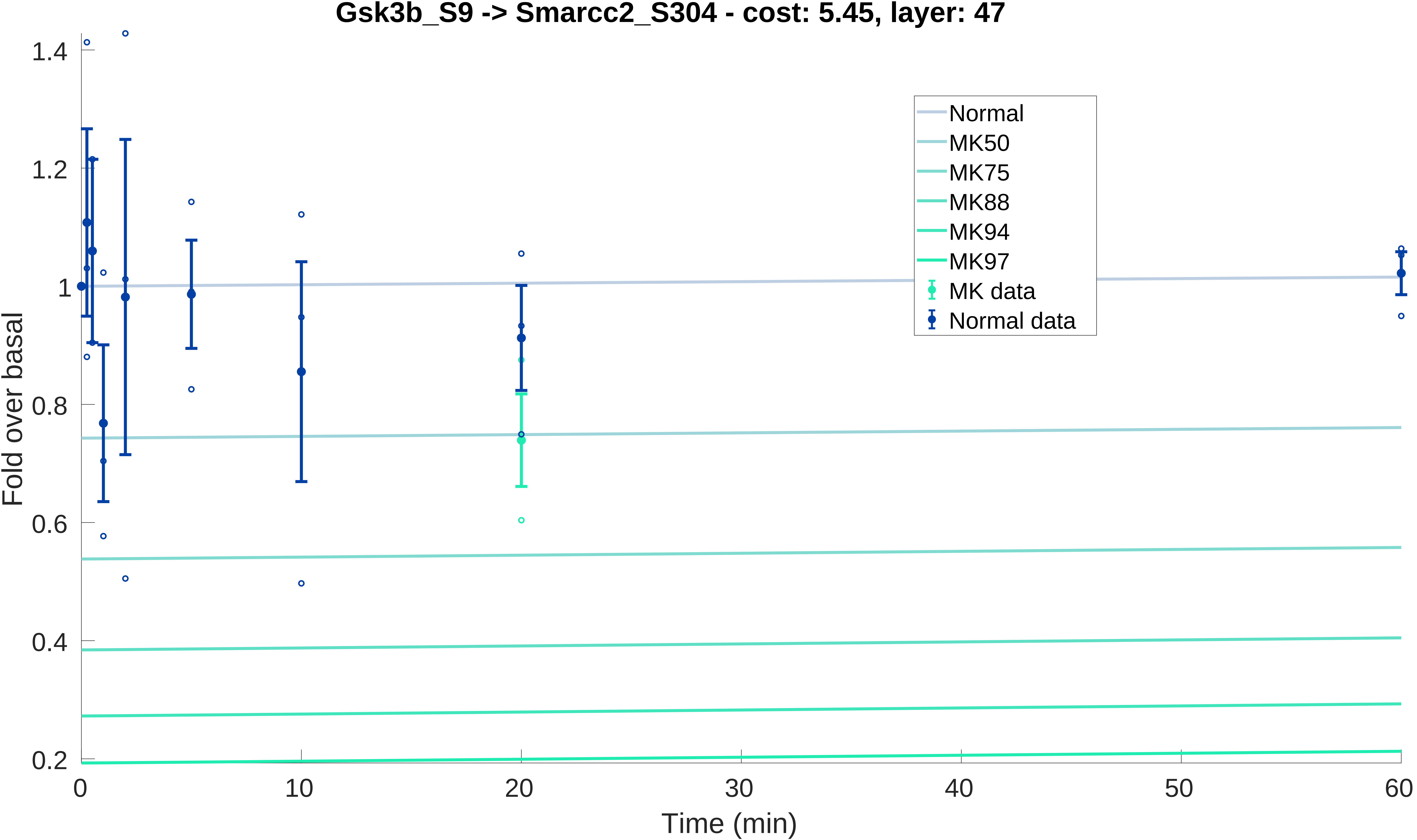
Gsk3b_S9 -> Smarcc2_S304 - cost: 5.45, layer: 47
Fold over basal
Time (min)
1.4
1.2
1
0.8
0.6
0.4
0.2
0
10
20
30
40
50
60
Normal
MK50
MK75
MK88
MK94
MK97
MK data
Normal data

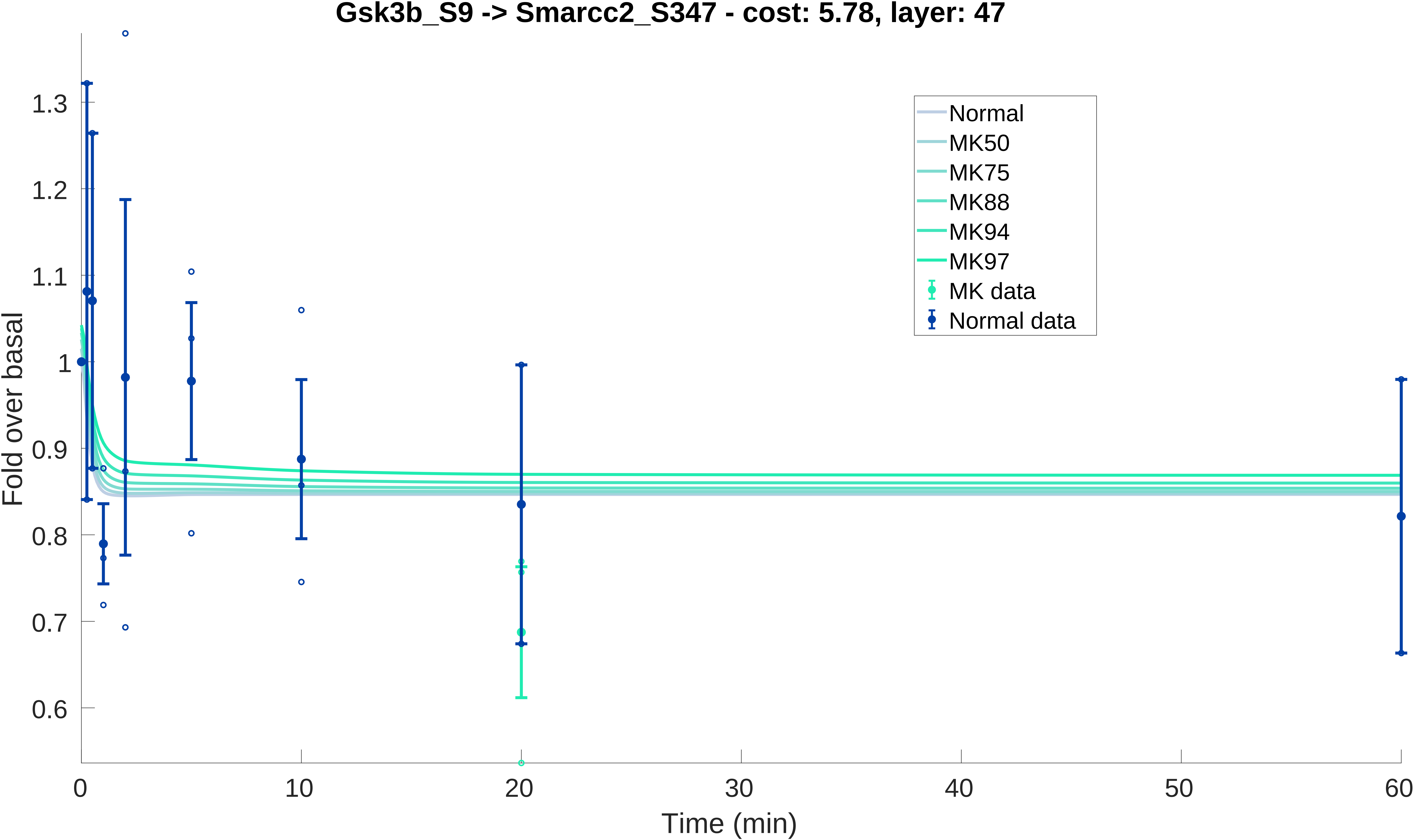

Gsk3b_S9 -> Smarcc2_S347 - cost: 5.78, layer: 47
Fold over basal
Time (min)
0.6
0.7
0.8
0.9
1
1.1
1.2
1.3
0
10
20
30
40
50
60
Normal
MK50
MK75
MK88
MK94
MK97
MK data
Normal data

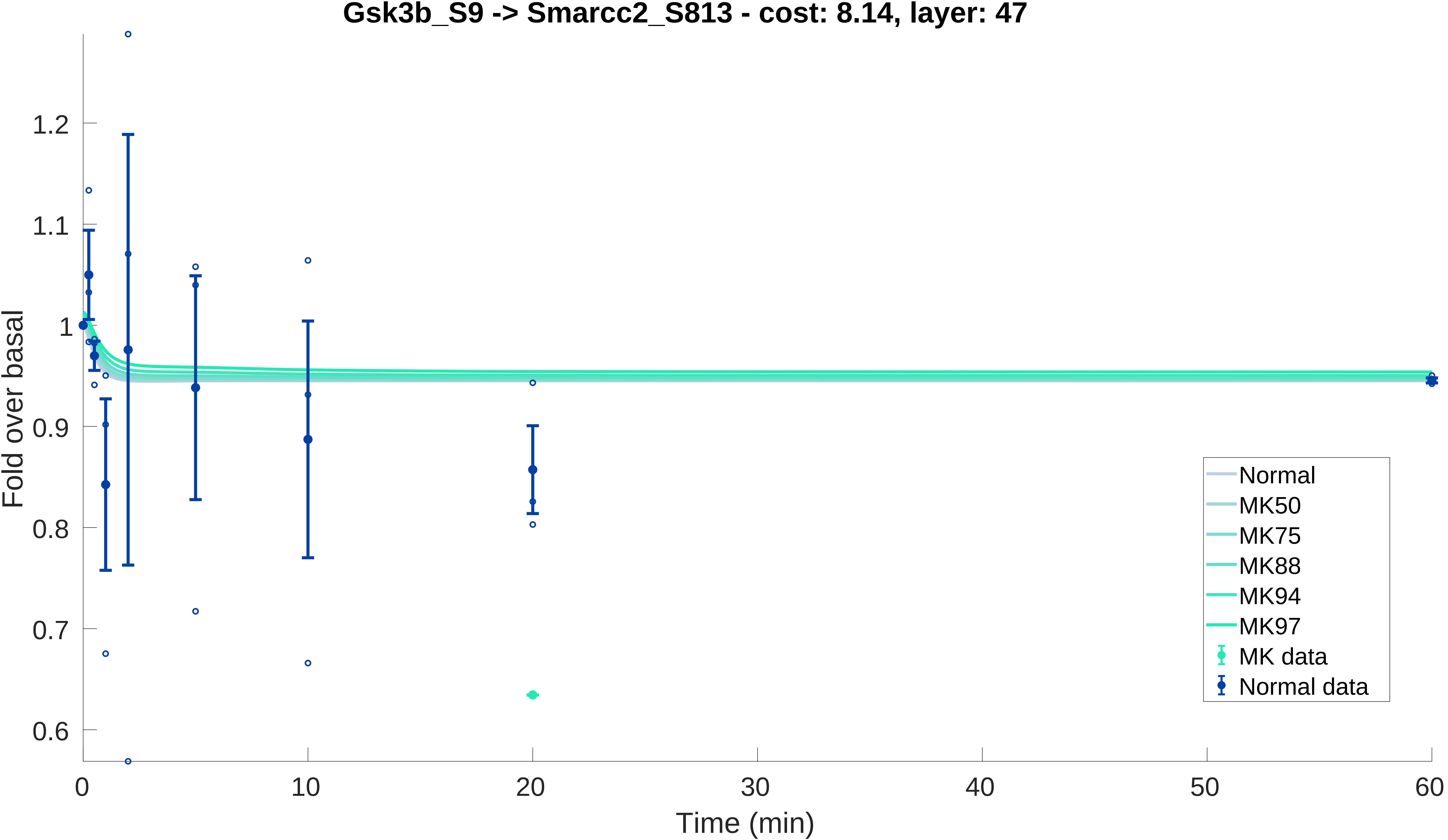

Gsk3b_S9 -> Smarcc2_S813 - cost: 8.14, layer: 47
Fold over basal
Time (min)
0.6
0.7
0.8
0.9
1
1.1
1.2
0
10
20
30
40
50
60
Normal
MK50
MK75
MK88
MK94
MK97
MK data
Normal data

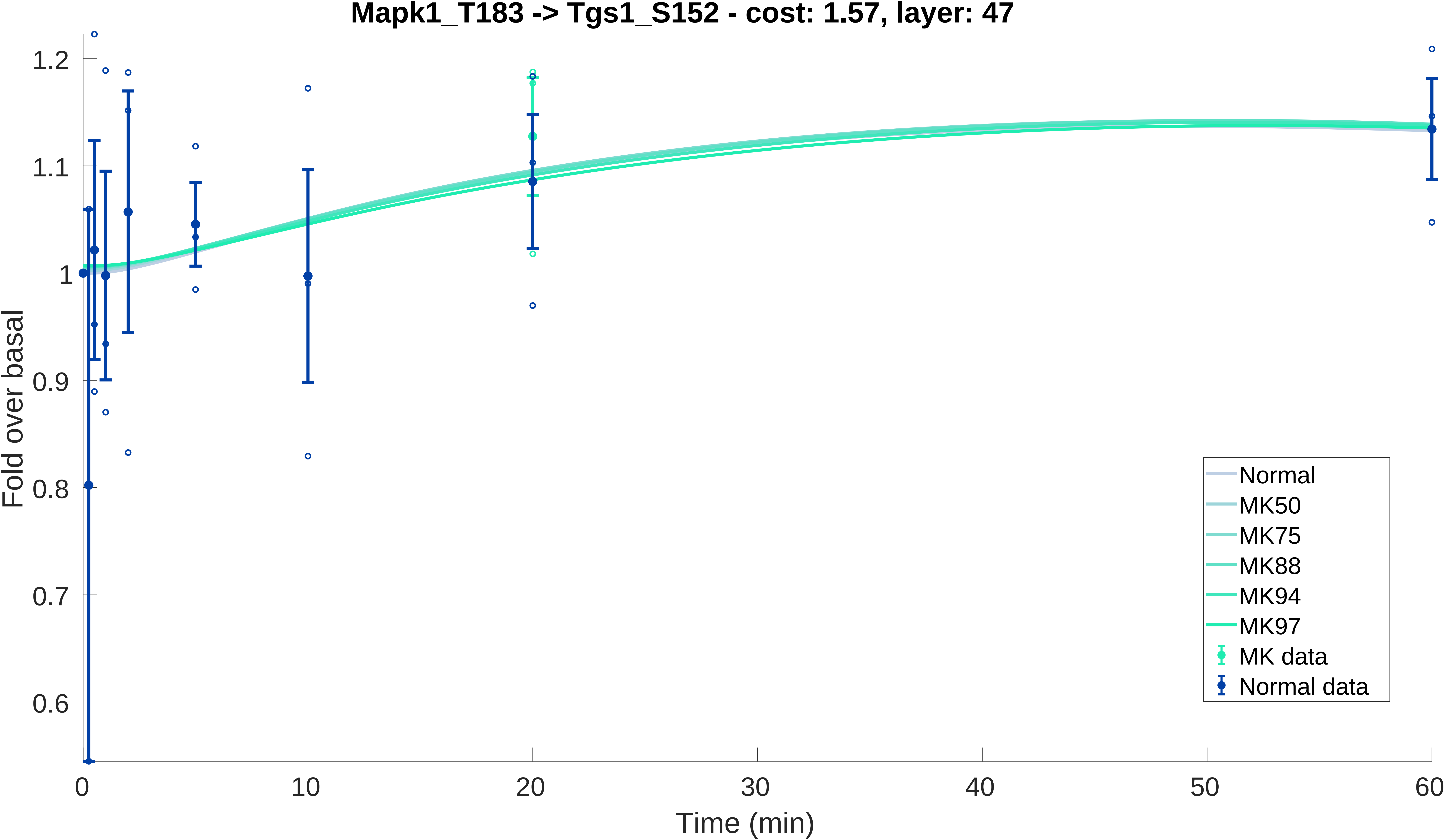

Mapk1_T183 -> Tgs1_S152 - cost: 1.57, layer: 47
Fold over basal
Time (min)
1.2
1.1
1
0.9
0.8
0.7
0.6
0
10
20
30
40
50
60
Normal
MK50
MK75
MK88
MK94
MK97
MK data
Normal data

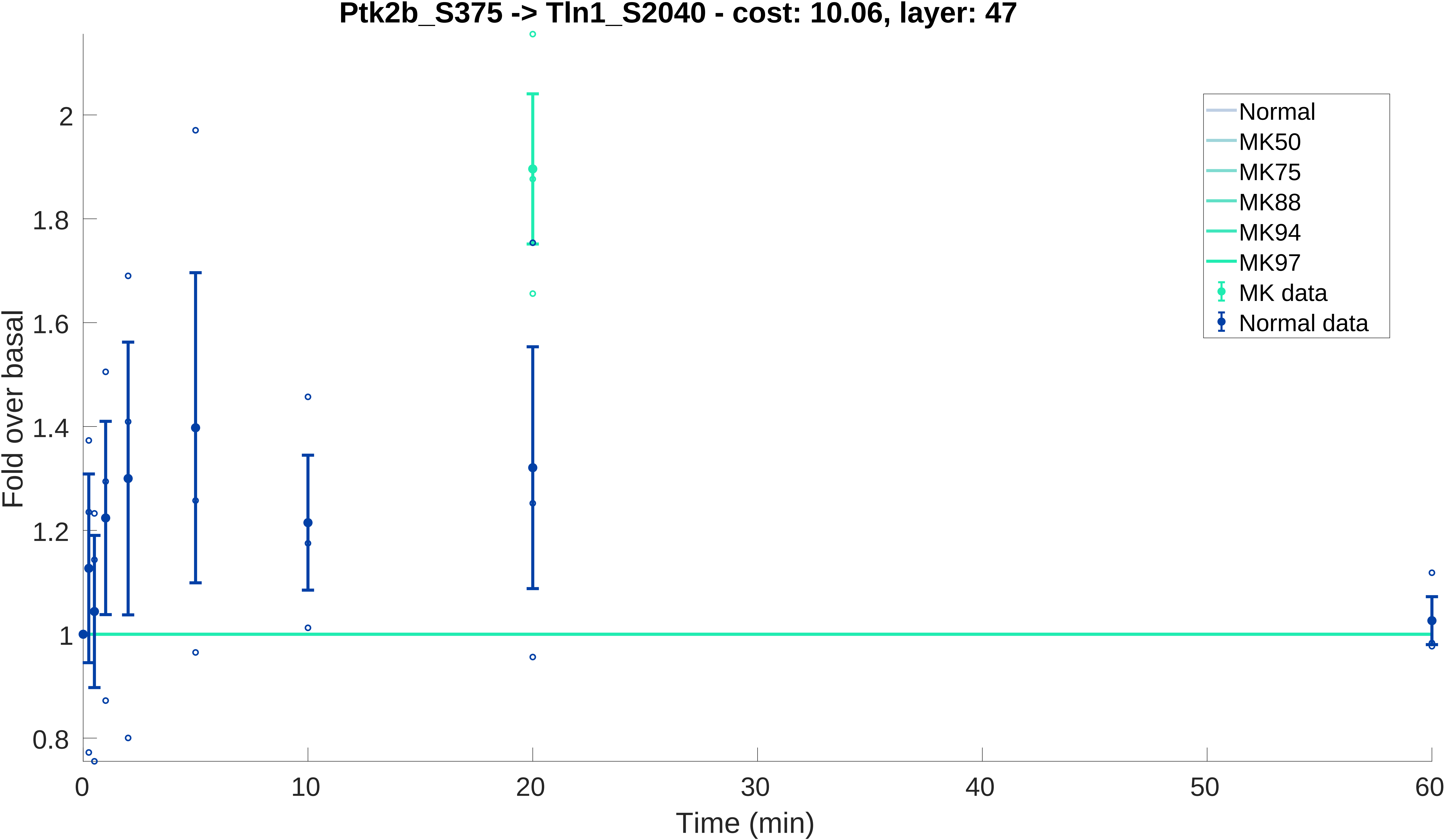

Ptk2b_S375 -> Tln1_S2040 - cost: 10.06, layer: 47
Fold over basal
Time (min)
0.8
1
1.2
1.4
1.6
1.8
2
0
10
20
30
40
50
60
Normal
MK50
MK75
MK88
MK94
MK97
MK data
Normal data

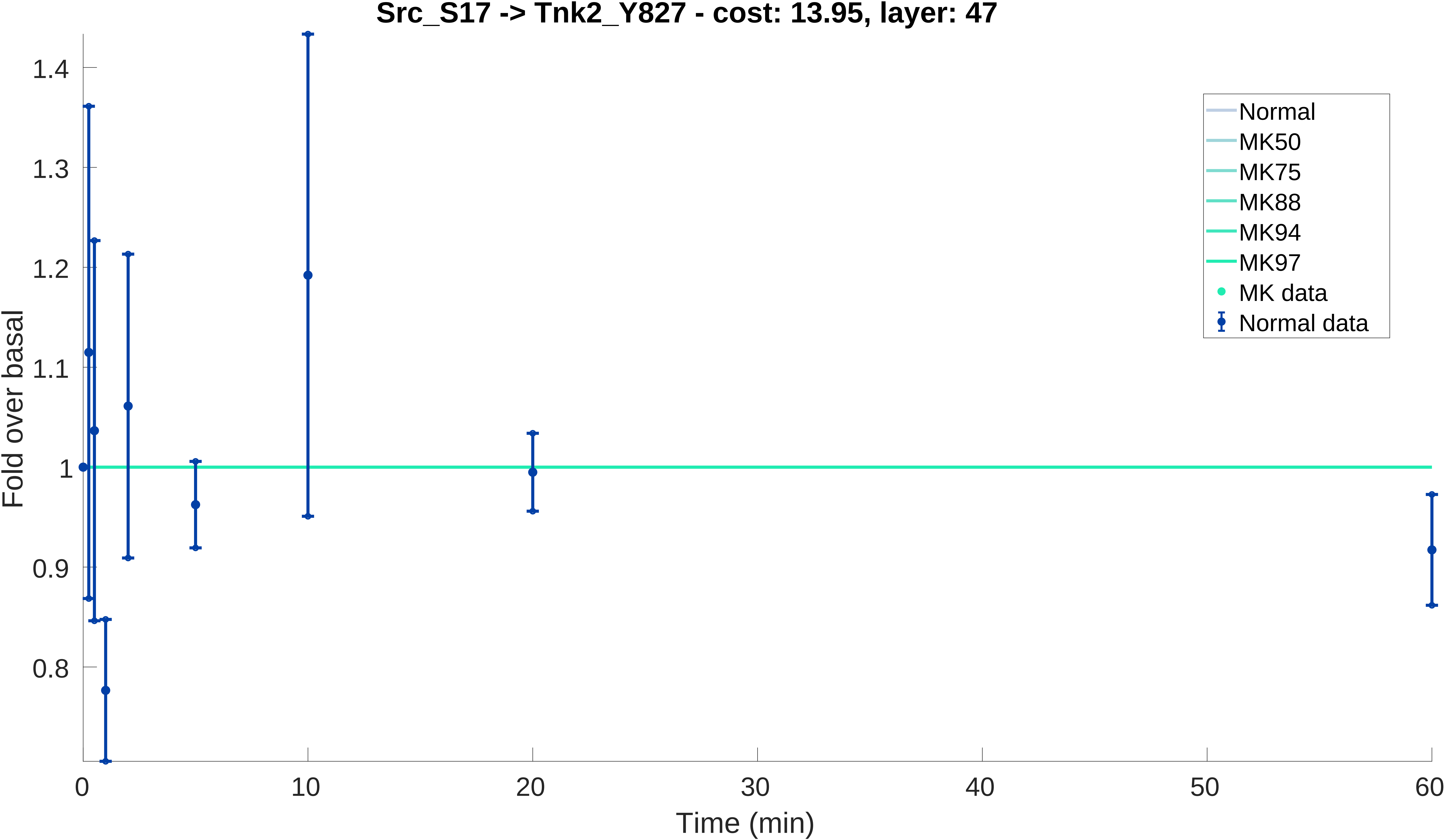

Src_S17 -> Tnk2_Y827 - cost: 13.95, layer: 47
Fold over basal
1.4
1.3
1.2
1.1
1
0.9
0.8
0
10
20
30
40
50
60
Time (min)
Normal
MK50
MK75
MK88
MK94
MK97
MK data
Normal data

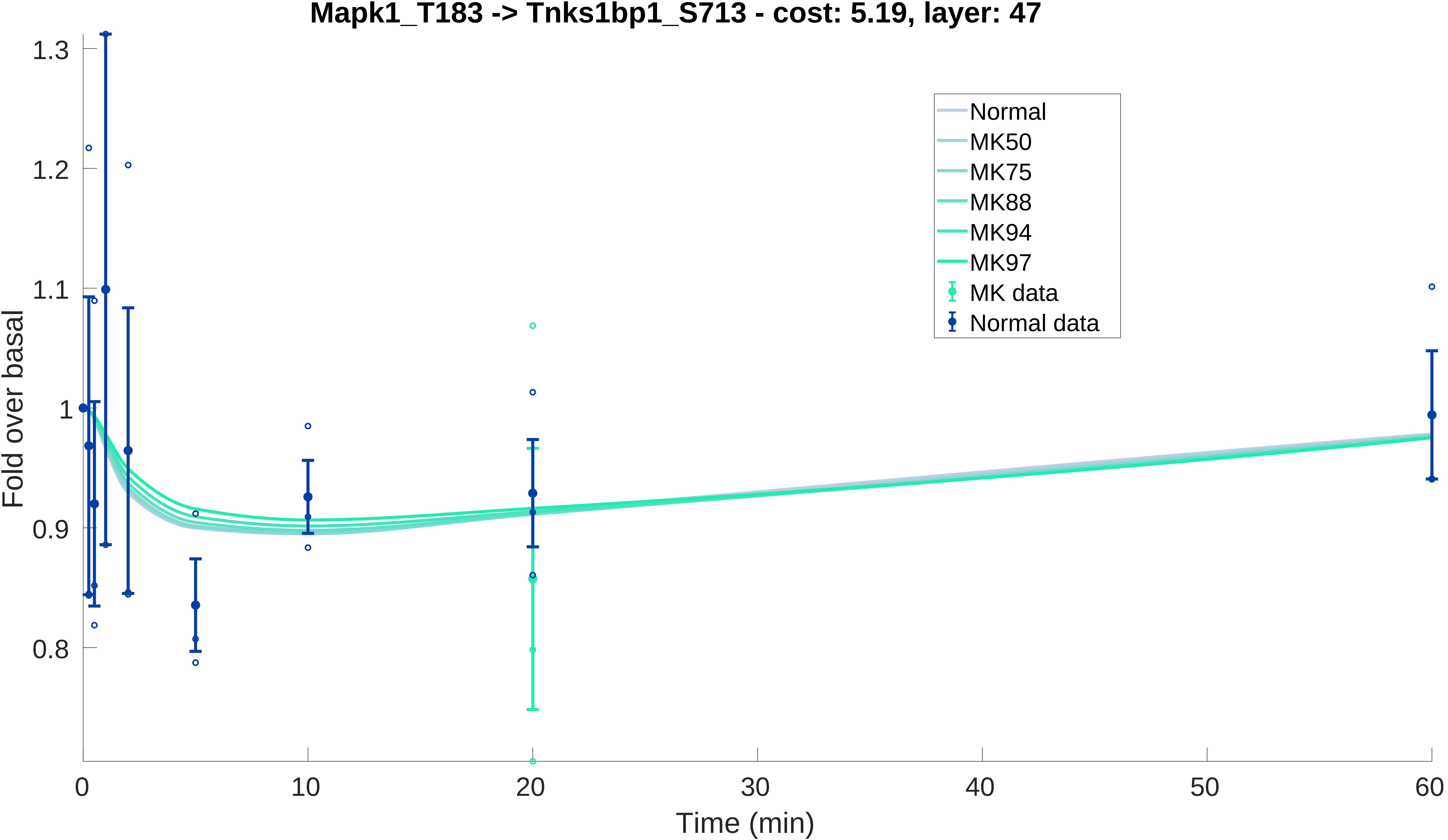

Mapk1_T183 -> Tnks1bp1_S713 - cost: 5.19, layer: 47
Fold over basal
Time (min)
1.3
1.2
1.1
1
0.9
0.8
0
10
20
30
40
50
60
Normal
MK50
MK75
MK88
MK94
MK97
MK data
Normal data

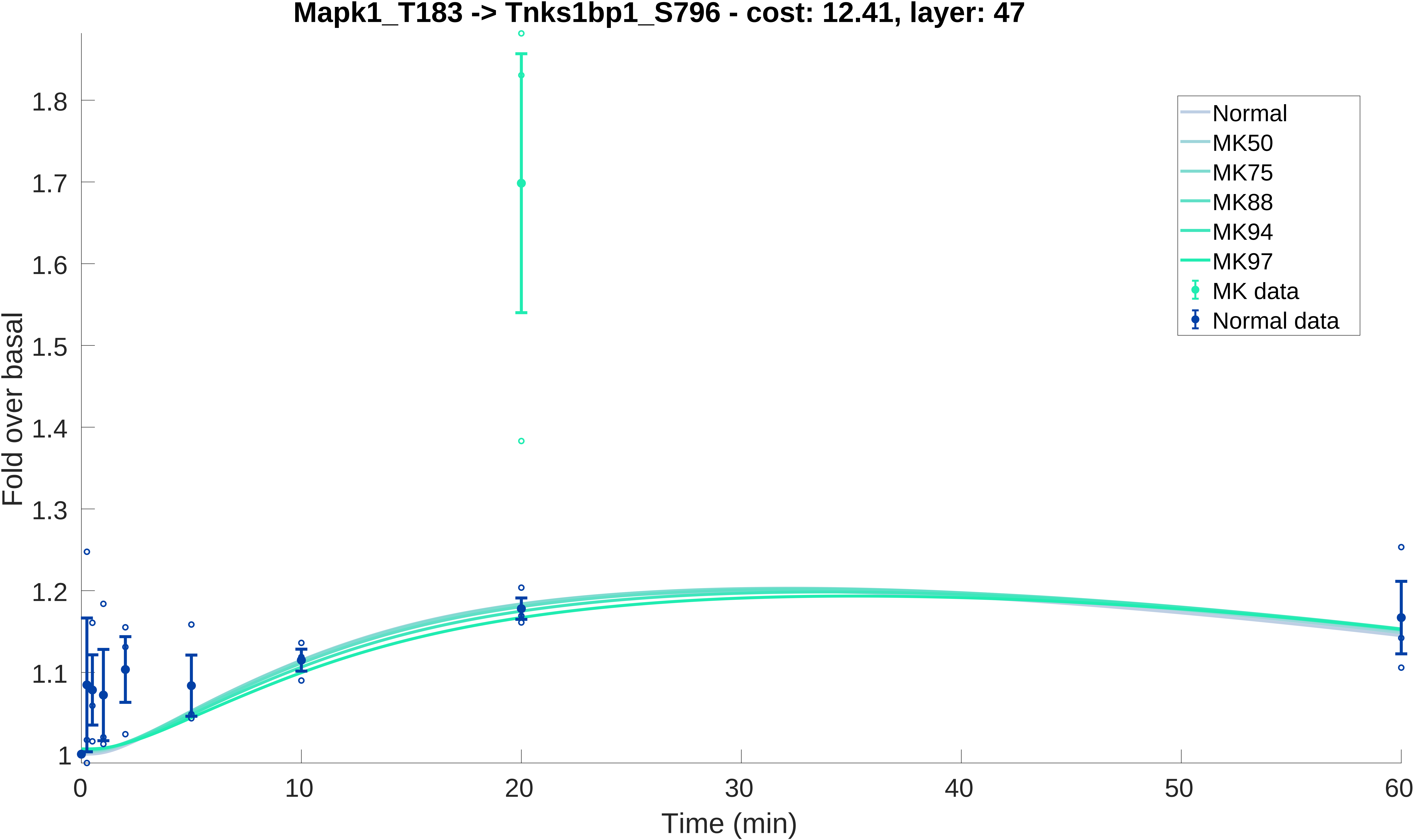
Mapk1_T183 -> Tnks1bp1_S796 - cost: 12.41, layer: 47
Fold over basal
Time (min)
1
1.1
1.2
1.3
1.4
1.5
1.6
1.7
1.8
0
10
20
30
40
50
60
Normal
MK50
MK75
MK88
MK94
MK97
MK data
Normal data

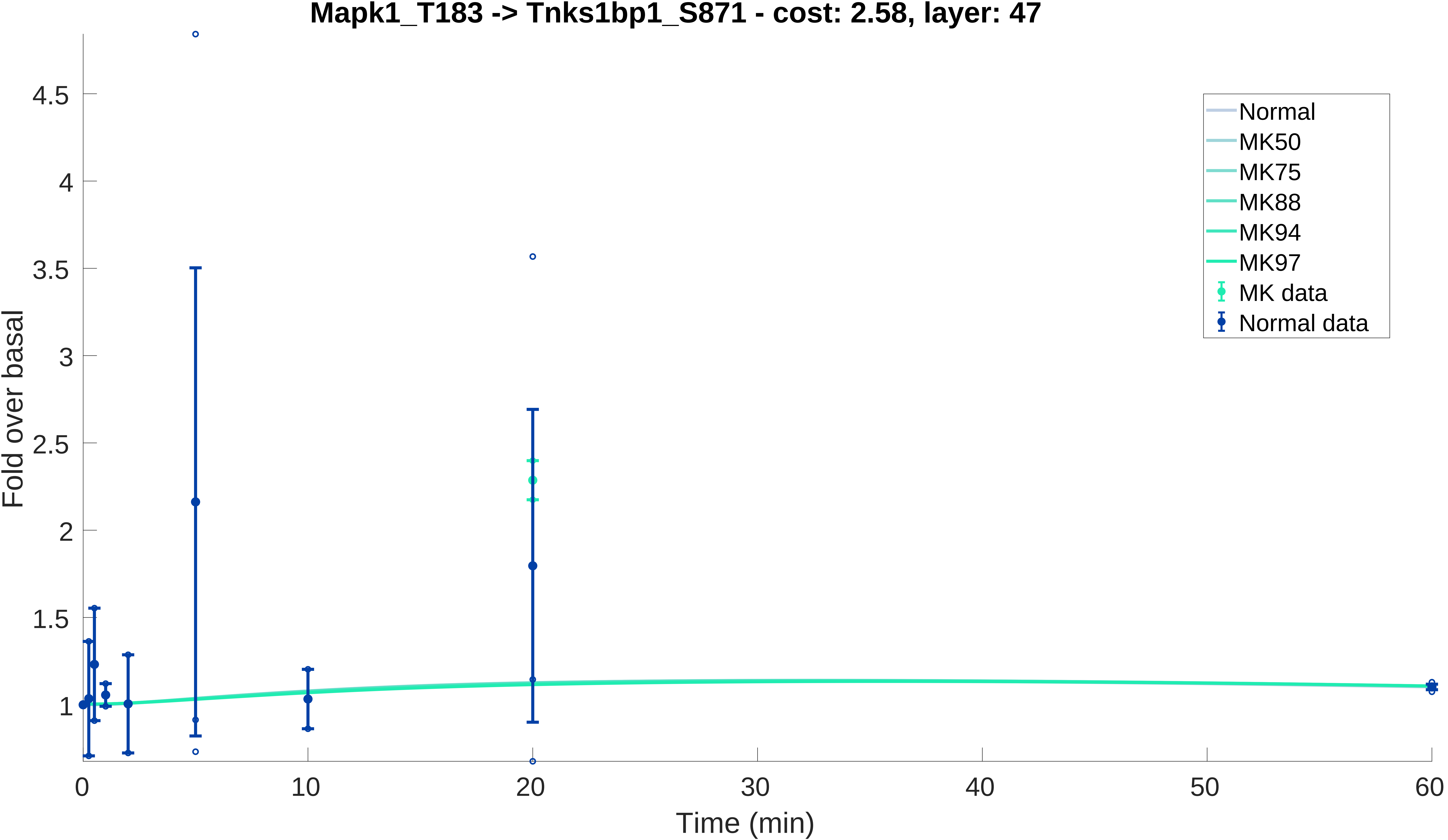

Mapk1_T183 -> Tnks1bp1_S871 - cost: 2.58, layer: 47
Fold over basal
Time (min)
Normal
MK50
MK75
MK88
MK94
MK97
MK data
Normal data
4.5
4
3.5
3
2.5
2
1.5
1
0
10
20
30
40
50
60

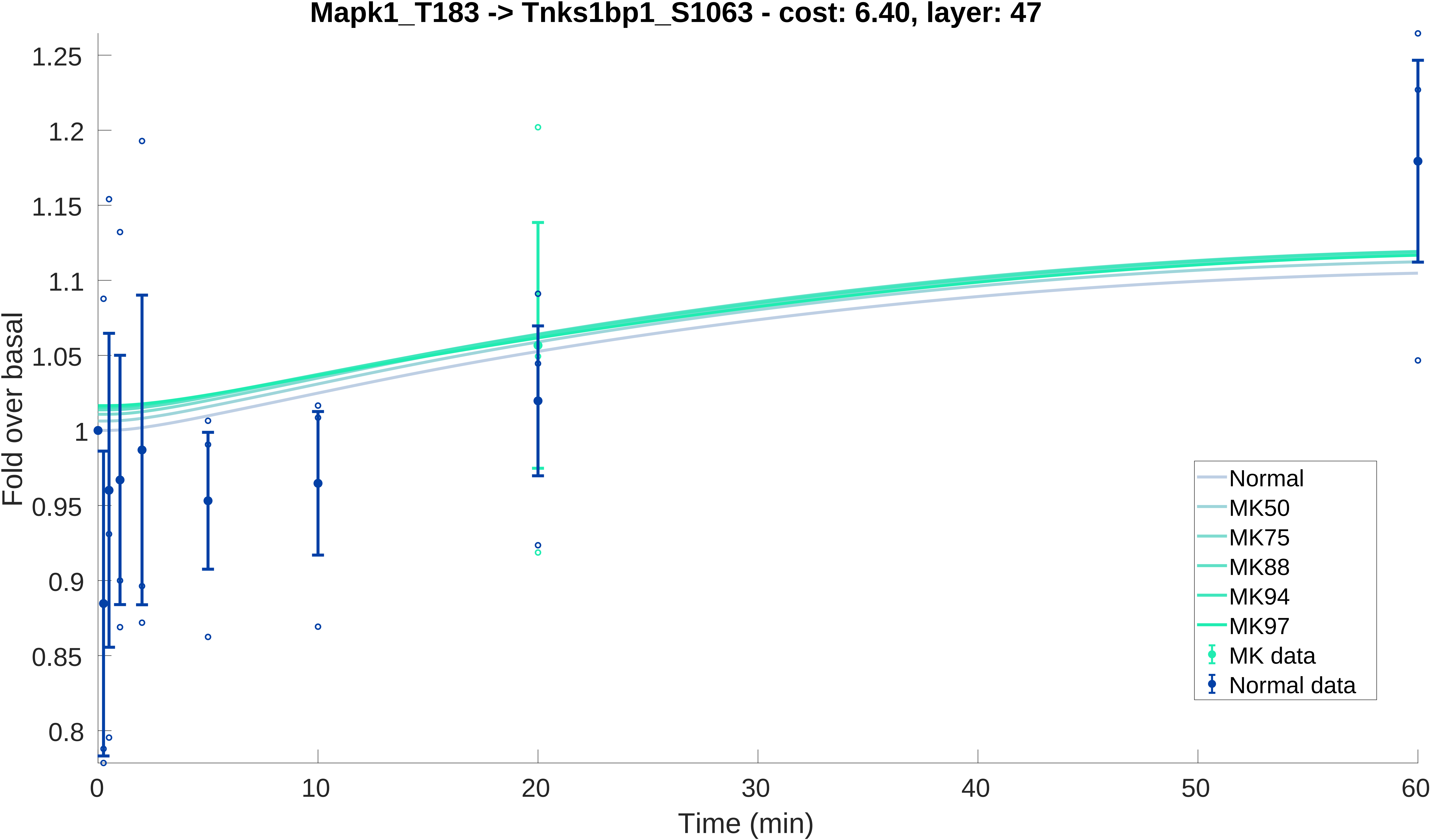

Mapk1_T183 -> Tnks1bp1_S1063 - cost: 6.40, layer: 47
Fold over basal
1.25
1.2
1.15
1.1
1.05
1
0.95
0.9
0.85
0.8
0
10
20
30
40
50
60
Time (min)
Normal
MK50
MK75
MK88
MK94
MK97
MK data
Normal data

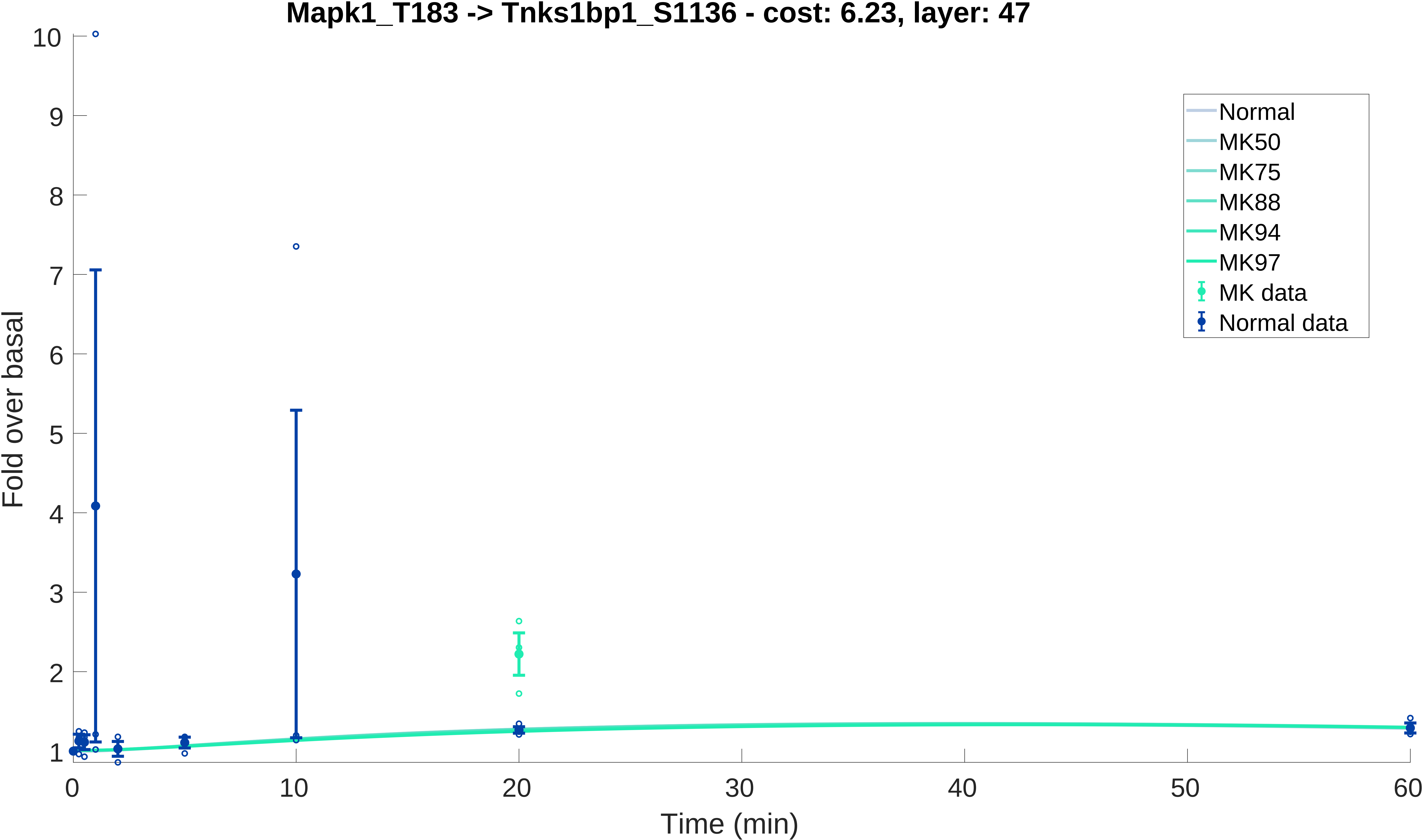

Mapk1_T183 -> Tnks1bp1_S1136 - cost: 6.23, layer: 47
Fold over basal
Time (min)
10
9
8
7
6
5
4
3
2
1
0
10
20
30
40
50
60
Normal
MK50
MK75
MK88
MK94
MK97
MK data
Normal data

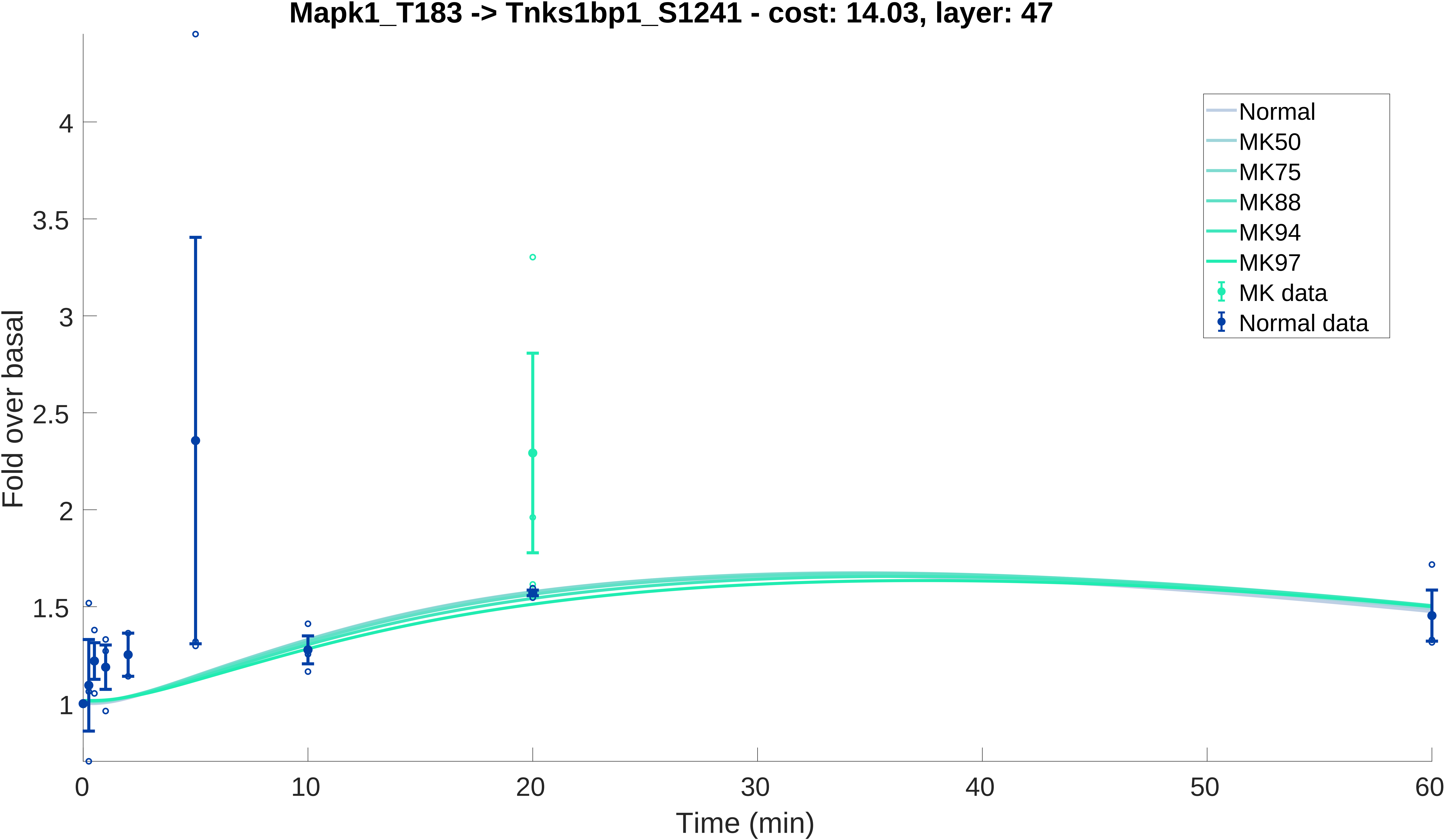

Mapk1_T183 -> Tnks1bp1_S1241 - cost: 14.03, layer: 47
Fold over basal
Time (min)
0
10
20
30
40
50
60
1
1.5
2
2.5
3
3.5
4
Normal
MK50
MK75
MK88
MK94
MK97
MK data
Normal data

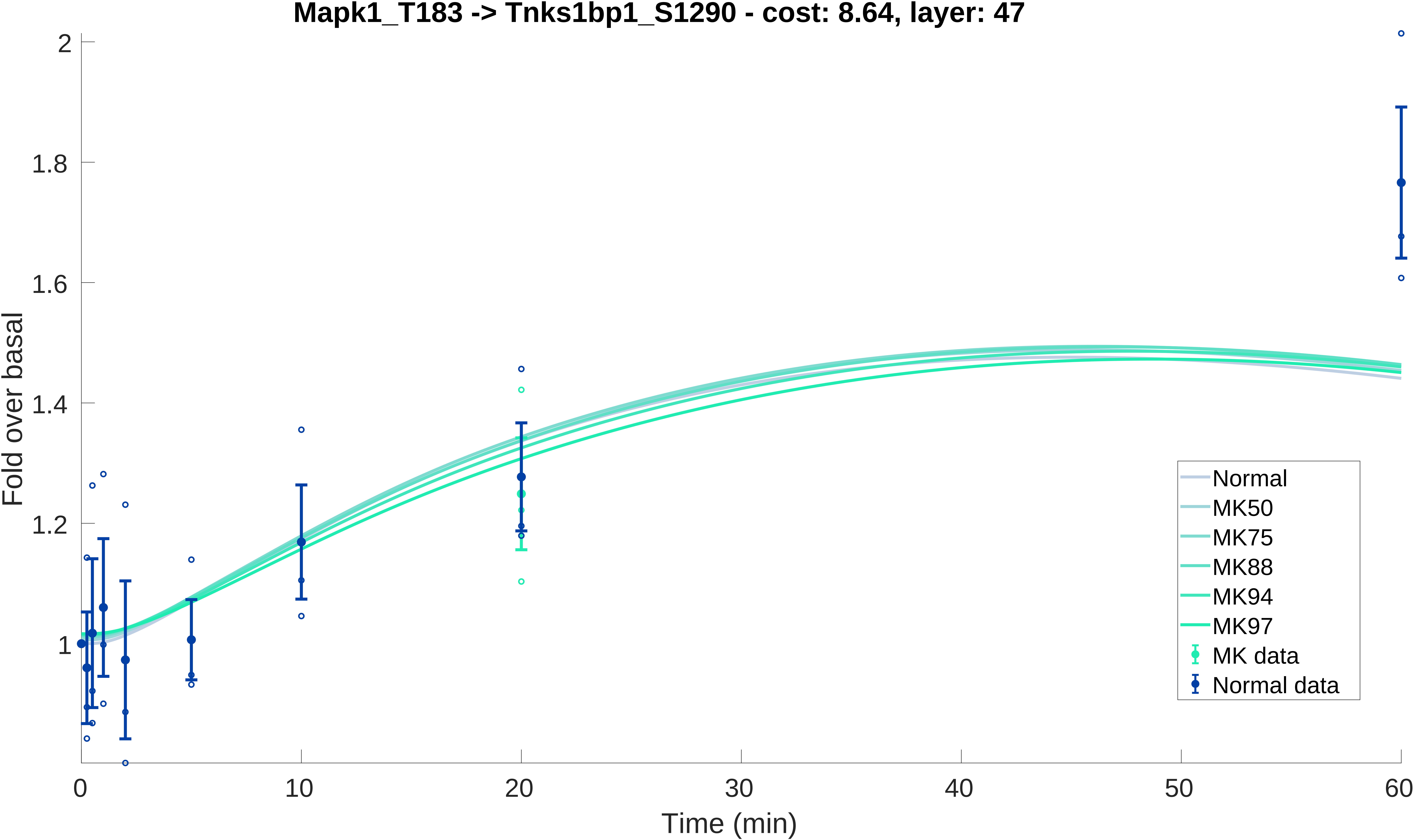

Mapk1_T183 -> Tnks1bp1_S1290 - cost: 8.64, layer: 47
Fold over basal
2
1.8
1.6
1.4
1.2
1
0
10
20
30
40
50
60
Time (min)
Normal
MK50
MK75
MK88
MK94
MK97
MK data
Normal data

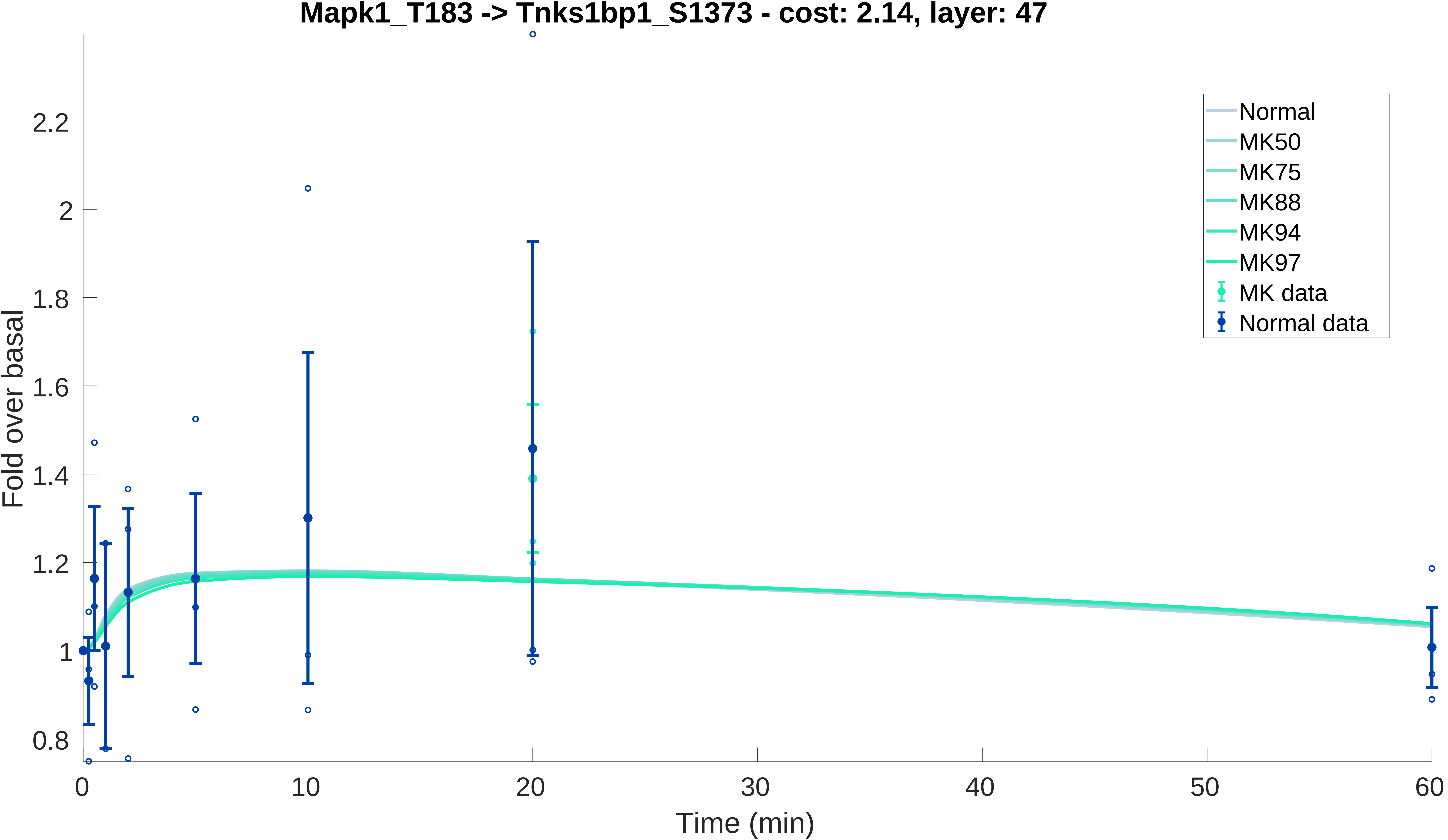
Mapk1_T183 -> Tnks1bp1_S1373 - cost: 2.14, layer: 47
Fold over basal
Time (min)
0.8
1
1.2
1.4
1.6
1.8
2
2.2
0
10
20
30
40
50
60
Normal
MK50
MK75
MK88
MK94
MK97
MK data
Normal data

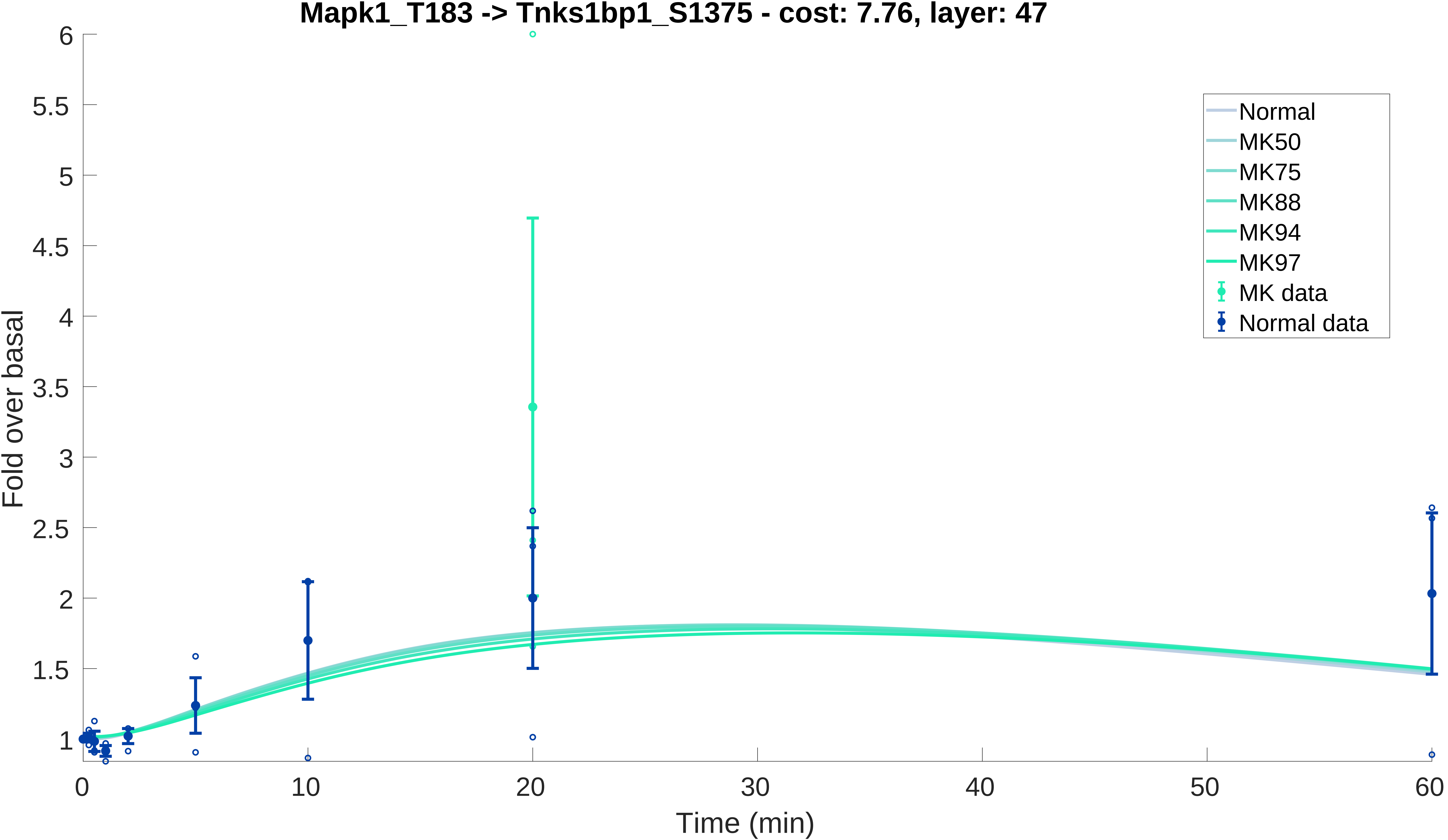
Mapk1_T183 -> Tnks1bp1_S1375 - cost: 7.76, layer: 47
Fold over basal
Time (min)
Normal
MK50
MK75
MK88
MK94
MK97
MK data
Normal data
0
10
20
30
40
50
60
1
1.5
2
2.5
3
3.5
4
4.5
5
5.5
6

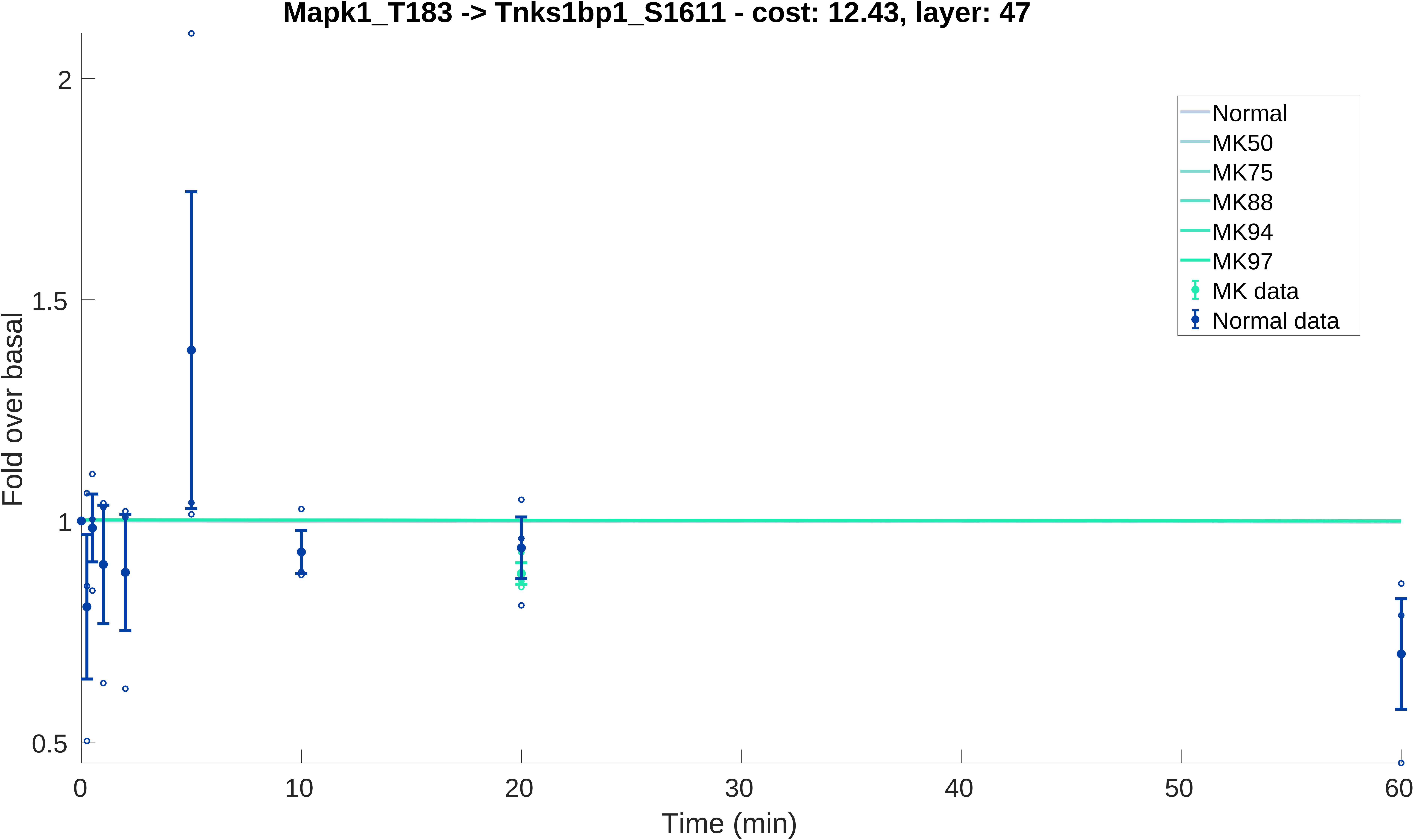
Mapk1_T183 -> Tnks1bp1_S1611 - cost: 12.43, layer: 47
Fold over basal
Time (min)
0.5
1
1.5
2
0
10
20
30
40
50
60
Normal
MK50
MK75
MK88
MK94
MK97
MK data
Normal data

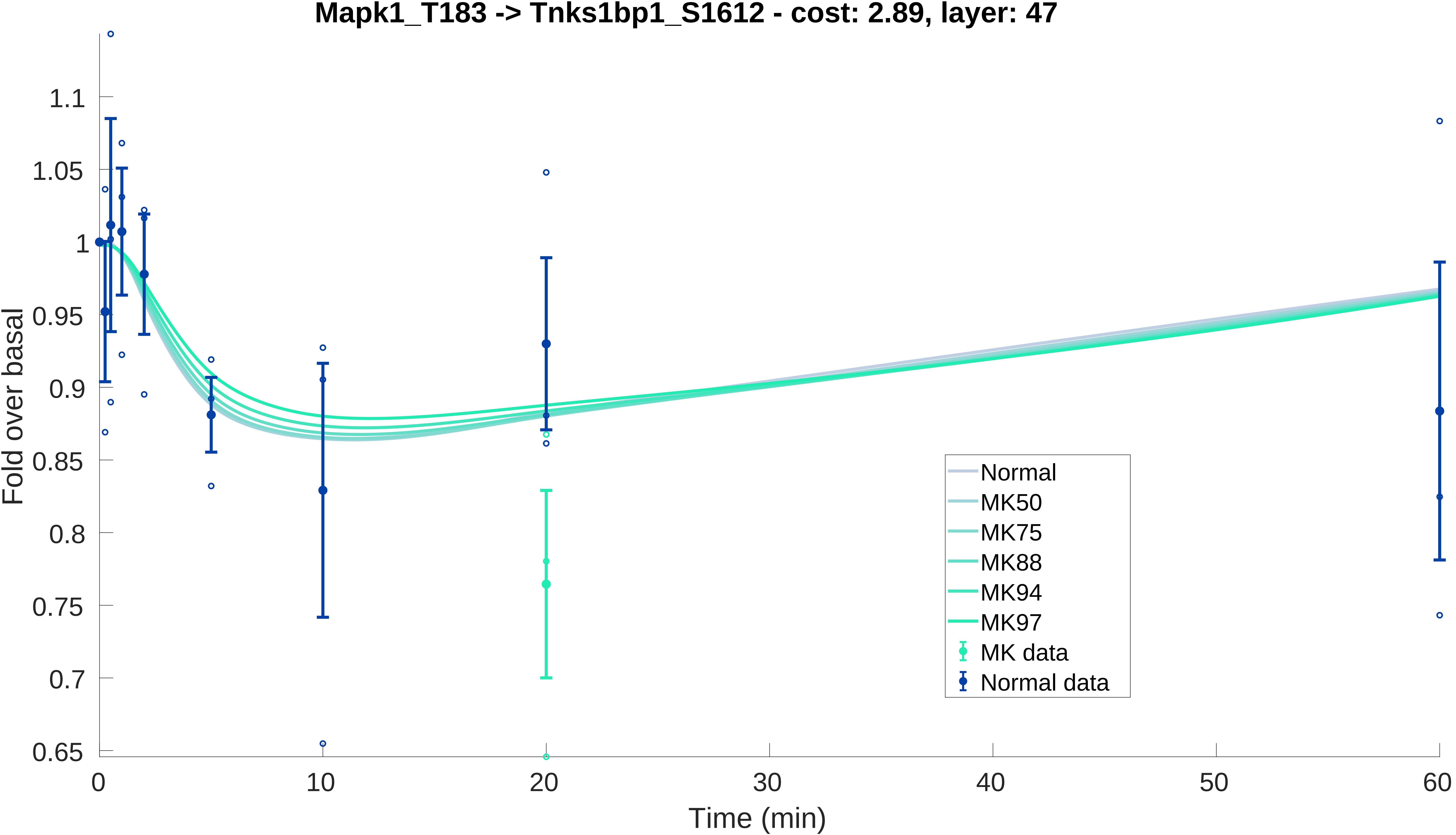

Mapk1_T183 -> Tnks1bp1_S1612 - cost: 2.89, layer: 47
Fold over basal
Time (min)
1.1
1.05
1
0.95
0.9
0.85
0.8
0.75
0.7
0.65
0
10
20
30
40
50
60
Normal
MK50
MK75
MK88
MK94
MK97
MK data
Normal data

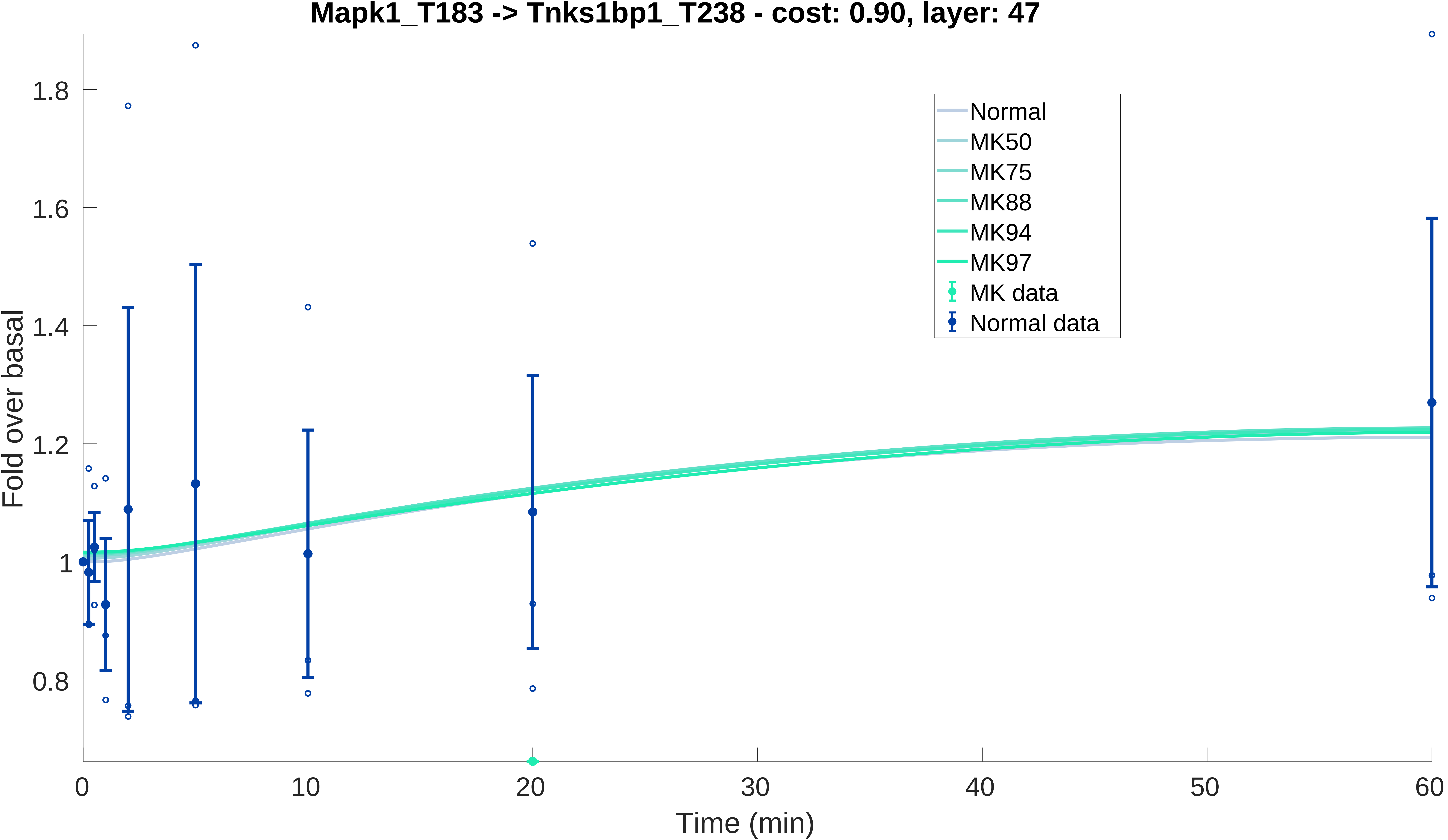

Mapk1_T183 -> Tnks1bp1_T238 - cost: 0.90, layer: 47
Fold over basal
Time (min)
0.8
1
1.2
1.4
1.6
1.8
0
10
20
30
40
50
60
Normal
MK50
MK75
MK88
MK94
MK97
MK data
Normal data

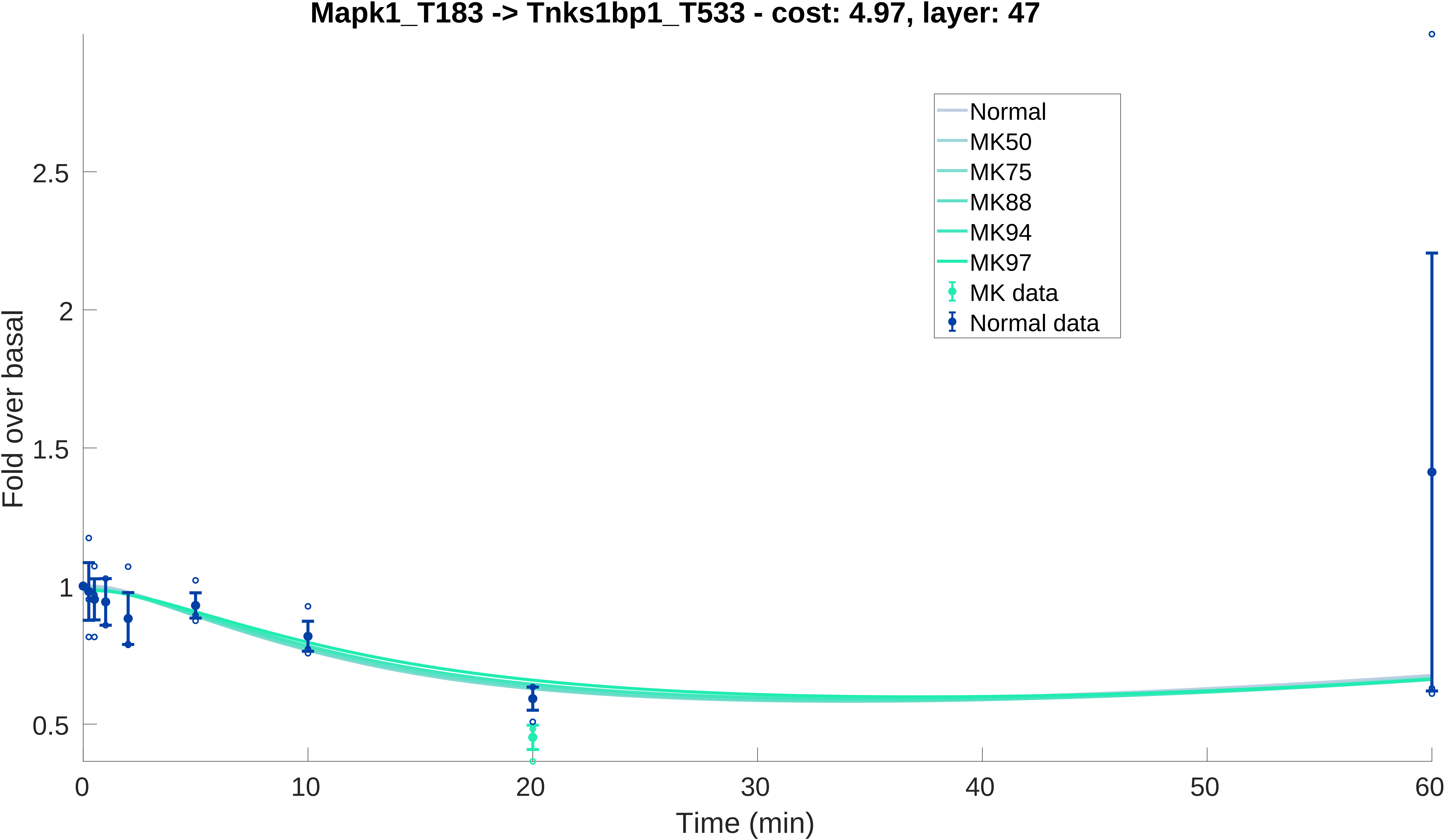
Mapk1_T183 -> Tnks1bp1_T533 - cost: 4.97, layer: 47
Normal
MK50
MK75
MK88
MK94
MK97
MK data
Normal data
Fold over basal
Time (min)
0.5
1
1.5
2
2.5
0
10
20
30
40
50
60

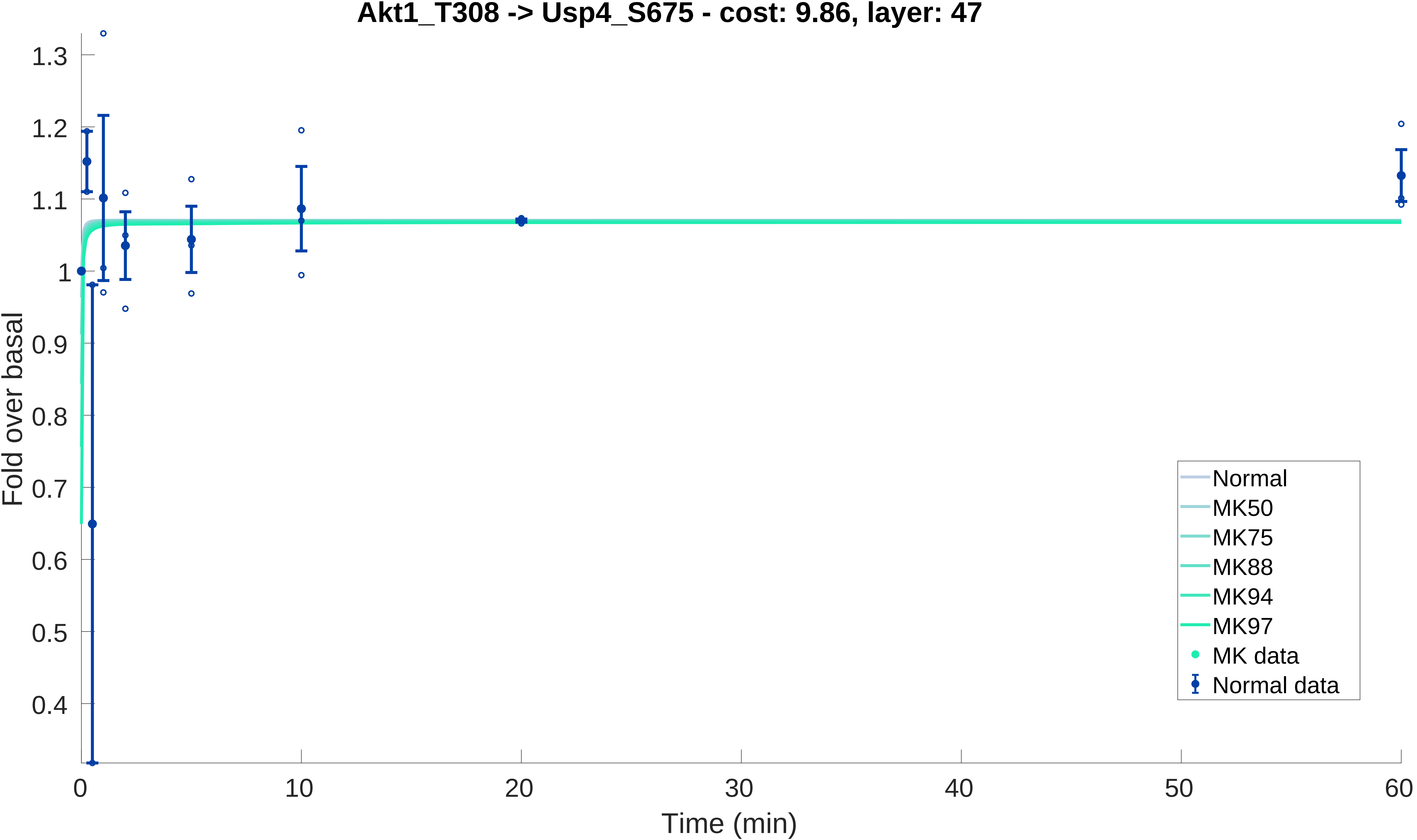

Akt1_T308 -> Usp4_S675 - cost: 9.86, layer: 47
Fold over basal
Time (min)
1.3
1.2
1.1
1
0.9
0.8
0.7
0.6
0.5
0.4
0
10
20
30
40
50
60
Normal
MK50
MK75
MK88
MK94
MK97
MK data
Normal data

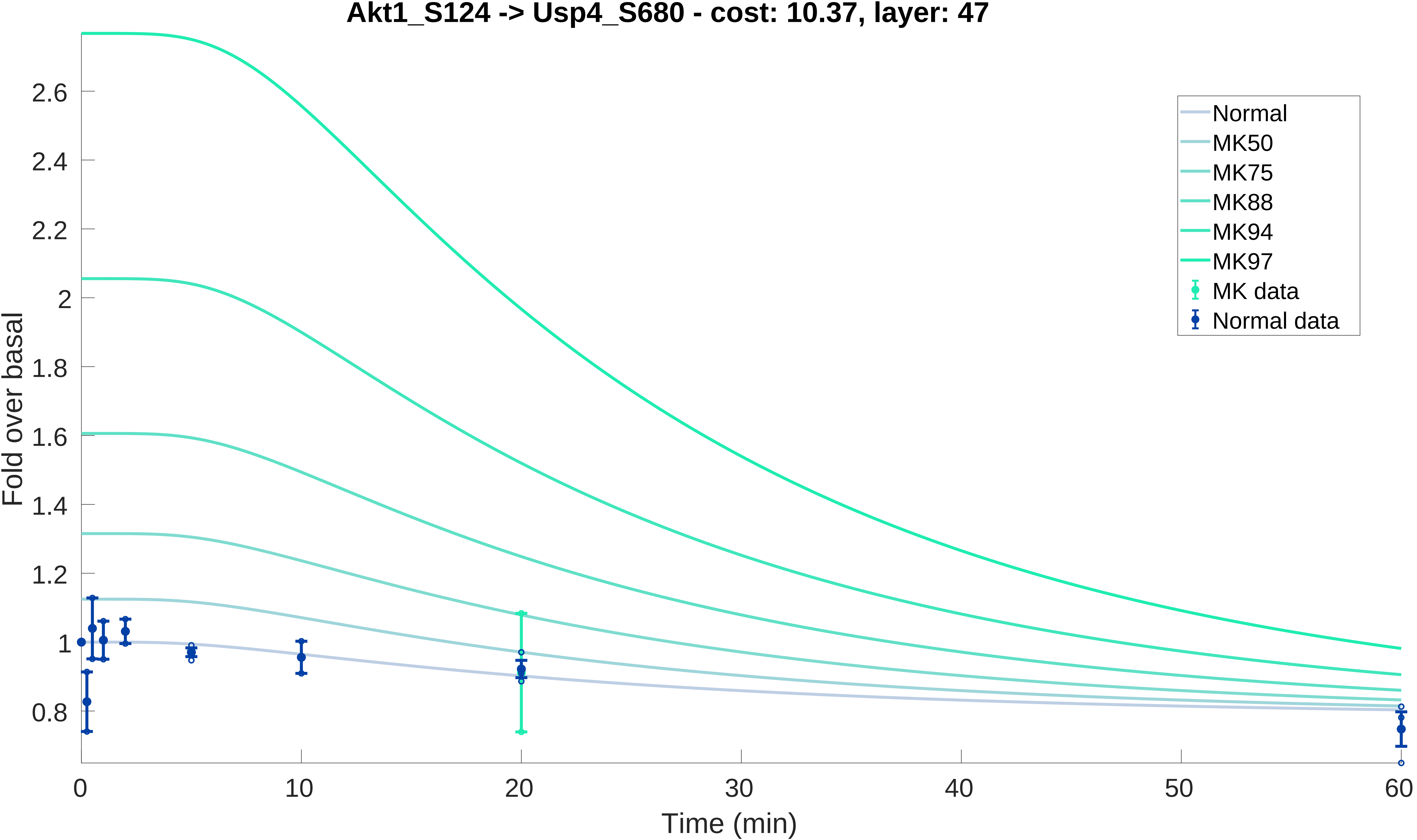

Akt1_S124 -> Usp4_S680 - cost: 10.37, layer: 47
Fold over basal
Time (min)
0.8
1
1.2
1.4
1.6
1.8
2
2.2
2.4
2.6
0
10
20
30
40
50
60
Normal
MK50
MK75
MK88
MK94
MK97
MK data
Normal data

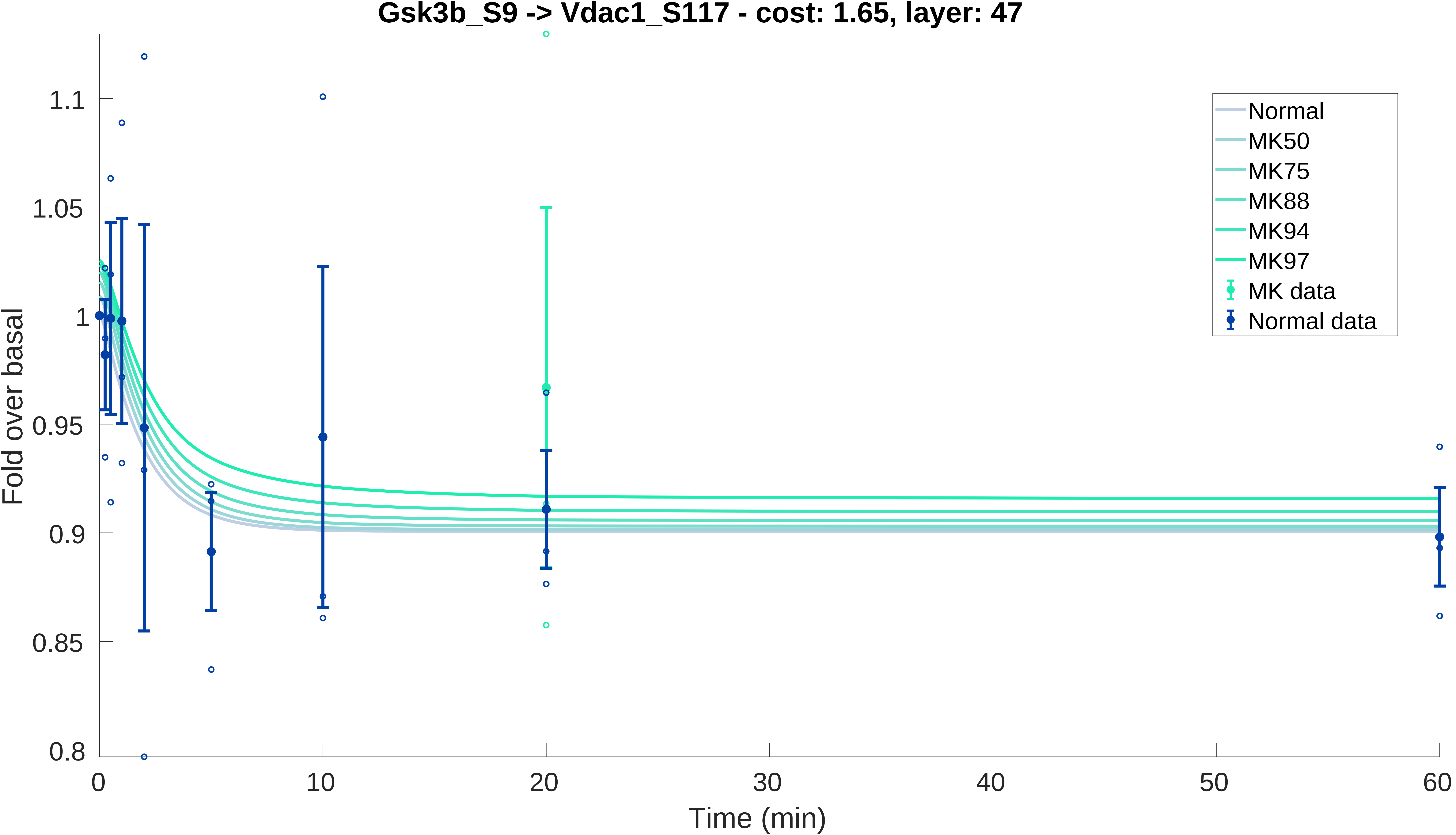
Gsk3b_S9 -> Vdac1_S117 - cost: 1.65, layer: 47
Fold over basal
Time (min)
1.1
1.05
1
0.95
0.9
0.85
0.8
0
10
20
30
40
50
60
Normal
MK50
MK75
MK88
MK94
MK97
MK data
Normal data

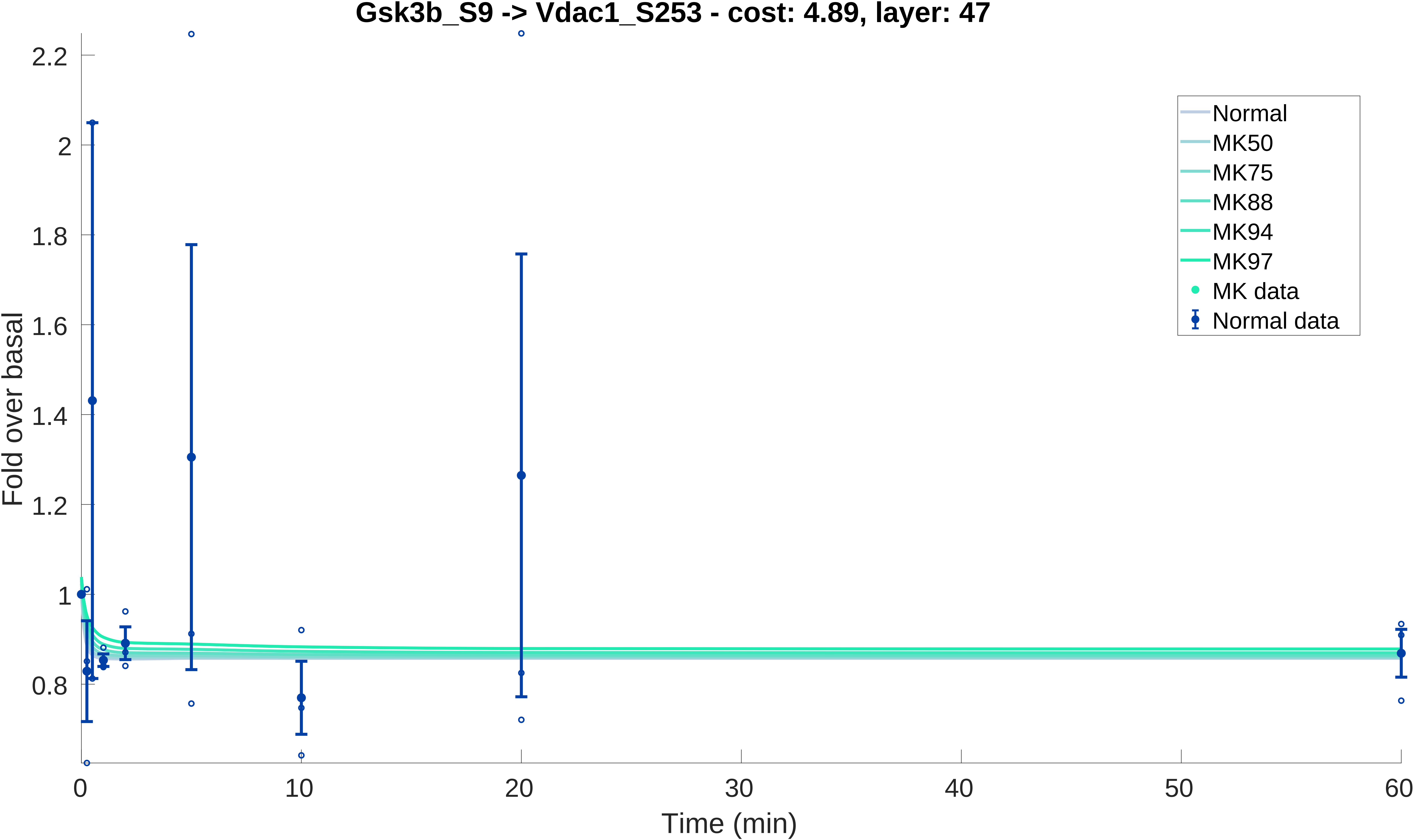
Gsk3b_S9 -> Vdac1_S253 - cost: 4.89, layer: 47
Fold over basal
Time (min)
0.8
1
1.2
1.4
1.6
1.8
2
2.2
0
10
20
30
40
50
60
Normal
MK50
MK75
MK88
MK94
MK97
MK data
Normal data

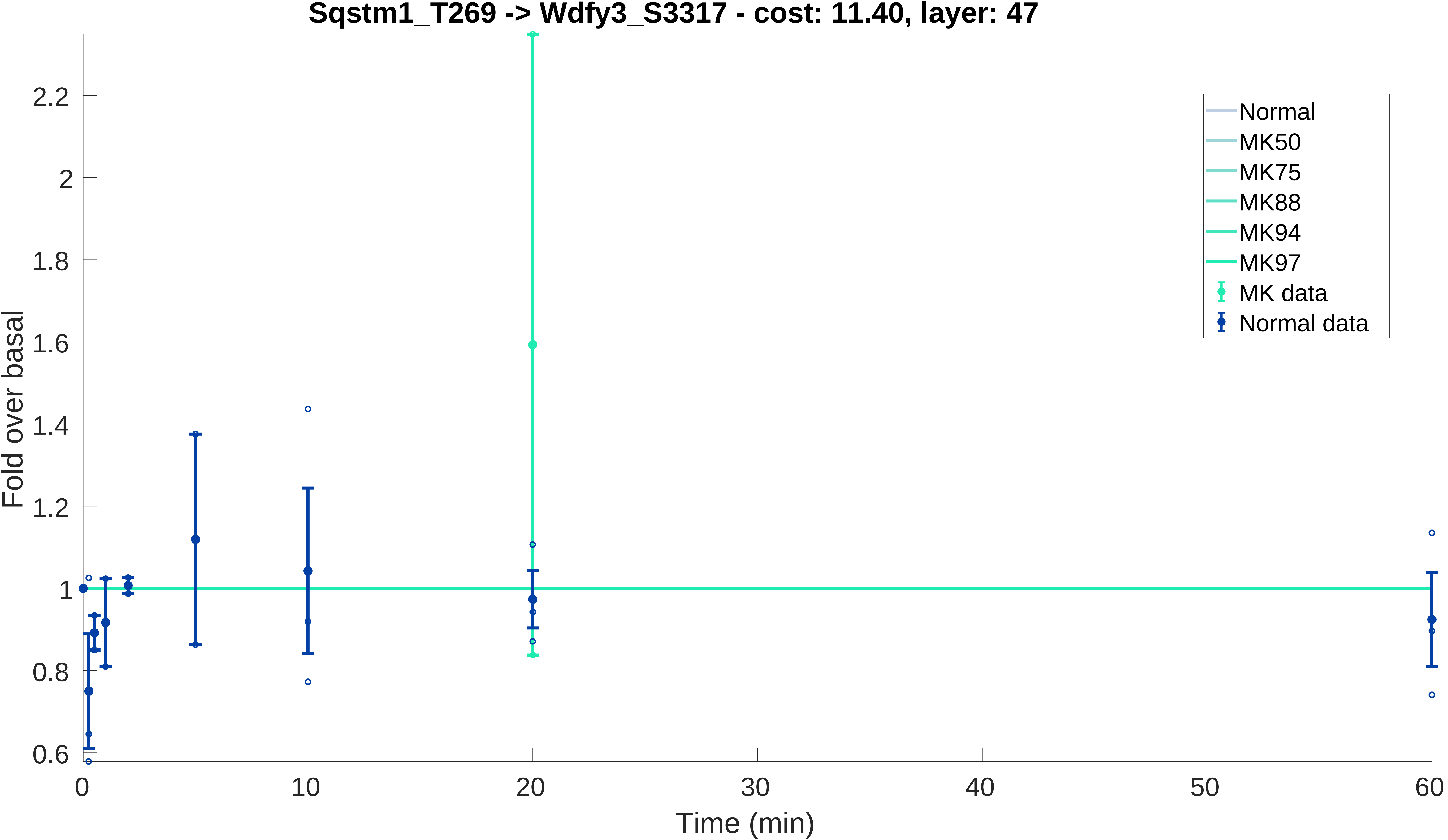

Sqstm1_T269 -> Wdfy3_S3317 - cost: 11.40, layer: 47
Fold over basal
0.6
0.8
1
1.2
1.4
1.6
1.8
2
2.2
Time (min)
0
10
20
30
40
50
60
Normal
MK50
MK75
MK88
MK94
MK97
MK data
Normal data

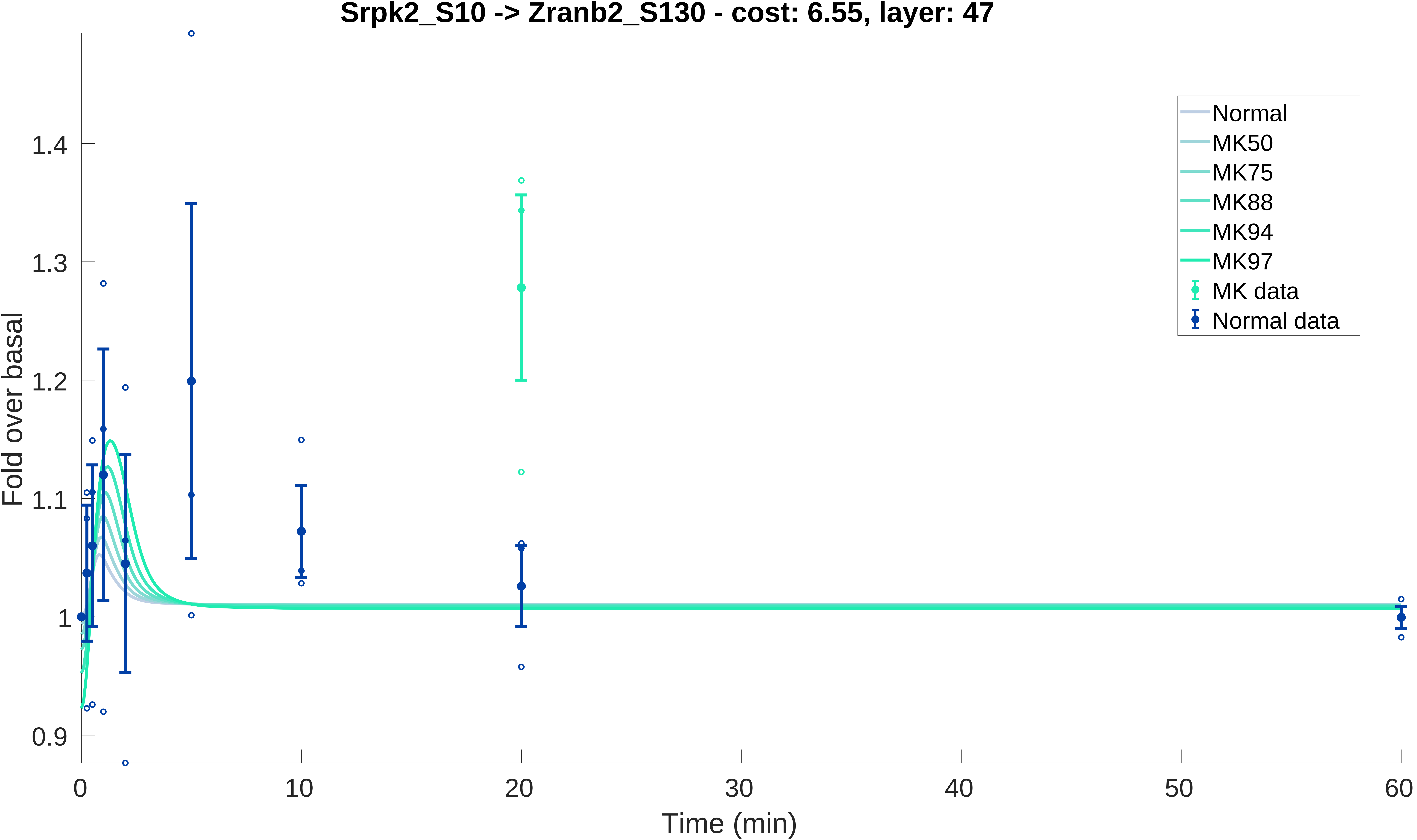
Srpk2_S10 -> Zranb2_S130 - cost: 6.55, layer: 47
Fold over basal
Time (min)
0.9
1
1.1
1.2
1.3
1.4
0
10
20
30
40
50
60
Normal
MK50
MK75
MK88
MK94
MK97
MK data
Normal data

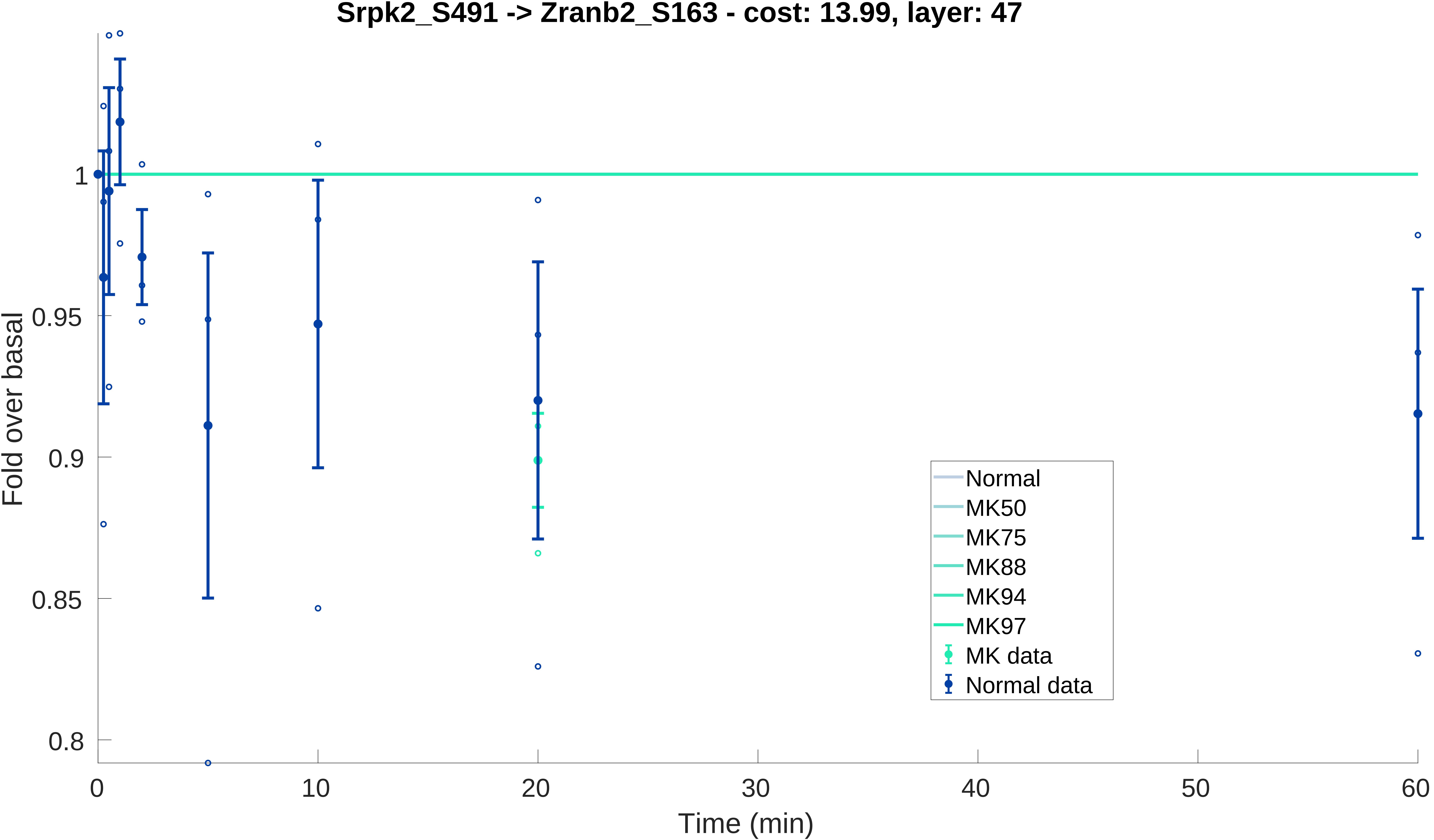

Srpk2_S491 -> Zranb2_S163 - cost: 13.99, layer: 47
Fold over basal
Time (min)
0.8
0.85
0.9
0.95
1
0
10
20
30
40
50
60
Normal
MK50
MK75
MK88
MK94
MK97
MK data
Normal data

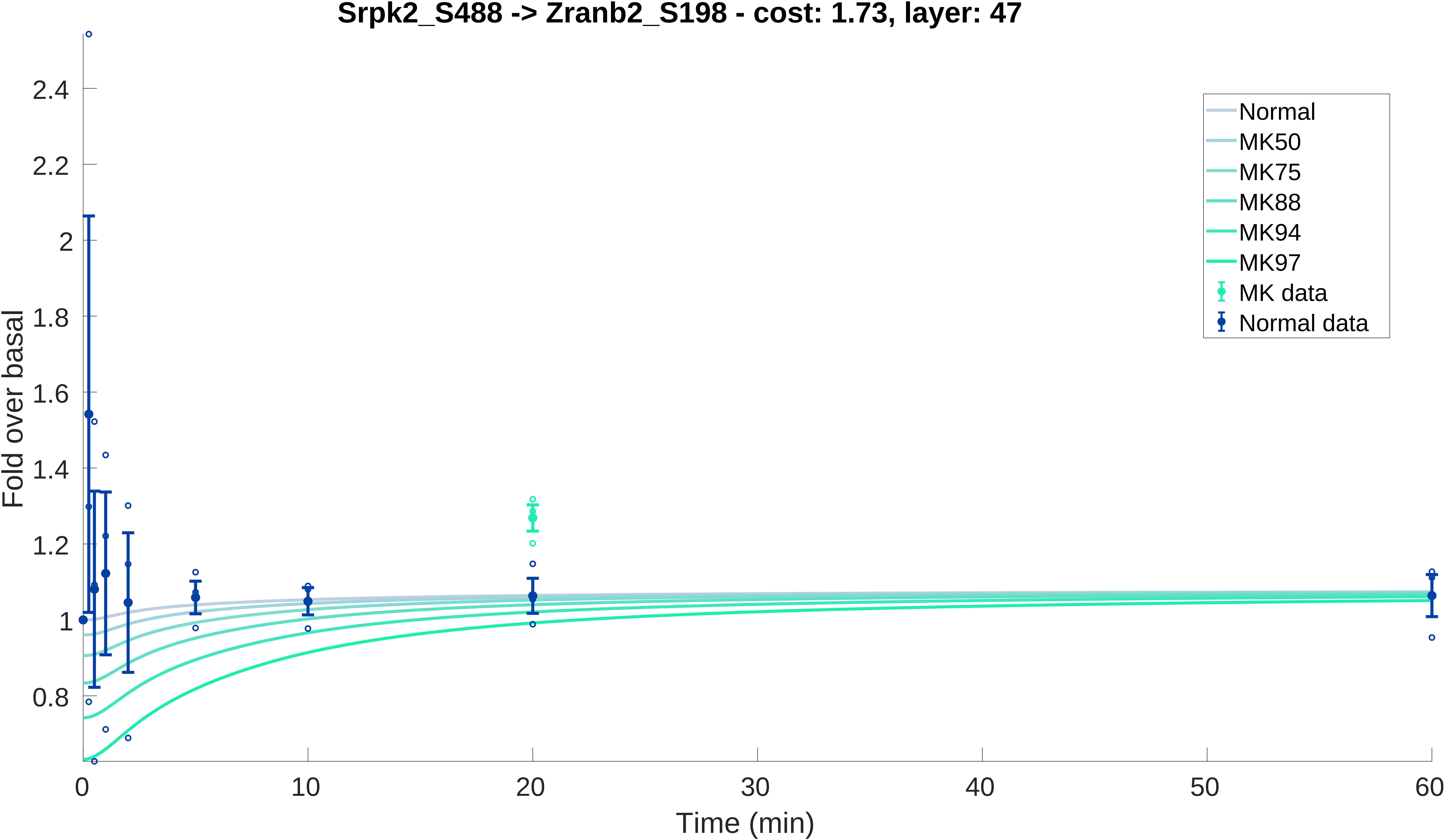

Srpk2_S488 -> Zranb2_S198 - cost: 1.73, layer: 47
Fold over basal
2.4
2.2
2
1.8
1.6
1.4
1.2
1
0.8
0
10
20
30
40
50
60
Time (min)
Normal
MK50
MK75
MK88
MK94
MK97
MK data
Normal data

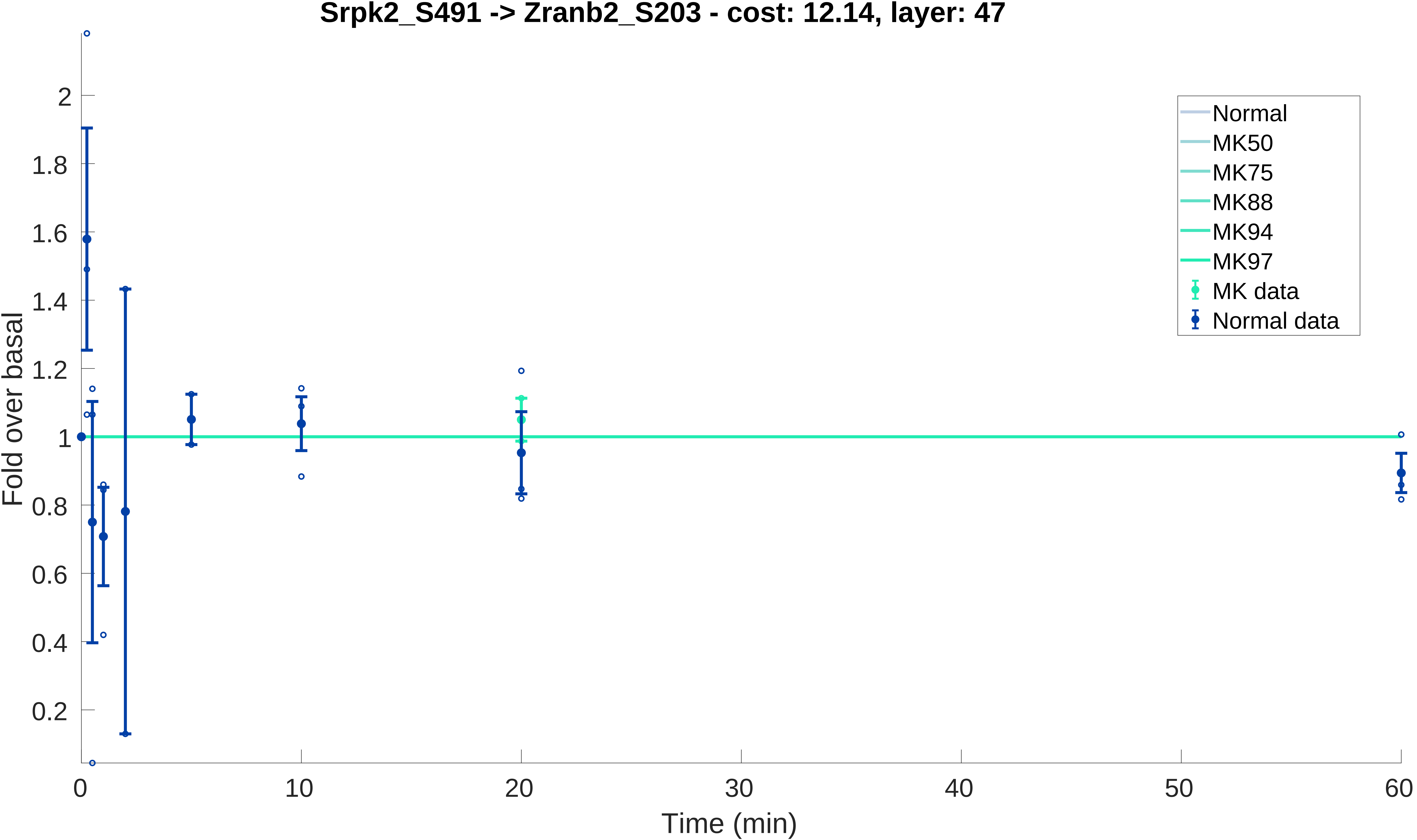
Srpk2_S491 -> Zranb2_S203 - cost: 12.14, layer: 47
Fold over basal
Time (min)
0
10
20
30
40
50
60
0.2
0.4
0.6
0.8
1
1.2
1.4
1.6
1.8
2
Normal
MK50
MK75
MK88
MK94
MK97
MK data
Normal data

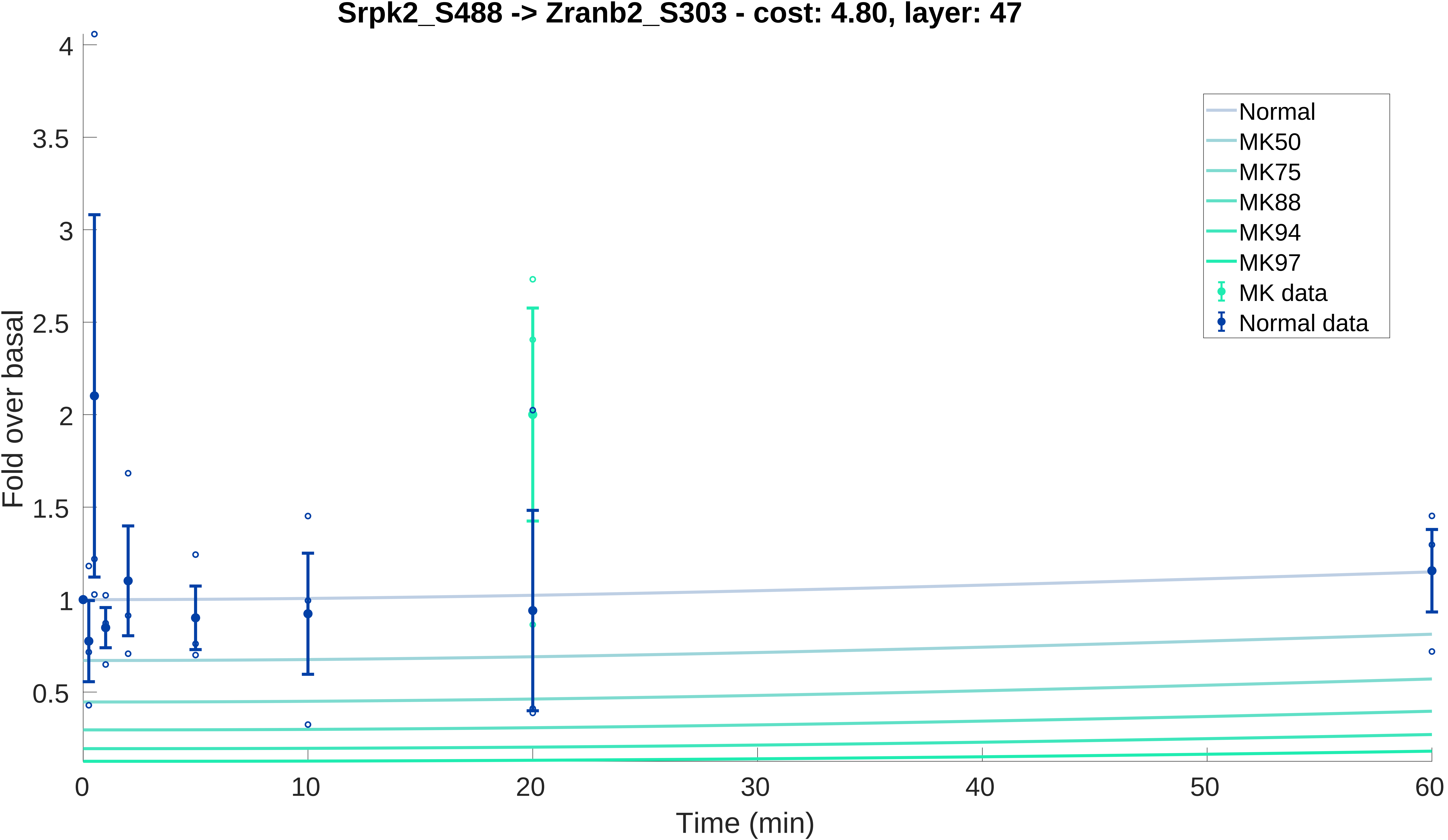

Srpk2_S488 -> Zranb2_S303 - cost: 4.80, layer: 47
Fold over basal
4
3.5
3
2.5
2
1.5
1
0.5
0
10
20
30
40
50
60
Time (min)
Normal
MK50
MK75
MK88
MK94
MK97
MK data
Normal data

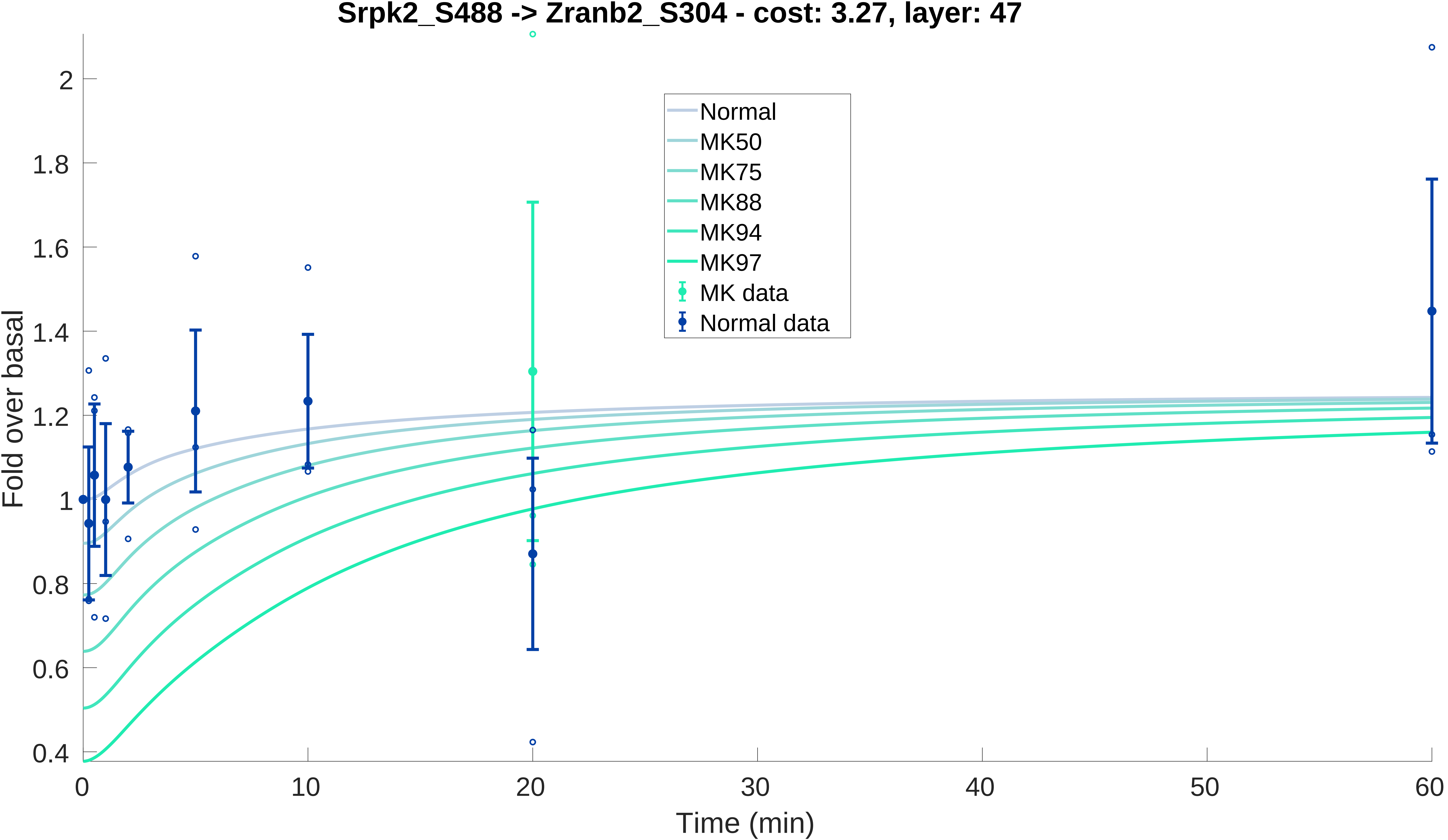
Srpk2_S488 -> Zranb2_S304 - cost: 3.27, layer: 47
Fold over basal
Time (min)
0
10
20
30
40
50
60
0.4
0.6
0.8
1
1.2
1.4
1.6
1.8
2
Normal
MK50
MK75
MK88
MK94
MK97
MK data
Normal data

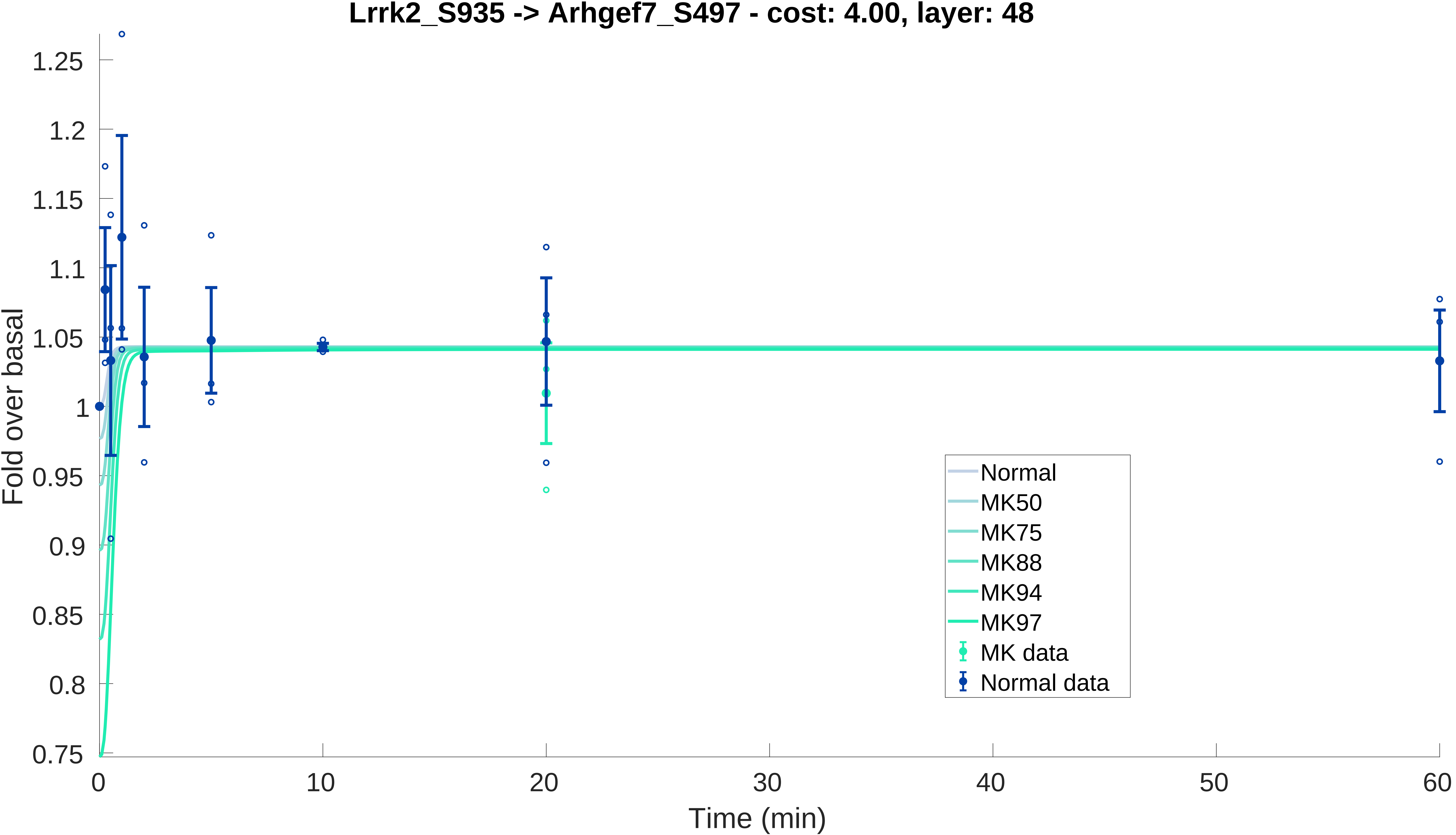

Lrrk2_S935 -> Arhgef7_S497 - cost: 4.00, layer: 48
Fold over basal
Time (min)
Normal
MK50
MK75
MK88
MK94
MK97
MK data
Normal data
1.25
1.2
1.15
1.1
1.05
1
0.95
0.9
0.85
0.8
0.75
0
10
20
30
40
50
60

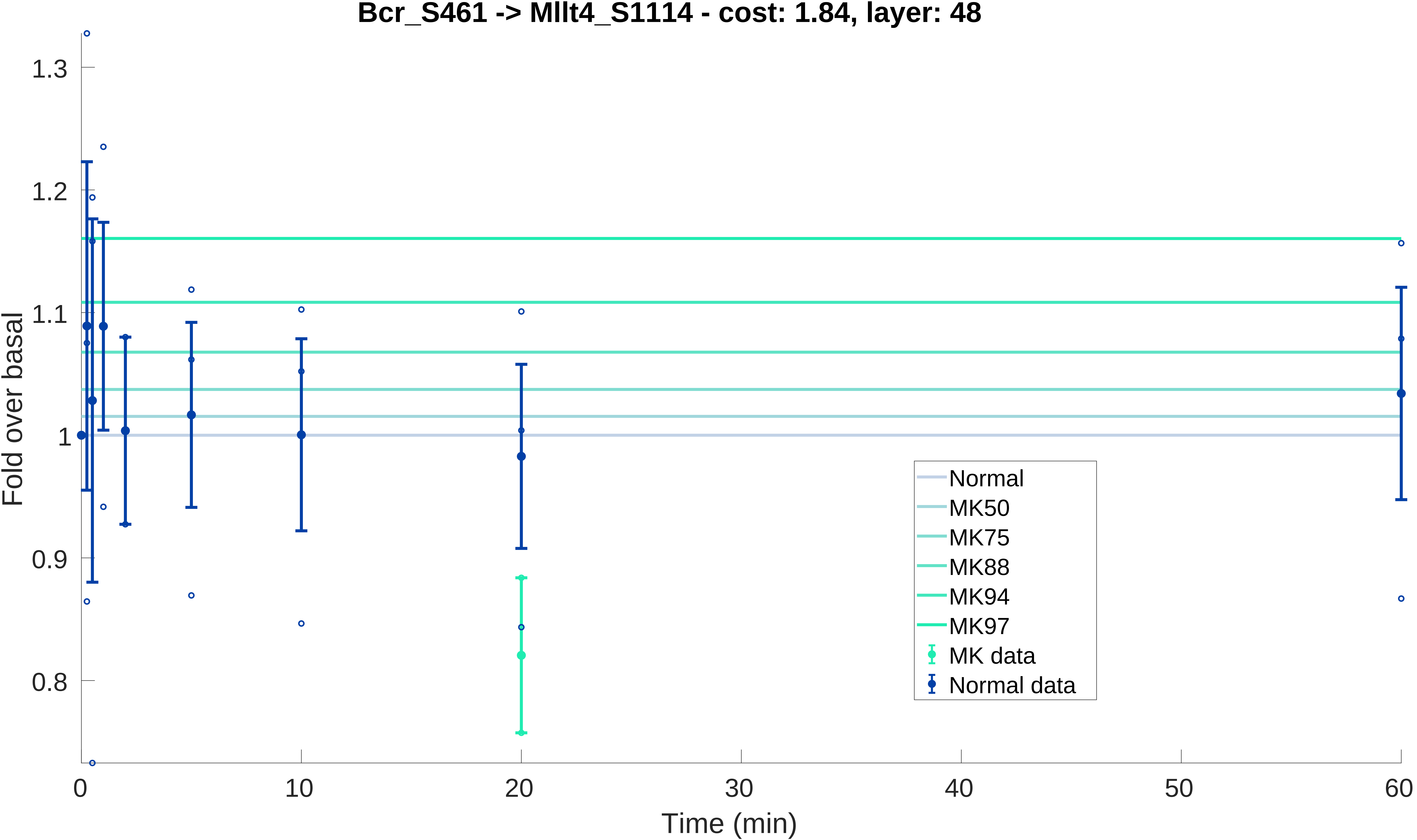

Bcr_S461 -> Mllt4_S1114 - cost: 1.84, layer: 48
Fold over basal
1.3
1.2
1.1
1
0.9
0.8
0
10
20
30
40
50
60
Time (min)
Normal
MK50
MK75
MK88
MK94
MK97
MK data
Normal data

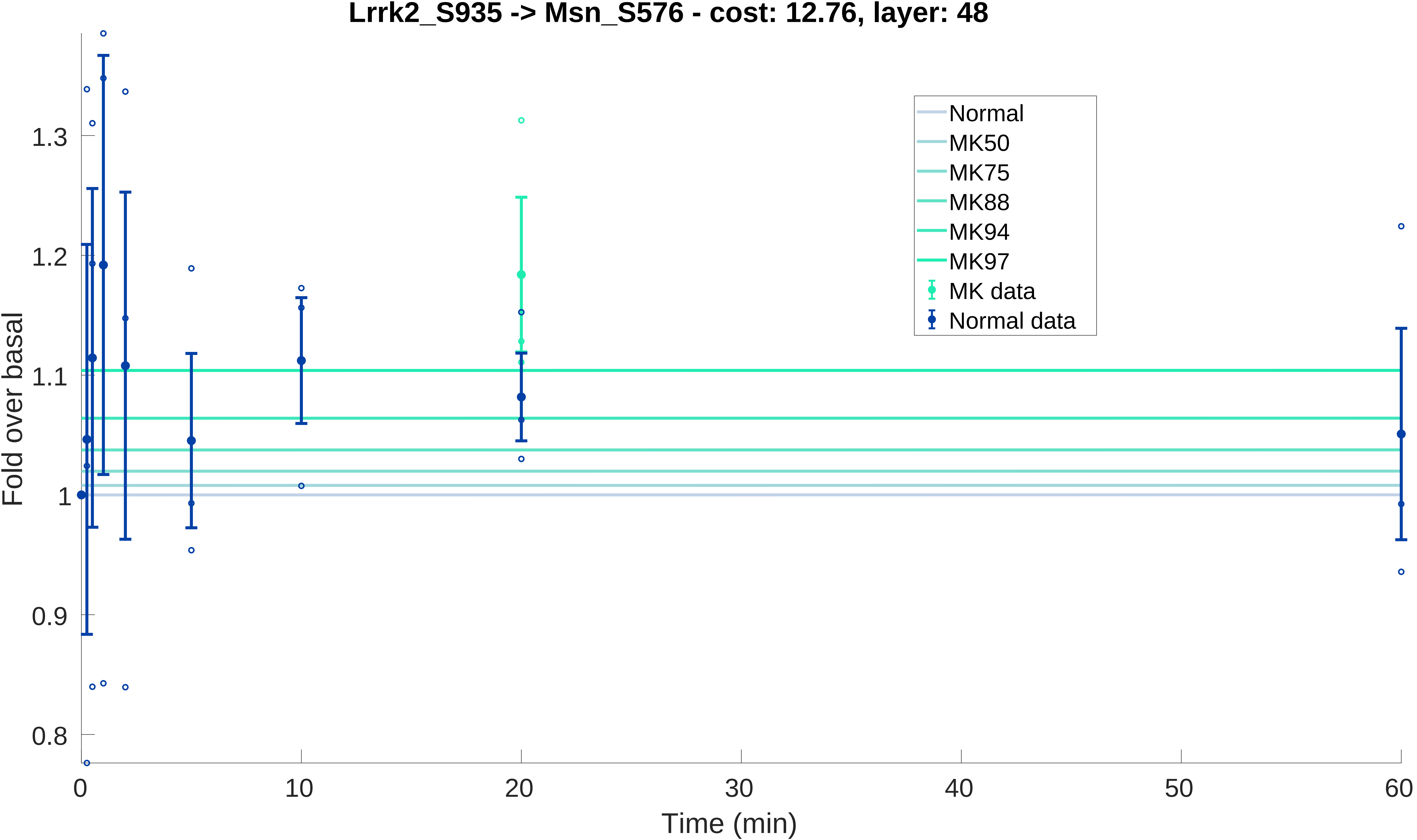

Lrrk2_S935 -> Msn_S576 - cost: 12.76, layer: 48
Normal
MK50
MK75
MK88
MK94
MK97
MK data
Normal data
Fold over basal
1.3
1.2
1.1
1
0.9
0.8
0
10
20
30
40
50
60
Time (min)

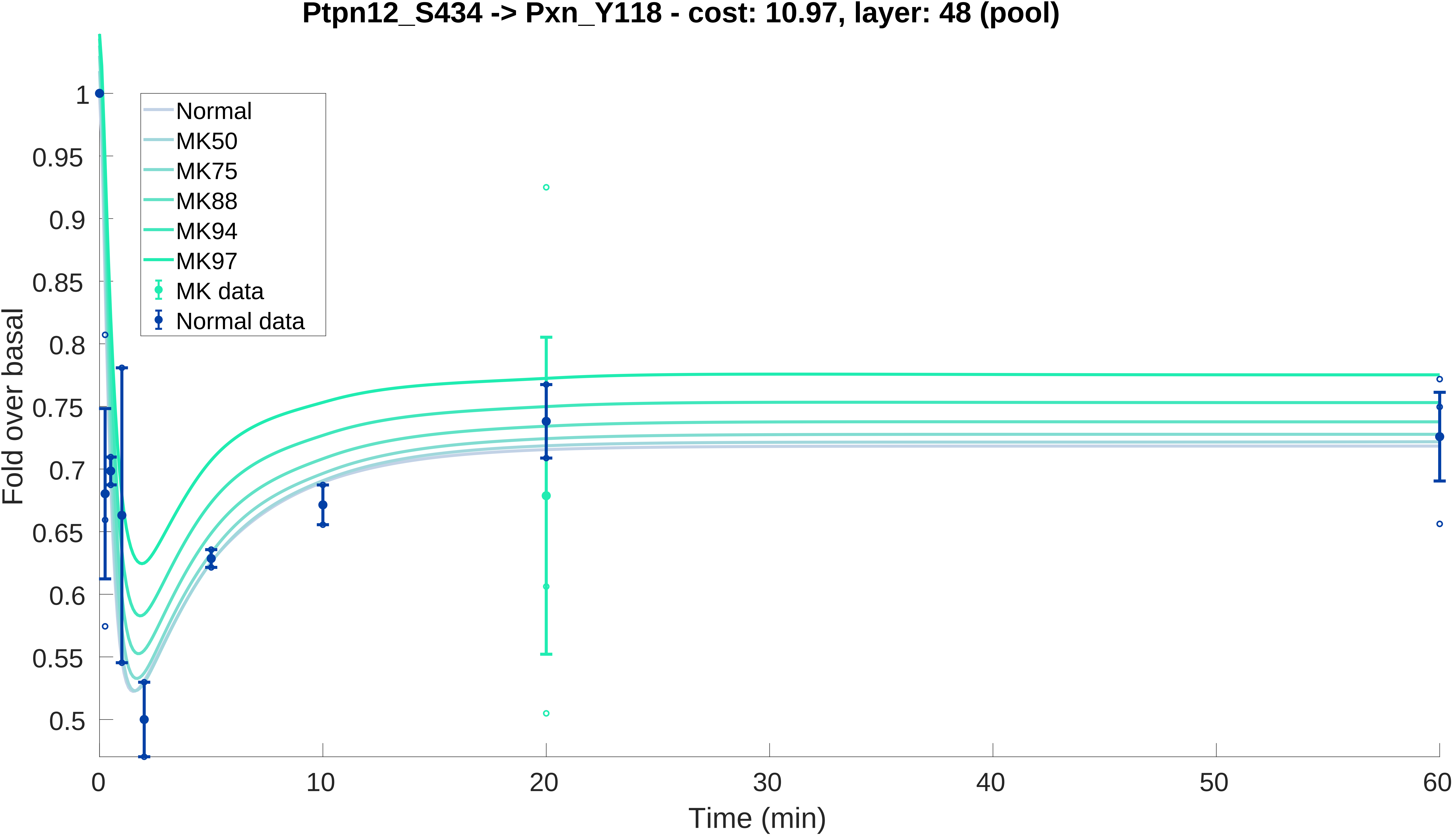

Ptpn12_S434 -> Pxn_Y118 - cost: 10.97, layer: 48 (pool)
Normal
MK50
MK75
MK88
MK94
MK97
MK data
Normal data
Fold over basal
Time (min)
1
0.95
0.9
0.85
0.8
0.75
0.7
0.65
0.6
0.55
0.5
0
10
20
30
40
50
60

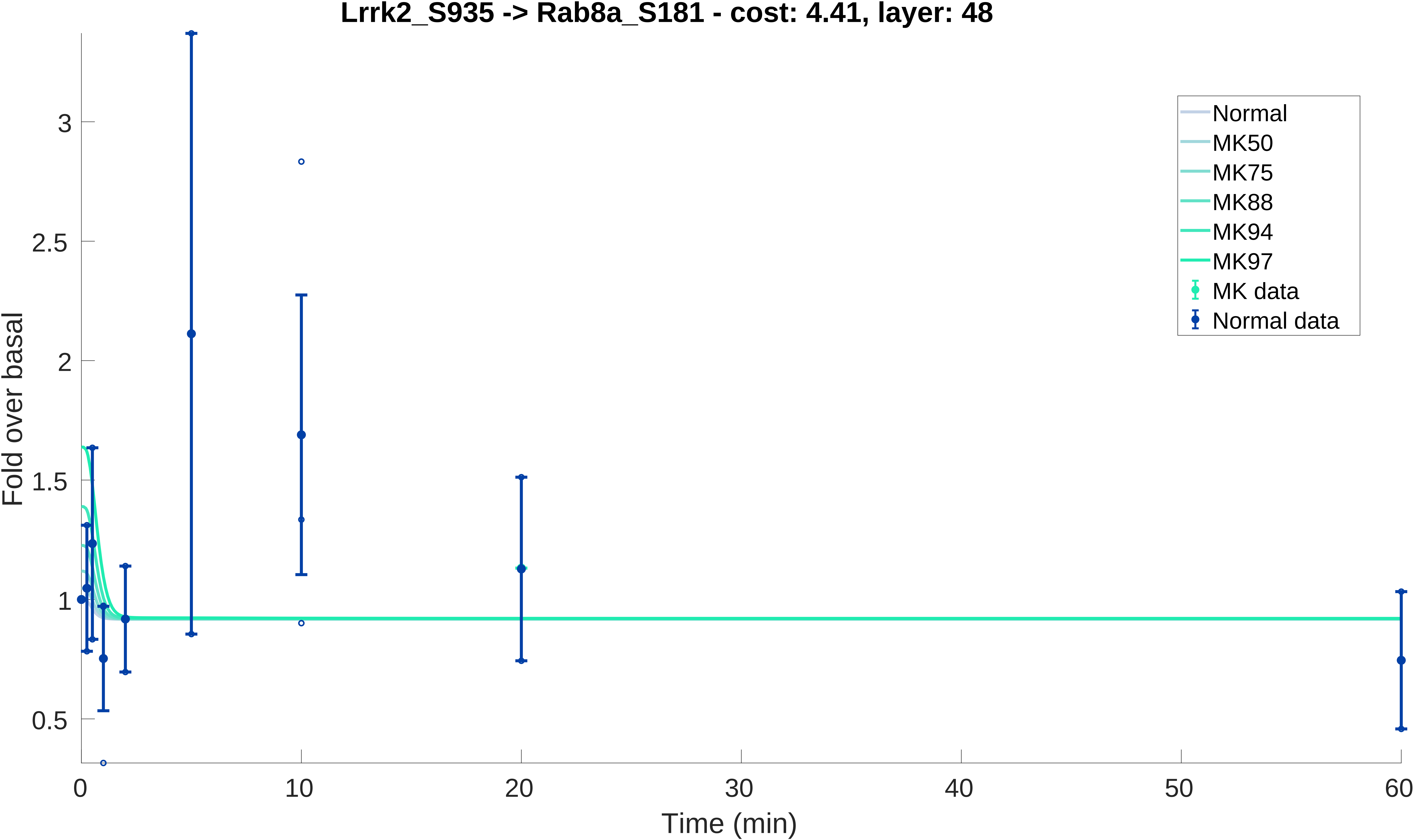

Lrrk2_S935 -> Rab8a_S181 - cost: 4.41, layer: 48
Fold over basal
Time (min)
0.5
1
1.5
2
2.5
3
0
10
20
30
40
50
60
Normal
MK50
MK75
MK88
MK94
MK97
MK data
Normal data

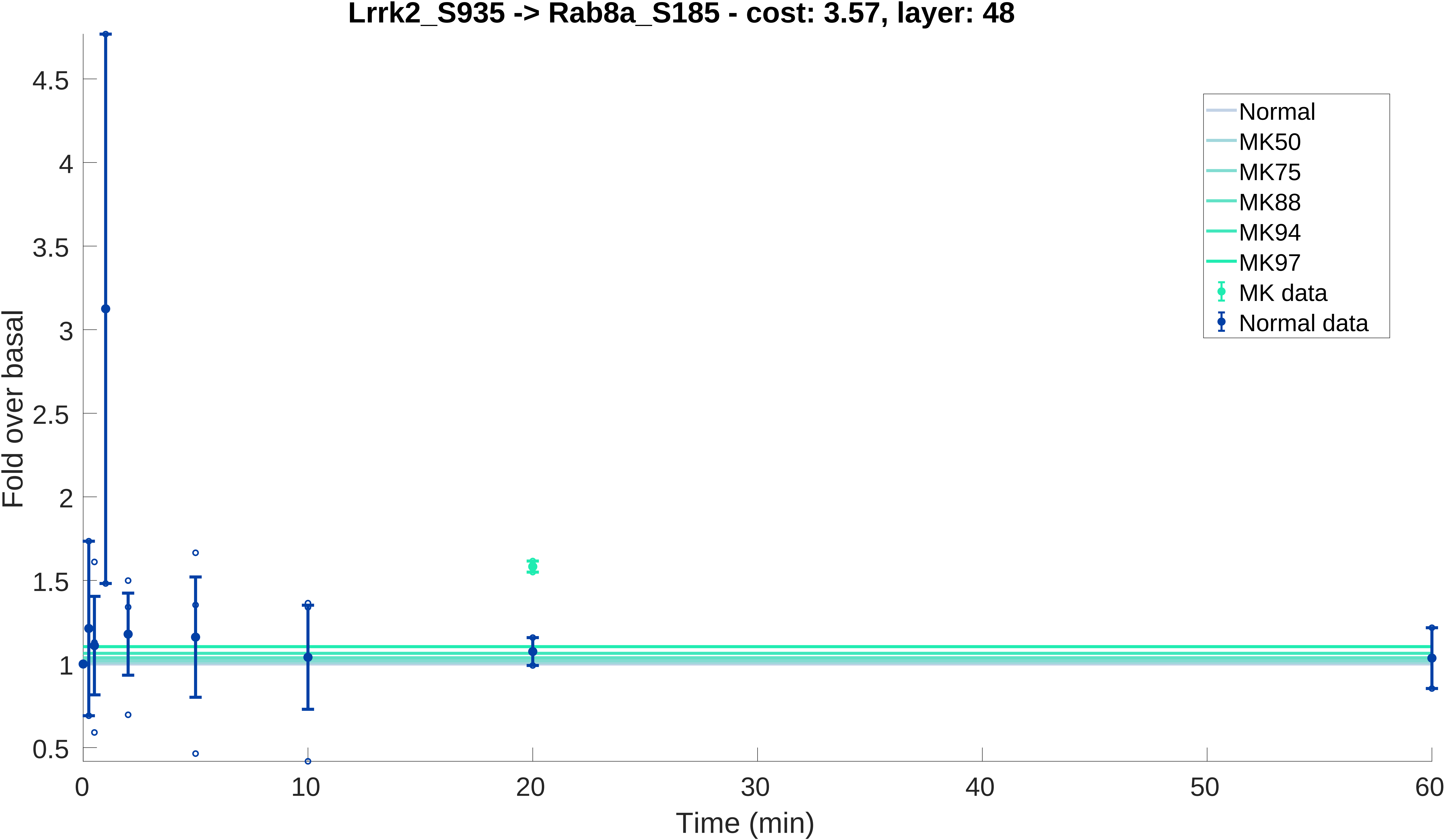
Lrrk2_S935 -> Rab8a_S185 - cost: 3.57, layer: 48
Fold over basal
Time (min)
Normal
MK50
MK75
MK88
MK94
MK97
MK data
Normal data
0.5
1
1.5
2
2.5
3
3.5
4
4.5
0
10
20
30
40
50
60

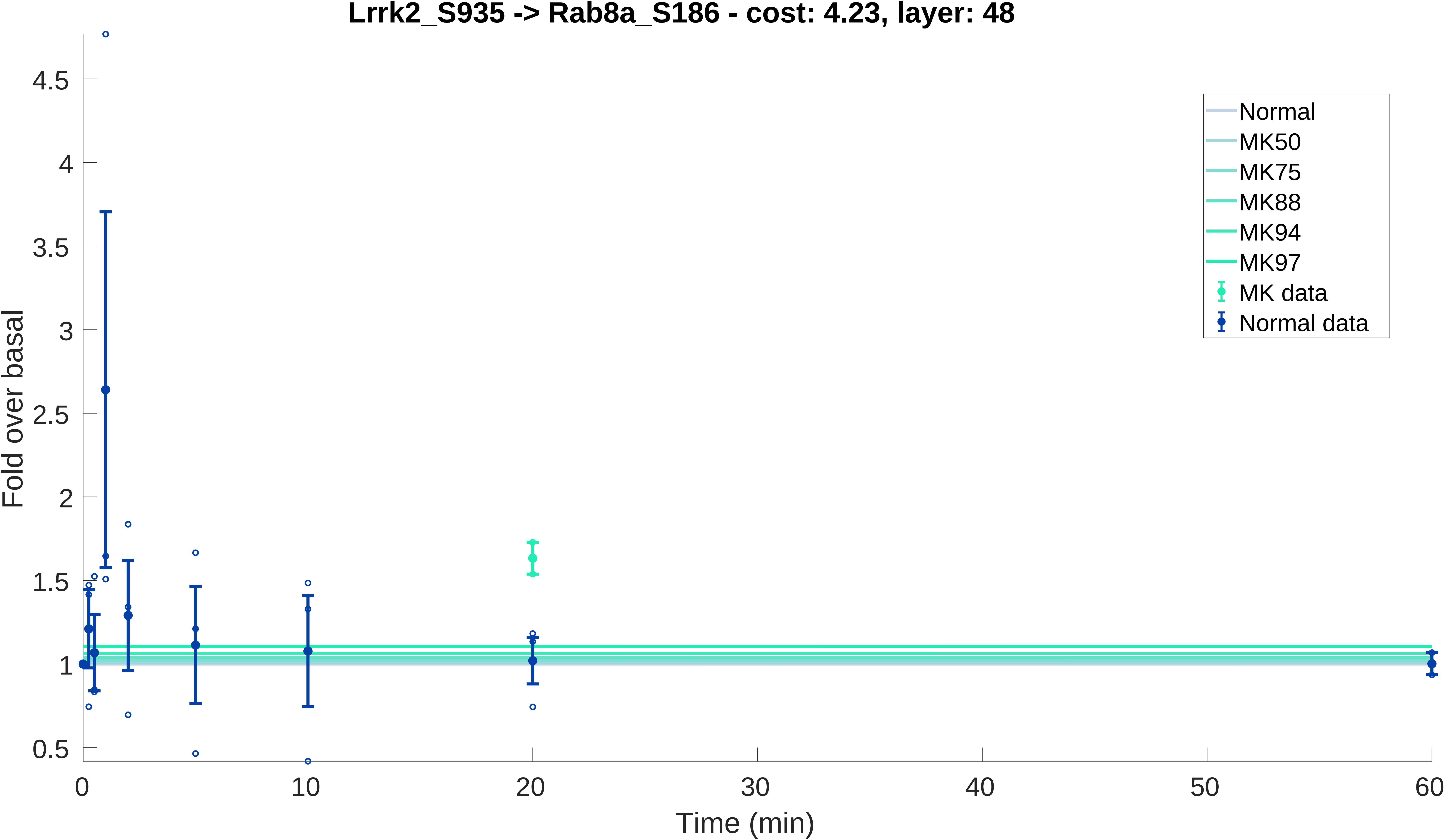

Lrrk2_S935 -> Rab8a_S186 - cost: 4.23, layer: 48
Fold over basal
Time (min)
0.5
1
1.5
2
2.5
3
3.5
4
4.5
0
10
20
30
40
50
60
Normal
MK50
MK75
MK88
MK94
MK97
MK data
Normal data

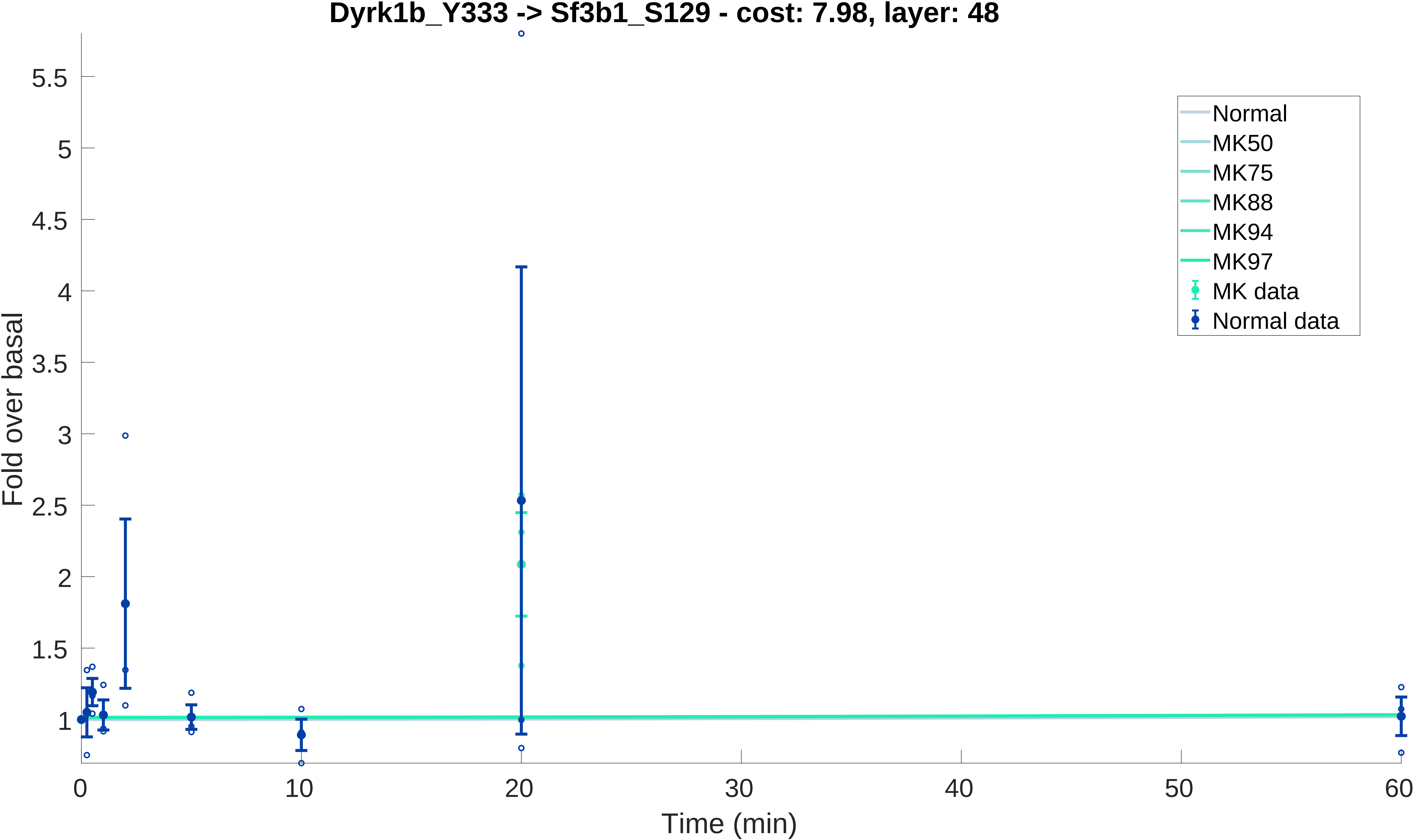

Dyrk1b_Y333 -> Sf3b1_S129 - cost: 7.98, layer: 48
Fold over basal
Time (min)
Normal
MK50
MK75
MK88
MK94
MK97
MK data
Normal data
5.5
5
4.5
4
3.5
3
2.5
2
1.5
1
0
10
20
30
40
50
60

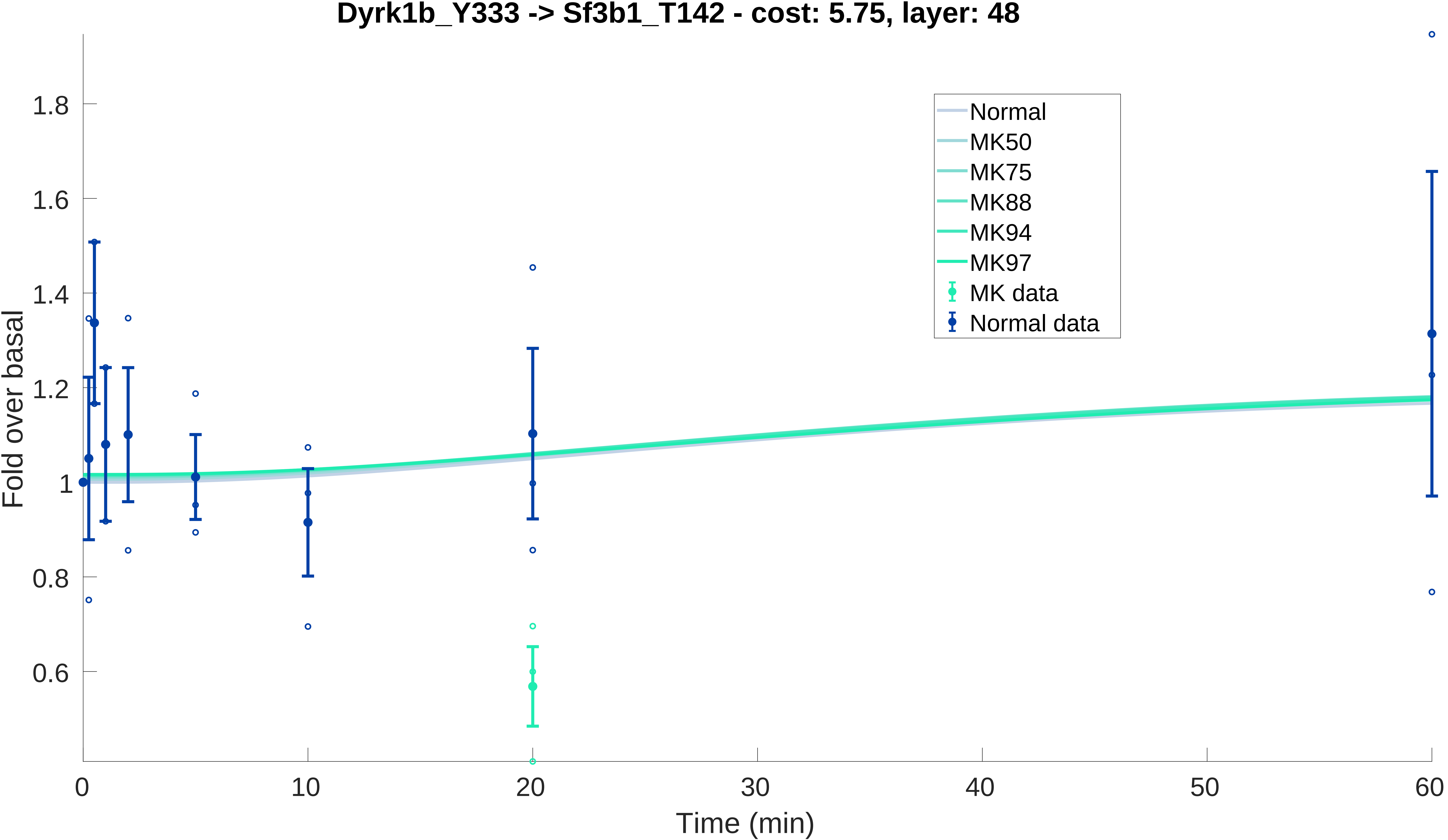
Dyrk1b_Y333 -> Sf3b1_T142 - cost: 5.75, layer: 48
Fold over basal
Time (min)
0.6
0.8
1
1.2
1.4
1.6
1.8
0
10
20
30
40
50
60
Normal
MK50
MK75
MK88
MK94
MK97
MK data
Normal data

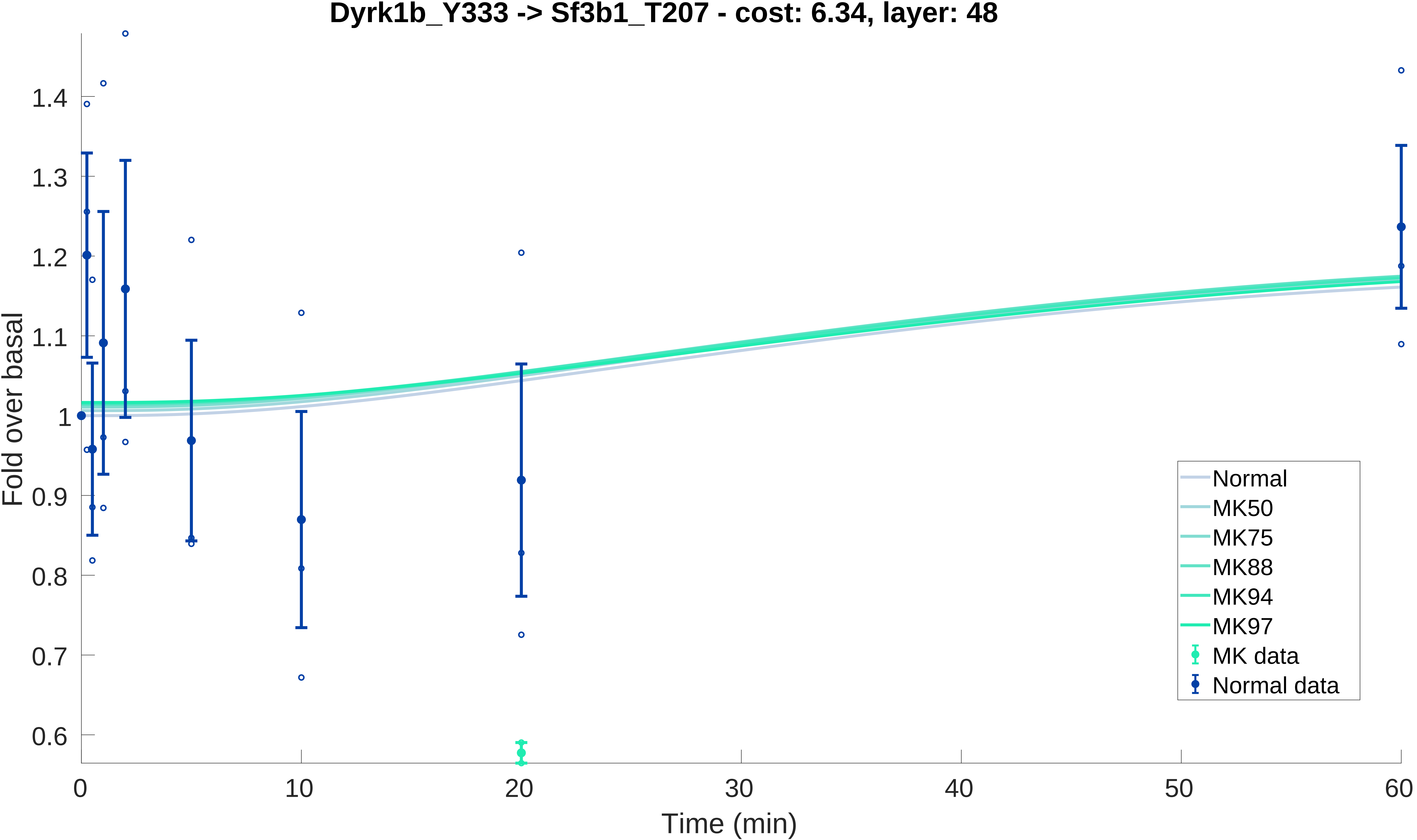

Dyrk1b_Y333 -> Sf3b1_T207 - cost: 6.34, layer: 48
Fold over basal
1.4
1.3
1.2
1.1
1
0.9
0.8
0.7
0.6
0
10
20
30
40
50
60
Time (min)
Normal
MK50
MK75
MK88
MK94
MK97
MK data
Normal data

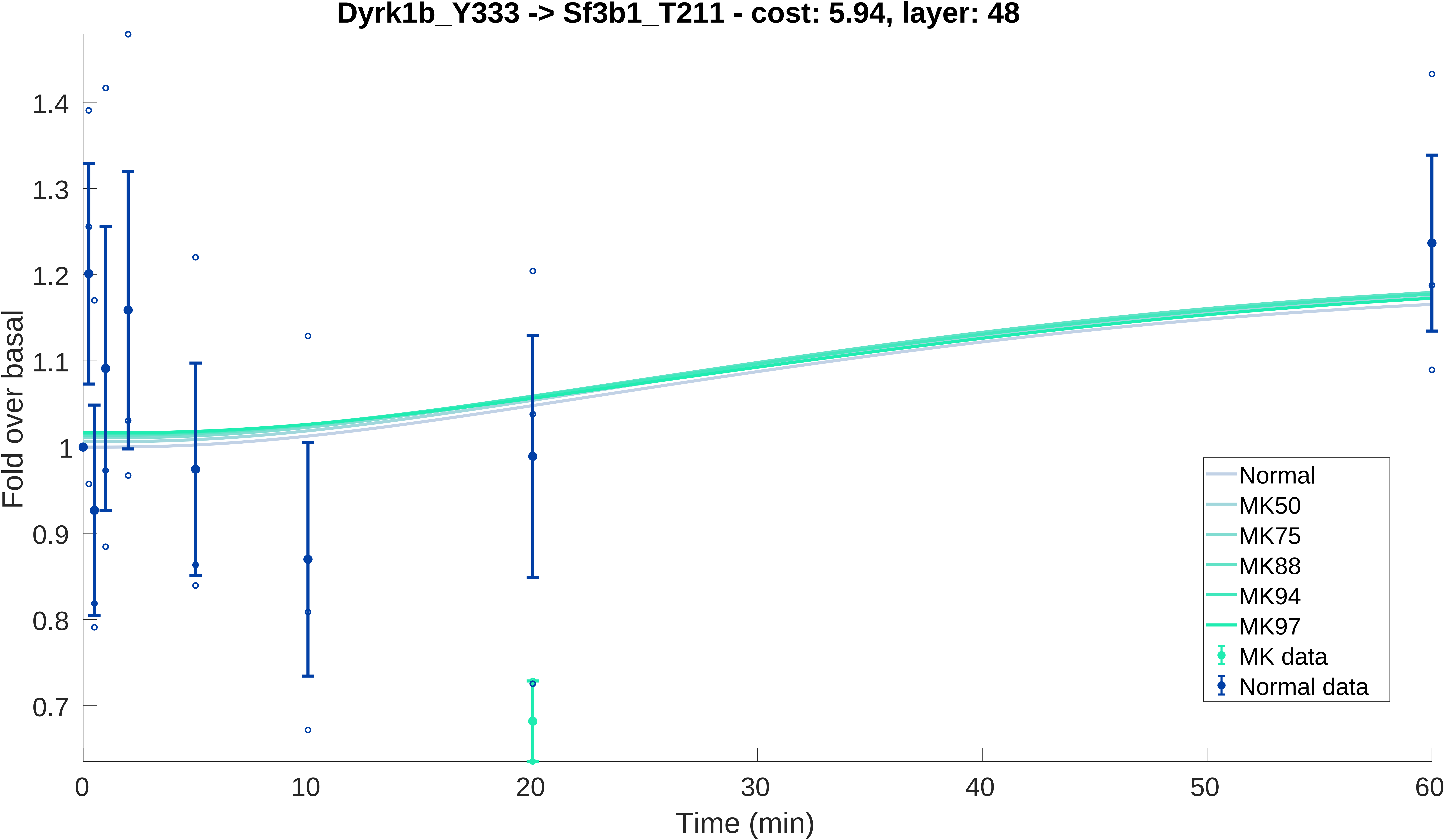

Dyrk1b_Y333 -> Sf3b1_T211 - cost: 5.94, layer: 48
Fold over basal
Time (min)
0.7
0.8
0.9
1
1.1
1.2
1.3
1.4
0
10
20
30
40
50
60
Normal
MK50
MK75
MK88
MK94
MK97
MK data
Normal data

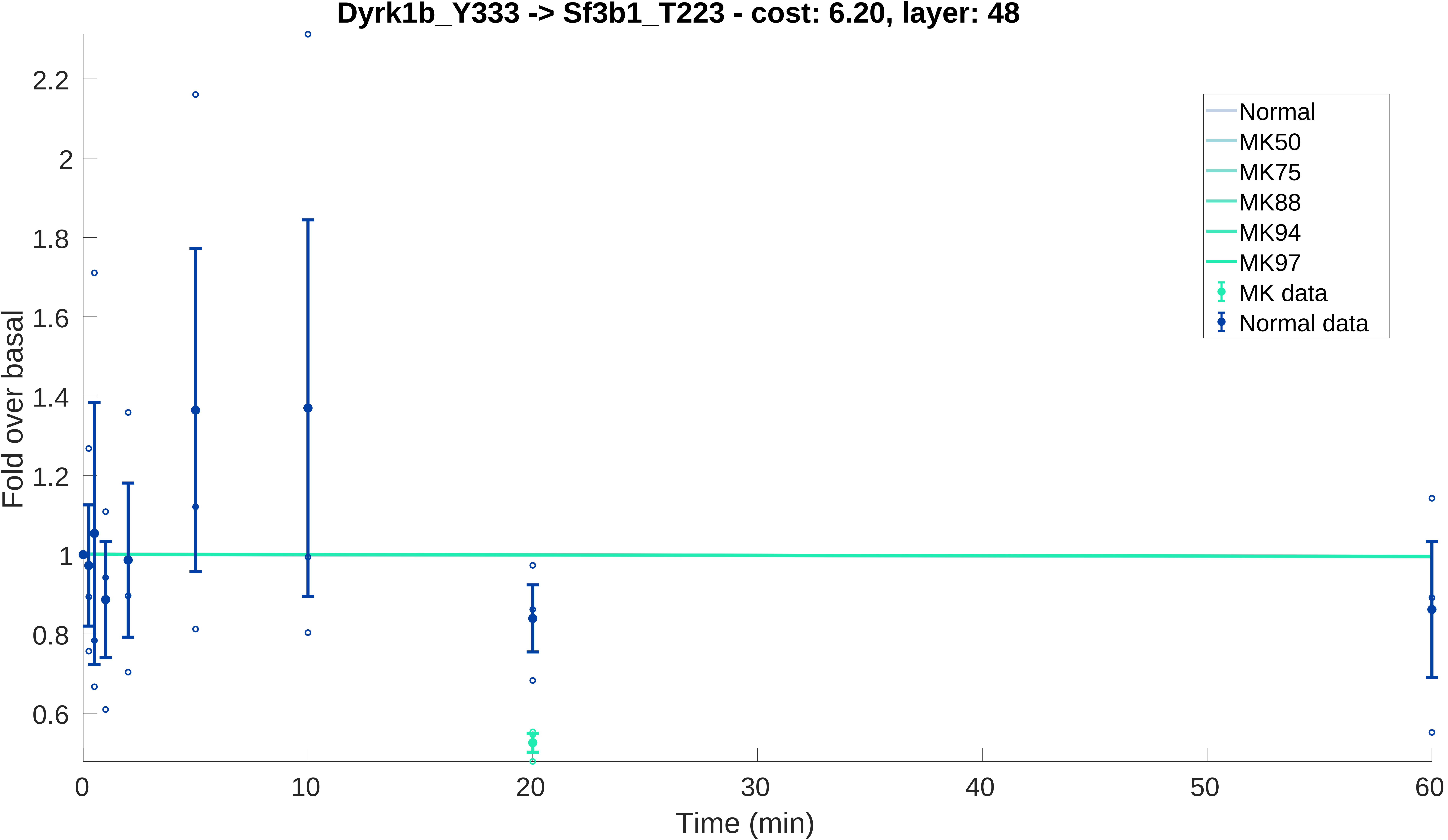

Dyrk1b_Y333 -> Sf3b1_T223 - cost: 6.20, layer: 48
Fold over basal
Time (min)
Normal
MK50
MK75
MK88
MK94
MK97
MK data
Normal data
0.6
0.8
1
1.2
1.4
1.6
1.8
2
2.2
0
10
20
30
40
50
60

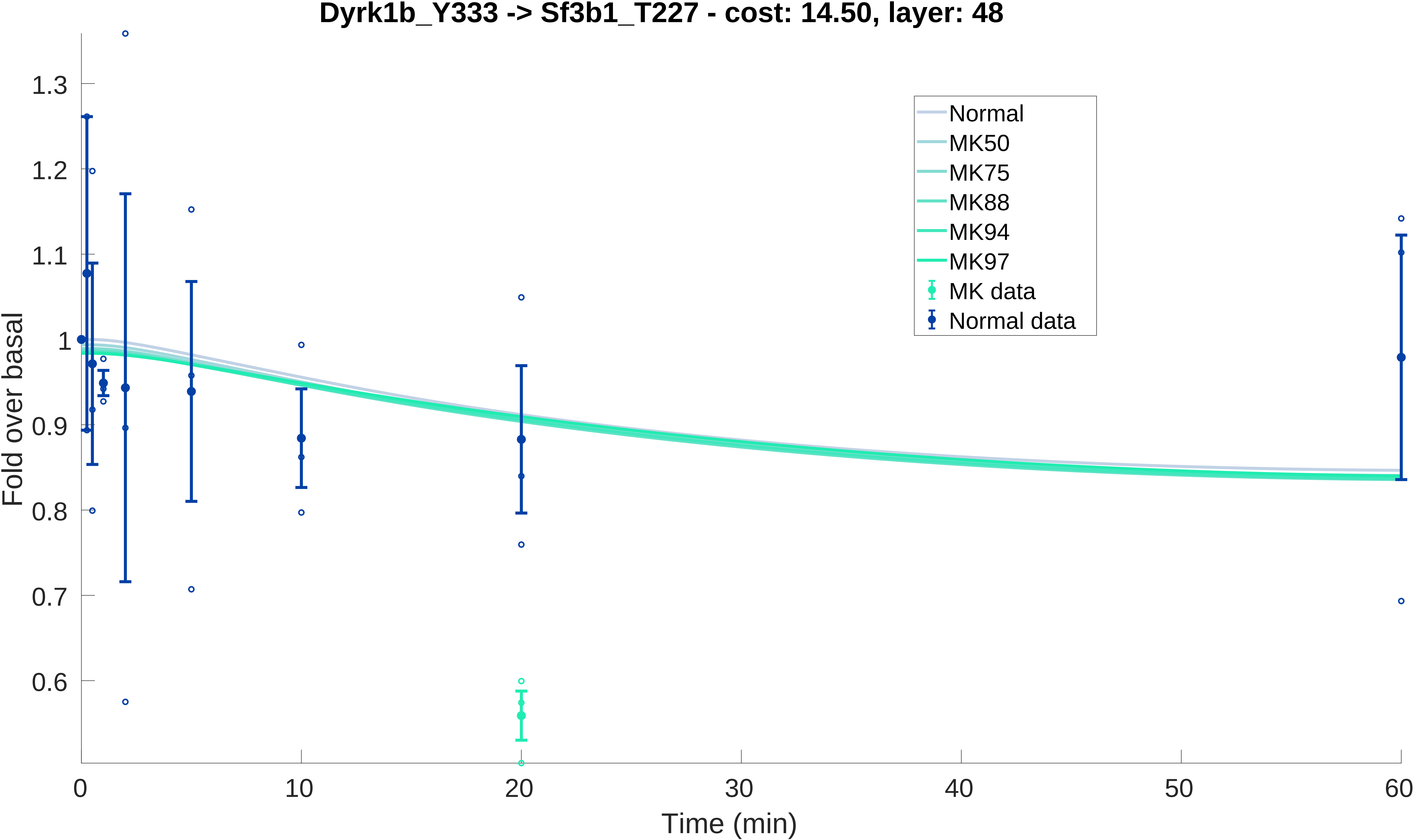

Dyrk1b_Y333 -> Sf3b1_T227 - cost: 14.50, layer: 48
Fold over basal
Time (min)
1.3
1.2
1.1
1
0.9
0.8
0.7
0.6
0
10
20
30
40
50
60
Normal
MK50
MK75
MK88
MK94
MK97
MK data
Normal data

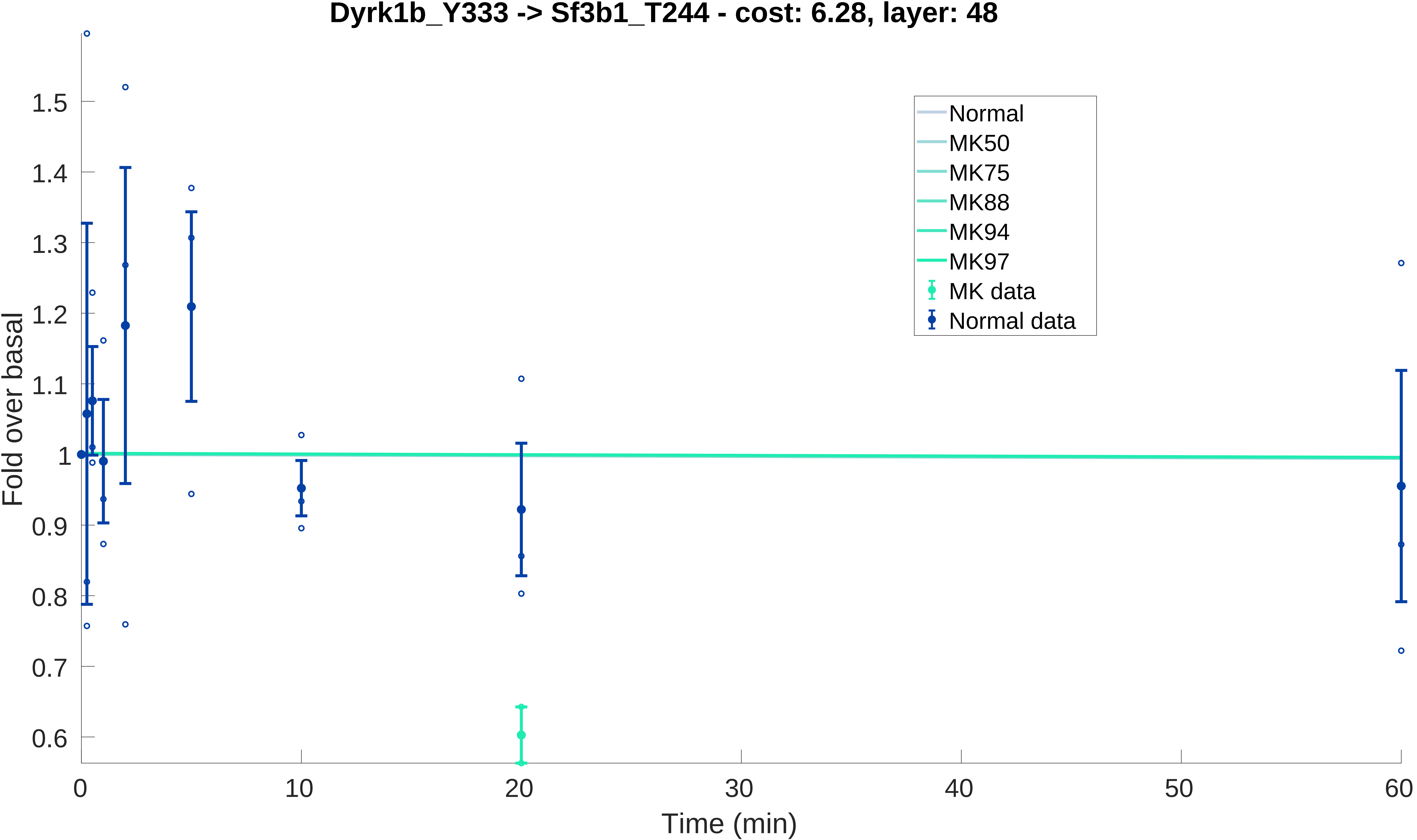

Dyrk1b_Y333 -> Sf3b1_T244 - cost: 6.28, layer: 48
Fold over basal
Time (min)
Normal
MK50
MK75
MK88
MK94
MK97
MK data
Normal data
1.5
1.4
1.3
1.2
1.1
1
0.9
0.8
0.7
0.6
0
10
20
30
40
50
60

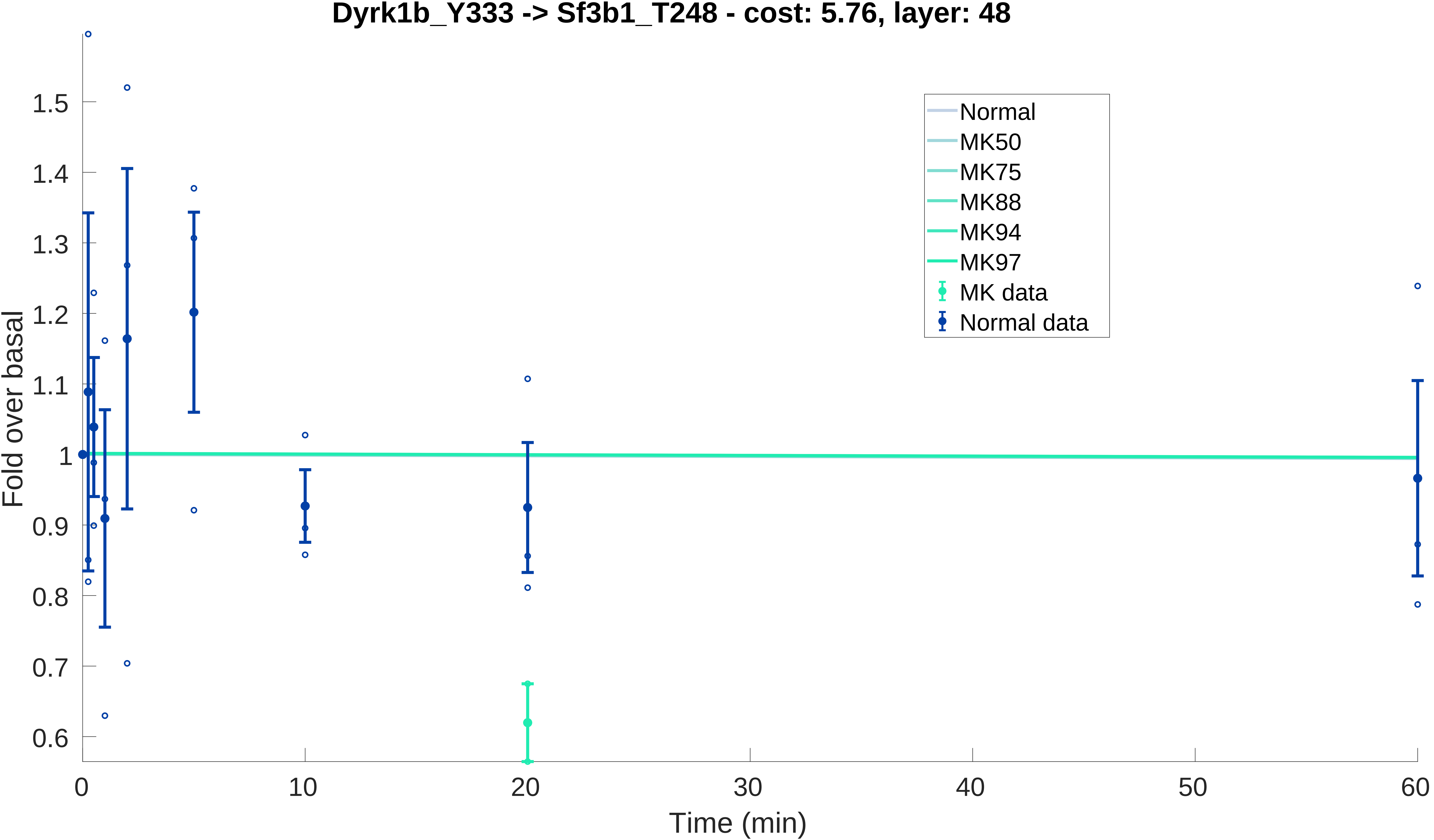
Dyrk1b_Y333 -> Sf3b1_T248 - cost: 5.76, layer: 48
Fold over basal
Time (min)
Normal
MK50
MK75
MK88
MK94
MK97
MK data
Normal data
1.5
1.4
1.3
1.2
1.1
1
0.9
0.8
0.7
0.6
0
10
20
30
40
50
60

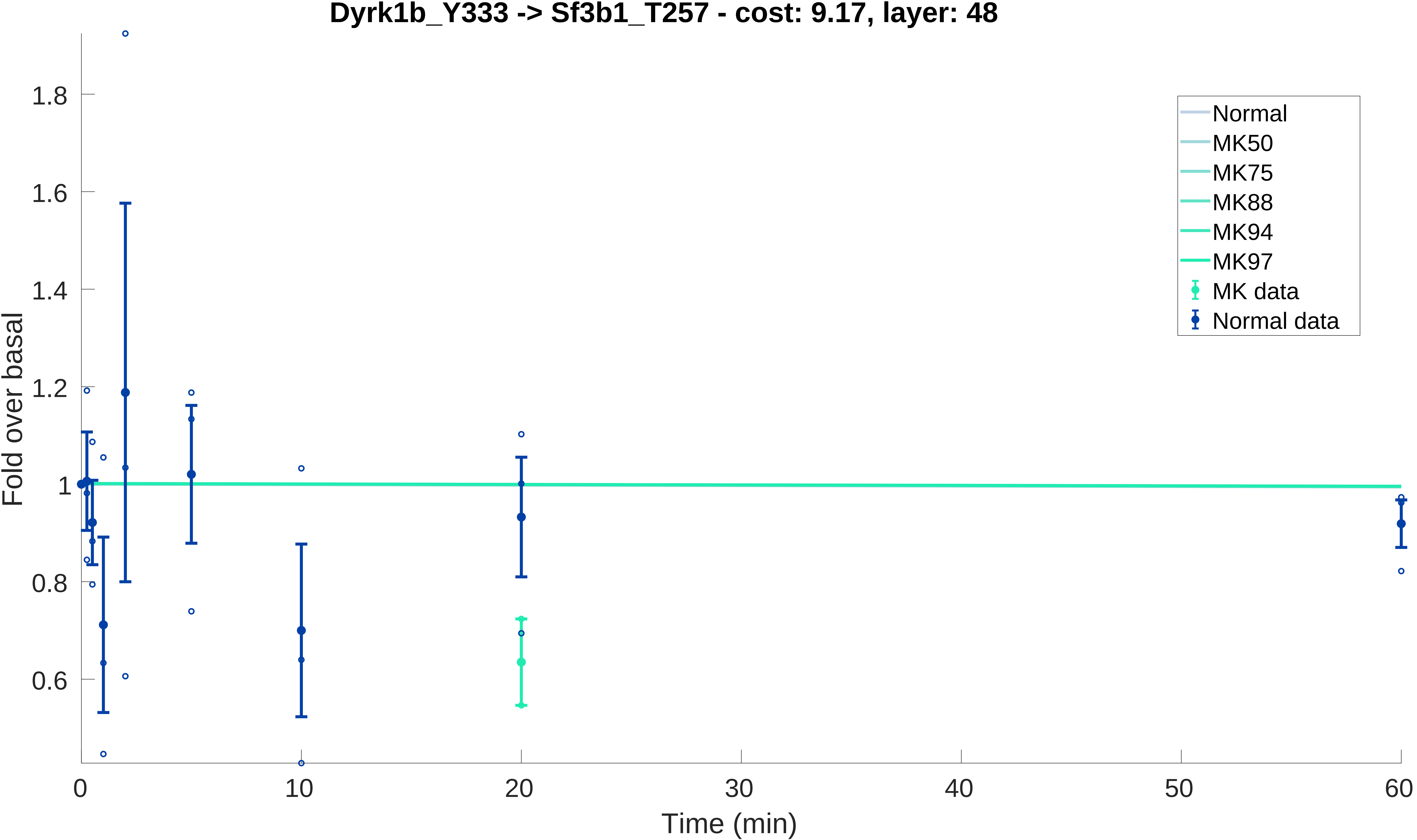

Dyrk1b_Y333 -> Sf3b1_T257 - cost: 9.17, layer: 48
Fold over basal
Time (min)
0.6
0.8
1
1.2
1.4
1.6
1.8
0
10
20
30
40
50
60
Normal
MK50
MK75
MK88
MK94
MK97
MK data
Normal data

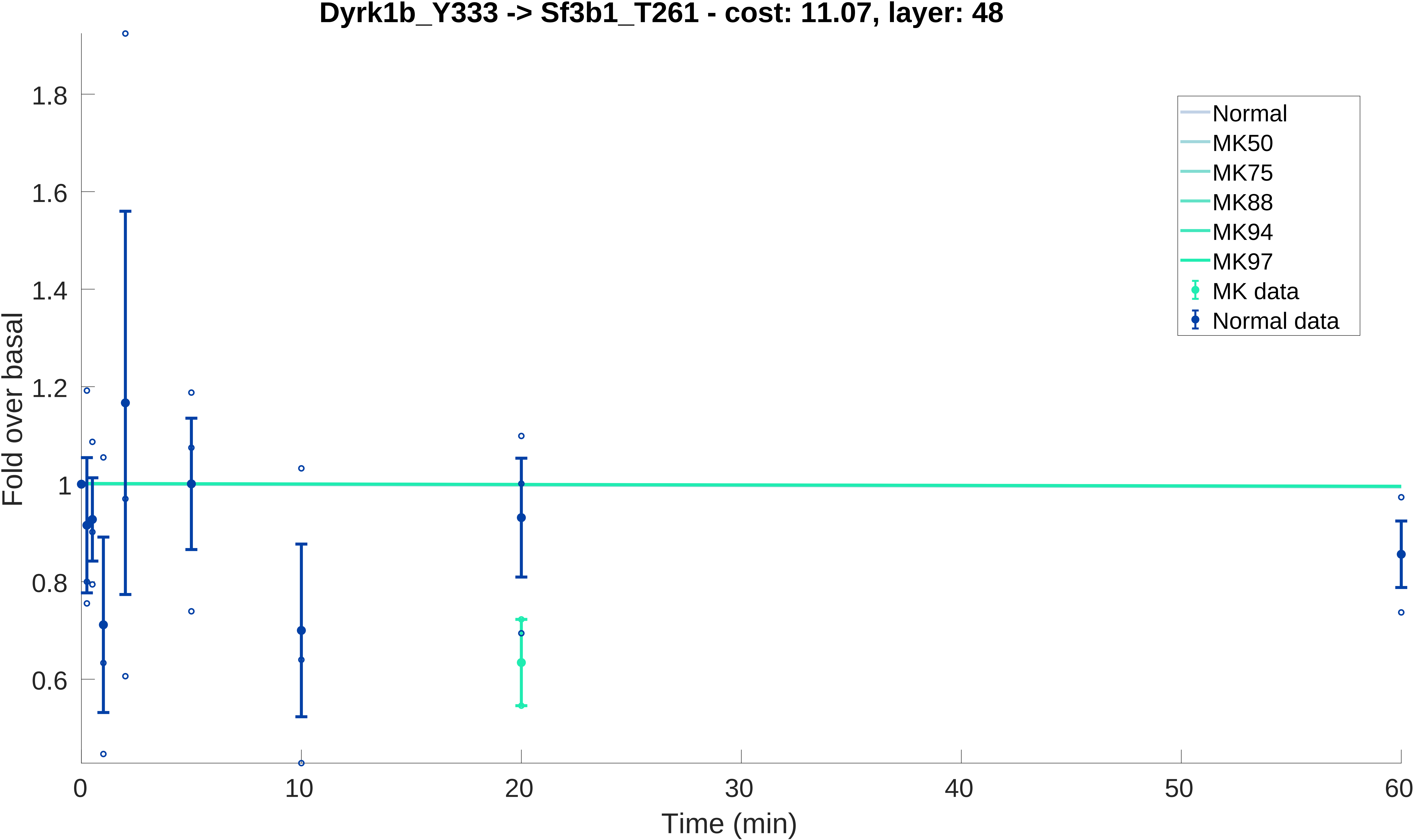
Dyrk1b_Y333 -> Sf3b1_T261 - cost: 11.07, layer: 48
Fold over basal
Time (min)
Normal
MK50
MK75
MK88
MK94
MK97
MK data
Normal data
0.6
0.8
1
1.2
1.4
1.6
1.8
0
10
20
30
40
50
60

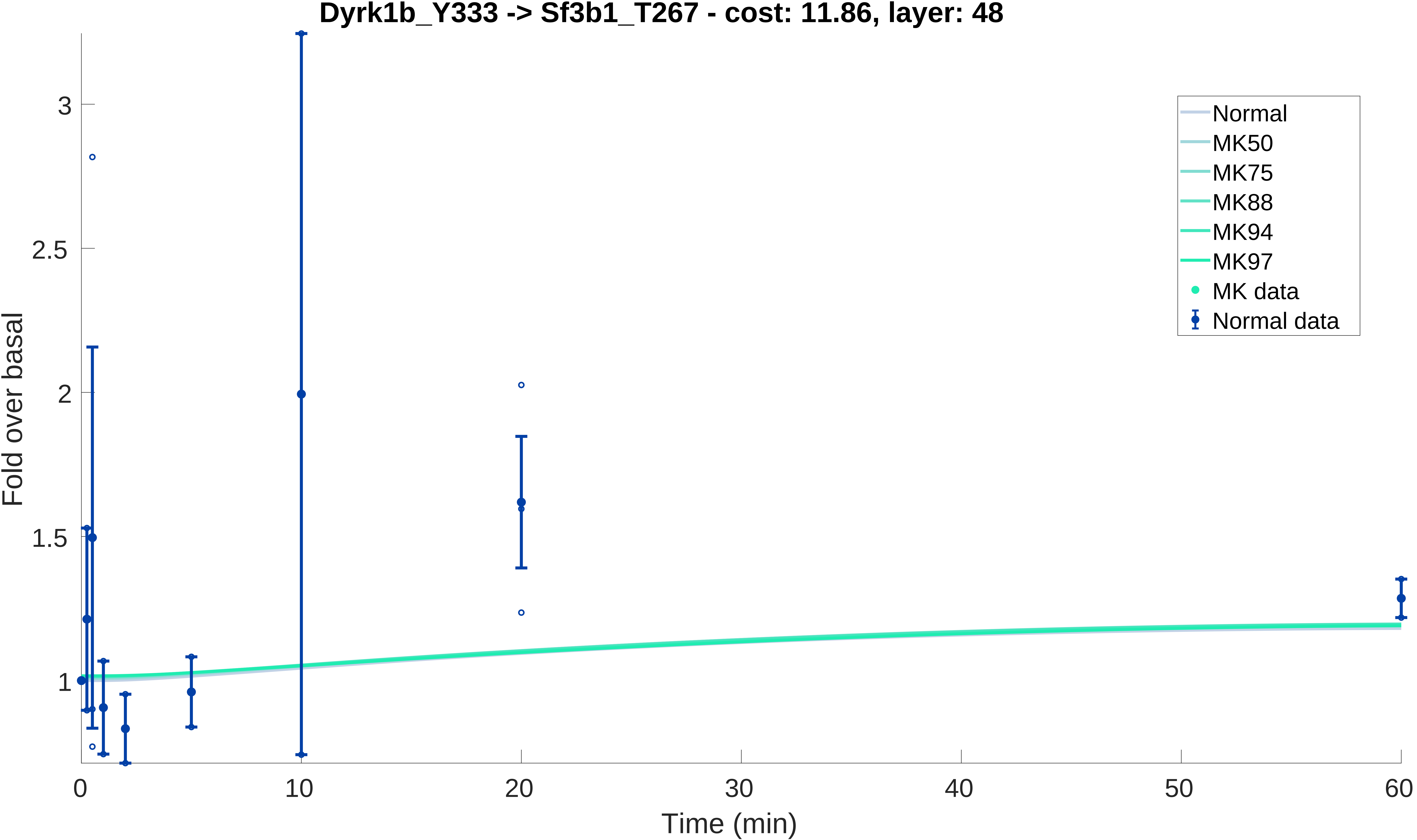
Dyrk1b_Y333 -> Sf3b1_T267 - cost: 11.86, layer: 48
Fold over basal
Time (min)
0
10
20
30
40
50
60
1
1.5
2
2.5
3
Normal
MK50
MK75
MK88
MK94
MK97
MK data
Normal data

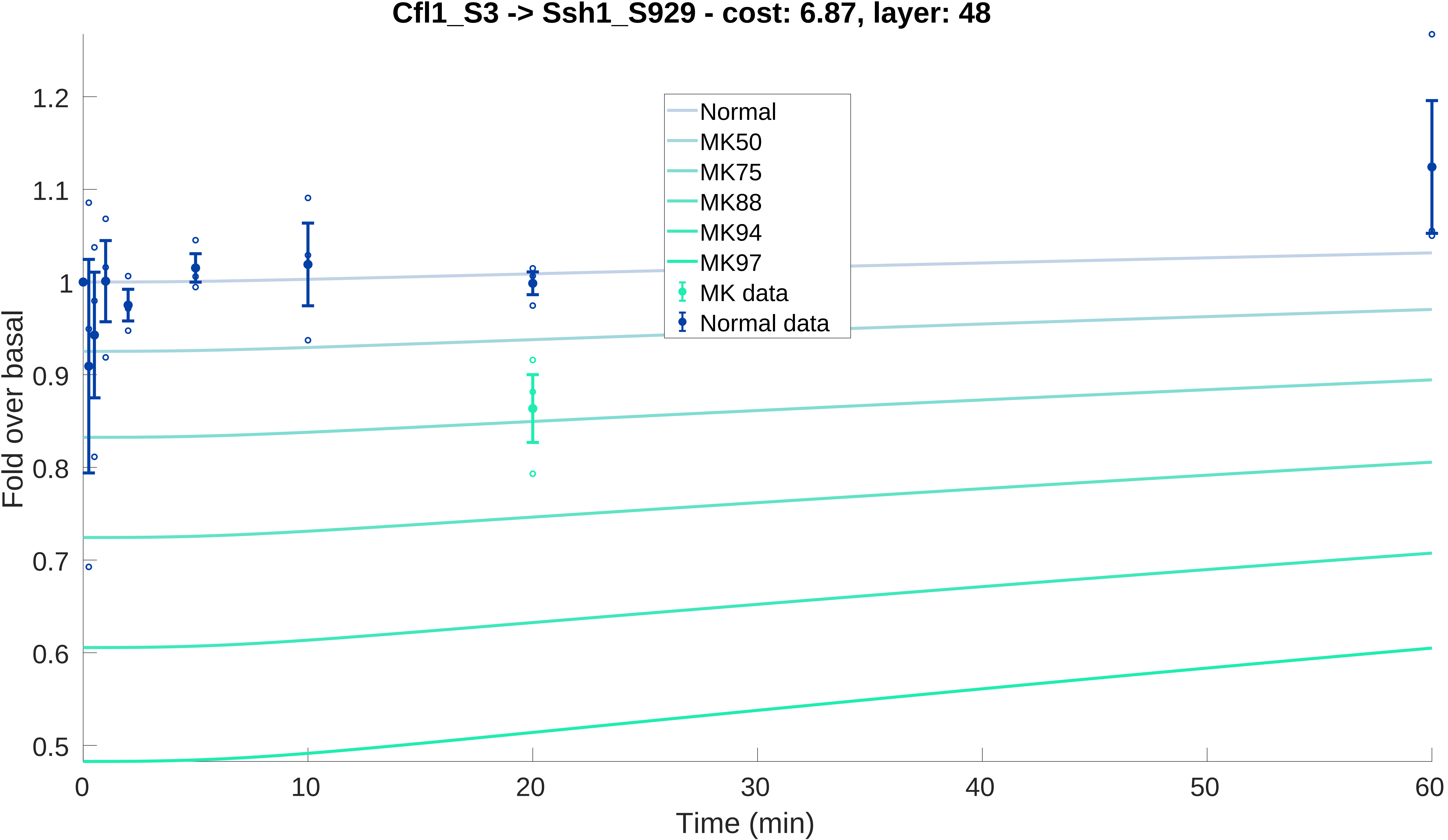

Cfl1_S3 -> Ssh1_S929 - cost: 6.87, layer: 48
Normal
MK50
MK75
MK88
MK94
MK97
MK data
Normal data
Fold over basal
Time (min)
1.2
1.1
1
0.9
0.8
0.7
0.6
0.5
0
10
20
30
40
50
60

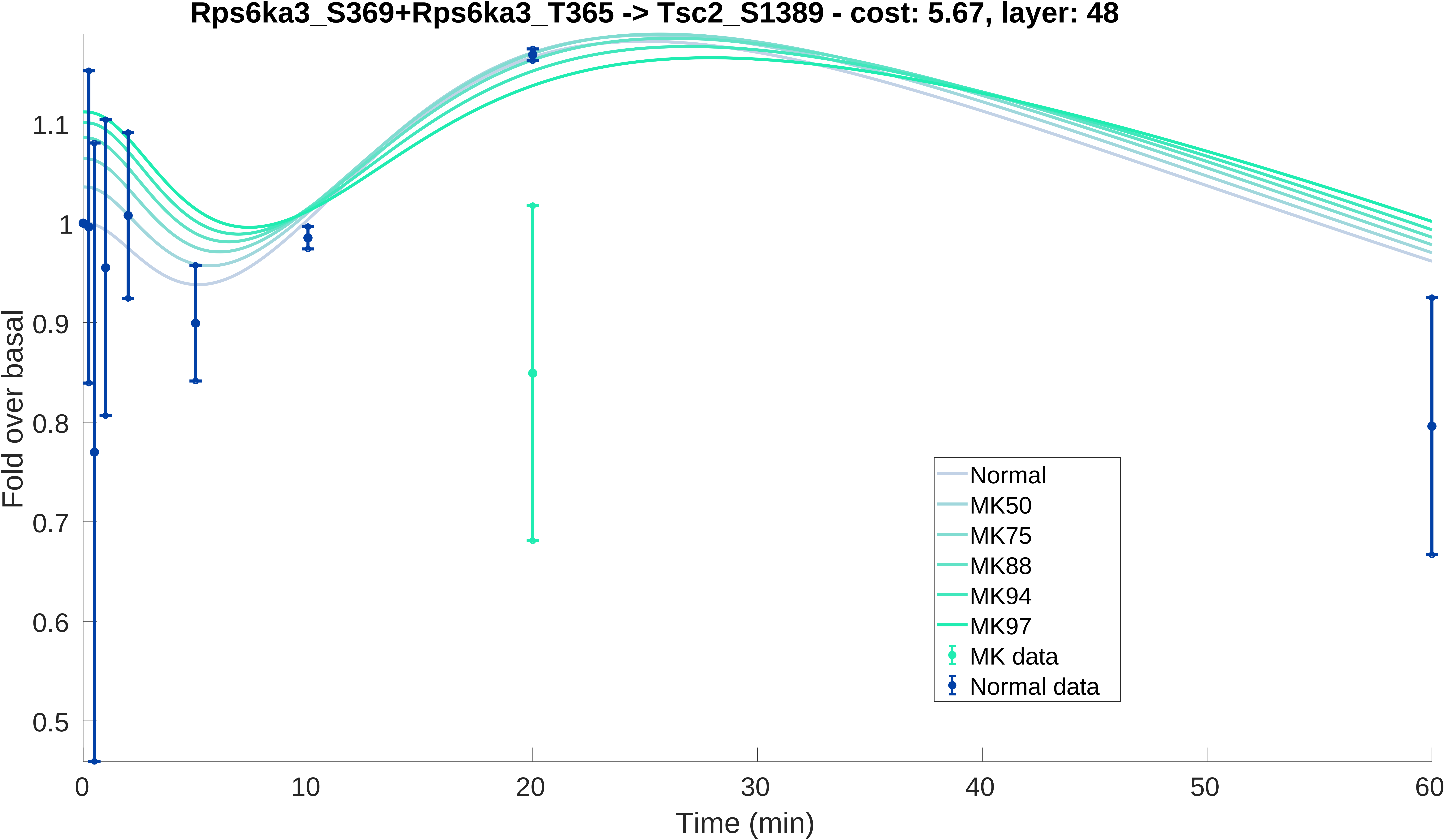

Rps6ka3_S369+Rps6ka3_T365 -> Tsc2_S1389 - cost: 5.67, layer: 48
Fold over basal
Time (min)
0
10
20
30
40
50
60
0.5
0.6
0.7
0.8
0.9
1
1.1
Normal
MK50
MK75
MK88
MK94
MK97
MK data
Normal data

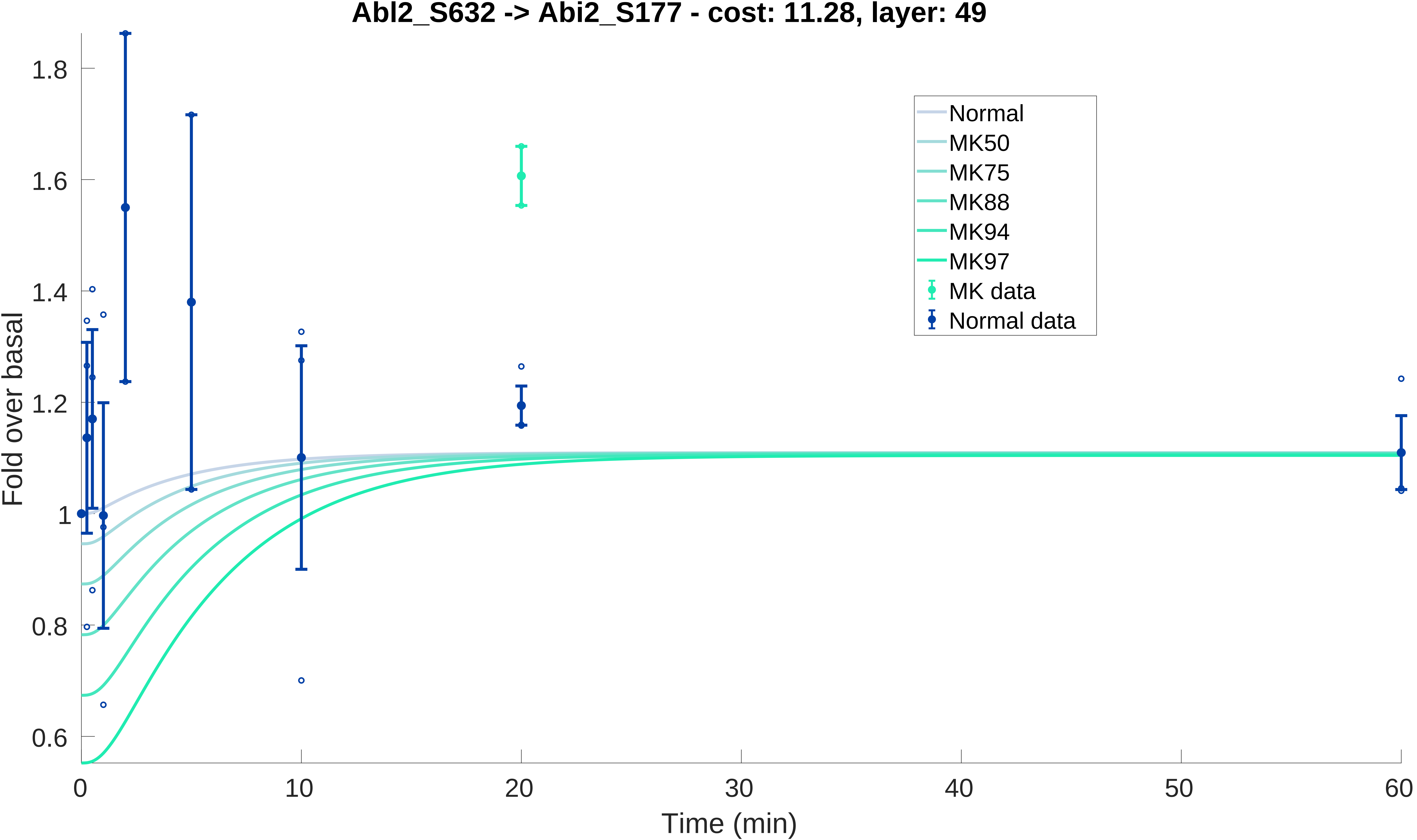

Abl2_S632 -> Abi2_S177 - cost: 11.28, layer: 49
Fold over basal
Time (min)
Normal
MK50
MK75
MK88
MK94
MK97
MK data
Normal data
1.8
1.6
1.4
1.2
1
0.8
0.6
0
10
20
30
40
50
60

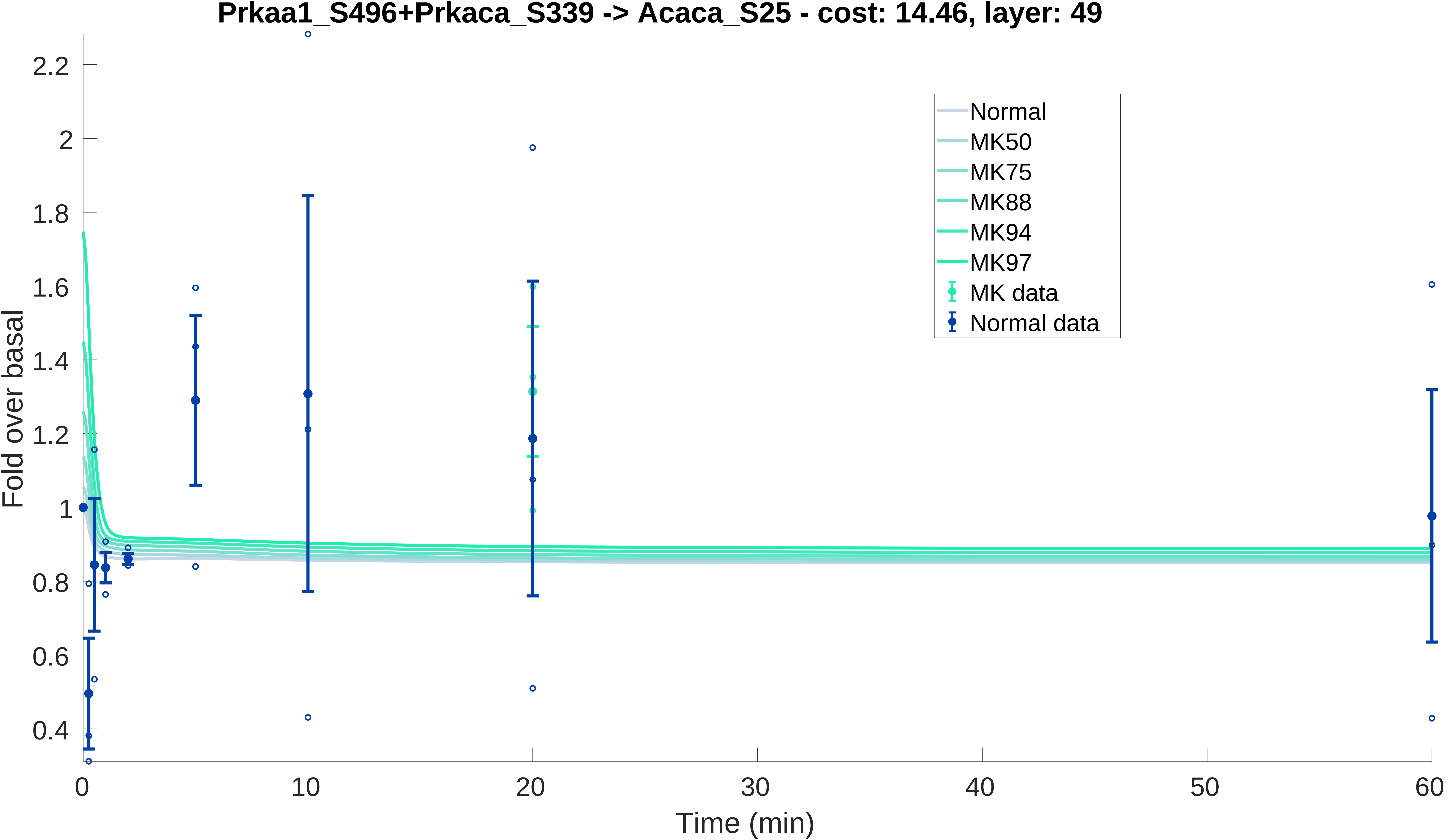

Prkaa1_S496+Prkaca_S339 -> Acaca_S25 - cost: 14.46, layer: 49
Fold over basal
Time (min)
2.2
2
1.8
1.6
1.4
1.2
1
0.8
0.6
0.4
0
10
20
30
40
50
60
Normal
MK50
MK75
MK88
MK94
MK97
MK data
Normal data

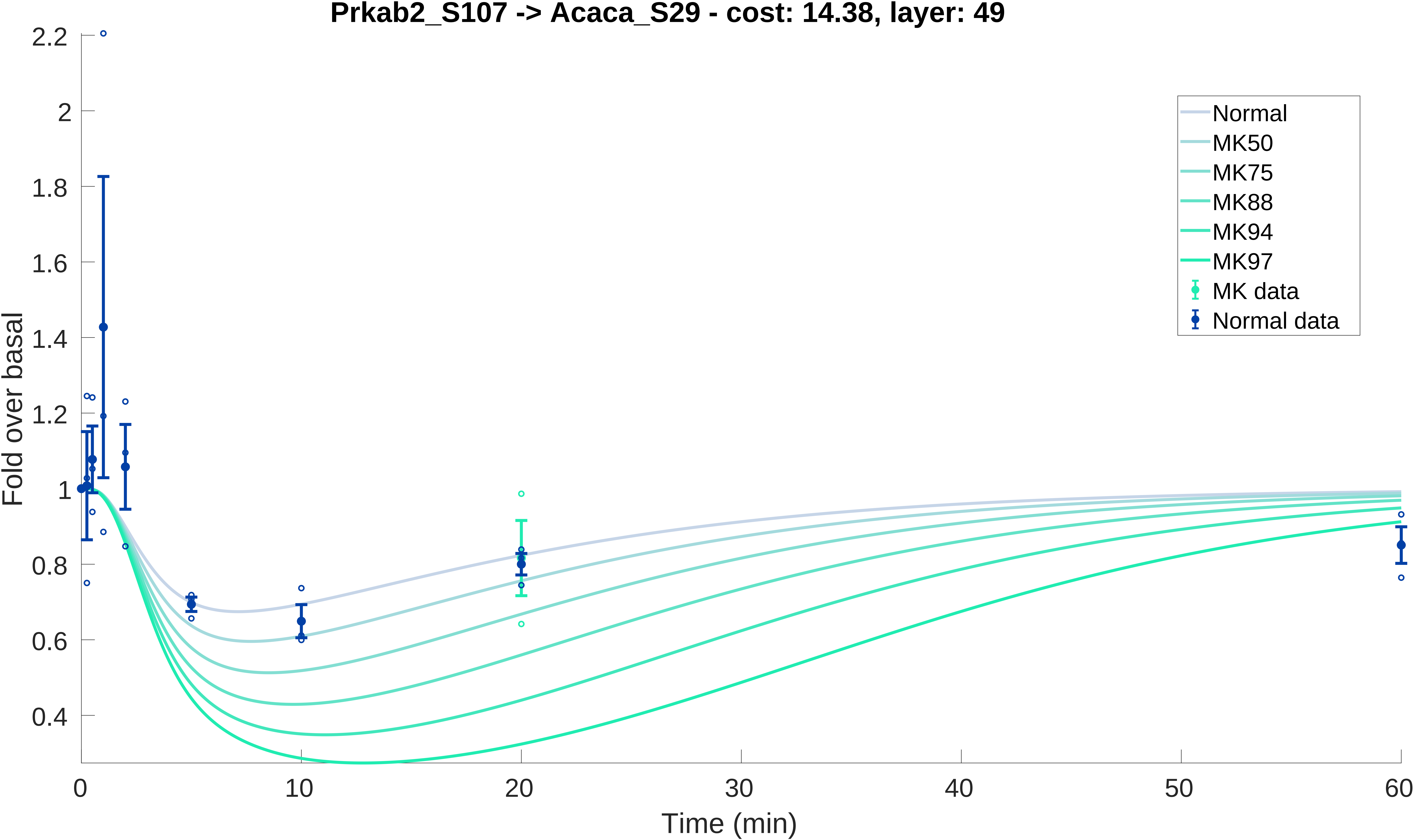
Prkab2_S107 -> Acaca_S29 - cost: 14.38, layer: 49
Fold over basal
Time (min)
0
10
20
30
40
50
60
0.4
0.6
0.8
1
1.2
1.4
1.6
1.8
2
2.2
Normal
MK50
MK75
MK88
MK94
MK97
MK data
Normal data

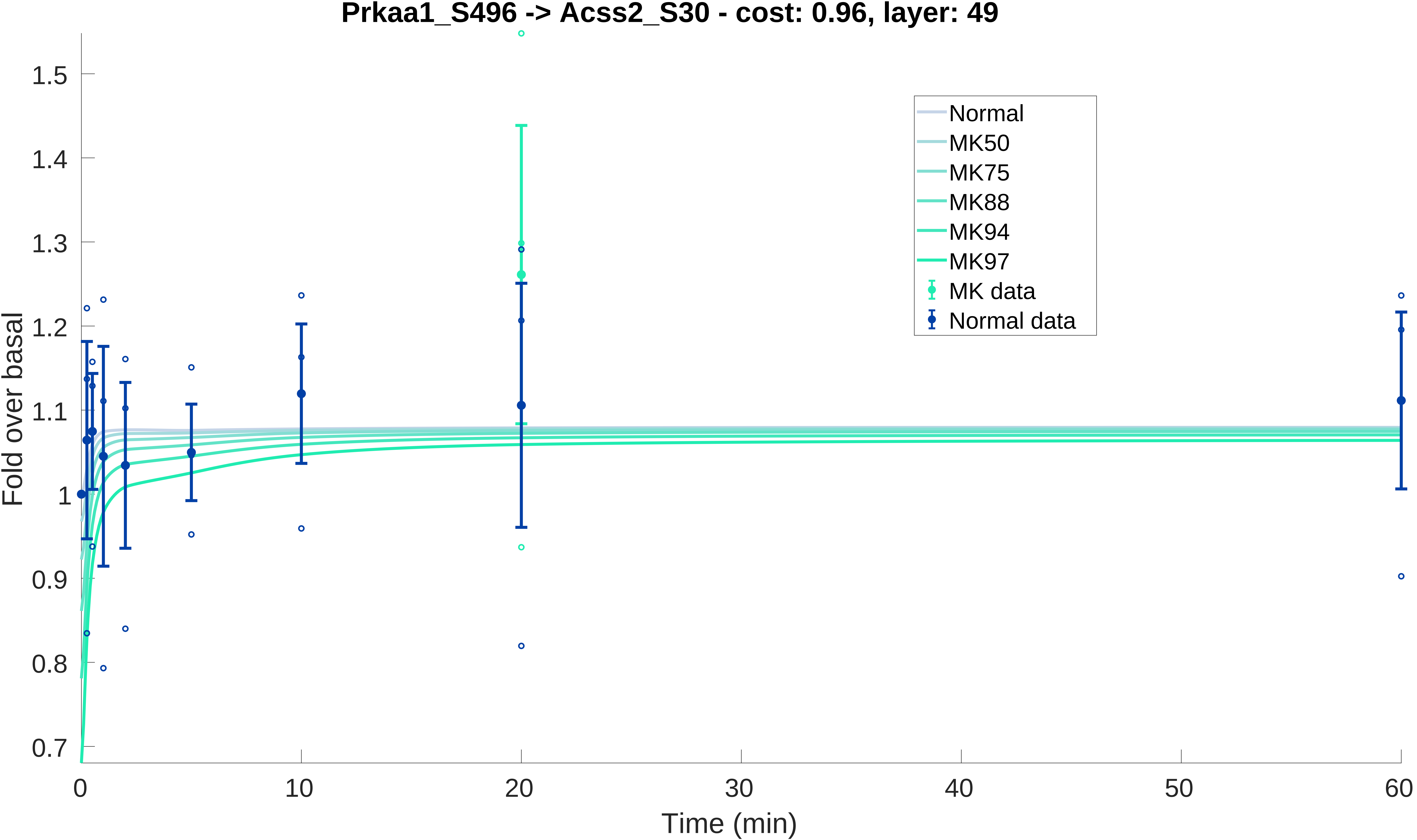

Prrkaa1_S496 -> Acss2_S30 - cost: 0.96, layer: 49
Fold over basal
Time (min)
Normal
MK50
MK75
MK88
MK94
MK97
MK data
Normal data
0.7
0.8
0.9
1
1.1
1.2
1.3
1.4
1.5
0
10
20
30
40
50
60

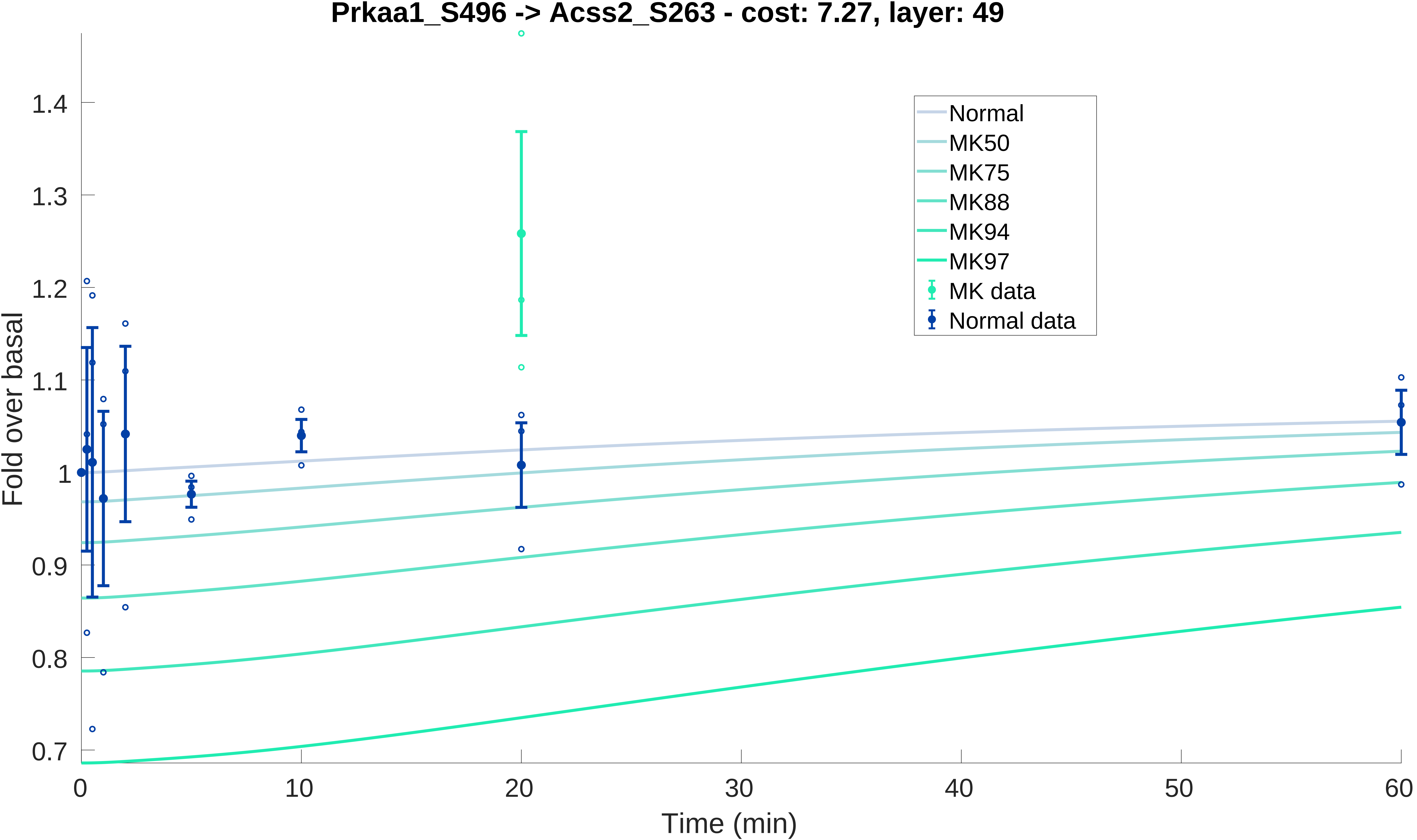
Prrkaa1_S496 -> Acss2_S263 - cost: 7.27, layer: 49
Fold over basal
Time (min)
0
10
20
30
40
50
60
0.7
0.8
0.9
1
1.1
1.2
1.3
1.4
Normal
MK50
MK75
MK88
MK94
MK97
MK data
Normal data

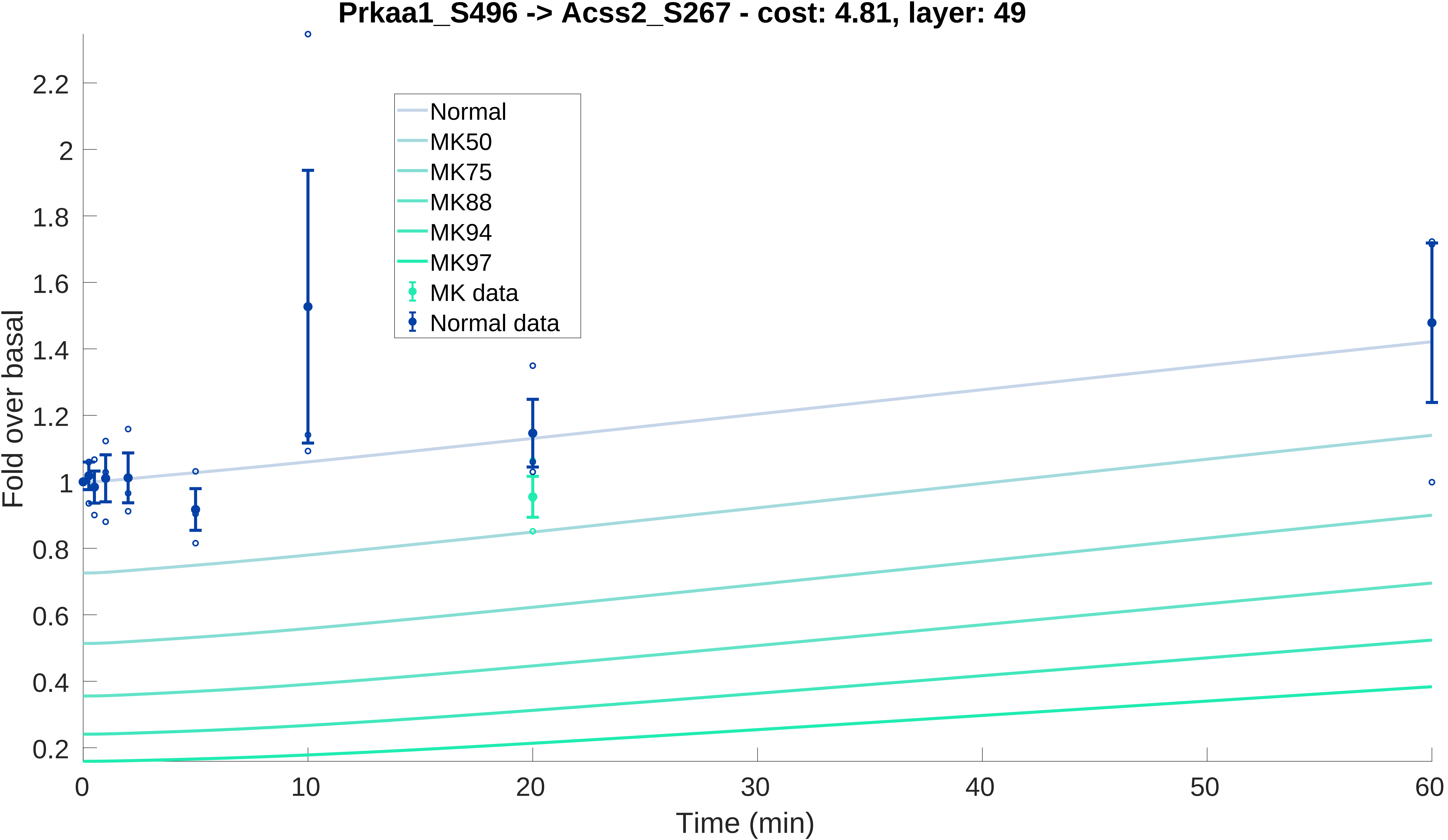
Prkaa1_S496 -> Acss2_S267 - cost: 4.81, layer: 49
Fold over basal
Time (min)
0
10
20
30
40
50
60
0.2
0.4
0.6
0.8
1
1.2
1.4
1.6
1.8
2
2.2
Normal
MK50
MK75
MK88
MK94
MK97
MK data
Normal data

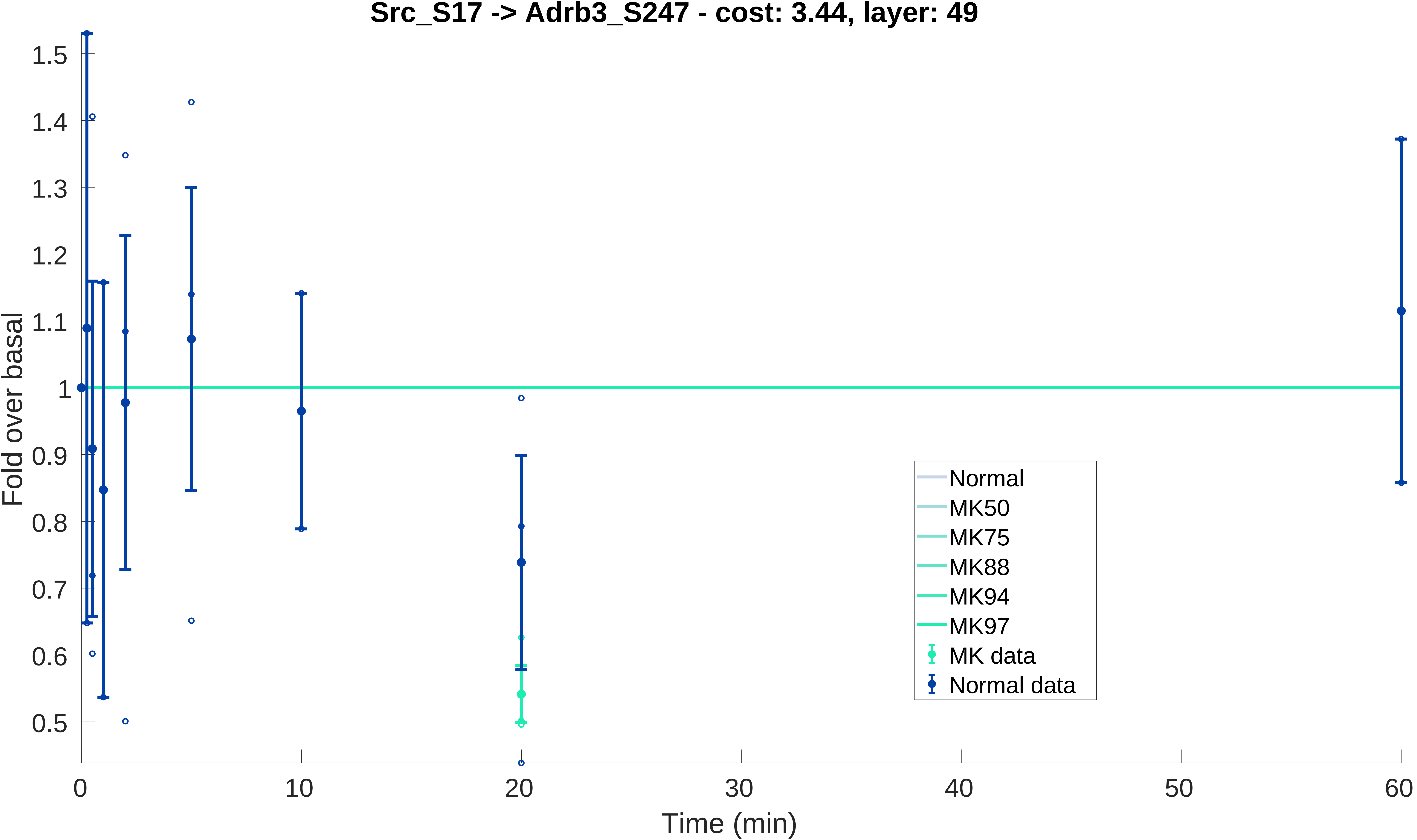

Src_S17 -> Adrb3_S247 - cost: 3.44, layer: 49
Fold over basal
1.5
1.4
1.3
1.2
1.1
1
0.9
0.8
0.7
0.6
0.5
0
10
20
30
40
50
60
Time (min)
Normal
MK50
MK75
MK88
MK94
MK97
MK data
Normal data

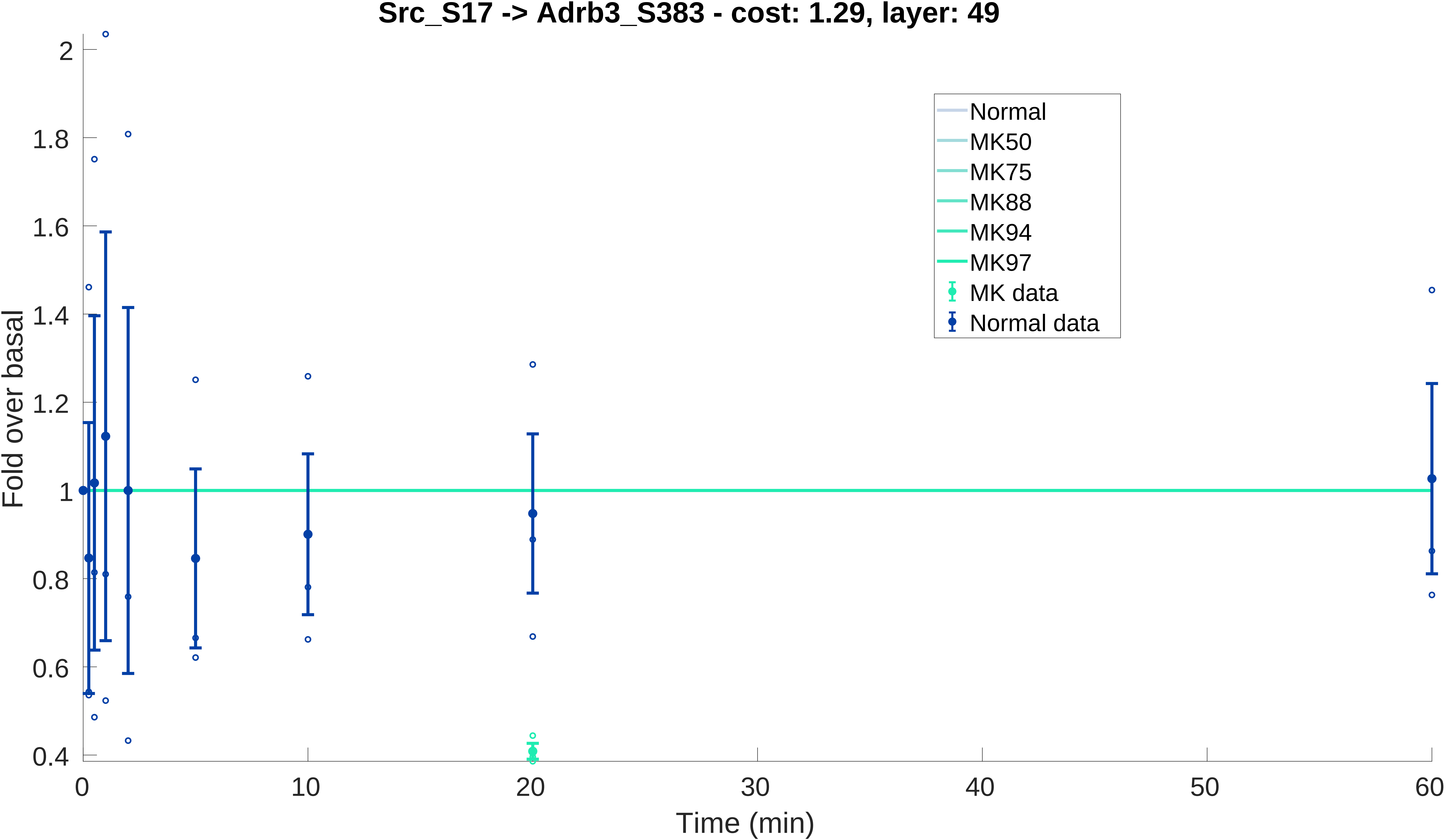

Src_S17 -> Adrb3_S383 - cost: 1.29, layer: 49
Fold over basal
Time (min)
0.4
0.6
0.8
1
1.2
1.4
1.6
1.8
2
0
10
20
30
40
50
60
Normal
MK50
MK75
MK88
MK94
MK97
MK data
Normal data

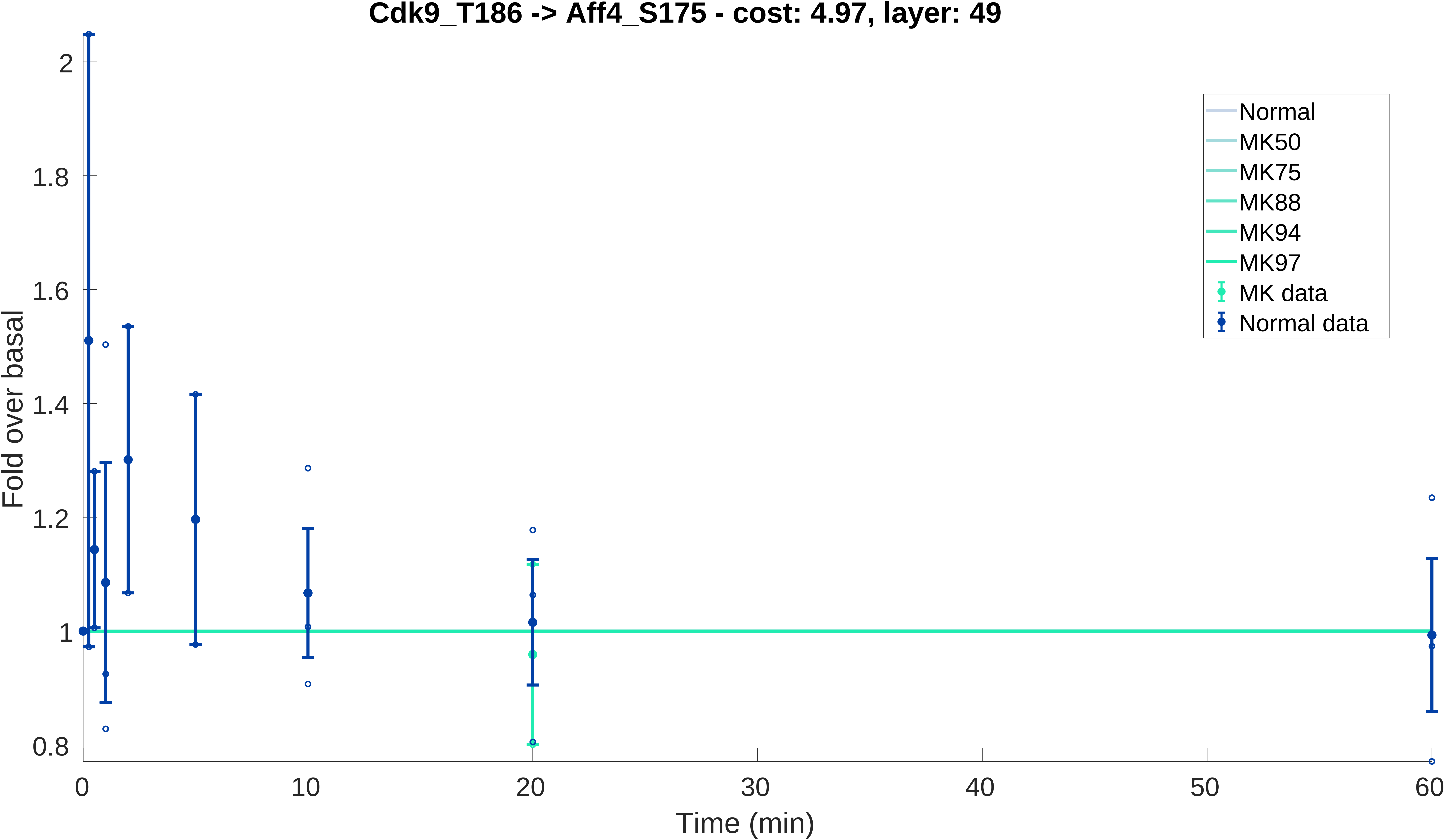

Cdk9_T186 -> Aff4_S175 - cost: 4.97, layer: 49
Fold over basal
Time (min)
Normal
MK50
MK75
MK88
MK94
MK97
MK data
Normal data
0.8
1
1.2
1.4
1.6
1.8
2
0
10
20
30
40
50
60

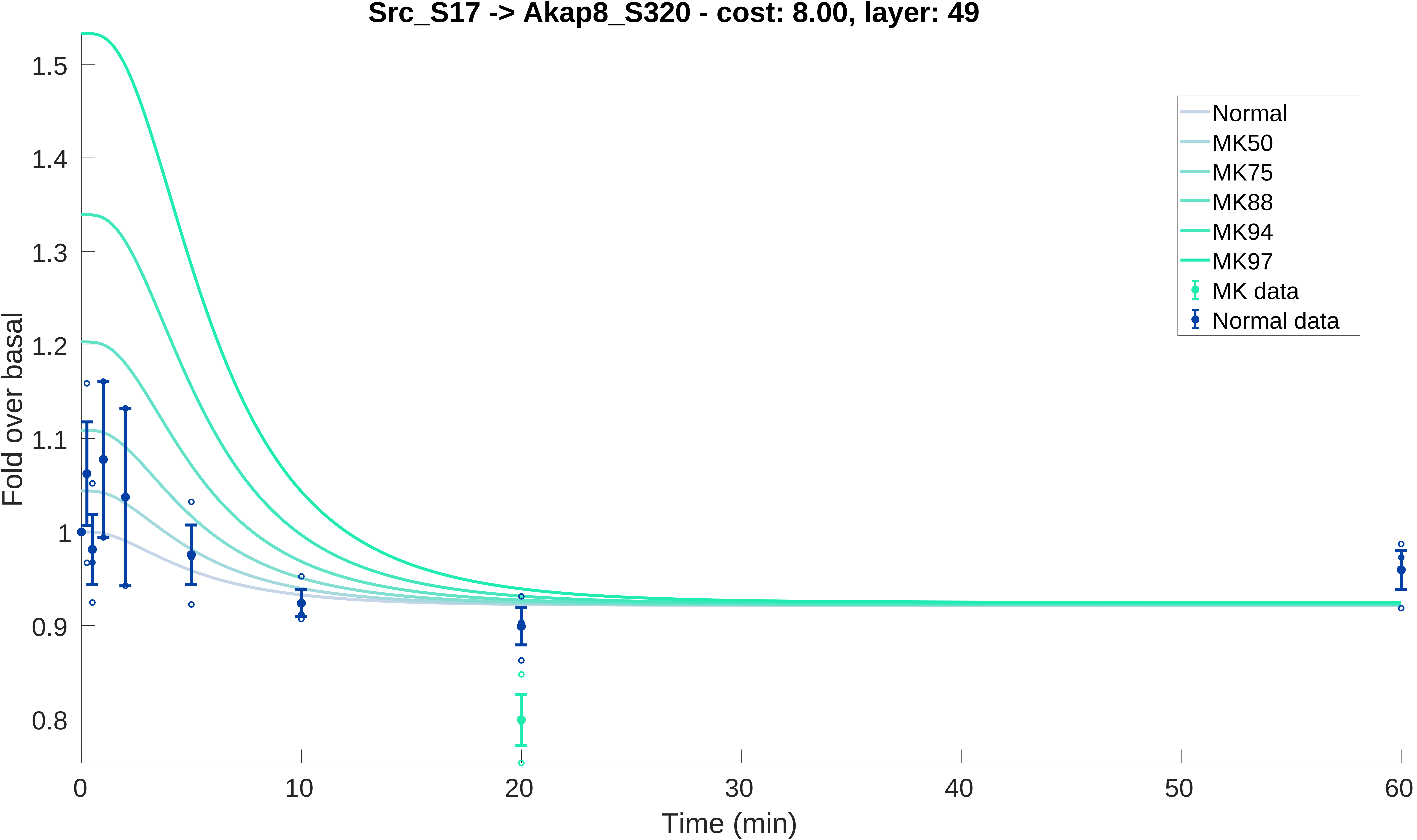

Src_S17 -> Akap8_S320 - cost: 8.00, layer: 49
Fold over basal
Time (min)
1.5
1.4
1.3
1.2
1.1
1
0.9
0.8
0
10
20
30
40
50
60
Normal
MK50
MK75
MK88
MK94
MK97
MK data
Normal data

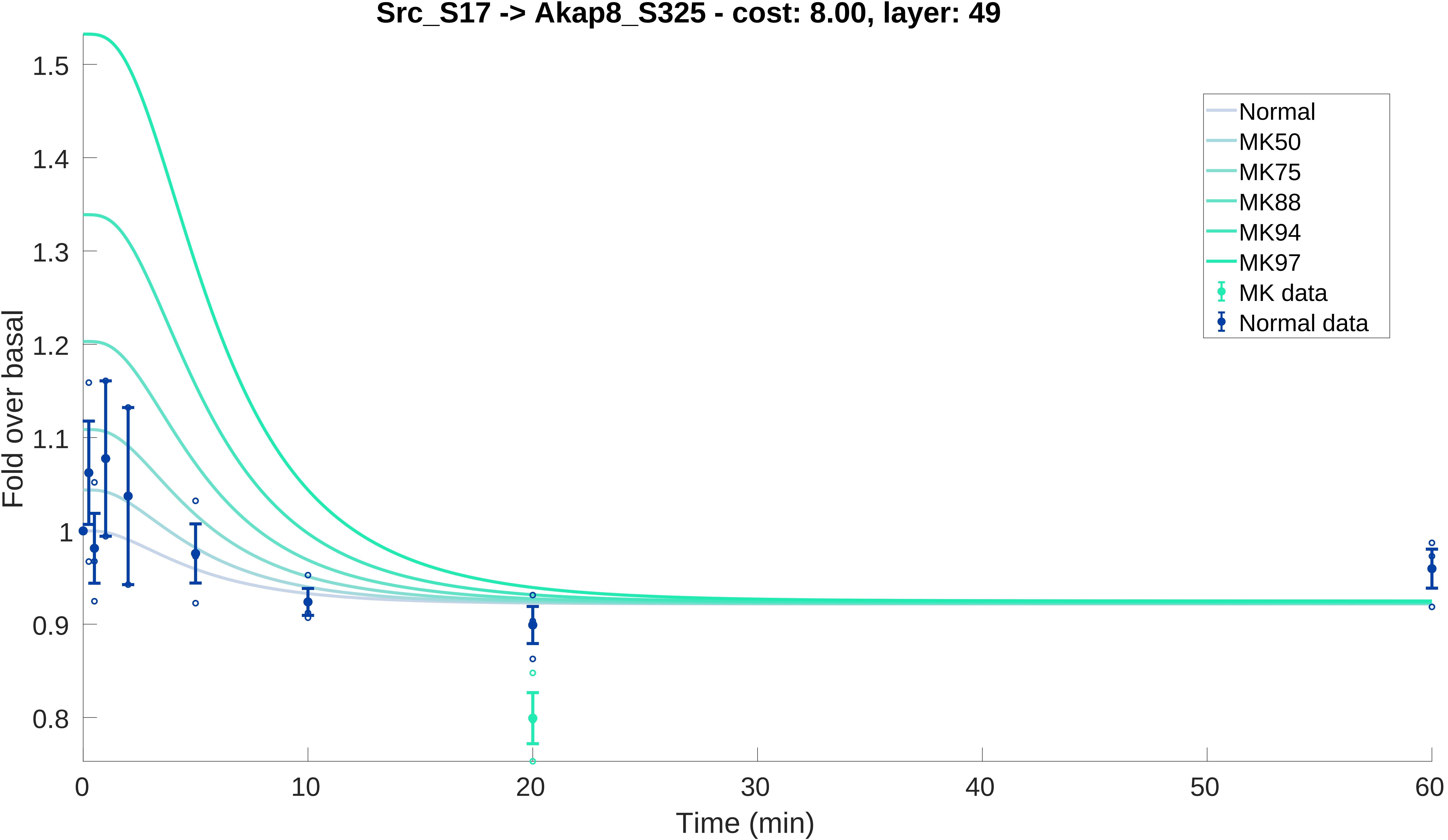

Src_S17 -> Akap8_S325 - cost: 8.00, layer: 49
Fold over basal
Time (min)
1.5
1.4
1.3
1.2
1.1
1
0.9
0.8
0
10
20
30
40
50
60
Normal
MK50
MK75
MK88
MK94
MK97
MK data
Normal data

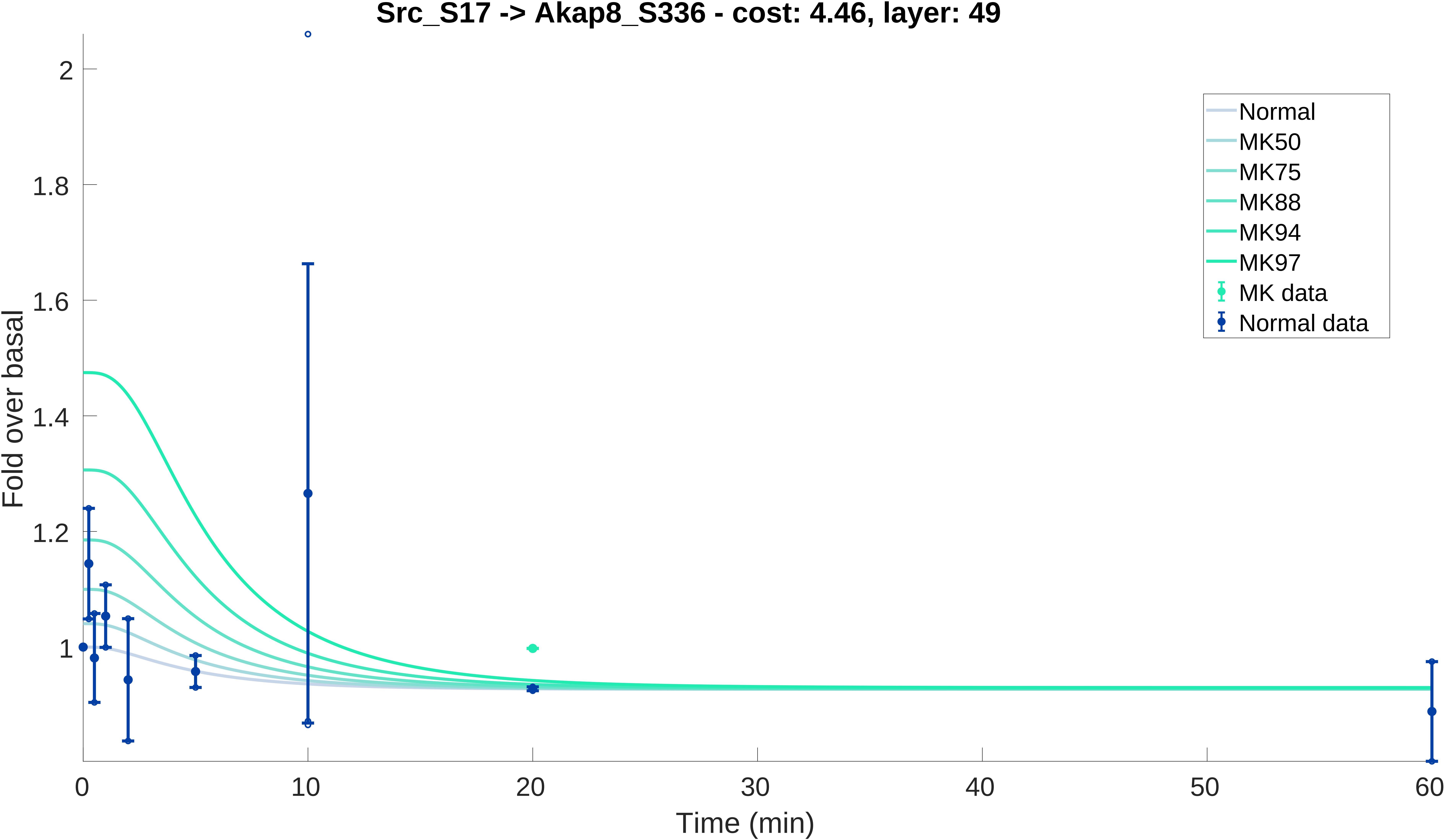

Src_S17 -> Akap8_S336 - cost: 4.46, layer: 49
Fold over basal
2
1.8
1.6
1.4
1.2
1
0
10
20
30
40
50
60
Time (min)
Normal
MK50
MK75
MK88
MK94
MK97
MK data
Normal data

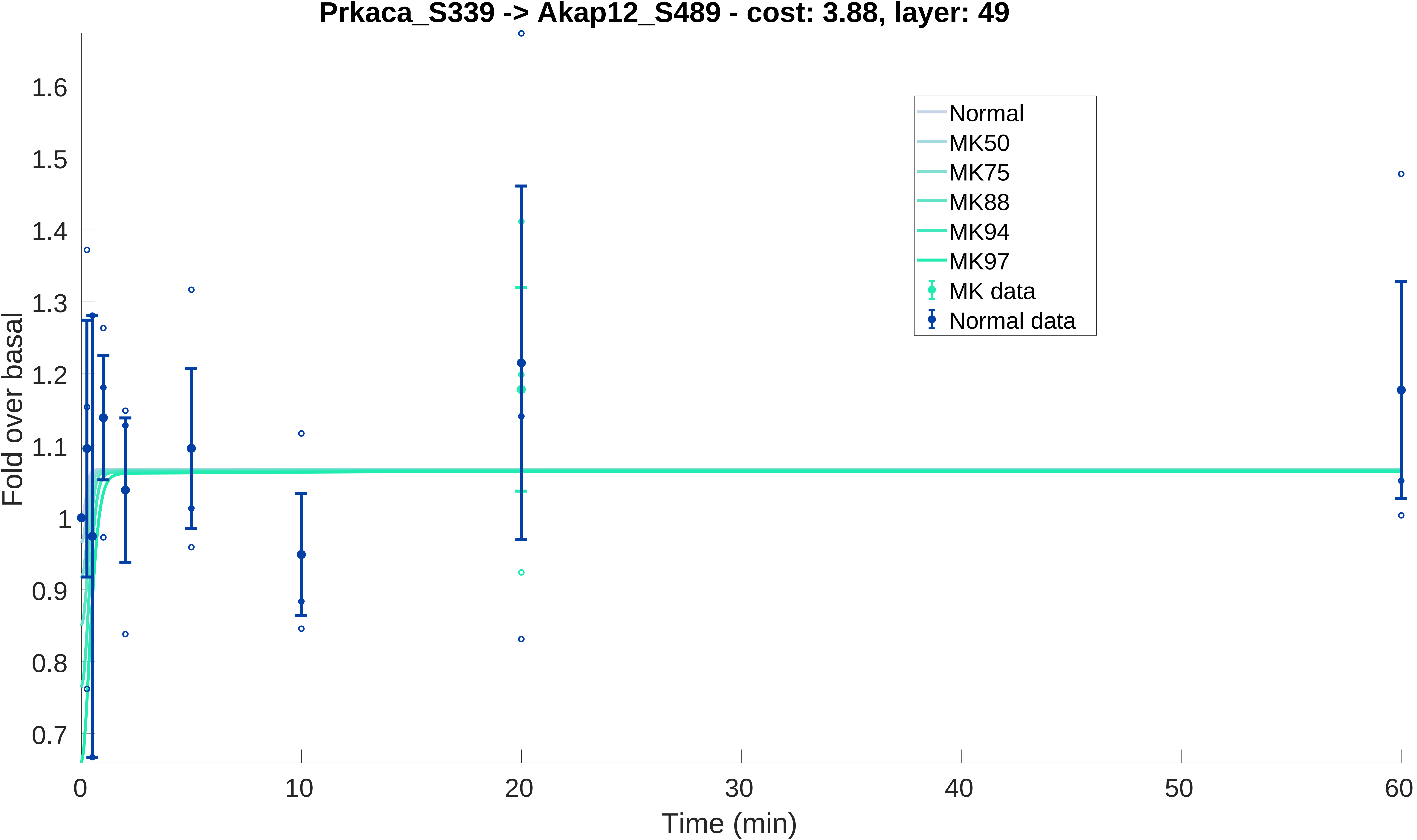
Prkaca_S339 -> Akap12_S489 - cost: 3.88, layer: 49
Fold over basal
Time (min)
0.7
0.8
0.9
1
1.1
1.2
1.3
1.4
1.5
1.6
0
10
20
30
40
50
60
Normal
MK50
MK75
MK88
MK94
MK97
MK data
Normal data

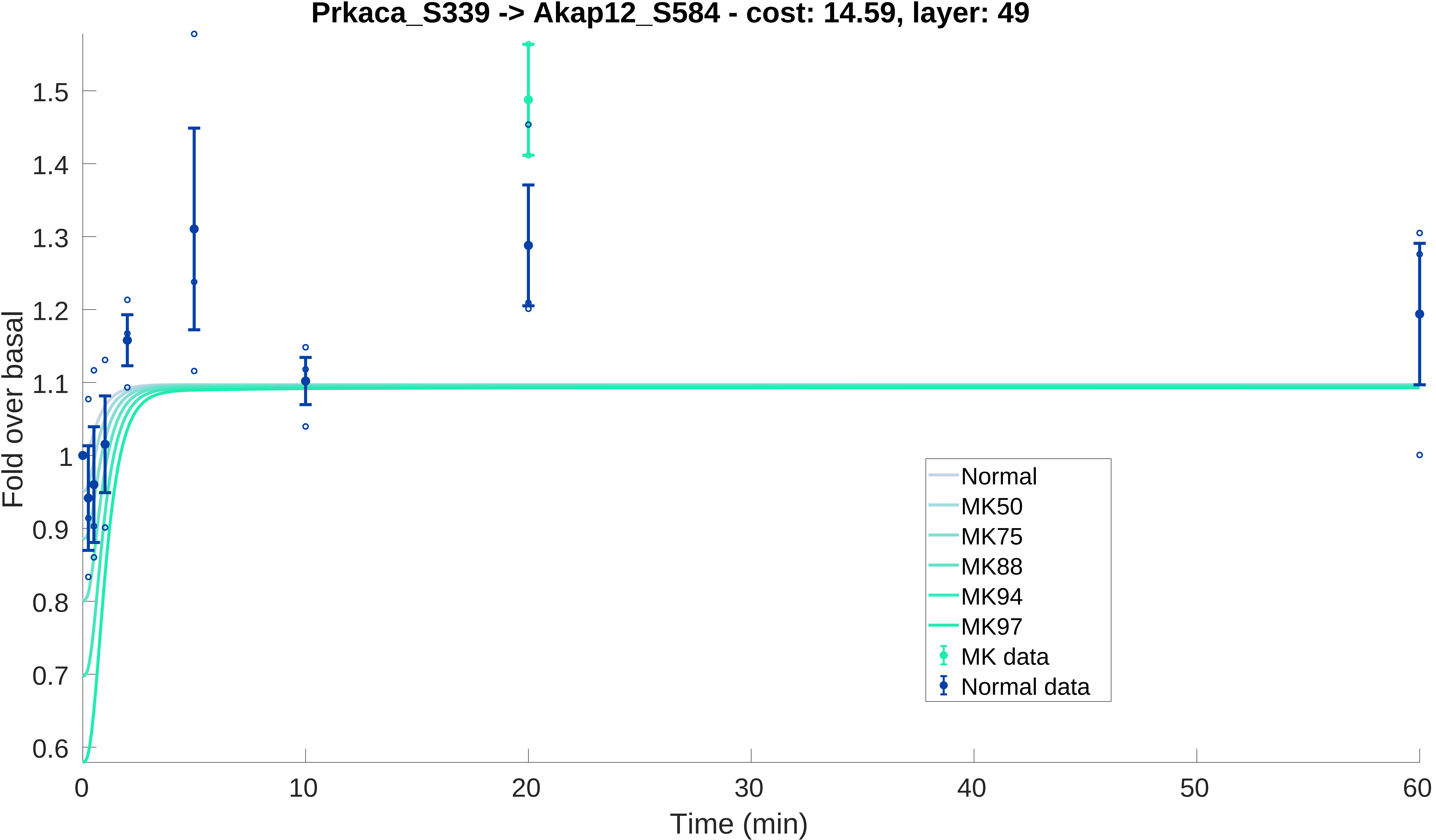

Prkaca_S339 -> Akap12_S584 - cost: 14.59, layer: 49
Fold over basal
Time (min)
Normal
MK50
MK75
MK88
MK94
MK97
MK data
Normal data

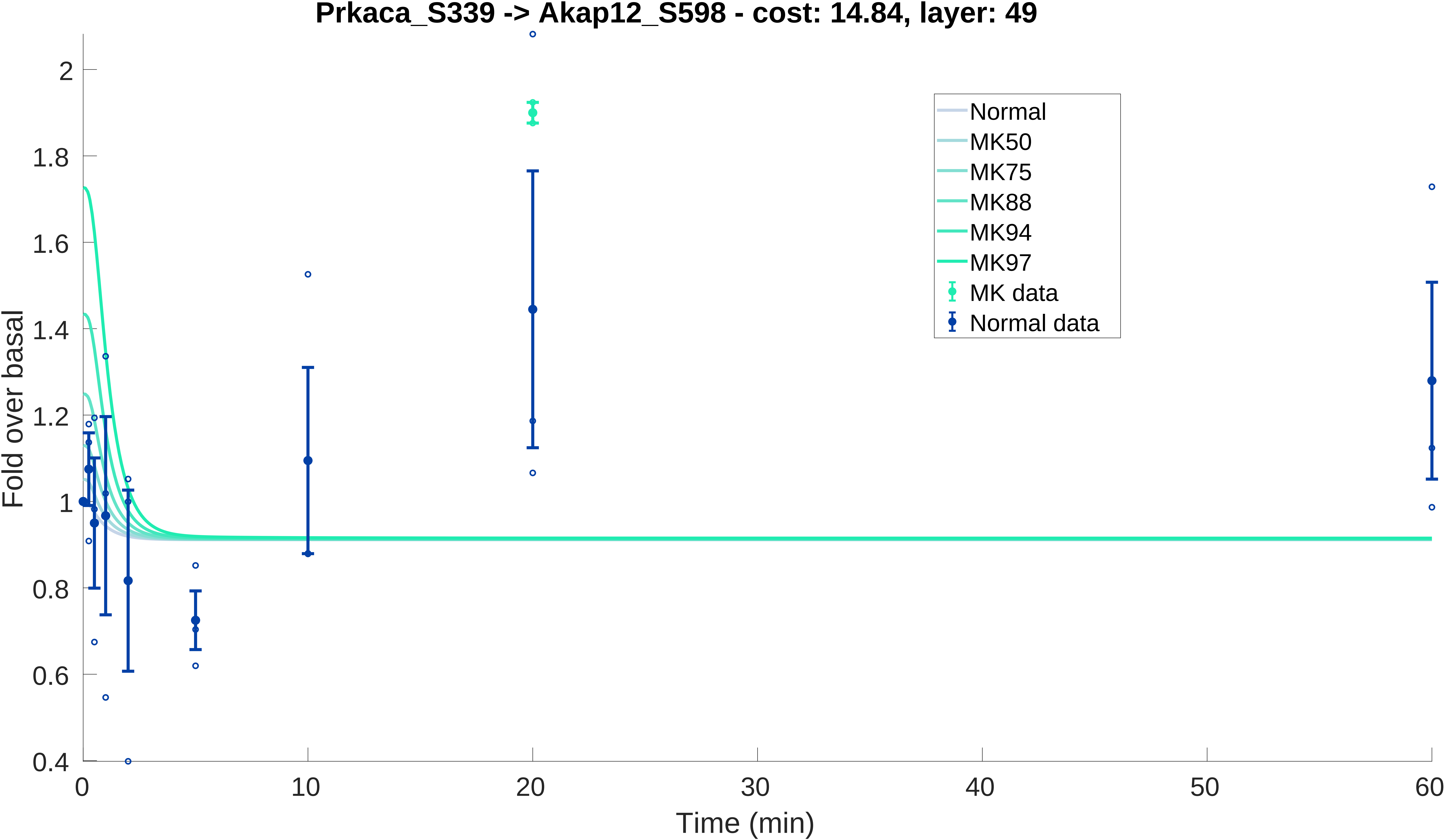
Prkaca_S339 -> Akap12_S598 - cost: 14.84, layer: 49
Fold over basal
Time (min)
0.4
0.6
0.8
1
1.2
1.4
1.6
1.8
2
0
10
20
30
40
50
60
Normal
MK50
MK75
MK88
MK94
MK97
MK data
Normal data

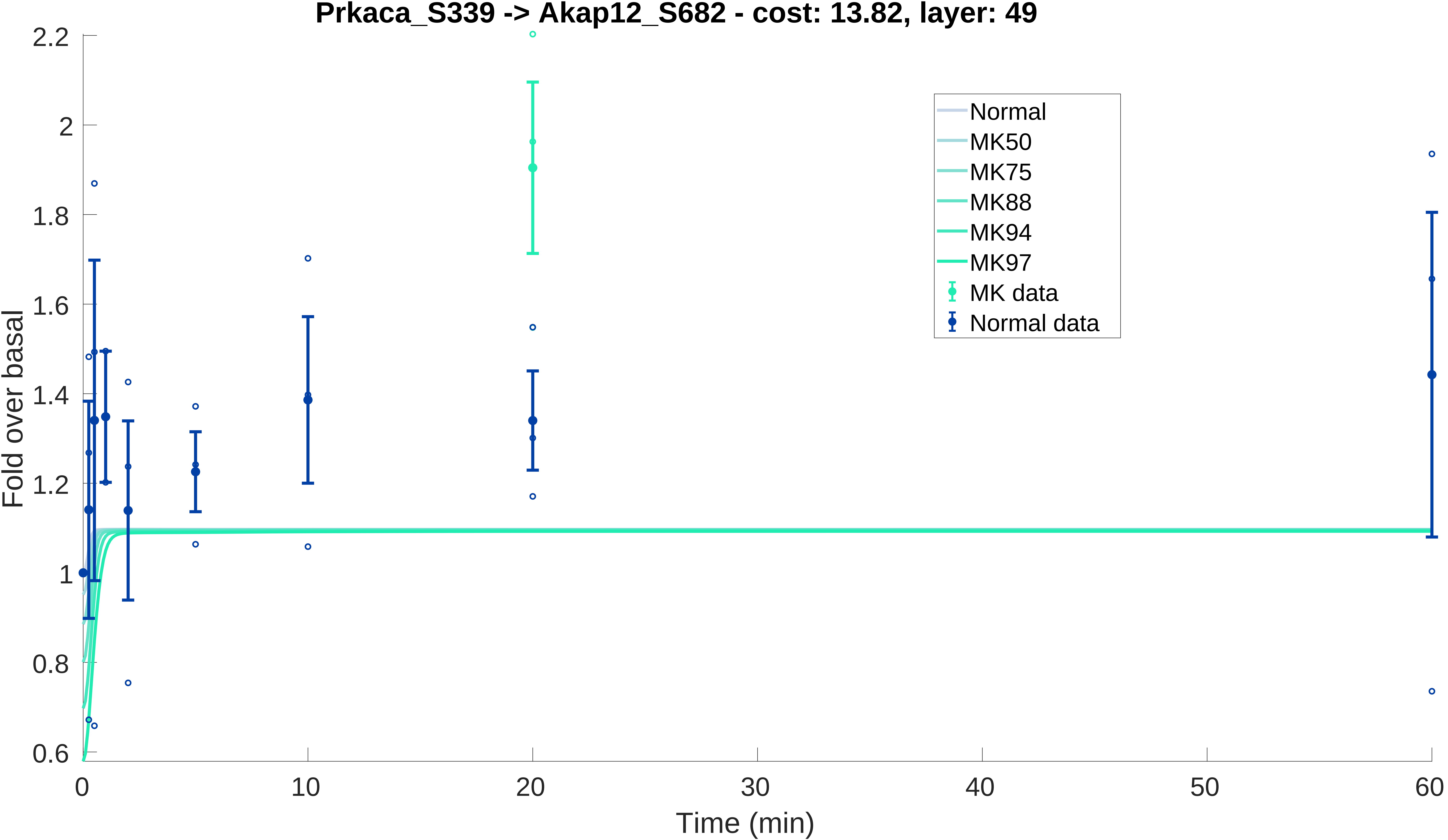
Prkaca_S339 -> Akap12_S682 - cost: 13.82, layer: 49
Fold over basal
Time (min)
0.6
0.8
1
1.2
1.4
1.6
1.8
2
2.2
0
10
20
30
40
50
60
Normal
MK50
MK75
MK88
MK94
MK97
MK data
Normal data

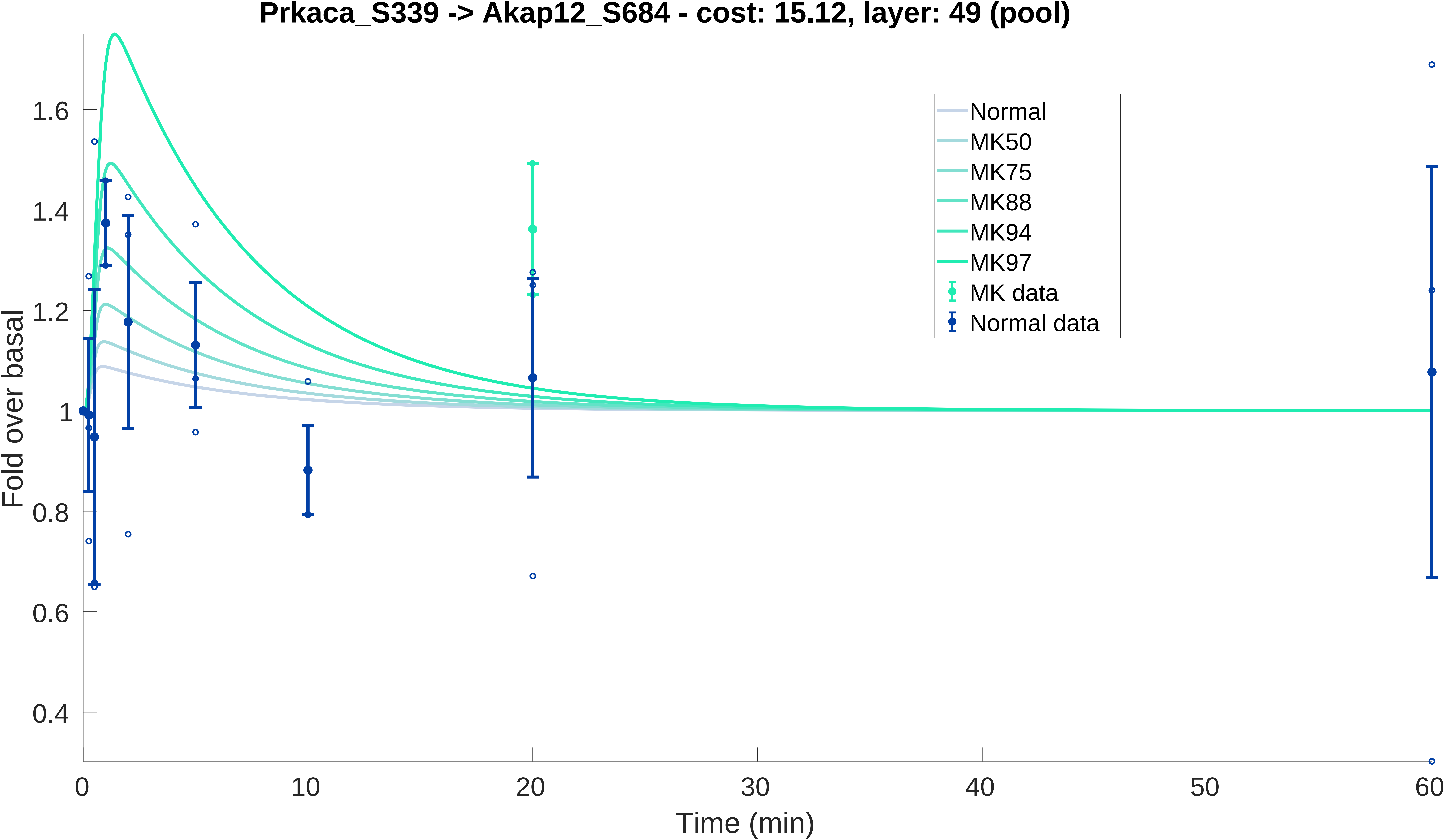
Prkaca_S339 -> Akap12_S684 - cost: 15.12, layer: 49 (pool)
Fold over basal
Time (min)
0.4
0.6
0.8
1
1.2
1.4
1.6
0
10
20
30
40
50
60
Normal
MK50
MK75
MK88
MK94
MK97
MK data
Normal data

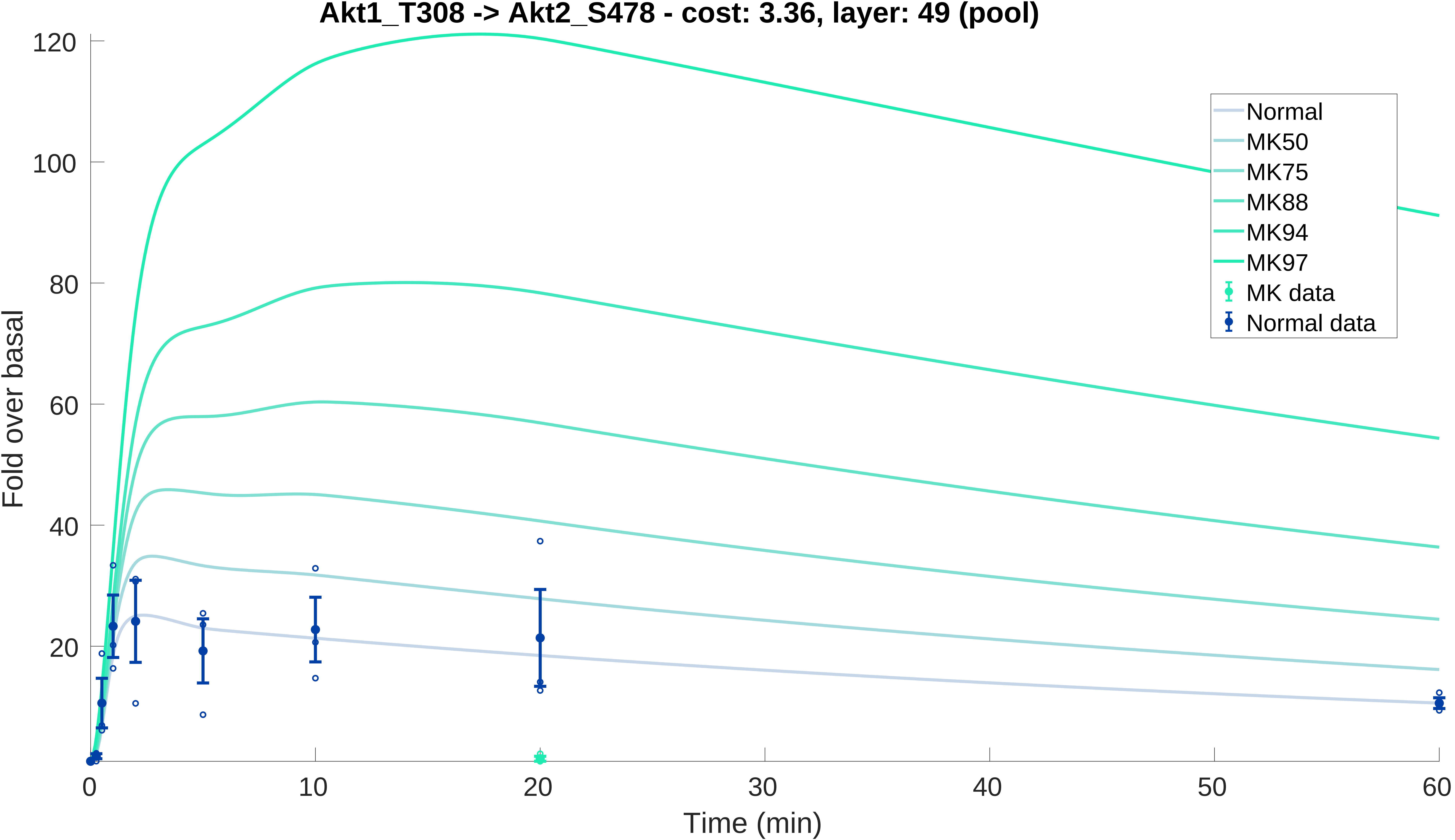
Akt1_T308 -> Akt2_S478 - cost: 3.36, layer: 49 (pool)
Fold over basal
Time (min)
0
10
20
30
40
50
60
20
40
60
80
100
120
Normal
MK50
MK75
MK88
MK94
MK97
MK data
Normal data

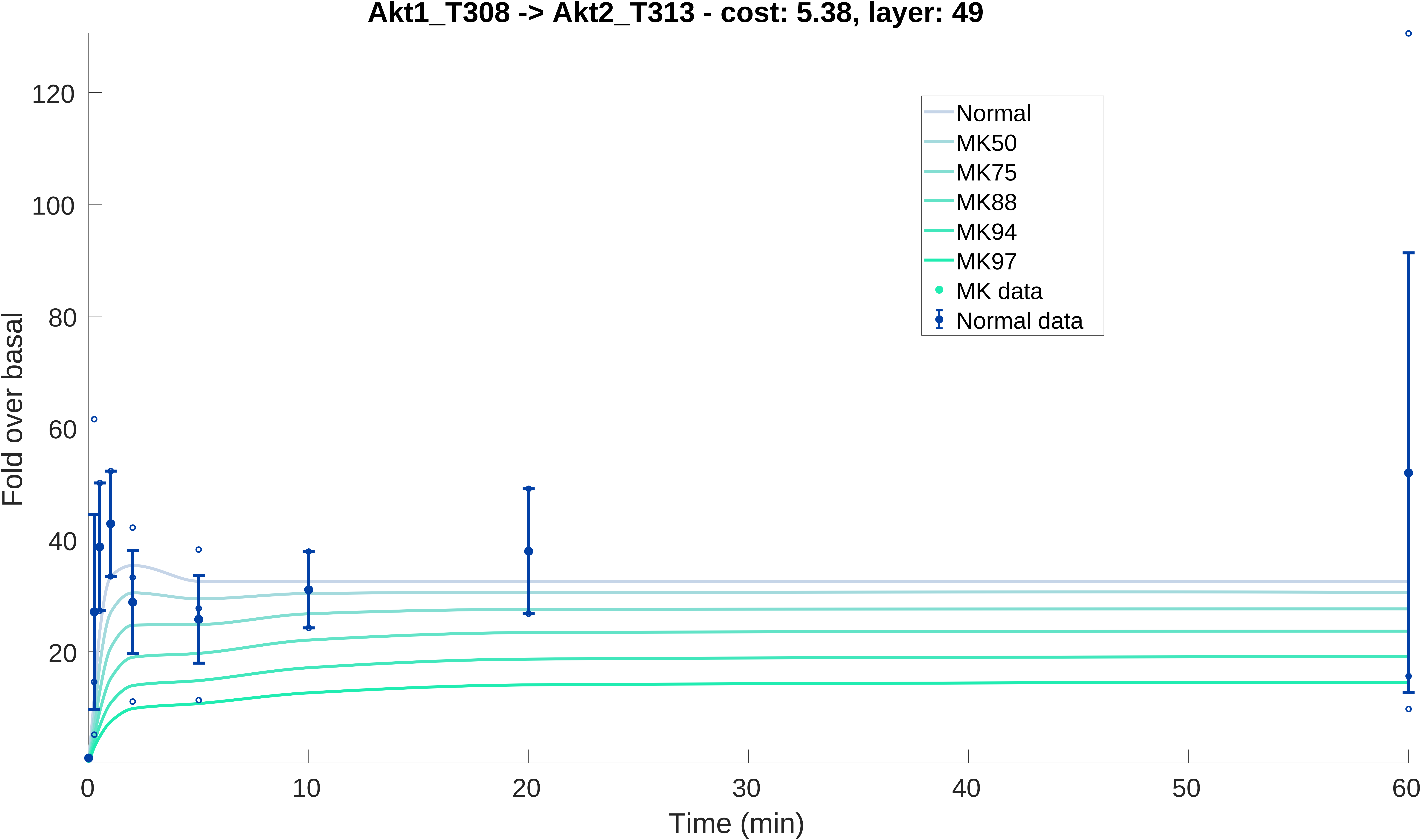
Akt1_T308 -> Akt2_T313 - cost: 5.38, layer: 49
Fold over basal
Time (min)
0
10
20
30
40
50
60
20
40
60
80
100
120
Normal
MK50
MK75
MK88
MK94
MK97
MK data
Normal data

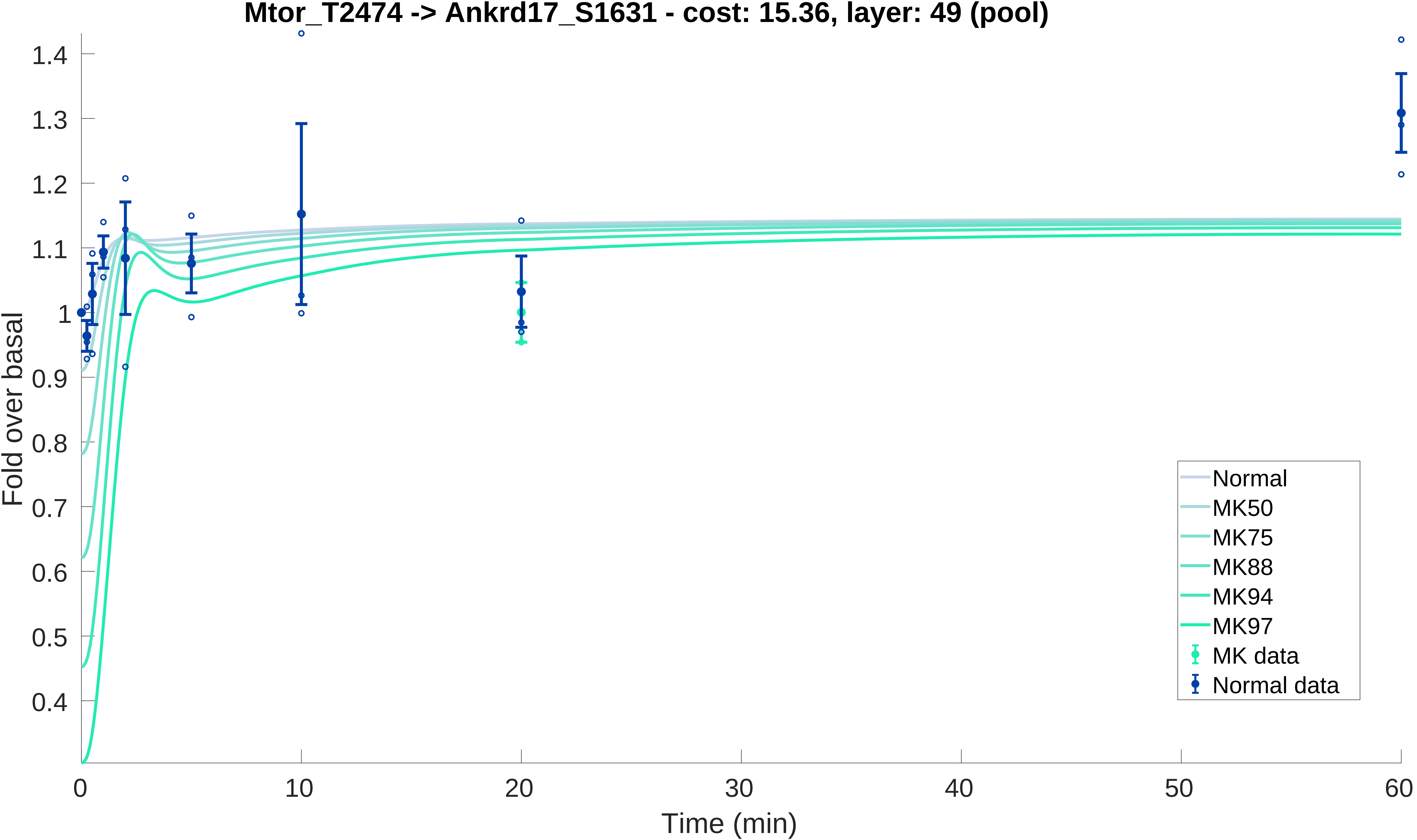

Mtor_T2474 -> Ankrd17_S1631 - cost: 15.36, layer: 49 (pool)
Fold over basal
Time (min)
1.4
1.3
1.2
1.1
1
0.9
0.8
0.7
0.6
0.5
0.4
0
10
20
30
40
50
60
Normal
MK50
MK75
MK88
MK94
MK97
MK data
Normal data

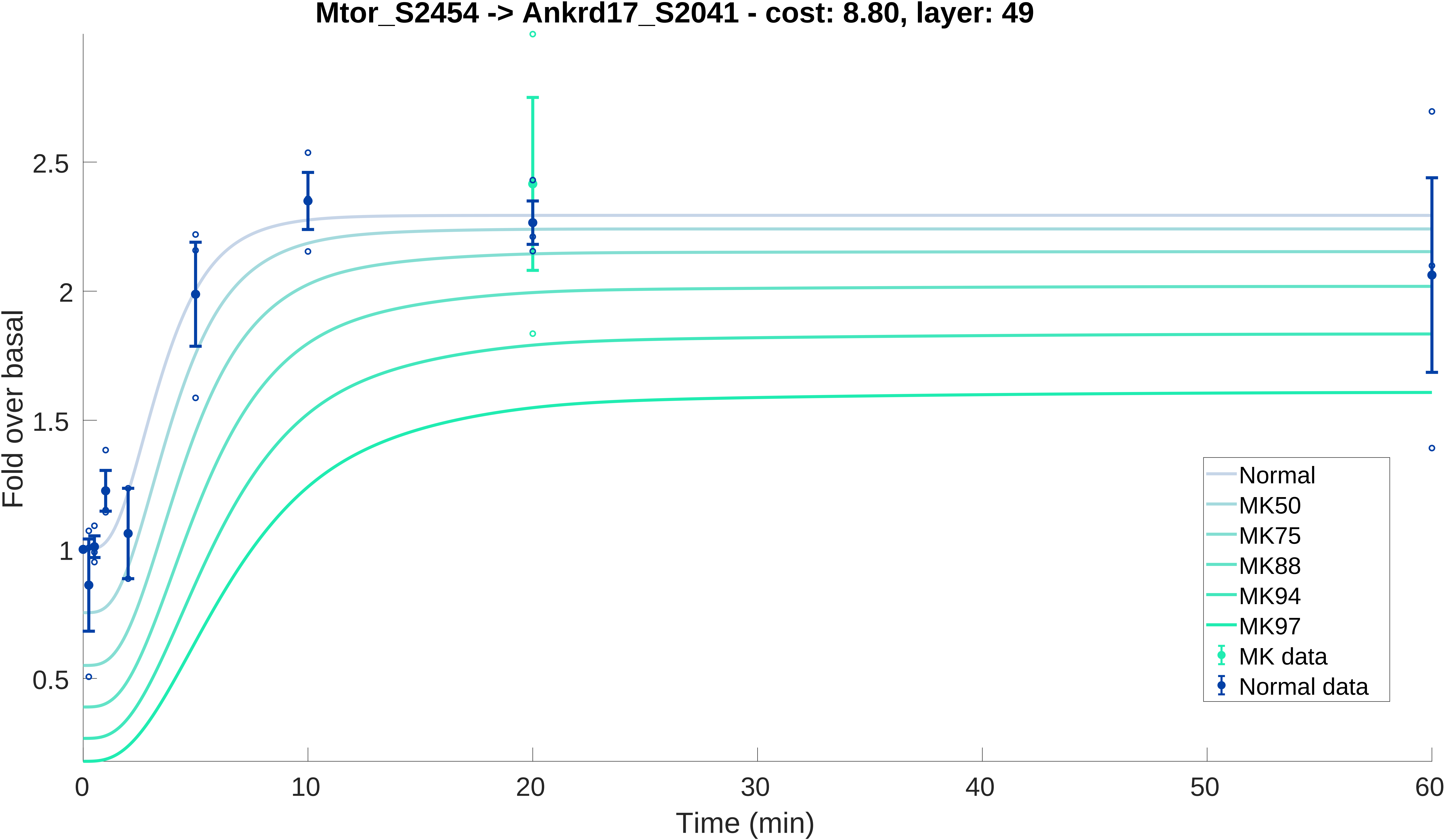

Mtor_S2454 -> Ankrd17_S2041 - cost: 8.80, layer: 49
Fold over basal
Time (min)
0.5
1
1.5
2
2.5
0
10
20
30
40
50
60
Normal
MK50
MK75
MK88
MK94
MK97
MK data
Normal data

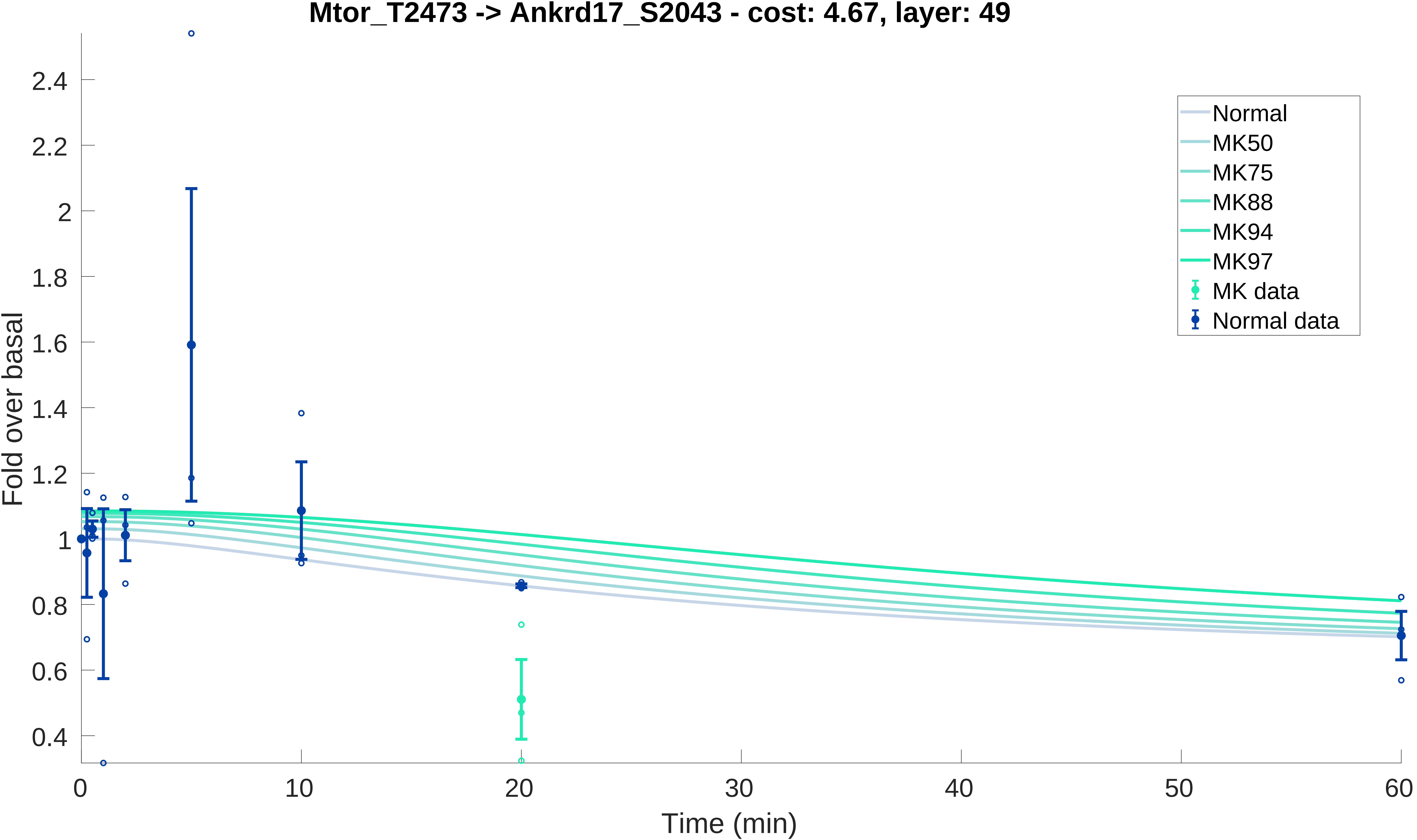

Mtor_T2473 -> Ankrd17_S2043 - cost: 4.67, layer: 49
Fold over basal
Time (min)
2.4
2.2
2
1.8
1.6
1.4
1.2
1
0.8
0.6
0.4
0
10
20
30
40
50
60
Normal
MK50
MK75
MK88
MK94
MK97
MK data
Normal data

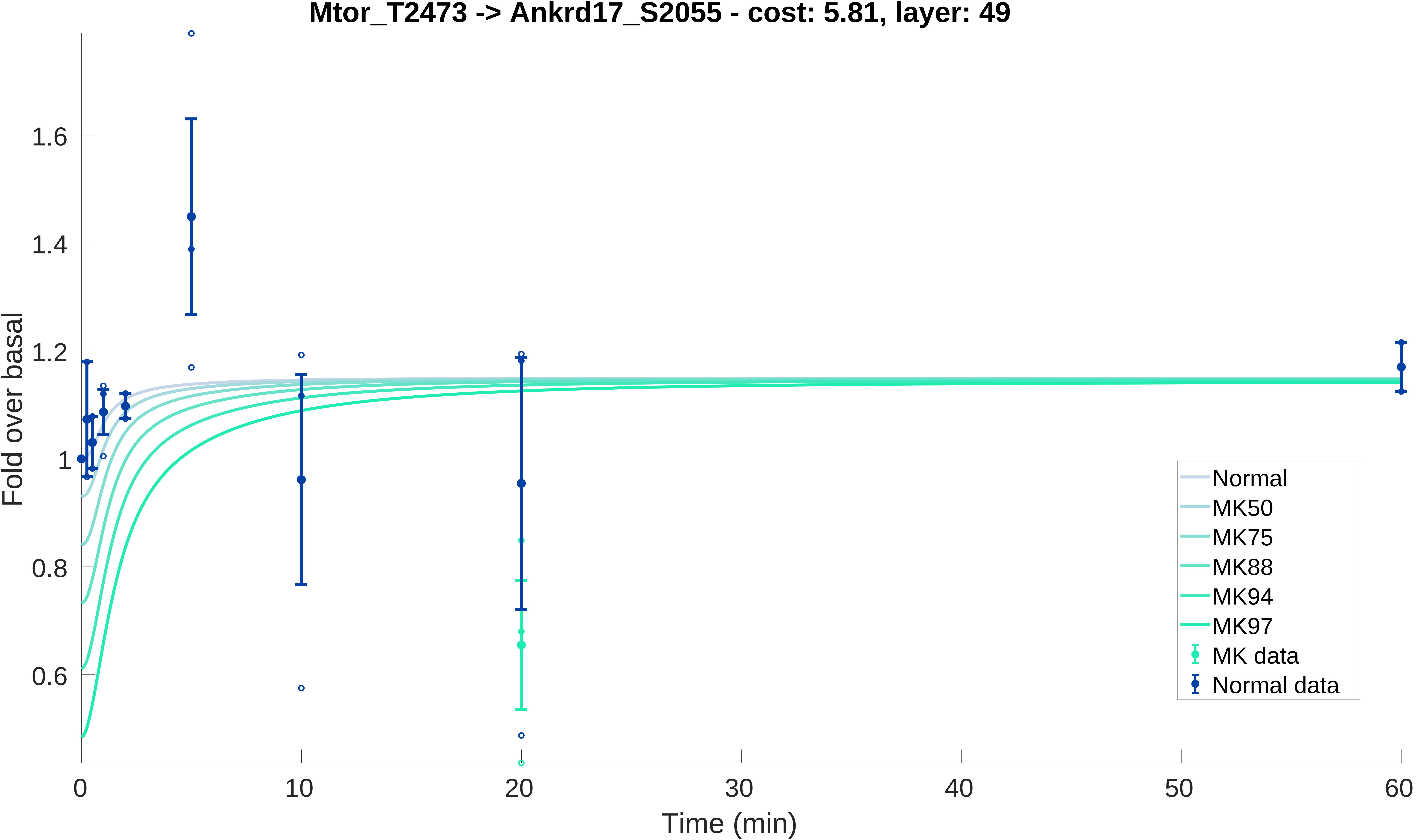

Mtor_T2473 -> Ankrd17_S2055 - cost: 5.81, layer: 49
Fold over basal
1.6
1.4
1.2
1
0.8
0.6
0
10
20
30
40
50
60
Time (min)
Normal
MK50
MK75
MK88
MK94
MK97
MK data
Normal data

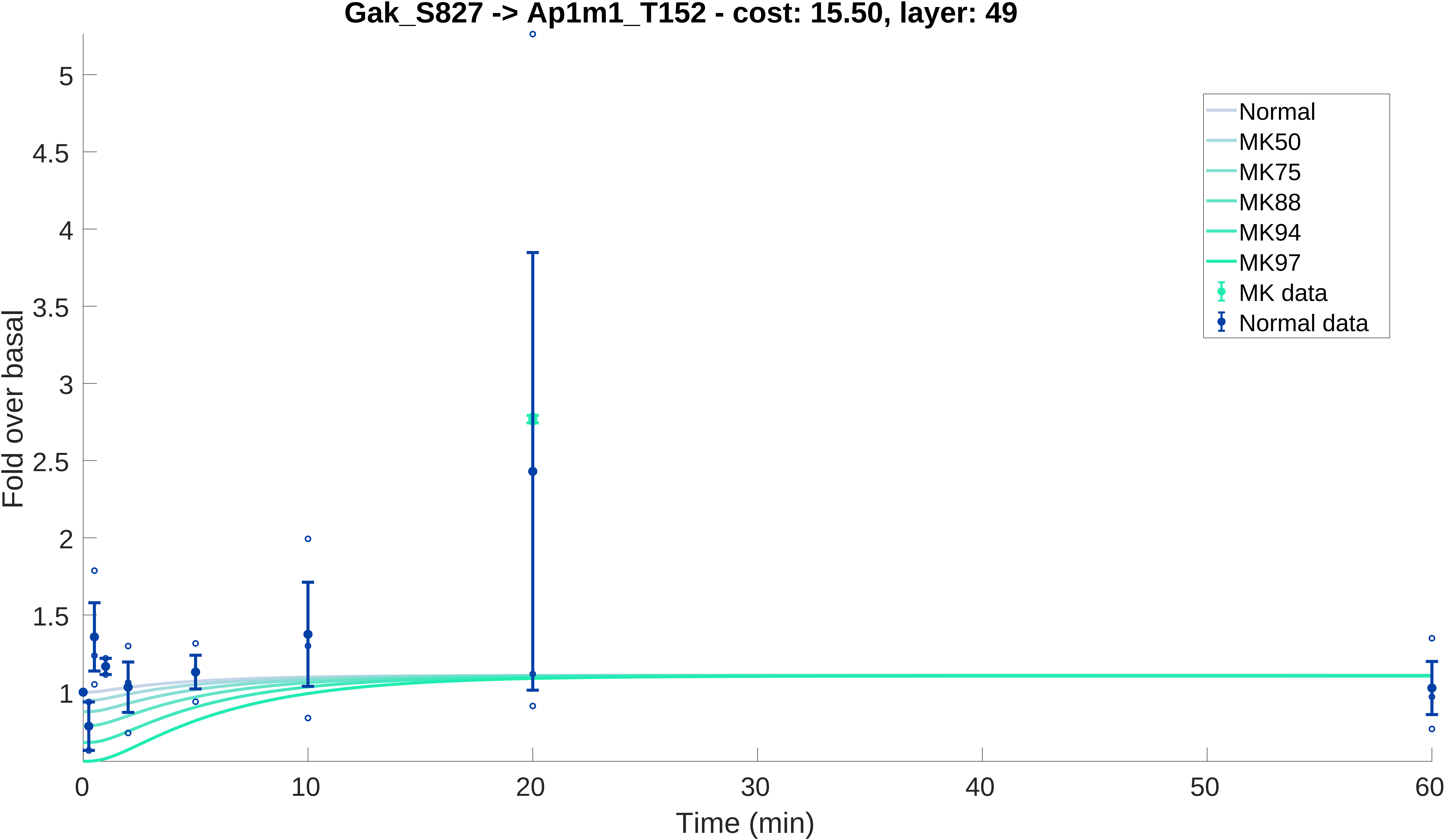

Gak_S827 -> Ap1m1_T152 - cost: 15.50, layer: 49
Fold over basal
Time (min)
Normal
MK50
MK75
MK88
MK94
MK97
MK data
Normal data
5
4.5
4
3.5
3
2.5
2
1.5
1
0
10
20
30
40
50
60

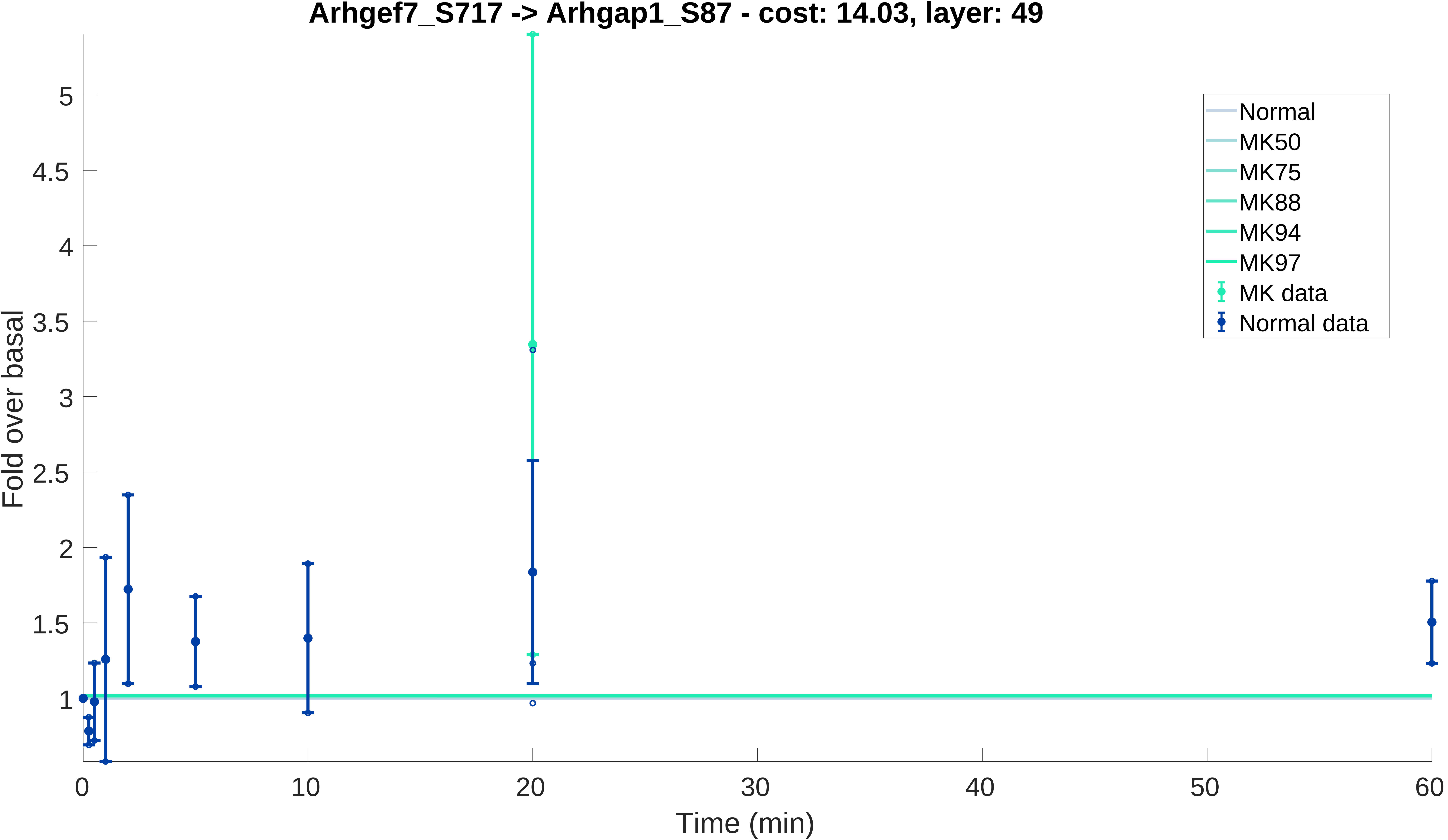
Arhgef7_S717 -> Arhgap1_S87 - cost: 14.03, layer: 49
Fold over basal
Time (min)
Normal
MK50
MK75
MK88
MK94
MK97
MK data
Normal data
0
10
20
30
40
50
60
1
1.5
2
2.5
3
3.5
4
4.5
5

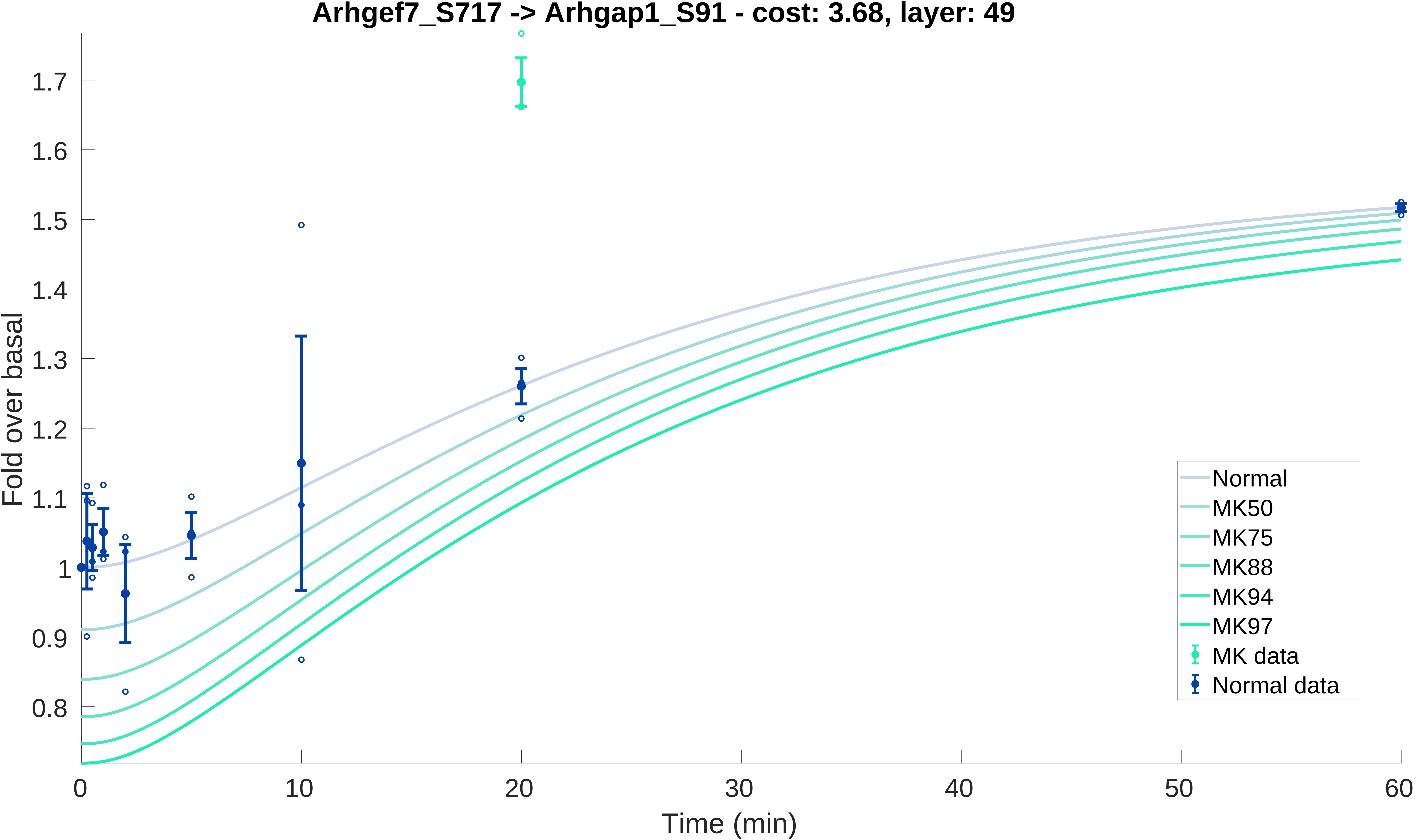

Arhgef7_S717 -> Arhgap1_S91 - cost: 3.68, layer: 49
Fold over basal
Time (min)
0
10
20
30
40
50
60
0.8
0.9
1
1.1
1.2
1.3
1.4
1.5
1.6
1.7
Normal
MK50
MK75
MK88
MK94
MK97
MK data
Normal data

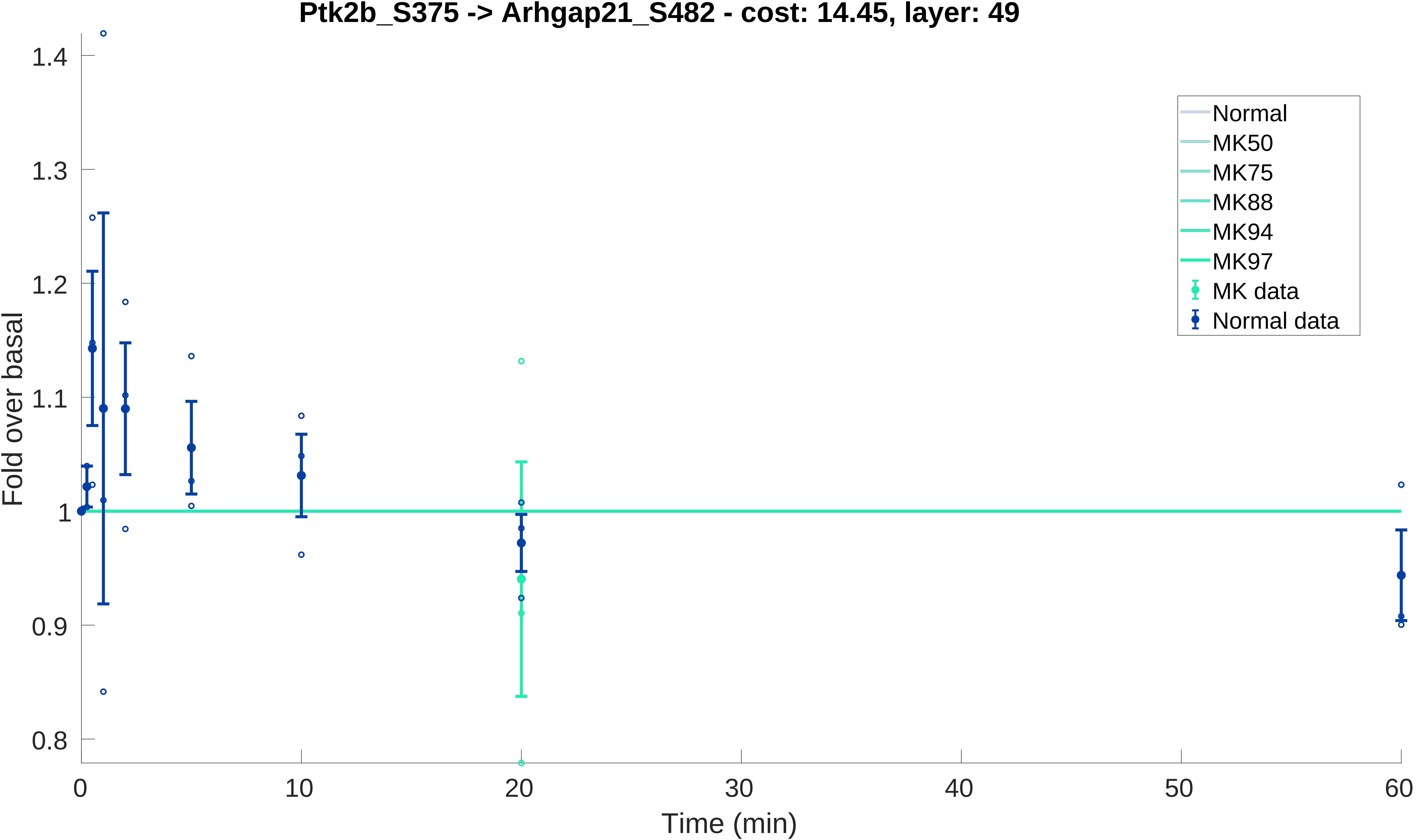

Ptk2b_S375 -> Arhgap21_S482 - cost: 14.45, layer: 49
Fold over basal
1.4
1.3
1.2
1.1
1
0.9
0.8
0
10
20
30
40
50
60
Time (min)
Normal
MK50
MK75
MK88
MK94
MK97
MK data
Normal data

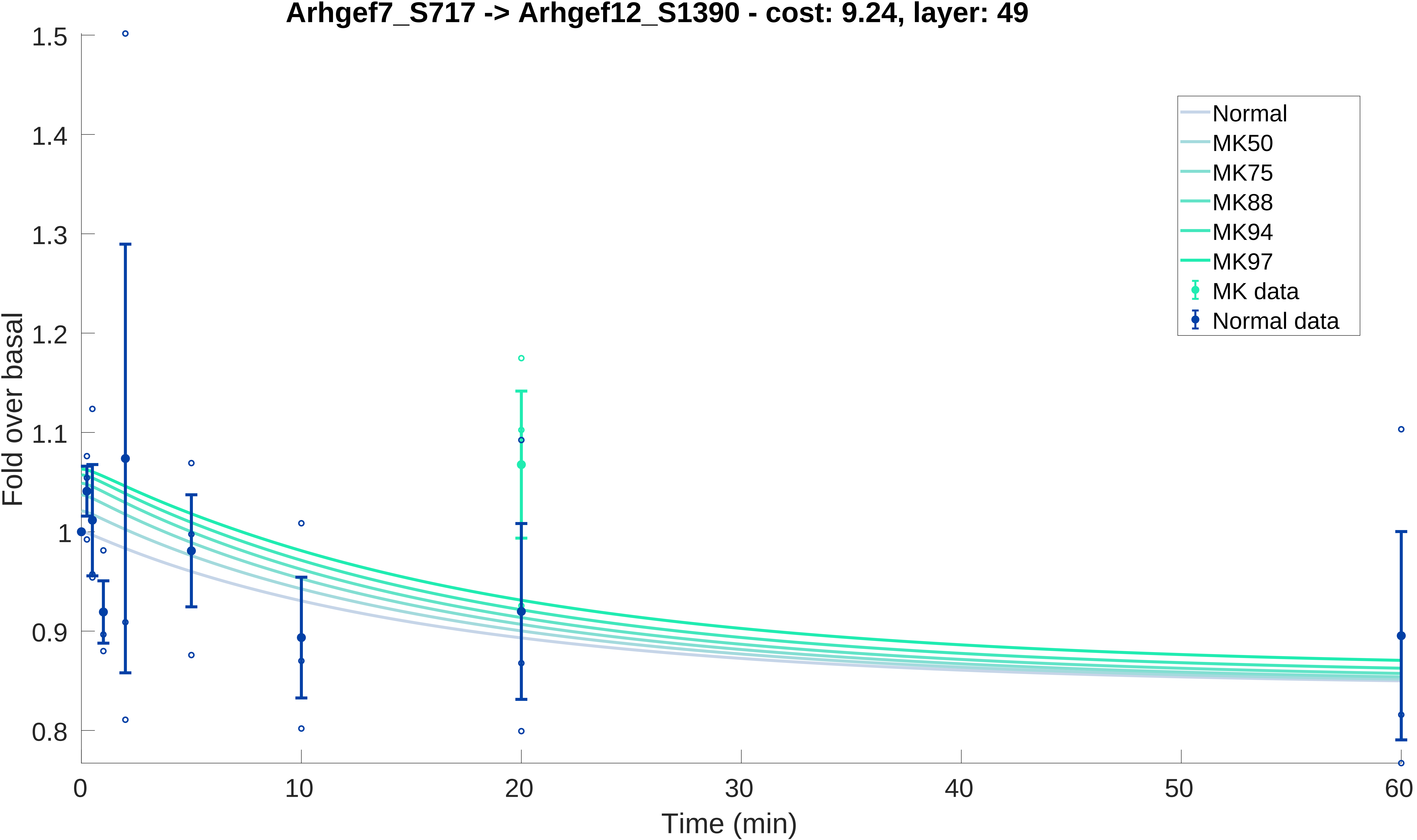

Arhgef7_S717 -> Arhgef12_S1390 - cost: 9.24, layer: 49
Fold over basal
Time (min)
1.5
1.4
1.3
1.2
1.1
1
0.9
0.8
0
10
20
30
40
50
60
Normal
MK50
MK75
MK88
MK94
MK97
MK data
Normal data

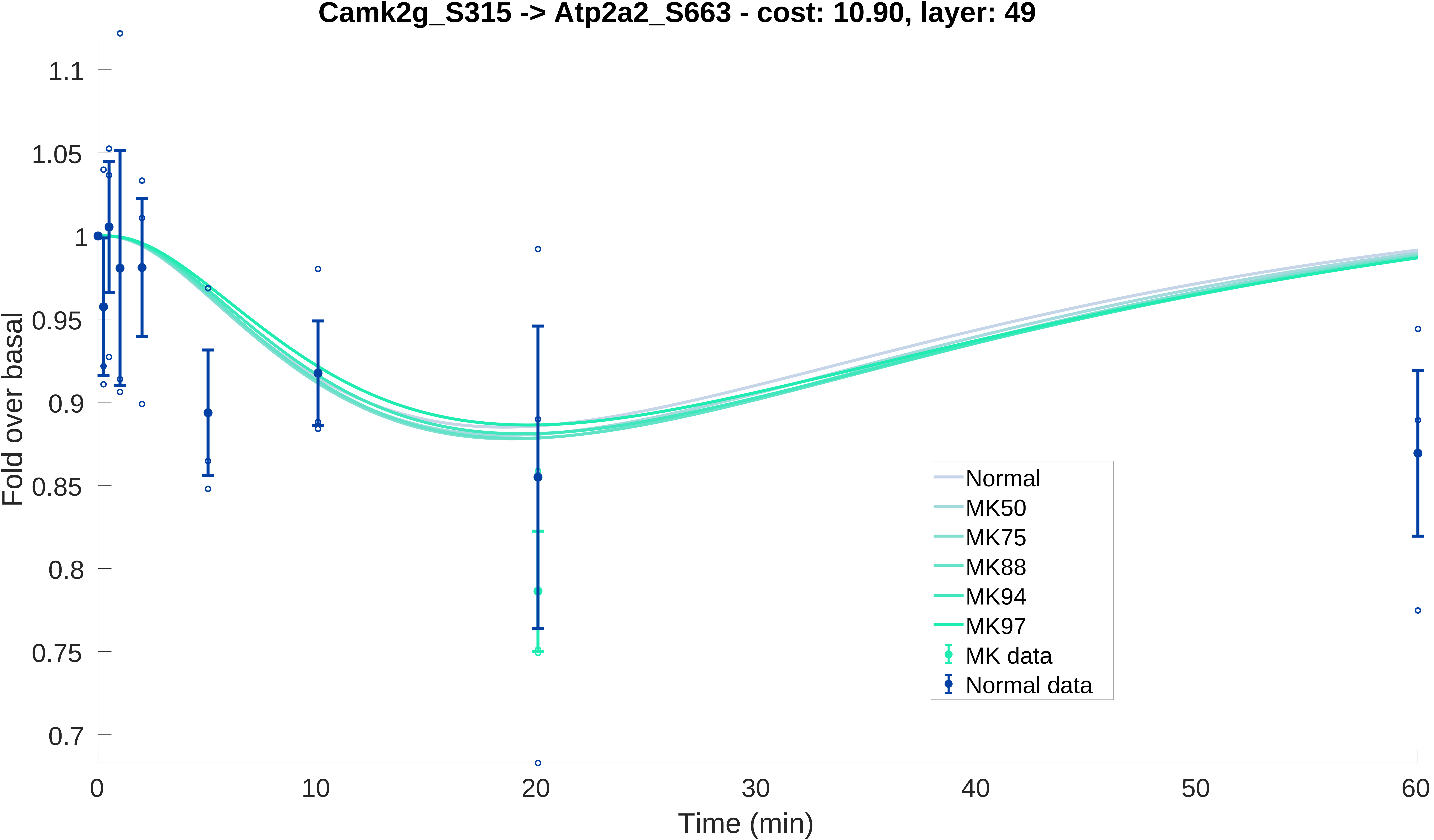

Camk2g_S315 -> Atp2a2_S663 - cost: 10.90, layer: 49
Fold over basal
1.1
1.05
1
0.95
0.9
0.85
0.8
0.75
0.7
0
10
20
30
40
50
60
Time (min)
Normal
MK50
MK75
MK88
MK94
MK97
MK data
Normal data

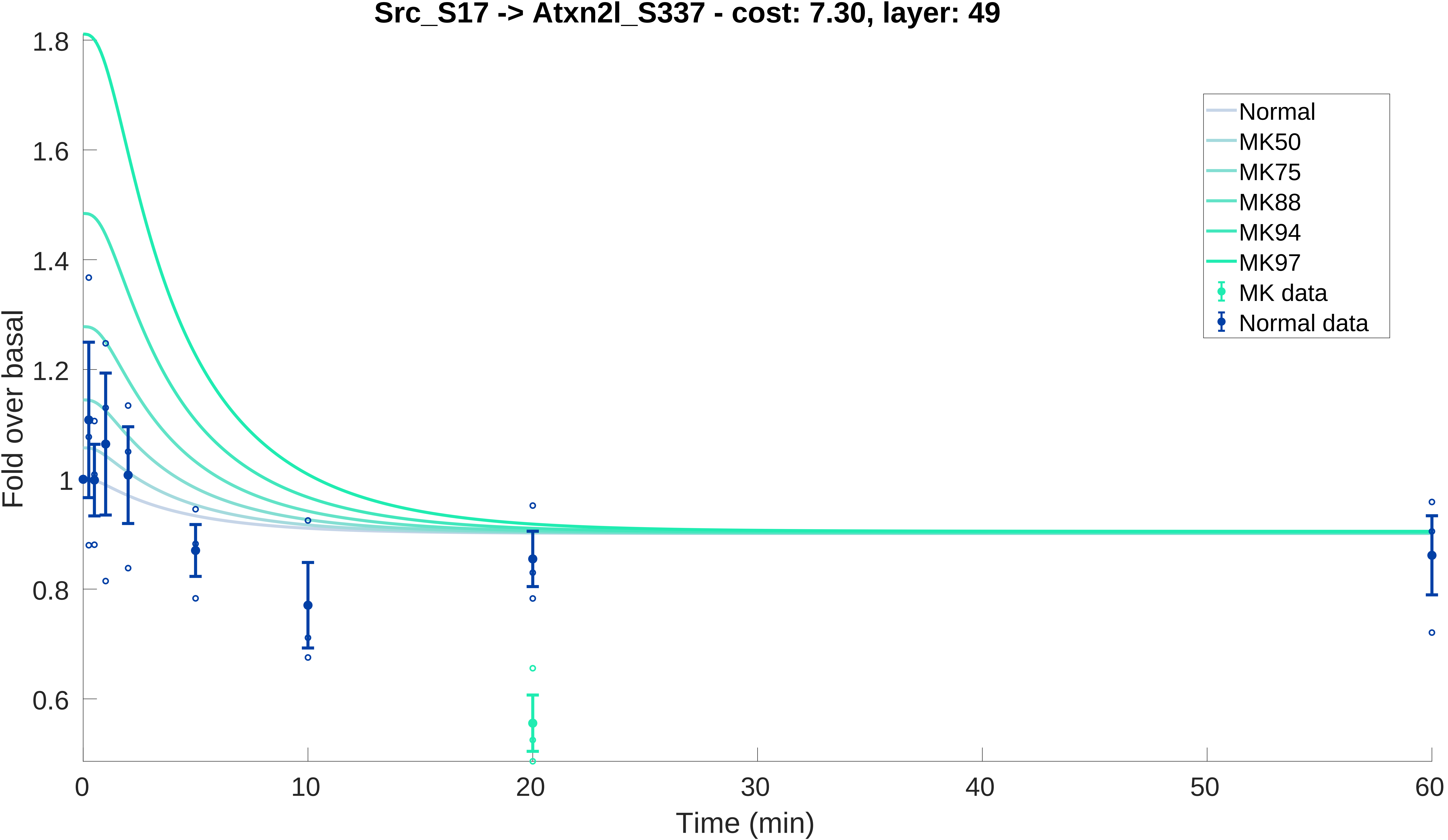

Src_S17 -> Atxn2l_S337 - cost: 7.30, layer: 49
Fold over basal
1.8
1.6
1.4
1.2
1
0.8
0.6
0
10
20
30
40
50
60
Time (min)
Normal
MK50
MK75
MK88
MK94
MK97
MK data
Normal data

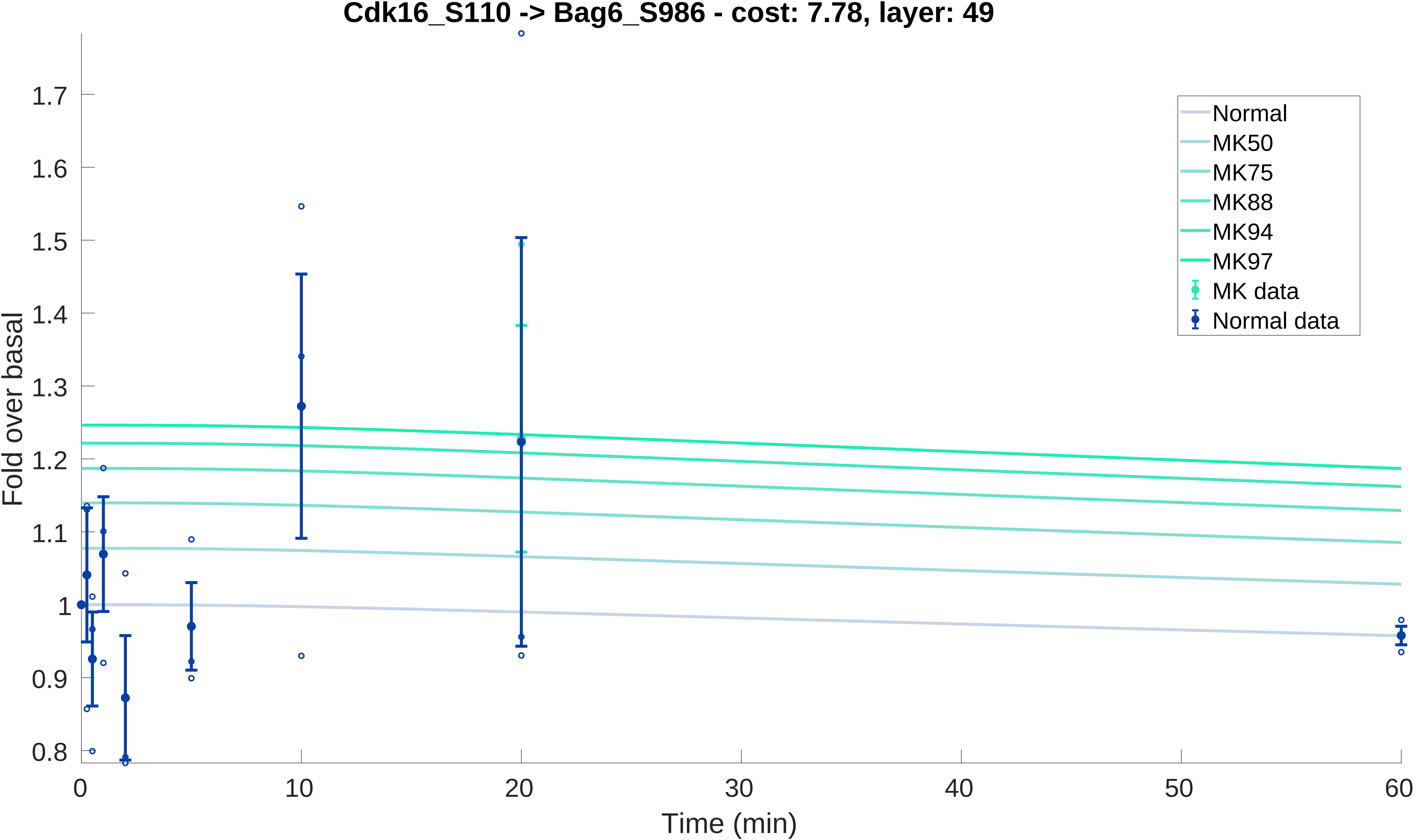

Cdk16_S110 -> Bag6_S986 - cost: 7.78, layer: 49
Fold over basal
Time (min)
Normal
MK50
MK75
MK88
MK94
MK97
MK data
Normal data
1.7
1.6
1.5
1.4
1.3
1.2
1.1
1
0.9
0.8
0
10
20
30
40
50
60

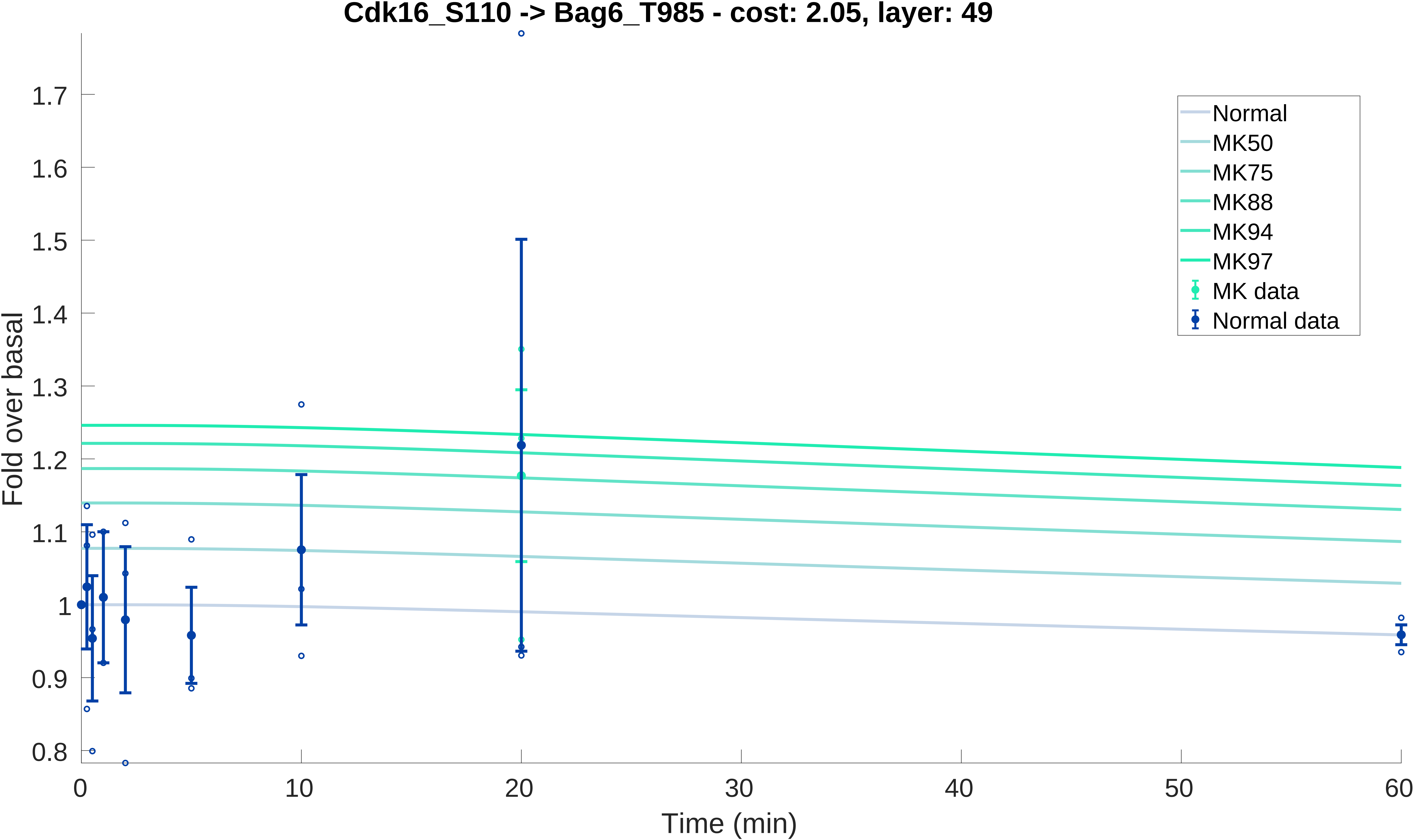

Cdk16_S110 -> Bag6_T985 - cost: 2.05, layer: 49
Fold over basal
Time (min)
0.8
0.9
1
1.1
1.2
1.3
1.4
1.5
1.6
1.7
0
10
20
30
40
50
60
Normal
MK50
MK75
MK88
MK94
MK97
MK data
Normal data

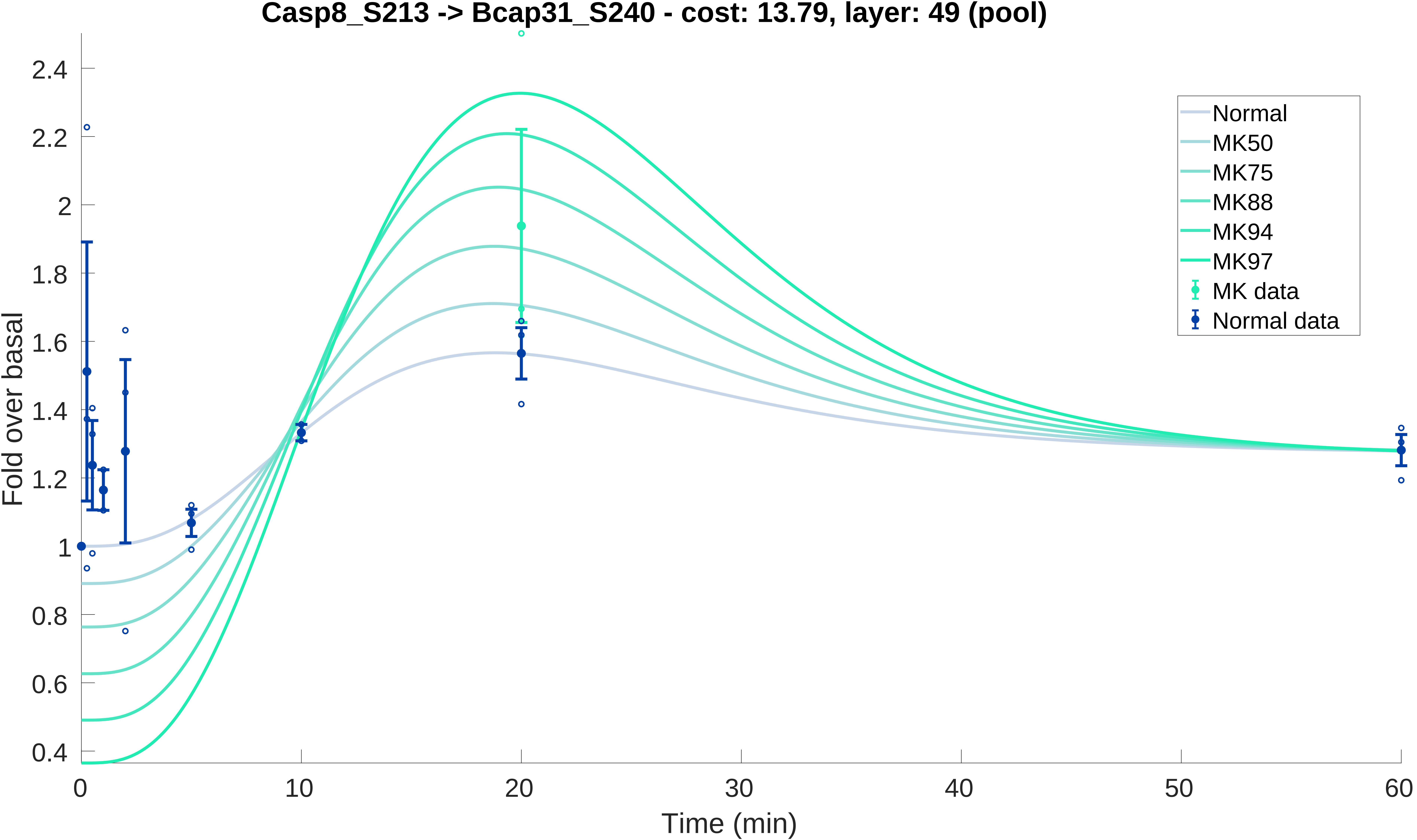
Casp8_S213 -> Bcap31_S240 - cost: 13.79, layer: 49 (pool)
Fold over basal
Time (min)
Normal
MK50
MK75
MK88
MK94
MK97
MK data
Normal data
0.4
0.6
0.8
1
1.2
1.4
1.6
1.8
2
2.2
2.4
0
10
20
30
40
50
60

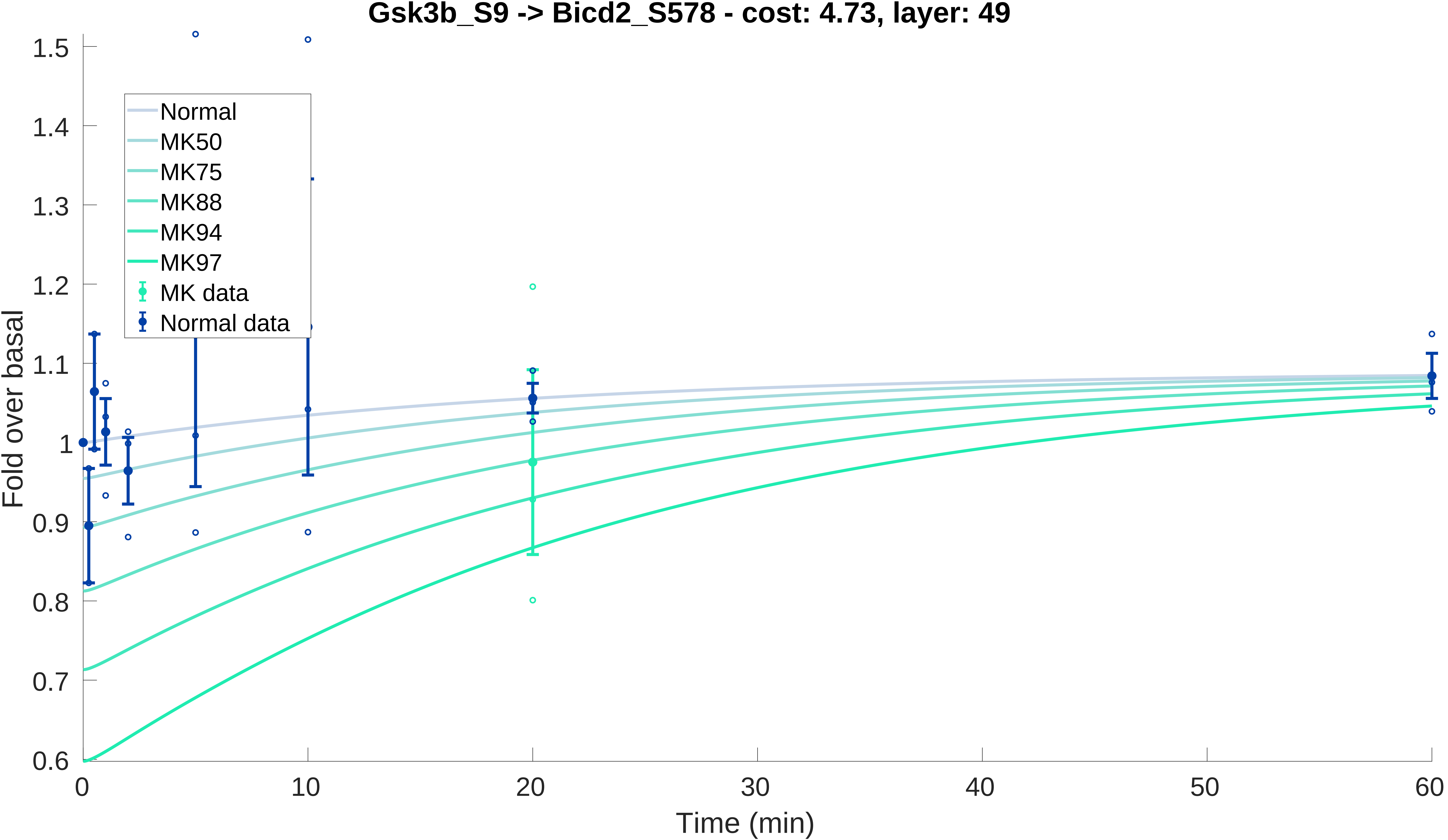

Gsk3b_S9 -> Bicd2_S578 - cost: 4.73, layer: 49
Normal
MK50
MK75
MK88
MK94
MK97
MK data
Normal data
Fold over basal
Time (min)
1.5
1.4
1.3
1.2
1.1
1
0.9
0.8
0.7
0.6
0
10
20
30
40
50
60

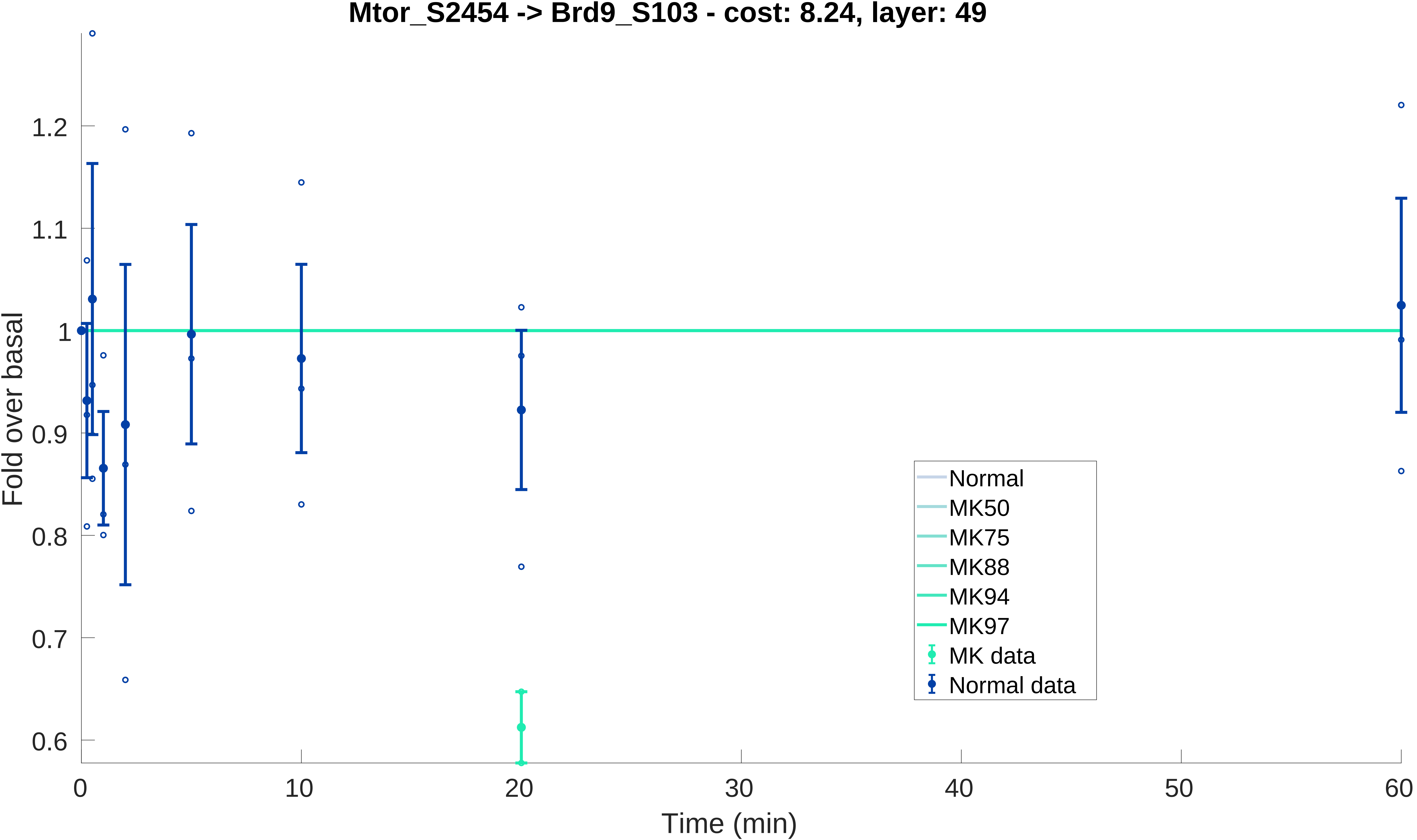
Mtor_S2454 -> Brd9_S103 - cost: 8.24, layer: 49
Fold over basal
Time (min)
0.6
0.7
0.8
0.9
1
1.1
1.2
0
10
20
30
40
50
60
Normal
MK50
MK75
MK88
MK94
MK97
MK data
Normal data

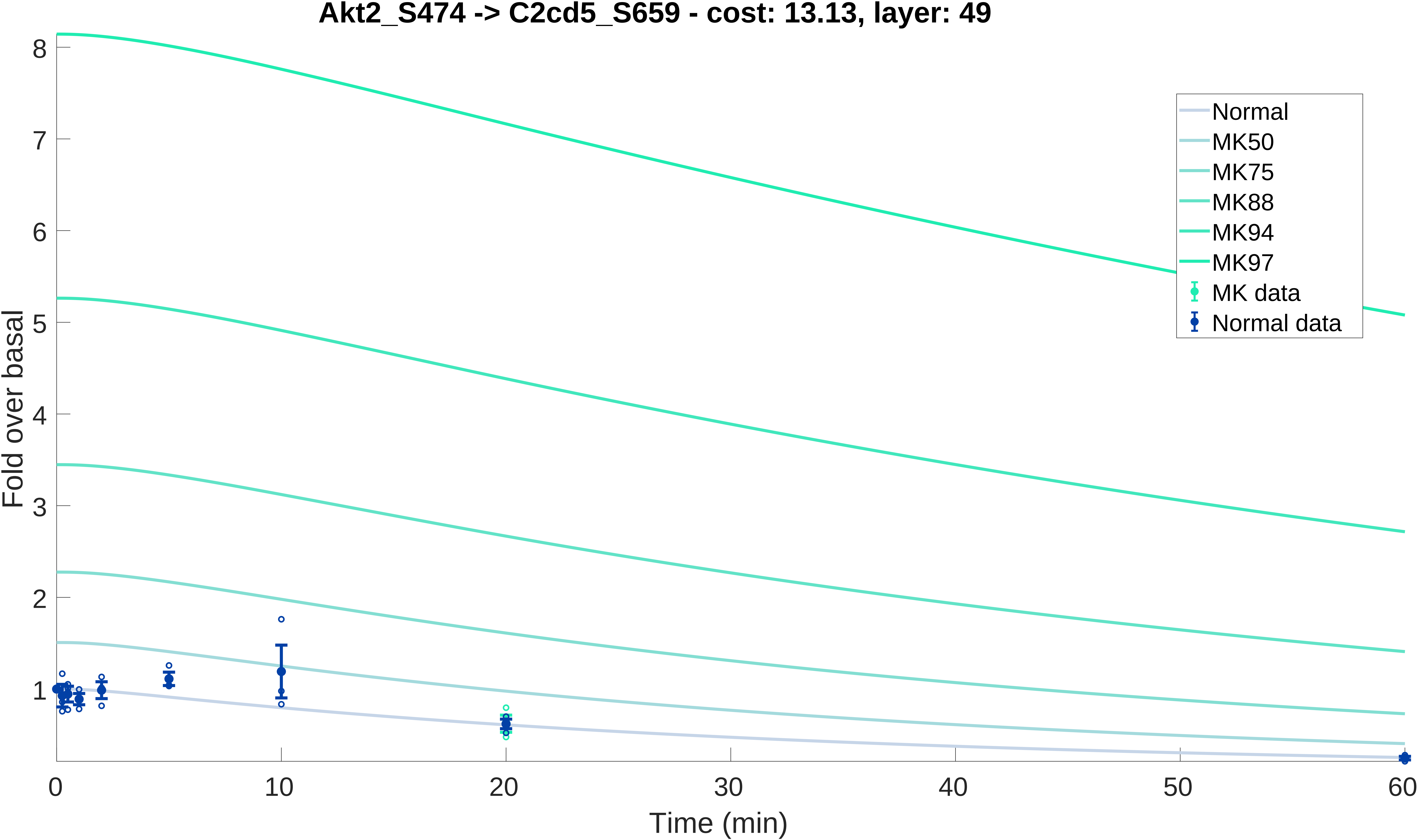
Akt2_S474 -> C2cd5_S659 - cost: 13.13, layer: 49
Fold over basal
Time (min)
Normal
MK50
MK75
MK88
MK94
MK97
MK data
Normal data
0
10
20
30
40
50
60
1
2
3
4
5
6
7
8

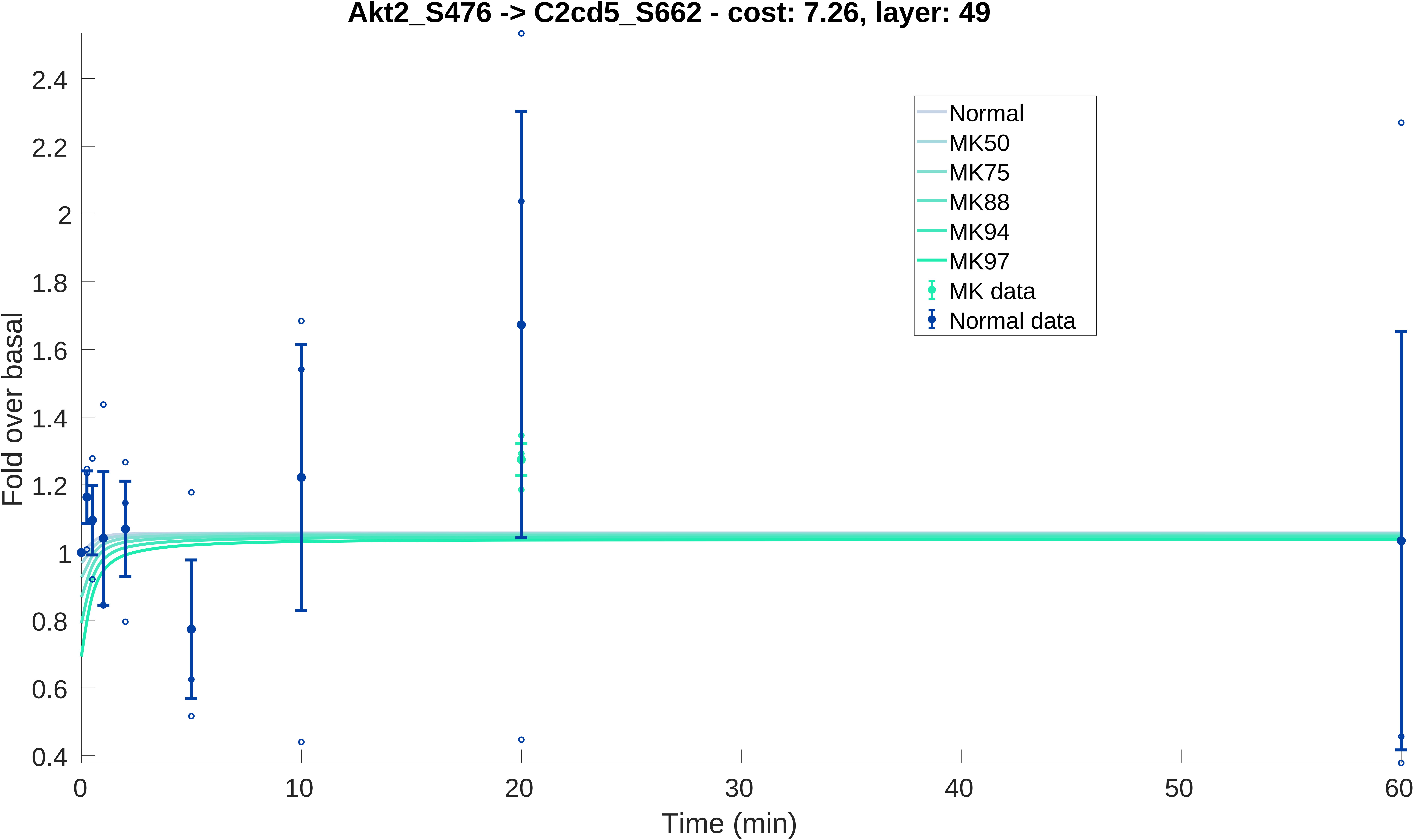

Akt2_S476 -> C2cd5_S662 - cost: 7.26, layer: 49
Fold over basal
Time (min)
Normal
MK50
MK75
MK88
MK94
MK97
MK data
Normal data
0.4
0.6
0.8
1
1.2
1.4
1.6
1.8
2
2.2
2.4
0
10
20
30
40
50
60

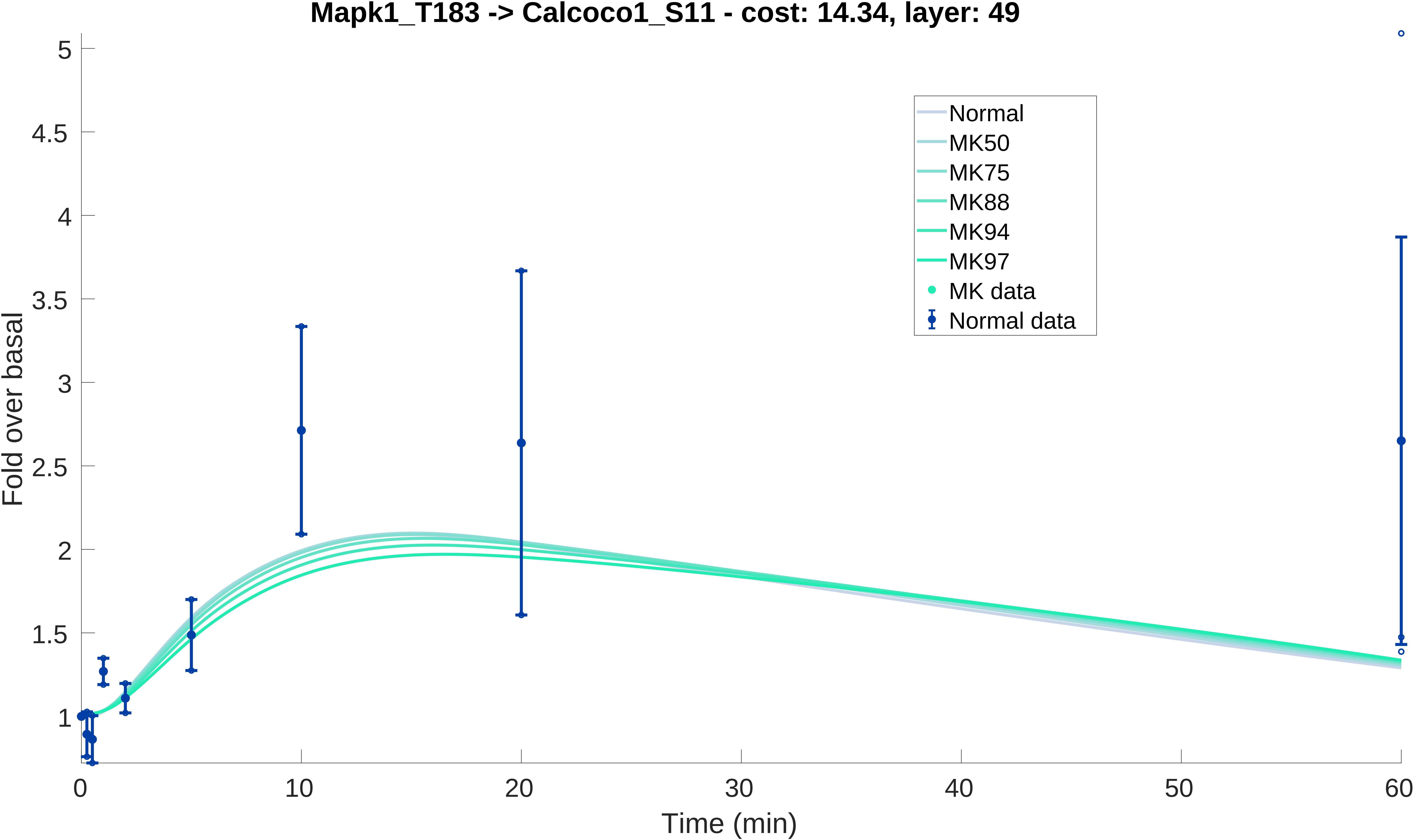
Mapk1_T183 -> Calcoco1_S11 - cost: 14.34, layer: 49
Fold over basal
Time (min)
Normal
MK50
MK75
MK88
MK94
MK97
MK data
Normal data
5
4.5
4
3.5
3
2.5
2
1.5
1
0
10
20
30
40
50
60

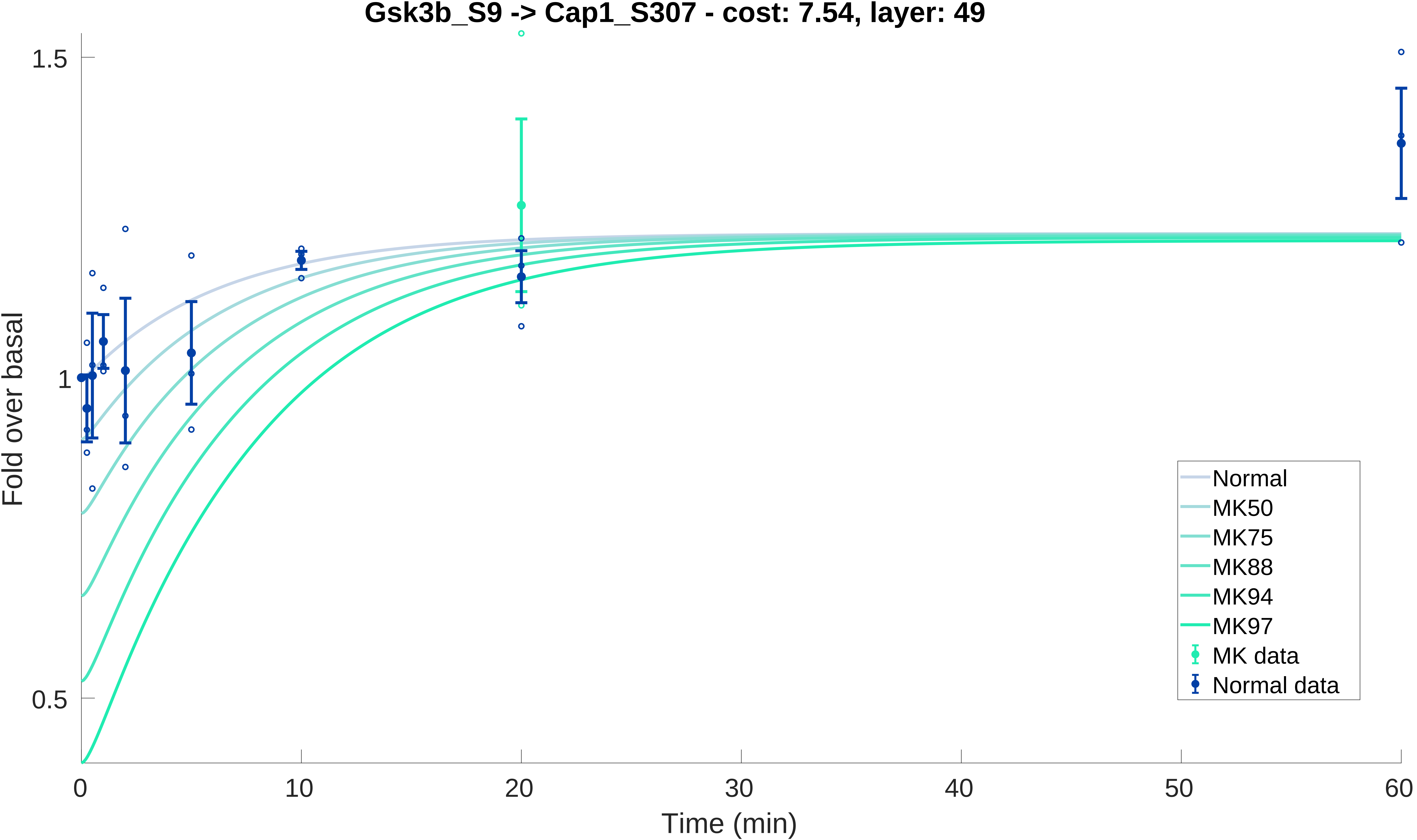

Gsk3b_S9 -> Cap1_S307 - cost: 7.54, layer: 49
Fold over basal
1.5
1
0.5
0
10
20
30
40
50
60
Time (min)
Normal
MK50
MK75
MK88
MK94
MK97
MK data
Normal data

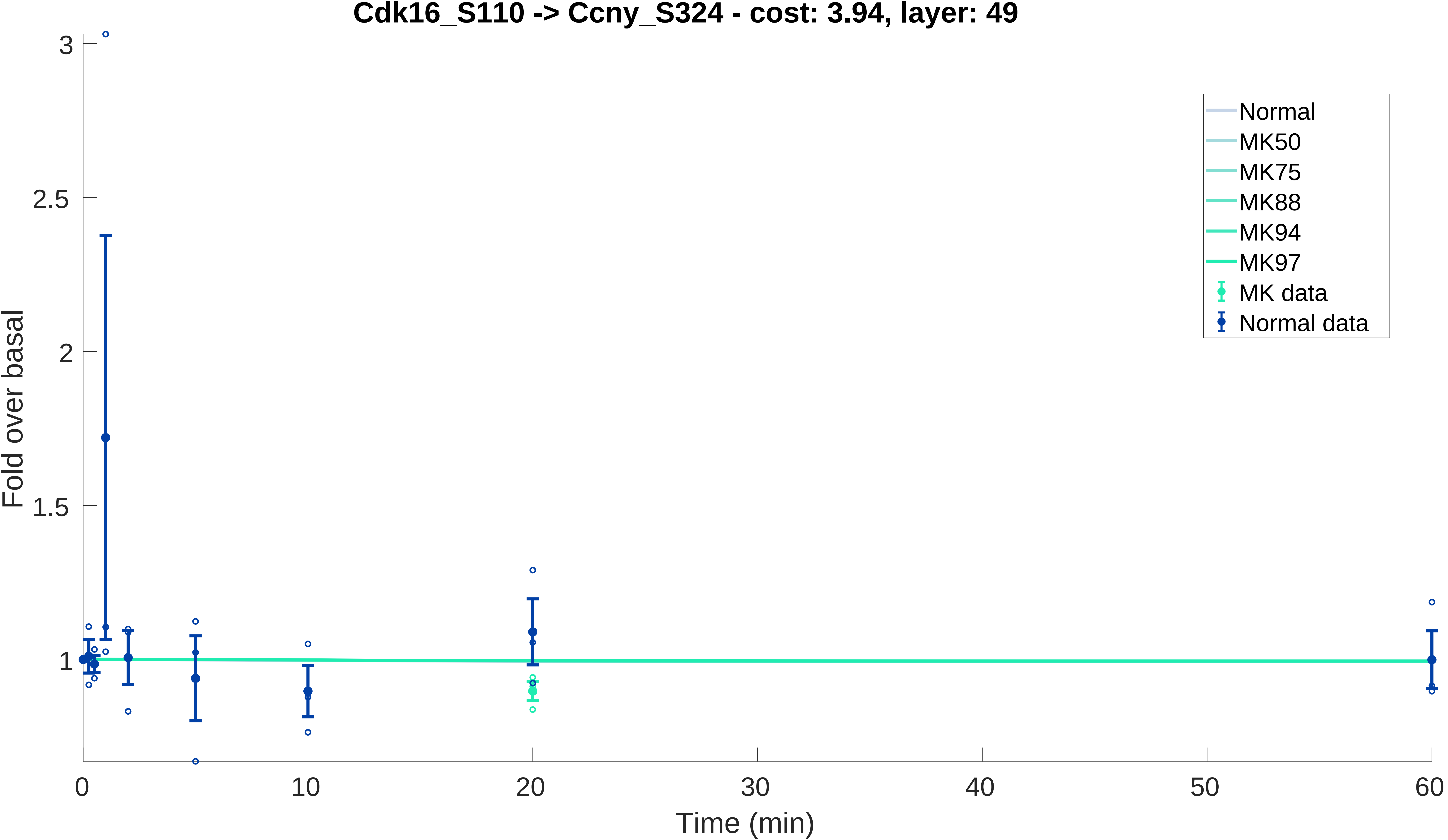

Cdk16_S110 -> Ccny_S324 - cost: 3.94, layer: 49
Fold over basal
Time (min)
3
2.5
2
1.5
1
0
10
20
30
40
50
60
Normal
MK50
MK75
MK88
MK94
MK97
MK data
Normal data

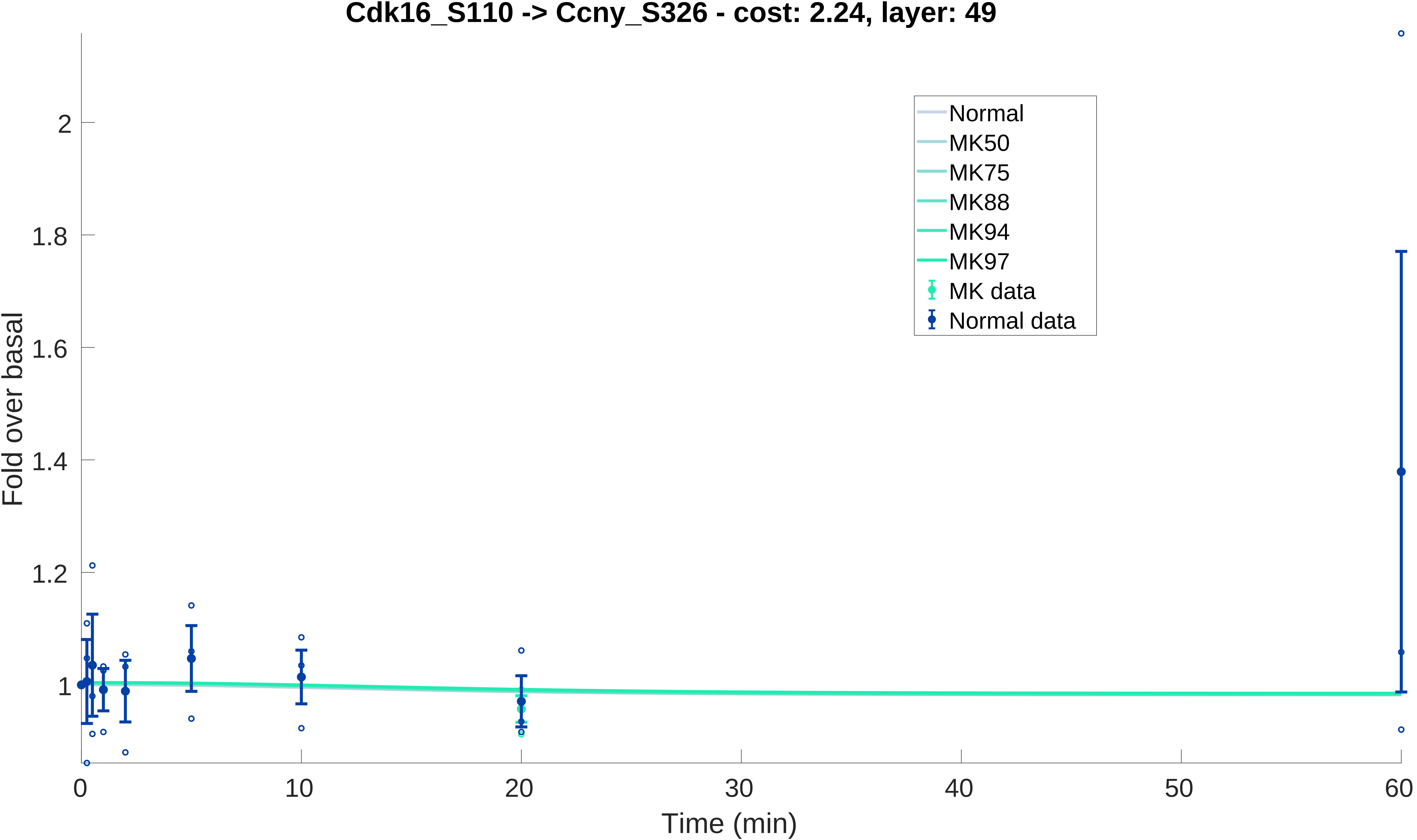
Cdk16_S110 -> Ccny_S326 - cost: 2.24, layer: 49
Fold over basal
Time (min)
Normal
MK50
MK75
MK88
MK94
MK97
MK data
Normal data
2
1.8
1.6
1.4
1.2
1
0
10
20
30
40
50
60

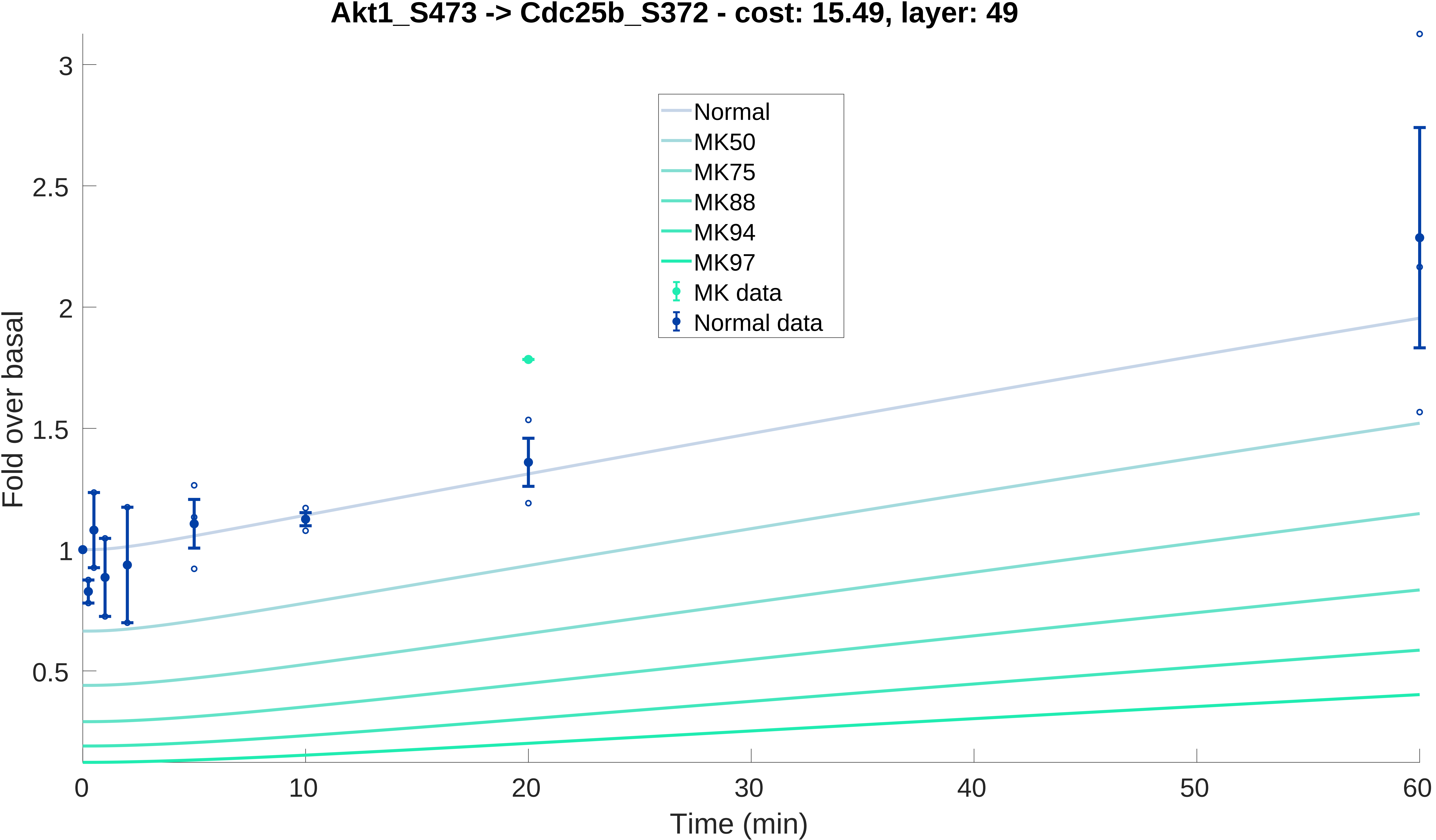

Akt1_S473 -> Cdc25b_S372 - cost: 15.49, layer: 49
Normal
MK50
MK75
MK88
MK94
MK97
MK data
Normal data
Fold over basal
Time (min)
0.5
1
1.5
2
2.5
3
0
10
20
30
40
50
60

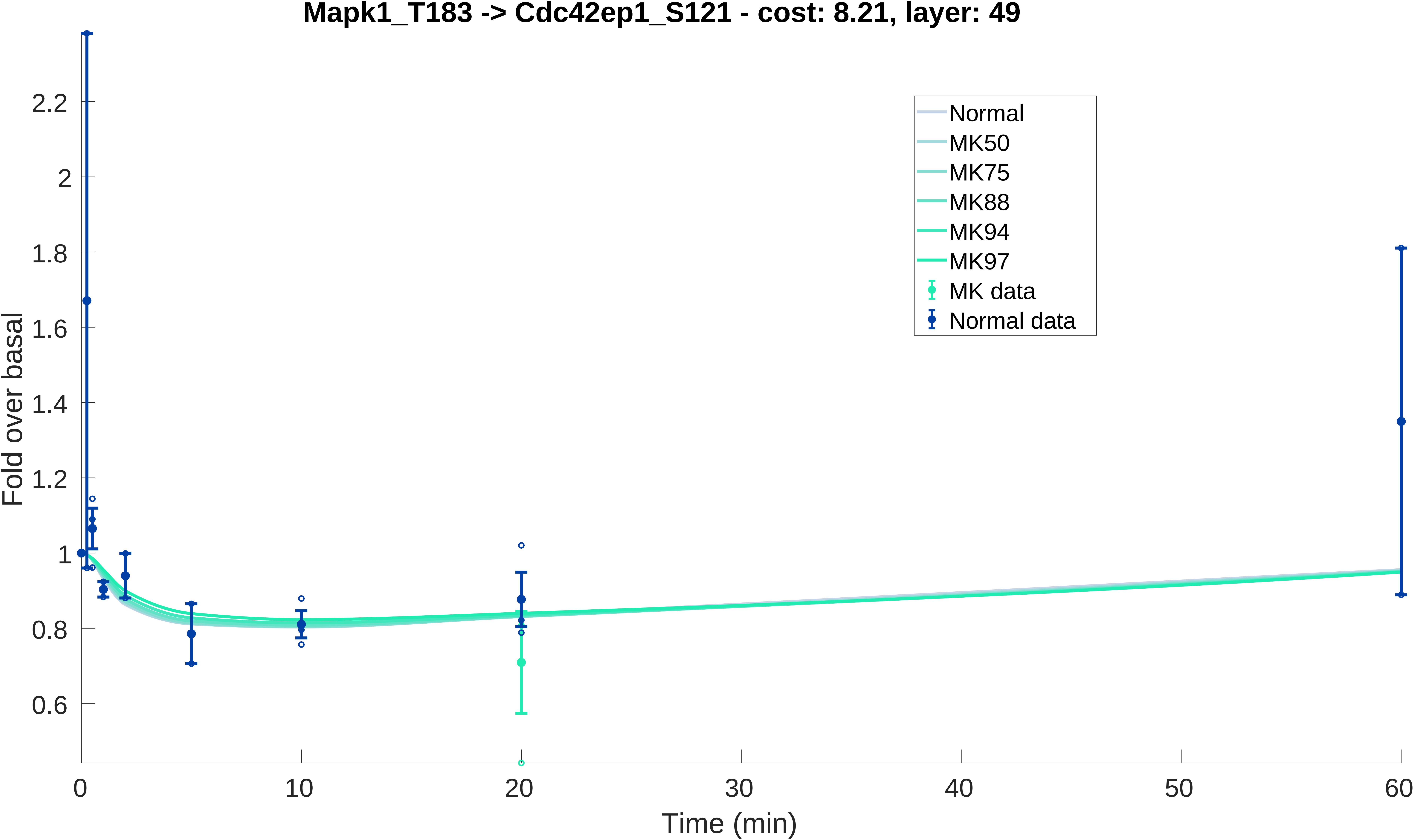
Mapk1_T183 -> Cdc42ep1_S121 - cost: 8.21, layer: 49
Fold over basal
Time (min)
0.6
0.8
1
1.2
1.4
1.6
1.8
2
2.2
0
10
20
30
40
50
60
Normal
MK50
MK75
MK88
MK94
MK97
MK data
Normal data

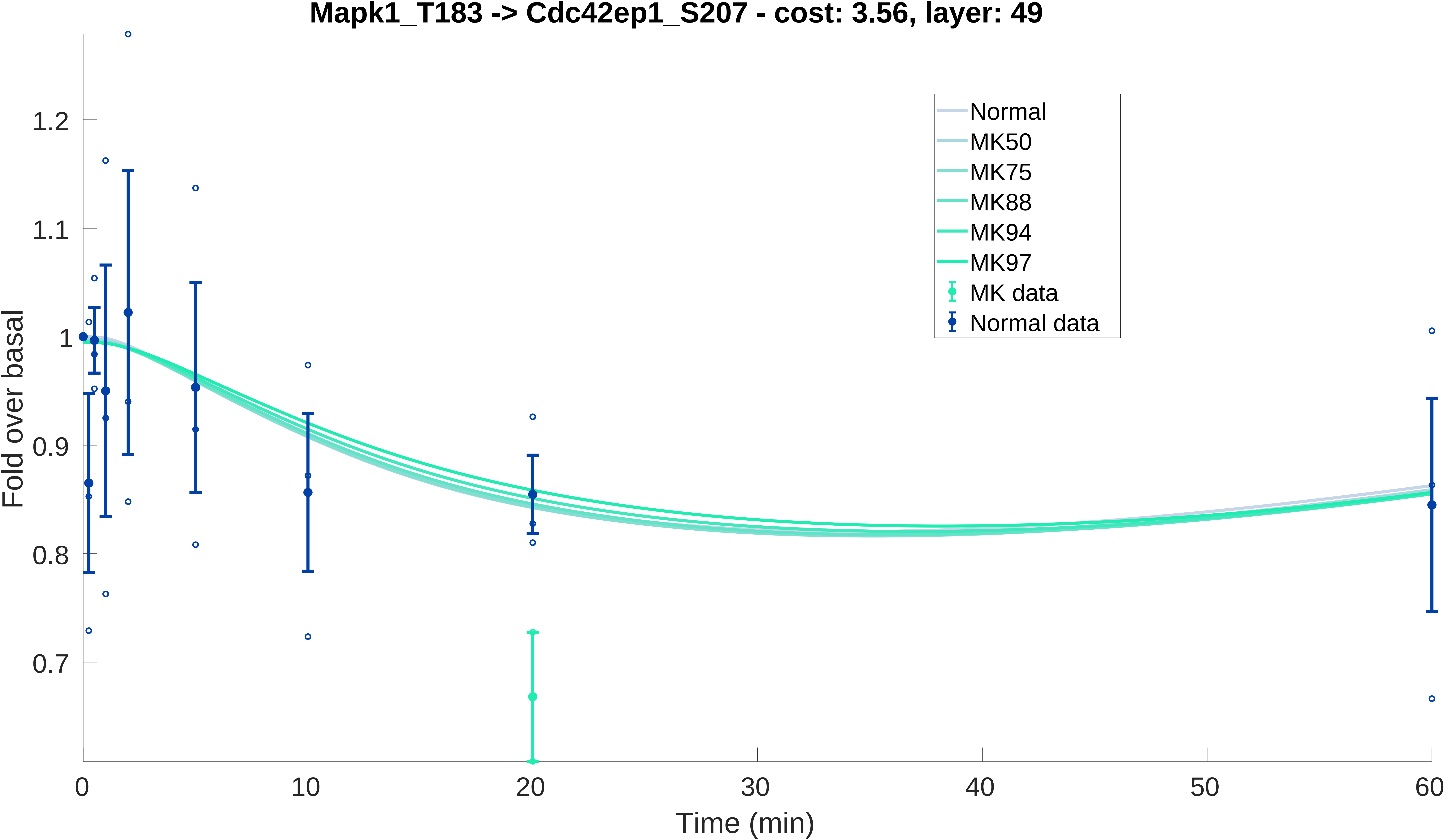

Mapk1_T183 -> Cdc42ep1_S207 - cost: 3.56, layer: 49
Fold over basal
Time (min)
0
10
20
30
40
50
60
0.7
0.8
0.9
1
1.1
1.2
Normal
MK50
MK75
MK88
MK94
MK97
MK data
Normal data

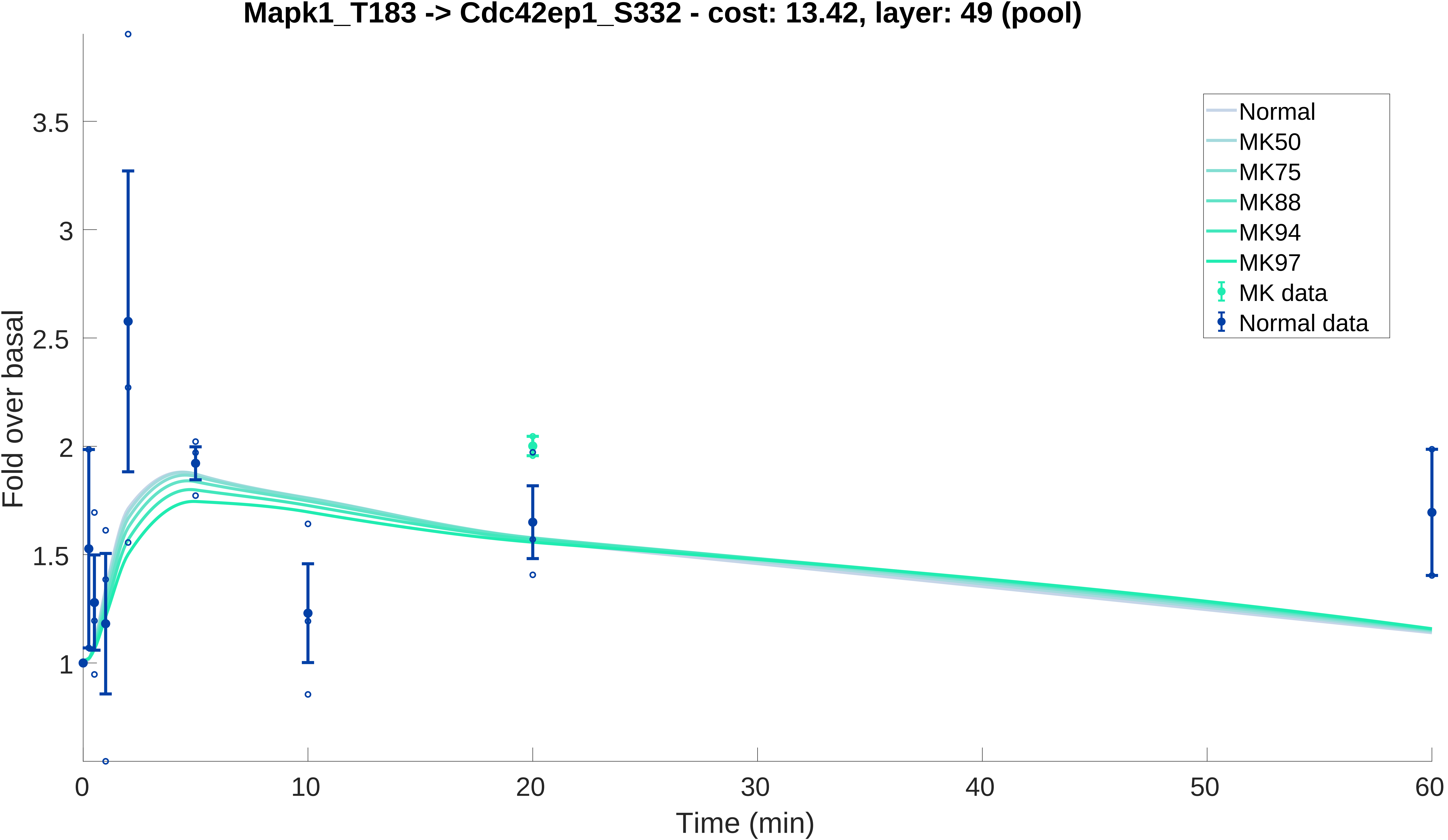
Mapk1_T183 -> Cdc42ep1_S332 - cost: 13.42, layer: 49 (pool)
Fold over basal
Time (min)
0
10
20
30
40
50
60
1
1.5
2
2.5
3
3.5
Normal
MK50
MK75
MK88
MK94
MK97
MK data
Normal data

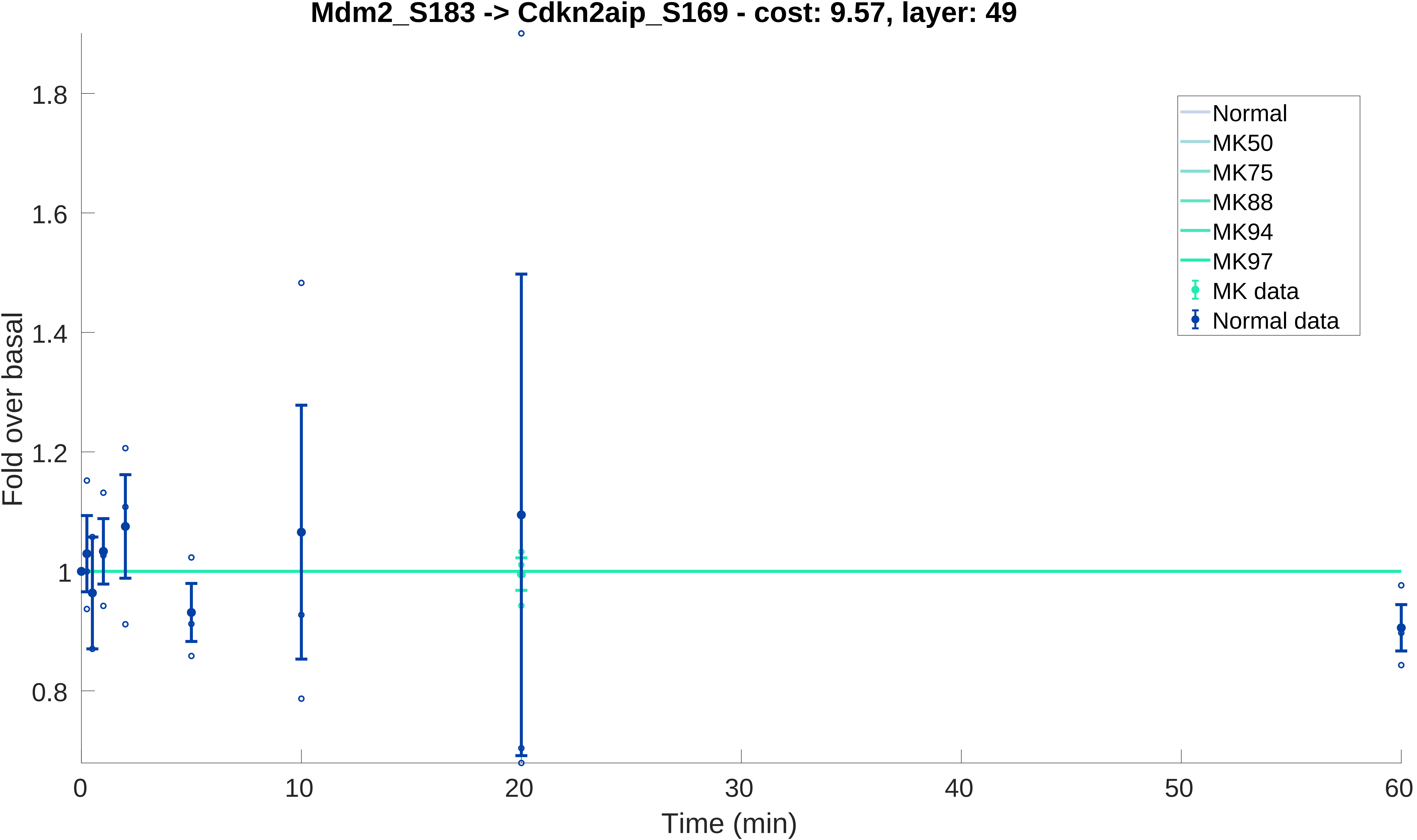

Mdm2_S183 -> Cdkn2aip_S169 - cost: 9.57, layer: 49
Fold over basal
Time (min)
0.8
1
1.2
1.4
1.6
1.8
0
10
20
30
40
50
60
Normal
MK50
MK75
MK88
MK94
MK97
MK data
Normal data

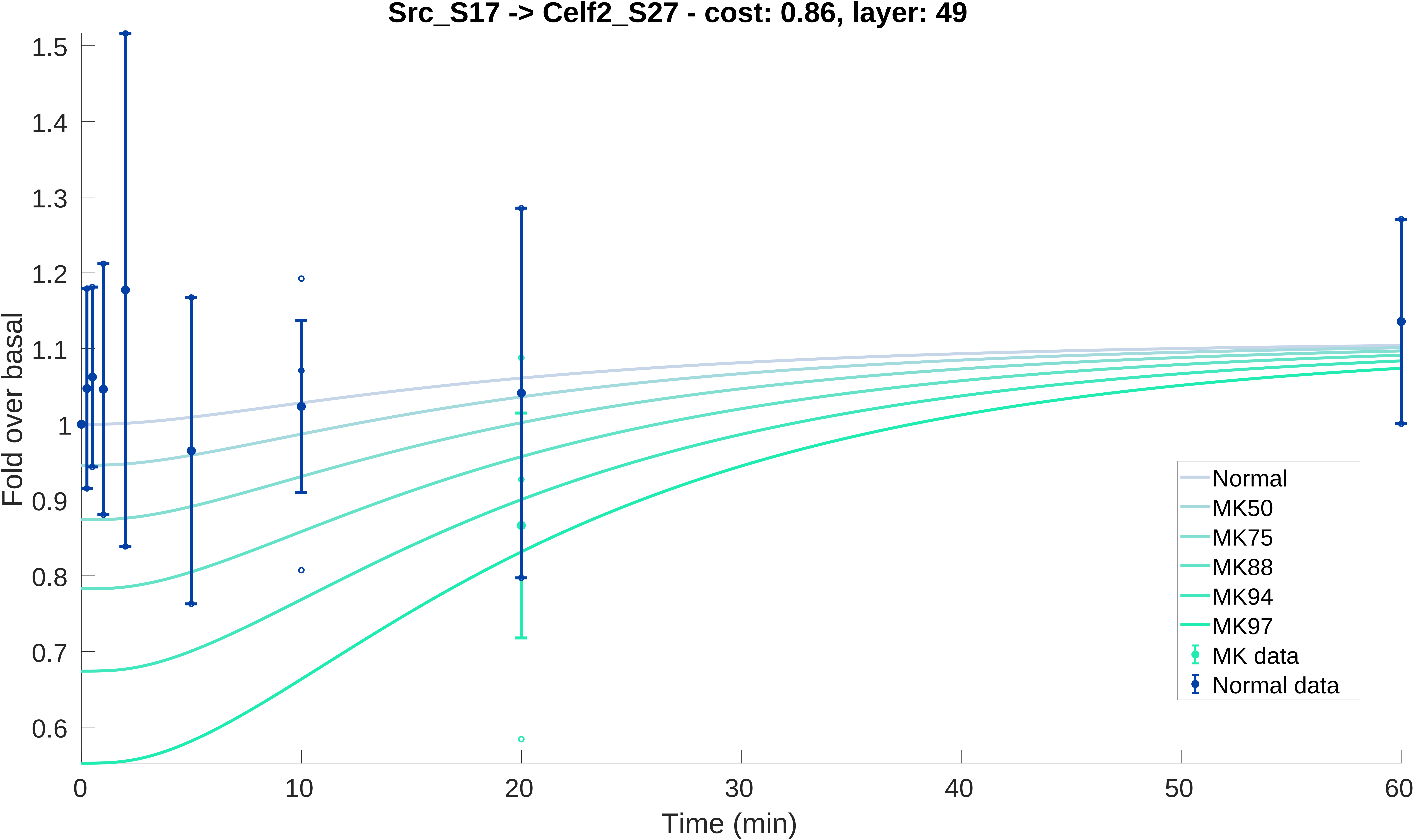

Src_S17 -> Celf2_S27 - cost: 0.86, layer: 49
Fold over basal
Time (min)
0
10
20
30
40
50
60
0.6
0.7
0.8
0.9
1
1.1
1.2
1.3
1.4
1.5
Normal
MK50
MK75
MK88
MK94
MK97
MK data
Normal data

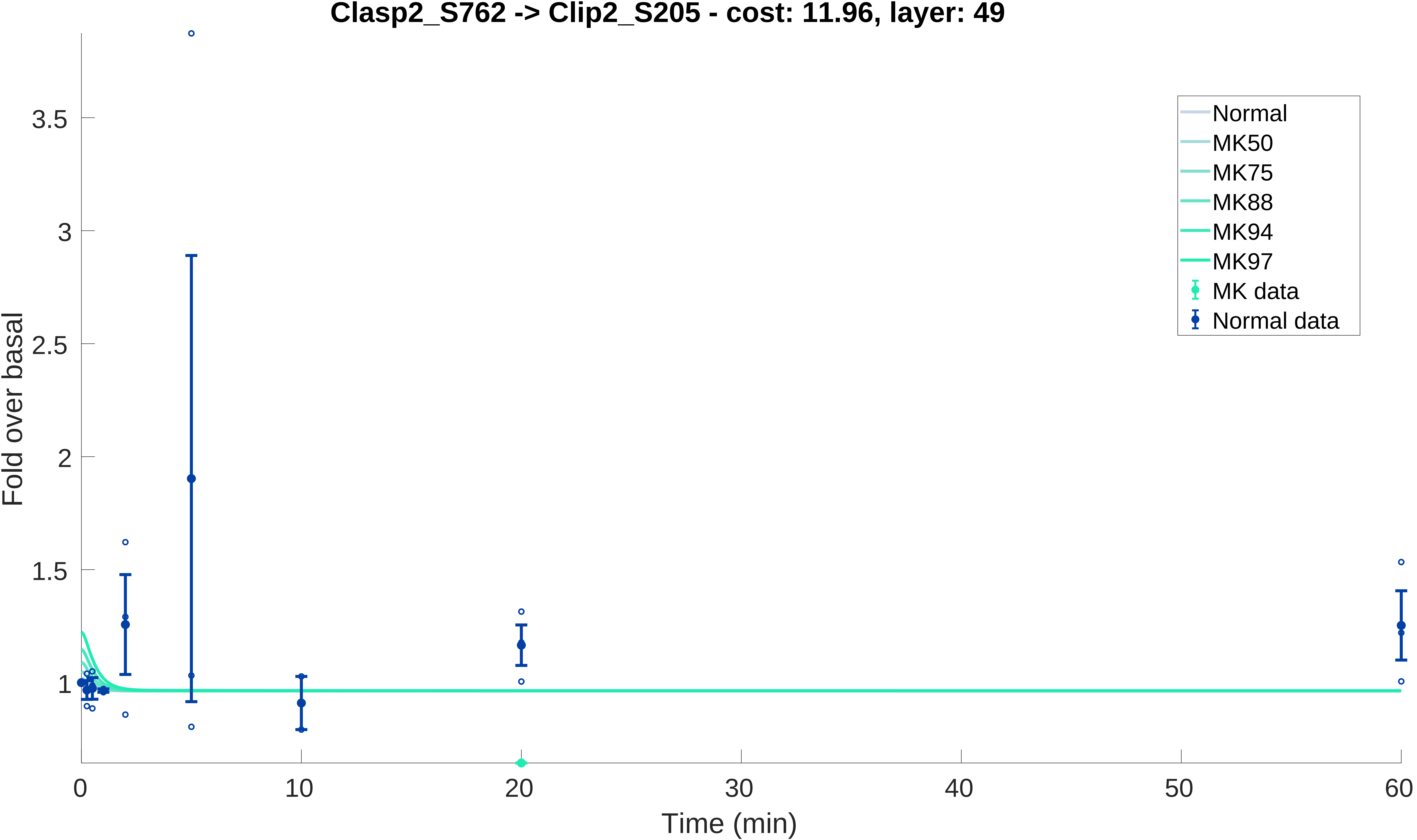

Clasp2_S762 -> Clip2_S205 - cost: 11.96, layer: 49
Fold over basal
Time (min)
Normal
MK50
MK75
MK88
MK94
MK97
MK data
Normal data
0
10
20
30
40
50
60
1
1.5
2
2.5
3
3.5

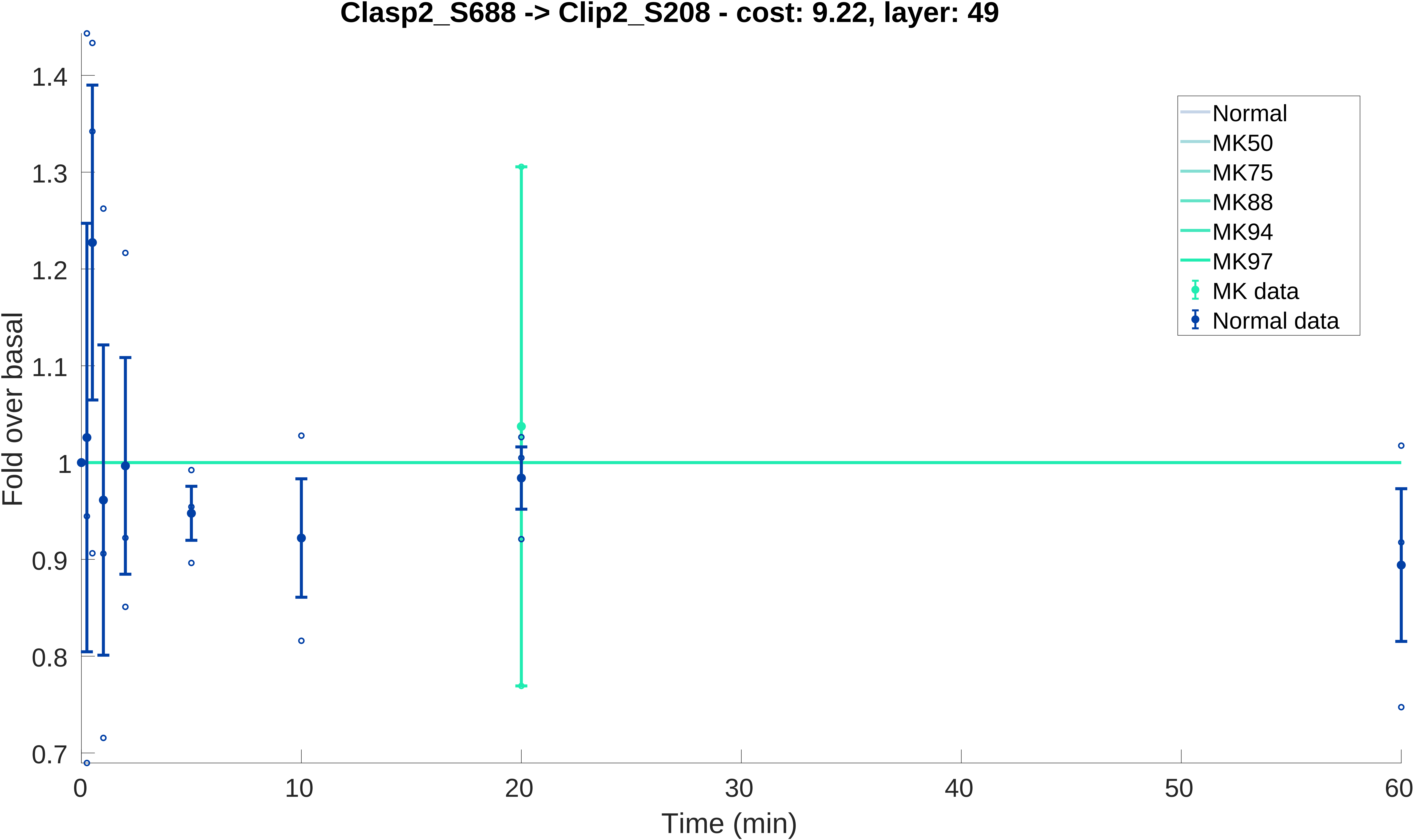

Clasp2_S688 -> Clip2_S208 - cost: 9.22, layer: 49
Fold over basal
Time (min)
1.4
1.3
1.2
1.1
1
0.9
0.8
0.7
0
10
20
30
40
50
60
Normal
MK50
MK75
MK88
MK94
MK97
MK data
Normal data

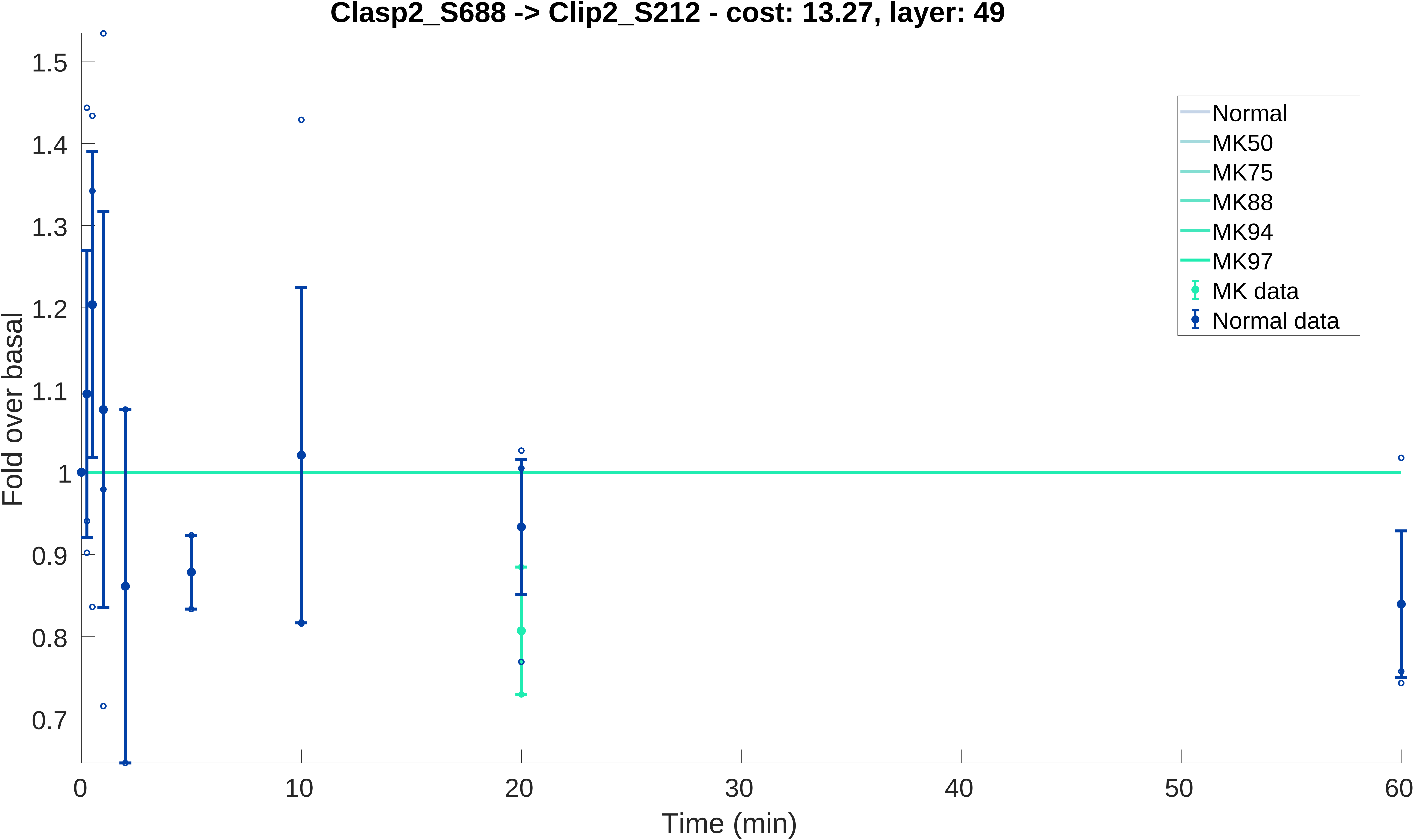
Clasp2_S688 -> Clip2_S212 - cost: 13.27, layer: 49
Fold over basal
Time (min)
Normal
MK50
MK75
MK88
MK94
MK97
MK data
Normal data
0
10
20
30
40
50
60
0.7
0.8
0.9
1
1.1
1.2
1.3
1.4
1.5

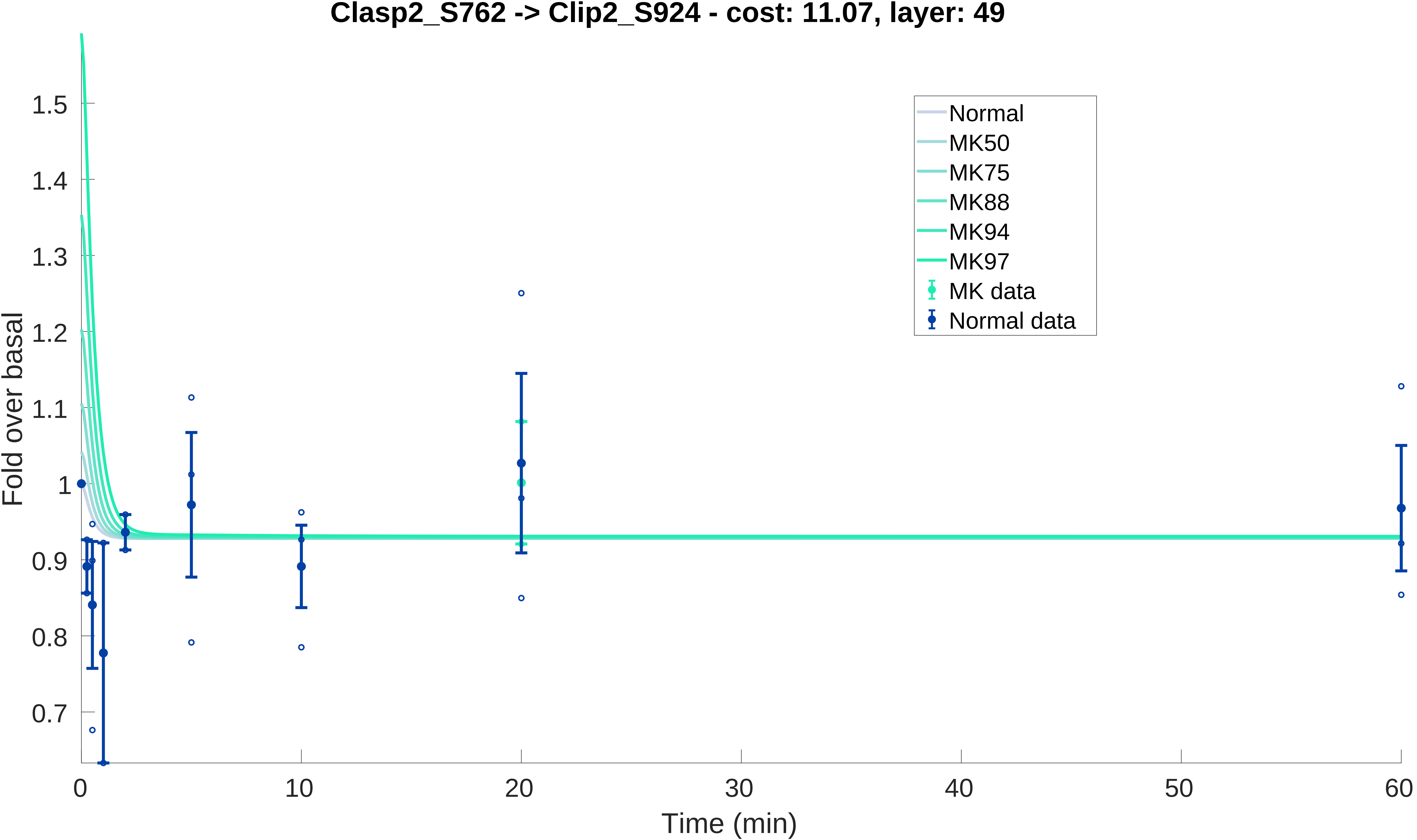
Clasp2_S762 -> Clip2_S924 - cost: 11.07, layer: 49
Fold over basal
1.5
1.4
1.3
1.2
1.1
1
0.9
0.8
0.7
0
10
20
30
40
50
60
Time (min)
Normal
MK50
MK75
MK88
MK94
MK97
MK data
Normal data

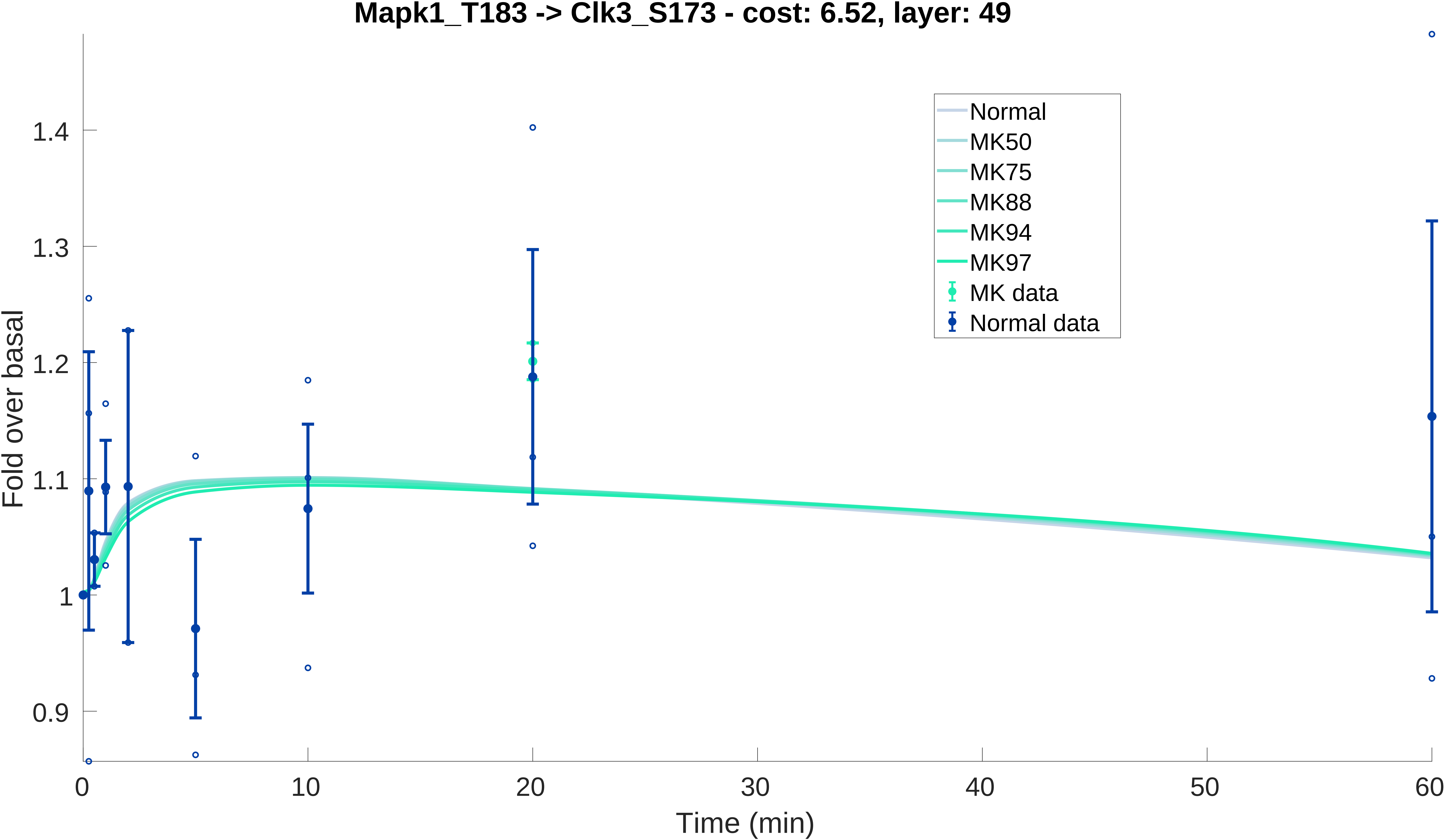

Mapk1_T183 -> Clk3_S173 - cost: 6.52, layer: 49
Fold over basal
Time (min)
0.9
1
1.1
1.2
1.3
1.4
0
10
20
30
40
50
60
Normal
MK50
MK75
MK88
MK94
MK97
MK data
Normal data

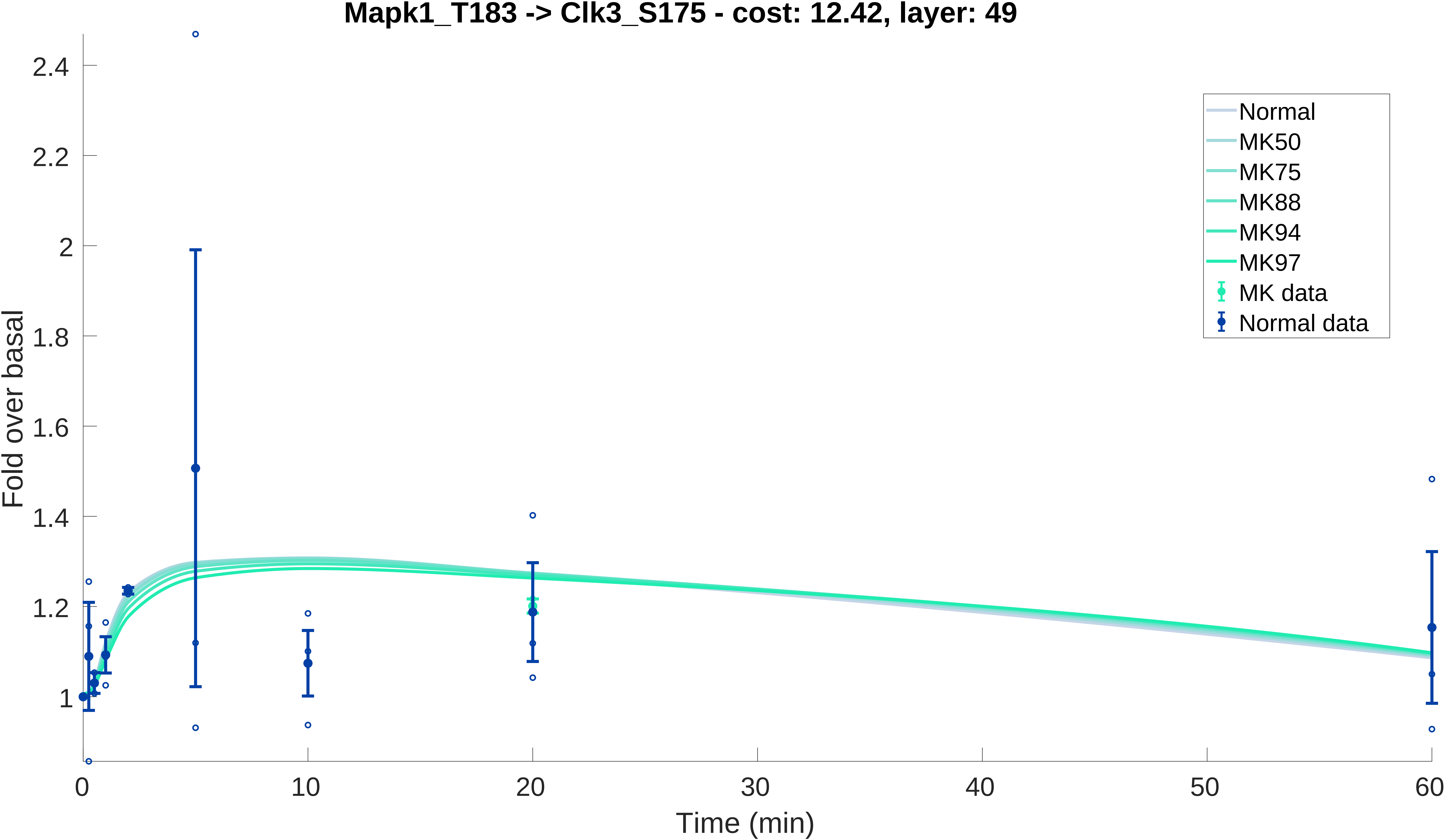

Mapk1_T183 -> Clk3_S175 - cost: 12.42, layer: 49
Fold over basal
Time (min)
2.4
2.2
2
1.8
1.6
1.4
1.2
1
0
10
20
30
40
50
60
Normal
MK50
MK75
MK88
MK94
MK97
MK data
Normal data

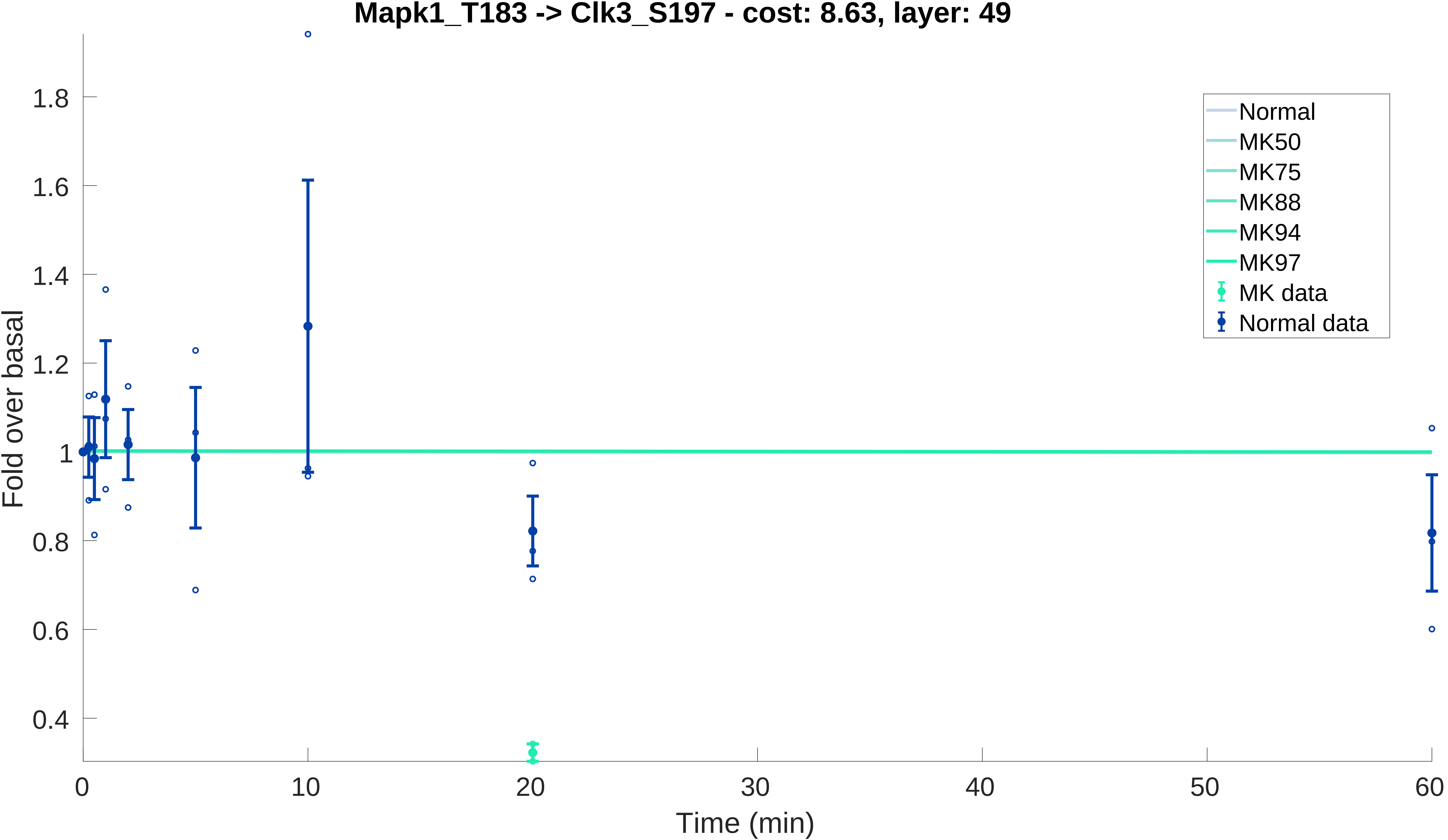
Mapk1_T183 -> Clk3_S197 - cost: 8.63, layer: 49
Fold over basal
Time (min)
Normal
MK50
MK75
MK88
MK94
MK97
MK data
Normal data
0
10
20
30
40
50
60
0.4
0.6
0.8
1
1.2
1.4
1.6
1.8

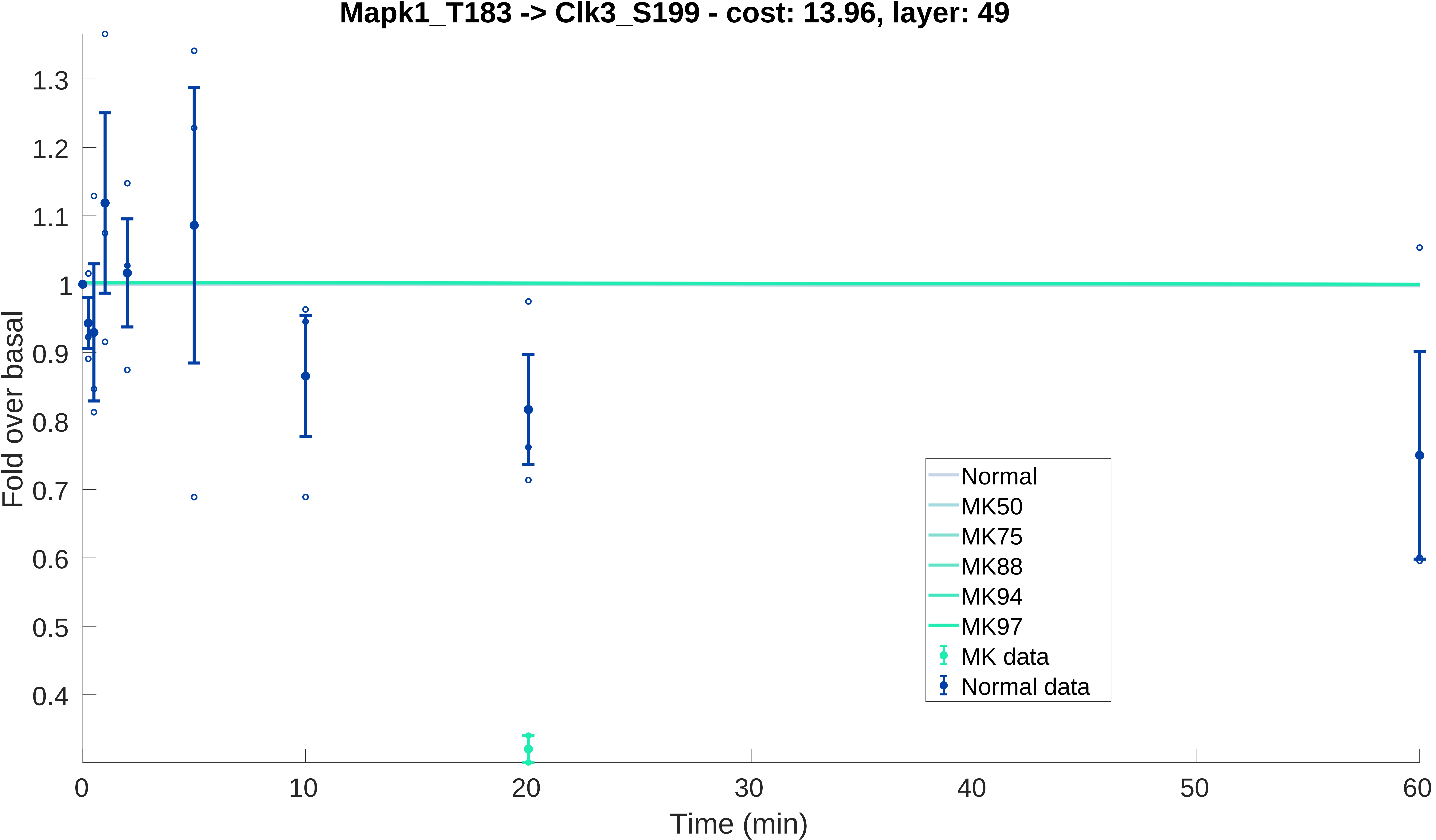

Mapk1_T183 -> Clk3_S199 - cost: 13.96, layer: 49
Fold over basal
Time (min)
1.3
1.2
1.1
1
0.9
0.8
0.7
0.6
0.5
0.4
0
10
20
30
40
50
60
Normal
MK50
MK75
MK88
MK94
MK97
MK data
Normal data

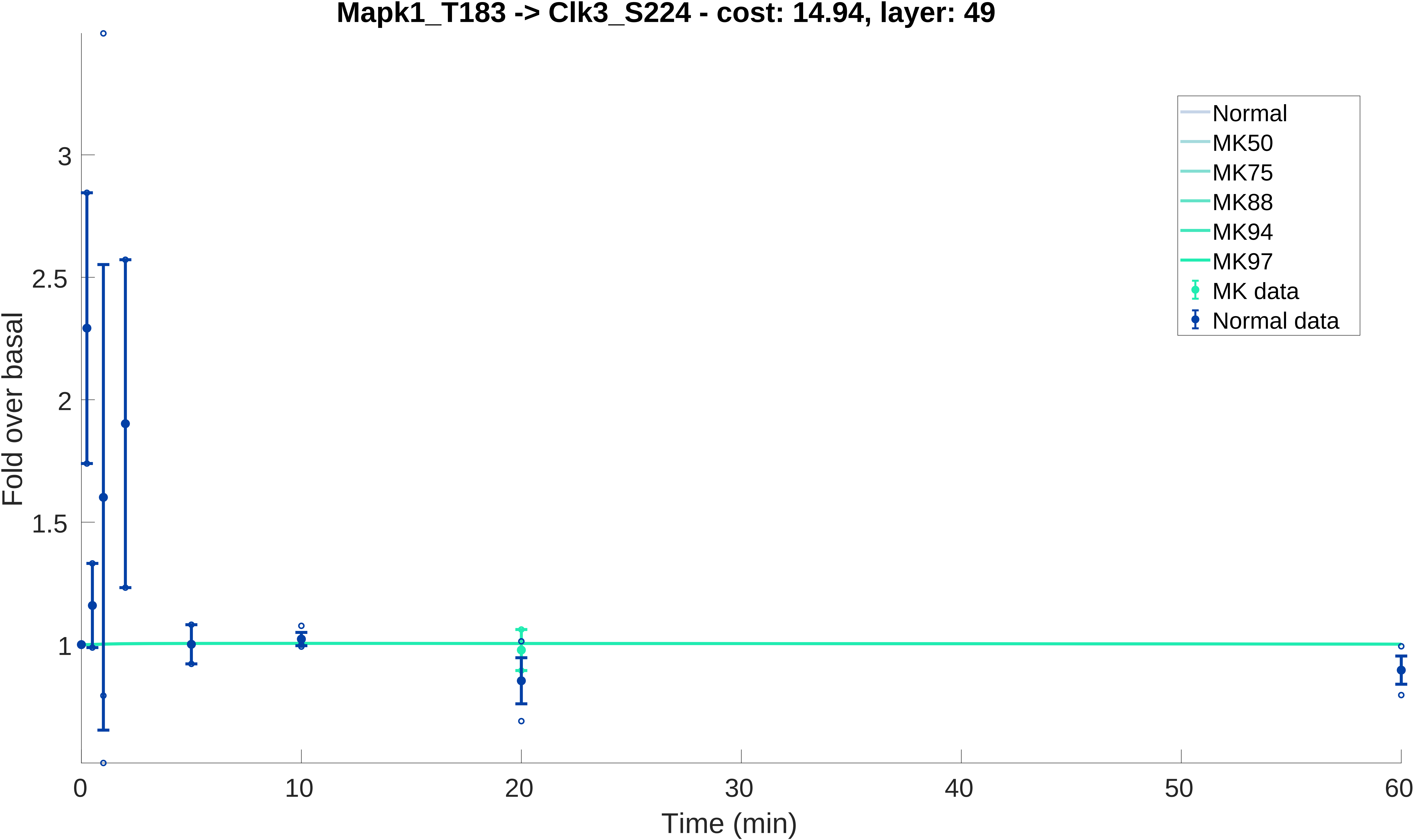

Mapk1_T183 -> Clk3_S224 - cost: 14.94, layer: 49
Fold over basal
3
2.5
2
1.5
1
0
10
20
30
40
50
60
Time (min)
Normal
MK50
MK75
MK88
MK94
MK97
MK data
Normal data

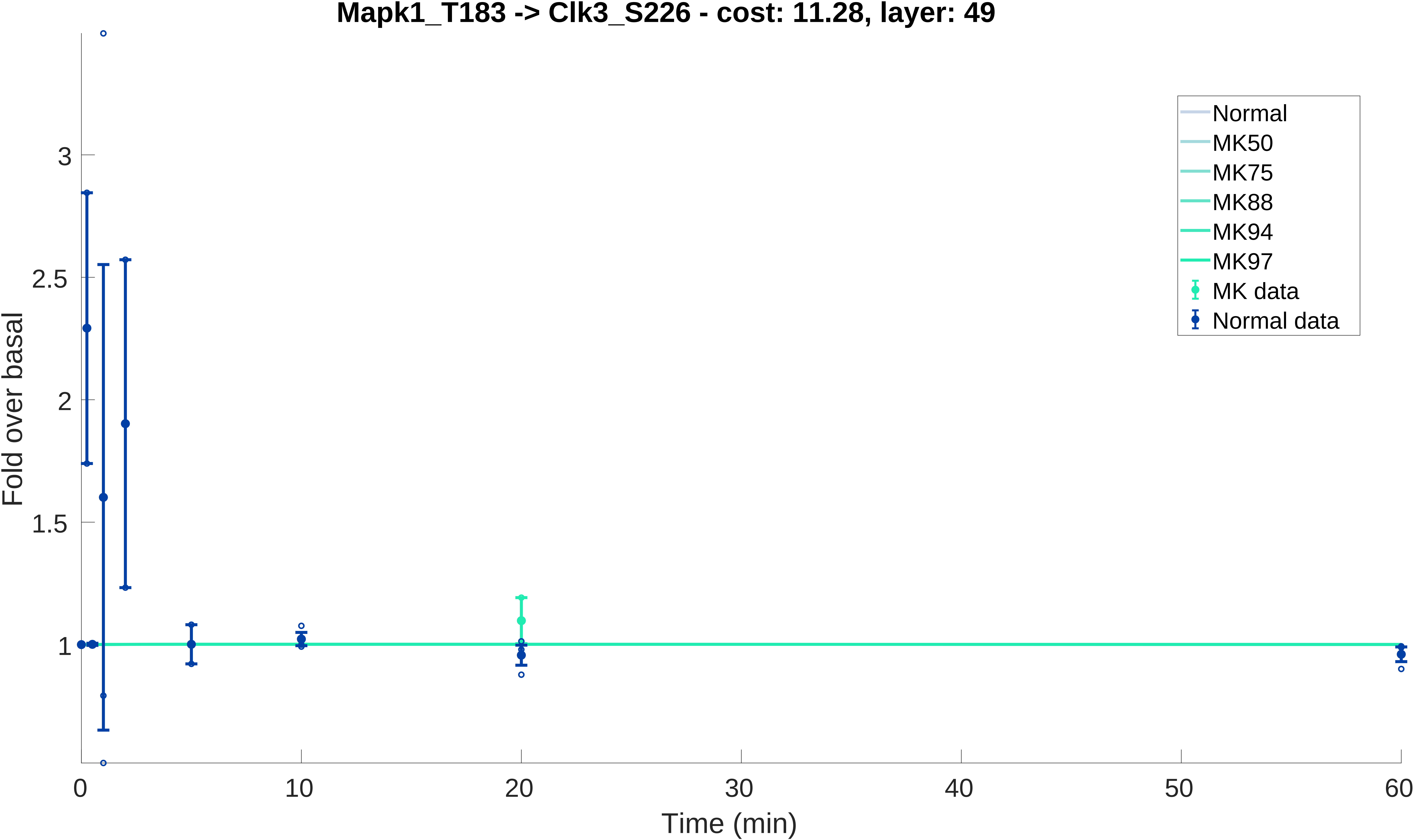

Mapk1_T183 -> Clk3_S226 - cost: 11.28, layer: 49
Fold over basal
Time (min)
3
2.5
2
1.5
1
0
10
20
30
40
50
60
Normal
MK50
MK75
MK88
MK94
MK97
MK data
Normal data

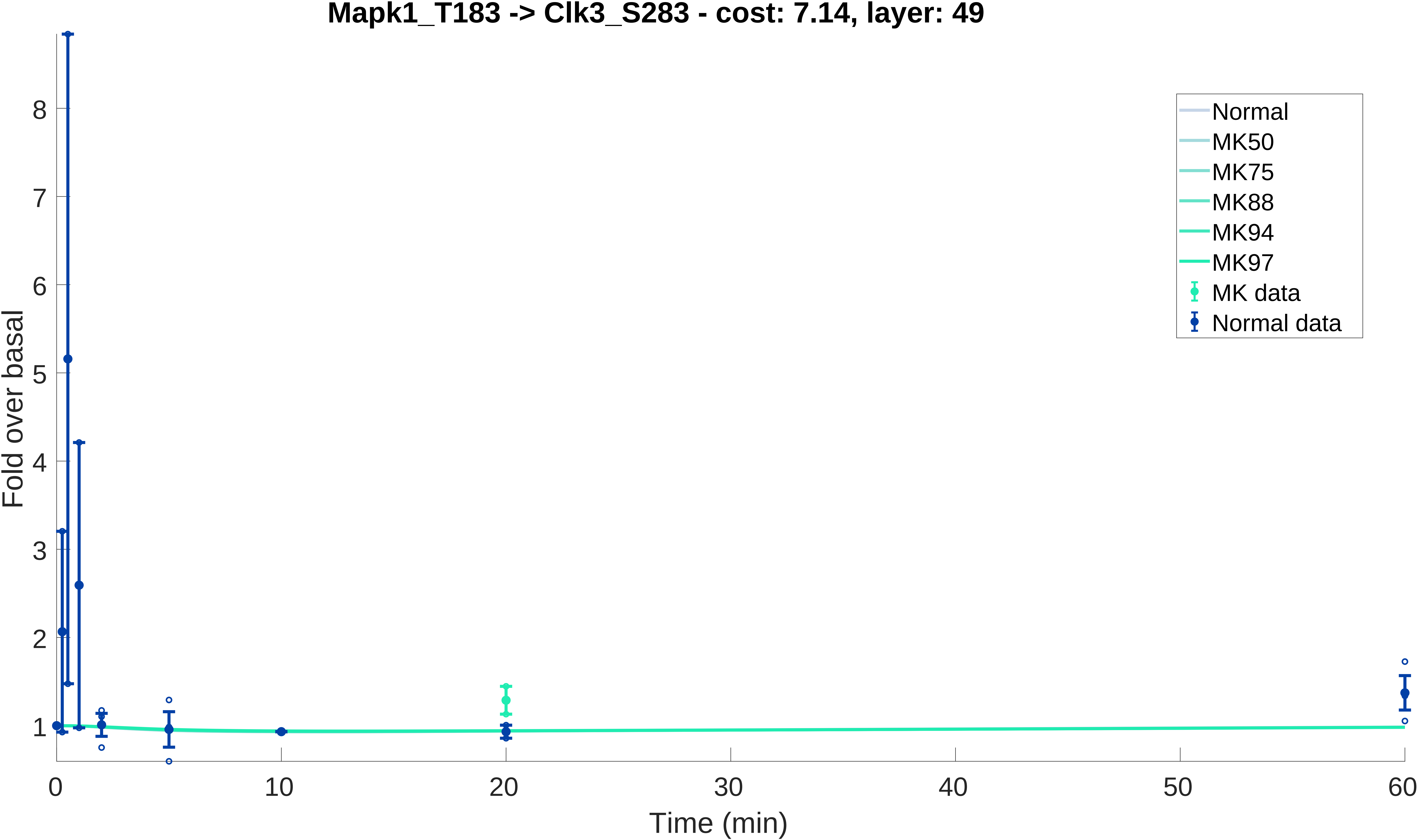
Mapk1_T183 -> Clk3_S283 - cost: 7.14, layer: 49
Fold over basal
Time (min)
Normal
MK50
MK75
MK88
MK94
MK97
MK data
Normal data
0
10
20
30
40
50
60
1
2
3
4
5
6
7
8

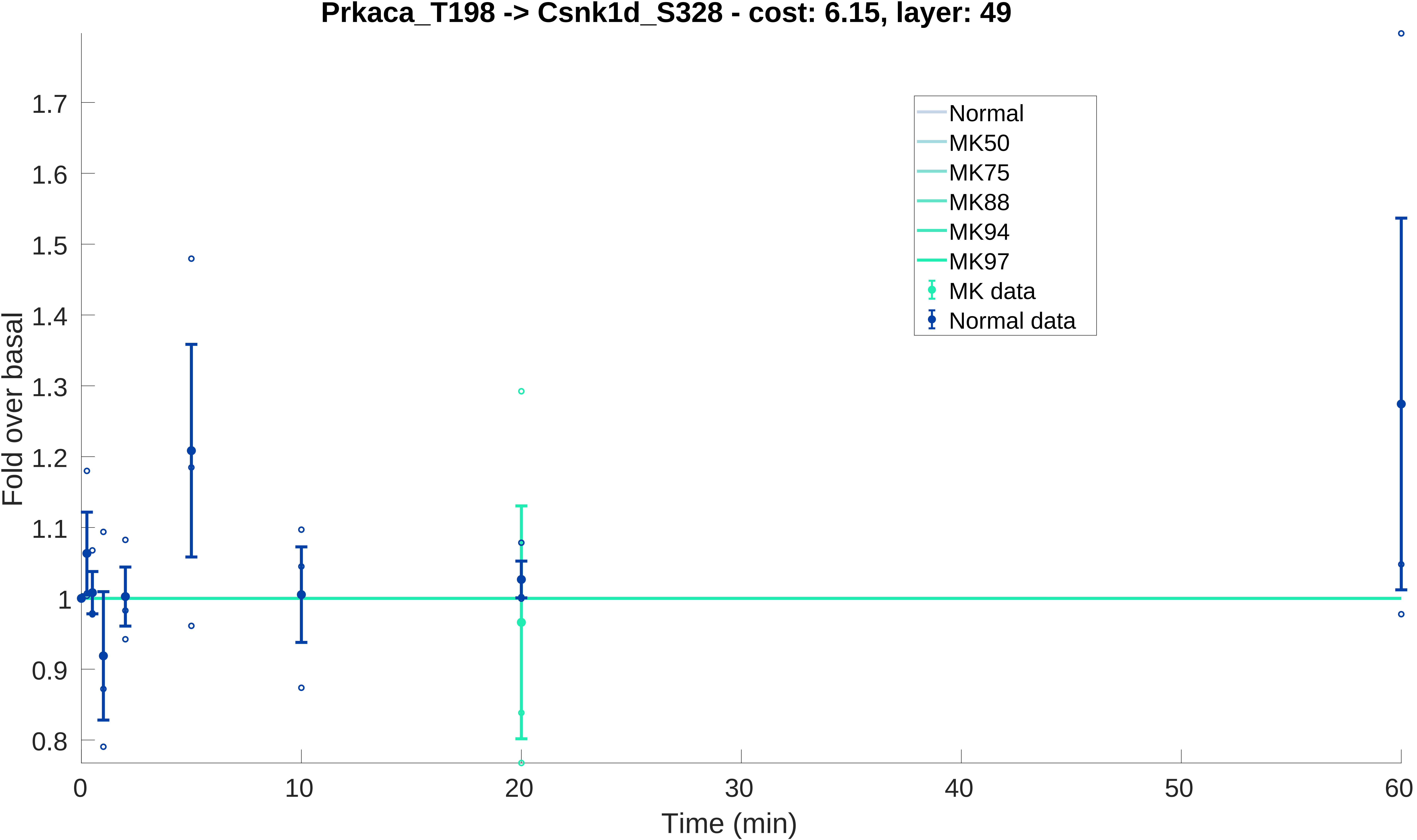

Prrkaca_T198 -> Csnk1d_S328 - cost: 6.15, layer: 49
Fold over basal
Time (min)
1.7
1.6
1.5
1.4
1.3
1.2
1.1
1
0.9
0.8
0
10
20
30
40
50
60
Normal
MK50
MK75
MK88
MK94
MK97
MK data
Normal data

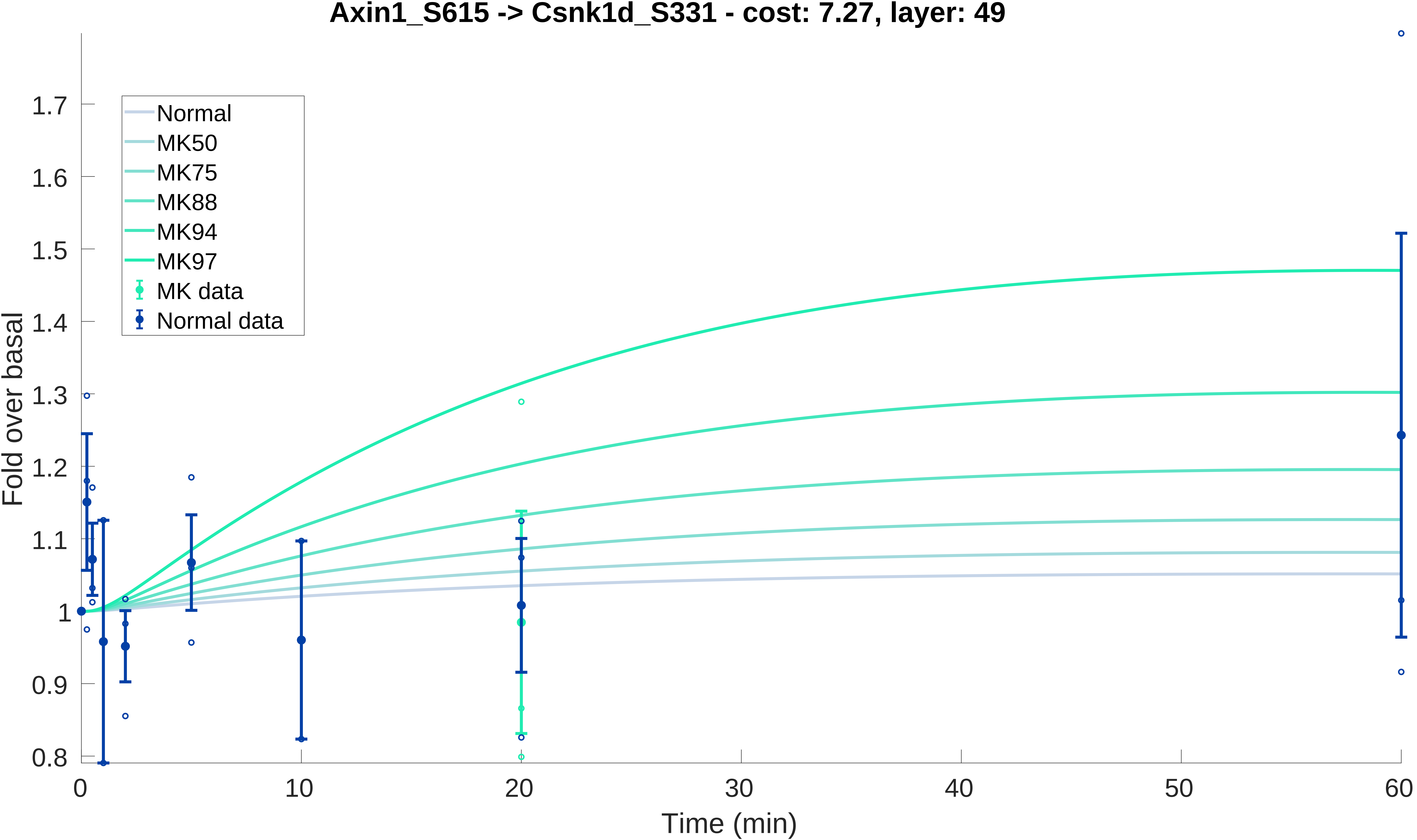
Axin1_S615 -> Csnk1d_S331 - cost: 7.27, layer: 49
Normal
MK50
MK75
MK88
MK94
MK97
MK data
Normal data
Fold over basal
Time (min)
0.8
0.9
1
1.1
1.2
1.3
1.4
1.5
1.6
1.7
0
10
20
30
40
50
60

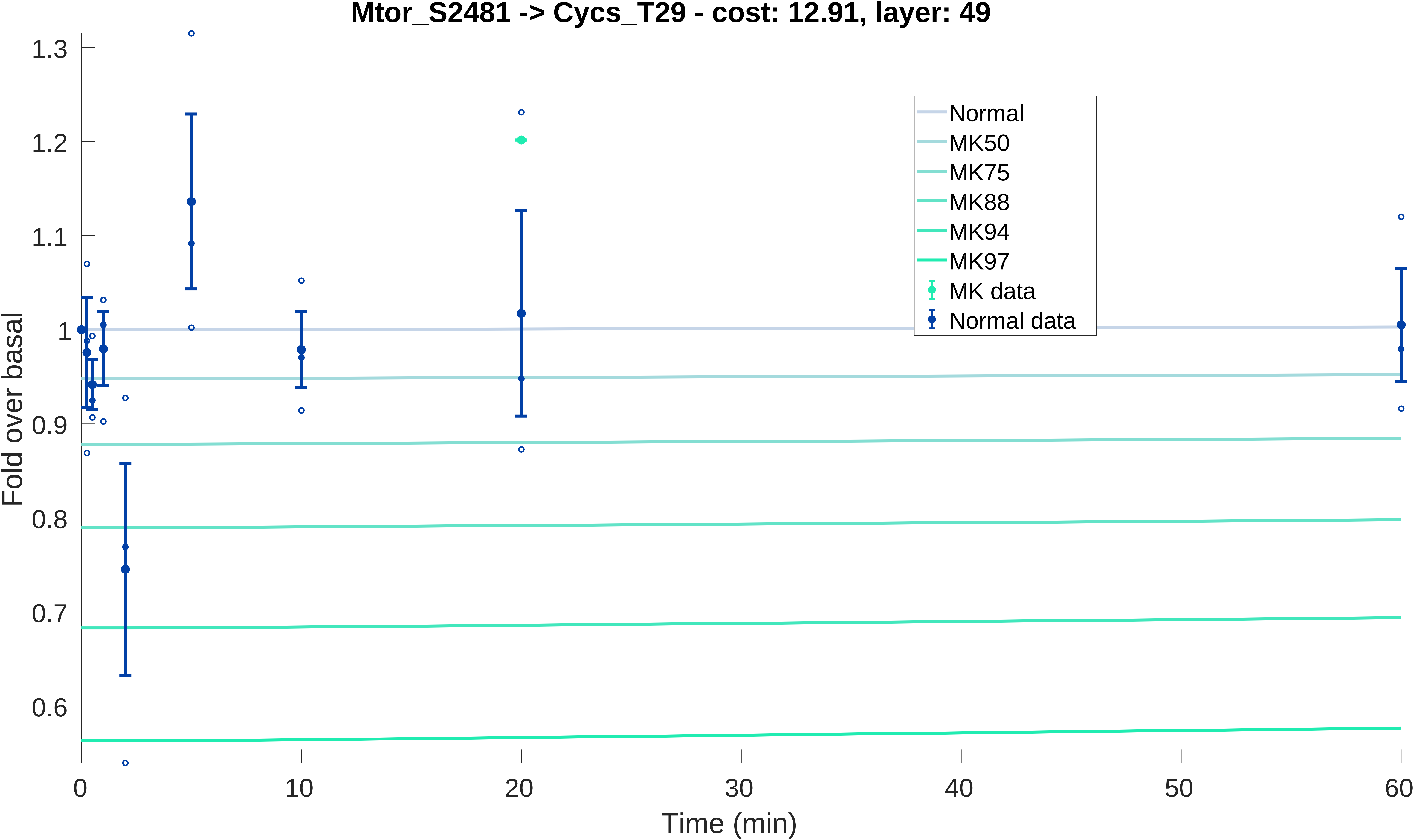
Mtor_S2481 -> Cycs_T29 - cost: 12.91, layer: 49
Fold over basal
1.3
1.2
1.1
1
0.9
0.8
0.7
0.6
Time (min)
0
10
20
30
40
50
60
Normal
MK50
MK75
MK88
MK94
MK97
MK data
Normal data

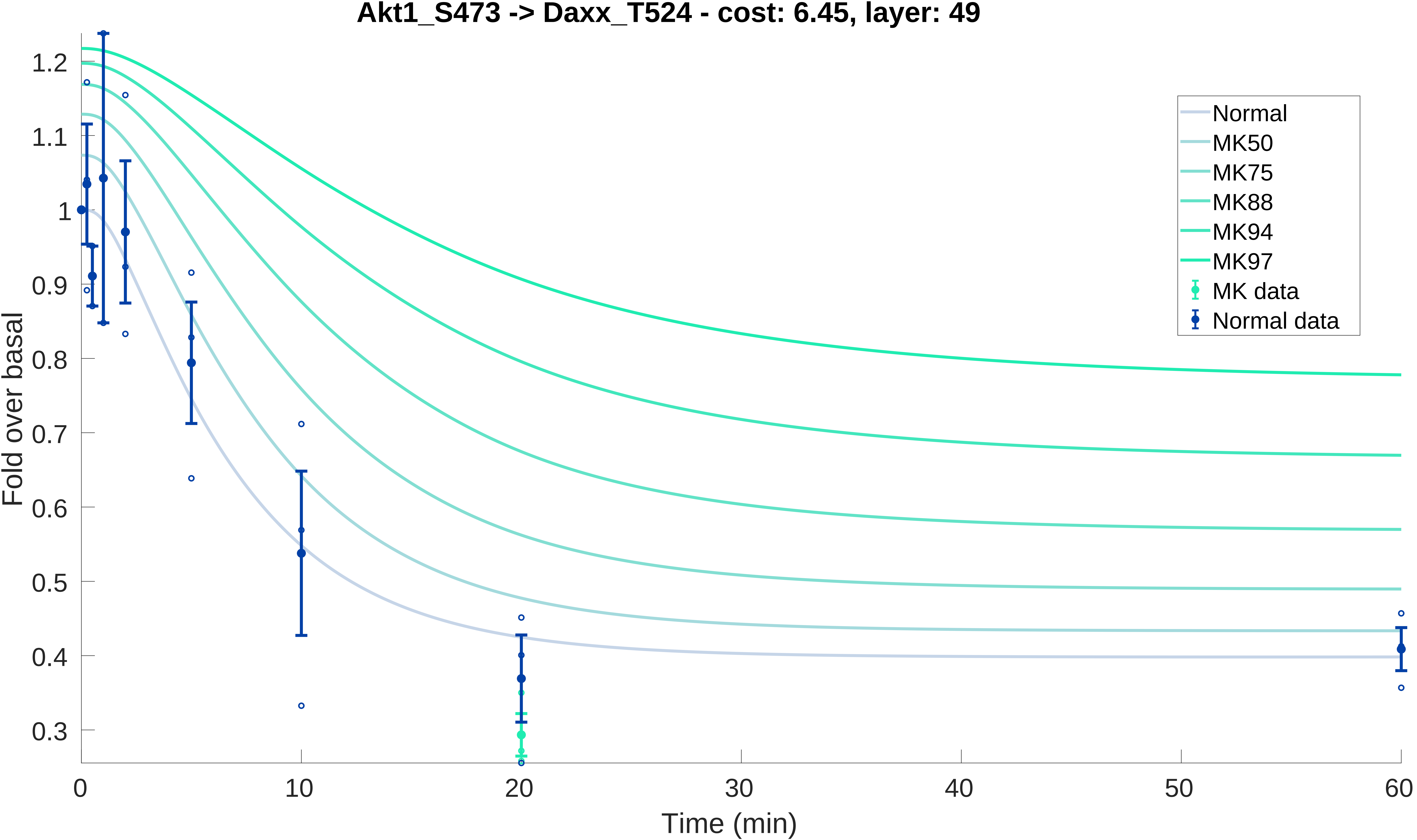

Akt1_S473 -> Daxx_T524 - cost: 6.45, layer: 49
Fold over basal
Time (min)
1.2
1.1
1
0.9
0.8
0.7
0.6
0.5
0.4
0.3
0
10
20
30
40
50
60
Normal
MK50
MK75
MK88
MK94
MK97
MK data
Normal data

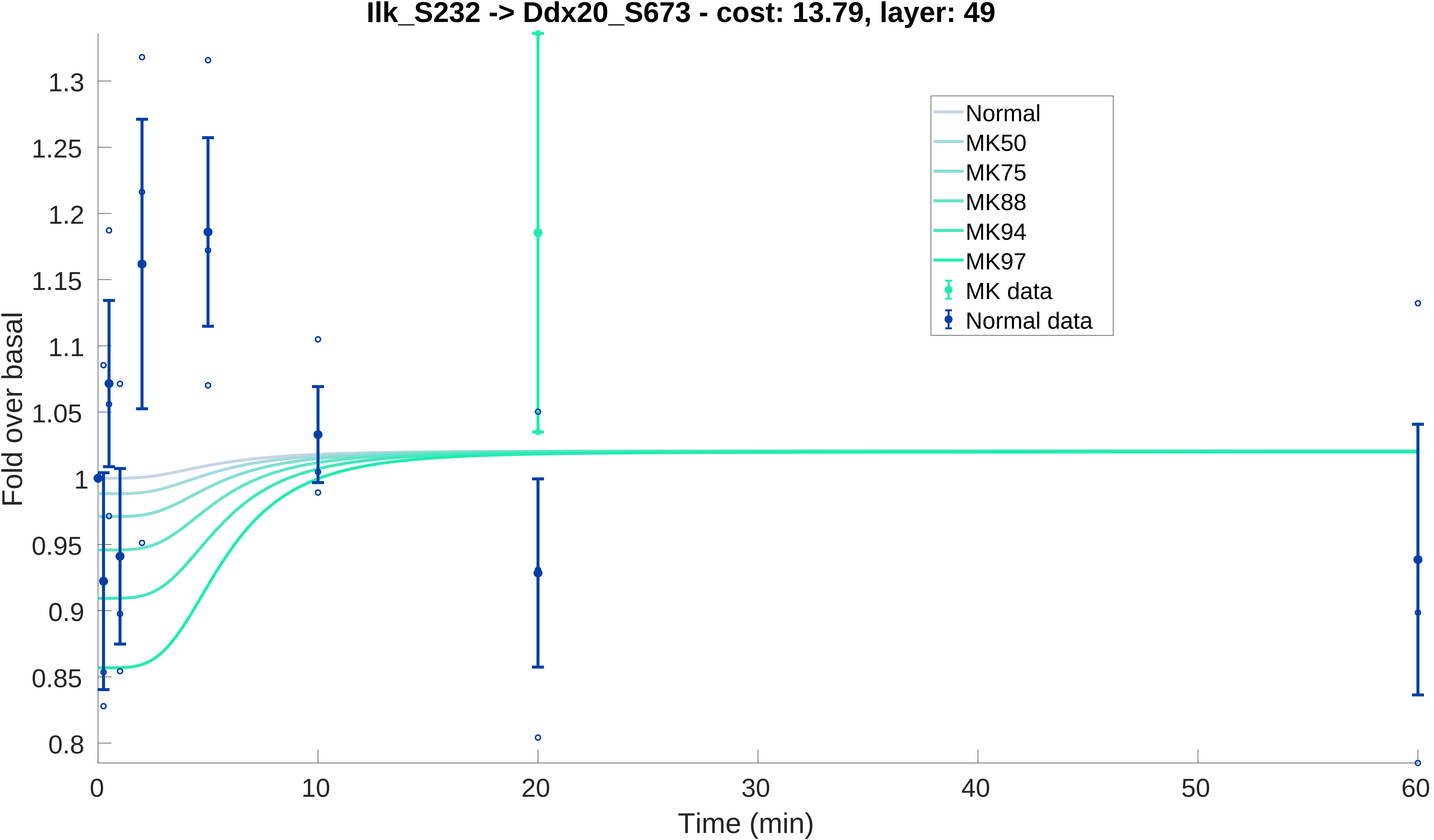

Ilk_S232 -> Ddx20_S673 - cost: 13.79, layer: 49
Fold over basal
Time (min)
Normal
MK50
MK75
MK88
MK94
MK97
MK data
Normal data
1.3
1.25
1.2
1.15
1.1
1.05
1
0.95
0.9
0.85
0.8
0
10
20
30
40
50
60

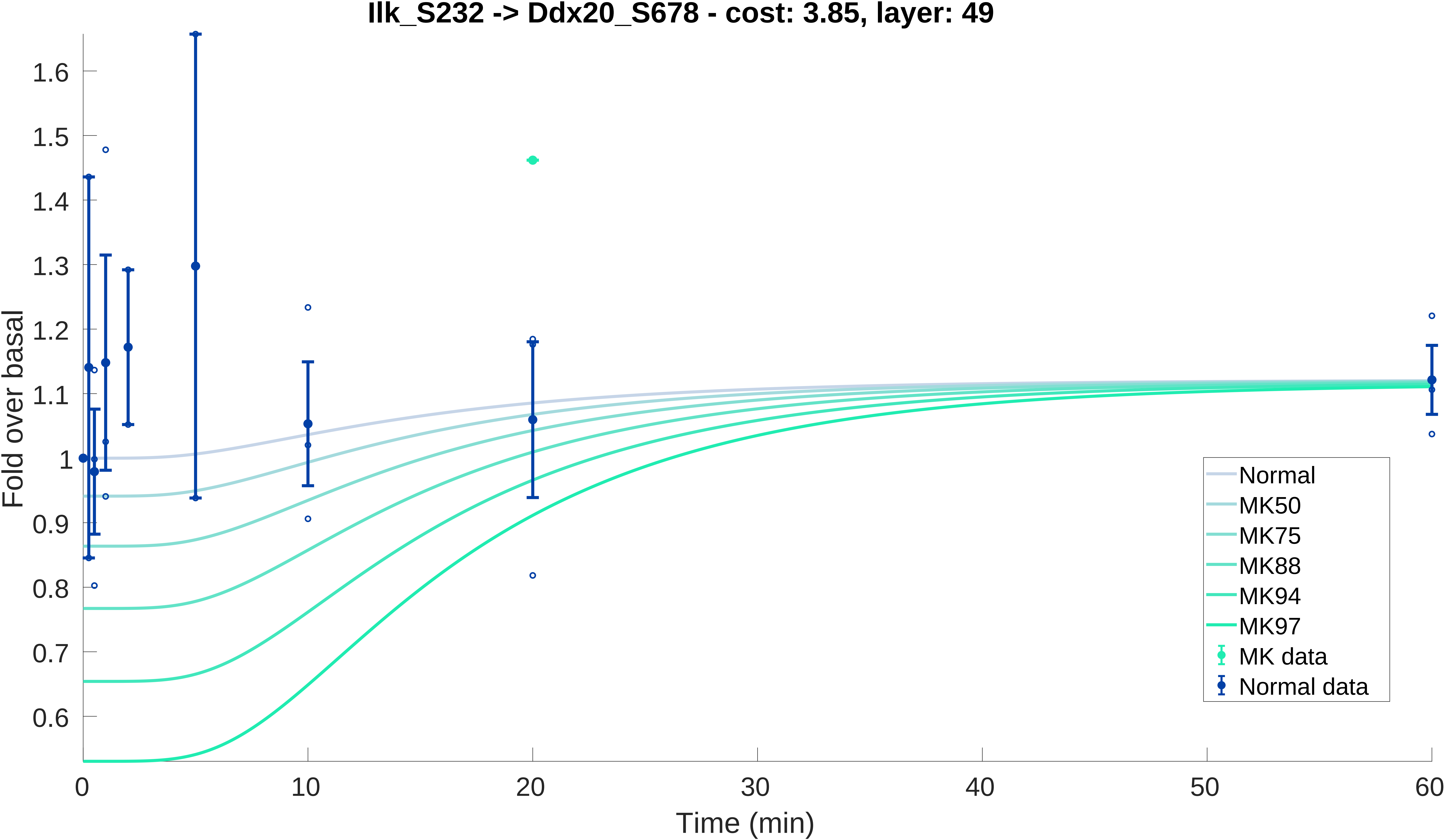
Ilk_S232 -> Ddx20_S678 - cost: 3.85, layer: 49
Fold over basal
1.6
1.5
1.4
1.3
1.2
1.1
1
0.9
0.8
0.7
0.6
0
10
20
30
40
50
60
Time (min)
Normal
MK50
MK75
MK88
MK94
MK97
MK data
Normal data

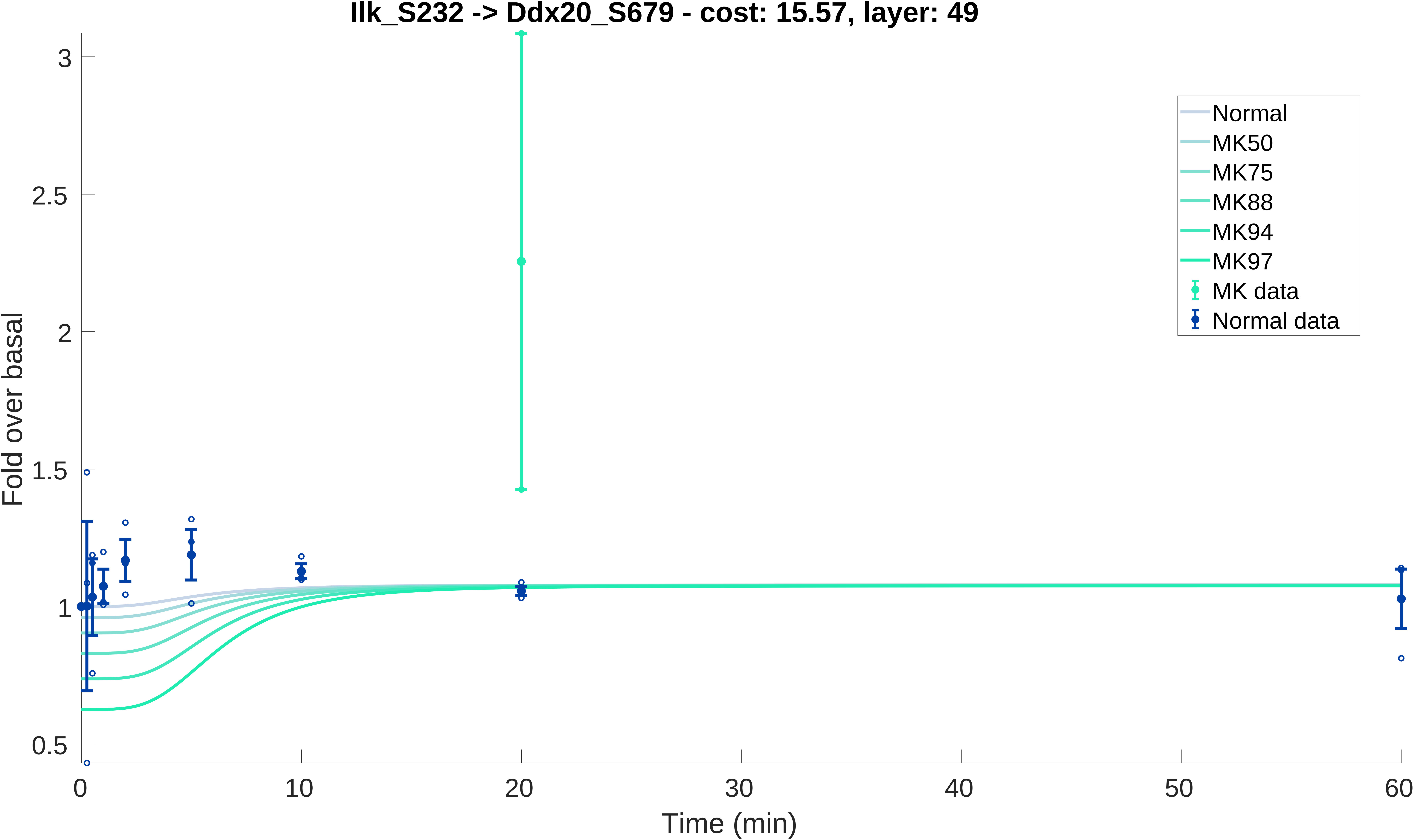

Ilk_S232 -> Ddx20_S679 - cost: 15.57, layer: 49
Fold over basal
Time (min)
0
10
20
30
40
50
60
0.5
1
1.5
2
2.5
3
Normal
MK50
MK75
MK88
MK94
MK97
MK data
Normal data

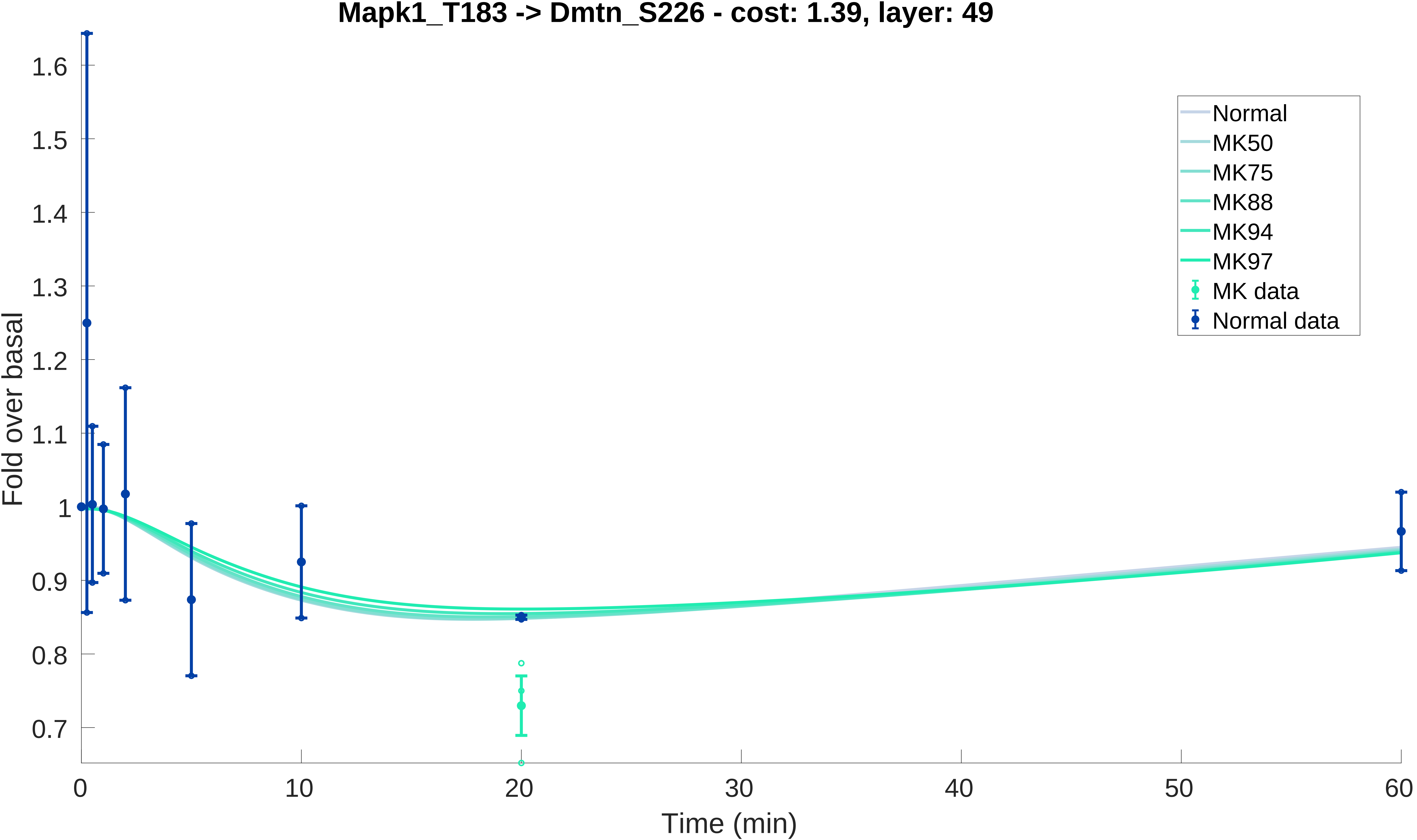

Mapk1_T183 -> Dmtn_S226 - cost: 1.39, layer: 49
Fold over basal
Time (min)
1.6
1.5
1.4
1.3
1.2
1.1
1
0.9
0.8
0.7
0
10
20
30
40
50
60
Normal
MK50
MK75
MK88
MK94
MK97
MK data
Normal data

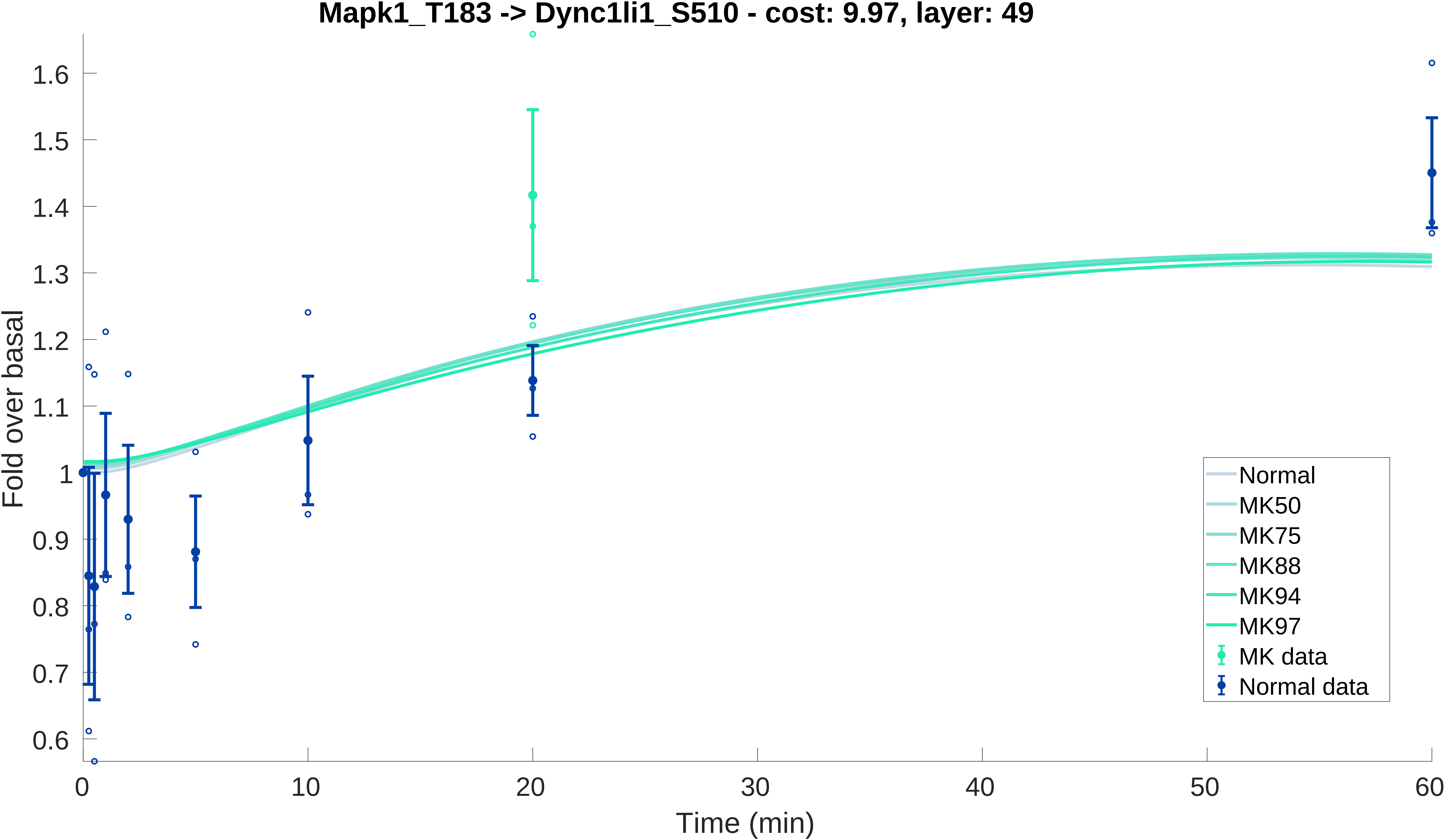

Mapk1_T183 -> Dync1li1_S510 - cost: 9.97, layer: 49
Fold over basal
1.6
1.5
1.4
1.3
1.2
1.1
1
0.9
0.8
0.7
0.6
0
10
20
30
40
50
60
Time (min)
Normal
MK50
MK75
MK88
MK94
MK97
MK data
Normal data

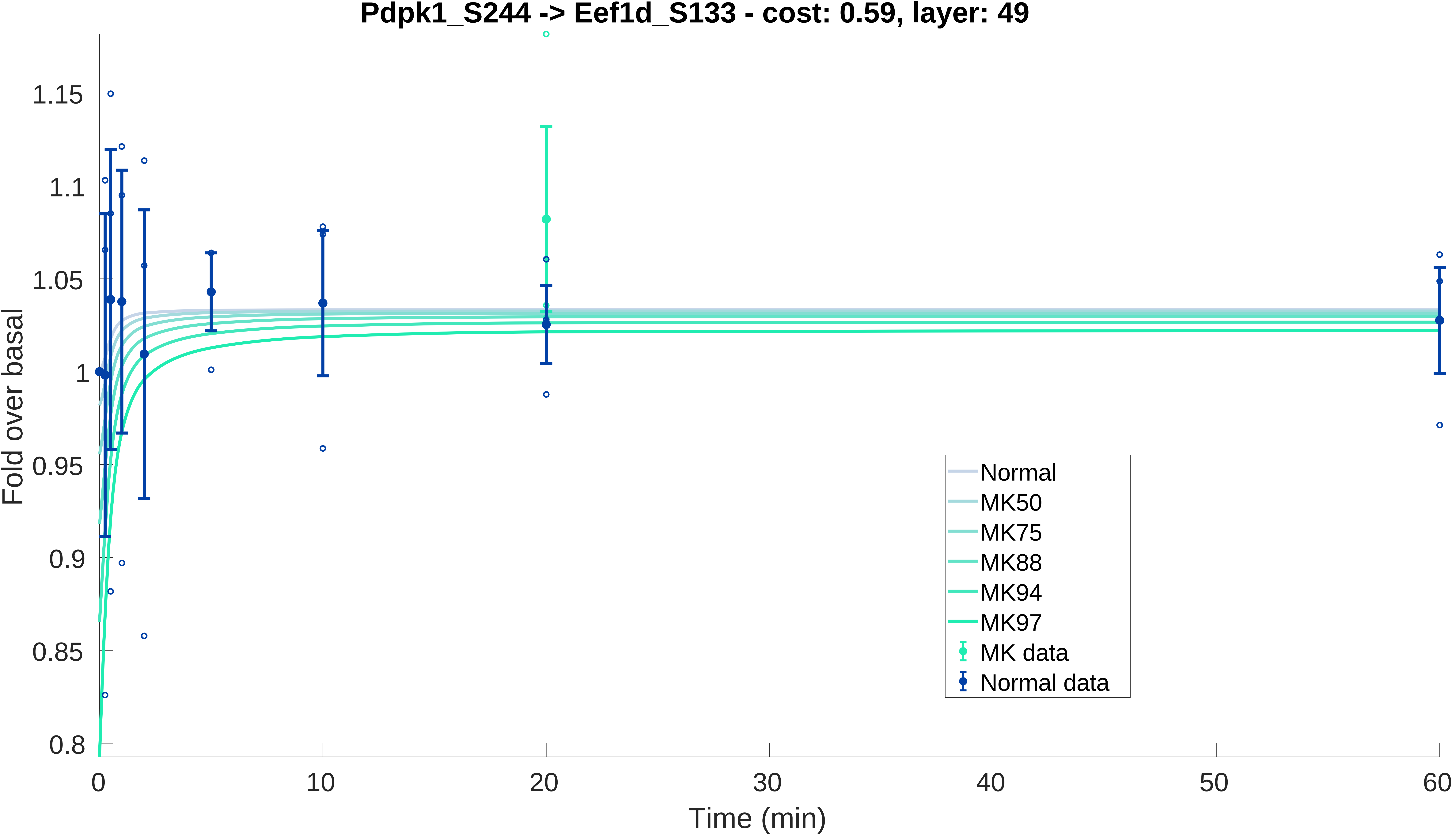

Pdpk1_S244 -> Eef1d_S133 - cost: 0.59, layer: 49
Fold over basal
Time (min)
0.8
0.85
0.9
0.95
1
1.05
1.1
1.15
0
10
20
30
40
50
60
Normal
MK50
MK75
MK88
MK94
MK97
MK data
Normal data

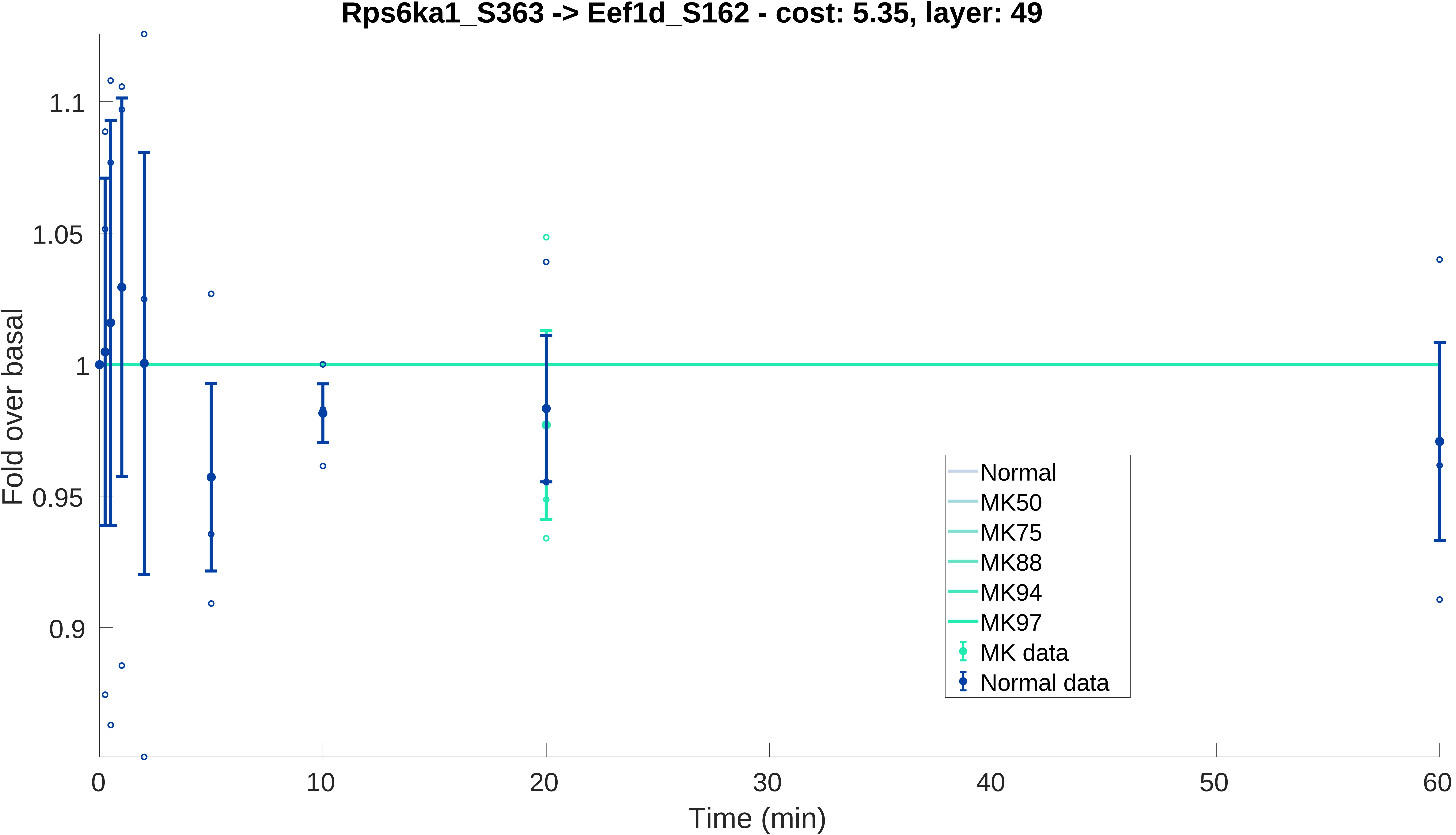

Rps6ka1_S363 -> Eef1d_S162 - cost: 5.35, layer: 49
Fold over basal
1.1
1.05
1
0.95
0.9
0
10
20
30
40
50
60
Time (min)
Normal
MK50
MK75
MK88
MK94
MK97
MK data
Normal data

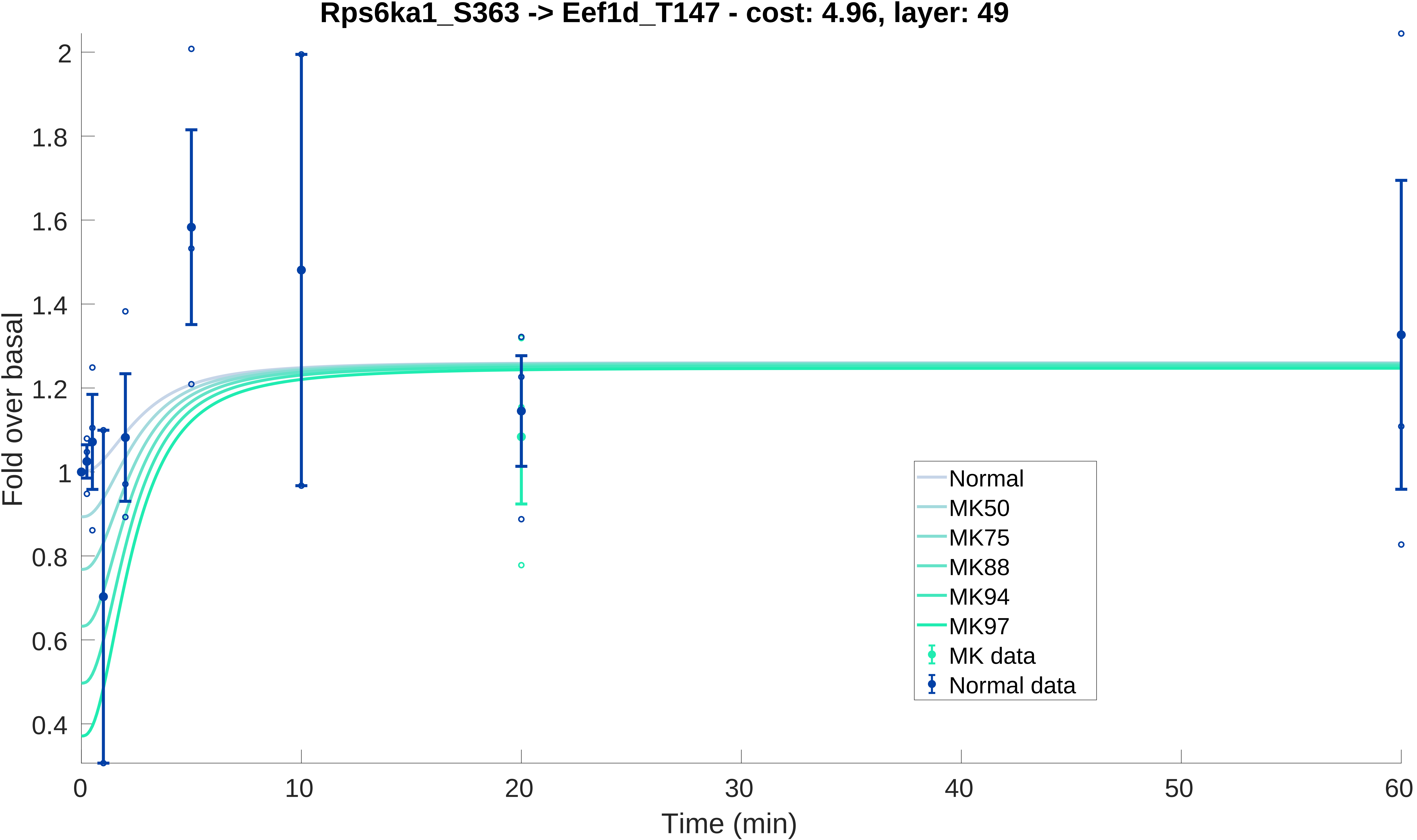

Rps6ka1_S363 -> Eef1d_T147 - cost: 4.96, layer: 49
Fold over basal
2
1.8
1.6
1.4
1.2
1
0.8
0.6
0.4
0
10
20
30
40
50
60
Time (min)
Normal
MK50
MK75
MK88
MK94
MK97
MK data
Normal data

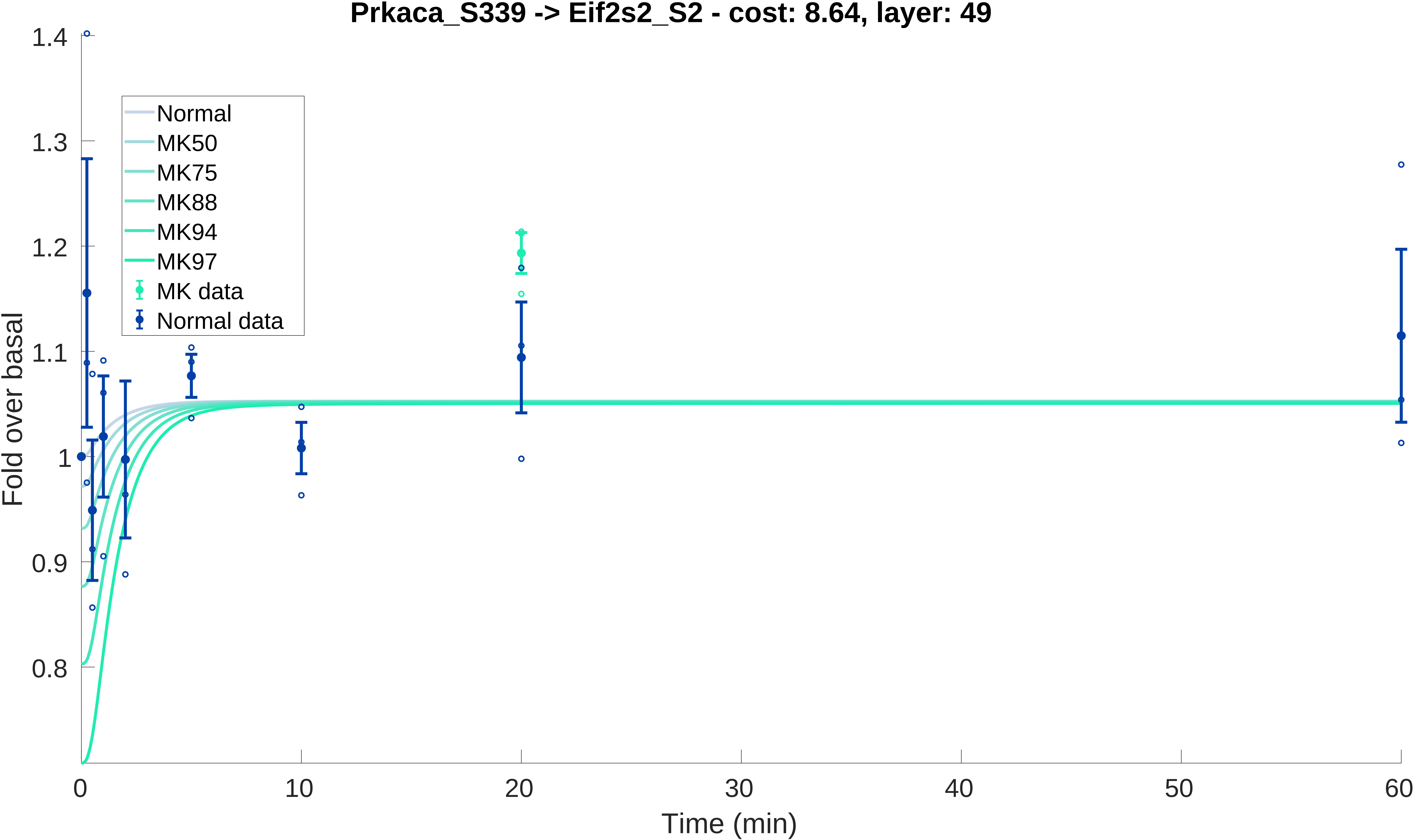

Prkaca_S339 -> Eif2s2_S2 - cost: 8.64, layer: 49
Normal
MK50
MK75
MK88
MK94
MK97
MK data
Normal data
Fold over basal
Time (min)
1.4
1.3
1.2
1.1
1
0.9
0.8
0
10
20
30
40
50
60

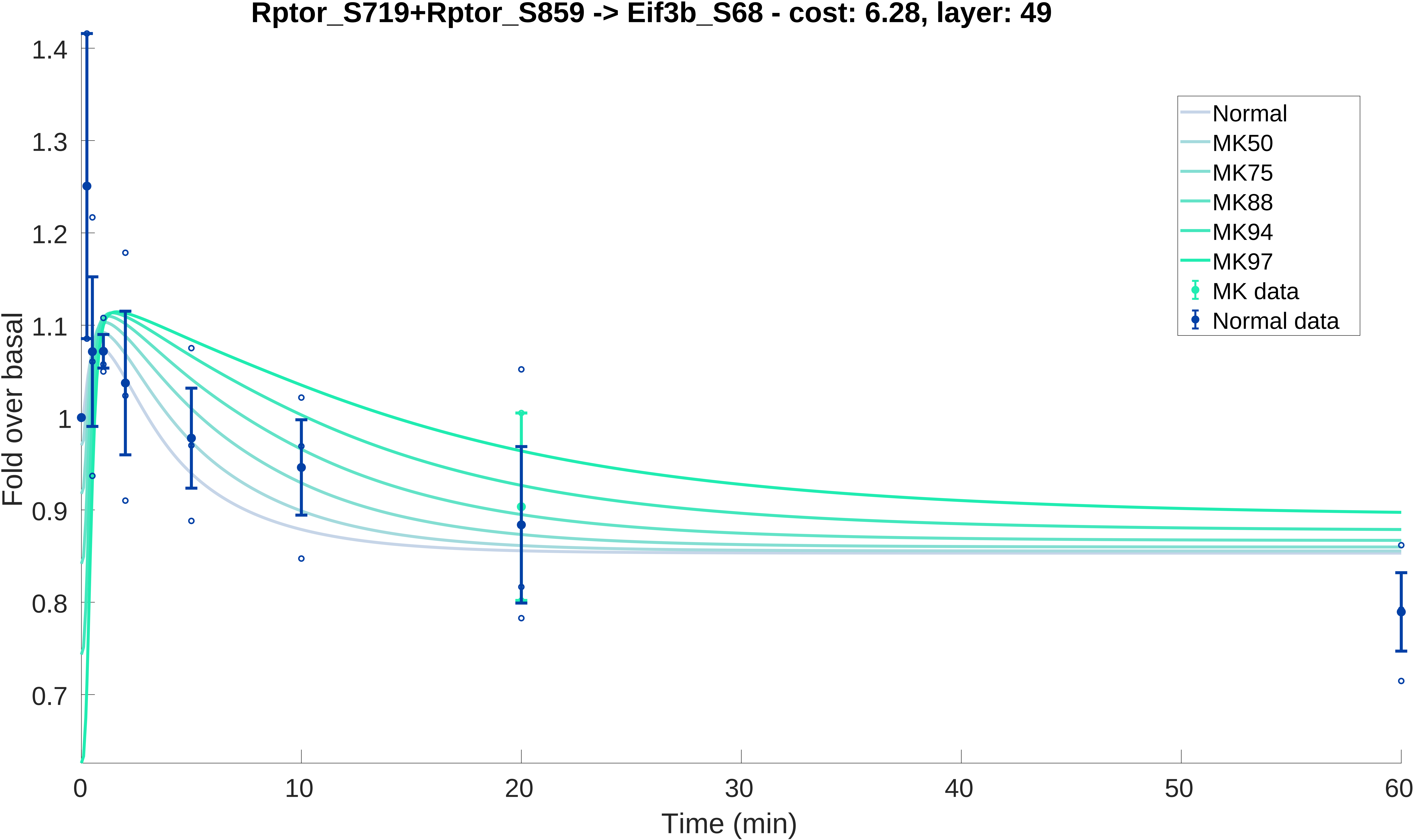
Rptor_S719+Rptor_S859 -> Eif3b_S68 - cost: 6.28, layer: 49
Fold over basal
1.4
1.3
1.2
1.1
1
0.9
0.8
0.7
Time (min)
0
10
20
30
40
50
60
Normal
MK50
MK75
MK88
MK94
MK97
MK data
Normal data

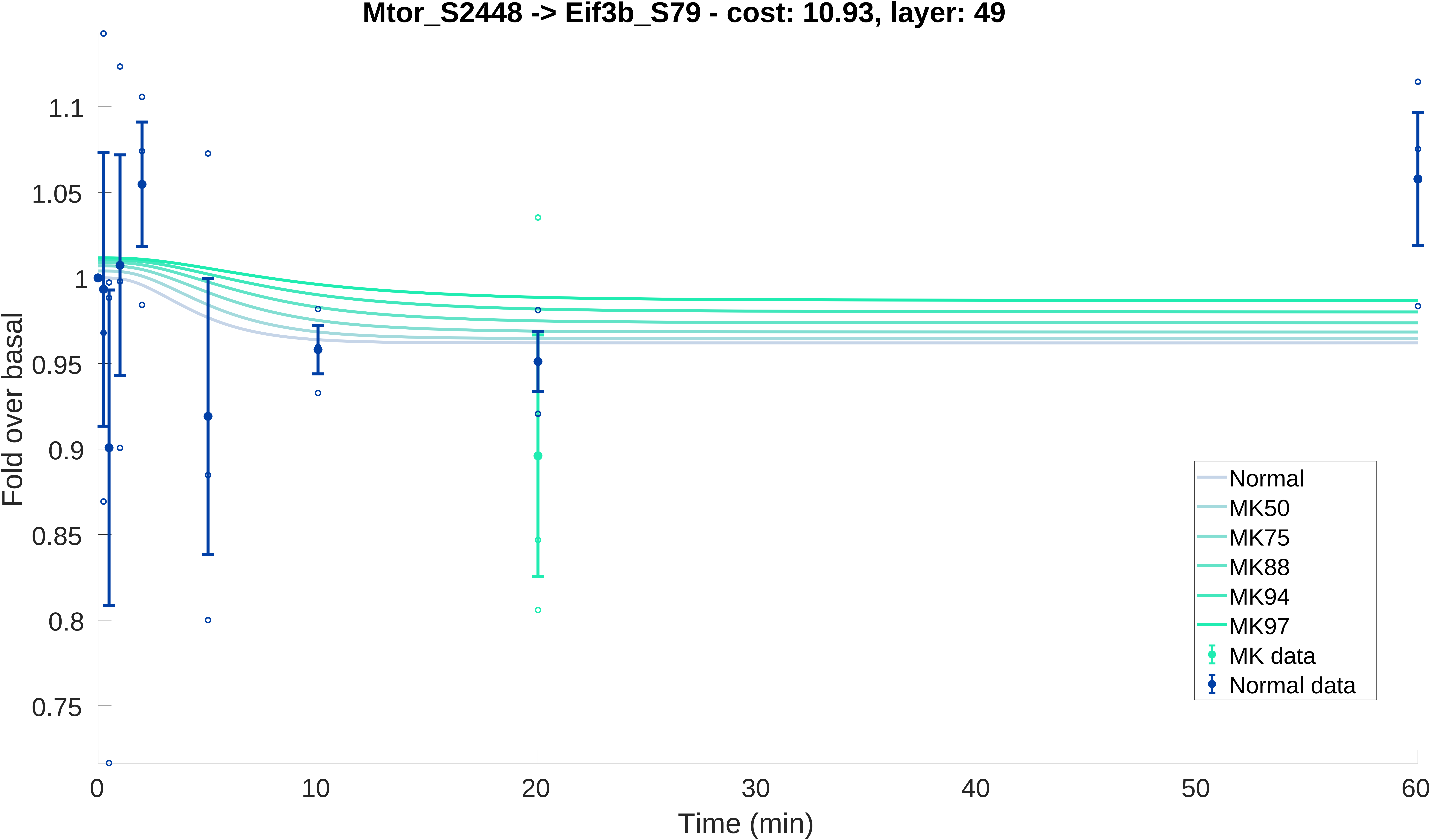

Mtor_S2448 -> Eif3b_S79 - cost: 10.93, layer: 49
Fold over basal
Time (min)
1.1
1.05
1
0.95
0.9
0.85
0.8
0.75
0
10
20
30
40
50
60
Normal
MK50
MK75
MK88
MK94
MK97
MK data
Normal data

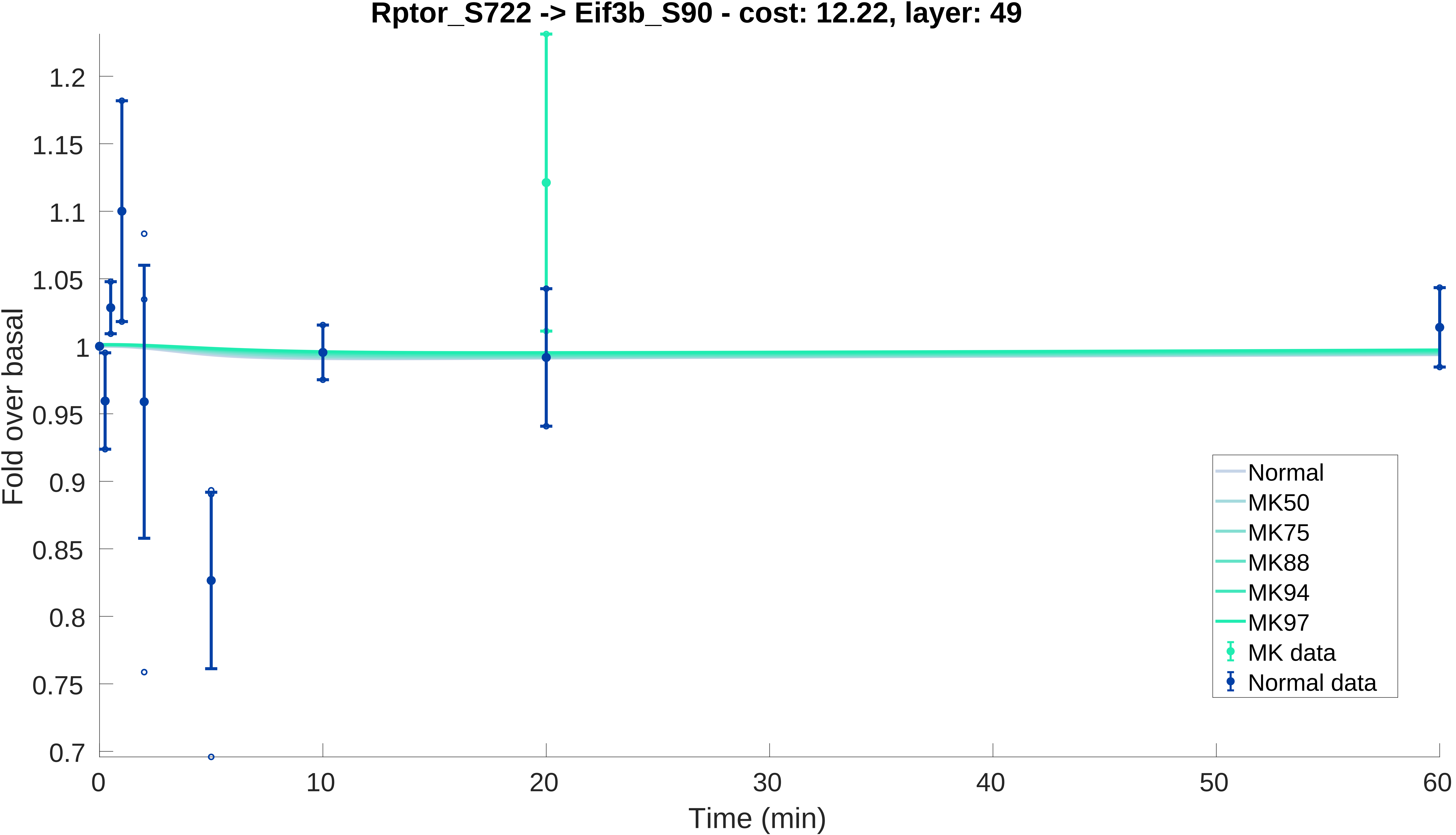

Rptor_S722 -> Eif3b_S90 - cost: 12.22, layer: 49
Fold over basal
Time (min)
1.2
1.15
1.1
1.05
1
0.95
0.9
0.85
0.8
0.75
0.7
0
10
20
30
40
50
60
Normal
MK50
MK75
MK88
MK94
MK97
MK data
Normal data

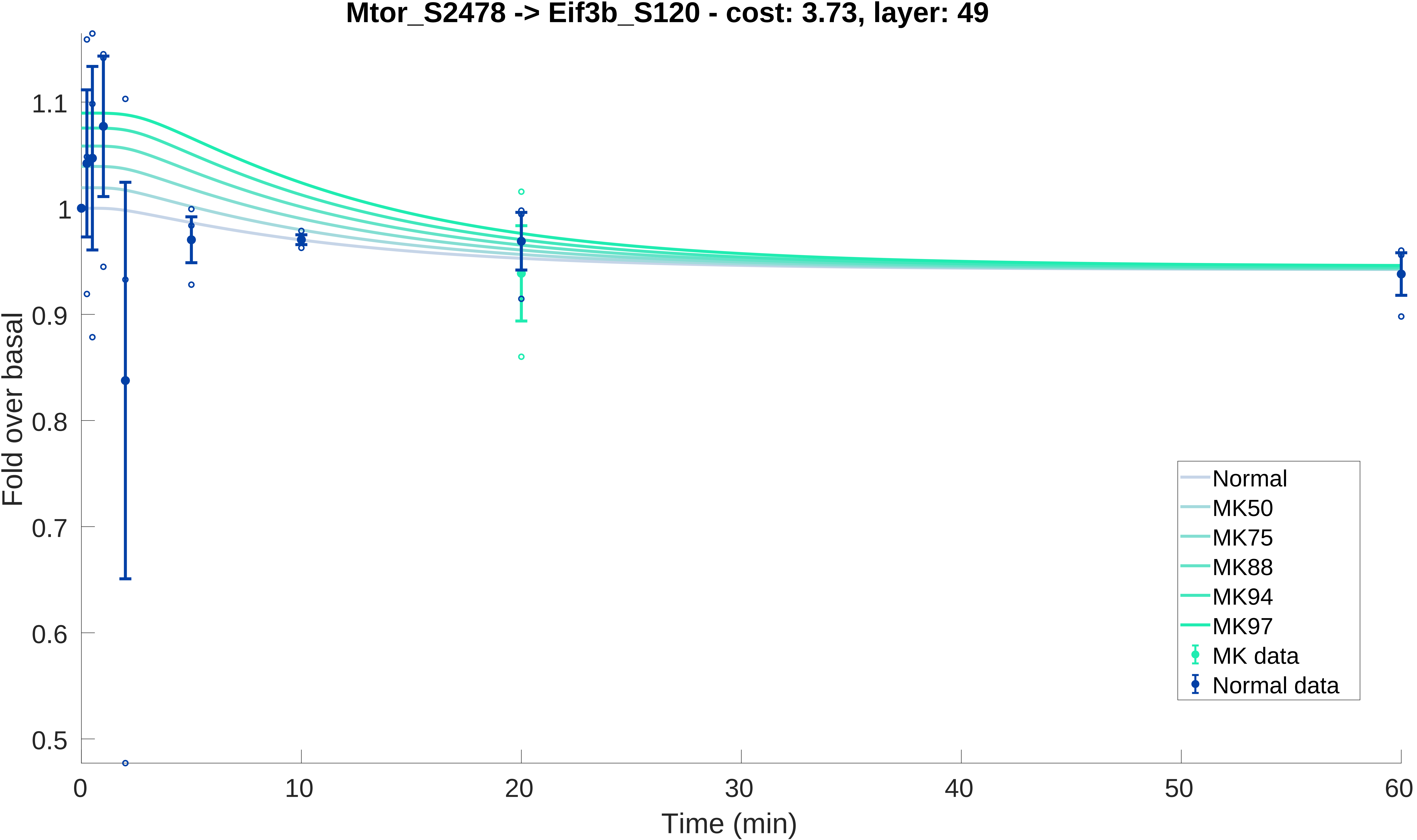

Mtor_S2478 -> Eif3b_S120 - cost: 3.73, layer: 49
Fold over basal
Time (min)
0.5
0.6
0.7
0.8
0.9
1
1.1
0
10
20
30
40
50
60
Normal
MK50
MK75
MK88
MK94
MK97
MK data
Normal data

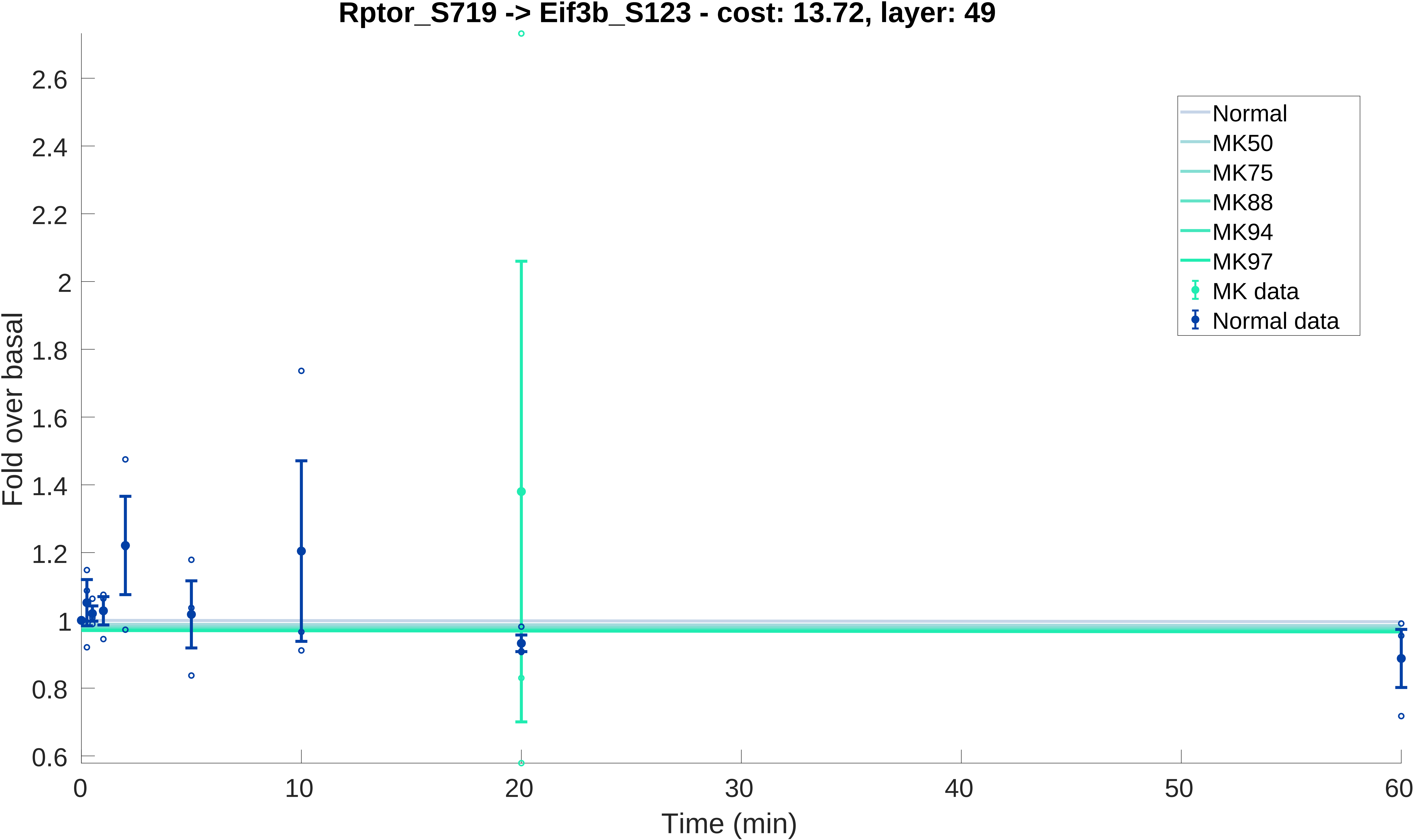

Rptor_S719 -> Eif3b_S123 - cost: 13.72, layer: 49
Fold over basal
Time (min)
0.6
0.8
1
1.2
1.4
1.6
1.8
2
2.2
2.4
2.6
0
10
20
30
40
50
60
Normal
MK50
MK75
MK88
MK94
MK97
MK data
Normal data

Akt1_S122+Rps6kb1_S452 -> Eif4ebp1_S2 - cost: 4.27, layer: 49

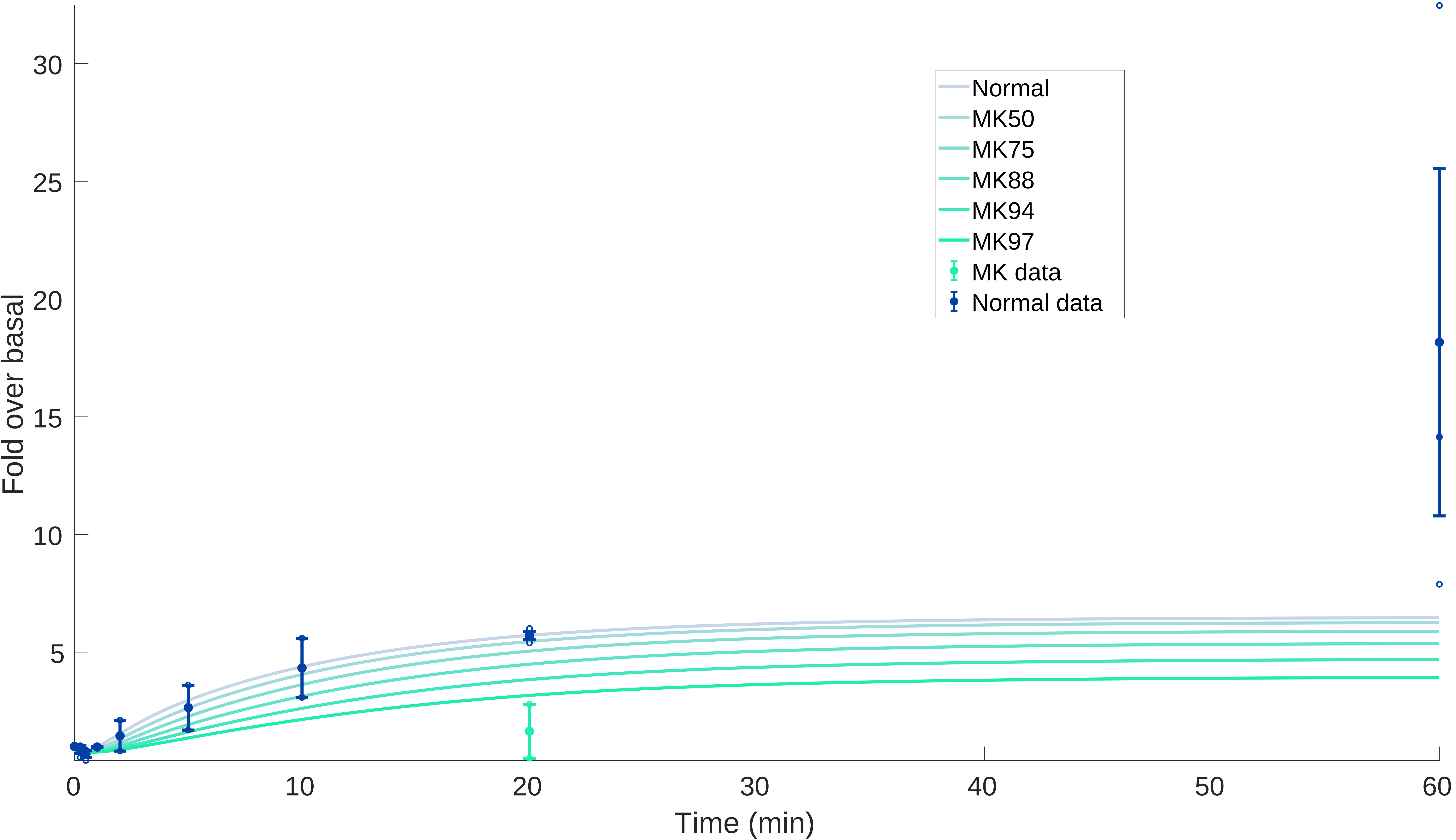

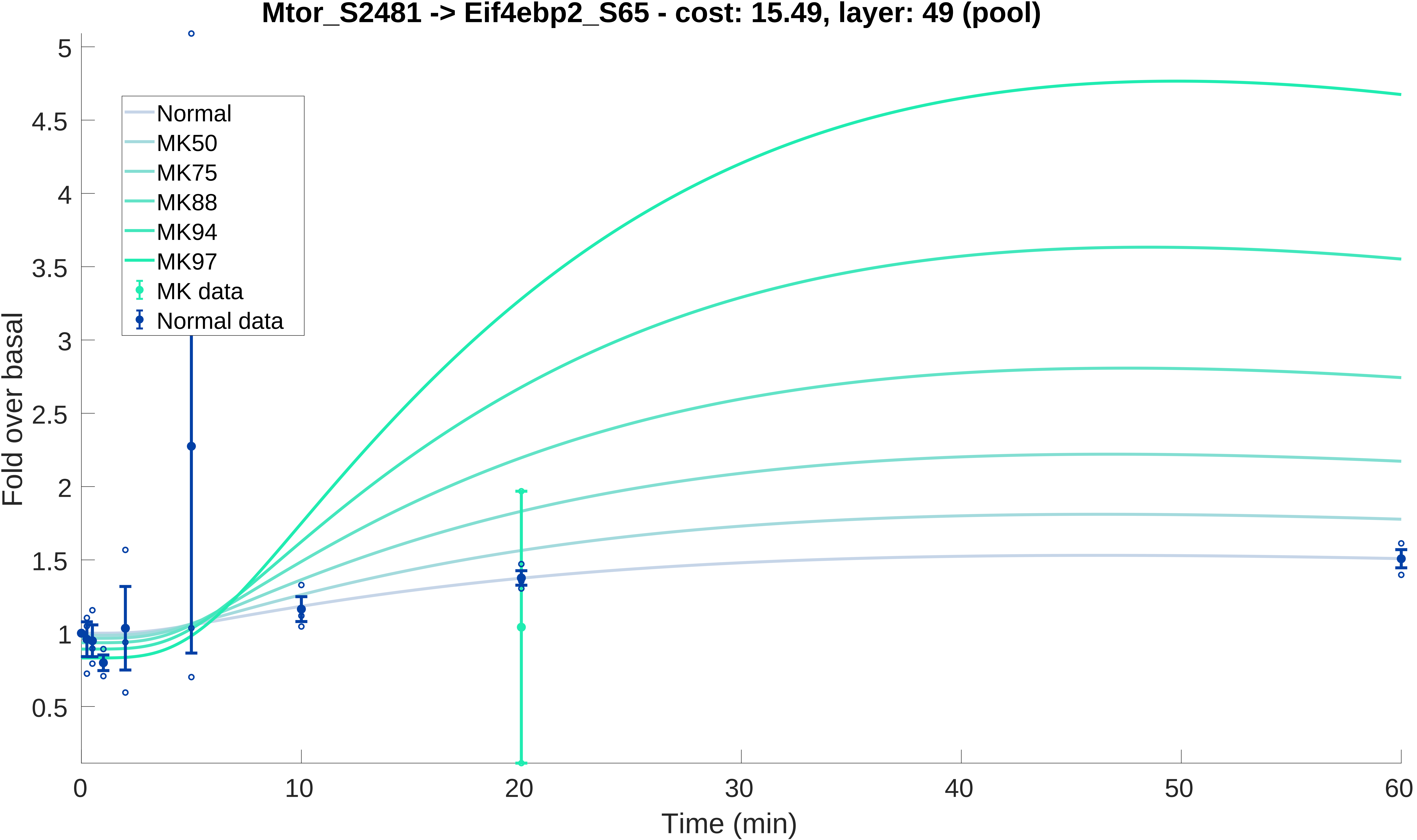

Mtor_S2481 -> Eif4ebp2_S65 - cost: 15.49, layer: 49 (pool)
Normal
MK50
MK75
MK88
MK94
MK97
MK data
Normal data
Fold over basal
Time (min)
0
10
20
30
40
50
60
0.5
1
1.5
2
2.5
3
3.5
4
4.5
5

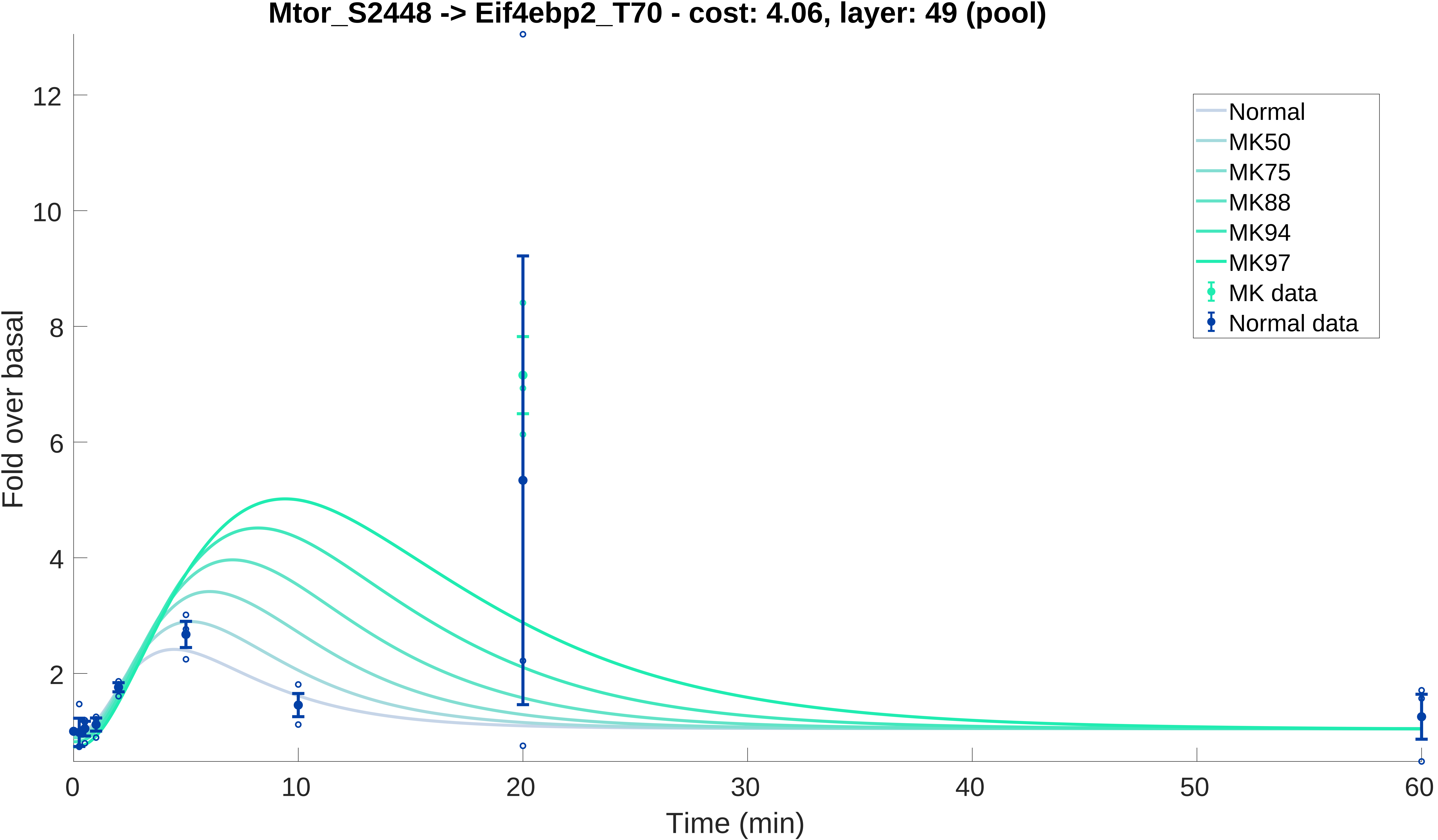

Mtor_S2448 -> Eif4ebp2_T70 - cost: 4.06, layer: 49 (pool)
Fold over basal
Time (min)
0
10
20
30
40
50
60
2
4
6
8
10
12
Normal
MK50
MK75
MK88
MK94
MK97
MK data
Normal data

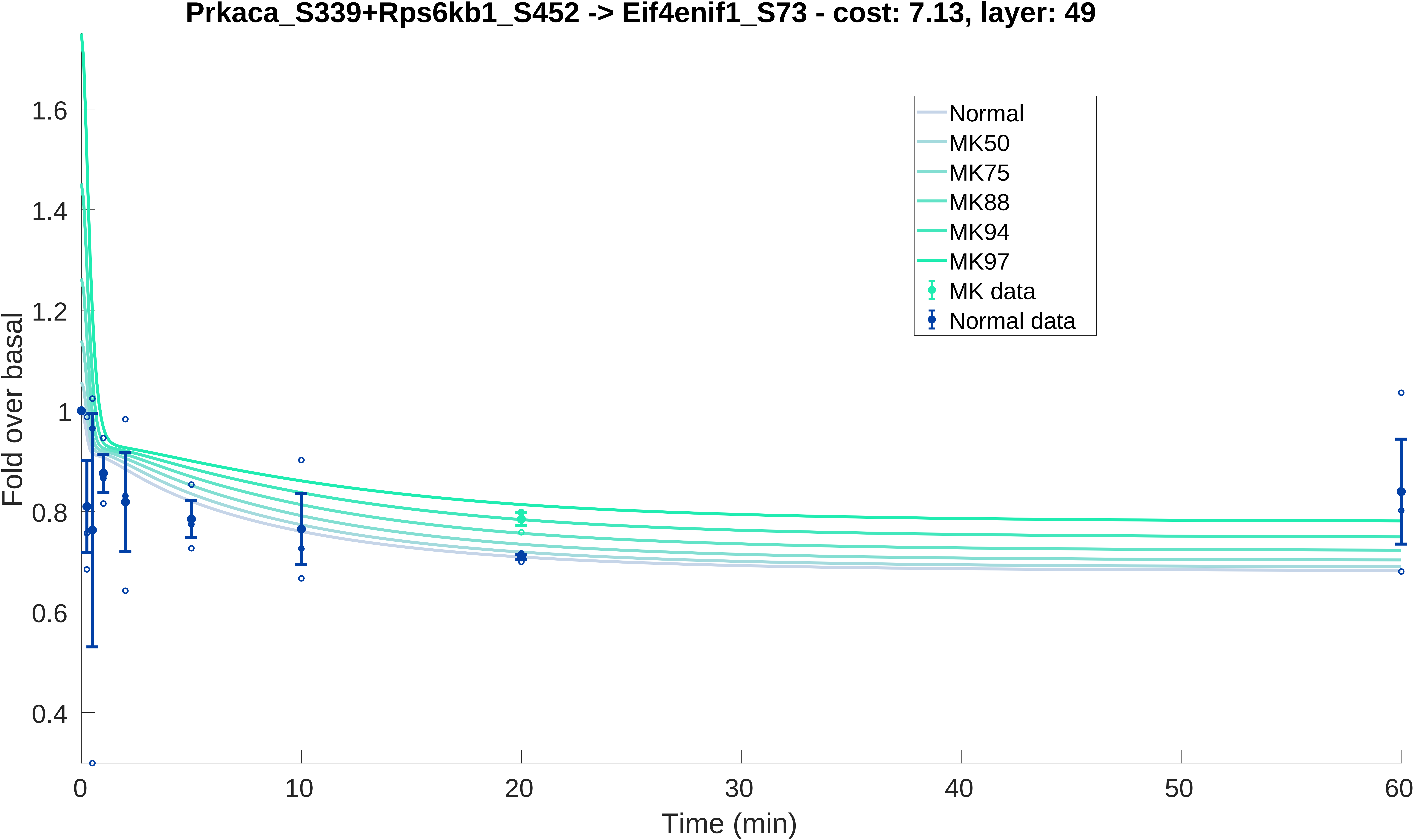
Prrkaca_S339+Rps6kb1_S452 -> Eif4enif1_S73 - cost: 7.13, layer: 49
Fold over basal
Time (min)
Normal
MK50
MK75
MK88
MK94
MK97
MK data
Normal data
0.4
0.6
0.8
1
1.2
1.4
1.6
0
10
20
30
40
50
60

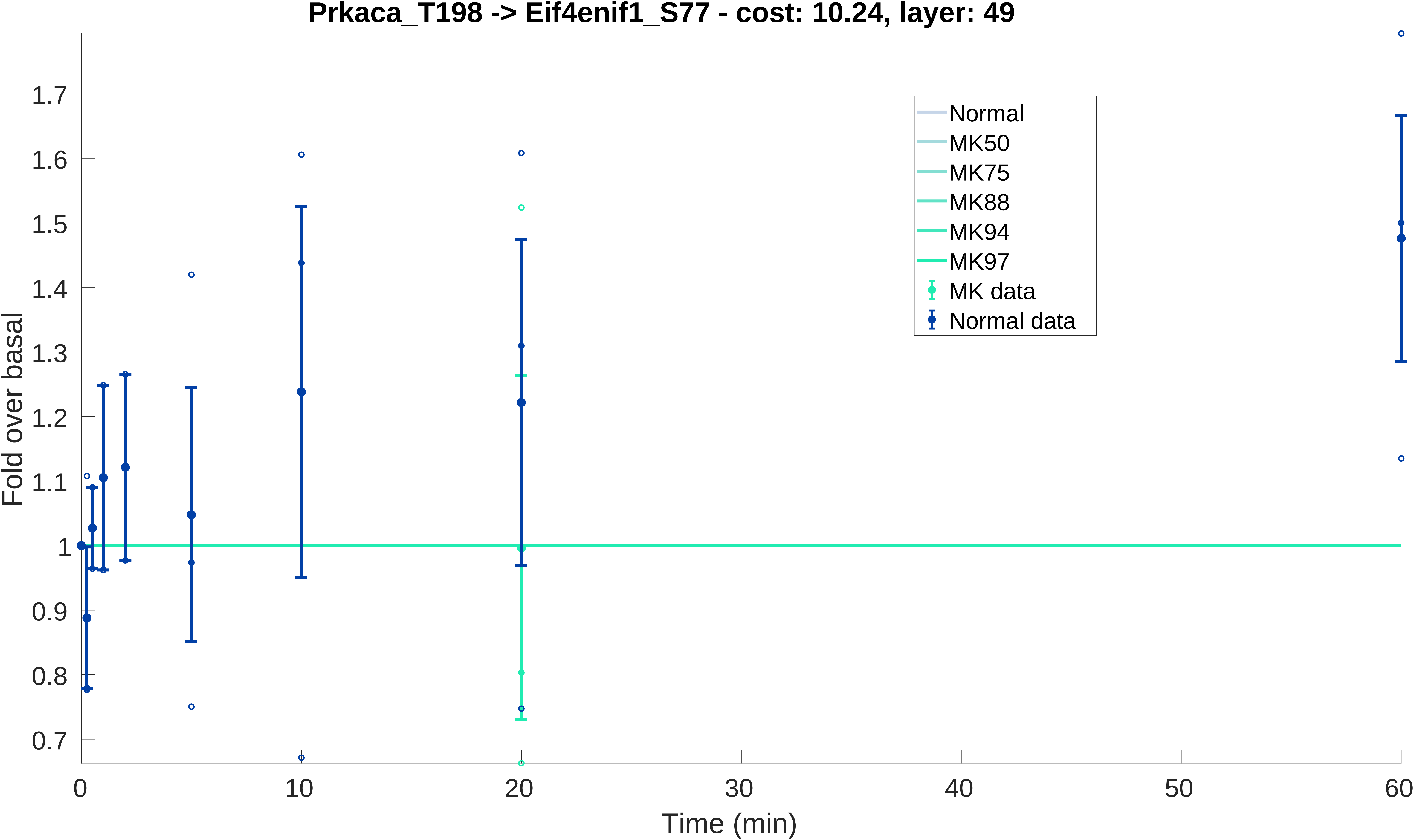

Prkaca_T198 -> Eif4enif1_S77 - cost: 10.24, layer: 49
Fold over basal
Time (min)
1.7
1.6
1.5
1.4
1.3
1.2
1.1
1
0.9
0.8
0.7
0
10
20
30
40
50
60
Normal
MK50
MK75
MK88
MK94
MK97
MK data
Normal data

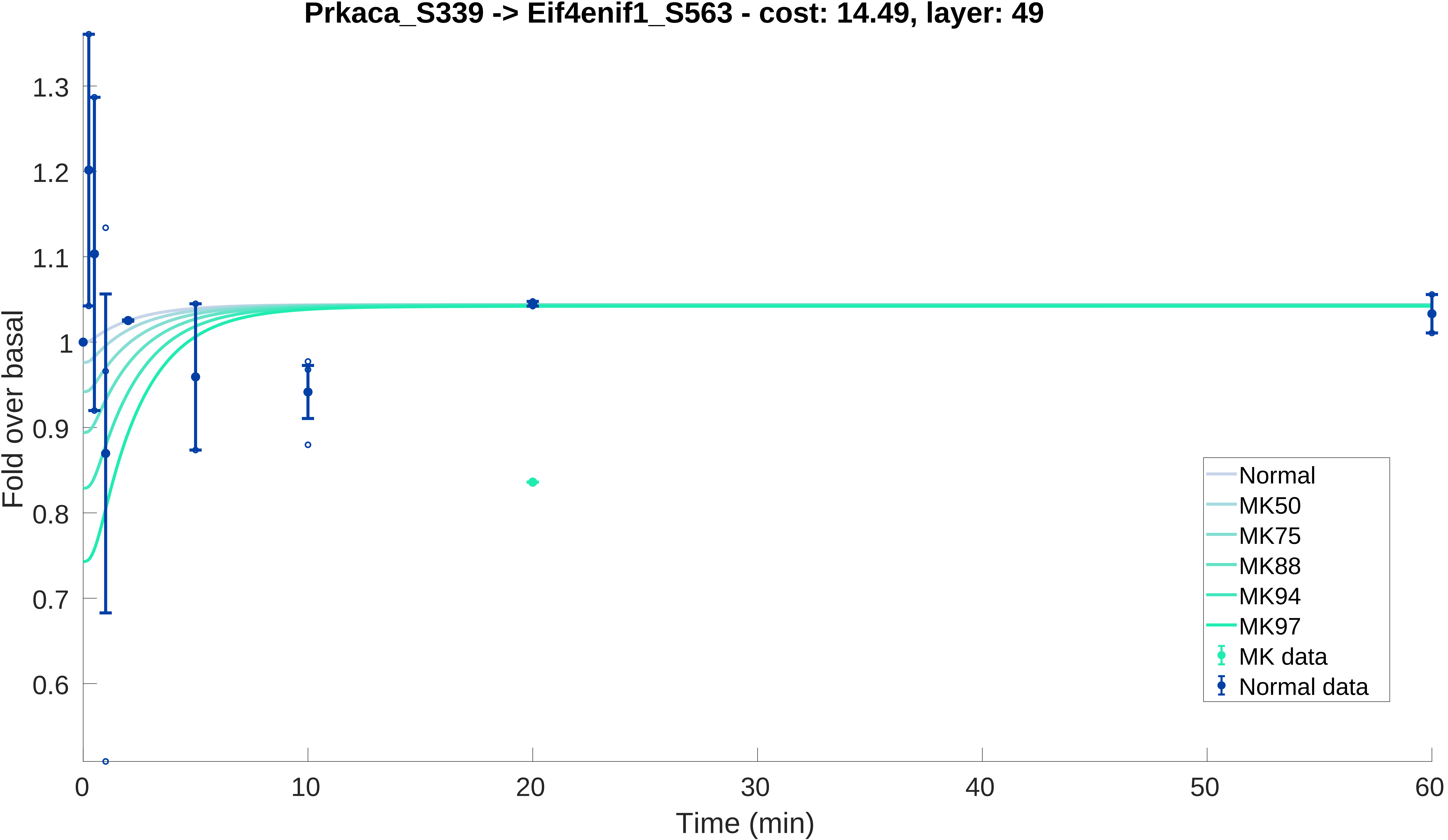

Prkaca_S339 -> Eif4enif1_S563 - cost: 14.49, layer: 49
Fold over basal
1.3
1.2
1.1
1
0.9
0.8
0.7
0.6
0
10
20
30
40
50
60
Time (min)
Normal
MK50
MK75
MK88
MK94
MK97
MK data
Normal data

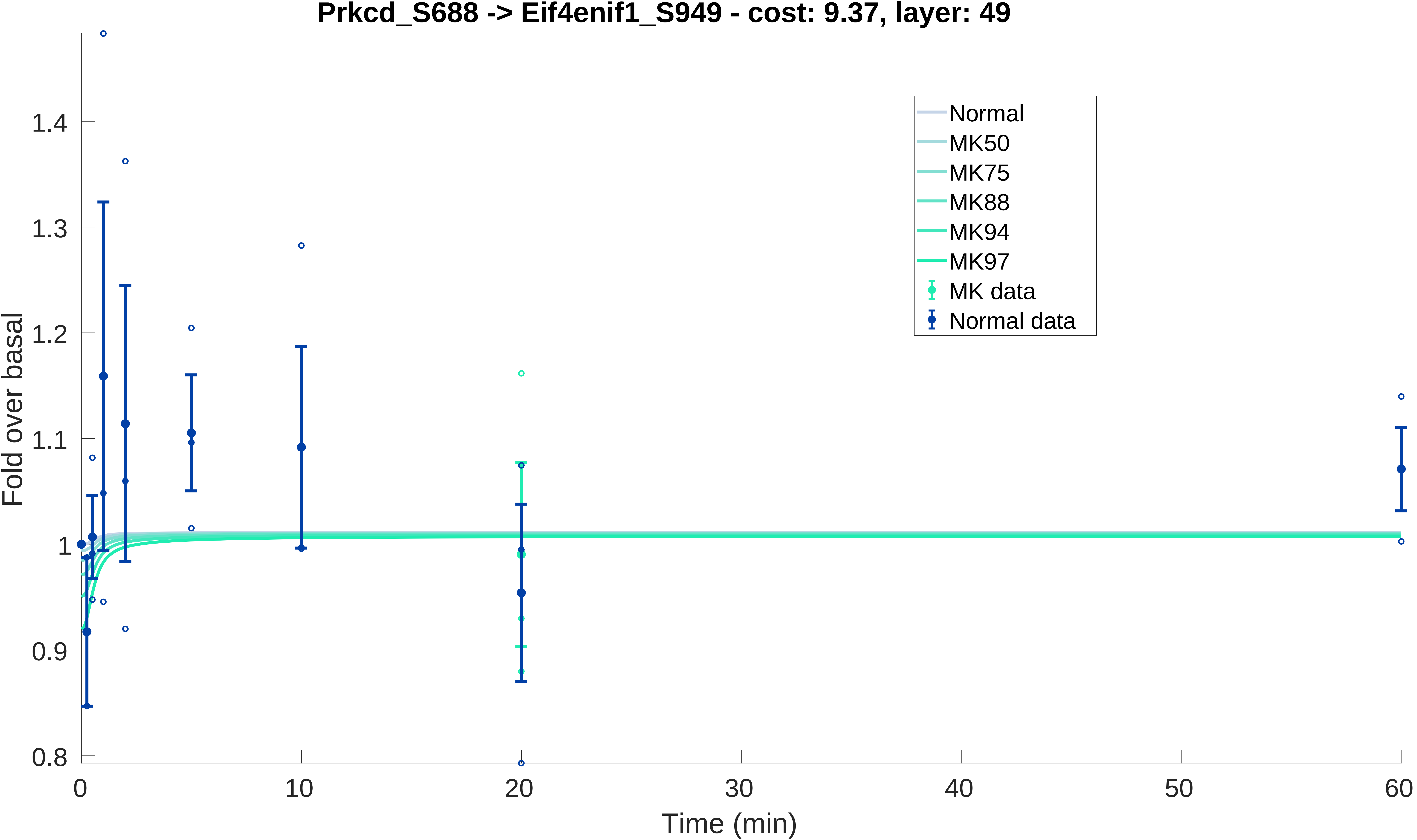

Prkcd_S688 -> Eif4enif1_S949 - cost: 9.37, layer: 49
Normal
MK50
MK75
MK88
MK94
MK97
MK data
Normal data
Fold over basal
Time (min)
0.8
0.9
1
1.1
1.2
1.3
1.4
0
10
20
30
40
50
60

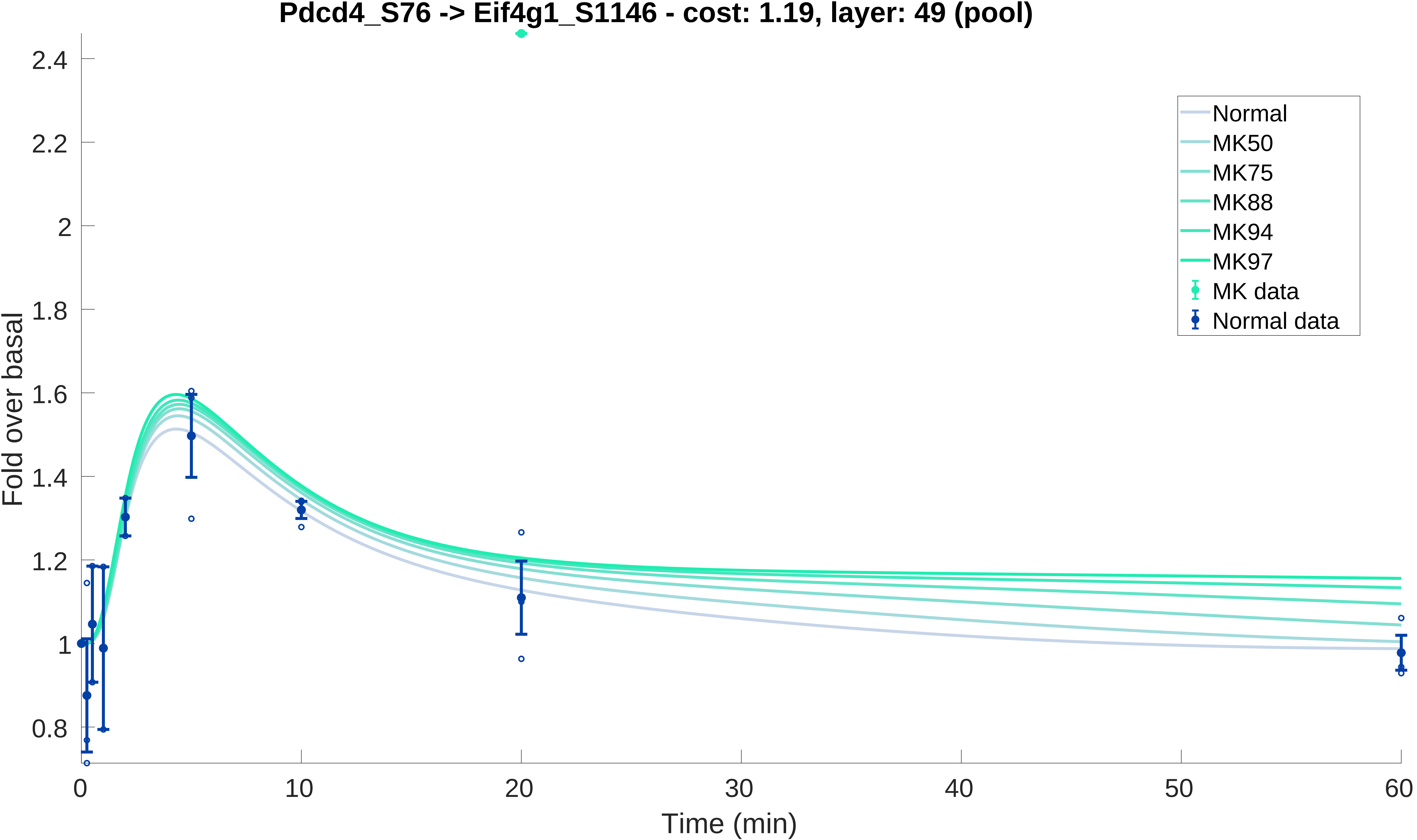

Pdcd4_S76 -> Eif4g1_S1146 - cost: 1.19, layer: 49 (pool)
Fold over basal
2.4
2.2
2
1.8
1.6
1.4
1.2
1
0.8
0
10
20
30
40
50
60
Time (min)
Normal
MK50
MK75
MK88
MK94
MK97
MK data
Normal data

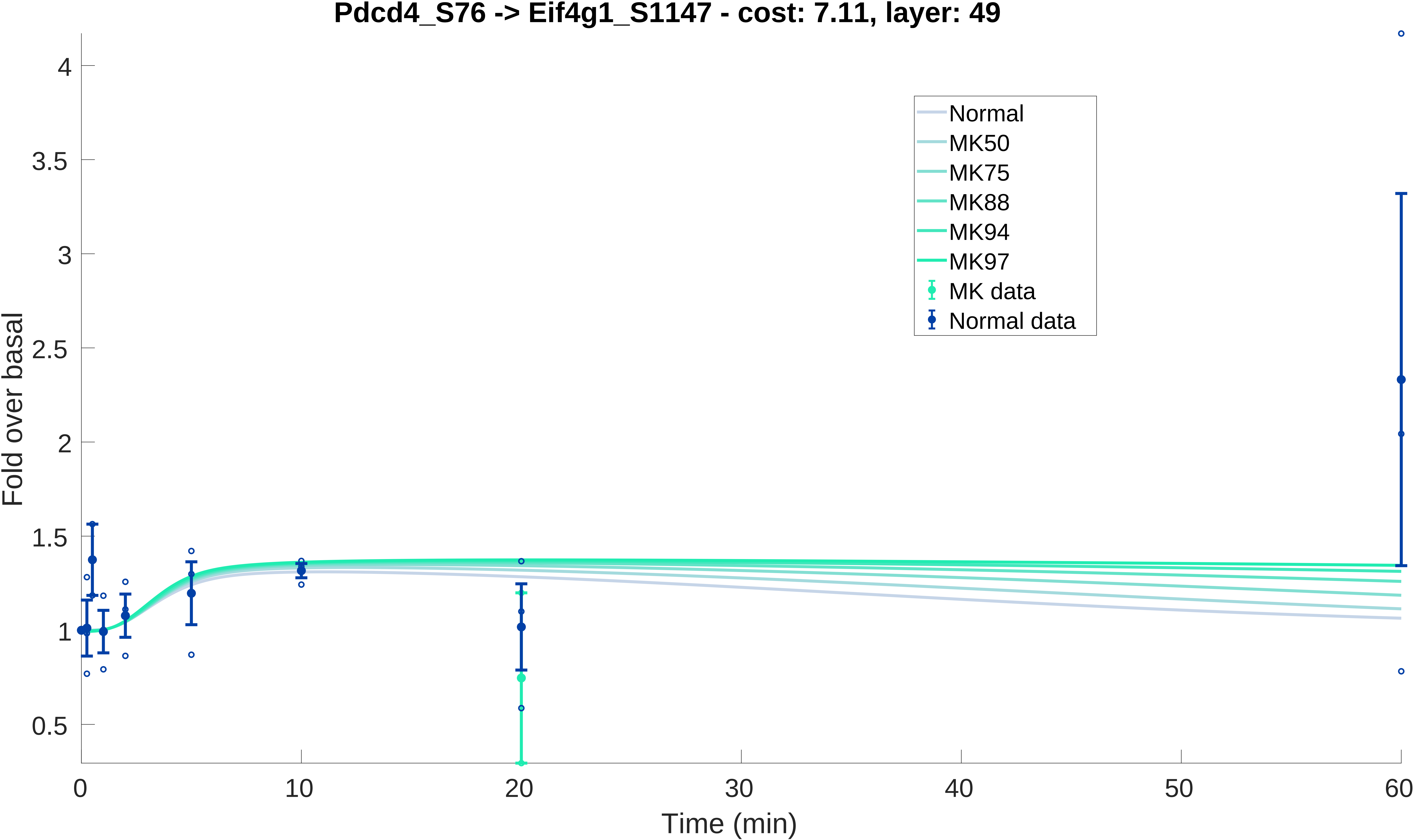
Pdcd4_S76 -> Eif4g1_S1147 - cost: 7.11, layer: 49
Fold over basal
Time (min)
0
10
20
30
40
50
60
0.5
1
1.5
2
2.5
3
3.5
4
Normal
MK50
MK75
MK88
MK94
MK97
MK data
Normal data

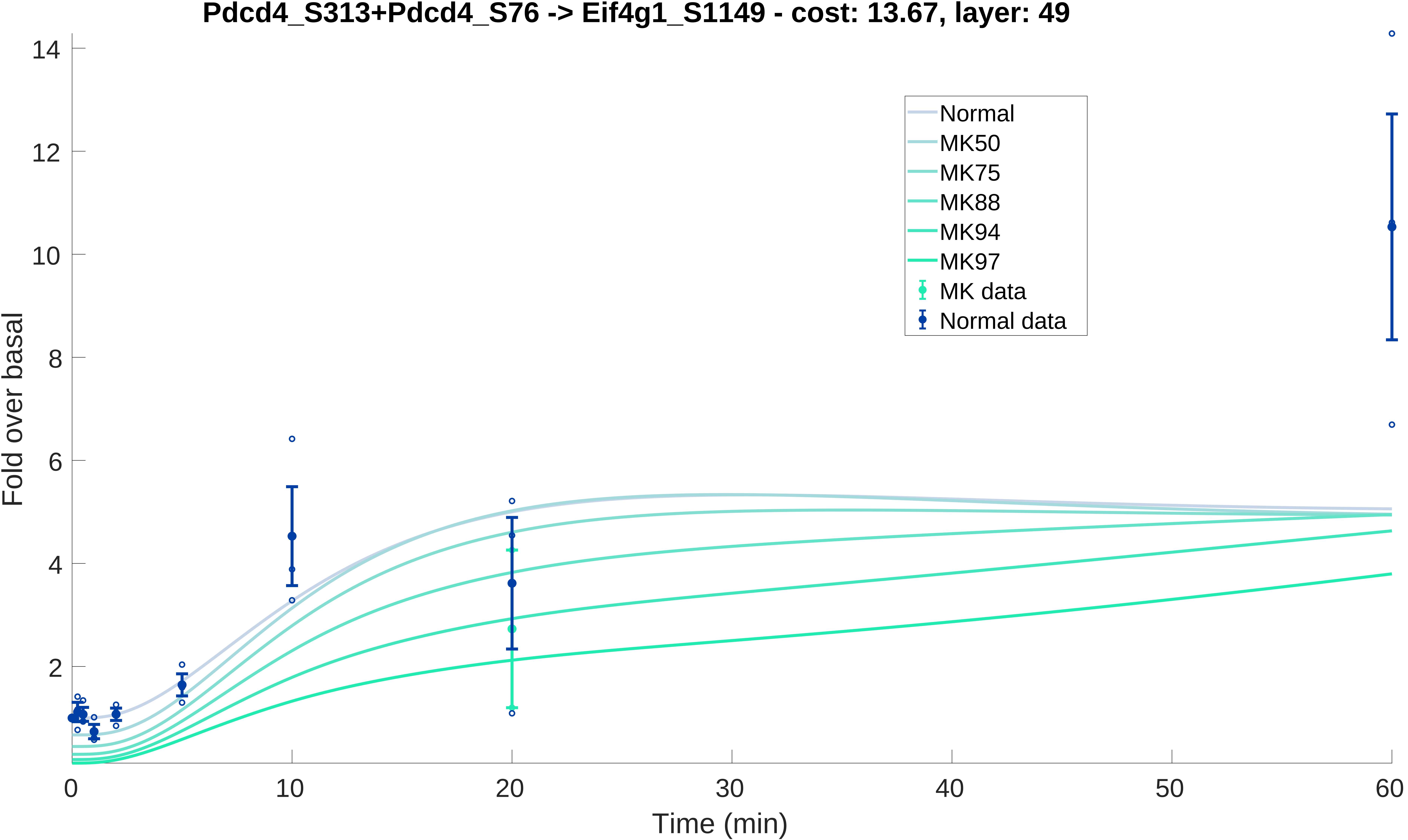

Pdcd4_S313+Pdcd4_S76 -> Eif4g1_S1149 - cost: 13.67, layer: 49
Fold over basal
Time (min)
0
10
20
30
40
50
60
2
4
6
8
10
12
14
Normal
MK50
MK75
MK88
MK94
MK97
MK data
Normal data

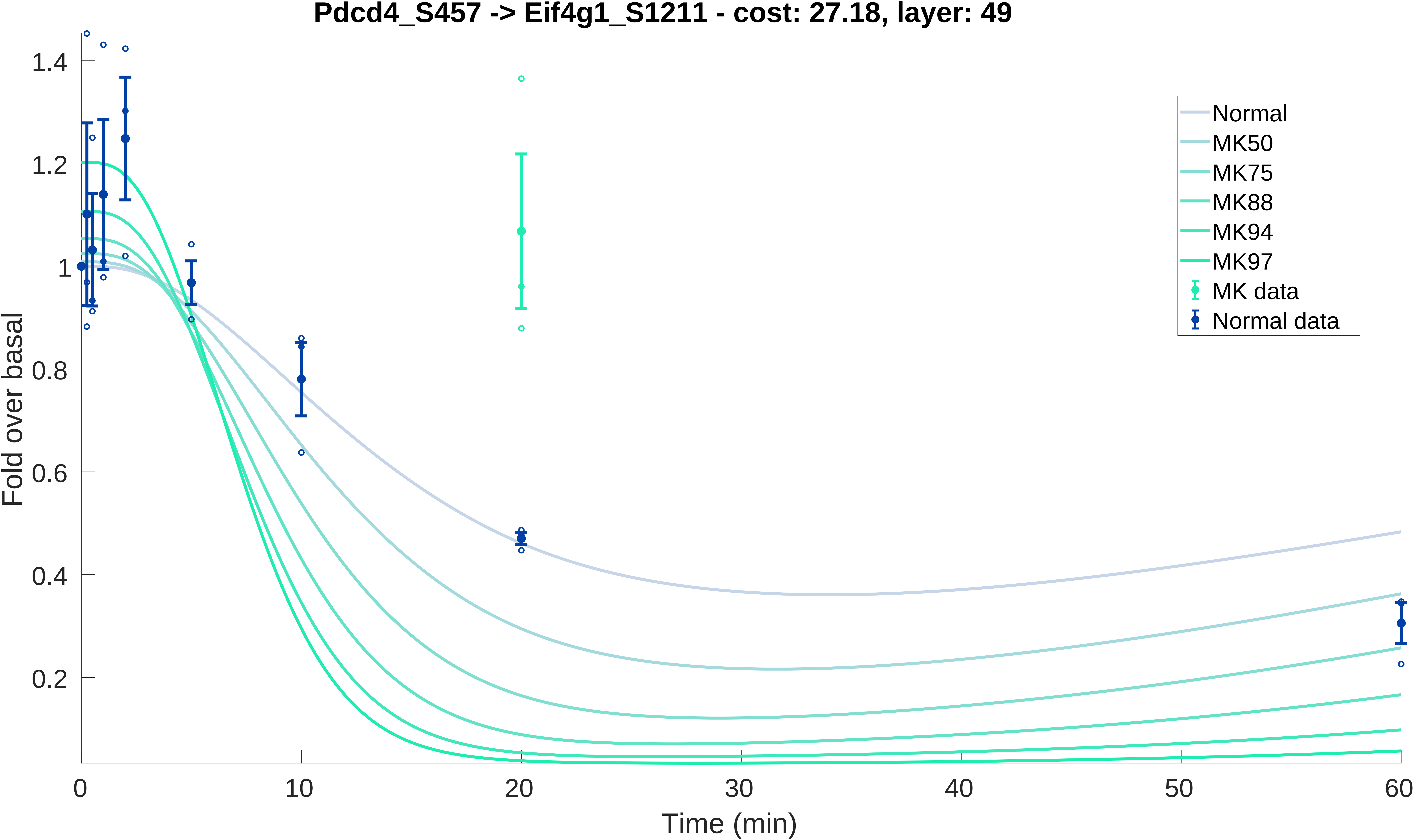

Pdcd4_S457 -> Eif4g1_S1211 - cost: 27.18, layer: 49
Fold over basal
Time (min)
0
10
20
30
40
50
60
0.2
0.4
0.6
0.8
1
1.2
1.4
Normal
MK50
MK75
MK88
MK94
MK97
MK data
Normal data

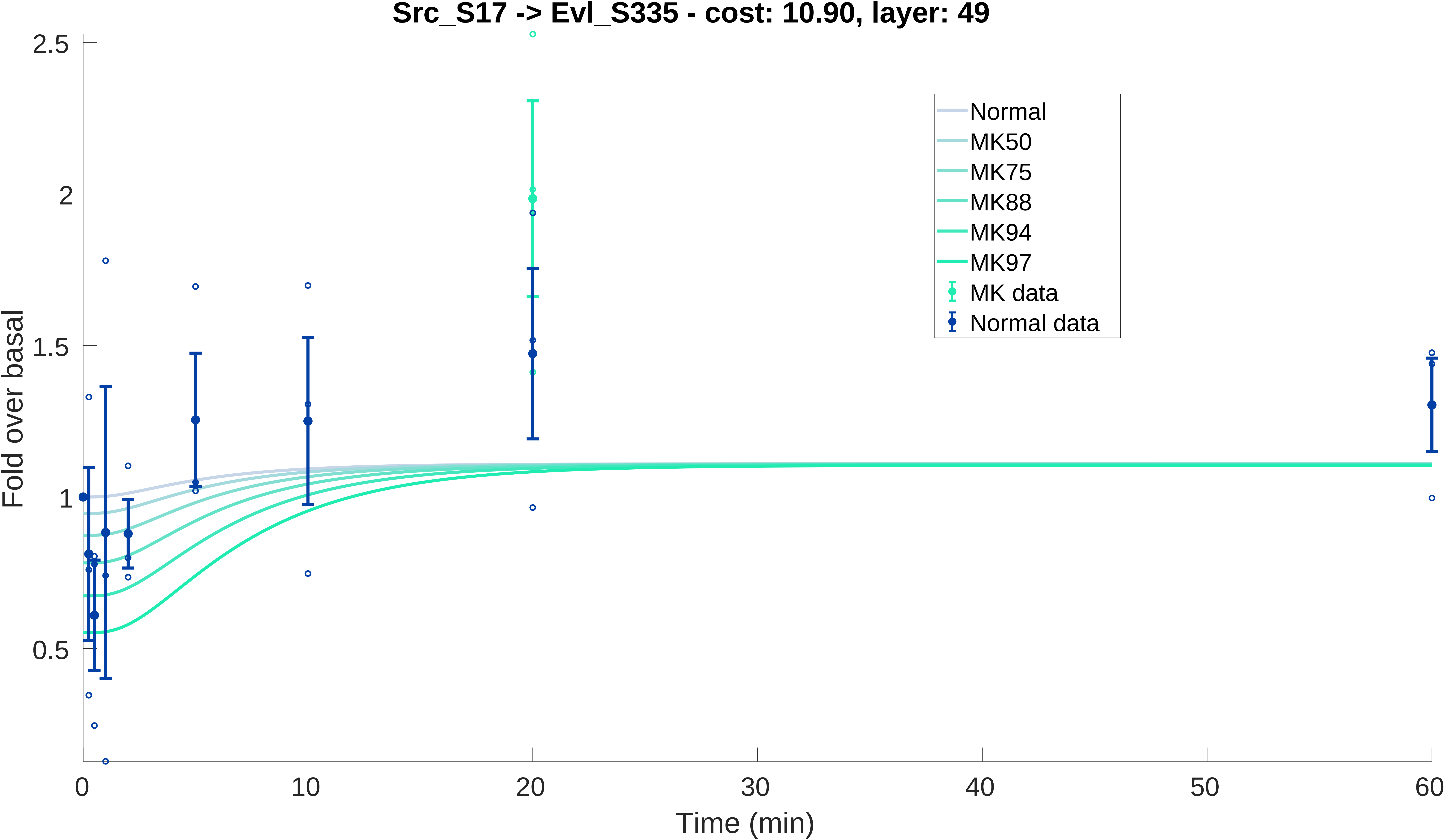
Src_S17 -> Evl_S335 - cost: 10.90, layer: 49
Fold over basal
Time (min)
2.5
2
1.5
1
0.5
0
10
20
30
40
50
60
Normal
MK50
MK75
MK88
MK94
MK97
MK data
Normal data

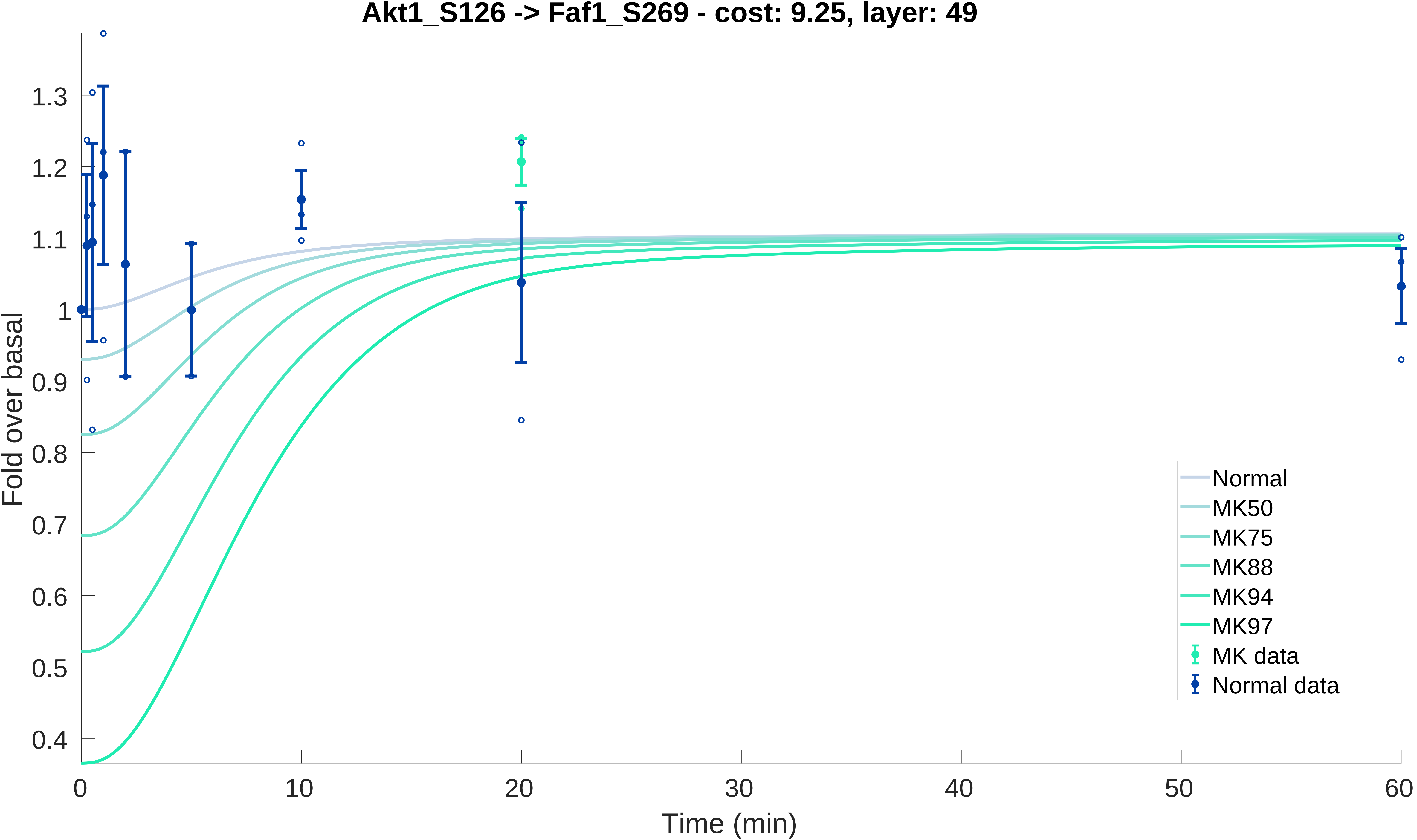

Akt1_S126 -> Faf1_S269 - cost: 9.25, layer: 49
Fold over basal
Time (min)
0
10
20
30
40
50
60
0.4
0.5
0.6
0.7
0.8
0.9
1
1.1
1.2
1.3
Normal
MK50
MK75
MK88
MK94
MK97
MK data
Normal data

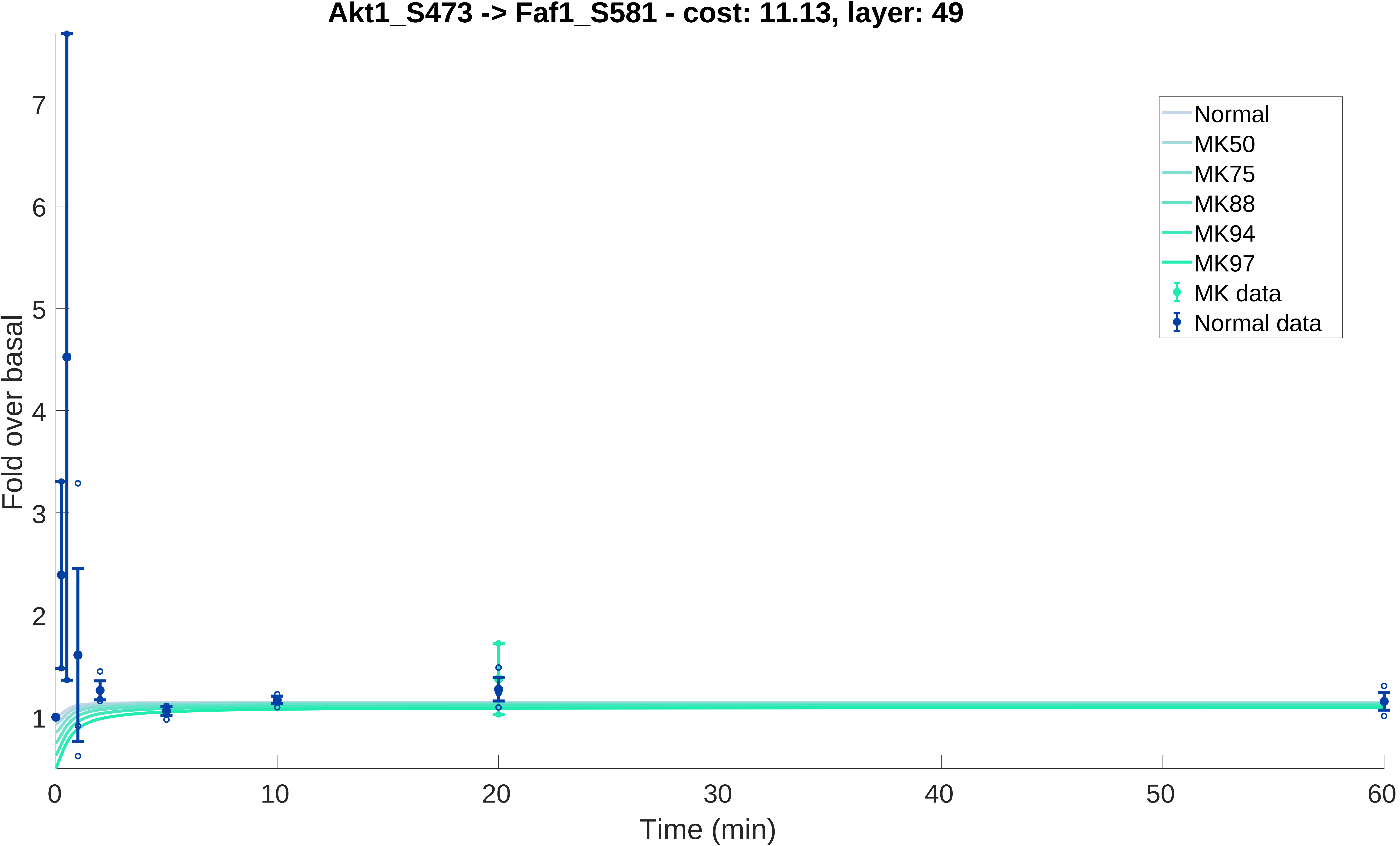

Akt1_S473 -> Faf1_S581 - cost: 11.13, layer: 49
Fold over basal
Time (min)
Normal
MK50
MK75
MK88
MK94
MK97
MK data
Normal data
0
10
20
30
40
50
60
1
2
3
4
5
6
7

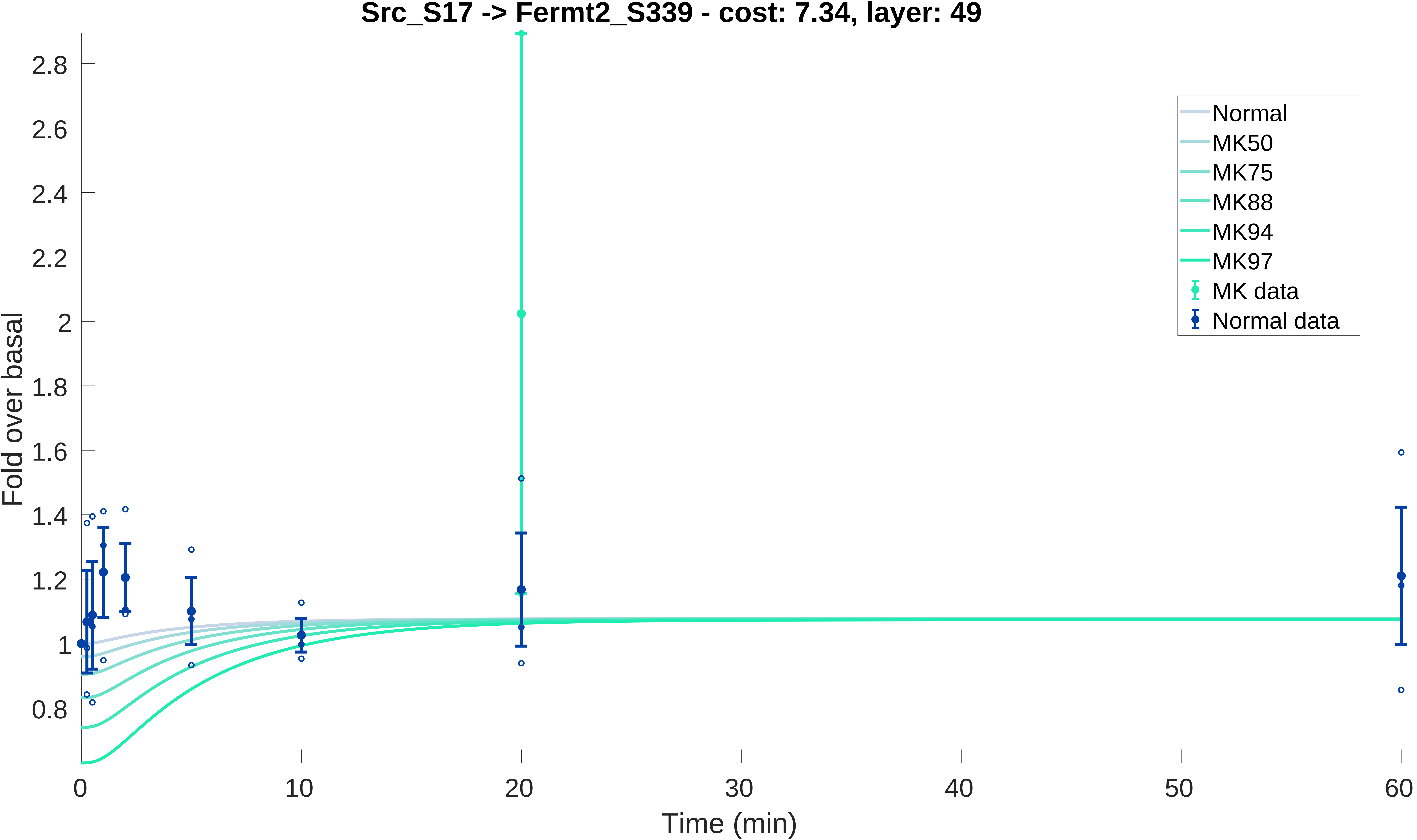

Src_S17 -> Fermt2_S339 - cost: 7.34, layer: 49
Fold over basal
Time (min)
0.8
1
1.2
1.4
1.6
1.8
2
2.2
2.4
2.6
2.8
0
10
20
30
40
50
60
Normal
MK50
MK75
MK88
MK94
MK97
MK data
Normal data

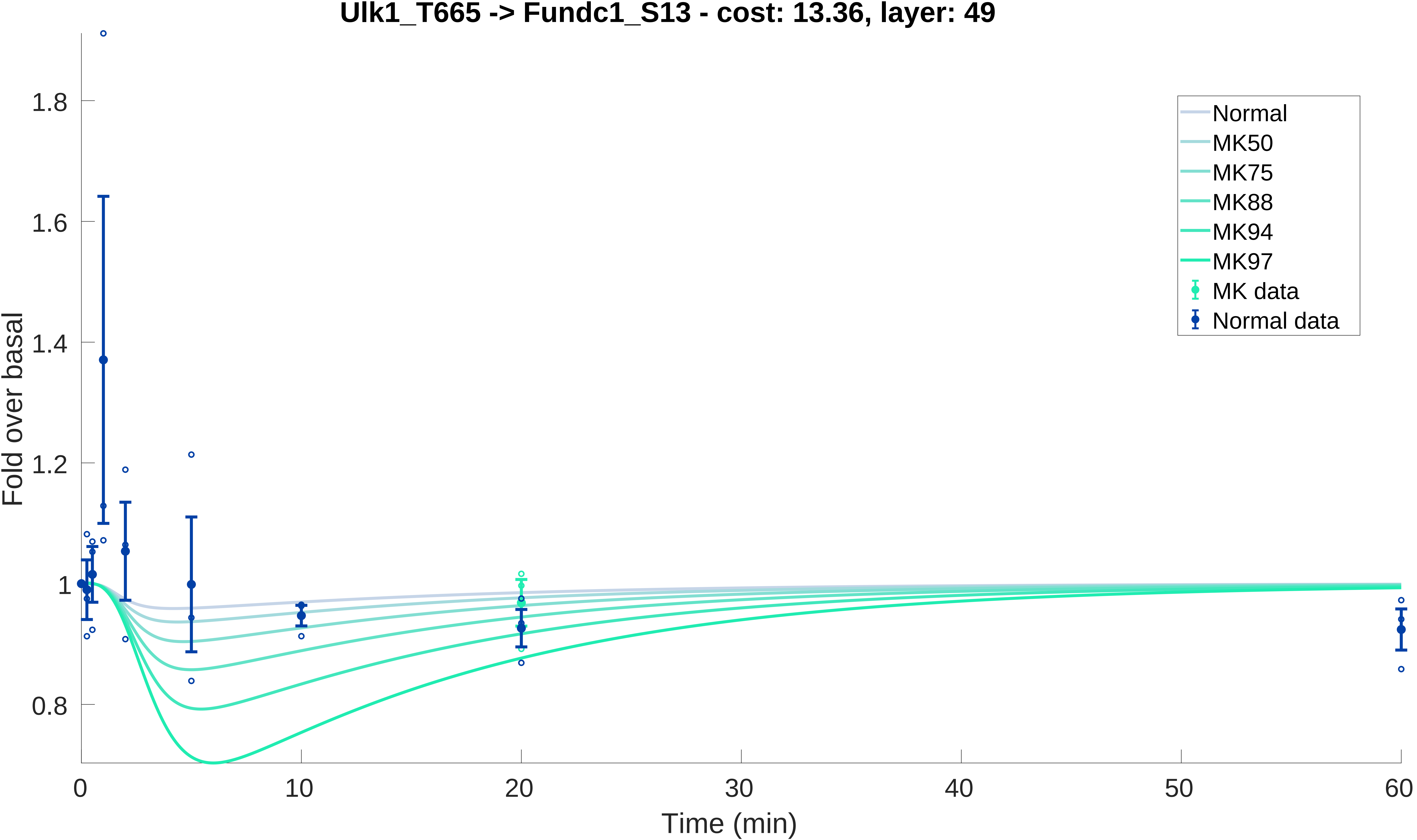
Ulk1_T665 -> Fundc1_S13 - cost: 13.36, layer: 49
Fold over basal
Time (min)
Normal
MK50
MK75
MK88
MK94
MK97
MK data
Normal data
1.8
1.6
1.4
1.2
1
0.8
0
10
20
30
40
50
60

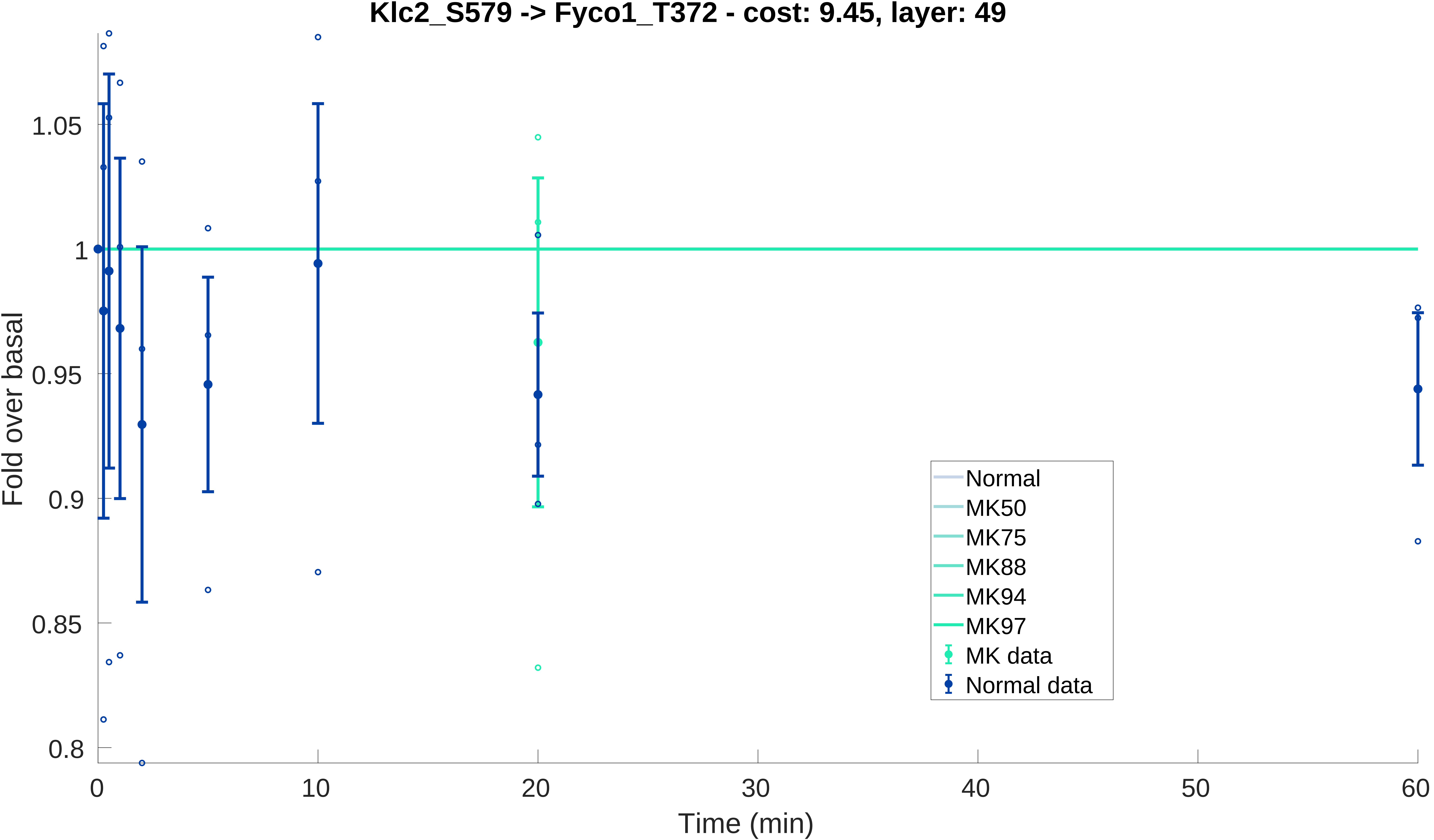

Klc2_S579 -> Fyco1_T372 - cost: 9.45, layer: 49
Fold over basal
1.05
1
0.95
0.9
0.85
0.8
0
10
20
30
40
50
60
Time (min)
Normal
MK50
MK75
MK88
MK94
MK97
MK data
Normal data

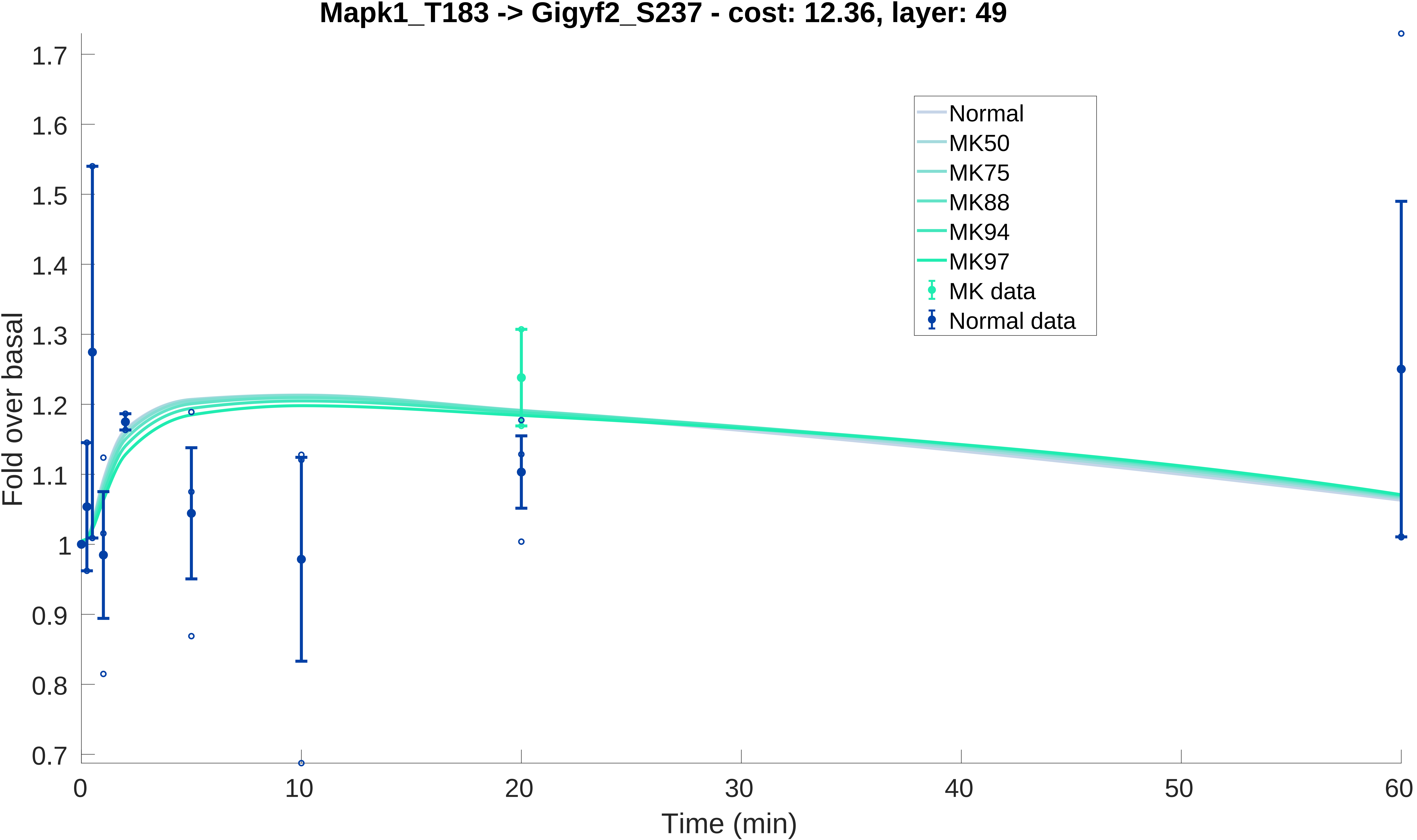

Mapk1_T183 -> Gigyf2_S237 - cost: 12.36, layer: 49
Fold over basal
1.7
1.6
1.5
1.4
1.3
1.2
1.1
1
0.9
0.8
0.7
0
10
20
30
40
50
60
Time (min)
Normal
MK50
MK75
MK88
MK94
MK97
MK data
Normal data

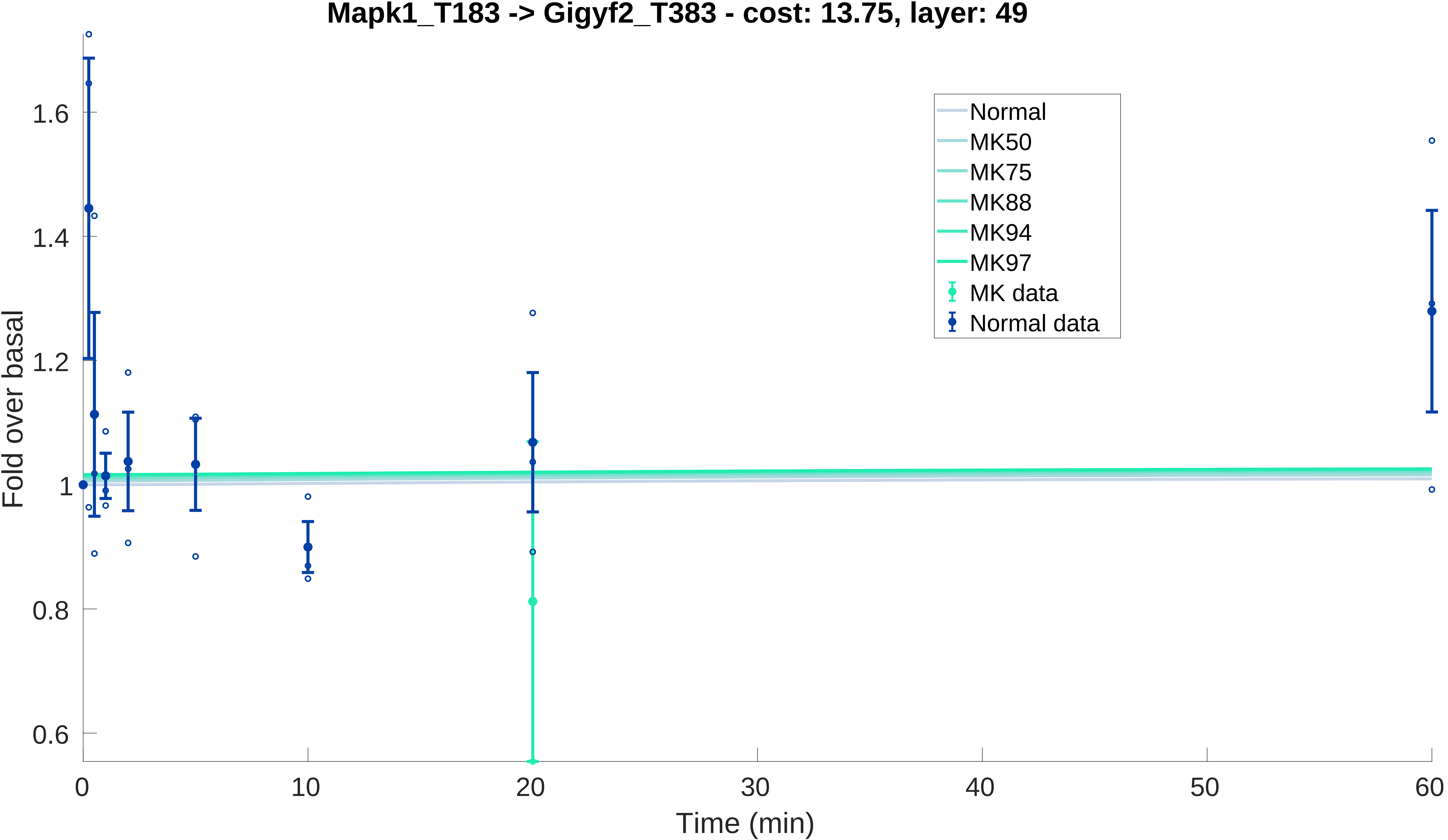

Mapk1_T183 -> Gigyf2_T383 - cost: 13.75, layer: 49
Fold over basal
Time (min)
Normal
MK50
MK75
MK88
MK94
MK97
MK data
Normal data

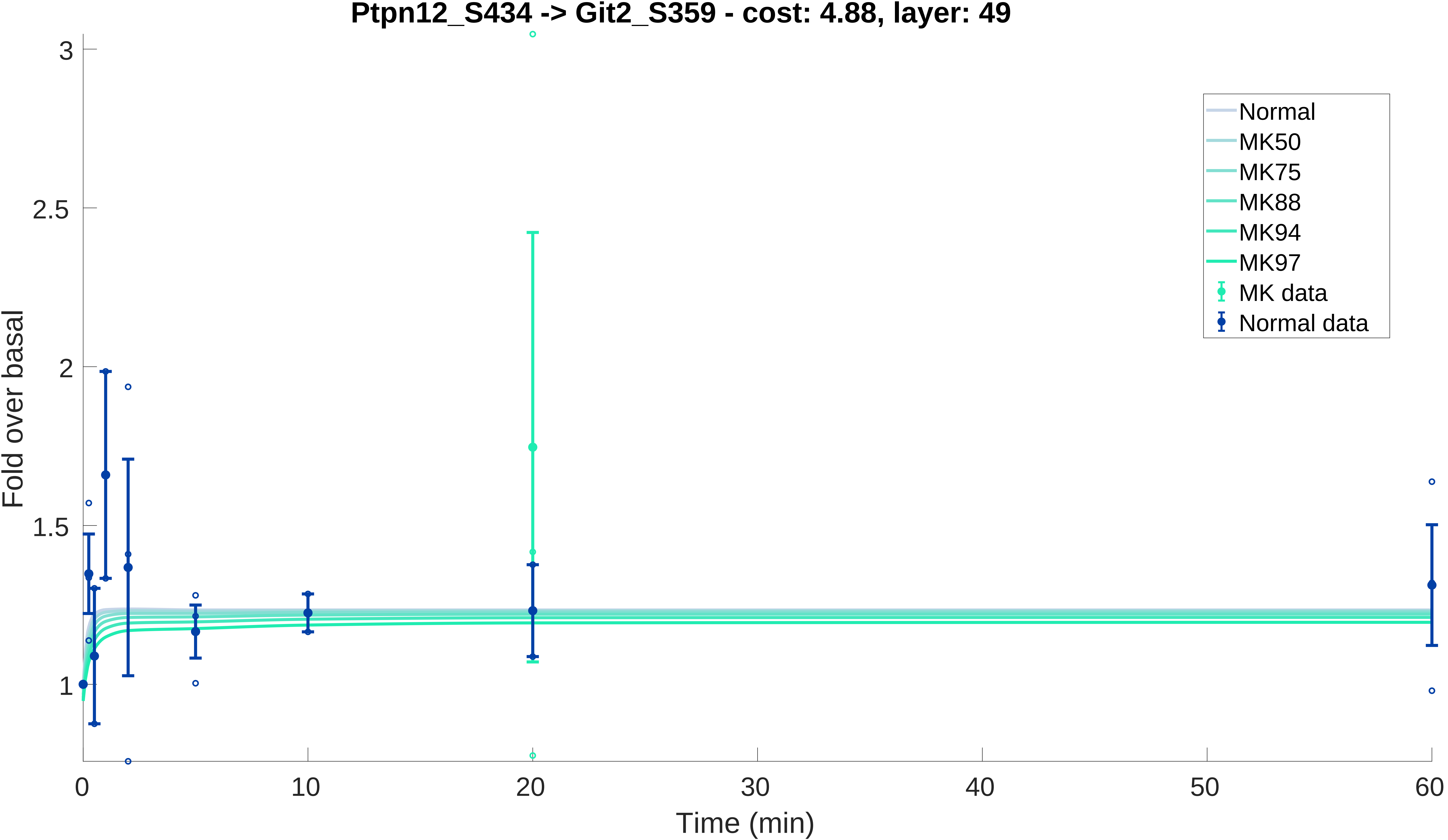

Ptpn12_S434 -> Git2_S359 - cost: 4.88, layer: 49
Fold over basal
Time (min)
3
2.5
2
1.5
1
0
10
20
30
40
50
60
Normal
MK50
MK75
MK88
MK94
MK97
MK data
Normal data

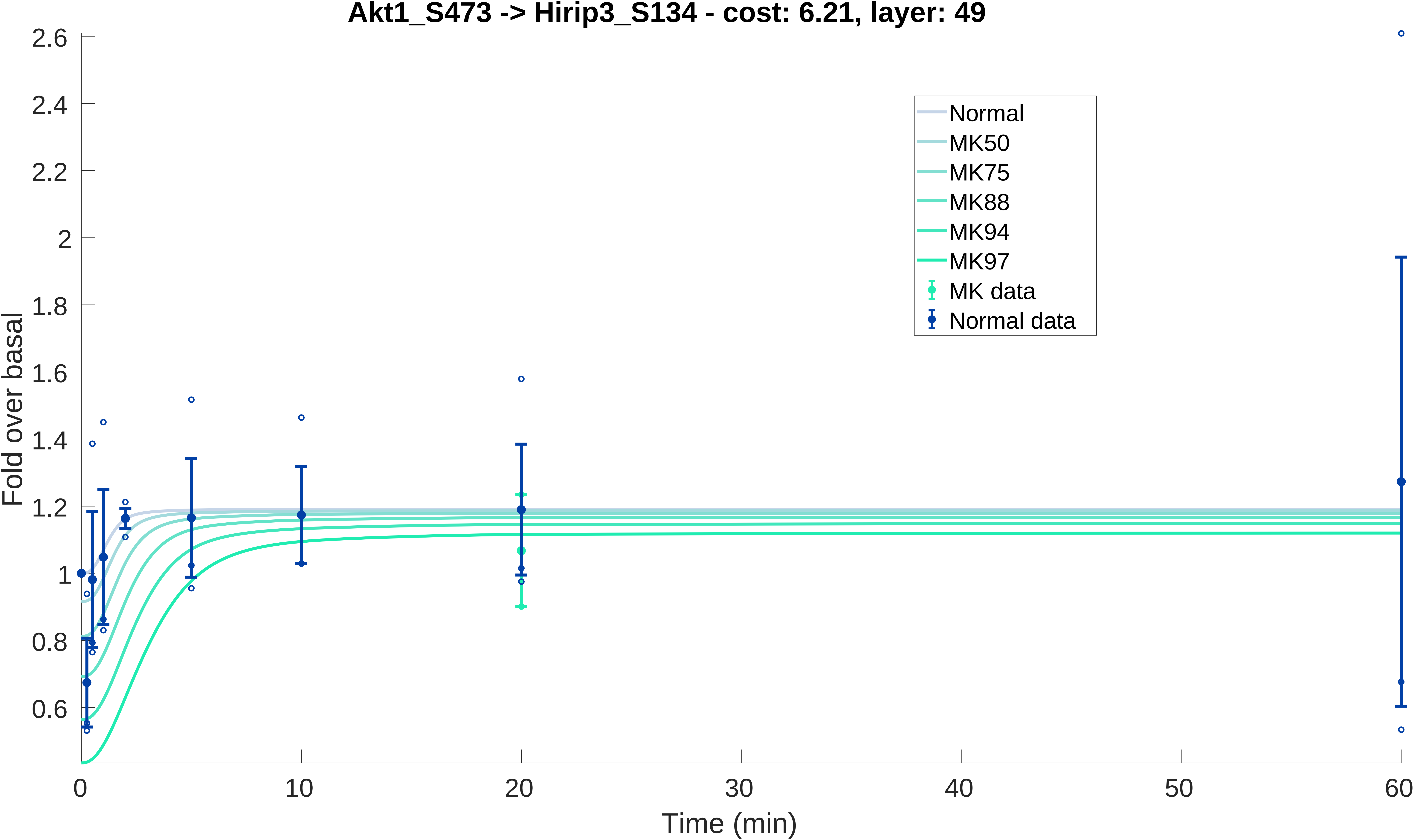

Akt1_S473 -> Hirip3_S134 - cost: 6.21, layer: 49
Fold over basal
Time (min)
2.6
2.4
2.2
2
1.8
1.6
1.4
1.2
1
0.8
0.6
0
10
20
30
40
50
60
Normal
MK50
MK75
MK88
MK94
MK97
MK data
Normal data

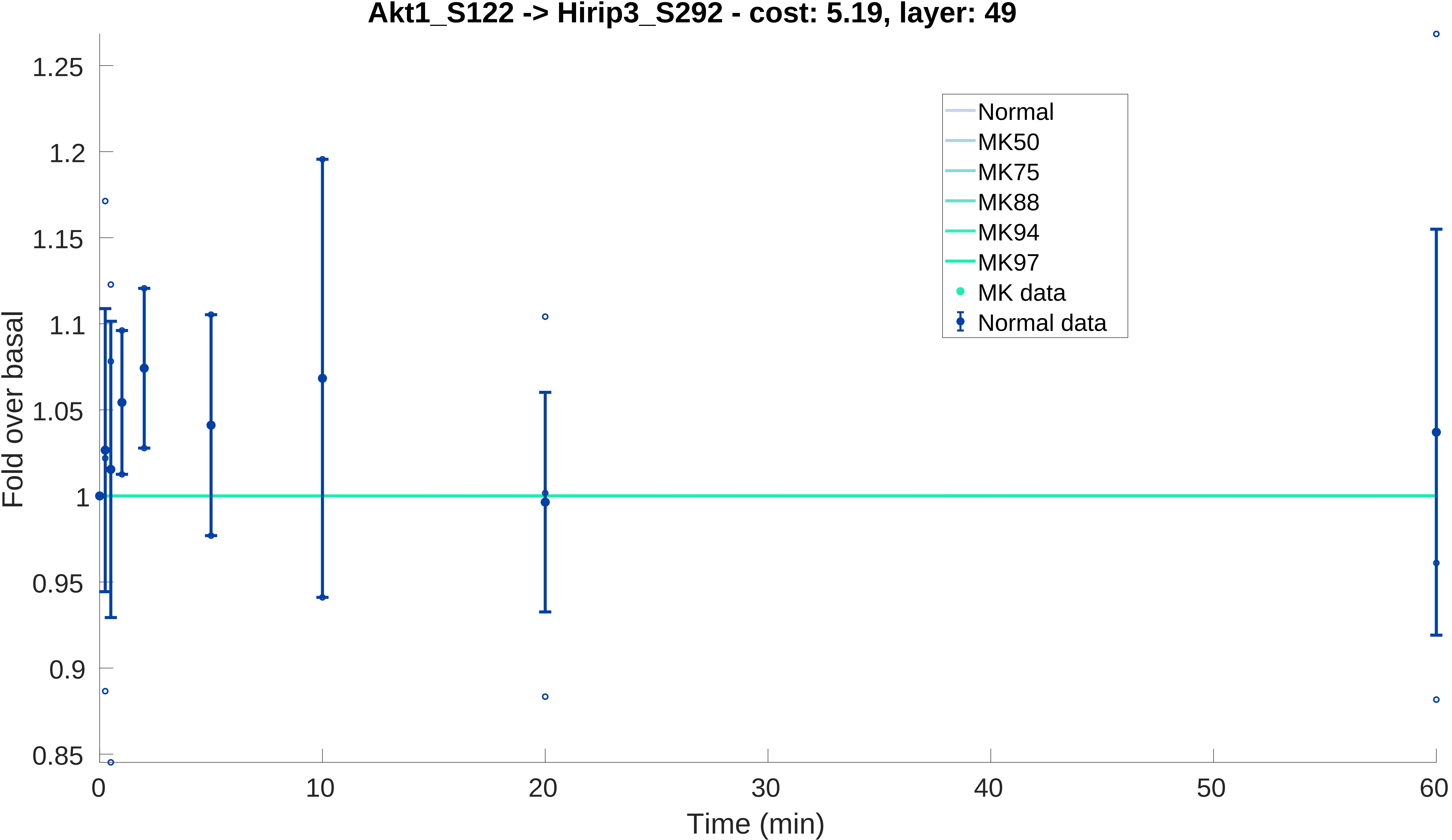

Akt1_S122 -> Hirip3_S292 - cost: 5.19, layer: 49
Fold over basal
Time (min)
1.25
1.2
1.15
1.1
1.05
1
0.95
0.9
0.85
0
10
20
30
40
50
60
Normal
MK50
MK75
MK88
MK94
MK97
MK data
Normal data

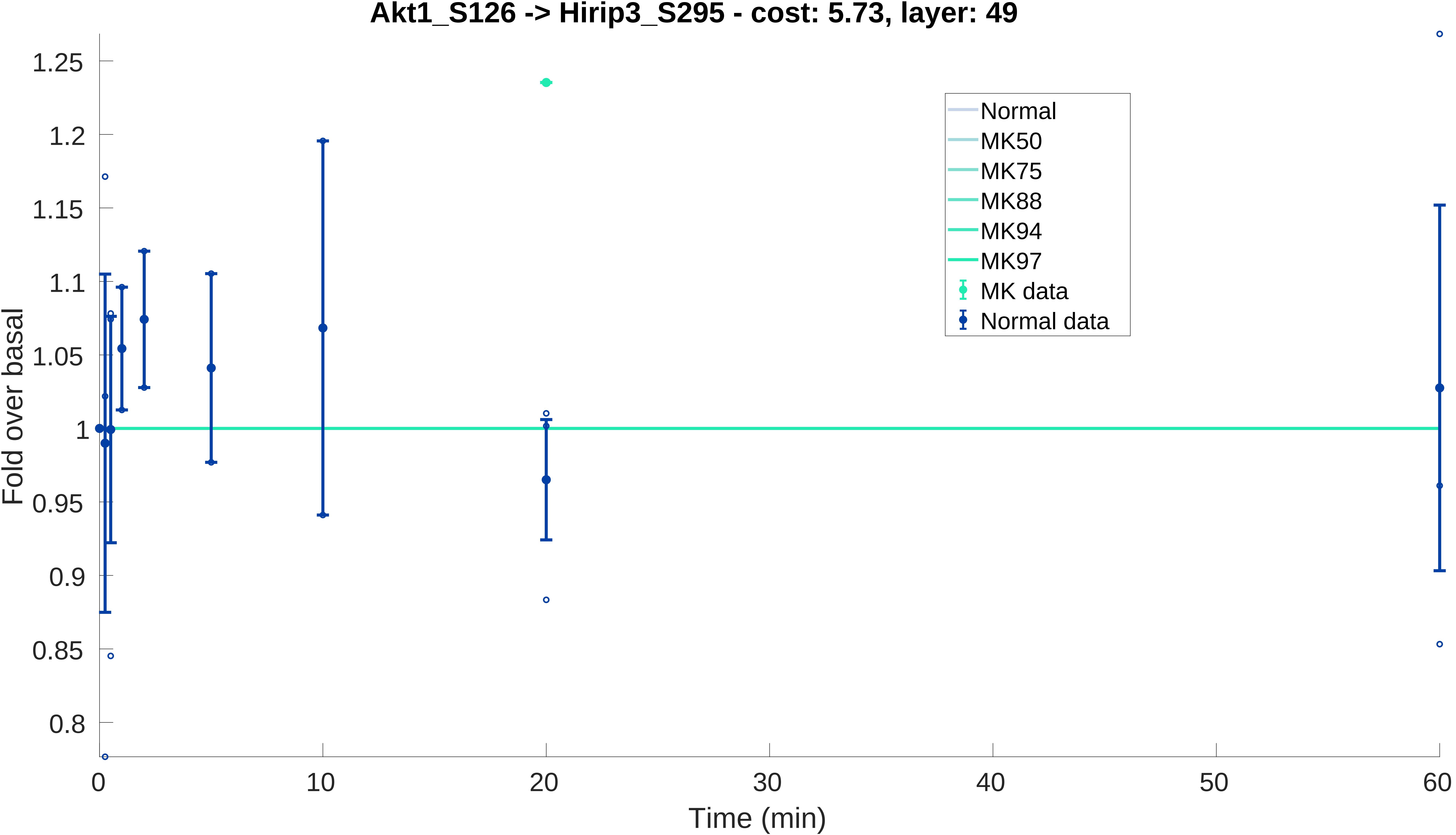

Akt1_S126 -> Hirip3_S295 - cost: 5.73, layer: 49
Fold over basal
Time (min)
1.25
1.2
1.15
1.1
1.05
1
0.95
0.9
0.85
0.8
0
10
20
30
40
50
60
Normal
MK50
MK75
MK88
MK94
MK97
MK data
Normal data

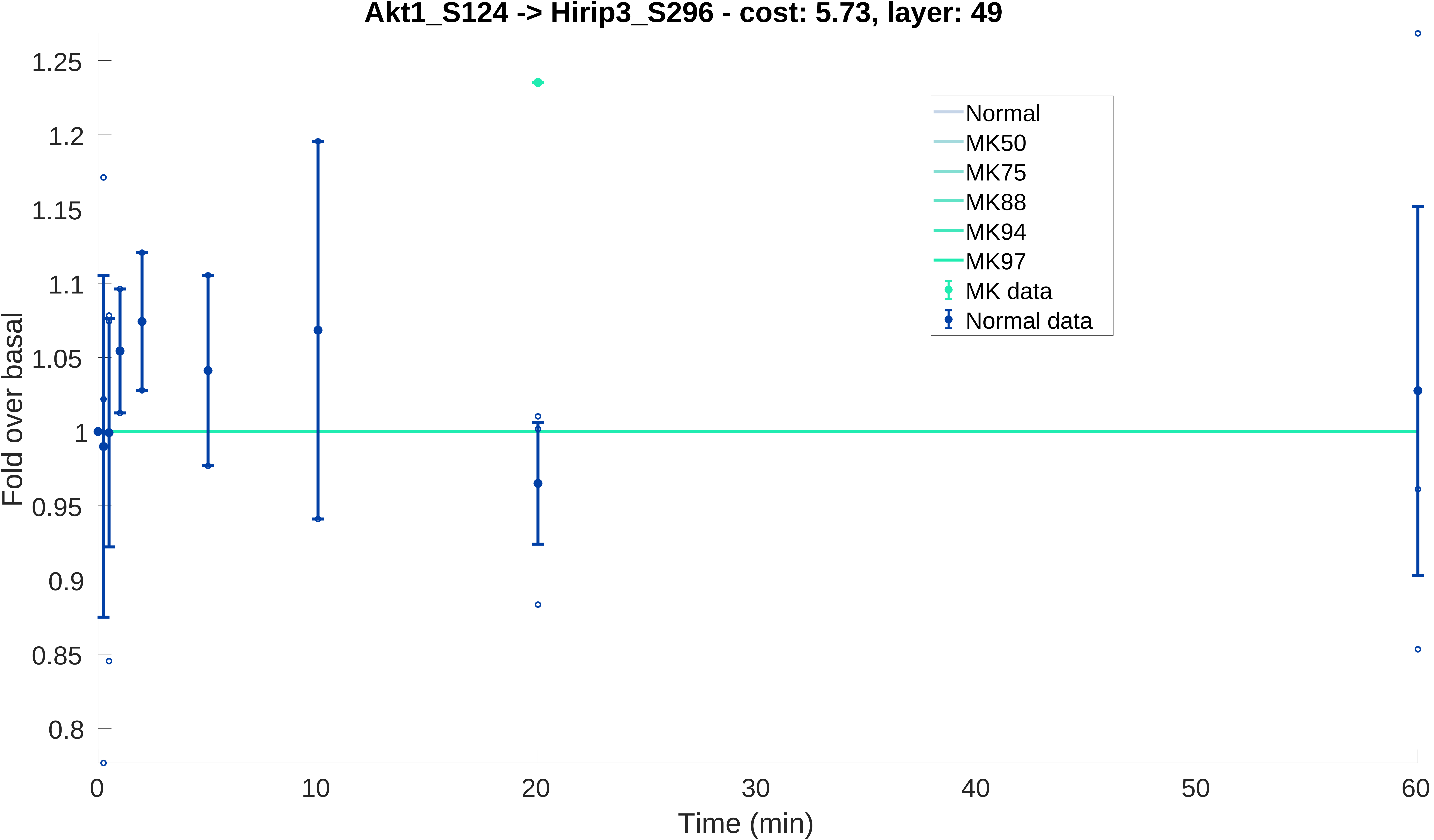
Akt1_S124 -> Hirip3_S296 - cost: 5.73, layer: 49
Fold over basal
Time (min)
1.25
1.2
1.15
1.1
1.05
1
0.95
0.9
0.85
0.8
0
10
20
30
40
50
60
Normal
MK50
MK75
MK88
MK94
MK97
MK data
Normal data

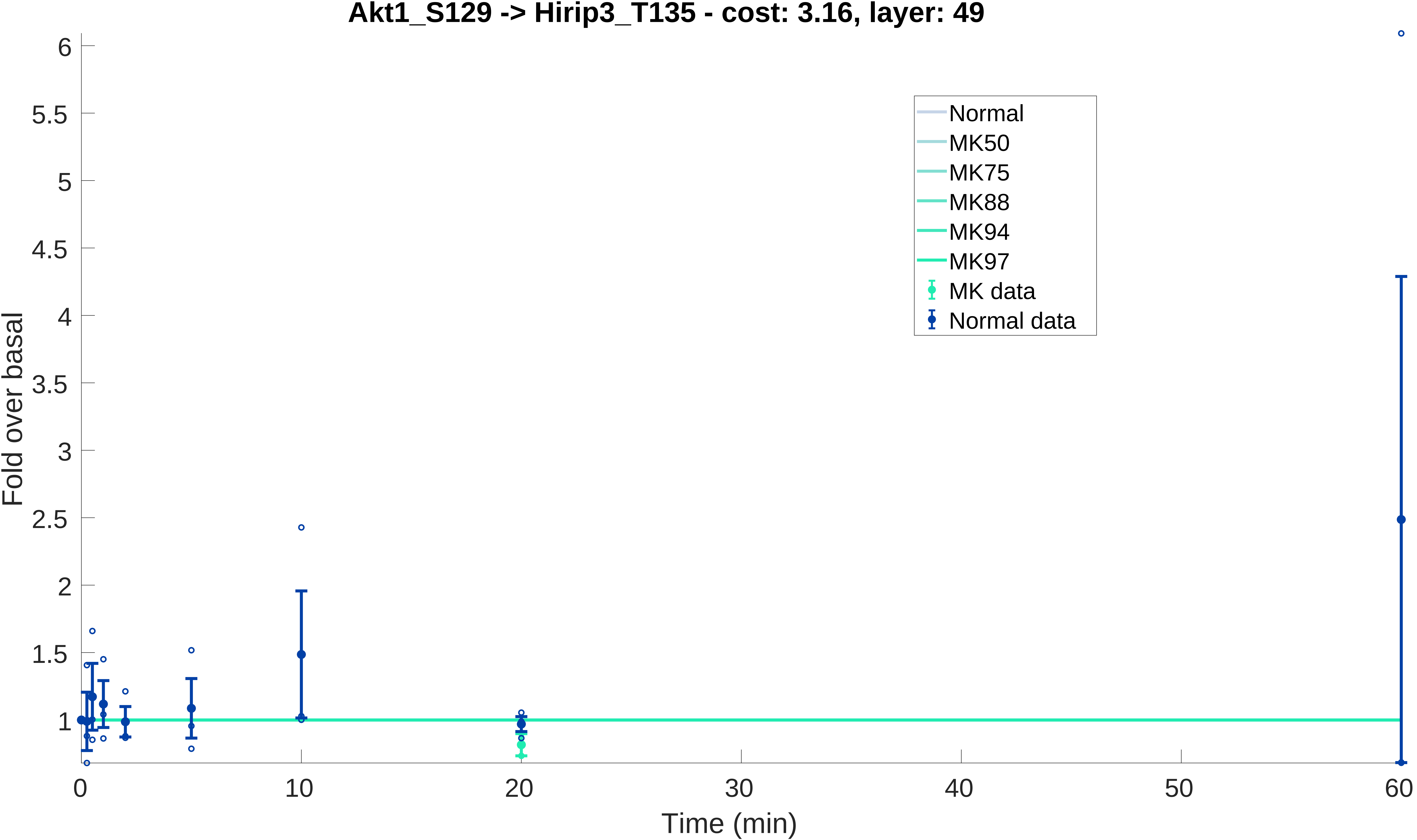

Akt1_S129 -> Hirip3_T135 - cost: 3.16, layer: 49
Fold over basal
Time (min)
6
5.5
5
4.5
4
3.5
3
2.5
2
1.5
1
0
10
20
30
40
50
60
Normal
MK50
MK75
MK88
MK94
MK97
MK data
Normal data

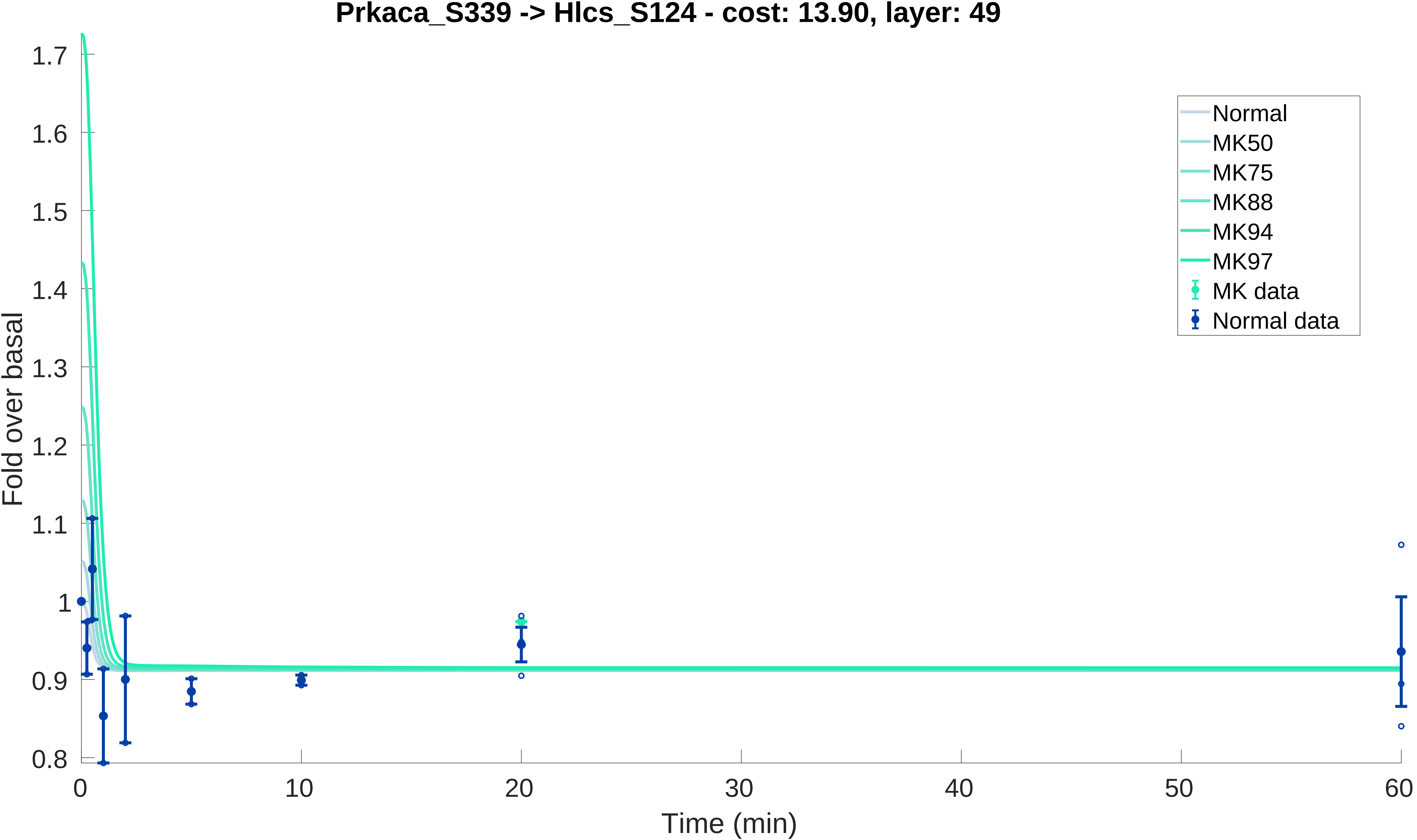

Prkaca_S339 -> Hlcs_S124 - cost: 13.90, layer: 49
Fold over basal
Time (min)
0.8
0.9
1
1.1
1.2
1.3
1.4
1.5
1.6
1.7
0
10
20
30
40
50
60
Normal
MK50
MK75
MK88
MK94
MK97
MK data
Normal data

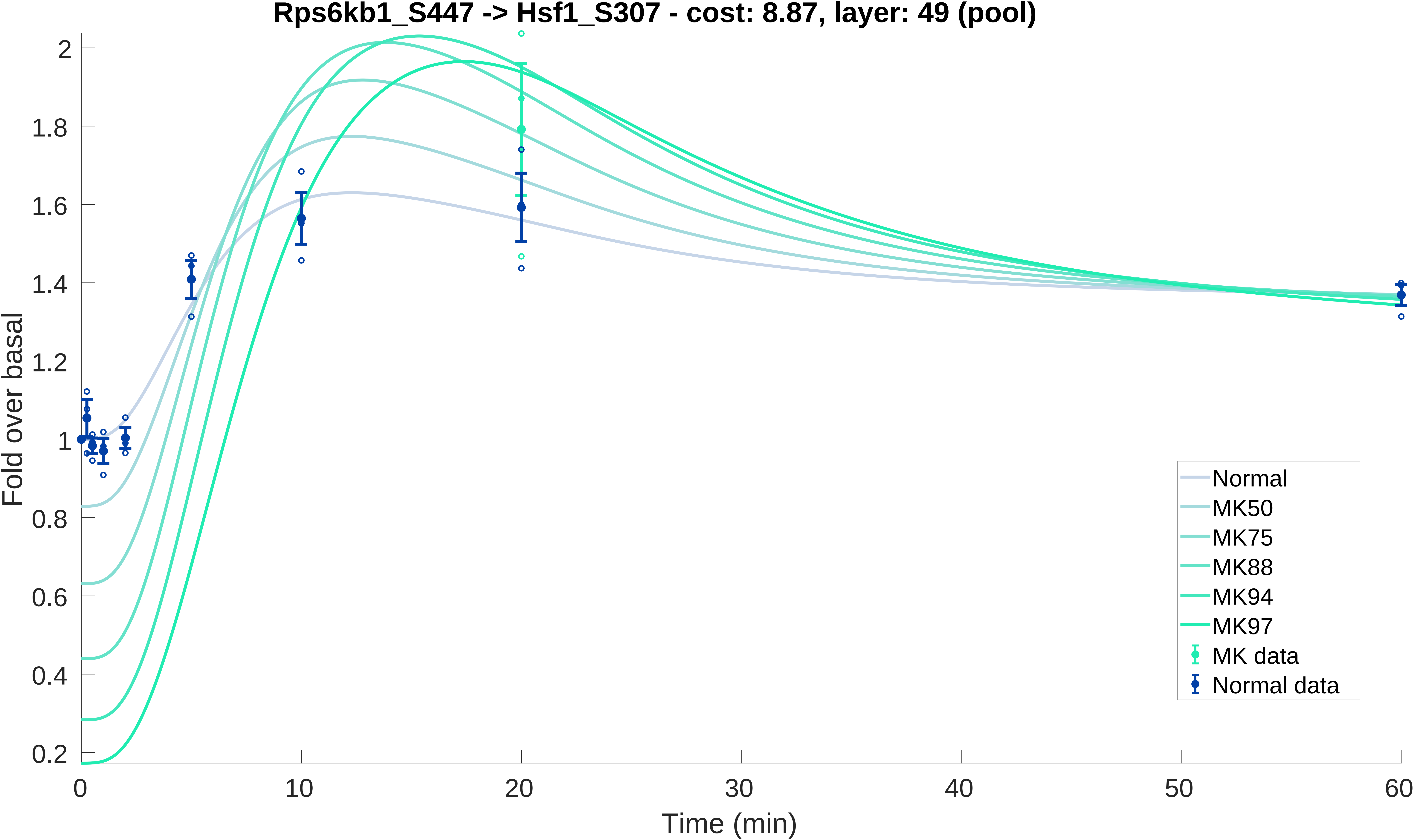

Rps6kb1_S447 -> Hsf1_S307 - cost: 8.87, layer: 49 (pool)
Fold over basal
0.2
0.4
0.6
0.8
1
1.2
1.4
1.6
1.8
2
Time (min)
0
10
20
30
40
50
60
Normal
MK50
MK75
MK88
MK94
MK97
MK data
Normal data

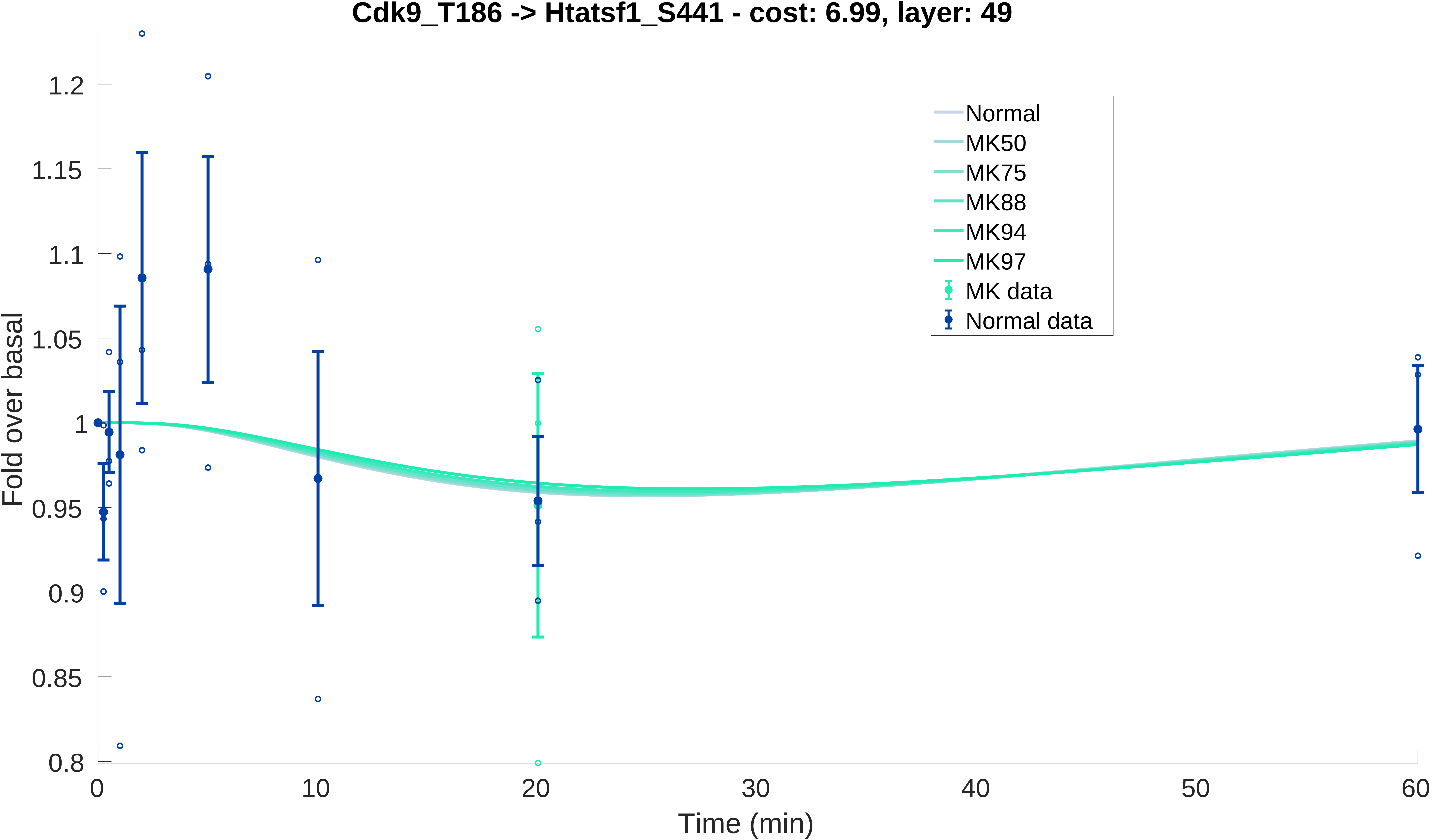

Cdk9_T186 -> Htatsf1_S441 - cost: 6.99, layer: 49
Fold over basal
Time (min)
Normal
MK50
MK75
MK88
MK94
MK97
MK data
Normal data
1.2
1.15
1.1
1.05
1
0.95
0.9
0.85
0.8
0
10
20
30
40
50
60

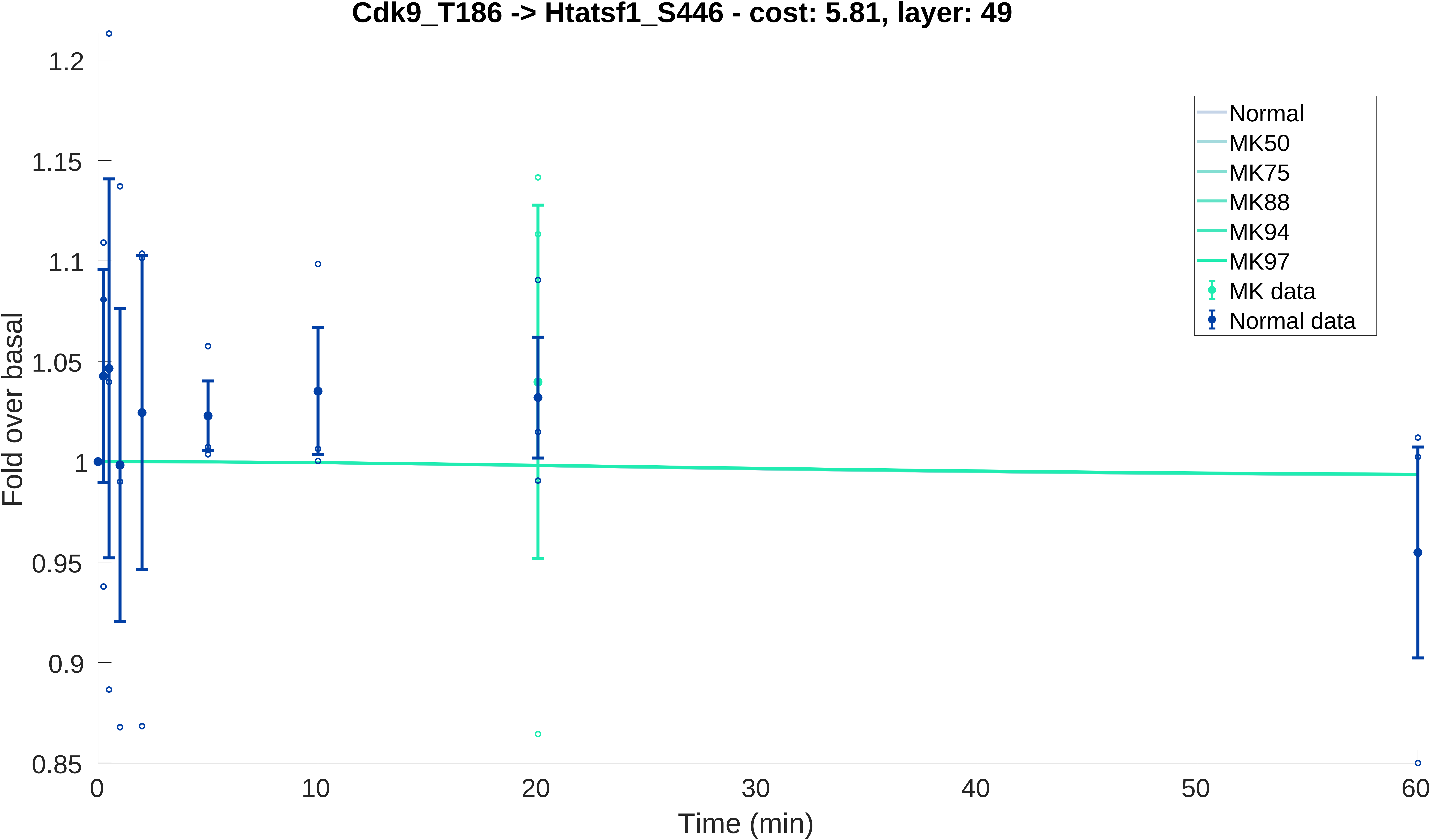

Cdk9_T186 -> Htatsf1_S446 - cost: 5.81, layer: 49
Fold over basal
Time (min)
1.2
1.15
1.1
1.05
1
0.95
0.9
0.85
0
10
20
30
40
50
60
Normal
MK50
MK75
MK88
MK94
MK97
MK data
Normal data

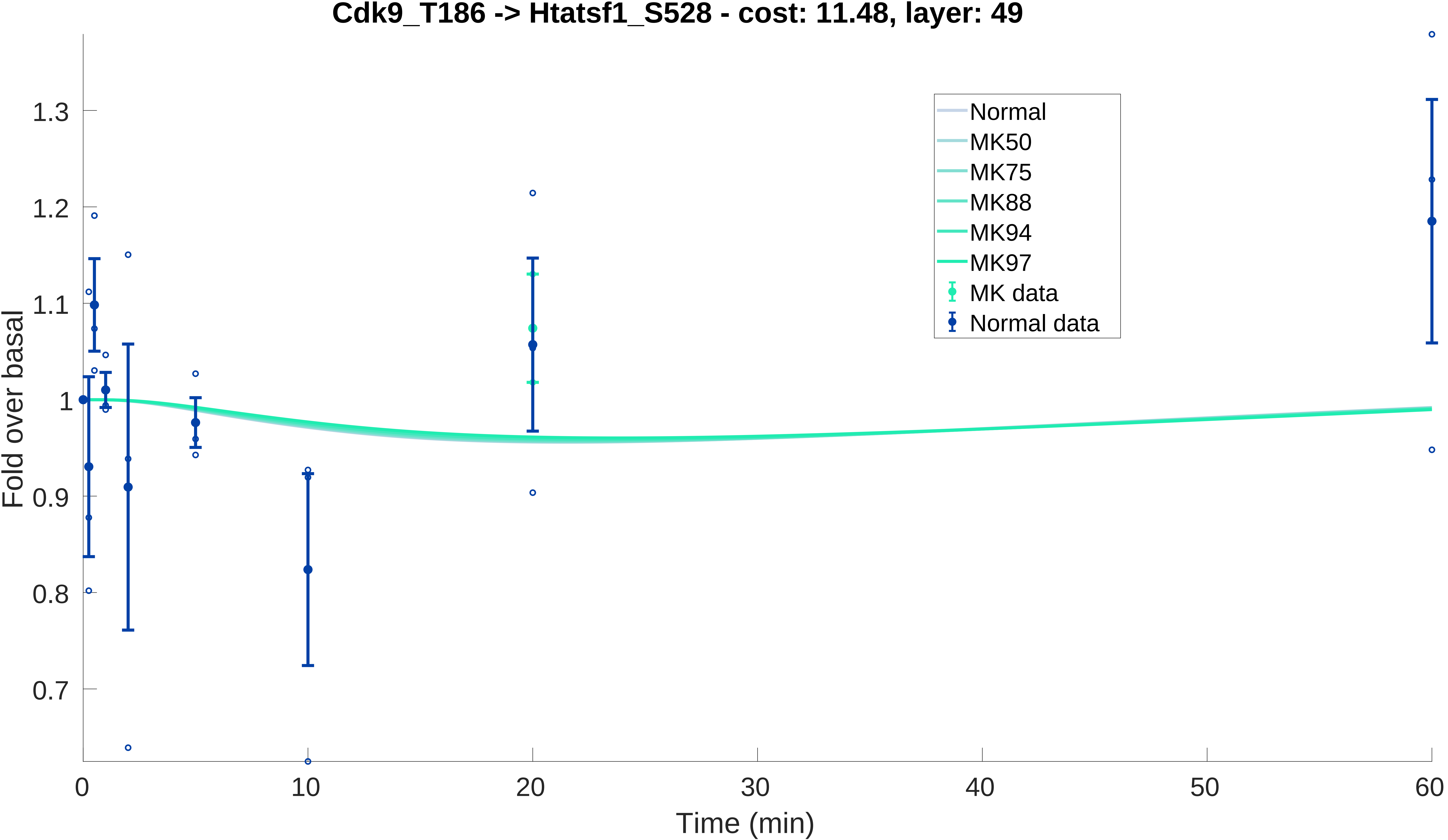

Cdk9_T186 -> Htatsf1_S528 - cost: 11.48, layer: 49
Fold over basal
1.3
1.2
1.1
1
0.9
0.8
0.7
0
10
20
30
40
50
60
Time (min)
Normal
MK50
MK75
MK88
MK94
MK97
MK data
Normal data

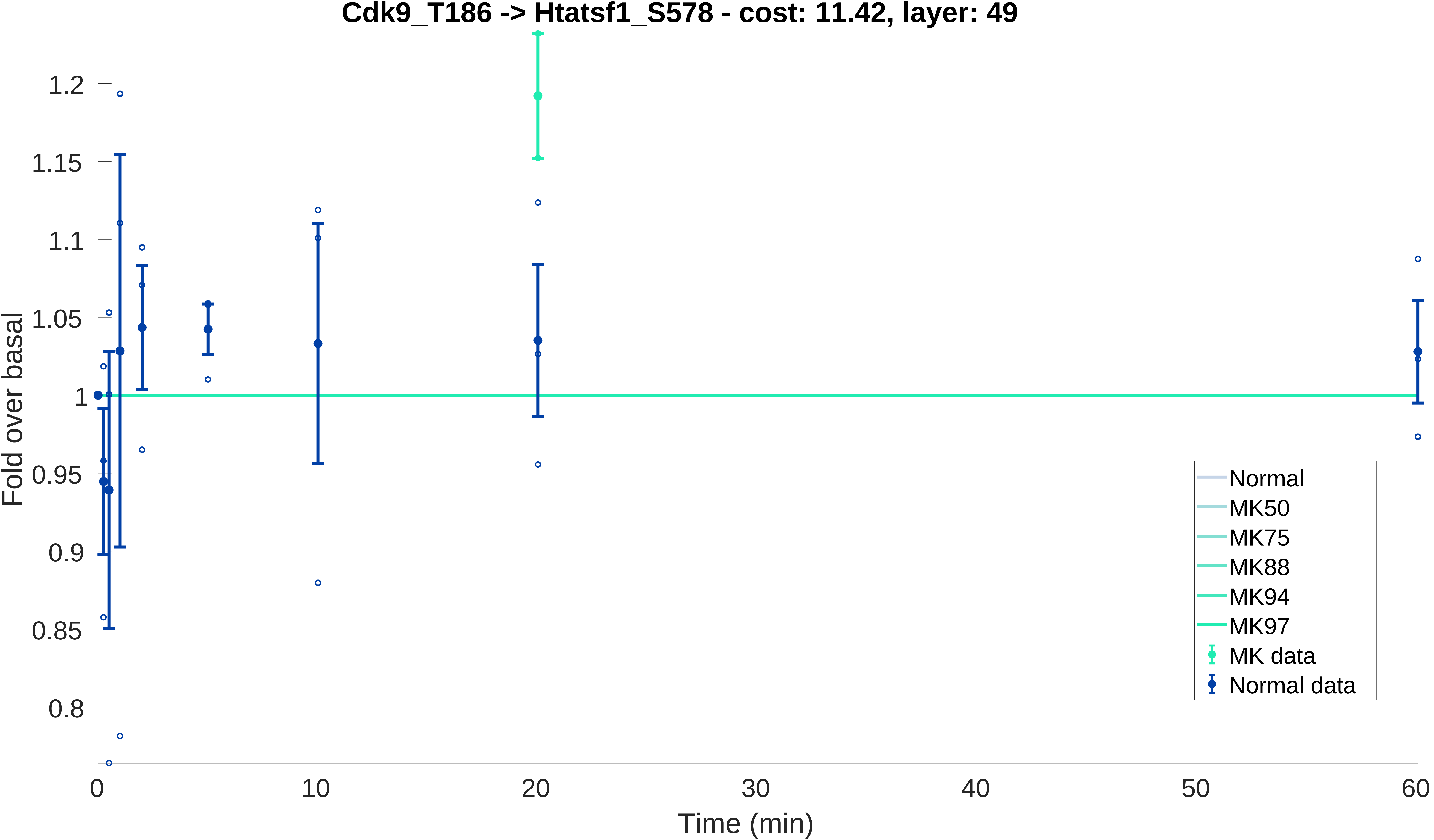

Cdk9_T186 -> Htatsf1_S578 - cost: 11.42, layer: 49
Fold over basal
Time (min)
0.8
0.85
0.9
0.95
1
1.05
1.1
1.15
1.2
0
10
20
30
40
50
60
Normal
MK50
MK75
MK88
MK94
MK97
MK data
Normal data

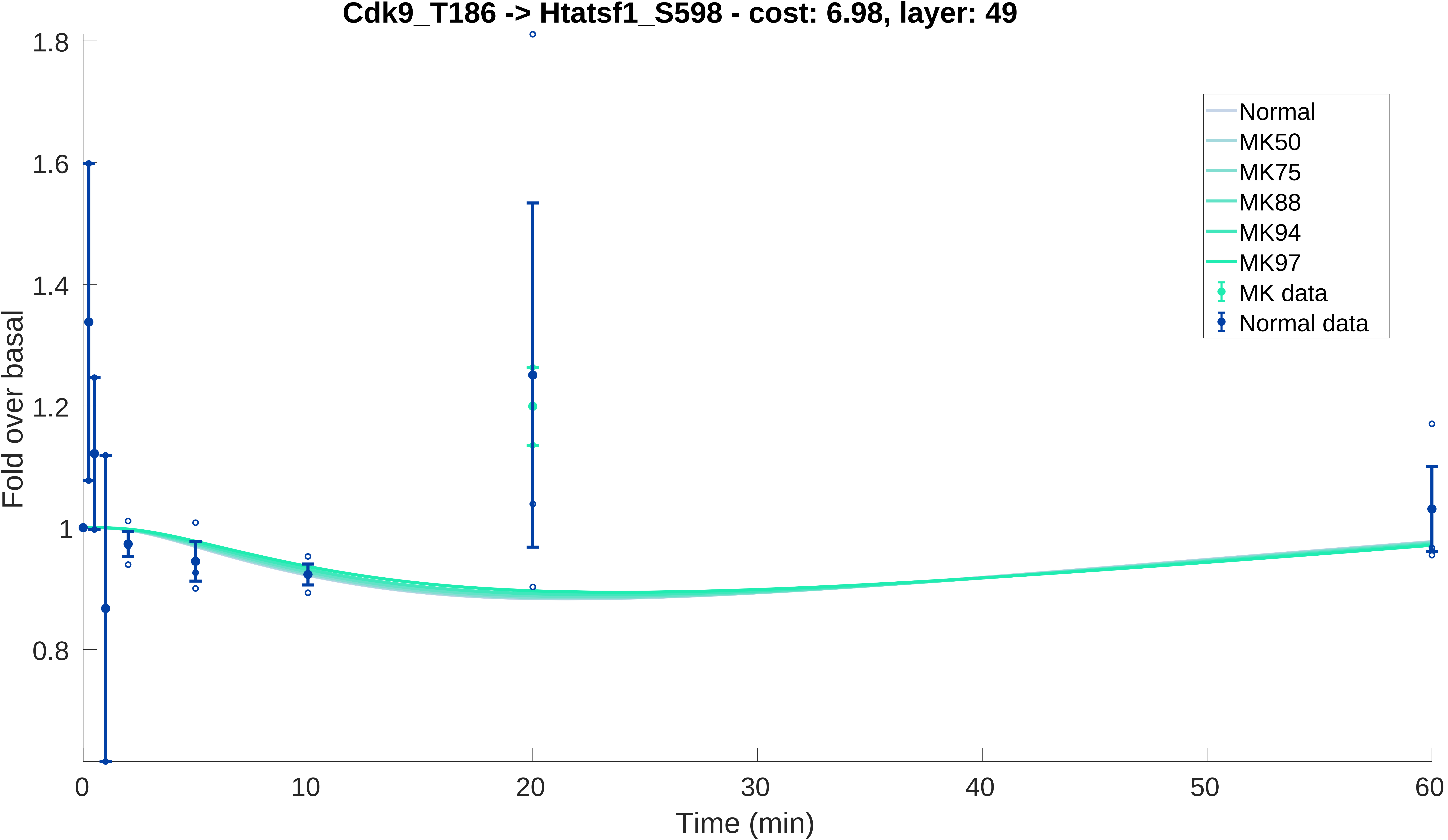

Cdk9_T186 -> Htatsf1_S598 - cost: 6.98, layer: 49
Fold over basal
Time (min)
1.8
1.6
1.4
1.2
1
0.8
0
10
20
30
40
50
60
Normal
MK50
MK75
MK88
MK94
MK97
MK data
Normal data

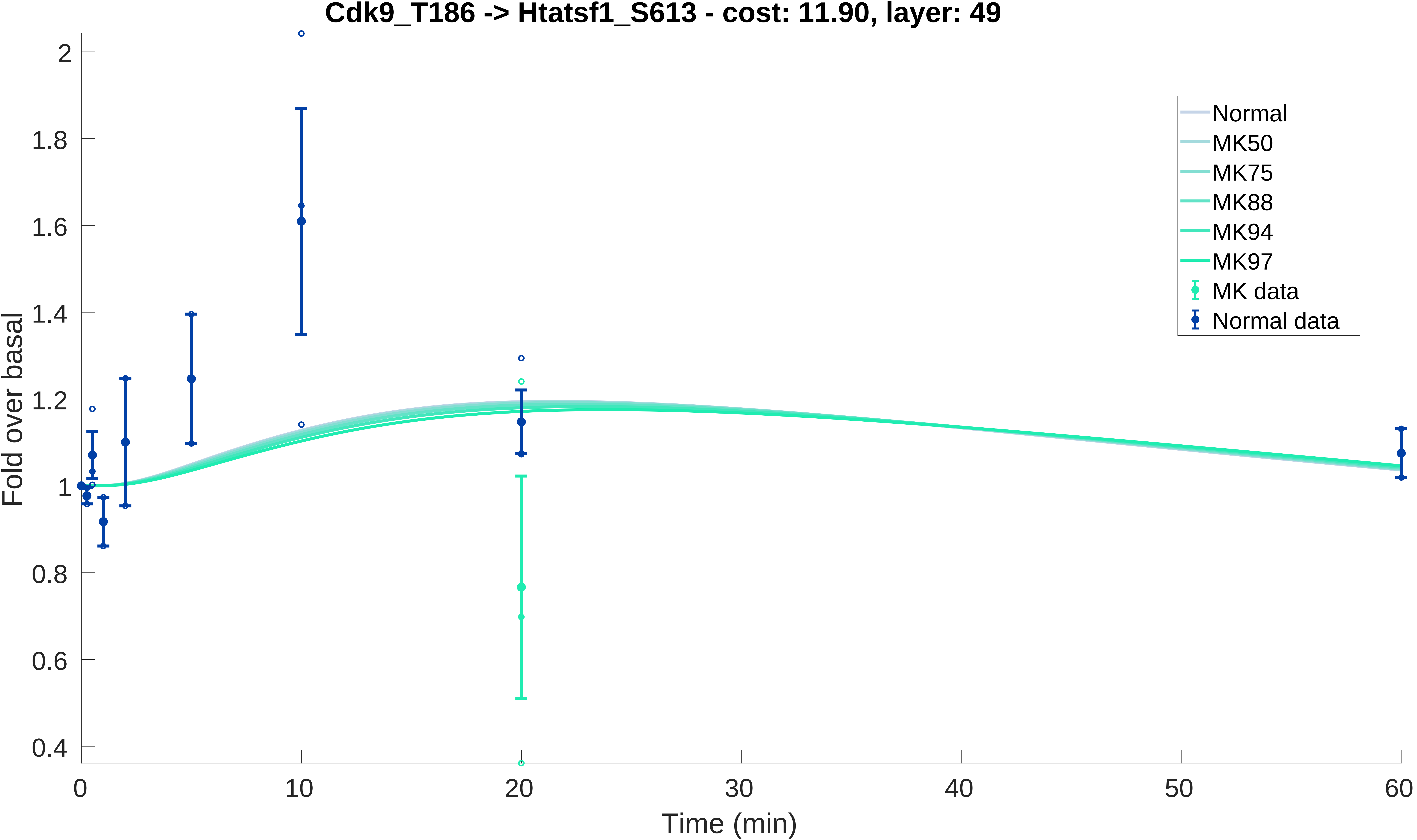
Cdk9_T186 -> Htatsf1_S613 - cost: 11.90, layer: 49
Fold over basal
Time (min)
0
10
20
30
40
50
60
0.4
0.6
0.8
1
1.2
1.4
1.6
1.8
2
Normal
MK50
MK75
MK88
MK94
MK97
MK data
Normal data

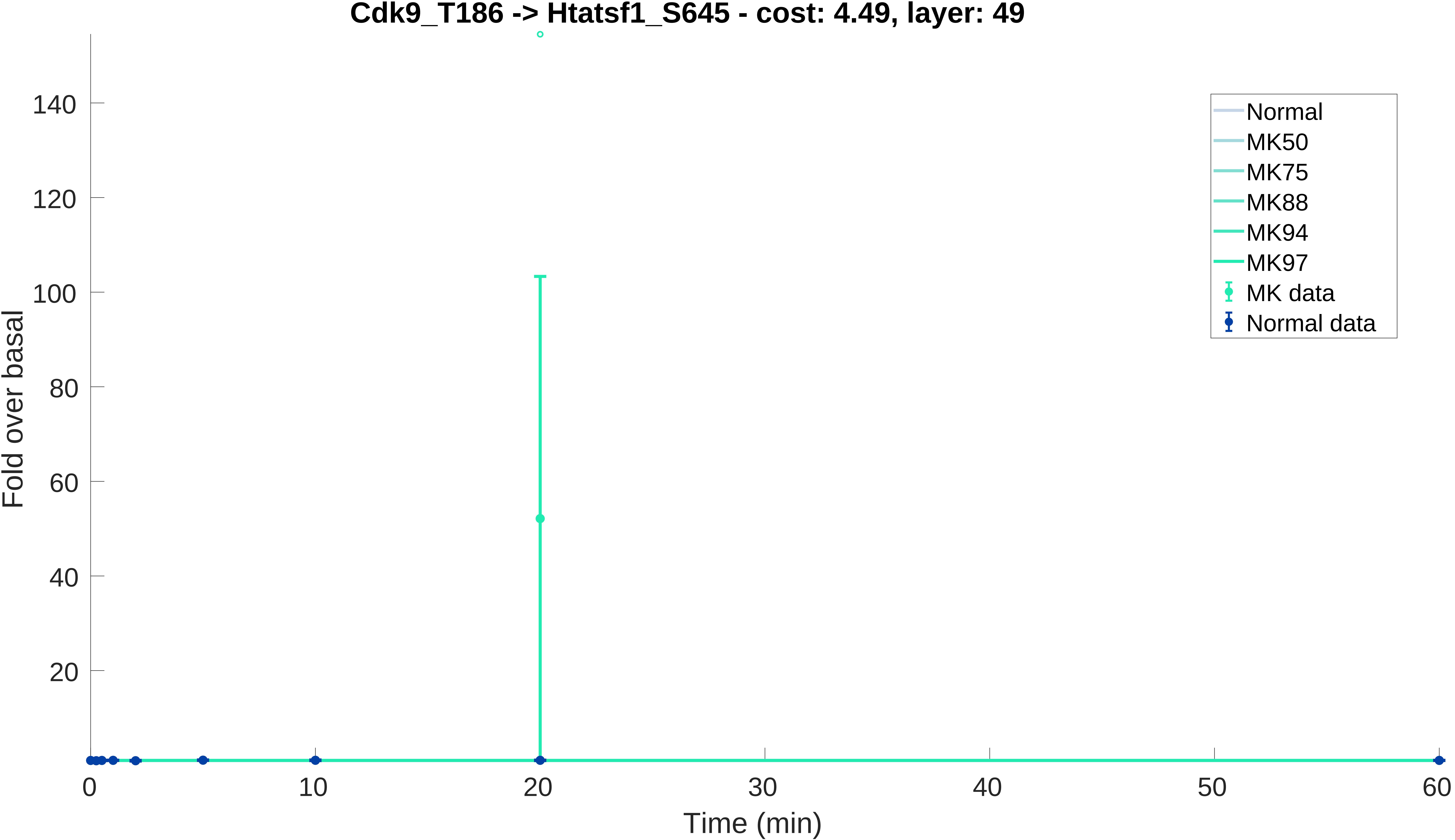

Cdk9_T186 -> Htatsf1_S645 - cost: 4.49, layer: 49
Fold over basal
Time (min)
20
40
60
80
100
120
140
0
10
20
30
40
50
60
Normal
MK50
MK75
MK88
MK94
MK97
MK data
Normal data

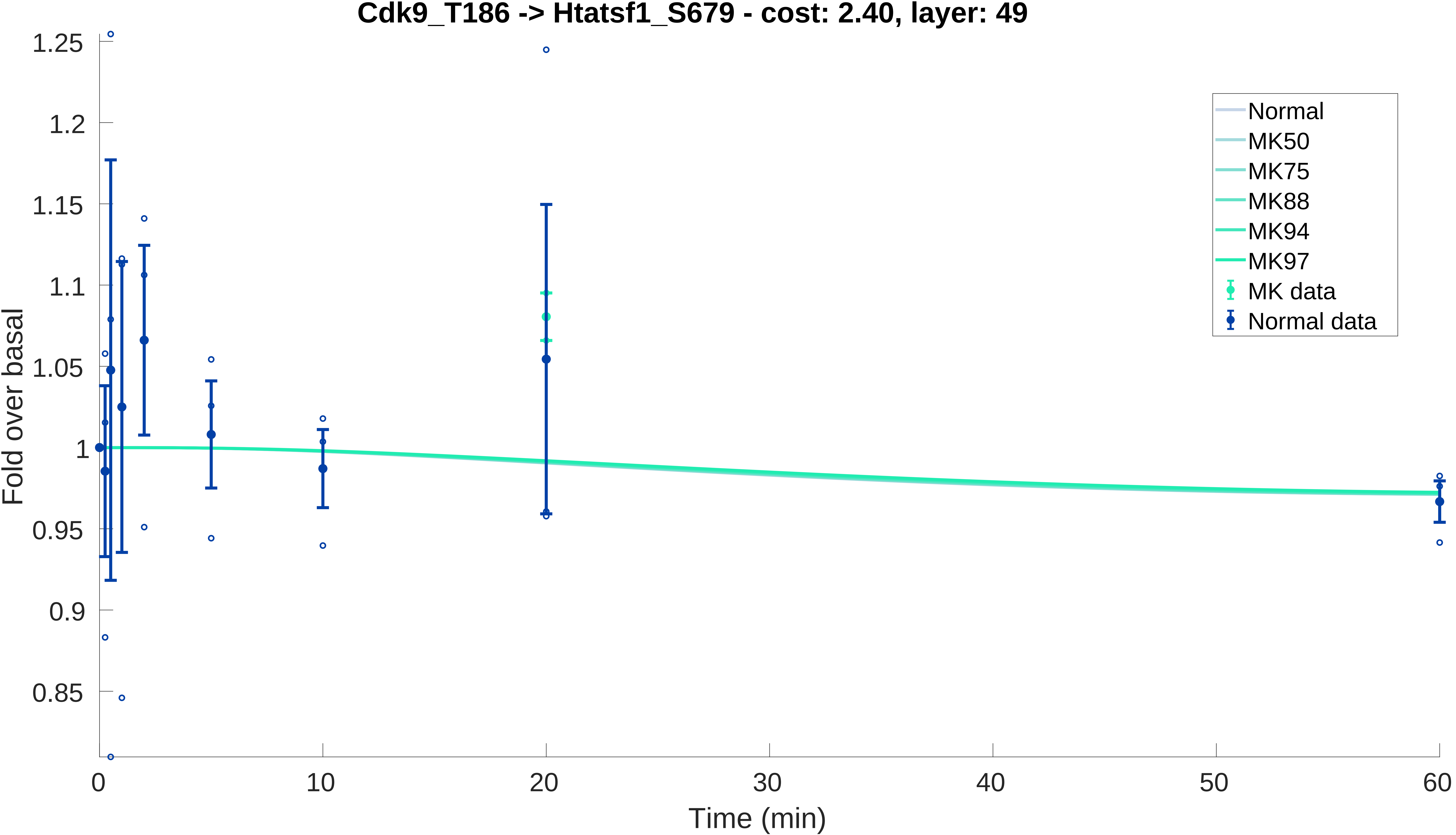

Cdk9_T186 -> Htatsf1_S679 - cost: 2.40, layer: 49
Fold over basal
1.25
1.2
1.15
1.1
1.05
1
0.95
0.9
0.85
0
10
20
30
40
50
60
Time (min)
Normal
MK50
MK75
MK88
MK94
MK97
MK data
Normal data

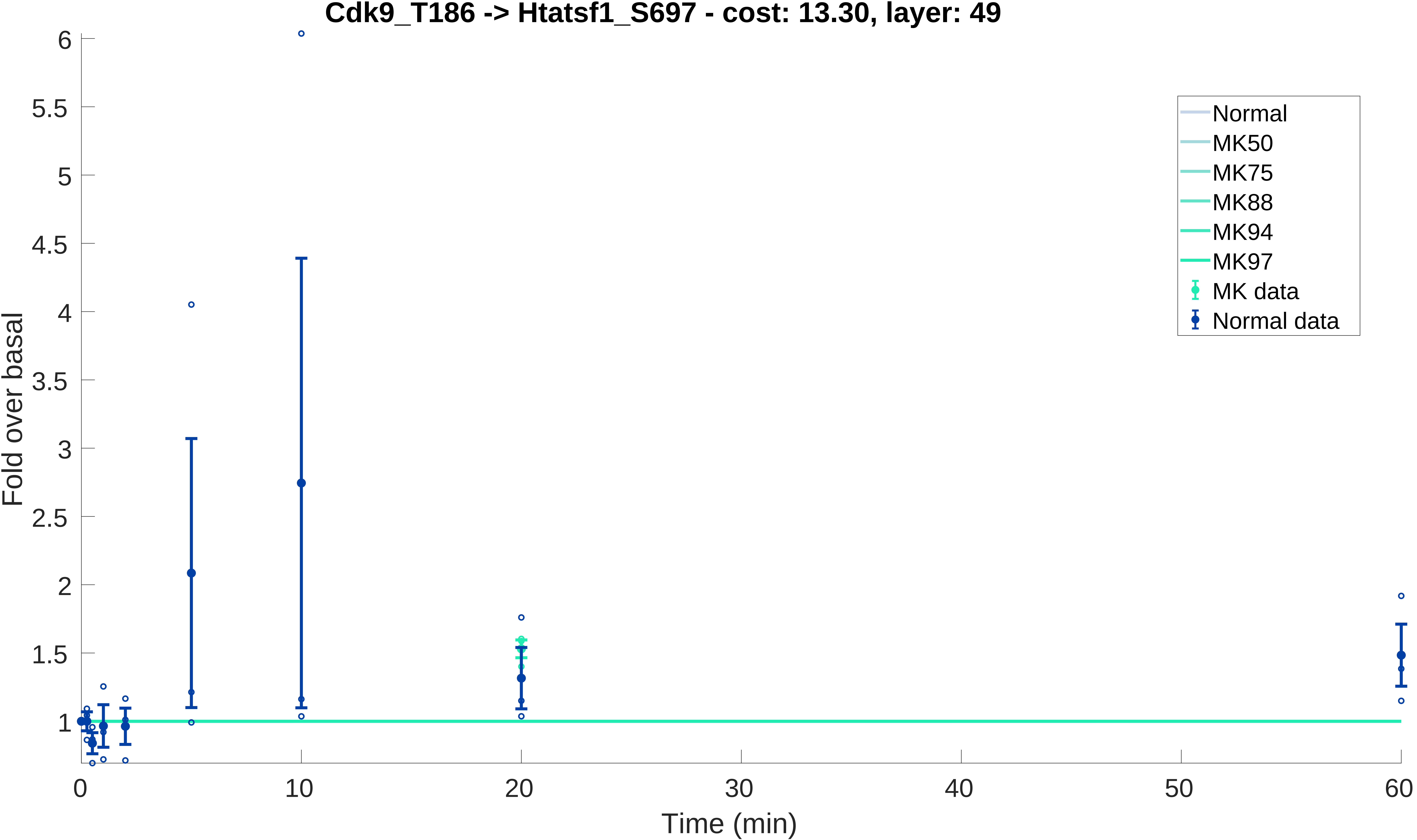

Cdk9_T186 -> Htatsf1_S697 - cost: 13.30, layer: 49
Fold over basal
Time (min)
6
5.5
5
4.5
4
3.5
3
2.5
2
1.5
1
0
10
20
30
40
50
60
Normal
MK50
MK75
MK88
MK94
MK97
MK data
Normal data

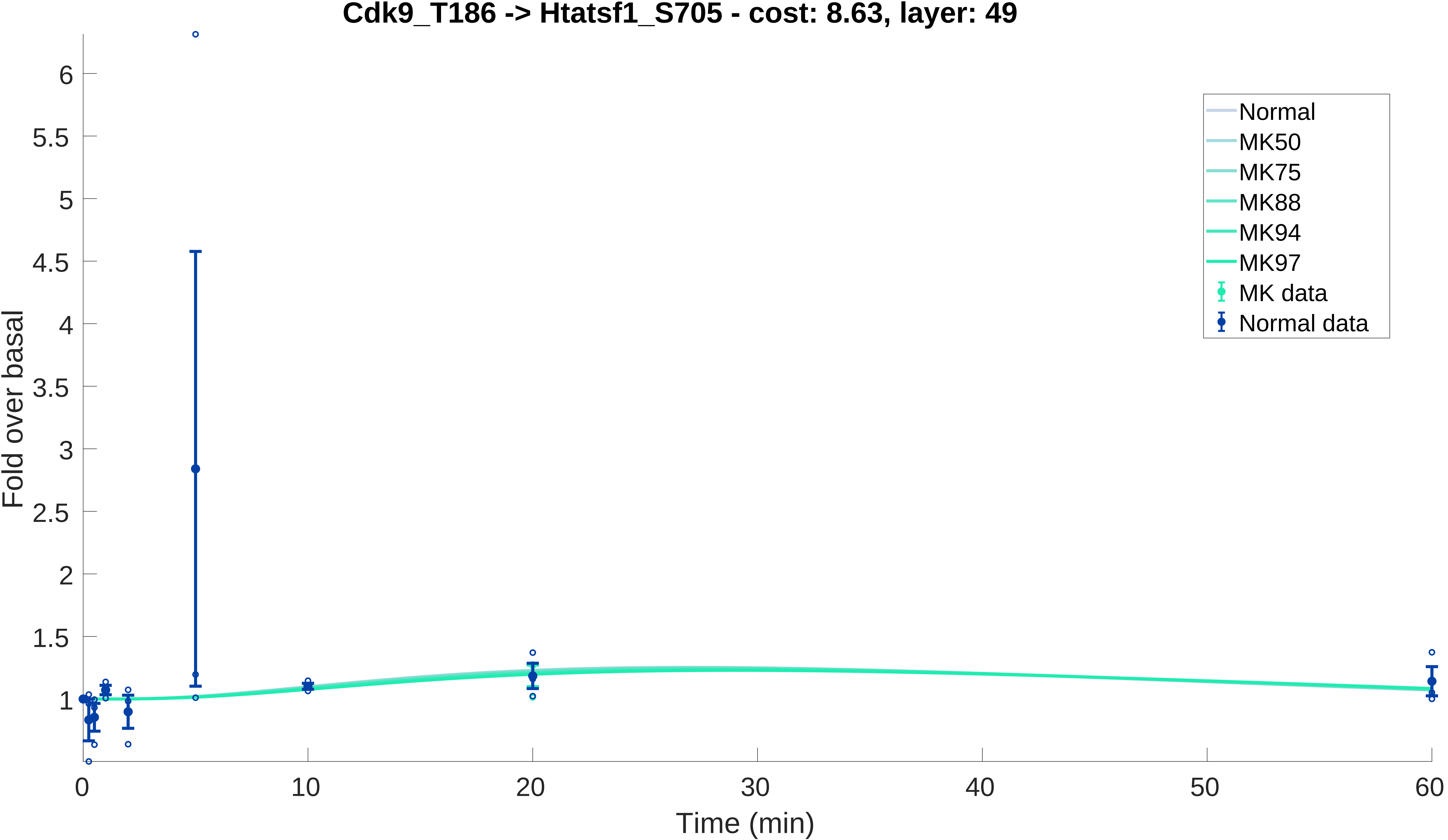

Cdk9_T186 -> Htatsf1_S705 - cost: 8.63, layer: 49
Fold over basal
Time (min)
0
10
20
30
40
50
60
1
1.5
2
2.5
3
3.5
4
4.5
5
5.5
6
Normal
MK50
MK75
MK88
MK94
MK97
MK data
Normal data

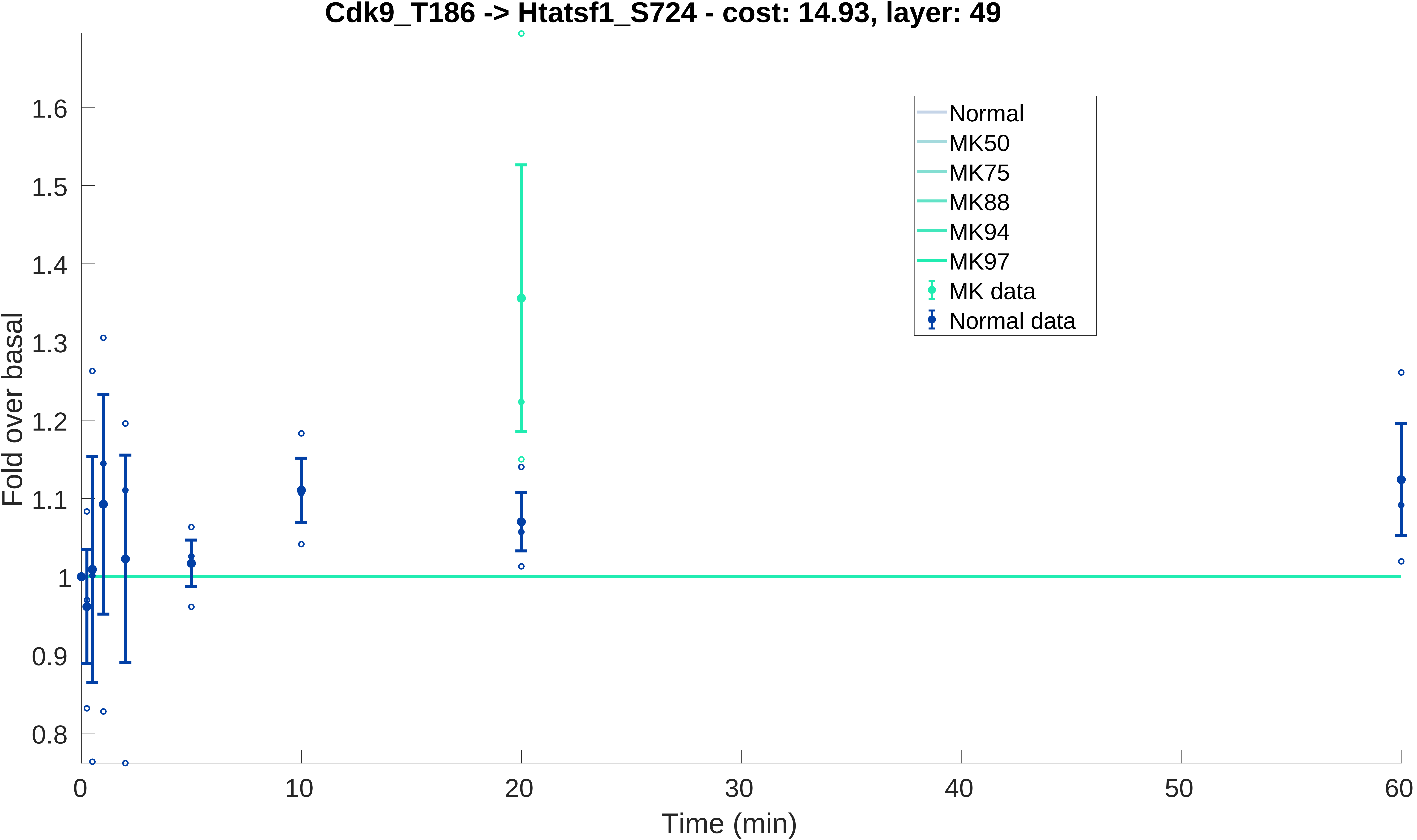

Cdk9_T186 -> Htatsf1_S724 - cost: 14.93, layer: 49
Fold over basal
Time (min)
1.6
1.5
1.4
1.3
1.2
1.1
1
0.9
0.8
0
10
20
30
40
50
60
Normal
MK50
MK75
MK88
MK94
MK97
MK data
Normal data

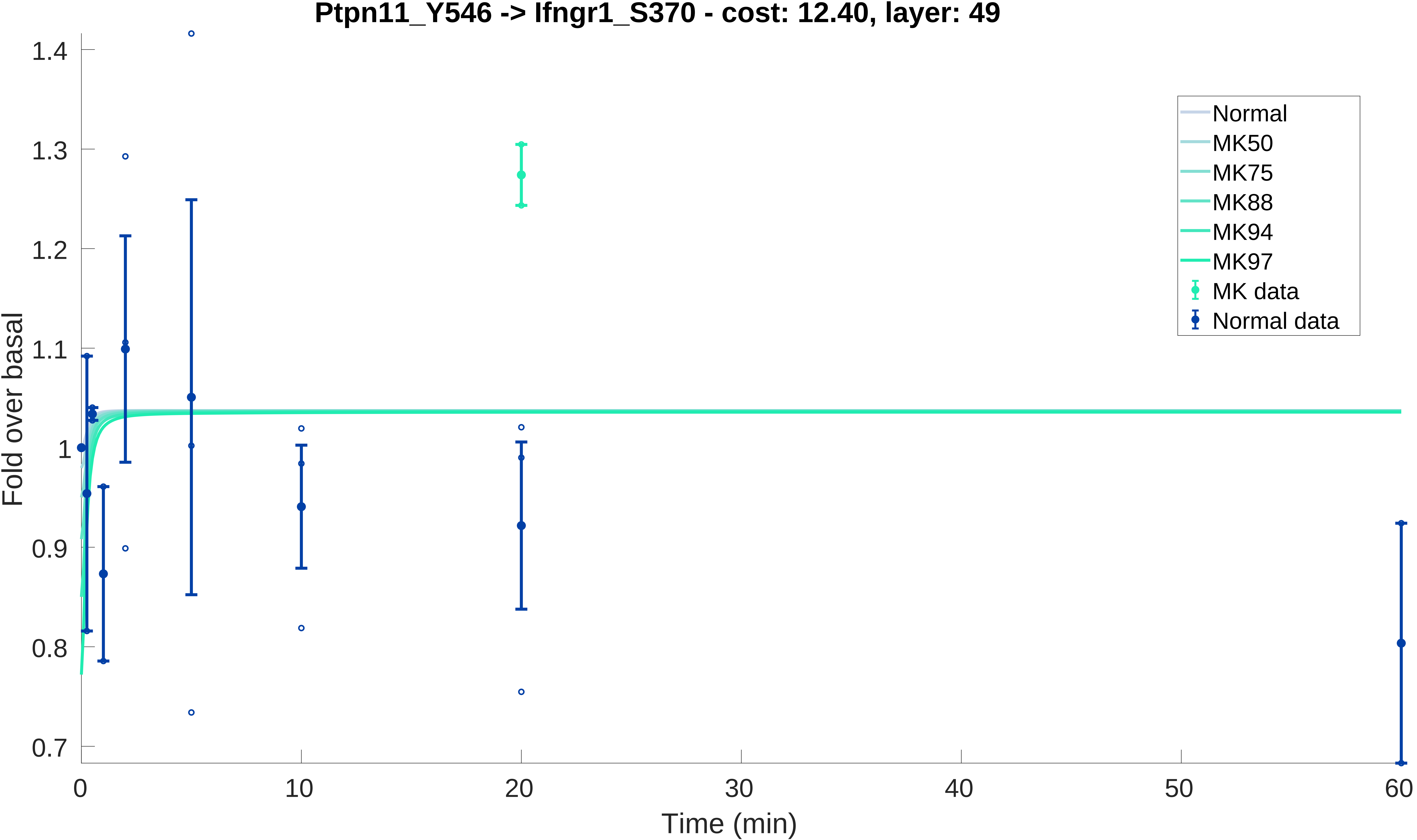

Ptpn11_Y546 -> Ifngr1_S370 - cost: 12.40, layer: 49
Fold over basal
1.4
1.3
1.2
1.1
1
0.9
0.8
0.7
0
10
20
30
40
50
60
Time (min)
Normal
MK50
MK75
MK88
MK94
MK97
MK data
Normal data

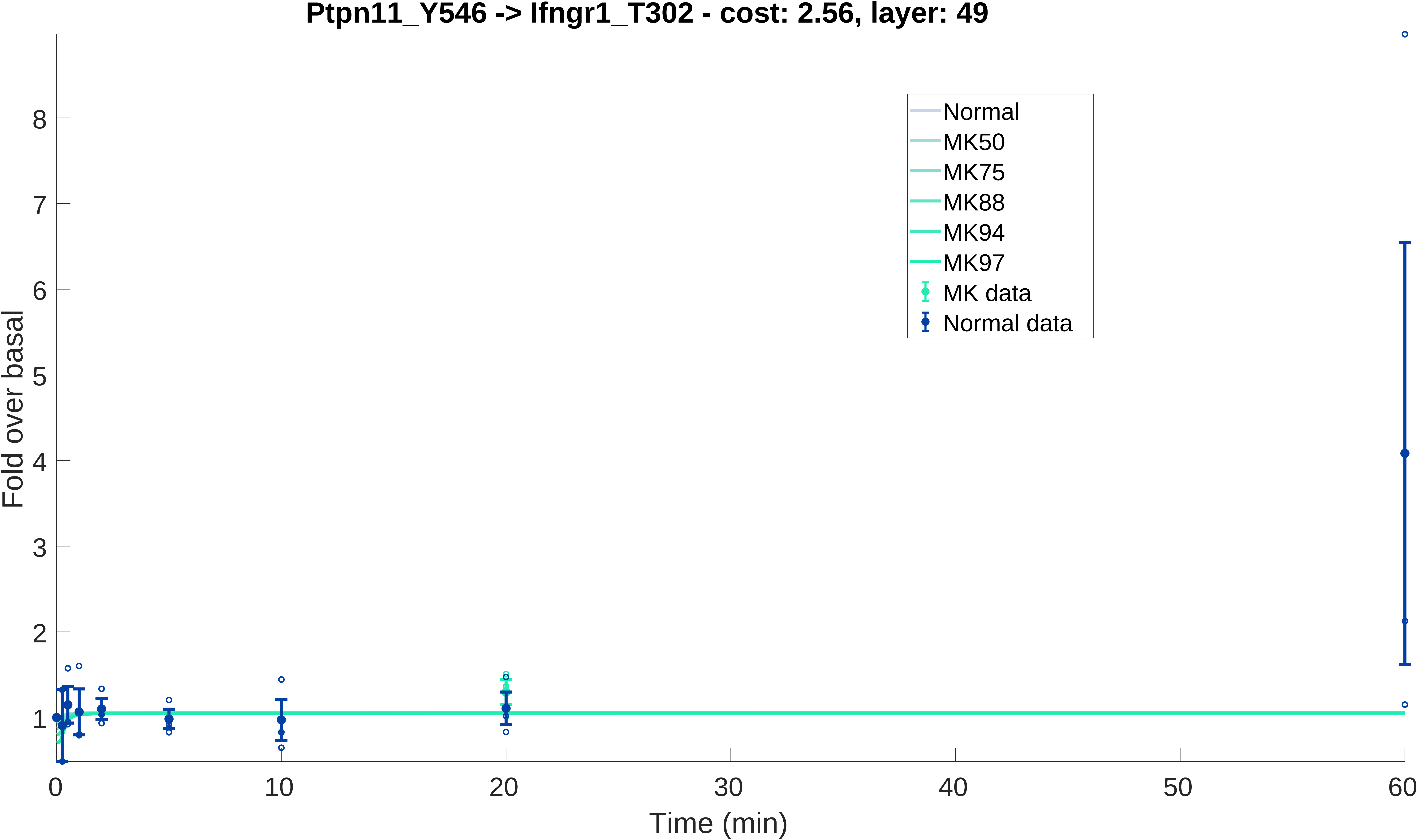

Ptpn11_Y546 -> Ifngr1_T302 - cost: 2.56, layer: 49
Fold over basal
Time (min)
Normal
MK50
MK75
MK88
MK94
MK97
MK data
Normal data
0
10
20
30
40
50
60
1
2
3
4
5
6
7
8

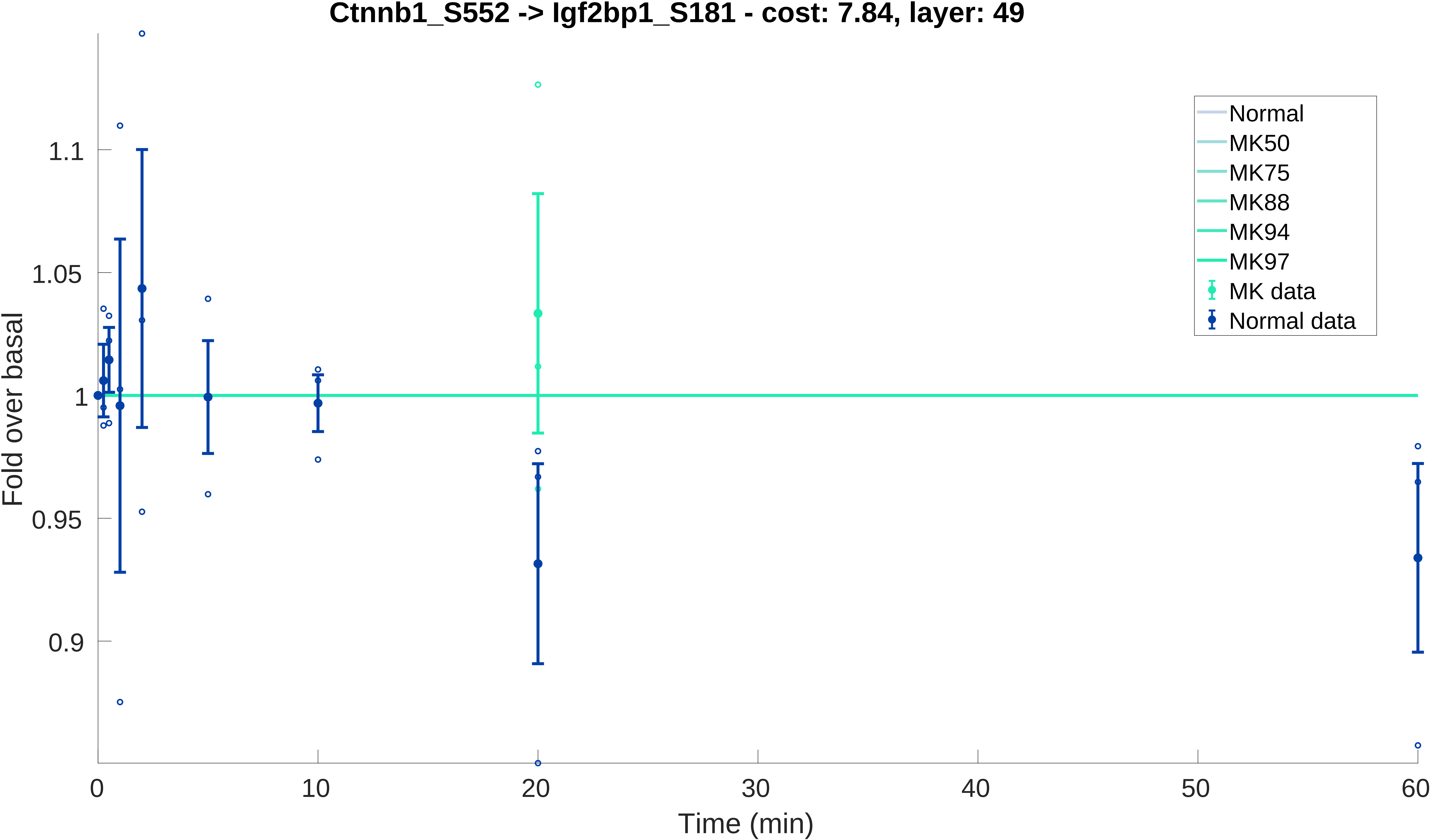
Ctnnb1_S552 -> Igf2bp1_S181 - cost: 7.84, layer: 49
Fold over basal
1.1
1.05
1
0.95
0.9
0
10
20
30
40
50
60
Time (min)
Normal
MK50
MK75
MK88
MK94
MK97
MK data
Normal data

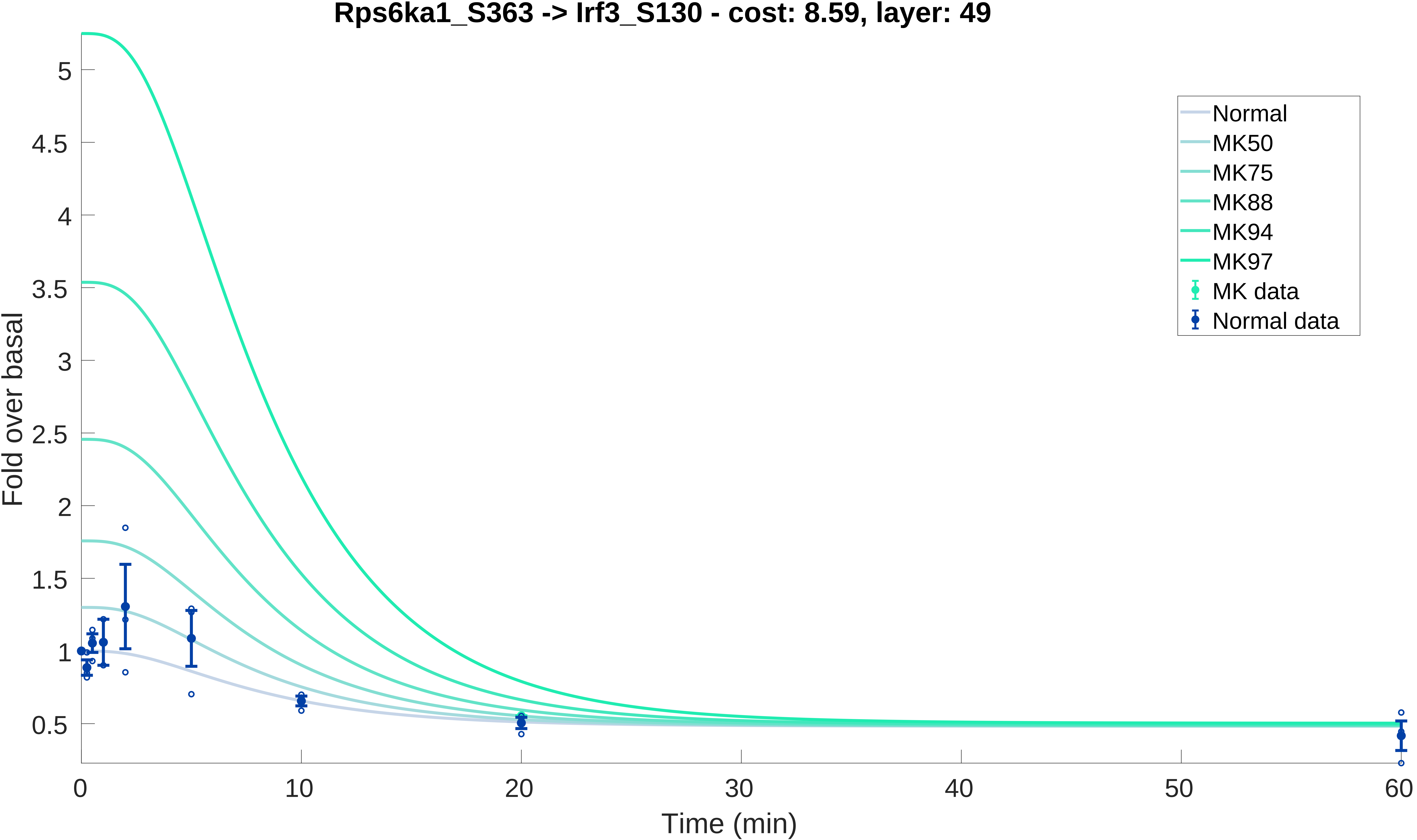
Rps6ka1_S363 -> Irf3_S130 - cost: 8.59, layer: 49
Fold over basal
Time (min)
0
10
20
30
40
50
60
0.5
1
1.5
2
2.5
3
3.5
4
4.5
5
Normal
MK50
MK75
MK88
MK94
MK97
MK data
Normal data

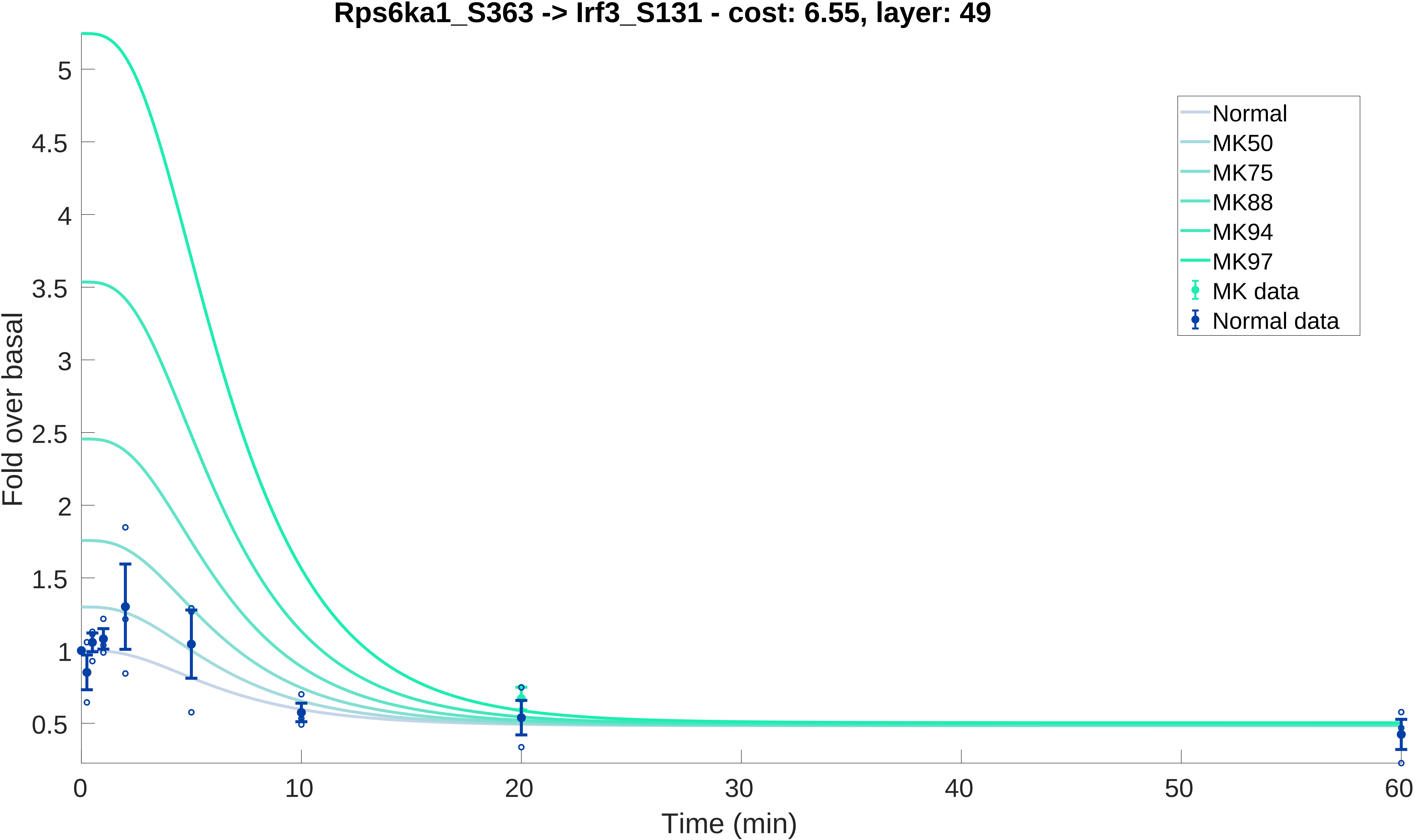

Rps6ka1_S363 -> Irf3_S131 - cost: 6.55, layer: 49
Fold over basal
Time (min)
0.5
1
1.5
2
2.5
3
3.5
4
4.5
5
0
10
20
30
40
50
60
Normal
MK50
MK75
MK88
MK94
MK97
MK data
Normal data

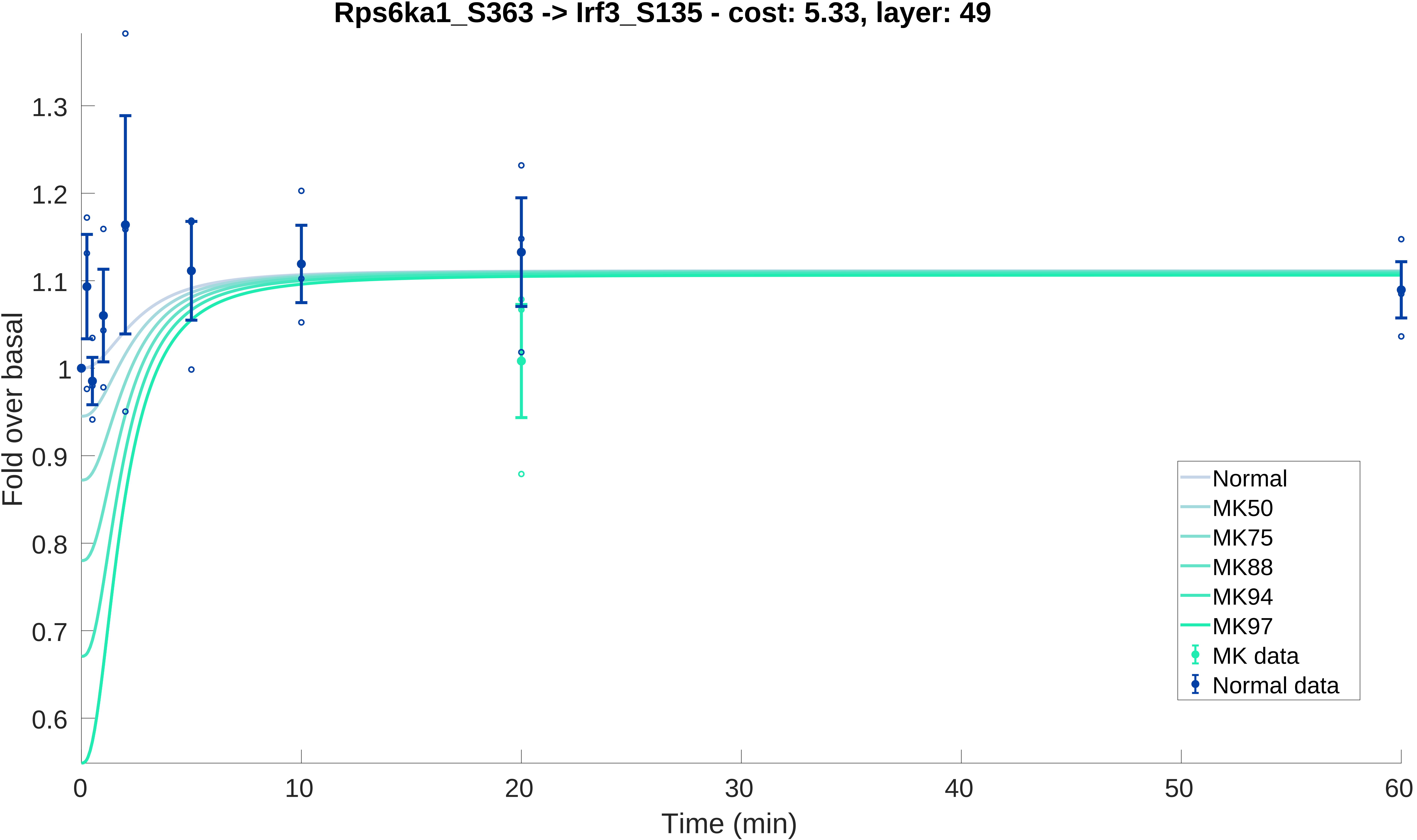

Rps6ka1_S363 -> Irf3_S135 - cost: 5.33, layer: 49
Fold over basal
Time (min)
0.6
0.7
0.8
0.9
1
1.1
1.2
1.3
0
10
20
30
40
50
60
Normal
MK50
MK75
MK88
MK94
MK97
MK data
Normal data

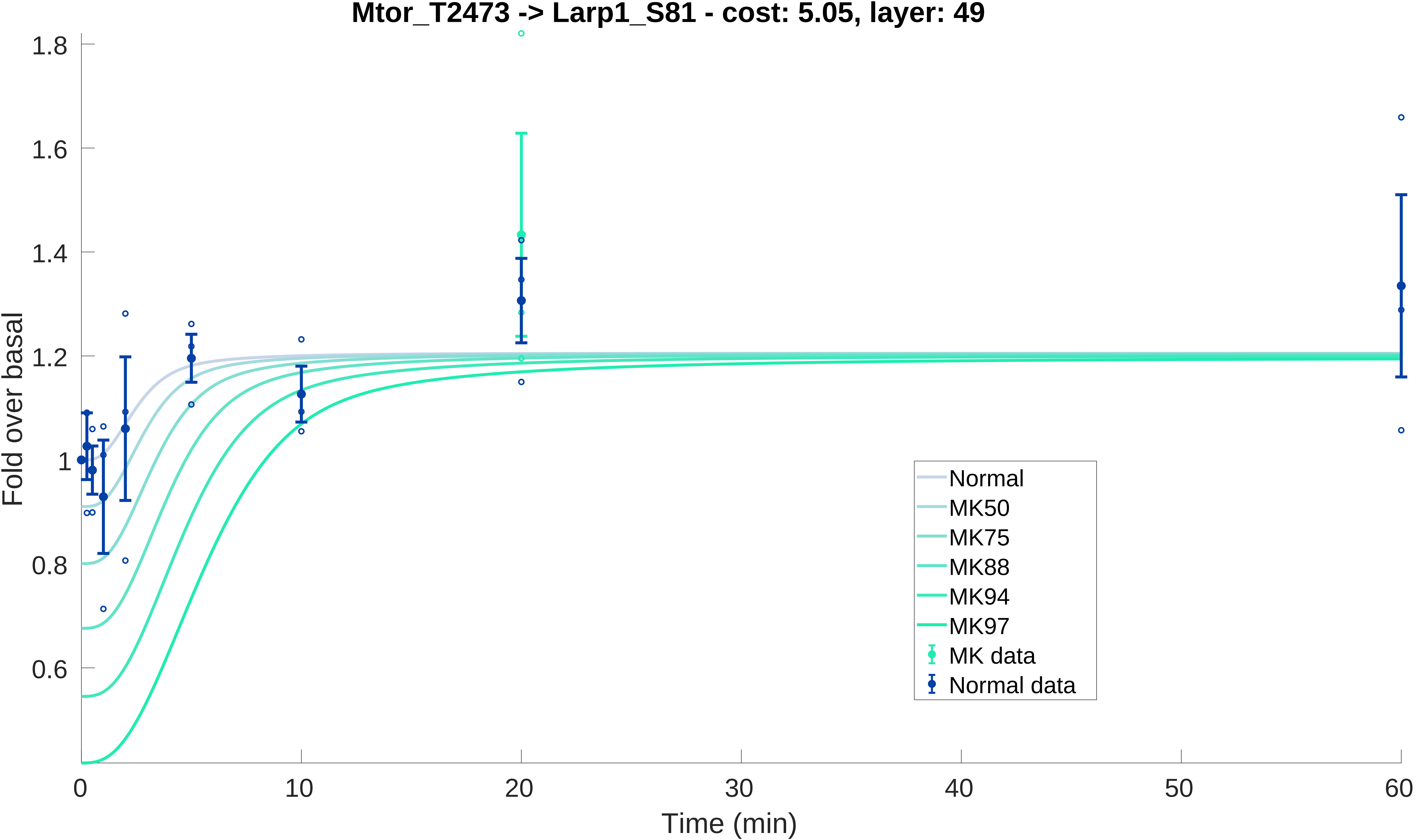
Mtor_T2473 -> Larp1_S81 - cost: 5.05, layer: 49
Fold over basal
1.8
1.6
1.4
1.2
1
0.8
0.6
0
10
20
30
40
50
60
Time (min)
Normal
MK50
MK75
MK88
MK94
MK97
MK data
Normal data

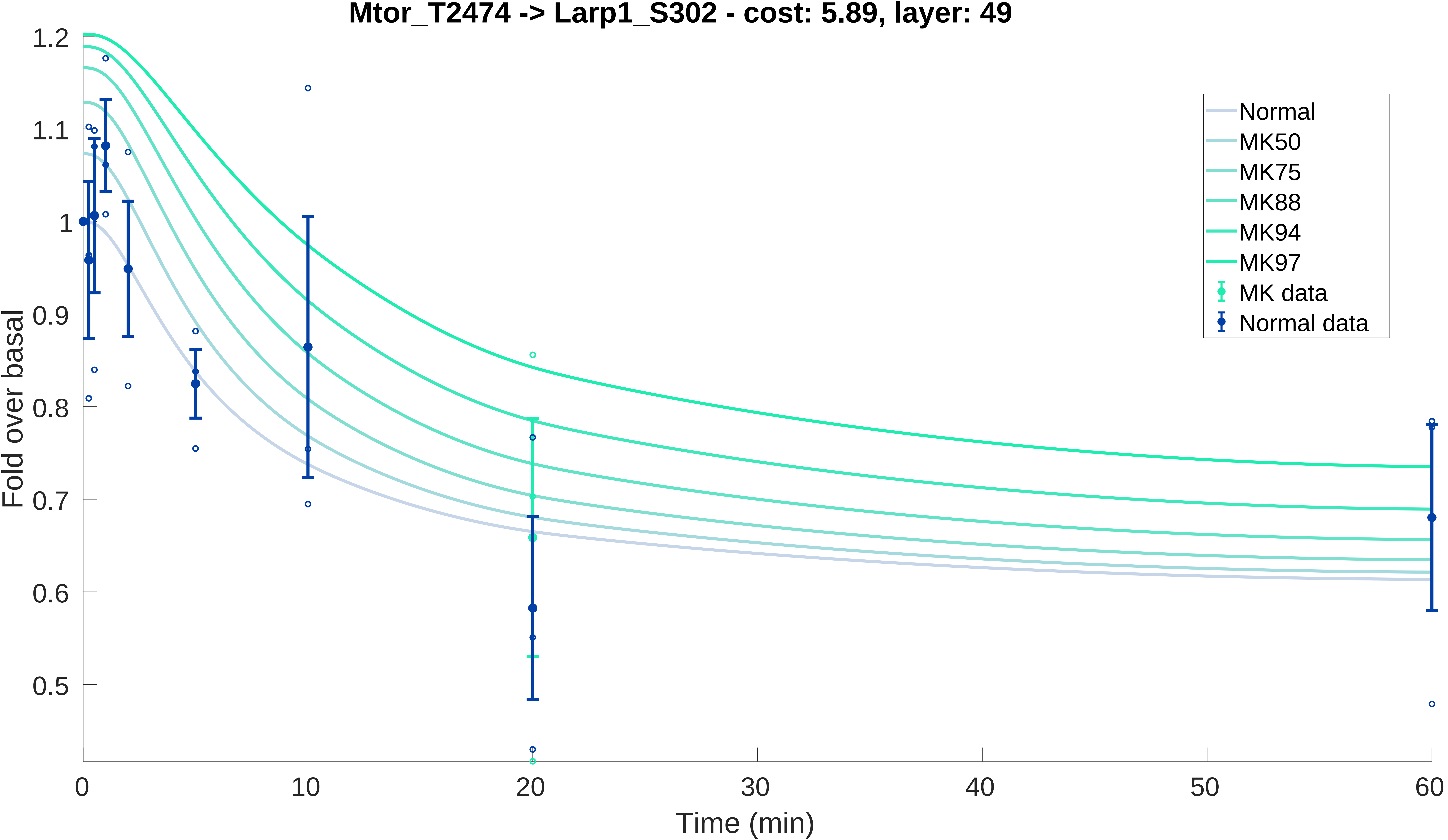

Mtor_T2474 -> Larp1_S302 - cost: 5.89, layer: 49
Fold over basal
Time (min)
1.2
1.1
1
0.9
0.8
0.7
0.6
0.5
0
10
20
30
40
50
60
Normal
MK50
MK75
MK88
MK94
MK97
MK data
Normal data

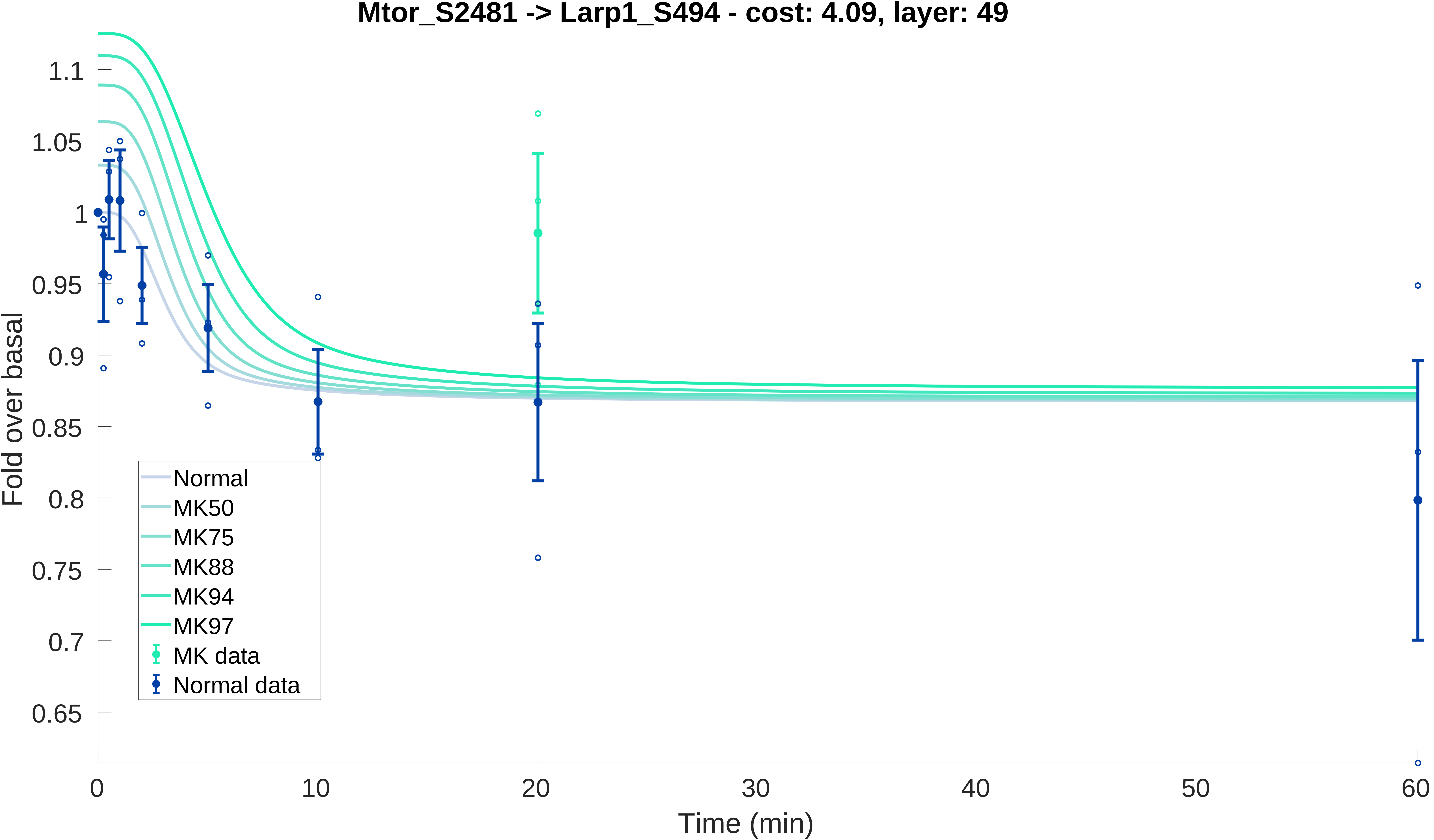

Mtor_S2481 -> Larp1_S494 - cost: 4.09, layer: 49
Fold over basal
Time (min)
1.1
1.05
1
0.95
0.9
0.85
0.8
0.75
0.7
0.65
0
10
20
30
40
50
60
Normal
MK50
MK75
MK88
MK94
MK97
MK data
Normal data

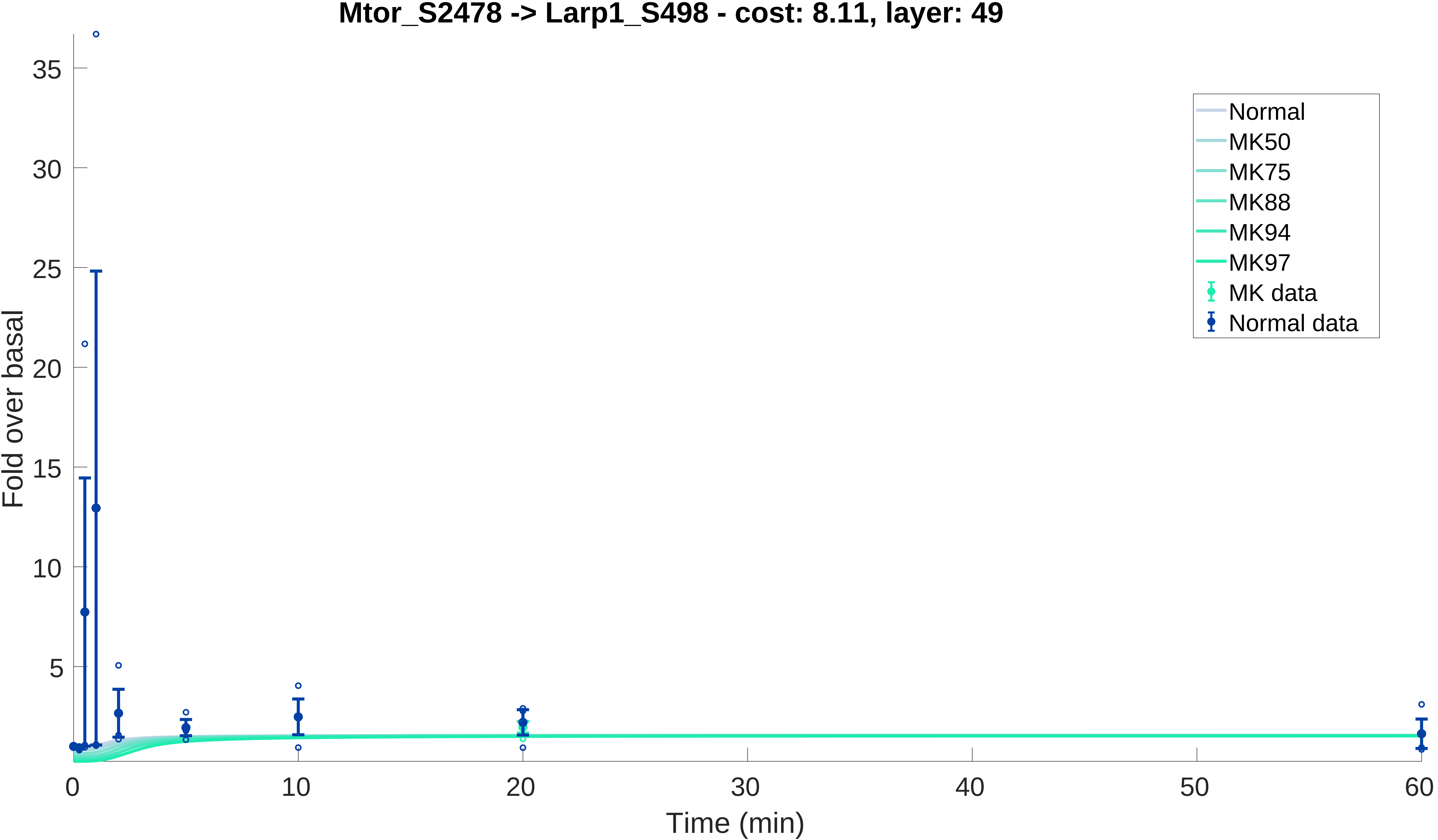
Mtor_S2478 -> Larp1_S498 - cost: 8.11, layer: 49
Fold over basal
Time (min)
0
5
10
15
20
25
30
35
0
10
20
30
40
50
60
Normal
MK50
MK75
MK88
MK94
MK97
MK data
Normal data

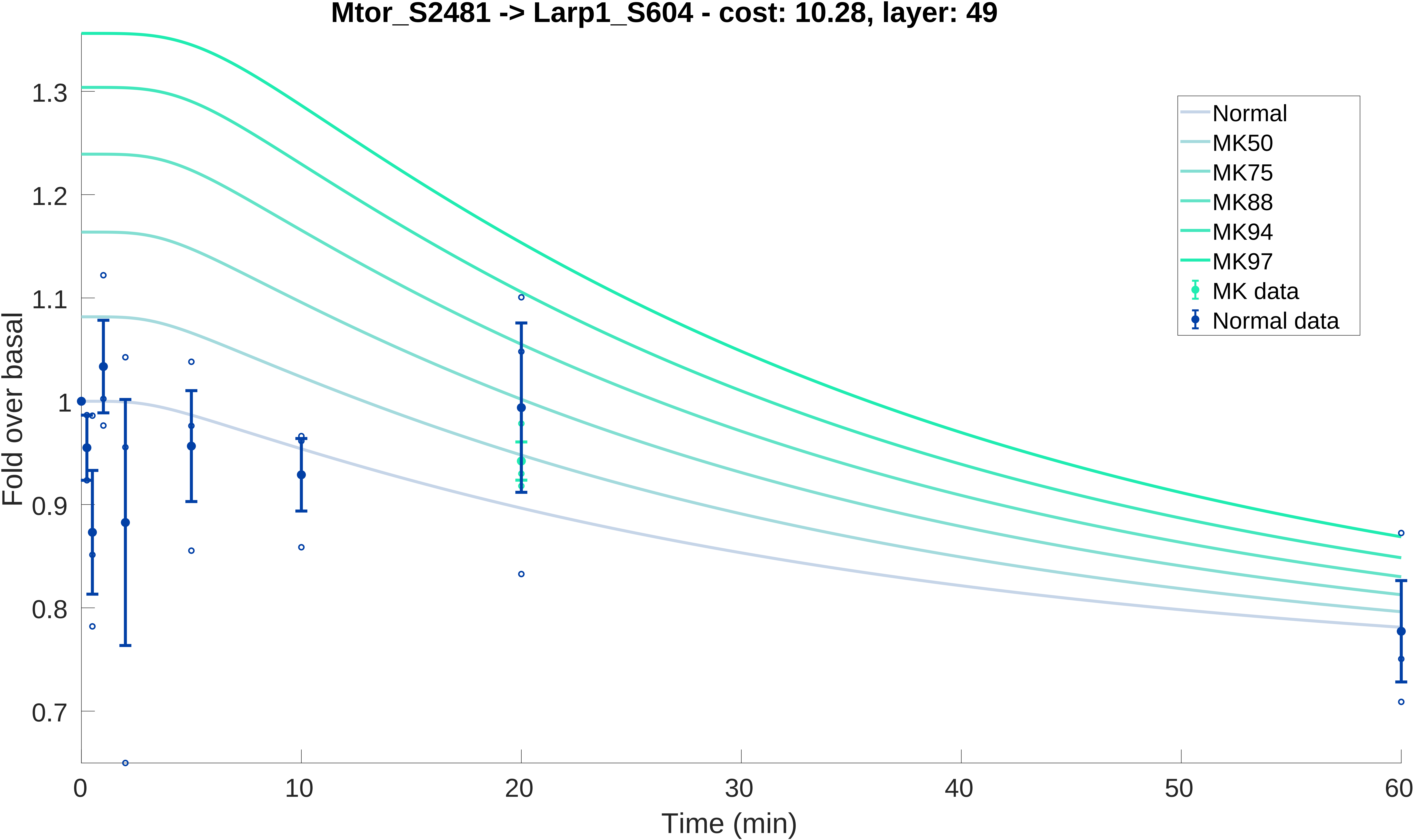

Mtor_S2481 -> Larp1_S604 - cost: 10.28, layer: 49
Normal
MK50
MK75
MK88
MK94
MK97
MK data
Normal data
Fold over basal
1.3
1.2
1.1
1
0.9
0.8
0.7
0
10
20
30
40
50
60
Time (min)

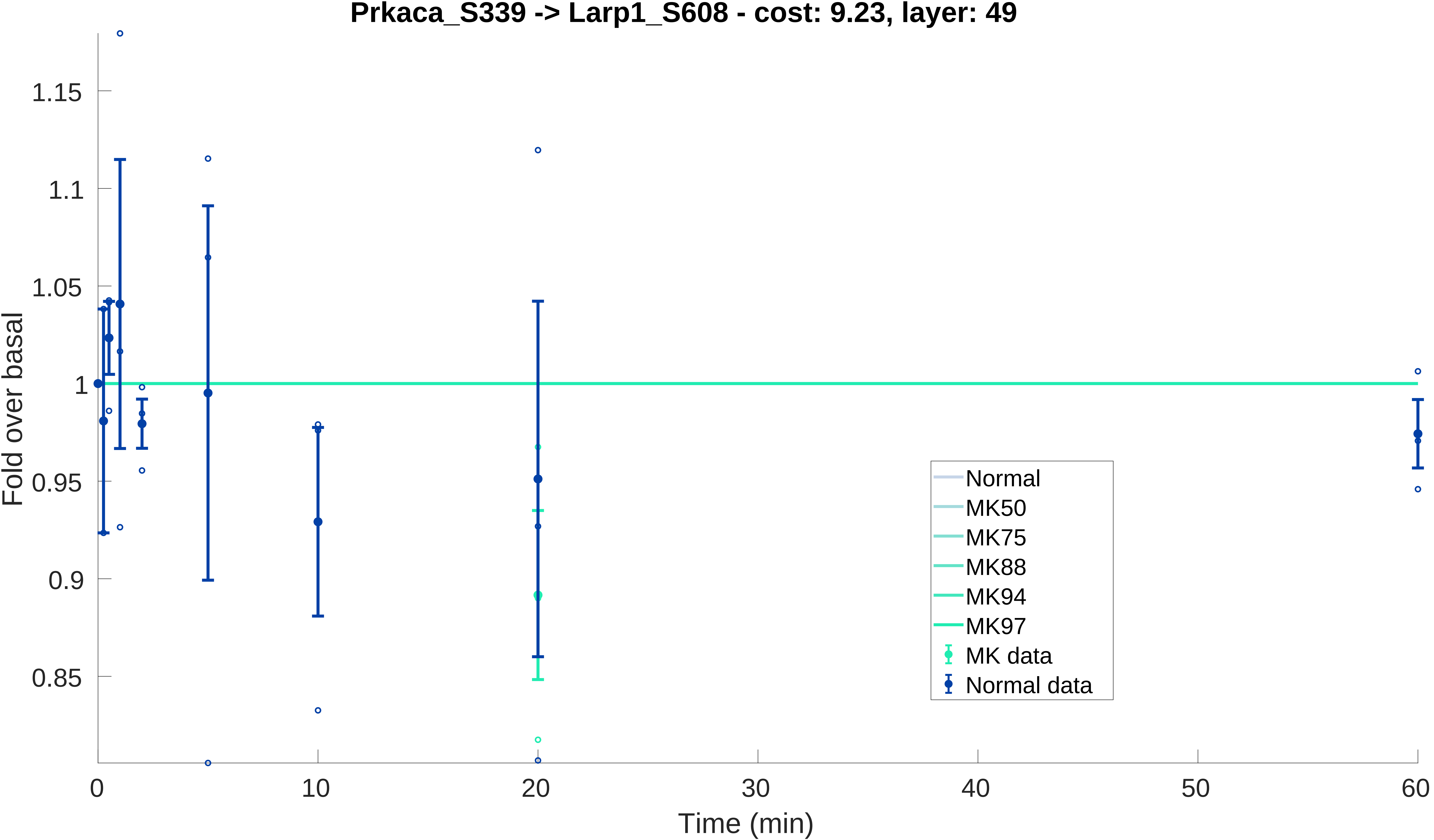
Prkaca_S339 -> Larp1_S608 - cost: 9.23, layer: 49
Fold over basal
1.15
1.1
1.05
1
0.95
0.9
0.85
0
10
20
30
40
50
60
Time (min)
Normal
MK50
MK75
MK88
MK94
MK97
MK data
Normal data

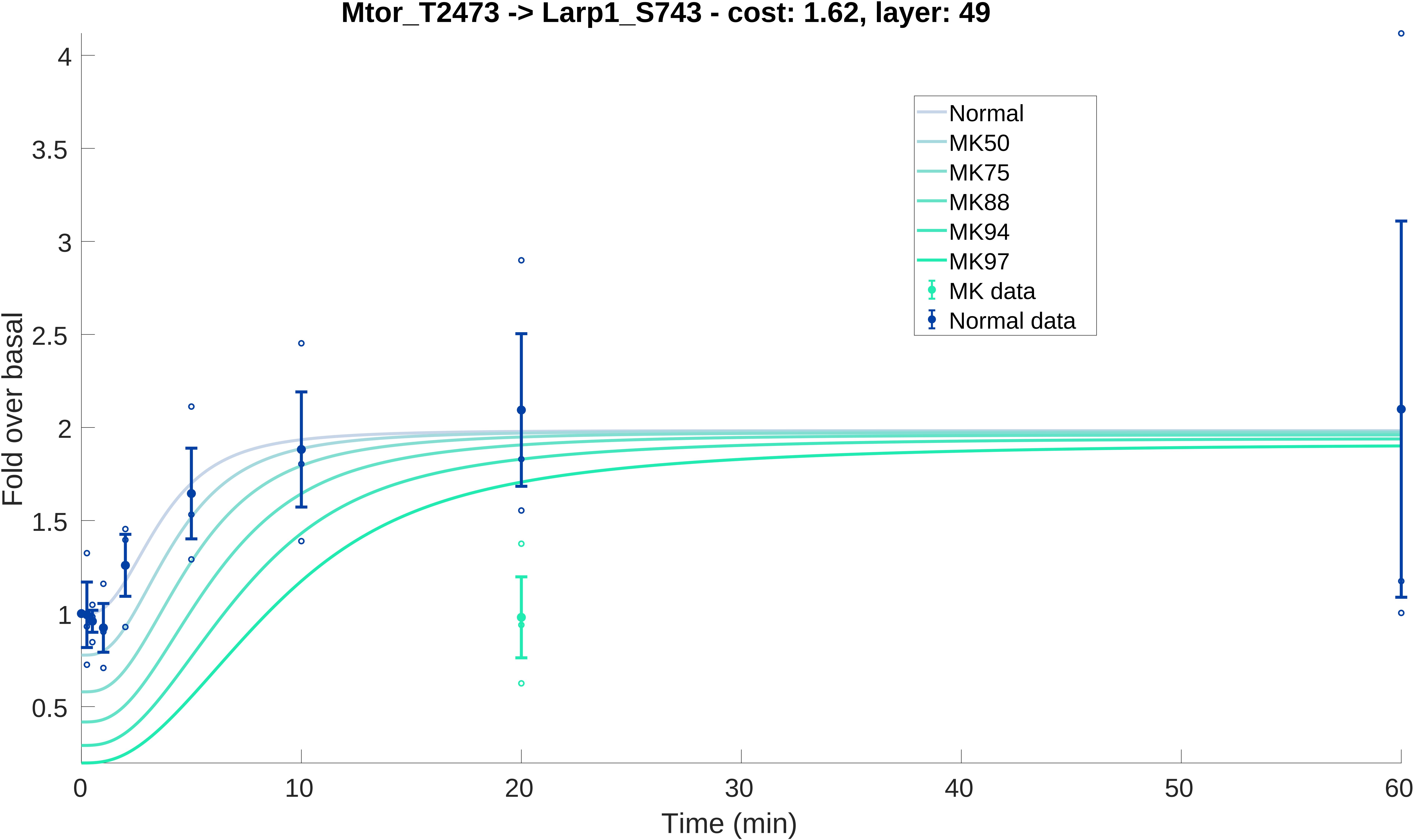

Mtor_T2473 -> Larp1_S743 - cost: 1.62, layer: 49
Fold over basal
Time (min)
Normal
MK50
MK75
MK88
MK94
MK97
MK data
Normal data
4
3.5
3
2.5
2
1.5
1
0.5
0
10
20
30
40
50
60

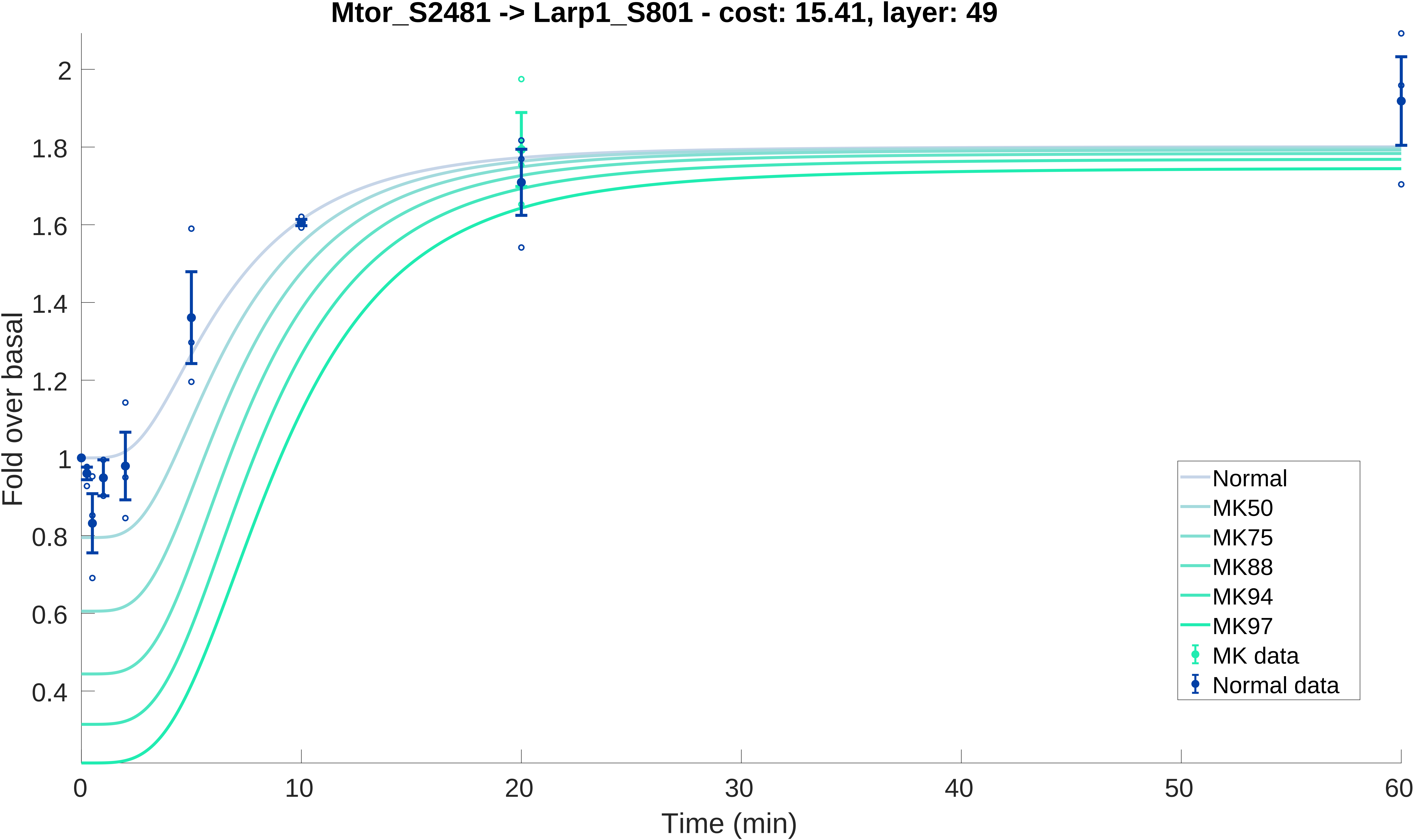

Mtor_S2481 -> Larp1_S801 - cost: 15.41, layer: 49
Fold over basal
Time (min)
0
10
20
30
40
50
60
0.4
0.6
0.8
1
1.2
1.4
1.6
1.8
2
Normal
MK50
MK75
MK88
MK94
MK97
MK data
Normal data

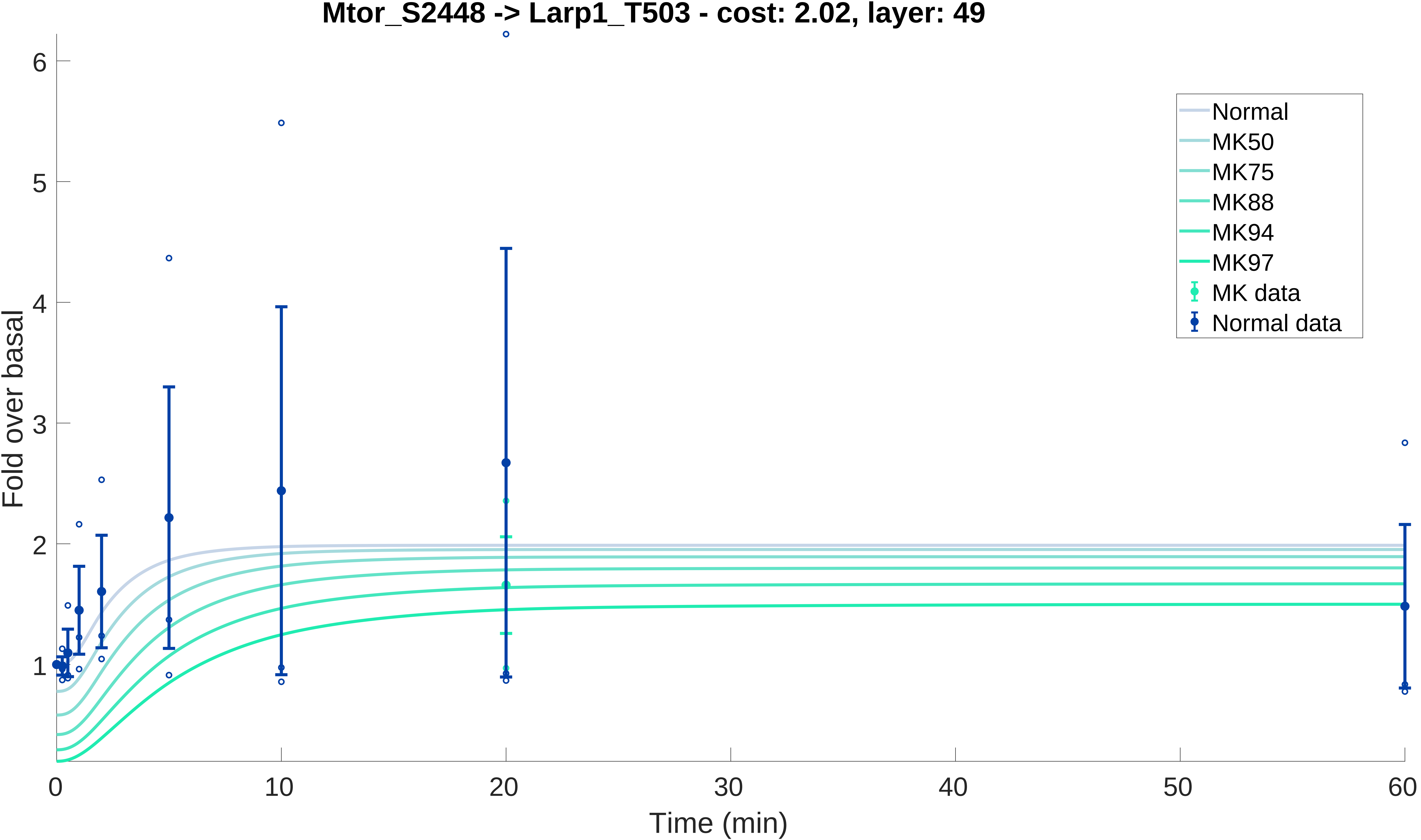

Mtor_S2448 -> Larp1_T503 - cost: 2.02, layer: 49
Fold over basal
Time (min)
0
10
20
30
40
50
60
1
2
3
4
5
6
Normal
MK50
MK75
MK88
MK94
MK97
MK data
Normal data

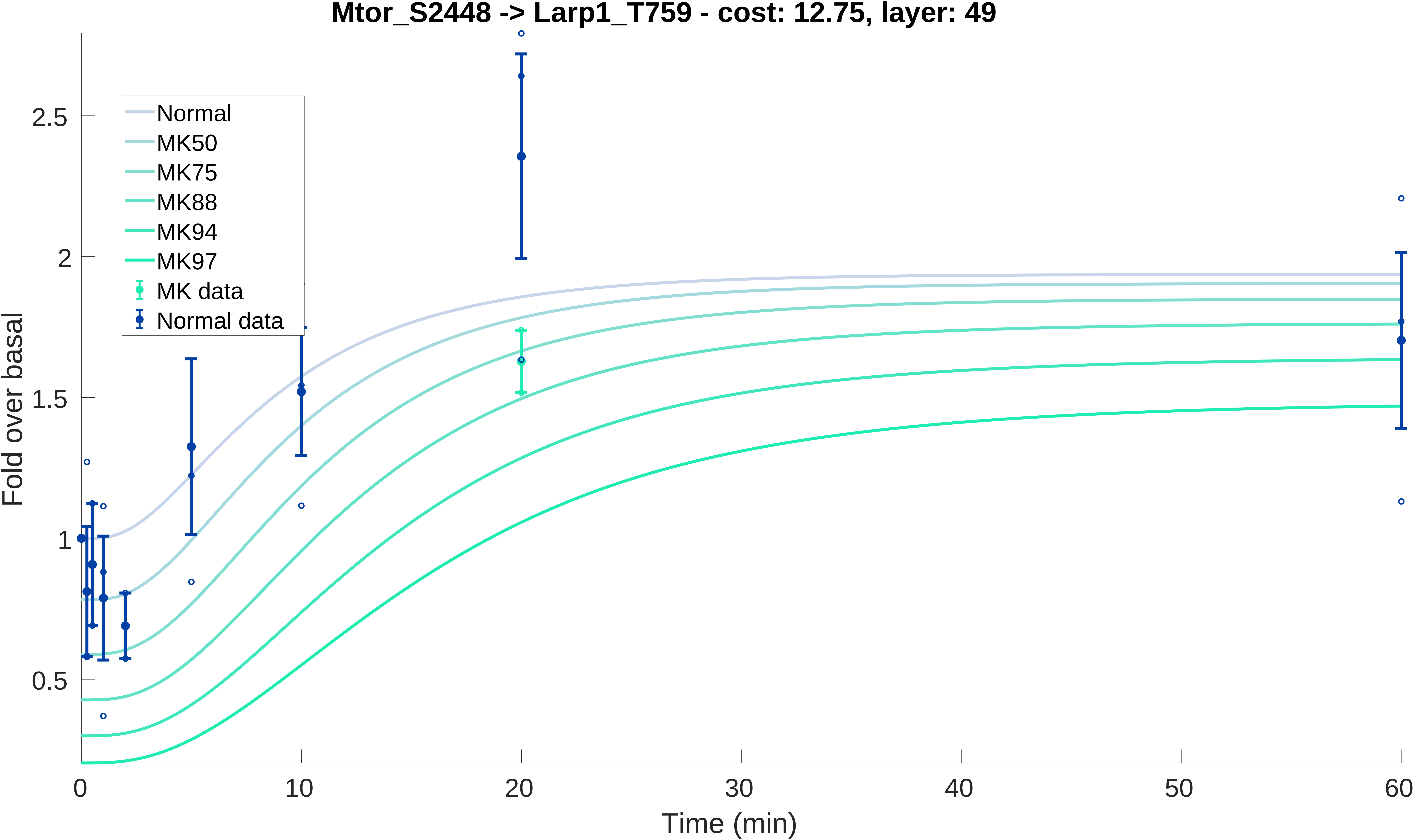

Mtor_S2448 -> Larp1_T759 - cost: 12.75, layer: 49
Normal
MK50
MK75
MK88
MK94
MK97
MK data
Normal data
Fold over basal
Time (min)
0.5
1
1.5
2
2.5
0
10
20
30
40
50
60

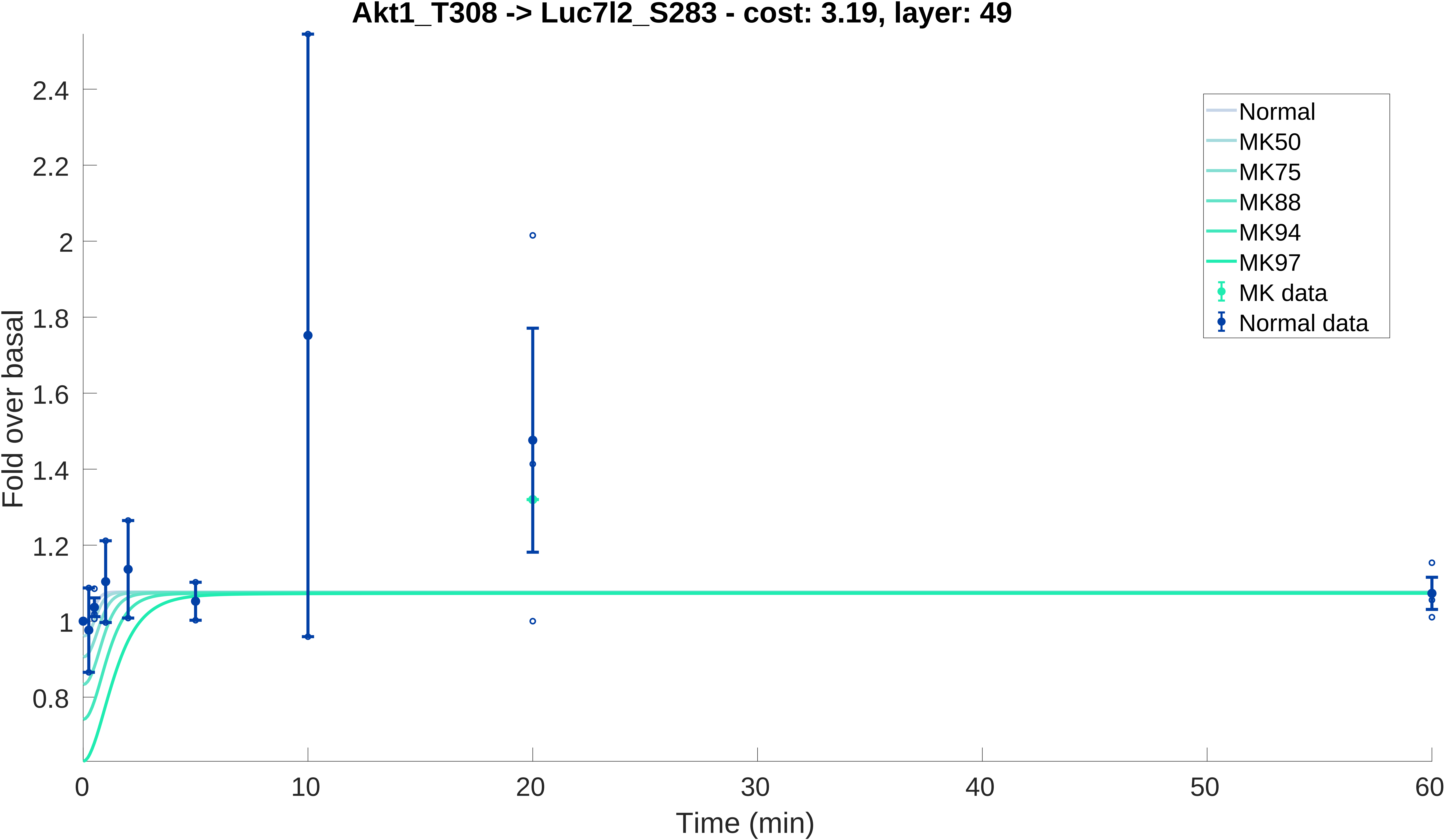

Akt1_T308 -> Luc7l2_S283 - cost: 3.19, layer: 49
Fold over basal
Time (min)
0.8
1
1.2
1.4
1.6
1.8
2
2.2
2.4
0
10
20
30
40
50
60
Normal
MK50
MK75
MK88
MK94
MK97
MK data
Normal data

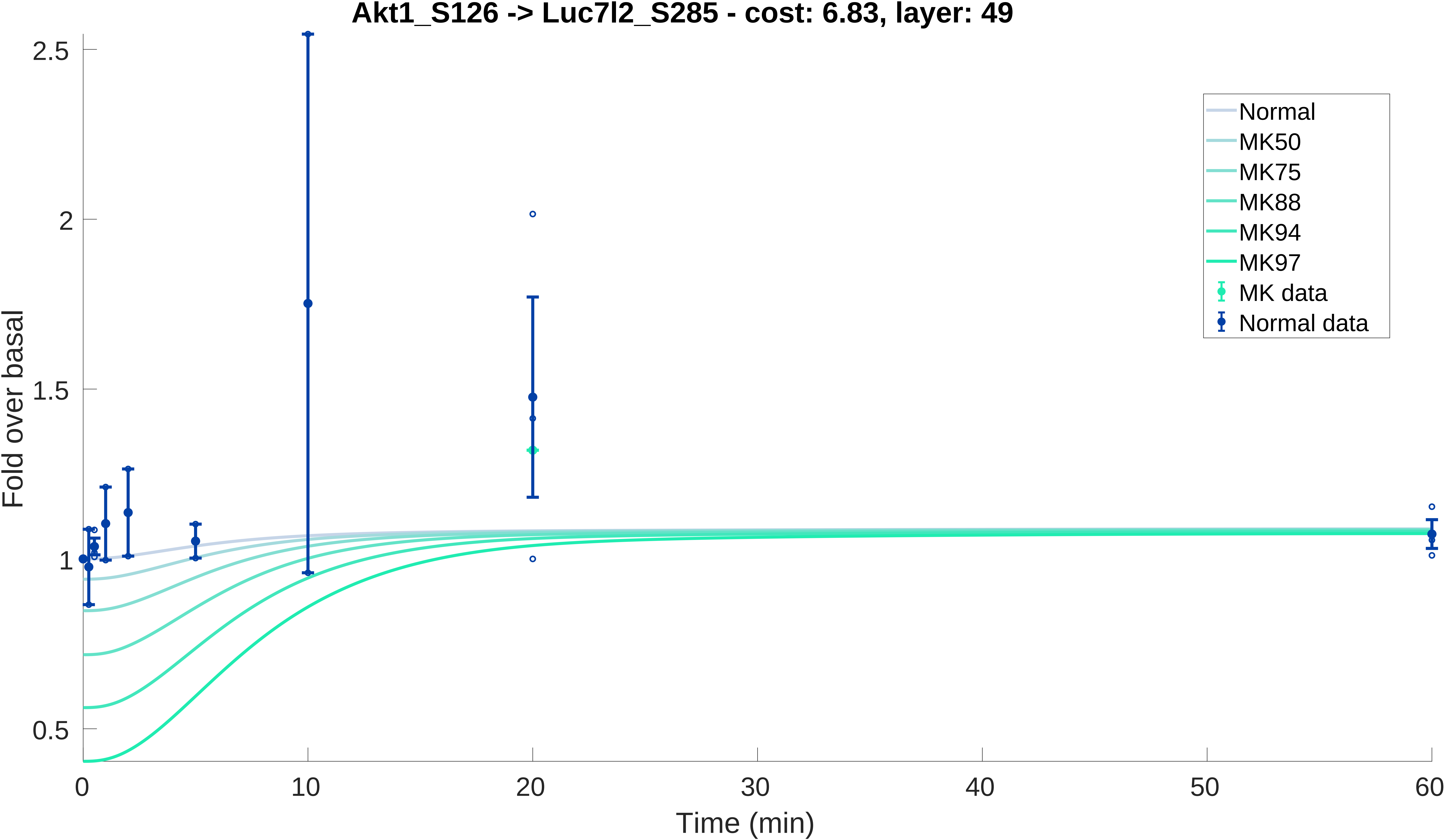

Akt1_S126 -> Luc7l2_S285 - cost: 6.83, layer: 49
Fold over basal
2.5
2
1.5
1
0.5
0
10
20
30
40
50
60
Time (min)
Normal
MK50
MK75
MK88
MK94
MK97
MK data
Normal data

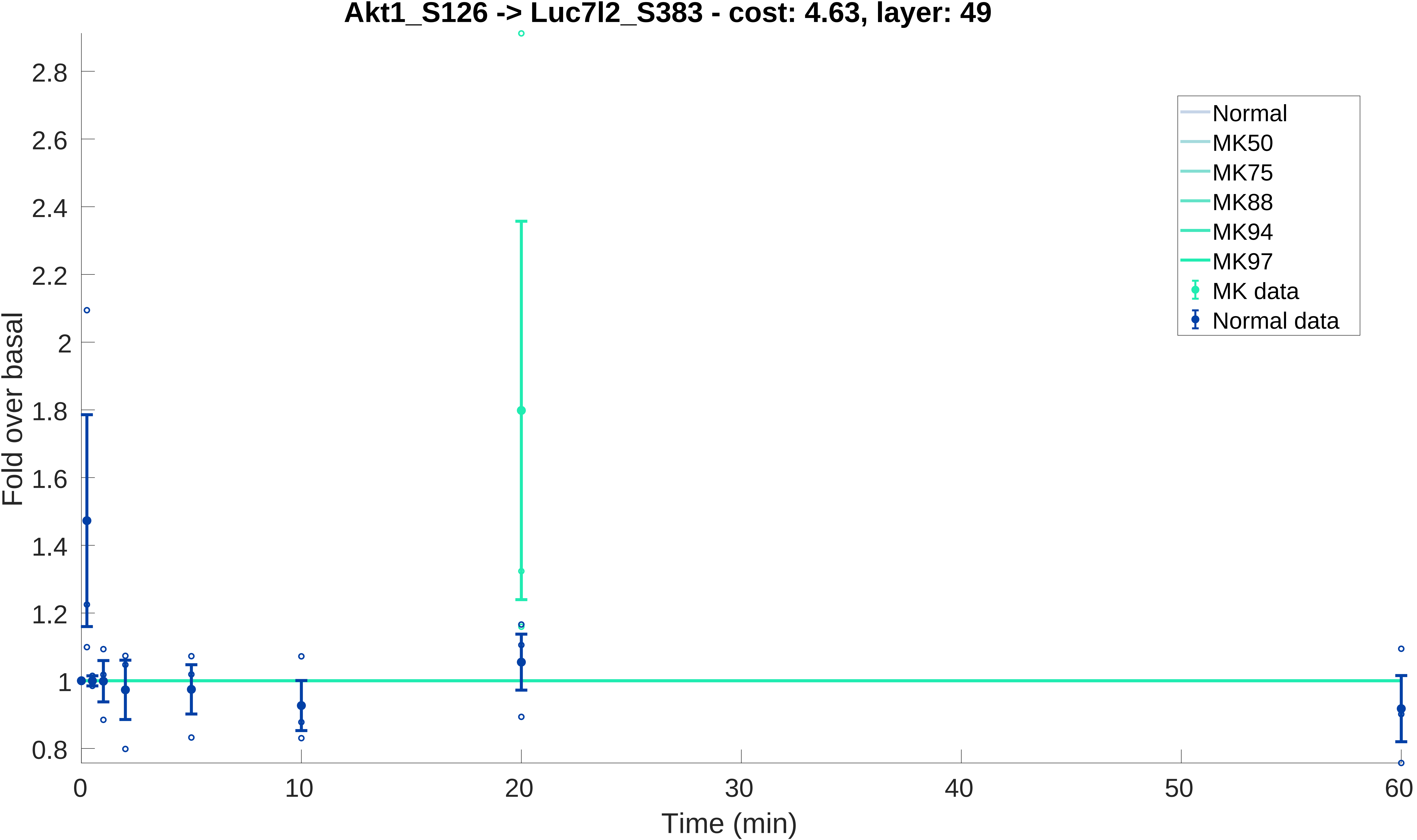

Akt1_S126 -> Luc7l2_S383 - cost: 4.63, layer: 49
Fold over basal
Time (min)
Normal
MK50
MK75
MK88
MK94
MK97
MK data
Normal data
0.8
1
1.2
1.4
1.6
1.8
2
2.2
2.4
2.6
2.8
0
10
20
30
40
50
60

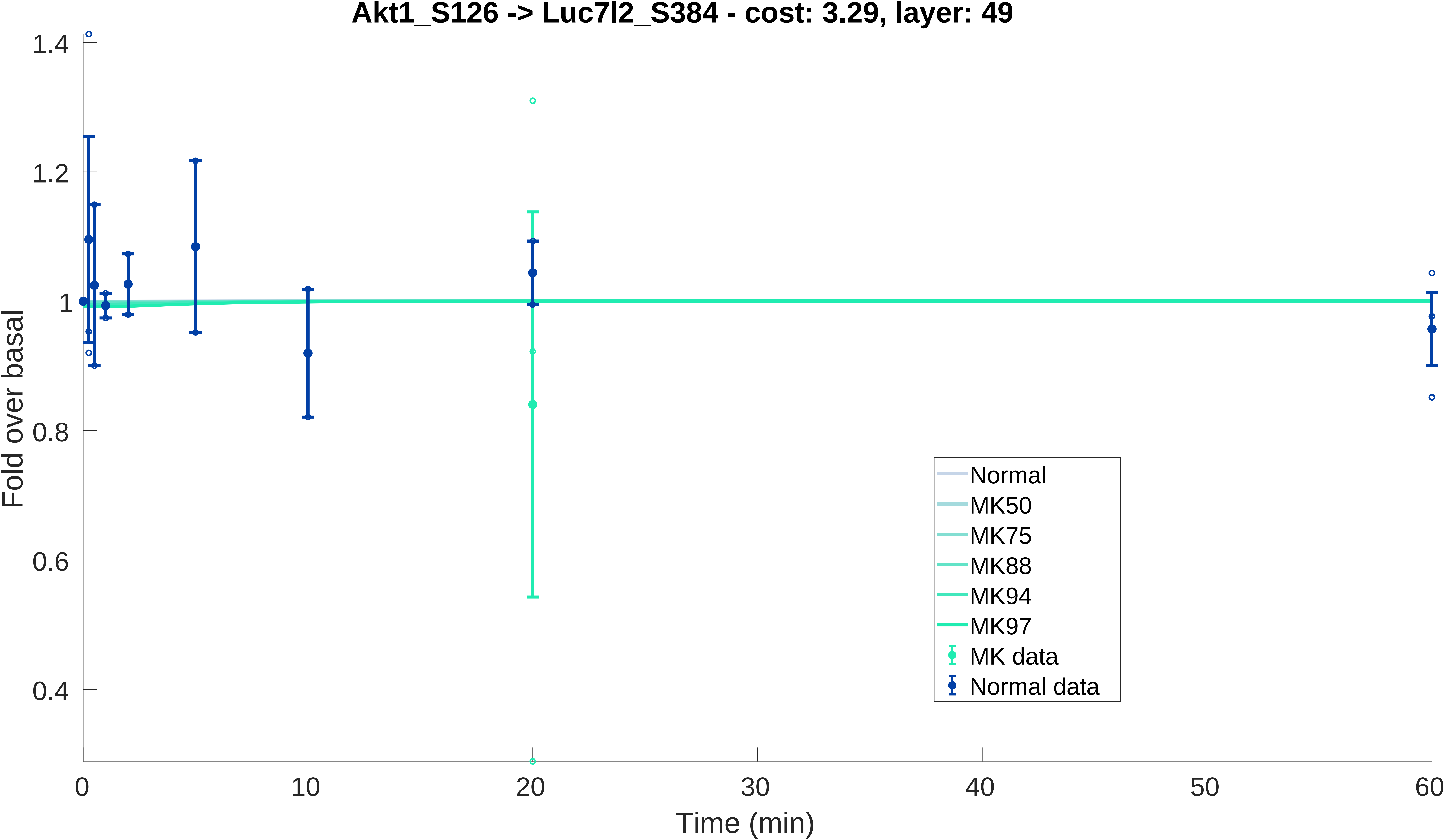

Akt1_S126 -> Luc7l2_S384 - cost: 3.29, layer: 49
Fold over basal
1.4
1.2
1
0.8
0.6
0.4
0
10
20
30
40
50
60
Time (min)
Normal
MK50
MK75
MK88
MK94
MK97
MK data
Normal data

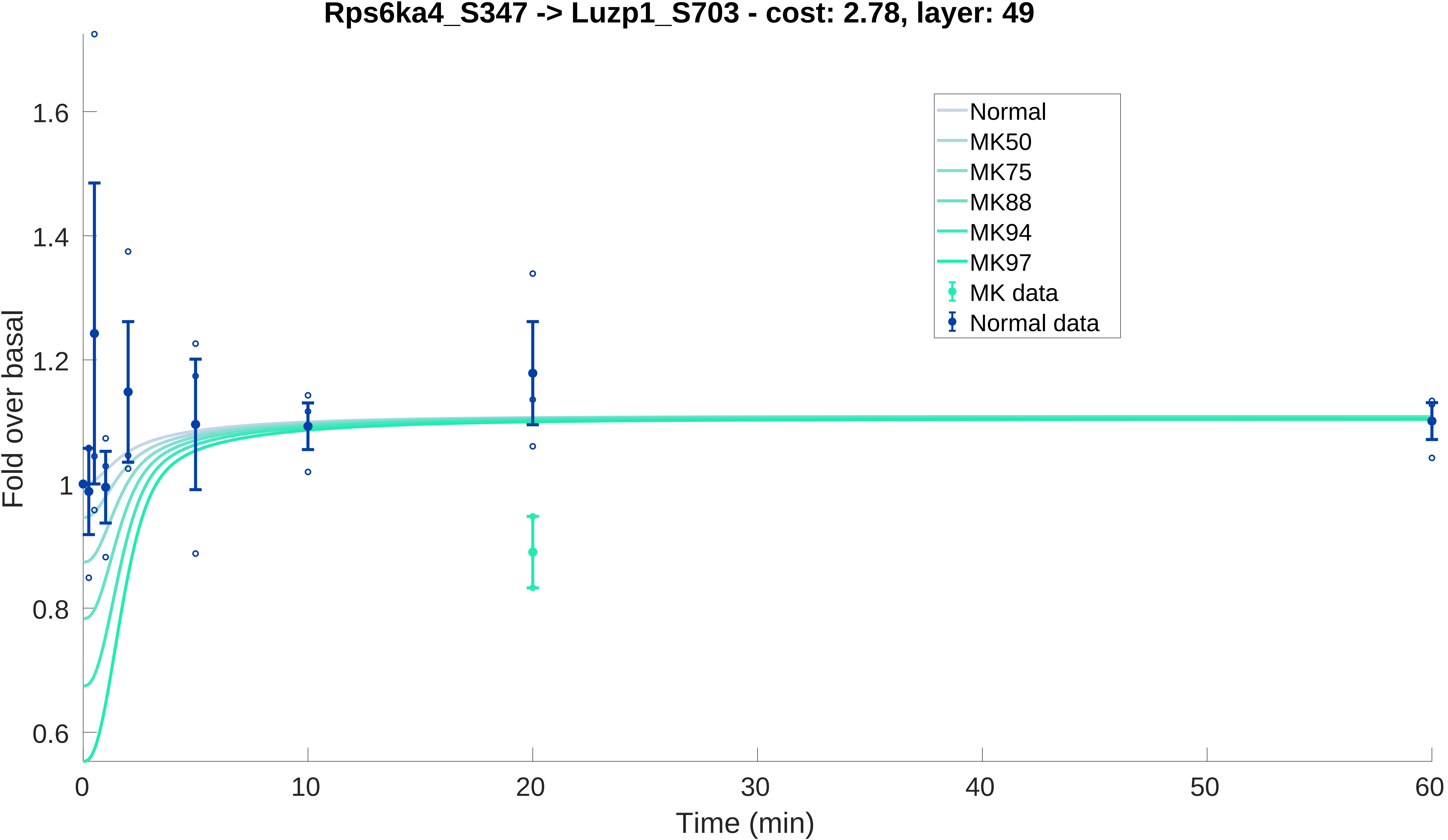

Rps6ka4_S347 -> Luzp1_S703 - cost: 2.78, layer: 49
Fold over basal
Time (min)
0.6
0.8
1
1.2
1.4
1.6
0
10
20
30
40
50
60
Normal
MK50
MK75
MK88
MK94
MK97
MK data
Normal data

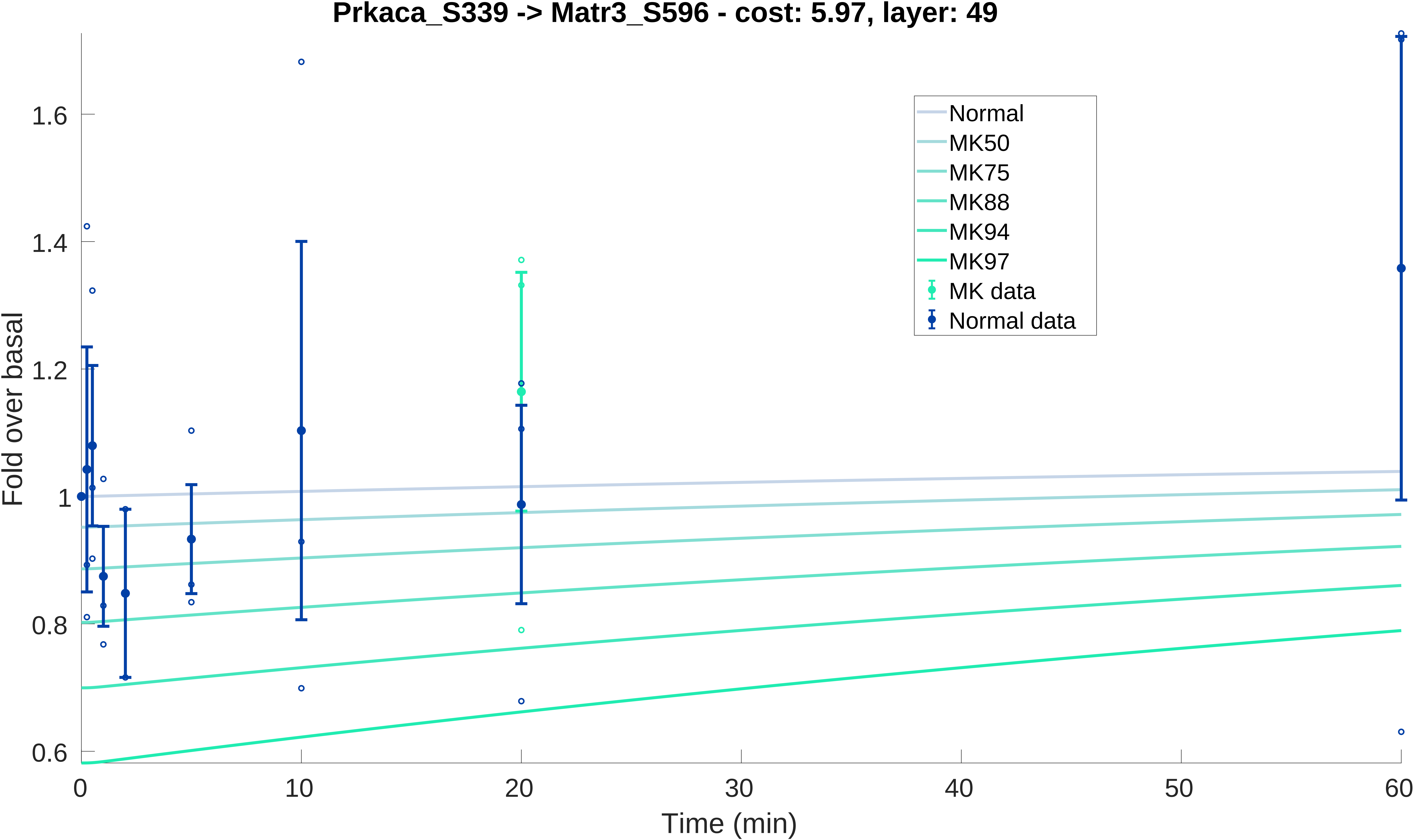

Prrkaca_S339 -> Matr3_S596 - cost: 5.97, layer: 49
Fold over basal
Time (min)
0.6
0.8
1
1.2
1.4
1.6
0
10
20
30
40
50
60
Normal
MK50
MK75
MK88
MK94
MK97
MK data
Normal data

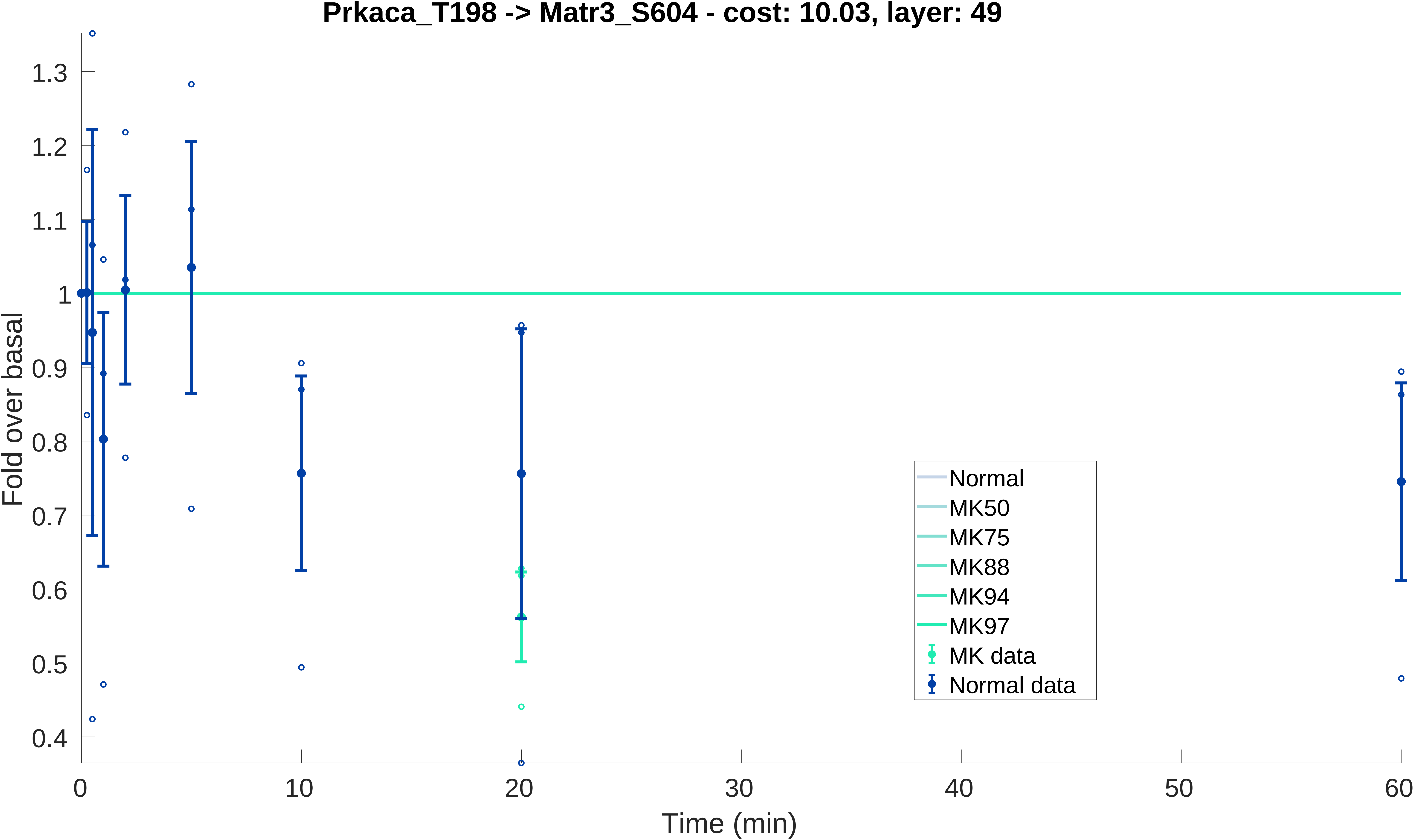

Prkaca_T198 -> Matr3_S604 - cost: 10.03, layer: 49
Fold over basal
1.3
1.2
1.1
1
0.9
0.8
0.7
0.6
0.5
0.4
0
10
20
30
40
50
60
Time (min)
Normal
MK50
MK75
MK88
MK94
MK97
MK data
Normal data

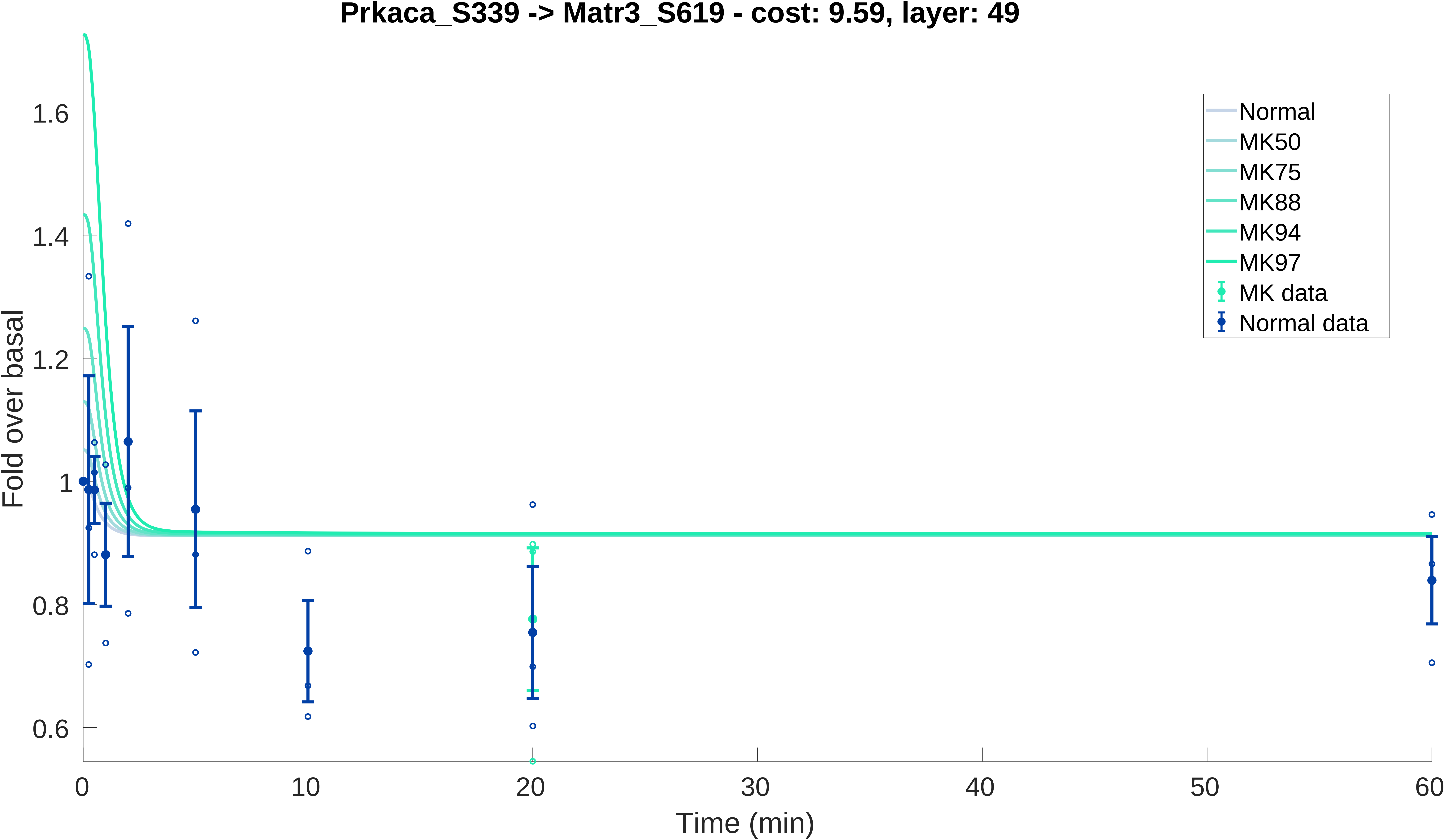
Prkaca_S339 -> Matr3_S619 - cost: 9.59, layer: 49
Fold over basal
1.6
1.4
1.2
1
0.8
0.6
0
10
20
30
40
50
60
Time (min)
Normal
MK50
MK75
MK88
MK94
MK97
MK data
Normal data

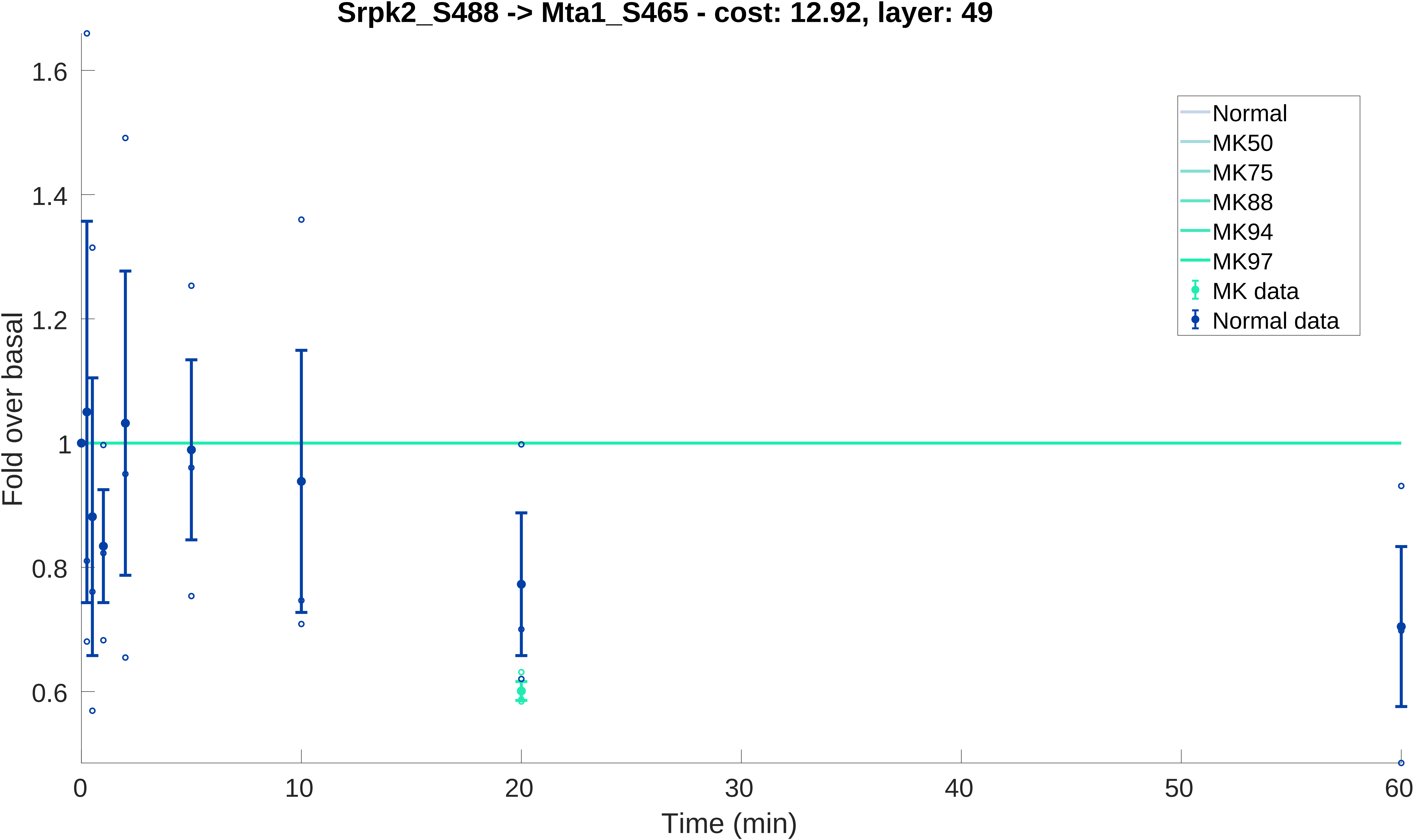
Srpk2_S488 -> Mta1_S465 - cost: 12.92, layer: 49
Fold over basal
Time (min)
0.6
0.8
1
1.2
1.4
1.6
0
10
20
30
40
50
60
Normal
MK50
MK75
MK88
MK94
MK97
MK data
Normal data

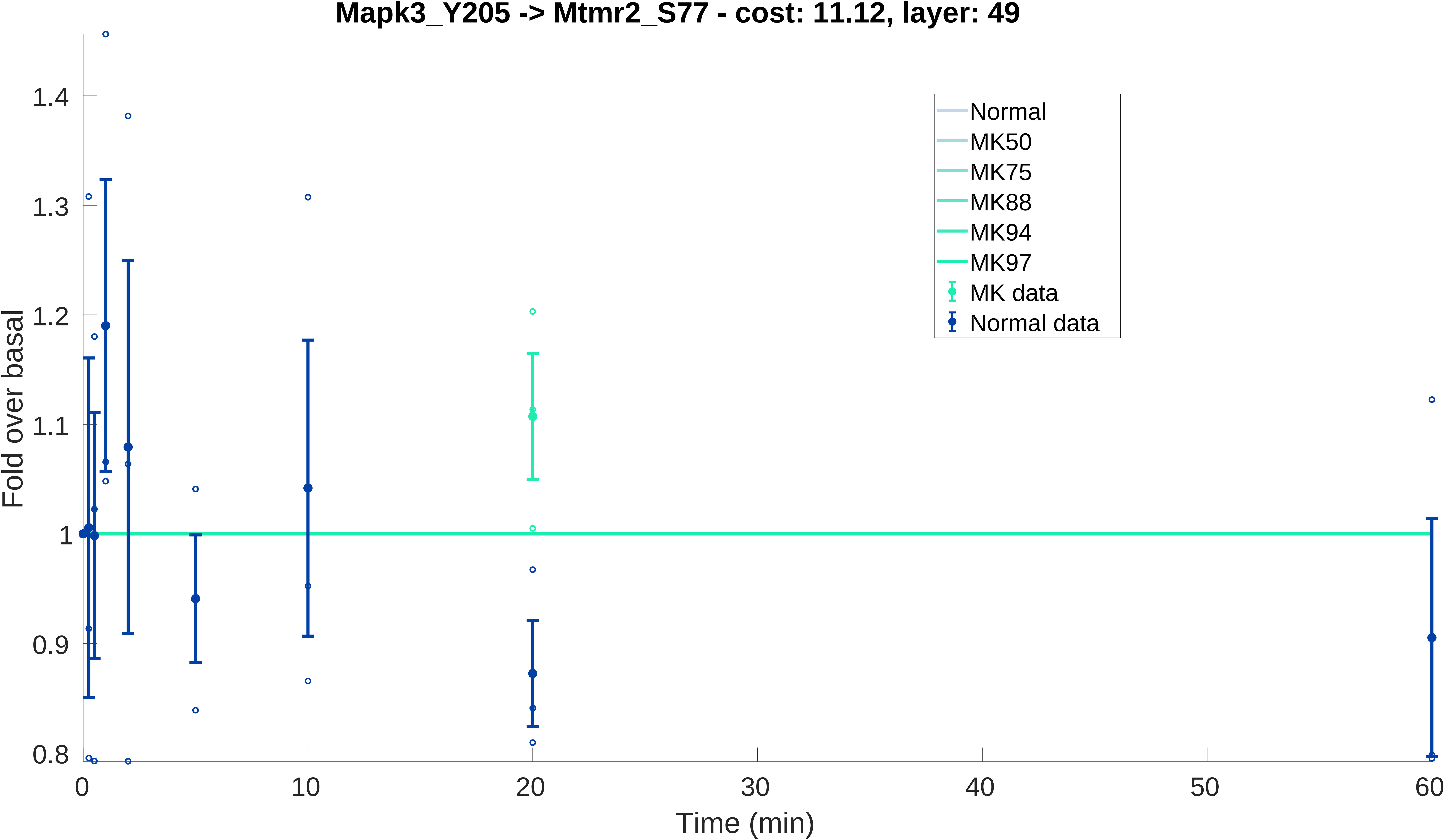

Mapk3_Y205 -> Mtmr2_S77 - cost: 11.12, layer: 49
Fold over basal
1.4
1.3
1.2
1.1
1
0.9
0.8
0
10
20
30
40
50
60
Time (min)
Normal
MK50
MK75
MK88
MK94
MK97
MK data
Normal data

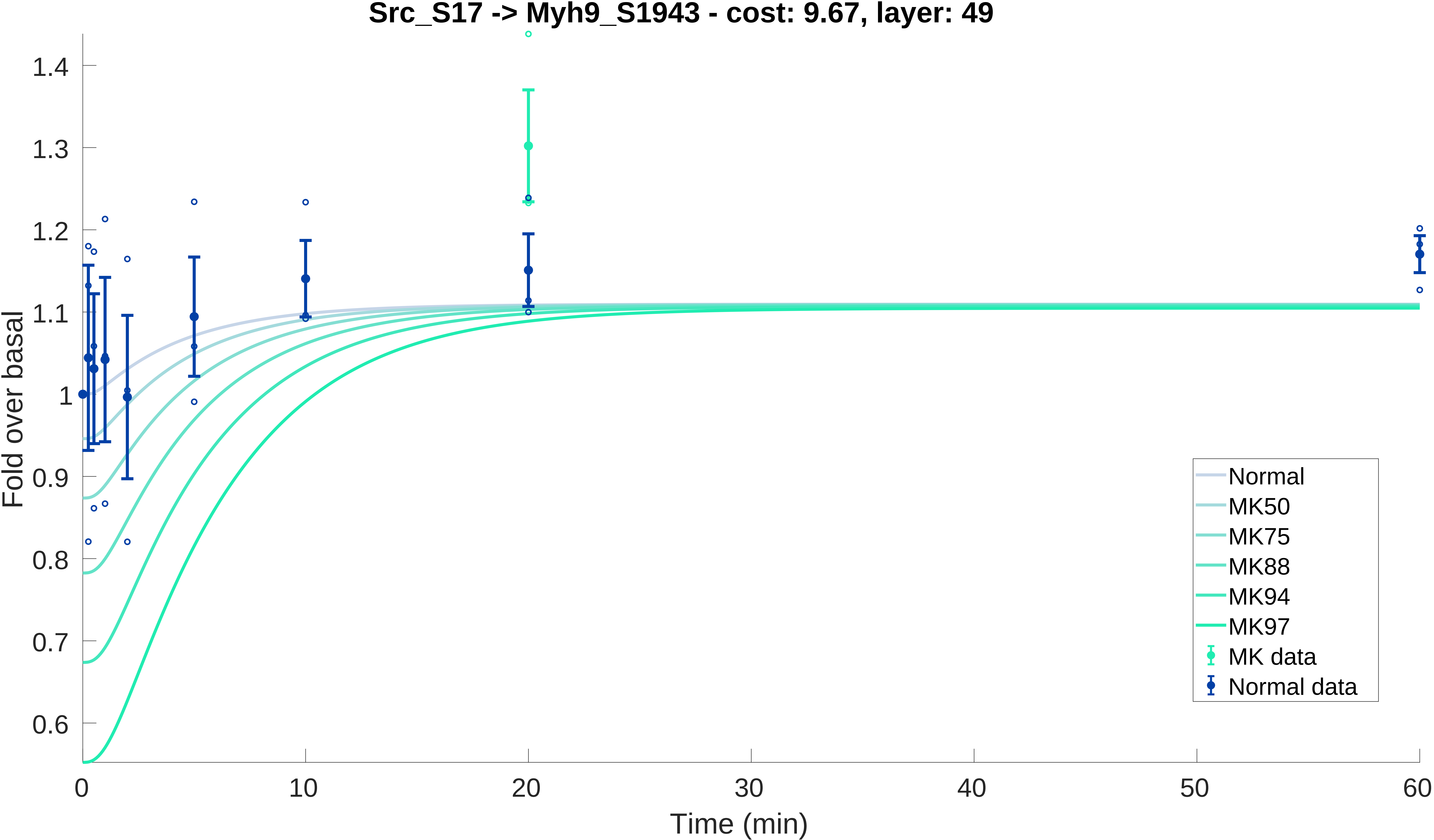
Src_S17 -> Myh9_S1943 - cost: 9.67, layer: 49
Fold over basal
Time (min)
1.4
1.3
1.2
1.1
1
0.9
0.8
0.7
0.6
0
10
20
30
40
50
60
Normal
MK50
MK75
MK88
MK94
MK97
MK data
Normal data

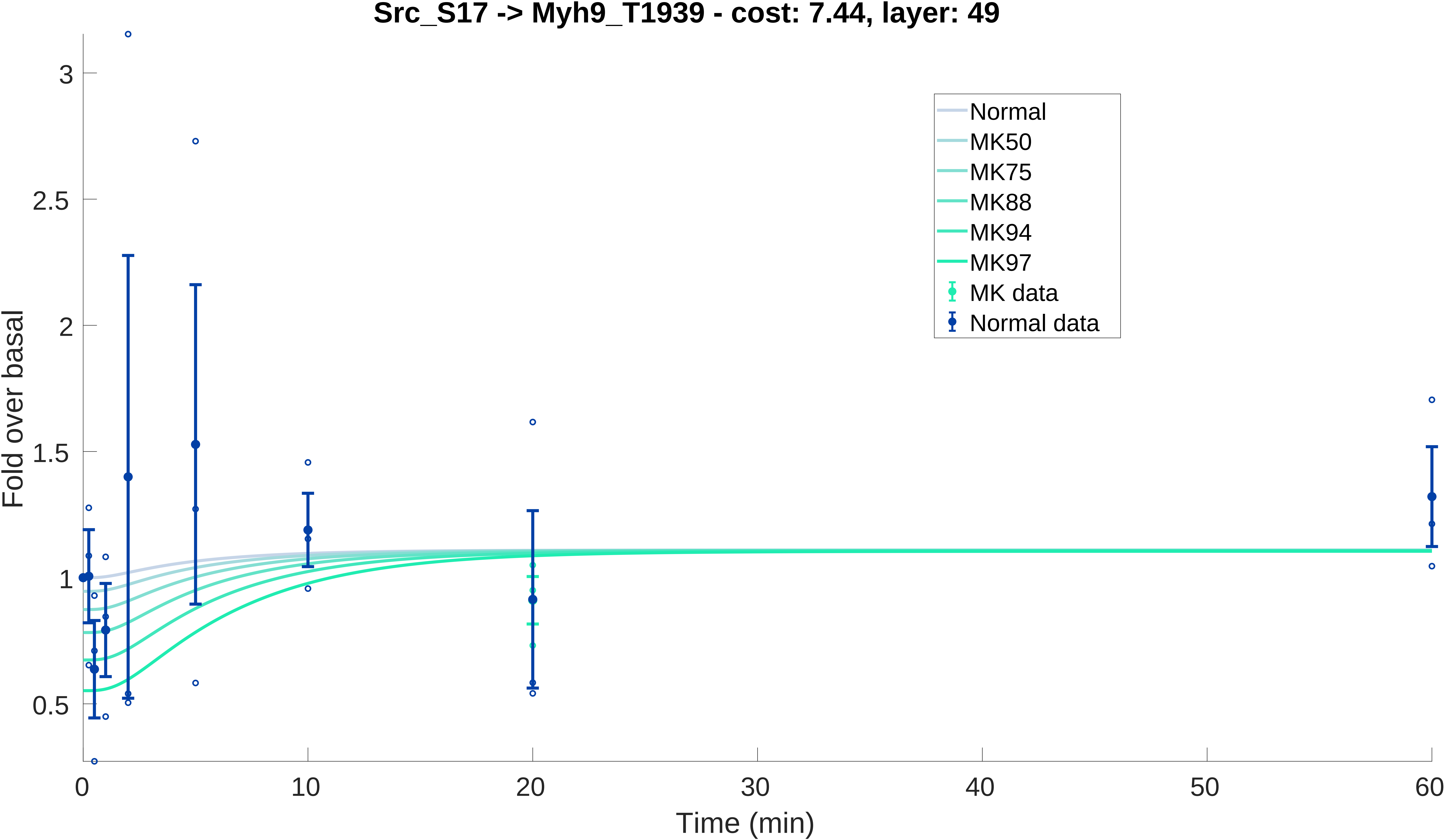

Src_S17 -> Myh9_T1939 - cost: 7.44, layer: 49
Fold over basal
Time (min)
0
10
20
30
40
50
60
0.5
1
1.5
2
2.5
3
Normal
MK50
MK75
MK88
MK94
MK97
MK data
Normal data

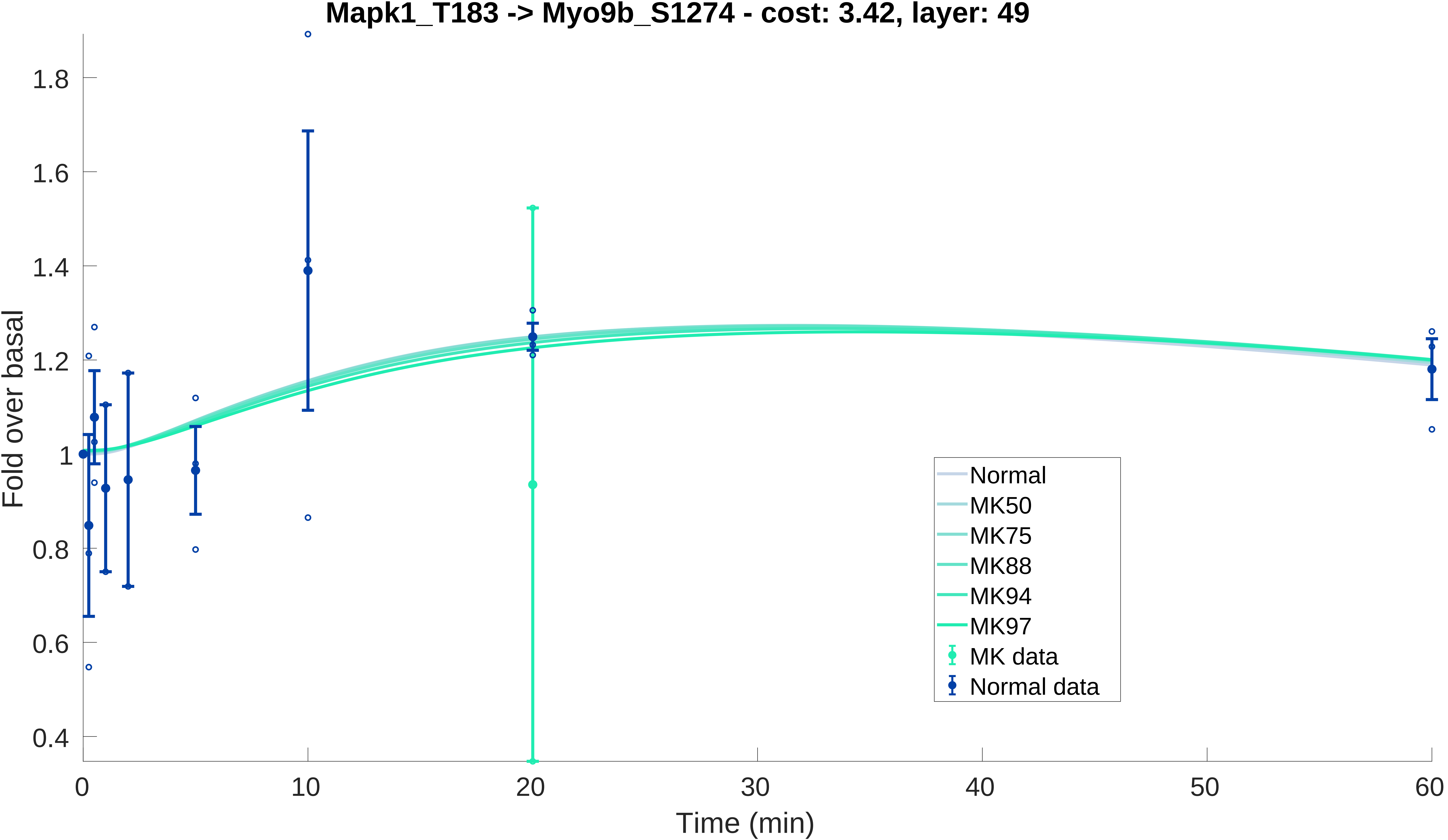

Mapk1_T183 -> Myo9b_S1274 - cost: 3.42, layer: 49
Fold over basal
1.8
1.6
1.4
1.2
1
0.8
0.6
0.4
0
10
20
30
40
50
60
Time (min)
Normal
MK50
MK75
MK88
MK94
MK97
MK data
Normal data

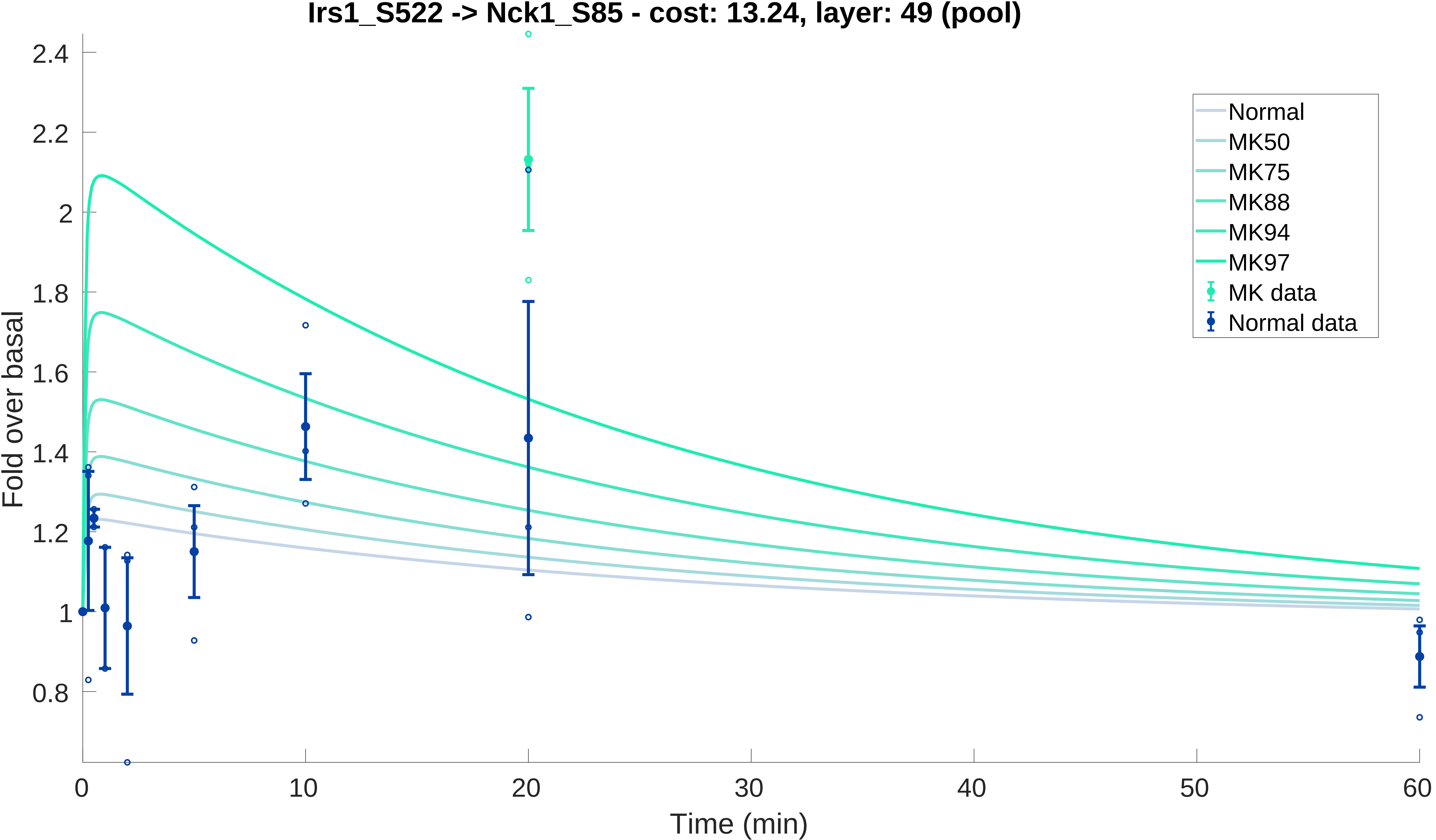

Irs1_S522 -> Nck1_S85 - cost: 13.24, layer: 49 (pool)
Fold over basal
Time (min)
0.8
1
1.2
1.4
1.6
1.8
2
2.2
2.4
0
10
20
30
40
50
60
Normal
MK50
MK75
MK88
MK94
MK97
MK data
Normal data

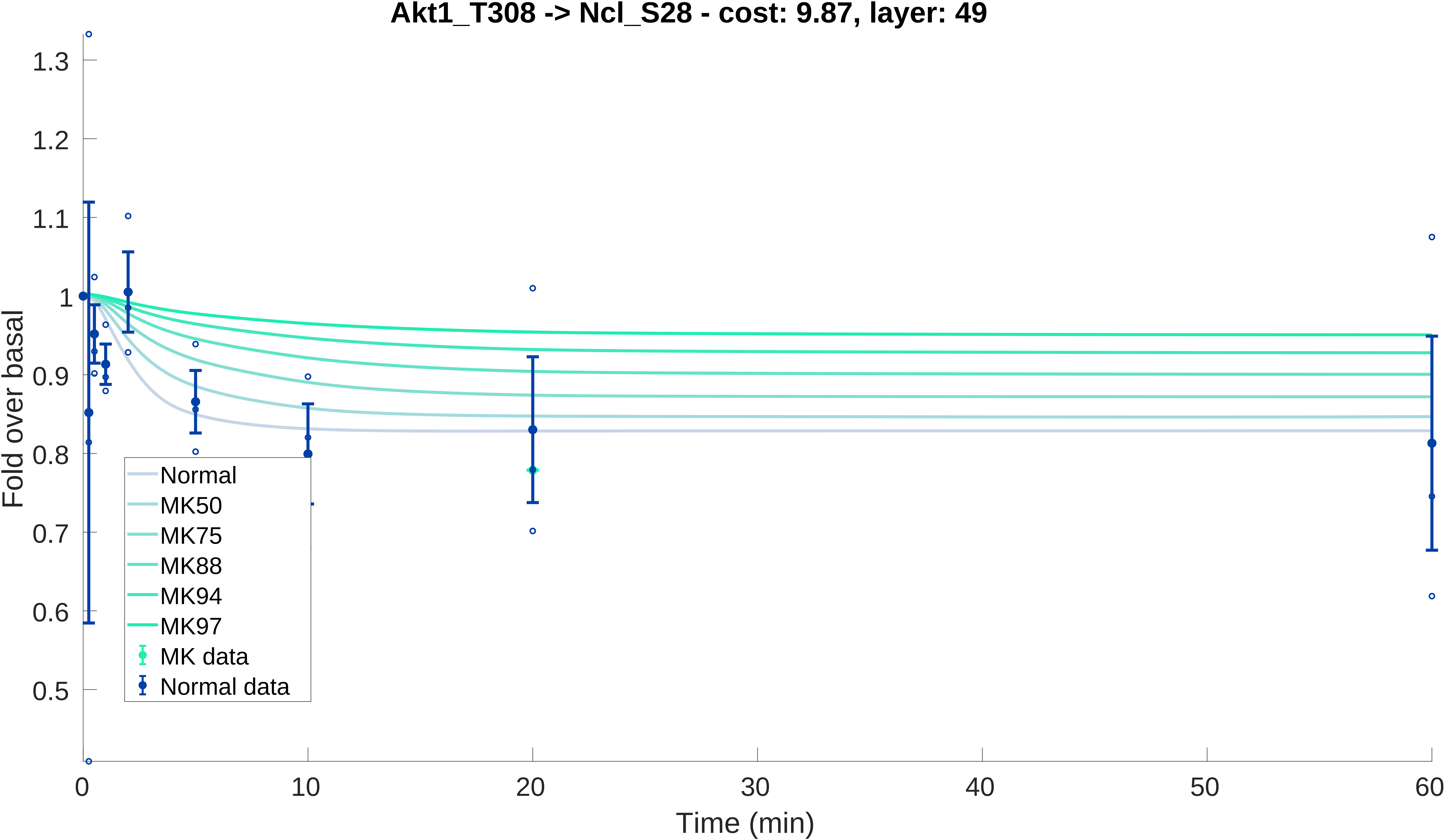
Akt1_T308 -> Ncl_S28 - cost: 9.87, layer: 49
Fold over basal
1.3
1.2
1.1
1
0.9
0.8
0.7
0.6
0.5
0
10
20
30
40
50
60
Time (min)
Normal
MK50
MK75
MK88
MK94
MK97
MK data
Normal data

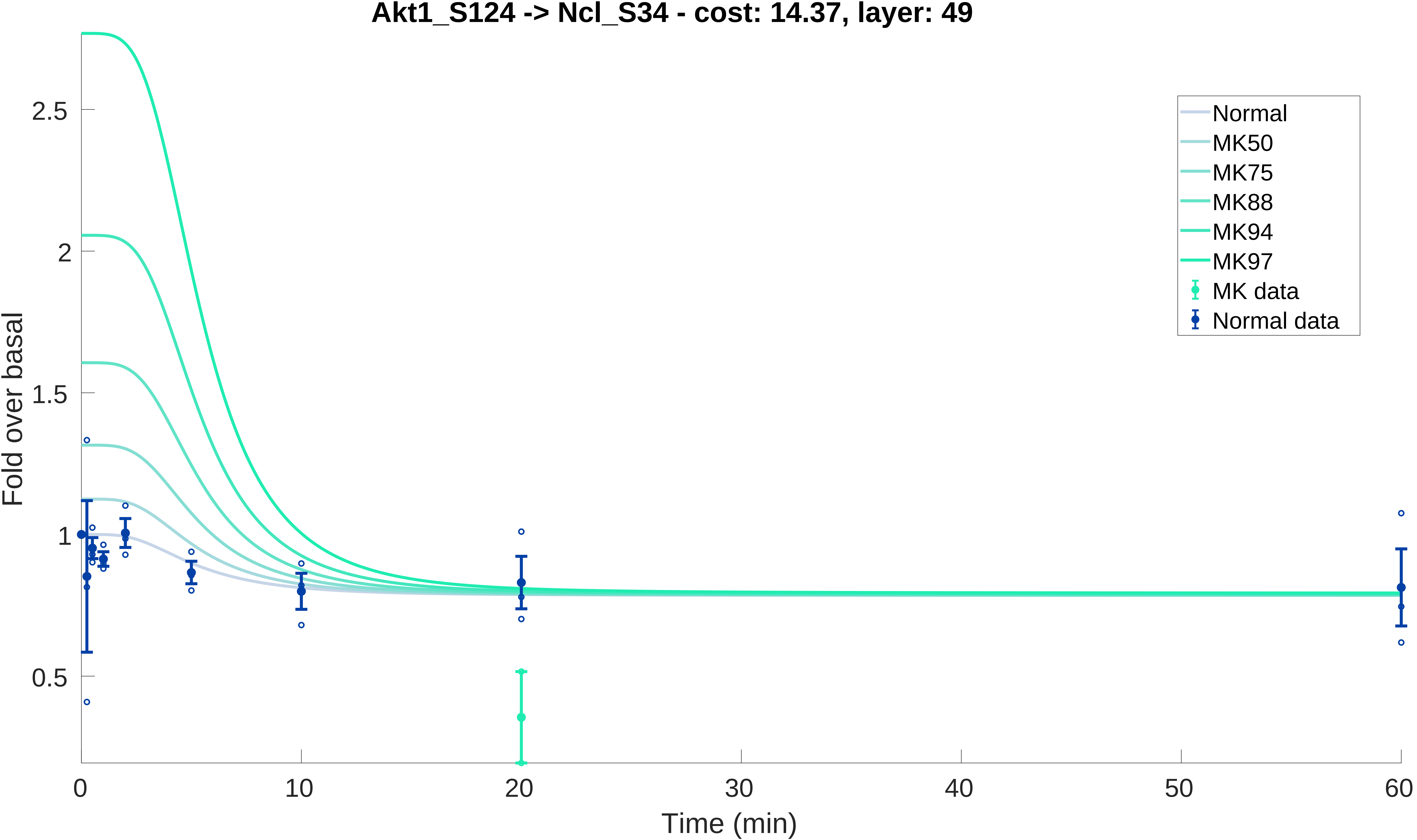

Akt1_S124 -> Ncl_S34 - cost: 14.37, layer: 49
Fold over basal
Time (min)
2.5
2
1.5
1
0.5
0
10
20
30
40
50
60
Normal
MK50
MK75
MK88
MK94
MK97
MK data
Normal data

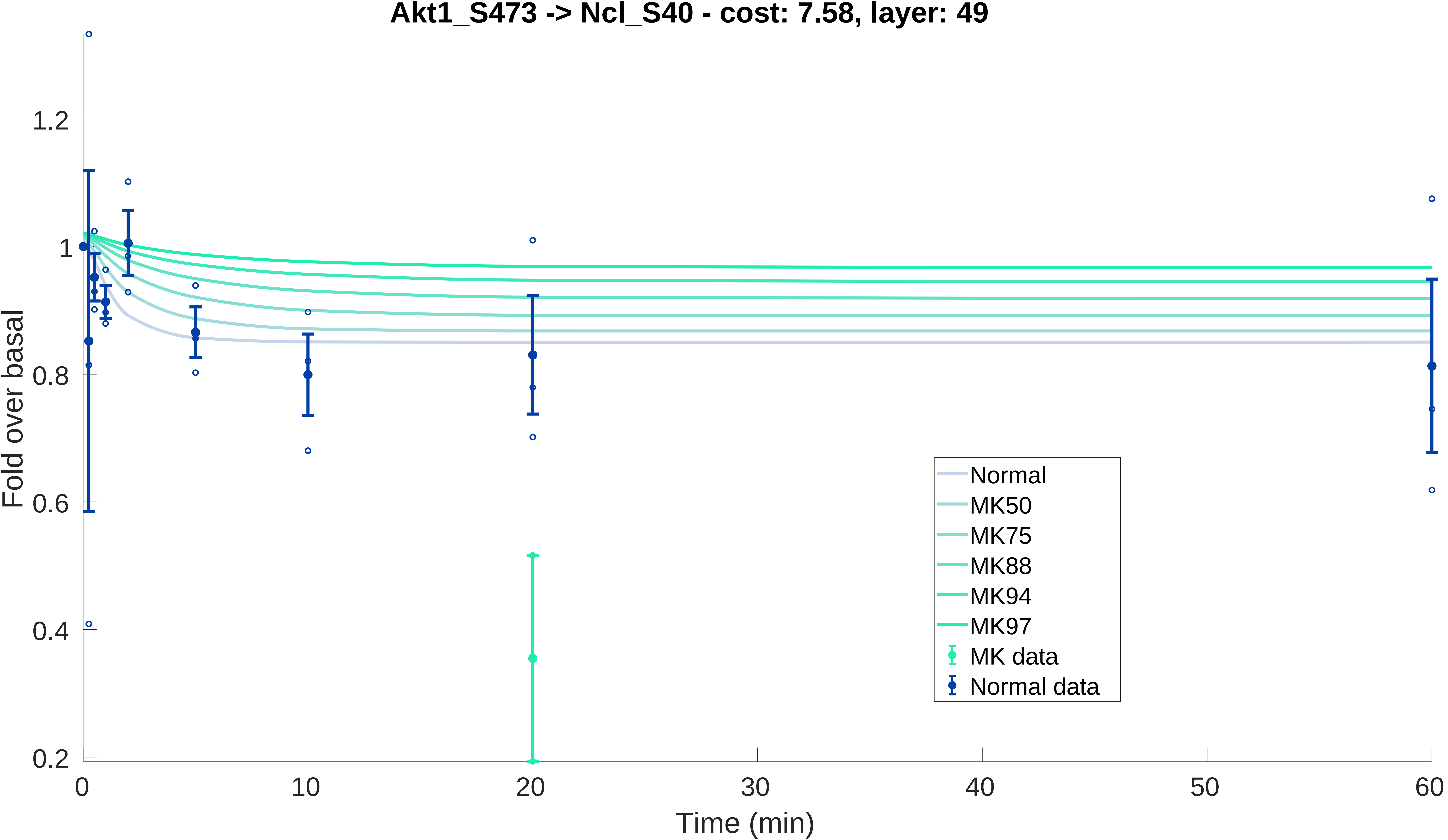

Akt1_S473 -> Ncl_S40 - cost: 7.58, layer: 49
Fold over basal
Time (min)
0.2
0.4
0.6
0.8
1
1.2
0
10
20
30
40
50
60
Normal
MK50
MK75
MK88
MK94
MK97
MK data
Normal data

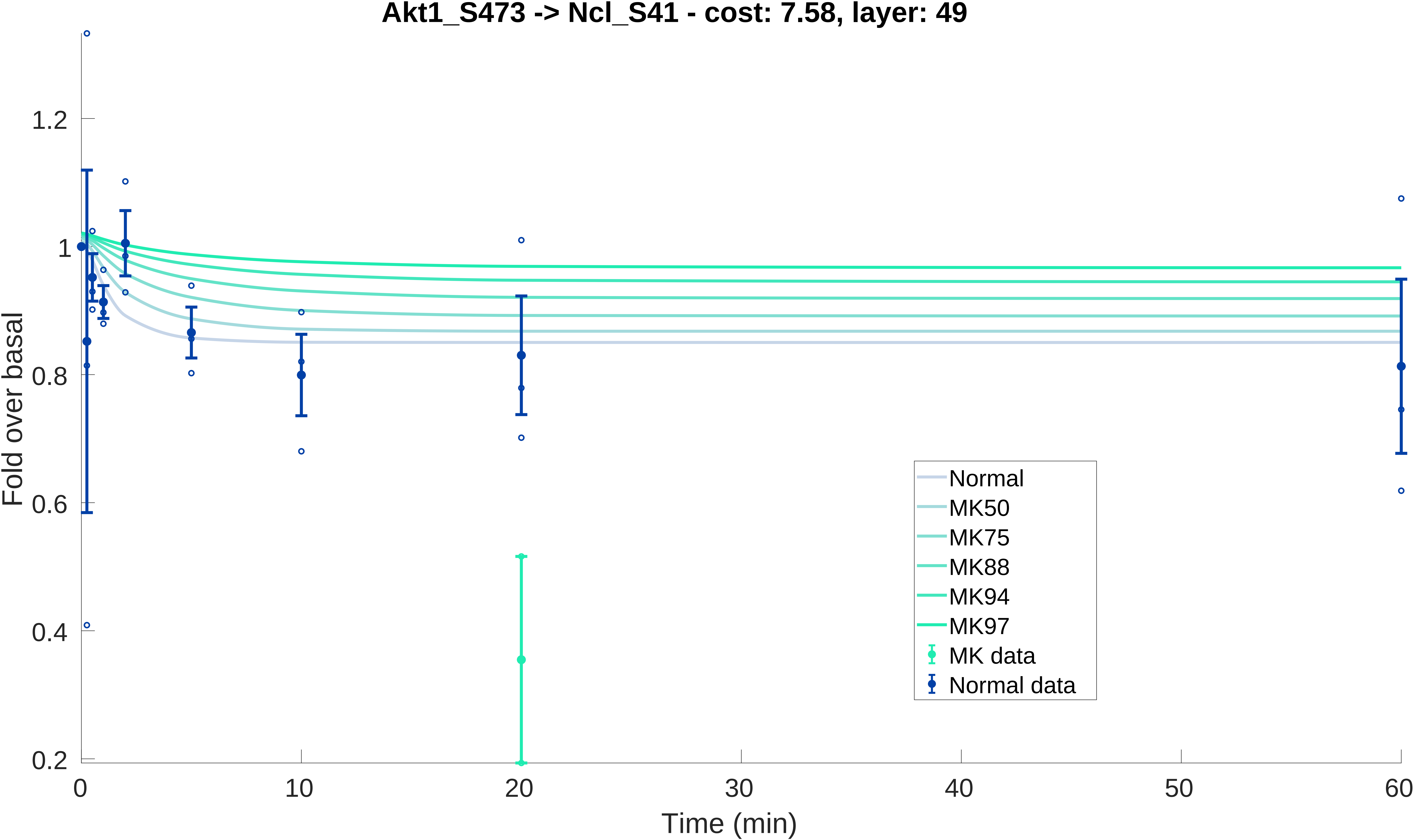

Akt1_S473 -> Ncl_S41 - cost: 7.58, layer: 49
Fold over basal
Time (min)
1.2
1
0.8
0.6
0.4
0.2
0
10
20
30
40
50
60
Normal
MK50
MK75
MK88
MK94
MK97
MK data
Normal data

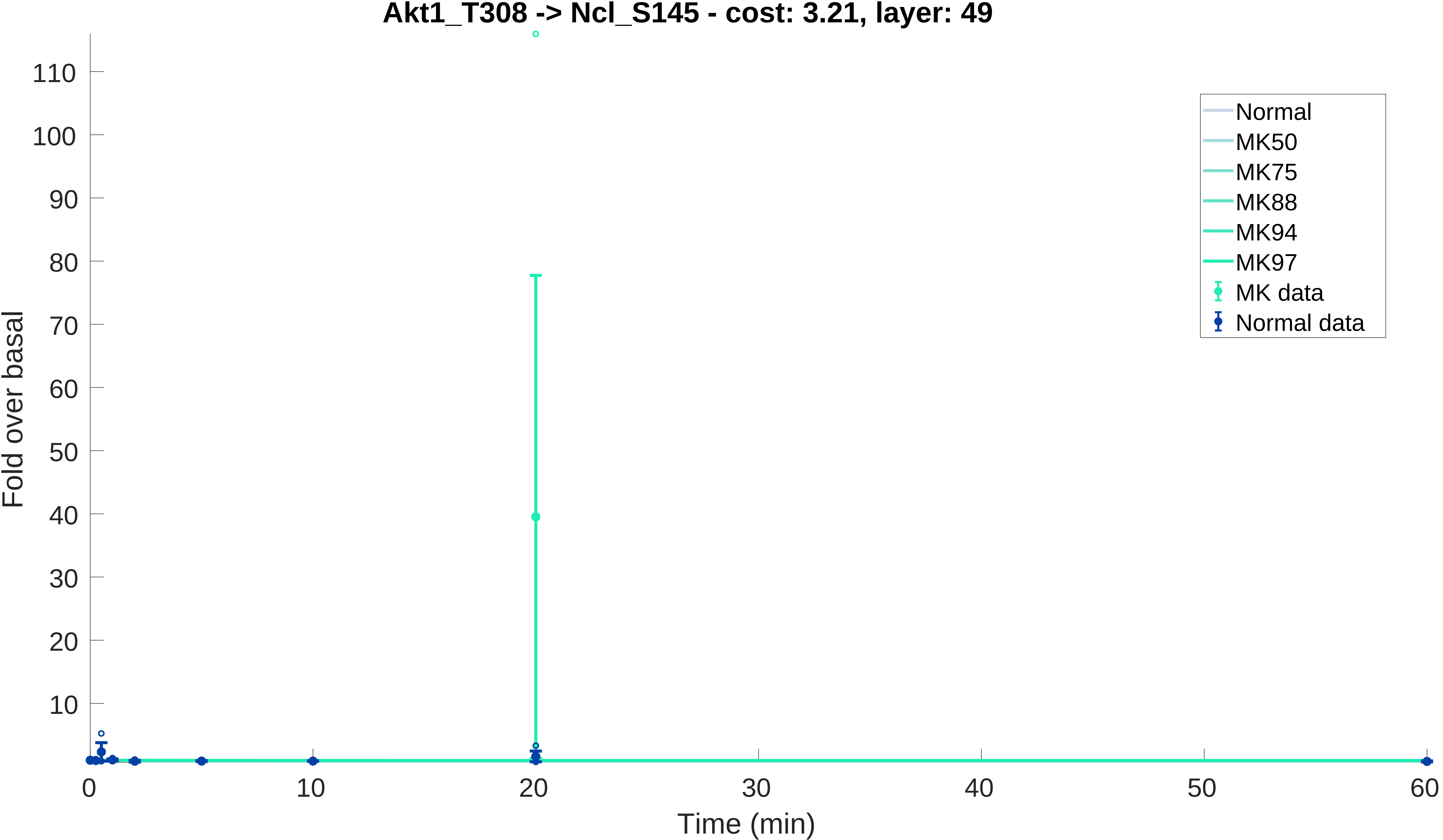

Akt1_T308 -> Ncl_S145 - cost: 3.21, layer: 49
Fold over basal
Time (min)
110
100
90
80
70
60
50
40
30
20
10
0
10
20
30
40
50
60
Normal
MK50
MK75
MK88
MK94
MK97
MK data
Normal data

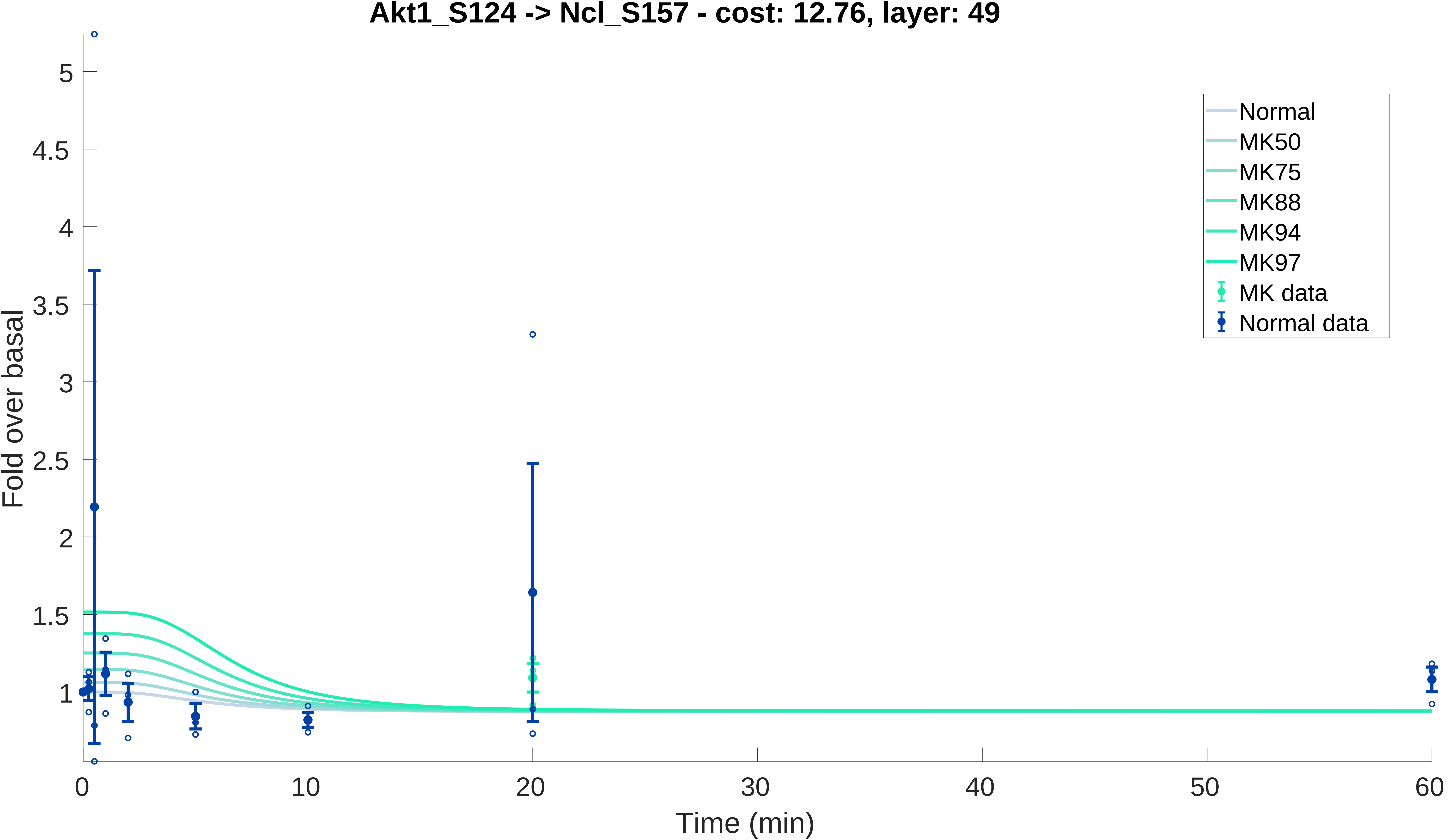
Akt1_S124 -> Ncl_S157 - cost: 12.76, layer: 49
Fold over basal
Time (min)
0
10
20
30
40
50
60
1
1.5
2
2.5
3
3.5
4
4.5
5
Normal
MK50
MK75
MK88
MK94
MK97
MK data
Normal data

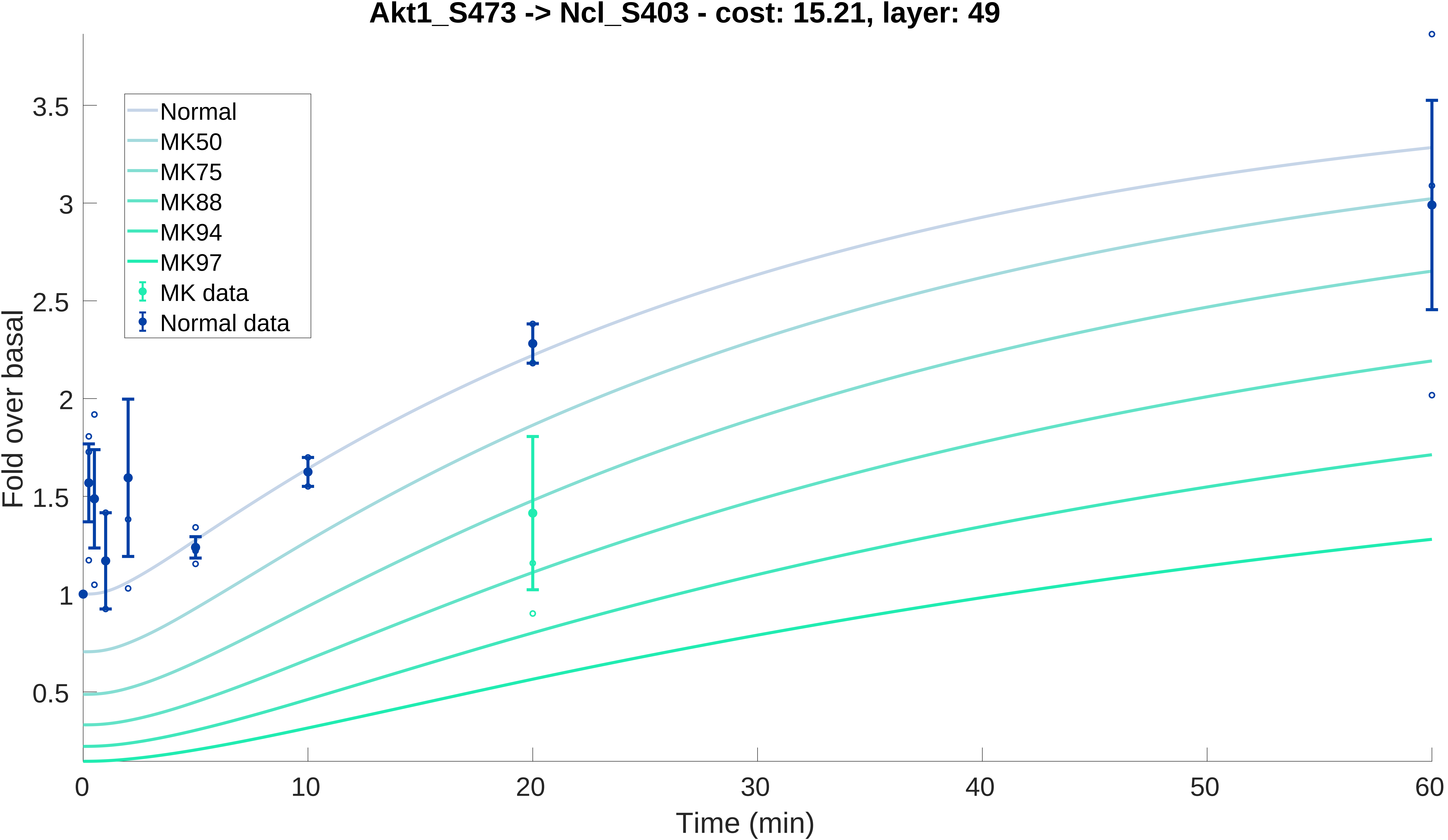

Akt1_S473 -> Ncl_S403 - cost: 15.21, layer: 49
Normal
MK50
MK75
MK88
MK94
MK97
MK data
Normal data
Fold over basal
Time (min)
0.5
1
1.5
2
2.5
3
3.5
0
10
20
30
40
50
60

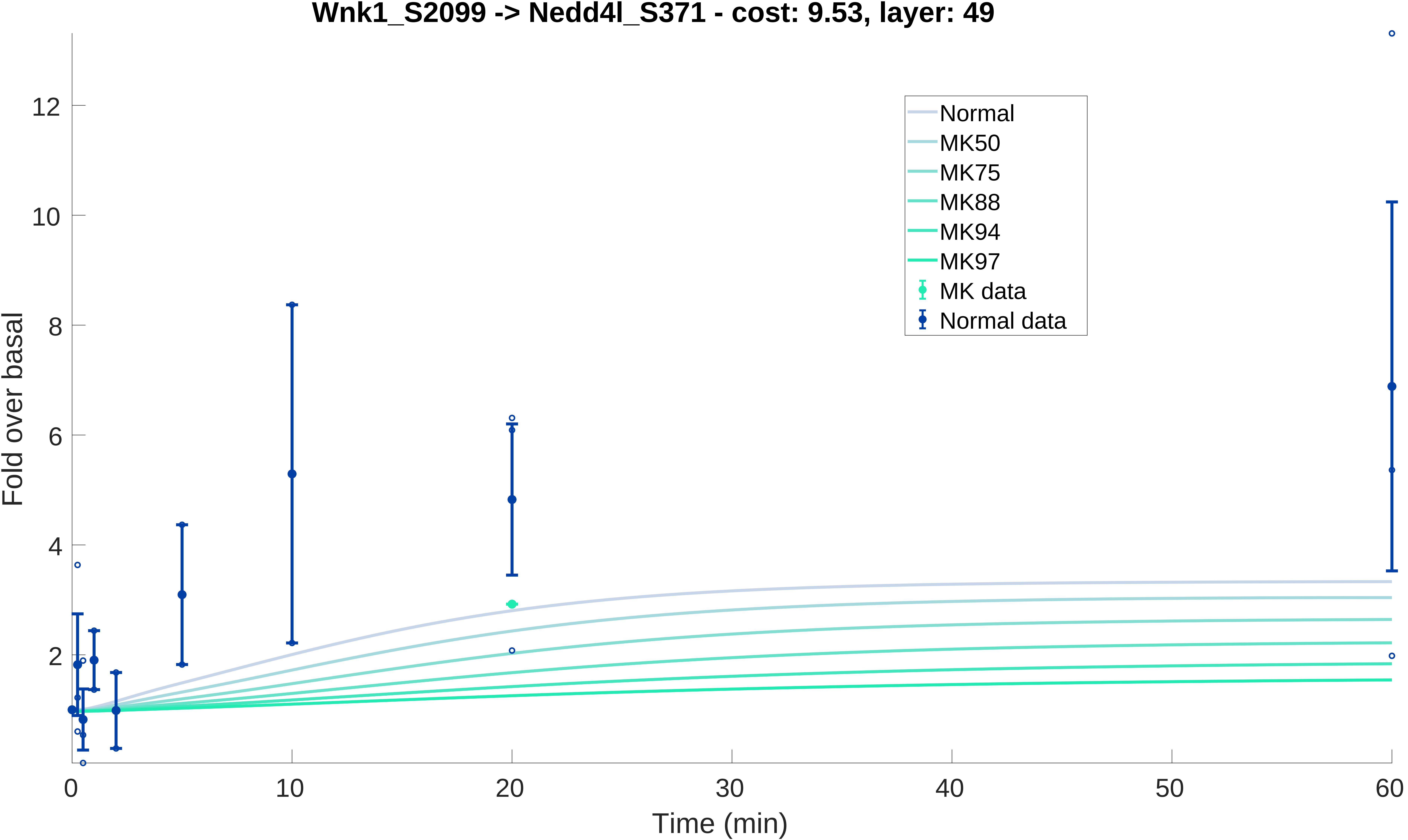

Wnk1_S2099 -> Nedd4l_S371 - cost: 9.53, layer: 49
Fold over basal
Time (min)
Normal
MK50
MK75
MK88
MK94
MK97
MK data
Normal data
0
10
20
30
40
50
60
2
4
6
8
10
12

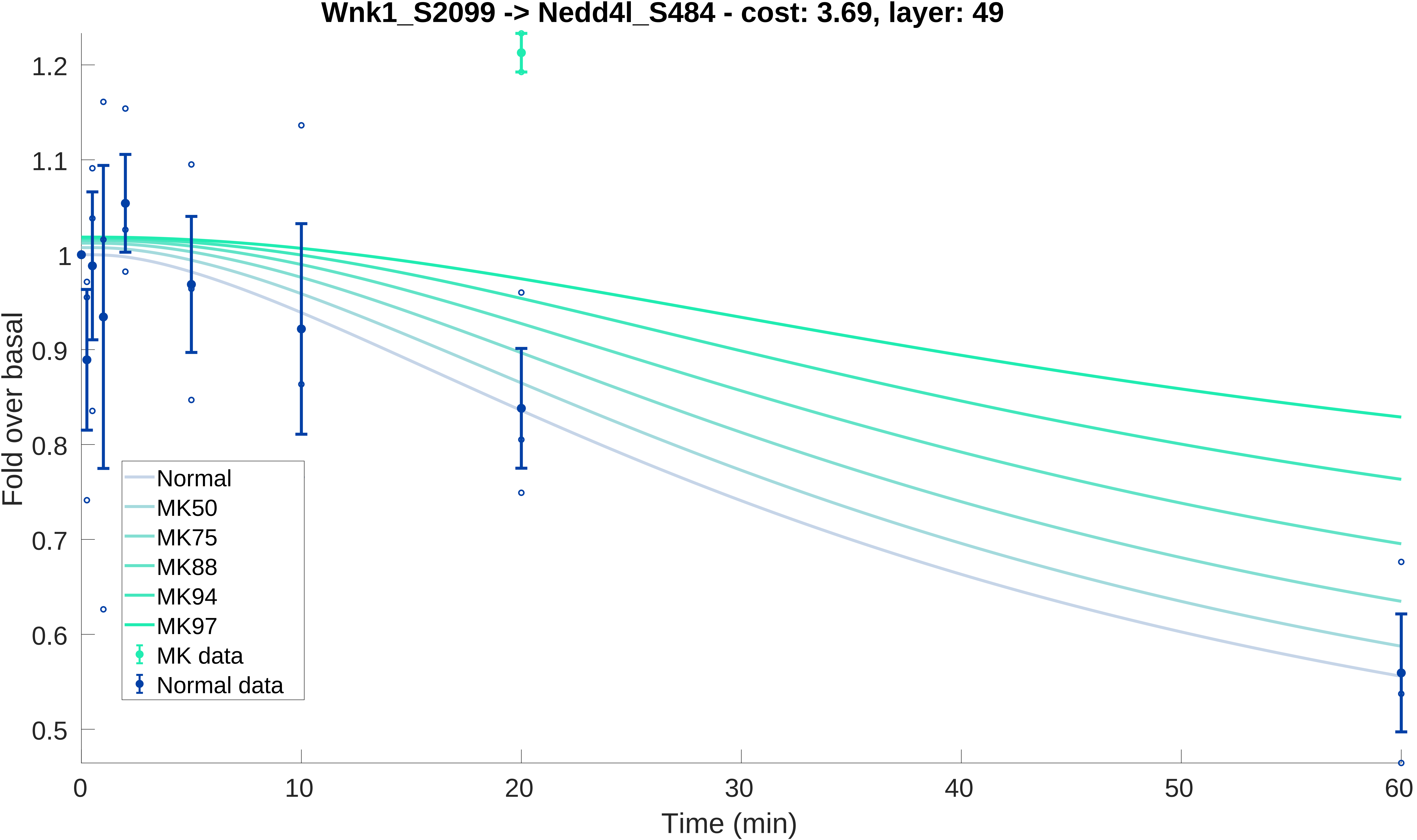

Wnk1_S2099 -> Nedd4l_S484 - cost: 3.69, layer: 49
Fold over basal
Time (min)
1.2
1.1
1
0.9
0.8
0.7
0.6
0.5
0
10
20
30
40
50
60
Normal
MK50
MK75
MK88
MK94
MK97
MK data
Normal data

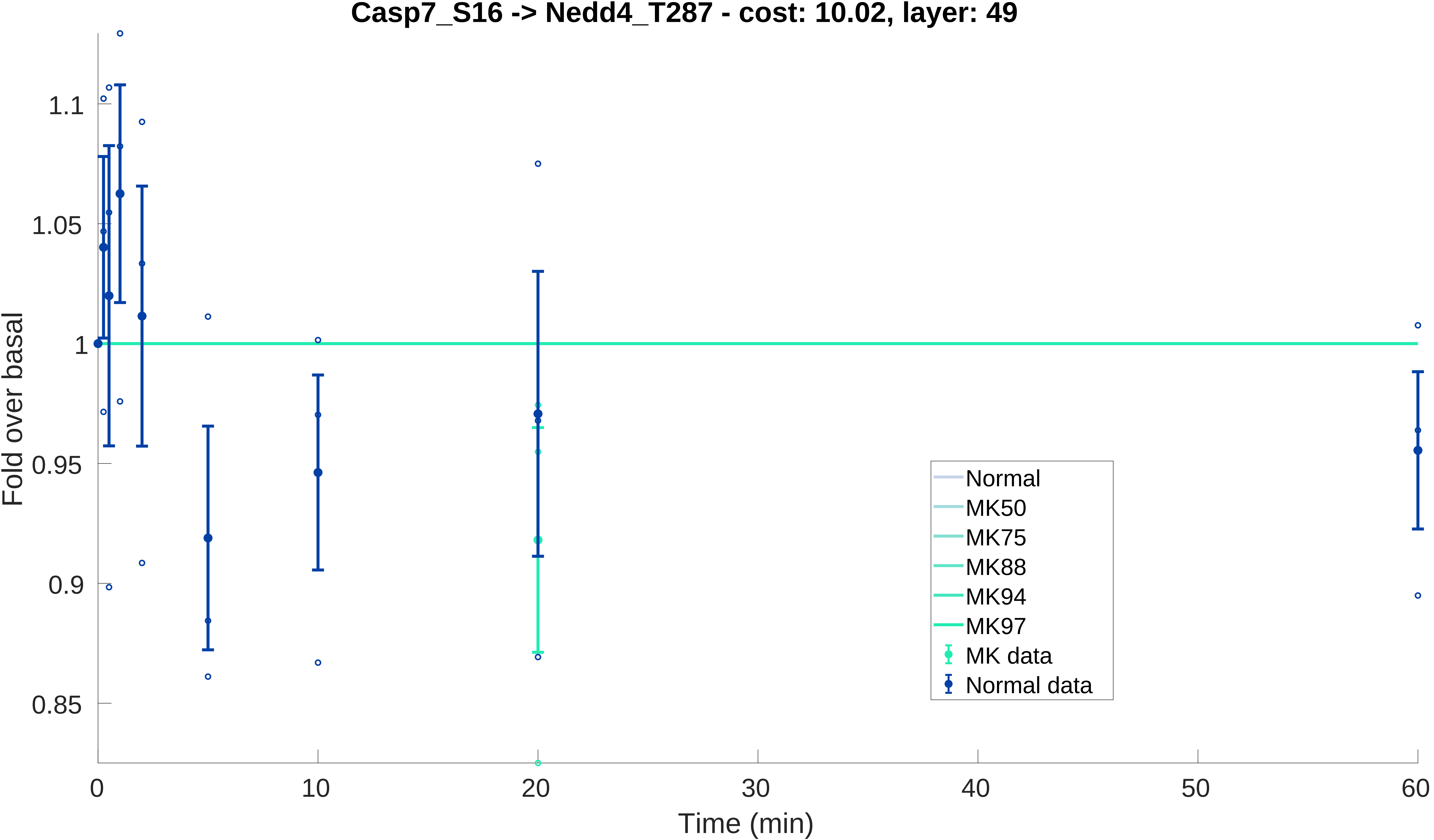

Casp7_S16 -> Nedd4_T287 - cost: 10.02, layer: 49
Fold over basal
1.1
1.05
1
0.95
0.9
0.85
0
10
20
30
40
50
60
Time (min)
Normal
MK50
MK75
MK88
MK94
MK97
MK data
Normal data

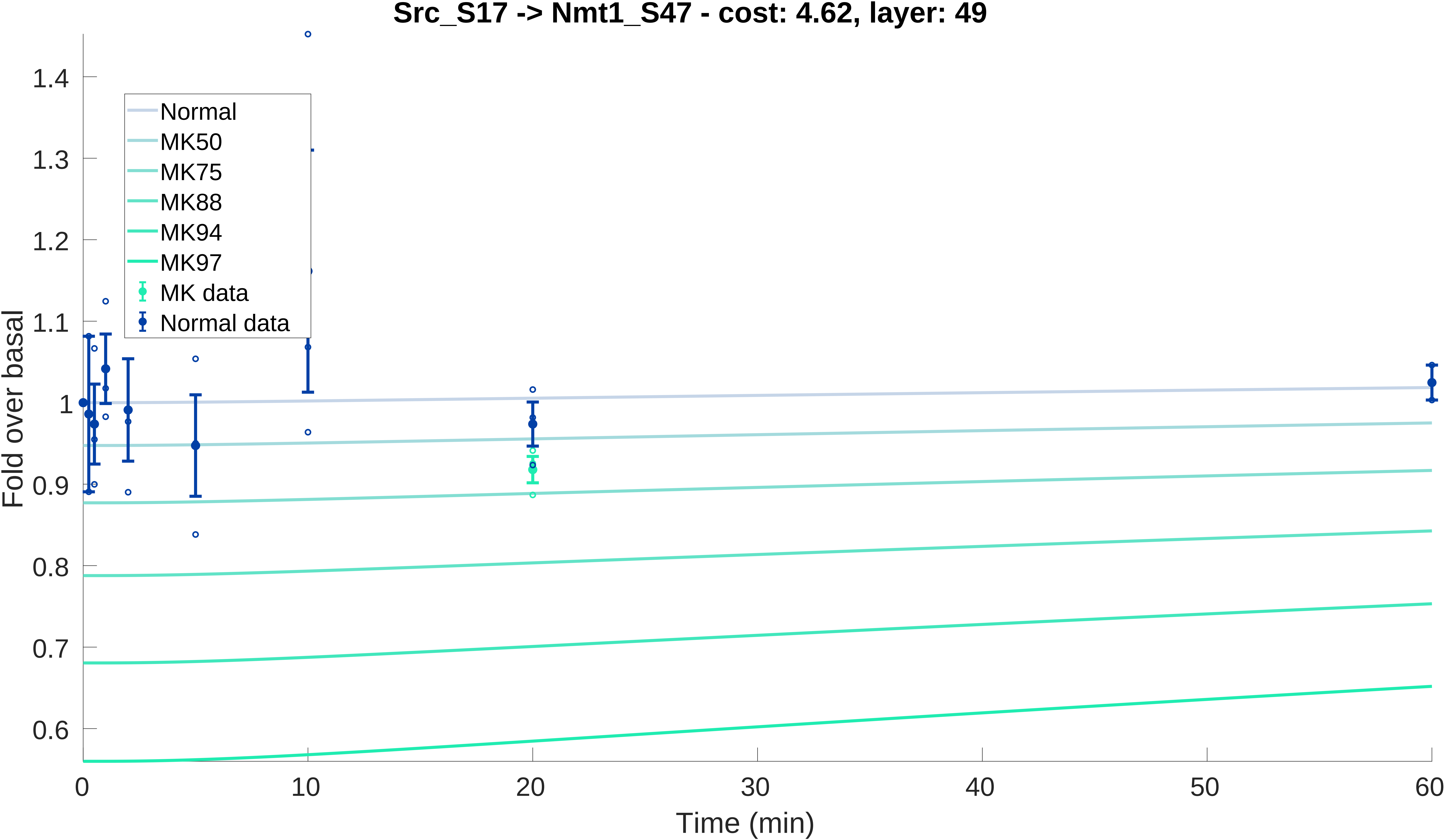

Src_S17 -> Nmt1_S47 - cost: 4.62, layer: 49
Normal
MK50
MK75
MK88
MK94
MK97
MK data
Normal data
Fold over basal
Time (min)
1.4
1.3
1.2
1.1
1
0.9
0.8
0.7
0.6
0
10
20
30
40
50
60

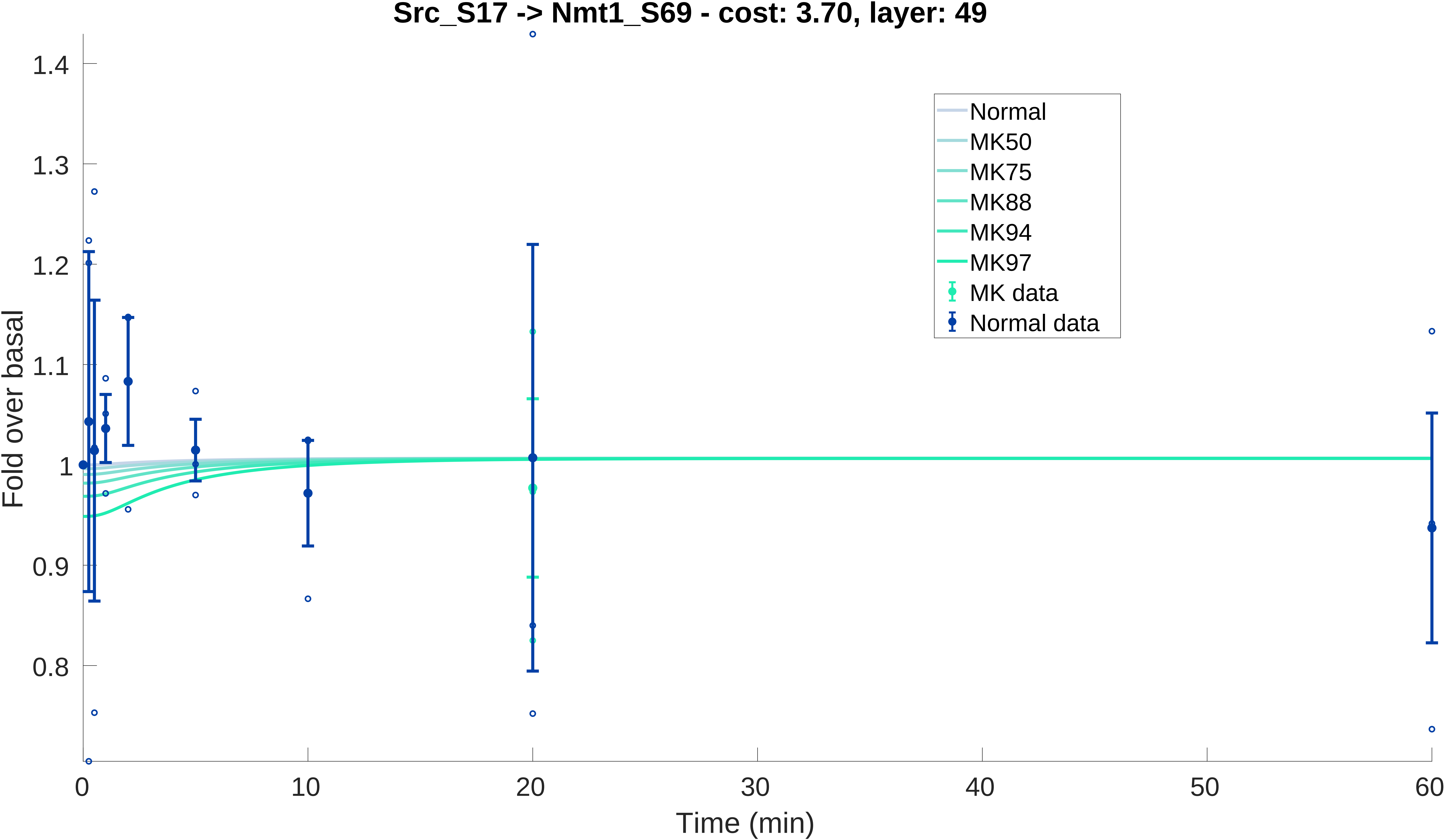

Src_S17 -> Nmt1_S69 - cost: 3.70, layer: 49
Fold over basal
Time (min)
1.4
1.3
1.2
1.1
1
0.9
0.8
0
10
20
30
40
50
60
Normal
MK50
MK75
MK88
MK94
MK97
MK data
Normal data

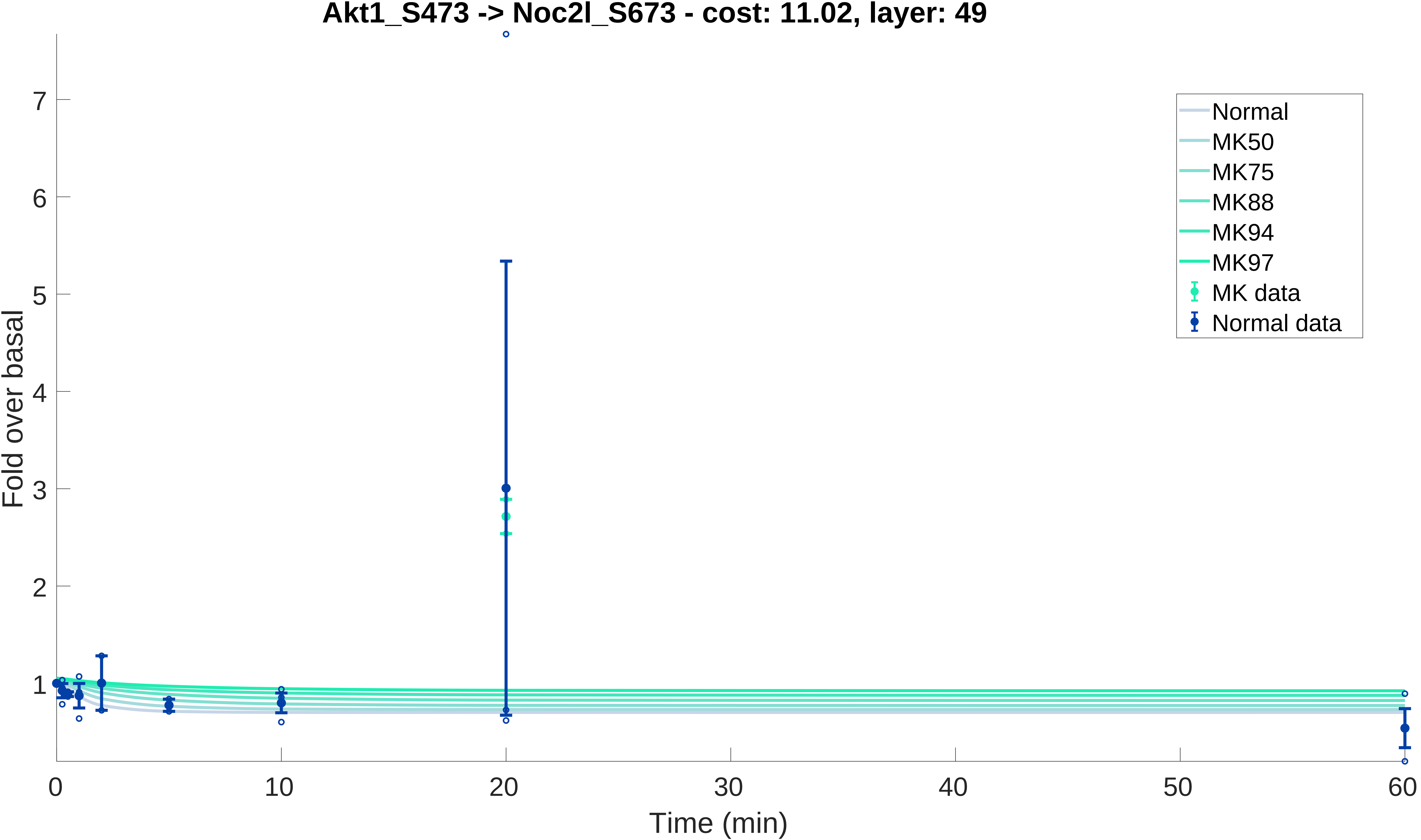

Akt1_S473 -> Noc2l_S673 - cost: 11.02, layer: 49
Fold over basal
Time (min)
0
10
20
30
40
50
60
1
2
3
4
5
6
7
Normal
MK50
MK75
MK88
MK94
MK97
MK data
Normal data

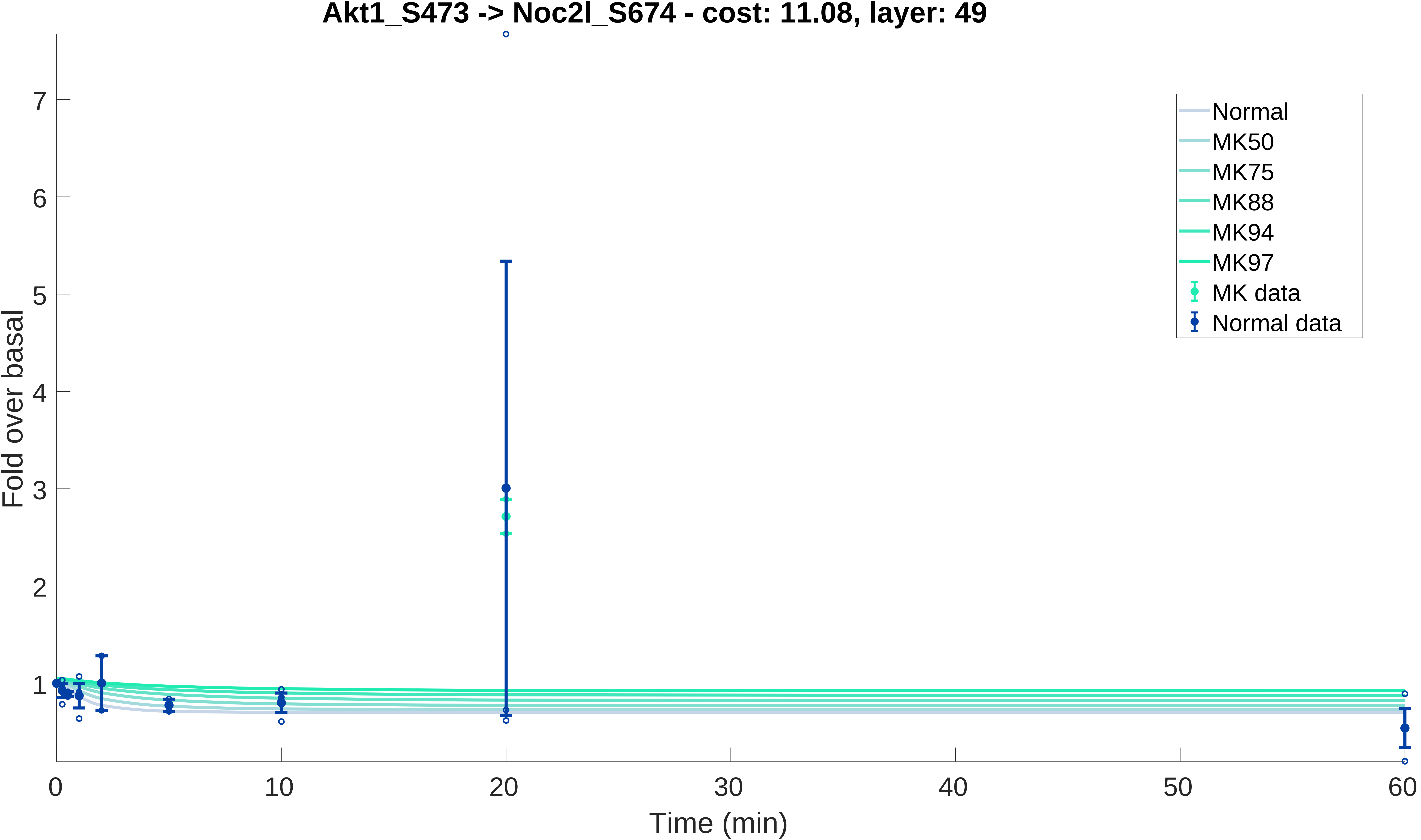

Akt1_S473 -> Noc2l_S674 - cost: 11.08, layer: 49
Fold over basal
Time (min)
0
10
20
30
40
50
60
1
2
3
4
5
6
7
Normal
MK50
MK75
MK88
MK94
MK97
MK data
Normal data

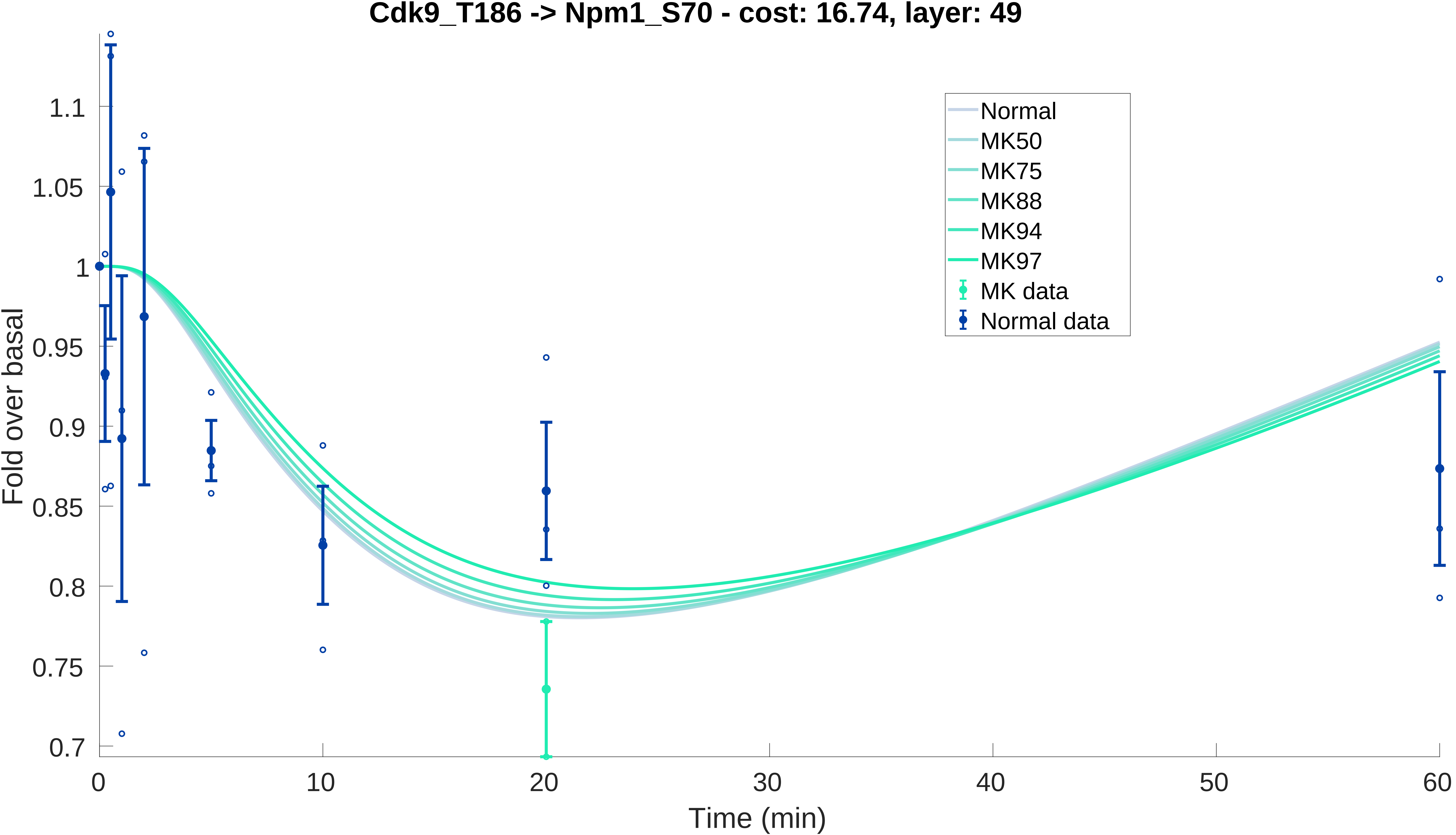

Cdk9_T186 -> Npm1_S70 - cost: 16.74, layer: 49
Fold over basal
Time (min)
Normal
MK50
MK75
MK88
MK94
MK97
MK data
Normal data
1.1
1.05
1
0.95
0.9
0.85
0.8
0.75
0.7
0
10
20
30
40
50
60

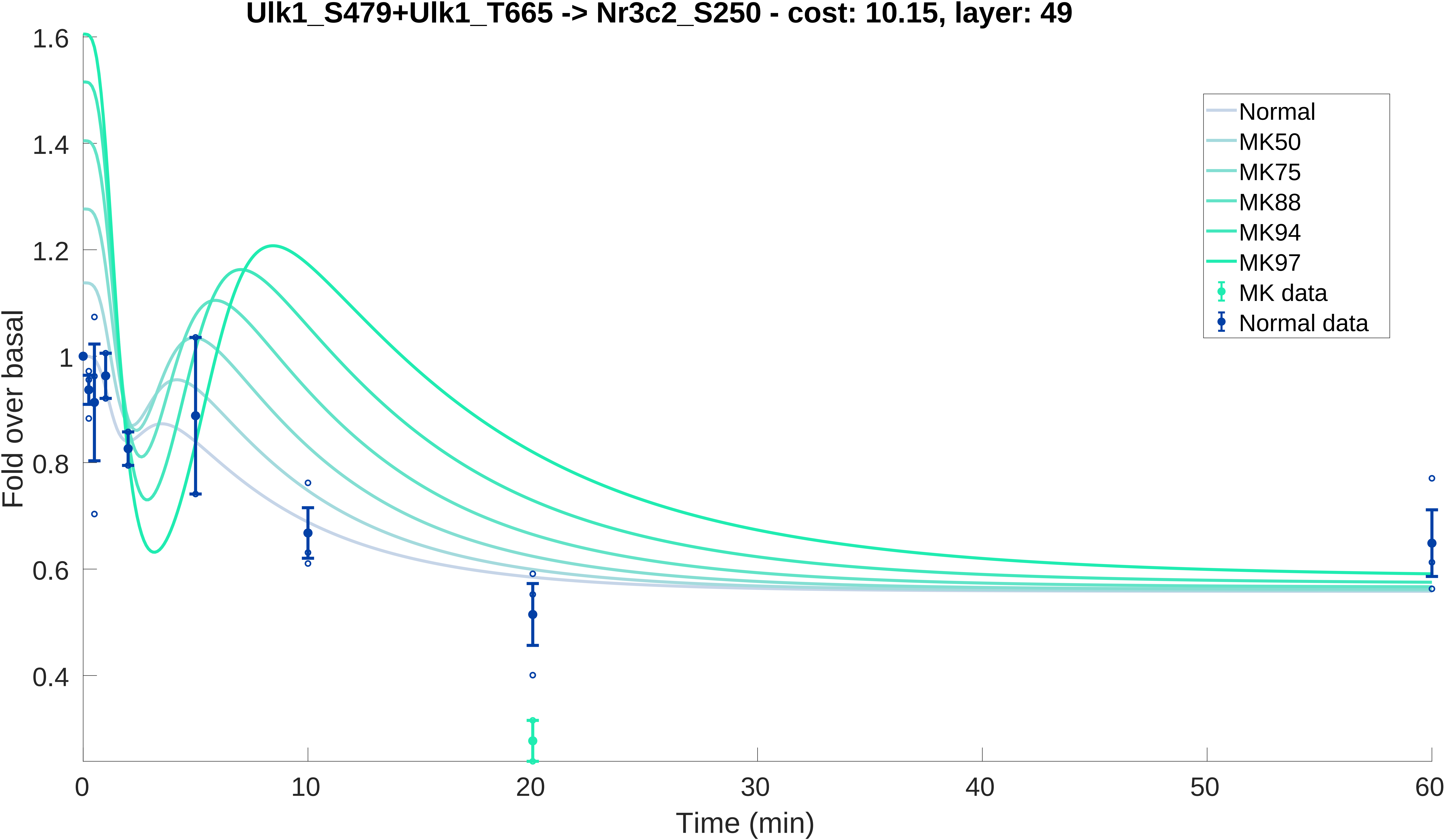

Ulk1_S479+Ulk1_T665 -> Nr3c2_S250 - cost: 10.15, layer: 49
Fold over basal
Time (min)
0
10
20
30
40
50
60
0.4
0.6
0.8
1
1.2
1.4
1.6
Normal
MK50
MK75
MK88
MK94
MK97
MK data
Normal data

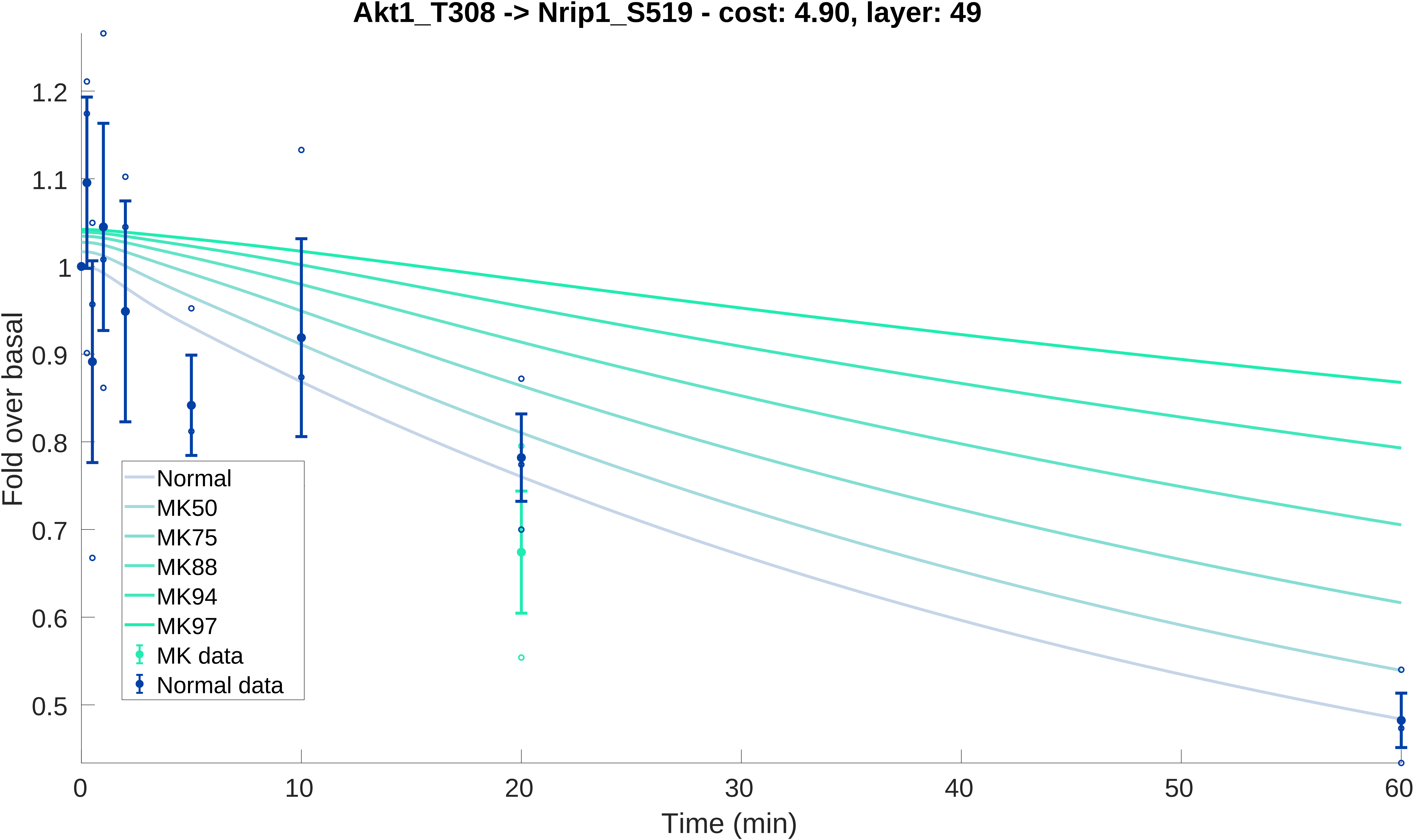

Akt1_T308 -> Nrip1_S519 - cost: 4.90, layer: 49
Fold over basal
Time (min)
1.2
1.1
1
0.9
0.8
0.7
0.6
0.5
0
10
20
30
40
50
60
Normal
MK50
MK75
MK88
MK94
MK97
MK data
Normal data

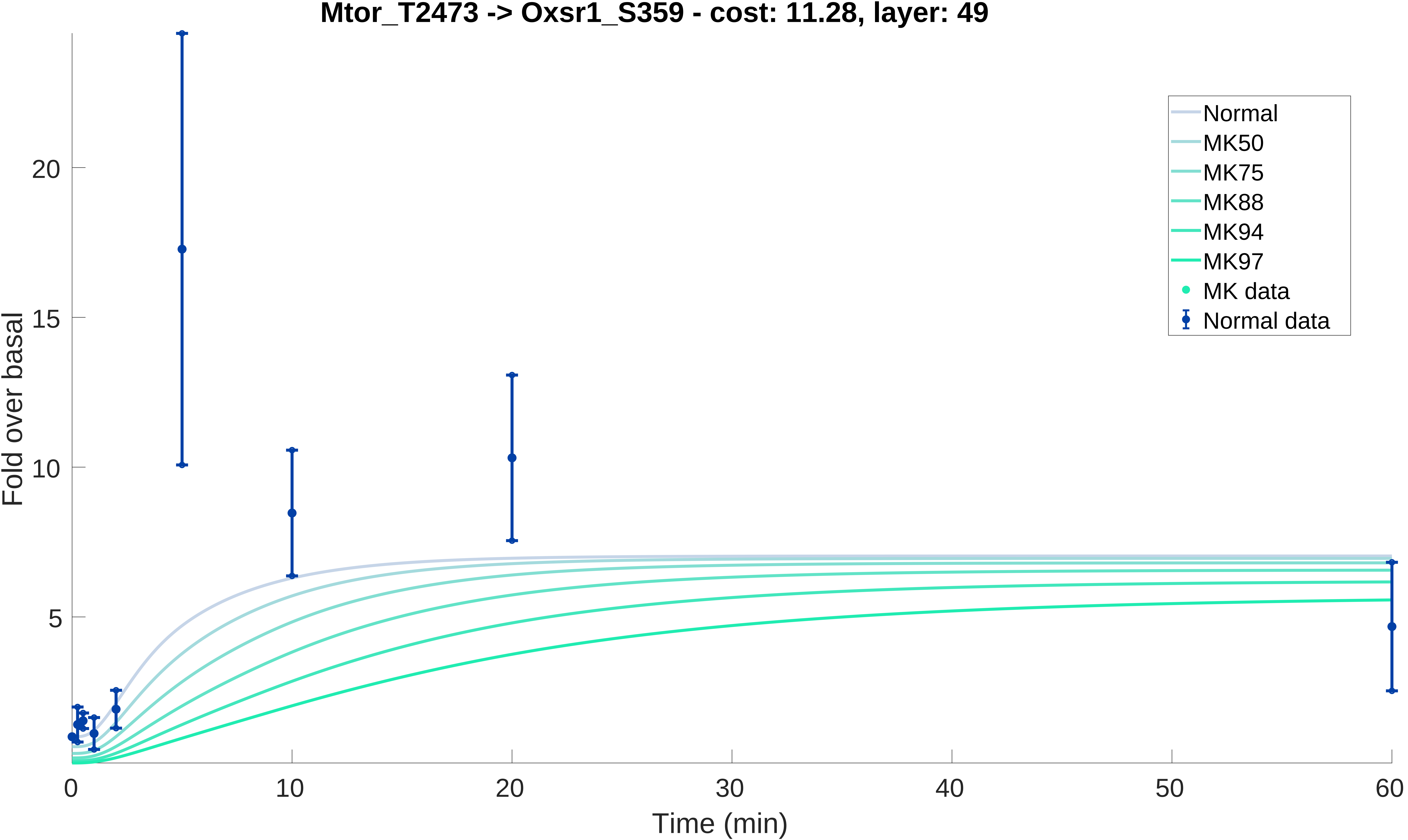

Mtor_T2473 -> Oxsr1_S359 - cost: 11.28, layer: 49
Fold over basal
Time (min)
0
10
20
30
40
50
60
5
10
15
20
Normal
MK50
MK75
MK88
MK94
MK97
MK data
Normal data

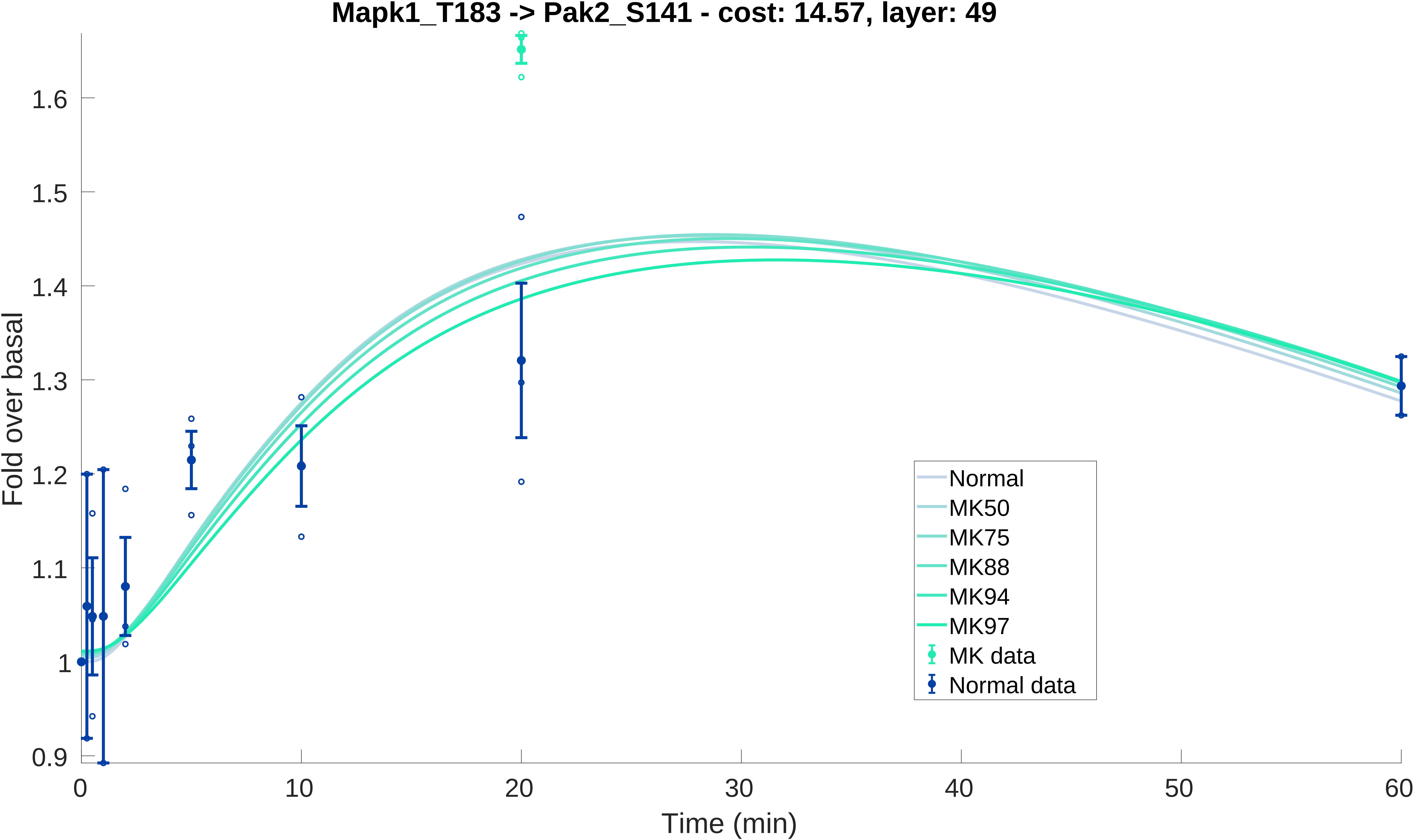
Mapk1_T183 -> Pak2_S141 - cost: 14.57, layer: 49
Fold over basal
Time (min)
0.9
1
1.1
1.2
1.3
1.4
1.5
1.6
0
10
20
30
40
50
60
Normal
MK50
MK75
MK88
MK94
MK97
MK data
Normal data

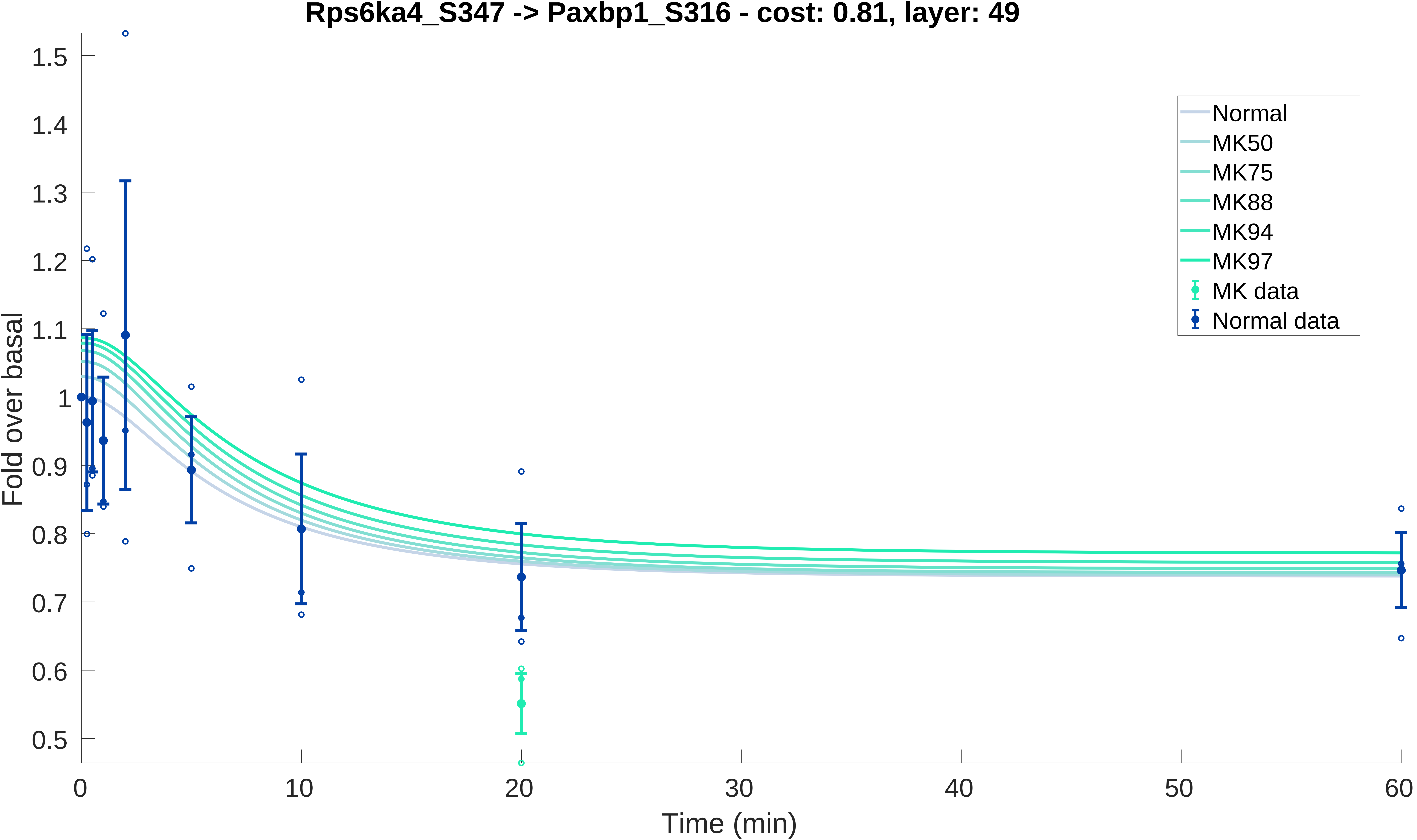

Rps6ka4_S347 -> Paxbp1_S316 - cost: 0.81, layer: 49
Fold over basal
Time (min)
1.5
1.4
1.3
1.2
1.1
1
0.9
0.8
0.7
0.6
0.5
0
10
20
30
40
50
60
Normal
MK50
MK75
MK88
MK94
MK97
MK data
Normal data

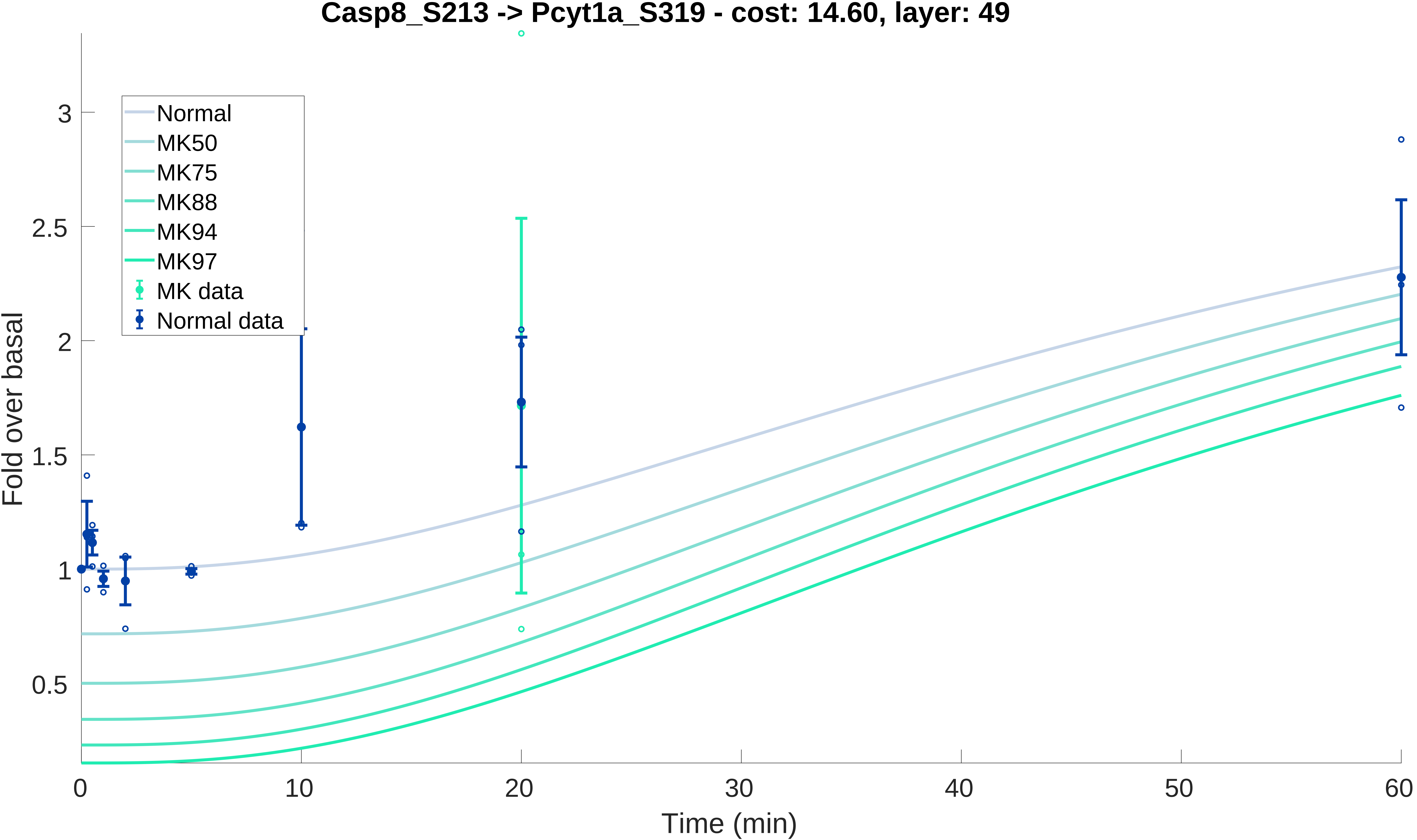

Casp8_S213 -> Pcyt1a_S319 - cost: 14.60, layer: 49
Normal
MK50
MK75
MK88
MK94
MK97
MK data
Normal data
Fold over basal
Time (min)
0.5
1
1.5
2
2.5
3
0
10
20
30
40
50
60

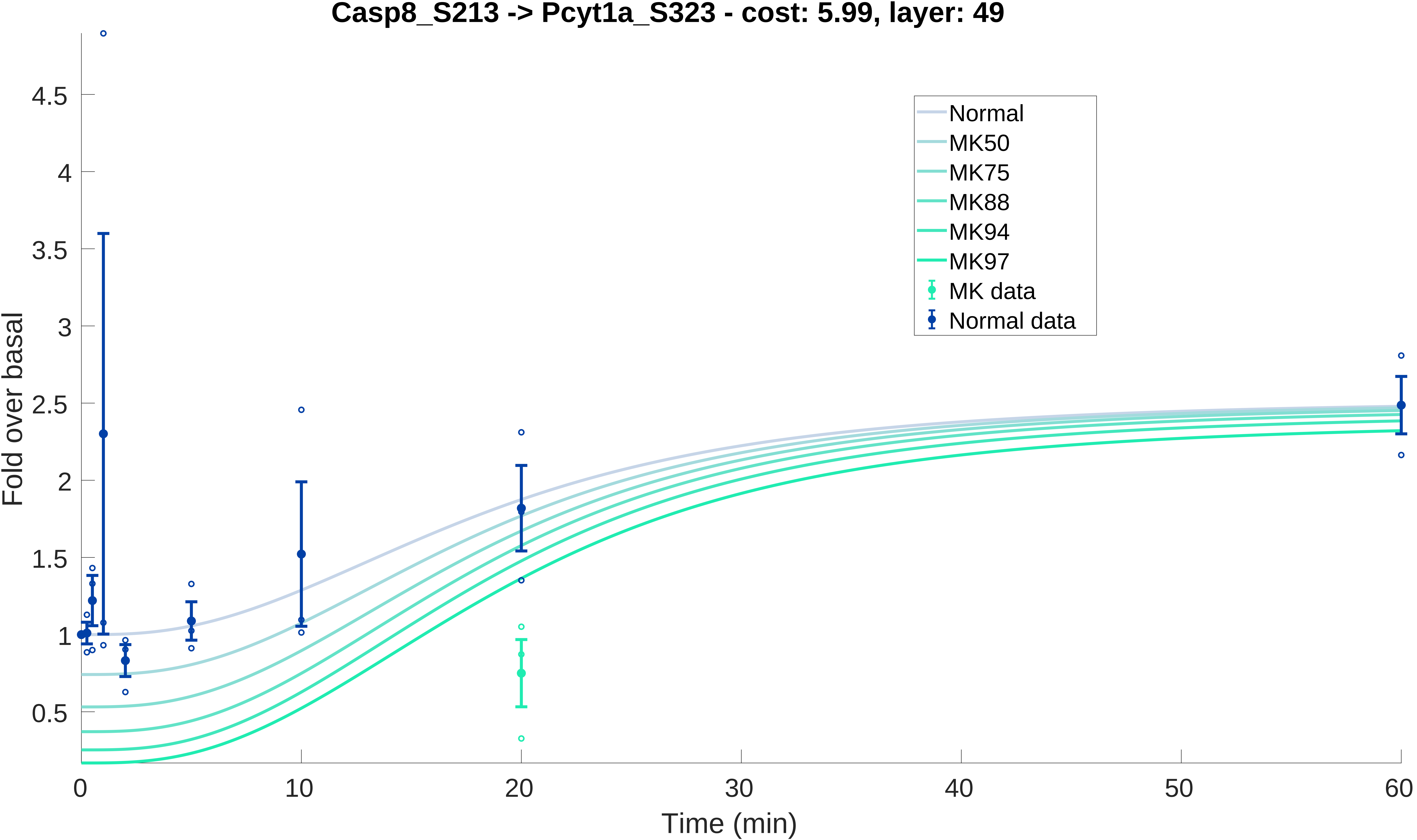

Casp8_S213 -> Pcyt1a_S323 - cost: 5.99, layer: 49
Fold over basal
Time (min)
0
10
20
30
40
50
60
0.5
1
1.5
2
2.5
3
3.5
4
4.5
Normal
MK50
MK75
MK88
MK94
MK97
MK data
Normal data

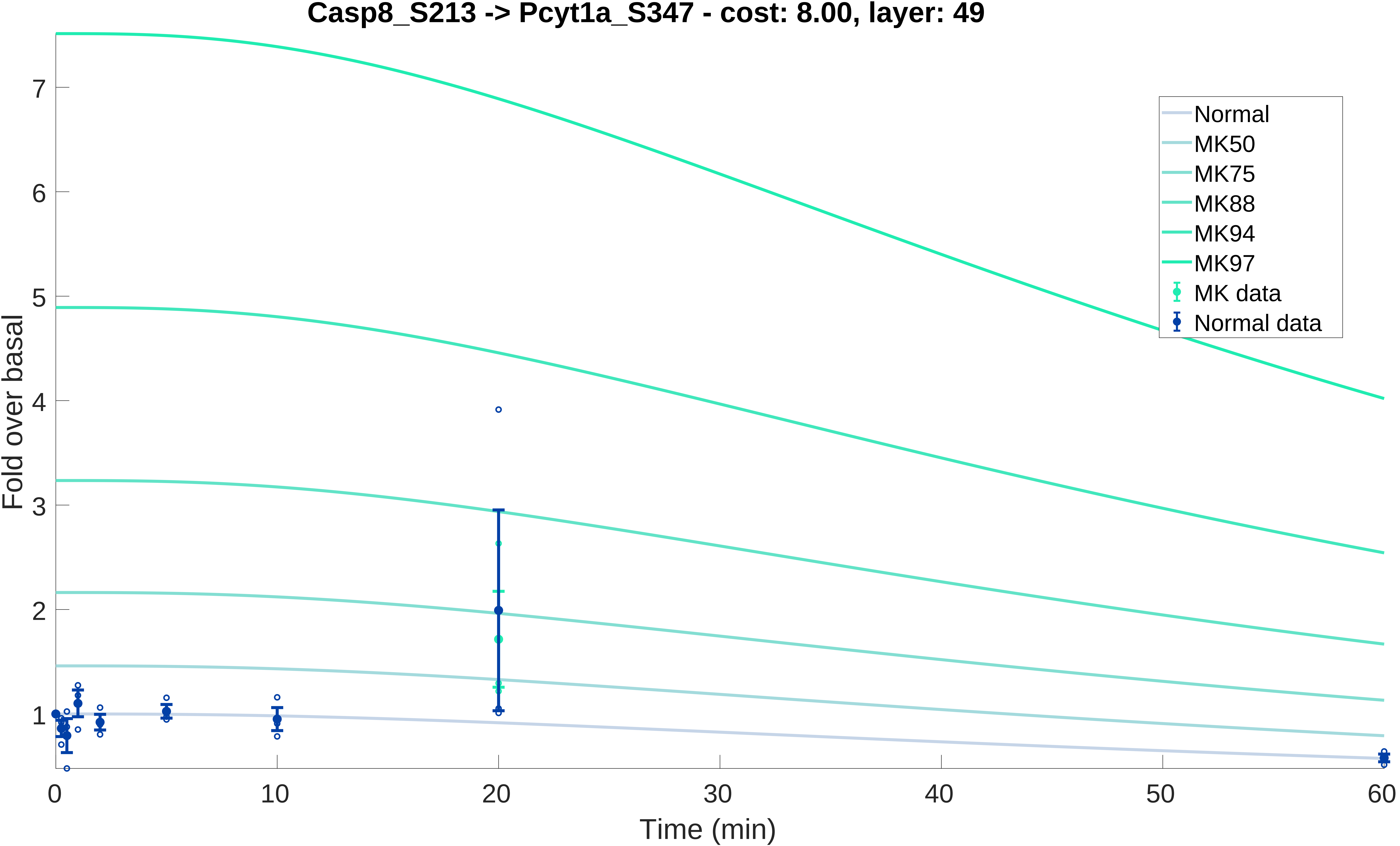

Casp8_S213 -> Pcyt1a_S347 - cost: 8.00, layer: 49
Fold over basal
Time (min)
0
10
20
30
40
50
60
1
2
3
4
5
6
7
Normal
MK50
MK75
MK88
MK94
MK97
MK data
Normal data

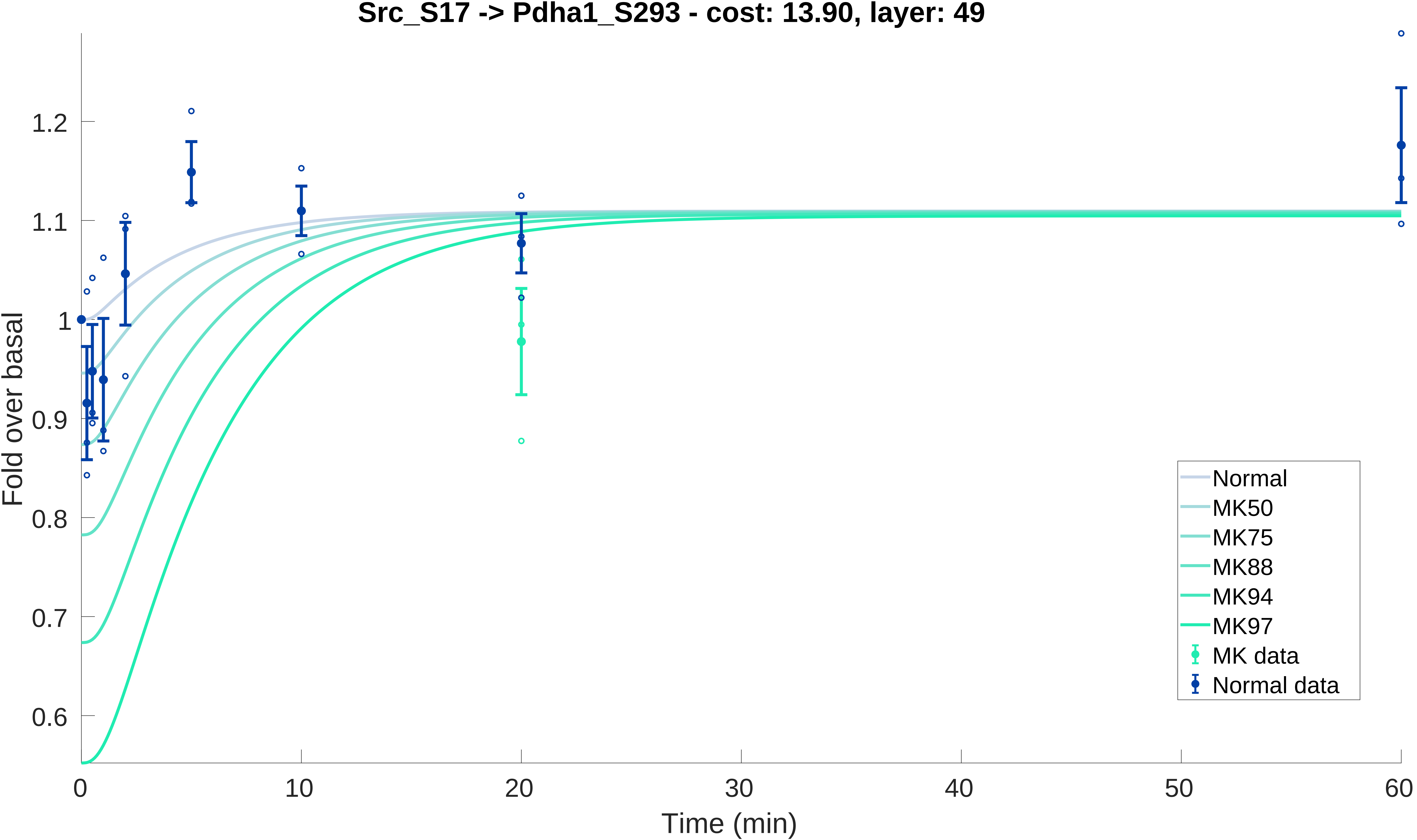

Src_S17 -> Pdha1_S293 - cost: 13.90, layer: 49
Fold over basal
Time (min)
0.6
0.7
0.8
0.9
1
1.1
1.2
0
10
20
30
40
50
60
Normal
MK50
MK75
MK88
MK94
MK97
MK data
Normal data

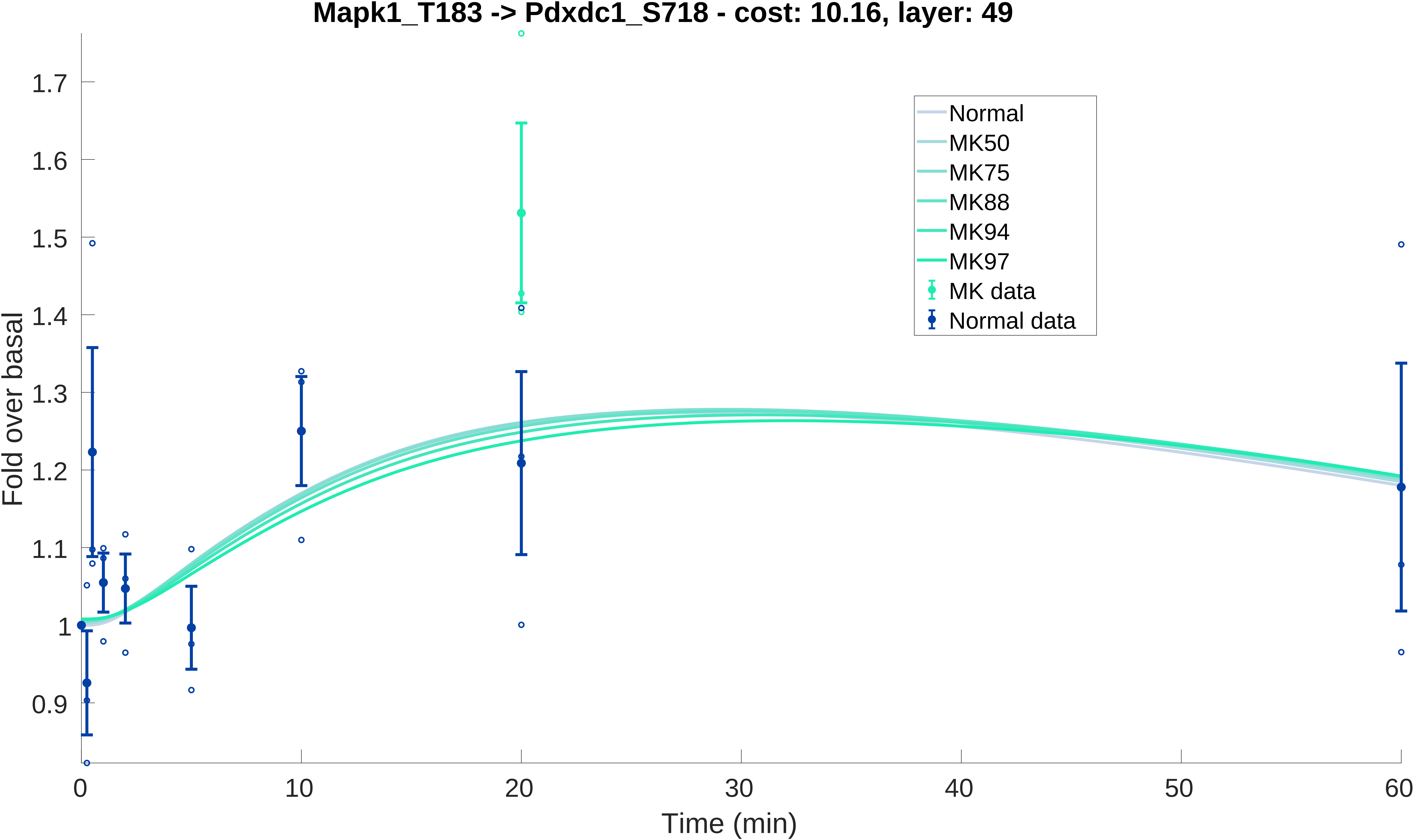

Mapk1_T183 -> Pdxdc1_S718 - cost: 10.16, layer: 49
Fold over basal
Time (min)
Normal
MK50
MK75
MK88
MK94
MK97
MK data
Normal data
0.9
1
1.1
1.2
1.3
1.4
1.5
1.6
1.7
0
10
20
30
40
50
60

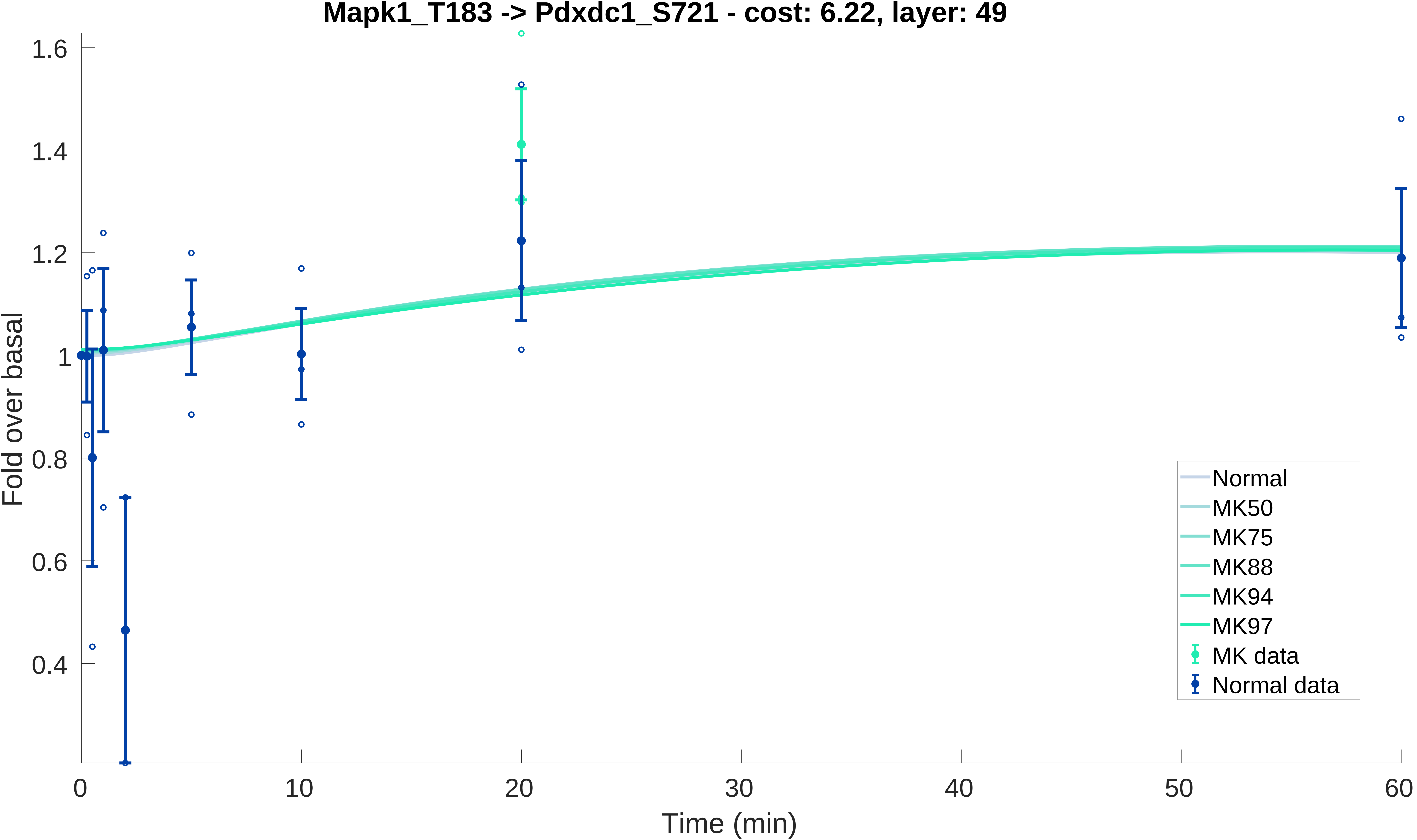

Mapk1_T183 -> Pdxdc1_S721 - cost: 6.22, layer: 49
Fold over basal
1.6
1.4
1.2
1
0.8
0.6
0.4
0
10
20
30
40
50
60
Time (min)
Normal
MK50
MK75
MK88
MK94
MK97
MK data
Normal data

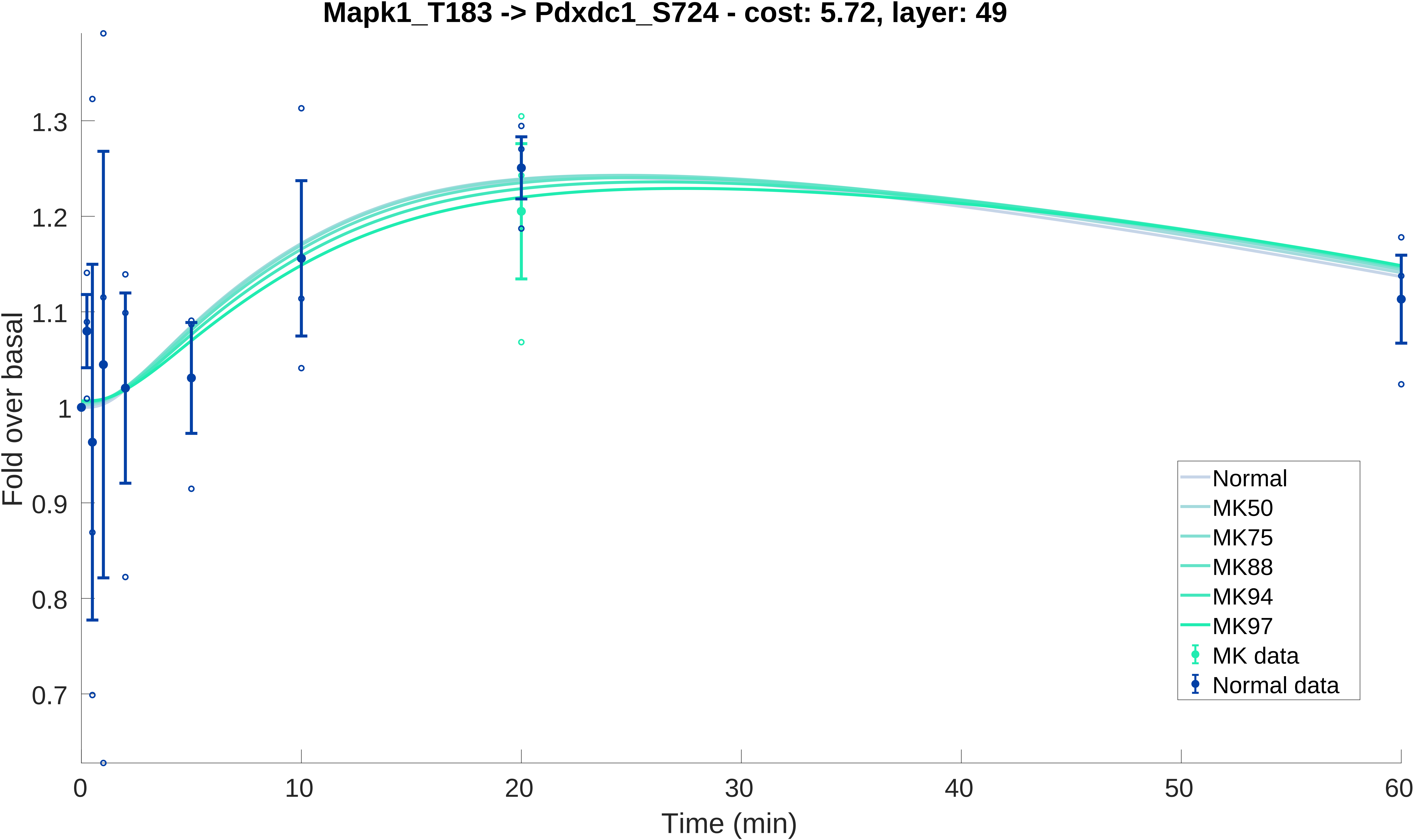

Mapk1_T183 -> Pdxdc1_S724 - cost: 5.72, layer: 49
Fold over basal
Time (min)
0.7
0.8
0.9
1
1.1
1.2
1.3
0
10
20
30
40
50
60
Normal
MK50
MK75
MK88
MK94
MK97
MK data
Normal data

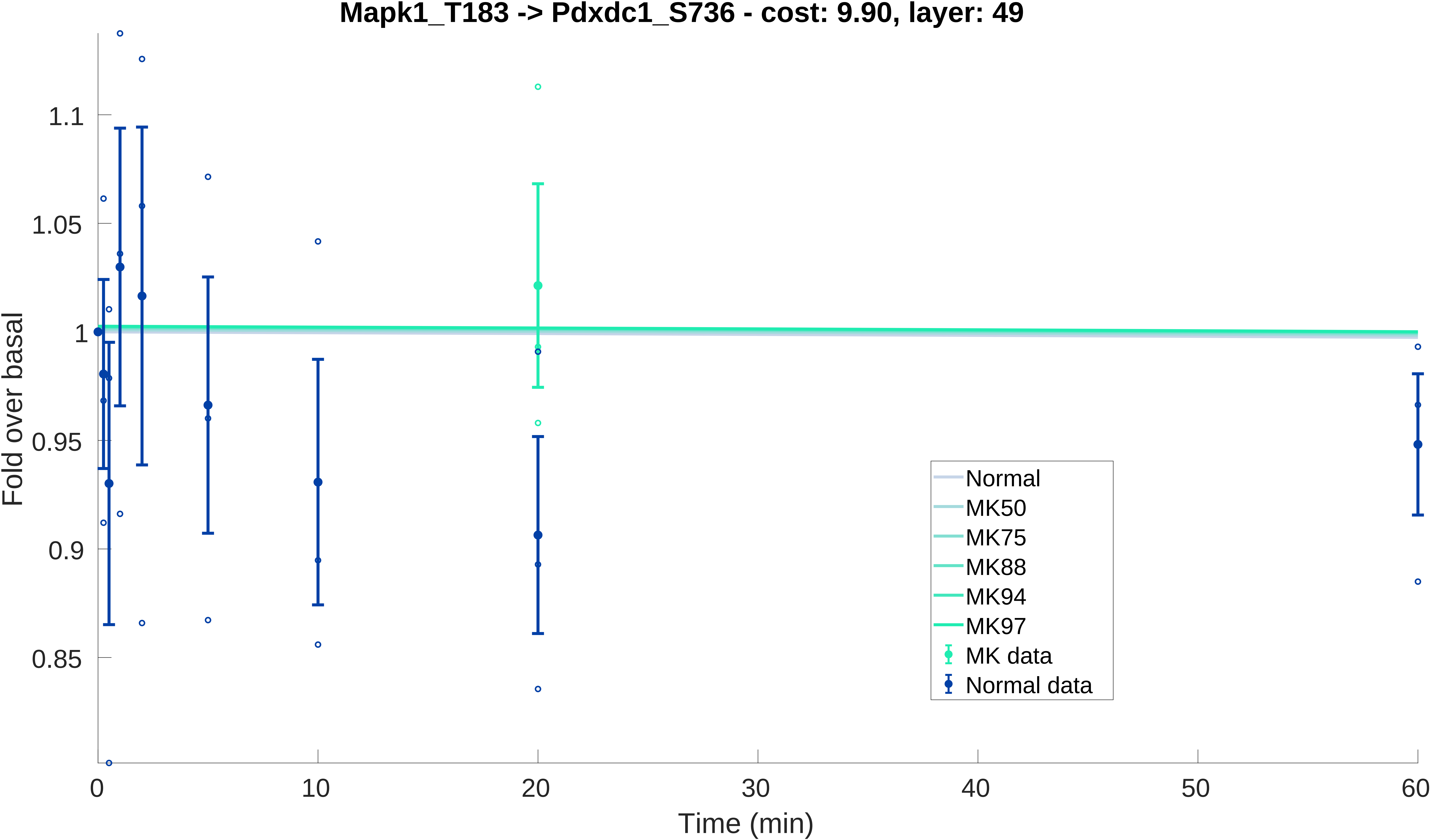

Mapk1_T183 -> Pdxdc1_S736 - cost: 9.90, layer: 49
Fold over basal
1.1
1.05
1
0.95
0.9
0.85
0
10
20
30
40
50
60
Time (min)
Normal
MK50
MK75
MK88
MK94
MK97
MK data
Normal data

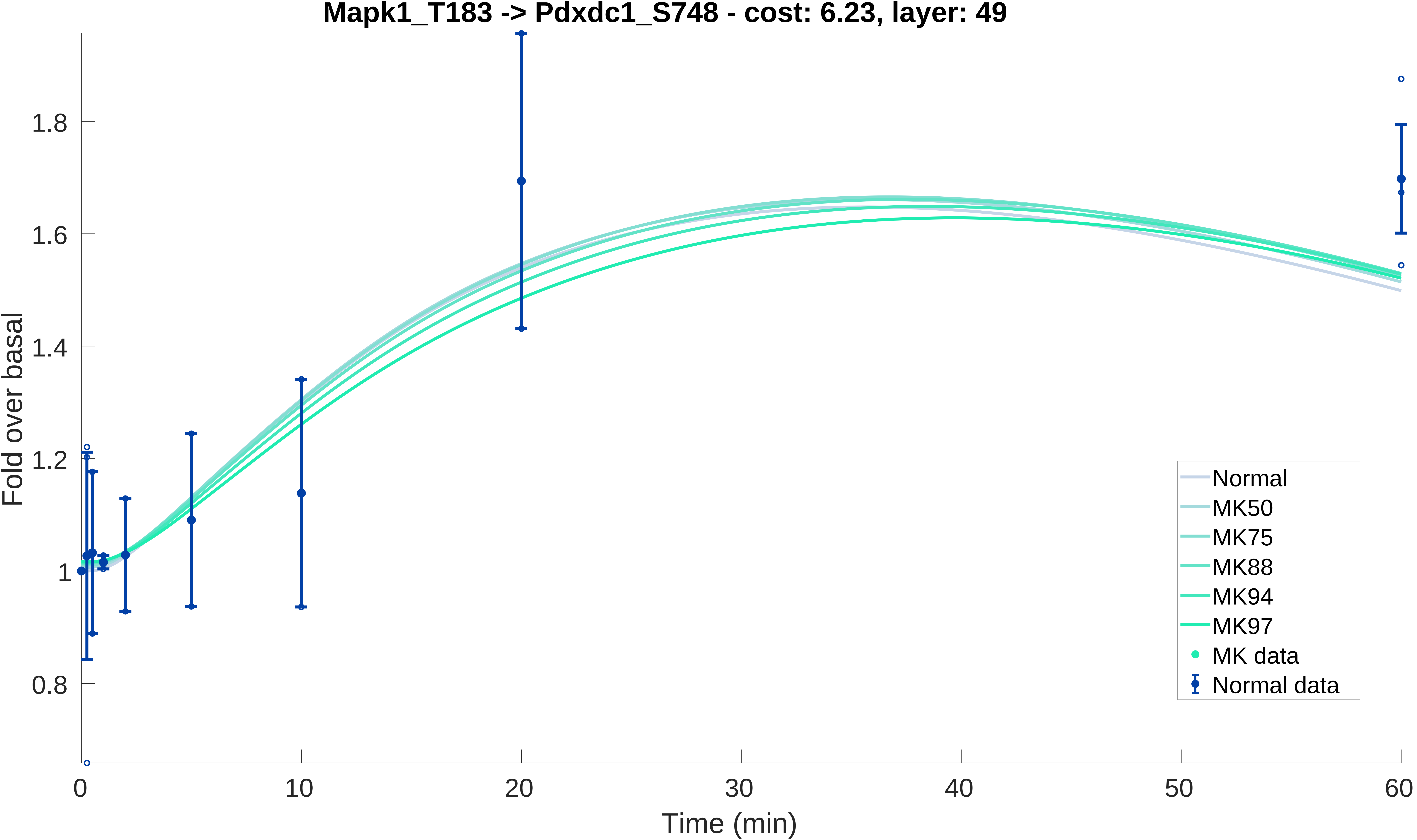
Mapk1_T183 -> Pdxdc1_S748 - cost: 6.23, layer: 49
Fold over basal
0.8
1
1.2
1.4
1.6
1.8
Time (min)
0
10
20
30
40
50
60
Normal
MK50
MK75
MK88
MK94
MK97
MK data
Normal data

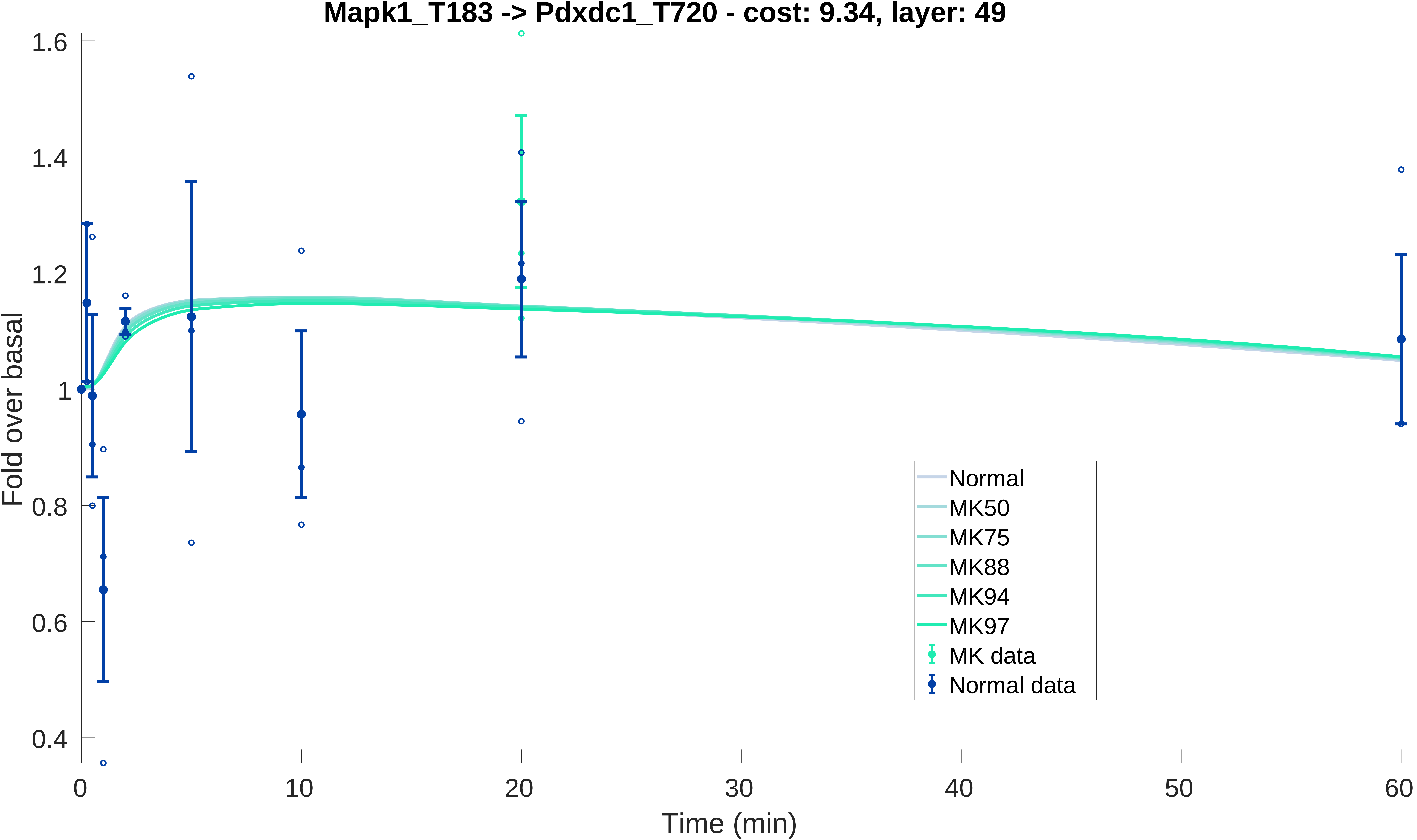

Mapk1_T183 -> Pdxdc1_T720 - cost: 9.34, layer: 49
Fold over basal
Time (min)
1.6
1.4
1.2
1
0.8
0.6
0.4
0
10
20
30
40
50
60
Normal
MK50
MK75
MK88
MK94
MK97
MK data
Normal data

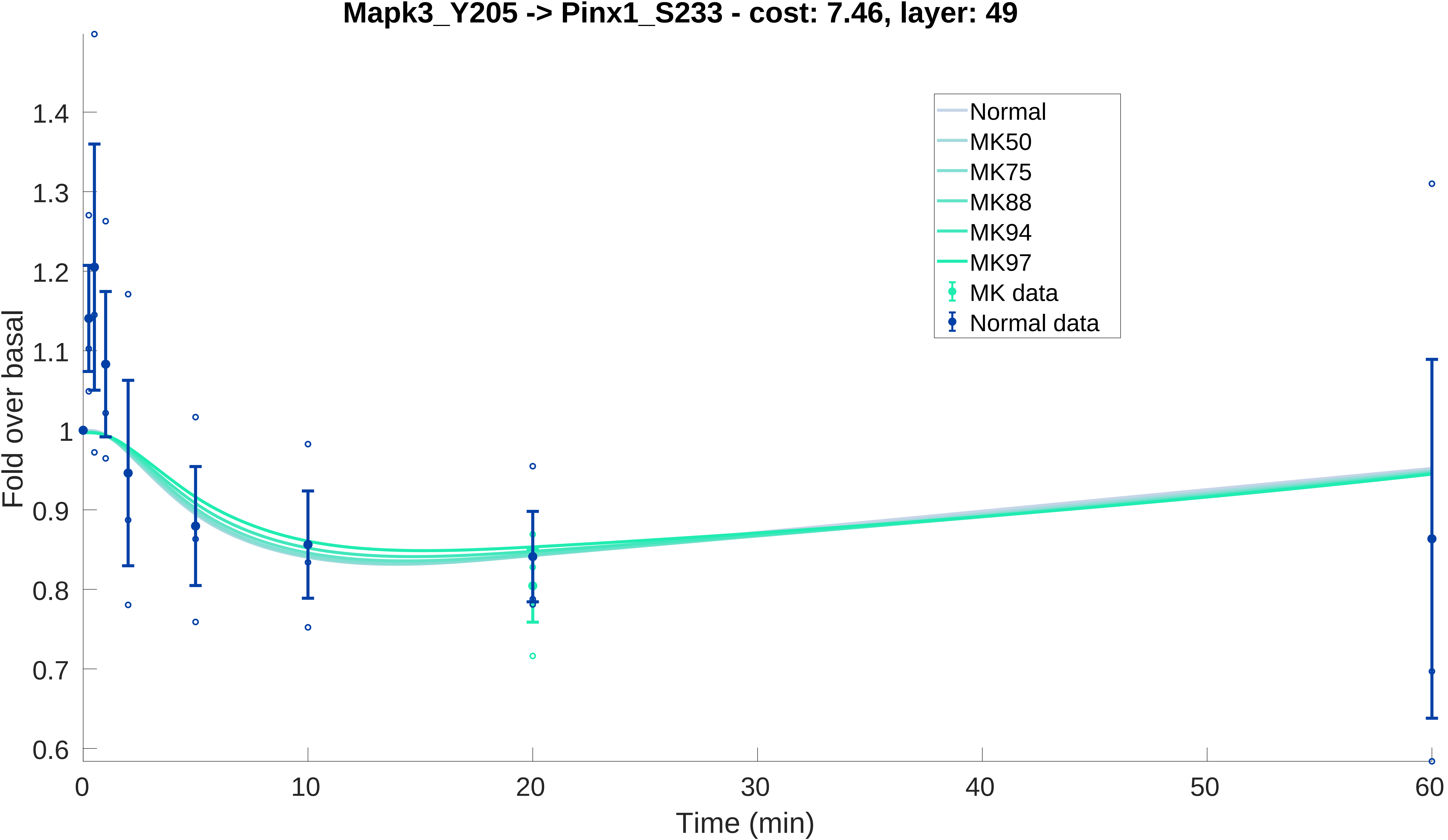
Mapk3_Y205 -> Pinx1_S233 - cost: 7.46, layer: 49
Fold over basal
Time (min)
0
10
20
30
40
50
60
0.6
0.7
0.8
0.9
1
1.1
1.2
1.3
1.4
Normal
MK50
MK75
MK88
MK94
MK97
MK data
Normal data

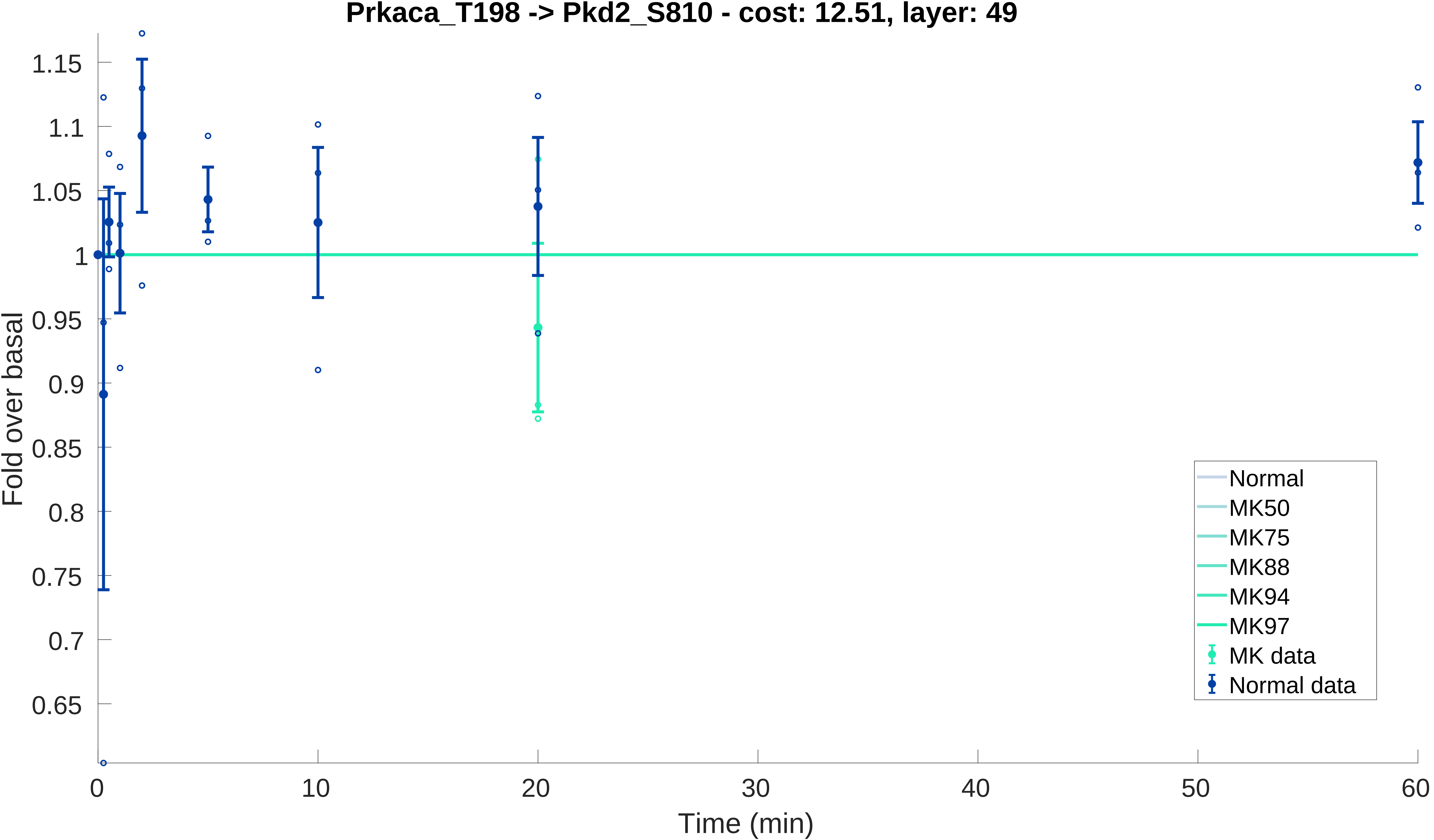
Prrkaca_T198 -> Pkd2_S810 - cost: 12.51, layer: 49
Fold over basal
1.15
1.1
1.05
1
0.95
0.9
0.85
0.8
0.75
0.7
0.65
0
10
20
30
40
50
60
Time (min)
Normal
MK50
MK75
MK88
MK94
MK97
MK data
Normal data

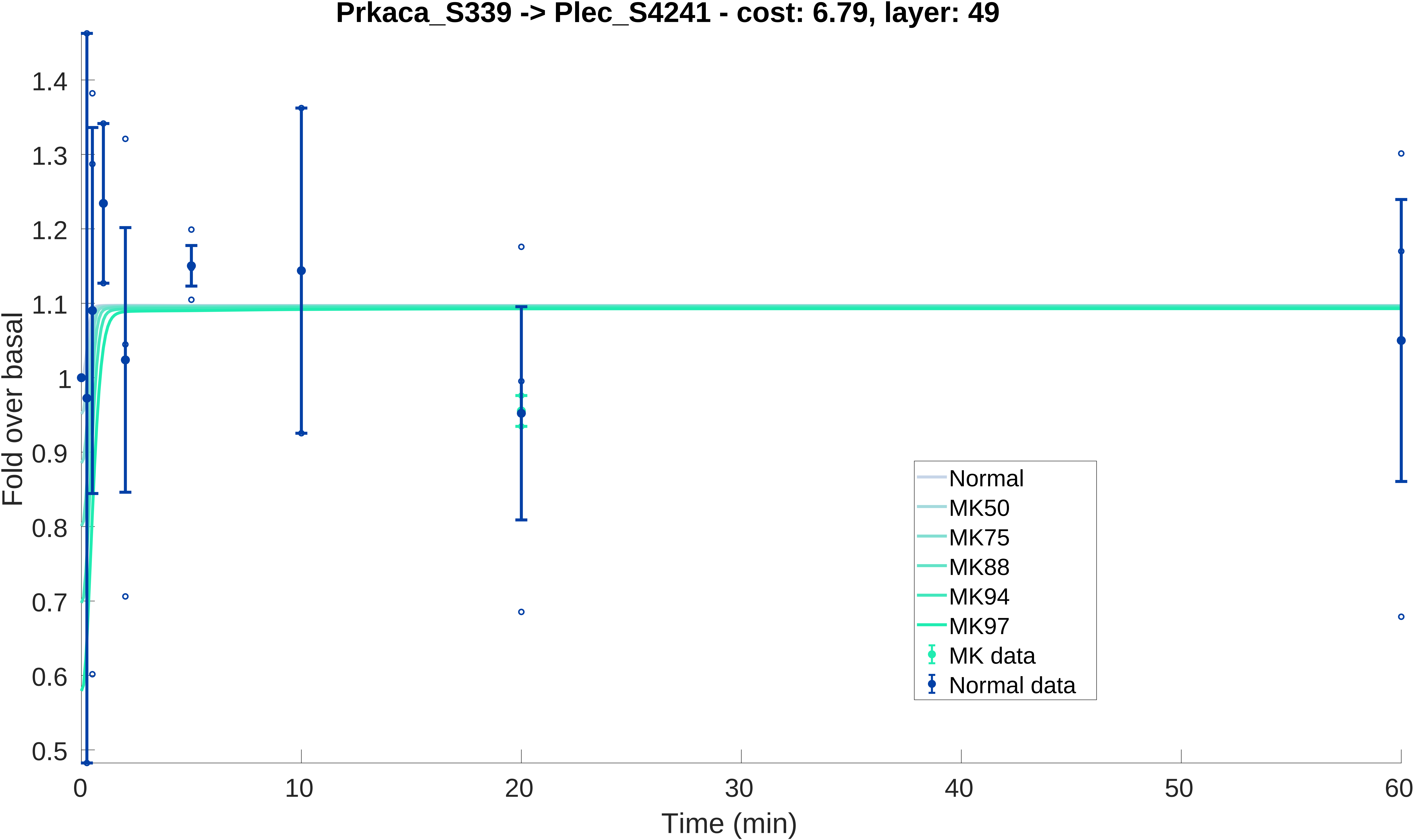
Prkaca_S339 -> Plec_S4241 - cost: 6.79, layer: 49
Fold over basal
Time (min)
0.5
0.6
0.7
0.8
0.9
1
1.1
1.2
1.3
1.4
0
10
20
30
40
50
60
Normal
MK50
MK75
MK88
MK94
MK97
MK data
Normal data

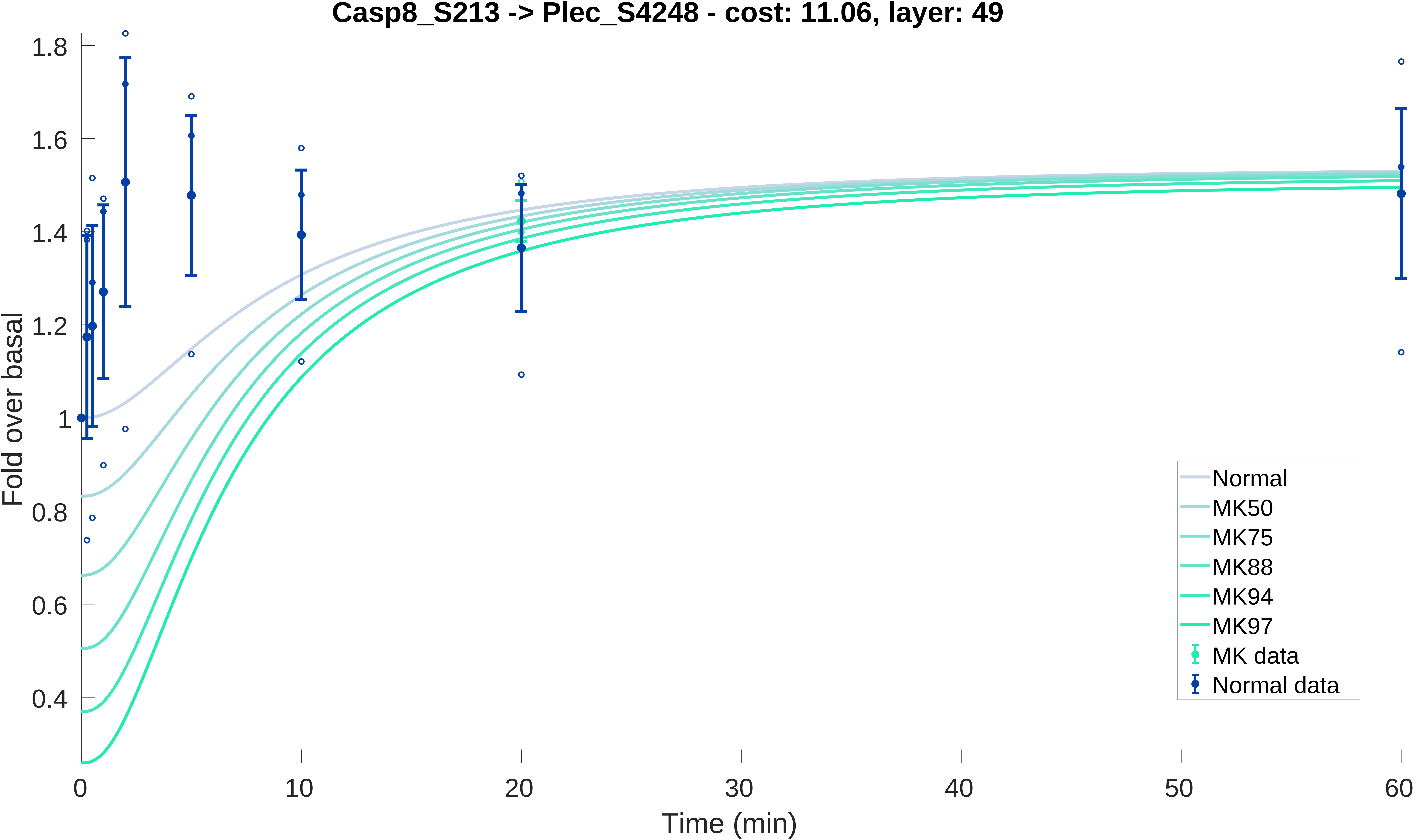
Casp8_S213 -> Plec_S4248 - cost: 11.06, layer: 49
Fold over basal
Time (min)
1.8
1.6
1.4
1.2
1
0.8
0.6
0.4
0
10
20
30
40
50
60
Normal
MK50
MK75
MK88
MK94
MK97
MK data
Normal data

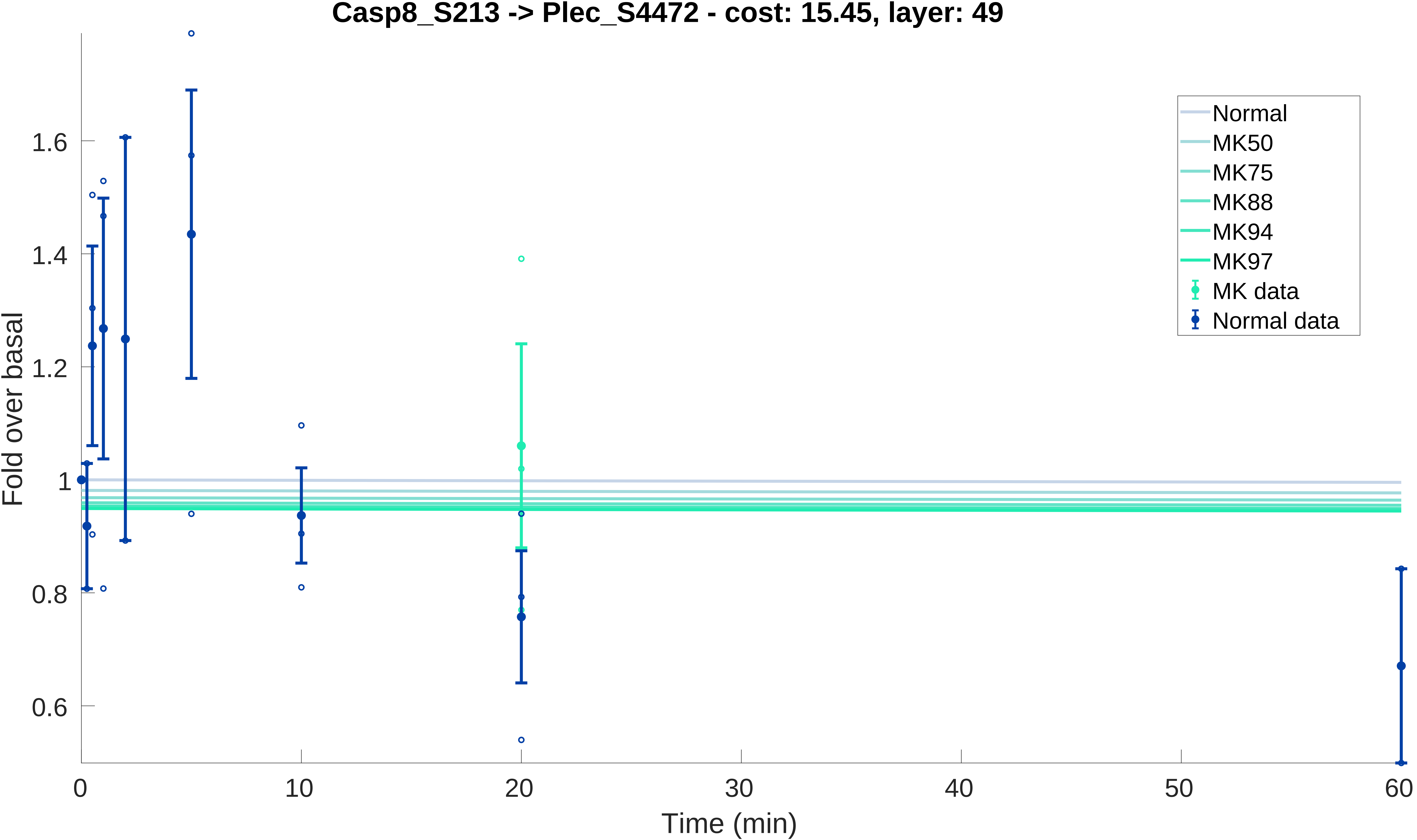
Casp8_S213 -> Plec_S4472 - cost: 15.45, layer: 49
Fold over basal
Time (min)
0
10
20
30
40
50
60
0.6
0.8
1
1.2
1.4
1.6
Normal
MK50
MK75
MK88
MK94
MK97
MK data
Normal data

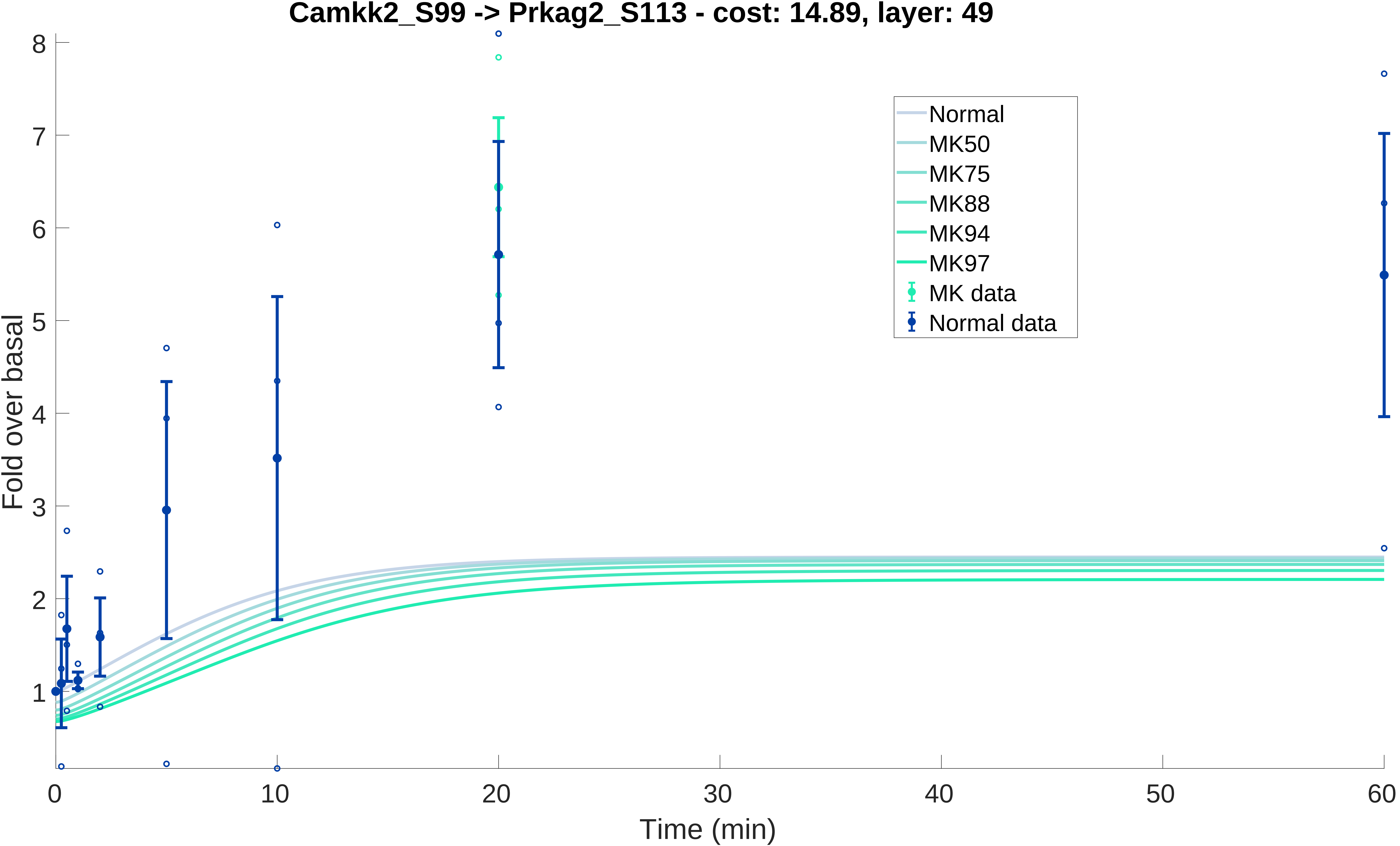

Camkk2_S99 -> Prkag2_S113 - cost: 14.89, layer: 49
Fold over basal
Time (min)
0
10
20
30
40
50
60
1
2
3
4
5
6
7
8
Normal
MK50
MK75
MK88
MK94
MK97
MK data
Normal data

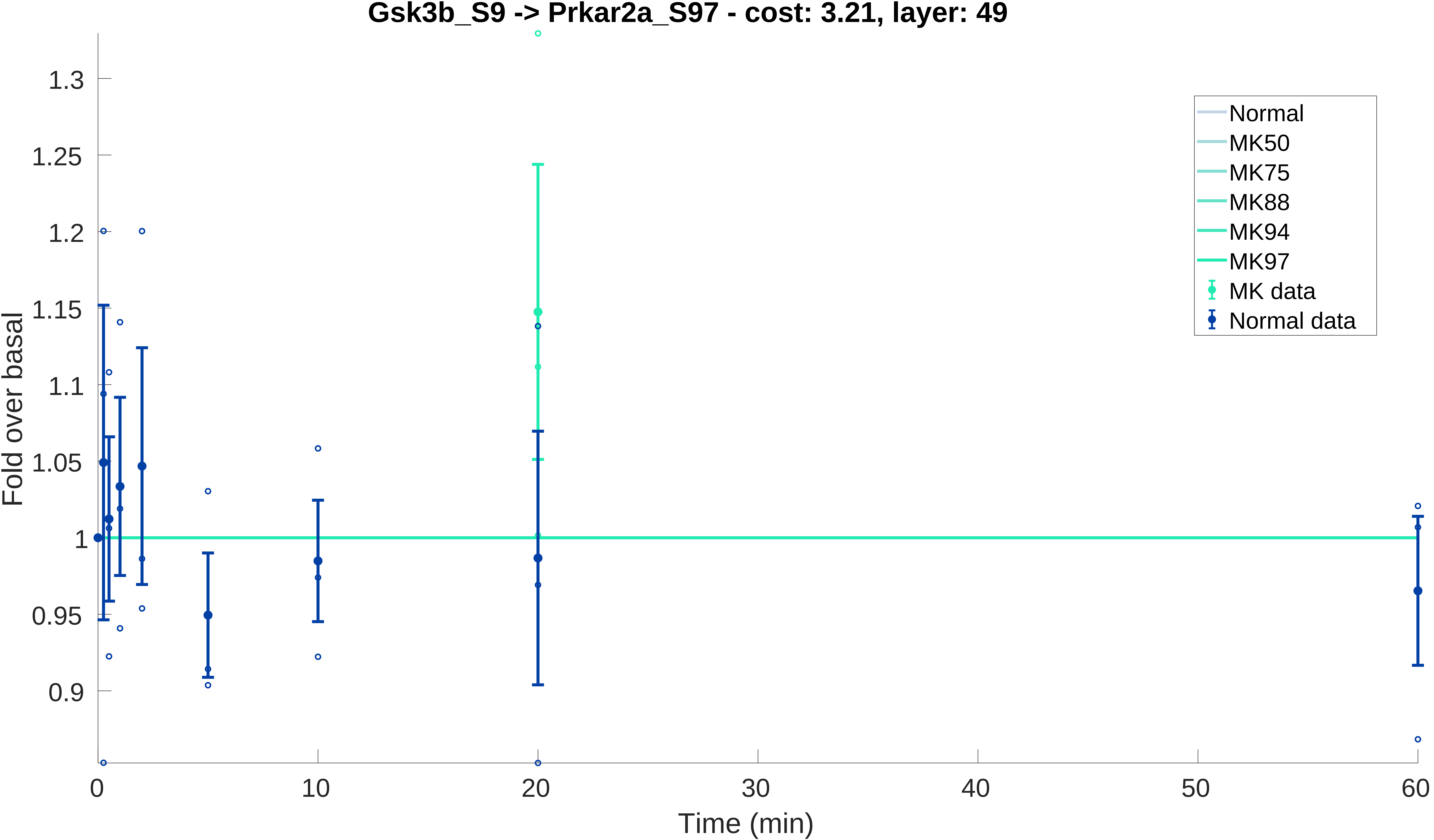

Gsk3b_S9 -> Prkar2a_S97 - cost: 3.21, layer: 49
Fold over basal
Time (min)
1.3
1.25
1.2
1.15
1.1
1.05
1
0.95
0.9
0
10
20
30
40
50
60
Normal
MK50
MK75
MK88
MK94
MK97
MK data
Normal data

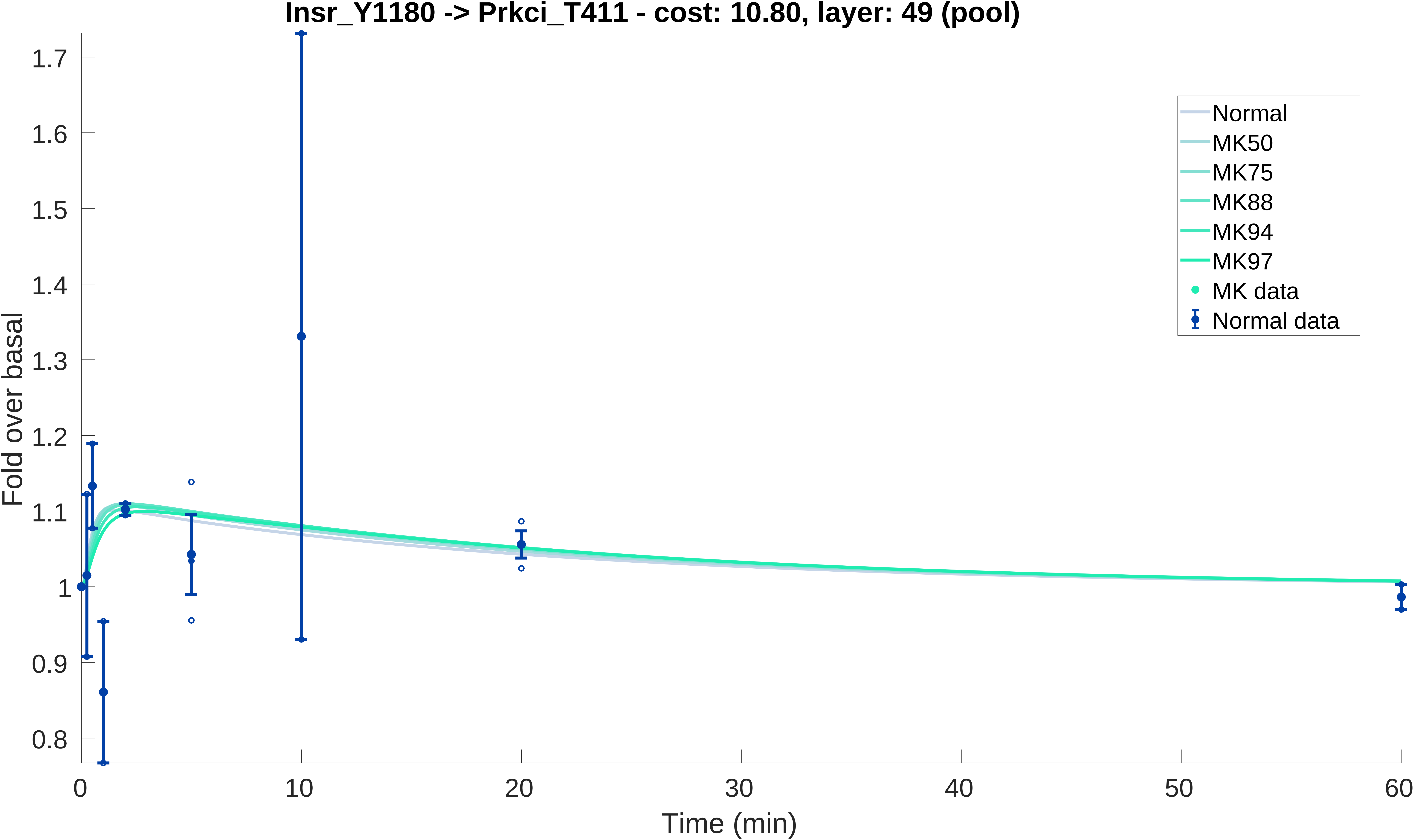
Insr_Y1180 -> Prkci_T411 - cost: 10.80, layer: 49 (pool)
Fold over basal
1.7
1.6
1.5
1.4
1.3
1.2
1.1
1
0.9
0.8
0
10
20
30
40
50
60
Time (min)
Normal
MK50
MK75
MK88
MK94
MK97
MK data
Normal data

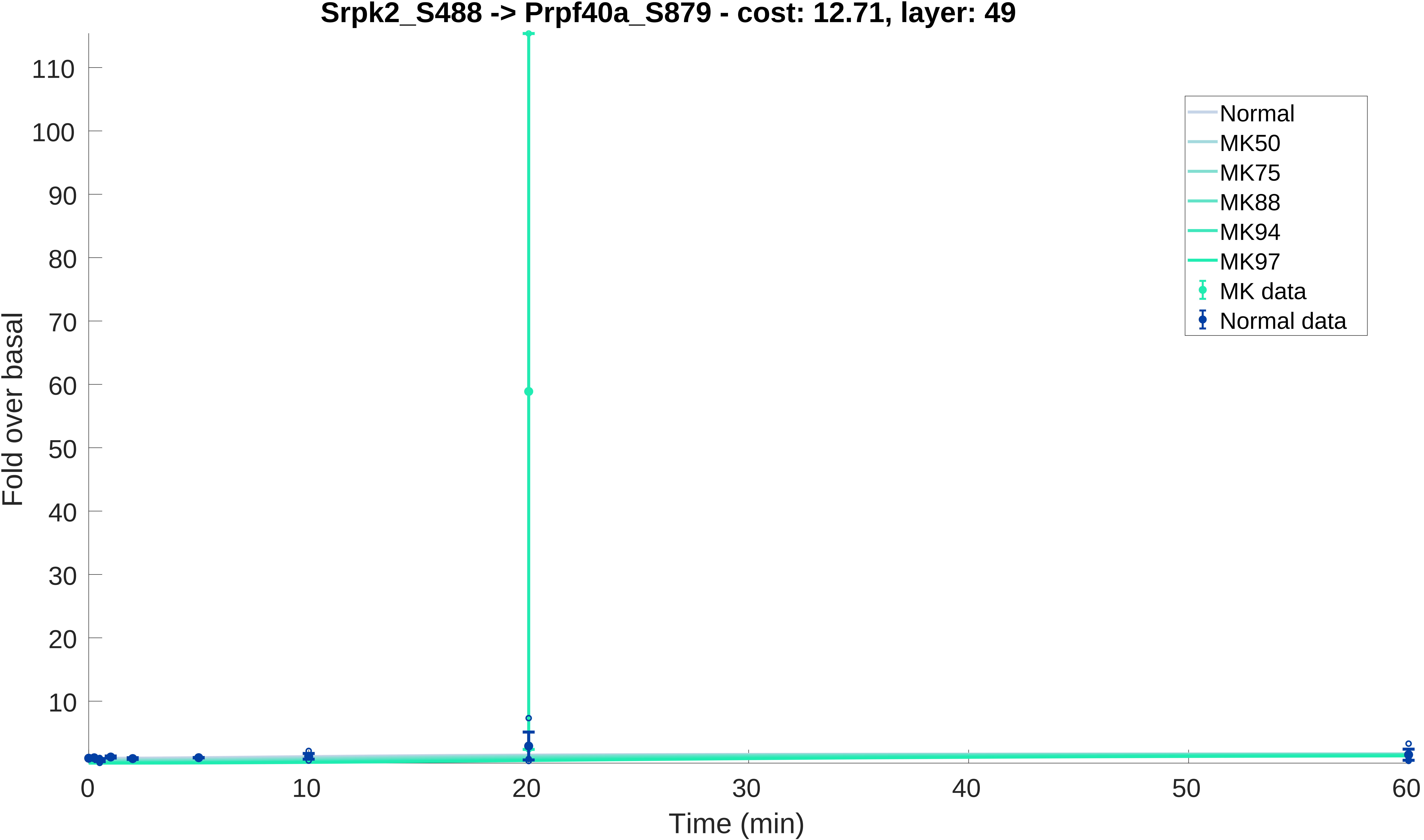
Srpk2_S488 -> Prpf40a_S879 - cost: 12.71, layer: 49
Fold over basal
110
100
90
80
70
60
50
40
30
20
10
0
10
20
30
40
50
60
Time (min)
Normal
MK50
MK75
MK88
MK94
MK97
MK data
Normal data

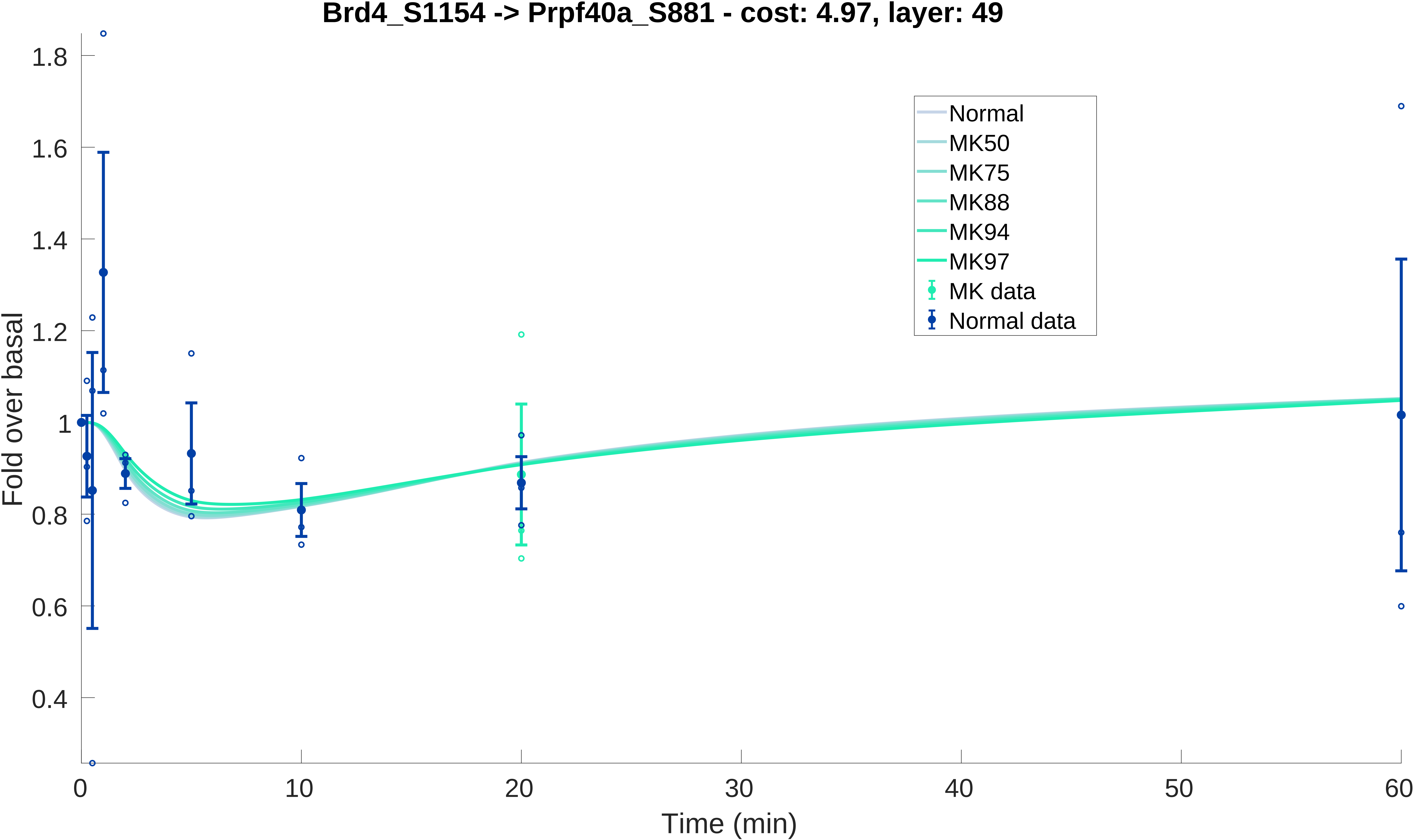
Brd4_S1154 -> Prpf40a_S881 - cost: 4.97, layer: 49
Fold over basal
Time (min)
0.4
0.6
0.8
1
1.2
1.4
1.6
1.8
0
10
20
30
40
50
60
Normal
MK50
MK75
MK88
MK94
MK97
MK data
Normal data

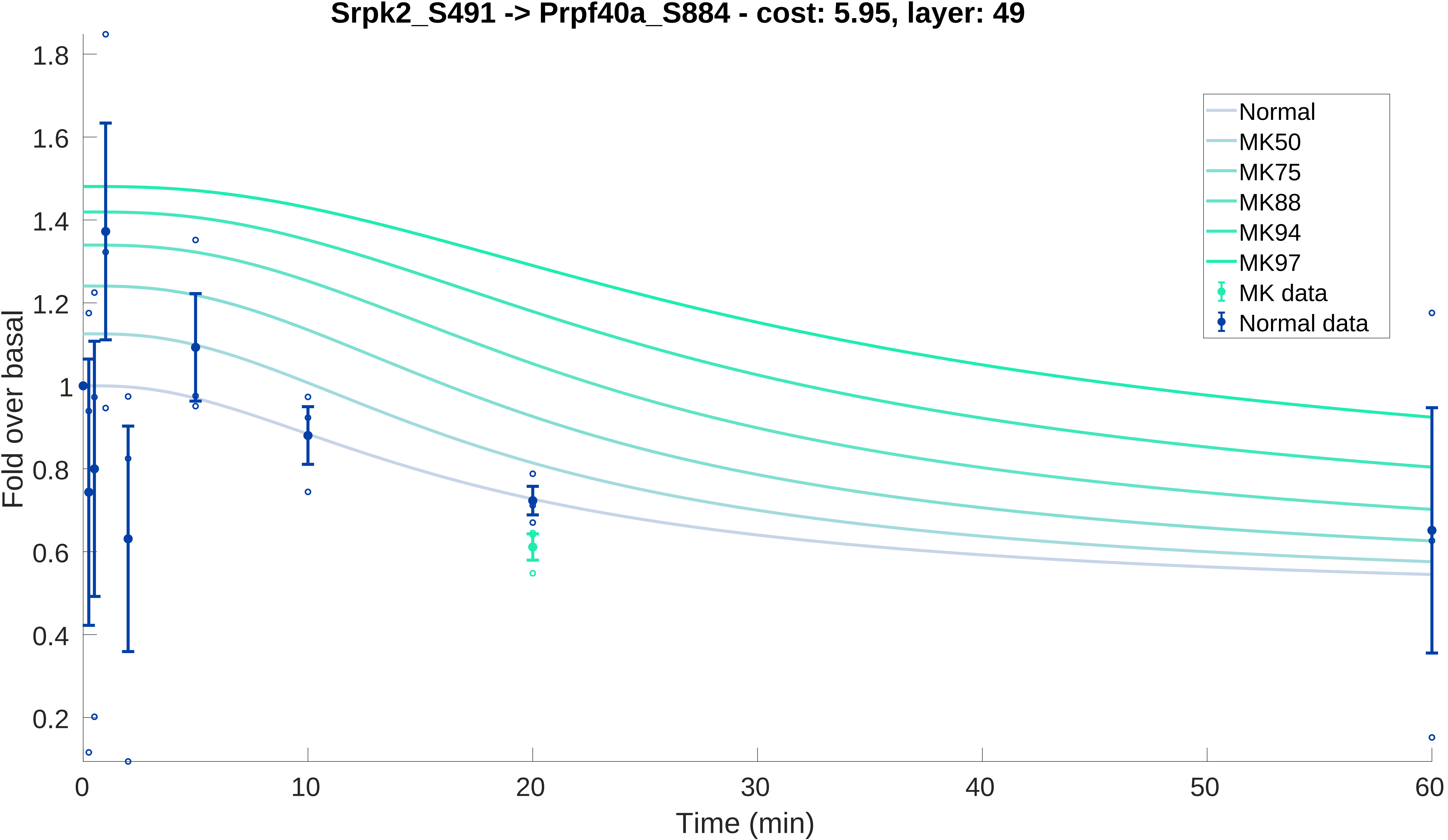

Srpk2_S491 -> Prpf40a_S884 - cost: 5.95, layer: 49
Fold over basal
Time (min)
0
10
20
30
40
50
60
0.2
0.4
0.6
0.8
1
1.2
1.4
1.6
1.8
Normal
MK50
MK75
MK88
MK94
MK97
MK data
Normal data

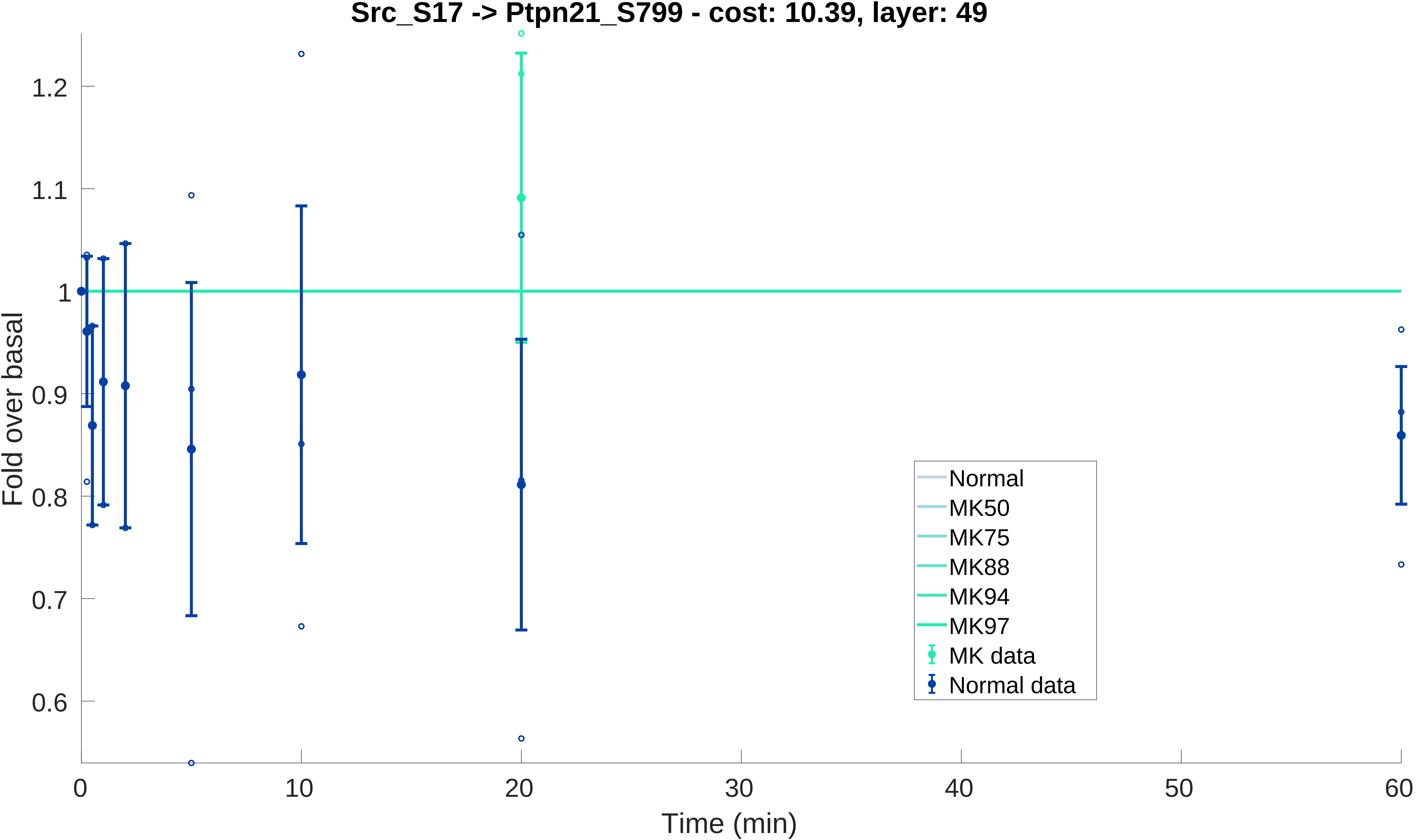

Src_S17 -> Ptpn21_S799 - cost: 10.39, layer: 49
Fold over basal
1.2
1.1
1
0.9
0.8
0.7
0.6
Time (min)
0
10
20
30
40
50
60
Normal
MK50
MK75
MK88
MK94
MK97
MK data
Normal data

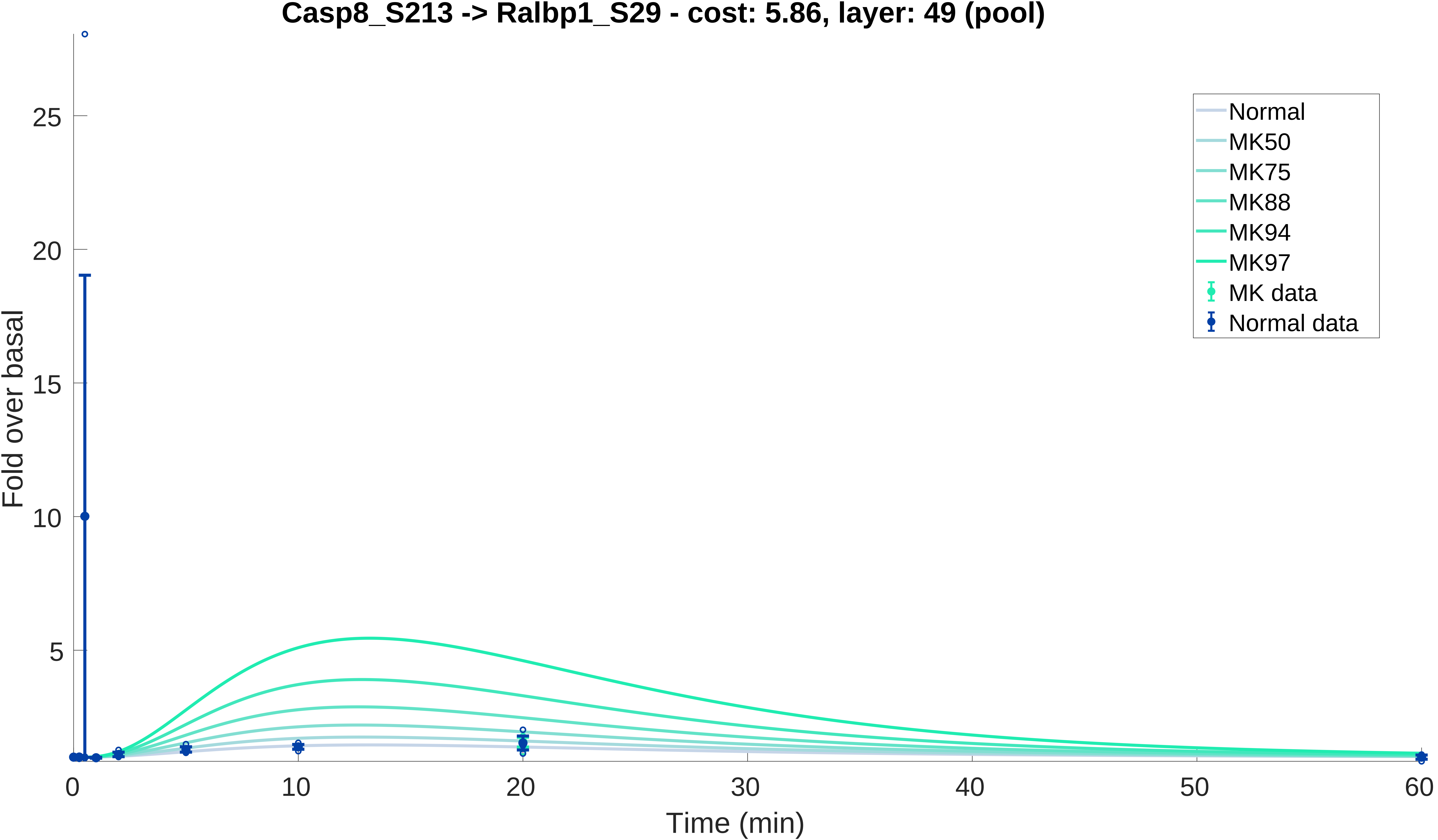

Casp8_S213 -> Ralbp1_S29 - cost: 5.86, layer: 49 (pool)
Fold over basal
Time (min)
0
5
10
15
20
25
0
10
20
30
40
50
60
Normal
MK50
MK75
MK88
MK94
MK97
MK data
Normal data

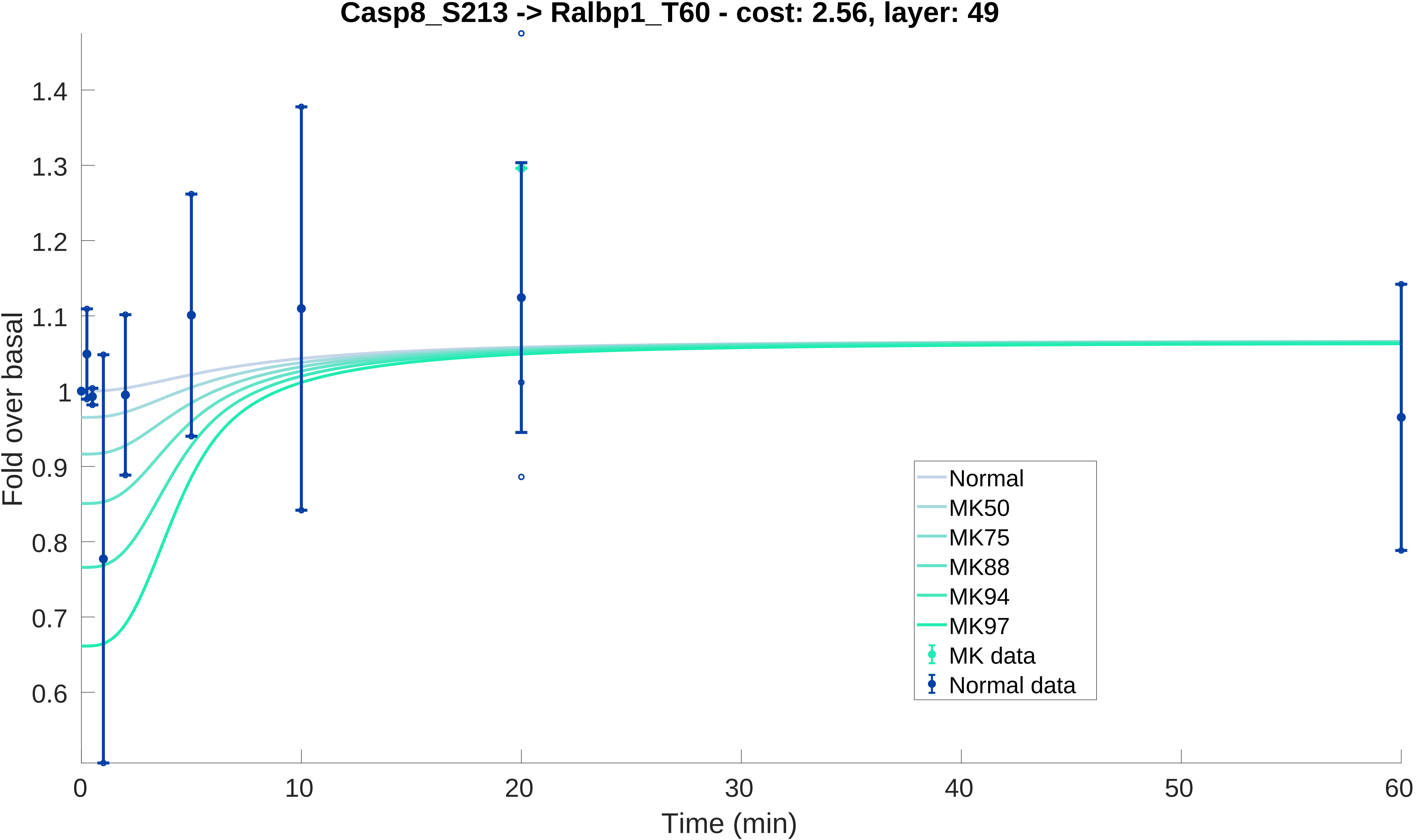

Casp8_S213 -> Ralbp1_T60 - cost: 2.56, layer: 49
Fold over basal
Time (min)
1.4
1.3
1.2
1.1
1
0.9
0.8
0.7
0.6
0
10
20
30
40
50
60
Normal
MK50
MK75
MK88
MK94
MK97
MK data
Normal data

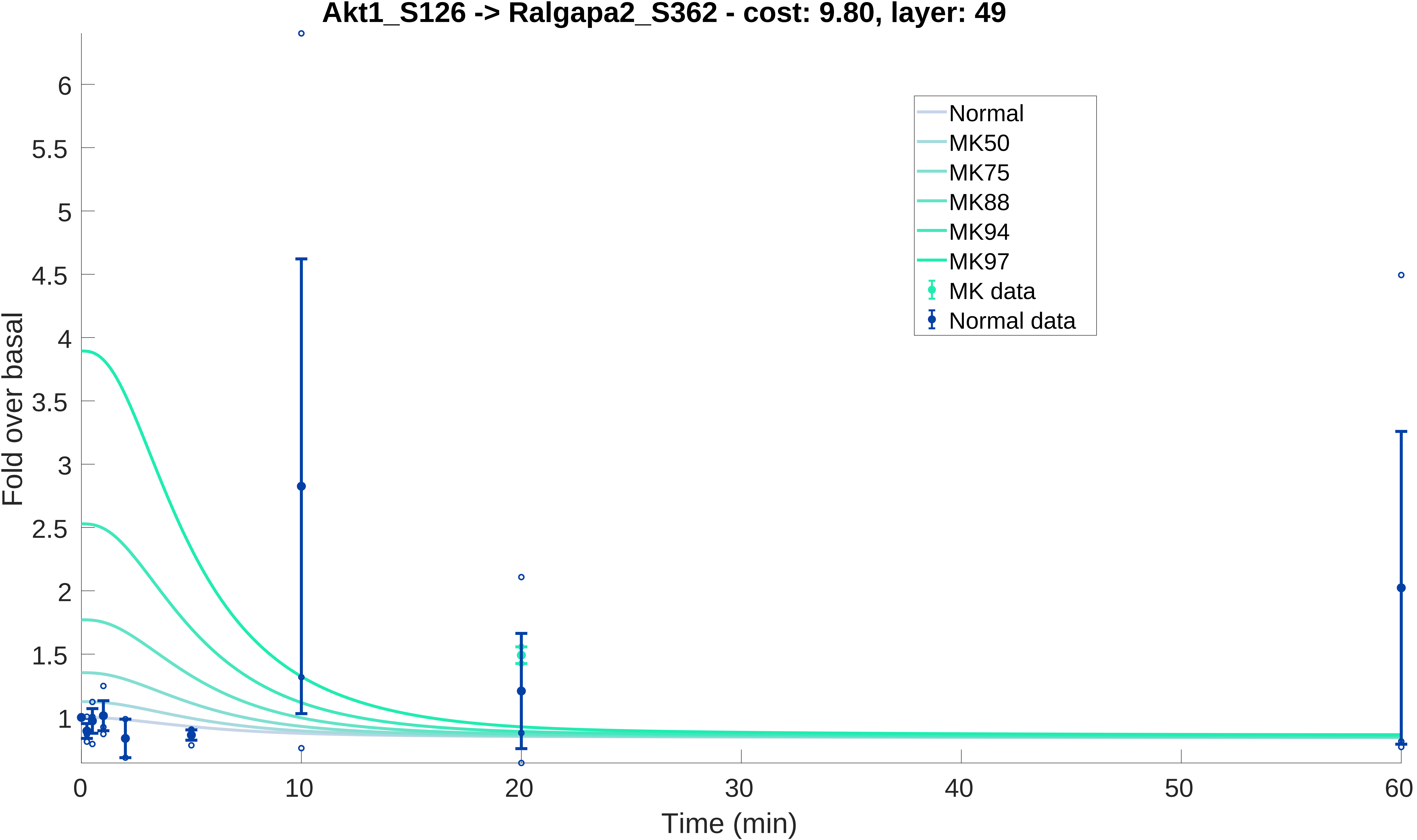
Akt1_S126 -> Ralgapa2_S362 - cost: 9.80, layer: 49
Fold over basal
Time (min)
Normal
MK50
MK75
MK88
MK94
MK97
MK data
Normal data
0
10
20
30
40
50
60
1
1.5
2
2.5
3
3.5
4
4.5
5
5.5
6

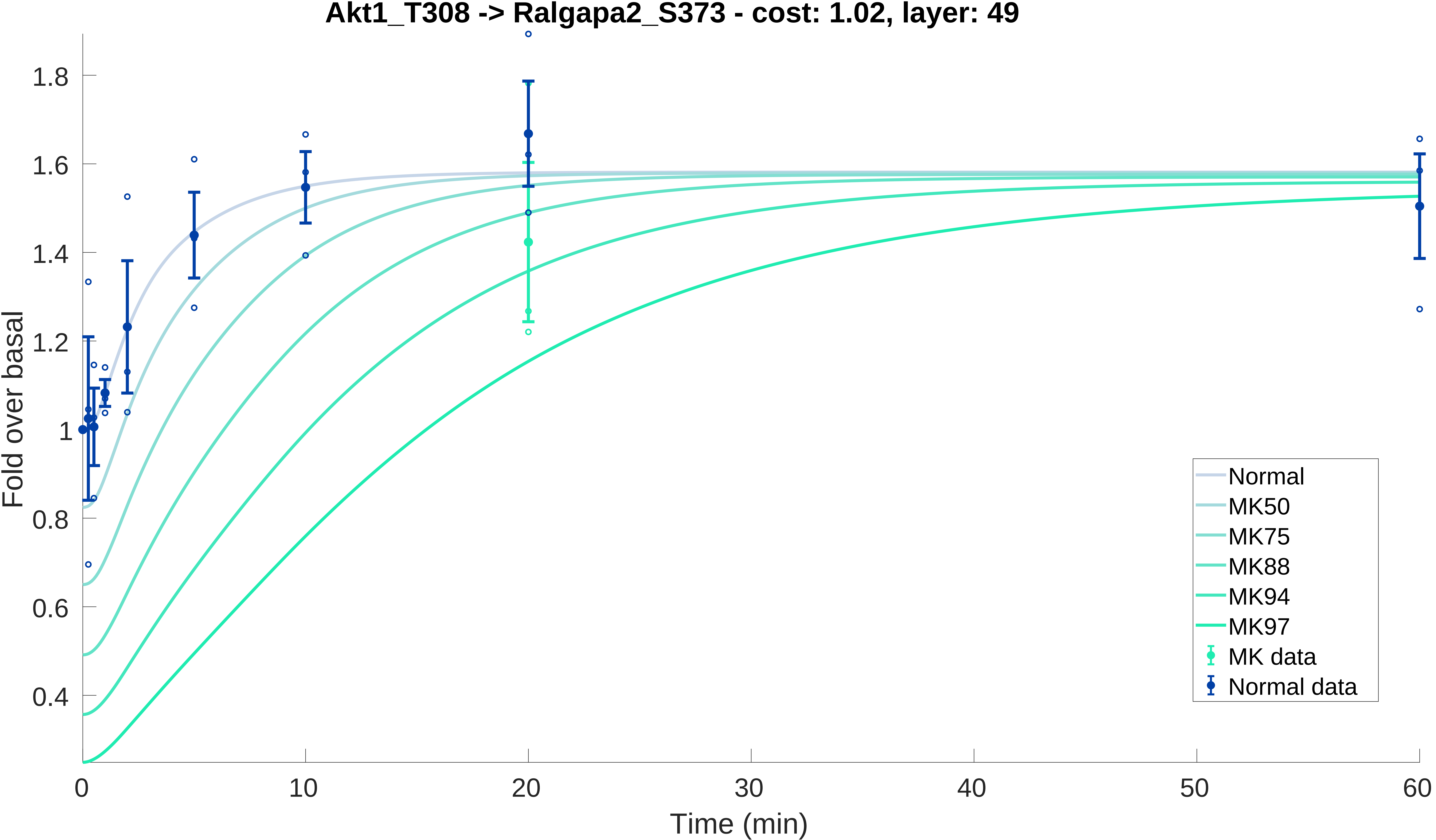

Akt1_T308 -> Ralgapa2_S373 - cost: 1.02, layer: 49
Fold over basal
Time (min)
0
10
20
30
40
50
60
0.4
0.6
0.8
1
1.2
1.4
1.6
1.8
Normal
MK50
MK75
MK88
MK94
MK97
MK data
Normal data

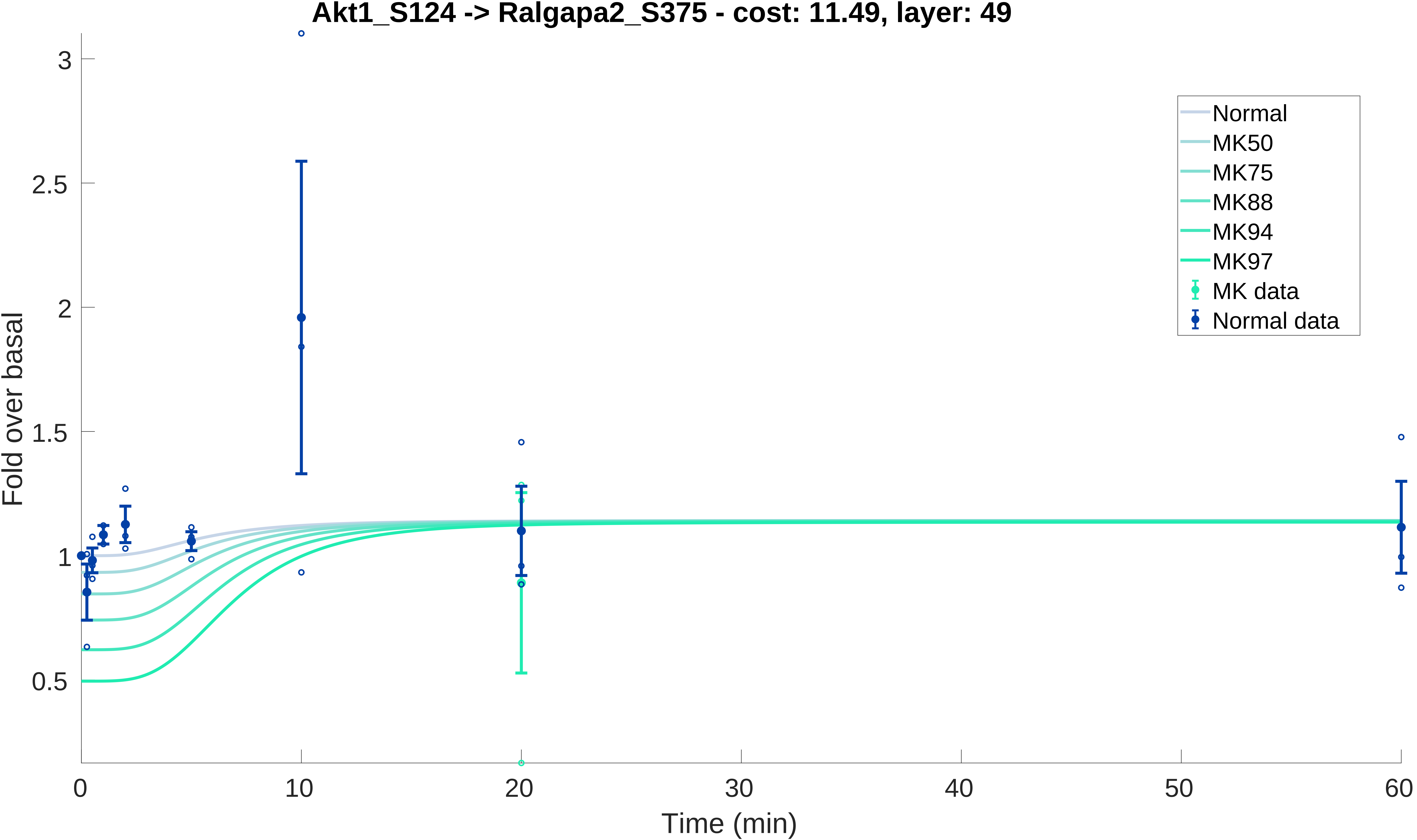

Akt1_S124 -> Ralgapa2_S375 - cost: 11.49, layer: 49
Fold over basal
Time (min)
0.5
1
1.5
2
2.5
3
0
10
20
30
40
50
60
Normal
MK50
MK75
MK88
MK94
MK97
MK data
Normal data

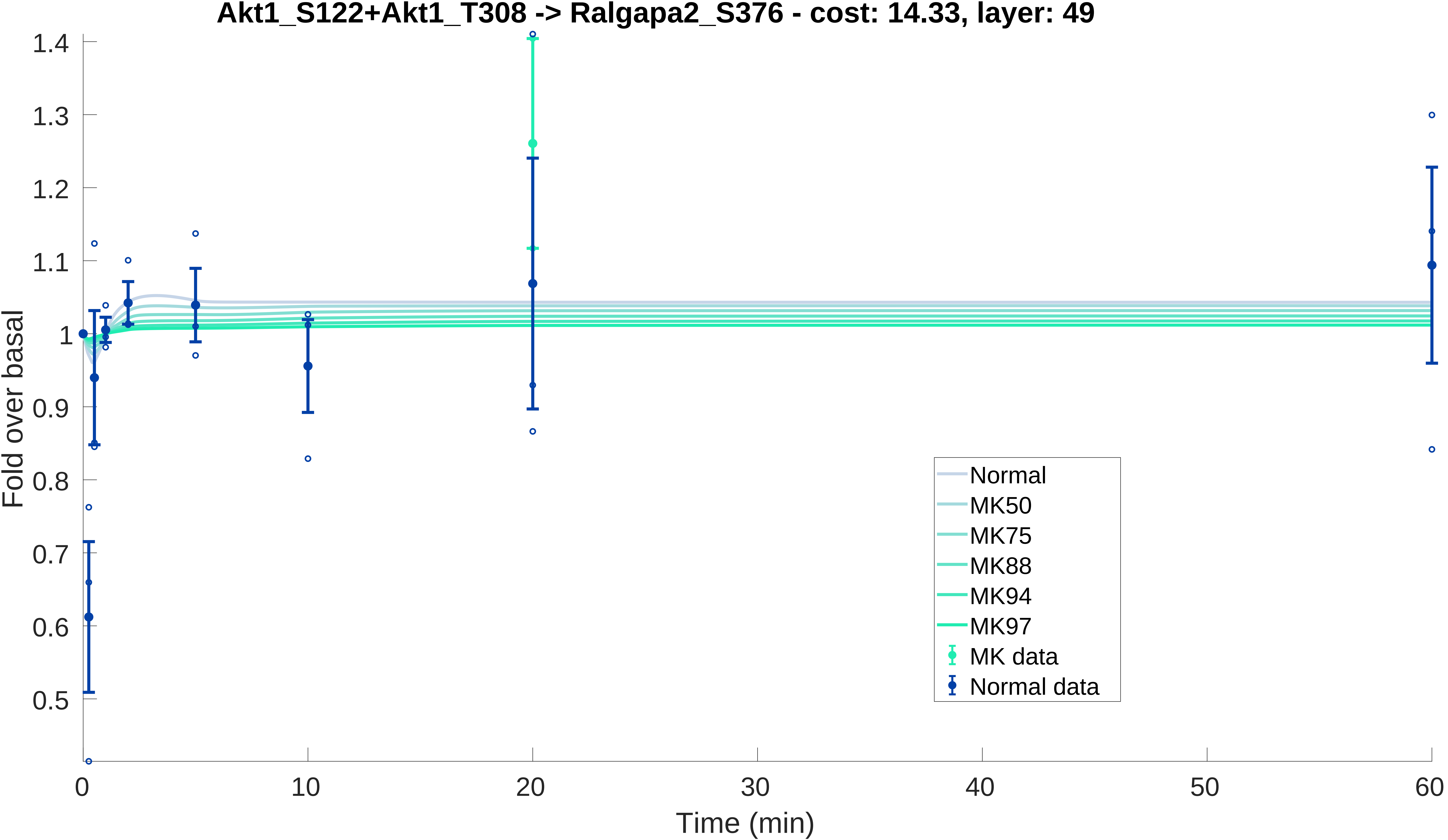

Akt1_S122+Akt1_T308 -> Ralgapa2_S376 - cost: 14.33, layer: 49
Fold over basal
Time (min)
1.4
1.3
1.2
1.1
1
0.9
0.8
0.7
0.6
0.5
0
10
20
30
40
50
60
Normal
MK50
MK75
MK88
MK94
MK97
MK data
Normal data

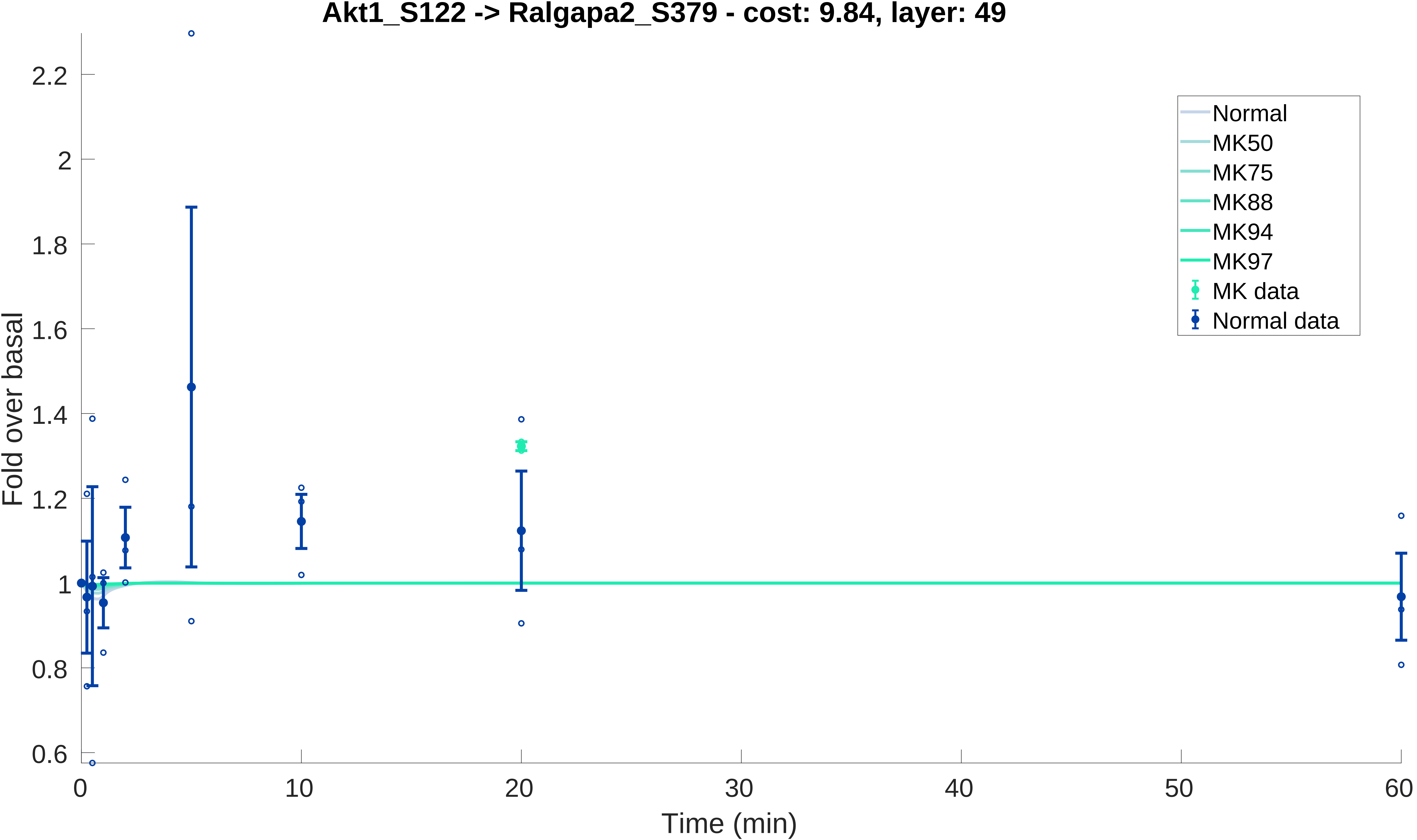

Akt1_S122 -> Ralgapa2_S379 - cost: 9.84, layer: 49
Fold over basal
Time (min)
0.6
0.8
1
1.2
1.4
1.6
1.8
2
2.2
0
10
20
30
40
50
60
Normal
MK50
MK75
MK88
MK94
MK97
MK data
Normal data

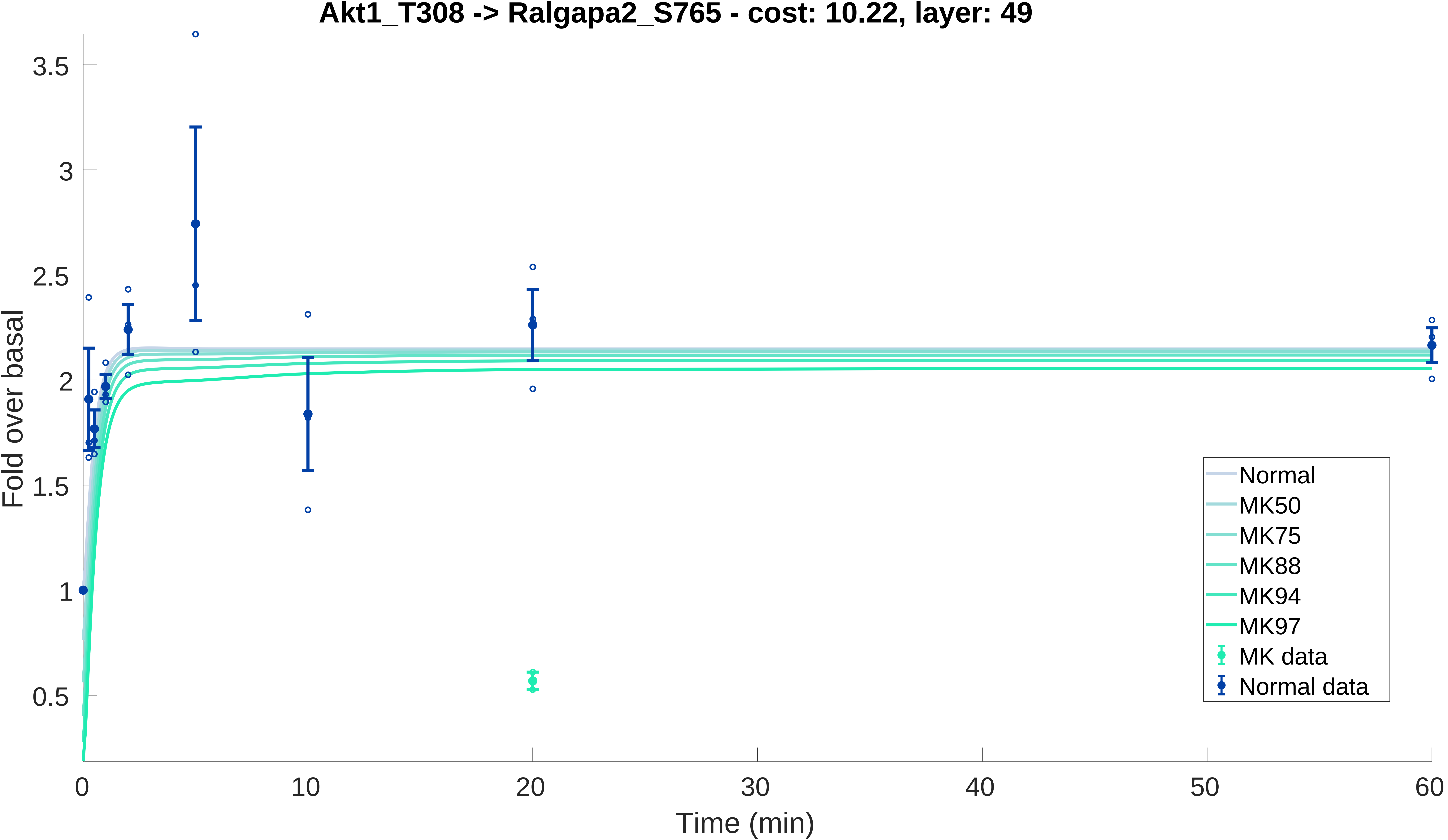

Akt1_T308 -> Ralgapa2_S765 - cost: 10.22, layer: 49
Fold over basal
3.5
3
2.5
2
1.5
1
0.5
0
10
20
30
40
50
60
Time (min)
Normal
MK50
MK75
MK88
MK94
MK97
MK data
Normal data

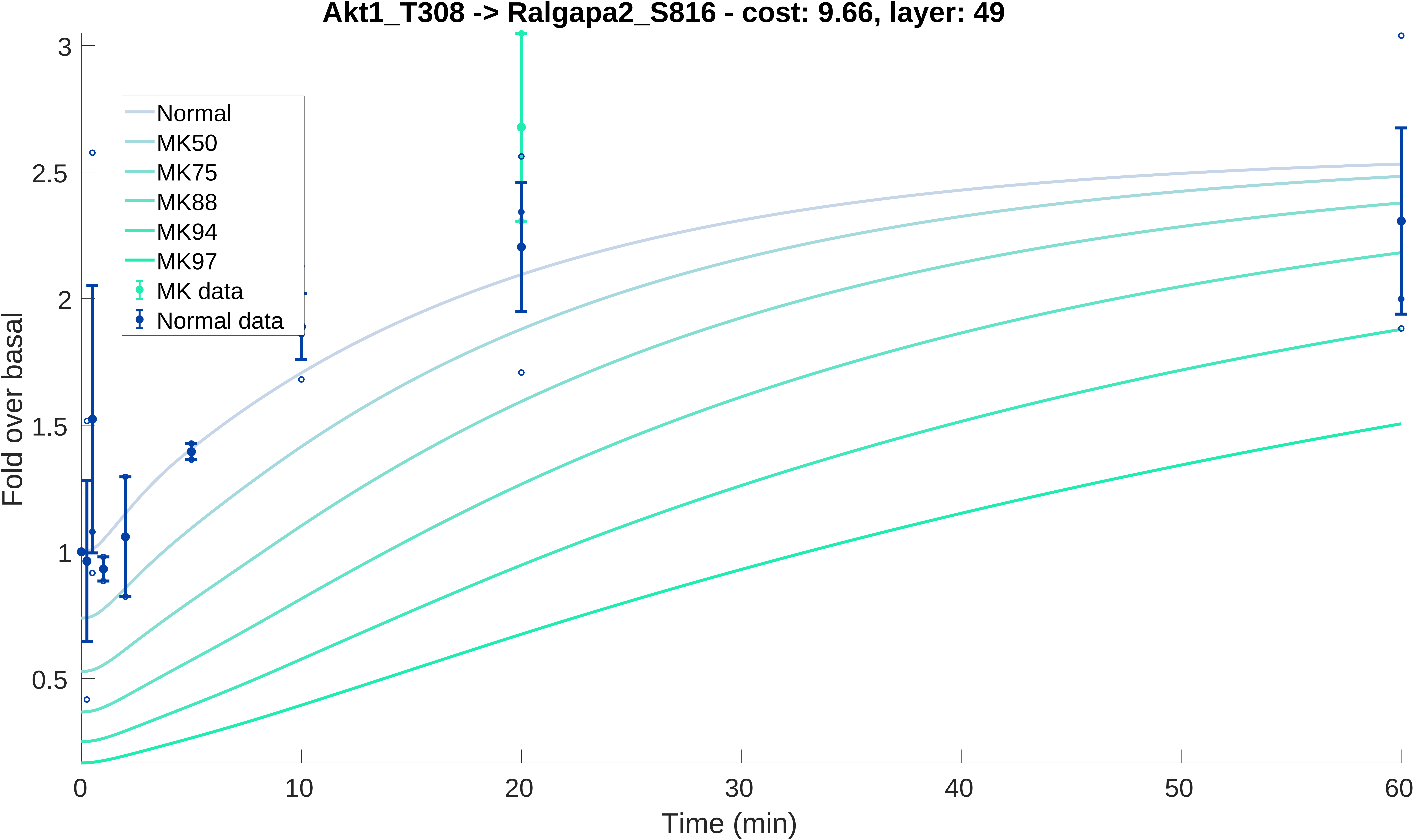
Akt1_T308 -> Ralgapa2_S816 - cost: 9.66, layer: 49
Fold over basal
Time (min)
Normal
MK50
MK75
MK88
MK94
MK97
MK data
Normal data
0.5
1
1.5
2
2.5
3
0
10
20
30
40
50
60

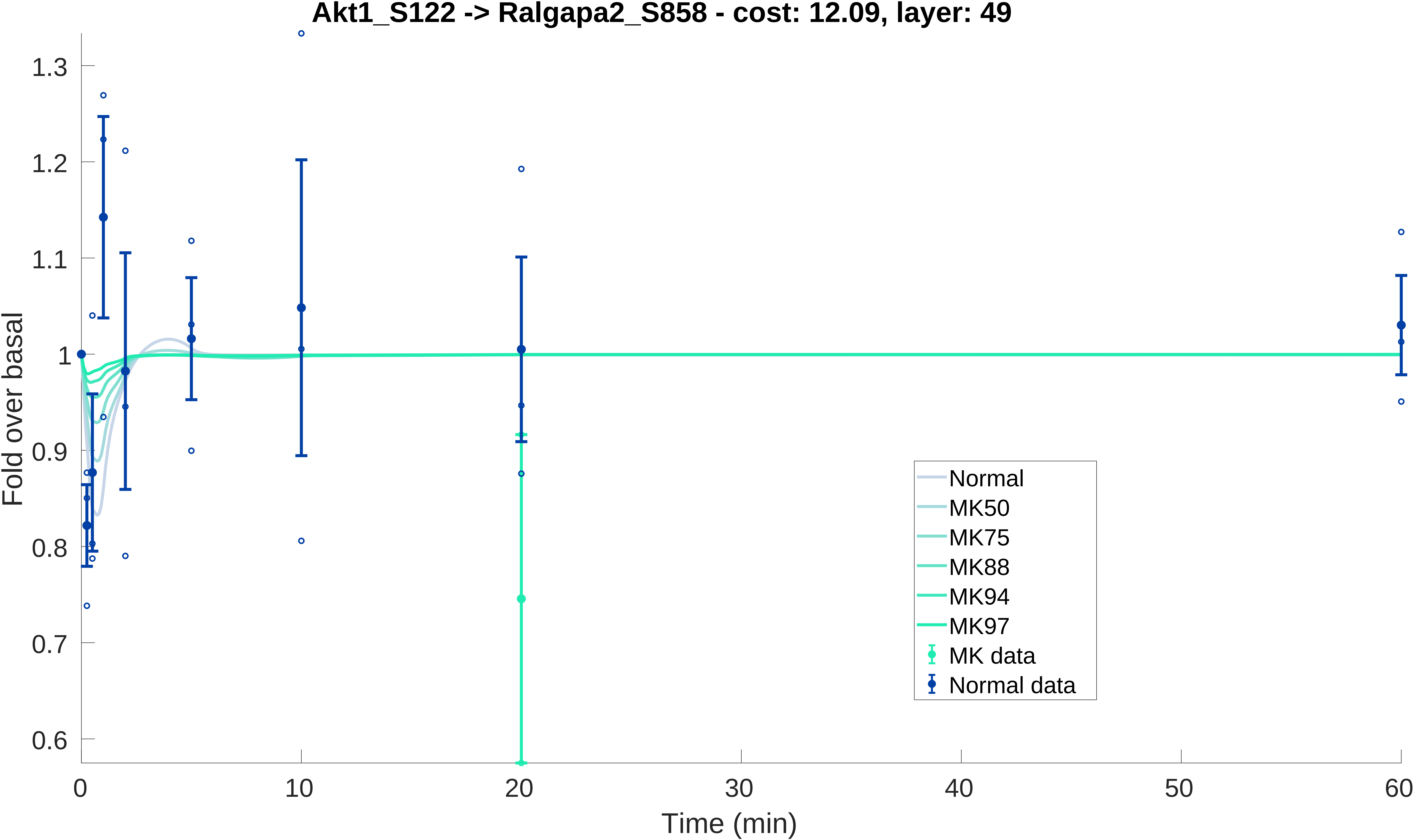

Akt1_S122 -> Ralgapa2_S858 - cost: 12.09, layer: 49
Fold over basal
Time (min)
0
10
20
30
40
50
60
0.6
0.7
0.8
0.9
1
1.1
1.2
1.3
Normal
MK50
MK75
MK88
MK94
MK97
MK data
Normal data

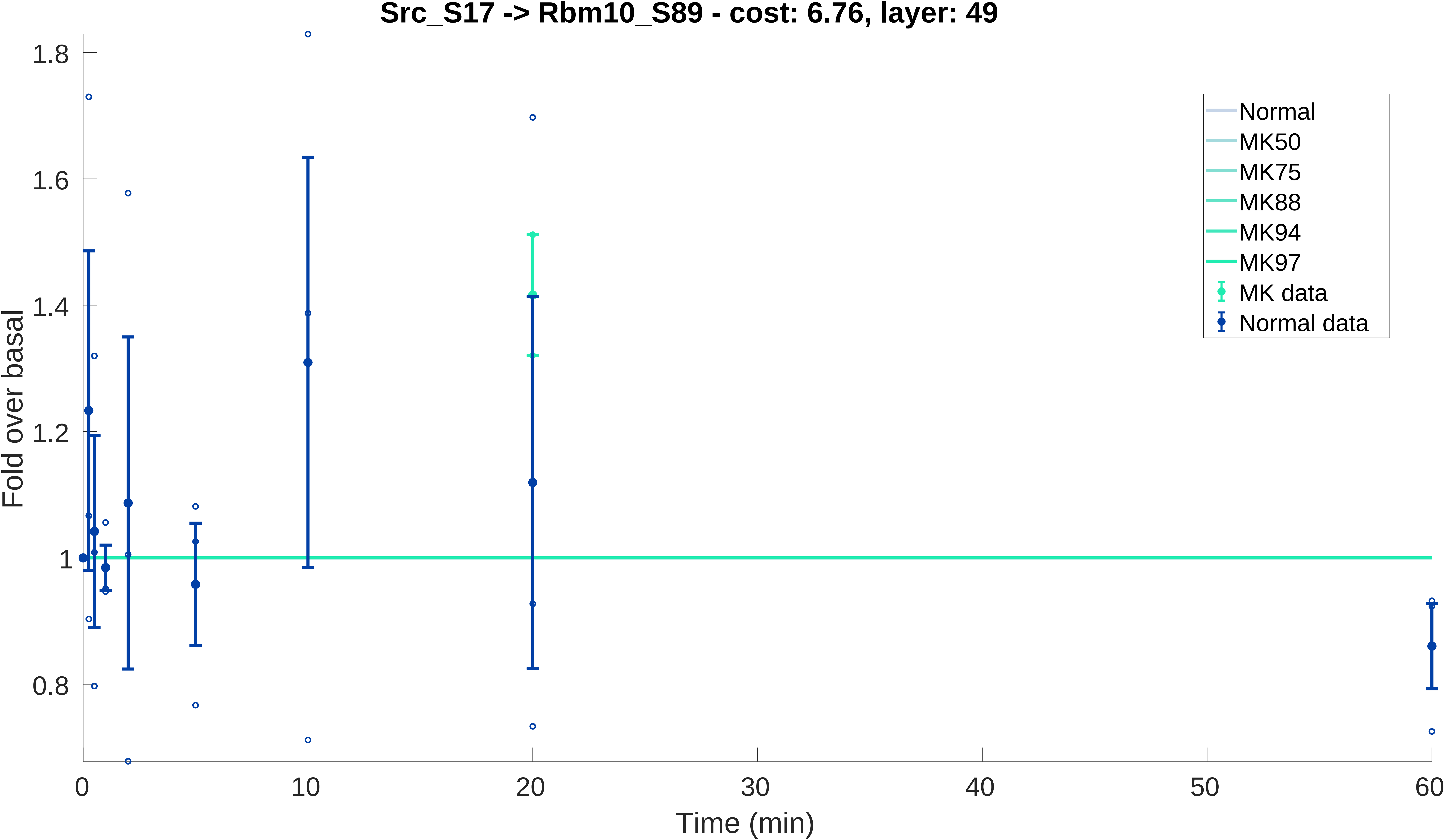
Src_S17 -> Rbm10_S89 - cost: 6.76, layer: 49
Fold over basal
1.8
1.6
1.4
1.2
1
0.8
0
10
20
30
40
50
60
Time (min)
Normal
MK50
MK75
MK88
MK94
MK97
MK data
Normal data

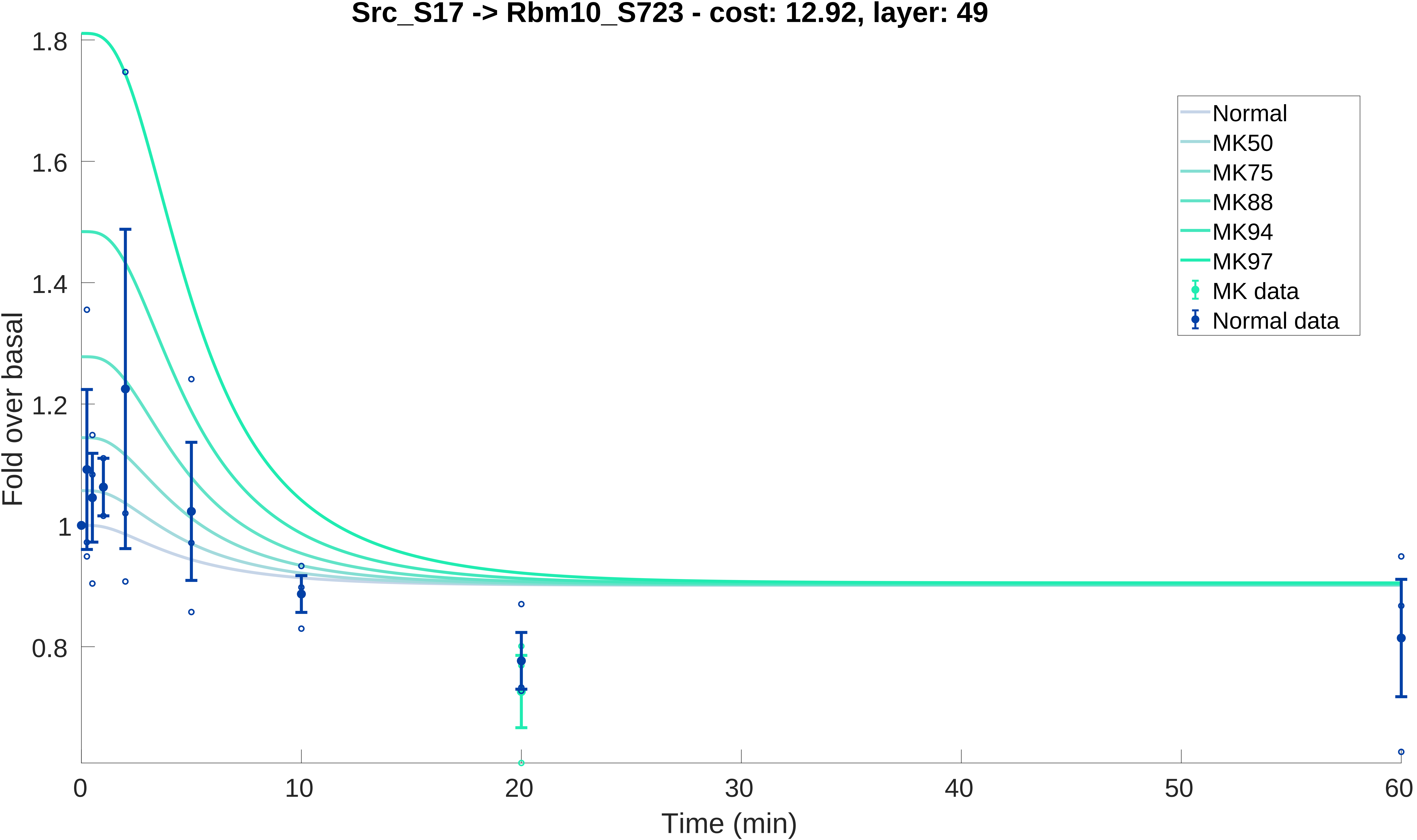

Src_S17 -> Rbm10_S723 - cost: 12.92, layer: 49
Fold over basal
Time (min)
0.8
1
1.2
1.4
1.6
1.8
0
10
20
30
40
50
60
Normal
MK50
MK75
MK88
MK94
MK97
MK data
Normal data

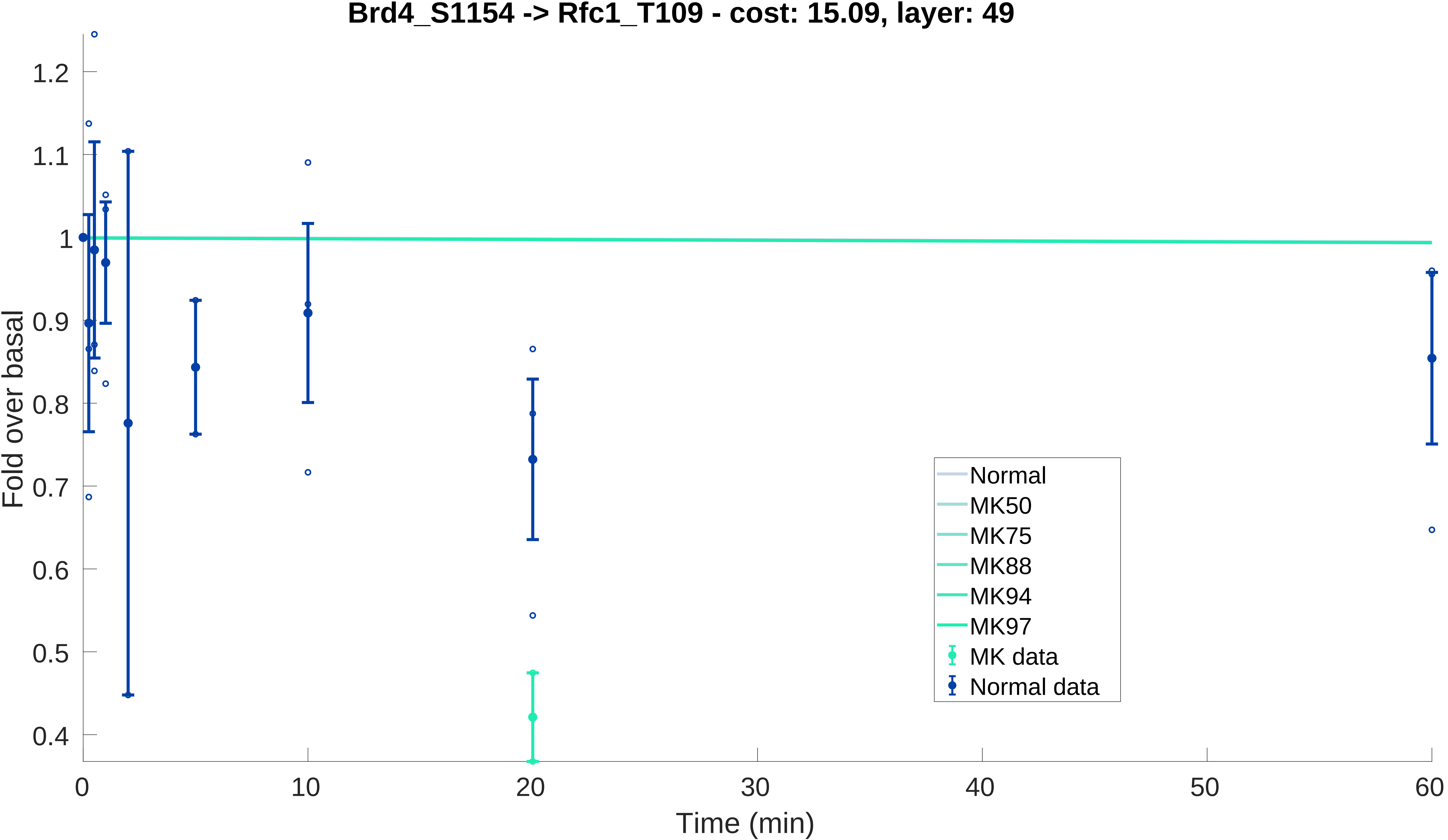
Brd4_S1154 -> Rfc1_T109 - cost: 15.09, layer: 49
Fold over basal
1.2
1.1
1
0.9
0.8
0.7
0.6
0.5
0.4
0
10
20
30
40
50
60
Time (min)
Normal
MK50
MK75
MK88
MK94
MK97
MK data
Normal data

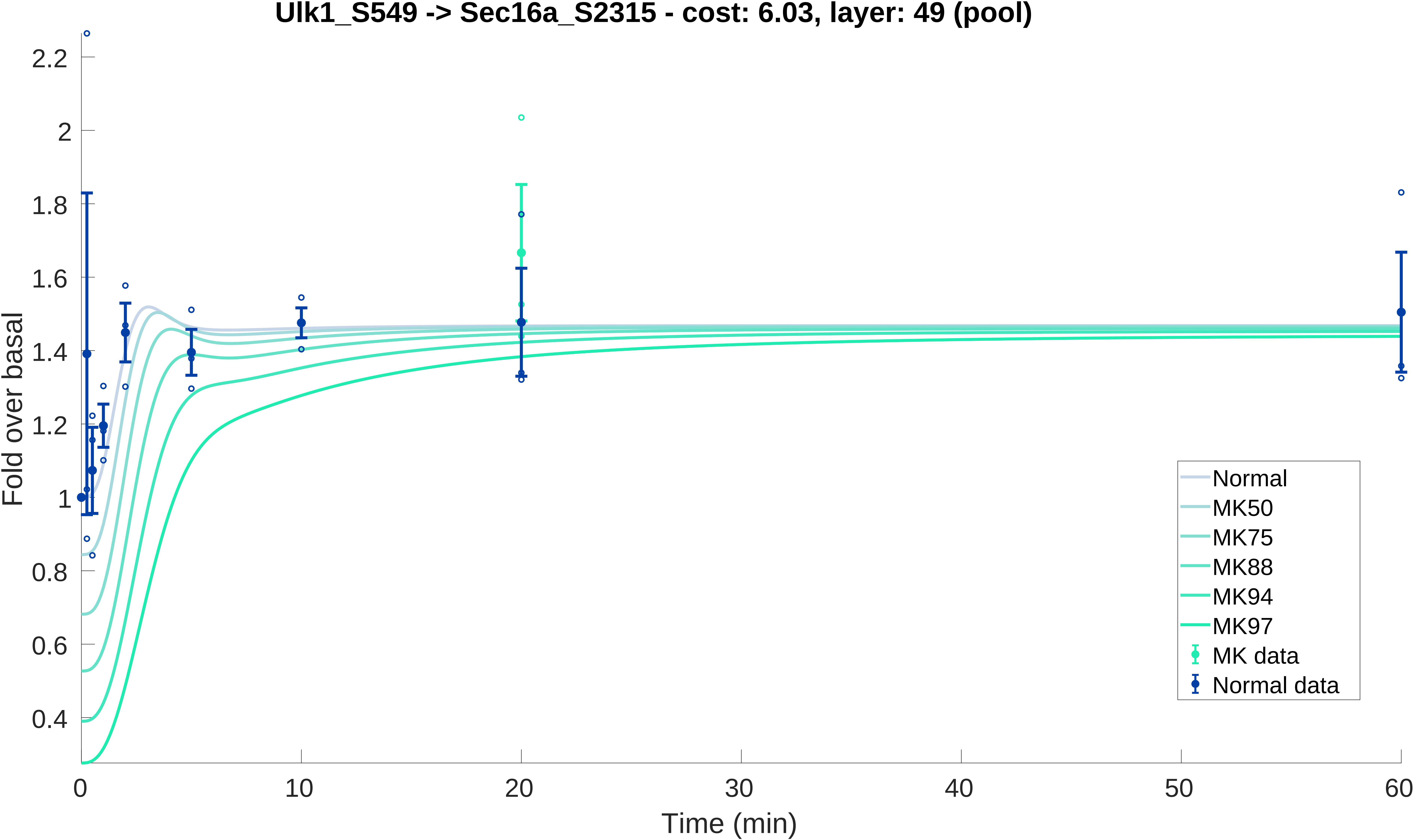

Ulk1_S549 -> Sec16a_S2315 - cost: 6.03, layer: 49 (pool)
Fold over basal
2.2
2
1.8
1.6
1.4
1.2
1
0.8
0.6
0.4
0
10
20
30
40
50
60
Time (min)
Normal
MK50
MK75
MK88
MK94
MK97
MK data
Normal data

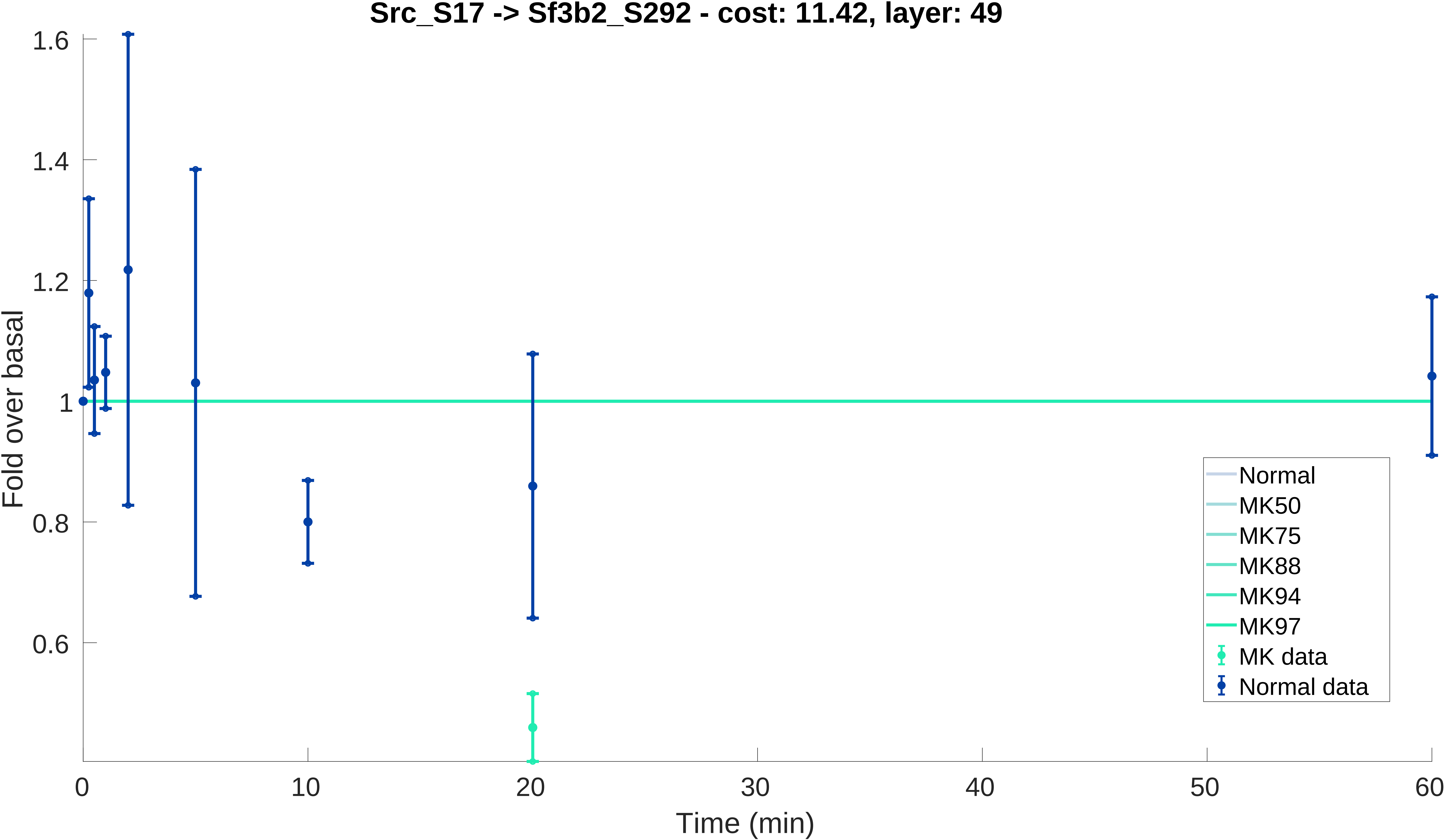
Src_S17 -> Sf3b2_S292 - cost: 11.42, layer: 49
Fold over basal
1.6
1.4
1.2
1
0.8
0.6
0
10
20
30
40
50
60
Time (min)
Normal
MK50
MK75
MK88
MK94
MK97
MK data
Normal data

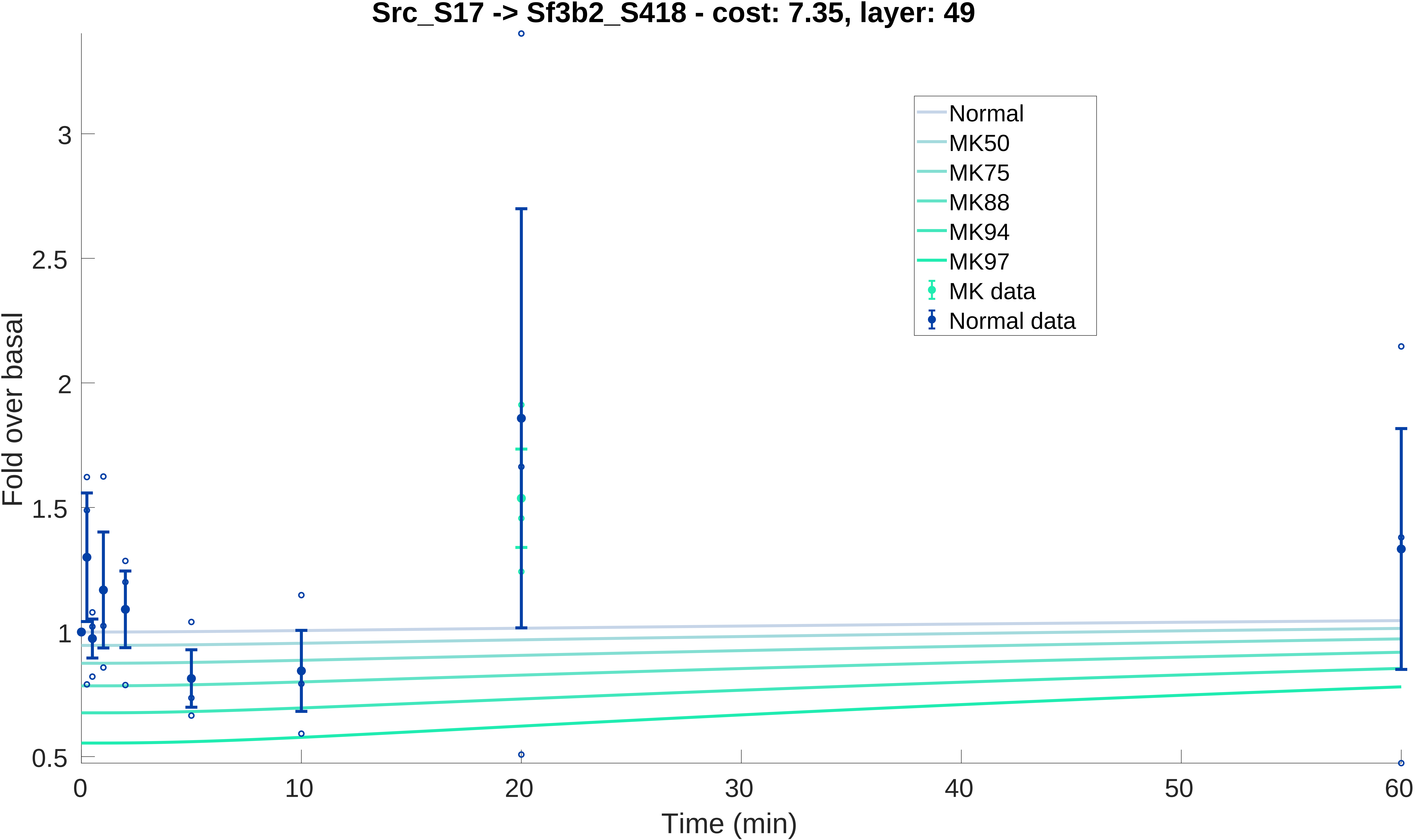

Src_S17 -> Sf3b2_S418 - cost: 7.35, layer: 49
Normal
MK50
MK75
MK88
MK94
MK97
MK data
Normal data
Fold over basal
Time (min)
0.5
1
1.5
2
2.5
3
0
10
20
30
40
50
60

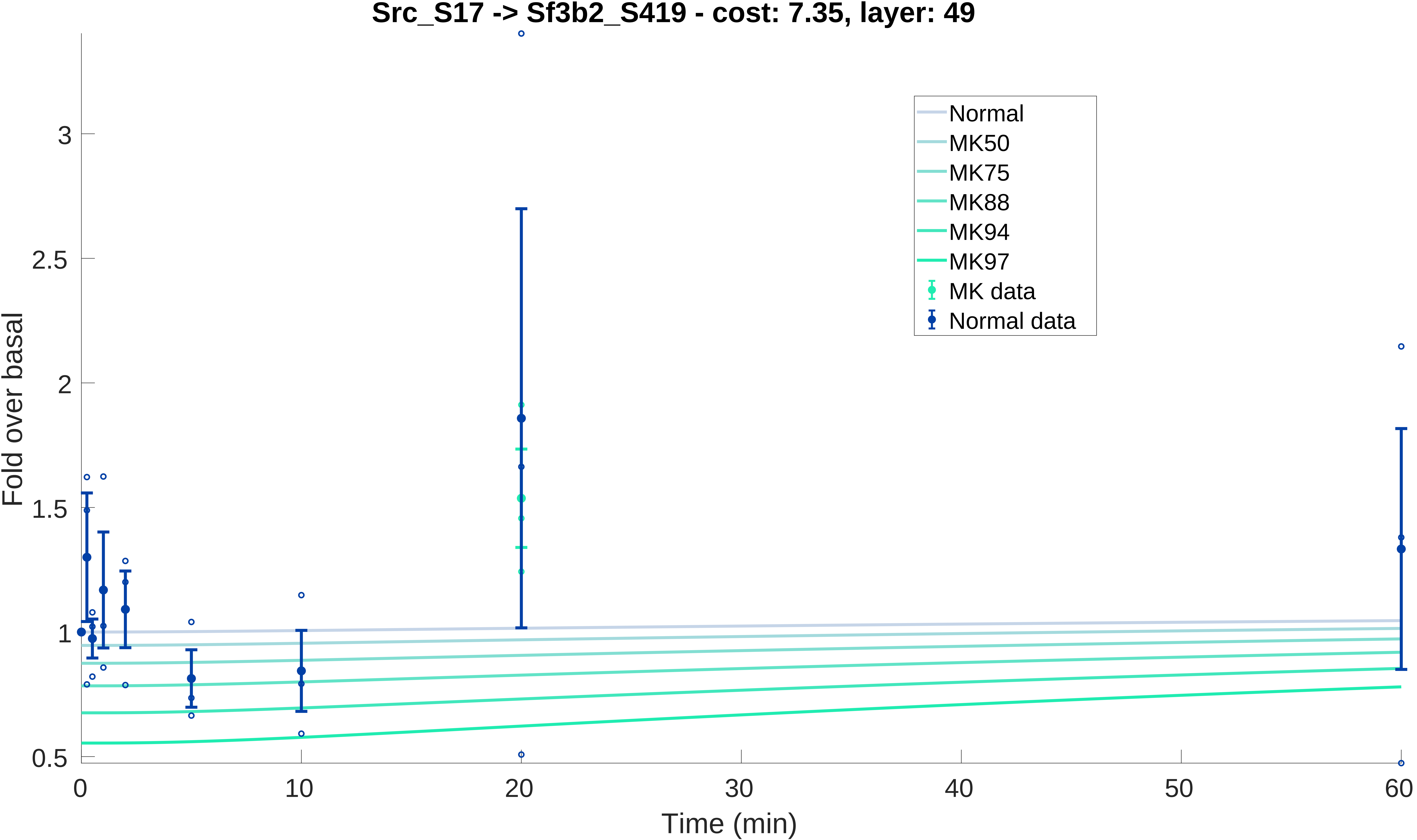

Src_S17 -> Sf3b2_S419 - cost: 7.35, layer: 49
Fold over basal
Time (min)
Normal
MK50
MK75
MK88
MK94
MK97
MK data
Normal data
0.5
1
1.5
2
2.5
3
0
10
20
30
40
50
60

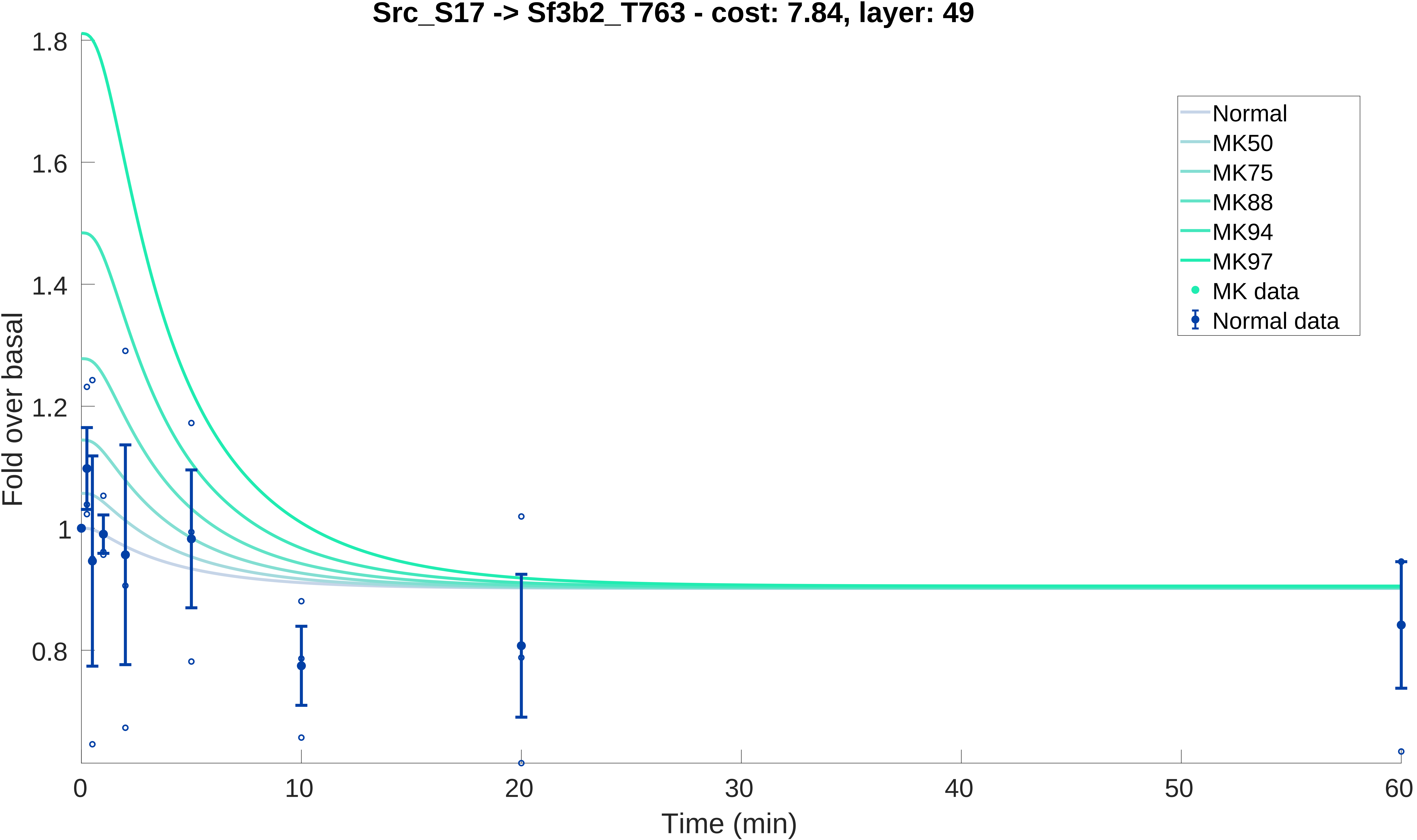
Src_S17 -> Sf3b2_T763 - cost: 7.84, layer: 49
Fold over basal
1.8
1.6
1.4
1.2
1
0.8
0
10
20
30
40
50
60
Time (min)
Normal
MK50
MK75
MK88
MK94
MK97
MK data
Normal data

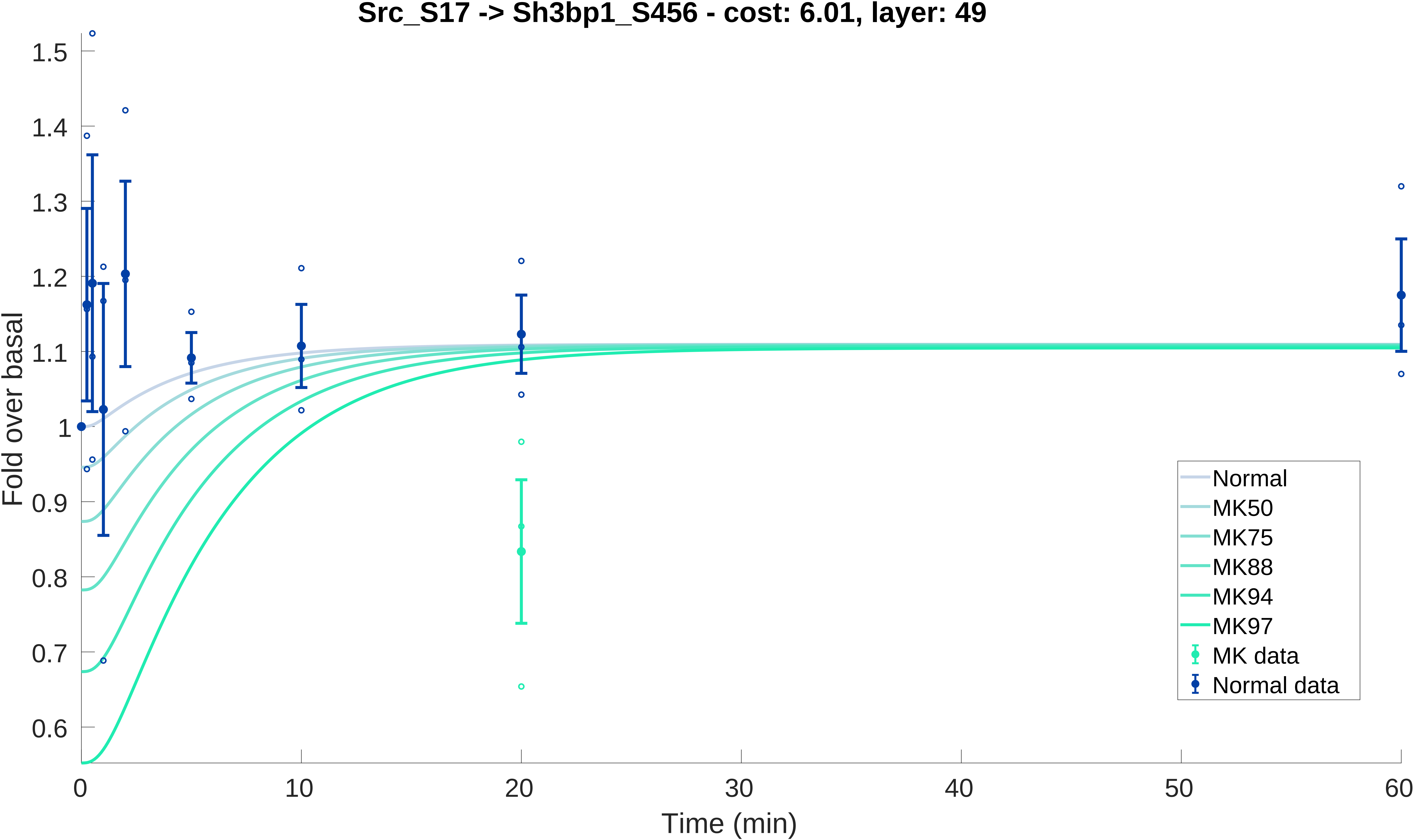

Src_S17 -> Sh3bp1_S456 - cost: 6.01, layer: 49
Fold over basal
1.5
1.4
1.3
1.2
1.1
1
0.9
0.8
0.7
0.6
0
10
20
30
40
50
60
Time (min)
Normal
MK50
MK75
MK88
MK94
MK97
MK data
Normal data

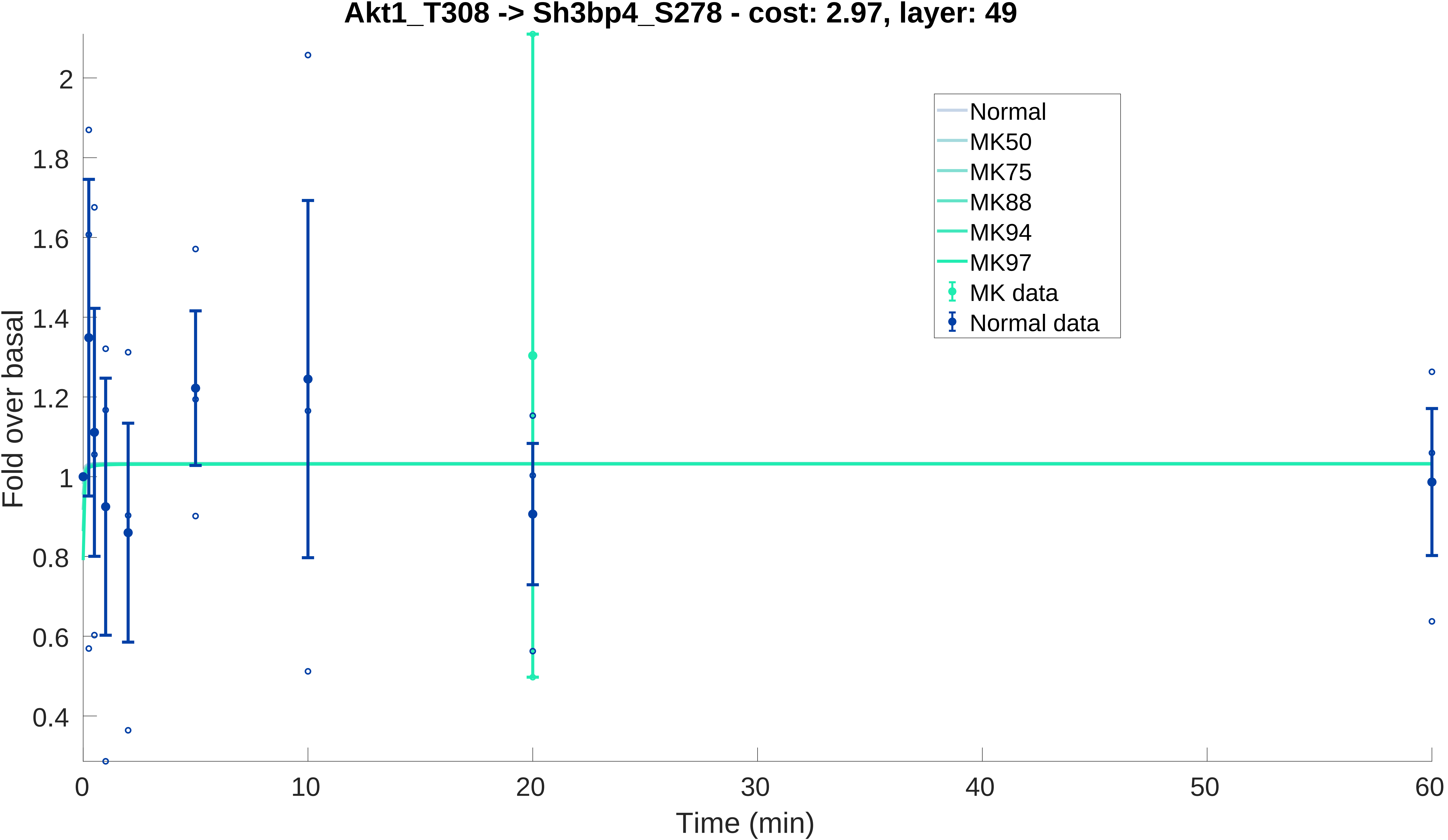

Akt1_T308 -> Sh3bp4_S278 - cost: 2.97, layer: 49
Fold over basal
Time (min)
0
10
20
30
40
50
60
0.4
0.6
0.8
1
1.2
1.4
1.6
1.8
2
Normal
MK50
MK75
MK88
MK94
MK97
MK data
Normal data

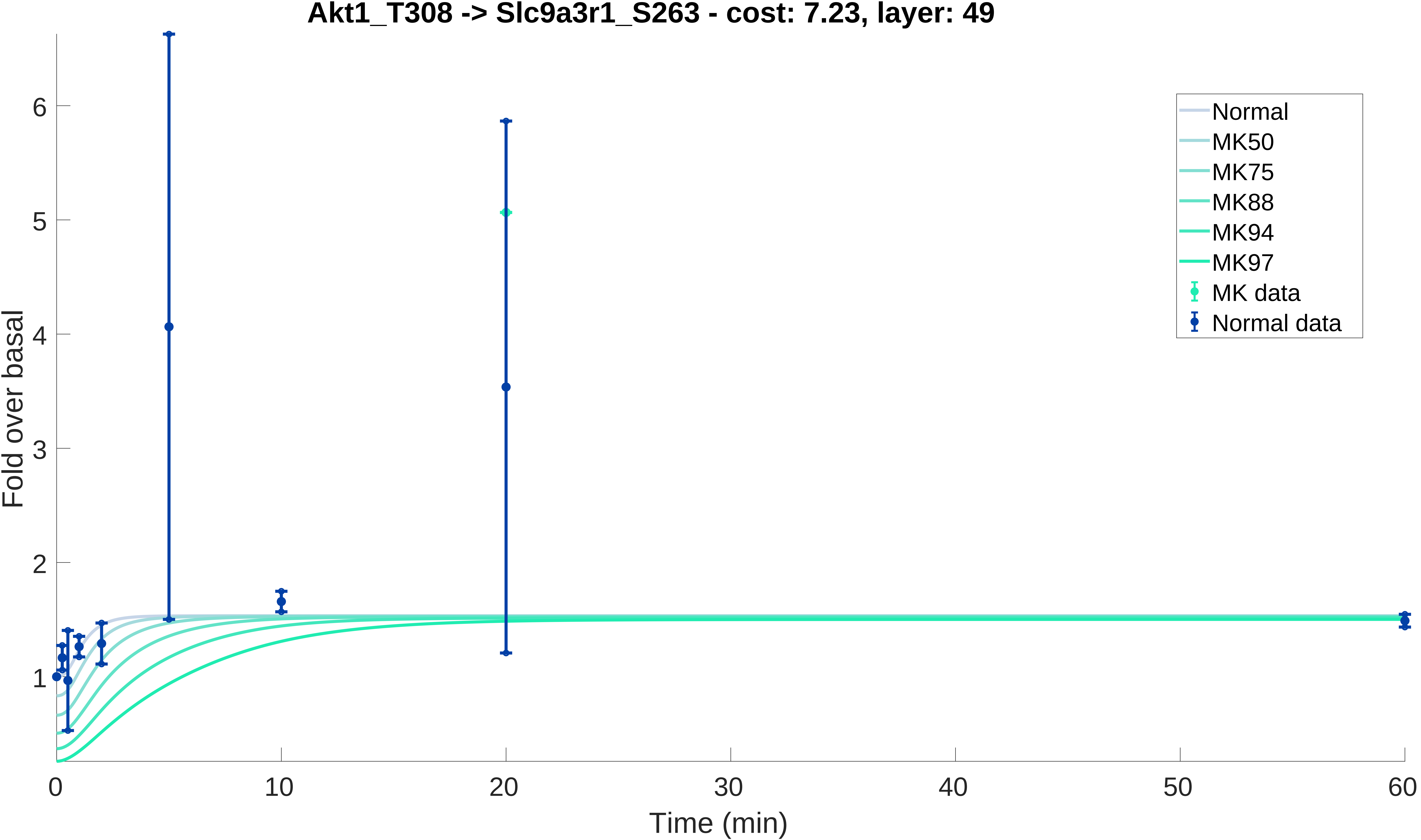

Akt1_T308 -> Slc9a3r1_S263 - cost: 7.23, layer: 49
Fold over basal
Time (min)
0
10
20
30
40
50
60
1
2
3
4
5
6
Normal
MK50
MK75
MK88
MK94
MK97
MK data
Normal data

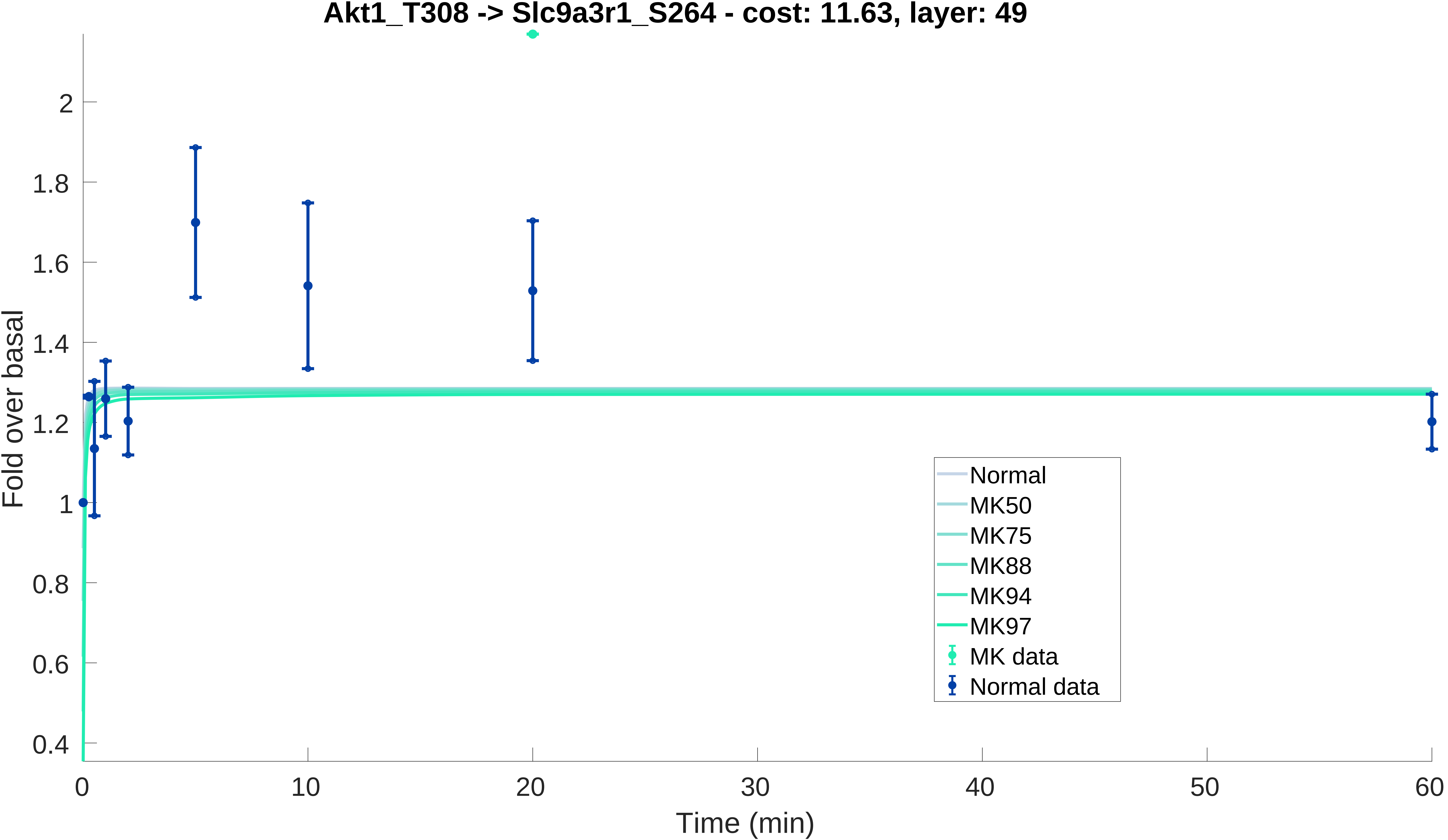

Akt1_T308 -> Slc9a3r1_S264 - cost: 11.63, layer: 49
Fold over basal
Time (min)
0.4
0.6
0.8
1
1.2
1.4
1.6
1.8
2
0
10
20
30
40
50
60
Normal
MK50
MK75
MK88
MK94
MK97
MK data
Normal data

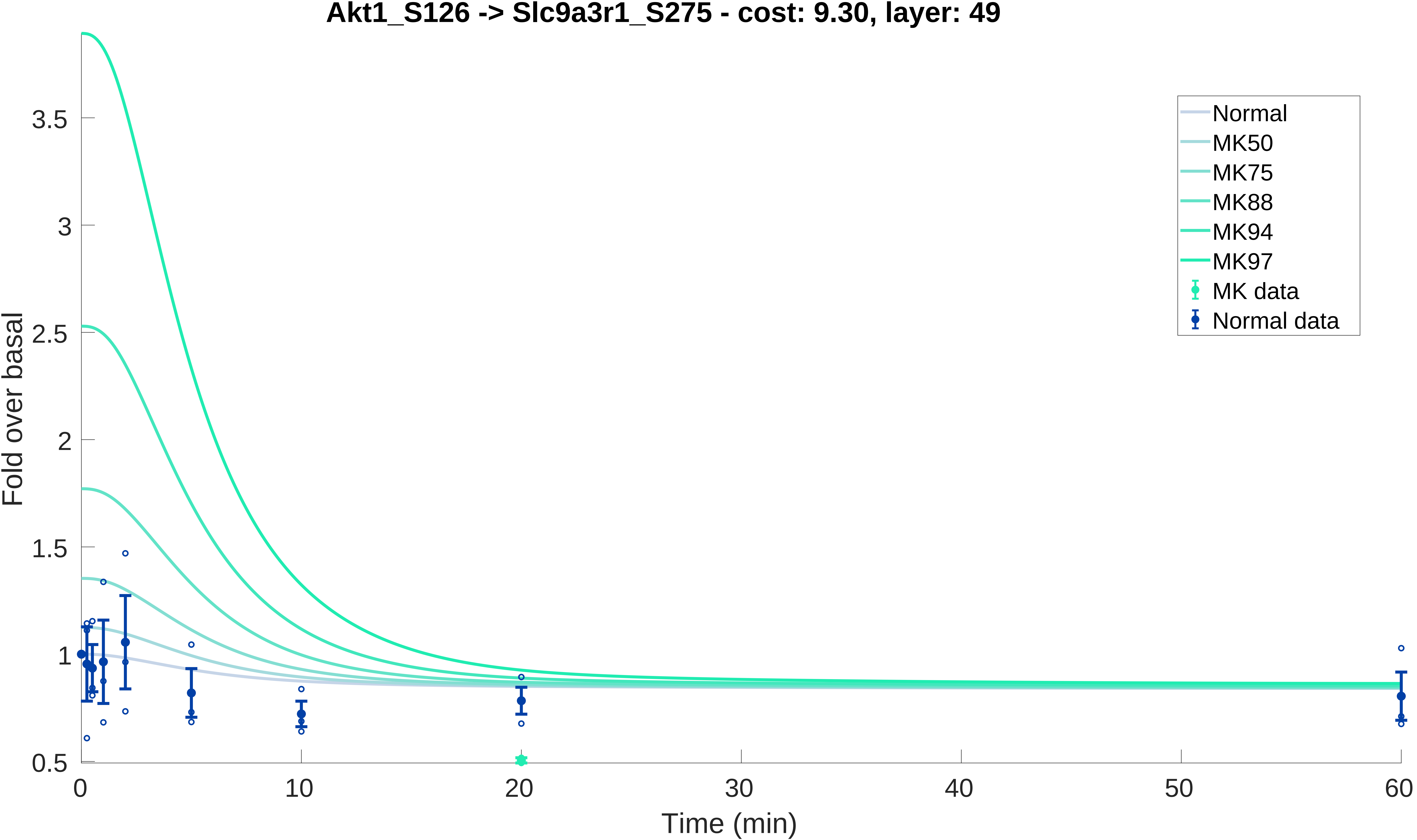

Akt1_S126 -> Slc9a3r1_S275 - cost: 9.30, layer: 49
Fold over basal
Time (min)
0.5
1
1.5
2
2.5
3
3.5
0
10
20
30
40
50
60
Normal
MK50
MK75
MK88
MK94
MK97
MK data
Normal data

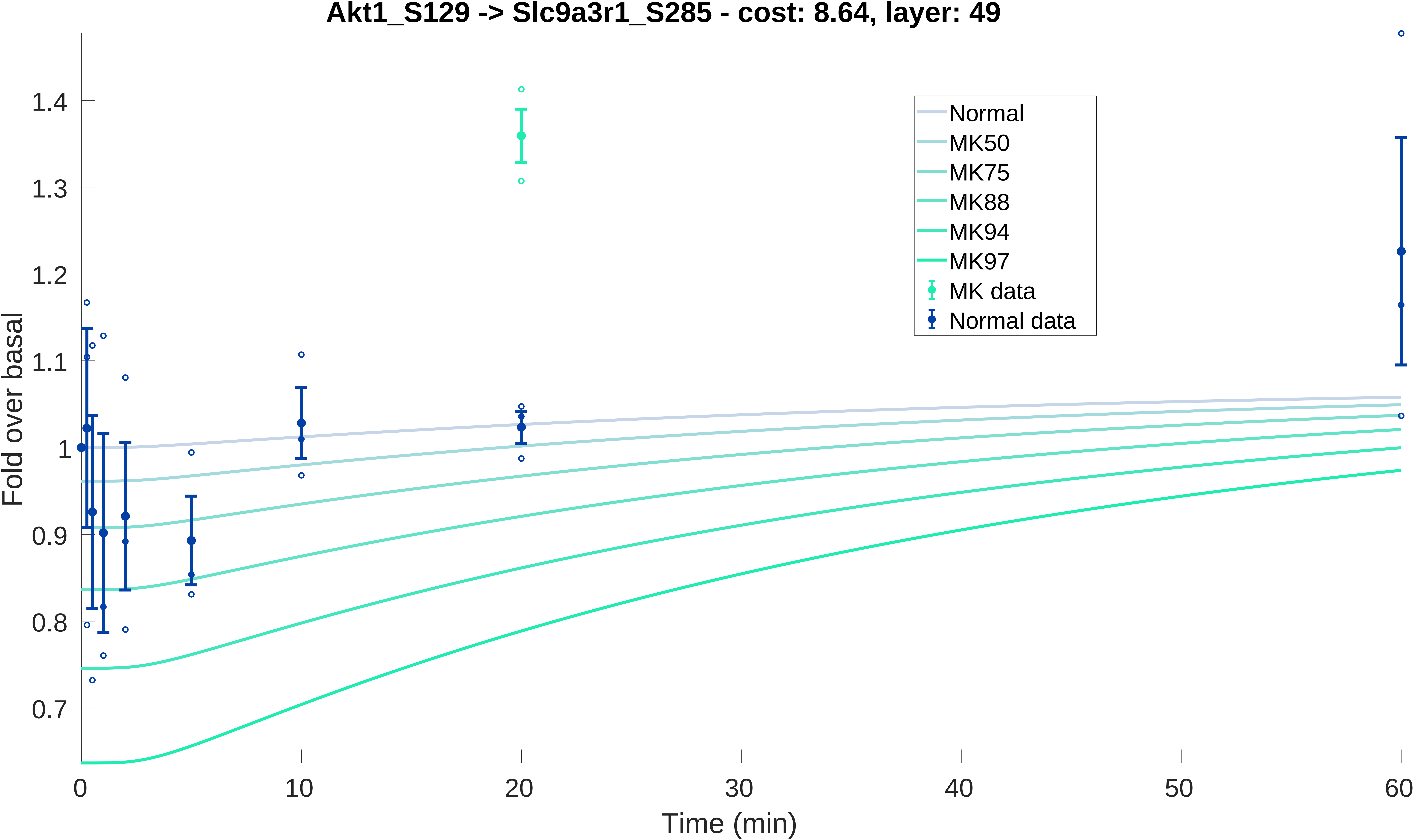
Akt1_S129 -> Slc9a3r1_S285 - cost: 8.64, layer: 49
Fold over basal
Time (min)
0.7
0.8
0.9
1
1.1
1.2
1.3
1.4
0
10
20
30
40
50
60
Normal
MK50
MK75
MK88
MK94
MK97
MK data
Normal data

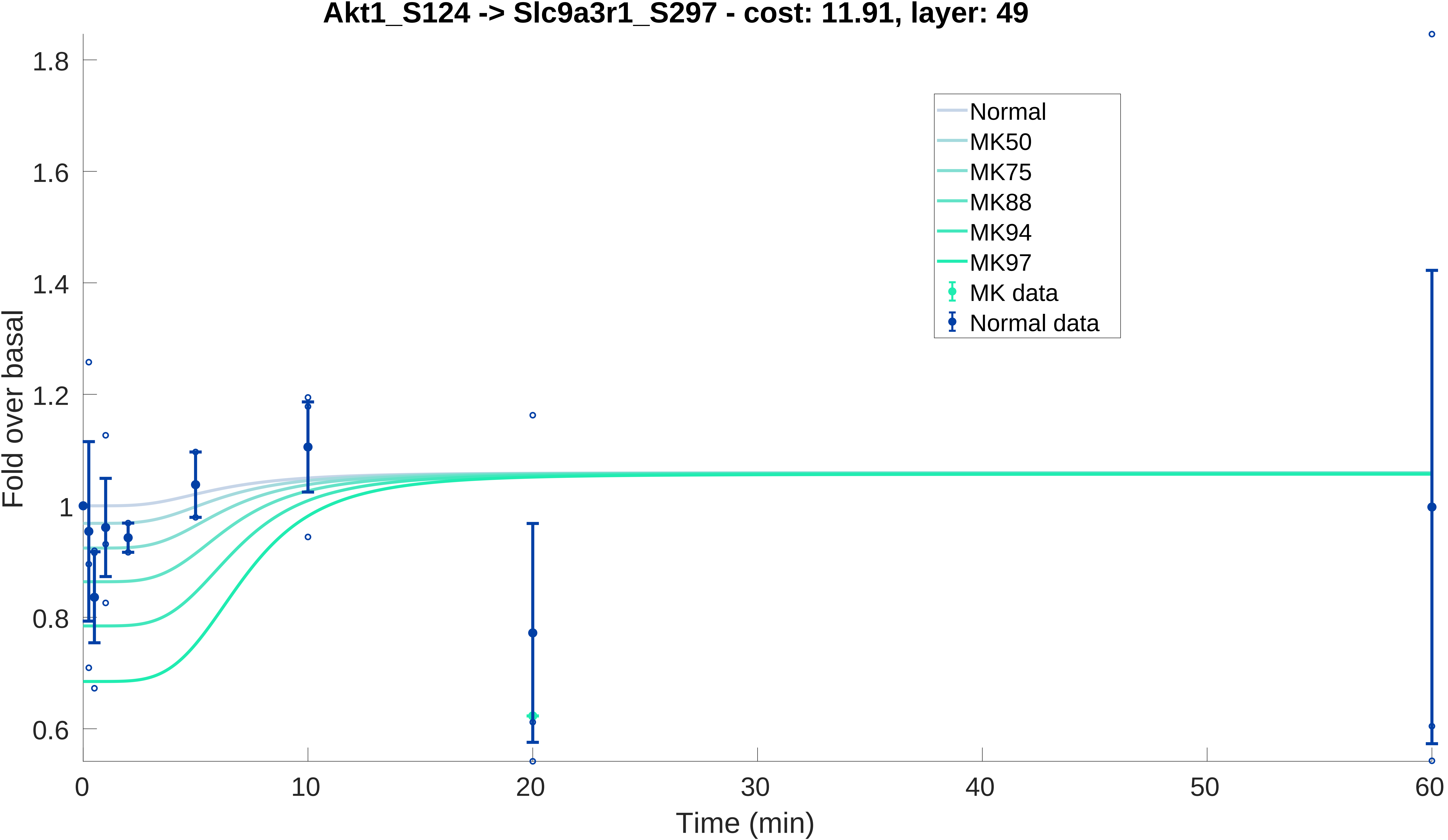

Akt1_S124 -> Slc9a3r1_S297 - cost: 11.91, layer: 49
Fold over basal
Time (min)
1.8
1.6
1.4
1.2
1
0.8
0.6
0
10
20
30
40
50
60
Normal
MK50
MK75
MK88
MK94
MK97
MK data
Normal data

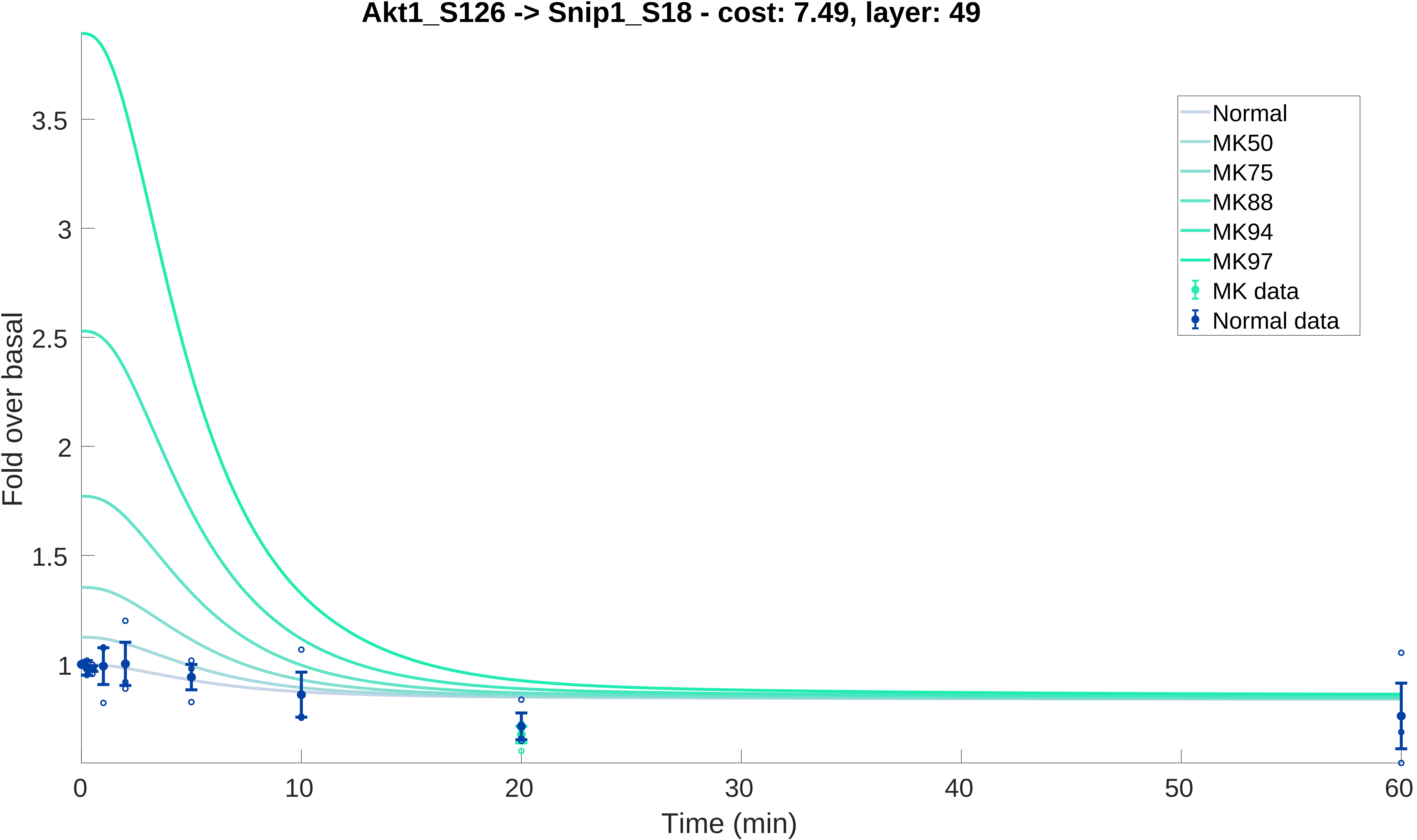
Akt1_S126 -> Snip1_S18 - cost: 7.49, layer: 49
Fold over basal
Time (min)
Normal
MK50
MK75
MK88
MK94
MK97
MK data
Normal data
3.5
3
2.5
2
1.5
1
0
10
20
30
40
50
60

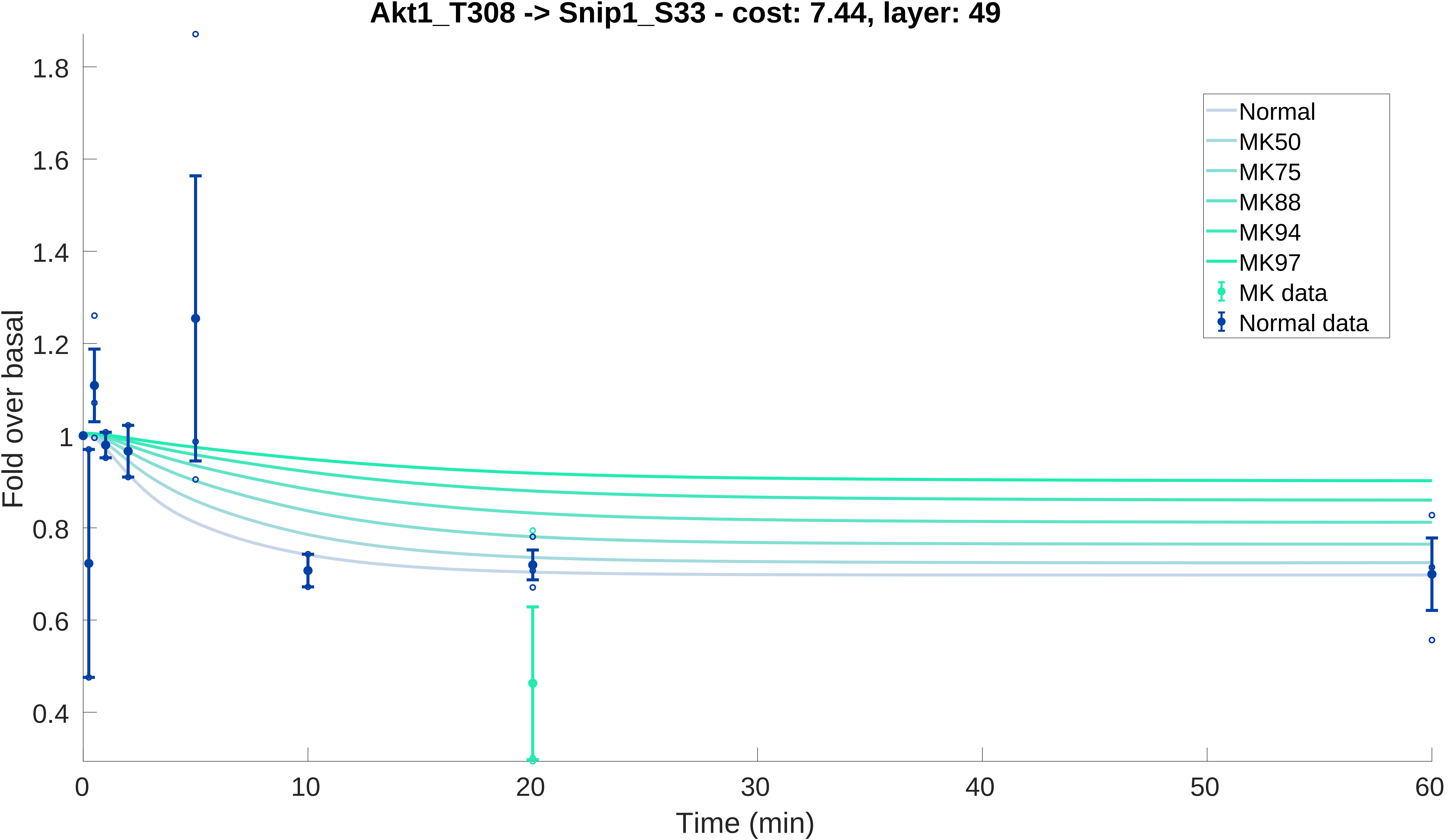

Akt1_T308 -> Snip1_S33 - cost: 7.44, layer: 49
Fold over basal
1.8
1.6
1.4
1.2
1
0.8
0.6
0.4
0
10
20
30
40
50
60
Time (min)
Normal
MK50
MK75
MK88
MK94
MK97
MK data
Normal data

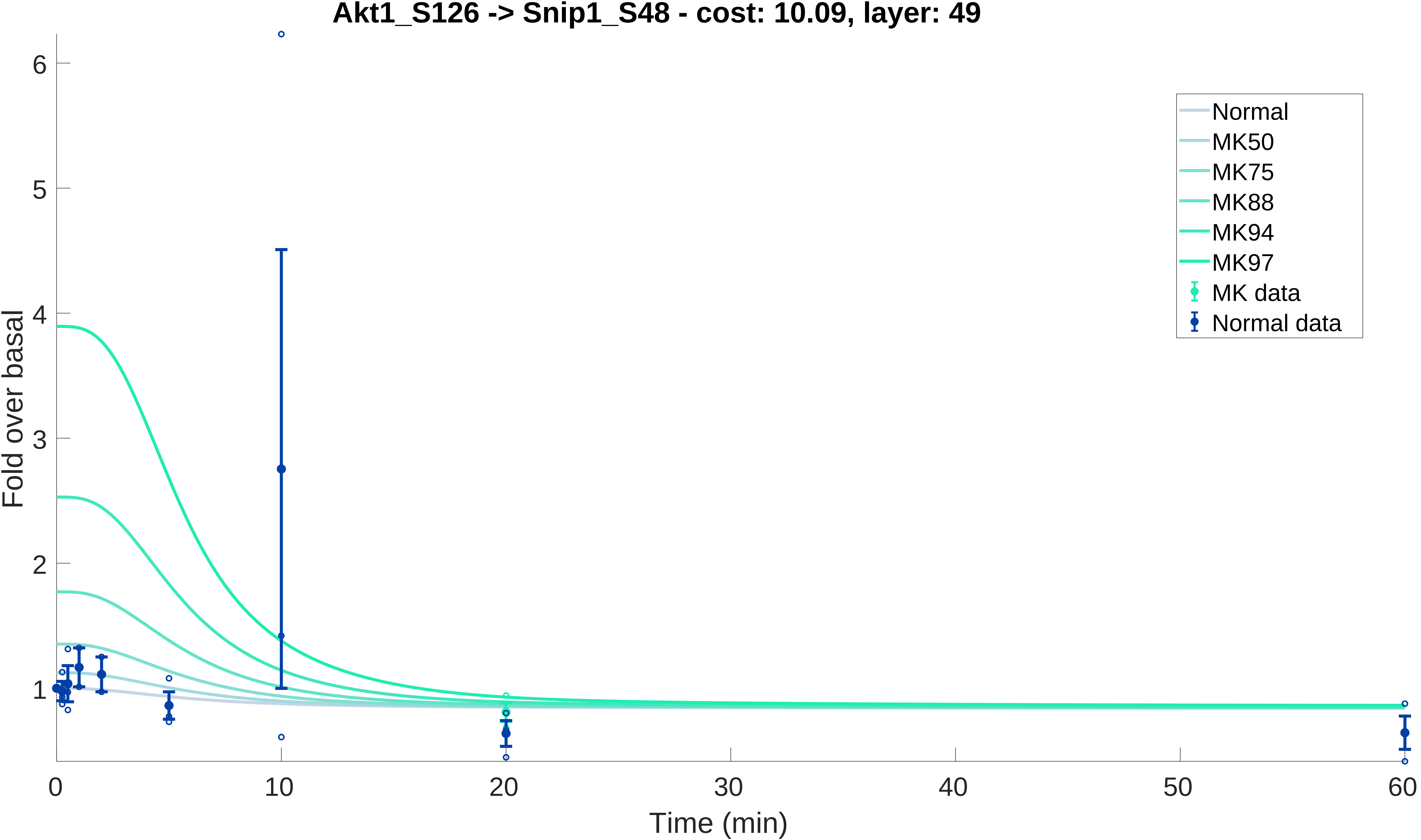
Akt1_S126 -> Snip1_S48 - cost: 10.09, layer: 49
Fold over basal
Time (min)
0
10
20
30
40
50
60
1
2
3
4
5
6
Normal
MK50
MK75
MK88
MK94
MK97
MK data
Normal data

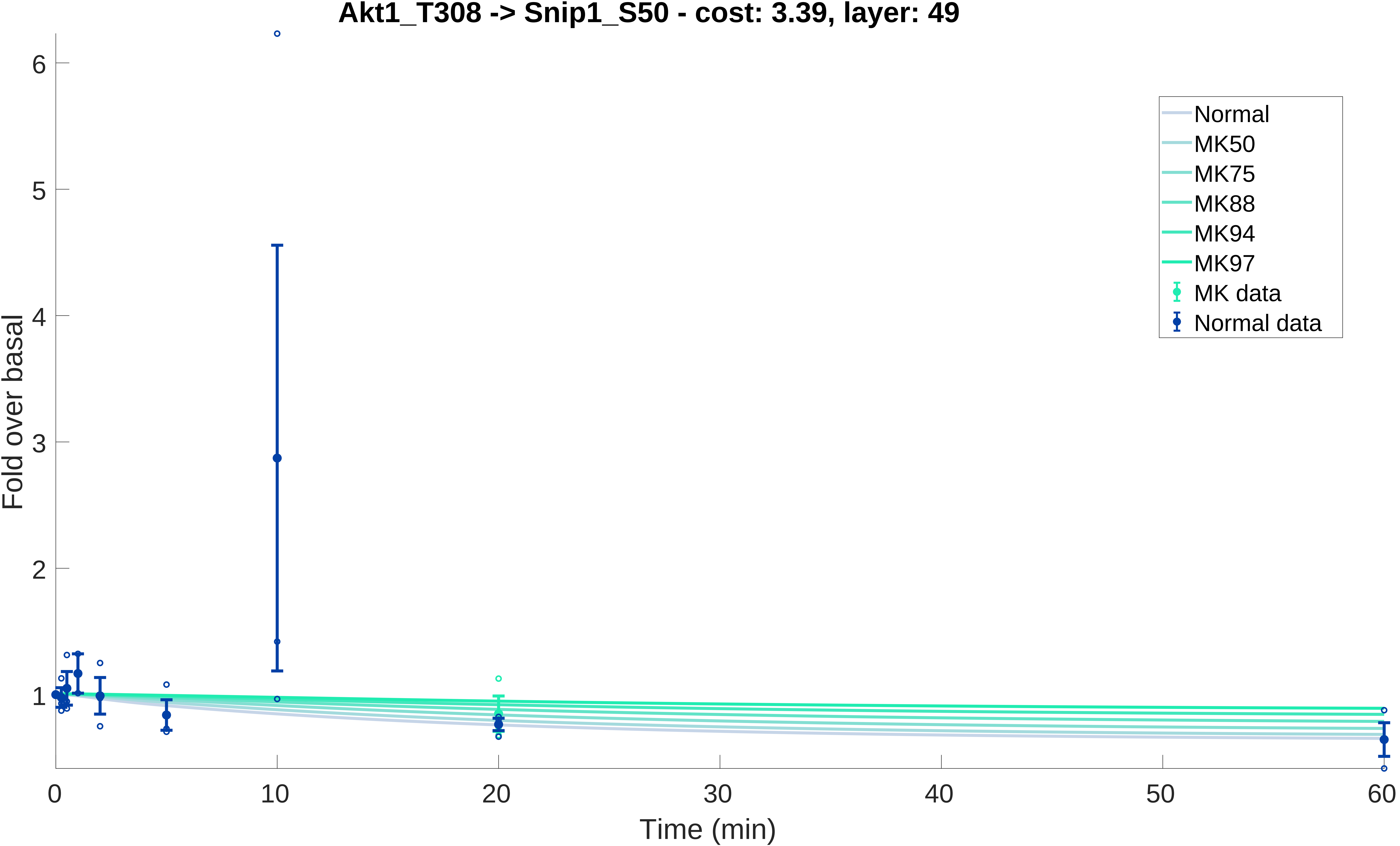

Akt1_T308 -> Snip1_S50 - cost: 3.39, layer: 49
Fold over basal
Time (min)
0
10
20
30
40
50
60
1
2
3
4
5
6
Normal
MK50
MK75
MK88
MK94
MK97
MK data
Normal data

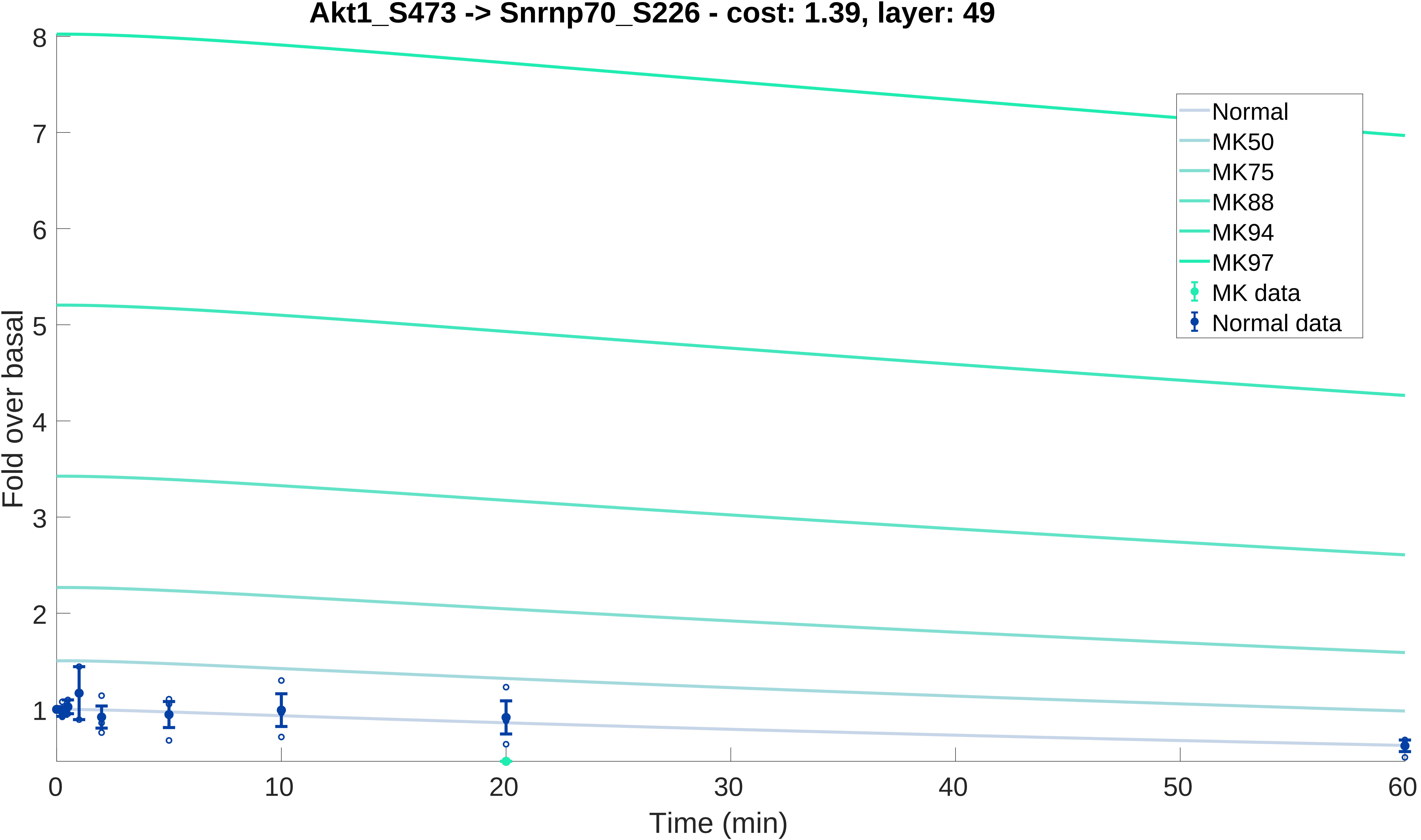

Akt1_S473 -> Snrnp70_S226 - cost: 1.39, layer: 49
Fold over basal
Time (min)
0
10
20
30
40
50
60
1
2
3
4
5
6
7
8
Normal
MK50
MK75
MK88
MK94
MK97
MK data
Normal data

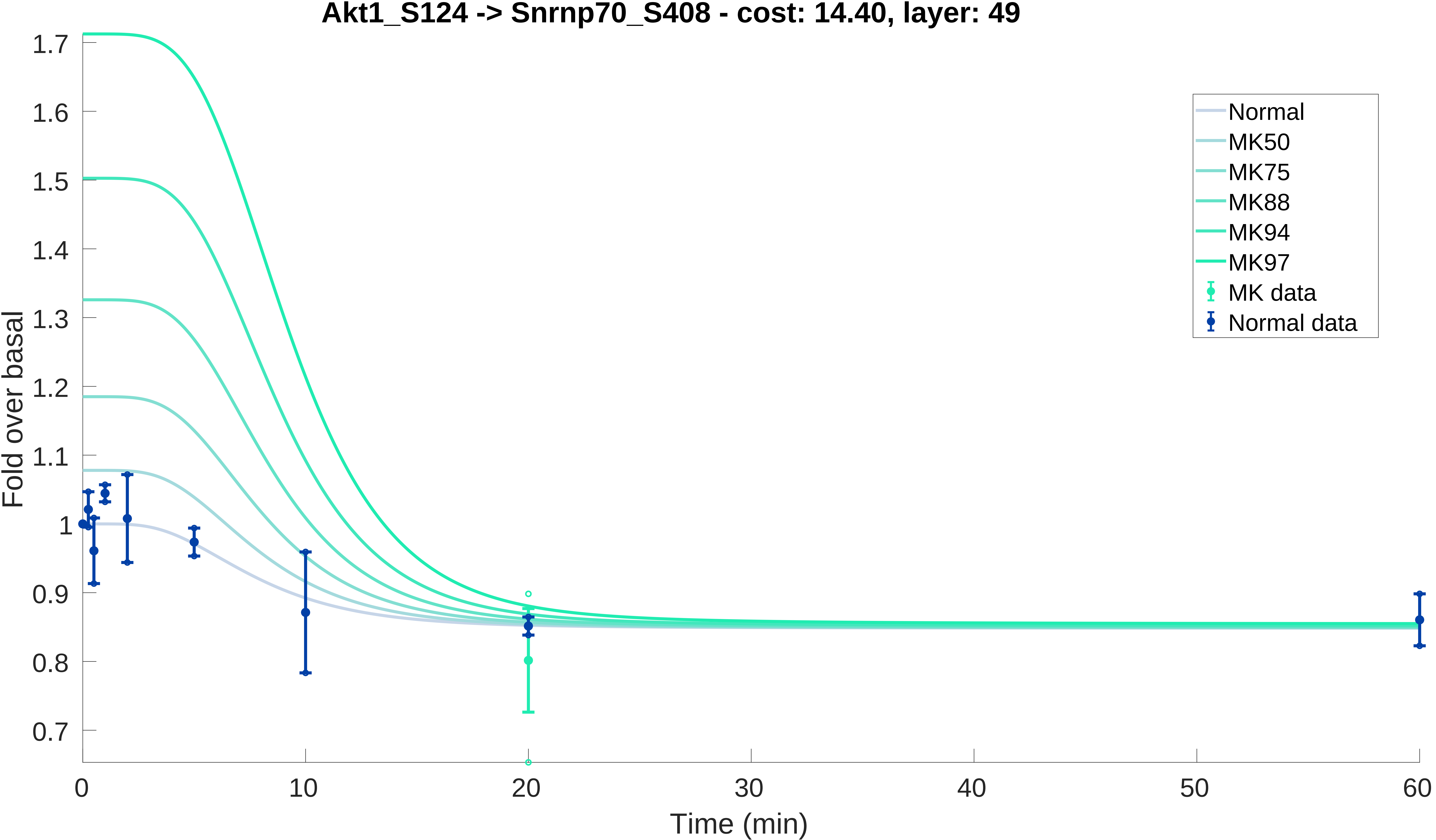

Akt1_S124 -> Snrnp70_S408 - cost: 14.40, layer: 49
Fold over basal
1.7
1.6
1.5
1.4
1.3
1.2
1.1
1
0.9
0.8
0.7
0
10
20
30
40
50
60
Time (min)
Normal
MK50
MK75
MK88
MK94
MK97
MK data
Normal data

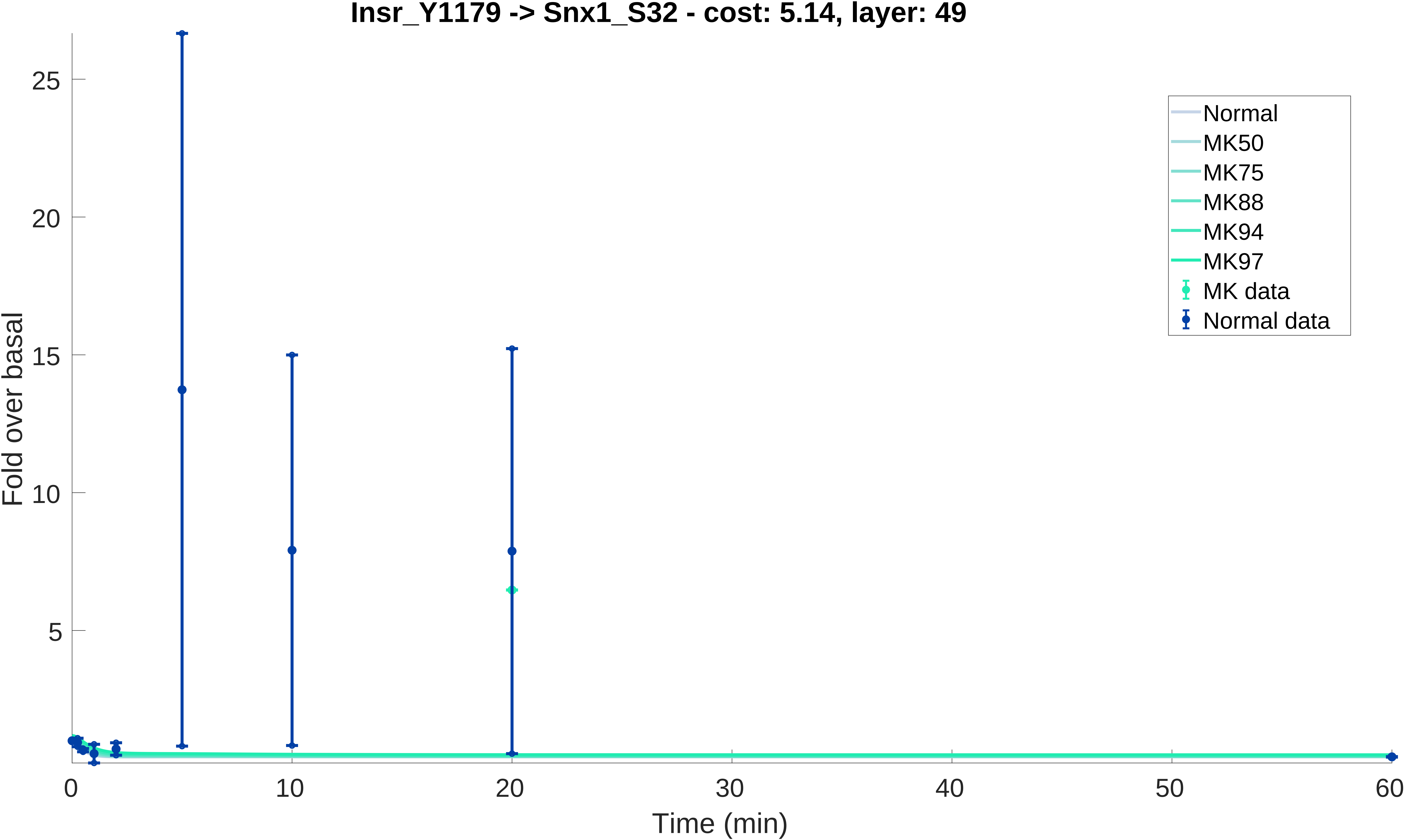

Insr_Y1179 -> Snx1_S32 - cost: 5.14, layer: 49
Fold over basal
Time (min)
0
10
20
30
40
50
60
5
10
15
20
25
Normal
MK50
MK75
MK88
MK94
MK97
MK data
Normal data

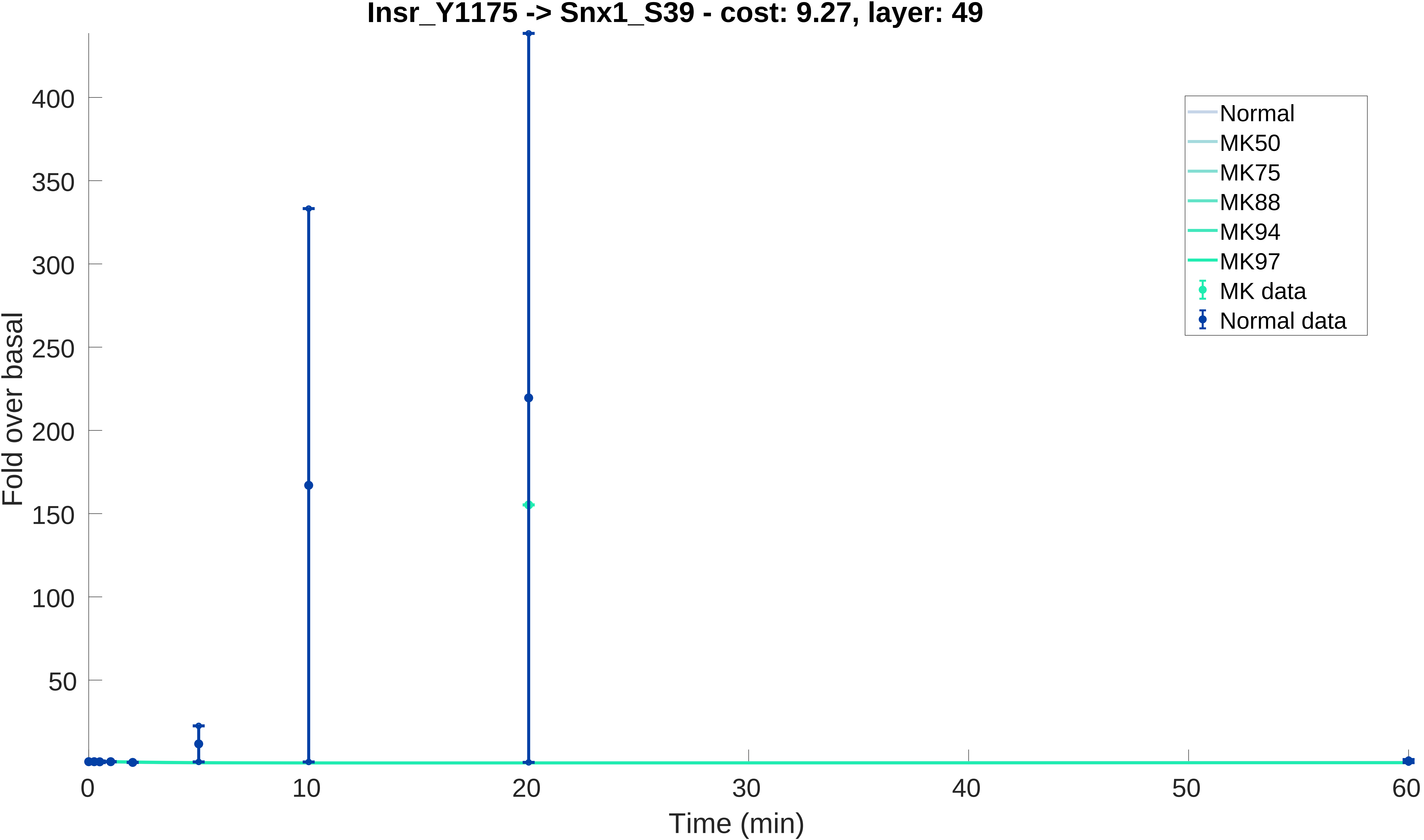
Insr_Y1175 -> Snx1_S39 - cost: 9.27, layer: 49
Fold over basal
Time (min)
400
350
300
250
200
150
100
50
0
10
20
30
40
50
60
Normal
MK50
MK75
MK88
MK94
MK97
MK data
Normal data

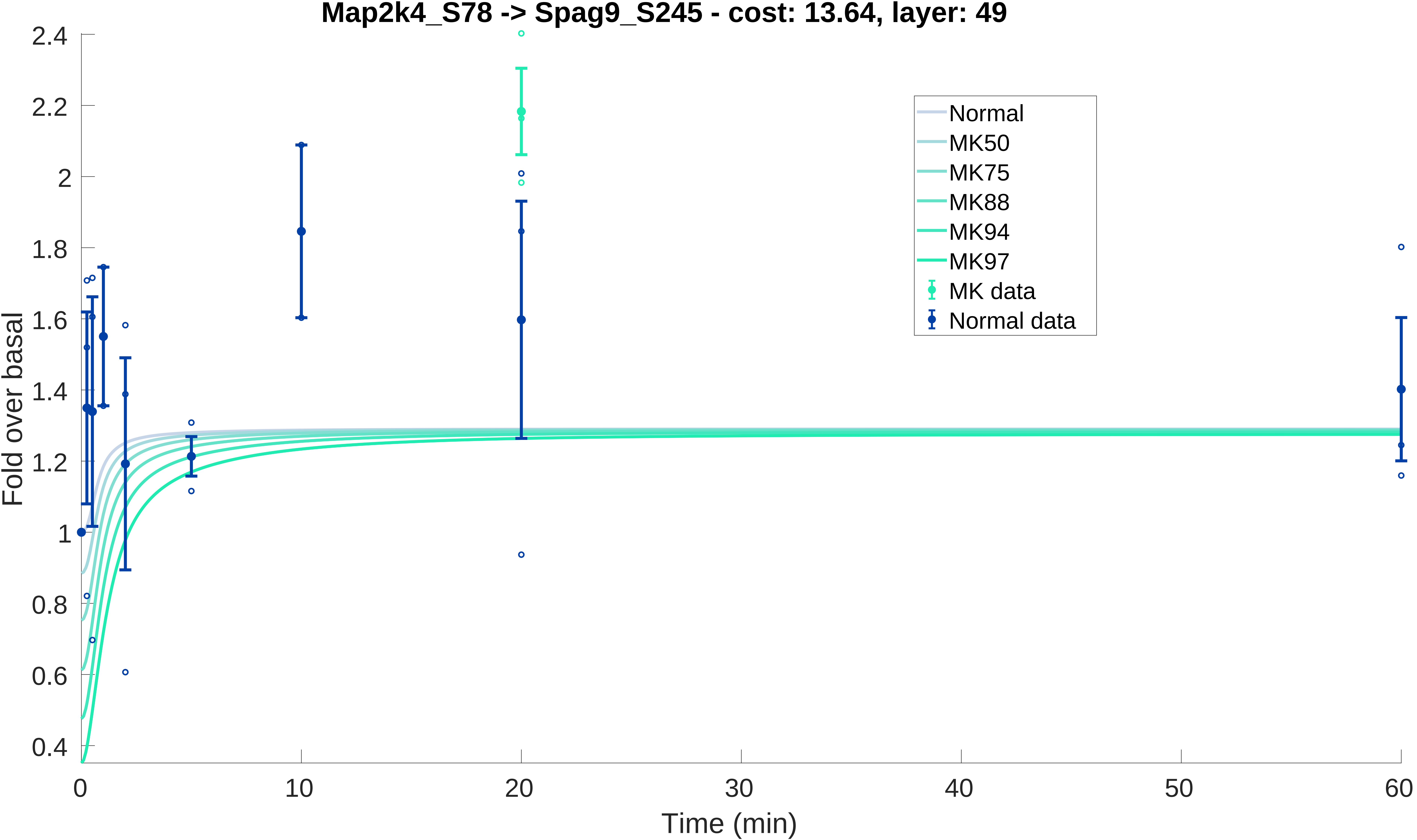

Map2k4_S78 -> Spag9_S245 - cost: 13.64, layer: 49
Fold over basal
Time (min)
Normal
MK50
MK75
MK88
MK94
MK97
MK data
Normal data
0.4
0.6
0.8
1
1.2
1.4
1.6
1.8
2
2.2
2.4
0
10
20
30
40
50
60

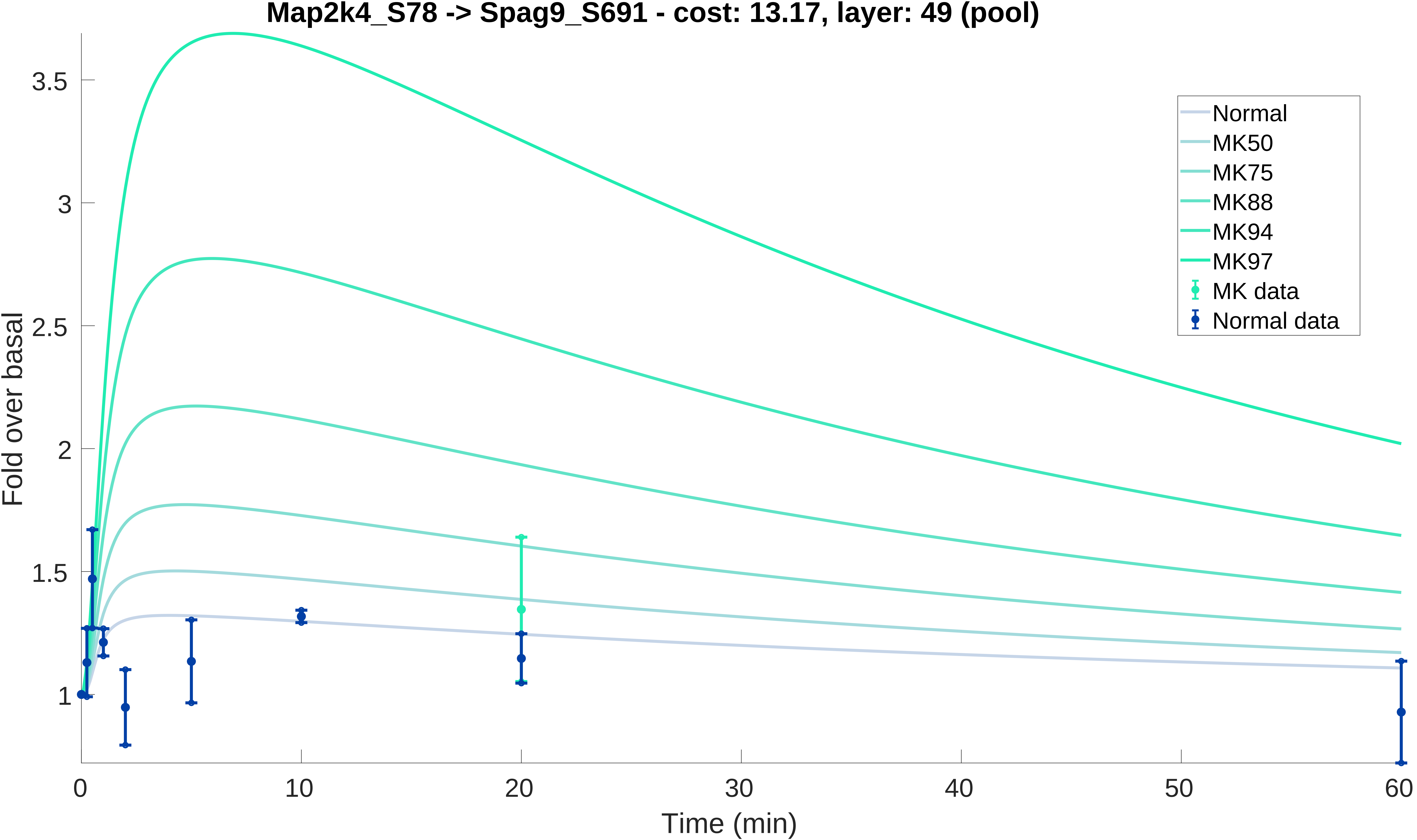

Map2k4_S78 -> Spag9_S691 - cost: 13.17, layer: 49 (pool)
Fold over basal
Time (min)
3.5
3
2.5
2
1.5
1
0
10
20
30
40
50
60
Normal
MK50
MK75
MK88
MK94
MK97
MK data
Normal data

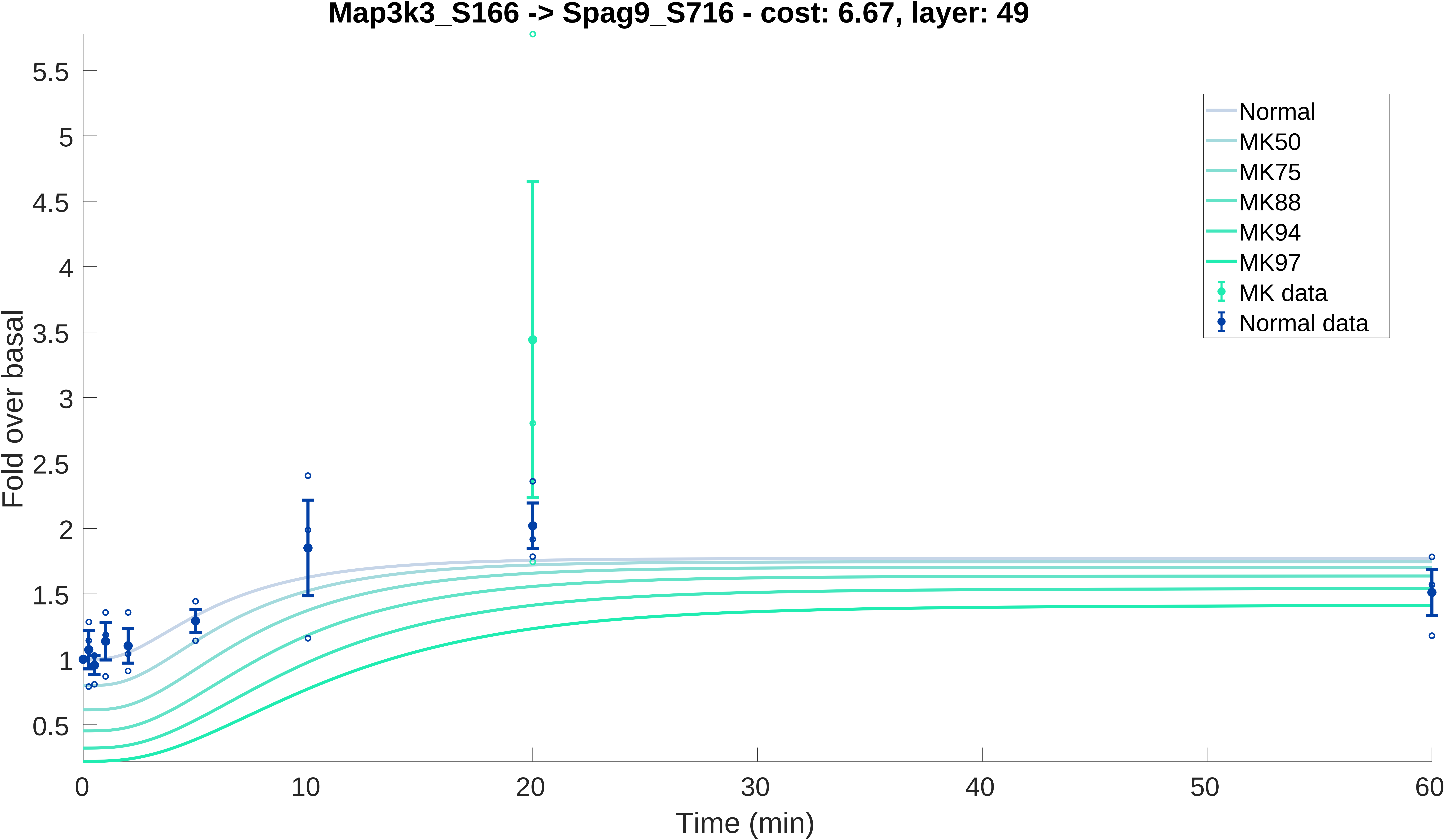

Map3k3_S166 -> Spag9_S716 - cost: 6.67, layer: 49
Fold over basal
Time (min)
0.5
1
1.5
2
2.5
3
3.5
4
4.5
5
5.5
0
10
20
30
40
50
60
Normal
MK50
MK75
MK88
MK94
MK97
MK data
Normal data

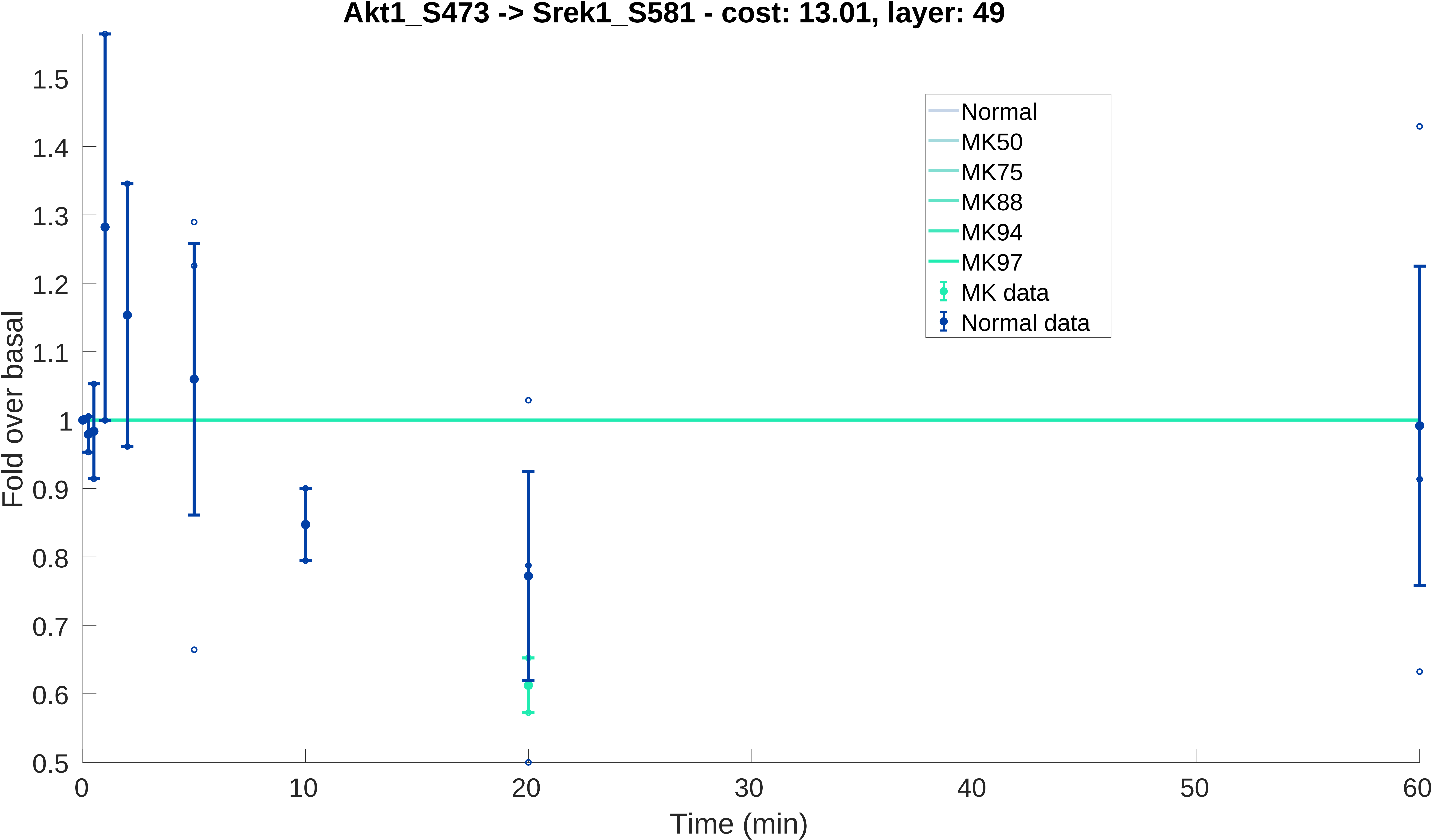

Akt1_S473 -> Srek1_S581 - cost: 13.01, layer: 49
Fold over basal
1.5
1.4
1.3
1.2
1.1
1
0.9
0.8
0.7
0.6
0.5
0
10
20
30
40
50
60
Time (min)
Normal
MK50
MK75
MK88
MK94
MK97
MK data
Normal data

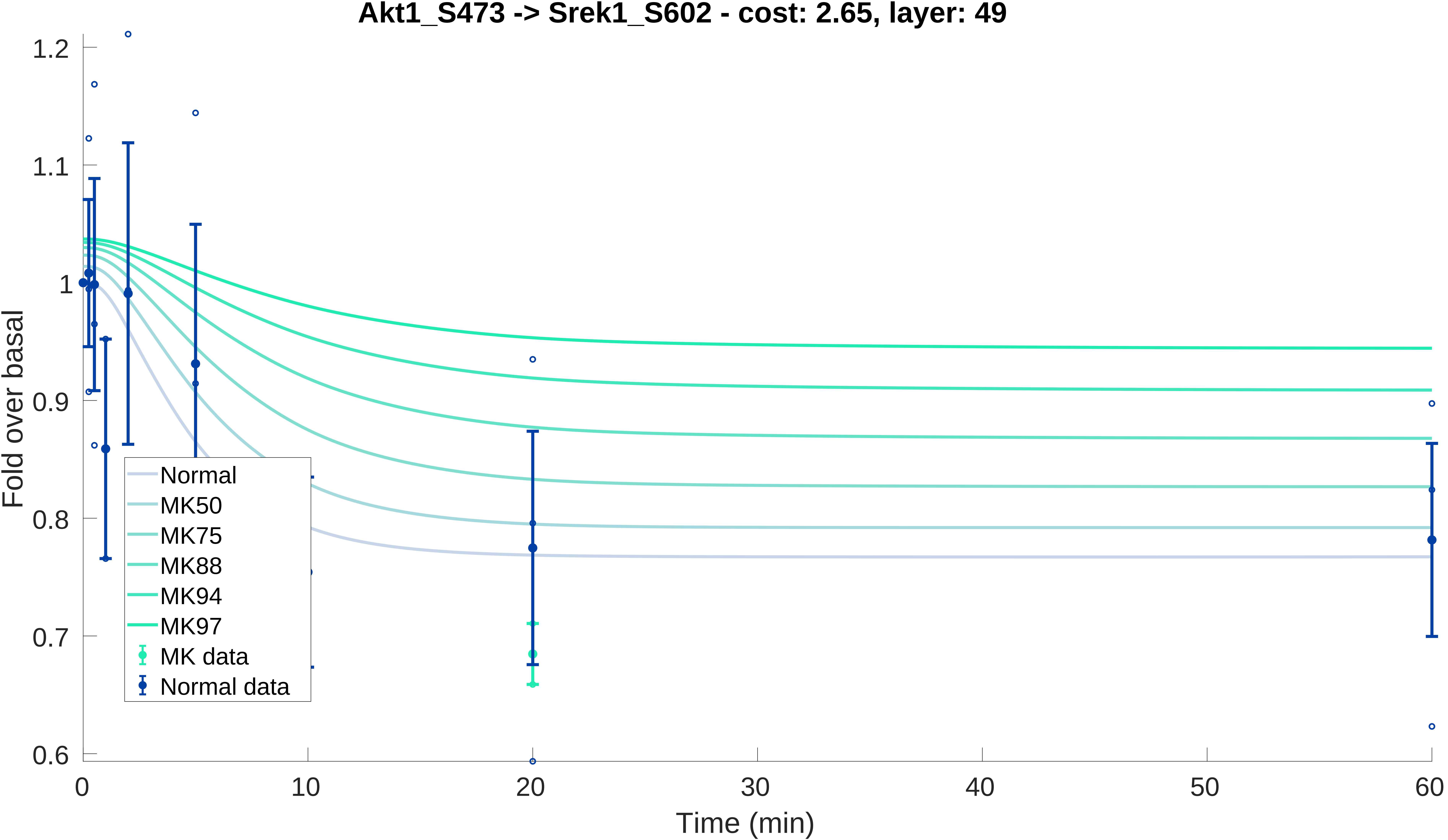
Akt1_S473 -> Srek1_S602 - cost: 2.65, layer: 49
Fold over basal
Time (min)
1.2
1.1
1
0.9
0.8
0.7
0.6
0
10
20
30
40
50
60
Normal
MK50
MK75
MK88
MK94
MK97
MK data
Normal data

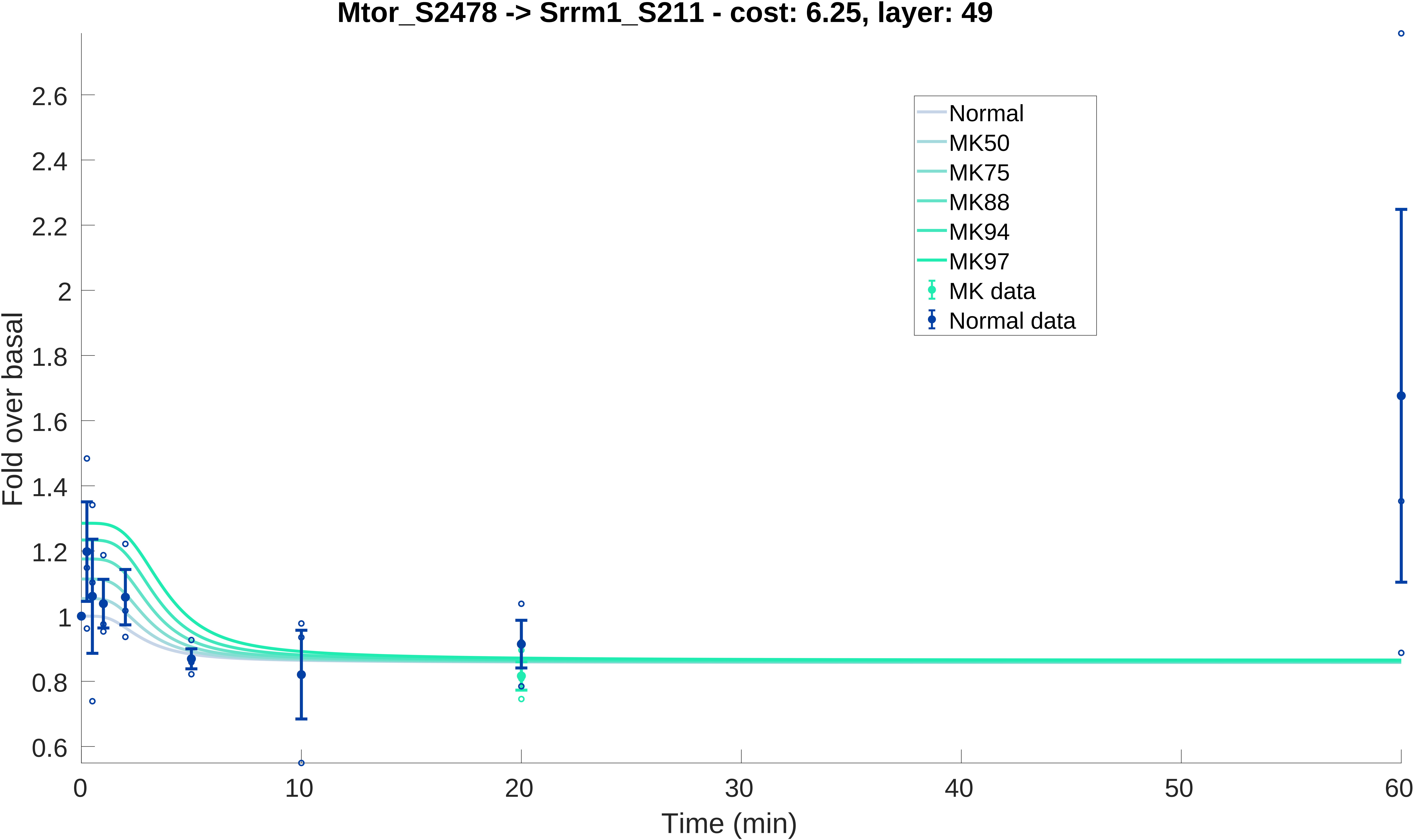

Mtor_S2478 -> Srrm1_S211 - cost: 6.25, layer: 49
Fold over basal
Time (min)
Normal
MK50
MK75
MK88
MK94
MK97
MK data
Normal data
0.6
0.8
1
1.2
1.4
1.6
1.8
2
2.2
2.4
2.6
0
10
20
30
40
50
60

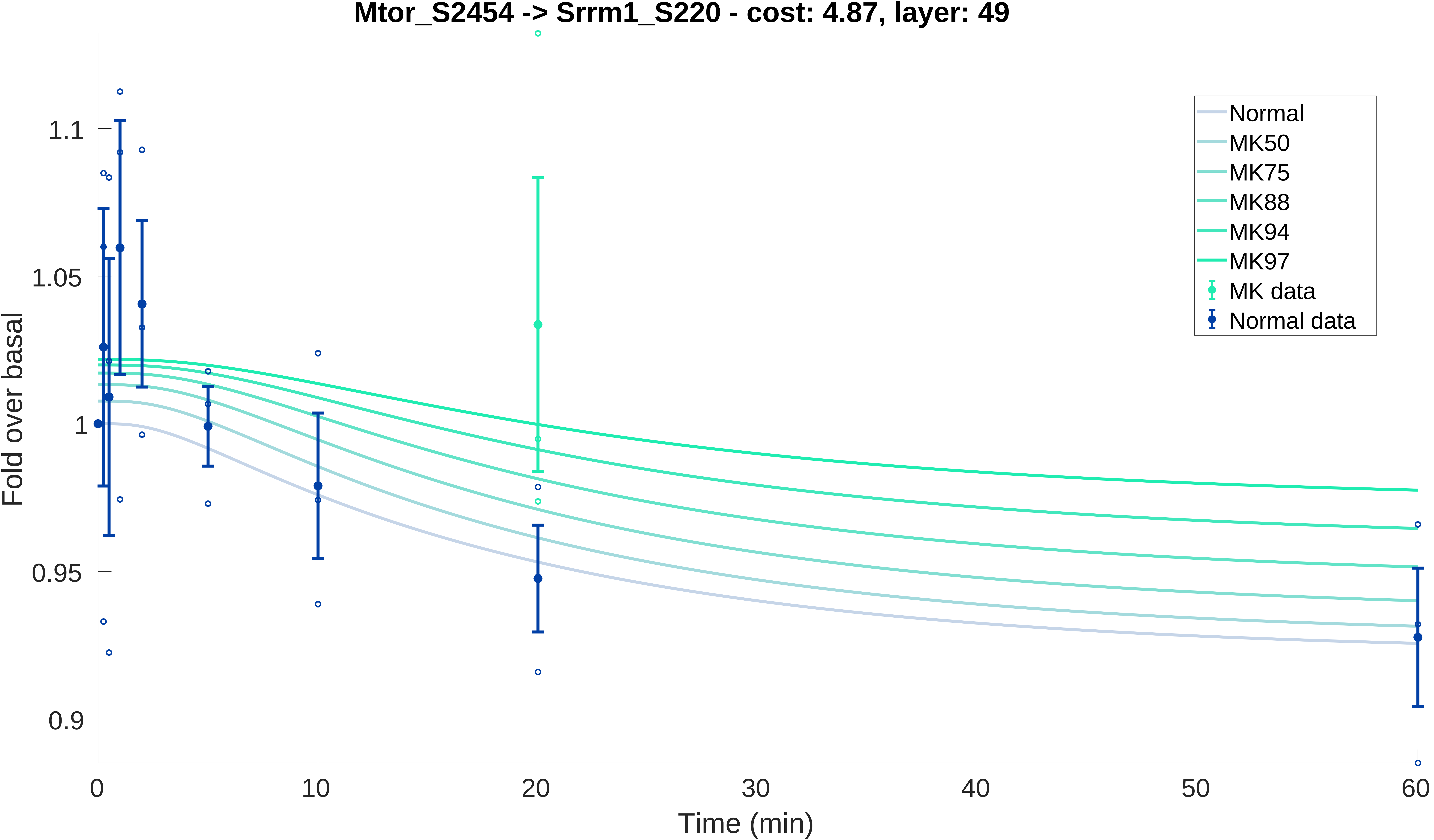

Mtor_S2454 -> Srrm1_S220 - cost: 4.87, layer: 49
Fold over basal
1.1
1.05
1
0.95
0.9
Time (min)
0
10
20
30
40
50
60
Normal
MK50
MK75
MK88
MK94
MK97
MK data
Normal data

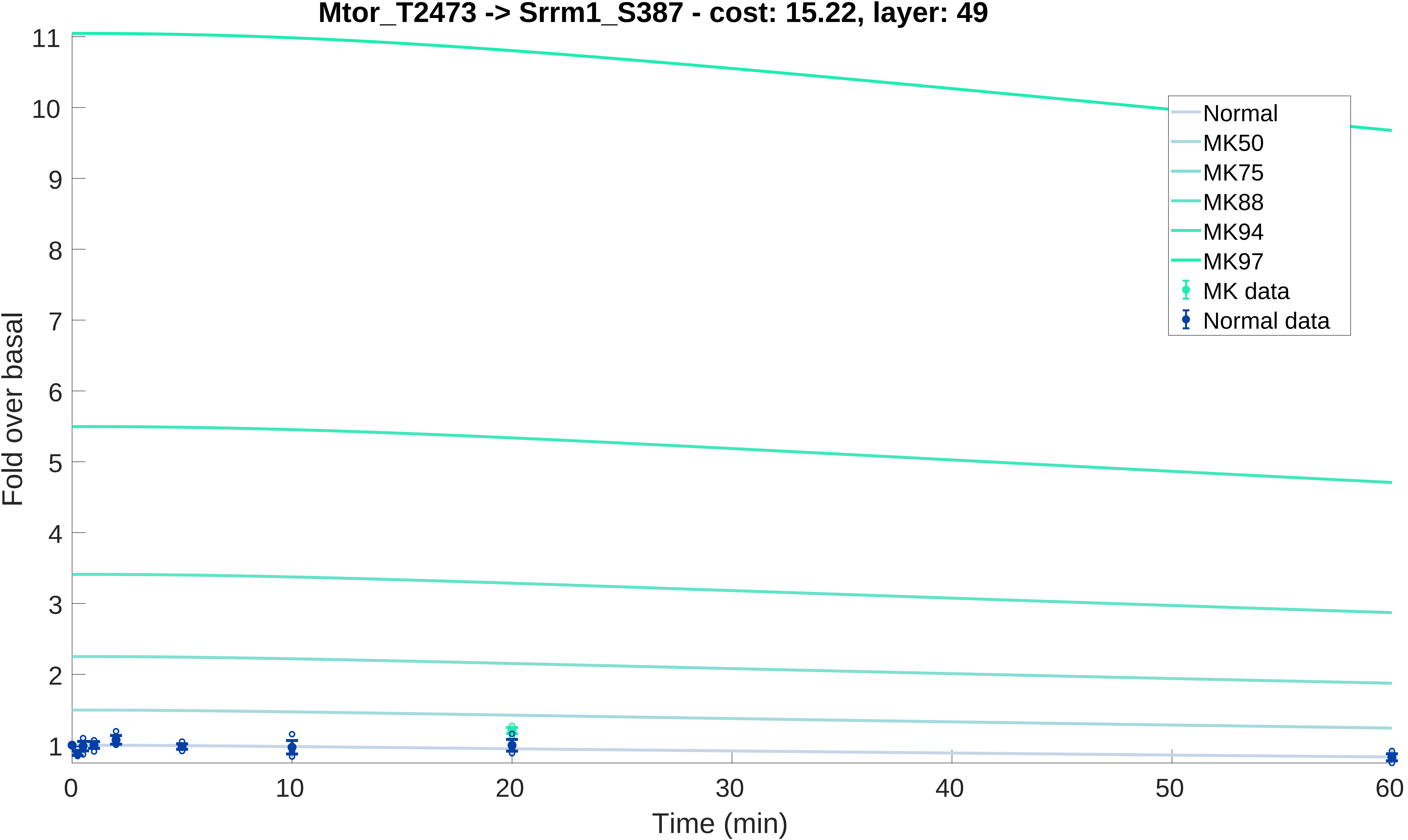

Mtor_T2473 -> Srrm1_S387 - cost: 15.22, layer: 49
Fold over basal
Time (min)
Normal
MK50
MK75
MK88
MK94
MK97
MK data
Normal data
0
10
20
30
40
50
60
1
2
3
4
5
6
7
8
9
10
11

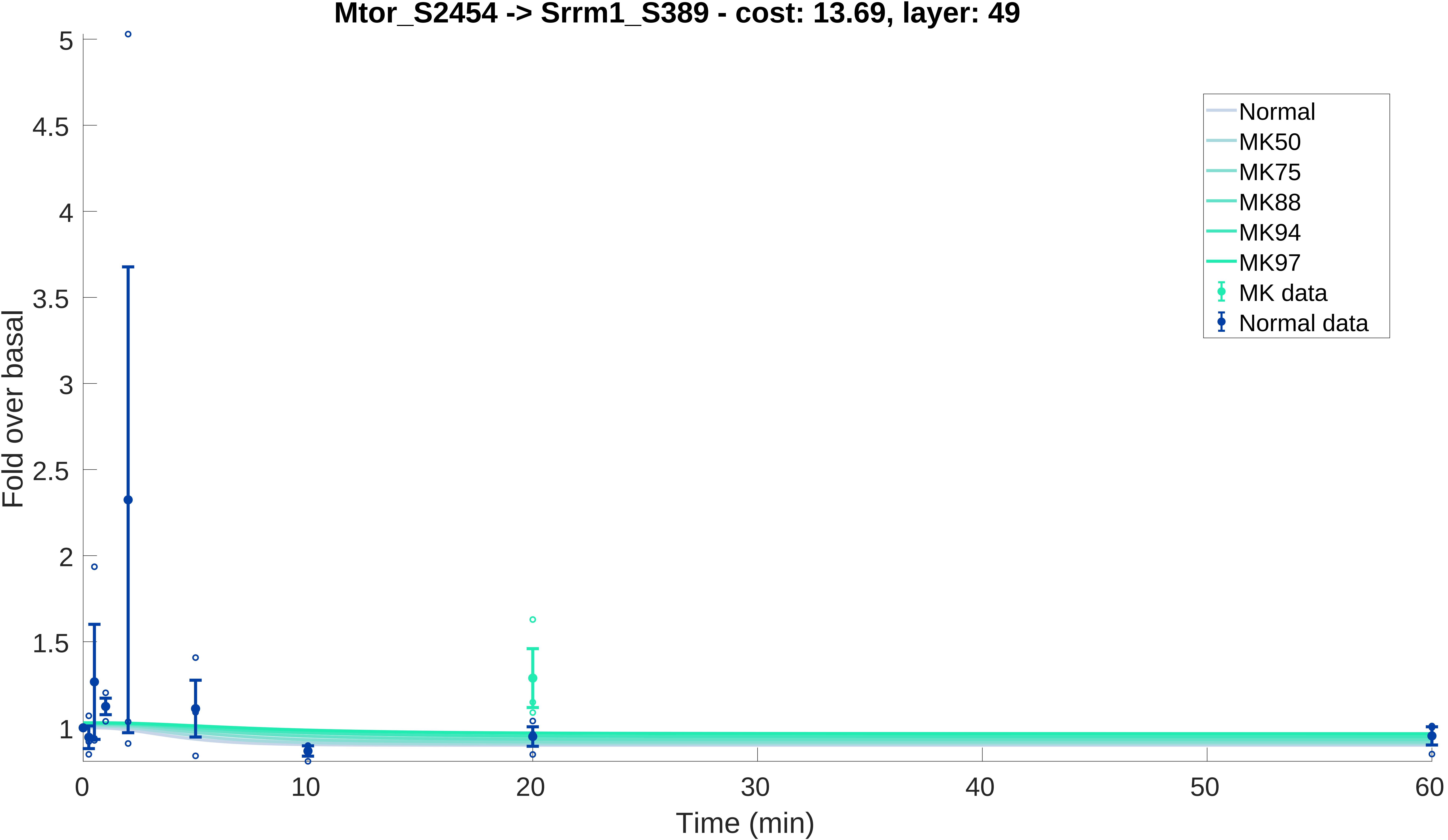

Mtor_S2454 -> Srrm1_S389 - cost: 13.69, layer: 49
Fold over basal
Time (min)
5
4.5
4
3.5
3
2.5
2
1.5
1
0
10
20
30
40
50
60
Normal
MK50
MK75
MK88
MK94
MK97
MK data
Normal data

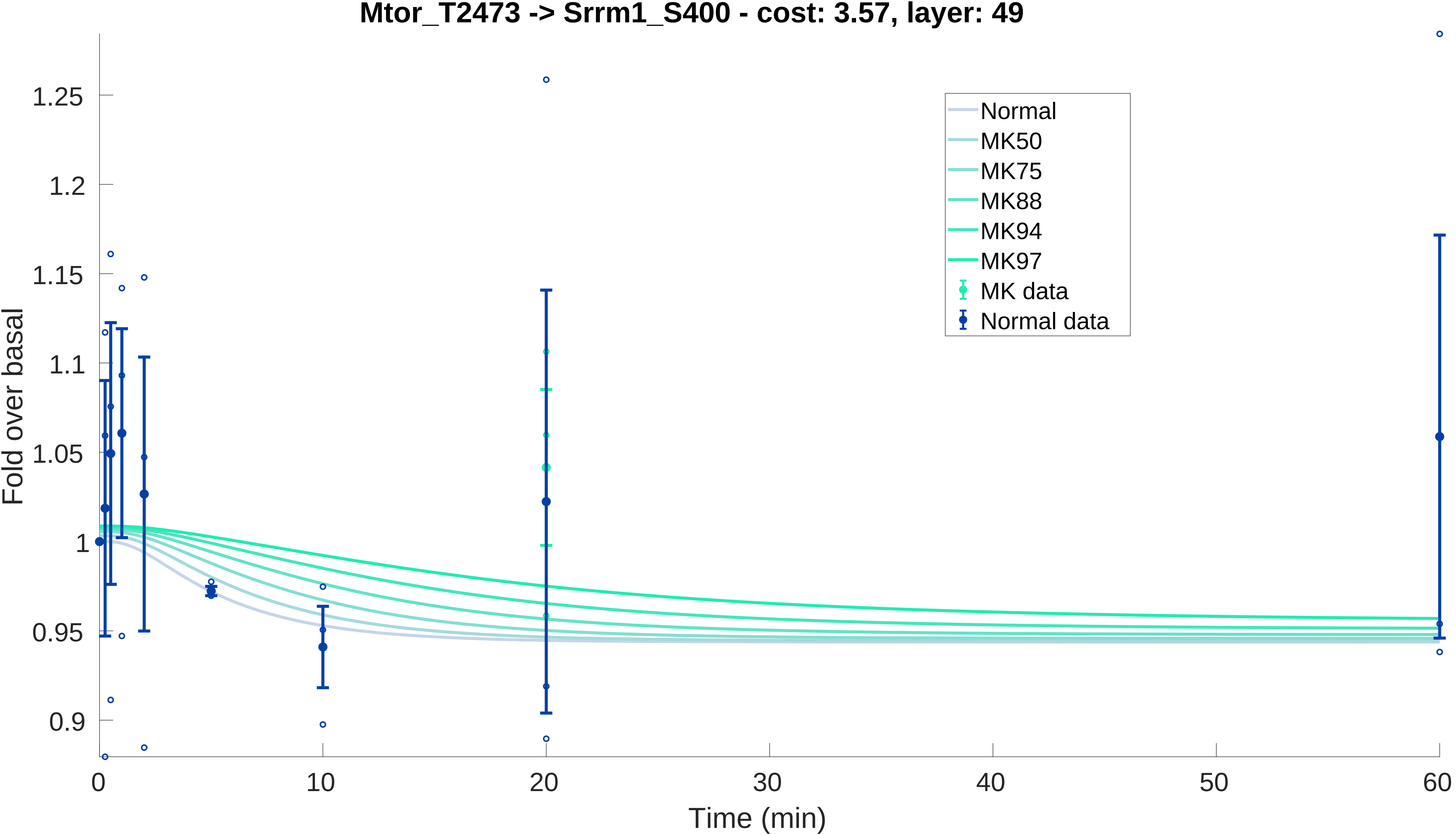

Mtor_T2473 -> Srrm1_S400 - cost: 3.57, layer: 49
Normal
MK50
MK75
MK88
MK94
MK97
MK data
Normal data
Fold over basal
1.25
1.2
1.15
1.1
1.05
1
0.95
0.9
0
10
20
30
40
50
60
Time (min)

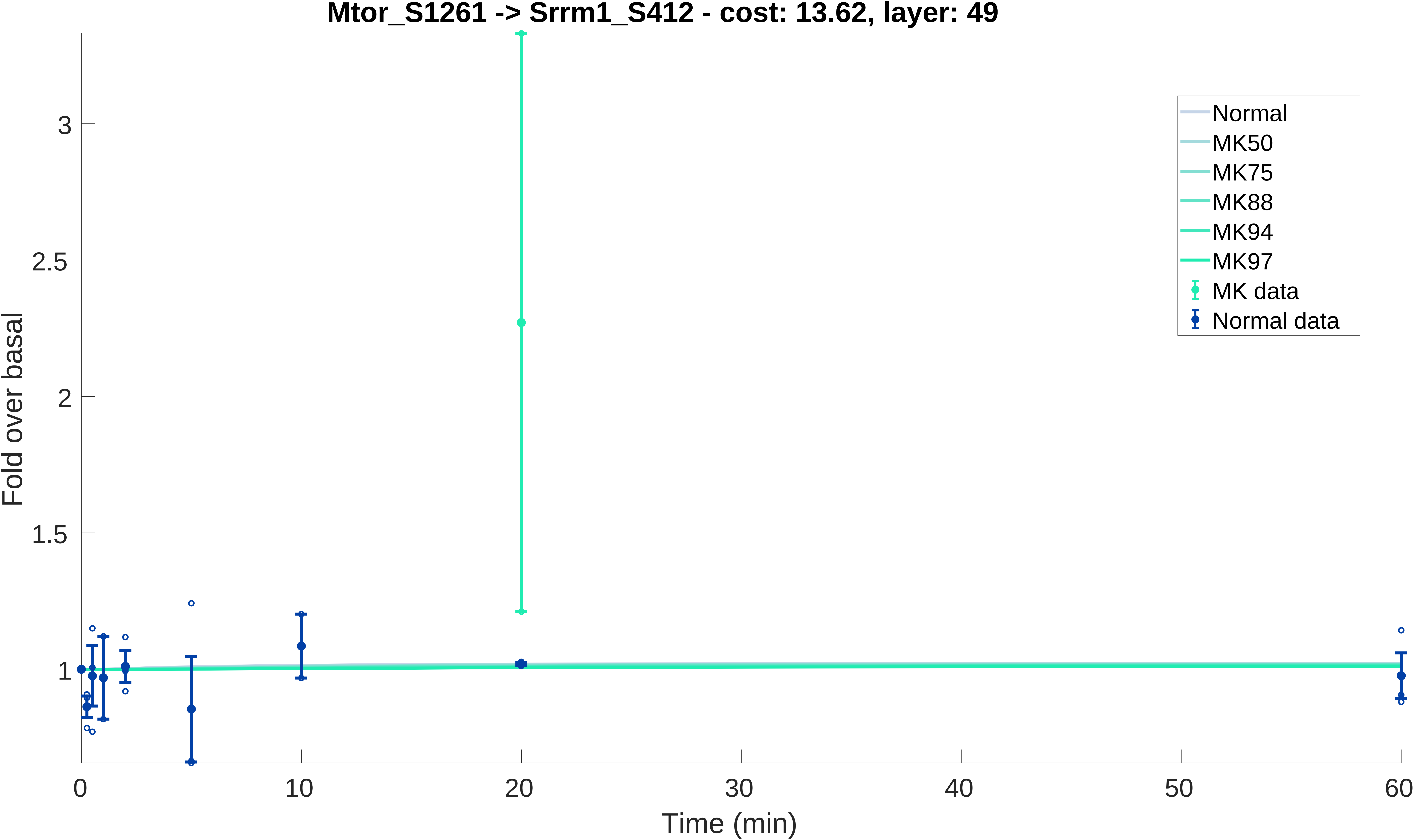

Mtor_S1261 -> Srrm1_S412 - cost: 13.62, layer: 49
Fold over basal
Time (min)
3
2.5
2
1.5
1
0
10
20
30
40
50
60
Normal
MK50
MK75
MK88
MK94
MK97
MK data
Normal data

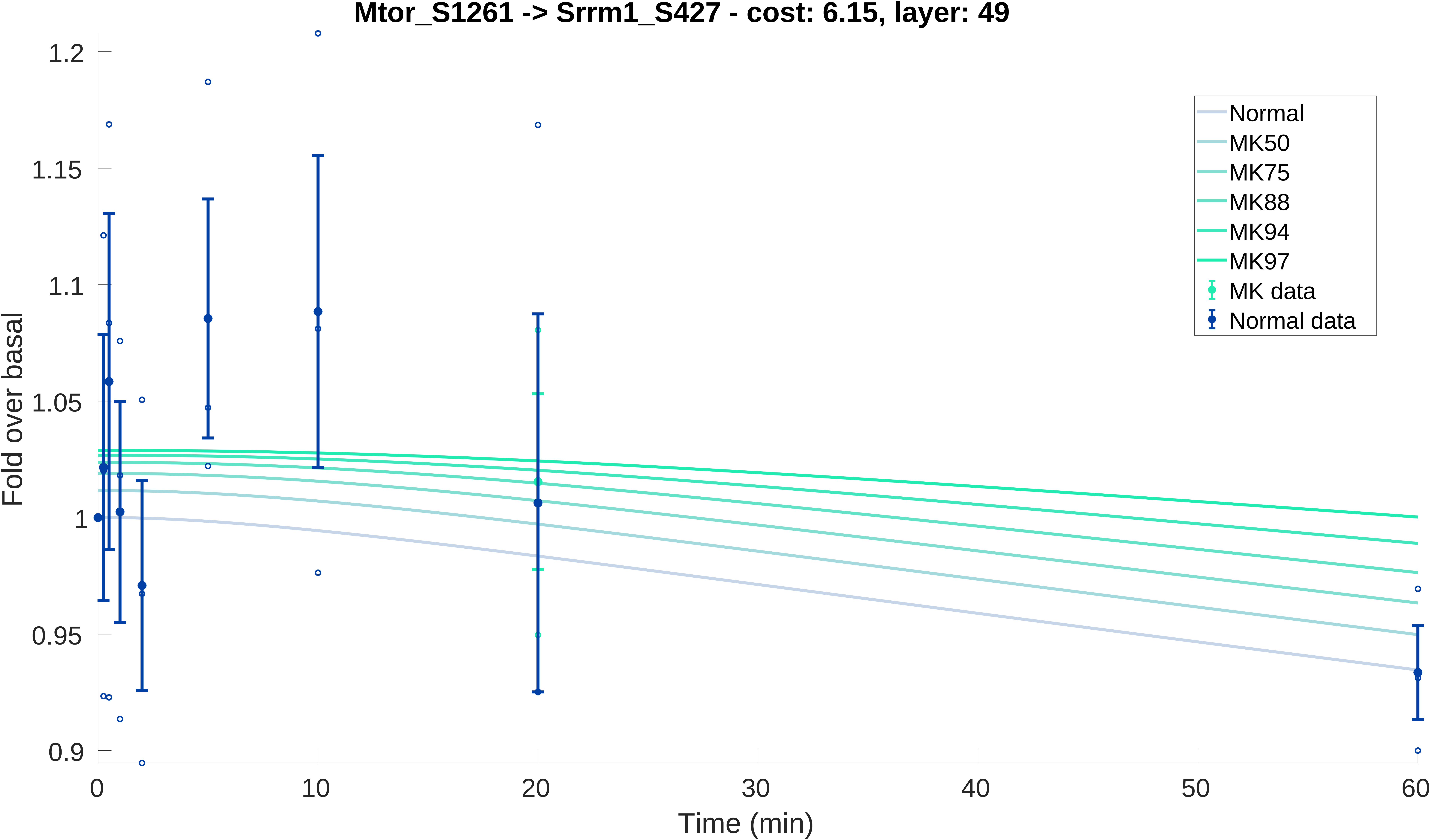
Mtor_S1261 -> Srrm1_S427 - cost: 6.15, layer: 49
Fold over basal
Time (min)
Normal
MK50
MK75
MK88
MK94
MK97
MK data
Normal data
1.2
1.15
1.1
1.05
1
0.95
0.9
0
10
20
30
40
50
60

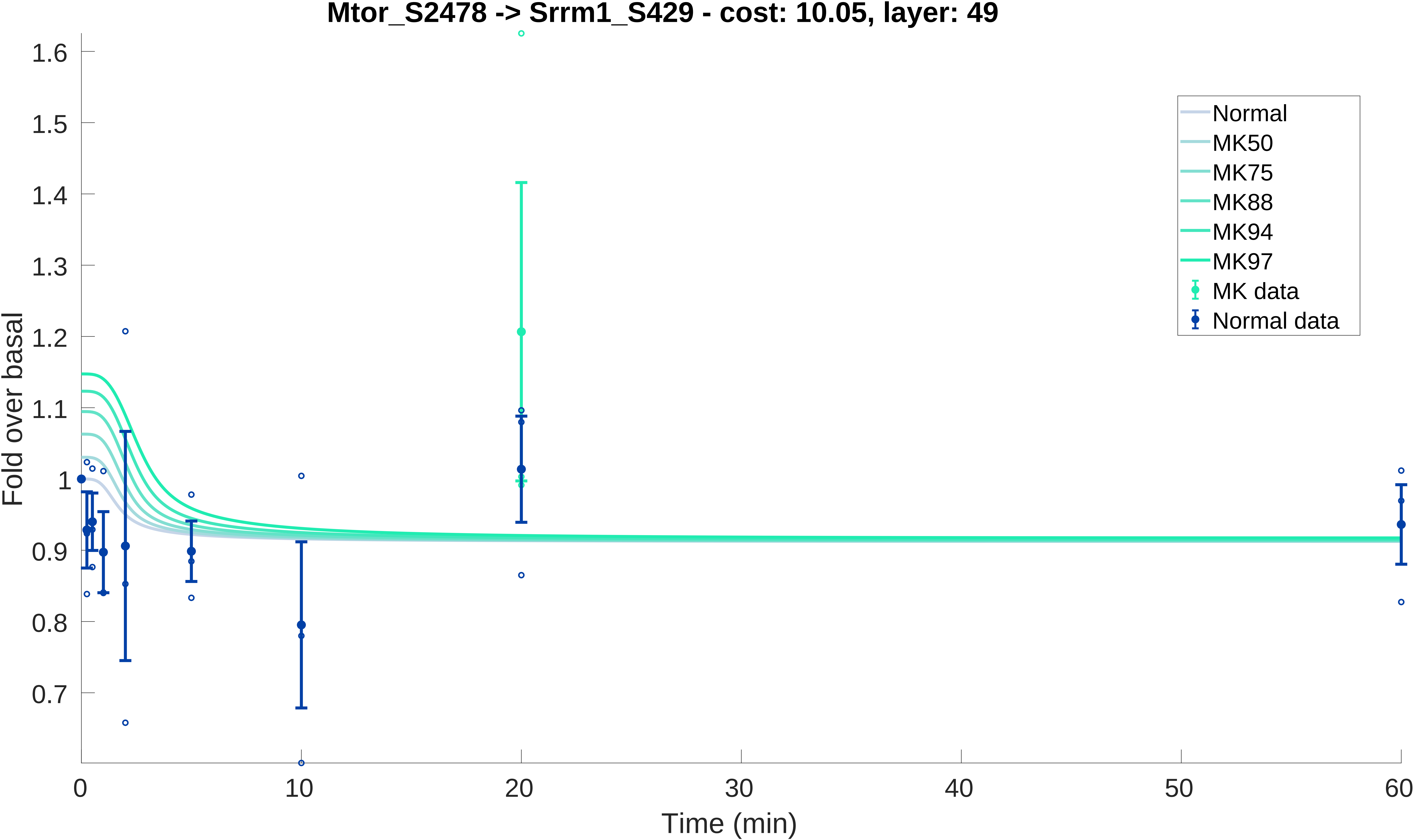
Mtor_S2478 -> Srrm1_S429 - cost: 10.05, layer: 49
Fold over basal
1.6
1.5
1.4
1.3
1.2
1.1
1
0.9
0.8
0.7
0
10
20
30
40
50
60
Time (min)
Normal
MK50
MK75
MK88
MK94
MK97
MK data
Normal data

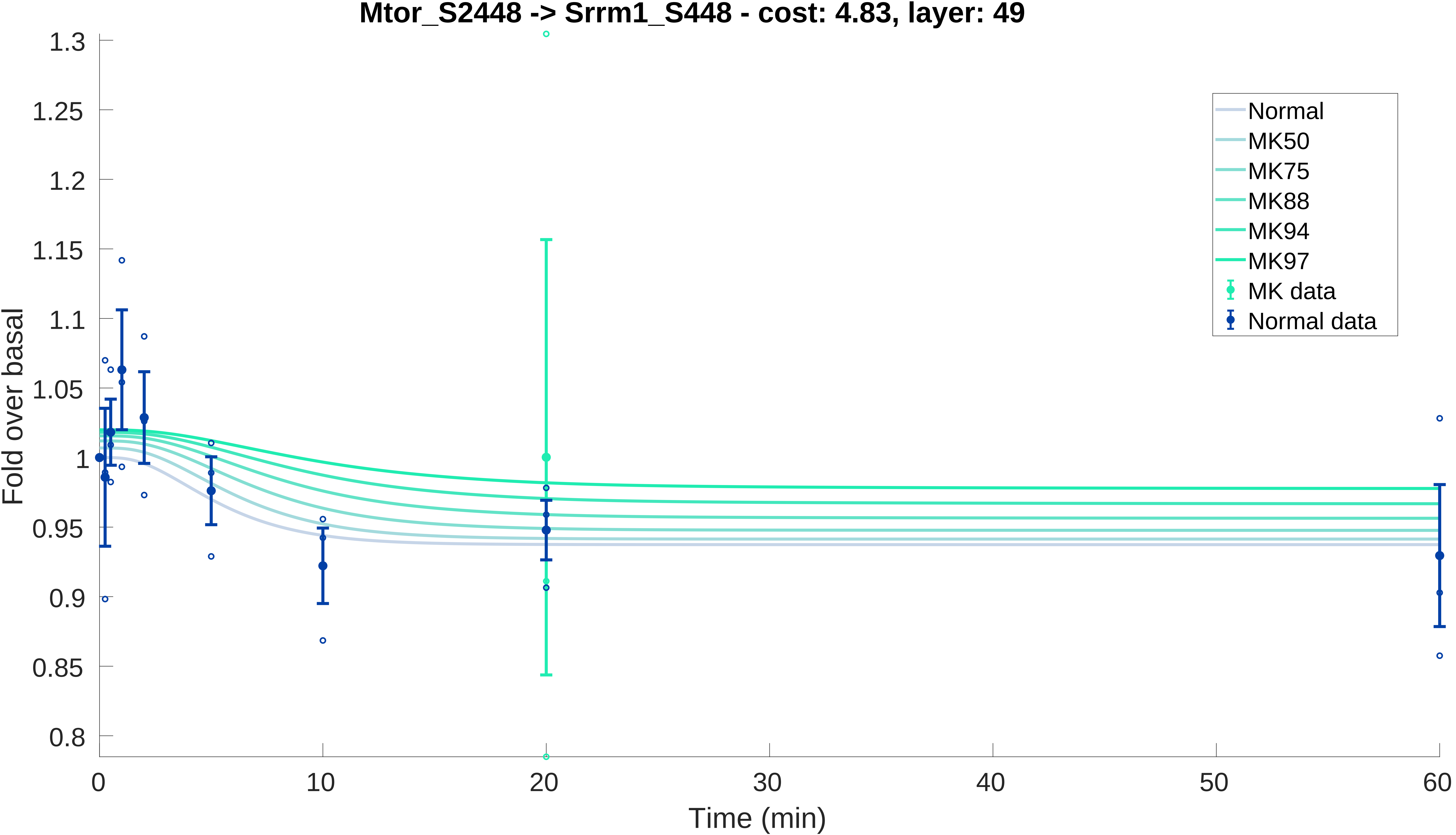

Mtor_S2448 -> Srrm1_S448 - cost: 4.83, layer: 49
Fold over basal
Time (min)
1.3
1.25
1.2
1.15
1.1
1.05
1
0.95
0.9
0.85
0.8
0
10
20
30
40
50
60
Normal
MK50
MK75
MK88
MK94
MK97
MK data
Normal data

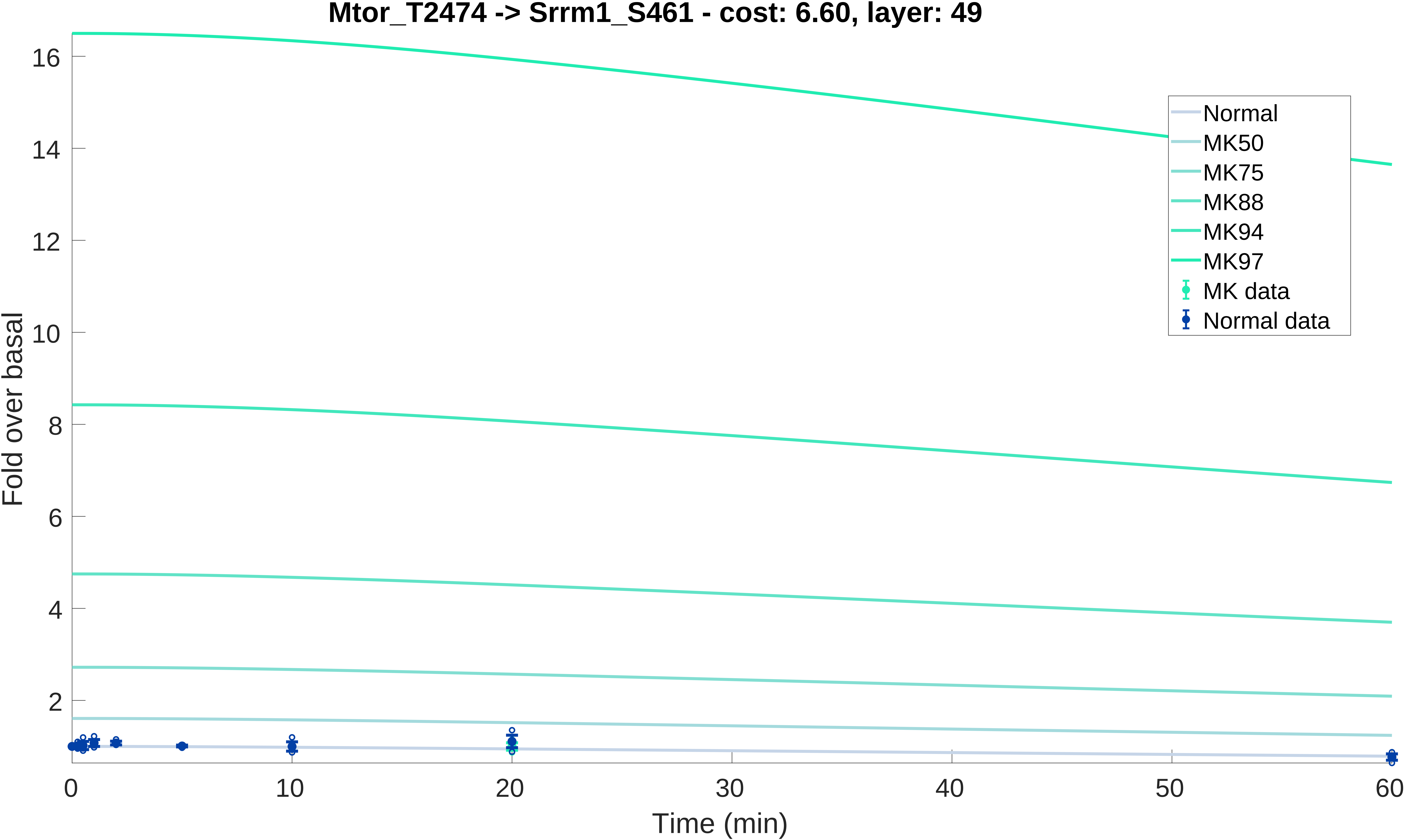
Mtor_T2474 -> Srrm1_S461 - cost: 6.60, layer: 49
Fold over basal
Time (min)
Normal
MK50
MK75
MK88
MK94
MK97
MK data
Normal data
0
10
20
30
40
50
60
2
4
6
8
10
12
14
16

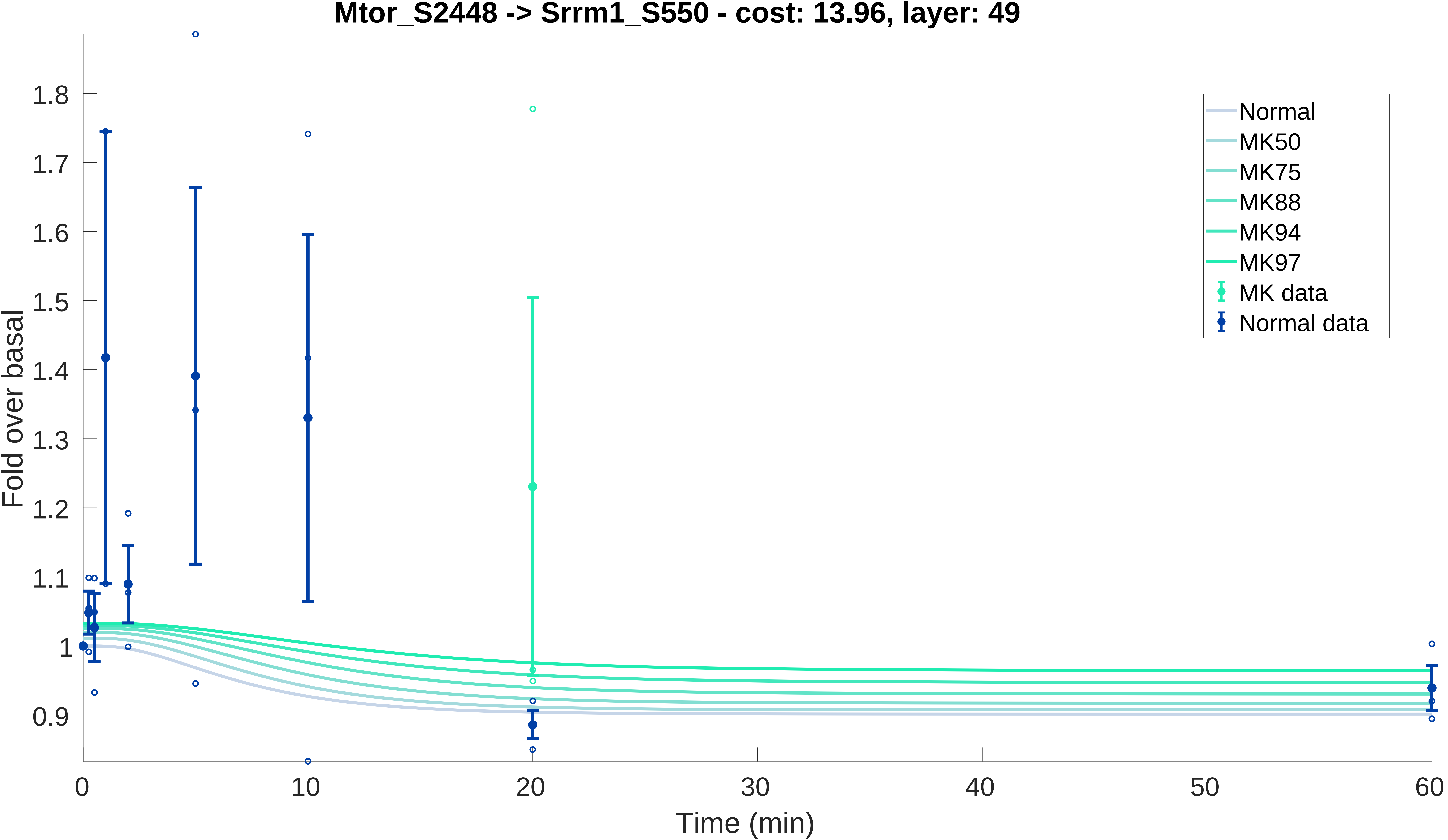

Mtor_S2448 -> Srrm1_S550 - cost: 13.96, layer: 49
Fold over basal
Time (min)
0
10
20
30
40
50
60
0.9
1
1.1
1.2
1.3
1.4
1.5
1.6
1.7
1.8
Normal
MK50
MK75
MK88
MK94
MK97
MK data
Normal data

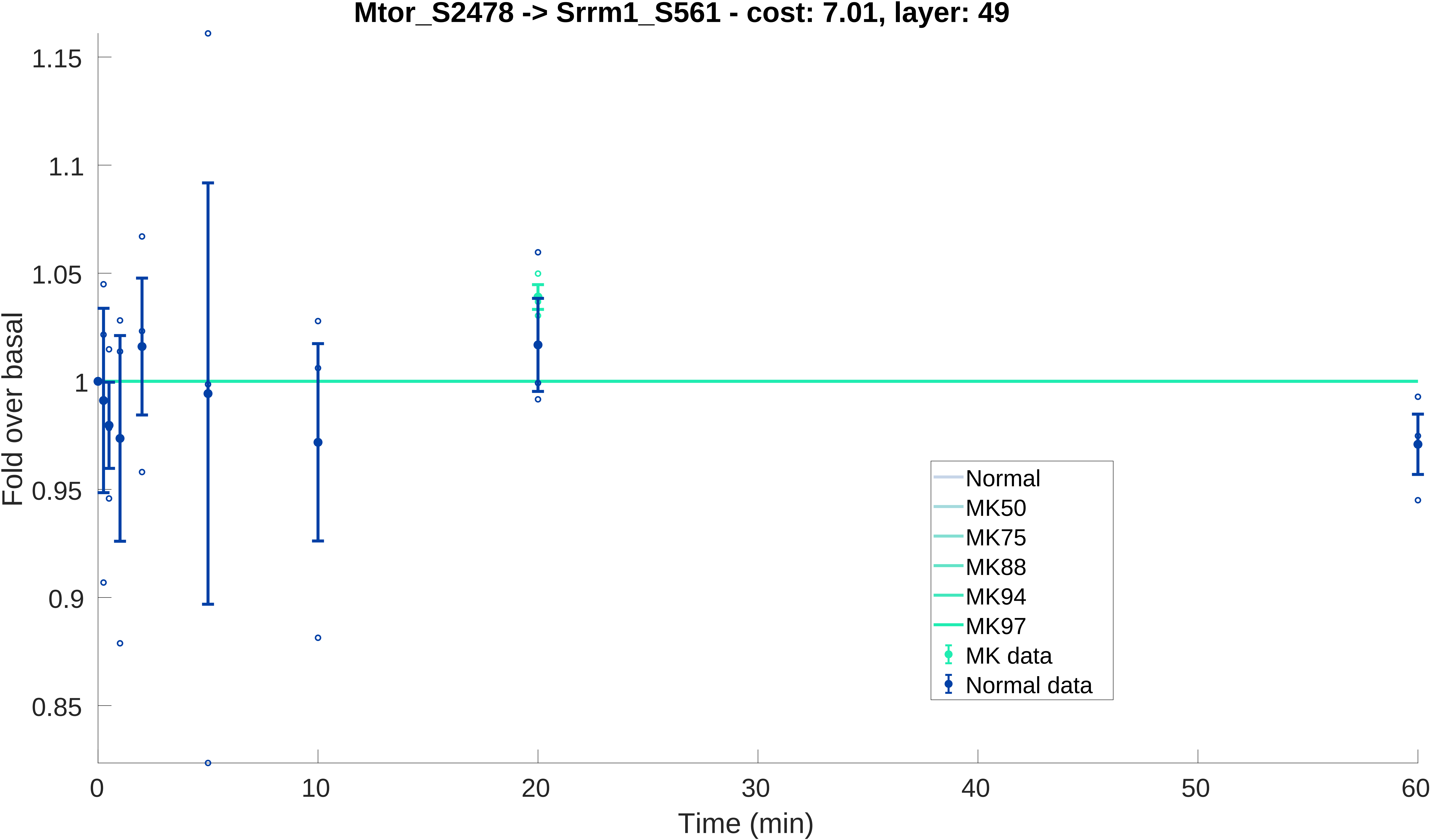

Mtor_S2478 -> Srrm1_S561 - cost: 7.01, layer: 49
Fold over basal
1.15
1.1
1.05
1
0.95
0.9
0.85
0
10
20
30
40
50
60
Time (min)
Normal
MK50
MK75
MK88
MK94
MK97
MK data
Normal data

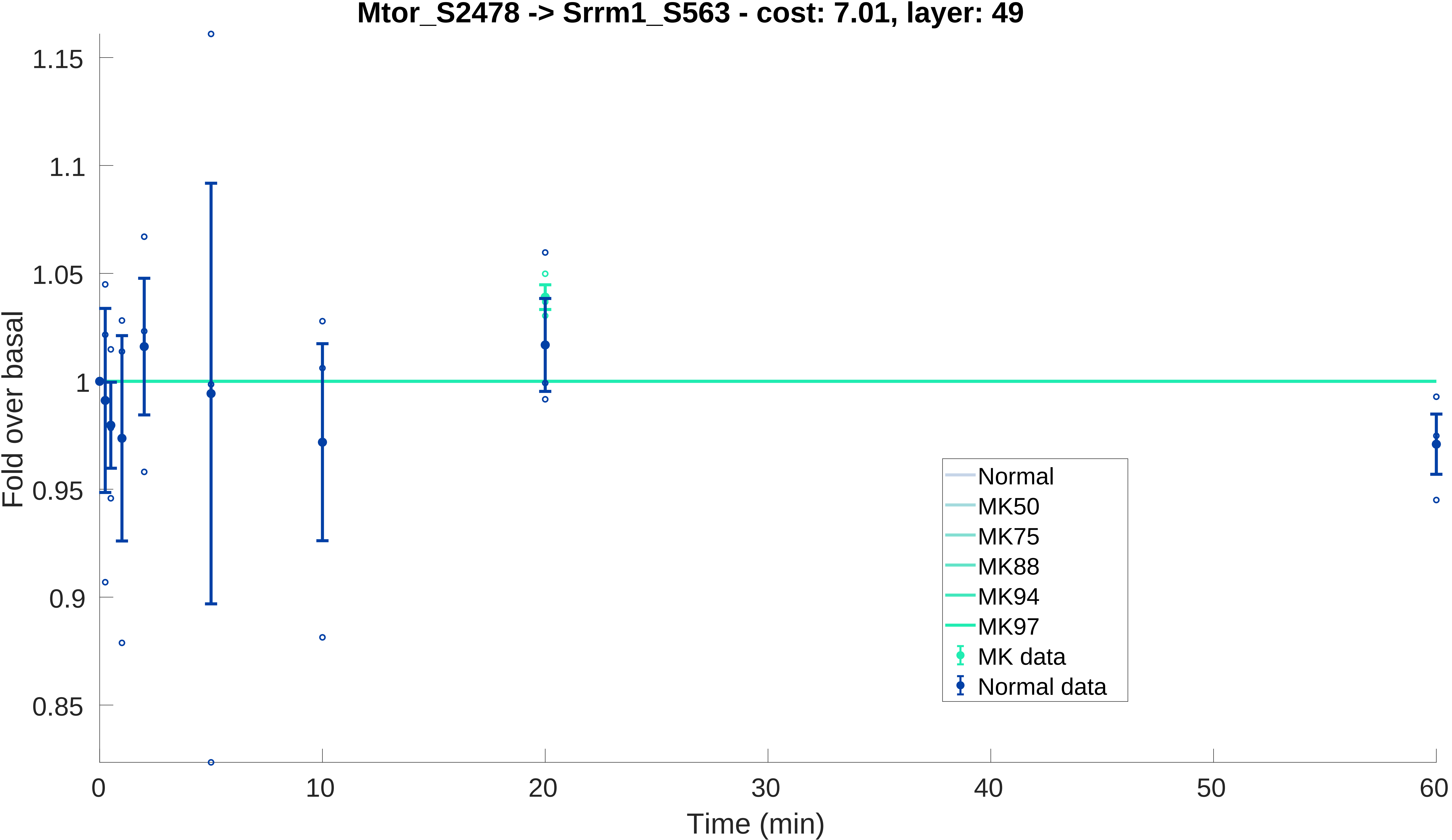

Mtor_S2478 -> Srrm1_S563 - cost: 7.01, layer: 49
Fold over basal
1.15
1.1
1.05
1
0.95
0.9
0.85
0
10
20
30
40
50
60
Time (min)
Normal
MK50
MK75
MK88
MK94
MK97
MK data
Normal data

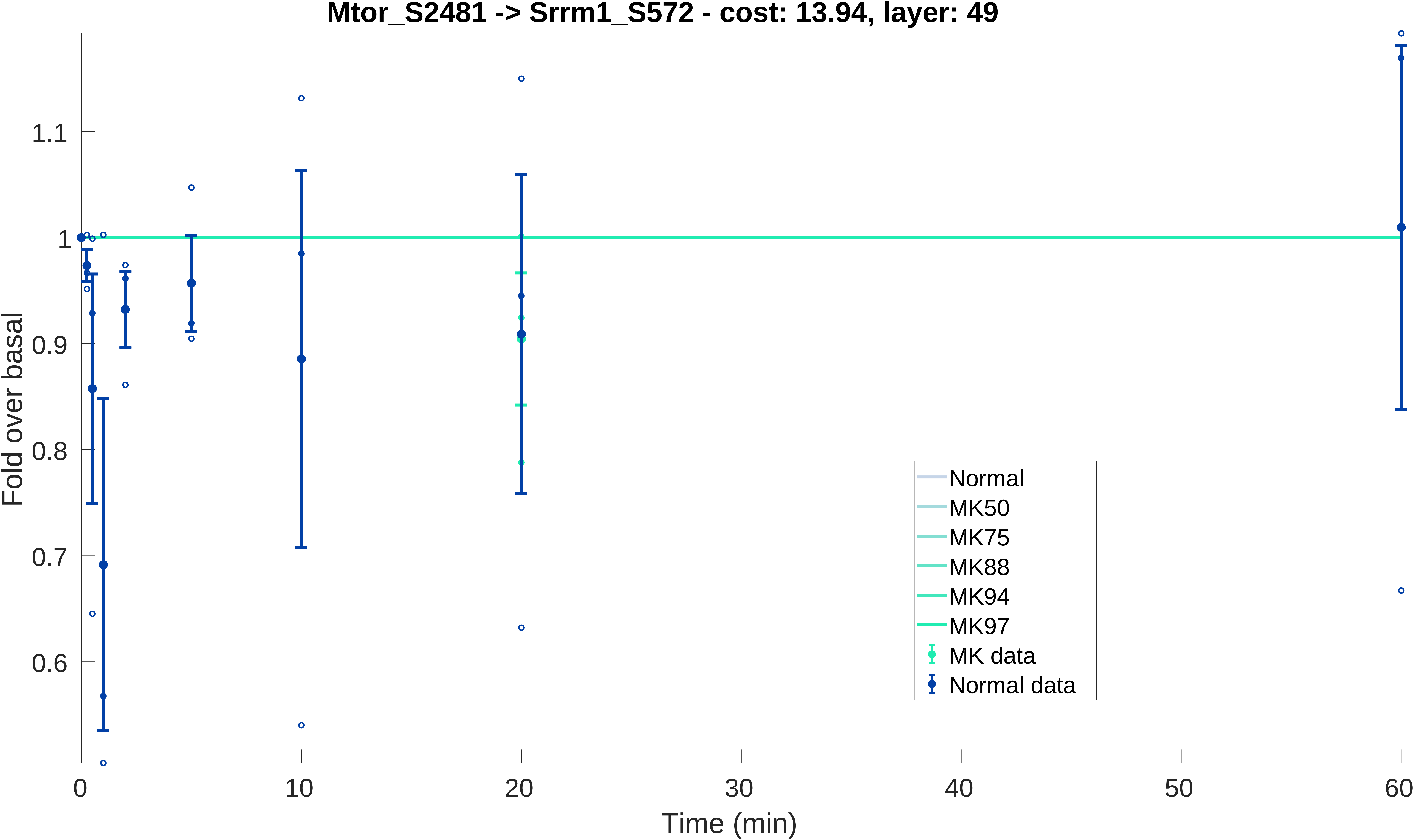

Mtor_S2481 -> Srrm1_S572 - cost: 13.94, layer: 49
Fold over basal
Time (min)
0.6
0.7
0.8
0.9
1
1.1
0
10
20
30
40
50
60
Normal
MK50
MK75
MK88
MK94
MK97
MK data
Normal data

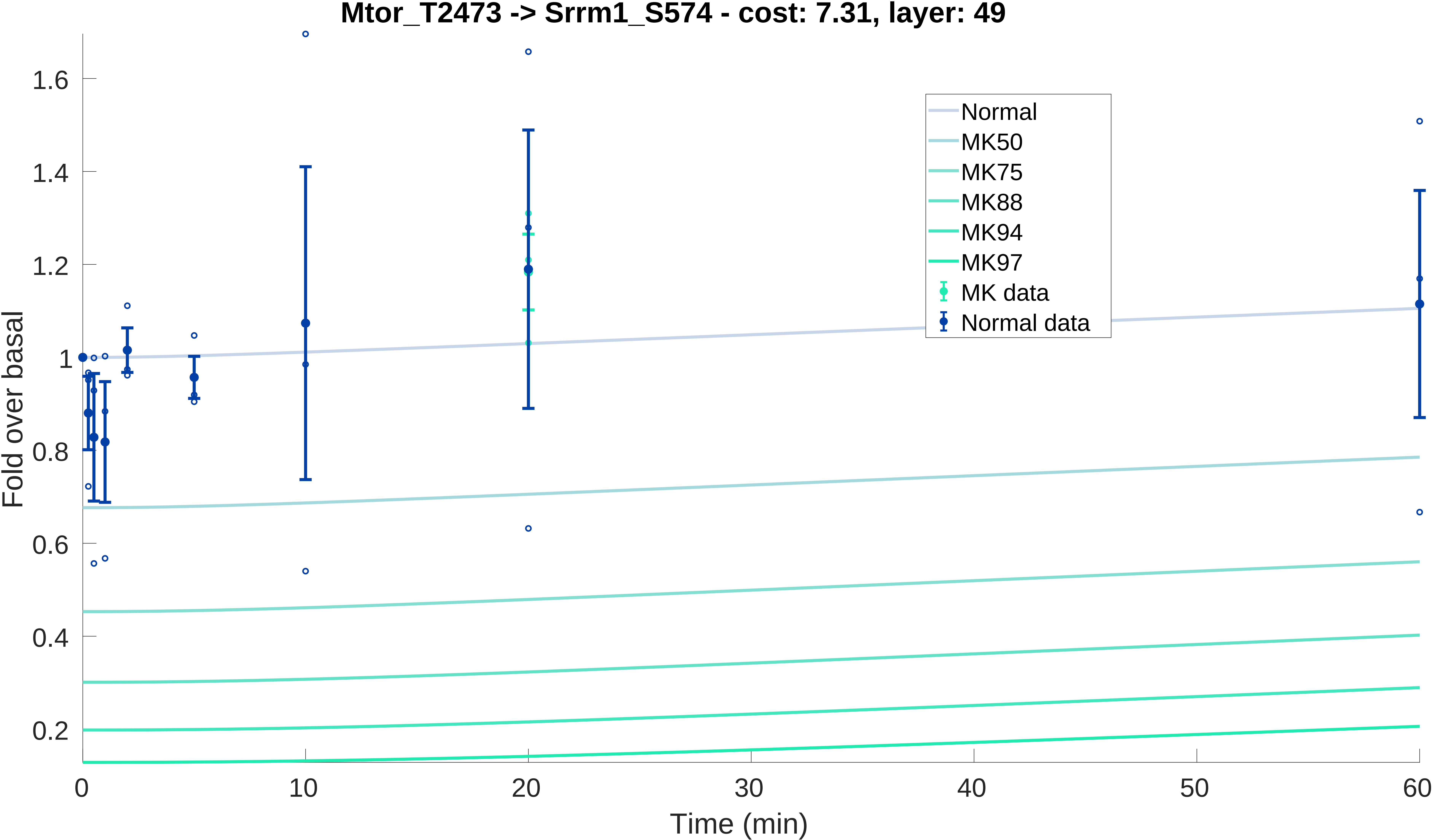

Mtor_T2473 -> Srrm1_S574 - cost: 7.31, layer: 49
Fold over basal
Time (min)
1.6
1.4
1.2
1
0.8
0.6
0.4
0.2
0
10
20
30
40
50
60
Normal
MK50
MK75
MK88
MK94
MK97
MK data
Normal data

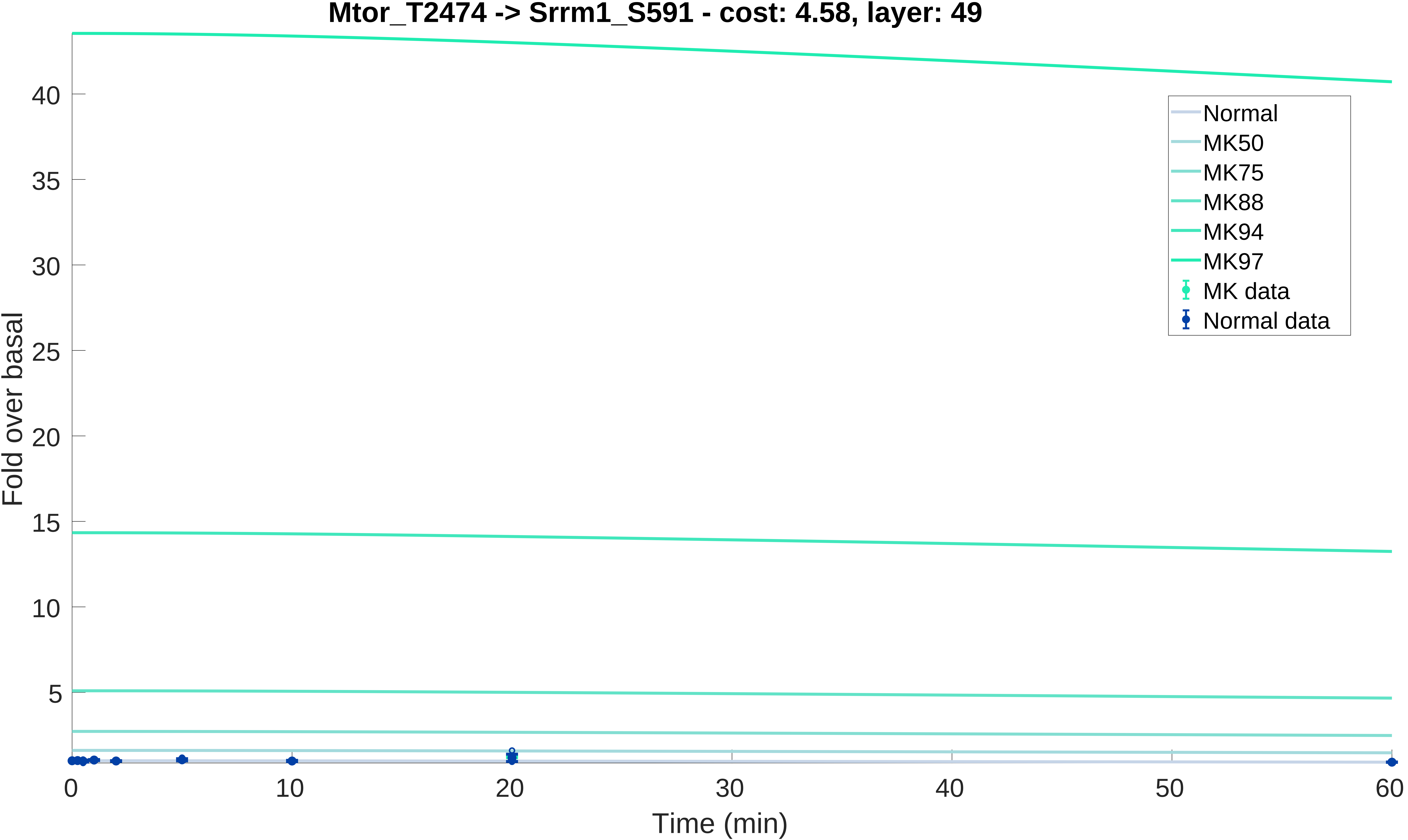

Mtor_T2474 -> Srrm1_S591 - cost: 4.58, layer: 49
Fold over basal
Time (min)
0
10
20
30
40
50
60
5
10
15
20
25
30
35
40
Normal
MK50
MK75
MK88
MK94
MK97
MK data
Normal data

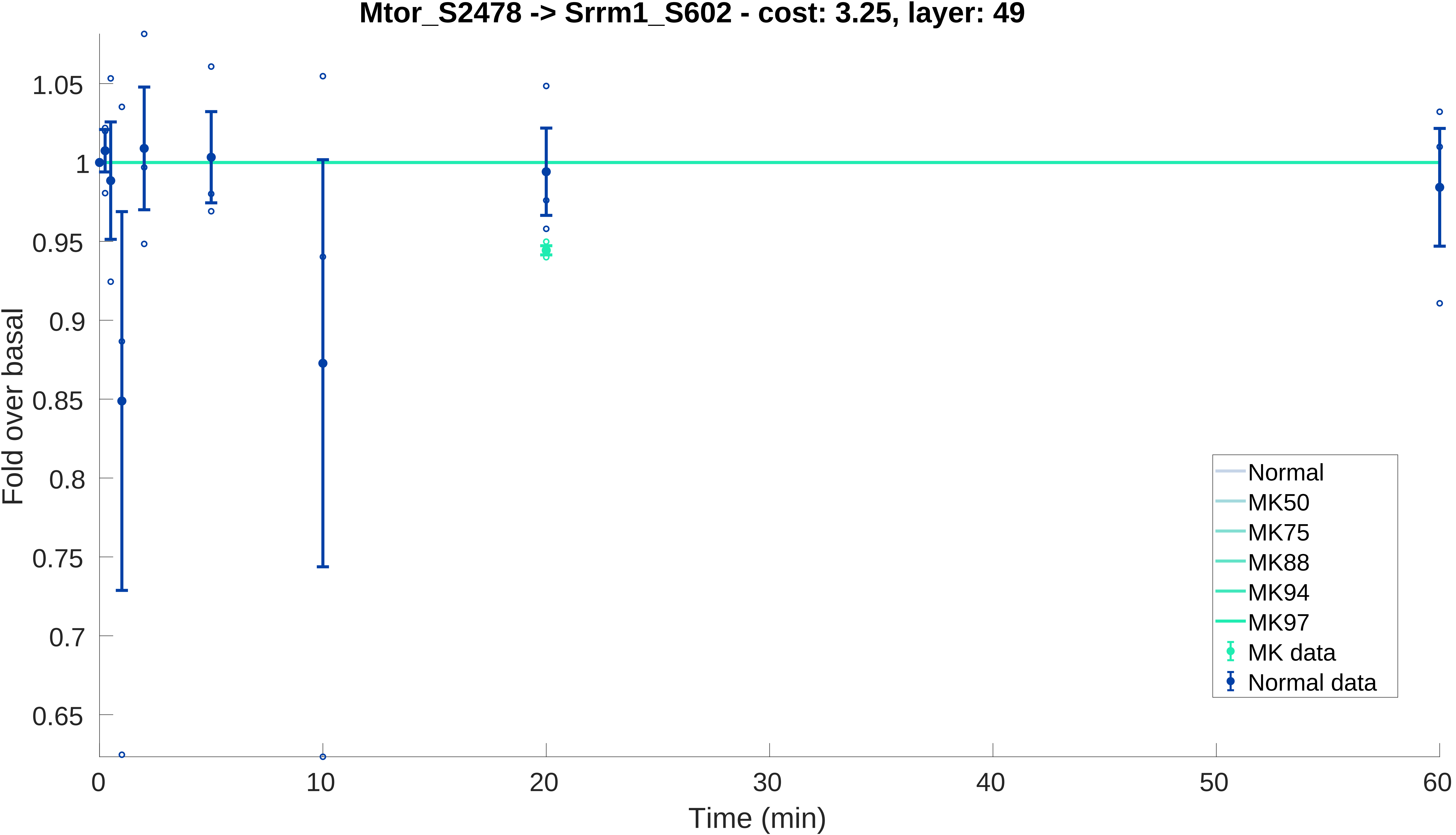

Mtor_S2478 -> Srrm1_S602 - cost: 3.25, layer: 49
Fold over basal
1.05
1
0.95
0.9
0.85
0.8
0.75
0.7
0.65
0
10
20
30
40
50
60
Time (min)
Normal
MK50
MK75
MK88
MK94
MK97
MK data
Normal data

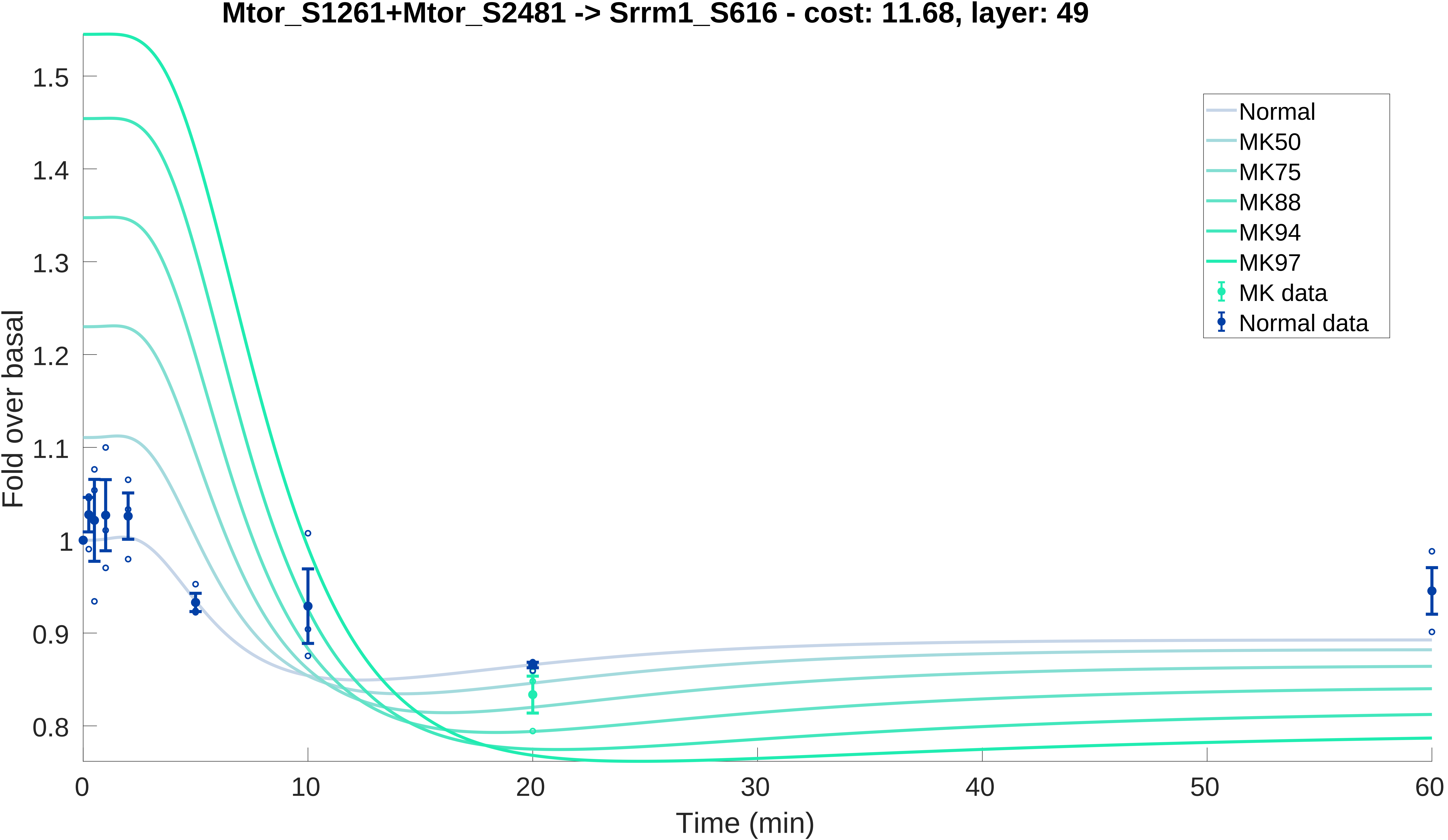

Mtor_S1261+Mtor_S2481 -> Srrm1_S616 - cost: 11.68, layer: 49
Fold over basal
Time (min)
1.5
1.4
1.3
1.2
1.1
1
0.9
0.8
0
10
20
30
40
50
60
Normal
MK50
MK75
MK88
MK94
MK97
MK data
Normal data

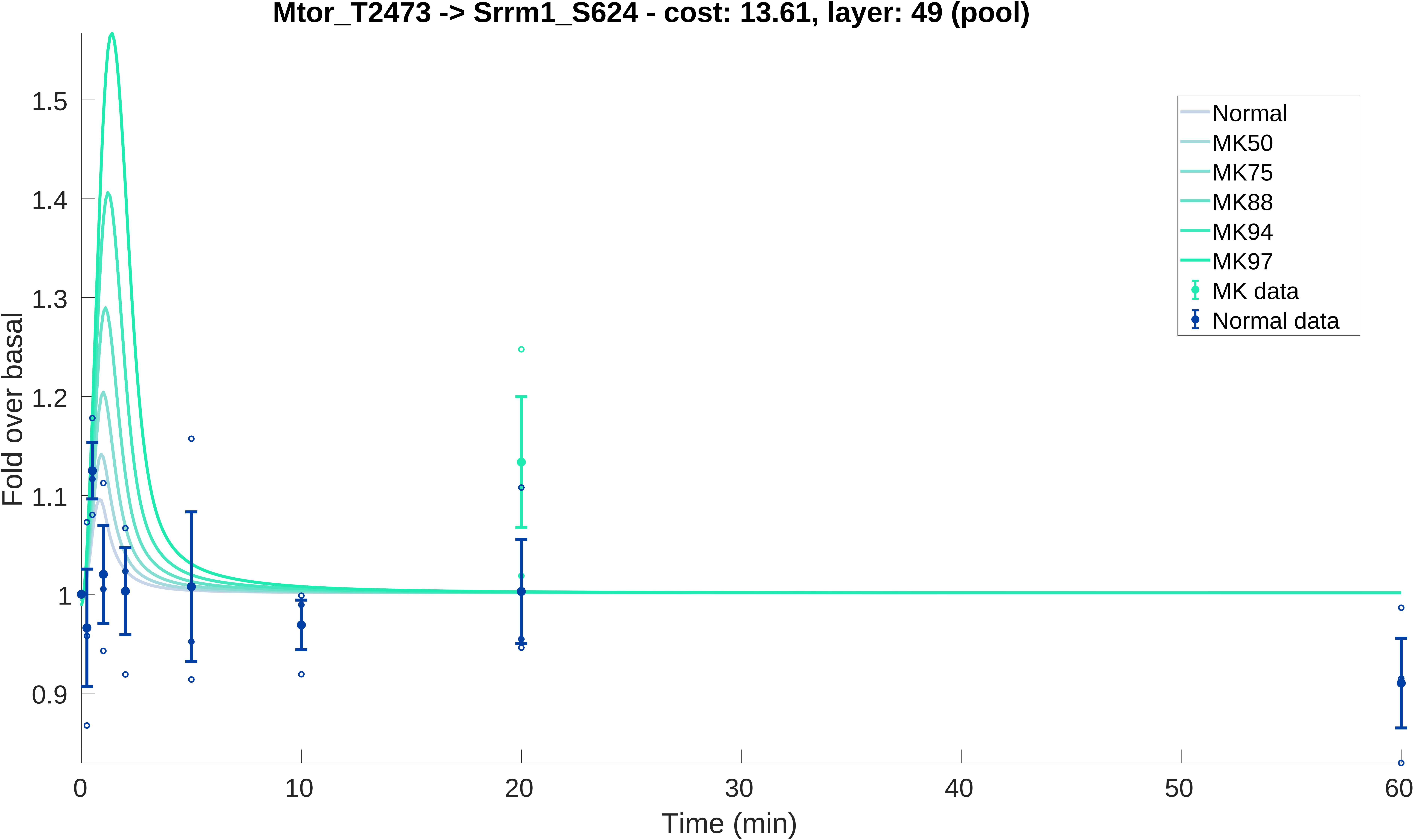

Mtor_T2473 -> Srrm1_S624 - cost: 13.61, layer: 49 (pool)
Fold over basal
Time (min)
Normal
MK50
MK75
MK88
MK94
MK97
MK data
Normal data
0.9
1
1.1
1.2
1.3
1.4
1.5
0
10
20
30
40
50
60

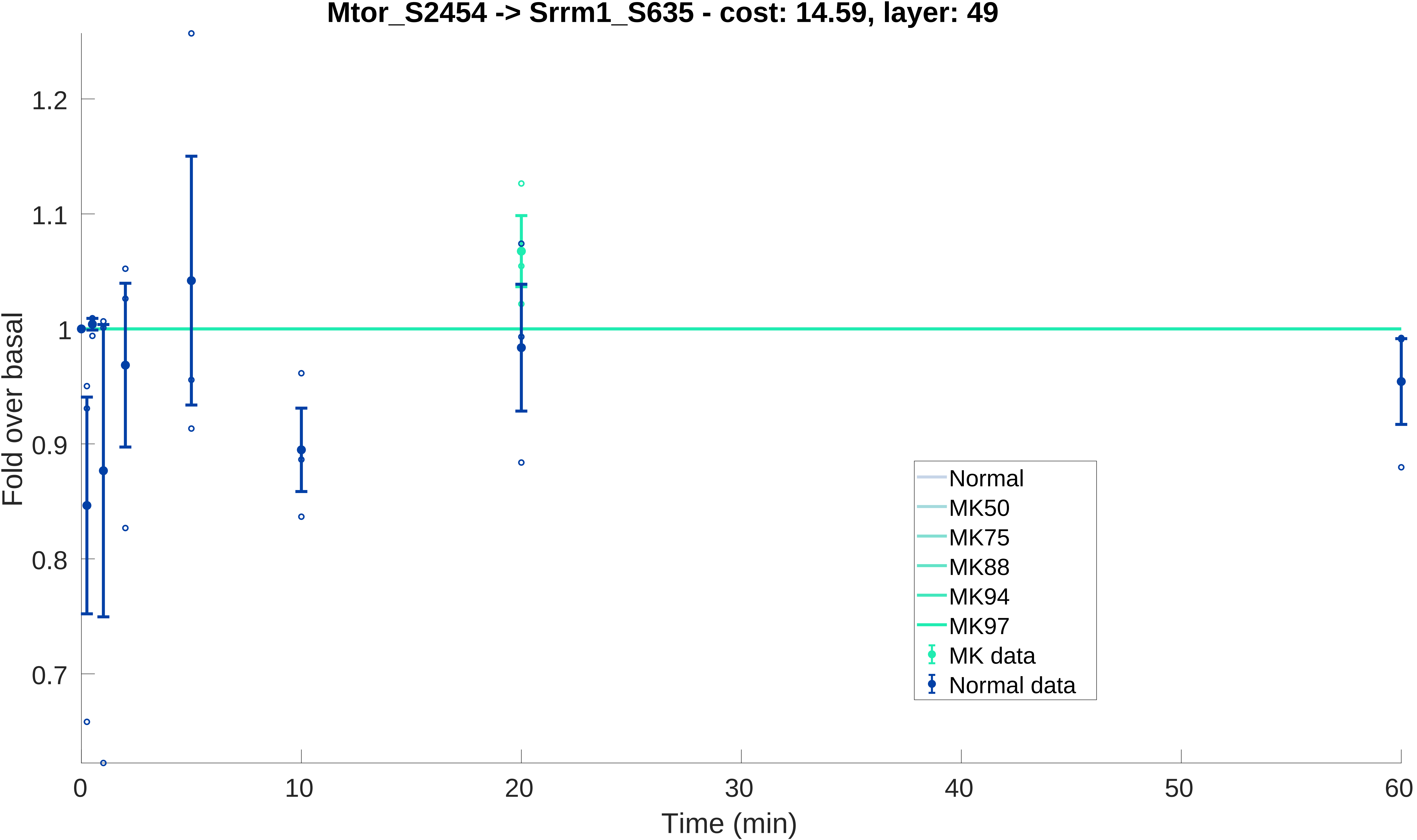

Mtor_S2454 -> Srrm1_S635 - cost: 14.59, layer: 49
Fold over basal
Time (min)
1.2
1.1
1
0.9
0.8
0.7
0
10
20
30
40
50
60
Normal
MK50
MK75
MK88
MK94
MK97
MK data
Normal data

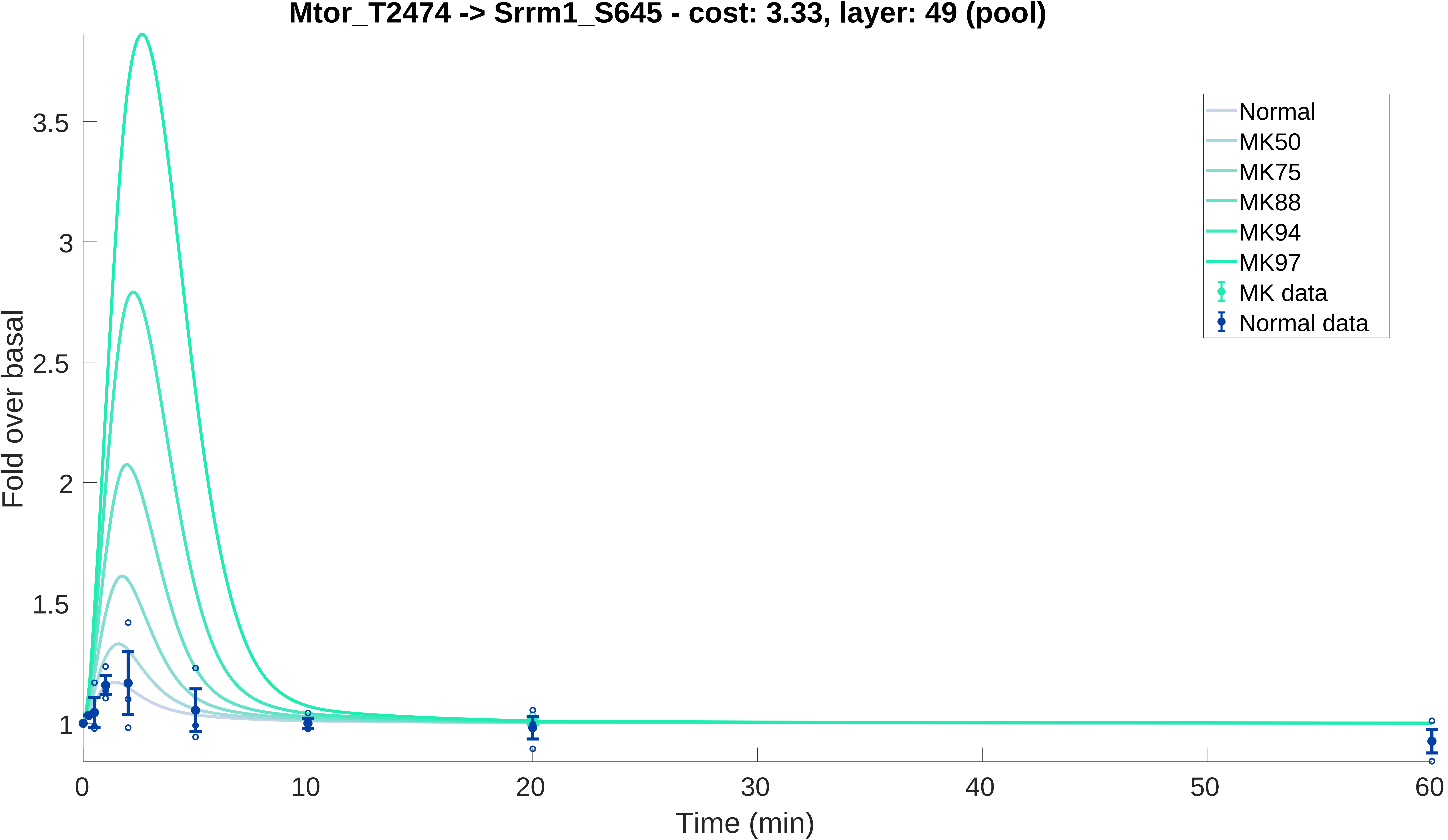

Mtor_T2474 -> Srrm1_S645 - cost: 3.33, layer: 49 (pool)
Fold over basal
Time (min)
0
10
20
30
40
50
60
1
1.5
2
2.5
3
3.5
Normal
MK50
MK75
MK88
MK94
MK97
MK data
Normal data

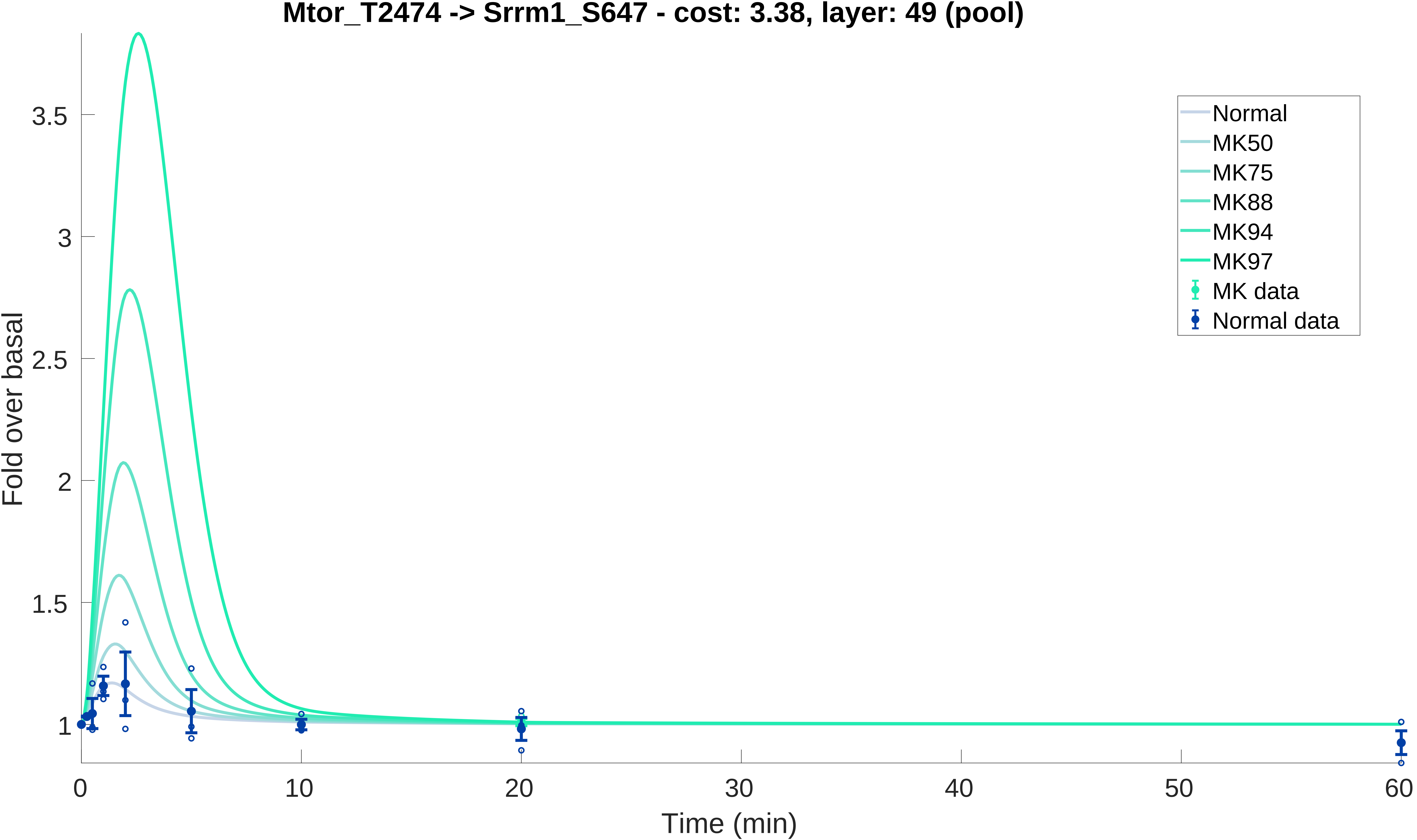

Mtor_T2474 -> Srrm1_S647 - cost: 3.38, layer: 49 (pool)
Fold over basal
Time (min)
3.5
3
2.5
2
1.5
1
0
10
20
30
40
50
60
Normal
MK50
MK75
MK88
MK94
MK97
MK data
Normal data

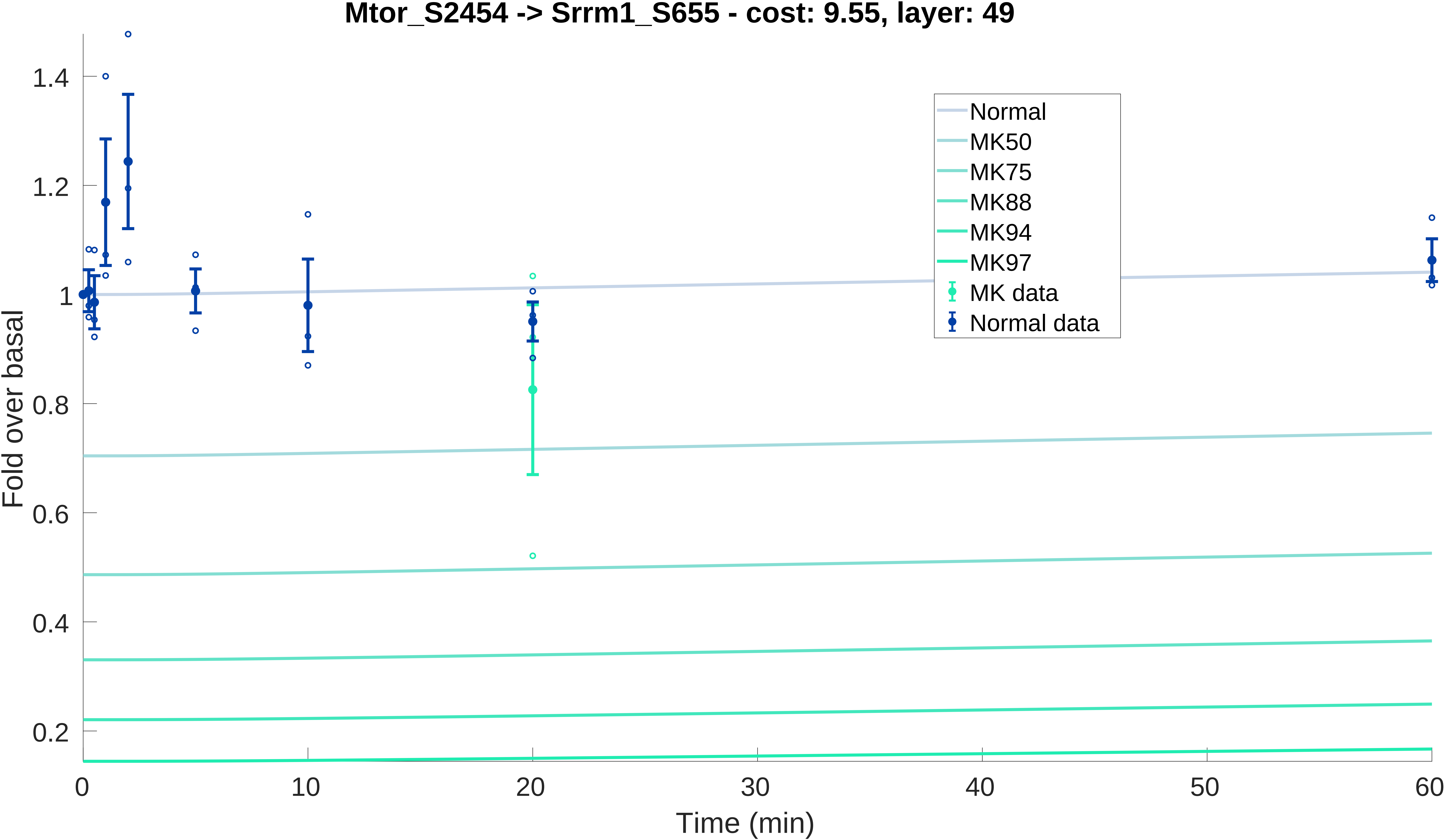

Mtor_S2454 -> Srrm1_S655 - cost: 9.55, layer: 49
Fold over basal
Time (min)
1.4
1.2
1
0.8
0.6
0.4
0.2
0
10
20
30
40
50
60
Normal
MK50
MK75
MK88
MK94
MK97
MK data
Normal data

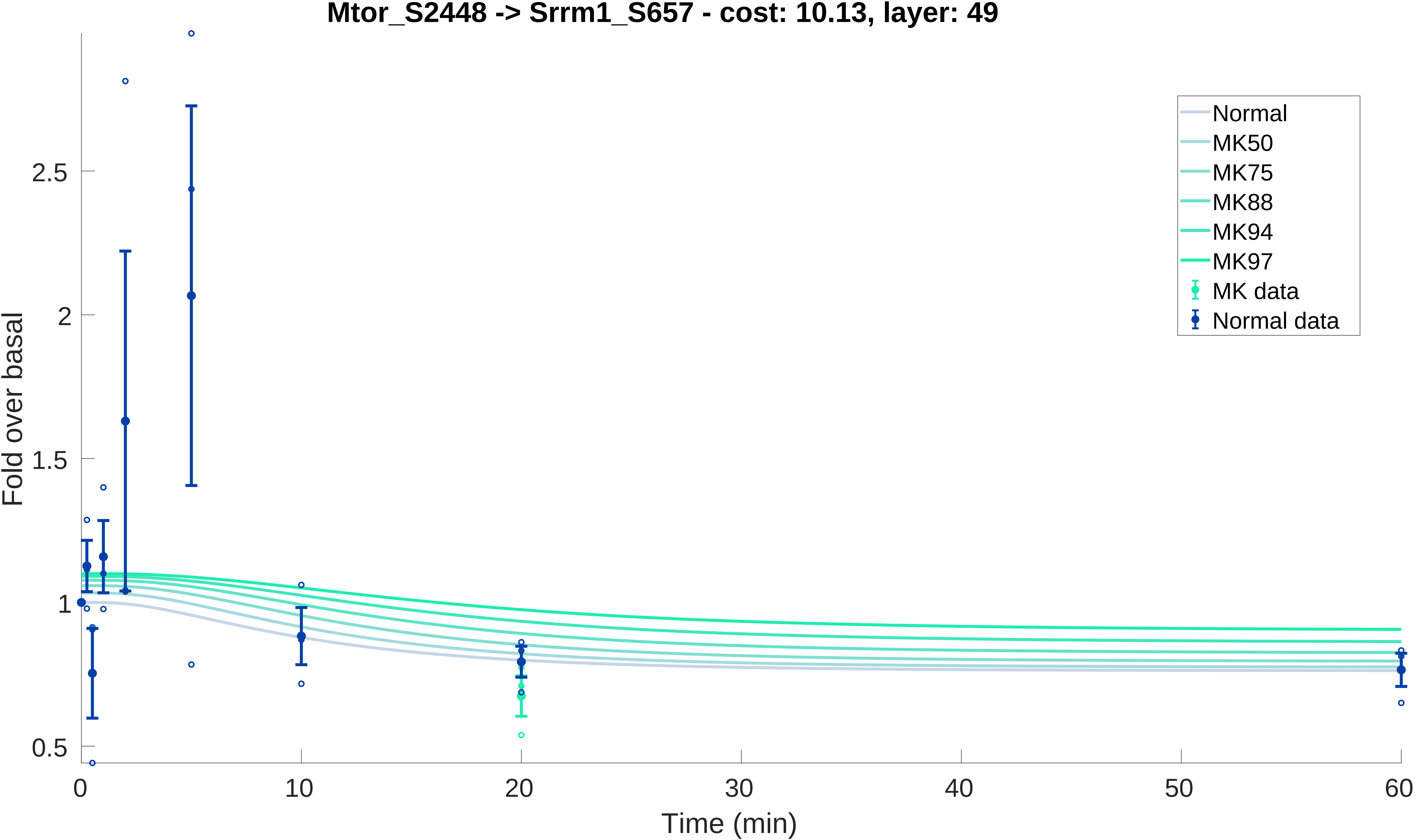

Mtor_S2448 -> Srrm1_S657 - cost: 10.13, layer: 49
Fold over basal
2.5
2
1.5
1
0.5
0
10
20
30
40
50
60
Time (min)
Normal
MK50
MK75
MK88
MK94
MK97
MK data
Normal data

Mtor_S2448 -> Srrm1_S664 - cost: 9.61, layer: 49 (pool)

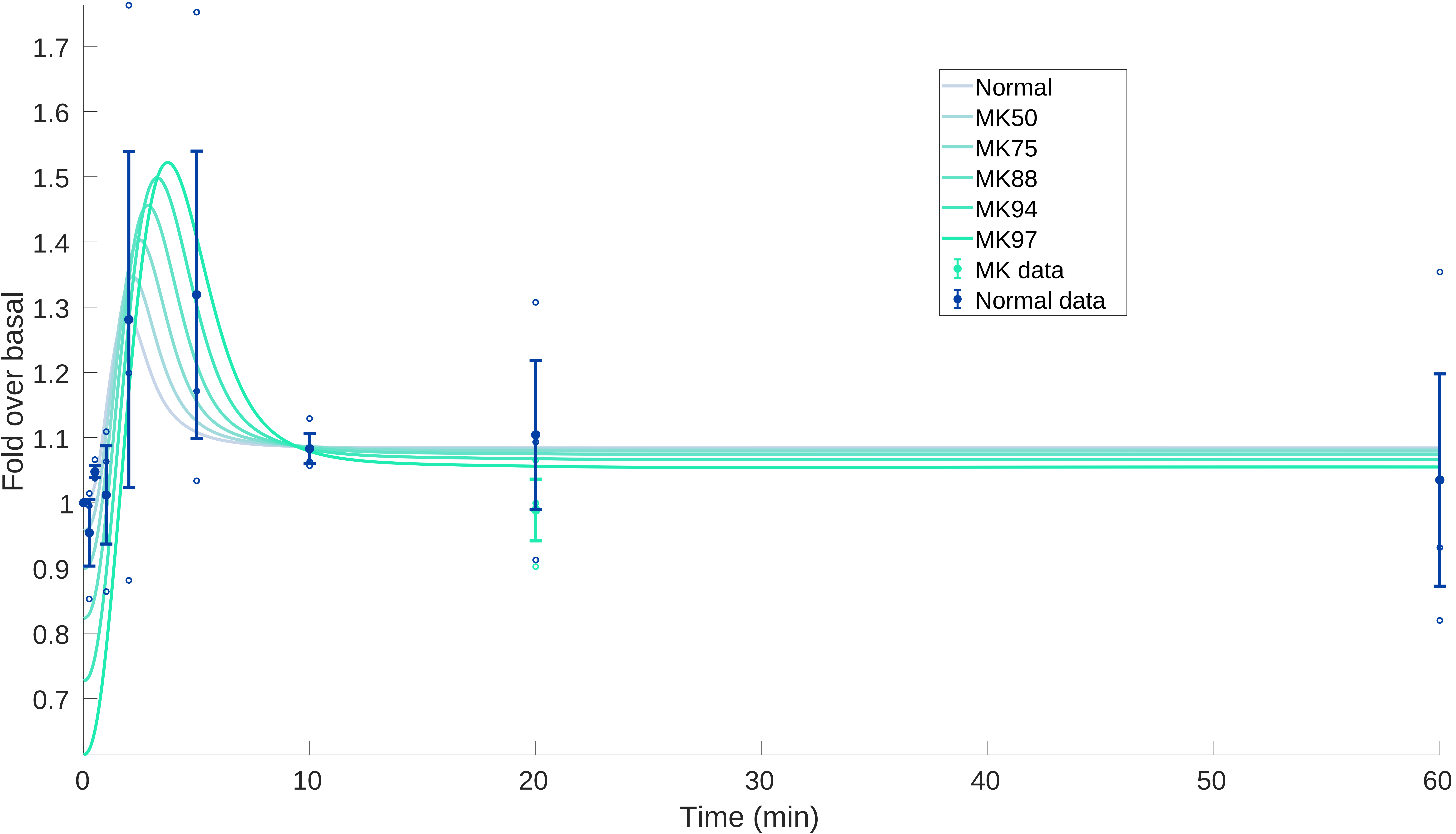

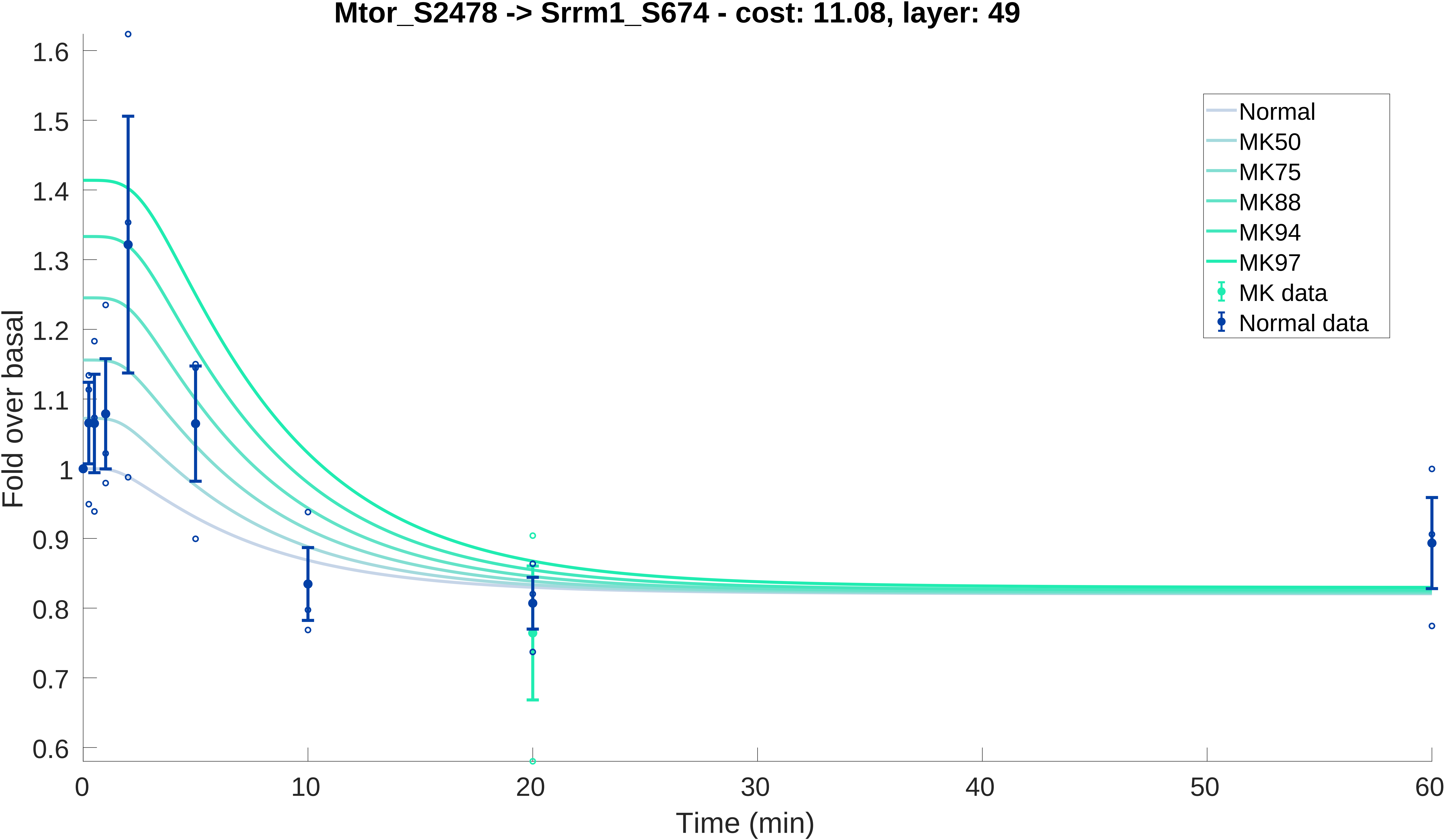
Mtor_S2478 -> Srrm1_S674 - cost: 11.08, layer: 49
Fold over basal
Time (min)
Normal
MK50
MK75
MK88
MK94
MK97
MK data
Normal data
1.6
1.5
1.4
1.3
1.2
1.1
1
0.9
0.8
0.7
0.6
0
10
20
30
40
50
60

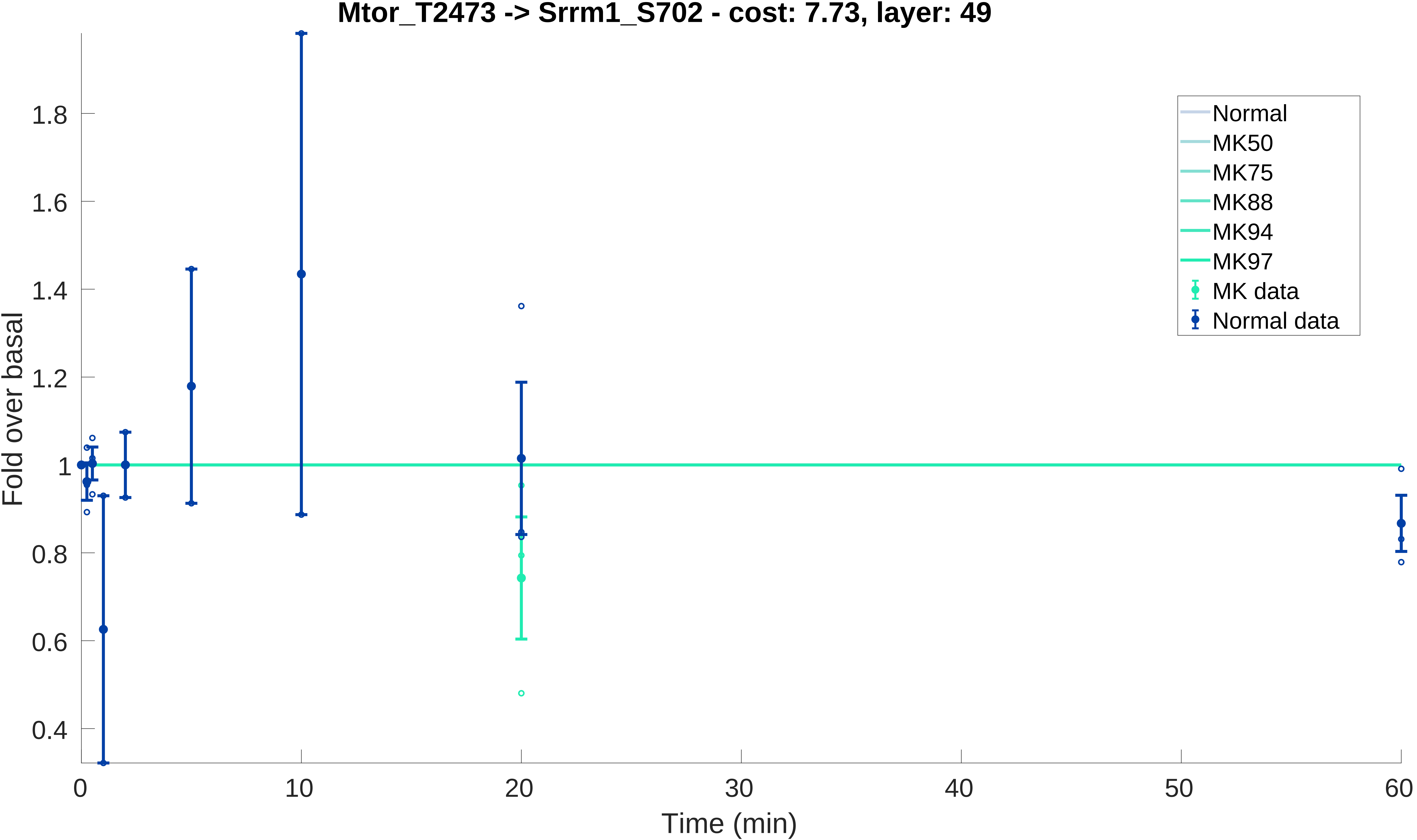

Mtor_T2473 -> Srrm1_S702 - cost: 7.73, layer: 49
Fold over basal
Time (min)
0.4
0.6
0.8
1
1.2
1.4
1.6
1.8
0
10
20
30
40
50
60
Normal
MK50
MK75
MK88
MK94
MK97
MK data
Normal data

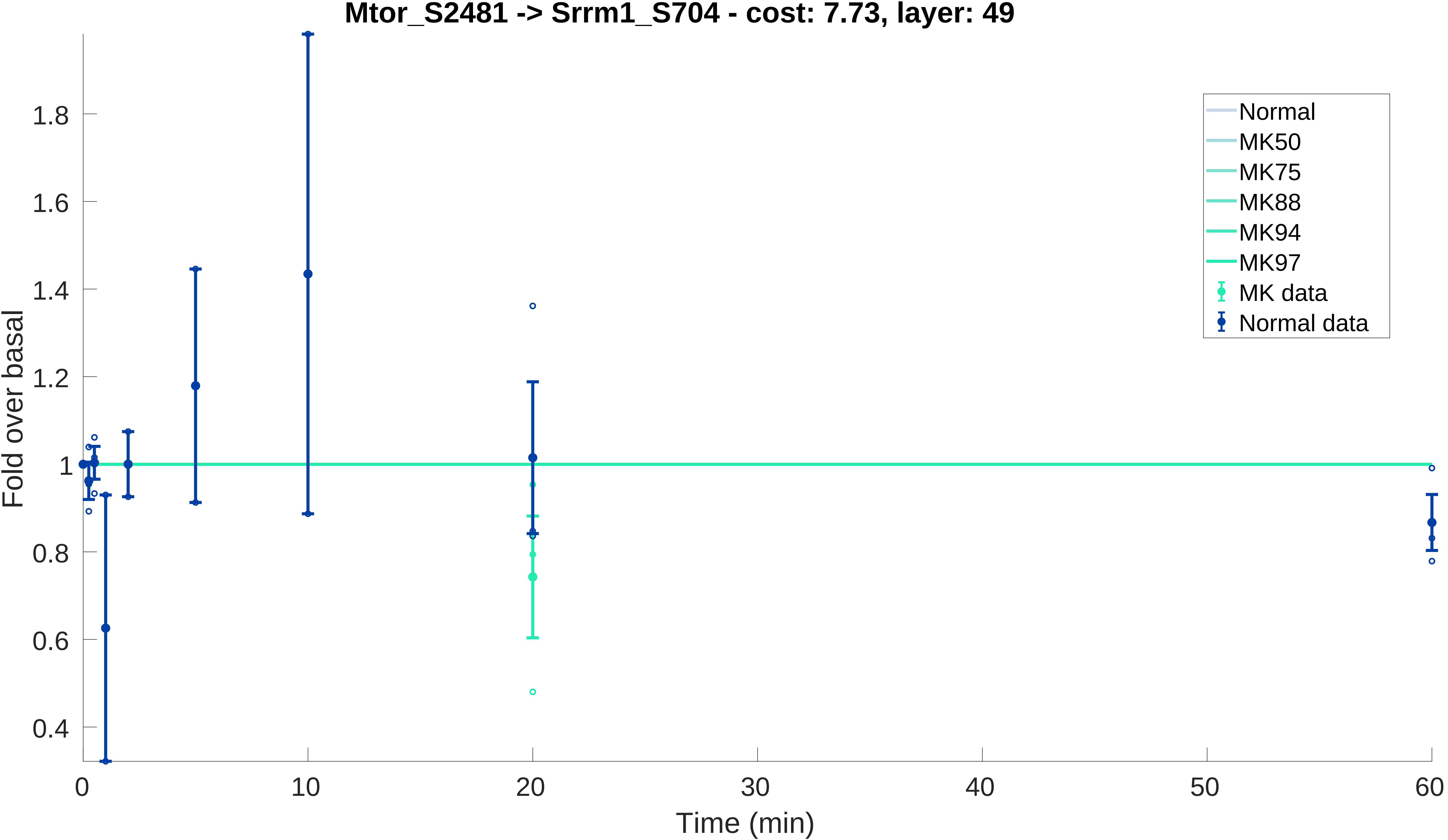
Mtor_S2481 -> Srrm1_S704 - cost: 7.73, layer: 49
Fold over basal
Time (min)
0.4
0.6
0.8
1
1.2
1.4
1.6
1.8
0
10
20
30
40
50
60
Normal
MK50
MK75
MK88
MK94
MK97
MK data
Normal data

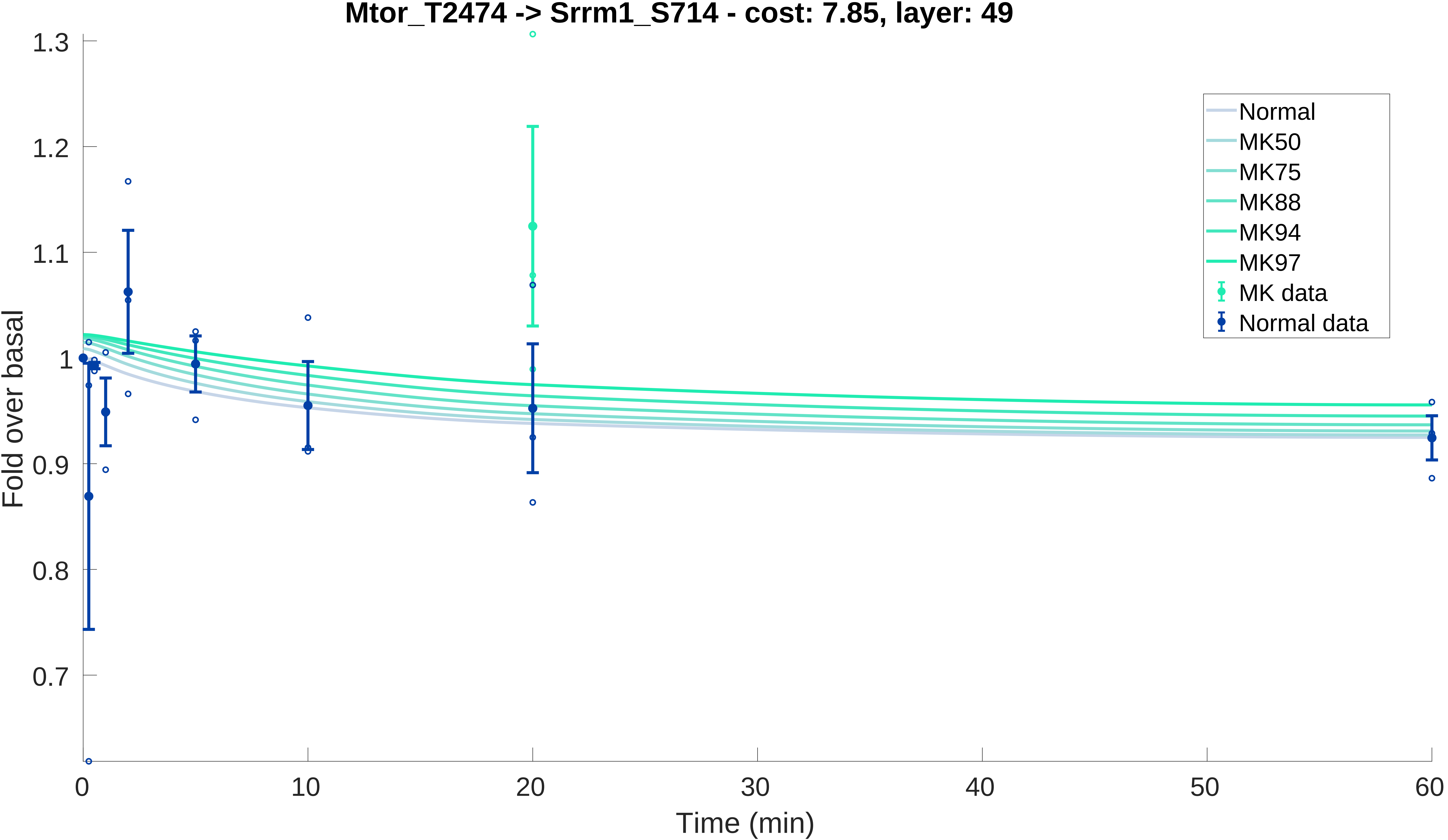

Mtor_T2474 -> Srrm1_S714 - cost: 7.85, layer: 49
Fold over basal
Time (min)
1.3
1.2
1.1
1
0.9
0.8
0.7
0
10
20
30
40
50
60
Normal
MK50
MK75
MK88
MK94
MK97
MK data
Normal data

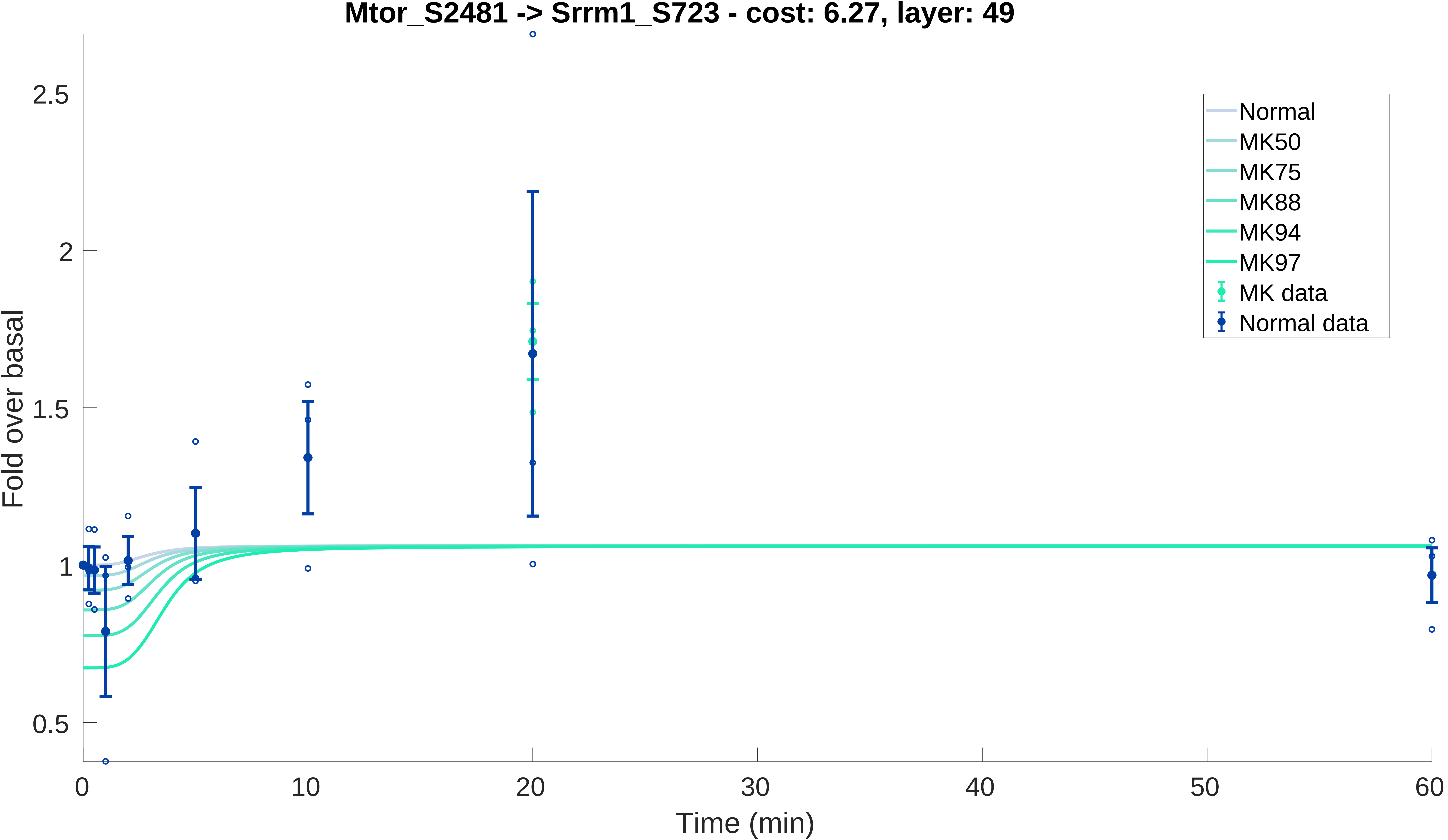

Mtor_S2481 -> Srrm1_S723 - cost: 6.27, layer: 49
Fold over basal
Time (min)
0.5
1
1.5
2
2.5
0
10
20
30
40
50
60
Normal
MK50
MK75
MK88
MK94
MK97
MK data
Normal data

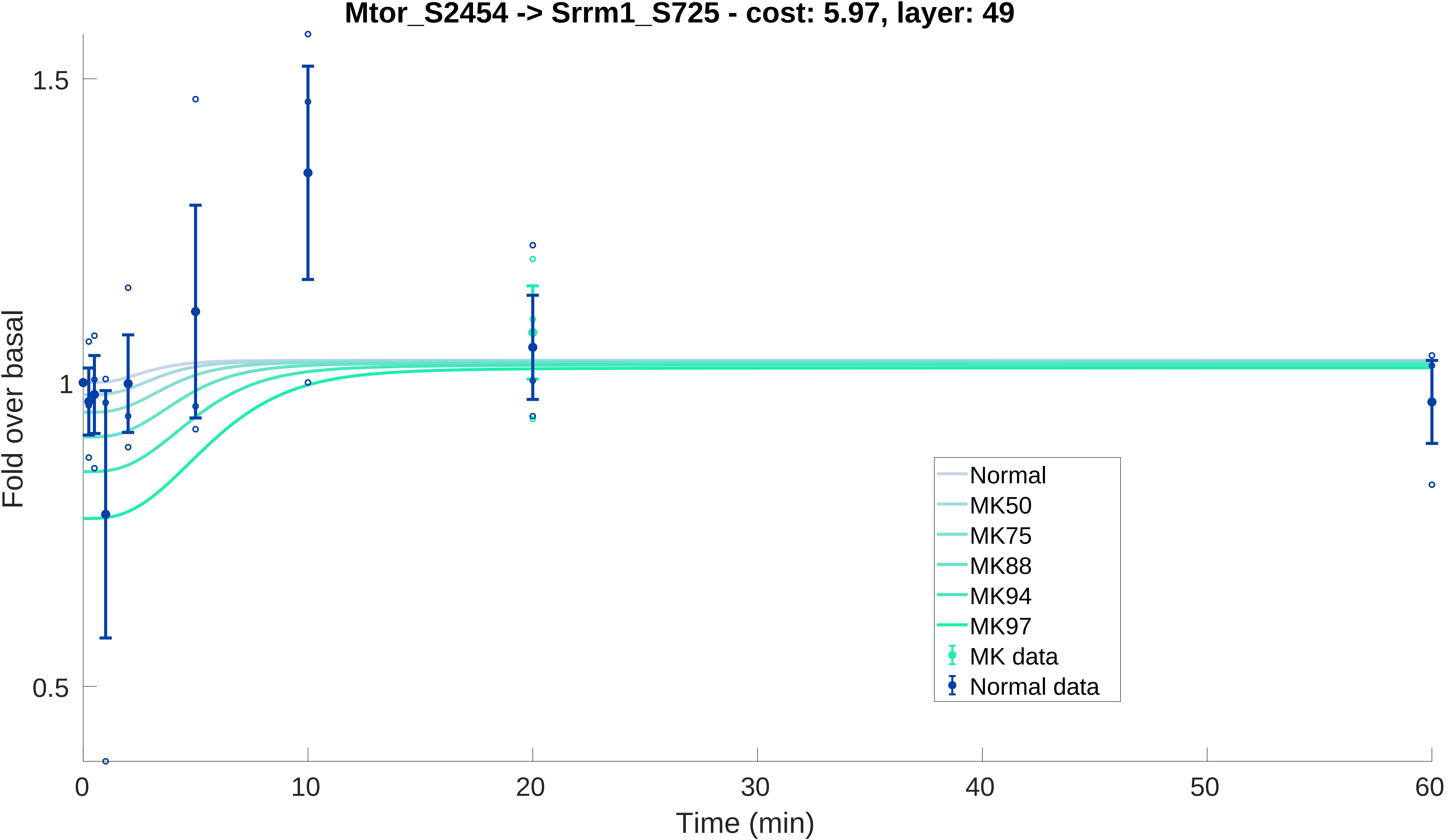

Mtor_S2454 -> Srrm1_S725 - cost: 5.97, layer: 49
Fold over basal
1.5
1
0.5
0
10
20
30
40
50
60
Time (min)
Normal
MK50
MK75
MK88
MK94
MK97
MK data
Normal data

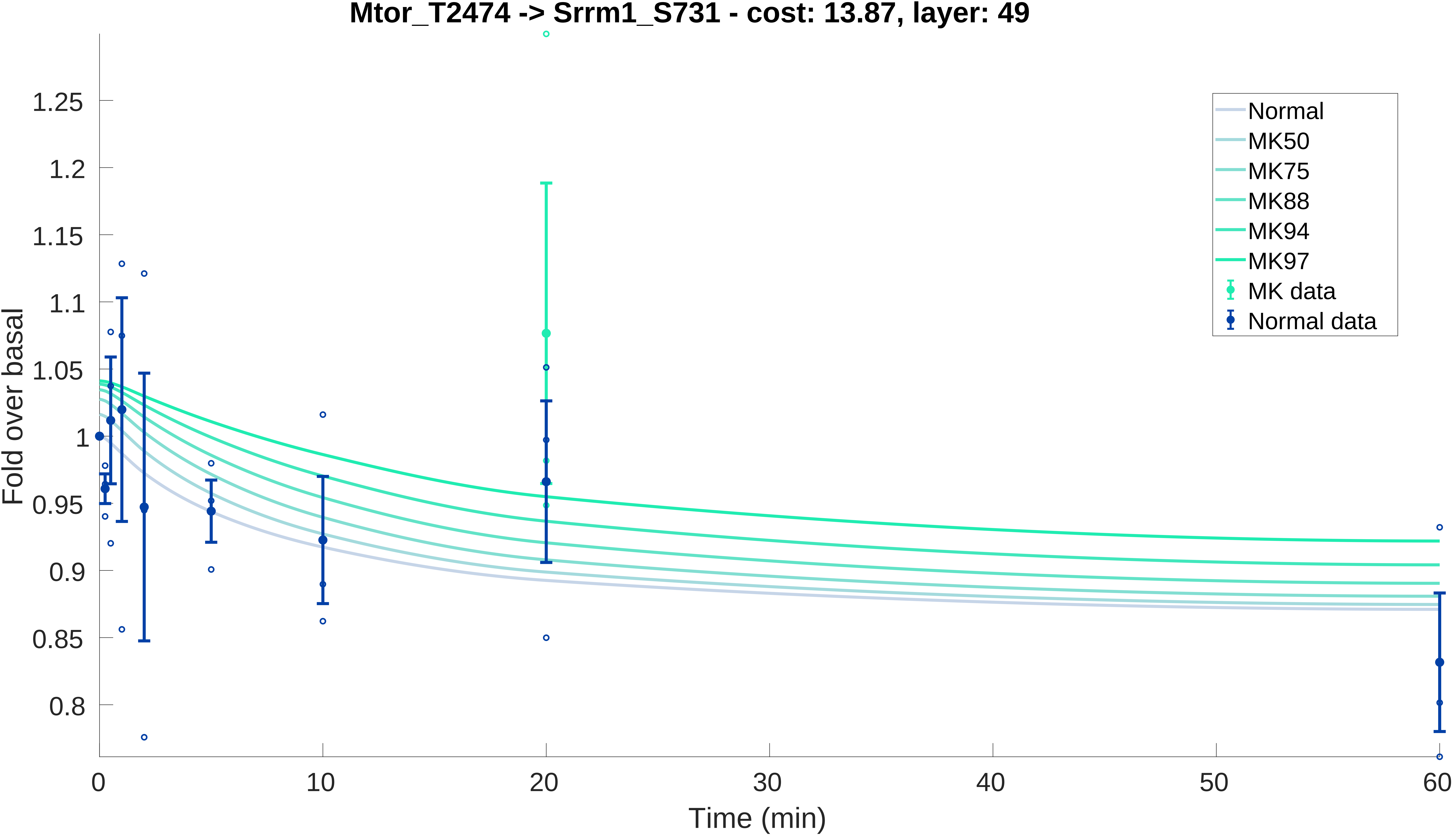

Mtor_T2474 -> Srrm1_S731 - cost: 13.87, layer: 49
Fold over basal
Time (min)
1.25
1.2
1.15
1.1
1.05
1
0.95
0.9
0.85
0.8
0
10
20
30
40
50
60
Normal
MK50
MK75
MK88
MK94
MK97
MK data
Normal data

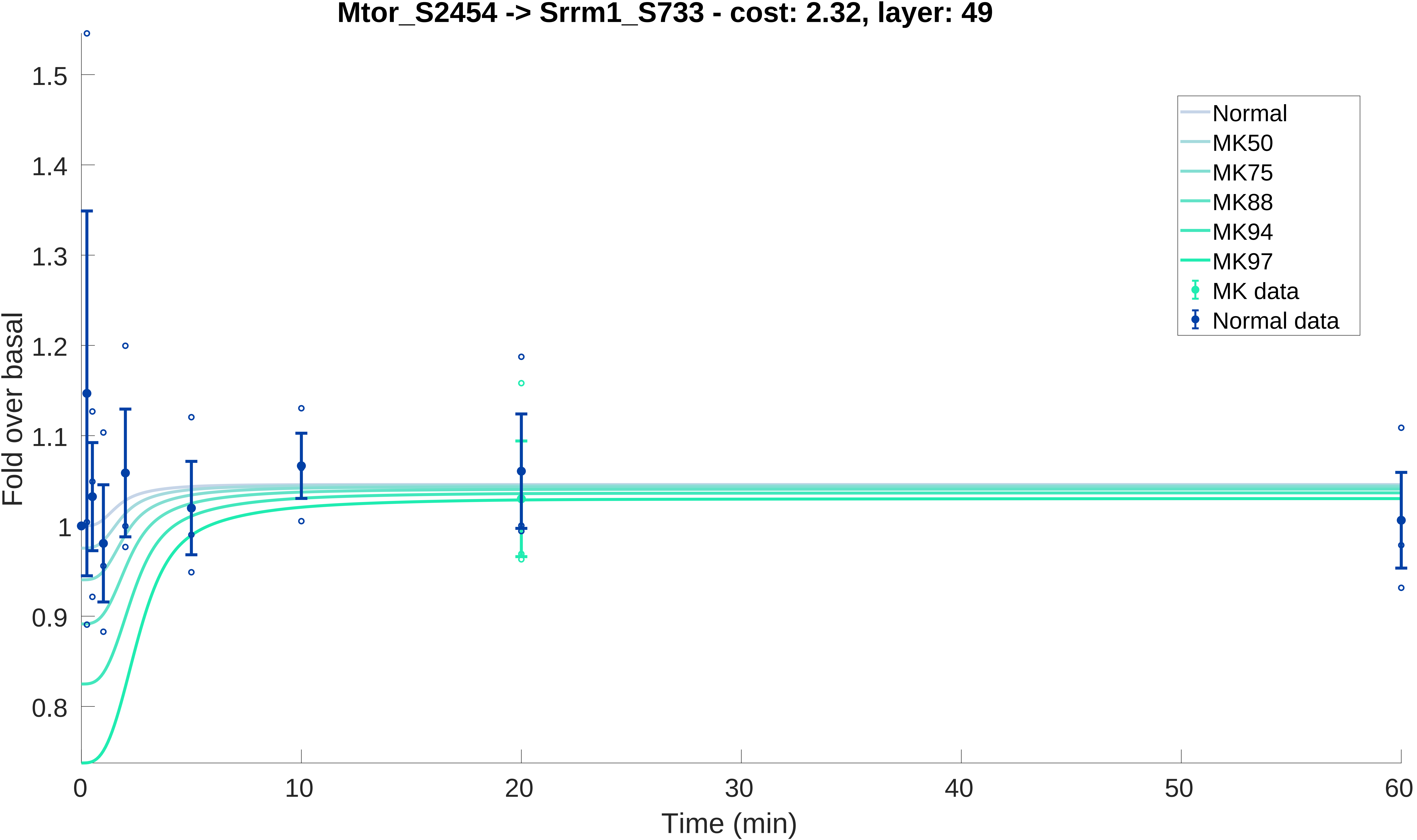

Mtor_S2454 -> Srrm1_S733 - cost: 2.32, layer: 49
Fold over basal
Time (min)
0.8
0.9
1
1.1
1.2
1.3
1.4
1.5
0
10
20
30
40
50
60
Normal
MK50
MK75
MK88
MK94
MK97
MK data
Normal data

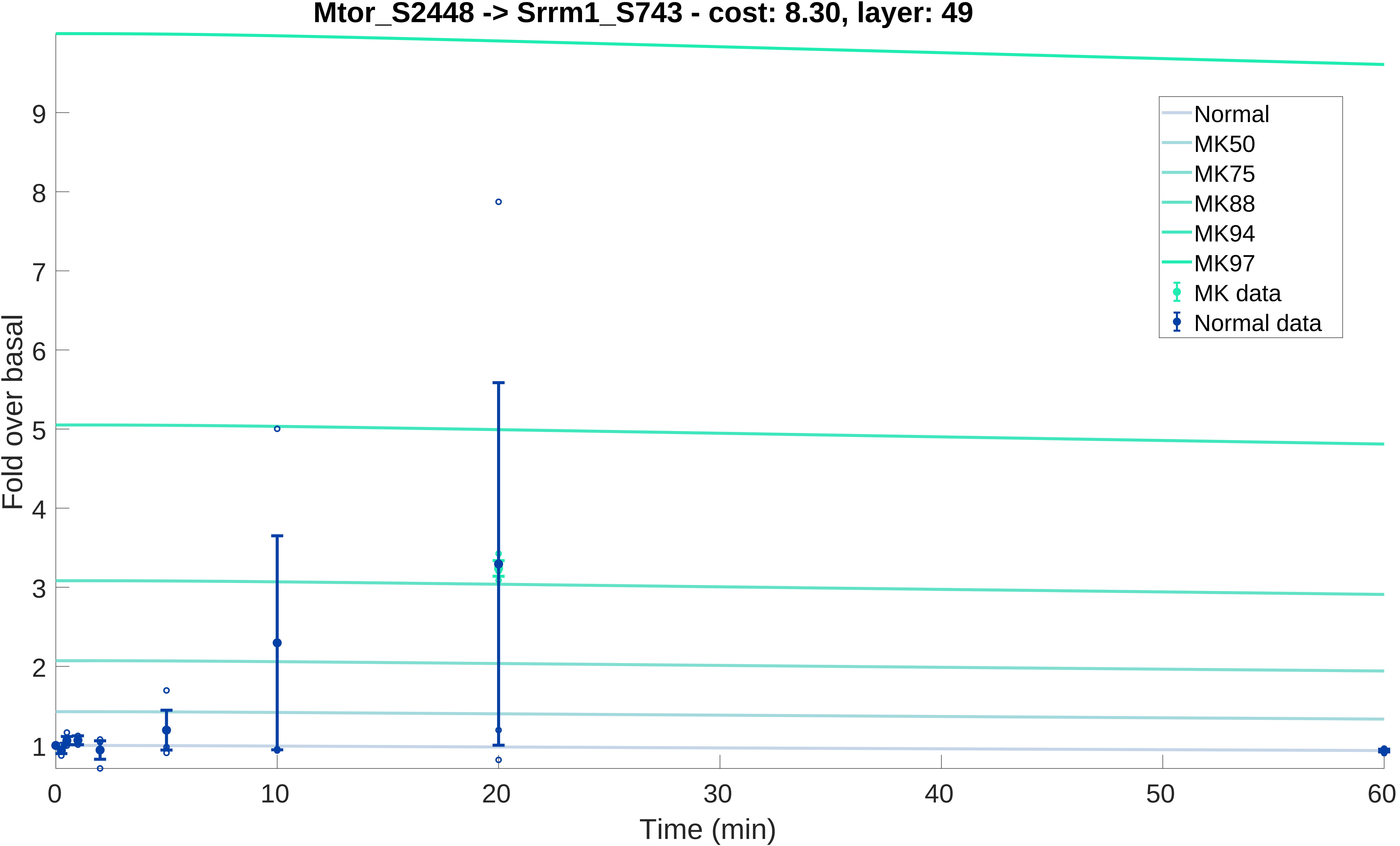

Mtor_S2448 -> Srrm1_S743 - cost: 8.30, layer: 49
Fold over basal
Time (min)
0
10
20
30
40
50
60
1
2
3
4
5
6
7
8
9
Normal
MK50
MK75
MK88
MK94
MK97
MK data
Normal data

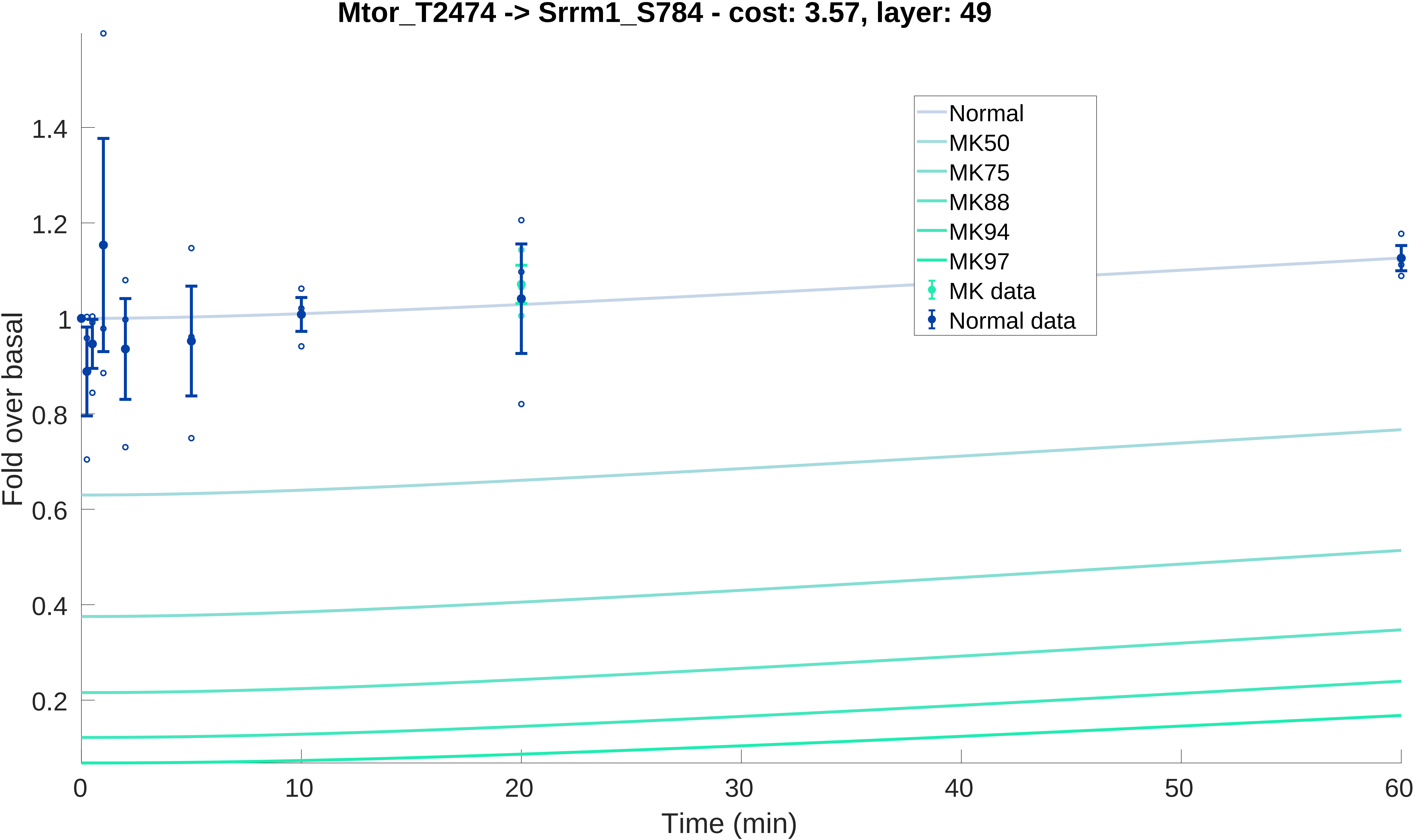

Mtor_T2474 -> Srrm1_S784 - cost: 3.57, layer: 49
Normal
MK50
MK75
MK88
MK94
MK97
MK data
Normal data
Fold over basal
Time (min)
0
10
20
30
40
50
60
0.2
0.4
0.6
0.8
1
1.2
1.4

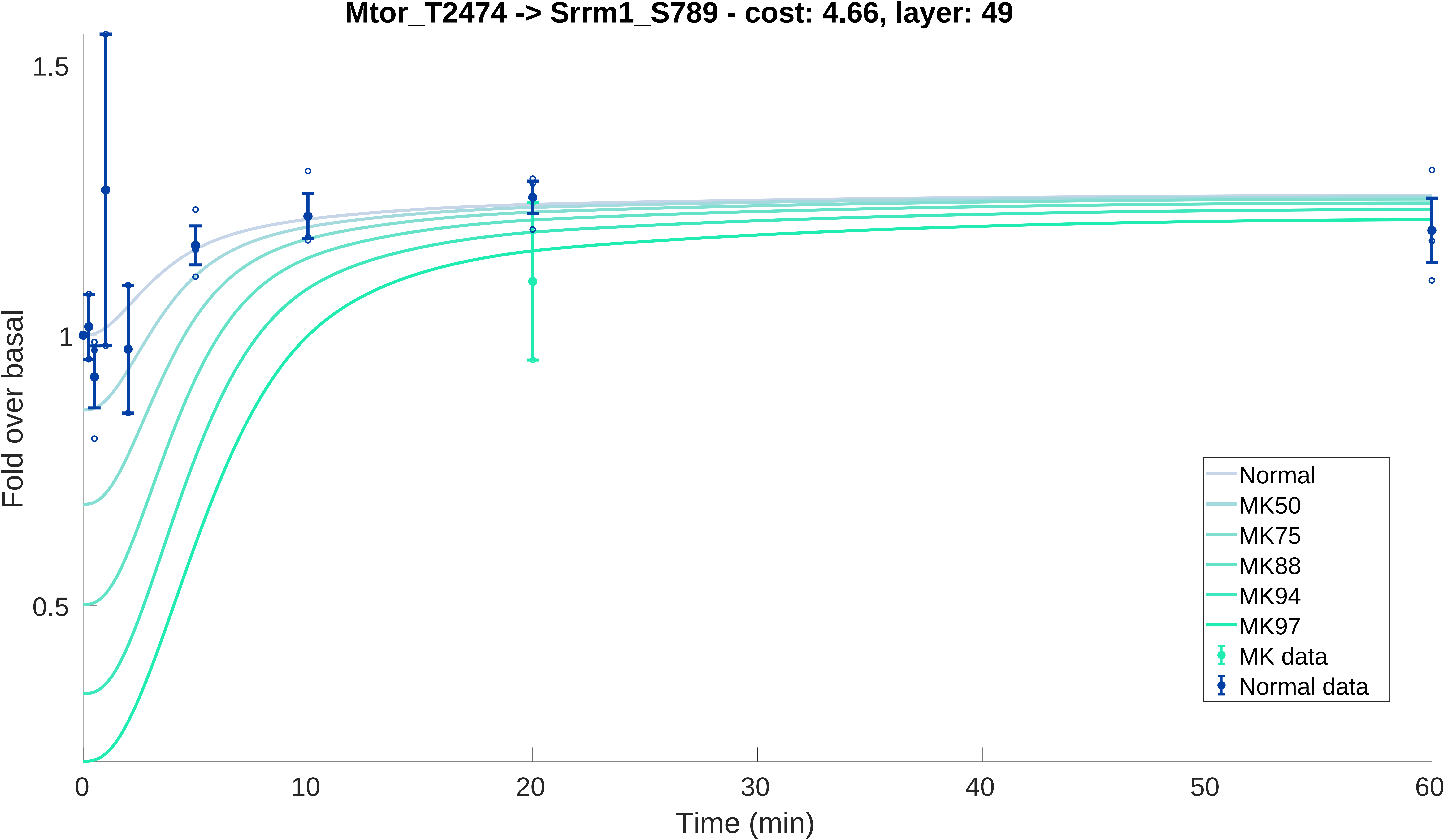

Mtor_T2474 -> Srrm1_S789 - cost: 4.66, layer: 49
Fold over basal
1.5
1
0.5
0
10
20
30
40
50
60
Time (min)
Normal
MK50
MK75
MK88
MK94
MK97
MK data
Normal data

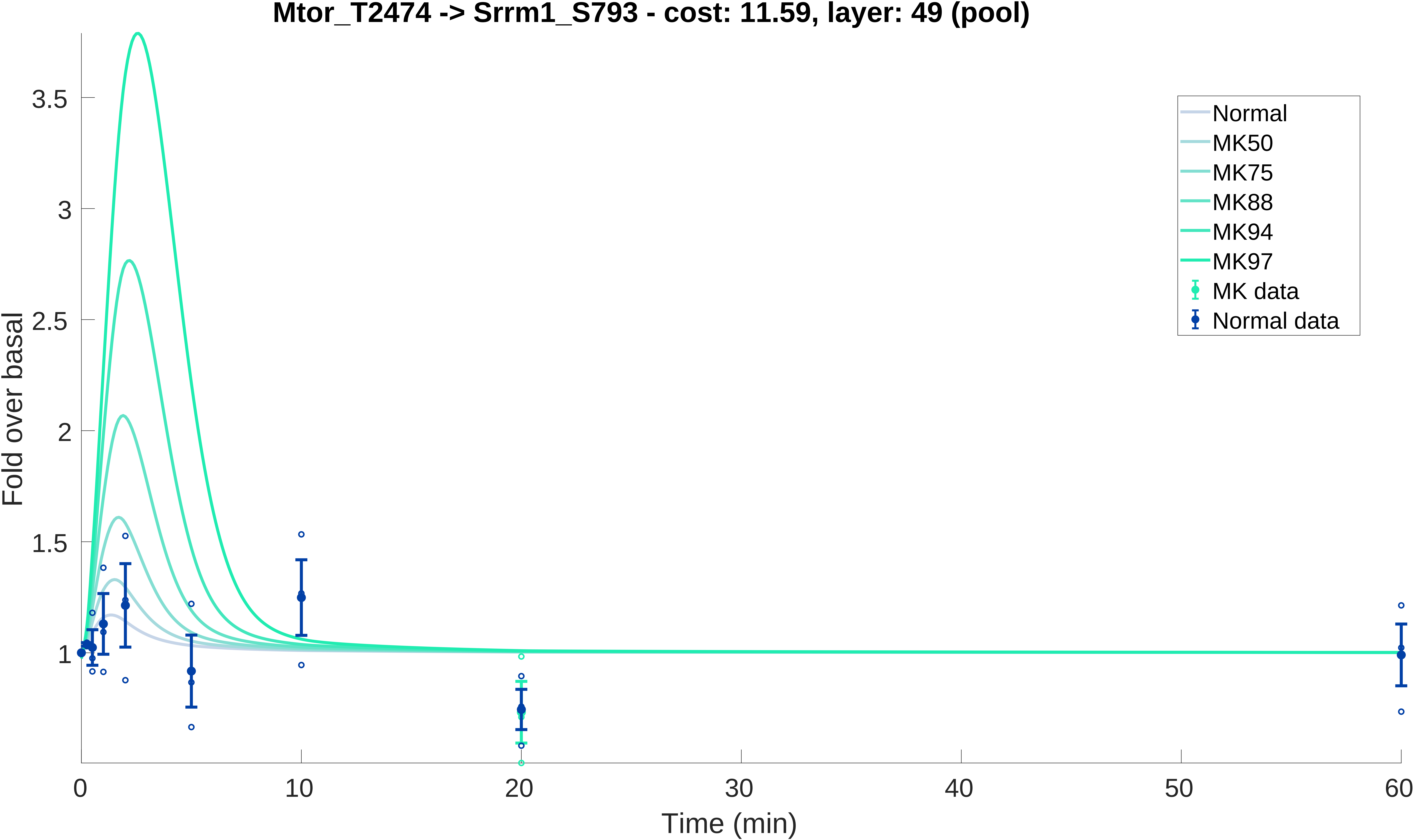
Mtor_T2474 -> Srrm1_S793 - cost: 11.59, layer: 49 (pool)
Fold over basal
3.5
3
2.5
2
1.5
1
0
10
20
30
40
50
60
Time (min)
Normal
MK50
MK75
MK88
MK94
MK97
MK data
Normal data

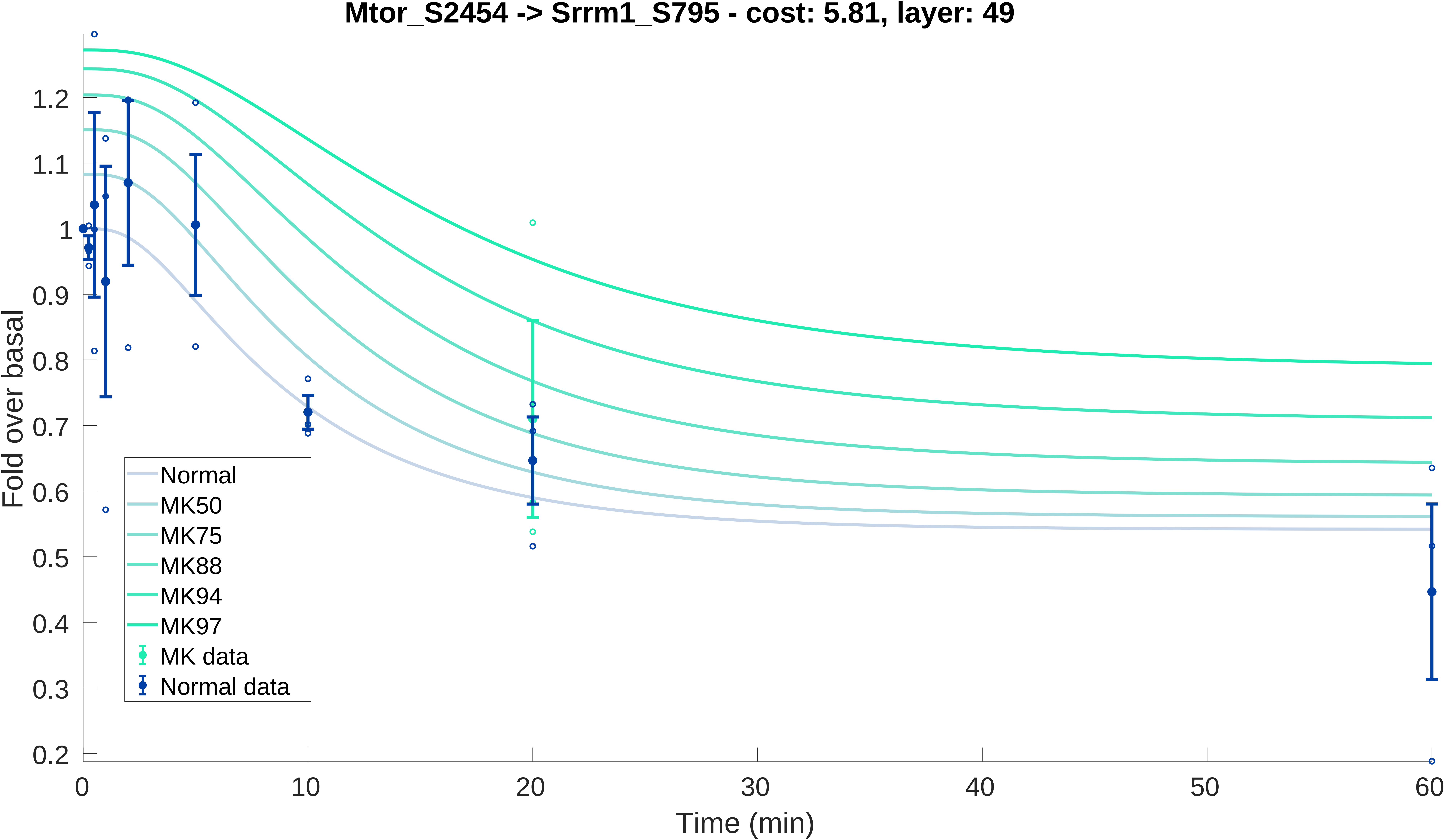
Mtor_S2454 -> Srrm1_S795 - cost: 5.81, layer: 49
Fold over basal
Time (min)
0.2
0.3
0.4
0.5
0.6
0.7
0.8
0.9
1
1.1
1.2
0
10
20
30
40
50
60
Normal
MK50
MK75
MK88
MK94
MK97
MK data
Normal data

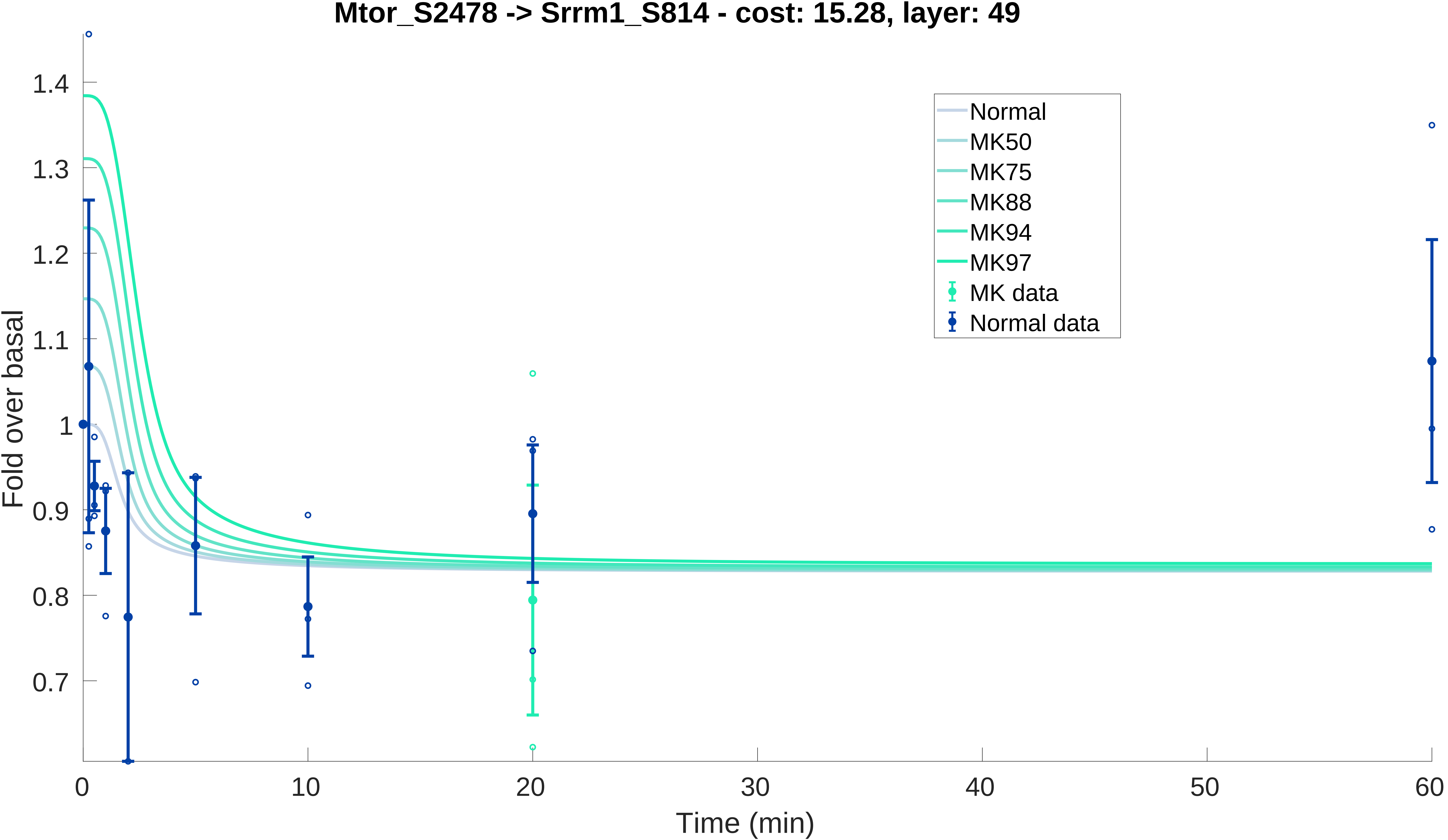
Mtor_S2478 -> Srrm1_S814 - cost: 15.28, layer: 49
Fold over basal
Time (min)
Normal
MK50
MK75
MK88
MK94
MK97
MK data
Normal data
1.4
1.3
1.2
1.1
1
0.9
0.8
0.7
0
10
20
30
40
50
60

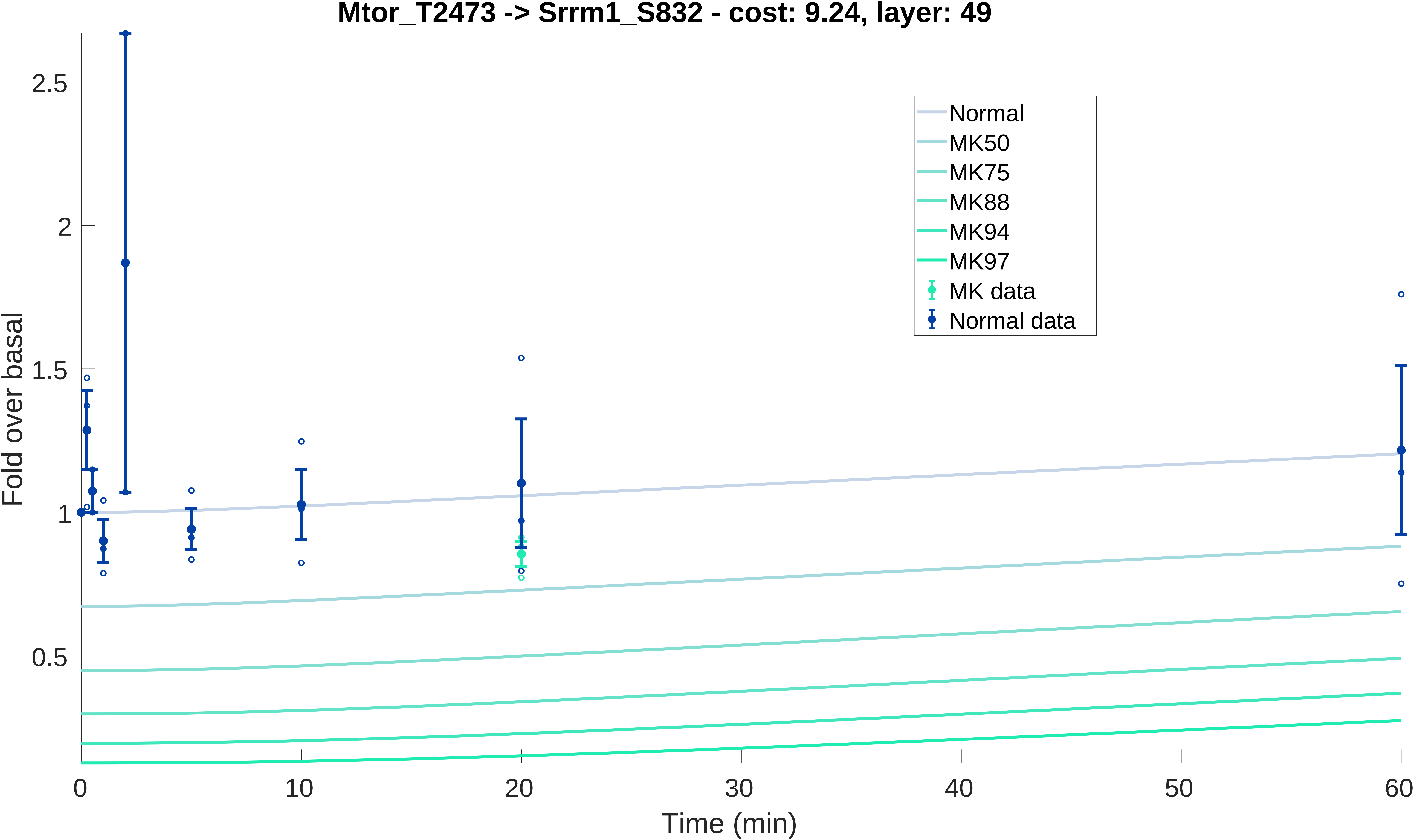
Mtor_T2473 -> Srrm1_S832 - cost: 9.24, layer: 49
Fold over basal
2.5
2
1.5
1
0.5
0
10
20
30
40
50
60
Time (min)
Normal
MK50
MK75
MK88
MK94
MK97
MK data
Normal data

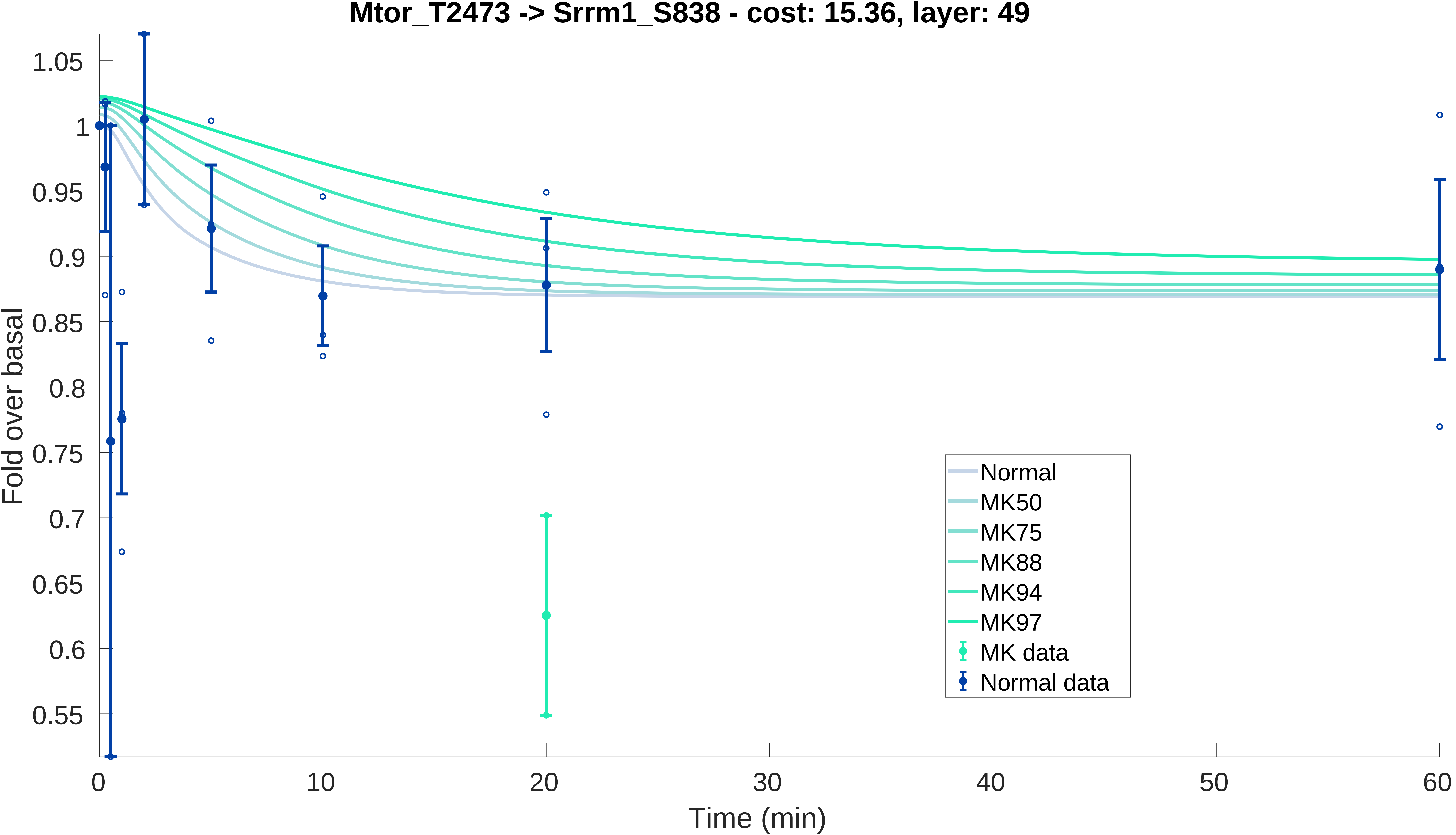

Mtor_T2473 -> Srrm1_S838 - cost: 15.36, layer: 49
Fold over basal
Time (min)
1.05
1
0.95
0.9
0.85
0.8
0.75
0.7
0.65
0.6
0.55
0
10
20
30
40
50
60
Normal
MK50
MK75
MK88
MK94
MK97
MK data
Normal data

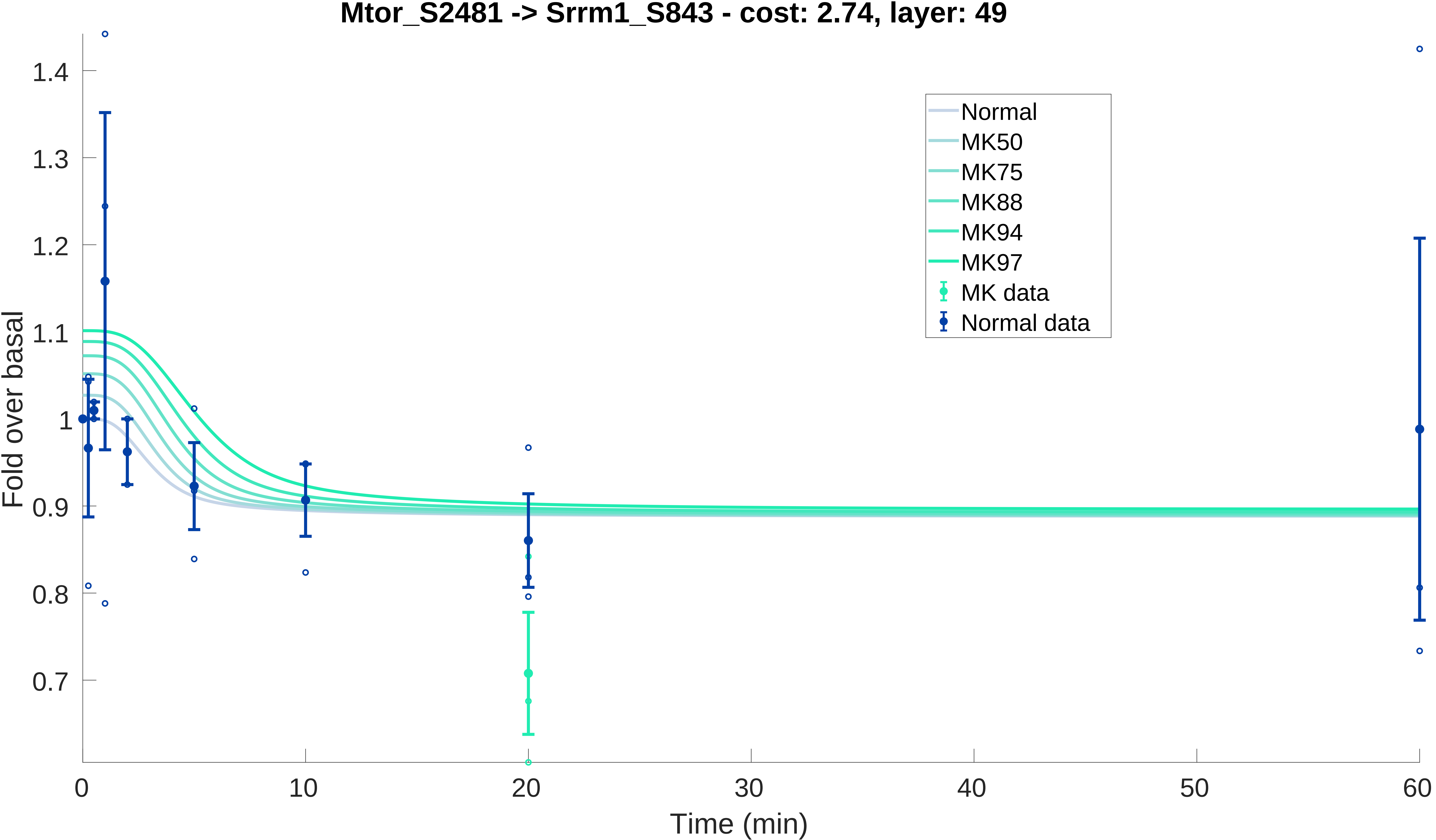

Mtor_S2481 -> Srrm1_S843 - cost: 2.74, layer: 49
Fold over basal
1.4
1.3
1.2
1.1
1
0.9
0.8
0.7
Time (min)
0
10
20
30
40
50
60
Normal
MK50
MK75
MK88
MK94
MK97
MK data
Normal data

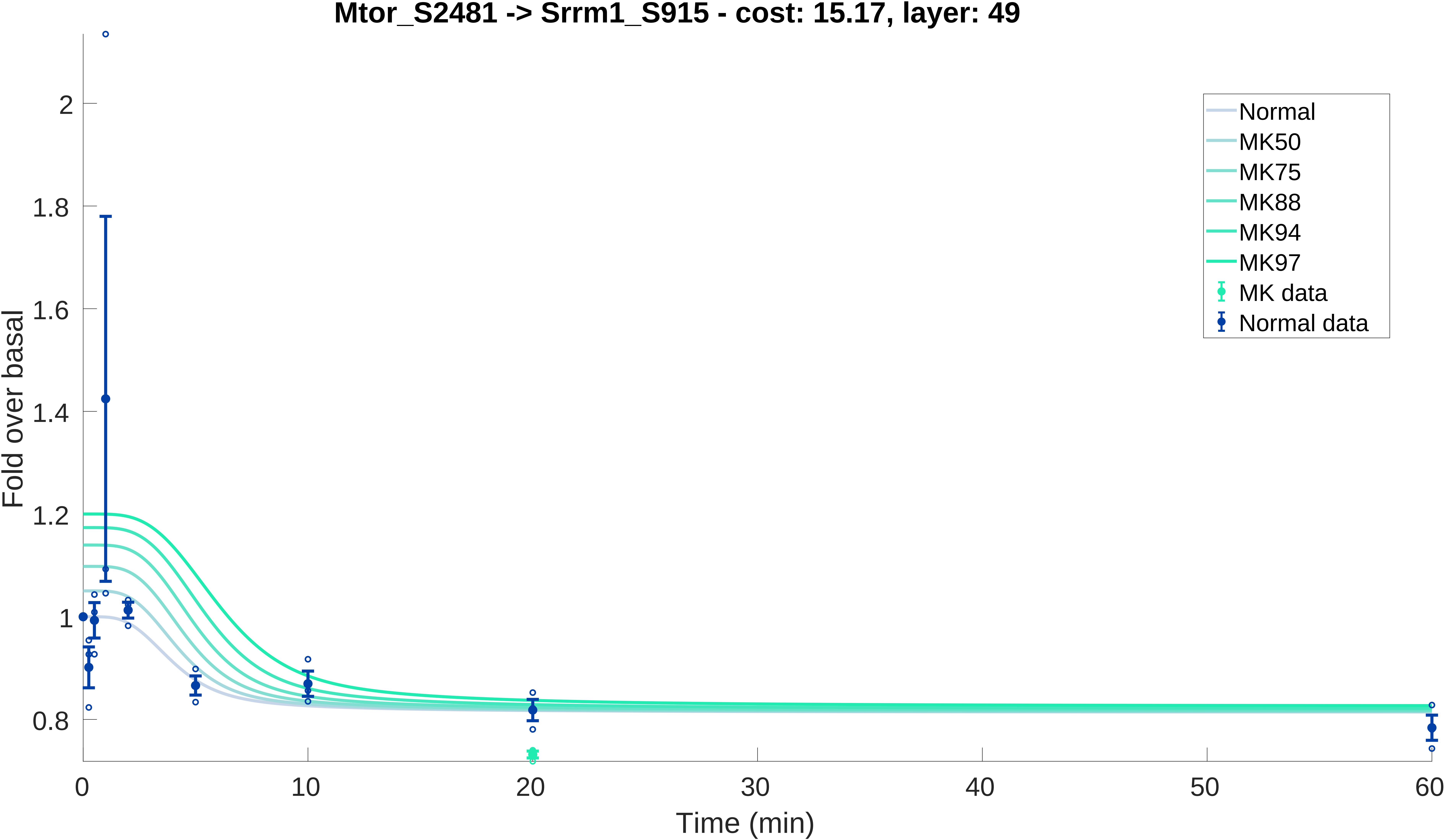

Mtor_S2481 -> Srrm1_S915 - cost: 15.17, layer: 49
Fold over basal
Time (min)
0.8
1
1.2
1.4
1.6
1.8
2
0
10
20
30
40
50
60
Normal
MK50
MK75
MK88
MK94
MK97
MK data
Normal data

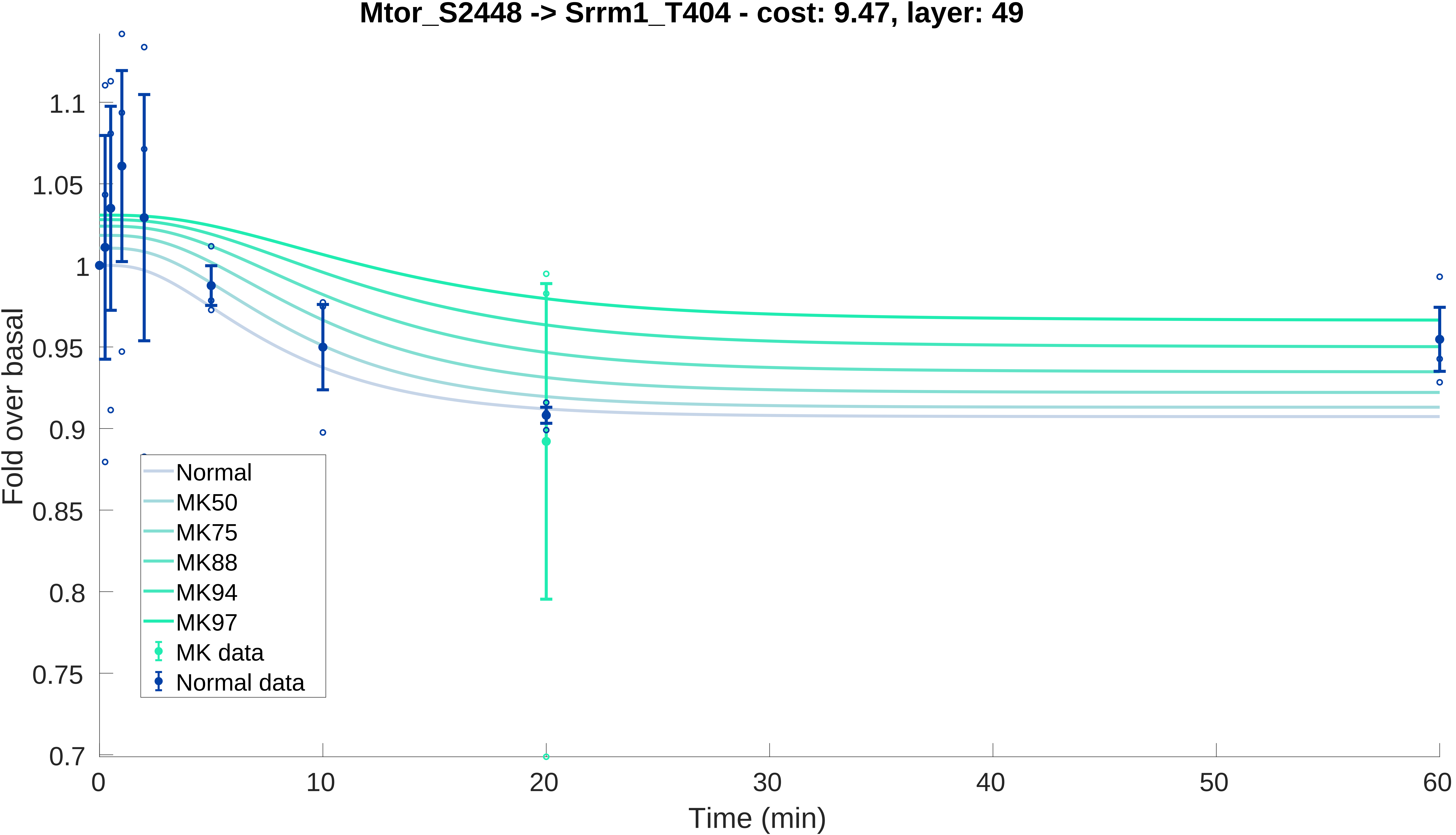

Mtor_S2448 -> Srrm1_T404 - cost: 9.47, layer: 49
Fold over basal
Time (min)
0.7
0.75
0.8
0.85
0.9
0.95
1
1.05
1.1
0
10
20
30
40
50
60
Normal
MK50
MK75
MK88
MK94
MK97
MK data
Normal data

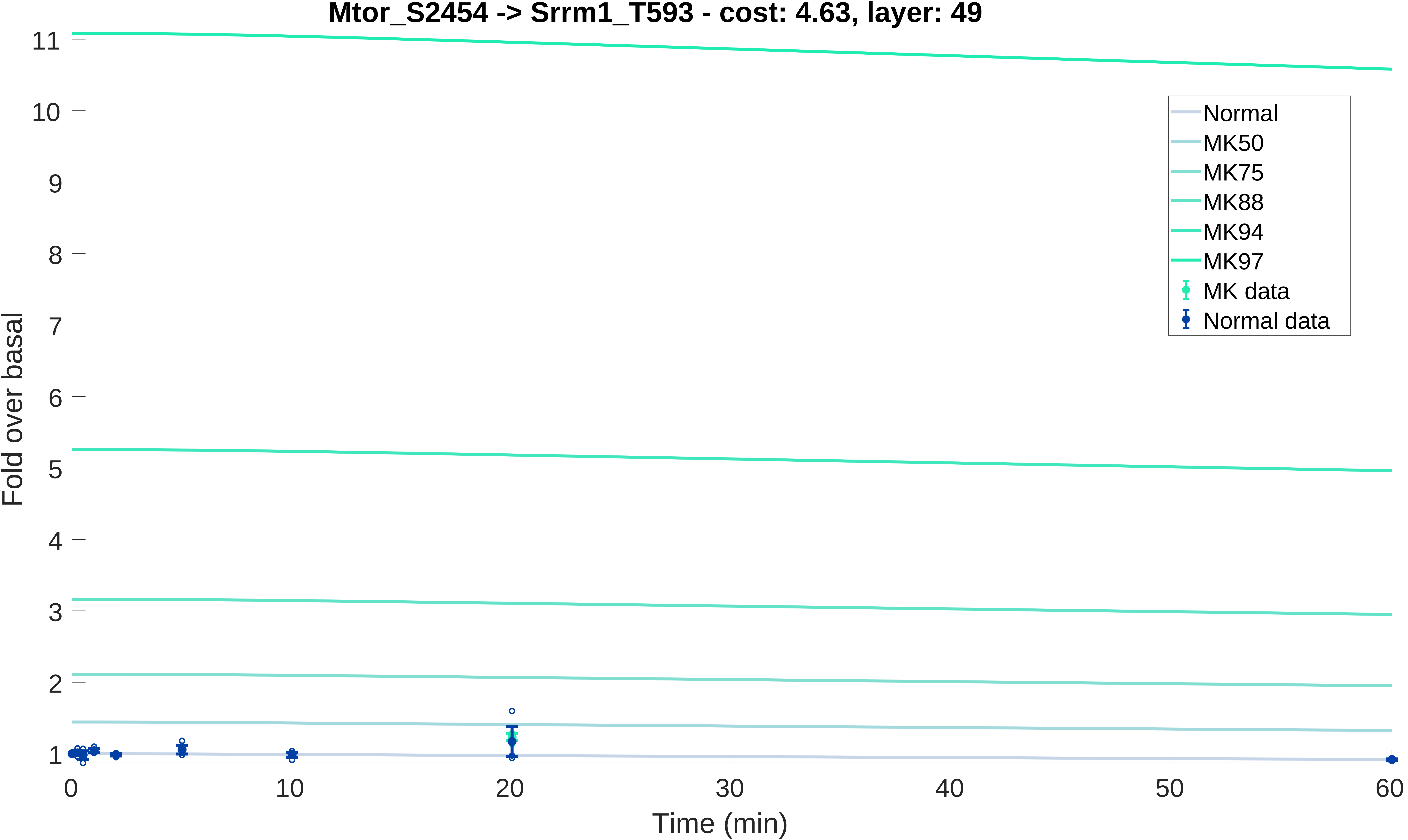
Mtor_S2454 -> Srrm1_T593 - cost: 4.63, layer: 49
Fold over basal
Time (min)
Normal
MK50
MK75
MK88
MK94
MK97
MK data
Normal data
0
10
20
30
40
50
60
1
2
3
4
5
6
7
8
9
10
11

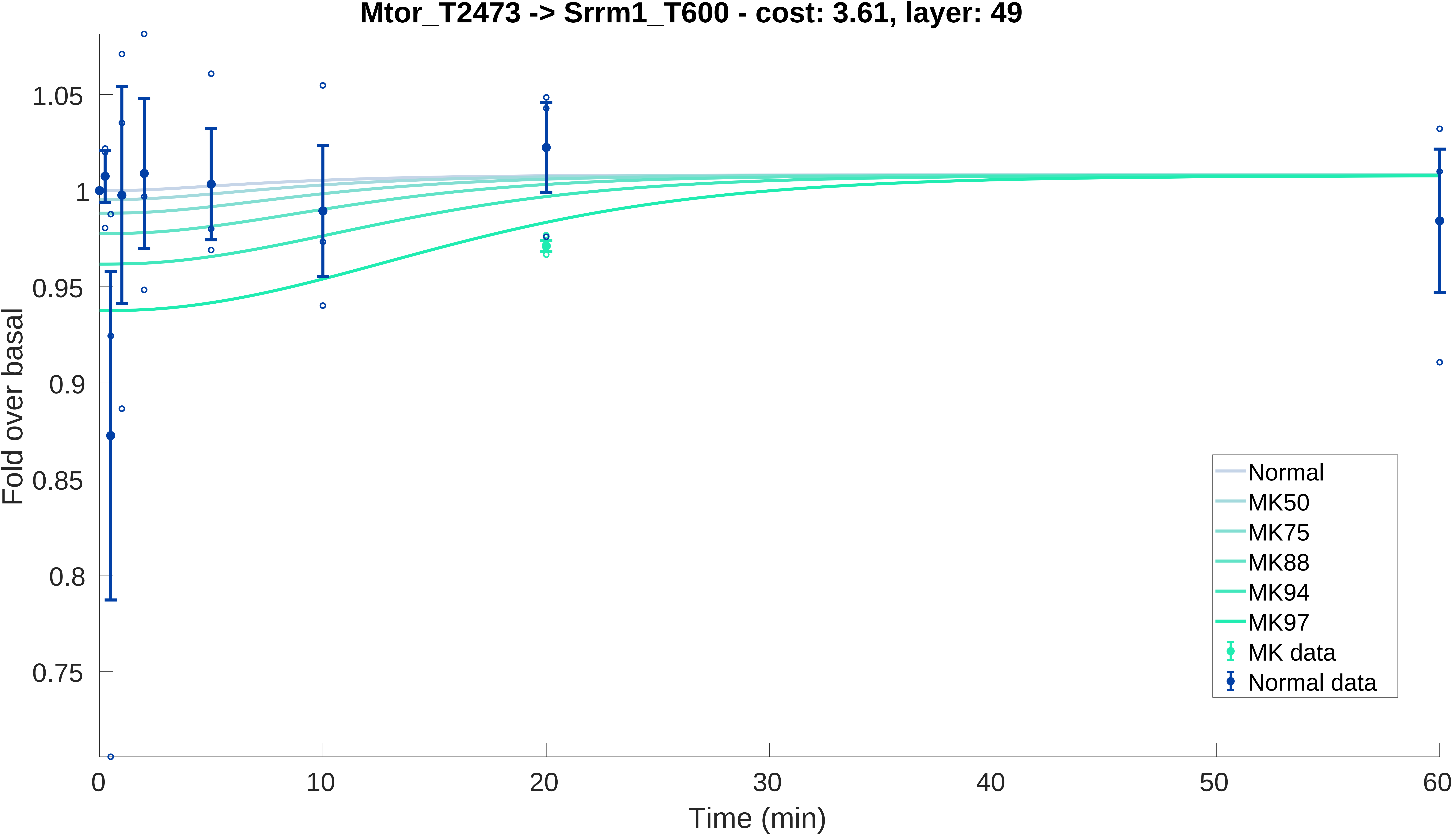

Mtor_T2473 -> Srrm1_T600 - cost: 3.61, layer: 49
Fold over basal
1.05
1
0.95
0.9
0.85
0.8
0.75
0
10
20
30
40
50
60
Time (min)
Normal
MK50
MK75
MK88
MK94
MK97
MK data
Normal data

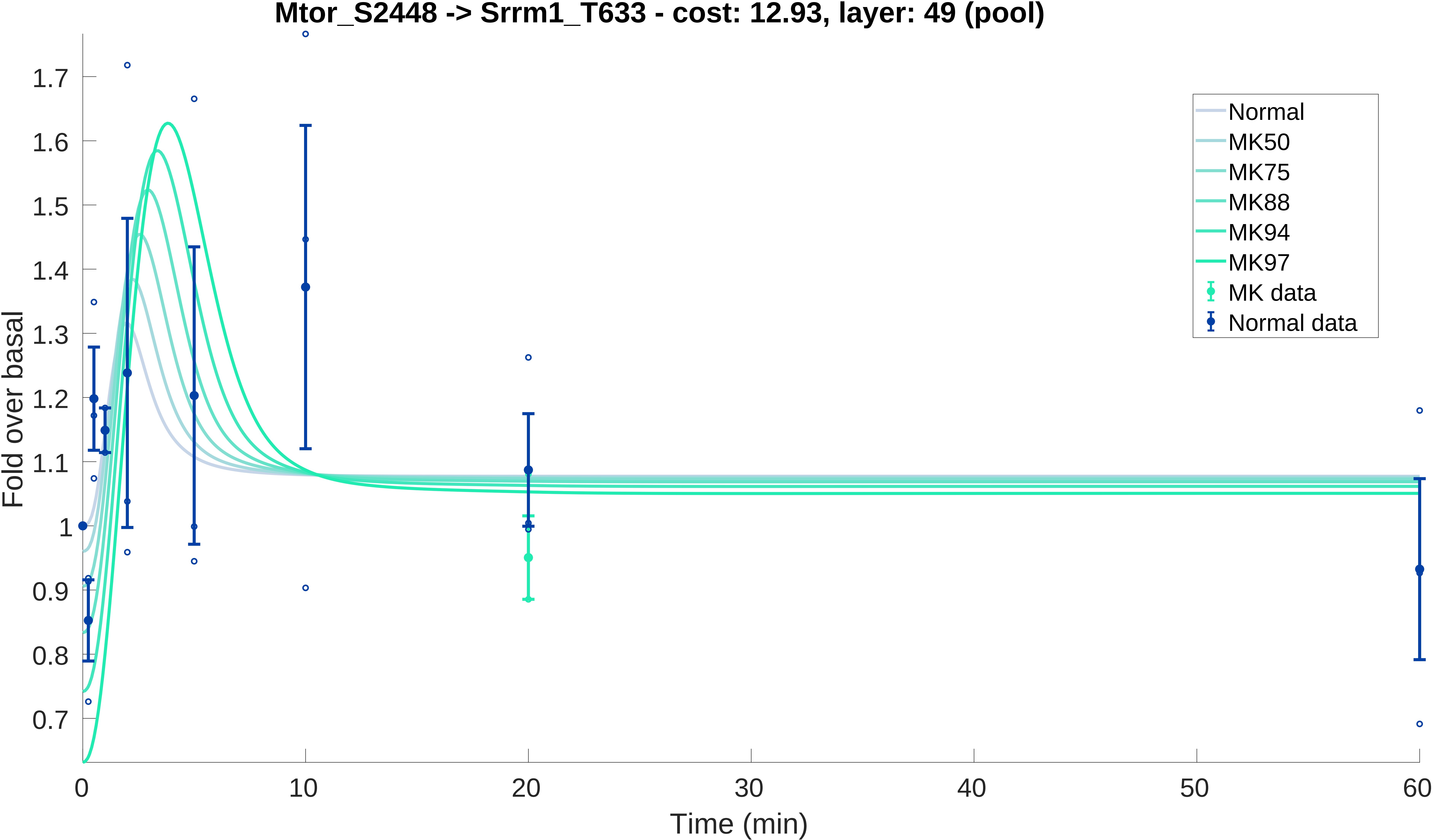
Mtor_S2448 -> Srrm1_T633 - cost: 12.93, layer: 49 (pool)
Fold over basal
Time (min)
0.7
0.8
0.9
1
1.1
1.2
1.3
1.4
1.5
1.6
1.7
0
10
20
30
40
50
60
Normal
MK50
MK75
MK88
MK94
MK97
MK data
Normal data

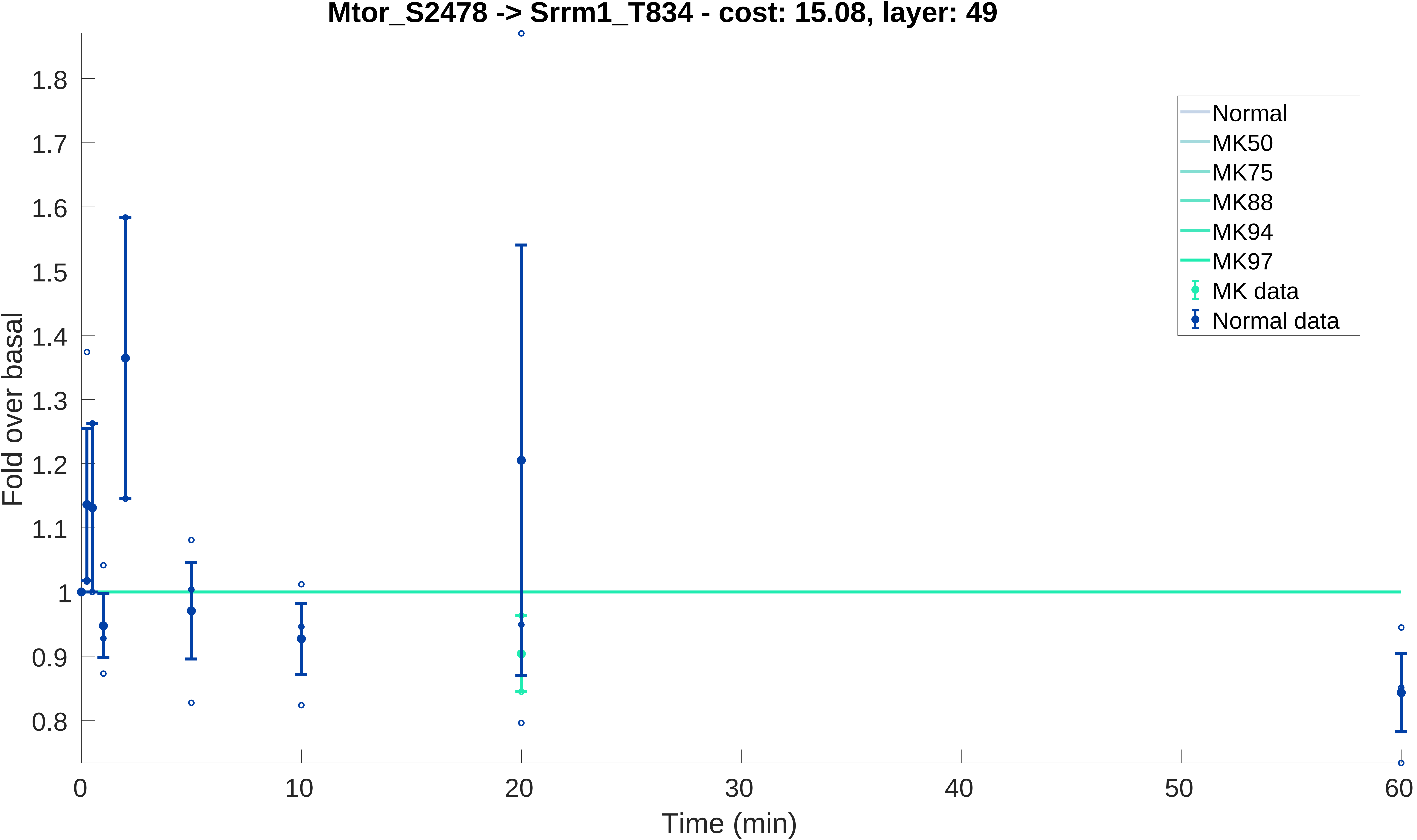

Mtor_S2478 -> Srrm1_T834 - cost: 15.08, layer: 49
Fold over basal
Time (min)
0.8
0.9
1
1.1
1.2
1.3
1.4
1.5
1.6
1.7
1.8
0
10
20
30
40
50
60
Normal
MK50
MK75
MK88
MK94
MK97
MK data
Normal data

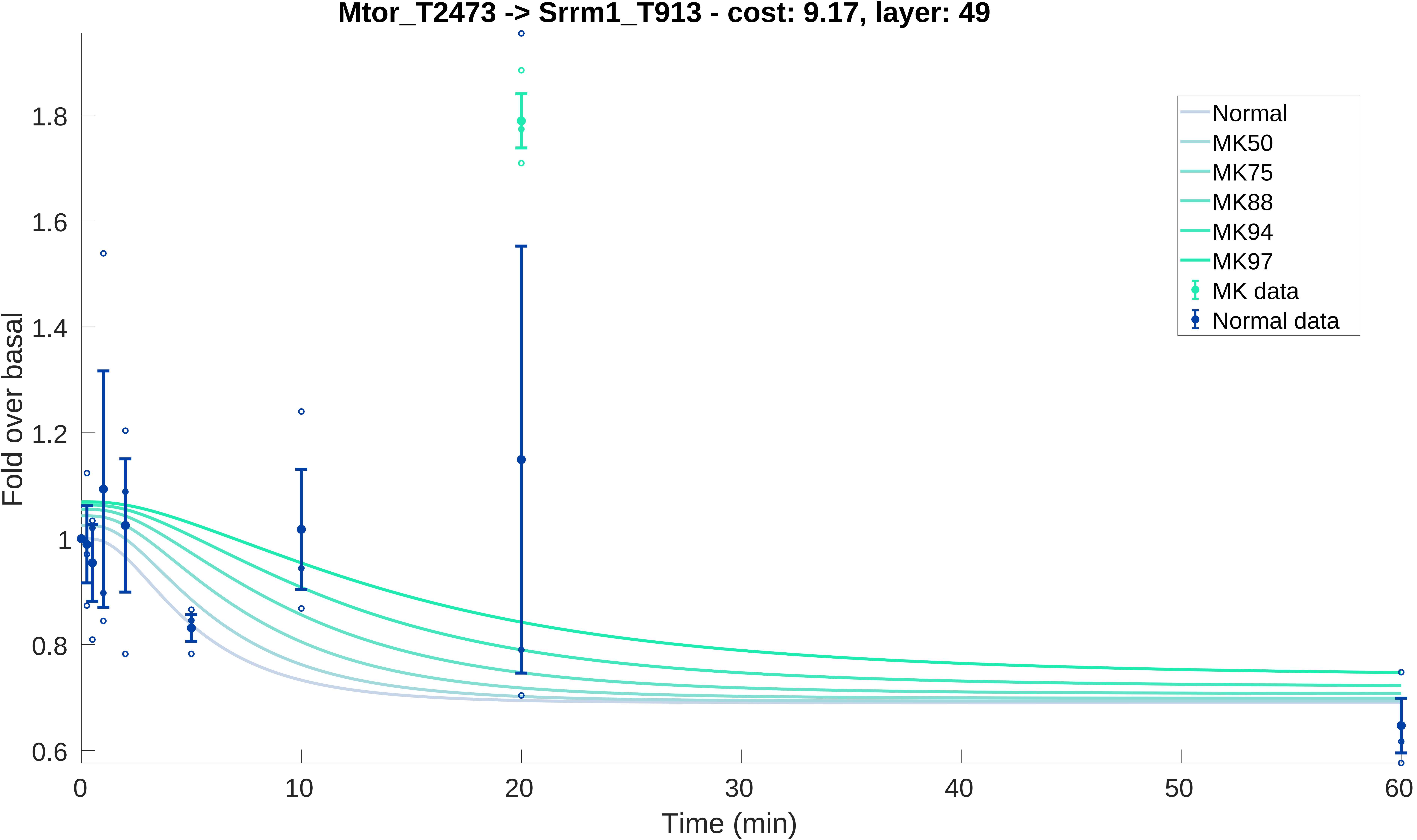
Mtor_T2473 -> Srrm1_T913 - cost: 9.17, layer: 49
Fold over basal
Time (min)
0.6
0.8
1
1.2
1.4
1.6
1.8
0
10
20
30
40
50
60
Normal
MK50
MK75
MK88
MK94
MK97
MK data
Normal data

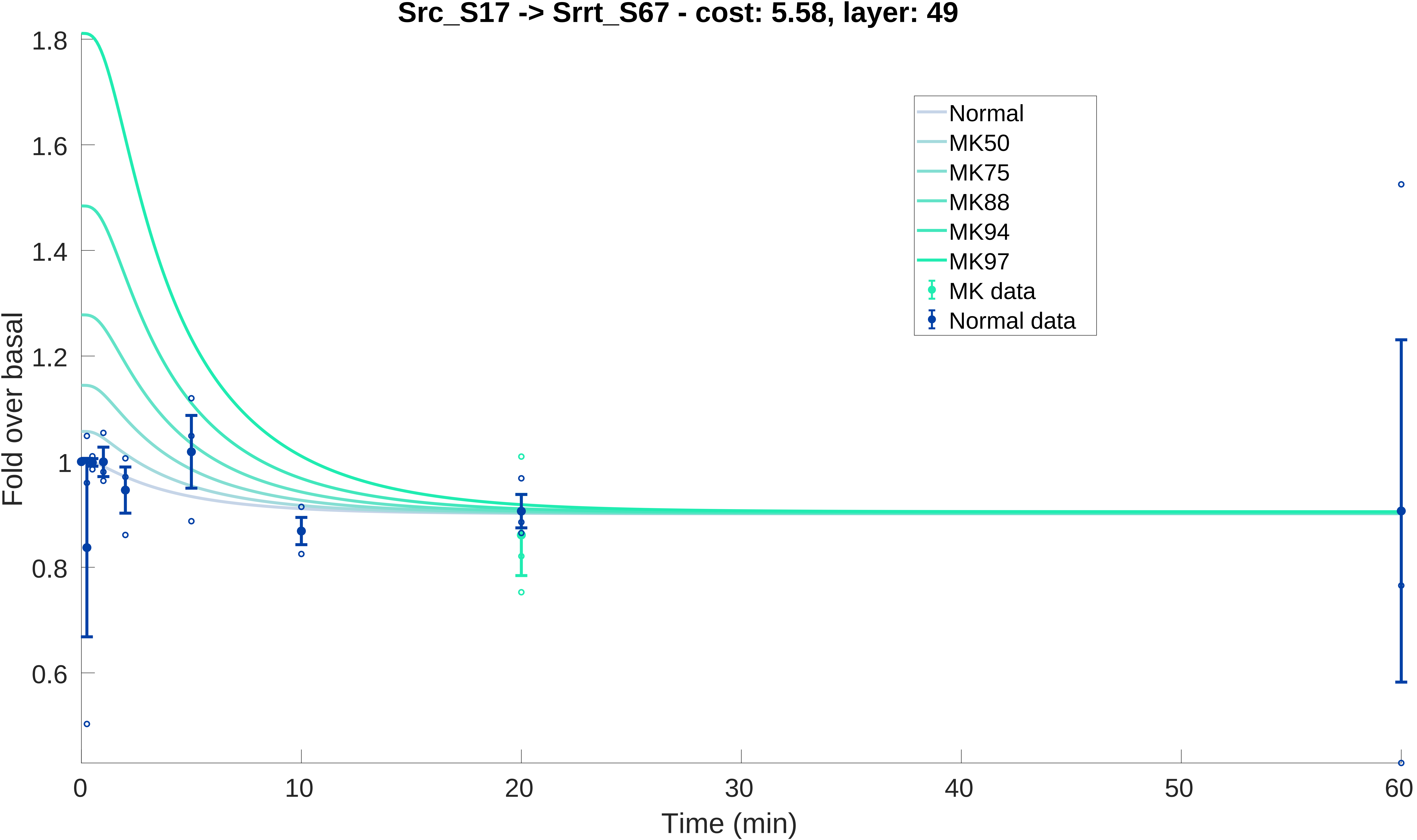

Src_S17 -> Srrt_S67 - cost: 5.58, layer: 49
Fold over basal
Time (min)
1.8
1.6
1.4
1.2
1
0.8
0.6
0
10
20
30
40
50
60
Normal
MK50
MK75
MK88
MK94
MK97
MK data
Normal data

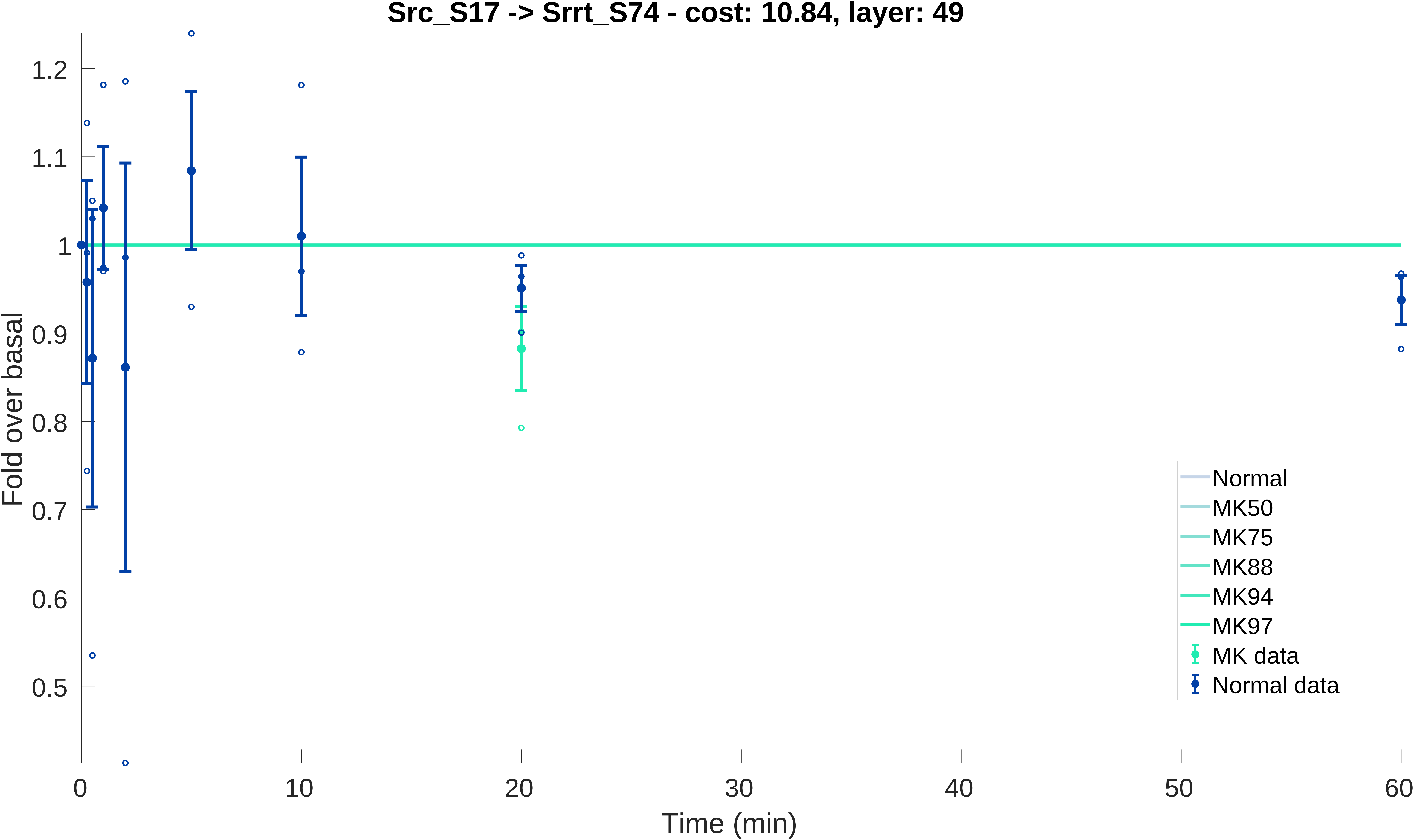

Src_S17 -> Srrt_S74 - cost: 10.84, layer: 49
Fold over basal
Time (min)
1.2
1.1
1
0.9
0.8
0.7
0.6
0.5
0
10
20
30
40
50
60
Normal
MK50
MK75
MK88
MK94
MK97
MK data
Normal data

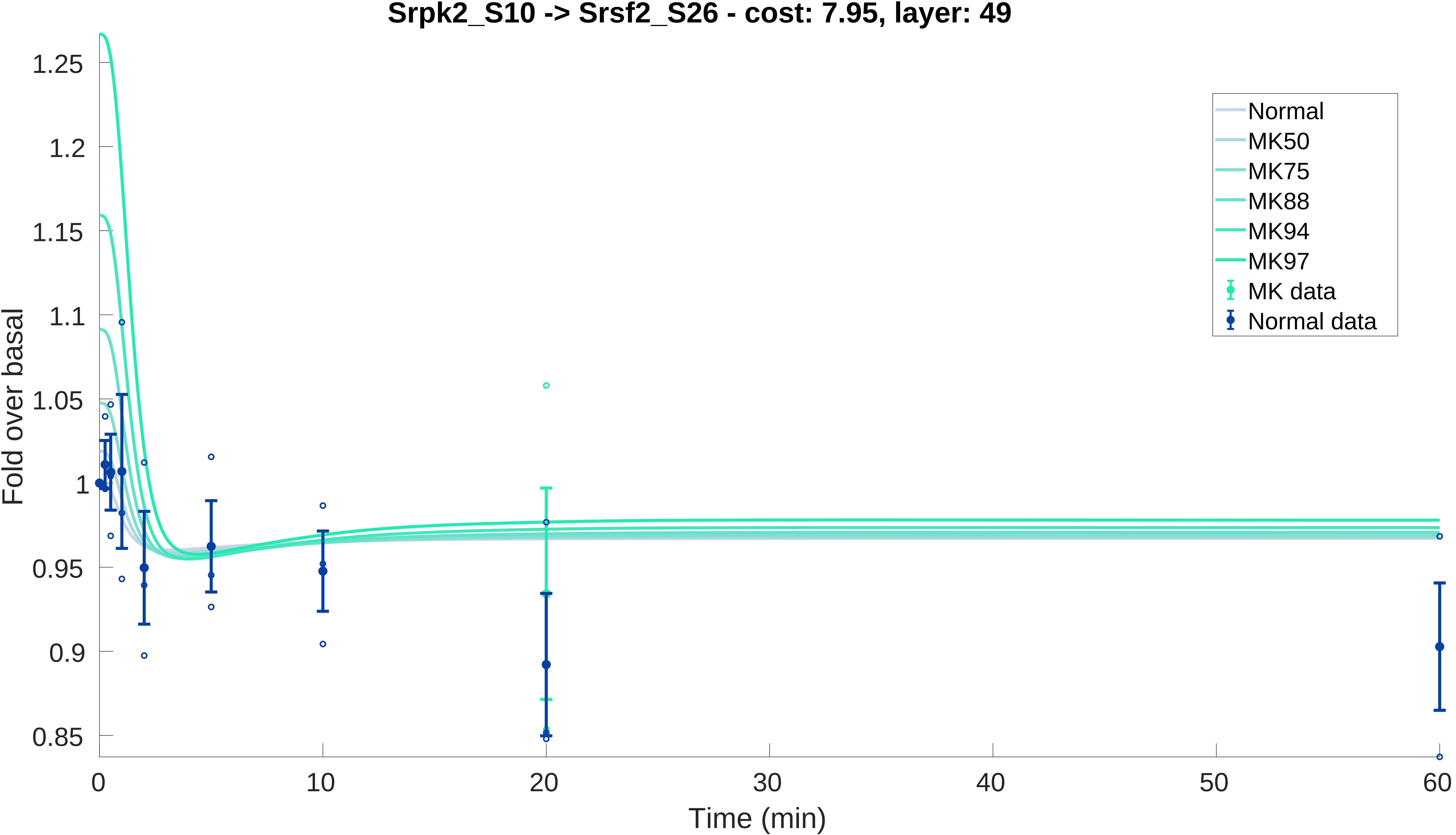

Srpk2_S10 -> Srsf2_S26 - cost: 7.95, layer: 49
Fold over basal
1.25
1.2
1.15
1.1
1.05
1
0.95
0.9
0.85
0
10
20
30
40
50
60
Time (min)
Normal
MK50
MK75
MK88
MK94
MK97
MK data
Normal data

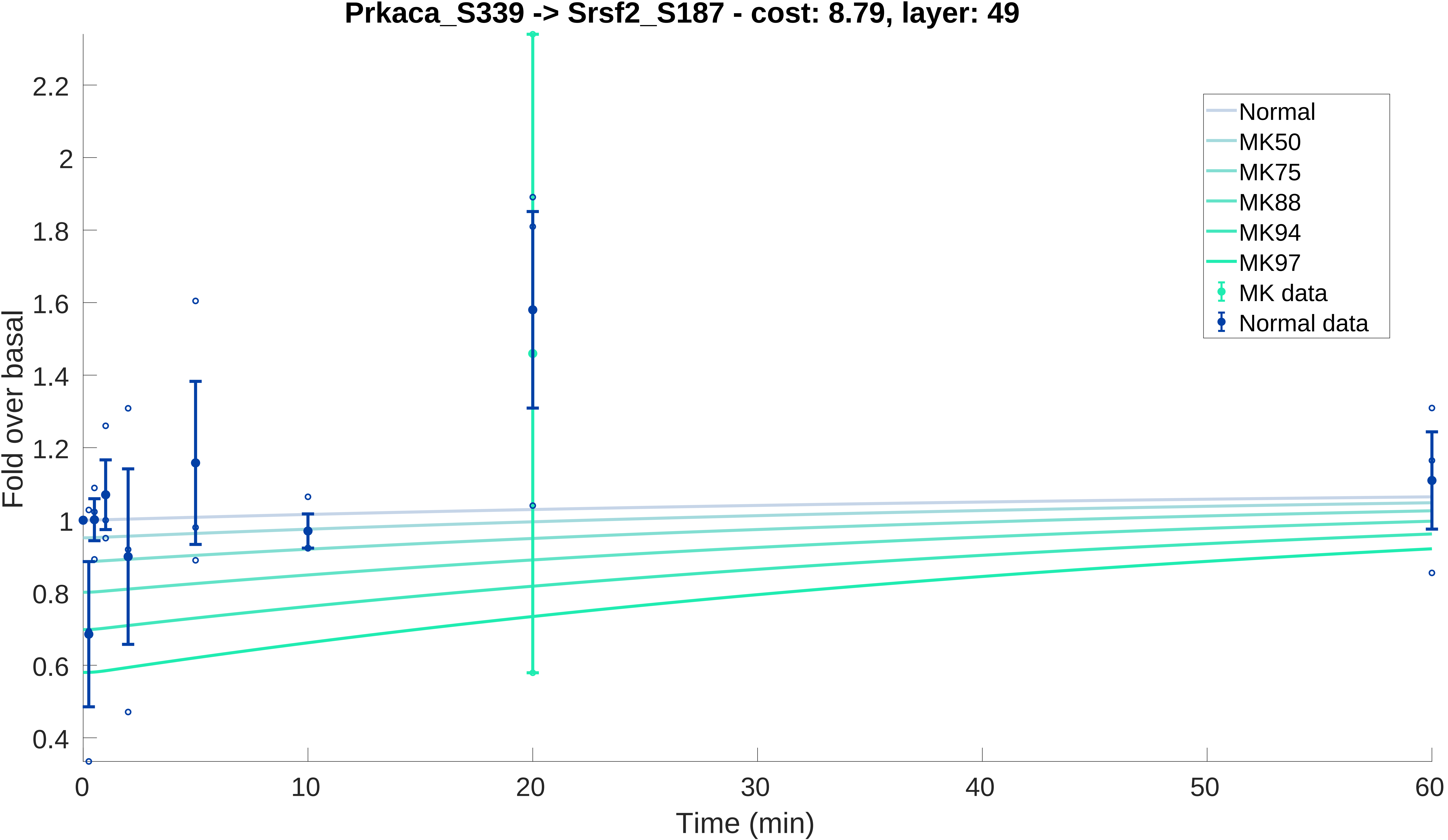

Prkaca_S339 -> Srsf2_S187 - cost: 8.79, layer: 49
Fold over basal
Time (min)
Normal
MK50
MK75
MK88
MK94
MK97
MK data
Normal data
0.4
0.6
0.8
1
1.2
1.4
1.6
1.8
2
2.2
0
10
20
30
40
50
60

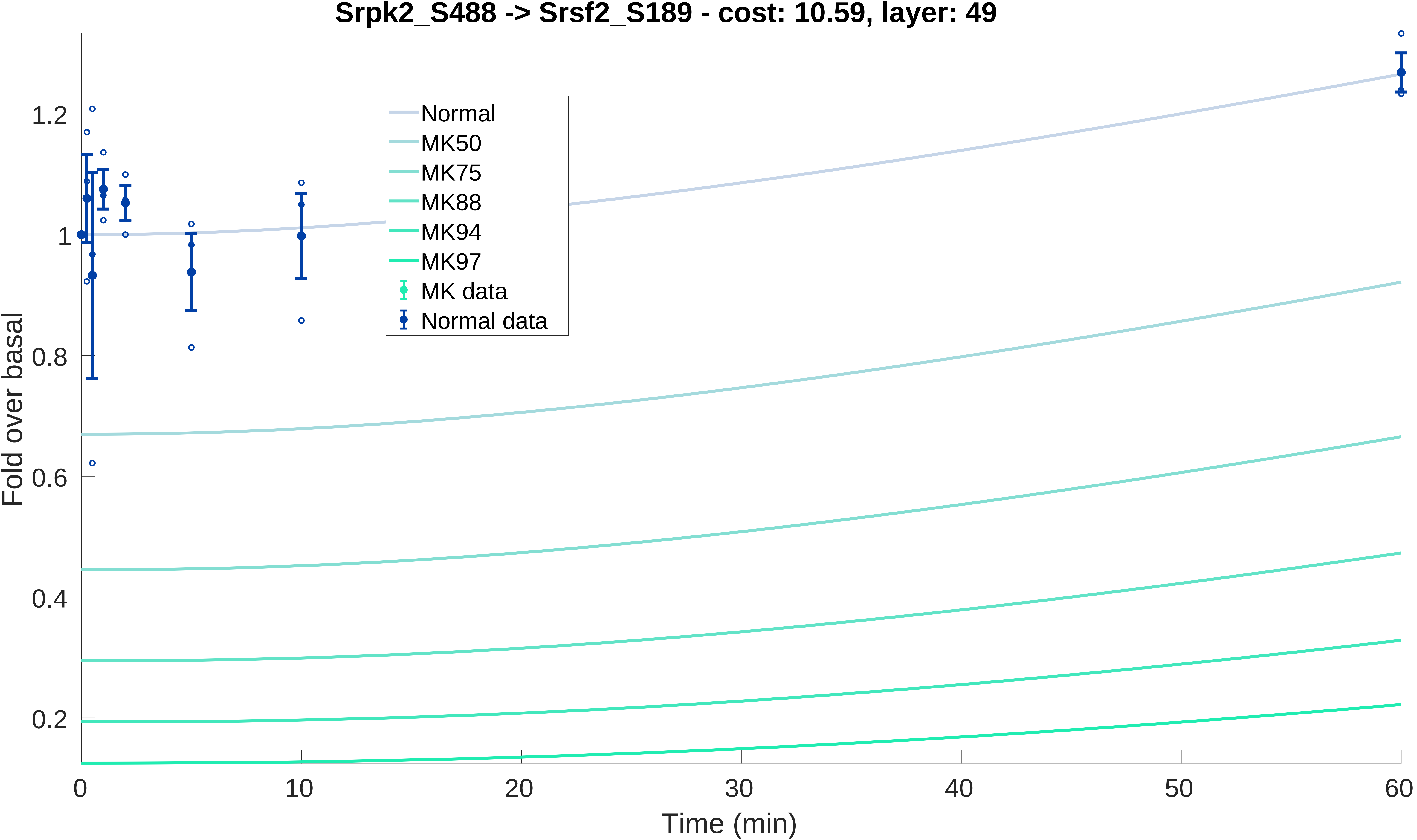

Srpk2_S488 -> Srsf2_S189 - cost: 10.59, layer: 49
Normal
MK50
MK75
MK88
MK94
MK97
MK data
Normal data
Fold over basal
0.2
0.4
0.6
0.8
1
1.2
0
10
20
30
40
50
60
Time (min)

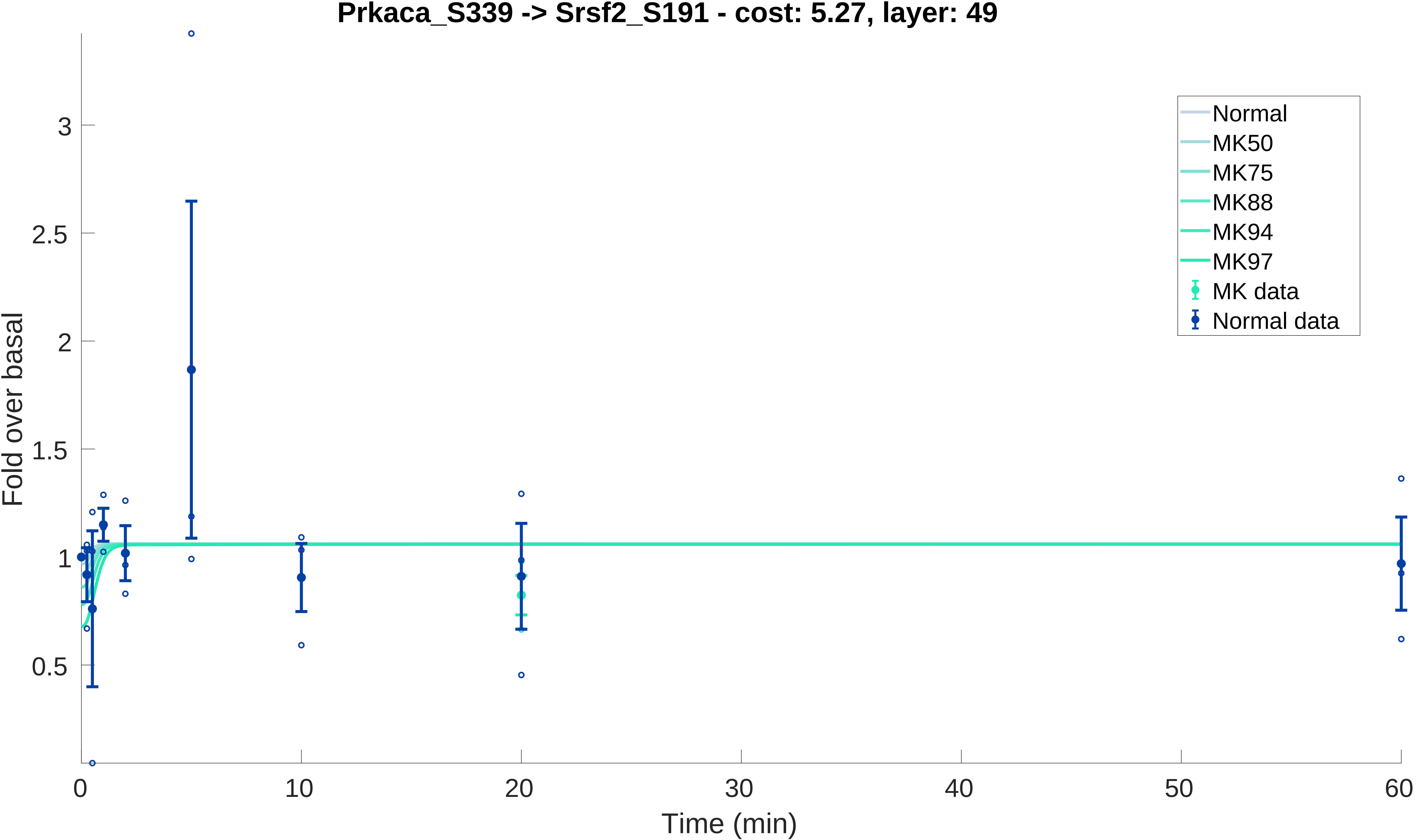

Prrkaca_S339 -> Srsf2_S191 - cost: 5.27, layer: 49
Fold over basal
Time (min)
0
10
20
30
40
50
60
0.5
1
1.5
2
2.5
3
Normal
MK50
MK75
MK88
MK94
MK97
MK data
Normal data

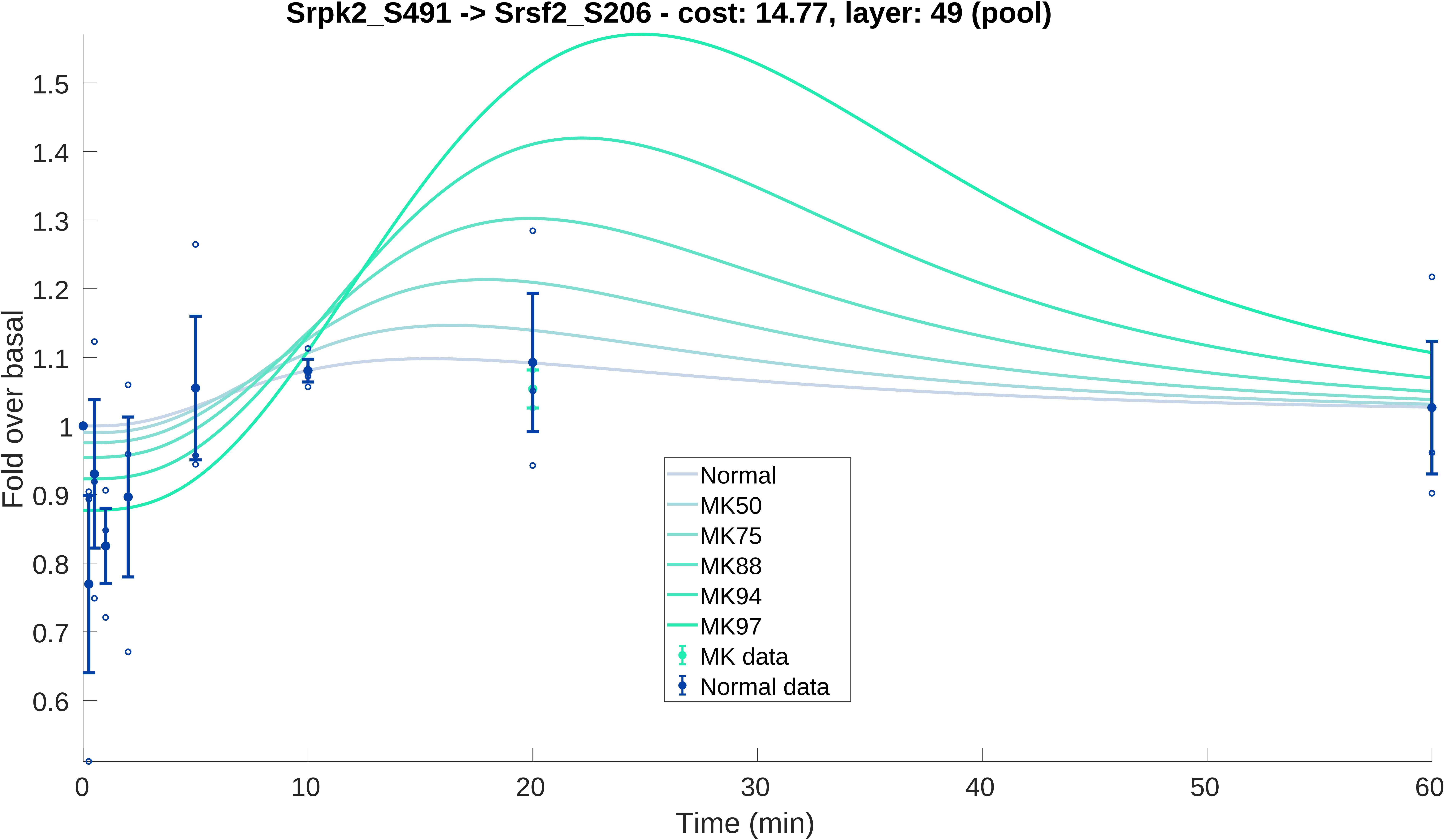

Srpk2_S491 -> Srsf2_S206 - cost: 14.77, layer: 49 (pool)
Fold over basal
1.5
1.4
1.3
1.2
1.1
1
0.9
0.8
0.7
0.6
0
10
20
30
40
50
60
Time (min)
Normal
MK50
MK75
MK88
MK94
MK97
MK data
Normal data

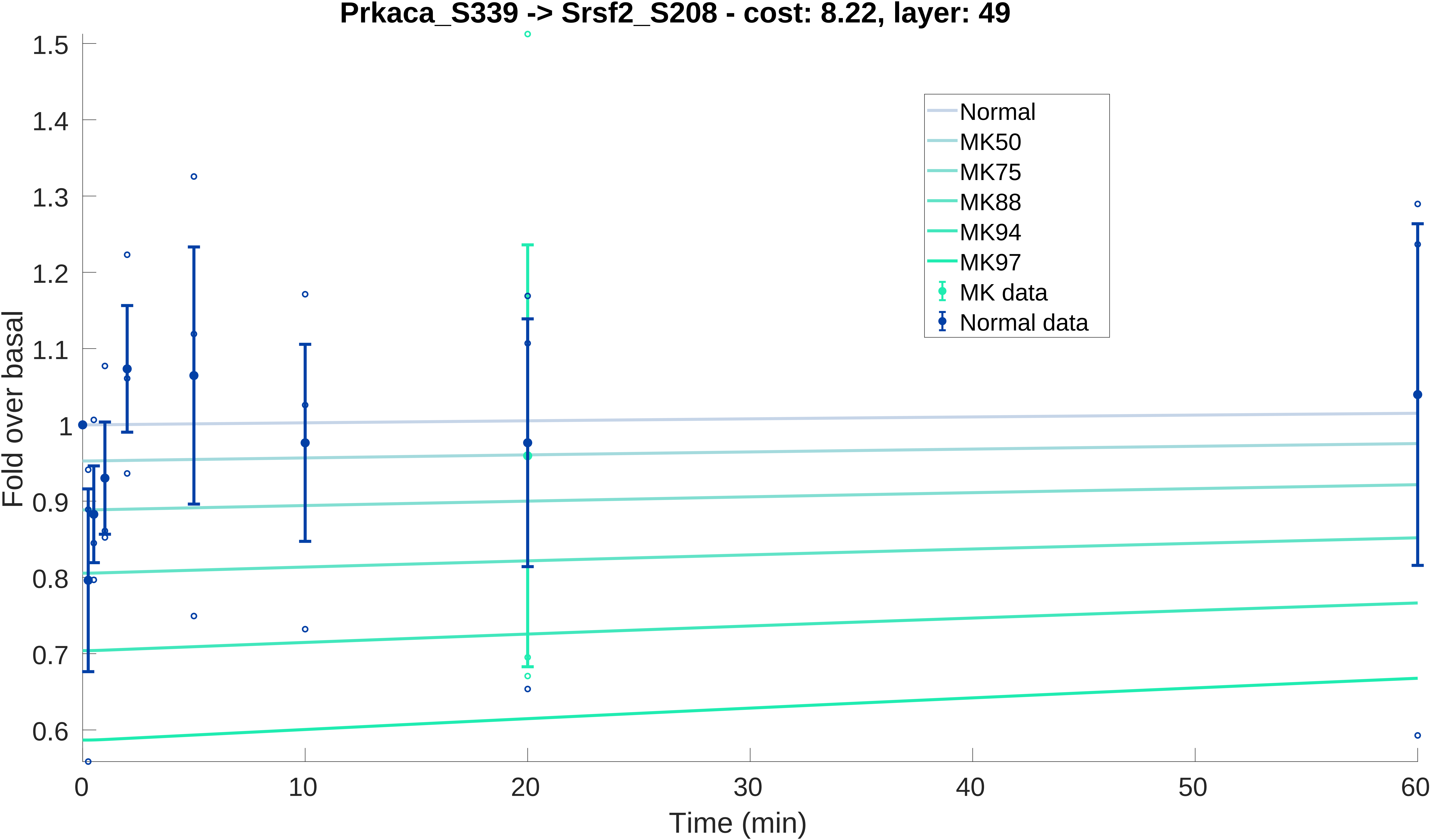

Prkaca_S339 -> Srsf2_S208 - cost: 8.22, layer: 49
Fold over basal
Time (min)
0
10
20
30
40
50
60
0.6
0.7
0.8
0.9
1
1.1
1.2
1.3
1.4
1.5
Normal
MK50
MK75
MK88
MK94
MK97
MK data
Normal data

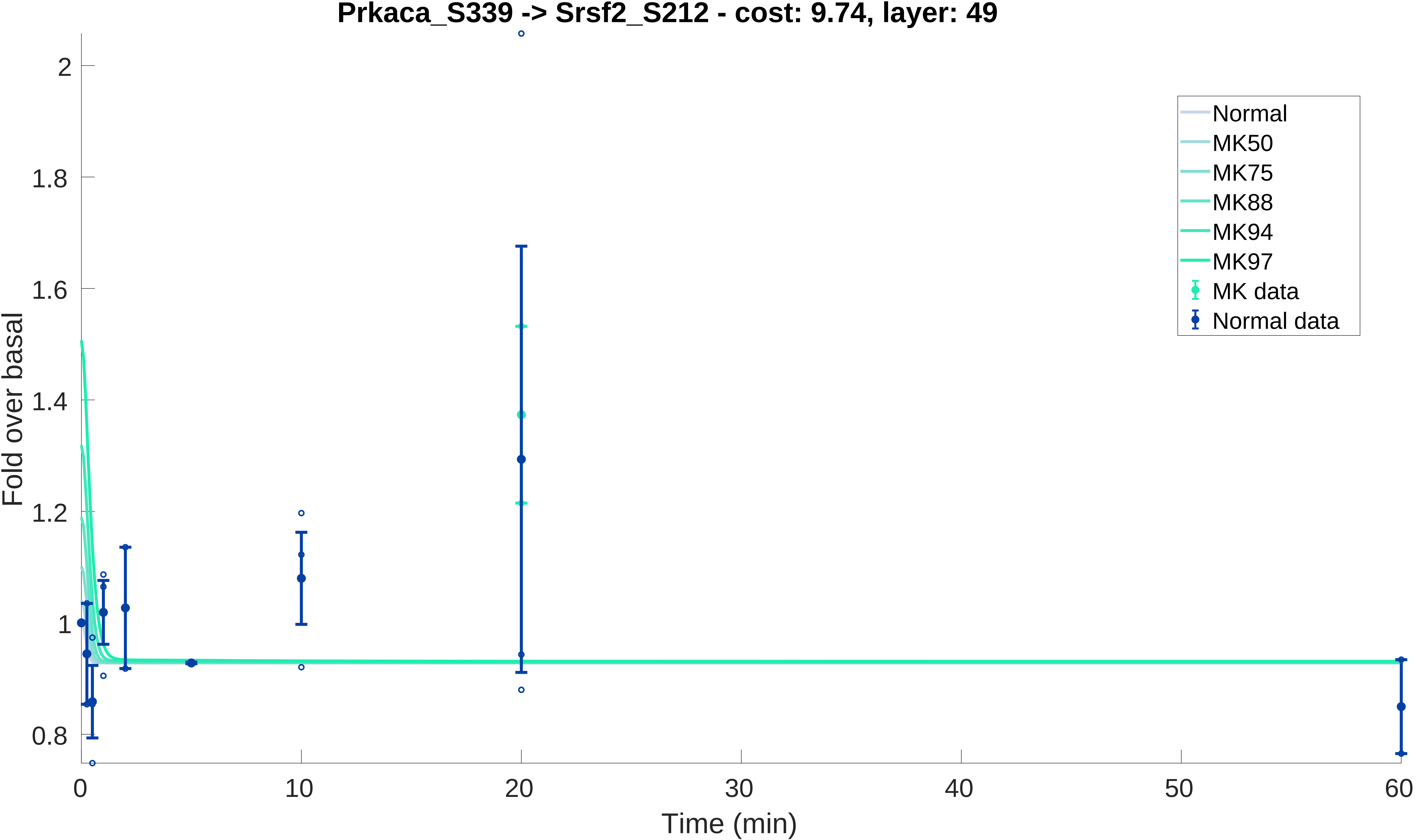

Prkaca_S339 -> Srsf2_S212 - cost: 9.74, layer: 49
Fold over basal
Time (min)
0
10
20
30
40
50
60
0.8
1
1.2
1.4
1.6
1.8
2
Normal
MK50
MK75
MK88
MK94
MK97
MK data
Normal data

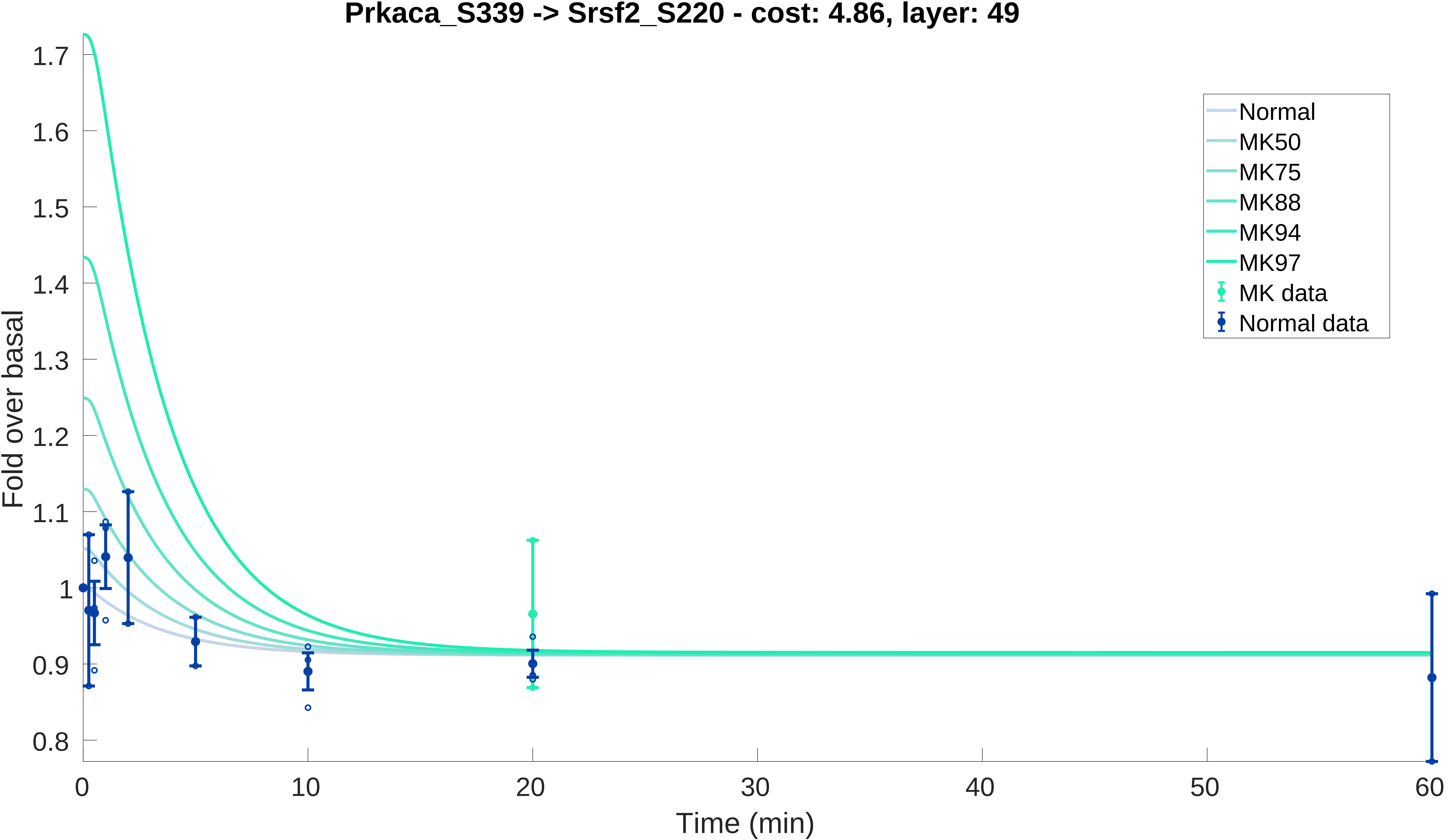

Prkaca_S339 -> Srsf2_S220 - cost: 4.86, layer: 49
Fold over basal
Time (min)
1.7
1.6
1.5
1.4
1.3
1.2
1.1
1
0.9
0.8
0
10
20
30
40
50
60
Normal
MK50
MK75
MK88
MK94
MK97
MK data
Normal data

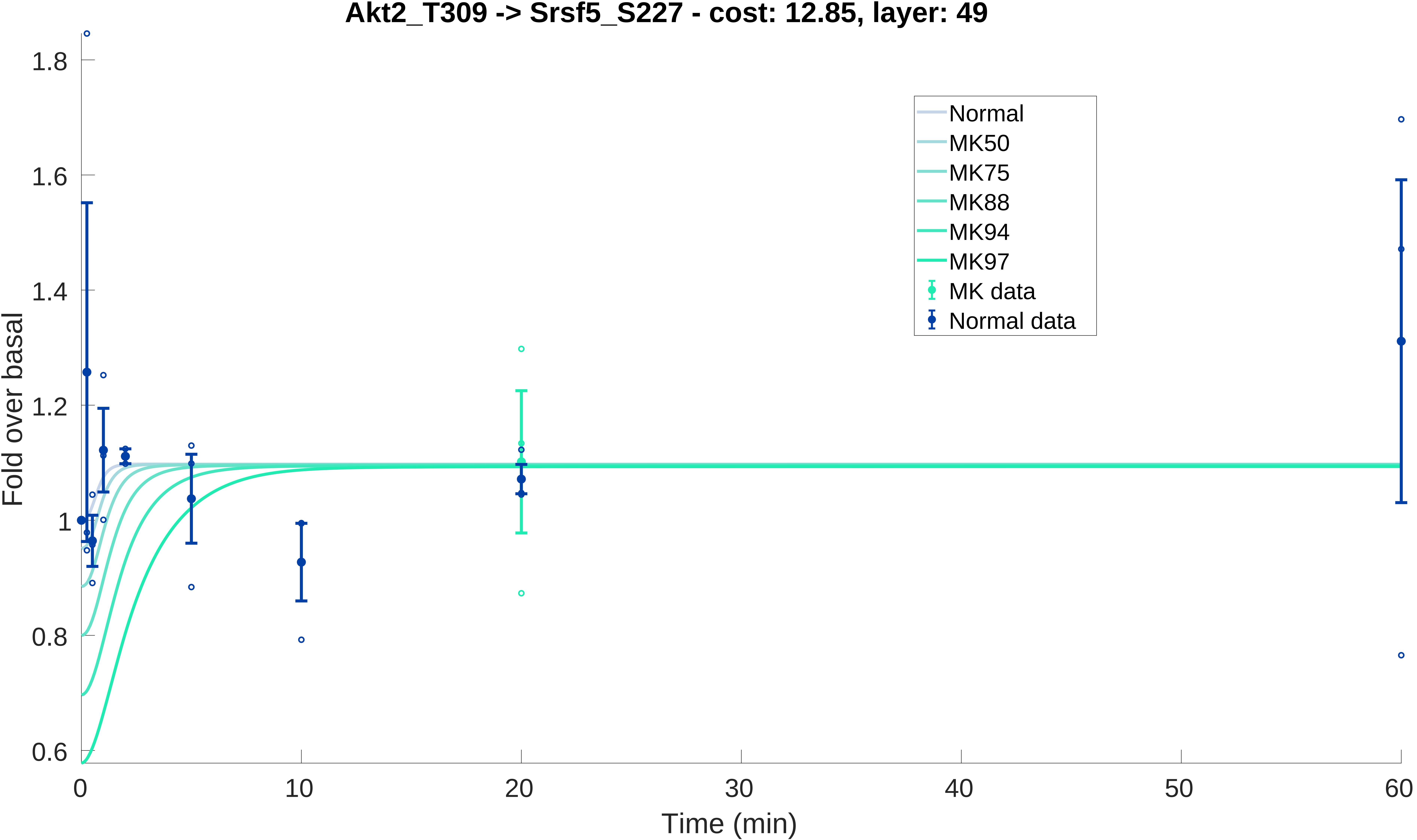

Akt2_T309 -> Srsf5_S227 - cost: 12.85, layer: 49
Fold over basal
Time (min)
1.8
1.6
1.4
1.2
1
0.8
0.6
0
10
20
30
40
50
60
Normal
MK50
MK75
MK88
MK94
MK97
MK data
Normal data

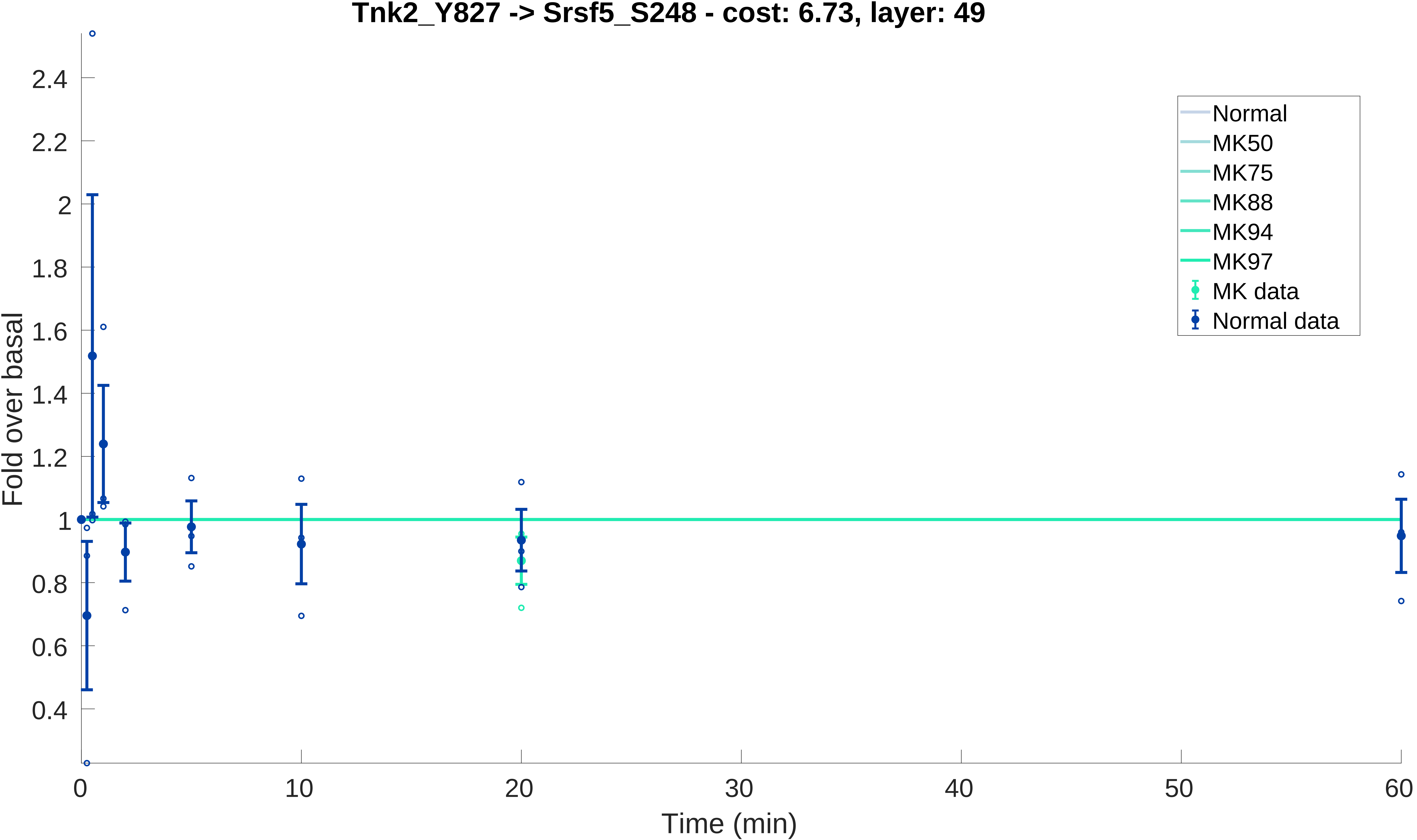
Tnk2_Y827 -> Srsf5_S248 - cost: 6.73, layer: 49
Fold over basal
Time (min)
Normal
MK50
MK75
MK88
MK94
MK97
MK data
Normal data
2.4
2.2
2
1.8
1.6
1.4
1.2
1
0.8
0.6
0.4
0
10
20
30
40
50
60

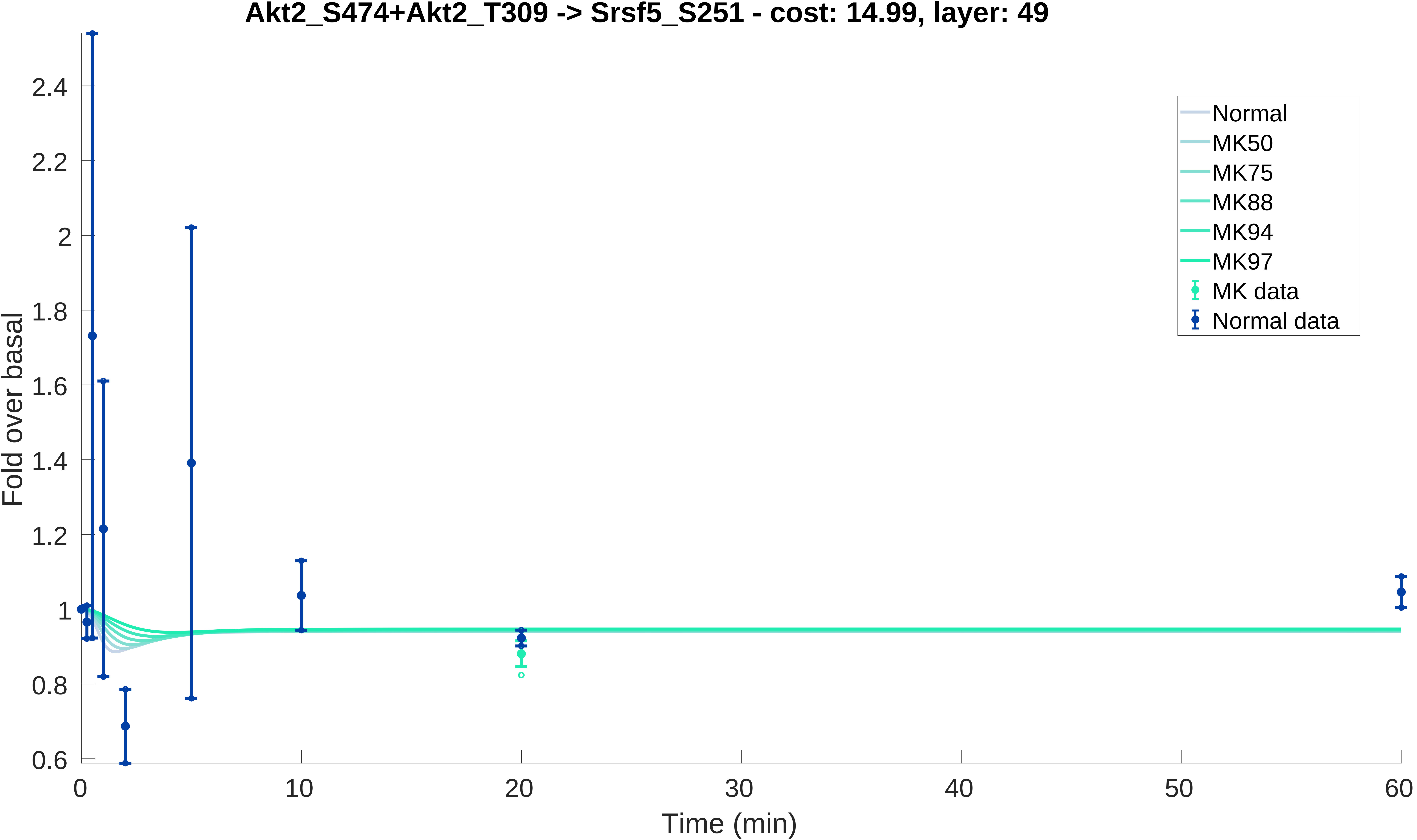

Akt2_S474+Akt2_T309 -> Srsf5_S251 - cost: 14.99, layer: 49
Fold over basal
Time (min)
Normal
MK50
MK75
MK88
MK94
MK97
MK data
Normal data
0.6
0.8
1
1.2
1.4
1.6
1.8
2
2.2
2.4
0
10
20
30
40
50
60

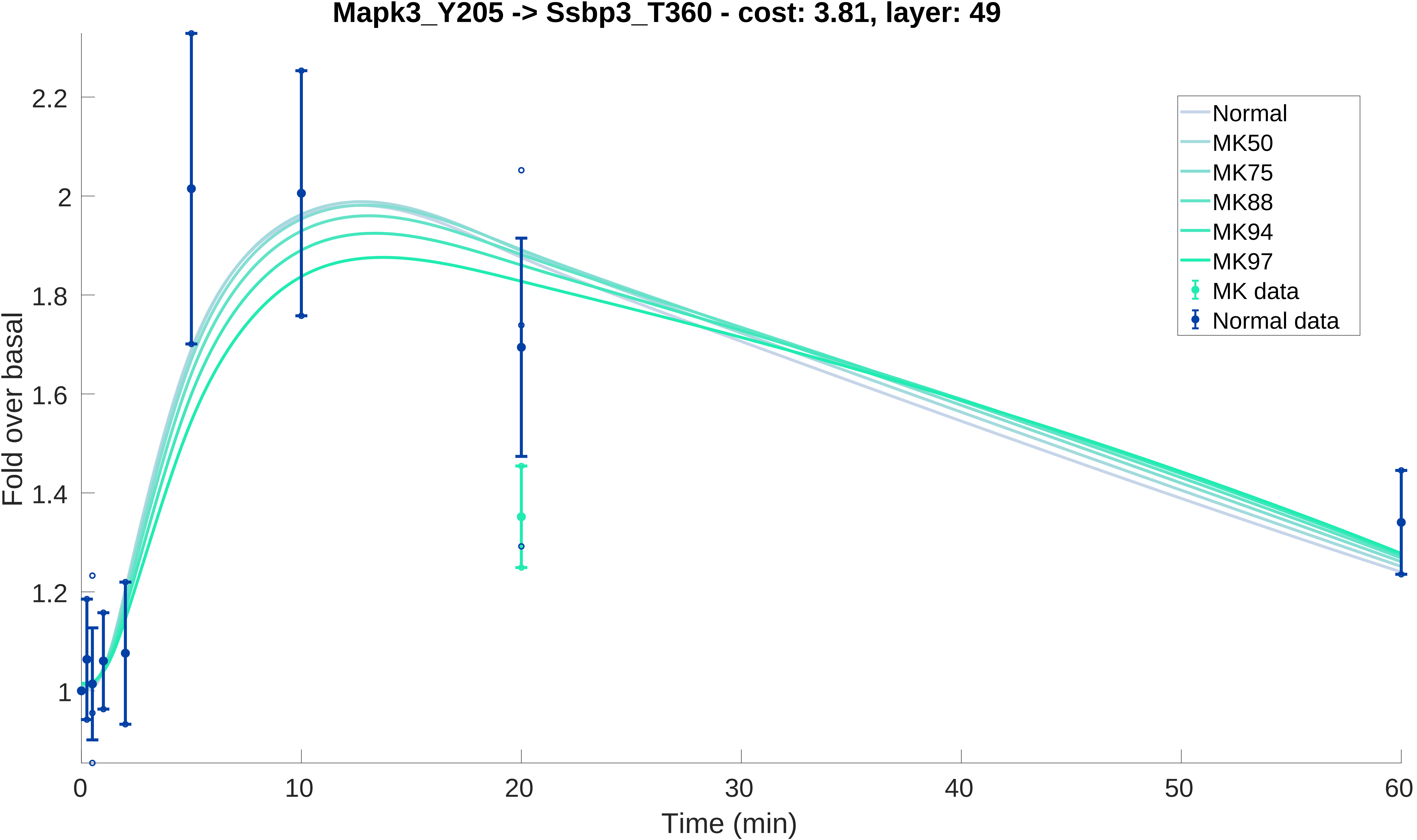
Mapk3_Y205 -> Ssbp3_T360 - cost: 3.81, layer: 49
Fold over basal
Time (min)
Normal
MK50
MK75
MK88
MK94
MK97
MK data
Normal data
2.2
2
1.8
1.6
1.4
1.2
1
0
10
20
30
40
50
60

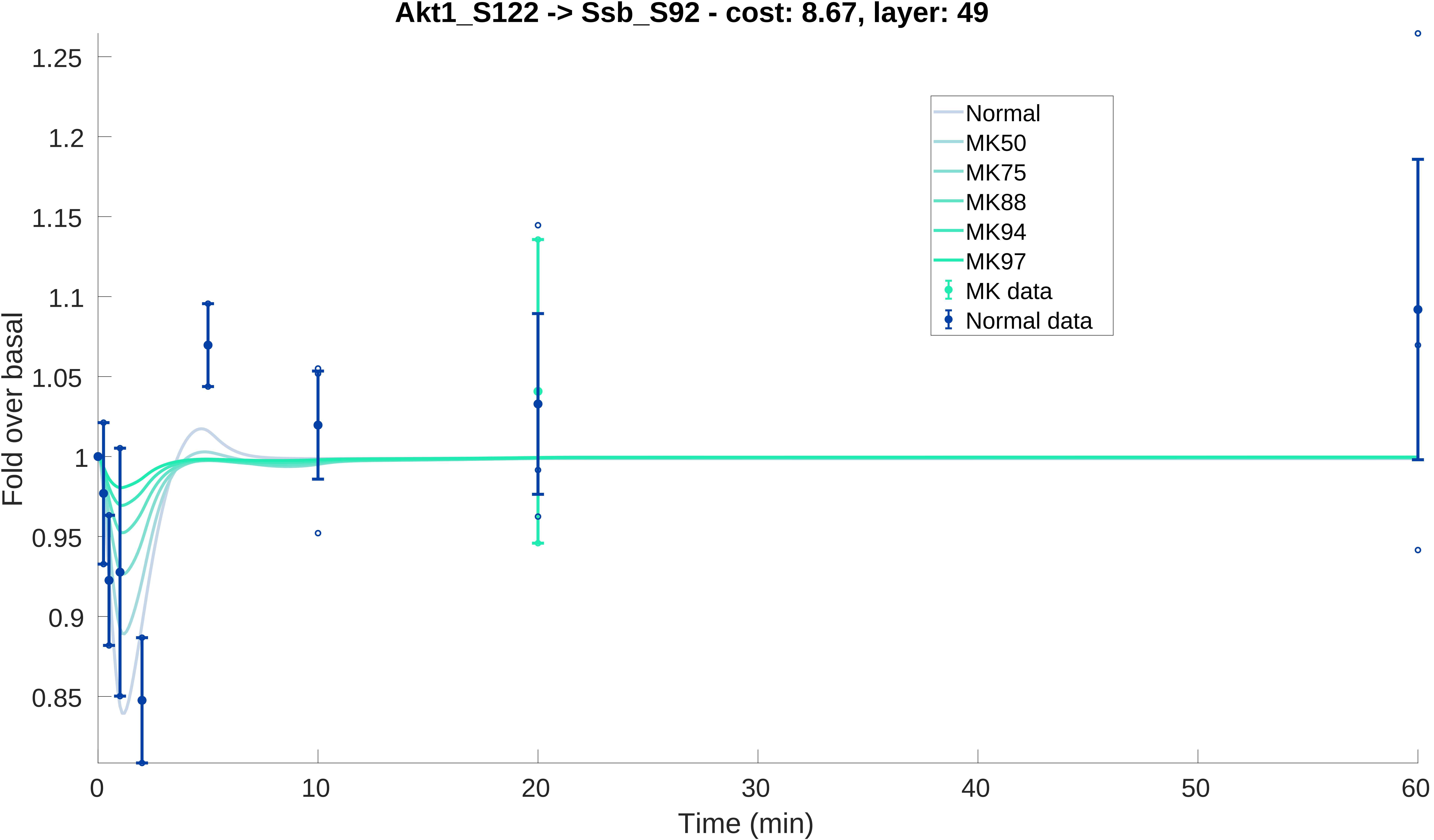

Akt1_S122 -> Ssb_S92 - cost: 8.67, layer: 49
Fold over basal
Time (min)
0
10
20
30
40
50
60
0.85
0.9
0.95
1
1.05
1.1
1.15
1.2
1.25
Normal
MK50
MK75
MK88
MK94
MK97
MK data
Normal data

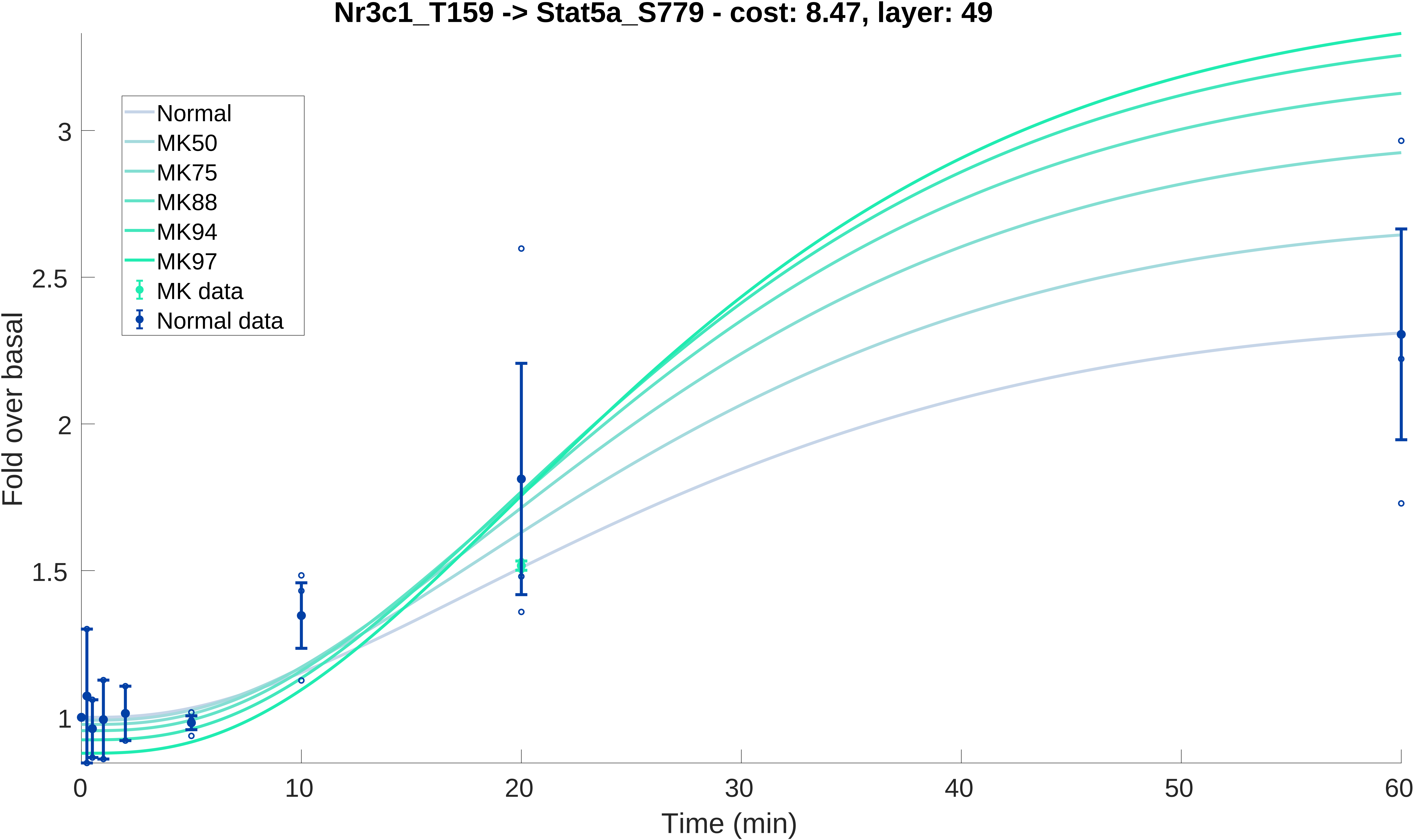

Nr3c1_T159 -> Stat5a_S779 - cost: 8.47, layer: 49
Normal
MK50
MK75
MK88
MK94
MK97
MK data
Normal data
Fold over basal
1
1.5
2
2.5
3
0
10
20
30
40
50
60
Time (min)

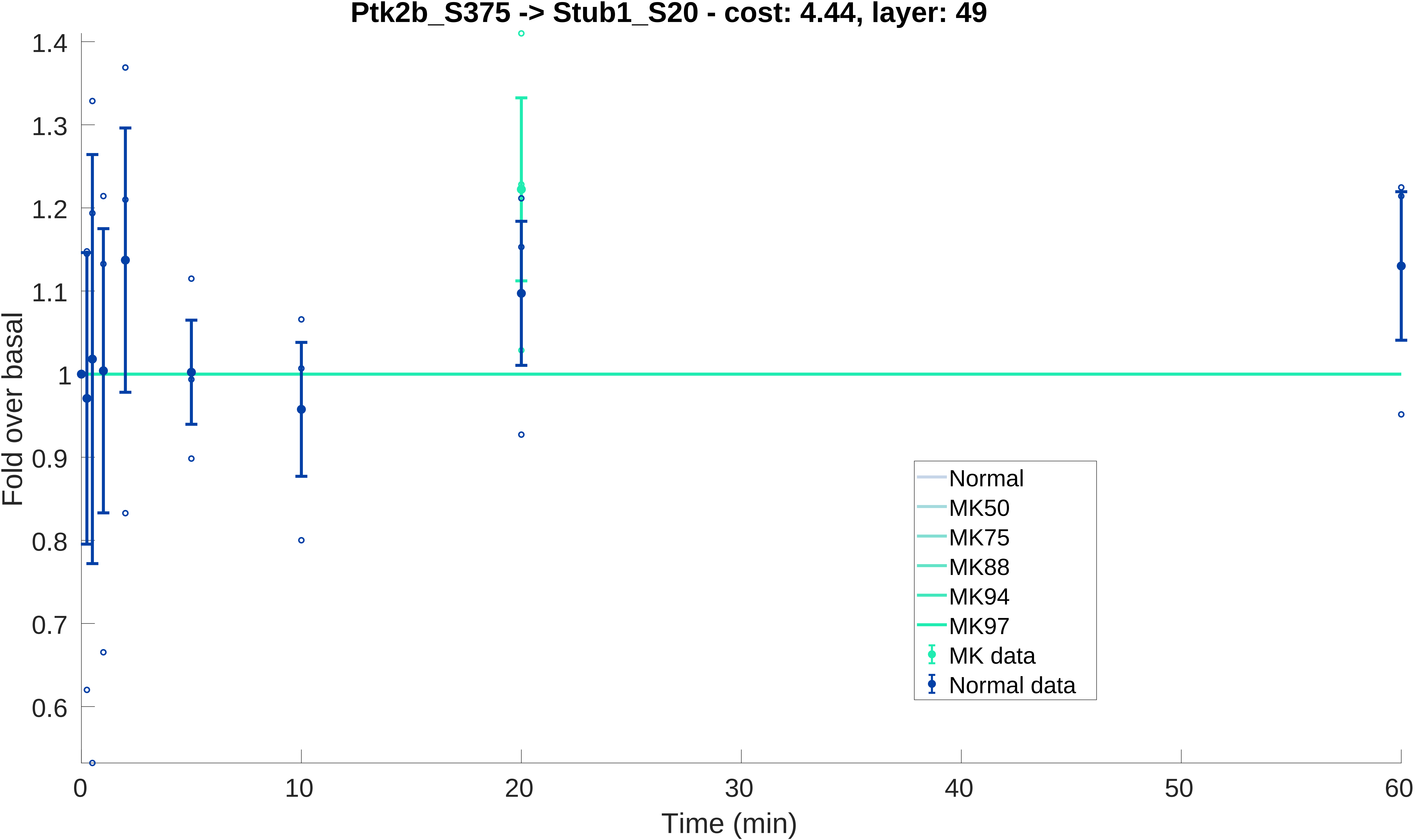

Ptk2b_S375 -> Stub1_S20 - cost: 4.44, layer: 49
Fold over basal
Time (min)
1.4
1.3
1.2
1.1
1
0.9
0.8
0.7
0.6
0
10
20
30
40
50
60
Normal
MK50
MK75
MK88
MK94
MK97
MK data
Normal data

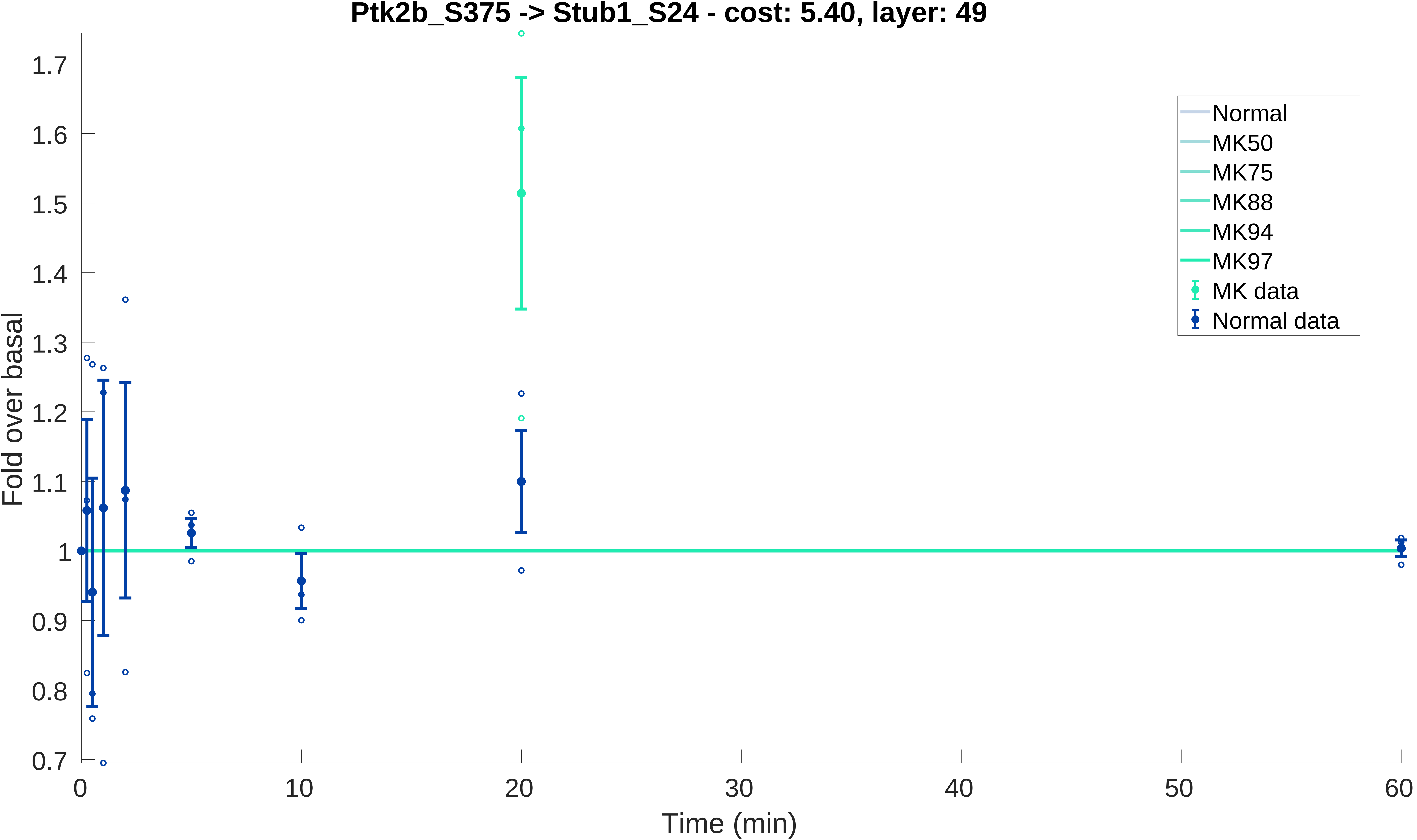

Ptk2b_S375 -> Stub1_S24 - cost: 5.40, layer: 49
Fold over basal
Time (min)
0.7
0.8
0.9
1
1.1
1.2
1.3
1.4
1.5
1.6
1.7
0
10
20
30
40
50
60
Normal
MK50
MK75
MK88
MK94
MK97
MK data
Normal data

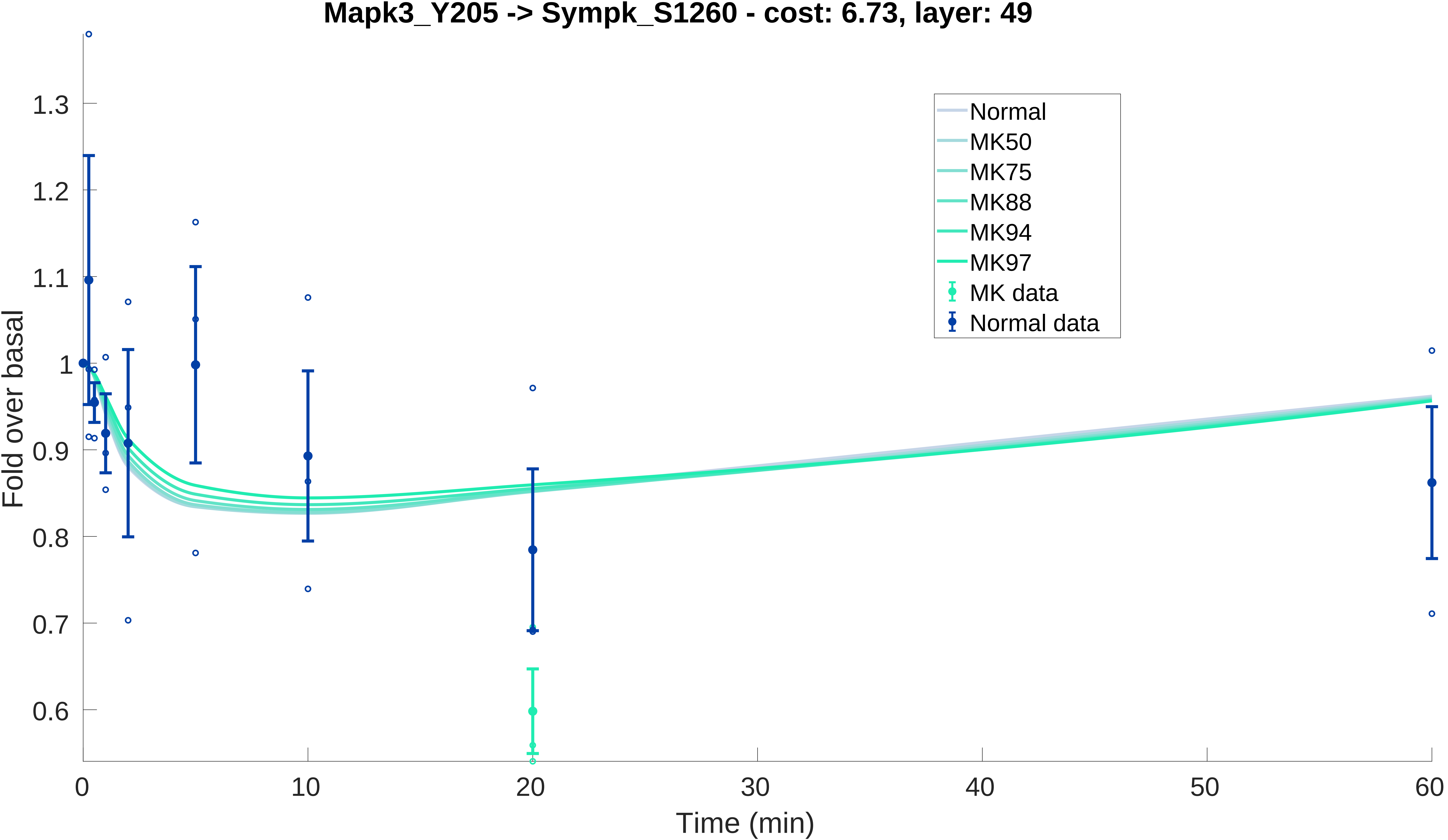

Mapk3_Y205 -> Sympk_S1260 - cost: 6.73, layer: 49
Fold over basal
Time (min)
1.3
1.2
1.1
1
0.9
0.8
0.7
0.6
0
10
20
30
40
50
60
Normal
MK50
MK75
MK88
MK94
MK97
MK data
Normal data

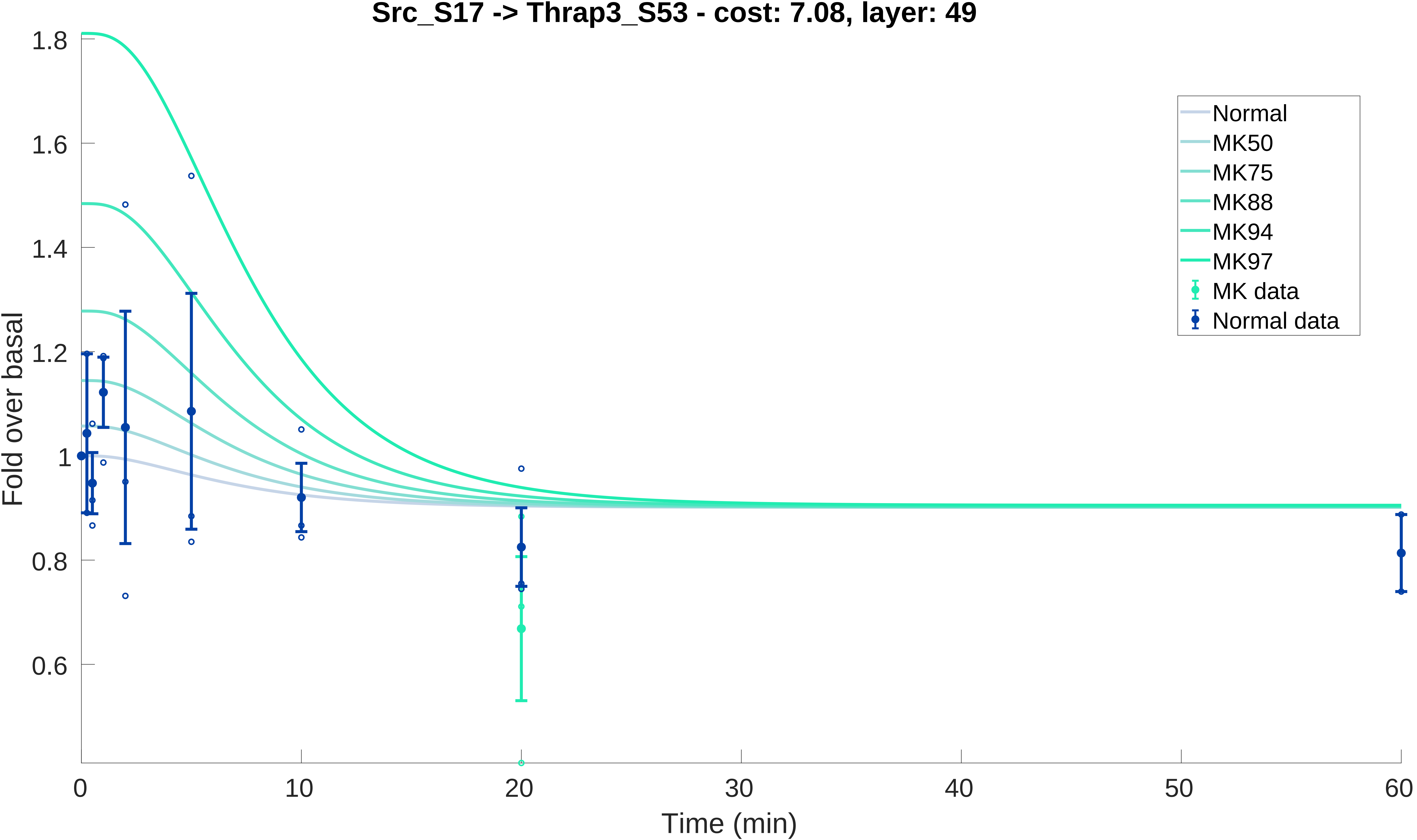
Src_S17 -> Thrap3_S53 - cost: 7.08, layer: 49
Fold over basal
Time (min)
1.8
1.6
1.4
1.2
1
0.8
0.6
0
10
20
30
40
50
60
Normal
MK50
MK75
MK88
MK94
MK97
MK data
Normal data

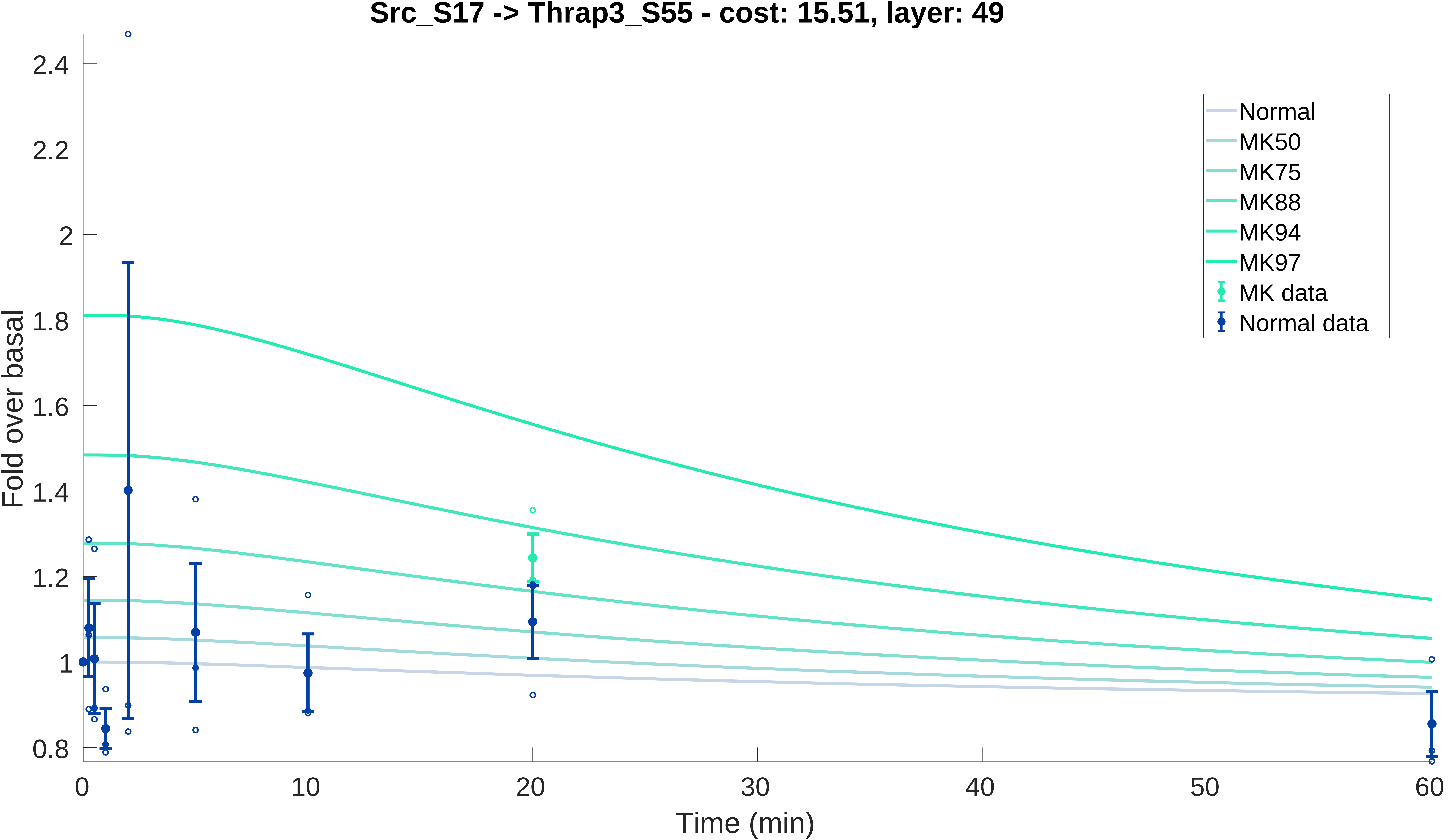

Src_S17 -> Thrap3_S55 - cost: 15.51, layer: 49
Fold over basal
Time (min)
0.8
1
1.2
1.4
1.6
1.8
2
2.2
2.4
0
10
20
30
40
50
60
Normal
MK50
MK75
MK88
MK94
MK97
MK data
Normal data

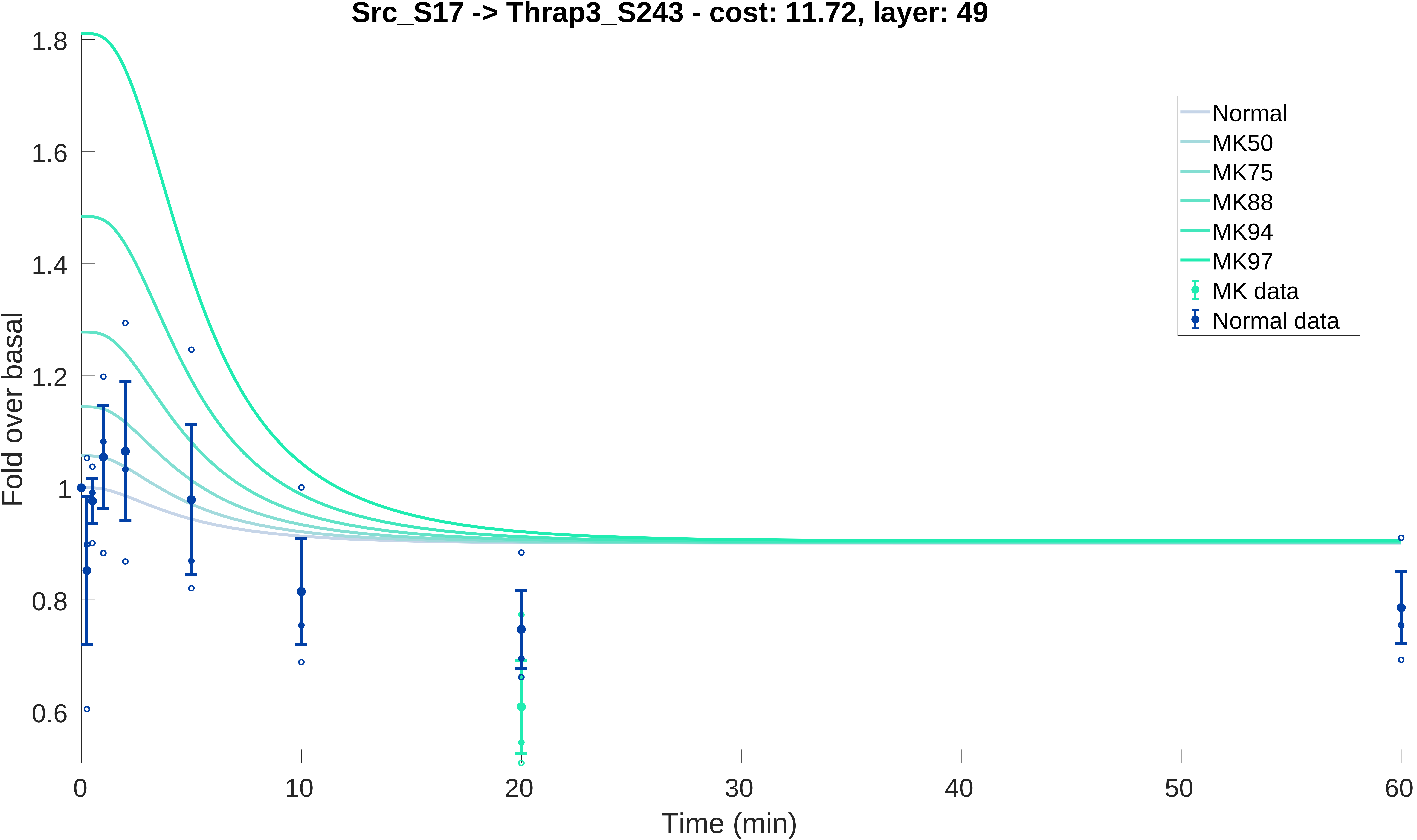

Src_S17 -> Thrap3_S243 - cost: 11.72, layer: 49
Fold over basal
Time (min)
1.8
1.6
1.4
1.2
1
0.8
0.6
0
10
20
30
40
50
60
Normal
MK50
MK75
MK88
MK94
MK97
MK data
Normal data

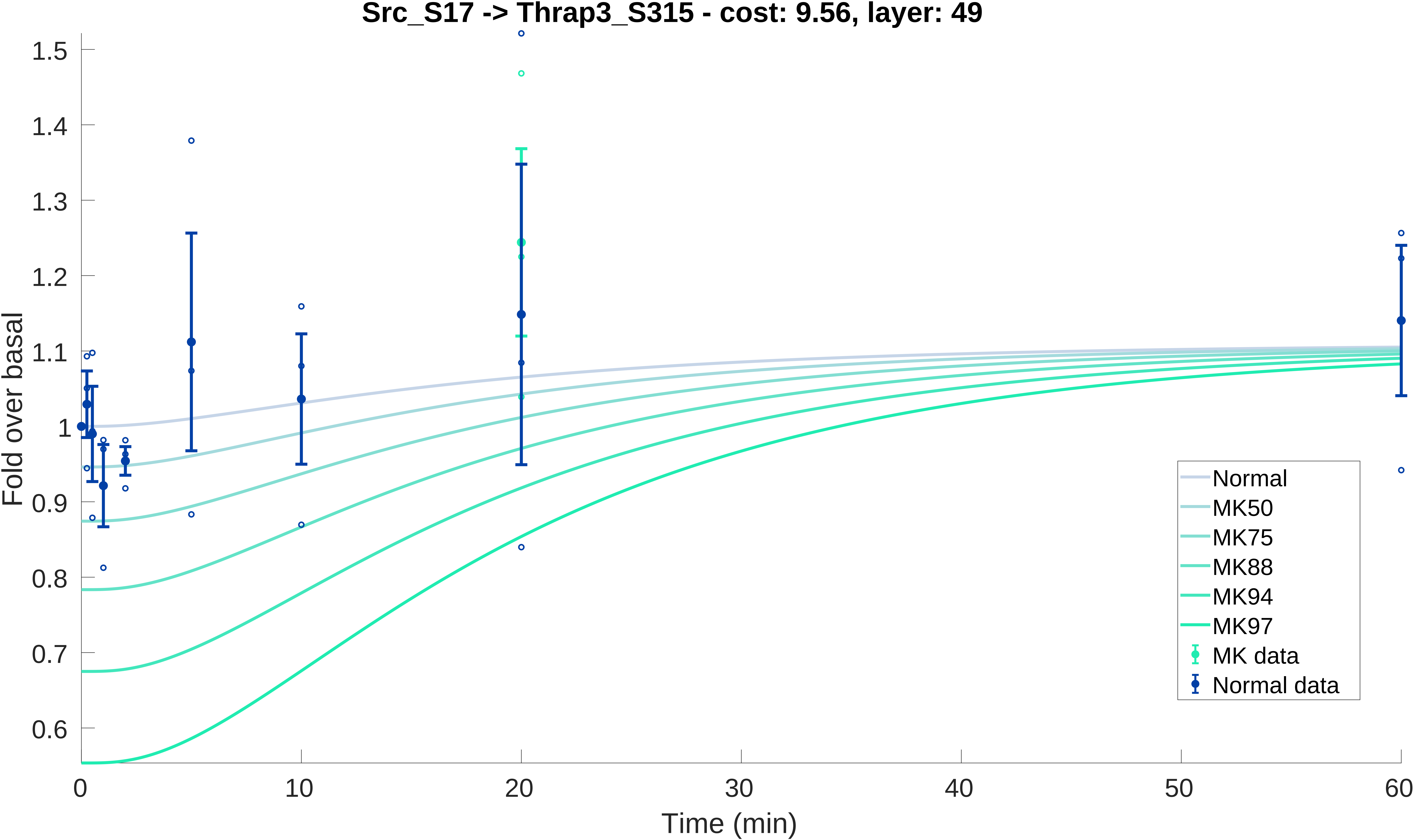
Src_S17 -> Thrap3_S315 - cost: 9.56, layer: 49
Fold over basal
Time (min)
0.6
0.7
0.8
0.9
1
1.1
1.2
1.3
1.4
1.5
0
10
20
30
40
50
60
Normal
MK50
MK75
MK88
MK94
MK97
MK data
Normal data

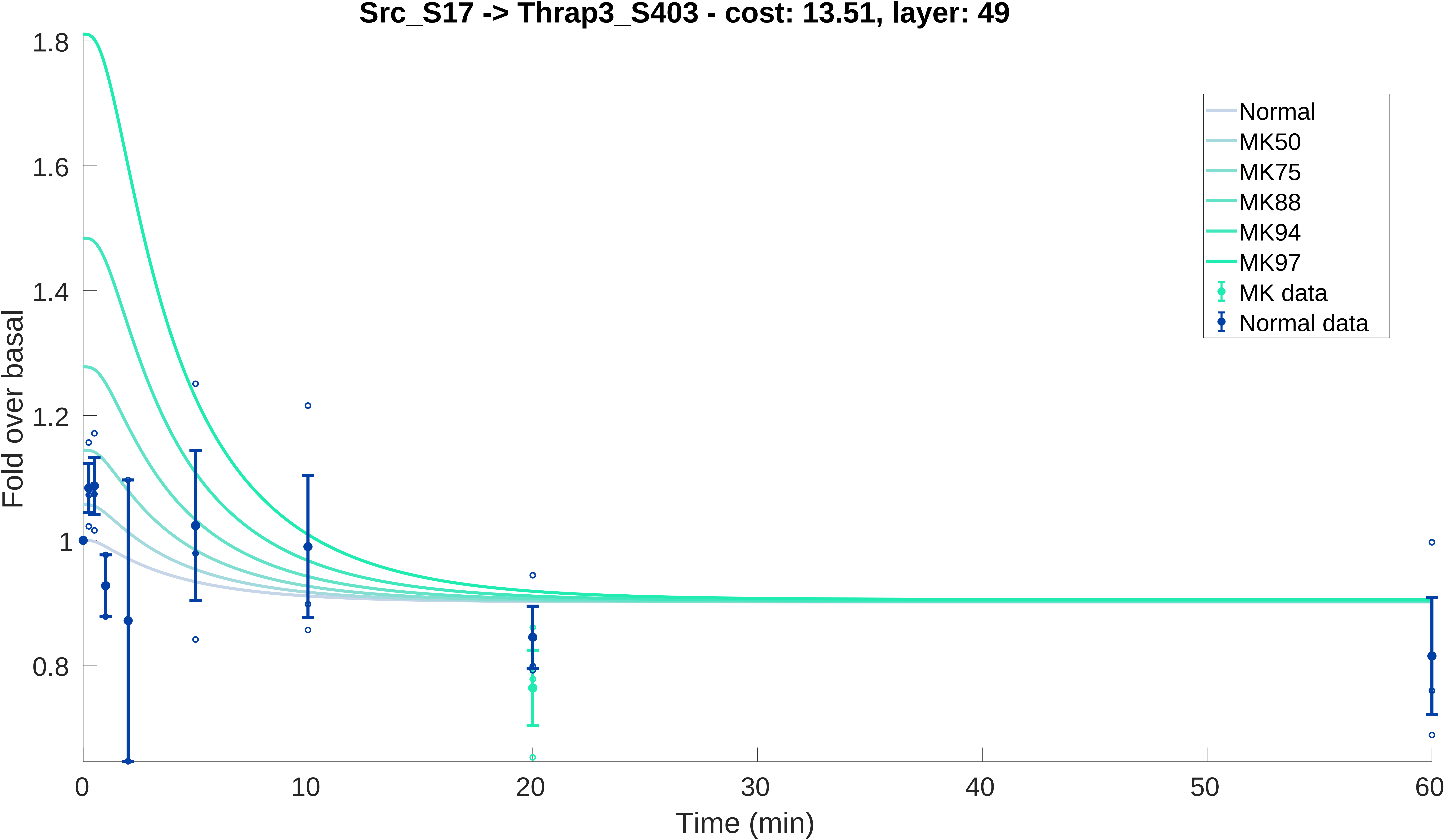

Src_S17 -> Thrap3_S403 - cost: 13.51, layer: 49
Fold over basal
1.8
1.6
1.4
1.2
1
0.8
0
10
20
30
40
50
60
Time (min)
Normal
MK50
MK75
MK88
MK94
MK97
MK data
Normal data

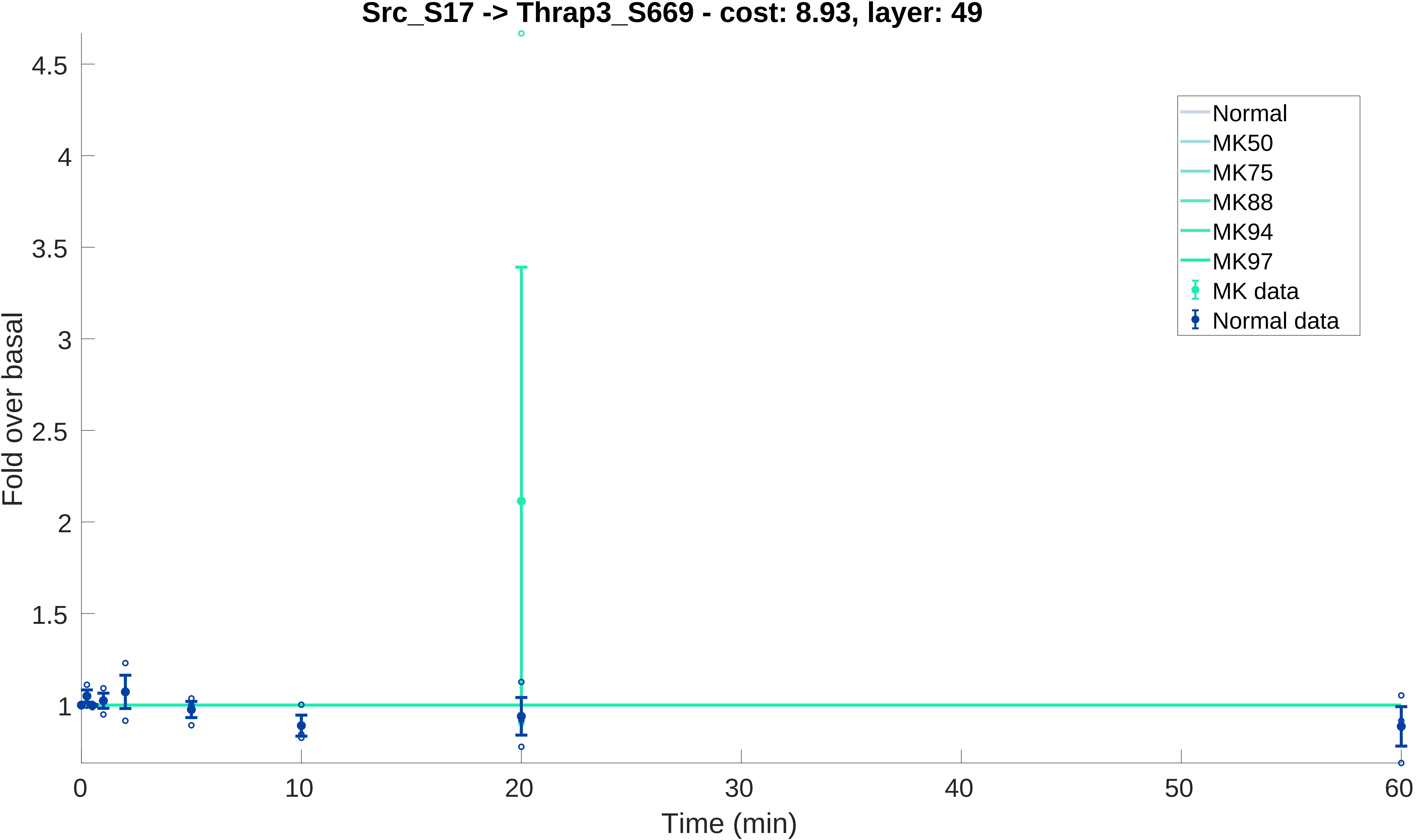

Src_S17 -> Thrap3_S669 - cost: 8.93, layer: 49
Fold over basal
Time (min)
4.5
4
3.5
3
2.5
2
1.5
1
0
10
20
30
40
50
60
Normal
MK50
MK75
MK88
MK94
MK97
MK data
Normal data

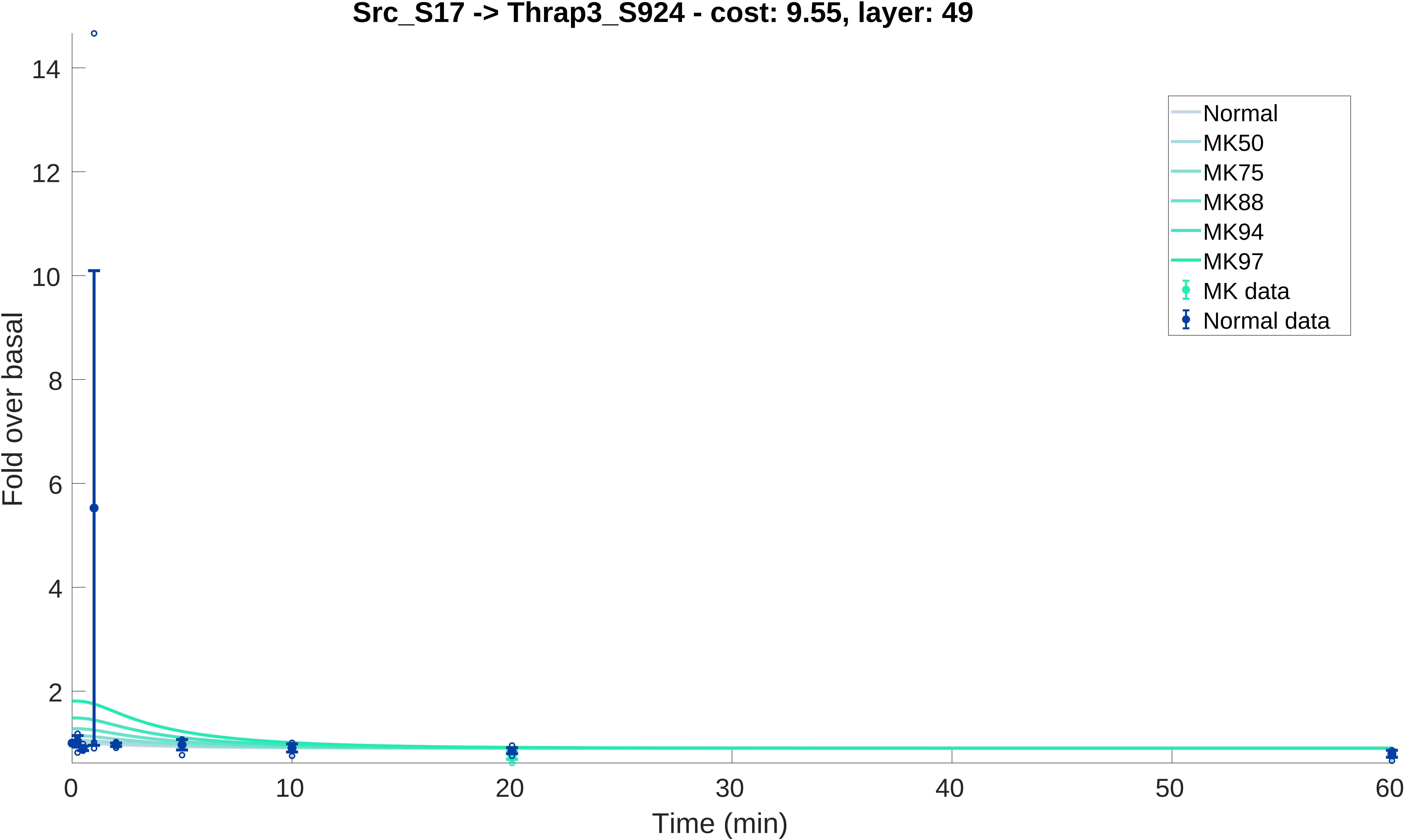

Src_S17 -> Thrap3_S924 - cost: 9.55, layer: 49
Fold over basal
Time (min)
0
10
20
30
40
50
60
2
4
6
8
10
12
14
Normal
MK50
MK75
MK88
MK94
MK97
MK data
Normal data

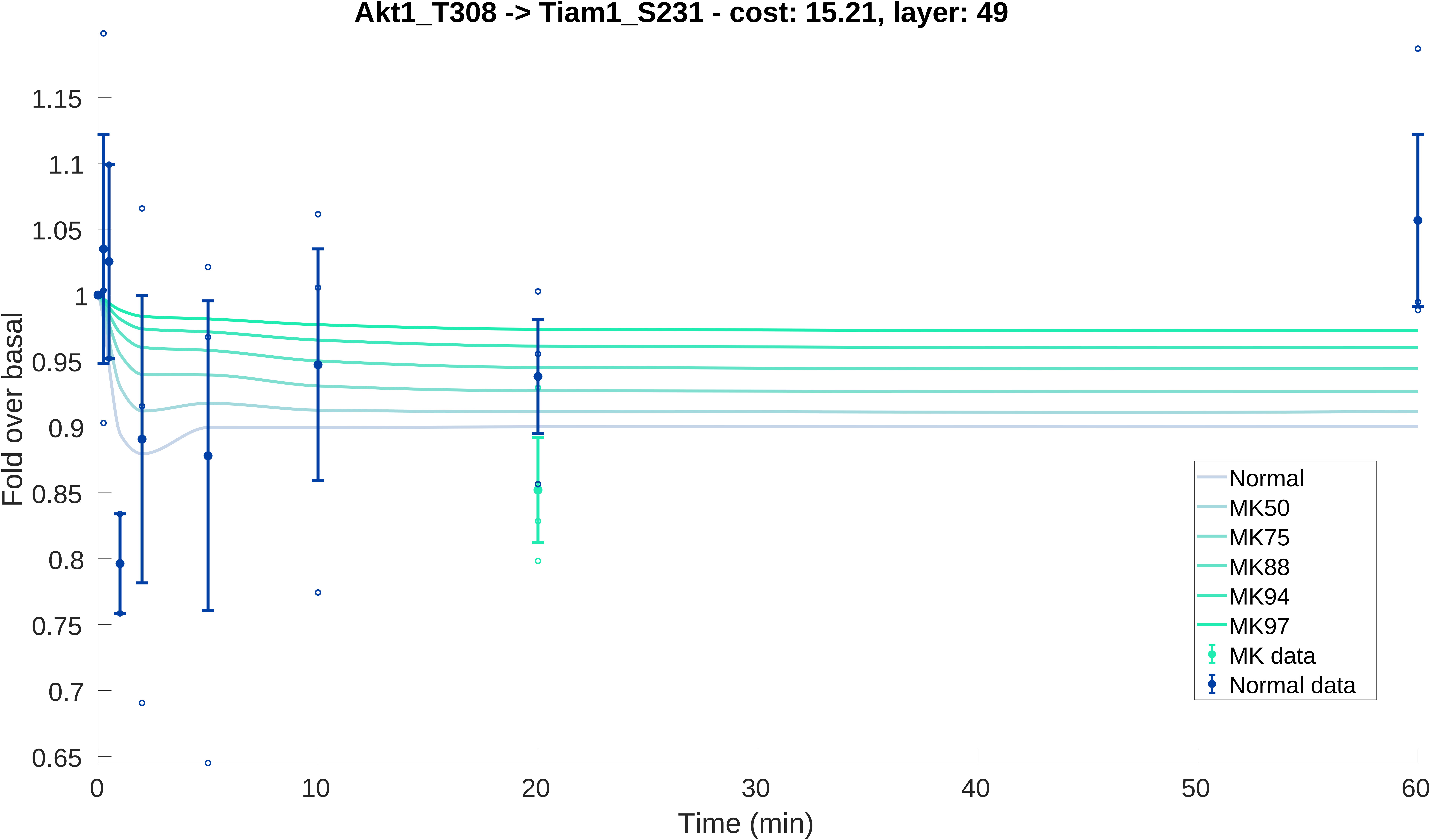

Akt1_T308 -> Tiam1_S231 - cost: 15.21, layer: 49
Fold over basal
Time (min)
1.15
1.1
1.05
1
0.95
0.9
0.85
0.8
0.75
0.7
0.65
0
10
20
30
40
50
60
Normal
MK50
MK75
MK88
MK94
MK97
MK data
Normal data

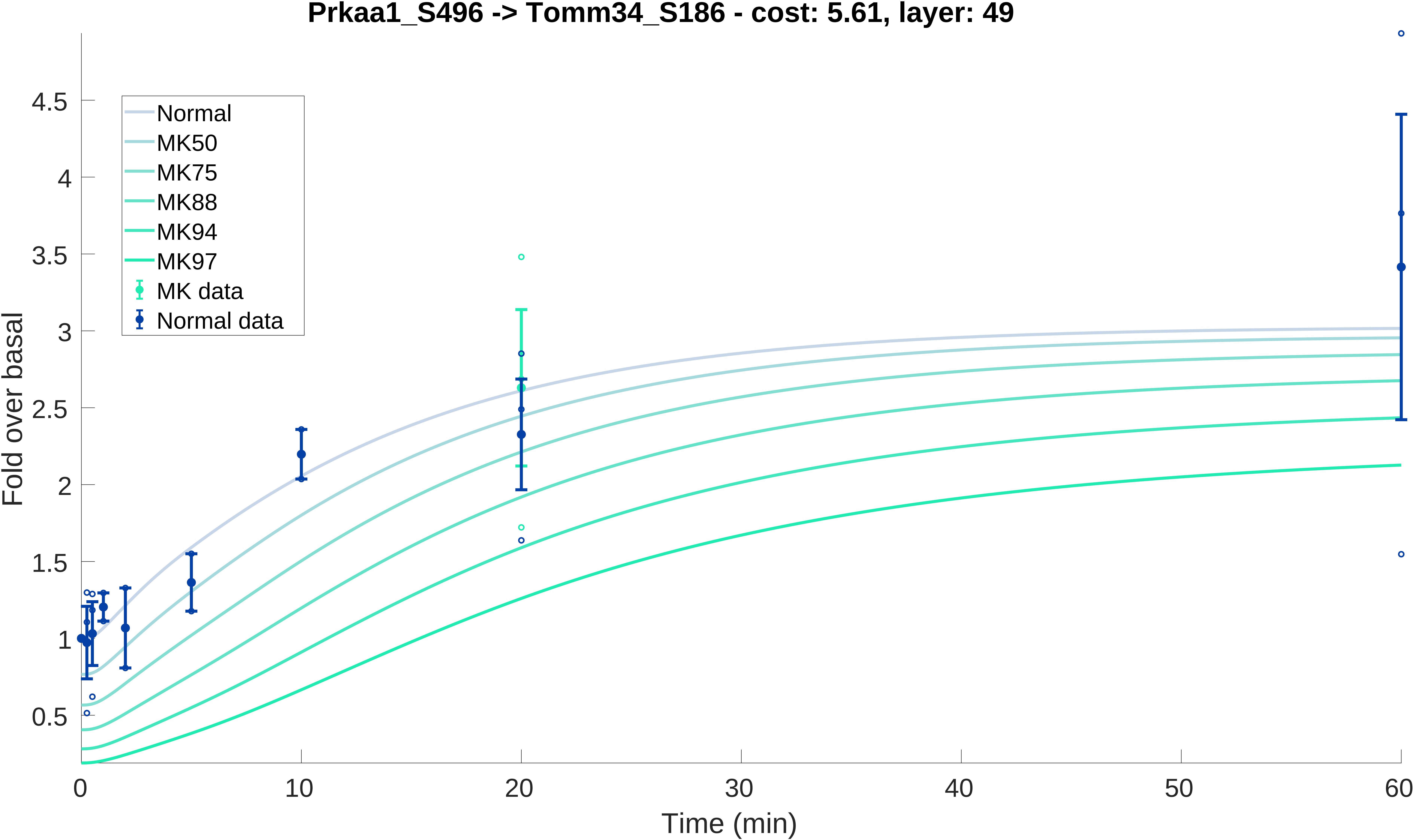
Prkaa1_S496 -> Tomm34_S186 - cost: 5.61, layer: 49
Normal
MK50
MK75
MK88
MK94
MK97
MK data
Normal data
Fold over basal
Time (min)
4.5
4
3.5
3
2.5
2
1.5
1
0.5
0
10
20
30
40
50
60

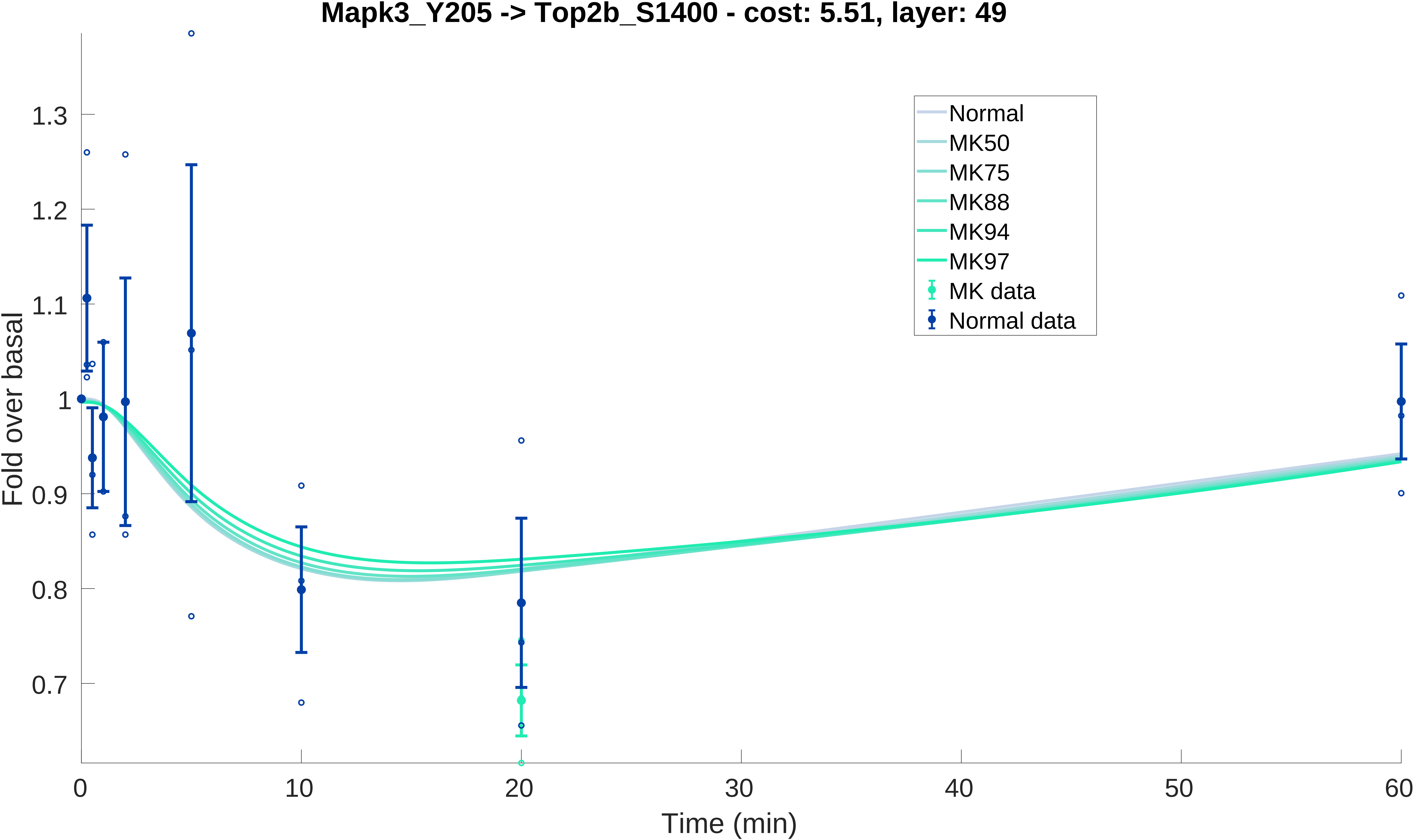

Mapk3_Y205 -> Top2b_S1400 - cost: 5.51, layer: 49
Fold over basal
Time (min)
0.7
0.8
0.9
1
1.1
1.2
1.3
0
10
20
30
40
50
60
Normal
MK50
MK75
MK88
MK94
MK97
MK data
Normal data

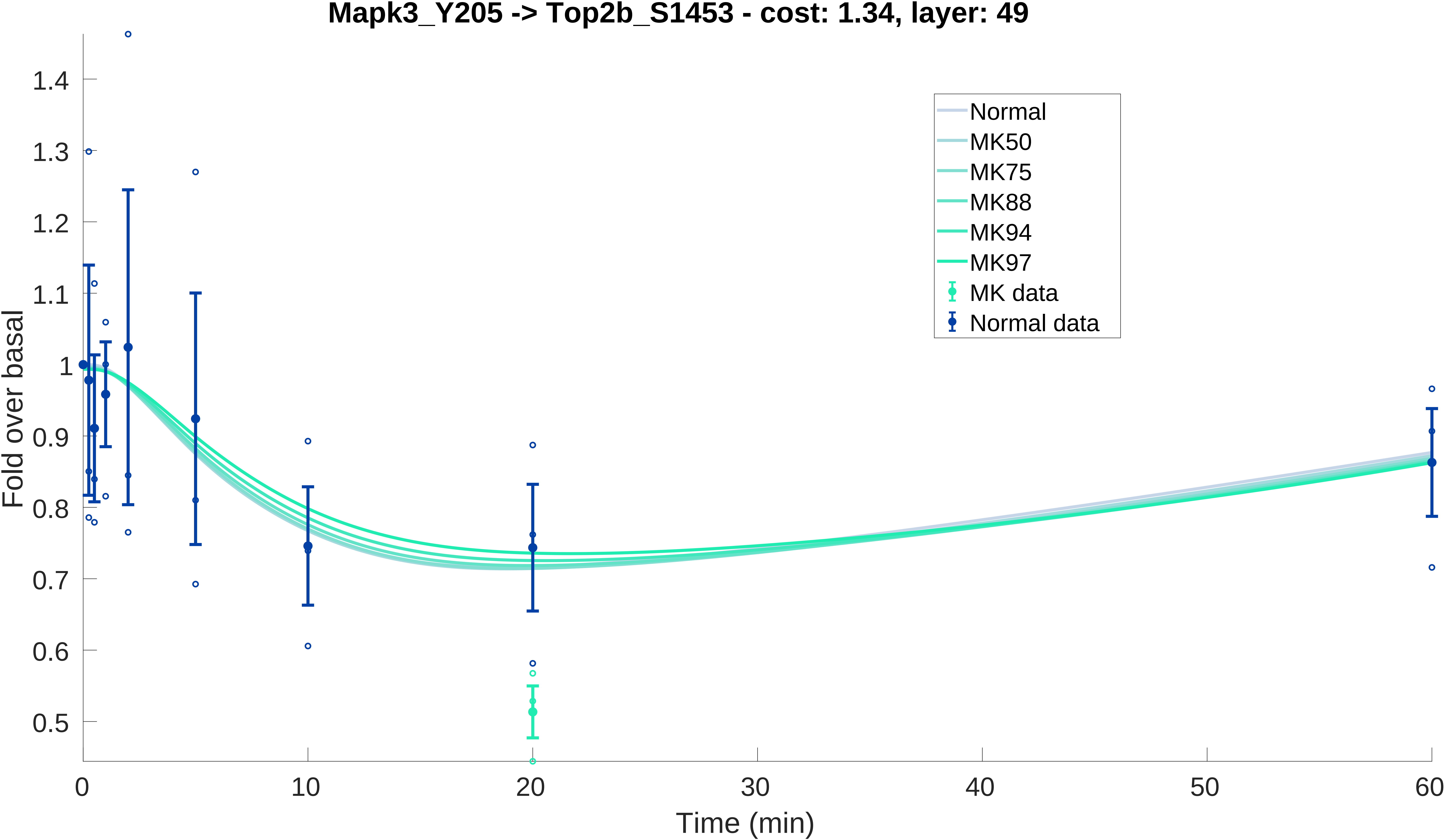
Mapk3_Y205 -> Top2b_S1453 - cost: 1.34, layer: 49
Fold over basal
Time (min)
0
10
20
30
40
50
60
0.5
0.6
0.7
0.8
0.9
1
1.1
1.2
1.3
1.4
Normal
MK50
MK75
MK88
MK94
MK97
MK data
Normal data

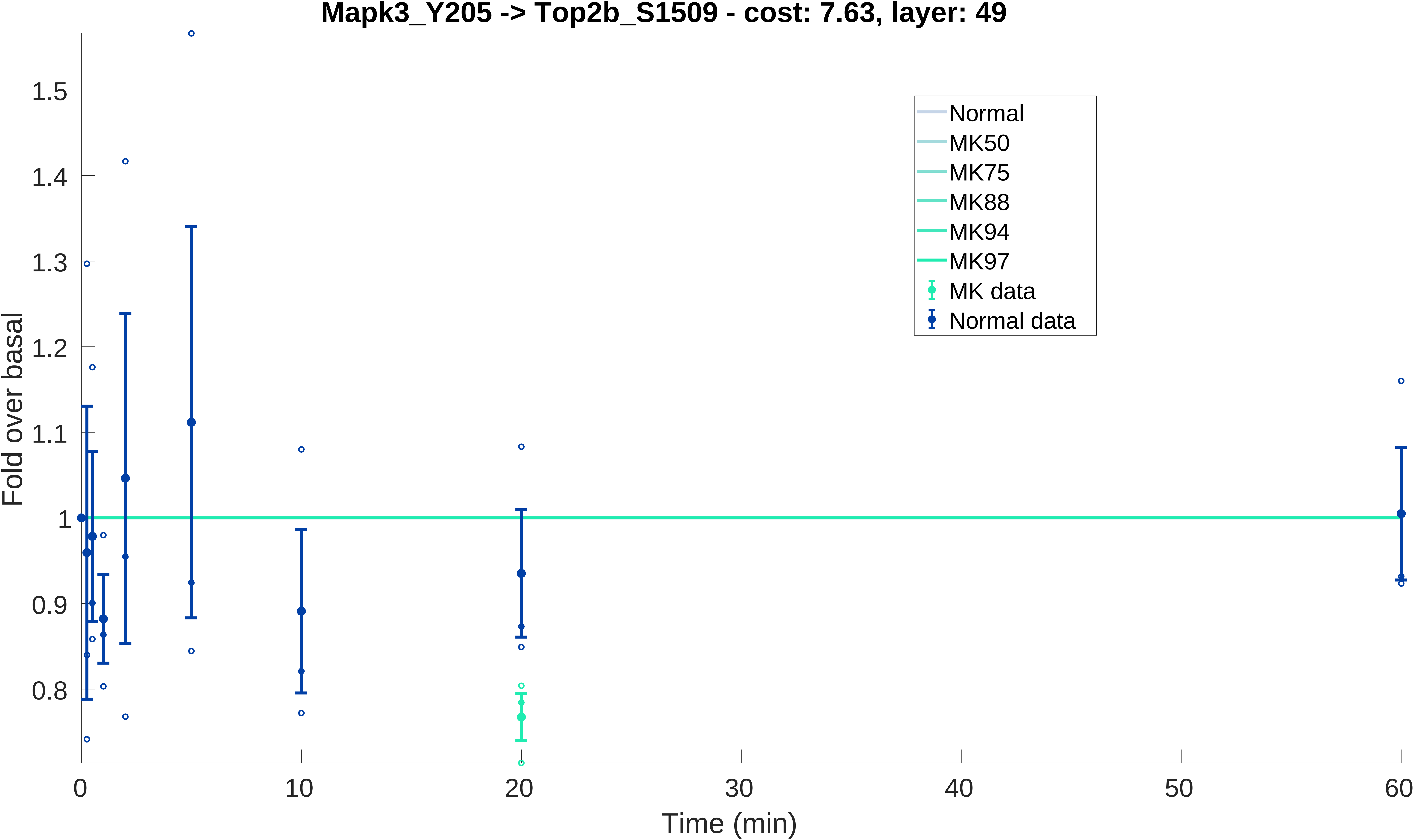

Mapk3_Y205 -> Top2b_S1509 - cost: 7.63, layer: 49
Fold over basal
1.5
1.4
1.3
1.2
1.1
1
0.9
0.8
0
10
20
30
40
50
60
Time (min)
Normal
MK50
MK75
MK88
MK94
MK97
MK data
Normal data

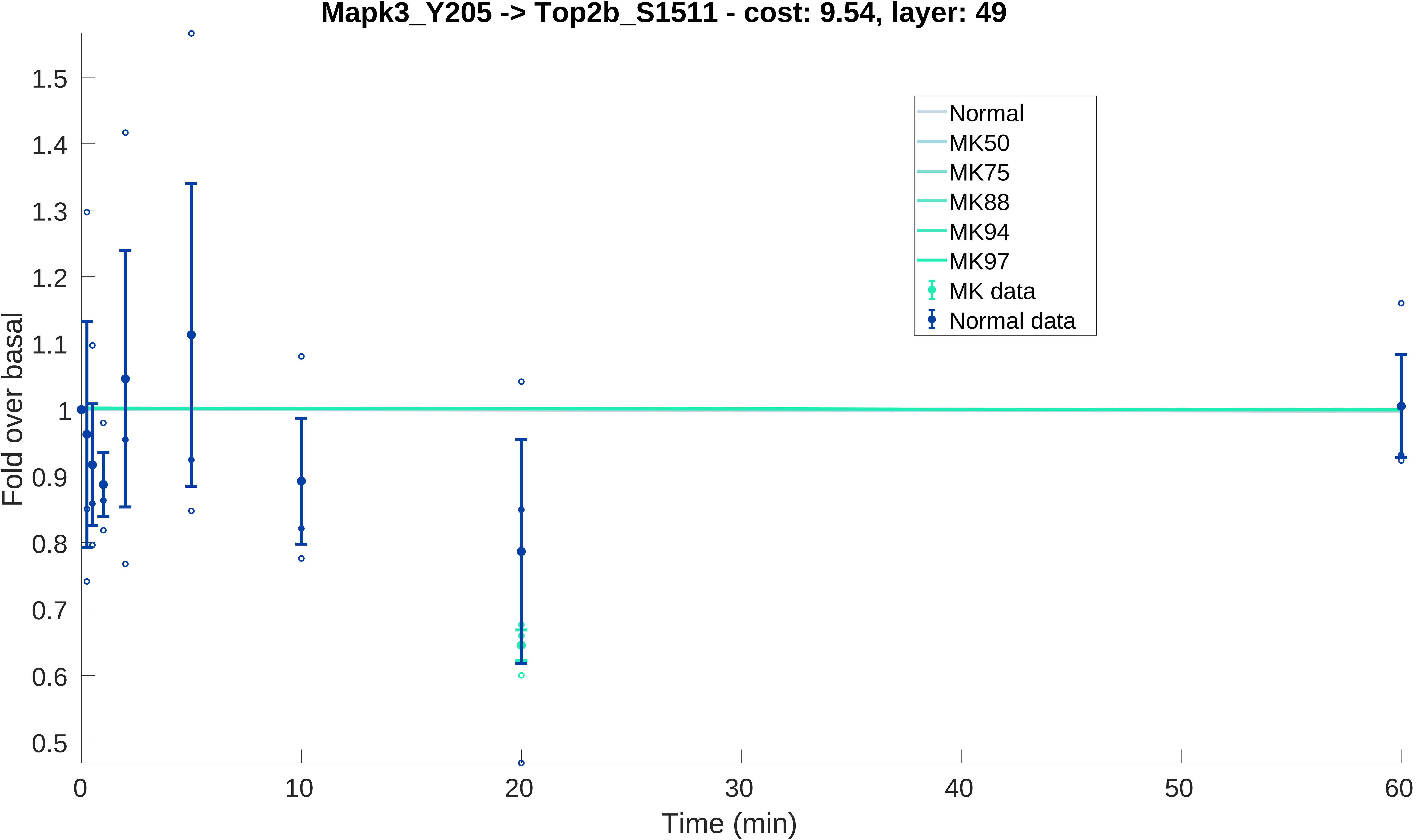

Mapk3_Y205 -> Top2b_S1511 - cost: 9.54, layer: 49
Fold over basal
Time (min)
Normal
MK50
MK75
MK88
MK94
MK97
MK data
Normal data
0.5
0.6
0.7
0.8
0.9
1
1.1
1.2
1.3
1.4
1.5
0
10
20
30
40
50
60

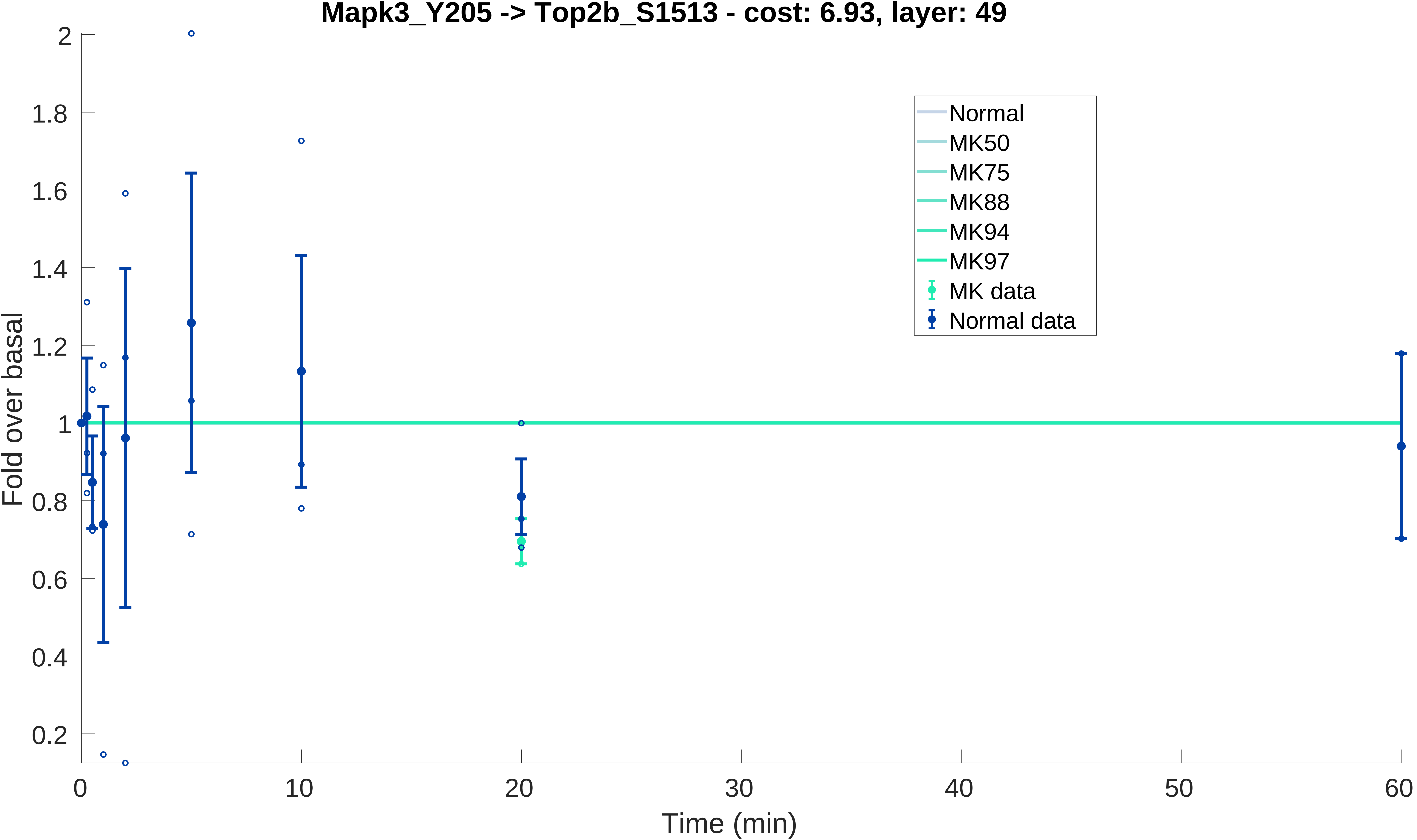

Mapk3_Y205 -> Top2b_S1513 - cost: 6.93, layer: 49
Fold over basal
Time (min)
0
10
20
30
40
50
60
0.2
0.4
0.6
0.8
1
1.2
1.4
1.6
1.8
2
Normal
MK50
MK75
MK88
MK94
MK97
MK data
Normal data

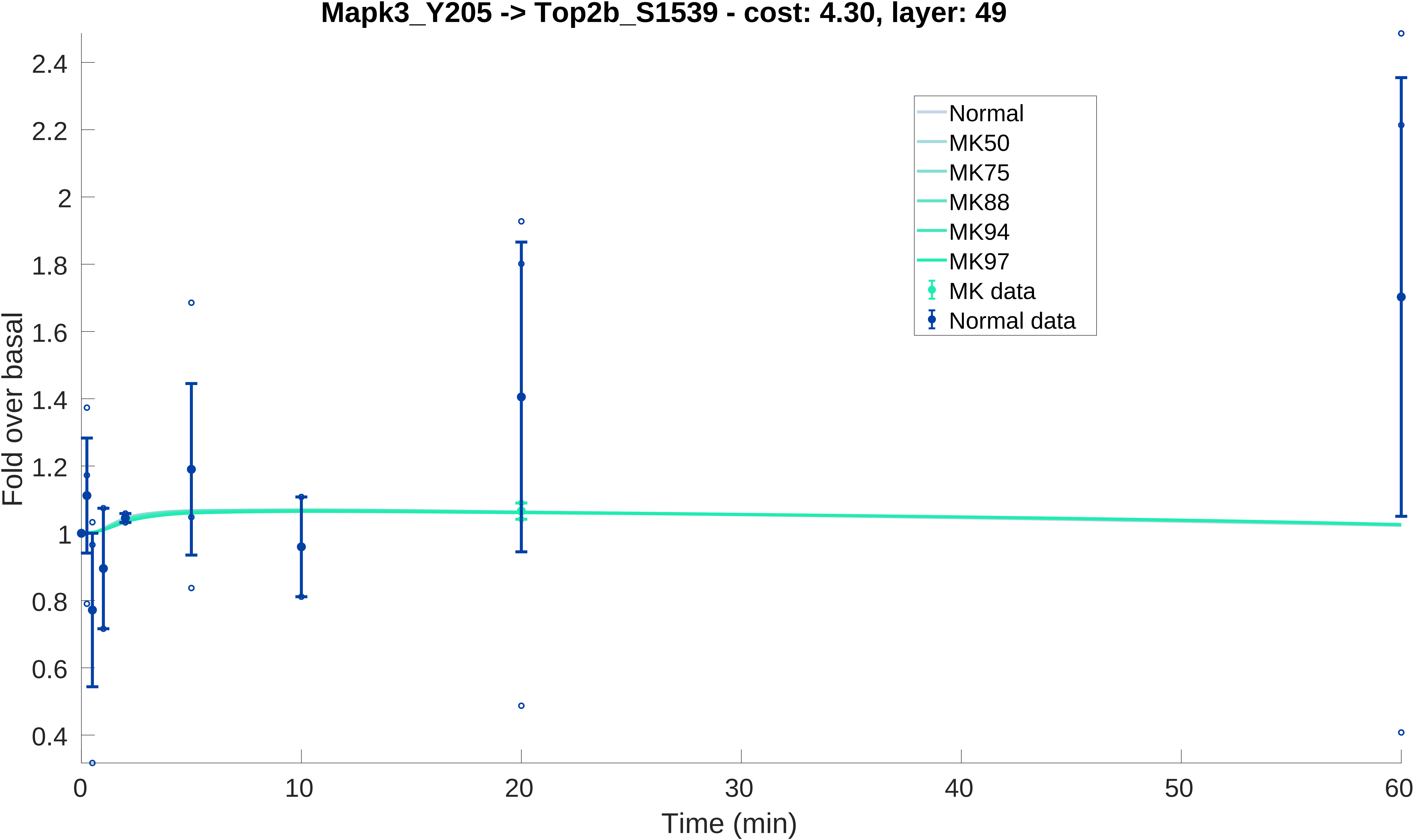

Mapk3_Y205 -> Top2b_S1539 - cost: 4.30, layer: 49
Fold over basal
Time (min)
2.4
2.2
2
1.8
1.6
1.4
1.2
1
0.8
0.6
0.4
0
10
20
30
40
50
60
Normal
MK50
MK75
MK88
MK94
MK97
MK data
Normal data

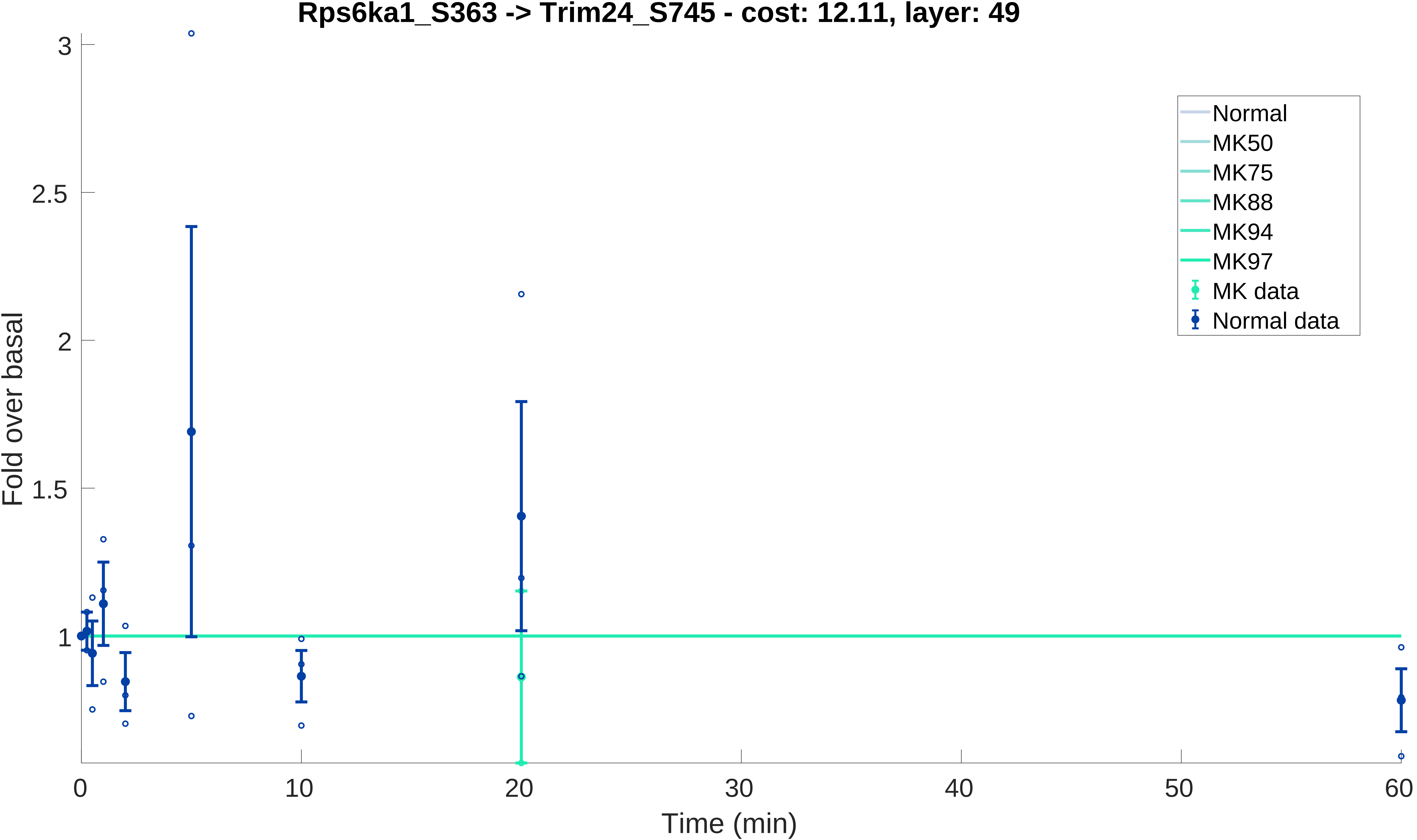

Rps6ka1_S363 -> Trim24_S745 - cost: 12.11, layer: 49
Fold over basal
Time (min)
3
2.5
2
1.5
1
0
10
20
30
40
50
60
Normal
MK50
MK75
MK88
MK94
MK97
MK data
Normal data

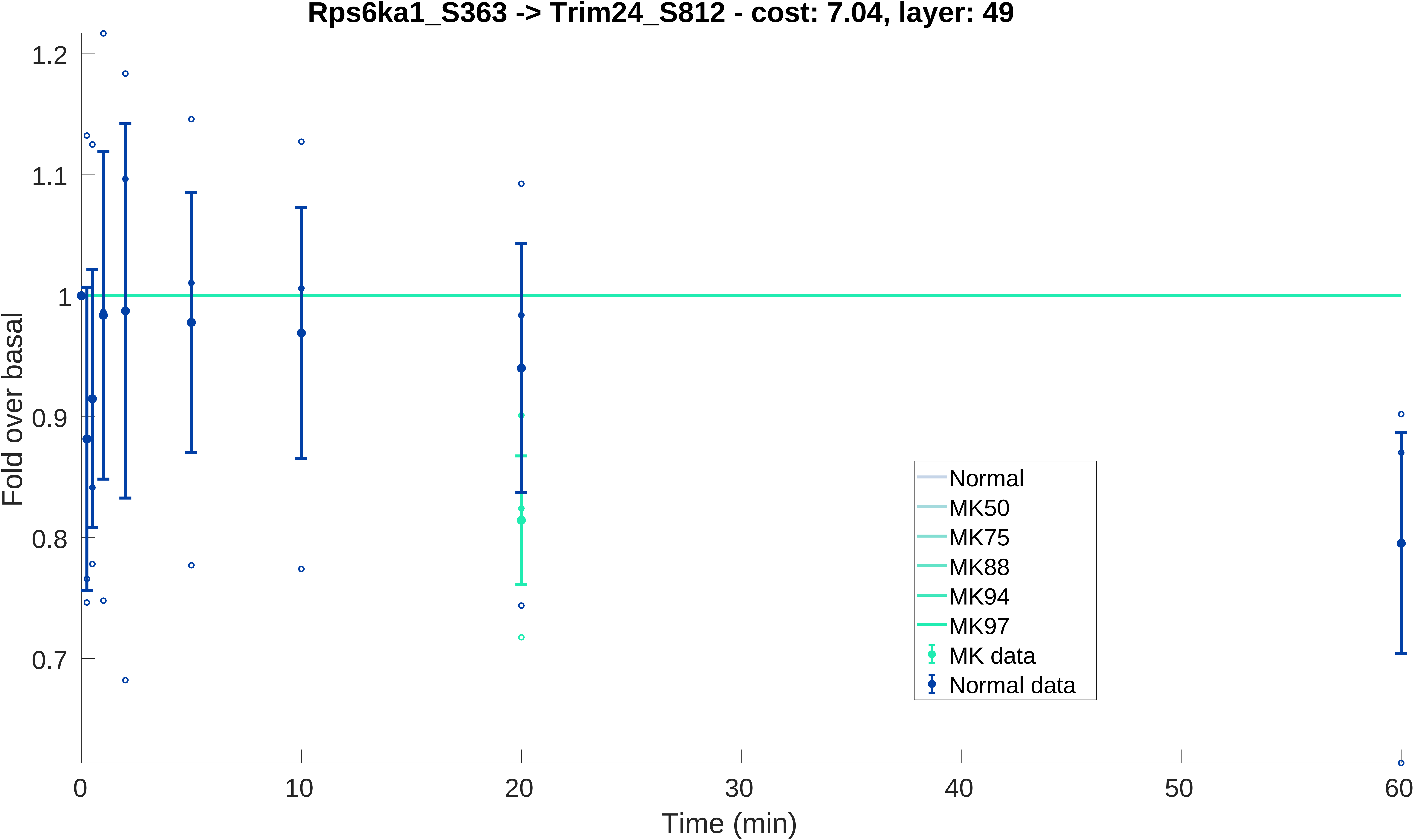

Rps6ka1_S363 -> Trim24_S812 - cost: 7.04, layer: 49
Fold over basal
Time (min)
0
10
20
30
40
50
60
0.7
0.8
0.9
1
1.1
1.2
Normal
MK50
MK75
MK88
MK94
MK97
MK data
Normal data

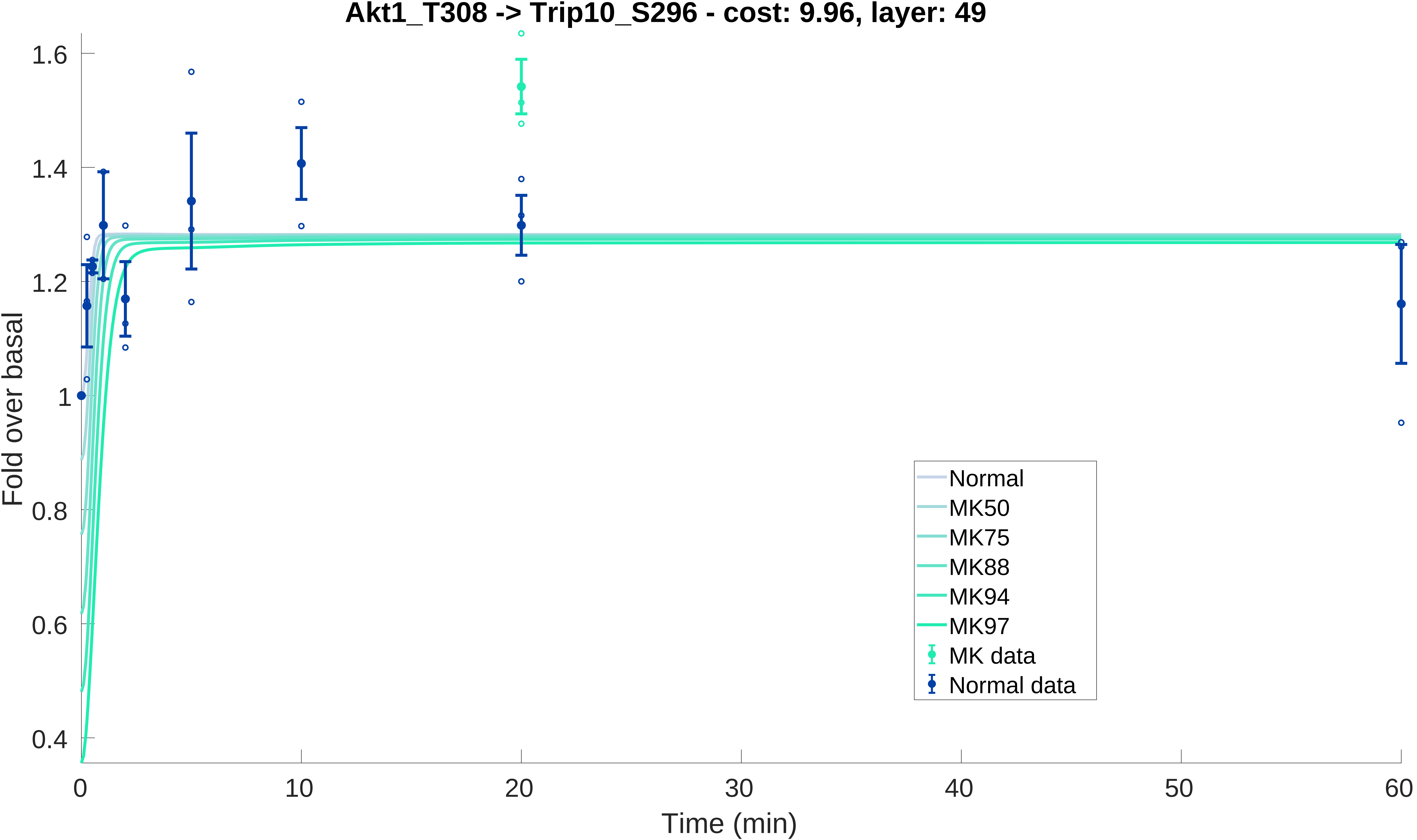

Akt1_T308 -> Trip10_S296 - cost: 9.96, layer: 49
Fold over basal
1.6
1.4
1.2
1
0.8
0.6
0.4
0
10
20
30
40
50
60
Time (min)
Normal
MK50
MK75
MK88
MK94
MK97
MK data
Normal data

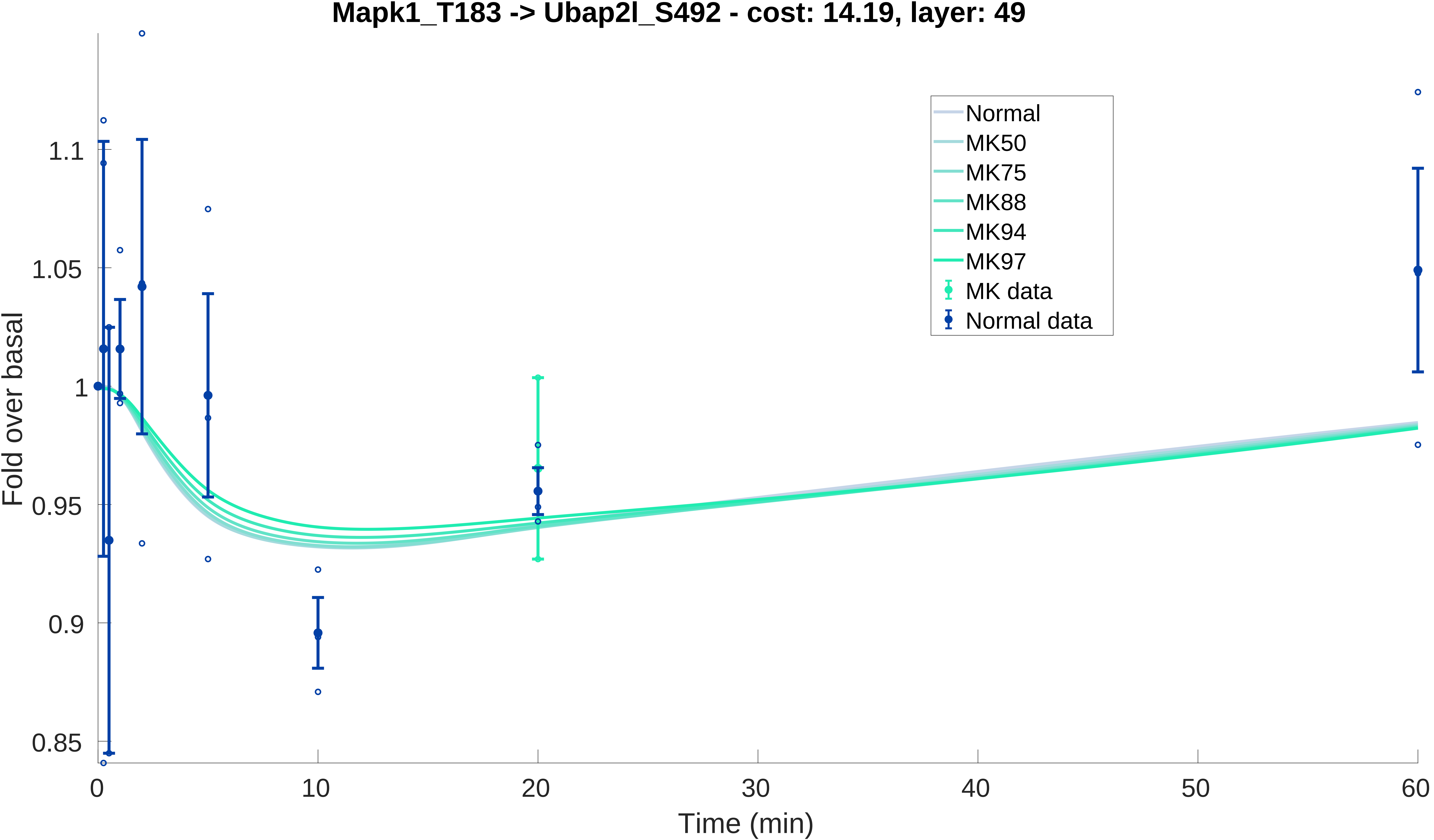
Mapk1_T183 -> Ubap2l_S492 - cost: 14.19, layer: 49
Fold over basal
Time (min)
0.85
0.9
0.95
1
1.05
1.1
0
10
20
30
40
50
60
Normal
MK50
MK75
MK88
MK94
MK97
MK data
Normal data

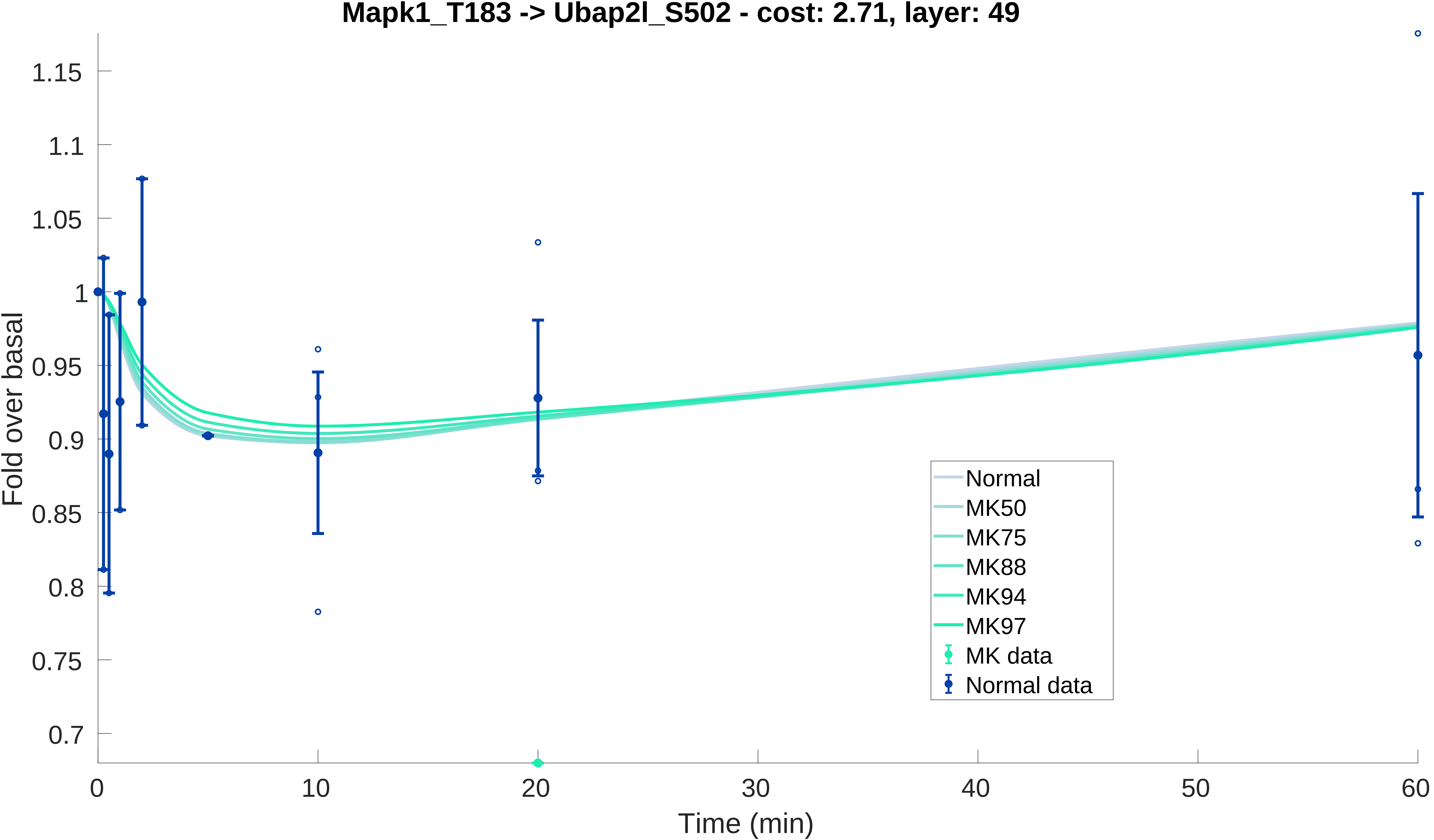
Mapk1_T183 -> Ubap2l_S502 - cost: 2.71, layer: 49
Fold over basal
1.15
1.1
1.05
1
0.95
0.9
0.85
0.8
0.75
0.7
0
10
20
30
40
50
60
Time (min)
Normal
MK50
MK75
MK88
MK94
MK97
MK data
Normal data

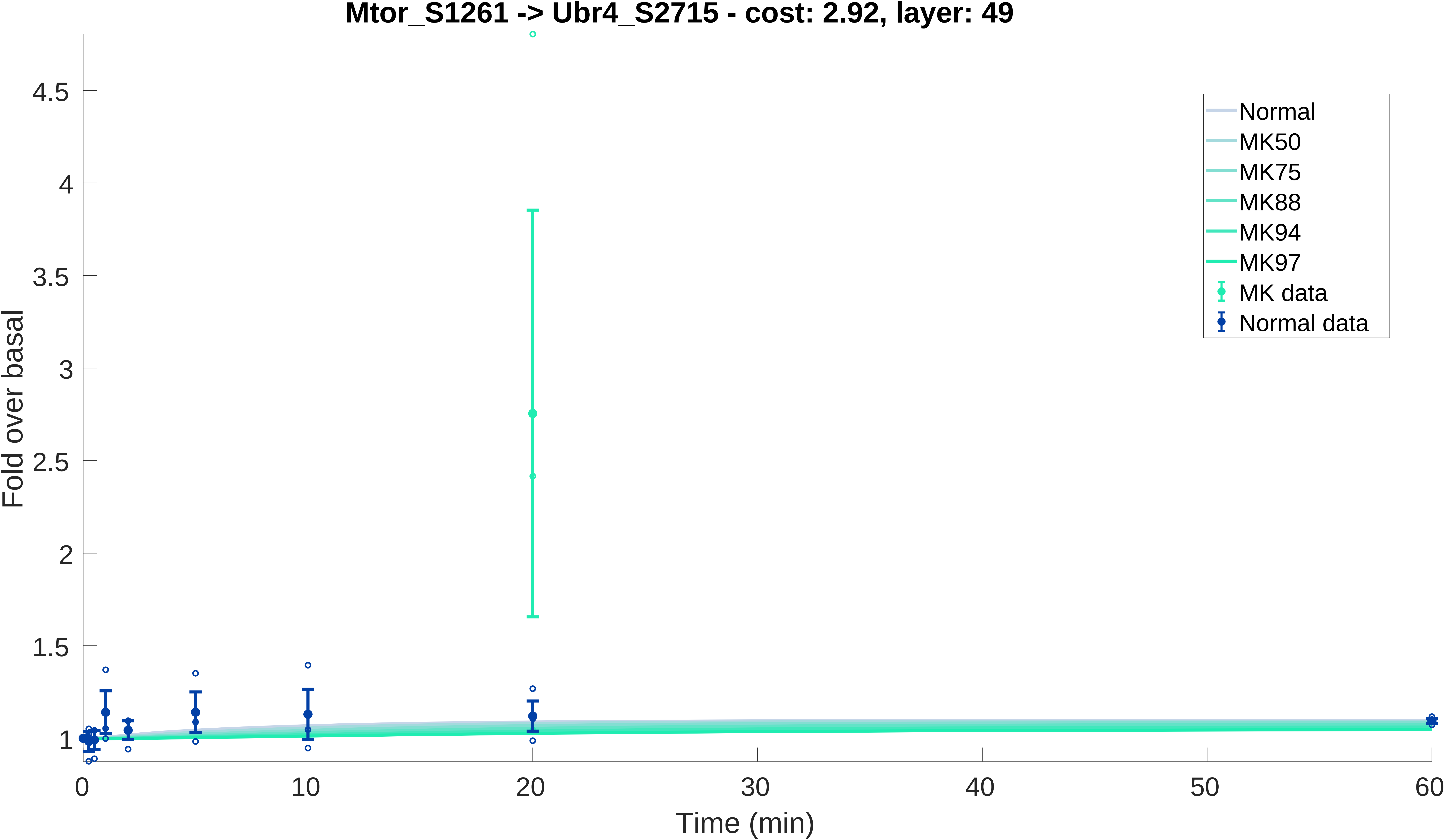

Mtor_S1261 -> Ubr4_S2715 - cost: 2.92, layer: 49
Fold over basal
4.5
4
3.5
3
2.5
2
1.5
1
0
10
20
30
40
50
60
Time (min)
Normal
MK50
MK75
MK88
MK94
MK97
MK data
Normal data

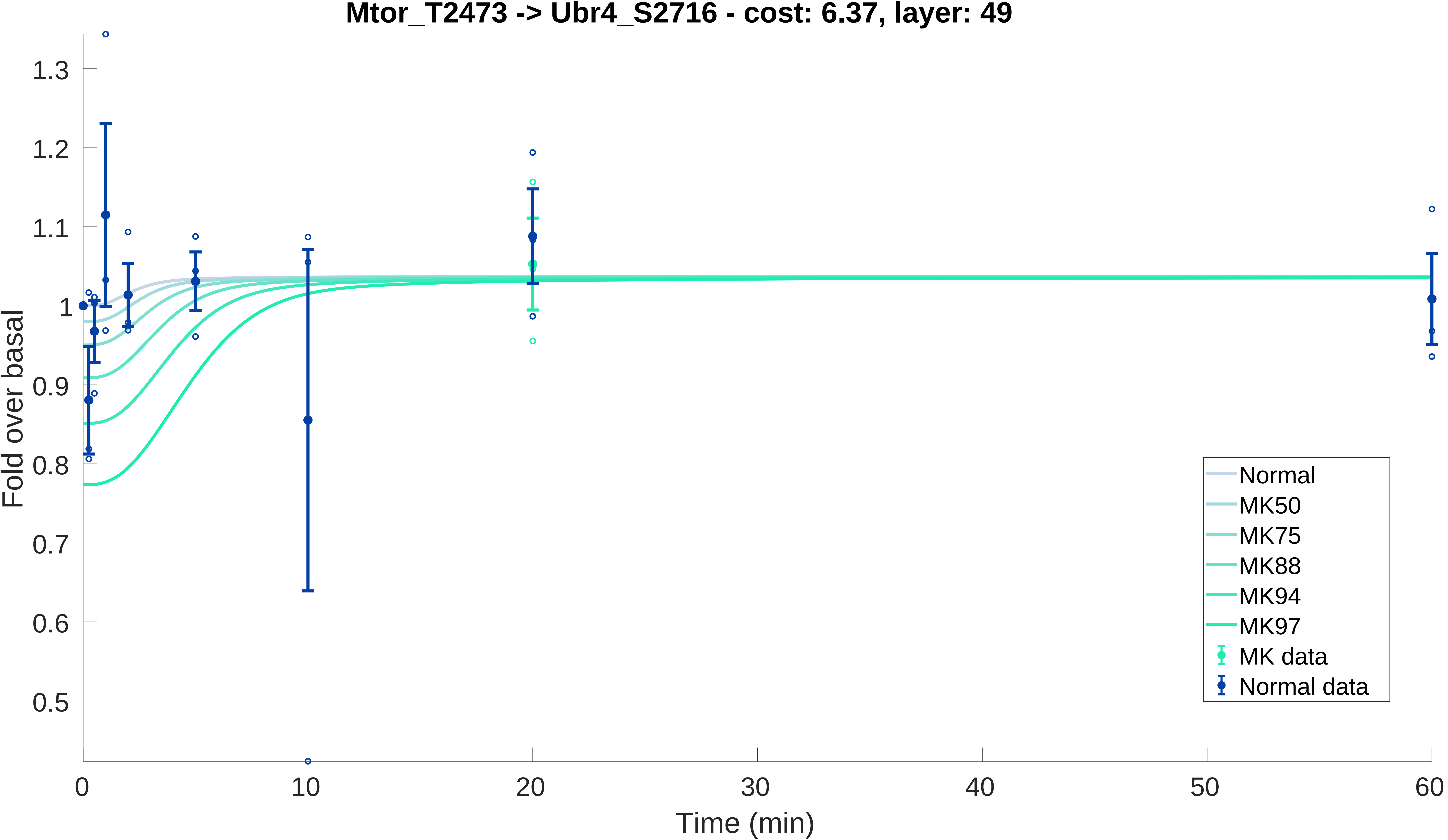

Mtor_T2473 -> Ubr4_S2716 - cost: 6.37, layer: 49
Fold over basal
Time (min)
1.3
1.2
1.1
1
0.9
0.8
0.7
0.6
0.5
0
10
20
30
40
50
60
Normal
MK50
MK75
MK88
MK94
MK97
MK data
Normal data

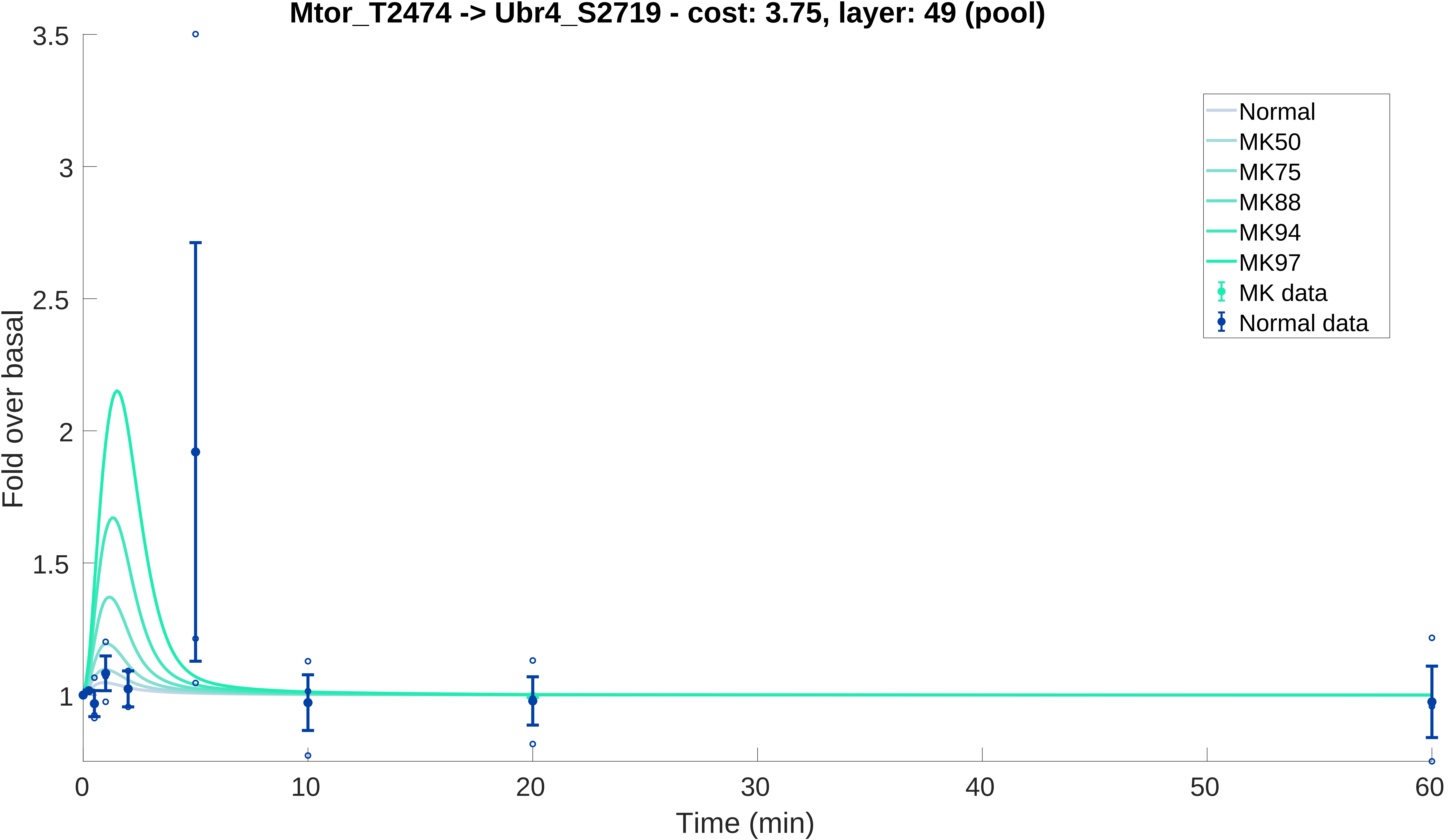

Mtor_T2474 -> Ubr4_S2719 - cost: 3.75, layer: 49 (pool)
Fold over basal
Time (min)
0
10
20
30
40
50
60
1
1.5
2
2.5
3
3.5
Normal
MK50
MK75
MK88
MK94
MK97
MK data
Normal data

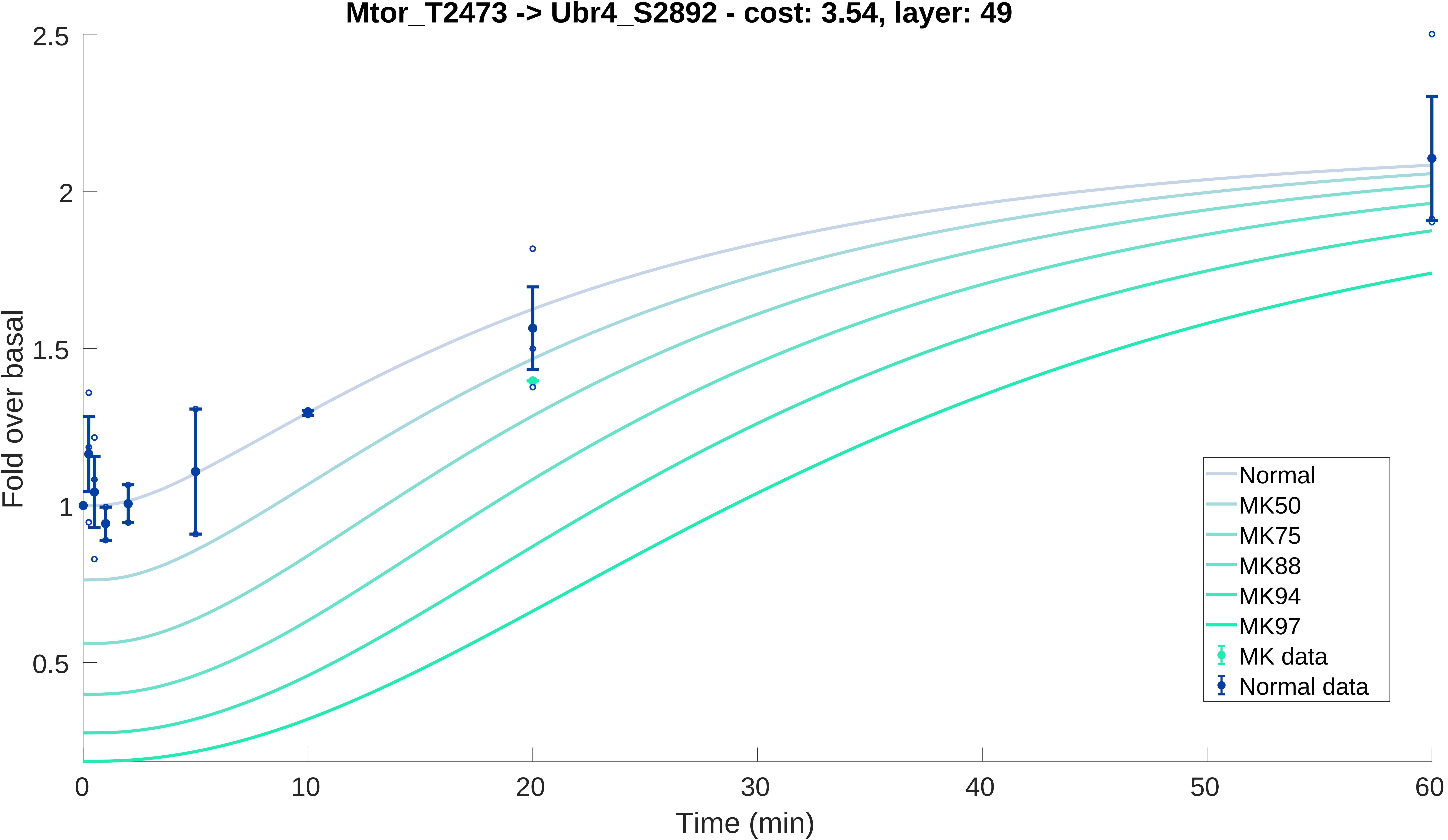
Mtor_T2473 -> Ubr4_S2892 - cost: 3.54, layer: 49
Fold over basal
Time (min)
0
10
20
30
40
50
60
0.5
1
1.5
2
2.5
Normal
MK50
MK75
MK88
MK94
MK97
MK data
Normal data

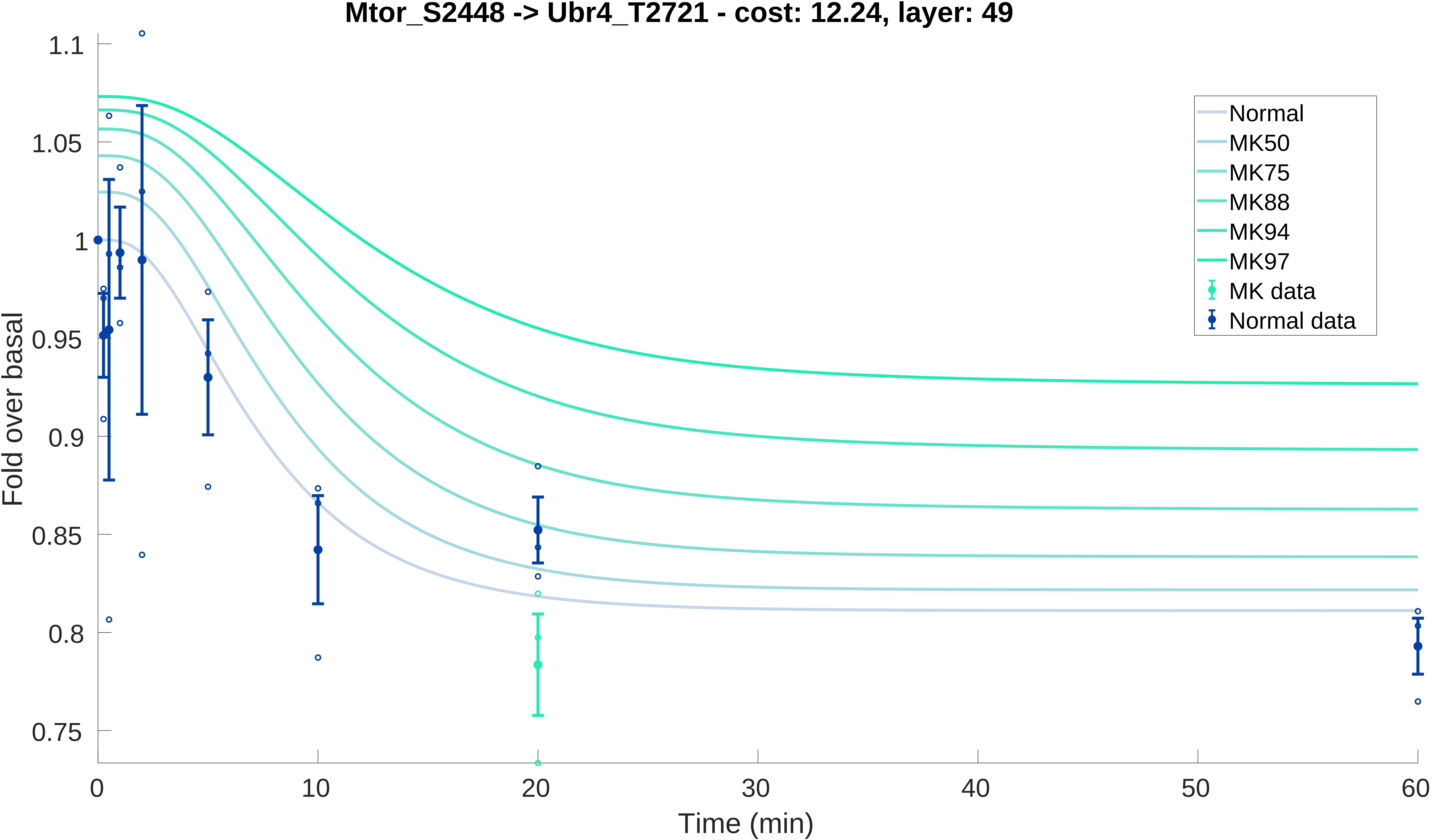

Mtor_S2448 -> Ubr4_T2721 - cost: 12.24, layer: 49
Fold over basal
Time (min)
1.1
1.05
1
0.95
0.9
0.85
0.8
0.75
0
10
20
30
40
50
60
Normal
MK50
MK75
MK88
MK94
MK97
MK data
Normal data

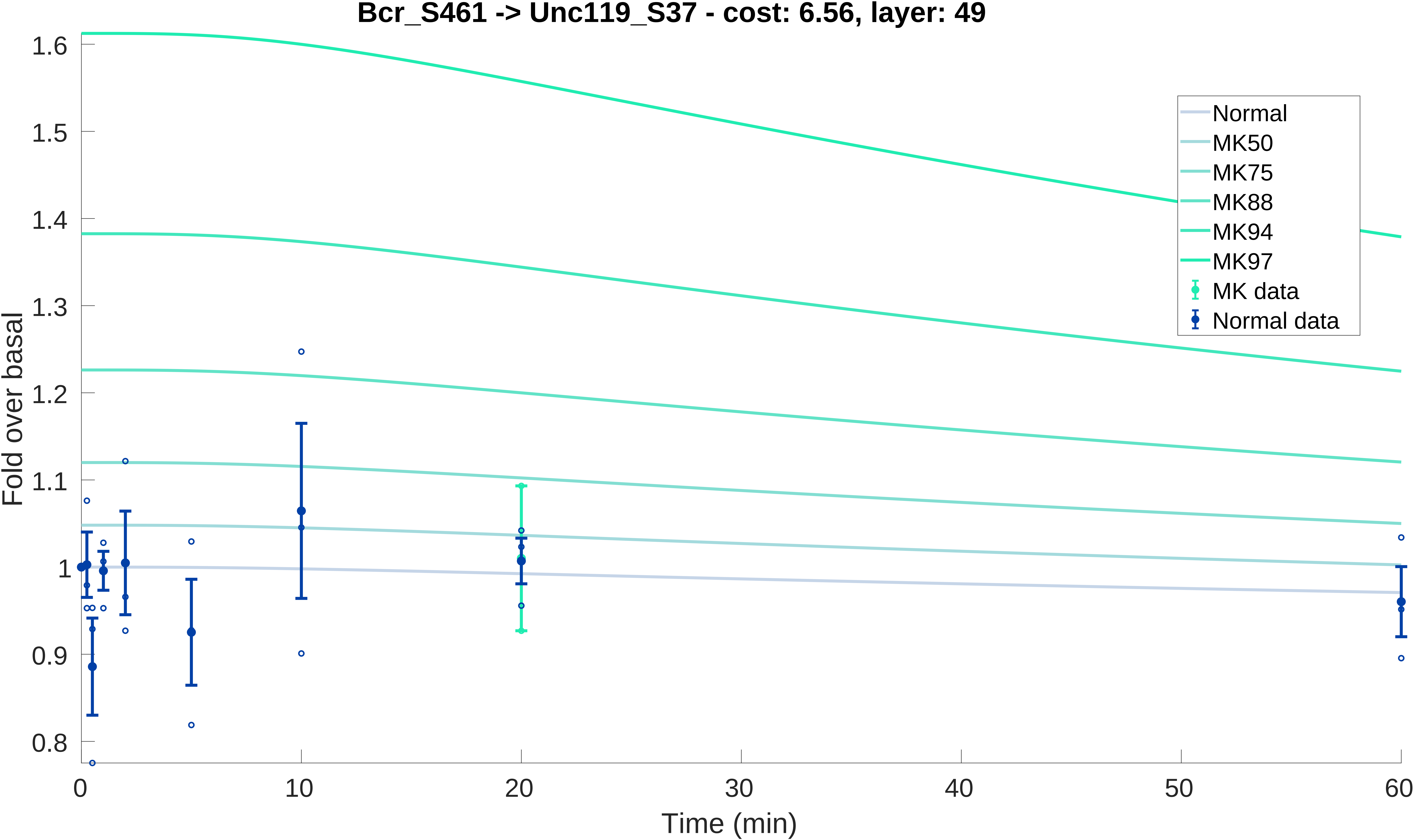
Bcr_S461 -> Unc119_S37 - cost: 6.56, layer: 49
Fold over basal
Time (min)
Normal
MK50
MK75
MK88
MK94
MK97
MK data
Normal data
0.8
0.9
1
1.1
1.2
1.3
1.4
1.5
1.6
0
10
20
30
40
50
60

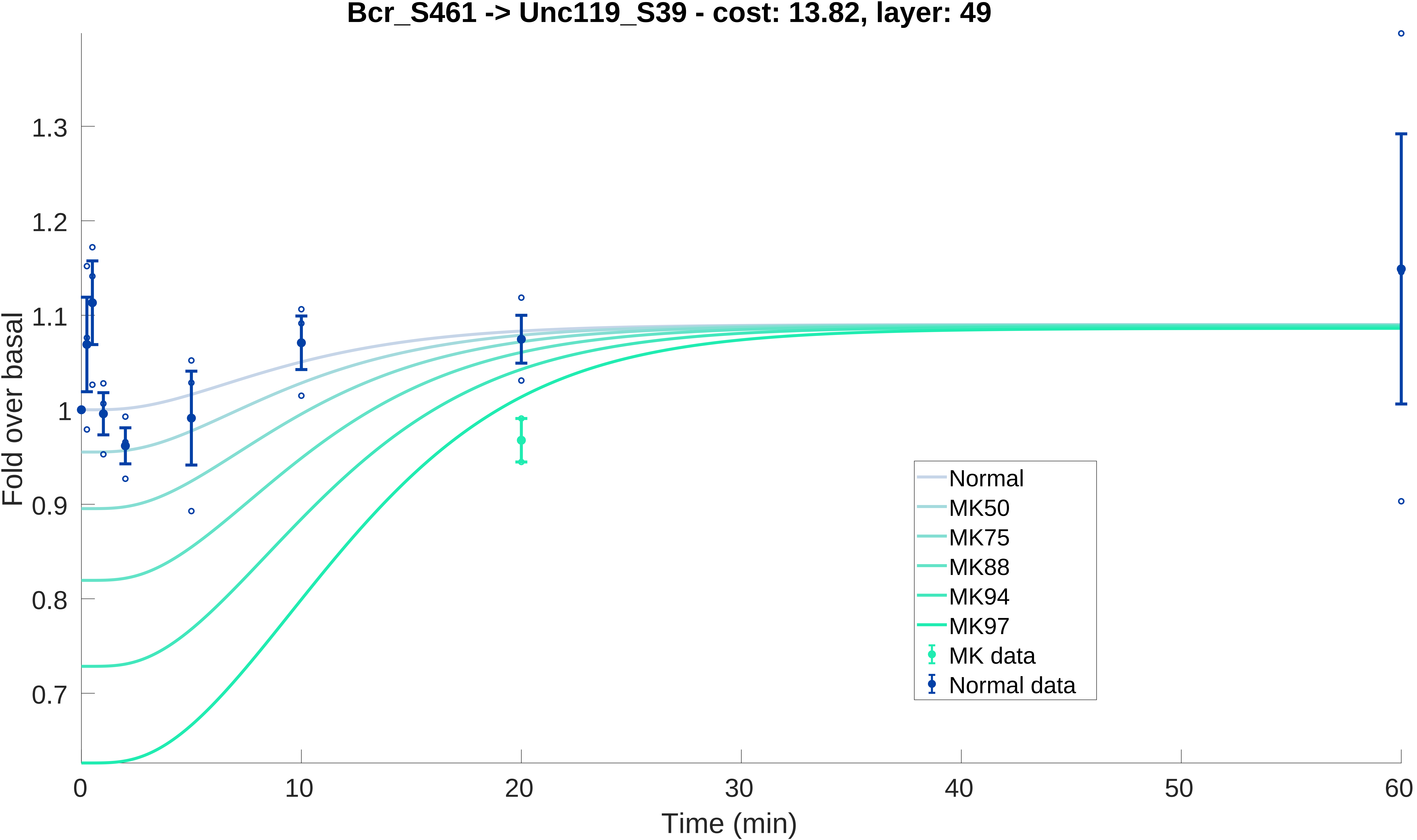

Bcr_S461 -> Unc119_S39 - cost: 13.82, layer: 49
Fold over basal
Time (min)
1.3
1.2
1.1
1
0.9
0.8
0.7
0
10
20
30
40
50
60
Normal
MK50
MK75
MK88
MK94
MK97
MK data
Normal data

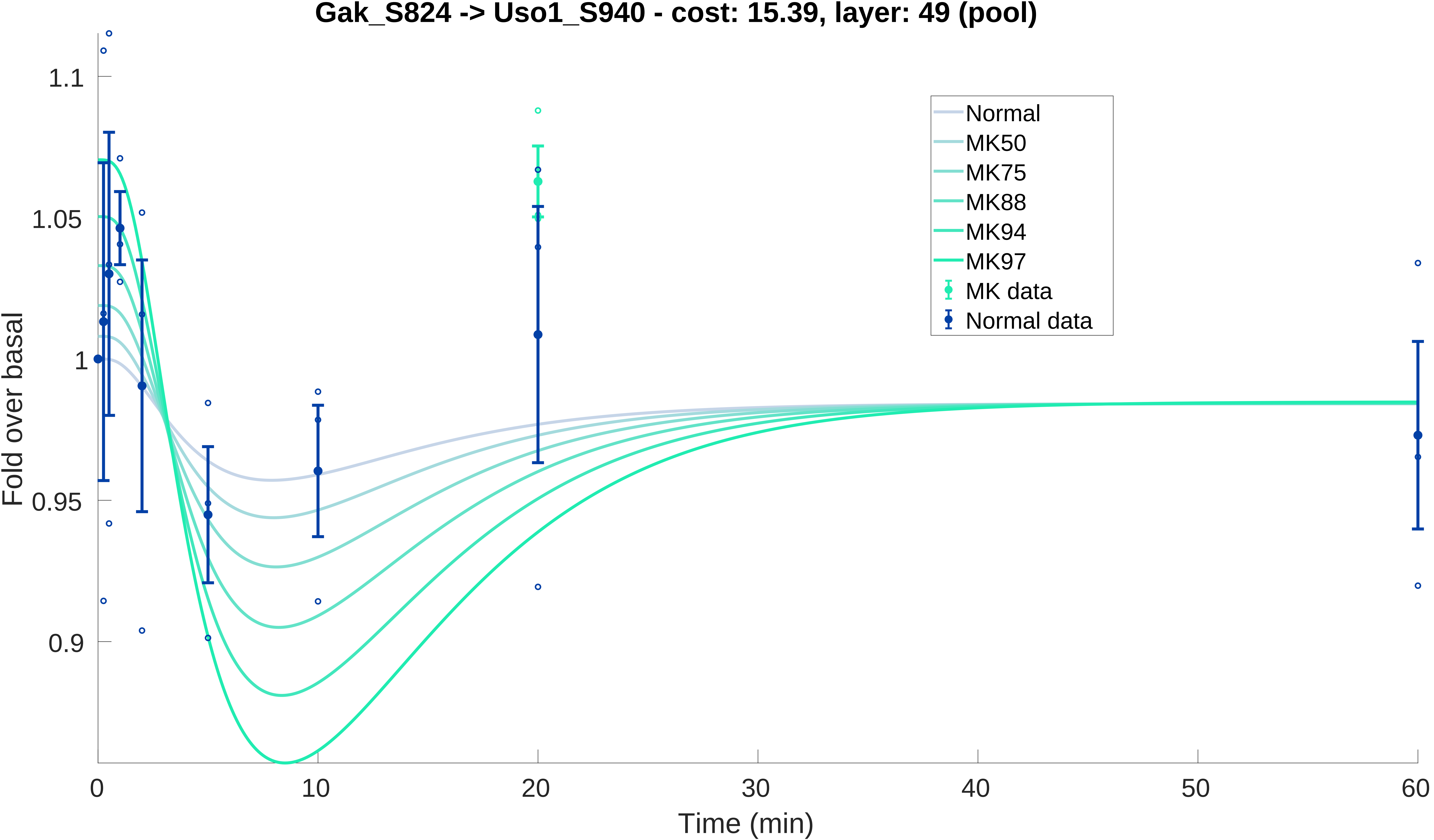

Gak_S824 -> Uso1_S940 - cost: 15.39, layer: 49 (pool)
Fold over basal
1.1
1.05
1
0.95
0.9
0
10
20
30
40
50
60
Time (min)
Normal
MK50
MK75
MK88
MK94
MK97
MK data
Normal data

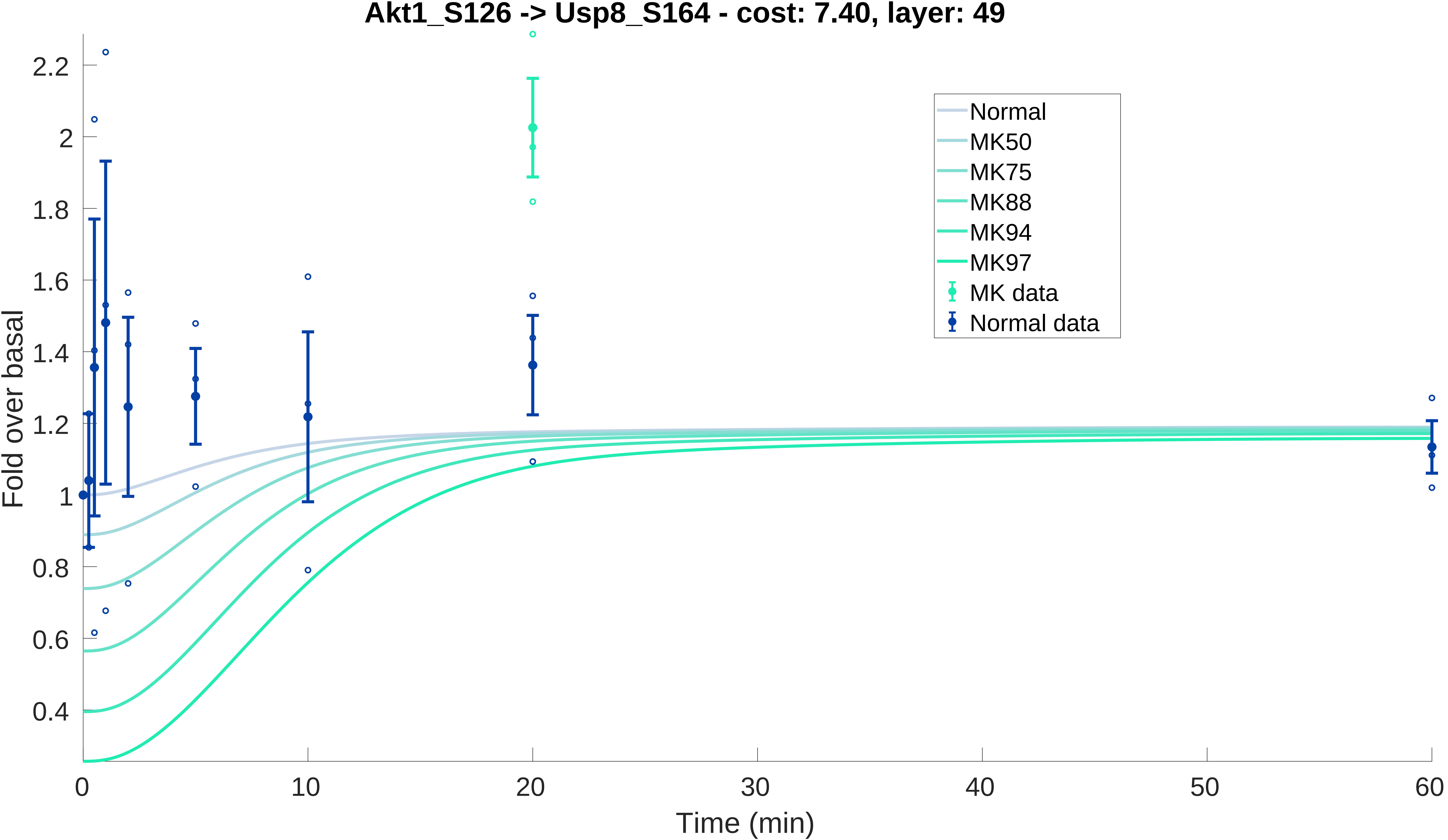
Akt1_S126 -> Usp8_S164 - cost: 7.40, layer: 49
Fold over basal
Time (min)
0
10
20
30
40
50
60
0.4
0.6
0.8
1
1.2
1.4
1.6
1.8
2
2.2
Normal
MK50
MK75
MK88
MK94
MK97
MK data
Normal data

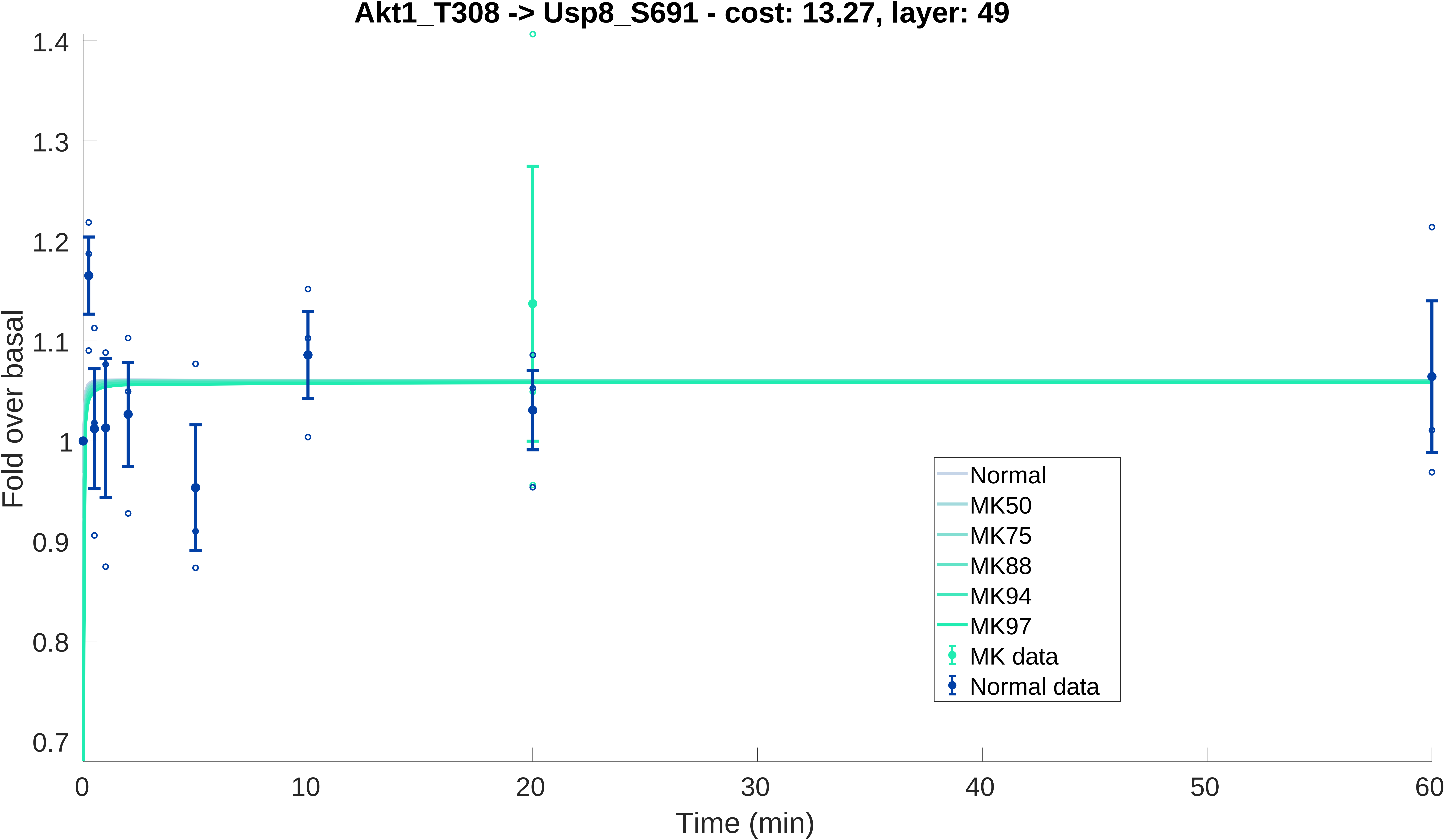

Akt1_T308 -> Usp8_S691 - cost: 13.27, layer: 49
Fold over basal
Time (min)
1.4
1.3
1.2
1.1
1
0.9
0.8
0.7
0
10
20
30
40
50
60
Normal
MK50
MK75
MK88
MK94
MK97
MK data
Normal data

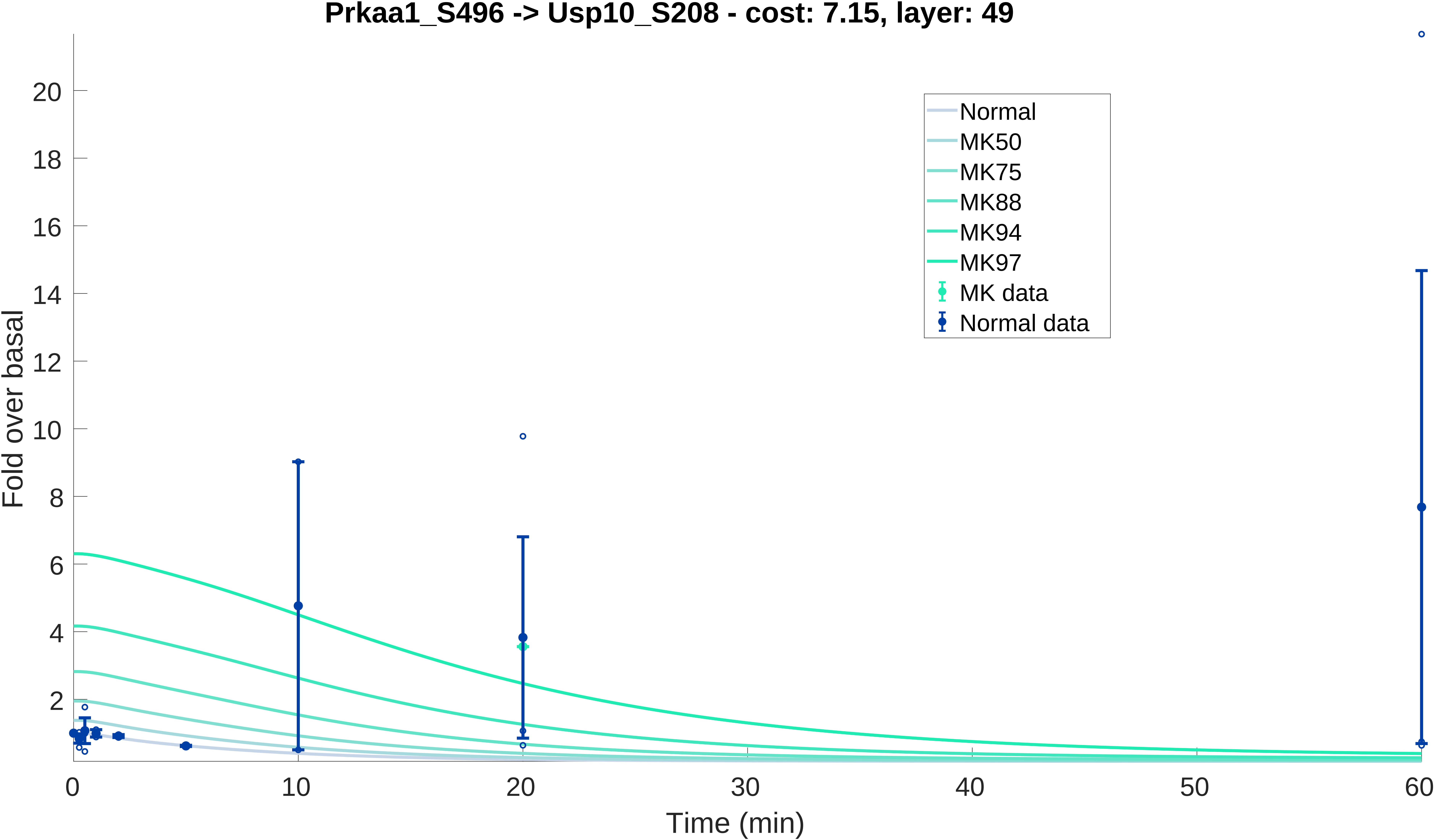

Prkaa1_S496 -> Usp10_S208 - cost: 7.15, layer: 49
Fold over basal
Time (min)
0
10
20
30
40
50
60
2
4
6
8
10
12
14
16
18
20
Normal
MK50
MK75
MK88
MK94
MK97
MK data
Normal data

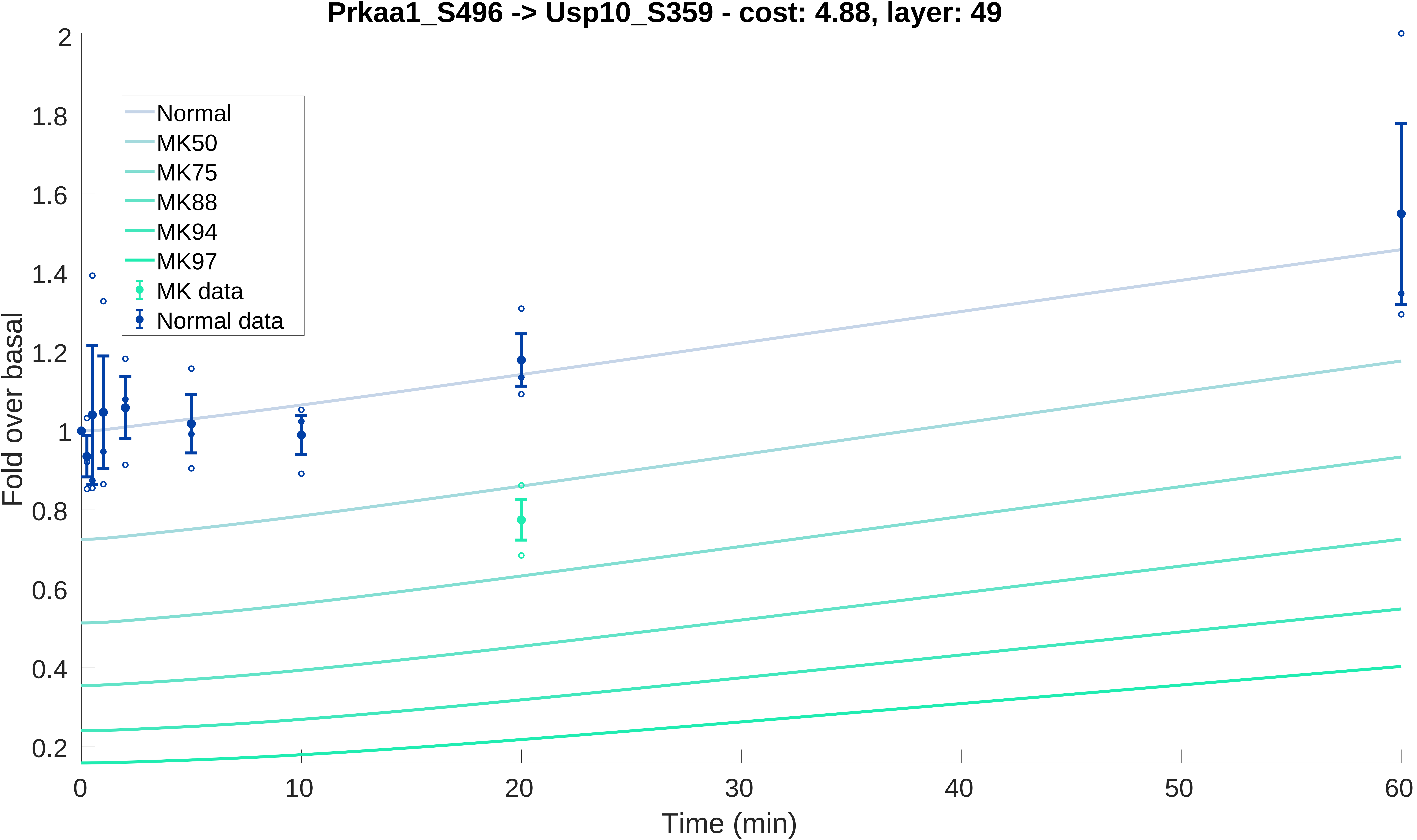
Prrkaa1_S496 -> Usp10_S359 - cost: 4.88, layer: 49
Normal
MK50
MK75
MK88
MK94
MK97
MK data
Normal data
Fold over basal
Time (min)
0
10
20
30
40
50
60
0.2
0.4
0.6
0.8
1
1.2
1.4
1.6
1.8
2

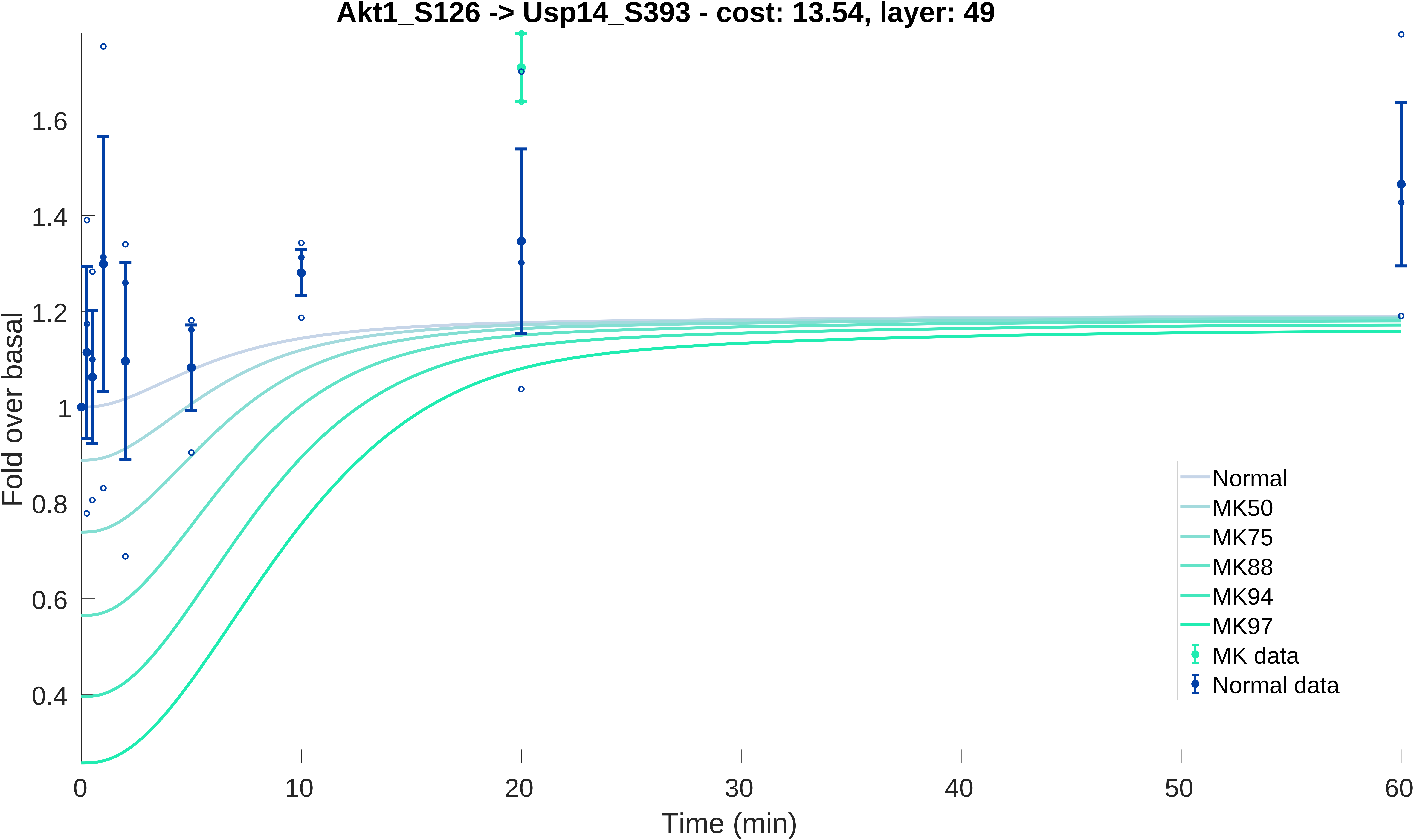
Akt1_S126 -> Usp14_S393 - cost: 13.54, layer: 49
Fold over basal
Time (min)
0.4
0.6
0.8
1
1.2
1.4
1.6
0
10
20
30
40
50
60
Normal
MK50
MK75
MK88
MK94
MK97
MK data
Normal data

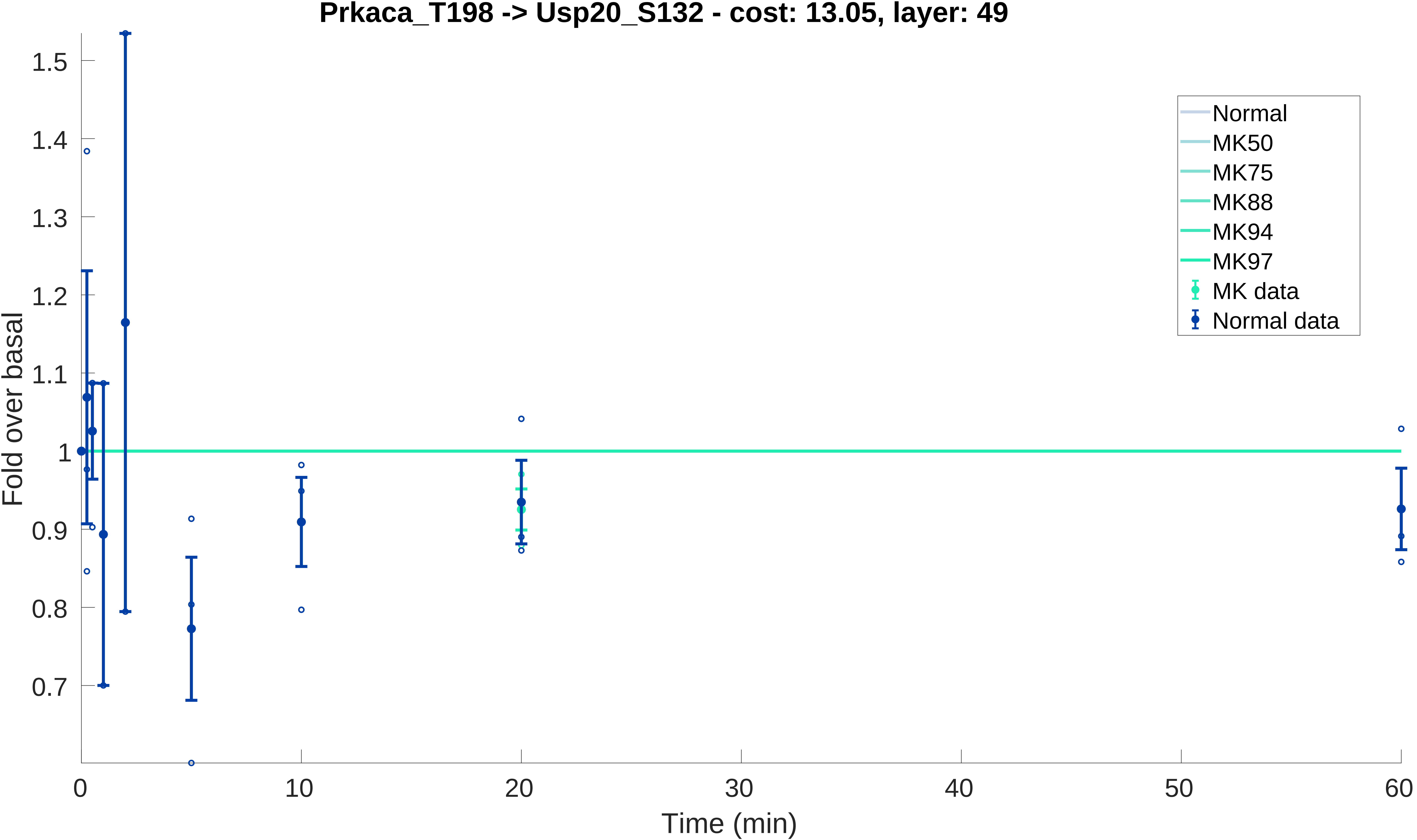

Prkaca_T198 -> Usp20_S132 - cost: 13.05, layer: 49
Fold over basal
1.5
1.4
1.3
1.2
1.1
1
0.9
0.8
0.7
0
10
20
30
40
50
60
Time (min)
Normal
MK50
MK75
MK88
MK94
MK97
MK data
Normal data

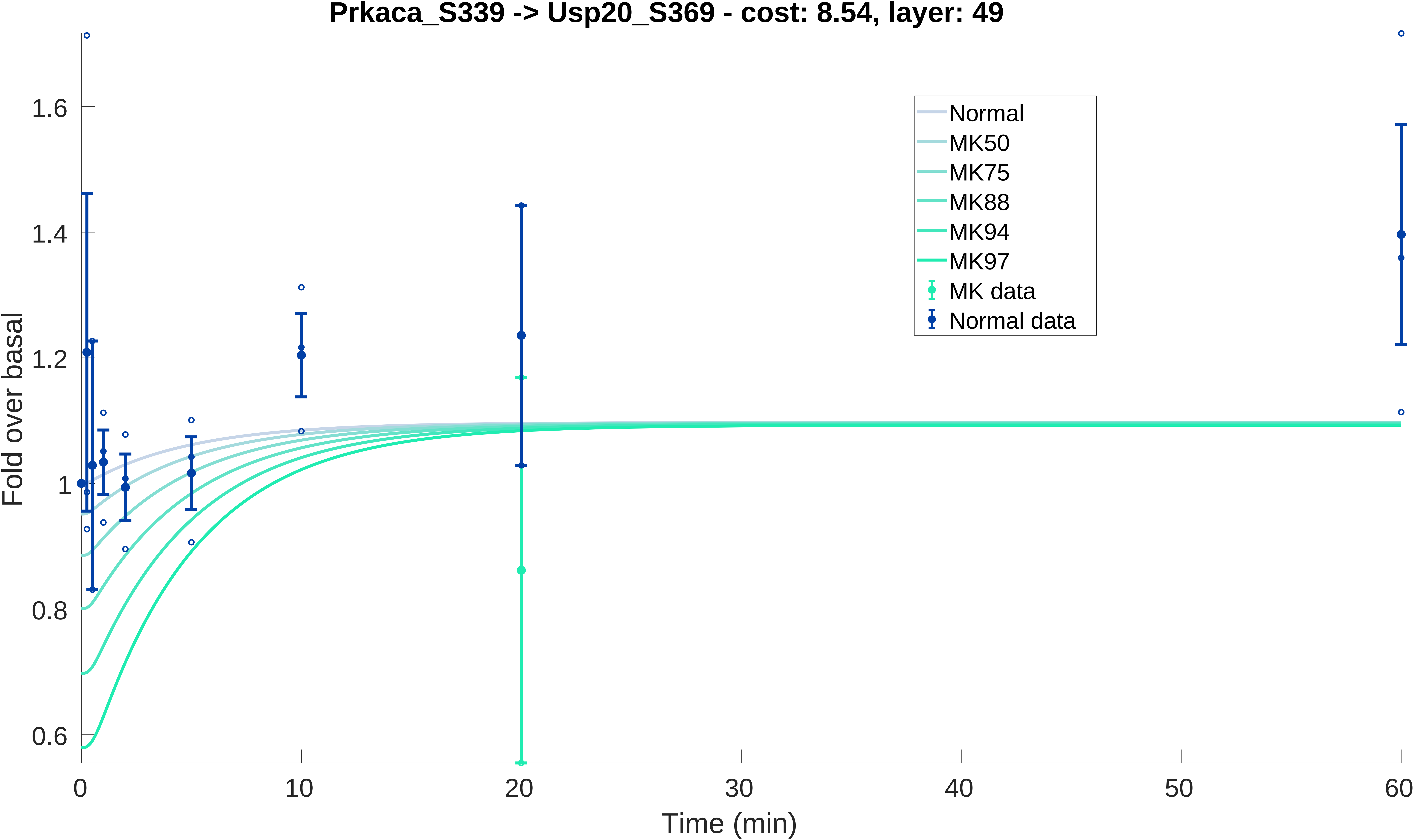

Prkaca_S339 -> Usp20_S369 - cost: 8.54, layer: 49
Fold over basal
Time (min)
Normal
MK50
MK75
MK88
MK94
MK97
MK data
Normal data
0.6
0.8
1
1.2
1.4
1.6
0
10
20
30
40
50
60

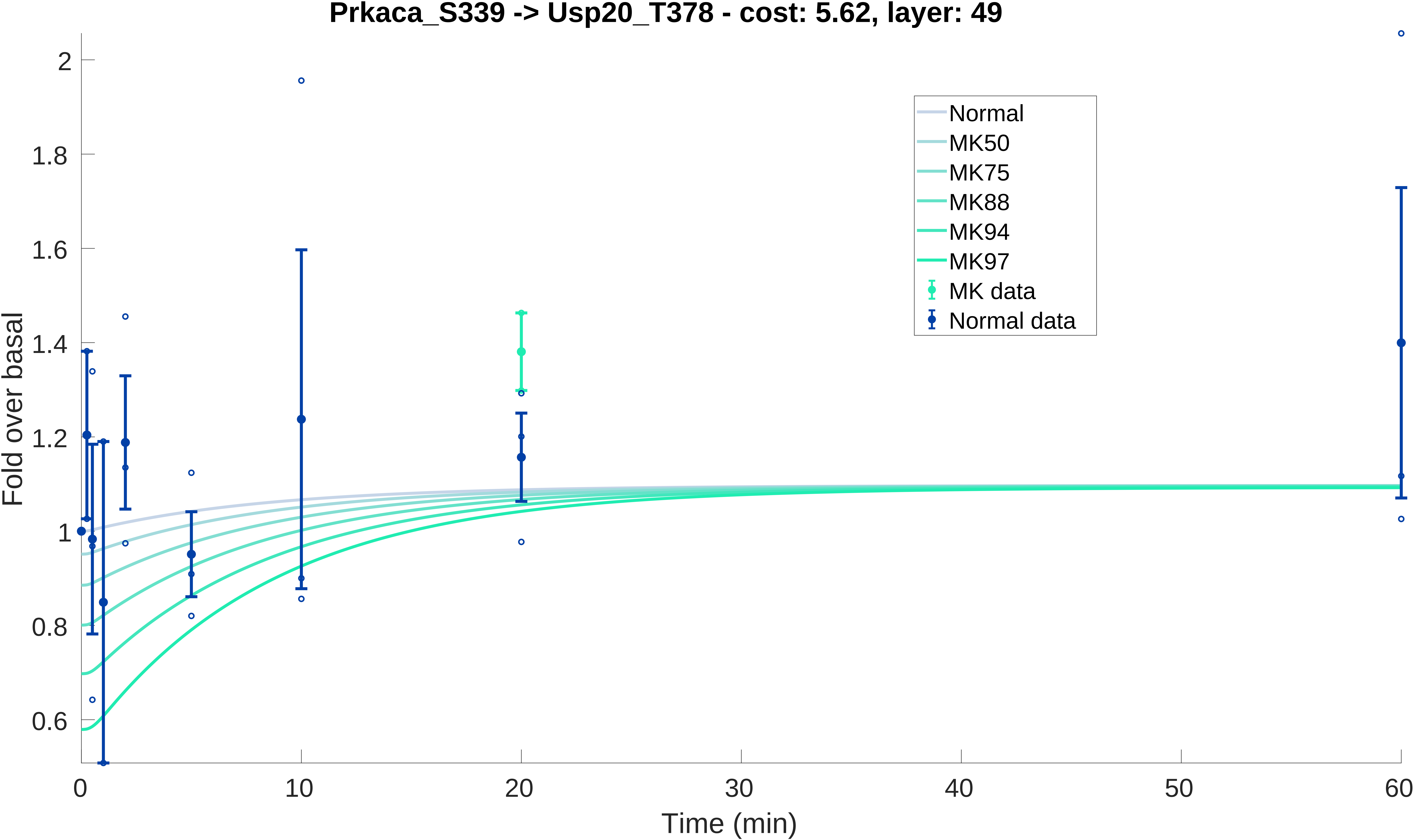

Prkaca_S339 -> Usp20_T378 - cost: 5.62, layer: 49
Fold over basal
Time (min)
0
10
20
30
40
50
60
0.6
0.8
1
1.2
1.4
1.6
1.8
2
Normal
MK50
MK75
MK88
MK94
MK97
MK data
Normal data

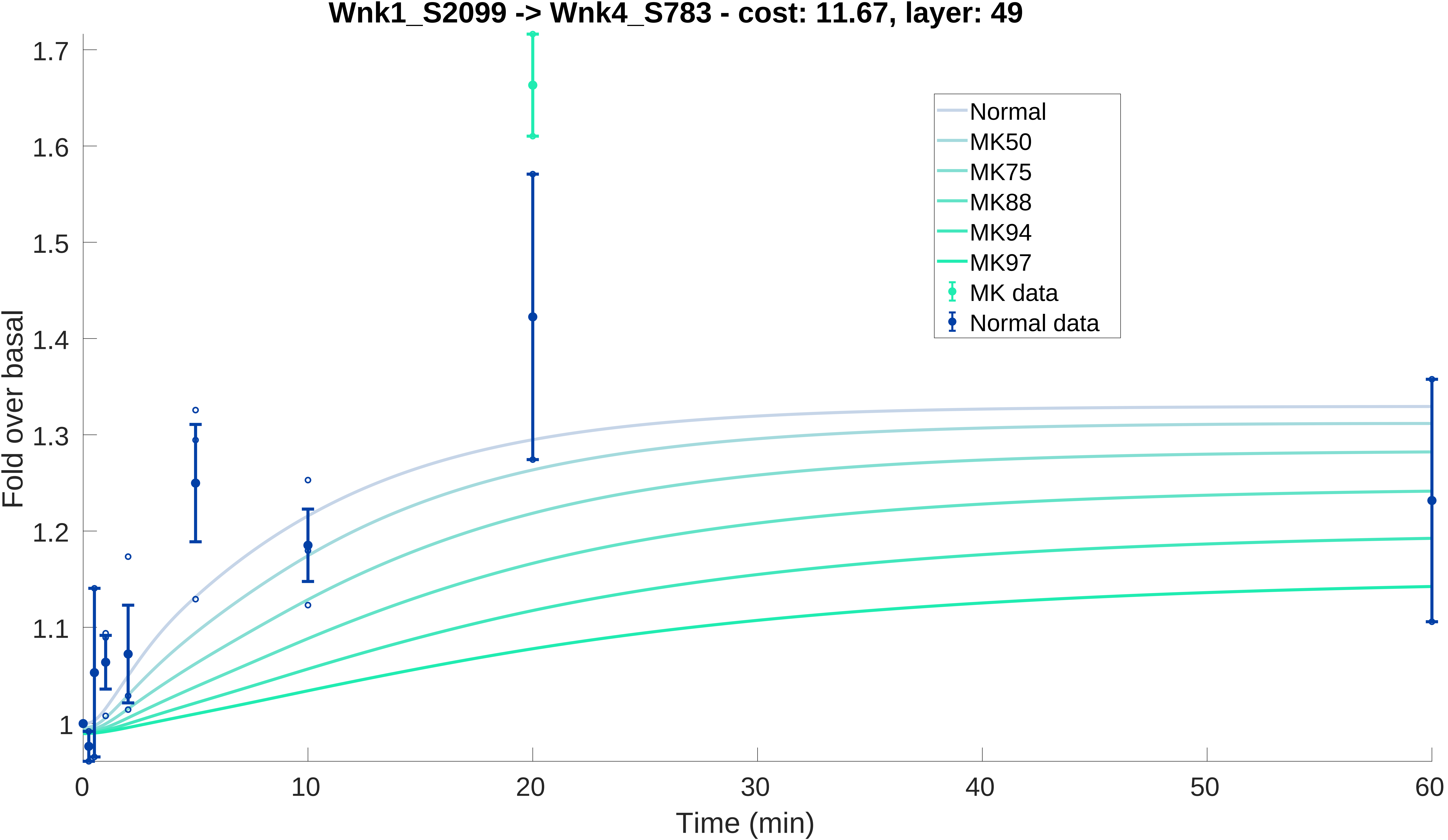
Wnk1_S2099 -> Wnk4_S783 - cost: 11.67, layer: 49
Fold over basal
Time (min)
Normal
MK50
MK75
MK88
MK94
MK97
MK data
Normal data
0
10
20
30
40
50
60
1
1.1
1.2
1.3
1.4
1.5
1.6
1.7

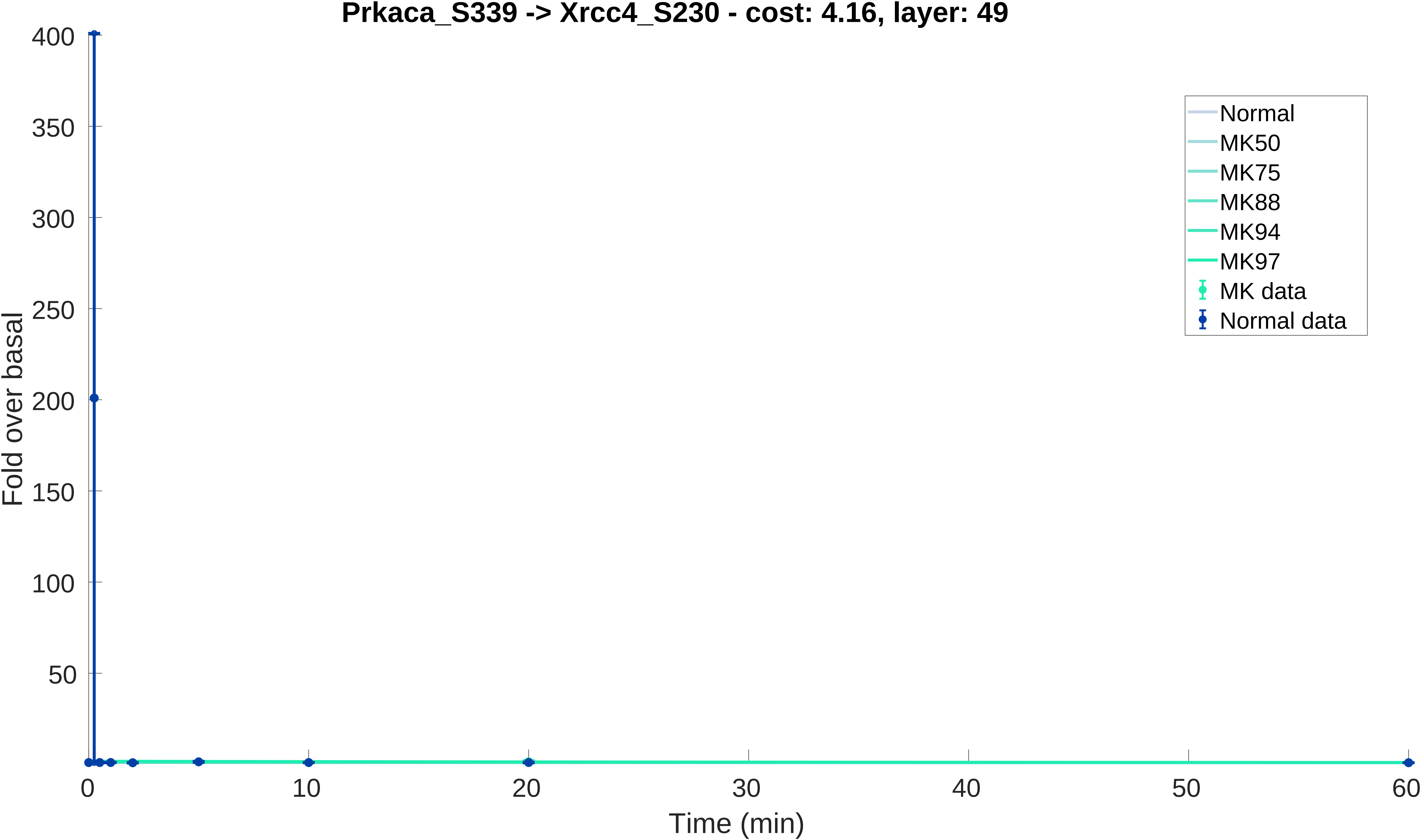
Prkaca_S339 -> Xrcc4_S230 - cost: 4.16, layer: 49
Fold over basal
400
350
300
250
200
150
100
50
0
10
20
30
40
50
60
Time (min)
Normal
MK50
MK75
MK88
MK94
MK97
MK data
Normal data

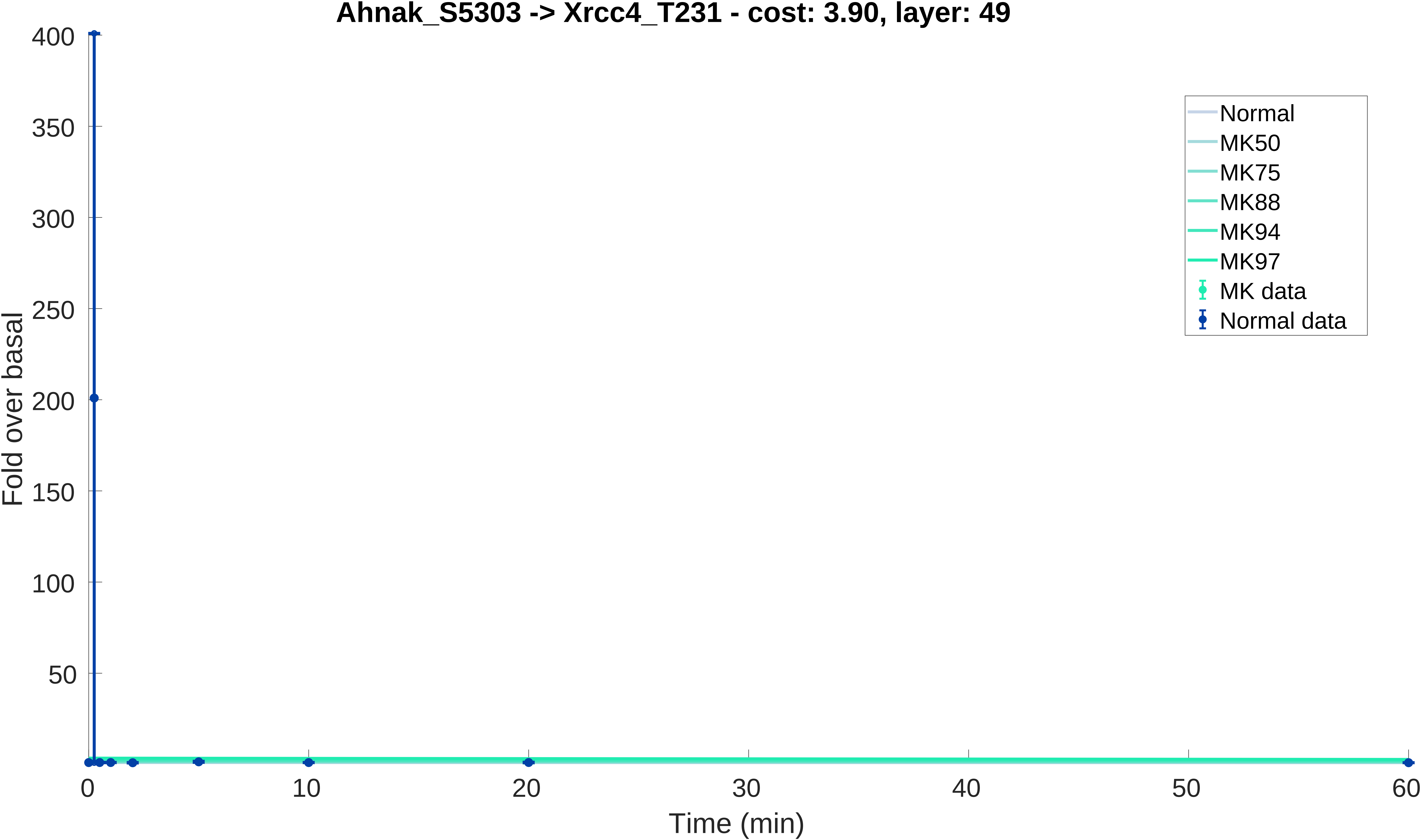

Ahnak_S5303 -> Xrcc4_T231 - cost: 3.90, layer: 49
Fold over basal
Time (min)
400
350
300
250
200
150
100
50
0
10
20
30
40
50
60
Normal
MK50
MK75
MK88
MK94
MK97
MK data
Normal data

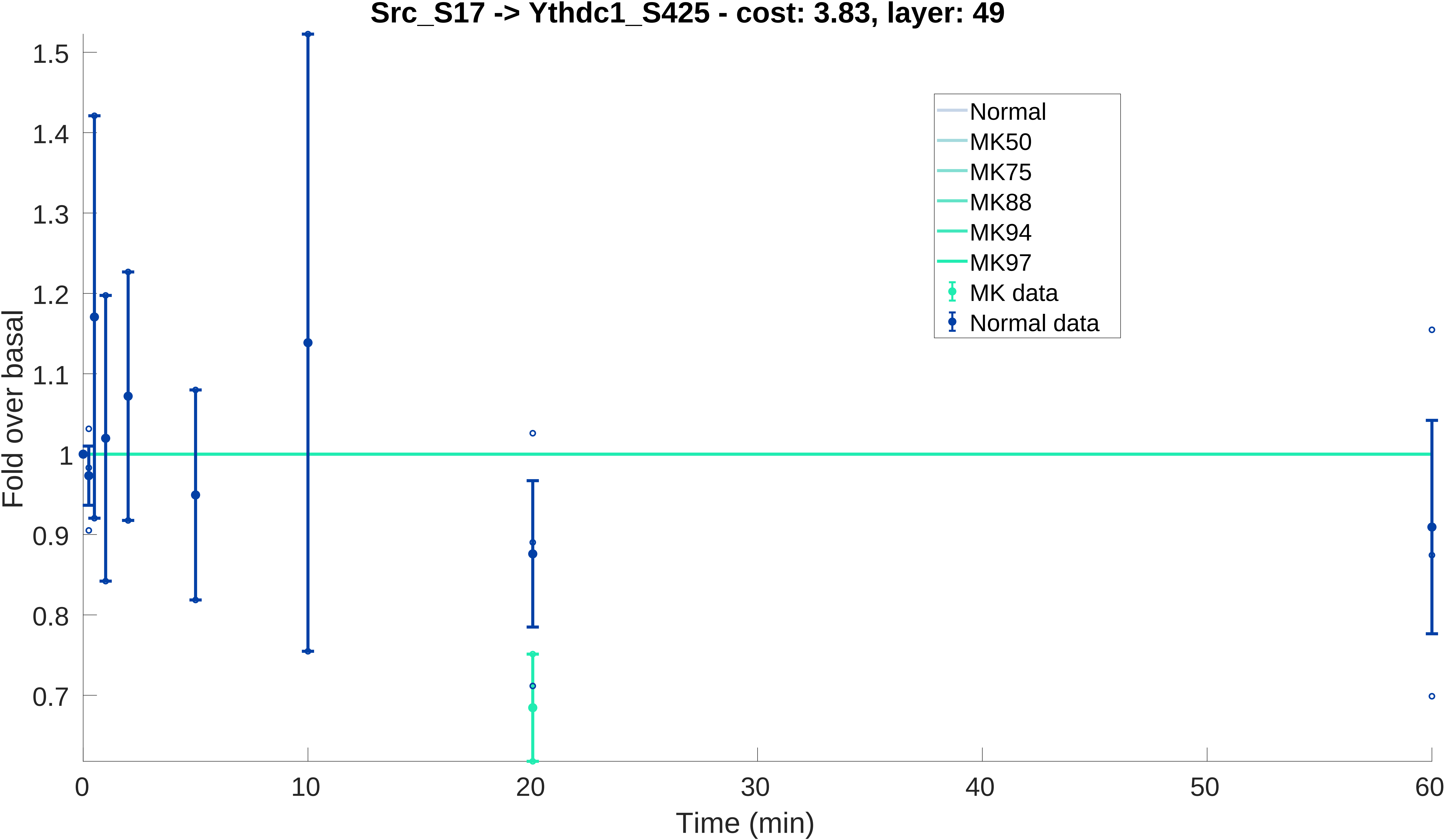

Src_S17 -> Ythdc1_S425 - cost: 3.83, layer: 49
Fold over basal
Time (min)
1.5
1.4
1.3
1.2
1.1
1
0.9
0.8
0.7
0
10
20
30
40
50
60
Normal
MK50
MK75
MK88
MK94
MK97
MK data
Normal data

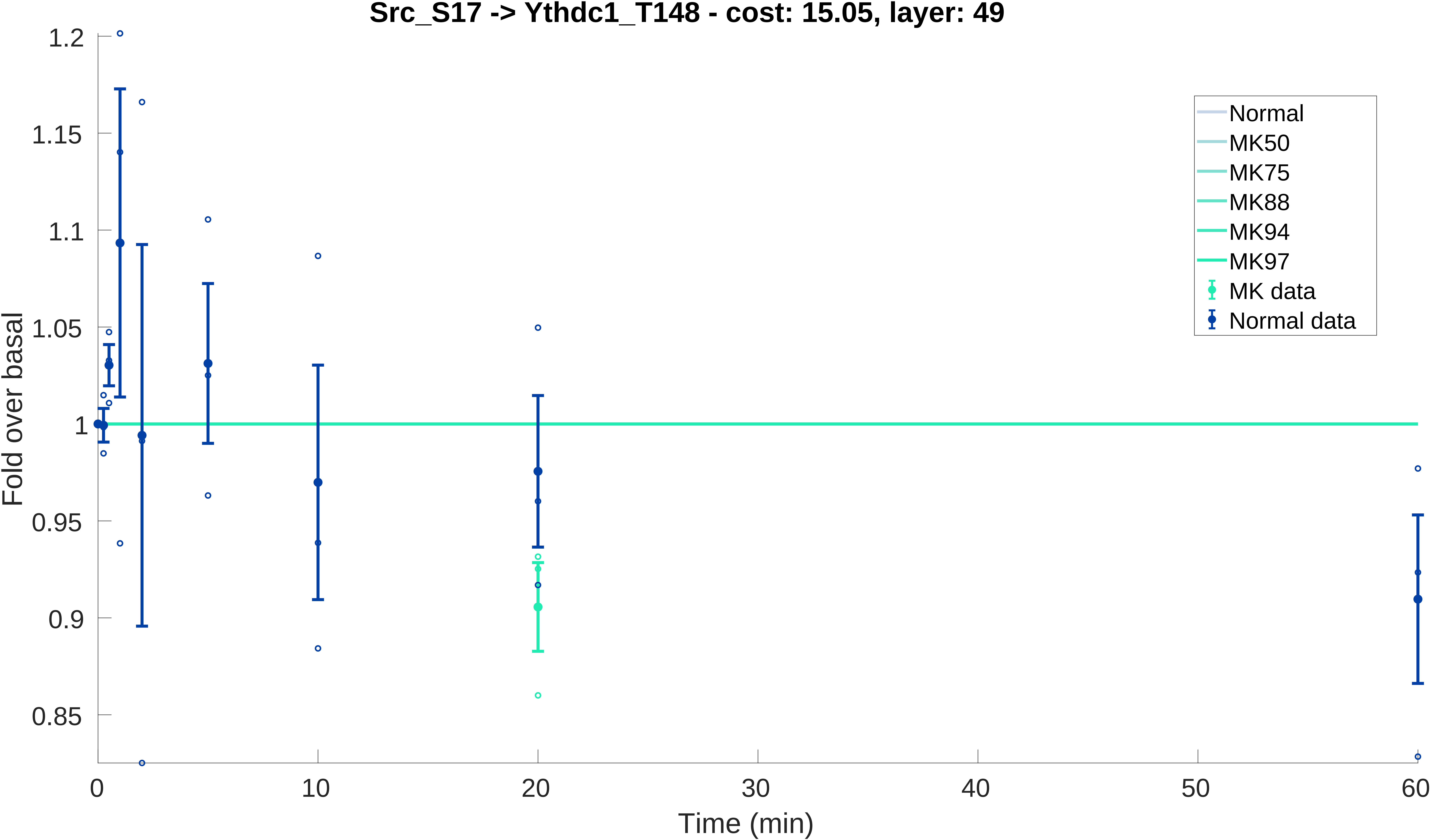

Src_S17 -> Ythdc1_T148 - cost: 15.05, layer: 49
Fold over basal
1.2
1.15
1.1
1.05
1
0.95
0.9
0.85
Time (min)
0
10
20
30
40
50
60
Normal
MK50
MK75
MK88
MK94
MK97
MK data
Normal data

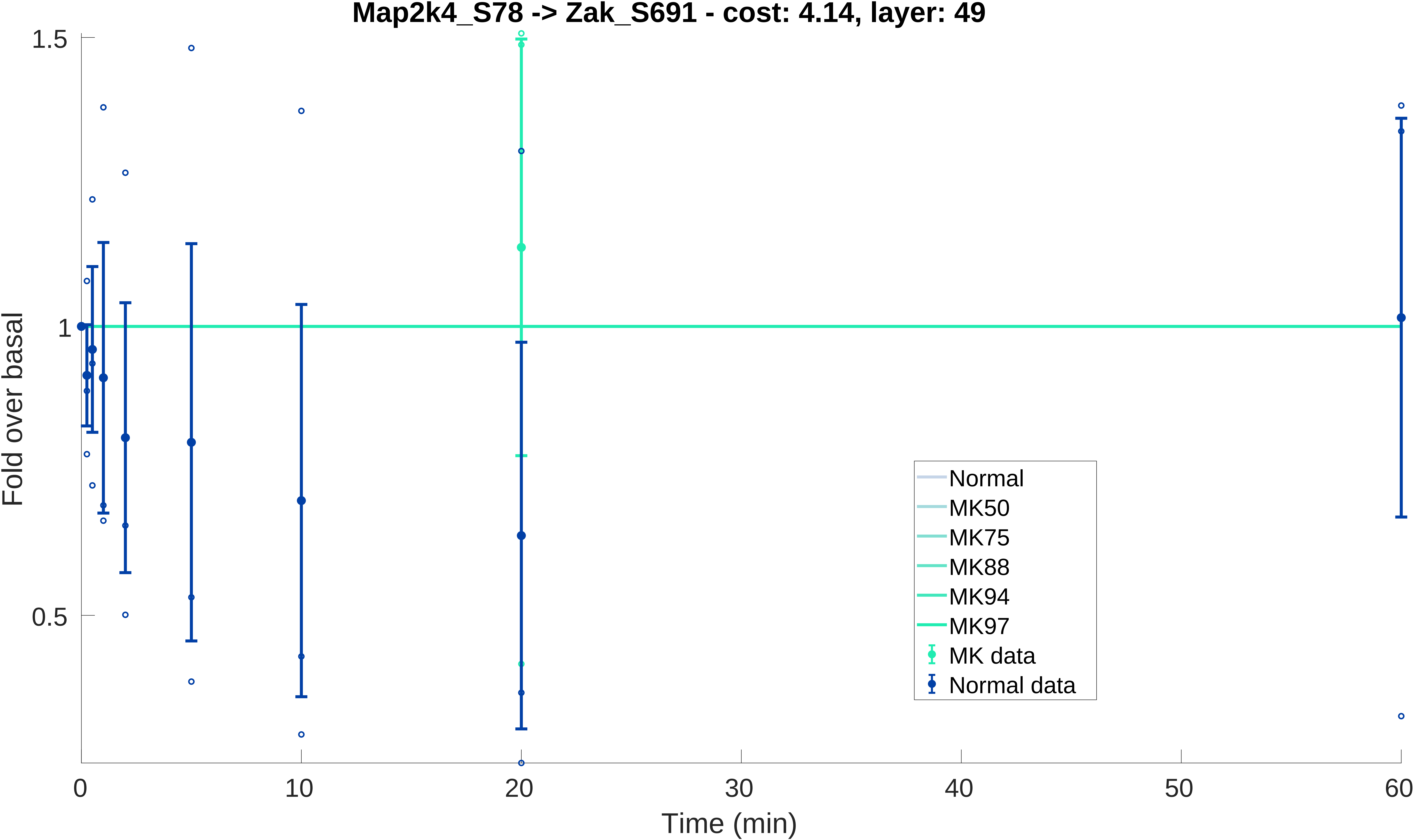

Map2k4_S78 -> Zak_S691 - cost: 4.14, layer: 49
Fold over basal
1.5
1
0.5
0
10
20
30
40
50
60
Time (min)
Normal
MK50
MK75
MK88
MK94
MK97
MK data
Normal data

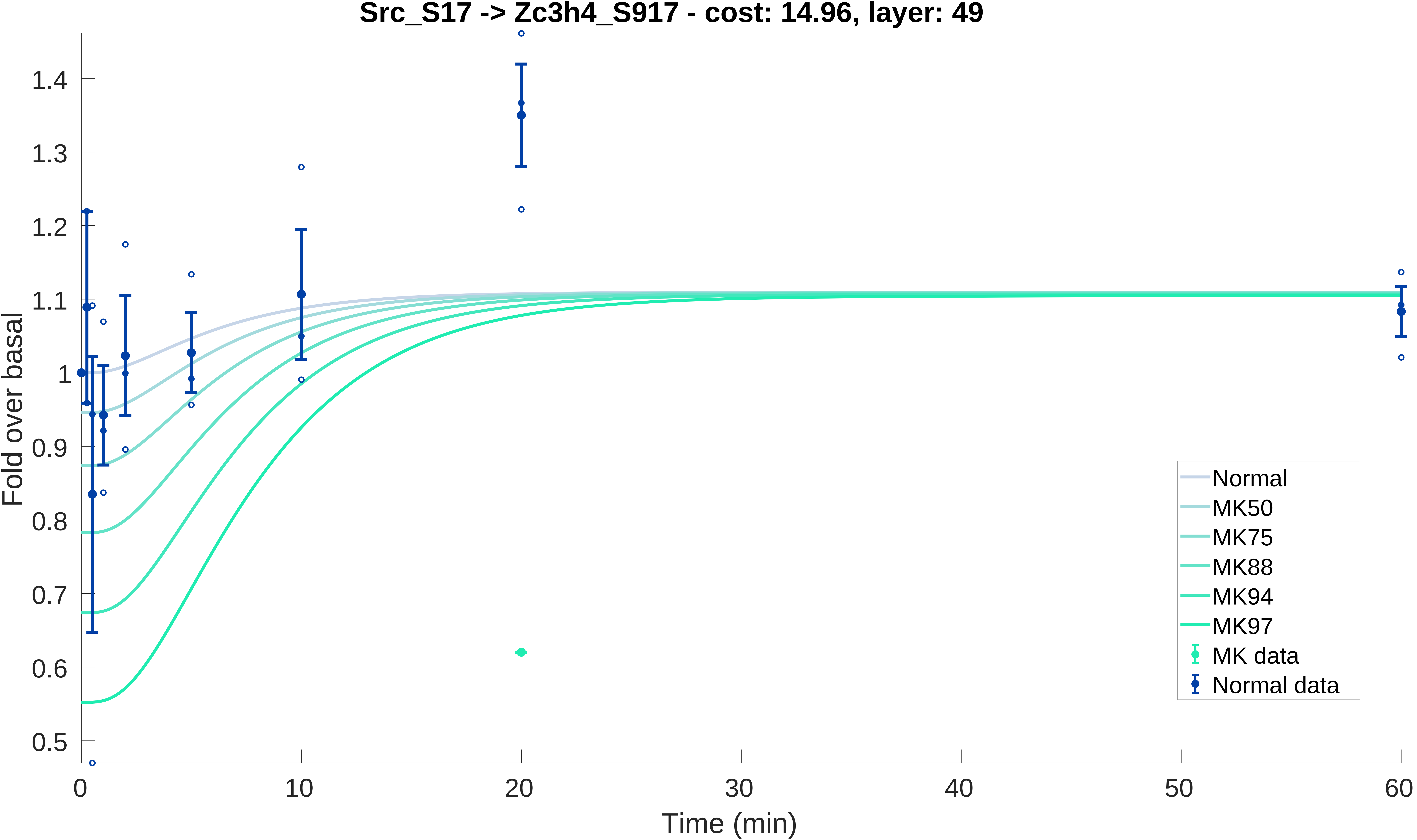

Src_S17 -> Zc3h4_S917 - cost: 14.96, layer: 49
Fold over basal
Time (min)
0.5
0.6
0.7
0.8
0.9
1
1.1
1.2
1.3
1.4
0
10
20
30
40
50
60
Normal
MK50
MK75
MK88
MK94
MK97
MK data
Normal data

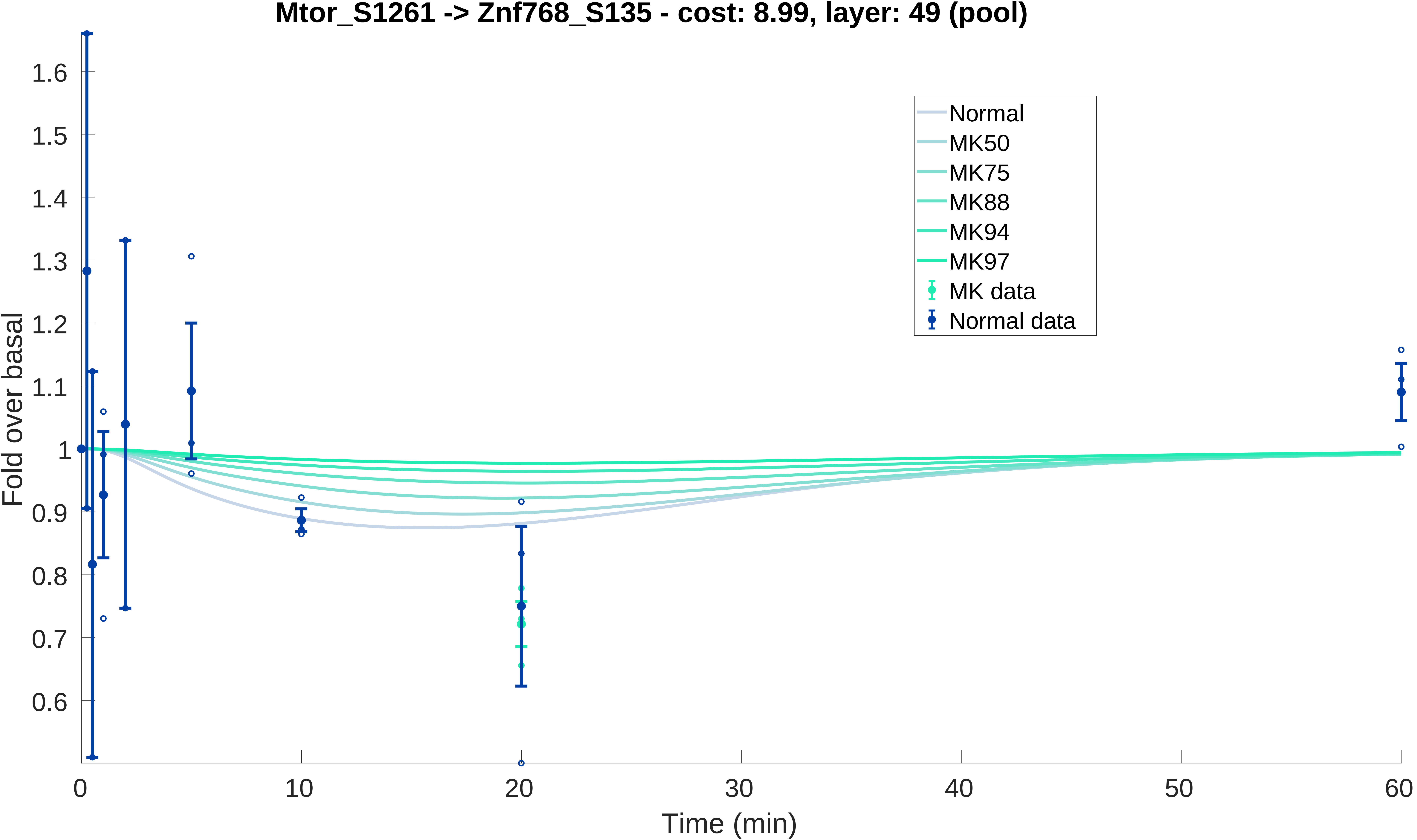
Mtor_S1261 -> Znf768_S135 - cost: 8.99, layer: 49 (pool)
Fold over basal
Time (min)
0
10
20
30
40
50
60
0.6
0.7
0.8
0.9
1
1.1
1.2
1.3
1.4
1.5
1.6
Normal
MK50
MK75
MK88
MK94
MK97
MK data
Normal data

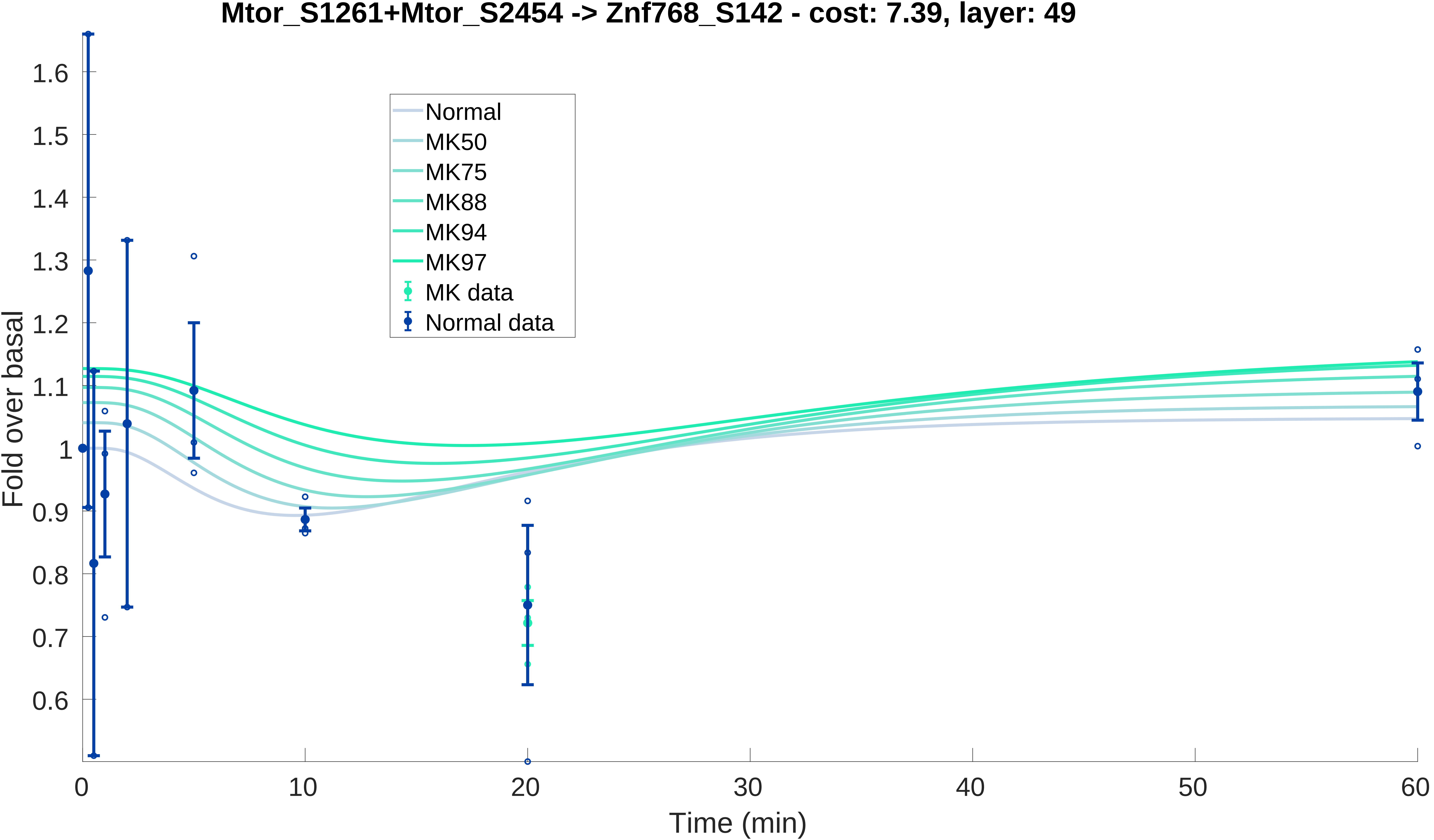
Mtor_S1261+Mtor_S2454 -> Znf768_S142 - cost: 7.39, layer: 49
Fold over basal
Time (min)
Normal
MK50
MK75
MK88
MK94
MK97
MK data
Normal data
0.6
0.7
0.8
0.9
1
1.1
1.2
1.3
1.4
1.5
1.6
0
10
20
30
40
50
60

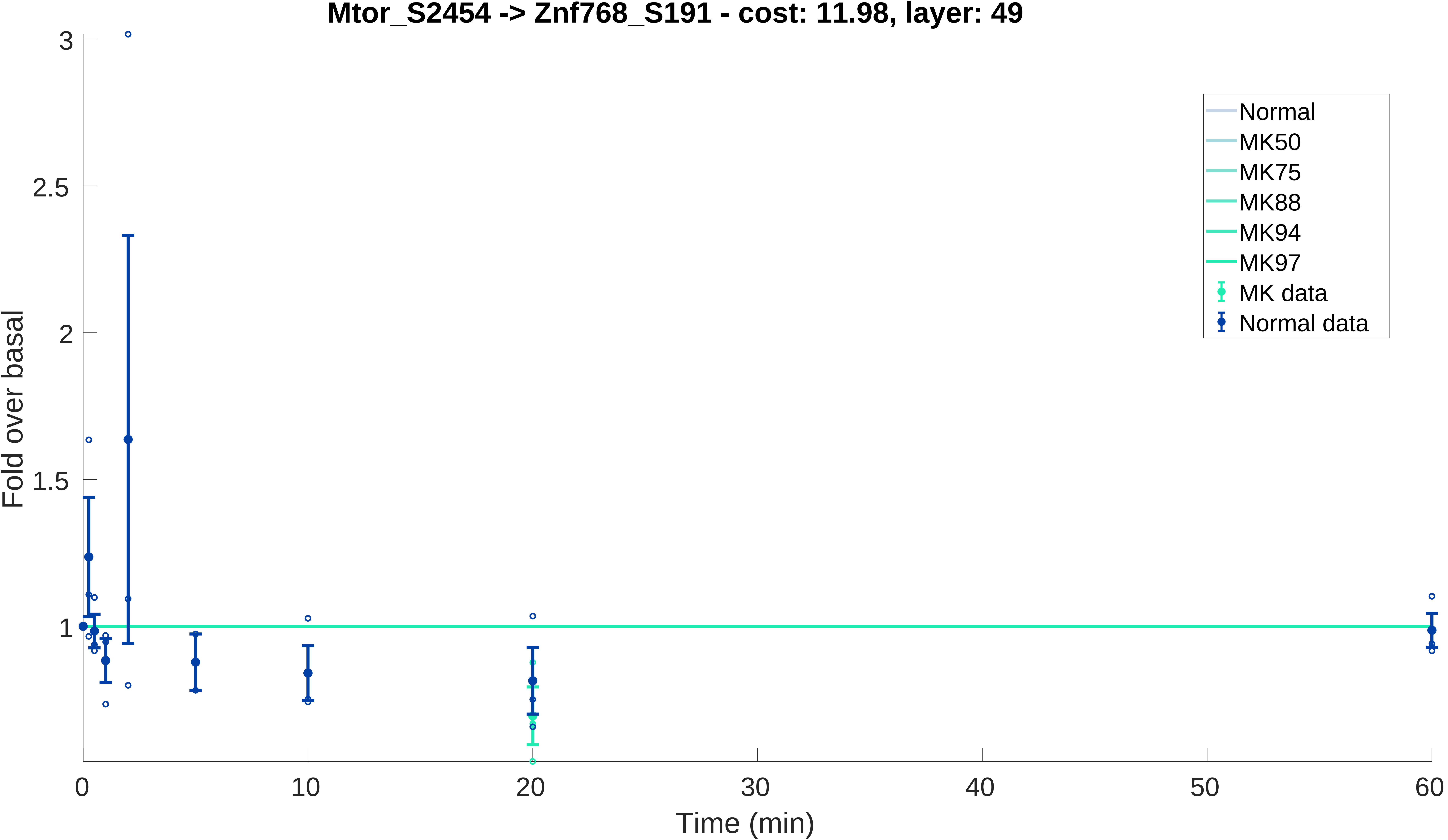

Mtor_S2454 -> Znf768_S191 - cost: 11.98, layer: 49
Fold over basal
Time (min)
3
2.5
2
1.5
1
0
10
20
30
40
50
60
Normal
MK50
MK75
MK88
MK94
MK97
MK data
Normal data

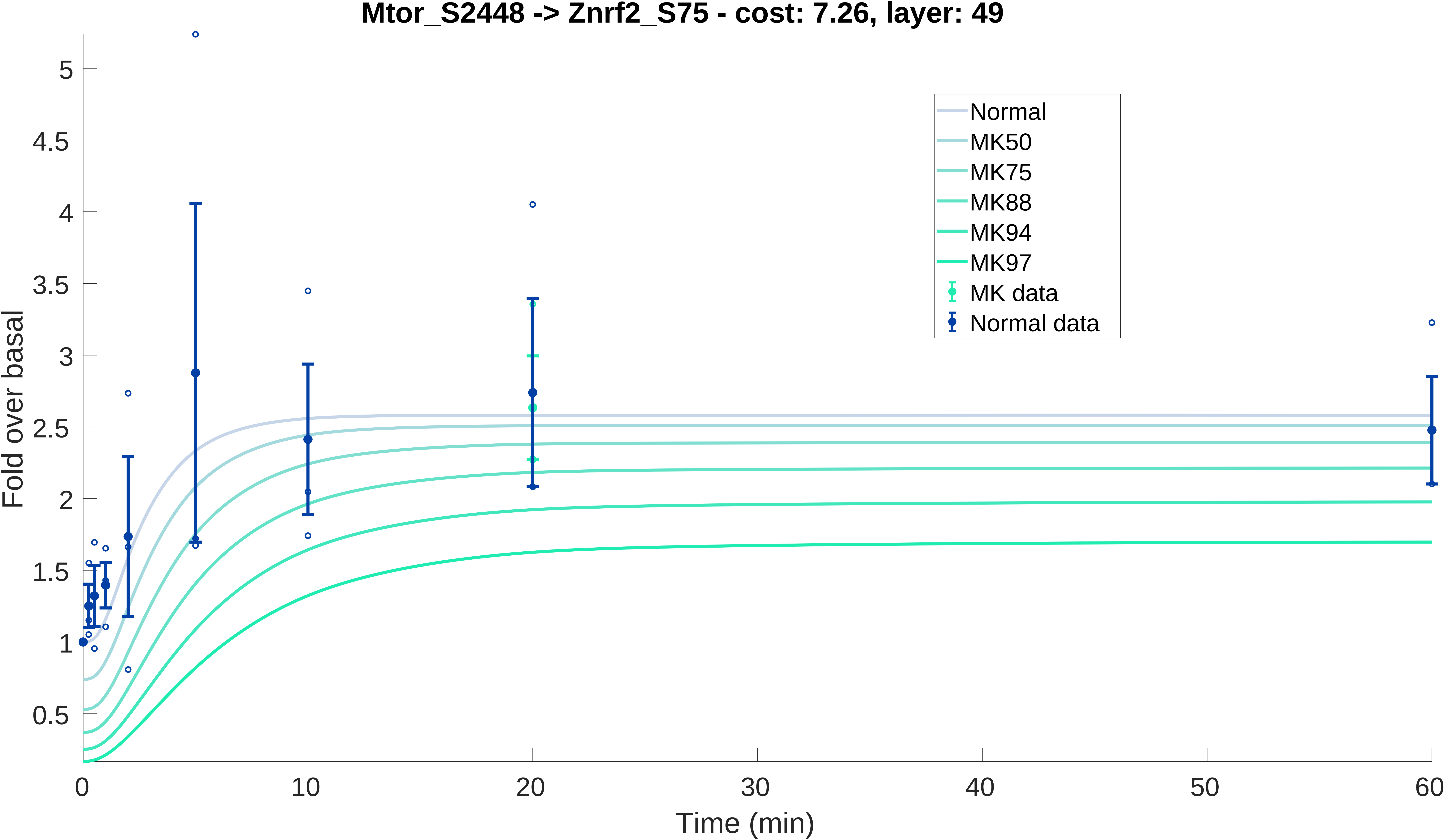
Mtor_S2448 -> Znrf2_S75 - cost: 7.26, layer: 49
Fold over basal
Time (min)
0.5
1
1.5
2
2.5
3
3.5
4
4.5
5
0
10
20
30
40
50
60
Normal
MK50
MK75
MK88
MK94
MK97
MK data
Normal data

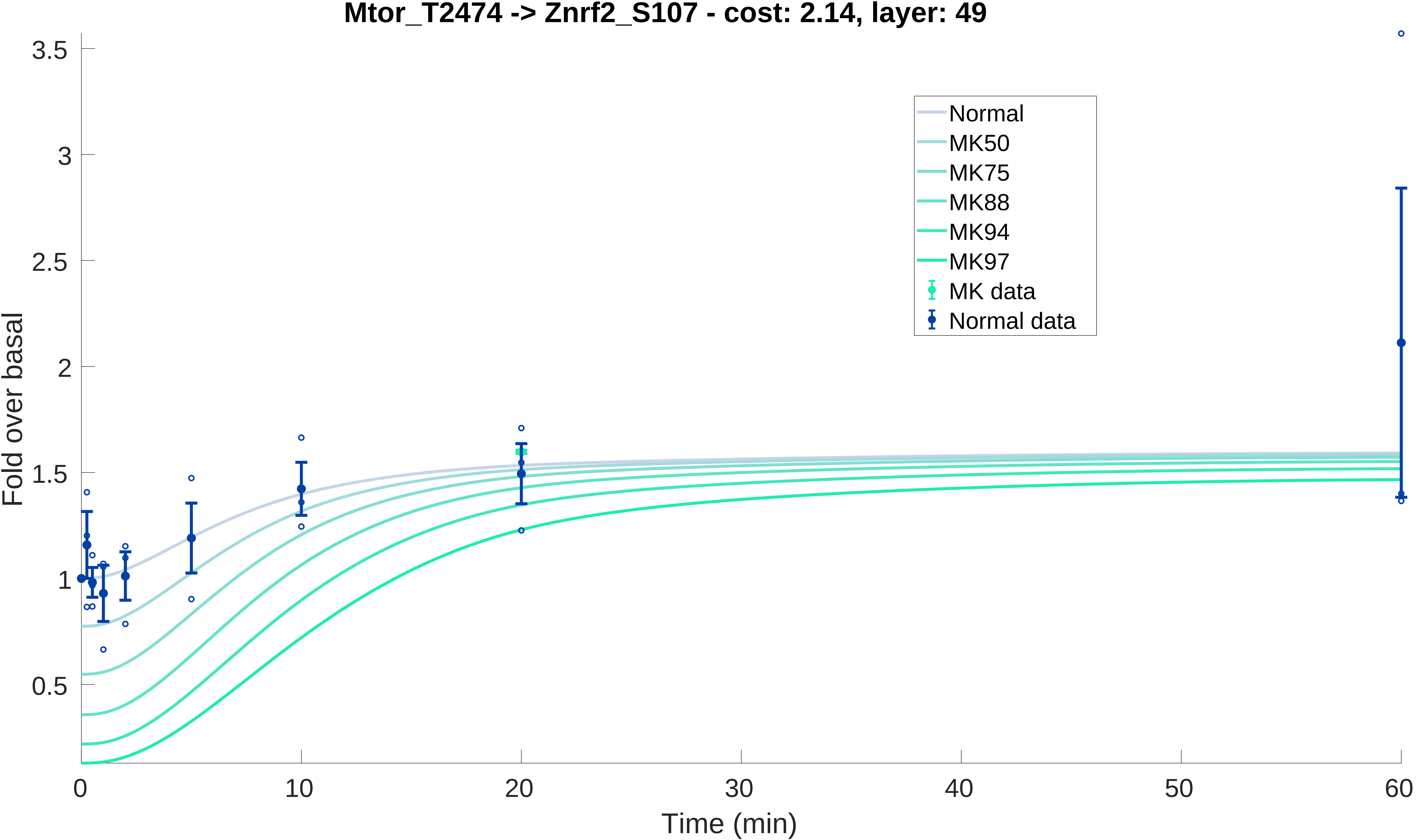
Mtor_T2474 -> Znrf2_S107 - cost: 2.14, layer: 49
Fold over basal
Time (min)
0
10
20
30
40
50
60
0.5
1
1.5
2
2.5
3
3.5
Normal
MK50
MK75
MK88
MK94
MK97
MK data
Normal data

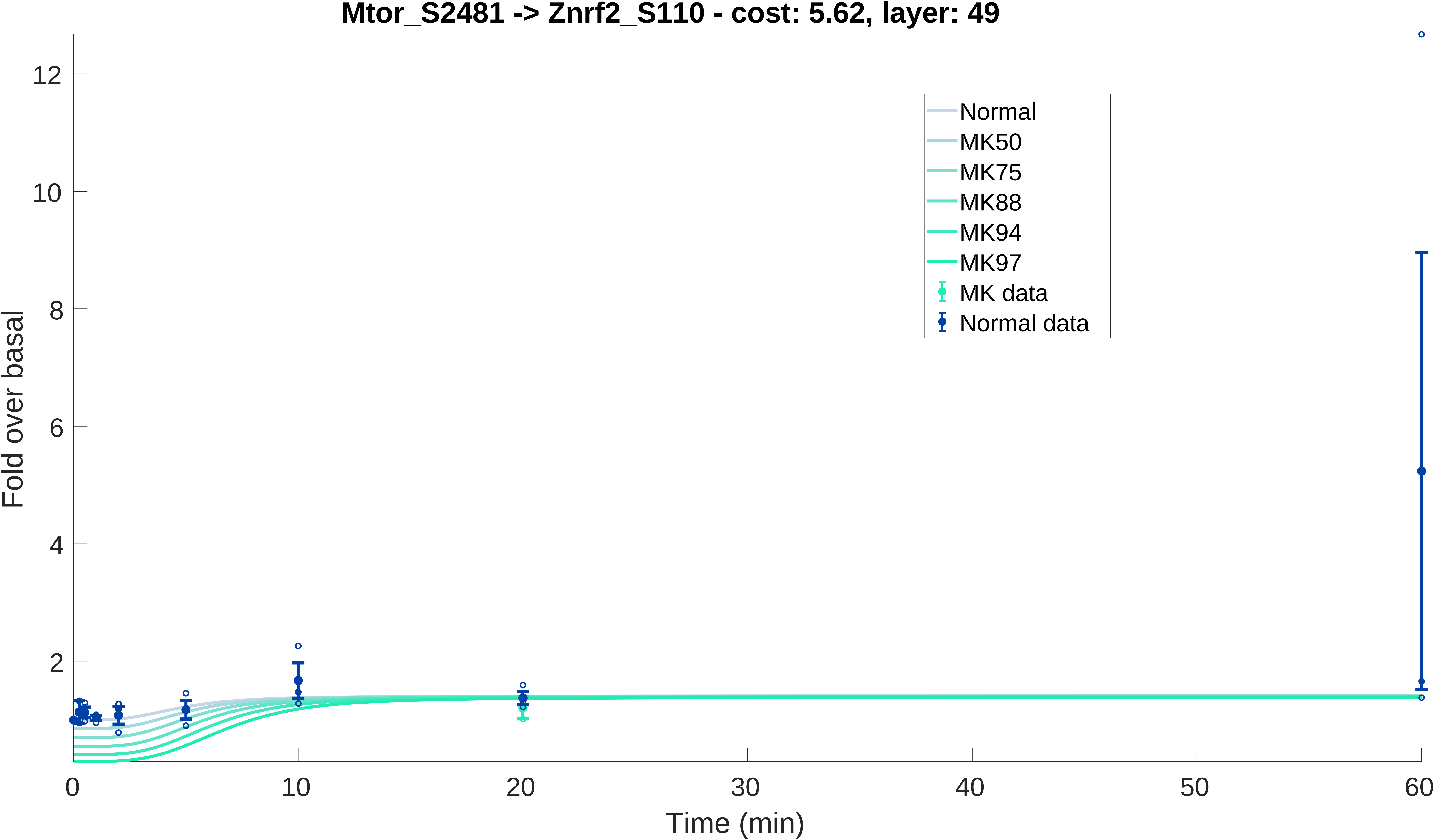

Mtor_S2481 -> Znrf2_S110 - cost: 5.62, layer: 49
Fold over basal
Time (min)
Normal
MK50
MK75
MK88
MK94
MK97
MK data
Normal data
0
10
20
30
40
50
60
2
4
6
8
10
12

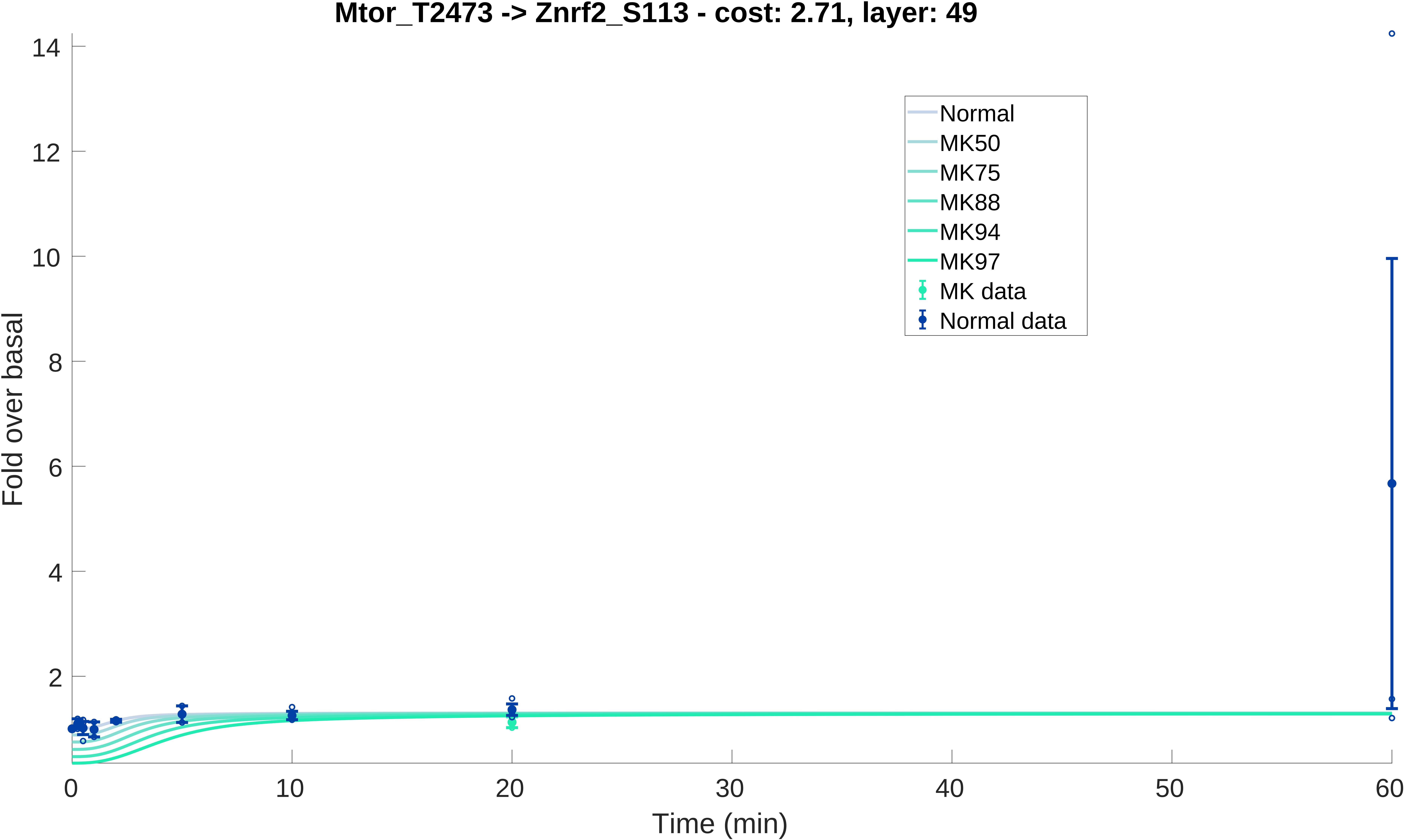
Mtor_T2473 -> Znrf2_S113 - cost: 2.71, layer: 49
Fold over basal
Time (min)
0
2
4
6
8
10
12
14
0
10
20
30
40
50
60
Normal
MK50
MK75
MK88
MK94
MK97
MK data
Normal data

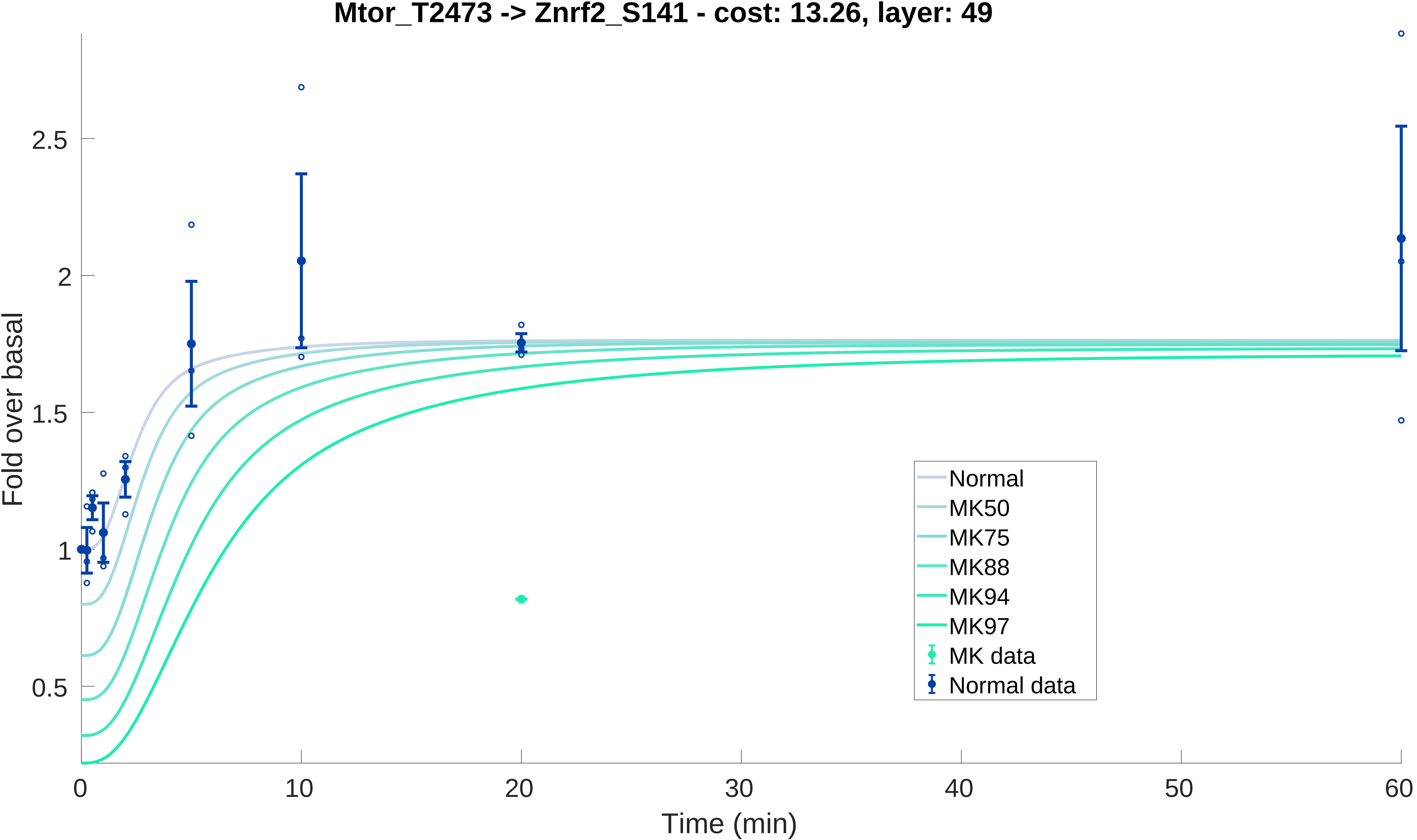

Mtor_T2473 -> Znrf2_S141 - cost: 13.26, layer: 49
Fold over basal
2.5
2
1.5
1
0.5
0
10
20
30
40
50
60
Time (min)
Normal
MK50
MK75
MK88
MK94
MK97
MK data
Normal data

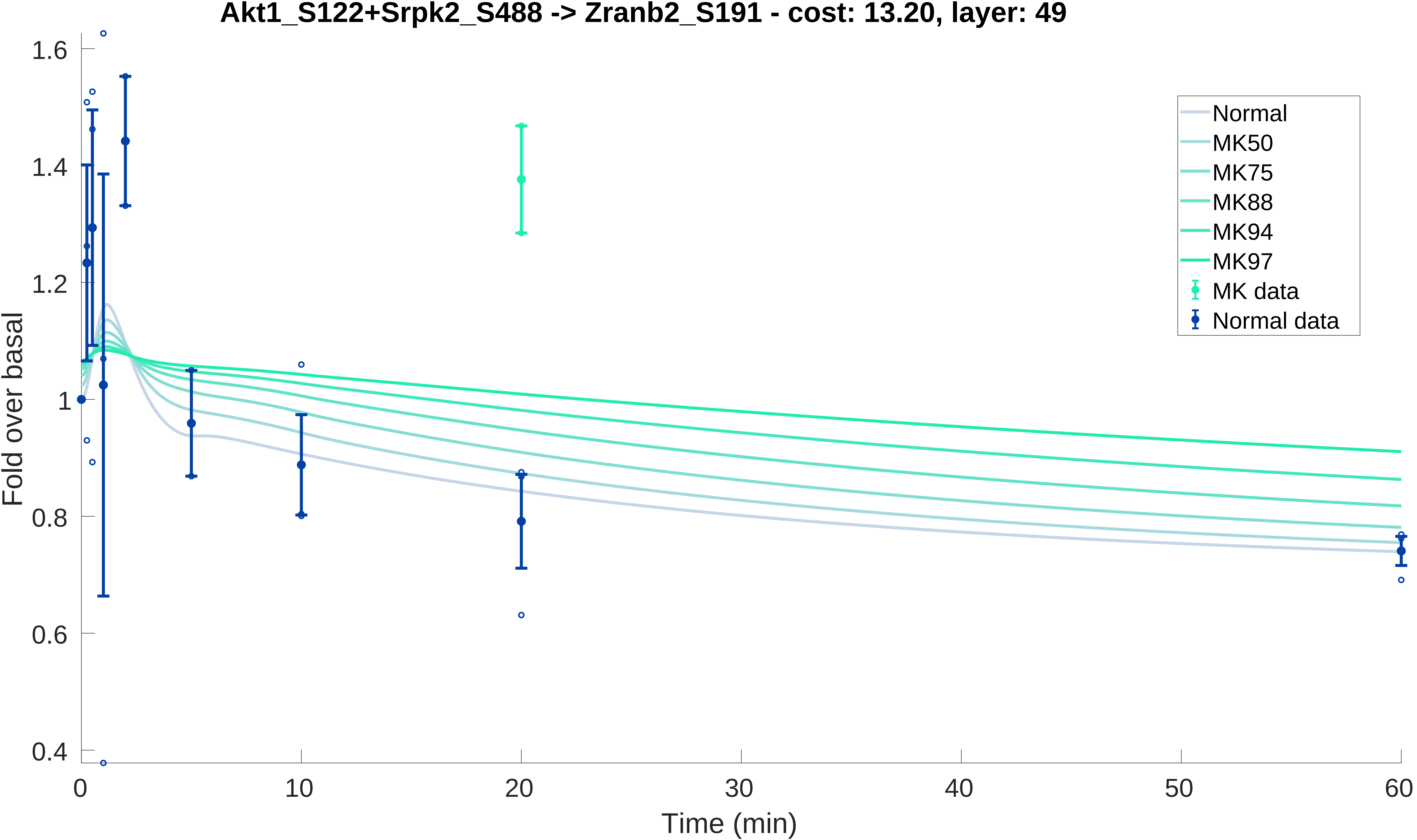
Akt1_S122+Srpk2_S488 -> Zranb2_S191 - cost: 13.20, layer: 49
Fold over basal
Time (min)
0.4
0.6
0.8
1
1.2
1.4
1.6
0
10
20
30
40
50
60
Normal
MK50
MK75
MK88
MK94
MK97
MK data
Normal data

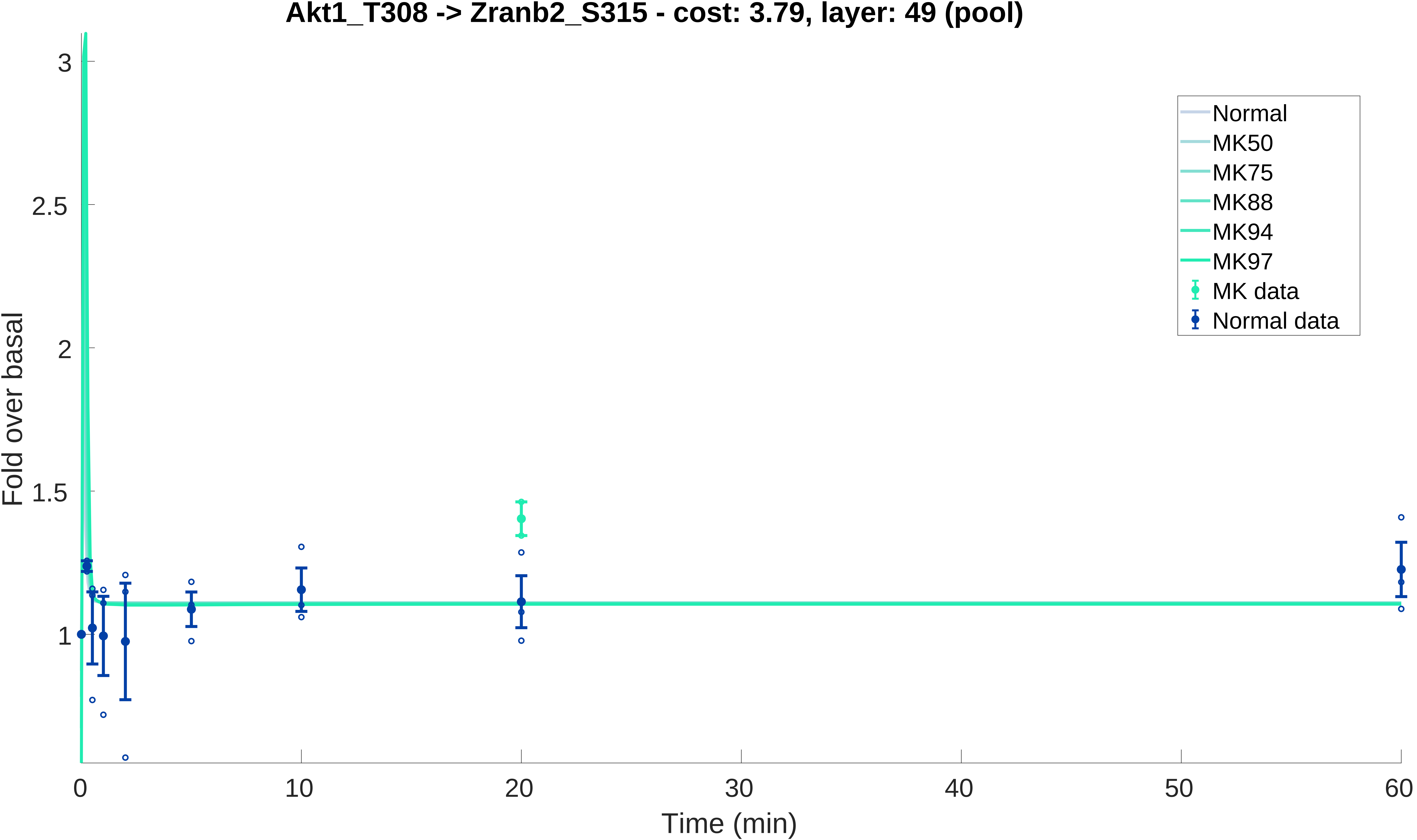

Akt1_T308 -> Zranb2_S315 - cost: 3.79, layer: 49 (pool)
Fold over basal
Time (min)
Normal
MK50
MK75
MK88
MK94
MK97
MK data
Normal data
3
2.5
2
1.5
1
0
10
20
30
40
50
60

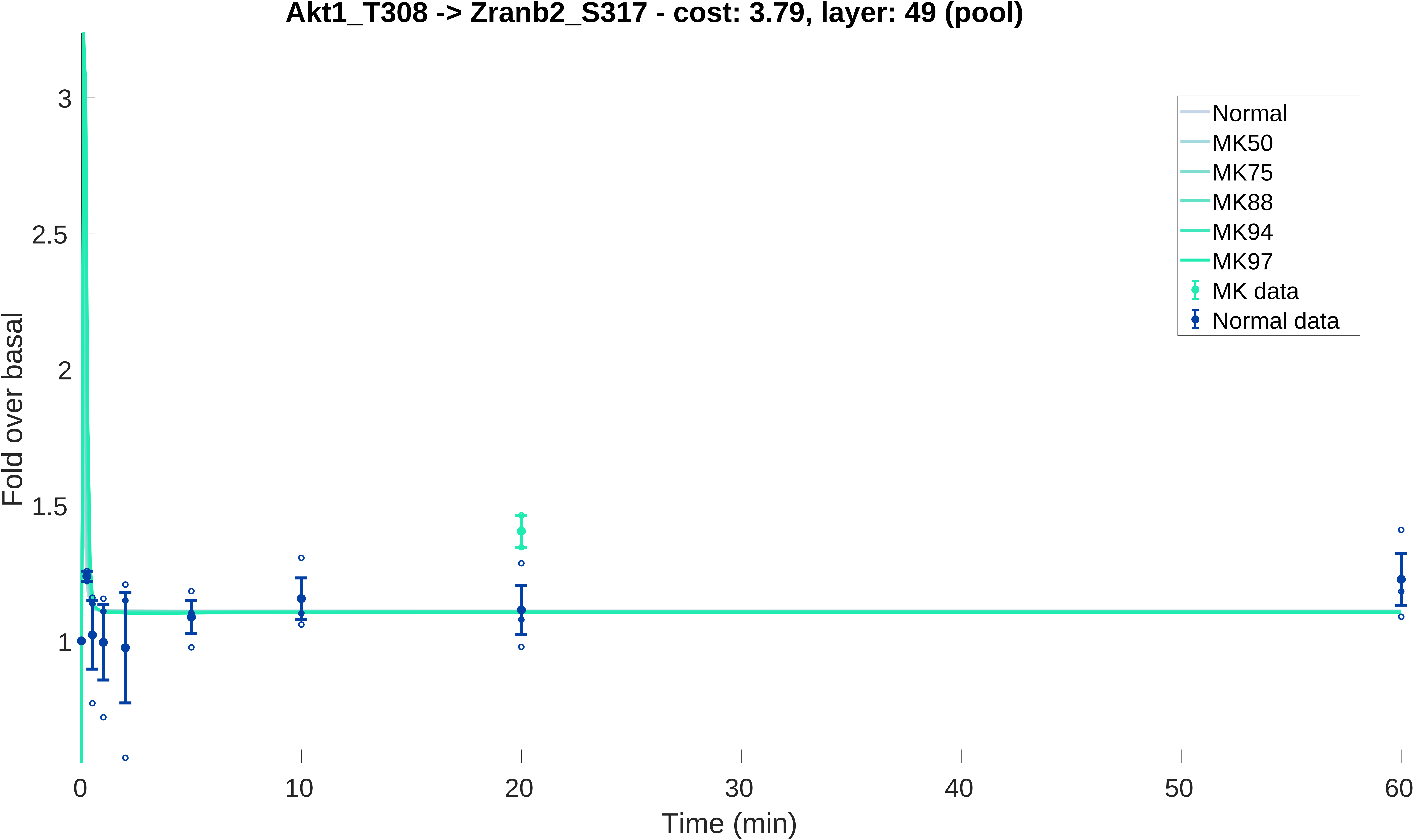

Akt1_T308 -> Zranb2_S317 - cost: 3.79, layer: 49 (pool)
Fold over basal
Time (min)
Normal
MK50
MK75
MK88
MK94
MK97
MK data
Normal data

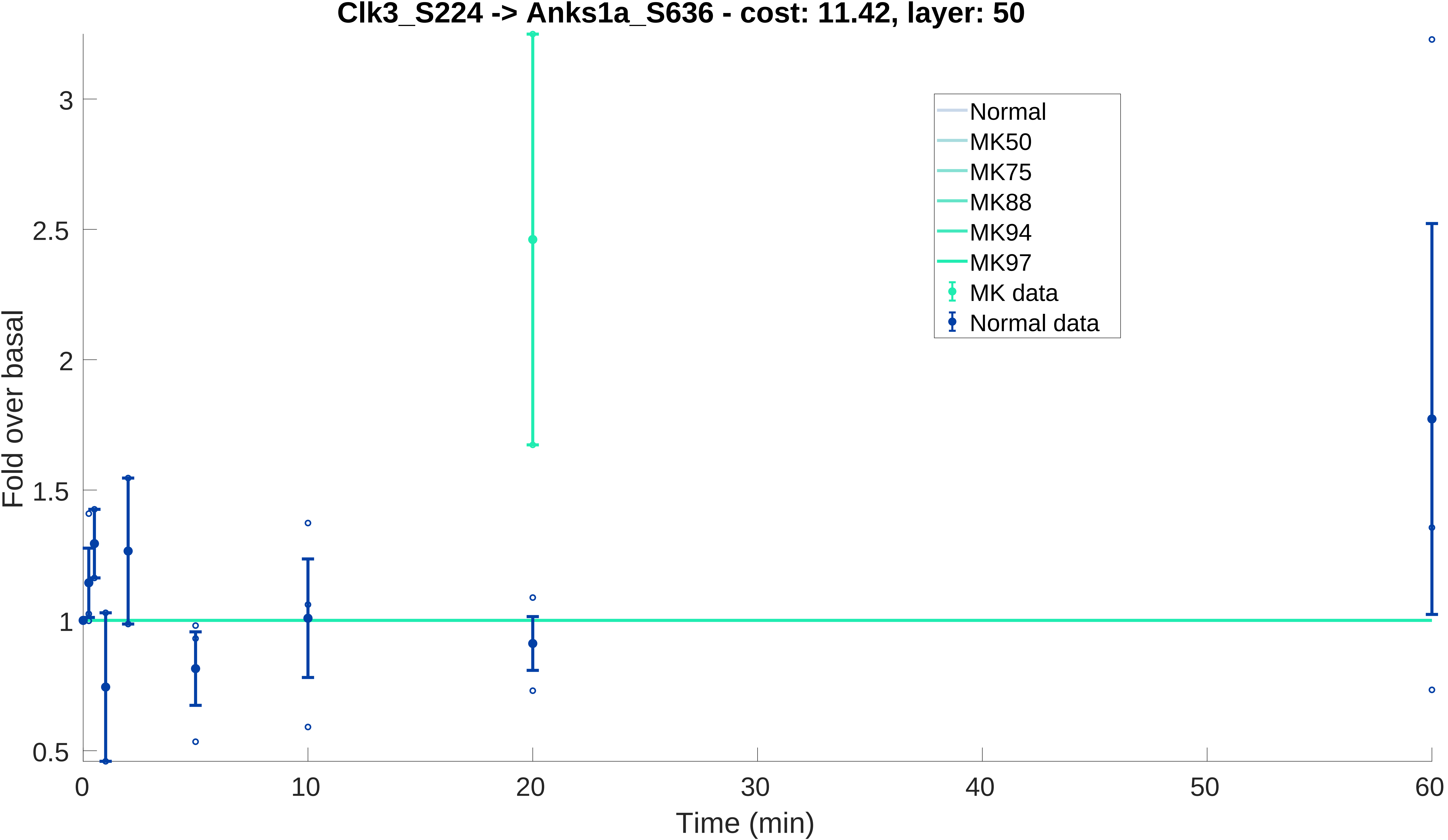

Clk3_S224 -> Anks1a_S636 - cost: 11.42, layer: 50
Fold over basal
Time (min)
0.5
1
1.5
2
2.5
3
0
10
20
30
40
50
60
Normal
MK50
MK75
MK88
MK94
MK97
MK data
Normal data

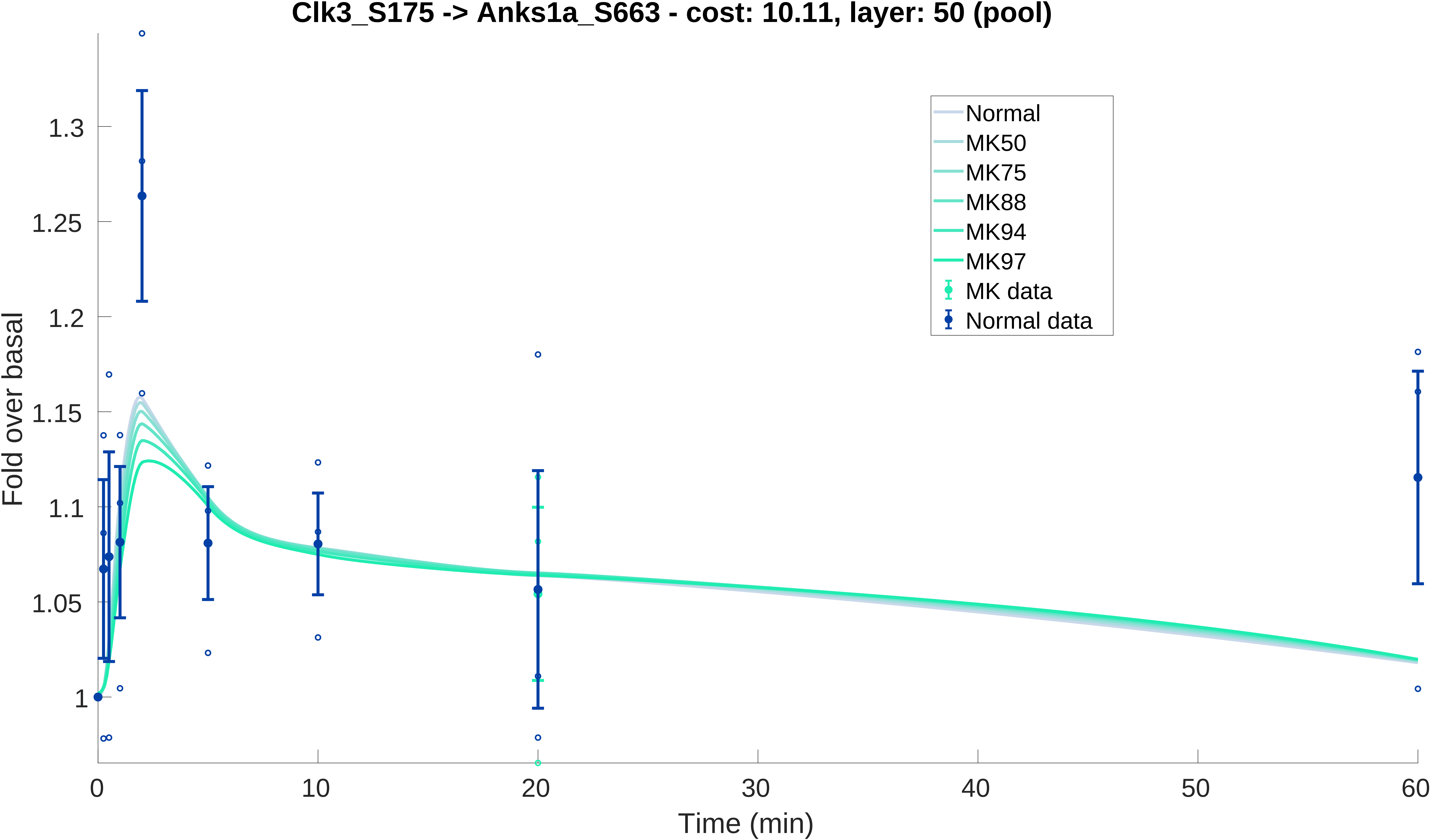

Clk3_S175 -> Anks1a_S663 - cost: 10.11, layer: 50 (pool)
Normal
MK50
MK75
MK88
MK94
MK97
MK data
Normal data
Fold over basal
1.3
1.25
1.2
1.15
1.1
1.05
1
0
10
20
30
40
50
60
Time (min)

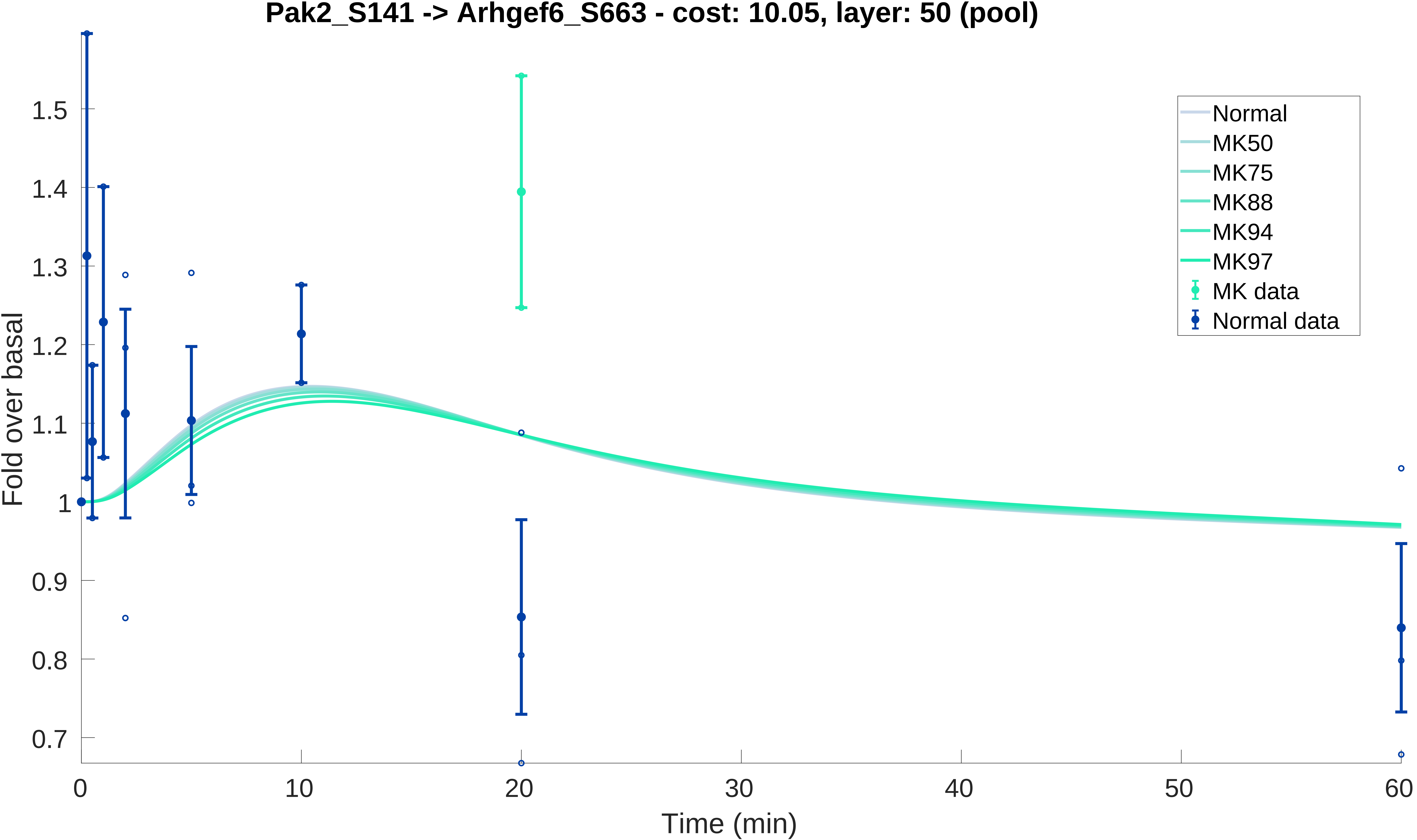

Pak2_S141 -> Arhgef6_S663 - cost: 10.05, layer: 50 (pool)
Fold over basal
Time (min)
0
10
20
30
40
50
60
0.7
0.8
0.9
1
1.1
1.2
1.3
1.4
1.5
Normal
MK50
MK75
MK88
MK94
MK97
MK data
Normal data

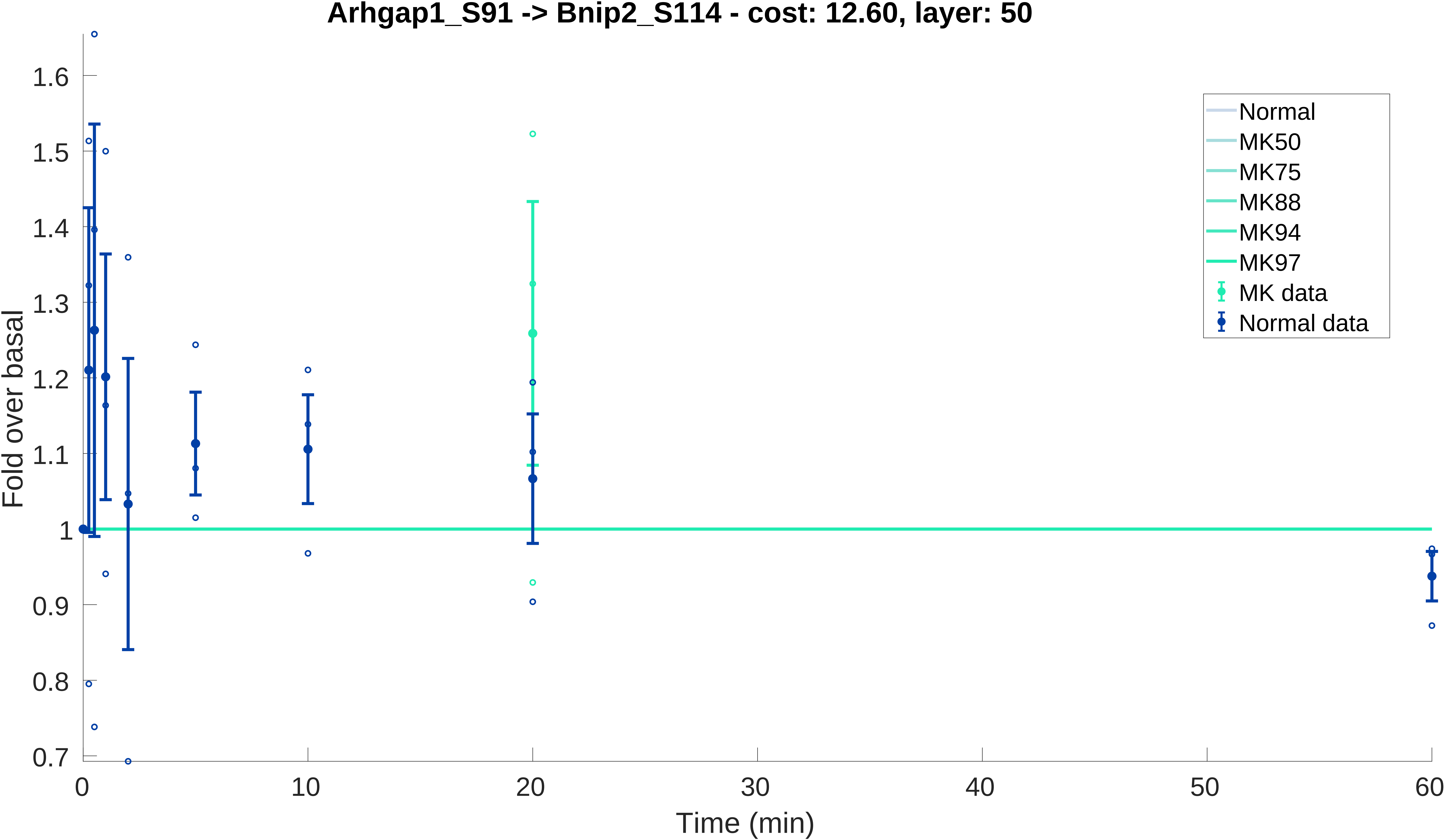

Arhgap1_S91 -> Bnip2_S114 - cost: 12.60, layer: 50
Fold over basal
Time (min)
0
10
20
30
40
50
60
0.7
0.8
0.9
1
1.1
1.2
1.3
1.4
1.5
1.6
Normal
MK50
MK75
MK88
MK94
MK97
MK data
Normal data

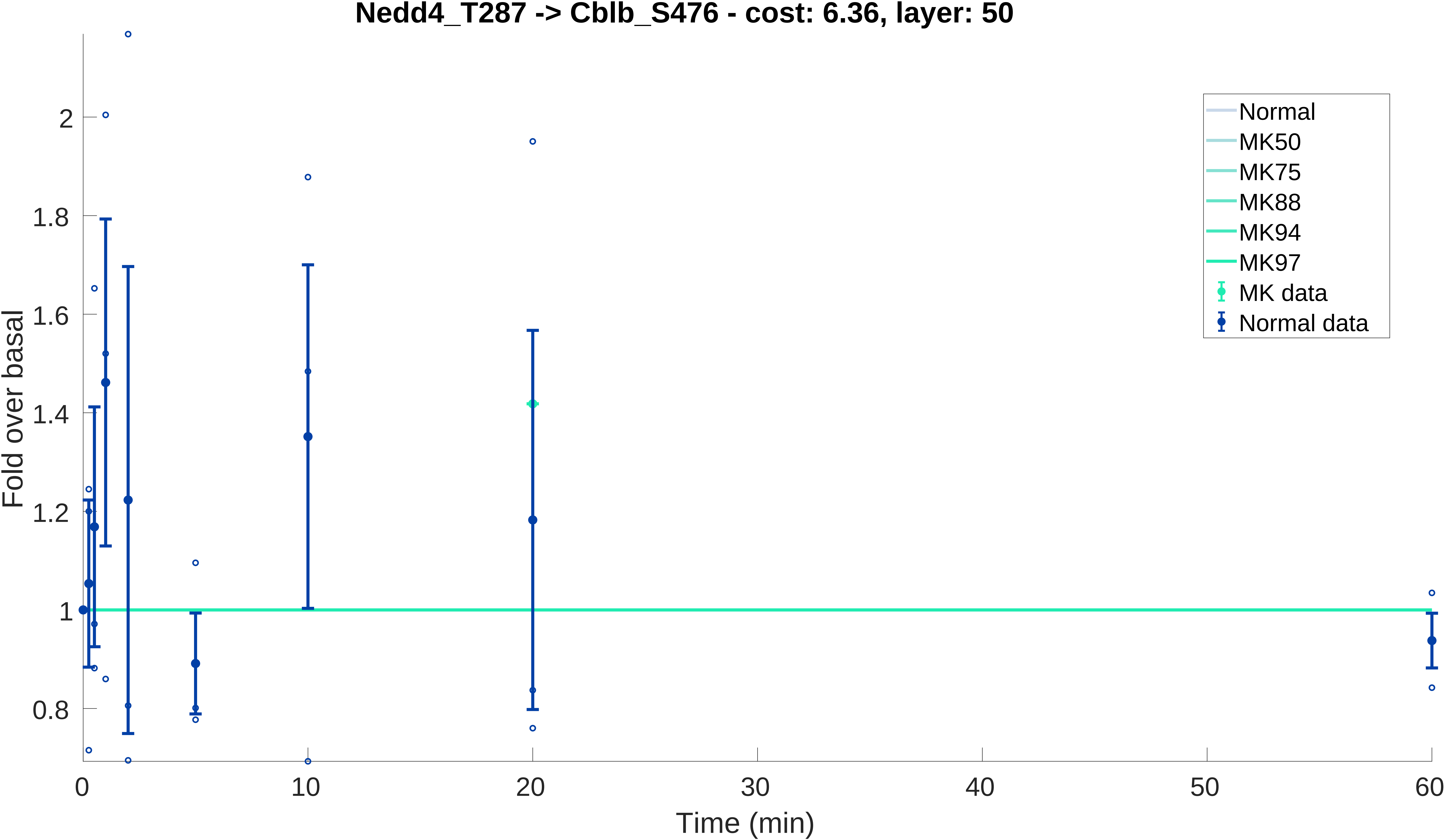

Nedd4_T287 -> Cblb_S476 - cost: 6.36, layer: 50
Fold over basal
Time (min)
0
10
20
30
40
50
60
0.8
1
1.2
1.4
1.6
1.8
2
Normal
MK50
MK75
MK88
MK94
MK97
MK data
Normal data

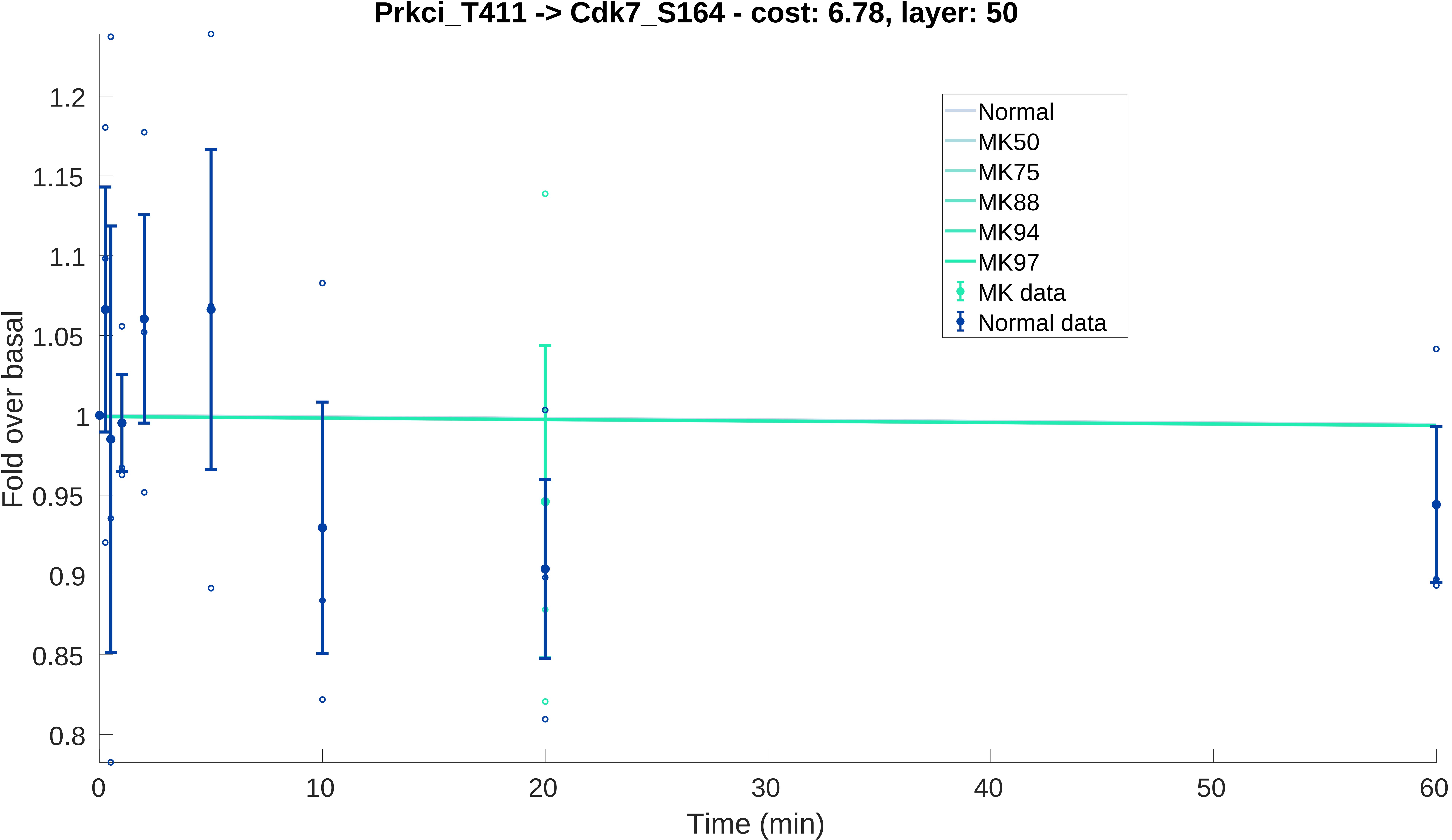

Prkci_T411 -> Cdk7_S164 - cost: 6.78, layer: 50
Fold over basal
Time (min)
Normal
MK50
MK75
MK88
MK94
MK97
MK data
Normal data
1.2
1.15
1.1
1.05
1
0.95
0.9
0.85
0.8
0
10
20
30
40
50
60

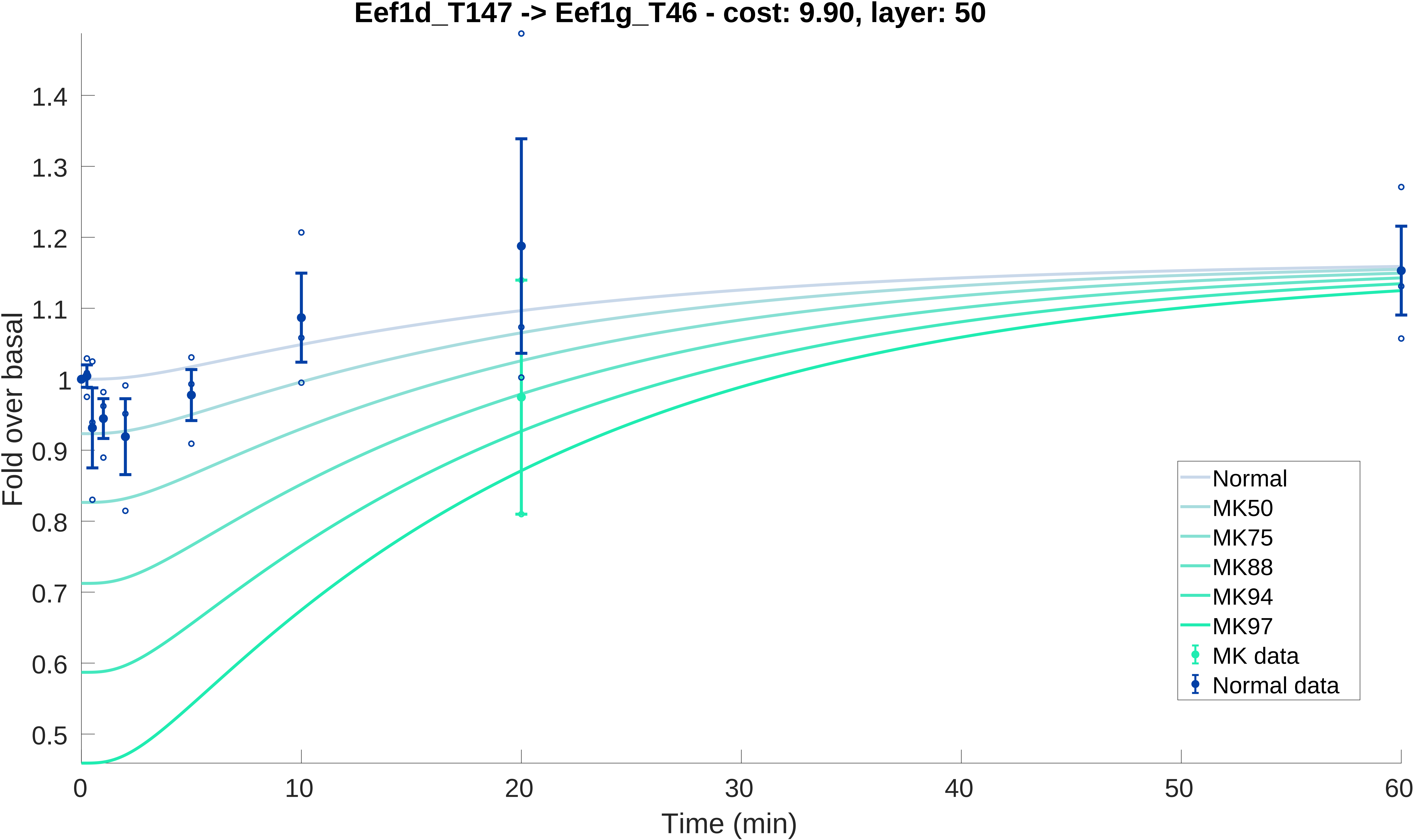

Eef1d_T147 -> Eef1g_T46 - cost: 9.90, layer: 50
Fold over basal
Time (min)
0
10
20
30
40
50
60
0.5
0.6
0.7
0.8
0.9
1
1.1
1.2
1.3
1.4
Normal
MK50
MK75
MK88
MK94
MK97
MK data
Normal data

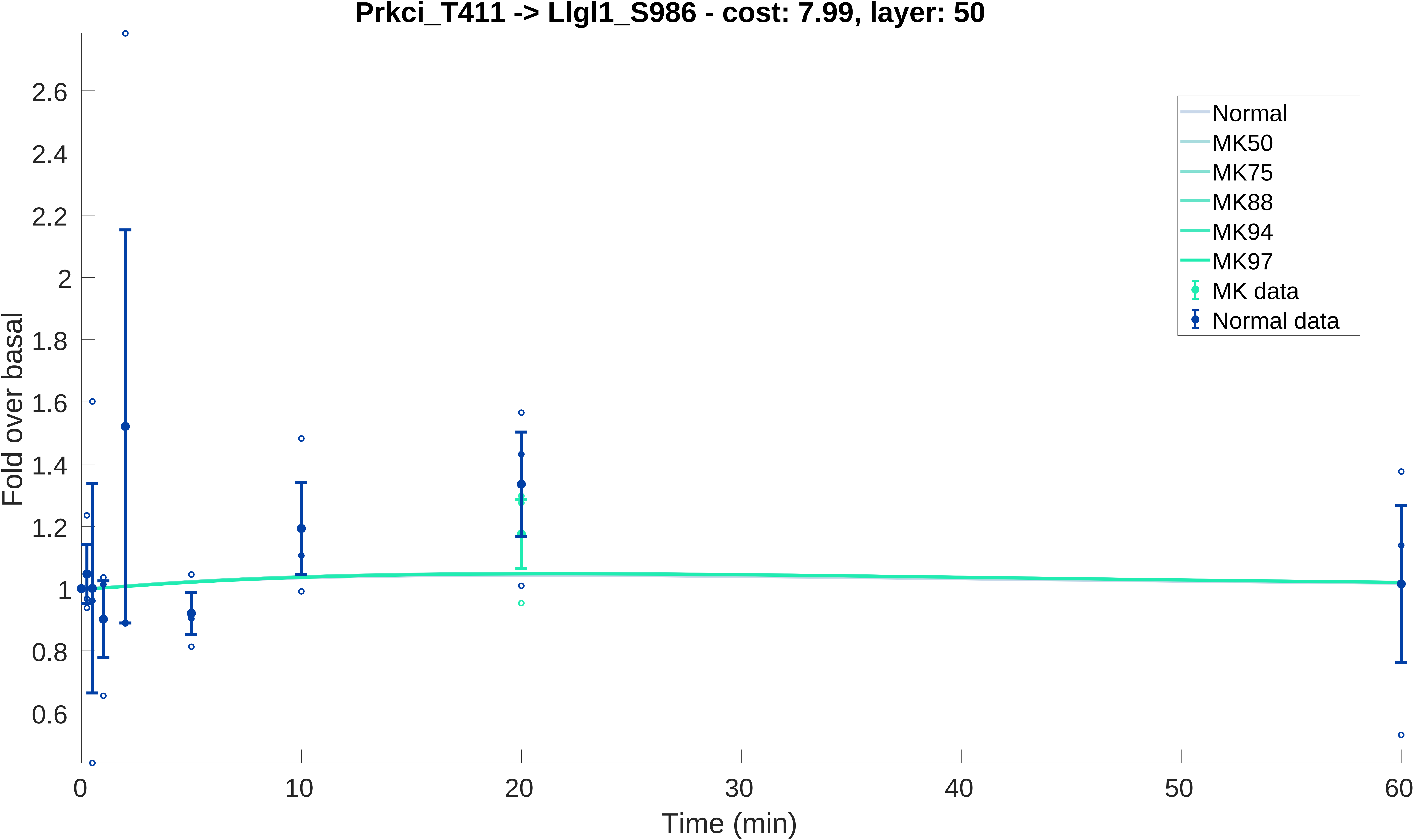
Prkci_T411 -> Llgl1_S986 - cost: 7.99, layer: 50
Fold over basal
Time (min)
0.6
0.8
1
1.2
1.4
1.6
1.8
2
2.2
2.4
2.6
0
10
20
30
40
50
60
Normal
MK50
MK75
MK88
MK94
MK97
MK data
Normal data

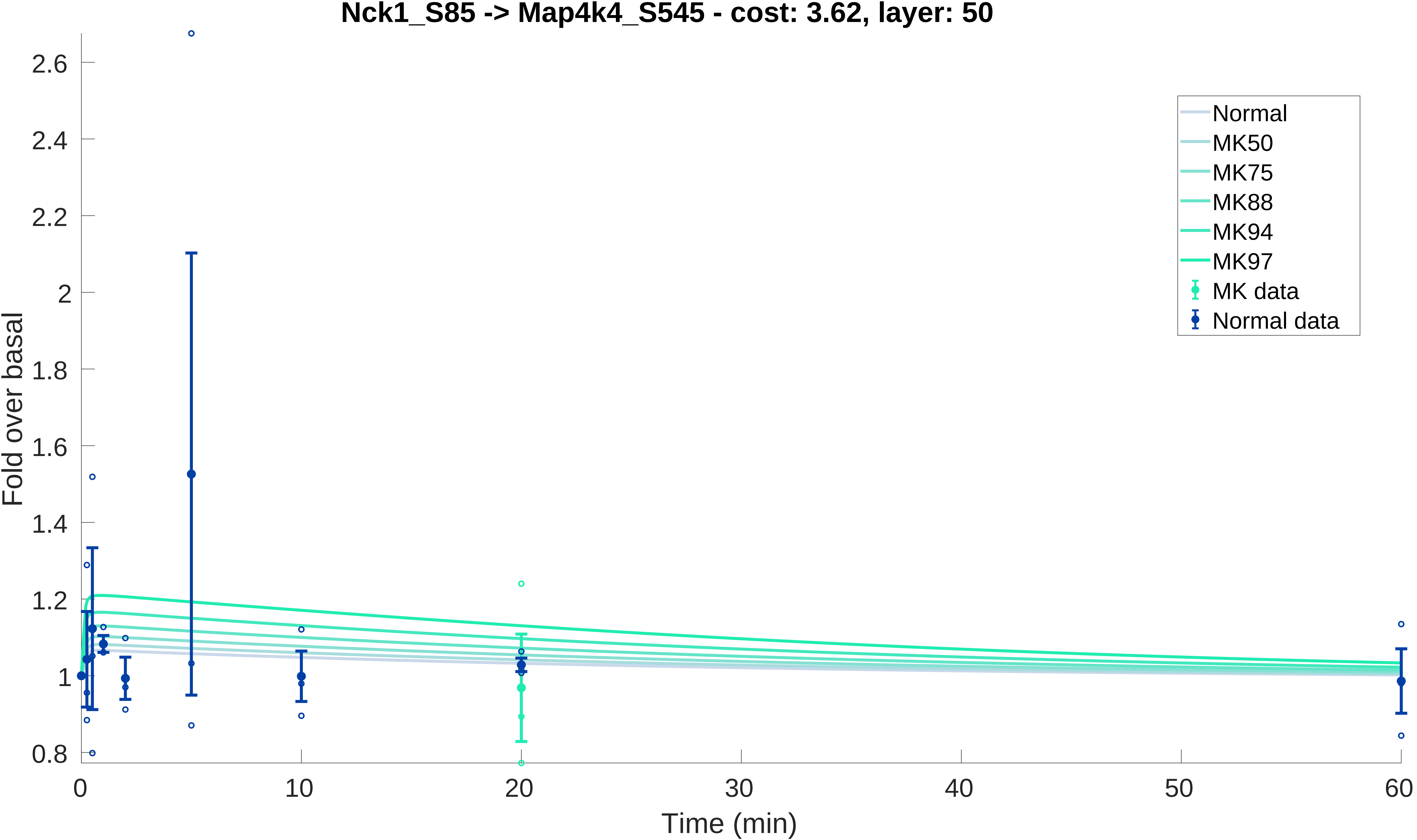

Nck1_S85 -> Map4k4_S545 - cost: 3.62, layer: 50
Fold over basal
2.6
2.4
2.2
2
1.8
1.6
1.4
1.2
1
0.8
0
10
20
30
40
50
60
Time (min)
Normal
MK50
MK75
MK88
MK94
MK97
MK data
Normal data

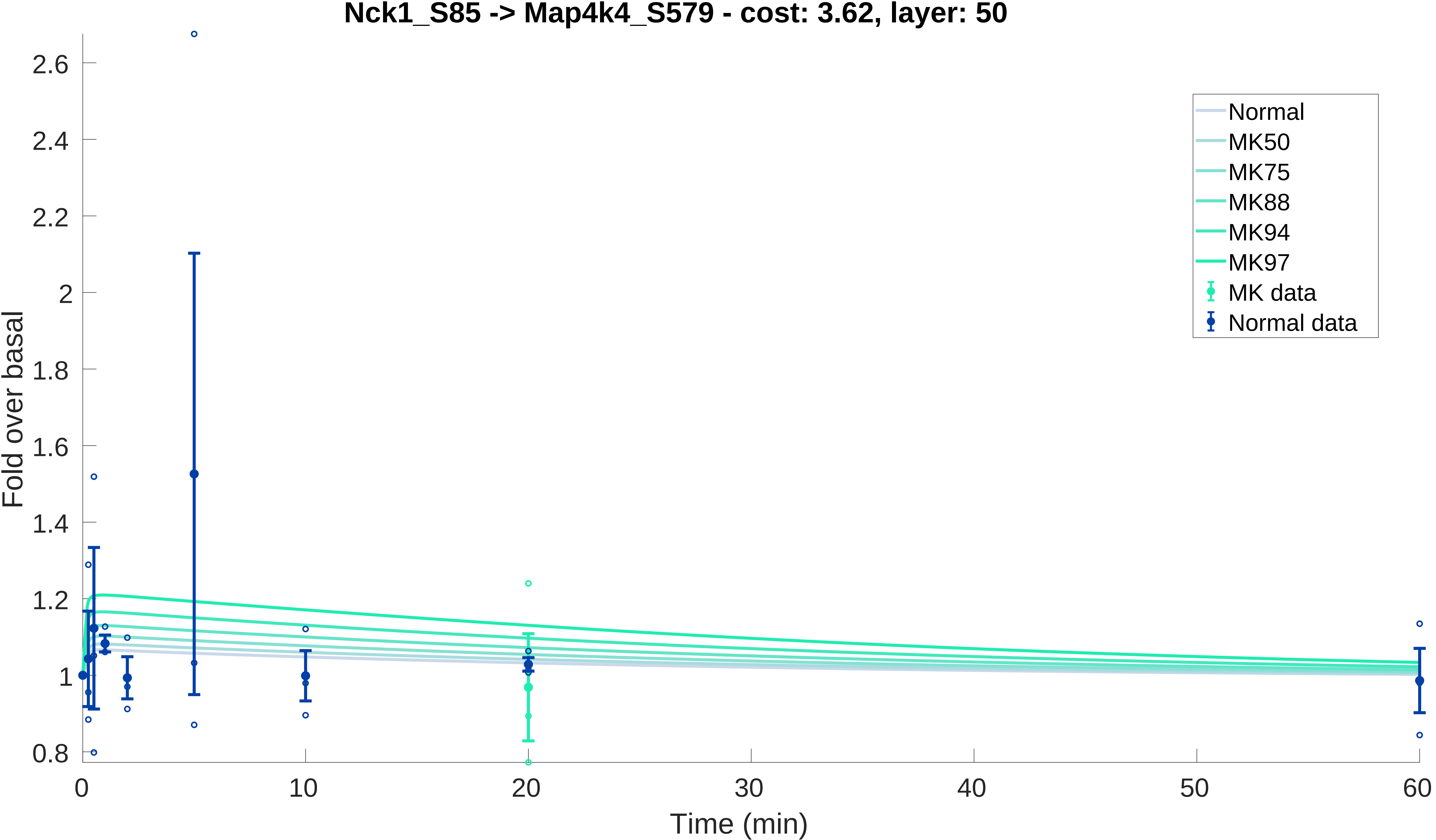

Nck1_S85 -> Map4k4_S579 - cost: 3.62, layer: 50
Fold over basal
Time (min)
0.8
1
1.2
1.4
1.6
1.8
2
2.2
2.4
2.6
0
10
20
30
40
50
60
Normal
MK50
MK75
MK88
MK94
MK97
MK data
Normal data

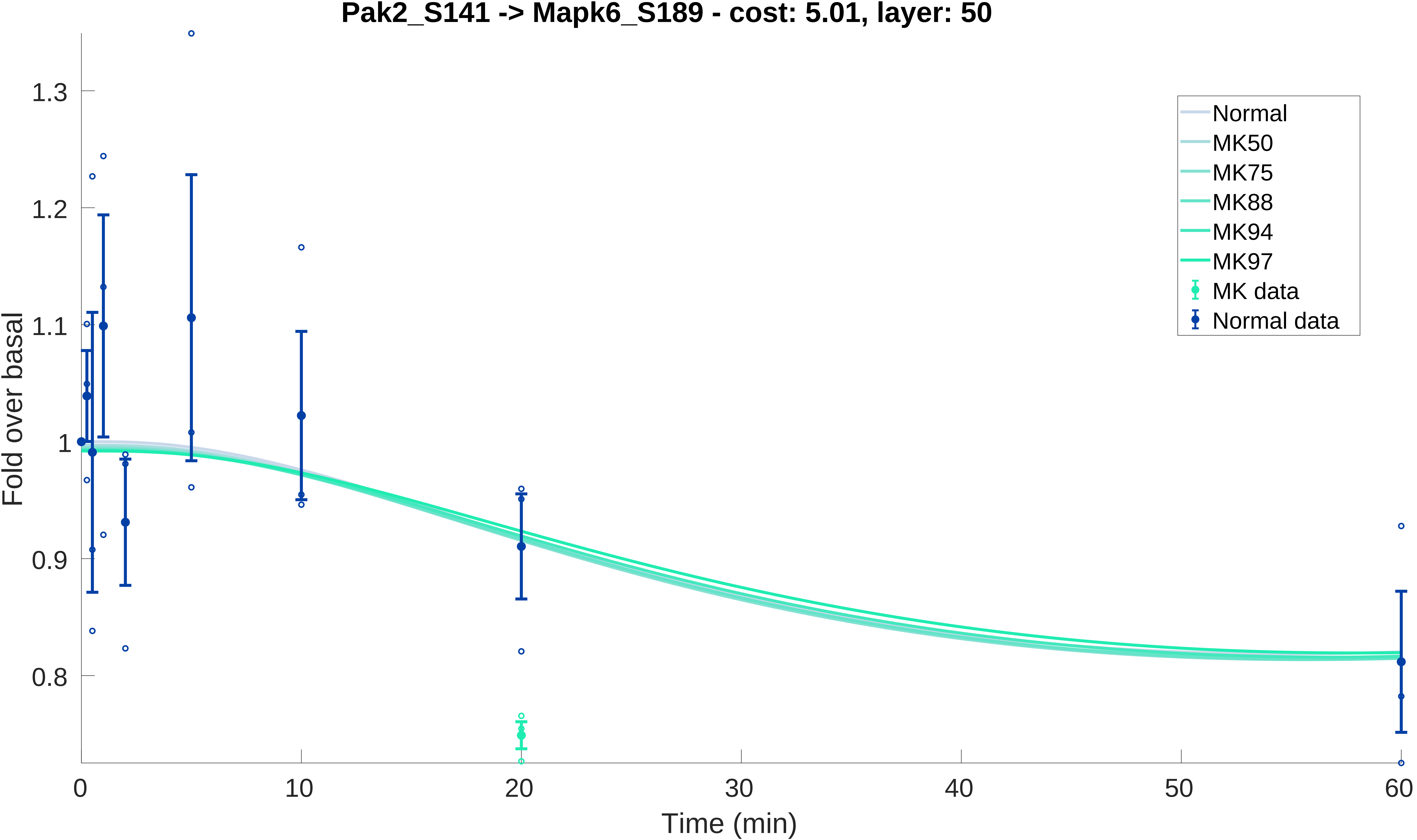

Pak2_S141 -> Mapk6_S189 - cost: 5.01, layer: 50
Fold over basal
Time (min)
1.3
1.2
1.1
1
0.9
0.8
0
10
20
30
40
50
60
Normal
MK50
MK75
MK88
MK94
MK97
MK data
Normal data

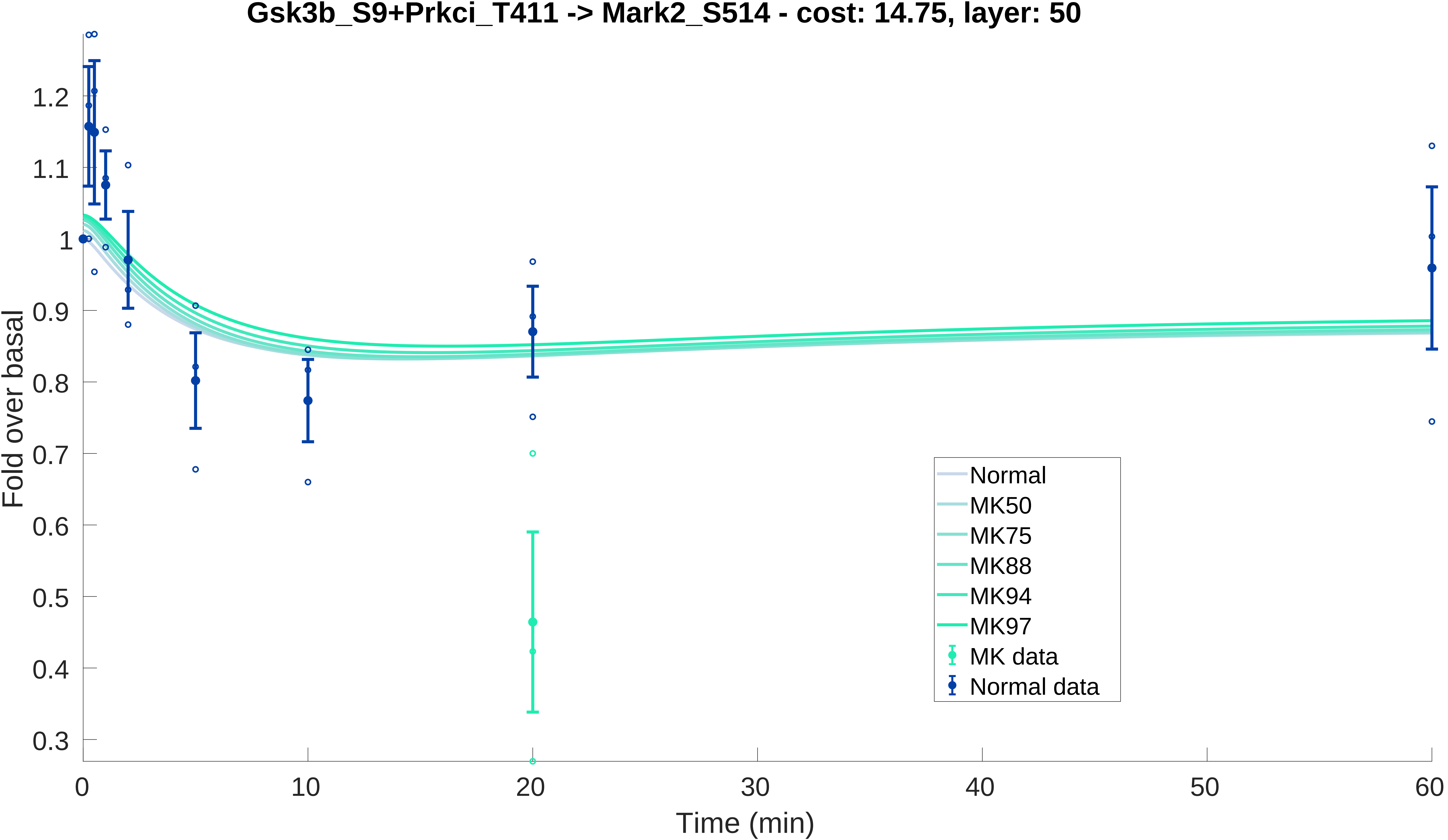

Gsk3b_S9+Prkci_T411 -> Mark2_S514 - cost: 14.75, layer: 50
Fold over basal
Time (min)
0.3
0.4
0.5
0.6
0.7
0.8
0.9
1
1.1
1.2
0
10
20
30
40
50
60
Normal
MK50
MK75
MK88
MK94
MK97
MK data
Normal data

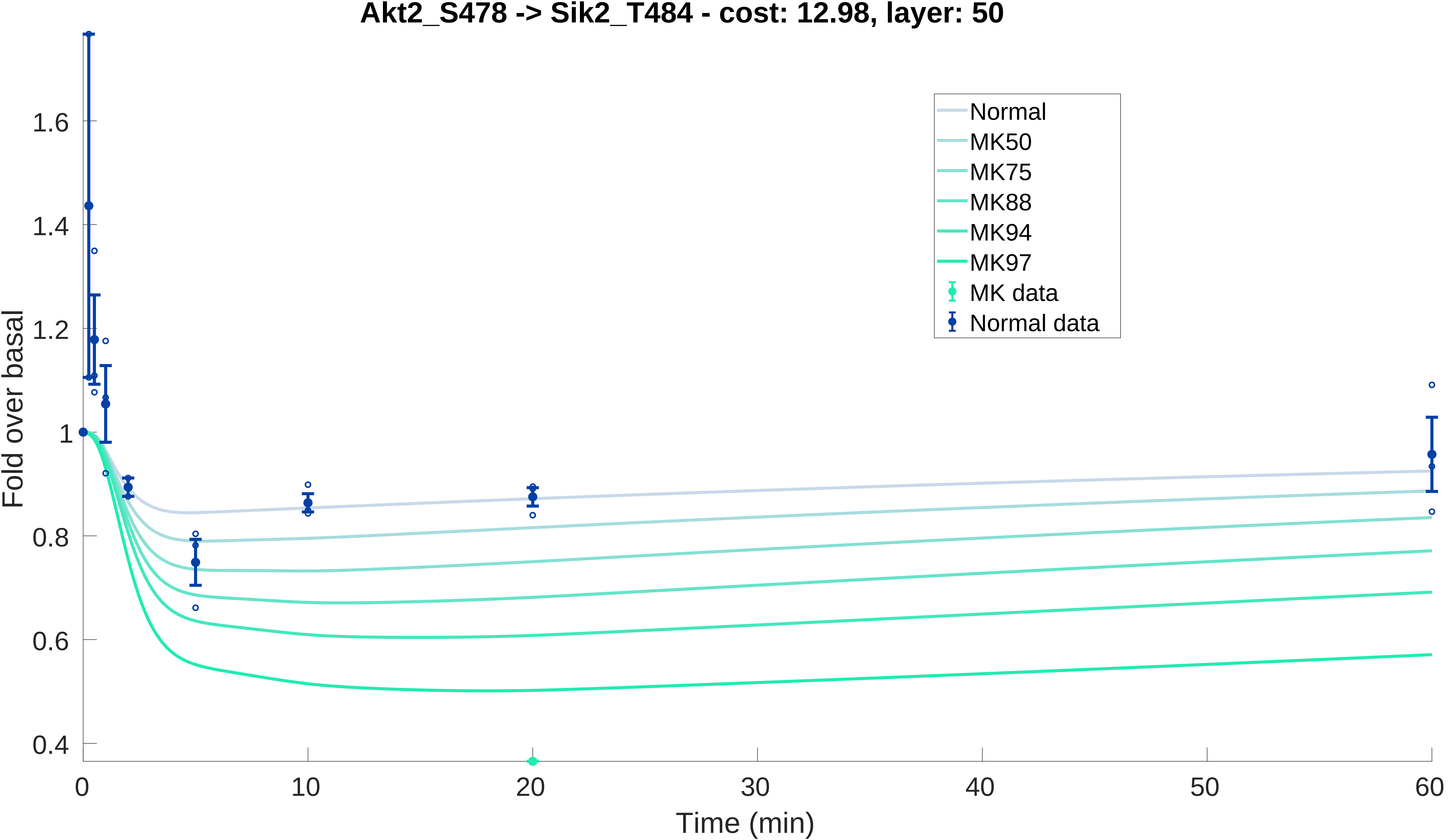

Akt2_S478 -> Sik2_T484 - cost: 12.98, layer: 50
Fold over basal
1.6
1.4
1.2
1
0.8
0.6
0.4
0
10
20
30
40
50
60
Time (min)
Normal
MK50
MK75
MK88
MK94
MK97
MK data
Normal data

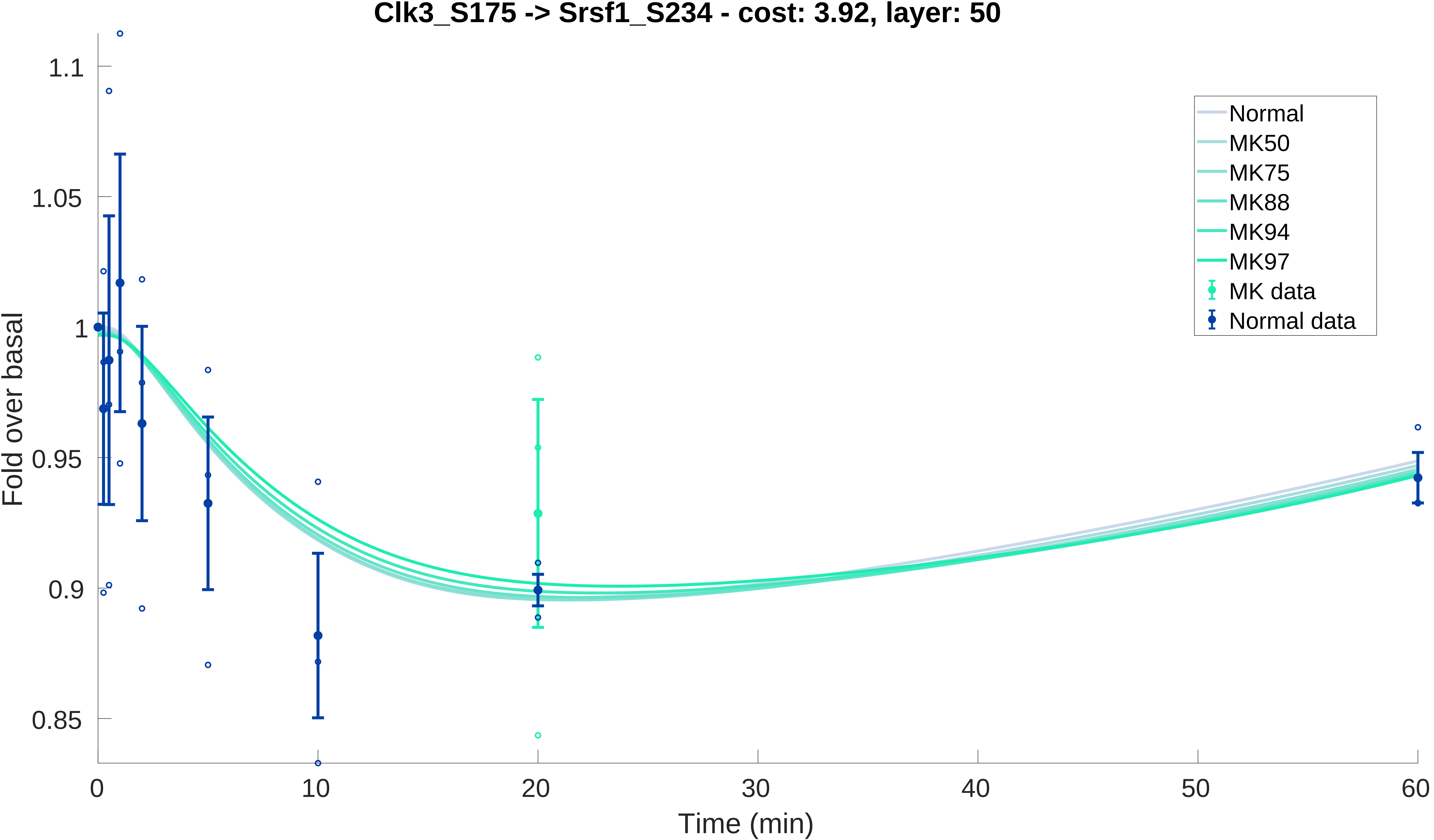
Clk3_S175 -> Srsf1_S234 - cost: 3.92, layer: 50
Fold over basal
1.1
1.05
1
0.95
0.9
0.85
Time (min)
0
10
20
30
40
50
60
Normal
MK50
MK75
MK88
MK94
MK97
MK data
Normal data

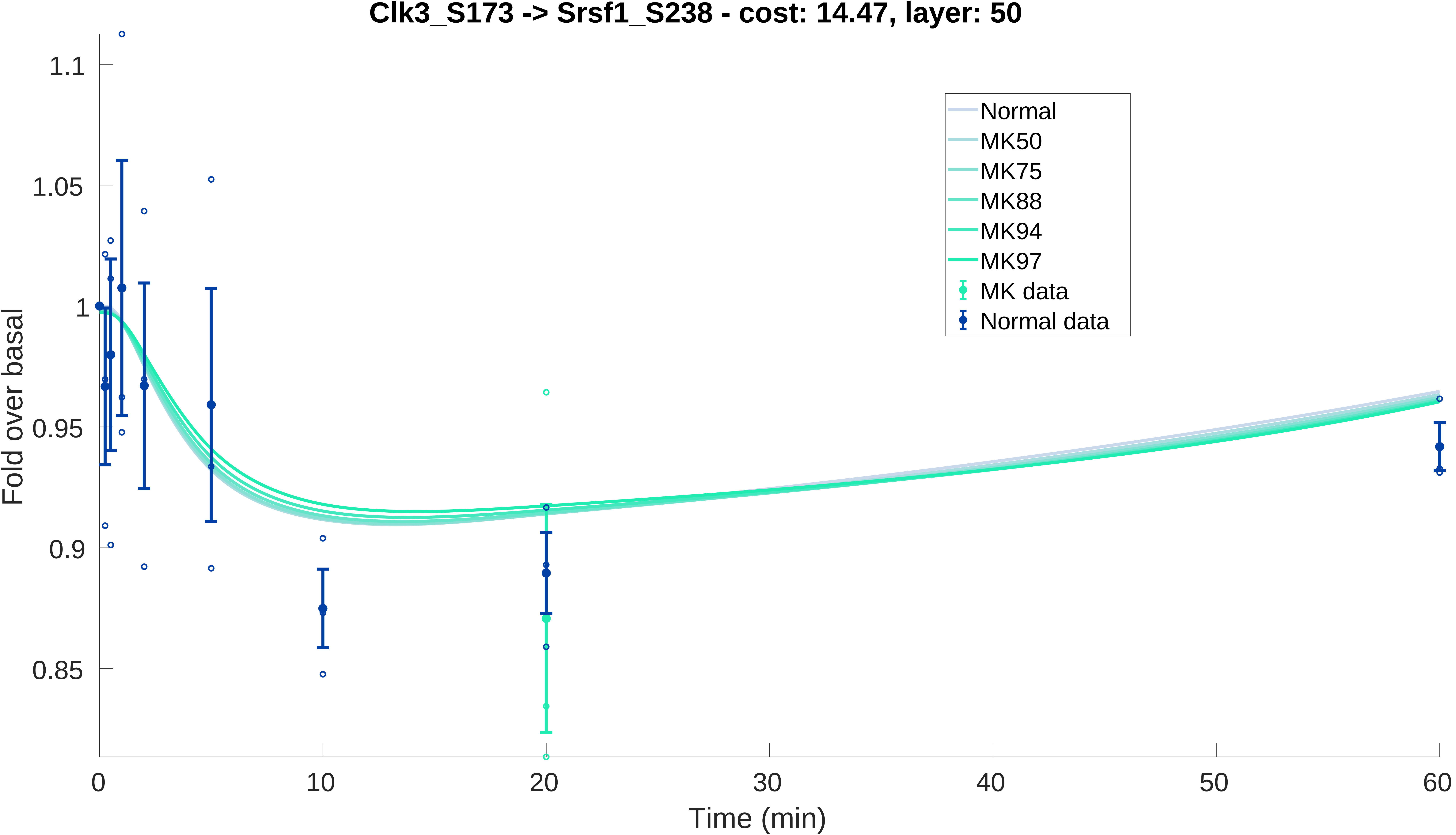

Clk3_S173 -> Srsf1_S238 - cost: 14.47, layer: 50
Fold over basal
Time (min)
1.1
1.05
1
0.95
0.9
0.85
0
10
20
30
40
50
60
Normal
MK50
MK75
MK88
MK94
MK97
MK data
Normal data

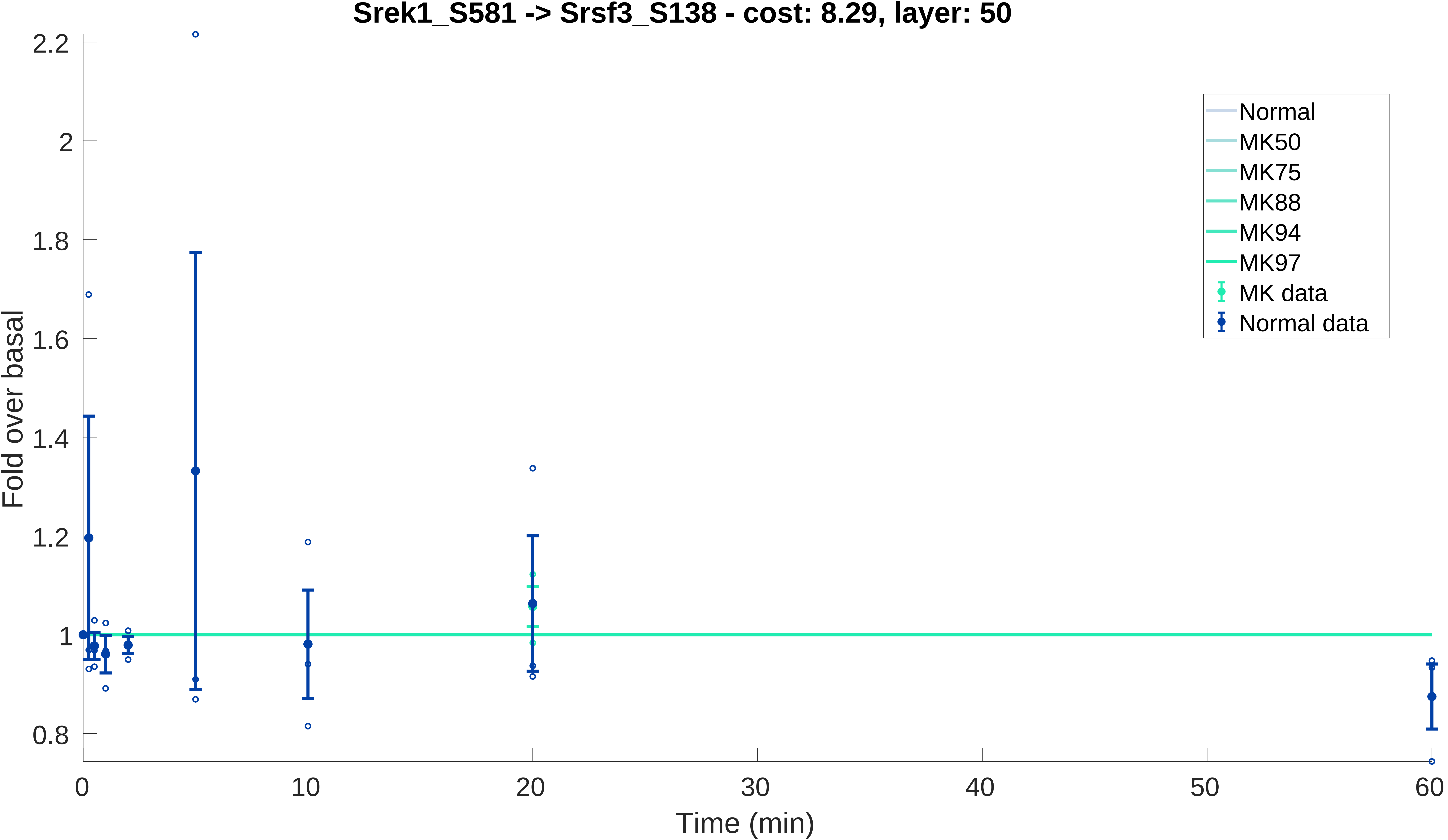

Srek1_S581 -> Srsf3_S138 - cost: 8.29, layer: 50
Fold over basal
Time (min)
2.2
2
1.8
1.6
1.4
1.2
1
0.8
0
10
20
30
40
50
60
Normal
MK50
MK75
MK88
MK94
MK97
MK data
Normal data

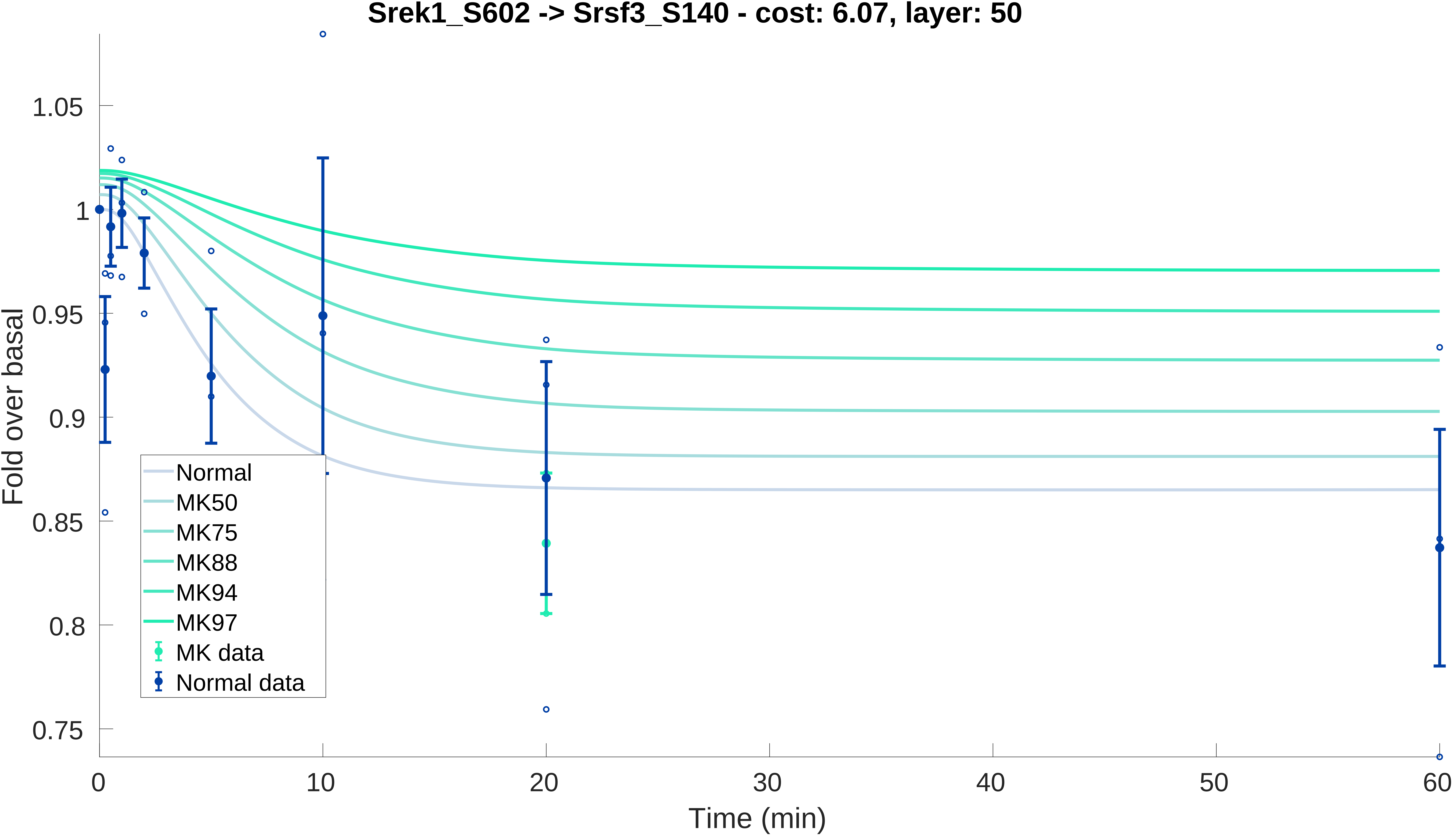

Srek1_S602 -> Srsf3_S140 - cost: 6.07, layer: 50
Fold over basal
1.05
1
0.95
0.9
0.85
0.8
0.75
0
10
20
30
40
50
60
Time (min)
Normal
MK50
MK75
MK88
MK94
MK97
MK data
Normal data

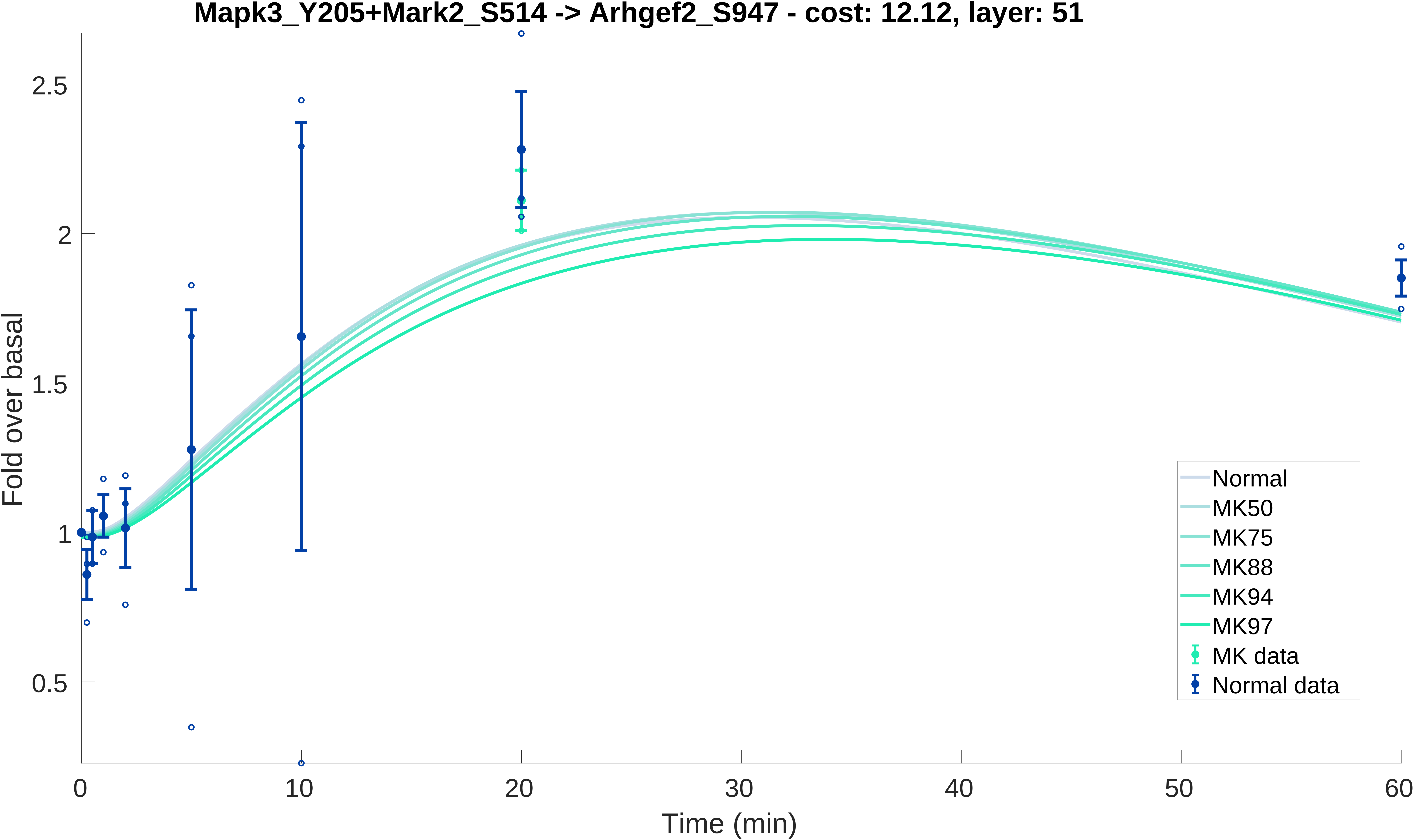
Mapk3_Y205+Mark2_S514 -> Arhgef2_S947 - cost: 12.12, layer: 51
Fold over basal
2.5
2
1.5
1
0.5
0
10
20
30
40
50
60
Time (min)
Normal
MK50
MK75
MK88
MK94
MK97
MK data
Normal data

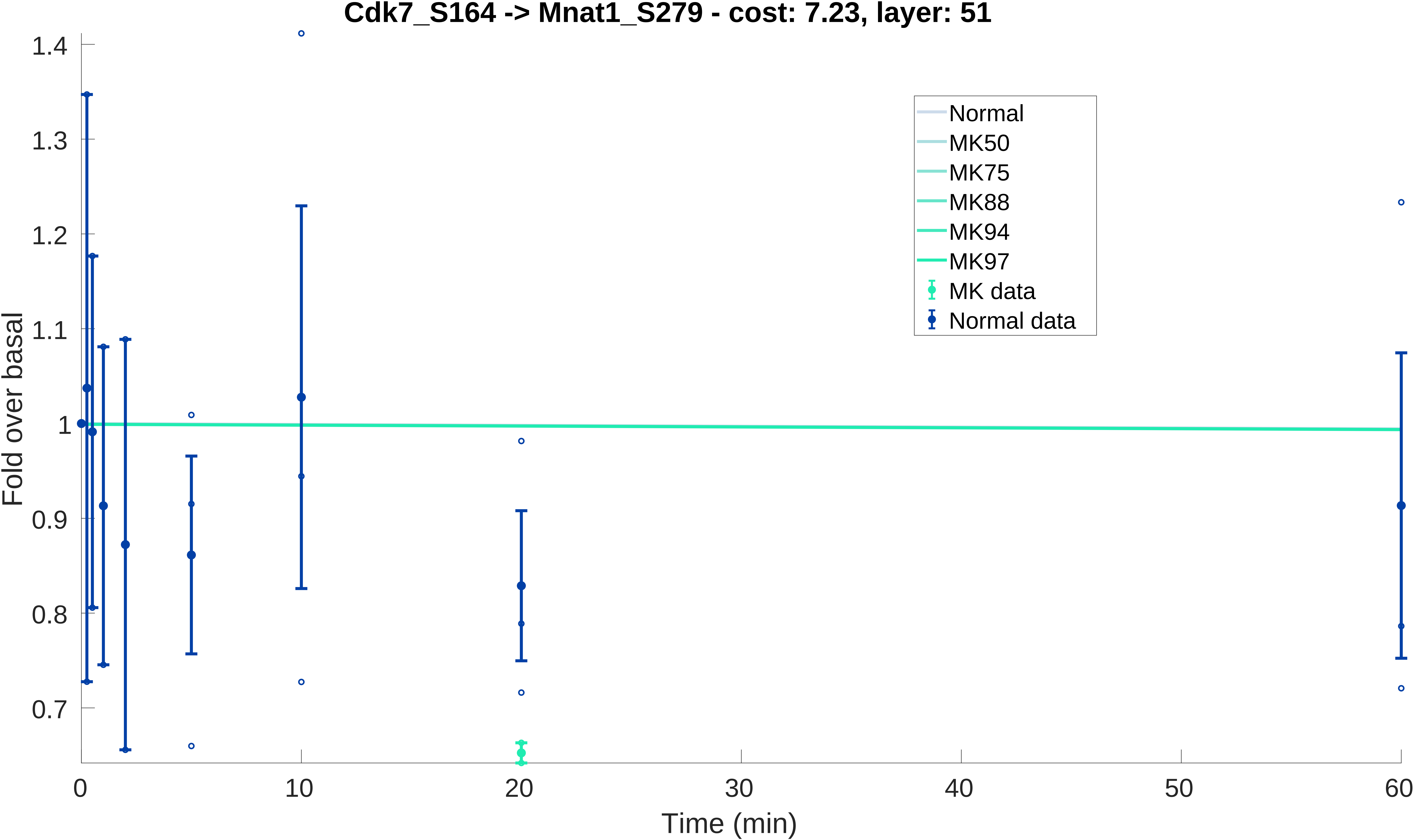

Cdk7_S164 -> Mnat1_S279 - cost: 7.23, layer: 51
Normal
MK50
MK75
MK88
MK94
MK97
MK data
Normal data
Fold over basal
Time (min)
1.4
1.3
1.2
1.1
1
0.9
0.8
0.7
0
10
20
30
40
50
60

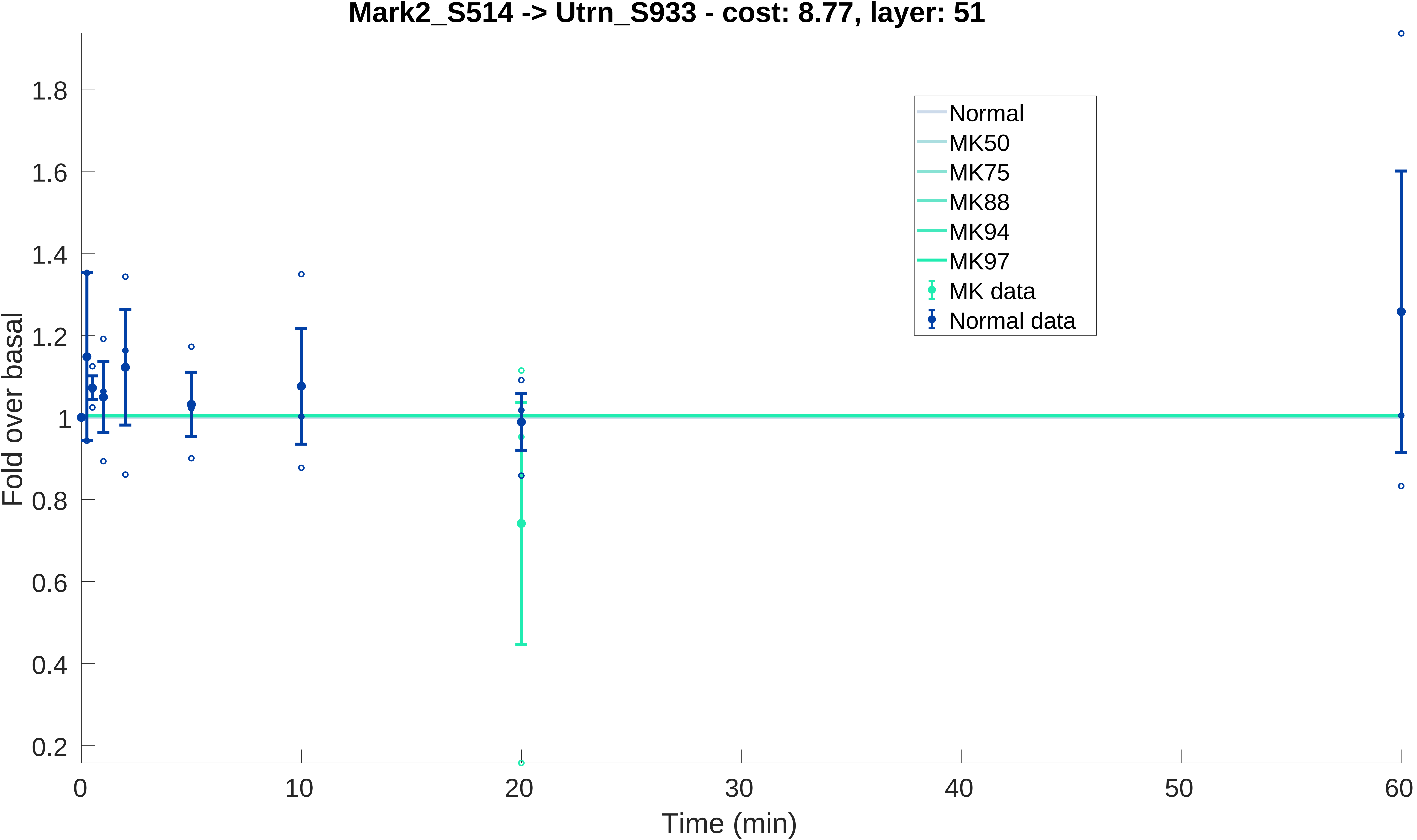

Mark2_S514 -> Utrn_S933 - cost: 8.77, layer: 51
Fold over basal
Time (min)
Normal
MK50
MK75
MK88
MK94
MK97
MK data
Normal data
0.2
0.4
0.6
0.8
1
1.2
1.4
1.6
1.8
0
10
20
30
40
50
60

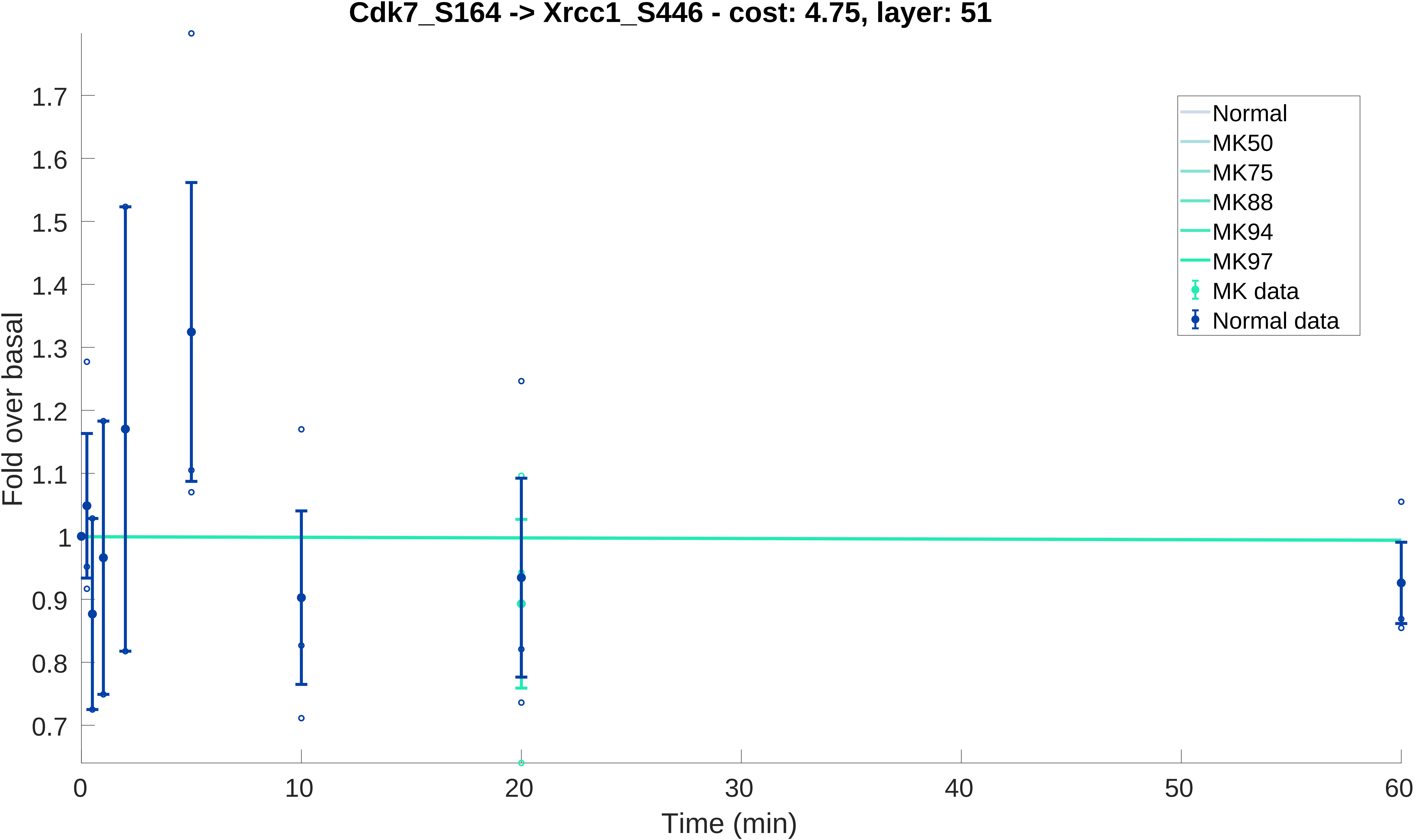

Cdk7_S164 -> Xrcc1_S446 - cost: 4.75, layer: 51
Fold over basal
Time (min)
0.7
0.8
0.9
1
1.1
1.2
1.3
1.4
1.5
1.6
1.7
0
10
20
30
40
50
60
Normal
MK50
MK75
MK88
MK94
MK97
MK data
Normal data

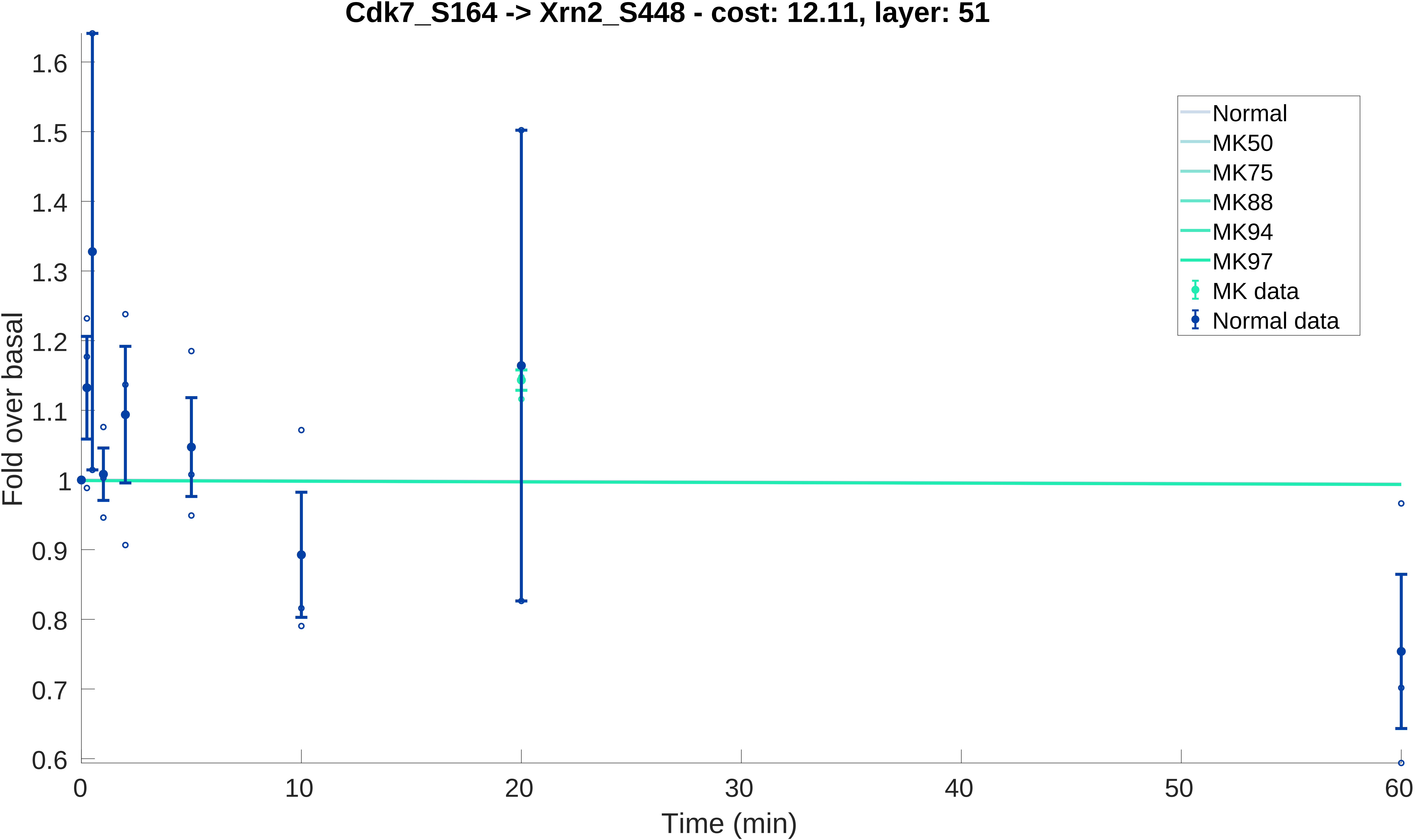

Cdk7_S164 -> Xrn2_S448 - cost: 12.11, layer: 51
Fold over basal
Time (min)
1.6
1.5
1.4
1.3
1.2
1.1
1
0.9
0.8
0.7
0.6
0
10
20
30
40
50
60
Normal
MK50
MK75
MK88
MK94
MK97
MK data
Normal data

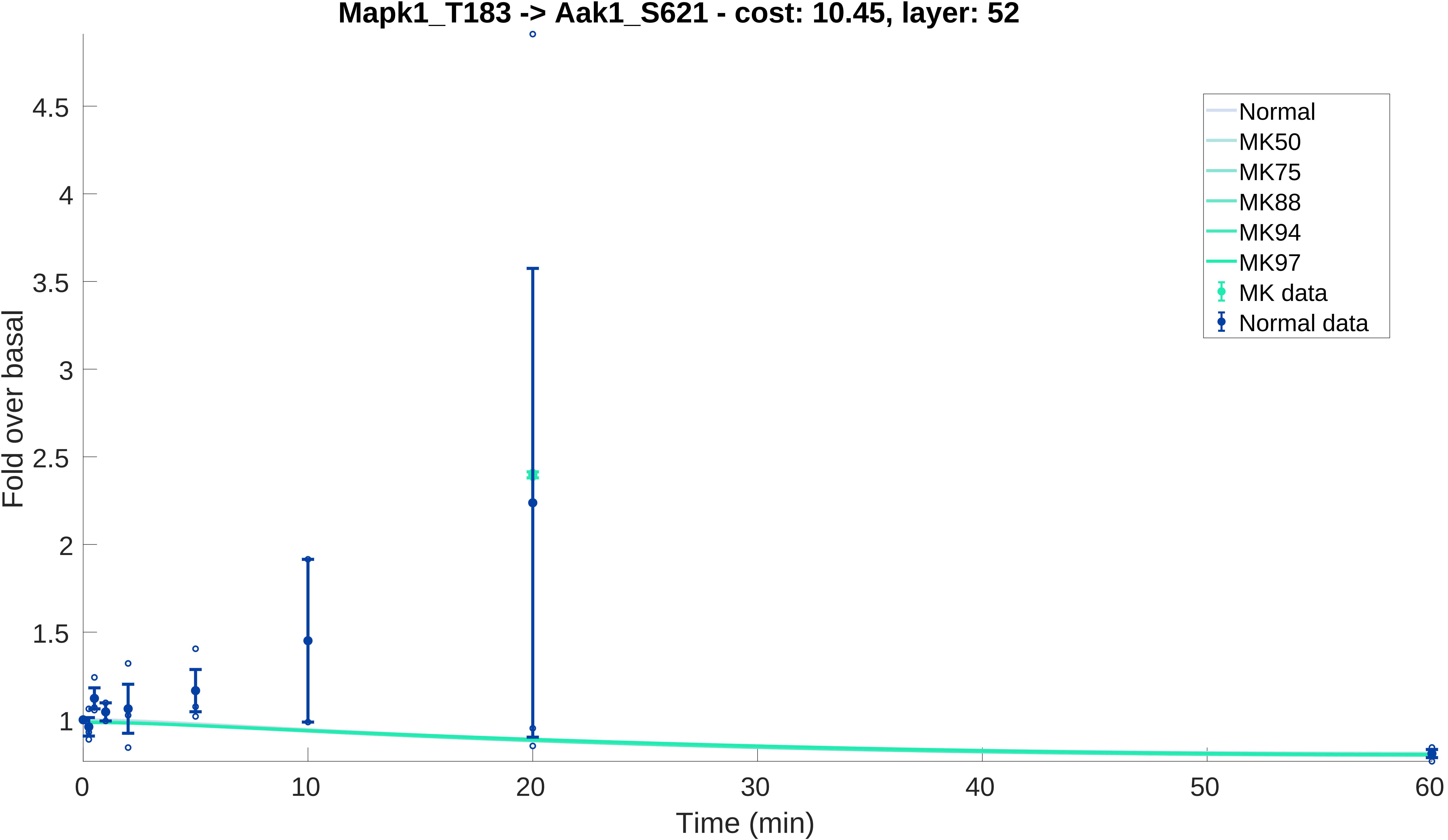

Mapk1_T183 -> Aak1_S621 - cost: 10.45, layer: 52
Fold over basal
4.5
4
3.5
3
2.5
2
1.5
1
0
10
20
30
40
50
60
Time (min)
Normal
MK50
MK75
MK88
MK94
MK97
MK data
Normal data

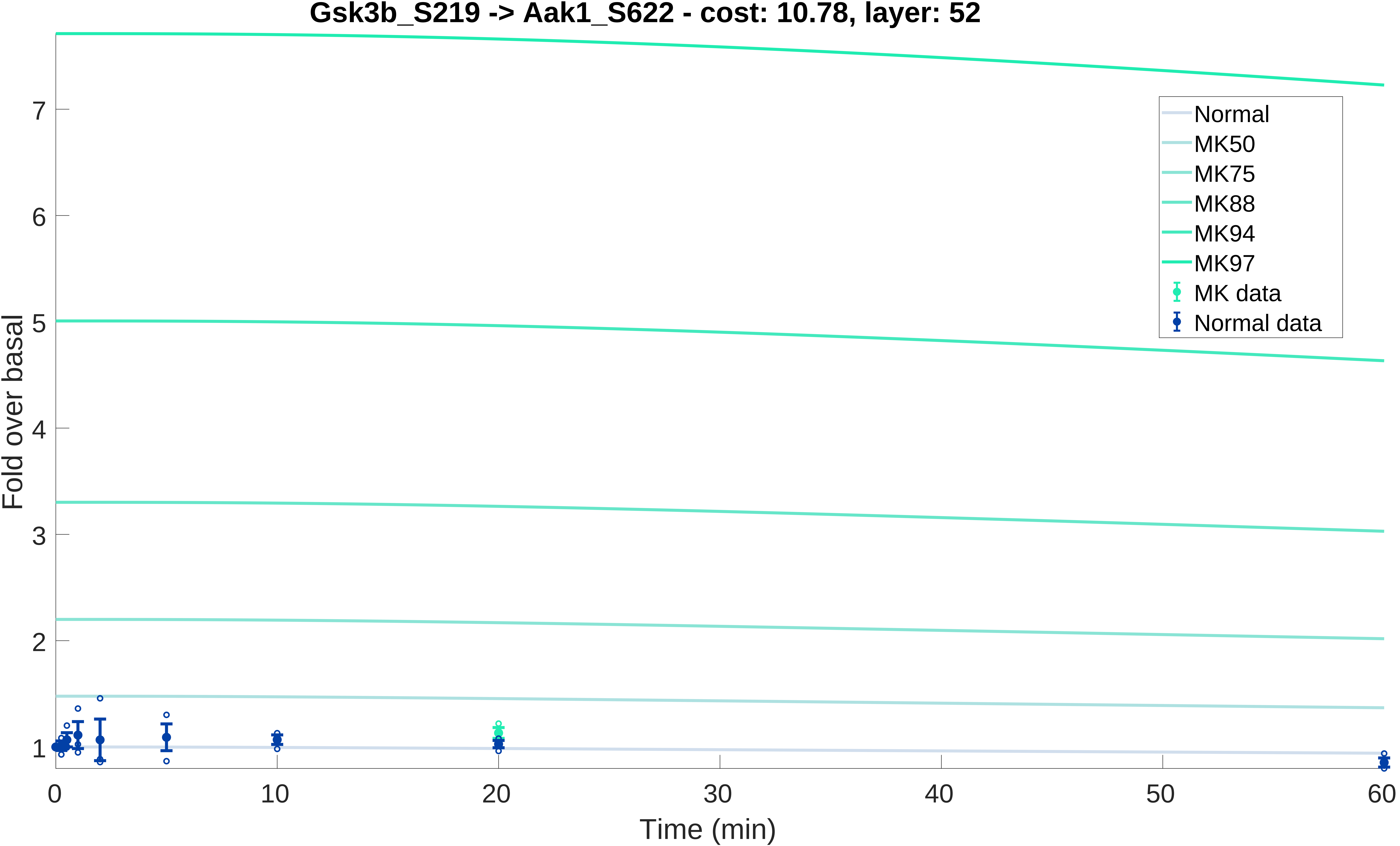

Gsk3b_S219 -> Aak1_S622 - cost: 10.78, layer: 52
Normal
MK50
MK75
MK88
MK94
MK97
MK data
Normal data
Fold over basal
1
2
3
4
5
6
7
0
10
20
30
40
50
60
Time (min)

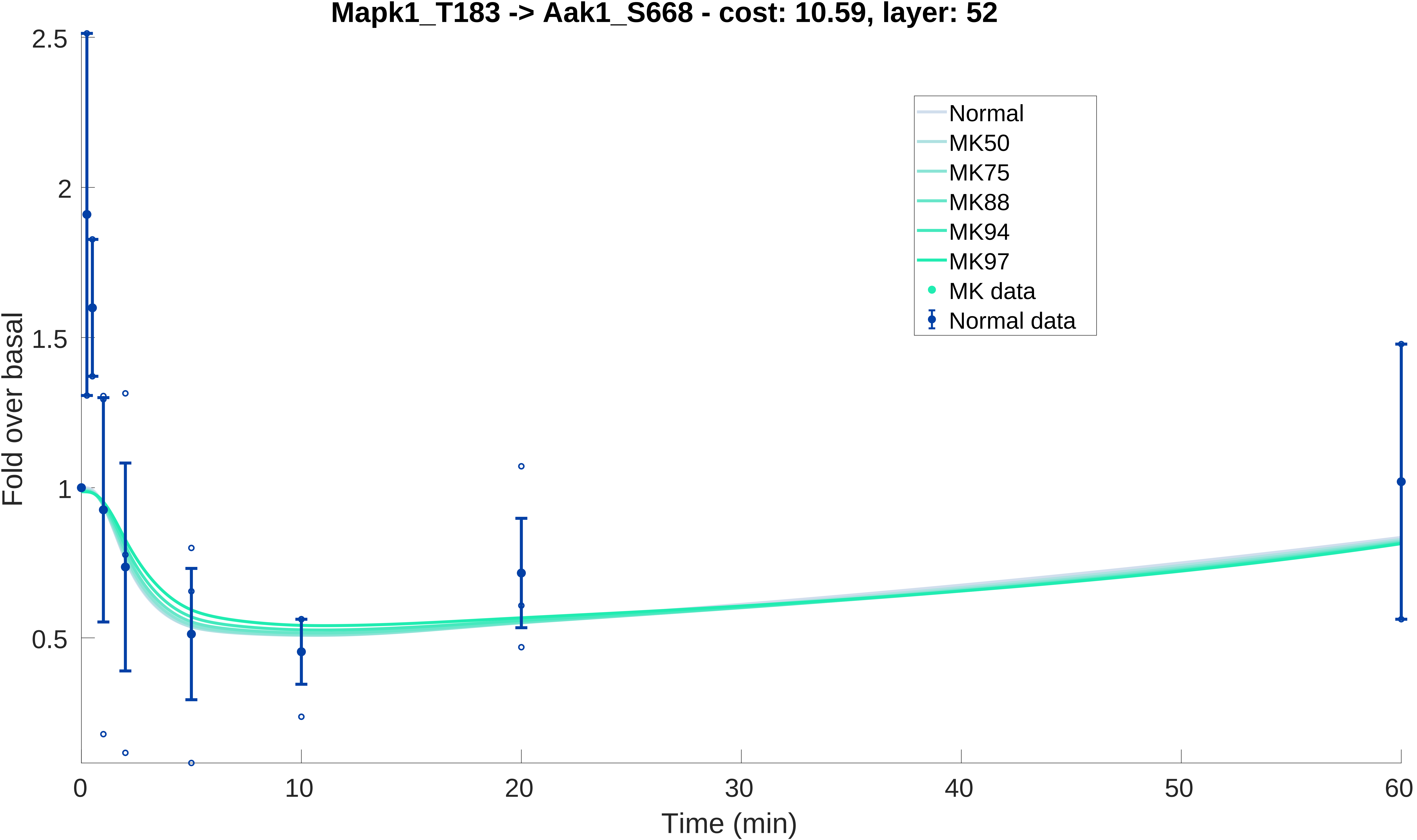

Mapk1_T183 -> Aak1_S668 - cost: 10.59, layer: 52
Fold over basal
Time (min)
0
10
20
30
40
50
60
0.5
1
1.5
2
2.5
Normal
MK50
MK75
MK88
MK94
MK97
MK data
Normal data

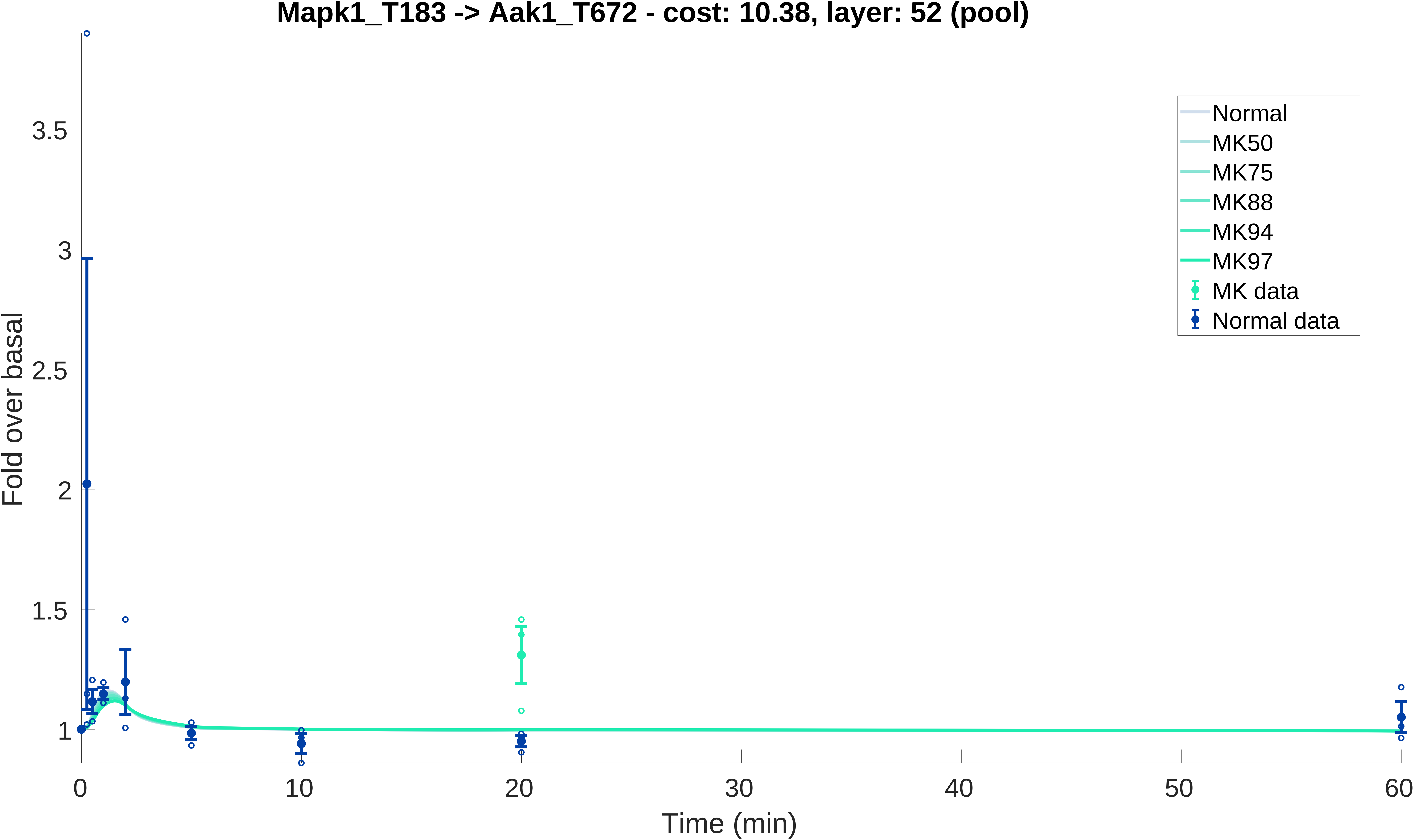
Mapk1_T183 -> Aak1_T672 - cost: 10.38, layer: 52 (pool)
Fold over basal
Time (min)
3.5
3
2.5
2
1.5
1
0
10
20
30
40
50
60
Normal
MK50
MK75
MK88
MK94
MK97
MK data
Normal data

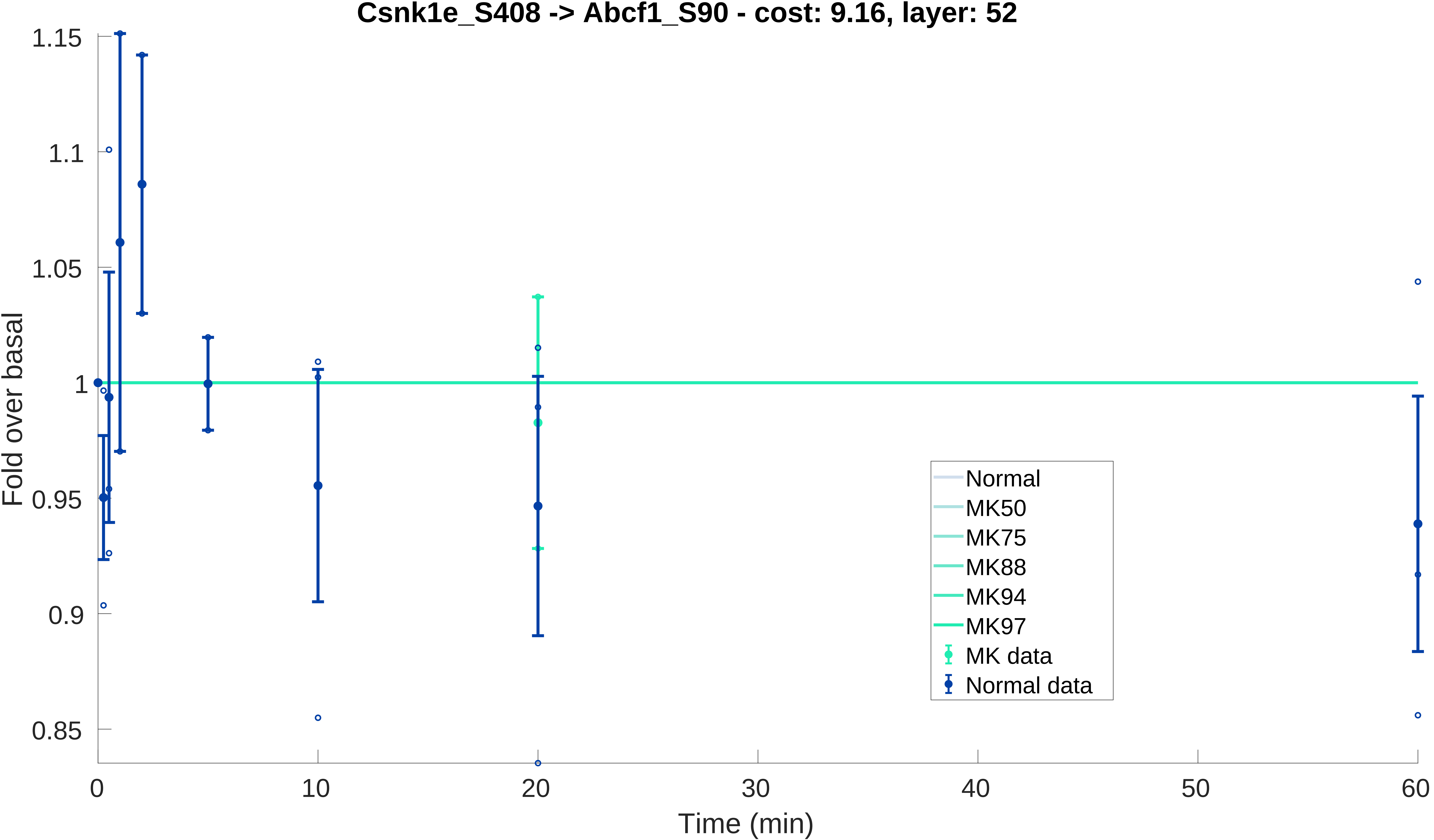
Csnk1e_S408 -> Abcf1_S90 - cost: 9.16, layer: 52
Fold over basal
Time (min)
1.15
1.1
1.05
1
0.95
0.9
0.85
0
10
20
30
40
50
60
Normal
MK50
MK75
MK88
MK94
MK97
MK data
Normal data

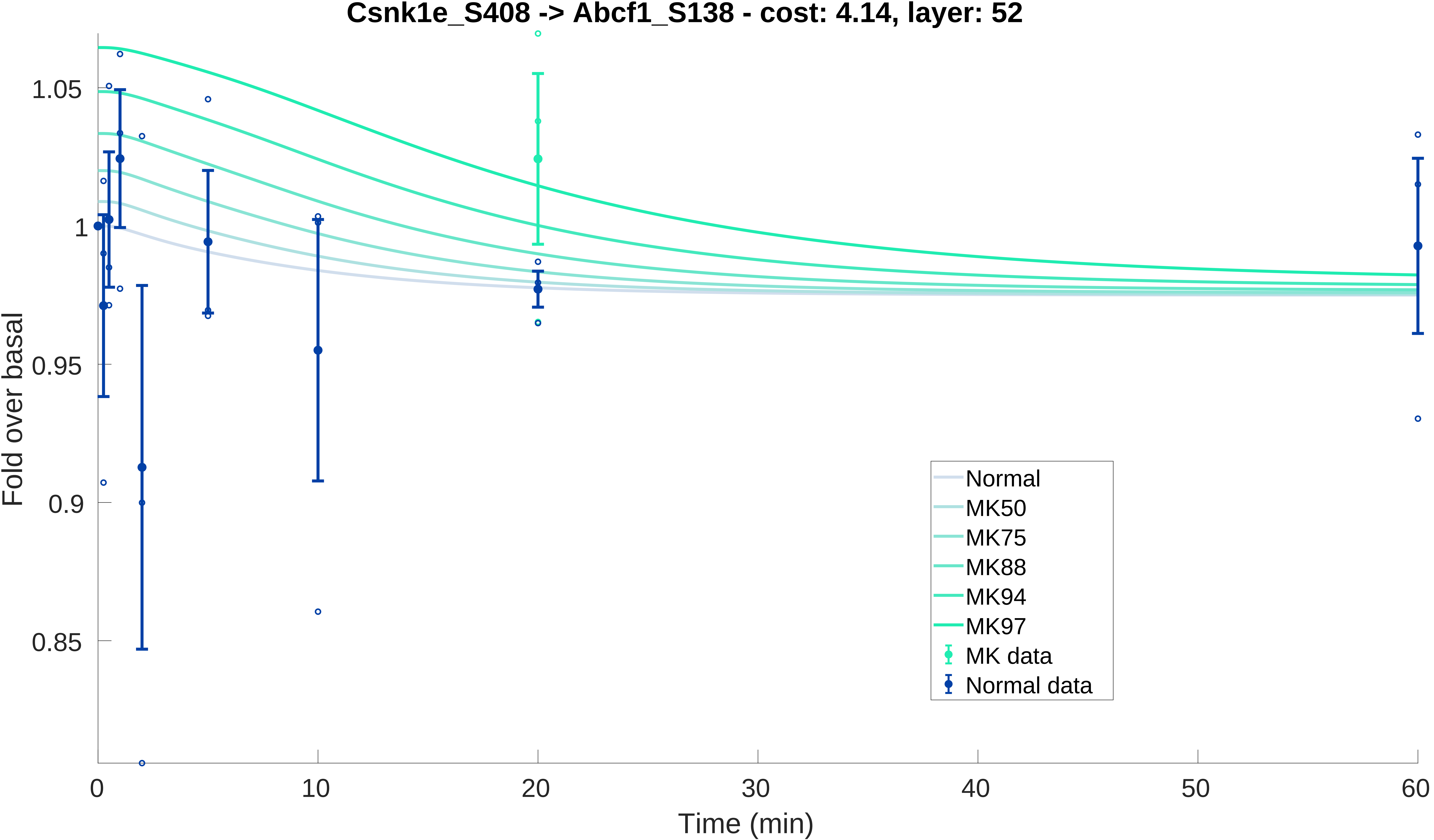

Csnk1e_S408 -> Abcf1_S138 - cost: 4.14, layer: 52
Fold over basal
1.05
1
0.95
0.9
0.85
0
10
20
30
40
50
60
Time (min)
Normal
MK50
MK75
MK88
MK94
MK97
MK data
Normal data

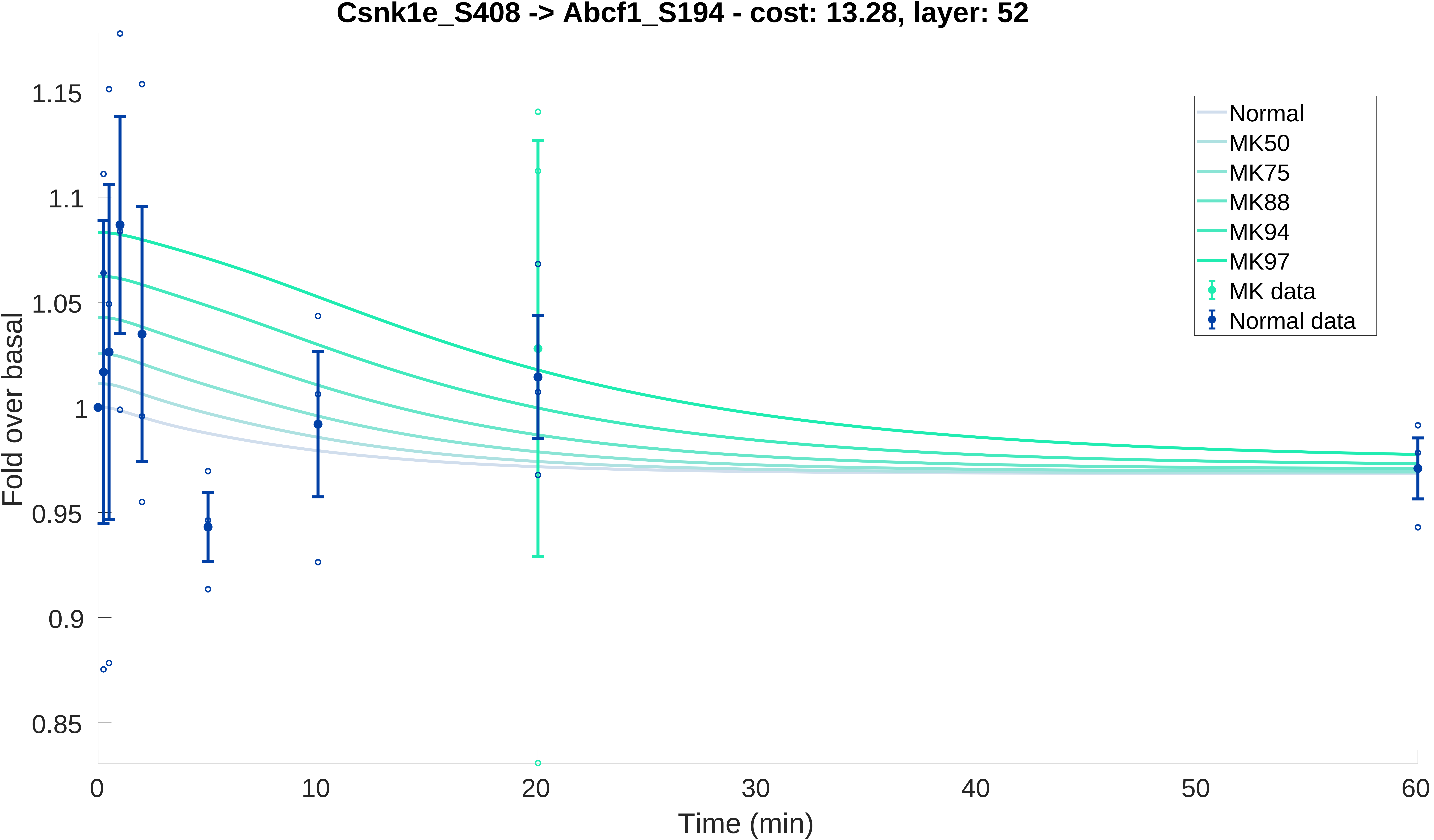

Csnk1e_S408 -> Abcf1_S194 - cost: 13.28, layer: 52
Fold over basal
1.15
1.1
1.05
1
0.95
0.9
0.85
Time (min)
0
10
20
30
40
50
60
Normal
MK50
MK75
MK88
MK94
MK97
MK data
Normal data

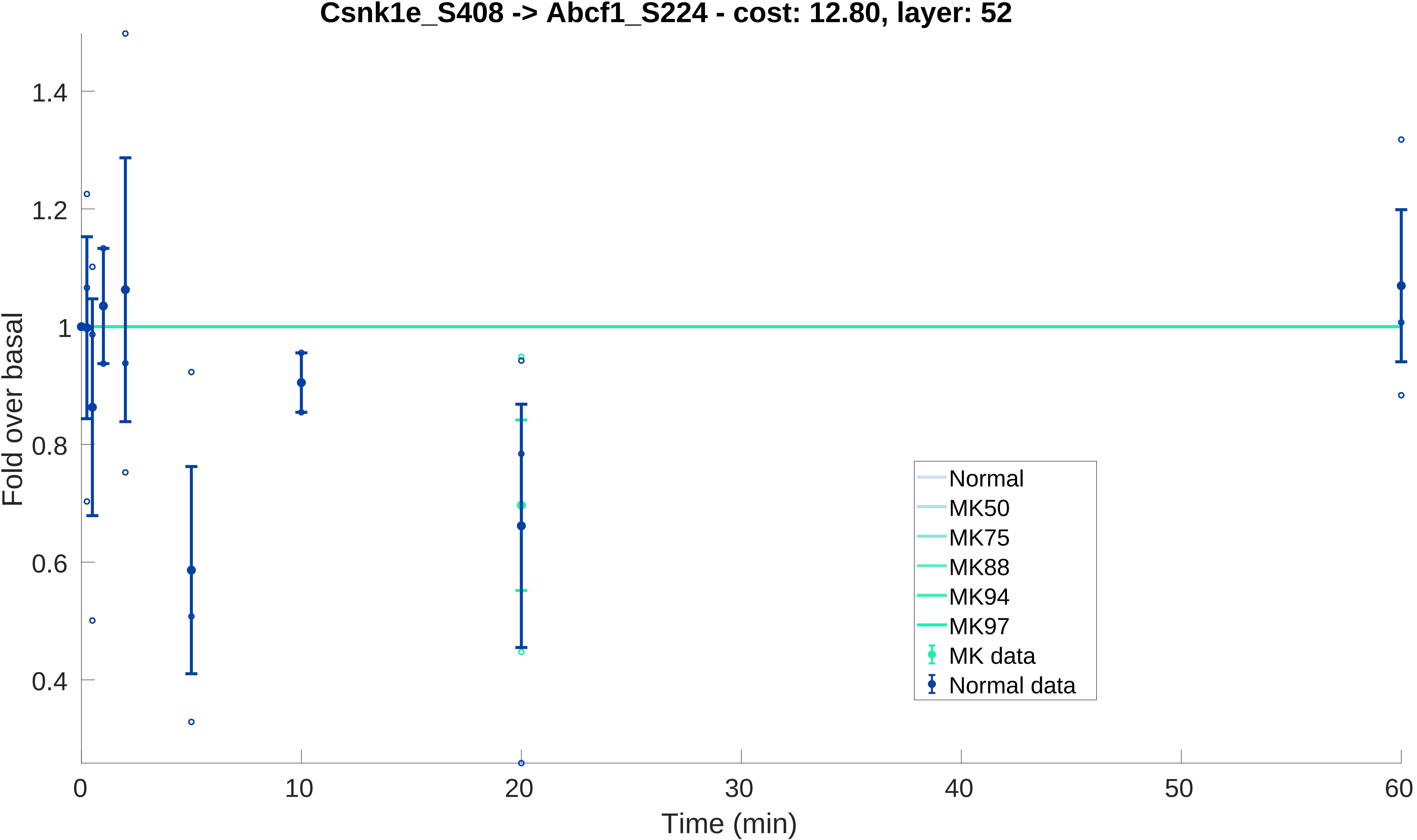

Csnk1e_S408 -> Abcf1_S224 - cost: 12.80, layer: 52
Fold over basal
1.4
1.2
1
0.8
0.6
0.4
0
10
20
30
40
50
60
Time (min)
Normal
MK50
MK75
MK88
MK94
MK97
MK data
Normal data

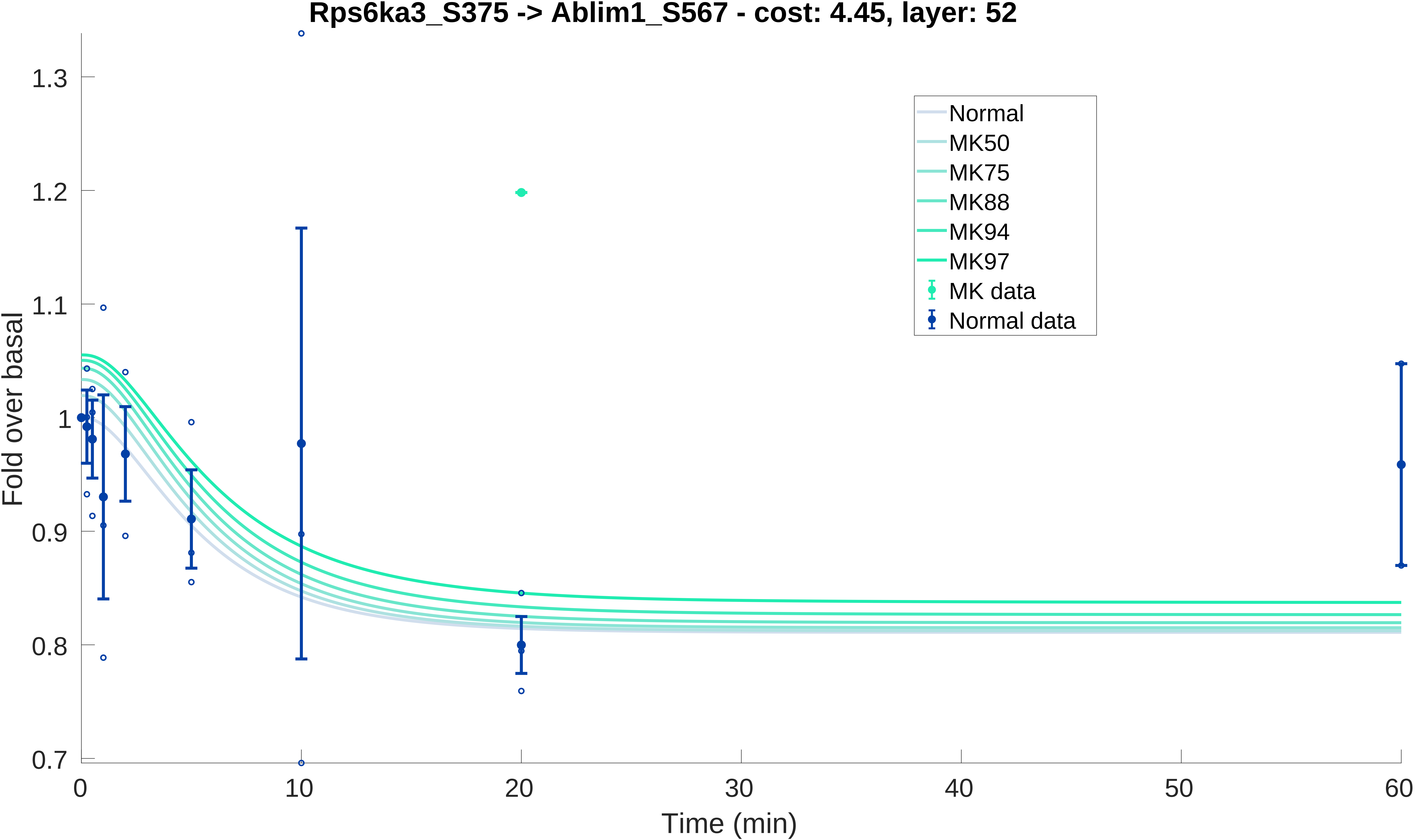

Rps6ka3_S375 -> Ablim1_S567 - cost: 4.45, layer: 52
Fold over basal
Time (min)
0.7
0.8
0.9
1
1.1
1.2
1.3
0
10
20
30
40
50
60
Normal
MK50
MK75
MK88
MK94
MK97
MK data
Normal data

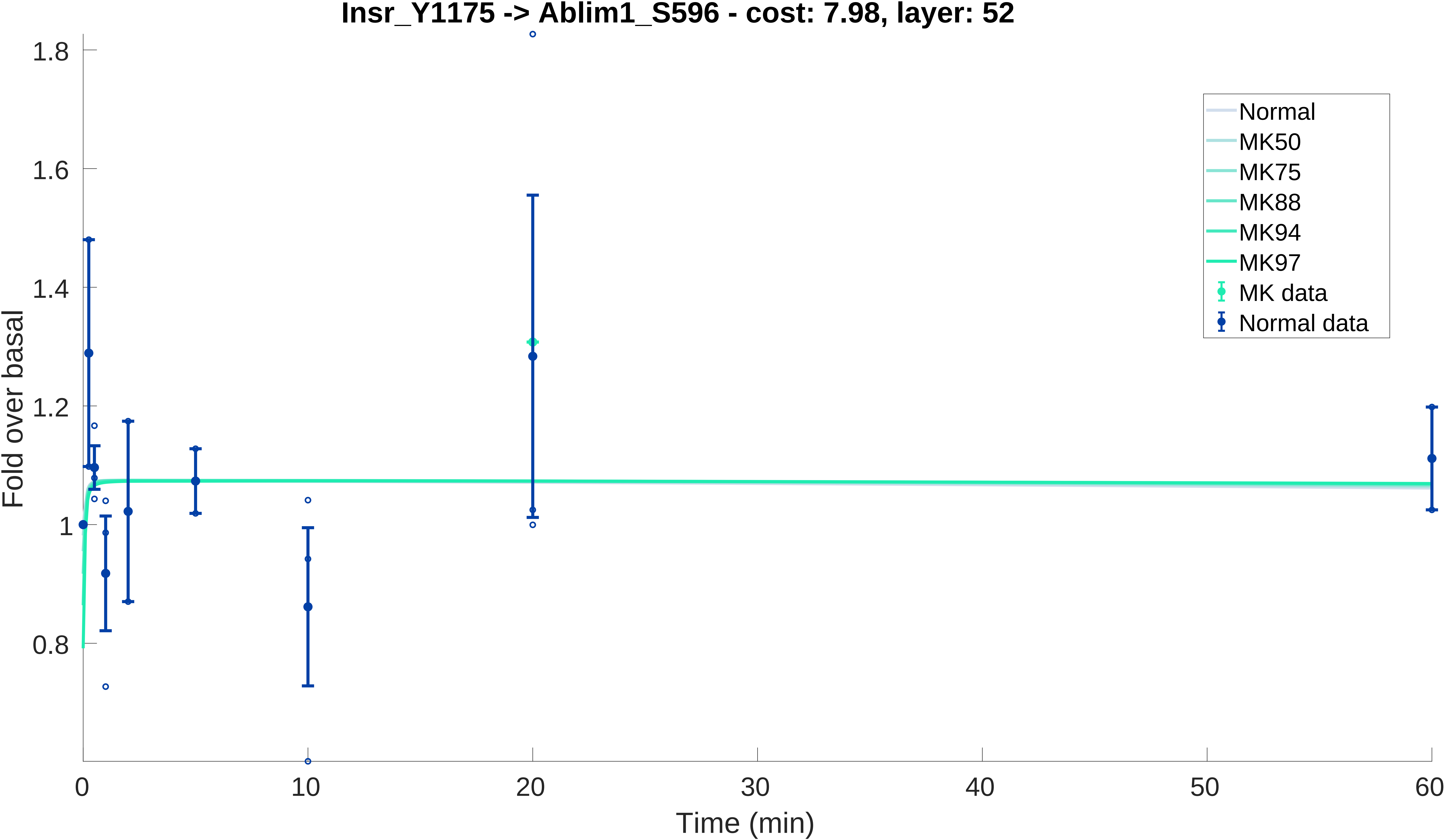
Insr_Y1175 -> Ablim1_S596 - cost: 7.98, layer: 52
Fold over basal
1.8
1.6
1.4
1.2
1
0.8
0
10
20
30
40
50
60
Time (min)
Normal
MK50
MK75
MK88
MK94
MK97
MK data
Normal data

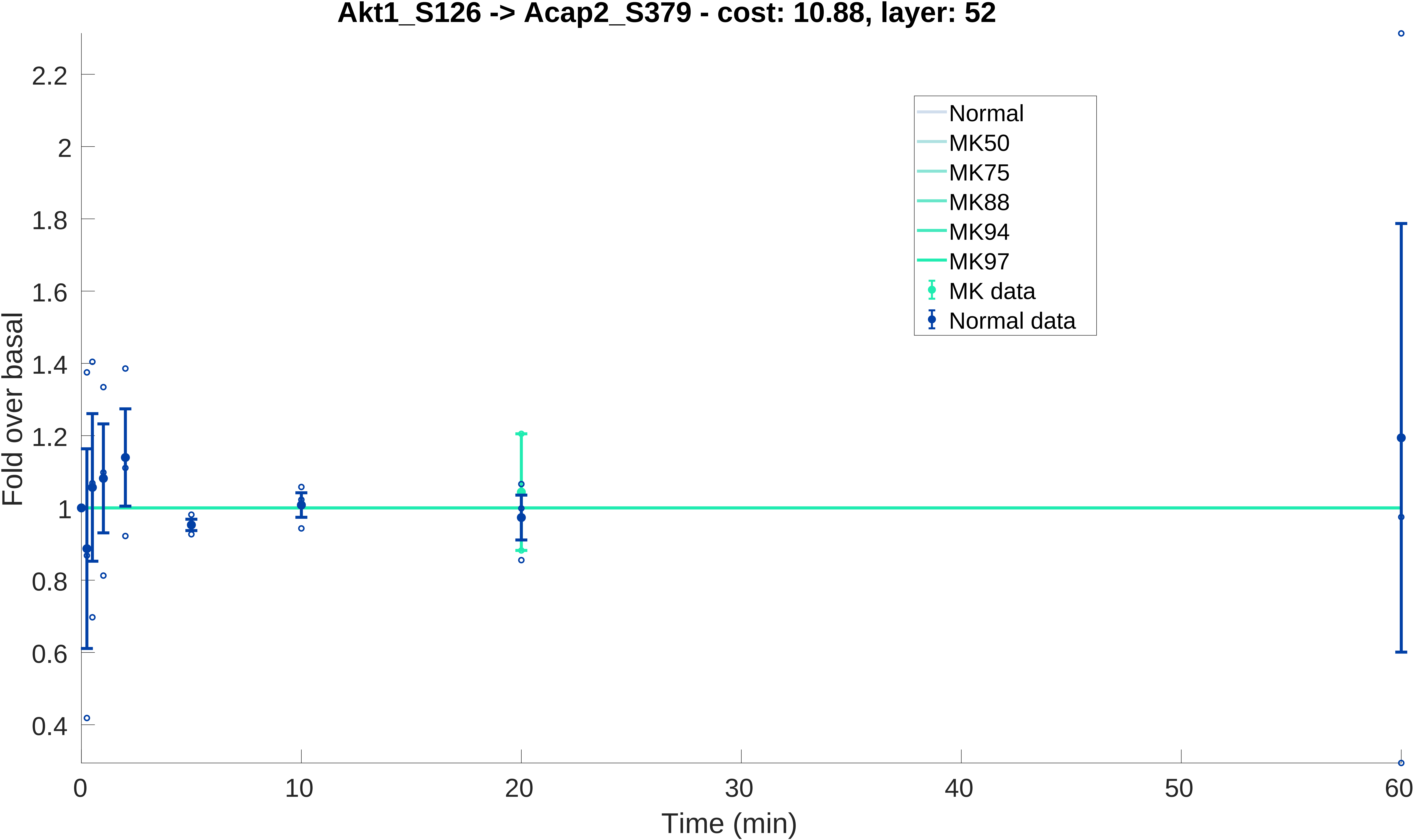

Akt1_S126 -> Acap2_S379 - cost: 10.88, layer: 52
Fold over basal
Time (min)
Normal
MK50
MK75
MK88
MK94
MK97
MK data
Normal data
0.4
0.6
0.8
1
1.2
1.4
1.6
1.8
2
2.2
0
10
20
30
40
50
60

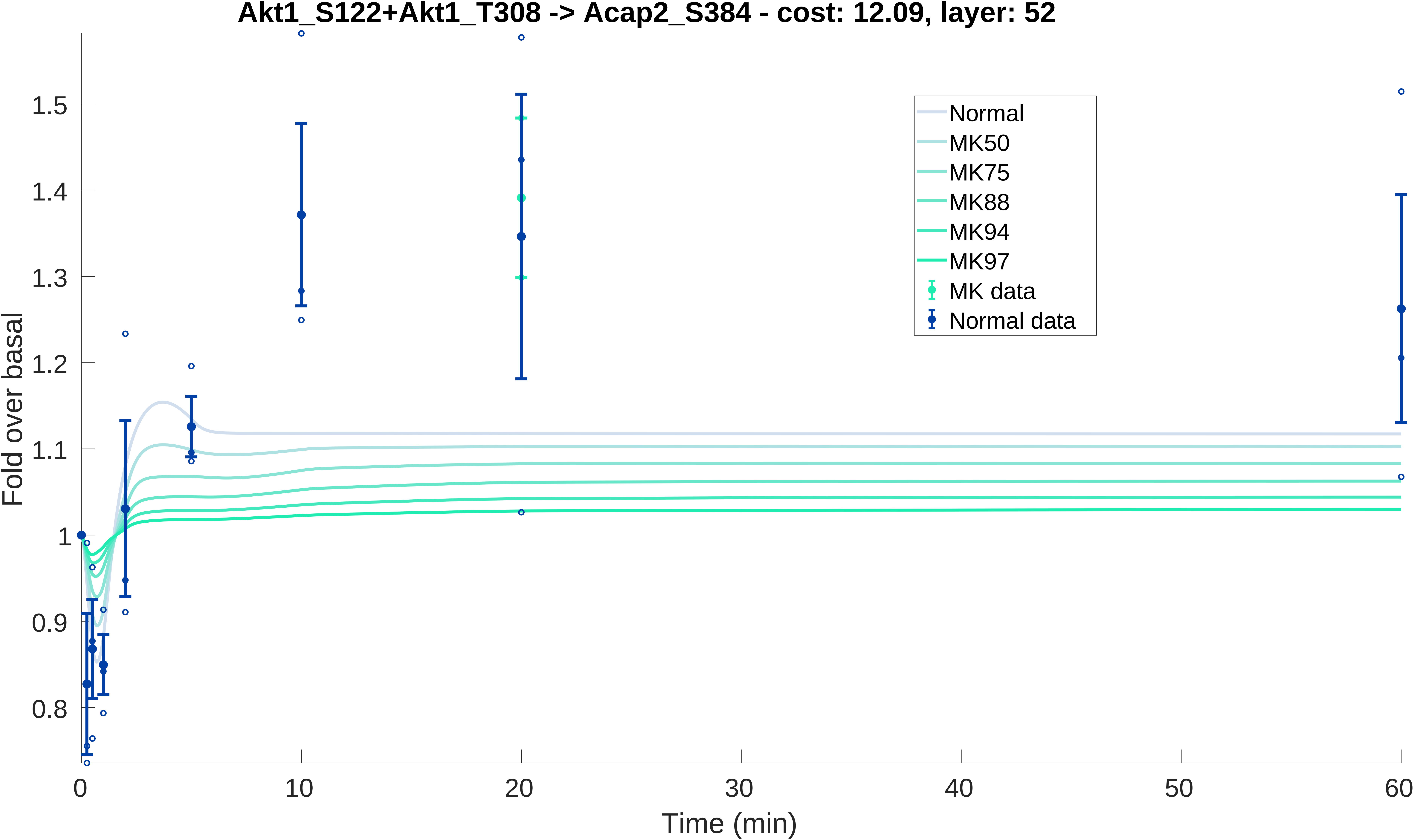

Akt1_S122+Akt1_T308 -> Acap2_S384 - cost: 12.09, layer: 52
Fold over basal
Time (min)
0.8
0.9
1
1.1
1.2
1.3
1.4
1.5
0
10
20
30
40
50
60
Normal
MK50
MK75
MK88
MK94
MK97
MK data
Normal data

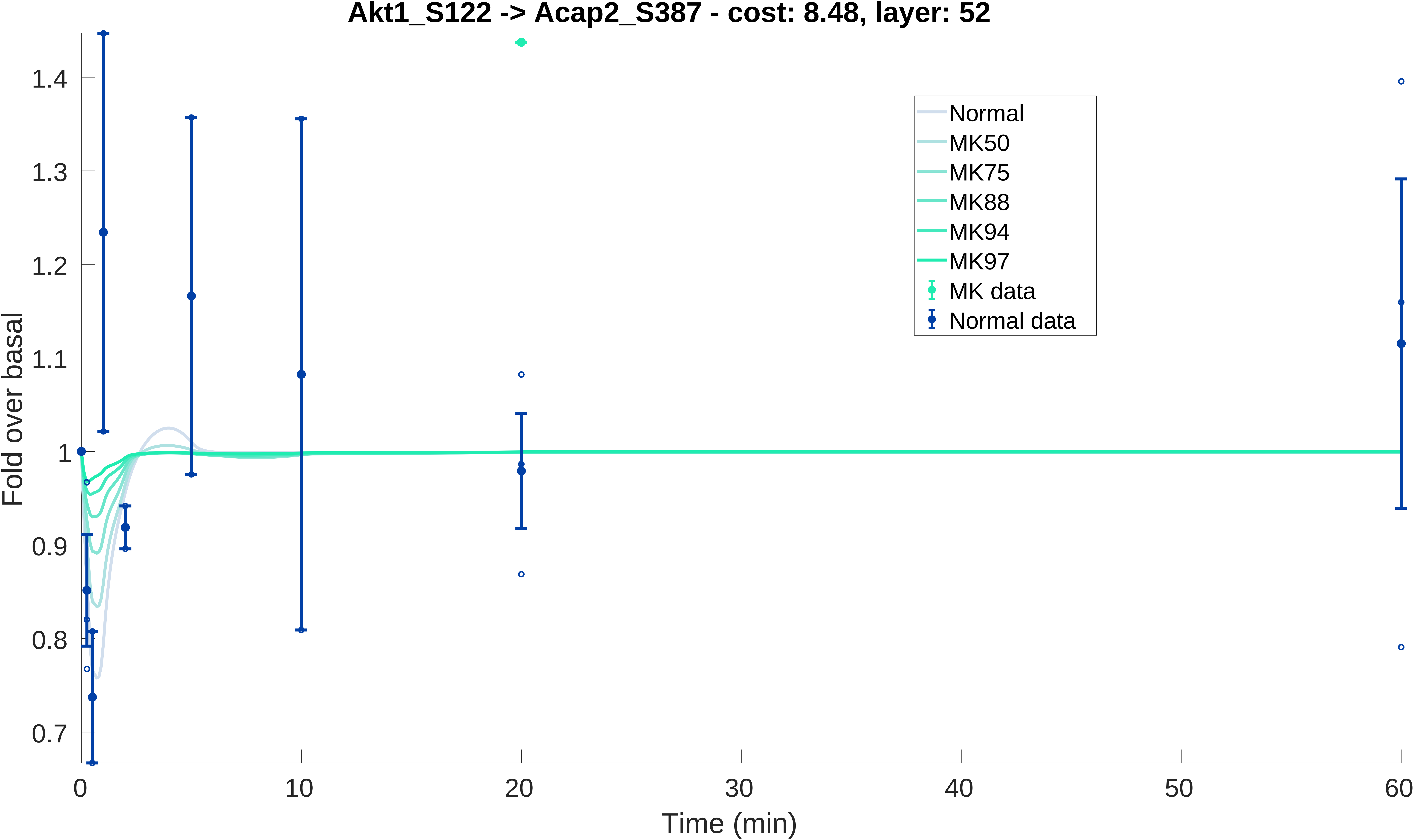
Akt1_S122 -> Acap2_S387 - cost: 8.48, layer: 52
Fold over basal
Time (min)
1.4
1.3
1.2
1.1
1
0.9
0.8
0.7
0
10
20
30
40
50
60
Normal
MK50
MK75
MK88
MK94
MK97
MK data
Normal data

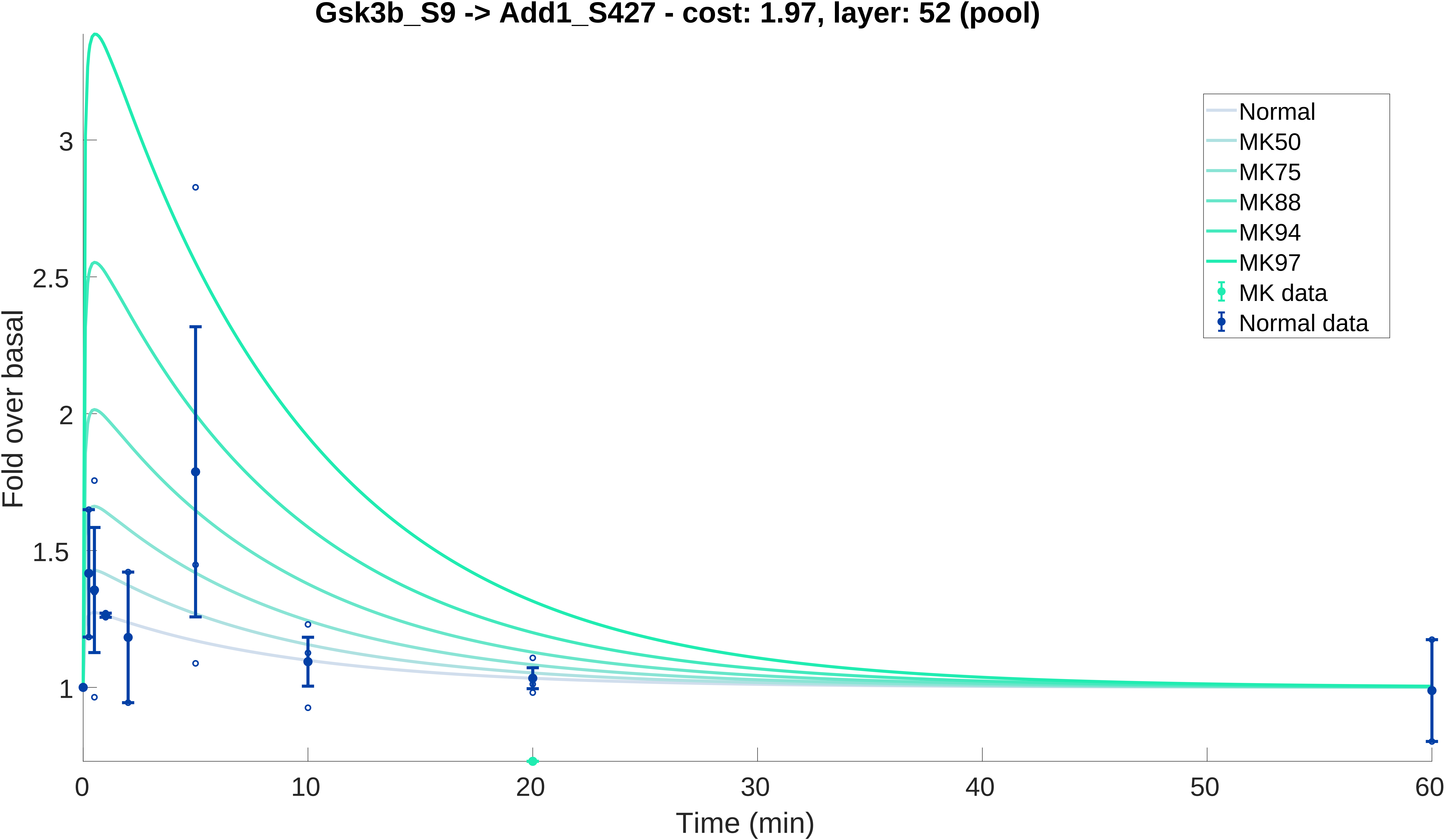
Gsk3b_S9 -> Add1_S427 - cost: 1.97, layer: 52 (pool)
Fold over basal
Time (min)
0
10
20
30
40
50
60
1
1.5
2
2.5
3
Normal
MK50
MK75
MK88
MK94
MK97
MK data
Normal data

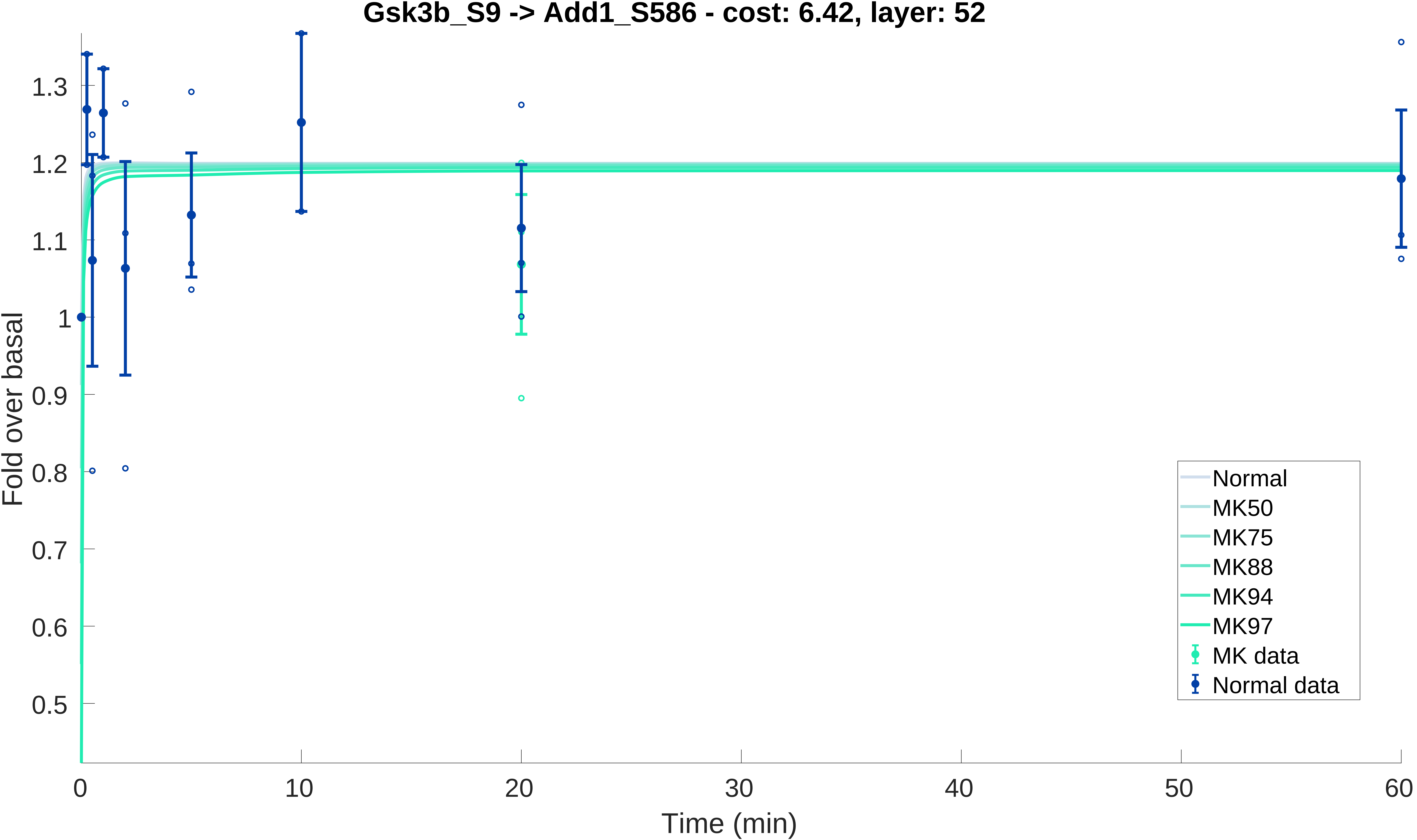

Gsk3b_S9 -> Add1_S586 - cost: 6.42, layer: 52
Fold over basal
Time (min)
1.3
1.2
1.1
1
0.9
0.8
0.7
0.6
0.5
0
10
20
30
40
50
60
Normal
MK50
MK75
MK88
MK94
MK97
MK data
Normal data

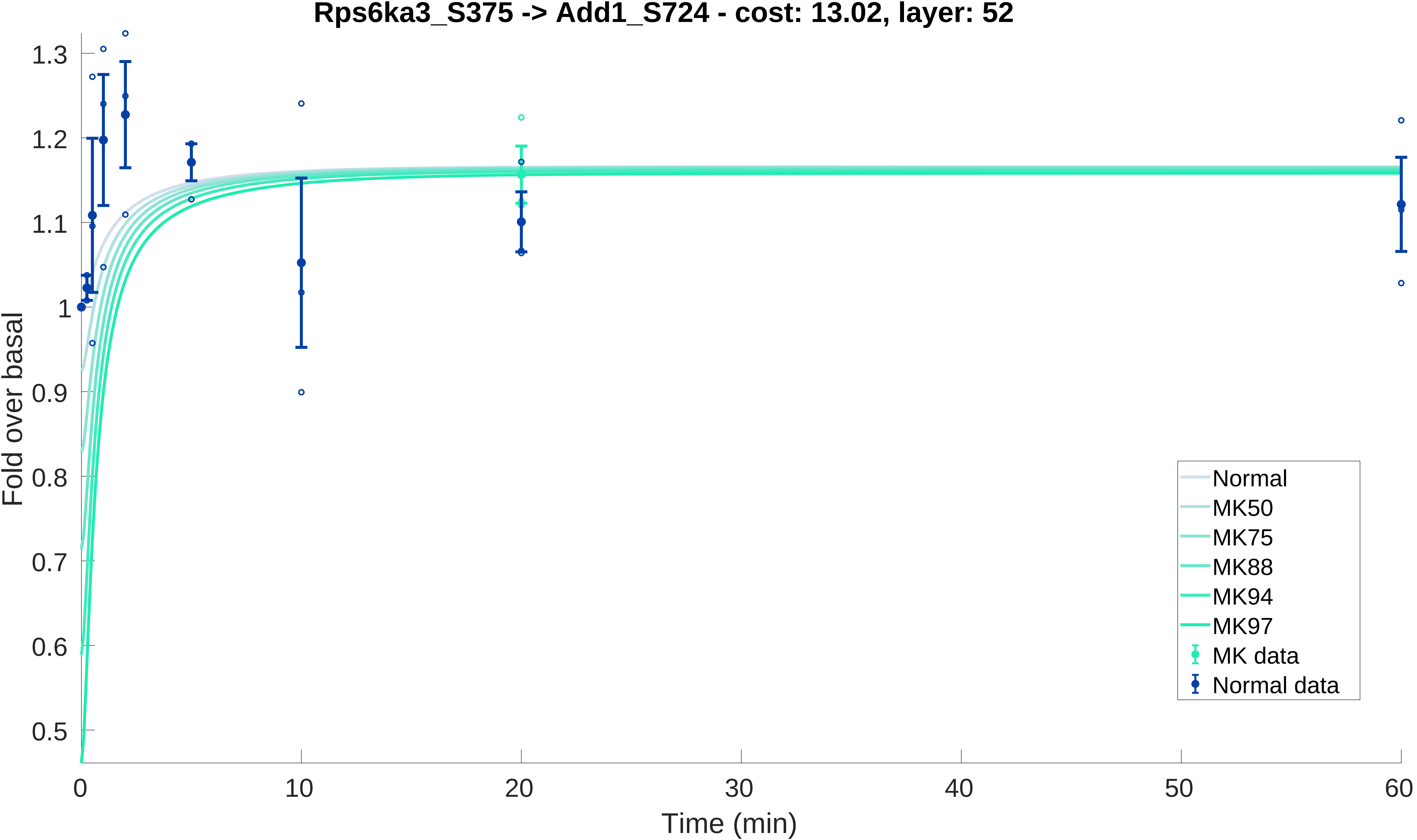

Rps6ka3_S375 -> Add1_S724 - cost: 13.02, layer: 52
Fold over basal
1.3
1.2
1.1
1
0.9
0.8
0.7
0.6
0.5
0
10
20
30
40
50
60
Time (min)
Normal
MK50
MK75
MK88
MK94
MK97
MK data
Normal data

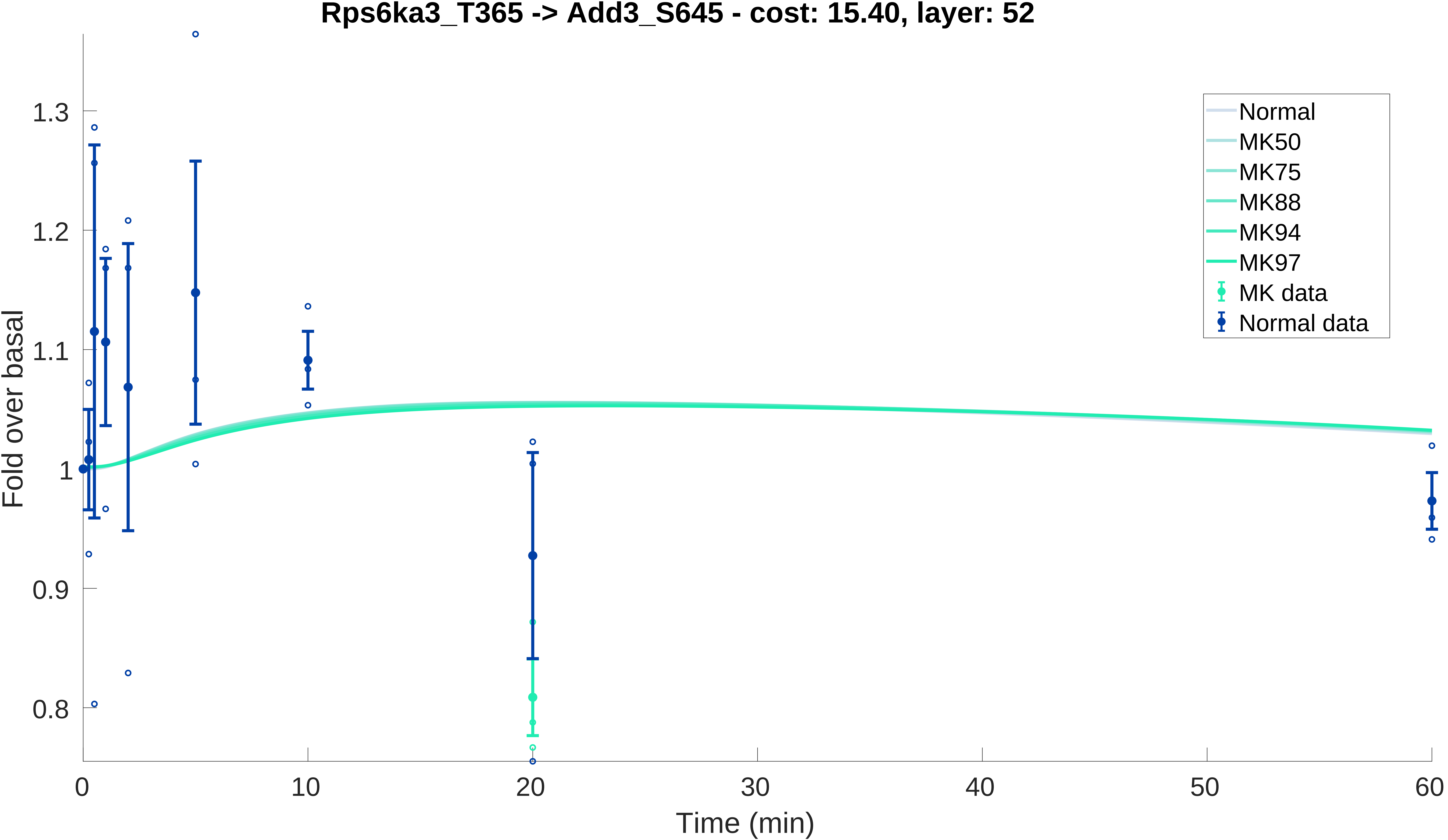
Rps6ka3_T365 -> Add3_S645 - cost: 15.40, layer: 52
Fold over basal
Time (min)
0
10
20
30
40
50
60
0.8
0.9
1
1.1
1.2
1.3
Normal
MK50
MK75
MK88
MK94
MK97
MK data
Normal data

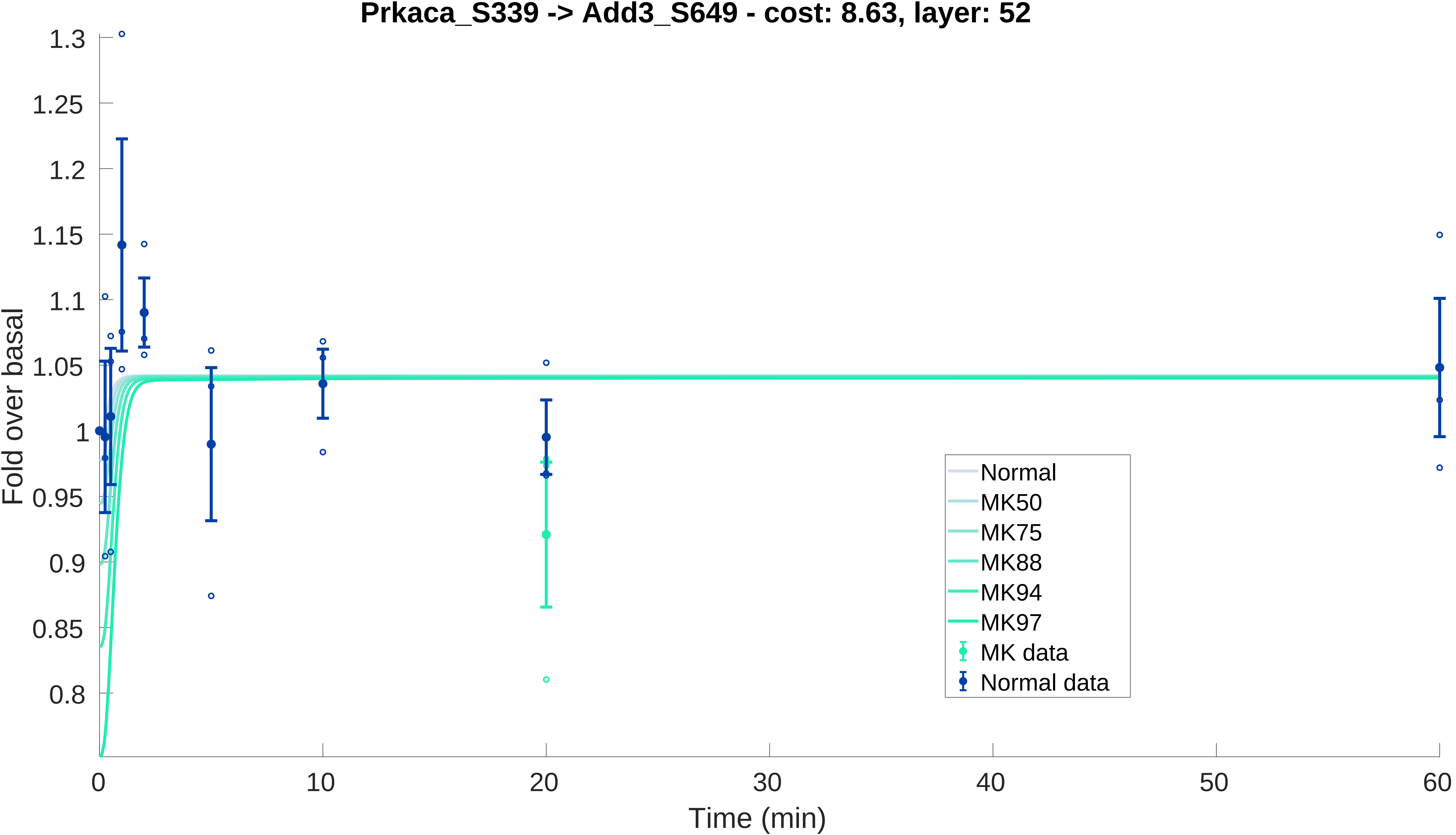
Prkaca_S339 -> Add3_S649 - cost: 8.63, layer: 52
Fold over basal
1.3
1.25
1.2
1.15
1.1
1.05
1
0.95
0.9
0.85
0.8
0
10
20
30
40
50
60
Time (min)
Normal
MK50
MK75
MK88
MK94
MK97
MK data
Normal data

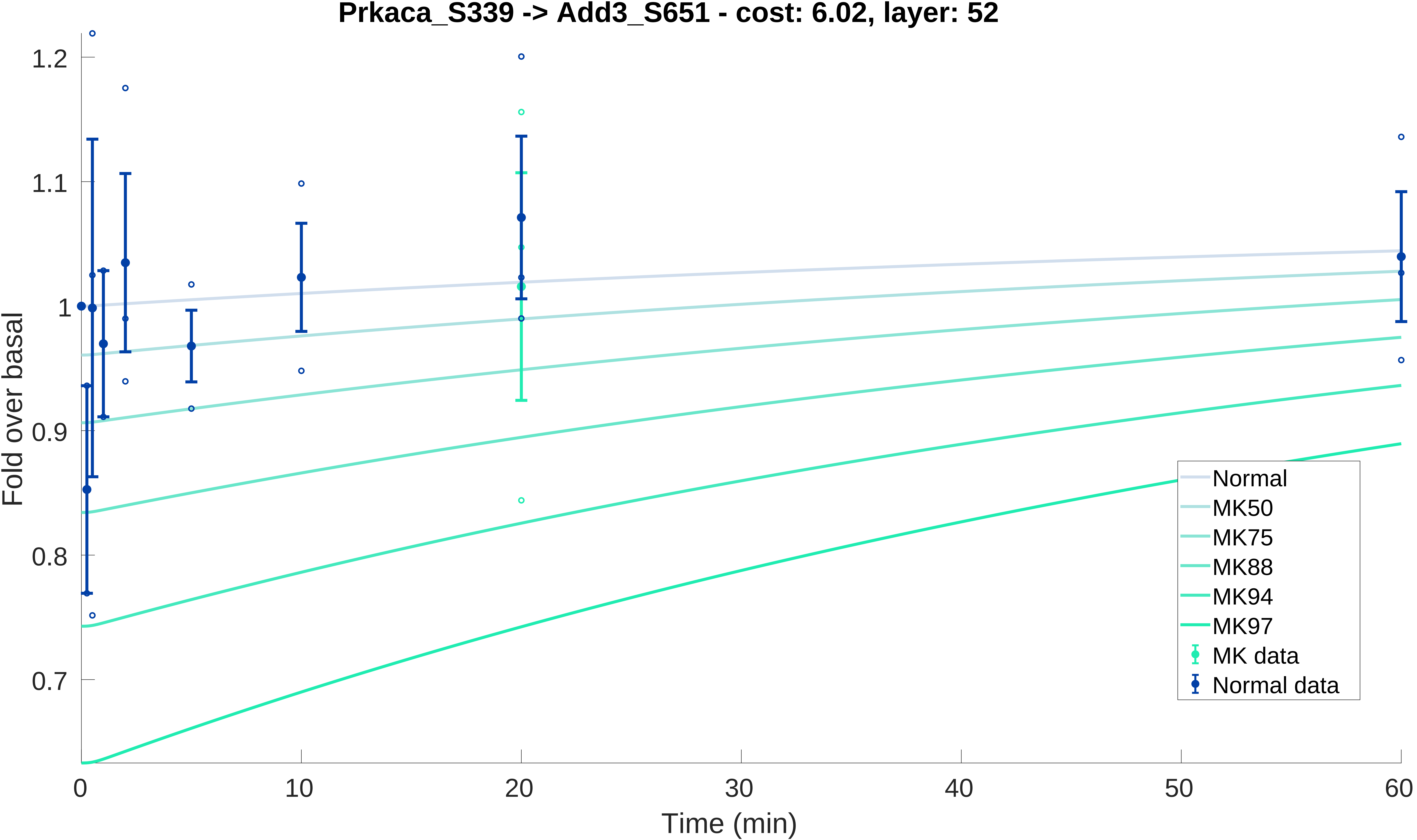
Prrkaca_S339 -> Add3_S651 - cost: 6.02, layer: 52
Fold over basal
Time (min)
1.2
1.1
1
0.9
0.8
0.7
0
10
20
30
40
50
60
Normal
MK50
MK75
MK88
MK94
MK97
MK data
Normal data

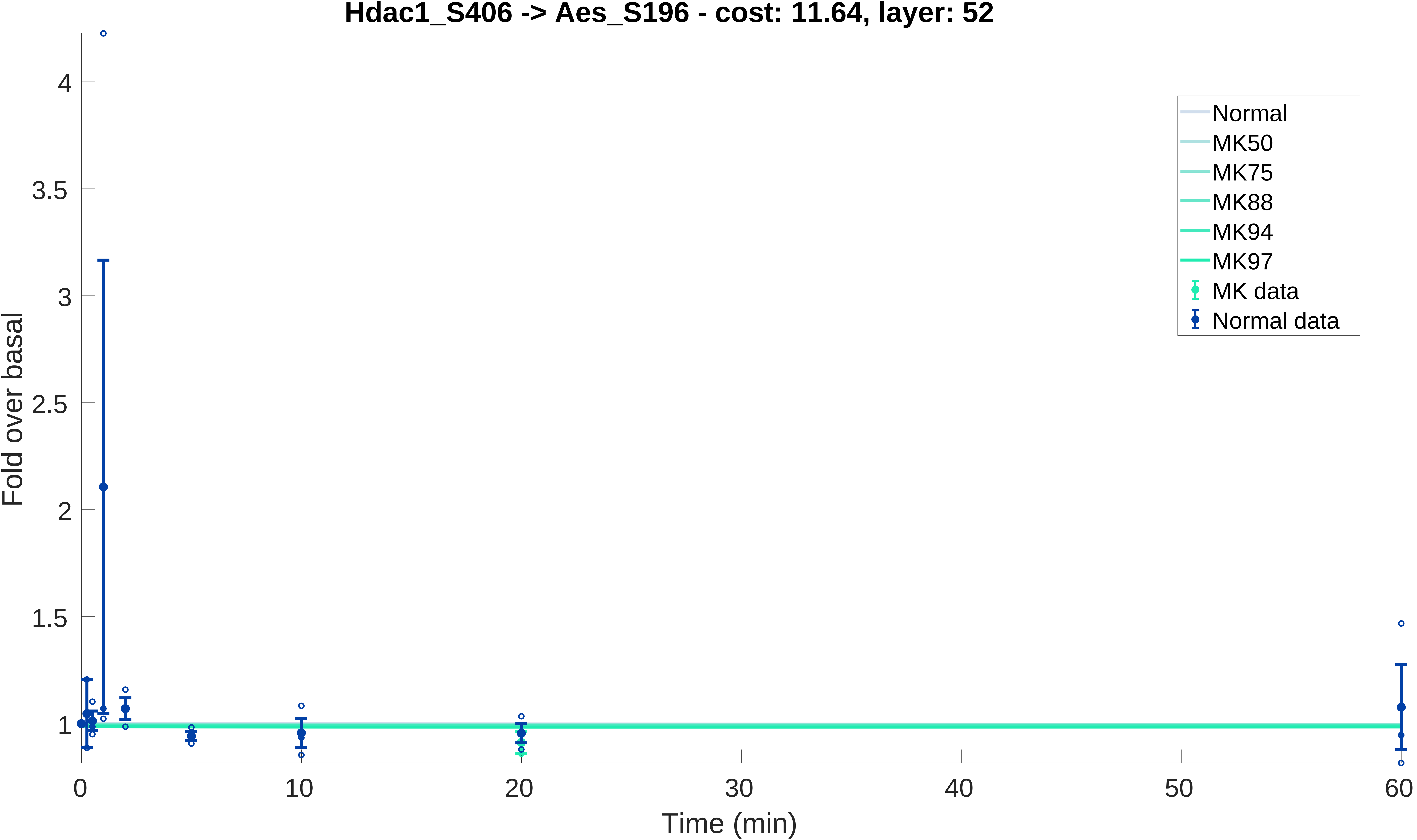

Hdac1_S406 -> Aes_S196 - cost: 11.64, layer: 52
Fold over basal
Time (min)
Normal
MK50
MK75
MK88
MK94
MK97
MK data
Normal data
4
3.5
3
2.5
2
1.5
1
0
10
20
30
40
50
60

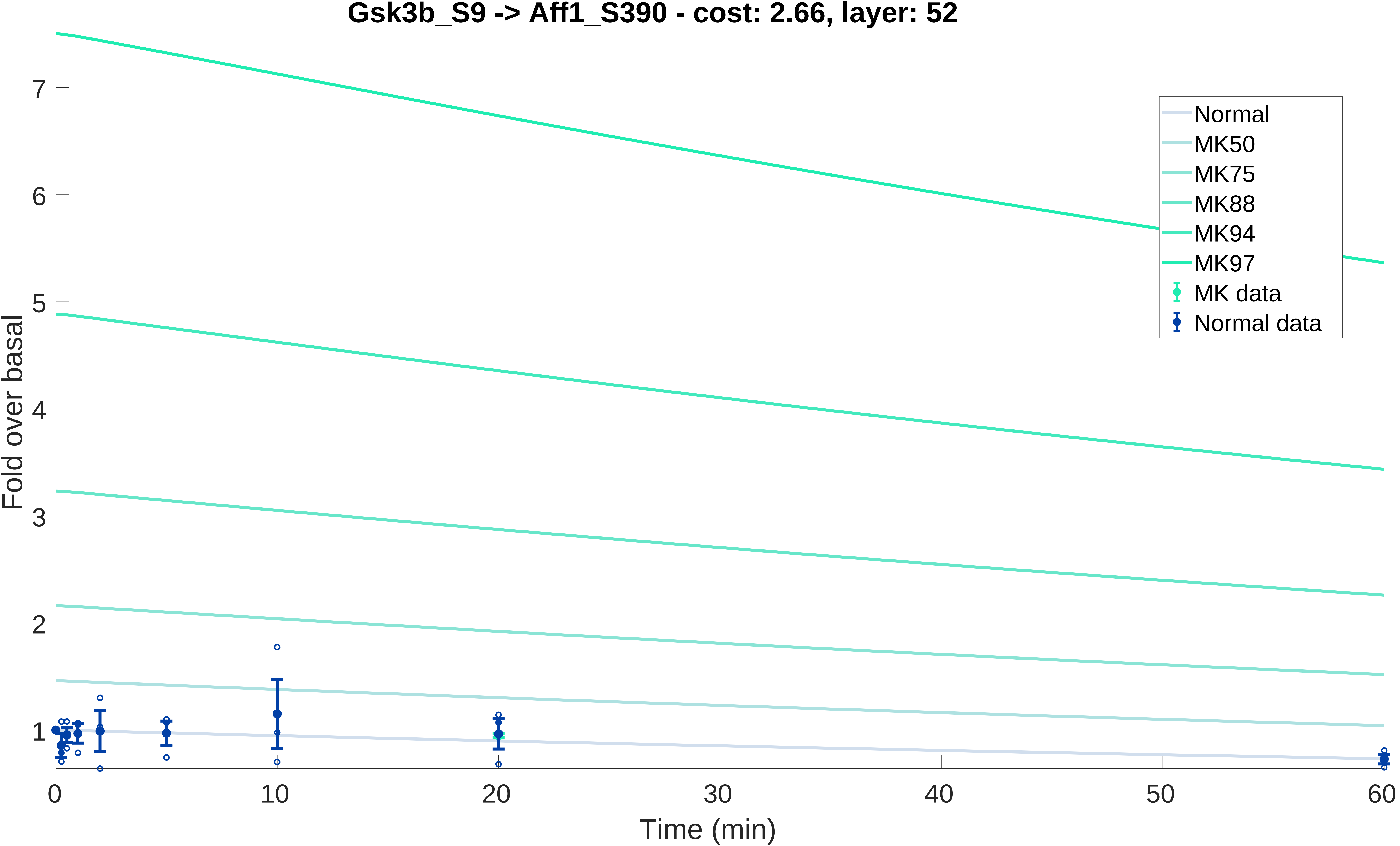

Gsk3b_S9 -> Aff1_S390 - cost: 2.66, layer: 52
Normal
MK50
MK75
MK88
MK94
MK97
MK data
Normal data
Fold over basal
Time (min)
1
2
3
4
5
6
7
0
10
20
30
40
50
60

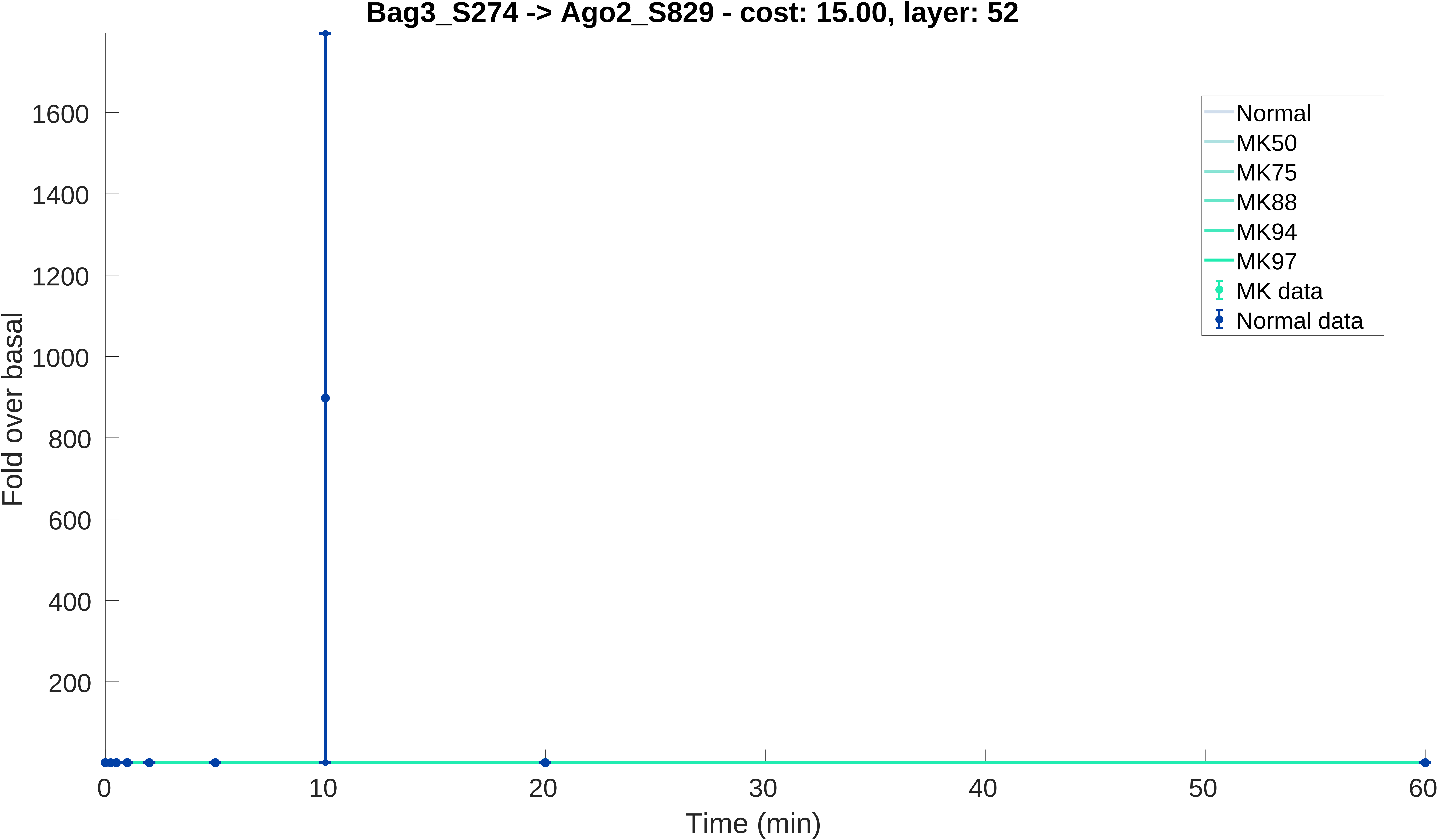

Bag3_S274 -> Ago2_S829 - cost: 15.00, layer: 52
Fold over basal
1600
1400
1200
1000
800
600
400
200
0
10
20
30
40
50
60
Time (min)
Normal
MK50
MK75
MK88
MK94
MK97
MK data
Normal data

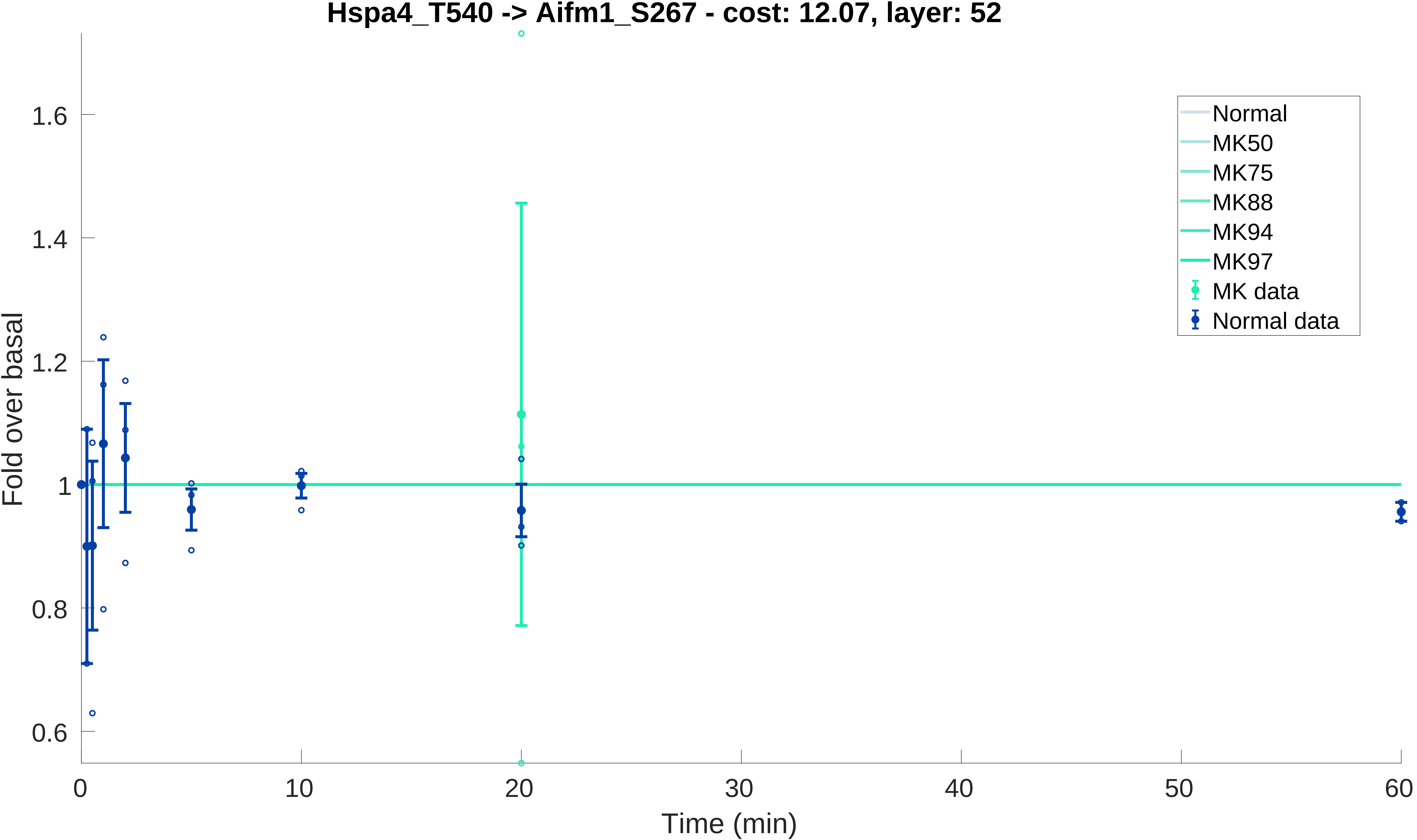
Hspa4_T540 -> Aifm1_S267 - cost: 12.07, layer: 52
Fold over basal
Time (min)
0.6
0.8
1
1.2
1.4
1.6
0
10
20
30
40
50
60
Normal
MK50
MK75
MK88
MK94
MK97
MK data
Normal data

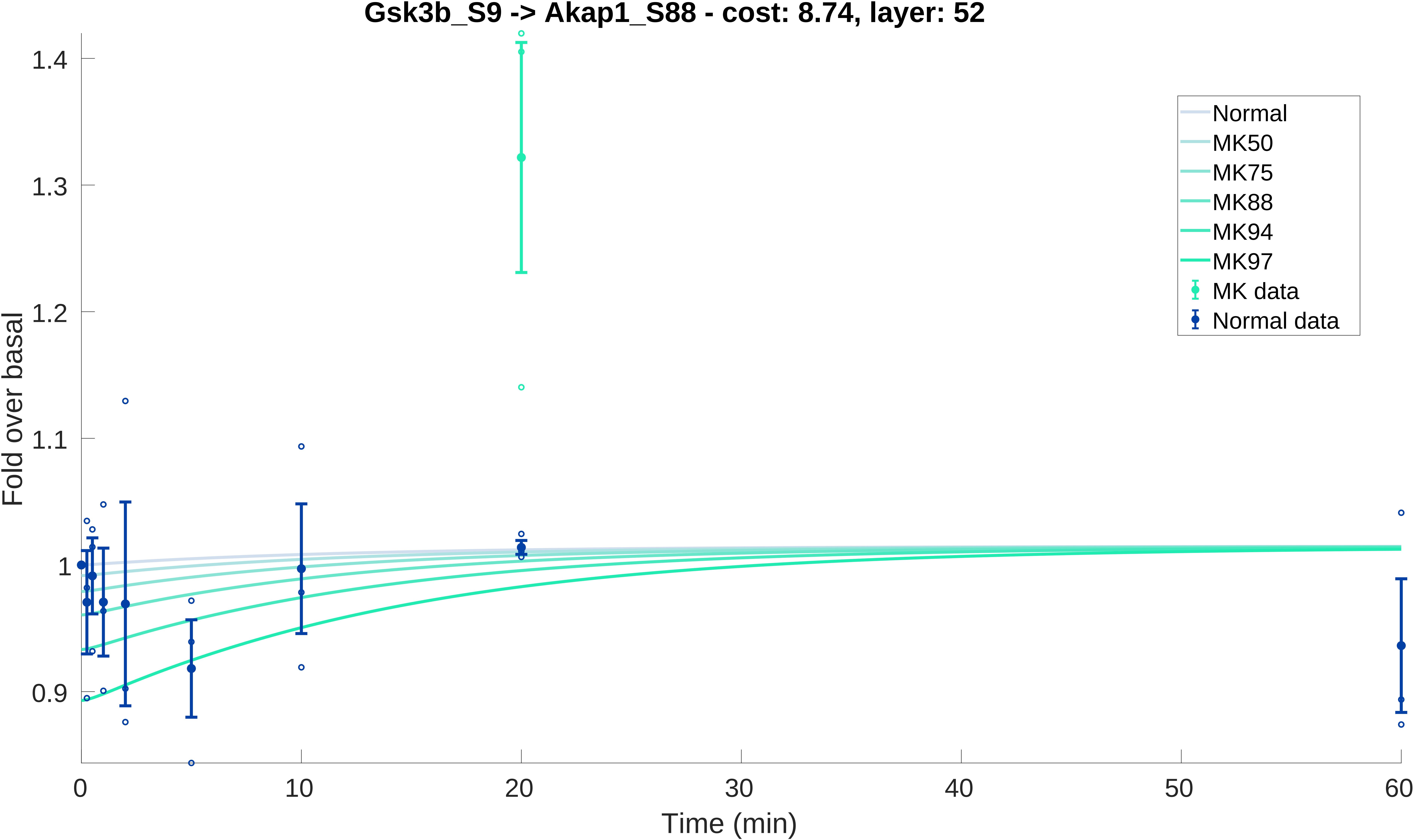

Gsk3b_S9 -> Akap1_S88 - cost: 8.74, layer: 52
Fold over basal
Time (min)
1.4
1.3
1.2
1.1
1
0.9
0
10
20
30
40
50
60
Normal
MK50
MK75
MK88
MK94
MK97
MK data
Normal data

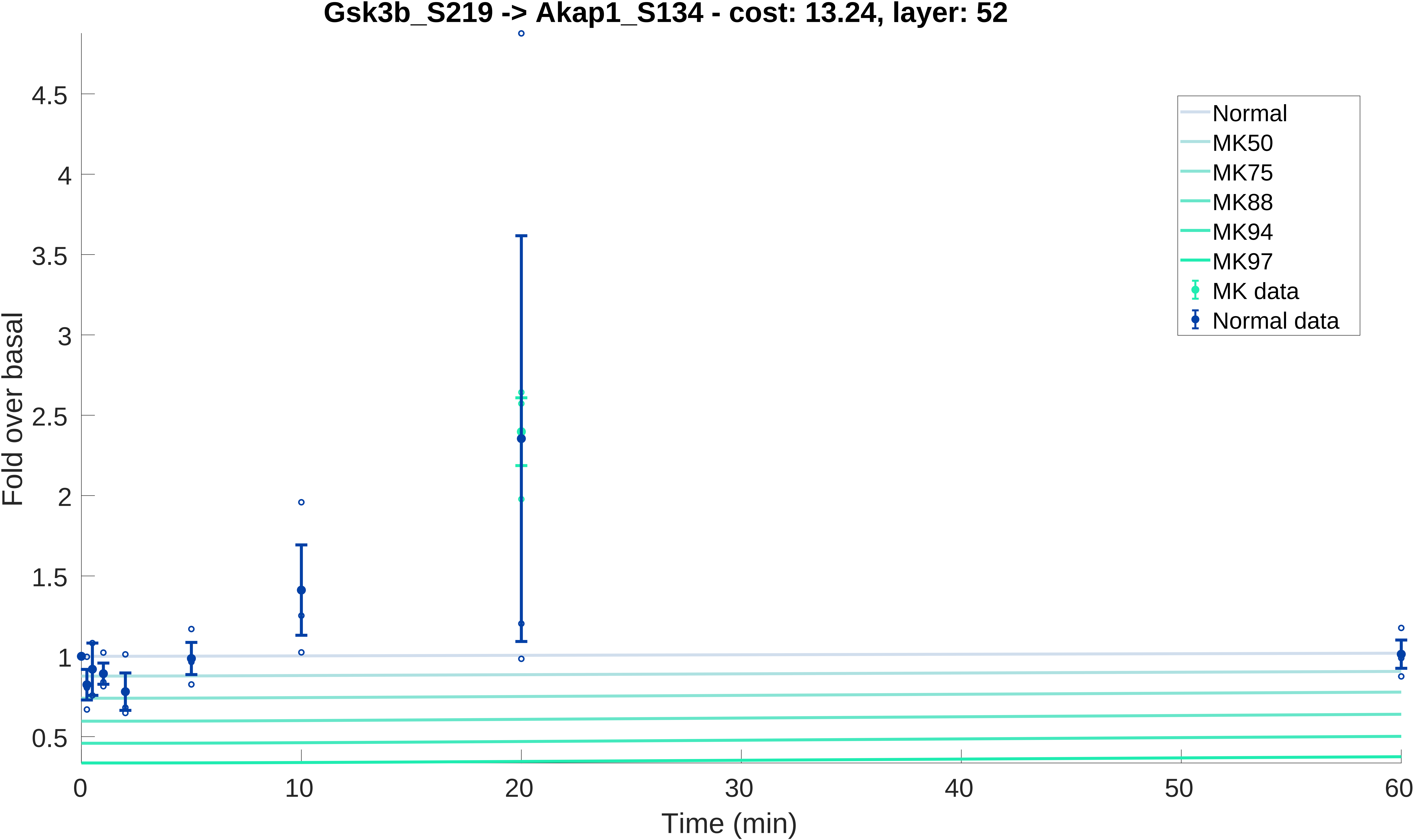

Gsk3b_S219 -> Akap1_S134 - cost: 13.24, layer: 52
Fold over basal
Time (min)
Normal
MK50
MK75
MK88
MK94
MK97
MK data
Normal data
0.5
1
1.5
2
2.5
3
3.5
4
4.5
0
10
20
30
40
50
60

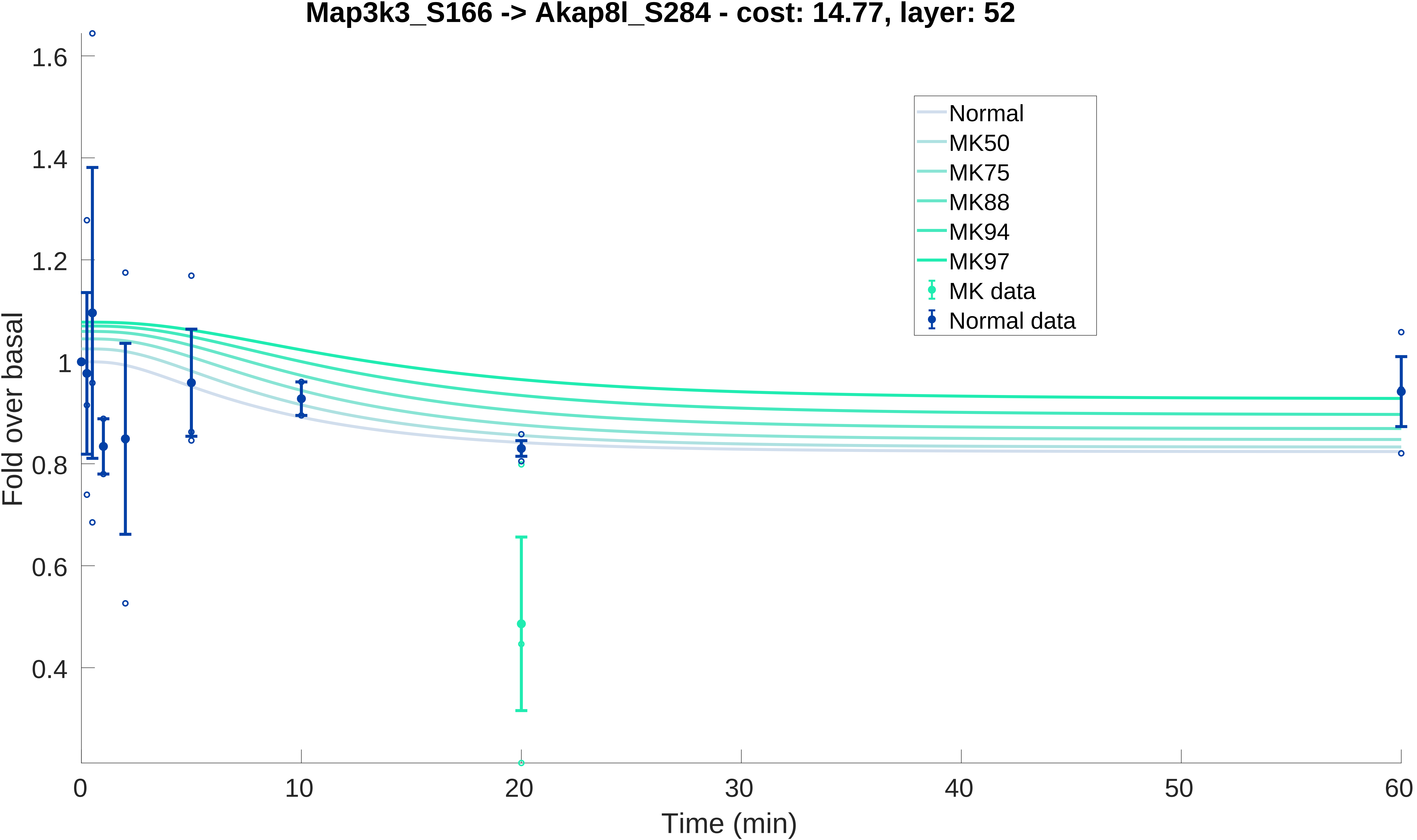

Map3k3_S166 -> Akap8l_S284 - cost: 14.77, layer: 52
Fold over basal
1.6
1.4
1.2
1
0.8
0.6
0.4
0
10
20
30
40
50
60
Time (min)
Normal
MK50
MK75
MK88
MK94
MK97
MK data
Normal data

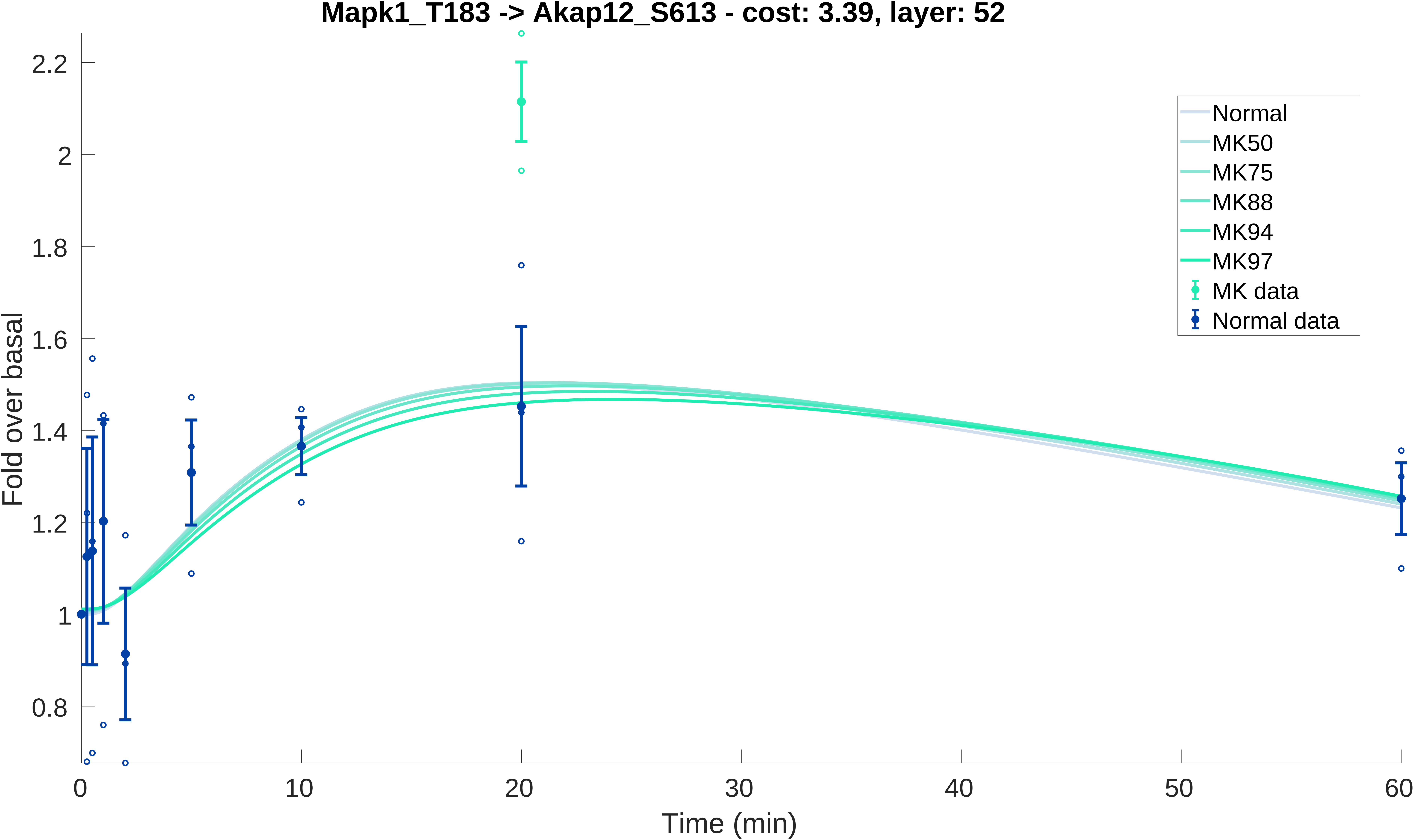

Mapk1_T183 -> Akap12_S613 - cost: 3.39, layer: 52
Fold over basal
Time (min)
0.8
1
1.2
1.4
1.6
1.8
2
2.2
0
10
20
30
40
50
60
Normal
MK50
MK75
MK88
MK94
MK97
MK data
Normal data

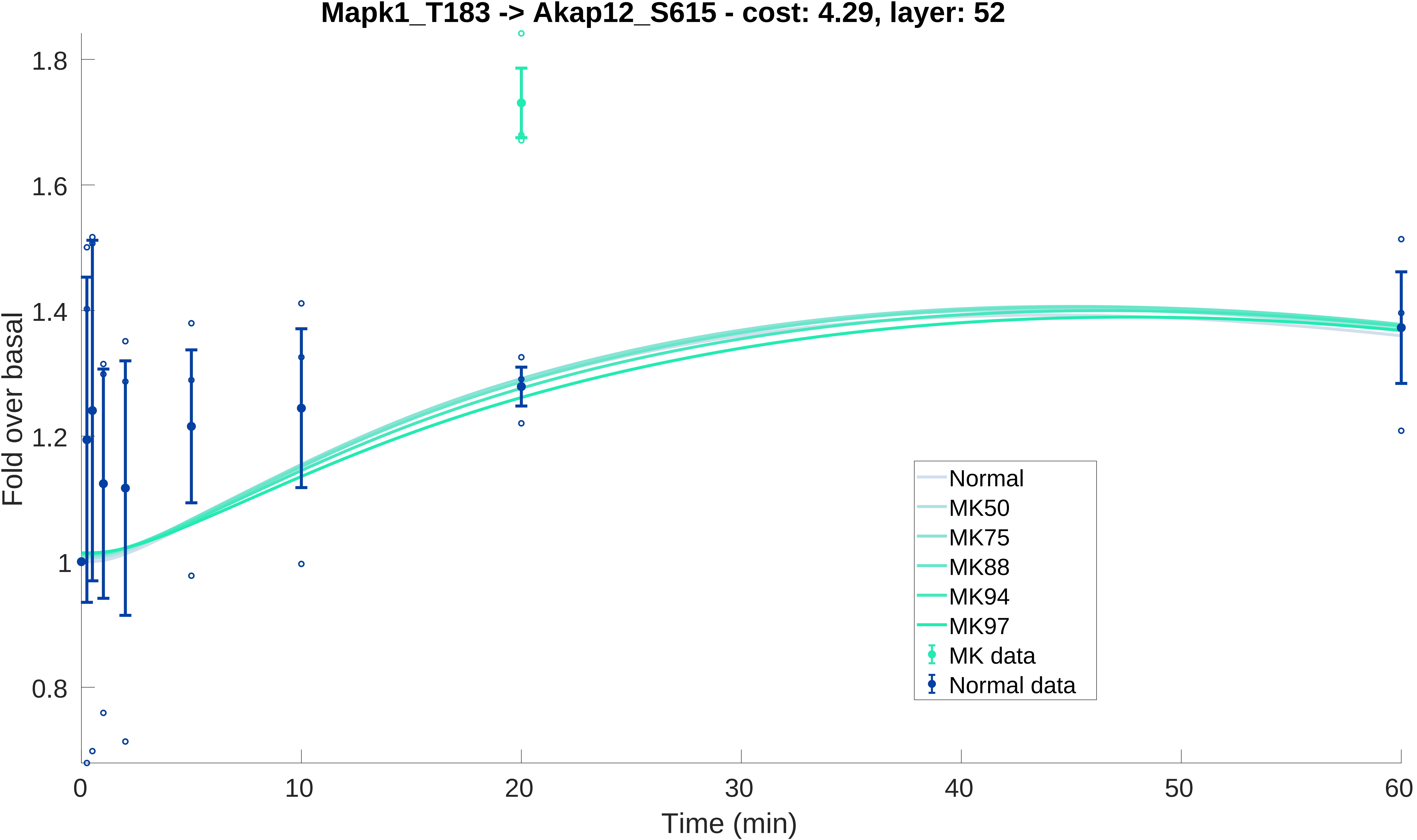
Mapk1_T183 -> Akap12_S615 - cost: 4.29, layer: 52
Fold over basal
1.8
1.6
1.4
1.2
1
0.8
0
10
20
30
40
50
60
Time (min)
Normal
MK50
MK75
MK88
MK94
MK97
MK data
Normal data

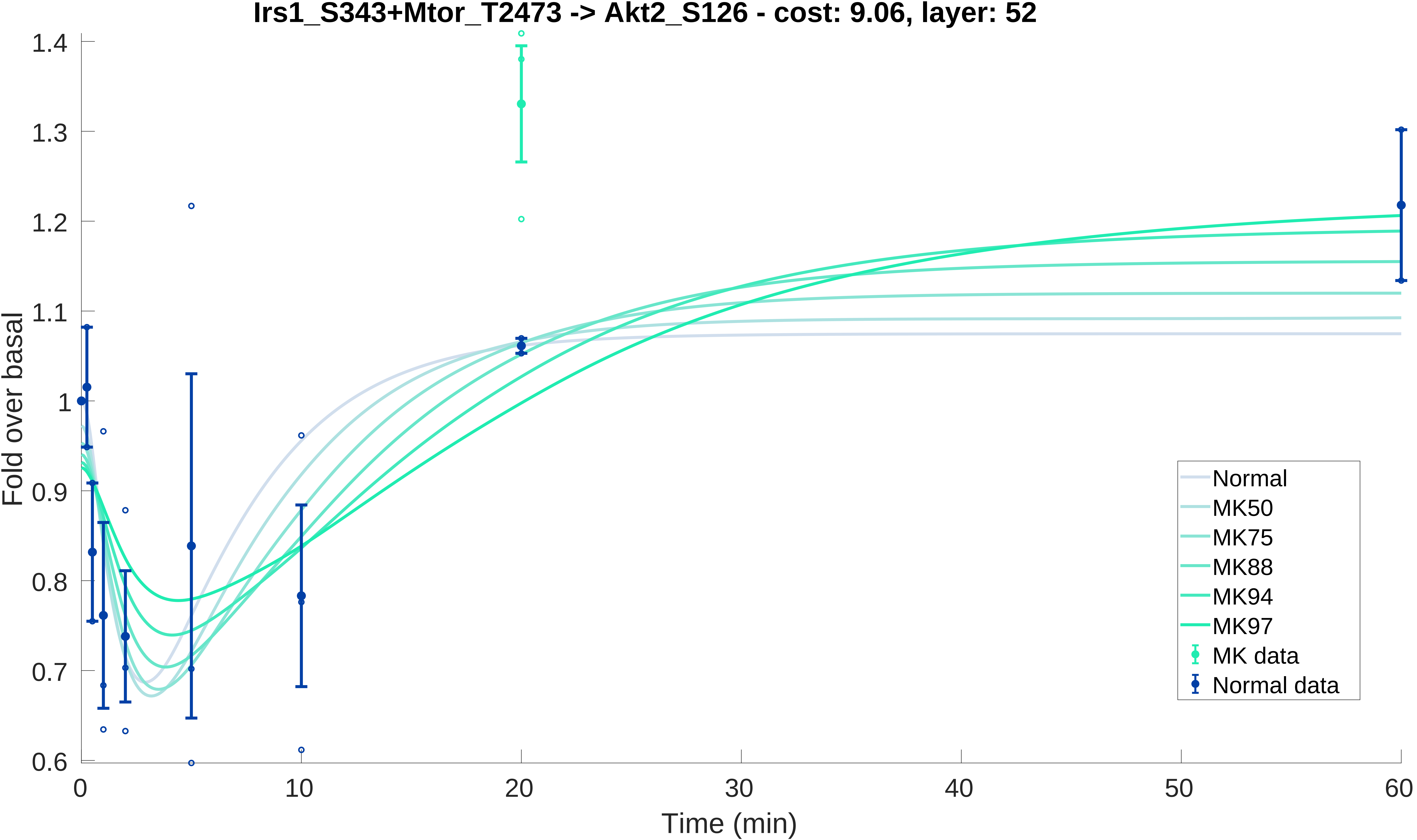

Irs1_S343+Mtor_T2473 -> Akt2_S126 - cost: 9.06, layer: 52
Fold over basal
Time (min)
1.4
1.3
1.2
1.1
1
0.9
0.8
0.7
0.6
0
10
20
30
40
50
60
Normal
MK50
MK75
MK88
MK94
MK97
MK data
Normal data

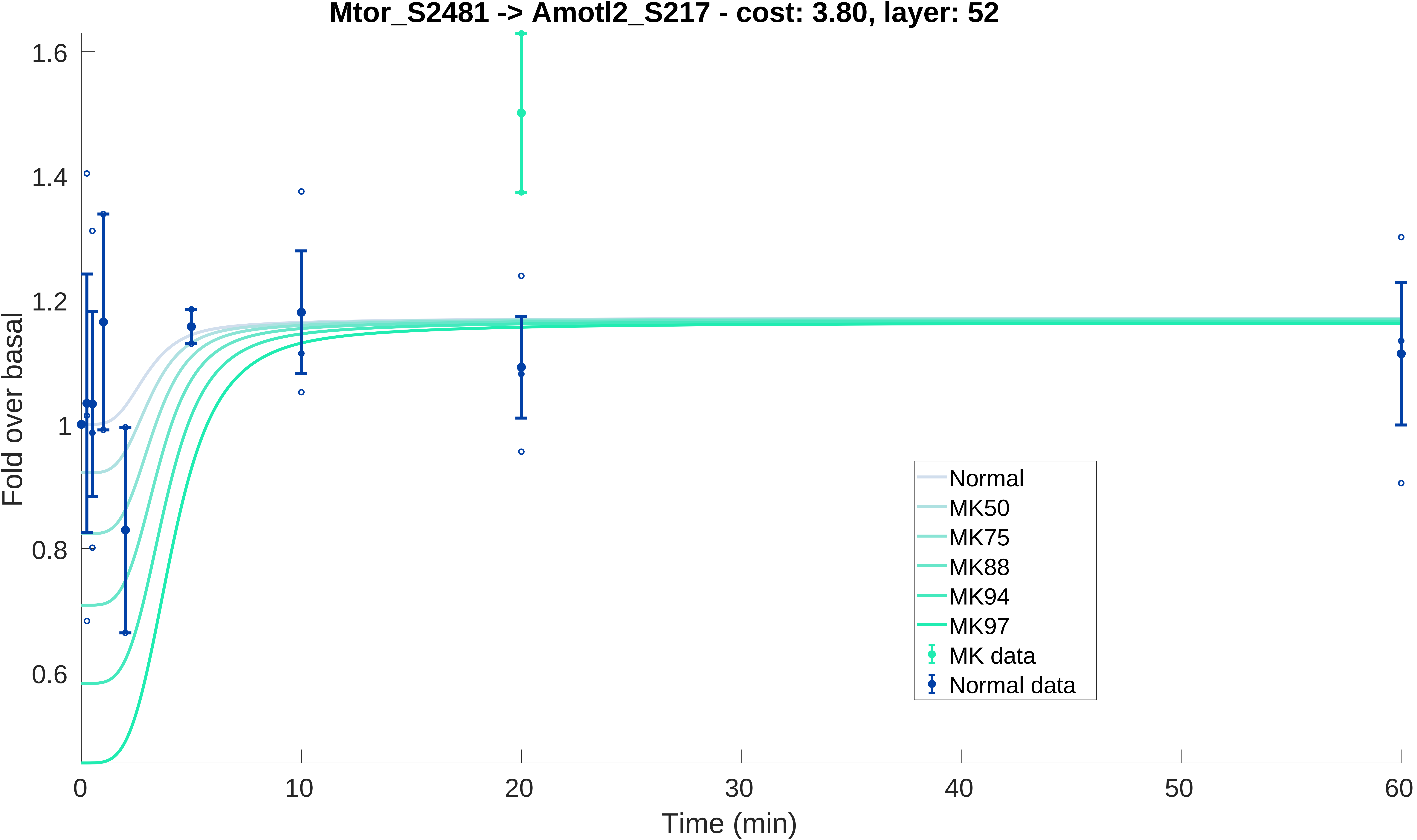

Mtor_S2481 -> Amotl2_S217 - cost: 3.80, layer: 52
Fold over basal
Time (min)
1.6
1.4
1.2
1
0.8
0.6
0
10
20
30
40
50
60
Normal
MK50
MK75
MK88
MK94
MK97
MK data
Normal data

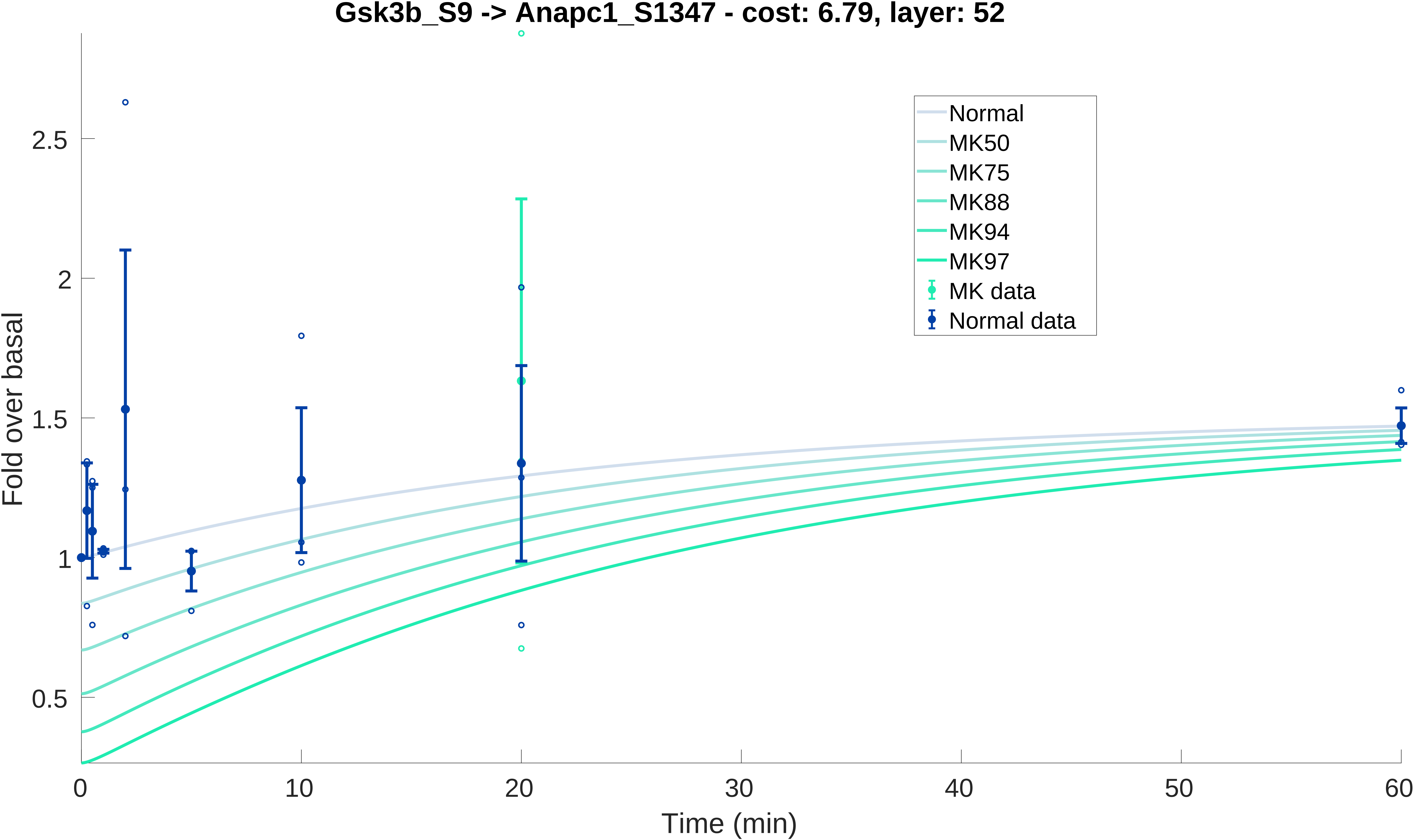

Gsk3b_S9 -> Anapc1_S1347 - cost: 6.79, layer: 52
Fold over basal
0.5
1
1.5
2
2.5
0
10
20
30
40
50
60
Time (min)
Normal
MK50
MK75
MK88
MK94
MK97
MK data
Normal data

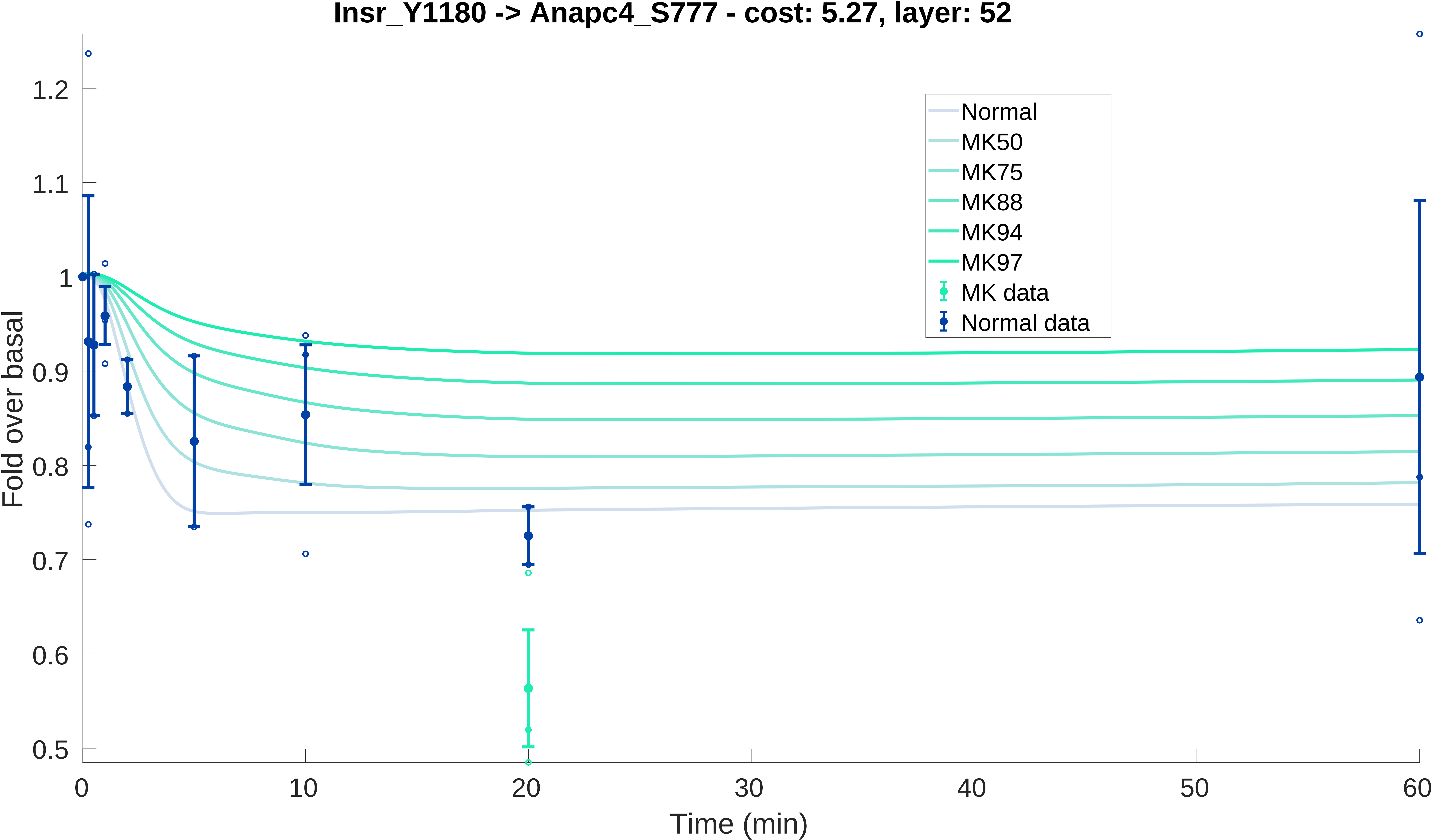
Insr_Y1180 -> Anapc4_S777 - cost: 5.27, layer: 52
Fold over basal
Time (min)
0
10
20
30
40
50
60
0.5
0.6
0.7
0.8
0.9
1
1.1
1.2
Normal
MK50
MK75
MK88
MK94
MK97
MK data
Normal data

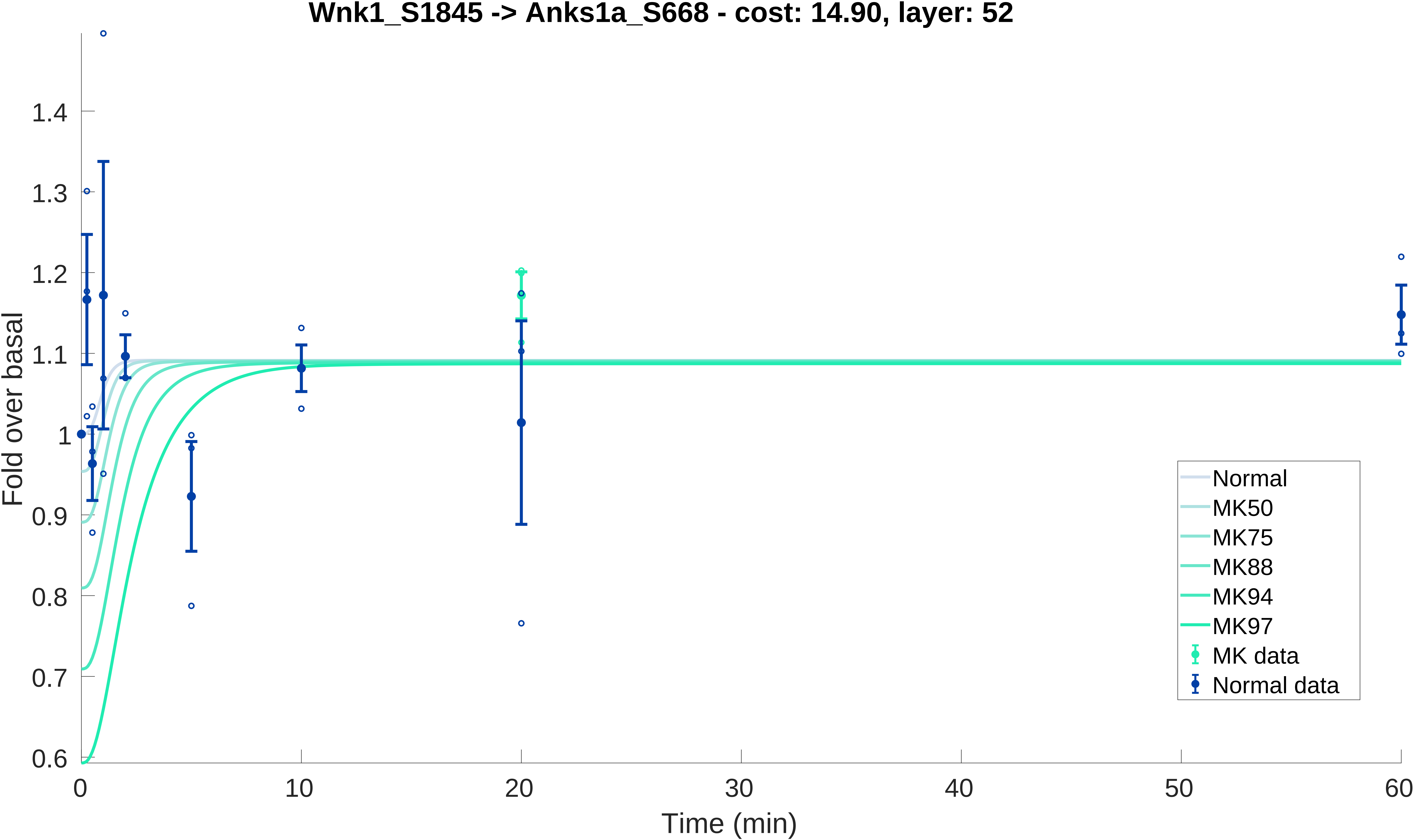
Wnk1_S1845 -> Anks1a_S668 - cost: 14.90, layer: 52
Fold over basal
1.4
1.3
1.2
1.1
1
0.9
0.8
0.7
0.6
0
10
20
30
40
50
60
Time (min)
Normal
MK50
MK75
MK88
MK94
MK97
MK data
Normal data

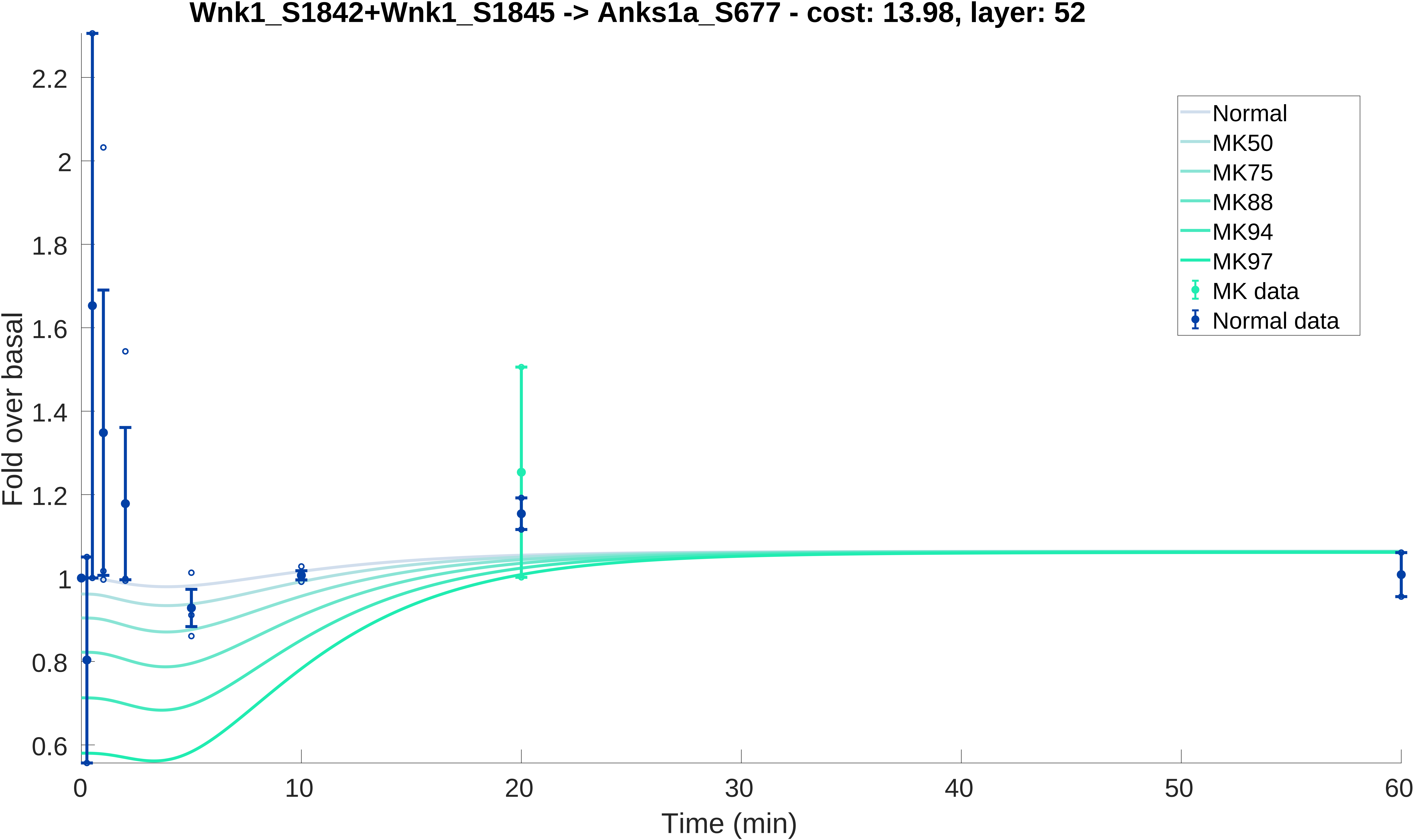
Wnk1_S1842+Wnk1_S1845 -> Anks1a_S677 - cost: 13.98, layer: 52
Fold over basal
Time (min)
2.2
2
1.8
1.6
1.4
1.2
1
0.8
0.6
0
10
20
30
40
50
60
Normal
MK50
MK75
MK88
MK94
MK97
MK data
Normal data

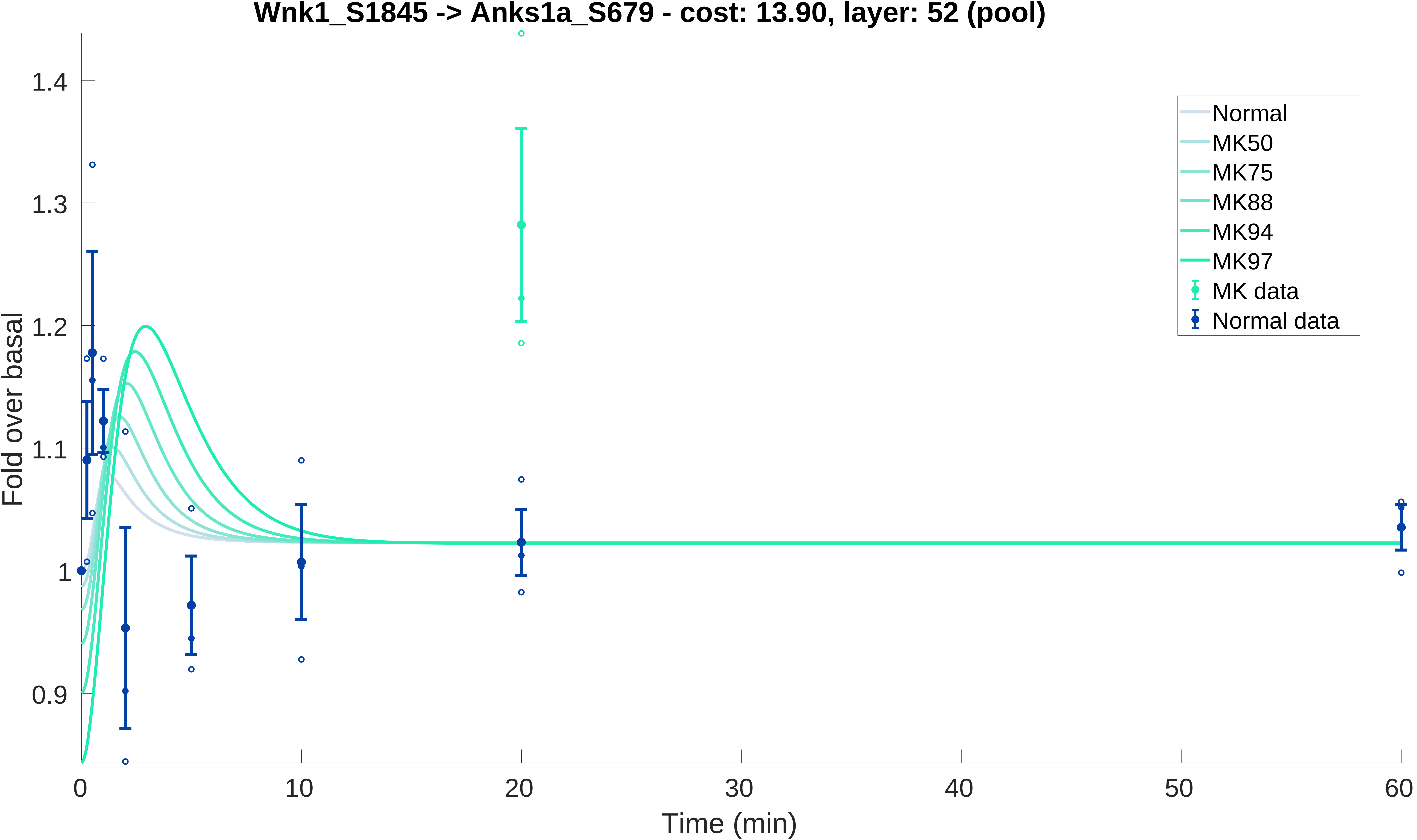
Wnk1_S1845 -> Anks1a_S679 - cost: 13.90, layer: 52 (pool)
Fold over basal
Time (min)
1.4
1.3
1.2
1.1
1
0.9
0
10
20
30
40
50
60
Normal
MK50
MK75
MK88
MK94
MK97
MK data
Normal data

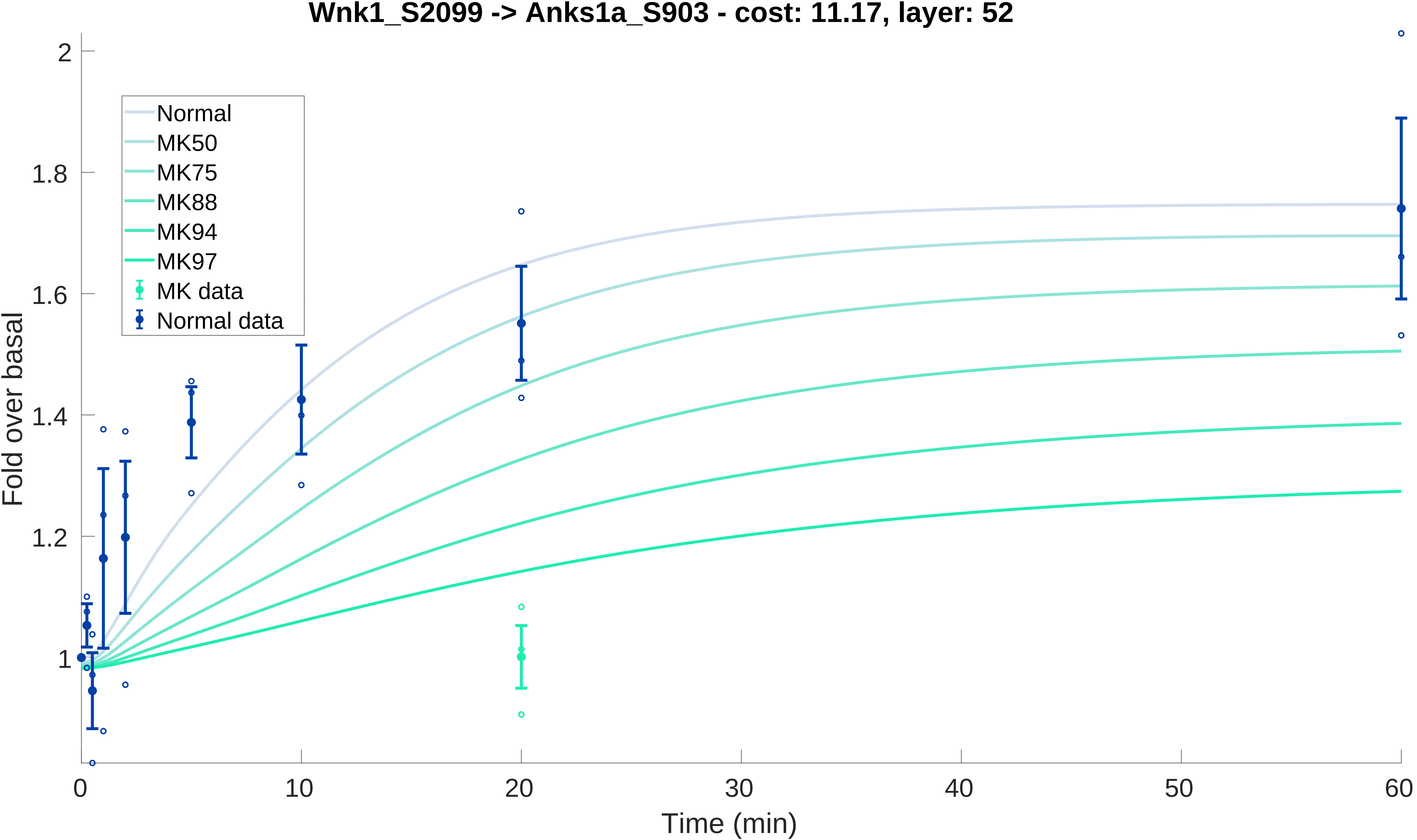

Wnk1_S2099 -> Anks1a_S903 - cost: 11.17, layer: 52
Normal
MK50
MK75
MK88
MK94
MK97
MK data
Normal data
Fold over basal
Time (min)
2
1.8
1.6
1.4
1.2
1
0
10
20
30
40
50
60

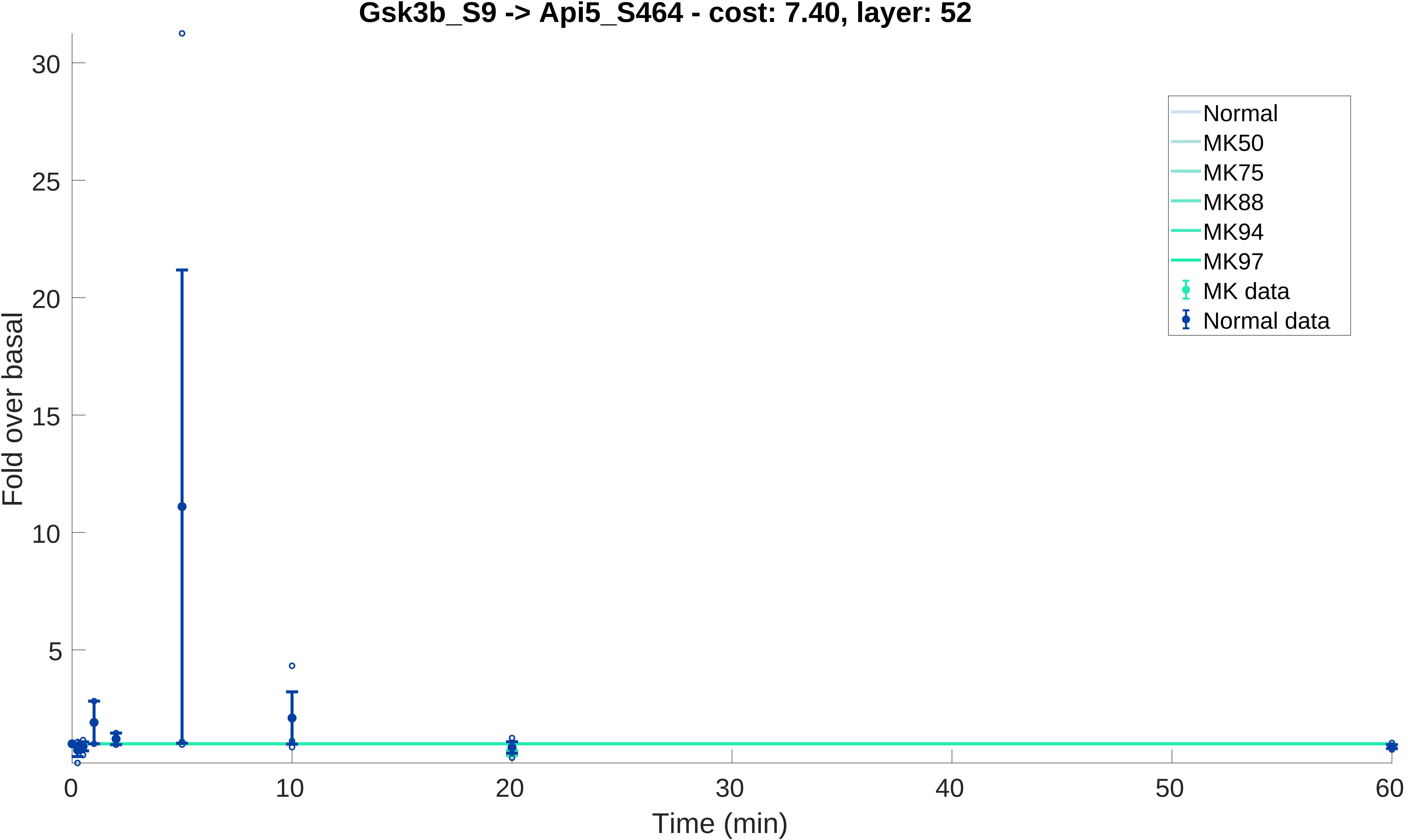

Gsk3b_S9 -> Api5_S464 - cost: 7.40, layer: 52
Fold over basal
Time (min)
0
5
10
15
20
25
30
0
10
20
30
40
50
60
Normal
MK50
MK75
MK88
MK94
MK97
MK data
Normal data

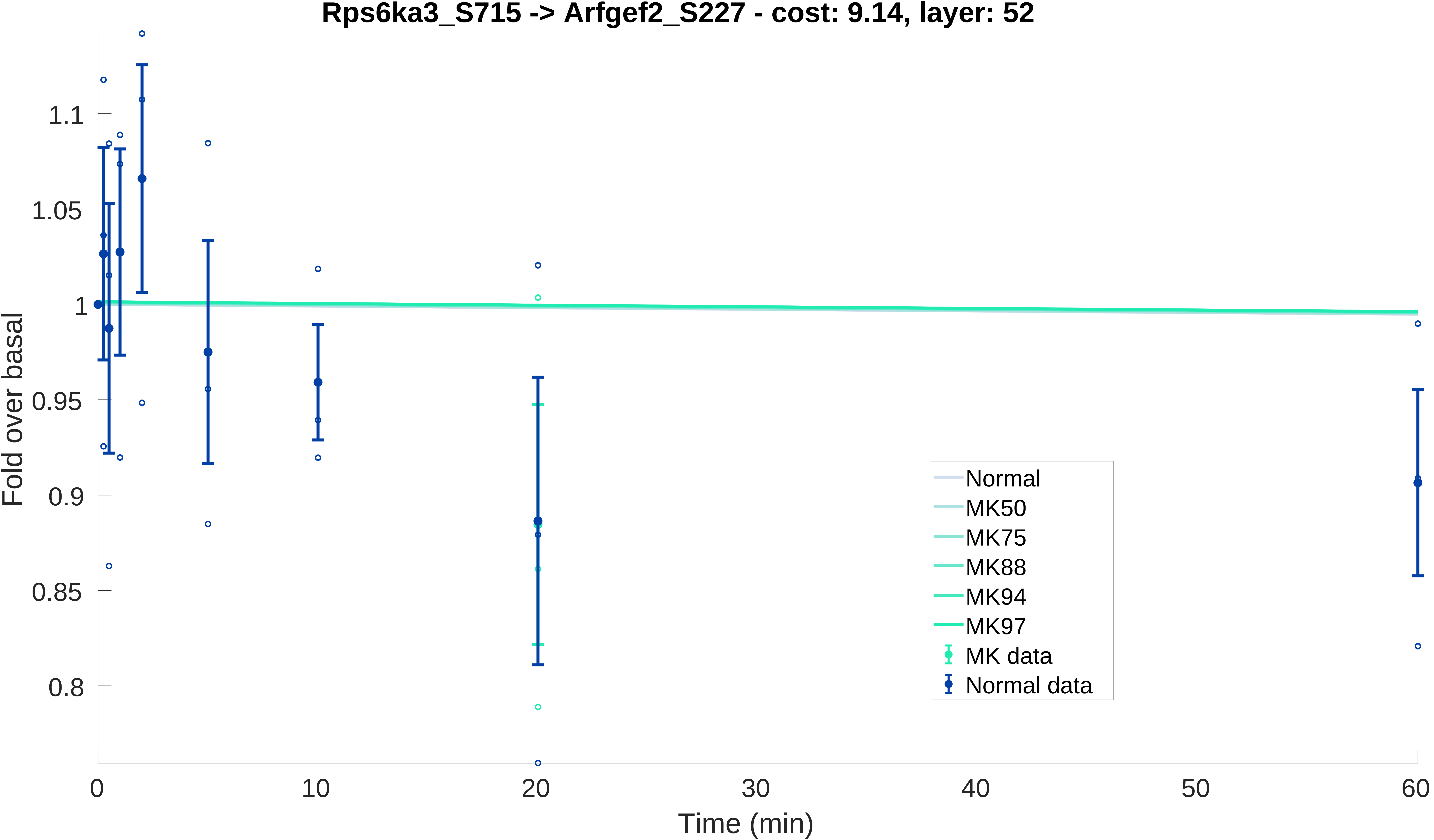

Rps6ka3_S715 -> Arfgef2_S227 - cost: 9.14, layer: 52
Fold over basal
1.1
1.05
1
0.95
0.9
0.85
0.8
0
10
20
30
40
50
60
Time (min)
Normal
MK50
MK75
MK88
MK94
MK97
MK data
Normal data

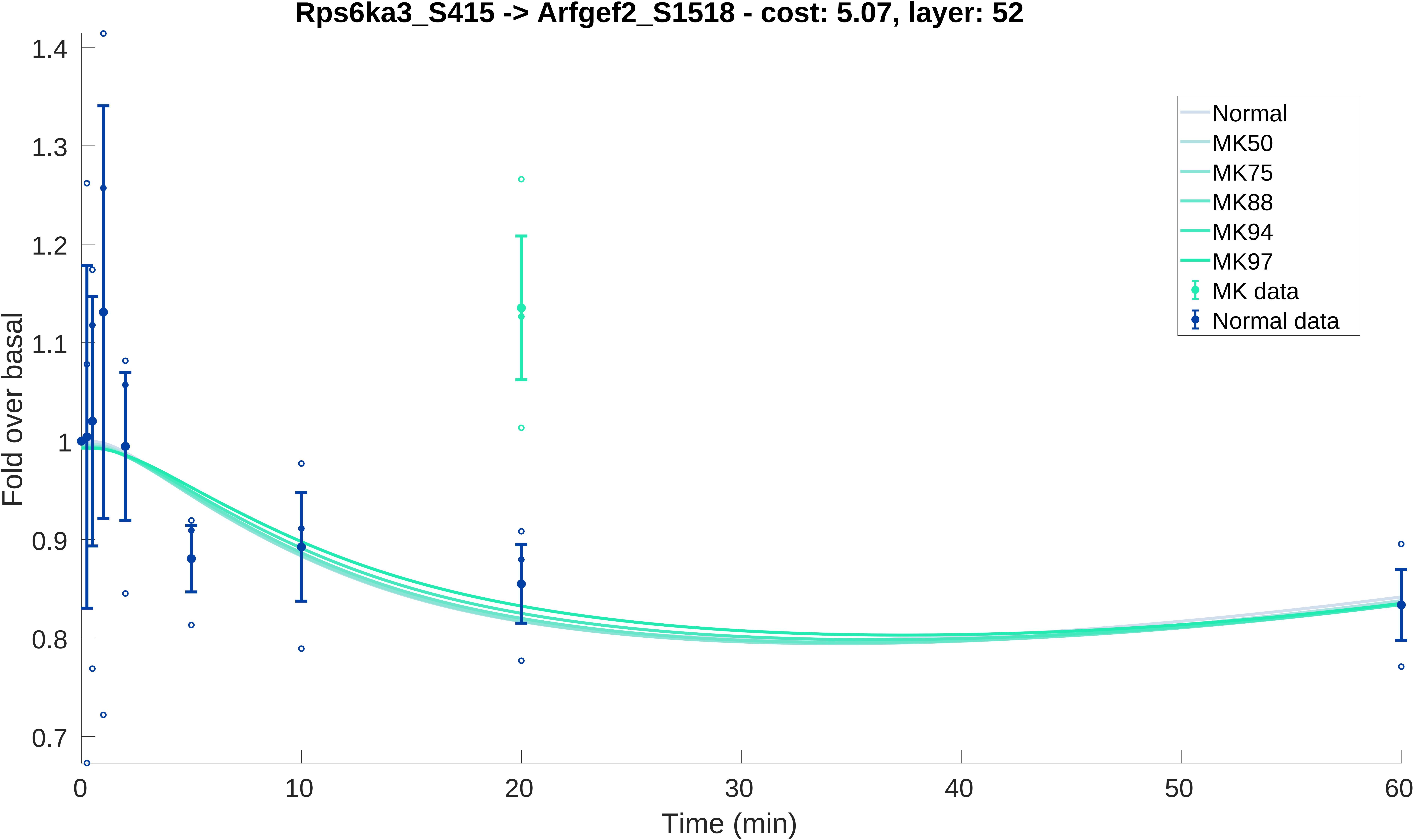

Rps6ka3_S415 -> Arfgef2_S1518 - cost: 5.07, layer: 52
Fold over basal
Time (min)
1.4
1.3
1.2
1.1
1
0.9
0.8
0.7
0
10
20
30
40
50
60
Normal
MK50
MK75
MK88
MK94
MK97
MK data
Normal data

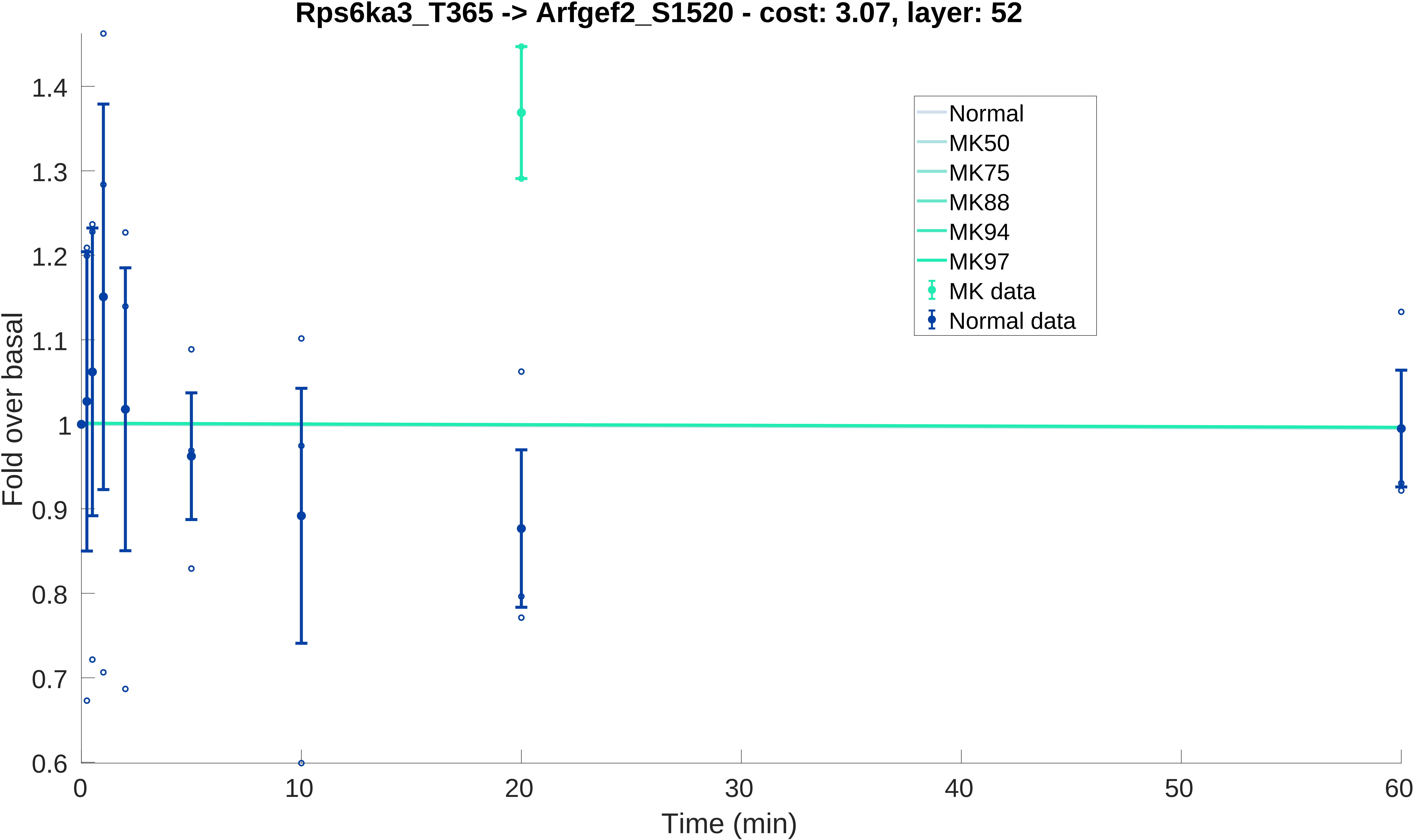
Rps6ka3_T365 -> Arfgef2_S1520 - cost: 3.07, layer: 52
Fold over basal
Time (min)
1.4
1.3
1.2
1.1
1
0.9
0.8
0.7
0.6
0
10
20
30
40
50
60
Normal
MK50
MK75
MK88
MK94
MK97
MK data
Normal data

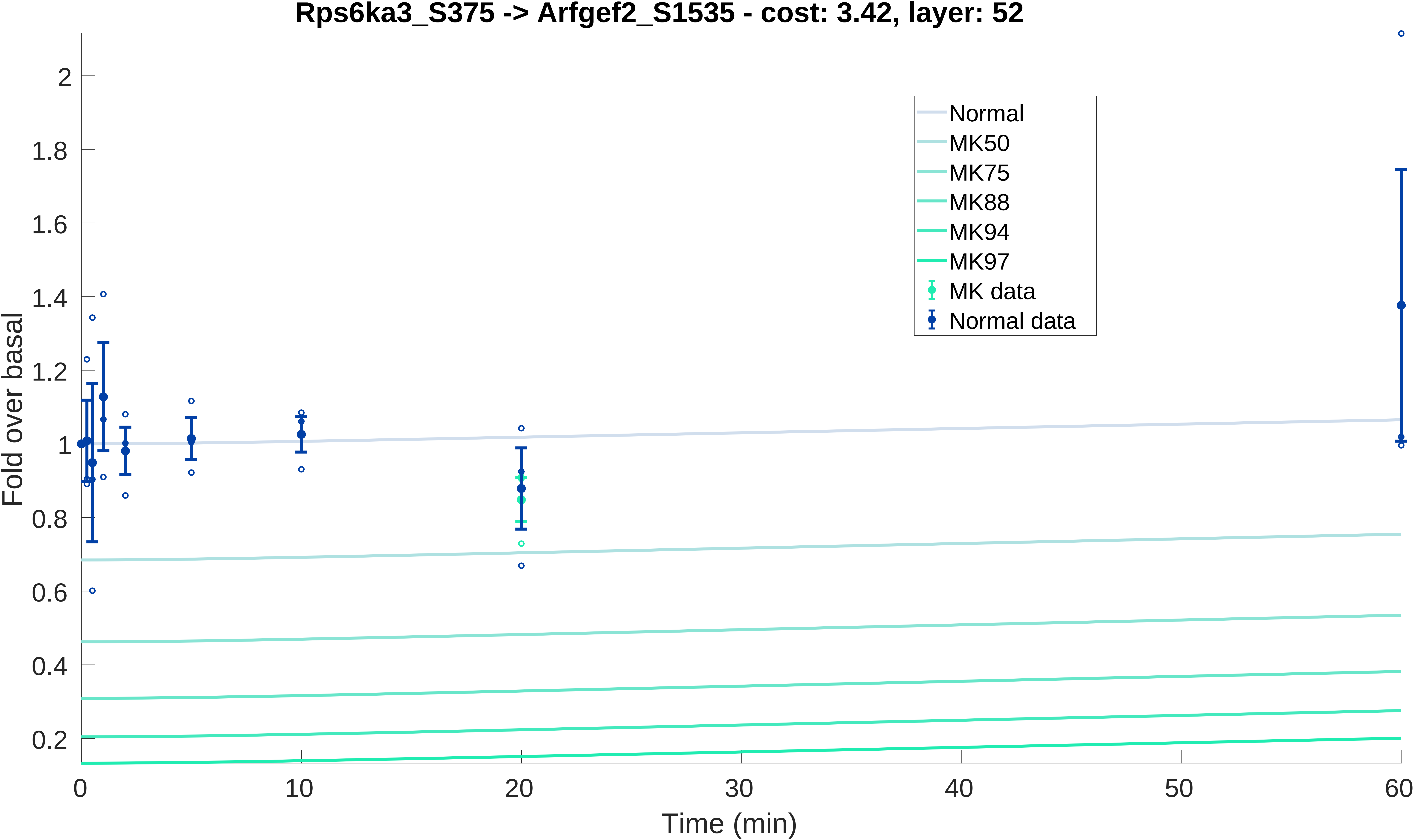

Rps6ka3_S375 -> Arfgef2_S1535 - cost: 3.42, layer: 52
Fold over basal
Time (min)
0
10
20
30
40
50
60
0.2
0.4
0.6
0.8
1
1.2
1.4
1.6
1.8
2
Normal
MK50
MK75
MK88
MK94
MK97
MK data
Normal data

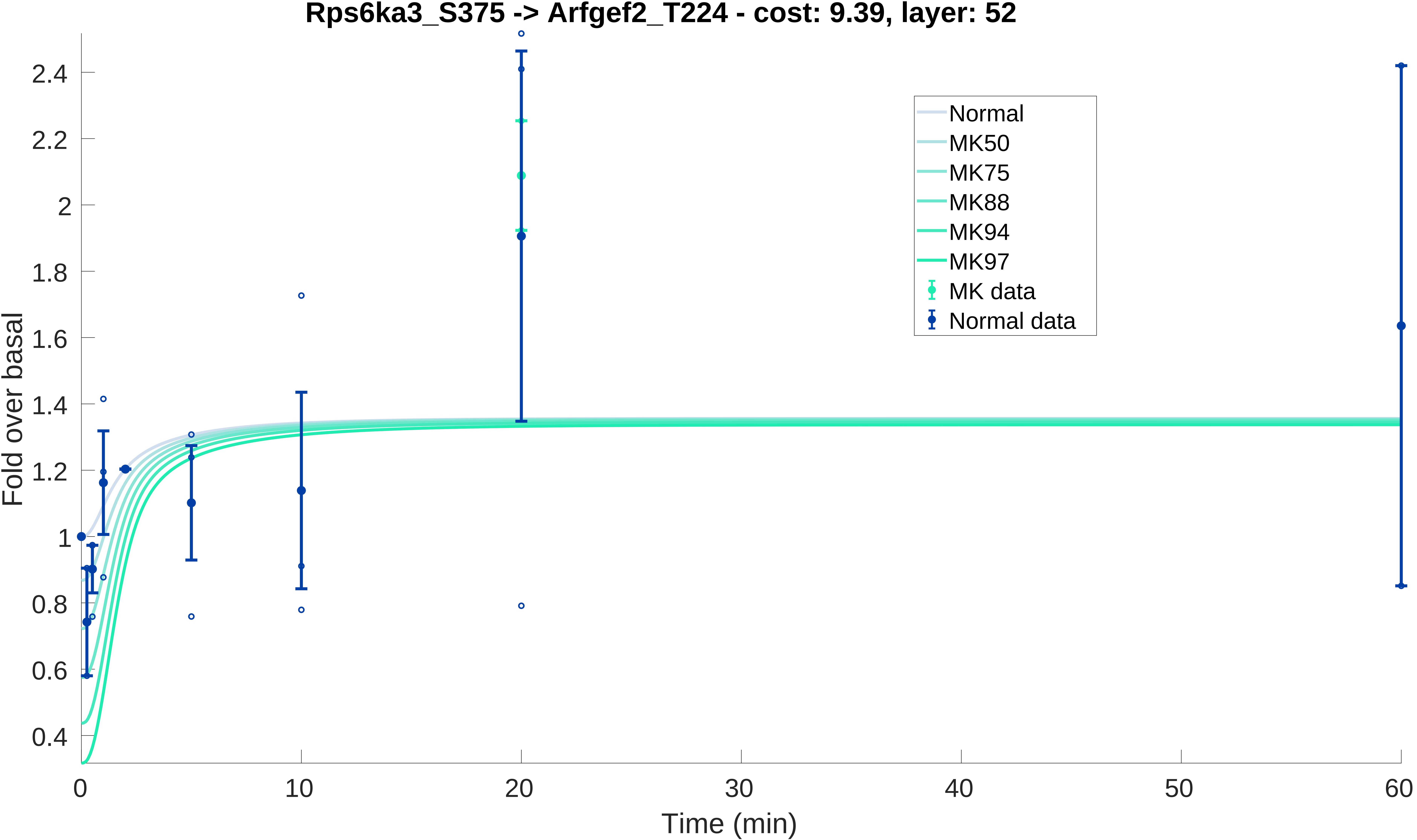

Rps6ka3_S375 -> Arfgef2_T224 - cost: 9.39, layer: 52
Fold over basal
Time (min)
0.4
0.6
0.8
1
1.2
1.4
1.6
1.8
2
2.2
2.4
0
10
20
30
40
50
60
Normal
MK50
MK75
MK88
MK94
MK97
MK data
Normal data

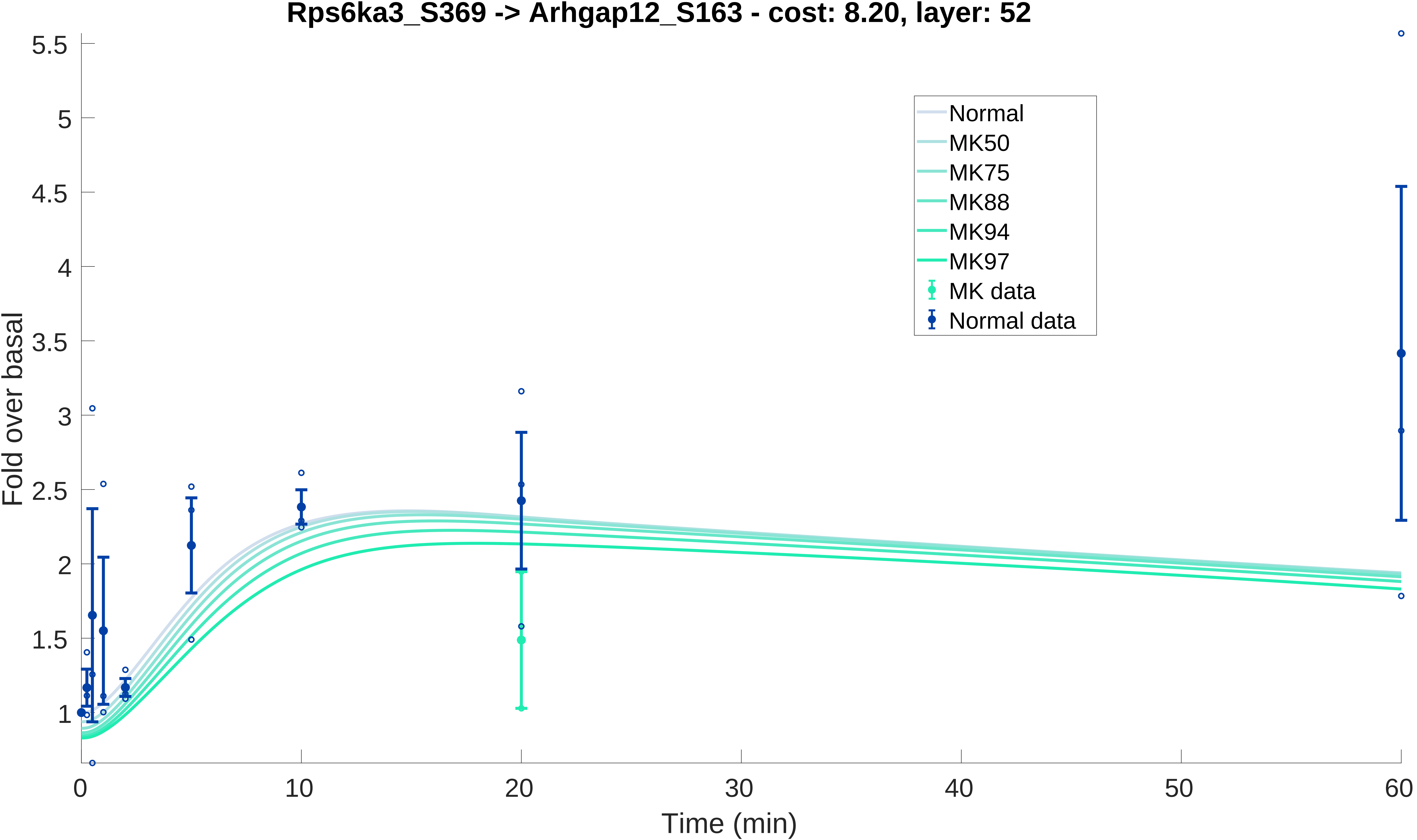

Rps6ka3_S369 -> Arhgap12_S163 - cost: 8.20, layer: 52
Fold over basal
Time (min)
Normal
MK50
MK75
MK88
MK94
MK97
MK data
Normal data
5.5
5
4.5
4
3.5
3
2.5
2
1.5
1
0
10
20
30
40
50
60

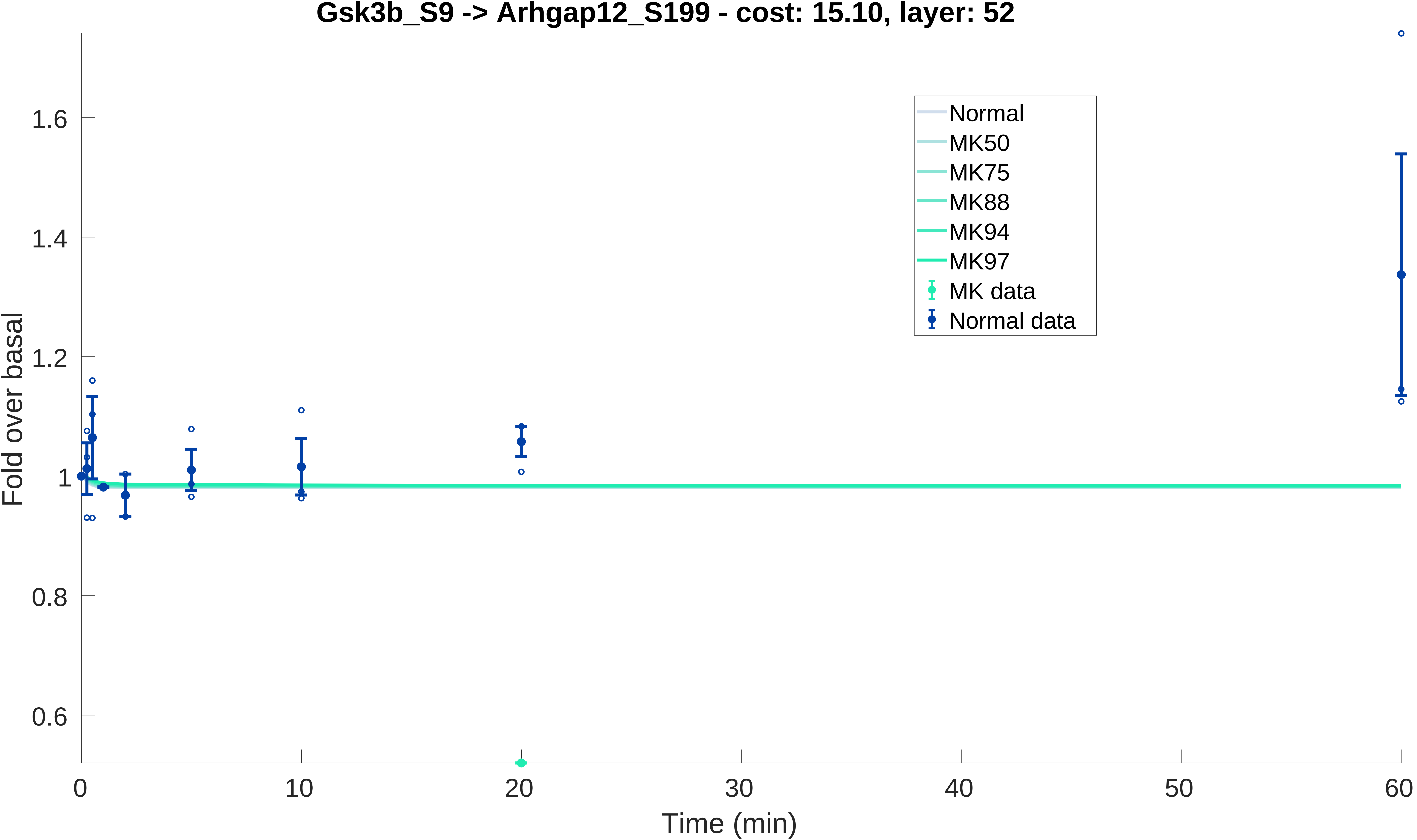

Gsk3b_S9 -> Arhgap12_S199 - cost: 15.10, layer: 52
Fold over basal
Time (min)
1.6
1.4
1.2
1
0.8
0.6
0
10
20
30
40
50
60
Normal
MK50
MK75
MK88
MK94
MK97
MK data
Normal data

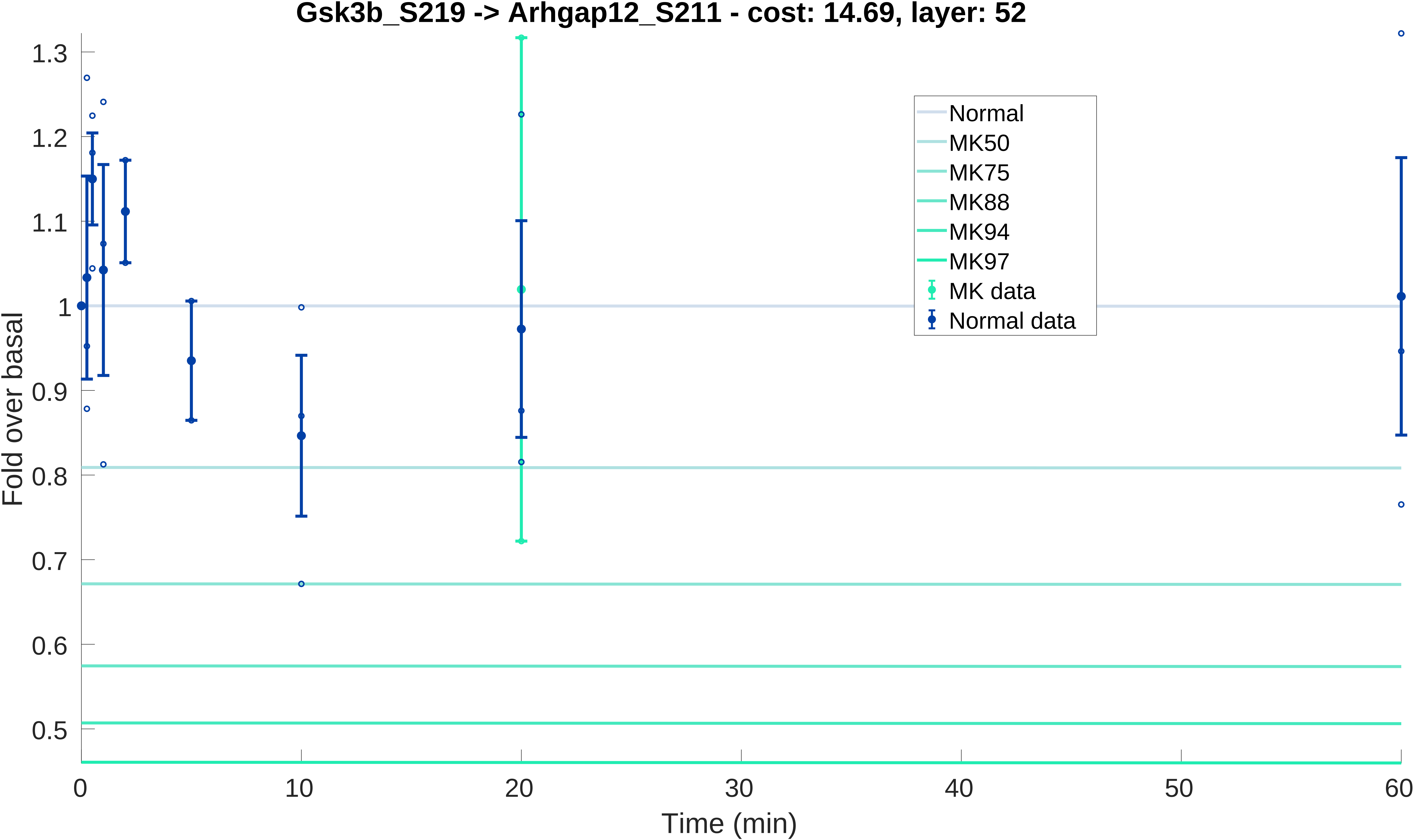
Gsk3b_S219 -> Arhgap12_S211 - cost: 14.69, layer: 52
Fold over basal
1.3
1.2
1.1
1
0.9
0.8
0.7
0.6
0.5
0
10
20
30
40
50
60
Time (min)
Normal
MK50
MK75
MK88
MK94
MK97
MK data
Normal data

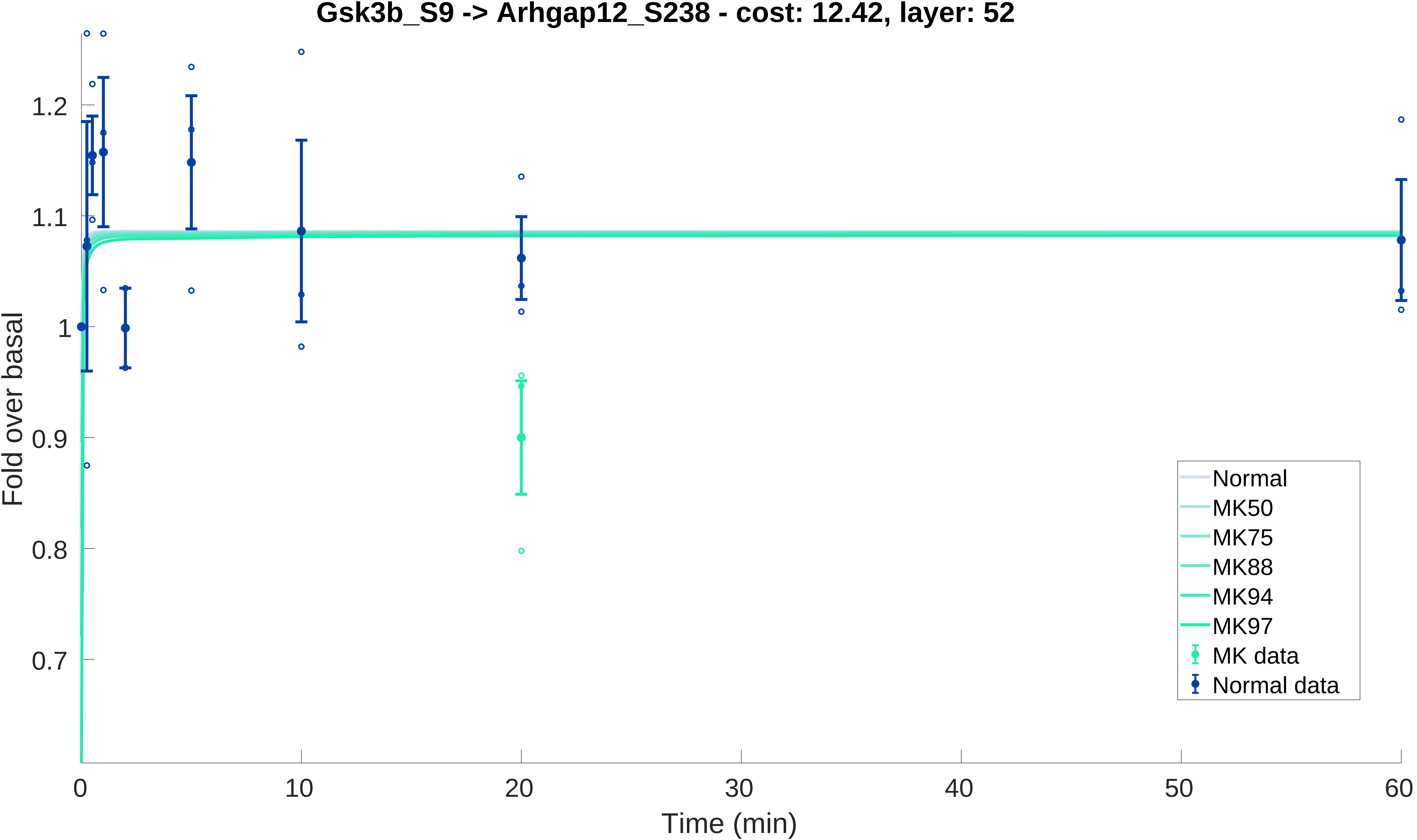

Gsk3b_S9 -> Arhgap12_S238 - cost: 12.42, layer: 52
Fold over basal
1.2
1.1
1
0.9
0.8
0.7
0
10
20
30
40
50
60
Time (min)
Normal
MK50
MK75
MK88
MK94
MK97
MK data
Normal data

Rps6ka3_S369 -> Arhgap12_T229 - cost: 5.21, layer: 52

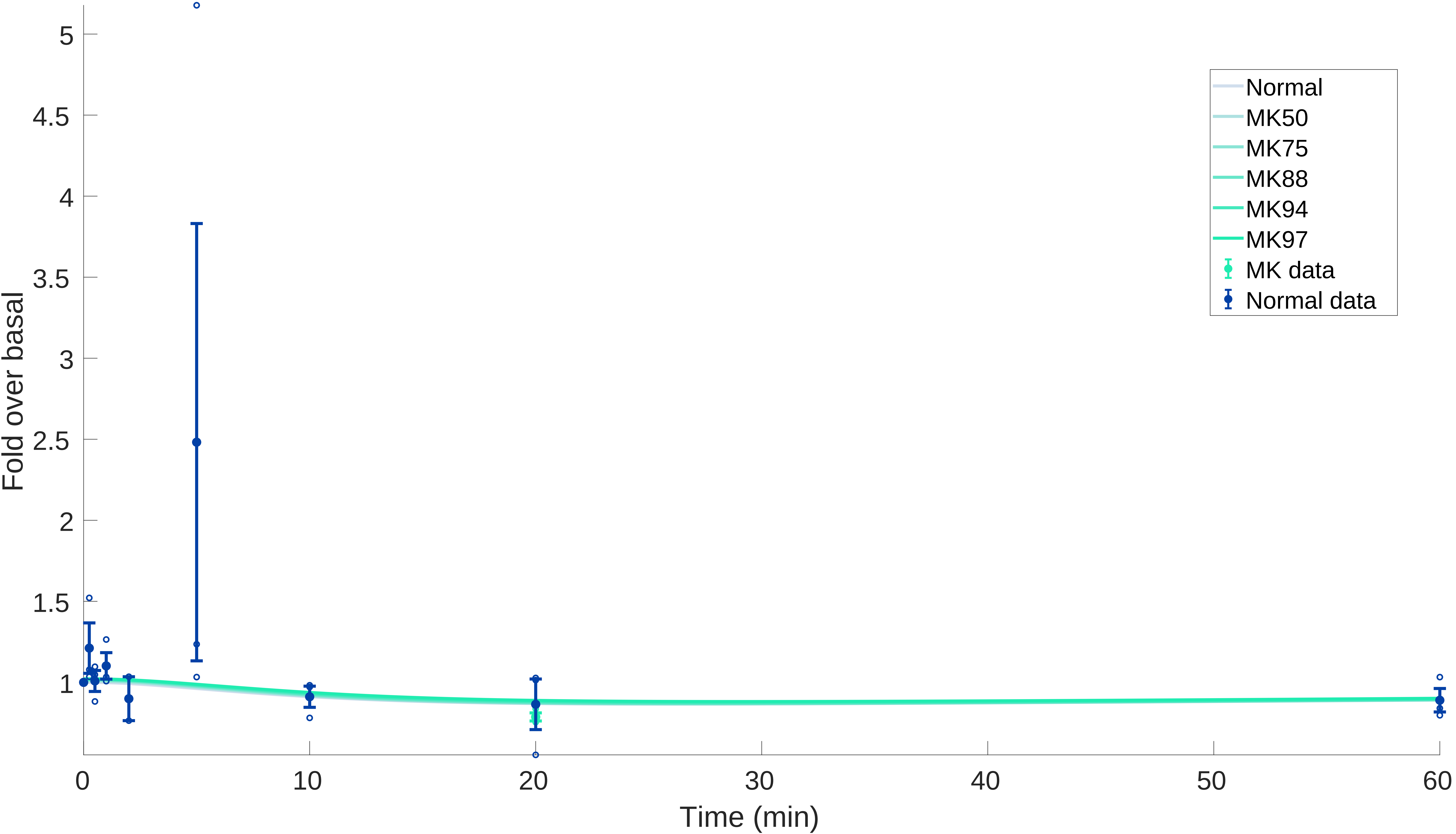

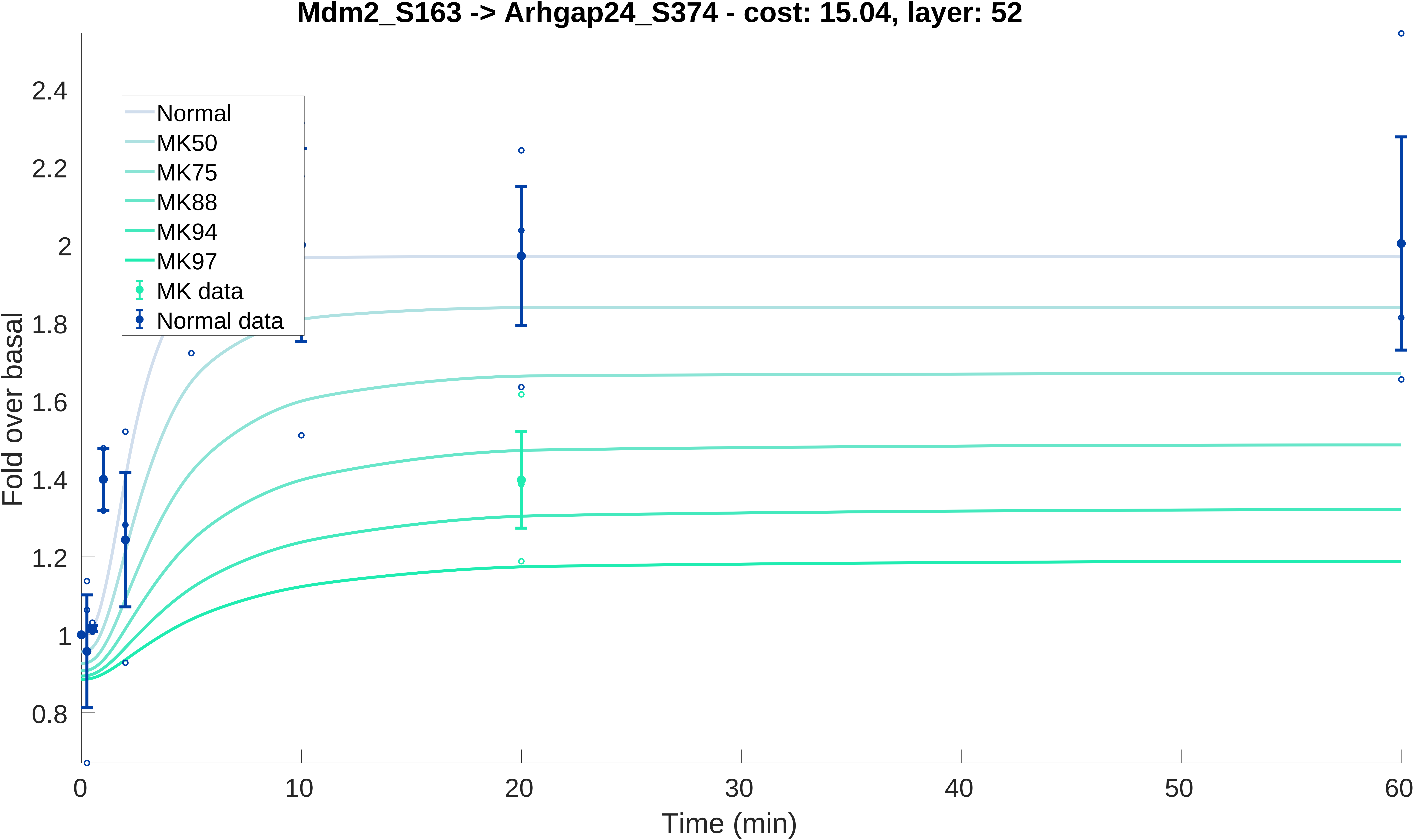

Mdm2_S163 -> Arhgap24_S374 - cost: 15.04, layer: 52
Normal
MK50
MK75
MK88
MK94
MK97
MK data
Normal data
Fold over basal
Time (min)
0.8
1
1.2
1.4
1.6
1.8
2
2.2
2.4
0
10
20
30
40
50
60

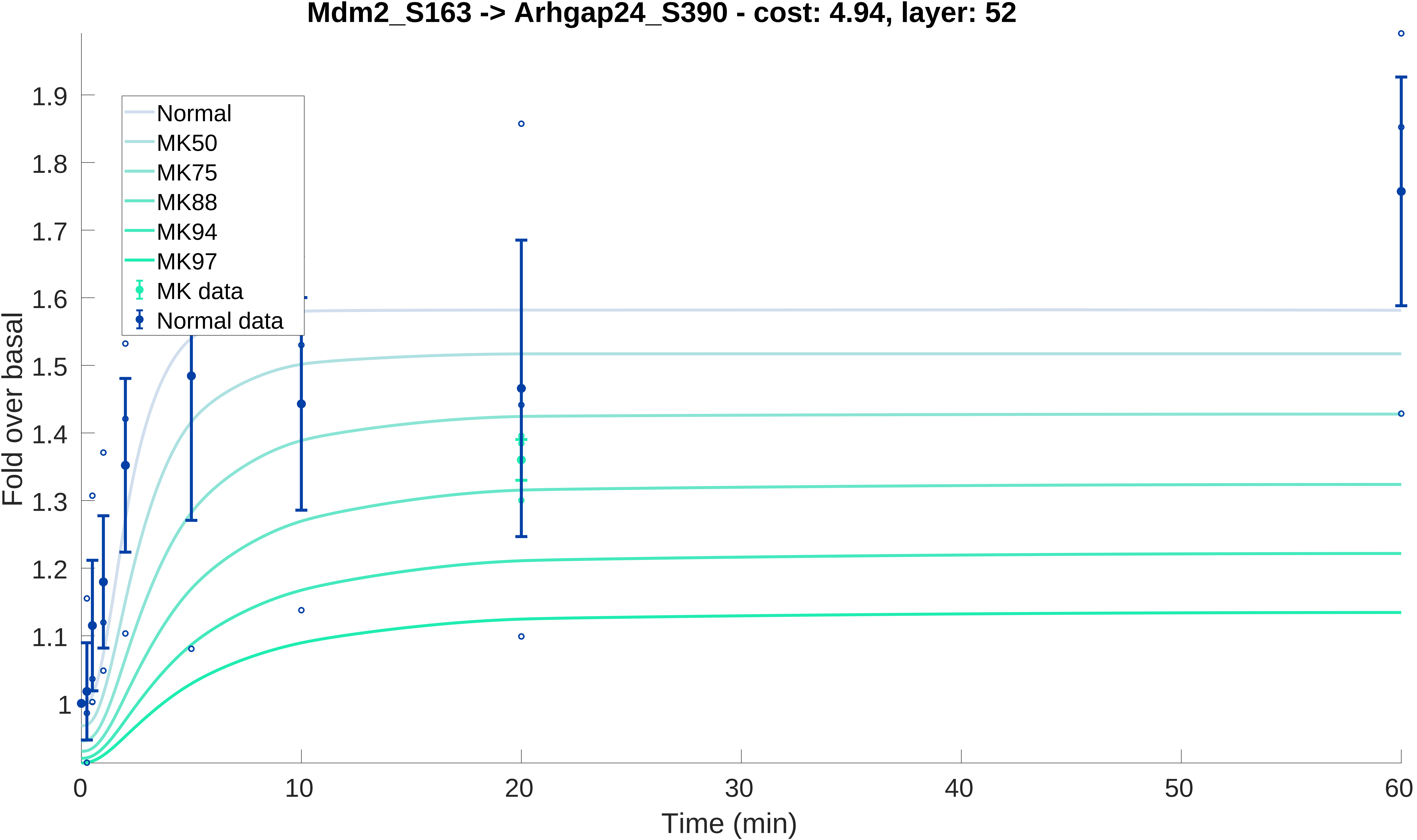

Mdm2_S163 -> Arhgap24_S390 - cost: 4.94, layer: 52
Normal
MK50
MK75
MK88
MK94
MK97
MK data
Normal data
Fold over basal
Time (min)
1
1.1
1.2
1.3
1.4
1.5
1.6
1.7
1.8
1.9
0
10
20
30
40
50
60

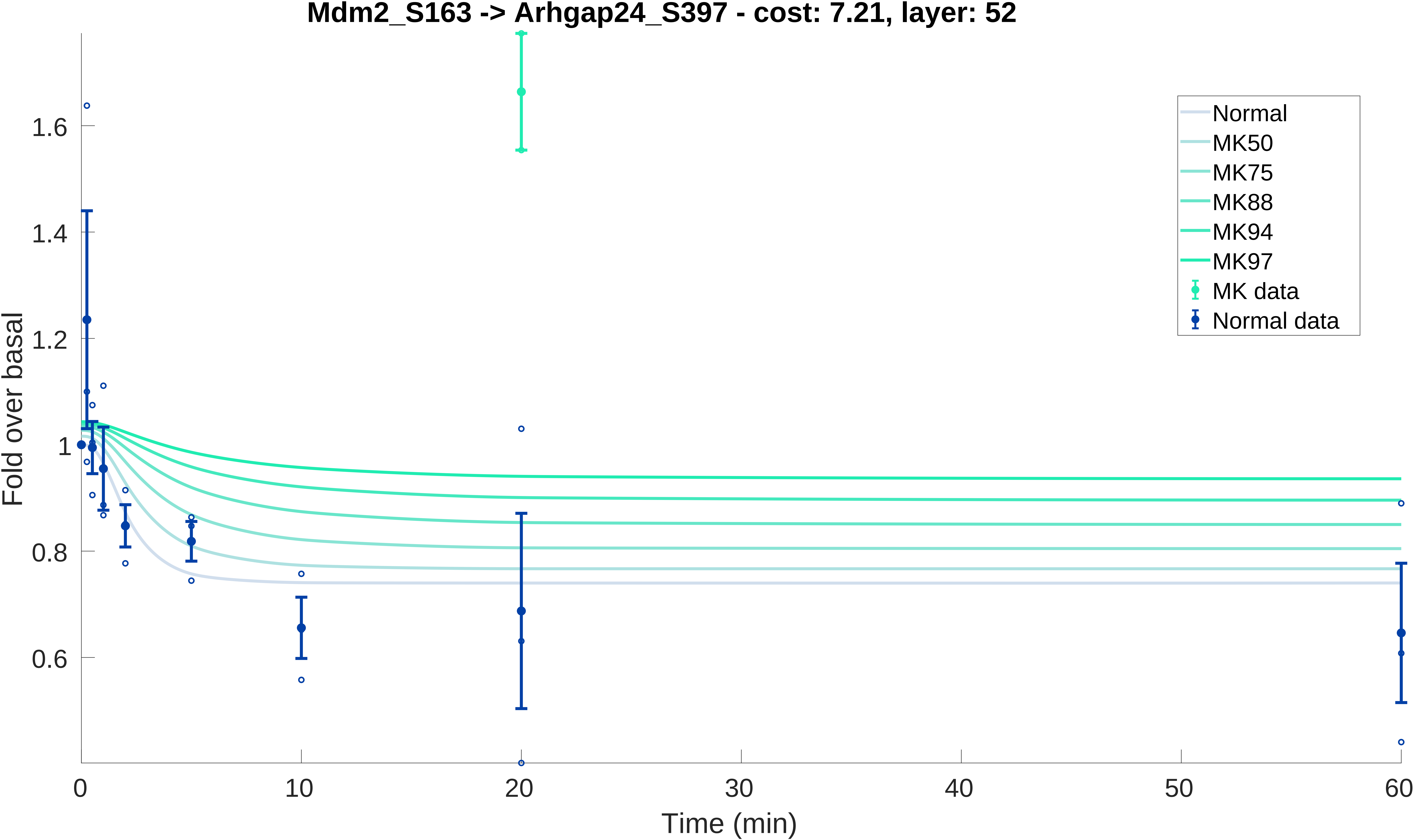
Mdm2_S163 -> Arhgap24_S397 - cost: 7.21, layer: 52
Fold over basal
1.6
1.4
1.2
1
0.8
0.6
0
10
20
30
40
50
60
Time (min)
Normal
MK50
MK75
MK88
MK94
MK97
MK data
Normal data

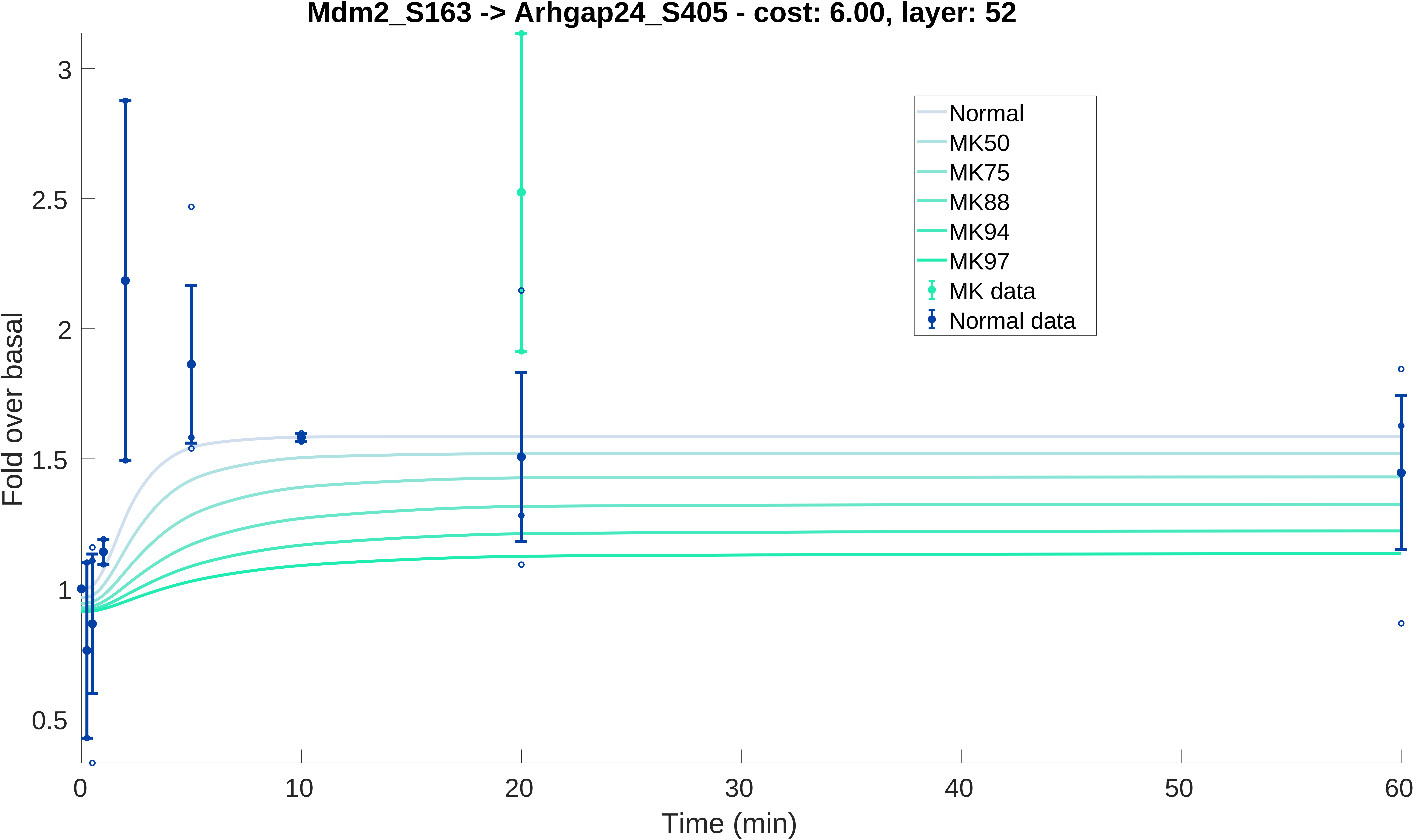

Mdm2_S163 -> Arhgap24_S405 - cost: 6.00, layer: 52
Fold over basal
Time (min)
0.5
1
1.5
2
2.5
3
0
10
20
30
40
50
60
Normal
MK50
MK75
MK88
MK94
MK97
MK data
Normal data

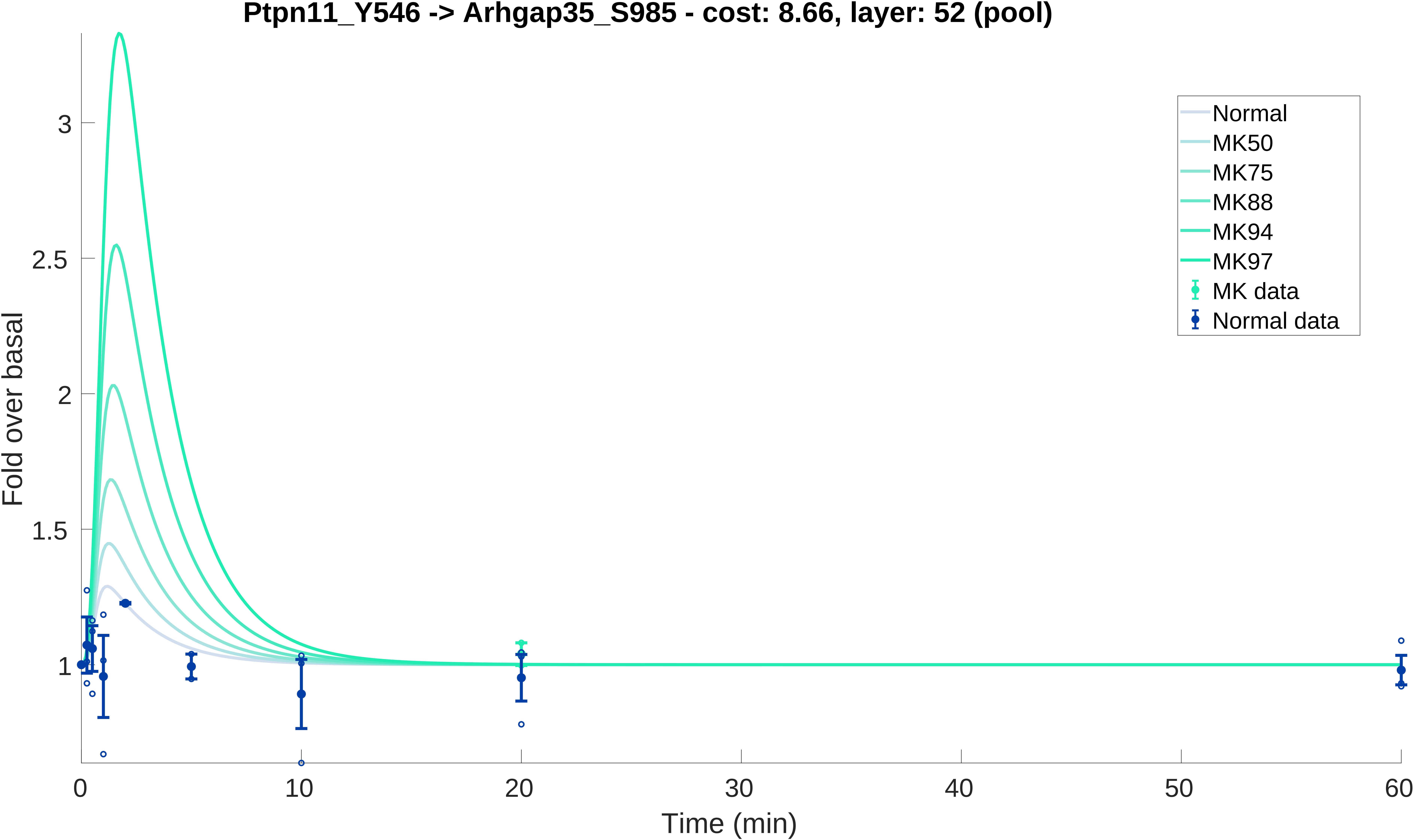

Ptpn11_Y546 -> Arhgap35_S985 - cost: 8.66, layer: 52 (pool)
Fold over basal
Time (min)
0
10
20
30
40
50
60
1
1.5
2
2.5
3
Normal
MK50
MK75
MK88
MK94
MK97
MK data
Normal data

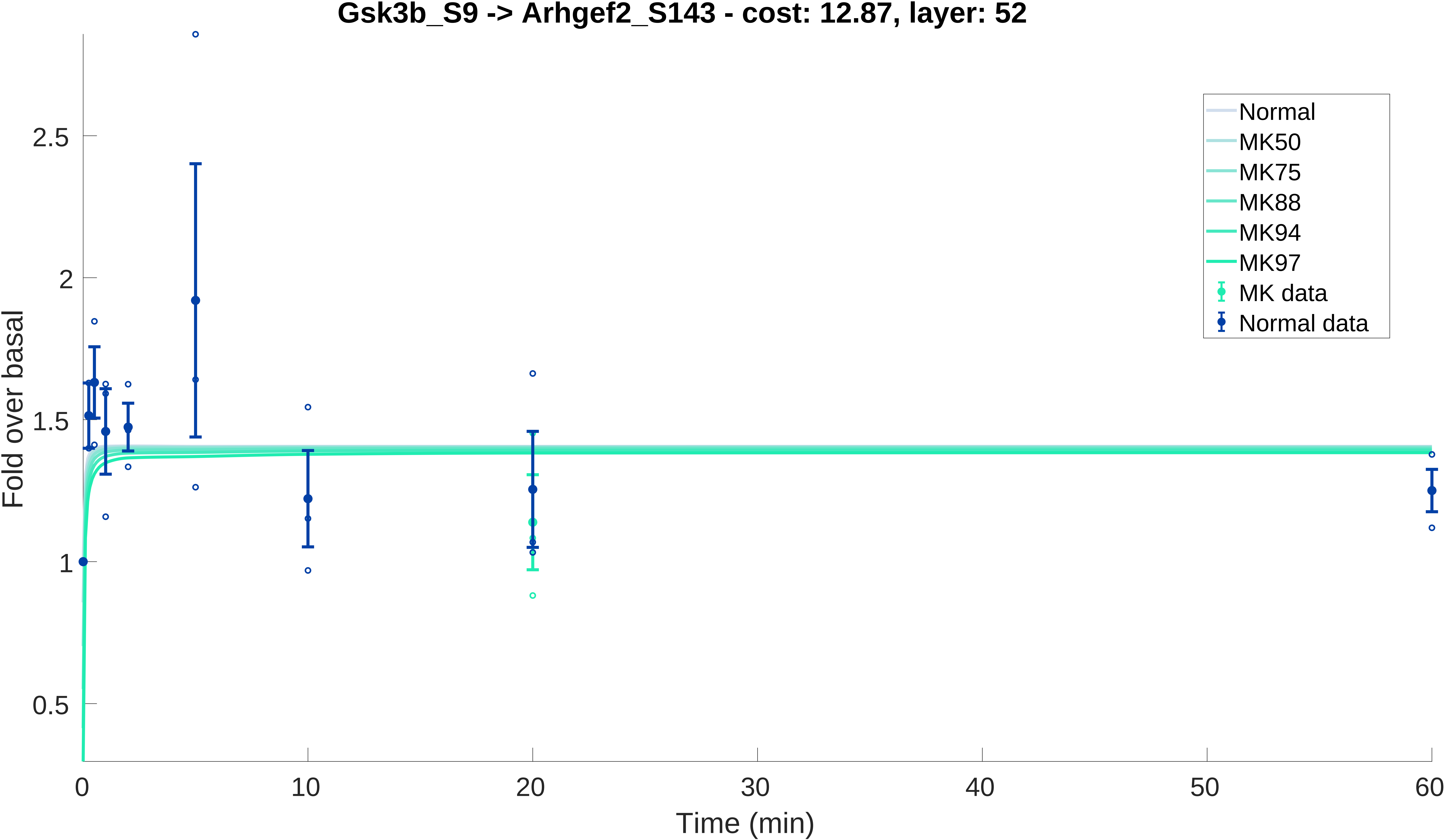

Gsk3b_S9 -> Arhgef2_S143 - cost: 12.87, layer: 52
Fold over basal
Time (min)
0.5
1
1.5
2
2.5
0
10
20
30
40
50
60
Normal
MK50
MK75
MK88
MK94
MK97
MK data
Normal data

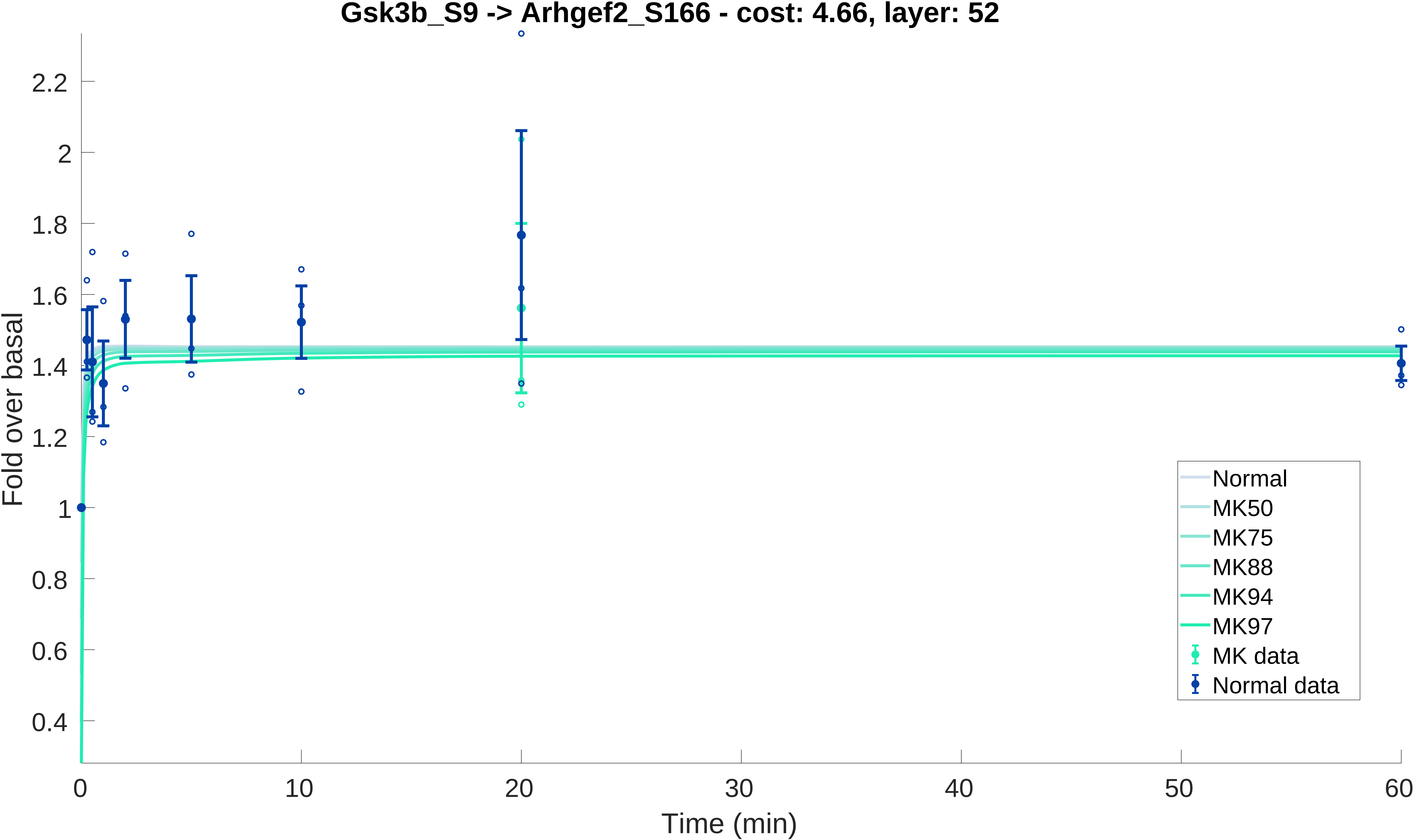

Gsk3b_S9 -> Arhgef2_S166 - cost: 4.66, layer: 52
Fold over basal
Time (min)
2.2
2
1.8
1.6
1.4
1.2
1
0.8
0.6
0.4
0
10
20
30
40
50
60
Normal
MK50
MK75
MK88
MK94
MK97
MK data
Normal data

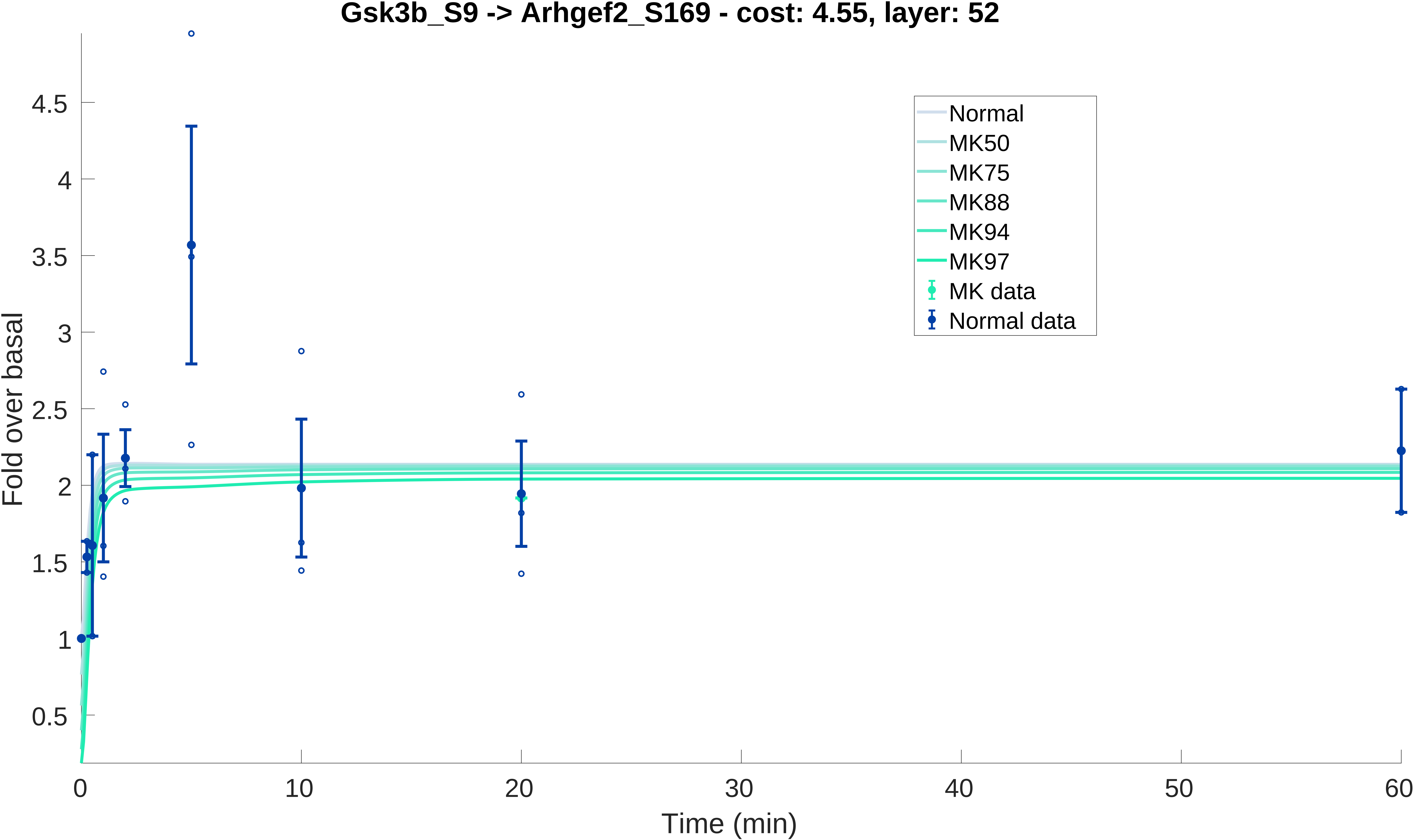
Gsk3b_S9 -> Arhgef2_S169 - cost: 4.55, layer: 52
Fold over basal
Time (min)
0.5
1
1.5
2
2.5
3
3.5
4
4.5
0
10
20
30
40
50
60
Normal
MK50
MK75
MK88
MK94
MK97
MK data
Normal data

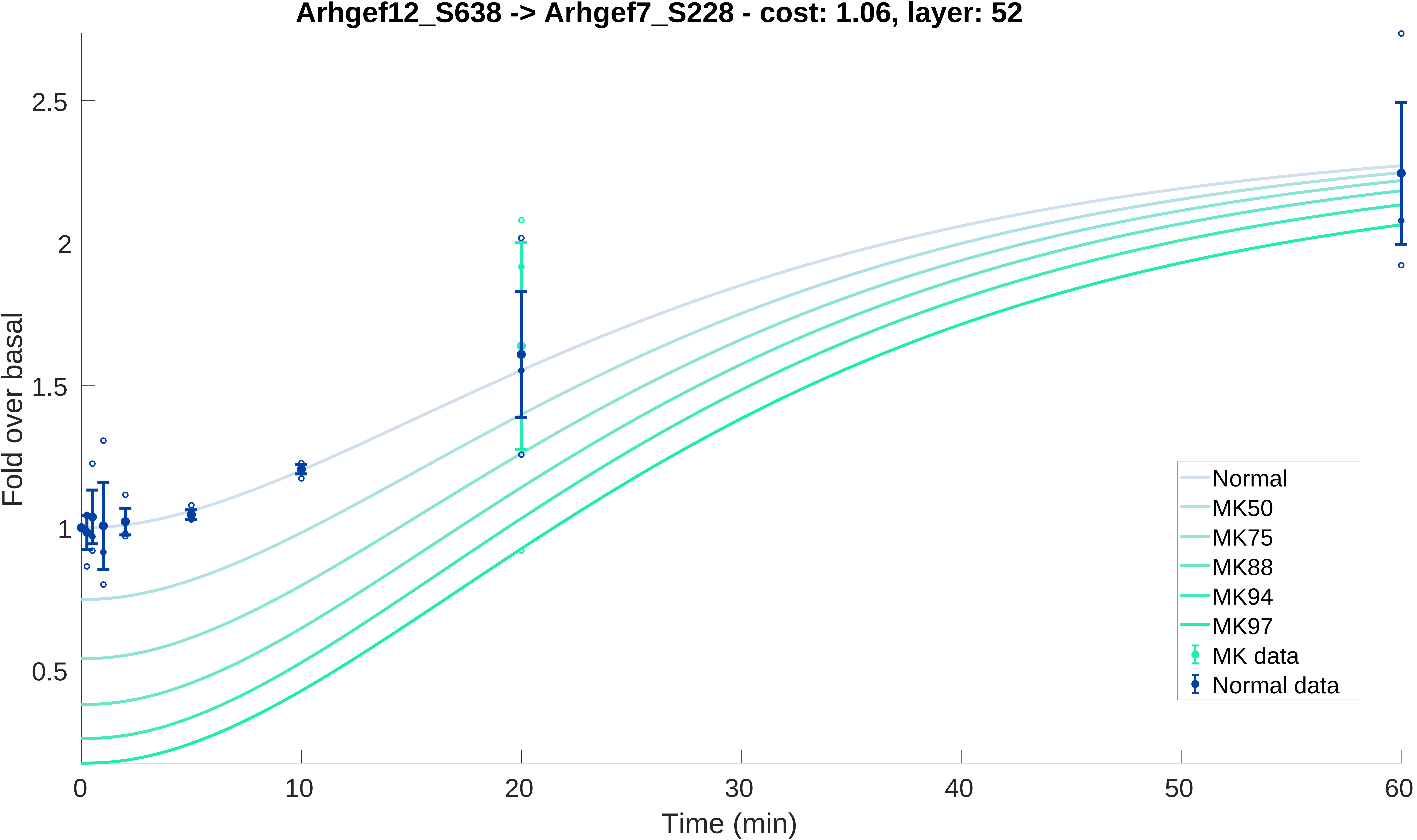

Arhgef12_S638 -> Arhgef7_S228 - cost: 1.06, layer: 52
Fold over basal
Time (min)
0
10
20
30
40
50
60
0.5
1
1.5
2
2.5
Normal
MK50
MK75
MK88
MK94
MK97
MK data
Normal data

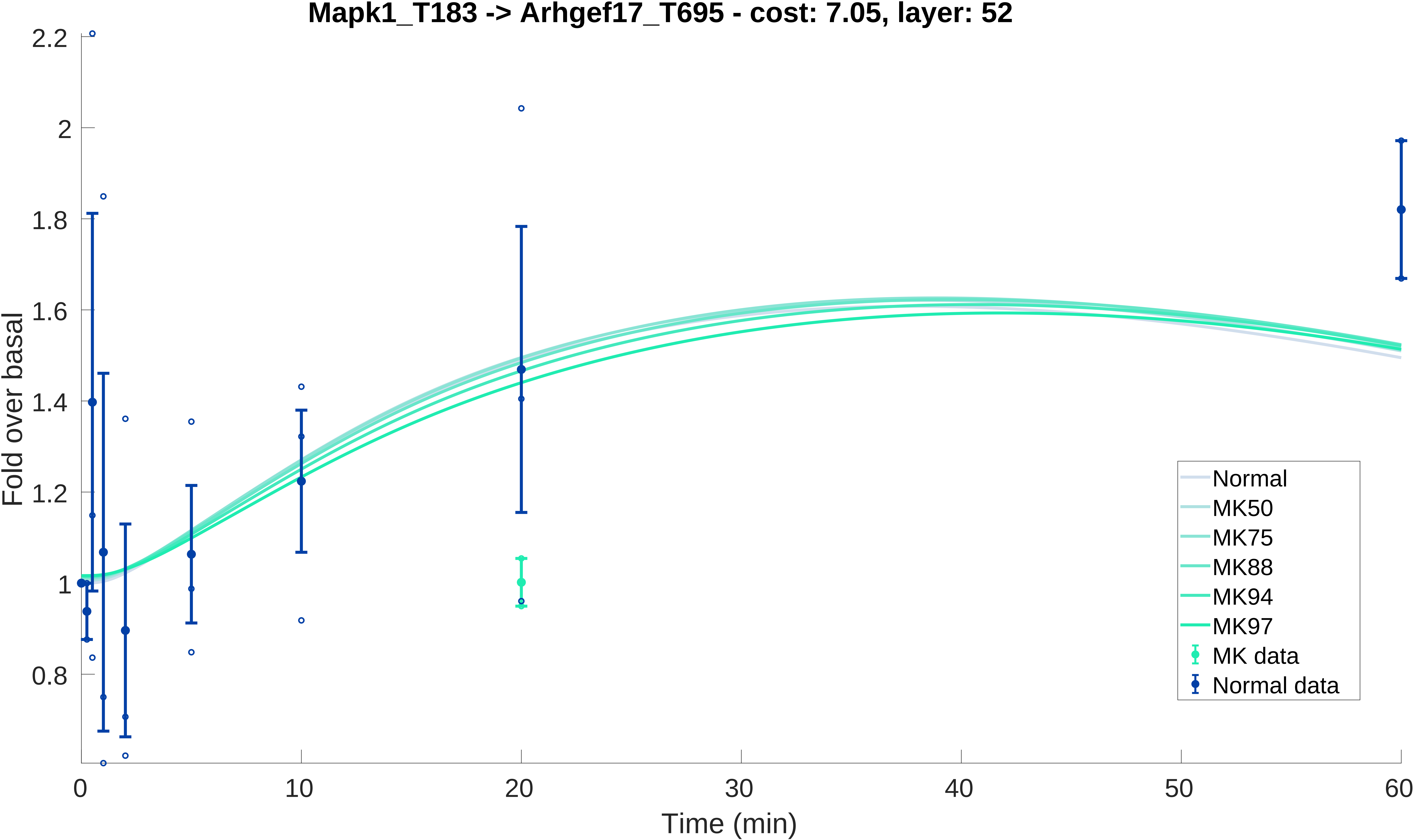
Mapk1_T183 -> Arhgef17_T695 - cost: 7.05, layer: 52
Fold over basal
Time (min)
Normal
MK50
MK75
MK88
MK94
MK97
MK data
Normal data

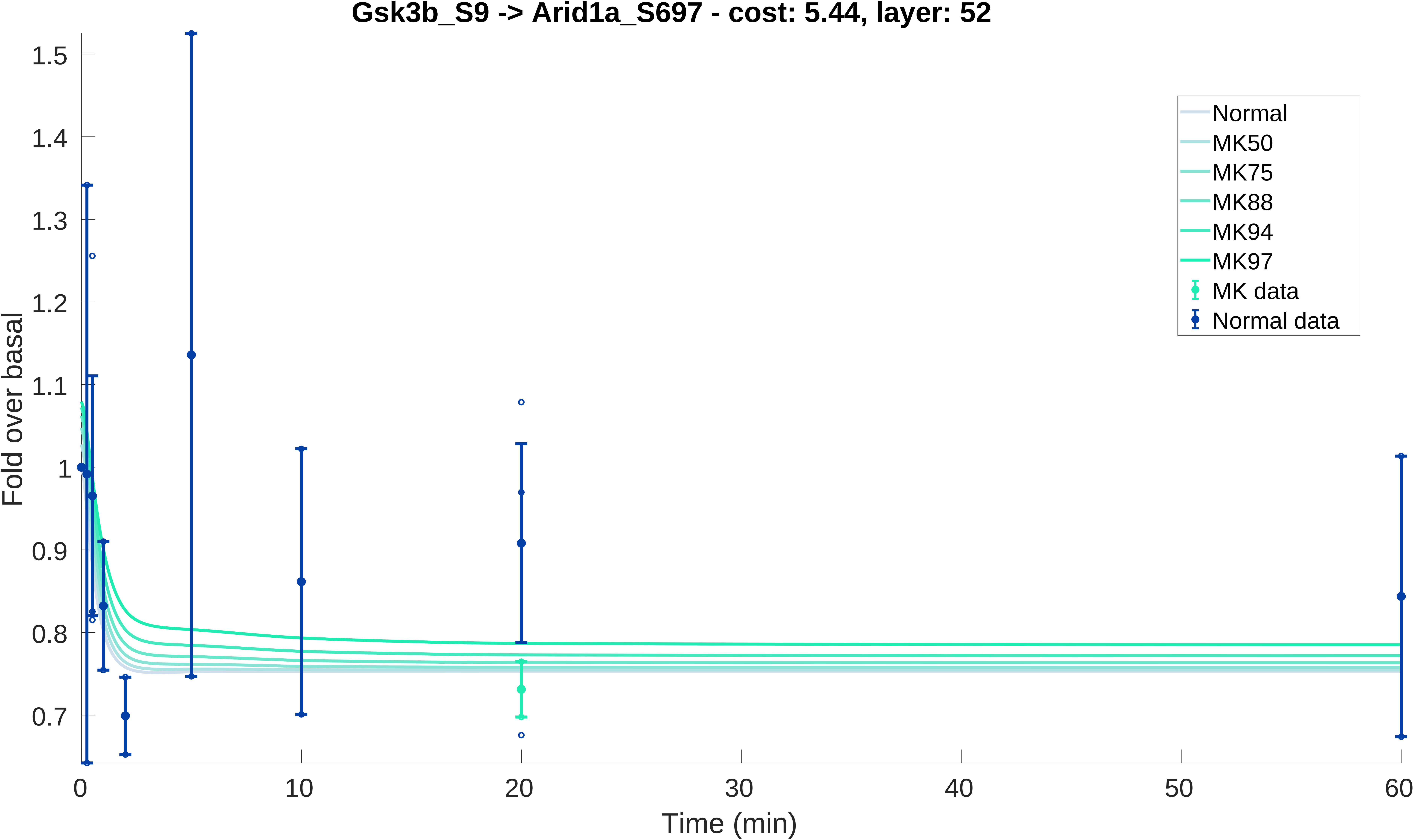

Gsk3b_S9 -> Arid1a_S697 - cost: 5.44, layer: 52
Fold over basal
Time (min)
1.5
1.4
1.3
1.2
1.1
1
0.9
0.8
0.7
0
10
20
30
40
50
60
Normal
MK50
MK75
MK88
MK94
MK97
MK data
Normal data

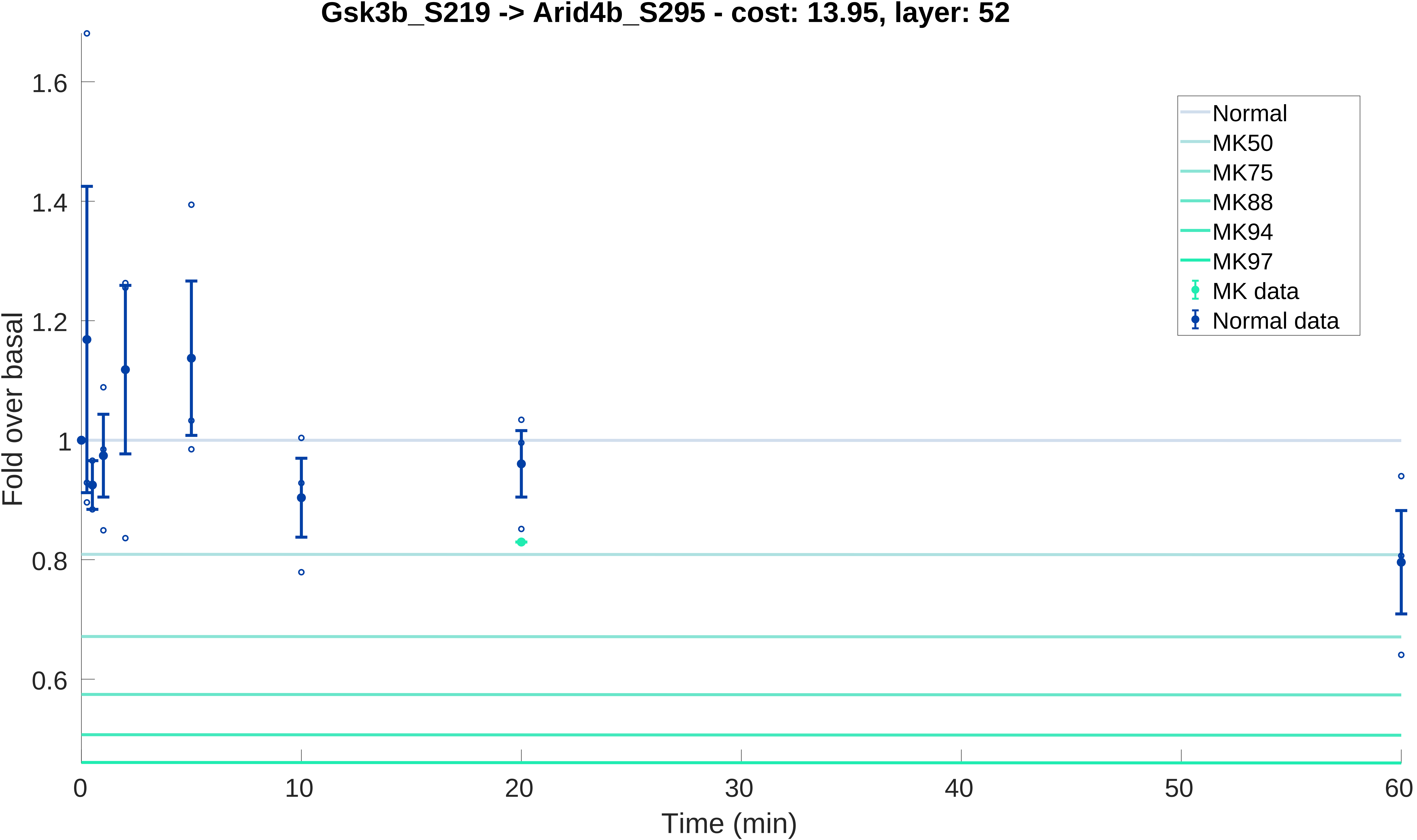
Gsk3b_S219 -> Arid4b_S295 - cost: 13.95, layer: 52
Fold over basal
1.6
1.4
1.2
1
0.8
0.6
0
10
20
30
40
50
60
Time (min)
Normal
MK50
MK75
MK88
MK94
MK97
MK data
Normal data

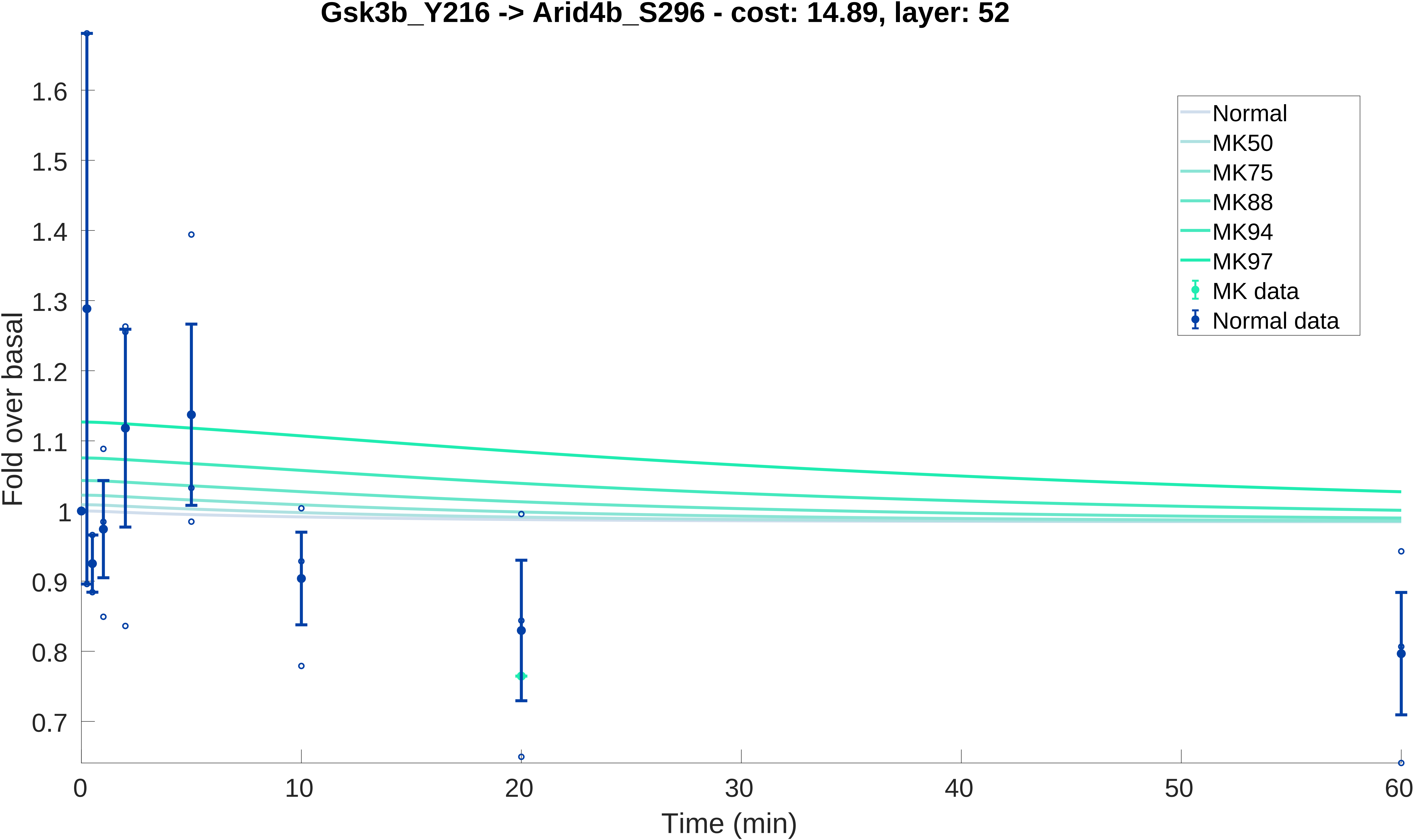

Gsk3b_Y216 -> Arid4b_S296 - cost: 14.89, layer: 52
Fold over basal
Time (min)
0.7
0.8
0.9
1
1.1
1.2
1.3
1.4
1.5
1.6
0
10
20
30
40
50
60
Normal
MK50
MK75
MK88
MK94
MK97
MK data
Normal data

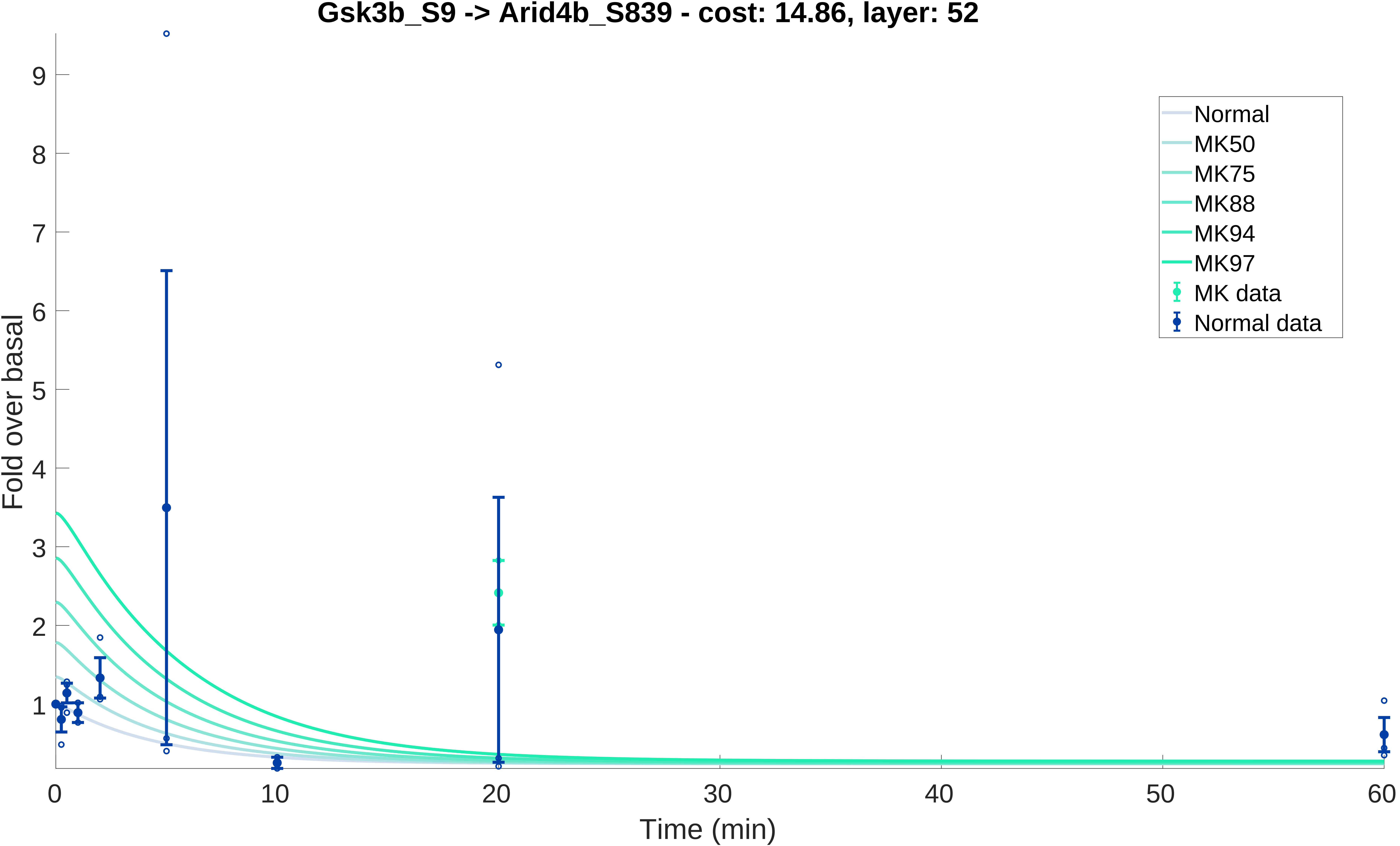

Gsk3b_S9 -> Arid4b_S839 - cost: 14.86, layer: 52
Fold over basal
Time (min)
0
10
20
30
40
50
60
1
2
3
4
5
6
7
8
9
Normal
MK50
MK75
MK88
MK94
MK97
MK data
Normal data

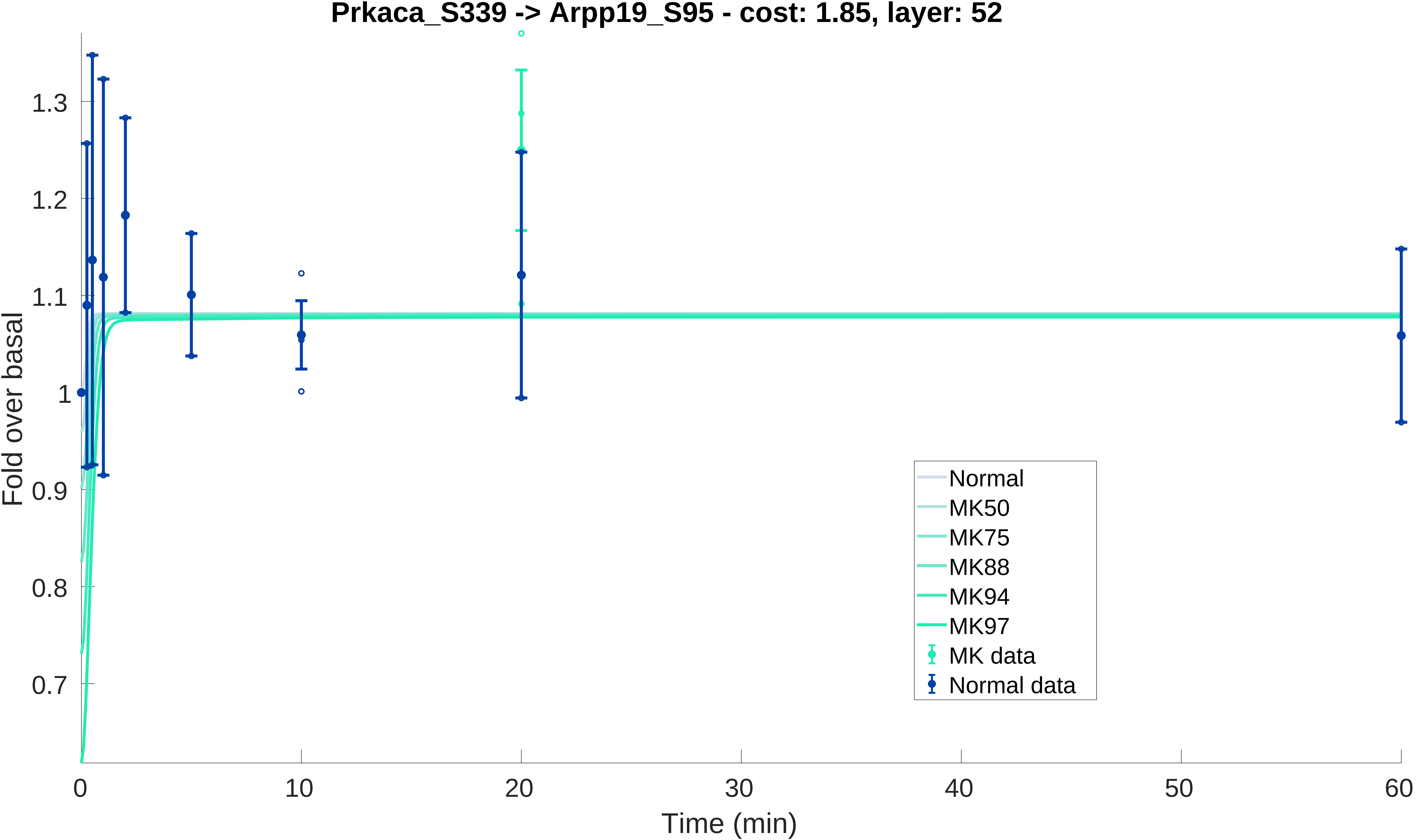

Prkaca_S339 -> Arpp19_S95 - cost: 1.85, layer: 52
Fold over basal
Time (min)
0.7
0.8
0.9
1
1.1
1.2
1.3
0
10
20
30
40
50
60
Normal
MK50
MK75
MK88
MK94
MK97
MK data
Normal data

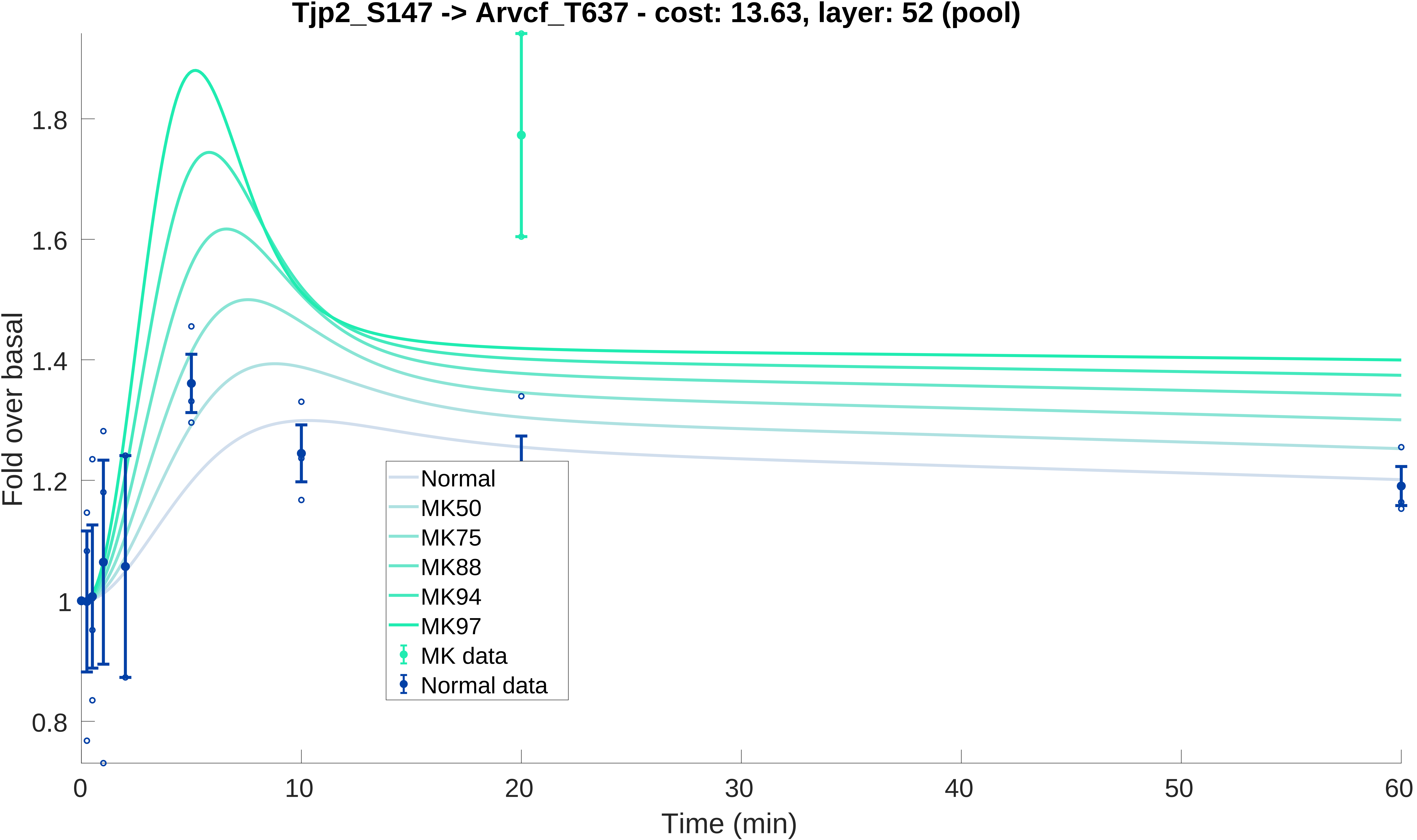

Tjp2_S147 -> Arvcf_T637 - cost: 13.63, layer: 52 (pool)
Fold over basal
Time (min)
0.8
1
1.2
1.4
1.6
1.8
0
10
20
30
40
50
60
Normal
MK50
MK75
MK88
MK94
MK97
MK data
Normal data

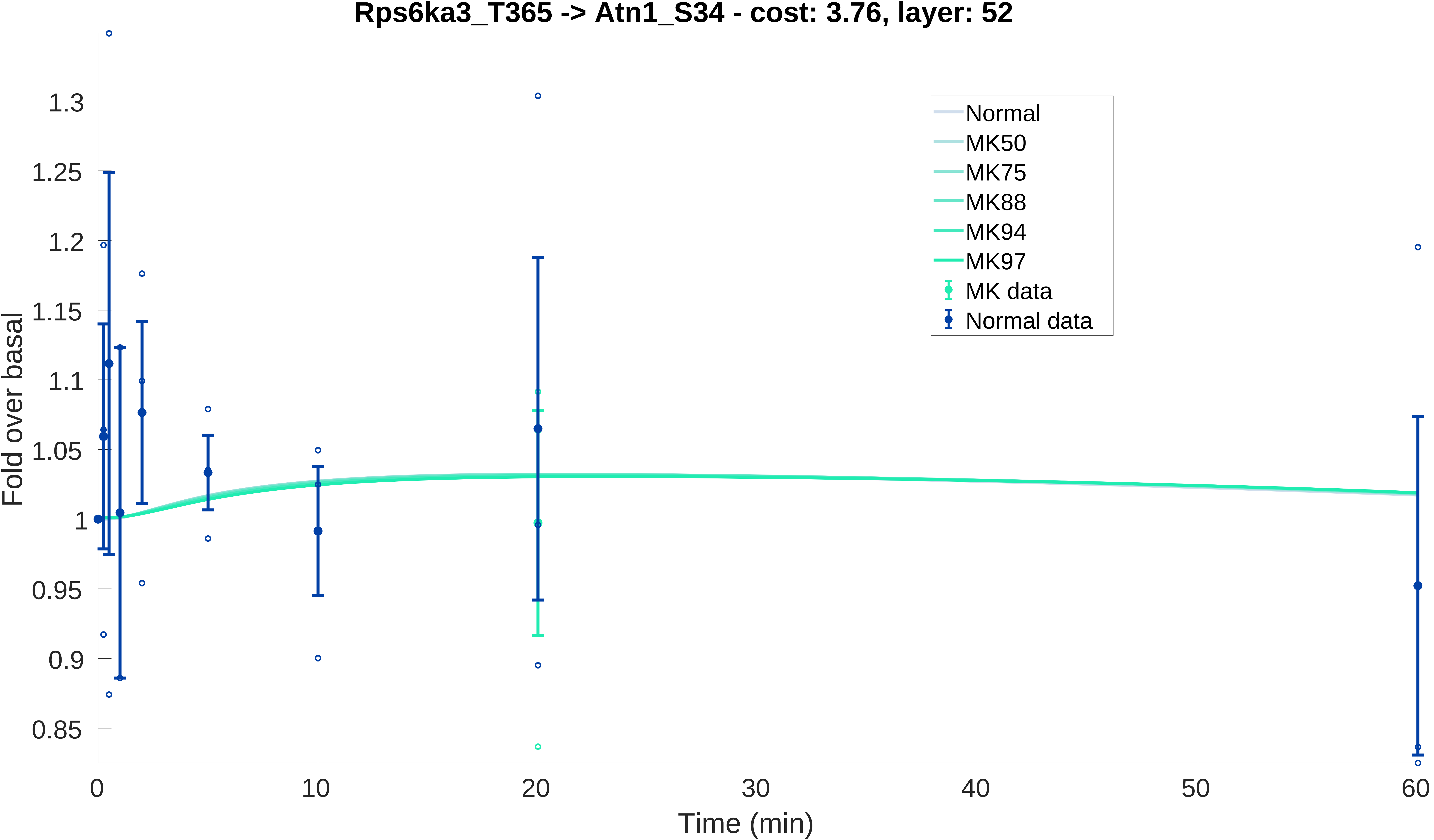

Rps6ka3_T365 -> Atn1_S34 - cost: 3.76, layer: 52
Fold over basal
1.3
1.25
1.2
1.15
1.1
1.05
1
0.95
0.9
0.85
0
10
20
30
40
50
60
Time (min)
Normal
MK50
MK75
MK88
MK94
MK97
MK data
Normal data

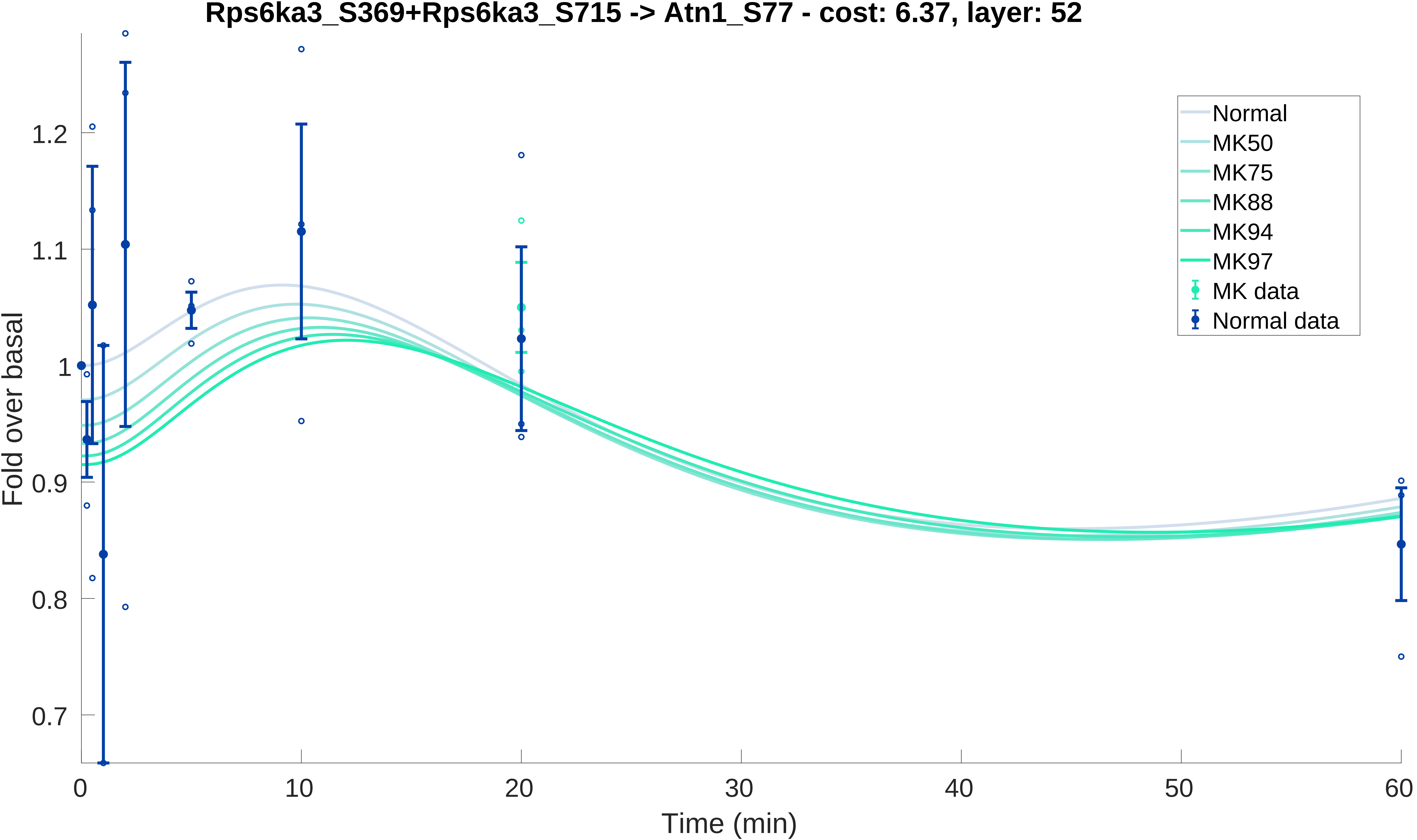

Rps6ka3_S369+Rps6ka3_S715 -> Atn1_S77 - cost: 6.37, layer: 52
Normal
MK50
MK75
MK88
MK94
MK97
MK data
Normal data
Fold over basal
1.2
1.1
1
0.9
0.8
0.7
0
10
20
30
40
50
60
Time (min)

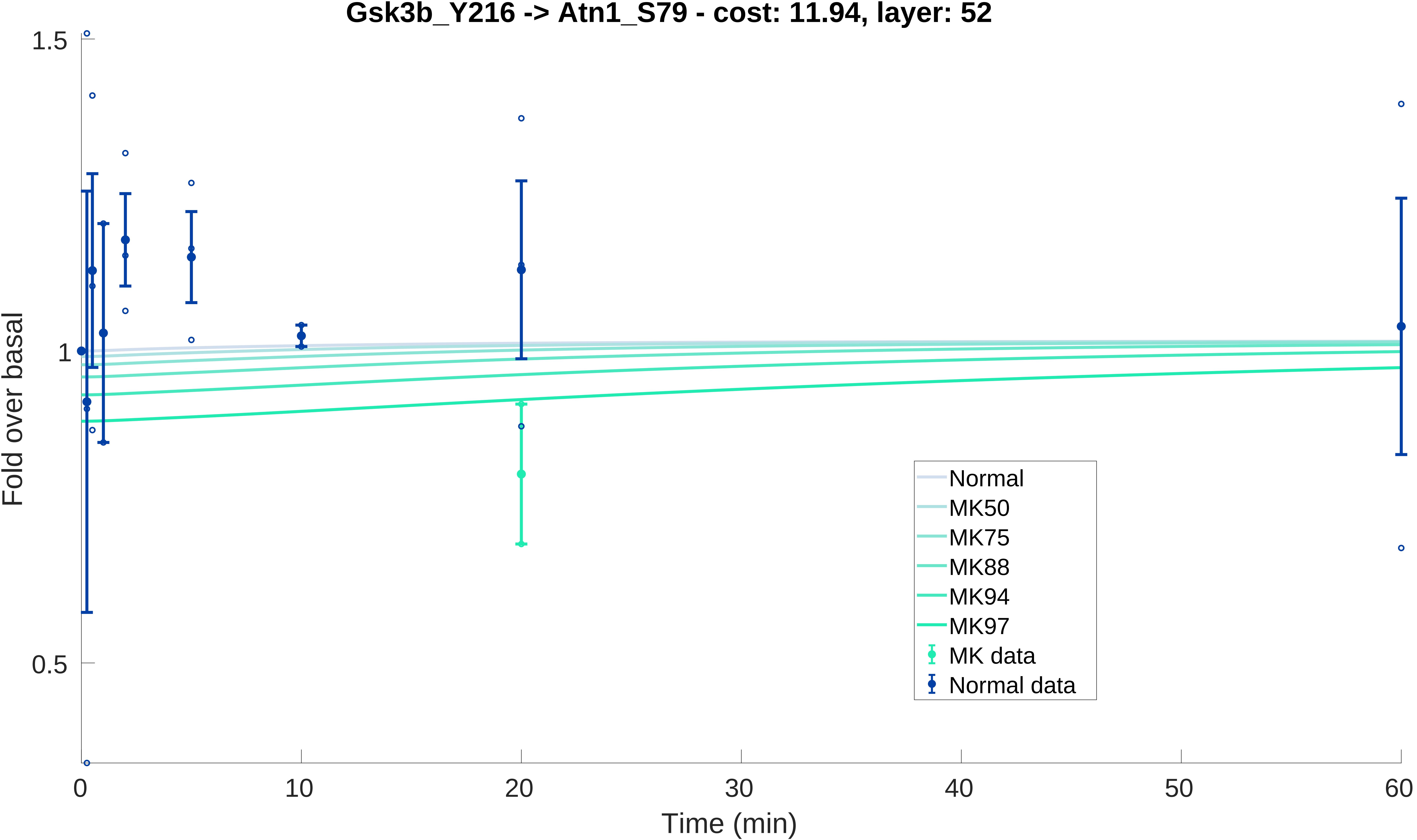

Gsk3b_Y216 -> Atn1_S79 - cost: 11.94, layer: 52
Fold over basal
1.5
1
0.5
0
10
20
30
40
50
60
Time (min)
Normal
MK50
MK75
MK88
MK94
MK97
MK data
Normal data

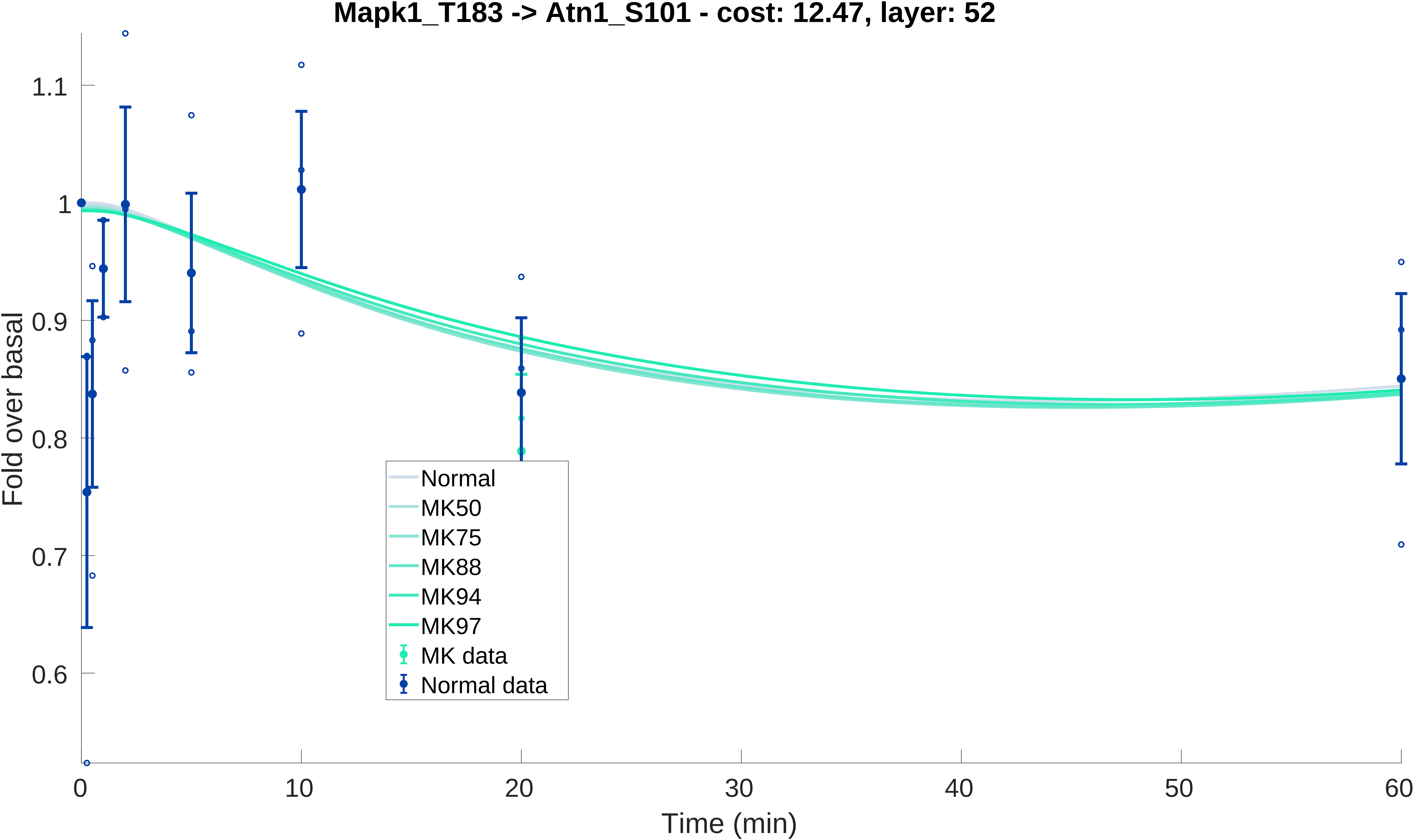

Mapk1_T183 -> Atn1_S101 - cost: 12.47, layer: 52
Fold over basal
Time (min)
1.1
1
0.9
0.8
0.7
0.6
0
10
20
30
40
50
60
Normal
MK50
MK75
MK88
MK94
MK97
MK data
Normal data

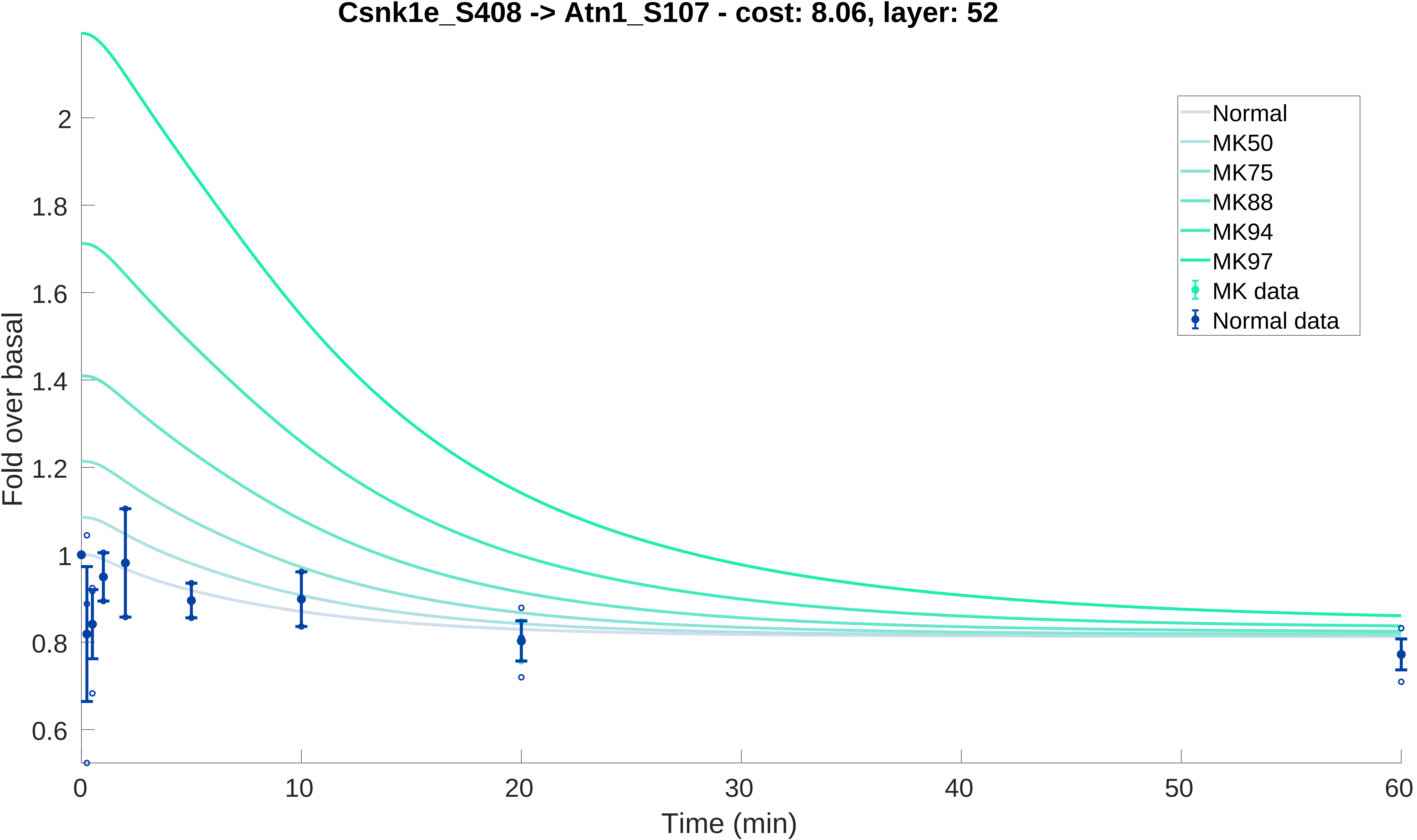

Csnk1e_S408 -> Atn1_S107 - cost: 8.06, layer: 52
Normal
MK50
MK75
MK88
MK94
MK97
MK data
Normal data
Fold over basal
0.6
0.8
1
1.2
1.4
1.6
1.8
2
0
10
20
30
40
50
60
Time (min)

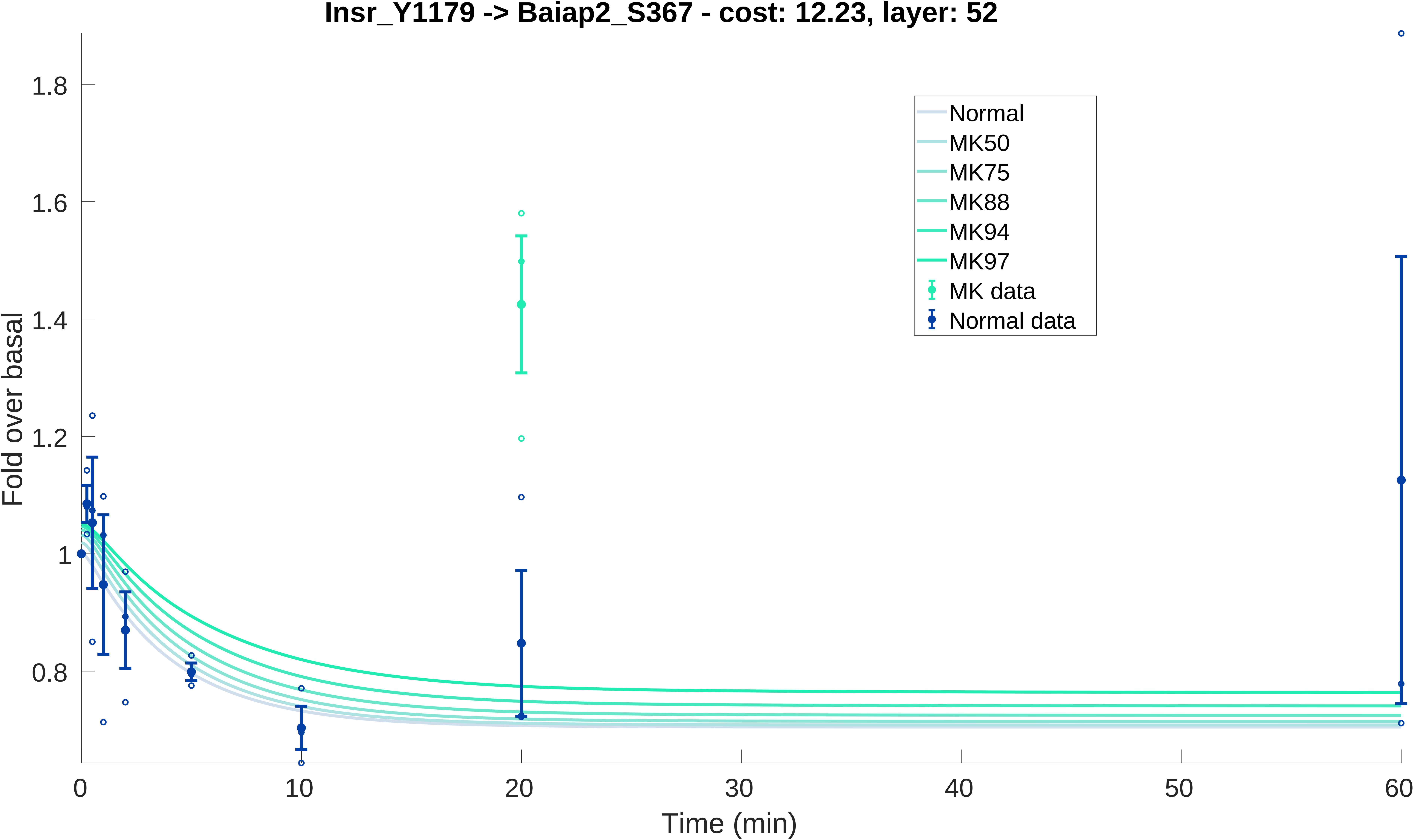

Insr_Y1179 -> Baiap2_S367 - cost: 12.23, layer: 52
Fold over basal
Time (min)
1.8
1.6
1.4
1.2
1
0.8
0
10
20
30
40
50
60
Normal
MK50
MK75
MK88
MK94
MK97
MK data
Normal data

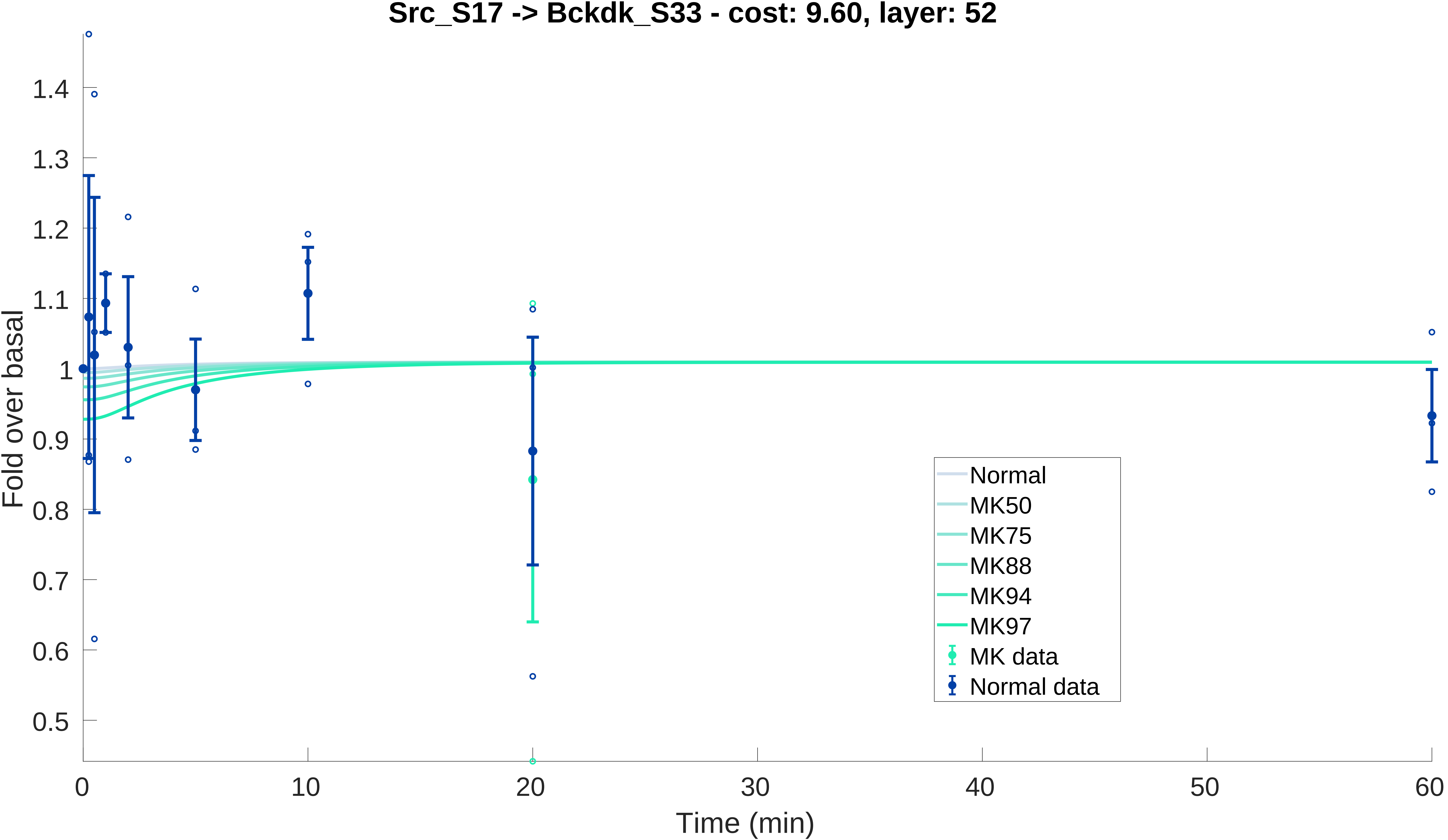

Src_S17 -> Bckdk_S33 - cost: 9.60, layer: 52
Fold over basal
Time (min)
1.4
1.3
1.2
1.1
1
0.9
0.8
0.7
0.6
0.5
0
10
20
30
40
50
60
Normal
MK50
MK75
MK88
MK94
MK97
MK data
Normal data

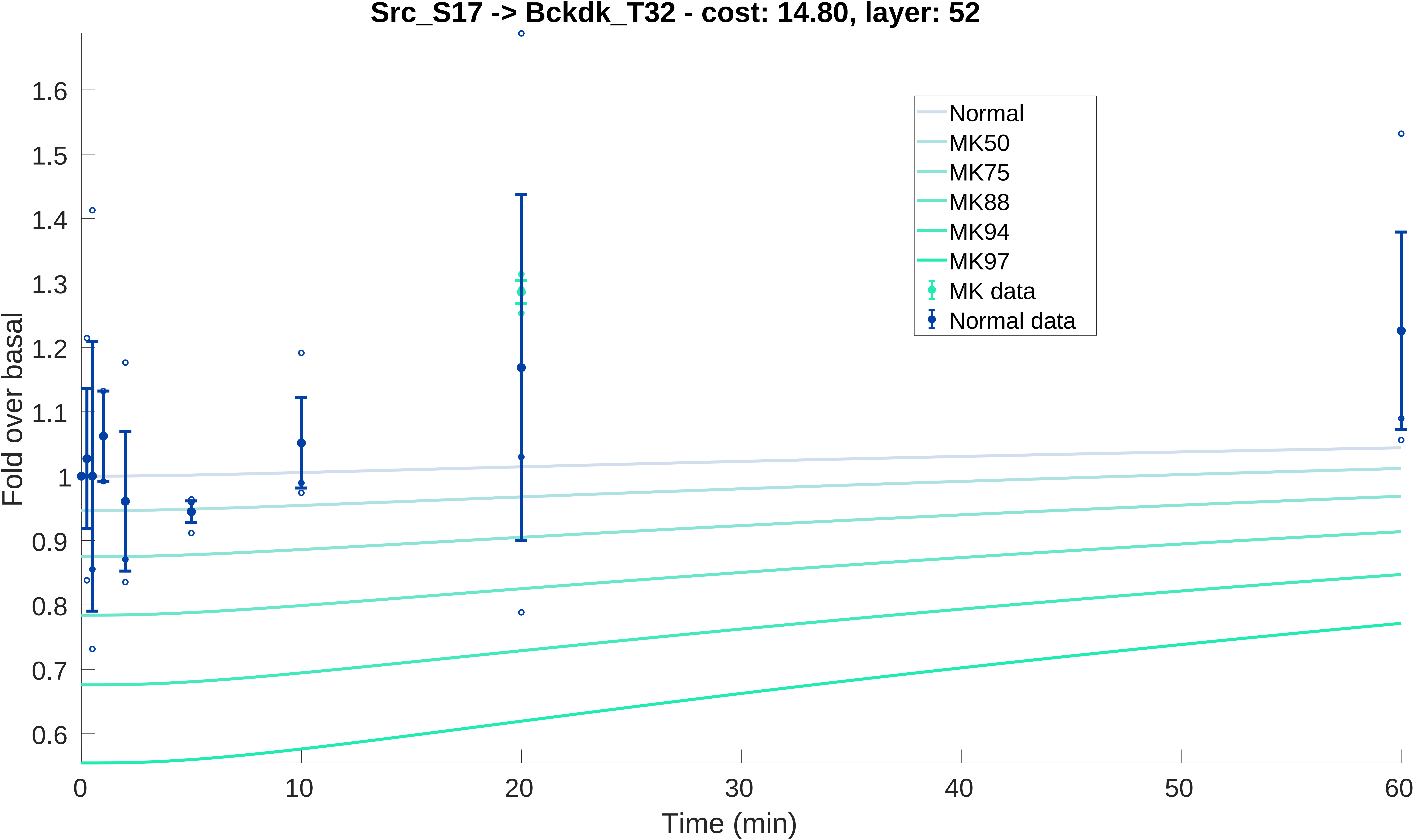

Src_S17 -> Bckdk_T32 - cost: 14.80, layer: 52
Fold over basal
Time (min)
0.6
0.7
0.8
0.9
1
1.1
1.2
1.3
1.4
1.5
1.6
0
10
20
30
40
50
60
Normal
MK50
MK75
MK88
MK94
MK97
MK data
Normal data

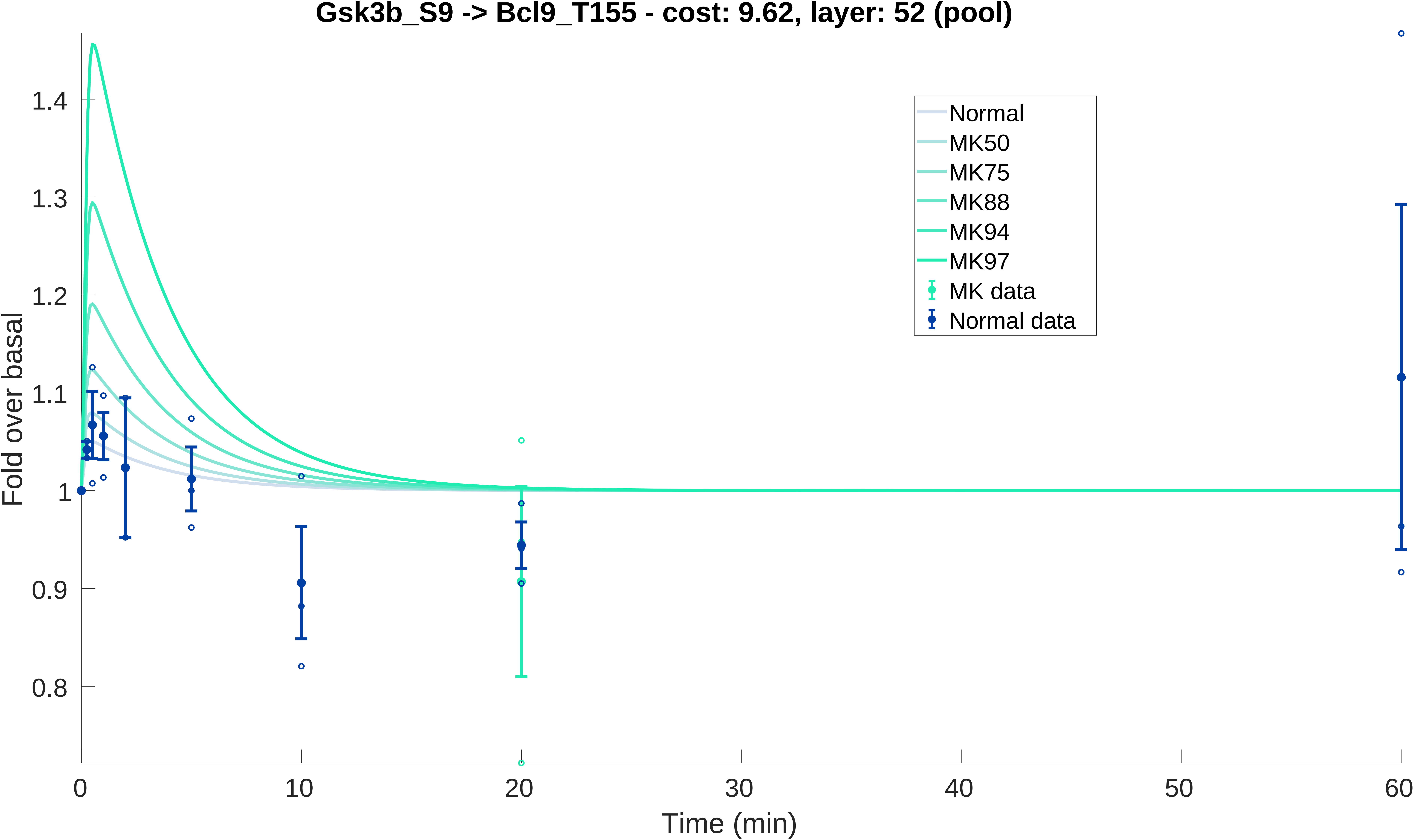

Gsk3b_S9 -> Bcl9_T155 - cost: 9.62, layer: 52 (pool)
Fold over basal
Time (min)
Normal
MK50
MK75
MK88
MK94
MK97
MK data
Normal data
1.4
1.3
1.2
1.1
1
0.9
0.8
0
10
20
30
40
50
60

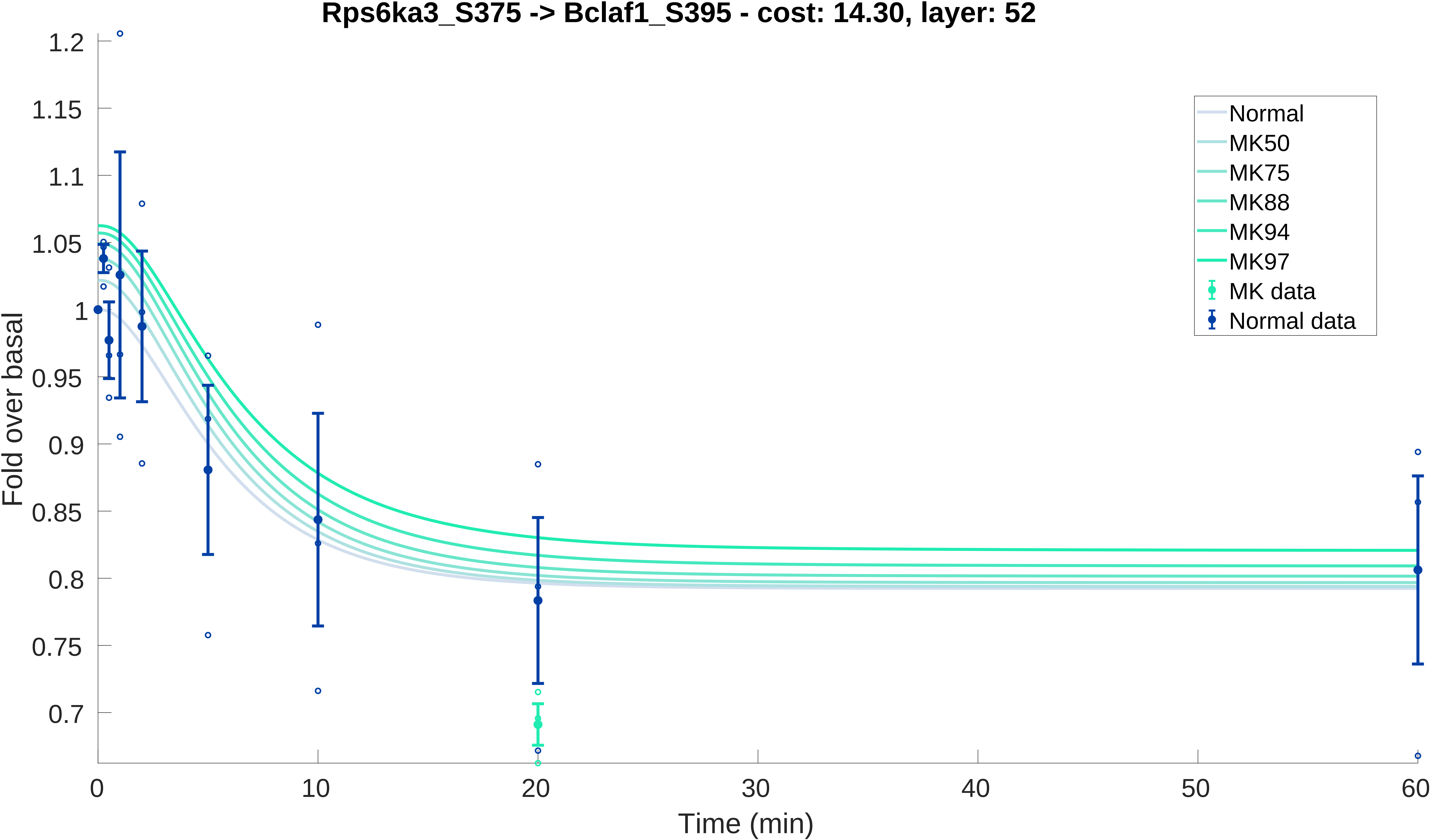

Rps6ka3_S375 -> Bclaf1_S395 - cost: 14.30, layer: 52
Fold over basal
Time (min)
1.2
1.15
1.1
1.05
1
0.95
0.9
0.85
0.8
0.75
0.7
0
10
20
30
40
50
60
Normal
MK50
MK75
MK88
MK94
MK97
MK data
Normal data

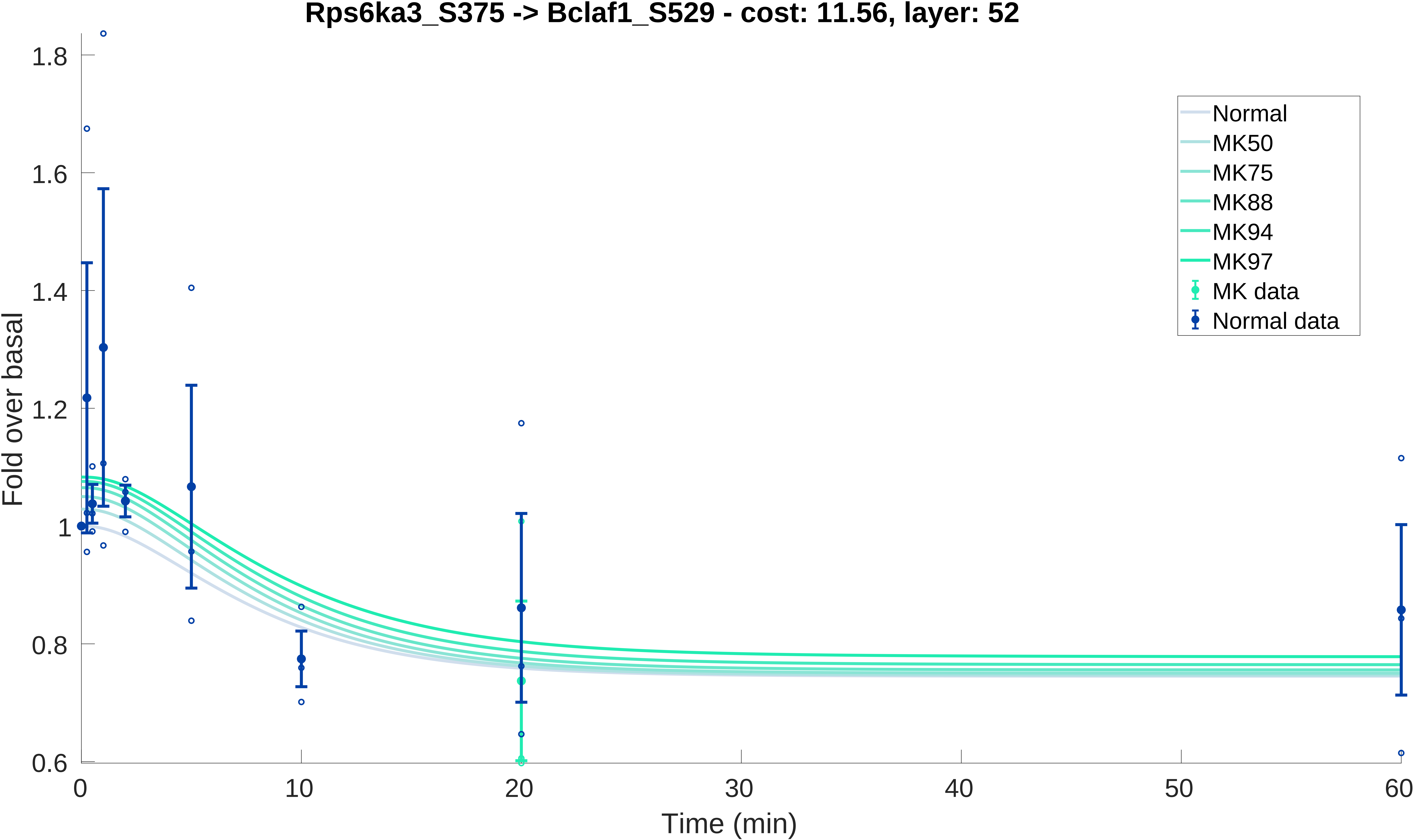
Rps6ka3_S375 -> Bclaf1_S529 - cost: 11.56, layer: 52
Fold over basal
Time (min)
0.6
0.8
1
1.2
1.4
1.6
1.8
0
10
20
30
40
50
60
Normal
MK50
MK75
MK88
MK94
MK97
MK data
Normal data

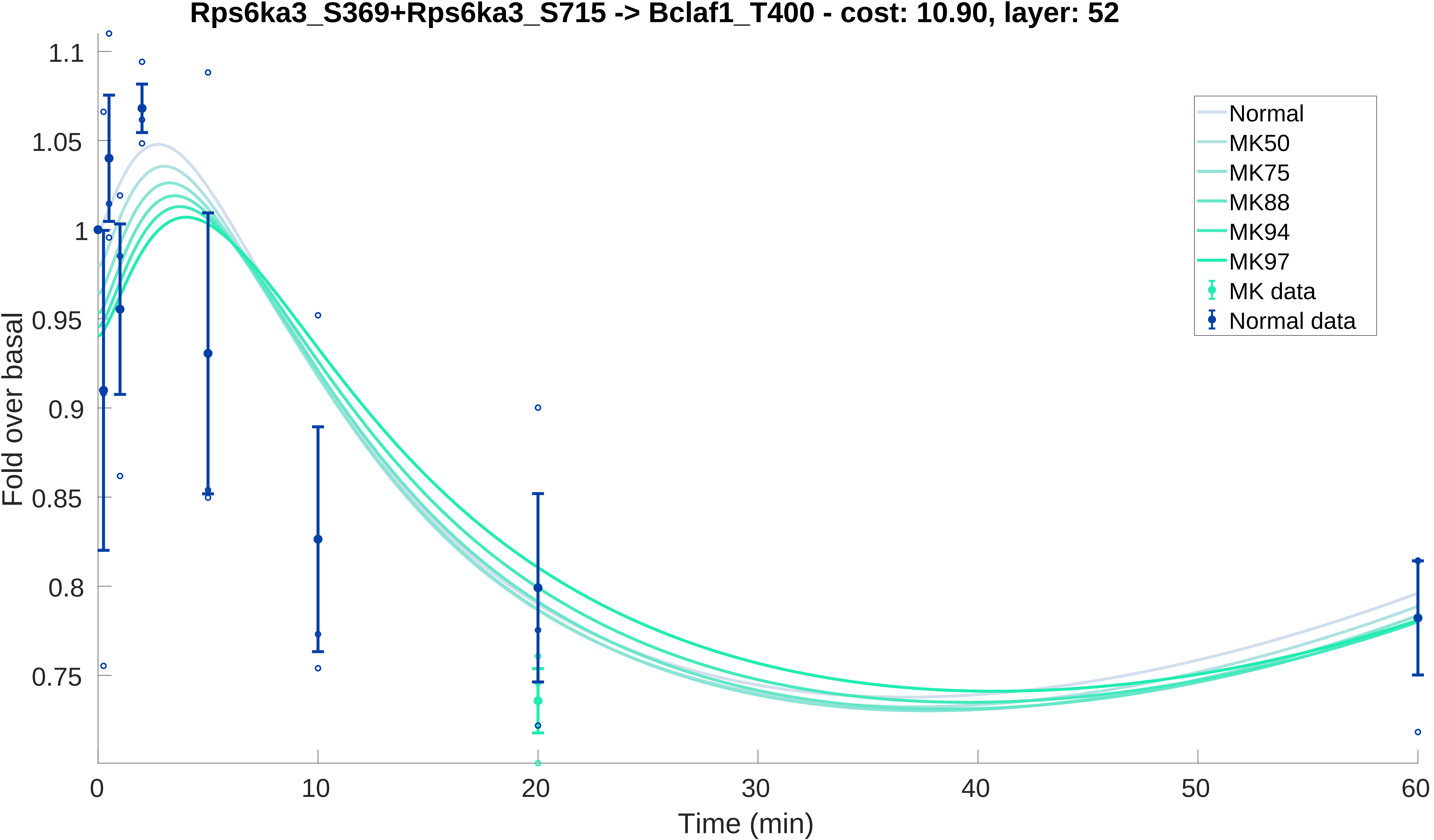
Rps6ka3_S369+Rps6ka3_S715 -> Bclaf1_T400 - cost: 10.90, layer: 52
Fold over basal
Time (min)
1.1
1.05
1
0.95
0.9
0.85
0.8
0.75
0
10
20
30
40
50
60
Normal
MK50
MK75
MK88
MK94
MK97
MK data
Normal data

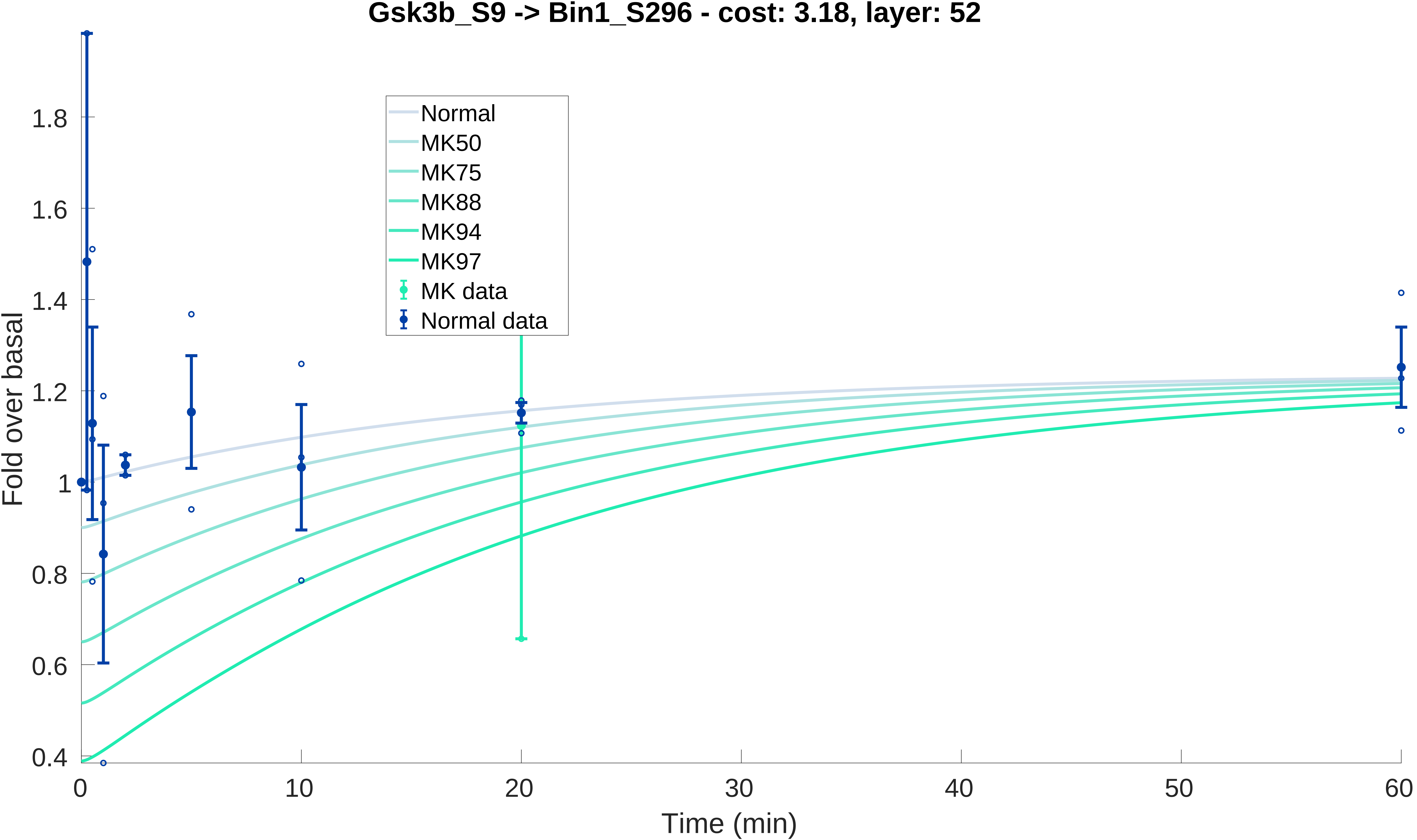
Gsk3b_S9 -> Bin1_S296 - cost: 3.18, layer: 52
Normal
MK50
MK75
MK88
MK94
MK97
MK data
Normal data
Fold over basal
Time (min)
0.4
0.6
0.8
1
1.2
1.4
1.6
1.8
0
10
20
30
40
50
60

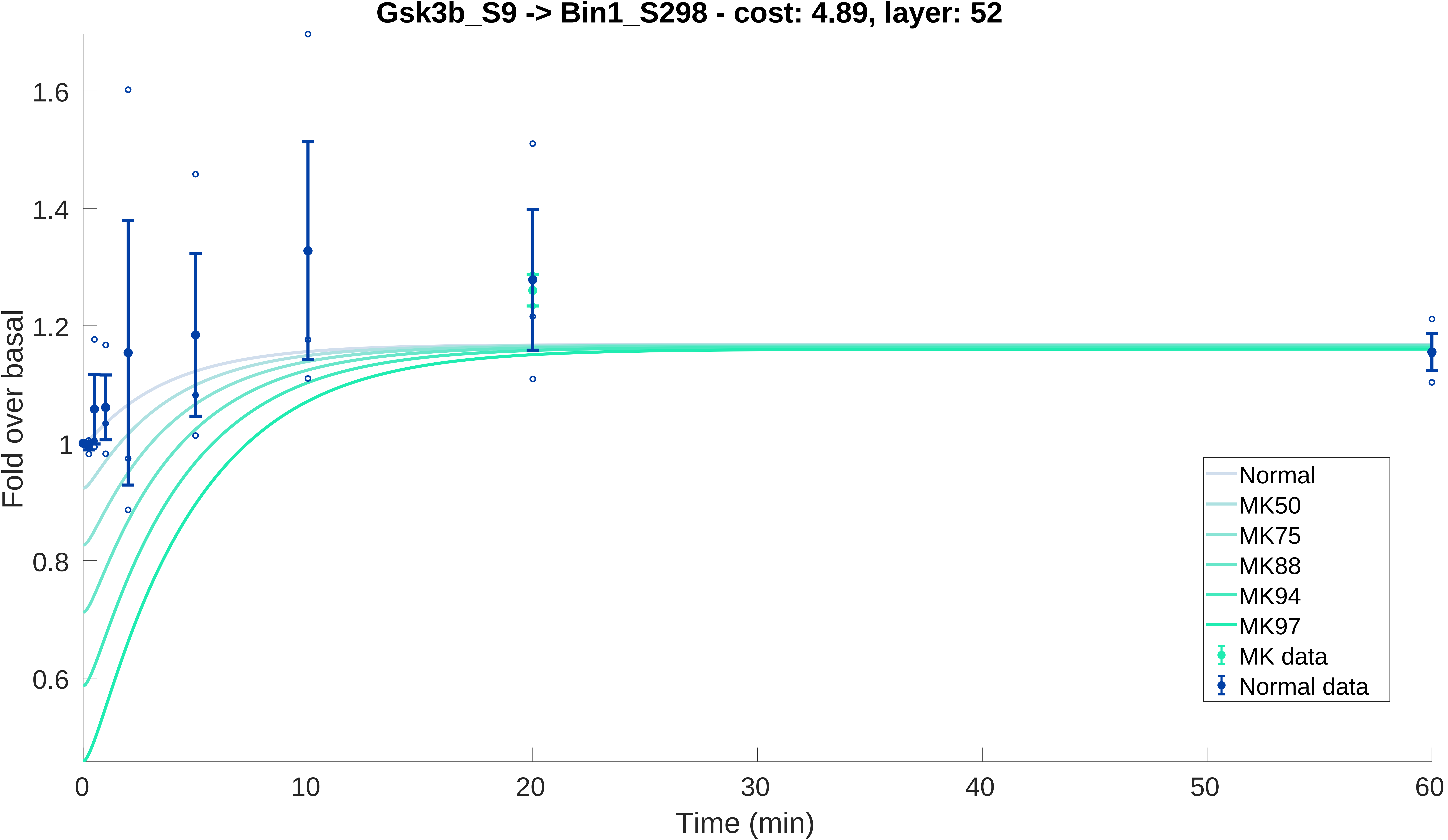

Gsk3b_S9 -> Bin1_S298 - cost: 4.89, layer: 52
1.6
1.4
1.2
1
0.8
0.6
Fold over basal
0
10
20
30
40
50
60
Time (min)
Normal
MK50
MK75
MK88
MK94
MK97
MK data
Normal data

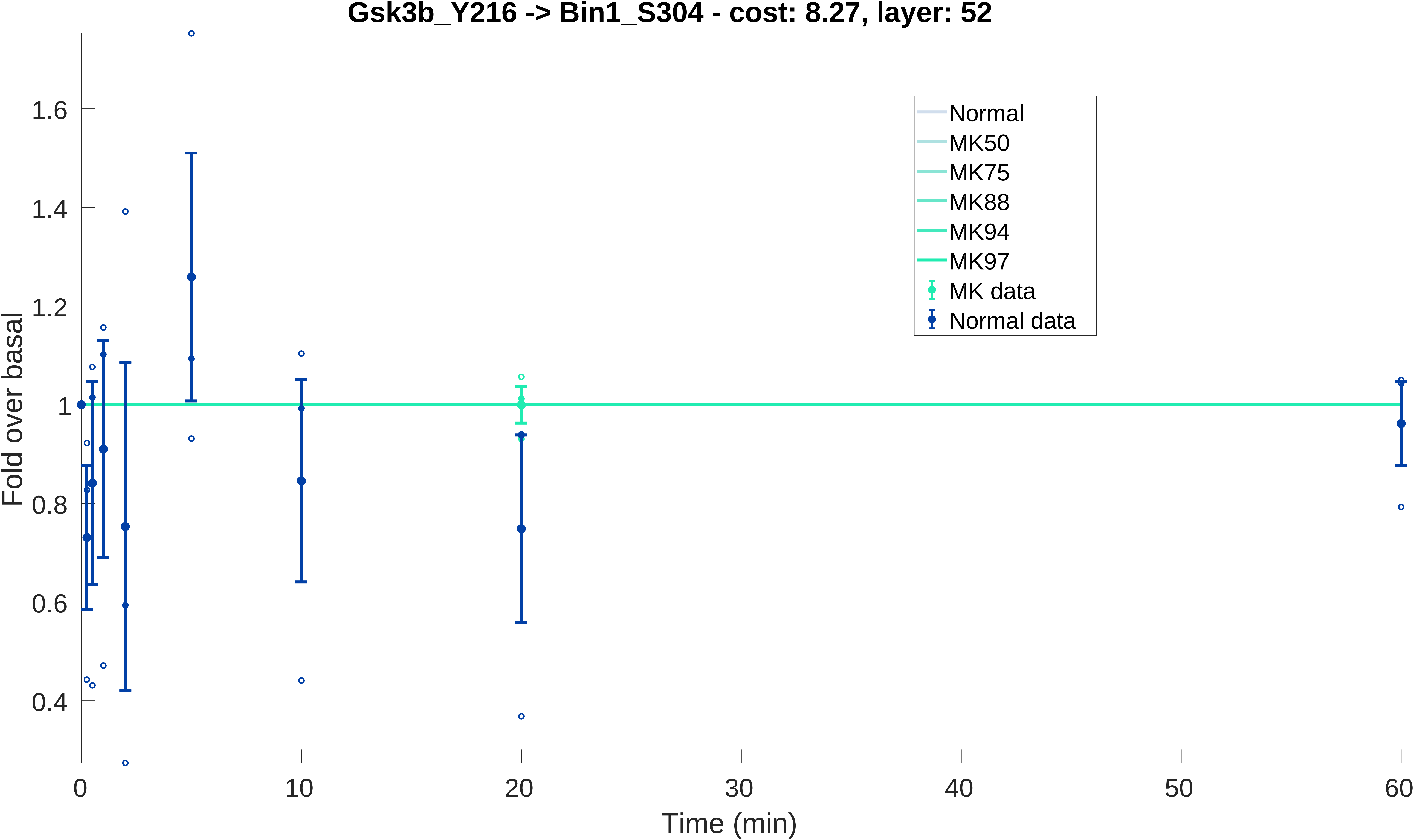

Gsk3b_Y216 -> Bin1_S304 - cost: 8.27, layer: 52
Fold over basal
1.6
1.4
1.2
1
0.8
0.6
0.4
Time (min)
0
10
20
30
40
50
60
Normal
MK50
MK75
MK88
MK94
MK97
MK data
Normal data

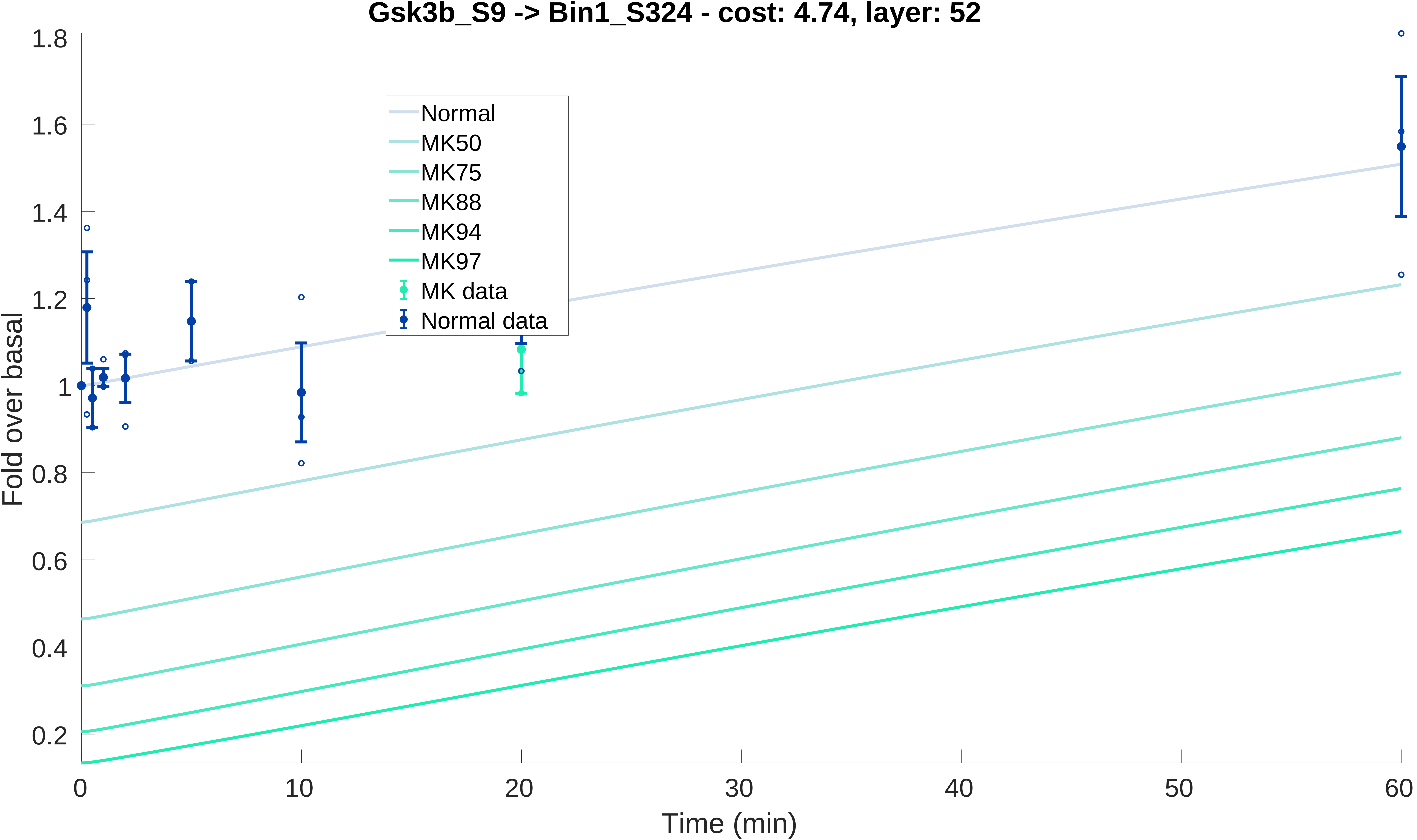

Gsk3b_S9 -> Bin1_S324 - cost: 4.74, layer: 52
Fold over basal
Time (min)
1.8
1.6
1.4
1.2
1
0.8
0.6
0.4
0.2
0
10
20
30
40
50
60
Normal
MK50
MK75
MK88
MK94
MK97
MK data
Normal data

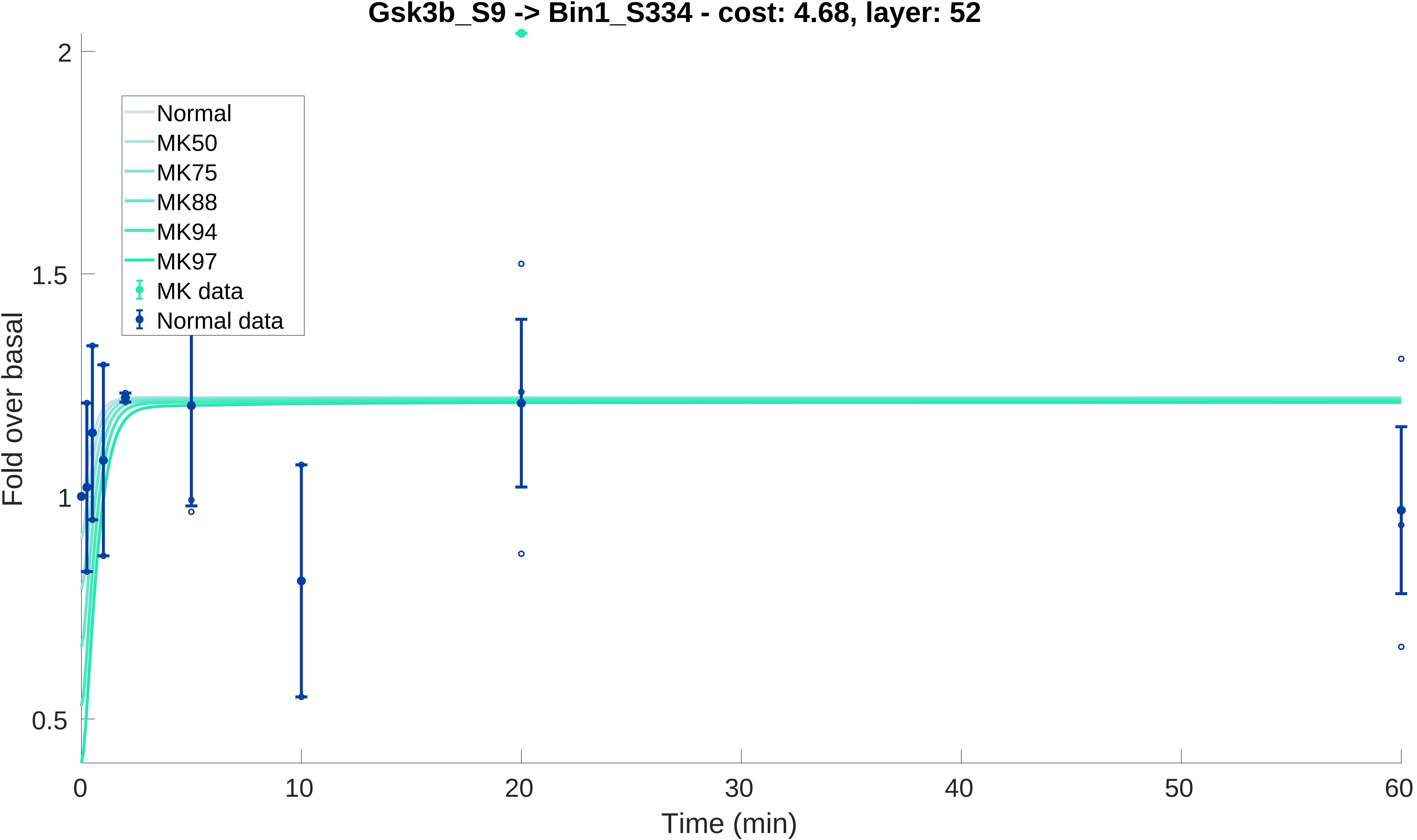

Gsk3b_S9 -> Bin1_S334 - cost: 4.68, layer: 52
Normal
MK50
MK75
MK88
MK94
MK97
MK data
Normal data
Fold over basal
Time (min)
0.5
1
1.5
2
0
10
20
30
40
50
60

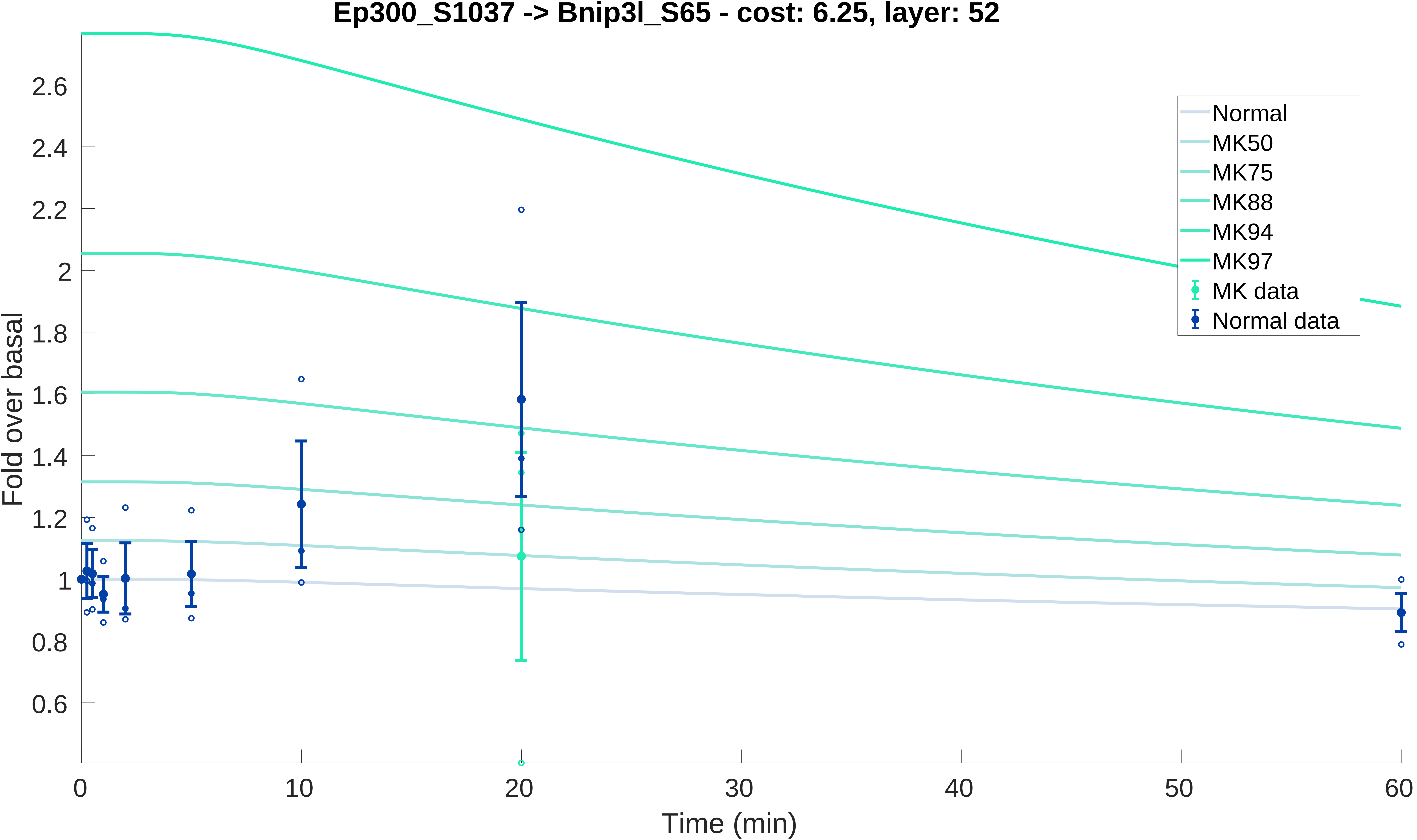

Ep300_S1037 -> Bnip3l_S65 - cost: 6.25, layer: 52
Fold over basal
Time (min)
Normal
MK50
MK75
MK88
MK94
MK97
MK data
Normal data
2.6
2.4
2.2
2
1.8
1.6
1.4
1.2
1
0.8
0.6
0
10
20
30
40
50
60

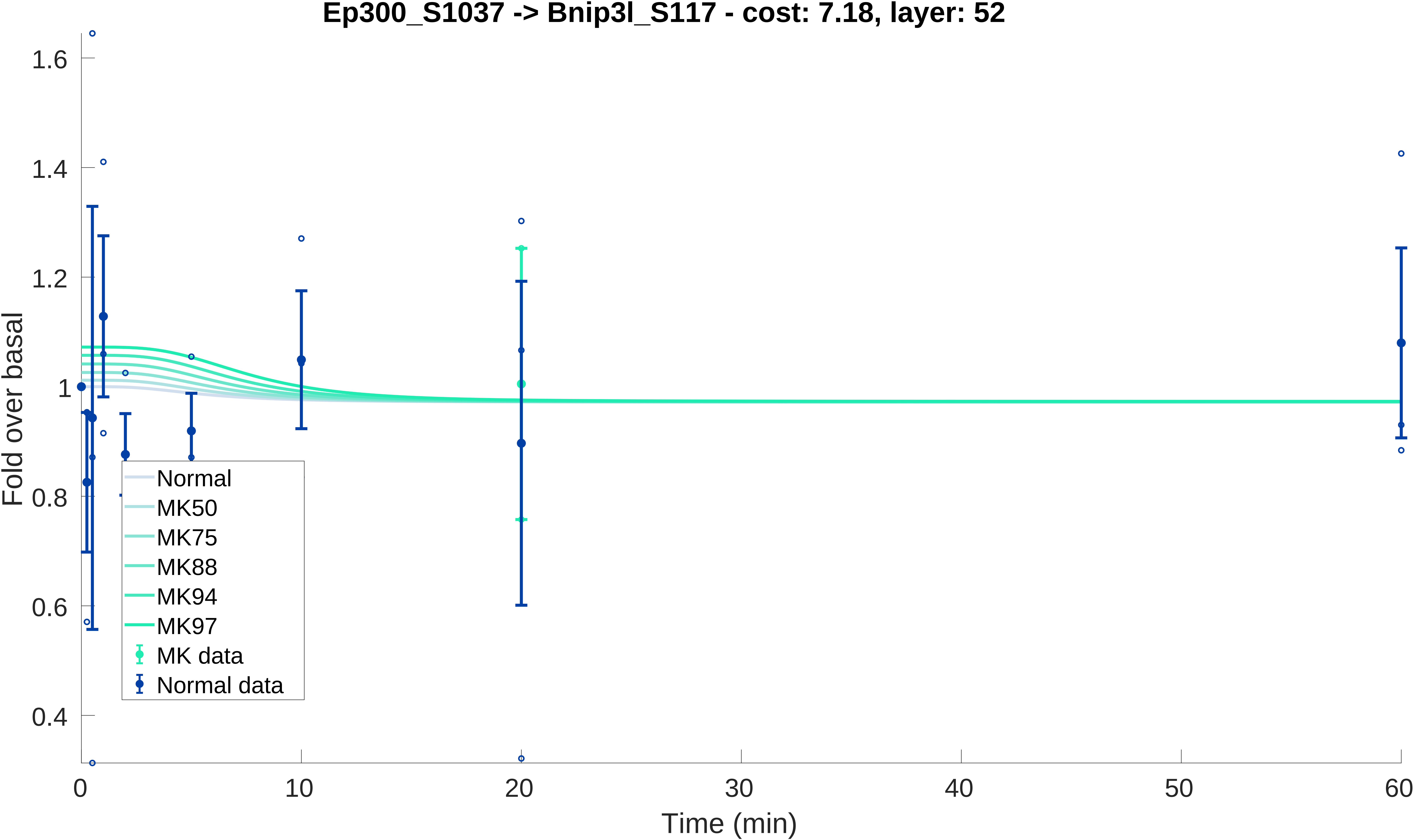

Ep300_S1037 -> Bnip3l_S117 - cost: 7.18, layer: 52
Fold over basal
1.6
1.4
1.2
1
0.8
0.6
0.4
0
10
20
30
40
50
60
Time (min)
Normal
MK50
MK75
MK88
MK94
MK97
MK data
Normal data

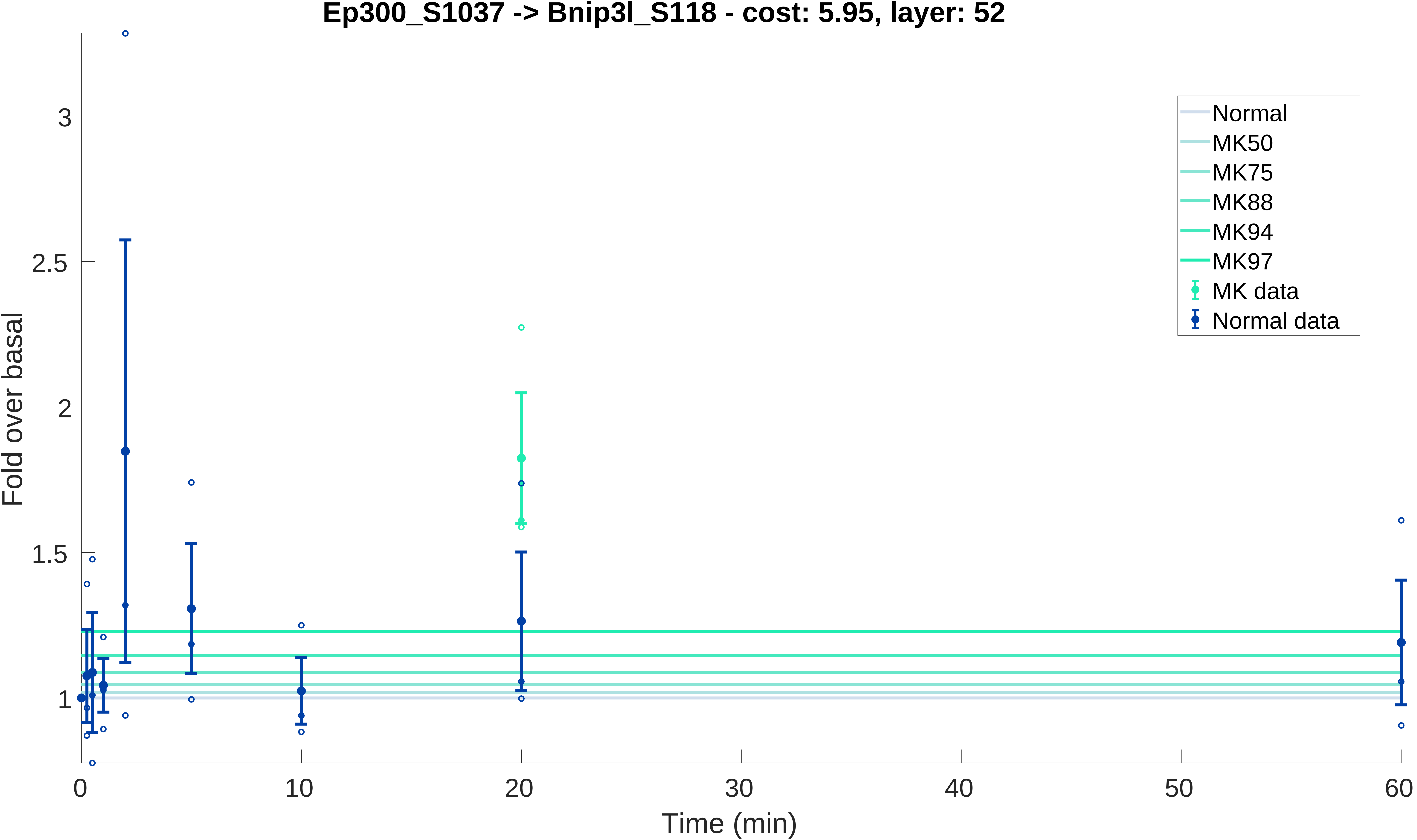
Ep300_S1037 -> Bnip3l_S118 - cost: 5.95, layer: 52
Fold over basal
Time (min)
3
2.5
2
1.5
1
0
10
20
30
40
50
60
Normal
MK50
MK75
MK88
MK94
MK97
MK data
Normal data

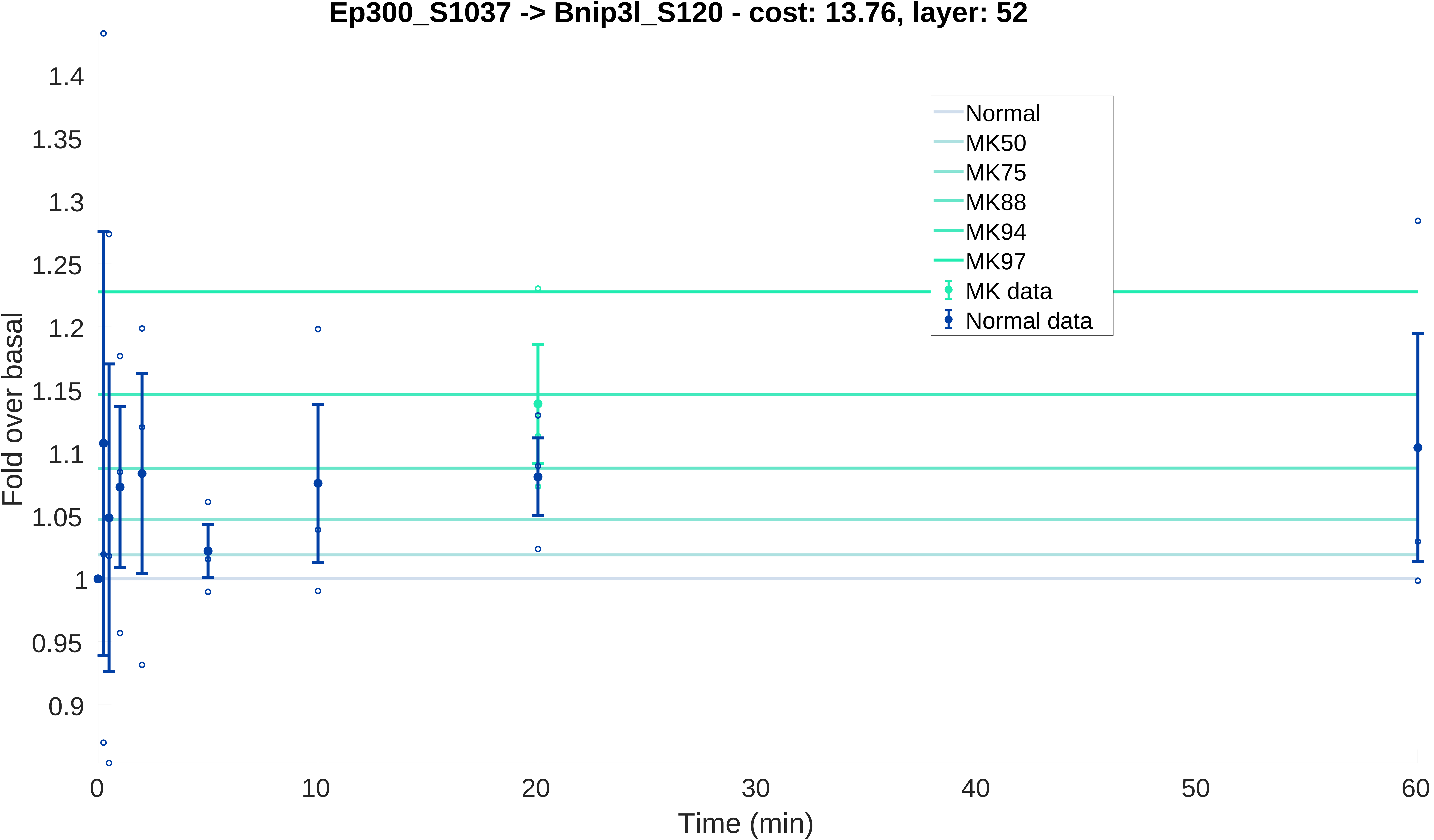

Ep300_S1037 -> Bnip3l_S120 - cost: 13.76, layer: 52
Fold over basal
1.4
1.35
1.3
1.25
1.2
1.15
1.1
1.05
1
0.95
0.9
Time (min)
0
10
20
30
40
50
60
Normal
MK50
MK75
MK88
MK94
MK97
MK data
Normal data

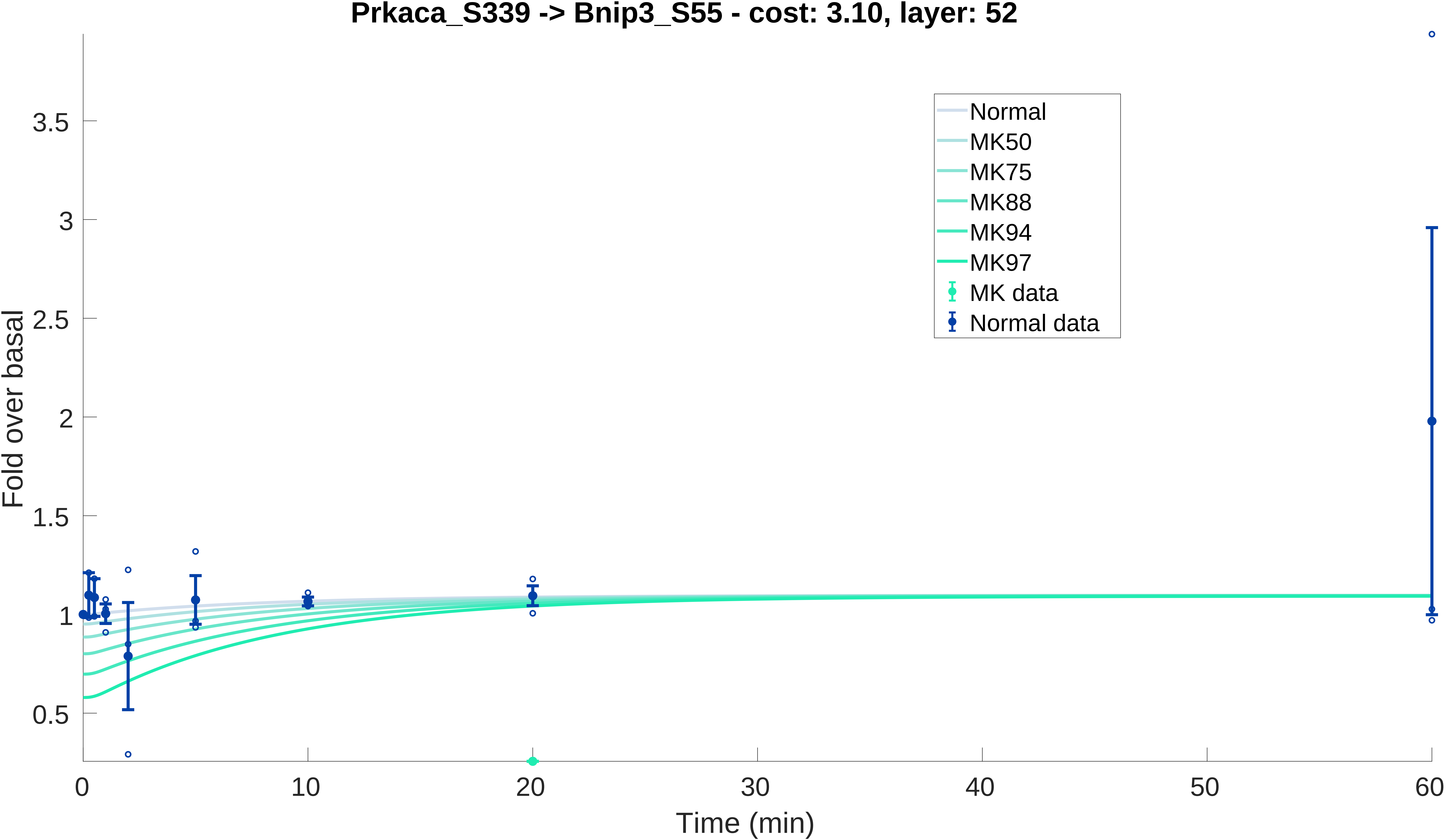

Prkaca_S339 -> Bnip3_S55 - cost: 3.10, layer: 52
Fold over basal
0.5
1
1.5
2
2.5
3
3.5
0
10
20
30
40
50
60
Time (min)
Normal
MK50
MK75
MK88
MK94
MK97
MK data
Normal data

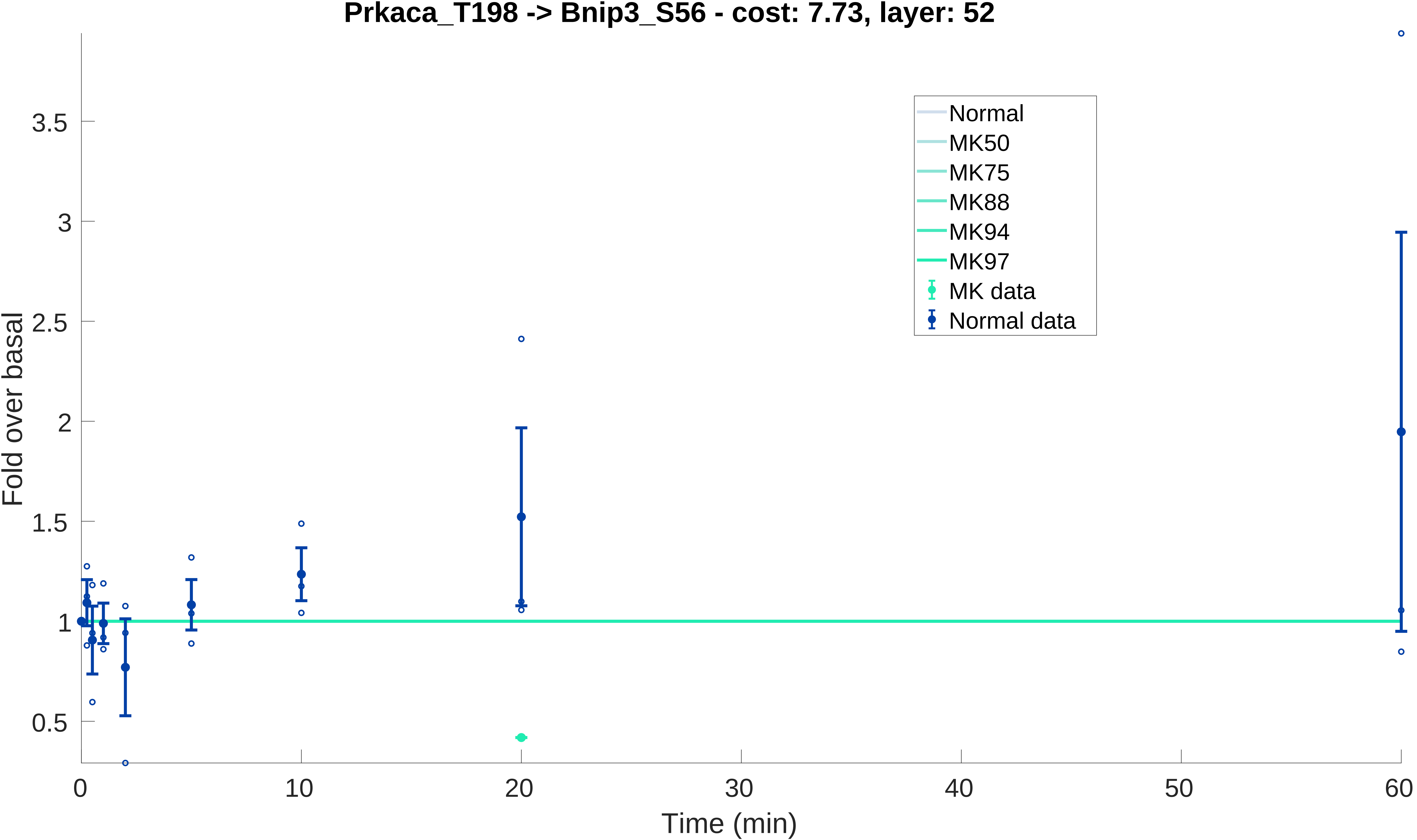
Prkaca_T198 -> Bnip3_S56 - cost: 7.73, layer: 52
Fold over basal
Time (min)
Normal
MK50
MK75
MK88
MK94
MK97
MK data
Normal data
0.5
1
1.5
2
2.5
3
3.5
0
10
20
30
40
50
60

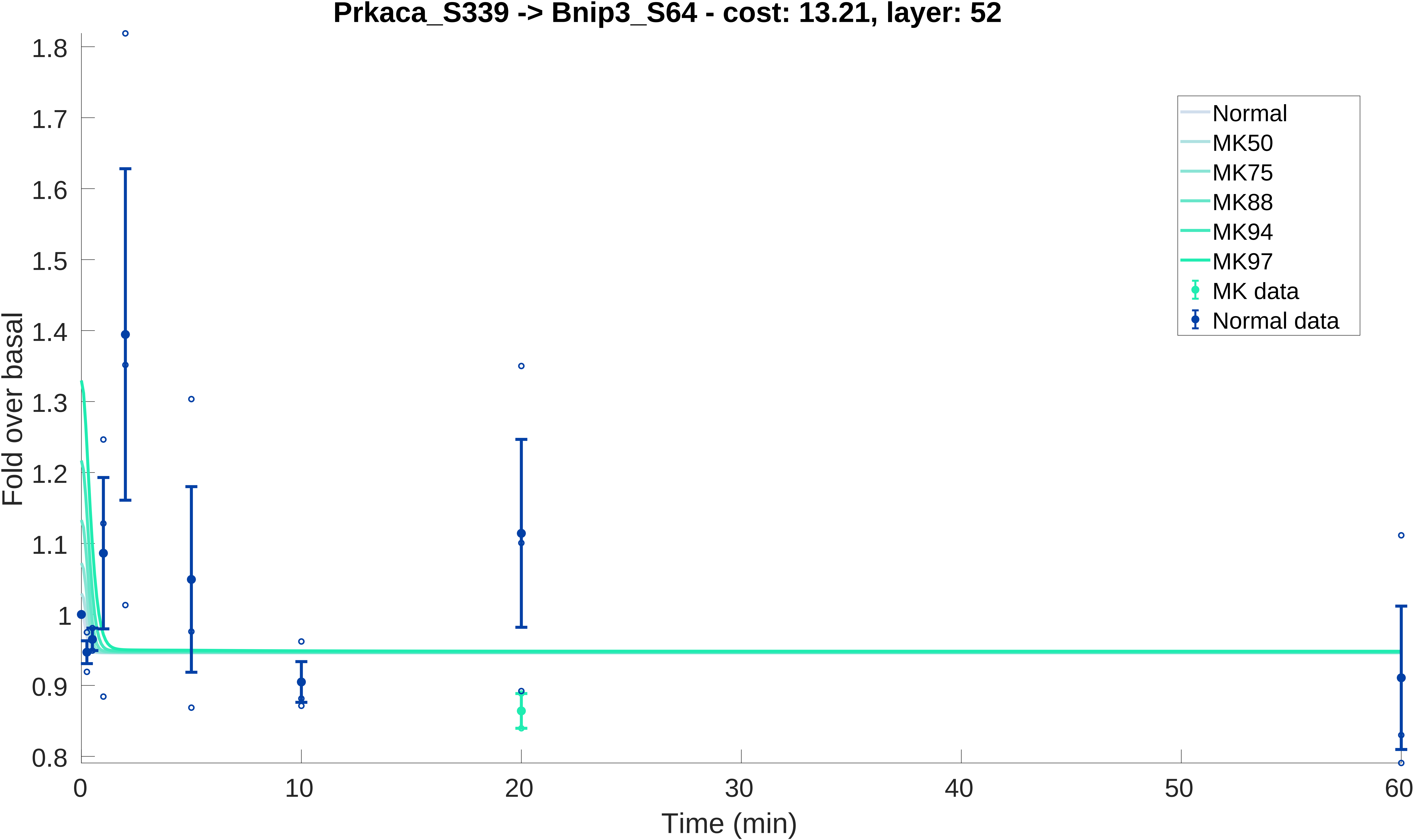

Prkaca_S339 -> Bnip3_S64 - cost: 13.21, layer: 52
Fold over basal
Time (min)
Normal
MK50
MK75
MK88
MK94
MK97
MK data
Normal data
1.8
1.7
1.6
1.5
1.4
1.3
1.2
1.1
1
0.9
0.8
0
10
20
30
40
50
60

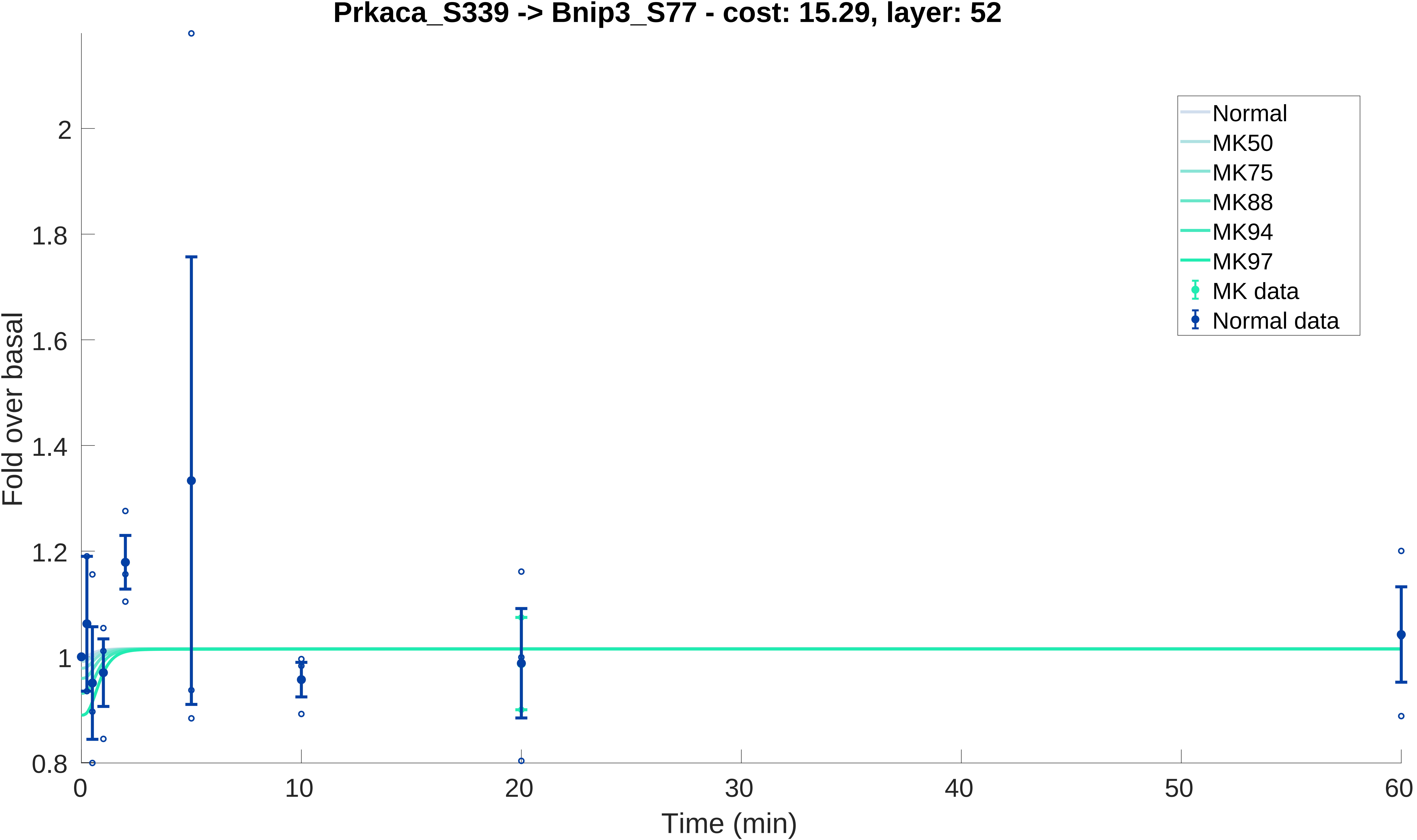

Prkaca_S339 -> Bnip3_S77 - cost: 15.29, layer: 52
Fold over basal
Time (min)
0.8
1
1.2
1.4
1.6
1.8
2
0
10
20
30
40
50
60
Normal
MK50
MK75
MK88
MK94
MK97
MK data
Normal data

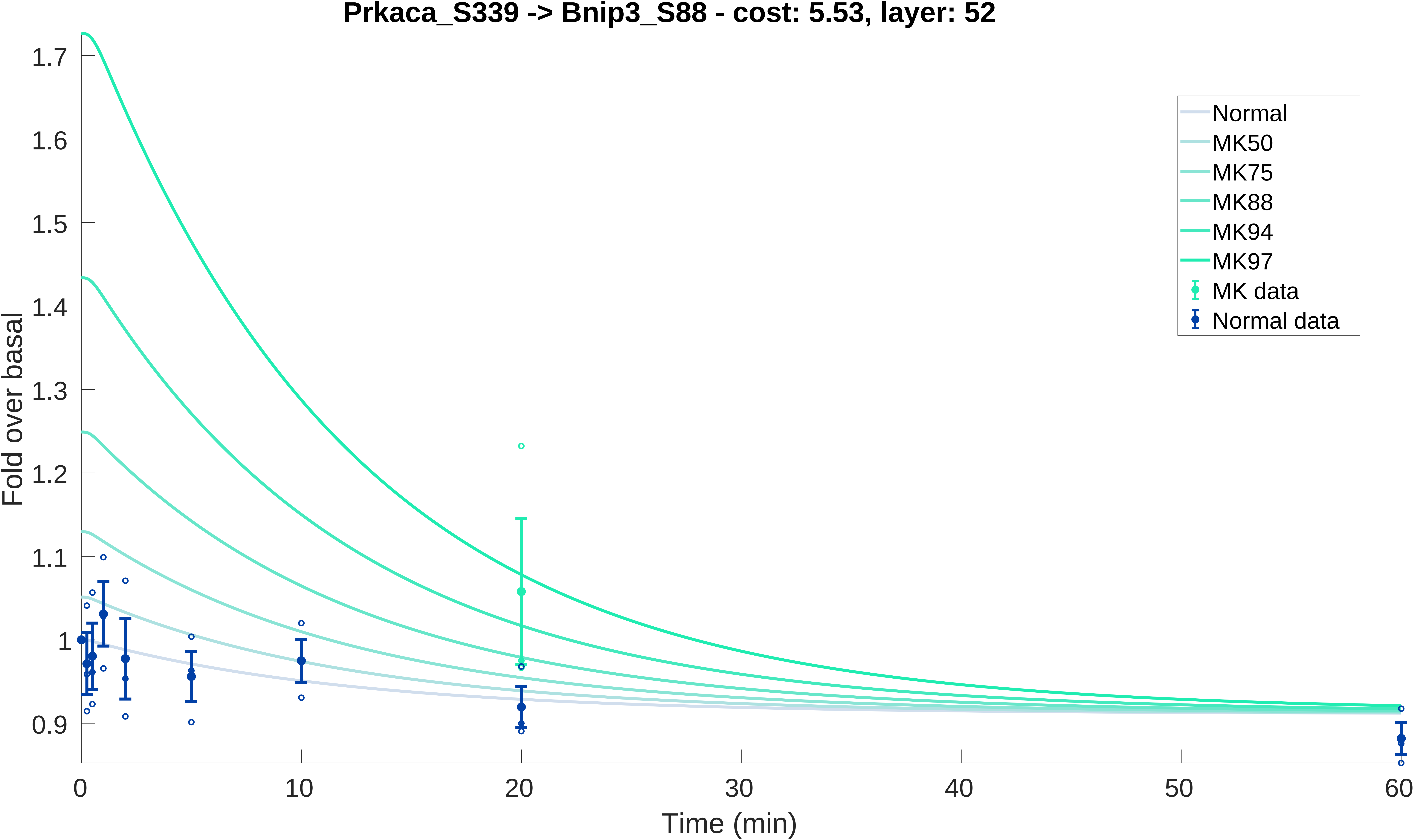

Prkaca_S339 -> Bnip3_S88 - cost: 5.53, layer: 52
Fold over basal
Time (min)
1.7
1.6
1.5
1.4
1.3
1.2
1.1
1
0.9
0
10
20
30
40
50
60
Normal
MK50
MK75
MK88
MK94
MK97
MK data
Normal data

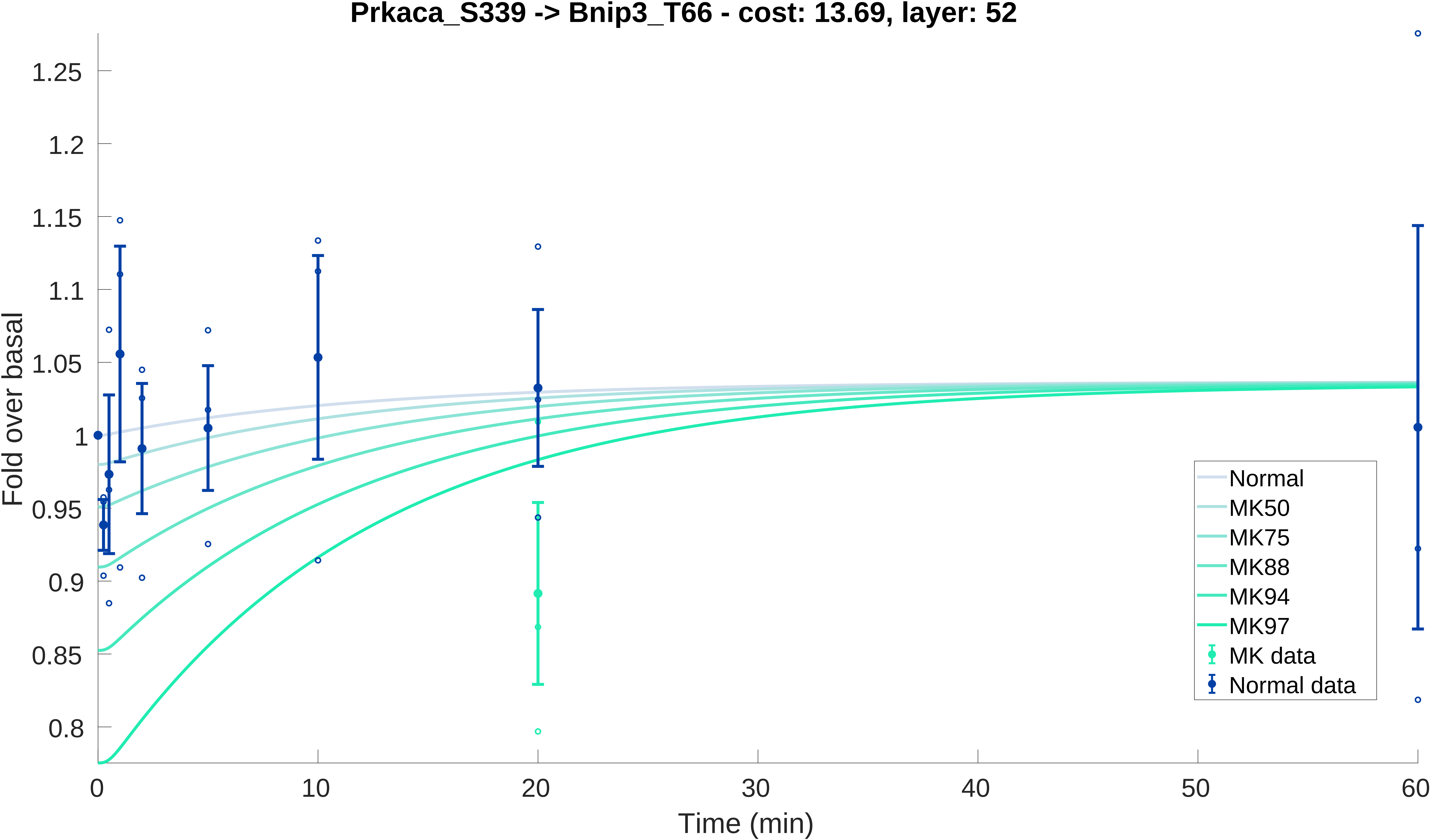

Prkaca_S339 -> Bnip3_T66 - cost: 13.69, layer: 52
Fold over basal
1.25
1.2
1.15
1.1
1.05
1
0.95
0.9
0.85
0.8
0
10
20
30
40
50
60
Time (min)
Normal
MK50
MK75
MK88
MK94
MK97
MK data
Normal data

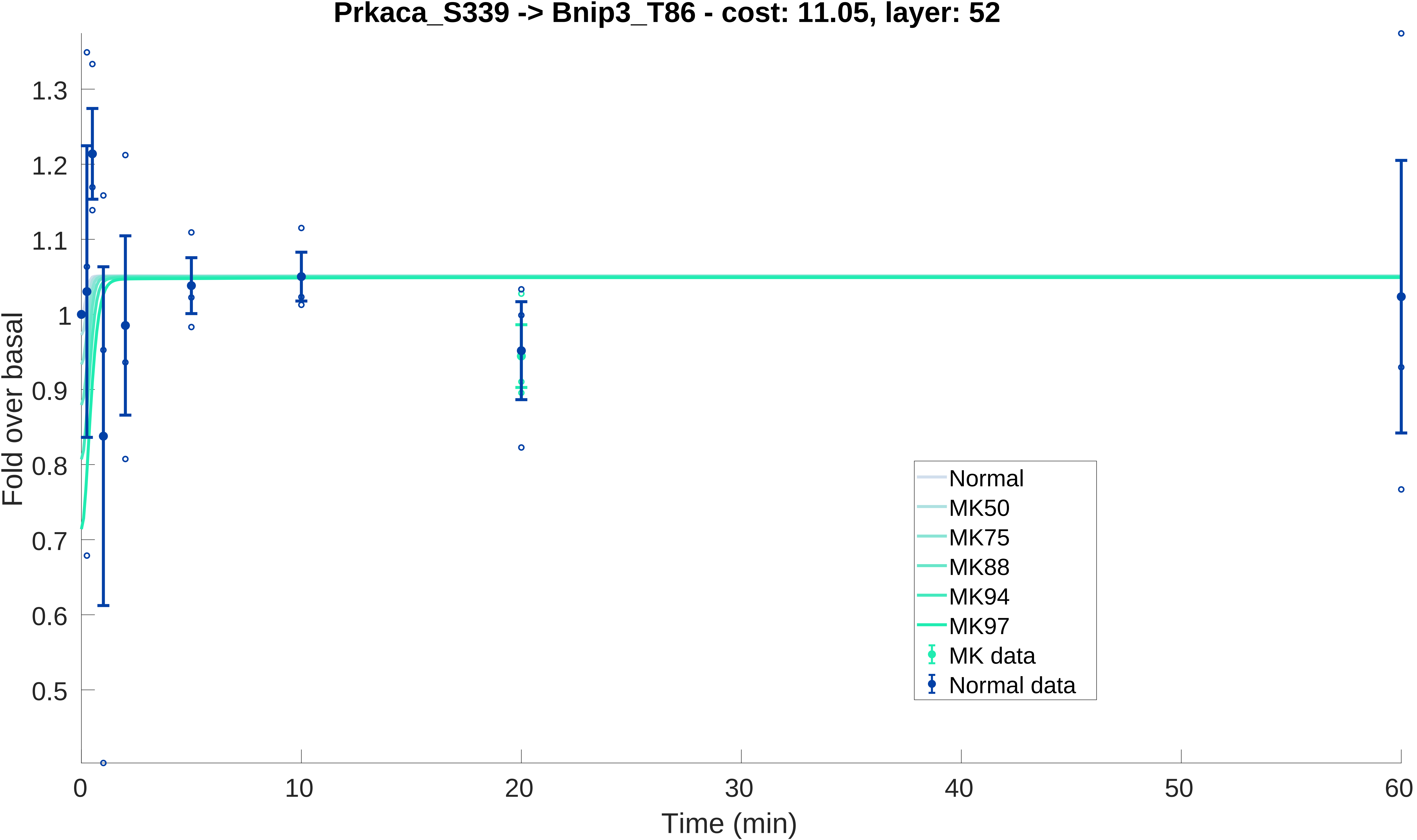
Prkaca_S339 -> Bnip3_T86 - cost: 11.05, layer: 52
Fold over basal
Time (min)
1.3
1.2
1.1
1
0.9
0.8
0.7
0.6
0.5
0
10
20
30
40
50
60
Normal
MK50
MK75
MK88
MK94
MK97
MK data
Normal data

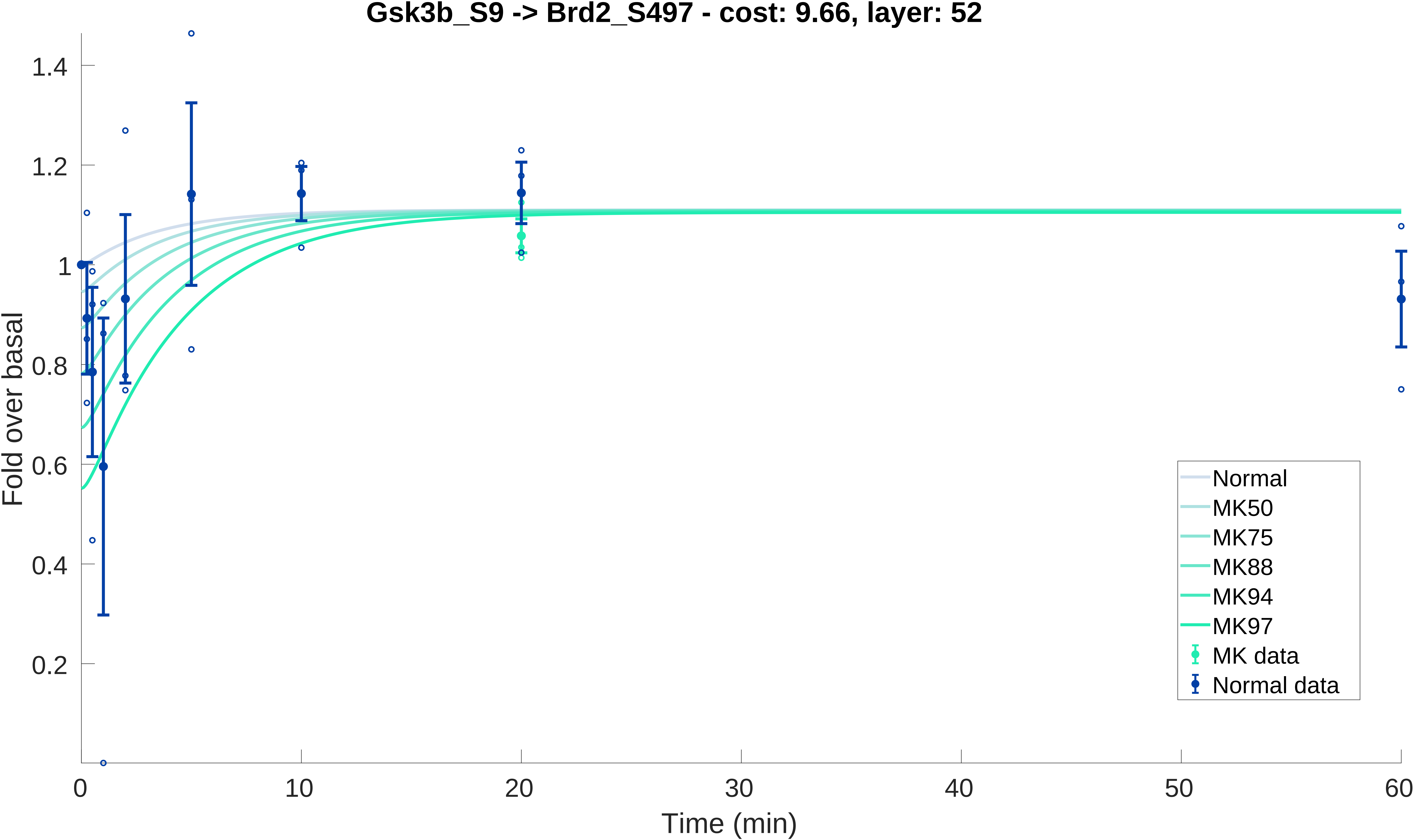

Gsk3b_S9 -> Brd2_S497 - cost: 9.66, layer: 52
Fold over basal
1.4
1.2
1
0.8
0.6
0.4
0.2
0
10
20
30
40
50
60
Time (min)
Normal
MK50
MK75
MK88
MK94
MK97
MK data
Normal data

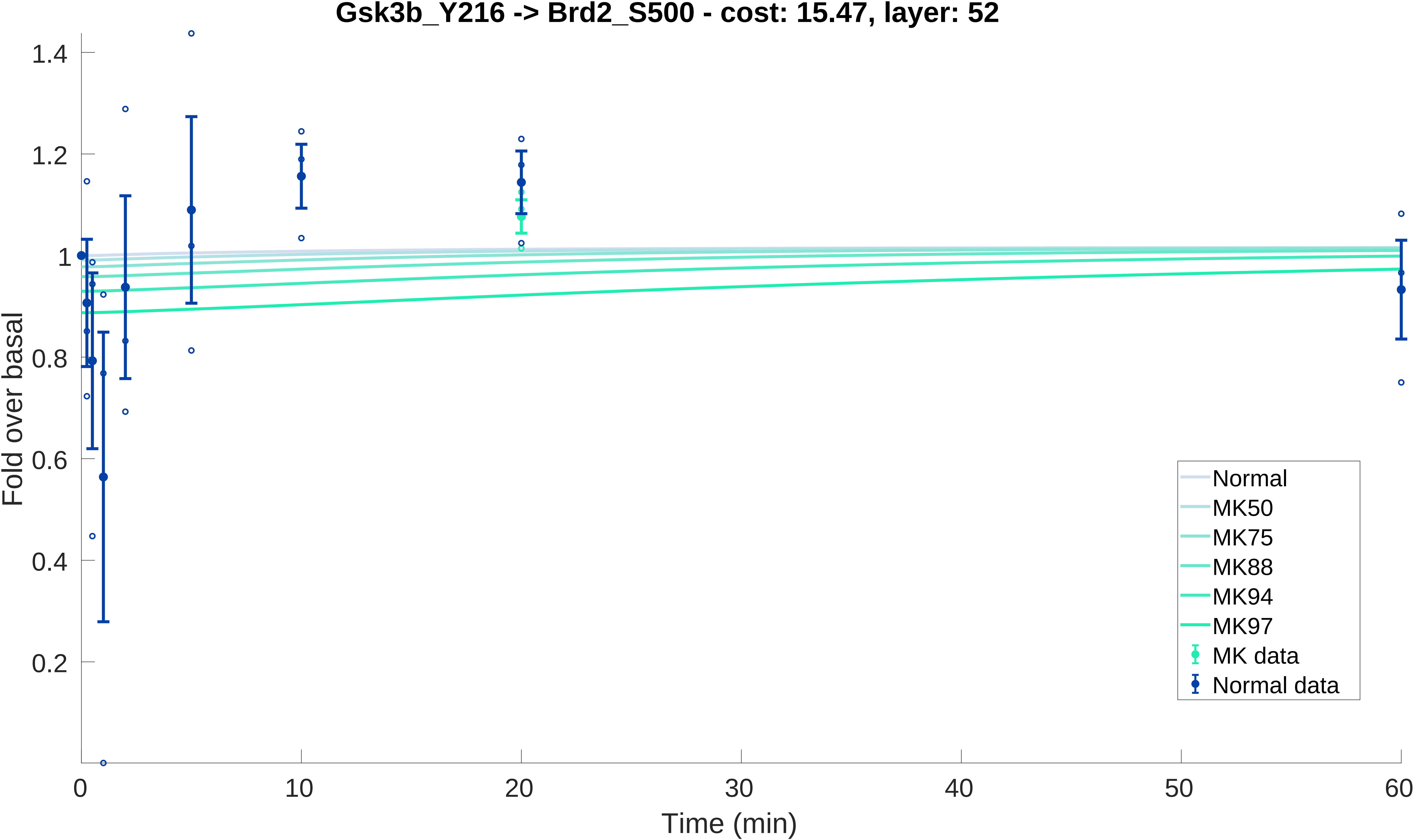

Gsk3b_Y216 -> Brd2_S500 - cost: 15.47, layer: 52
Fold over basal
1.4
1.2
1
0.8
0.6
0.4
0.2
0
10
20
30
40
50
60
Time (min)
Normal
MK50
MK75
MK88
MK94
MK97
MK data
Normal data

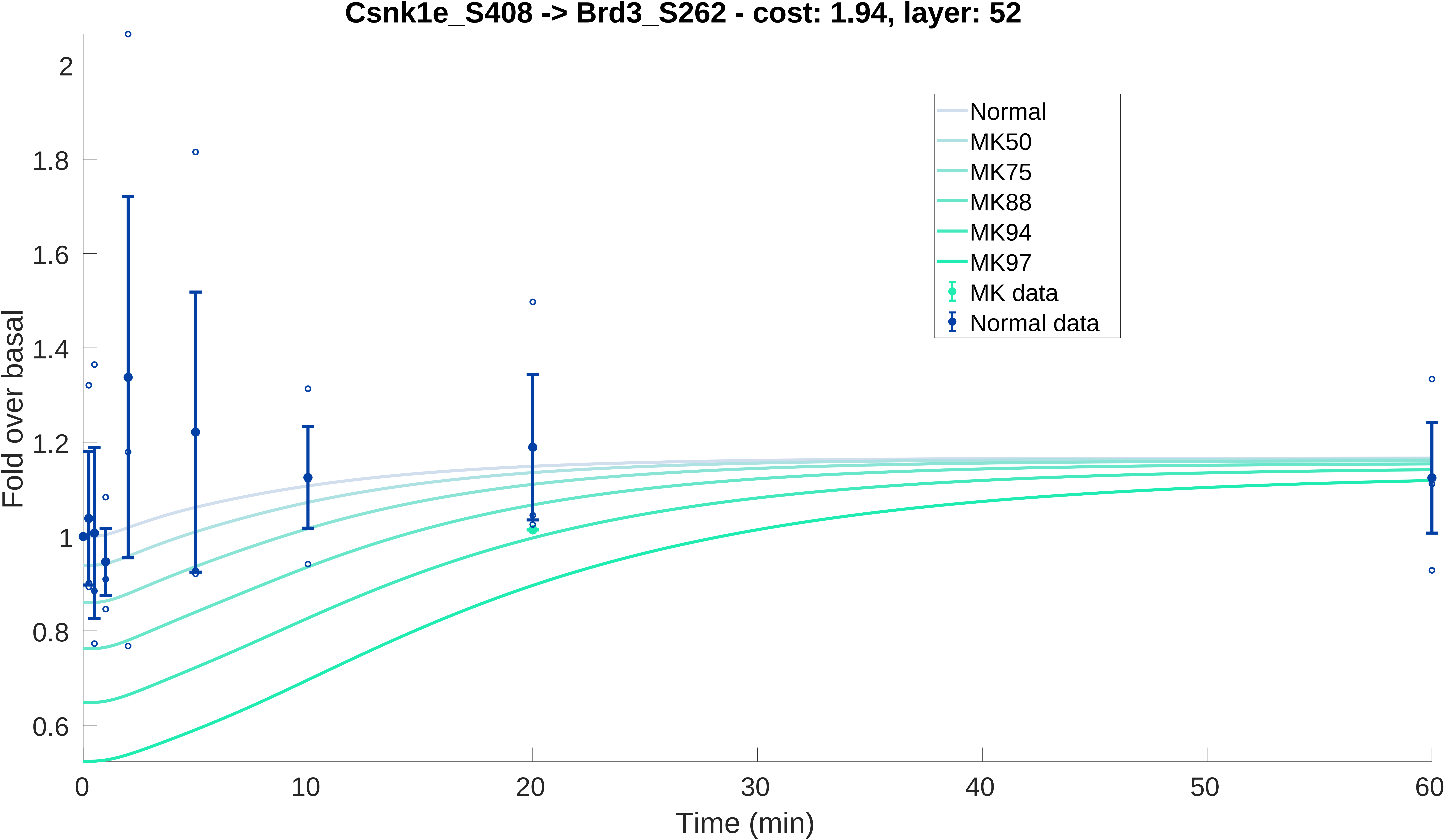
Csnk1e_S408 -> Brd3_S262 - cost: 1.94, layer: 52
Fold over basal
Time (min)
0.6
0.8
1
1.2
1.4
1.6
1.8
2
0
10
20
30
40
50
60
Normal
MK50
MK75
MK88
MK94
MK97
MK data
Normal data

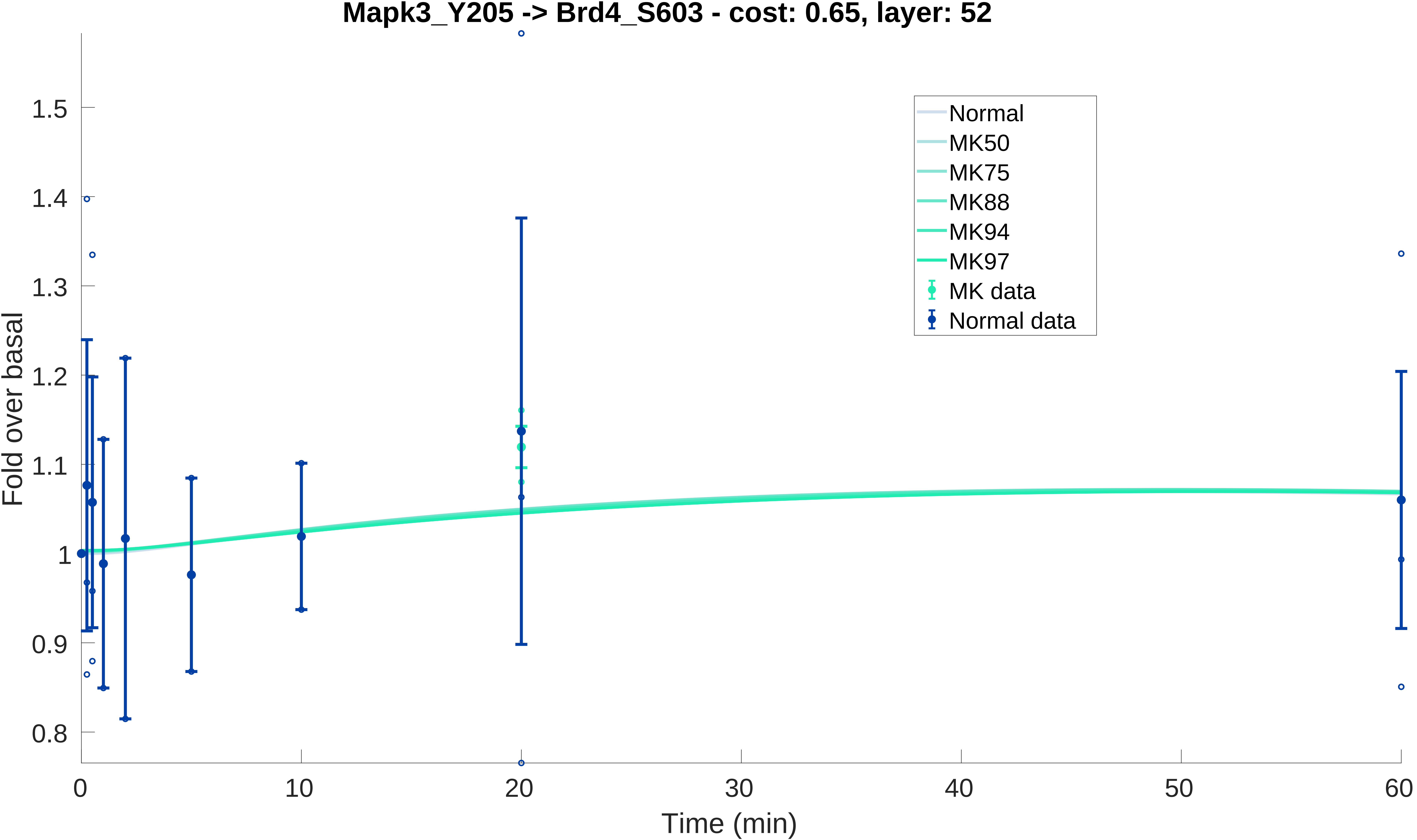

Mapk3_Y205 -> Brd4_S603 - cost: 0.65, layer: 52
Fold over basal
1.5
1.4
1.3
1.2
1.1
1
0.9
0.8
0
10
20
30
40
50
60
Time (min)
Normal
MK50
MK75
MK88
MK94
MK97
MK data
Normal data

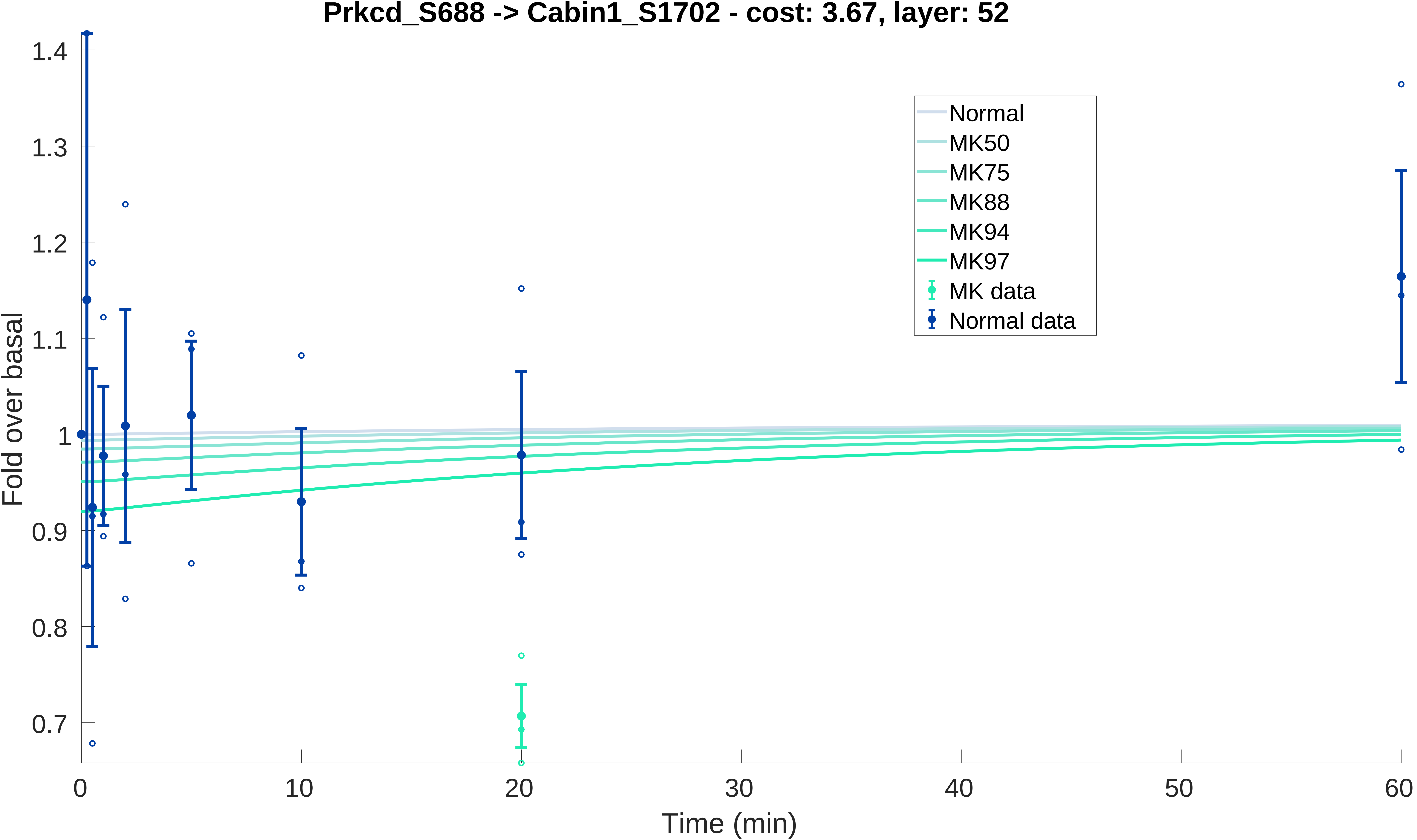

Prkcd_S688 -> Cabin1_S1702 - cost: 3.67, layer: 52
Fold over basal
Time (min)
1.4
1.3
1.2
1.1
1
0.9
0.8
0.7
0
10
20
30
40
50
60
Normal
MK50
MK75
MK88
MK94
MK97
MK data
Normal data

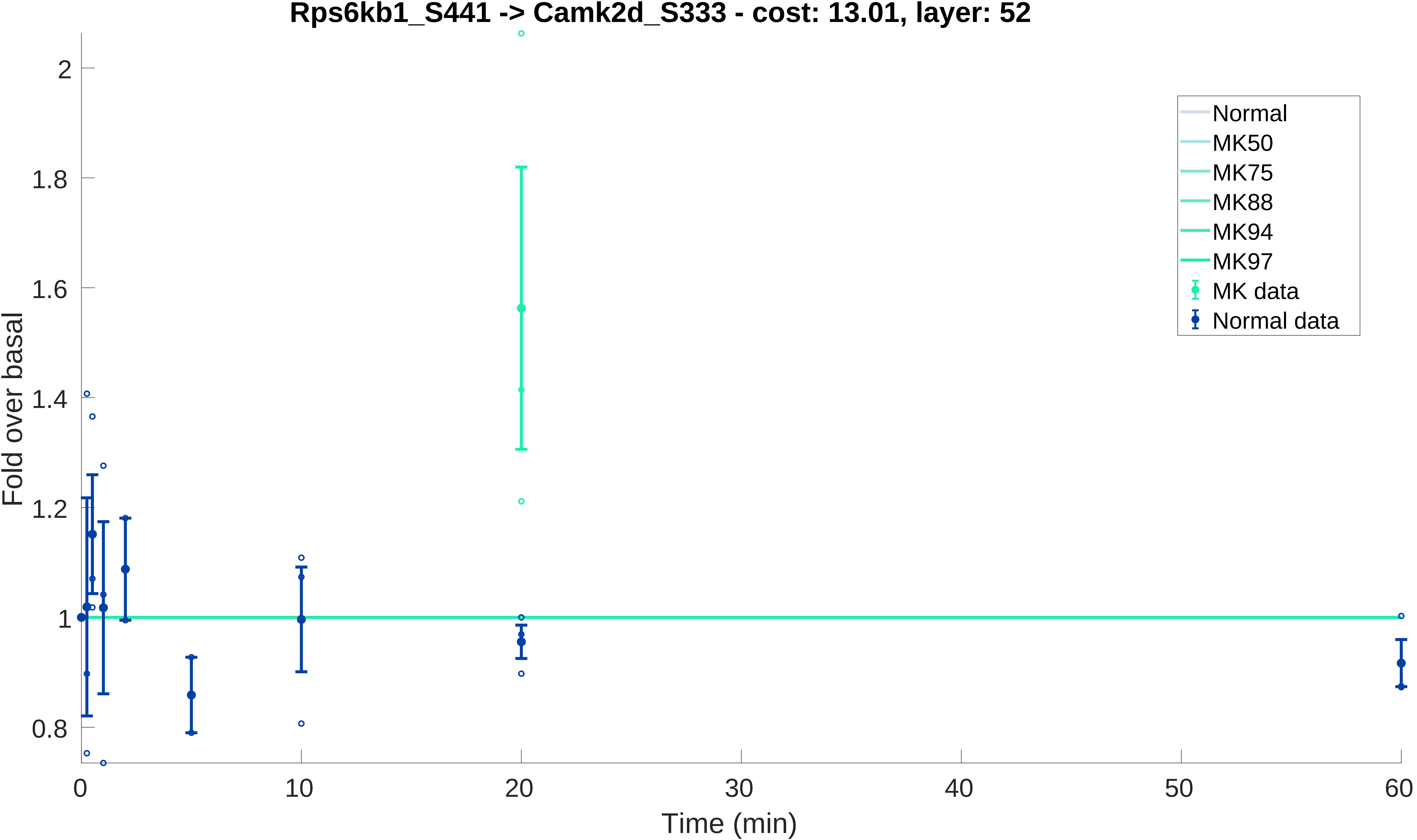

Rps6kb1_S441 -> Camk2d_S333 - cost: 13.01, layer: 52
Fold over basal
Time (min)
0.8
1
1.2
1.4
1.6
1.8
2
0
10
20
30
40
50
60
Normal
MK50
MK75
MK88
MK94
MK97
MK data
Normal data

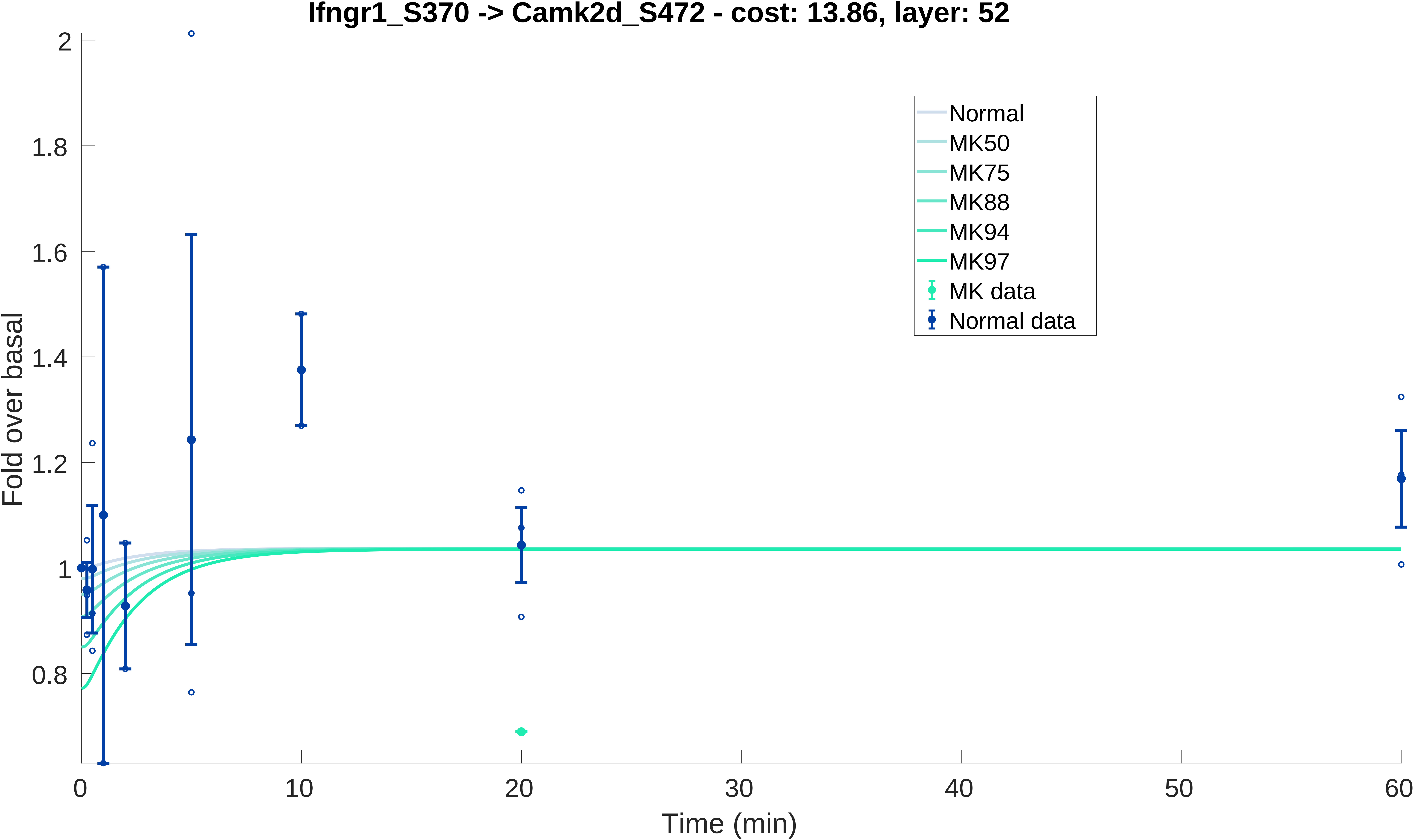
Ifngr1_S370 -> Camk2d_S472 - cost: 13.86, layer: 52
Fold over basal
Time (min)
0
10
20
30
40
50
60
0.8
1
1.2
1.4
1.6
1.8
2
Normal
MK50
MK75
MK88
MK94
MK97
MK data
Normal data

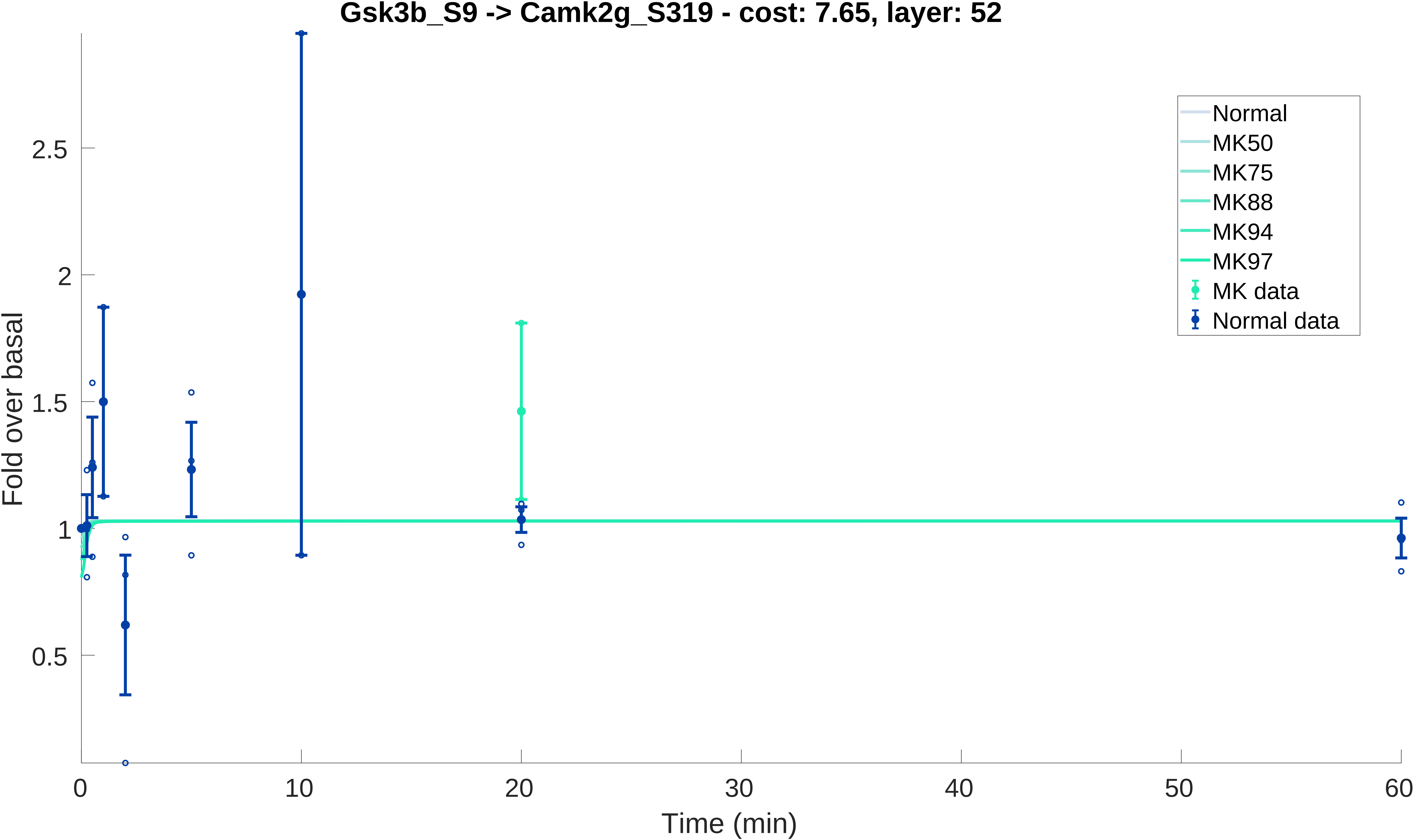

Gsk3b_S9 -> Camk2g_S319 - cost: 7.65, layer: 52
Fold over basal
Time (min)
0.5
1
1.5
2
2.5
0
10
20
30
40
50
60
Normal
MK50
MK75
MK88
MK94
MK97
MK data
Normal data

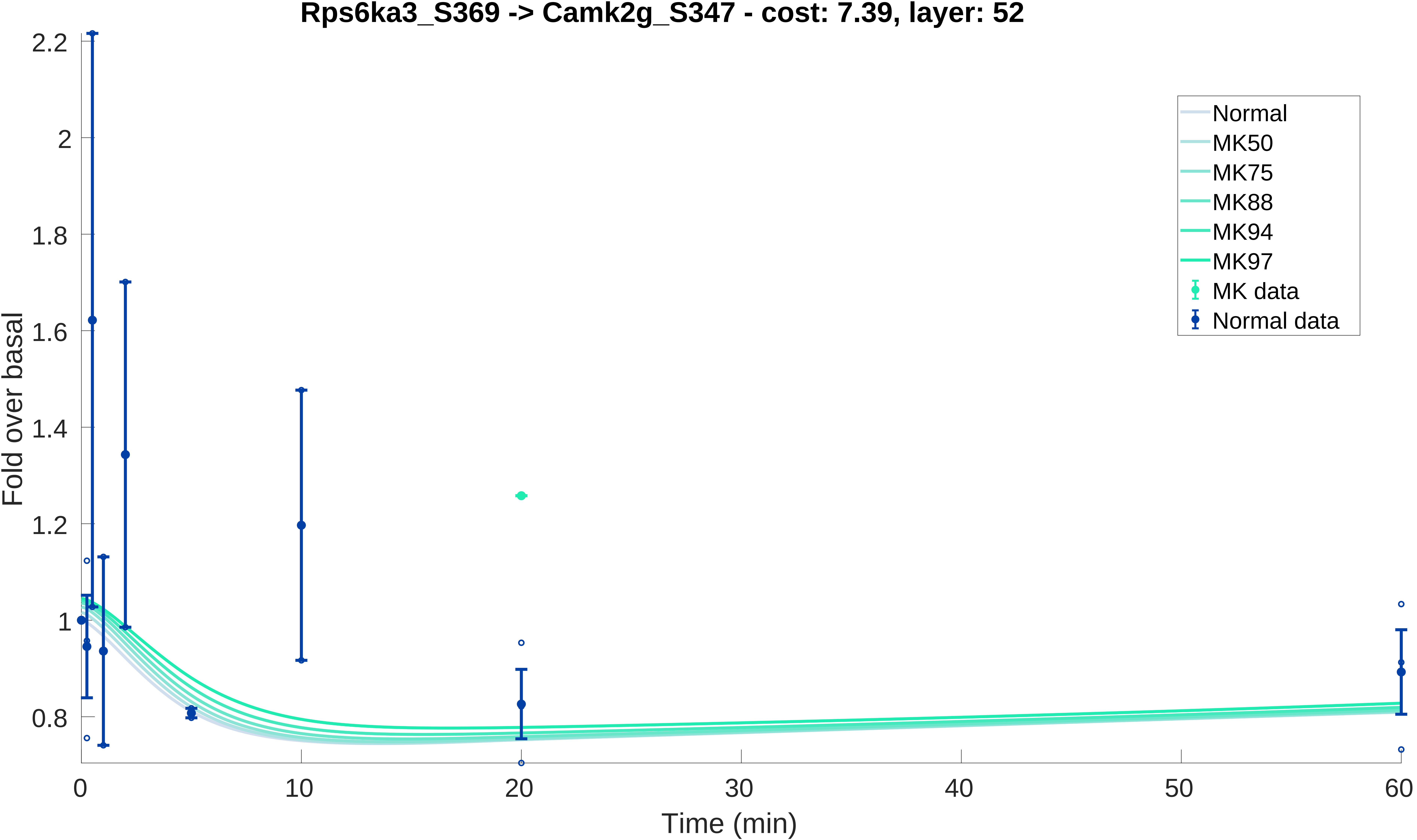

Rps6ka3_S369 -> Camk2g_S347 - cost: 7.39, layer: 52
Fold over basal
Time (min)
0
10
20
30
40
50
60
0.8
1
1.2
1.4
1.6
1.8
2
2.2
Normal
MK50
MK75
MK88
MK94
MK97
MK data
Normal data

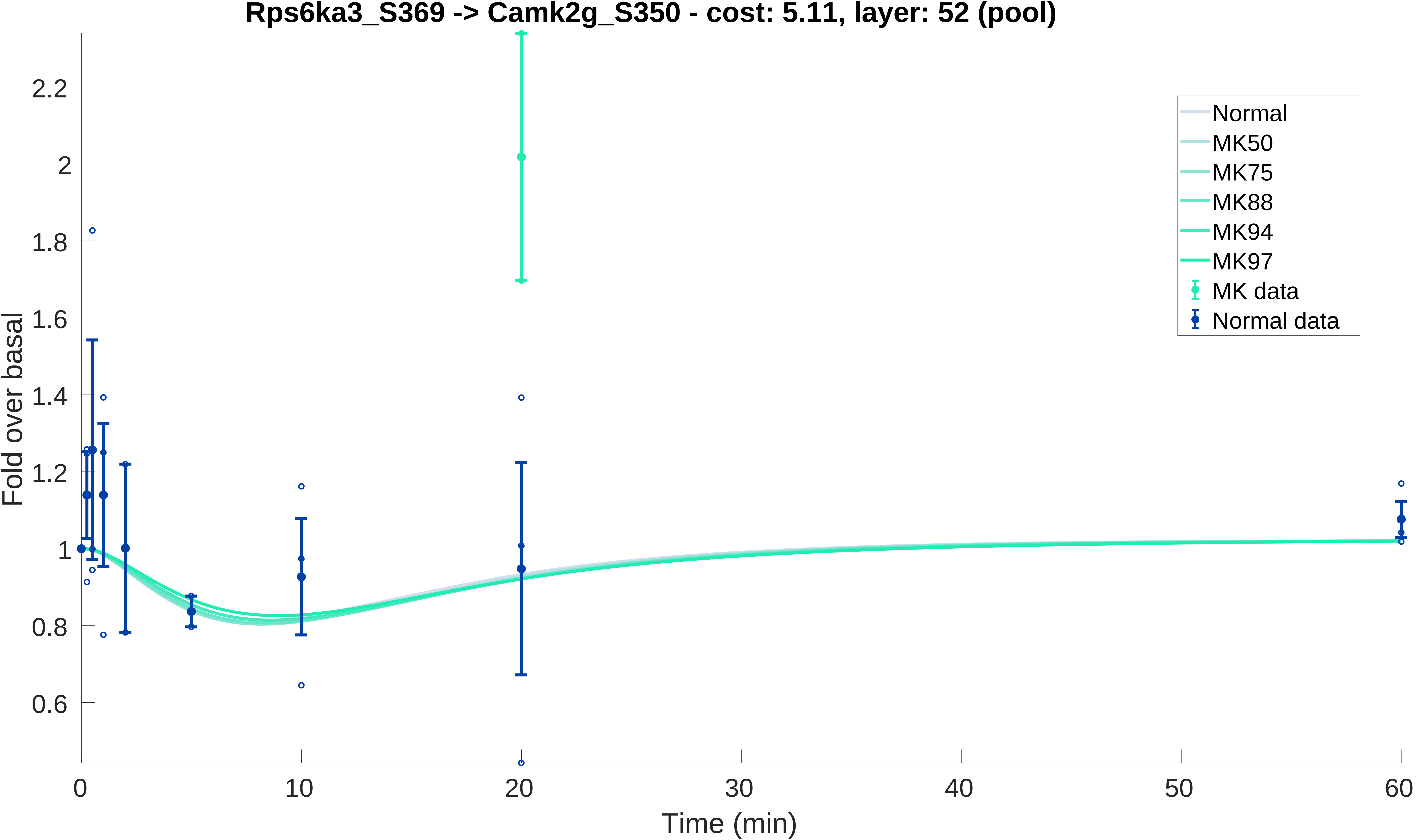

Rps6ka3_S369 -> Camk2g_S350 - cost: 5.11, layer: 52 (pool)
Fold over basal
Time (min)
0.6
0.8
1
1.2
1.4
1.6
1.8
2
2.2
0
10
20
30
40
50
60
Normal
MK50
MK75
MK88
MK94
MK97
MK data
Normal data

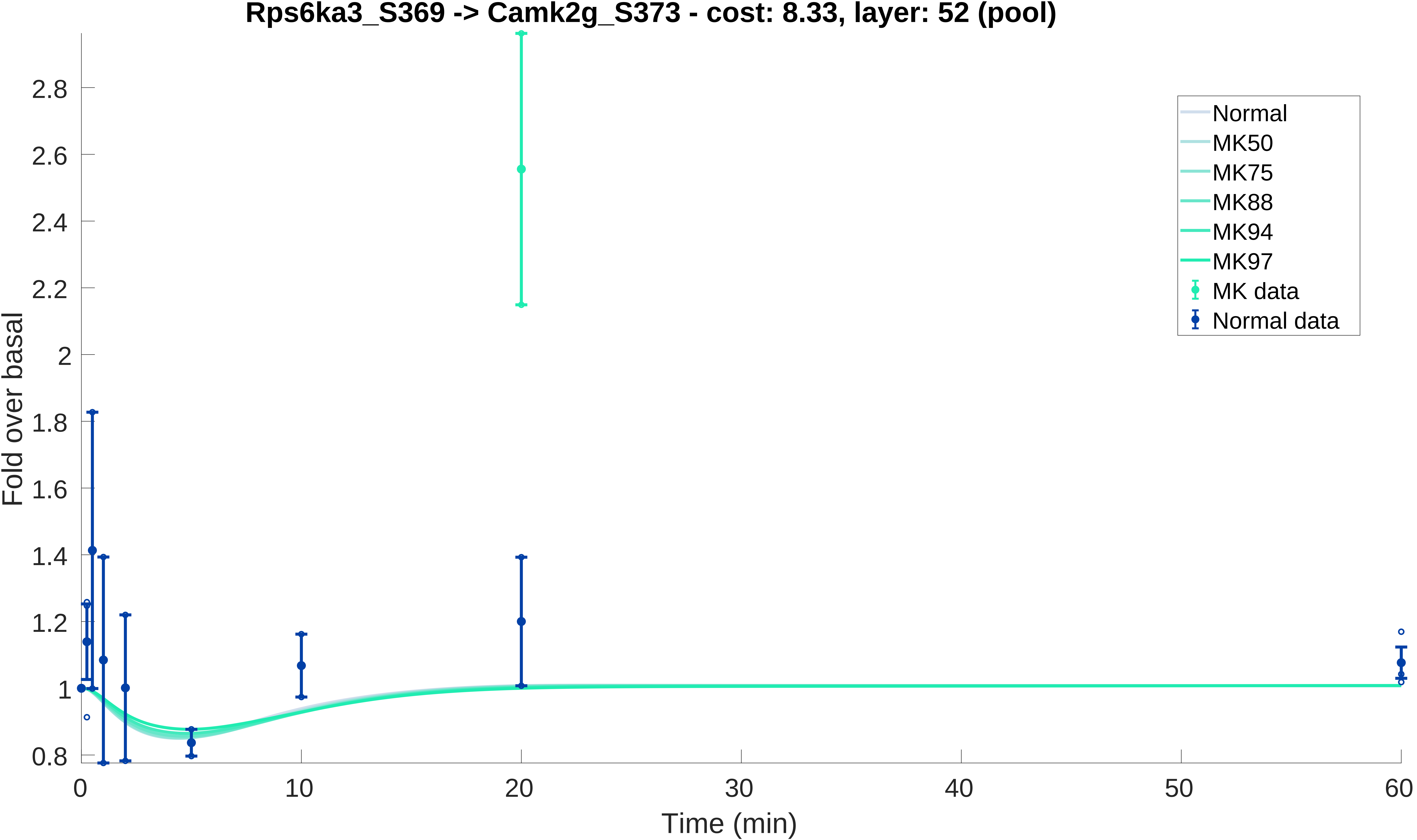

Rps6ka3_S369 -> Camk2g_S373 - cost: 8.33, layer: 52 (pool)
Fold over basal
Time (min)
0.8
1
1.2
1.4
1.6
1.8
2
2.2
2.4
2.6
2.8
0
10
20
30
40
50
60
Normal
MK50
MK75
MK88
MK94
MK97
MK data
Normal data

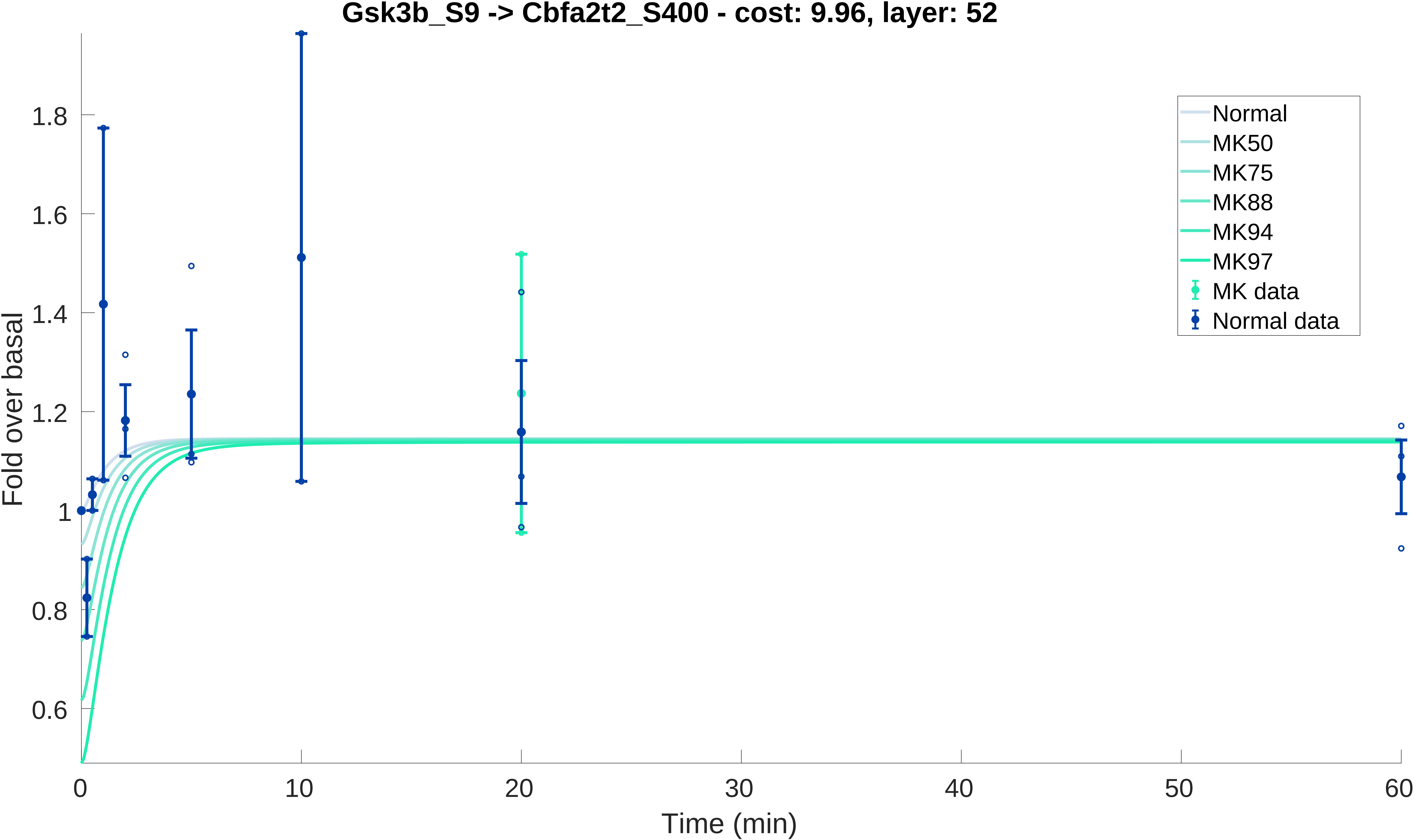
Gsk3b_S9 -> Cbfa2t2_S400 - cost: 9.96, layer: 52
Fold over basal
Time (min)
0.6
0.8
1
1.2
1.4
1.6
1.8
0
10
20
30
40
50
60
Normal
MK50
MK75
MK88
MK94
MK97
MK data
Normal data

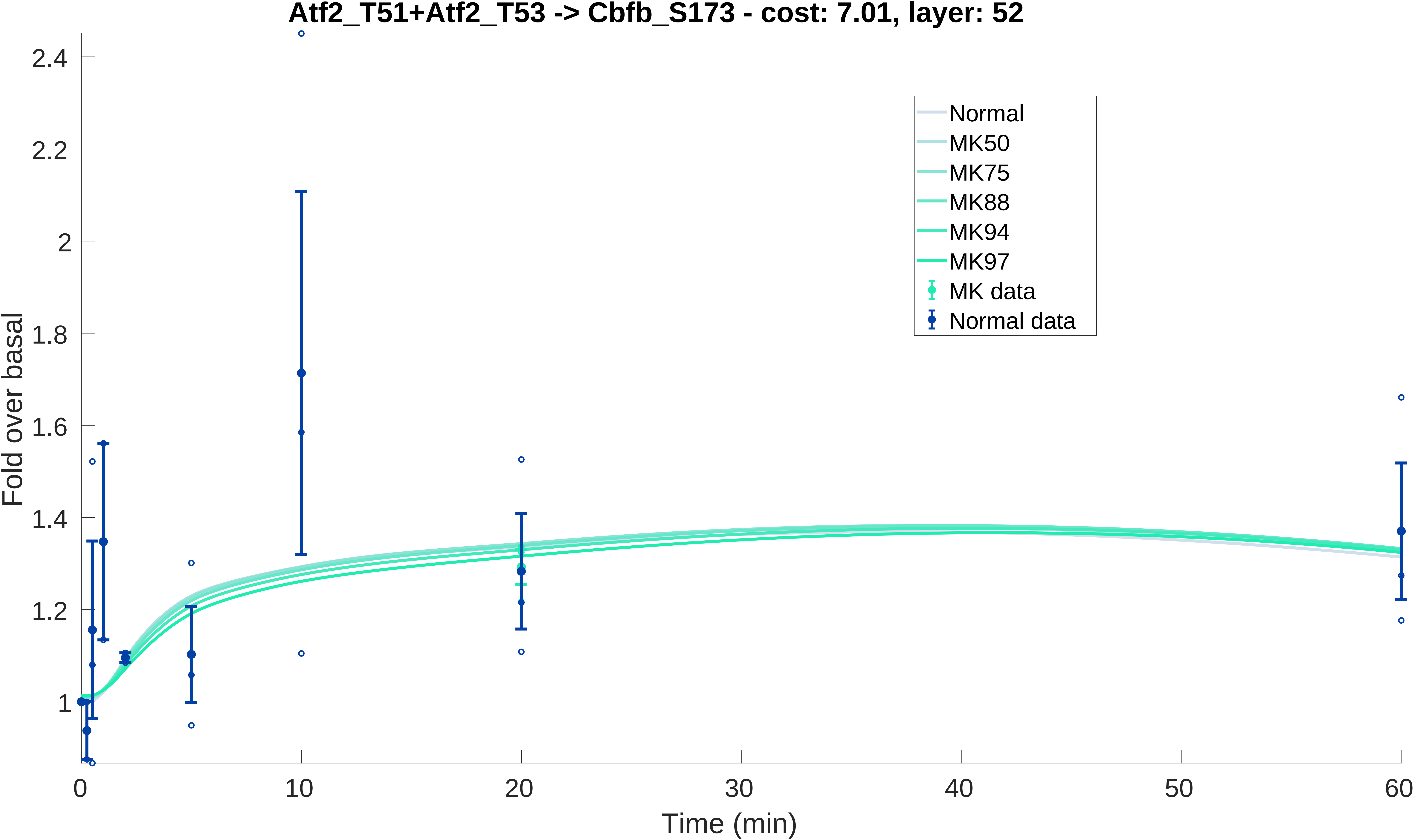

Atf2_T51+Atf2_T53 -> Cbfb_S173 - cost: 7.01, layer: 52
Fold over basal
Time (min)
Normal
MK50
MK75
MK88
MK94
MK97
MK data
Normal data
2.4
2.2
2
1.8
1.6
1.4
1.2
1
0
10
20
30
40
50
60

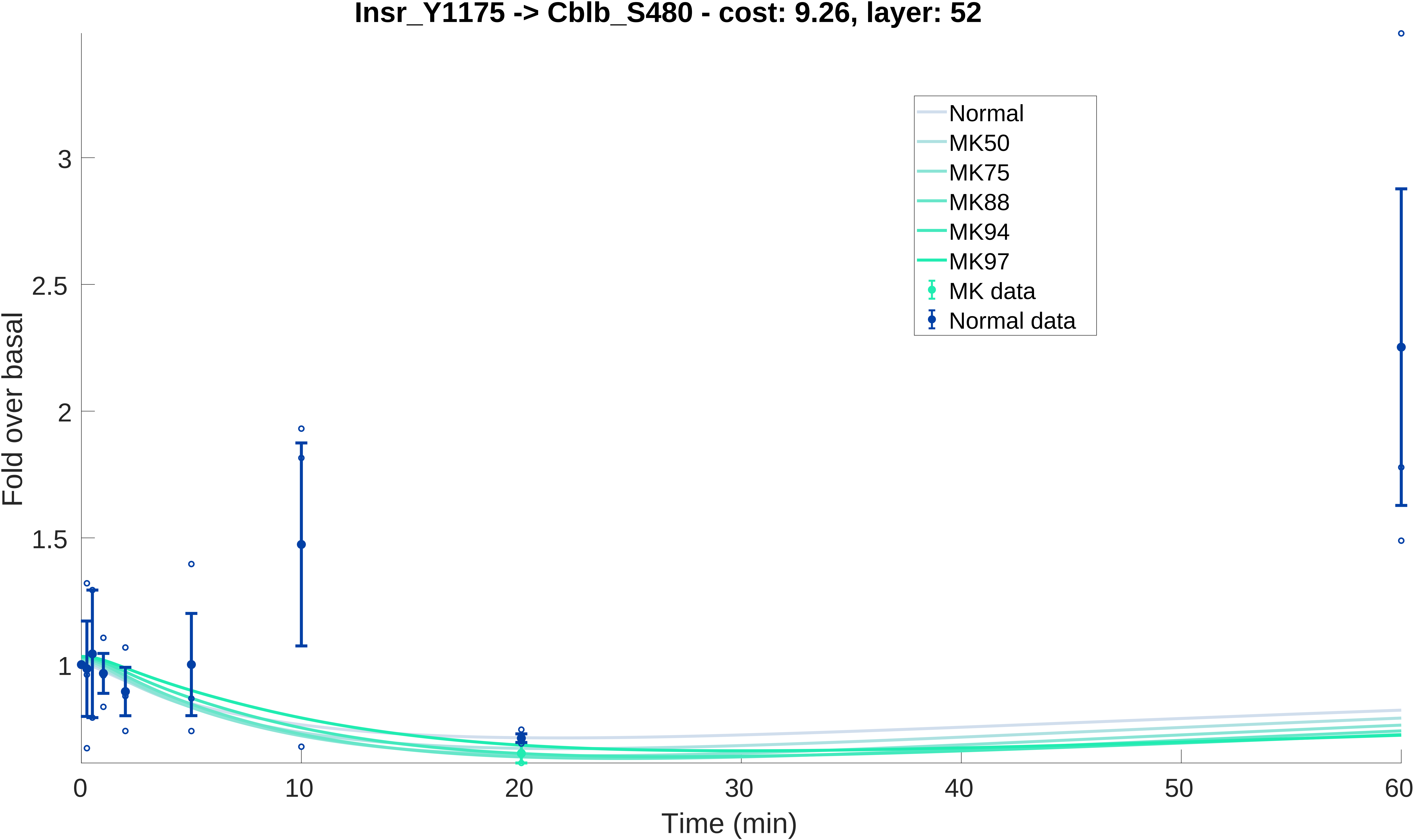

Insr_Y1175 -> Cblb_S480 - cost: 9.26, layer: 52
Fold over basal
Time (min)
0
10
20
30
40
50
60
1
1.5
2
2.5
3
Normal
MK50
MK75
MK88
MK94
MK97
MK data
Normal data

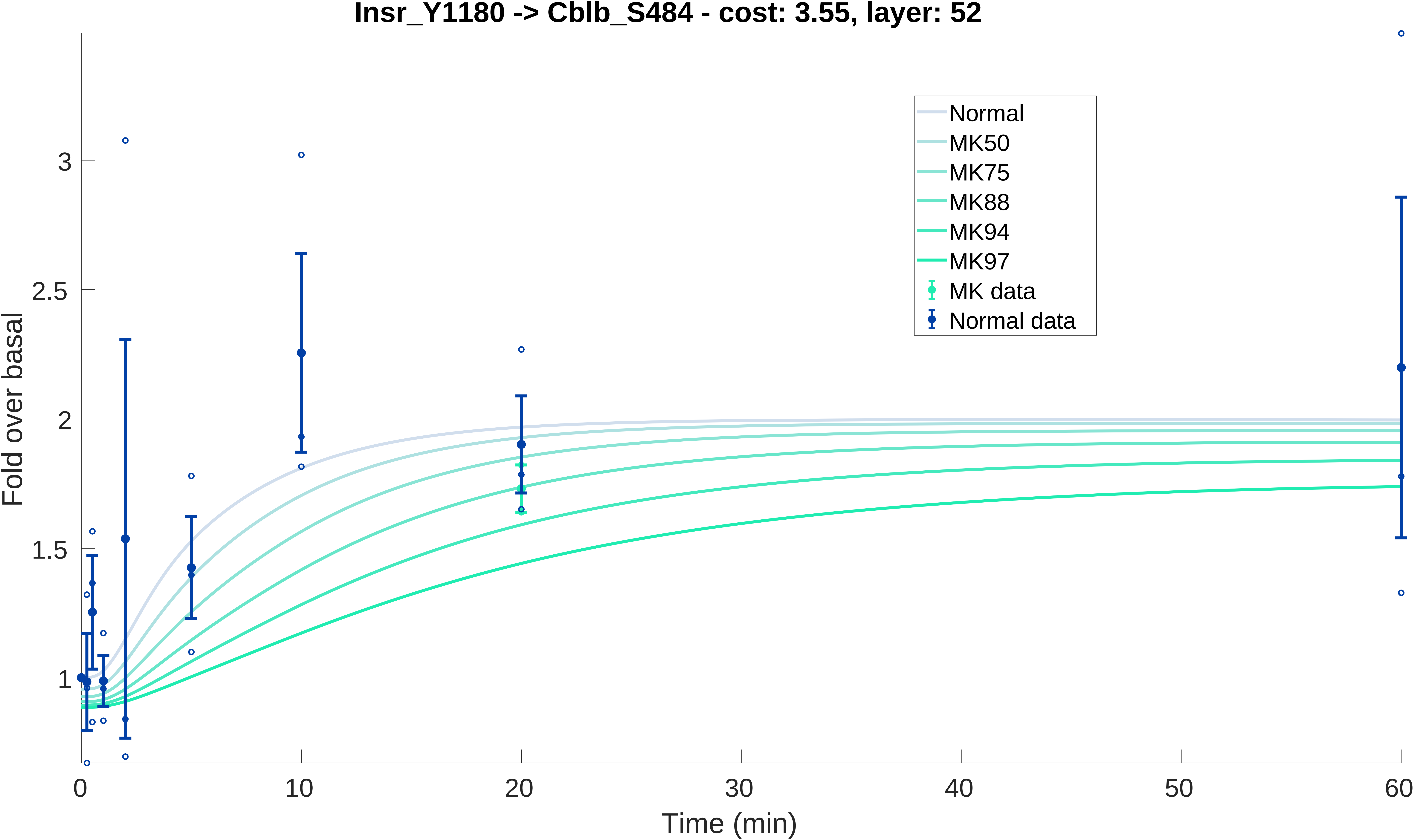

Insr_Y1180 -> Cblb_S484 - cost: 3.55, layer: 52
Normal
MK50
MK75
MK88
MK94
MK97
MK data
Normal data
Fold over basal
Time (min)
0
10
20
30
40
50
60
1
1.5
2
2.5
3

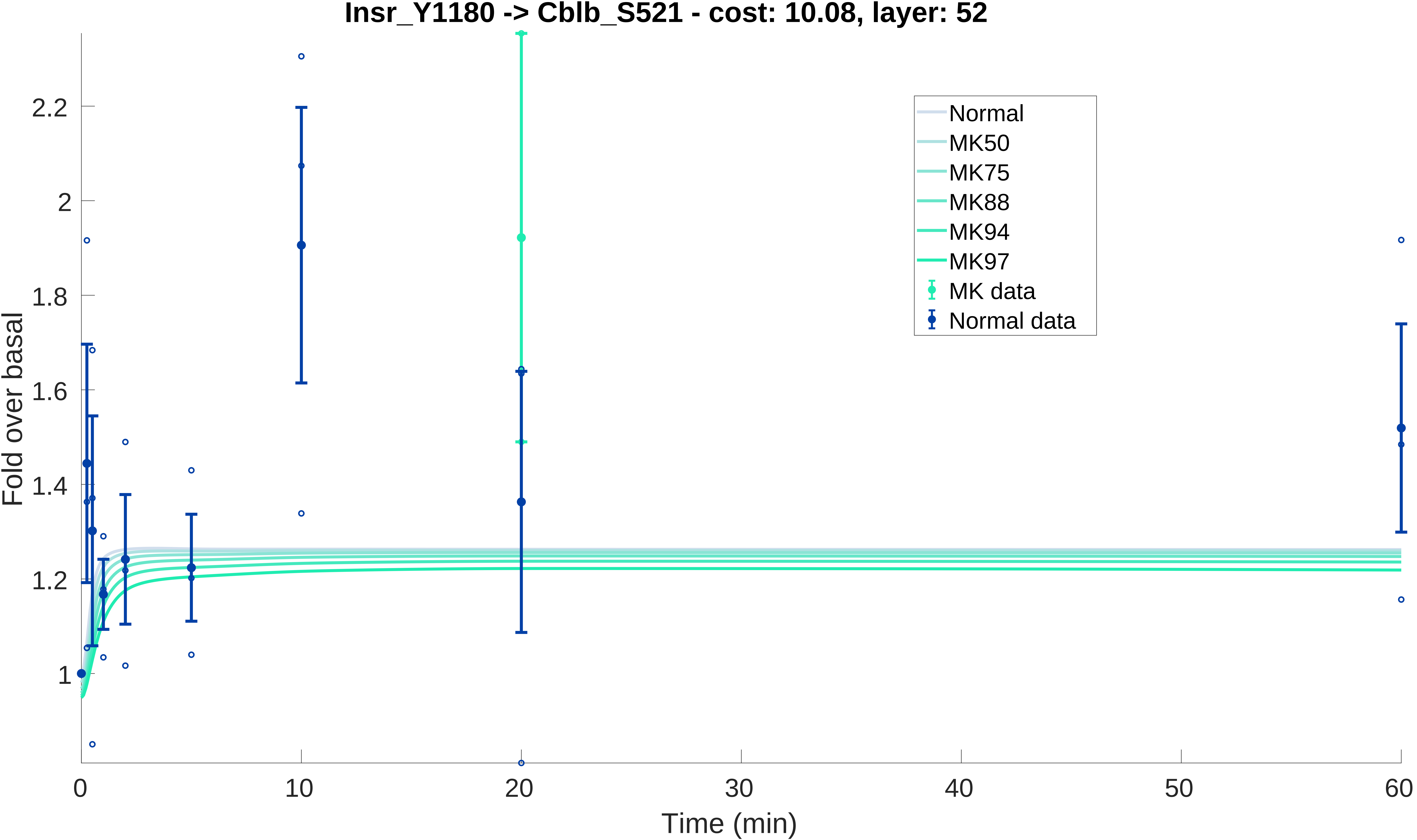
Insr_Y1180 -> Cblb_S521 - cost: 10.08, layer: 52
Fold over basal
Time (min)
Normal
MK50
MK75
MK88
MK94
MK97
MK data
Normal data
0
10
20
30
40
50
60
1
1.2
1.4
1.6
1.8
2
2.2

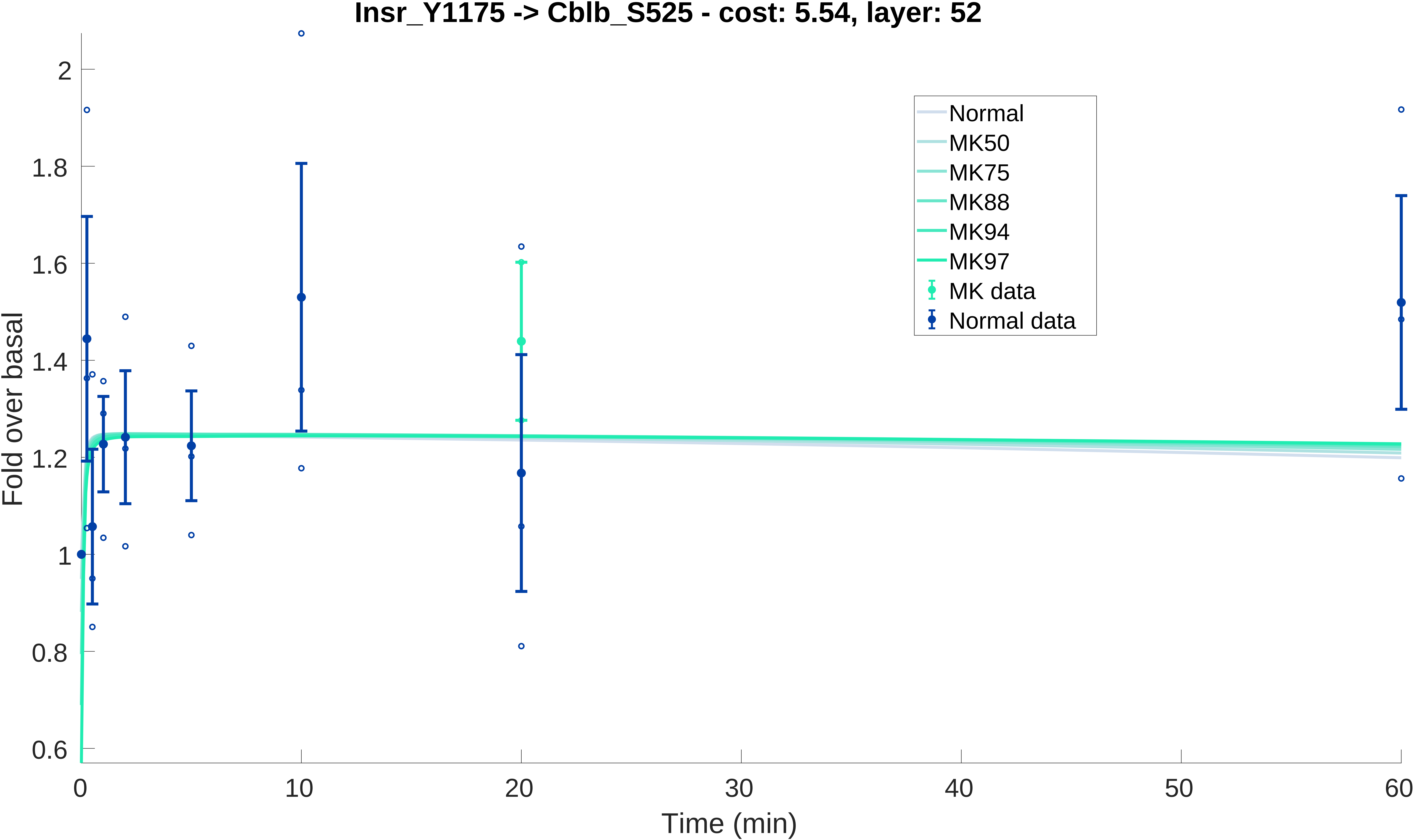
Insr_Y1175 -> Cblb_S525 - cost: 5.54, layer: 52
Fold over basal
Time (min)
Normal
MK50
MK75
MK88
MK94
MK97
MK data
Normal data
0.6
0.8
1
1.2
1.4
1.6
1.8
2
0
10
20
30
40
50
60

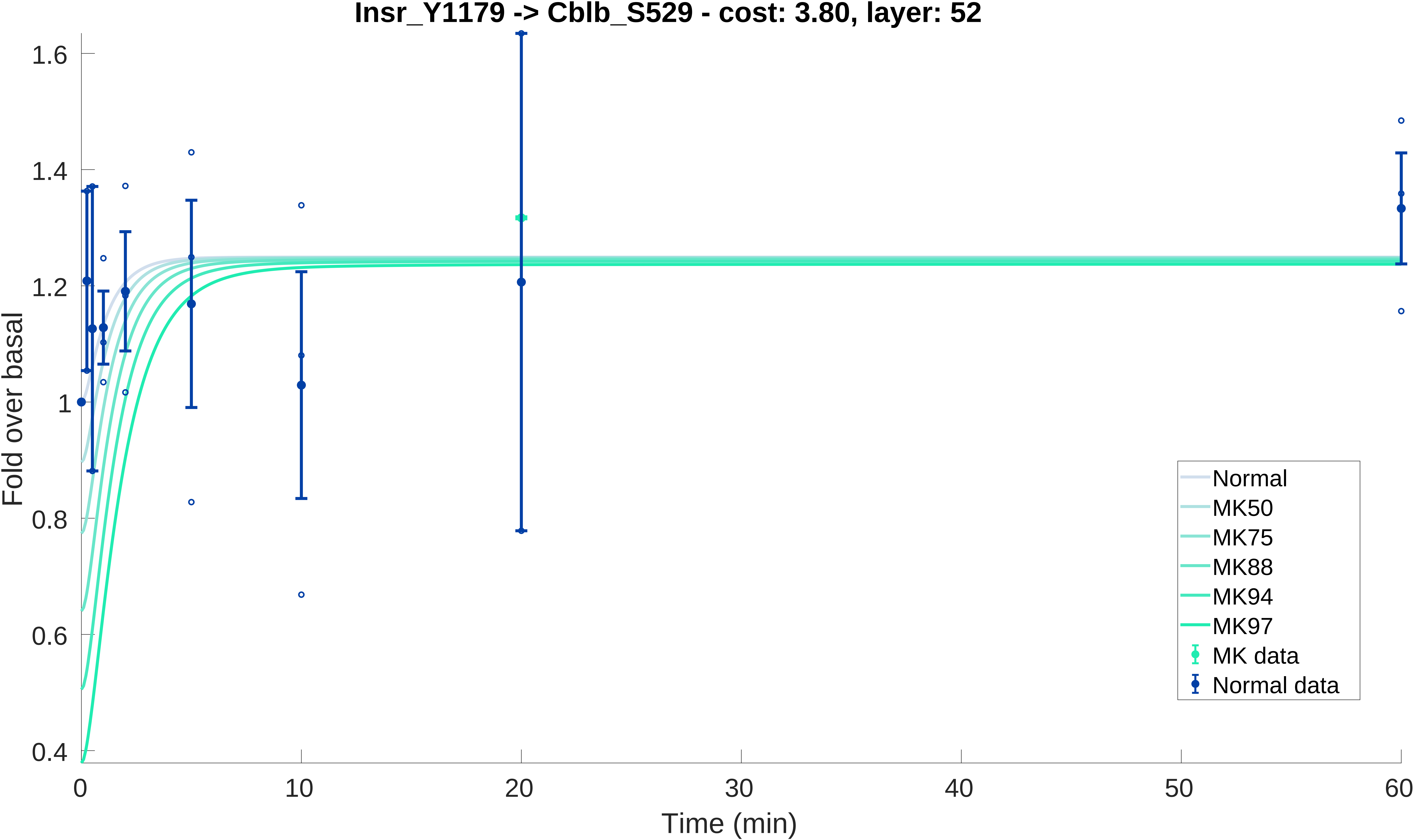

Insr_Y1179 -> Cblb_S529 - cost: 3.80, layer: 52
Fold over basal
1.6
1.4
1.2
1
0.8
0.6
0.4
0
10
20
30
40
50
60
Time (min)
Normal
MK50
MK75
MK88
MK94
MK97
MK data
Normal data

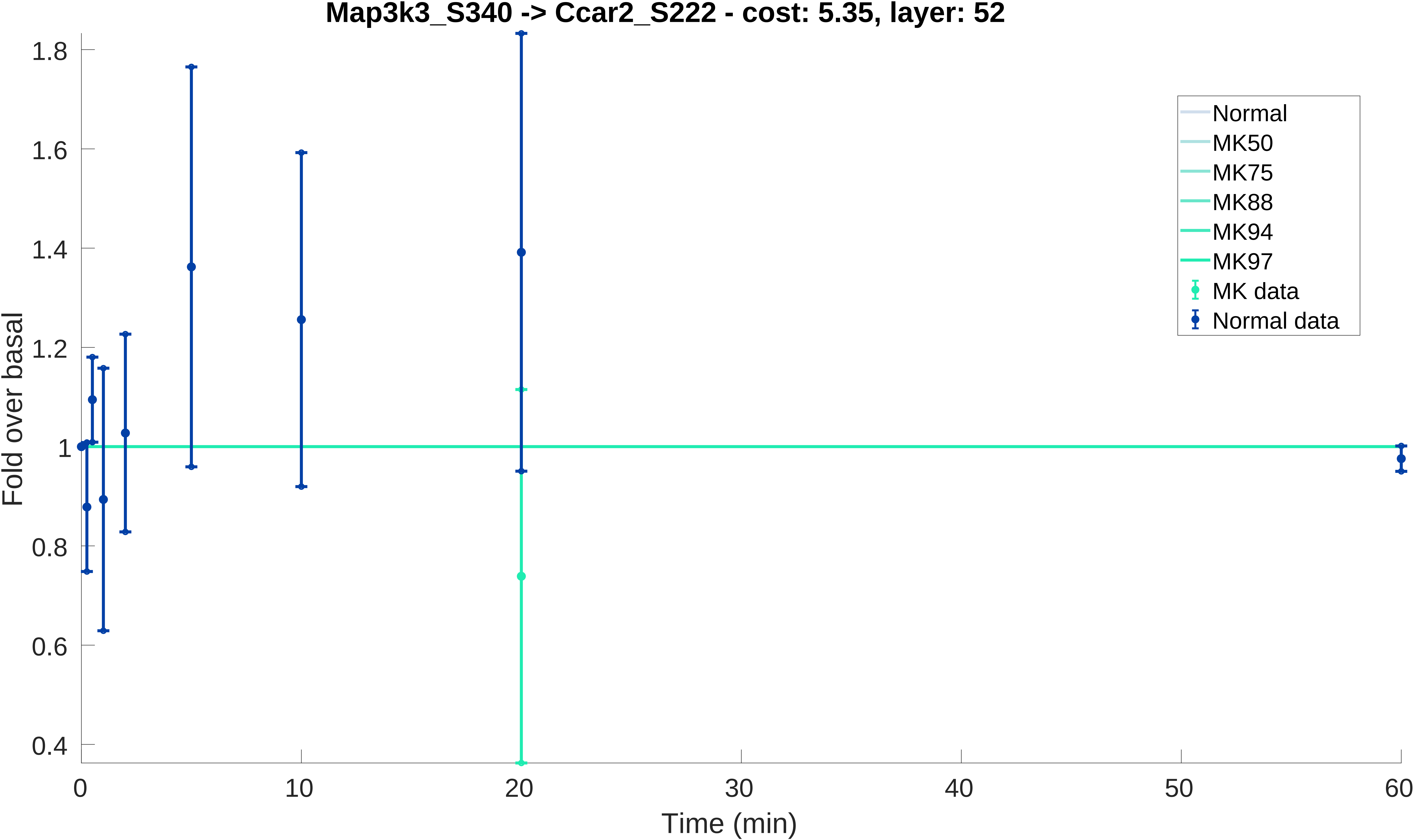

Map3k3_S340 -> Ccar2_S222 - cost: 5.35, layer: 52
Fold over basal
Time (min)
1.8
1.6
1.4
1.2
1
0.8
0.6
0.4
0
10
20
30
40
50
60
Normal
MK50
MK75
MK88
MK94
MK97
MK data
Normal data

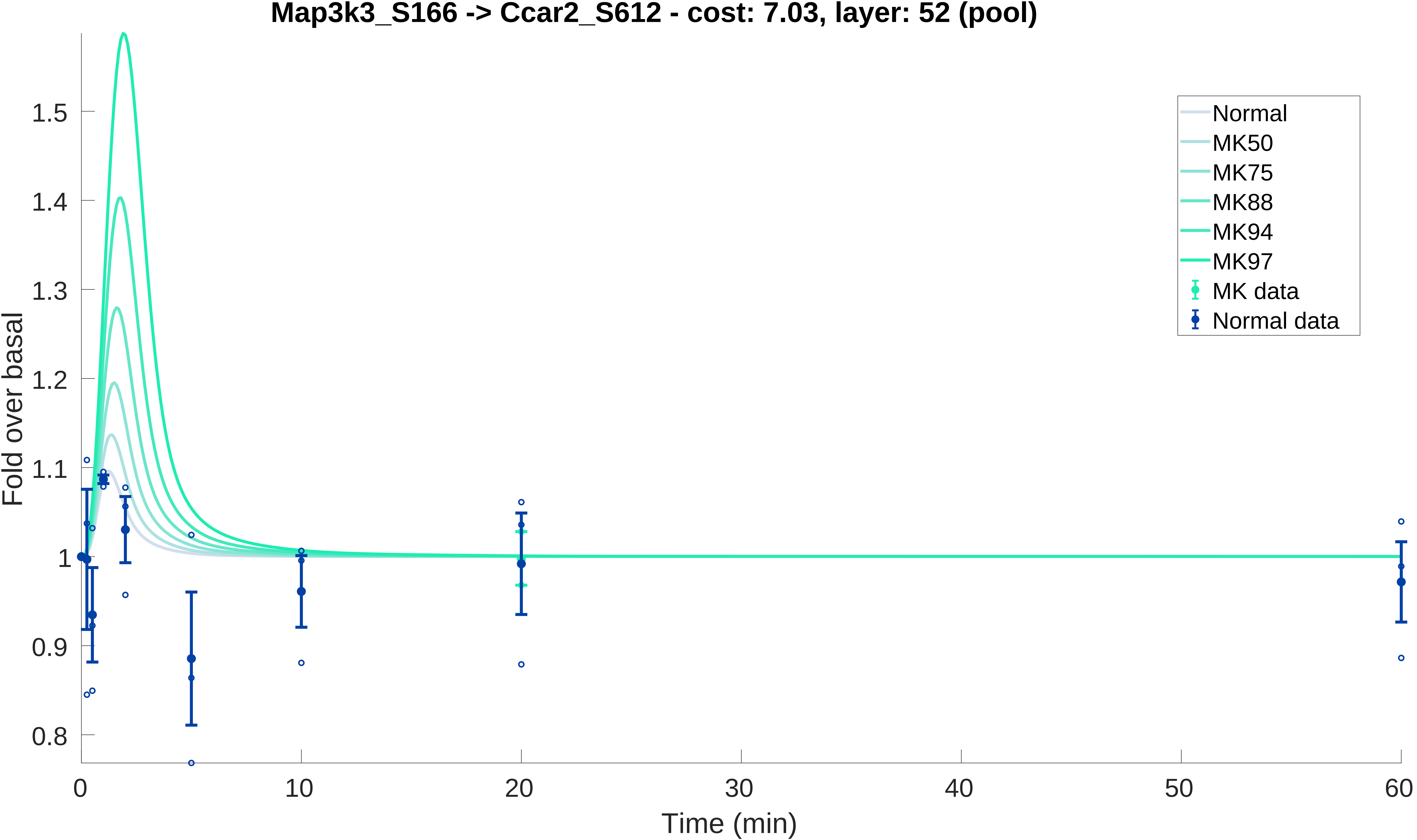
Map3k3_S166 -> Ccar2_S612 - cost: 7.03, layer: 52 (pool)
Fold over basal
Time (min)
0
10
20
30
40
50
60
0.8
0.9
1
1.1
1.2
1.3
1.4
1.5
Normal
MK50
MK75
MK88
MK94
MK97
MK data
Normal data

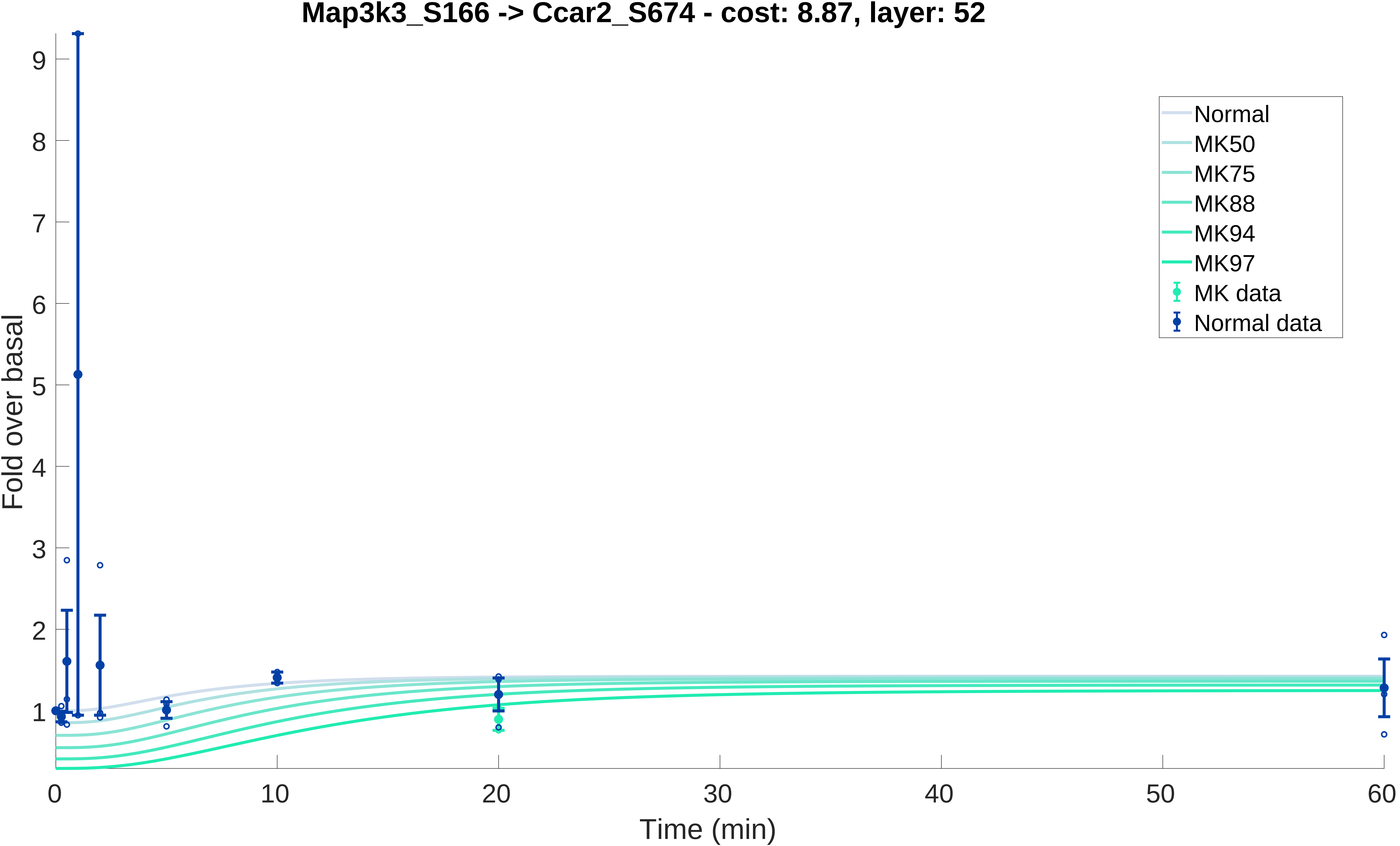
Map3k3_S166 -> Ccar2_S674 - cost: 8.87, layer: 52
Fold over basal
Time (min)
Normal
MK50
MK75
MK88
MK94
MK97
MK data
Normal data
0
10
20
30
40
50
60
1
2
3
4
5
6
7
8
9

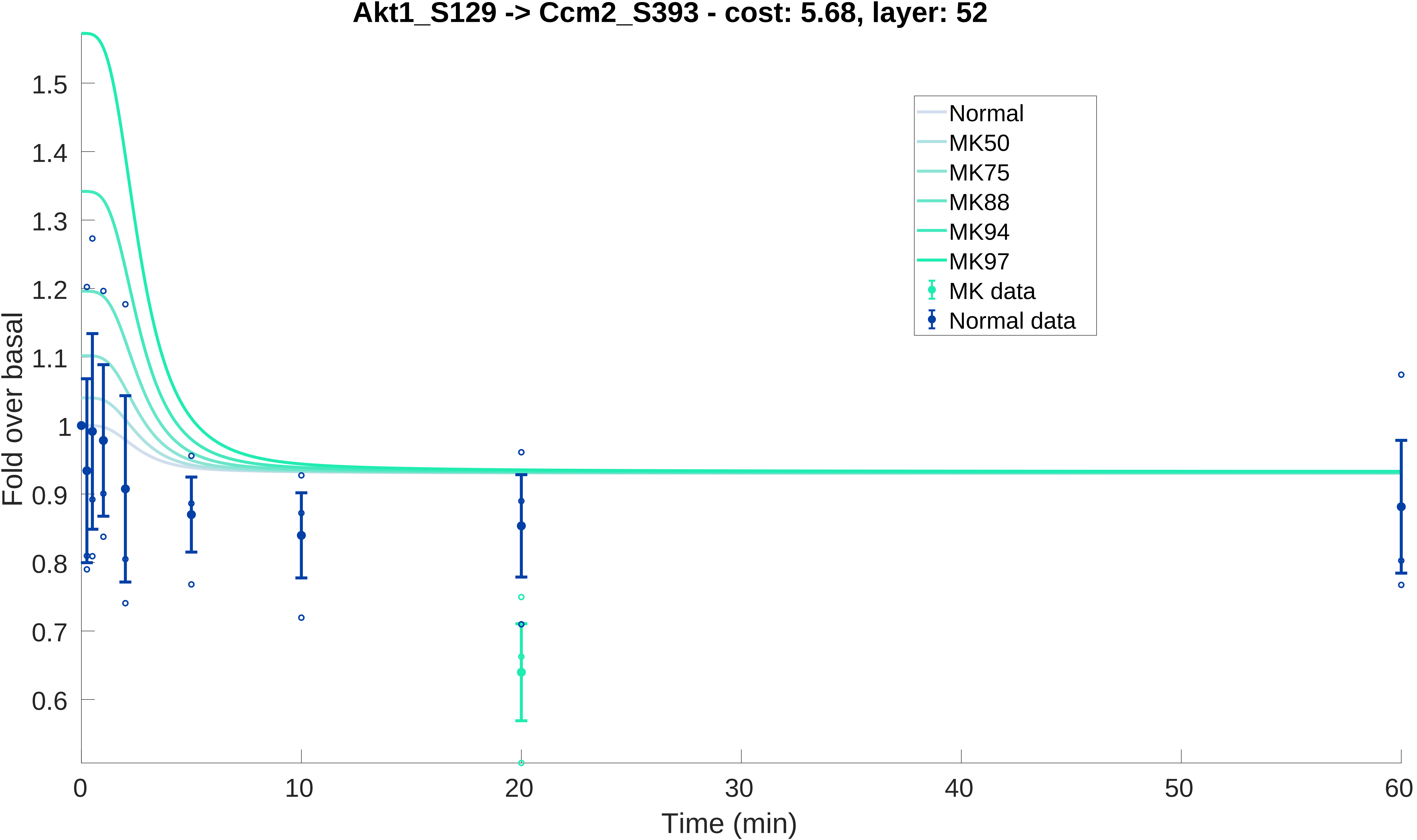

Akt1_S129 -> Ccm2_S393 - cost: 5.68, layer: 52
Normal
MK50
MK75
MK88
MK94
MK97
MK data
Normal data
Fold over basal
Time (min)
1.5
1.4
1.3
1.2
1.1
1
0.9
0.8
0.7
0.6
0
10
20
30
40
50
60

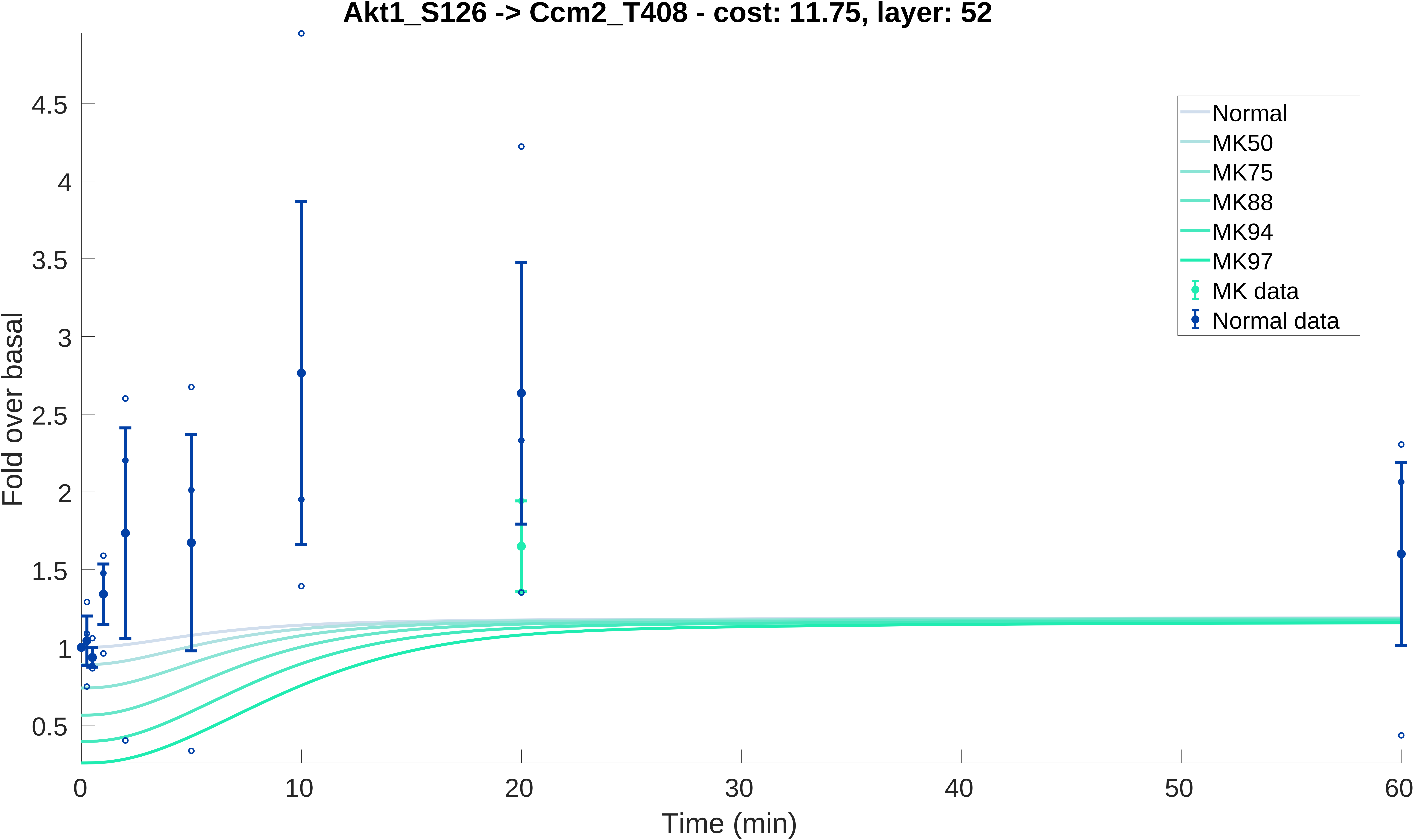

Akt1_S126 -> Ccm2_T408 - cost: 11.75, layer: 52
Fold over basal
Time (min)
0.5
1
1.5
2
2.5
3
3.5
4
4.5
0
10
20
30
40
50
60
Normal
MK50
MK75
MK88
MK94
MK97
MK data
Normal data

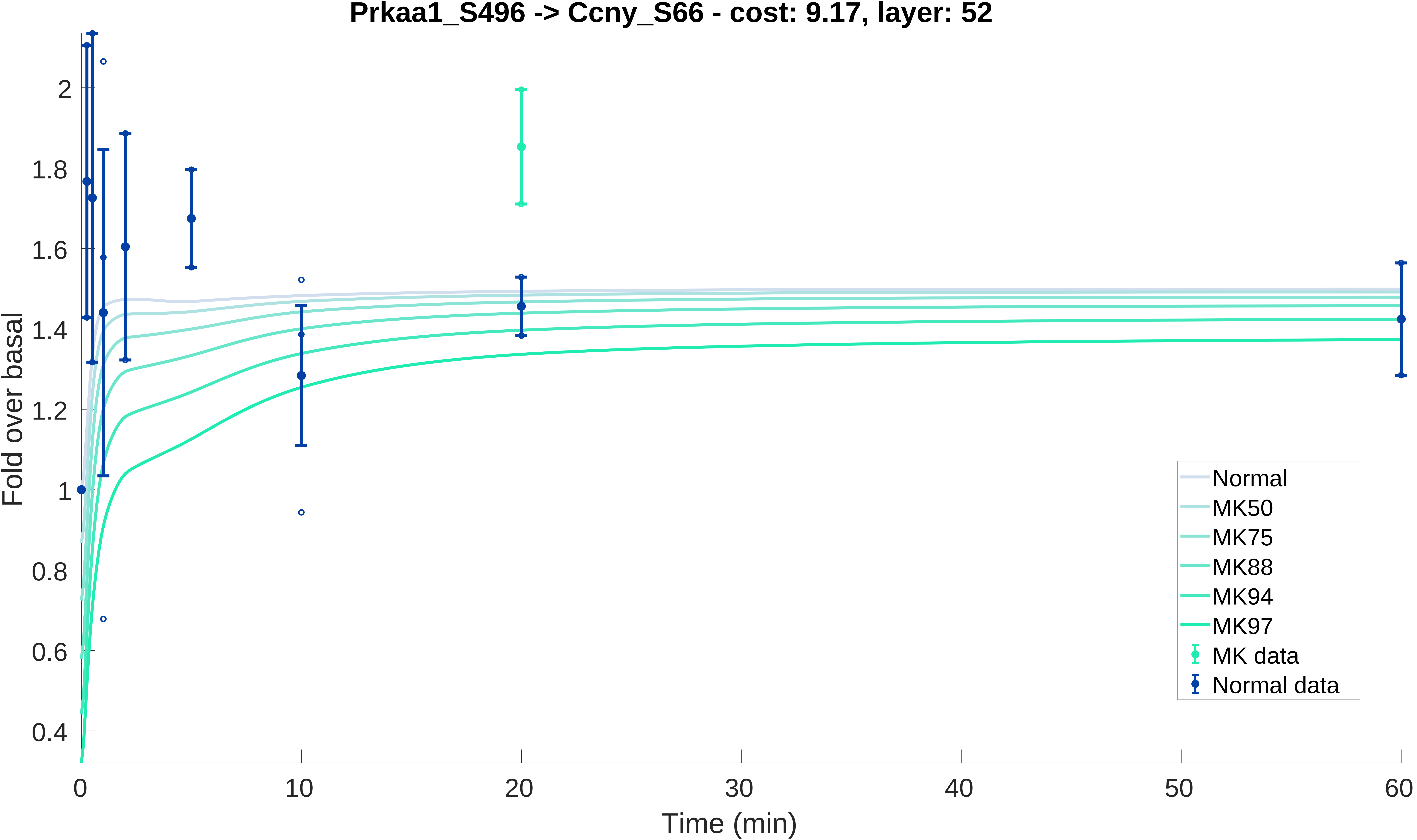

Prkaa1_S496 -> Ccny_S66 - cost: 9.17, layer: 52
Fold over basal
Time (min)
0.4
0.6
0.8
1
1.2
1.4
1.6
1.8
2
0
10
20
30
40
50
60
Normal
MK50
MK75
MK88
MK94
MK97
MK data
Normal data

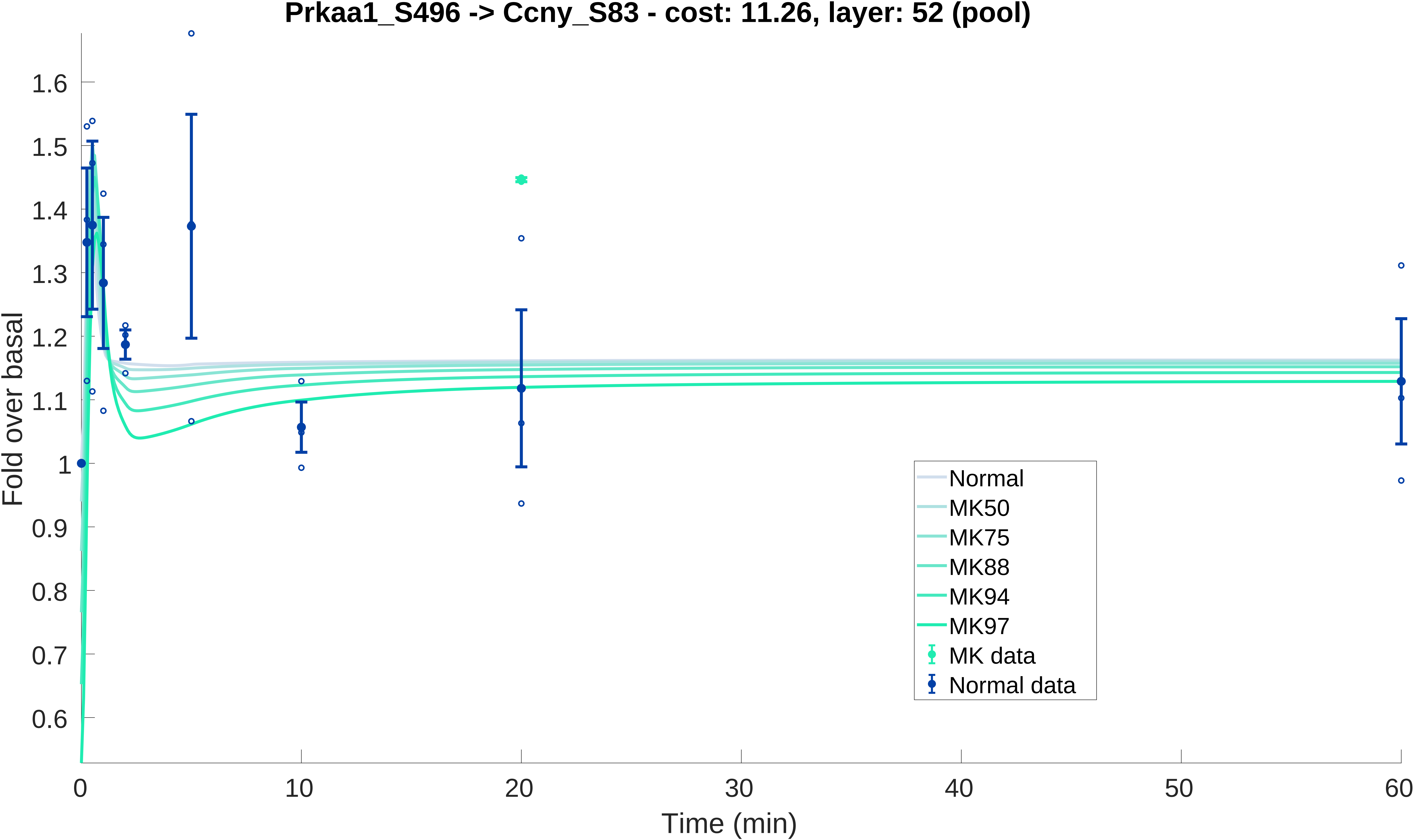

Prkaa1_S496 -> Ccny_S83 - cost: 11.26, layer: 52 (pool)
Fold over basal
Time (min)
1.6
1.5
1.4
1.3
1.2
1.1
1
0.9
0.8
0.7
0.6
0
10
20
30
40
50
60
Normal
MK50
MK75
MK88
MK94
MK97
MK data
Normal data

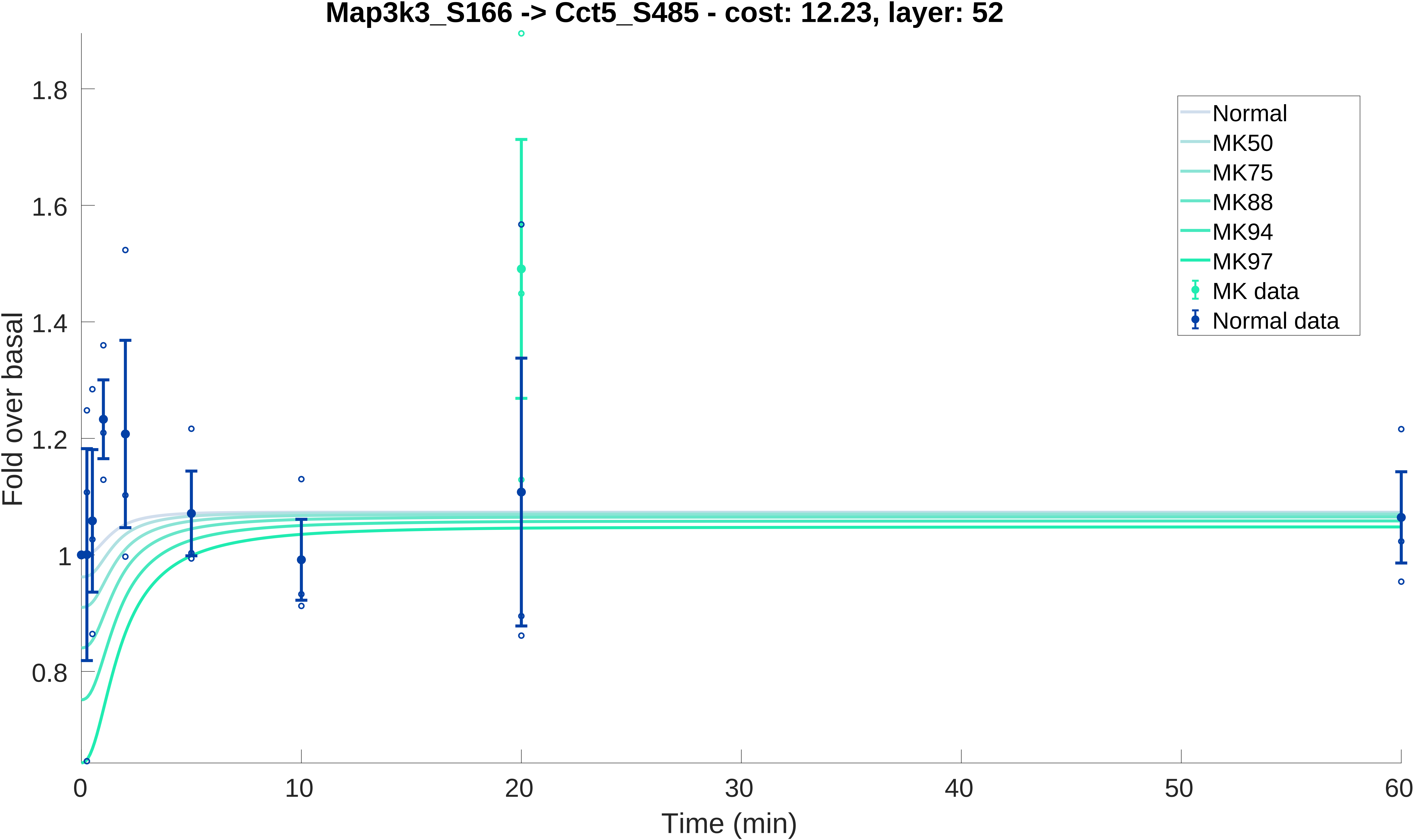

Map3k3_S166 -> Cct5_S485 - cost: 12.23, layer: 52
Fold over basal
Time (min)
0.8
1
1.2
1.4
1.6
1.8
0
10
20
30
40
50
60
Normal
MK50
MK75
MK88
MK94
MK97
MK data
Normal data

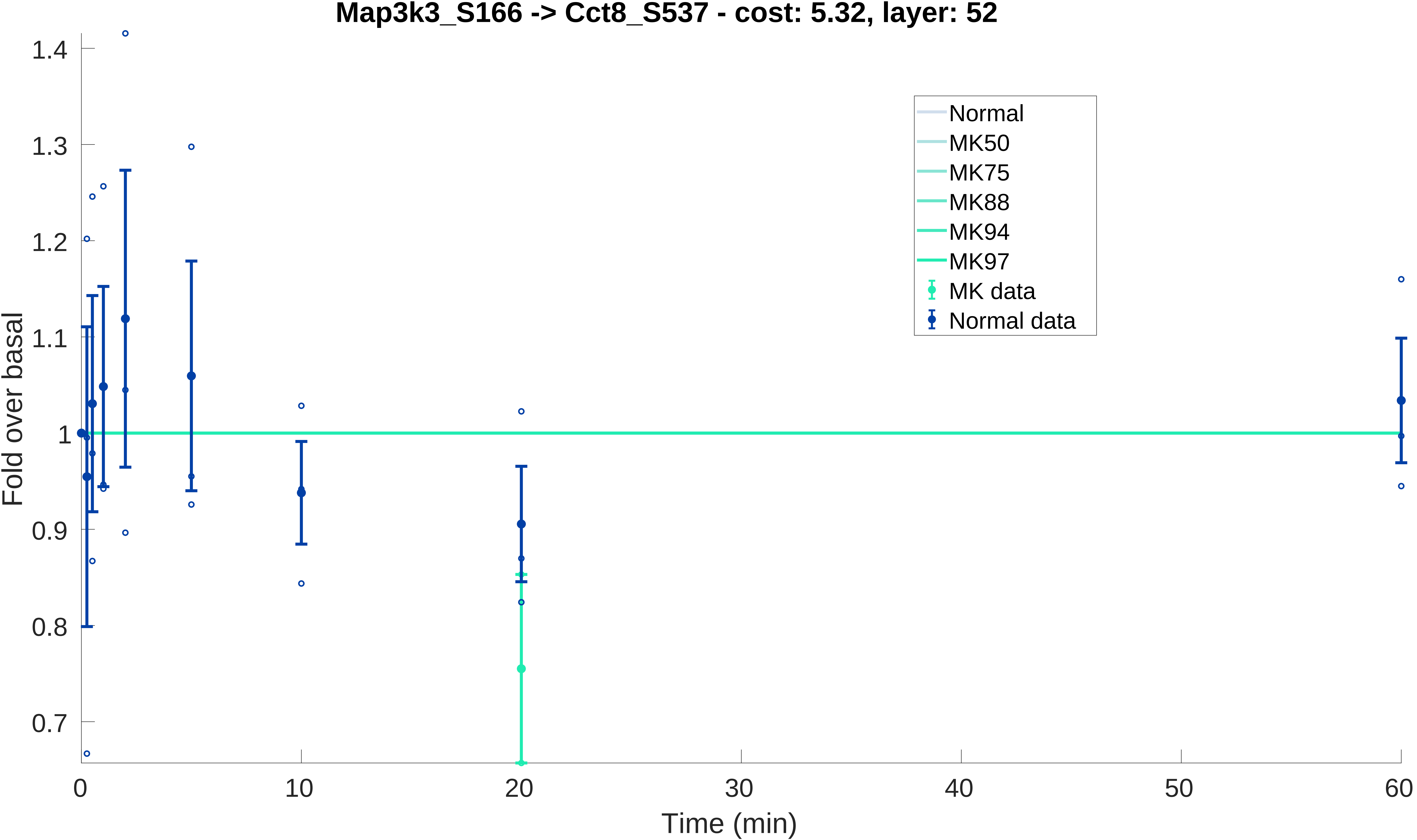

Map3k3_S166 -> Cct8_S537 - cost: 5.32, layer: 52
Fold over basal
Time (min)
Normal
MK50
MK75
MK88
MK94
MK97
MK data
Normal data
1.4
1.3
1.2
1.1
1
0.9
0.8
0.7
0
10
20
30
40
50
60

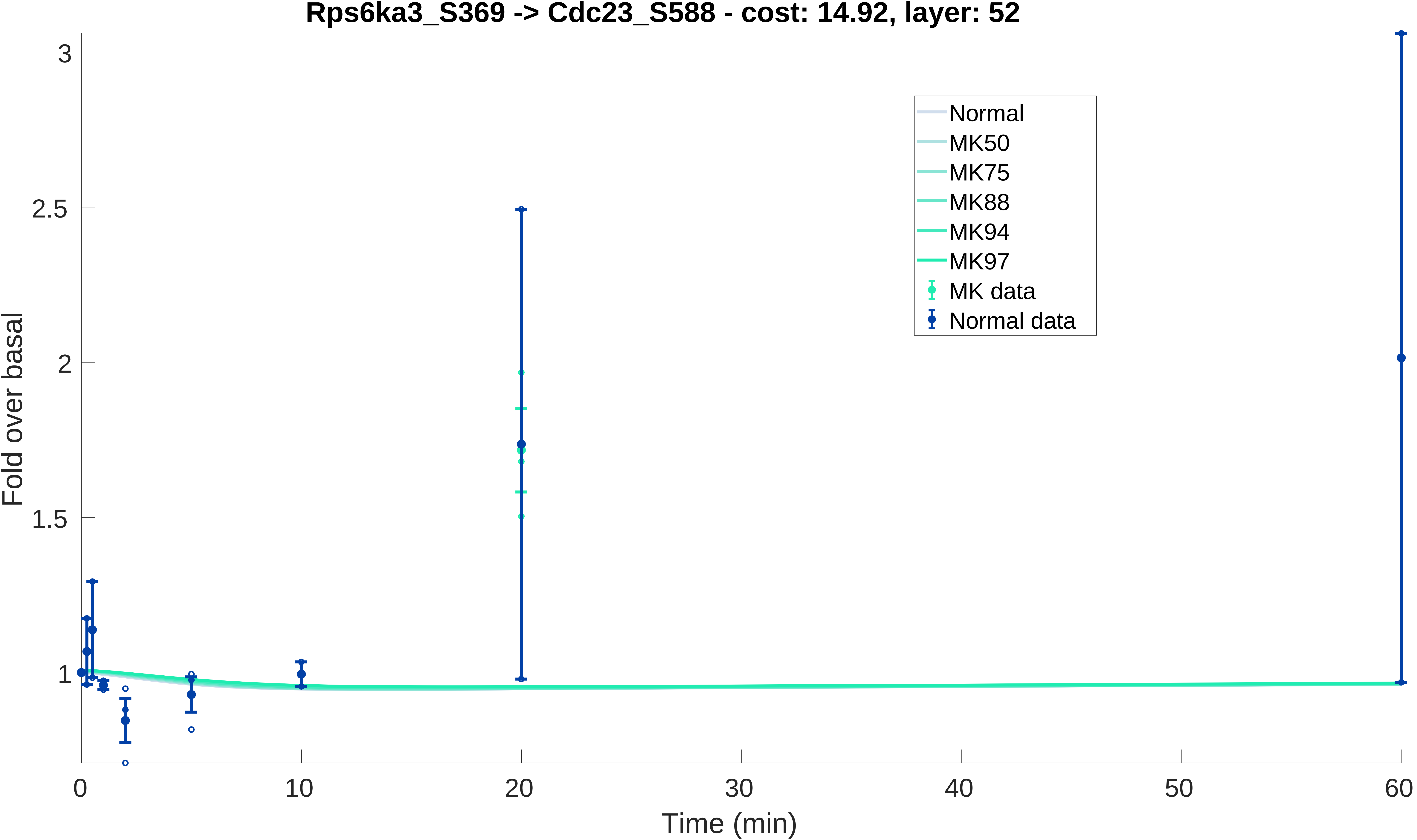

Rps6ka3_S369 -> Cdc23_S588 - cost: 14.92, layer: 52
Fold over basal
Time (min)
3
2.5
2
1.5
1
0
10
20
30
40
50
60
Normal
MK50
MK75
MK88
MK94
MK97
MK data
Normal data

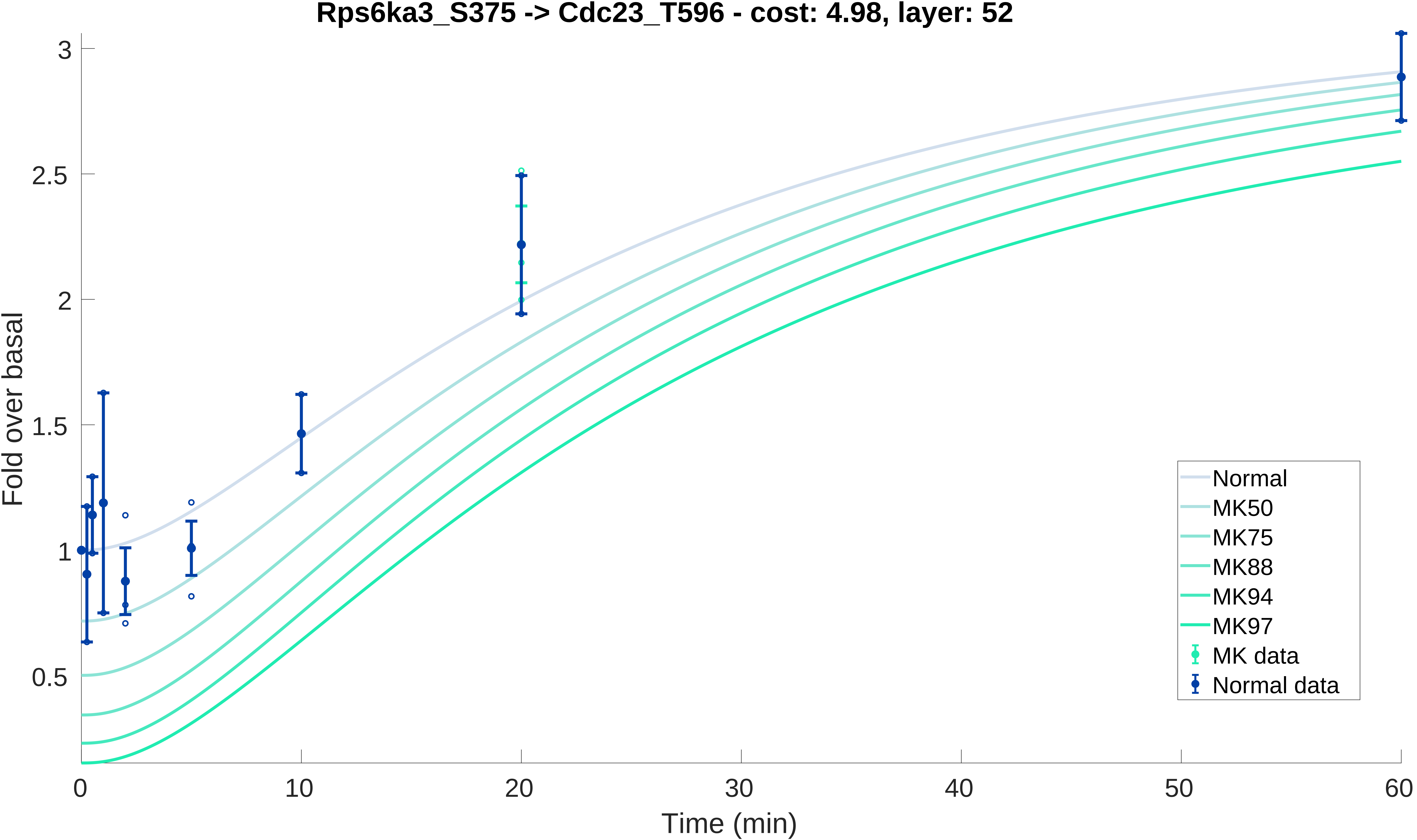

Rps6ka3_S375 -> Cdc23_T596 - cost: 4.98, layer: 52
Fold over basal
Time (min)
0
10
20
30
40
50
60
0.5
1
1.5
2
2.5
3
Normal
MK50
MK75
MK88
MK94
MK97
MK data
Normal data

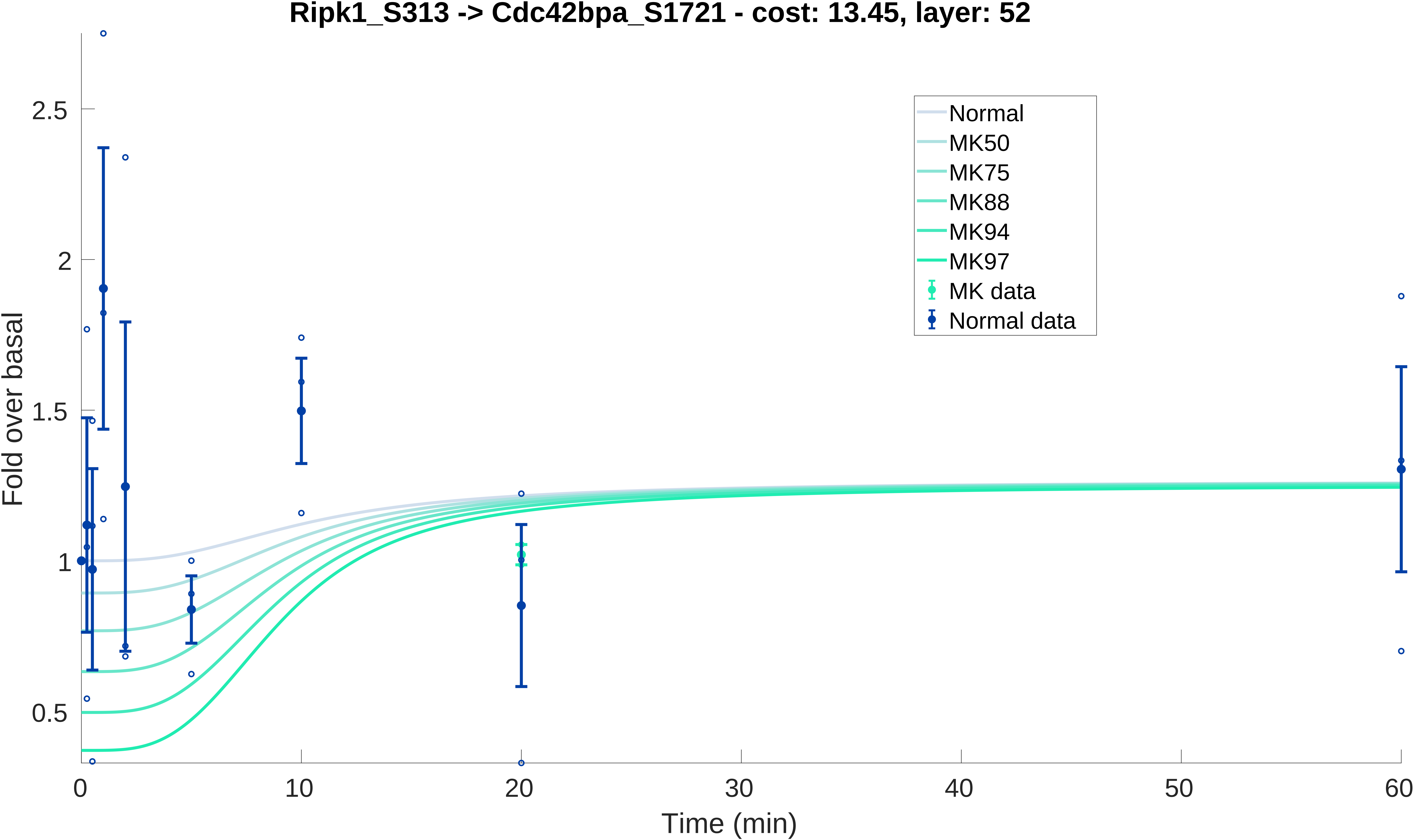

Ripk1_S313 -> Cdc42bpa_S1721 - cost: 13.45, layer: 52
Normal
MK50
MK75
MK88
MK94
MK97
MK data
Normal data
Fold over basal
2.5
2
1.5
1
0.5
0
10
20
30
40
50
60
Time (min)

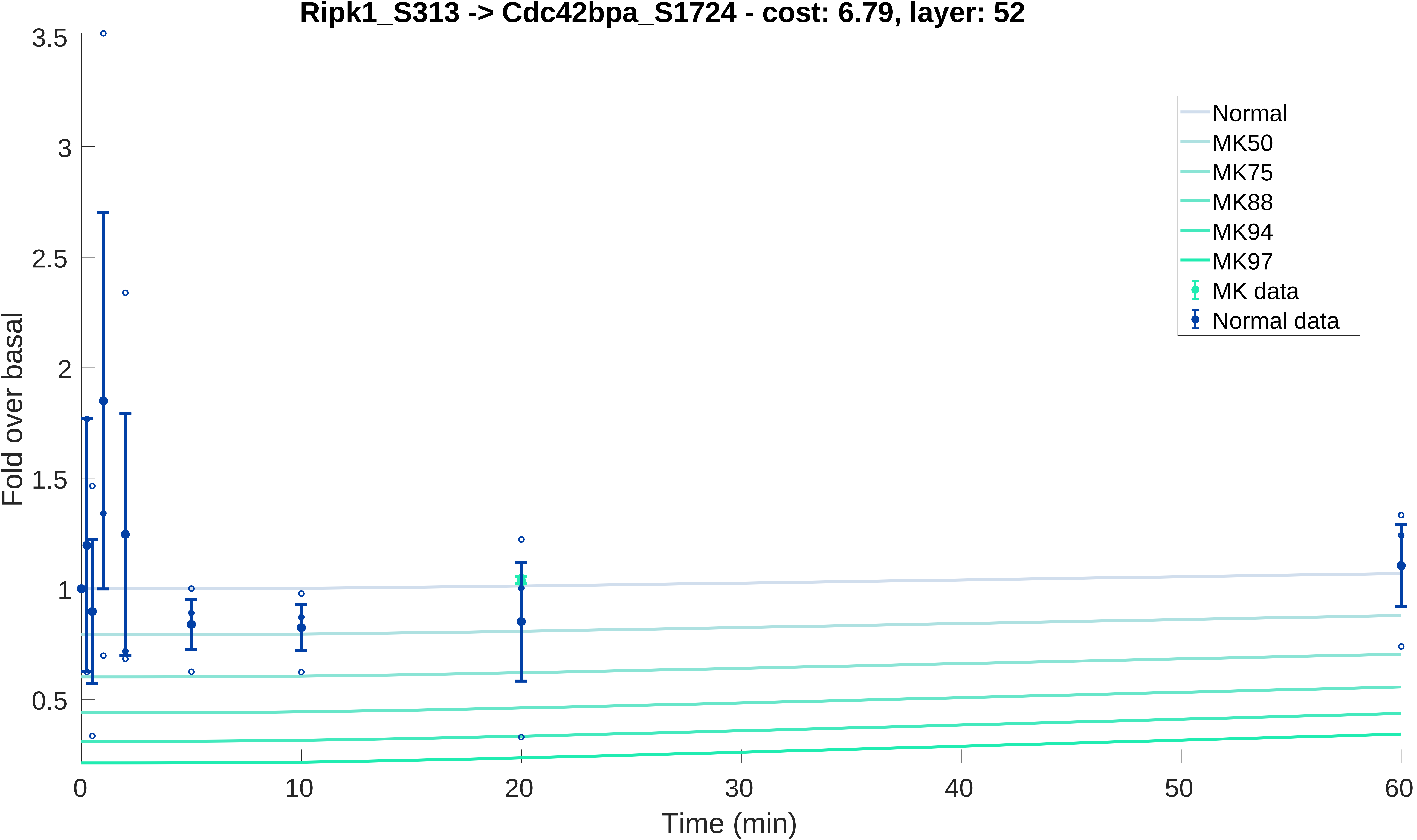

Ripk1_S313 -> Cdc42bpa_S1724 - cost: 6.79, layer: 52
Fold over basal
Time (min)
0.5
1
1.5
2
2.5
3
3.5
0
10
20
30
40
50
60
Normal
MK50
MK75
MK88
MK94
MK97
MK data
Normal data

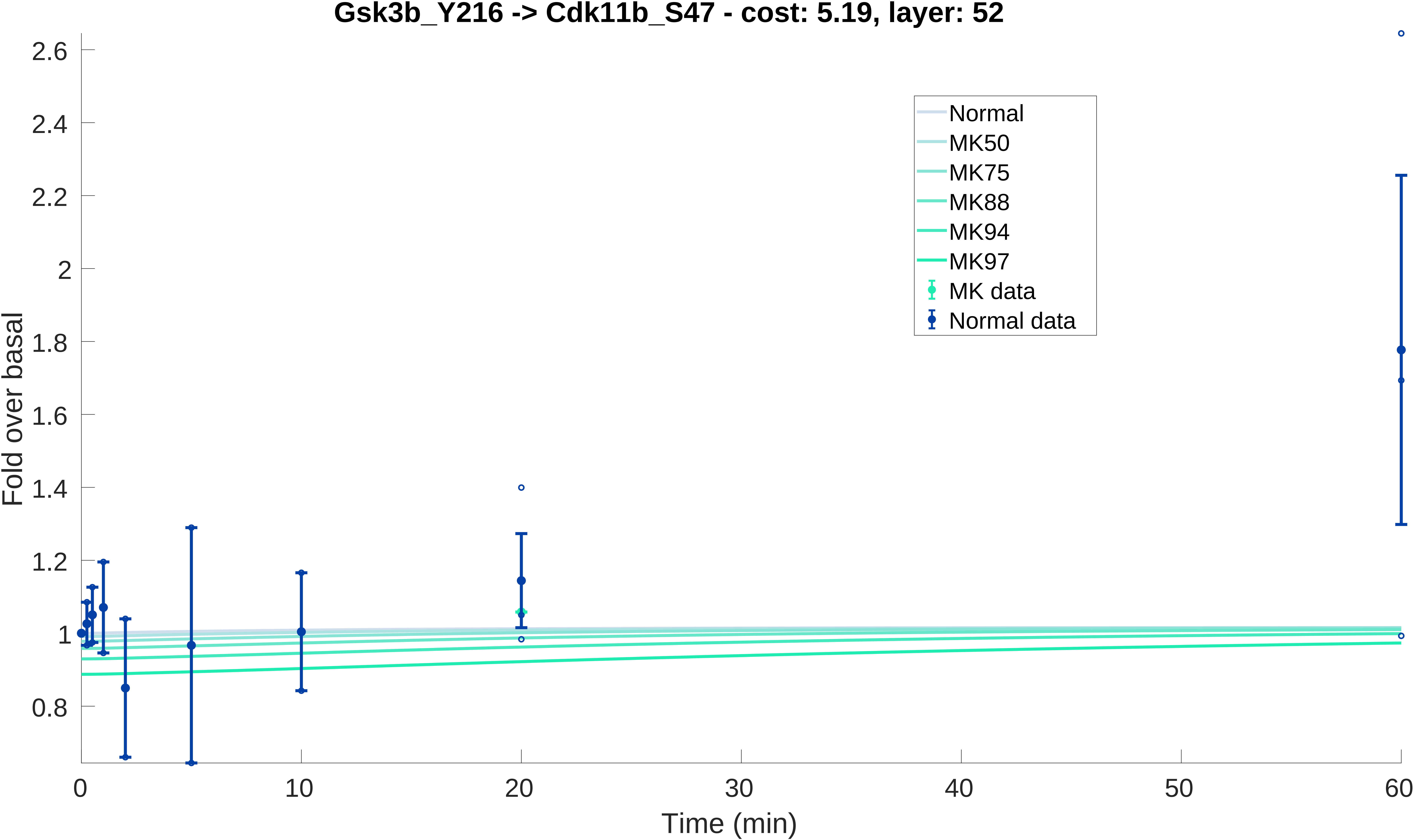

Gsk3b_Y216 -> Cdk11b_S47 - cost: 5.19, layer: 52
Normal
MK50
MK75
MK88
MK94
MK97
MK data
Normal data
Fold over basal
2.6
2.4
2.2
2
1.8
1.6
1.4
1.2
1
0.8
0
10
20
30
40
50
60
Time (min)

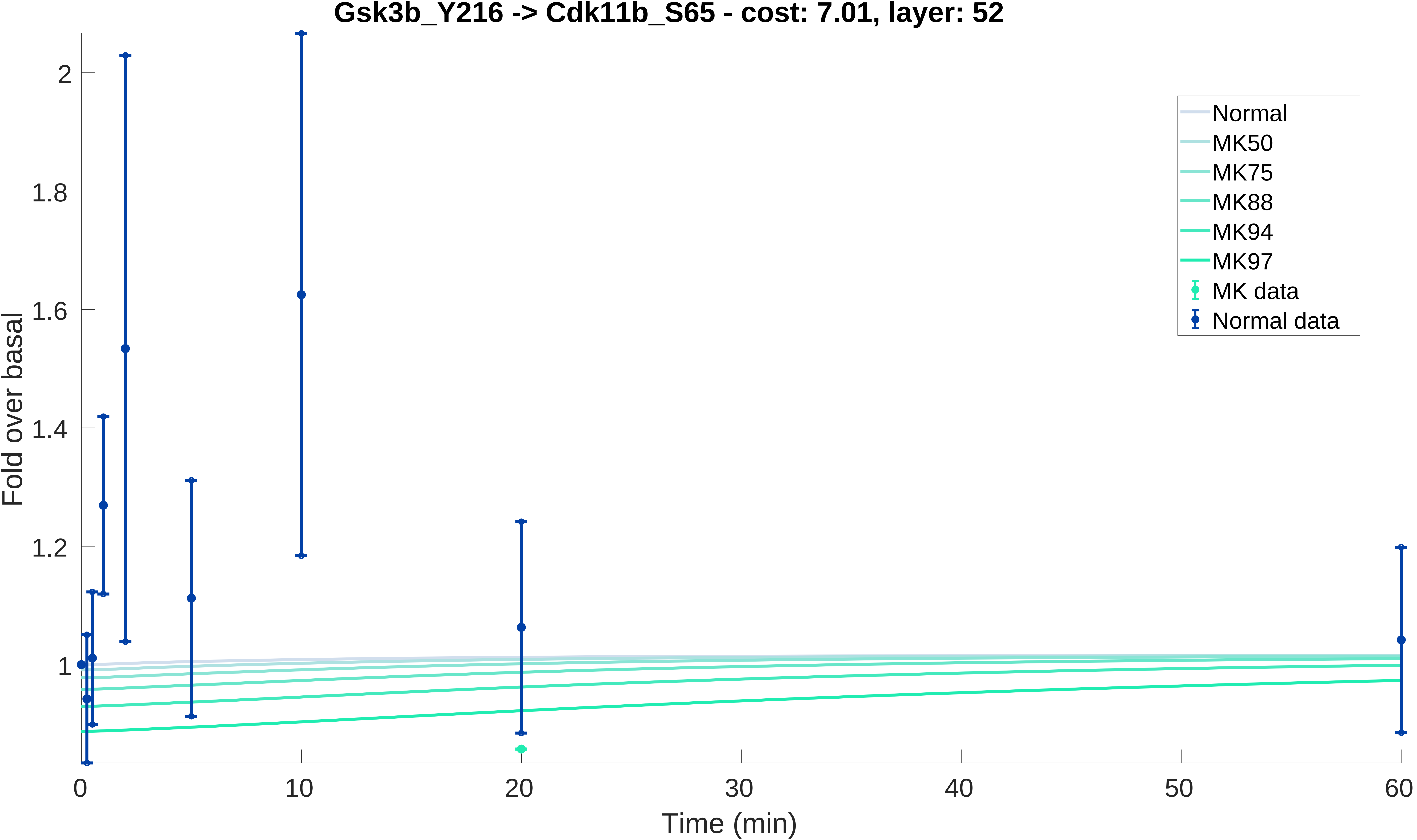

Gsk3b_Y216 -> Cdk11b_S65 - cost: 7.01, layer: 52
Fold over basal
2
1.8
1.6
1.4
1.2
1
0
10
20
30
40
50
60
Time (min)
Normal
MK50
MK75
MK88
MK94
MK97
MK data
Normal data

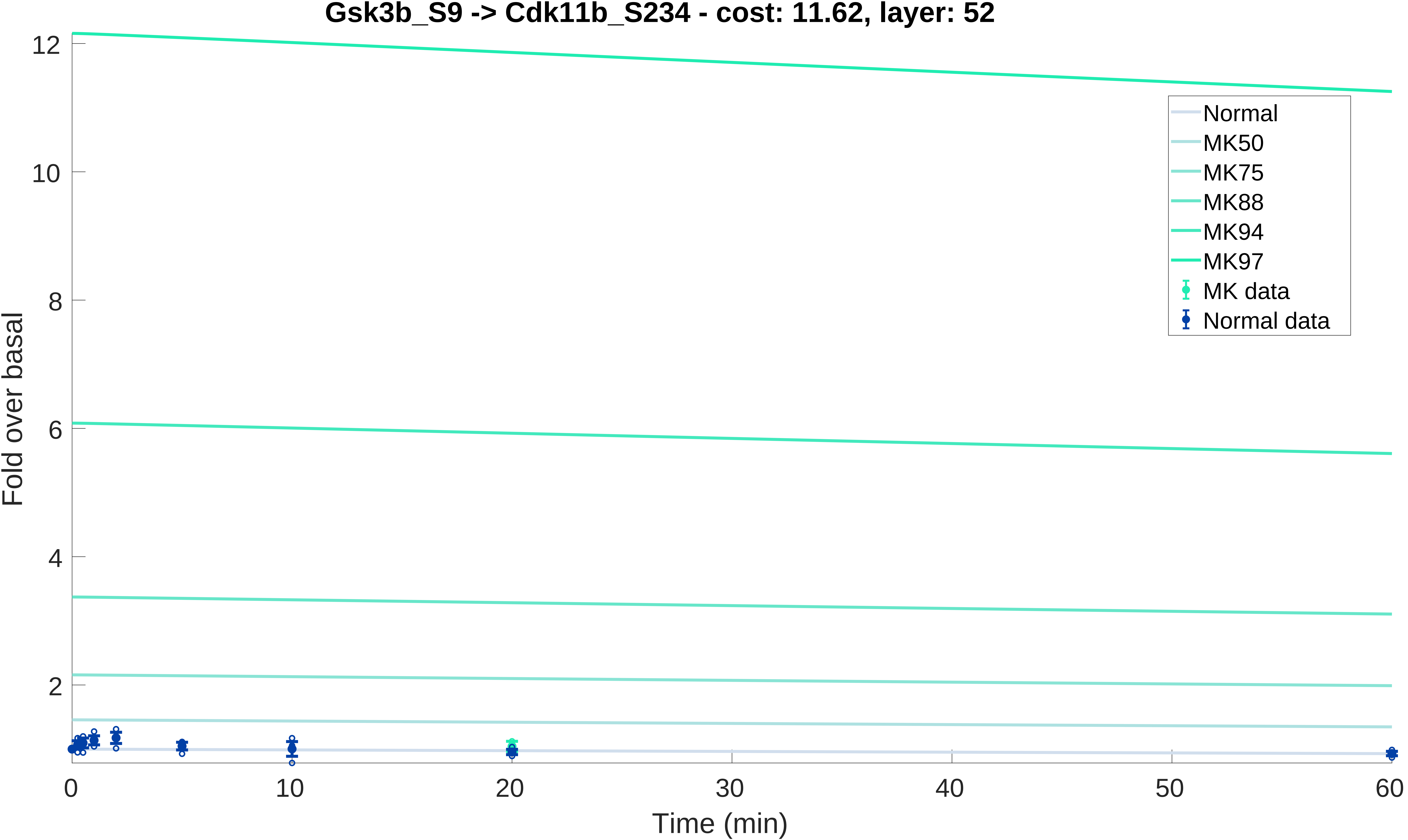

Gsk3b_S9 -> Cdk11b_S234 - cost: 11.62, layer: 52
Fold over basal
Time (min)
0
10
20
30
40
50
60
2
4
6
8
10
12
Normal
MK50
MK75
MK88
MK94
MK97
MK data
Normal data

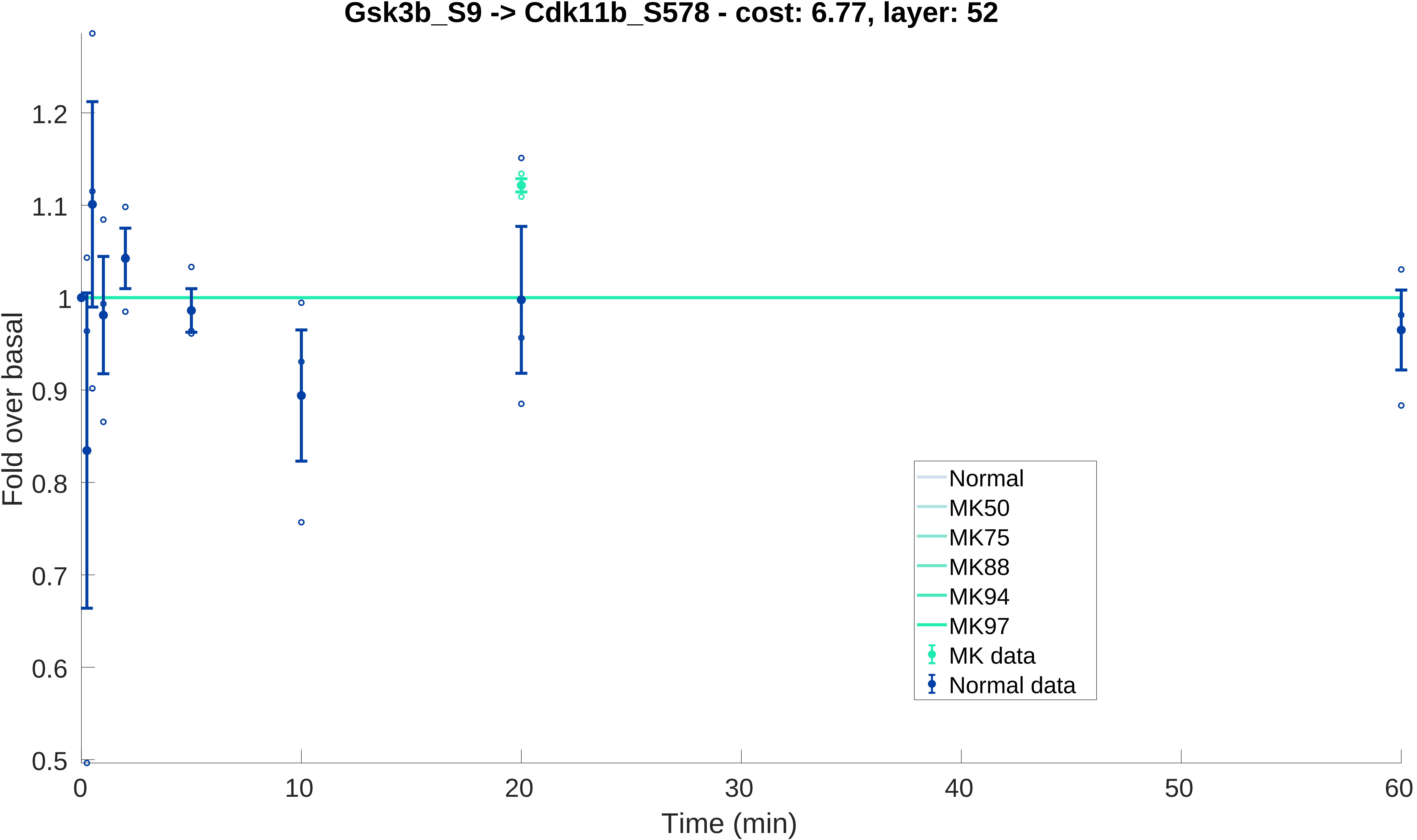
Gsk3b_S9 -> Cdk11b_S578 - cost: 6.77, layer: 52
Fold over basal
1.2
1.1
1
0.9
0.8
0.7
0.6
0.5
0
10
20
30
40
50
60
Time (min)
Normal
MK50
MK75
MK88
MK94
MK97
MK data
Normal data

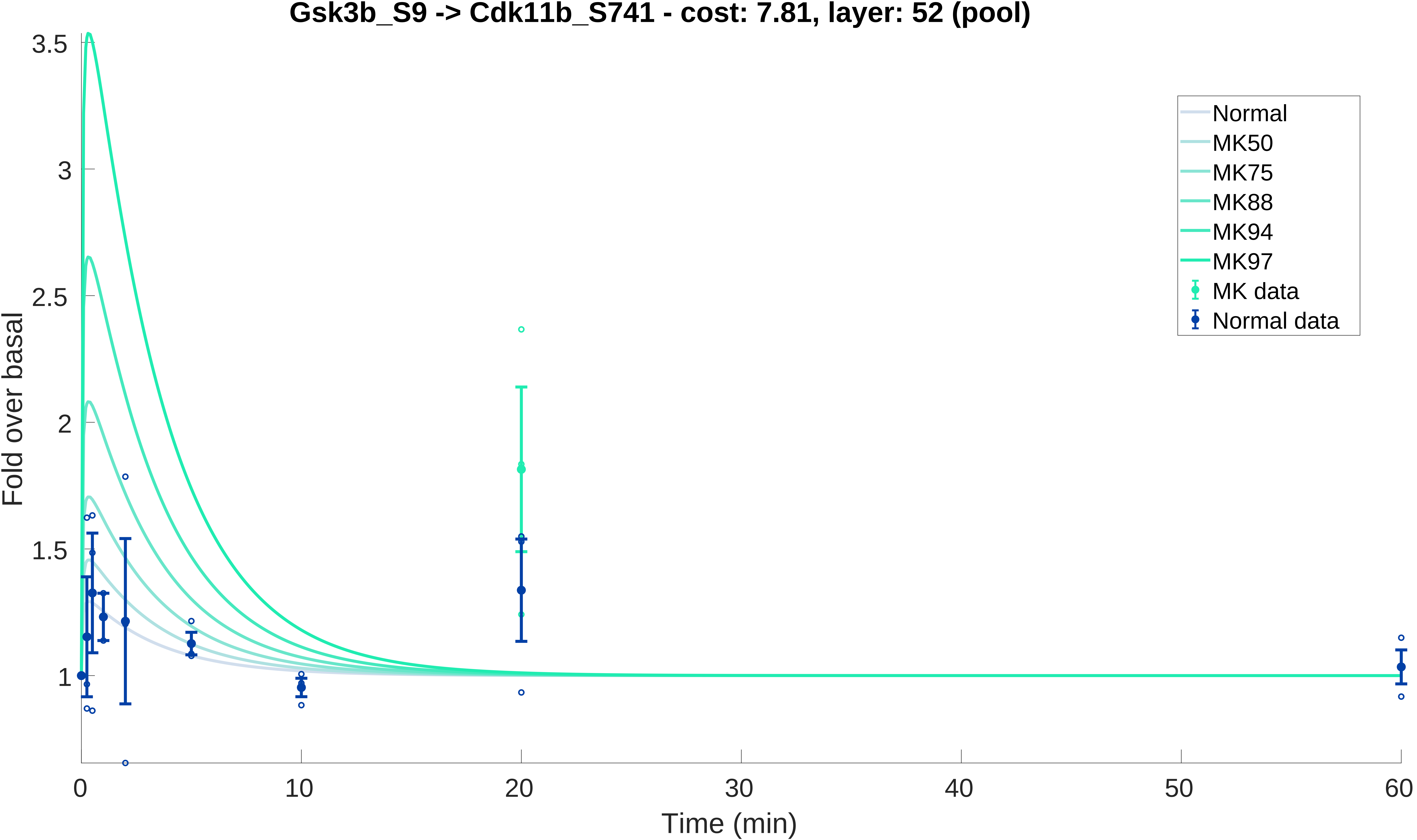

Gsk3b_S9 -> Cdk11b_S741 - cost: 7.81, layer: 52 (pool)
Fold over basal
Time (min)
Normal
MK50
MK75
MK88
MK94
MK97
MK data
Normal data
0
10
20
30
40
50
60
1
1.5
2
2.5
3
3.5

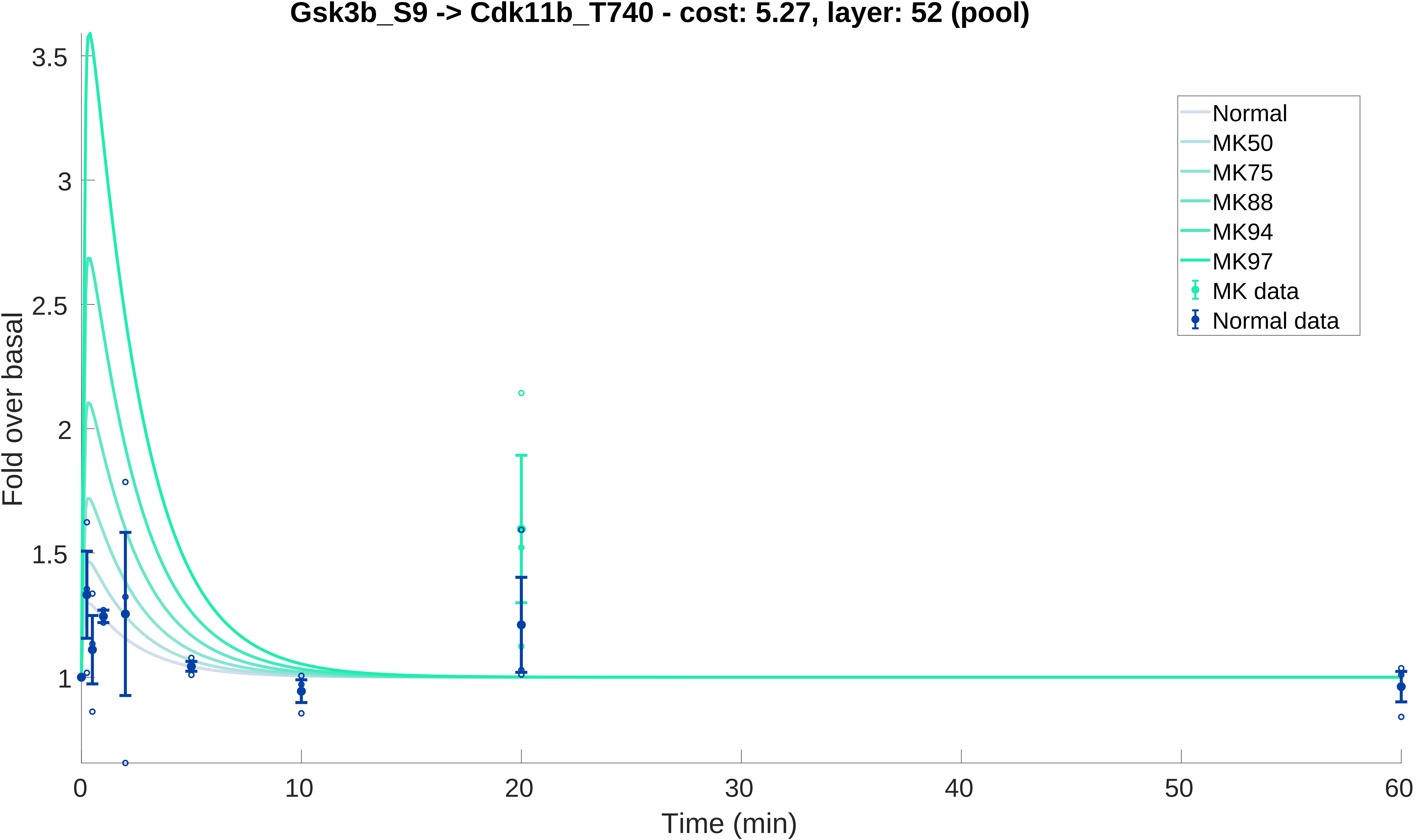
Gsk3b_S9 -> Cdk11b_T740 - cost: 5.27, layer: 52 (pool)
Fold over basal
Time (min)
Normal
MK50
MK75
MK88
MK94
MK97
MK data
Normal data
0
10
20
30
40
50
60
1
1.5
2
2.5
3
3.5

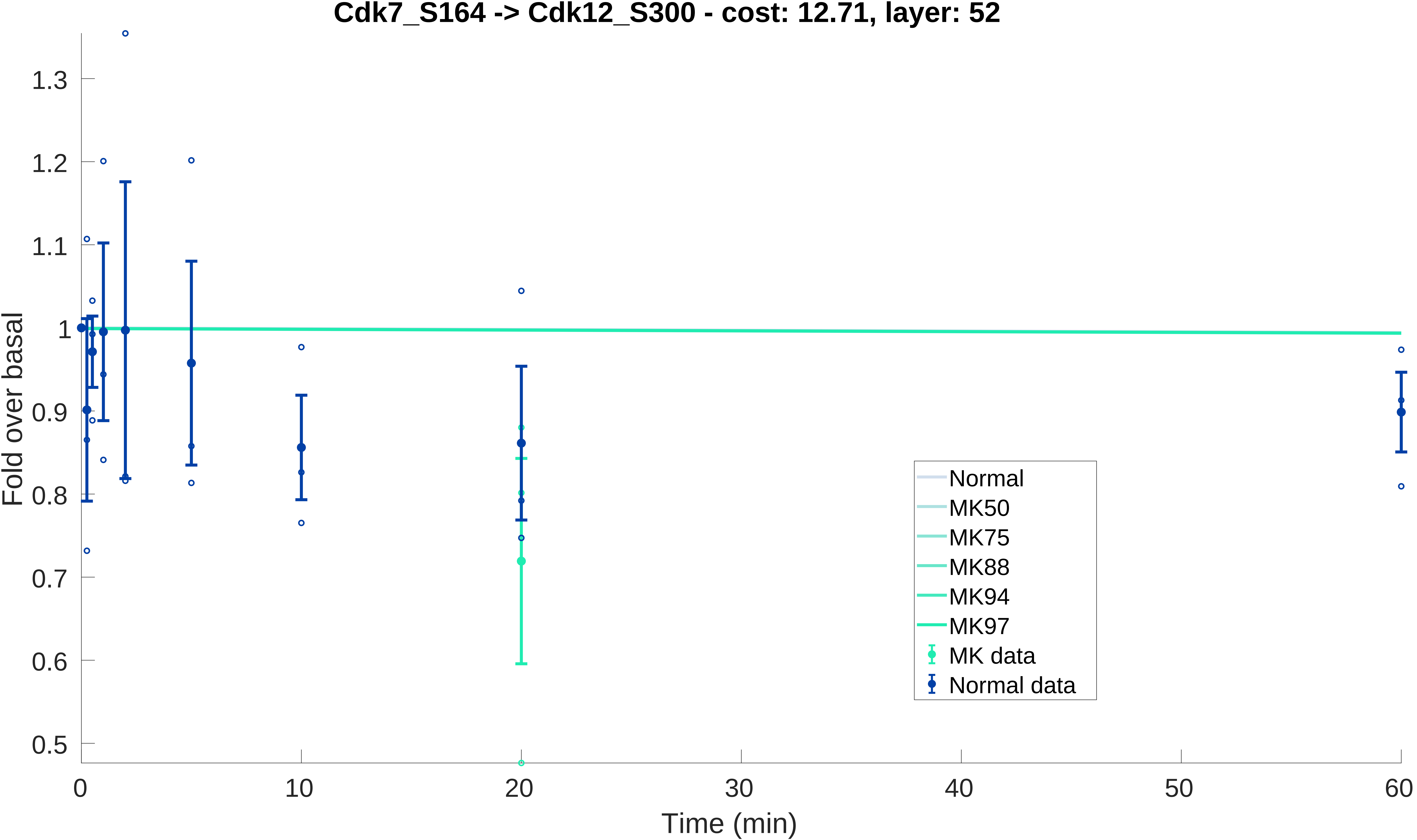
Cdk7_S164 -> Cdk12_S300 - cost: 12.71, layer: 52
Fold over basal
Time (min)
0
10
20
30
40
50
60
0.5
0.6
0.7
0.8
0.9
1
1.1
1.2
1.3
Normal
MK50
MK75
MK88
MK94
MK97
MK data
Normal data

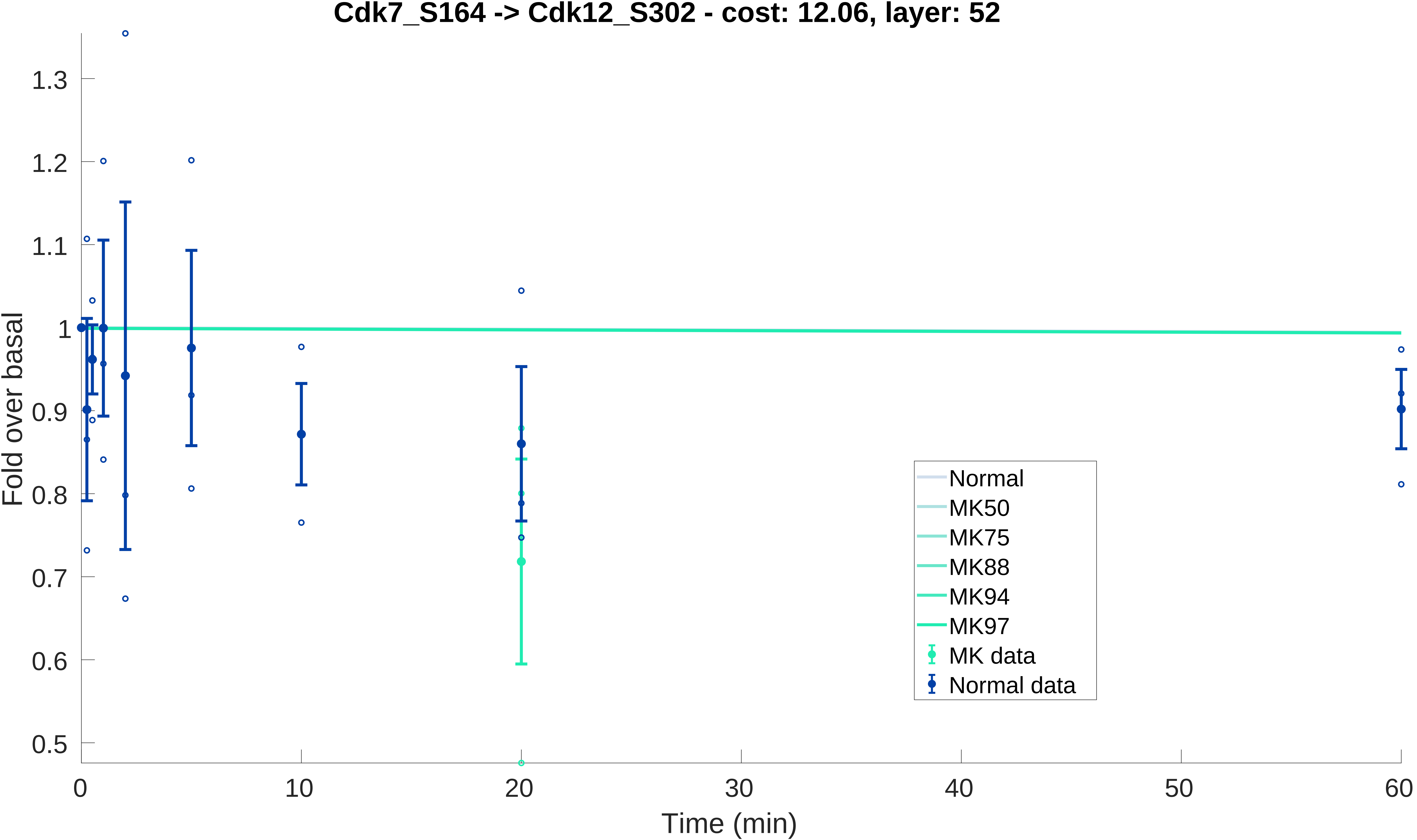
Cdk7_S164 -> Cdk12_S302 - cost: 12.06, layer: 52
Fold over basal
1.3
1.2
1.1
1
0.9
0.8
0.7
0.6
0.5
0
10
20
30
40
50
60
Time (min)
Normal
MK50
MK75
MK88
MK94
MK97
MK data
Normal data

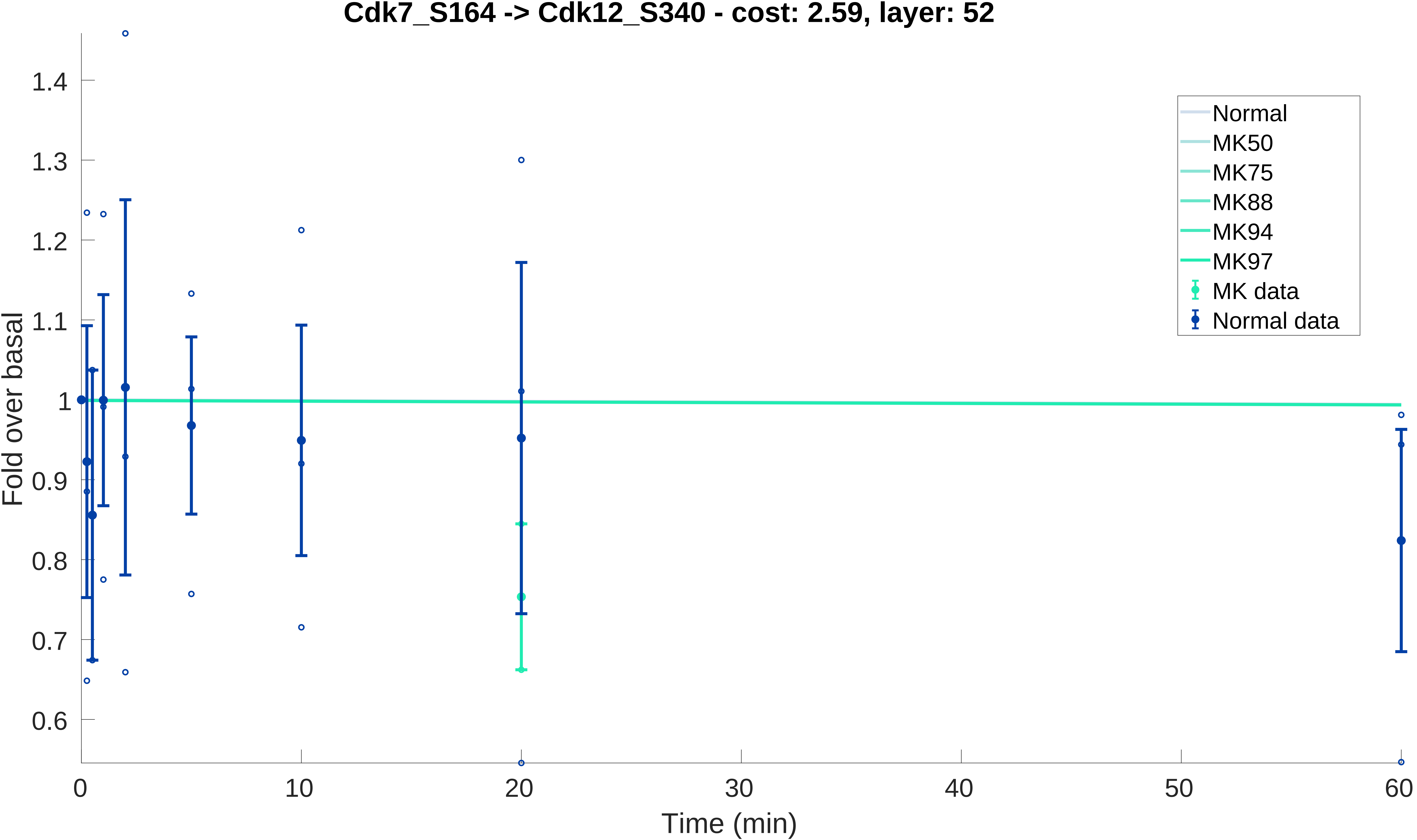

Cdk7_S164 -> Cdk12_S340 - cost: 2.59, layer: 52
Fold over basal
Time (min)
0.6
0.7
0.8
0.9
1
1.1
1.2
1.3
1.4
0
10
20
30
40
50
60
Normal
MK50
MK75
MK88
MK94
MK97
MK data
Normal data

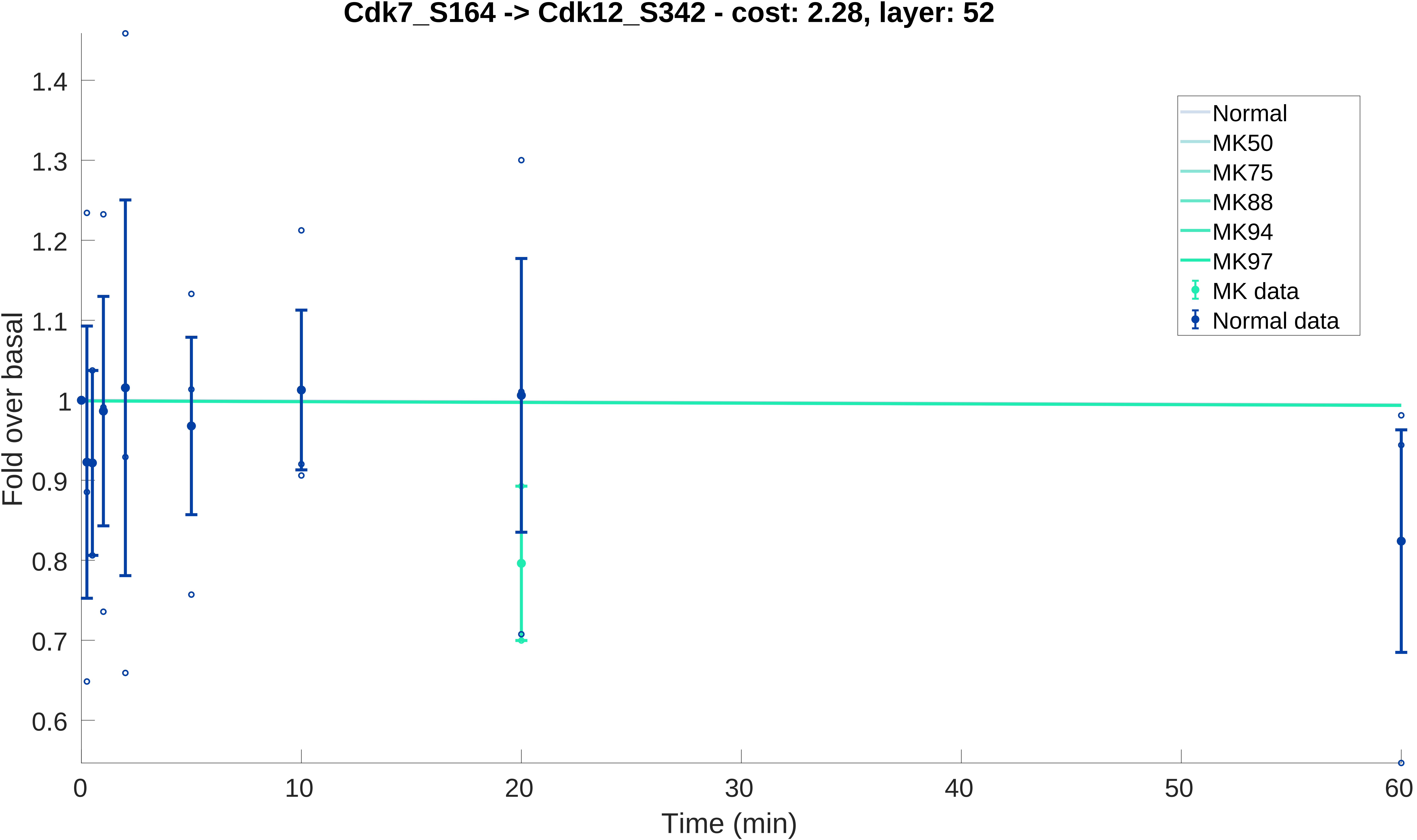

Cdk7_S164 -> Cdk12_S342 - cost: 2.28, layer: 52
Fold over basal
Time (min)
Normal
MK50
MK75
MK88
MK94
MK97
MK data
Normal data
1.4
1.3
1.2
1.1
1
0.9
0.8
0.7
0.6
0
10
20
30
40
50
60

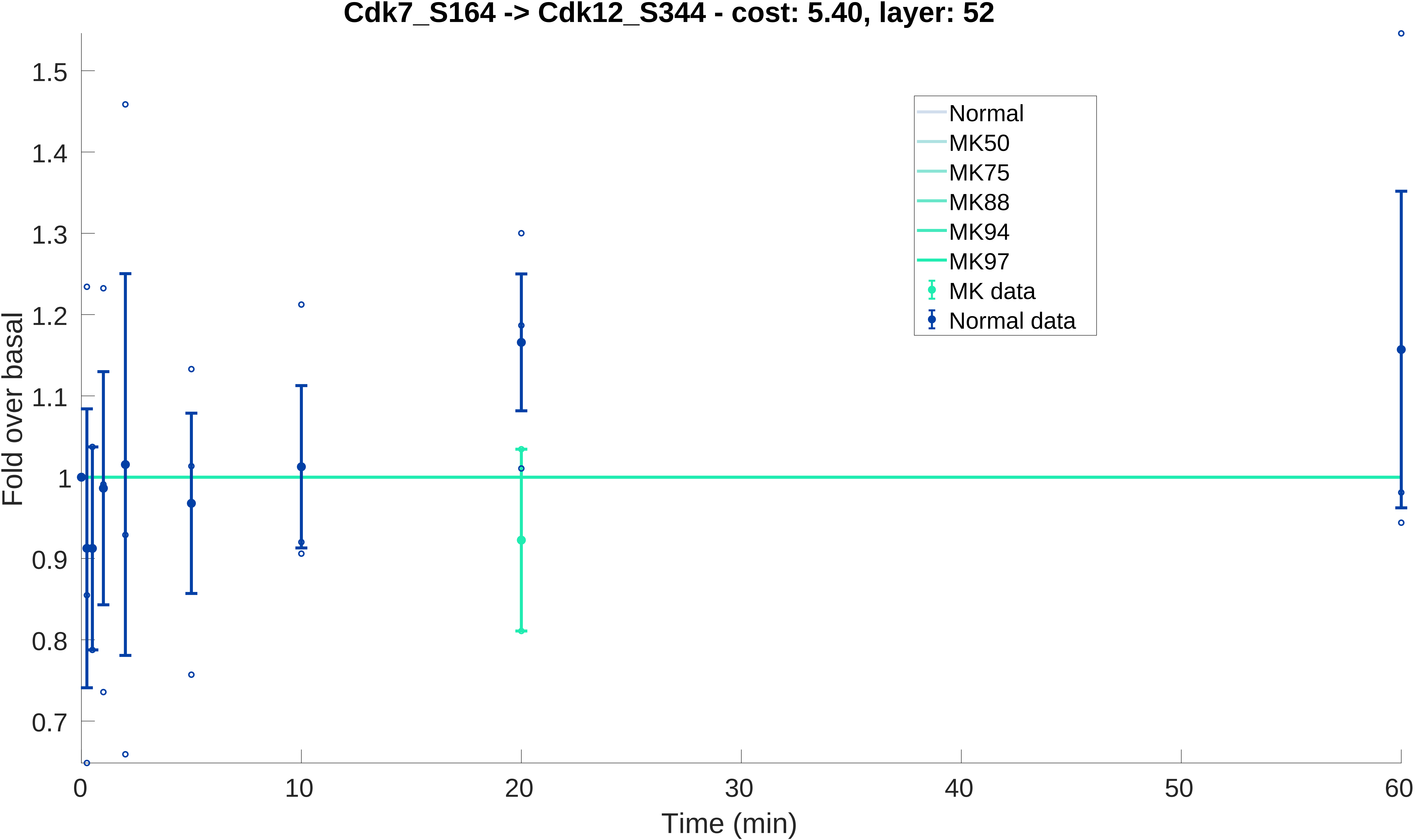

Cdk7_S164 -> Cdk12_S344 - cost: 5.40, layer: 52
Normal
MK50
MK75
MK88
MK94
MK97
MK data
Normal data
Fold over basal
Time (min)
1.5
1.4
1.3
1.2
1.1
1
0.9
0.8
0.7
0
10
20
30
40
50
60

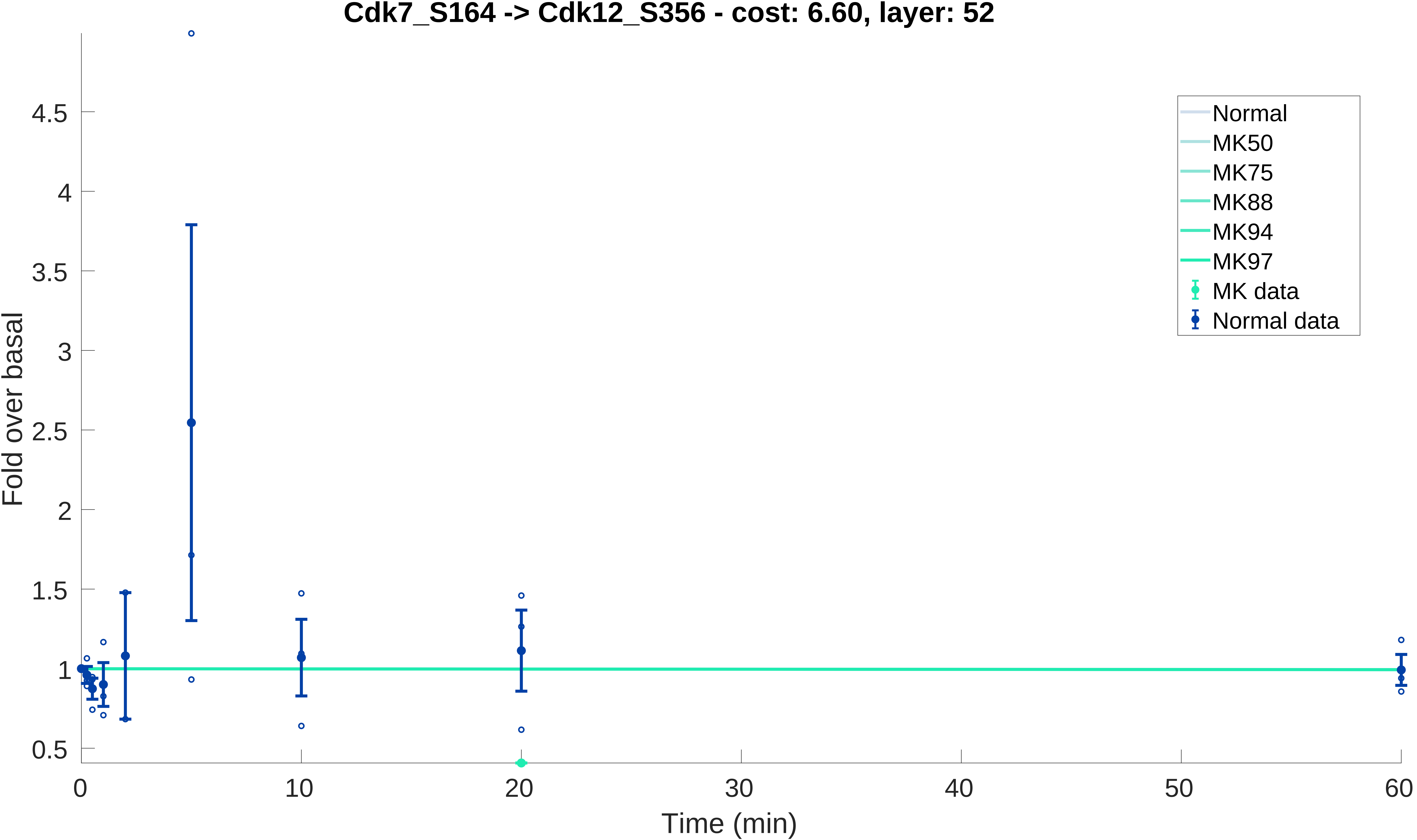
Cdk7_S164 -> Cdk12_S356 - cost: 6.60, layer: 52
Fold over basal
Time (min)
Normal
MK50
MK75
MK88
MK94
MK97
MK data
Normal data
0.5
1
1.5
2
2.5
3
3.5
4
4.5
0
10
20
30
40
50
60

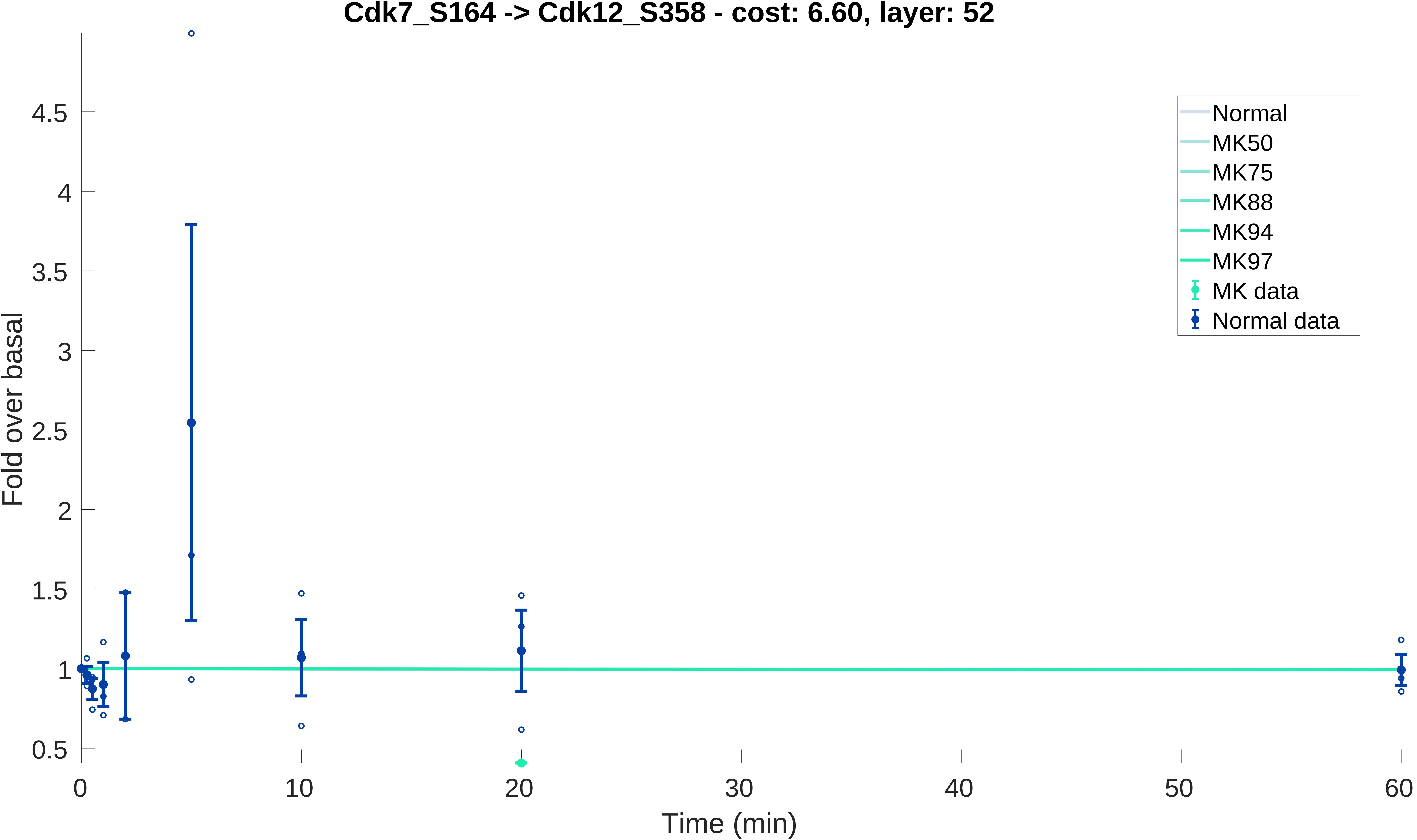
Cdk7_S164 -> Cdk12_S358 - cost: 6.60, layer: 52
Fold over basal
Time (min)
0.5
1
1.5
2
2.5
3
3.5
4
4.5
0
10
20
30
40
50
60
Normal
MK50
MK75
MK88
MK94
MK97
MK data
Normal data

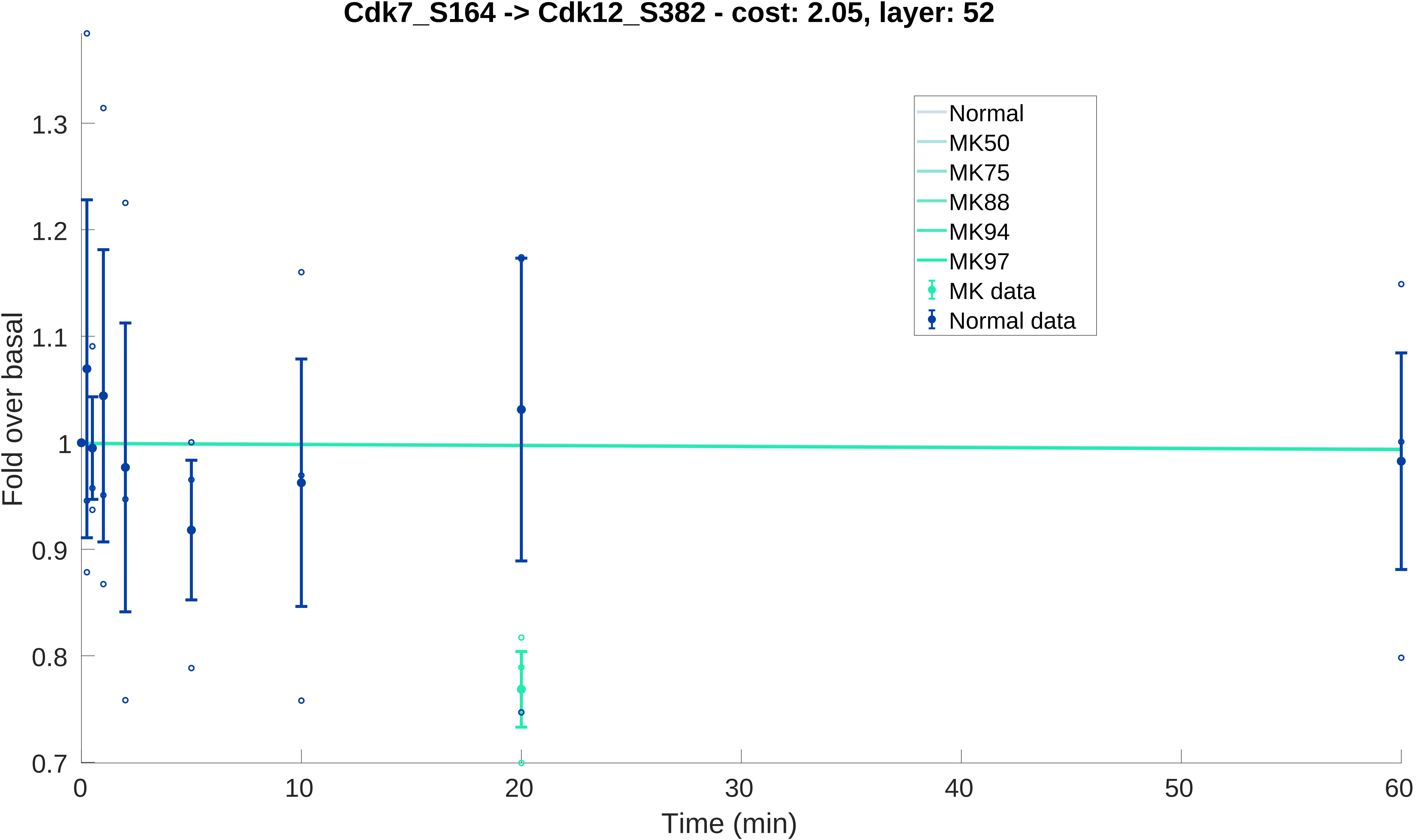
Cdk7_S164 -> Cdk12_S382 - cost: 2.05, layer: 52
Fold over basal
Time (min)
Normal
MK50
MK75
MK88
MK94
MK97
MK data
Normal data
1.3
1.2
1.1
1
0.9
0.8
0.7
0
10
20
30
40
50
60

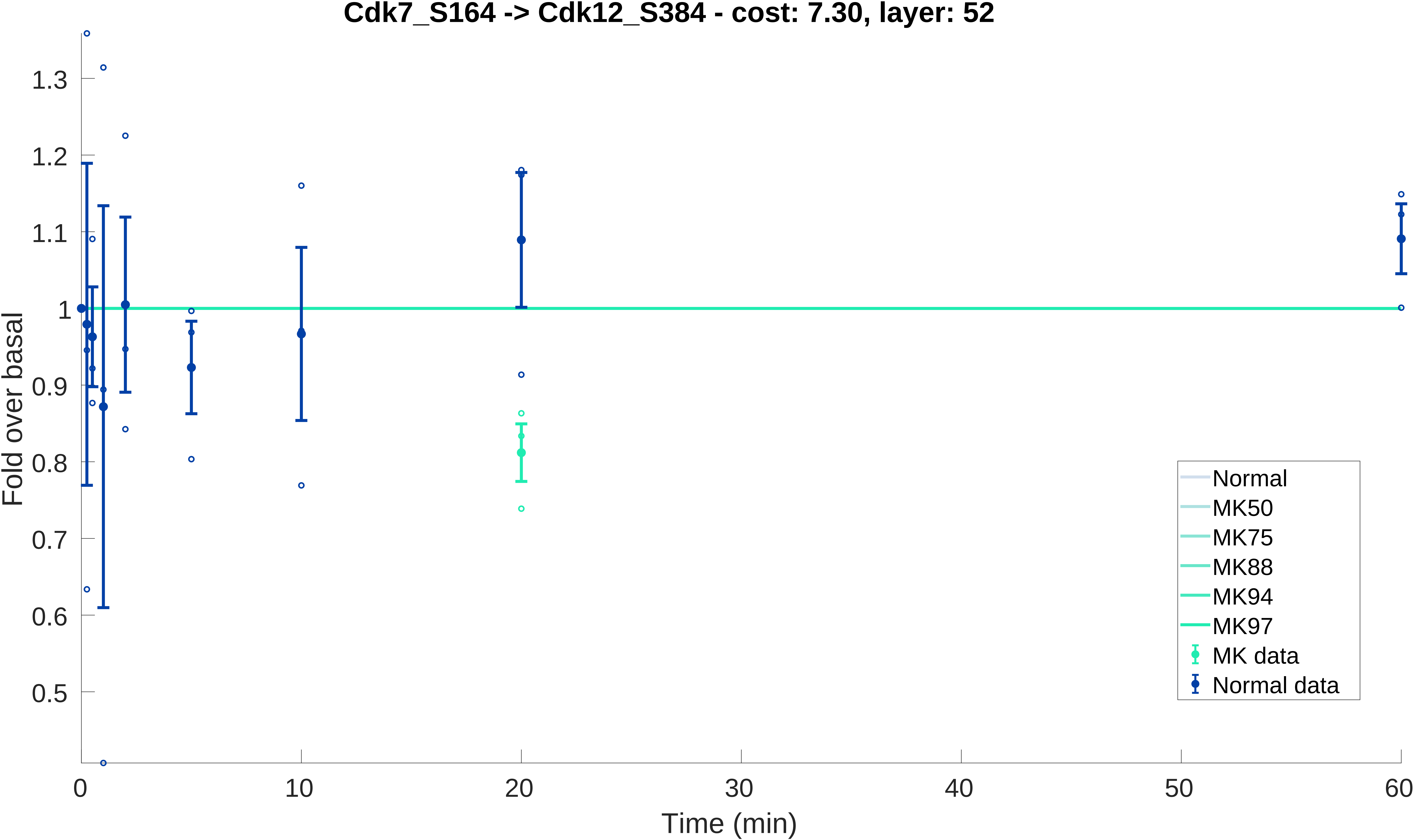
Cdk7_S164 -> Cdk12_S384 - cost: 7.30, layer: 52
Fold over basal
Time (min)
1.3
1.2
1.1
1
0.9
0.8
0.7
0.6
0.5
0
10
20
30
40
50
60
Normal
MK50
MK75
MK88
MK94
MK97
MK data
Normal data

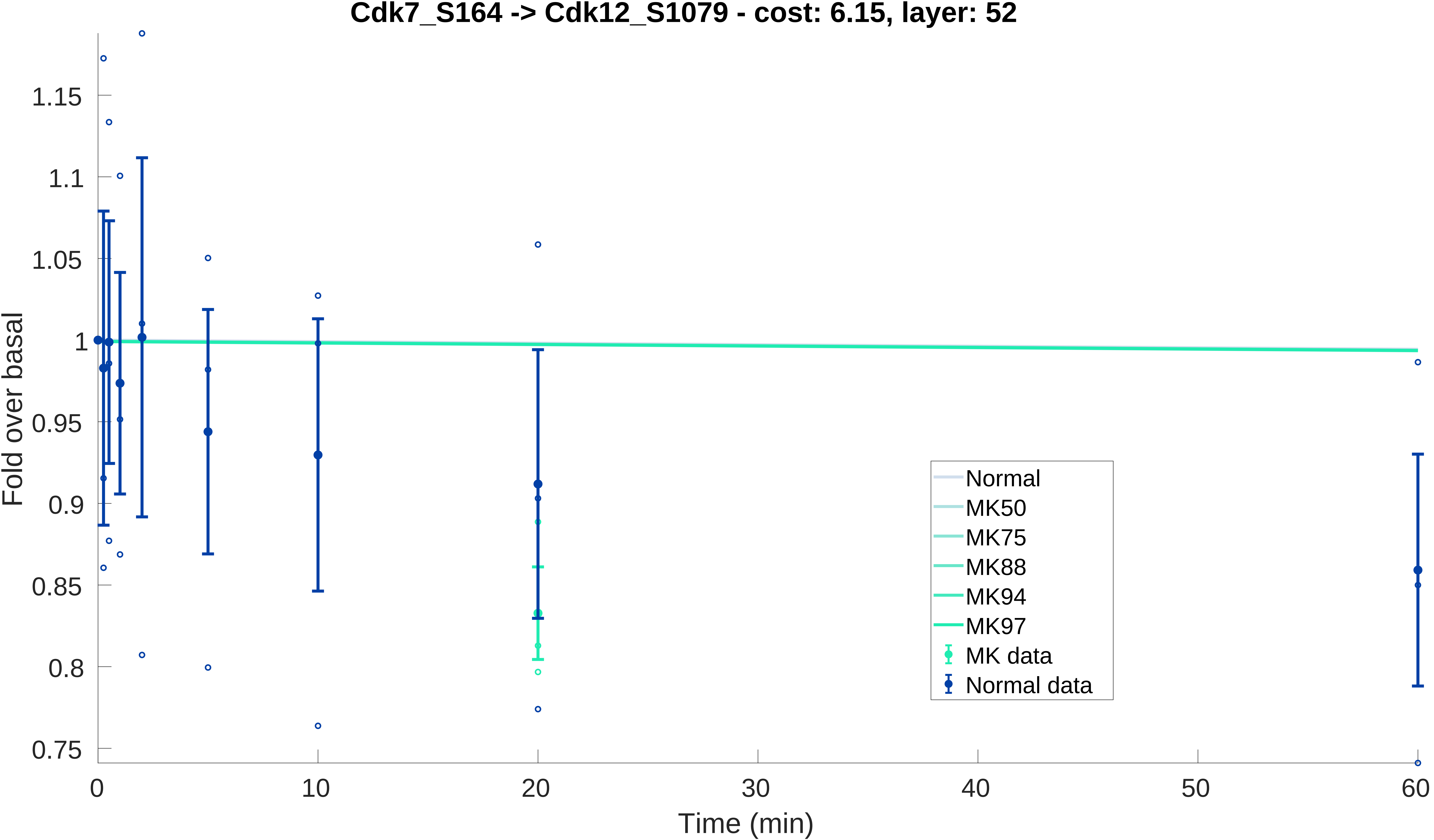

Cdk7_S164 -> Cdk12_S1079 - cost: 6.15, layer: 52
Fold over basal
1.15
1.1
1.05
1
0.95
0.9
0.85
0.8
0.75
0
10
20
30
40
50
60
Time (min)
Normal
MK50
MK75
MK88
MK94
MK97
MK data
Normal data

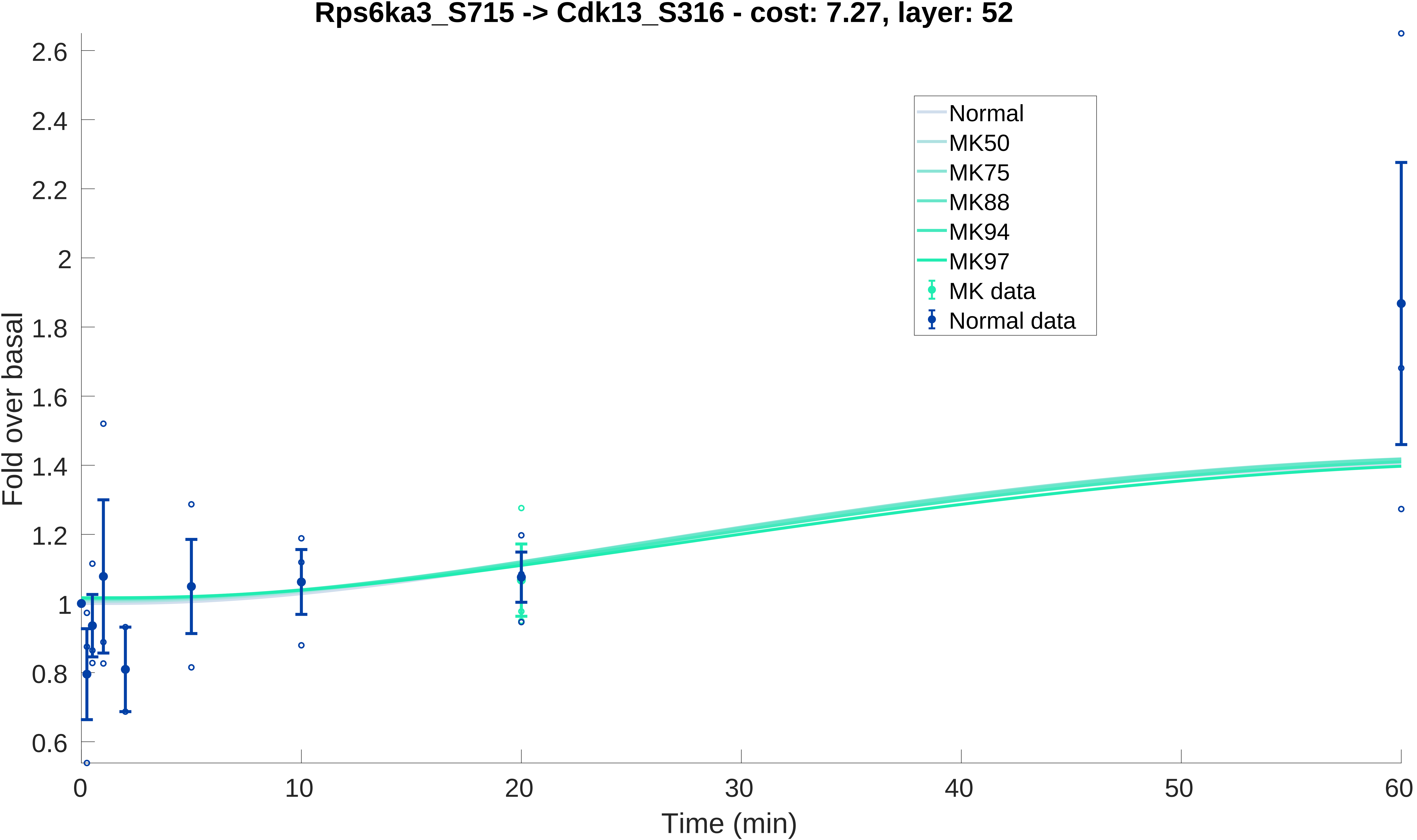

Rps6ka3_S715 -> Cdk13_S316 - cost: 7.27, layer: 52
Fold over basal
Time (min)
0.6
0.8
1
1.2
1.4
1.6
1.8
2
2.2
2.4
2.6
0
10
20
30
40
50
60
Normal
MK50
MK75
MK88
MK94
MK97
MK data
Normal data

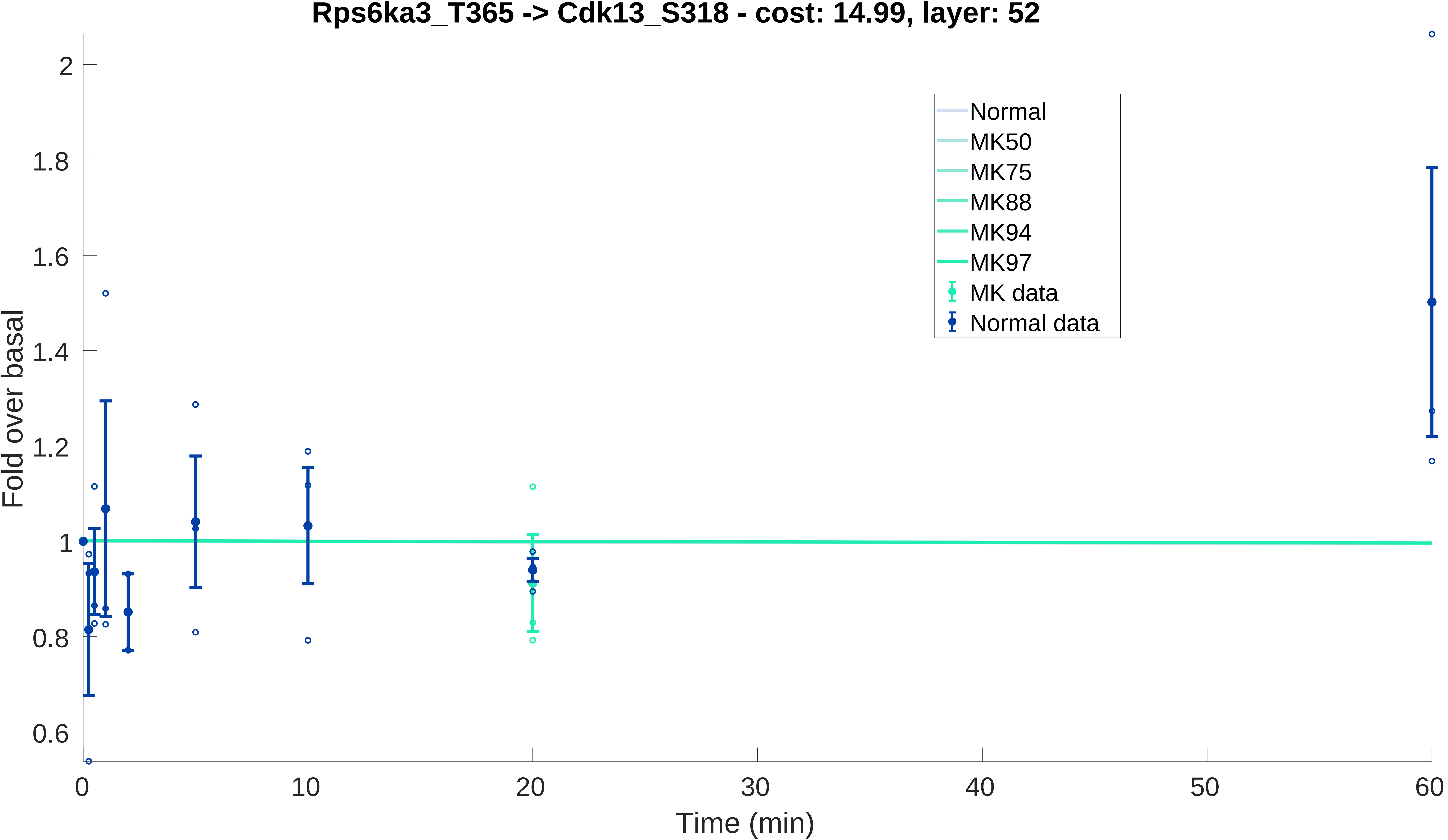
Rps6ka3_T365 -> Cdk13_S318 - cost: 14.99, layer: 52
Fold over basal
2
1.8
1.6
1.4
1.2
1
0.8
0.6
0
10
20
30
40
50
60
Time (min)
Normal
MK50
MK75
MK88
MK94
MK97
MK data
Normal data

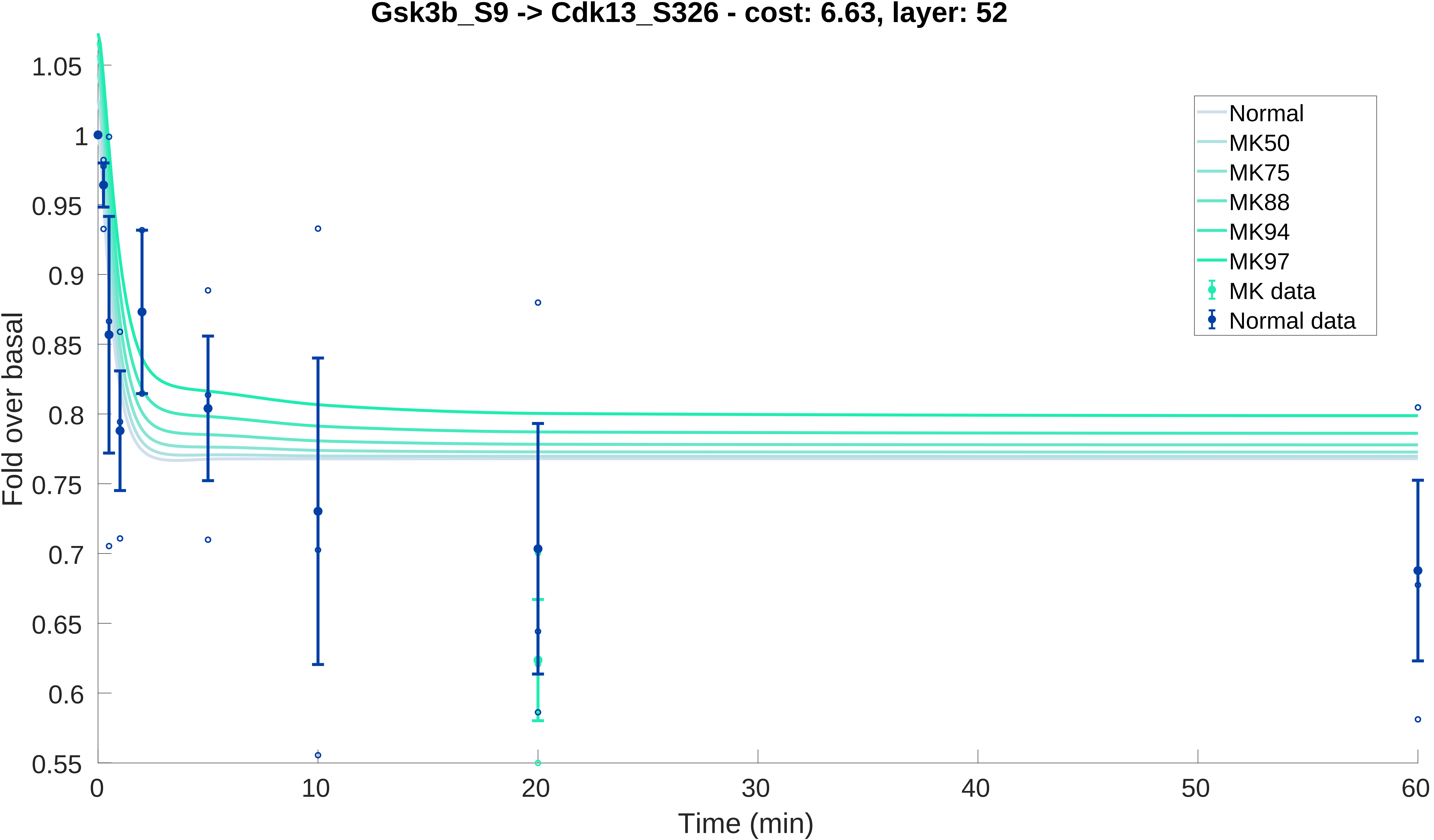
Gsk3b_S9 -> Cdk13_S326 - cost: 6.63, layer: 52
Fold over basal
Time (min)
1.05
1
0.95
0.9
0.85
0.8
0.75
0.7
0.65
0.6
0.55
0
10
20
30
40
50
60
Normal
MK50
MK75
MK88
MK94
MK97
MK data
Normal data

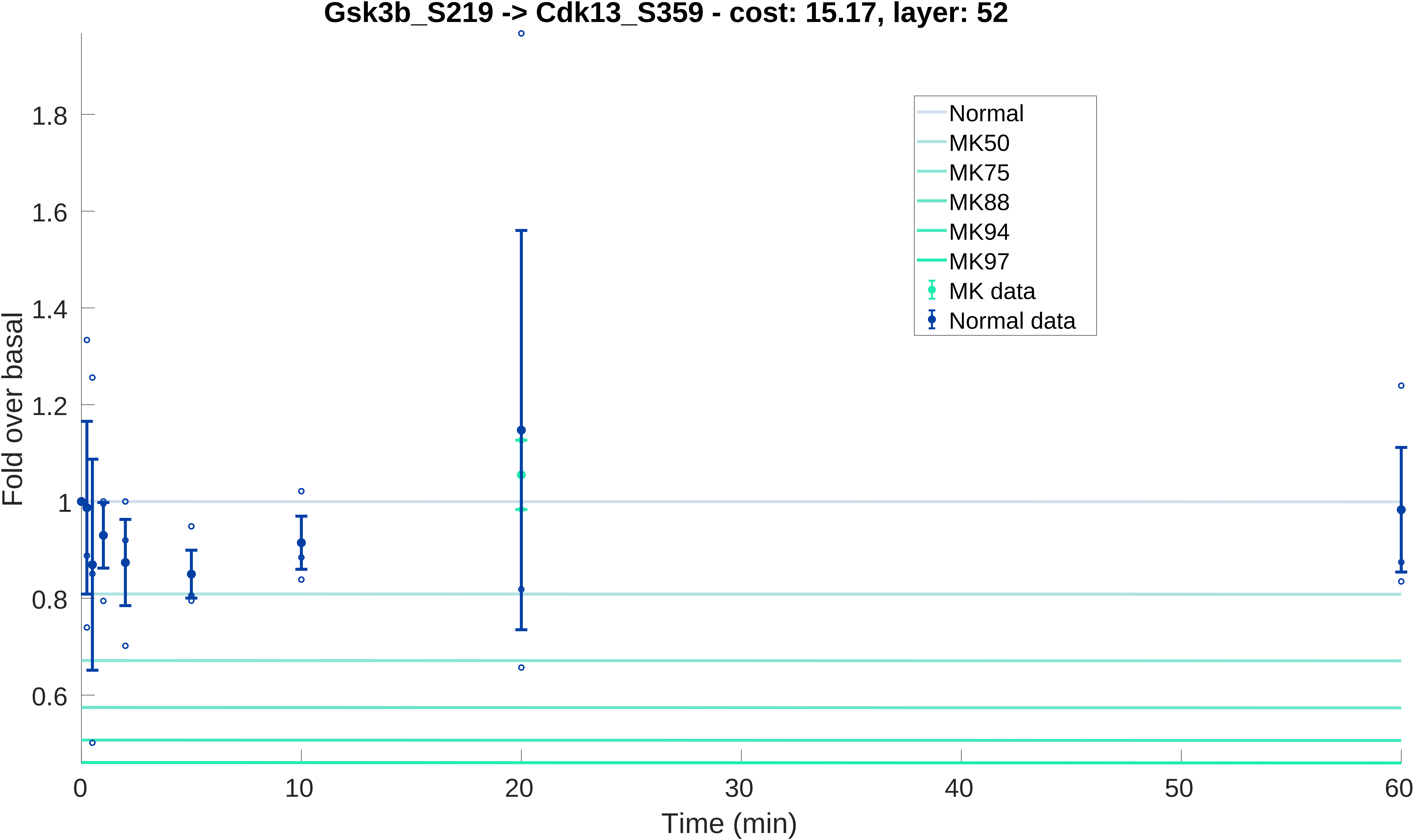

Gsk3b_S219 -> Cdk13_S359 - cost: 15.17, layer: 52
Fold over basal
Time (min)
0.6
0.8
1
1.2
1.4
1.6
1.8
0
10
20
30
40
50
60
Normal
MK50
MK75
MK88
MK94
MK97
MK data
Normal data

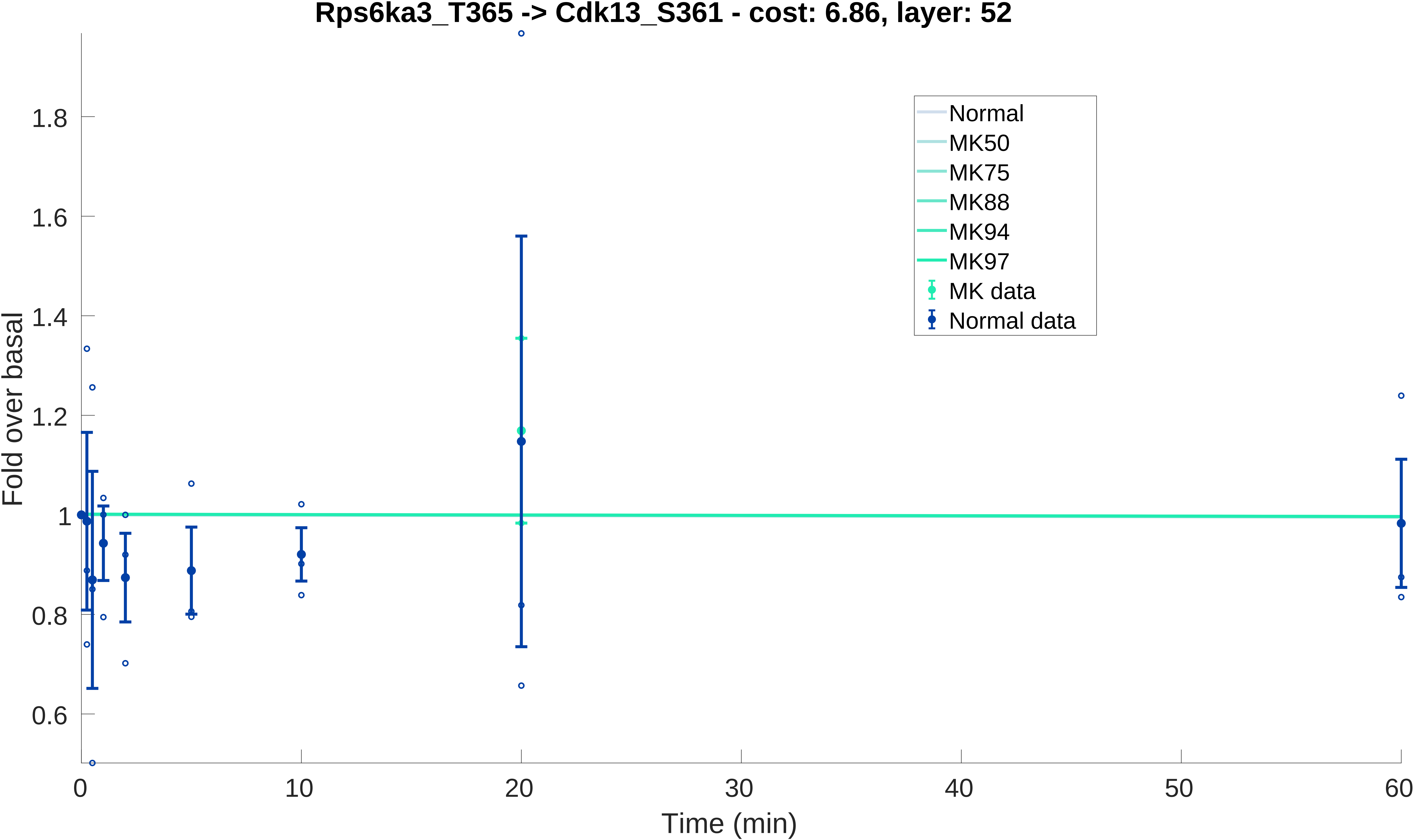

Rps6ka3_T365 -> Cdk13_S361 - cost: 6.86, layer: 52
Fold over basal
Time (min)
0.6
0.8
1
1.2
1.4
1.6
1.8
0
10
20
30
40
50
60
Normal
MK50
MK75
MK88
MK94
MK97
MK data
Normal data

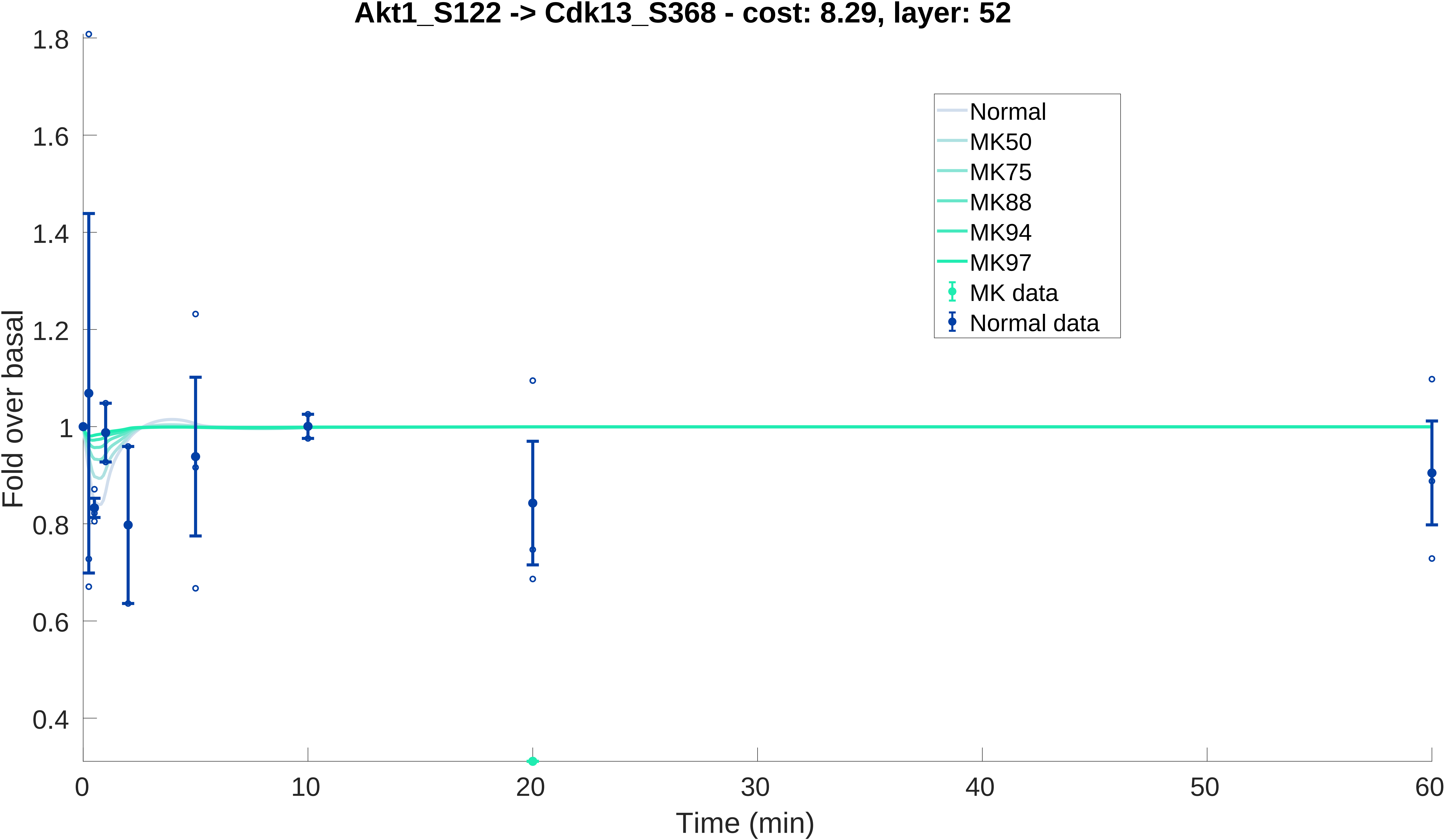
Akt1_S122 -> Cdk13_S368 - cost: 8.29, layer: 52
Fold over basal
Time (min)
1.8
1.6
1.4
1.2
1
0.8
0.6
0.4
0
10
20
30
40
50
60
Normal
MK50
MK75
MK88
MK94
MK97
MK data
Normal data

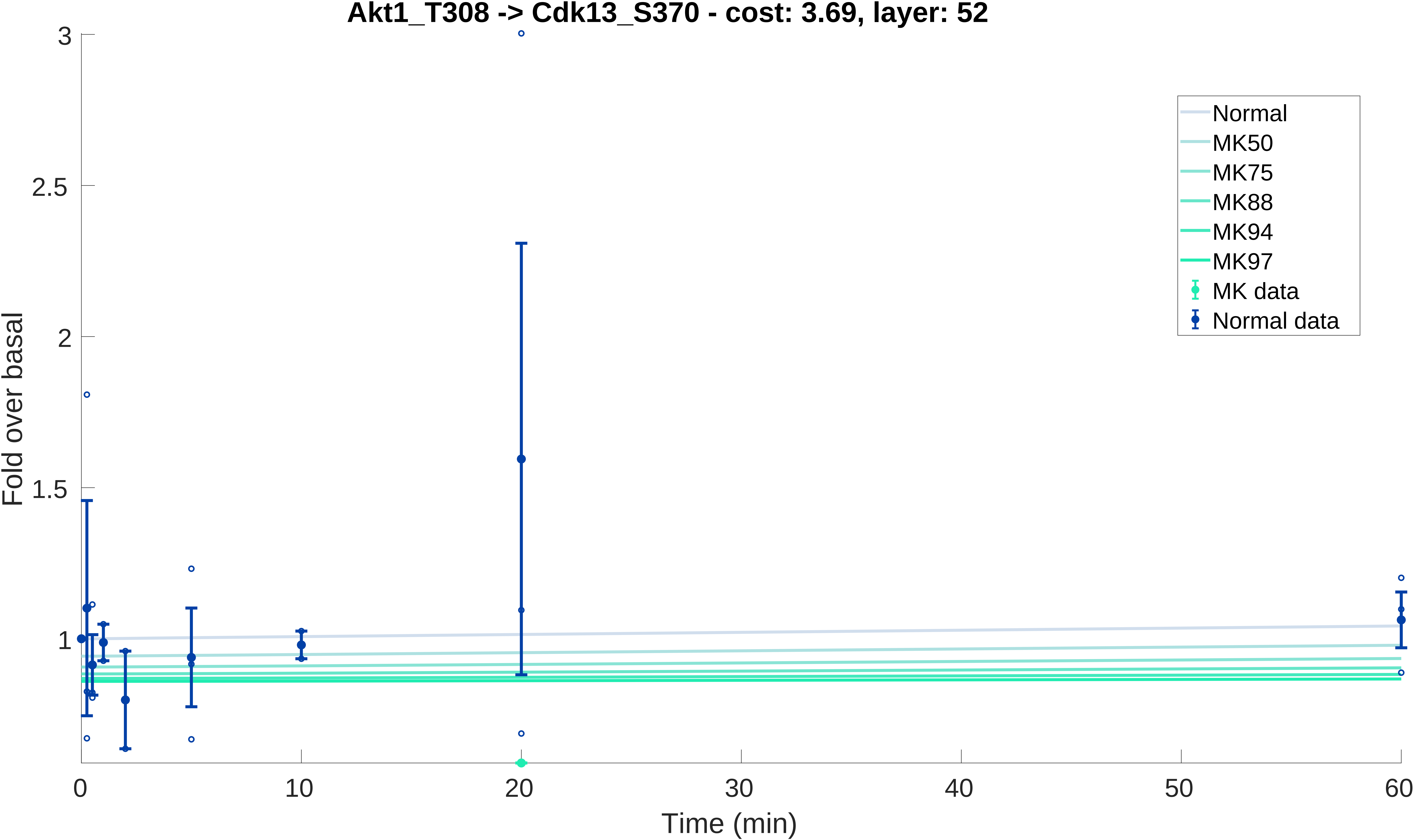
Akt1_T308 -> Cdk13_S370 - cost: 3.69, layer: 52
Fold over basal
Time (min)
3
2.5
2
1.5
1
0
10
20
30
40
50
60
Normal
MK50
MK75
MK88
MK94
MK97
MK data
Normal data

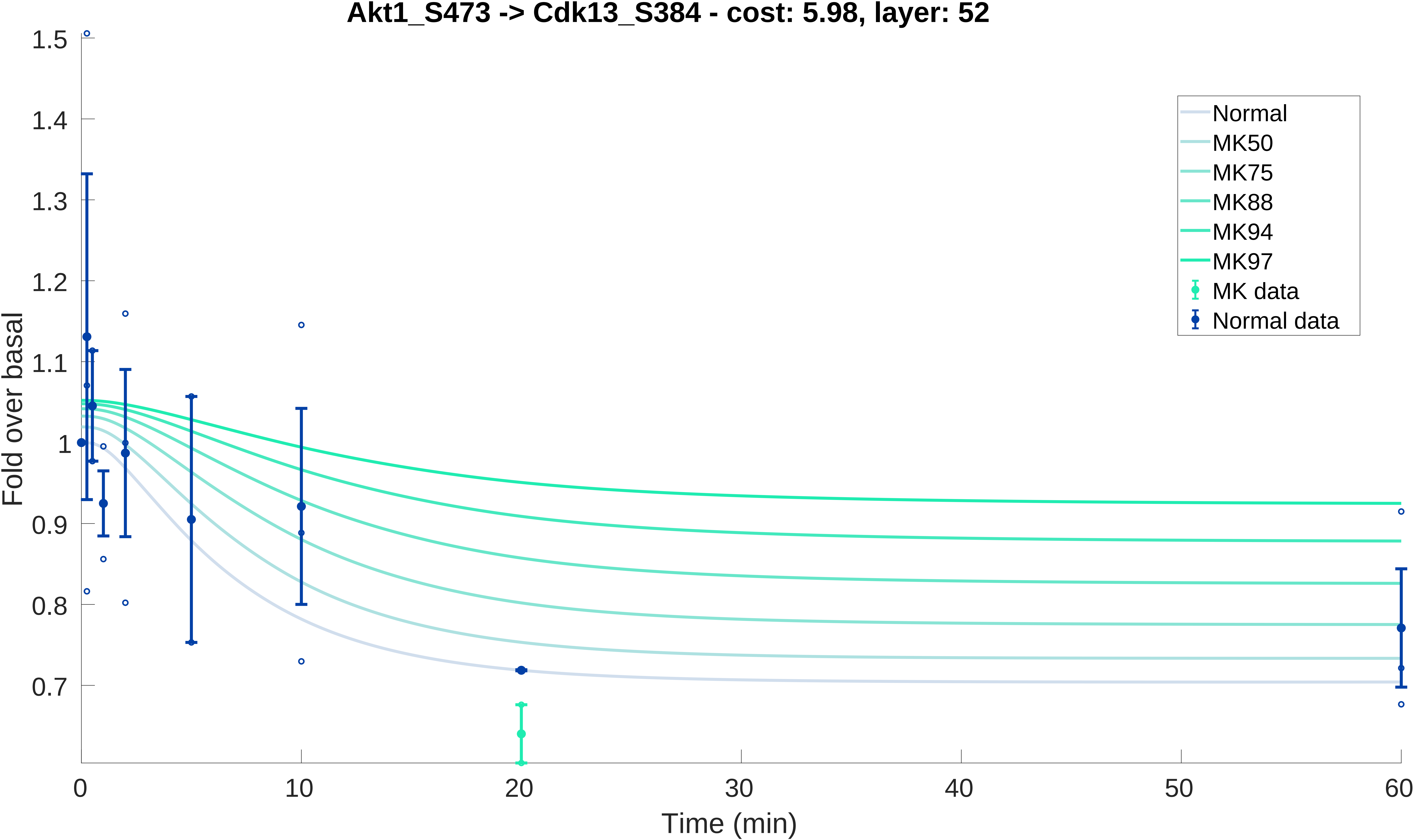
Akt1_S473 -> Cdk13_S384 - cost: 5.98, layer: 52
1.5
1.4
1.3
1.2
1.1
1
0.9
0.8
0.7
Fold over basal
0
10
20
30
40
50
60
Time (min)
Normal
MK50
MK75
MK88
MK94
MK97
MK data
Normal data

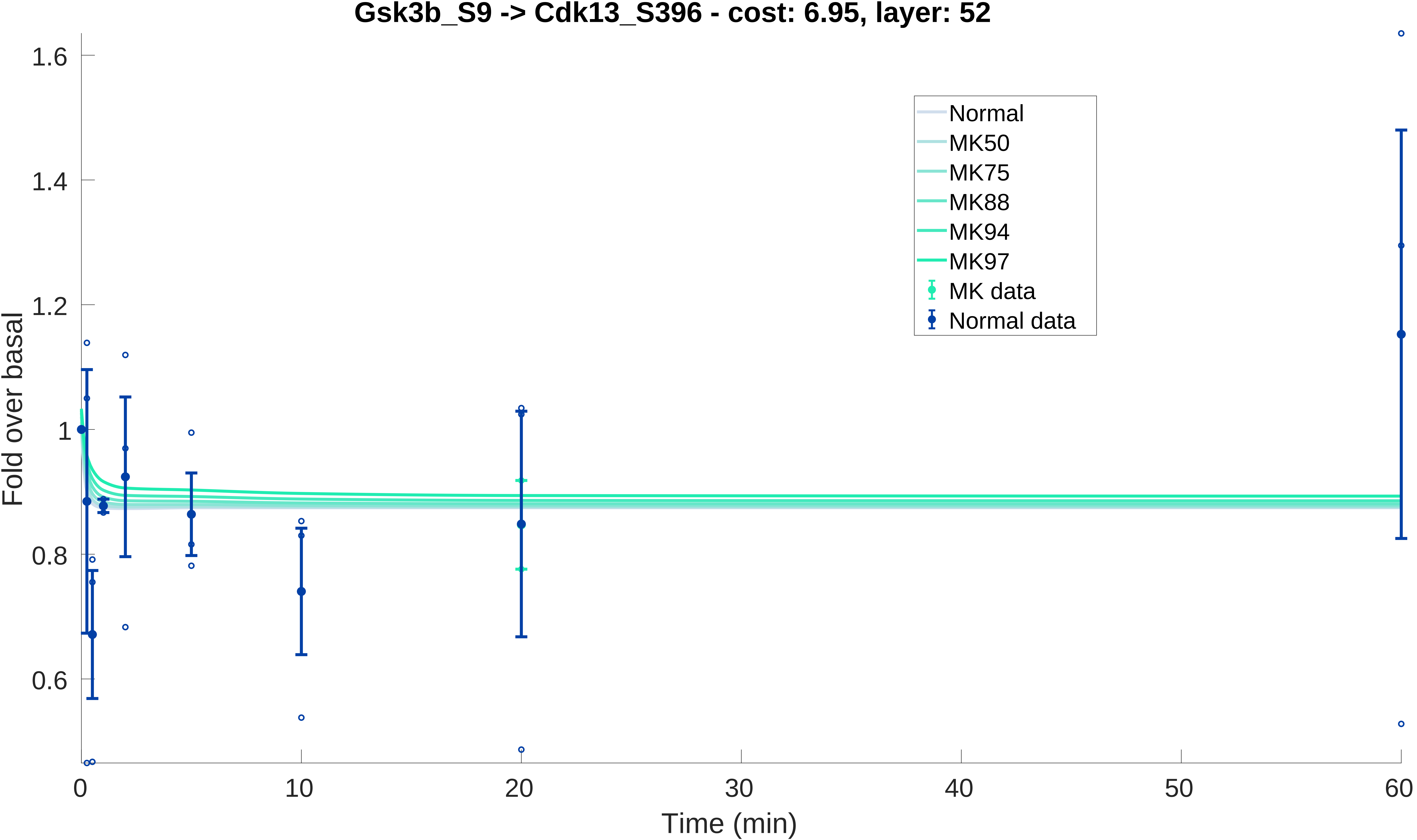

Gsk3b_S9 -> Cdk13_S396 - cost: 6.95, layer: 52
Fold over basal
Time (min)
1.6
1.4
1.2
1
0.8
0.6
0
10
20
30
40
50
60
Normal
MK50
MK75
MK88
MK94
MK97
MK data
Normal data

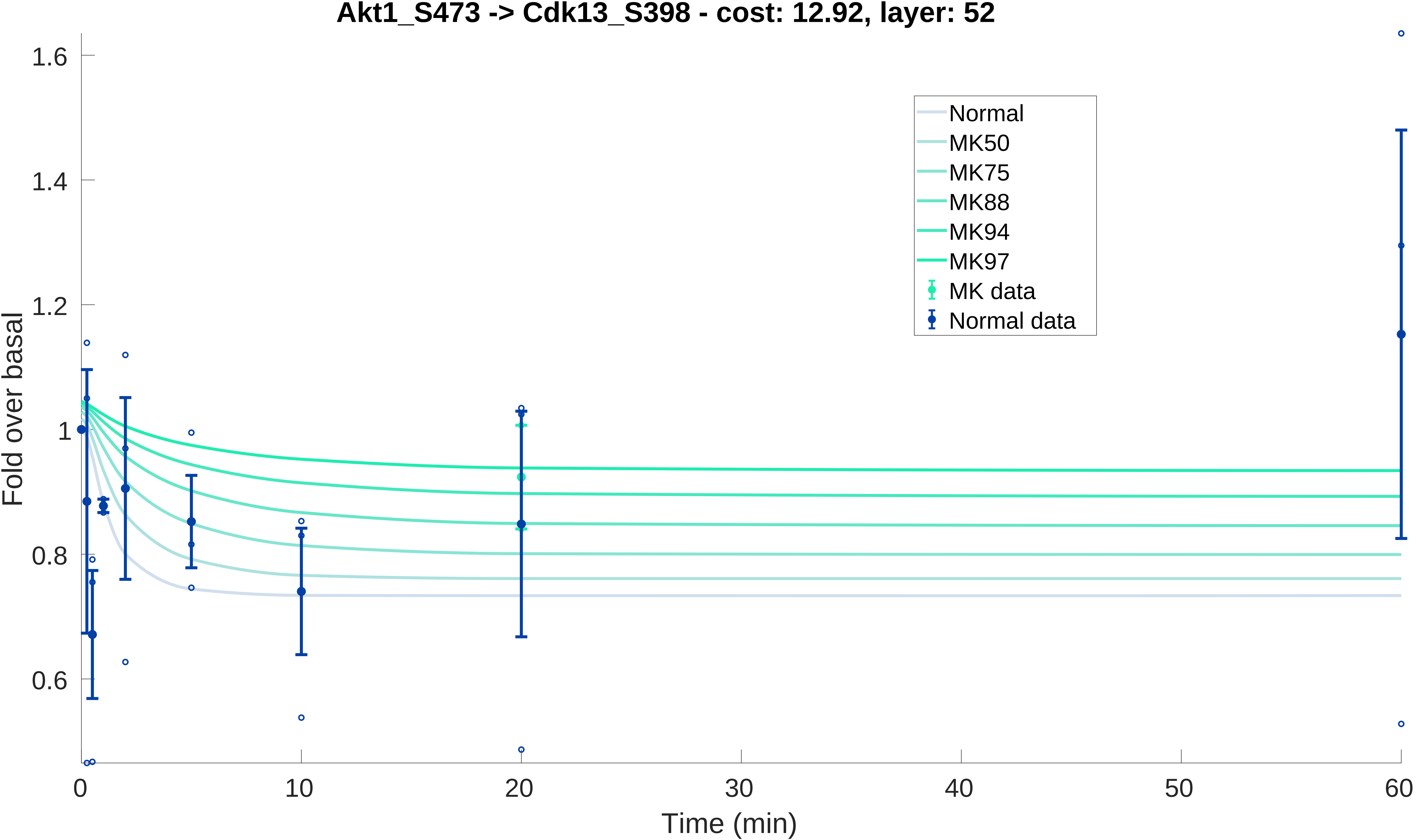

Akt1_S473 -> Cdk13_S398 - cost: 12.92, layer: 52
Fold over basal
Time (min)
1.6
1.4
1.2
1
0.8
0.6
0
10
20
30
40
50
60
Normal
MK50
MK75
MK88
MK94
MK97
MK data
Normal data

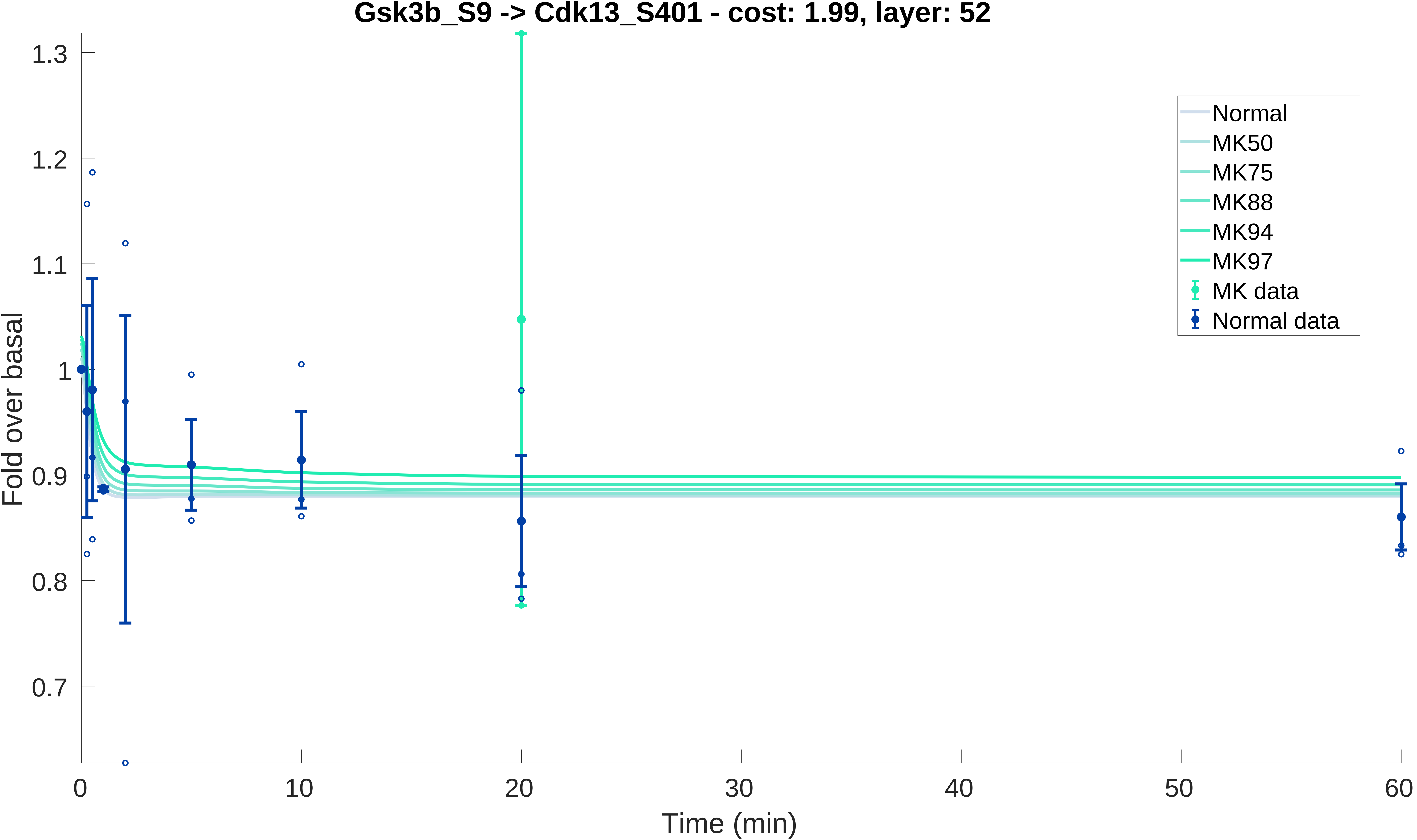

Gsk3b_S9 -> Cdk13_S401 - cost: 1.99, layer: 52
Fold over basal
Time (min)
Normal
MK50
MK75
MK88
MK94
MK97
MK data
Normal data
1.3
1.2
1.1
1
0.9
0.8
0.7
0
10
20
30
40
50
60

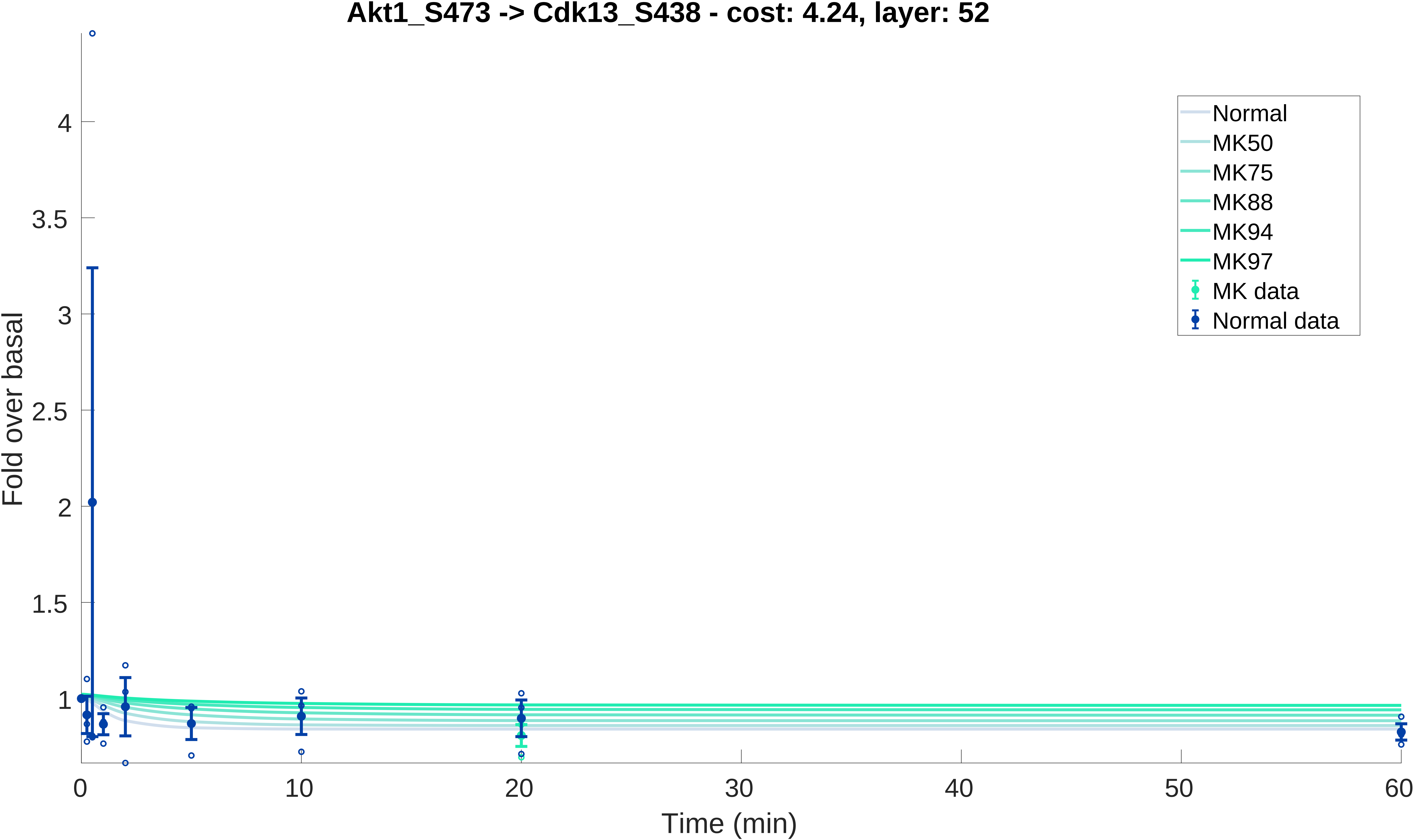

Akt1_S473 -> Cdk13_S438 - cost: 4.24, layer: 52
Fold over basal
Time (min)
4
3.5
3
2.5
2
1.5
1
0
10
20
30
40
50
60
Normal
MK50
MK75
MK88
MK94
MK97
MK data
Normal data

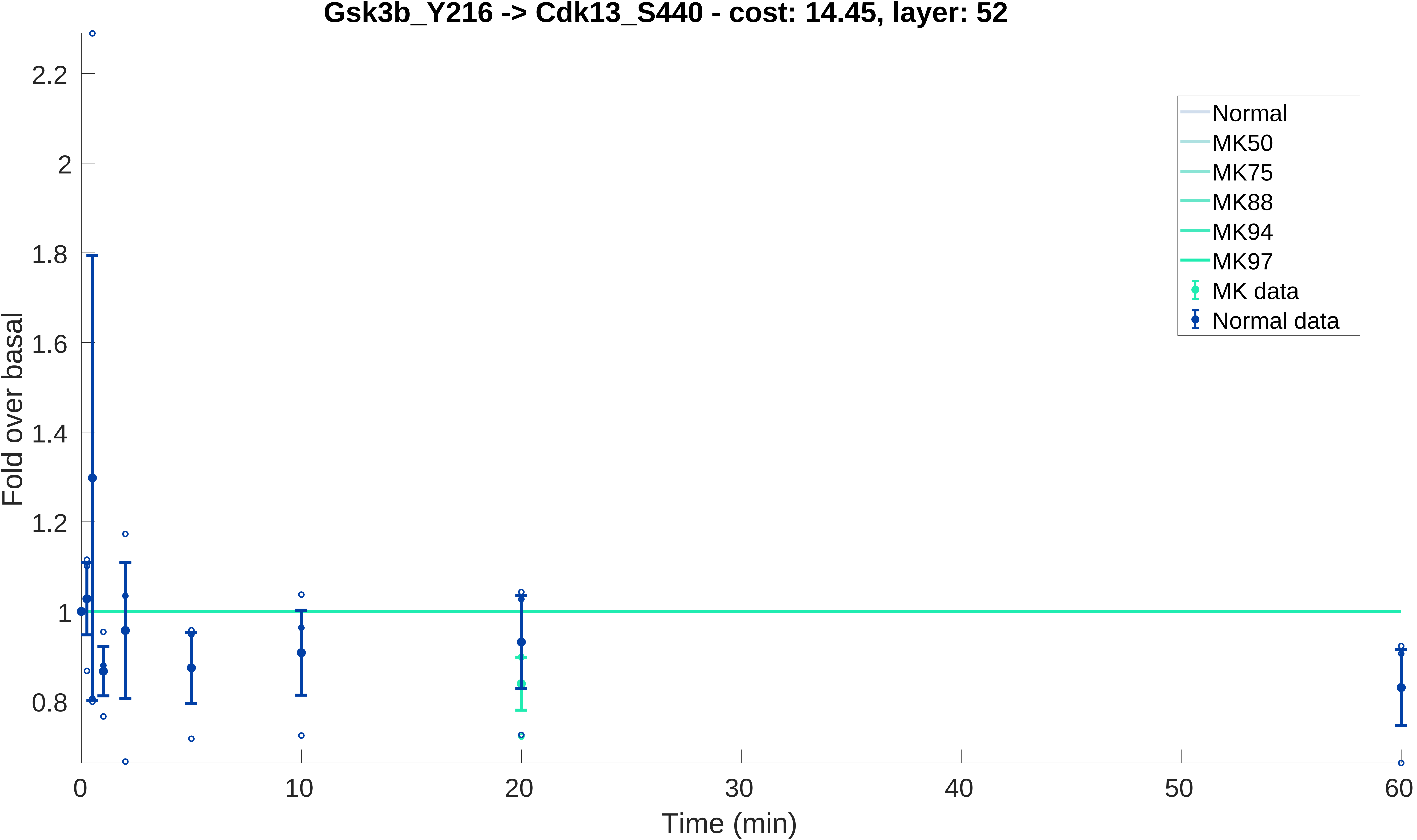

Gsk3b_Y216 -> Cdk13_S440 - cost: 14.45, layer: 52
Fold over basal
Time (min)
0.8
1
1.2
1.4
1.6
1.8
2
2.2
0
10
20
30
40
50
60
Normal
MK50
MK75
MK88
MK94
MK97
MK data
Normal data

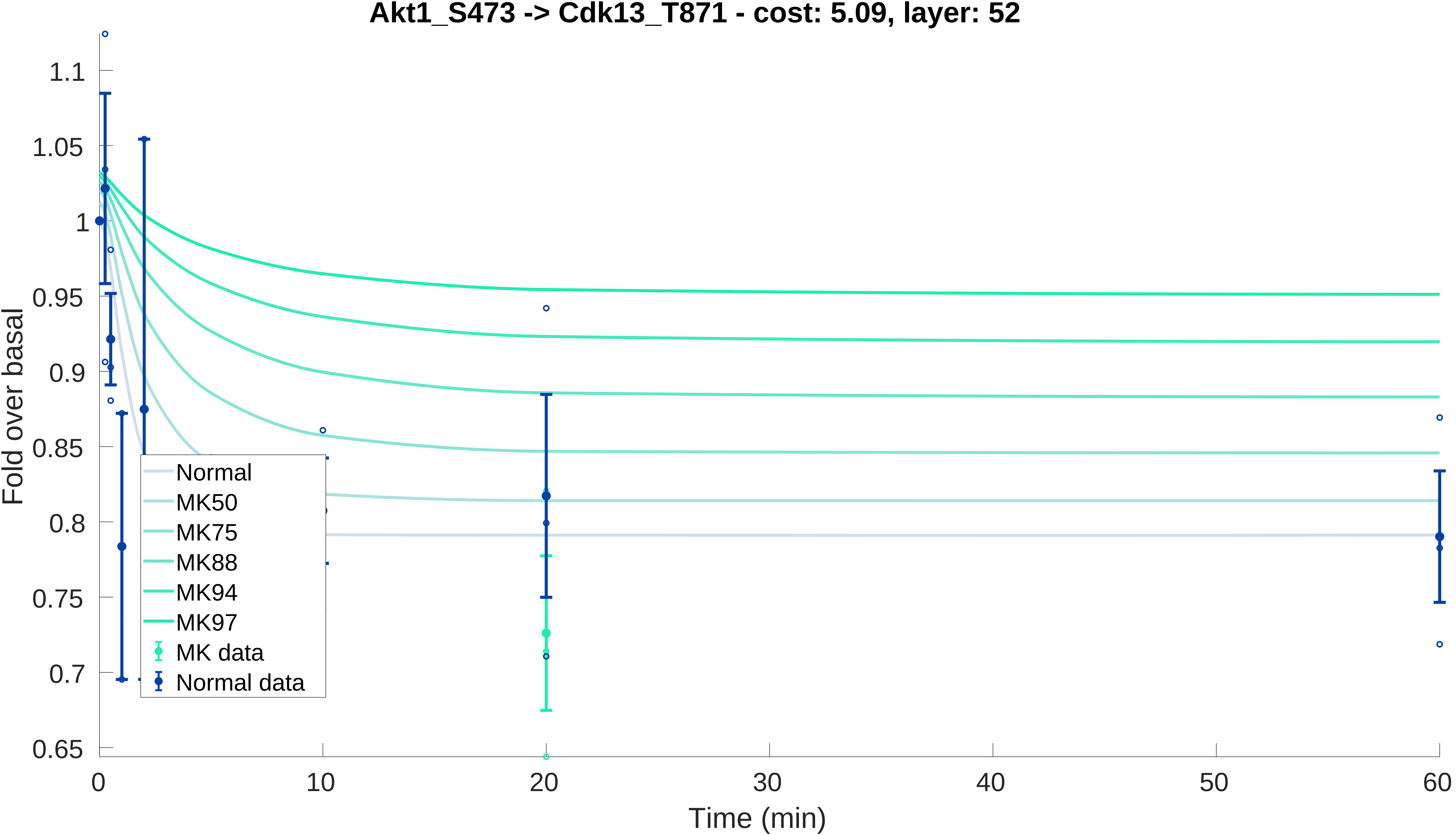

Akt1_S473 -> Cdk13_T871 - cost: 5.09, layer: 52
Fold over basal
Time (min)
1.1
1.05
1
0.95
0.9
0.85
0.8
0.75
0.7
0.65
0
10
20
30
40
50
60
Normal
MK50
MK75
MK88
MK94
MK97
MK data
Normal data

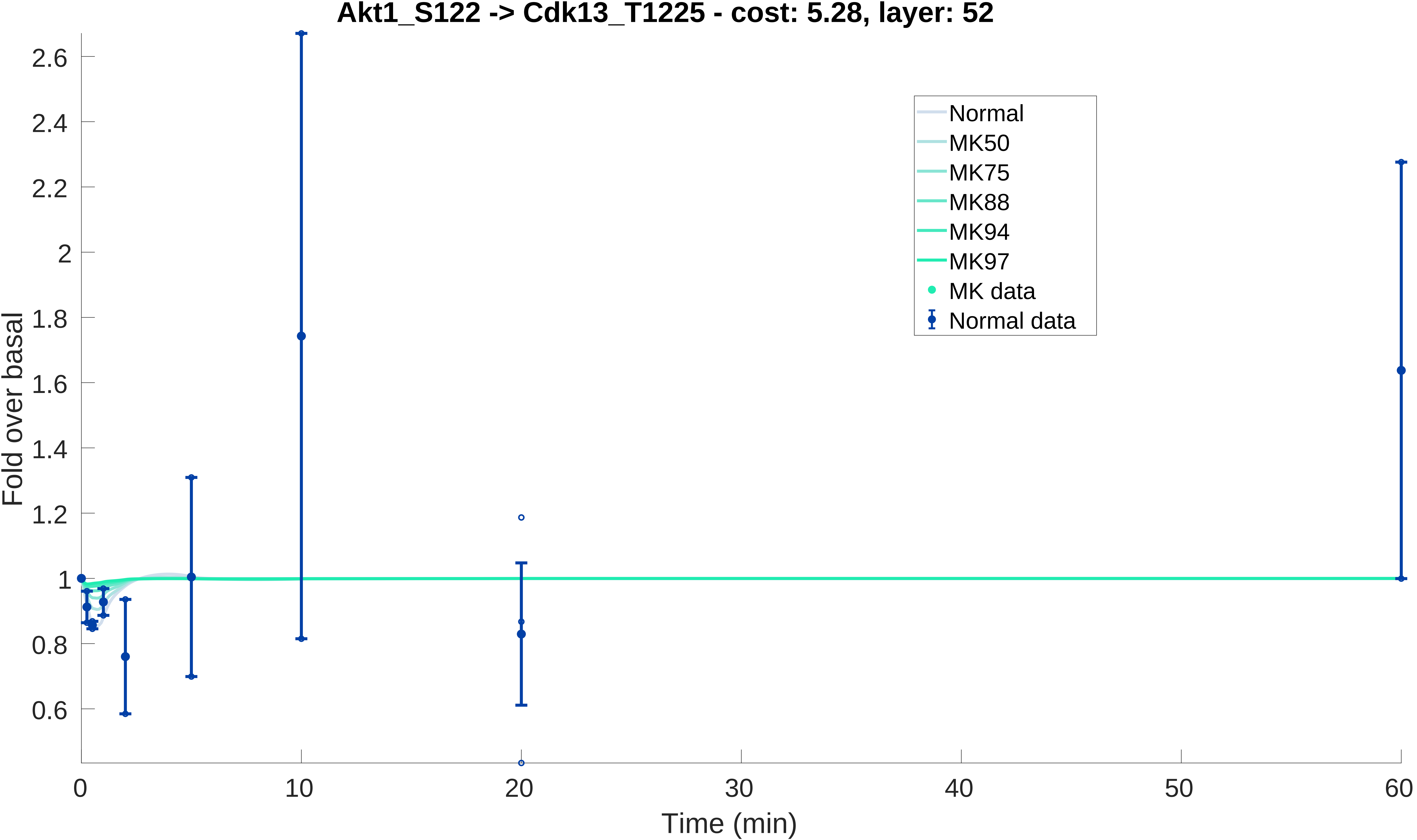

Akt1_S122 -> Cdk13_T1225 - cost: 5.28, layer: 52
Fold over basal
Time (min)
0.6
0.8
1
1.2
1.4
1.6
1.8
2
2.2
2.4
2.6
0
10
20
30
40
50
60
Normal
MK50
MK75
MK88
MK94
MK97
MK data
Normal data

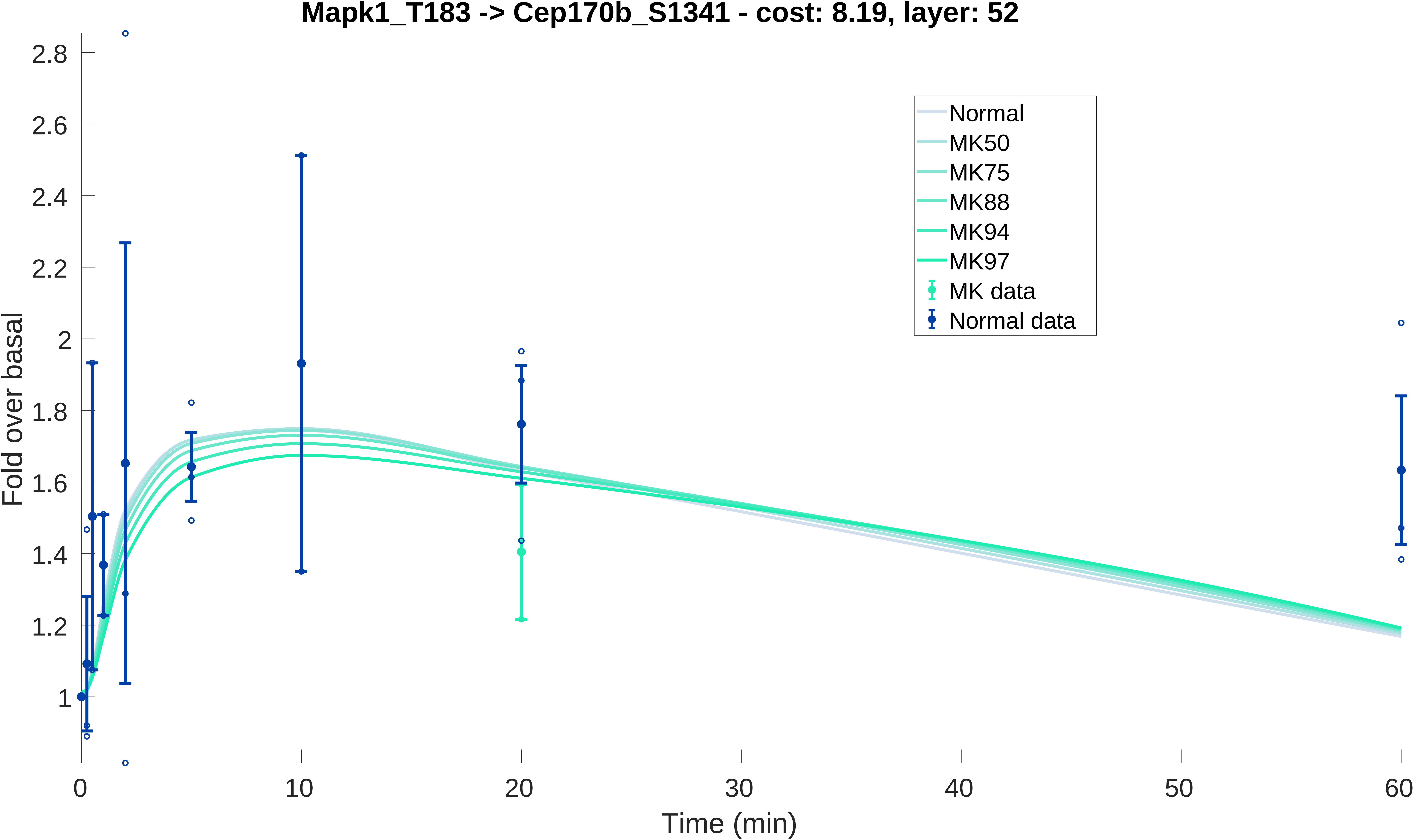

Mapk1_T183 -> Cep170b_S1341 - cost: 8.19, layer: 52
Fold over basal
Time (min)
2.8
2.6
2.4
2.2
2
1.8
1.6
1.4
1.2
1
0
10
20
30
40
50
60
Normal
MK50
MK75
MK88
MK94
MK97
MK data
Normal data

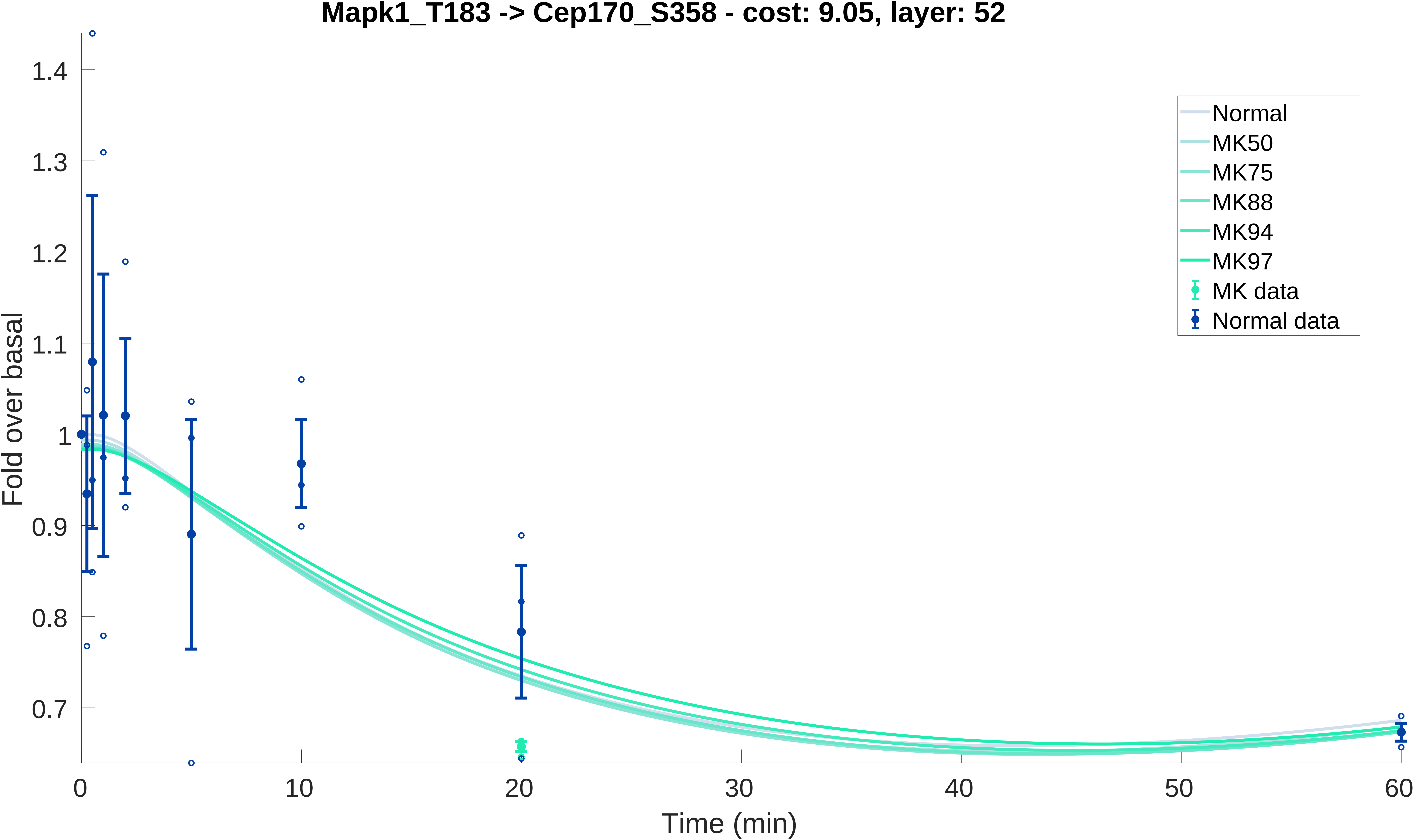
Mapk1_T183 -> Cep170_S358 - cost: 9.05, layer: 52
Fold over basal
Time (min)
Normal
MK50
MK75
MK88
MK94
MK97
MK data
Normal data
0.7
0.8
0.9
1
1.1
1.2
1.3
1.4
0
10
20
30
40
50
60

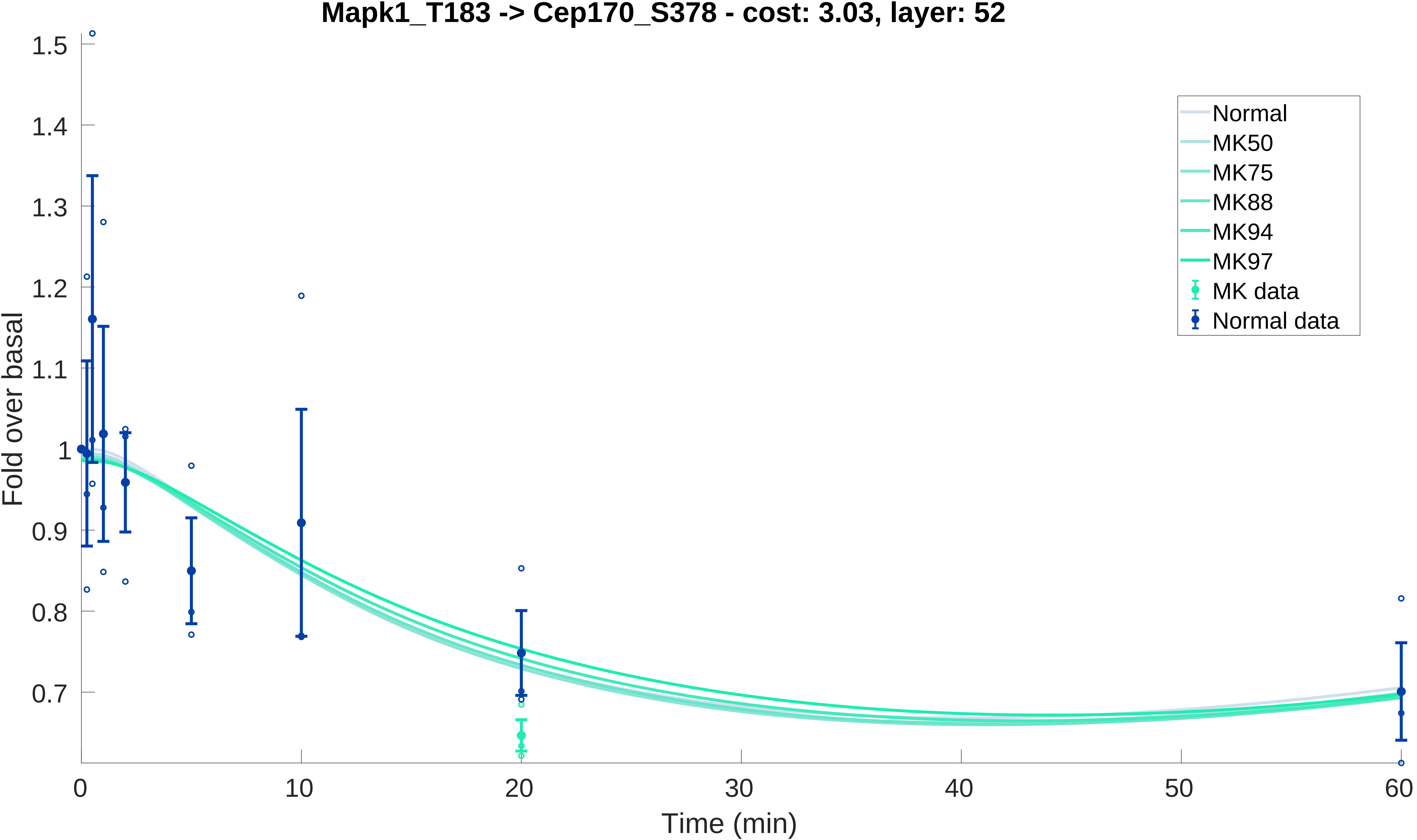

Mapk1_T183 -> Cep170_S378 - cost: 3.03, layer: 52
Fold over basal
Time (min)
1.5
1.4
1.3
1.2
1.1
1
0.9
0.8
0.7
0
10
20
30
40
50
60
Normal
MK50
MK75
MK88
MK94
MK97
MK data
Normal data

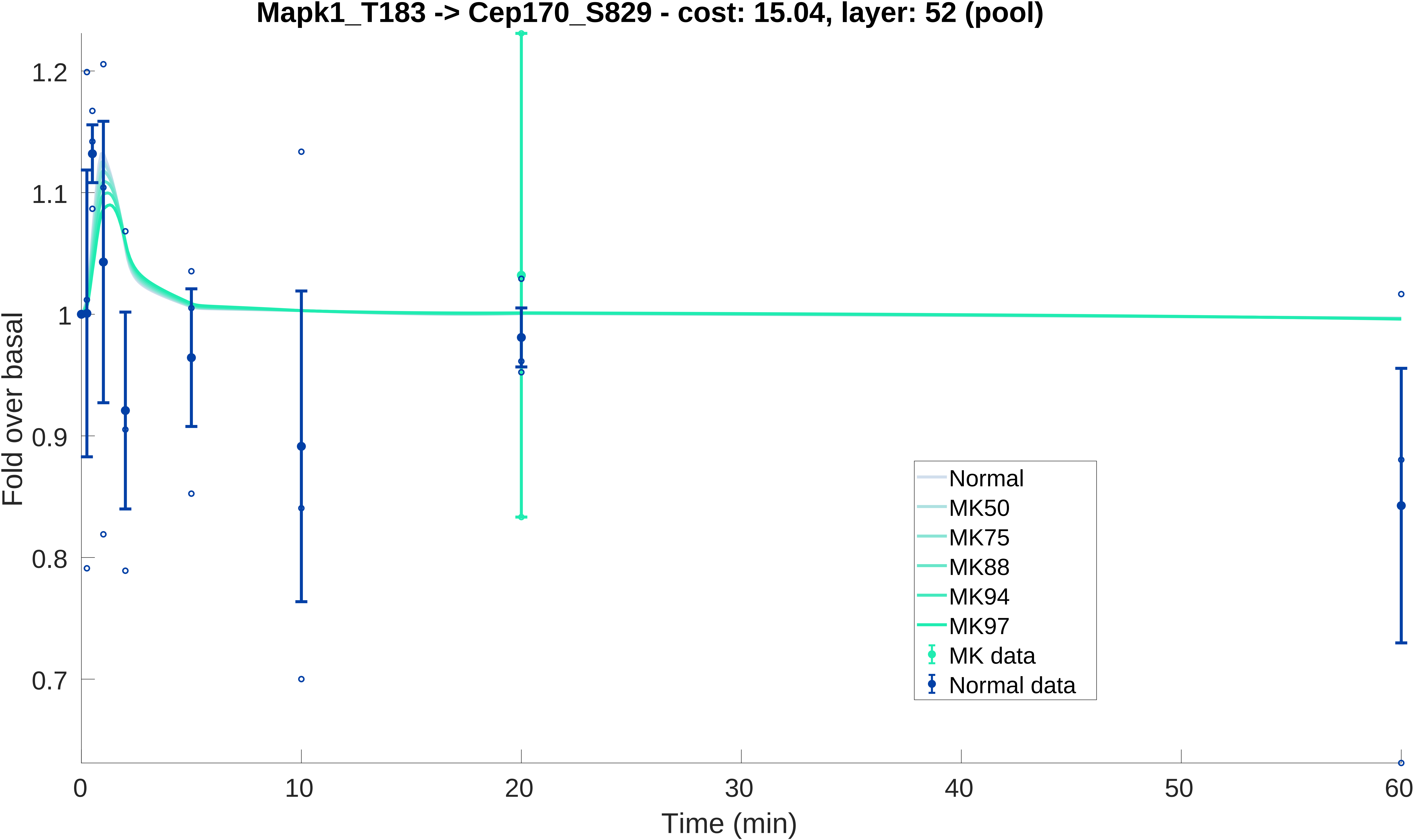

Mapk1_T183 -> Cep170_S829 - cost: 15.04, layer: 52 (pool)
Fold over basal
1.2
1.1
1
0.9
0.8
0.7
0
10
20
30
40
50
60
Time (min)
Normal
MK50
MK75
MK88
MK94
MK97
MK data
Normal data

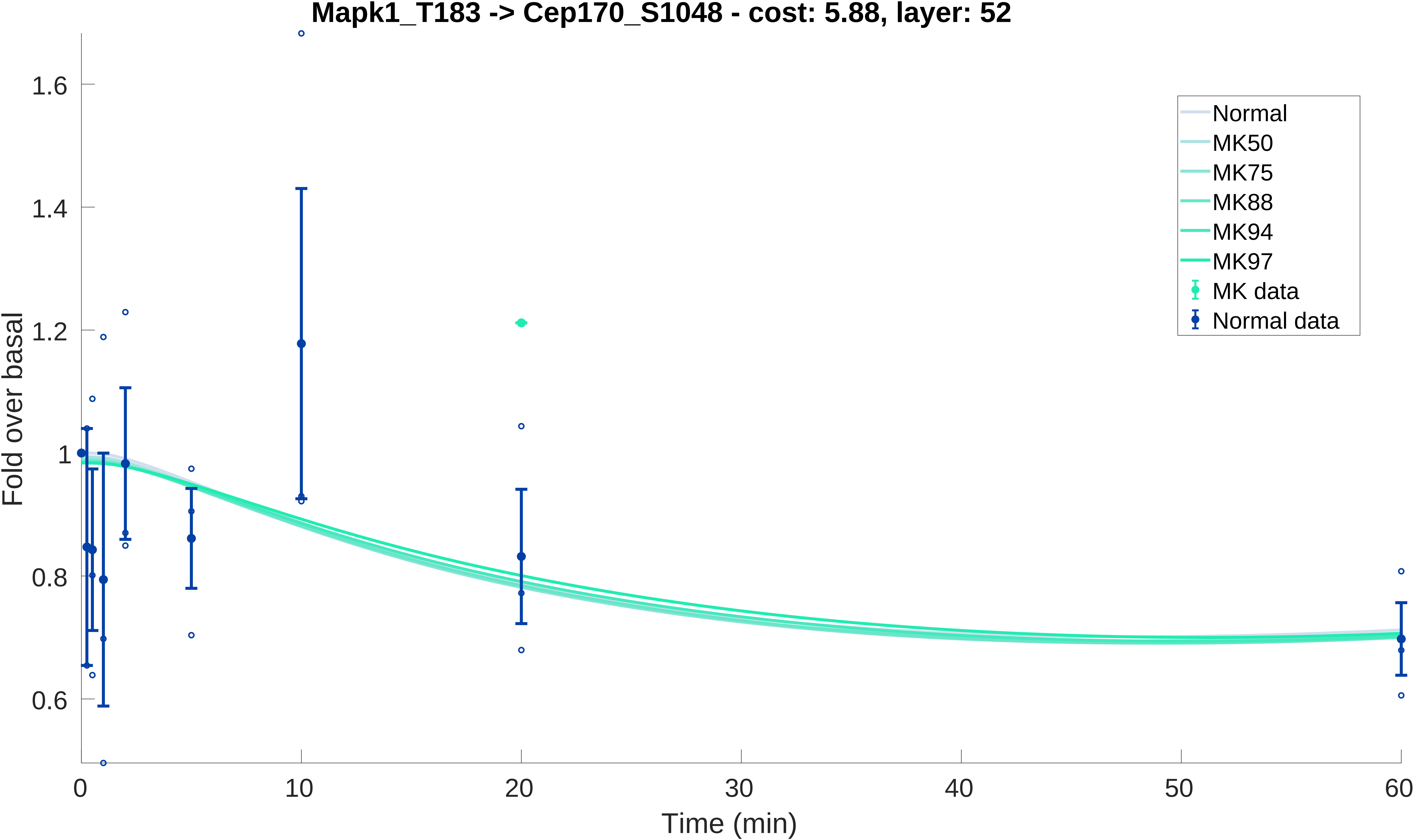

Mapk1_T183 -> Cep170_S1048 - cost: 5.88, layer: 52
Fold over basal
Time (min)
0.6
0.8
1
1.2
1.4
1.6
0
10
20
30
40
50
60
Normal
MK50
MK75
MK88
MK94
MK97
MK data
Normal data

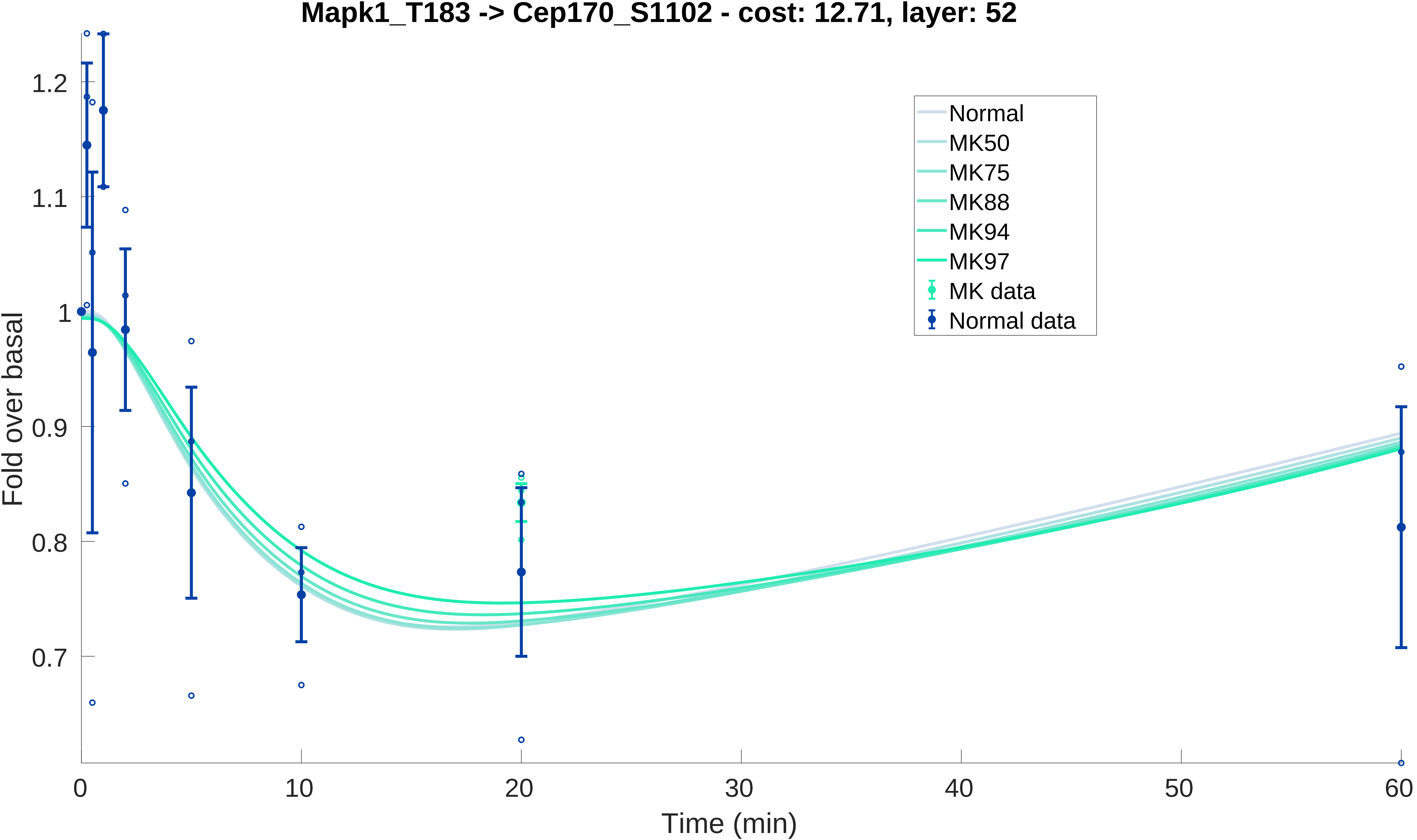

Mapk1_T183 -> Cep170_S1102 - cost: 12.71, layer: 52
Fold over basal
Time (min)
0.7
0.8
0.9
1
1.1
1.2
0
10
20
30
40
50
60
Normal
MK50
MK75
MK88
MK94
MK97
MK data
Normal data

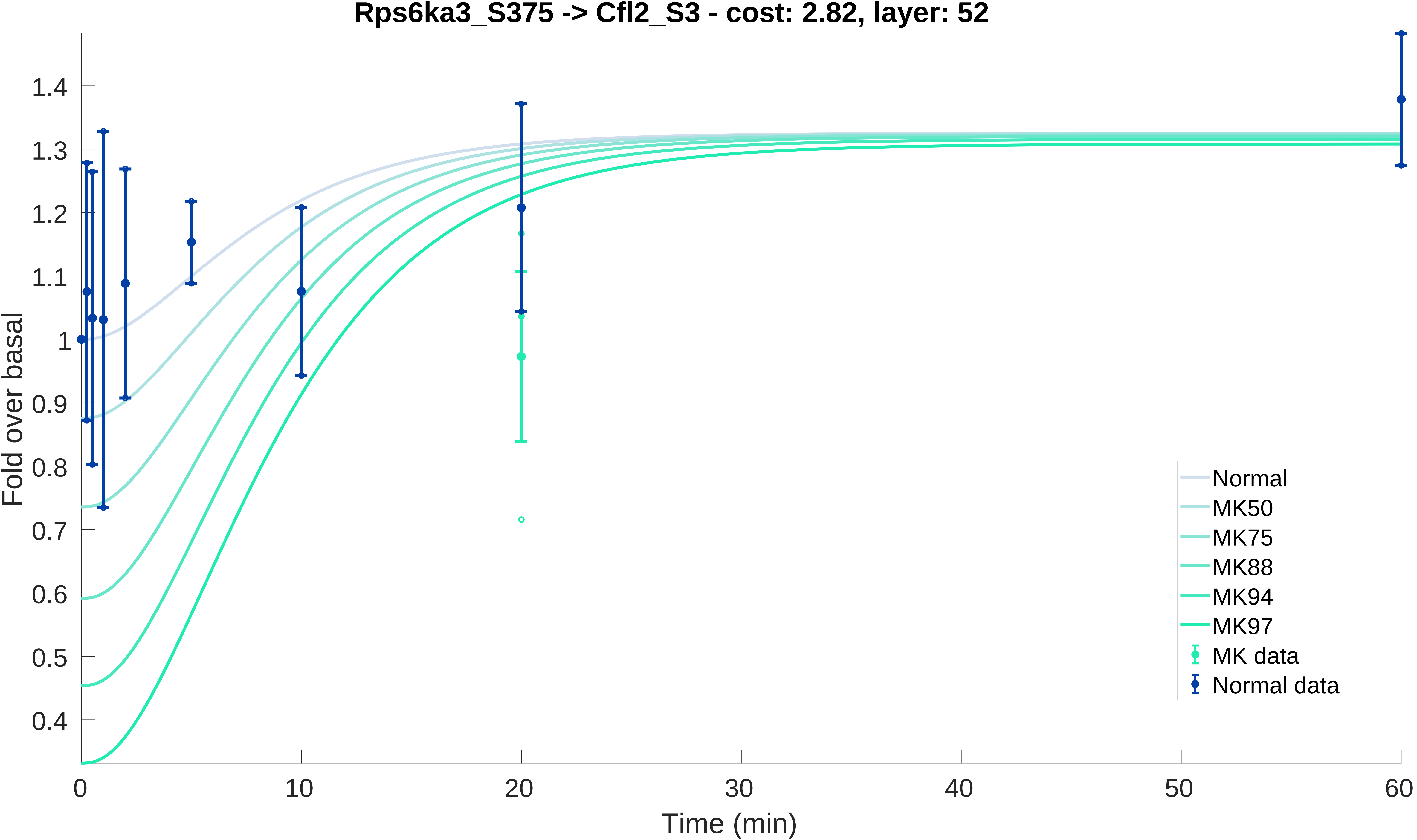

Rps6ka3_S375 -> Cfl2_S3 - cost: 2.82, layer: 52
1.4
1.3
1.2
1.1
1
0.9
0.8
0.7
0.6
0.5
0.4
Fold over basal
0
10
20
30
40
50
60
Time (min)
Normal
MK50
MK75
MK88
MK94
MK97
MK data
Normal data

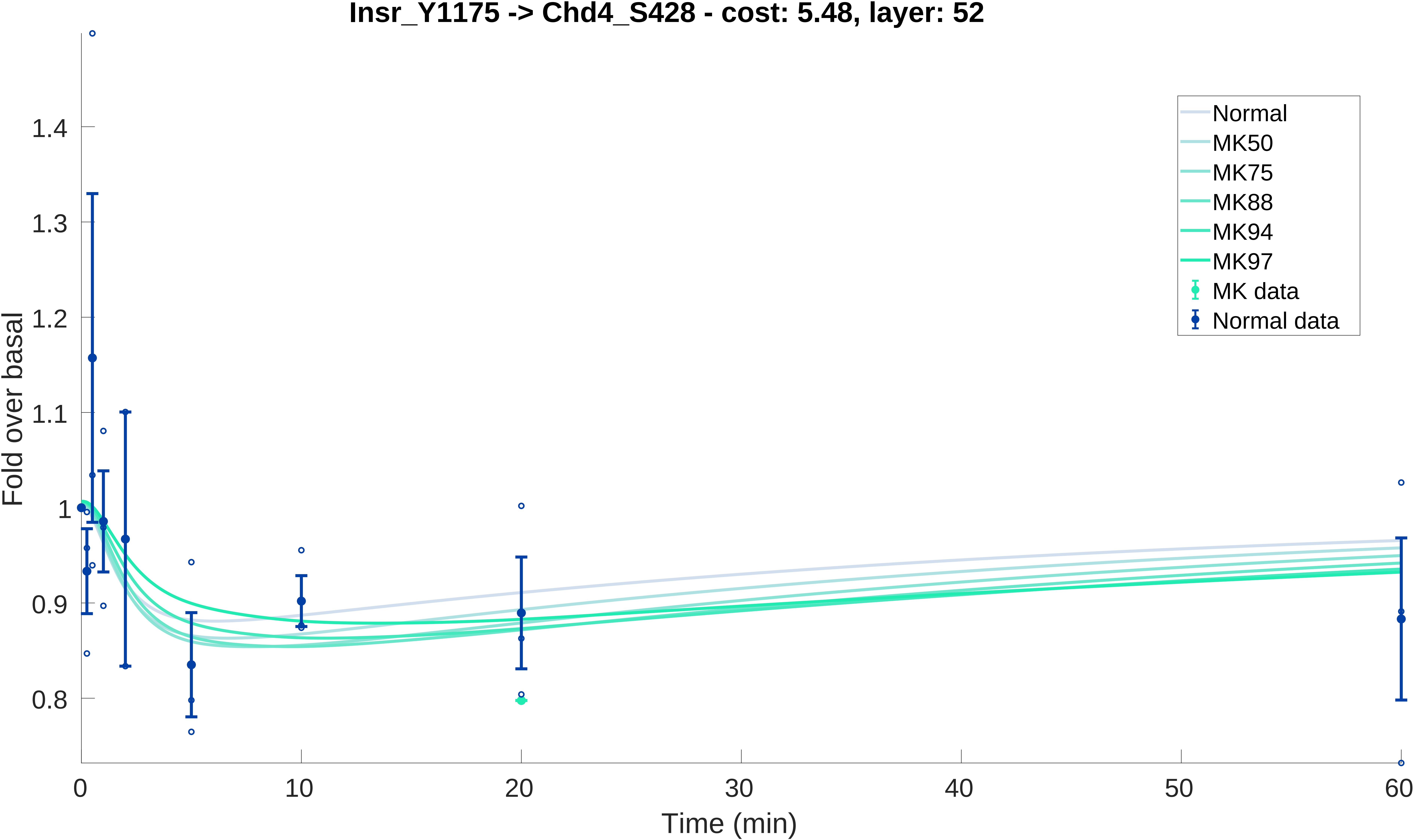

Insr_Y1175 -> Chd4_S428 - cost: 5.48, layer: 52
Fold over basal
Time (min)
0.8
0.9
1
1.1
1.2
1.3
1.4
0
10
20
30
40
50
60
Normal
MK50
MK75
MK88
MK94
MK97
MK data
Normal data

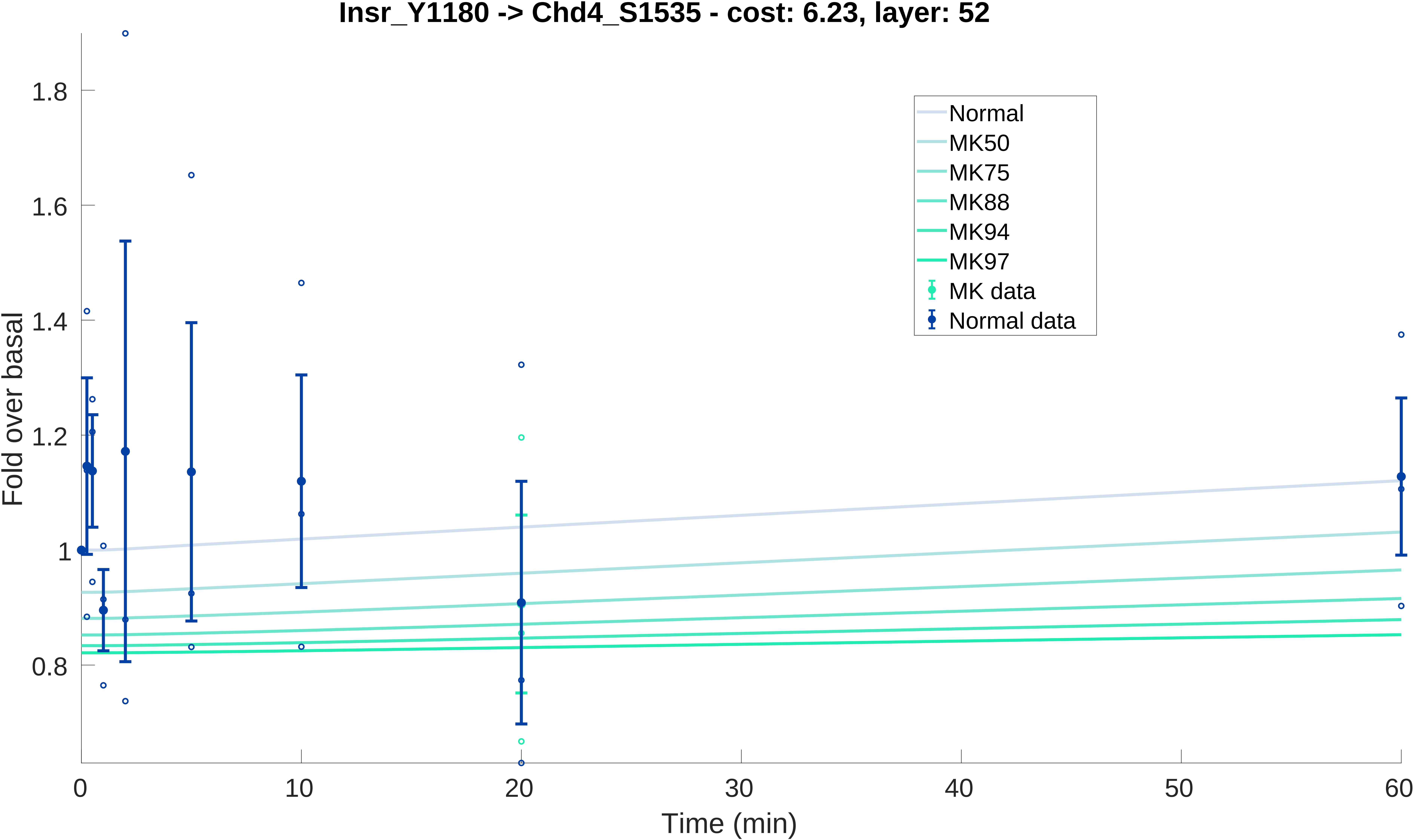
Insr_Y1180 -> Chd4_S1535 - cost: 6.23, layer: 52
Fold over basal
Time (min)
0.8
1
1.2
1.4
1.6
1.8
0
10
20
30
40
50
60
Normal
MK50
MK75
MK88
MK94
MK97
MK data
Normal data

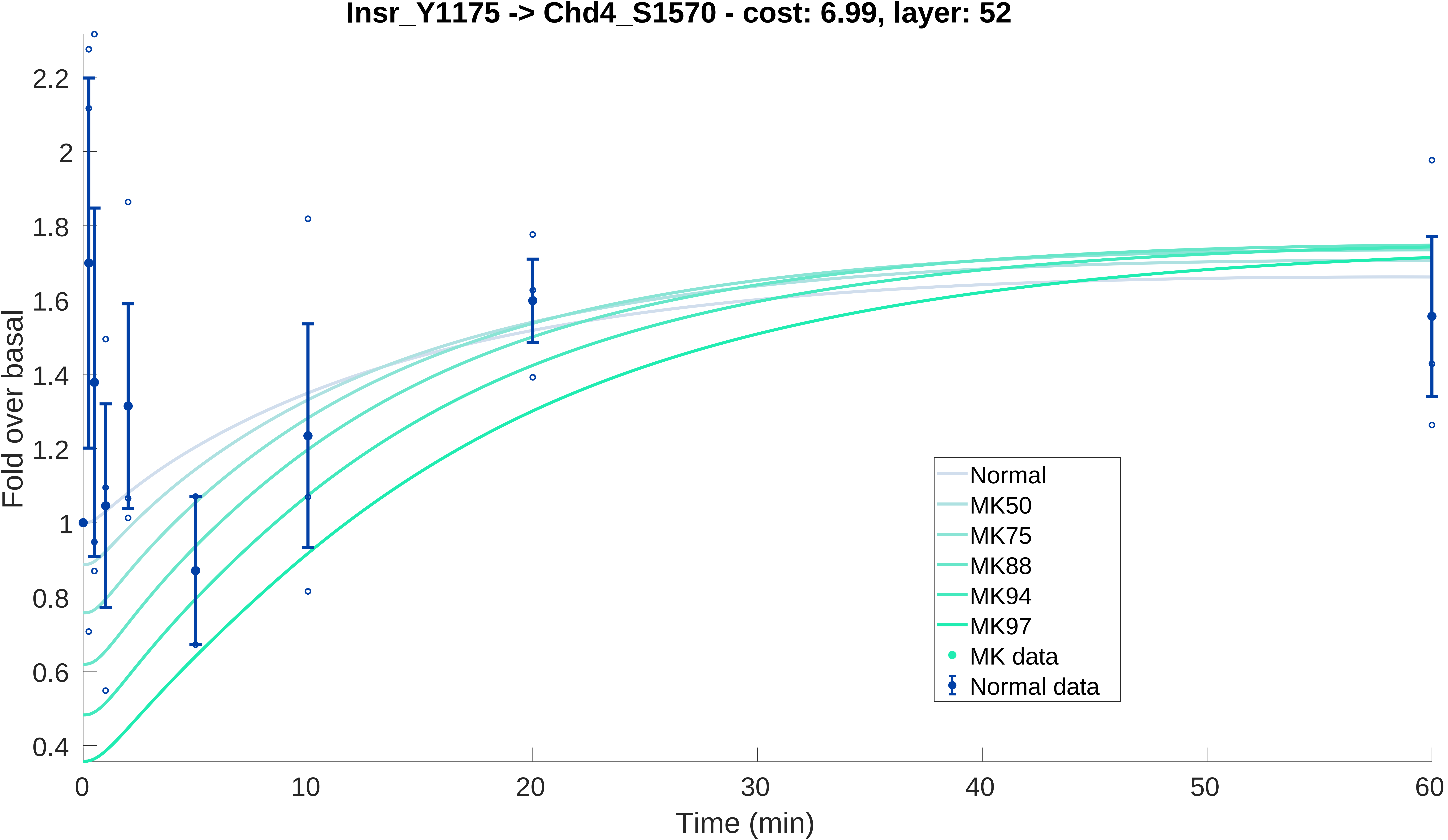

Insr_Y1175 -> Chd4_S1570 - cost: 6.99, layer: 52
Fold over basal
Time (min)
0
10
20
30
40
50
60
0.4
0.6
0.8
1
1.2
1.4
1.6
1.8
2
2.2
Normal
MK50
MK75
MK88
MK94
MK97
MK data
Normal data

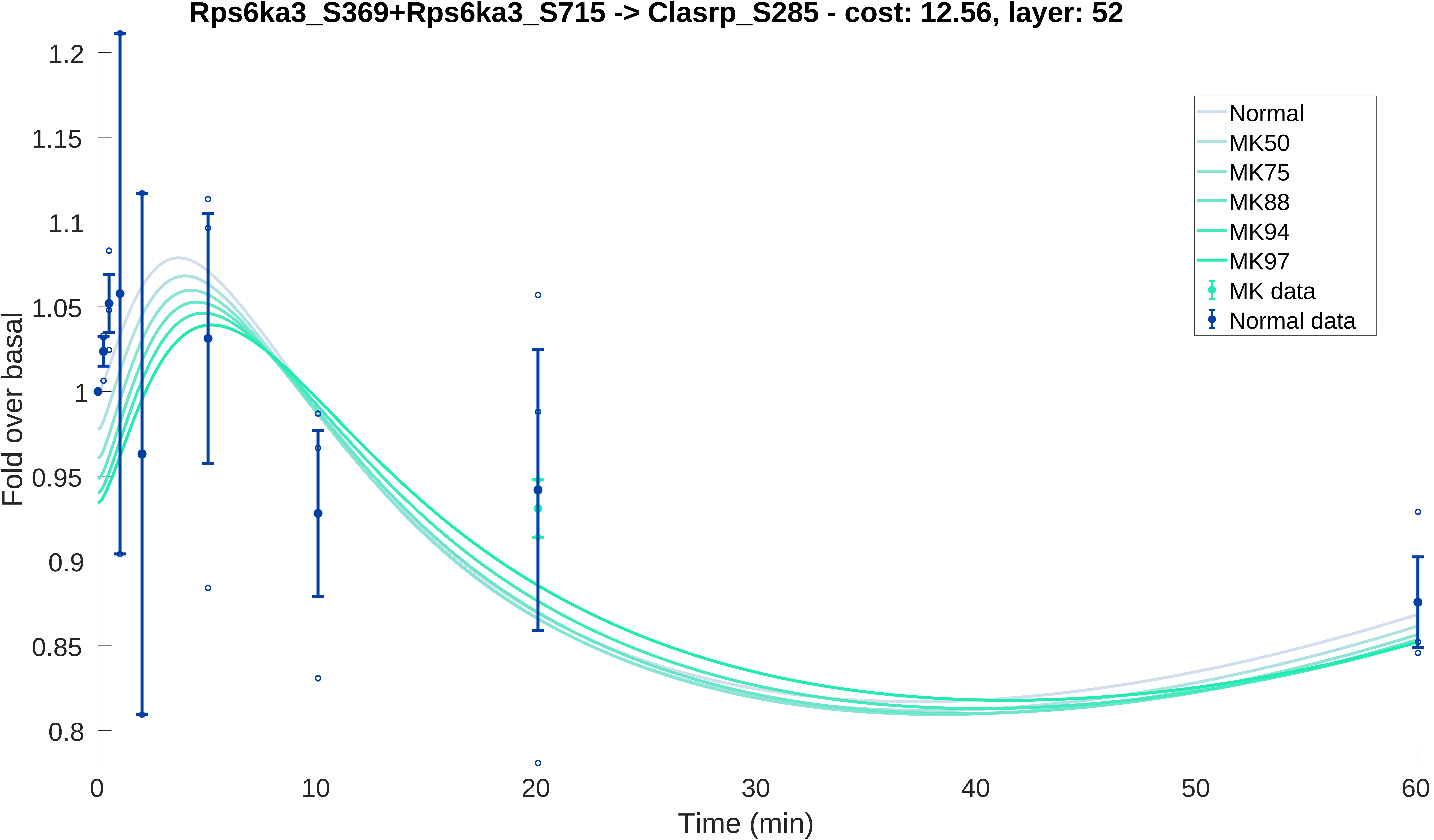

Rps6ka3_S369+Rps6ka3_S715 -> Clasrp_S285 - cost: 12.56, layer: 52
Fold over basal
1.2
1.15
1.1
1.05
1
0.95
0.9
0.85
0.8
0
10
20
30
40
50
60
Time (min)
Normal
MK50
MK75
MK88
MK94
MK97
MK data
Normal data

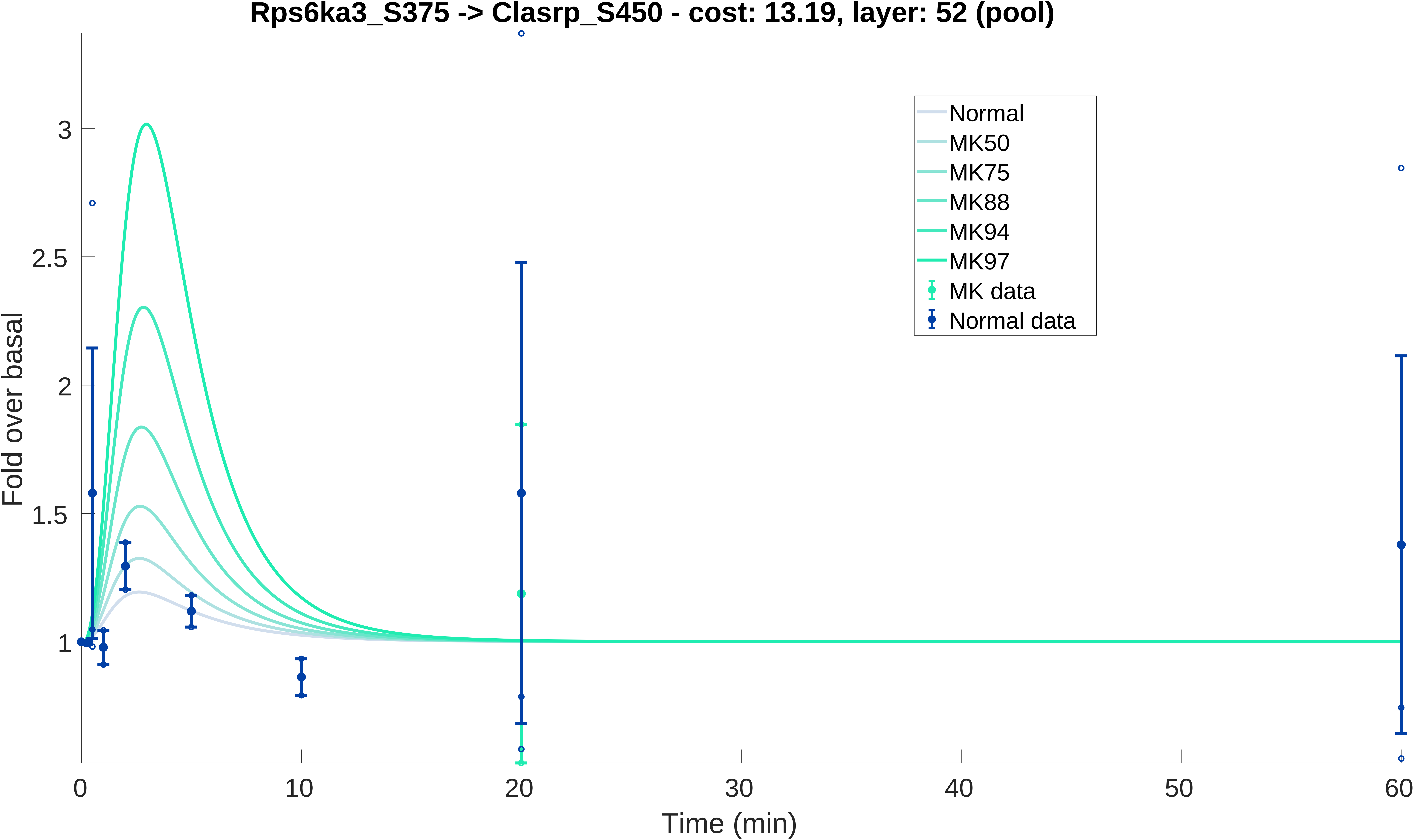

Rps6ka3_S375 -> Clasrp_S450 - cost: 13.19, layer: 52 (pool)
Fold over basal
Time (min)
0
10
20
30
40
50
60
1
1.5
2
2.5
3
Normal
MK50
MK75
MK88
MK94
MK97
MK data
Normal data

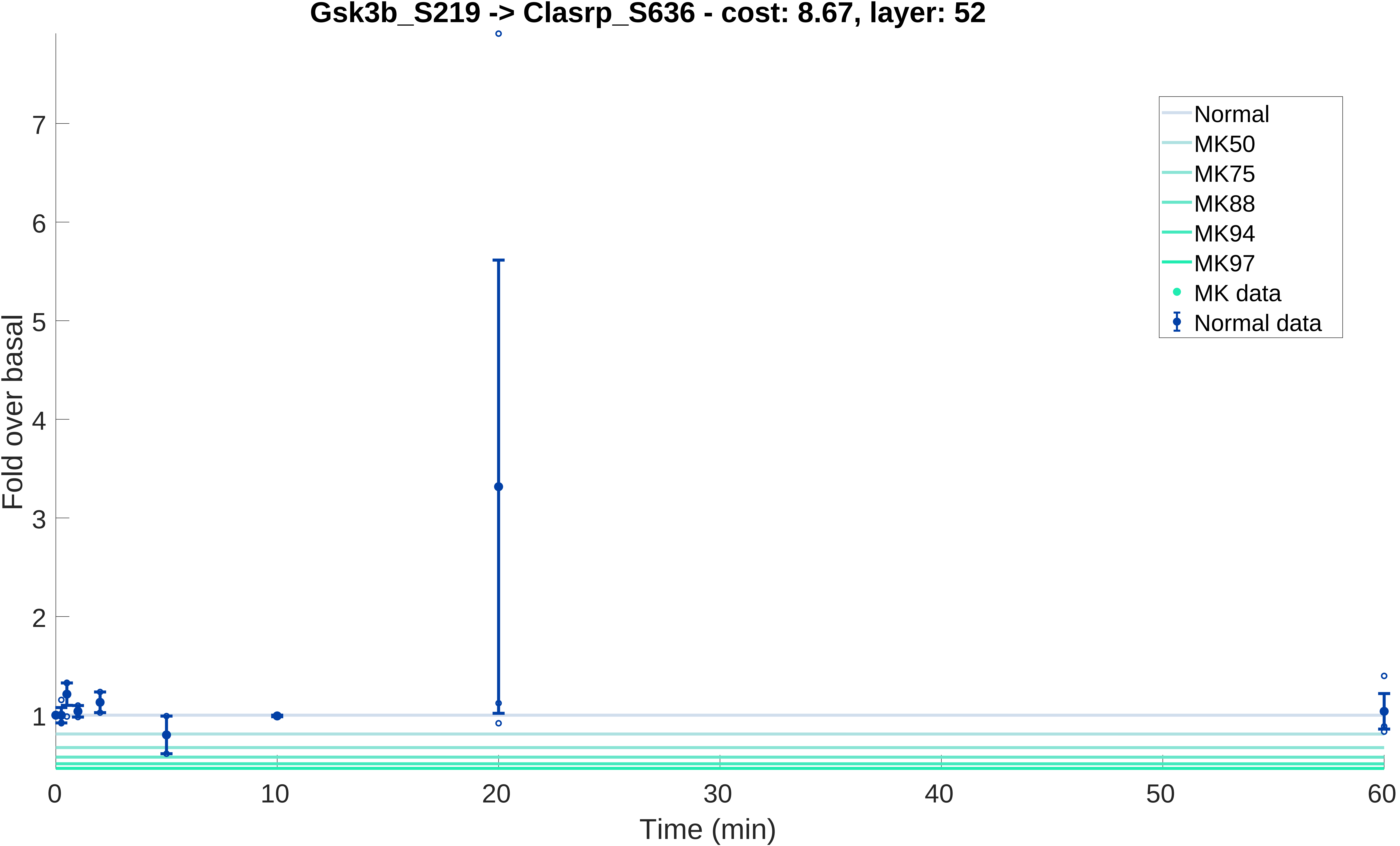

Gsk3b_S219 -> Clasrp_S636 - cost: 8.67, layer: 52
Fold over basal
Time (min)
0
10
20
30
40
50
60
1
2
3
4
5
6
7
Normal
MK50
MK75
MK88
MK94
MK97
MK data
Normal data

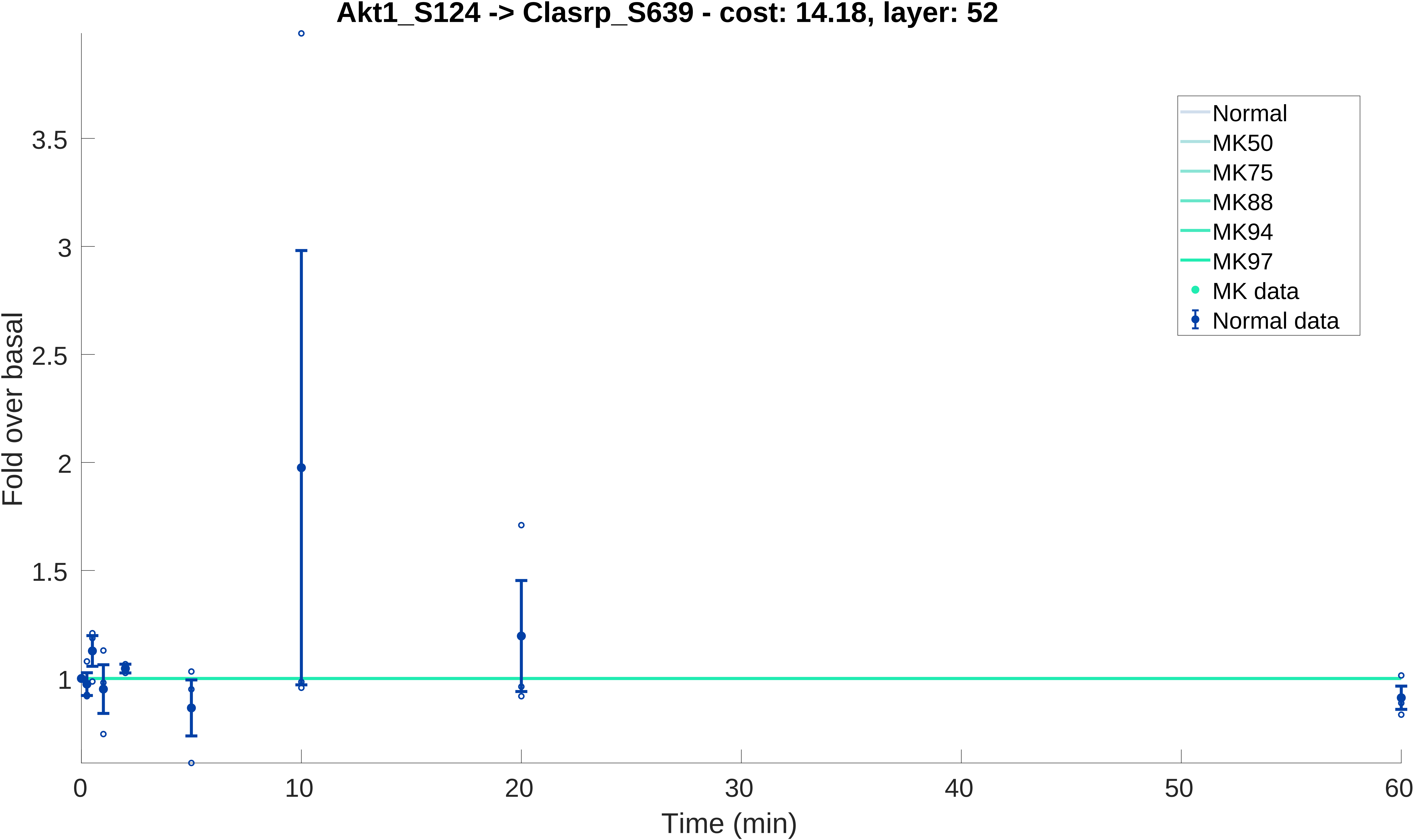

Akt1_S124 -> Clasrp_S639 - cost: 14.18, layer: 52
Fold over basal
3.5
3
2.5
2
1.5
1
0
10
20
30
40
50
60
Time (min)
Normal
MK50
MK75
MK88
MK94
MK97
MK data
Normal data

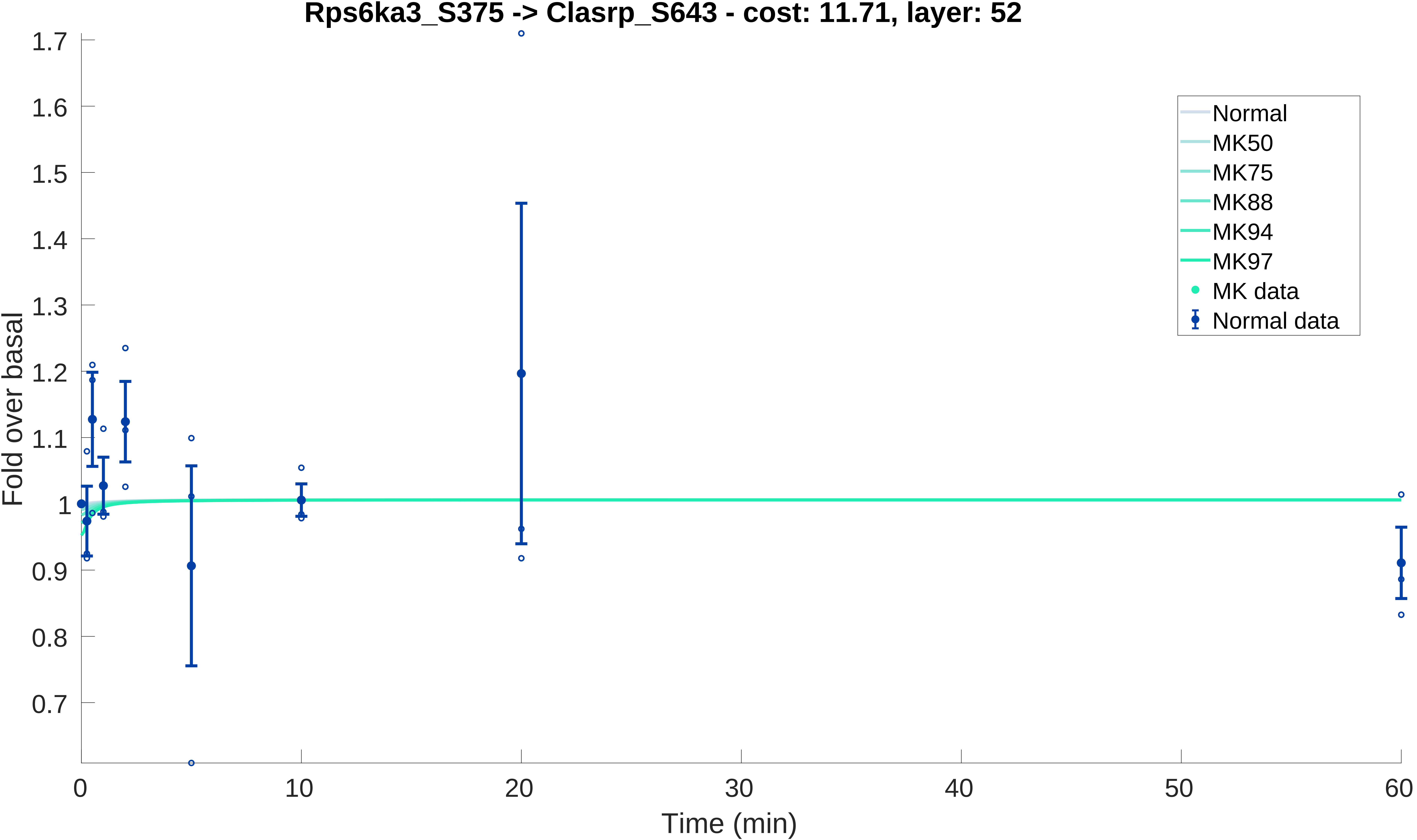

Rps6ka3_S375 -> Clasrp_S643 - cost: 11.71, layer: 52
Fold over basal
Time (min)
Normal
MK50
MK75
MK88
MK94
MK97
MK data
Normal data
0.7
0.8
0.9
1
1.1
1.2
1.3
1.4
1.5
1.6
1.7
0
10
20
30
40
50
60

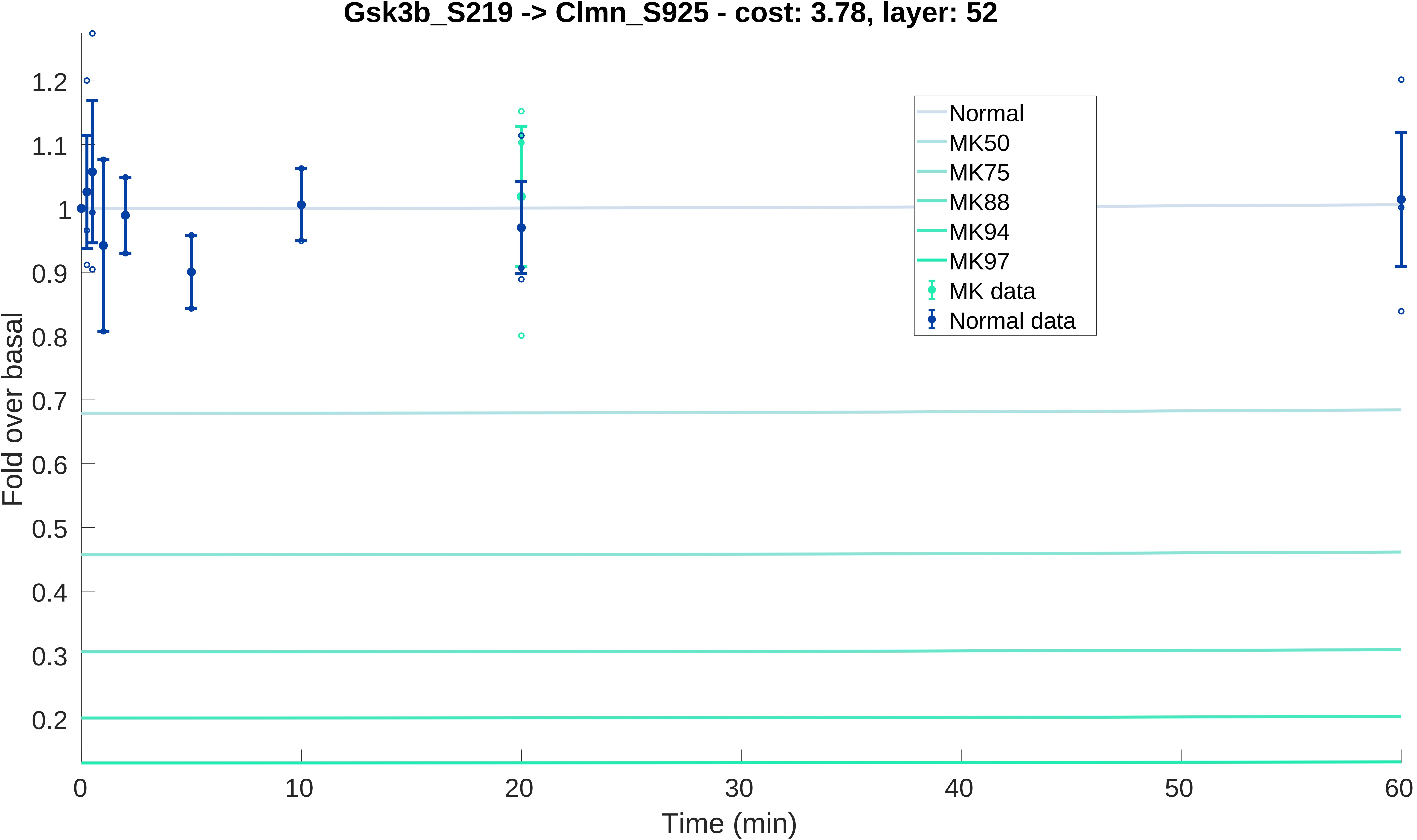
Gsk3b_S219 -> Clmn_S925 - cost: 3.78, layer: 52
Fold over basal
Time (min)
1.2
1.1
1
0.9
0.8
0.7
0.6
0.5
0.4
0.3
0.2
0
10
20
30
40
50
60
Normal
MK50
MK75
MK88
MK94
MK97
MK data
Normal data

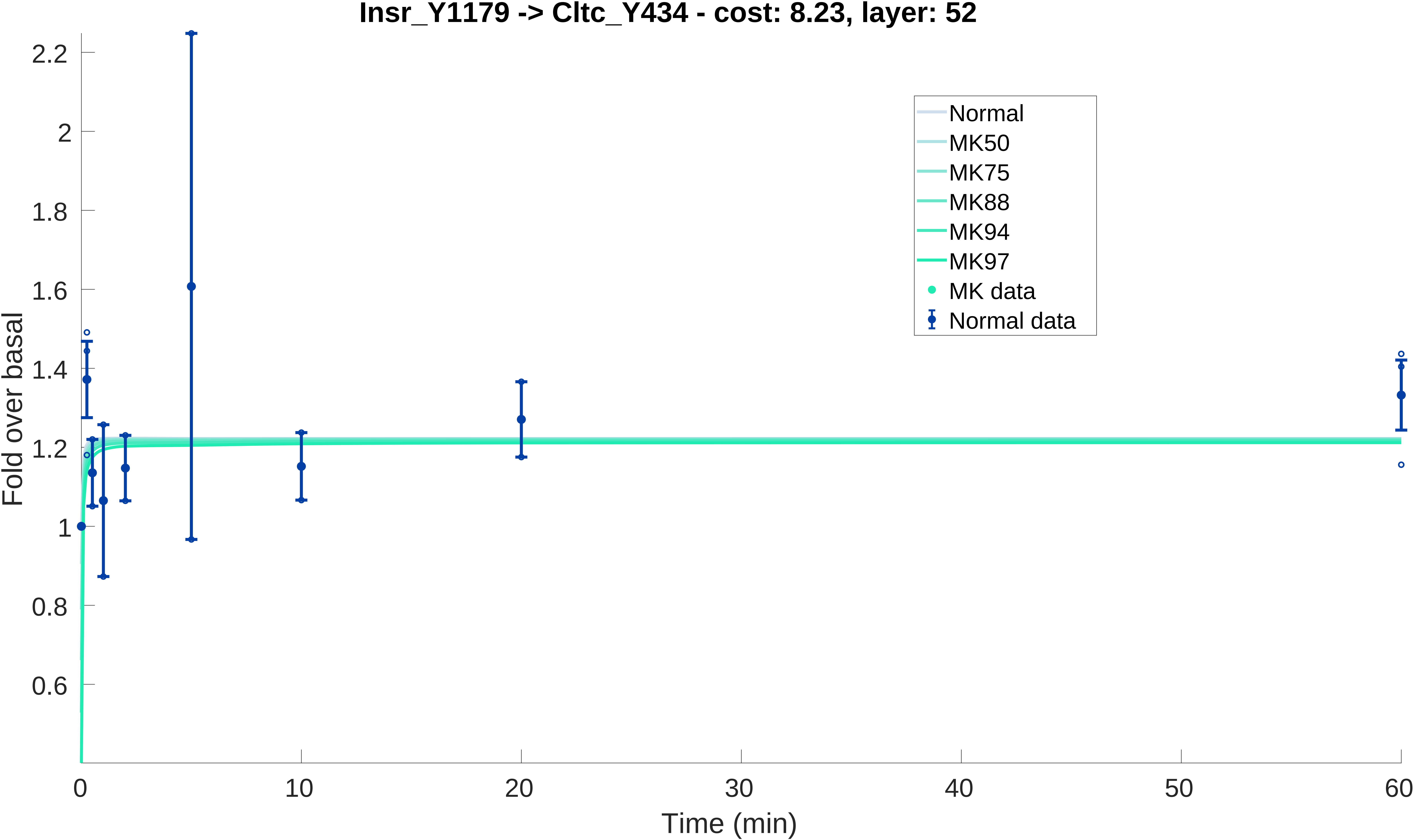

Insr_Y1179 -> Cltc_Y434 - cost: 8.23, layer: 52
Fold over basal
Time (min)
0.6
0.8
1
1.2
1.4
1.6
1.8
2
2.2
0
10
20
30
40
50
60
Normal
MK50
MK75
MK88
MK94
MK97
MK data
Normal data

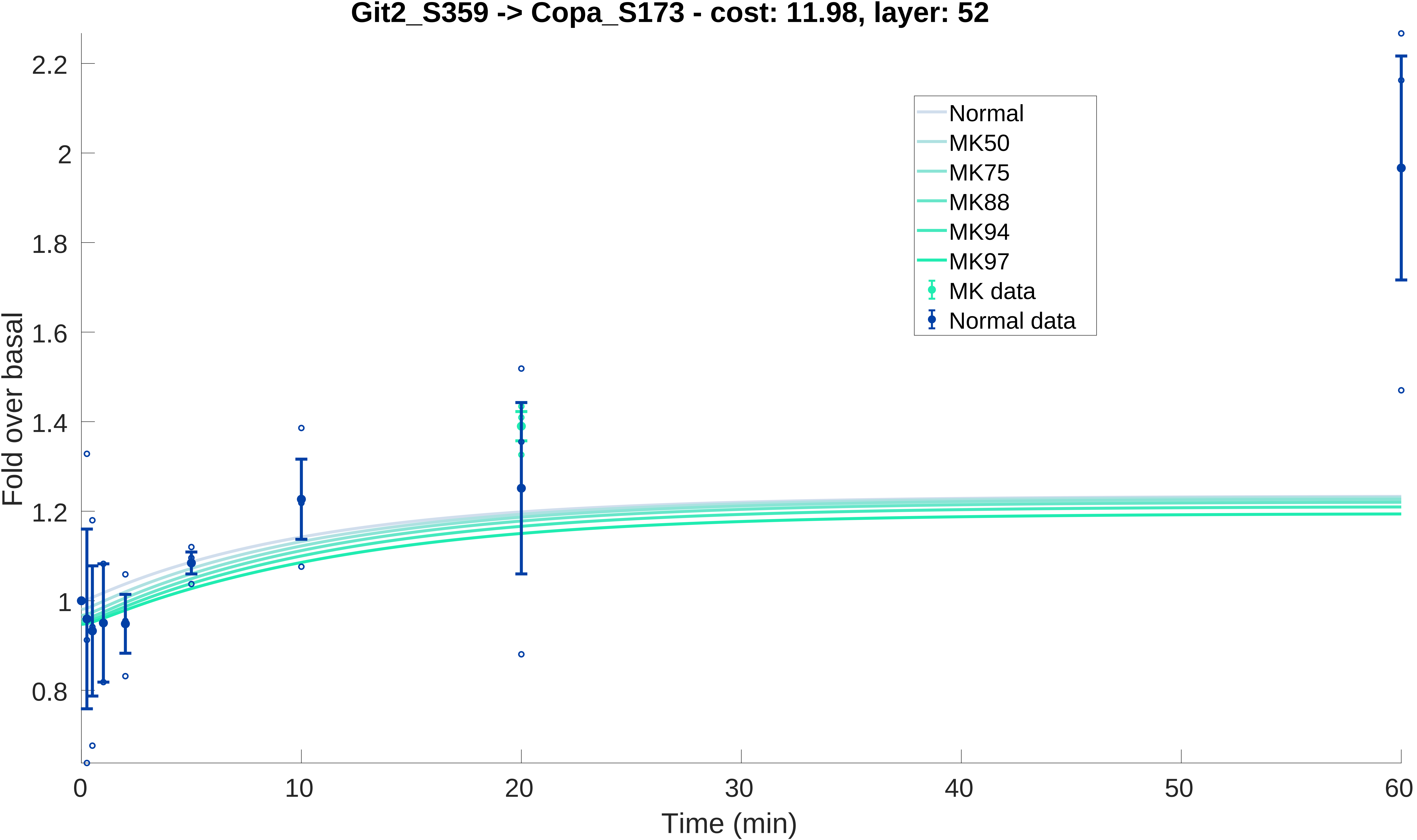

Git2_S359 -> Copa_S173 - cost: 11.98, layer: 52
Normal
MK50
MK75
MK88
MK94
MK97
MK data
Normal data
Fold over basal
2.2
2
1.8
1.6
1.4
1.2
1
0.8
0
10
20
30
40
50
60
Time (min)

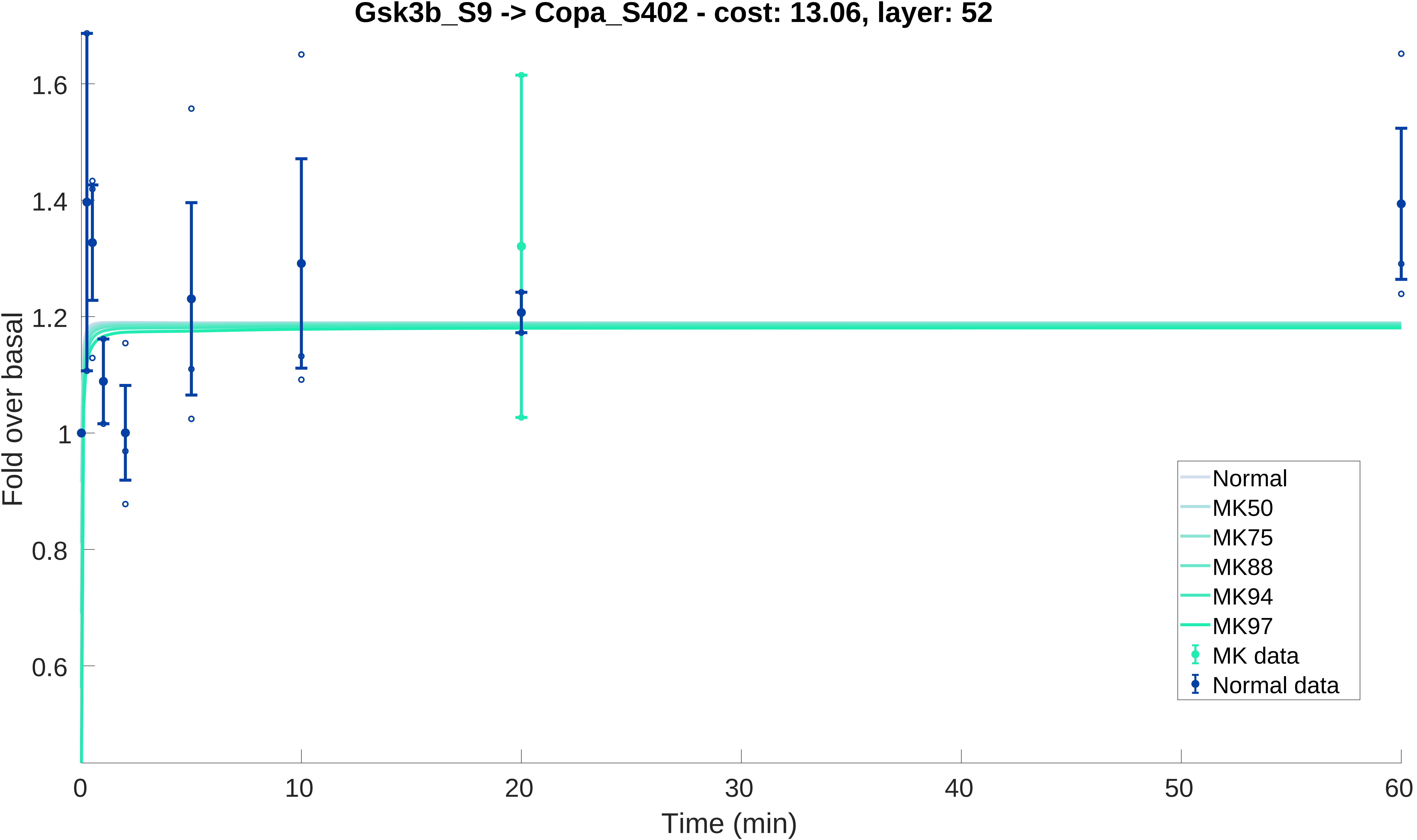

Gsk3b_S9 -> Copa_S402 - cost: 13.06, layer: 52
Fold over basal
Time (min)
0.6
0.8
1
1.2
1.4
1.6
0
10
20
30
40
50
60
Normal
MK50
MK75
MK88
MK94
MK97
MK data
Normal data

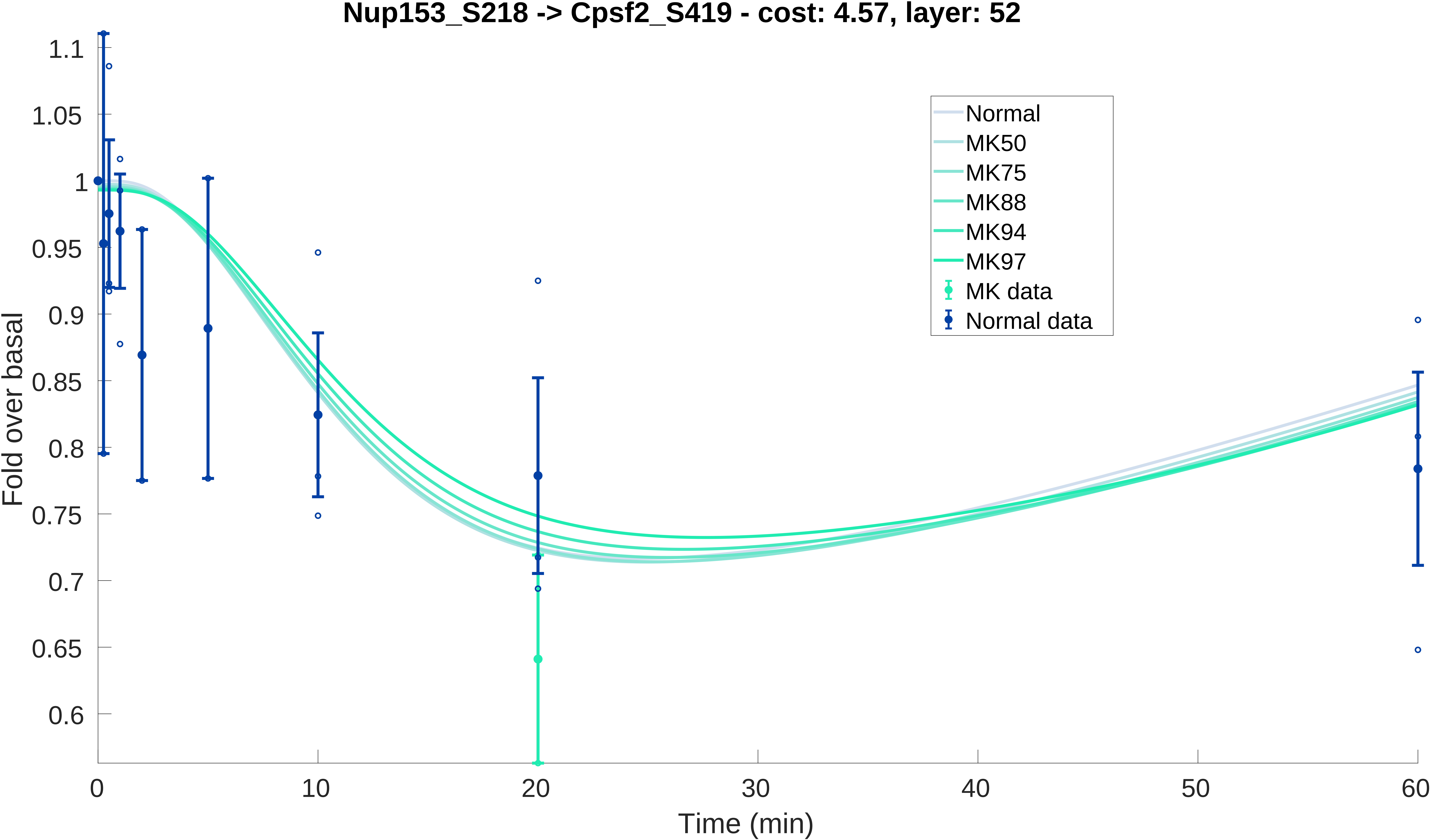

Nup153_S218 -> Cpsf2_S419 - cost: 4.57, layer: 52
Fold over basal
Time (min)
1.1
1.05
1
0.95
0.9
0.85
0.8
0.75
0.7
0.65
0.6
0
10
20
30
40
50
60
Normal
MK50
MK75
MK88
MK94
MK97
MK data
Normal data

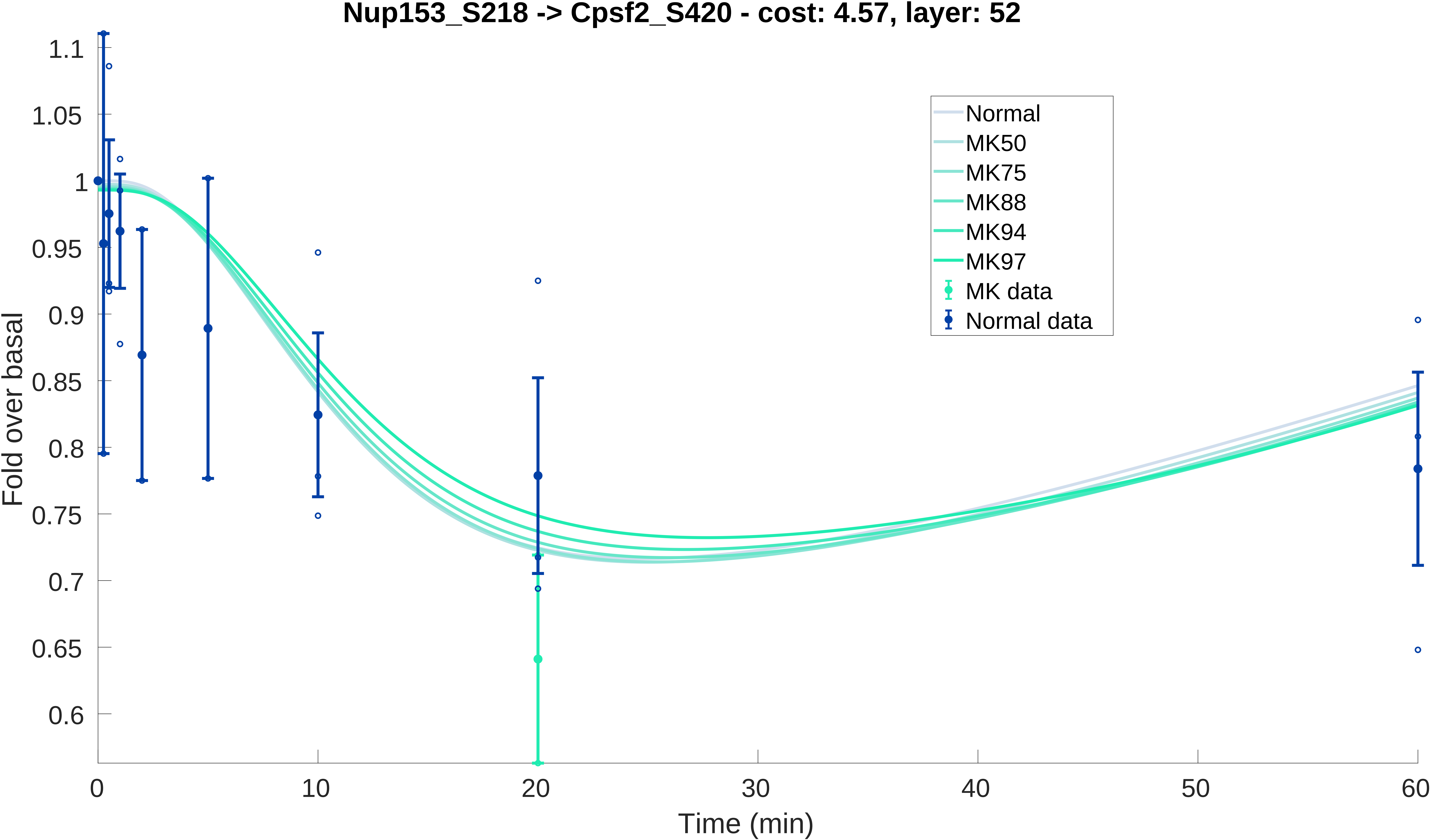

Nup153_S218 -> Cpsf2_S420 - cost: 4.57, layer: 52
Fold over basal
Time (min)
Normal
MK50
MK75
MK88
MK94
MK97
MK data
Normal data

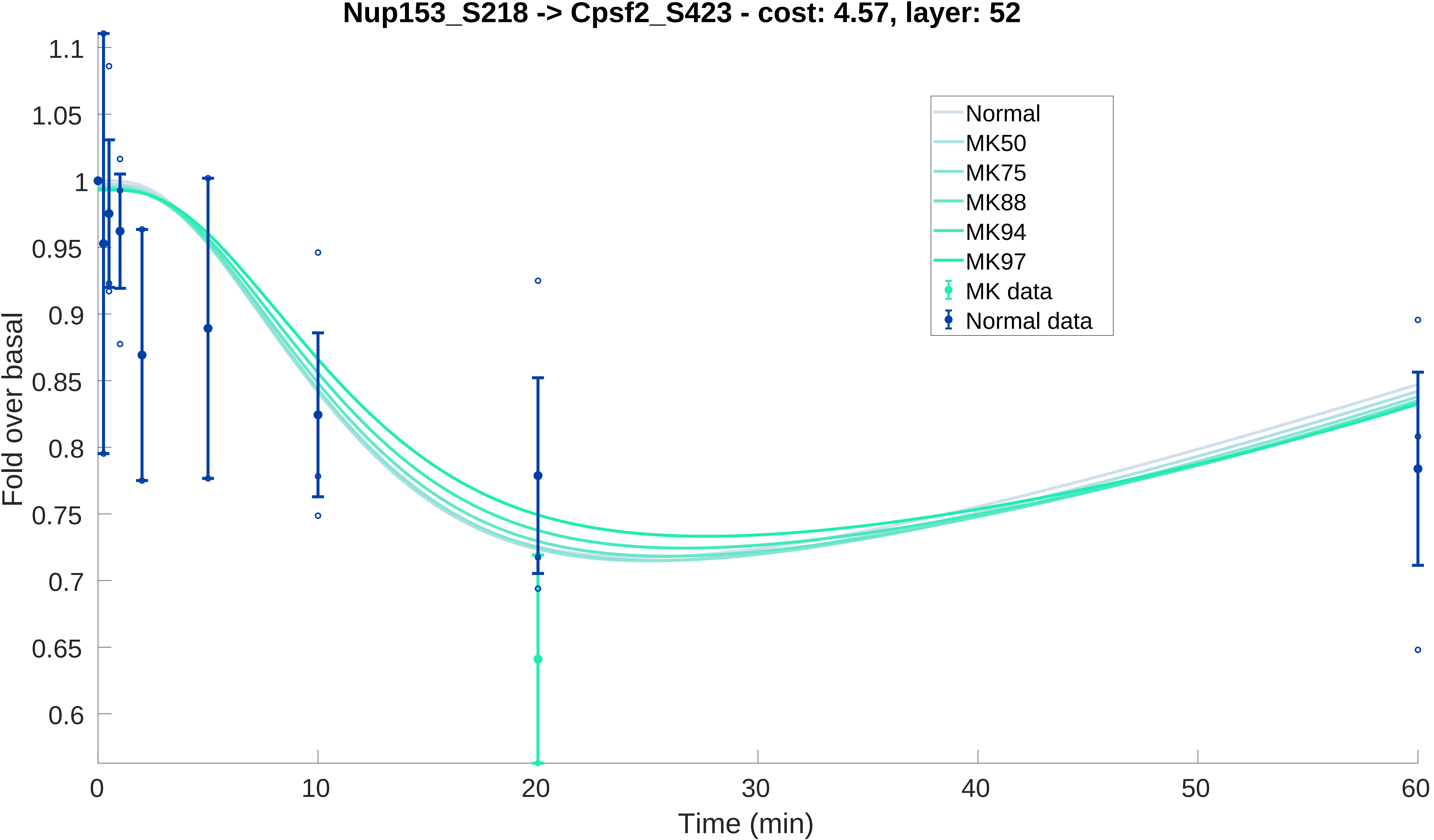
Nup153_S218 -> Cpsf2_S423 - cost: 4.57, layer: 52
Fold over basal
1.1
1.05
1
0.95
0.9
0.85
0.8
0.75
0.7
0.65
0.6
0
10
20
30
40
50
60
Time (min)
Normal
MK50
MK75
MK88
MK94
MK97
MK data
Normal data

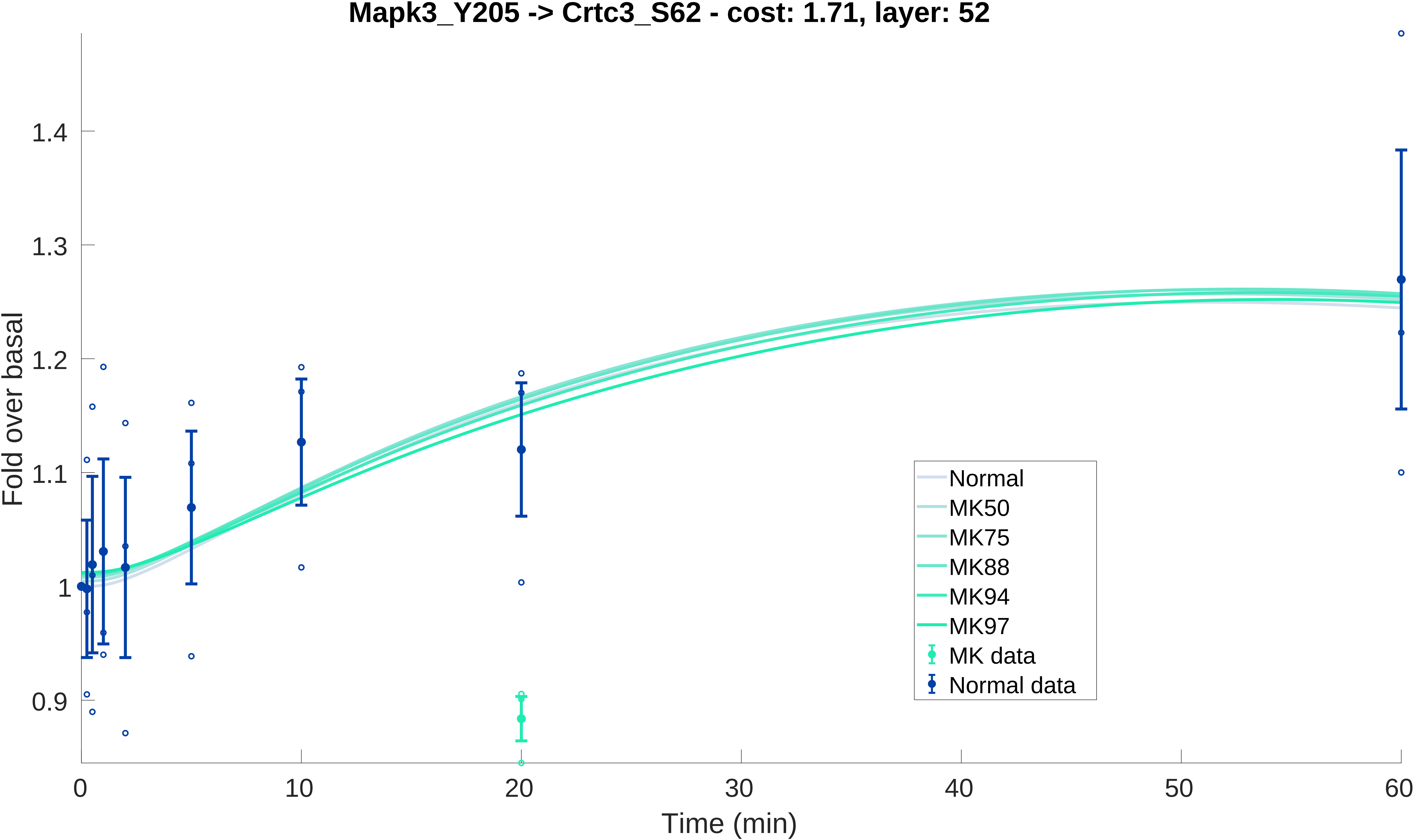

Mapk3_Y205 -> Crtc3_S62 - cost: 1.71, layer: 52
Fold over basal
Time (min)
0.9
1
1.1
1.2
1.3
1.4
0
10
20
30
40
50
60
Normal
MK50
MK75
MK88
MK94
MK97
MK data
Normal data

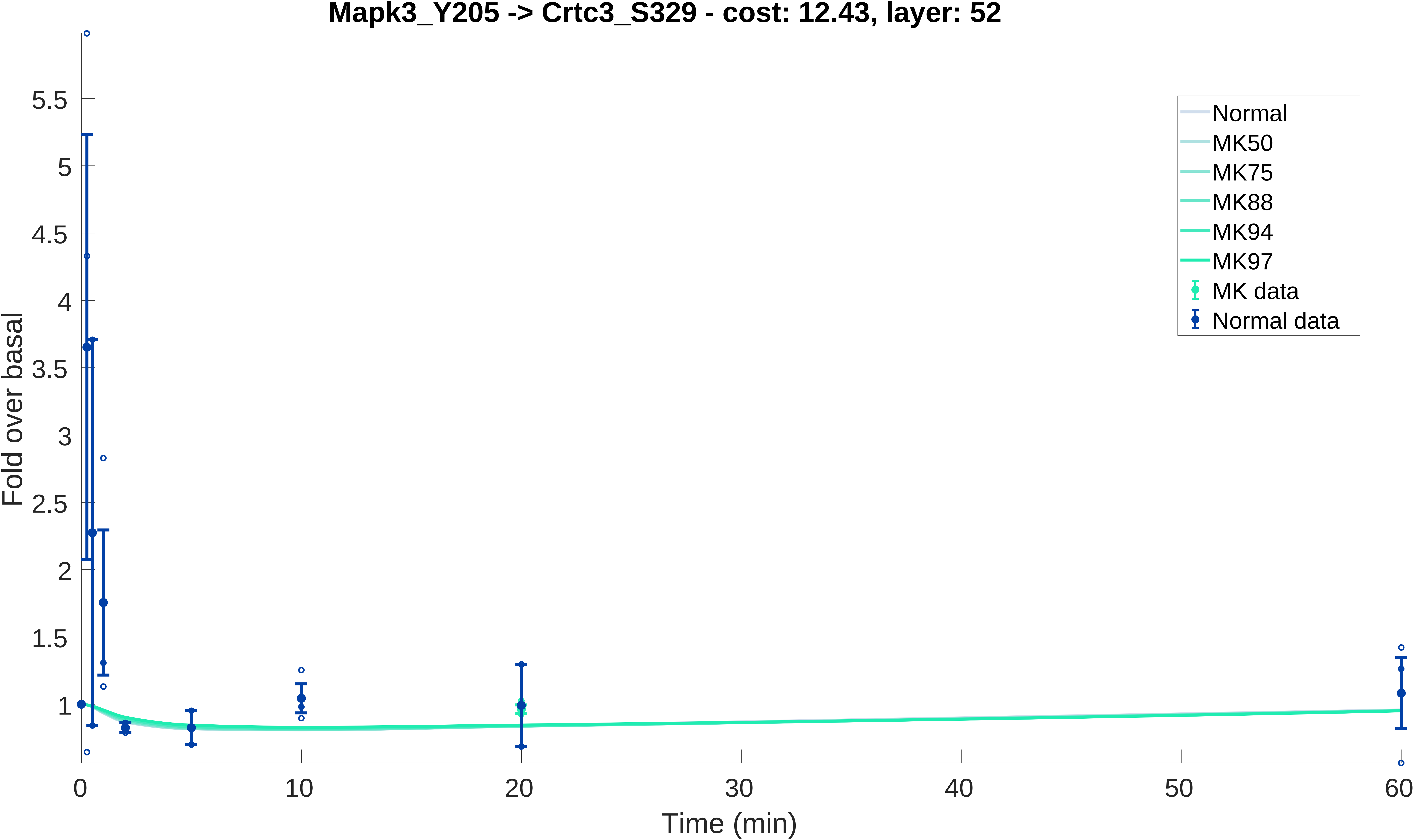

Mapk3_Y205 -> Crtc3_S329 - cost: 12.43, layer: 52
Fold over basal
Time (min)
0
10
20
30
40
50
60
1
1.5
2
2.5
3
3.5
4
4.5
5
5.5
Normal
MK50
MK75
MK88
MK94
MK97
MK data
Normal data

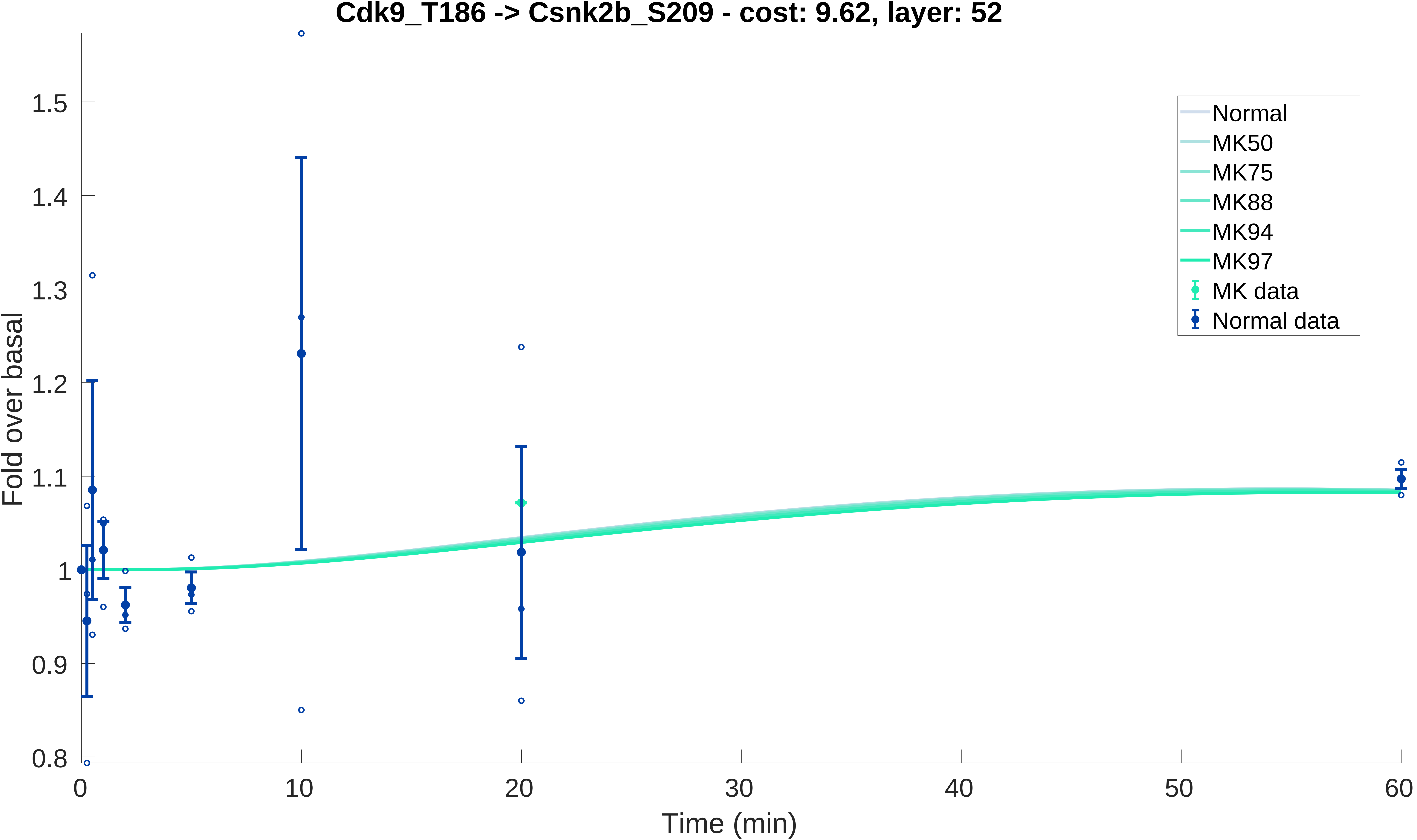

Cdk9_T186 -> Csnk2b_S209 - cost: 9.62, layer: 52
Fold over basal
Time (min)
0.8
0.9
1
1.1
1.2
1.3
1.4
1.5
0
10
20
30
40
50
60
Normal
MK50
MK75
MK88
MK94
MK97
MK data
Normal data

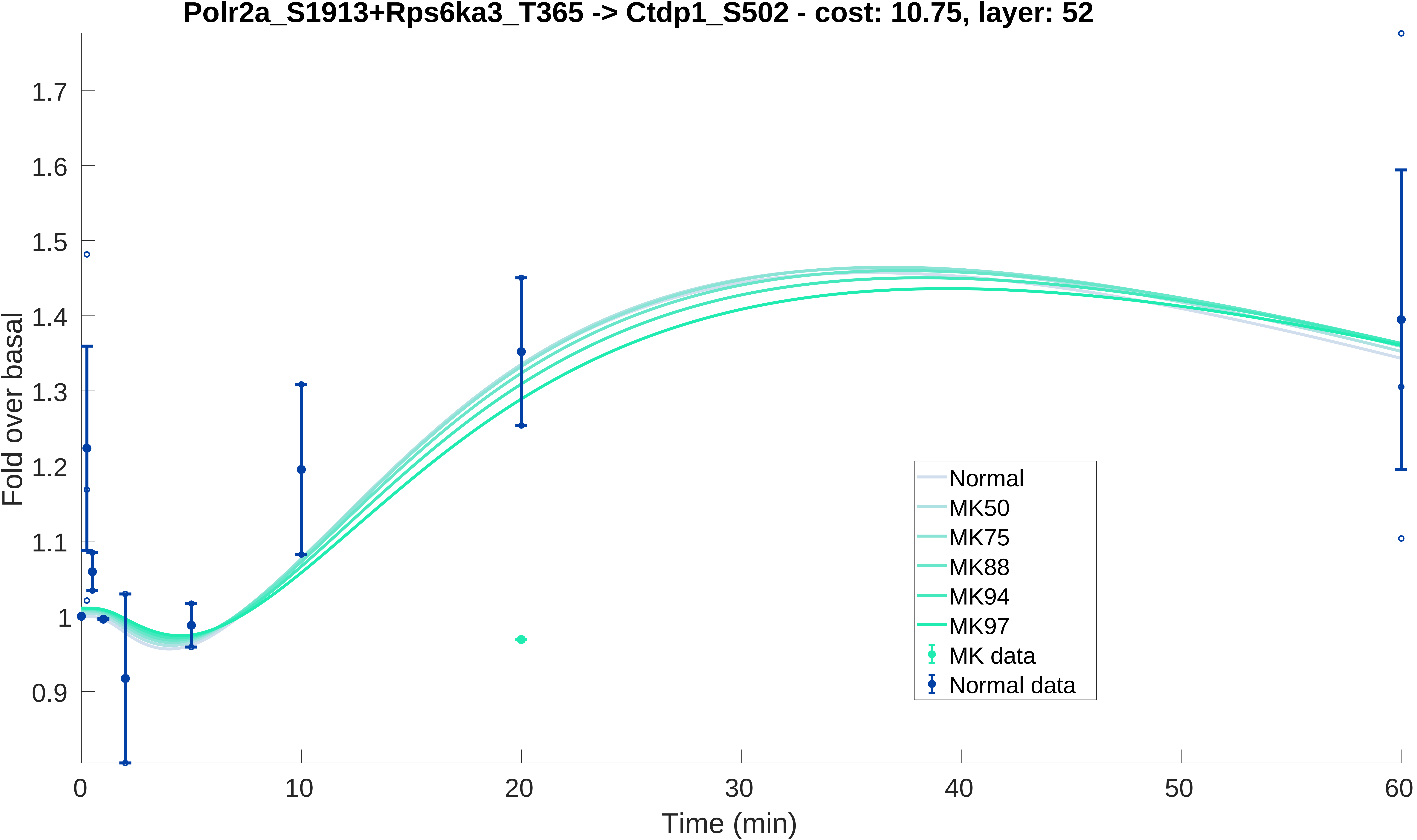

Polr2a_S1913+Rps6ka3_T365 -> Ctdp1_S502 - cost: 10.75, layer: 52
Fold over basal
Time (min)
0.9
1
1.1
1.2
1.3
1.4
1.5
1.6
1.7
0
10
20
30
40
50
60
Normal
MK50
MK75
MK88
MK94
MK97
MK data
Normal data

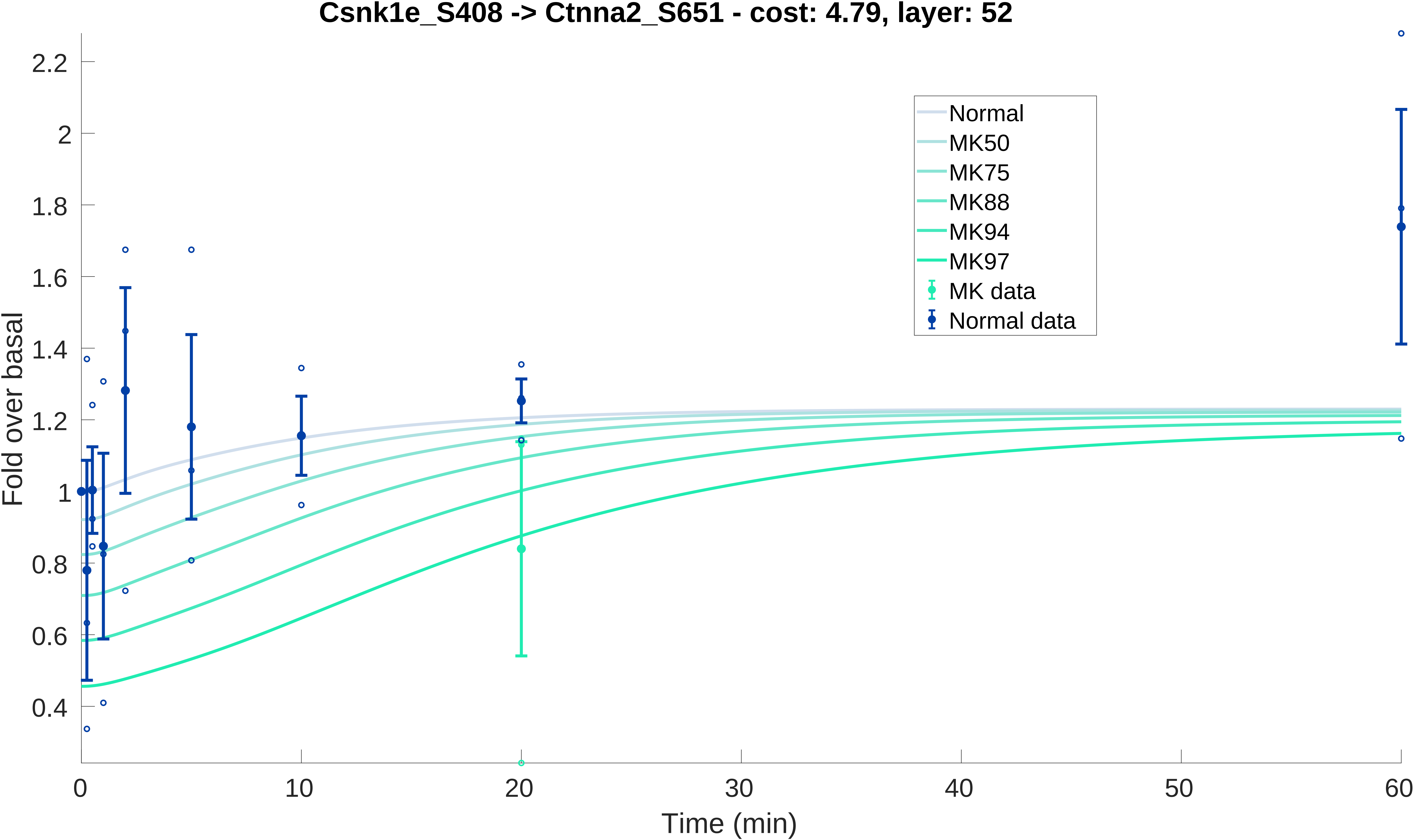

Csnk1e_S408 -> Ctnna2_S651 - cost: 4.79, layer: 52
Fold over basal
Time (min)
0.4
0.6
0.8
1
1.2
1.4
1.6
1.8
2
2.2
0
10
20
30
40
50
60
Normal
MK50
MK75
MK88
MK94
MK97
MK data
Normal data

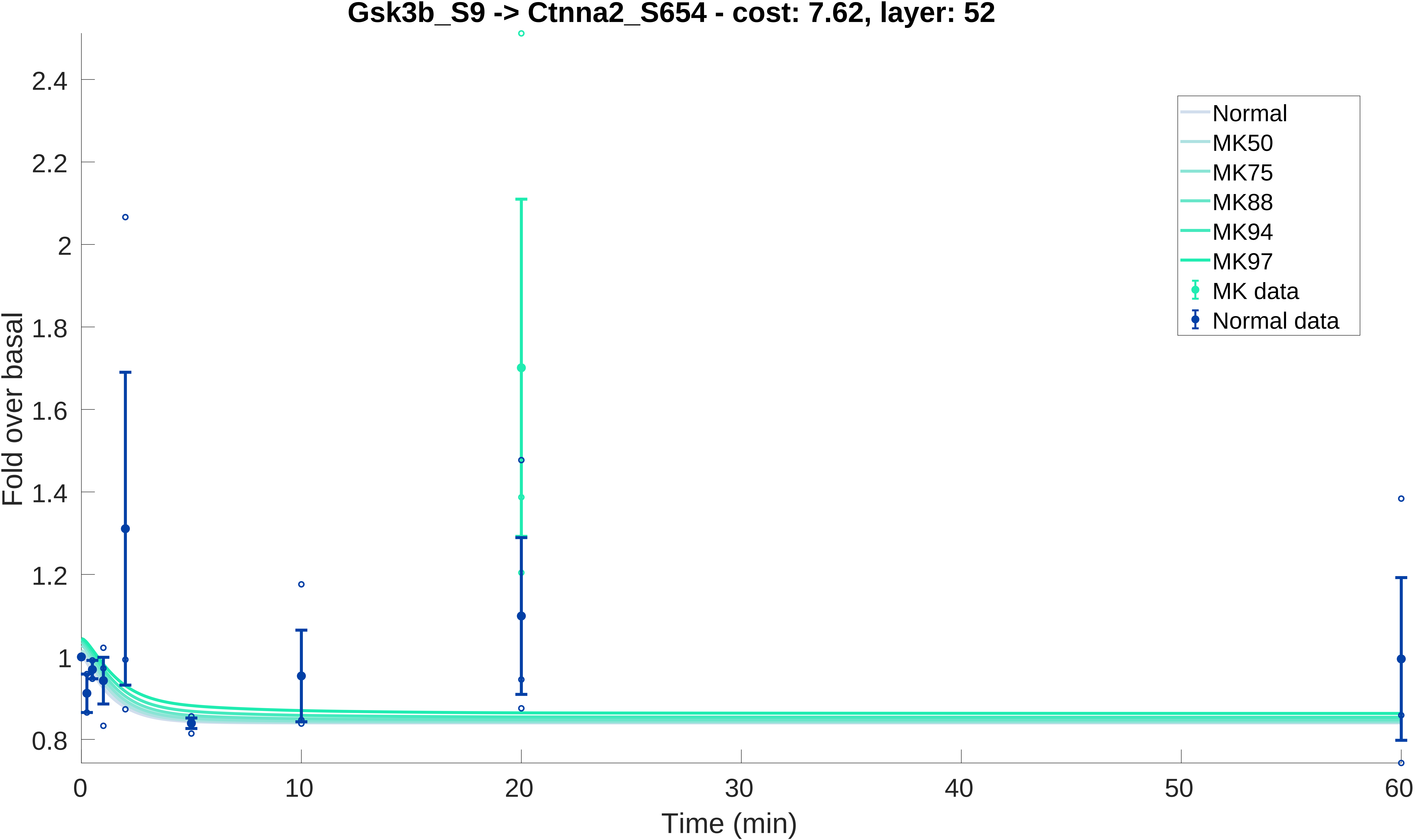
Gsk3b_S9 -> Ctnna2_S654 - cost: 7.62, layer: 52
Fold over basal
Time (min)
Normal
MK50
MK75
MK88
MK94
MK97
MK data
Normal data
2.4
2.2
2
1.8
1.6
1.4
1.2
1
0.8
0
10
20
30
40
50
60

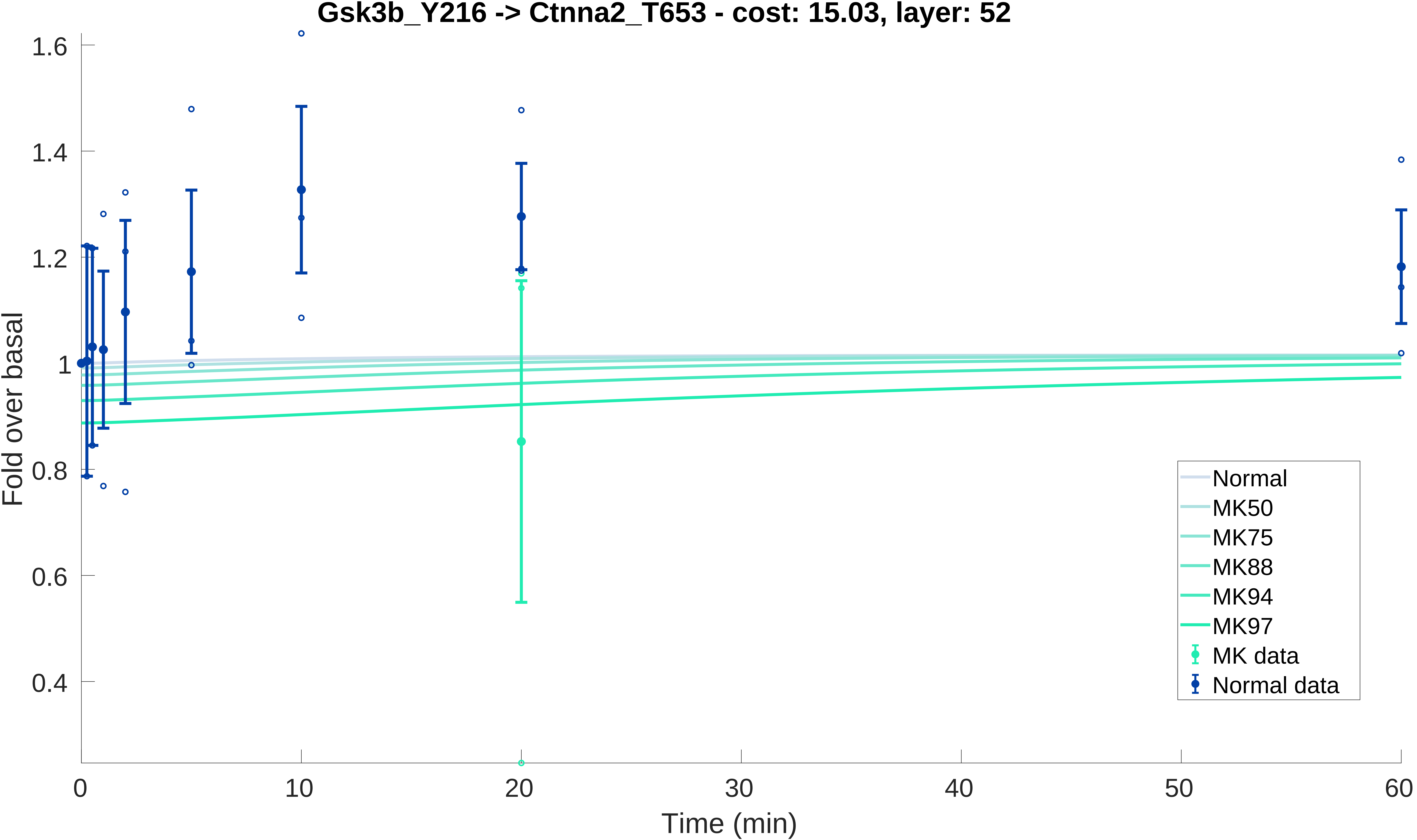

Gsk3b_Y216 -> Ctnna2_T653 - cost: 15.03, layer: 52
Fold over basal
1.6
1.4
1.2
1
0.8
0.6
0.4
0
10
20
30
40
50
60
Time (min)
Normal
MK50
MK75
MK88
MK94
MK97
MK data
Normal data

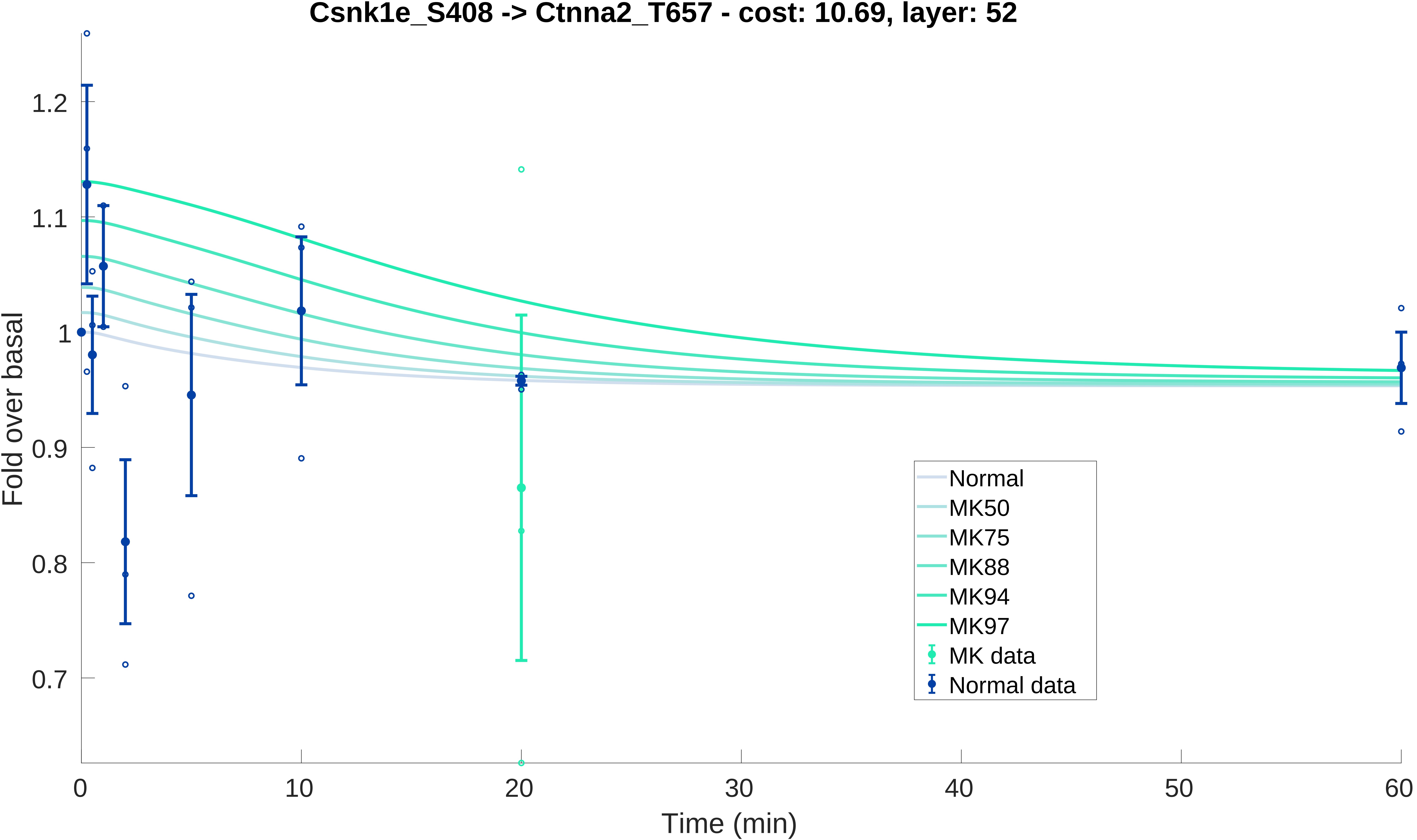

Csnk1e_S408 -> Ctnna2_T657 - cost: 10.69, layer: 52
Fold over basal
Time (min)
1.2
1.1
1
0.9
0.8
0.7
0
10
20
30
40
50
60
Normal
MK50
MK75
MK88
MK94
MK97
MK data
Normal data

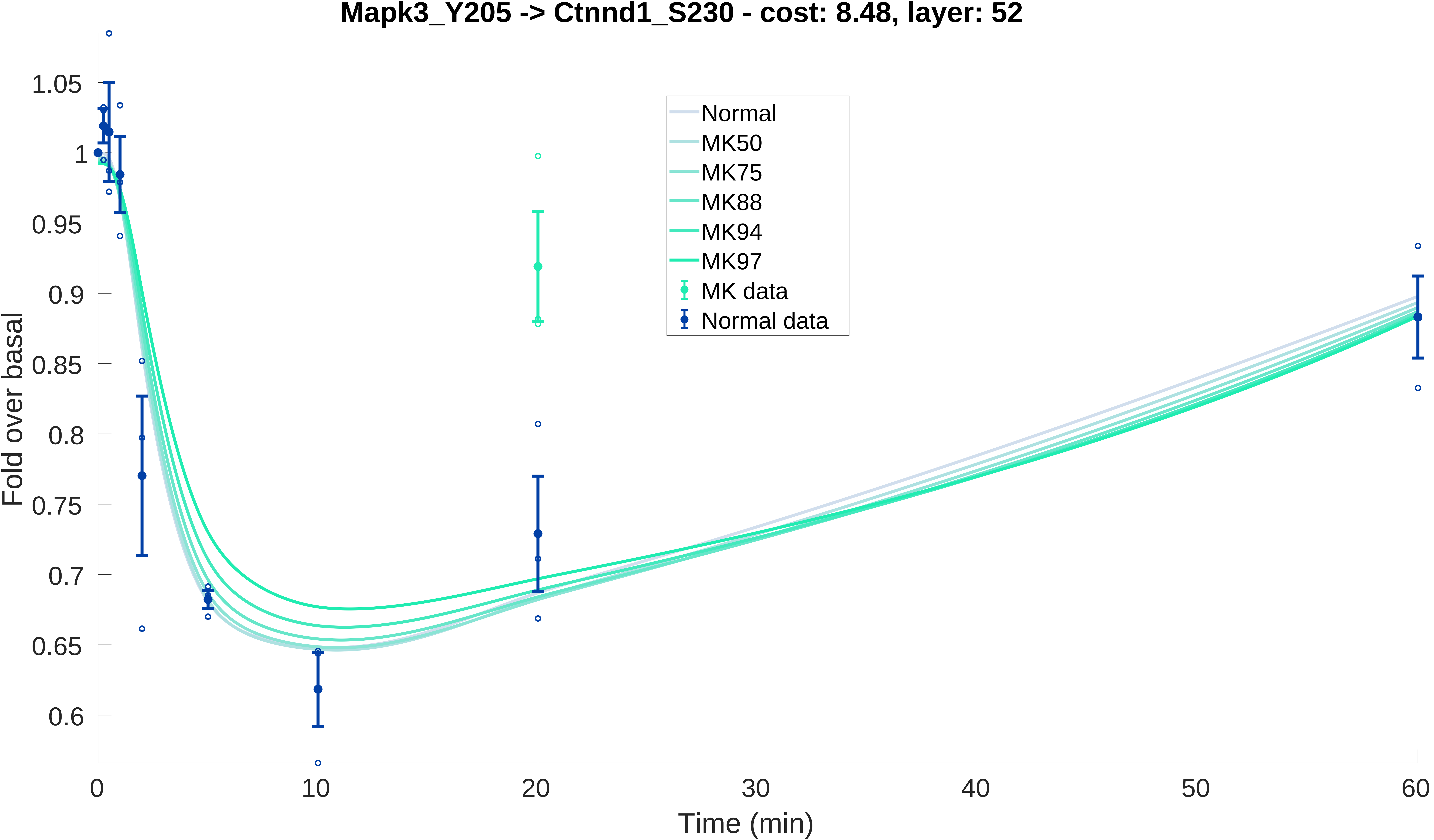

Mapk3_Y205 -> Ctnnd1_S230 - cost: 8.48, layer: 52
Fold over basal
Time (min)
Normal
MK50
MK75
MK88
MK94
MK97
MK data
Normal data
1.05
1
0.95
0.9
0.85
0.8
0.75
0.7
0.65
0.6
0
10
20
30
40
50
60

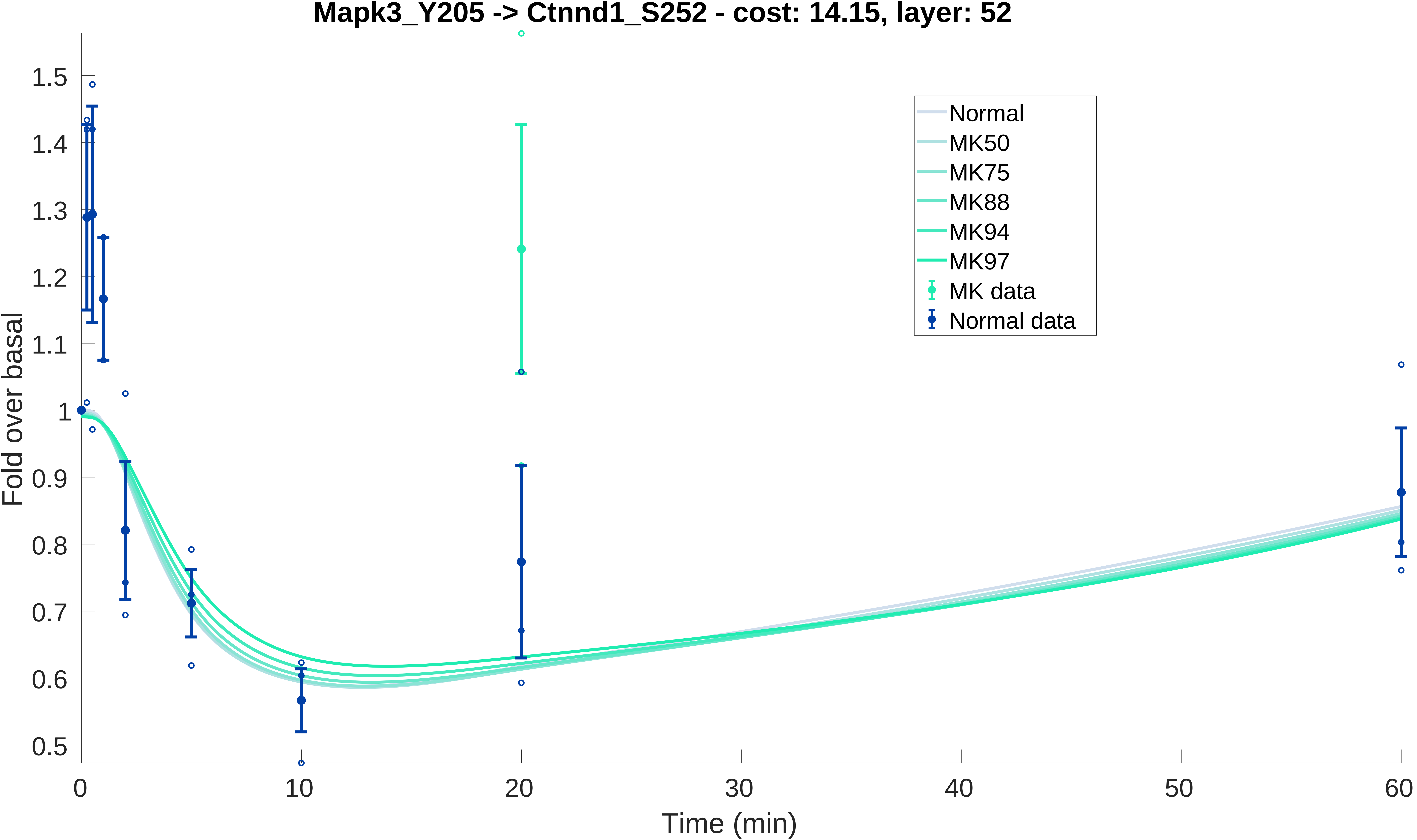

Mapk3_Y205 -> Ctnnd1_S252 - cost: 14.15, layer: 52
Fold over basal
Time (min)
0
10
20
30
40
50
60
0.5
0.6
0.7
0.8
0.9
1
1.1
1.2
1.3
1.4
1.5
Normal
MK50
MK75
MK88
MK94
MK97
MK data
Normal data

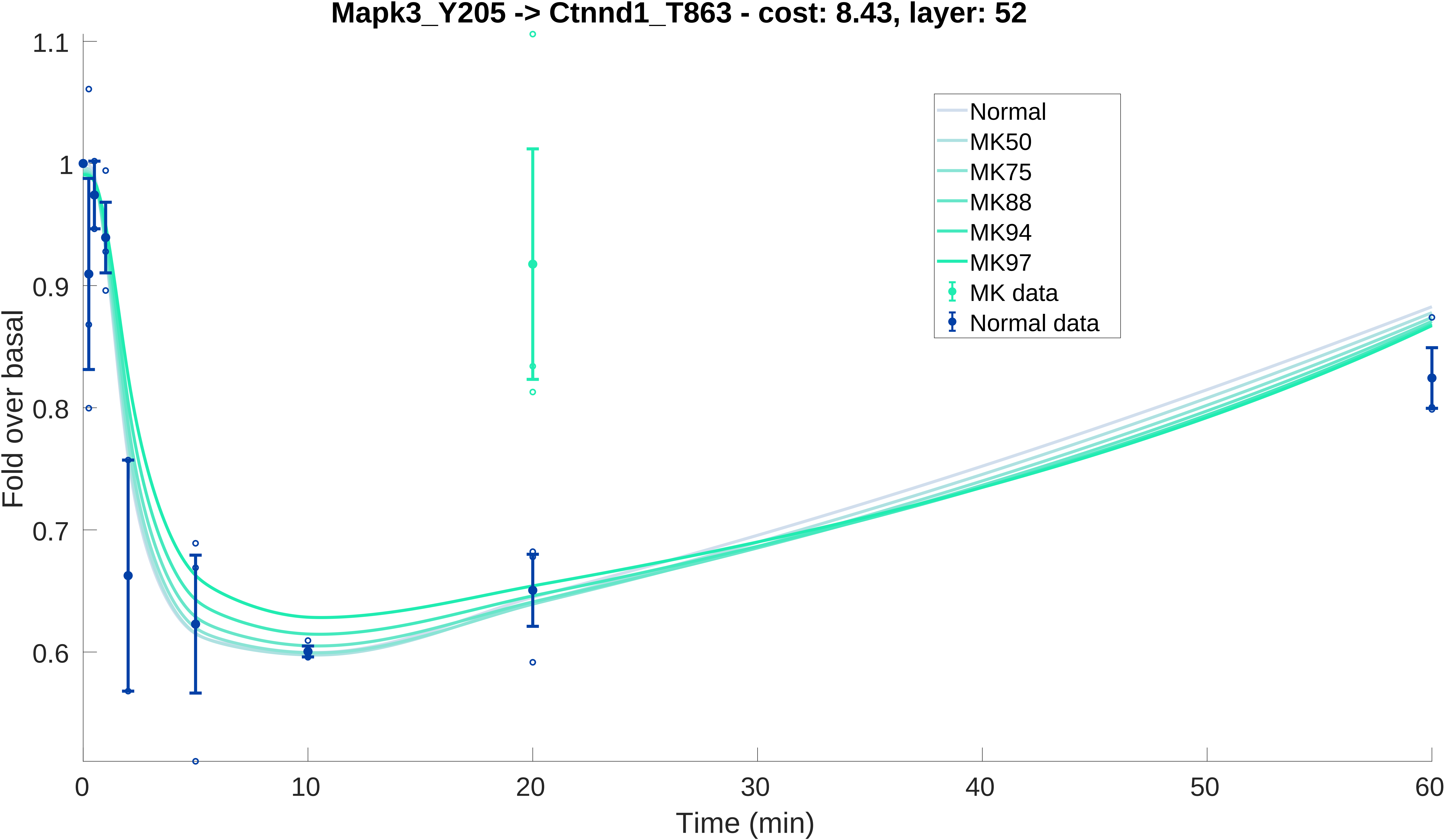

Mapk3_Y205 -> Ctnnd1_T863 - cost: 8.43, layer: 52
Fold over basal
Time (min)
1.1
1
0.9
0.8
0.7
0.6
0
10
20
30
40
50
60
Normal
MK50
MK75
MK88
MK94
MK97
MK data
Normal data

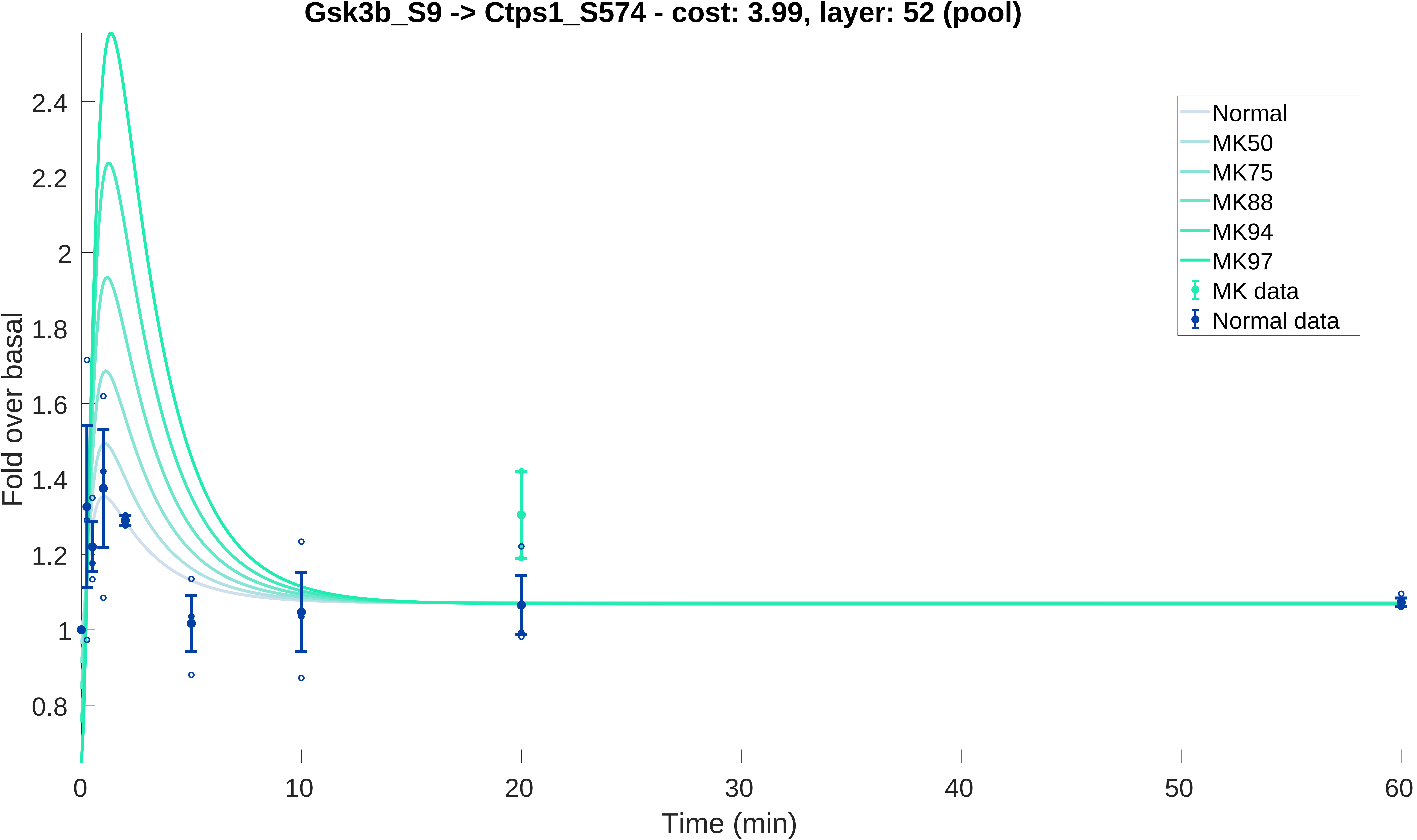

Gsk3b_S9 -> Ctps1_S574 - cost: 3.99, layer: 52 (pool)
Fold over basal
Time (min)
0.8
1
1.2
1.4
1.6
1.8
2
2.2
2.4
0
10
20
30
40
50
60
Normal
MK50
MK75
MK88
MK94
MK97
MK data
Normal data

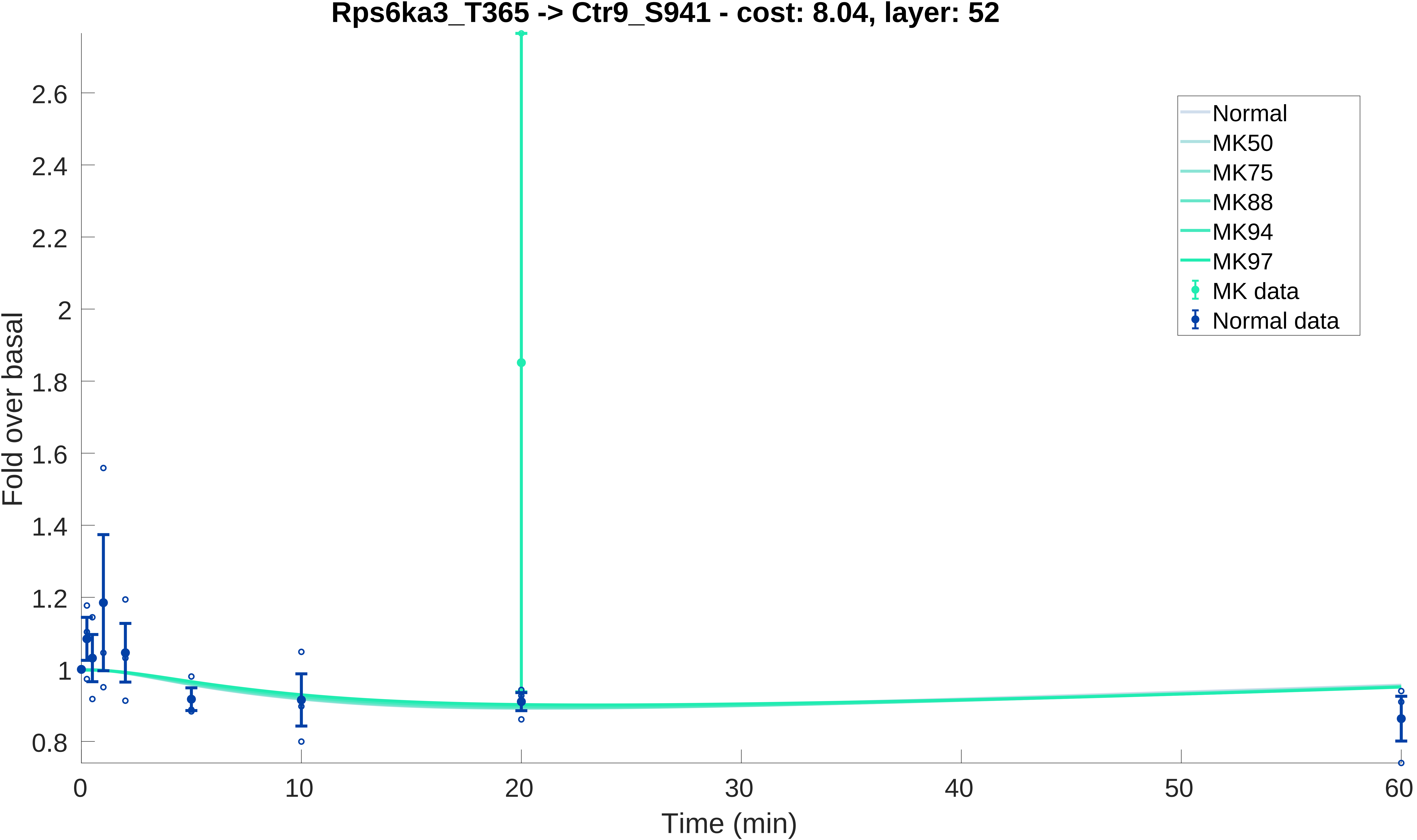
Rps6ka3_T365 -> Ctr9_S941 - cost: 8.04, layer: 52
Fold over basal
2.6
2.4
2.2
2
1.8
1.6
1.4
1.2
1
0.8
Time (min)
0
10
20
30
40
50
60
Normal
MK50
MK75
MK88
MK94
MK97
MK data
Normal data

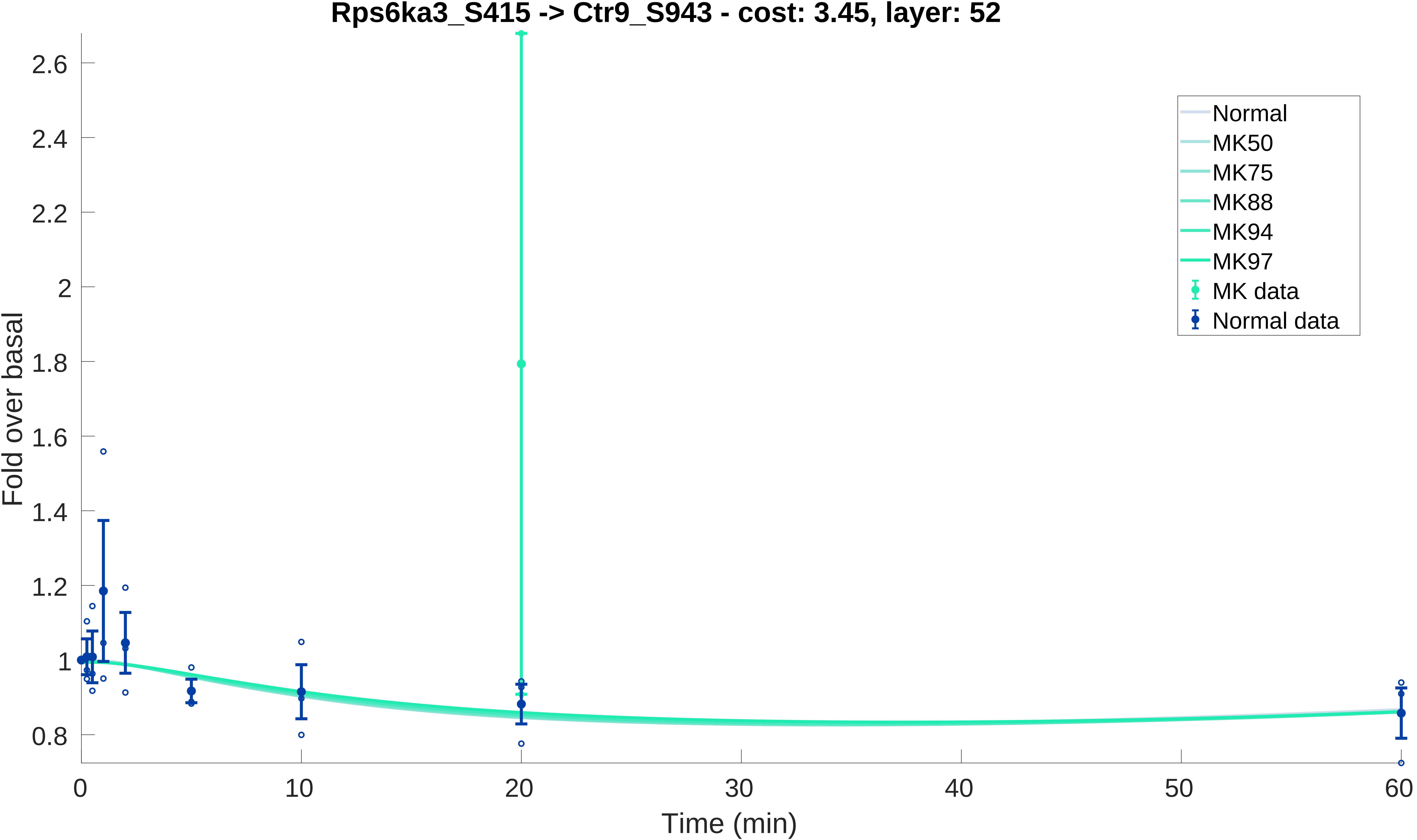

Rps6ka3_S415 -> Ctr9_S943 - cost: 3.45, layer: 52
Fold over basal
Time (min)
0.8
1
1.2
1.4
1.6
1.8
2
2.2
2.4
2.6
0
10
20
30
40
50
60
Normal
MK50
MK75
MK88
MK94
MK97
MK data
Normal data

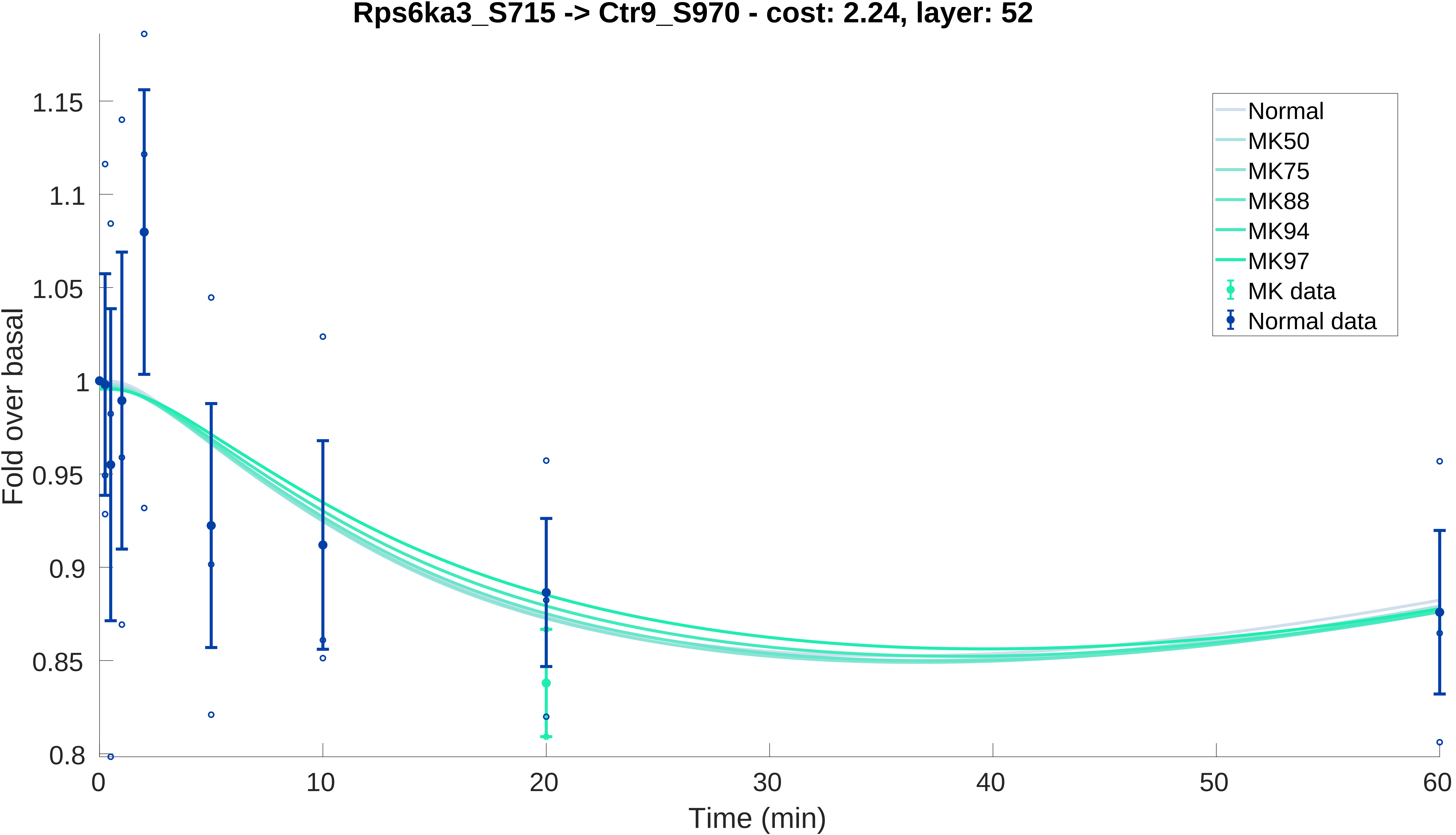
Rps6ka3_S715 -> Ctr9_S970 - cost: 2.24, layer: 52
Fold over basal
Time (min)
1.15
1.1
1.05
1
0.95
0.9
0.85
0.8
0
10
20
30
40
50
60
Normal
MK50
MK75
MK88
MK94
MK97
MK data
Normal data

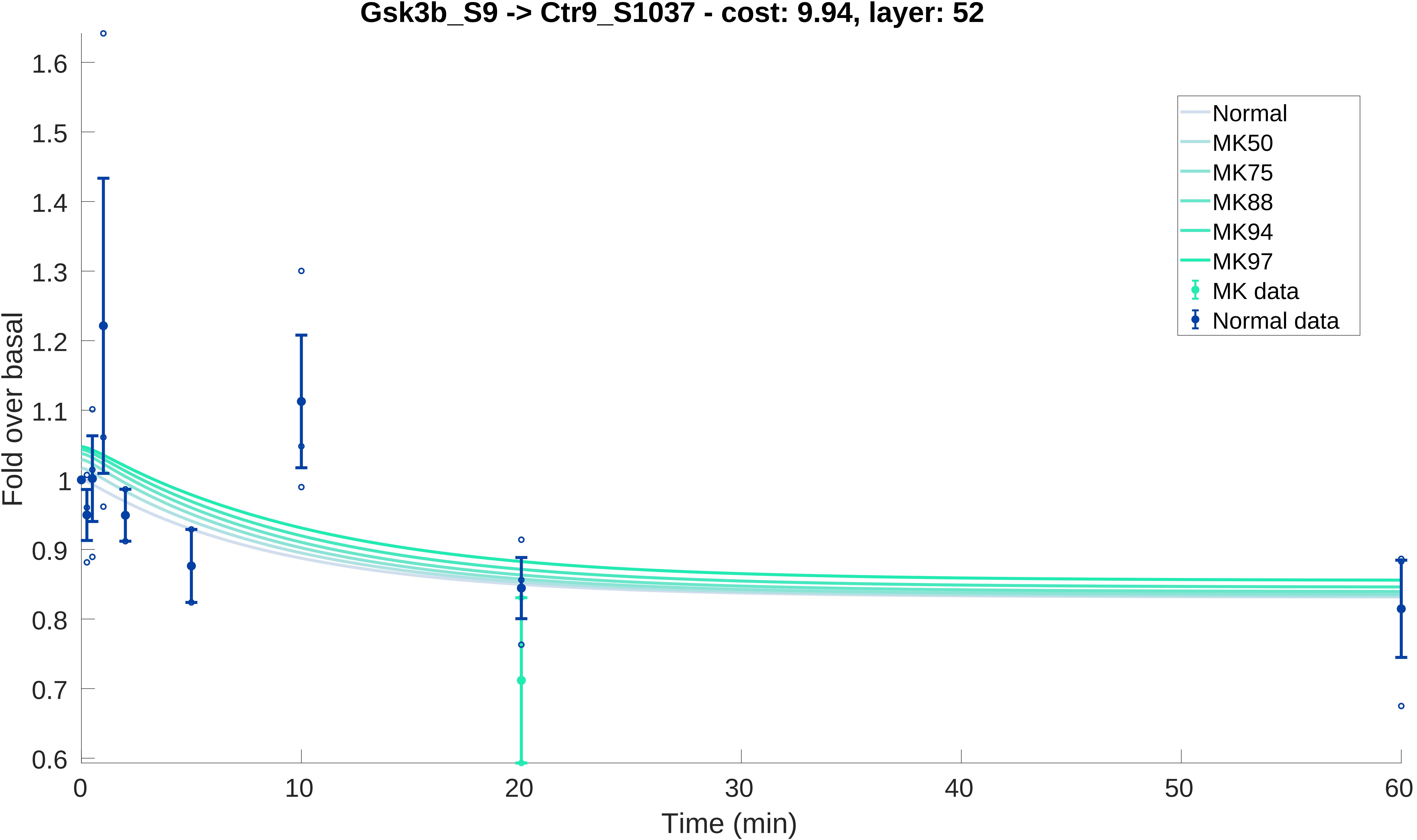
Gsk3b_S9 -> Ctr9_S1037 - cost: 9.94, layer: 52
Fold over basal
Time (min)
Normal
MK50
MK75
MK88
MK94
MK97
MK data
Normal data
1.6
1.5
1.4
1.3
1.2
1.1
1
0.9
0.8
0.7
0.6
0
10
20
30
40
50
60

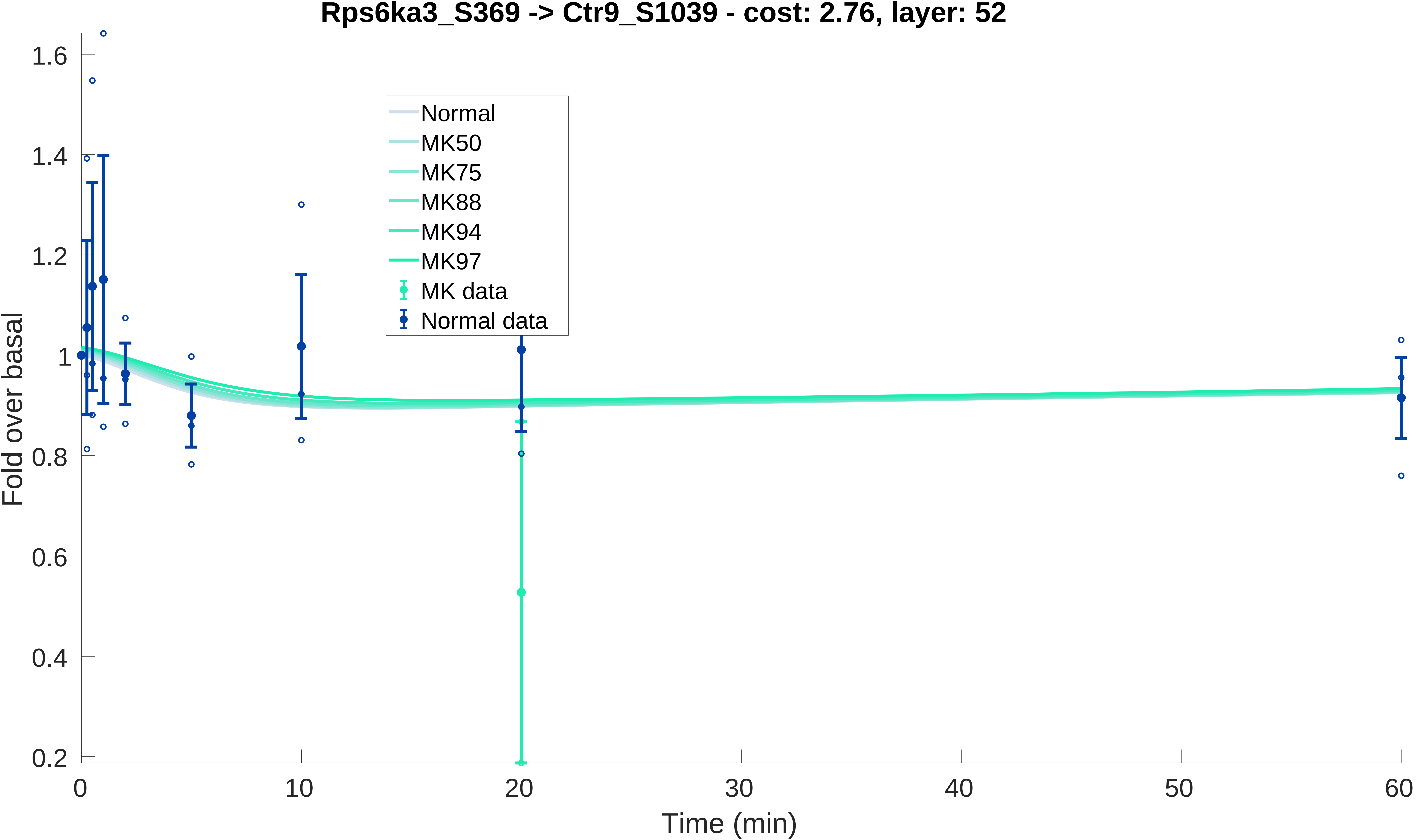
Rps6ka3_S369 -> Ctr9_S1039 - cost: 2.76, layer: 52
Fold over basal
Time (min)
Normal
MK50
MK75
MK88
MK94
MK97
MK data
Normal data
1.6
1.4
1.2
1
0.8
0.6
0.4
0.2
0
10
20
30
40
50
60

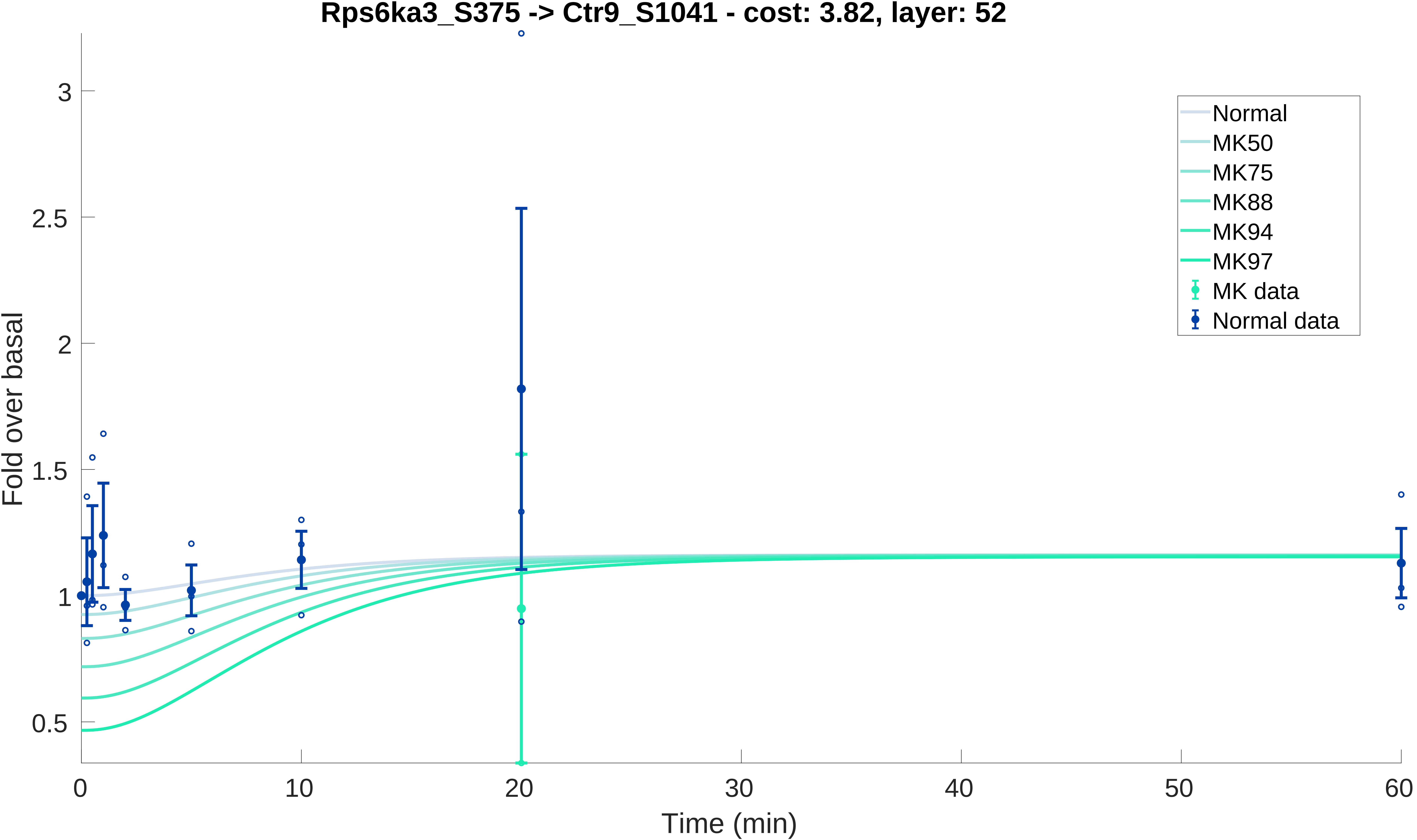

Rps6ka3_S375 -> Ctr9_S1041 - cost: 3.82, layer: 52
Fold over basal
Time (min)
Normal
MK50
MK75
MK88
MK94
MK97
MK data
Normal data
0.5
1
1.5
2
2.5
3
0
10
20
30
40
50
60

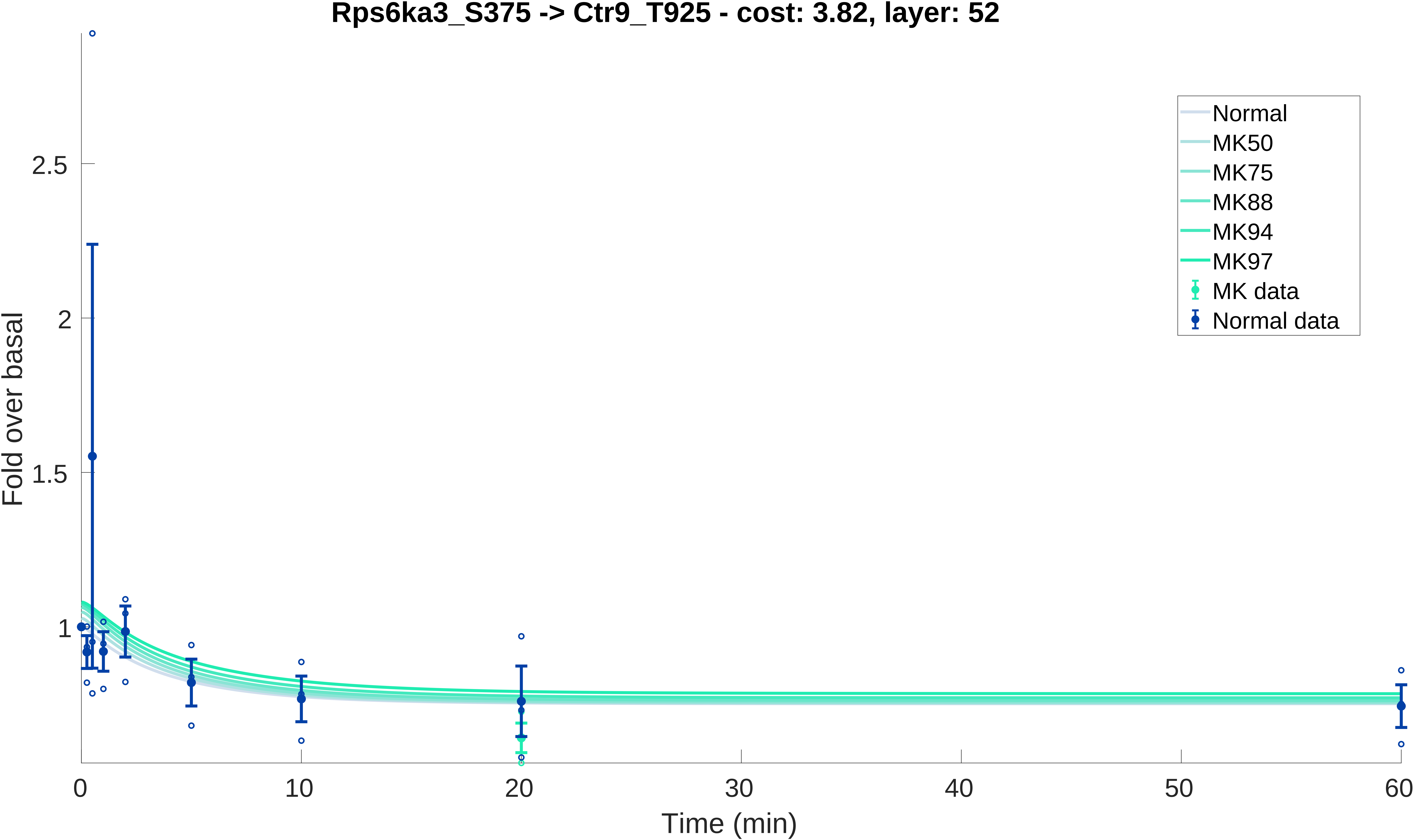
Rps6ka3_S375 -> Ctr9_T925 - cost: 3.82, layer: 52
Fold over basal
Time (min)
0
10
20
30
40
50
60
1
1.5
2
2.5
Normal
MK50
MK75
MK88
MK94
MK97
MK data
Normal data

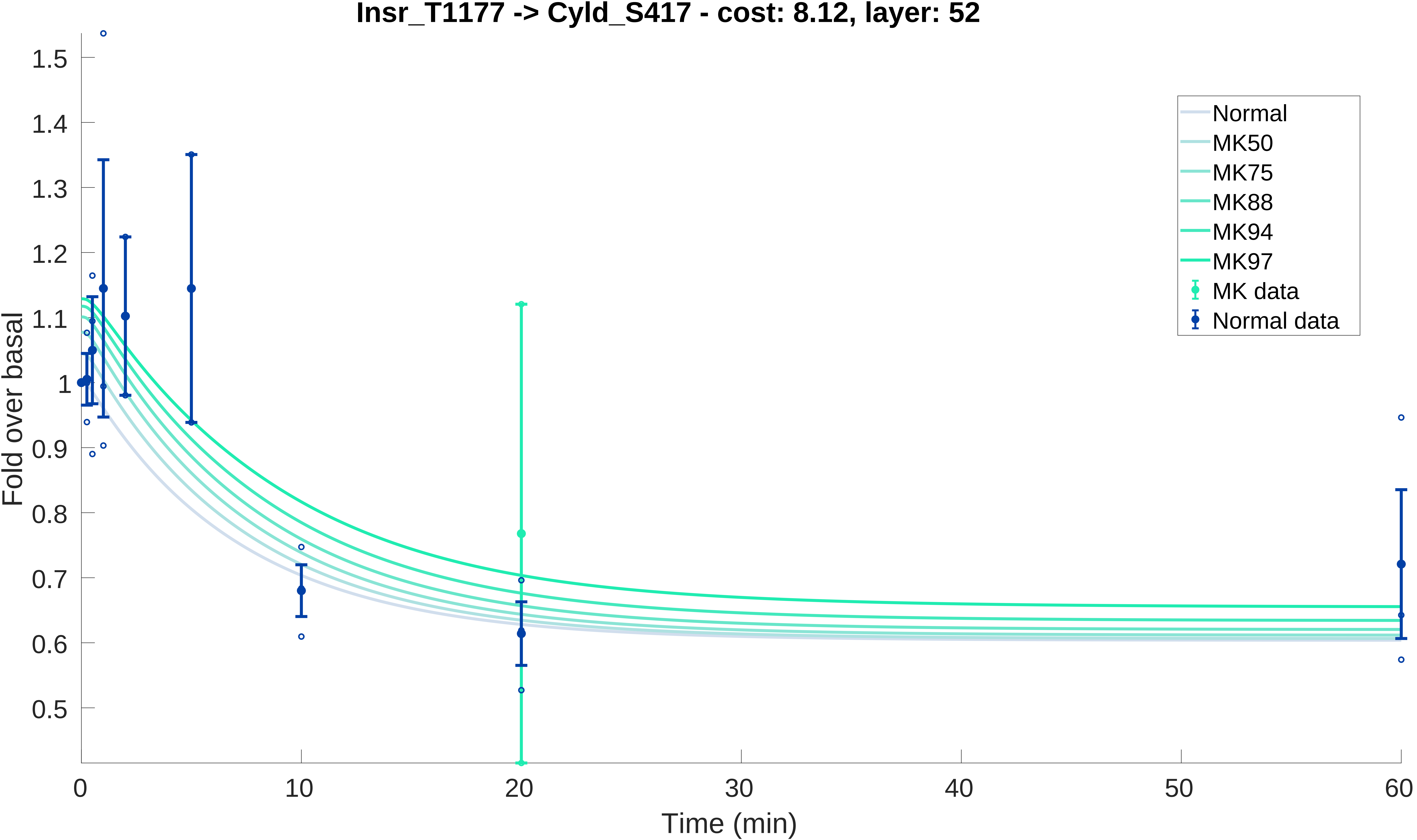

Insr_T1177 -> Cyld_S417 - cost: 8.12, layer: 52
Normal
MK50
MK75
MK88
MK94
MK97
MK data
Normal data
Fold over basal
Time (min)
1.5
1.4
1.3
1.2
1.1
1
0.9
0.8
0.7
0.6
0.5
0
10
20
30
40
50
60

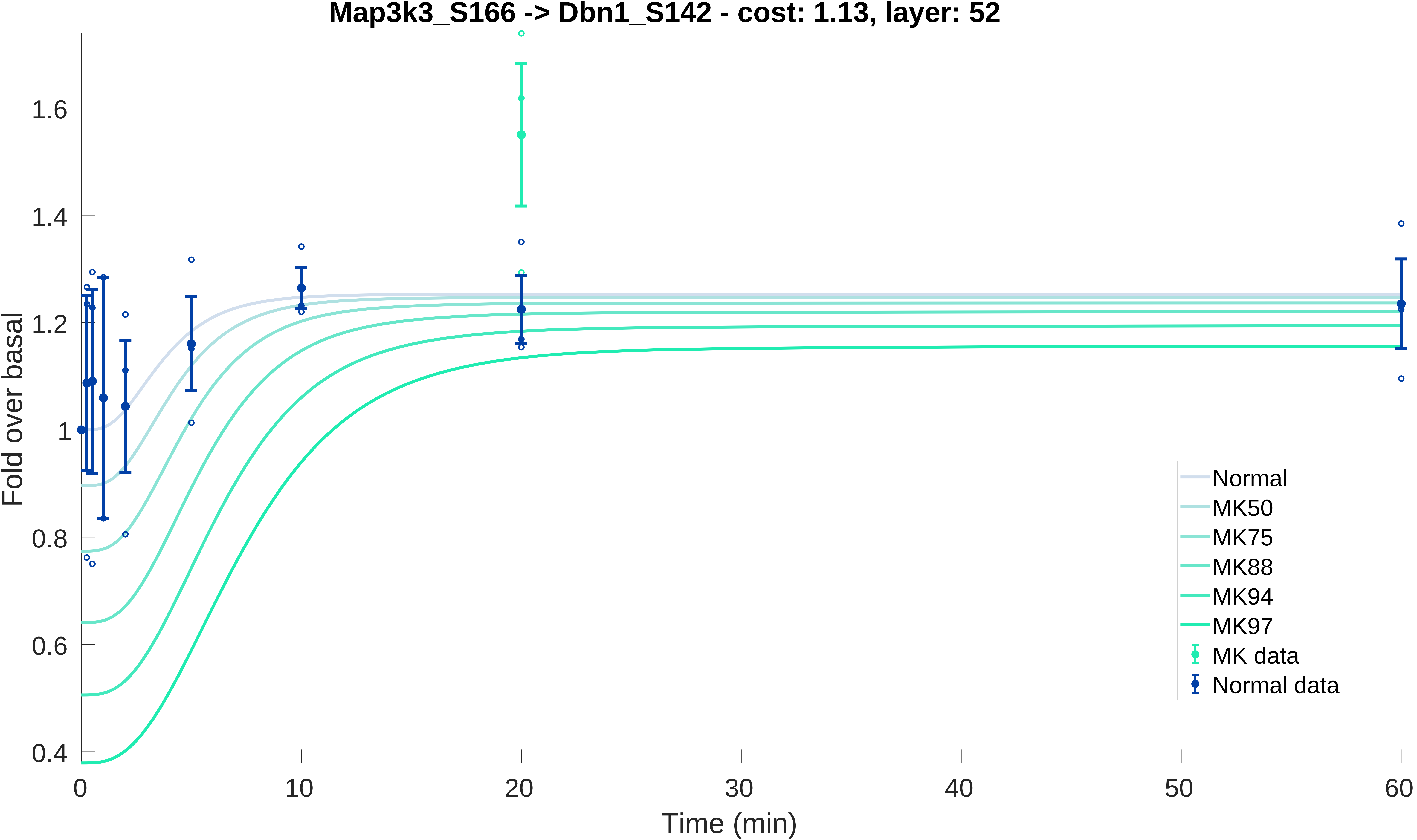

Map3k3_S166 -> Dbn1_S142 - cost: 1.13, layer: 52
Fold over basal
Time (min)
1.6
1.4
1.2
1
0.8
0.6
0.4
0
10
20
30
40
50
60
Normal
MK50
MK75
MK88
MK94
MK97
MK data
Normal data

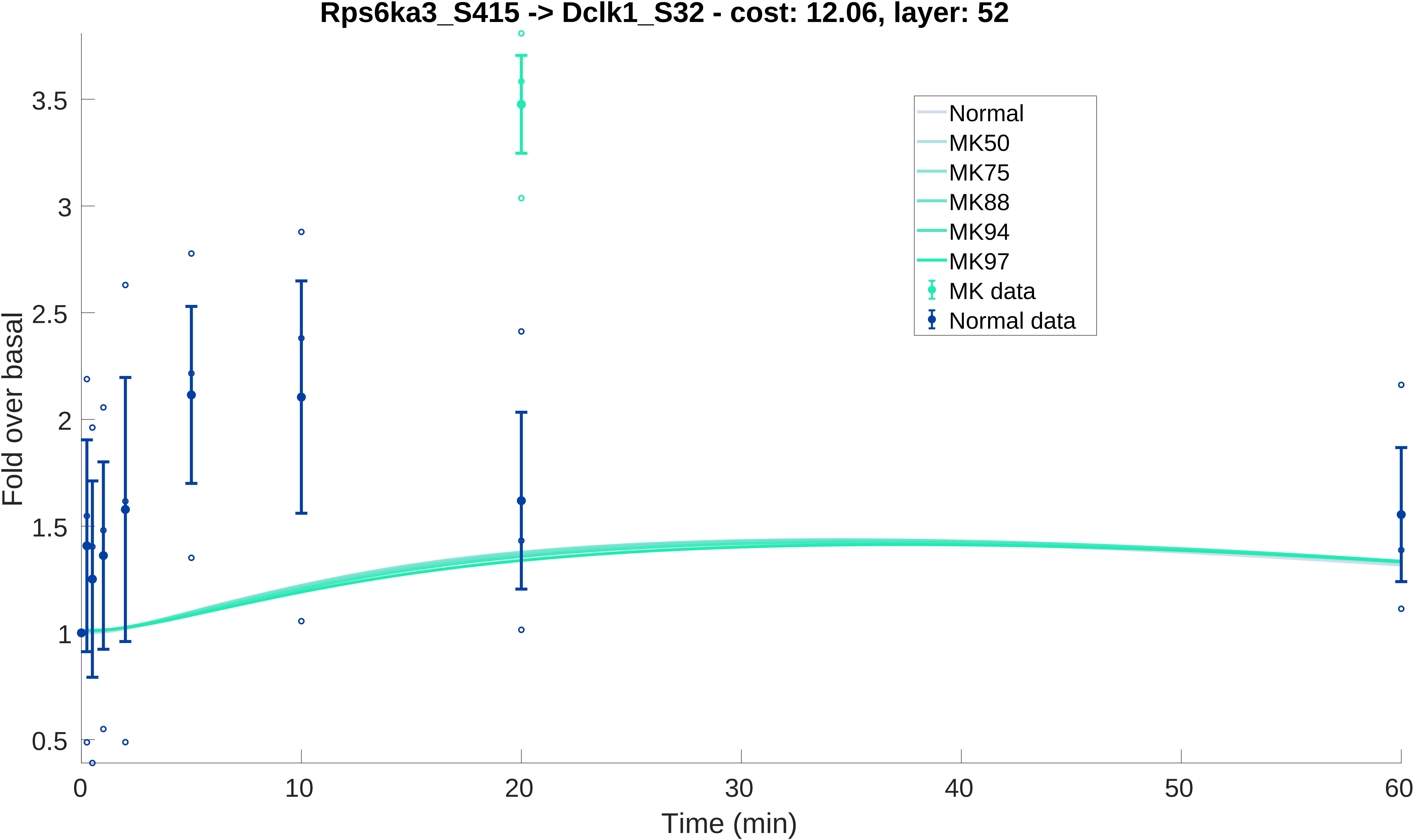
Rps6ka3_S415 -> Dclk1_S32 - cost: 12.06, layer: 52
Normal
MK50
MK75
MK88
MK94
MK97
MK data
Normal data
Fold over basal
Time (min)
0.5
1
1.5
2
2.5
3
3.5
0
10
20
30
40
50
60

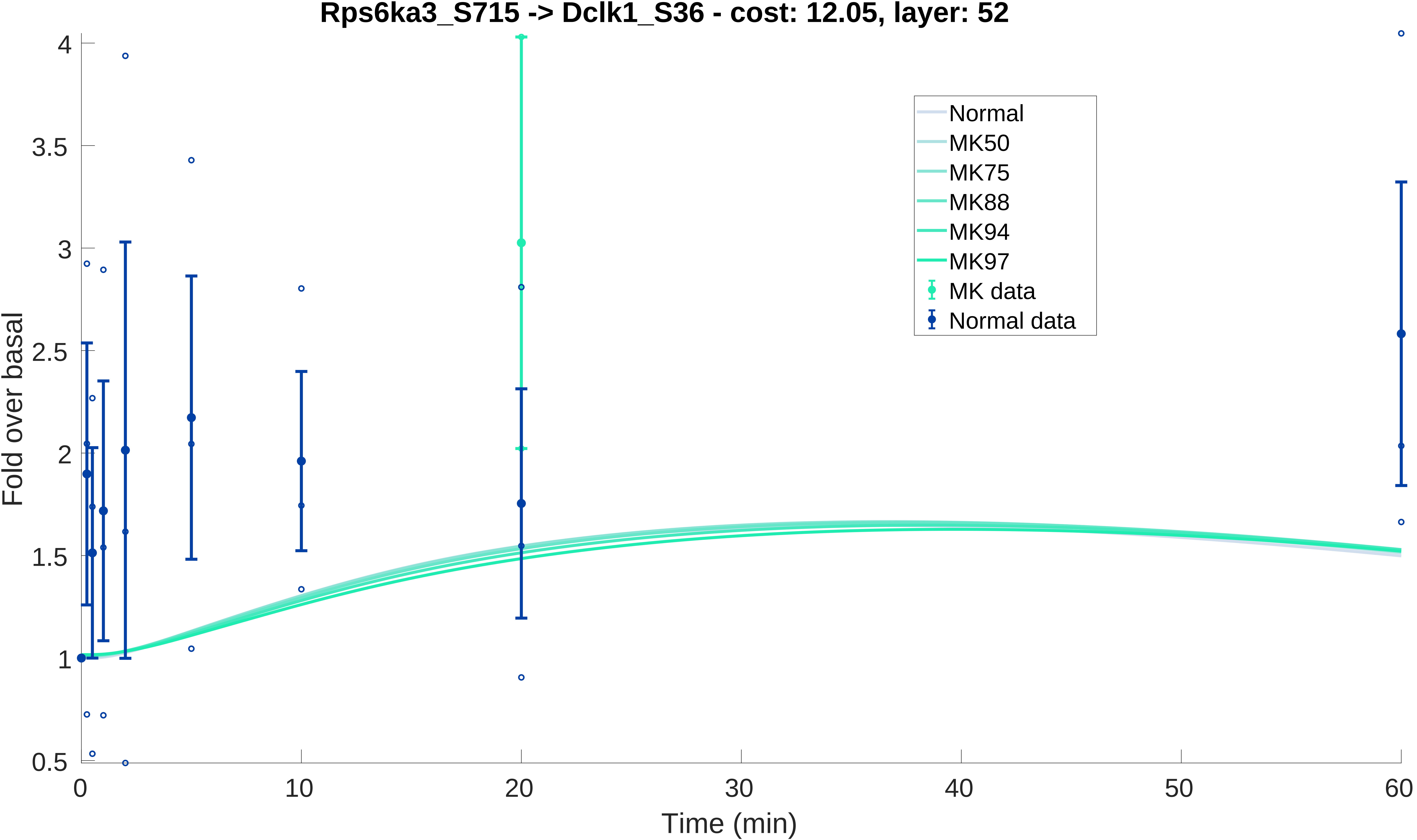

Rps6ka3_S715 -> Dclk1_S36 - cost: 12.05, layer: 52
Fold over basal
Time (min)
0.5
1
1.5
2
2.5
3
3.5
4
0
10
20
30
40
50
60
Normal
MK50
MK75
MK88
MK94
MK97
MK data
Normal data

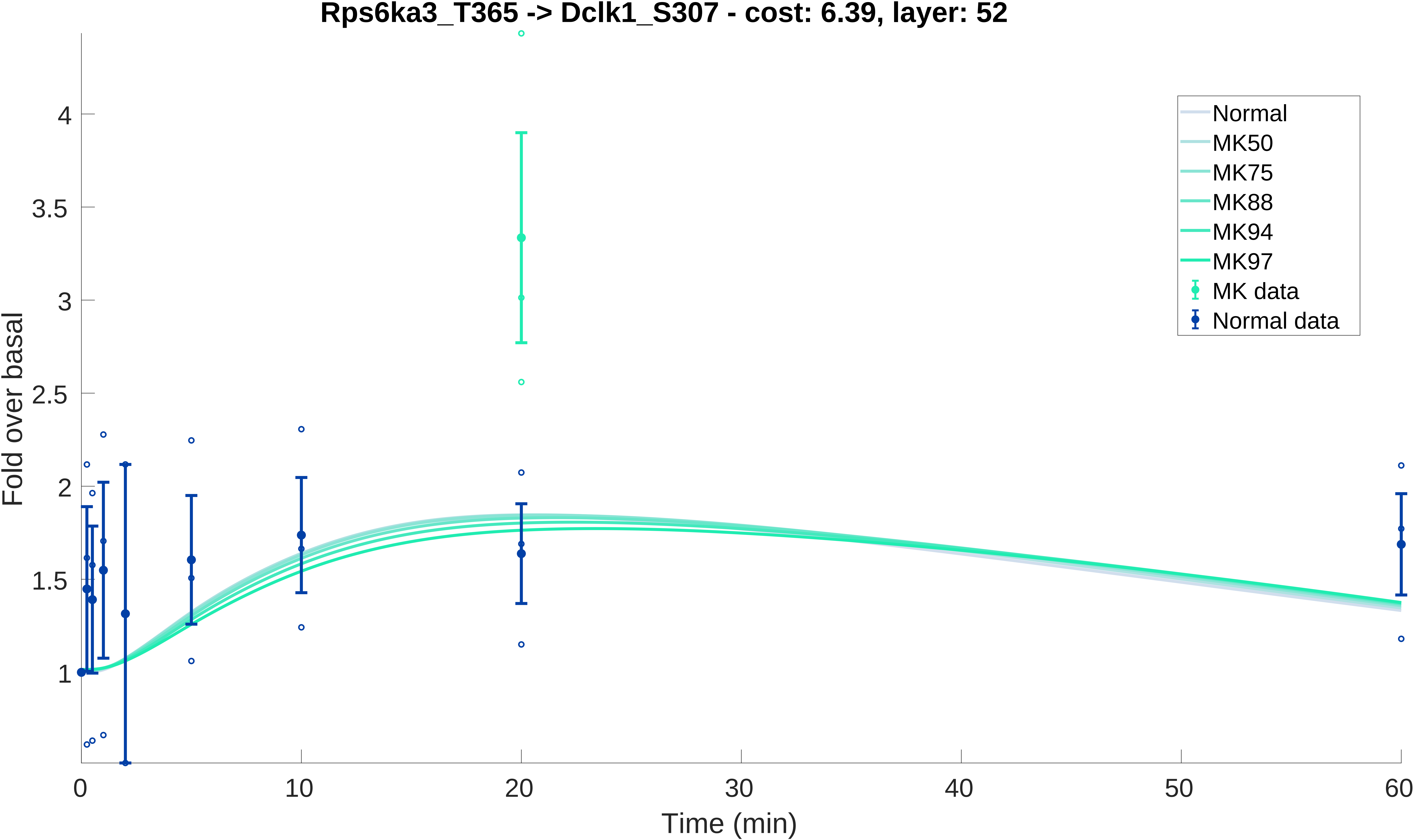

Rps6ka3_T365 -> Dclk1_S307 - cost: 6.39, layer: 52
Fold over basal
Time (min)
0
10
20
30
40
50
60
1
1.5
2
2.5
3
3.5
4
Normal
MK50
MK75
MK88
MK94
MK97
MK data
Normal data

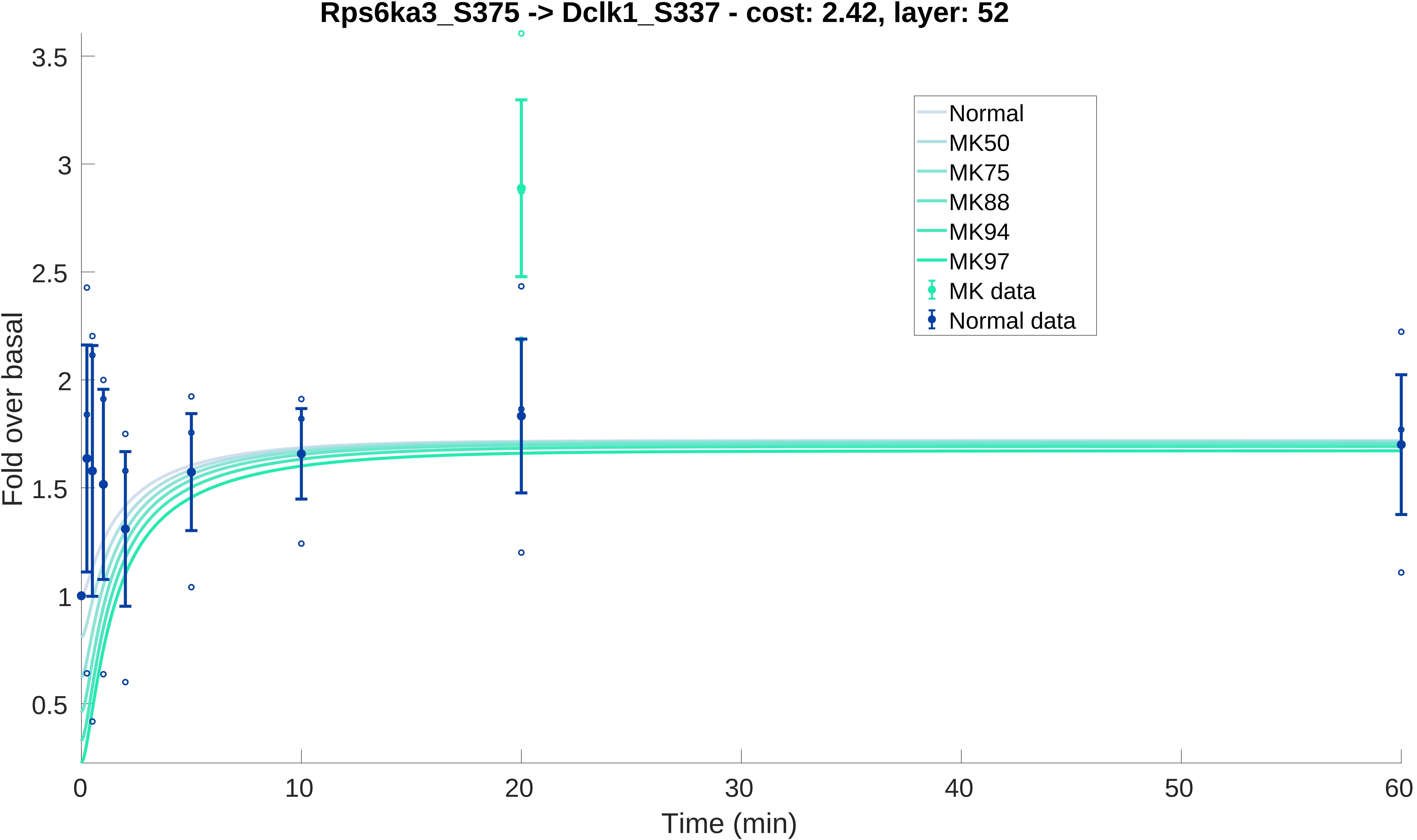

Rps6ka3_S375 -> Dclk1_S337 - cost: 2.42, layer: 52
Fold over basal
Time (min)
3.5
3
2.5
2
1.5
1
0.5
0
10
20
30
40
50
60
Normal
MK50
MK75
MK88
MK94
MK97
MK data
Normal data

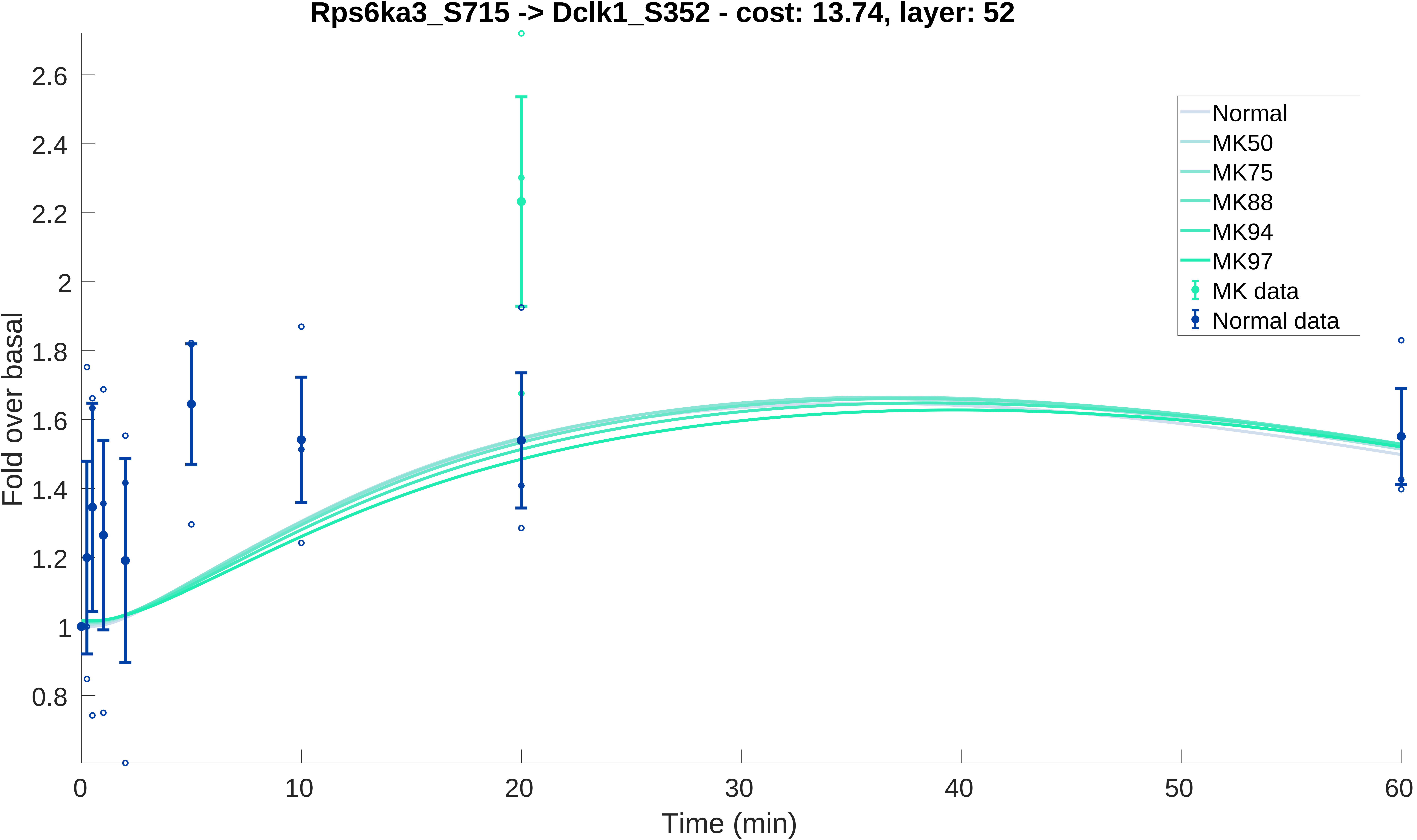

Rps6ka3_S715 -> Dclk1_S352 - cost: 13.74, layer: 52
Fold over basal
Time (min)
Normal
MK50
MK75
MK88
MK94
MK97
MK data
Normal data
0.8
1
1.2
1.4
1.6
1.8
2
2.2
2.4
2.6
0
10
20
30
40
50
60

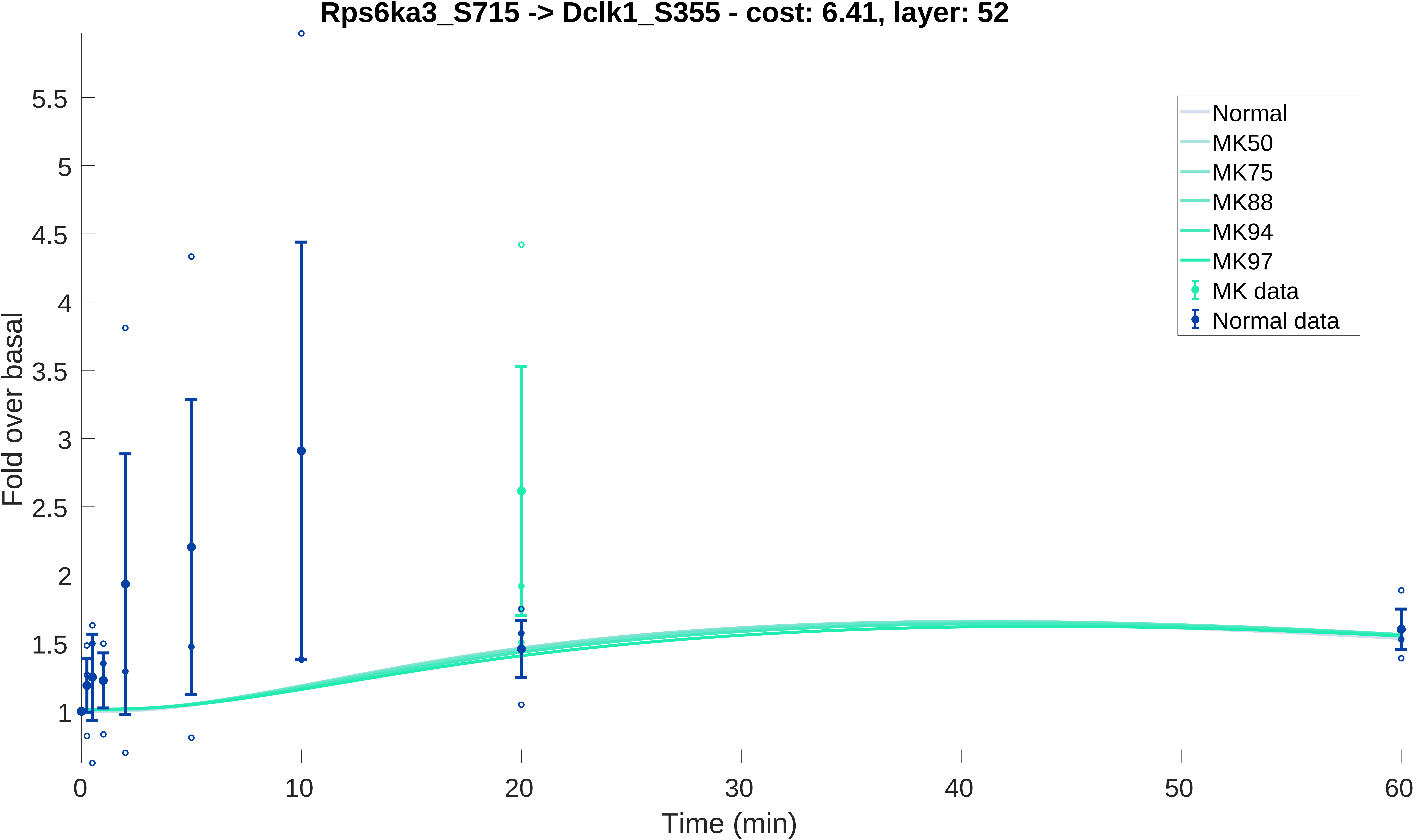

Rps6ka3_S715 -> Dclk1_S355 - cost: 6.41, layer: 52
Fold over basal
Time (min)
0
10
20
30
40
50
60
1
1.5
2
2.5
3
3.5
4
4.5
5
5.5
Normal
MK50
MK75
MK88
MK94
MK97
MK data
Normal data

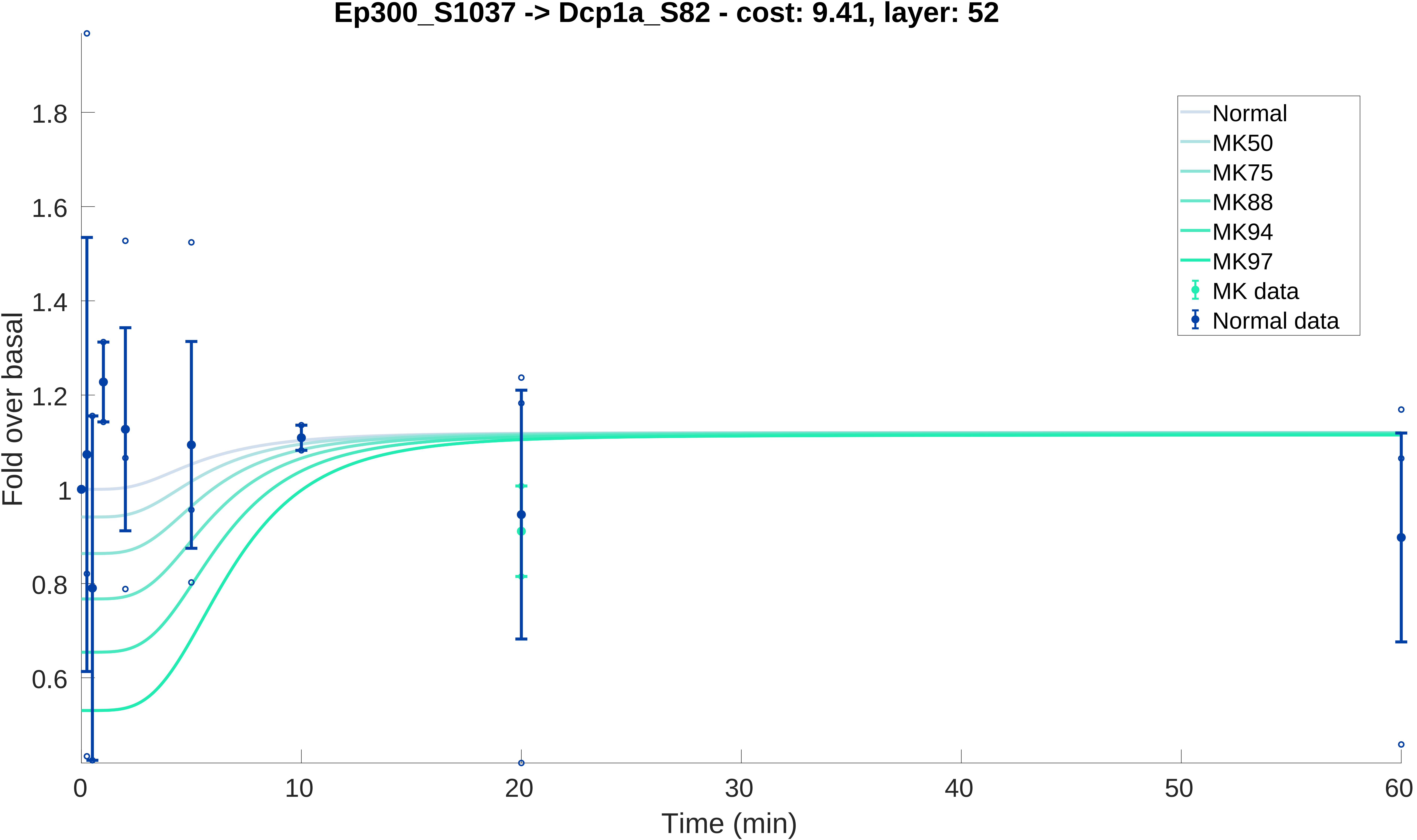

Ep300_S1037 -> Dcp1a_S82 - cost: 9.41, layer: 52
Fold over basal
Time (min)
0.6
0.8
1
1.2
1.4
1.6
1.8
0
10
20
30
40
50
60
Normal
MK50
MK75
MK88
MK94
MK97
MK data
Normal data

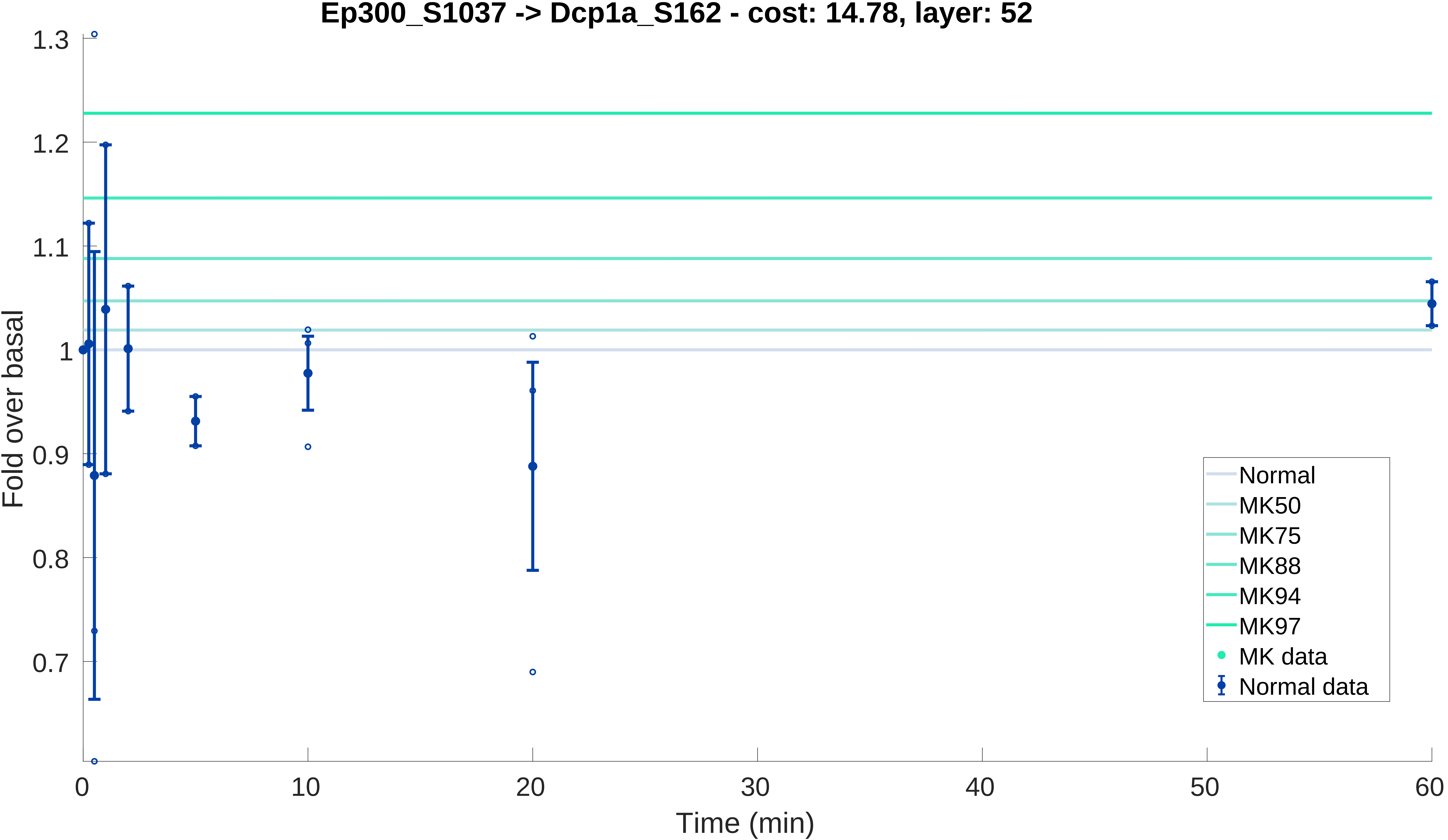

Ep300_S1037 -> Dcp1a_S162 - cost: 14.78, layer: 52
Fold over basal
Time (min)
1.3
1.2
1.1
1
0.9
0.8
0.7
0
10
20
30
40
50
60
Normal
MK50
MK75
MK88
MK94
MK97
MK data
Normal data

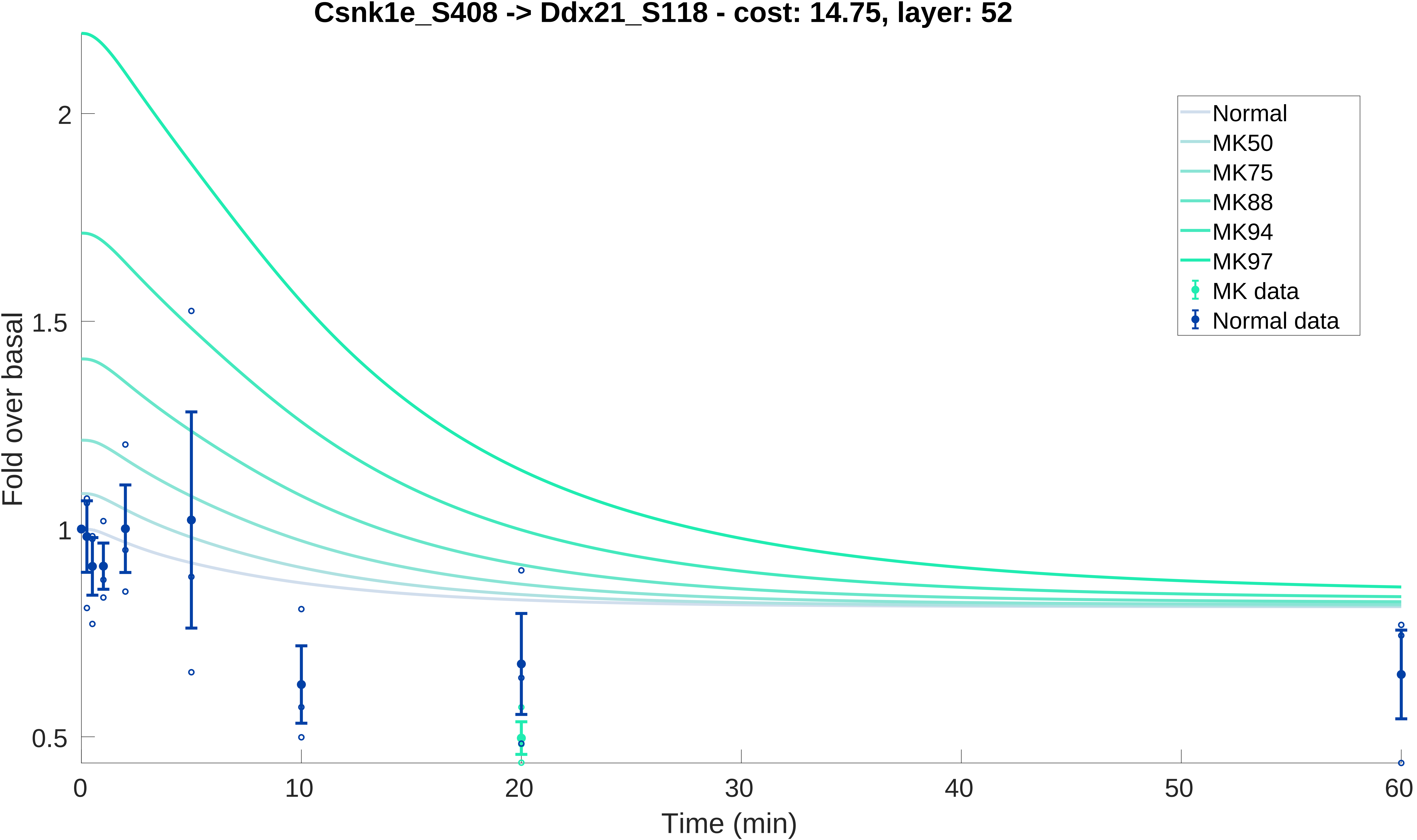

Csnk1e_S408 -> Ddx21_S118 - cost: 14.75, layer: 52
Fold over basal
Time (min)
Normal
MK50
MK75
MK88
MK94
MK97
MK data
Normal data
0.5
1
1.5
2
0
10
20
30
40
50
60

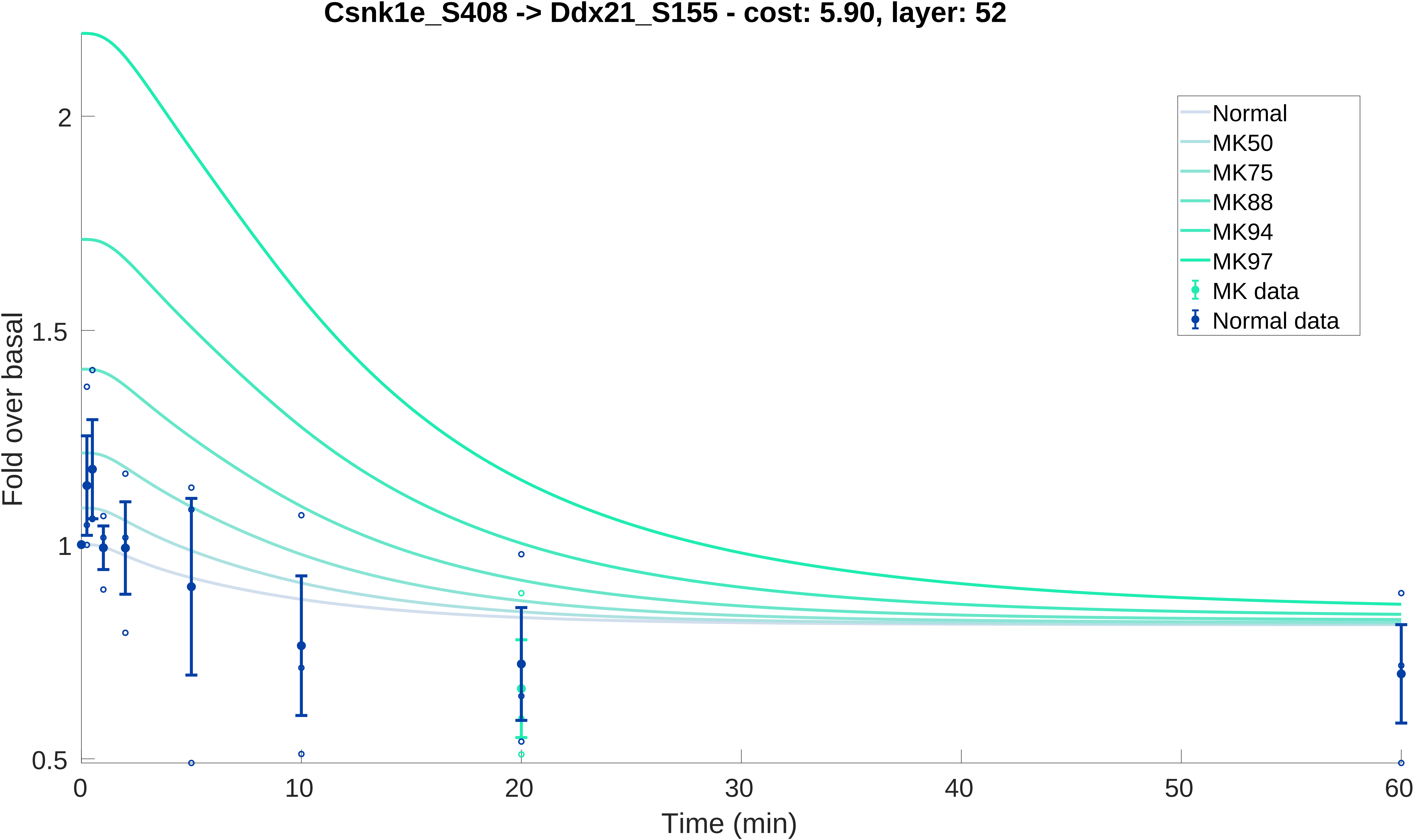
Csnk1e_S408 -> Ddx21_S155 - cost: 5.90, layer: 52
Fold over basal
Time (min)
0.5
1
1.5
2
0
10
20
30
40
50
60
Normal
MK50
MK75
MK88
MK94
MK97
MK data
Normal data

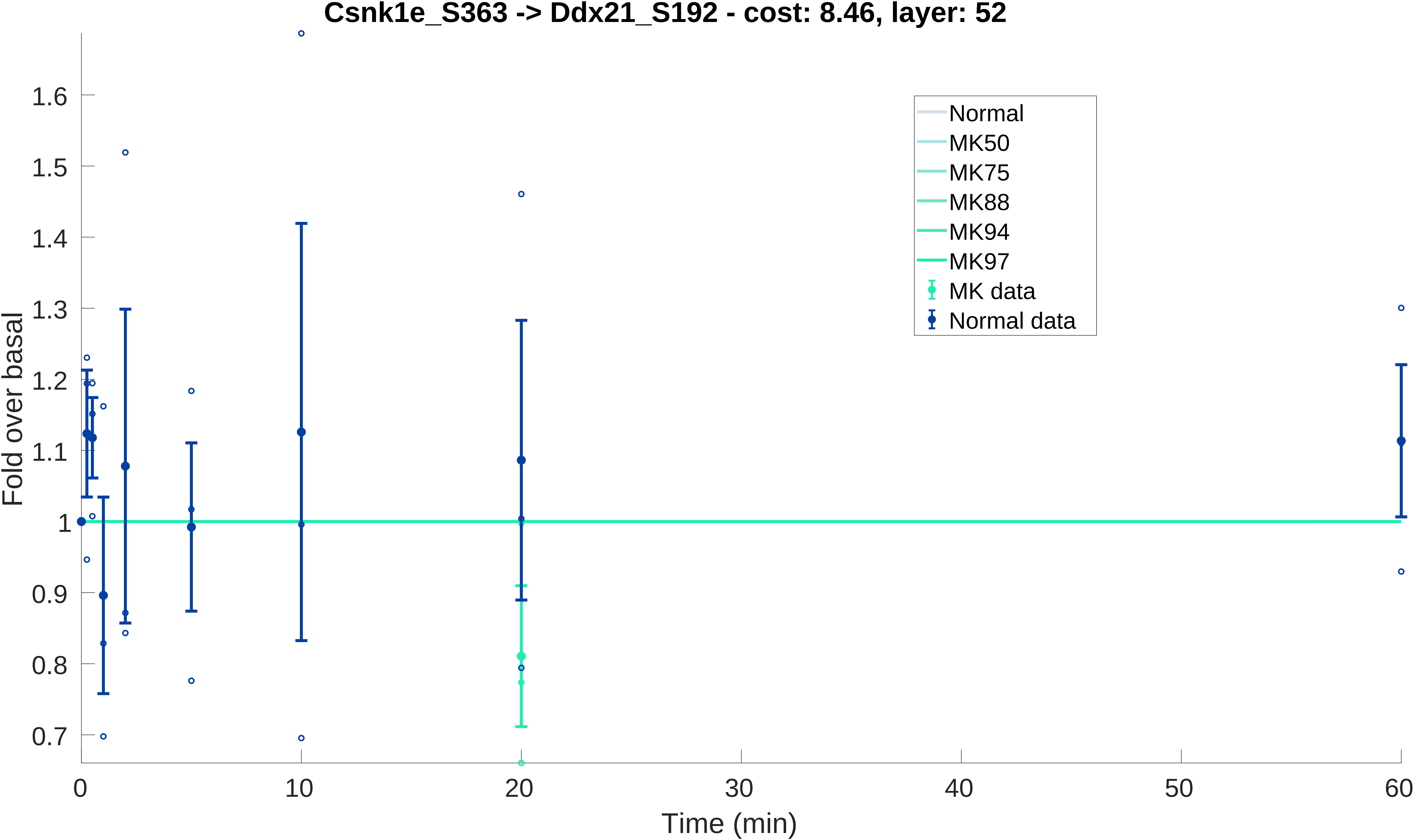

Csnk1e_S363 -> Ddx21_S192 - cost: 8.46, layer: 52
Fold over basal
1.6
1.5
1.4
1.3
1.2
1.1
1
0.9
0.8
0.7
0
10
20
30
40
50
60
Time (min)
Normal
MK50
MK75
MK88
MK94
MK97
MK data
Normal data

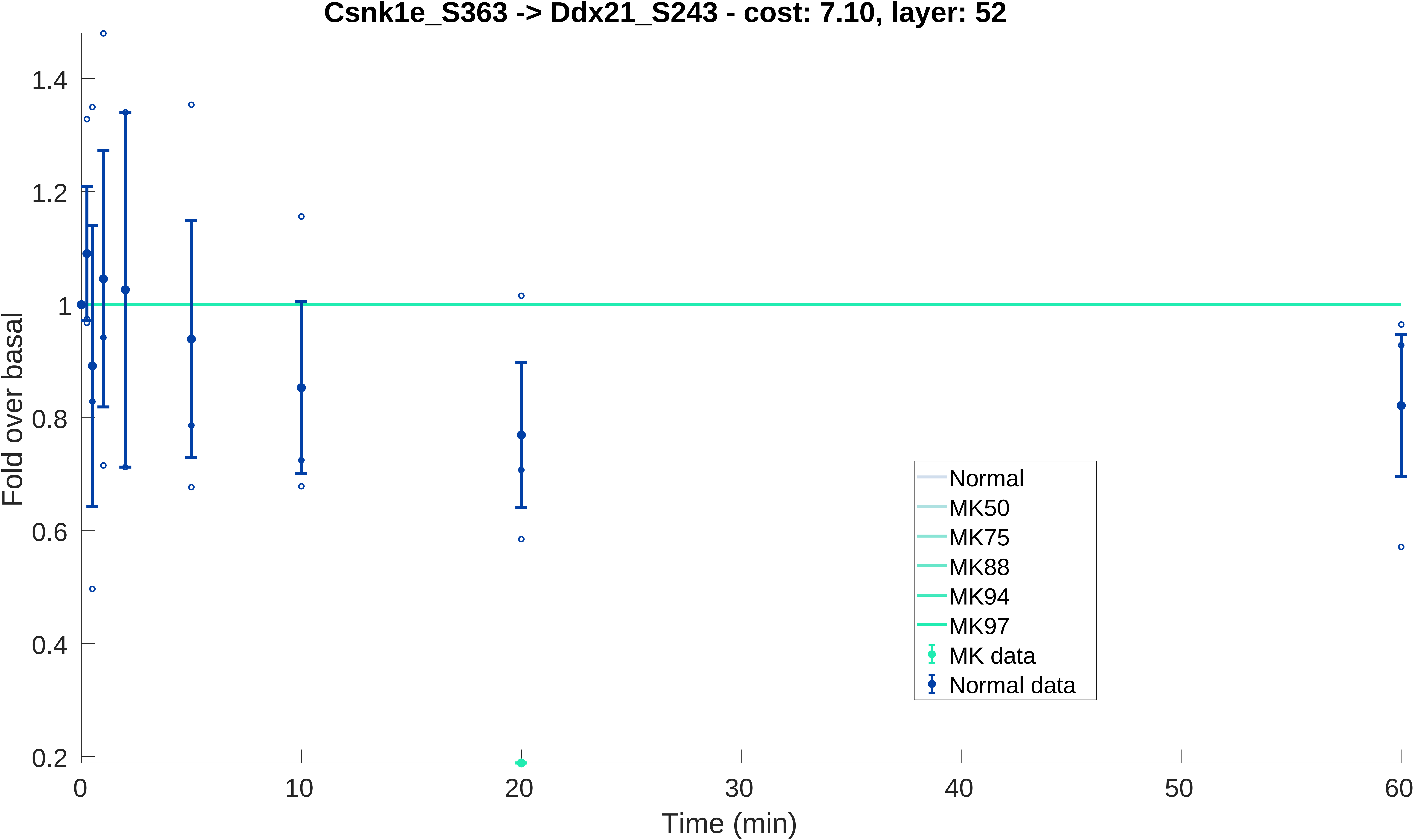

Csnk1e_S363 -> Ddx21_S243 - cost: 7.10, layer: 52
Fold over basal
Time (min)
1.4
1.2
1
0.8
0.6
0.4
0.2
0
10
20
30
40
50
60
Normal
MK50
MK75
MK88
MK94
MK97
MK data
Normal data

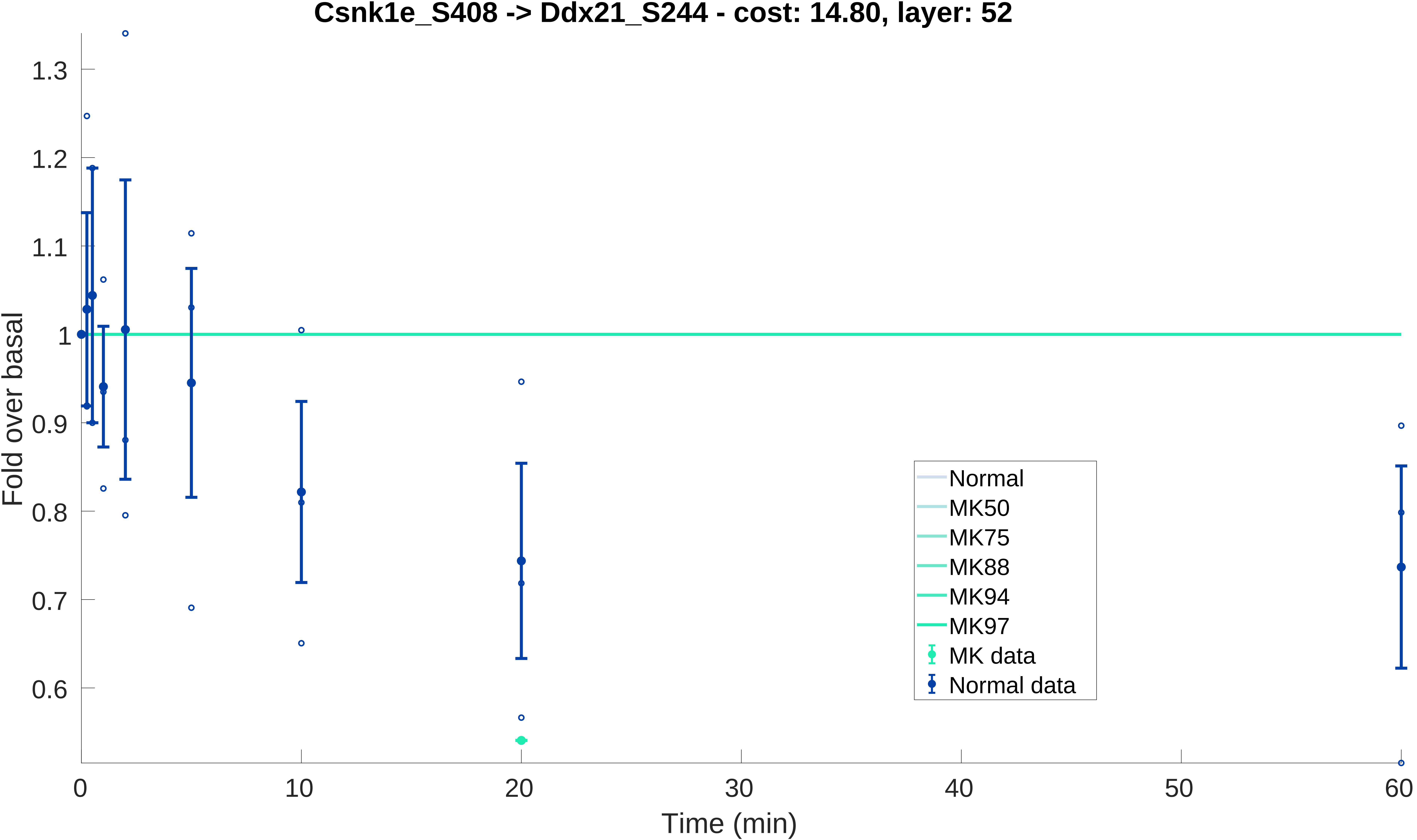

Csnk1e_S408 -> Ddx21_S244 - cost: 14.80, layer: 52
Fold over basal
Time (min)
0
10
20
30
40
50
60
0.6
0.7
0.8
0.9
1
1.1
1.2
1.3
Normal
MK50
MK75
MK88
MK94
MK97
MK data
Normal data

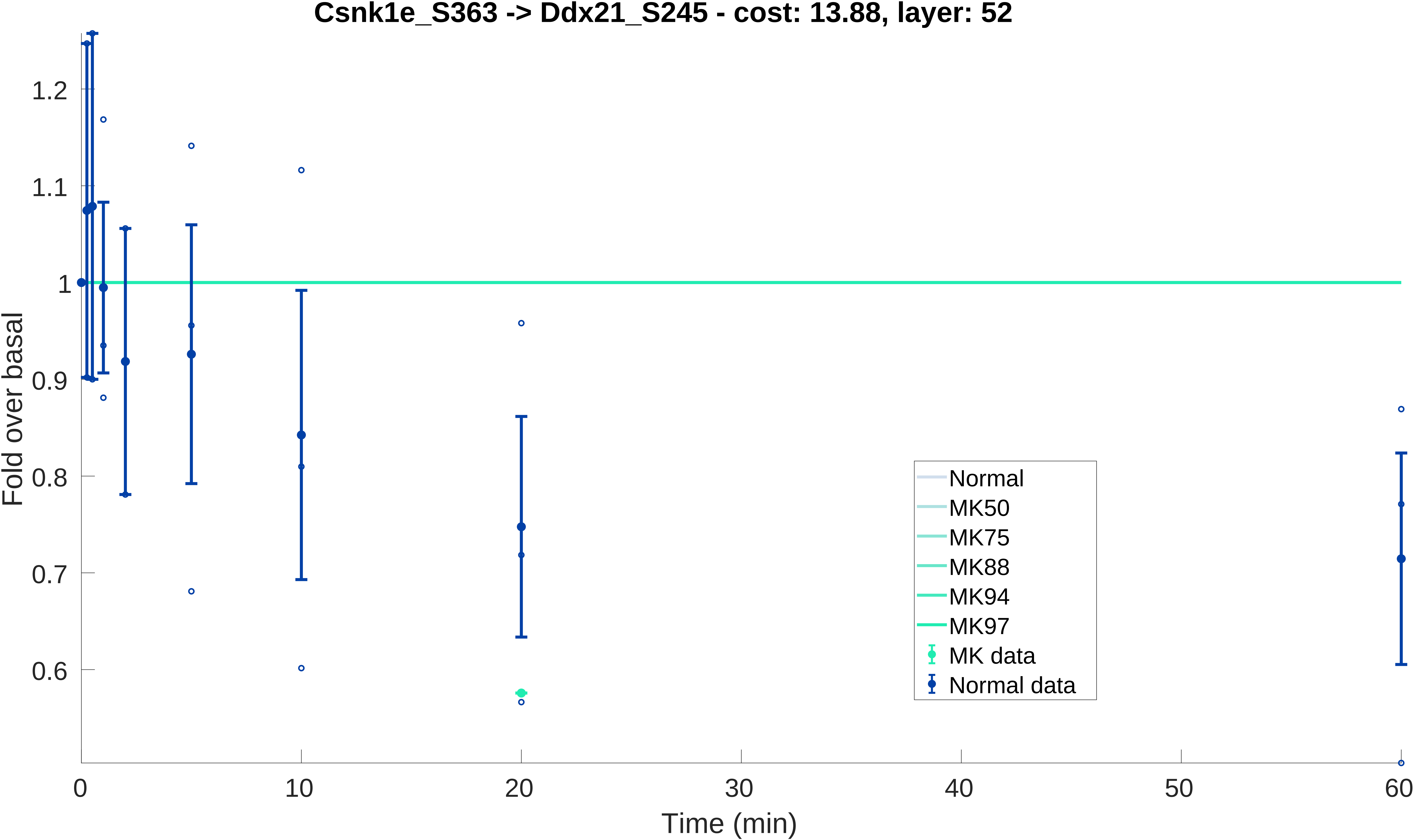

Csnk1e_S363 -> Ddx21_S245 - cost: 13.88, layer: 52
Fold over basal
1.2
1.1
1
0.9
0.8
0.7
0.6
0
10
20
30
40
50
60
Time (min)
Normal
MK50
MK75
MK88
MK94
MK97
MK data
Normal data

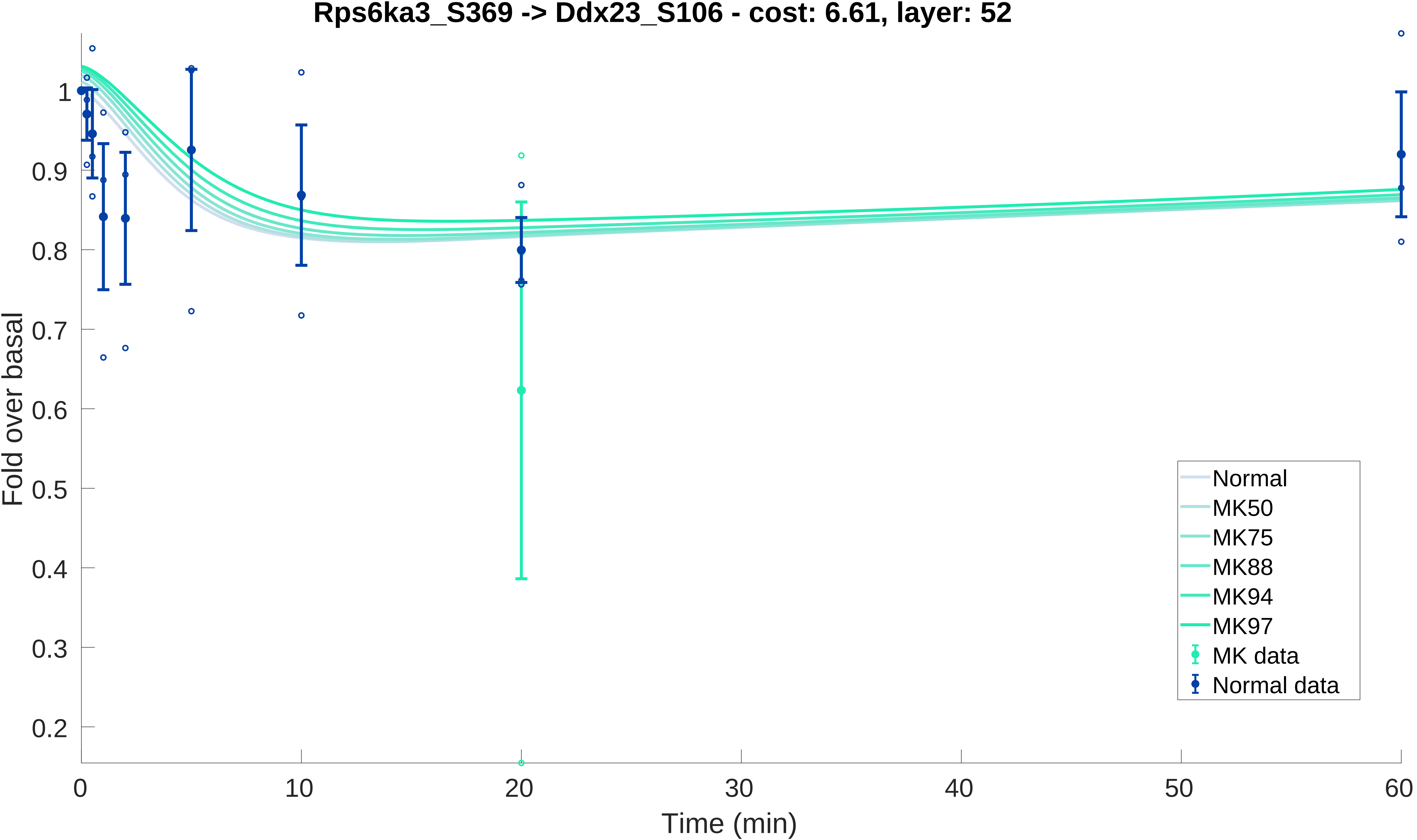

Rps6ka3_S369 -> Ddx23_S106 - cost: 6.61, layer: 52
Fold over basal
Time (min)
1
0.9
0.8
0.7
0.6
0.5
0.4
0.3
0.2
0
10
20
30
40
50
60
Normal
MK50
MK75
MK88
MK94
MK97
MK data
Normal data

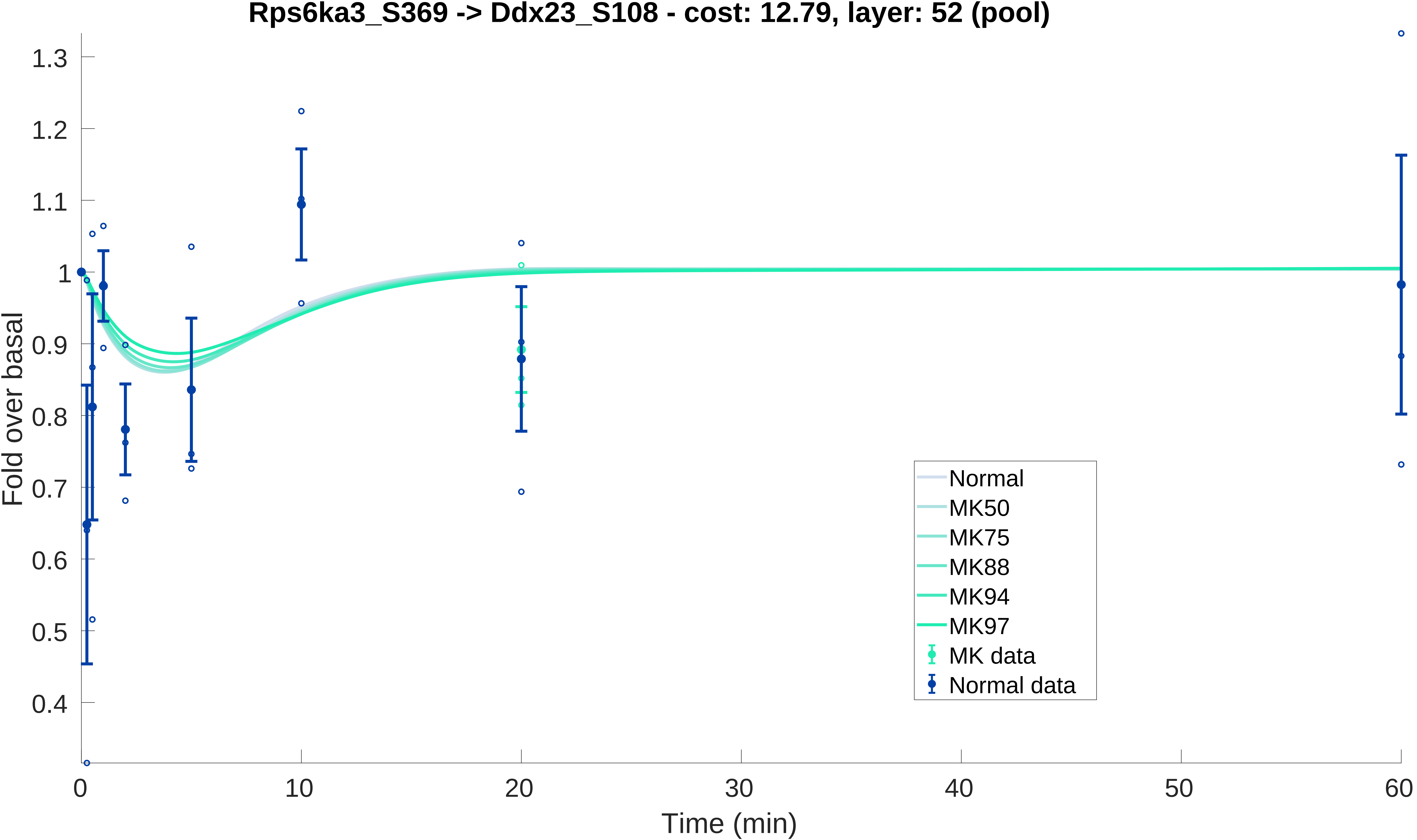
Rps6ka3_S369 -> Ddx23_S108 - cost: 12.79, layer: 52 (pool)
Fold over basal
Time (min)
1.3
1.2
1.1
1
0.9
0.8
0.7
0.6
0.5
0.4
0
10
20
30
40
50
60
Normal
MK50
MK75
MK88
MK94
MK97
MK data
Normal data

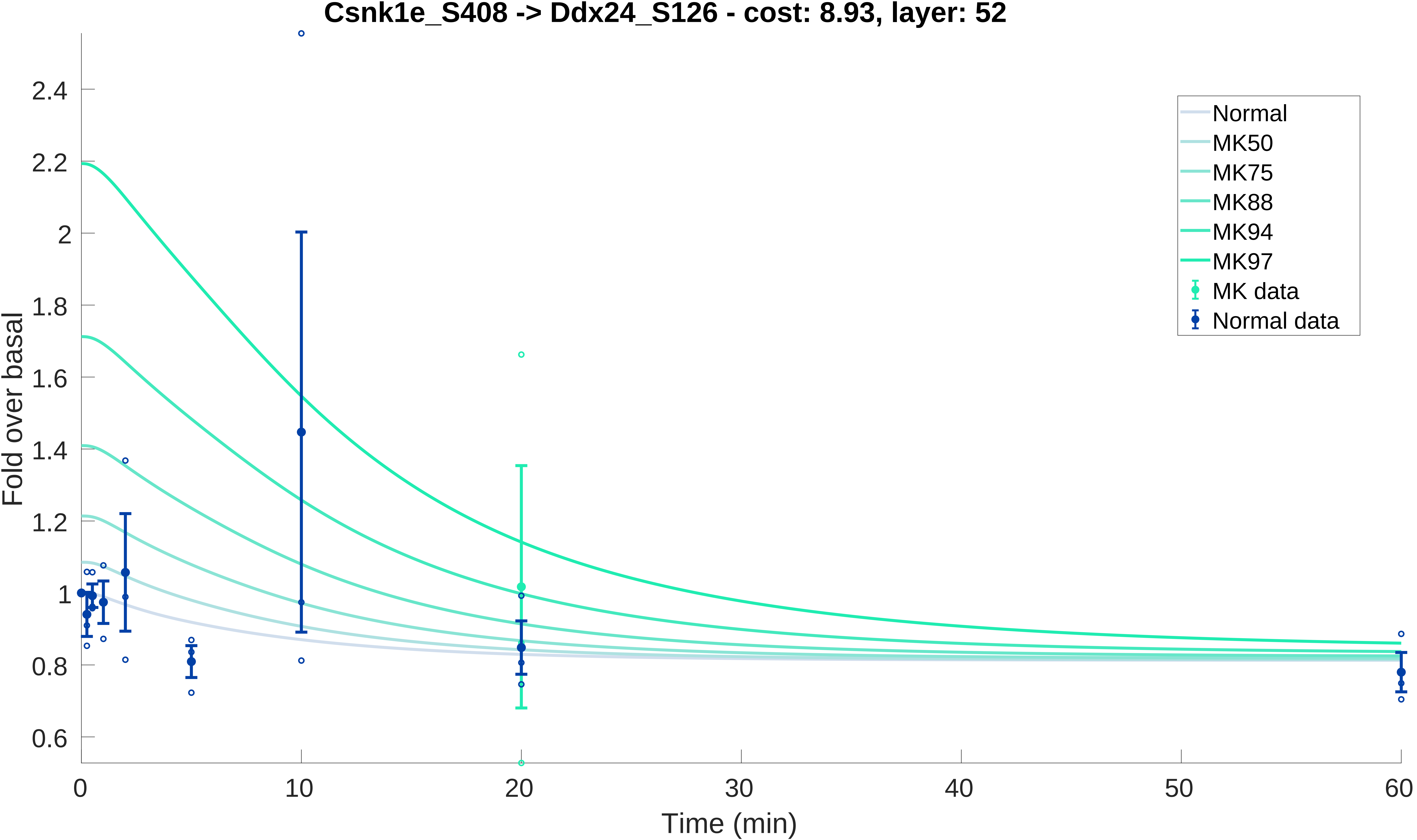
Csnk1e_S408 -> Ddx24_S126 - cost: 8.93, layer: 52
Fold over basal
Time (min)
0.6
0.8
1
1.2
1.4
1.6
1.8
2
2.2
2.4
0
10
20
30
40
50
60
Normal
MK50
MK75
MK88
MK94
MK97
MK data
Normal data

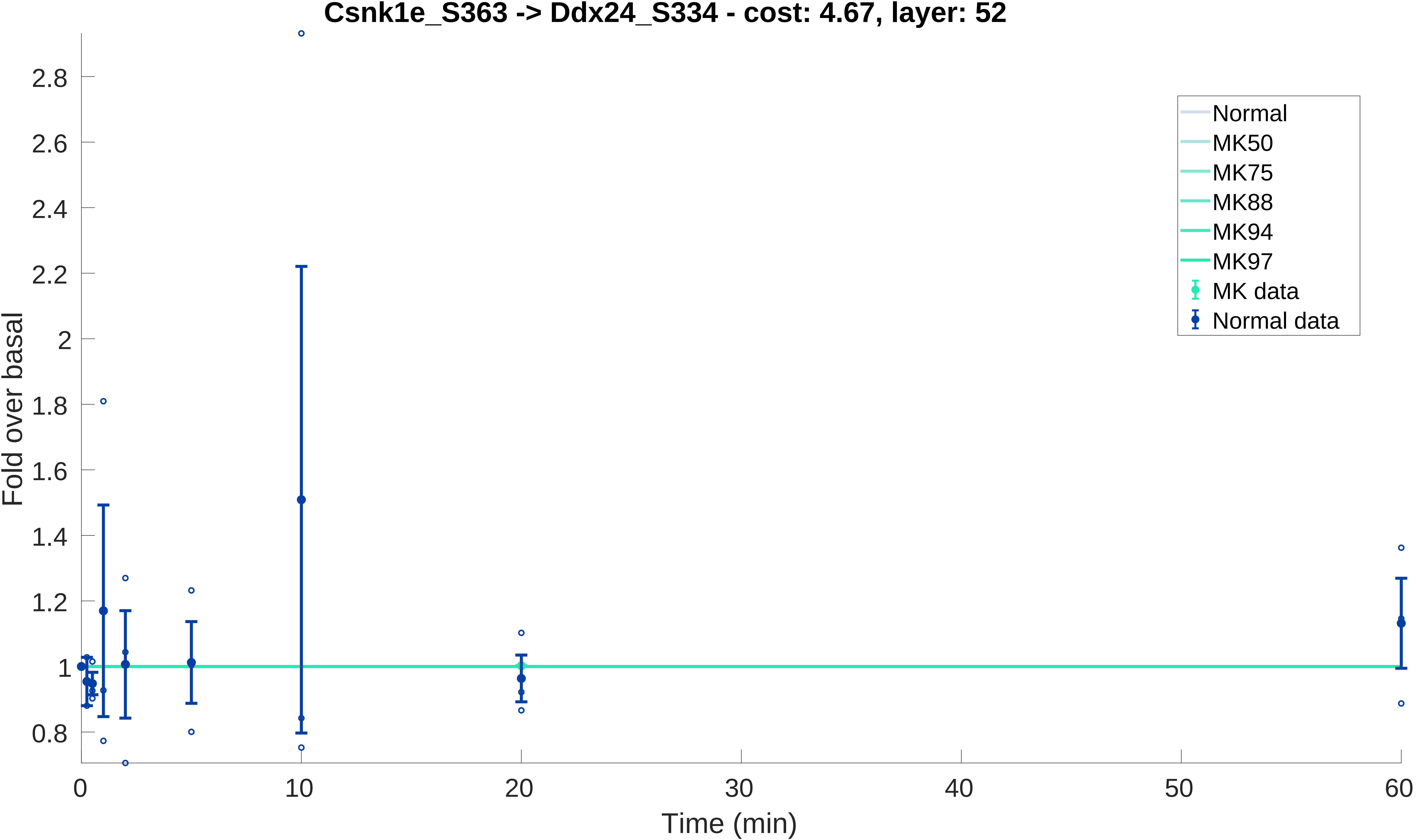

Csnk1e_S363 -> Ddx24_S334 - cost: 4.67, layer: 52
Fold over basal
Time (min)
Normal
MK50
MK75
MK88
MK94
MK97
MK data
Normal data
2.8
2.6
2.4
2.2
2
1.8
1.6
1.4
1.2
1
0.8
0
10
20
30
40
50
60

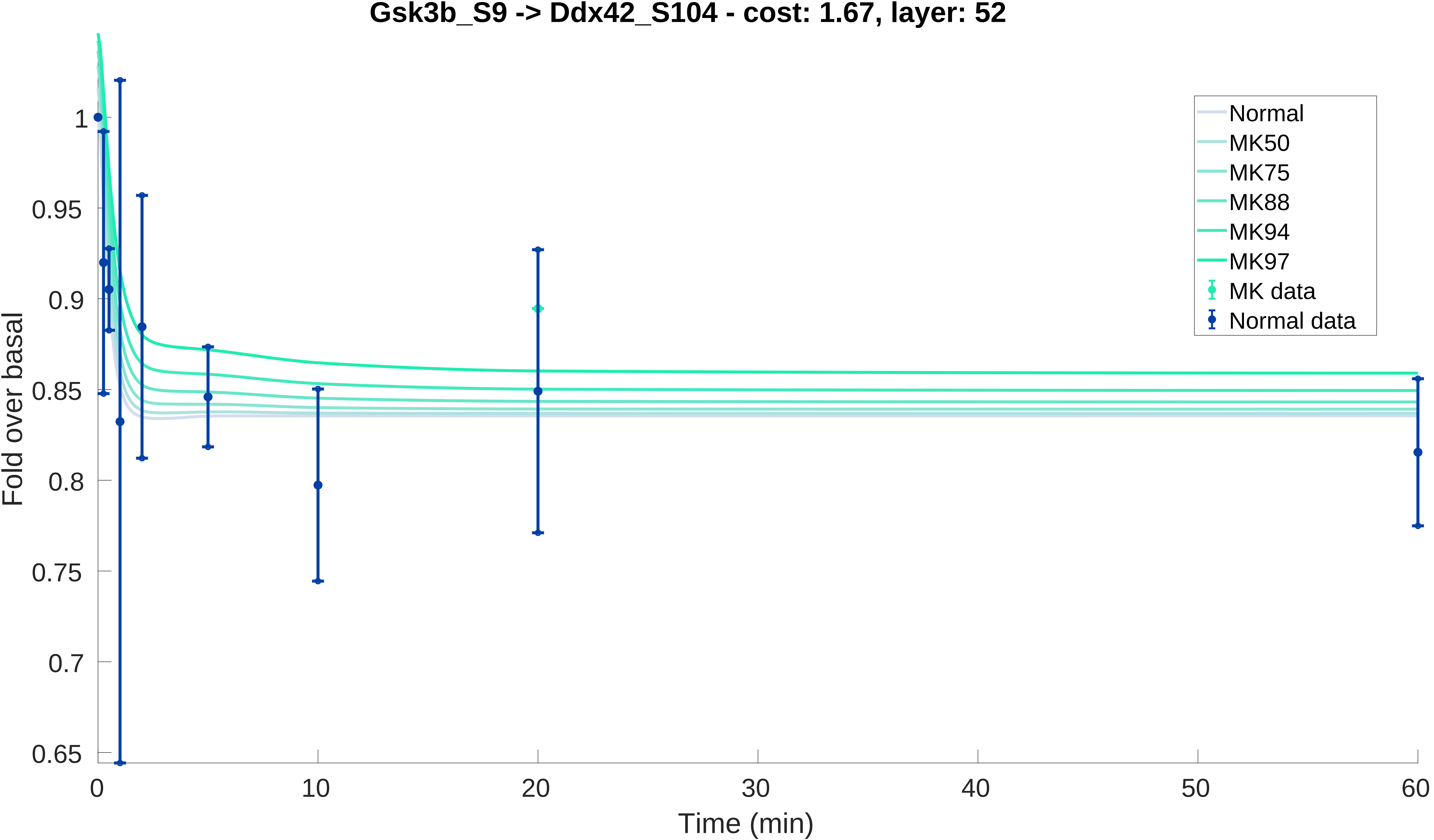

Gsk3b_S9 -> Ddx42_S104 - cost: 1.67, layer: 52
Fold over basal
Time (min)
1
0.95
0.9
0.85
0.8
0.75
0.7
0.65
0
10
20
30
40
50
60
Normal
MK50
MK75
MK88
MK94
MK97
MK data
Normal data

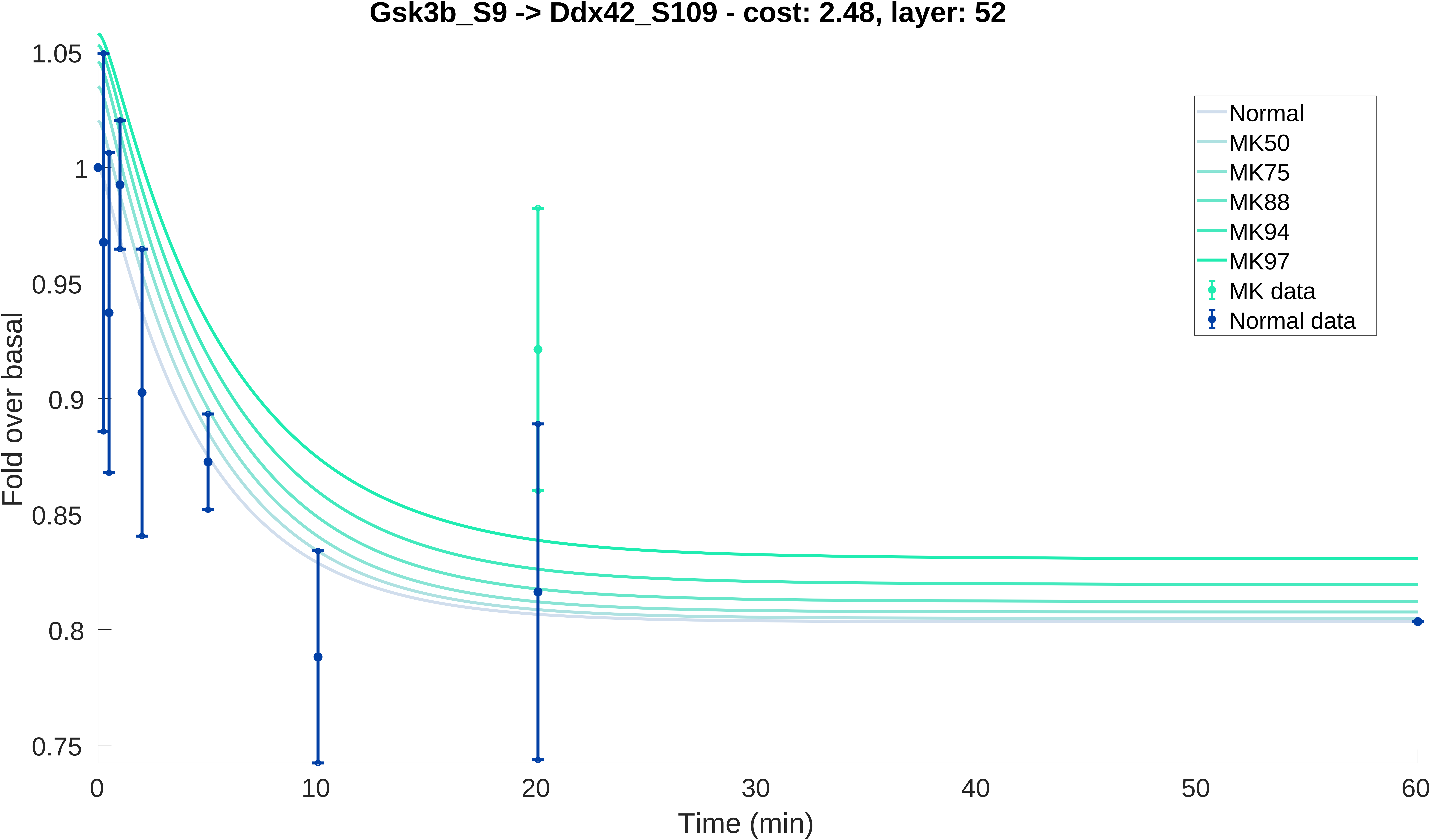

Gsk3b_S9 -> Ddx42_S109 - cost: 2.48, layer: 52
Fold over basal
Time (min)
1.05
1
0.95
0.9
0.85
0.8
0.75
0
10
20
30
40
50
60
Normal
MK50
MK75
MK88
MK94
MK97
MK data
Normal data

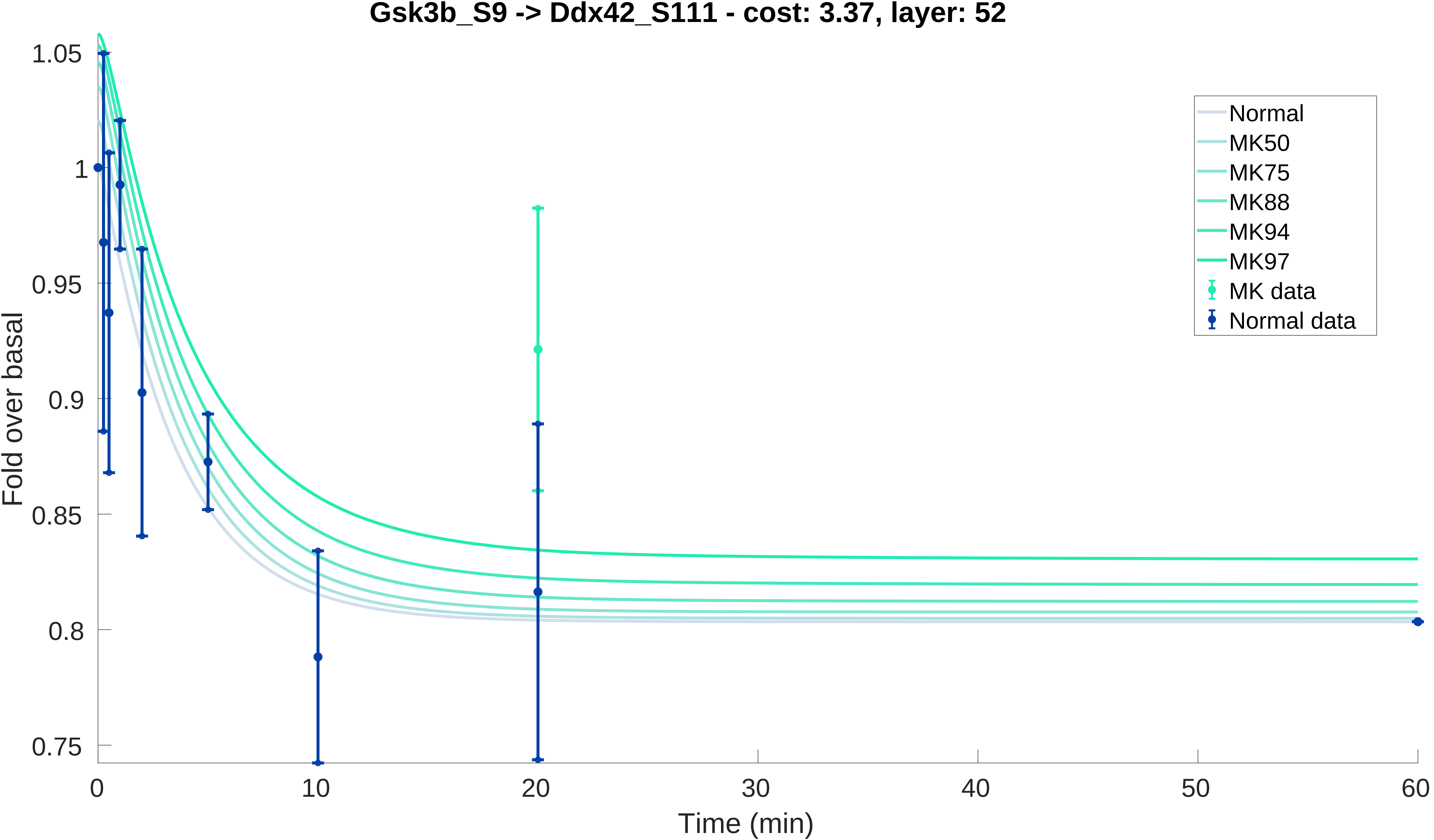

Gsk3b_S9 -> Ddx42_S111 - cost: 3.37, layer: 52
Fold over basal
1.05
1
0.95
0.9
0.85
0.8
0.75
0
10
20
30
40
50
60
Time (min)
Normal
MK50
MK75
MK88
MK94
MK97
MK data
Normal data

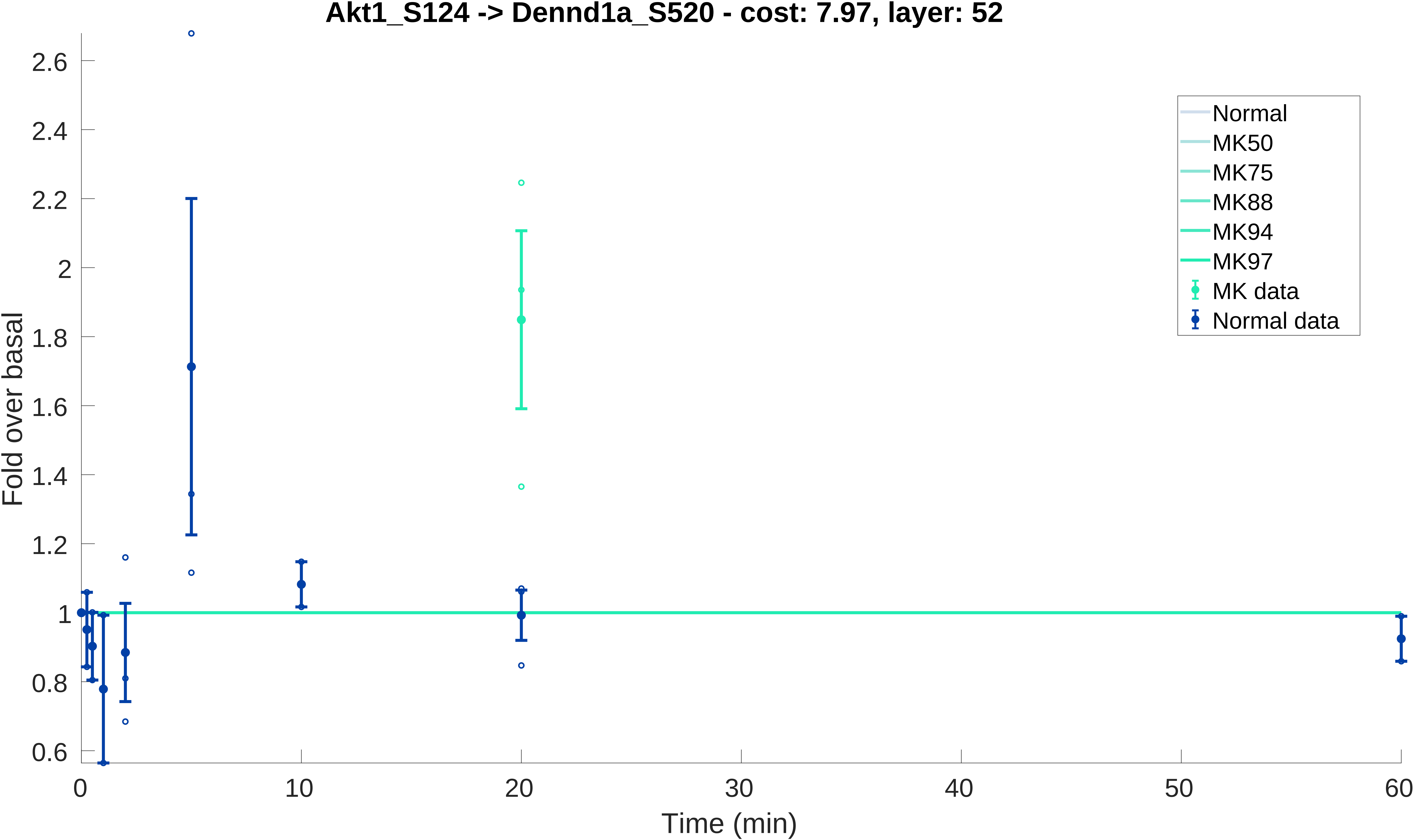

Akt1_S124 -> Dennd1a_S520 - cost: 7.97, layer: 52
Fold over basal
Time (min)
2.6
2.4
2.2
2
1.8
1.6
1.4
1.2
1
0.8
0.6
0
10
20
30
40
50
60
Normal
MK50
MK75
MK88
MK94
MK97
MK data
Normal data

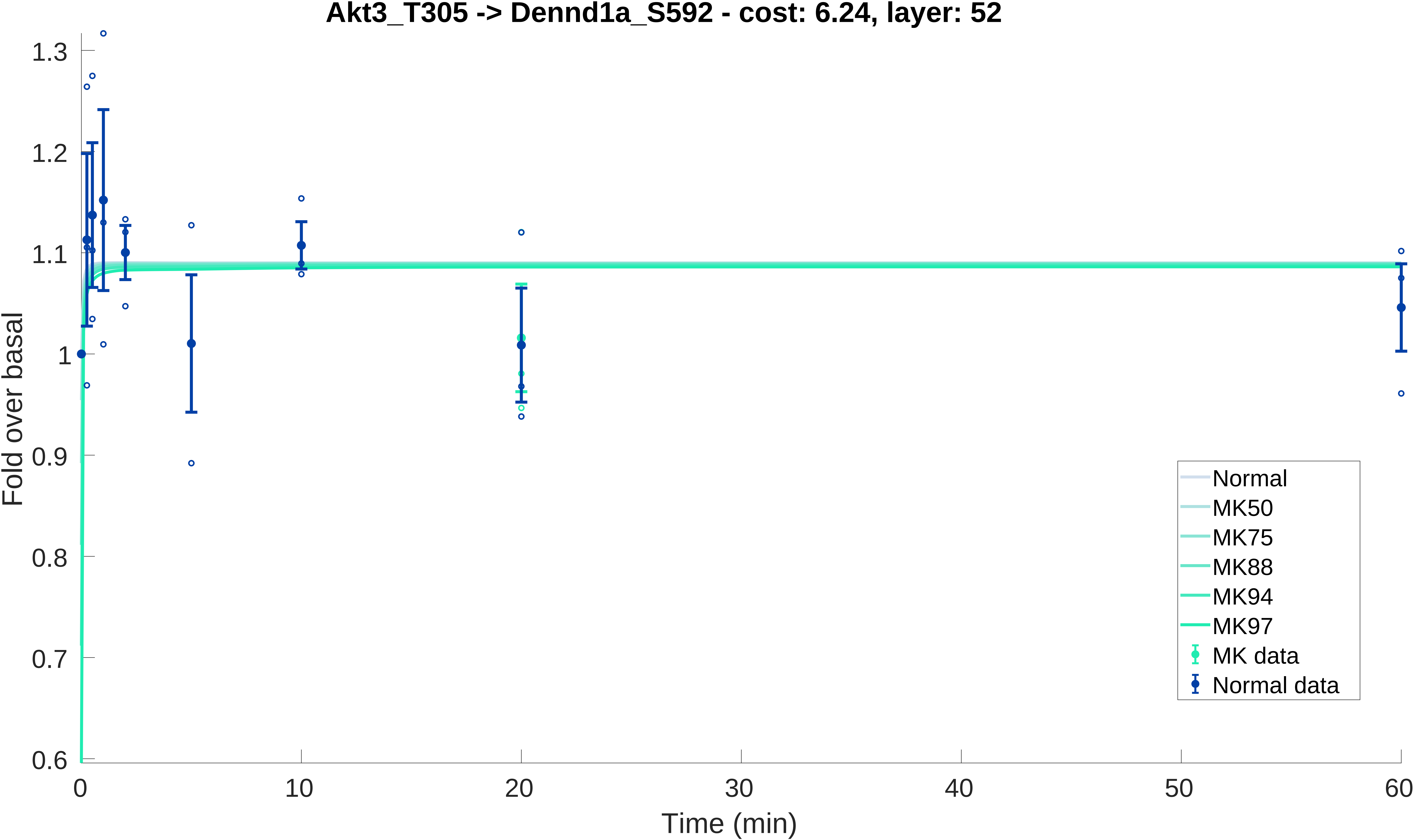
Akt3_T305 -> Dennd1a_S592 - cost: 6.24, layer: 52
Fold over basal
Time (min)
1.3
1.2
1.1
1
0.9
0.8
0.7
0.6
0
10
20
30
40
50
60
Normal
MK50
MK75
MK88
MK94
MK97
MK data
Normal data

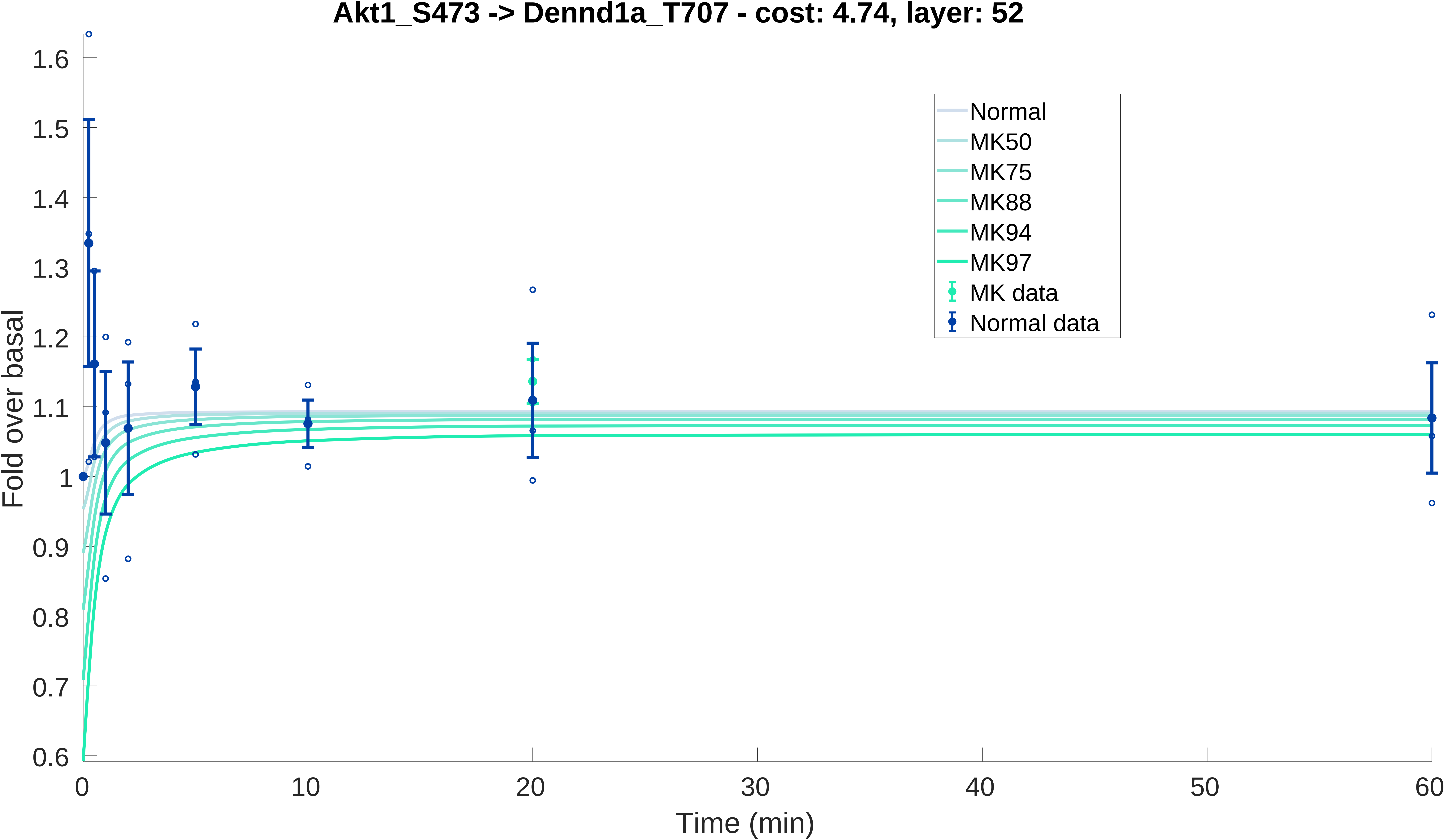

Akt1_S473 -> Dennd1a_T707 - cost: 4.74, layer: 52
Fold over basal
Time (min)
1.6
1.5
1.4
1.3
1.2
1.1
1
0.9
0.8
0.7
0.6
0
10
20
30
40
50
60
Normal
MK50
MK75
MK88
MK94
MK97
MK data
Normal data

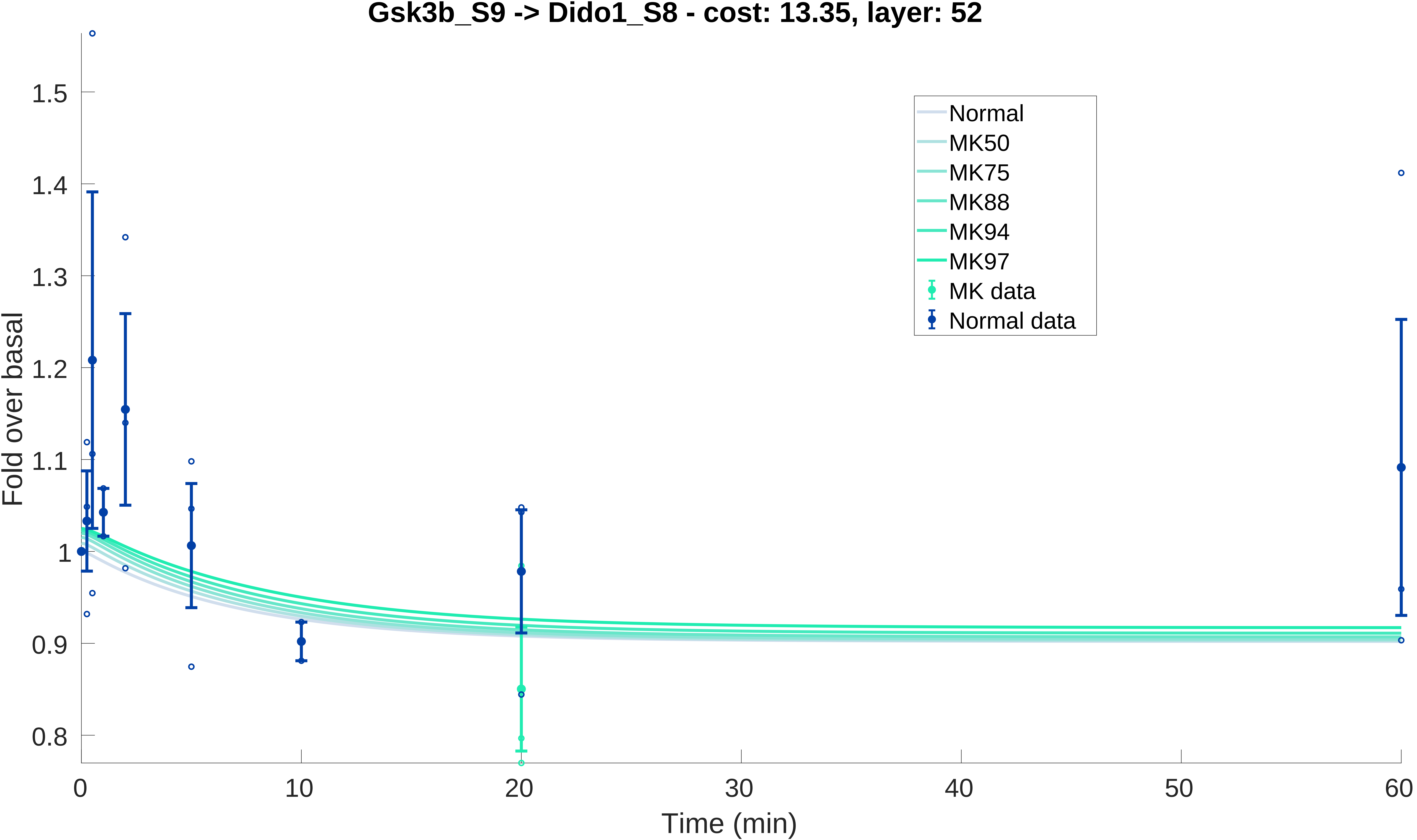

Gsk3b_S9 -> Dido1_S8 - cost: 13.35, layer: 52
Fold over basal
Time (min)
0
10
20
30
40
50
60
0.8
0.9
1
1.1
1.2
1.3
1.4
1.5
Normal
MK50
MK75
MK88
MK94
MK97
MK data
Normal data

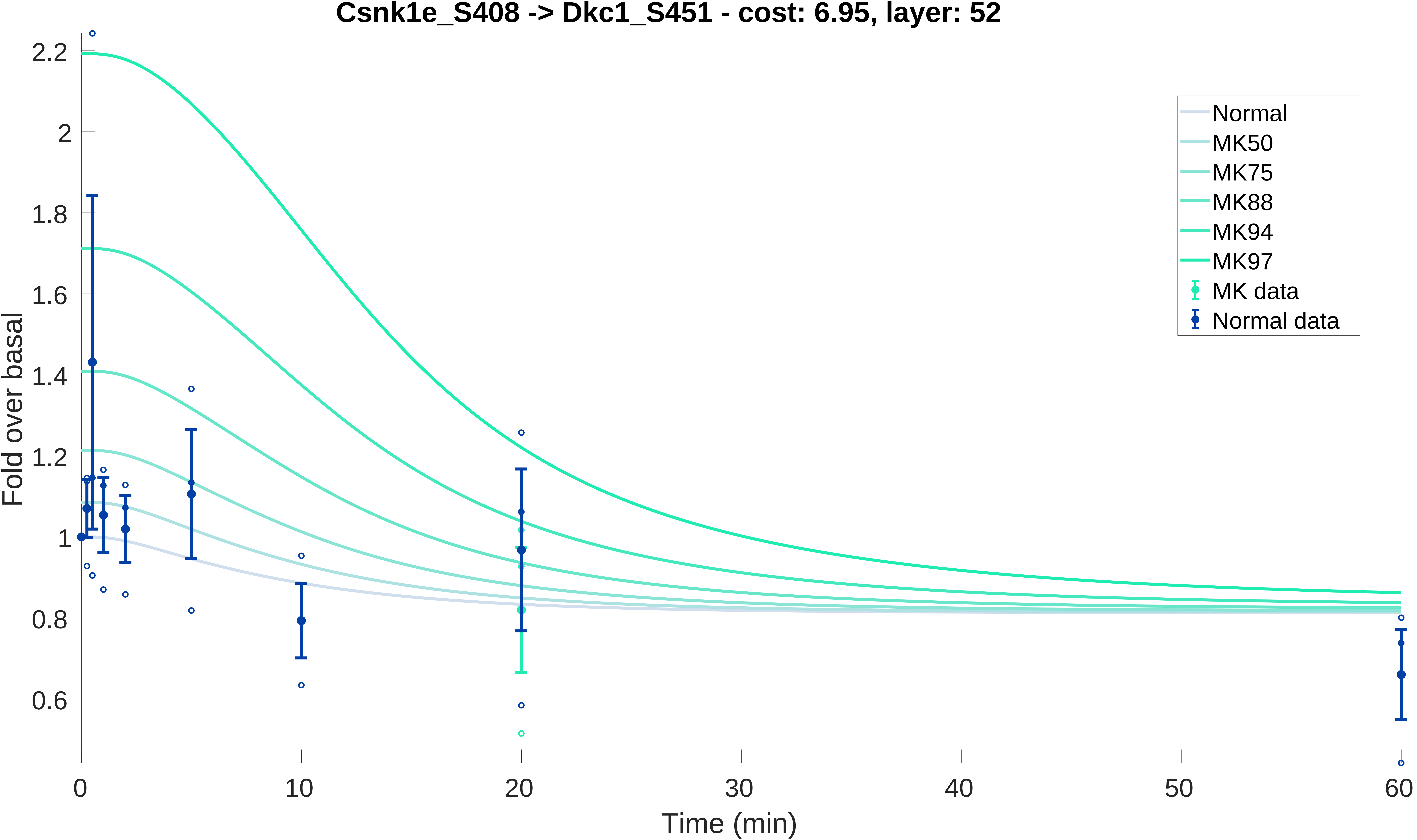

Csnk1e_S408 -> Dkc1_S451 - cost: 6.95, layer: 52
Fold over basal
Time (min)
0.6
0.8
1
1.2
1.4
1.6
1.8
2
2.2
0
10
20
30
40
50
60
Normal
MK50
MK75
MK88
MK94
MK97
MK data
Normal data

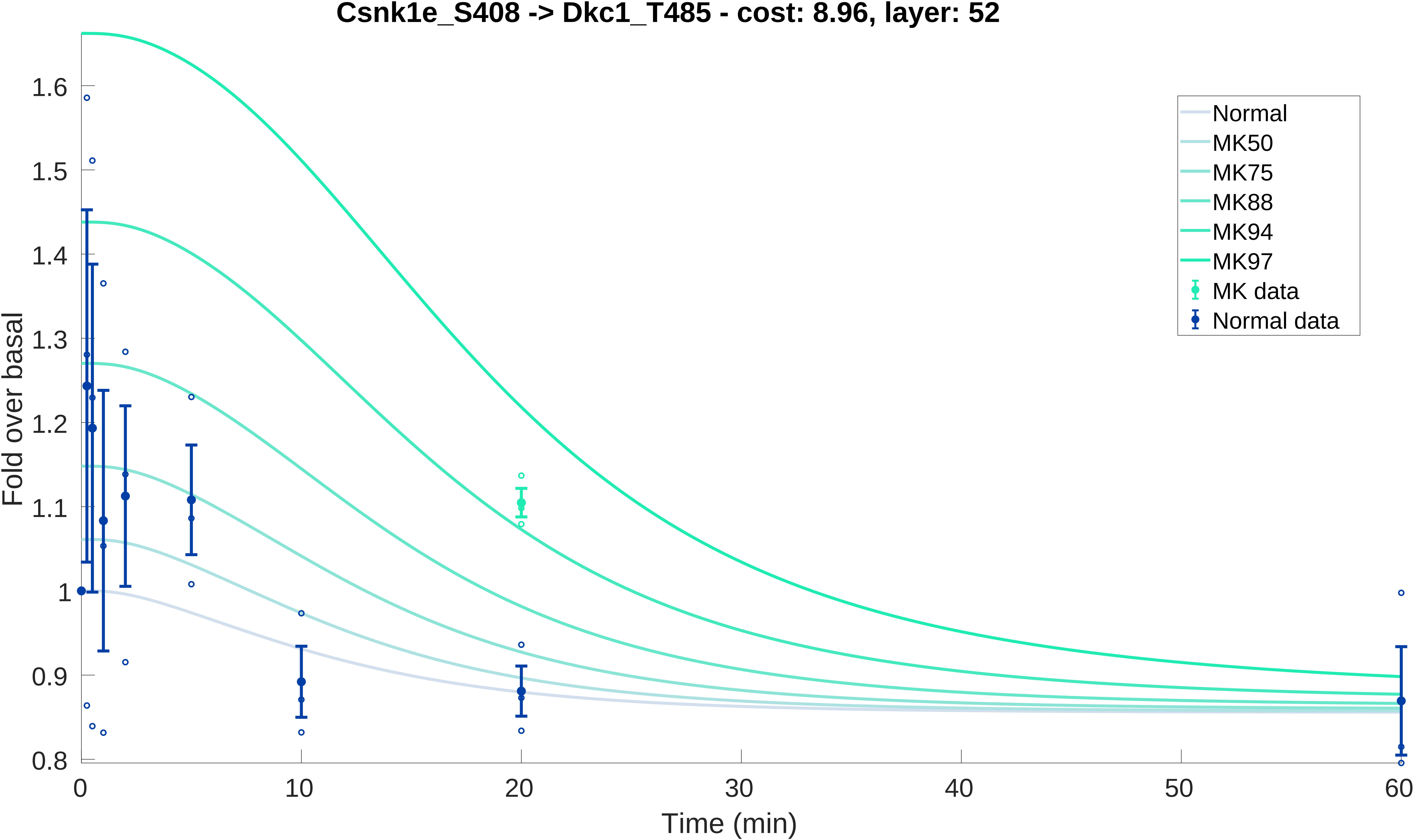

Csnk1e_S408 -> Dkc1_T485 - cost: 8.96, layer: 52
Fold over basal
Time (min)
0.8
0.9
1
1.1
1.2
1.3
1.4
1.5
1.6
0
10
20
30
40
50
60
Normal
MK50
MK75
MK88
MK94
MK97
MK data
Normal data

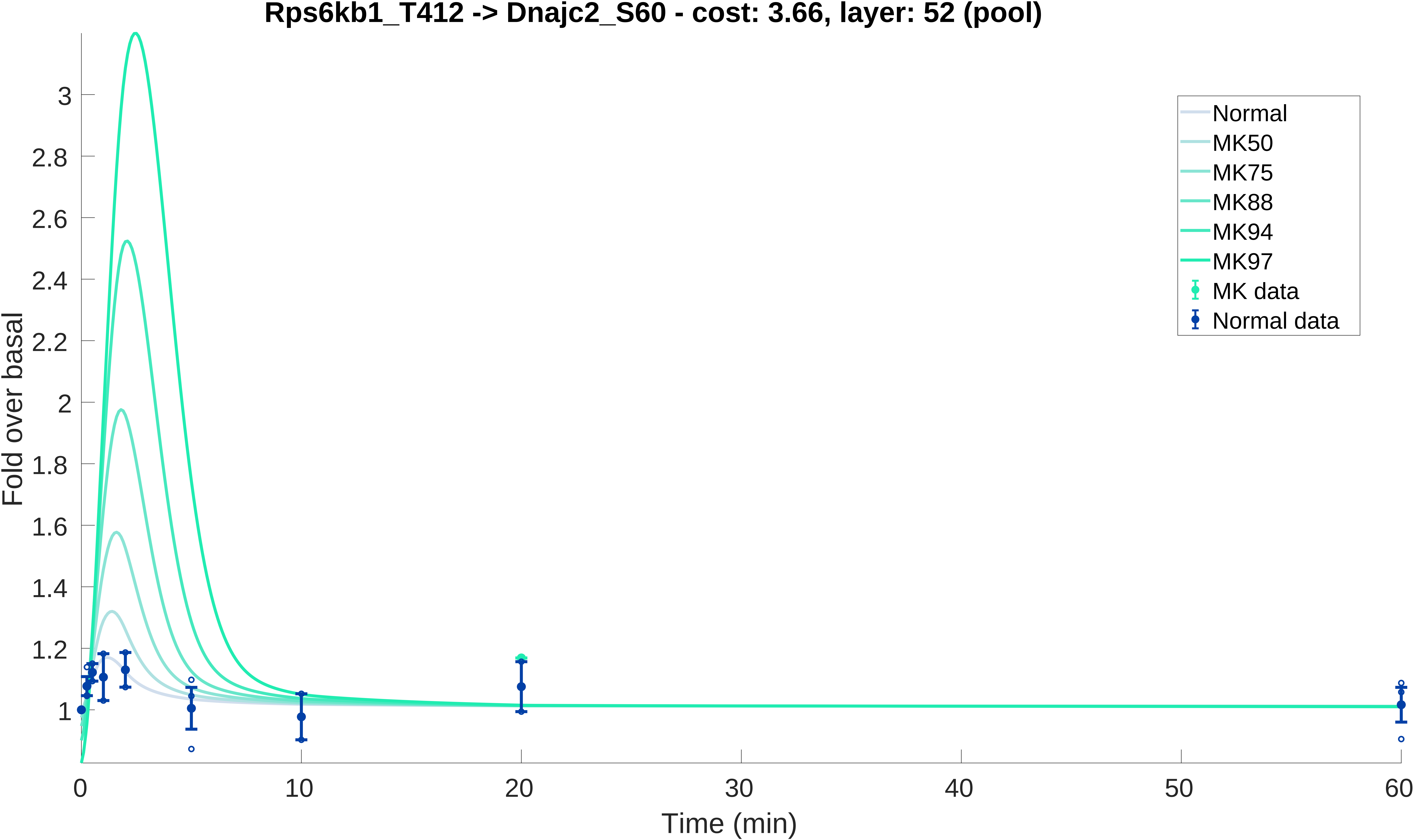
Rps6kb1_T412 -> Dnajc2_S60 - cost: 3.66, layer: 52 (pool)
Fold over basal
Time (min)
3
2.8
2.6
2.4
2.2
2
1.8
1.6
1.4
1.2
1
0
10
20
30
40
50
60
Normal
MK50
MK75
MK88
MK94
MK97
MK data
Normal data

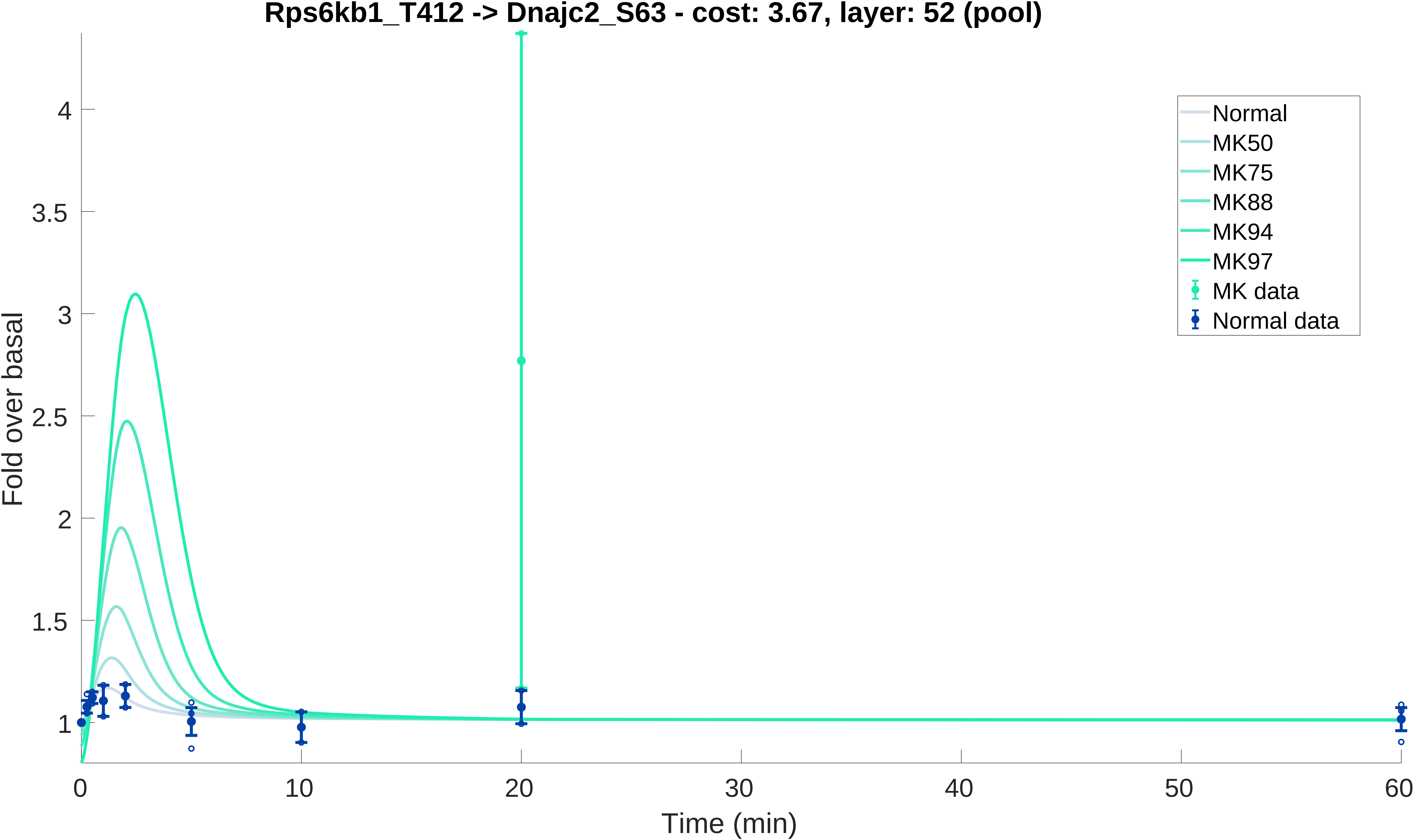

Rps6kb1_T412 -> Dnajc2_S63 - cost: 3.67, layer: 52 (pool)
Fold over basal
Time (min)
4
3.5
3
2.5
2
1.5
1
0
10
20
30
40
50
60
Normal
MK50
MK75
MK88
MK94
MK97
MK data
Normal data

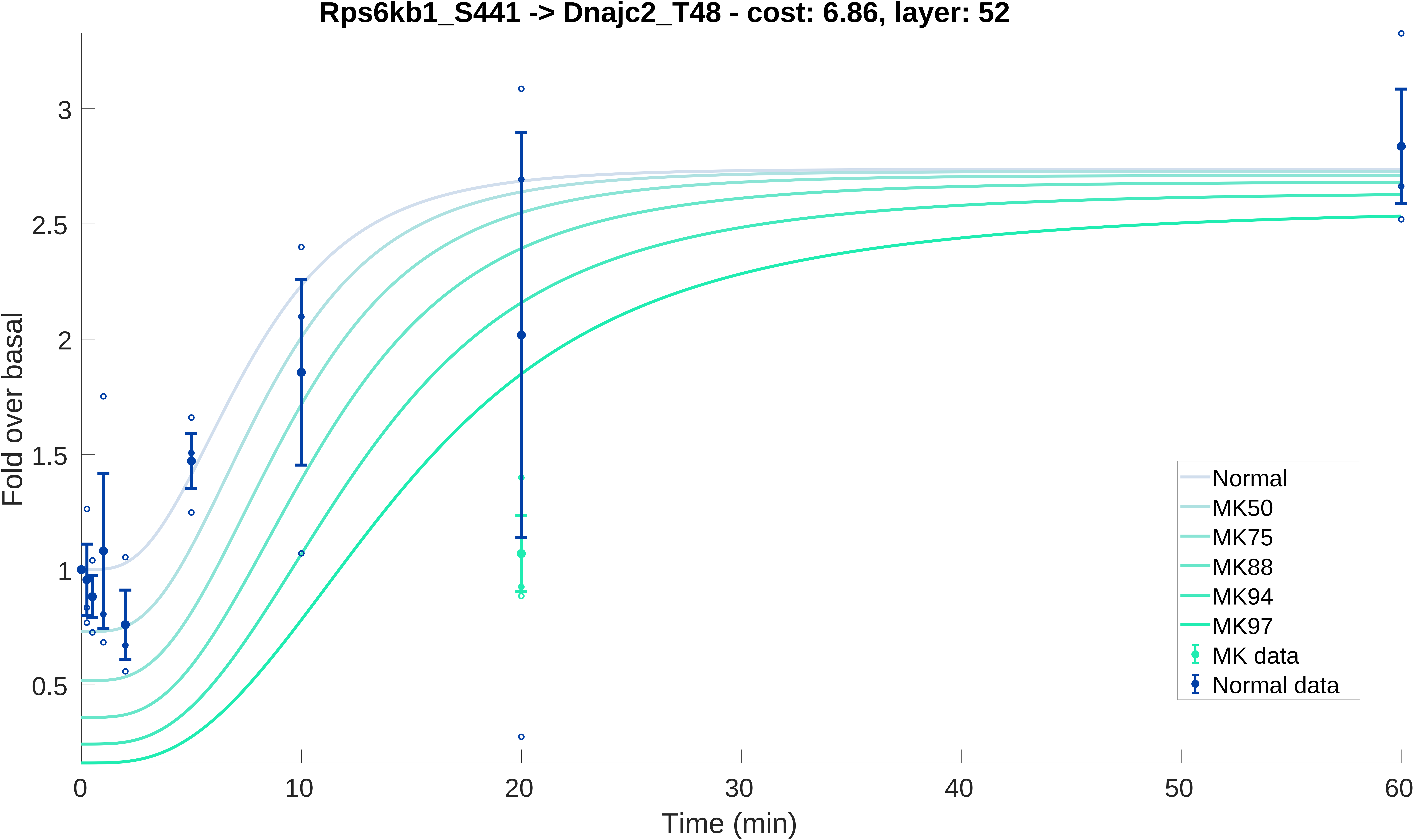

Rps6kb1_S441 -> Dnajc2_T48 - cost: 6.86, layer: 52
Fold over basal
0.5
1
1.5
2
2.5
3
0
10
20
30
40
50
60
Time (min)
Normal
MK50
MK75
MK88
MK94
MK97
MK data
Normal data

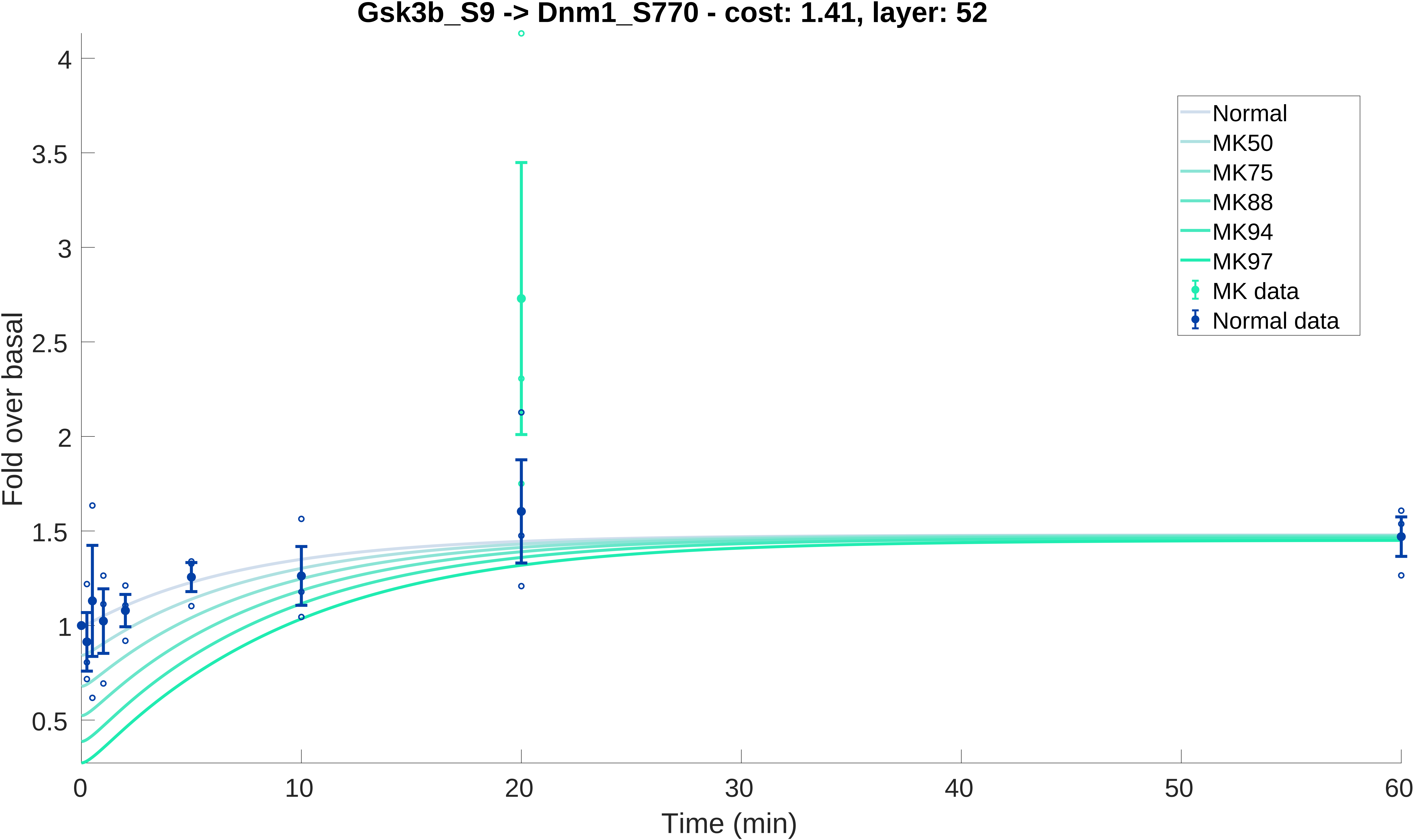

Gsk3b_S9 -> Dnm1_S770 - cost: 1.41, layer: 52
Fold over basal
Time (min)
0.5
1
1.5
2
2.5
3
3.5
4
0
10
20
30
40
50
60
Normal
MK50
MK75
MK88
MK94
MK97
MK data
Normal data

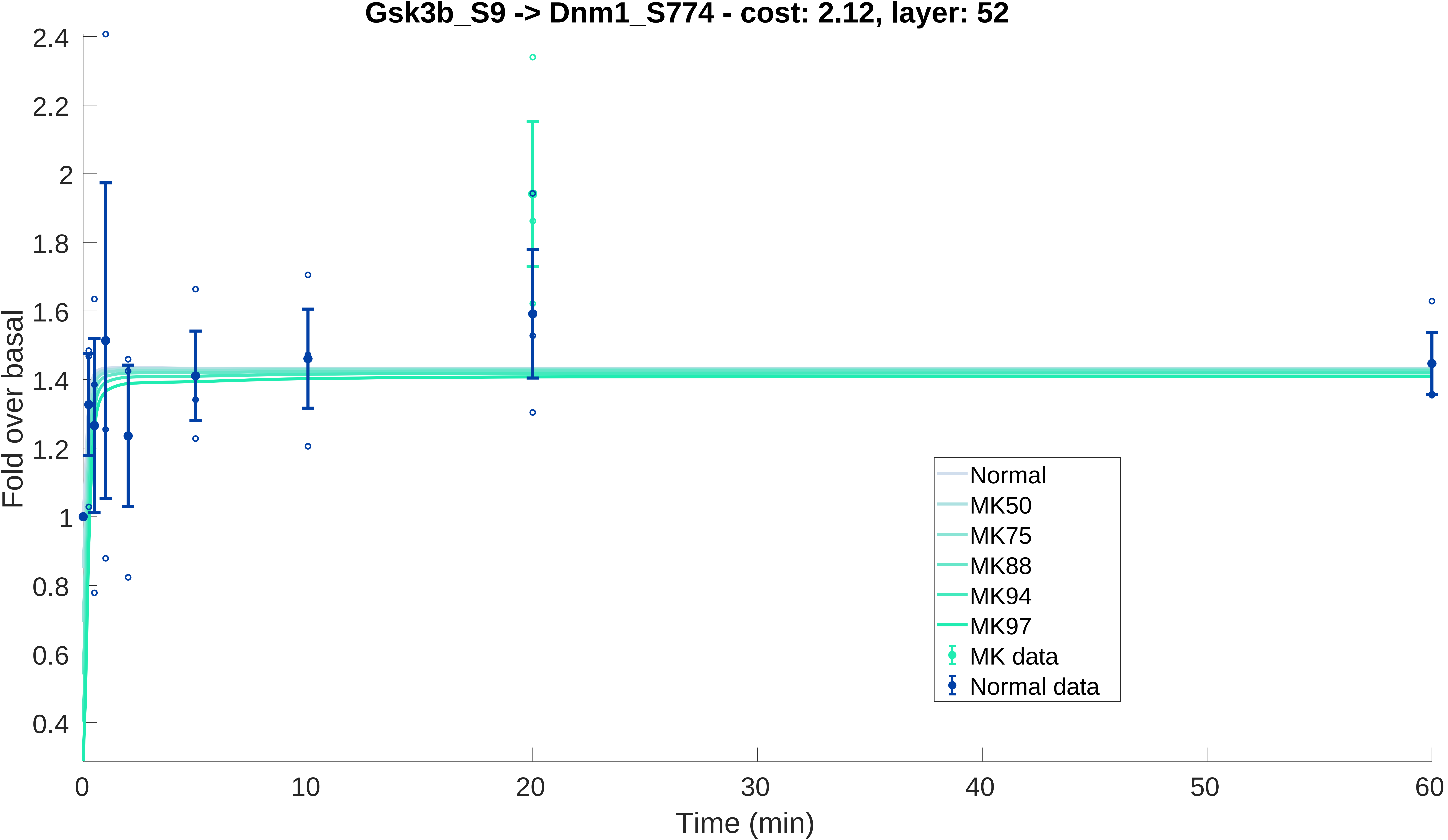

Gsk3b_S9 -> Dnm1_S774 - cost: 2.12, layer: 52
Fold over basal
2.4
2.2
2
1.8
1.6
1.4
1.2
1
0.8
0.6
0.4
0
10
20
30
40
50
60
Time (min)
Normal
MK50
MK75
MK88
MK94
MK97
MK data
Normal data

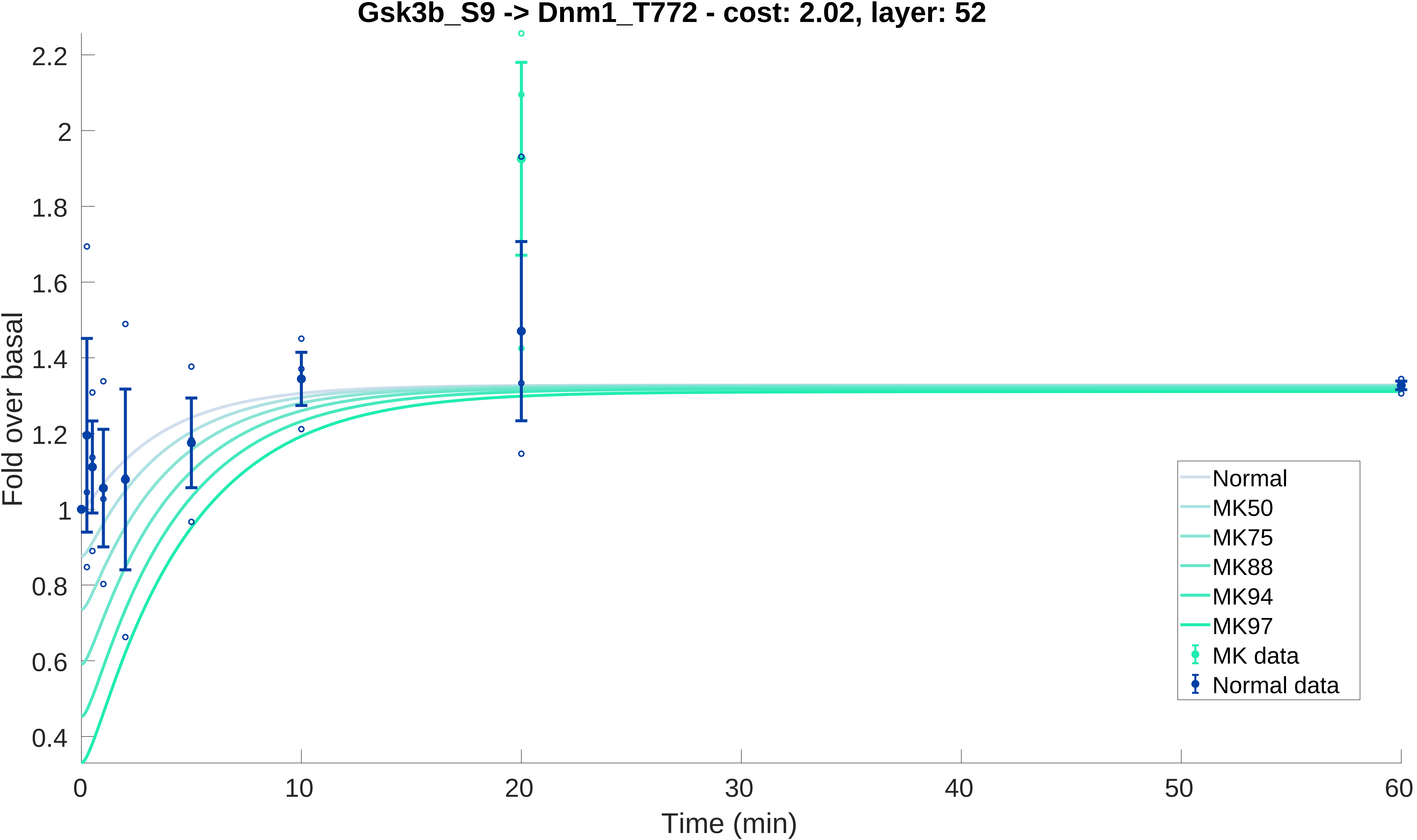

Gsk3b_S9 -> Dnm1_T772 - cost: 2.02, layer: 52
Fold over basal
Time (min)
2.2
2
1.8
1.6
1.4
1.2
1
0.8
0.6
0.4
0
10
20
30
40
50
60
Normal
MK50
MK75
MK88
MK94
MK97
MK data
Normal data

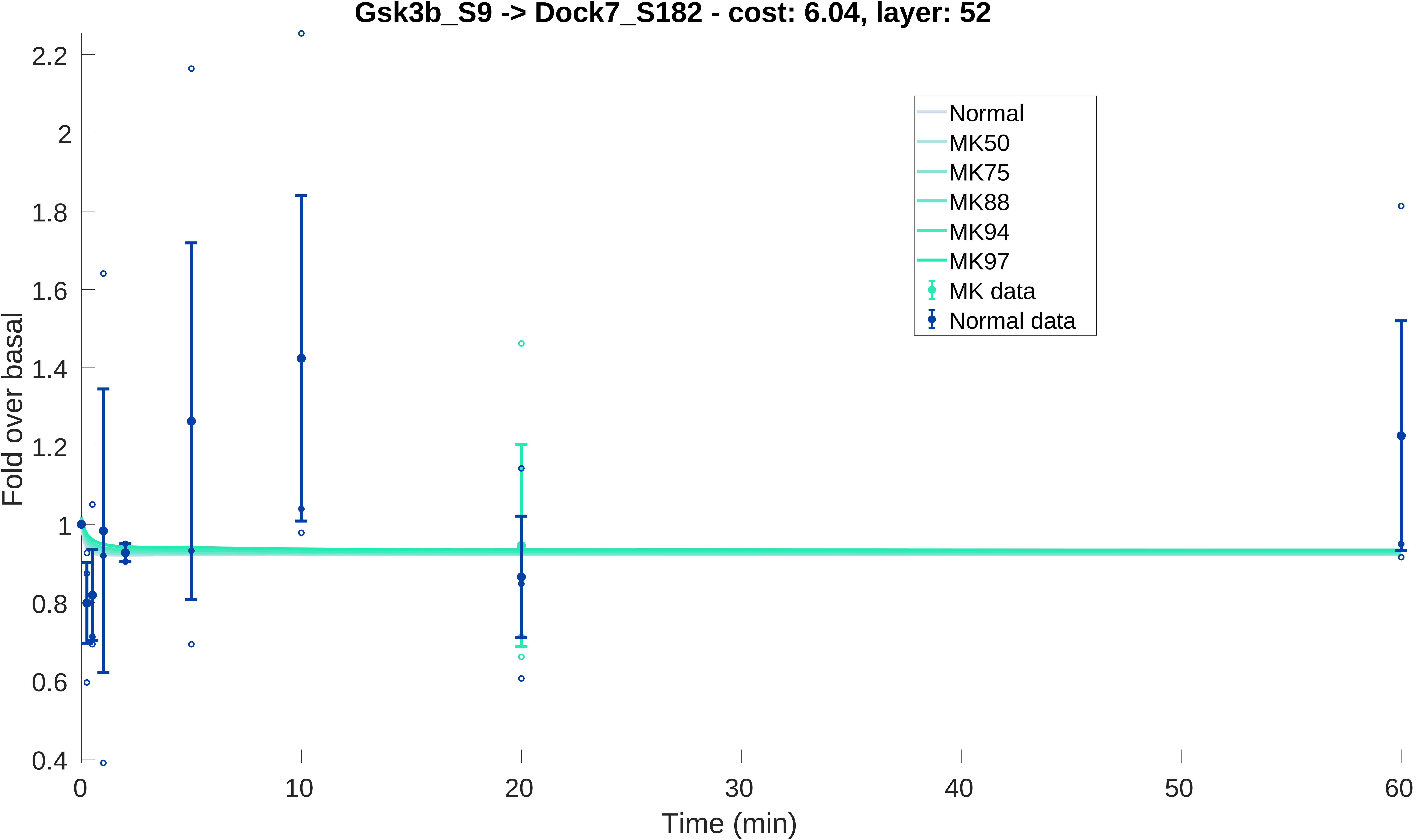
Gsk3b_S9 -> Dock7_S182 - cost: 6.04, layer: 52
Fold over basal
Time (min)
0
10
20
30
40
50
60
0.4
0.6
0.8
1
1.2
1.4
1.6
1.8
2
2.2
Normal
MK50
MK75
MK88
MK94
MK97
MK data
Normal data

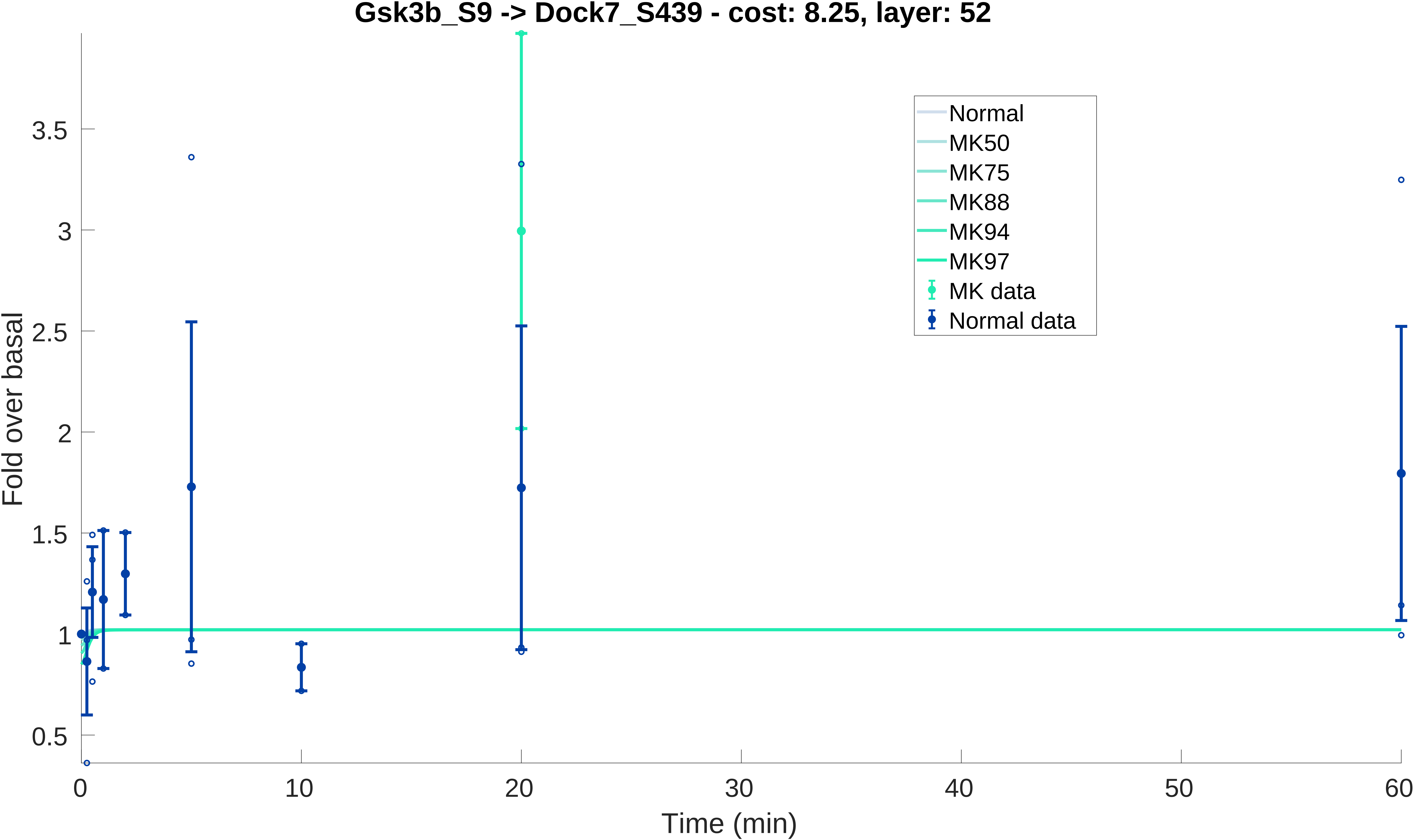

Gsk3b_S9 -> Dock7_S439 - cost: 8.25, layer: 52
Fold over basal
Time (min)
Normal
MK50
MK75
MK88
MK94
MK97
MK data
Normal data
0.5
1
1.5
2
2.5
3
3.5
0
10
20
30
40
50
60

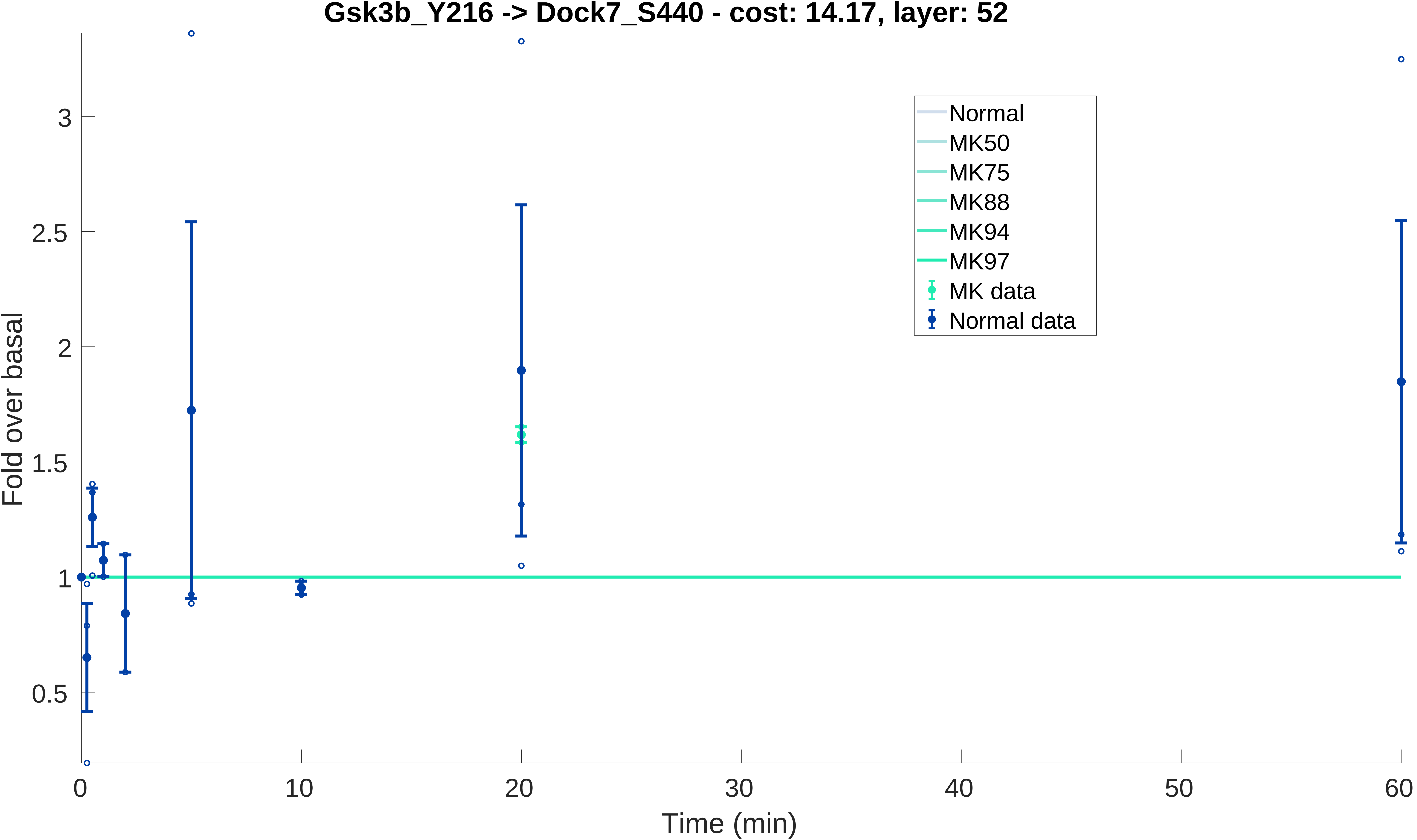

Gsk3b_Y216 -> Dock7_S440 - cost: 14.17, layer: 52
Normal
MK50
MK75
MK88
MK94
MK97
MK data
Normal data
Fold over basal
Time (min)
0.5
1
1.5
2
2.5
3
0
10
20
30
40
50
60

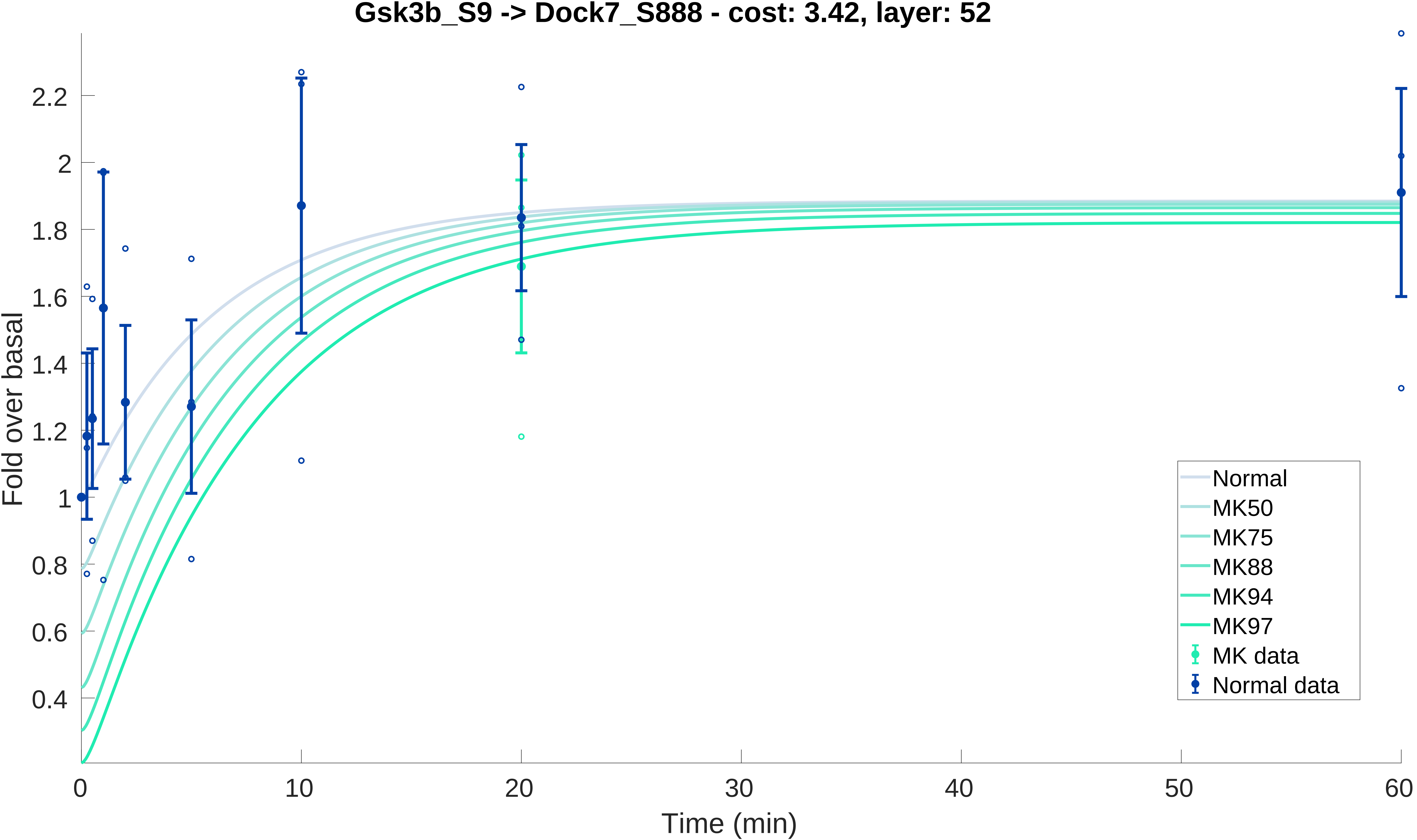

Gsk3b_S9 -> Dock7_S888 - cost: 3.42, layer: 52
Fold over basal
Time (min)
0.4
0.6
0.8
1
1.2
1.4
1.6
1.8
2
2.2
0
10
20
30
40
50
60
Normal
MK50
MK75
MK88
MK94
MK97
MK data
Normal data

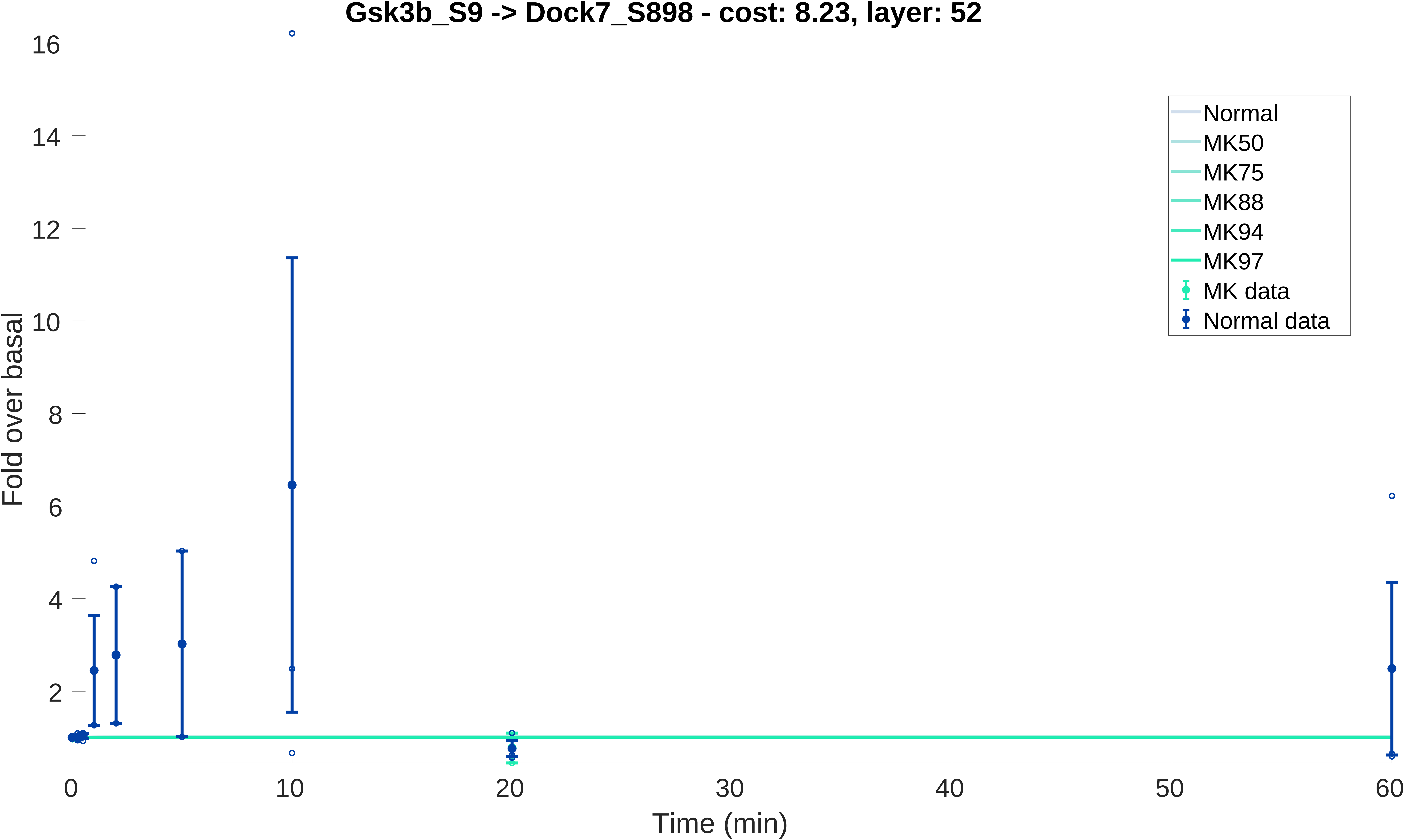

Gsk3b_S9 -> Dock7_S898 - cost: 8.23, layer: 52
Fold over basal
Time (min)
Normal
MK50
MK75
MK88
MK94
MK97
MK data
Normal data
0
10
20
30
40
50
60
2
4
6
8
10
12
14
16

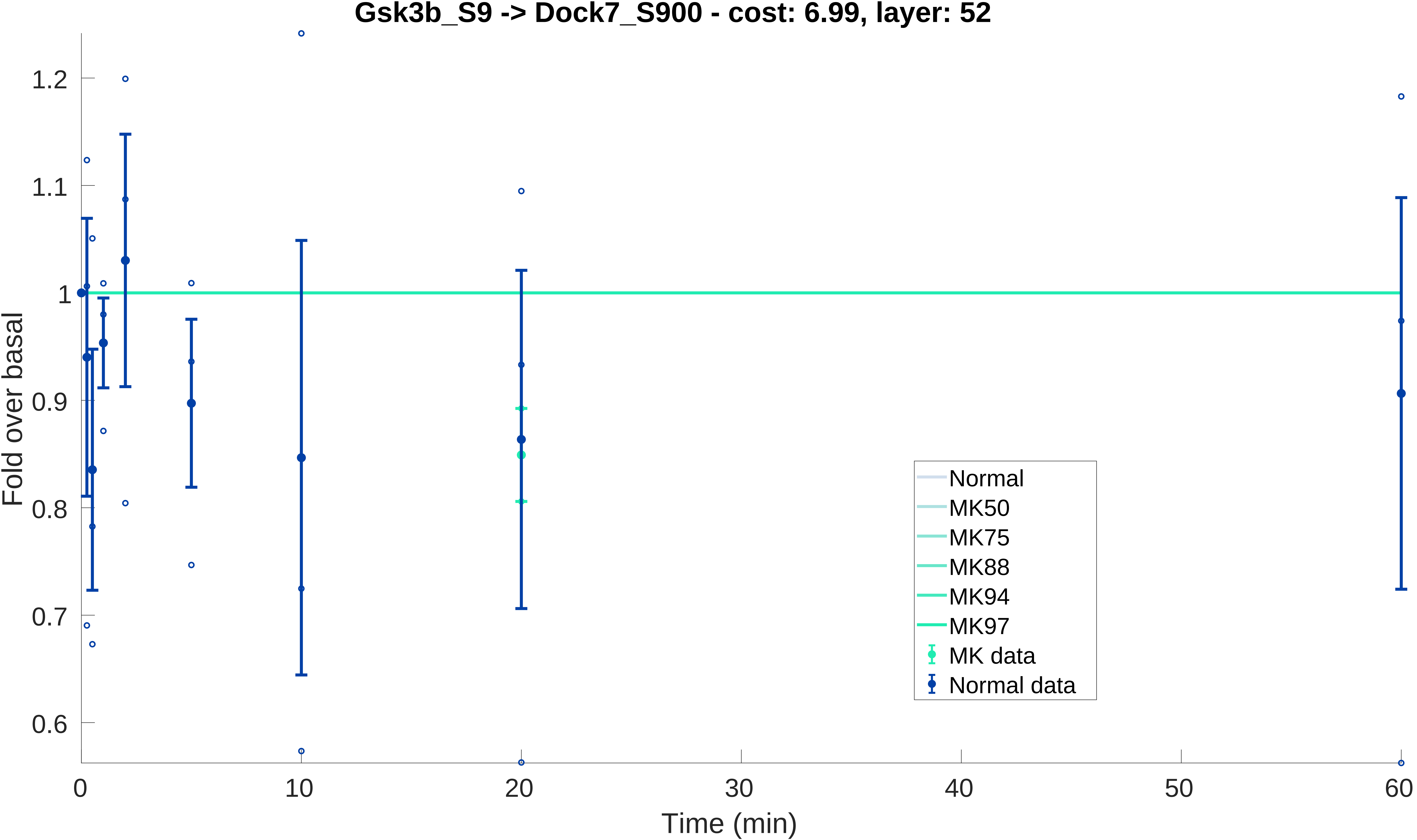
Gsk3b_S9 -> Dock7_S900 - cost: 6.99, layer: 52
Fold over basal
Time (min)
1.2
1.1
1
0.9
0.8
0.7
0.6
0
10
20
30
40
50
60
Normal
MK50
MK75
MK88
MK94
MK97
MK data
Normal data

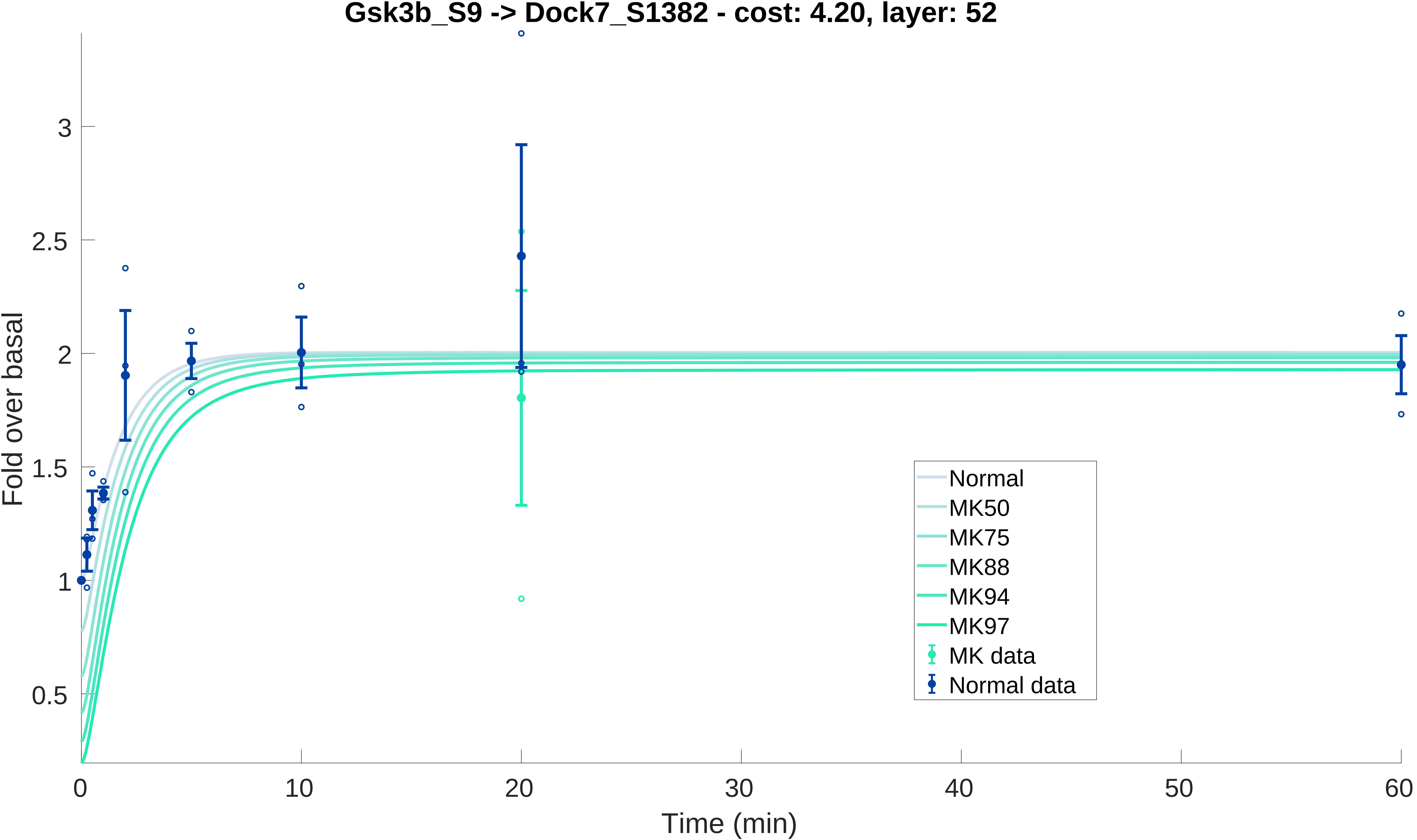

Gsk3b_S9 -> Dock7_S1382 - cost: 4.20, layer: 52
Fold over basal
Time (min)
0.5
1
1.5
2
2.5
3
0
10
20
30
40
50
60
Normal
MK50
MK75
MK88
MK94
MK97
MK data
Normal data

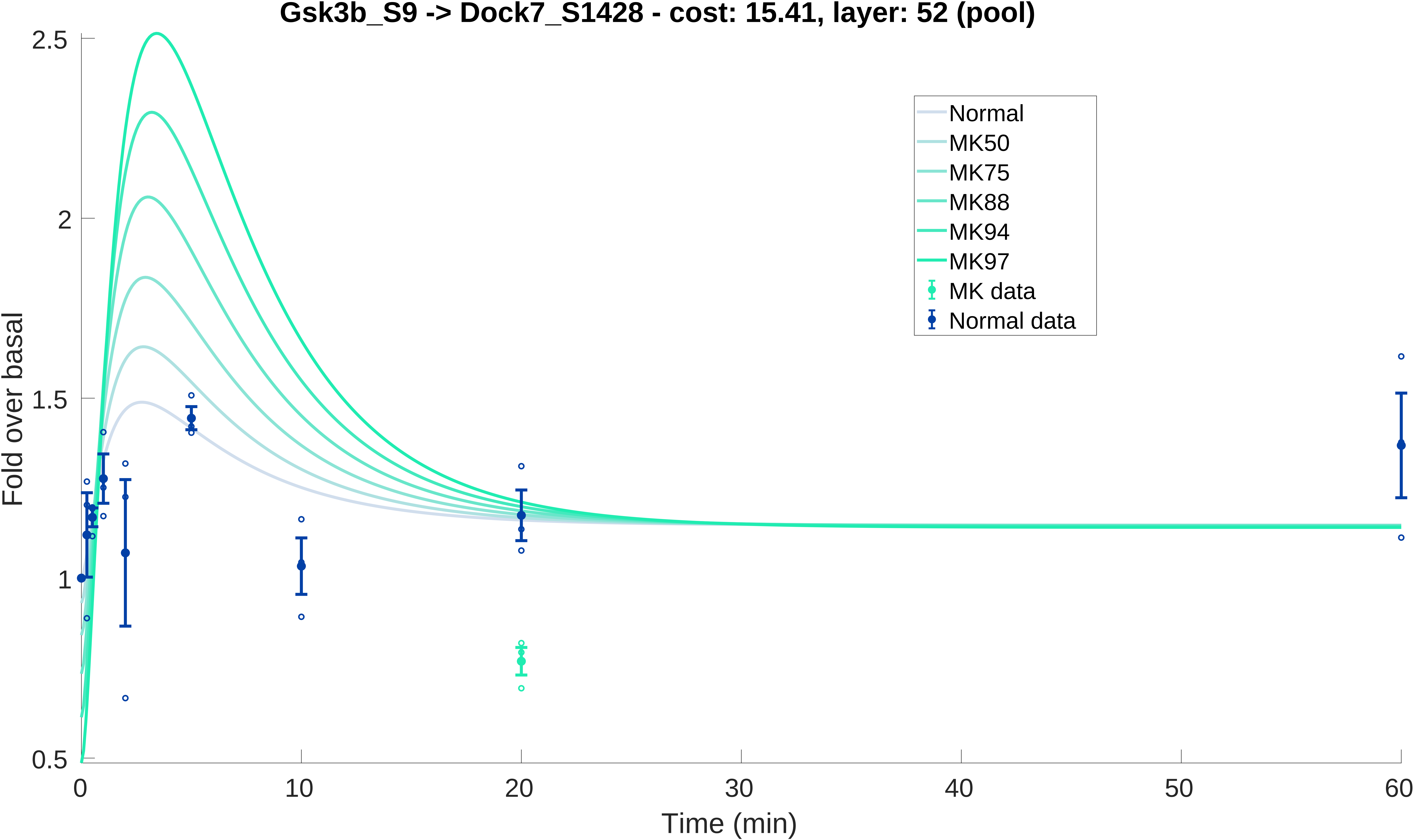

Gsk3b_S9 -> Dock7_S1428 - cost: 15.41, layer: 52 (pool)
Normal
MK50
MK75
MK88
MK94
MK97
MK data
Normal data
Fold over basal
Time (min)
0.5
1
1.5
2
2.5
0
10
20
30
40
50
60

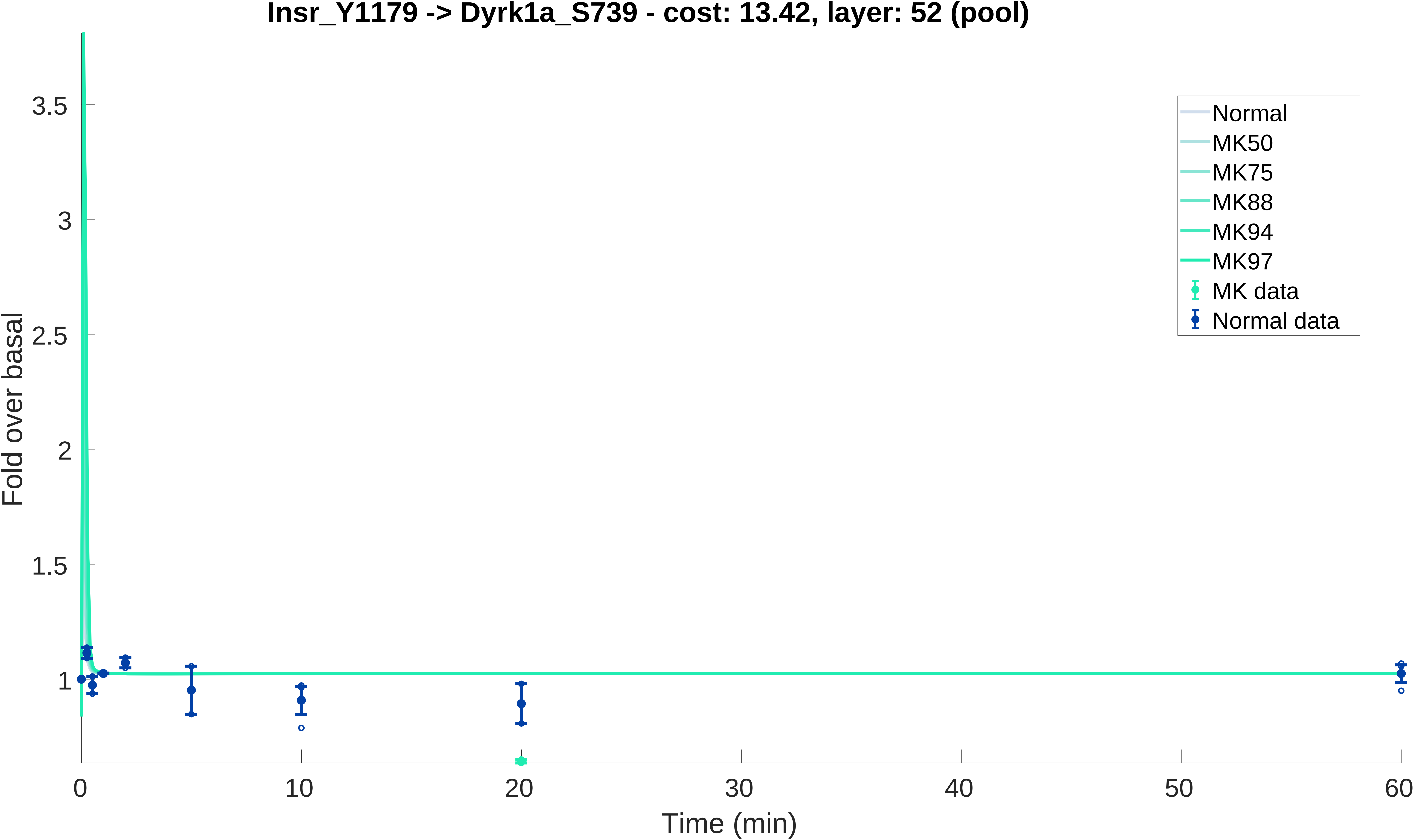

Insr_Y1179 -> Dyrk1a_S739 - cost: 13.42, layer: 52 (pool)
Fold over basal
3.5
3
2.5
2
1.5
1
0
10
20
30
40
50
60
Time (min)
Normal
MK50
MK75
MK88
MK94
MK97
MK data
Normal data

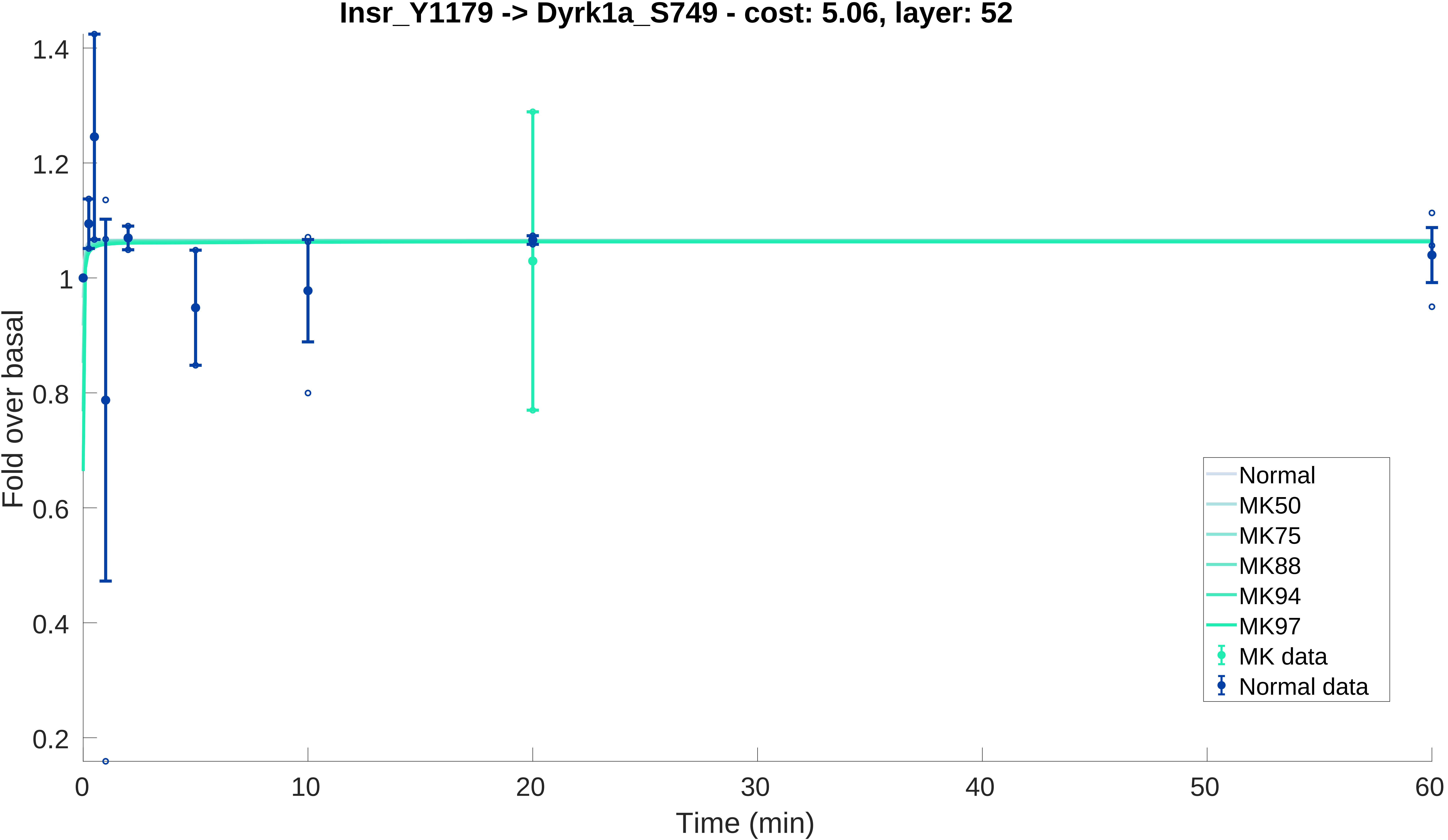
Insr_Y1179 -> Dyrk1a_S749 - cost: 5.06, layer: 52
Fold over basal
1.4
1.2
1
0.8
0.6
0.4
0.2
0
10
20
30
40
50
60
Time (min)
Normal
MK50
MK75
MK88
MK94
MK97
MK data
Normal data

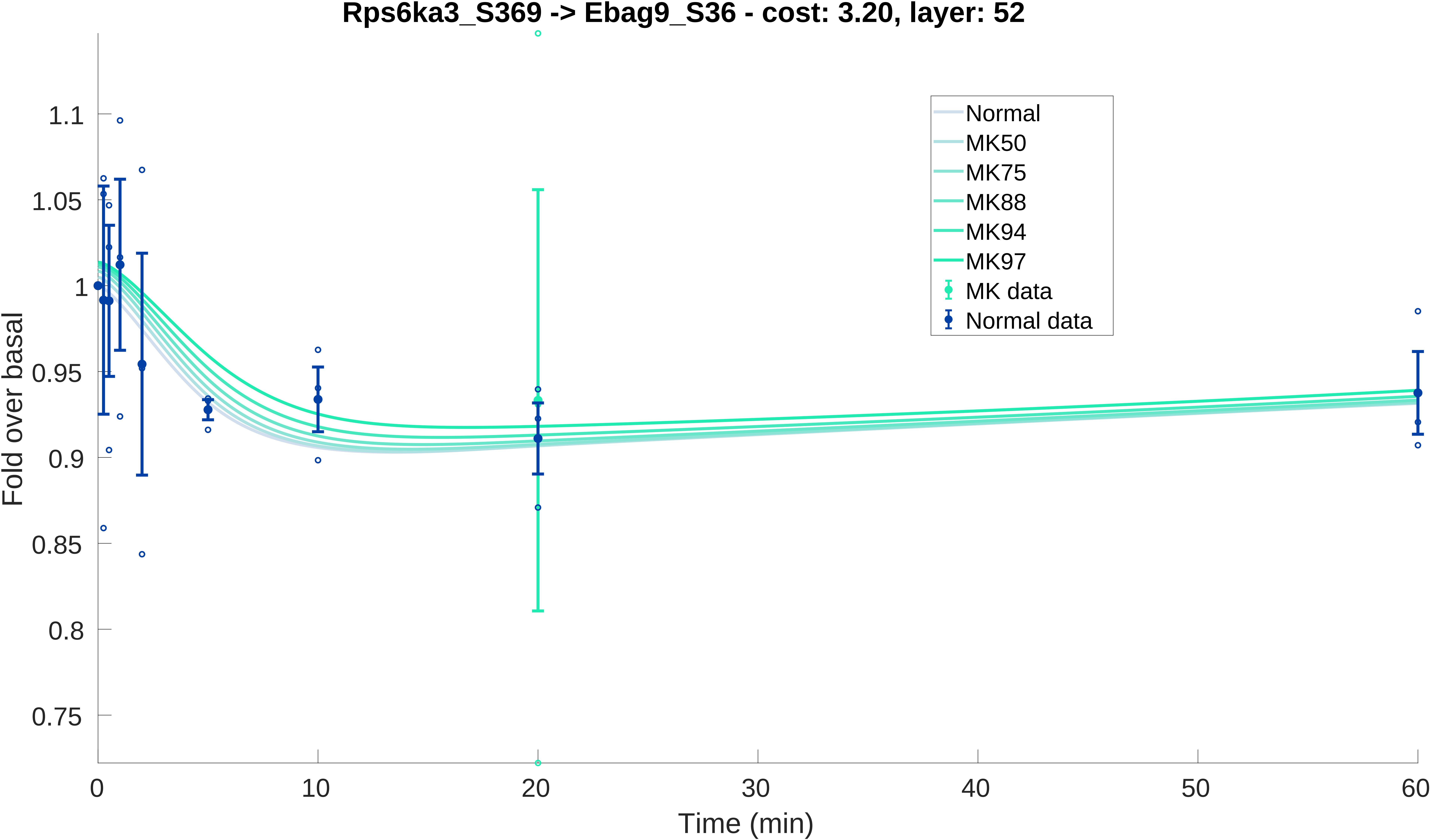

Rps6ka3_S369 -> Ebag9_S36 - cost: 3.20, layer: 52
Fold over basal
1.1
1.05
1
0.95
0.9
0.85
0.8
0.75
0
10
20
30
40
50
60
Time (min)
Normal
MK50
MK75
MK88
MK94
MK97
MK data
Normal data

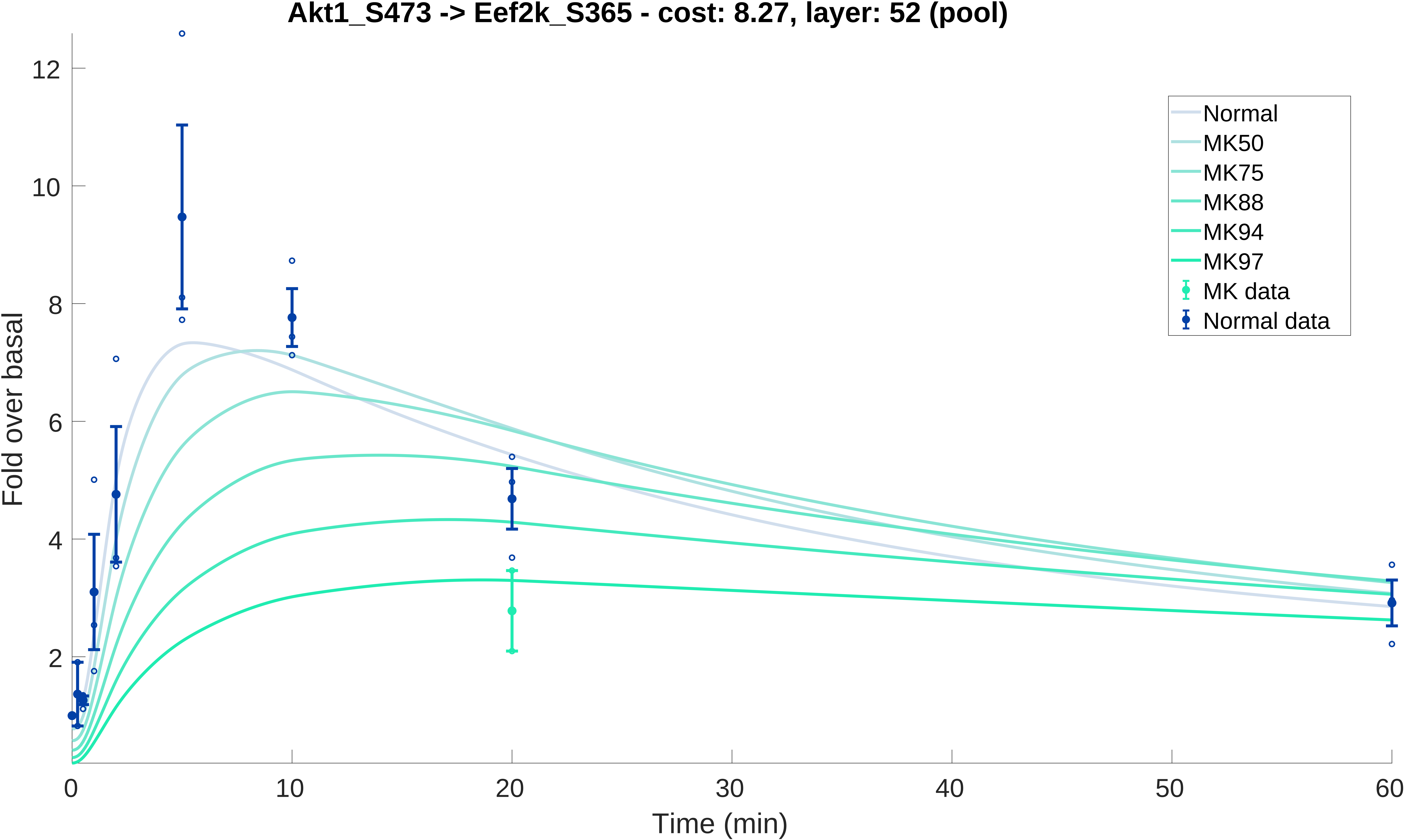

Akt1_S473 -> Eef2k_S365 - cost: 8.27, layer: 52 (pool)
Fold over basal
Time (min)
0
10
20
30
40
50
60
2
4
6
8
10
12
Normal
MK50
MK75
MK88
MK94
MK97
MK data
Normal data

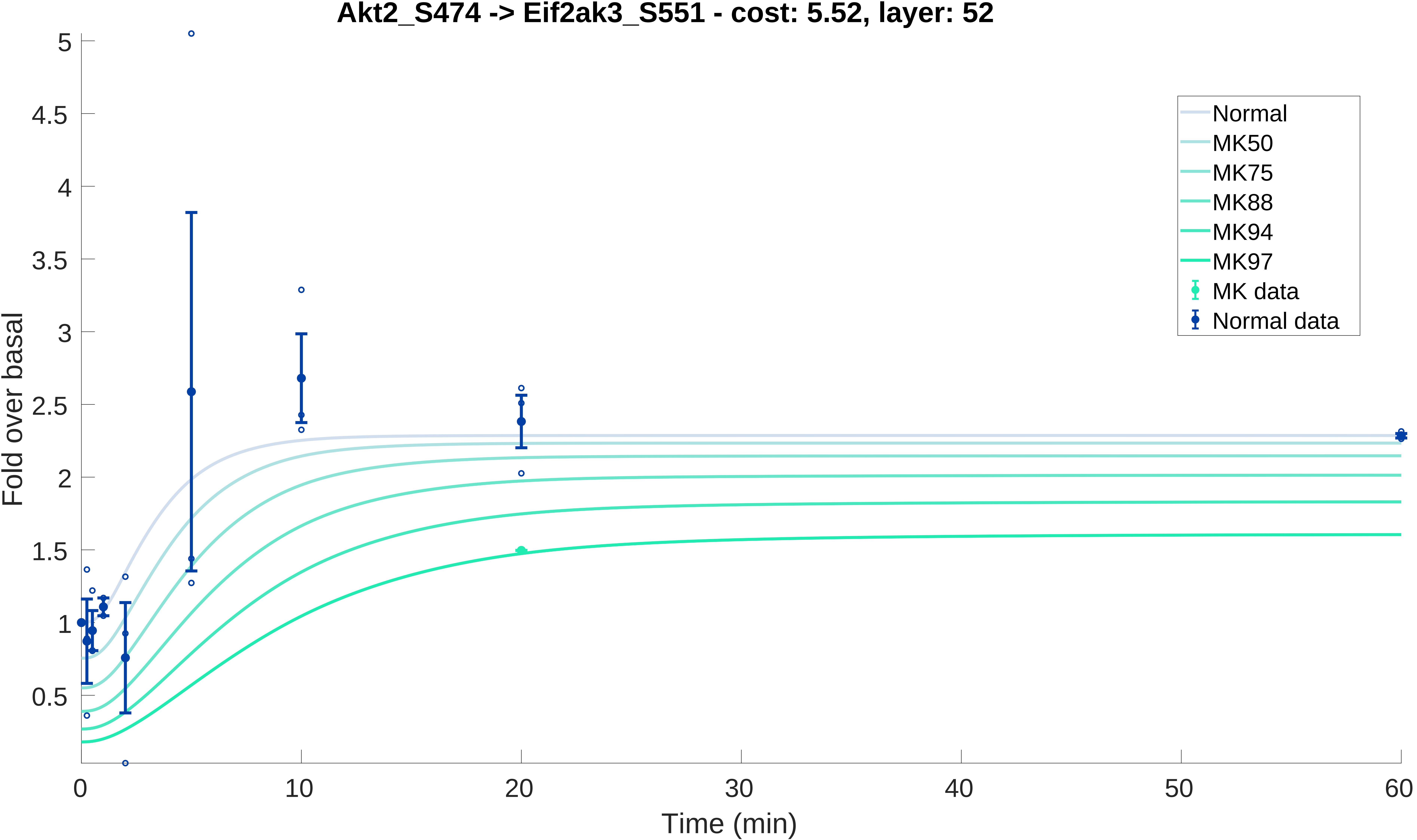

Akt2_S474 -> Eif2ak3_S551 - cost: 5.52, layer: 52
Fold over basal
Time (min)
0
10
20
30
40
50
60
0.5
1
1.5
2
2.5
3
3.5
4
4.5
5
Normal
MK50
MK75
MK88
MK94
MK97
MK data
Normal data

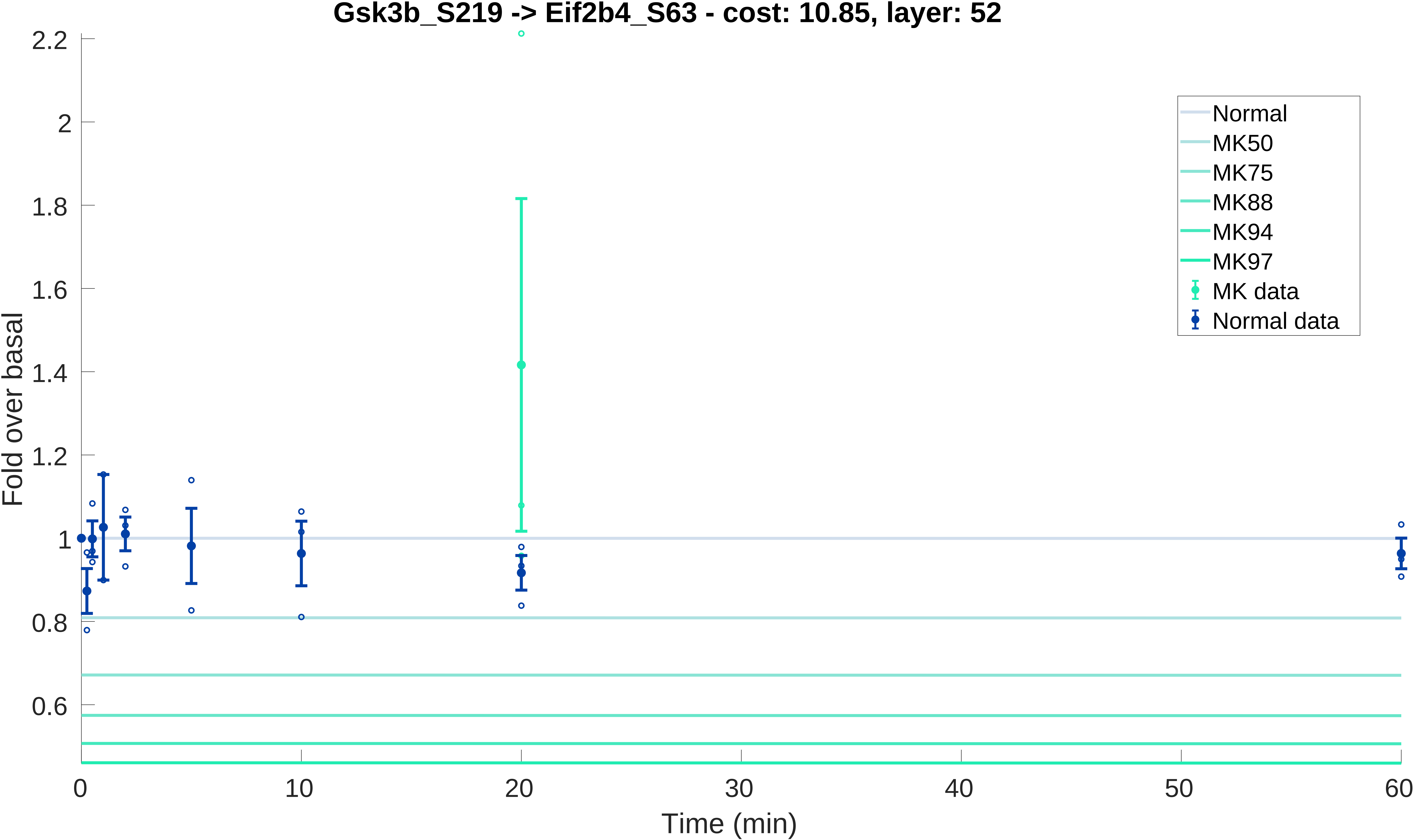

Gsk3b_S219 -> Eif2b4_S63 - cost: 10.85, layer: 52
Fold over basal
Time (min)
Normal
MK50
MK75
MK88
MK94
MK97
MK data
Normal data
2.2
2
1.8
1.6
1.4
1.2
1
0.8
0.6
0
10
20
30
40
50
60

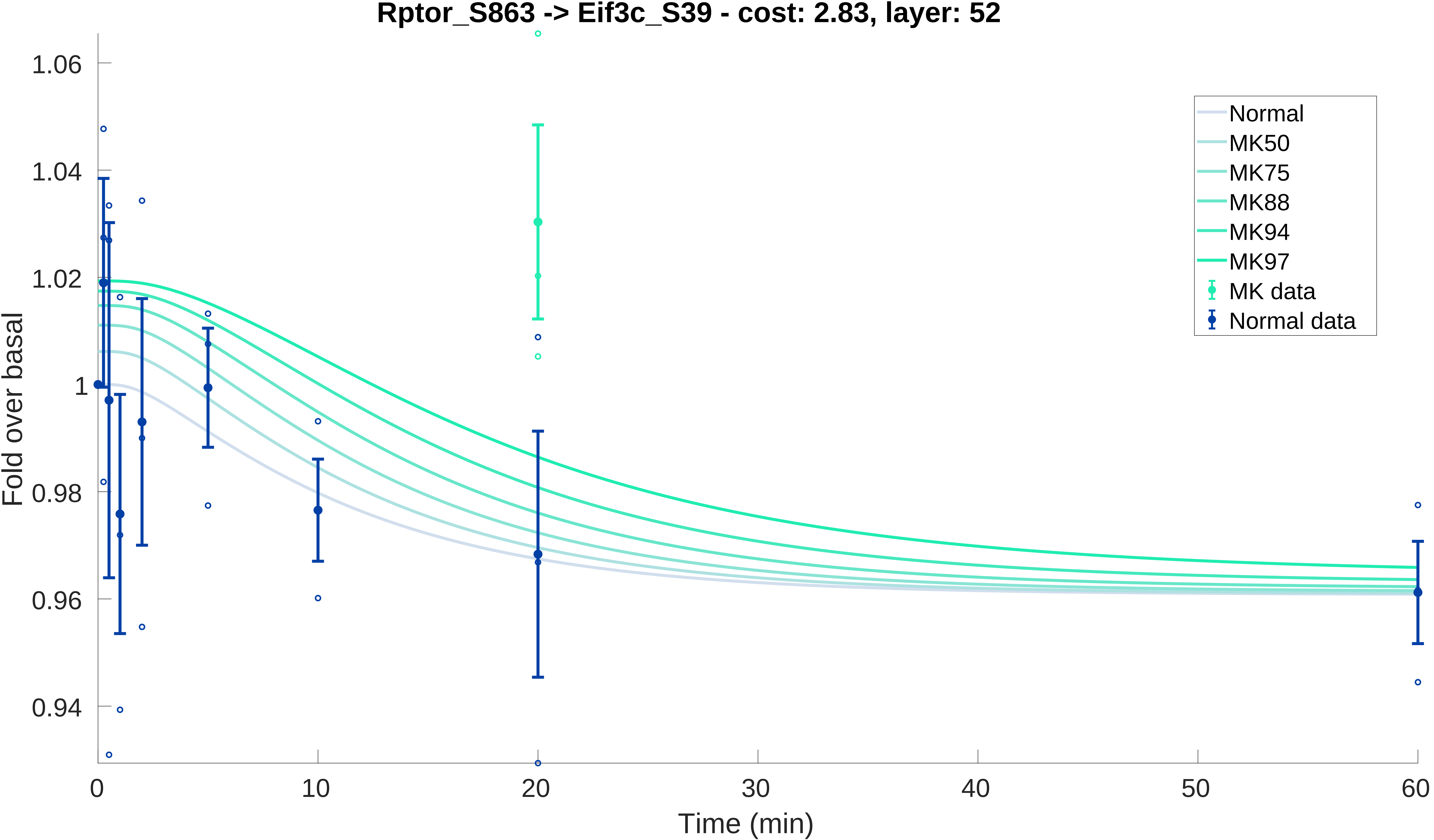

Rptor_S863 -> Eif3c_S39 - cost: 2.83, layer: 52
Fold over basal
1.06
1.04
1.02
1
0.98
0.96
0.94
0
10
20
30
40
50
60
Time (min)
Normal
MK50
MK75
MK88
MK94
MK97
MK data
Normal data

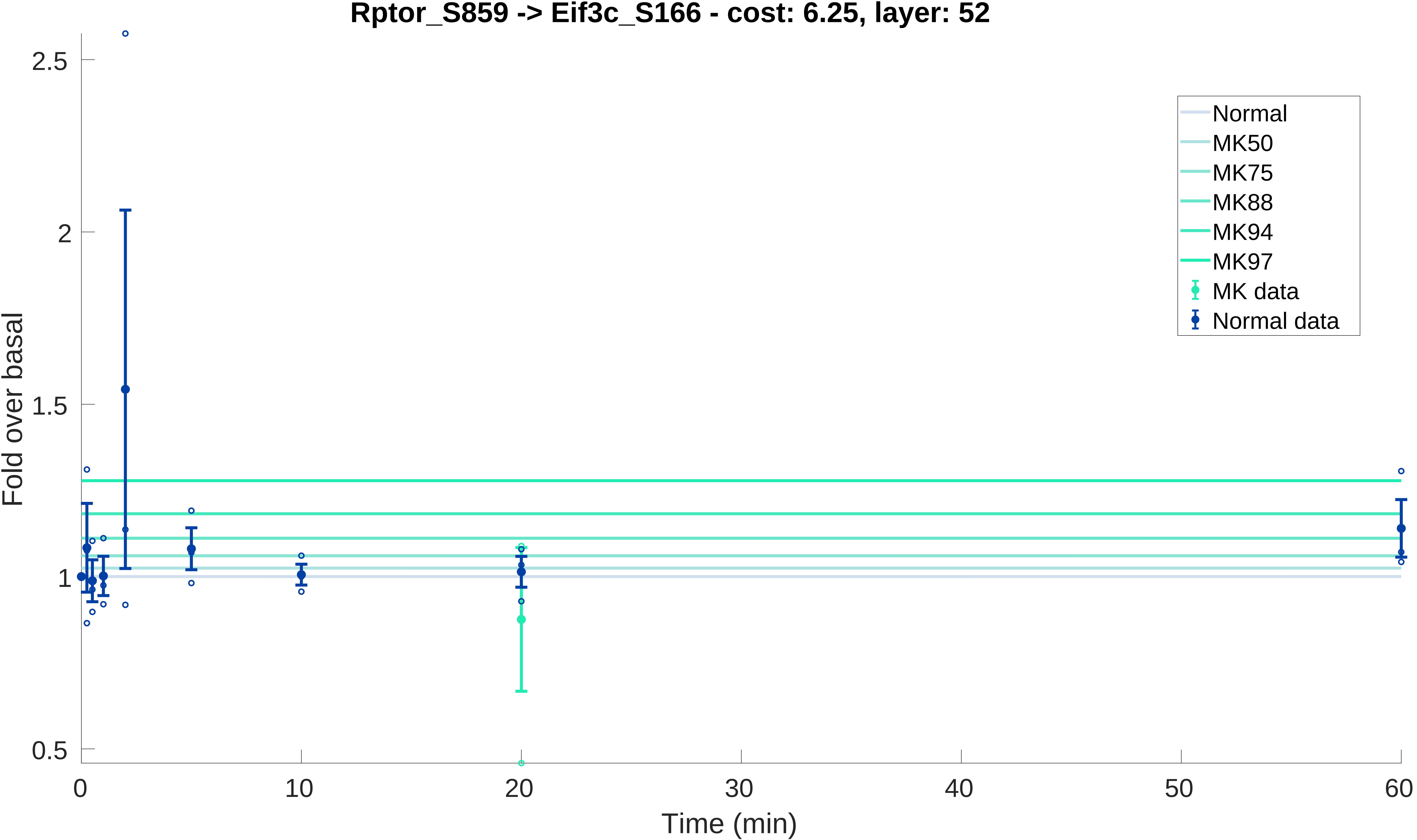
Rptor_S859 -> Eif3c_S166 - cost: 6.25, layer: 52
Fold over basal
Time (min)
0.5
1
1.5
2
2.5
0
10
20
30
40
50
60
Normal
MK50
MK75
MK88
MK94
MK97
MK data
Normal data

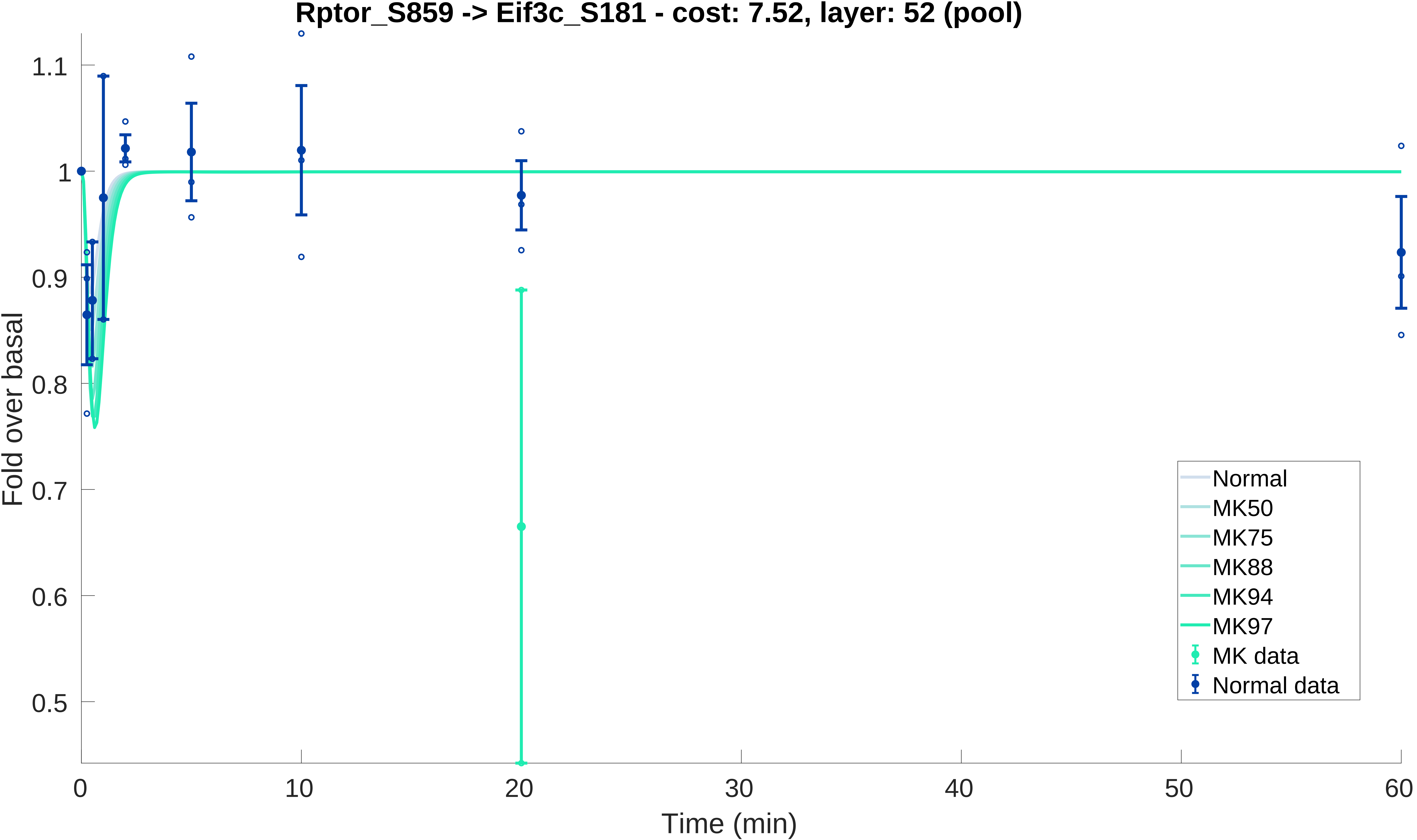

Rptor_S859 -> Eif3c_S181 - cost: 7.52, layer: 52 (pool)
Fold over basal
Time (min)
1.1
1
0.9
0.8
0.7
0.6
0.5
0
10
20
30
40
50
60
Normal
MK50
MK75
MK88
MK94
MK97
MK data
Normal data

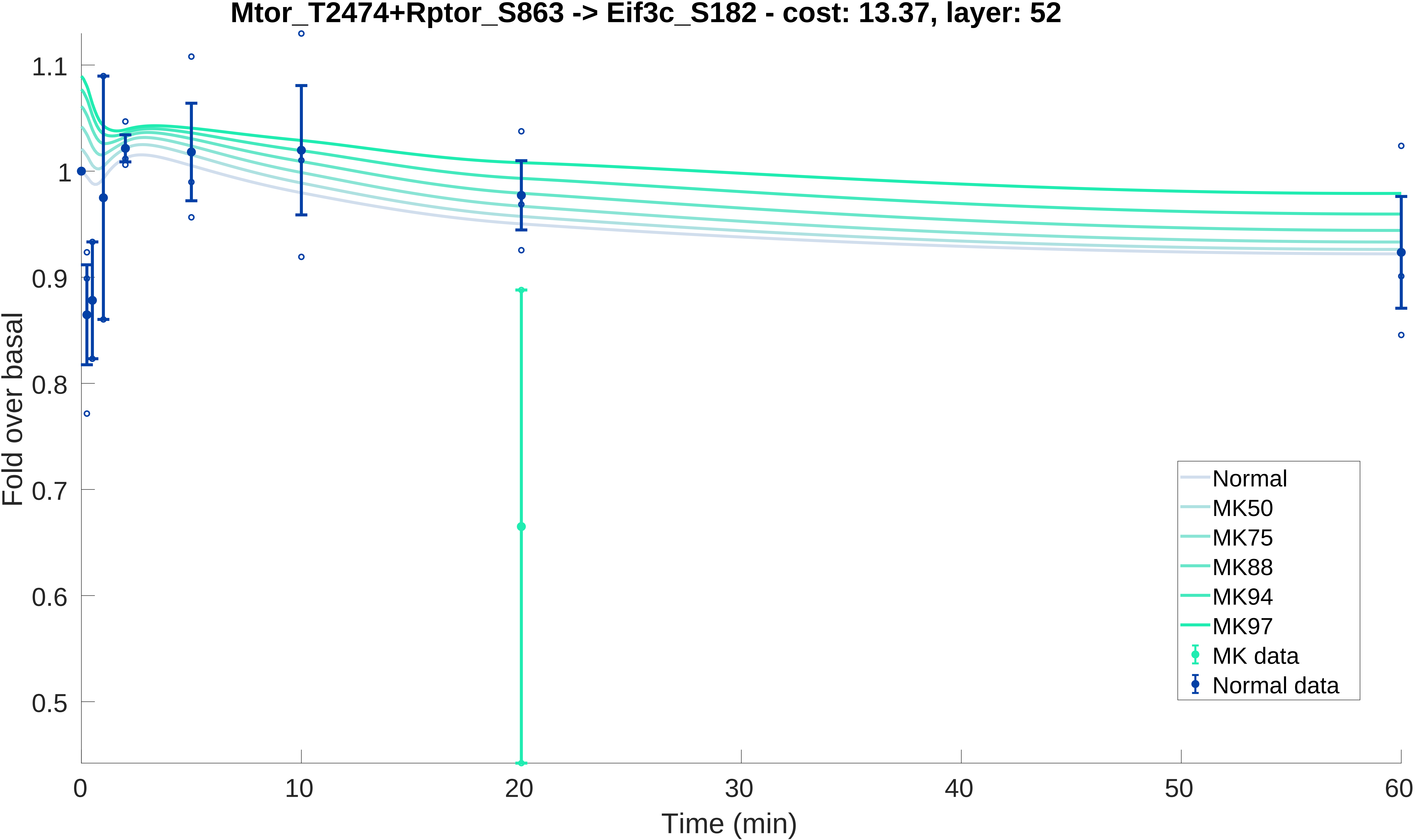

Mtor_T2474+Rptor_S863 -> Eif3c_S182 - cost: 13.37, layer: 52
Fold over basal
Time (min)
1.1
1
0.9
0.8
0.7
0.6
0.5
0
10
20
30
40
50
60
Normal
MK50
MK75
MK88
MK94
MK97
MK data
Normal data

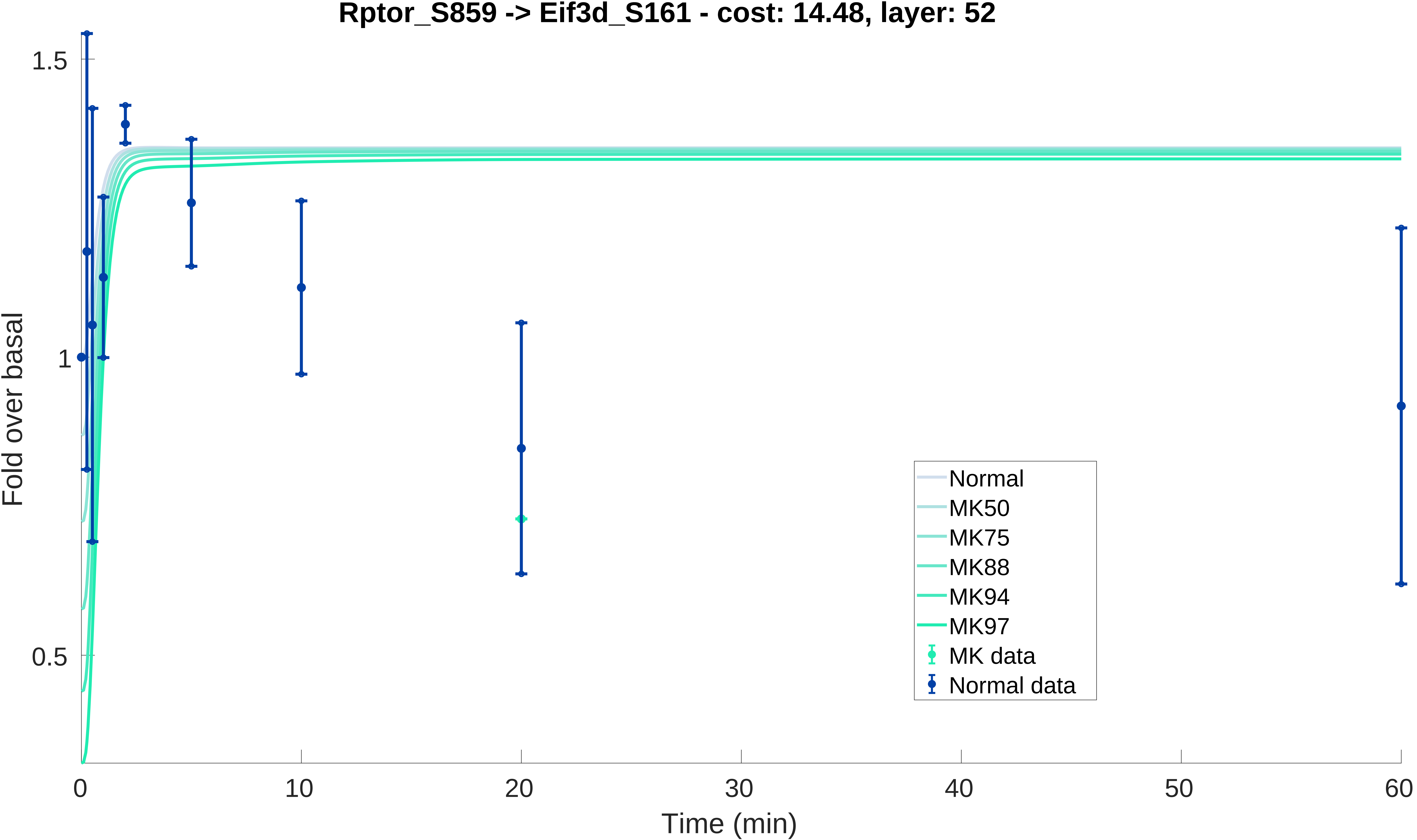
Rptor_S859 -> Eif3d_S161 - cost: 14.48, layer: 52
Fold over basal
1.5
1
0.5
0
10
20
30
40
50
60
Time (min)
Normal
MK50
MK75
MK88
MK94
MK97
MK data
Normal data

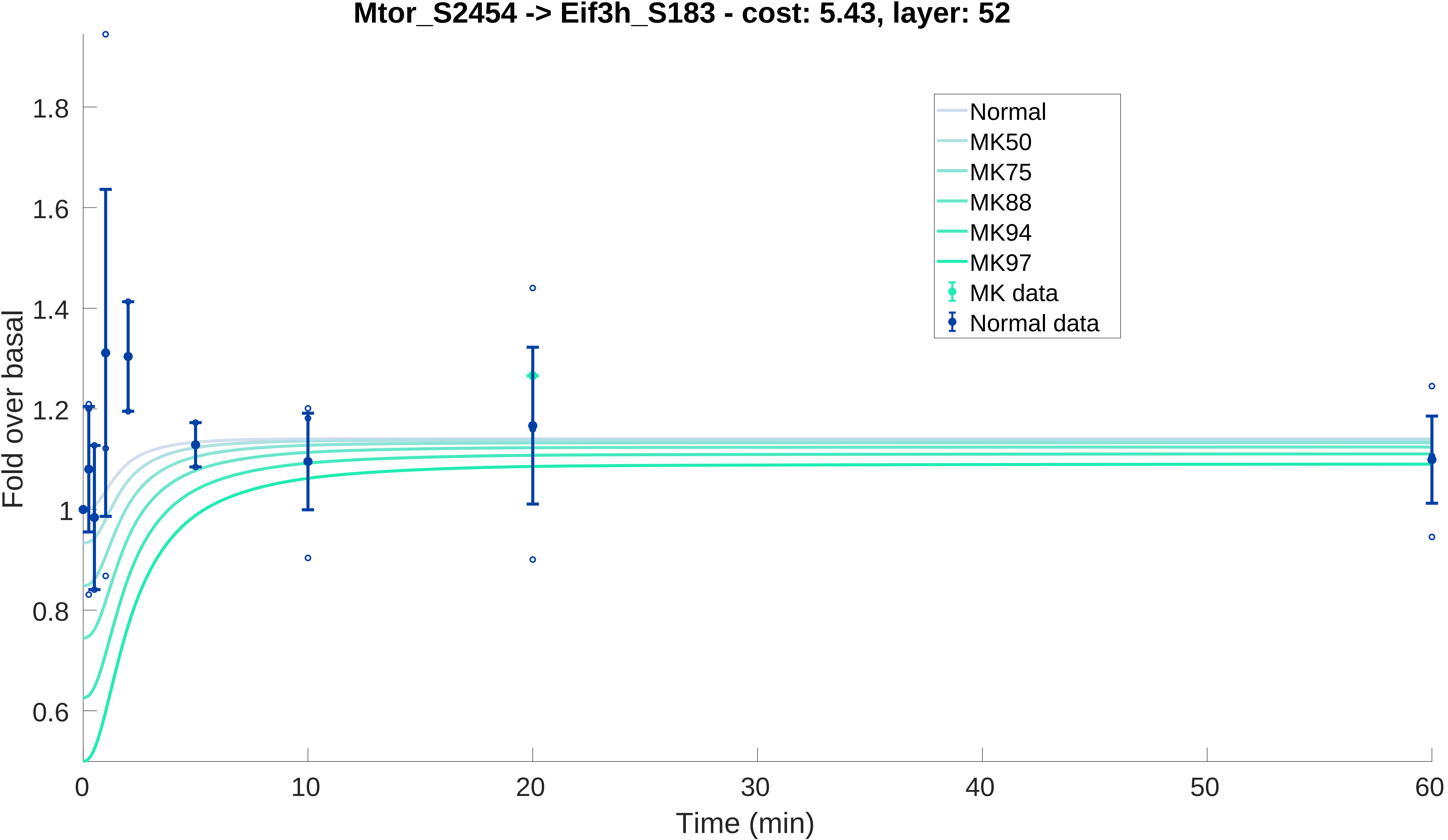

Mtor_S2454 -> Eif3h_S183 - cost: 5.43, layer: 52
Fold over basal
Time (min)
0.6
0.8
1
1.2
1.4
1.6
1.8
0
10
20
30
40
50
60
Normal
MK50
MK75
MK88
MK94
MK97
MK data
Normal data

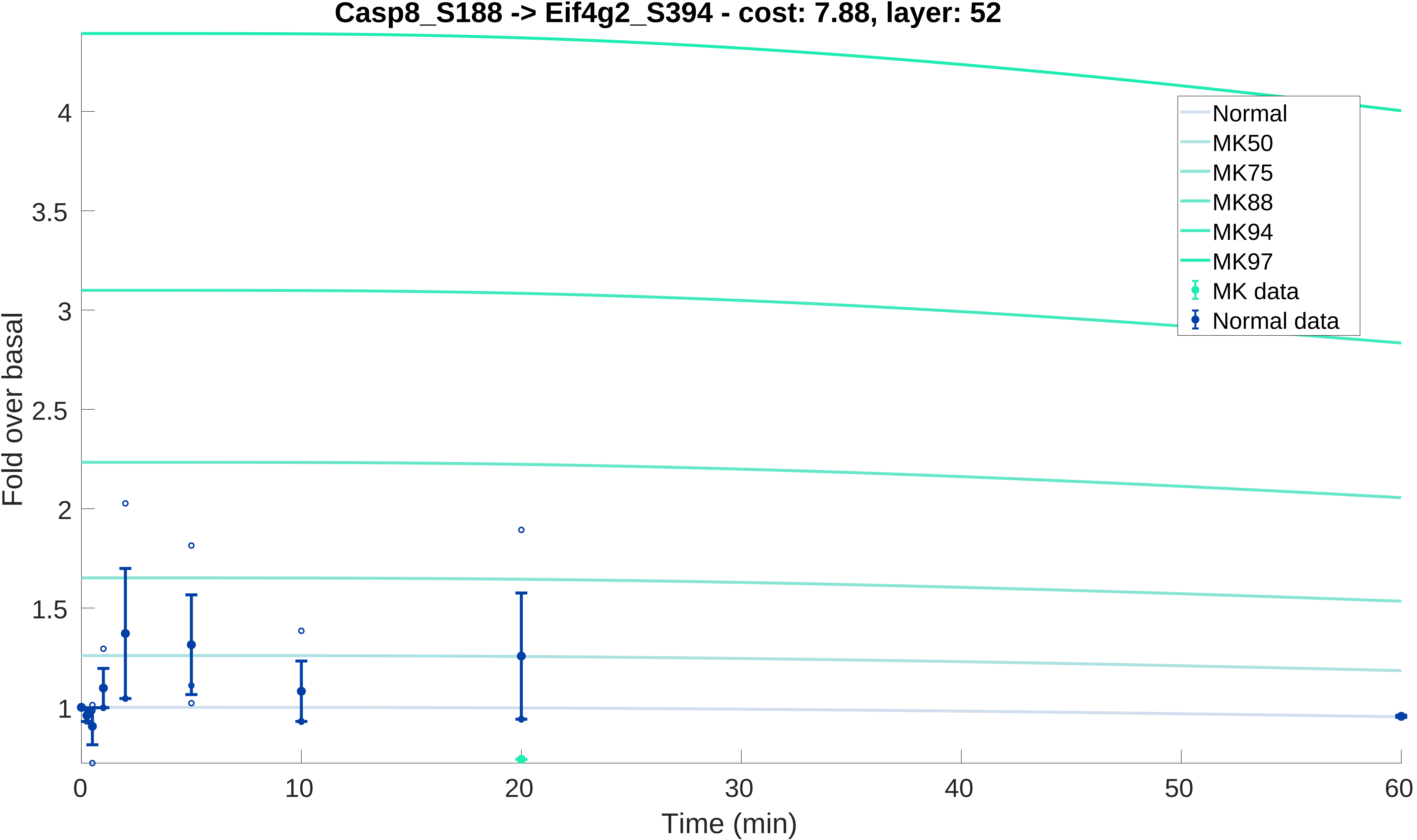

Casp8_S188 -> Eif4g2_S394 - cost: 7.88, layer: 52
Fold over basal
4
3.5
3
2.5
2
1.5
1
0
10
20
30
40
50
60
Time (min)
Normal
MK50
MK75
MK88
MK94
MK97
MK data
Normal data

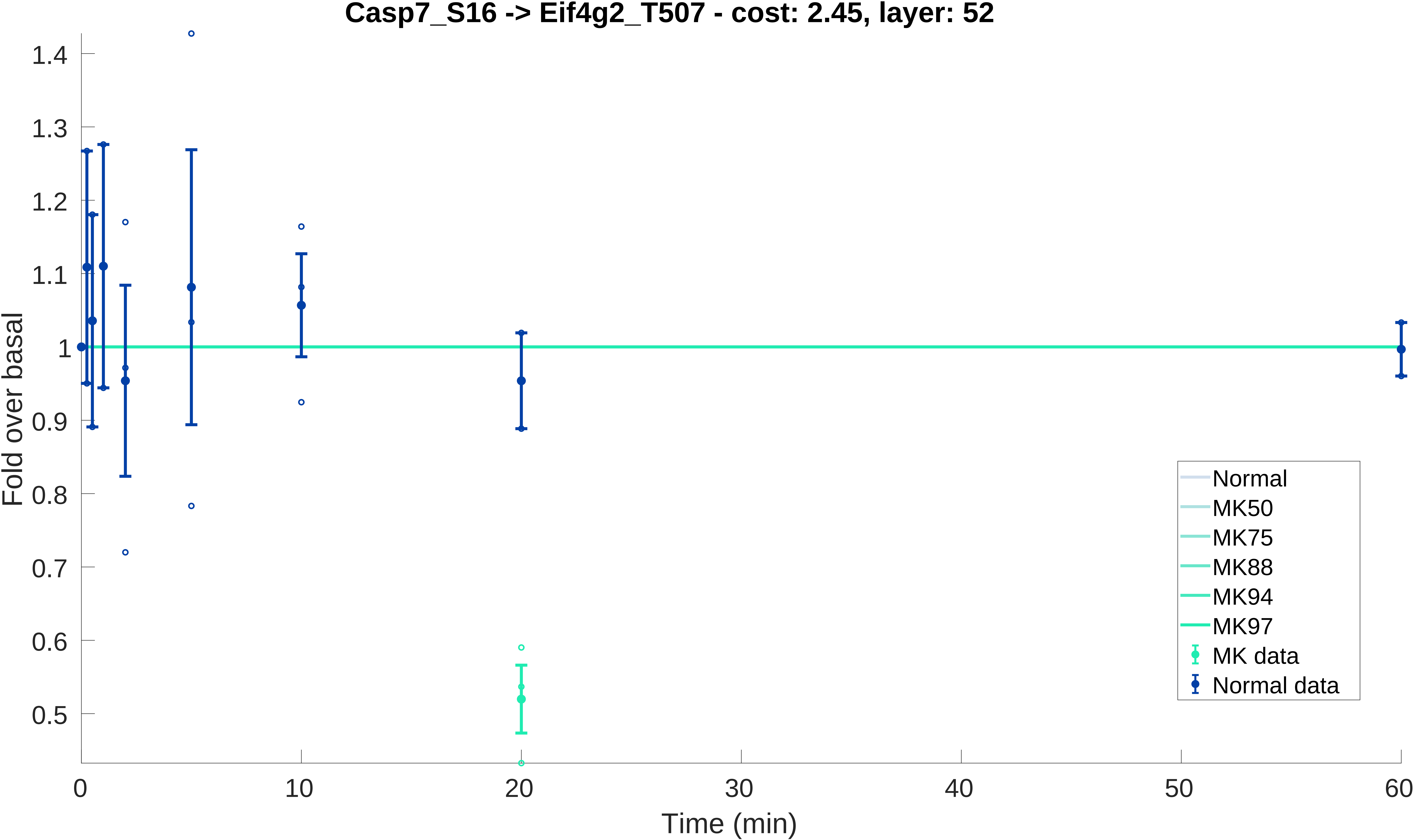

Casp7_S16 -> Eif4g2_T507 - cost: 2.45, layer: 52
Fold over basal
Time (min)
1.4
1.3
1.2
1.1
1
0.9
0.8
0.7
0.6
0.5
0
10
20
30
40
50
60
Normal
MK50
MK75
MK88
MK94
MK97
MK data
Normal data

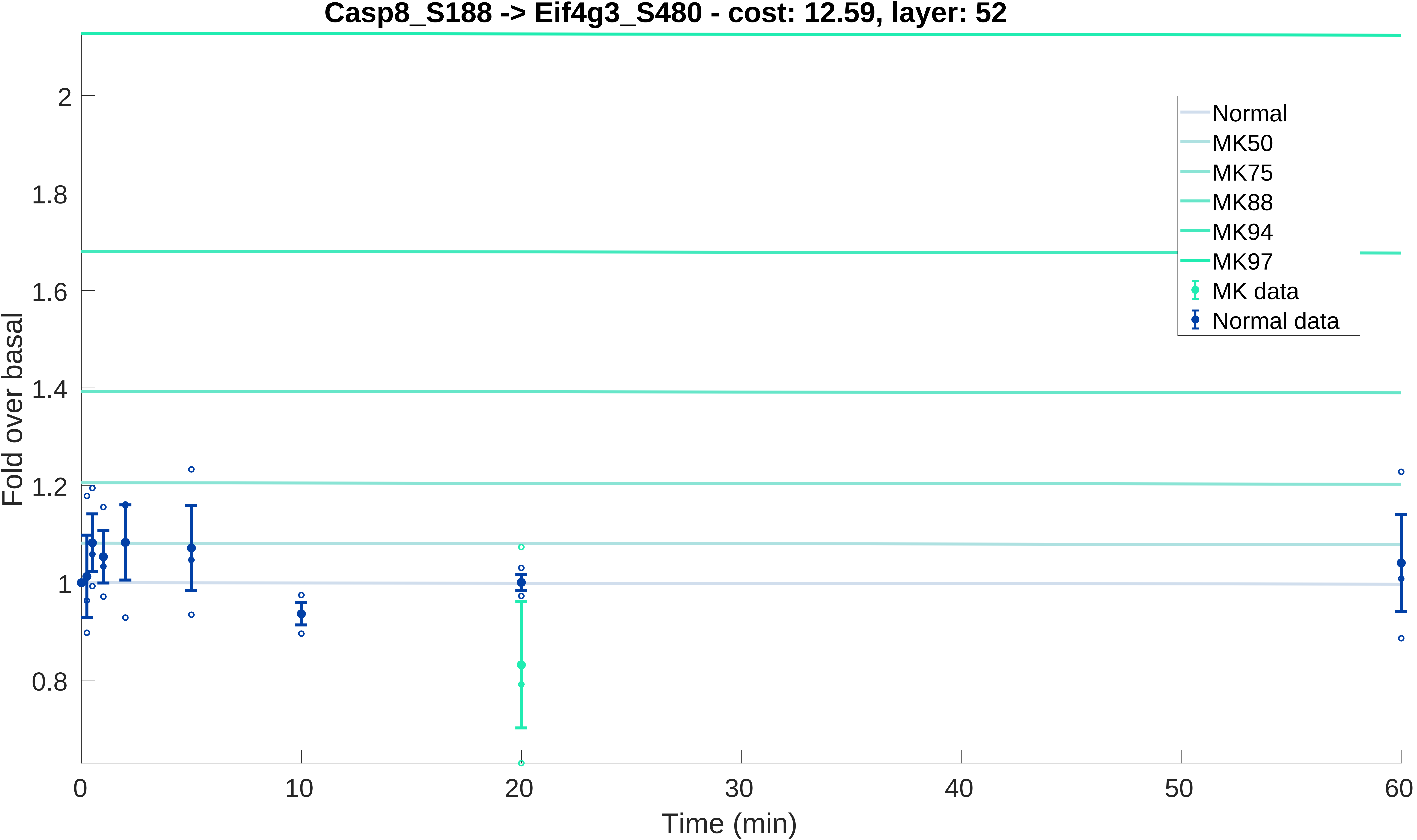

Casp8_S188 -> Eif4g3_S480 - cost: 12.59, layer: 52
Fold over basal
Time (min)
0.8
1
1.2
1.4
1.6
1.8
2
0
10
20
30
40
50
60
Normal
MK50
MK75
MK88
MK94
MK97
MK data
Normal data

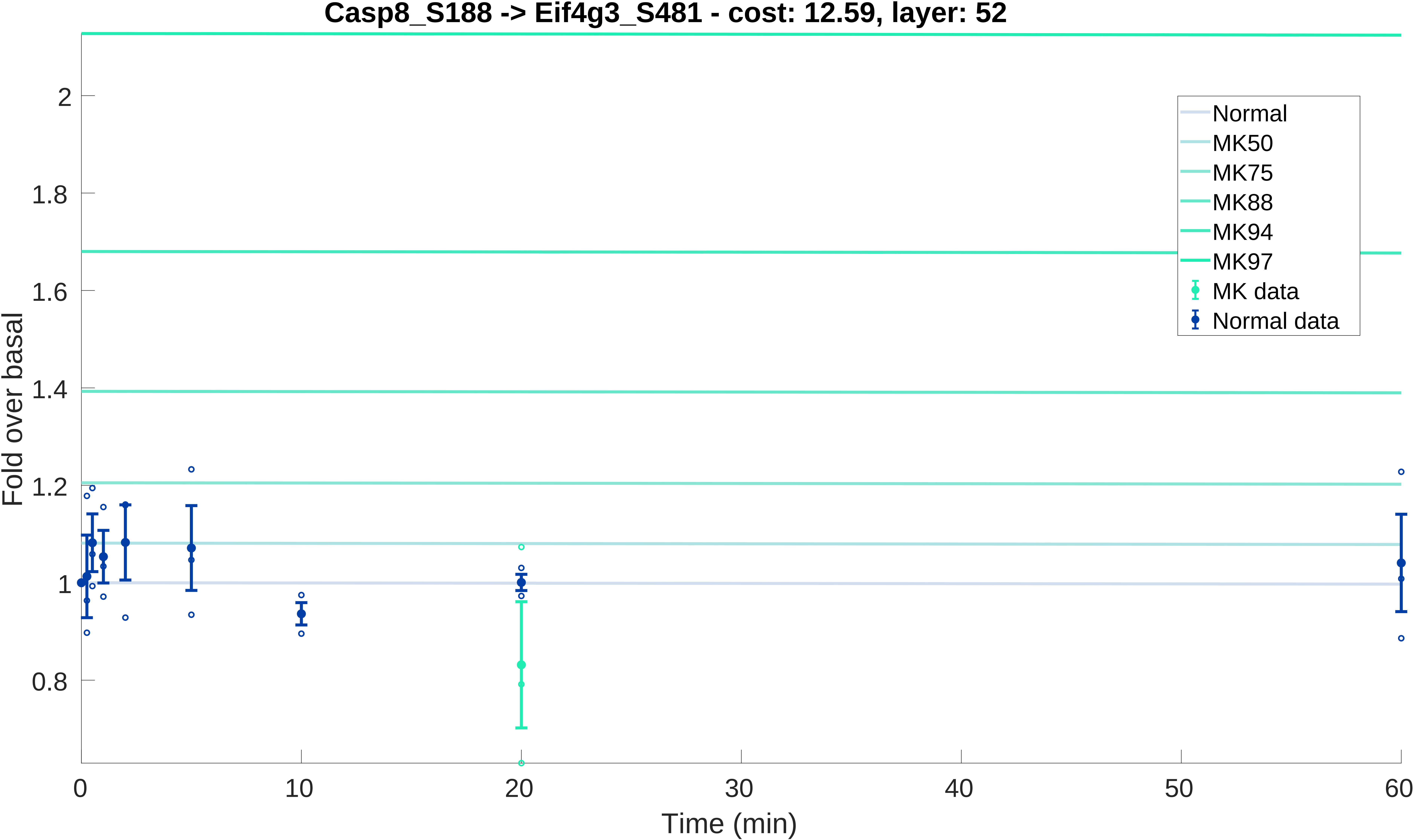
Casp8_S188 -> Eif4g3_S481 - cost: 12.59, layer: 52
Fold over basal
Time (min)
0.8
1
1.2
1.4
1.6
1.8
2
0
10
20
30
40
50
60
Normal
MK50
MK75
MK88
MK94
MK97
MK data
Normal data

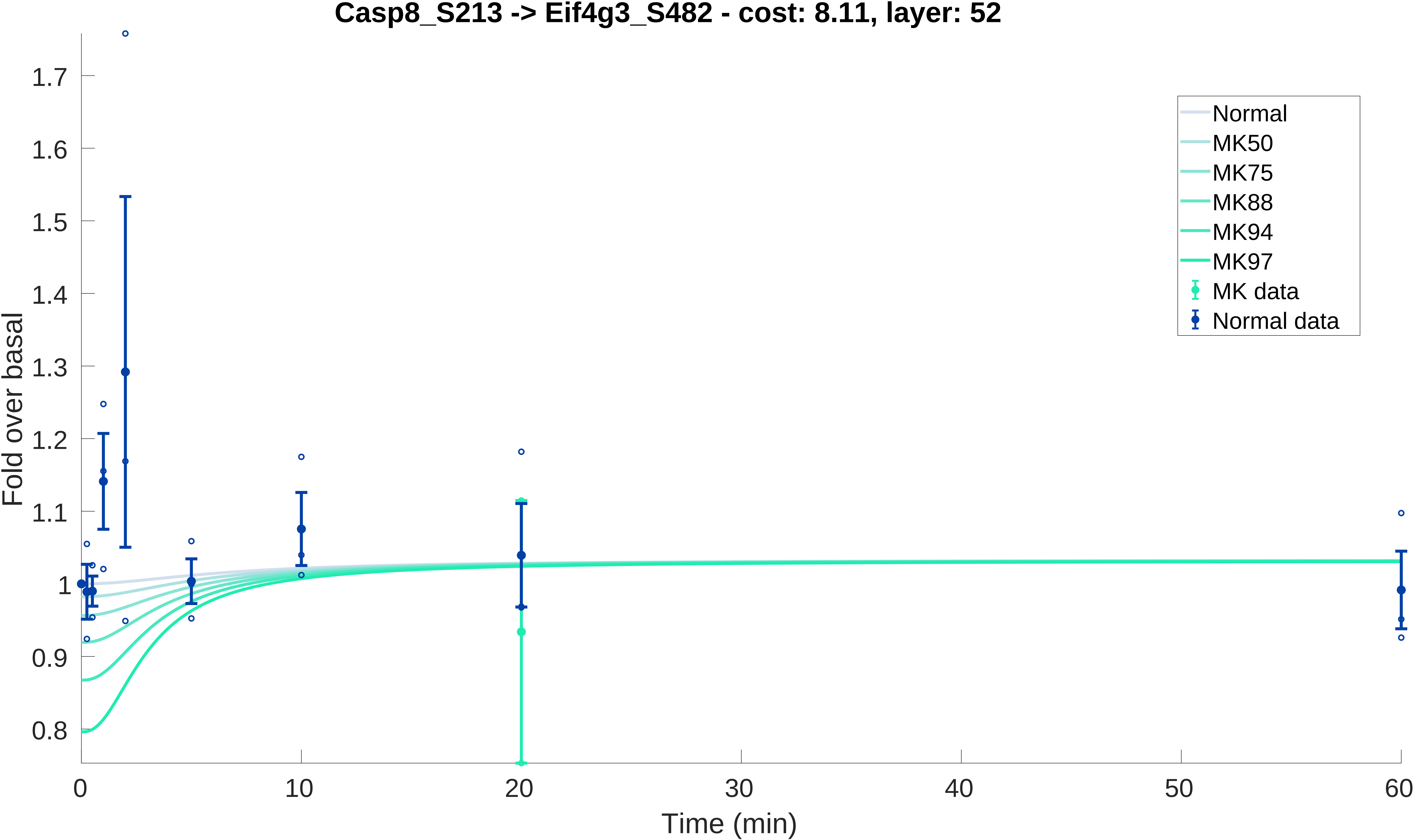

Casp8_S213 -> Eif4g3_S482 - cost: 8.11, layer: 52
Fold over basal
Time (min)
0
10
20
30
40
50
60
0.8
0.9
1
1.1
1.2
1.3
1.4
1.5
1.6
1.7
Normal
MK50
MK75
MK88
MK94
MK97
MK data
Normal data

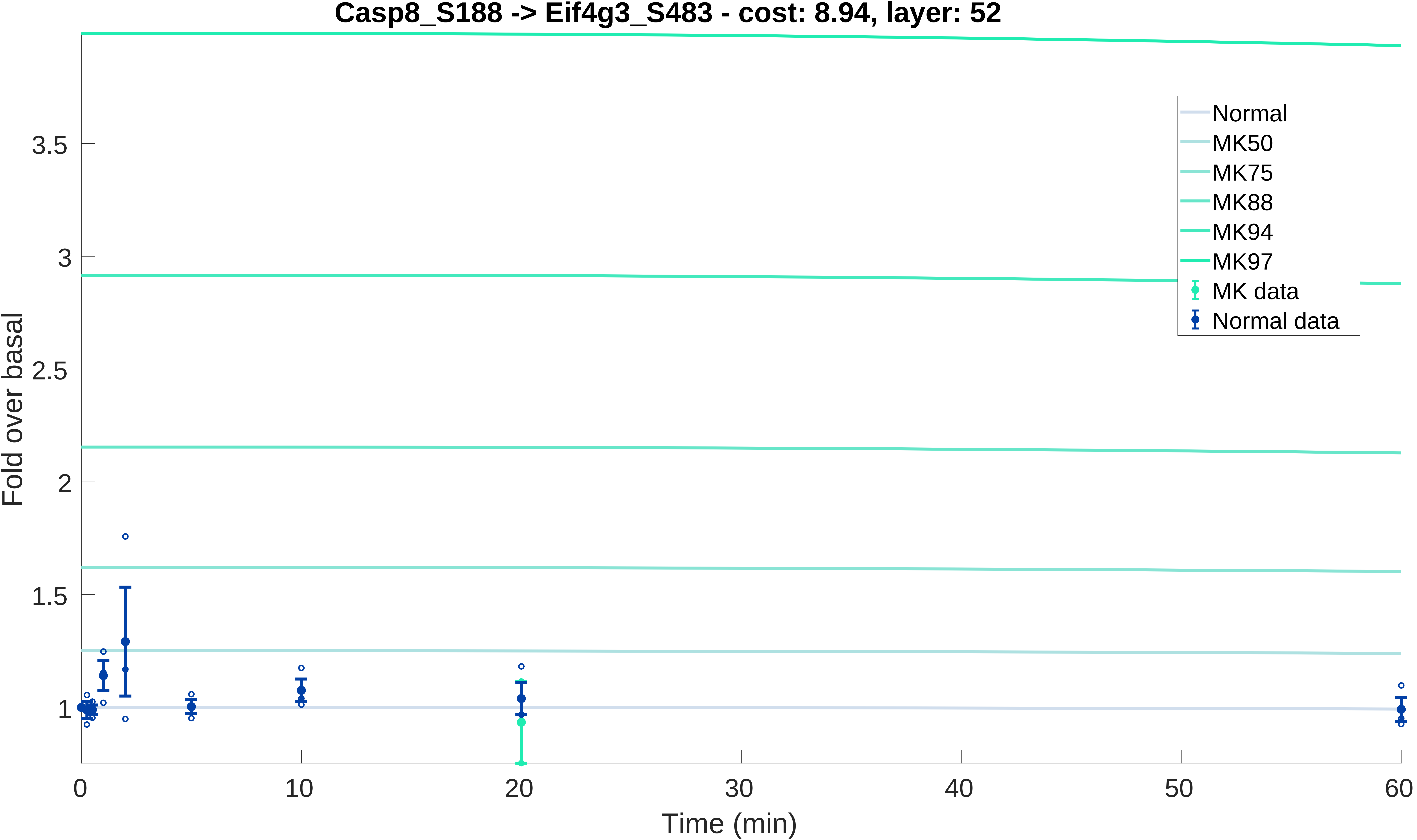

Casp8_S188 -> Eif4g3_S483 - cost: 8.94, layer: 52
Fold over basal
Time (min)
Normal
MK50
MK75
MK88
MK94
MK97
MK data
Normal data
3.5
3
2.5
2
1.5
1
0
10
20
30
40
50
60

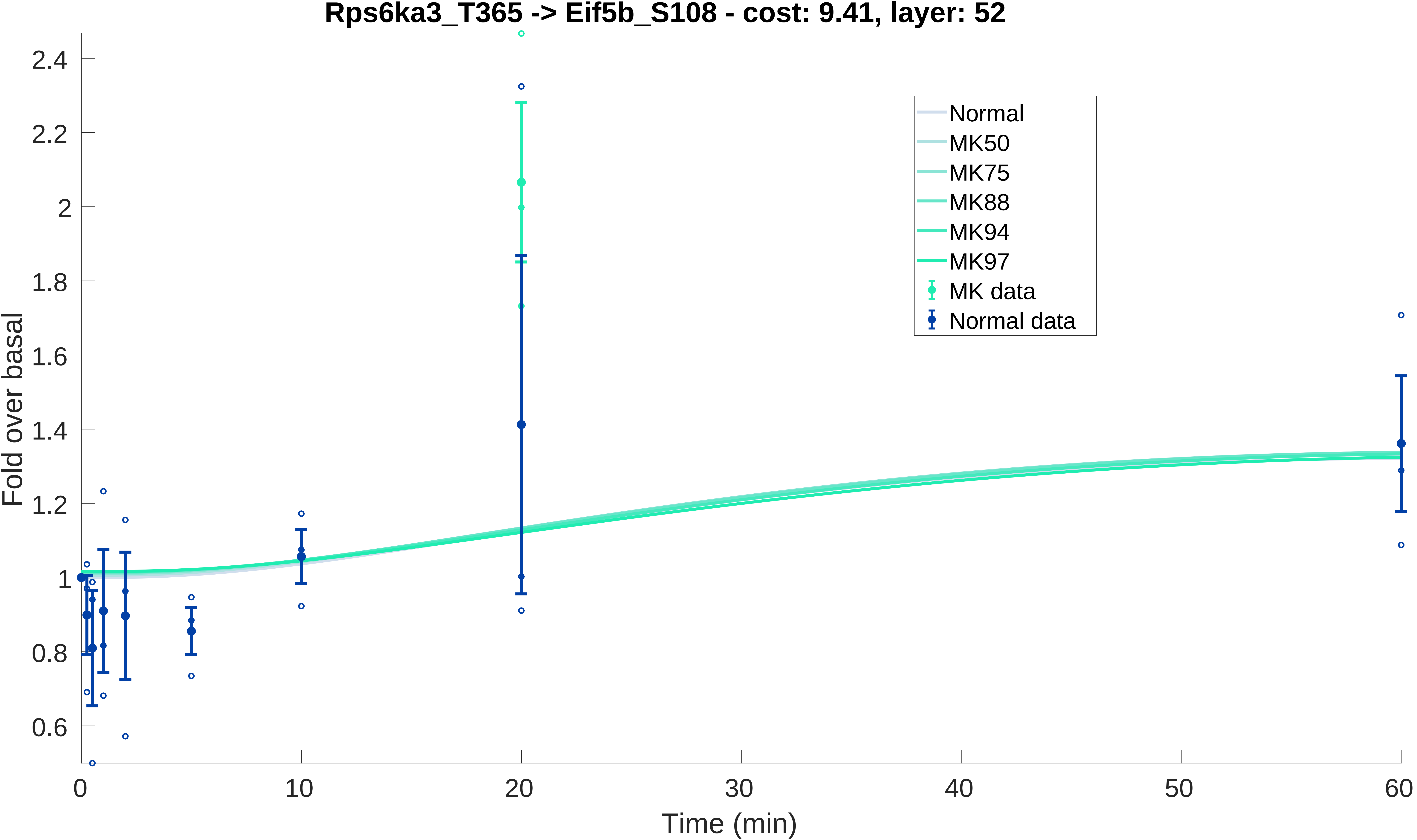
Rps6ka3_T365 -> Eif5b_S108 - cost: 9.41, layer: 52
Fold over basal
2.4
2.2
2
1.8
1.6
1.4
1.2
1
0.8
0.6
0
10
20
30
40
50
60
Time (min)
Normal
MK50
MK75
MK88
MK94
MK97
MK data
Normal data

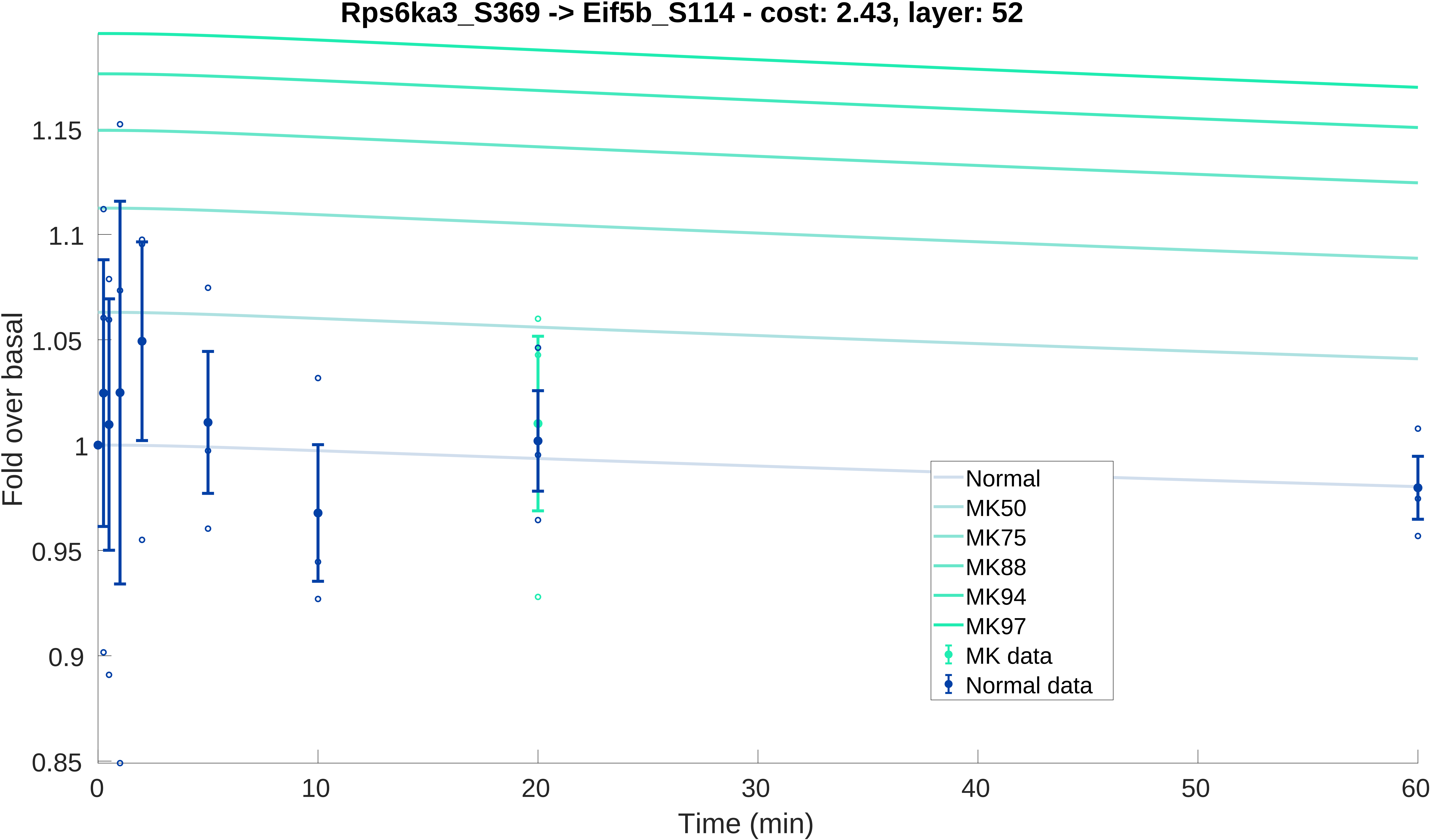

Rps6ka3_S369 -> Eif5b_S114 - cost: 2.43, layer: 52
Fold over basal
1.15
1.1
1.05
1
0.95
0.9
0.85
0
10
20
30
40
50
60
Time (min)
Normal
MK50
MK75
MK88
MK94
MK97
MK data
Normal data

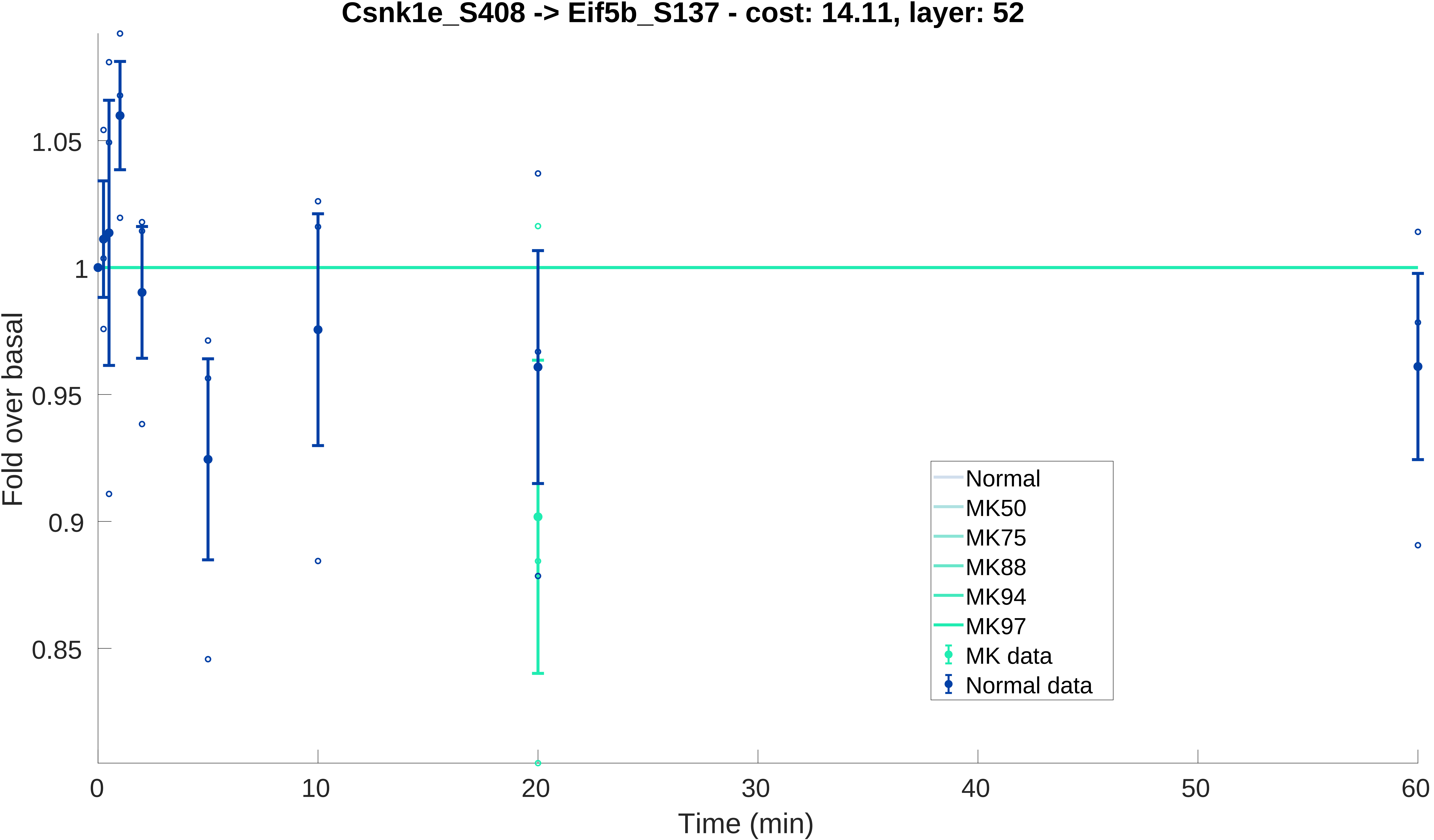

Csnk1e_S408 -> Eif5b_S137 - cost: 14.11, layer: 52
Fold over basal
1.05
1
0.95
0.9
0.85
0
10
20
30
40
50
60
Time (min)
Normal
MK50
MK75
MK88
MK94
MK97
MK data
Normal data

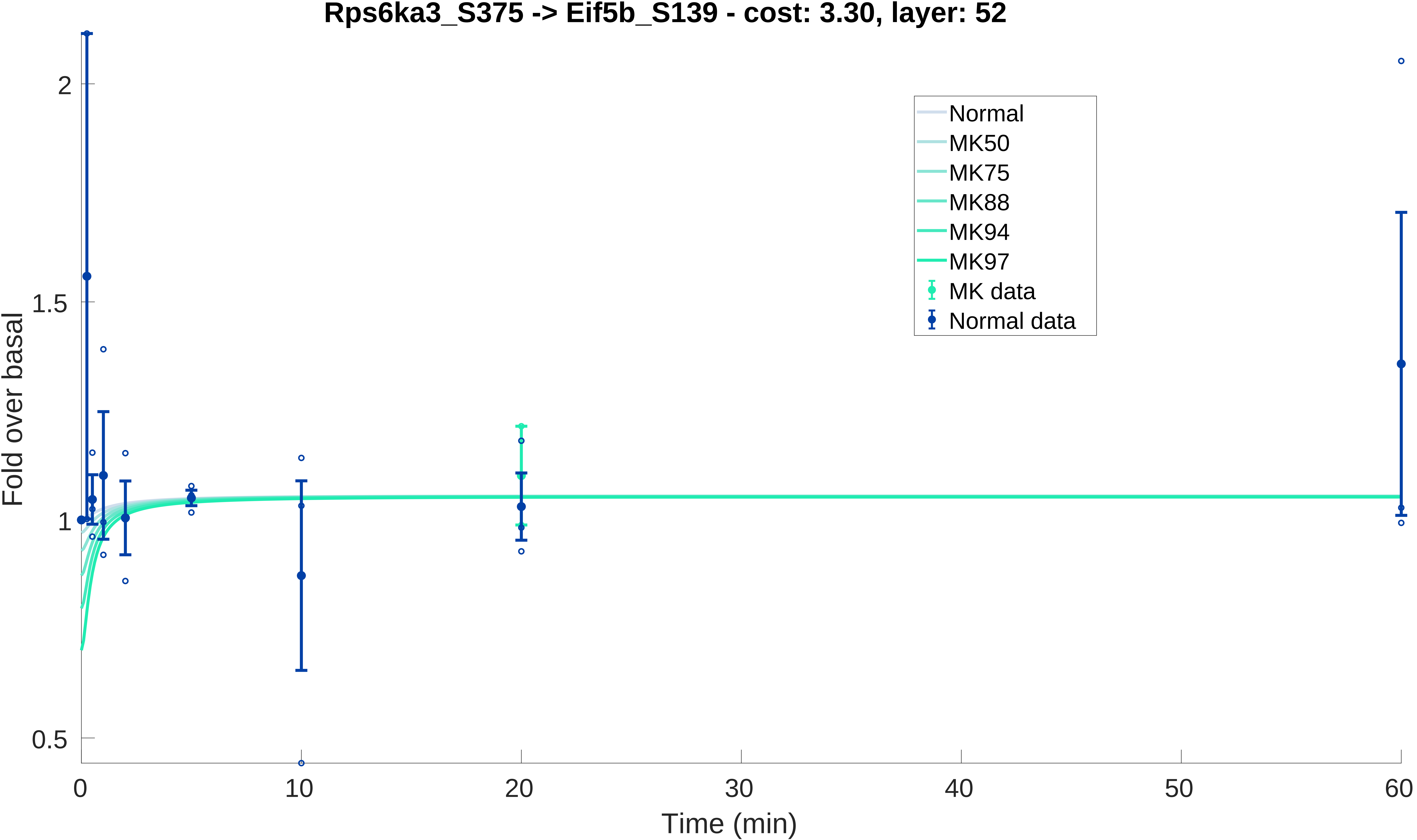

Rps6ka3_S375 -> Eif5b_S139 - cost: 3.30, layer: 52
Fold over basal
Time (min)
0.5
1
1.5
2
0
10
20
30
40
50
60
Normal
MK50
MK75
MK88
MK94
MK97
MK data
Normal data

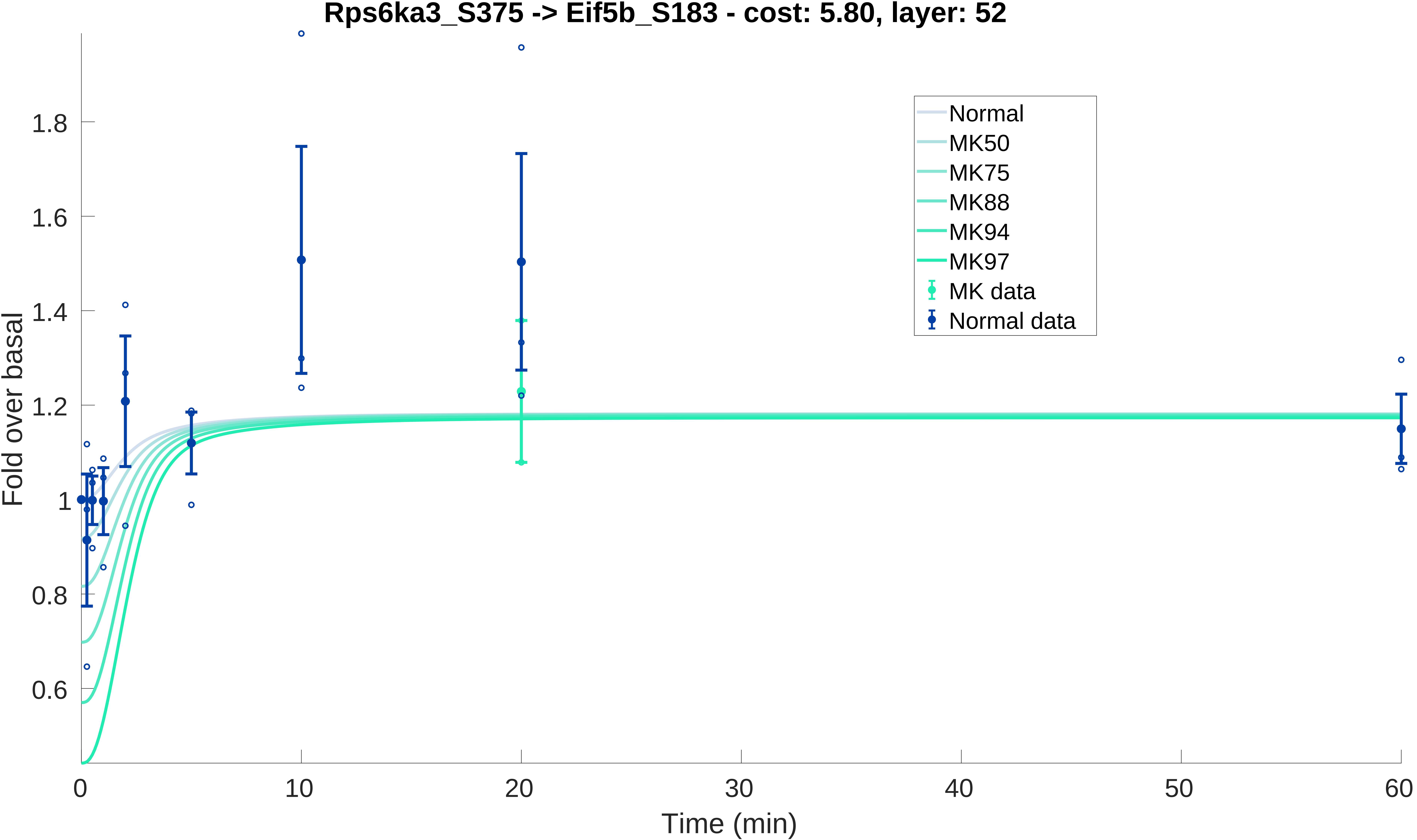

Rps6ka3_S375 -> Eif5b_S183 - cost: 5.80, layer: 52
Fold over basal
Time (min)
0.6
0.8
1
1.2
1.4
1.6
1.8
0
10
20
30
40
50
60
Normal
MK50
MK75
MK88
MK94
MK97
MK data
Normal data

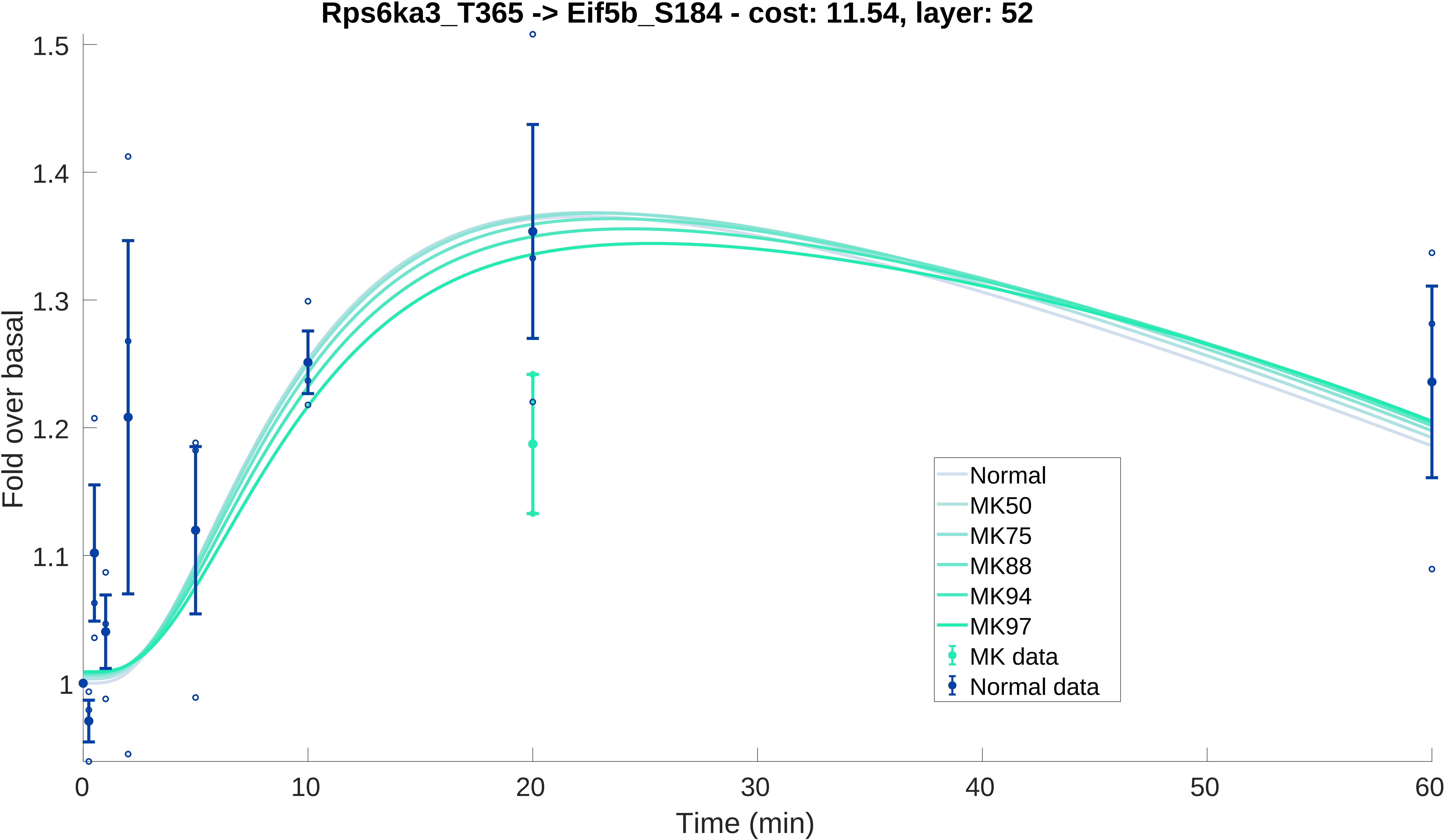

Rps6ka3_T365 -> Eif5b_S184 - cost: 11.54, layer: 52
Fold over basal
Time (min)
1.5
1.4
1.3
1.2
1.1
1
0
10
20
30
40
50
60
Normal
MK50
MK75
MK88
MK94
MK97
MK data
Normal data

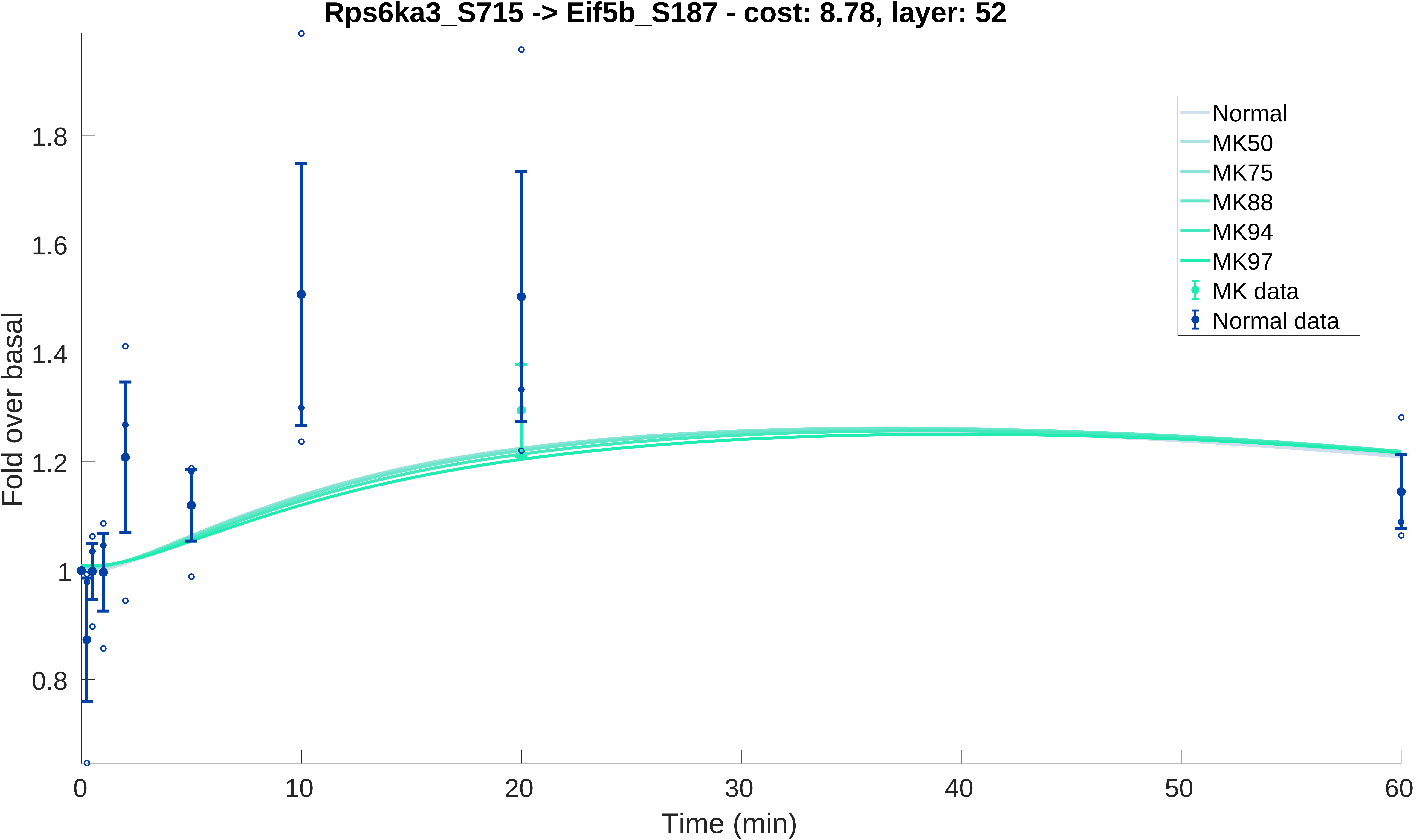
Rps6ka3_S715 -> Eif5b_S187 - cost: 8.78, layer: 52
Fold over basal
Time (min)
0.8
1
1.2
1.4
1.6
1.8
0
10
20
30
40
50
60
Normal
MK50
MK75
MK88
MK94
MK97
MK data
Normal data

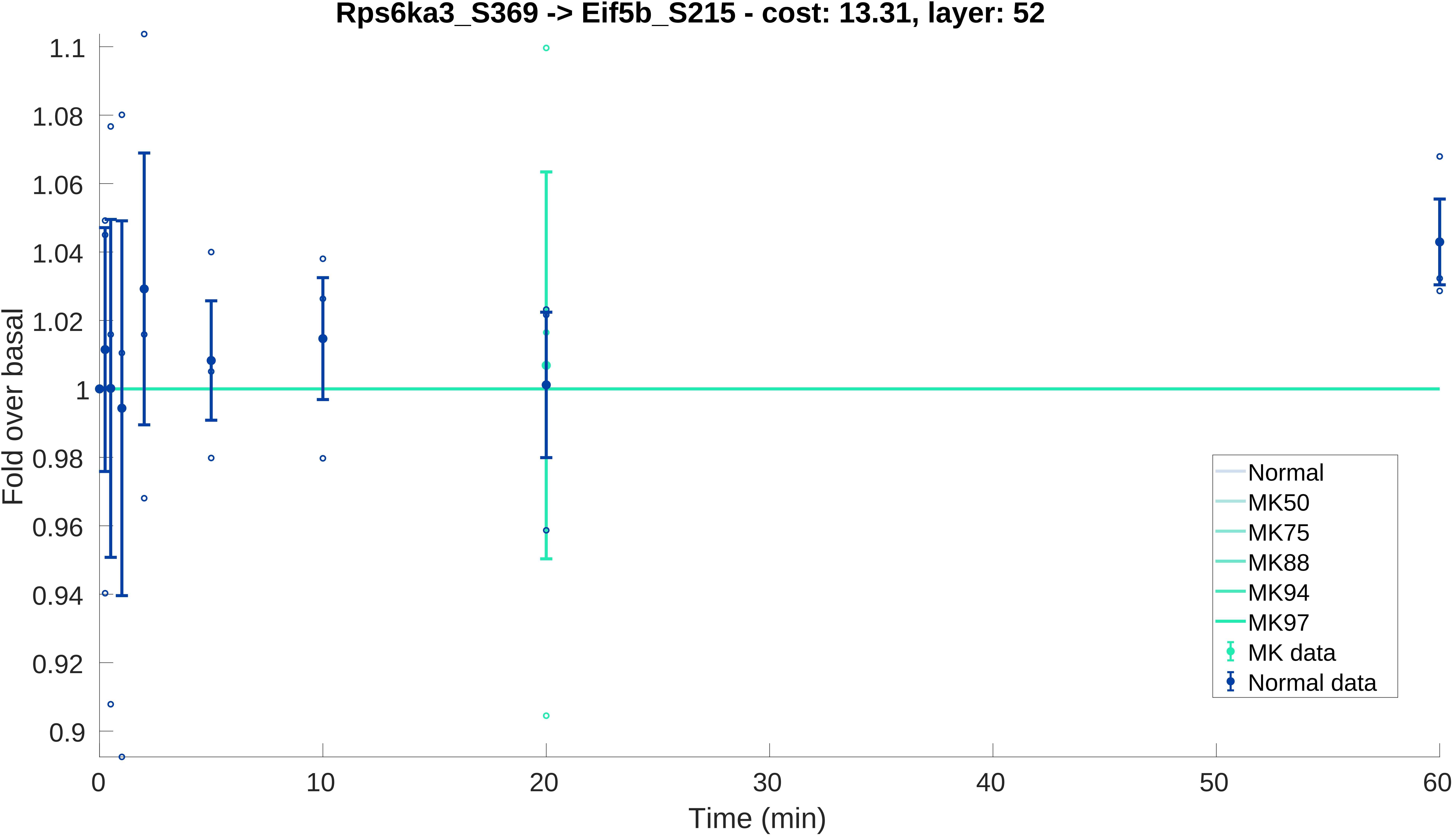
Rps6ka3_S369 -> Eif5b_S215 - cost: 13.31, layer: 52
Fold over basal
Time (min)
1.1
1.08
1.06
1.04
1.02
1
0.98
0.96
0.94
0.92
0.9
0
10
20
30
40
50
60
Normal
MK50
MK75
MK88
MK94
MK97
MK data
Normal data

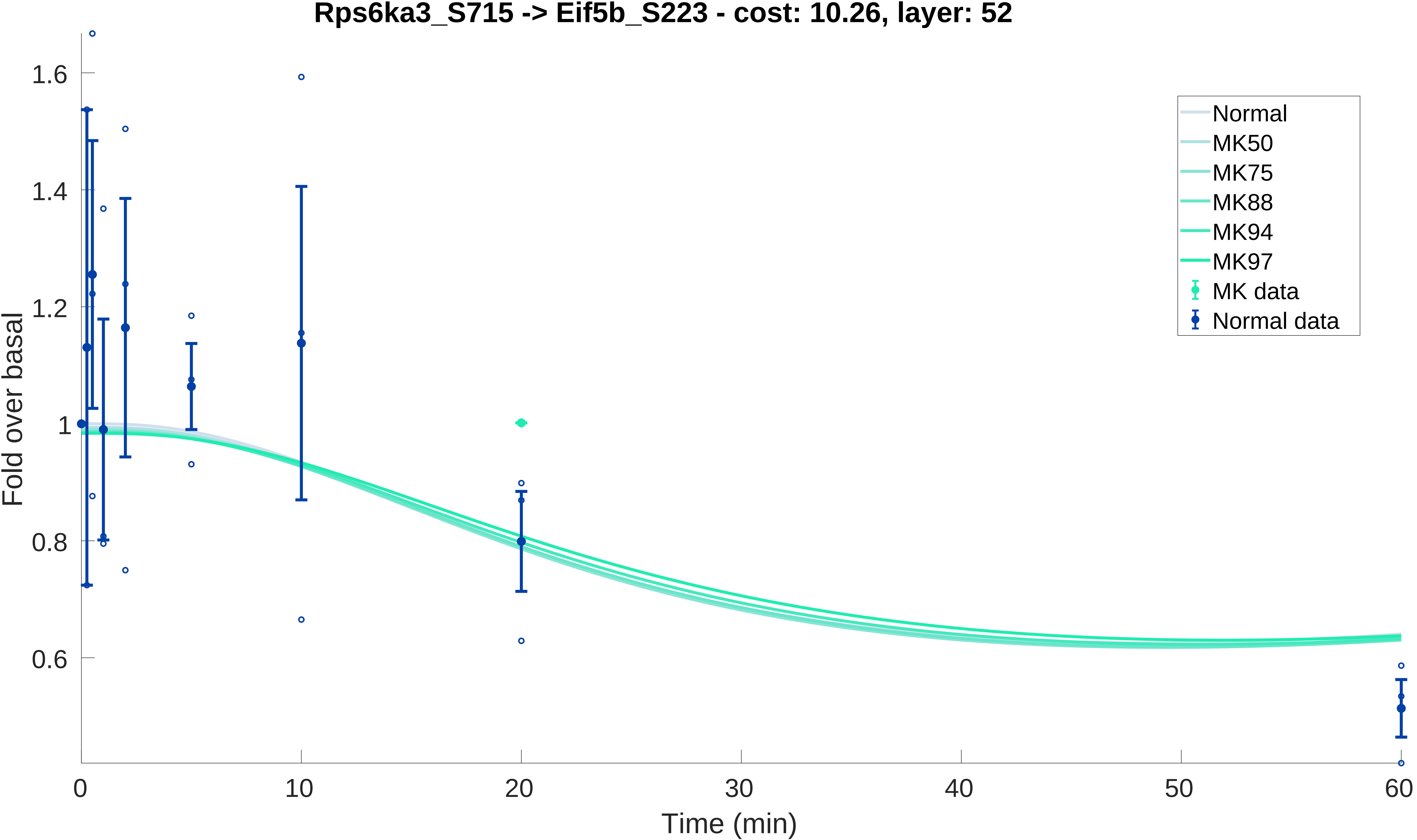

Rps6ka3_S715 -> Eif5b_S223 - cost: 10.26, layer: 52
Fold over basal
Time (min)
1.6
1.4
1.2
1
0.8
0.6
0
10
20
30
40
50
60
Normal
MK50
MK75
MK88
MK94
MK97
MK data
Normal data

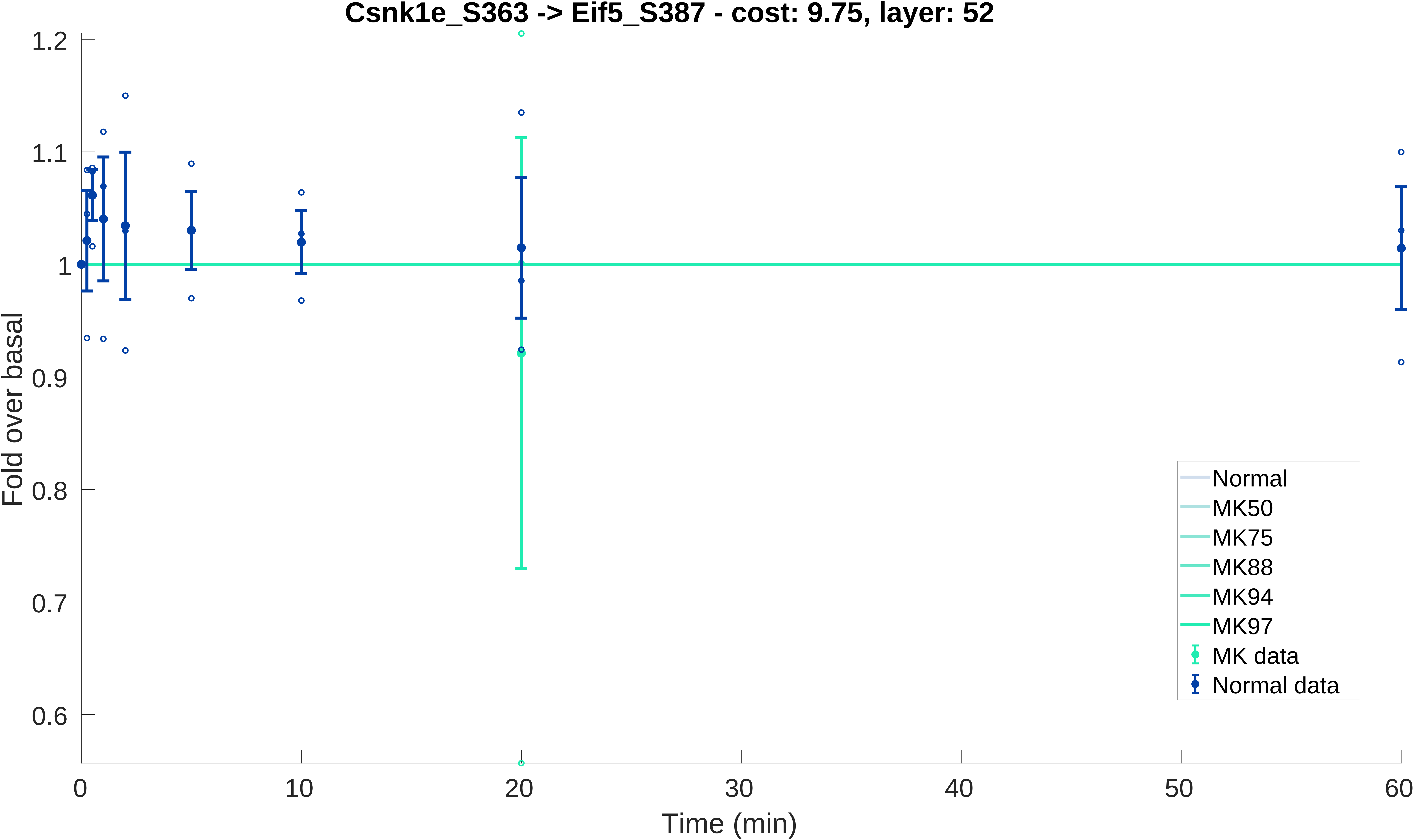

Csnk1e_S363 -> Eif5_S387 - cost: 9.75, layer: 52
Fold over basal
Time (min)
1.2
1.1
1
0.9
0.8
0.7
0.6
0
10
20
30
40
50
60
Normal
MK50
MK75
MK88
MK94
MK97
MK data
Normal data

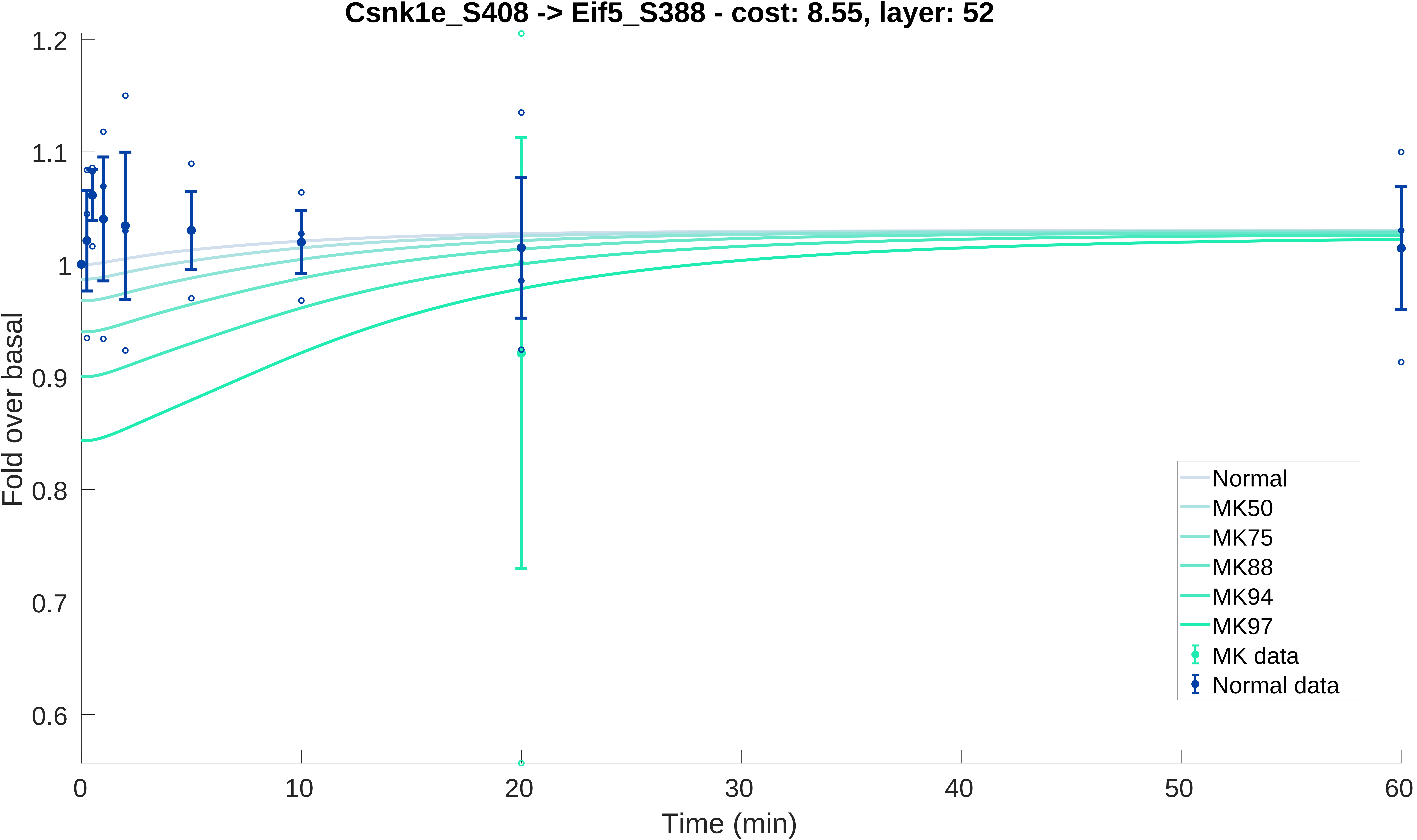

Csnk1e_S408 -> Eif5_S388 - cost: 8.55, layer: 52
Fold over basal
1.2
1.1
1
0.9
0.8
0.7
0.6
0
10
20
30
40
50
60
Time (min)
Normal
MK50
MK75
MK88
MK94
MK97
MK data
Normal data

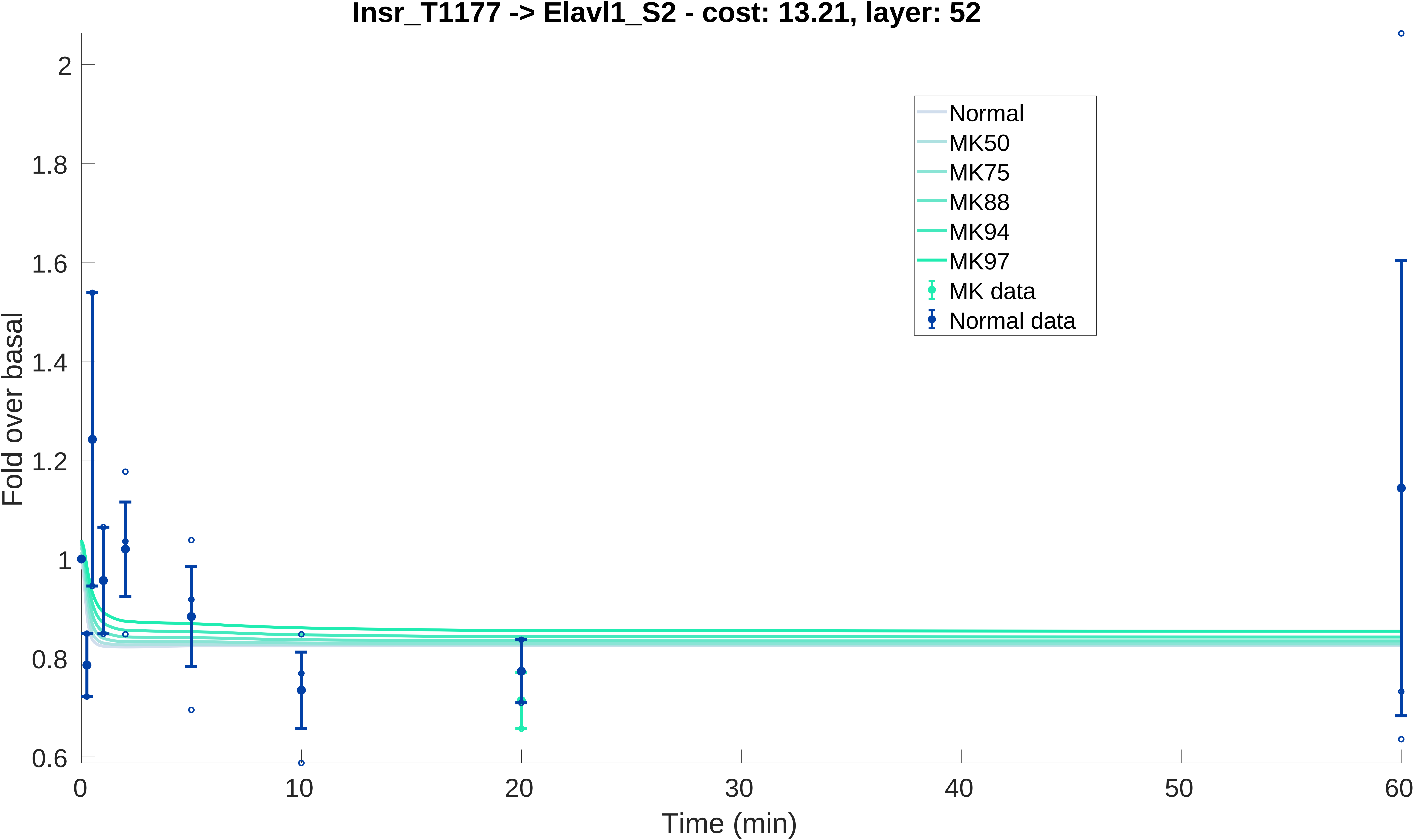

Insr_T1177 -> Elavl1_S2 - cost: 13.21, layer: 52
Fold over basal
Time (min)
Normal
MK50
MK75
MK88
MK94
MK97
MK data
Normal data
0
10
20
30
40
50
60
0.6
0.8
1
1.2
1.4
1.6
1.8
2

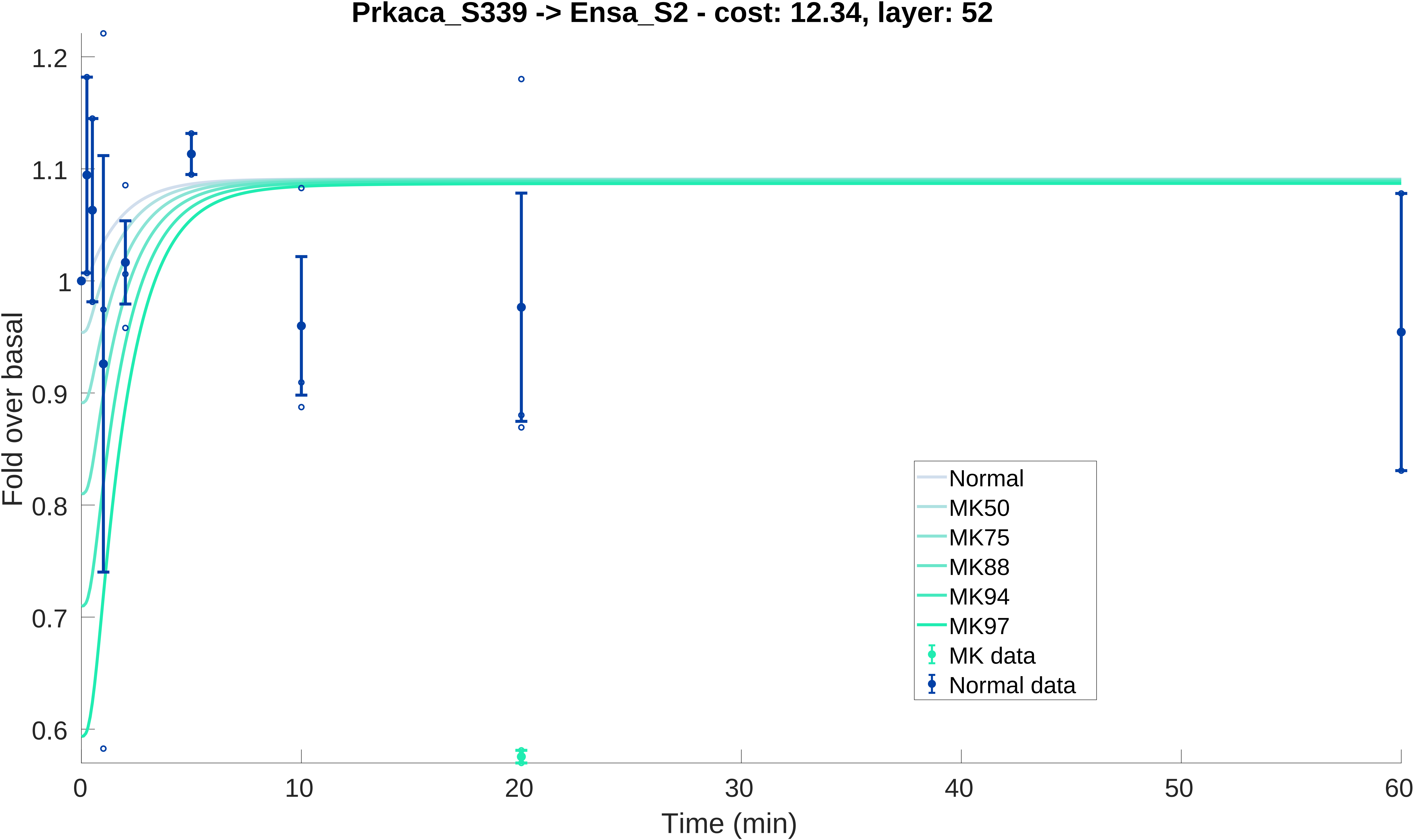

Prkaca_S339 -> Ensa_S2 - cost: 12.34, layer: 52
Fold over basal
Time (min)
0.6
0.7
0.8
0.9
1
1.1
1.2
0
10
20
30
40
50
60
Normal
MK50
MK75
MK88
MK94
MK97
MK data
Normal data

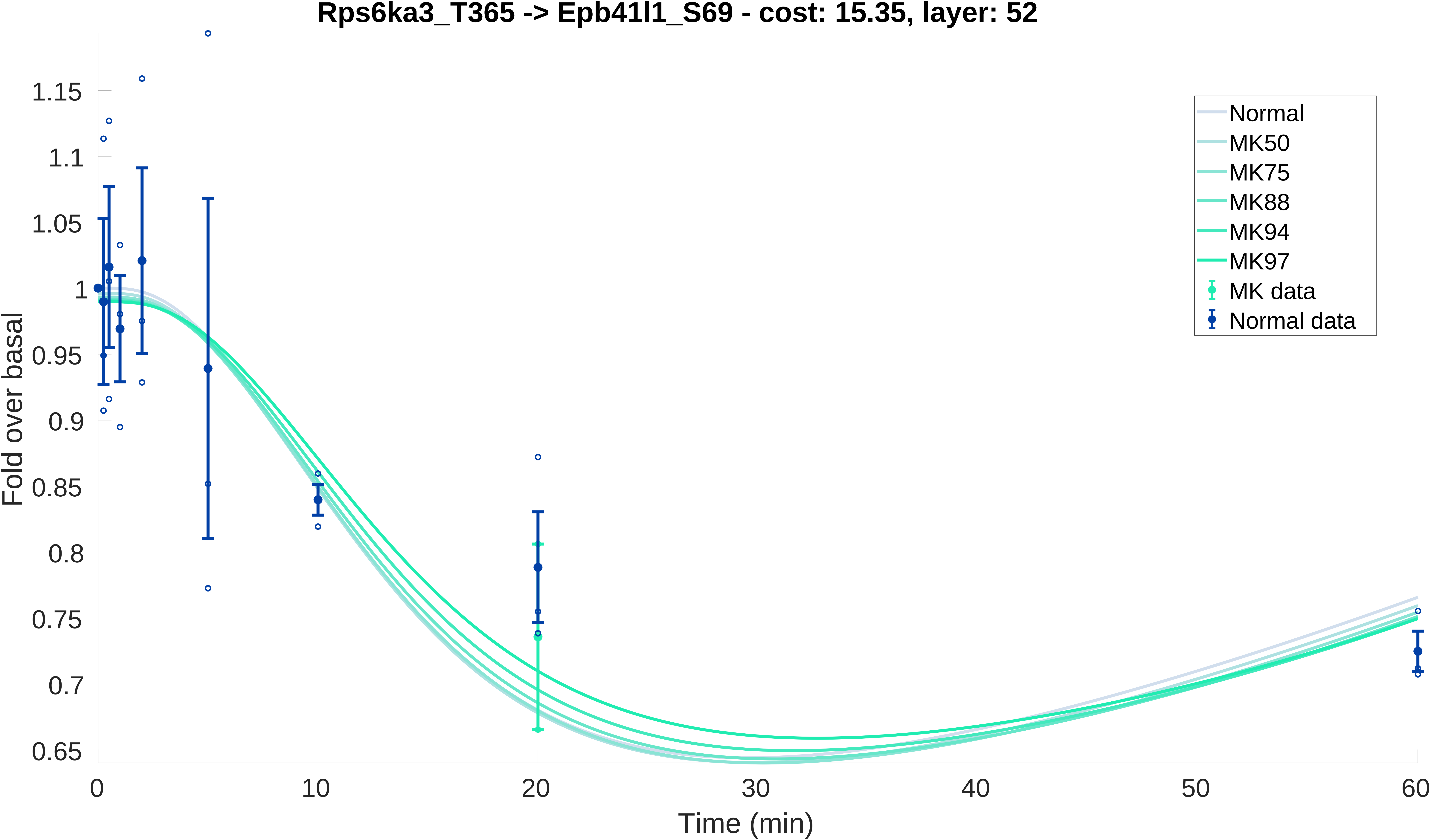
Rps6ka3_T365 -> Epb41l1_S69 - cost: 15.35, layer: 52
Fold over basal
Time (min)
1.15
1.1
1.05
1
0.95
0.9
0.85
0.8
0.75
0.7
0.65
0
10
20
30
40
50
60
Normal
MK50
MK75
MK88
MK94
MK97
MK data
Normal data

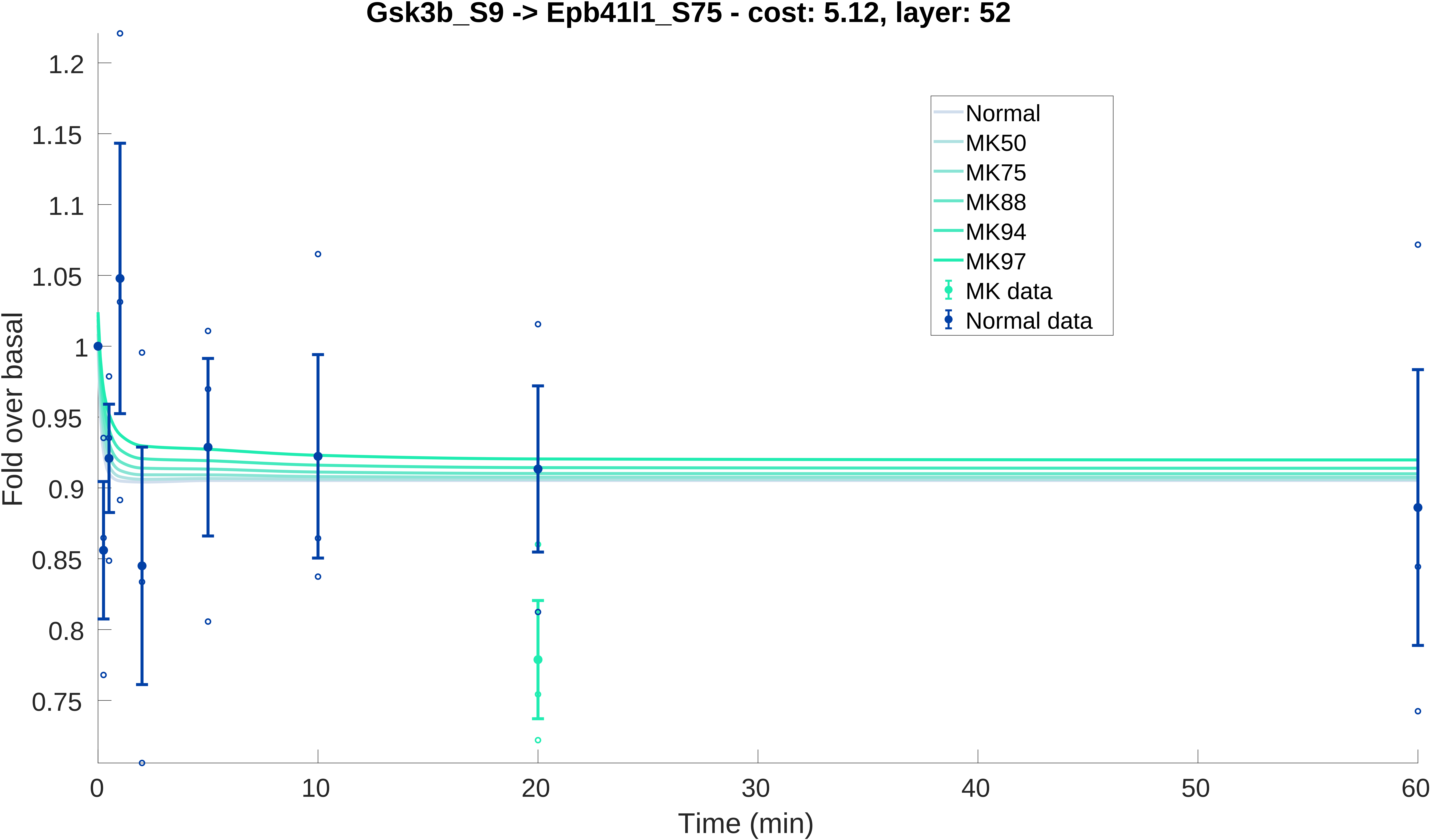

Gsk3b_S9 -> Epb41l1_S75 - cost: 5.12, layer: 52
Fold over basal
Time (min)
1.2
1.15
1.1
1.05
1
0.95
0.9
0.85
0.8
0.75
0
10
20
30
40
50
60
Normal
MK50
MK75
MK88
MK94
MK97
MK data
Normal data

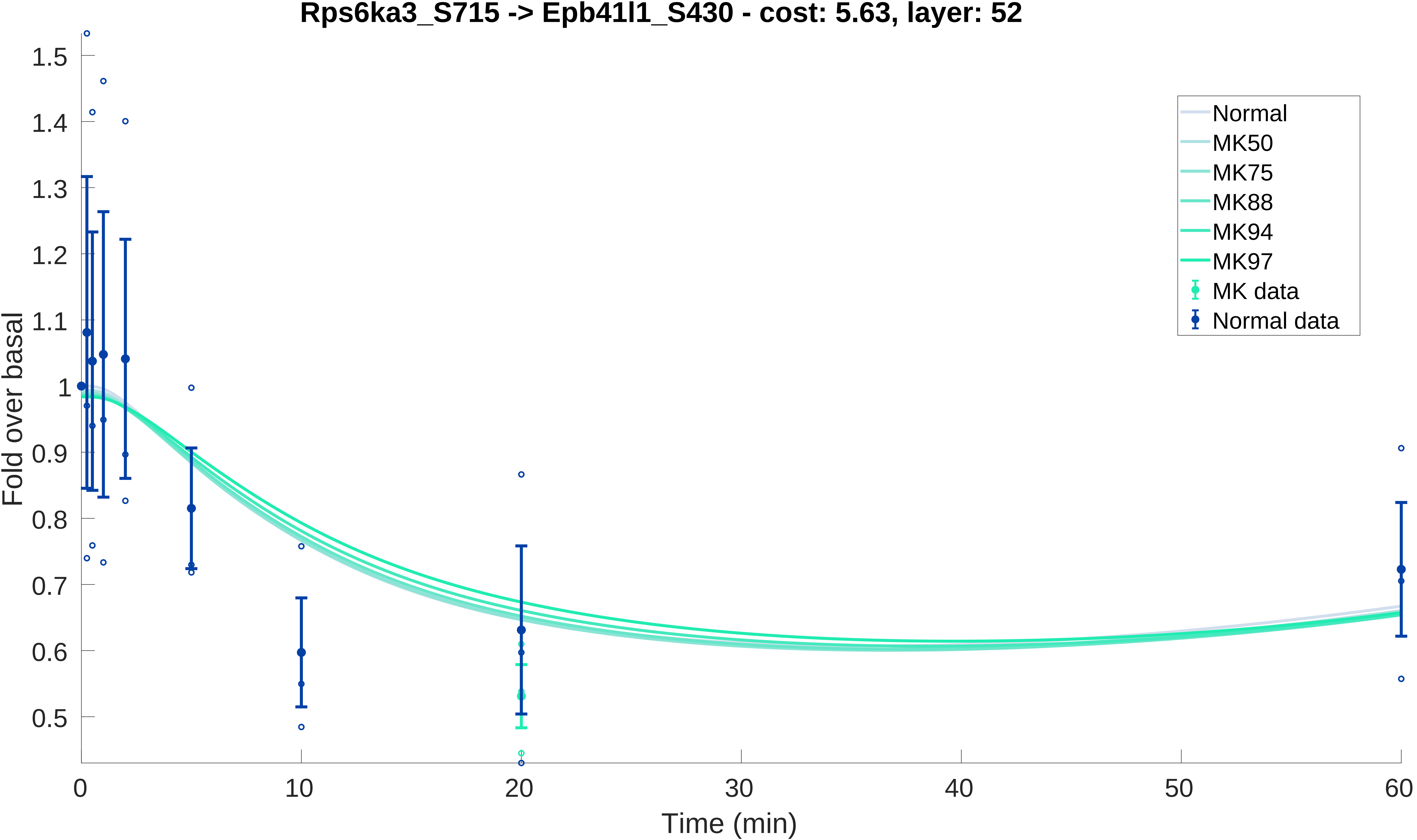

Rps6ka3_S715 -> Epb41l1_S430 - cost: 5.63, layer: 52
Fold over basal
Time (min)
1.5
1.4
1.3
1.2
1.1
1
0.9
0.8
0.7
0.6
0.5
0
10
20
30
40
50
60
Normal
MK50
MK75
MK88
MK94
MK97
MK data
Normal data

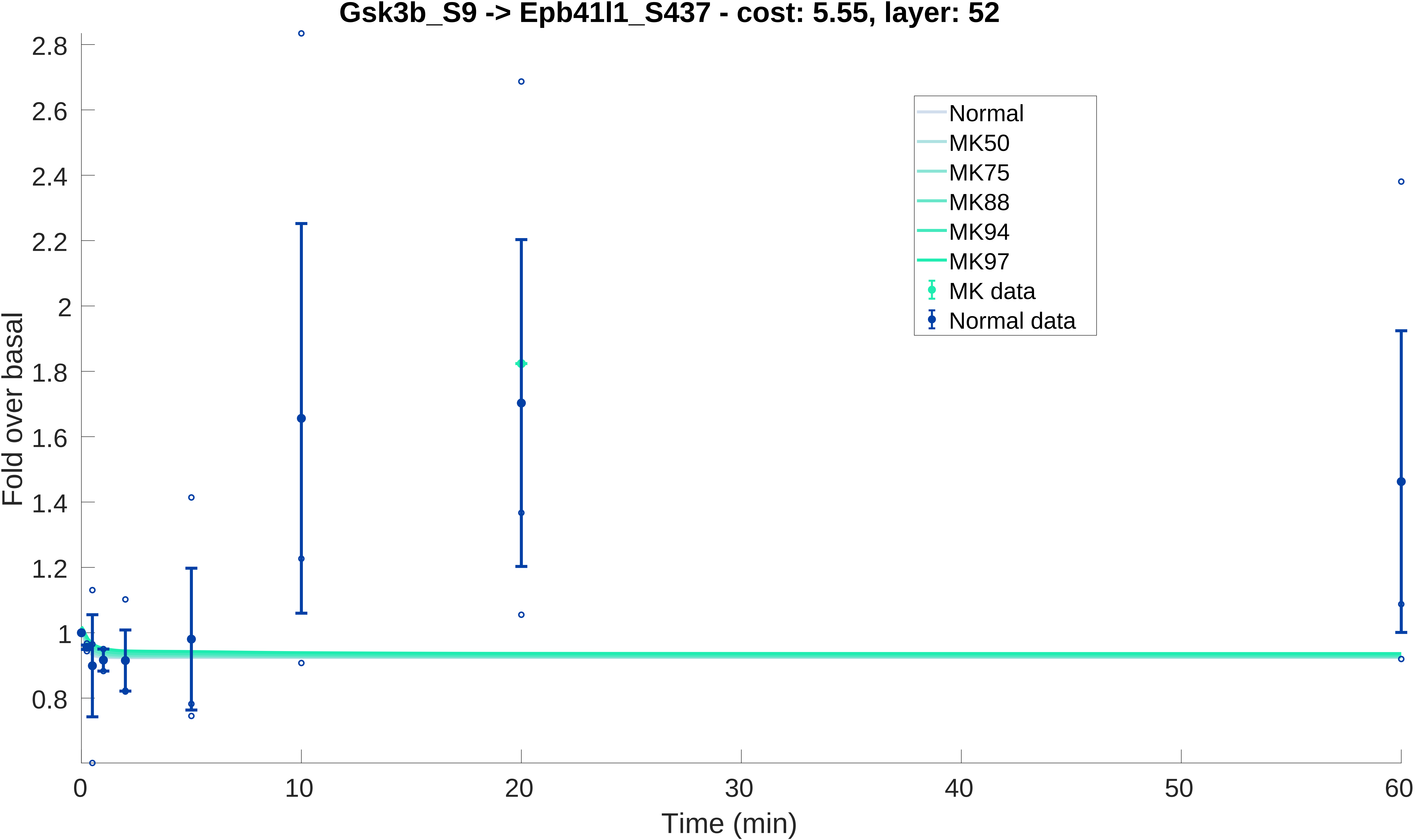

Gsk3b_S9 -> Epb41l1_S437 - cost: 5.55, layer: 52
Fold over basal
Time (min)
2.8
2.6
2.4
2.2
2
1.8
1.6
1.4
1.2
1
0.8
0
10
20
30
40
50
60
Normal
MK50
MK75
MK88
MK94
MK97
MK data
Normal data

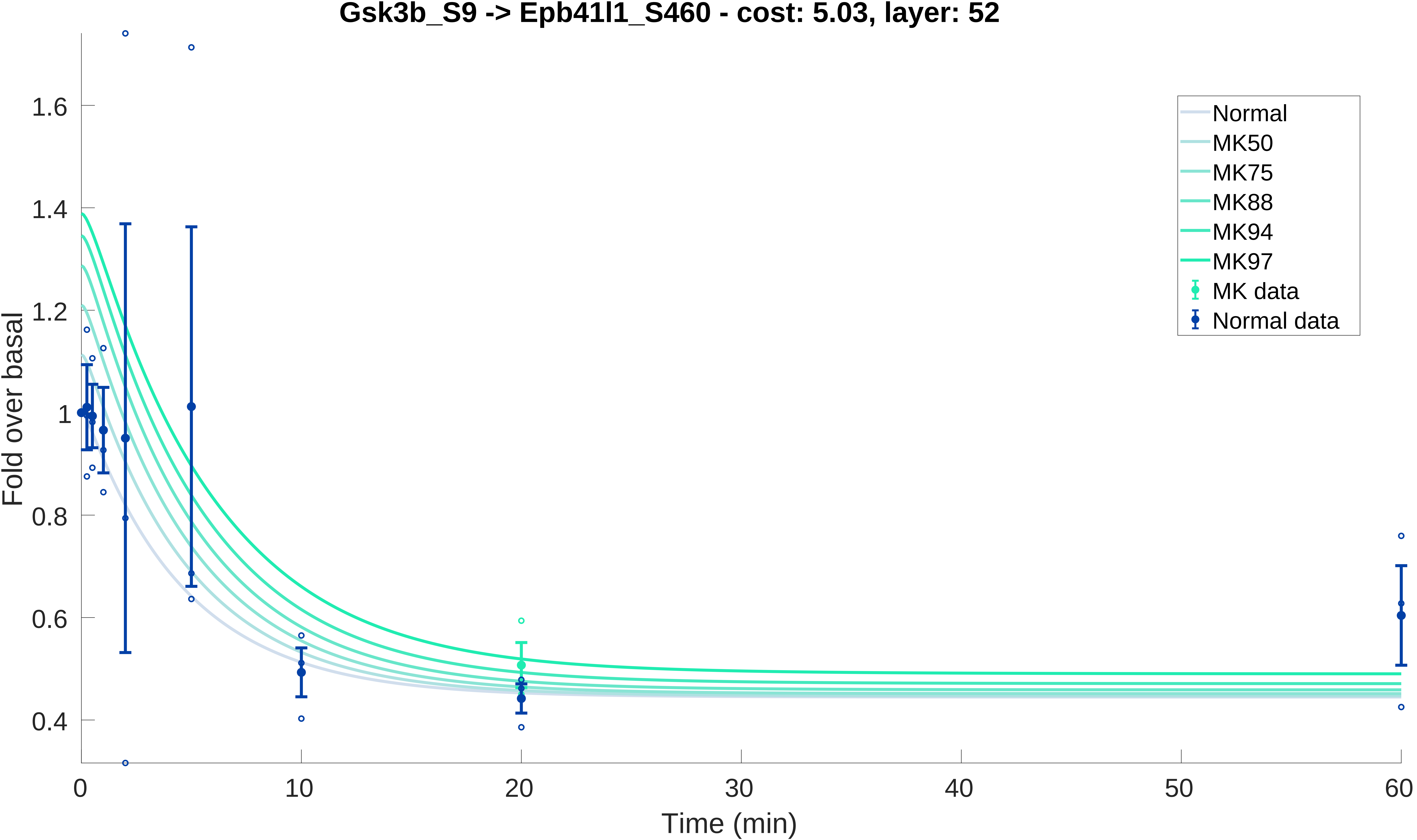
Gsk3b_S9 -> Epb41l1_S460 - cost: 5.03, layer: 52
Fold over basal
Time (min)
1.6
1.4
1.2
1
0.8
0.6
0.4
0
10
20
30
40
50
60
Normal
MK50
MK75
MK88
MK94
MK97
MK data
Normal data

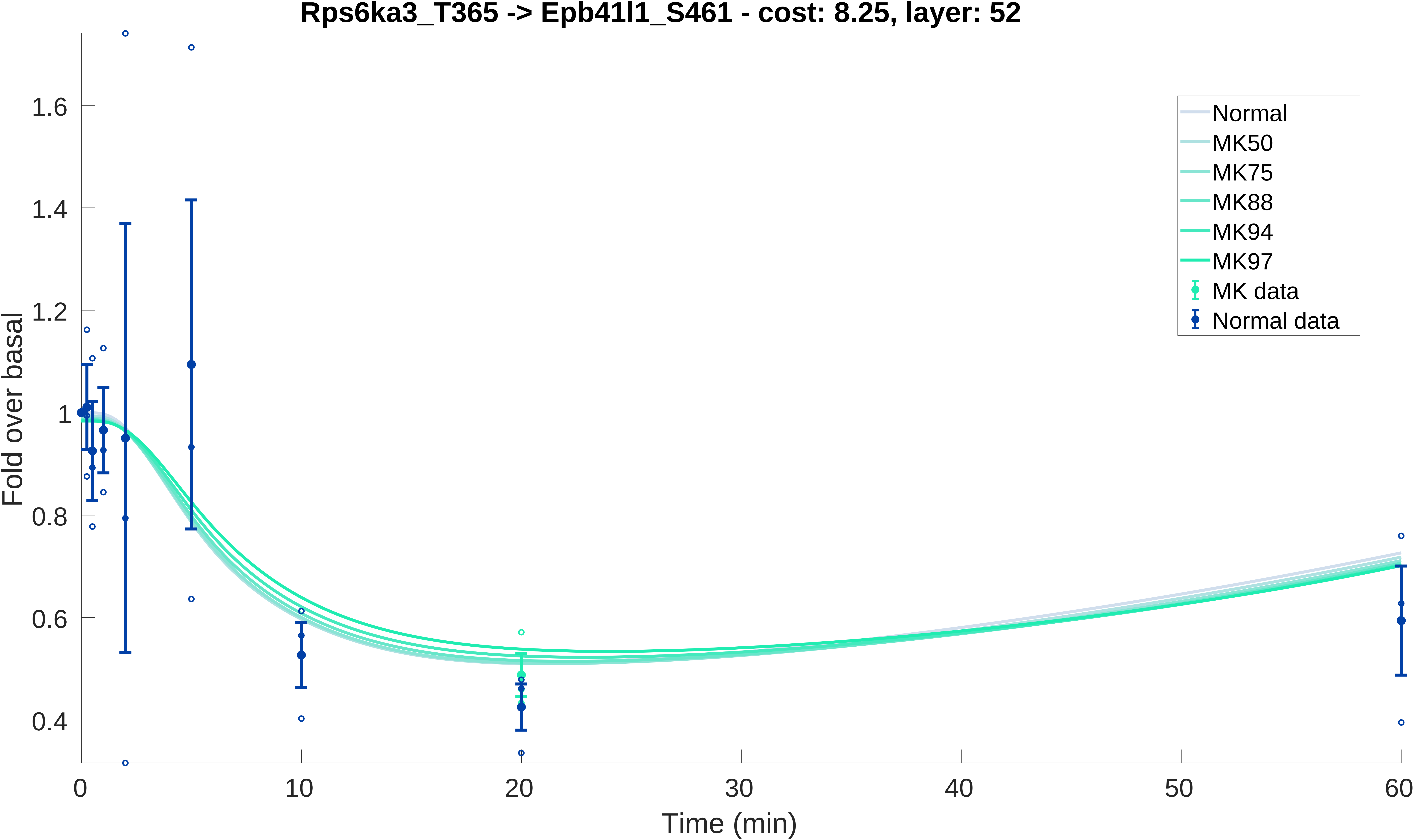

Rps6ka3_T365 -> Epb41l1_S461 - cost: 8.25, layer: 52
Fold over basal
Time (min)
0.4
0.6
0.8
1
1.2
1.4
1.6
0
10
20
30
40
50
60
Normal
MK50
MK75
MK88
MK94
MK97
MK data
Normal data

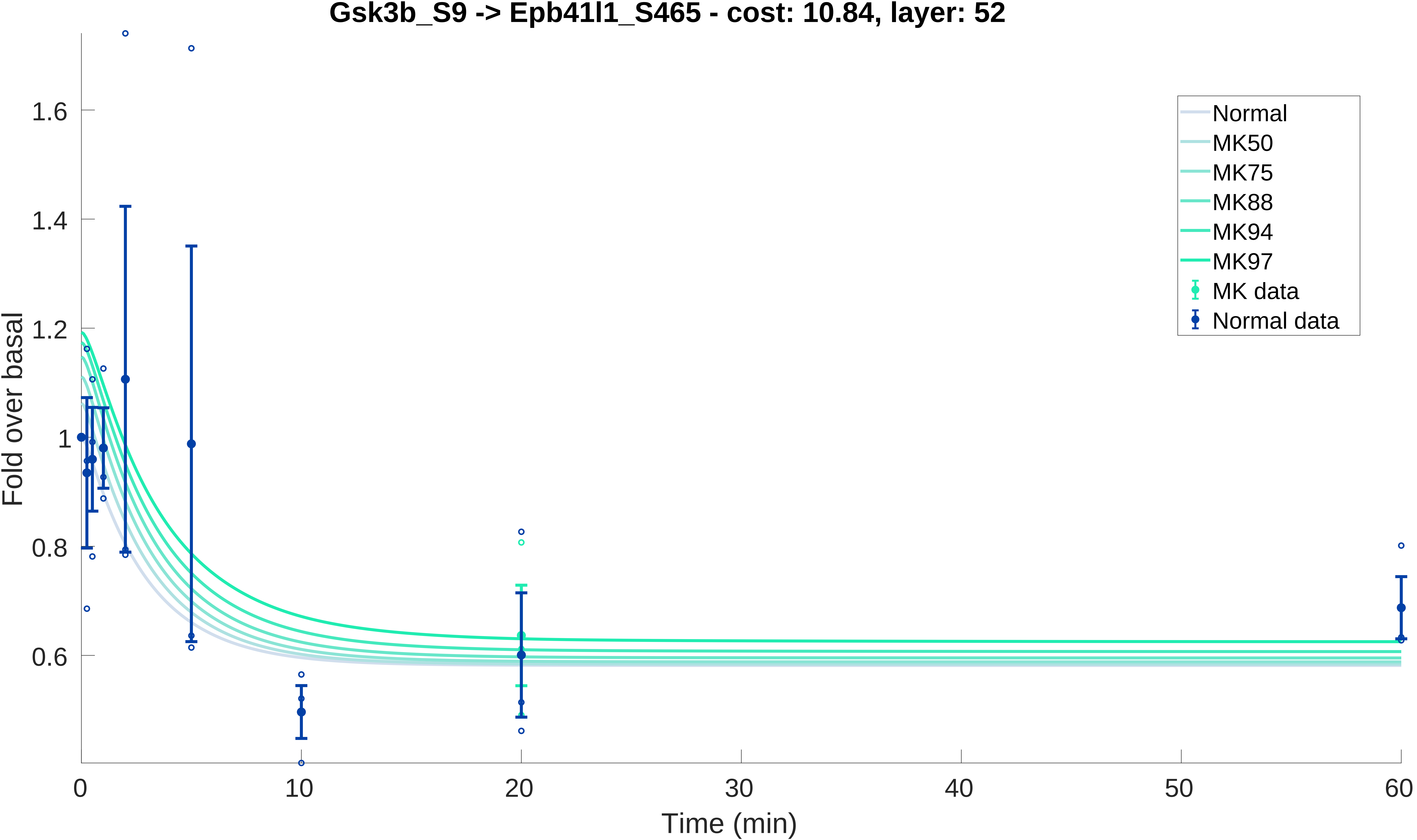

Gsk3b_S9 -> Epb41l1_S465 - cost: 10.84, layer: 52
Fold over basal
Time (min)
0
10
20
30
40
50
60
0.6
0.8
1
1.2
1.4
1.6
Normal
MK50
MK75
MK88
MK94
MK97
MK data
Normal data

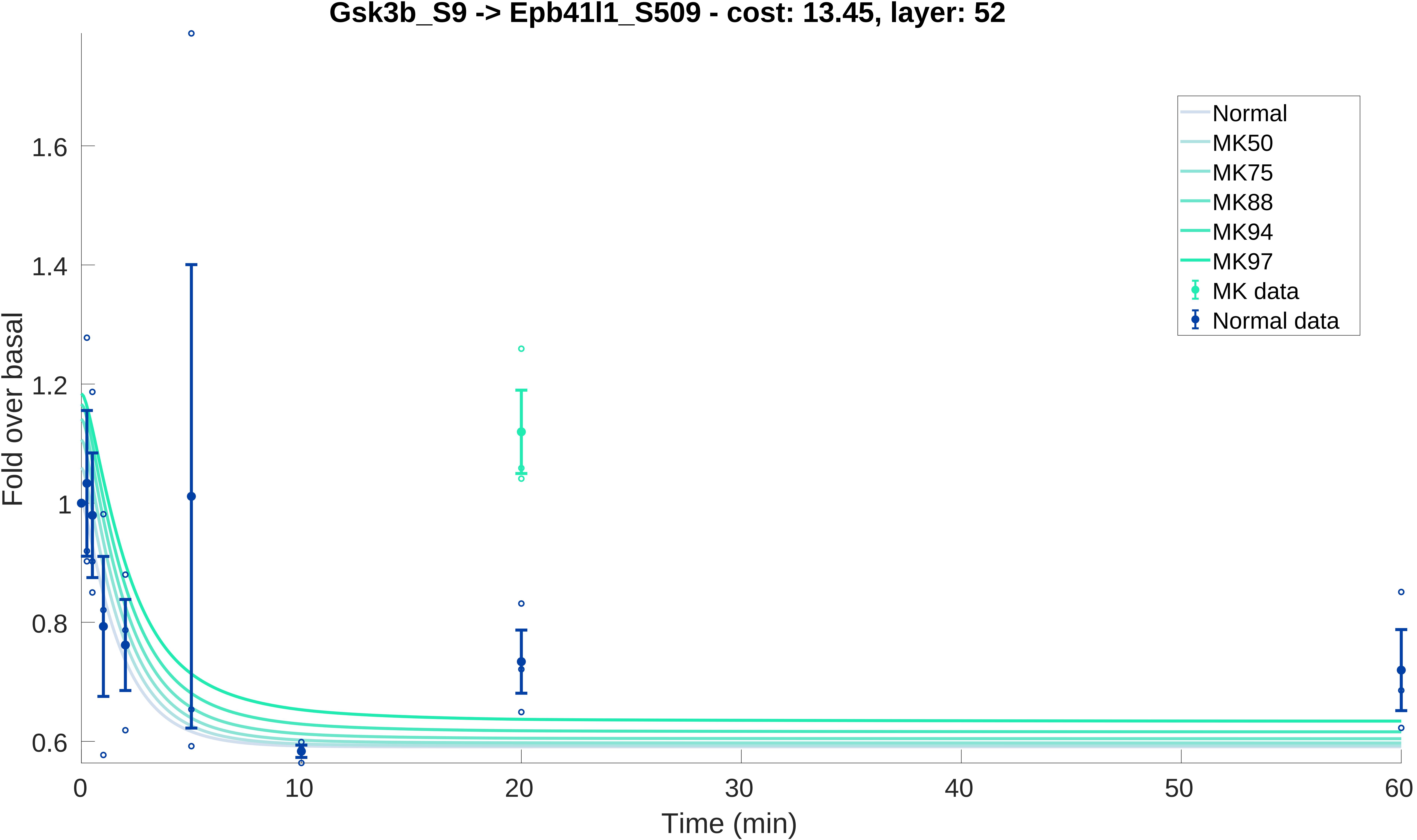

Gsk3b_S9 -> Epb41l1_S509 - cost: 13.45, layer: 52
Fold over basal
Time (min)
0.6
0.8
1
1.2
1.4
1.6
0
10
20
30
40
50
60
Normal
MK50
MK75
MK88
MK94
MK97
MK data
Normal data

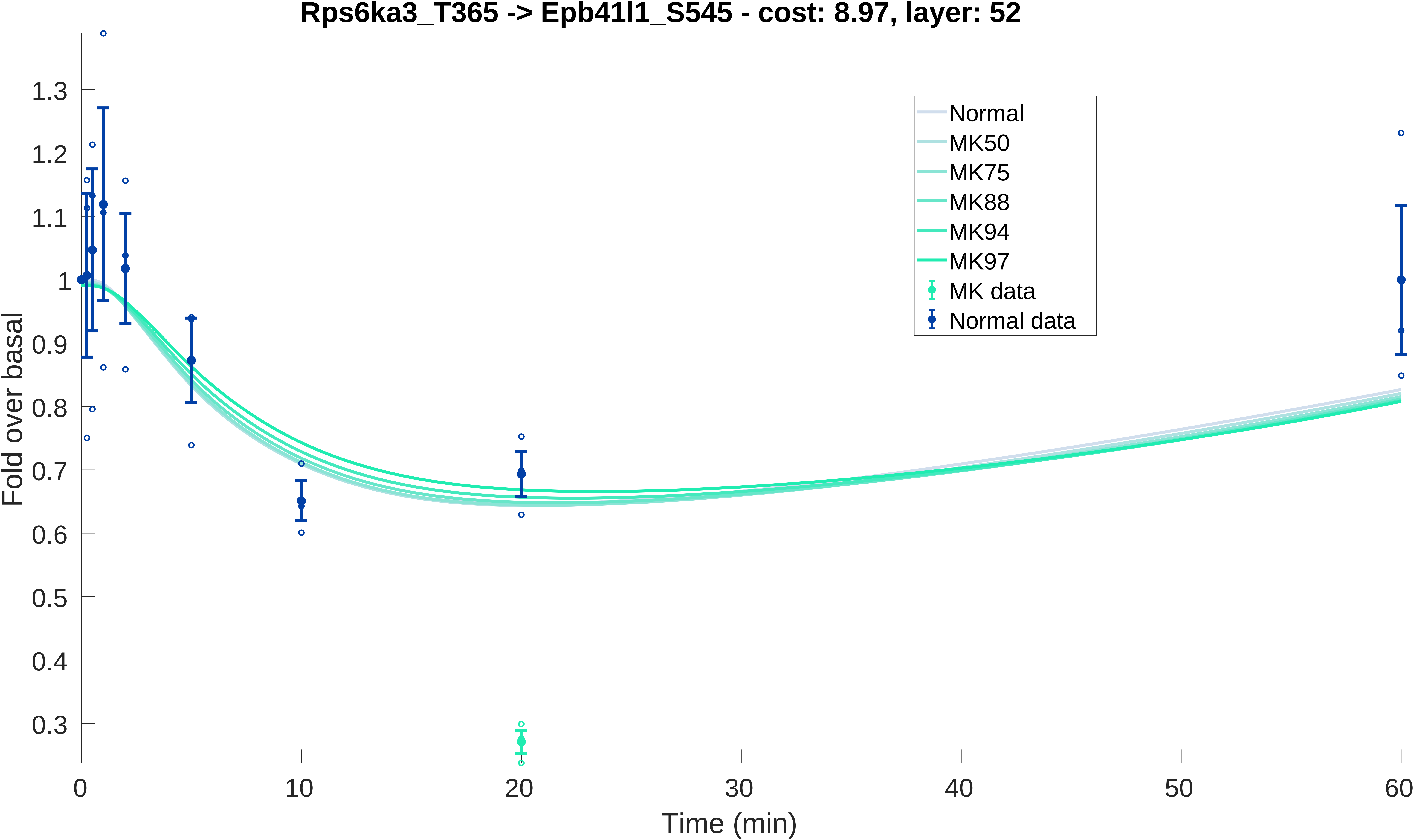

Rps6ka3_T365 -> Epb41l1_S545 - cost: 8.97, layer: 52
Fold over basal
Time (min)
Normal
MK50
MK75
MK88
MK94
MK97
MK data
Normal data
1.3
1.2
1.1
1
0.9
0.8
0.7
0.6
0.5
0.4
0.3
0
10
20
30
40
50
60

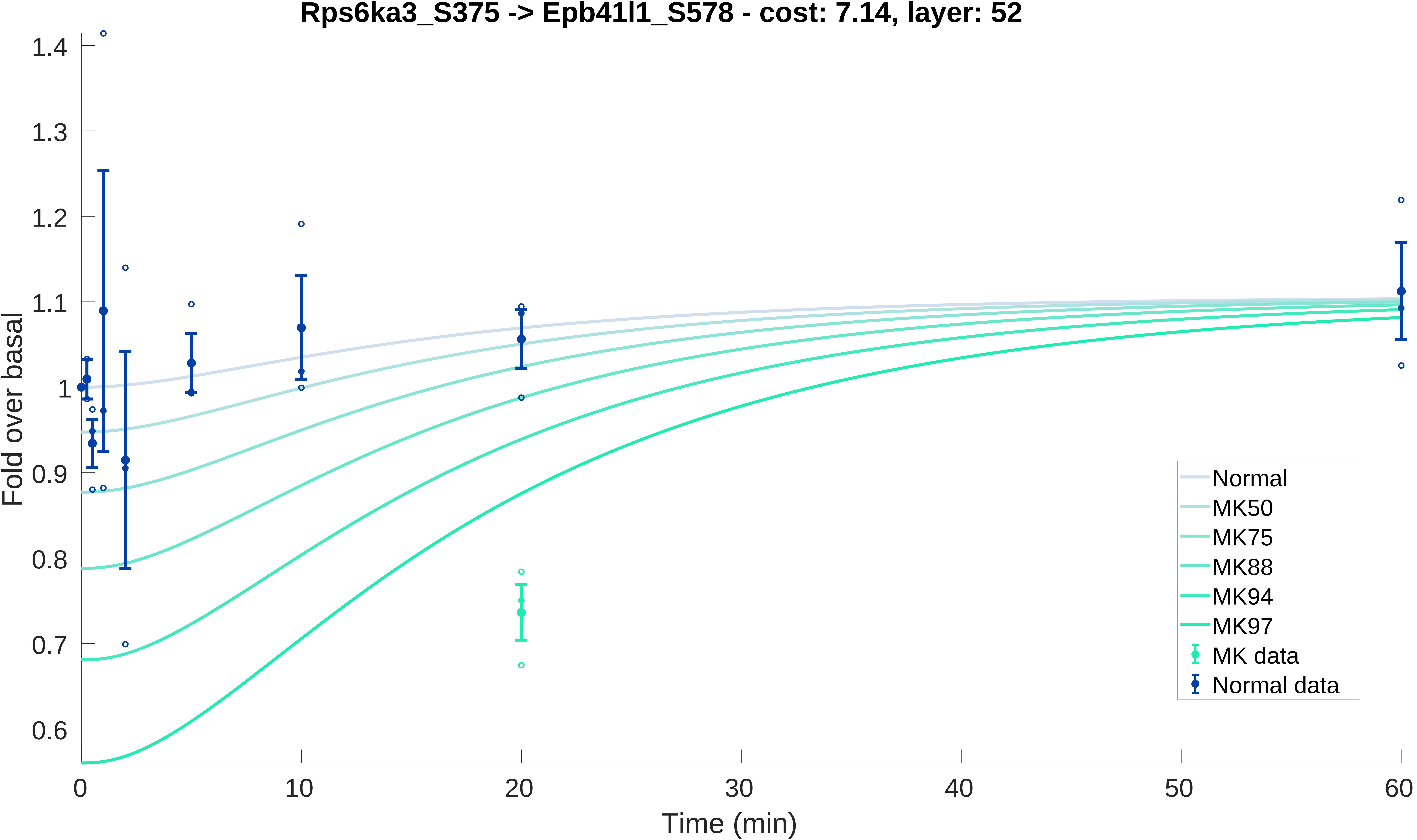
Rps6ka3_S375 -> Epb41l1_S578 - cost: 7.14, layer: 52
Fold over basal
1.4
1.3
1.2
1.1
1
0.9
0.8
0.7
0.6
0
10
20
30
40
50
60
Time (min)
Normal
MK50
MK75
MK88
MK94
MK97
MK data
Normal data

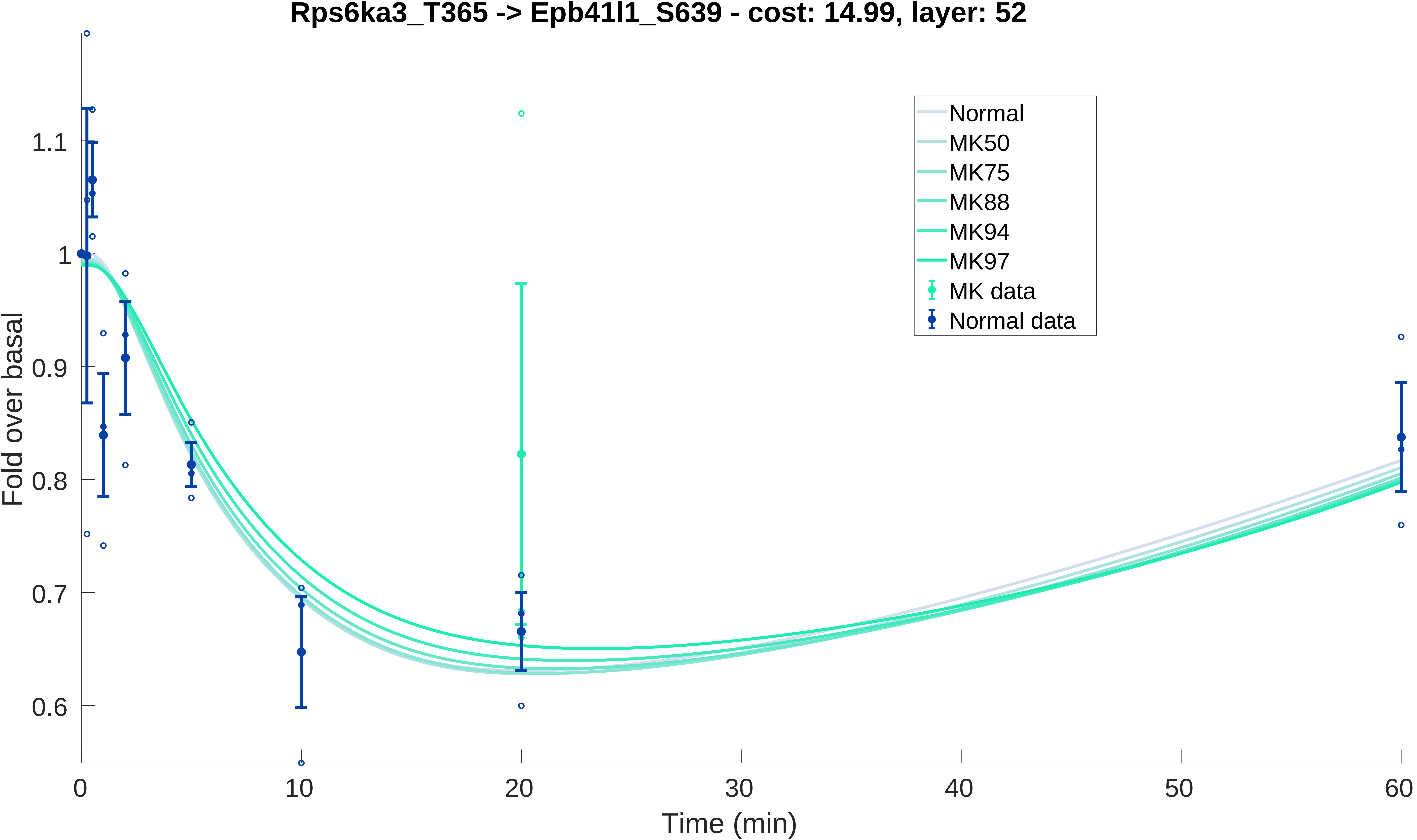

Rps6ka3_T365 -> Epb41l1_S639 - cost: 14.99, layer: 52
Fold over basal
Time (min)
0.6
0.7
0.8
0.9
1
1.1
0
10
20
30
40
50
60
Normal
MK50
MK75
MK88
MK94
MK97
MK data
Normal data

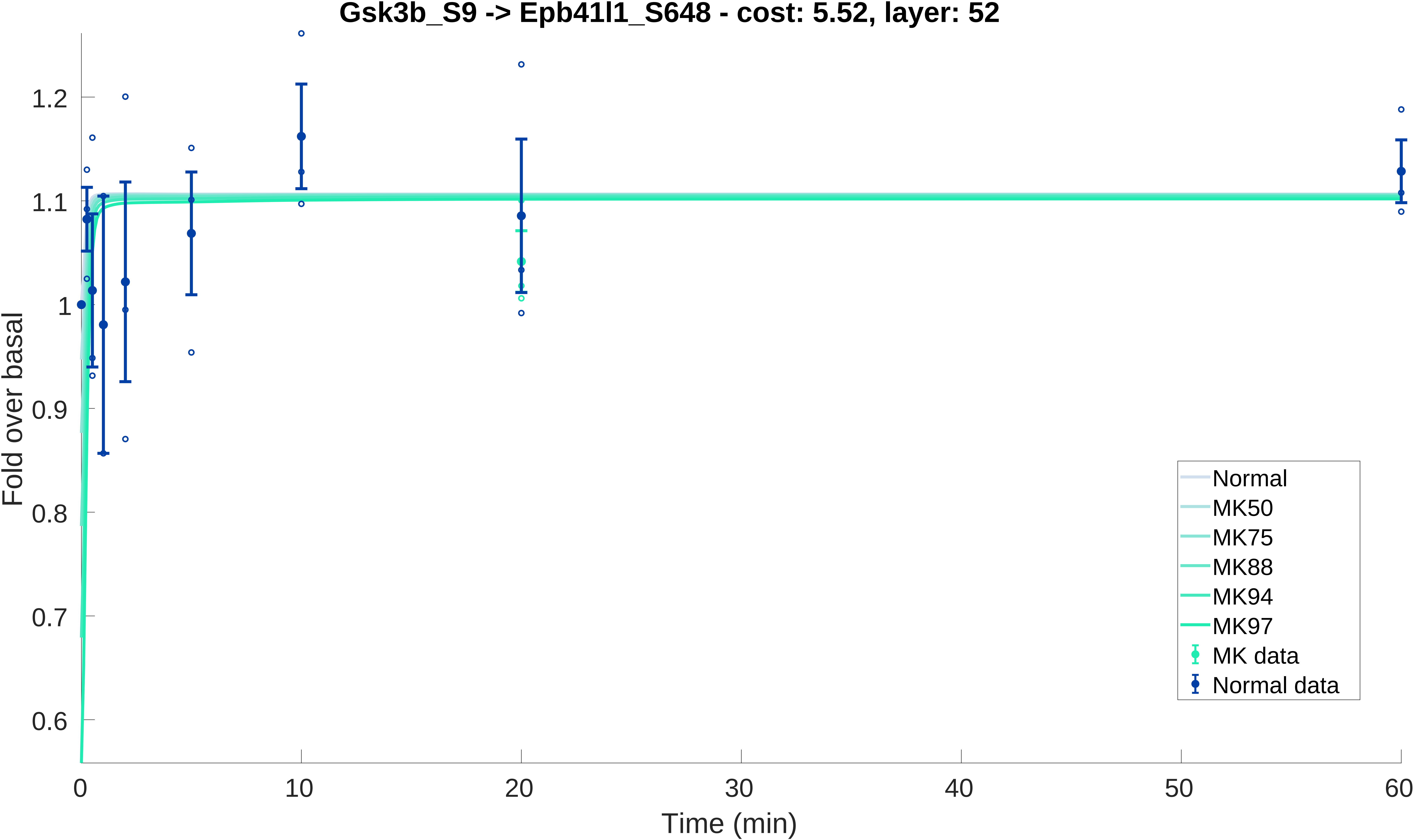
Gsk3b_S9 -> Epb41l1_S648 - cost: 5.52, layer: 52
Fold over basal
Time (min)
0.6
0.7
0.8
0.9
1
1.1
1.2
0
10
20
30
40
50
60
Normal
MK50
MK75
MK88
MK94
MK97
MK data
Normal data

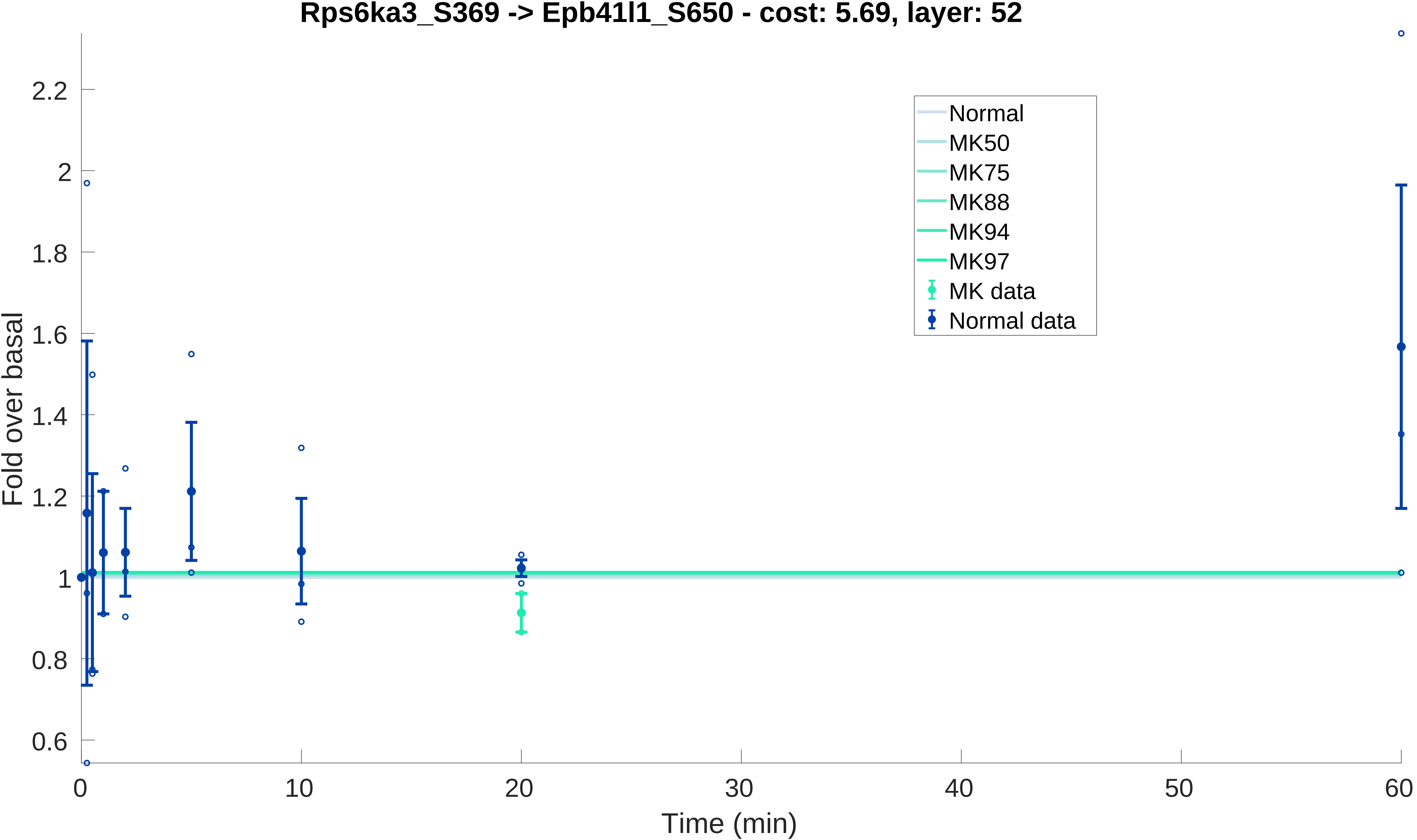

Rps6ka3_S369 -> Epb41l1_S650 - cost: 5.69, layer: 52
Fold over basal
Time (min)
2.2
2
1.8
1.6
1.4
1.2
1
0.8
0.6
0
10
20
30
40
50
60
Normal
MK50
MK75
MK88
MK94
MK97
MK data
Normal data

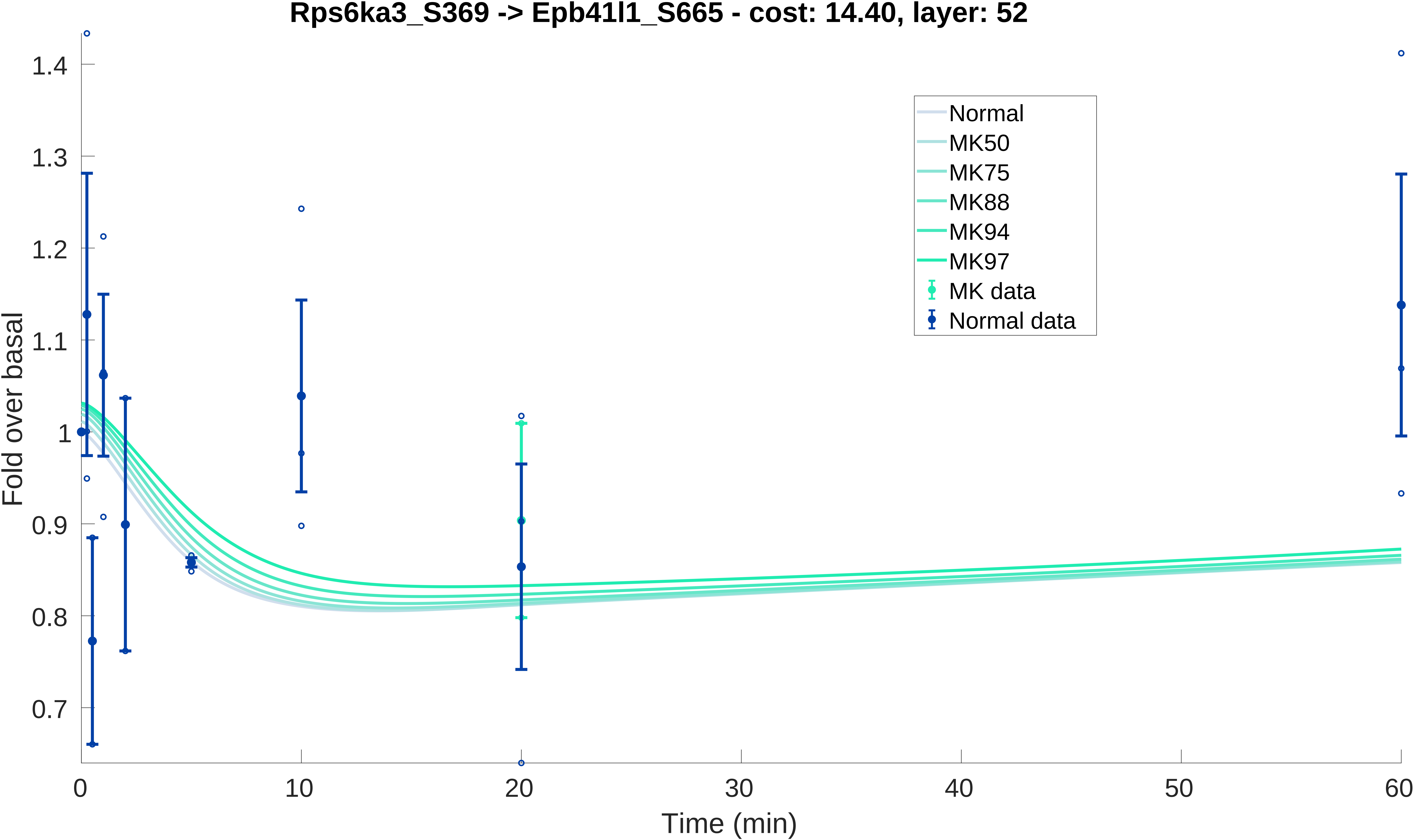

Rps6ka3_S369 -> Epb41l1_S665 - cost: 14.40, layer: 52
Fold over basal
Time (min)
Normal
MK50
MK75
MK88
MK94
MK97
MK data
Normal data
0.7
0.8
0.9
1
1.1
1.2
1.3
1.4
0
10
20
30
40
50
60

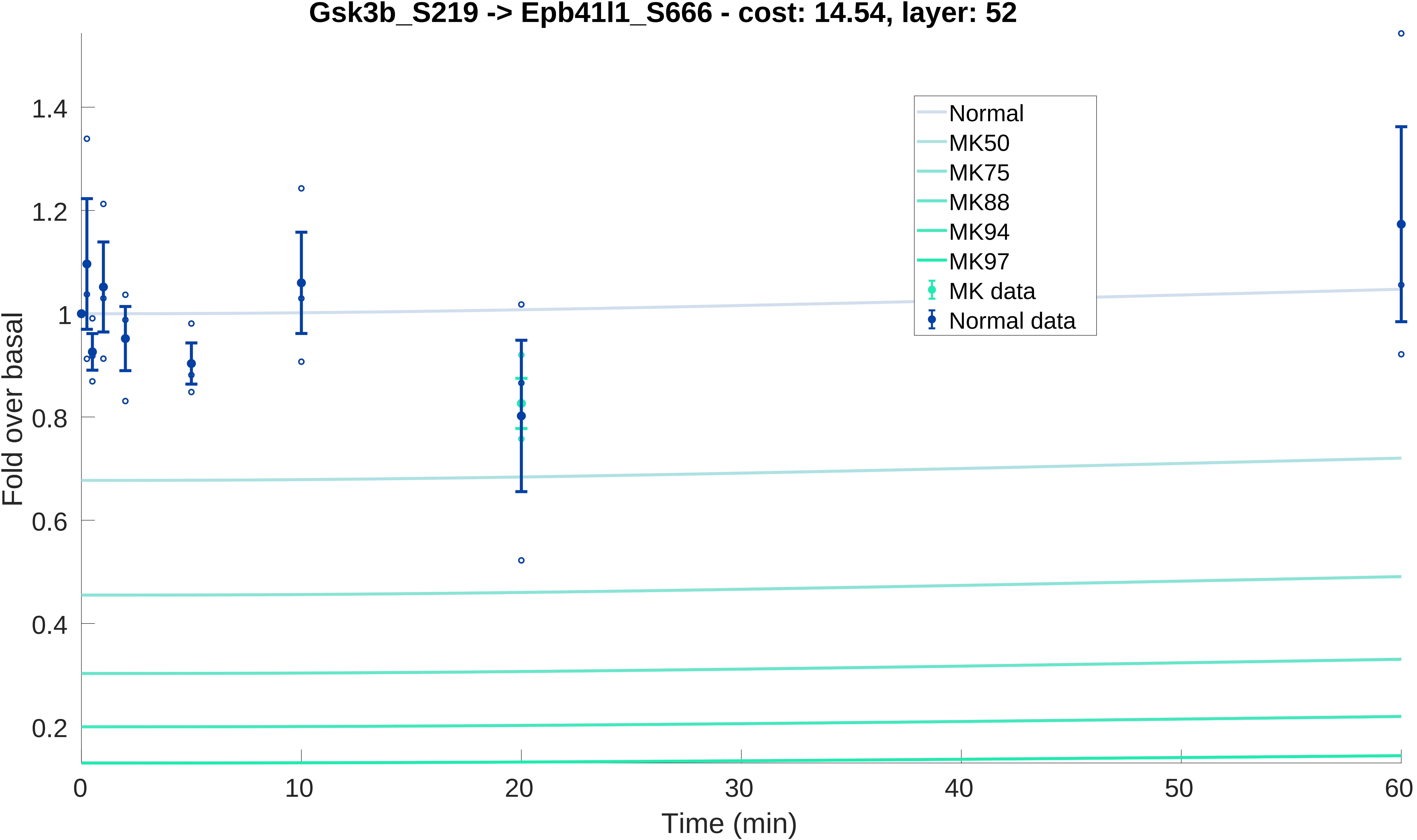
Gsk3b_S219 -> Epb41l1_S666 - cost: 14.54, layer: 52
Fold over basal
Time (min)
Normal
MK50
MK75
MK88
MK94
MK97
MK data
Normal data
0
10
20
30
40
50
60
0.2
0.4
0.6
0.8
1
1.2
1.4

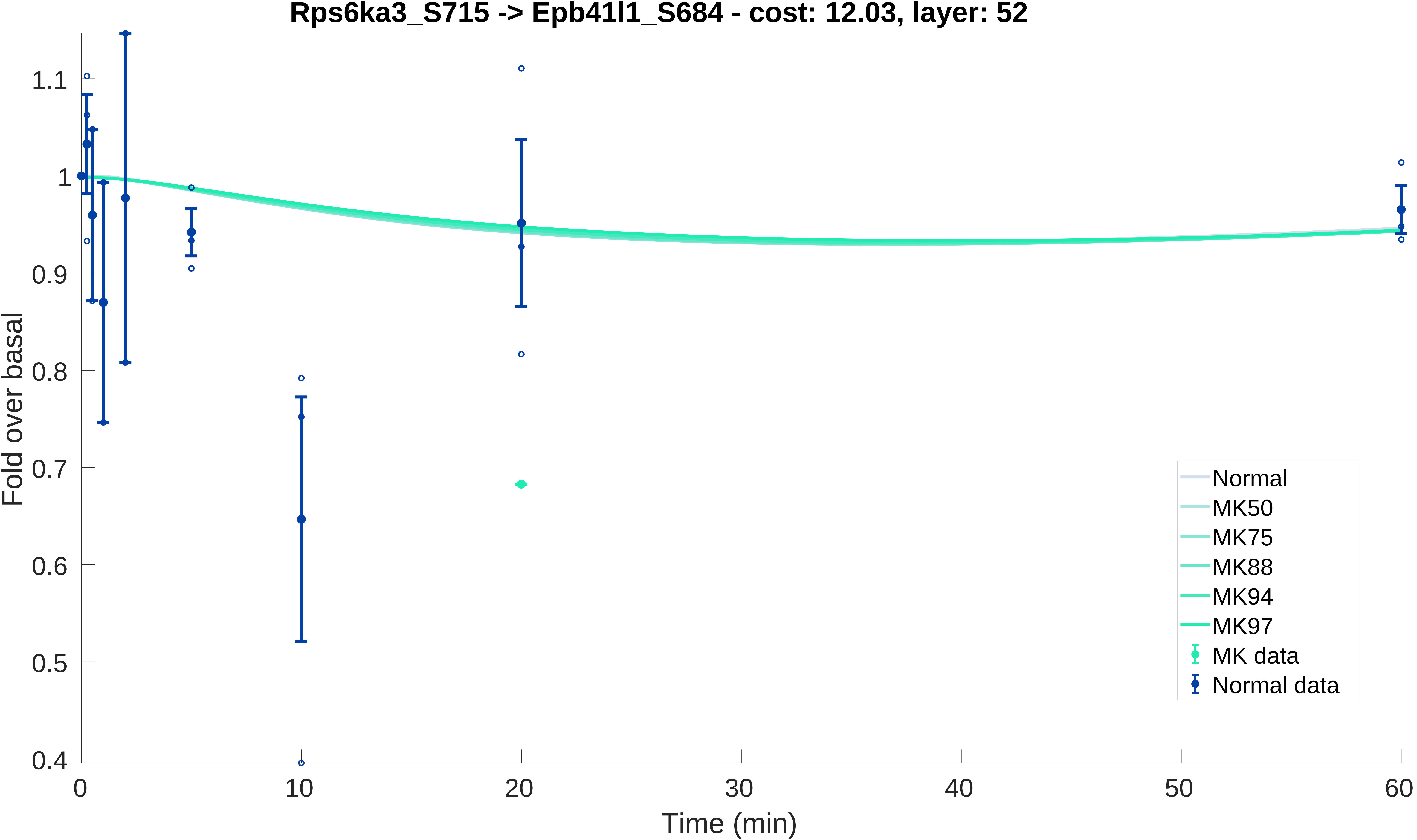

Rps6ka3_S715 -> Epb41l1_S684 - cost: 12.03, layer: 52
Fold over basal
Time (min)
0.4
0.5
0.6
0.7
0.8
0.9
1
1.1
0
10
20
30
40
50
60
Normal
MK50
MK75
MK88
MK94
MK97
MK data
Normal data

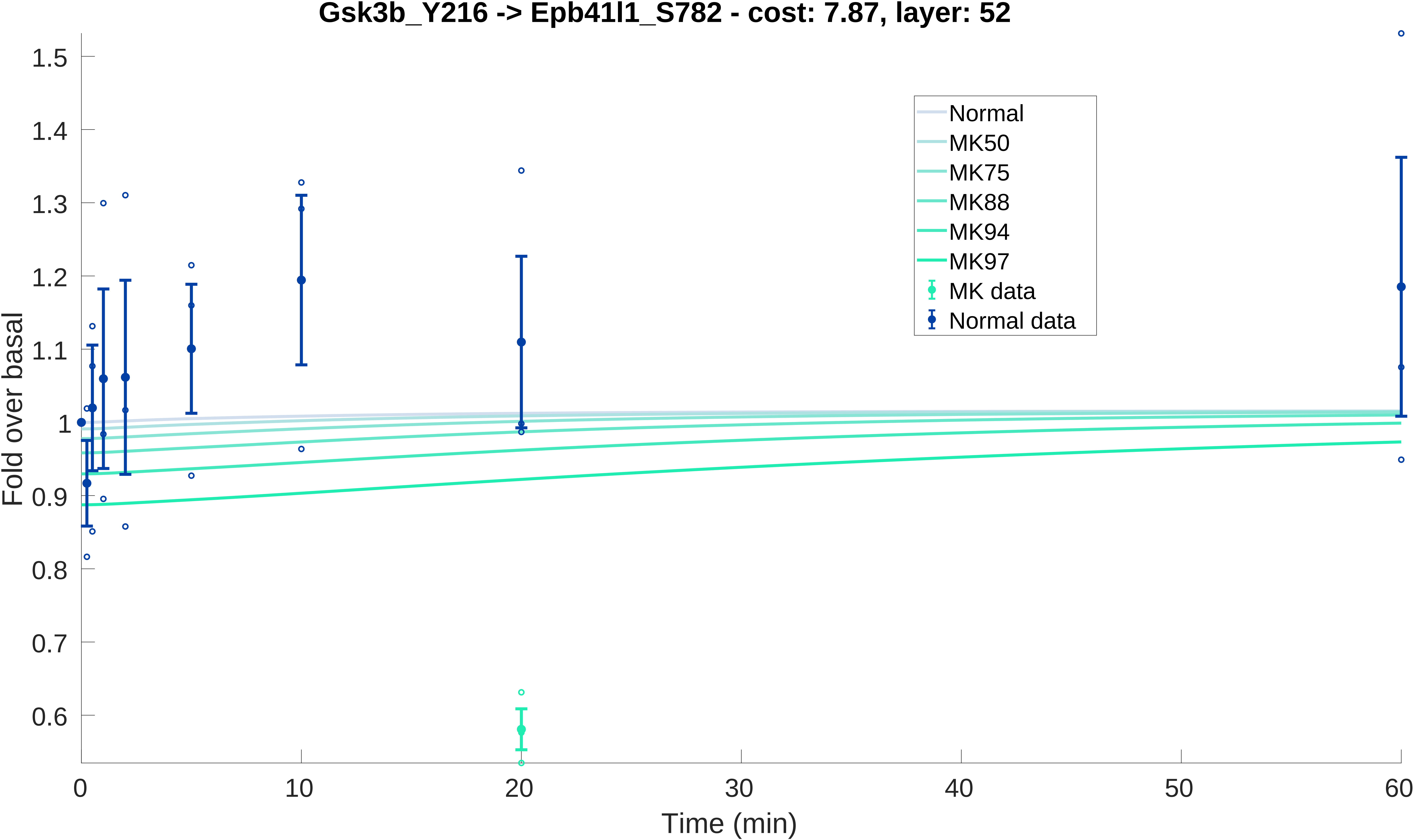

Gsk3b_Y216 -> Epb41l1_S782 - cost: 7.87, layer: 52
Fold over basal
Time (min)
Normal
MK50
MK75
MK88
MK94
MK97
MK data
Normal data
1.5
1.4
1.3
1.2
1.1
1
0.9
0.8
0.7
0.6
0
10
20
30
40
50
60

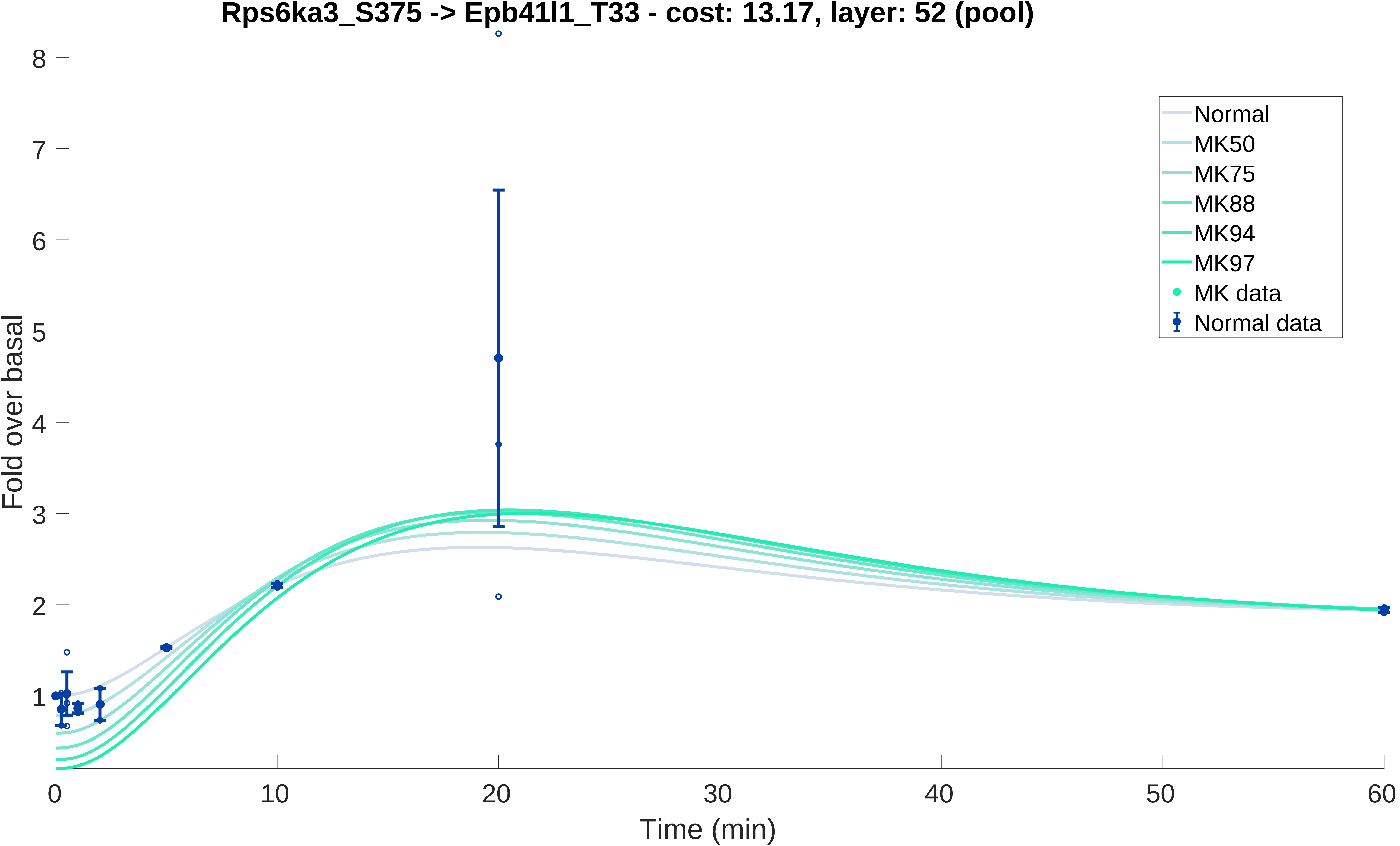

Rps6ka3_S375 -> Epb41l1_T33 - cost: 13.17, layer: 52 (pool)
Fold over basal
Time (min)
0
10
20
30
40
50
60
1
2
3
4
5
6
7
8
Normal
MK50
MK75
MK88
MK94
MK97
MK data
Normal data

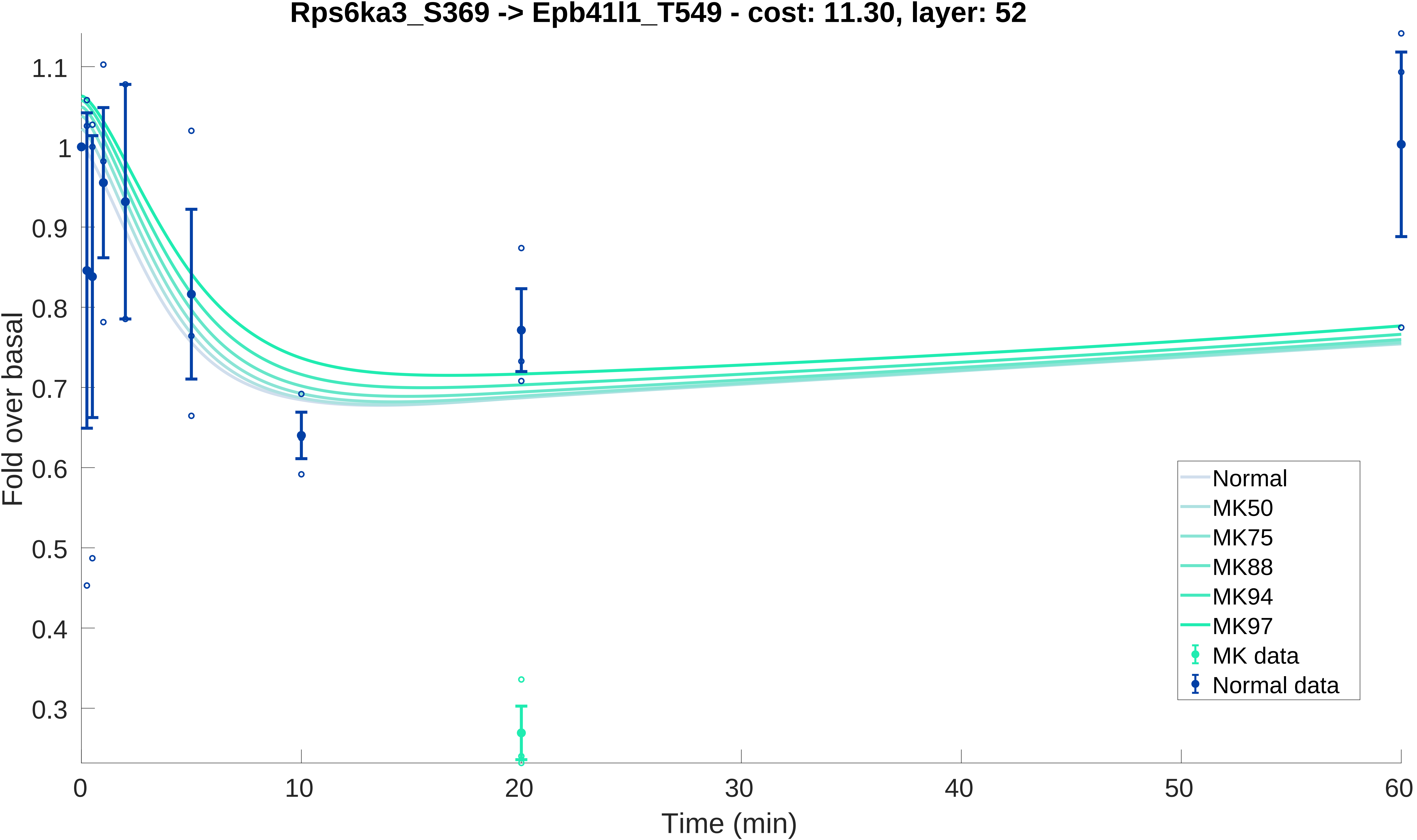

Rps6ka3_S369 -> Epb41l1_T549 - cost: 11.30, layer: 52
Fold over basal
Time (min)
0
10
20
30
40
50
60
0.3
0.4
0.5
0.6
0.7
0.8
0.9
1
1.1
Normal
MK50
MK75
MK88
MK94
MK97
MK data
Normal data

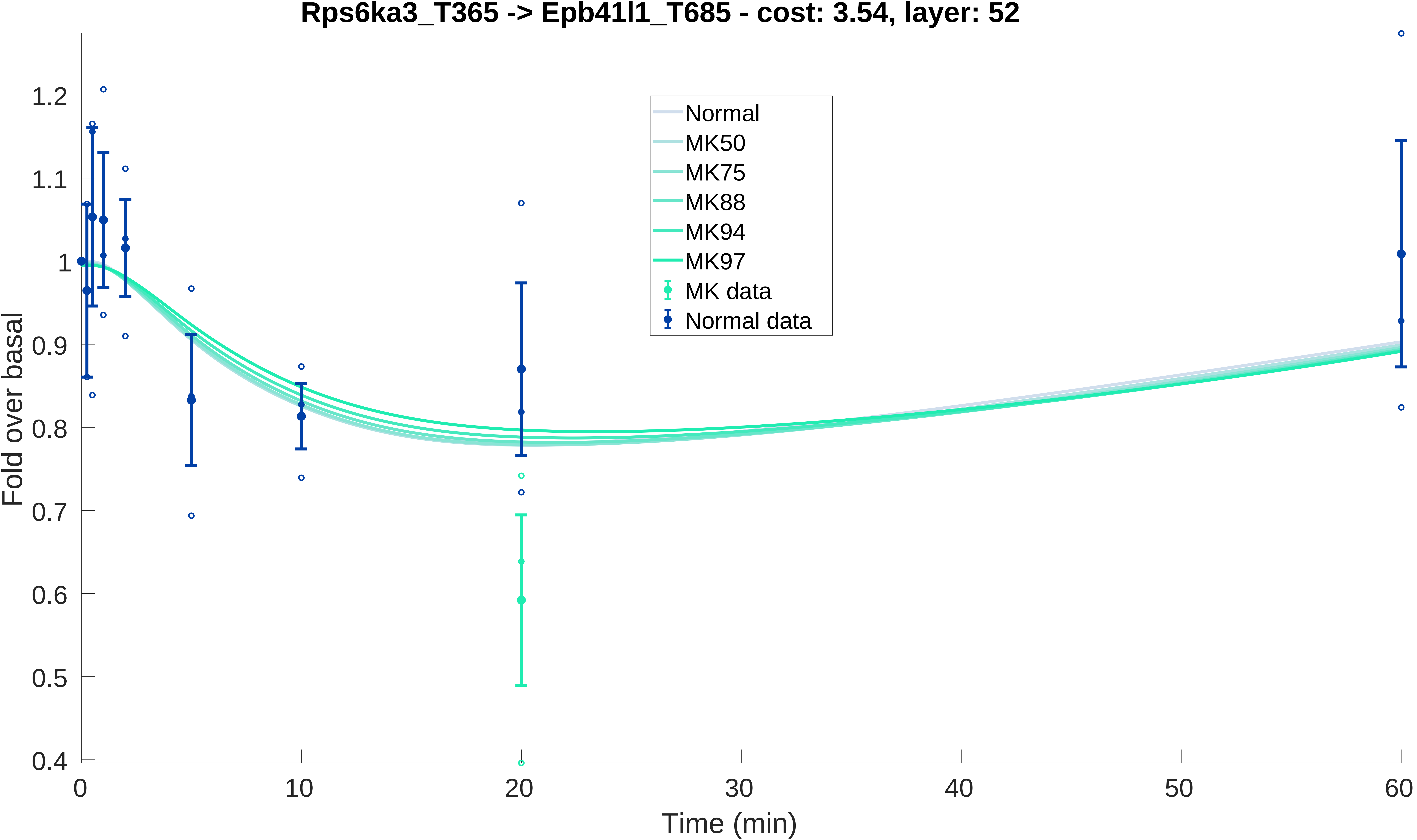

Rps6ka3_T365 -> Epb41l1_T685 - cost: 3.54, layer: 52
Fold over basal
Time (min)
Normal
MK50
MK75
MK88
MK94
MK97
MK data
Normal data
1.2
1.1
1
0.9
0.8
0.7
0.6
0.5
0.4
0
10
20
30
40
50
60

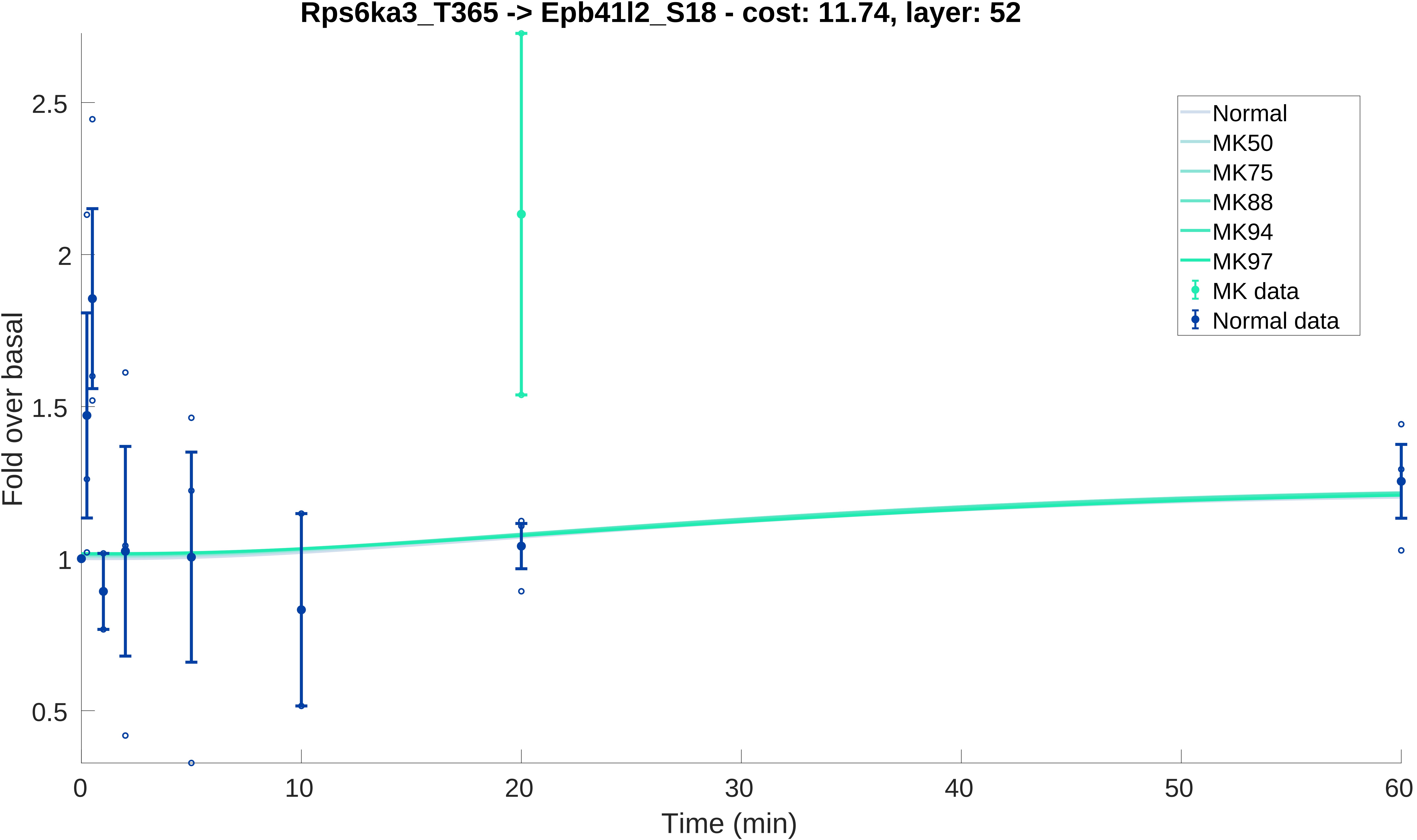

Rps6ka3_T365 -> Epb41l2_S18 - cost: 11.74, layer: 52
Fold over basal
Time (min)
2.5
2
1.5
1
0.5
0
10
20
30
40
50
60
Normal
MK50
MK75
MK88
MK94
MK97
MK data
Normal data

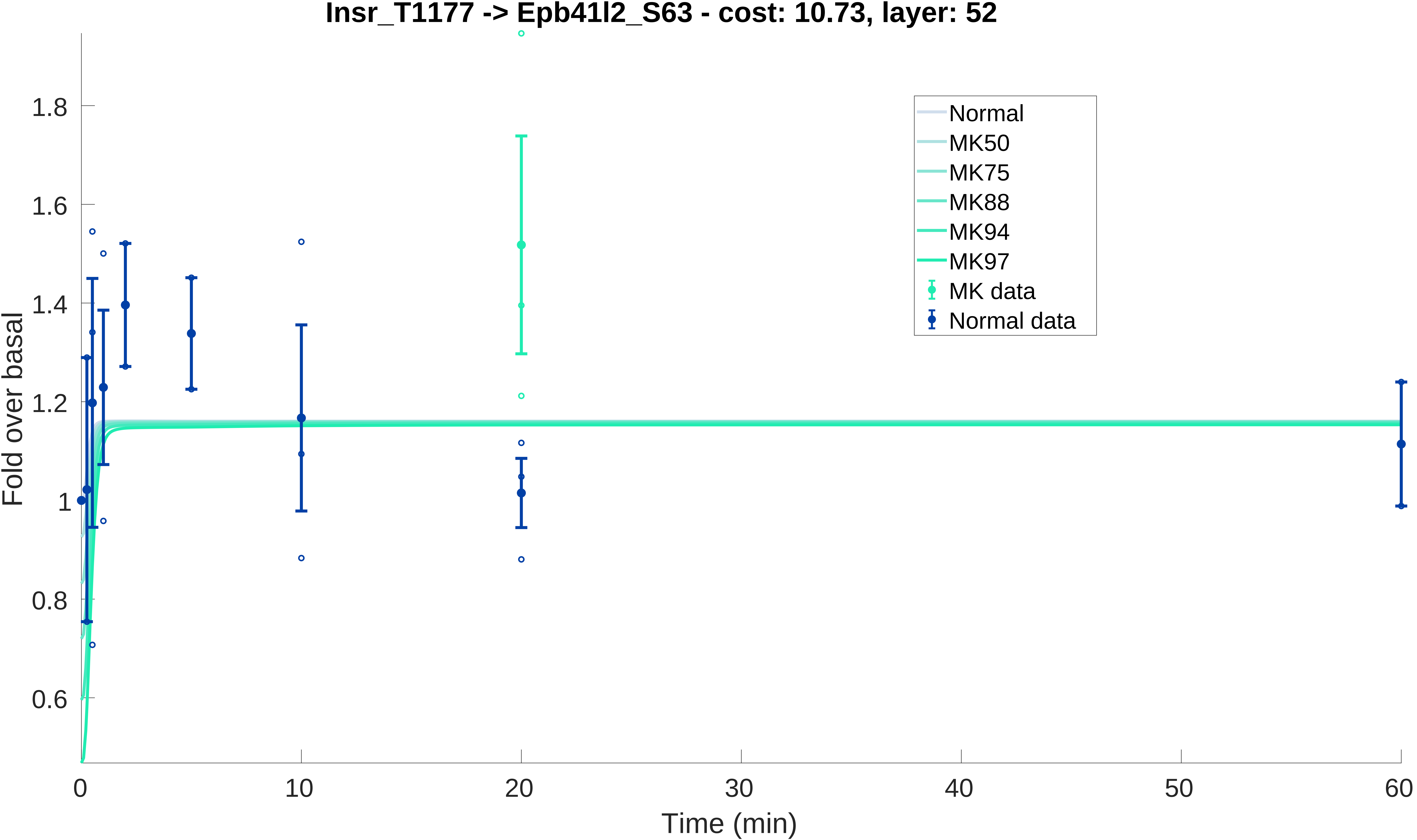

Insr_T1177 -> Epb41l2_S63 - cost: 10.73, layer: 52
Fold over basal
1.8
1.6
1.4
1.2
1
0.8
0.6
0
10
20
30
40
50
60
Time (min)
Normal
MK50
MK75
MK88
MK94
MK97
MK data
Normal data

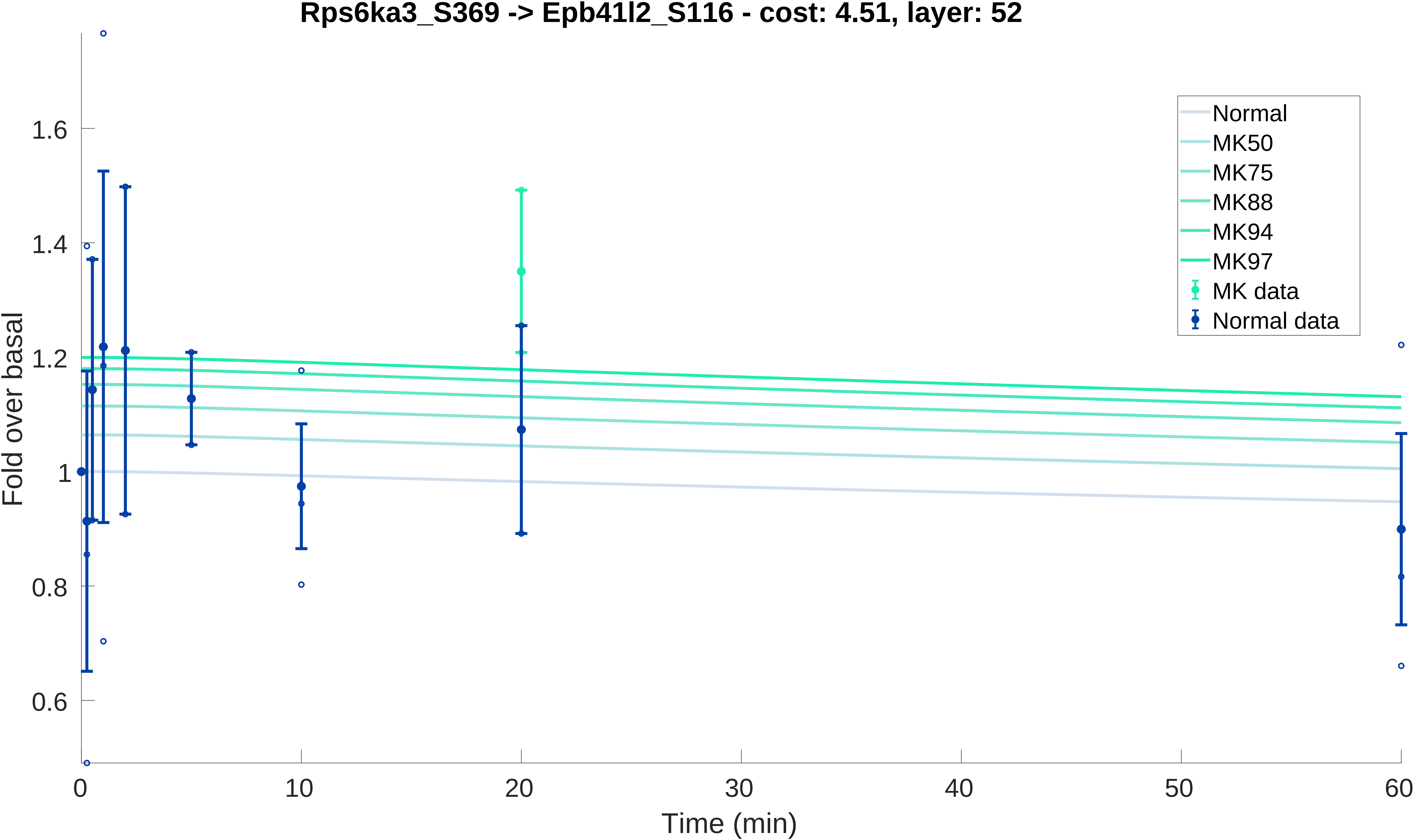

Rps6ka3_S369 -> Epb41l2_S116 - cost: 4.51, layer: 52
Fold over basal
Time (min)
0.6
0.8
1
1.2
1.4
1.6
0
10
20
30
40
50
60
Normal
MK50
MK75
MK88
MK94
MK97
MK data
Normal data

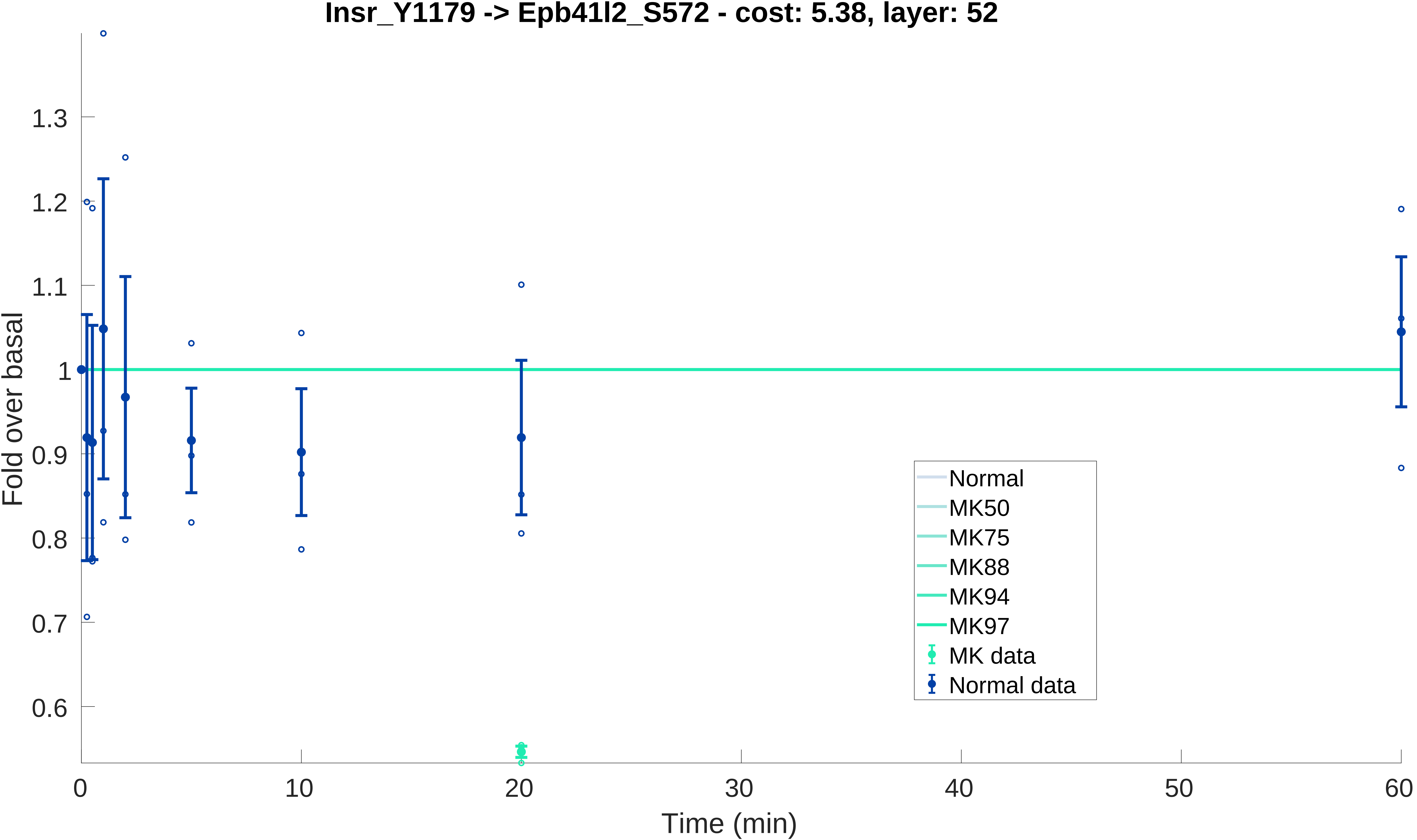

Insr_Y1179 -> Epb41l2_S572 - cost: 5.38, layer: 52
Fold over basal
Time (min)
1.3
1.2
1.1
1
0.9
0.8
0.7
0.6
0
10
20
30
40
50
60
Normal
MK50
MK75
MK88
MK94
MK97
MK data
Normal data

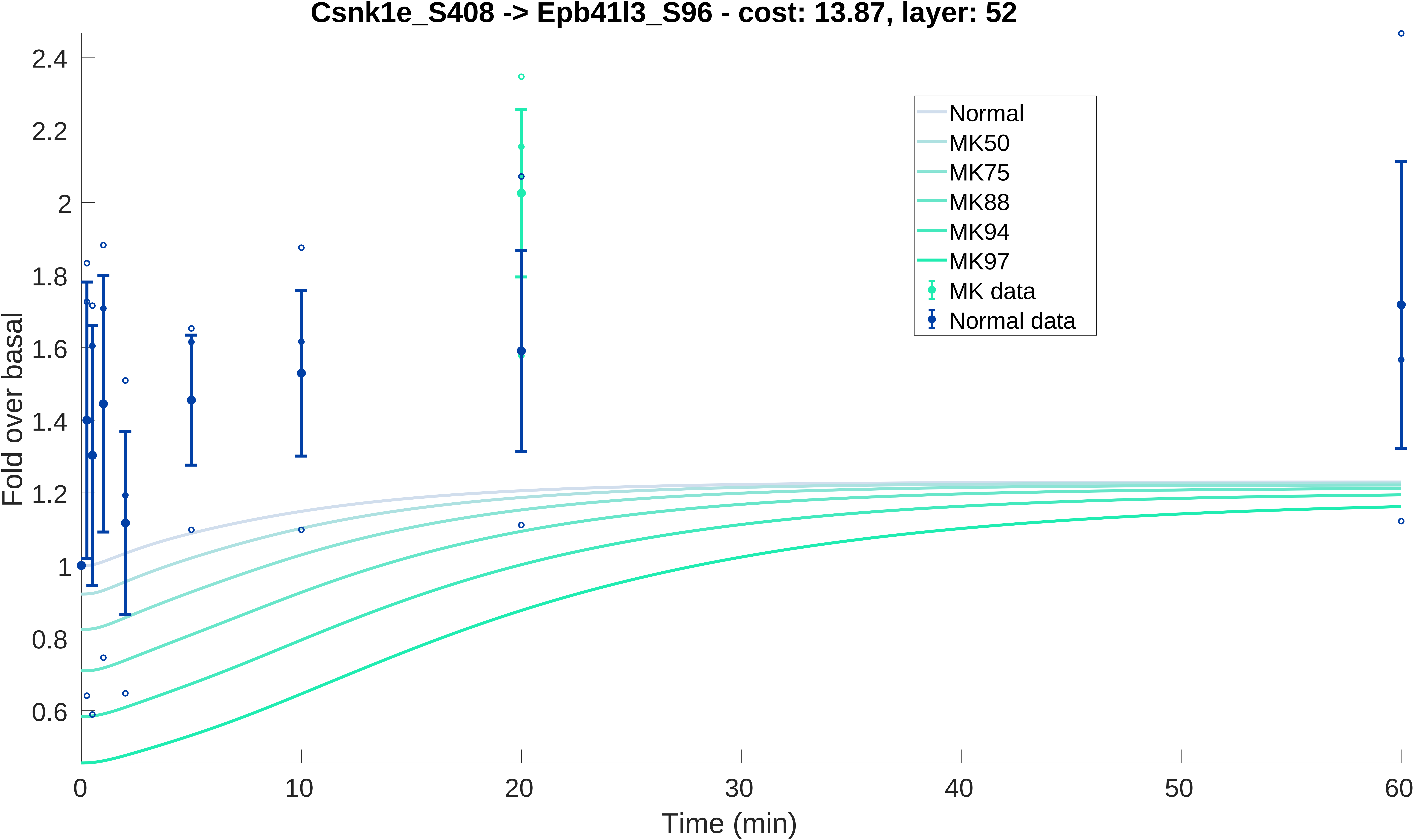

Csnk1e_S408 -> Epb41l3_S96 - cost: 13.87, layer: 52
Fold over basal
Time (min)
0.6
0.8
1
1.2
1.4
1.6
1.8
2
2.2
2.4
0
10
20
30
40
50
60
Normal
MK50
MK75
MK88
MK94
MK97
MK data
Normal data

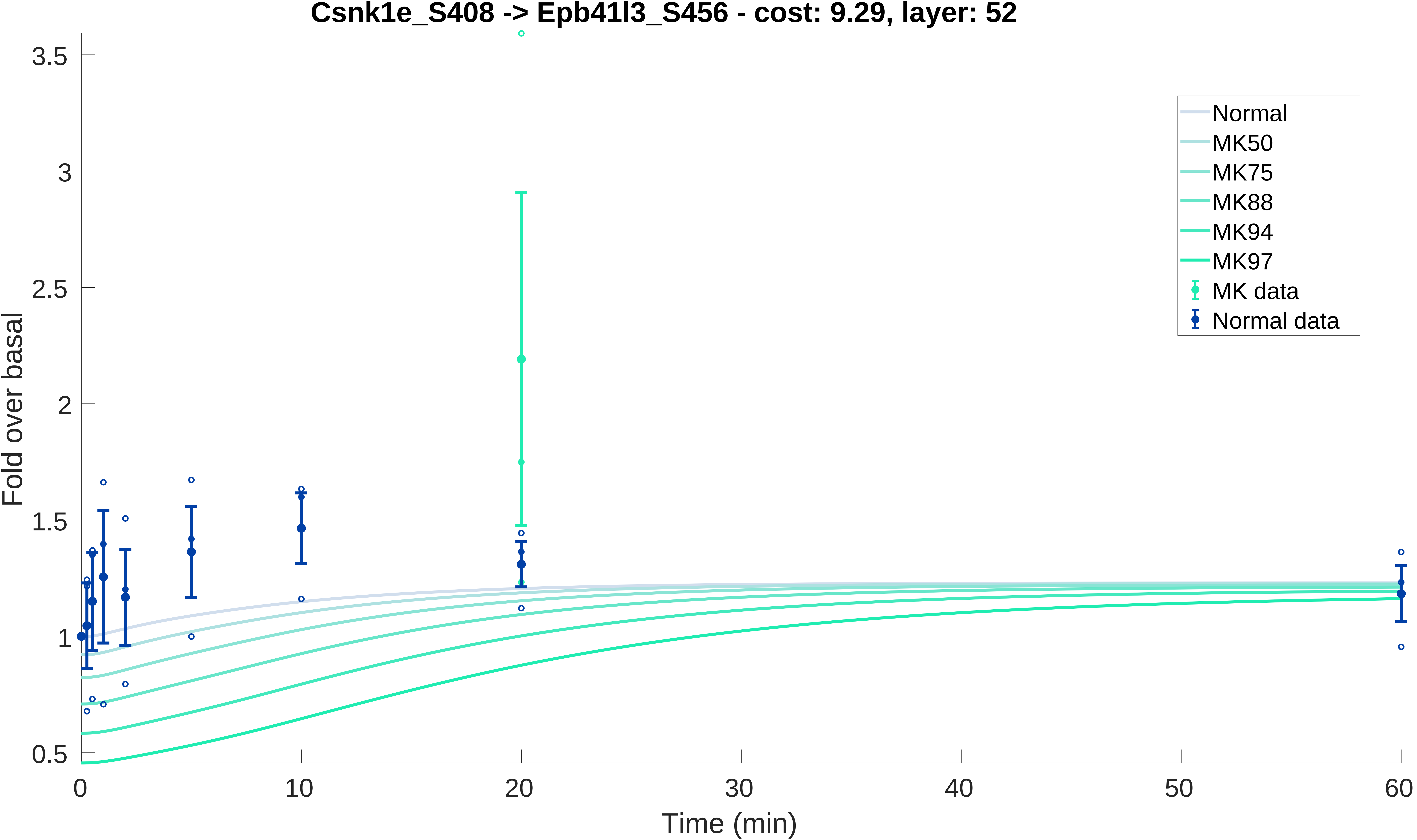

Csnk1e_S408 -> Epb41l3_S456 - cost: 9.29, layer: 52
Fold over basal
Time (min)
Normal
MK50
MK75
MK88
MK94
MK97
MK data
Normal data
3.5
3
2.5
2
1.5
1
0.5
0
10
20
30
40
50
60

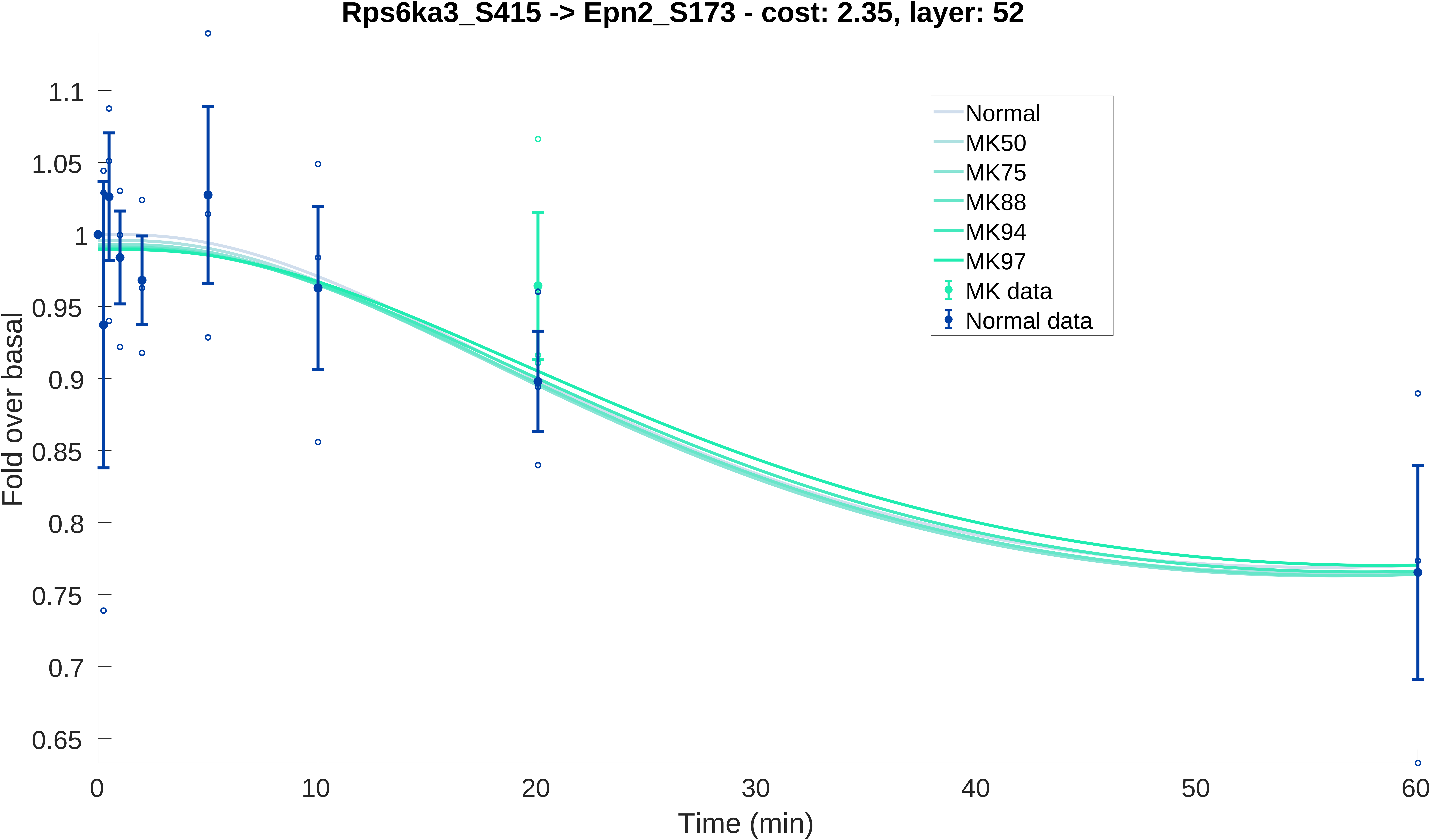

Rps6ka3_S415 -> Epn2_S173 - cost: 2.35, layer: 52
Fold over basal
Time (min)
1.1
1.05
1
0.95
0.9
0.85
0.8
0.75
0.7
0.65
0
10
20
30
40
50
60
Normal
MK50
MK75
MK88
MK94
MK97
MK data
Normal data

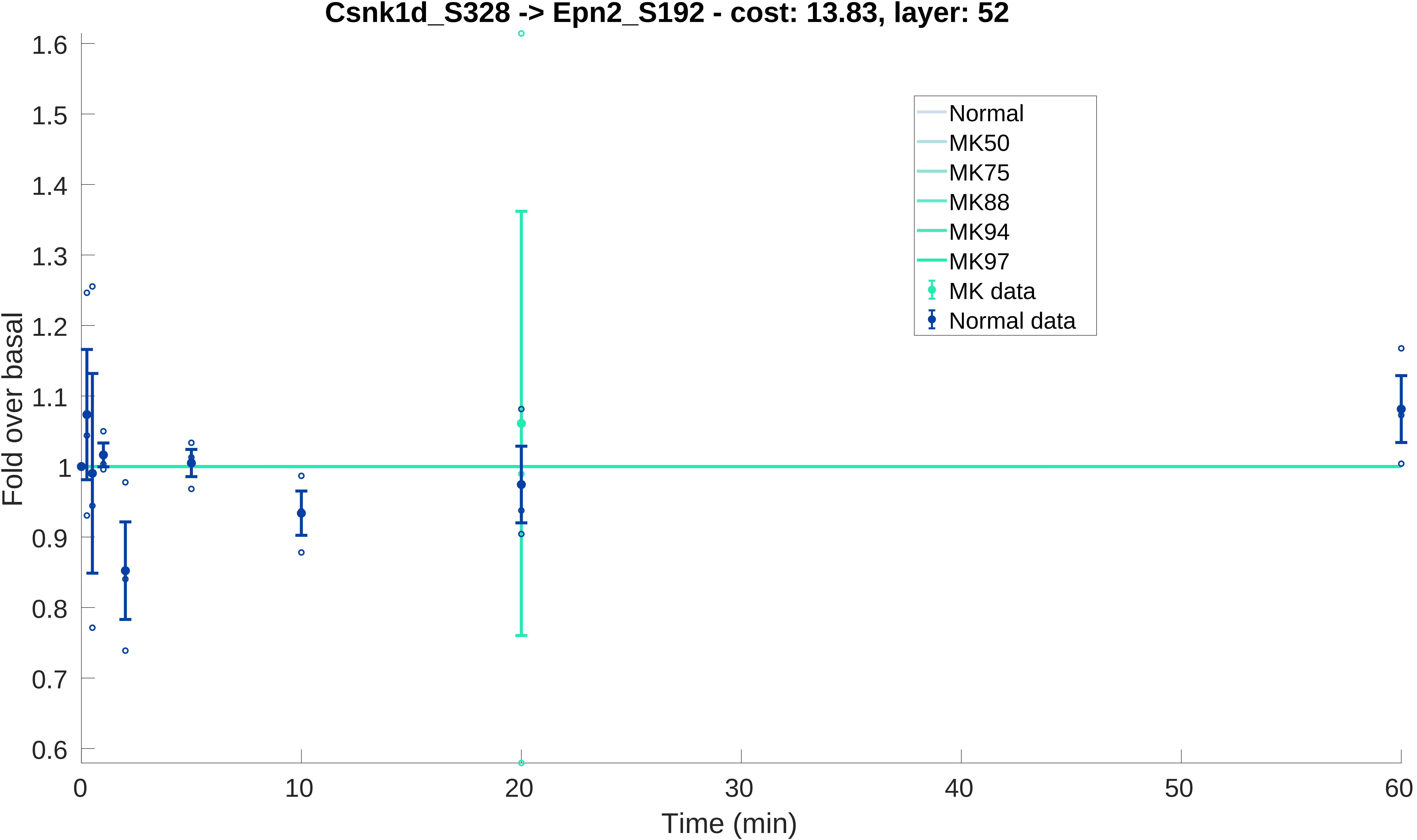

Csnk1d_S328 -> Epn2_S192 - cost: 13.83, layer: 52
Fold over basal
Time (min)
1.6
1.5
1.4
1.3
1.2
1.1
1
0.9
0.8
0.7
0.6
0
10
20
30
40
50
60
Normal
MK50
MK75
MK88
MK94
MK97
MK data
Normal data

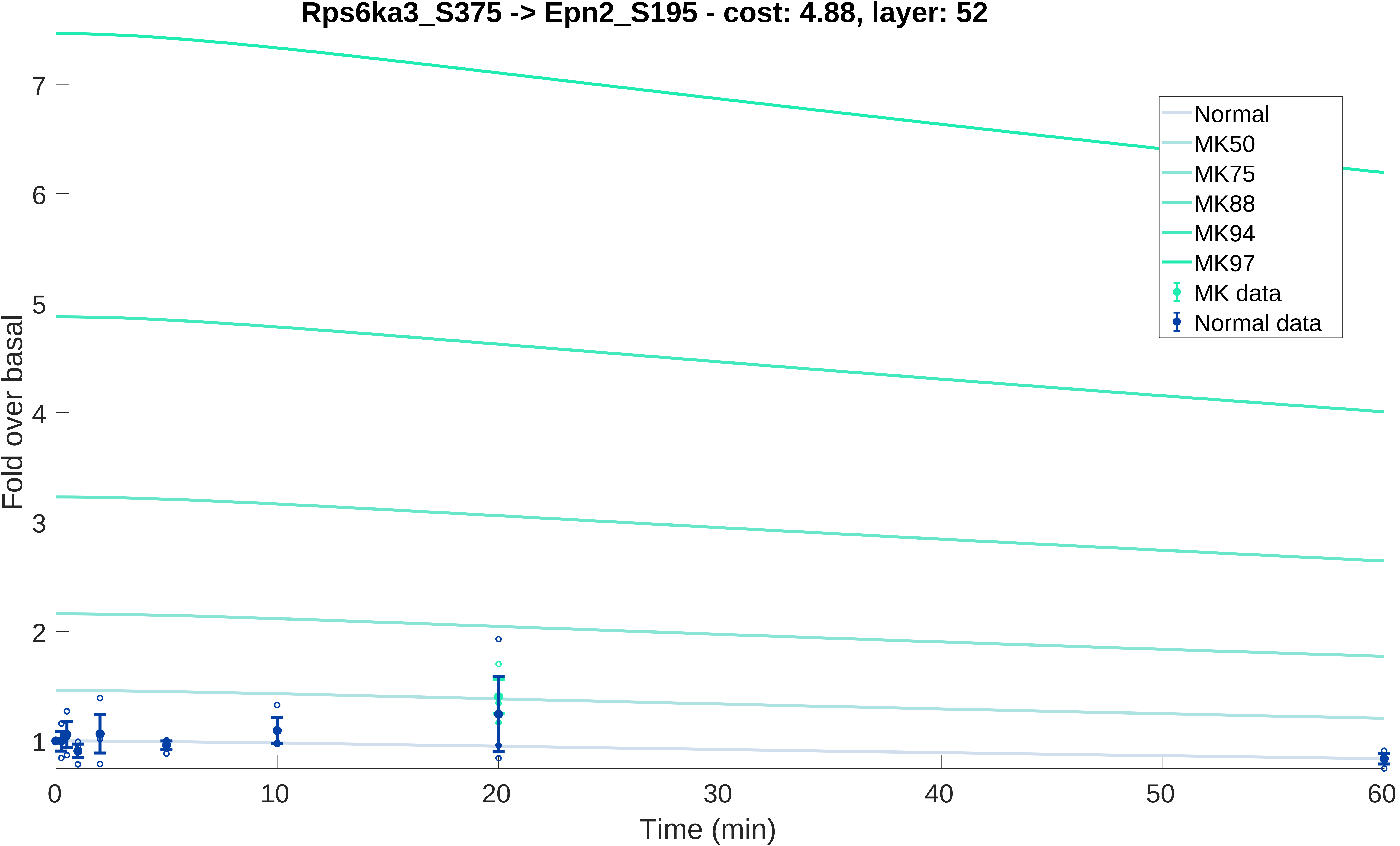
Rps6ka3_S375 -> Epn2_S195 - cost: 4.88, layer: 52
Fold over basal
Time (min)
0
10
20
30
40
50
60
1
2
3
4
5
6
7
Normal
MK50
MK75
MK88
MK94
MK97
MK data
Normal data

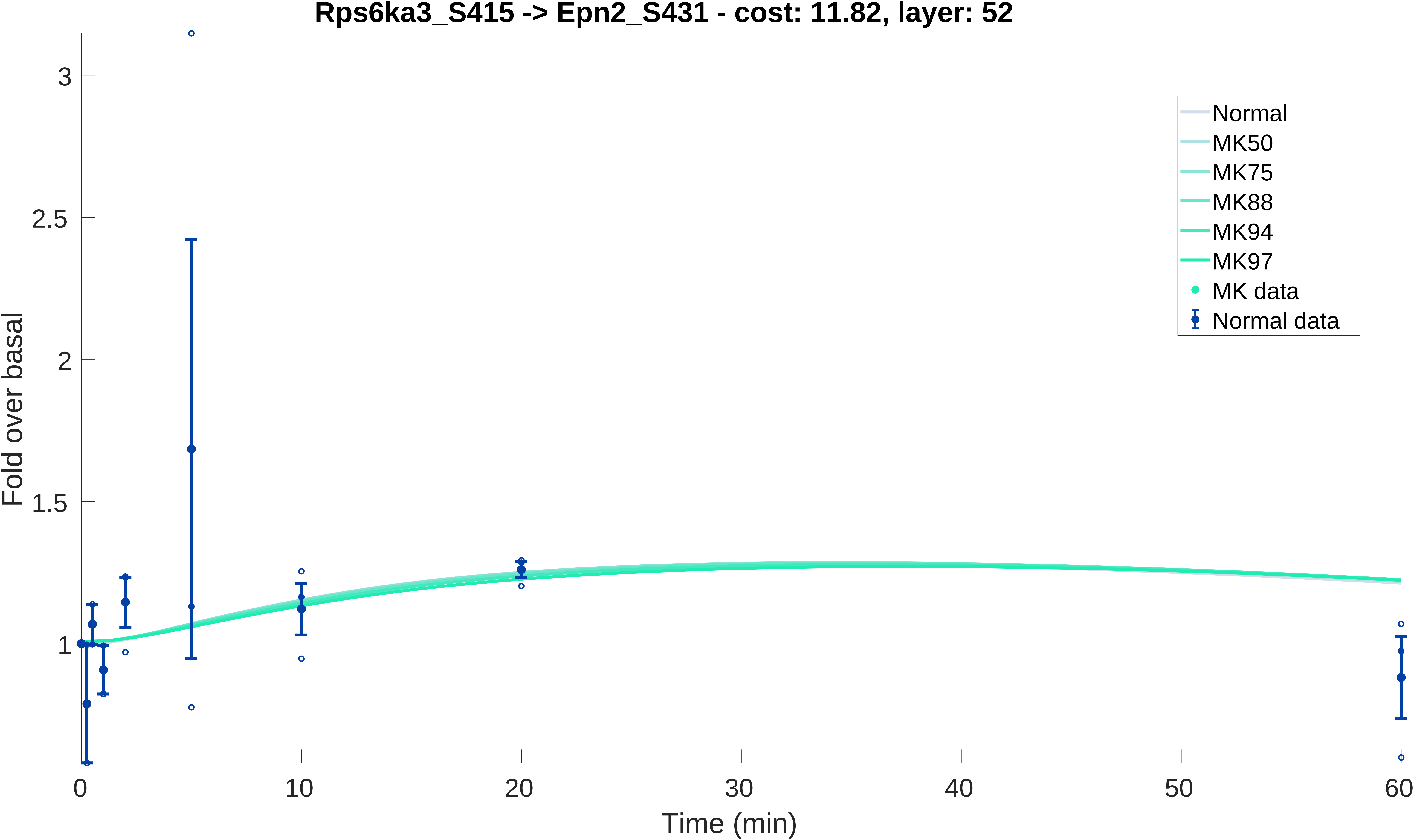
Rps6ka3_S415 -> Epn2_S431 - cost: 11.82, layer: 52
Fold over basal
Time (min)
3
2.5
2
1.5
1
0
10
20
30
40
50
60
Normal
MK50
MK75
MK88
MK94
MK97
MK data
Normal data

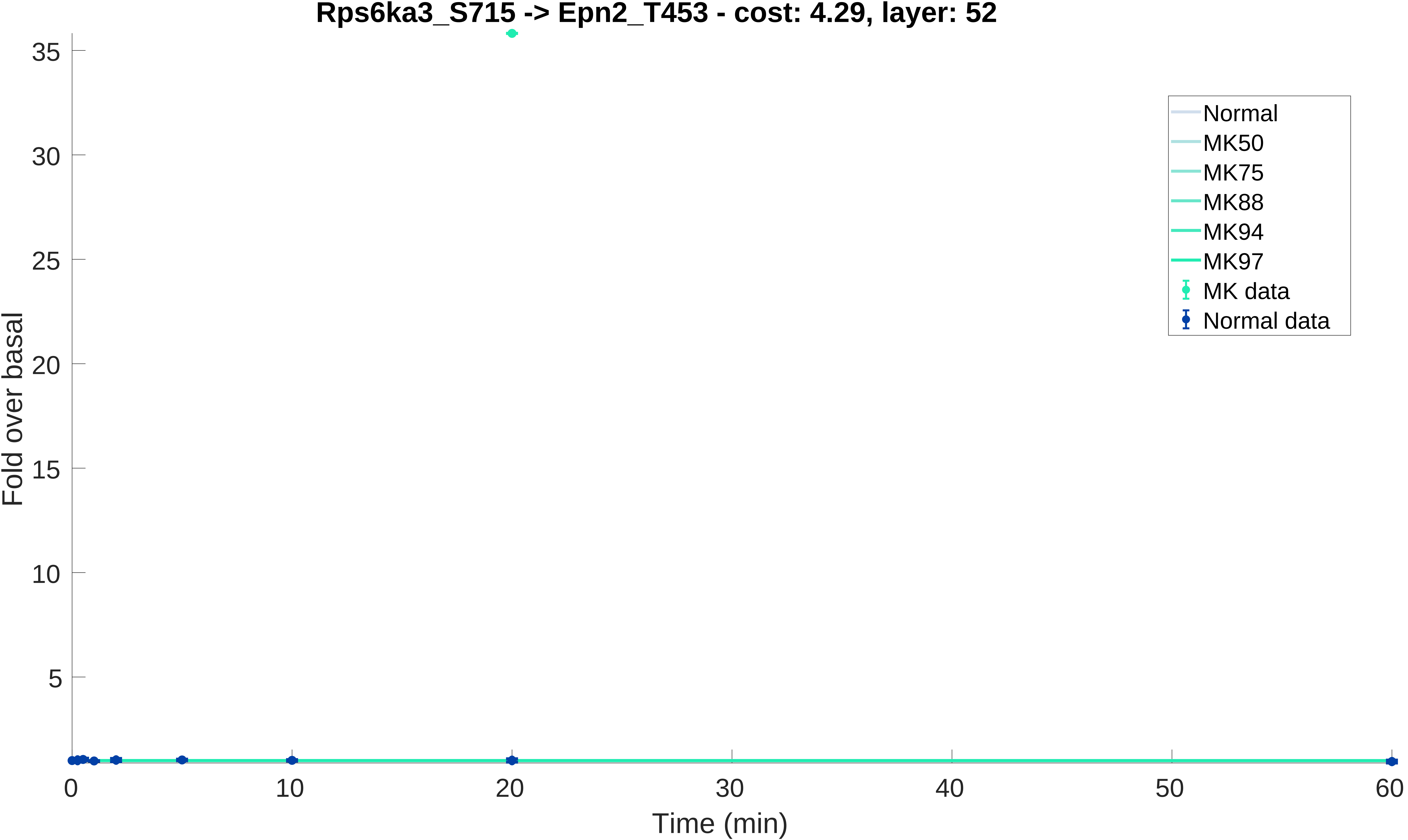
Rps6ka3_S715 -> Epn2_T453 - cost: 4.29, layer: 52
Fold over basal
Time (min)
35
30
25
20
15
10
5
0
10
20
30
40
50
60
Normal
MK50
MK75
MK88
MK94
MK97
MK data
Normal data

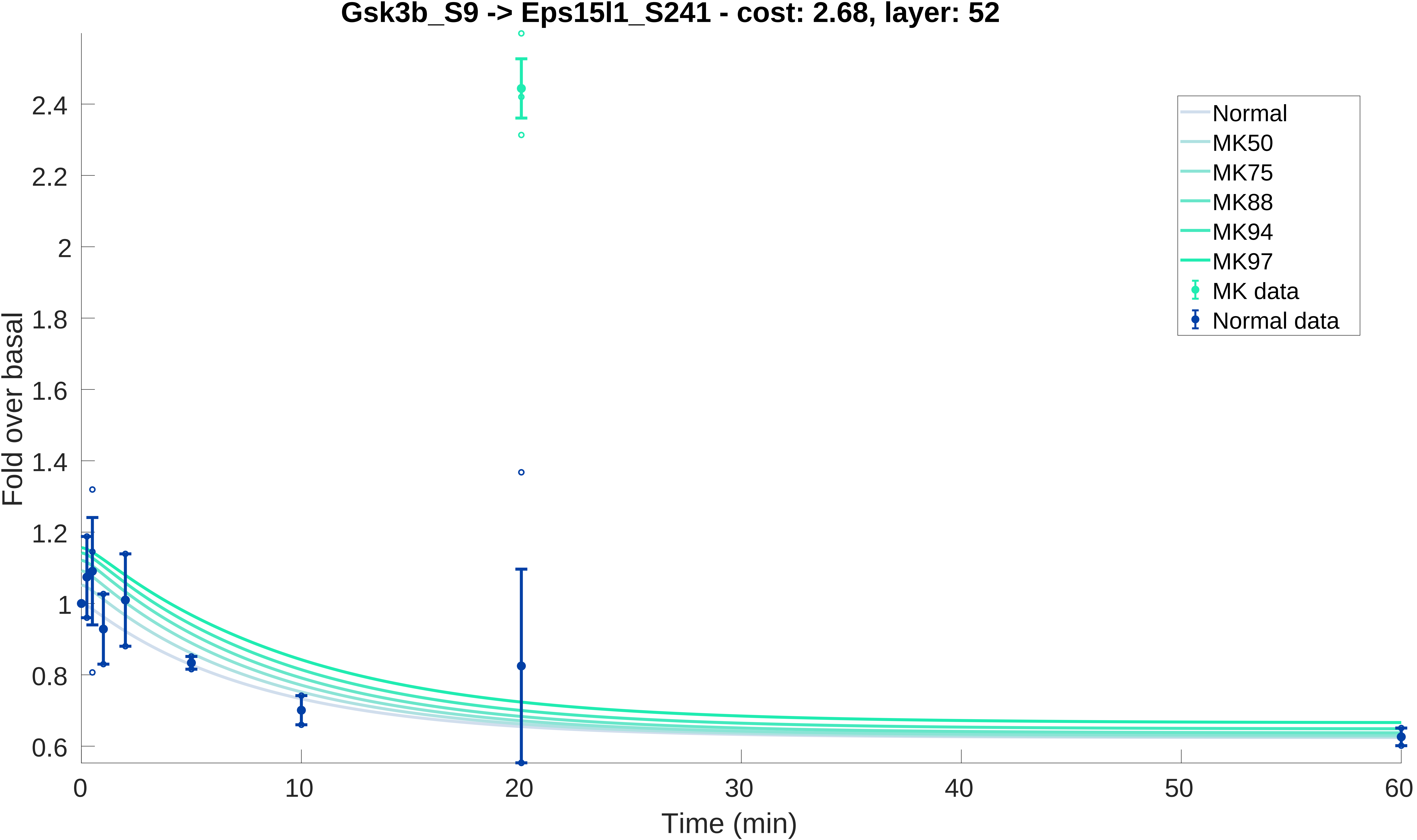

Gsk3b_S9 -> Eps15l1_S241 - cost: 2.68, layer: 52
Fold over basal
Time (min)
0
10
20
30
40
50
60
0.6
0.8
1
1.2
1.4
1.6
1.8
2
2.2
2.4
Normal
MK50
MK75
MK88
MK94
MK97
MK data
Normal data

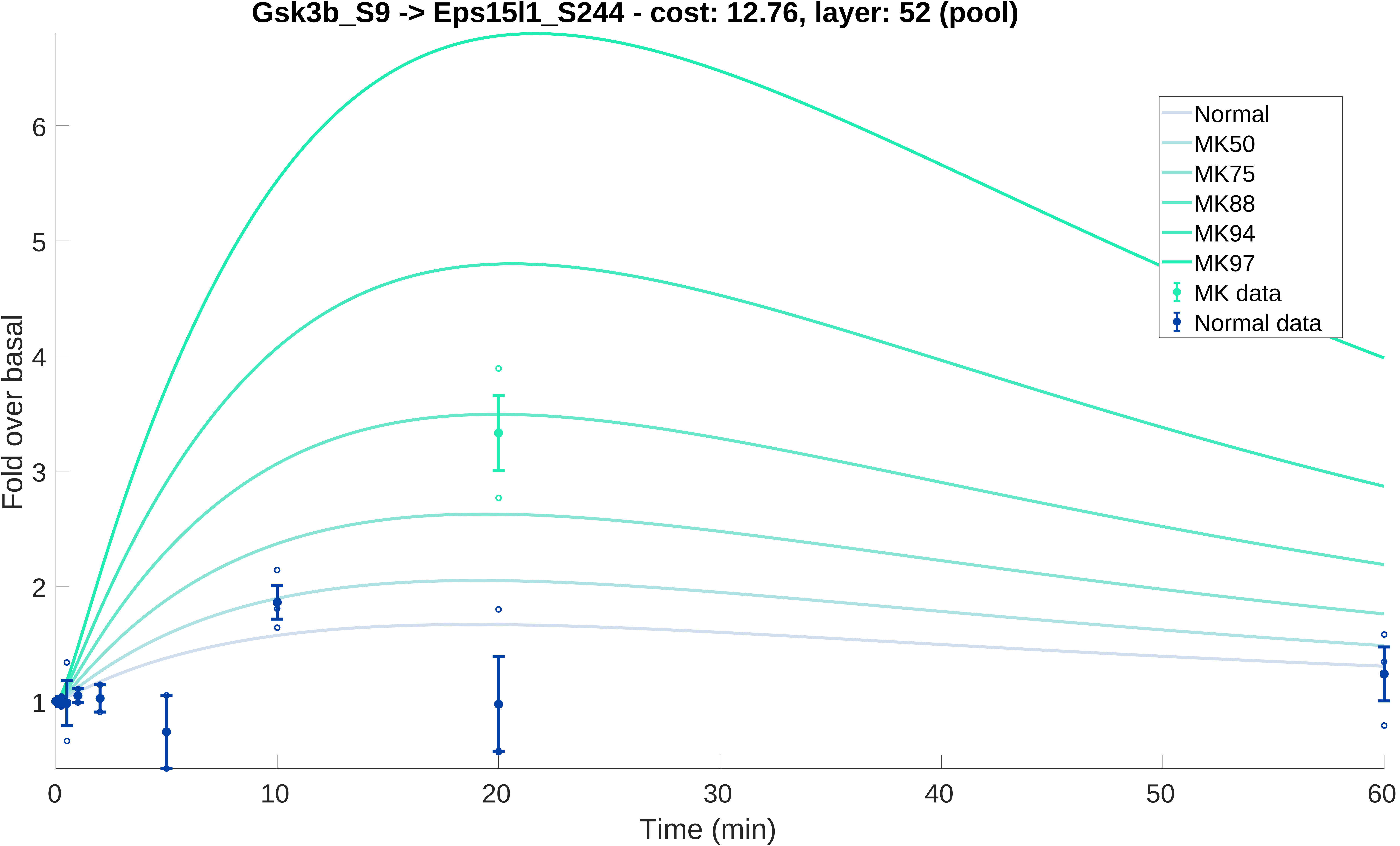

Gsk3b_S9 -> Eps15l1_S244 - cost: 12.76, layer: 52 (pool)
Normal
MK50
MK75
MK88
MK94
MK97
MK data
Normal data
Fold over basal
Time (min)
1
2
3
4
5
6
0
10
20
30
40
50
60

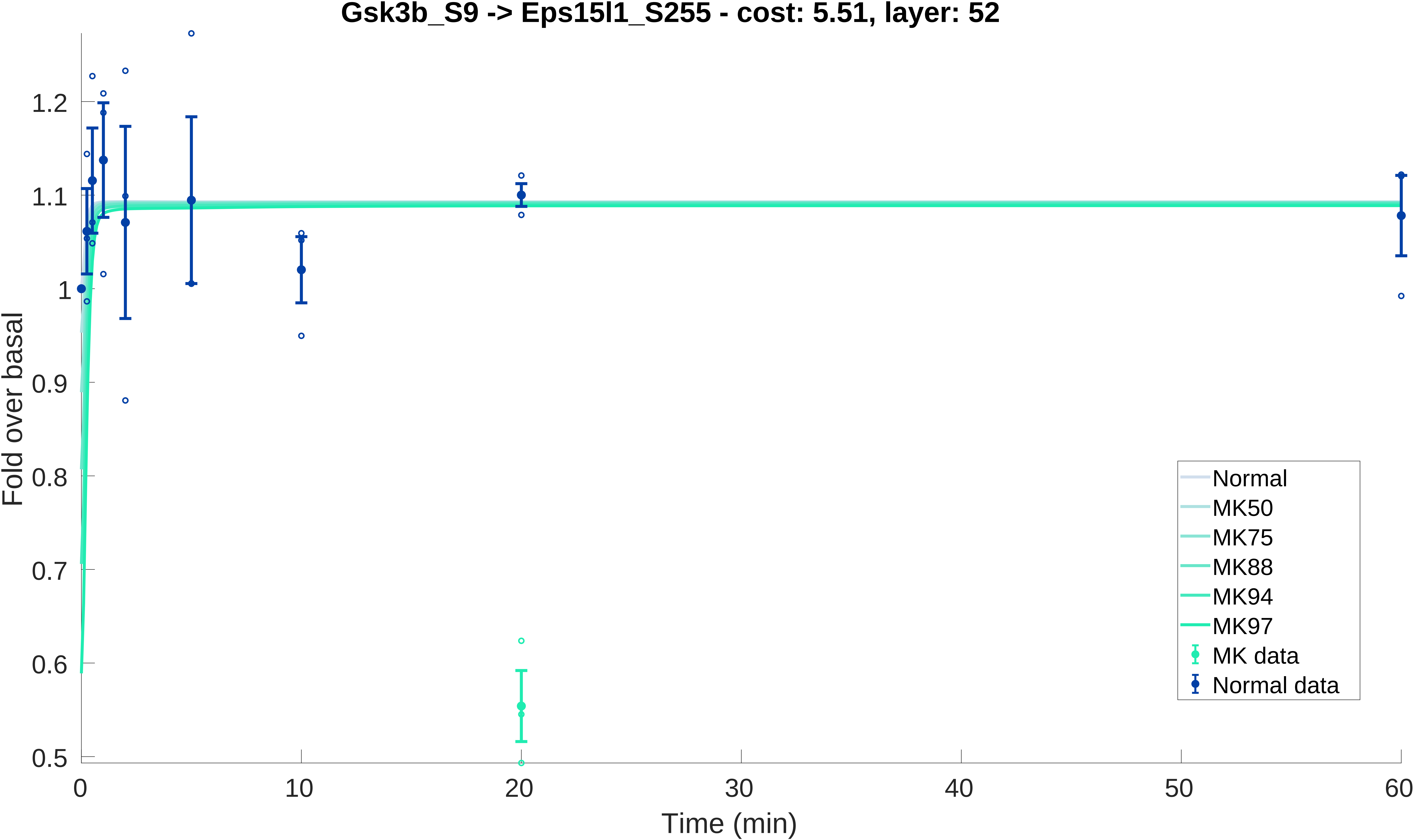

Gsk3b_S9 -> Eps15l1_S255 - cost: 5.51, layer: 52
Fold over basal
Time (min)
0.5
0.6
0.7
0.8
0.9
1
1.1
1.2
0
10
20
30
40
50
60
Normal
MK50
MK75
MK88
MK94
MK97
MK data
Normal data

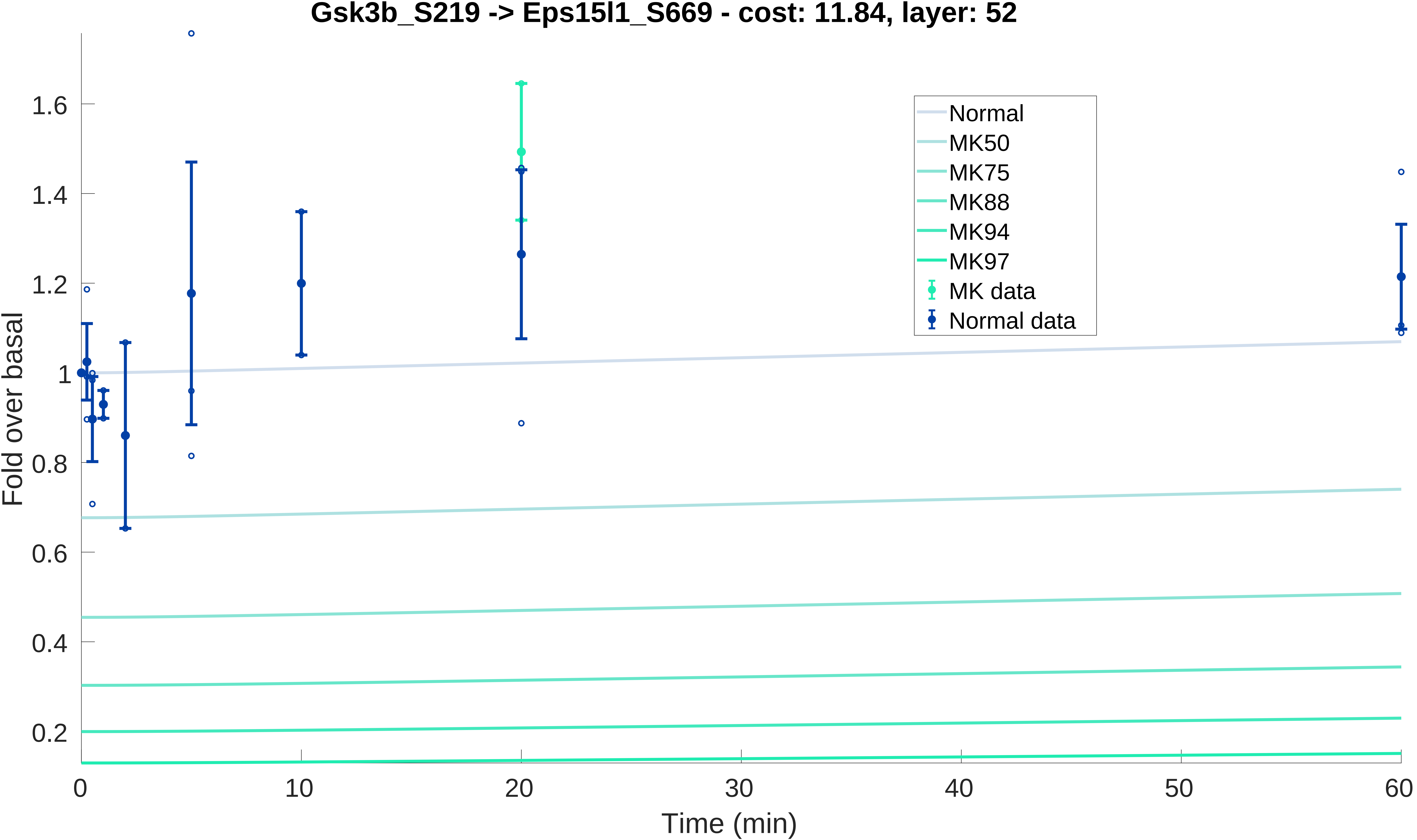

Gsk3b_S219 -> Eps15l1_S669 - cost: 11.84, layer: 52
Fold over basal
Time (min)
0
10
20
30
40
50
60
0.2
0.4
0.6
0.8
1
1.2
1.4
1.6
Normal
MK50
MK75
MK88
MK94
MK97
MK data
Normal data

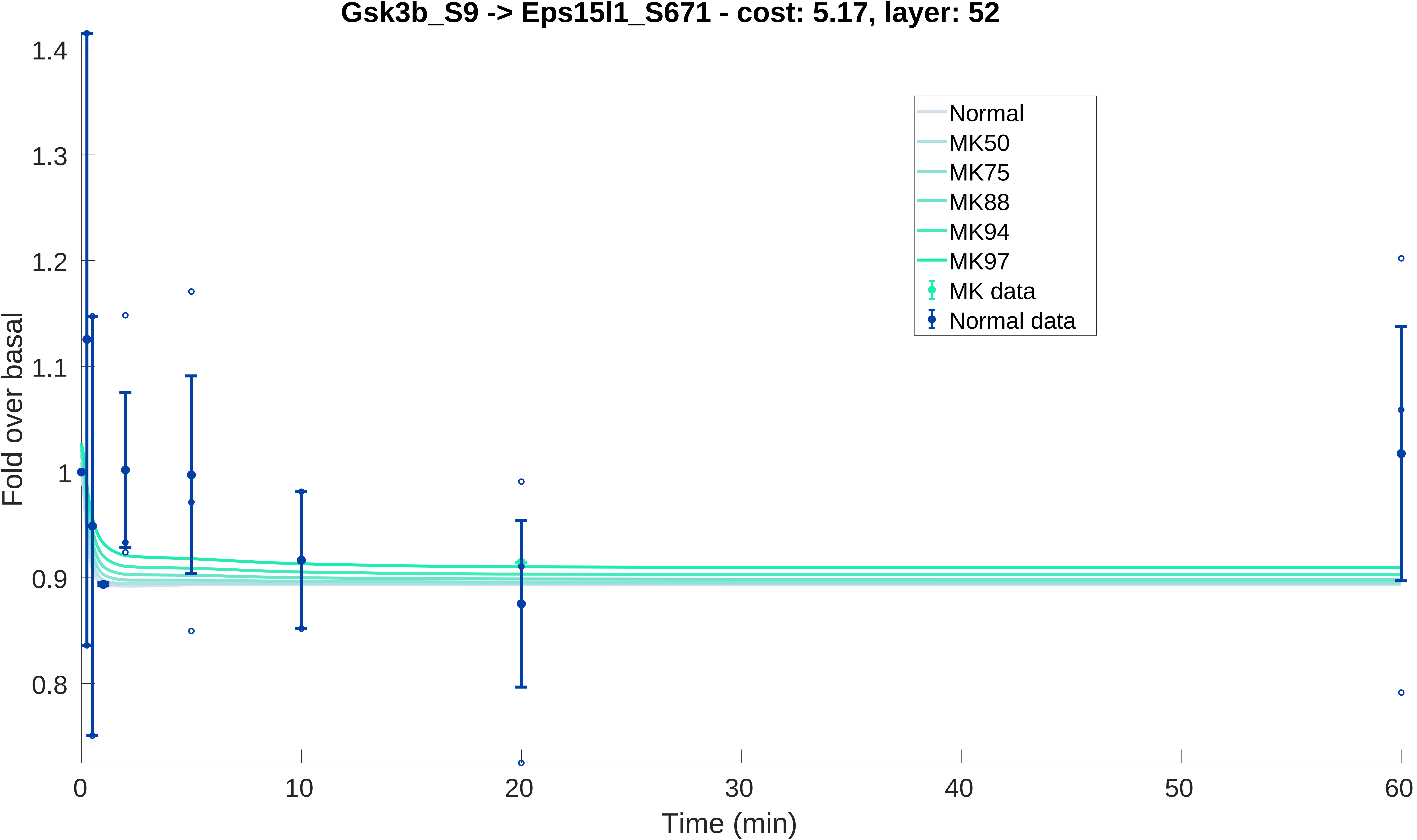

Gsk3b_S9 -> Eps15l1_S671 - cost: 5.17, layer: 52
Fold over basal
Time (min)
1.4
1.3
1.2
1.1
1
0.9
0.8
0
10
20
30
40
50
60
Normal
MK50
MK75
MK88
MK94
MK97
MK data
Normal data

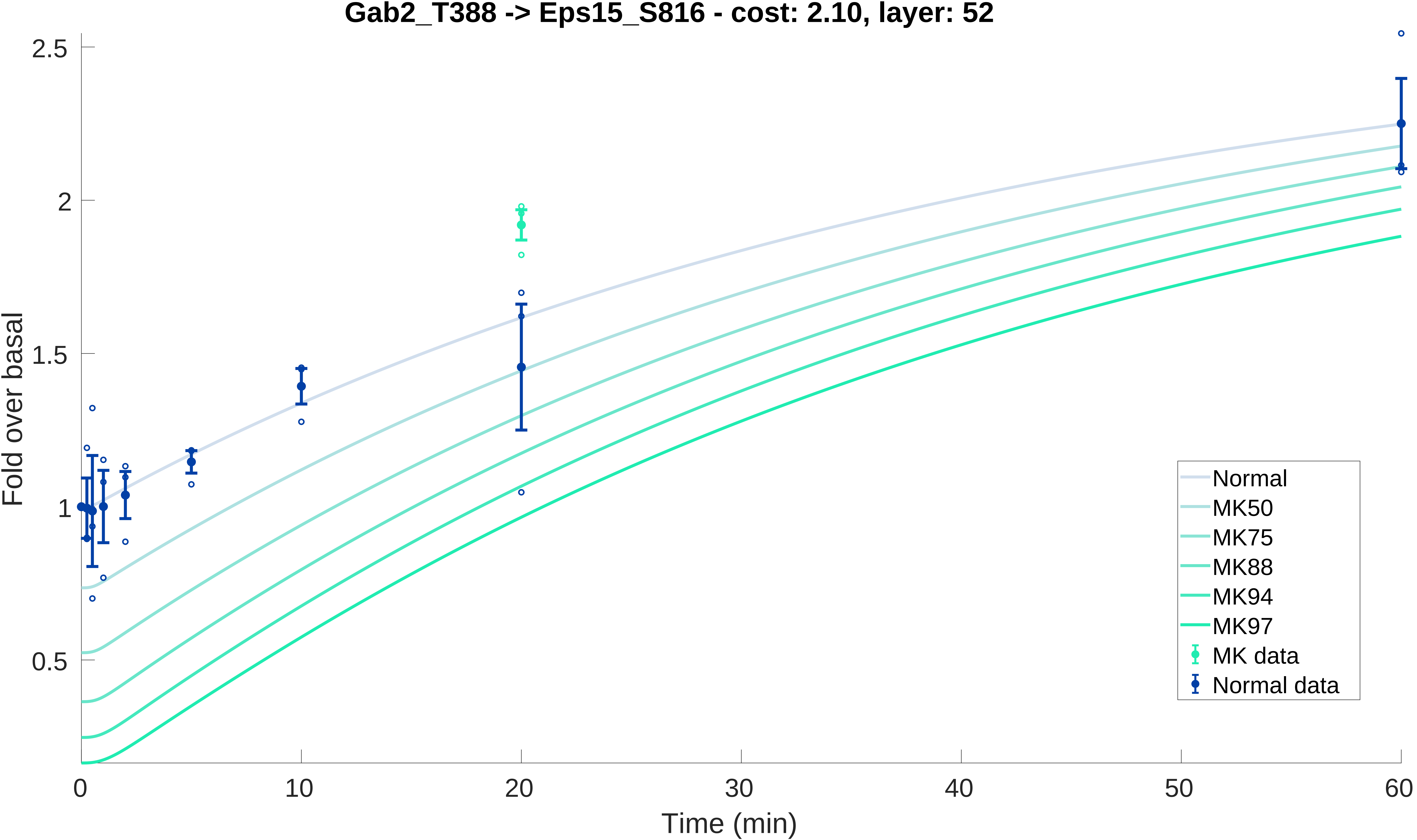

Gab2_T388 -> Eps15_S816 - cost: 2.10, layer: 52
Fold over basal
2.5
2
1.5
1
0.5
0
10
20
30
40
50
60
Time (min)
Normal
MK50
MK75
MK88
MK94
MK97
MK data
Normal data

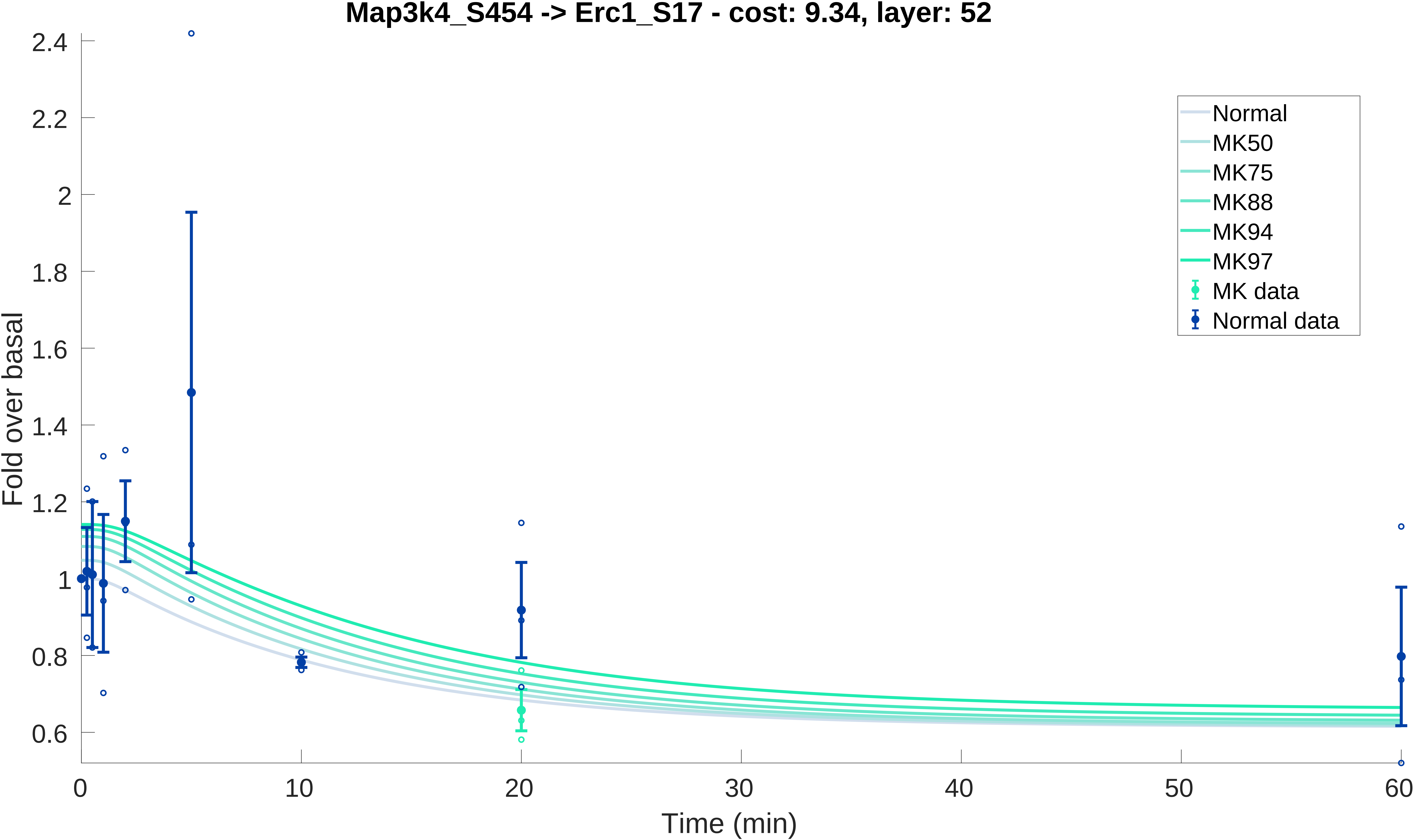

Map3k4_S454 -> Erc1_S17 - cost: 9.34, layer: 52
Fold over basal
Time (min)
0.6
0.8
1
1.2
1.4
1.6
1.8
2
2.2
2.4
0
10
20
30
40
50
60
Normal
MK50
MK75
MK88
MK94
MK97
MK data
Normal data

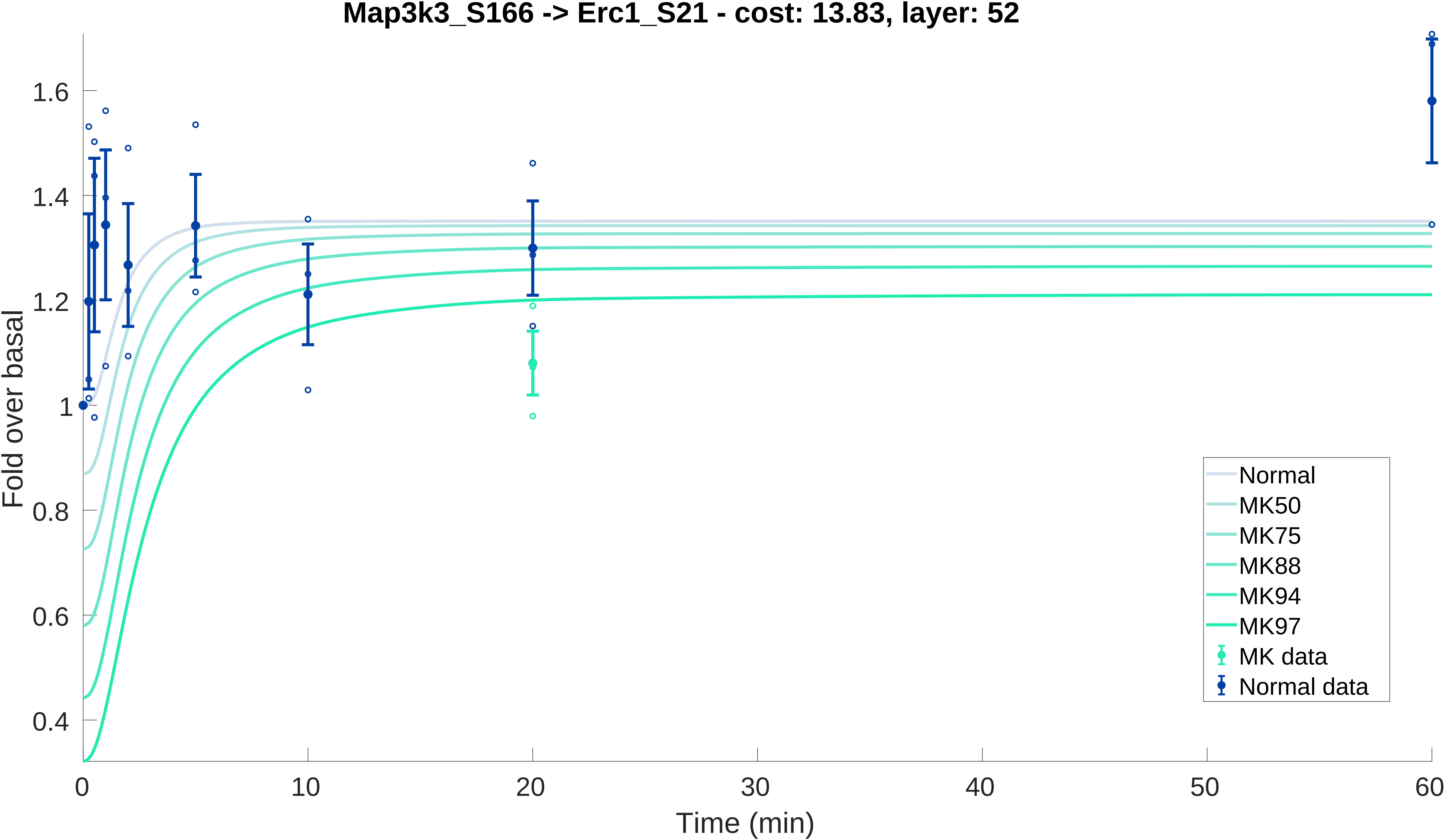

Map3k3_S166 -> Erc1_S21 - cost: 13.83, layer: 52
Fold over basal
Time (min)
1.6
1.4
1.2
1
0.8
0.6
0.4
0
10
20
30
40
50
60
Normal
MK50
MK75
MK88
MK94
MK97
MK data
Normal data

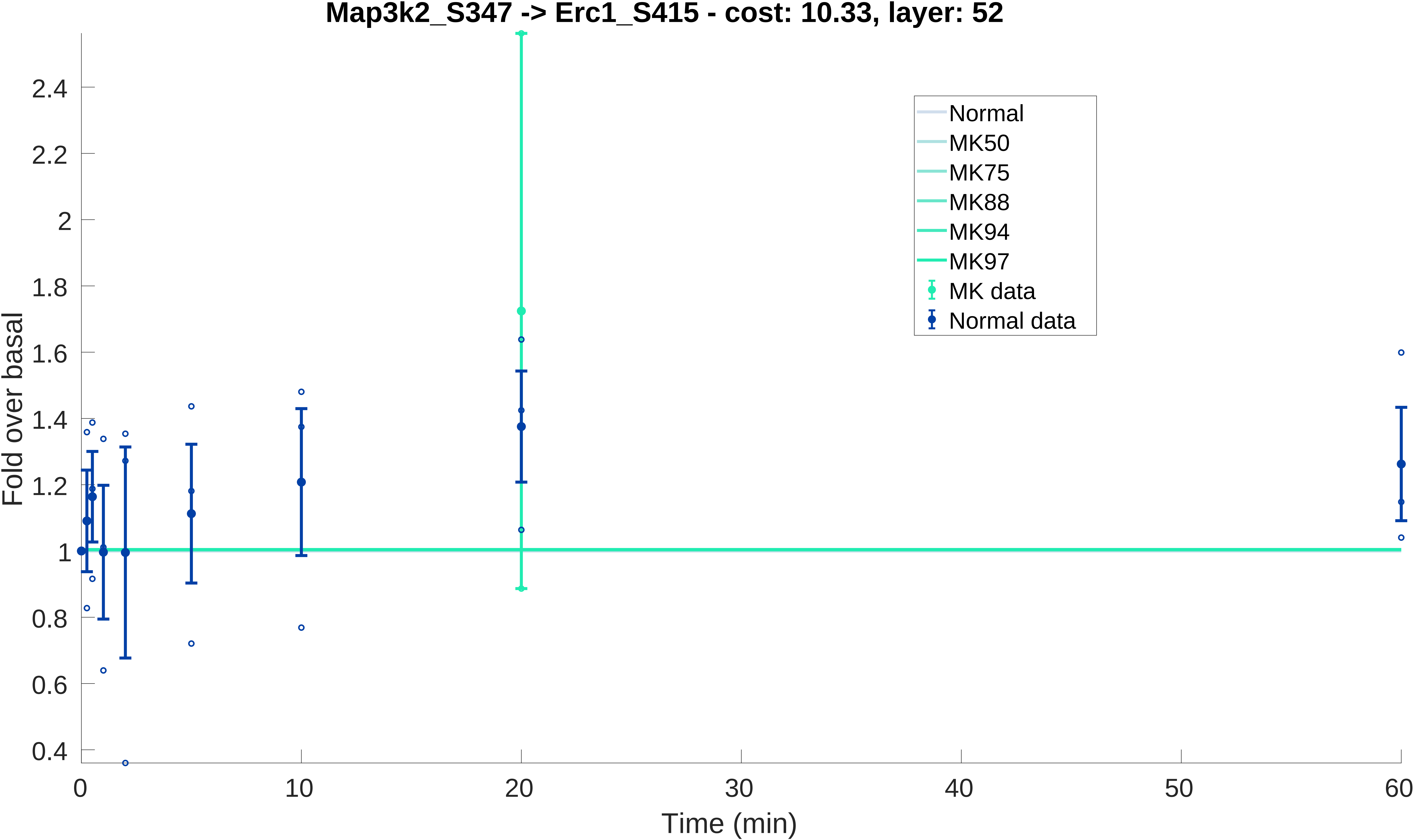

Map3k2_S347 -> Erc1_S415 - cost: 10.33, layer: 52
Fold over basal
Time (min)
Normal
MK50
MK75
MK88
MK94
MK97
MK data
Normal data
0.4
0.6
0.8
1
1.2
1.4
1.6
1.8
2
2.2
2.4
0
10
20
30
40
50
60

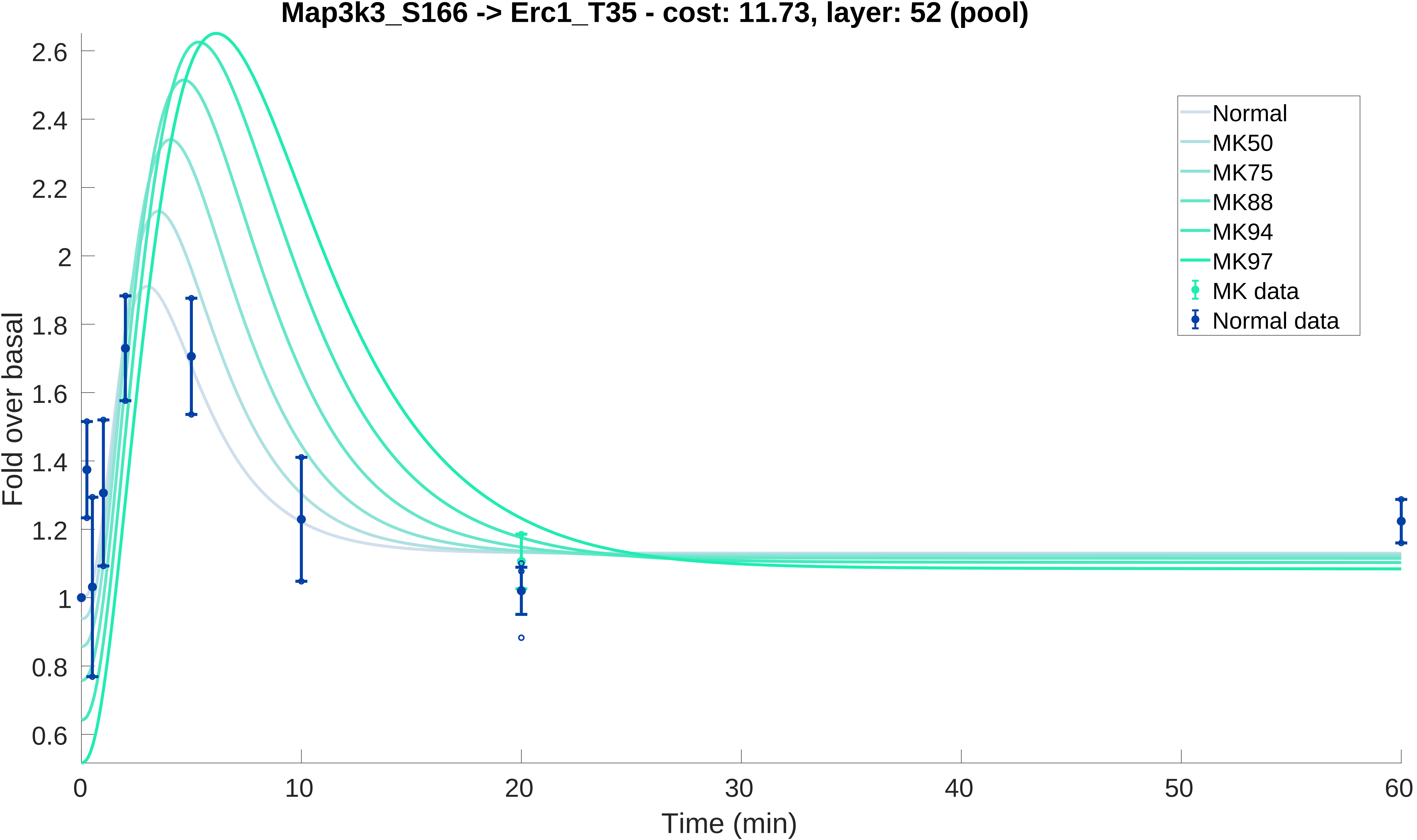

Map3k3_S166 -> Erc1_T35 - cost: 11.73, layer: 52 (pool)
Fold over basal
Time (min)
2.6
2.4
2.2
2
1.8
1.6
1.4
1.2
1
0.8
0.6
0
10
20
30
40
50
60
Normal
MK50
MK75
MK88
MK94
MK97
MK data
Normal data

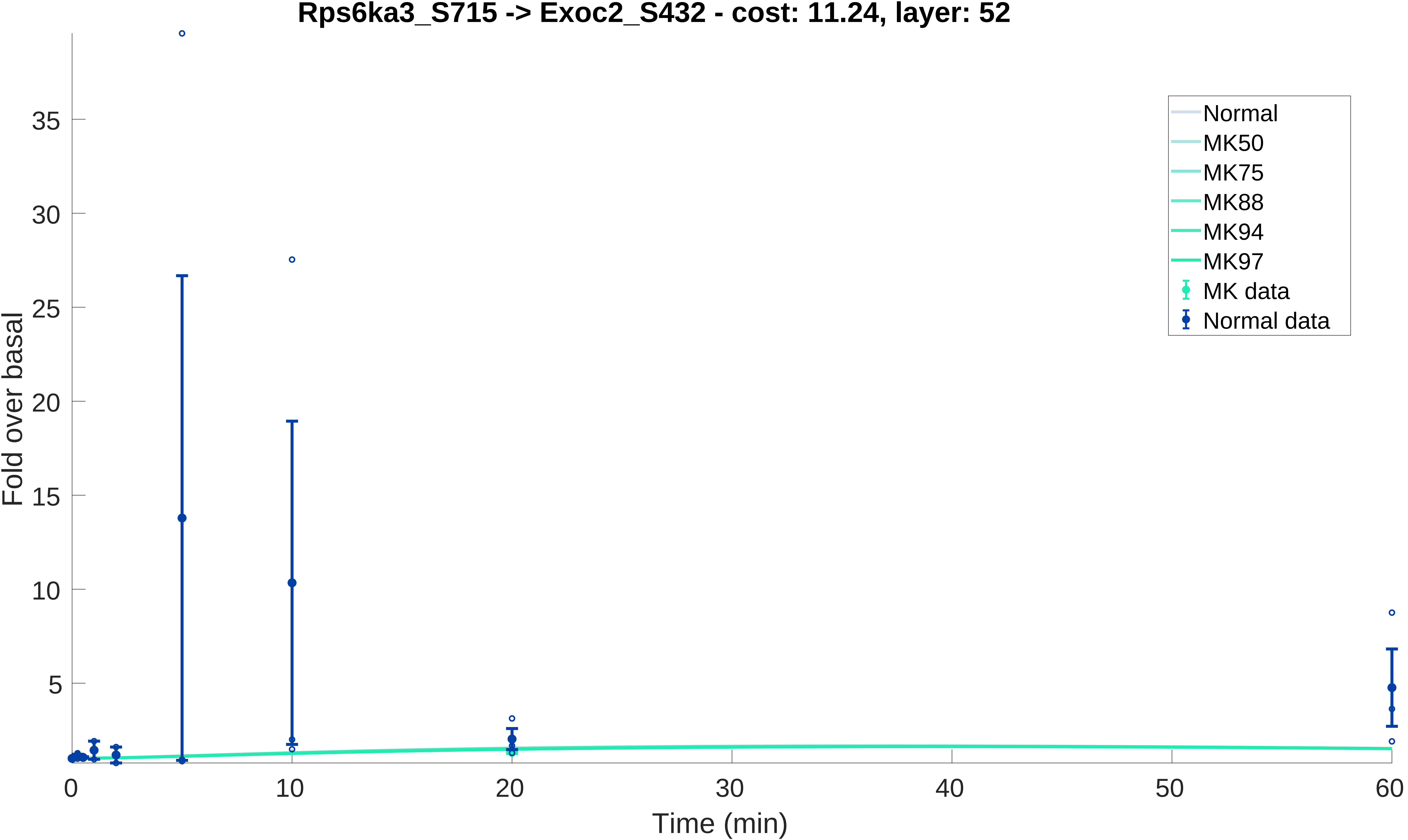
Rps6ka3_S715 -> Exoc2_S432 - cost: 11.24, layer: 52
Fold over basal
Time (min)
0
10
20
30
40
50
60
5
10
15
20
25
30
35
Normal
MK50
MK75
MK88
MK94
MK97
MK data
Normal data

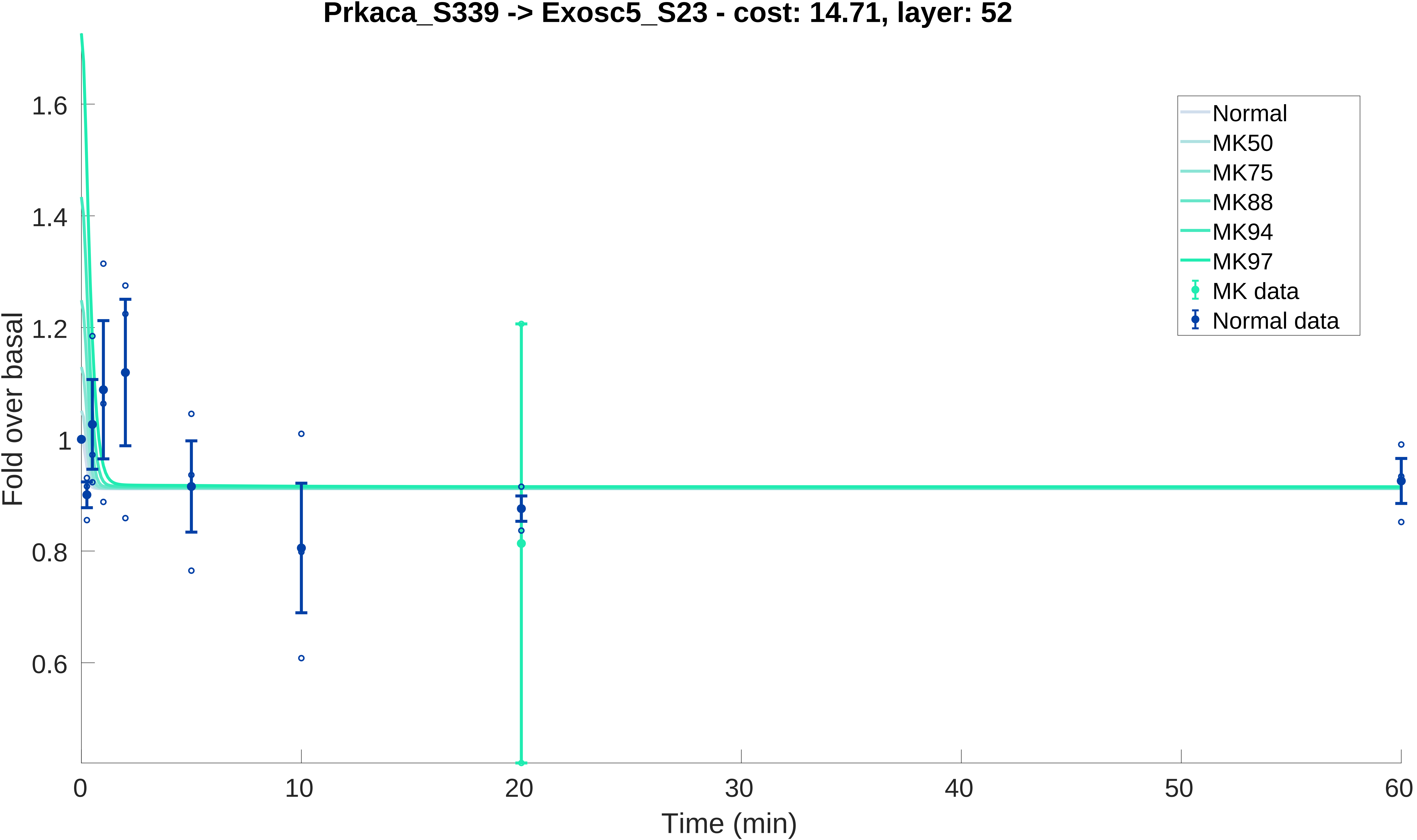

Prkaca_S339 -> Exosc5_S23 - cost: 14.71, layer: 52
Fold over basal
1.6
1.4
1.2
1
0.8
0.6
0
10
20
30
40
50
60
Time (min)
Normal
MK50
MK75
MK88
MK94
MK97
MK data
Normal data

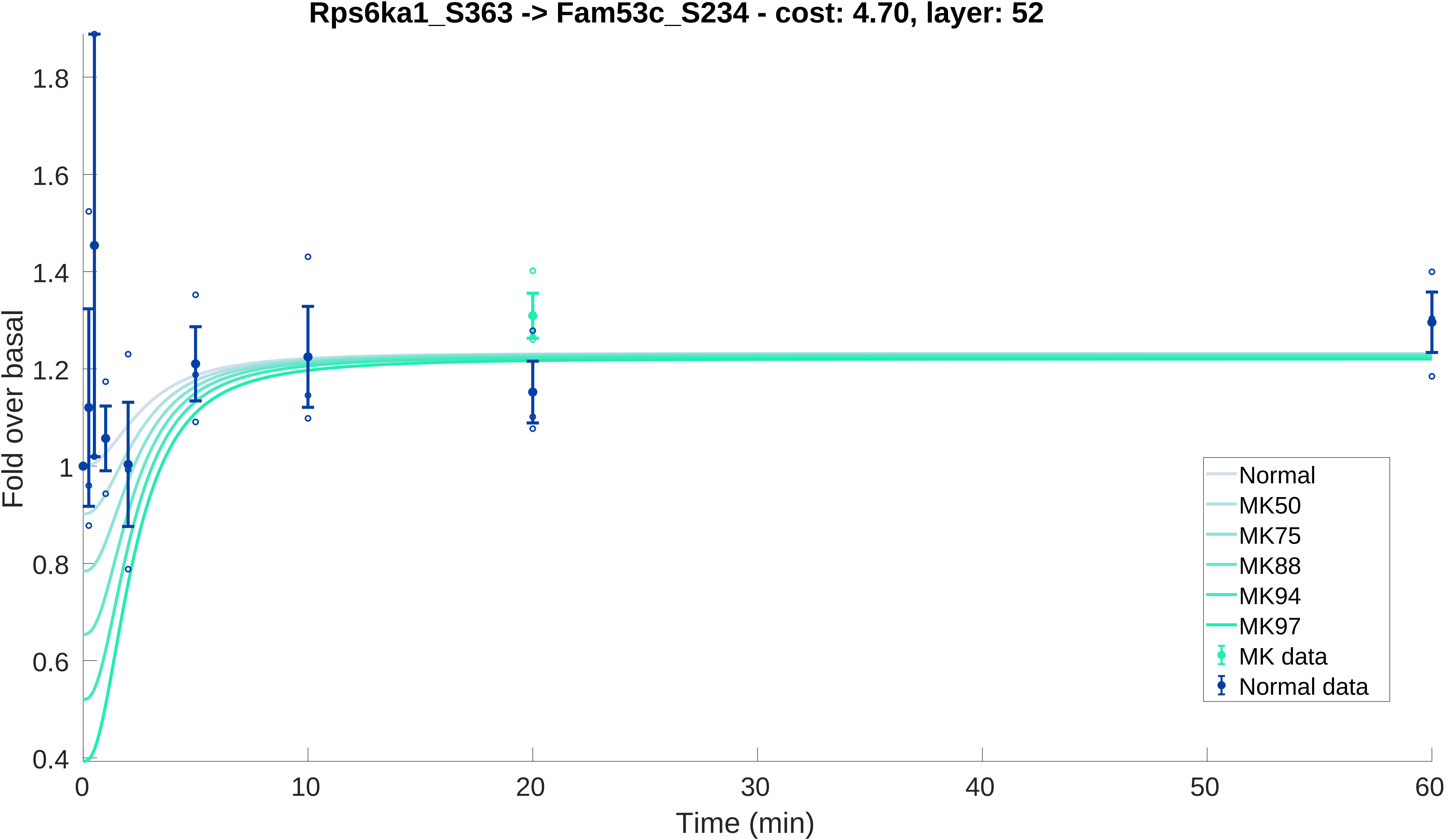

Rps6ka1_S363 -> Fam53c_S234 - cost: 4.70, layer: 52
Fold over basal
1.8
1.6
1.4
1.2
1
0.8
0.6
0.4
0
10
20
30
40
50
60
Time (min)
Normal
MK50
MK75
MK88
MK94
MK97
MK data
Normal data

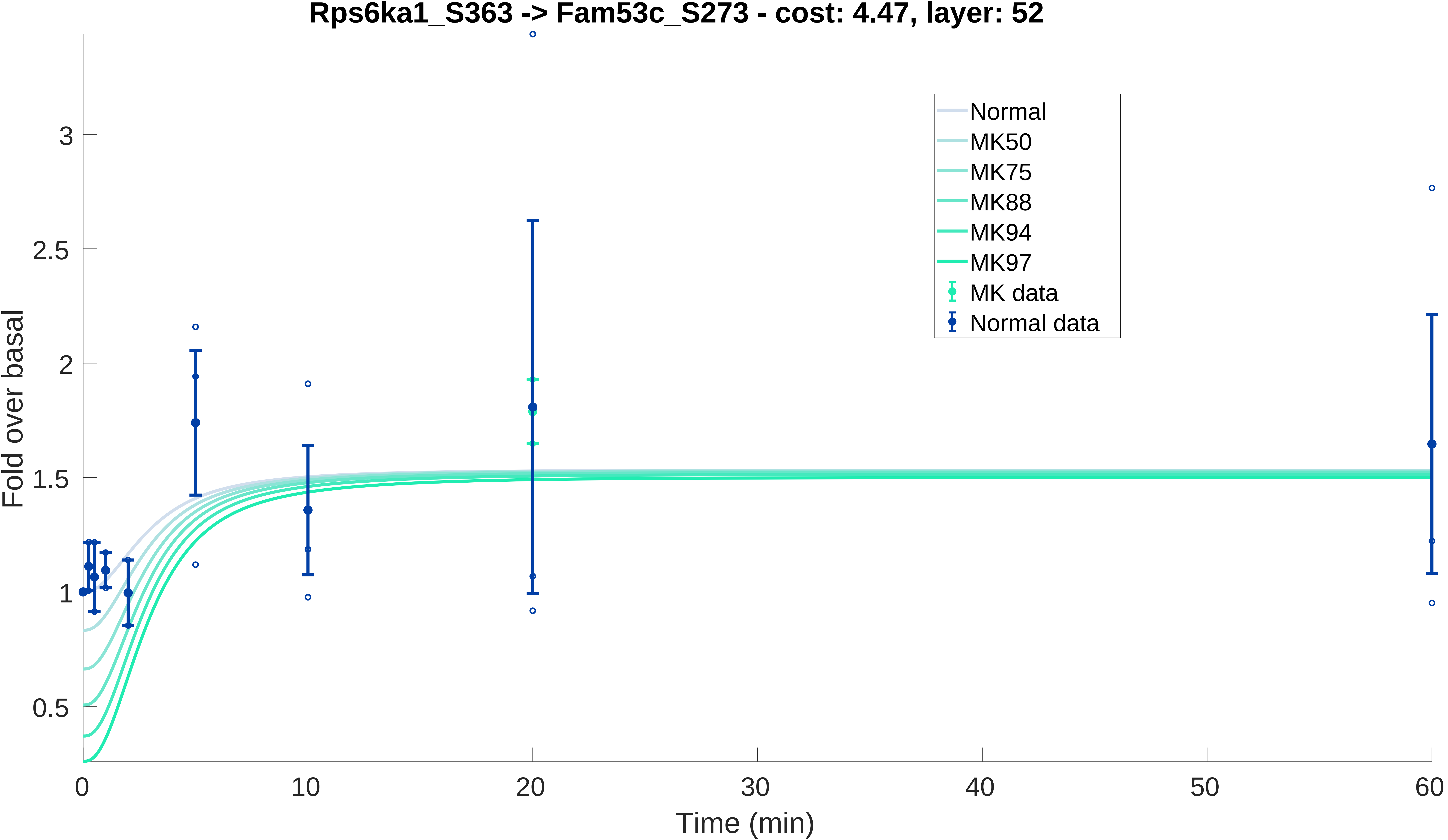

Rps6ka1_S363 -> Fam53c_S273 - cost: 4.47, layer: 52
Fold over basal
Time (min)
0.5
1
1.5
2
2.5
3
0
10
20
30
40
50
60
Normal
MK50
MK75
MK88
MK94
MK97
MK data
Normal data

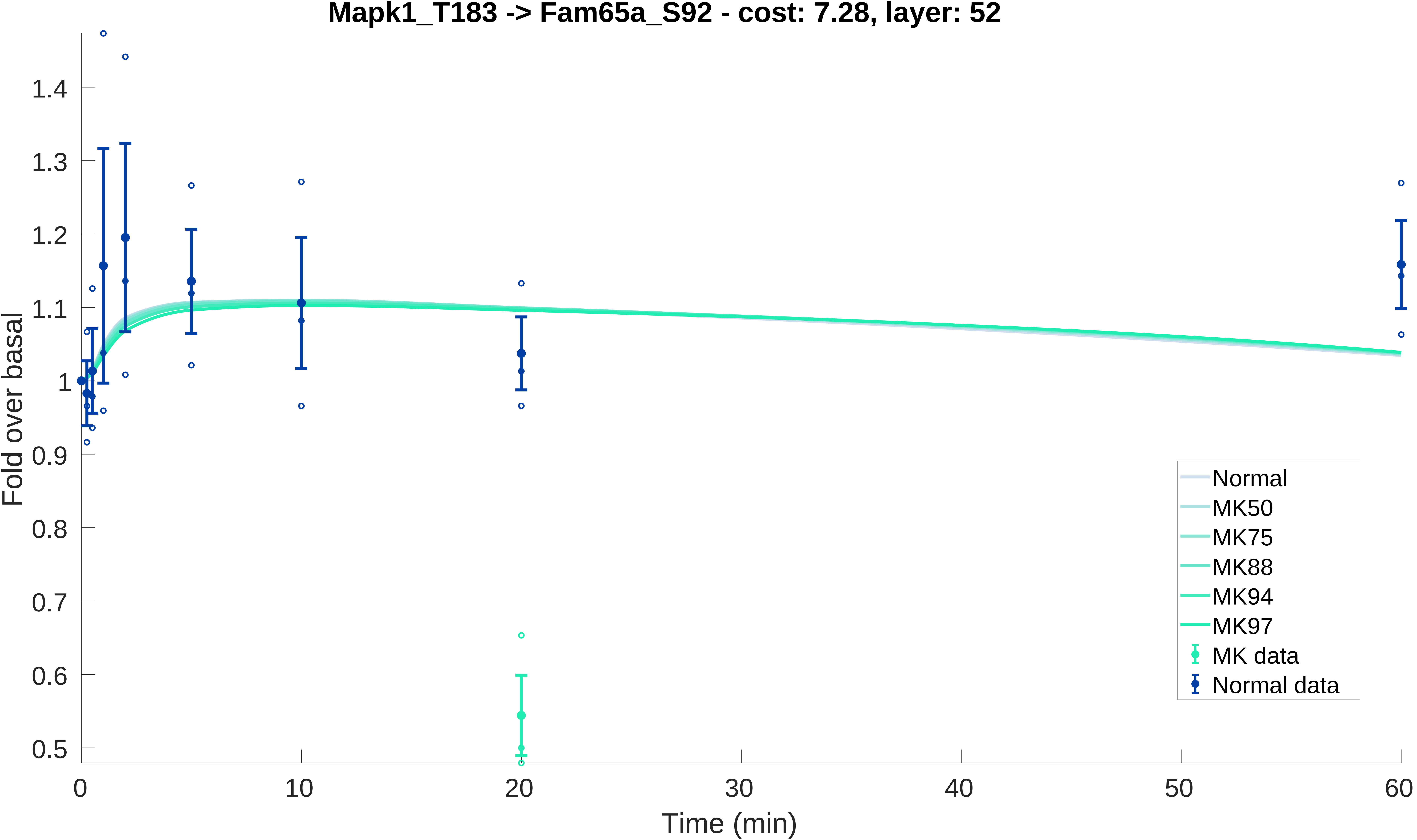
Mapk1_T183 -> Fam65a_S92 - cost: 7.28, layer: 52
Fold over basal
Time (min)
0.5
0.6
0.7
0.8
0.9
1
1.1
1.2
1.3
1.4
0
10
20
30
40
50
60
Normal
MK50
MK75
MK88
MK94
MK97
MK data
Normal data

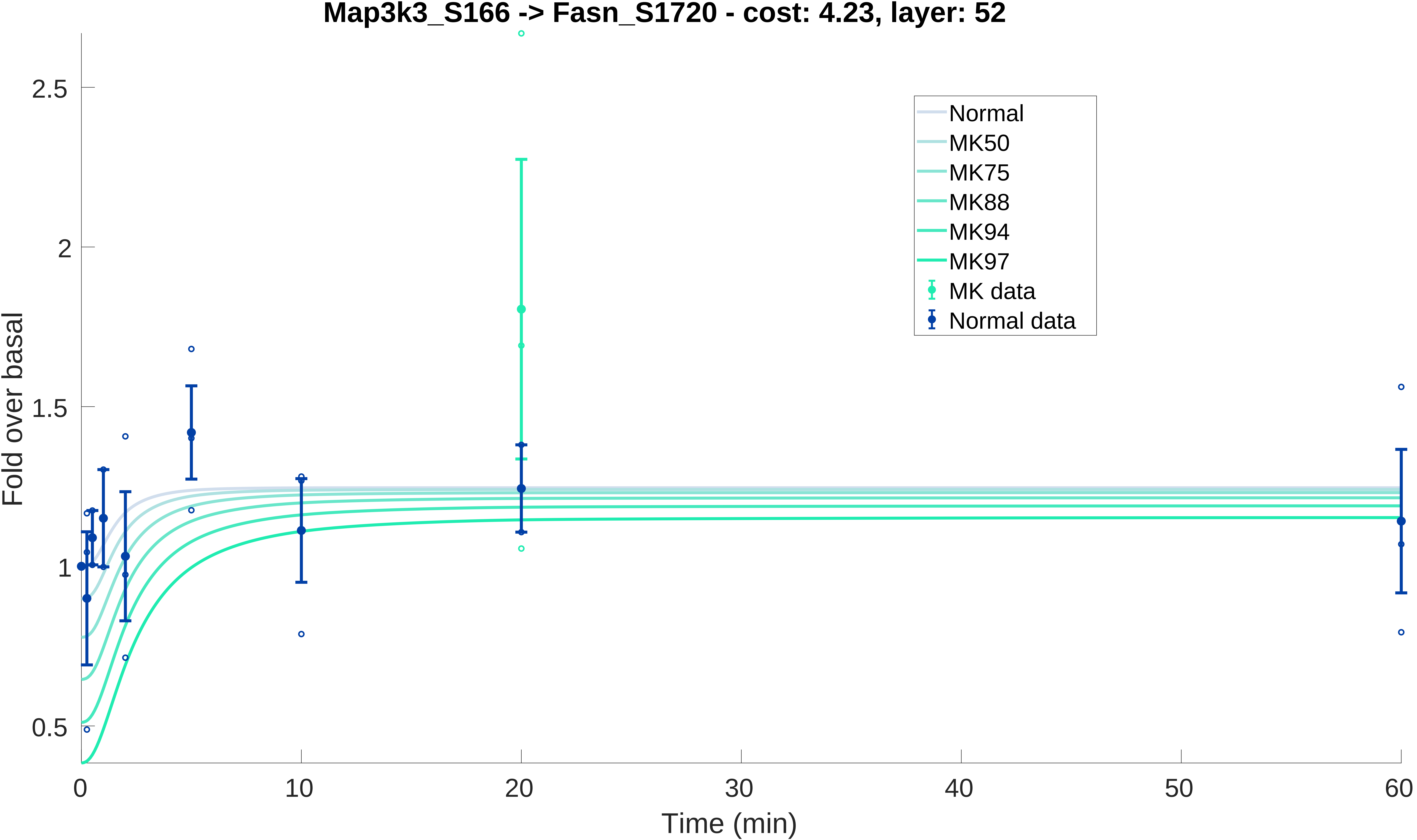
Map3k3_S166 -> Fasn_S1720 - cost: 4.23, layer: 52
Fold over basal
2.5
2
1.5
1
0.5
0
10
20
30
40
50
60
Time (min)
Normal
MK50
MK75
MK88
MK94
MK97
MK data
Normal data

Map3k3_S166 -> Fasn_S2199 - cost: 13.51, layer: 52 (pool)

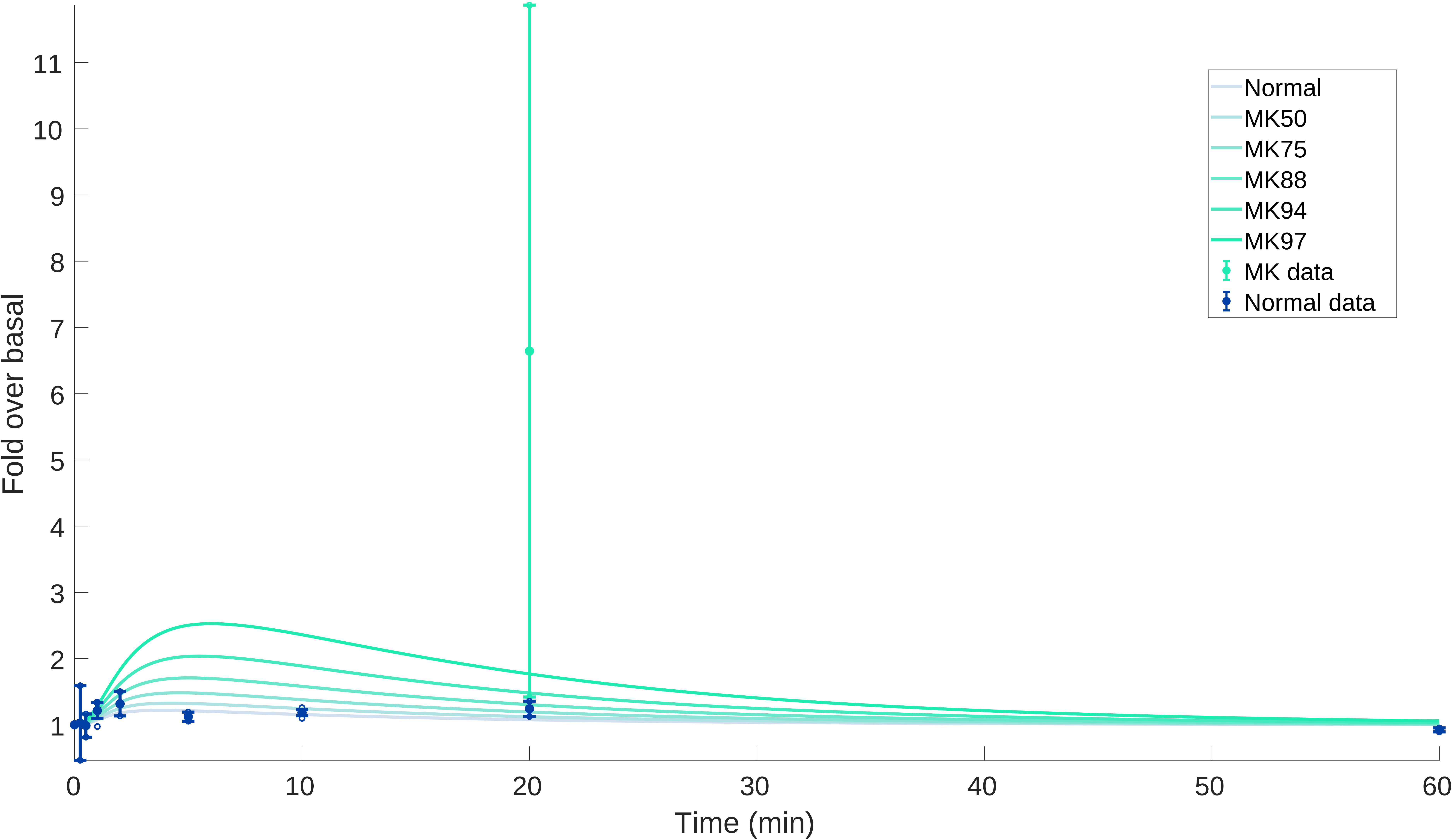

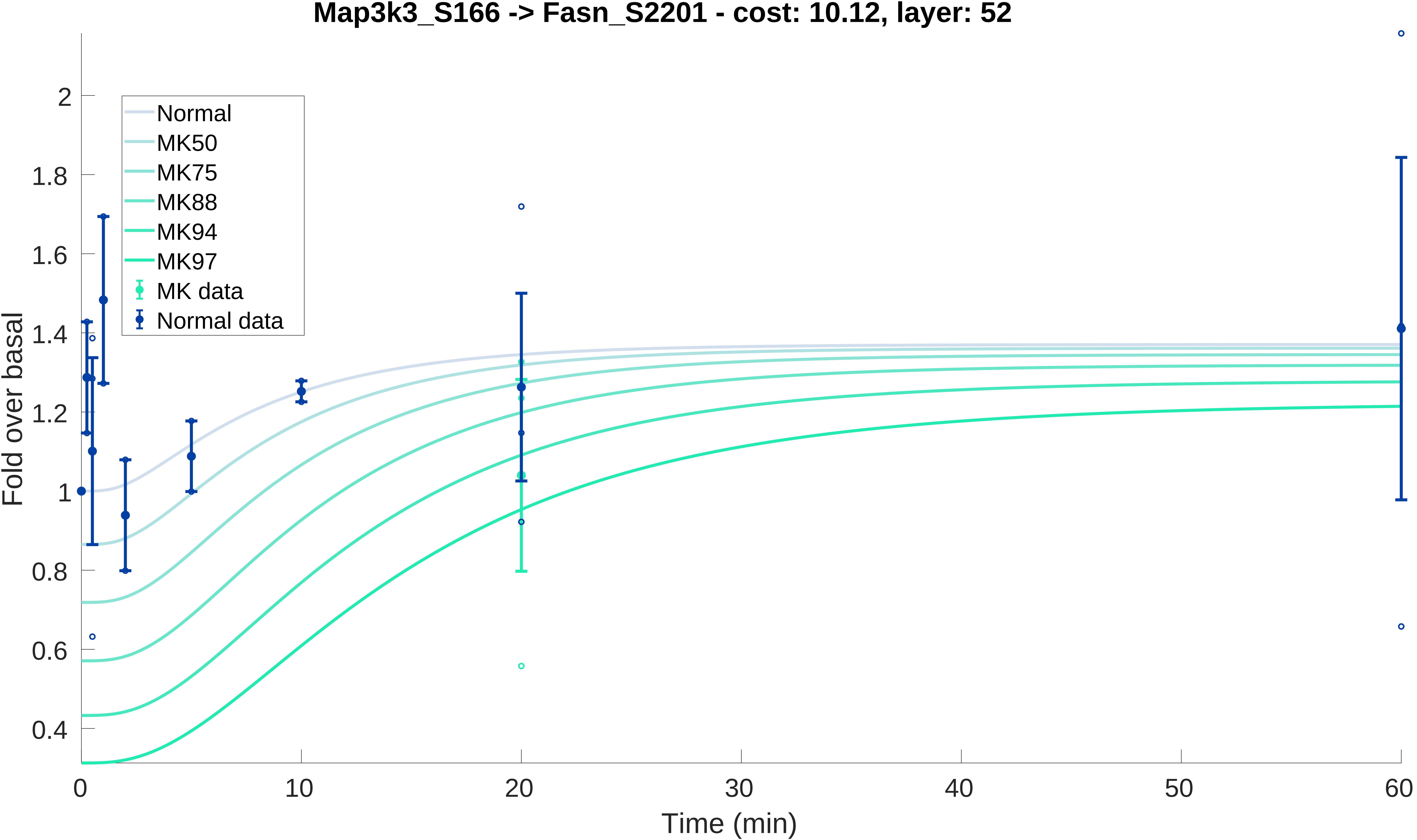
Map3k3_S166 -> Fasn_S2201 - cost: 10.12, layer: 52
Normal
MK50
MK75
MK88
MK94
MK97
MK data
Normal data
Fold over basal
Time (min)
0
10
20
30
40
50
60
0.4
0.6
0.8
1
1.2
1.4
1.6
1.8
2

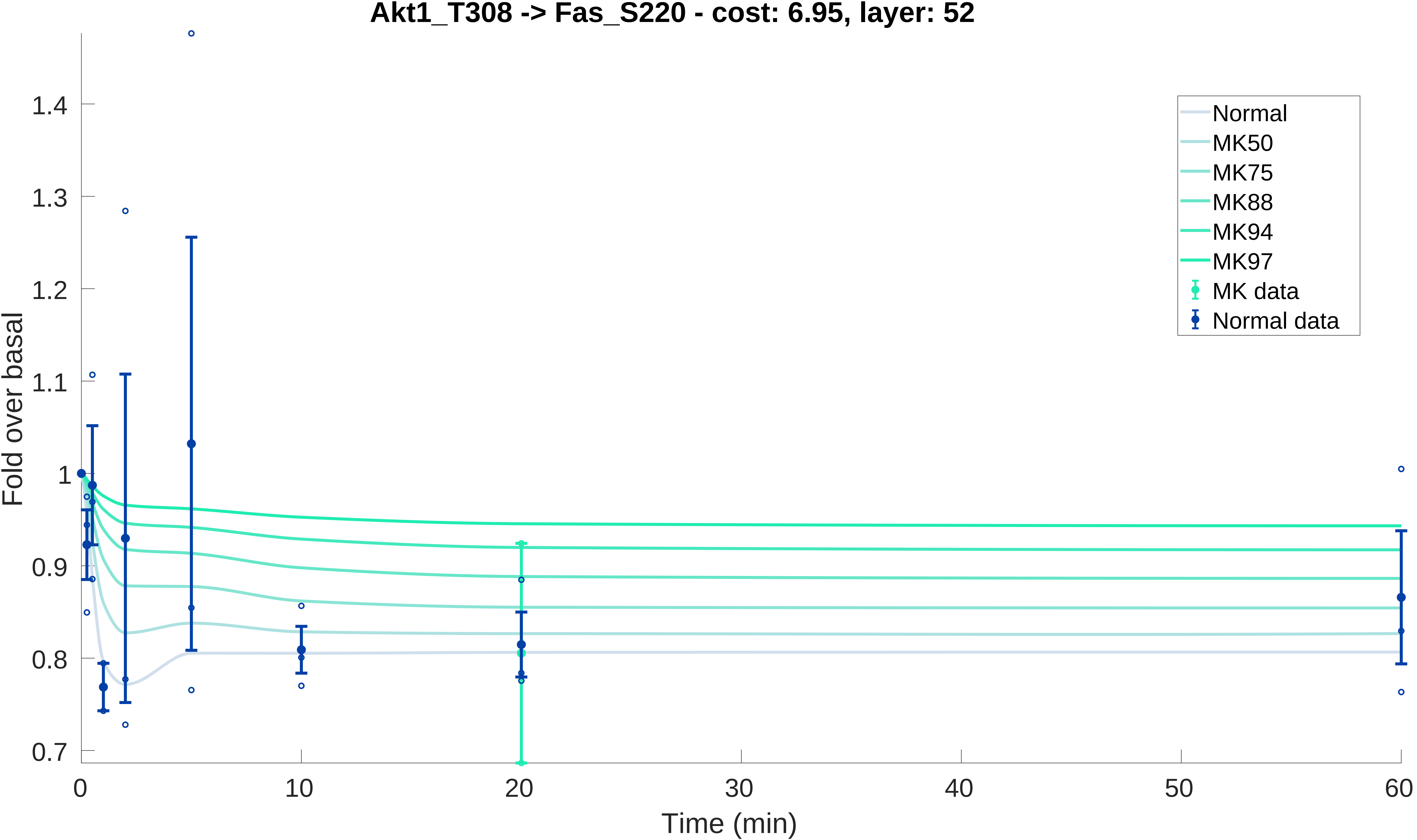
Akt1_T308 -> Fas_S220 - cost: 6.95, layer: 52
Fold over basal
Time (min)
Normal
MK50
MK75
MK88
MK94
MK97
MK data
Normal data
1.4
1.3
1.2
1.1
1
0.9
0.8
0.7
0
10
20
30
40
50
60

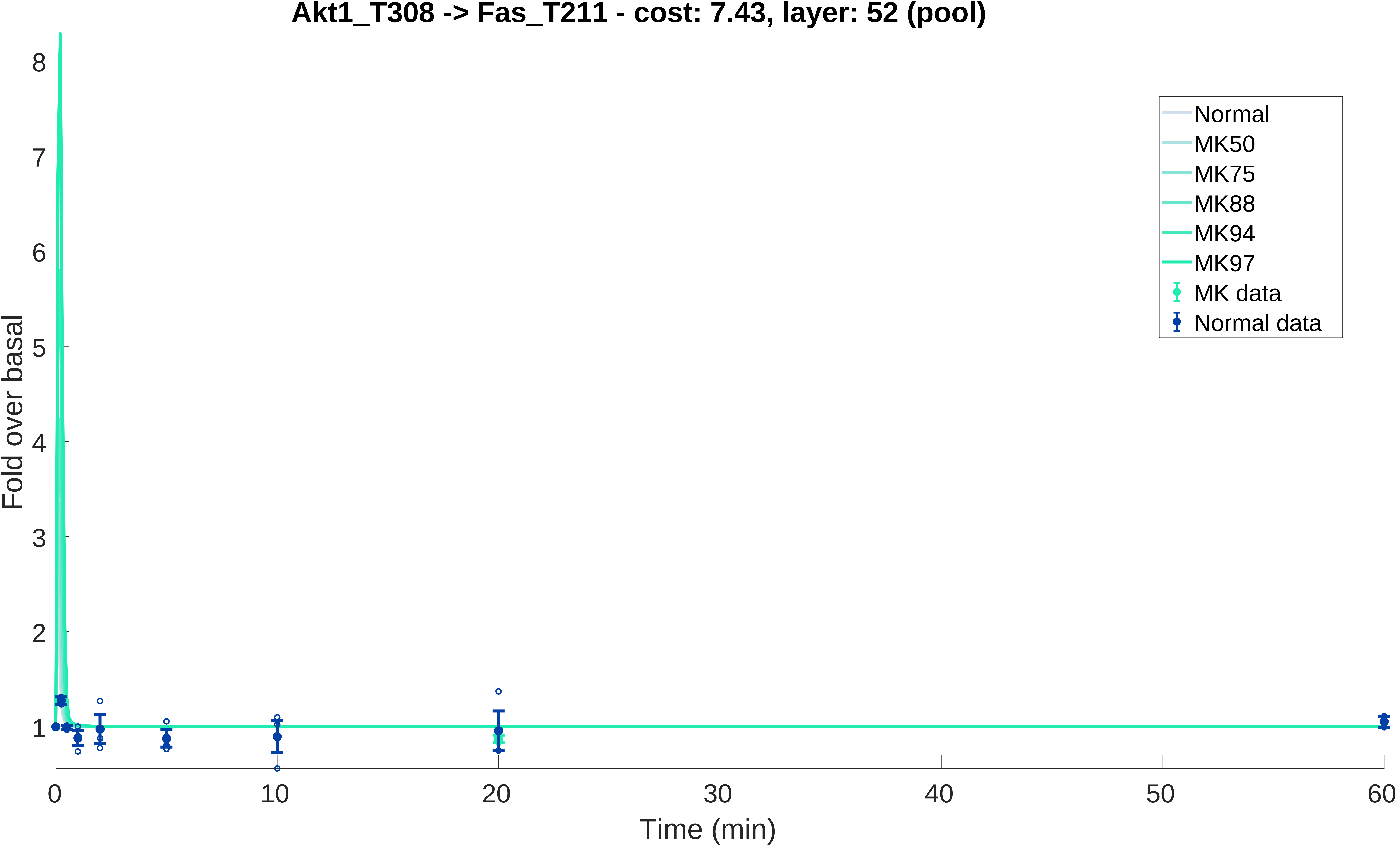

Akt1_T308 -> Fas_T211 - cost: 7.43, layer: 52 (pool)
Fold over basal
Time (min)
Normal
MK50
MK75
MK88
MK94
MK97
MK data
Normal data
0
10
20
30
40
50
60
1
2
3
4
5
6
7
8

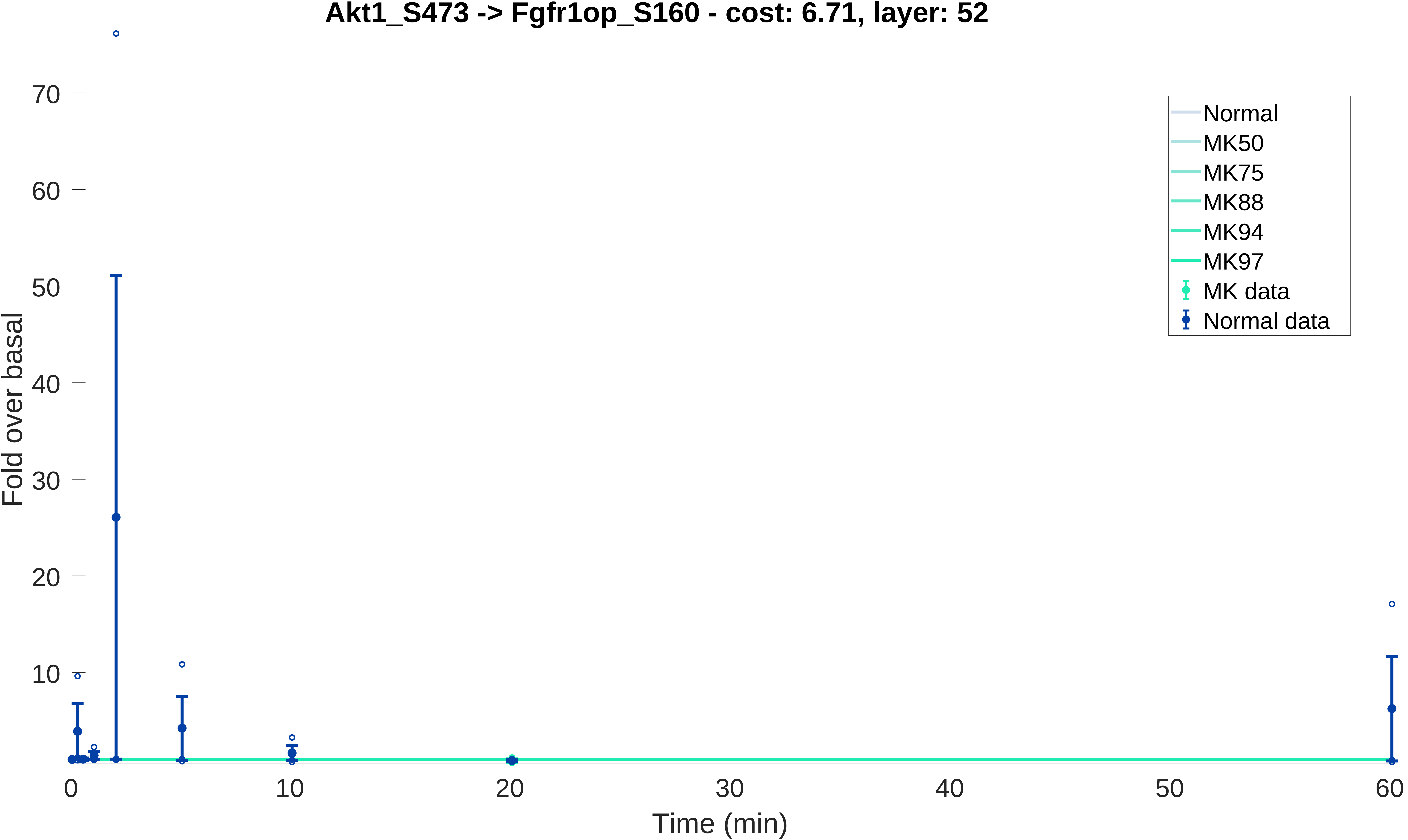
Akt1_S473 -> Fgfr1op_S160 - cost: 6.71, layer: 52
Fold over basal
Time (min)
0
10
20
30
40
50
60
70
Normal
MK50
MK75
MK88
MK94
MK97
MK data
Normal data

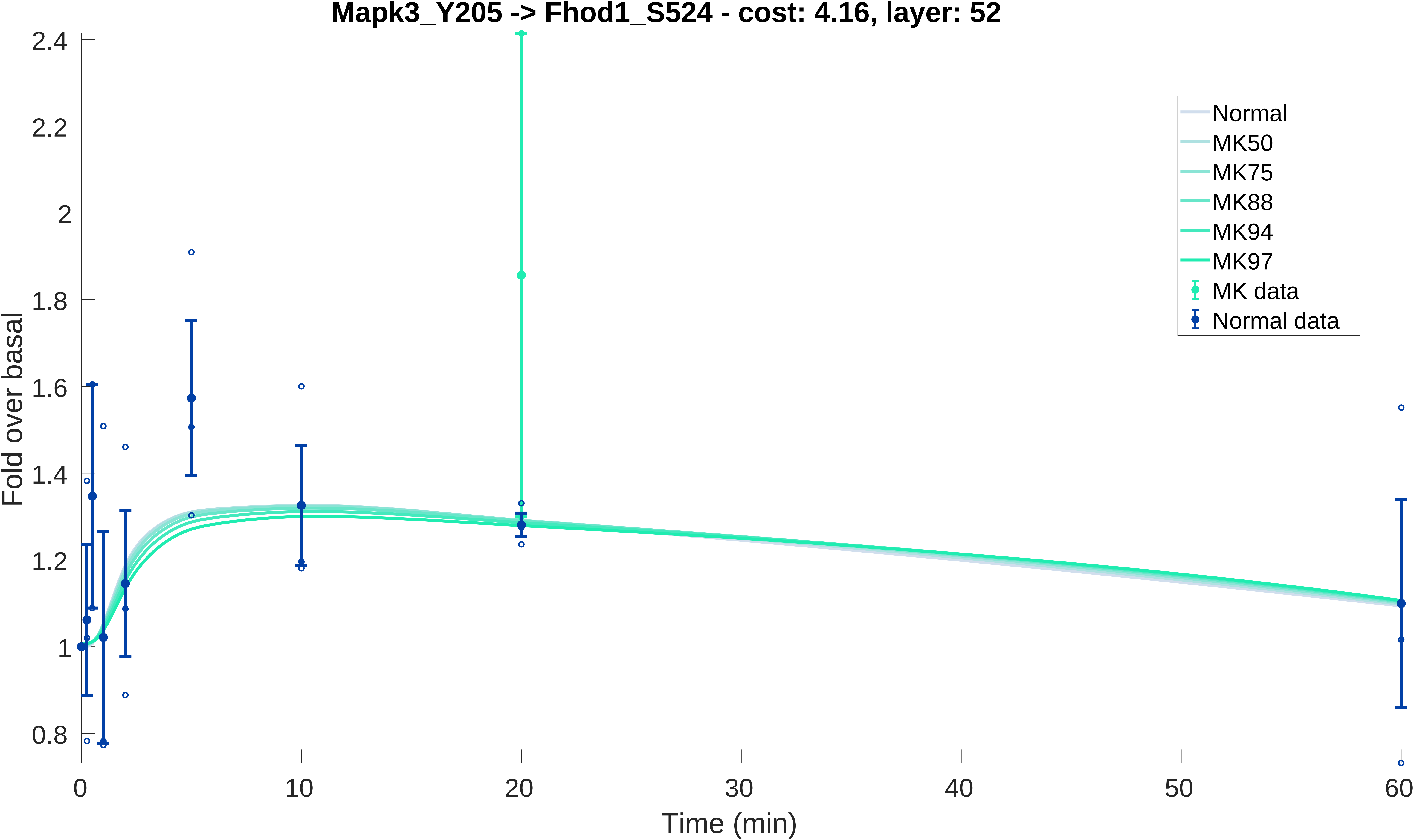
Mapk3_Y205 -> Fhod1_S524 - cost: 4.16, layer: 52
Fold over basal
Time (min)
Normal
MK50
MK75
MK88
MK94
MK97
MK data
Normal data
2.4
2.2
2
1.8
1.6
1.4
1.2
1
0.8
0
10
20
30
40
50
60

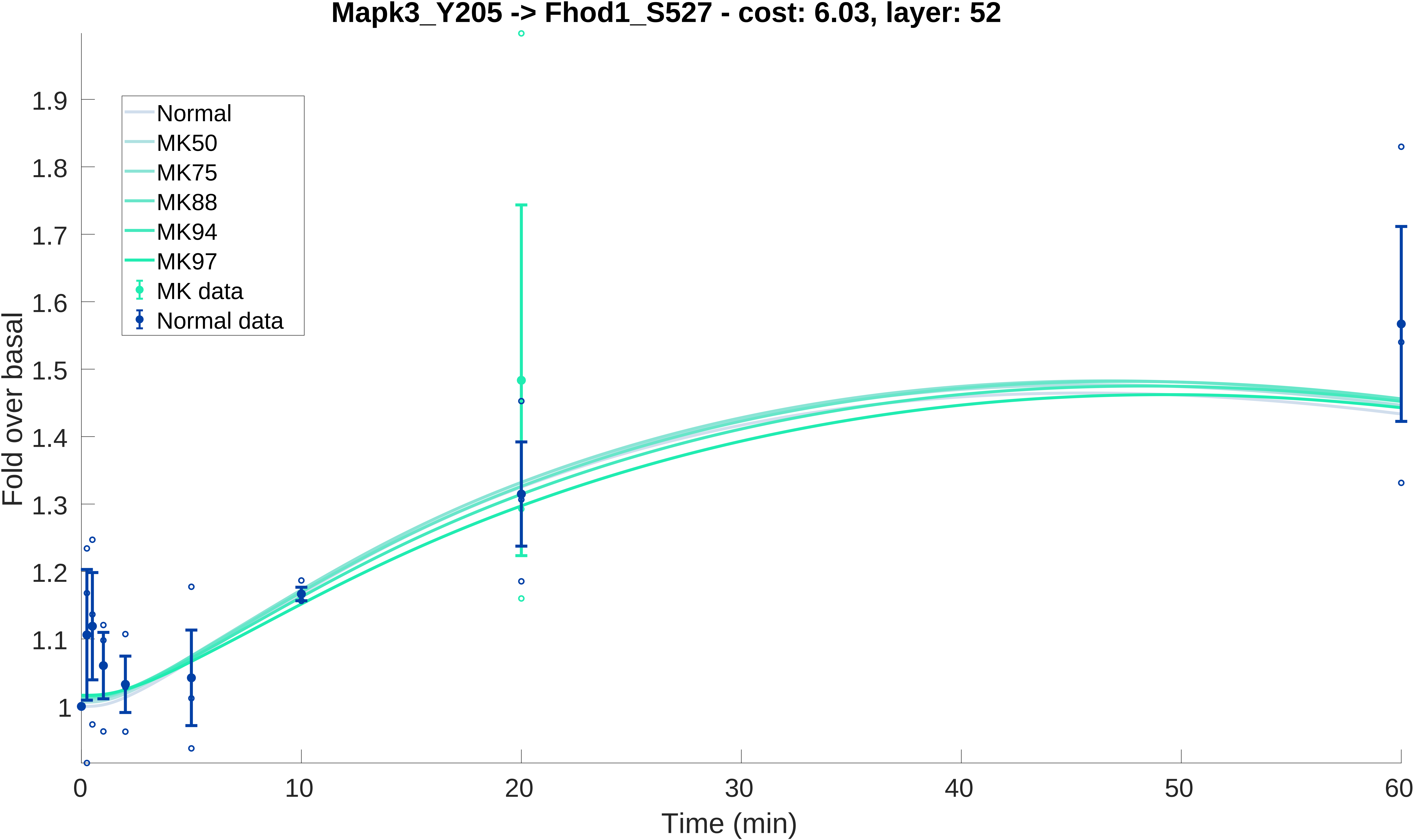

Mapk3_Y205 -> Fhod1_S527 - cost: 6.03, layer: 52
Fold over basal
Time (min)
Normal
MK50
MK75
MK88
MK94
MK97
MK data
Normal data
1
1.1
1.2
1.3
1.4
1.5
1.6
1.7
1.8
1.9
0
10
20
30
40
50
60

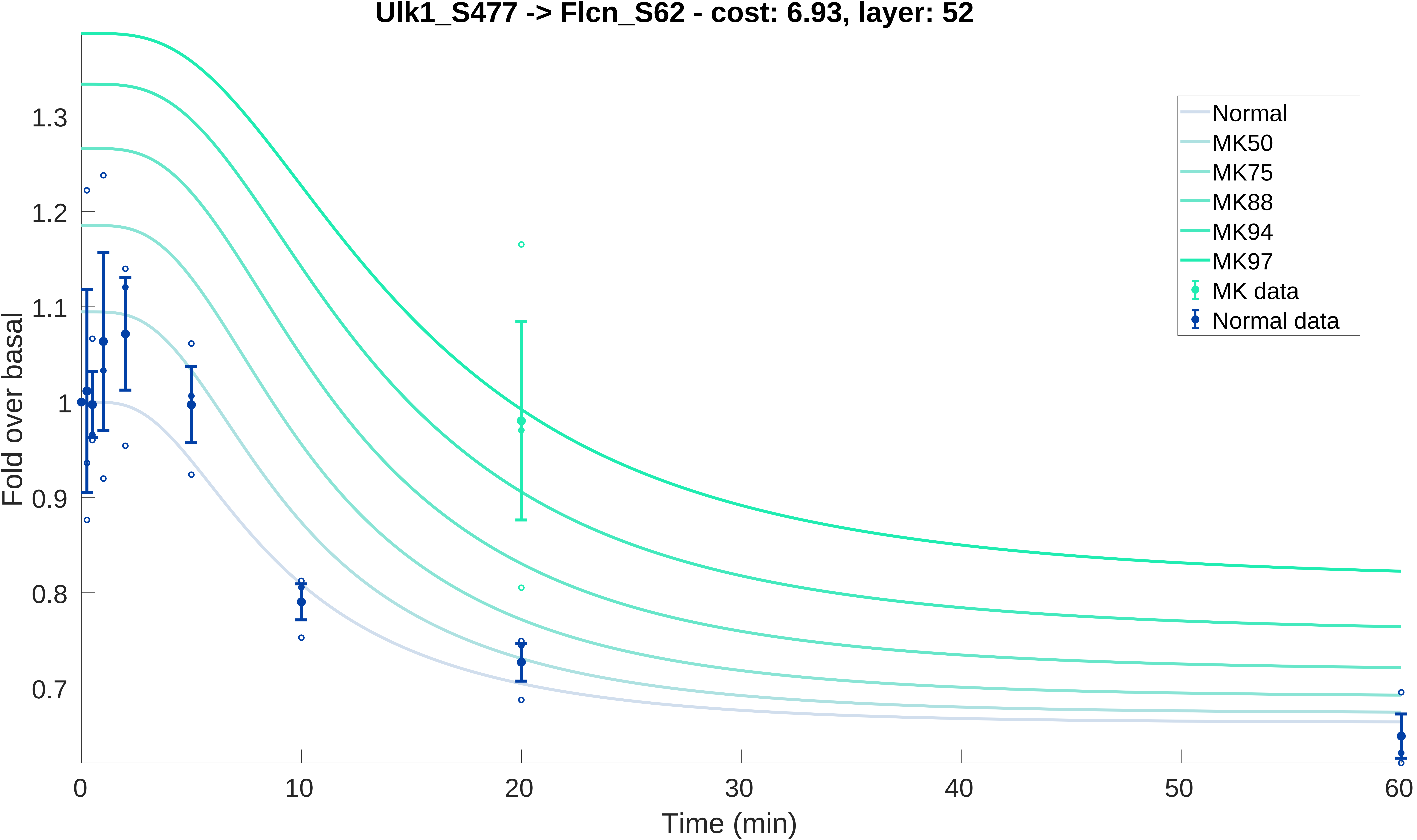
Ulk1_S477 -> Flcn_S62 - cost: 6.93, layer: 52
Fold over basal
Time (min)
1.3
1.2
1.1
1
0.9
0.8
0.7
0
10
20
30
40
50
60
Normal
MK50
MK75
MK88
MK94
MK97
MK data
Normal data

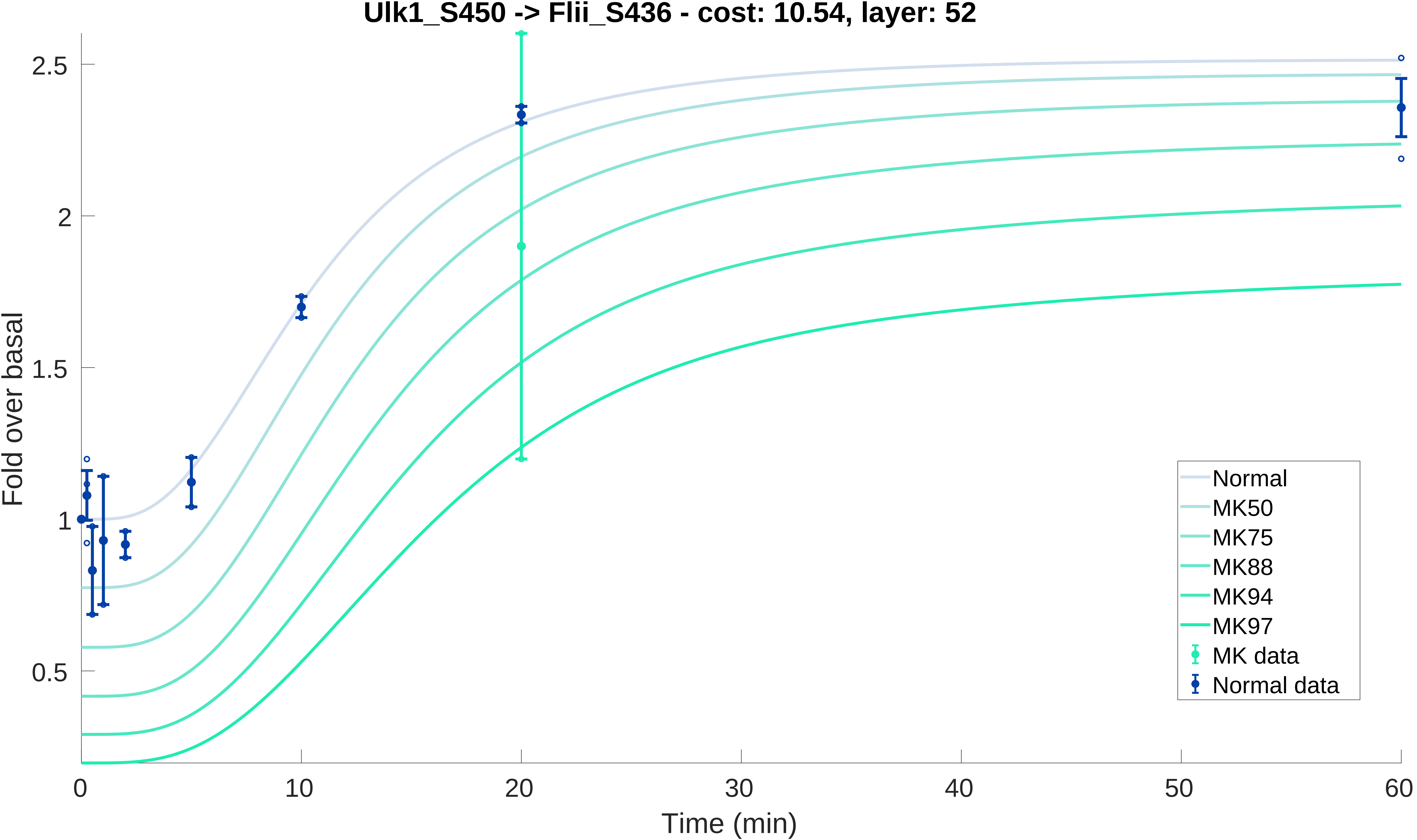

Ulk1_S450 -> Flii_S436 - cost: 10.54, layer: 52
Fold over basal
2.5
2
1.5
1
0.5
0
10
20
30
40
50
60
Time (min)
Normal
MK50
MK75
MK88
MK94
MK97
MK data
Normal data

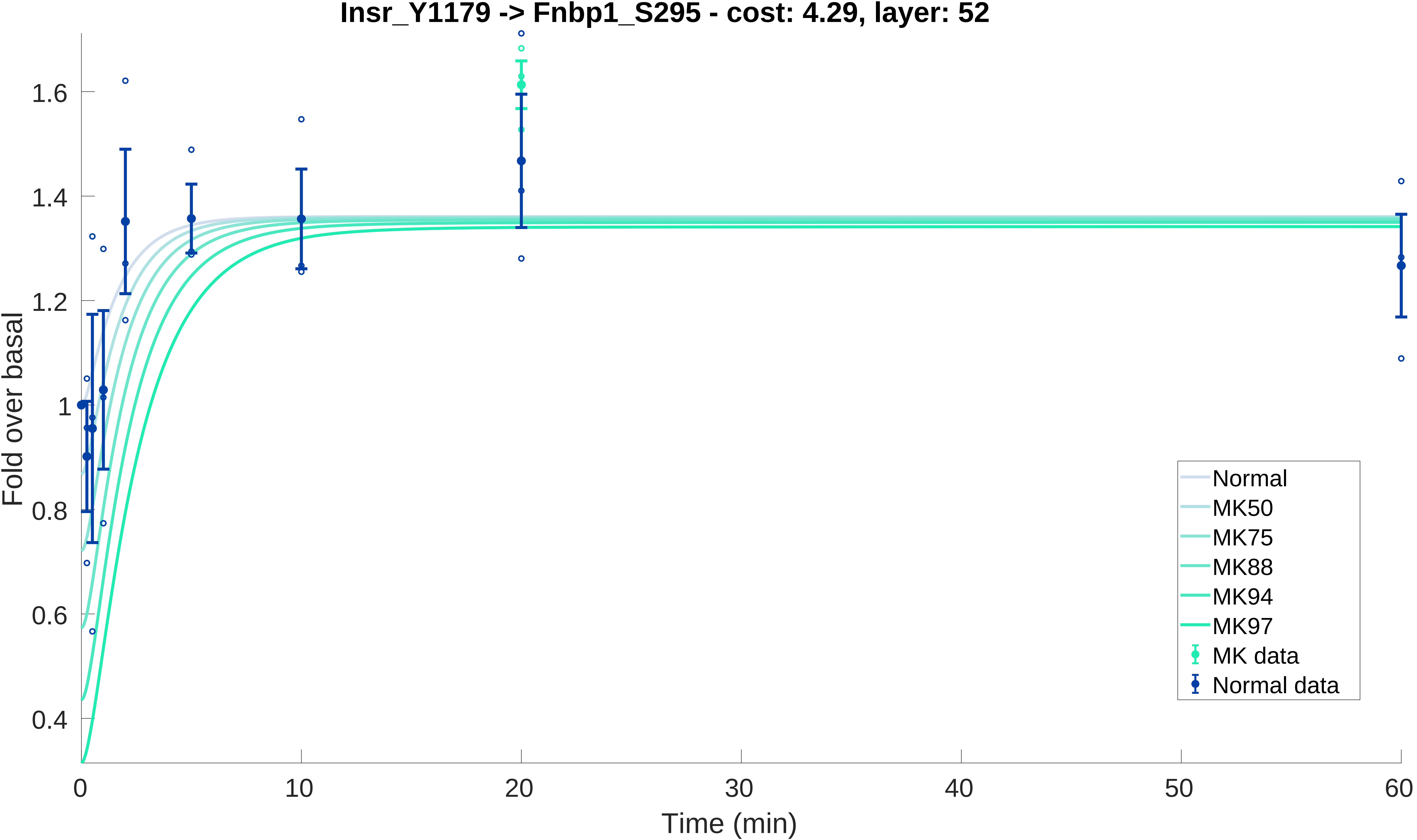

Insr_Y1179 -> Fnbp1_S295 - cost: 4.29, layer: 52
Fold over basal
Time (min)
1.6
1.4
1.2
1
0.8
0.6
0.4
0
10
20
30
40
50
60
Normal
MK50
MK75
MK88
MK94
MK97
MK data
Normal data

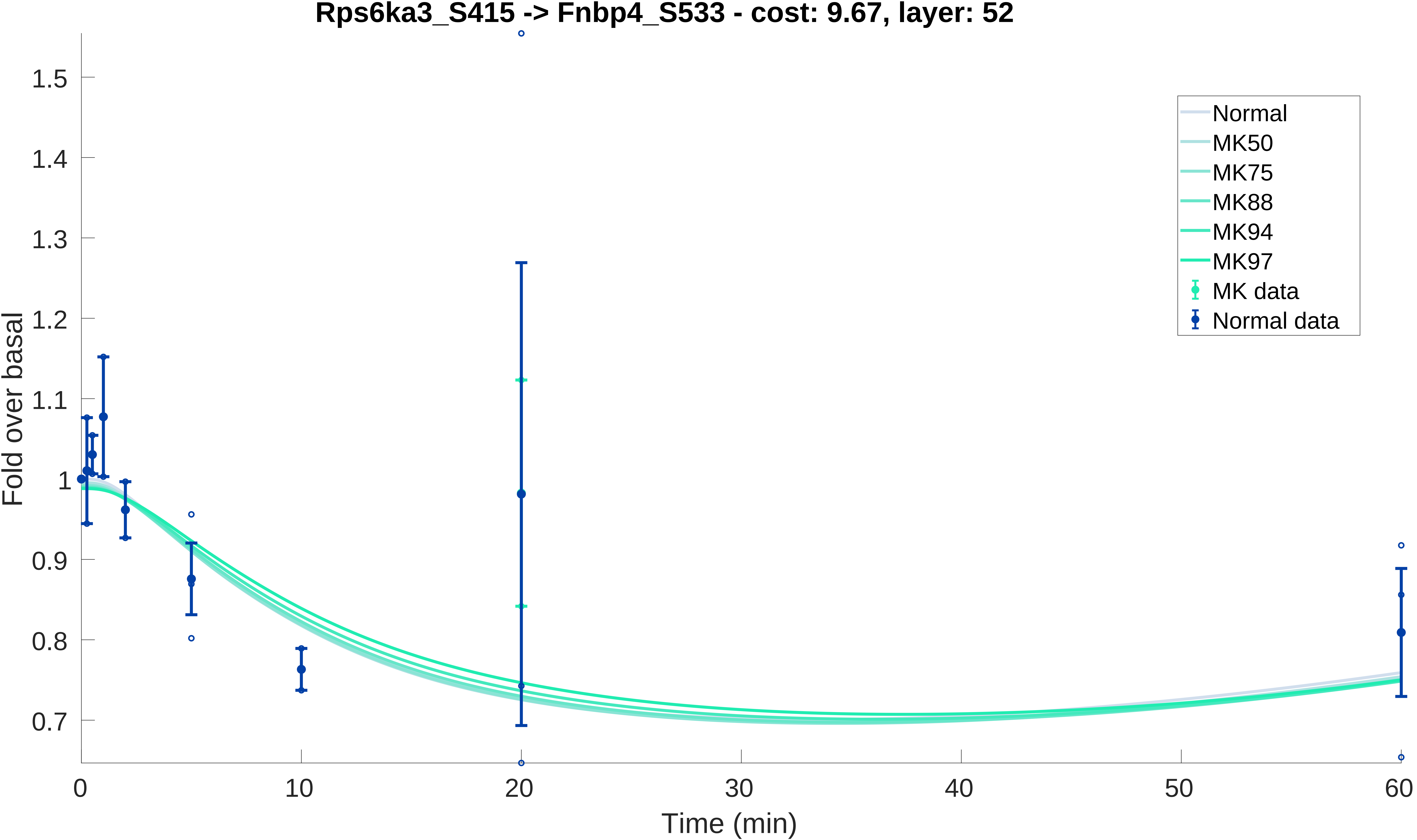
Rps6ka3_S415 -> Fnbp4_S533 - cost: 9.67, layer: 52
Fold over basal
Time (min)
Normal
MK50
MK75
MK88
MK94
MK97
MK data
Normal data
1.5
1.4
1.3
1.2
1.1
1
0.9
0.8
0.7
0
10
20
30
40
50
60

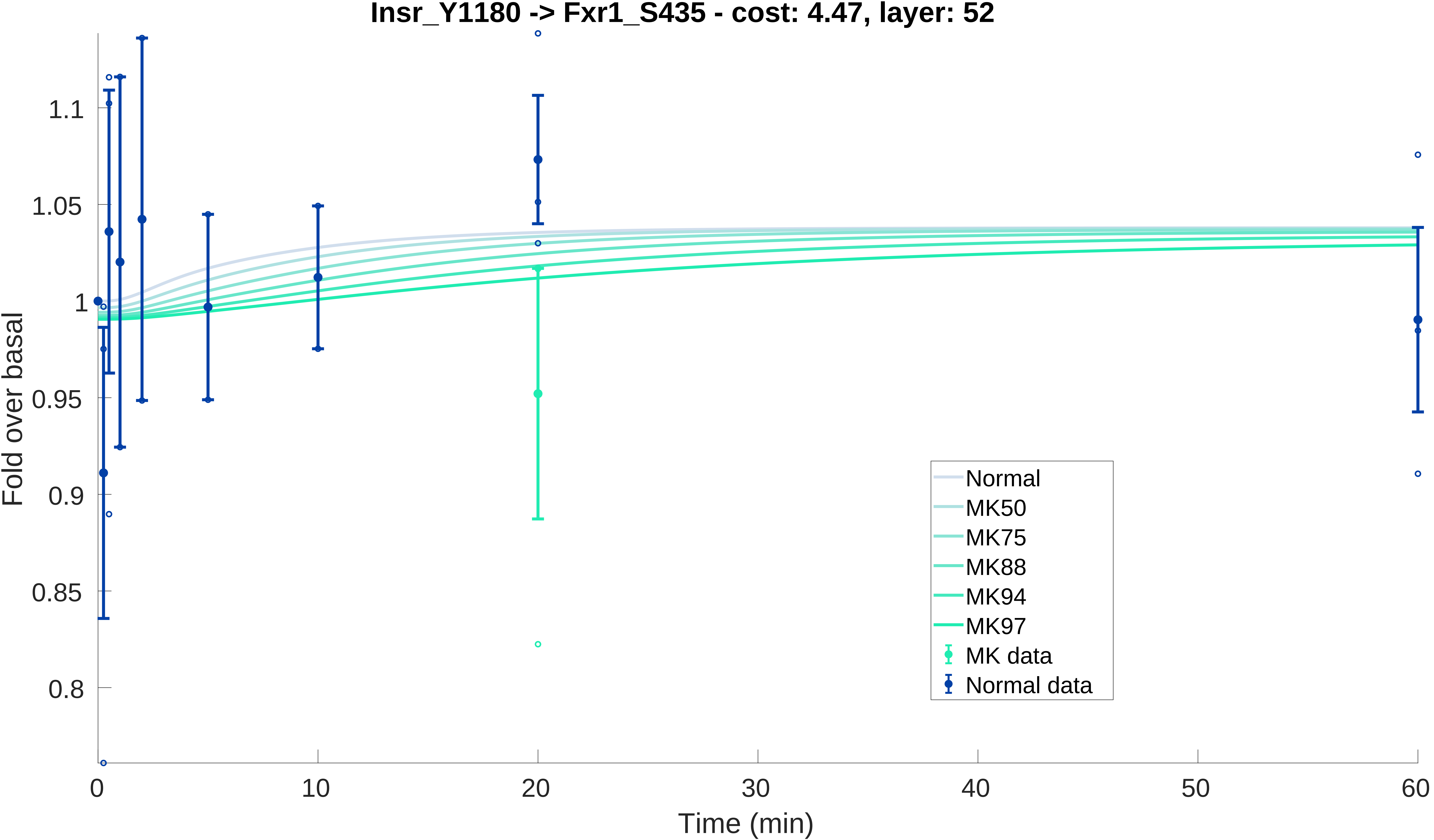

Insr_Y1180 -> Fxr1_S435 - cost: 4.47, layer: 52
Fold over basal
Time (min)
0.8
0.85
0.9
0.95
1
1.05
1.1
0
10
20
30
40
50
60
Normal
MK50
MK75
MK88
MK94
MK97
MK data
Normal data

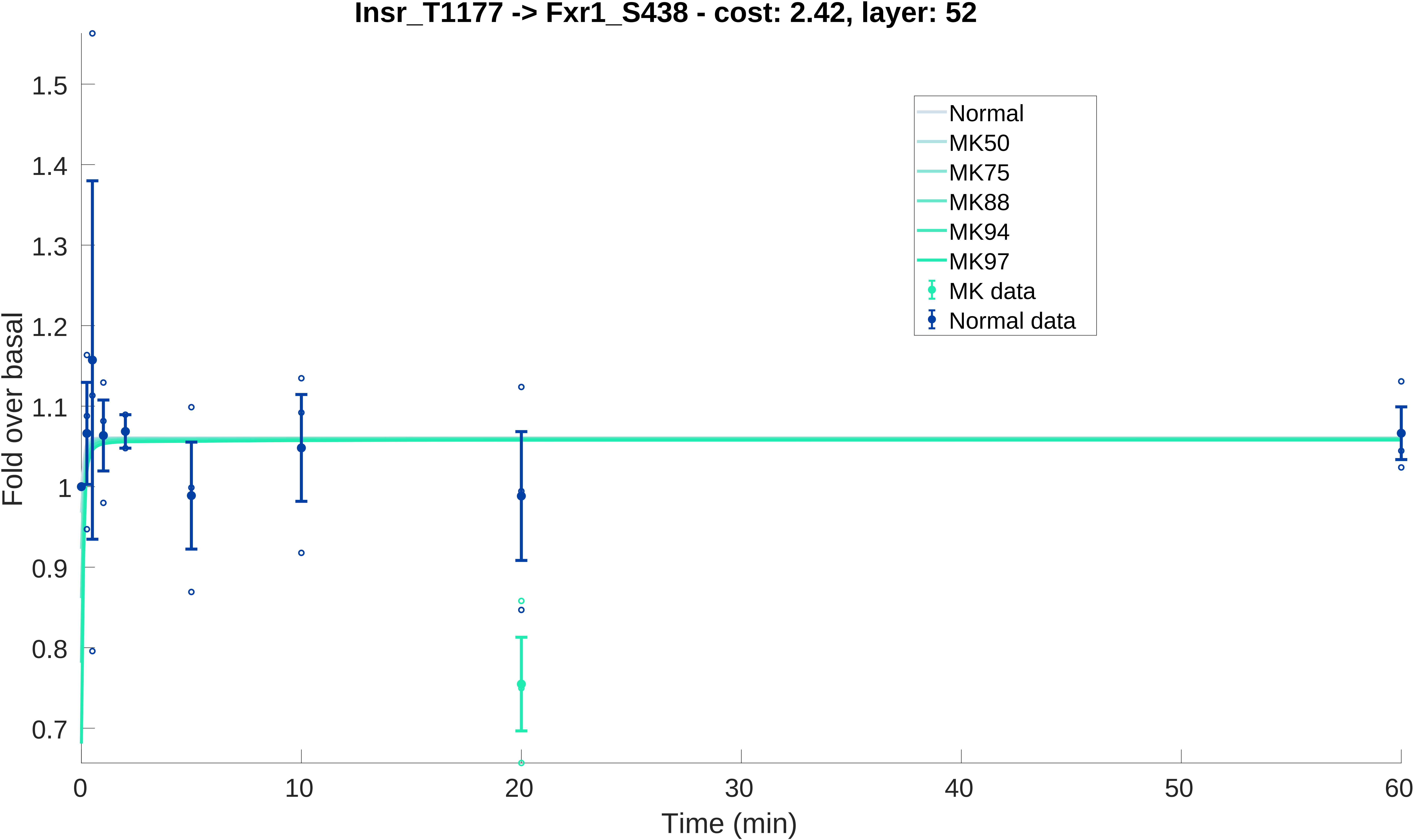
Insr_T1177 -> Fxr1_S438 - cost: 2.42, layer: 52
Fold over basal
1.5
1.4
1.3
1.2
1.1
1
0.9
0.8
0.7
0
10
20
30
40
50
60
Time (min)
Normal
MK50
MK75
MK88
MK94
MK97
MK data
Normal data

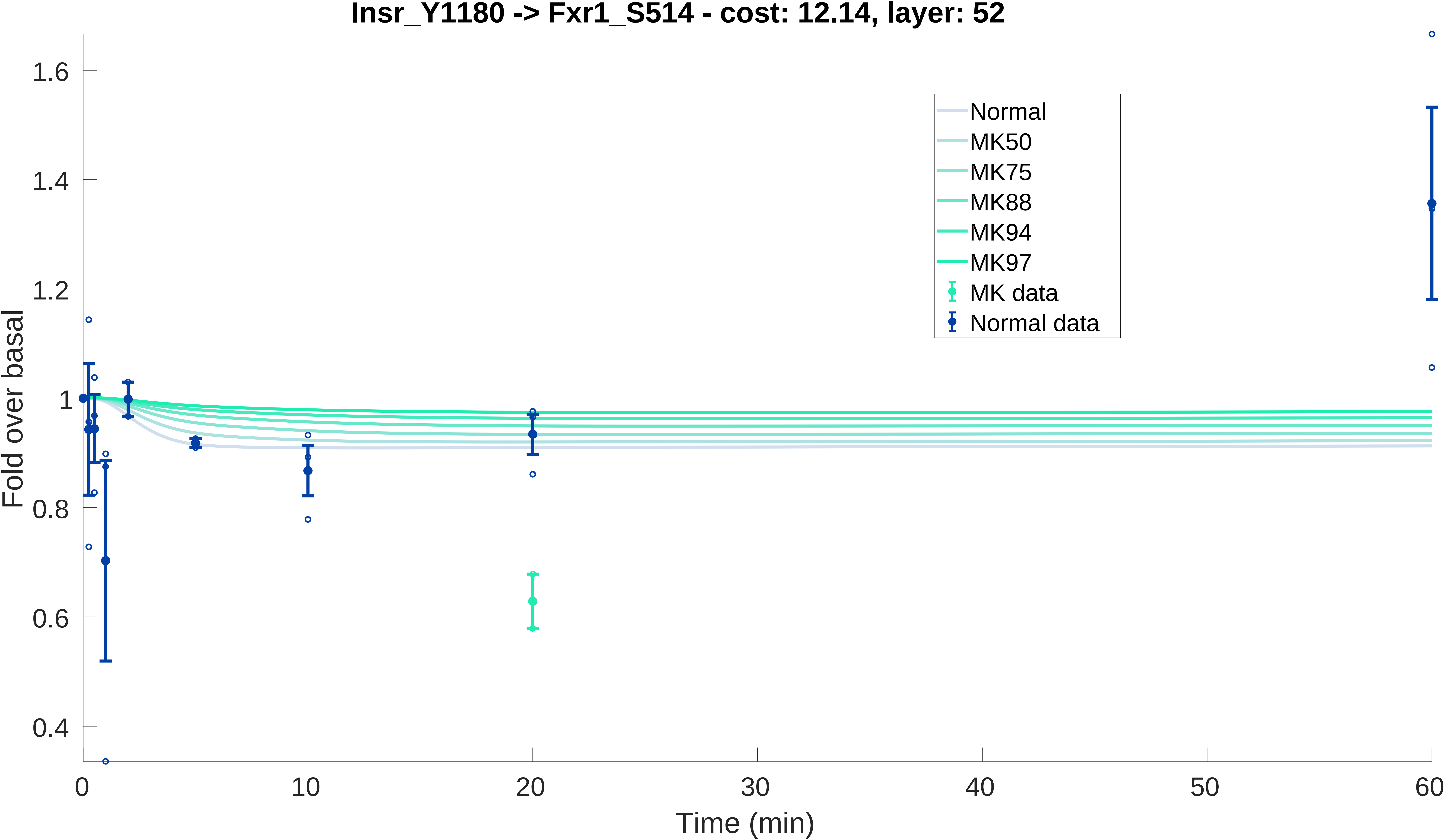
Insr_Y1180 -> Fxr1_S514 - cost: 12.14, layer: 52
Fold over basal
1.6
1.4
1.2
1
0.8
0.6
0.4
0
10
20
30
40
50
60
Time (min)
Normal
MK50
MK75
MK88
MK94
MK97
MK data
Normal data

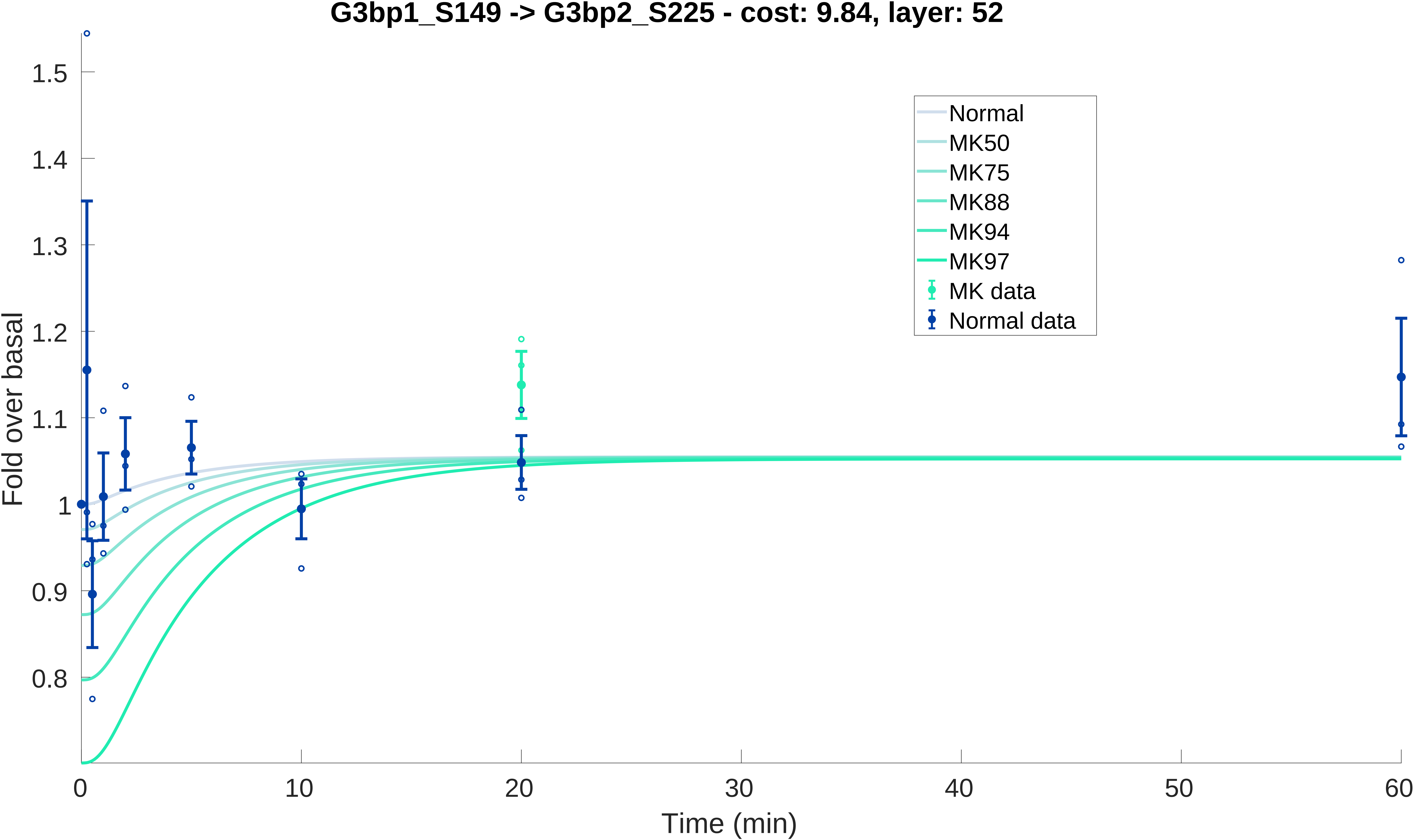
G3bp1_S149 -> G3bp2_S225 - cost: 9.84, layer: 52
Normal
MK50
MK75
MK88
MK94
MK97
MK data
Normal data
Fold over basal
Time (min)
1.5
1.4
1.3
1.2
1.1
1
0.9
0.8
0
10
20
30
40
50
60

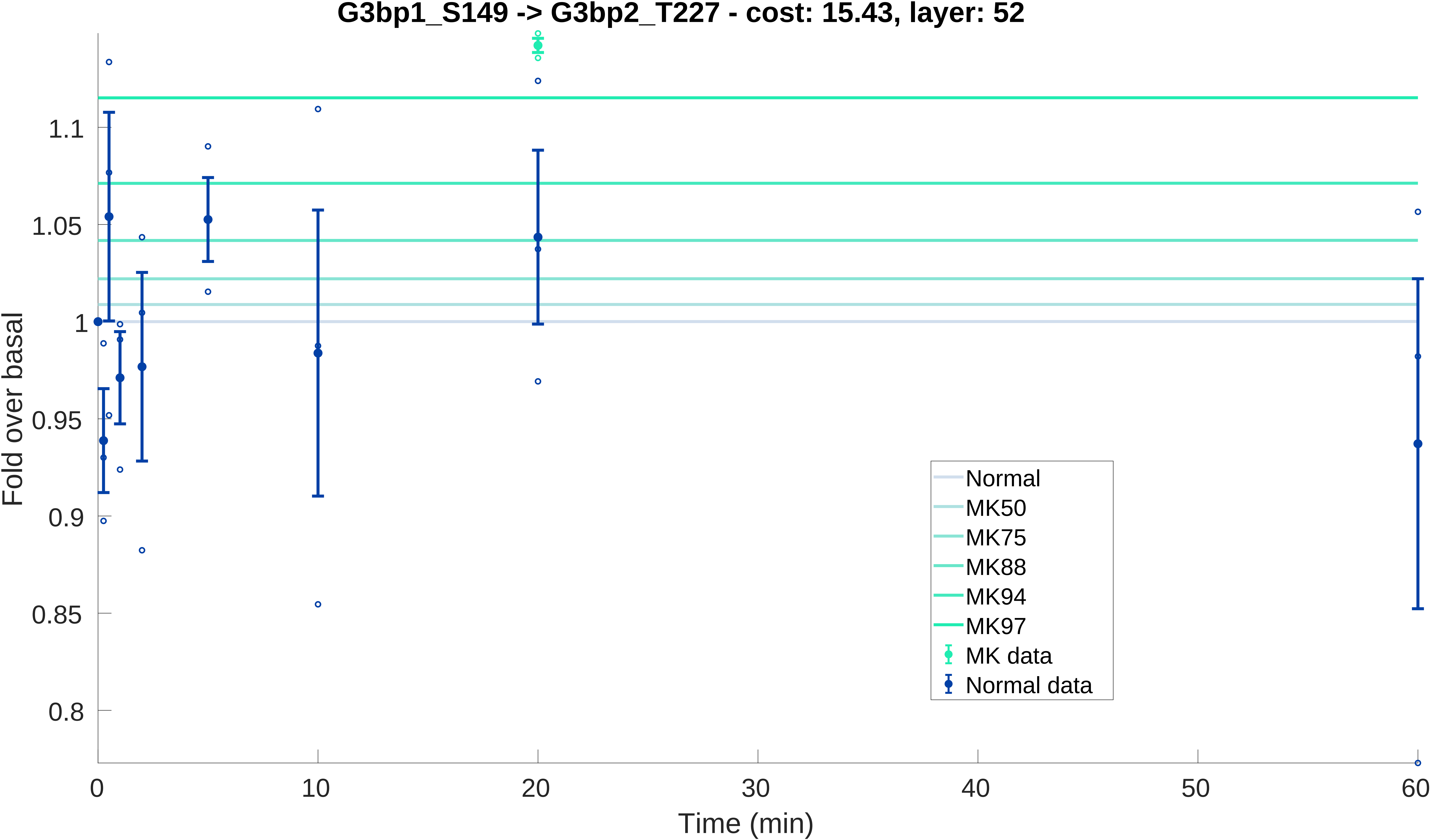

G3bp1_S149 -> G3bp2_T227 - cost: 15.43, layer: 52
Fold over basal
Time (min)
0.8
0.85
0.9
0.95
1
1.05
1.1
0
10
20
30
40
50
60
Normal
MK50
MK75
MK88
MK94
MK97
MK data
Normal data

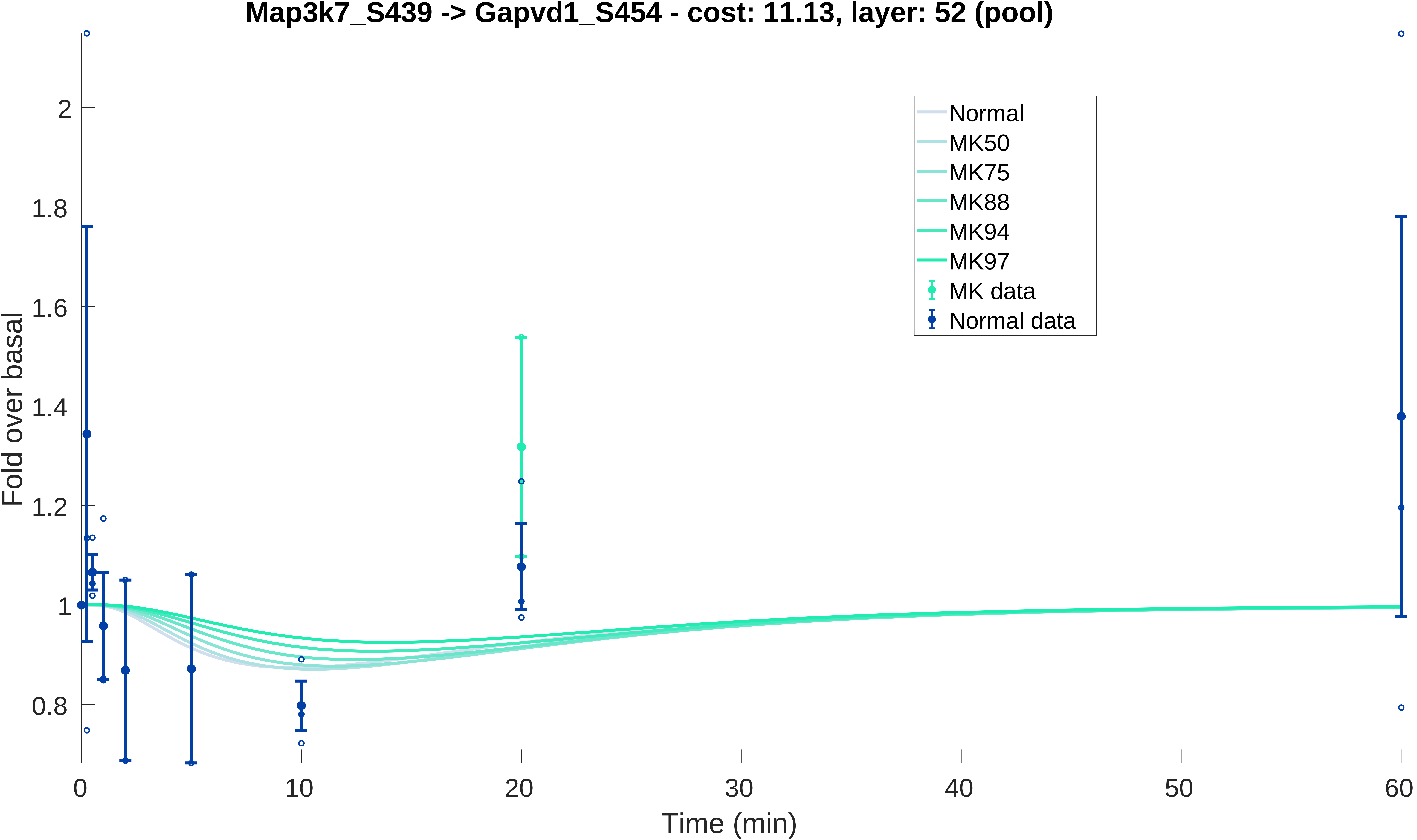

Map3k7_S439 -> Gapvd1_S454 - cost: 11.13, layer: 52 (pool)
Fold over basal
Time (min)
0.8
1
1.2
1.4
1.6
1.8
2
0
10
20
30
40
50
60
Normal
MK50
MK75
MK88
MK94
MK97
MK data
Normal data

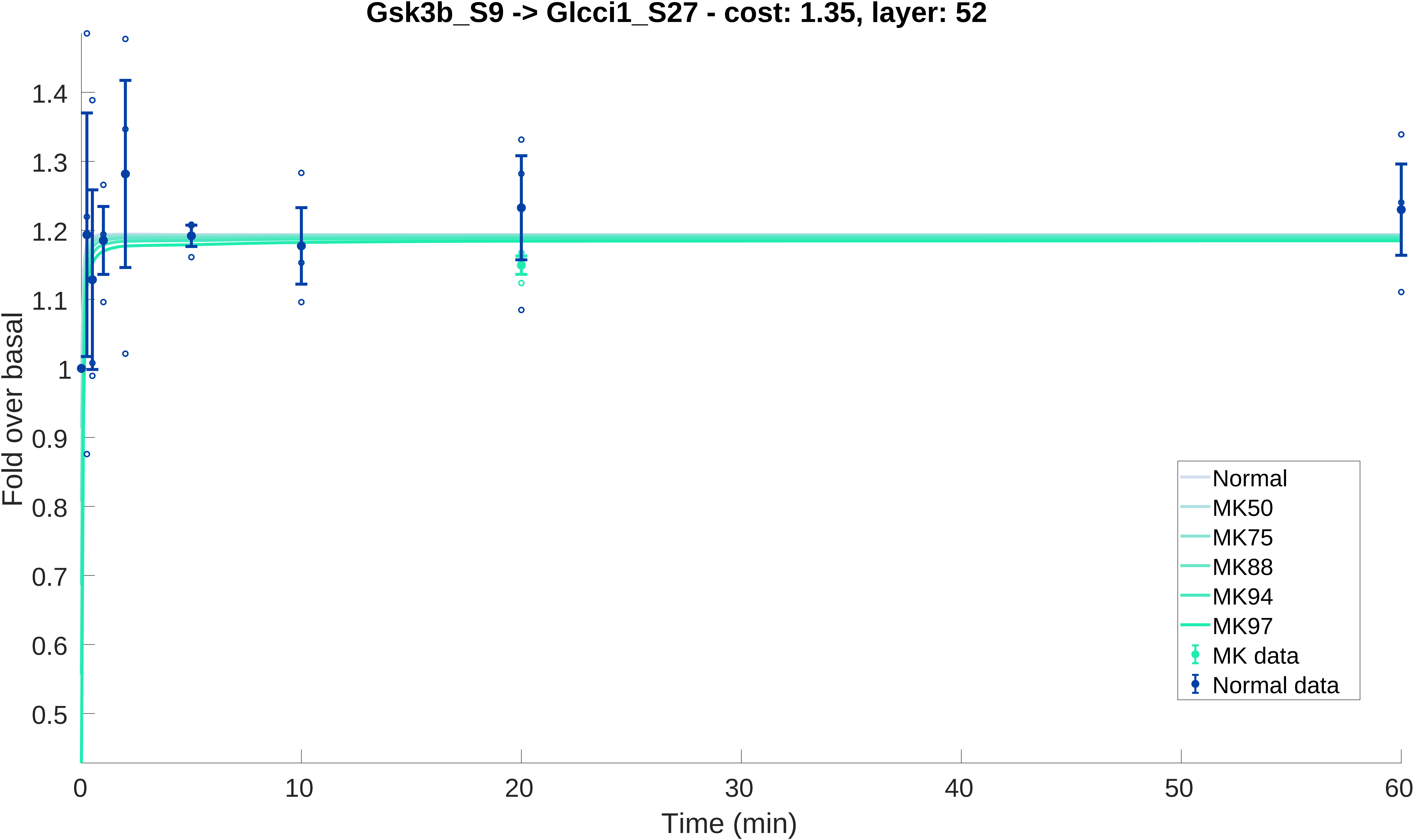

Gsk3b_S9 -> Glcci1_S27 - cost: 1.35, layer: 52
Fold over basal
Time (min)
0
10
20
30
40
50
60
0.5
0.6
0.7
0.8
0.9
1
1.1
1.2
1.3
1.4
Normal
MK50
MK75
MK88
MK94
MK97
MK data
Normal data

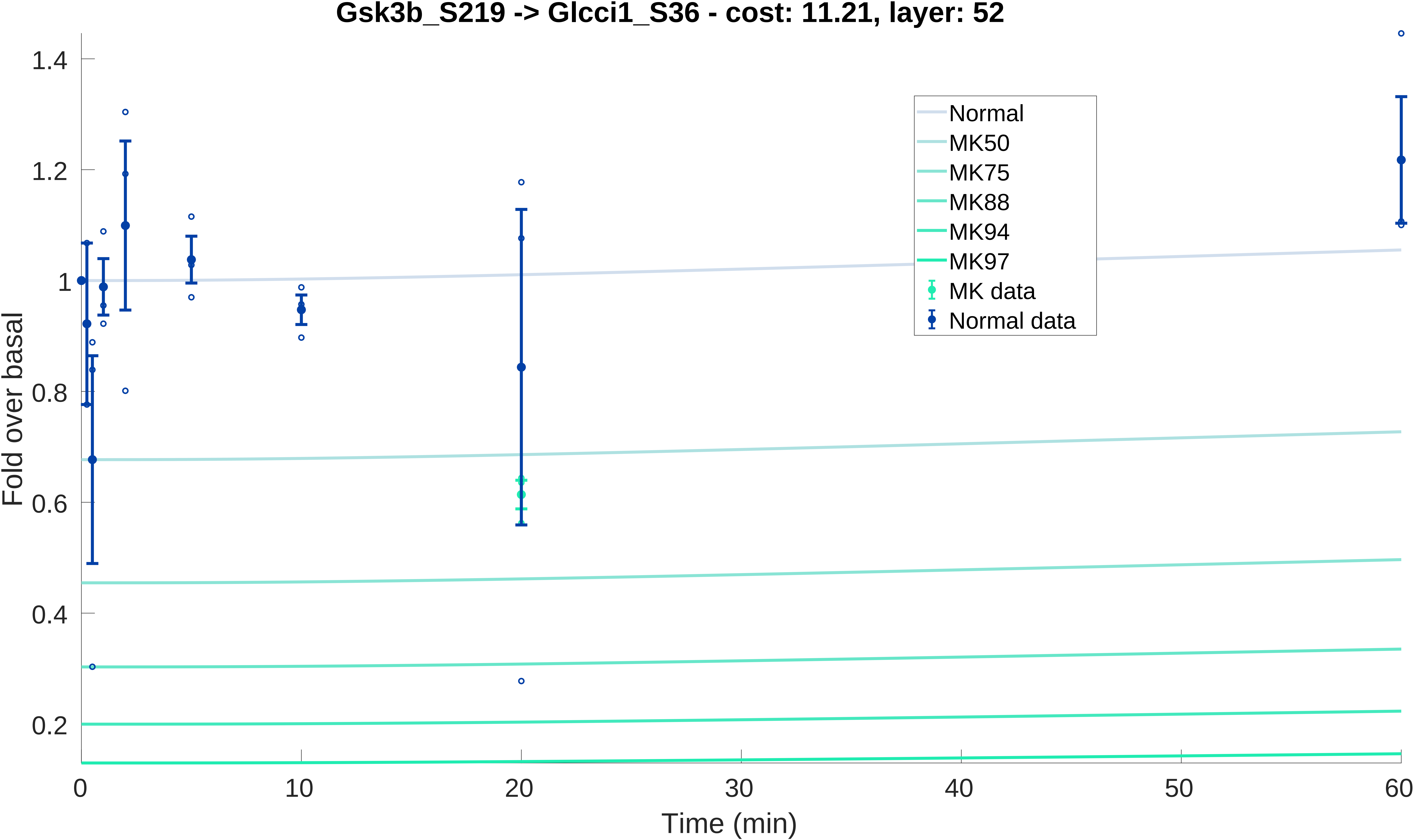

Gsk3b_S219 -> Glcci1_S36 - cost: 11.21, layer: 52
Normal
MK50
MK75
MK88
MK94
MK97
MK data
Normal data
Fold over basal
1.4
1.2
1
0.8
0.6
0.4
0.2
0
10
20
30
40
50
60
Time (min)

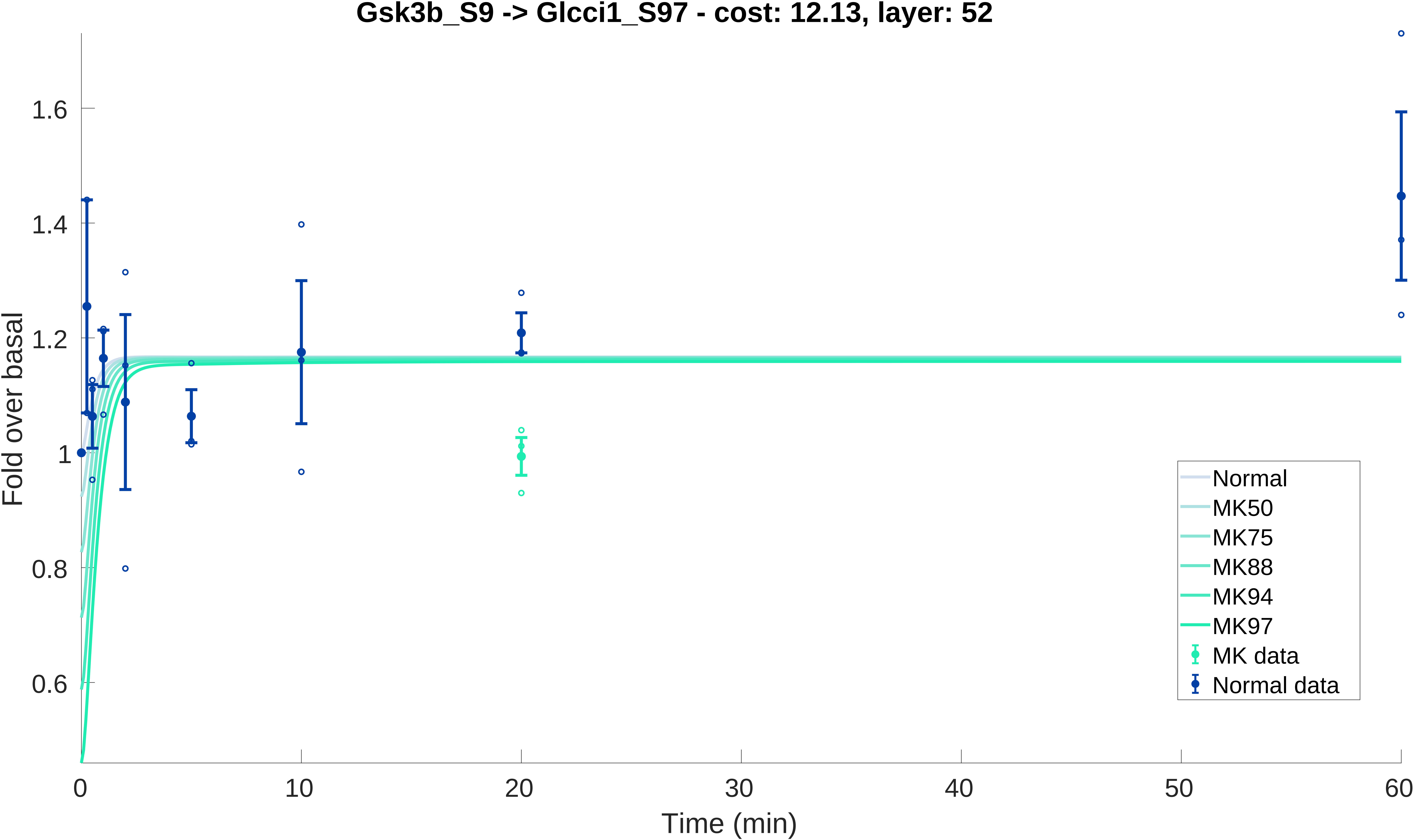
Gsk3b_S9 -> Glcci1_S97 - cost: 12.13, layer: 52
Fold over basal
Time (min)
1.6
1.4
1.2
1
0.8
0.6
0
10
20
30
40
50
60
Normal
MK50
MK75
MK88
MK94
MK97
MK data
Normal data

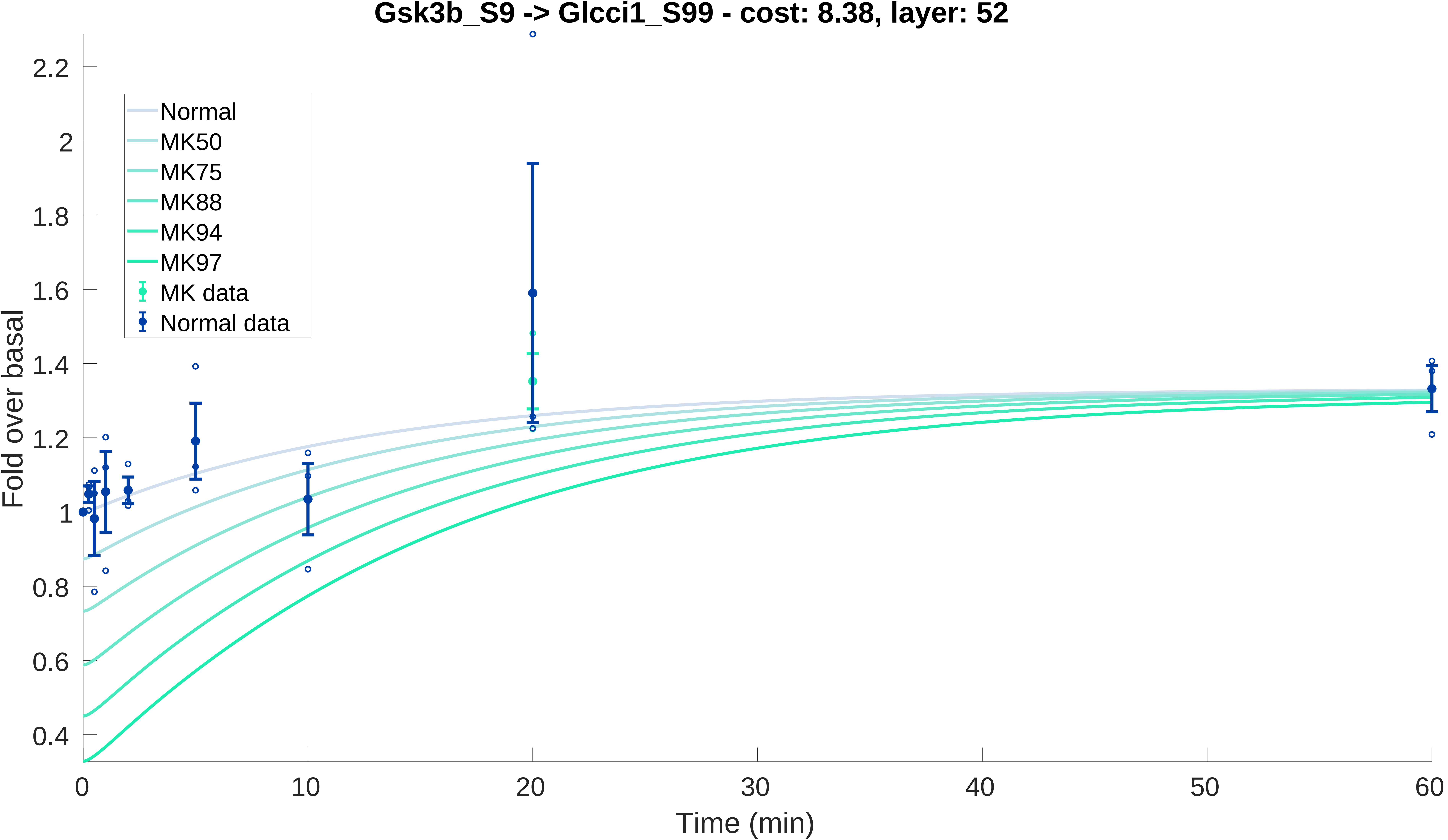
Gsk3b_S9 -> Glcci1_S99 - cost: 8.38, layer: 52
Normal
MK50
MK75
MK88
MK94
MK97
MK data
Normal data
Fold over basal
Time (min)
2.2
2
1.8
1.6
1.4
1.2
1
0.8
0.6
0.4
0
10
20
30
40
50
60

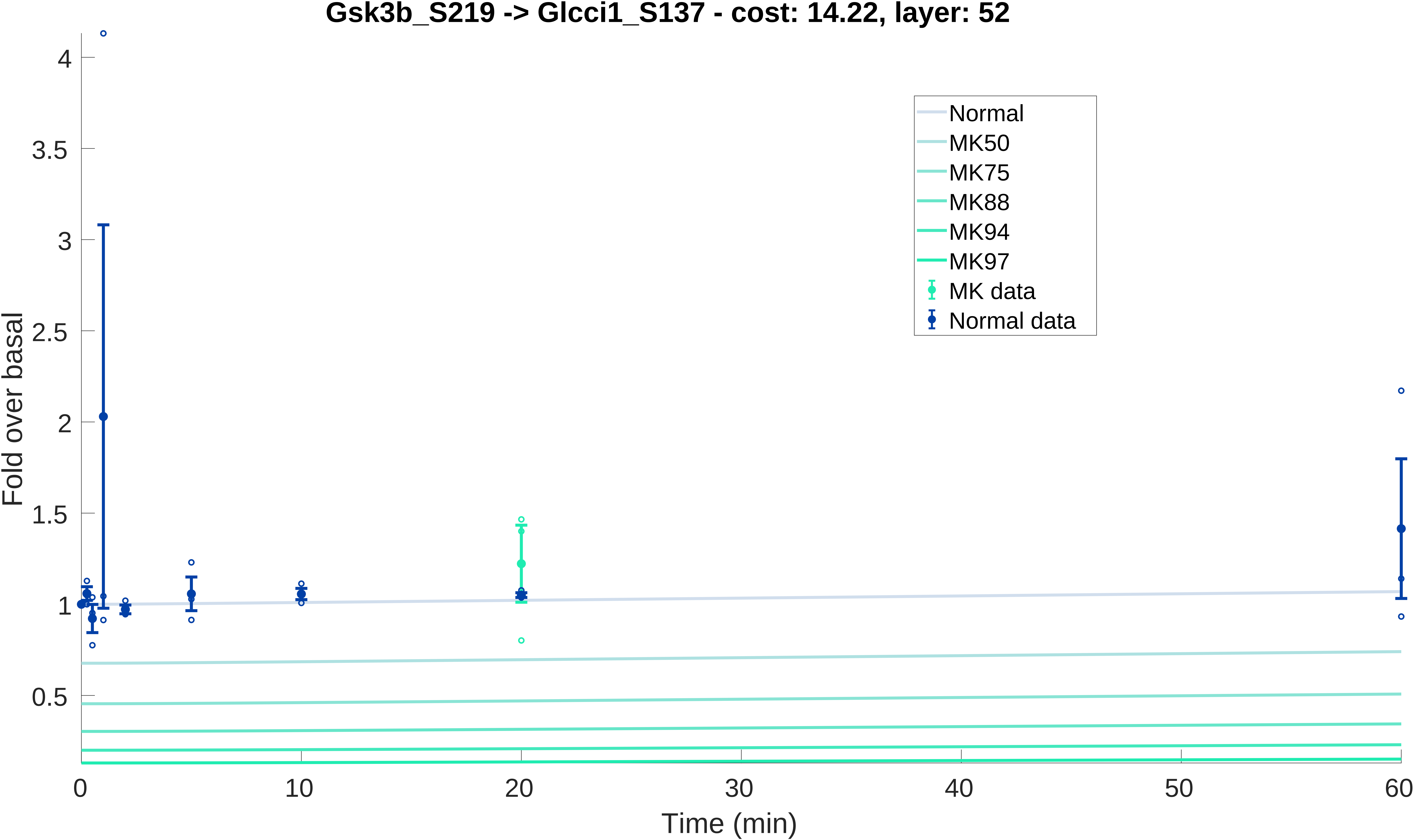

Gsk3b_S219 -> Glcci1_S137 - cost: 14.22, layer: 52
Fold over basal
Time (min)
0
10
20
30
40
50
60
0.5
1
1.5
2
2.5
3
3.5
4
Normal
MK50
MK75
MK88
MK94
MK97
MK data
Normal data

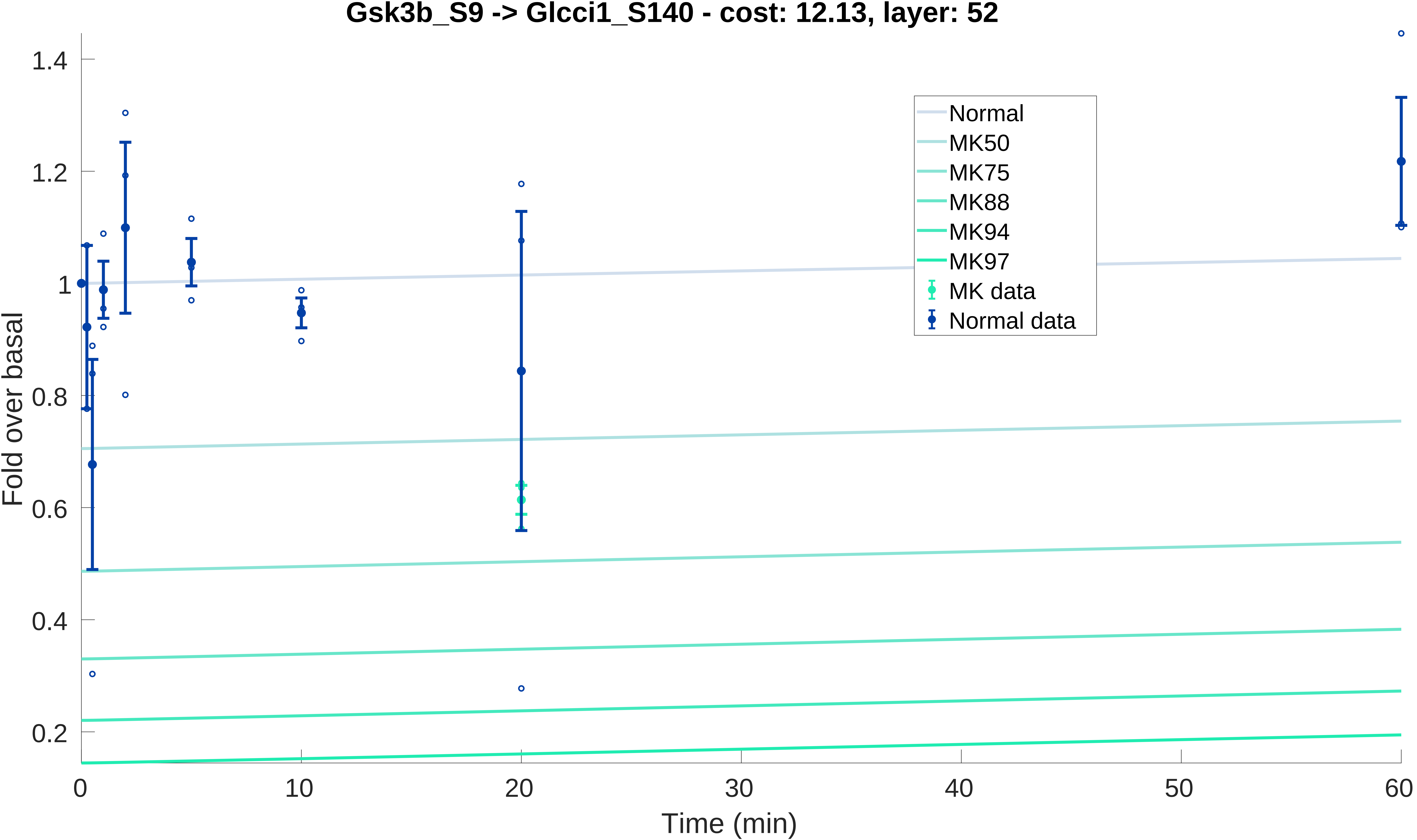

Gsk3b_S9 -> Glcci1_S140 - cost: 12.13, layer: 52
Fold over basal
Time (min)
1.4
1.2
1
0.8
0.6
0.4
0.2
0
10
20
30
40
50
60
Normal
MK50
MK75
MK88
MK94
MK97
MK data
Normal data

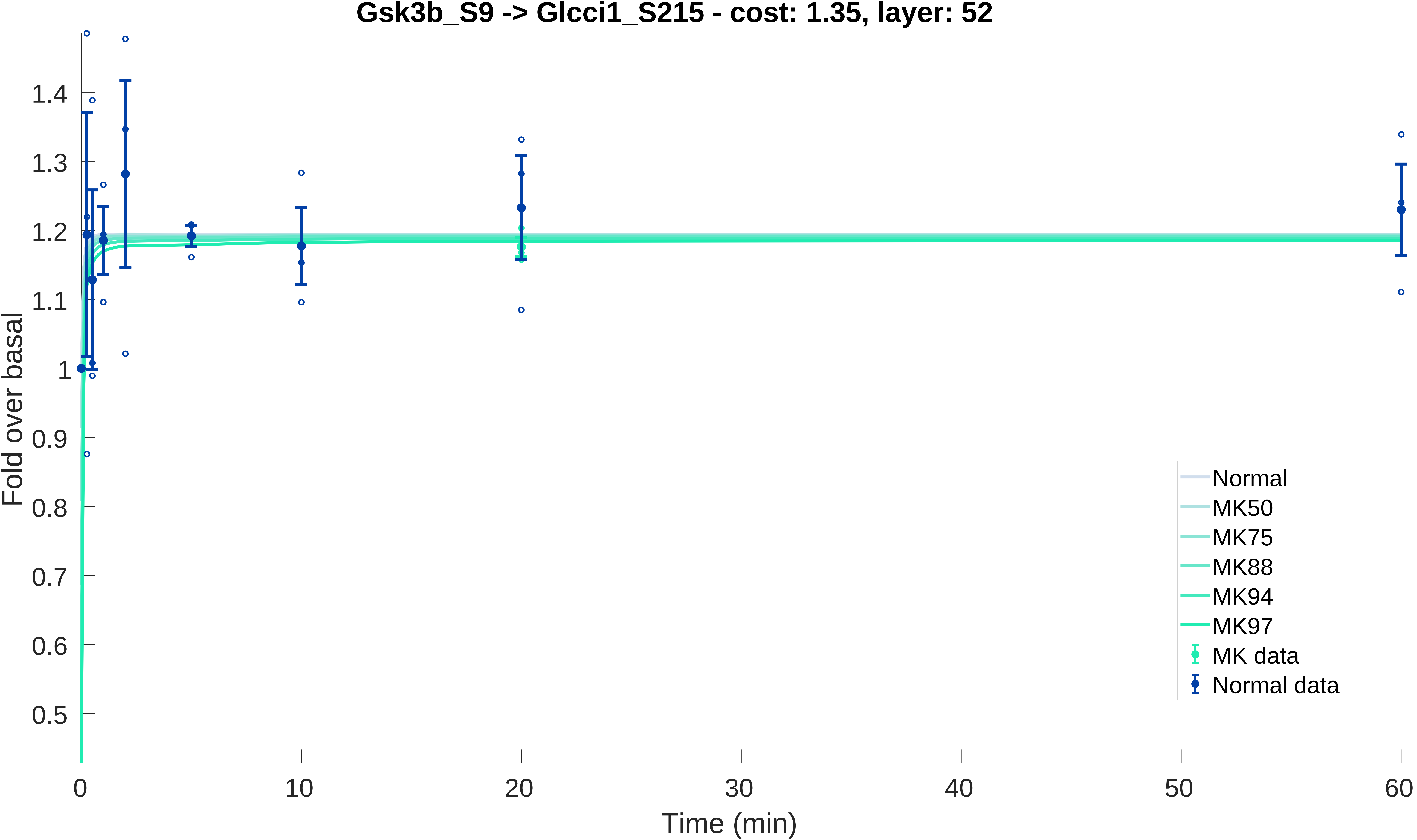

Gsk3b_S9 -> Glcci1_S215 - cost: 1.35, layer: 52
Fold over basal
Time (min)
1.4
1.3
1.2
1.1
1
0.9
0.8
0.7
0.6
0.5
0
10
20
30
40
50
60
Normal
MK50
MK75
MK88
MK94
MK97
MK data
Normal data

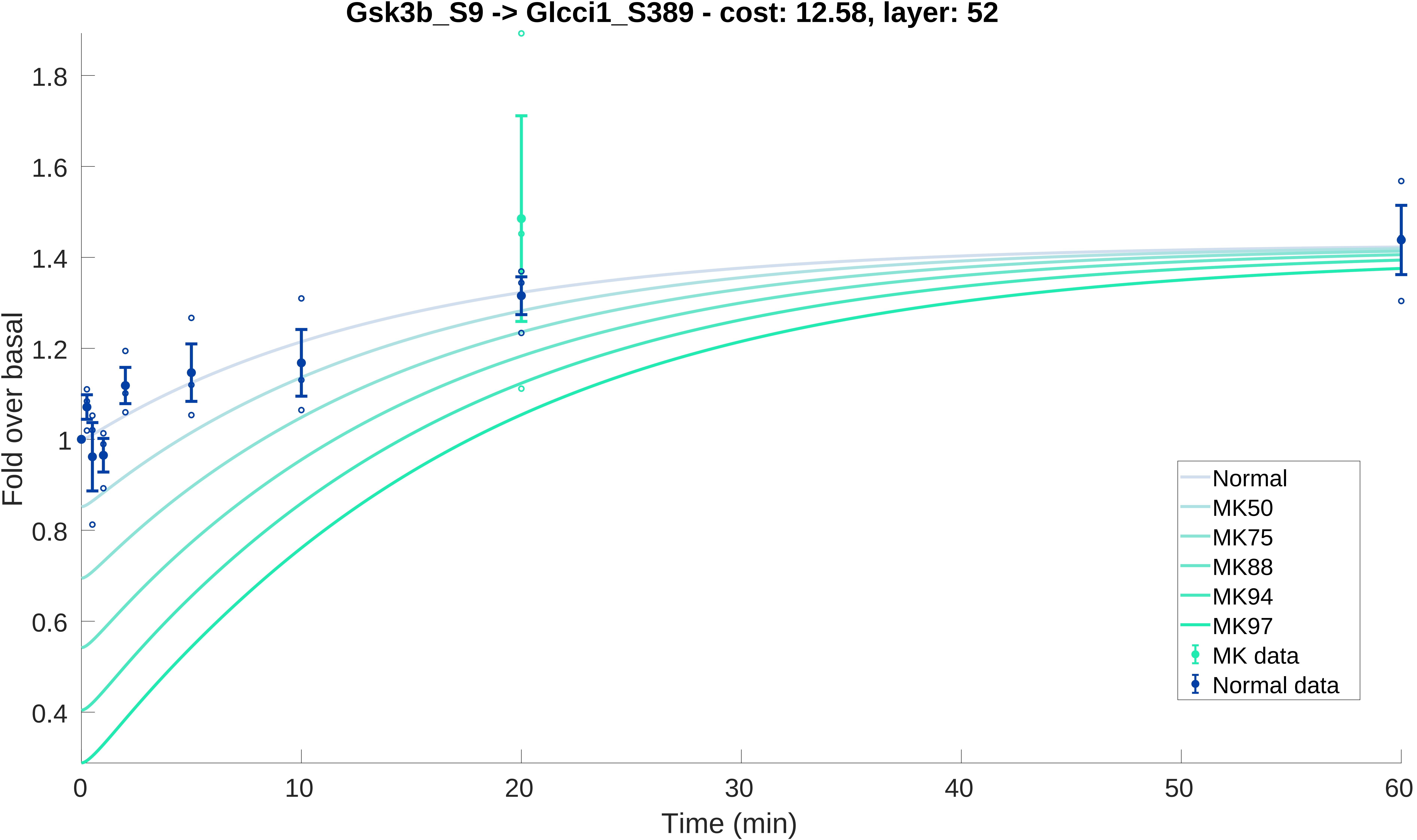

Gsk3b_S9 -> Glcci1_S389 - cost: 12.58, layer: 52
Fold over basal
Time (min)
1.8
1.6
1.4
1.2
1
0.8
0.6
0.4
0
10
20
30
40
50
60
Normal
MK50
MK75
MK88
MK94
MK97
MK data
Normal data

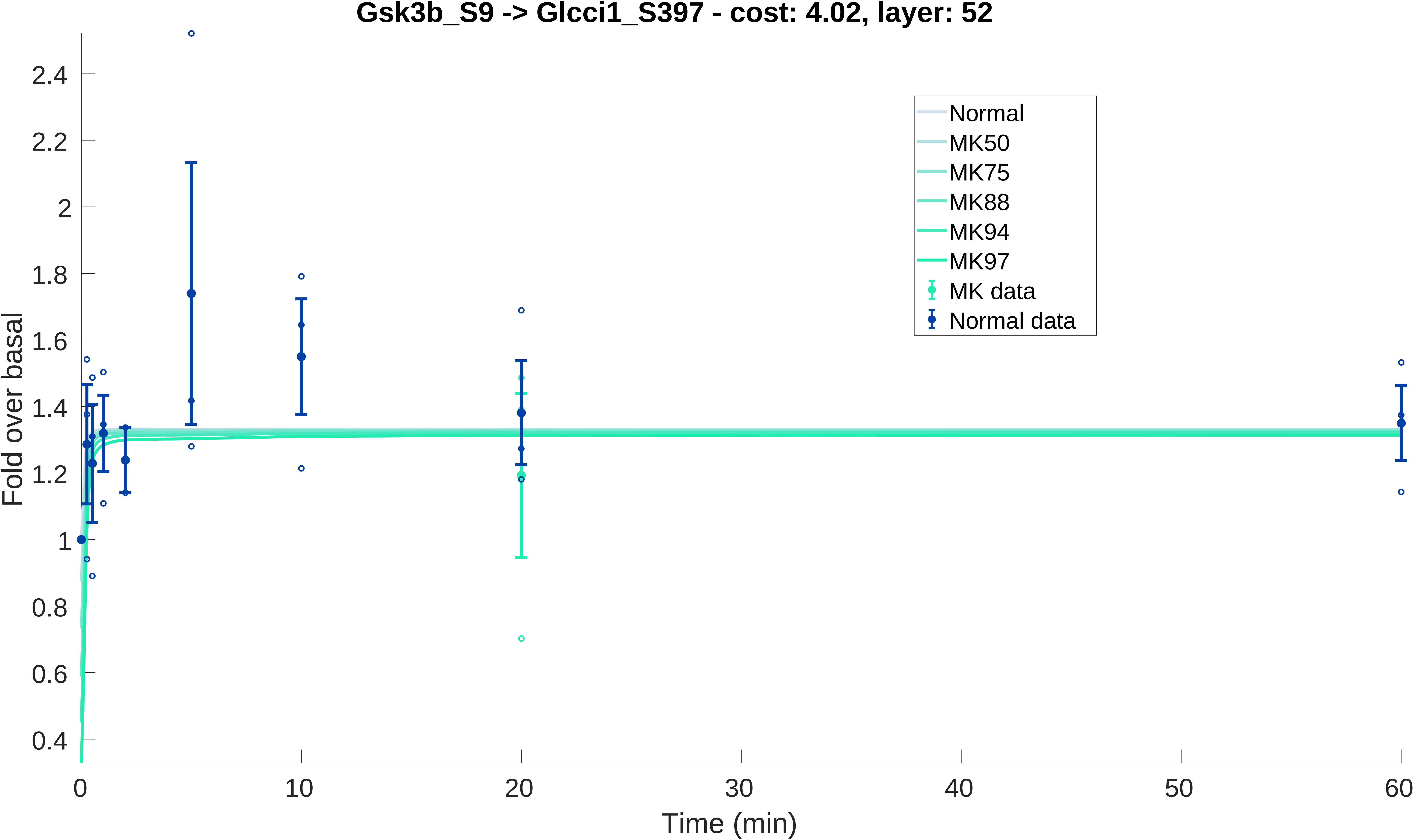
Gsk3b_S9 -> Glcci1_S397 - cost: 4.02, layer: 52
Fold over basal
Time (min)
Normal
MK50
MK75
MK88
MK94
MK97
MK data
Normal data
0.4
0.6
0.8
1
1.2
1.4
1.6
1.8
2
2.2
2.4
0
10
20
30
40
50
60

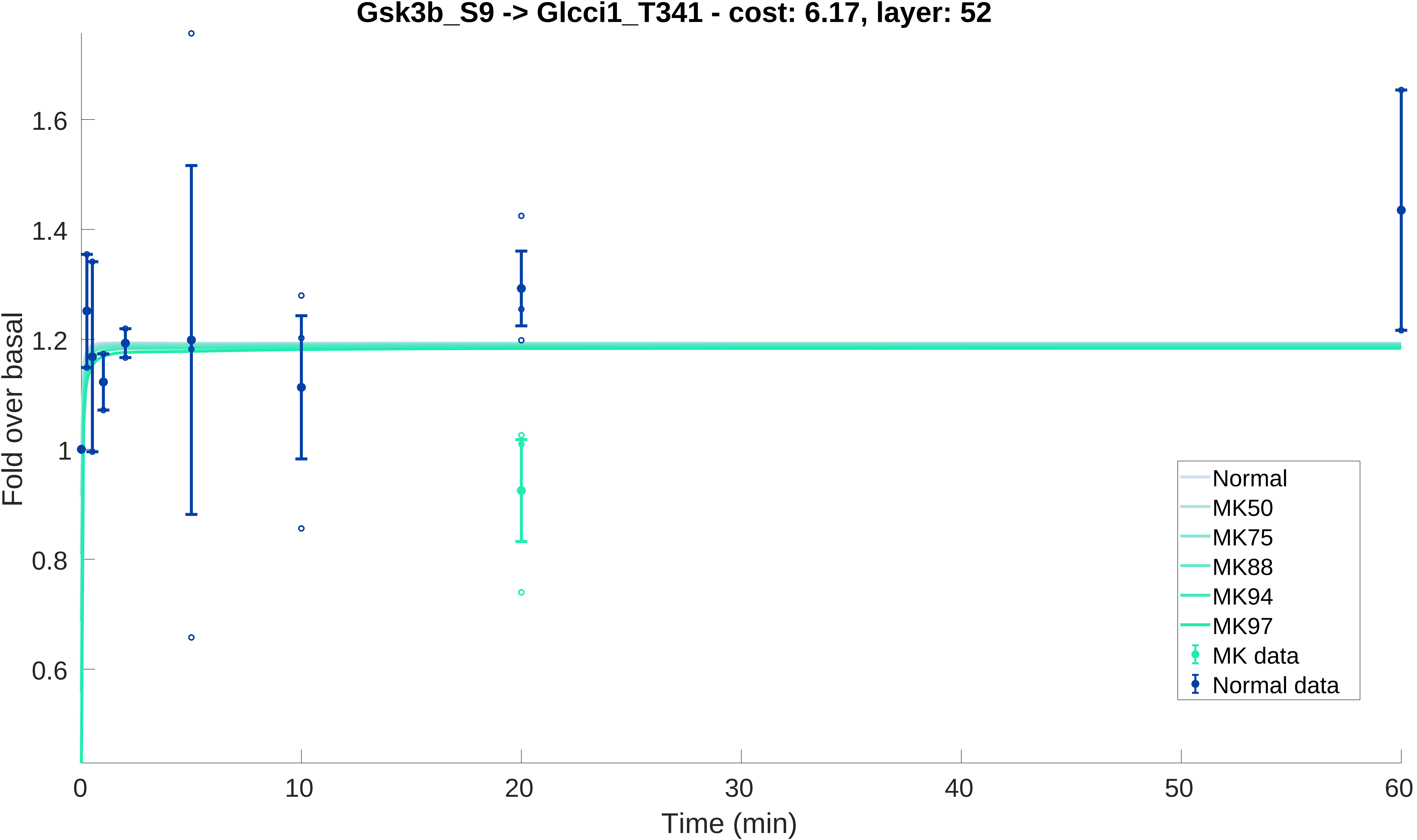

Gsk3b_S9 -> Glcci1_T341 - cost: 6.17, layer: 52
Fold over basal
1.6
1.4
1.2
1
0.8
0.6
0
10
20
30
40
50
60
Time (min)
Normal
MK50
MK75
MK88
MK94
MK97
MK data
Normal data

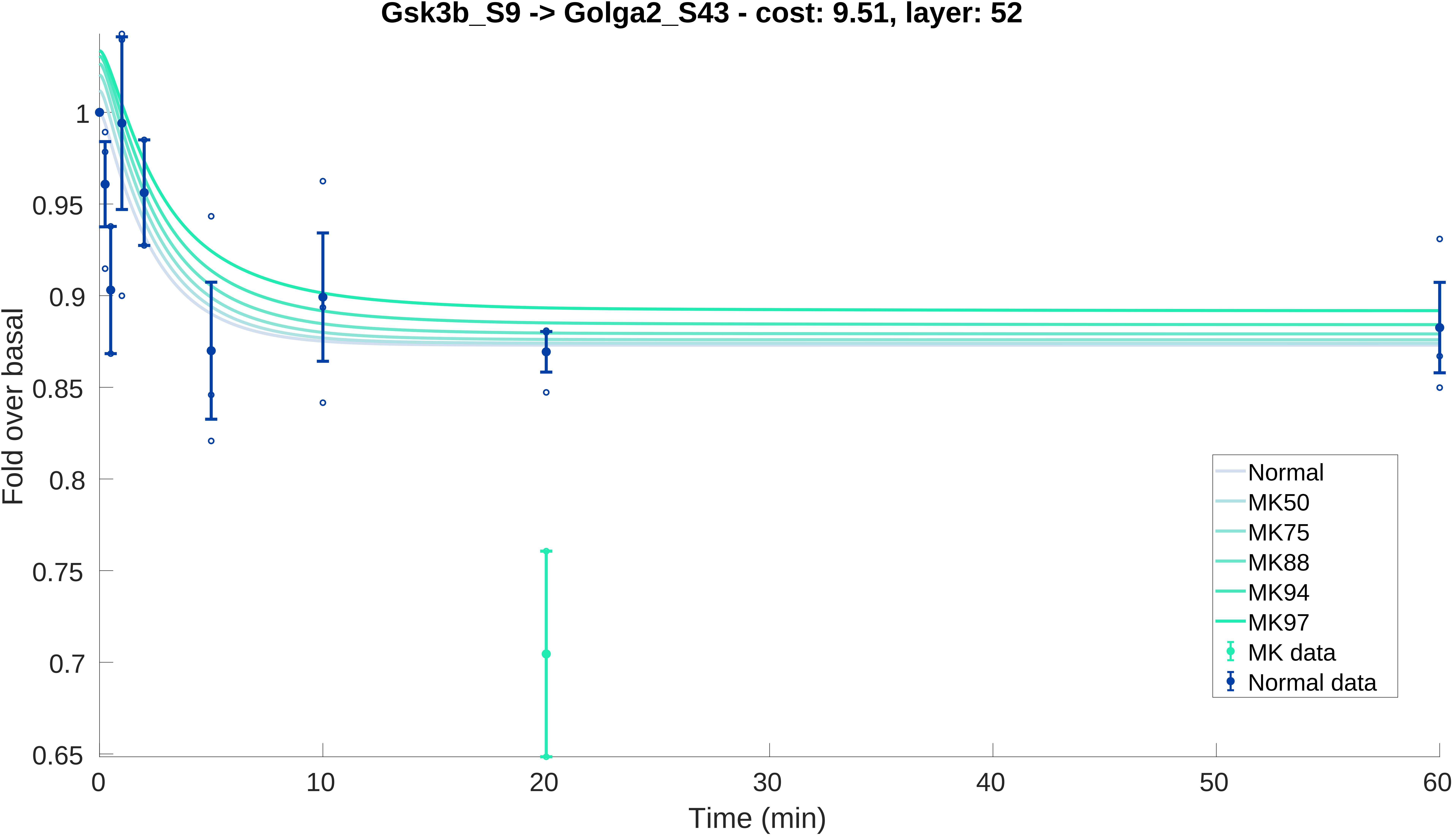

Gsk3b_S9 -> Golga2_S43 - cost: 9.51, layer: 52
Fold over basal
Time (min)
1
0.95
0.9
0.85
0.8
0.75
0.7
0.65
0
10
20
30
40
50
60
Normal
MK50
MK75
MK88
MK94
MK97
MK data
Normal data

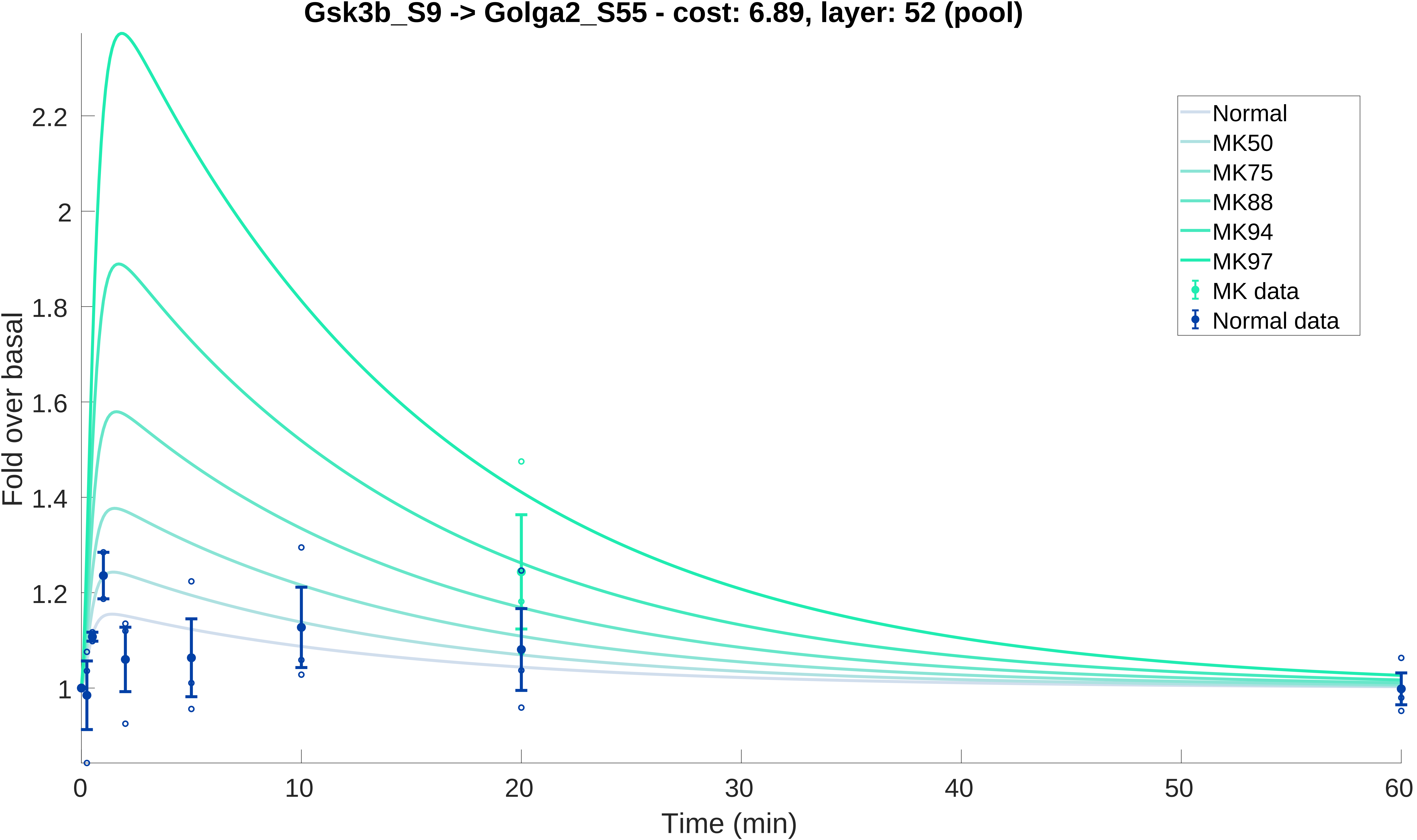
Gsk3b_S9 -> Golga2_S55 - cost: 6.89, layer: 52 (pool)
Fold over basal
Time (min)
2.2
2
1.8
1.6
1.4
1.2
1
0
10
20
30
40
50
60
Normal
MK50
MK75
MK88
MK94
MK97
MK data
Normal data

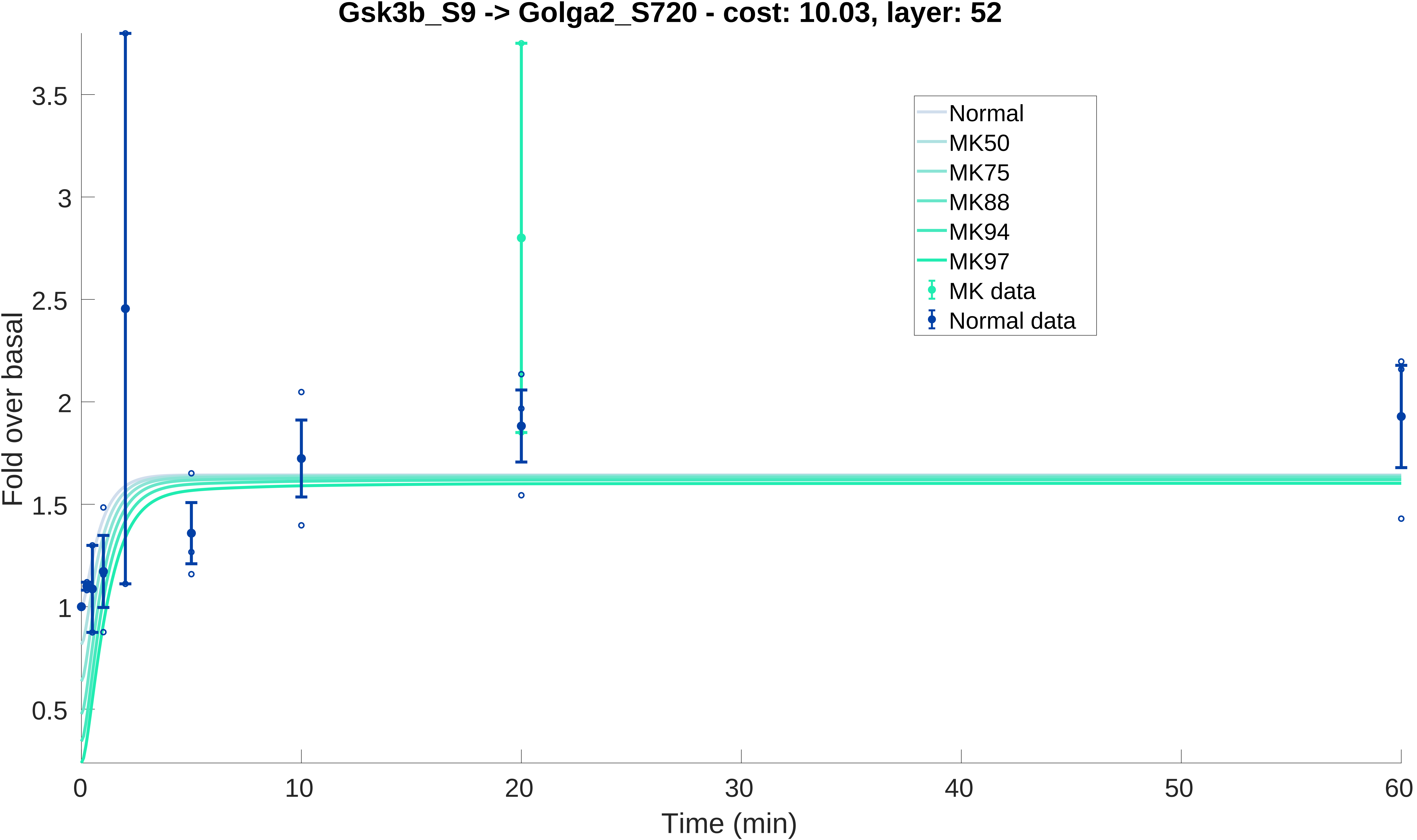

Gsk3b_S9 -> Golga2_S720 - cost: 10.03, layer: 52
Fold over basal
Time (min)
0.5
1
1.5
2
2.5
3
3.5
0
10
20
30
40
50
60
Normal
MK50
MK75
MK88
MK94
MK97
MK data
Normal data

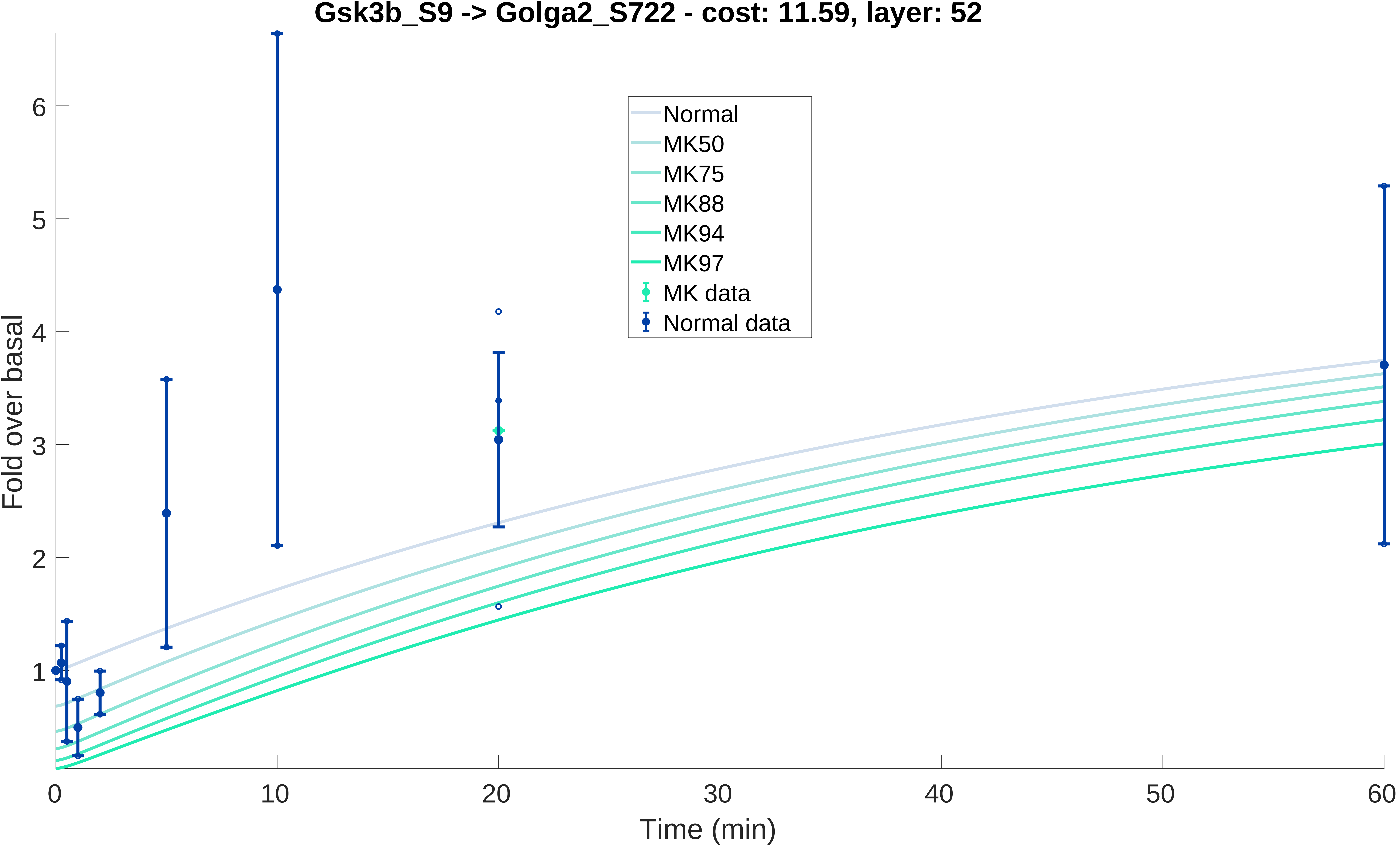

Gsk3b_S9 -> Golga2_S722 - cost: 11.59, layer: 52
Fold over basal
Time (min)
0
10
20
30
40
50
60
1
2
3
4
5
6
Normal
MK50
MK75
MK88
MK94
MK97
MK data
Normal data

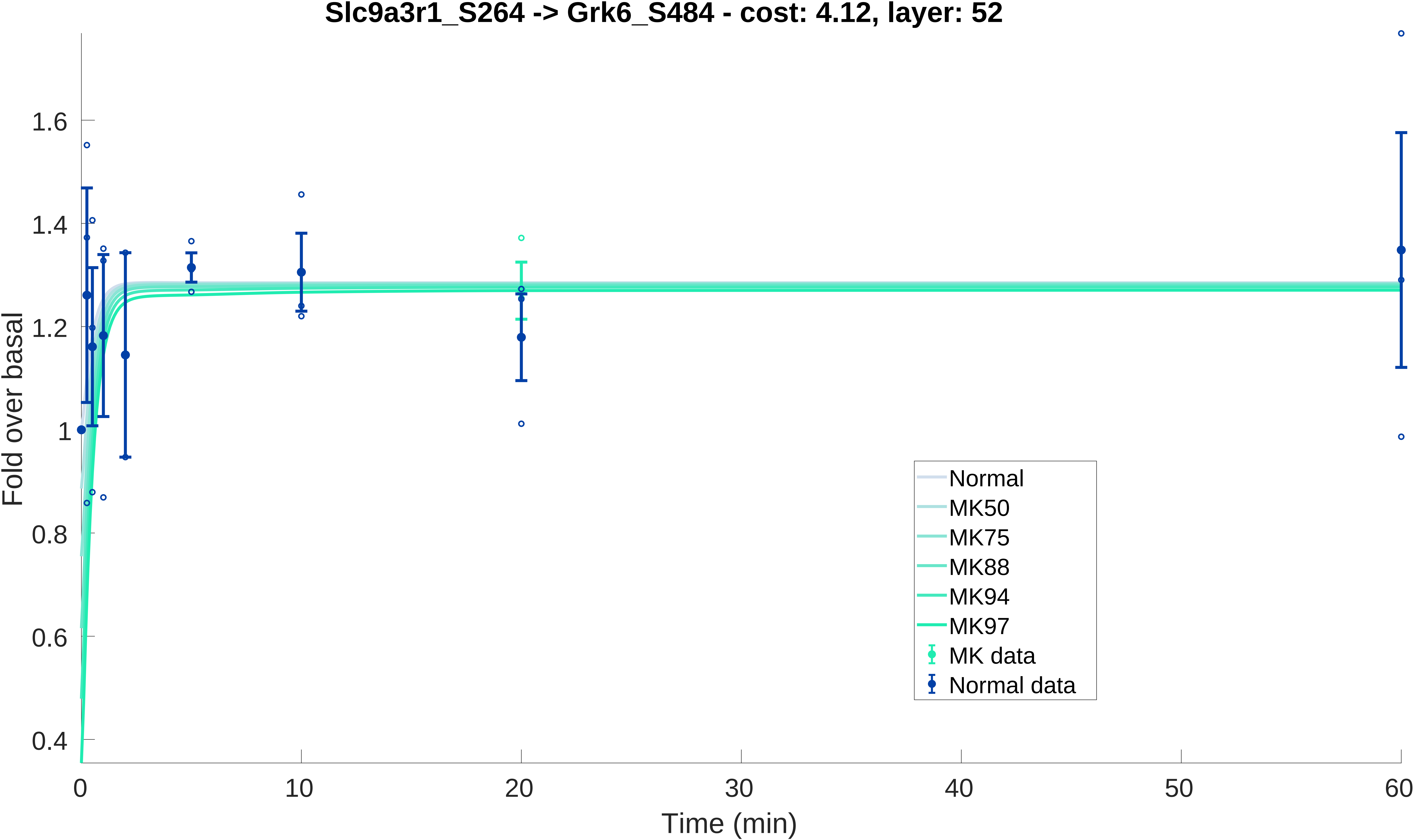
Slc9a3r1_S264 -> Grk6_S484 - cost: 4.12, layer: 52
Fold over basal
1.6
1.4
1.2
1
0.8
0.6
0.4
0
10
20
30
40
50
60
Time (min)
Normal
MK50
MK75
MK88
MK94
MK97
MK data
Normal data

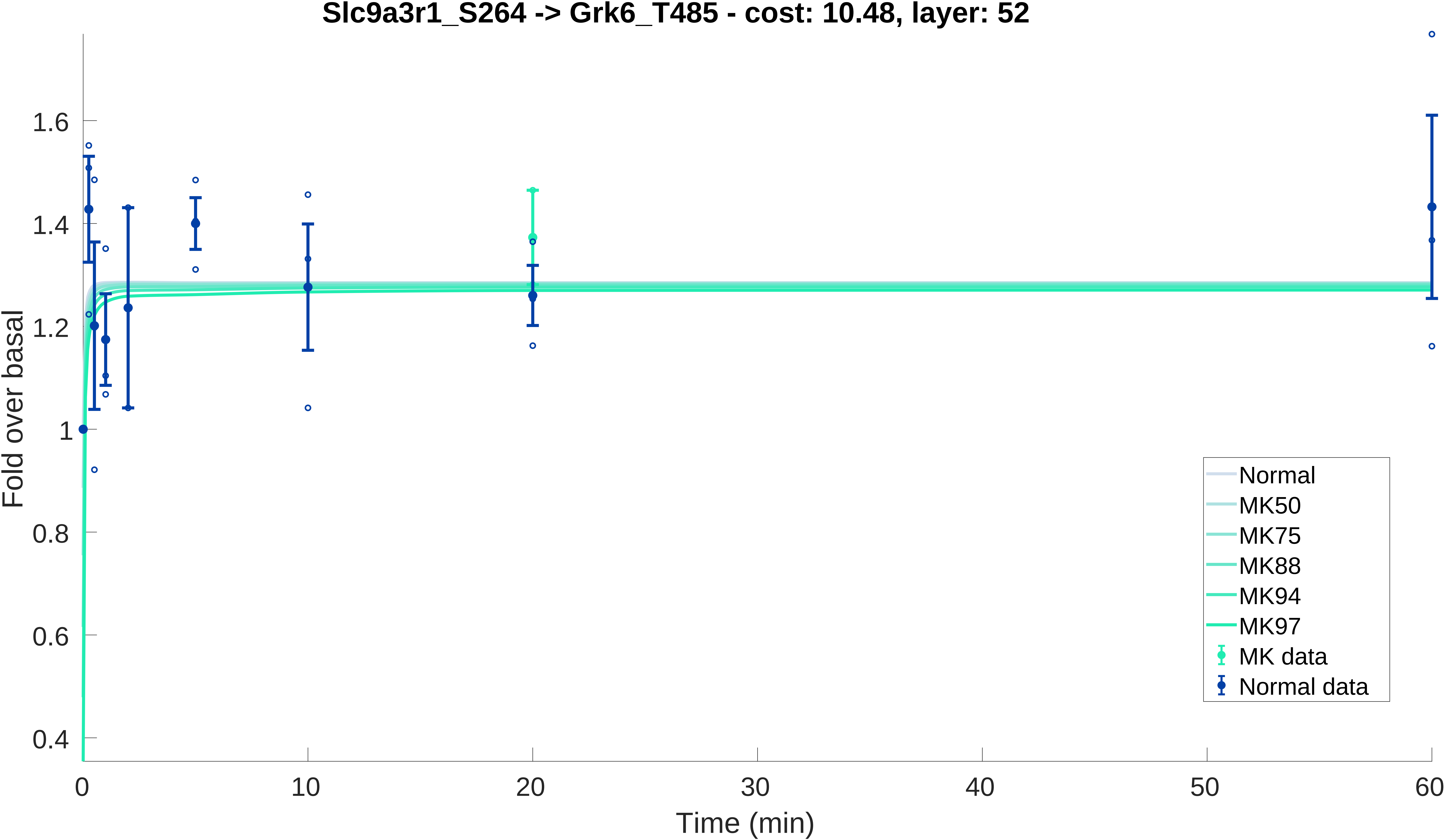

Slc9a3r1_S264 -> Grk6_T485 - cost: 10.48, layer: 52
Fold over basal
Time (min)
Normal
MK50
MK75
MK88
MK94
MK97
MK data
Normal data
1.6
1.4
1.2
1
0.8
0.6
0.4
0
10
20
30
40
50
60

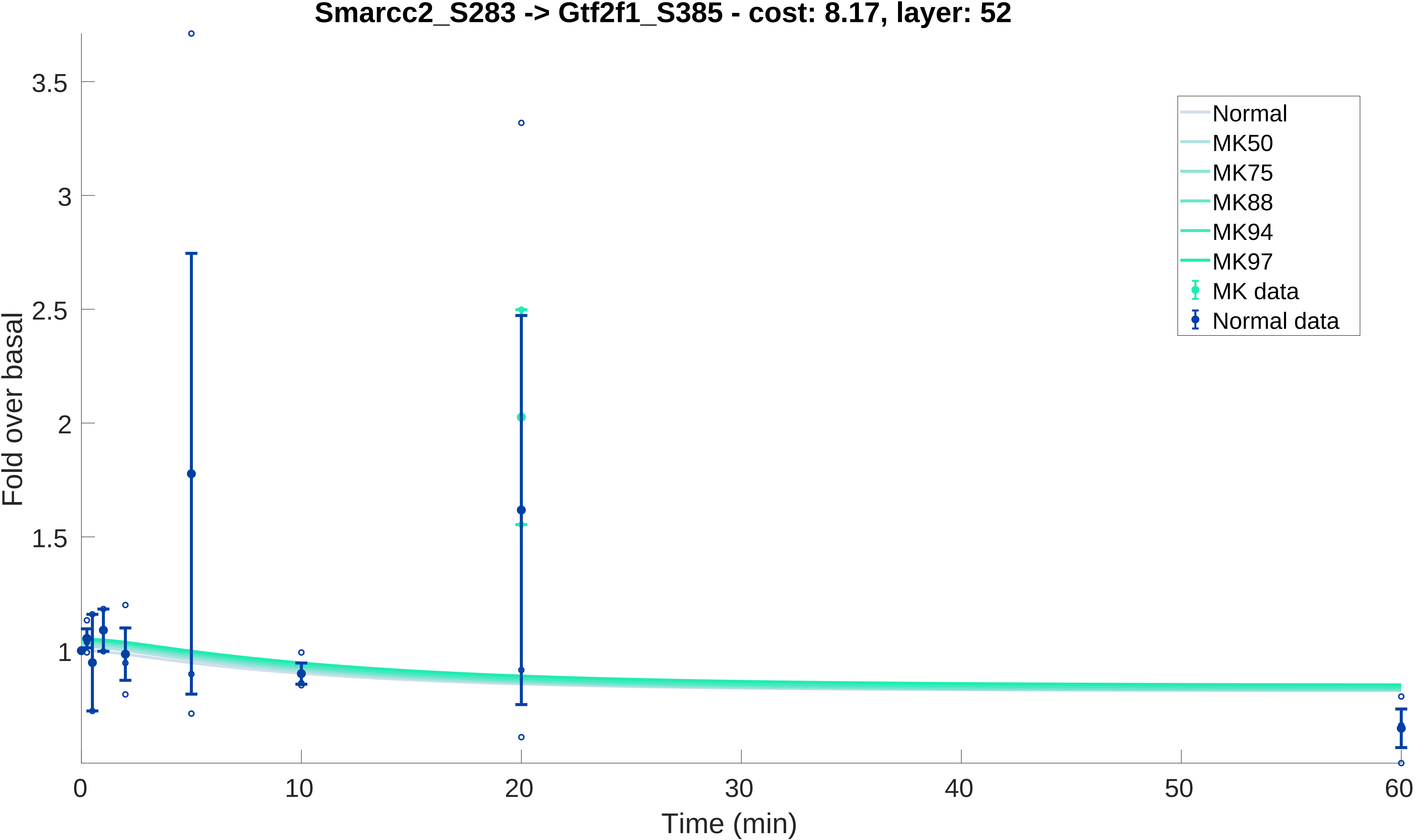
Smarcc2_S283 -> Gtf2f1_S385 - cost: 8.17, layer: 52
Fold over basal
Time (min)
0
10
20
30
40
50
60
1
1.5
2
2.5
3
3.5
Normal
MK50
MK75
MK88
MK94
MK97
MK data
Normal data

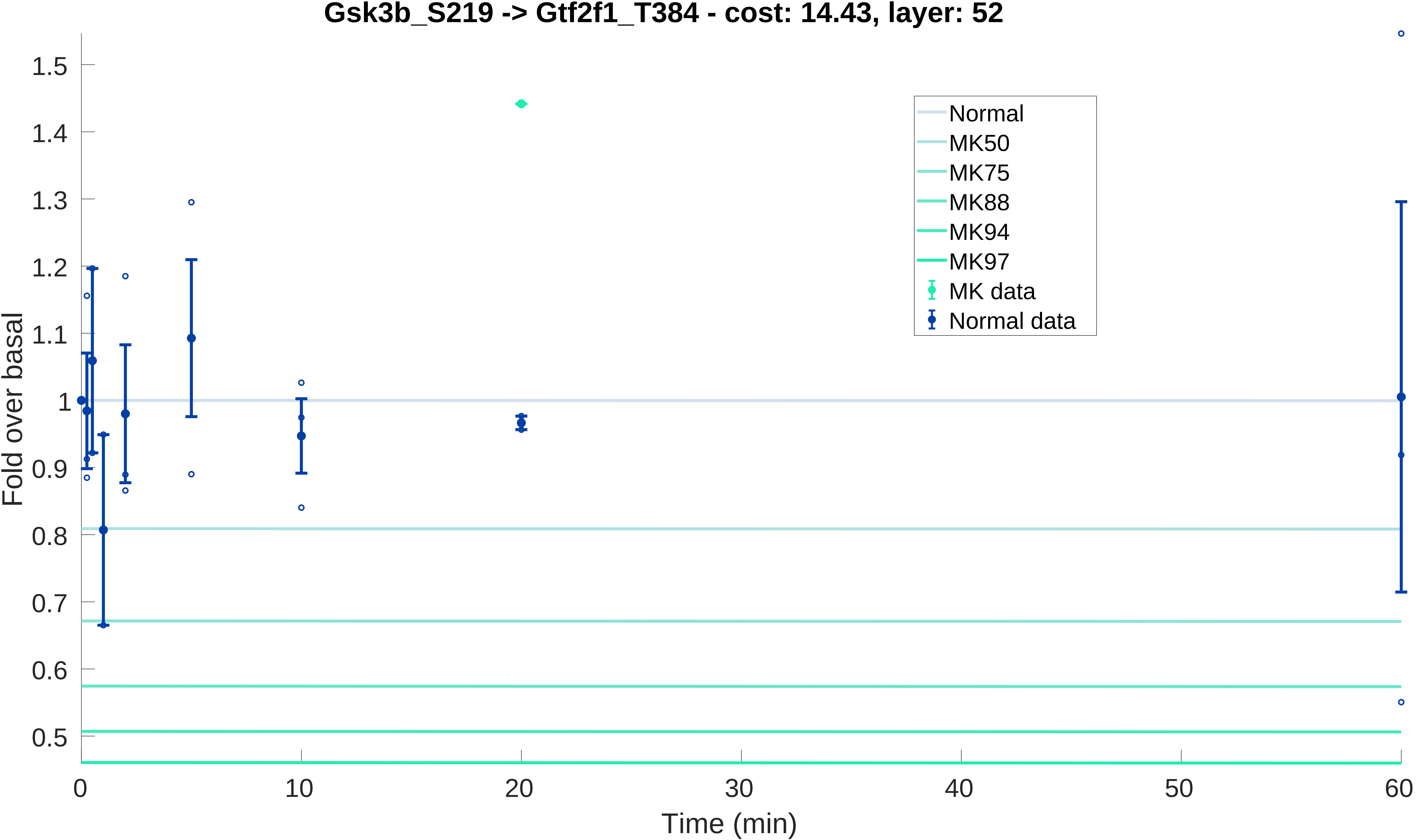

Gsk3b_S219 -> Gtf2f1_T384 - cost: 14.43, layer: 52
Fold over basal
Time (min)
1.5
1.4
1.3
1.2
1.1
1
0.9
0.8
0.7
0.6
0.5
0
10
20
30
40
50
60
Normal
MK50
MK75
MK88
MK94
MK97
MK data
Normal data

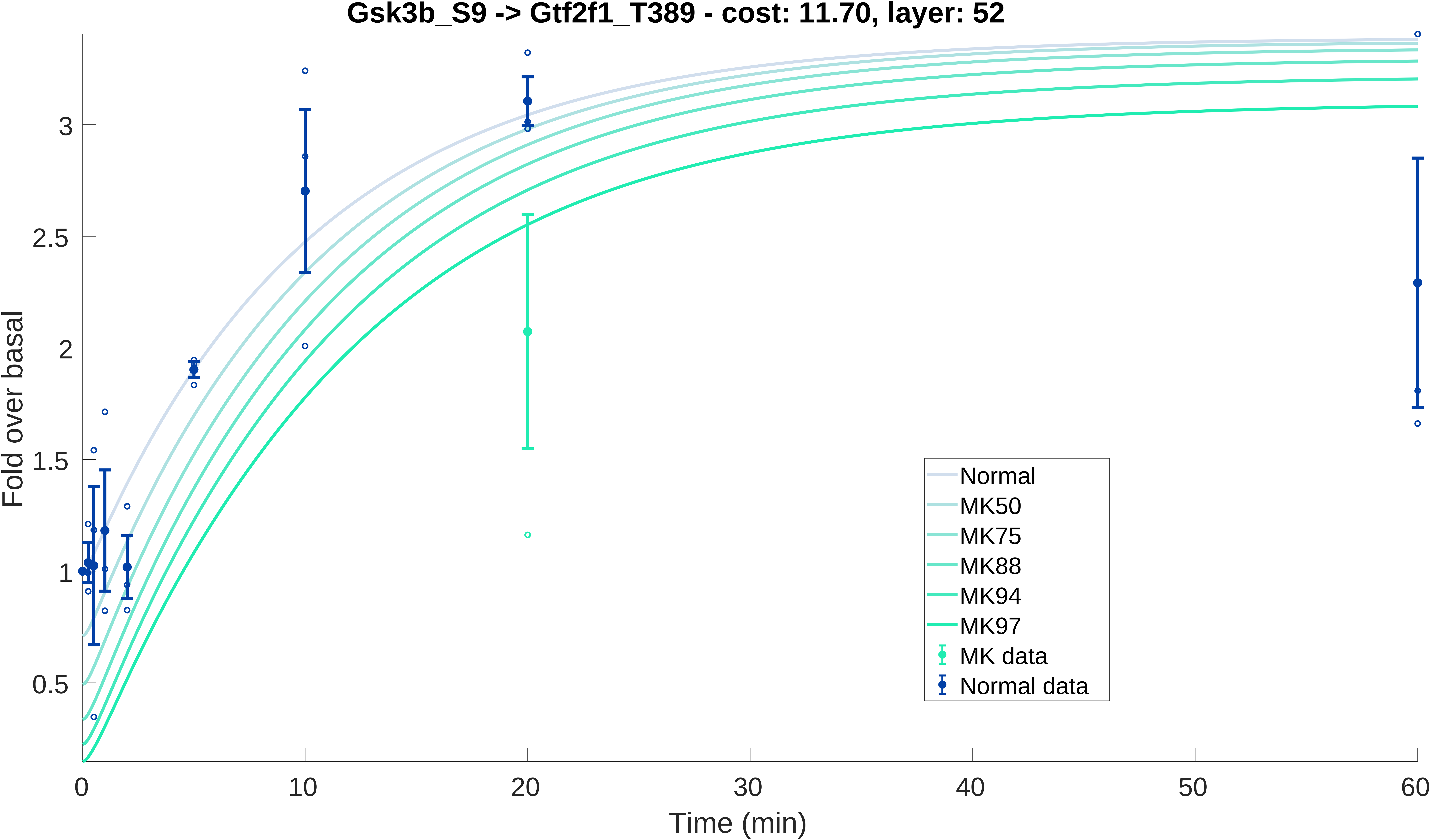

Gsk3b_S9 -> Gtf2f1_T389 - cost: 11.70, layer: 52
Fold over basal
Time (min)
0
10
20
30
40
50
60
0.5
1
1.5
2
2.5
3
Normal
MK50
MK75
MK88
MK94
MK97
MK data
Normal data

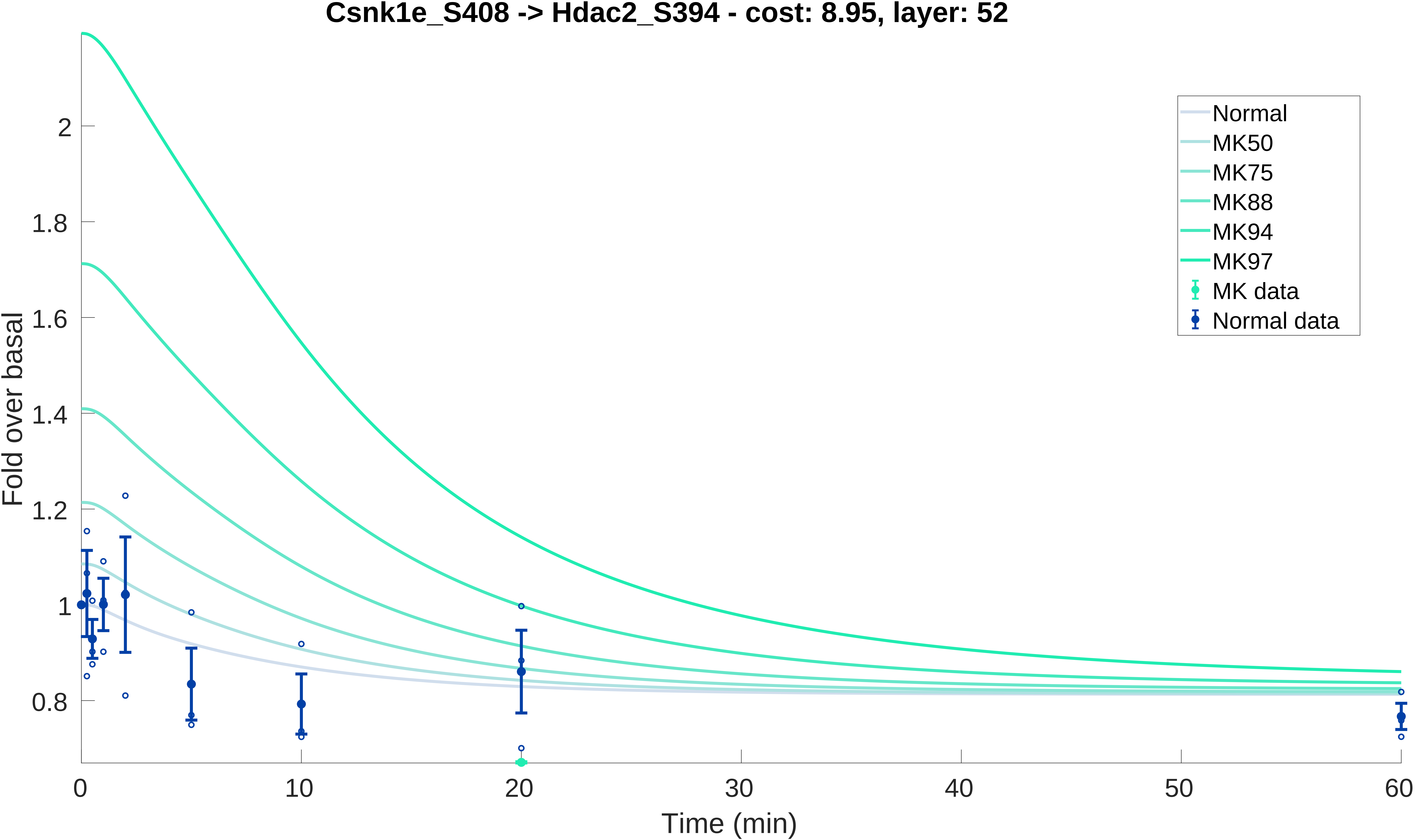
Csnk1e_S408 -> Hdac2_S394 - cost: 8.95, layer: 52
Fold over basal
Time (min)
0.8
1
1.2
1.4
1.6
1.8
2
0
10
20
30
40
50
60
Normal
MK50
MK75
MK88
MK94
MK97
MK data
Normal data

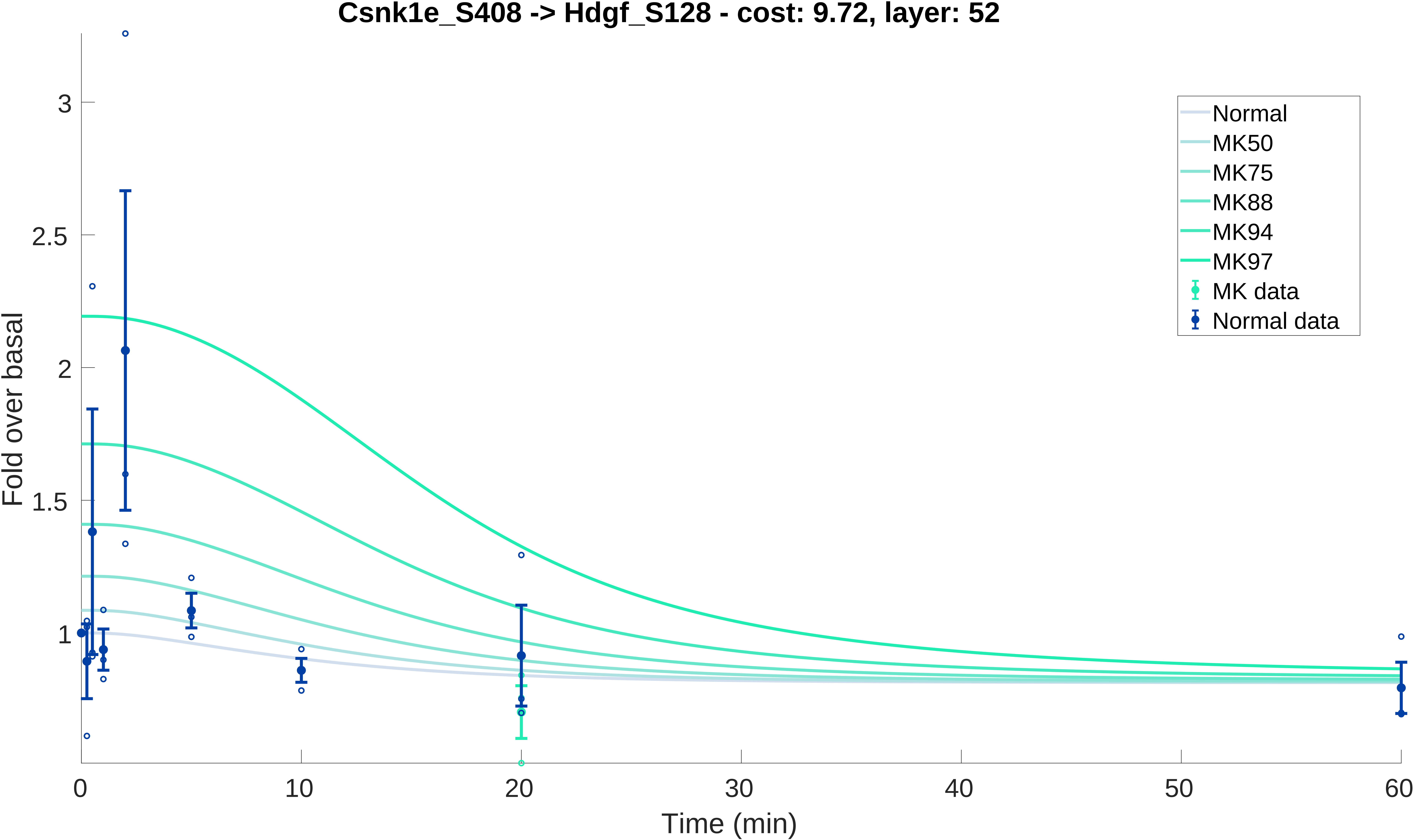

Csnk1e_S408 -> Hdgf_S128 - cost: 9.72, layer: 52
Fold over basal
Time (min)
Normal
MK50
MK75
MK88
MK94
MK97
MK data
Normal data
3
2.5
2
1.5
1
0
10
20
30
40
50
60

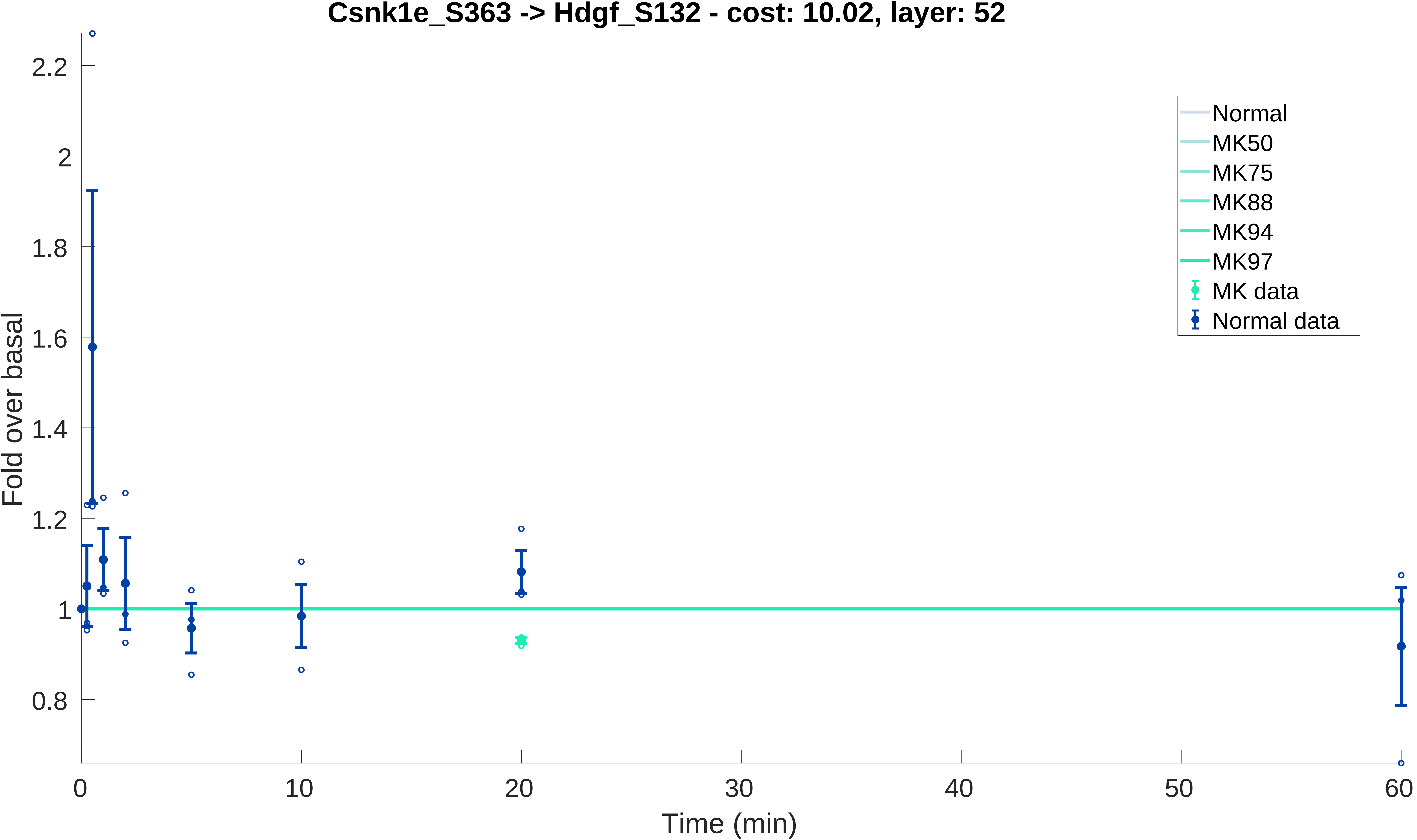

Csnk1e_S363 -> Hdgf_S132 - cost: 10.02, layer: 52
Fold over basal
Time (min)
0.8
1
1.2
1.4
1.6
1.8
2
2.2
0
10
20
30
40
50
60
Normal
MK50
MK75
MK88
MK94
MK97
MK data
Normal data

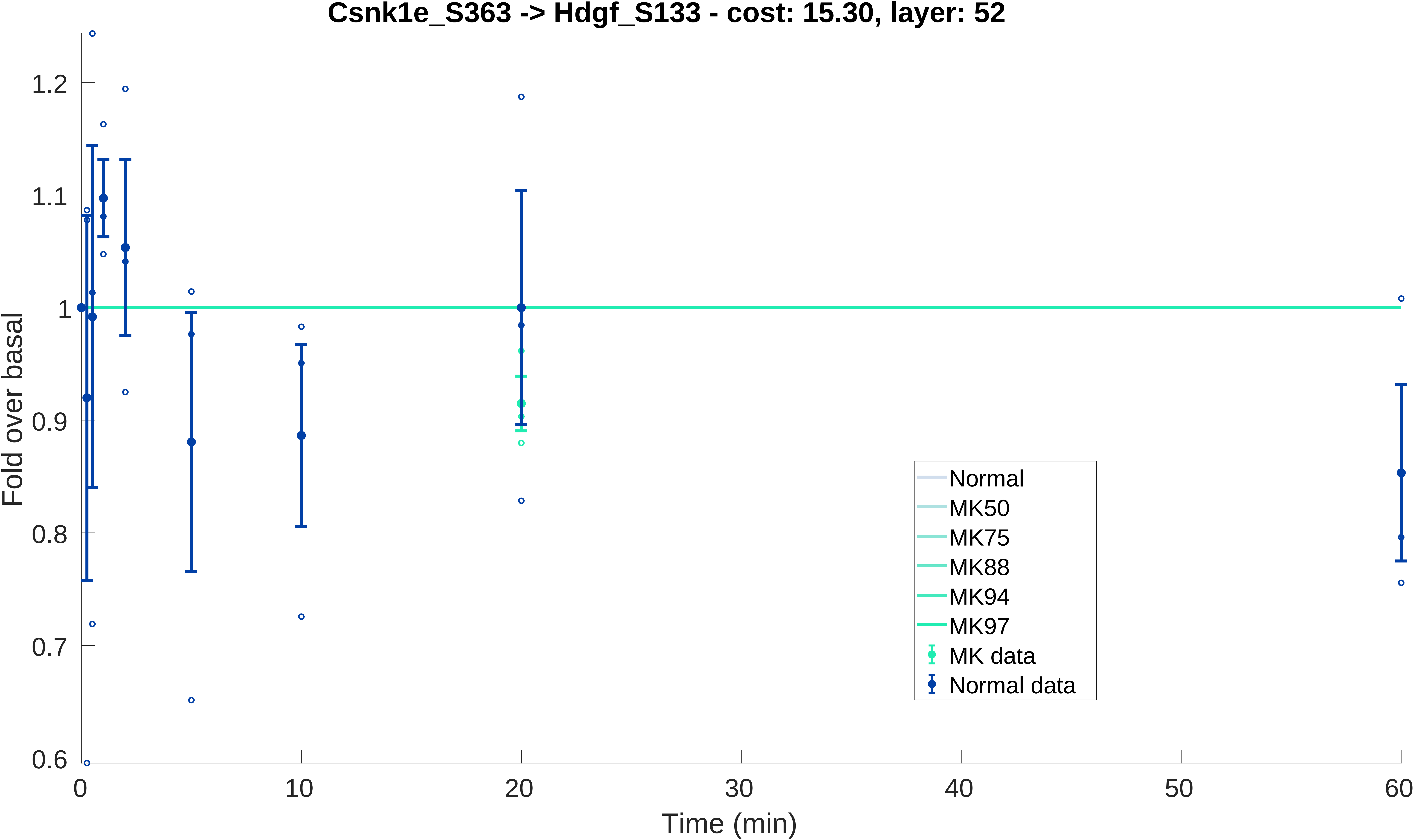

Csnk1e_S363 -> Hdgf_S133 - cost: 15.30, layer: 52
Fold over basal
1.2
1.1
1
0.9
0.8
0.7
0.6
0
10
20
30
40
50
60
Time (min)
Normal
MK50
MK75
MK88
MK94
MK97
MK data
Normal data

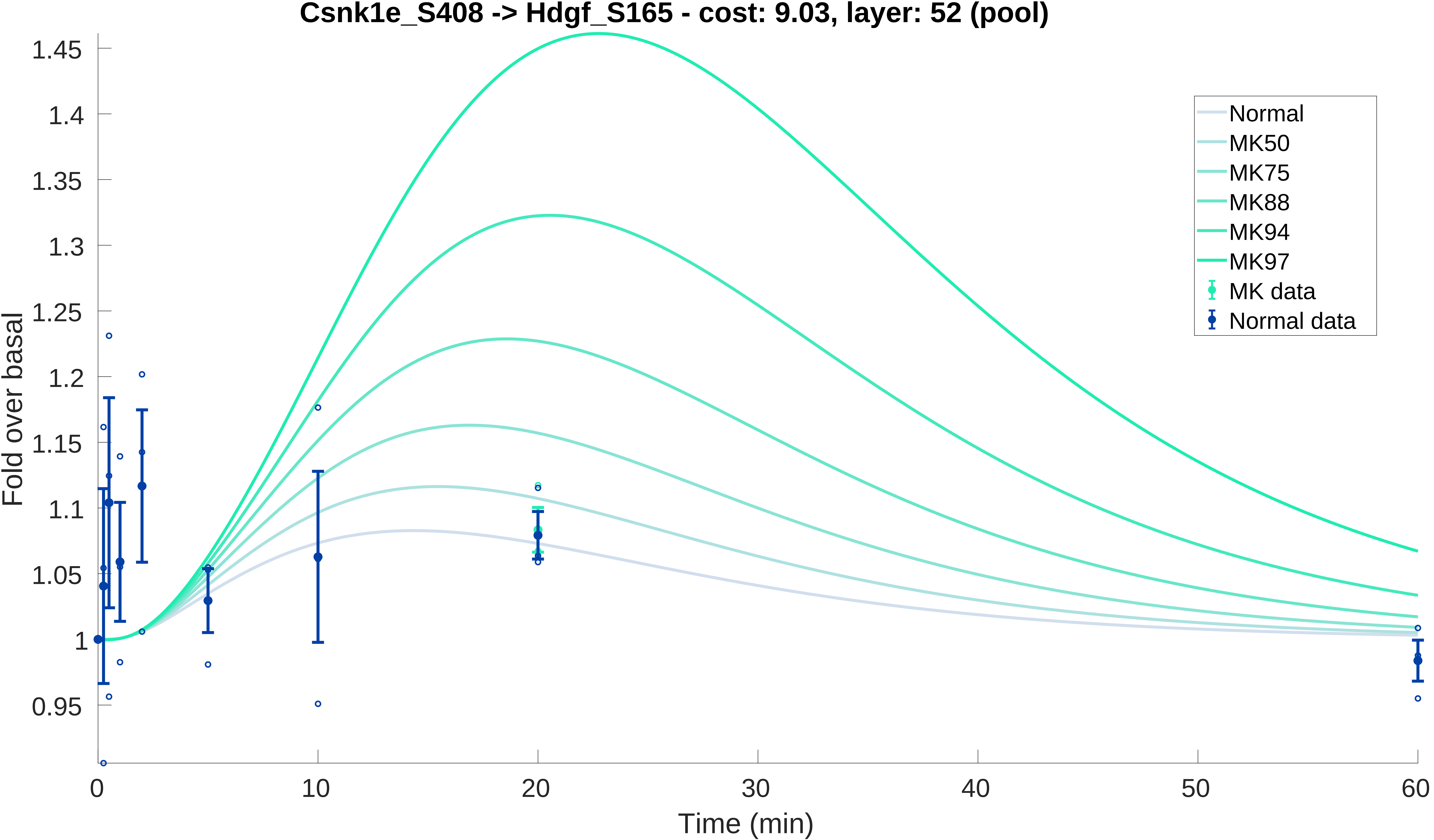

Csnk1e_S408 -> Hdgf_S165 - cost: 9.03, layer: 52 (pool)
Fold over basal
1.45
1.4
1.35
1.3
1.25
1.2
1.15
1.1
1.05
1
0.95
0
10
20
30
40
50
60
Time (min)
Normal
MK50
MK75
MK88
MK94
MK97
MK data
Normal data

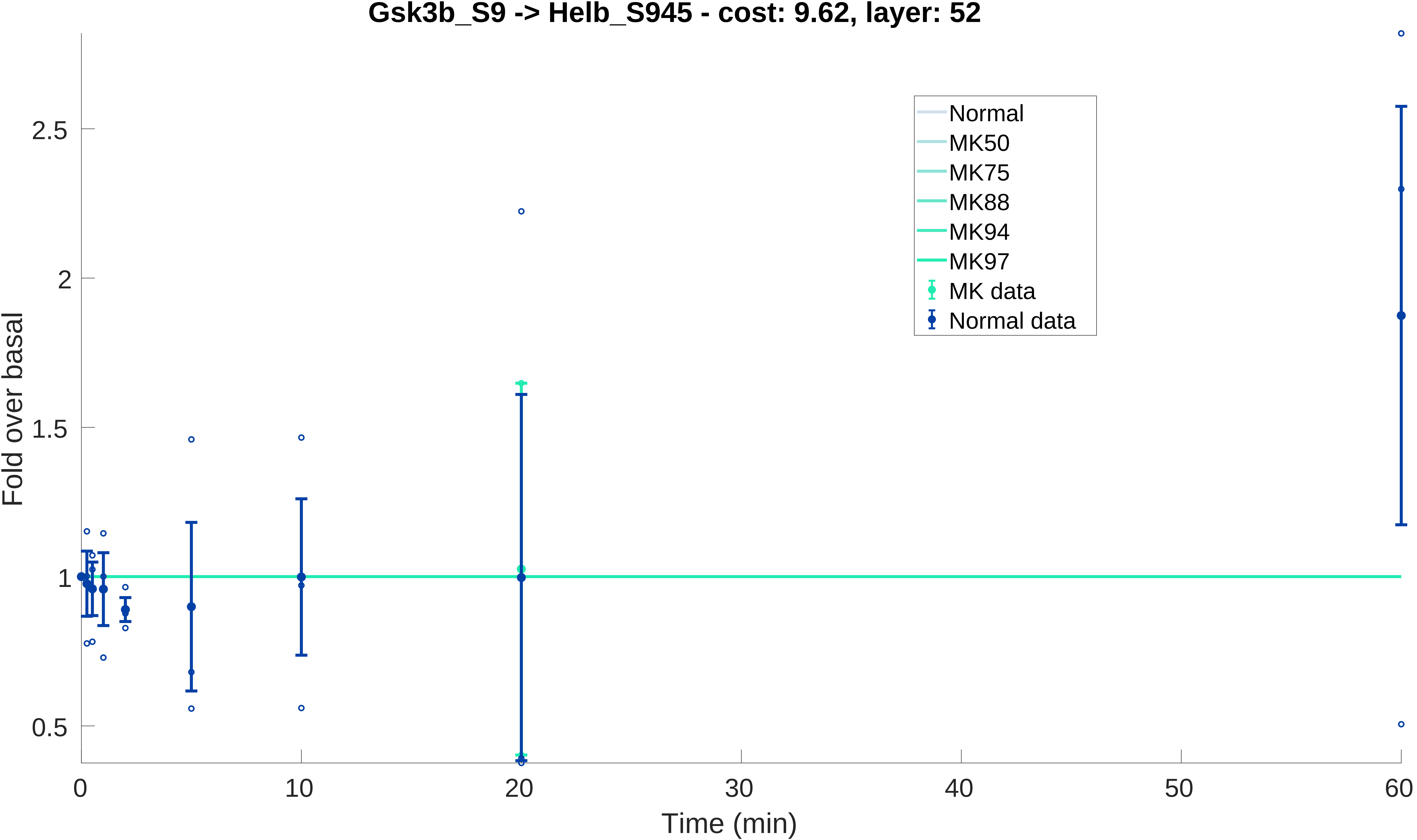

Gsk3b_S9 -> Helb_S945 - cost: 9.62, layer: 52
Fold over basal
2.5
2
1.5
1
0.5
0
10
20
30
40
50
60
Time (min)
Normal
MK50
MK75
MK88
MK94
MK97
MK data
Normal data

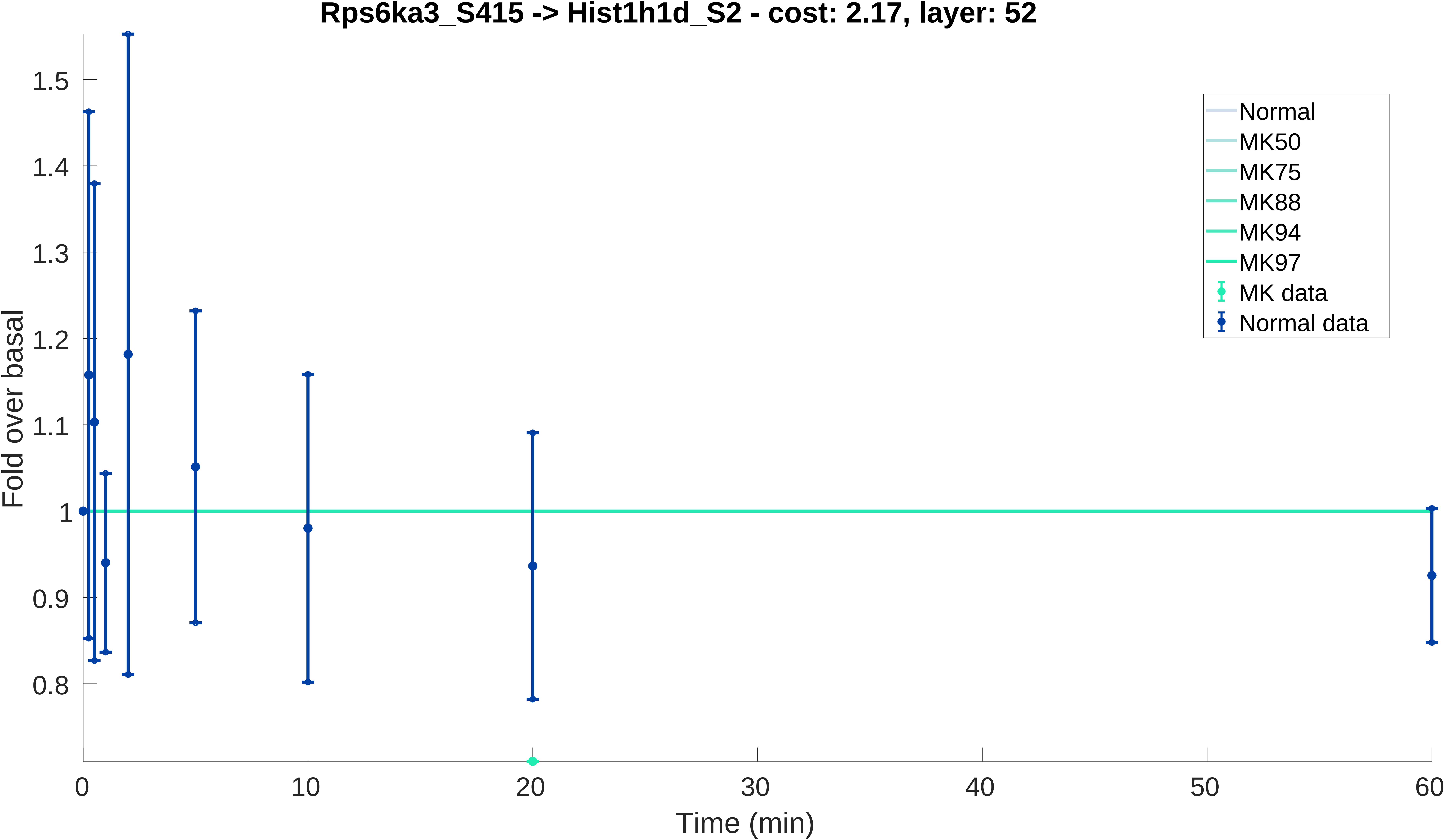

Rps6ka3_S415 -> Hist1h1d_S2 - cost: 2.17, layer: 52
Fold over basal
Time (min)
0.8
0.9
1
1.1
1.2
1.3
1.4
1.5
0
10
20
30
40
50
60
Normal
MK50
MK75
MK88
MK94
MK97
MK data
Normal data

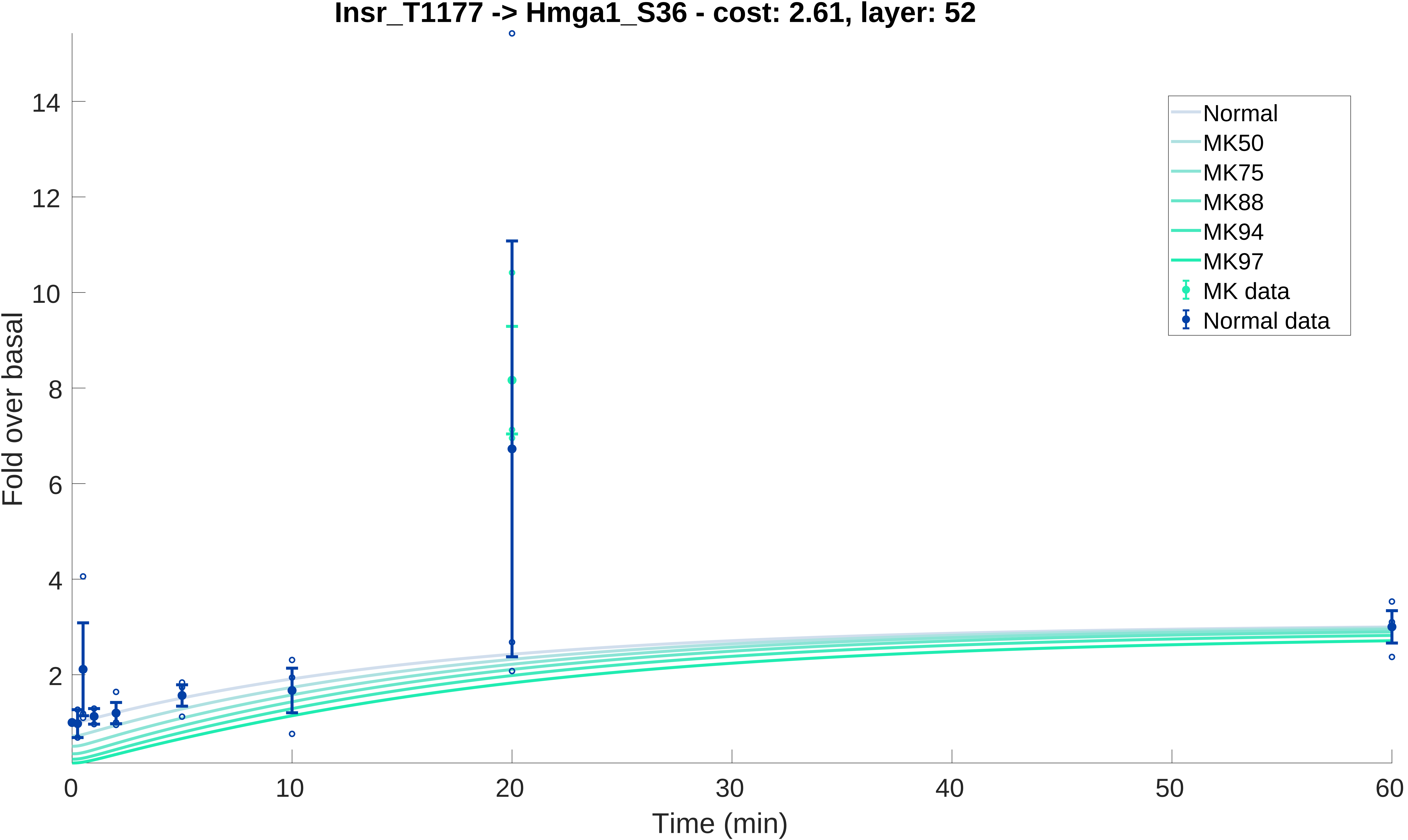

Insr_T1177 -> Hmga1_S36 - cost: 2.61, layer: 52
Fold over basal
Time (min)
Normal
MK50
MK75
MK88
MK94
MK97
MK data
Normal data
0
10
20
30
40
50
60
2
4
6
8
10
12
14

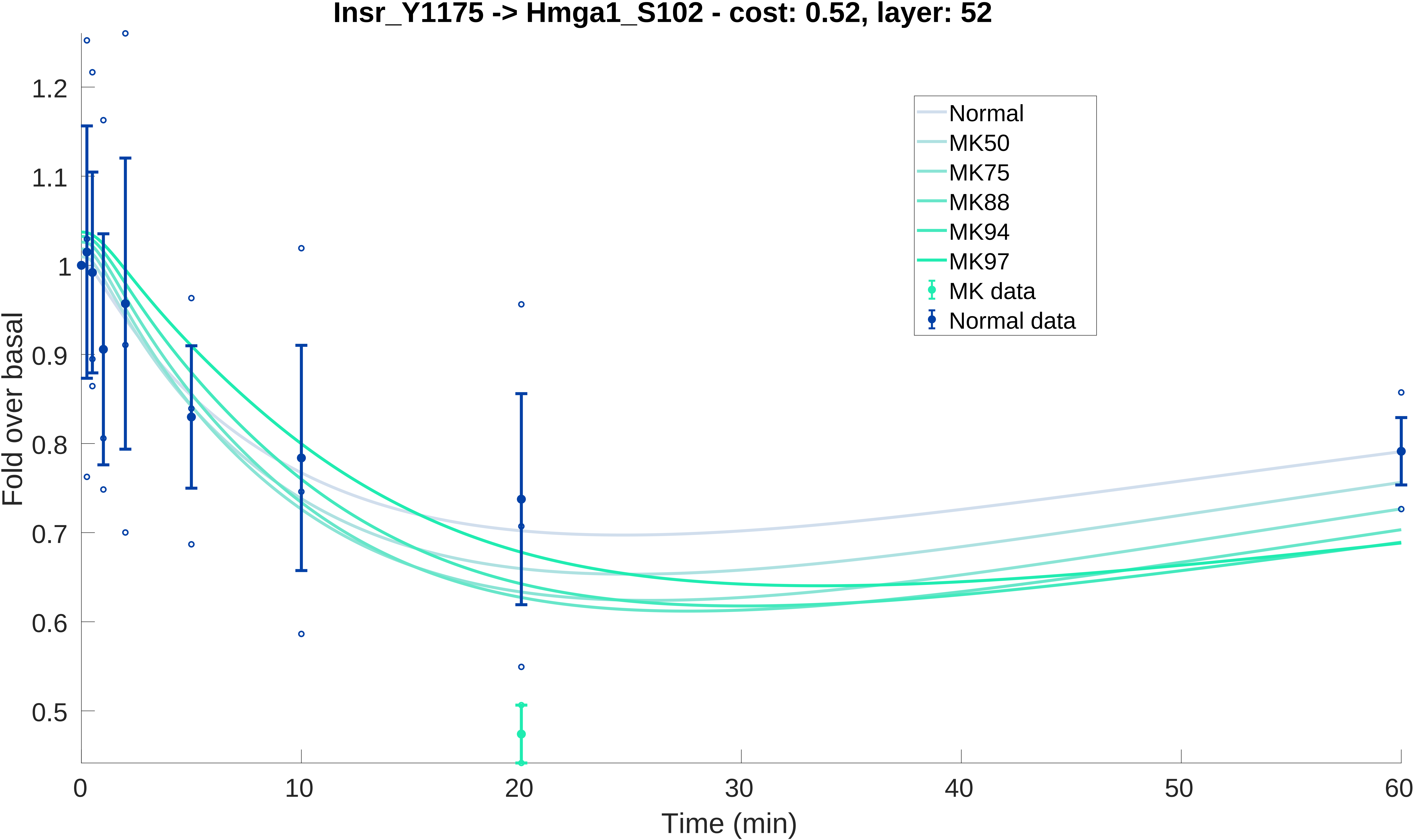

Insr_Y1175 -> Hmga1_S102 - cost: 0.52, layer: 52
Fold over basal
1.2
1.1
1
0.9
0.8
0.7
0.6
0.5
0
10
20
30
40
50
60
Time (min)
Normal
MK50
MK75
MK88
MK94
MK97
MK data
Normal data

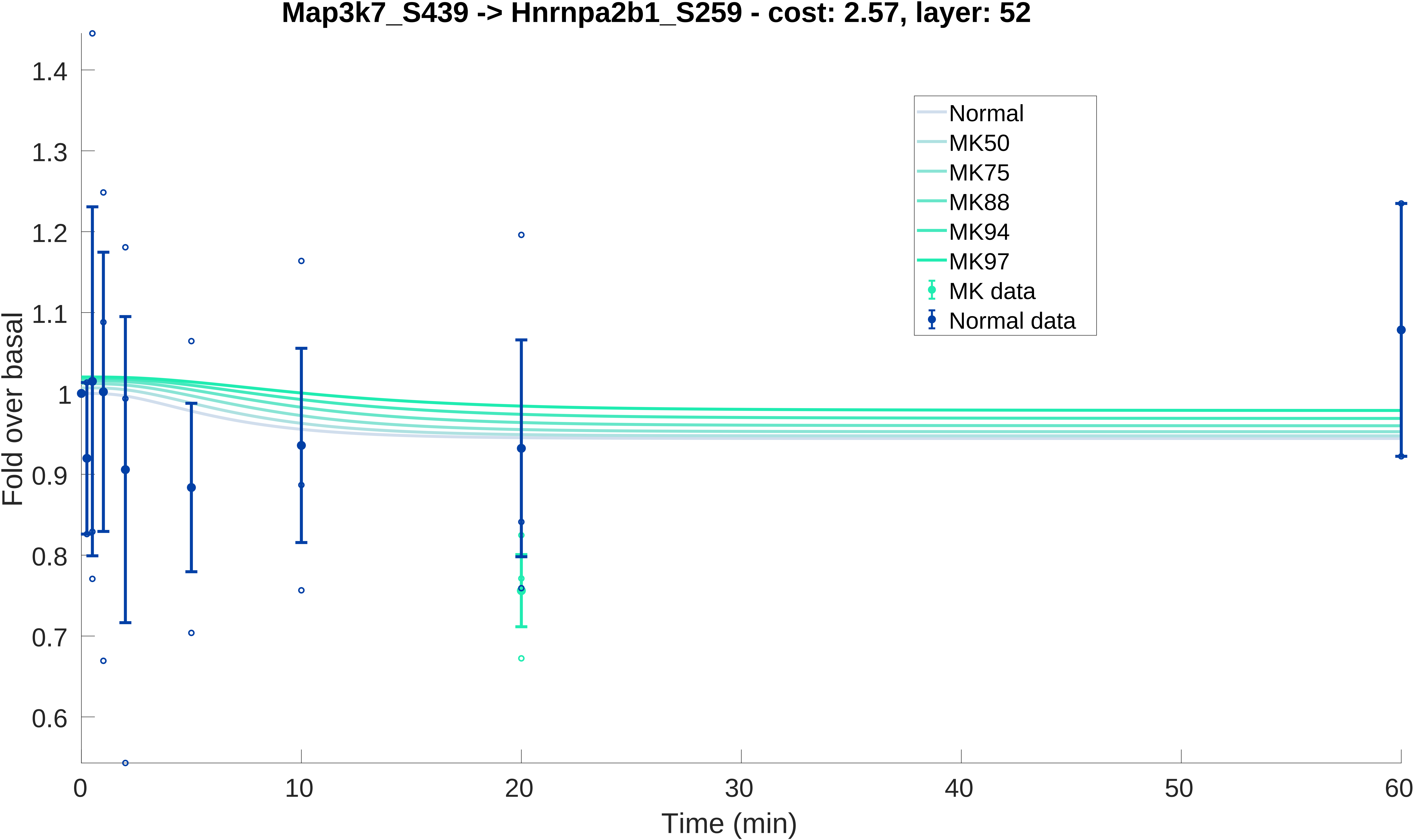

Map3k7_S439 -> Hnrnpa2b1_S259 - cost: 2.57, layer: 52
Fold over basal
Time (min)
Normal
MK50
MK75
MK88
MK94
MK97
MK data
Normal data
0.6
0.7
0.8
0.9
1
1.1
1.2
1.3
1.4
0
10
20
30
40
50
60

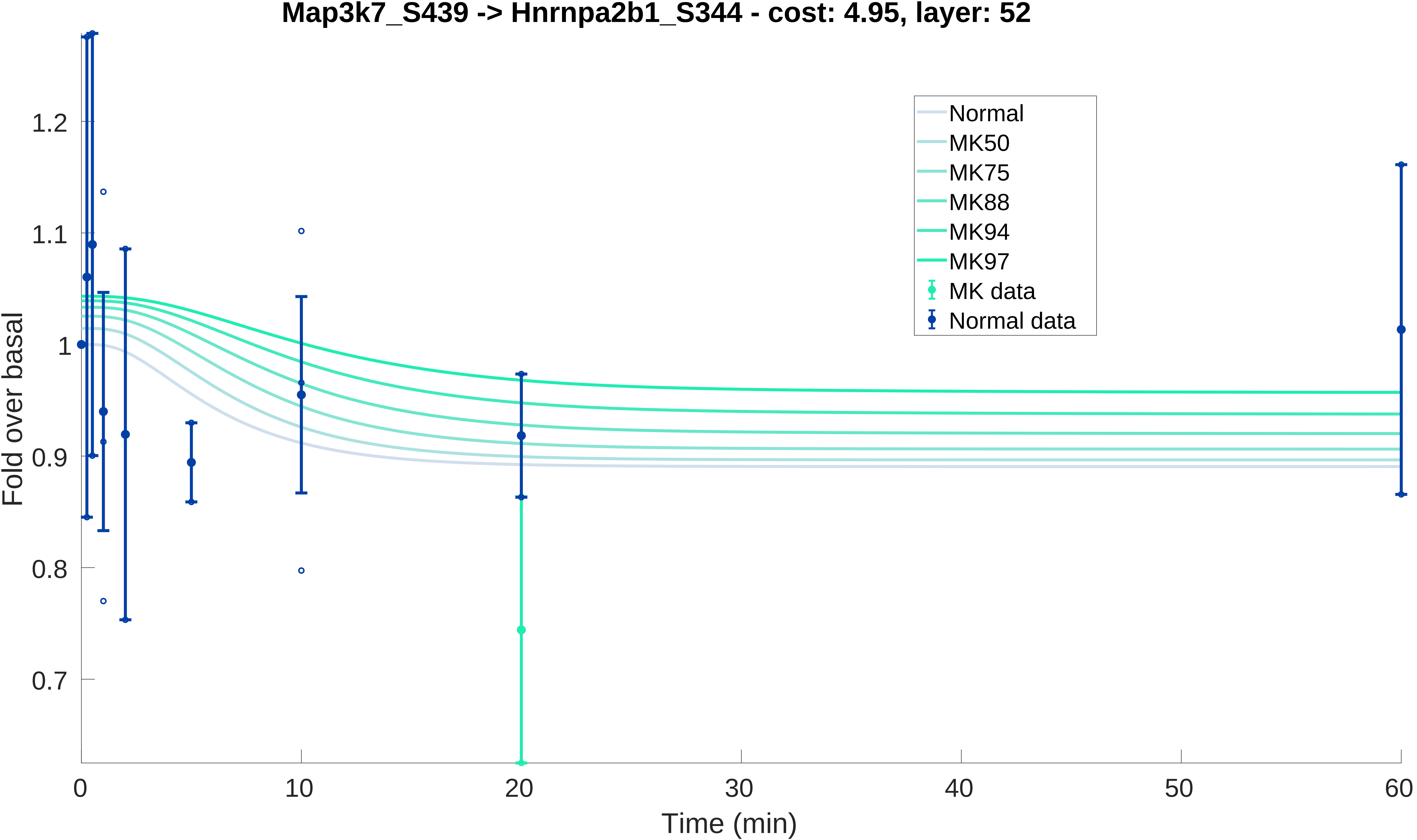

Map3k7_S439 -> Hnrnpa2b1_S344 - cost: 4.95, layer: 52
Fold over basal
Time (min)
0.7
0.8
0.9
1
1.1
1.2
0
10
20
30
40
50
60
Normal
MK50
MK75
MK88
MK94
MK97
MK data
Normal data

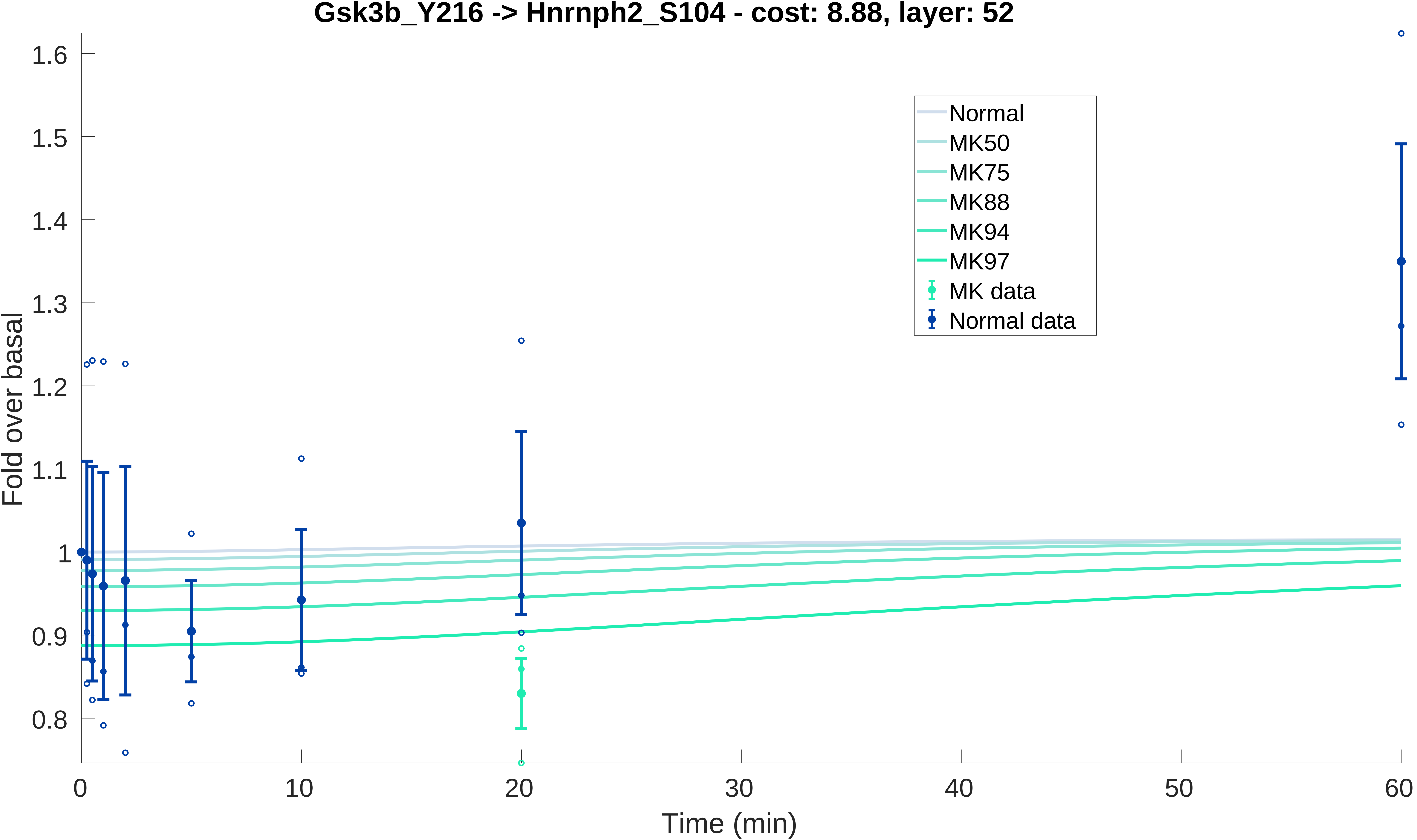

Gsk3b_Y216 -> Hnrnph2_S104 - cost: 8.88, layer: 52
Fold over basal
Time (min)
1.6
1.5
1.4
1.3
1.2
1.1
1
0.9
0.8
0
10
20
30
40
50
60
Normal
MK50
MK75
MK88
MK94
MK97
MK data
Normal data

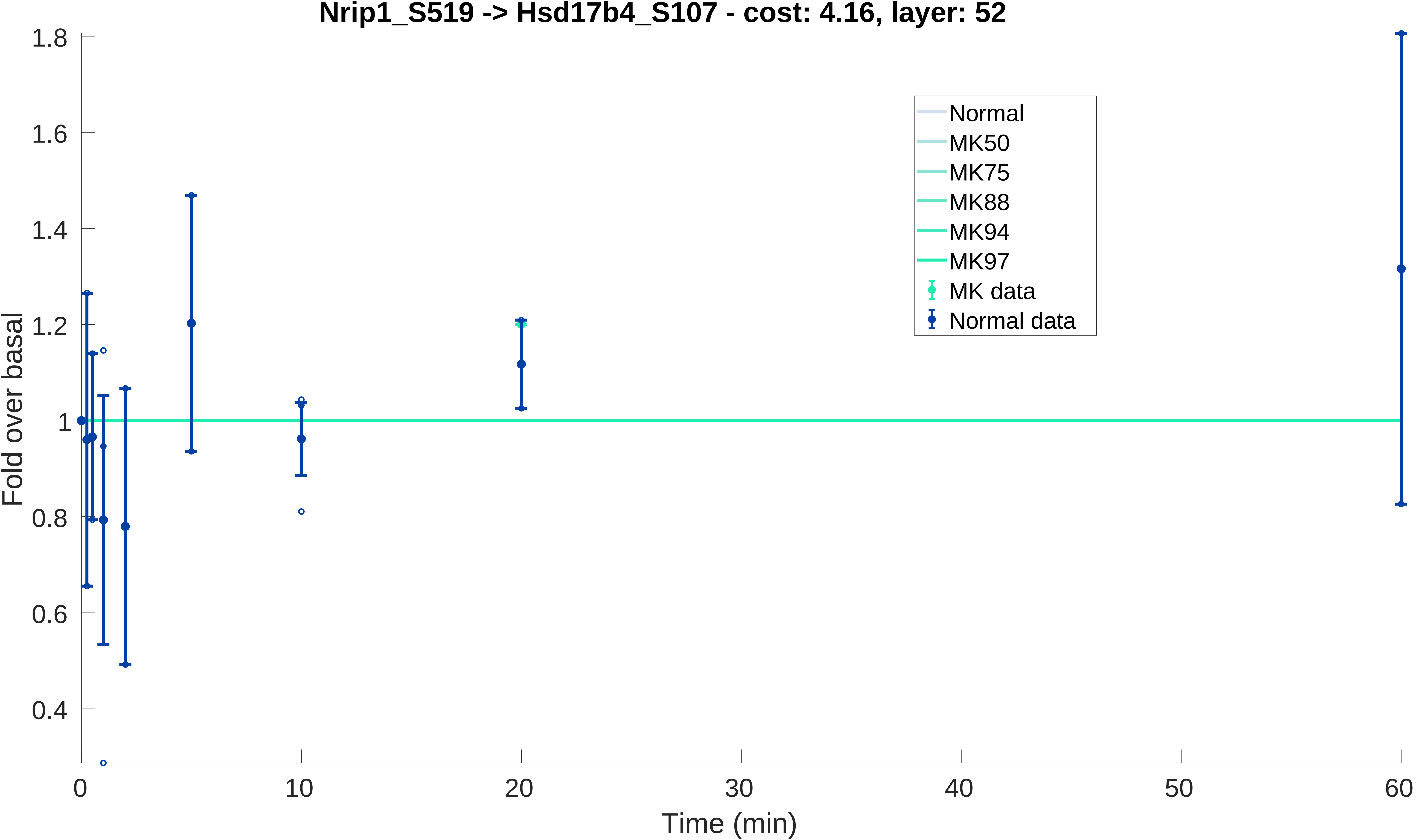

Nrip1_S519 -> Hsd17b4_S107 - cost: 4.16, layer: 52
Fold over basal
Time (min)
0.4
0.6
0.8
1
1.2
1.4
1.6
1.8
0
10
20
30
40
50
60
Normal
MK50
MK75
MK88
MK94
MK97
MK data
Normal data

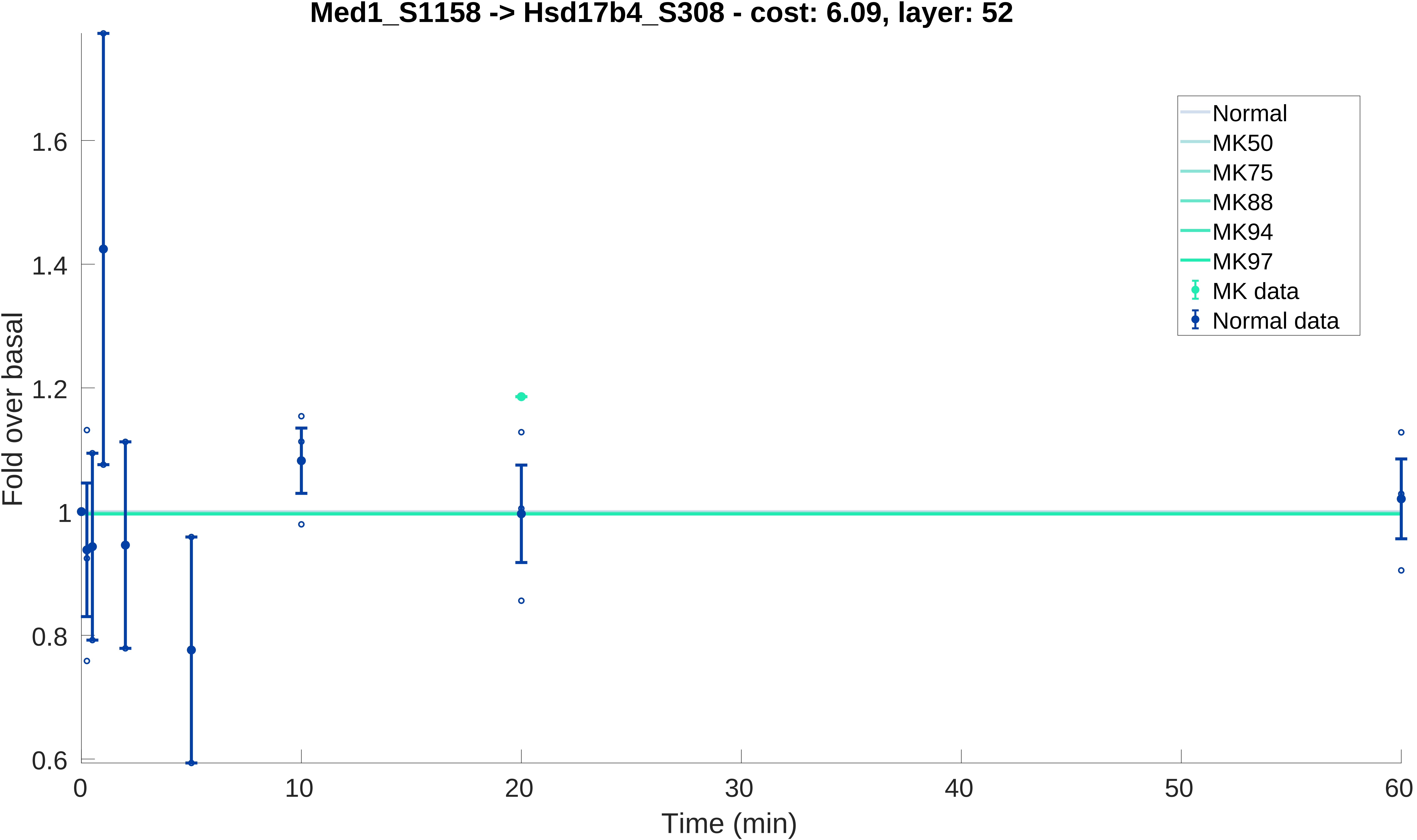
Med1_S1158 -> Hsd17b4_S308 - cost: 6.09, layer: 52
Fold over basal
Time (min)
1.6
1.4
1.2
1
0.8
0.6
0
10
20
30
40
50
60
Normal
MK50
MK75
MK88
MK94
MK97
MK data
Normal data

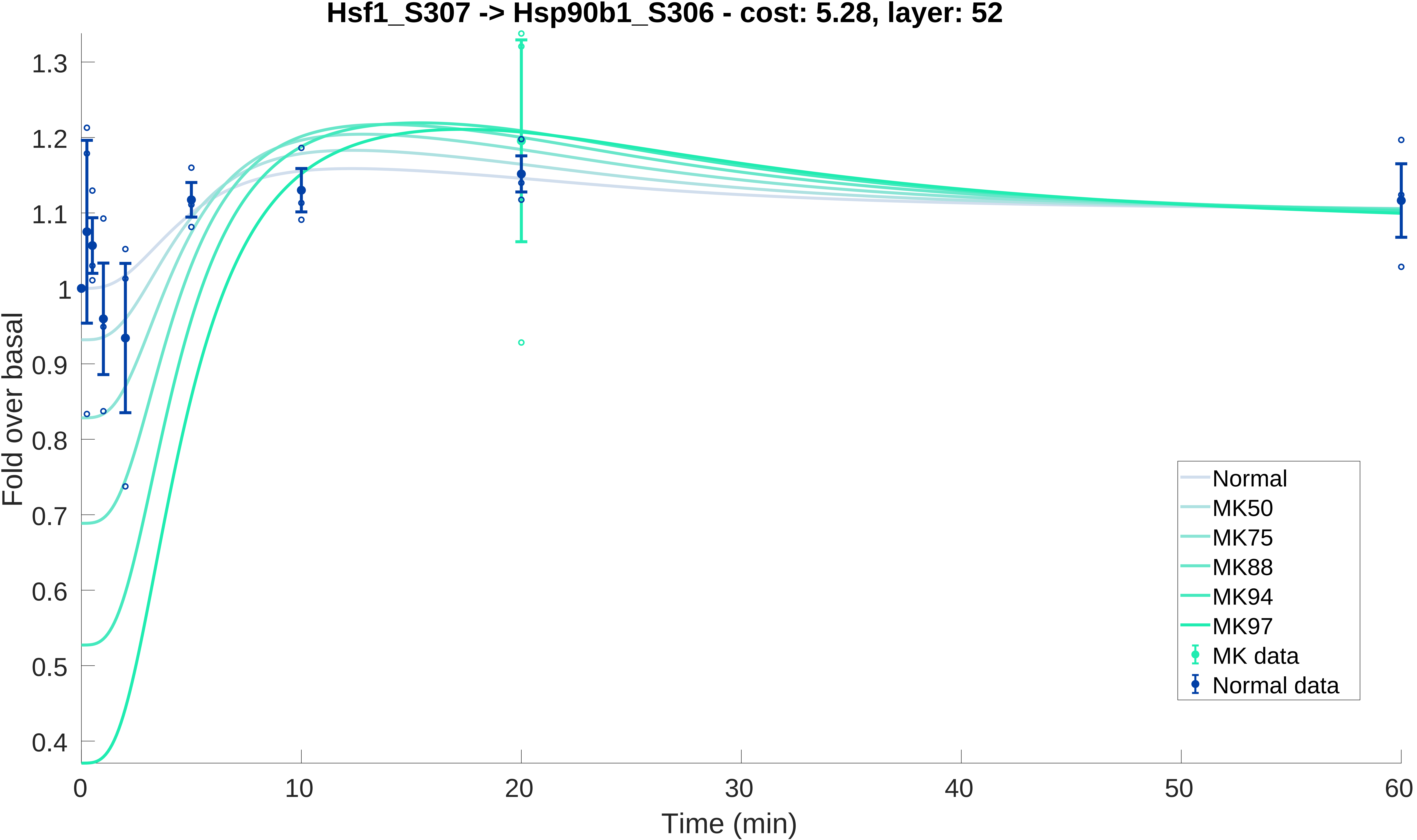

Hsf1_S307 -> Hsp90b1_S306 - cost: 5.28, layer: 52
Fold over basal
Time (min)
0
10
20
30
40
50
60
0.4
0.5
0.6
0.7
0.8
0.9
1
1.1
1.2
1.3
Normal
MK50
MK75
MK88
MK94
MK97
MK data
Normal data

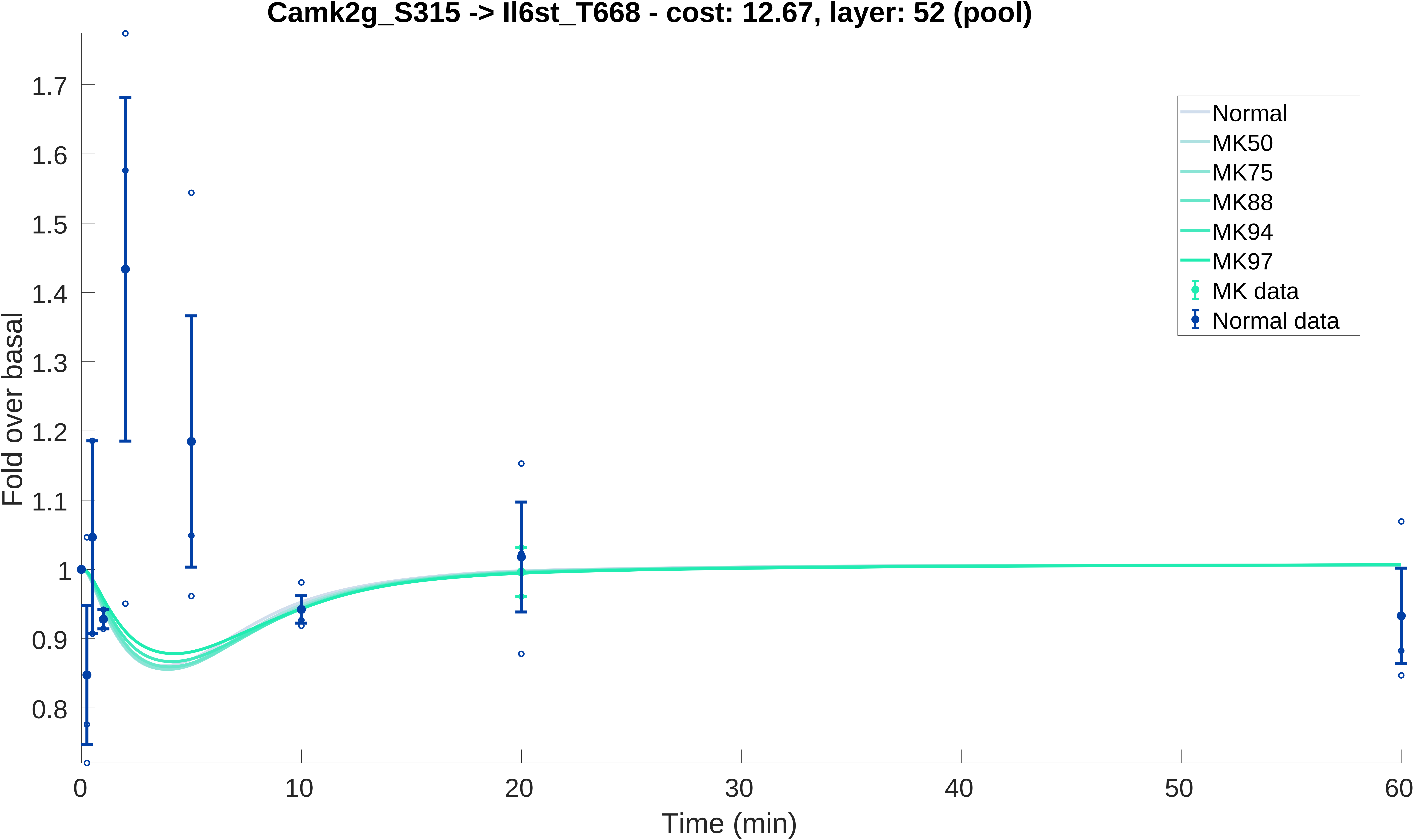

Camk2g_S315 -> Il6st_T668 - cost: 12.67, layer: 52 (pool)
Fold over basal
Time (min)
1.7
1.6
1.5
1.4
1.3
1.2
1.1
1
0.9
0.8
0
10
20
30
40
50
60
Normal
MK50
MK75
MK88
MK94
MK97
MK data
Normal data

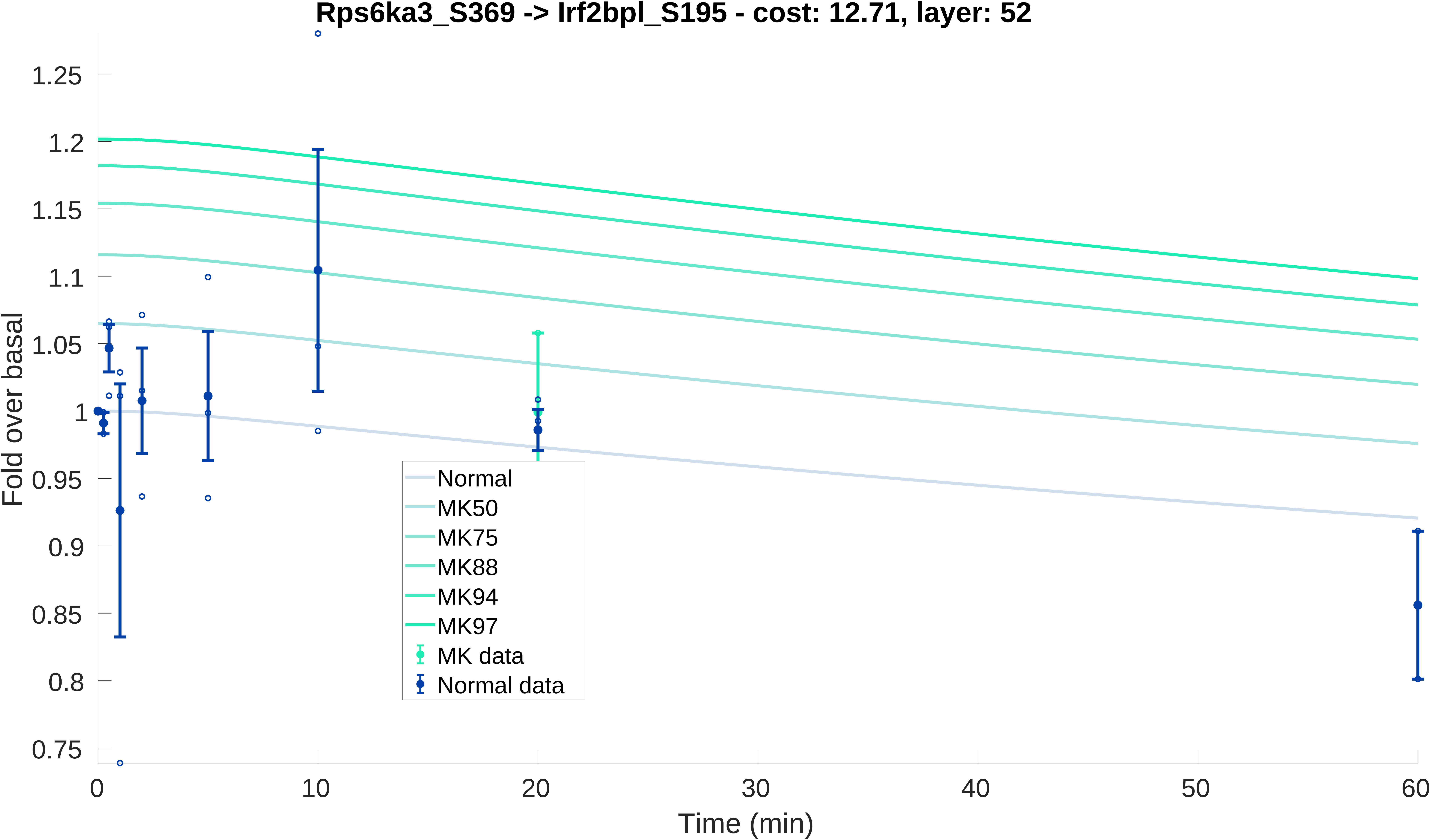
Rps6ka3_S369 -> Irf2bpl_S195 - cost: 12.71, layer: 52
Fold over basal
Time (min)
1.25
1.2
1.15
1.1
1.05
1
0.95
0.9
0.85
0.8
0.75
0
10
20
30
40
50
60
Normal
MK50
MK75
MK88
MK94
MK97
MK data
Normal data

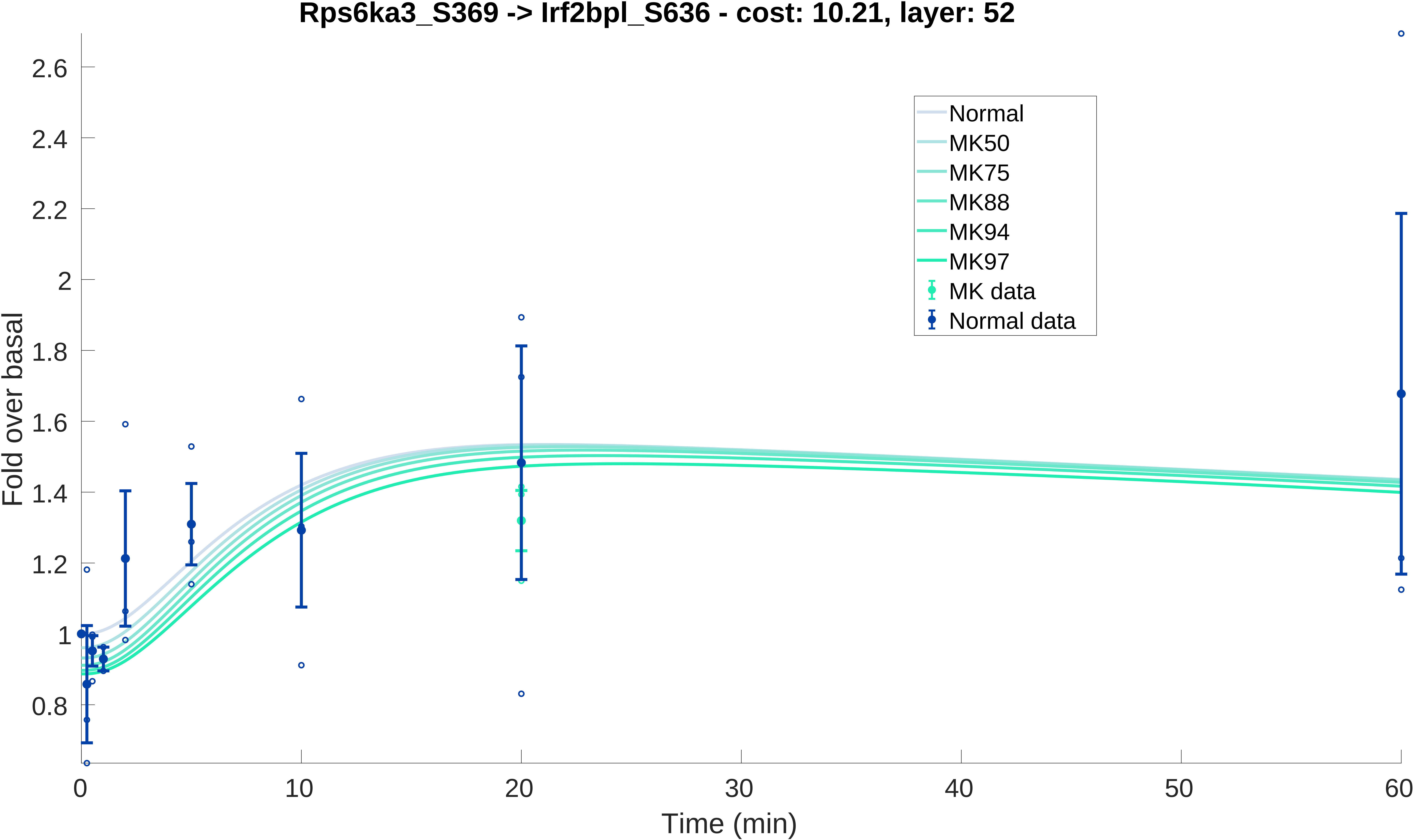

Rps6ka3_S369 -> Irf2bpl_S636 - cost: 10.21, layer: 52
Fold over basal
Time (min)
0.8
1
1.2
1.4
1.6
1.8
2
2.2
2.4
2.6
0
10
20
30
40
50
60
Normal
MK50
MK75
MK88
MK94
MK97
MK data
Normal data

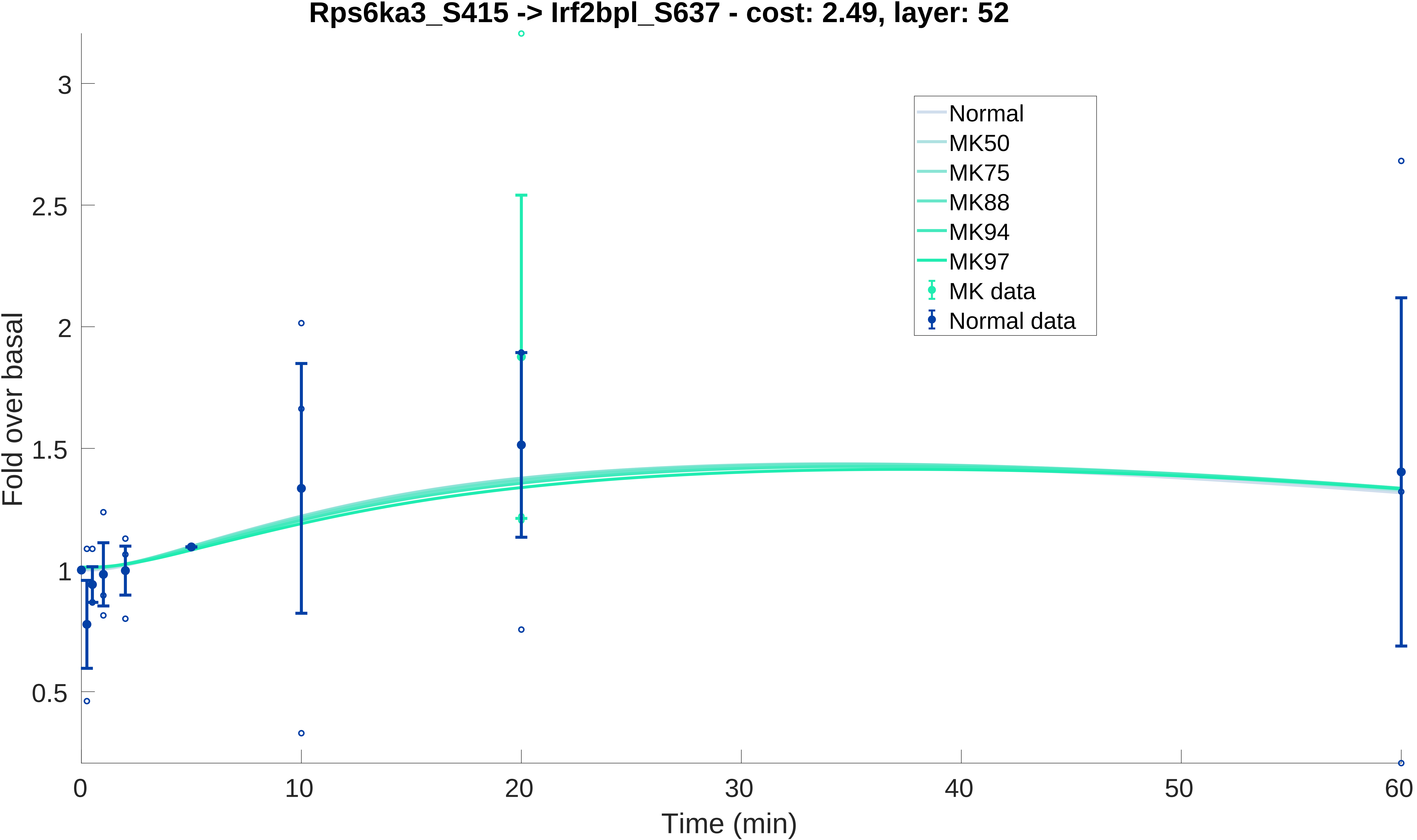

Rps6ka3_S415 -> Irf2bpl_S637 - cost: 2.49, layer: 52
Normal
MK50
MK75
MK88
MK94
MK97
MK data
Normal data
Fold over basal
Time (min)
0.5
1
1.5
2
2.5
3
0
10
20
30
40
50
60

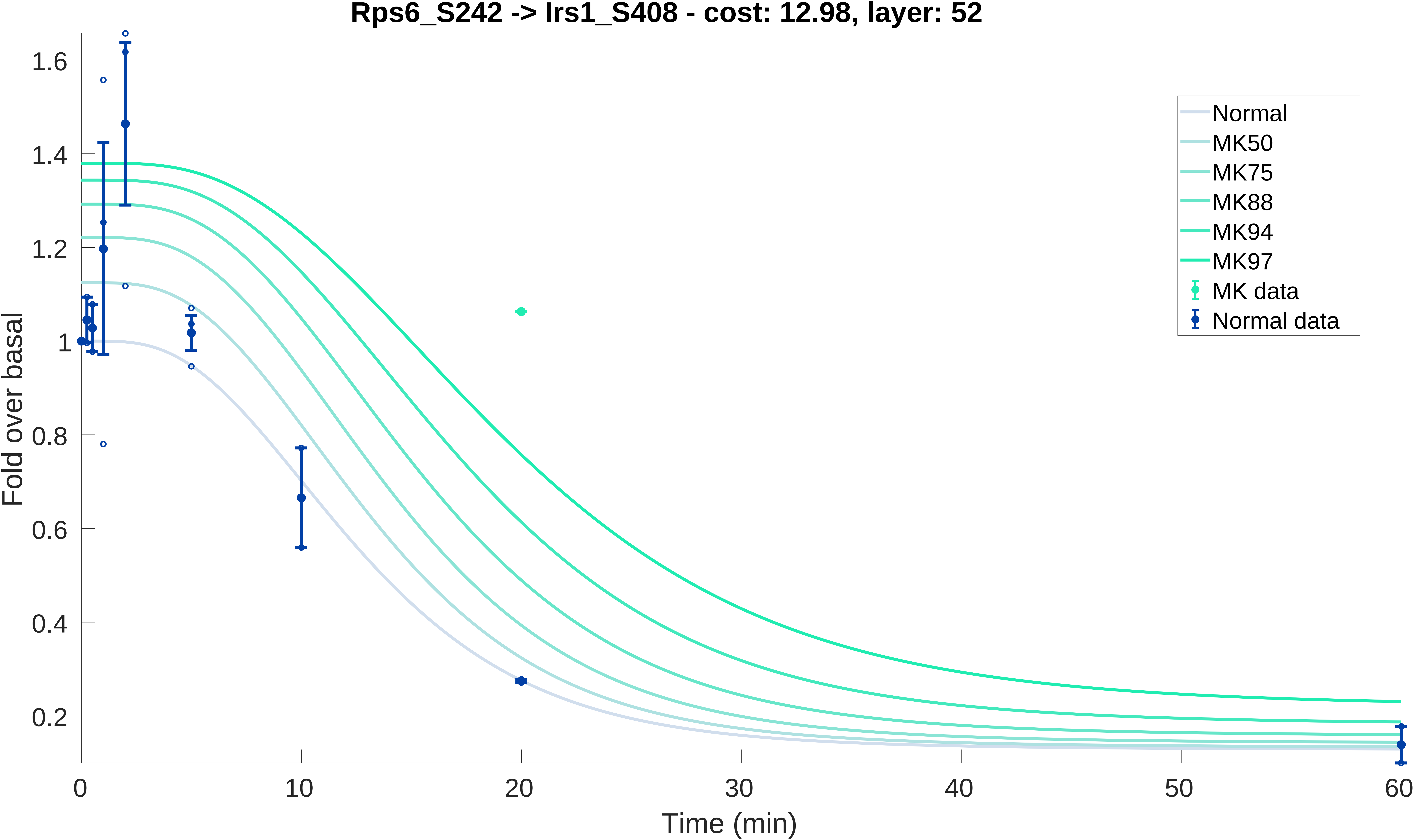

Rps6_S242 -> Irs1_S408 - cost: 12.98, layer: 52
Fold over basal
Time (min)
0
10
20
30
40
50
60
0.2
0.4
0.6
0.8
1
1.2
1.4
1.6
Normal
MK50
MK75
MK88
MK94
MK97
MK data
Normal data

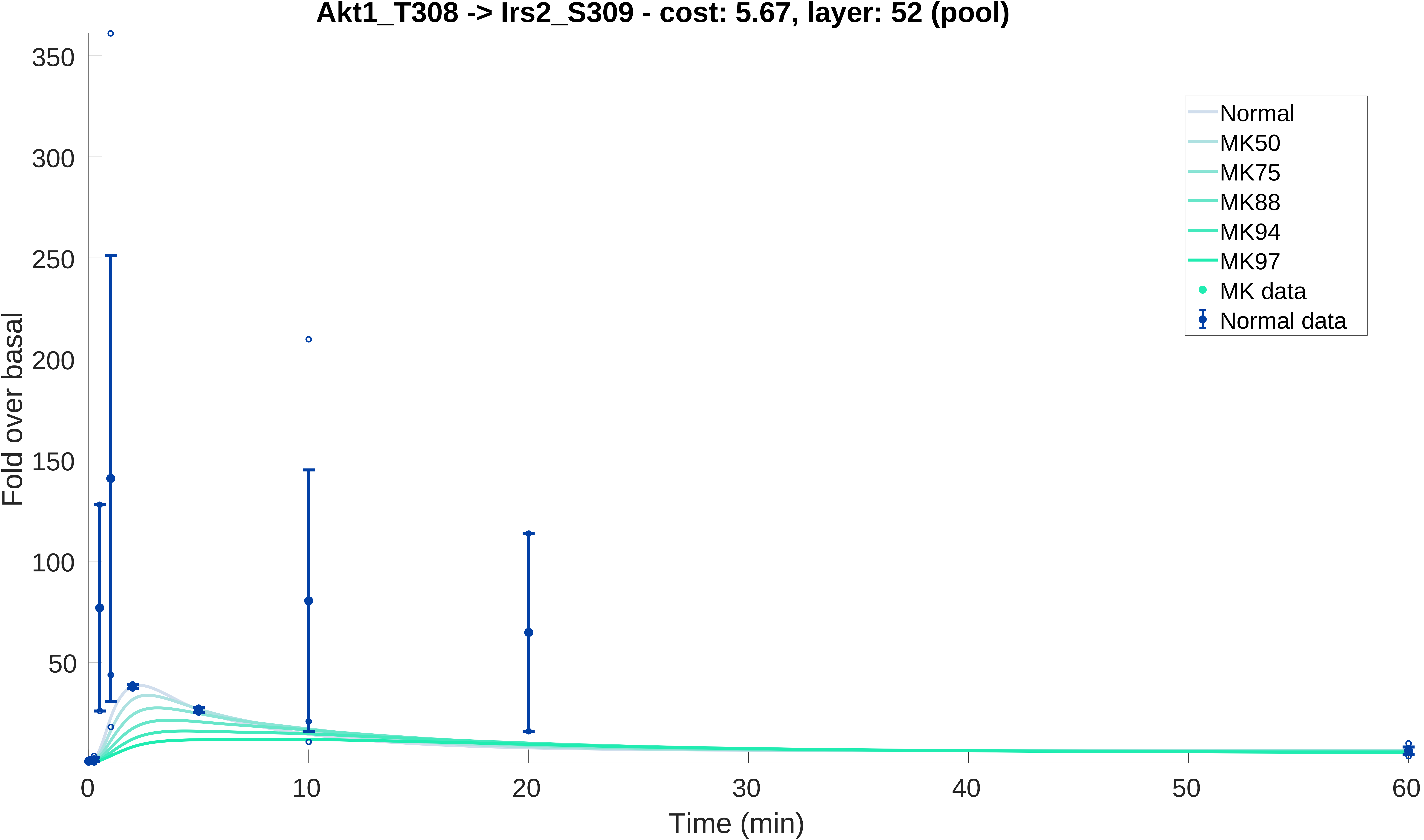

Akt1_T308 -> Irs2_S309 - cost: 5.67, layer: 52 (pool)
Fold over basal
Time (min)
350
300
250
200
150
100
50
0
10
20
30
40
50
60
Normal
MK50
MK75
MK88
MK94
MK97
MK data
Normal data

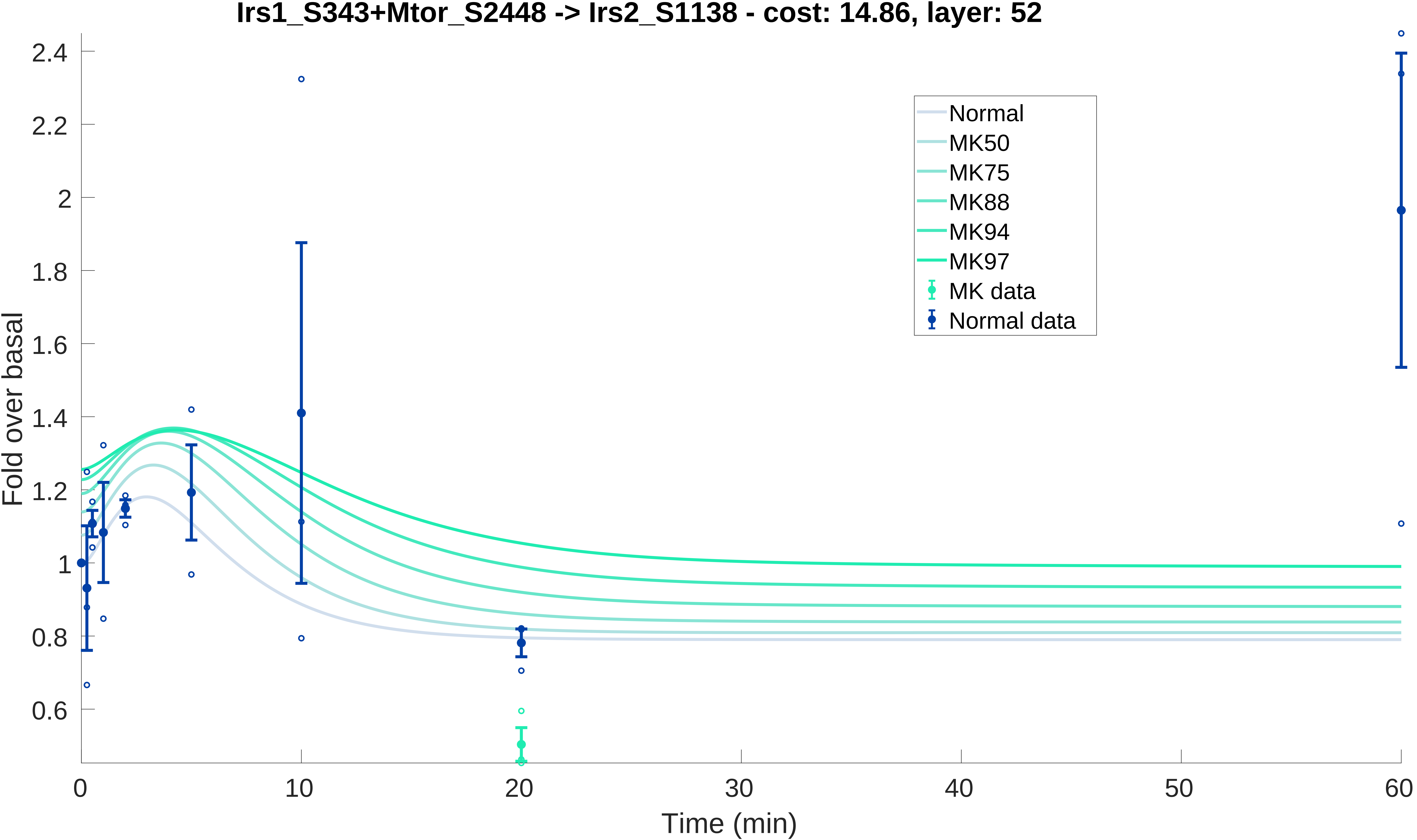

Irs1_S343+Mtor_S2448 -> Irs2_S1138 - cost: 14.86, layer: 52
Fold over basal
Time (min)
0.6
0.8
1
1.2
1.4
1.6
1.8
2
2.2
2.4
0
10
20
30
40
50
60
Normal
MK50
MK75
MK88
MK94
MK97
MK data
Normal data

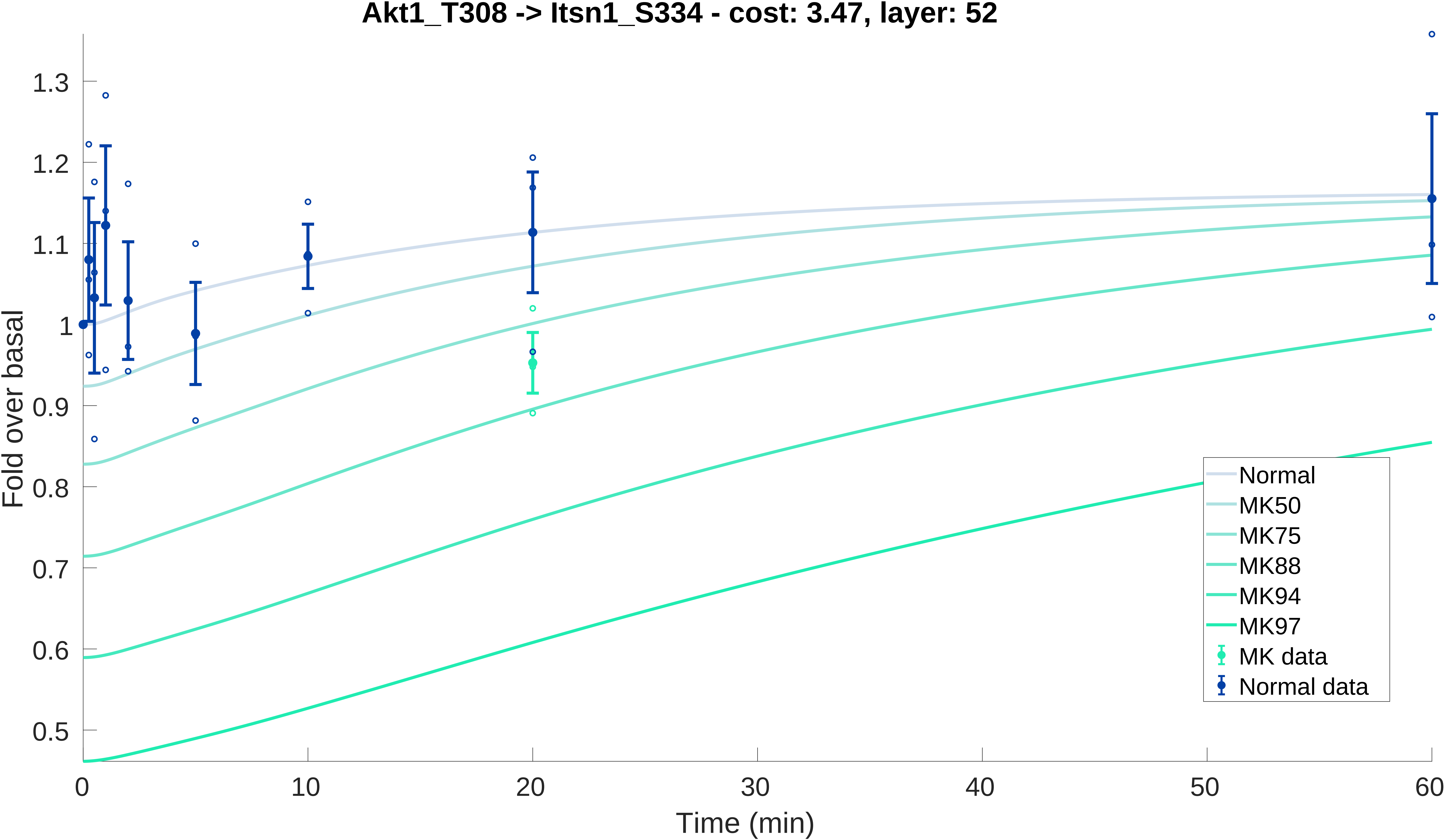
Akt1_T308 -> Itsn1_S334 - cost: 3.47, layer: 52
Fold over basal
Time (min)
0
10
20
30
40
50
60
0.5
0.6
0.7
0.8
0.9
1
1.1
1.2
1.3
Normal
MK50
MK75
MK88
MK94
MK97
MK data
Normal data

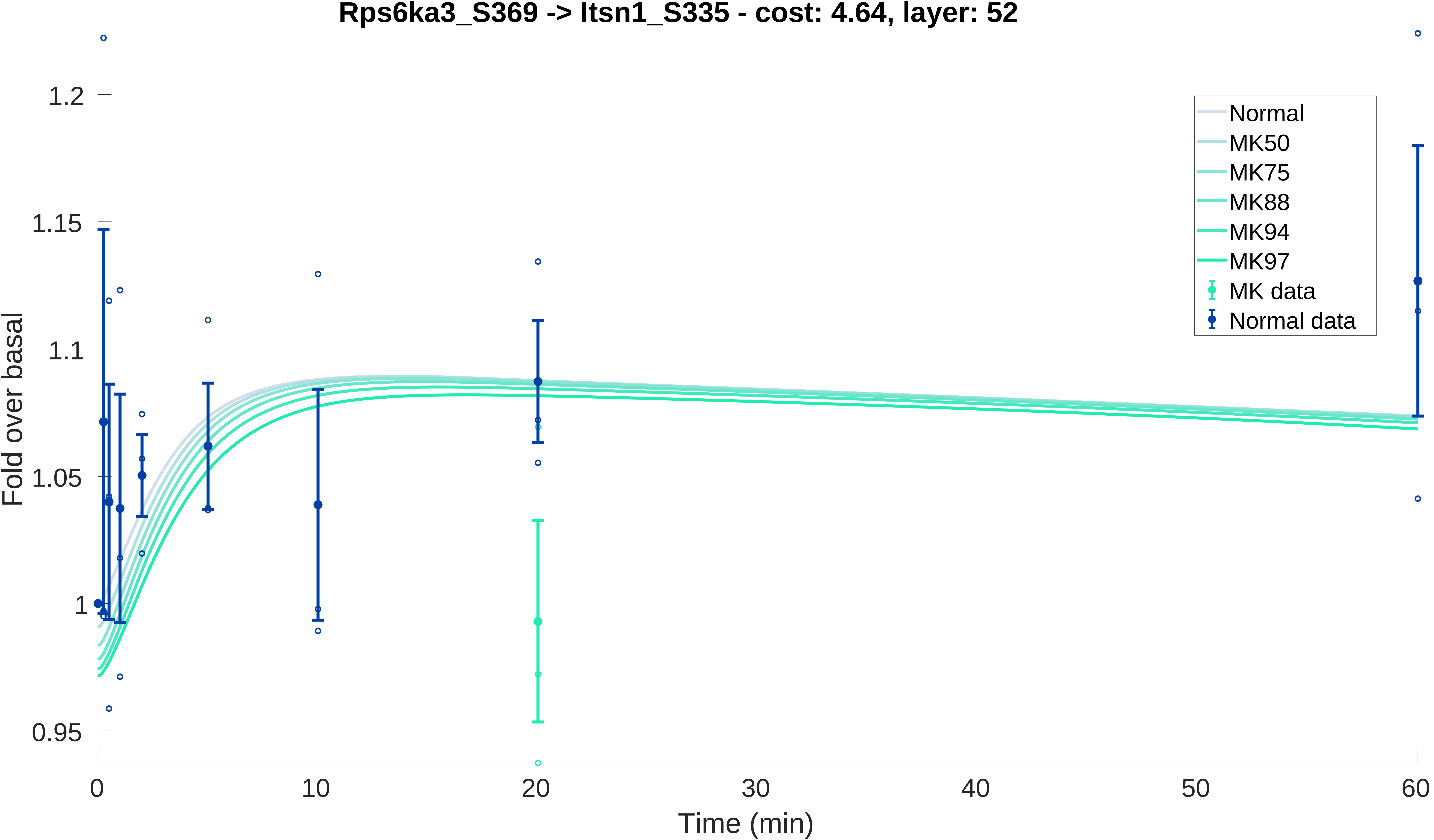

Rps6ka3_S369 -> Itsn1_S335 - cost: 4.64, layer: 52
Fold over basal
Time (min)
0.95
1
1.05
1.1
1.15
1.2
0
10
20
30
40
50
60
Normal
MK50
MK75
MK88
MK94
MK97
MK data
Normal data

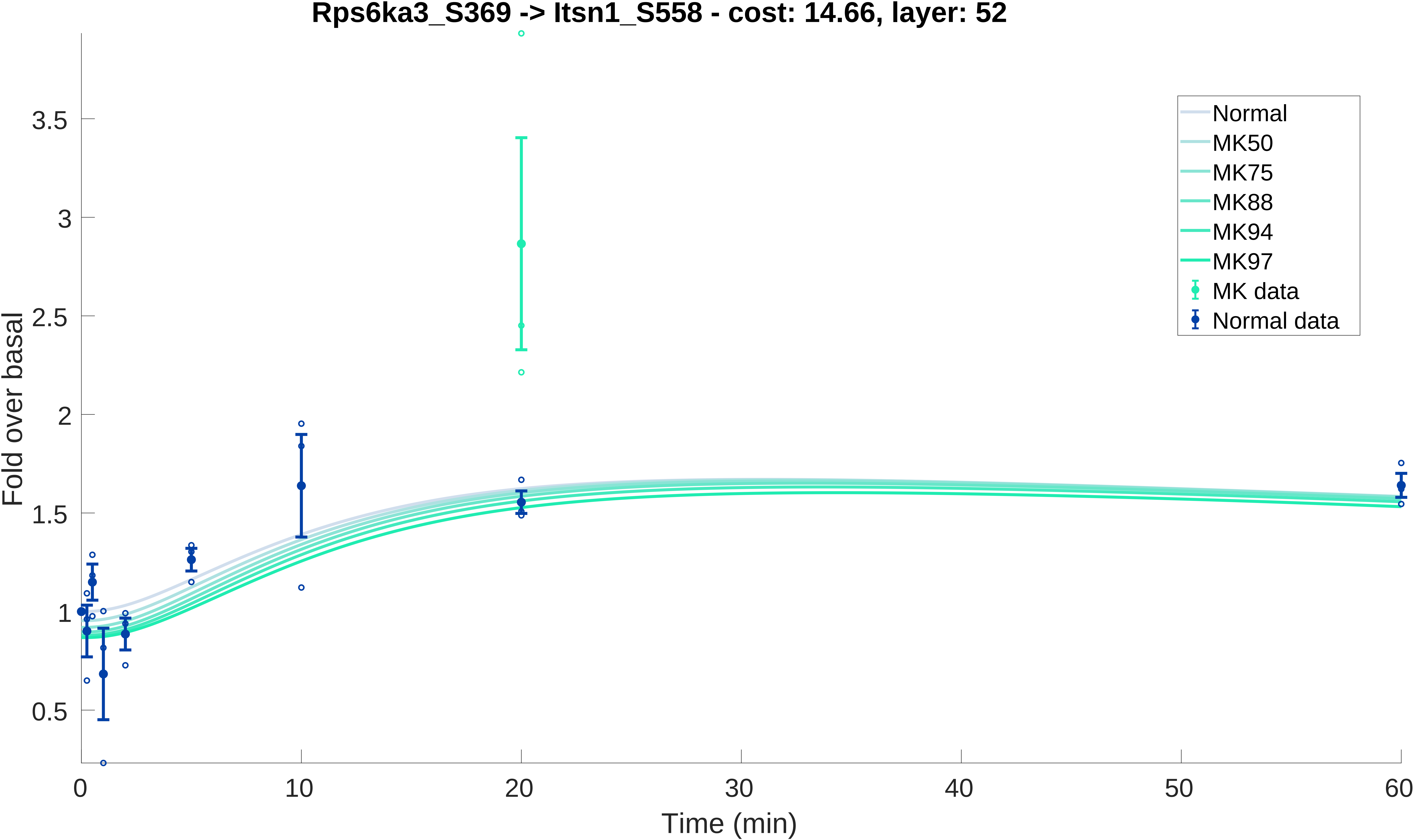
Rps6ka3_S369 -> Itsn1_S558 - cost: 14.66, layer: 52
Fold over basal
0.5
1
1.5
2
2.5
3
3.5
0
10
20
30
40
50
60
Time (min)
Normal
MK50
MK75
MK88
MK94
MK97
MK data
Normal data

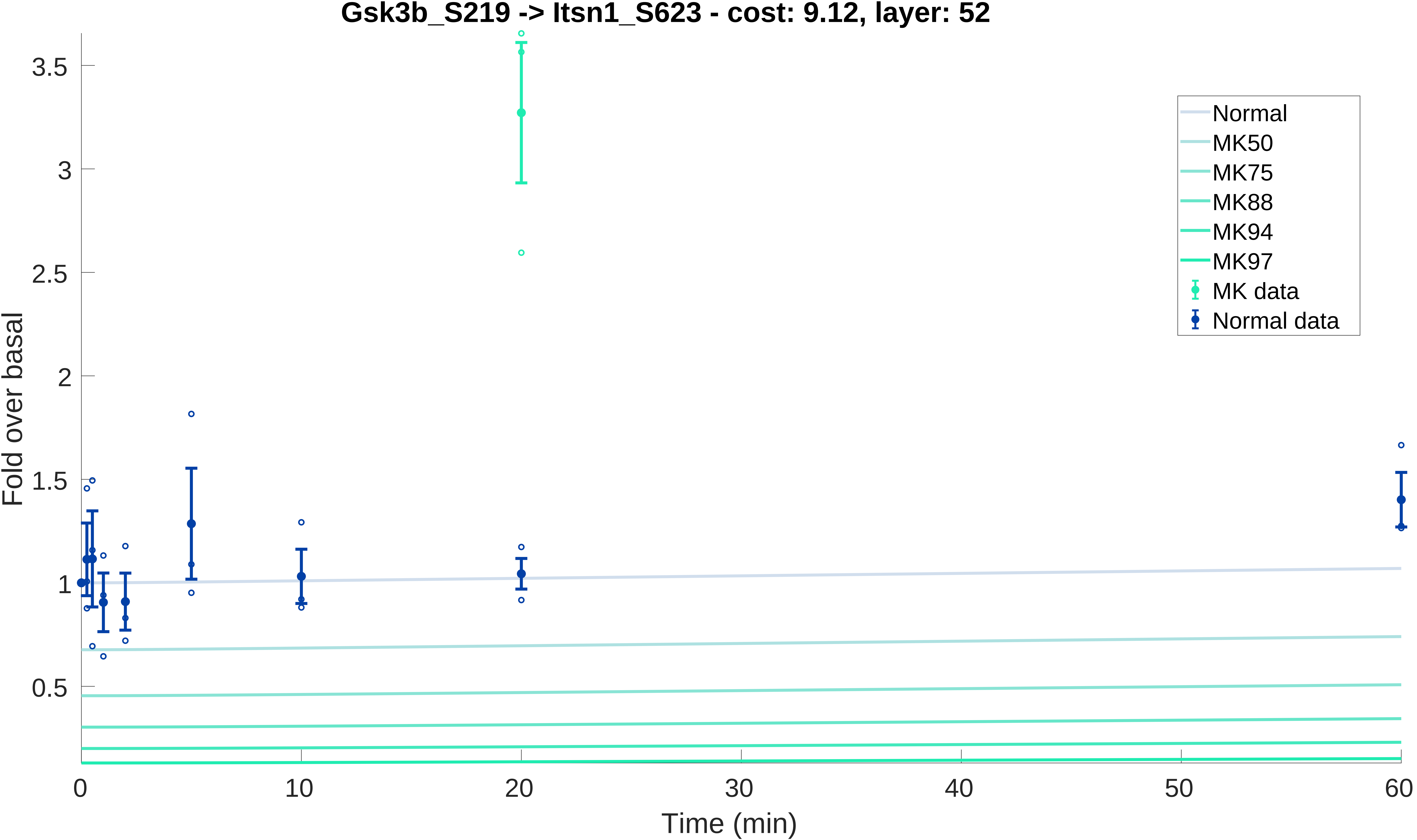

Gsk3b_S219 -> Itsn1_S623 - cost: 9.12, layer: 52
Fold over basal
3.5
3
2.5
2
1.5
1
0.5
0
10
20
30
40
50
60
Time (min)
Normal
MK50
MK75
MK88
MK94
MK97
MK data
Normal data

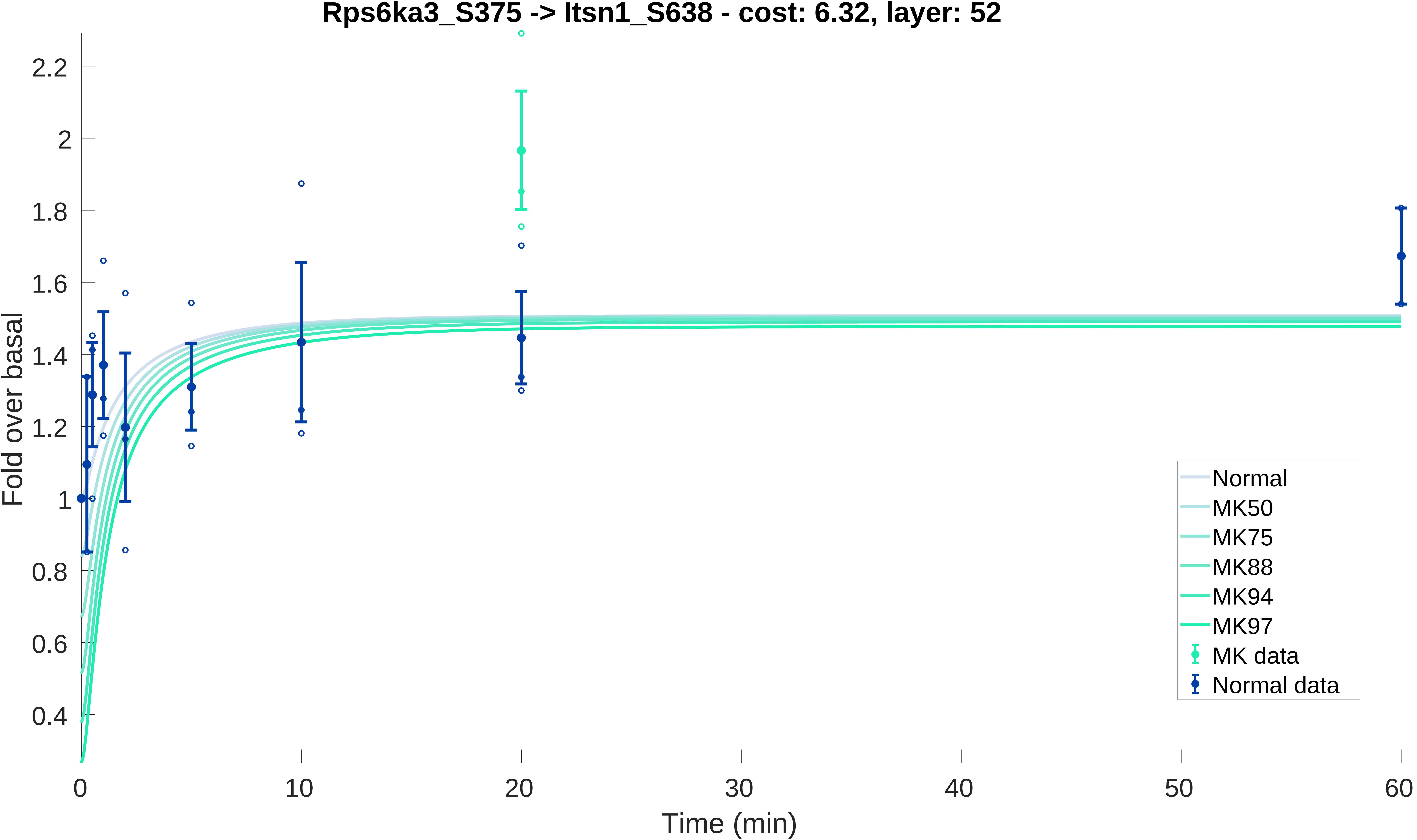

Rps6ka3_S375 -> Itsn1_S638 - cost: 6.32, layer: 52
Fold over basal
Time (min)
0.4
0.6
0.8
1
1.2
1.4
1.6
1.8
2
2.2
0
10
20
30
40
50
60
Normal
MK50
MK75
MK88
MK94
MK97
MK data
Normal data

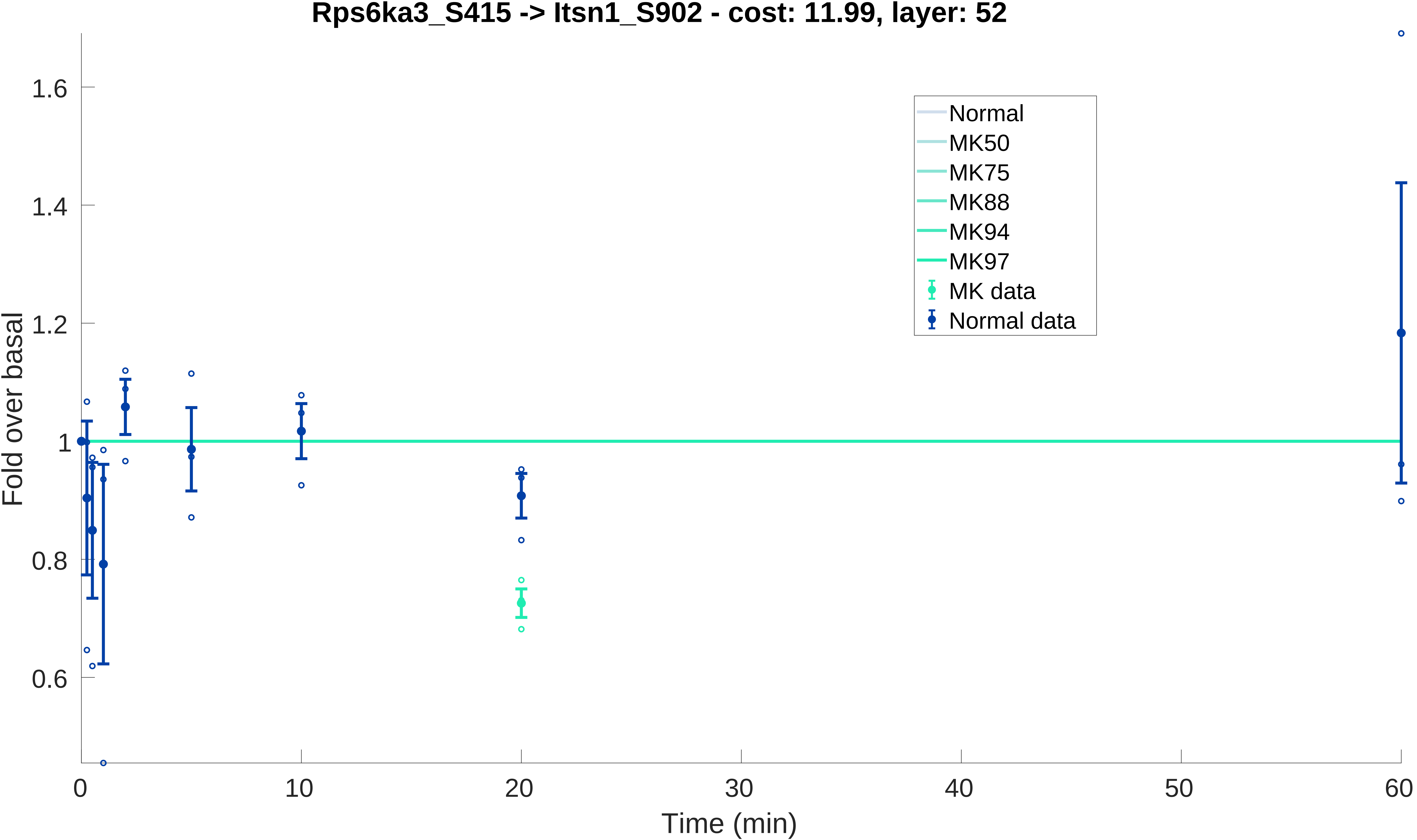

Rps6ka3_S415 -> Itsn1_S902 - cost: 11.99, layer: 52
Fold over basal
Time (min)
0.6
0.8
1
1.2
1.4
1.6
0
10
20
30
40
50
60
Normal
MK50
MK75
MK88
MK94
MK97
MK data
Normal data

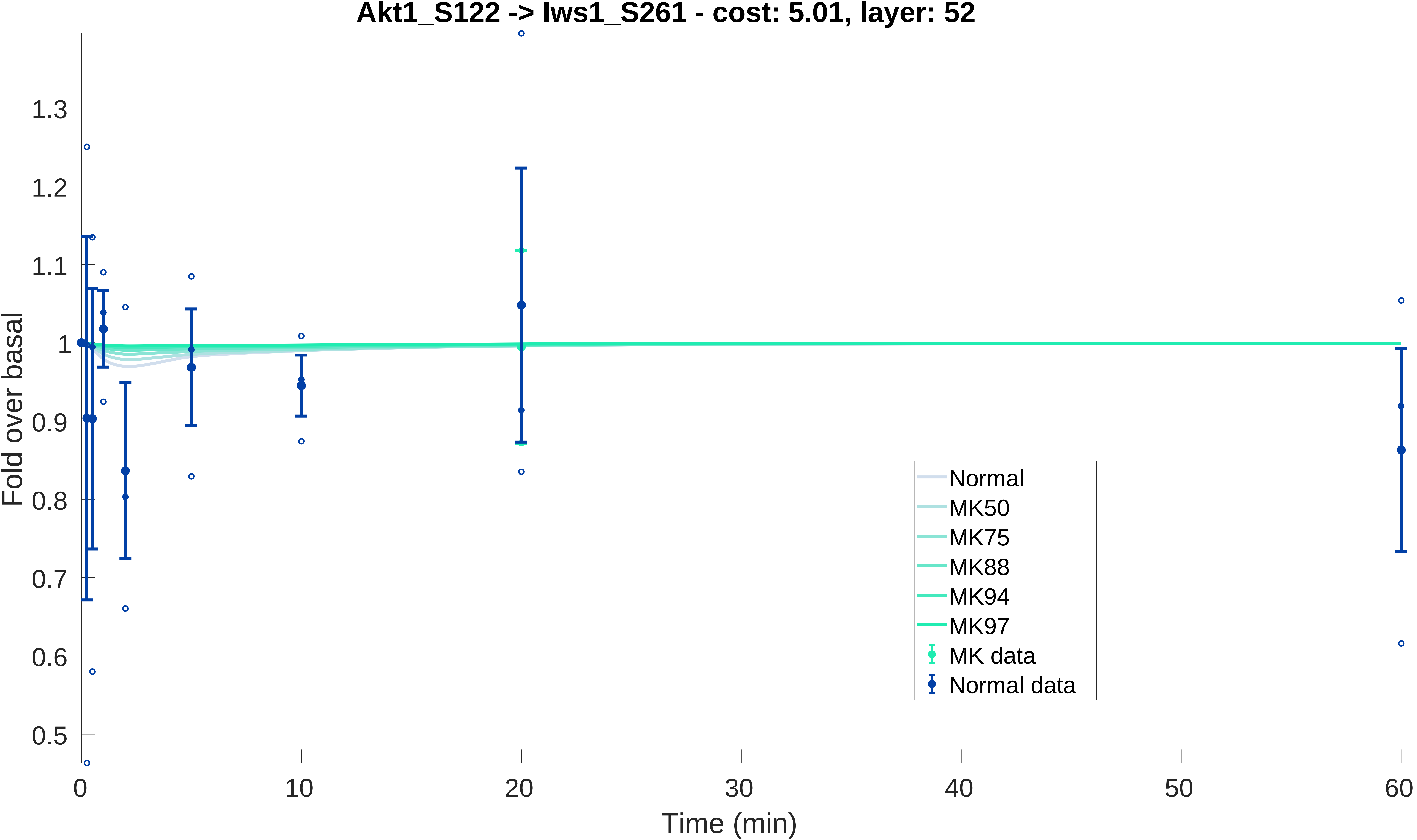
Akt1_S122 -> Iws1_S261 - cost: 5.01, layer: 52
Fold over basal
Time (min)
1.3
1.2
1.1
1
0.9
0.8
0.7
0.6
0.5
0
10
20
30
40
50
60
Normal
MK50
MK75
MK88
MK94
MK97
MK data
Normal data

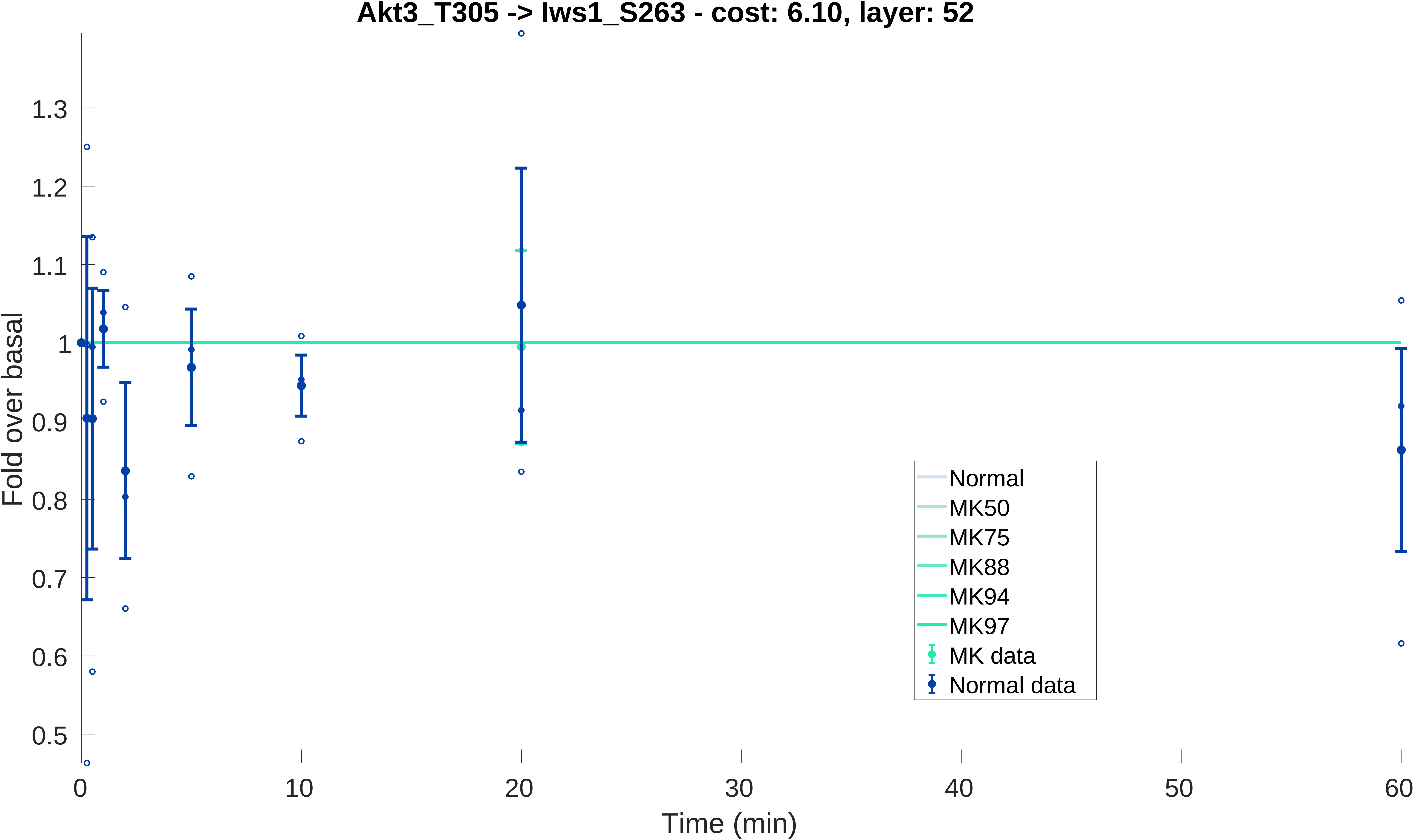

Akt3_T305 -> Iws1_S263 - cost: 6.10, layer: 52
Fold over basal
1.3
1.2
1.1
1
0.9
0.8
0.7
0.6
0.5
0
10
20
30
40
50
60
Time (min)
Normal
MK50
MK75
MK88
MK94
MK97
MK data
Normal data

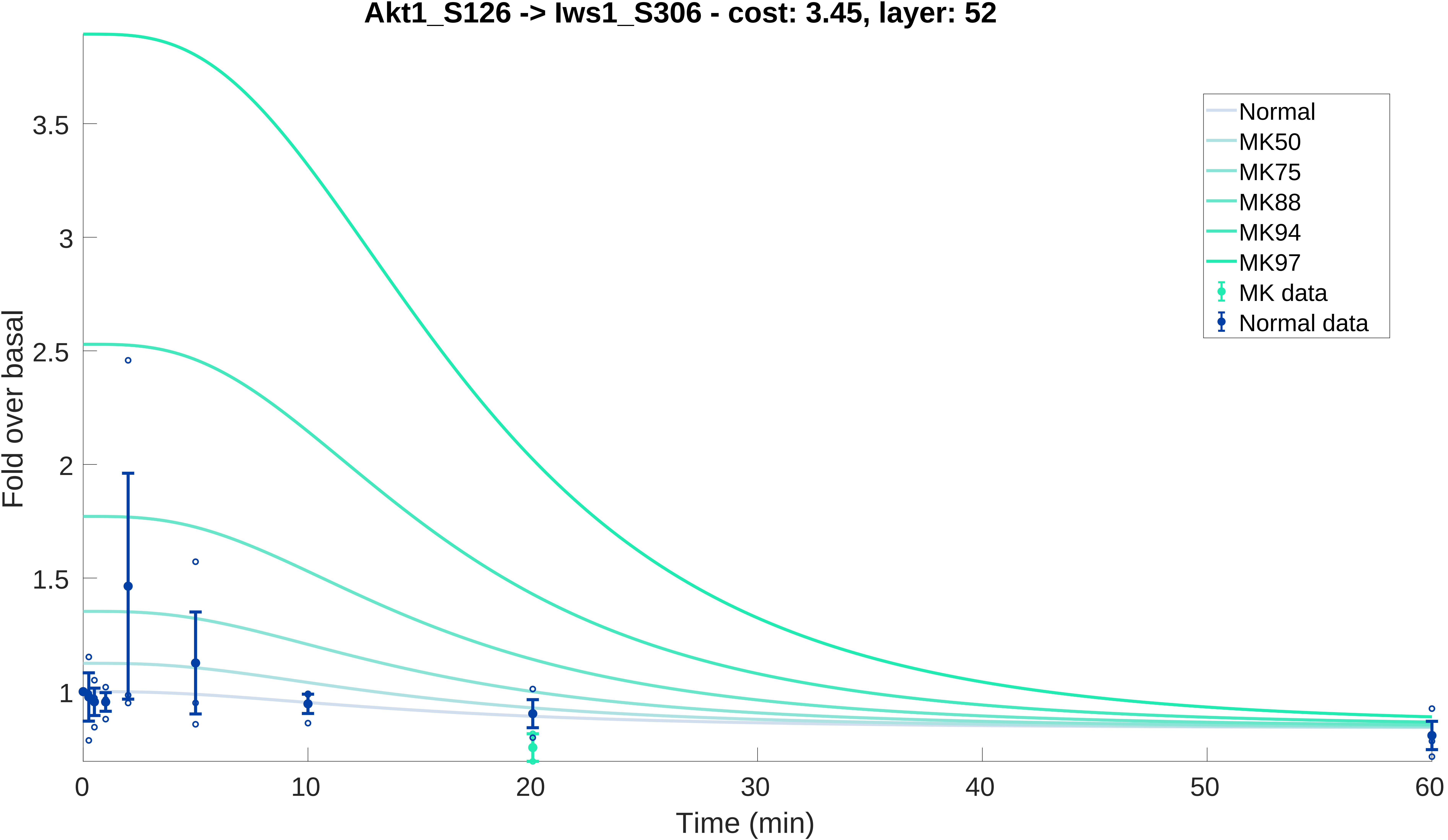
Akt1_S126 -> Iws1_S306 - cost: 3.45, layer: 52
Fold over basal
3.5
3
2.5
2
1.5
1
0
10
20
30
40
50
60
Time (min)
Normal
MK50
MK75
MK88
MK94
MK97
MK data
Normal data

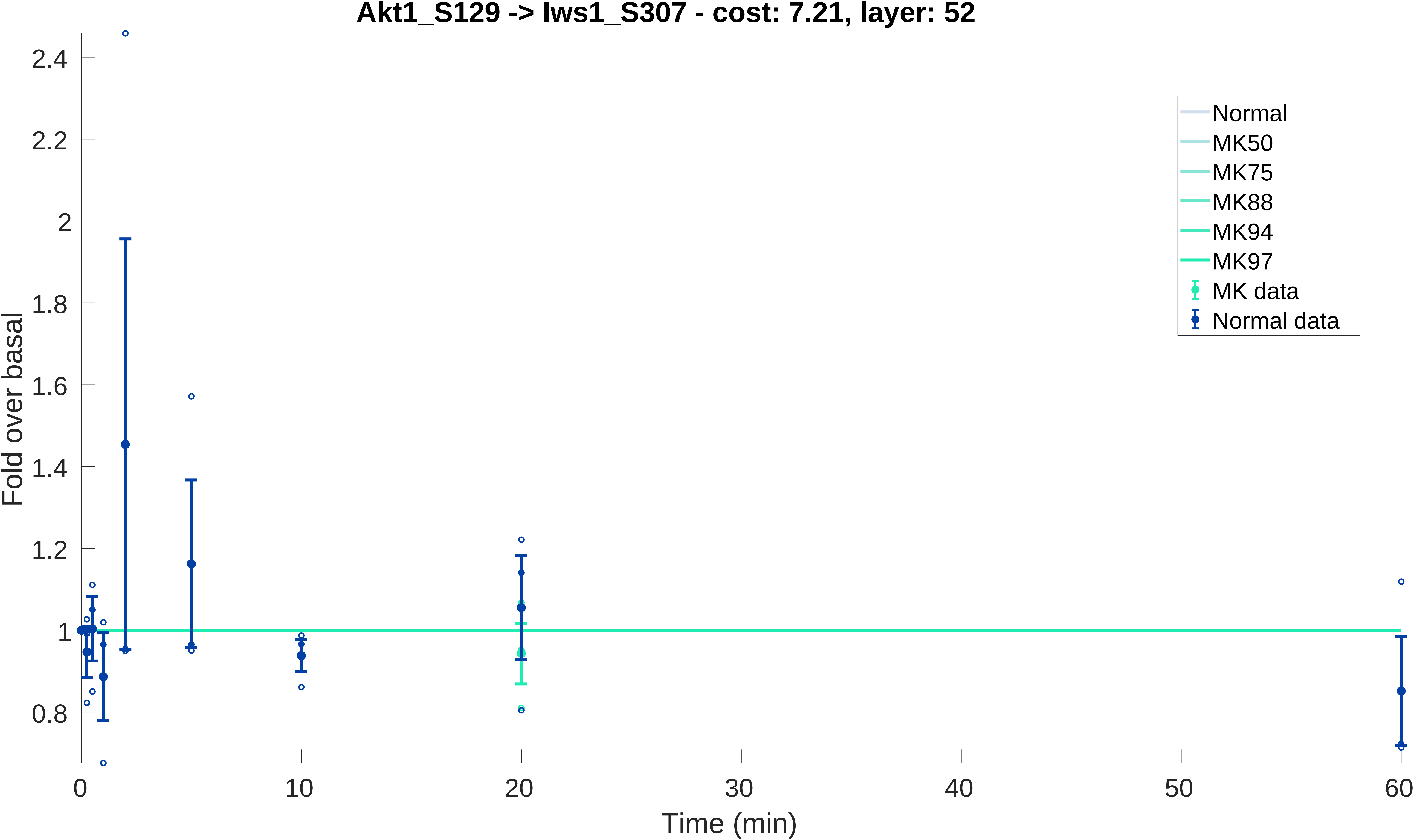

Akt1_S129 -> Iws1_S307 - cost: 7.21, layer: 52
Fold over basal
Time (min)
2.4
2.2
2
1.8
1.6
1.4
1.2
1
0.8
0
10
20
30
40
50
60
Normal
MK50
MK75
MK88
MK94
MK97
MK data
Normal data

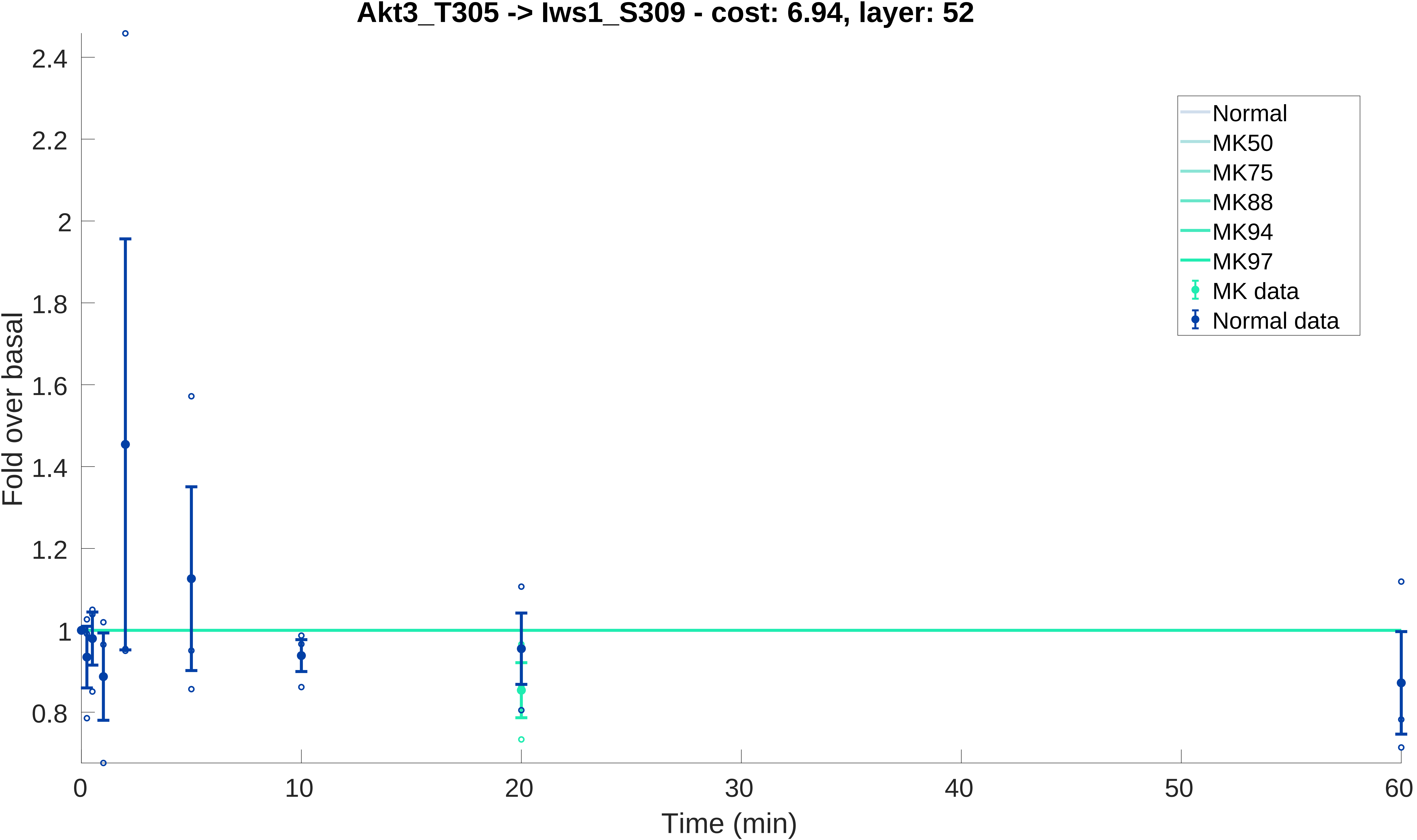

Akt3_T305 -> Iws1_S309 - cost: 6.94, layer: 52
Fold over basal
Time (min)
2.4
2.2
2
1.8
1.6
1.4
1.2
1
0.8
0
10
20
30
40
50
60
Normal
MK50
MK75
MK88
MK94
MK97
MK data
Normal data

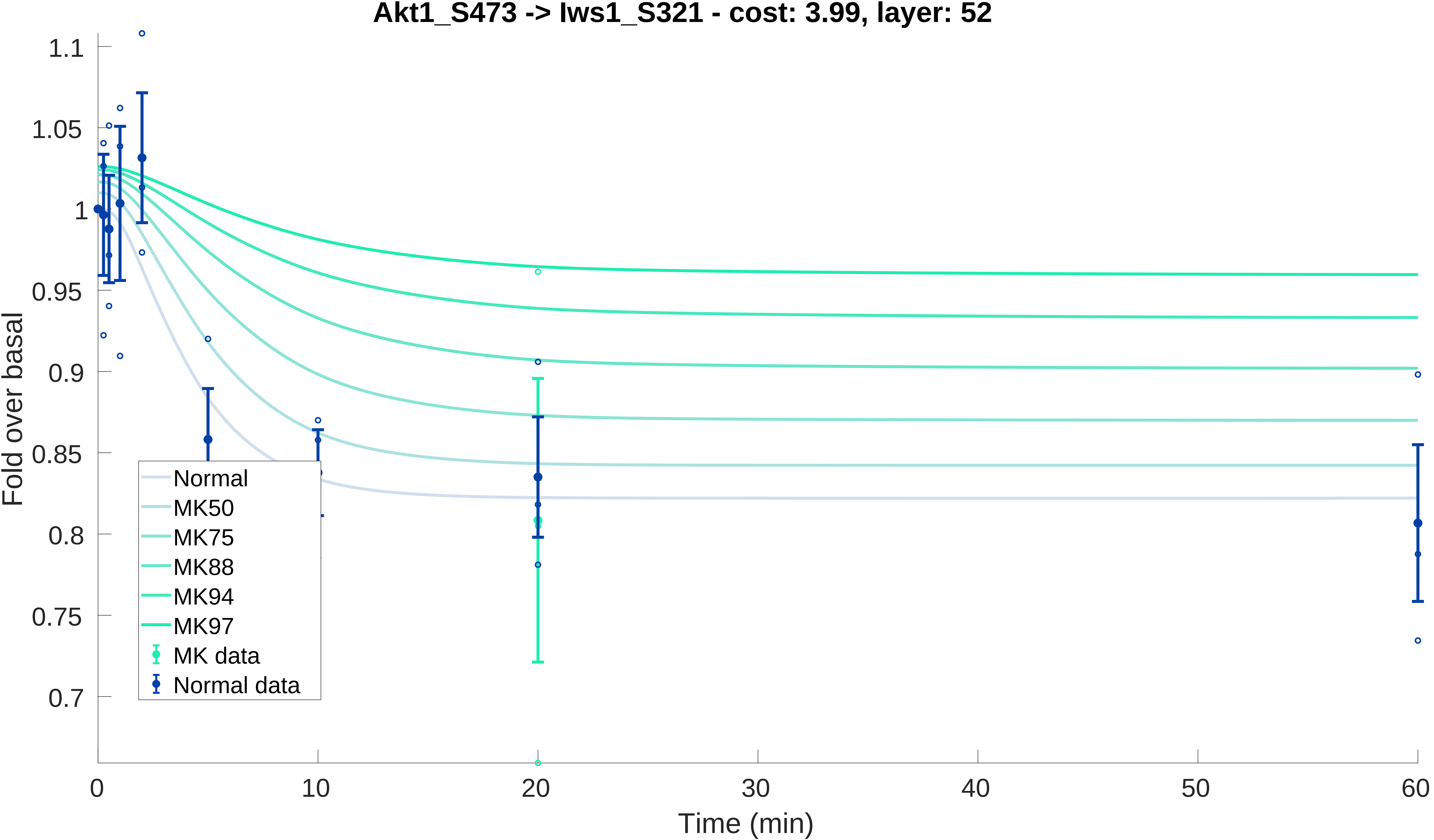

Akt1_S473 -> Iws1_S321 - cost: 3.99, layer: 52
Fold over basal
1.1
1.05
1
0.95
0.9
0.85
0.8
0.75
0.7
Time (min)
0
10
20
30
40
50
60
Normal
MK50
MK75
MK88
MK94
MK97
MK data
Normal data

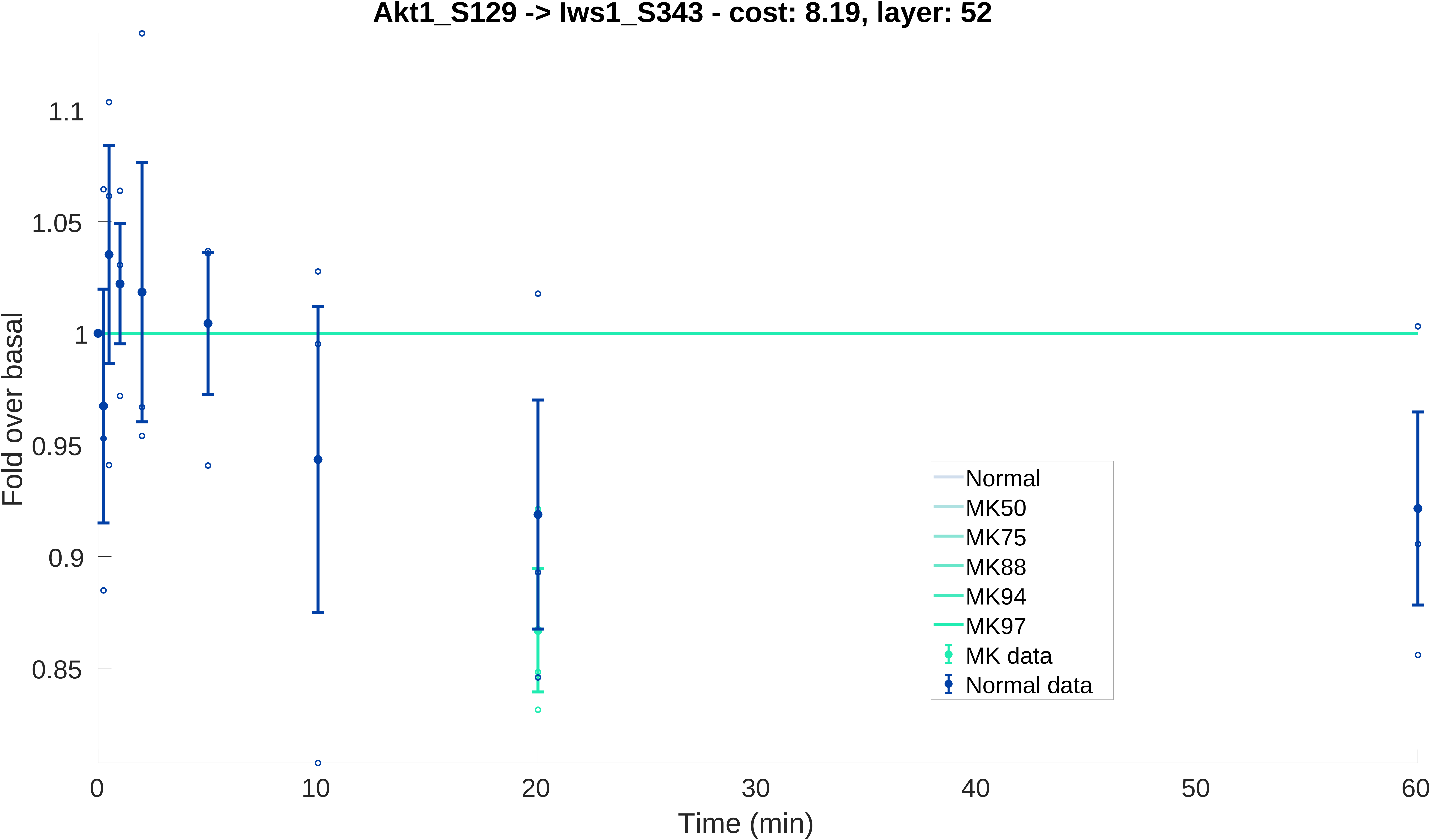

Akt1_S129 -> Iws1_S343 - cost: 8.19, layer: 52
Fold over basal
1.1
1.05
1
0.95
0.9
0.85
0
10
20
30
40
50
60
Time (min)
Normal
MK50
MK75
MK88
MK94
MK97
MK data
Normal data

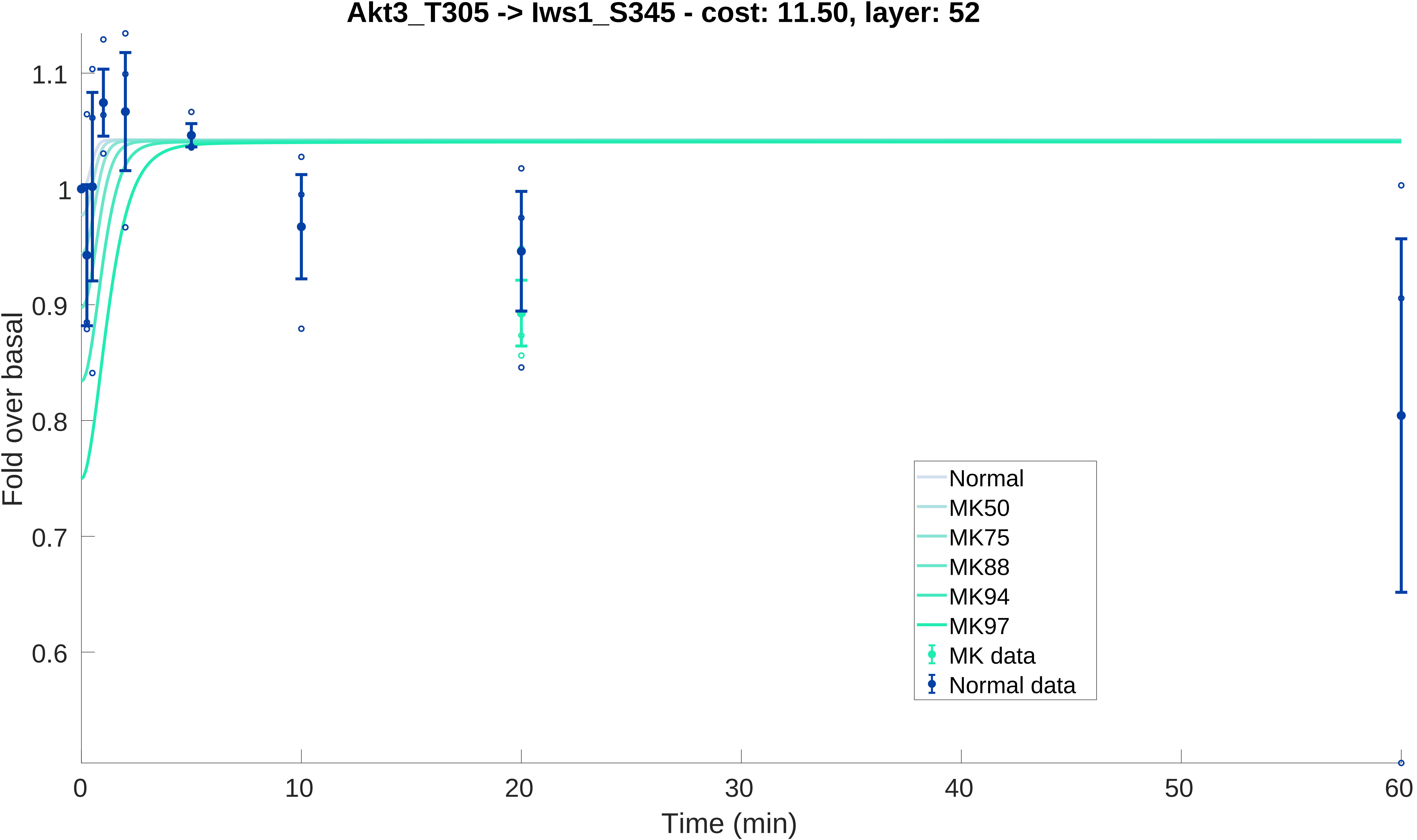

Akt3_T305 -> Iws1_S345 - cost: 11.50, layer: 52
Fold over basal
Time (min)
0.6
0.7
0.8
0.9
1
1.1
0
10
20
30
40
50
60
Normal
MK50
MK75
MK88
MK94
MK97
MK data
Normal data

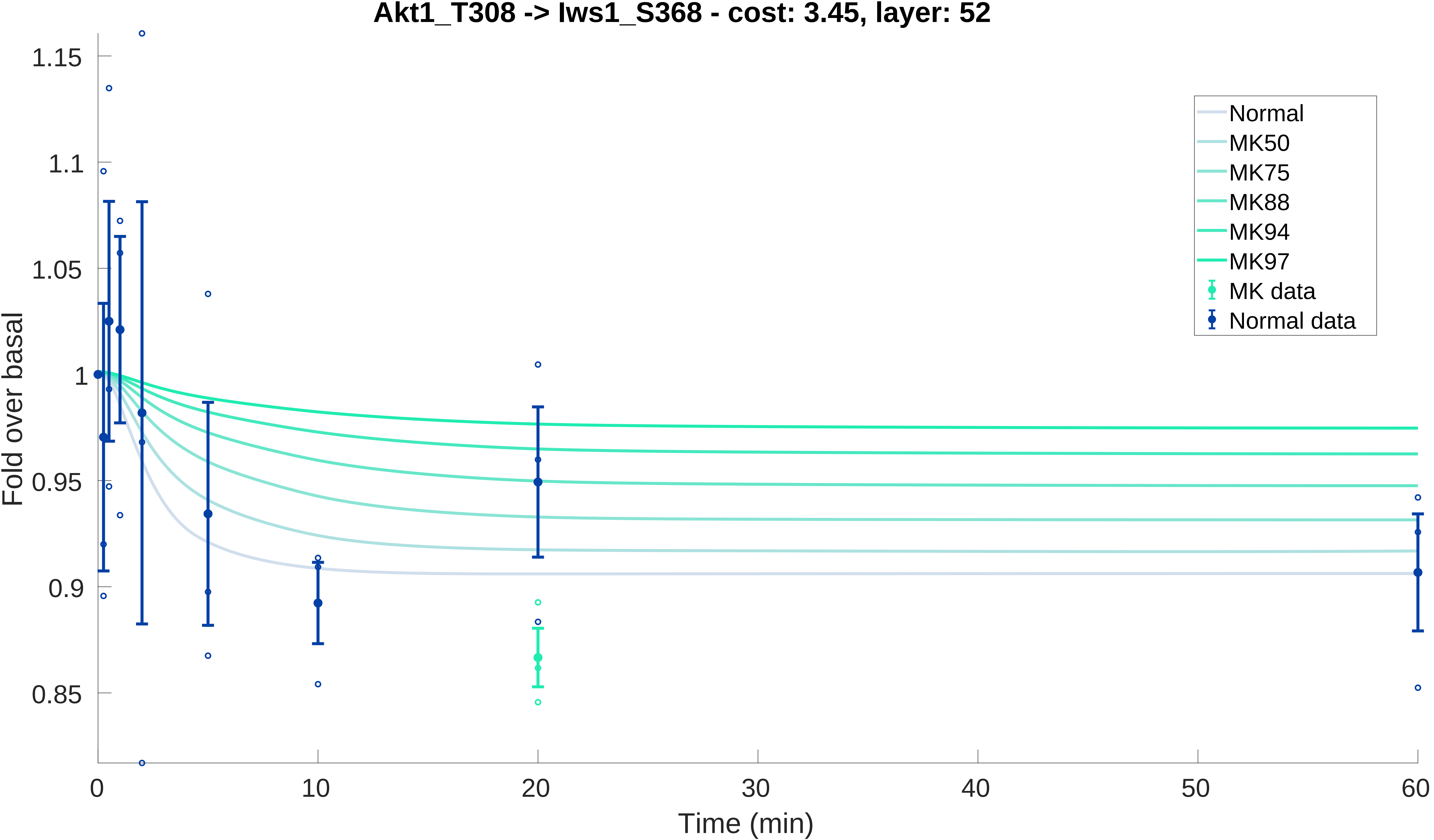
Akt1_T308 -> Iws1_S368 - cost: 3.45, layer: 52
Fold over basal
Time (min)
0
10
20
30
40
50
60
0.85
0.9
0.95
1
1.05
1.1
1.15
Normal
MK50
MK75
MK88
MK94
MK97
MK data
Normal data

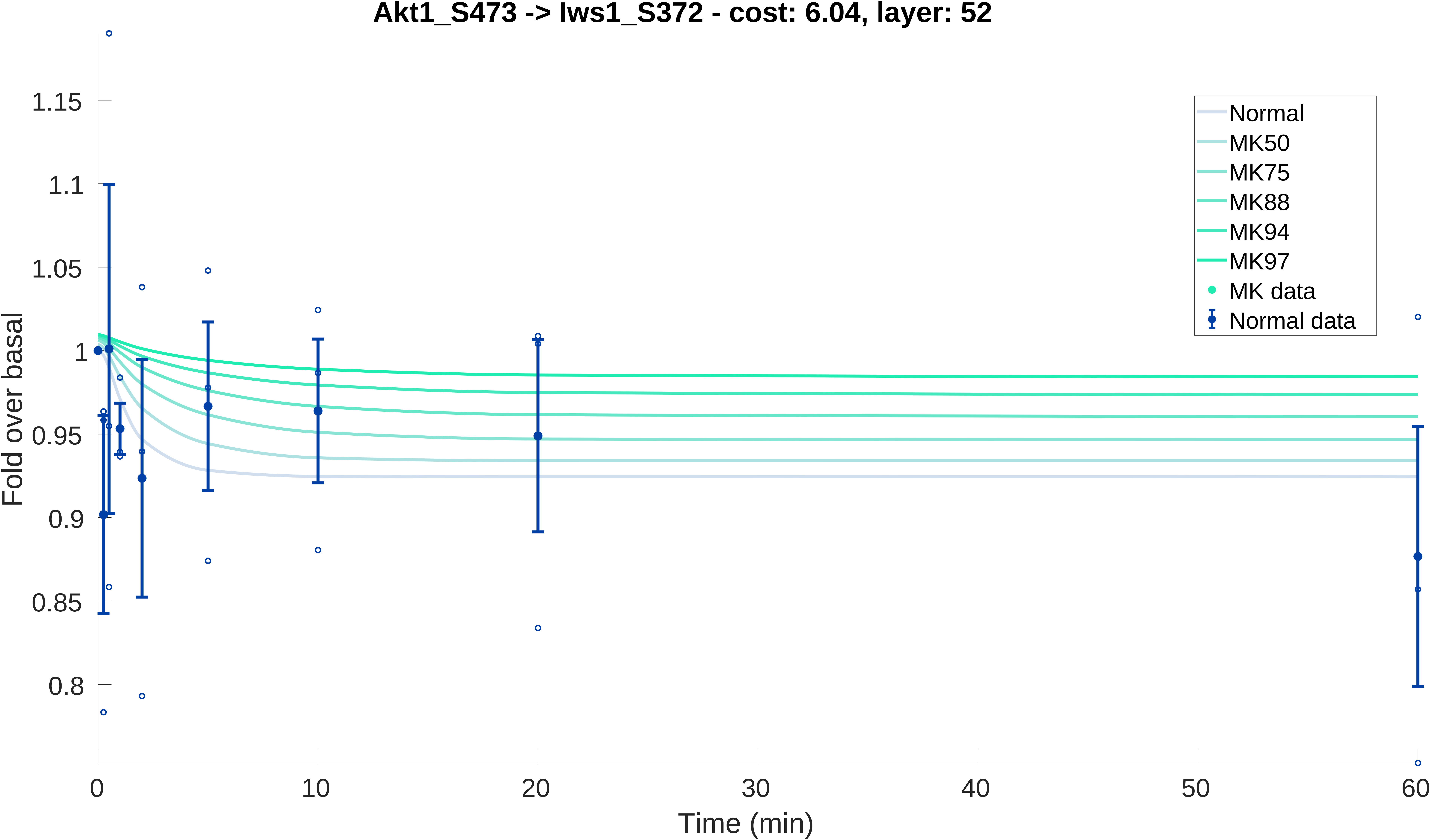

Akt1_S473 -> Iws1_S372 - cost: 6.04, layer: 52
Fold over basal
1.15
1.1
1.05
1
0.95
0.9
0.85
0.8
Time (min)
0
10
20
30
40
50
60
Normal
MK50
MK75
MK88
MK94
MK97
MK data
Normal data

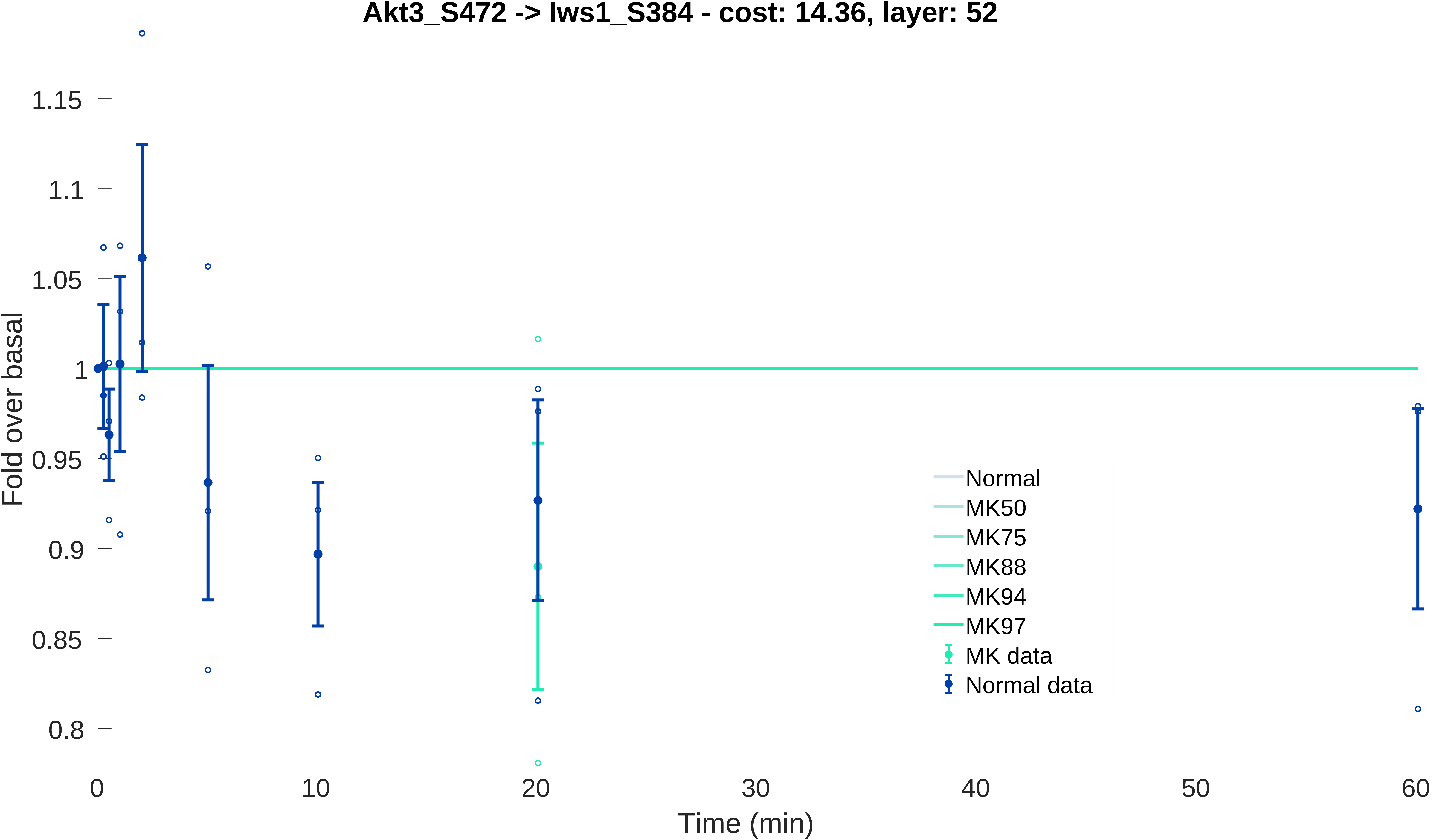
Akt3_S472 -> Iws1_S384 - cost: 14.36, layer: 52
Fold over basal
1.15
1.1
1.05
1
0.95
0.9
0.85
0.8
0
10
20
30
40
50
60
Time (min)
Normal
MK50
MK75
MK88
MK94
MK97
MK data
Normal data

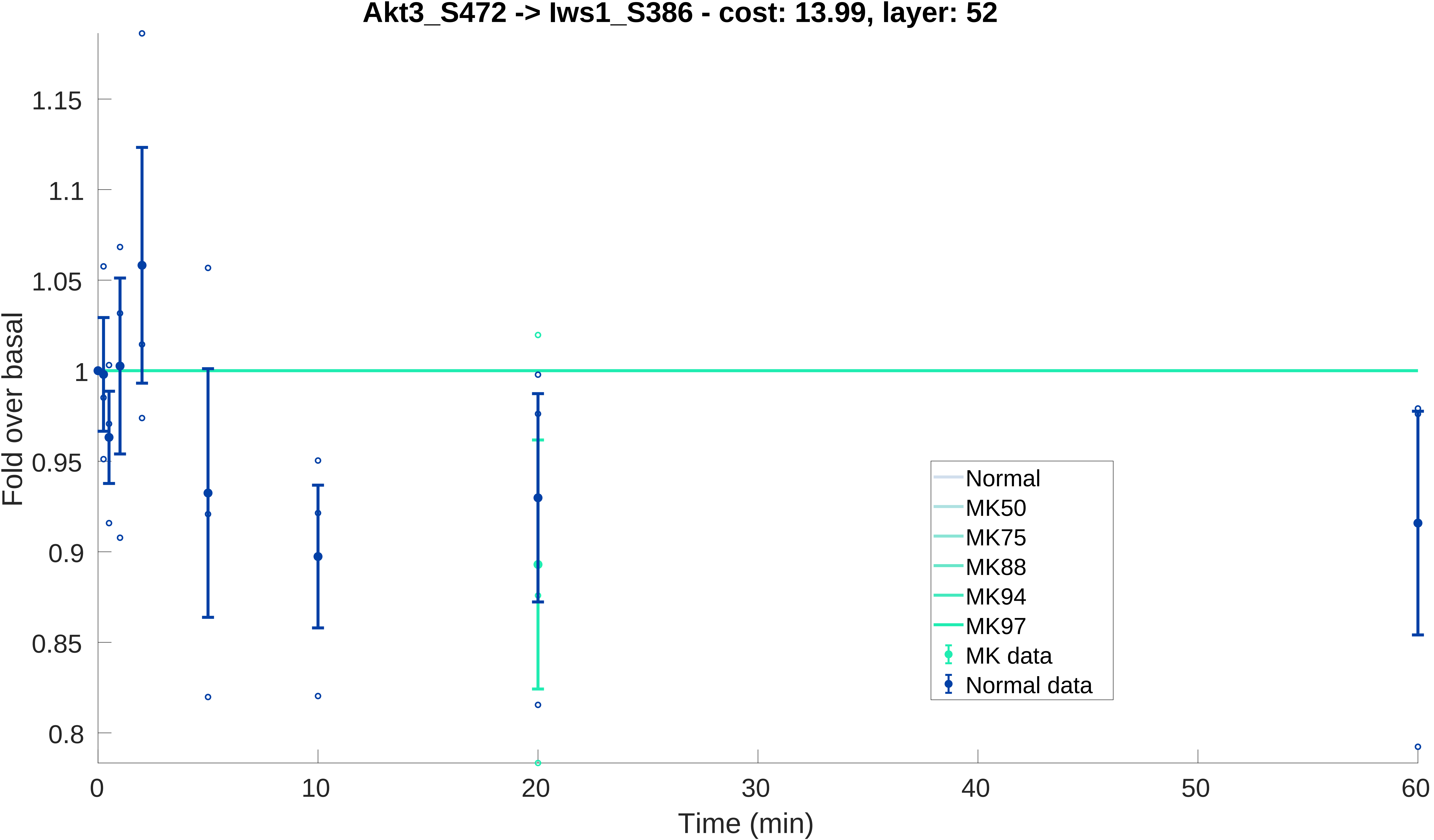

Akt3_S472 -> Iws1_S386 - cost: 13.99, layer: 52
Fold over basal
Time (min)
1.15
1.1
1.05
1
0.95
0.9
0.85
0.8
0
10
20
30
40
50
60
Normal
MK50
MK75
MK88
MK94
MK97
MK data
Normal data

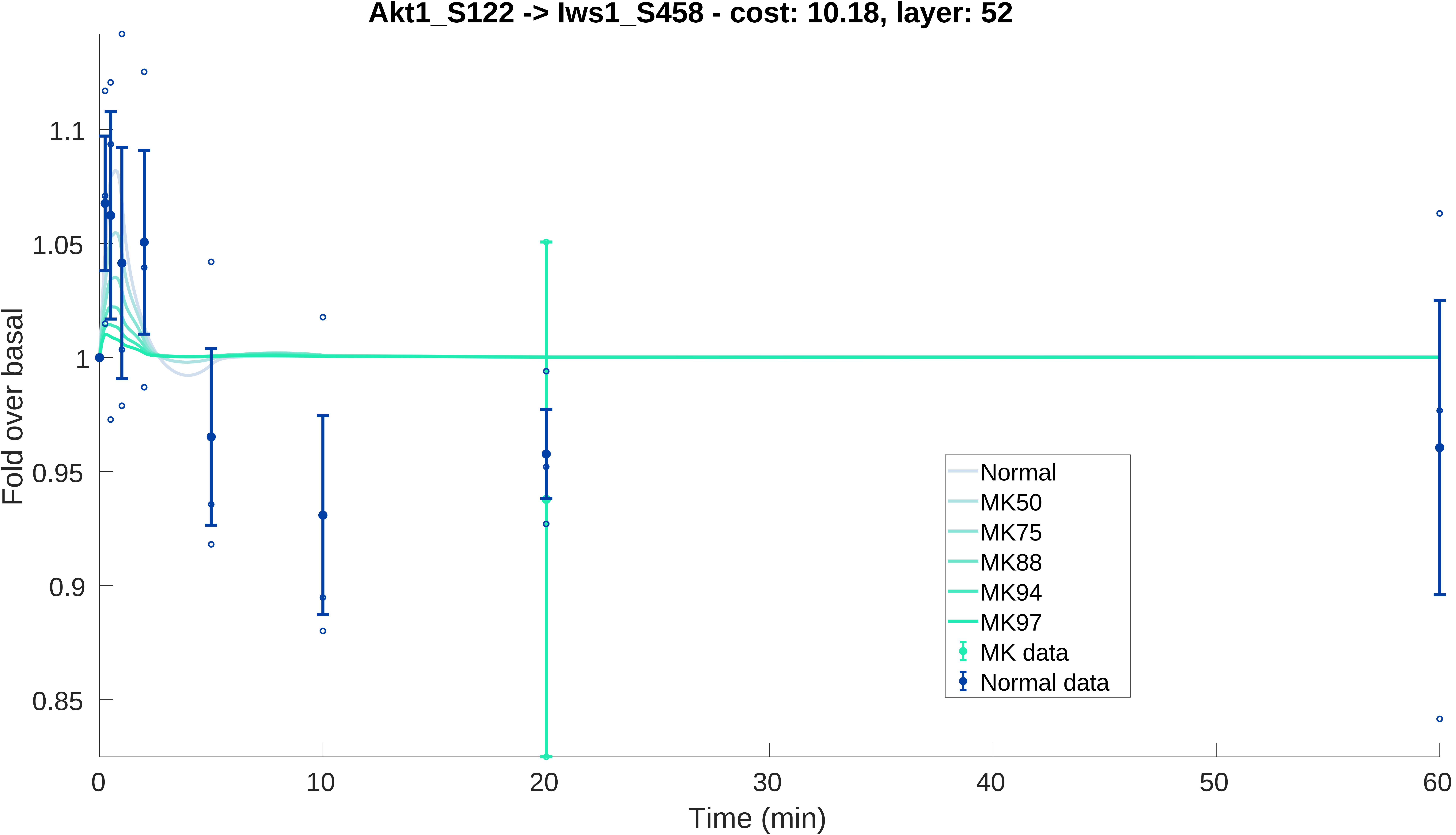

Akt1_S122 -> Iws1_S458 - cost: 10.18, layer: 52
1.1
1.05
1
0.95
0.9
0.85
Fold over basal
0
10
20
30
40
50
60
Time (min)
Normal
MK50
MK75
MK88
MK94
MK97
MK data
Normal data

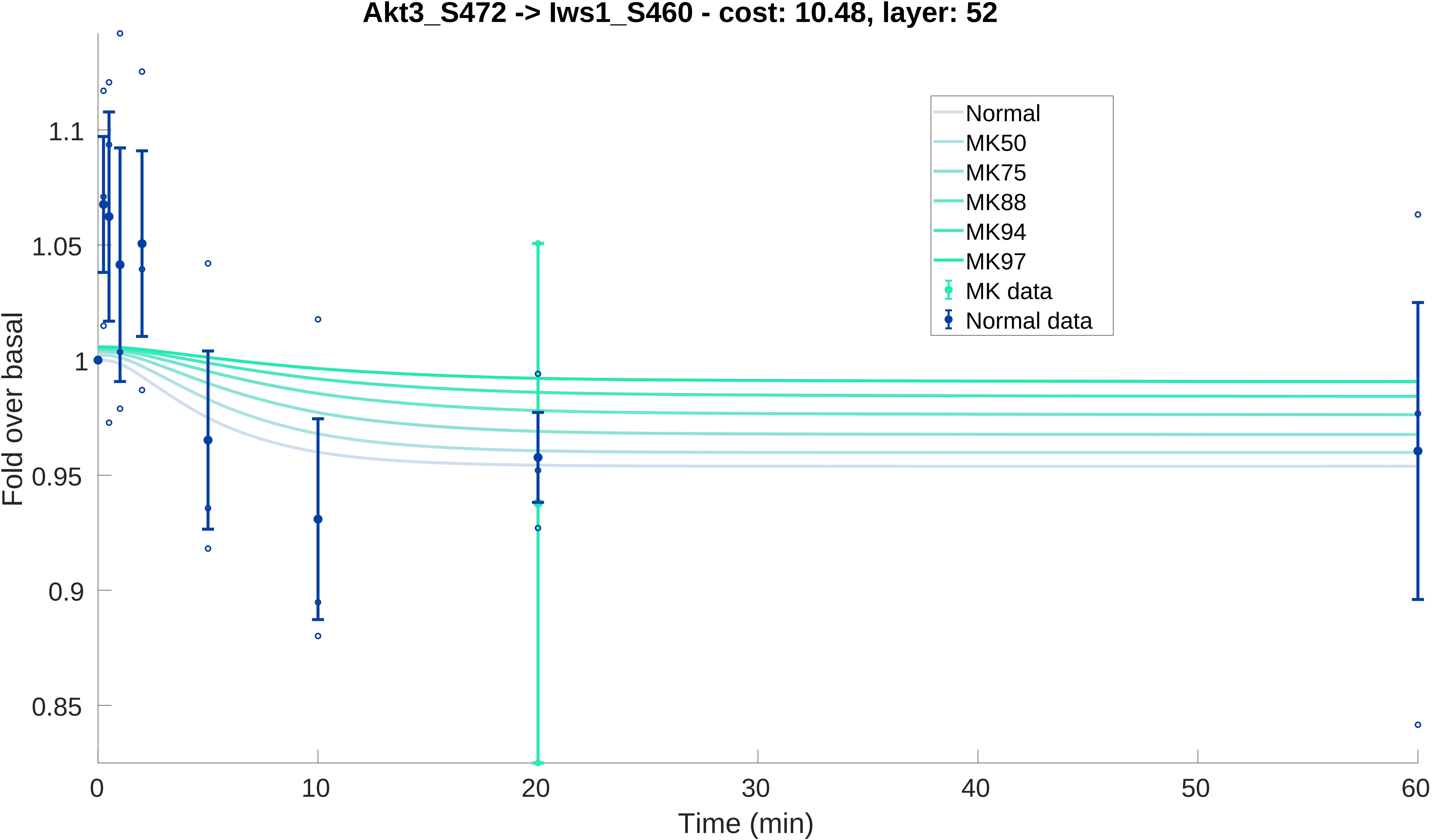

Akt3_S472 -> Iws1_S460 - cost: 10.48, layer: 52
Normal
MK50
MK75
MK88
MK94
MK97
MK data
Normal data
Fold over basal
1.1
1.05
1
0.95
0.9
0.85
0
10
20
30
40
50
60
Time (min)

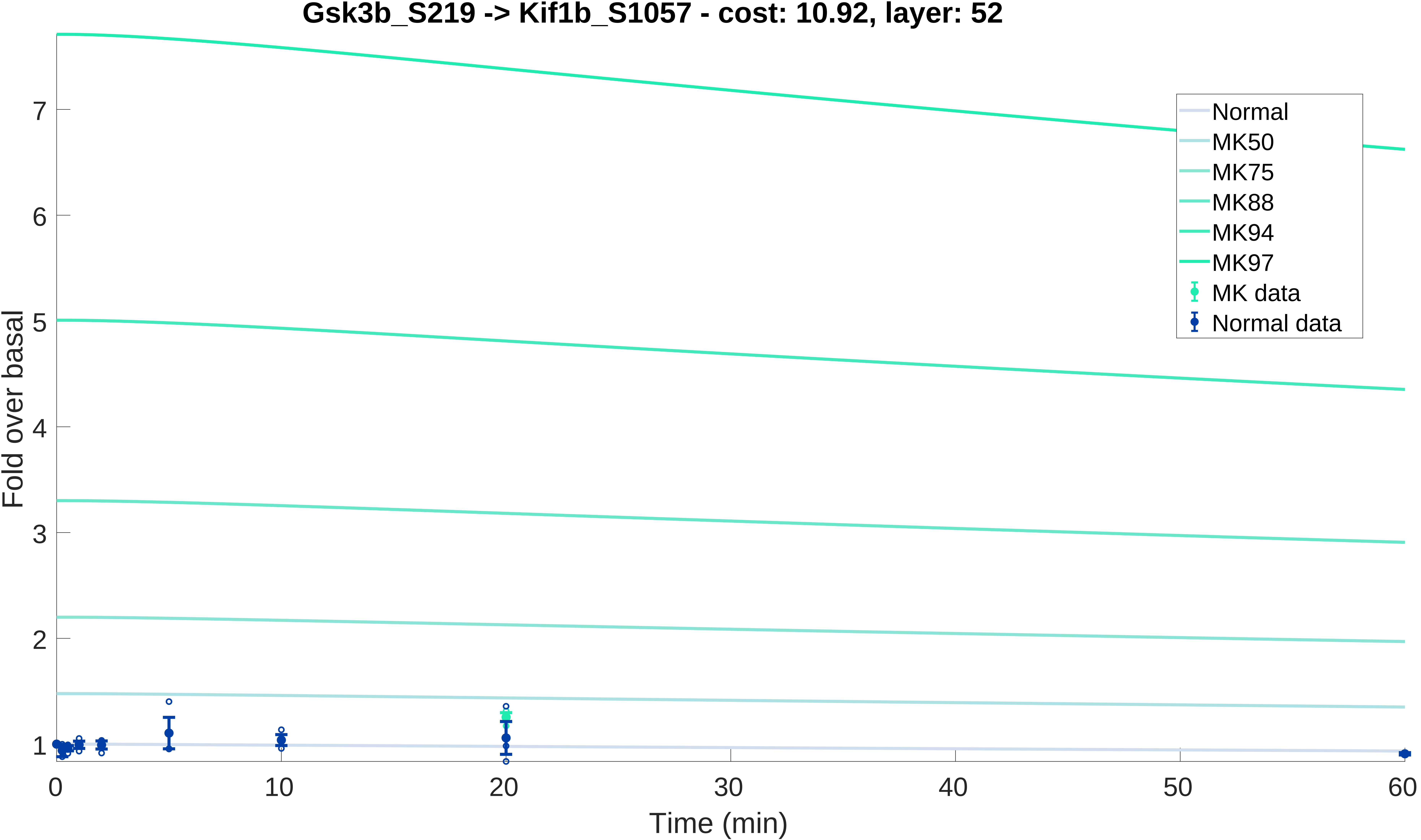

Gsk3b_S219 -> Kif1b_S1057 - cost: 10.92, layer: 52
Fold over basal
Time (min)
1
2
3
4
5
6
7
0
10
20
30
40
50
60
Normal
MK50
MK75
MK88
MK94
MK97
MK data
Normal data

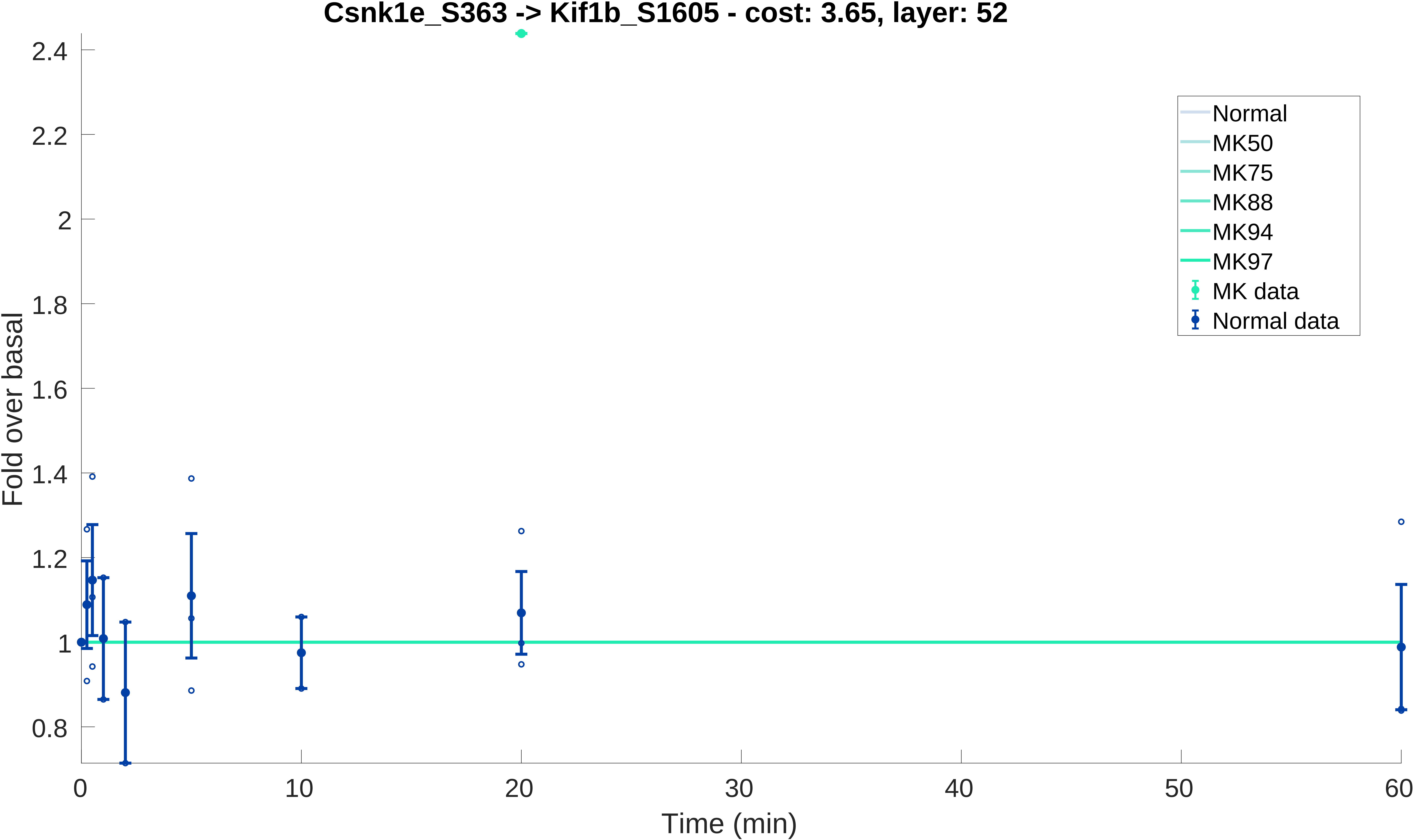

Csnk1e_S363 -> Kif1b_S1605 - cost: 3.65, layer: 52
Fold over basal
Time (min)
0
10
20
30
40
50
60
0.8
1
1.2
1.4
1.6
1.8
2
2.2
2.4
Normal
MK50
MK75
MK88
MK94
MK97
MK data
Normal data

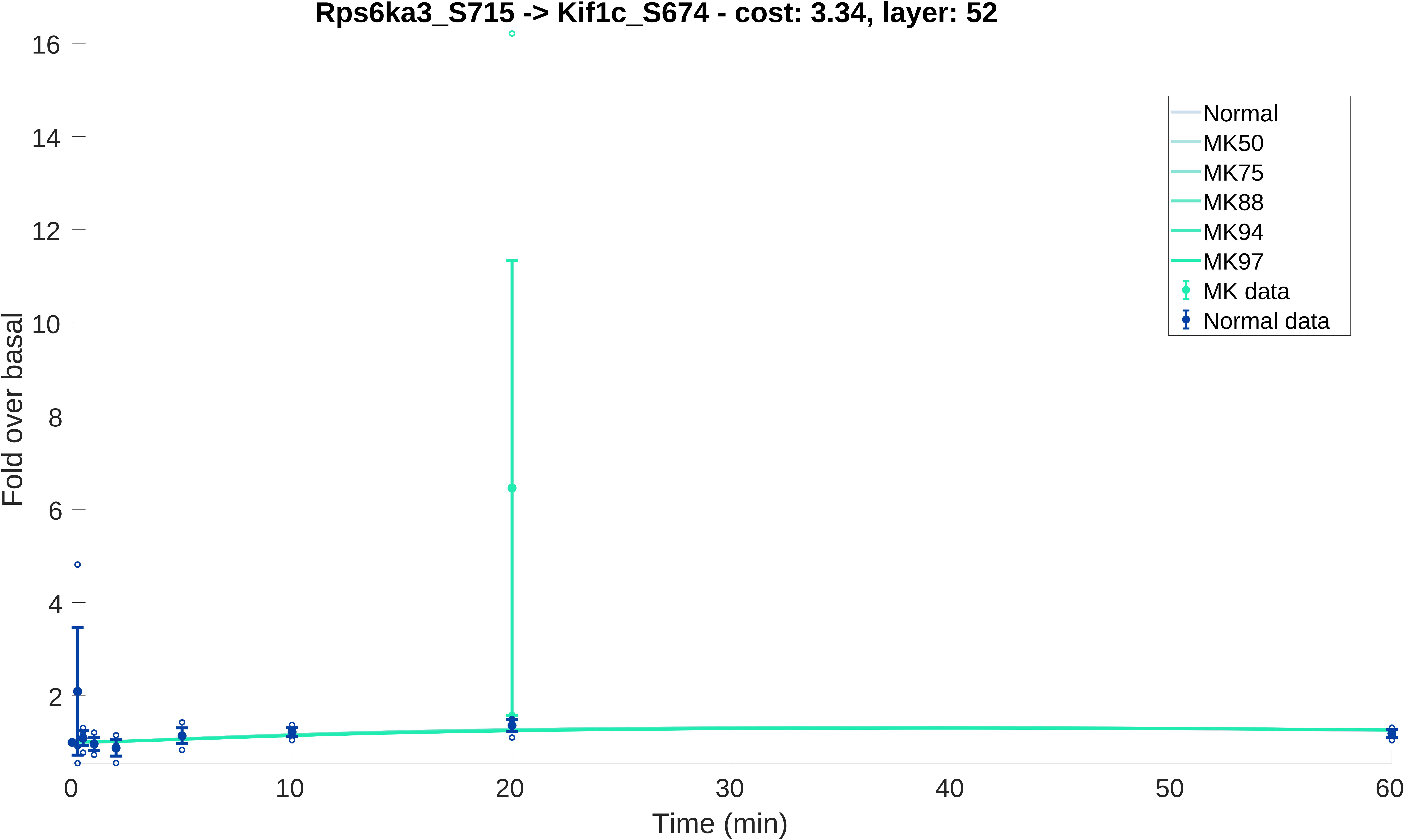

Rps6ka3_S715 -> Kif1c_S674 - cost: 3.34, layer: 52
Fold over basal
Time (min)
0
10
20
30
40
50
60
2
4
6
8
10
12
14
16
Normal
MK50
MK75
MK88
MK94
MK97
MK data
Normal data

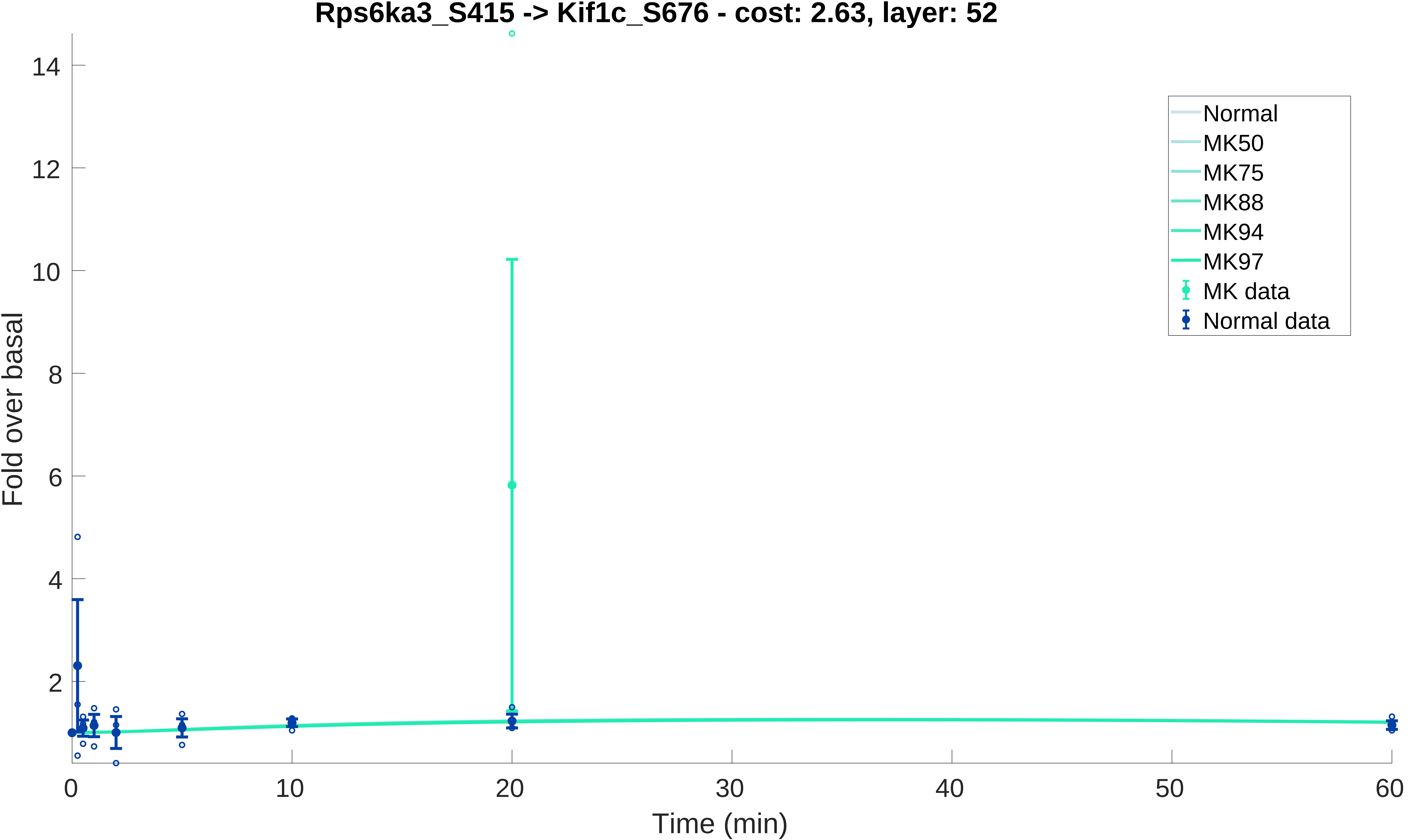

Rps6ka3_S415 -> Kif1c_S676 - cost: 2.63, layer: 52
Fold over basal
Time (min)
0
10
20
30
40
50
60
2
4
6
8
10
12
14
Normal
MK50
MK75
MK88
MK94
MK97
MK data
Normal data

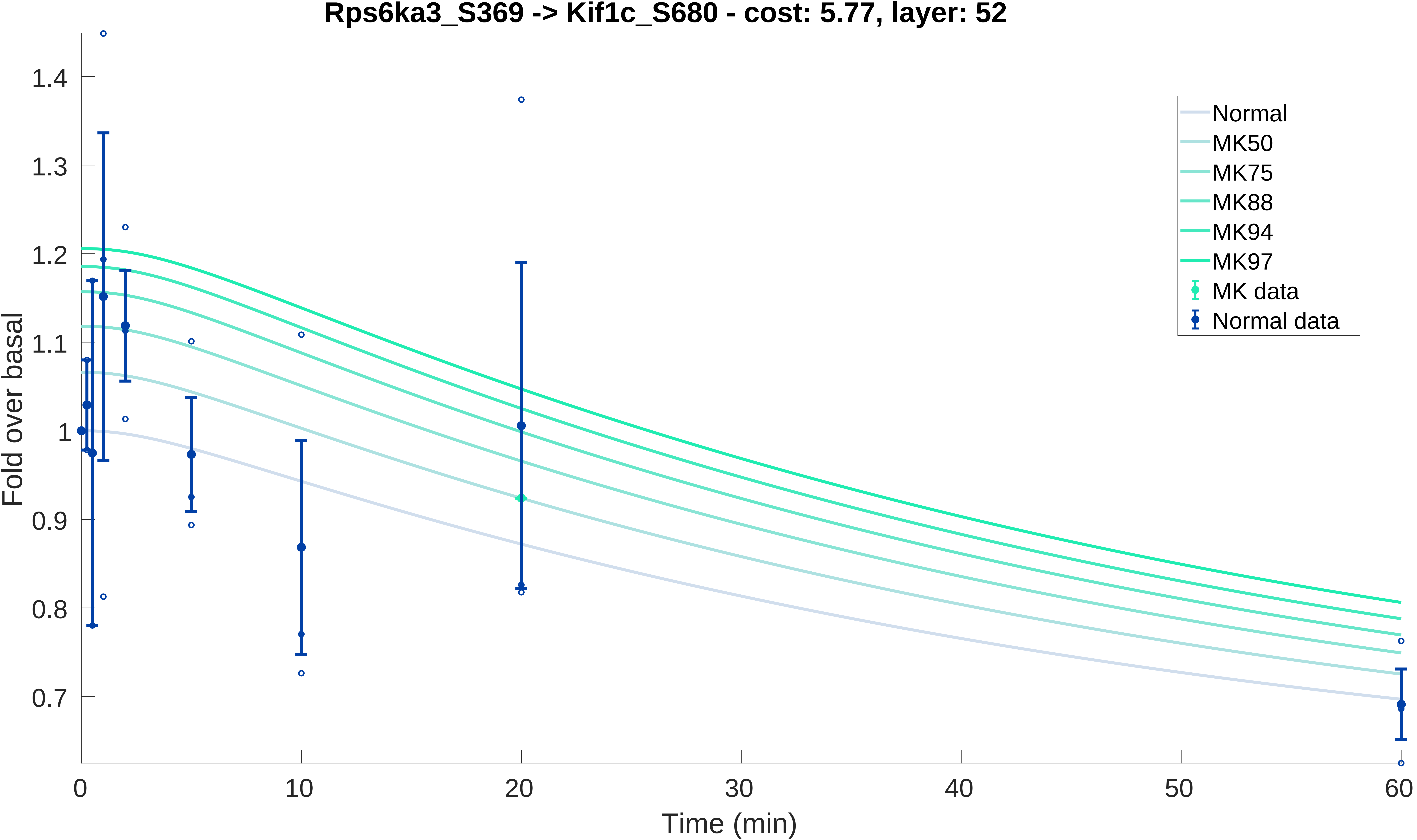

Rps6ka3_S369 -> Kif1c_S680 - cost: 5.77, layer: 52
Fold over basal
Time (min)
1.4
1.3
1.2
1.1
1
0.9
0.8
0.7
0
10
20
30
40
50
60
Normal
MK50
MK75
MK88
MK94
MK97
MK data
Normal data

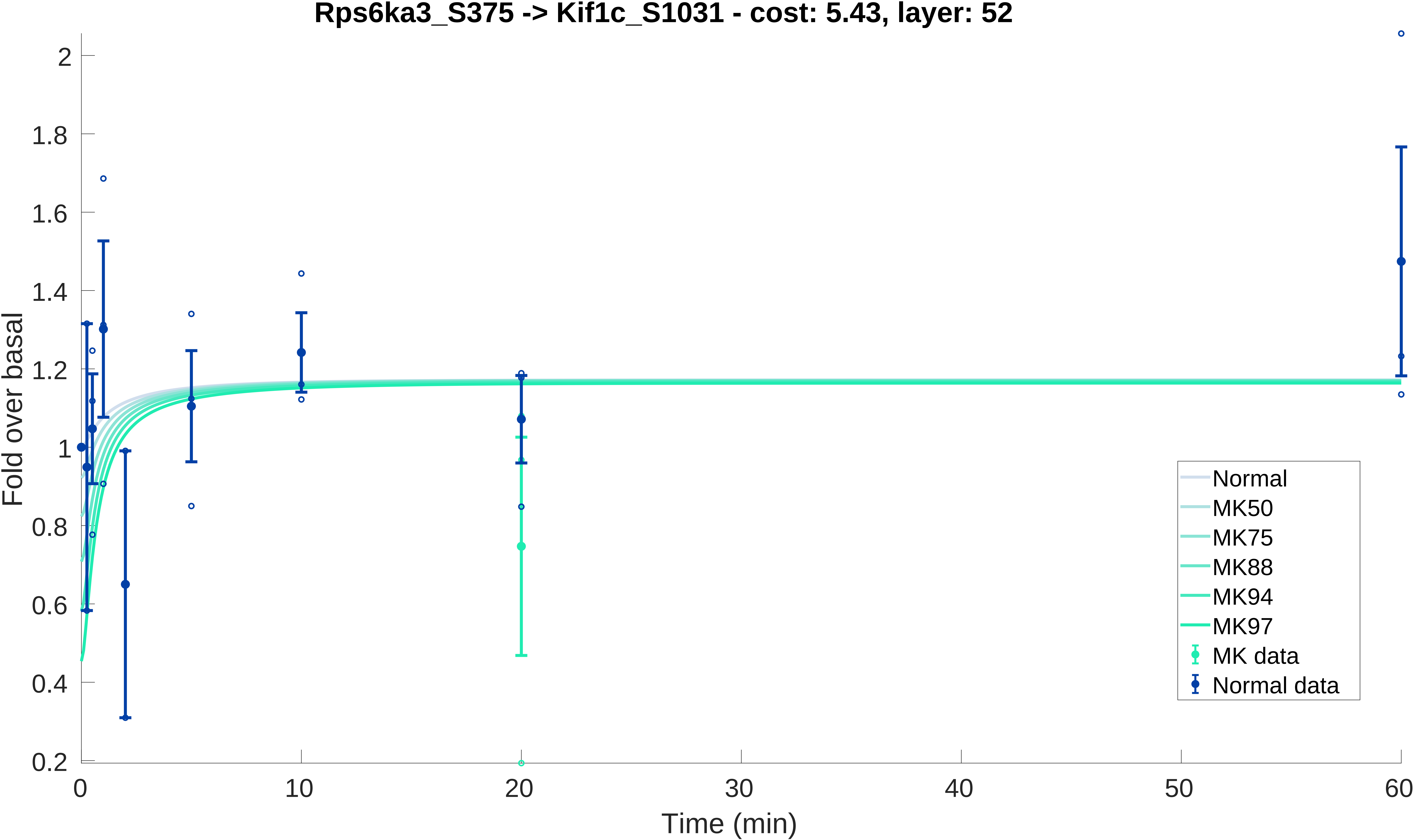

Rps6ka3_S375 -> Kif1c_S1031 - cost: 5.43, layer: 52
Fold over basal
Time (min)
2
1.8
1.6
1.4
1.2
1
0.8
0.6
0.4
0.2
0
10
20
30
40
50
60
Normal
MK50
MK75
MK88
MK94
MK97
MK data
Normal data

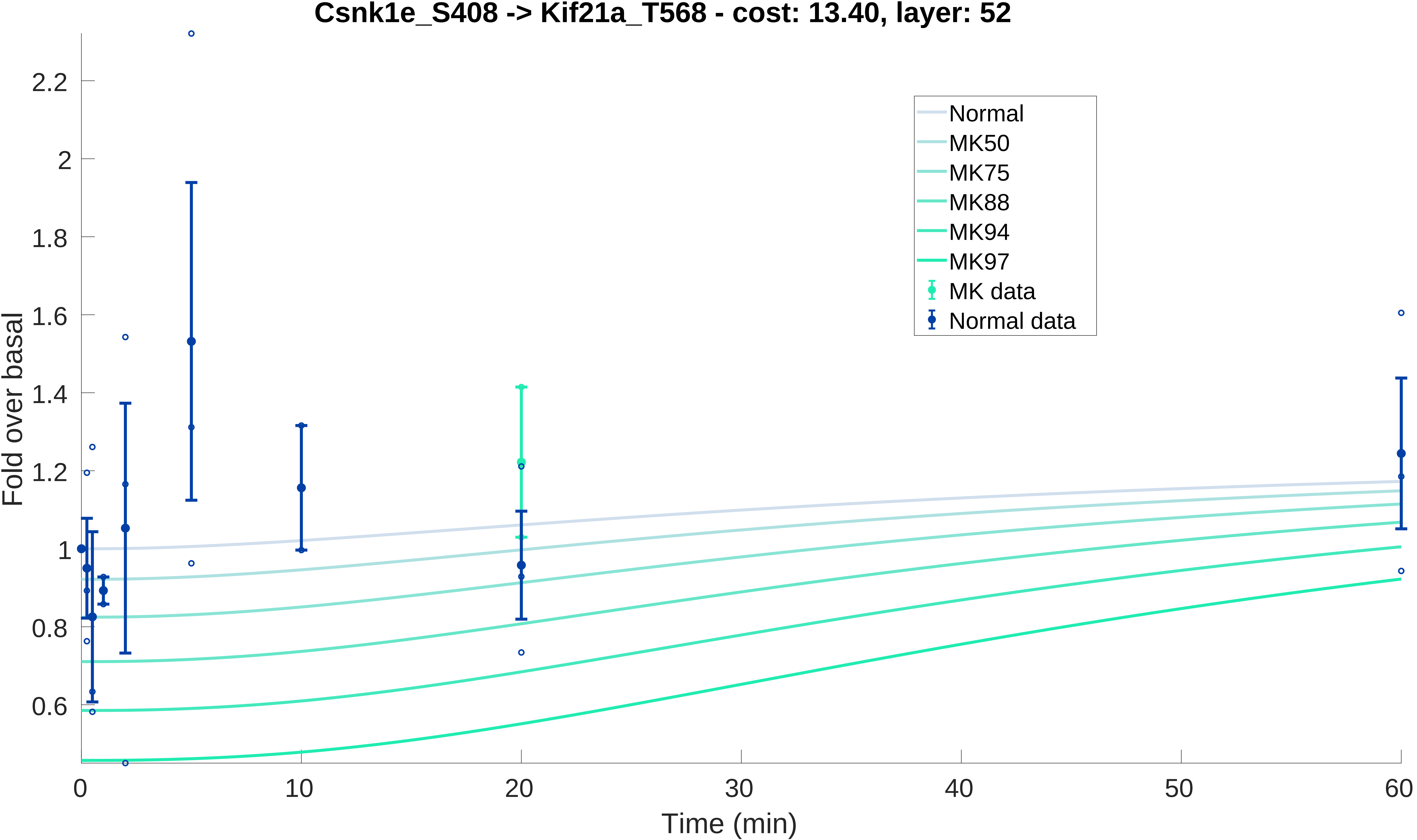

Csnk1e_S408 -> Kif21a_T568 - cost: 13.40, layer: 52
Fold over basal
Time (min)
Normal
MK50
MK75
MK88
MK94
MK97
MK data
Normal data
2.2
2
1.8
1.6
1.4
1.2
1
0.8
0.6
0
10
20
30
40
50
60

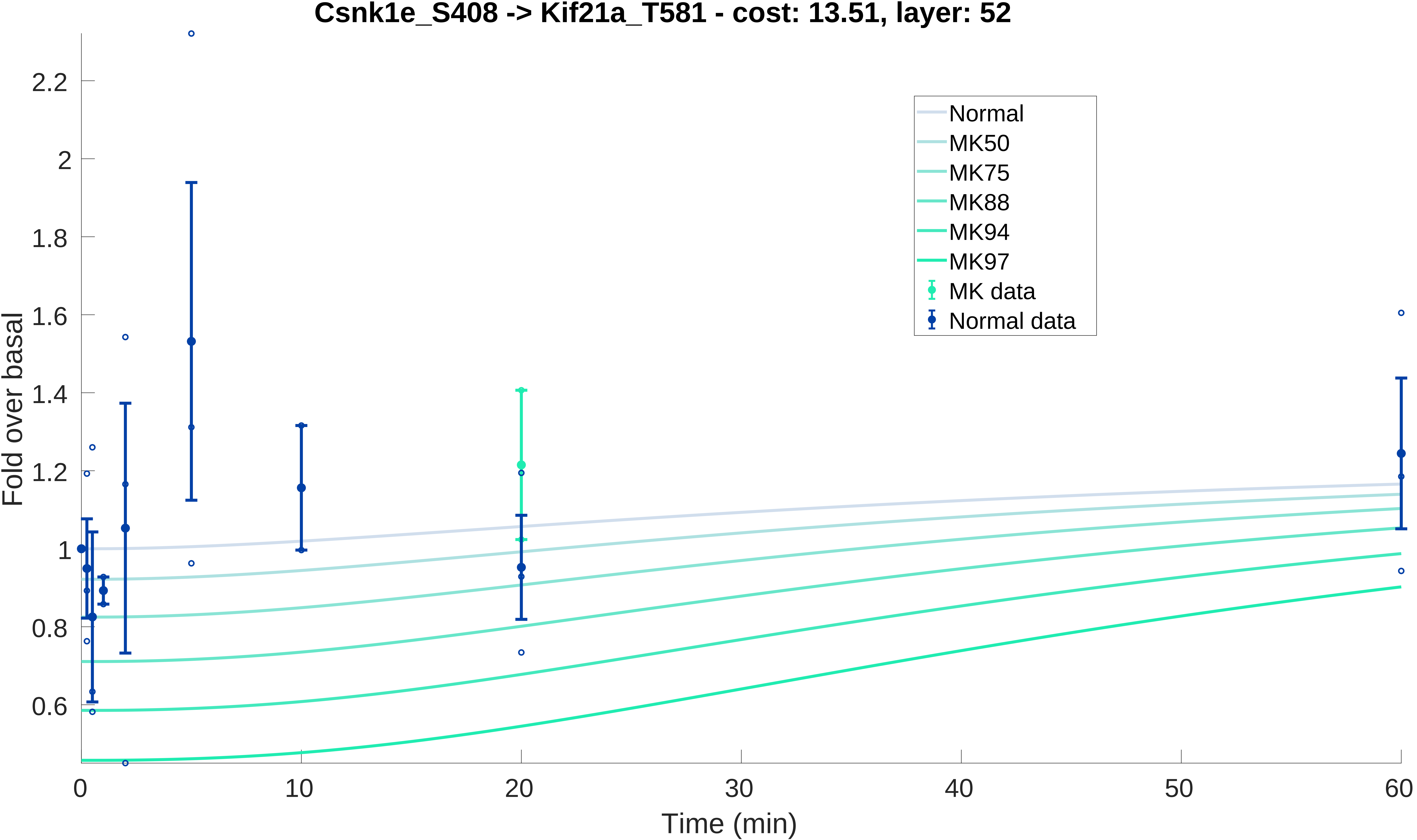
Csnk1e_S408 -> Kif21a_T581 - cost: 13.51, layer: 52
Fold over basal
Time (min)
2.2
2
1.8
1.6
1.4
1.2
1
0.8
0.6
0
10
20
30
40
50
60
Normal
MK50
MK75
MK88
MK94
MK97
MK data
Normal data

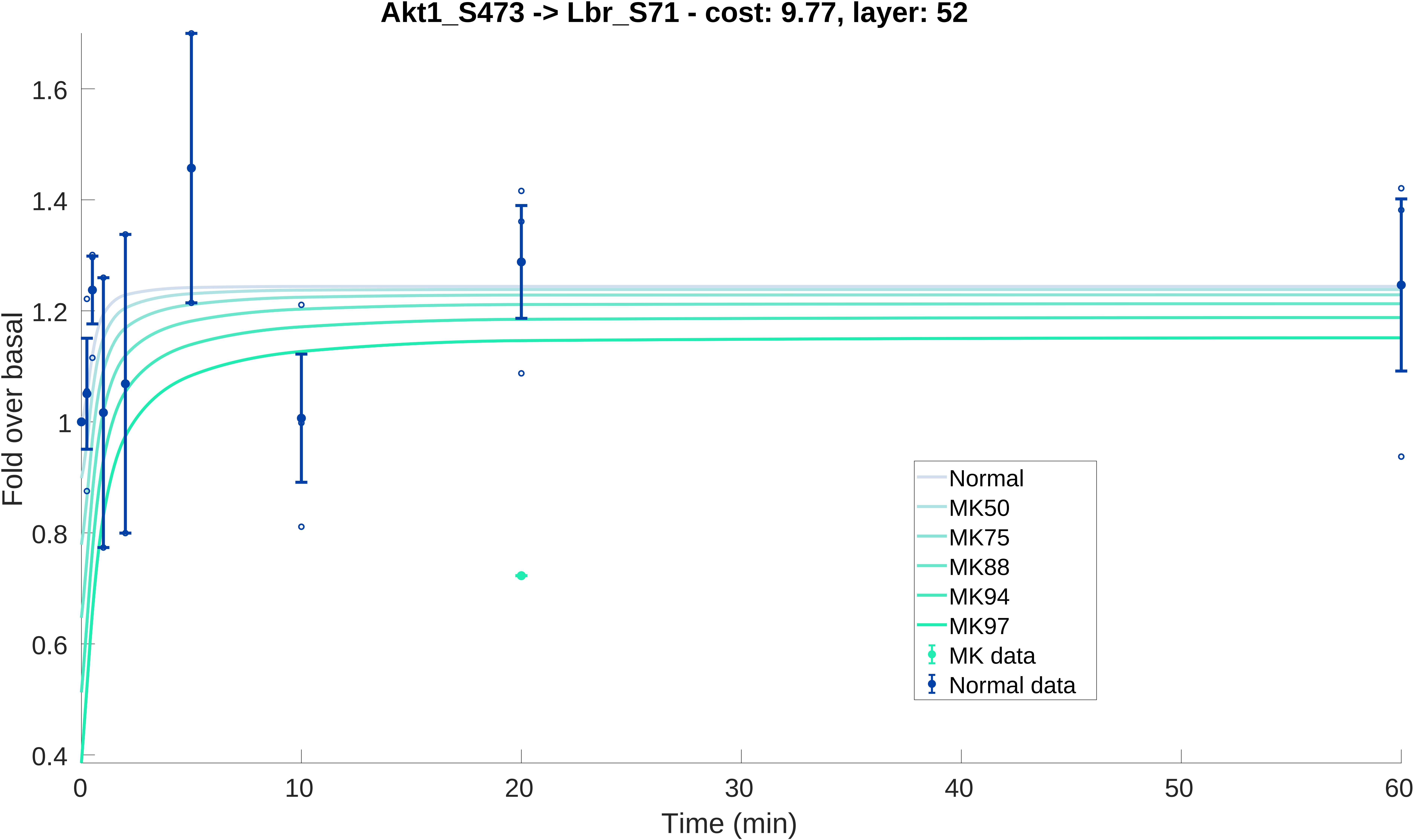
Akt1_S473 -> Lbr_S71 - cost: 9.77, layer: 52
Fold over basal
Time (min)
0
10
20
30
40
50
60
0.4
0.6
0.8
1
1.2
1.4
1.6
Normal
MK50
MK75
MK88
MK94
MK97
MK data
Normal data

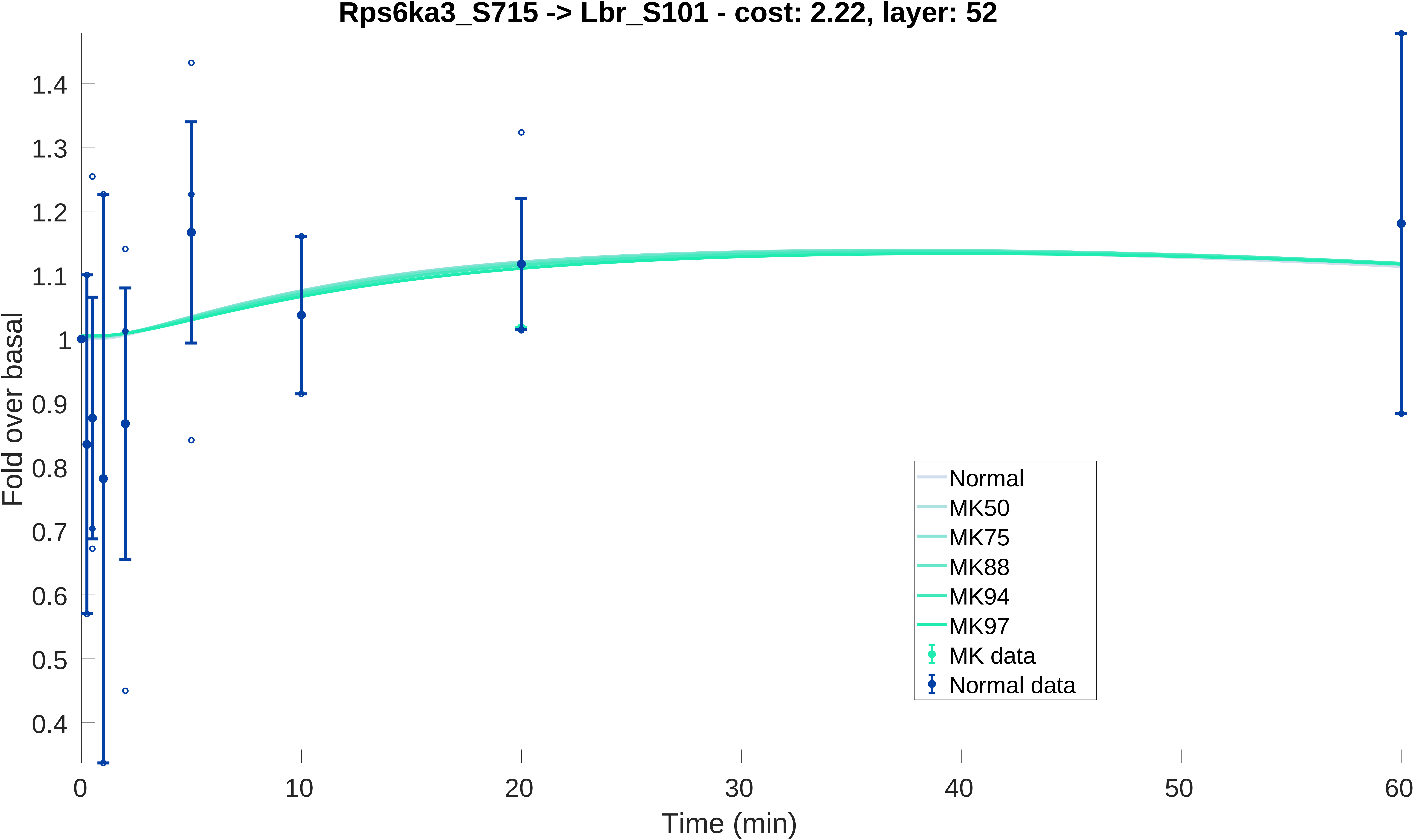
Rps6ka3_S715 -> Lbr_S101 - cost: 2.22, layer: 52
Fold over basal
Time (min)
1.4
1.3
1.2
1.1
1
0.9
0.8
0.7
0.6
0.5
0.4
0
10
20
30
40
50
60
Normal
MK50
MK75
MK88
MK94
MK97
MK data
Normal data

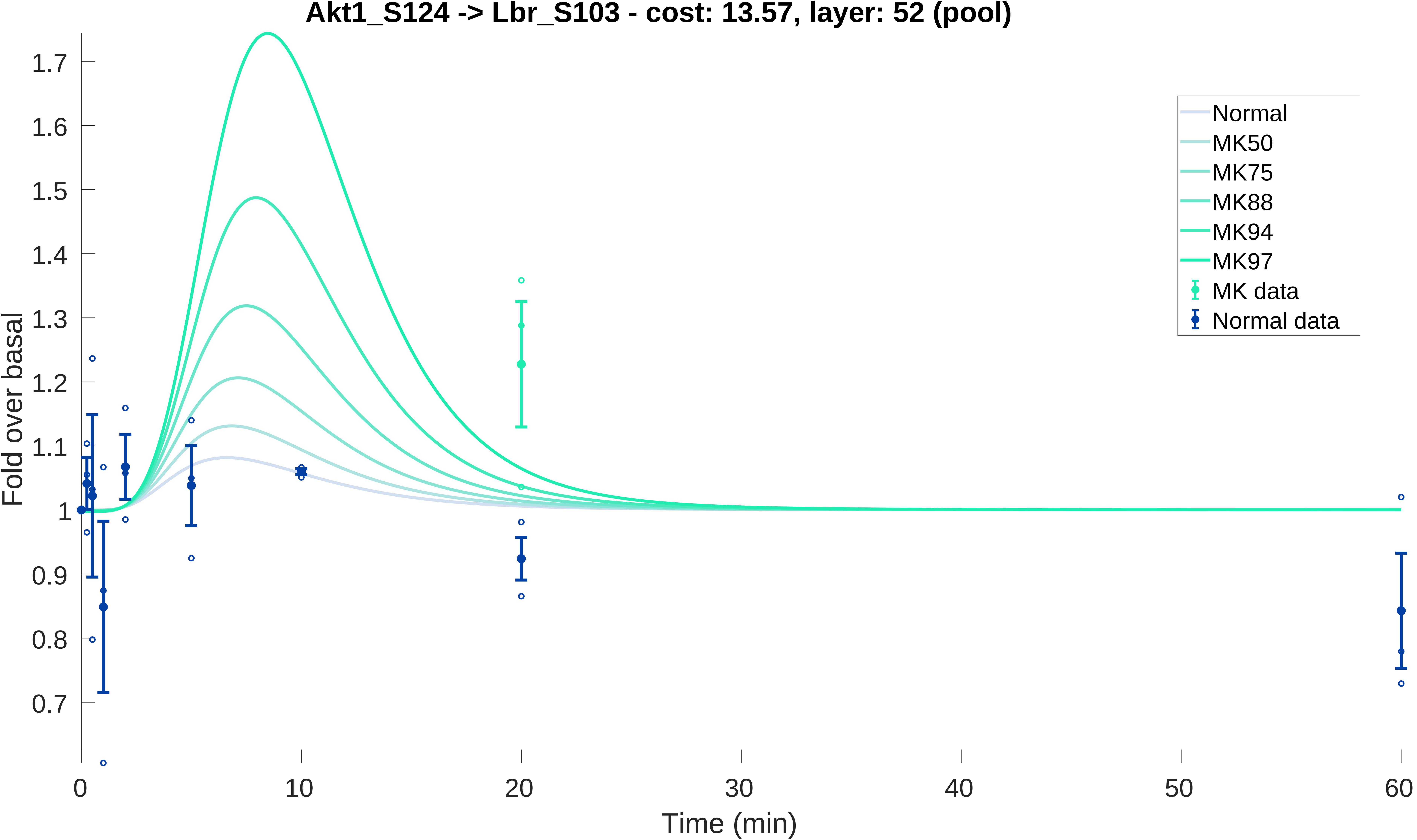

Akt1_S124 -> Lbr_S103 - cost: 13.57, layer: 52 (pool)
Fold over basal
Time (min)
0
10
20
30
40
50
60
0.7
0.8
0.9
1
1.1
1.2
1.3
1.4
1.5
1.6
1.7
Normal
MK50
MK75
MK88
MK94
MK97
MK data
Normal data

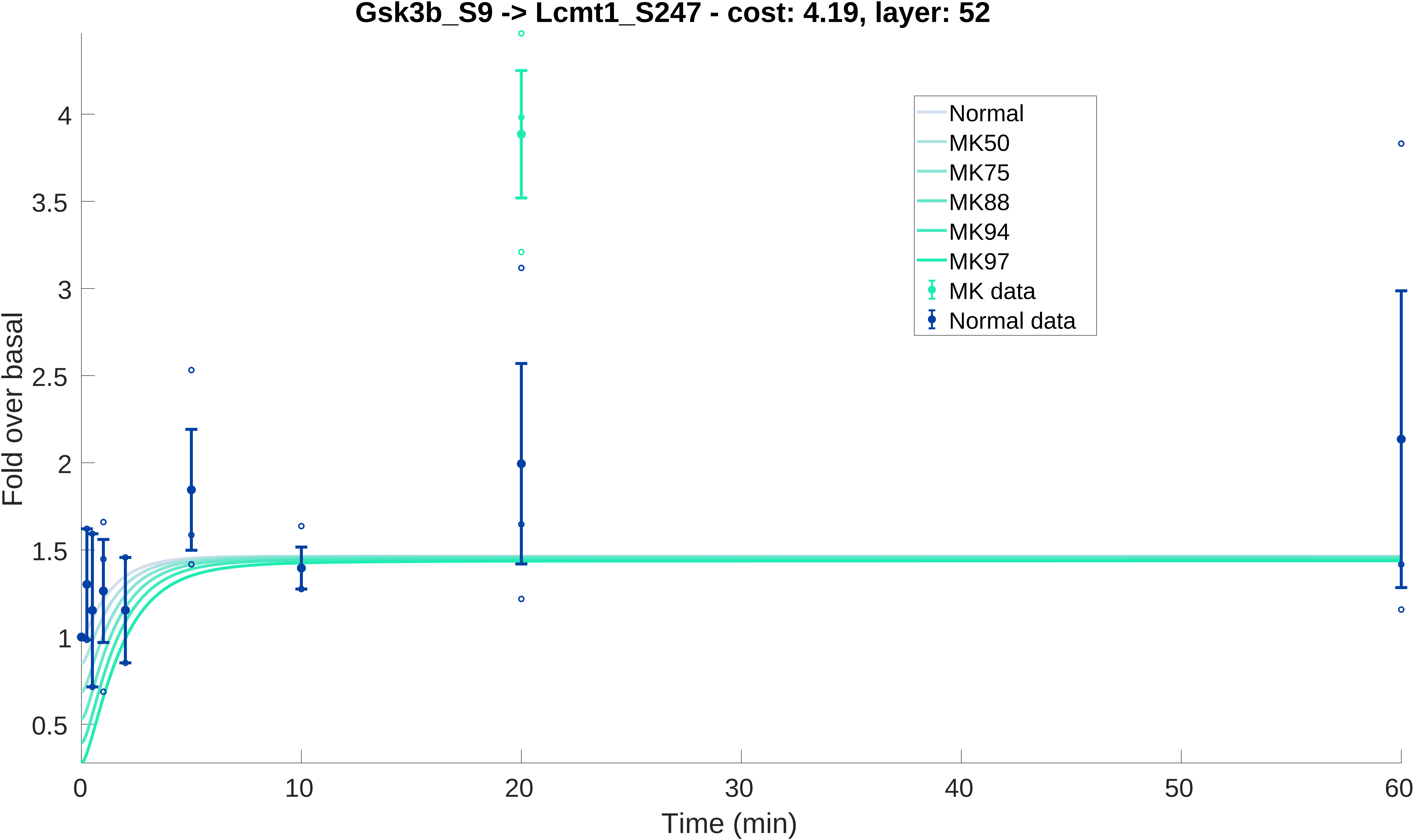

Gsk3b_S9 -> Lcmt1_S247 - cost: 4.19, layer: 52
Fold over basal
Time (min)
0
10
20
30
40
50
60
0.5
1
1.5
2
2.5
3
3.5
4
Normal
MK50
MK75
MK88
MK94
MK97
MK data
Normal data

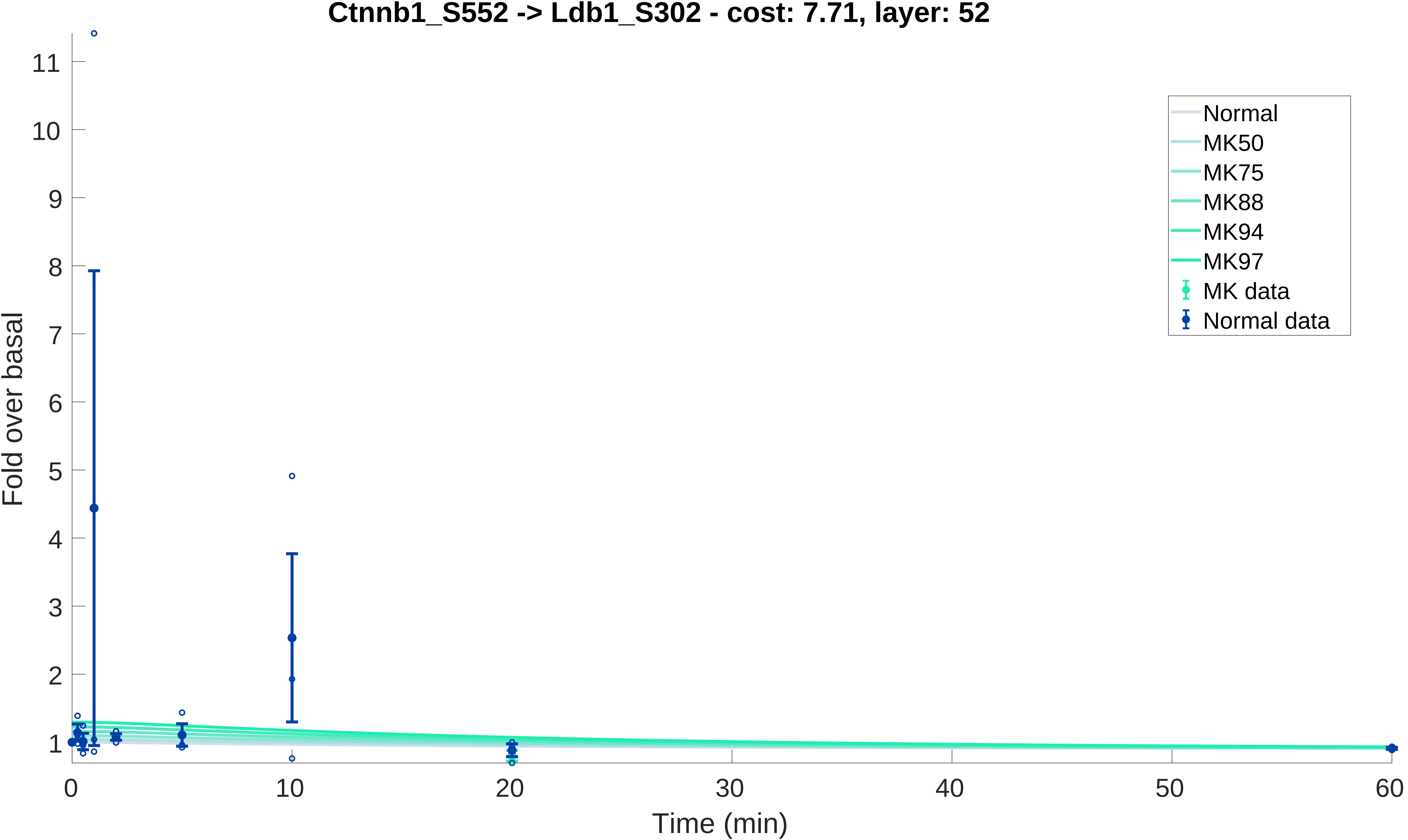

Ctnnb1_S552 -> Ldb1_S302 - cost: 7.71, layer: 52
Fold over basal
Time (min)
Normal
MK50
MK75
MK88
MK94
MK97
MK data
Normal data
0
10
20
30
40
50
60
1
2
3
4
5
6
7
8
9
10
11

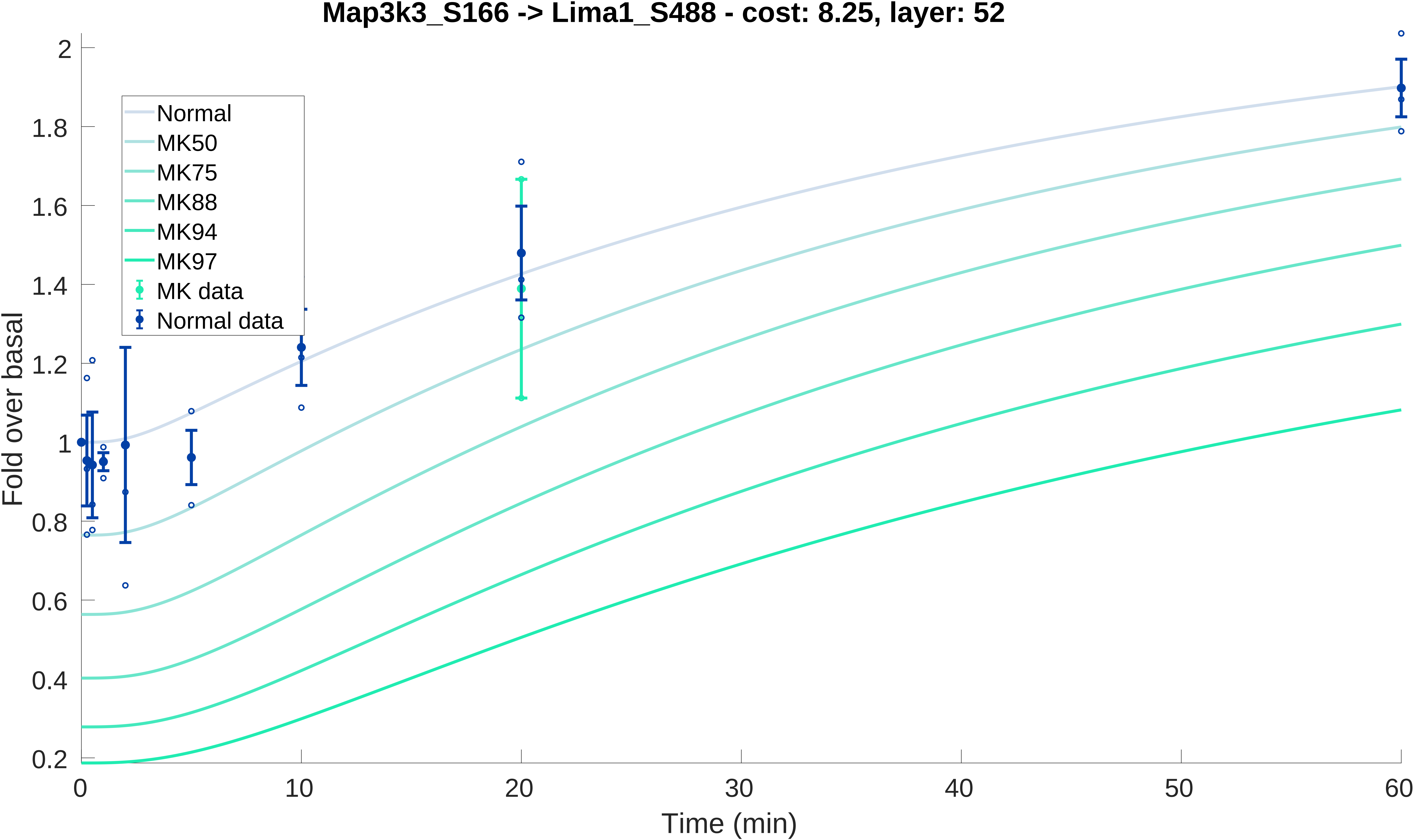
Map3k3_S166 -> Lima1_S488 - cost: 8.25, layer: 52
Normal
MK50
MK75
MK88
MK94
MK97
MK data
Normal data
Fold over basal
Time (min)
0.2
0.4
0.6
0.8
1
1.2
1.4
1.6
1.8
2
0
10
20
30
40
50
60

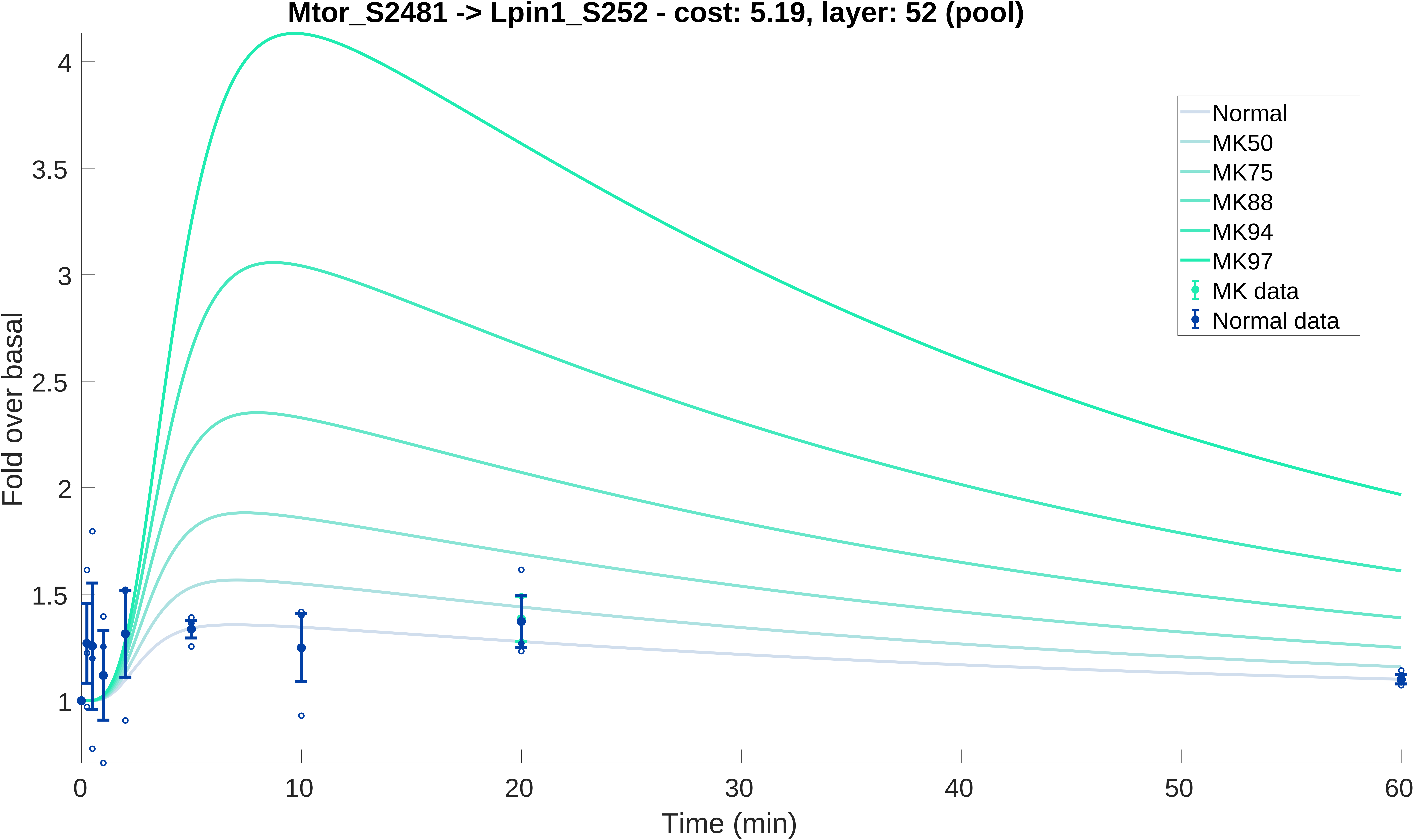
Mtor_S2481 -> Lpin1_S252 - cost: 5.19, layer: 52 (pool)
Fold over basal
Time (min)
Normal
MK50
MK75
MK88
MK94
MK97
MK data
Normal data
4
3.5
3
2.5
2
1.5
1
0
10
20
30
40
50
60

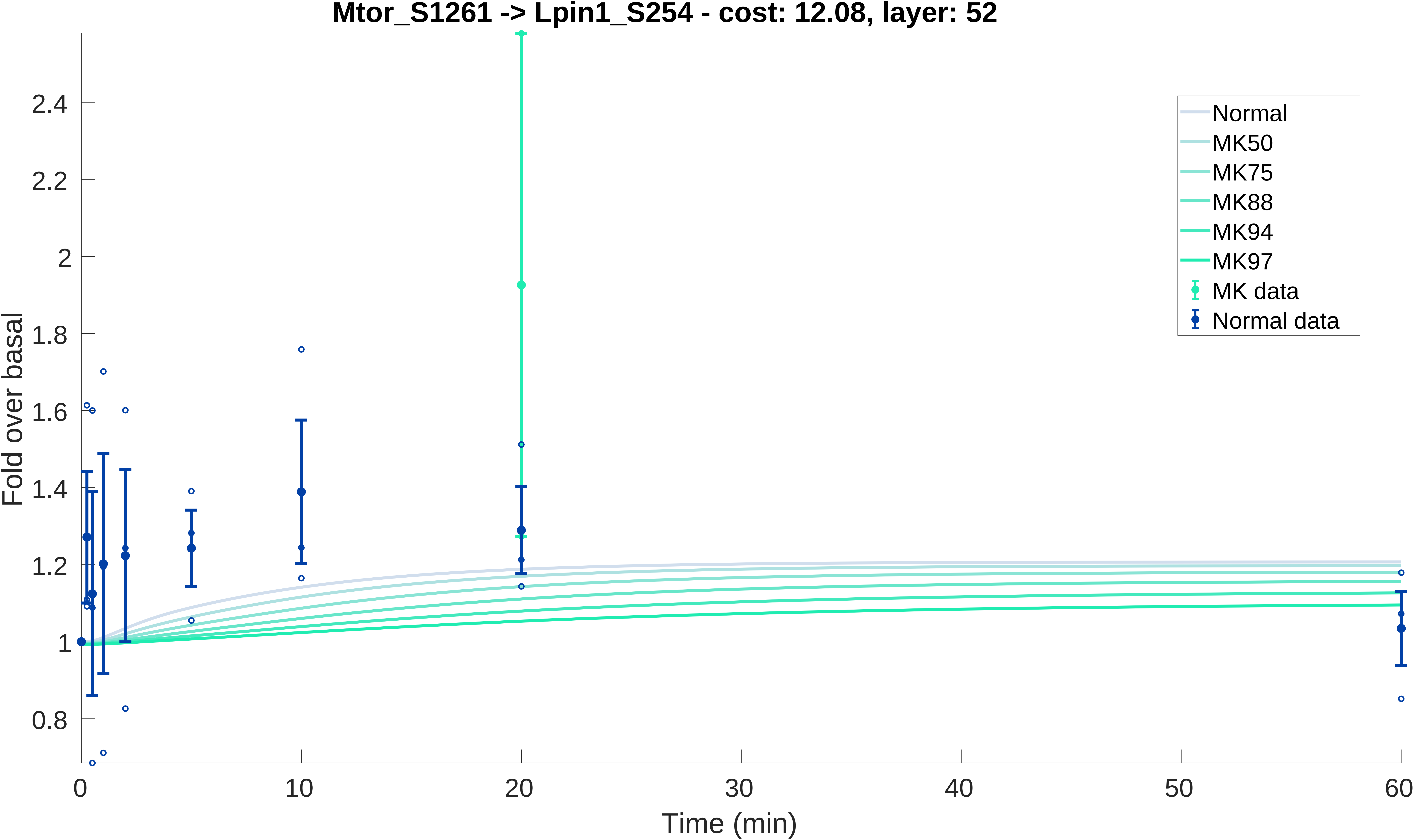

Mtor_S1261 -> Lpin1_S254 - cost: 12.08, layer: 52
Fold over basal
Time (min)
Normal
MK50
MK75
MK88
MK94
MK97
MK data
Normal data
2.4
2.2
2
1.8
1.6
1.4
1.2
1
0.8
0
10
20
30
40
50
60

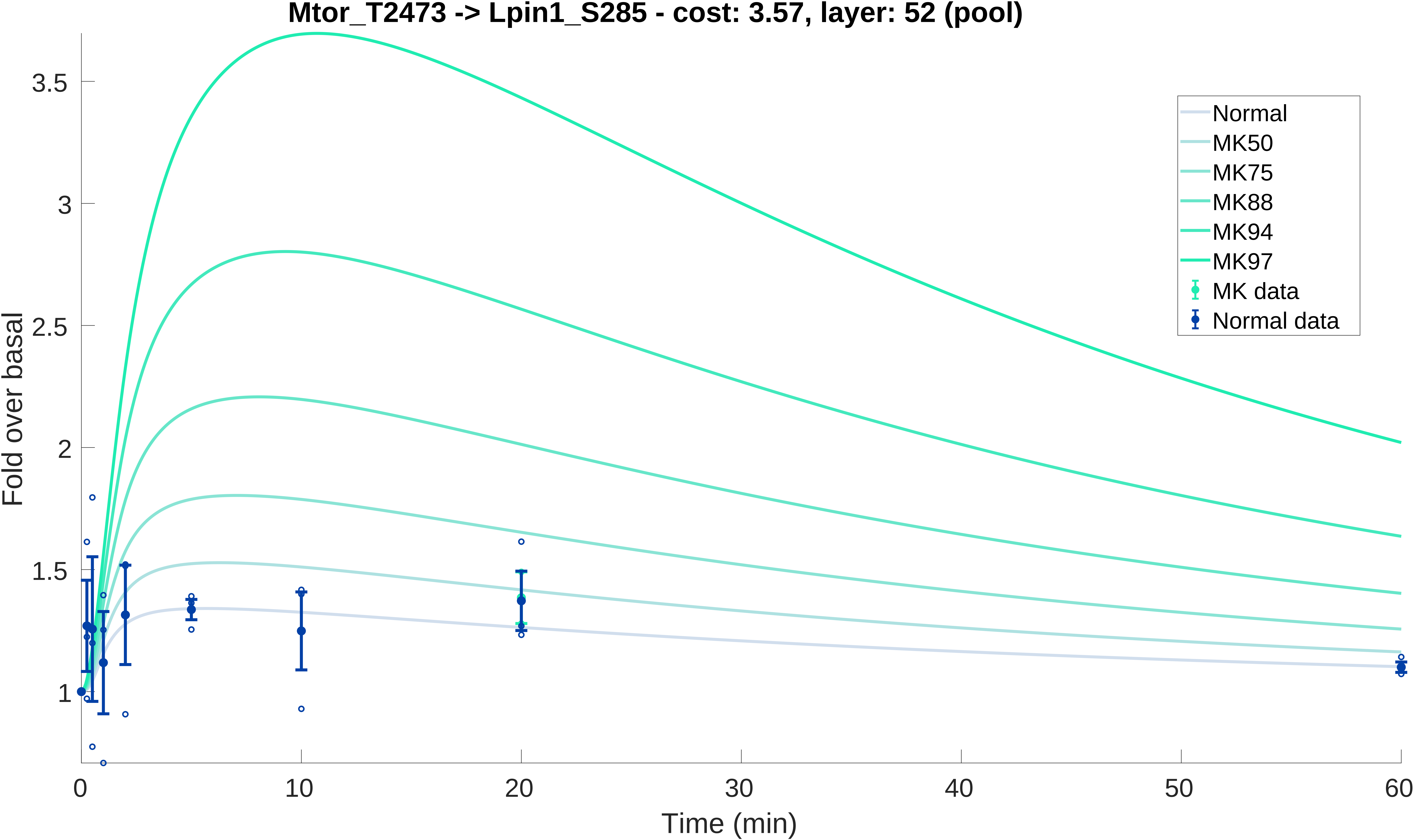

Mtor_T2473 -> Lpin1_S285 - cost: 3.57, layer: 52 (pool)
Fold over basal
Time (min)
3.5
3
2.5
2
1.5
1
0
10
20
30
40
50
60
Normal
MK50
MK75
MK88
MK94
MK97
MK data
Normal data

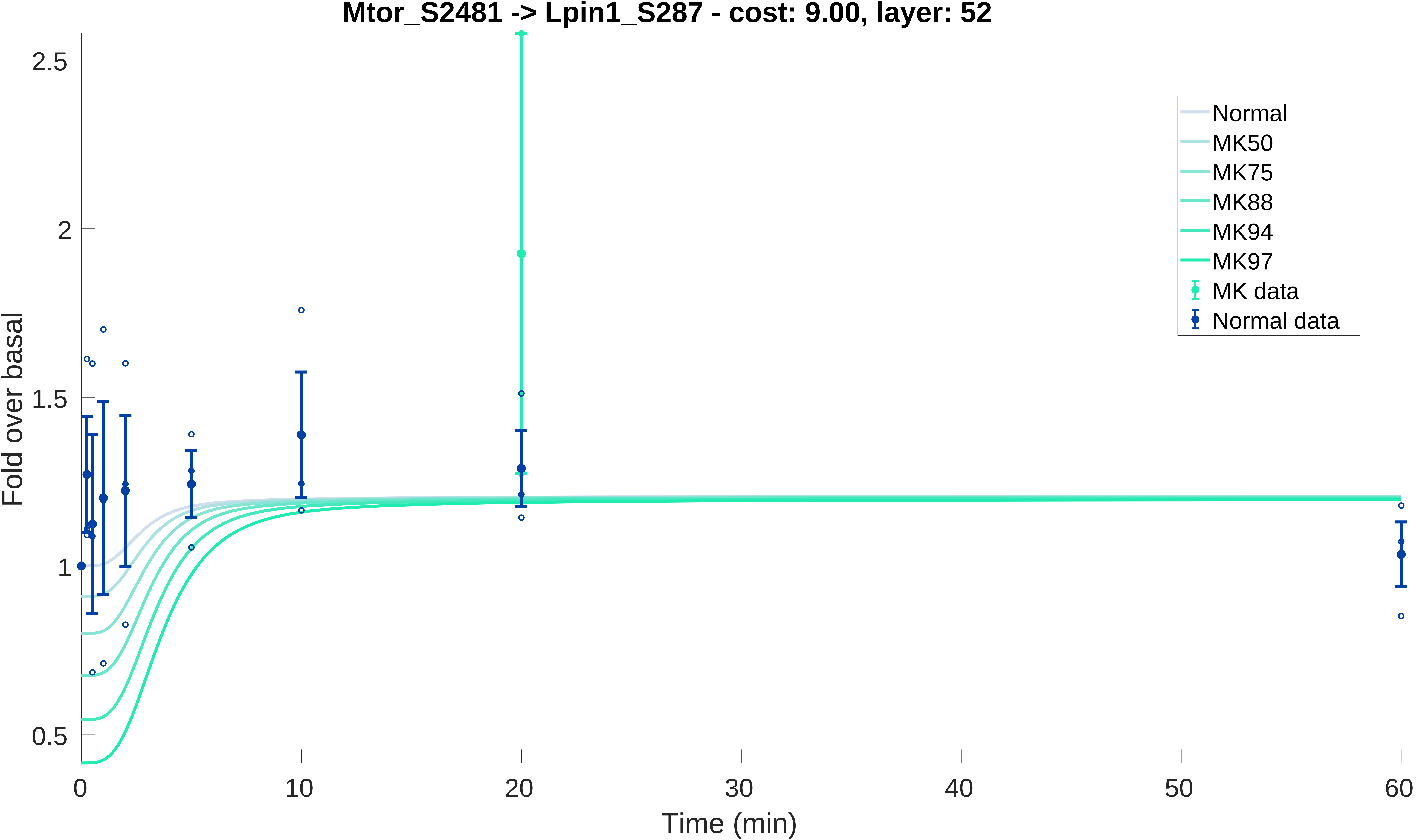

Mtor_S2481 -> Lpin1_S287 - cost: 9.00, layer: 52
Fold over basal
Time (min)
0.5
1
1.5
2
2.5
0
10
20
30
40
50
60
Normal
MK50
MK75
MK88
MK94
MK97
MK data
Normal data

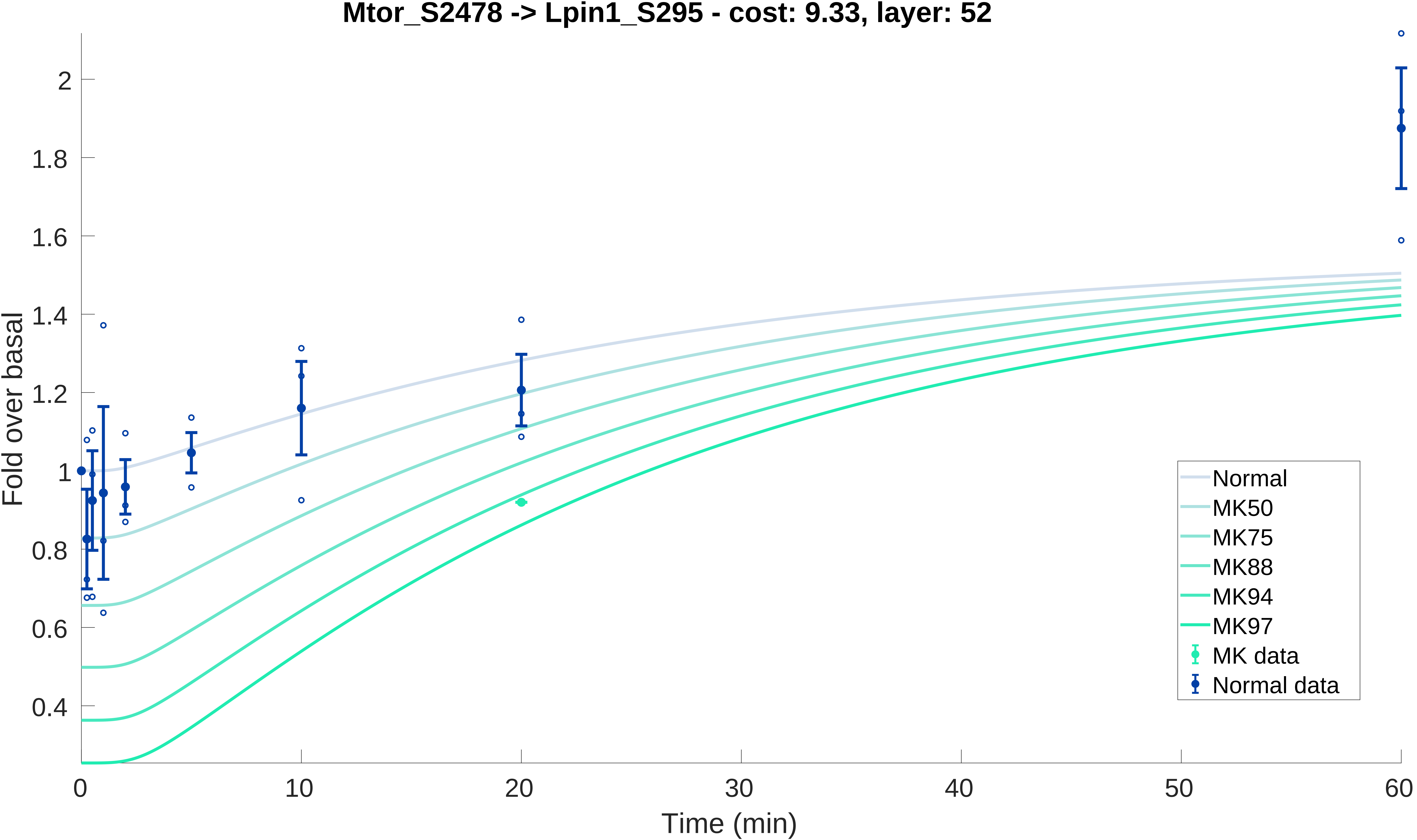
Mtor_S2478 -> Lpin1_S295 - cost: 9.33, layer: 52
Fold over basal
Time (min)
0.4
0.6
0.8
1
1.2
1.4
1.6
1.8
2
0
10
20
30
40
50
60
Normal
MK50
MK75
MK88
MK94
MK97
MK data
Normal data

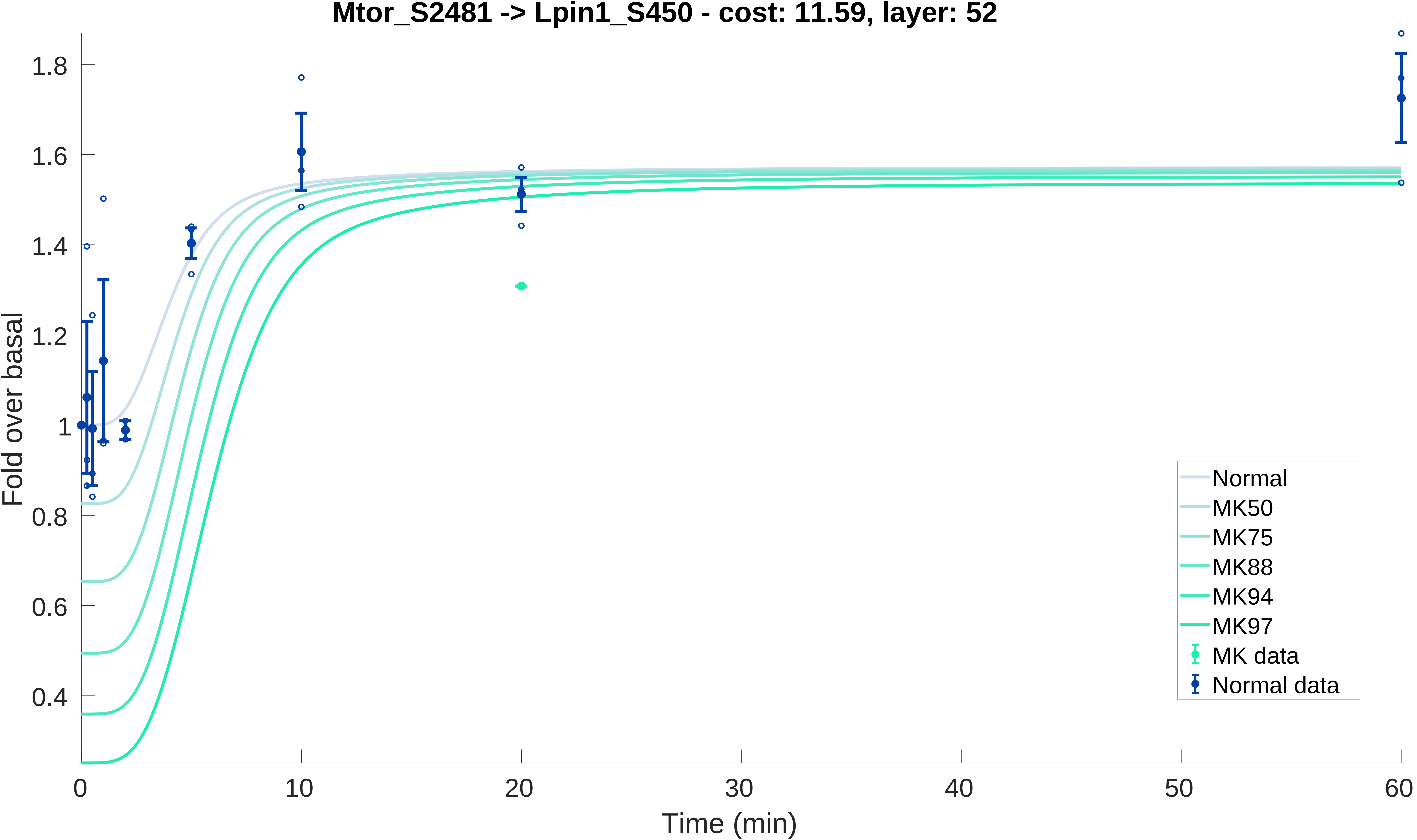
Mtor_S2481 -> Lpin1_S450 - cost: 11.59, layer: 52
Fold over basal
Time (min)
0.4
0.6
0.8
1
1.2
1.4
1.6
1.8
0
10
20
30
40
50
60
Normal
MK50
MK75
MK88
MK94
MK97
MK data
Normal data

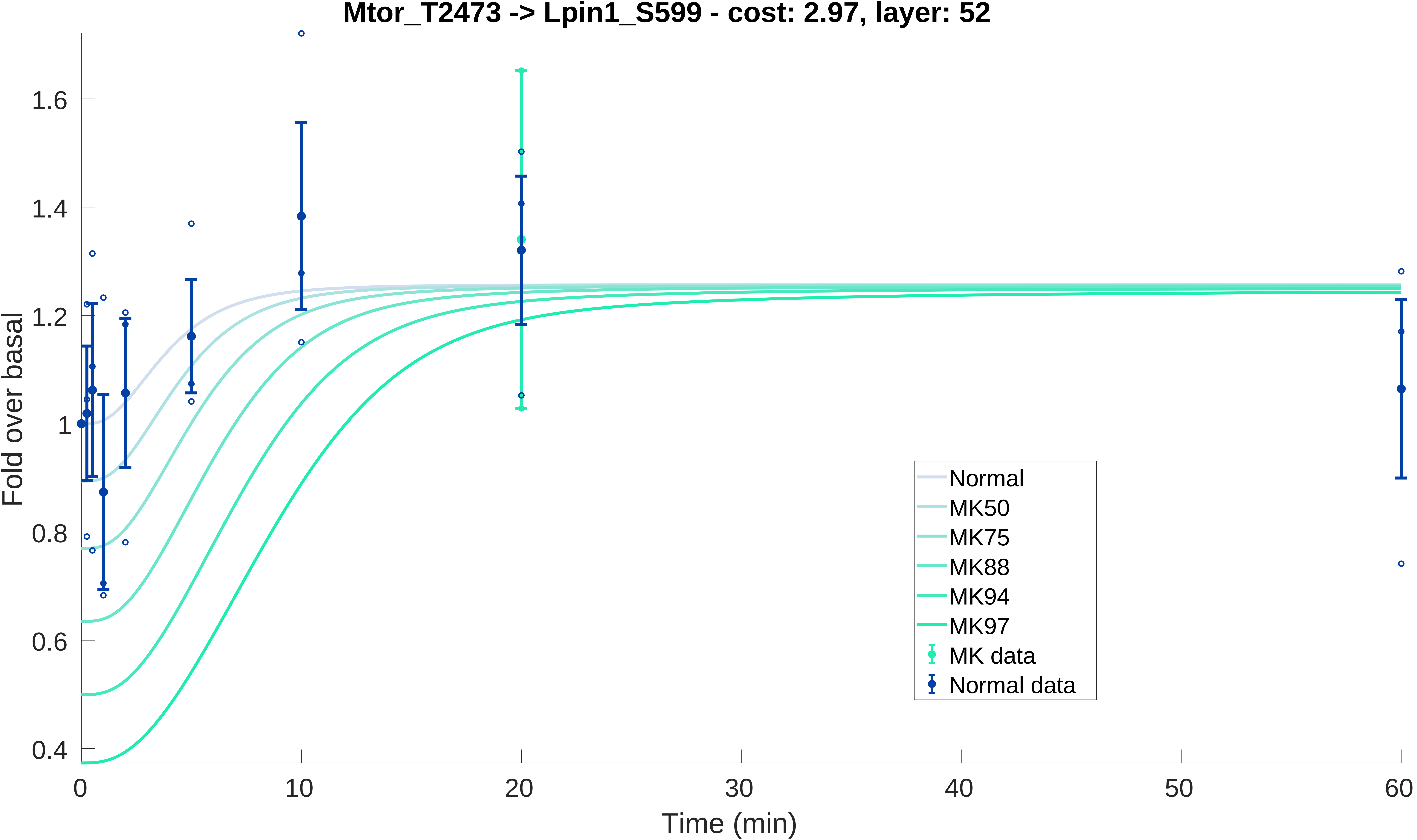

Mtor_T2473 -> Lpin1_S599 - cost: 2.97, layer: 52
Fold over basal
0.4
0.6
0.8
1
1.2
1.4
1.6
Time (min)
0
10
20
30
40
50
60
Normal
MK50
MK75
MK88
MK94
MK97
MK data
Normal data

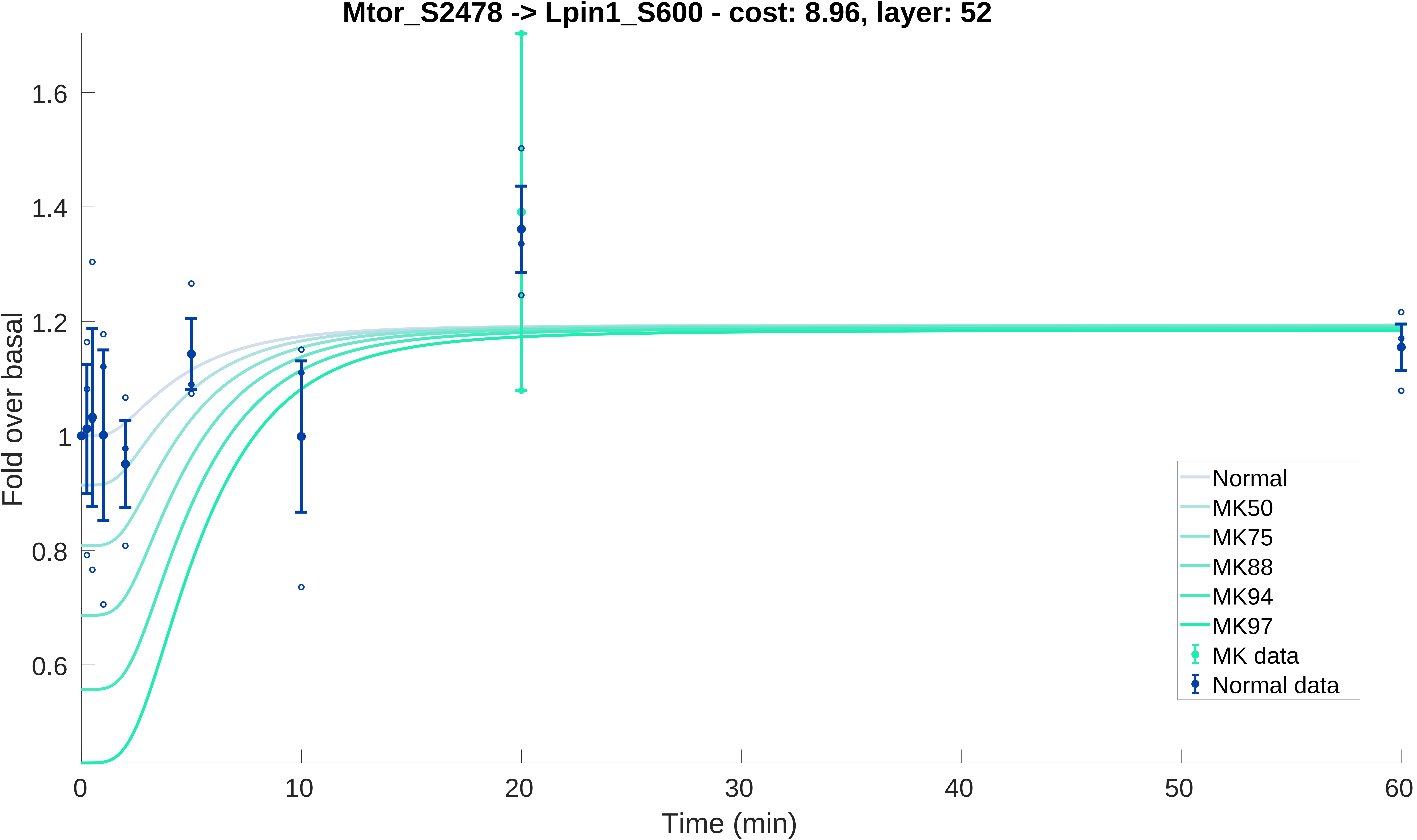

Mtor_S2478 -> Lpin1_S600 - cost: 8.96, layer: 52
Fold over basal
1.6
1.4
1.2
1
0.8
0.6
0
10
20
30
40
50
60
Time (min)
Normal
MK50
MK75
MK88
MK94
MK97
MK data
Normal data

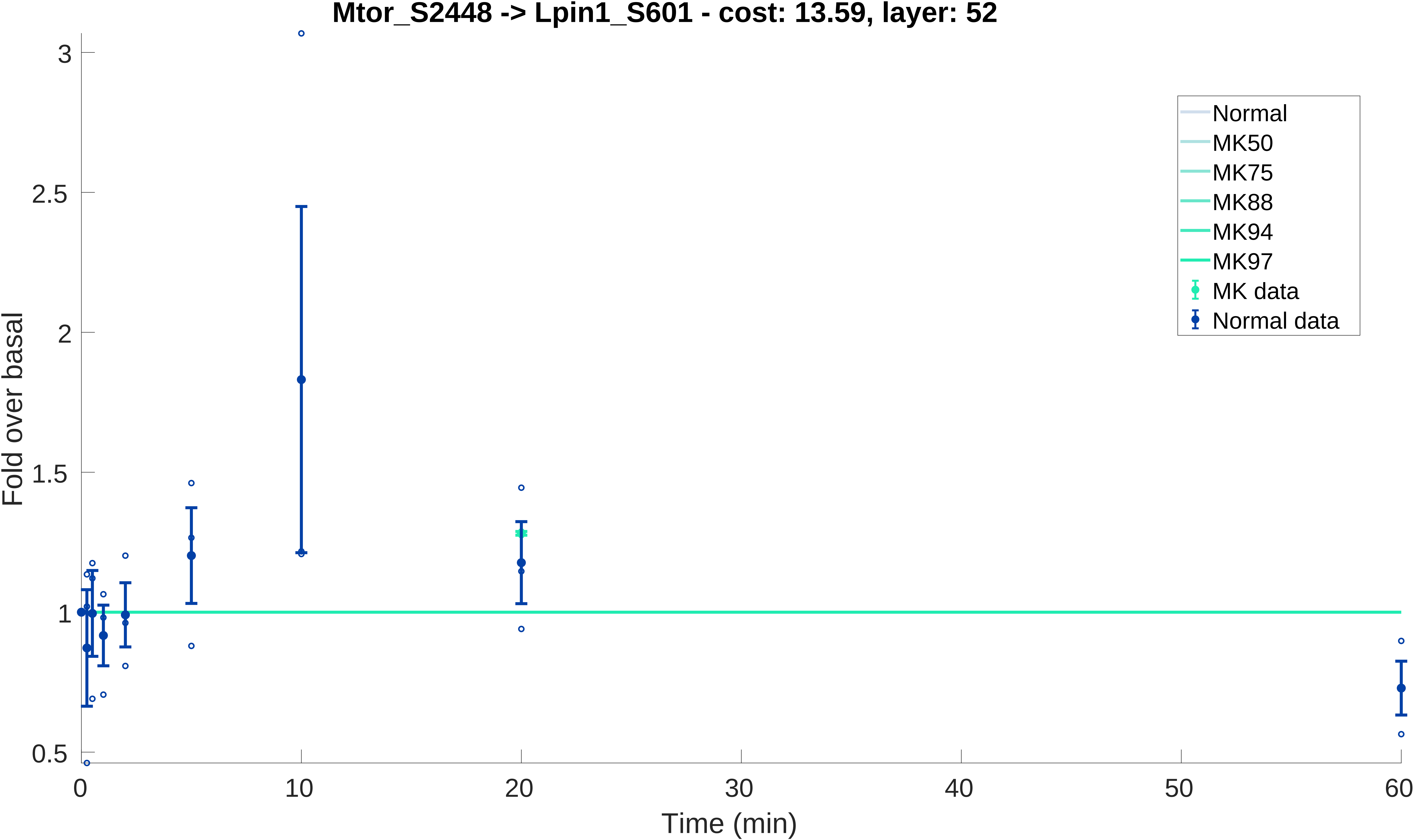

Mtor_S2448 -> Lpin1_S601 - cost: 13.59, layer: 52
Fold over basal
Time (min)
0.5
1
1.5
2
2.5
3
0
10
20
30
40
50
60
Normal
MK50
MK75
MK88
MK94
MK97
MK data
Normal data

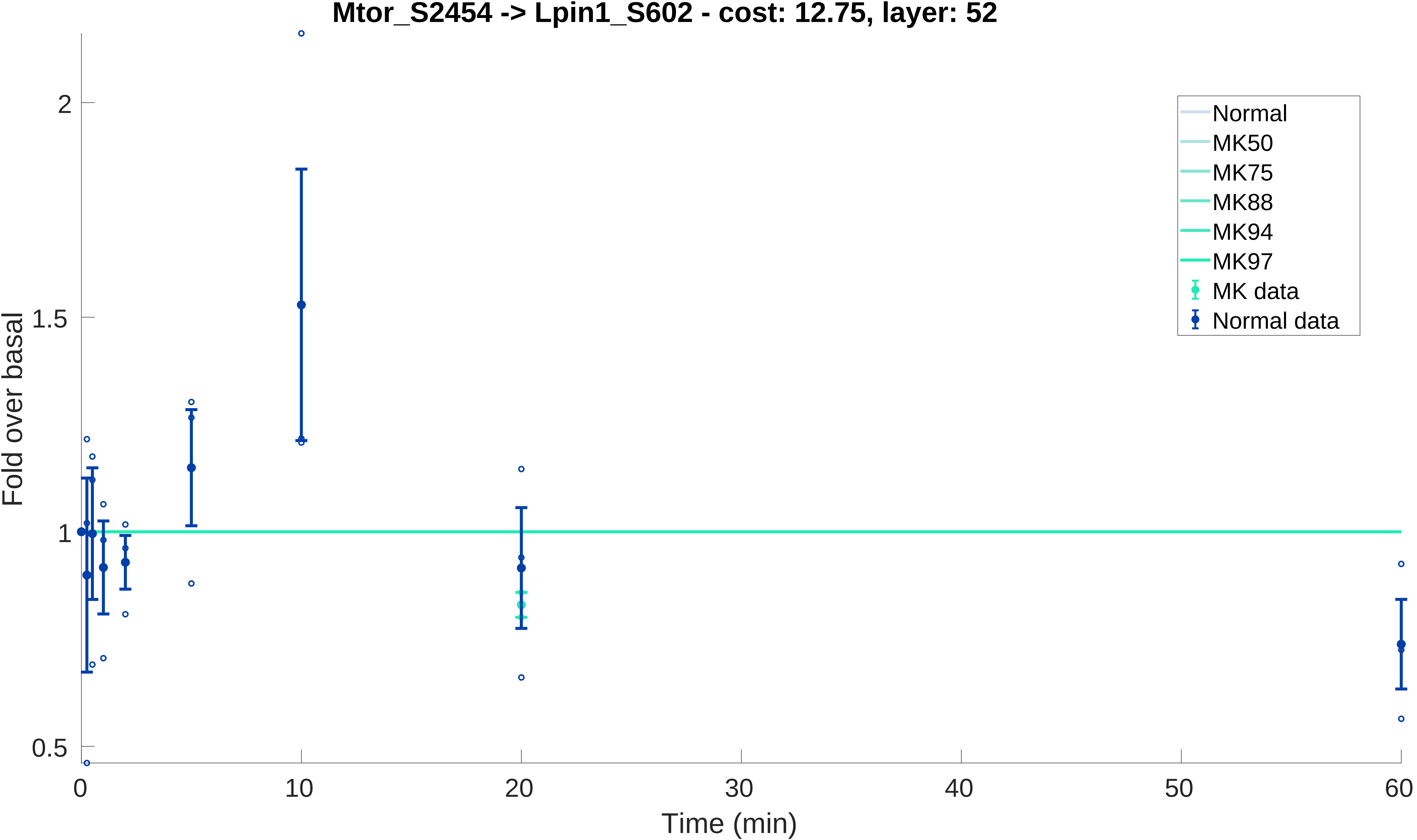

Mtor_S2454 -> Lpin1_S602 - cost: 12.75, layer: 52
Fold over basal
Time (min)
0.5
1
1.5
2
0
10
20
30
40
50
60
Normal
MK50
MK75
MK88
MK94
MK97
MK data
Normal data

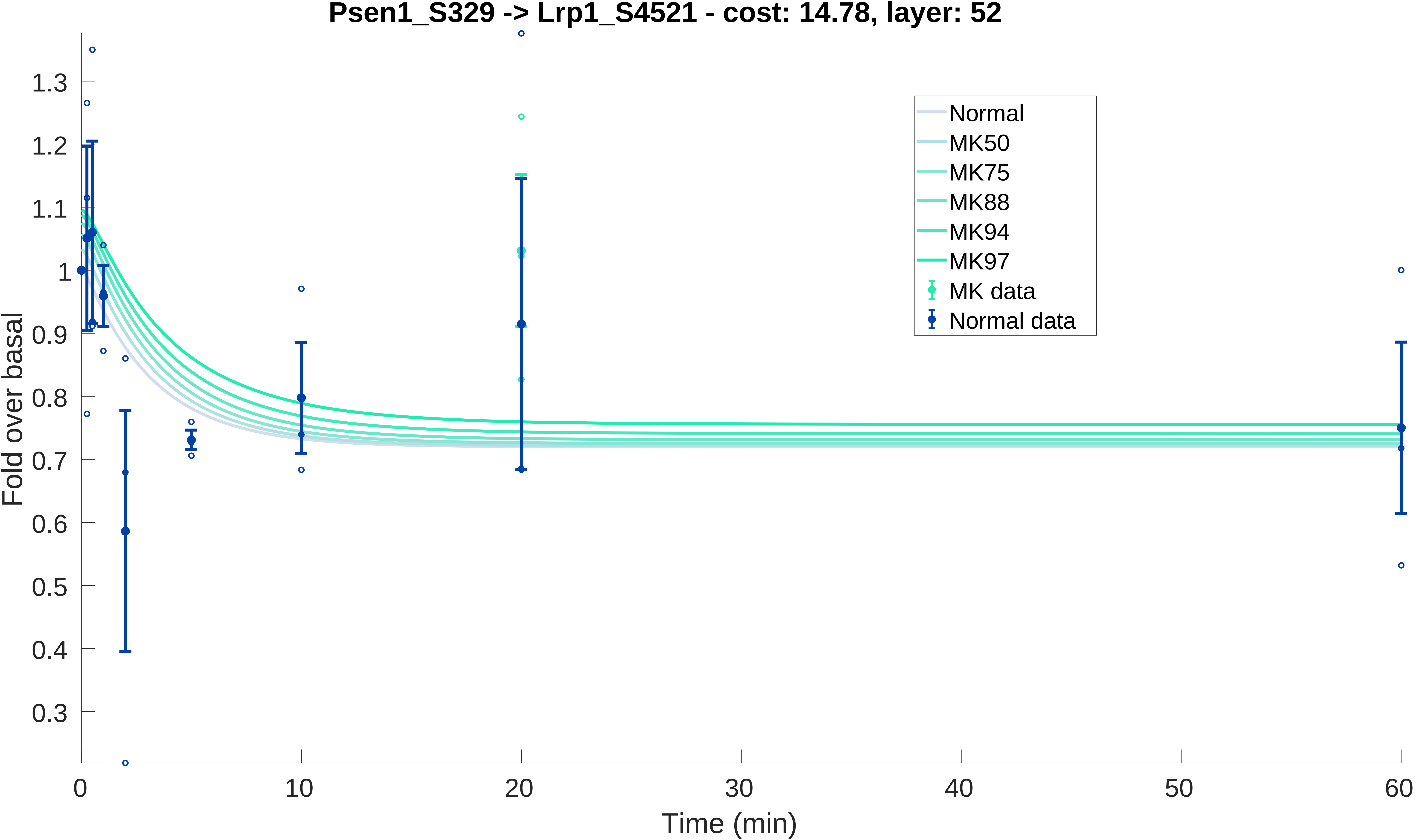
Psen1_S329 -> Lrp1_S4521 - cost: 14.78, layer: 52
Fold over basal
Time (min)
Normal
MK50
MK75
MK88
MK94
MK97
MK data
Normal data
0
10
20
30
40
50
60
0.3
0.4
0.5
0.6
0.7
0.8
0.9
1
1.1
1.2
1.3

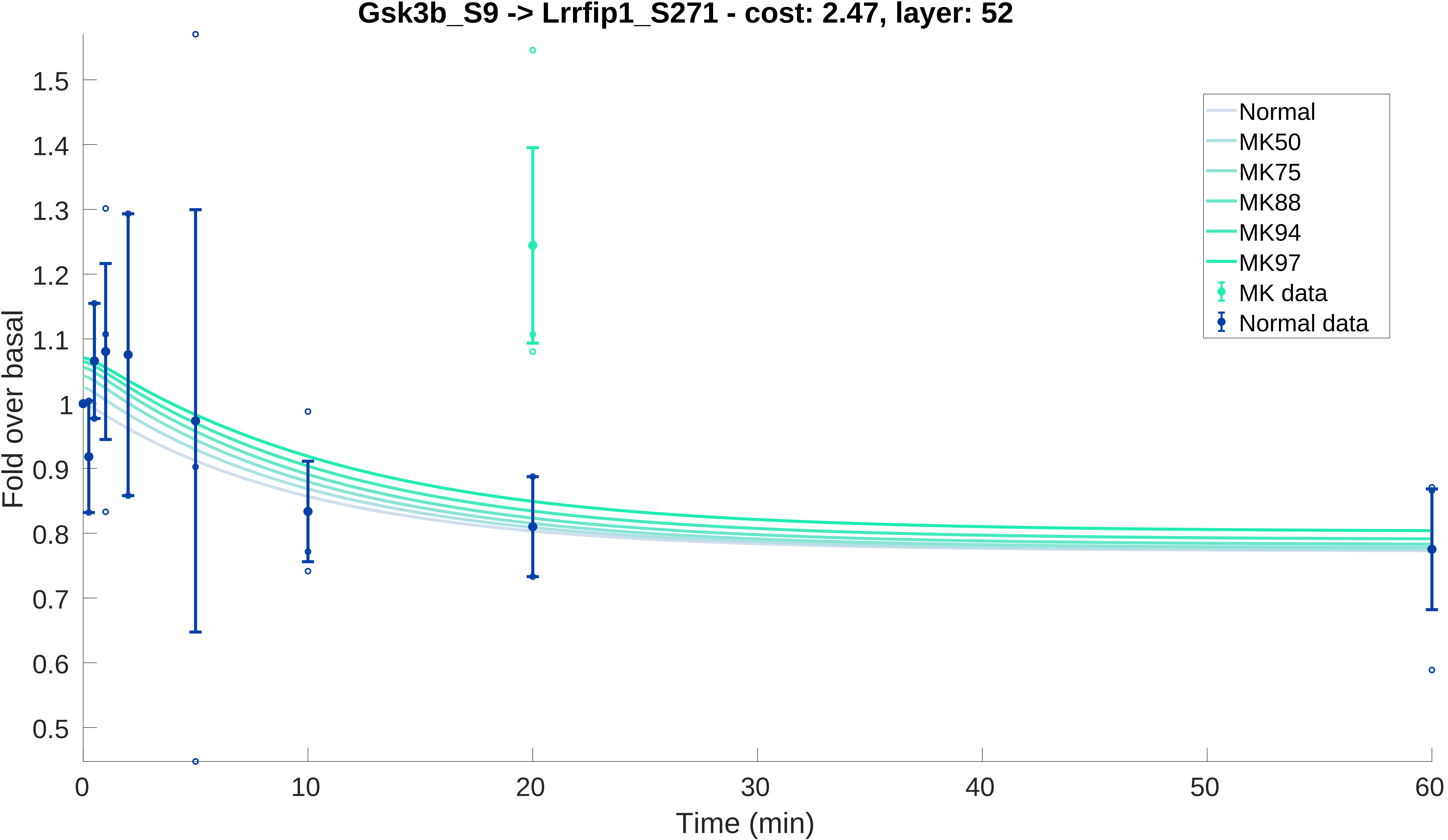

Gsk3b_S9 -> Lrrfip1_S271 - cost: 2.47, layer: 52
Fold over basal
Time (min)
0
10
20
30
40
50
60
0.5
0.6
0.7
0.8
0.9
1
1.1
1.2
1.3
1.4
1.5
Normal
MK50
MK75
MK88
MK94
MK97
MK data
Normal data

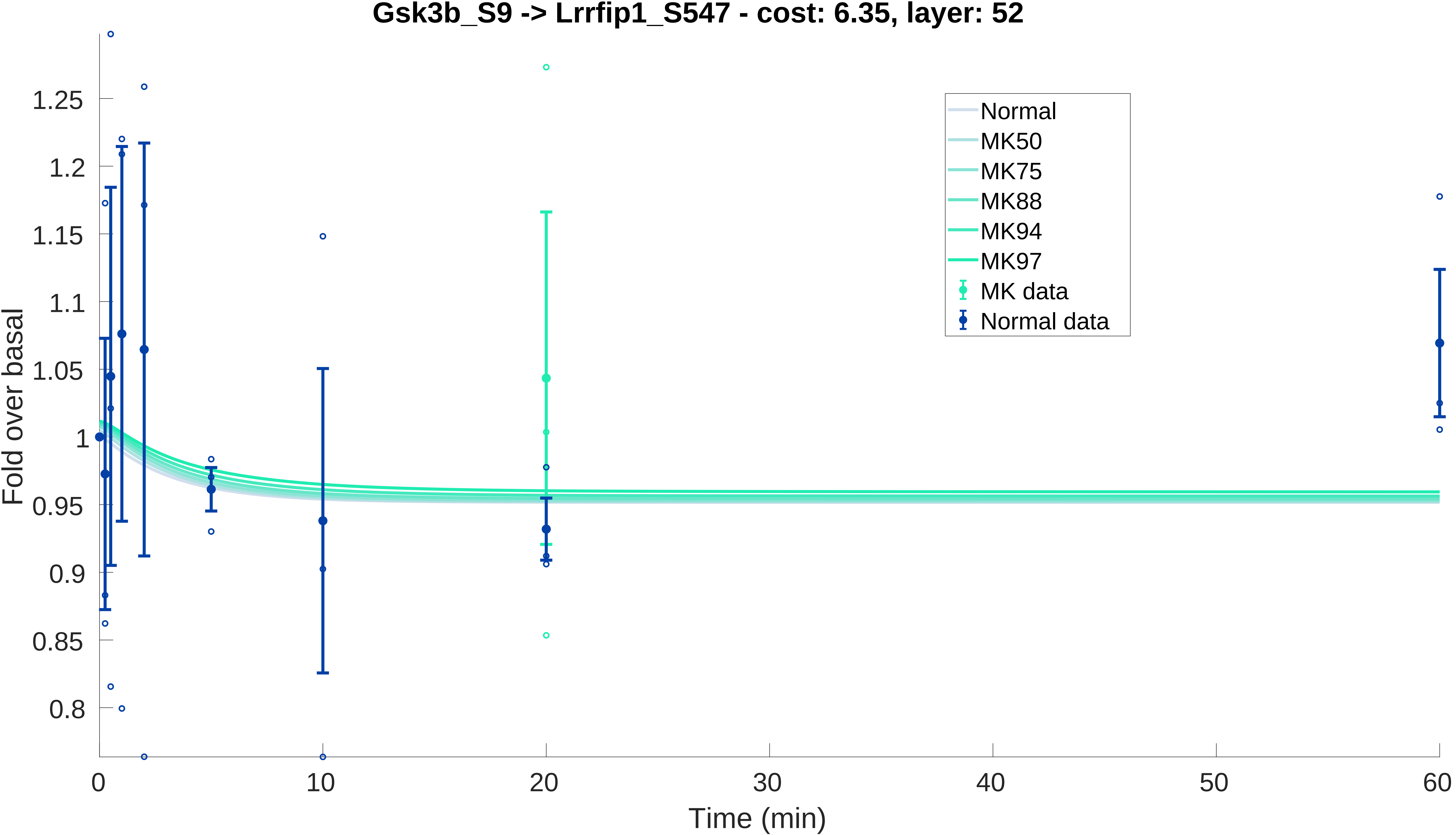

Gsk3b_S9 -> Lrrfip1_S547 - cost: 6.35, layer: 52
Fold over basal
Time (min)
1.25
1.2
1.15
1.1
1.05
1
0.95
0.9
0.85
0.8
0
10
20
30
40
50
60
Normal
MK50
MK75
MK88
MK94
MK97
MK data
Normal data

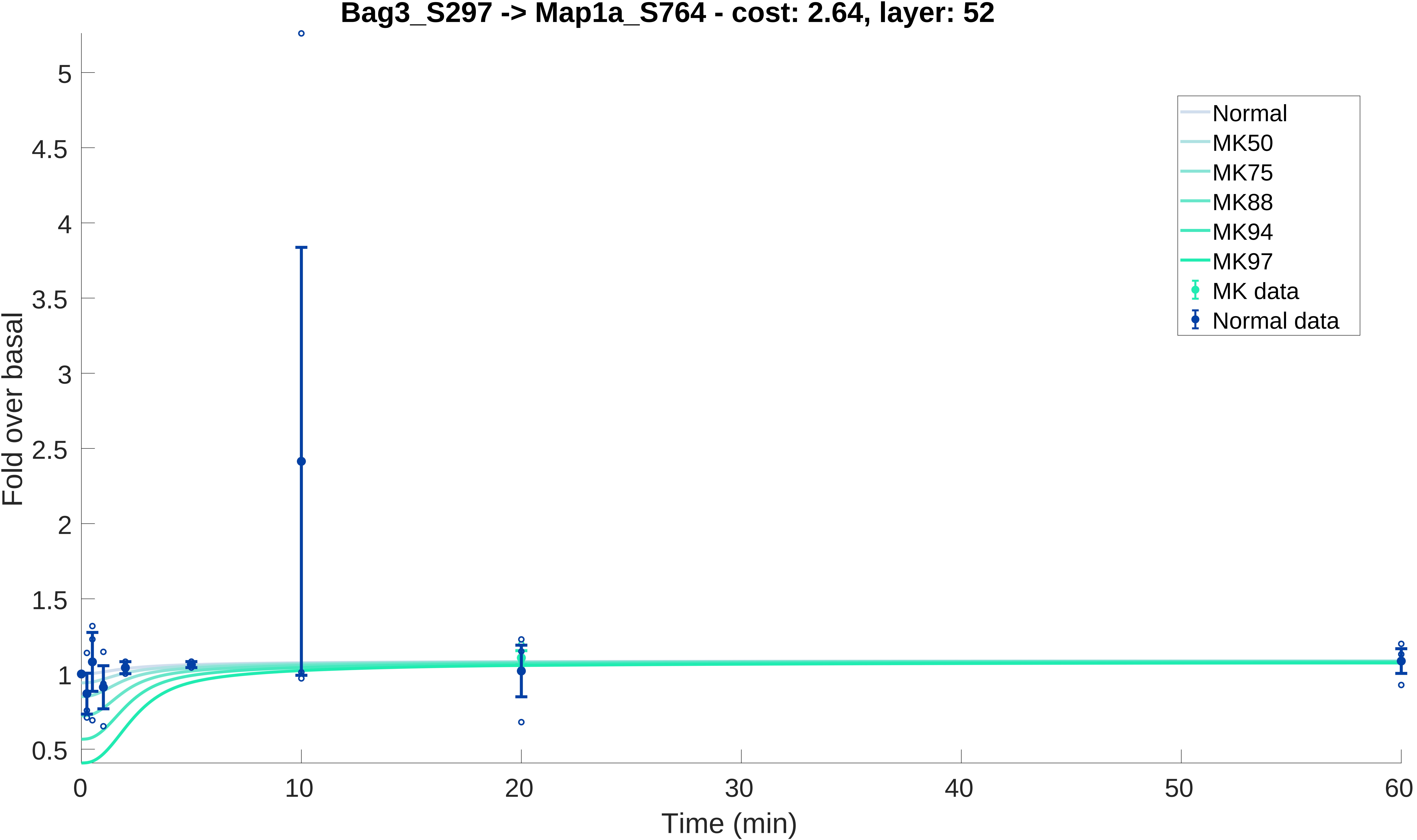

Bag3_S297 -> Map1a_S764 - cost: 2.64, layer: 52
Fold over basal
Time (min)
0.5
1
1.5
2
2.5
3
3.5
4
4.5
5
0
10
20
30
40
50
60
Normal
MK50
MK75
MK88
MK94
MK97
MK data
Normal data

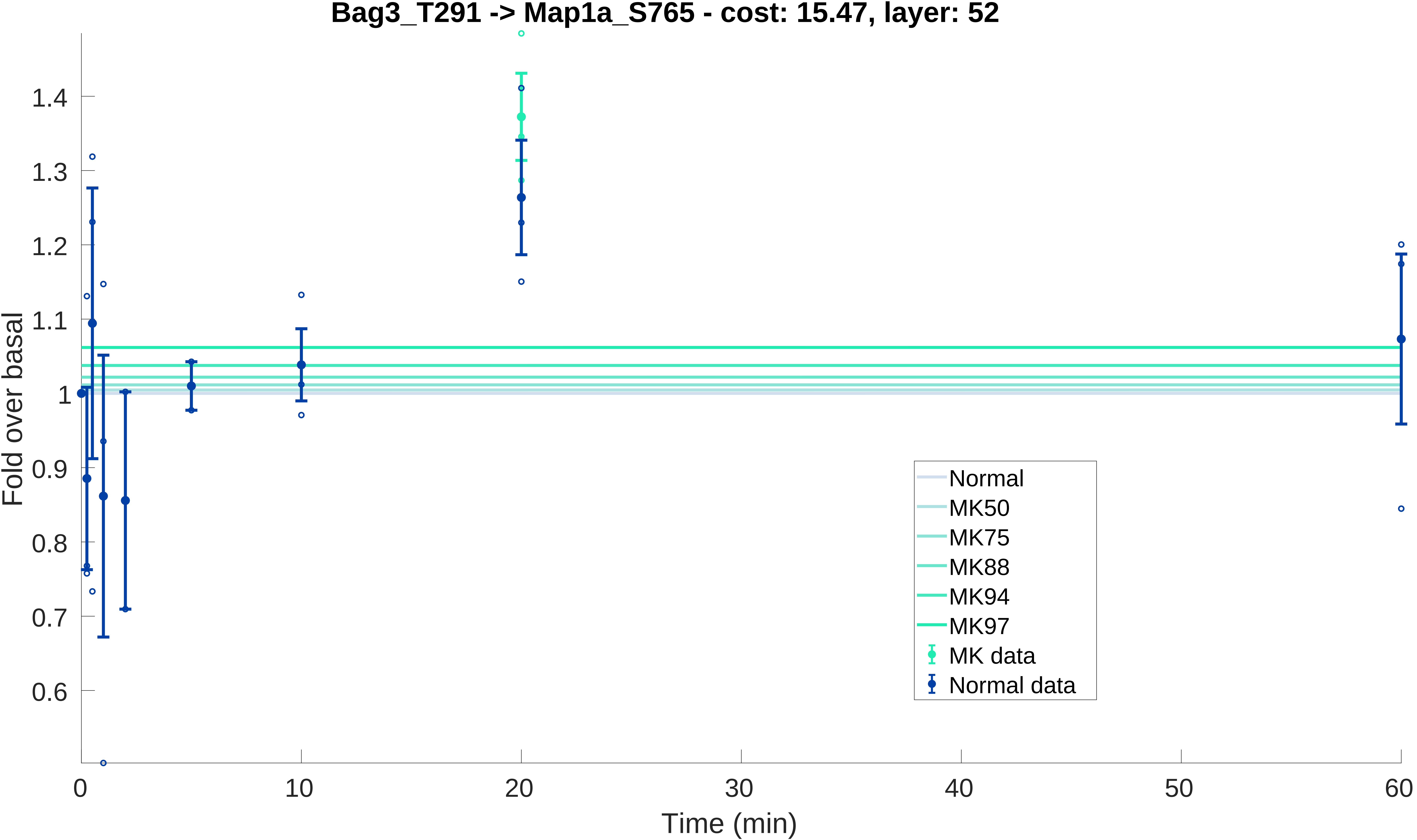
Bag3_T291 -> Map1a_S765 - cost: 15.47, layer: 52
Fold over basal
1.4
1.3
1.2
1.1
1
0.9
0.8
0.7
0.6
0
10
20
30
40
50
60
Time (min)
Normal
MK50
MK75
MK88
MK94
MK97
MK data
Normal data

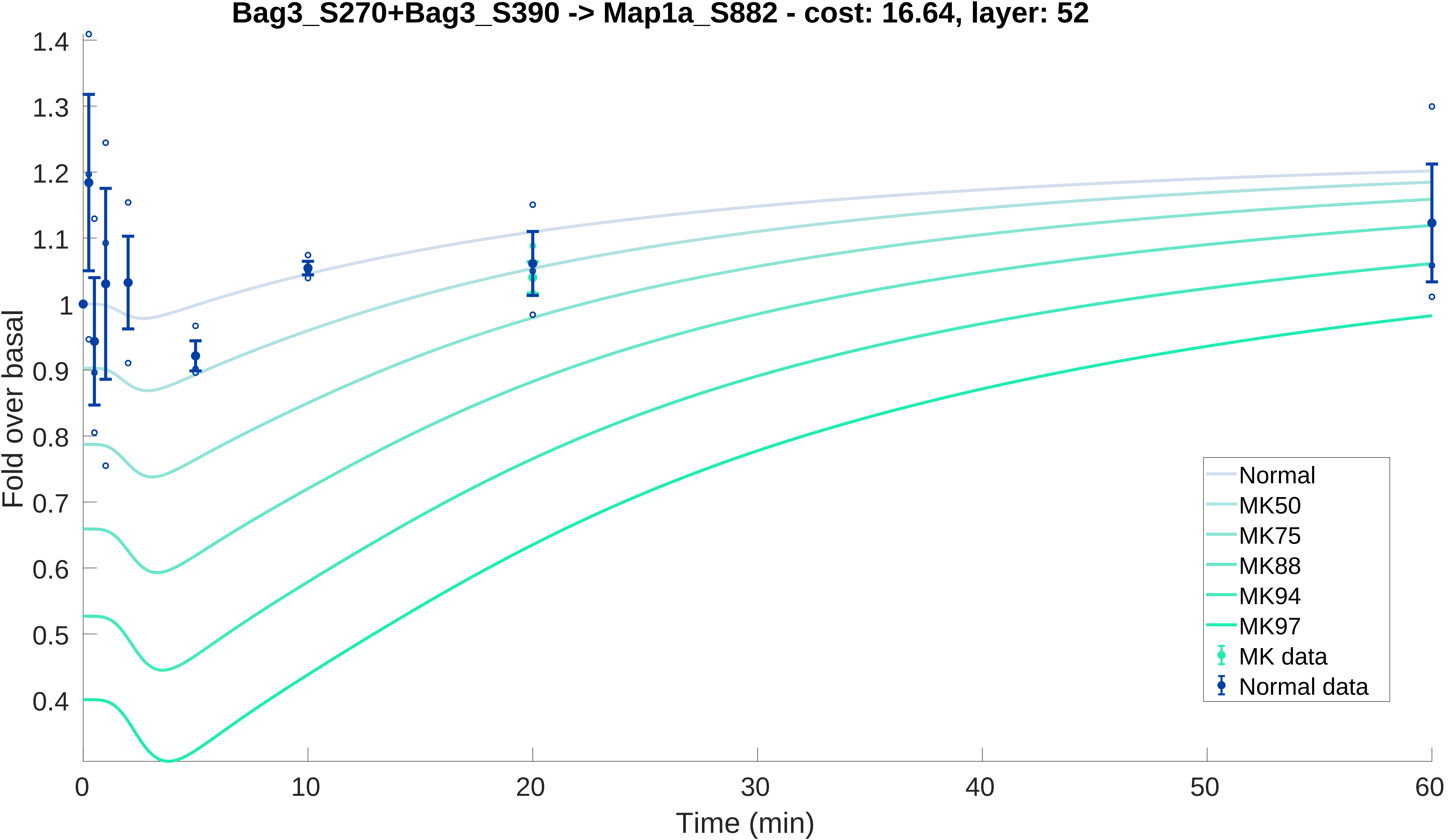

Bag3_S270+Bag3_S390 -> Map1a_S882 - cost: 16.64, layer: 52
Fold over basal
1.4
1.3
1.2
1.1
1
0.9
0.8
0.7
0.6
0.5
0.4
0
10
20
30
40
50
60
Time (min)
Normal
MK50
MK75
MK88
MK94
MK97
MK data
Normal data

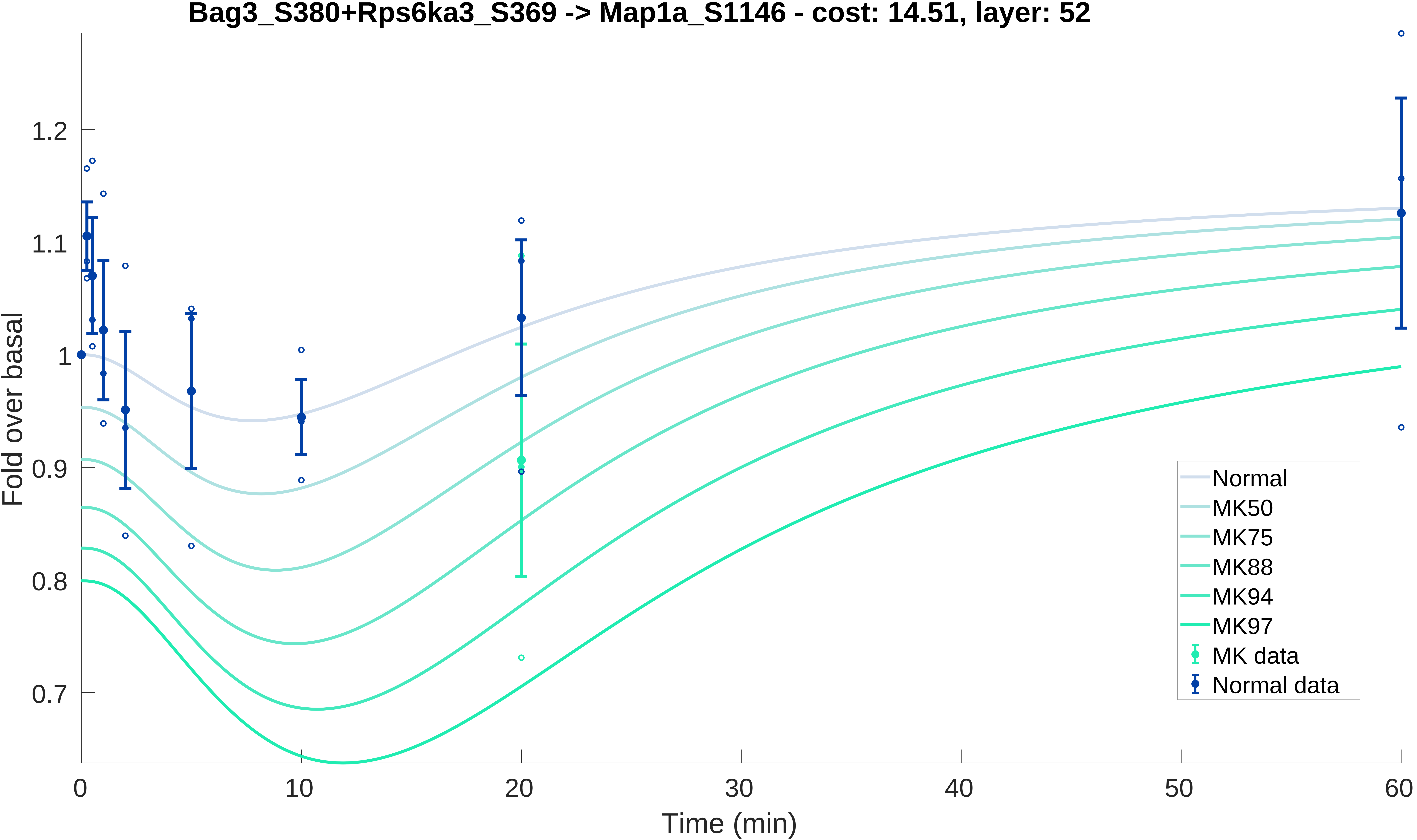

Bag3_S380+Rps6ka3_S369 -> Map1a_S1146 - cost: 14.51, layer: 52
Fold over basal
Time (min)
1.2
1.1
1
0.9
0.8
0.7
0
10
20
30
40
50
60
Normal
MK50
MK75
MK88
MK94
MK97
MK data
Normal data

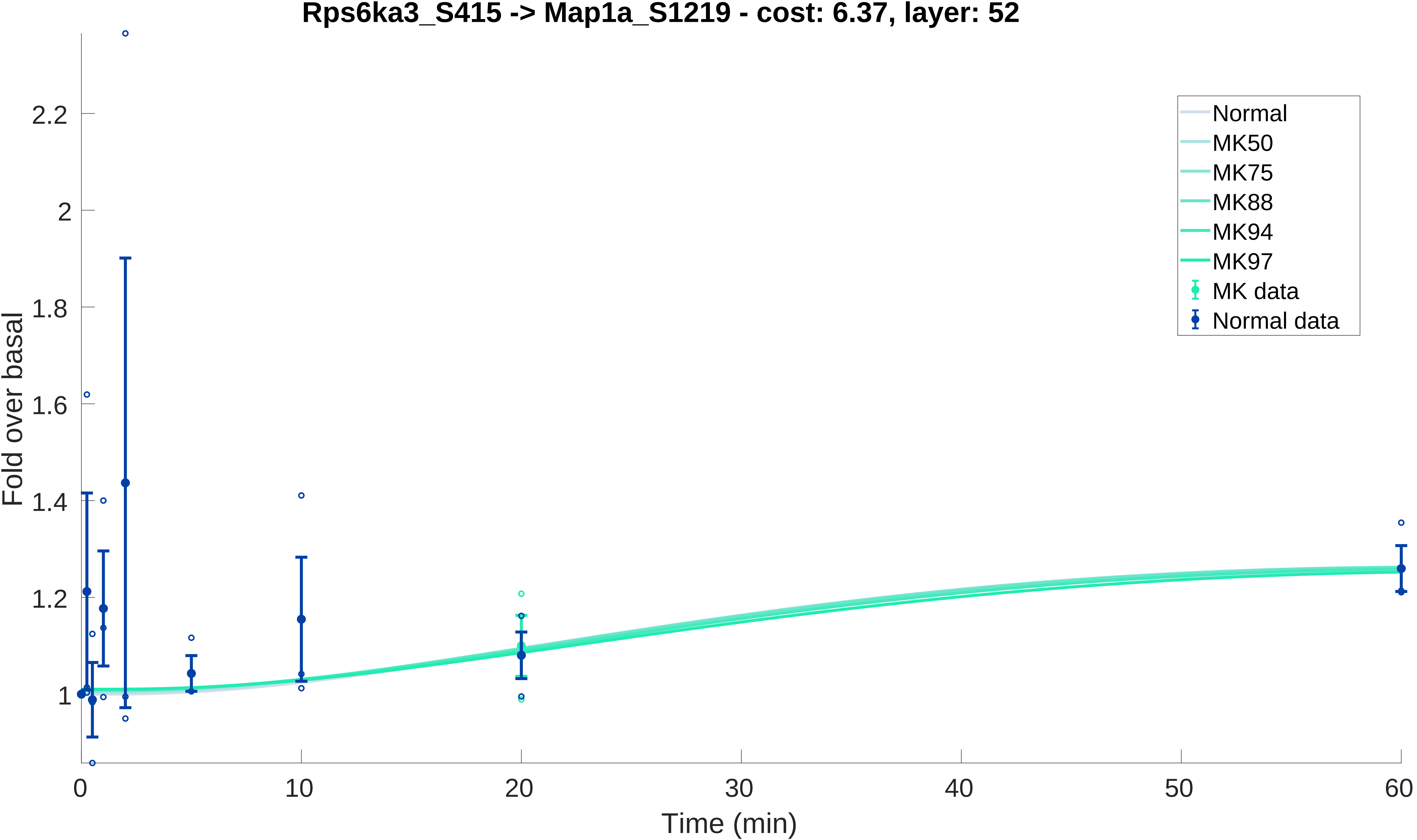
Rps6ka3_S415 -> Map1a_S1219 - cost: 6.37, layer: 52
Fold over basal
Time (min)
0
10
20
30
40
50
60
1
1.2
1.4
1.6
1.8
2
2.2
Normal
MK50
MK75
MK88
MK94
MK97
MK data
Normal data

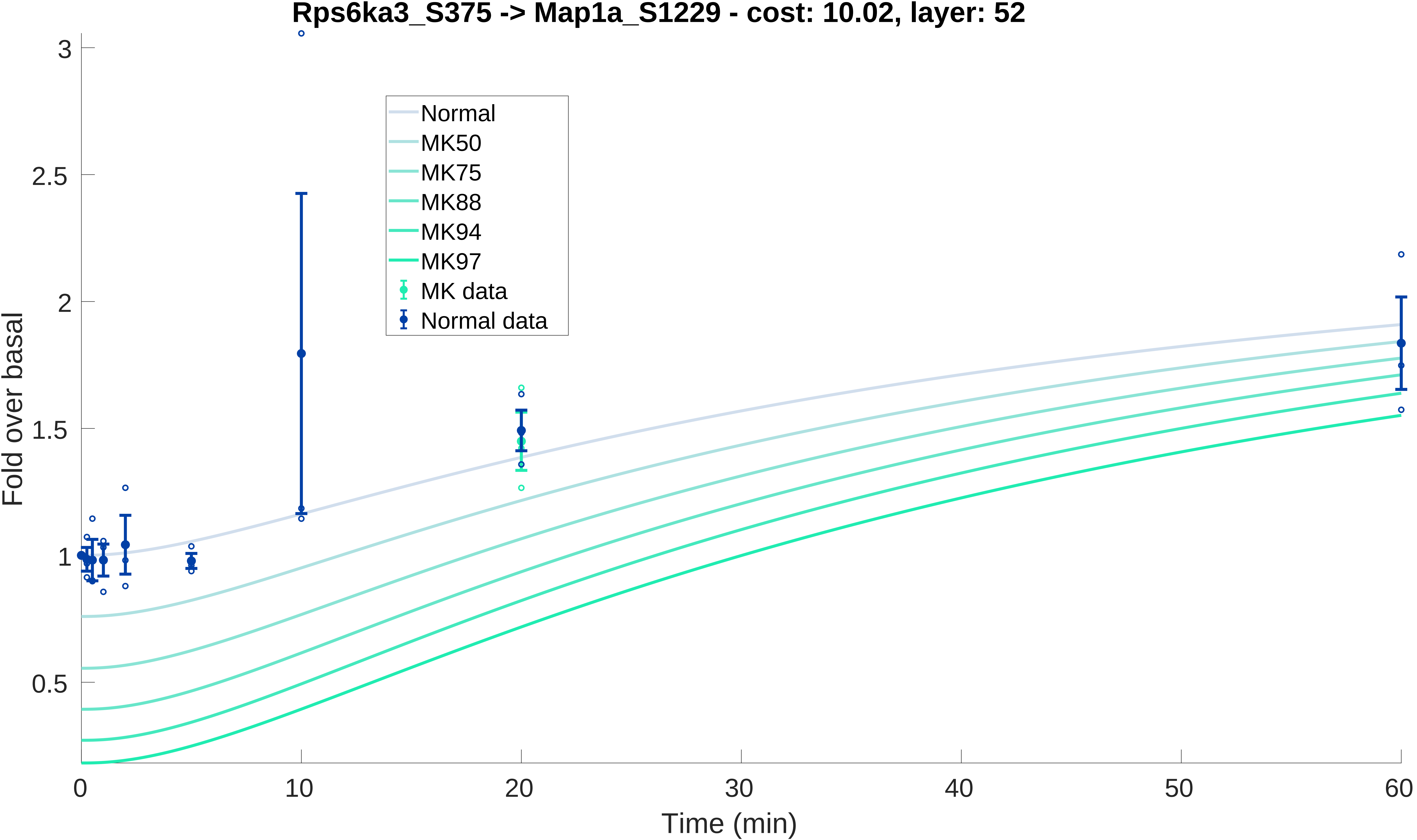

Rps6ka3_S375 -> Map1a_S1229 - cost: 10.02, layer: 52
Fold over basal
Time (min)
Normal
MK50
MK75
MK88
MK94
MK97
MK data
Normal data
0.5
1
1.5
2
2.5
3
0
10
20
30
40
50
60

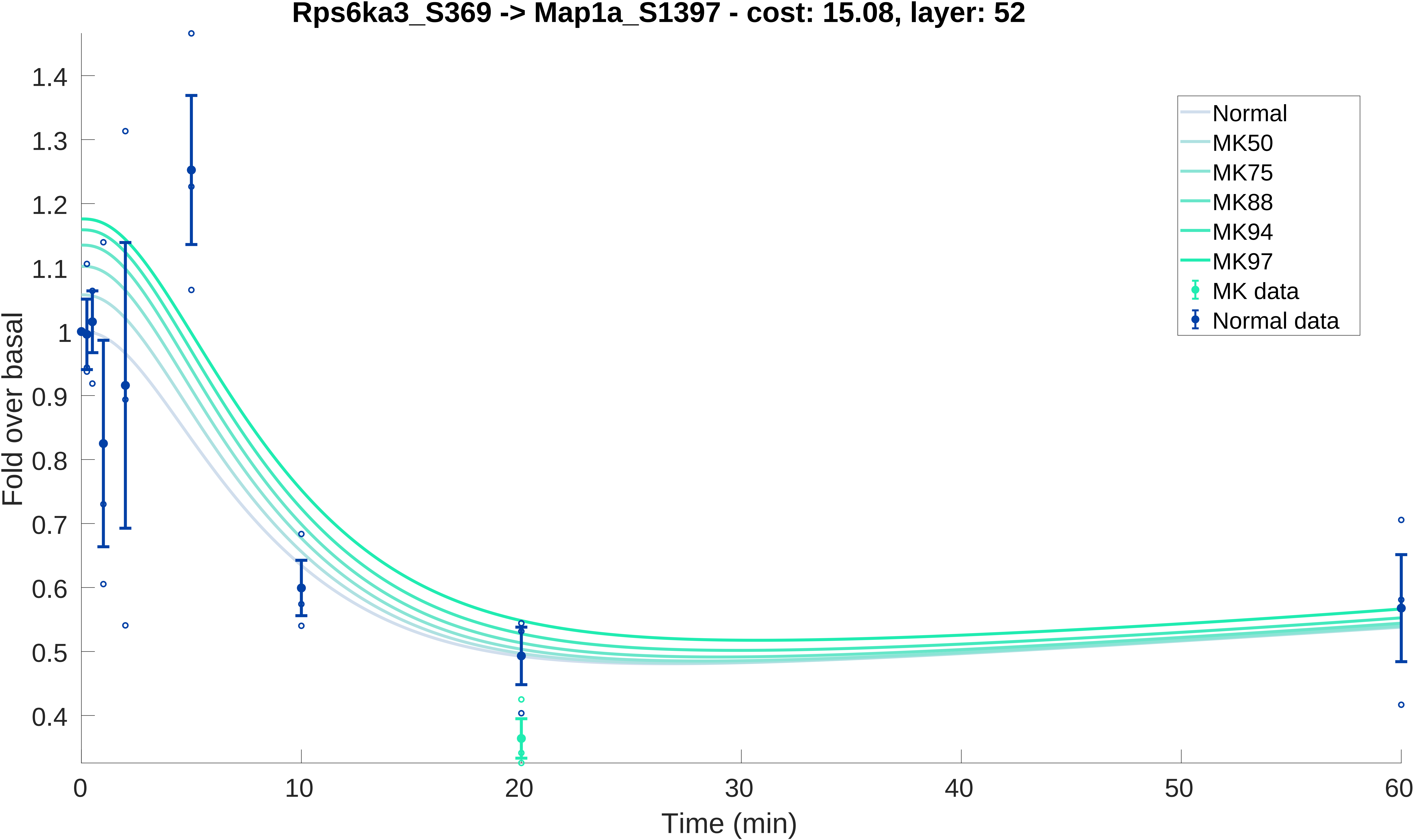
Rps6ka3_S369 -> Map1a_S1397 - cost: 15.08, layer: 52
Fold over basal
Time (min)
1.4
1.3
1.2
1.1
1
0.9
0.8
0.7
0.6
0.5
0.4
0
10
20
30
40
50
60
Normal
MK50
MK75
MK88
MK94
MK97
MK data
Normal data

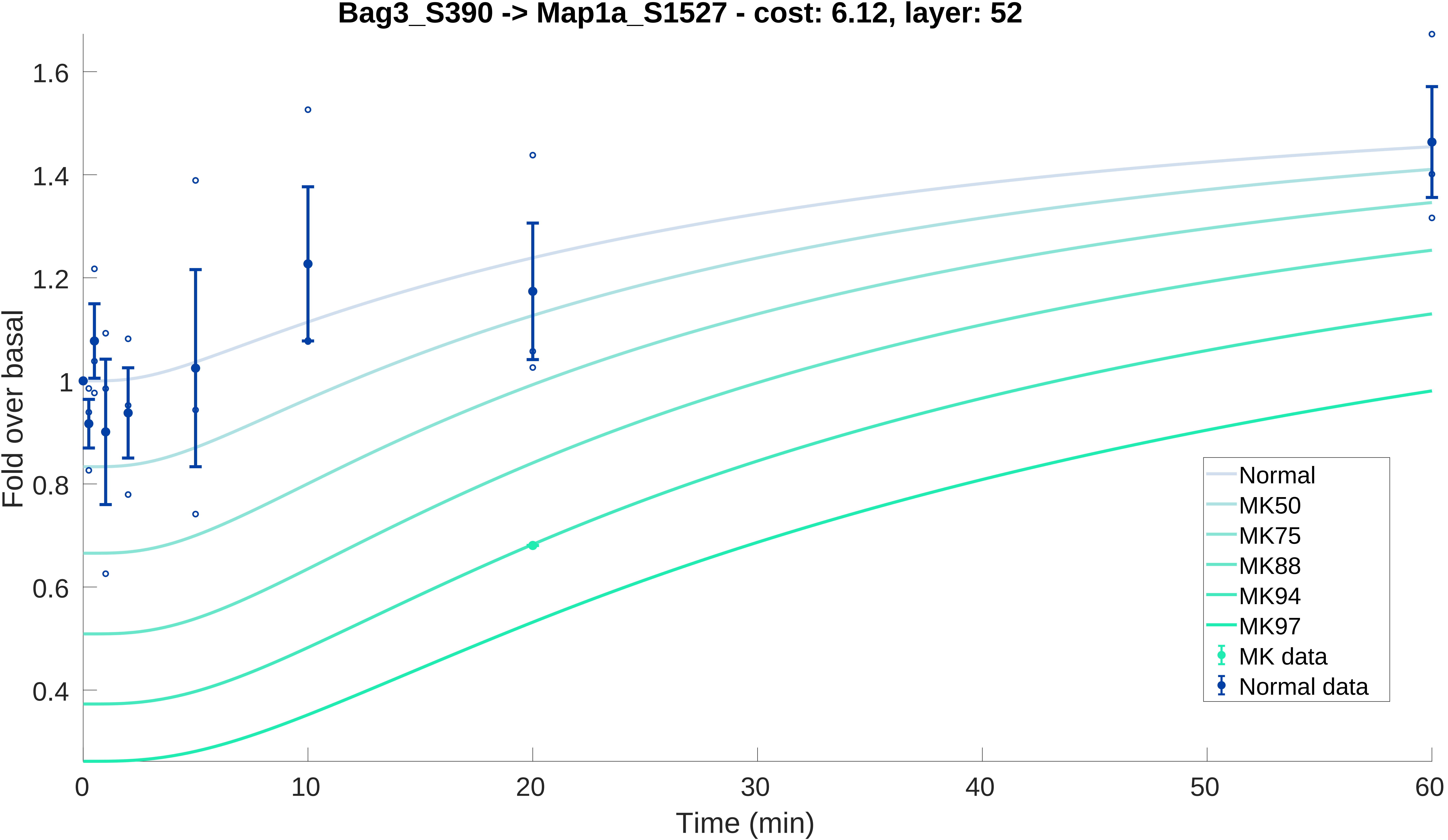

Bag3_S390 -> Map1a_S1527 - cost: 6.12, layer: 52
Fold over basal
1.6
1.4
1.2
1
0.8
0.6
0.4
0
10
20
30
40
50
60
Time (min)
Normal
MK50
MK75
MK88
MK94
MK97
MK data
Normal data

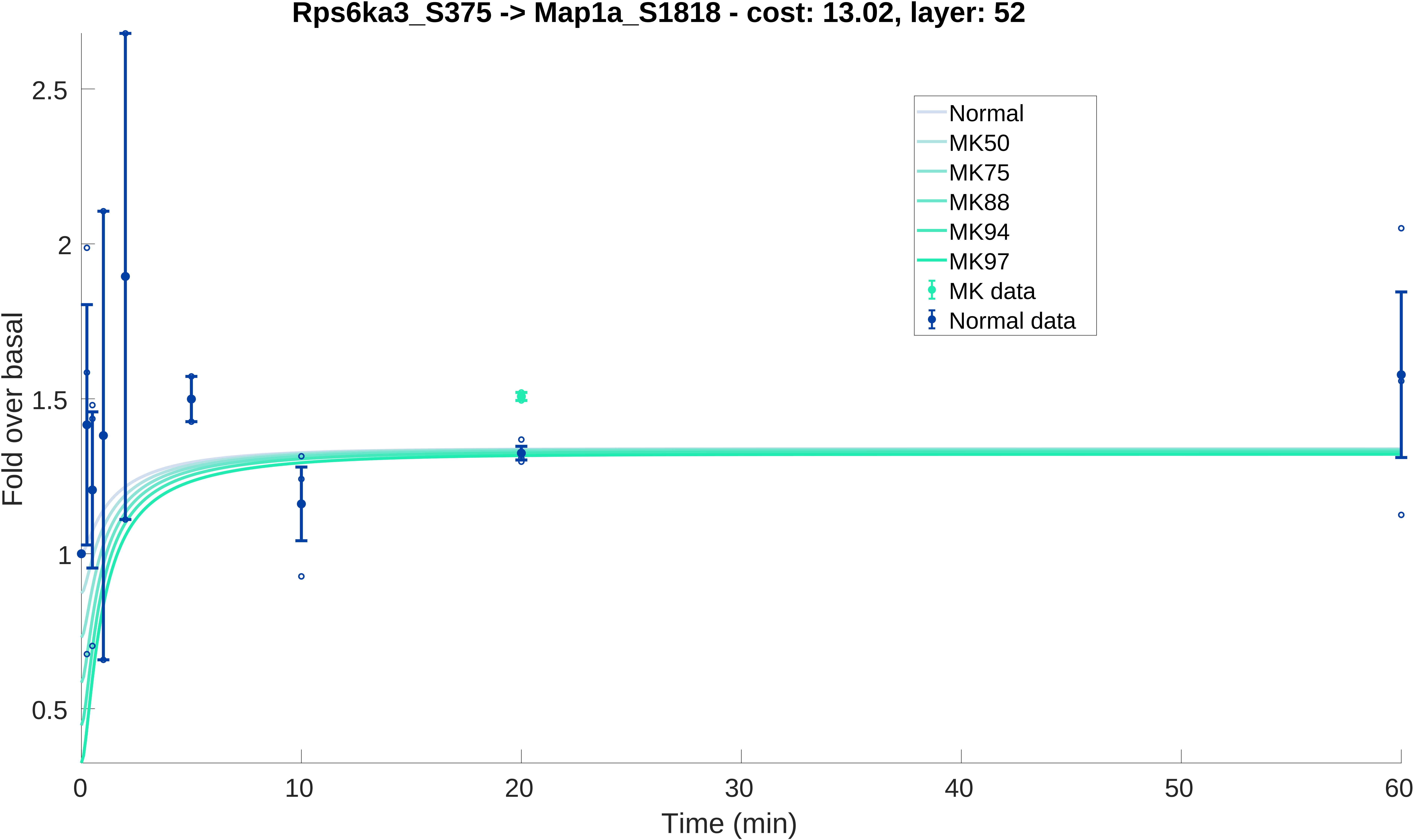

Rps6ka3_S375 -> Map1a_S1818 - cost: 13.02, layer: 52
Fold over basal
0.5
1
1.5
2
2.5
0
10
20
30
40
50
60
Time (min)
Normal
MK50
MK75
MK88
MK94
MK97
MK data
Normal data

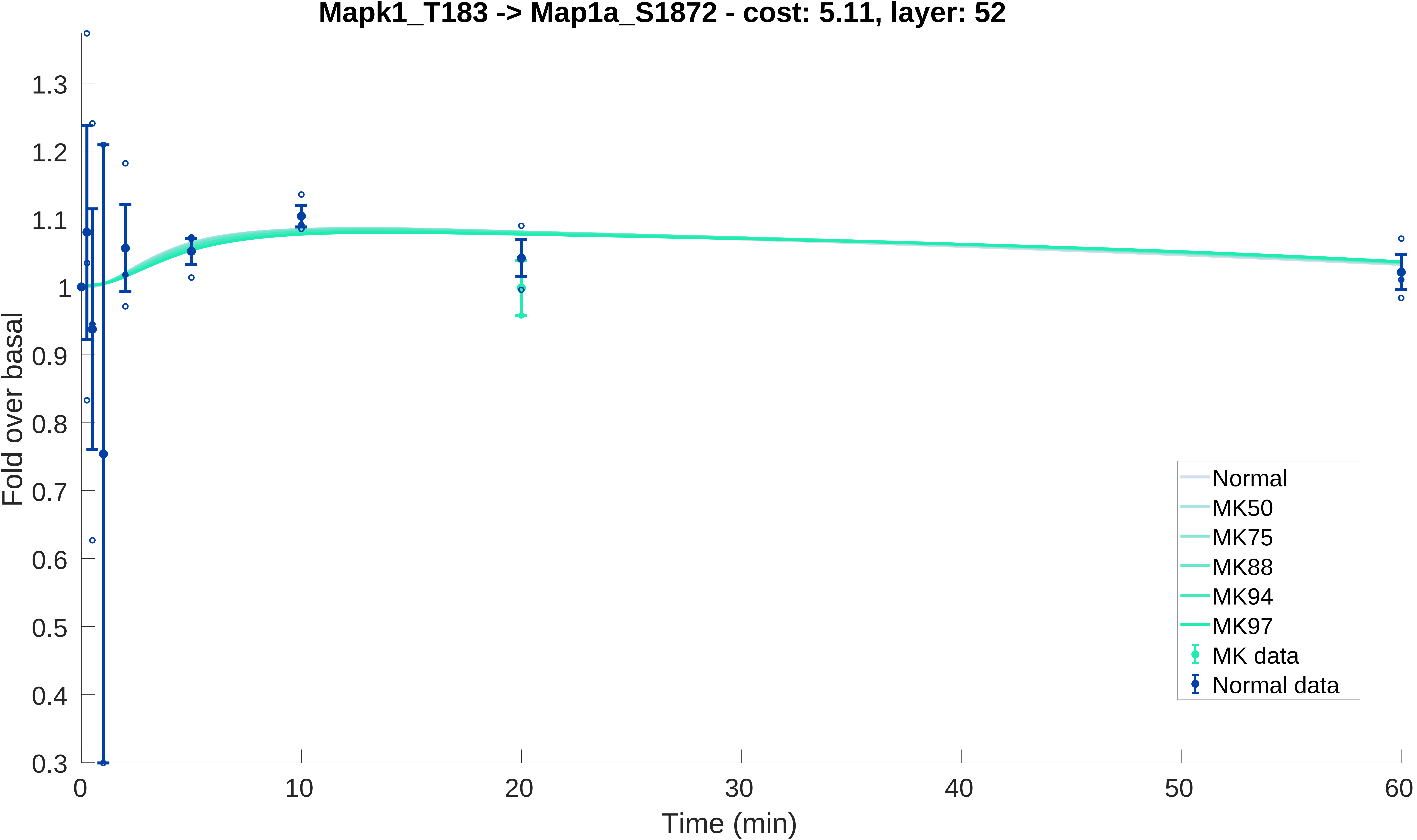
Mapk1_T183 -> Map1a_S1872 - cost: 5.11, layer: 52
Fold over basal
1.3
1.2
1.1
1
0.9
0.8
0.7
0.6
0.5
0.4
0.3
0
10
20
30
40
50
60
Time (min)
Normal
MK50
MK75
MK88
MK94
MK97
MK data
Normal data

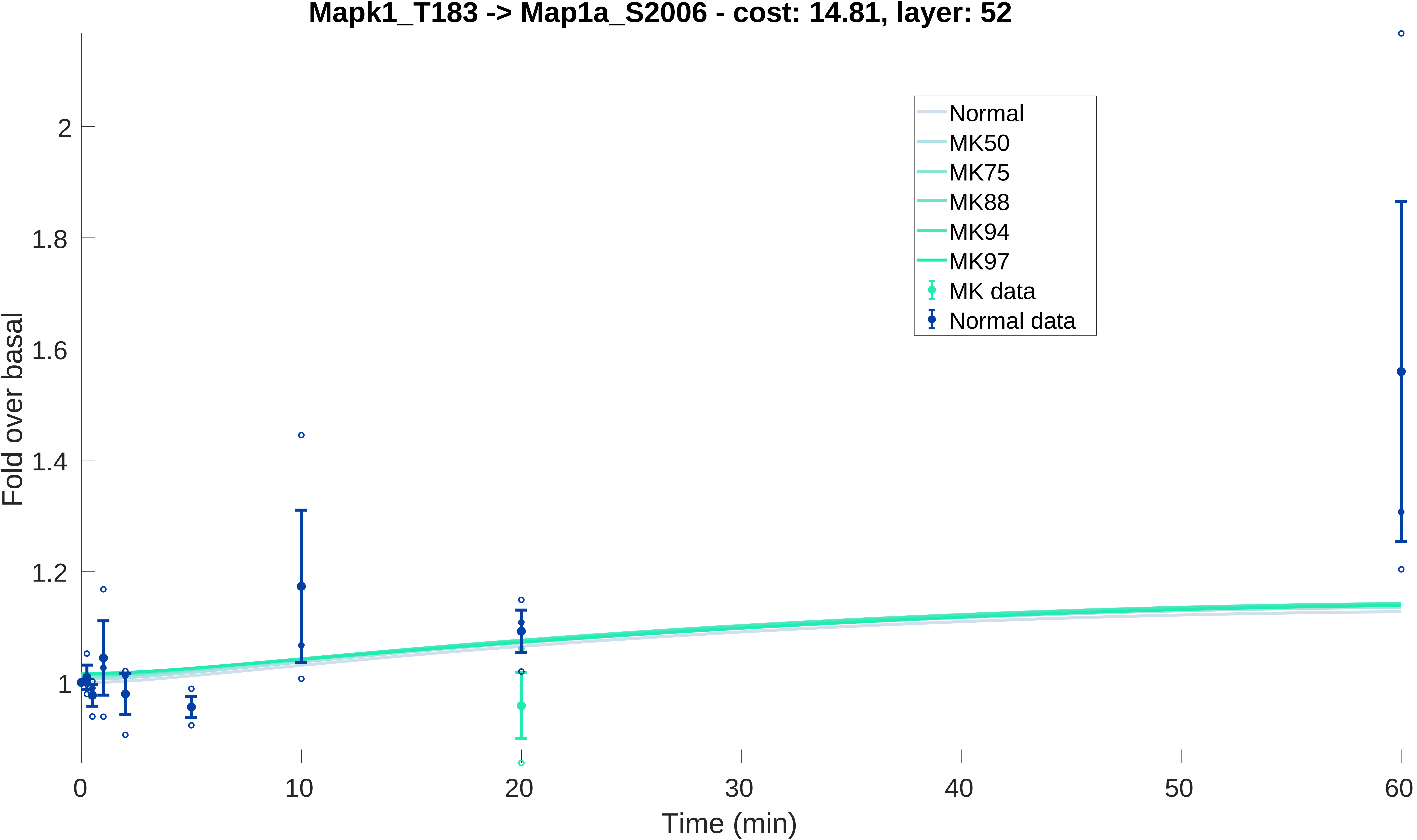
Mapk1_T183 -> Map1a_S2006 - cost: 14.81, layer: 52
Fold over basal
Time (min)
2
1.8
1.6
1.4
1.2
1
0
10
20
30
40
50
60
Normal
MK50
MK75
MK88
MK94
MK97
MK data
Normal data

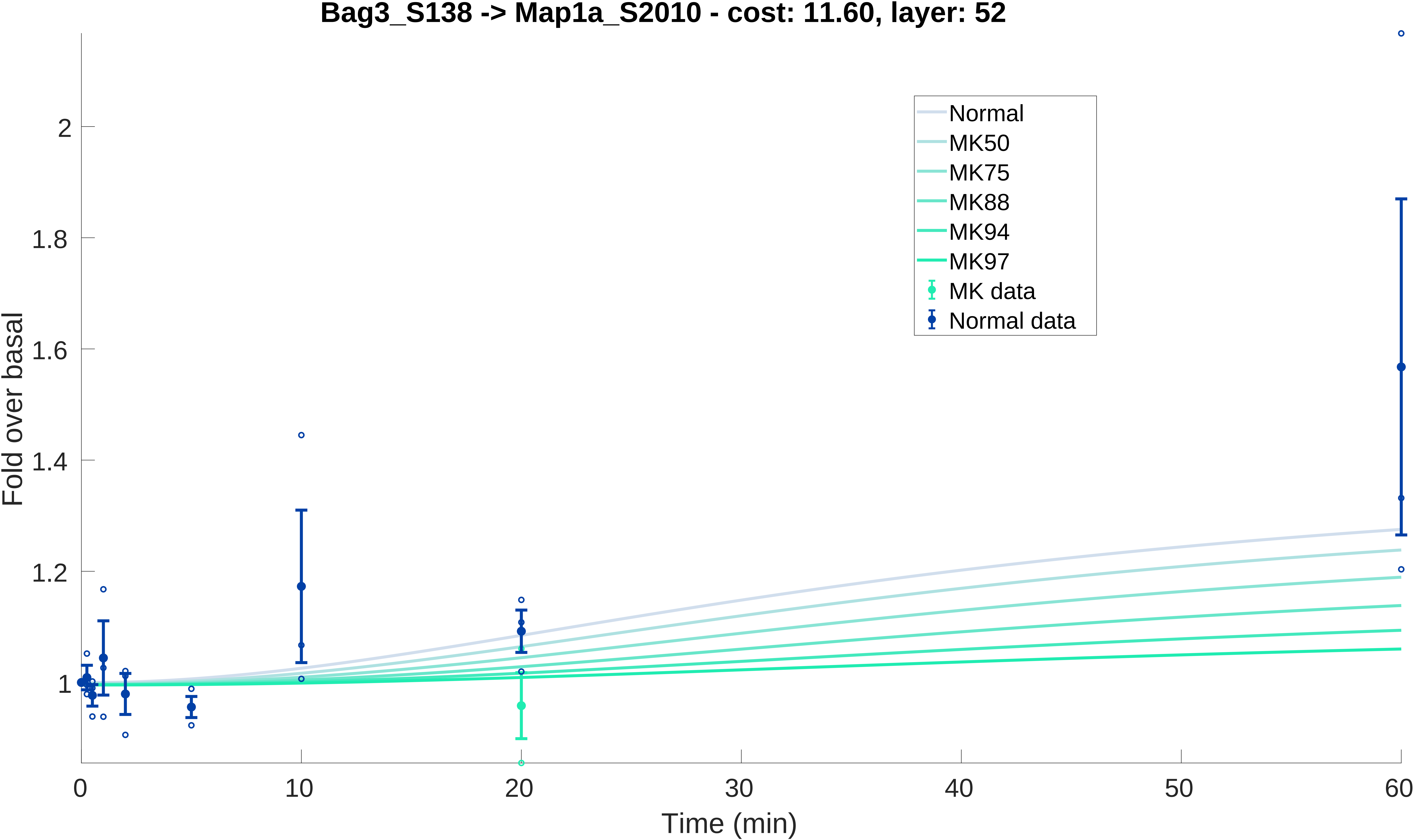
Bag3_S138 -> Map1a_S2010 - cost: 11.60, layer: 52
Normal
MK50
MK75
MK88
MK94
MK97
MK data
Normal data
Fold over basal
Time (min)
2
1.8
1.6
1.4
1.2
1
0
10
20
30
40
50
60

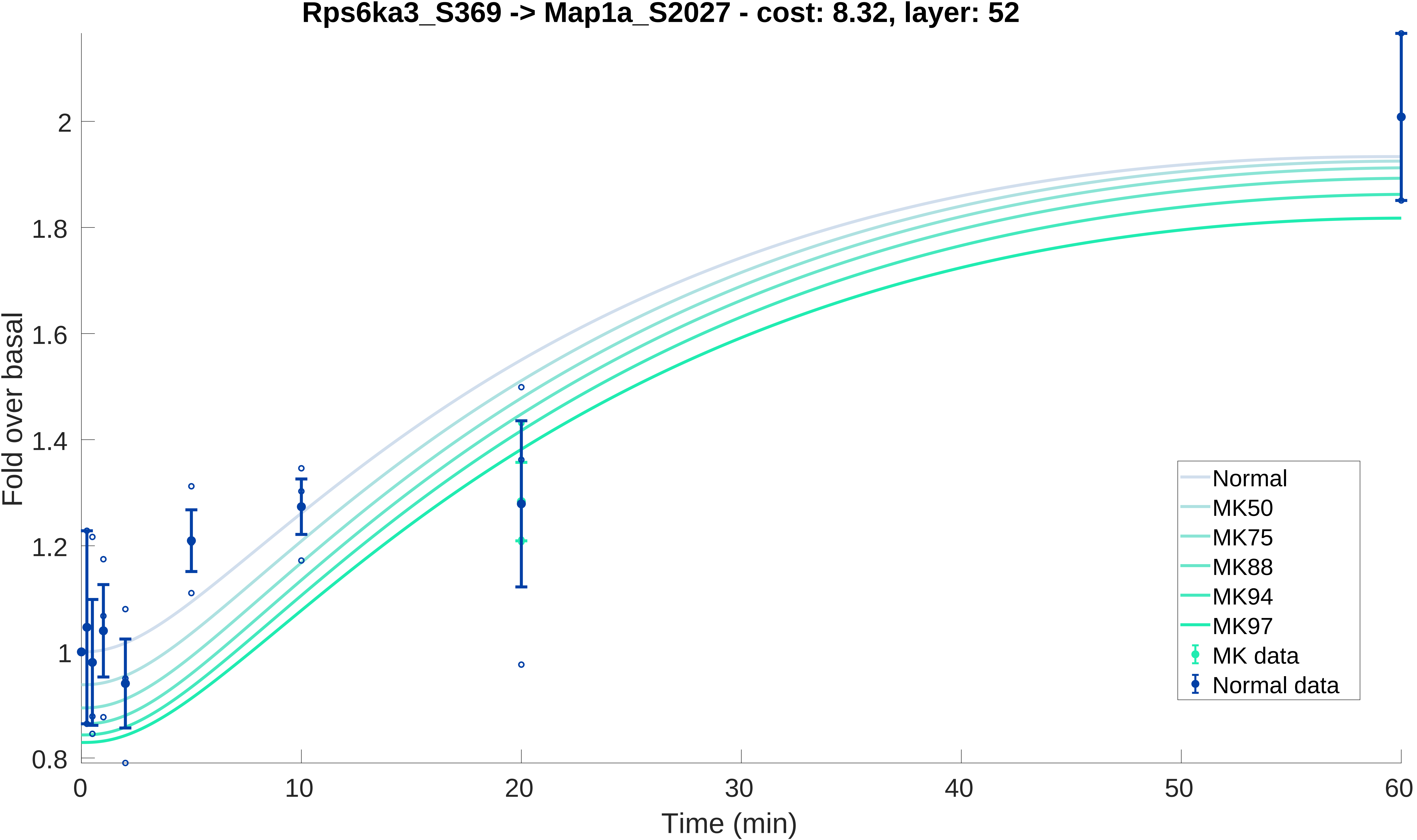

Rps6ka3_S369 -> Map1a_S2027 - cost: 8.32, layer: 52
Fold over basal
Time (min)
Normal
MK50
MK75
MK88
MK94
MK97
MK data
Normal data
0.8
1
1.2
1.4
1.6
1.8
2
0
10
20
30
40
50
60

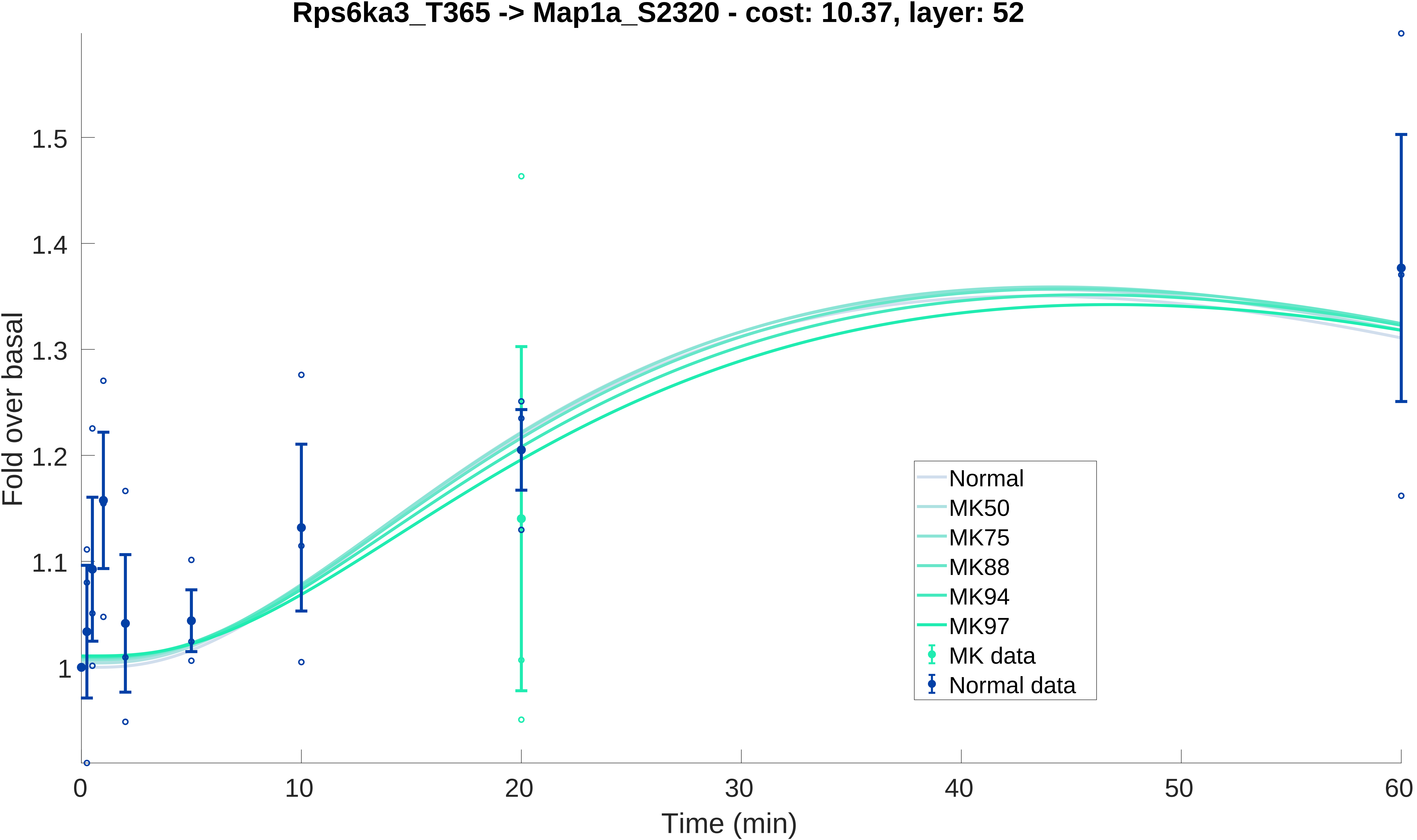

Rps6ka3_T365 -> Map1a_S2320 - cost: 10.37, layer: 52
Fold over basal
Time (min)
1
1.1
1.2
1.3
1.4
1.5
0
10
20
30
40
50
60
Normal
MK50
MK75
MK88
MK94
MK97
MK data
Normal data

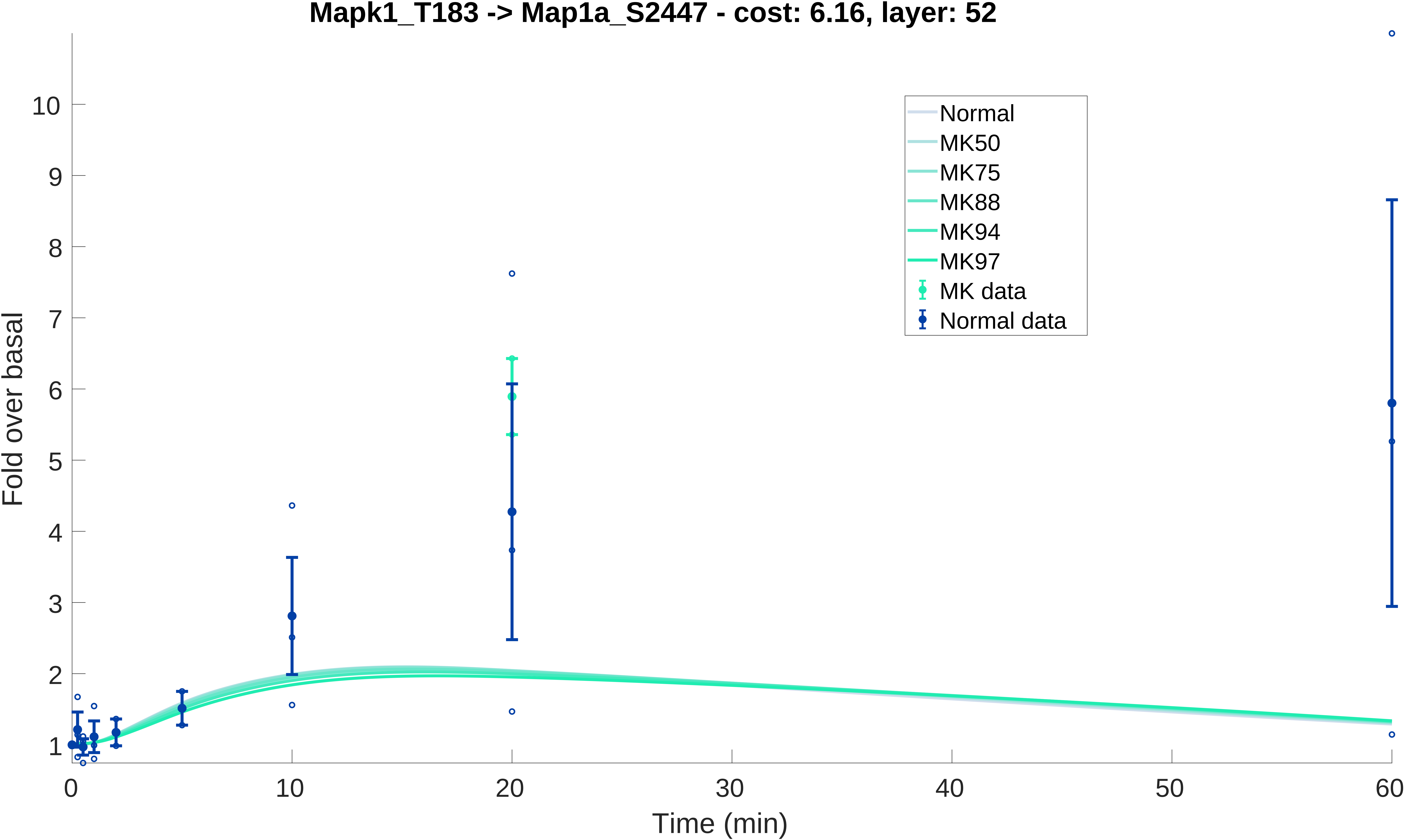

Mapk1_T183 -> Map1a_S2447 - cost: 6.16, layer: 52
Fold over basal
Time (min)
Normal
MK50
MK75
MK88
MK94
MK97
MK data
Normal data
0
10
20
30
40
50
60
1
2
3
4
5
6
7
8
9
10

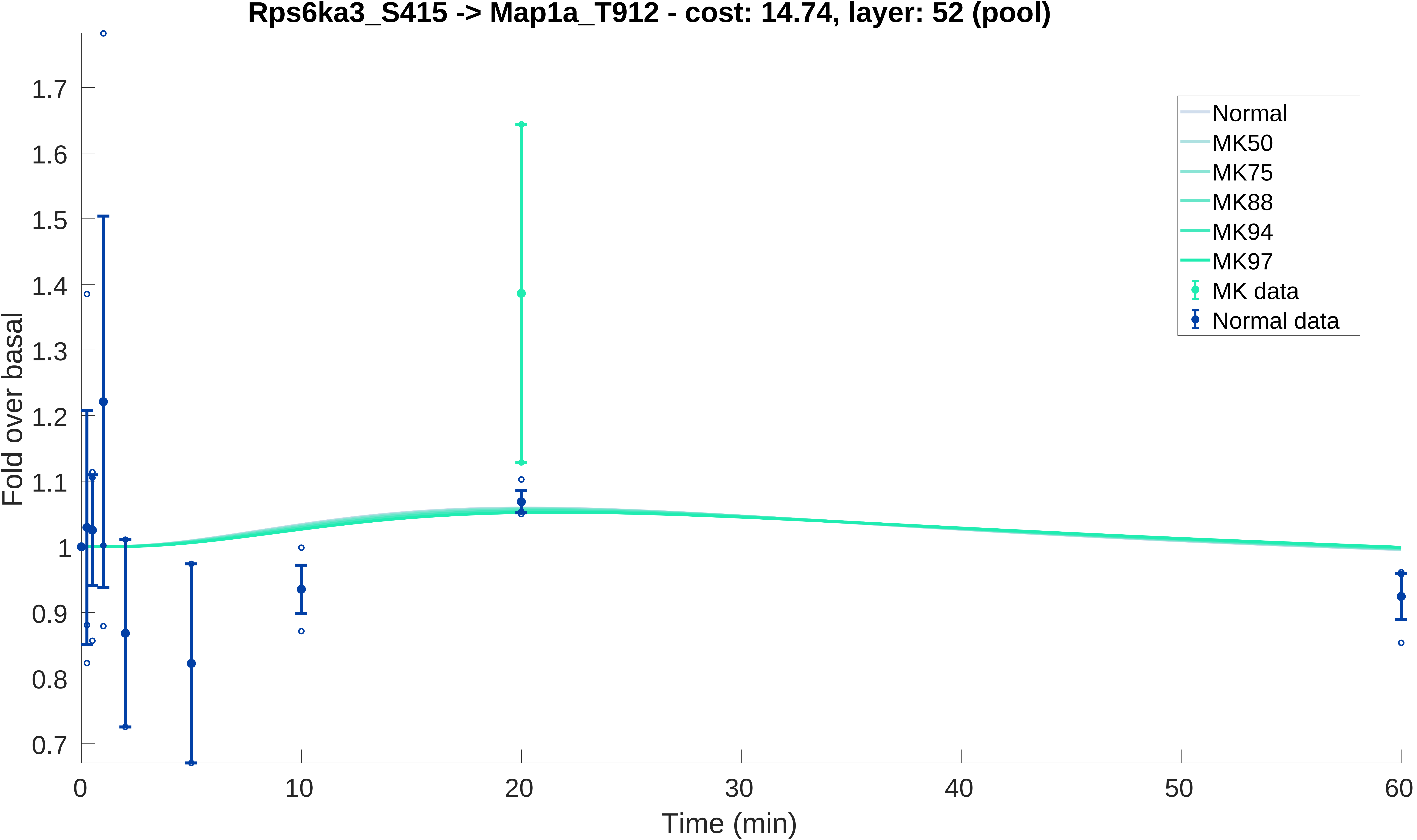

Rps6ka3_S415 -> Map1a_T912 - cost: 14.74, layer: 52 (pool)
Fold over basal
Time (min)
0.7
0.8
0.9
1
1.1
1.2
1.3
1.4
1.5
1.6
1.7
0
10
20
30
40
50
60
Normal
MK50
MK75
MK88
MK94
MK97
MK data
Normal data

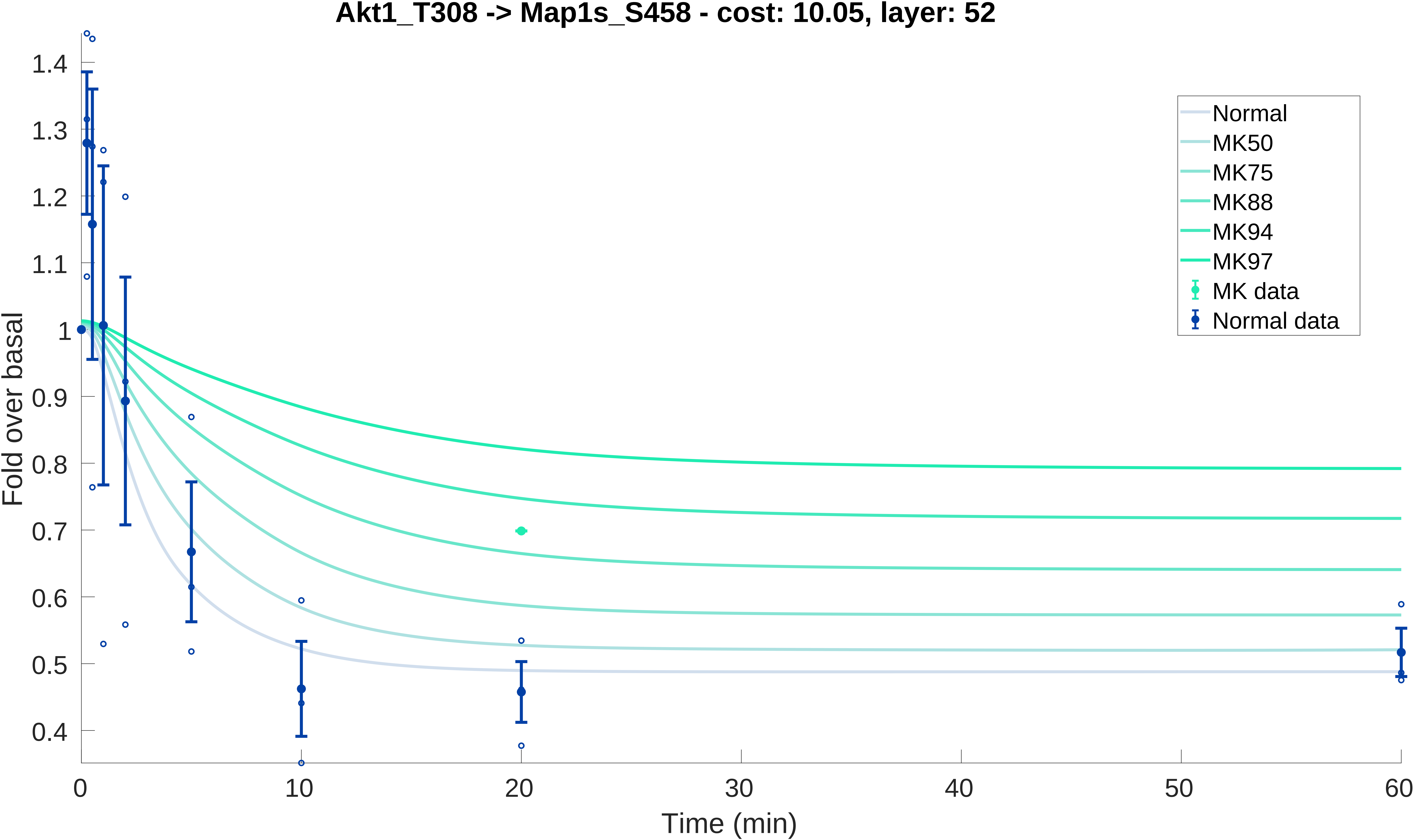

Akt1_T308 -> Map1s_S458 - cost: 10.05, layer: 52
Fold over basal
Time (min)
0
10
20
30
40
50
60
0.4
0.5
0.6
0.7
0.8
0.9
1
1.1
1.2
1.3
1.4
Normal
MK50
MK75
MK88
MK94
MK97
MK data
Normal data

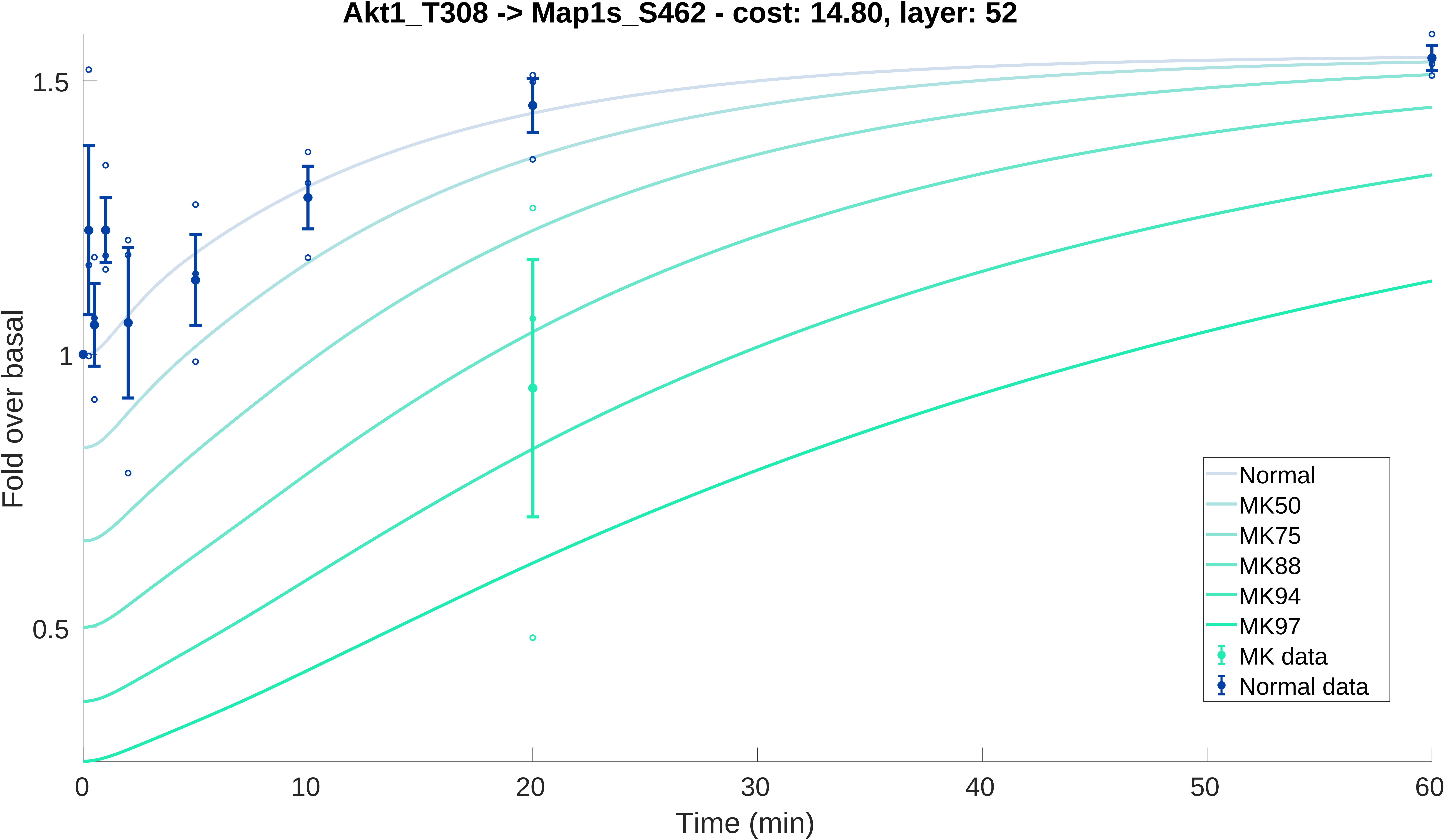
Akt1_T308 -> Map1s_S462 - cost: 14.80, layer: 52
Fold over basal
1.5
1
0.5
0
10
20
30
40
50
60
Time (min)
Normal
MK50
MK75
MK88
MK94
MK97
MK data
Normal data

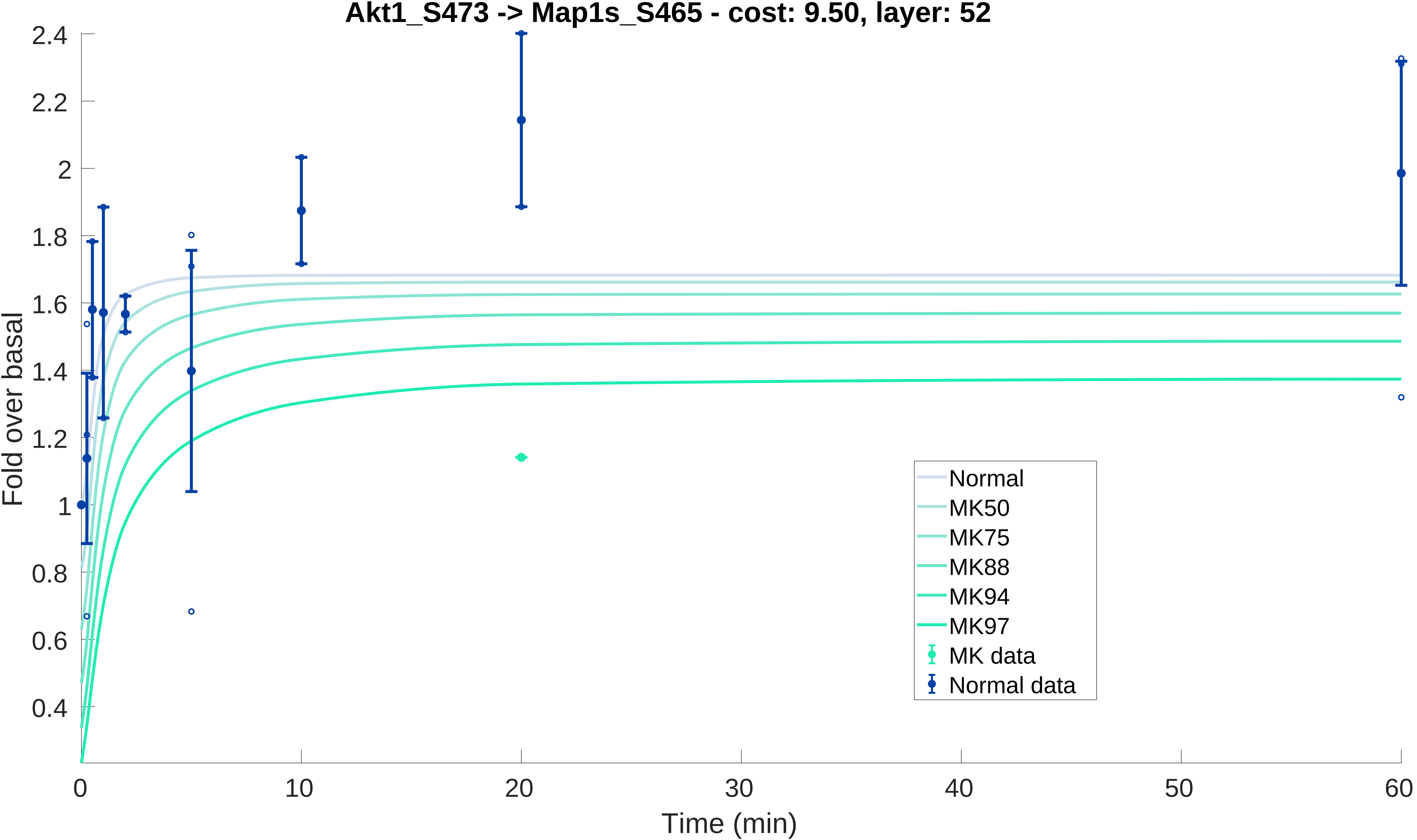

Akt1_S473 -> Map1s_S465 - cost: 9.50, layer: 52
Fold over basal
Time (min)
2.4
2.2
2
1.8
1.6
1.4
1.2
1
0.8
0.6
0.4
0
10
20
30
40
50
60
Normal
MK50
MK75
MK88
MK94
MK97
MK data
Normal data

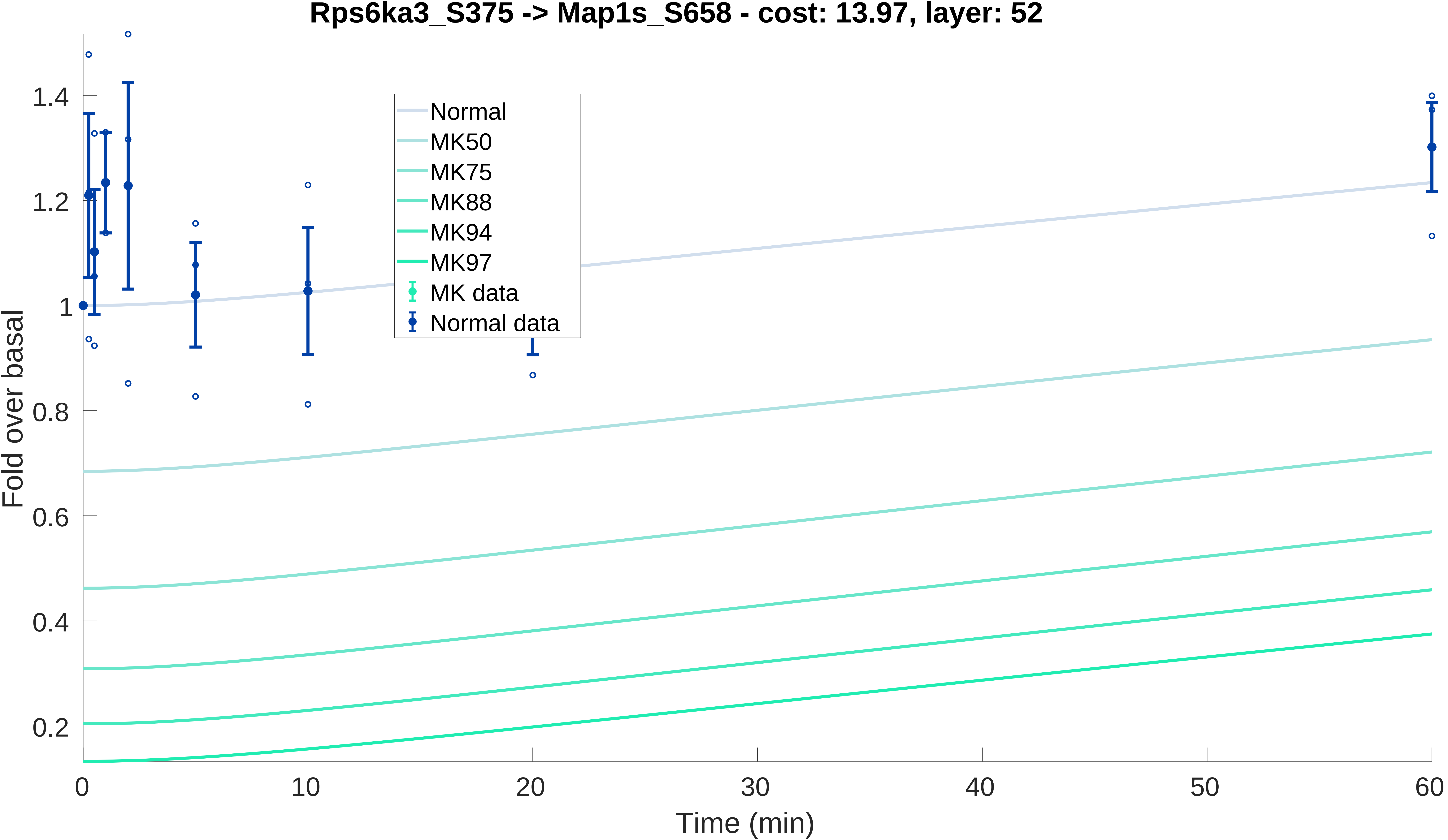

Rps6ka3_S375 -> Map1s_S658 - cost: 13.97, layer: 52
Fold over basal
1.4
1.2
1
0.8
0.6
0.4
0.2
0
10
20
30
40
50
60
Time (min)
Normal
MK50
MK75
MK88
MK94
MK97
MK data
Normal data

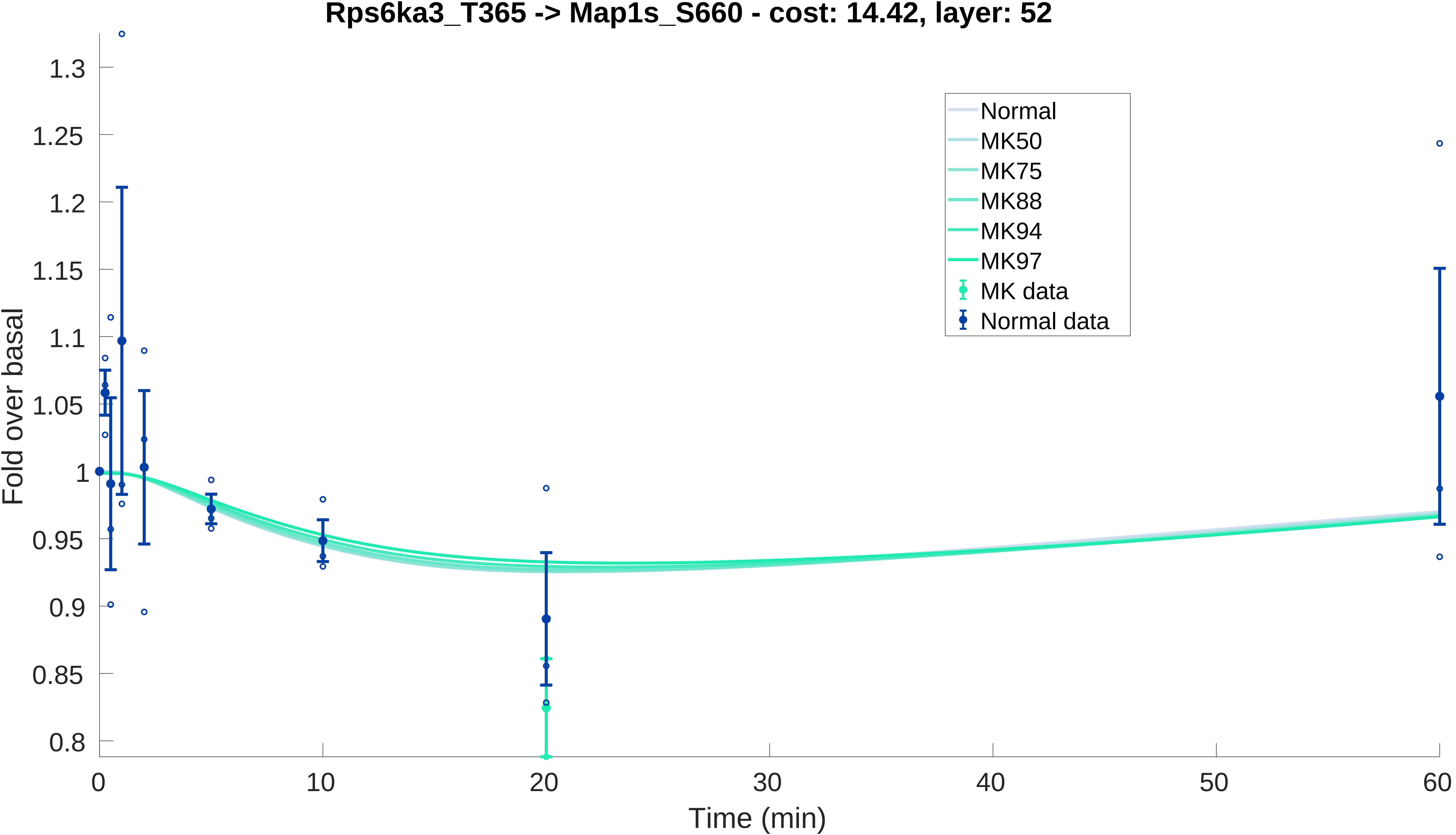
Rps6ka3_T365 -> Map1s_S660 - cost: 14.42, layer: 52
Fold over basal
Time (min)
Normal
MK50
MK75
MK88
MK94
MK97
MK data
Normal data
1.3
1.25
1.2
1.15
1.1
1.05
1
0.95
0.9
0.85
0.8
0
10
20
30
40
50
60

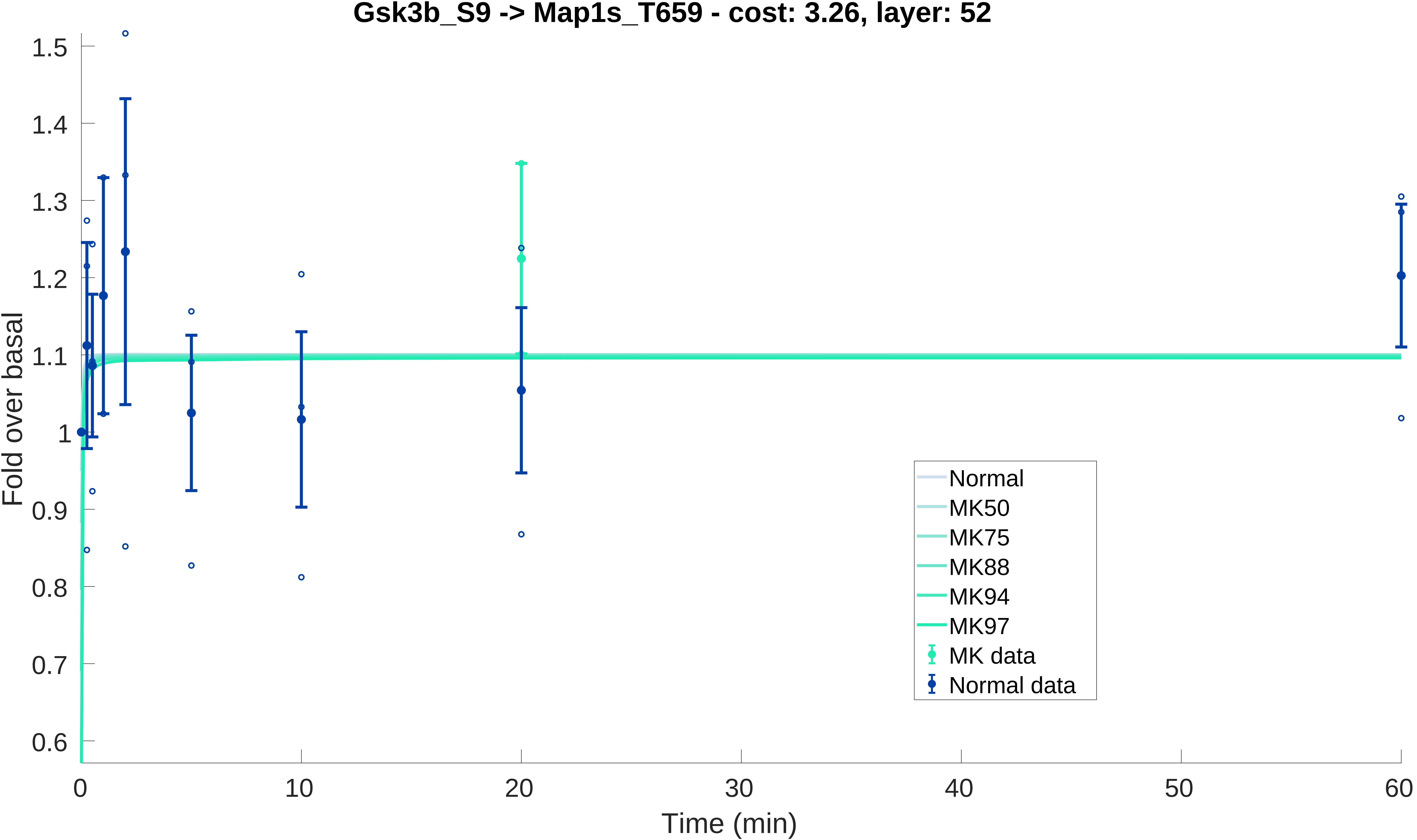

Gsk3b_S9 -> Map1s_T659 - cost: 3.26, layer: 52
Fold over basal
1.5
1.4
1.3
1.2
1.1
1
0.9
0.8
0.7
0.6
0
10
20
30
40
50
60
Time (min)
Normal
MK50
MK75
MK88
MK94
MK97
MK data
Normal data

Gsk3b_S9 -> Map2_S1783 - cost: 14.38, layer: 52

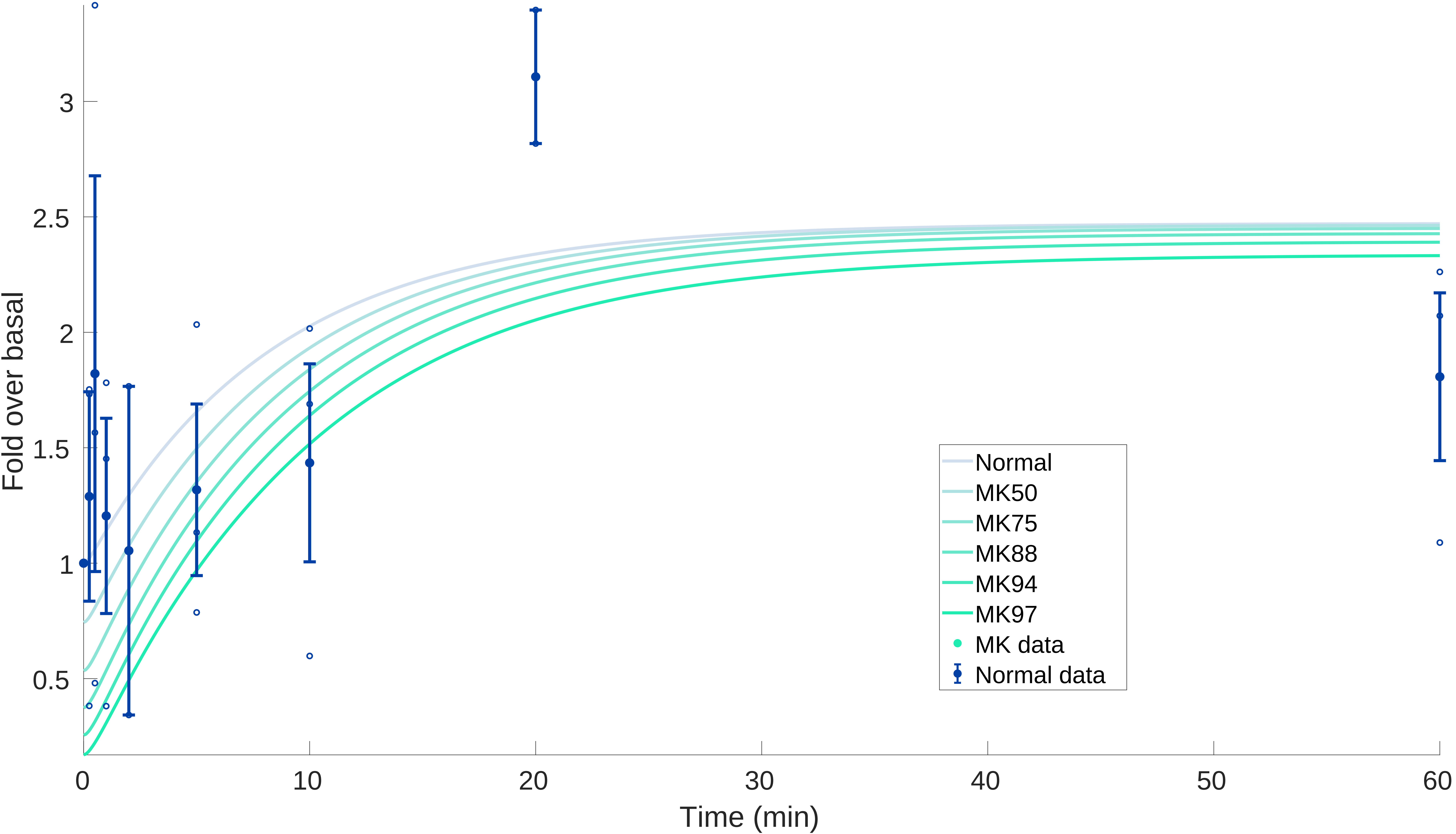

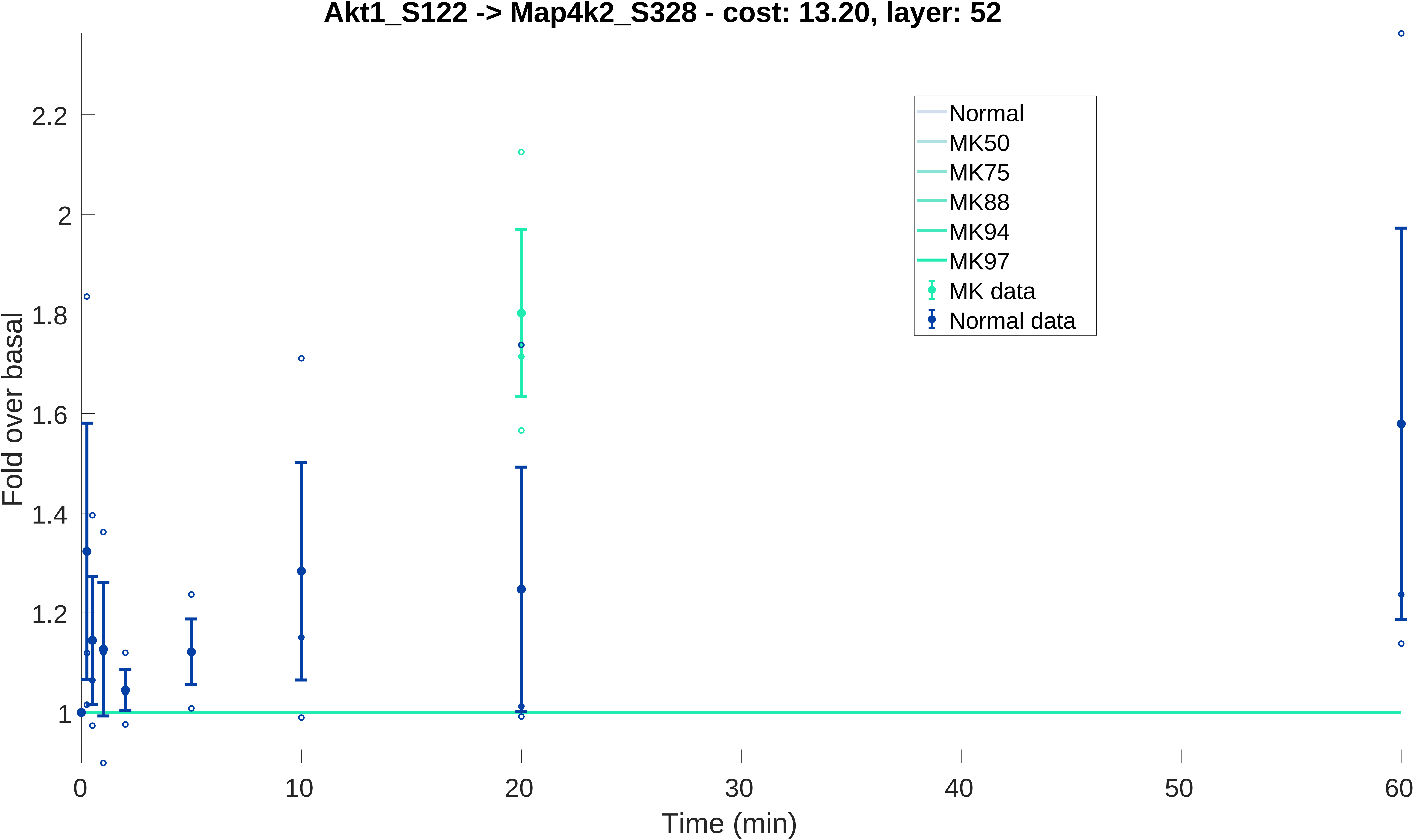

Akt1_S122 -> Map4k2_S328 - cost: 13.20, layer: 52
Fold over basal
Time (min)
Normal
MK50
MK75
MK88
MK94
MK97
MK data
Normal data
2.2
2
1.8
1.6
1.4
1.2
1
0
10
20
30
40
50
60

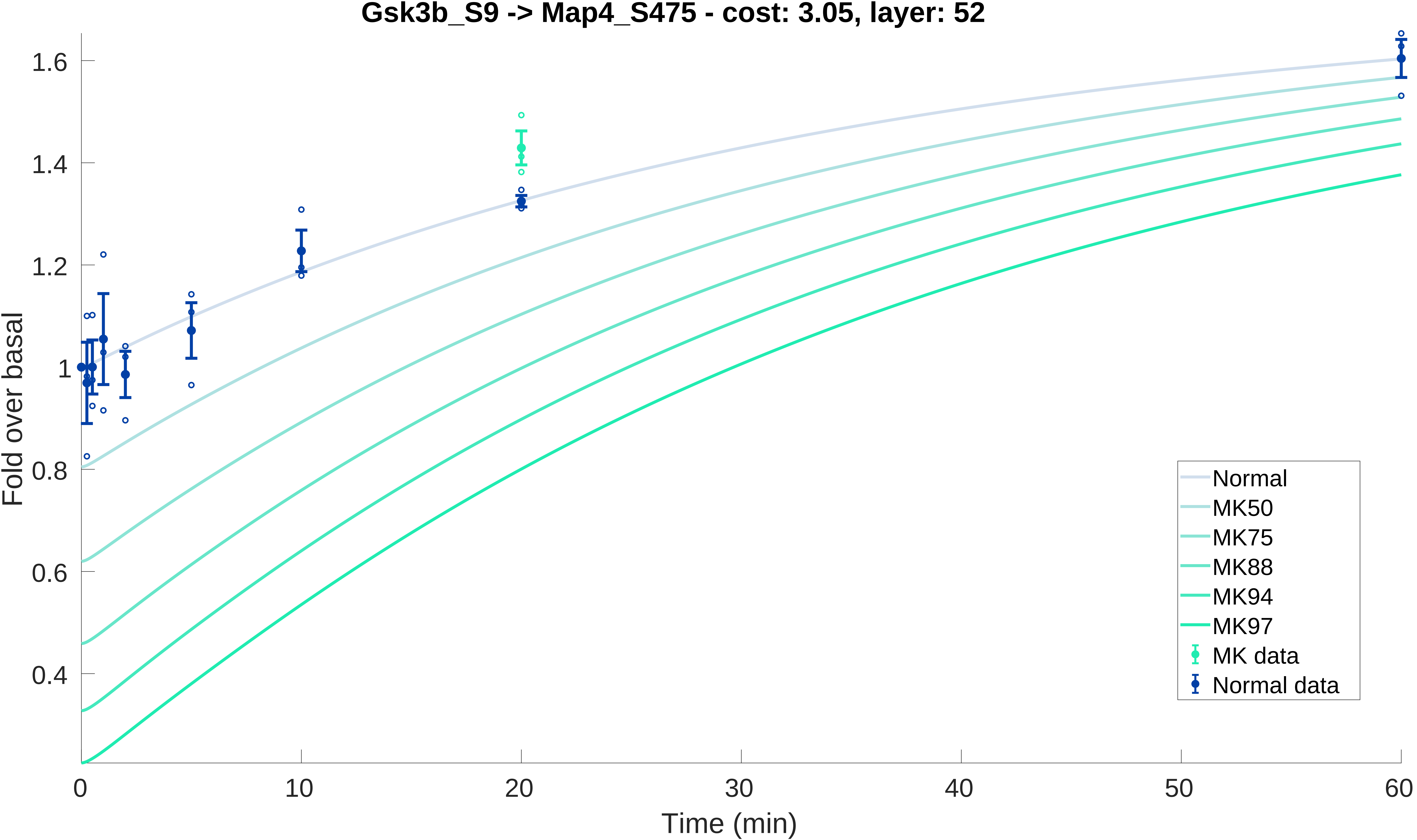
Gsk3b_S9 -> Map4_S475 - cost: 3.05, layer: 52
Fold over basal
Time (min)
0
10
20
30
40
50
60
0.4
0.6
0.8
1
1.2
1.4
1.6
Normal
MK50
MK75
MK88
MK94
MK97
MK data
Normal data

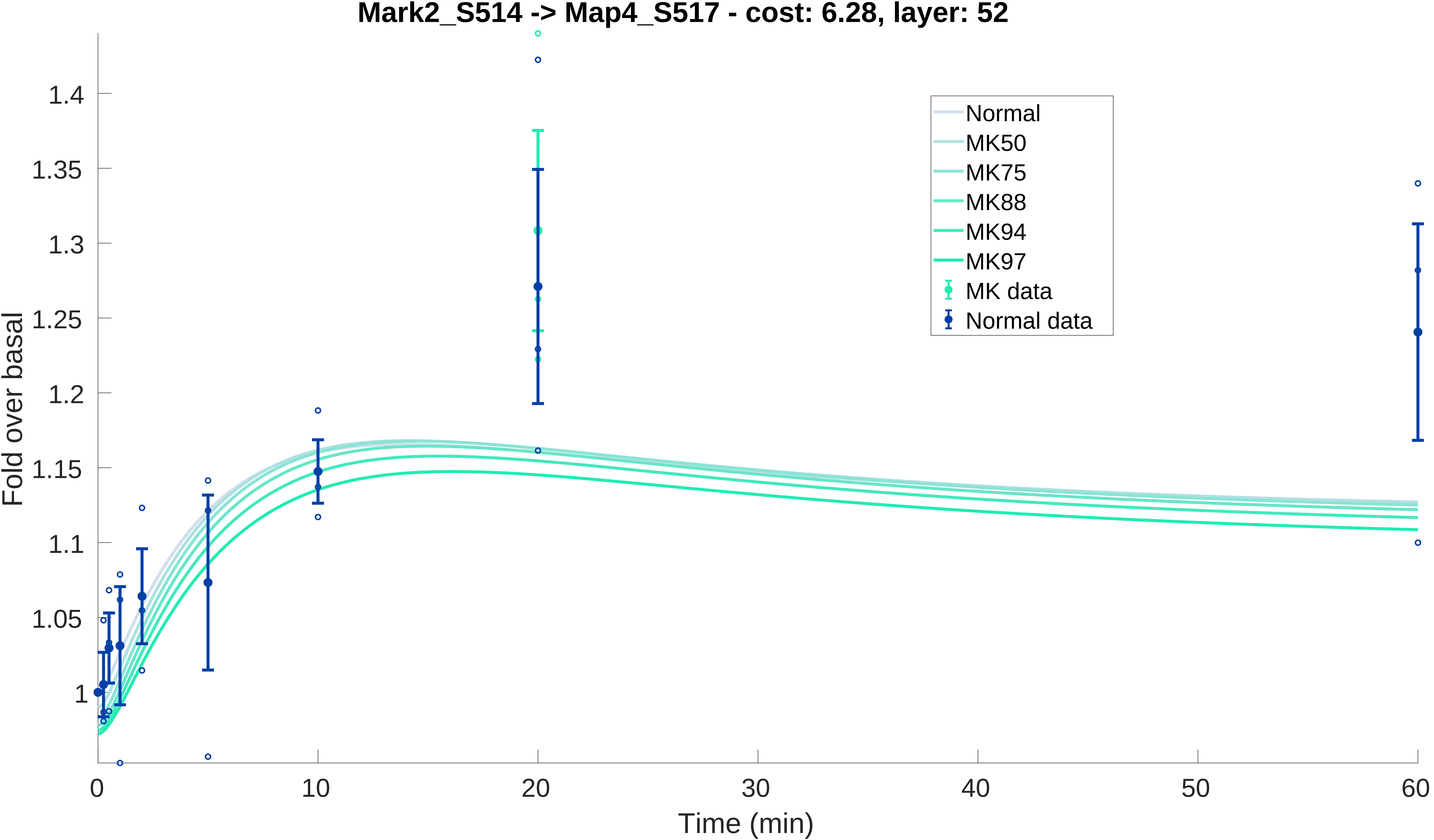

Mark2_S514 -> Map4_S517 - cost: 6.28, layer: 52
Fold over basal
1.4
1.35
1.3
1.25
1.2
1.15
1.1
1.05
1
Time (min)
0
10
20
30
40
50
60
Normal
MK50
MK75
MK88
MK94
MK97
MK data
Normal data

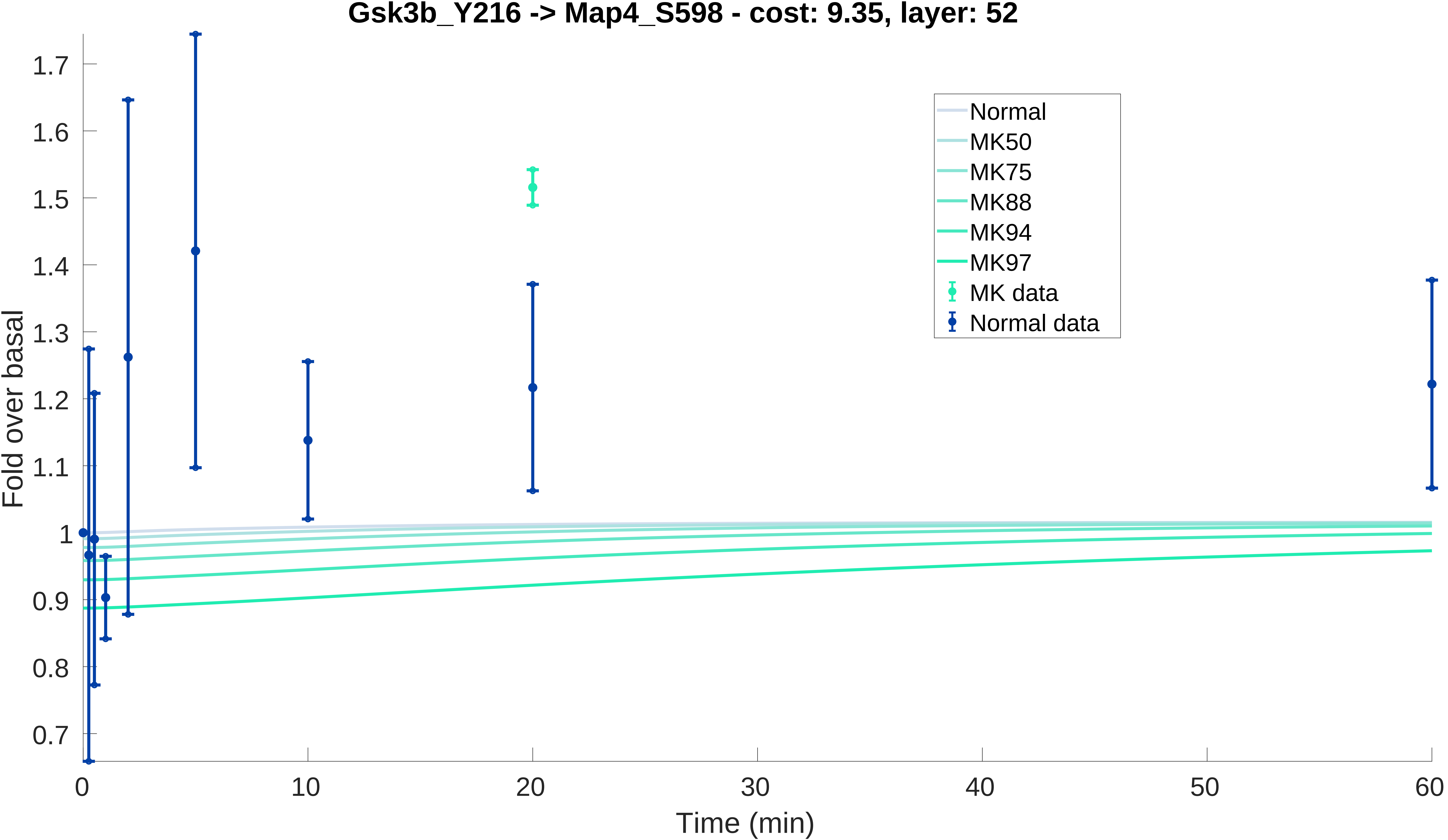
Gsk3b_Y216 -> Map4_S598 - cost: 9.35, layer: 52
Fold over basal
Time (min)
Normal
MK50
MK75
MK88
MK94
MK97
MK data
Normal data
0
10
20
30
40
50
60
0.7
0.8
0.9
1
1.1
1.2
1.3
1.4
1.5
1.6
1.7

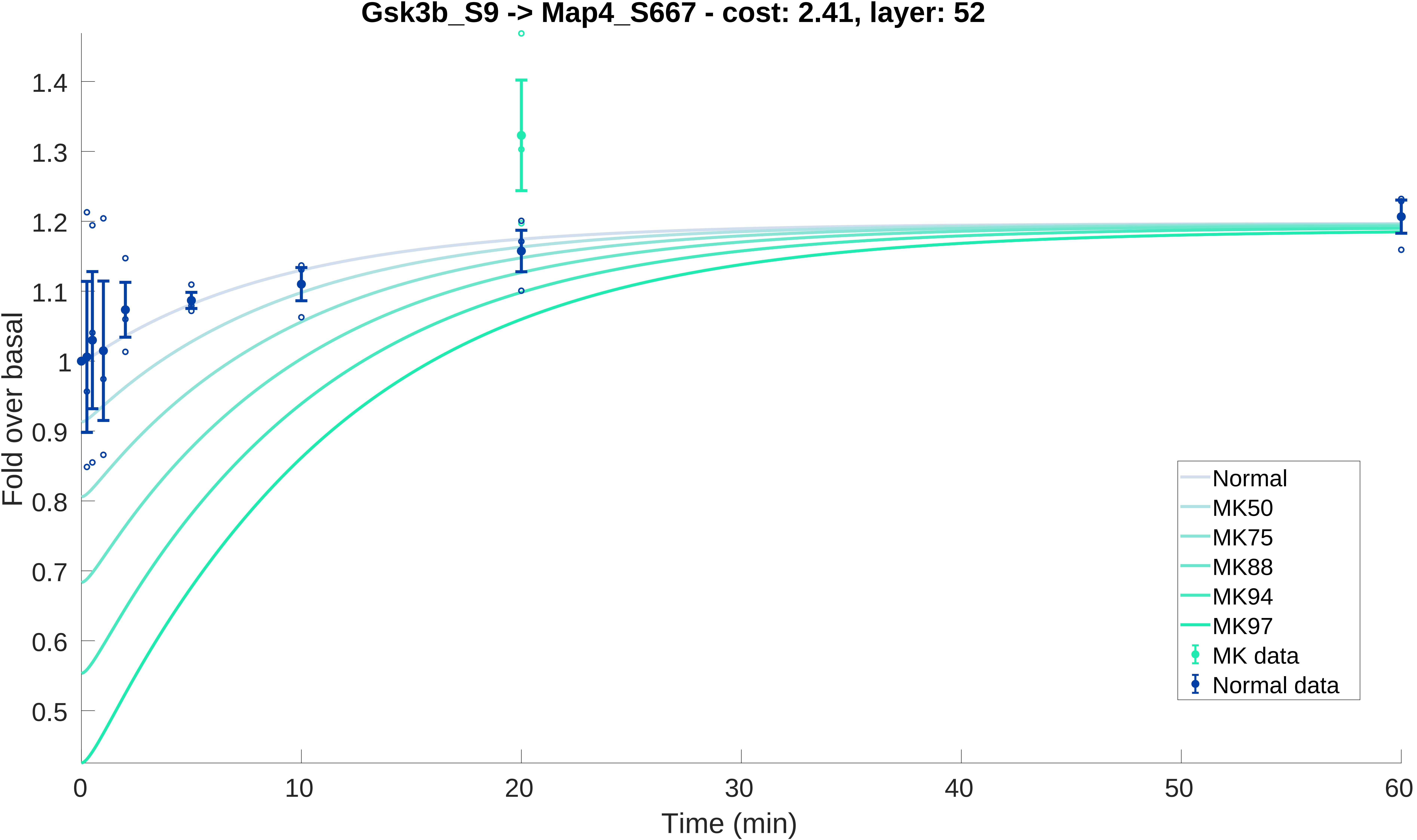

Gsk3b_S9 -> Map4_S667 - cost: 2.41, layer: 52
Fold over basal
Time (min)
1.4
1.3
1.2
1.1
1
0.9
0.8
0.7
0.6
0.5
0
10
20
30
40
50
60
Normal
MK50
MK75
MK88
MK94
MK97
MK data
Normal data

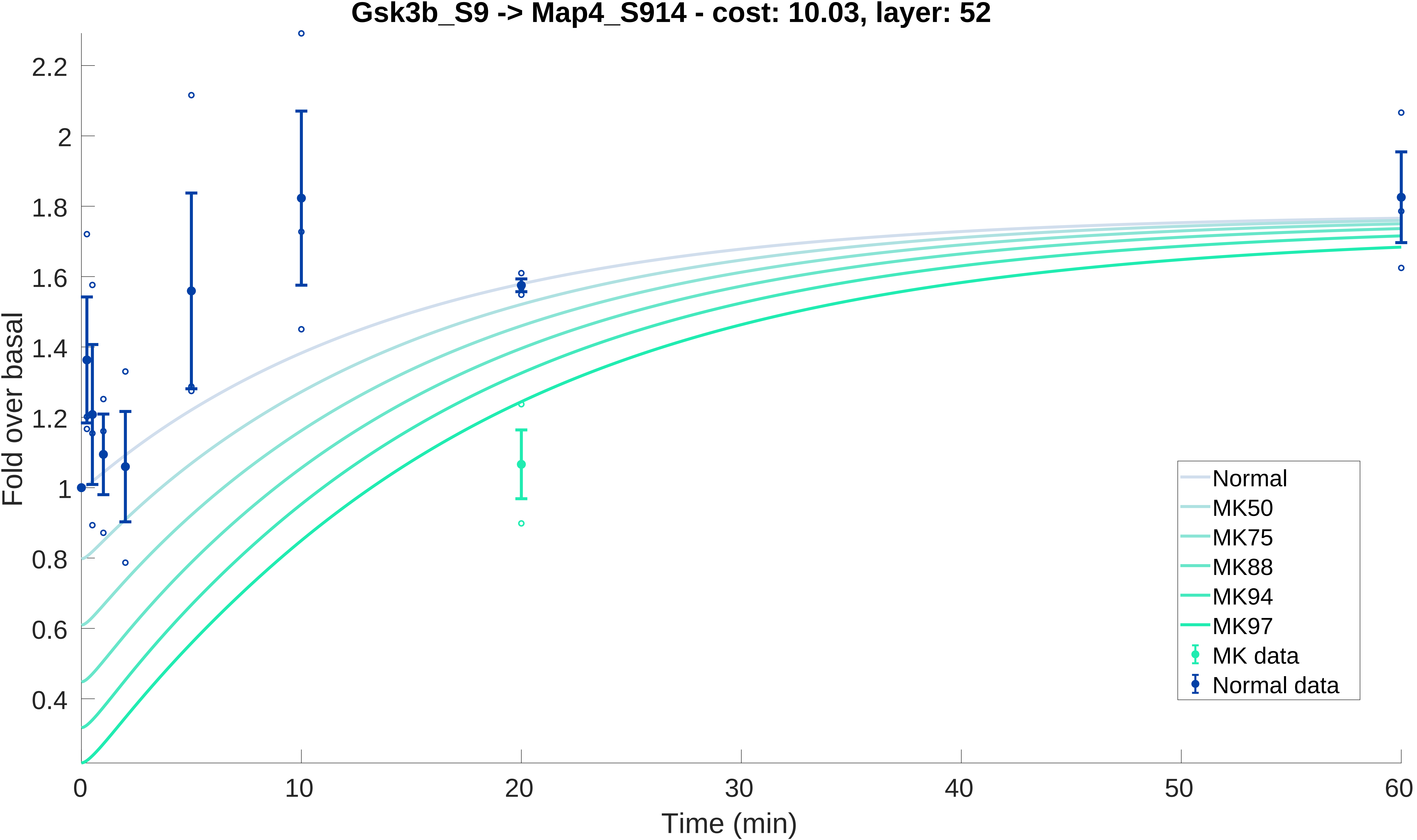
Gsk3b_S9 -> Map4_S914 - cost: 10.03, layer: 52
Fold over basal
Time (min)
2.2
2
1.8
1.6
1.4
1.2
1
0.8
0.6
0.4
0
10
20
30
40
50
60
Normal
MK50
MK75
MK88
MK94
MK97
MK data
Normal data

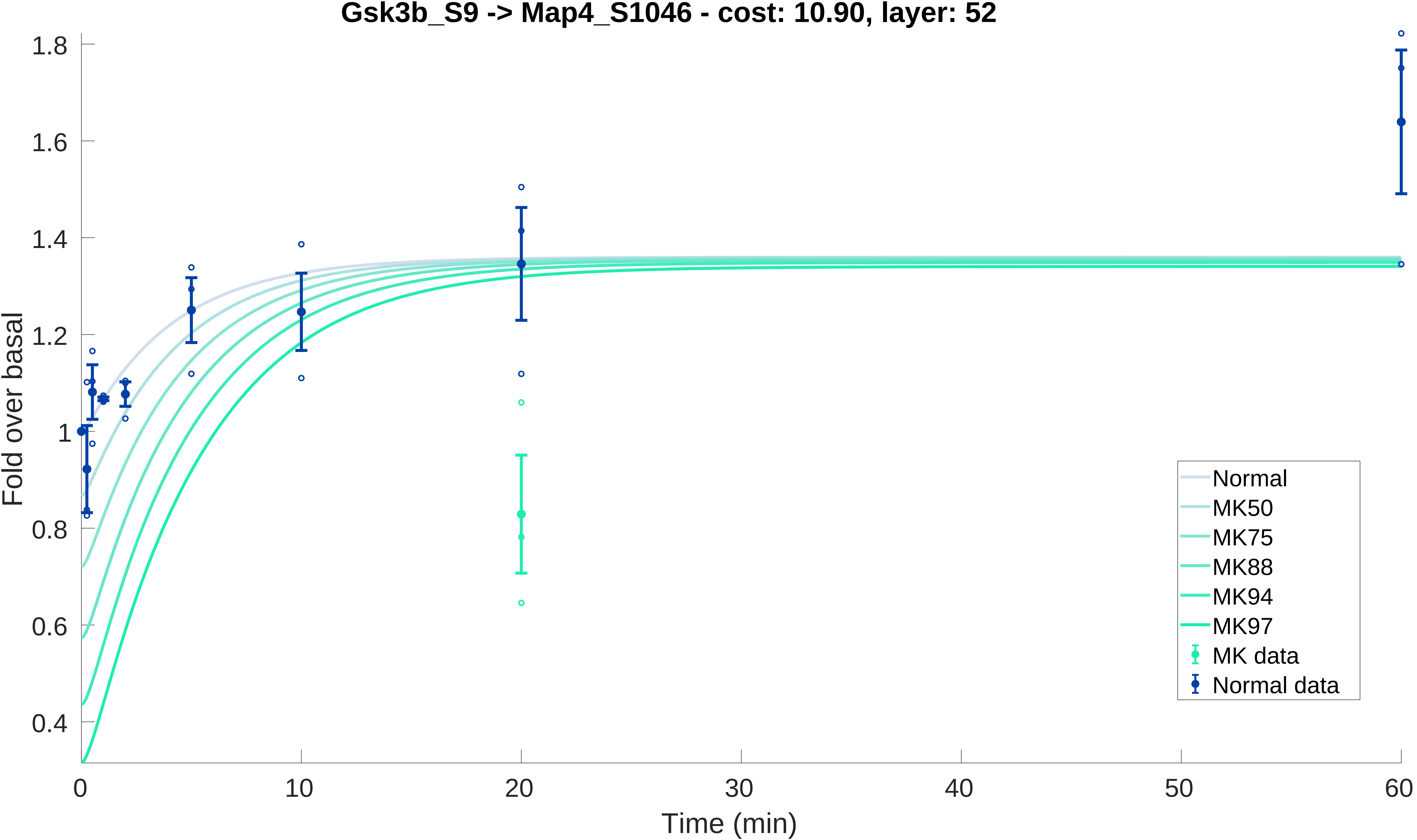

Gsk3b_S9 -> Map4_S1046 - cost: 10.90, layer: 52
Fold over basal
1.8
1.6
1.4
1.2
1
0.8
0.6
0.4
0
10
20
30
40
50
60
Time (min)
Normal
MK50
MK75
MK88
MK94
MK97
MK data
Normal data

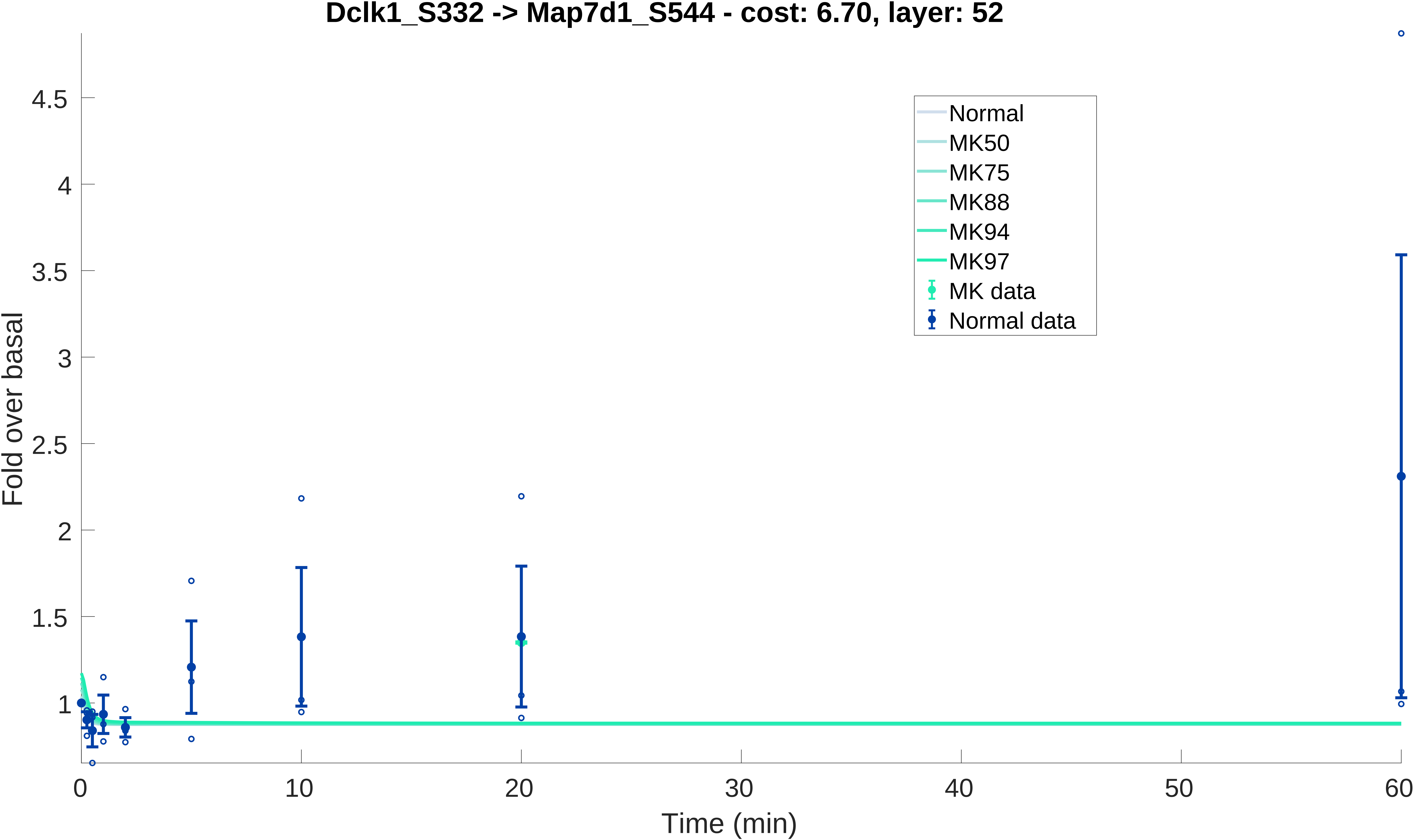

Dclk1_S332 -> Map7d1_S544 - cost: 6.70, layer: 52
Fold over basal
Time (min)
0
10
20
30
40
50
60
1
1.5
2
2.5
3
3.5
4
4.5
Normal
MK50
MK75
MK88
MK94
MK97
MK data
Normal data

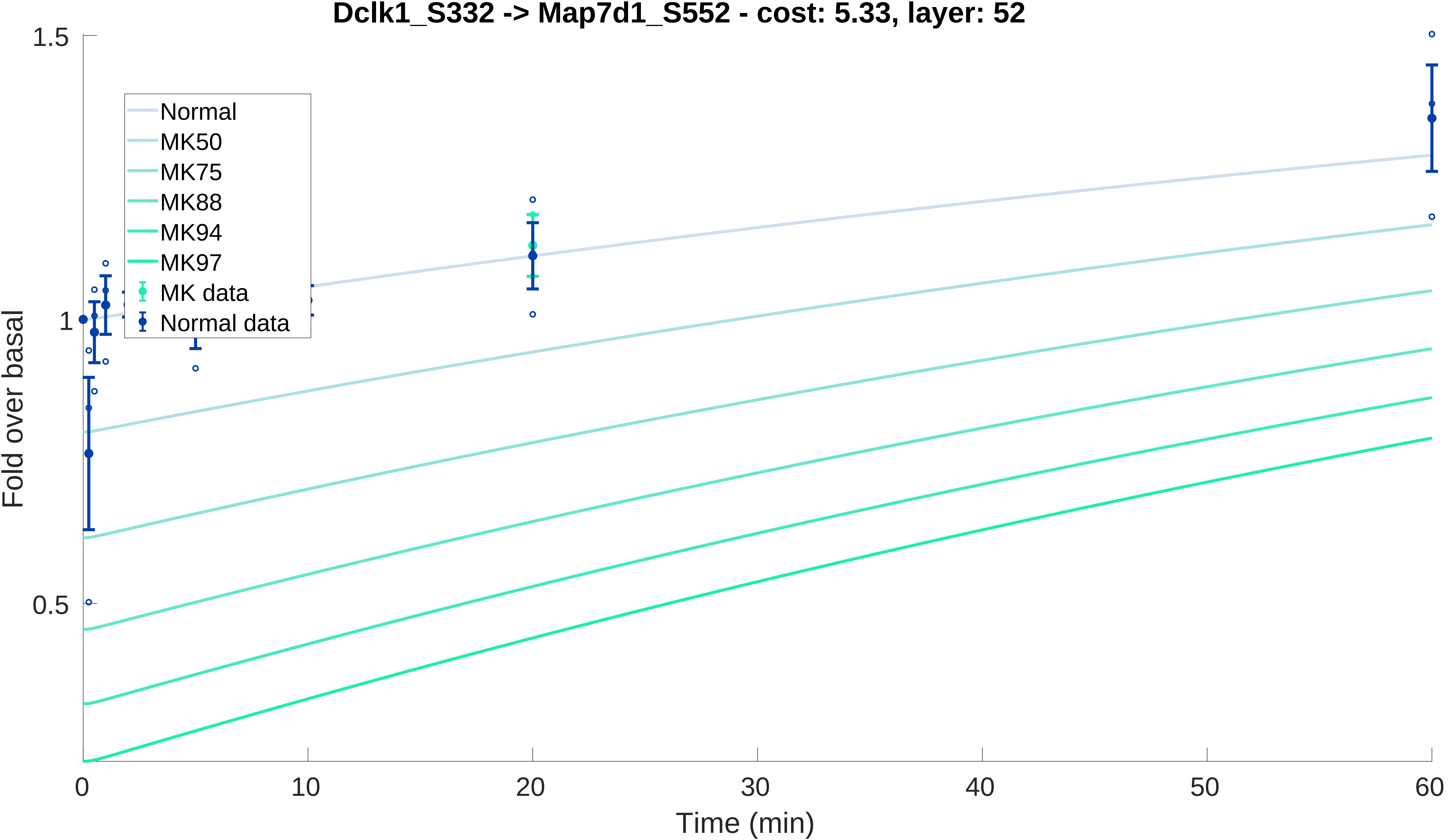

Dclk1_S332 -> Map7d1_S552 - cost: 5.33, layer: 52
1.5
1
0.5
Fold over basal
0
10
20
30
40
50
60
Time (min)
Normal
MK50
MK75
MK88
MK94
MK97
MK data
Normal data

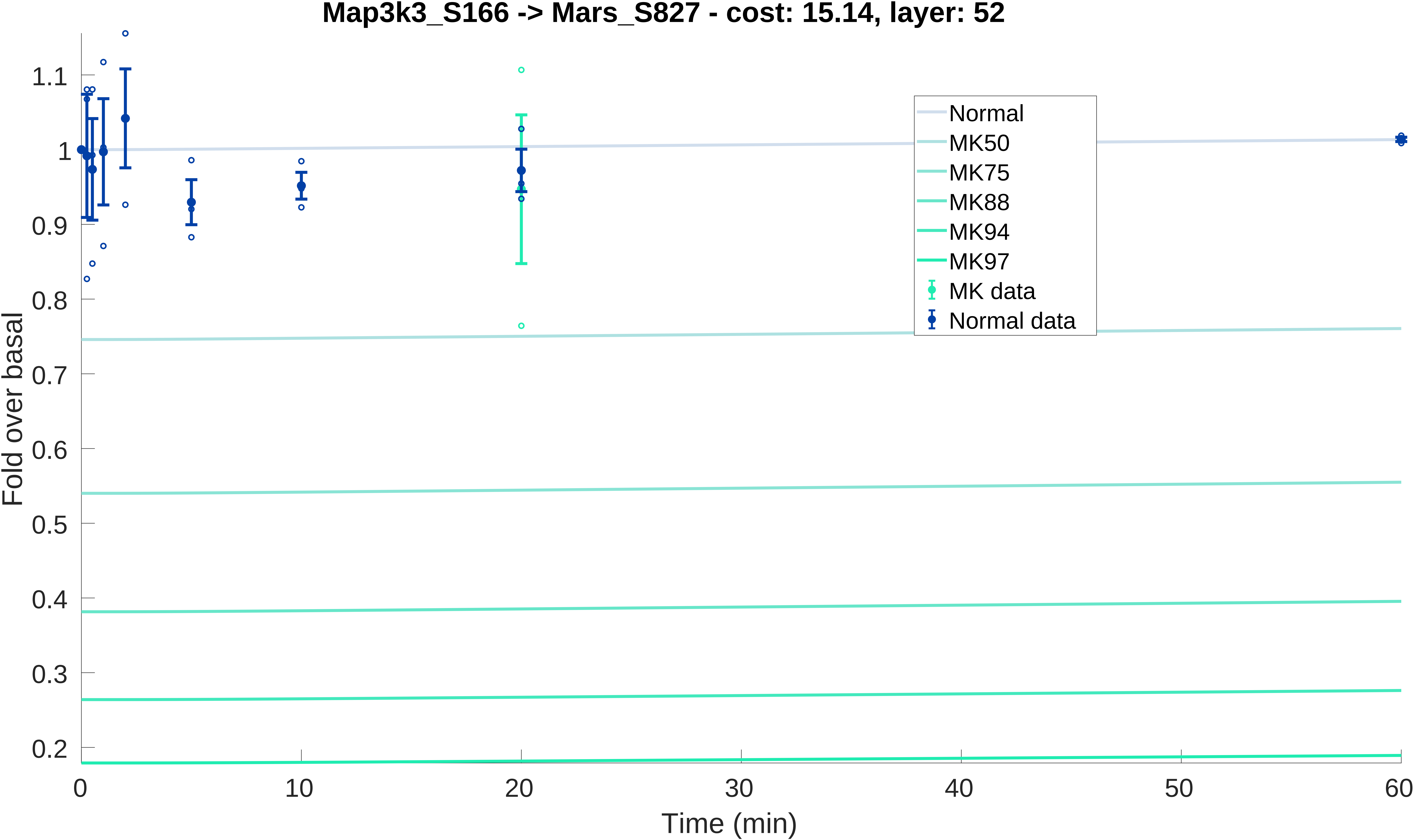

Map3k3_S166 -> Mars_S827 - cost: 15.14, layer: 52
Fold over basal
Time (min)
Normal
MK50
MK75
MK88
MK94
MK97
MK data
Normal data
1.1
1
0.9
0.8
0.7
0.6
0.5
0.4
0.3
0.2
0
10
20
30
40
50
60

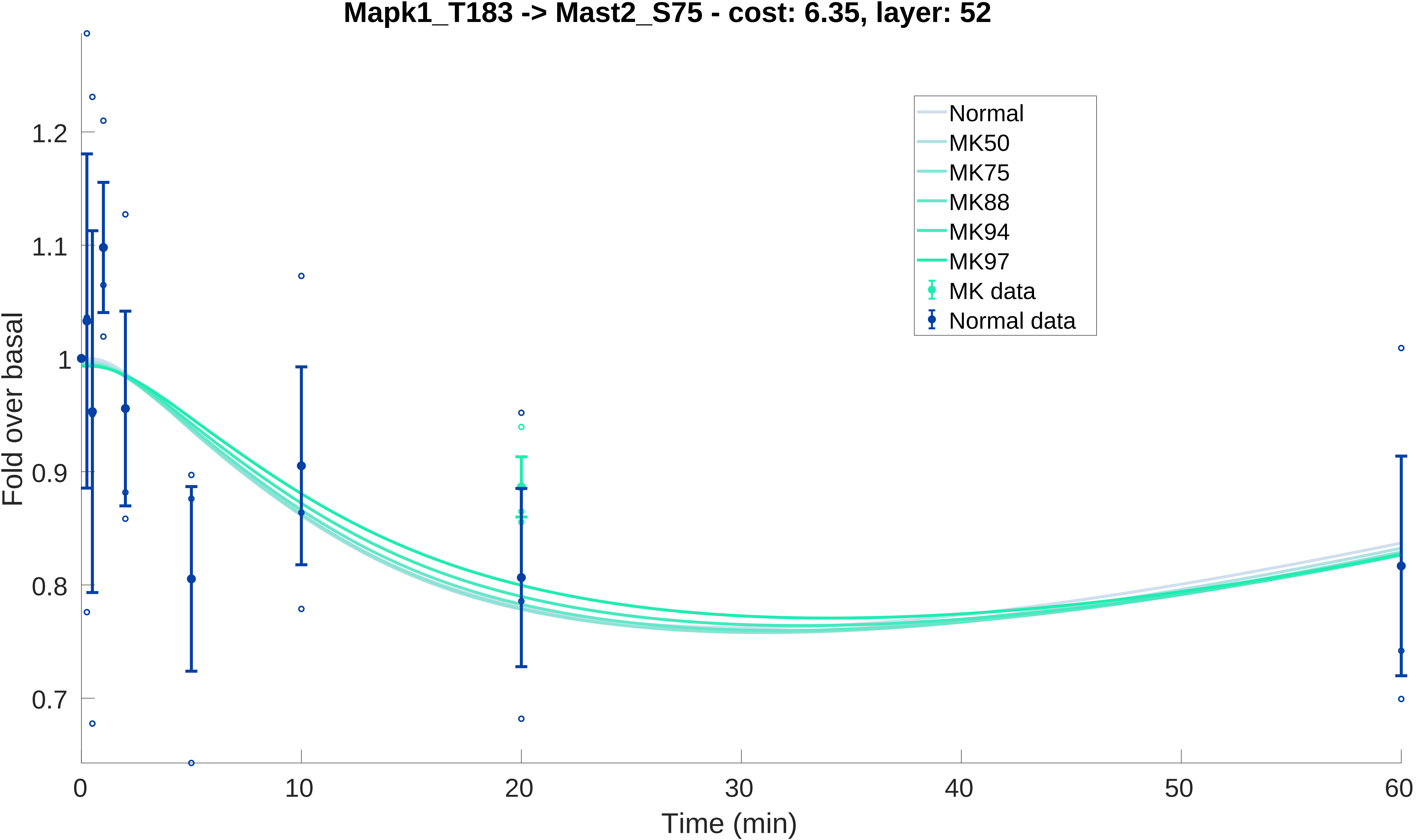

Mapk1_T183 -> Mast2_S75 - cost: 6.35, layer: 52
Fold over basal
Time (min)
0.7
0.8
0.9
1
1.1
1.2
0
10
20
30
40
50
60
Normal
MK50
MK75
MK88
MK94
MK97
MK data
Normal data

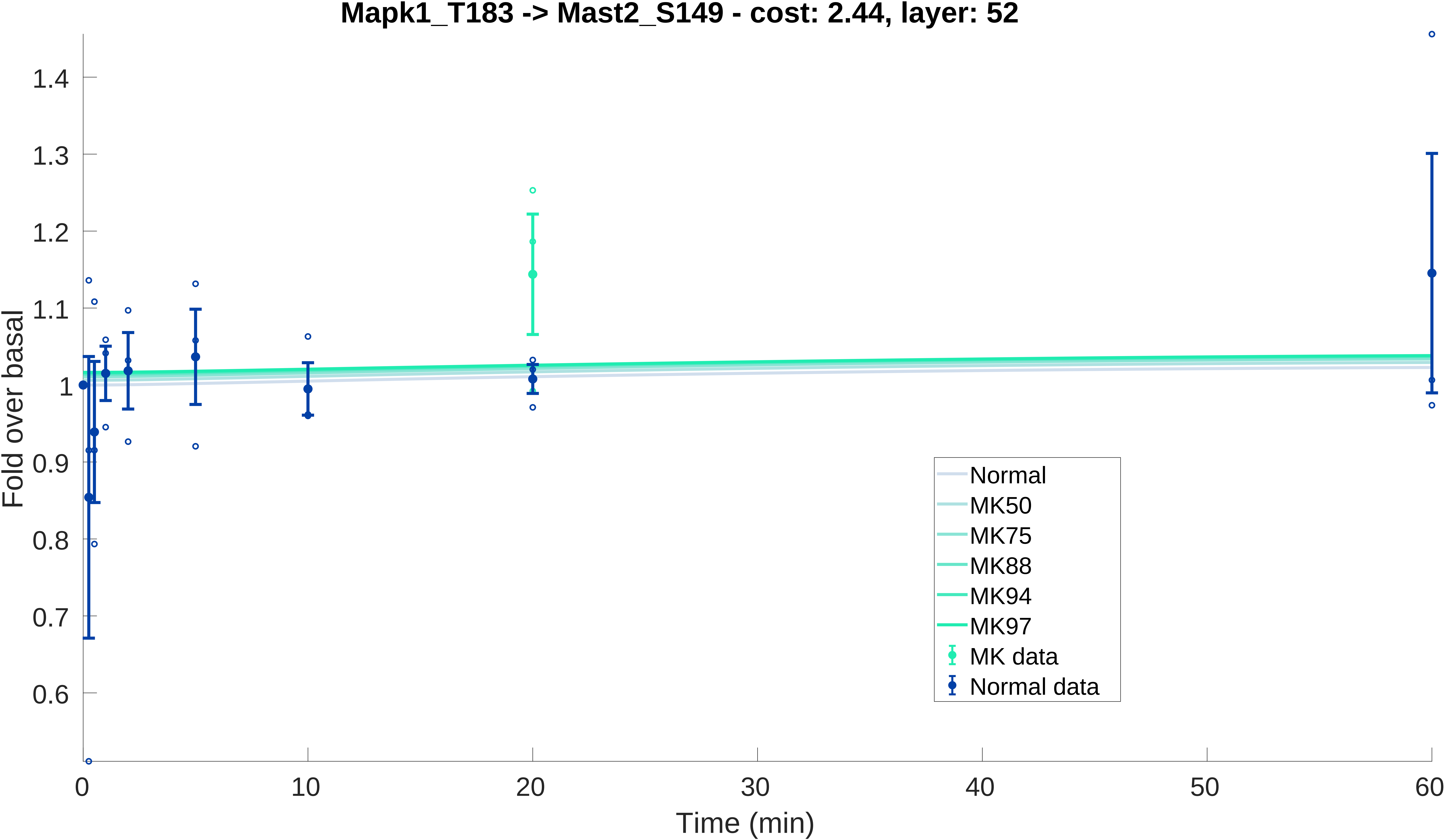

Mapk1_T183 -> Mast2_S149 - cost: 2.44, layer: 52
Fold over basal
Time (min)
1.4
1.3
1.2
1.1
1
0.9
0.8
0.7
0.6
0
10
20
30
40
50
60
Normal
MK50
MK75
MK88
MK94
MK97
MK data
Normal data

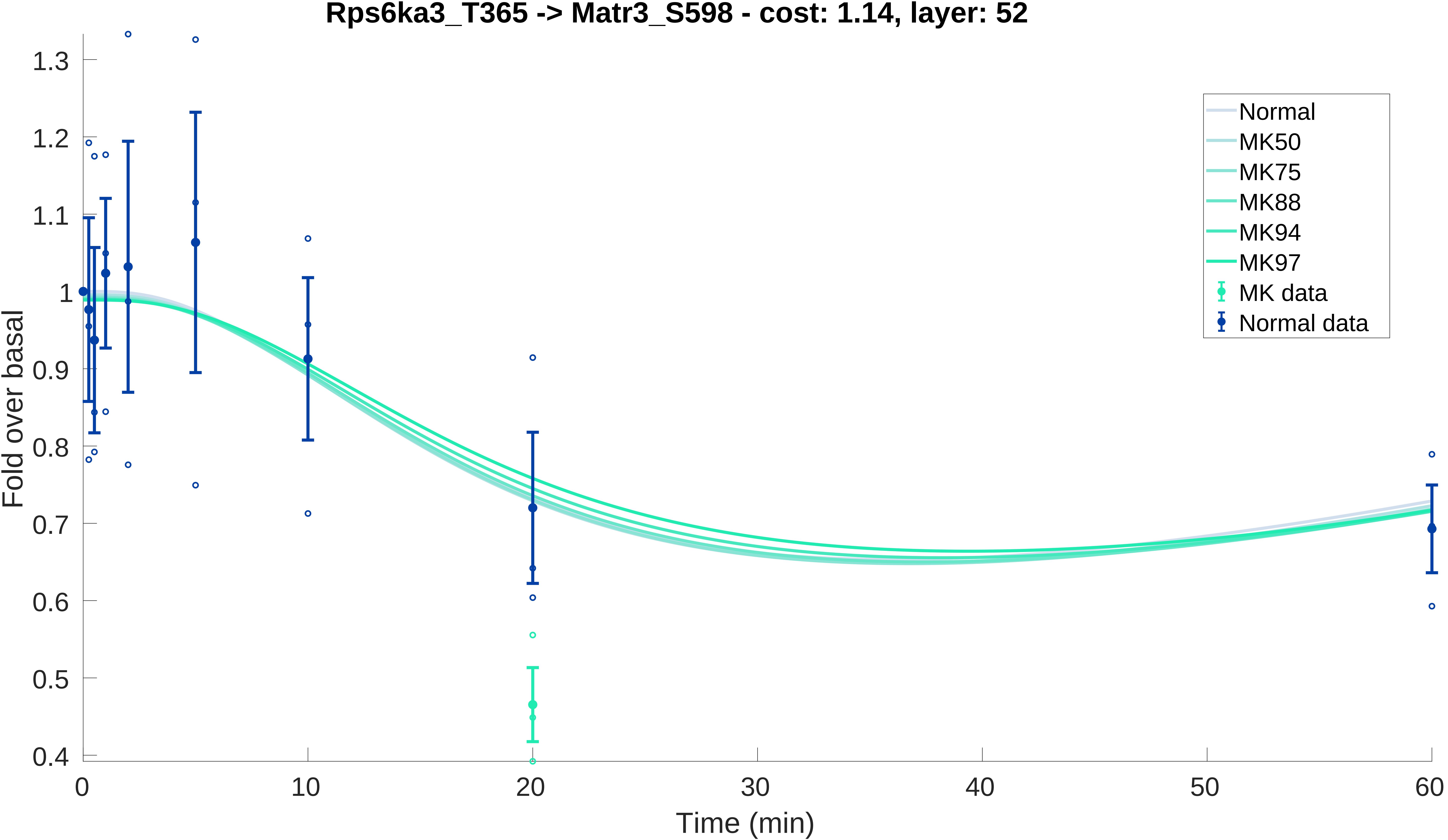
Rps6ka3_T365 -> Matr3_S598 - cost: 1.14, layer: 52
Fold over basal
Time (min)
1.3
1.2
1.1
1
0.9
0.8
0.7
0.6
0.5
0.4
0
10
20
30
40
50
60
Normal
MK50
MK75
MK88
MK94
MK97
MK data
Normal data

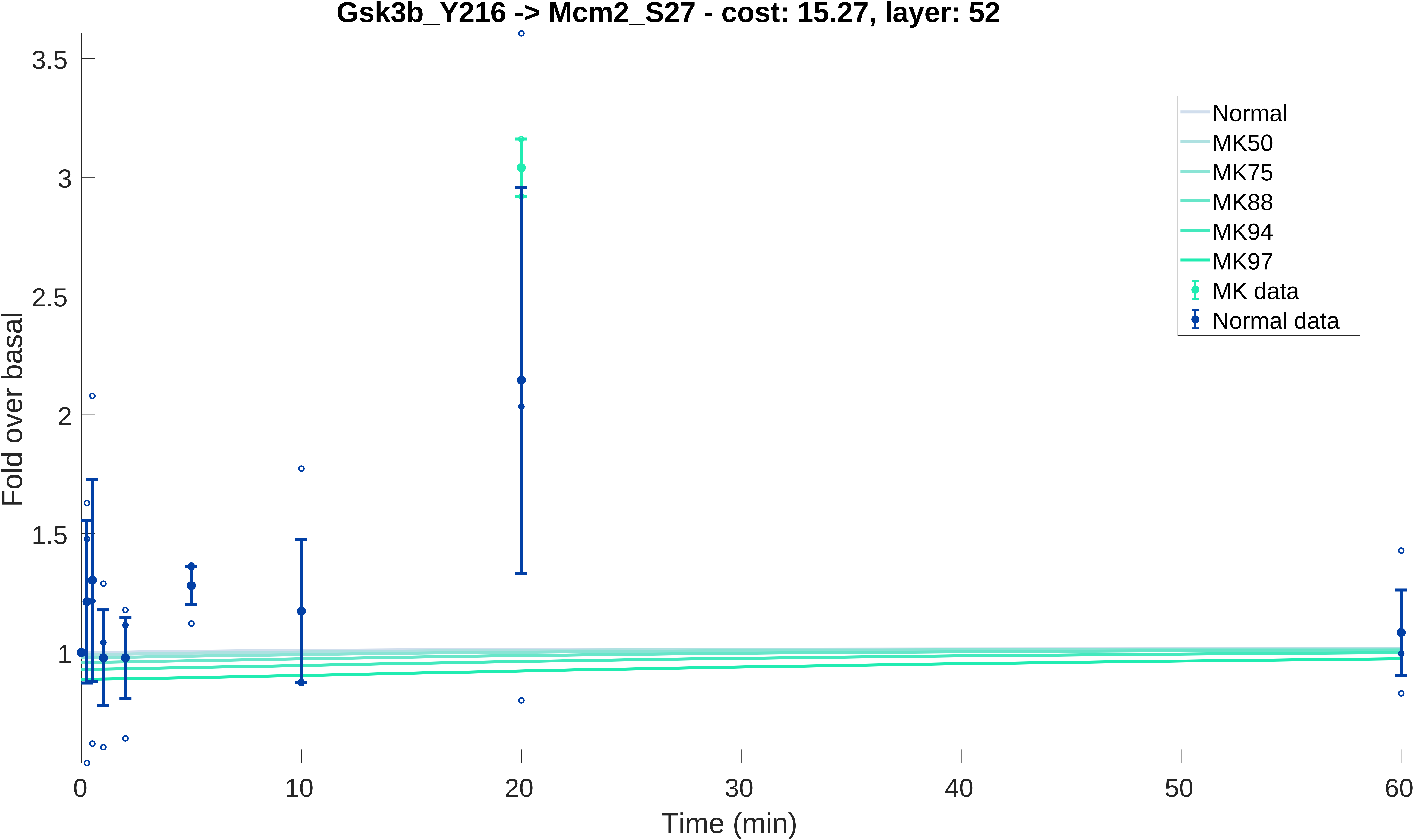
Gsk3b_Y216 -> Mcm2_S27 - cost: 15.27, layer: 52
Fold over basal
Time (min)
3.5
3
2.5
2
1.5
1
0
10
20
30
40
50
60
Normal
MK50
MK75
MK88
MK94
MK97
MK data
Normal data

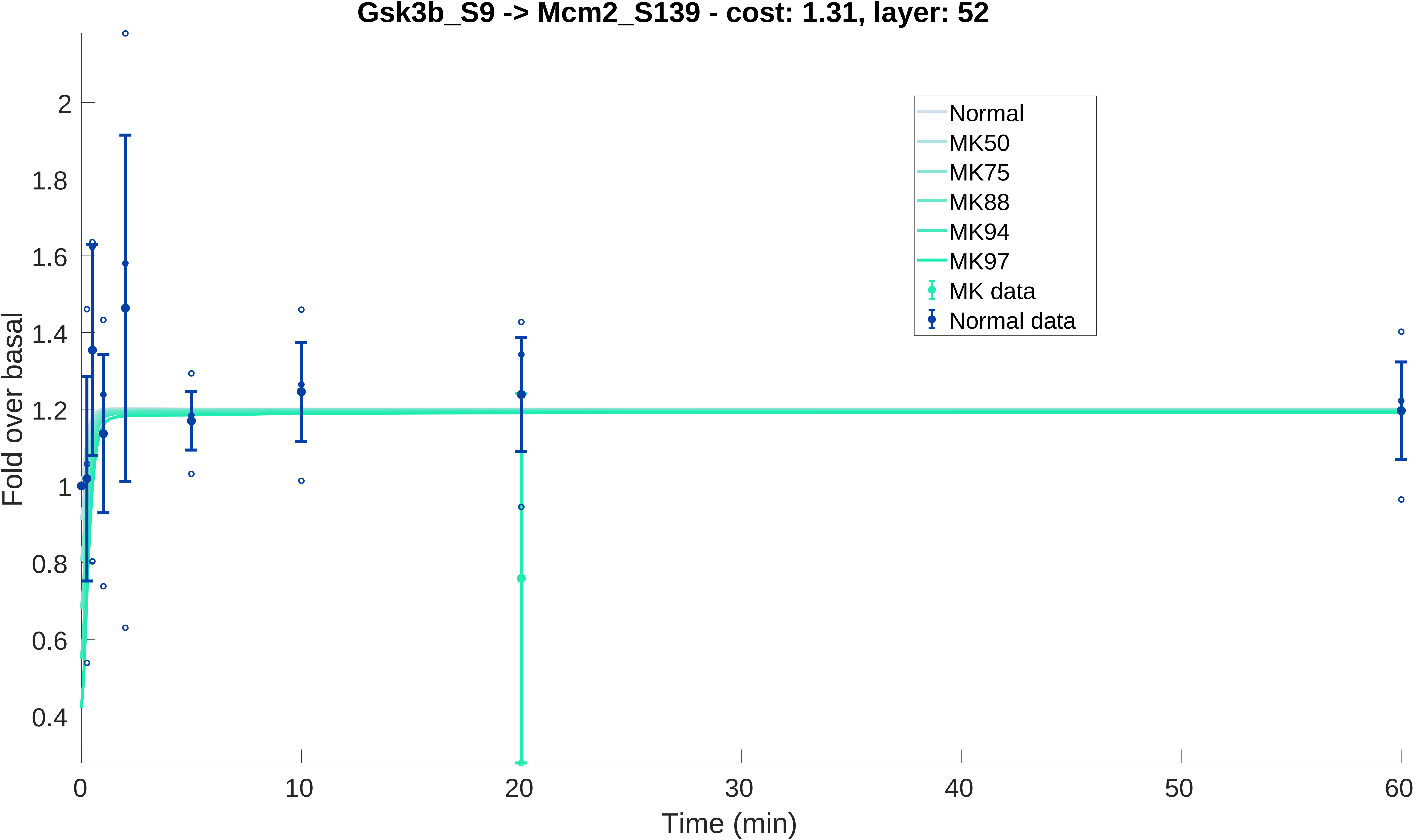

Gsk3b_S9 -> Mcm2_S139 - cost: 1.31, layer: 52
Normal
MK50
MK75
MK88
MK94
MK97
MK data
Normal data
Fold over basal
Time (min)
0.4
0.6
0.8
1
1.2
1.4
1.6
1.8
2
0
10
20
30
40
50
60

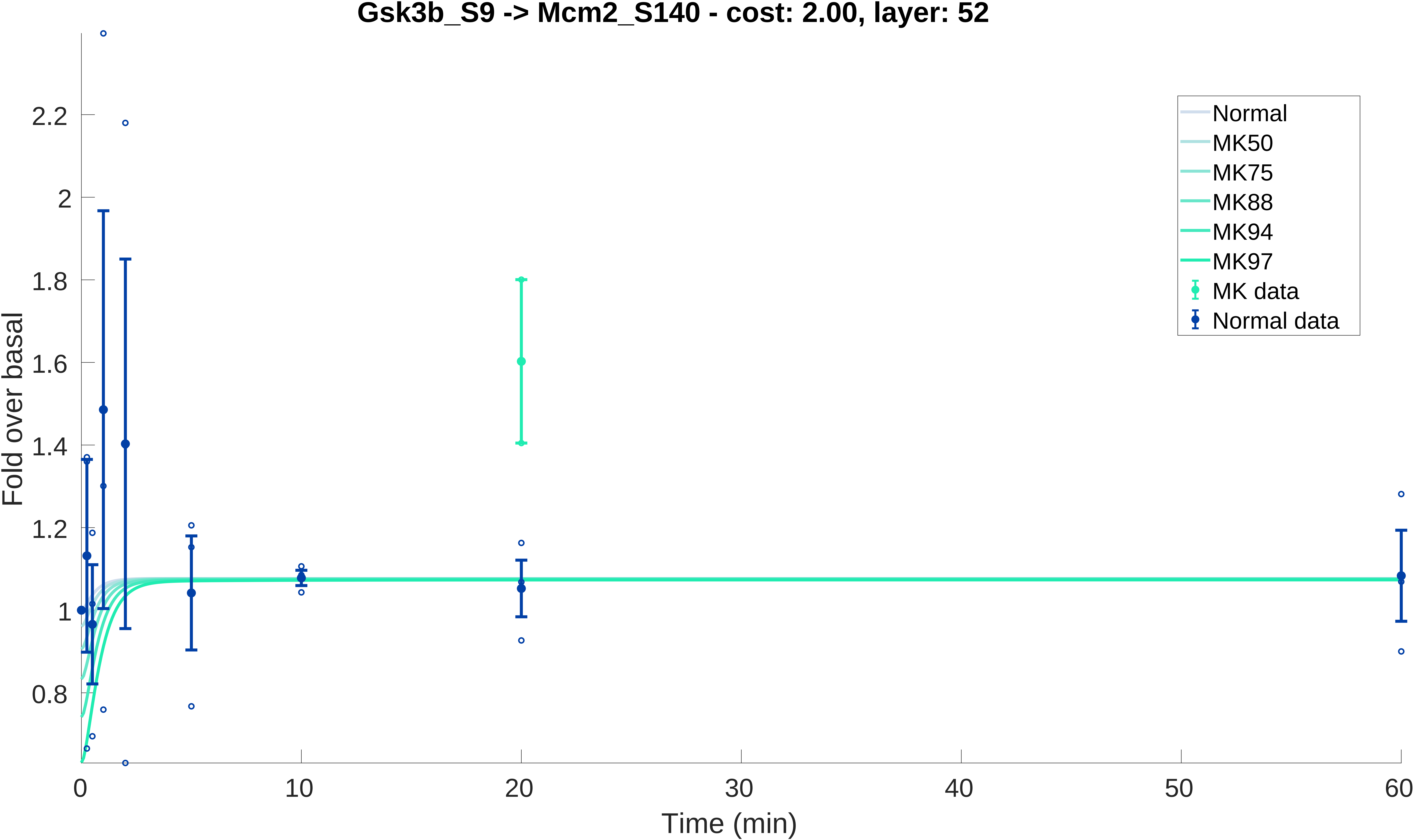

Gsk3b_S9 -> Mcm2_S140 - cost: 2.00, layer: 52
Fold over basal
Time (min)
Normal
MK50
MK75
MK88
MK94
MK97
MK data
Normal data
0.8
1
1.2
1.4
1.6
1.8
2
2.2
0
10
20
30
40
50
60

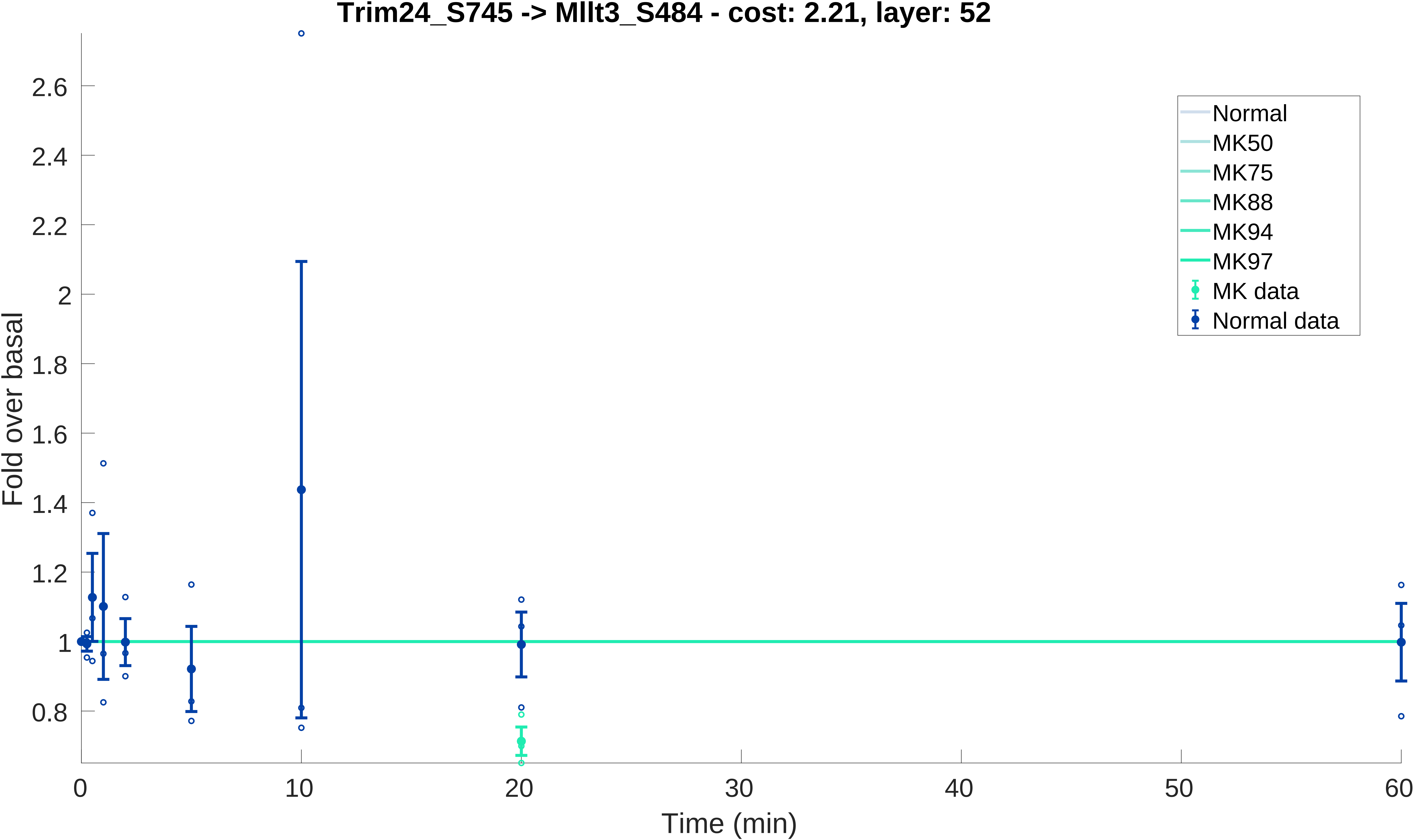

Trim24_S745 -> Mllt3_S484 - cost: 2.21, layer: 52
Fold over basal
Time (min)
0
10
20
30
40
50
60
0.8
1
1.2
1.4
1.6
1.8
2
2.2
2.4
2.6
Normal
MK50
MK75
MK88
MK94
MK97
MK data
Normal data

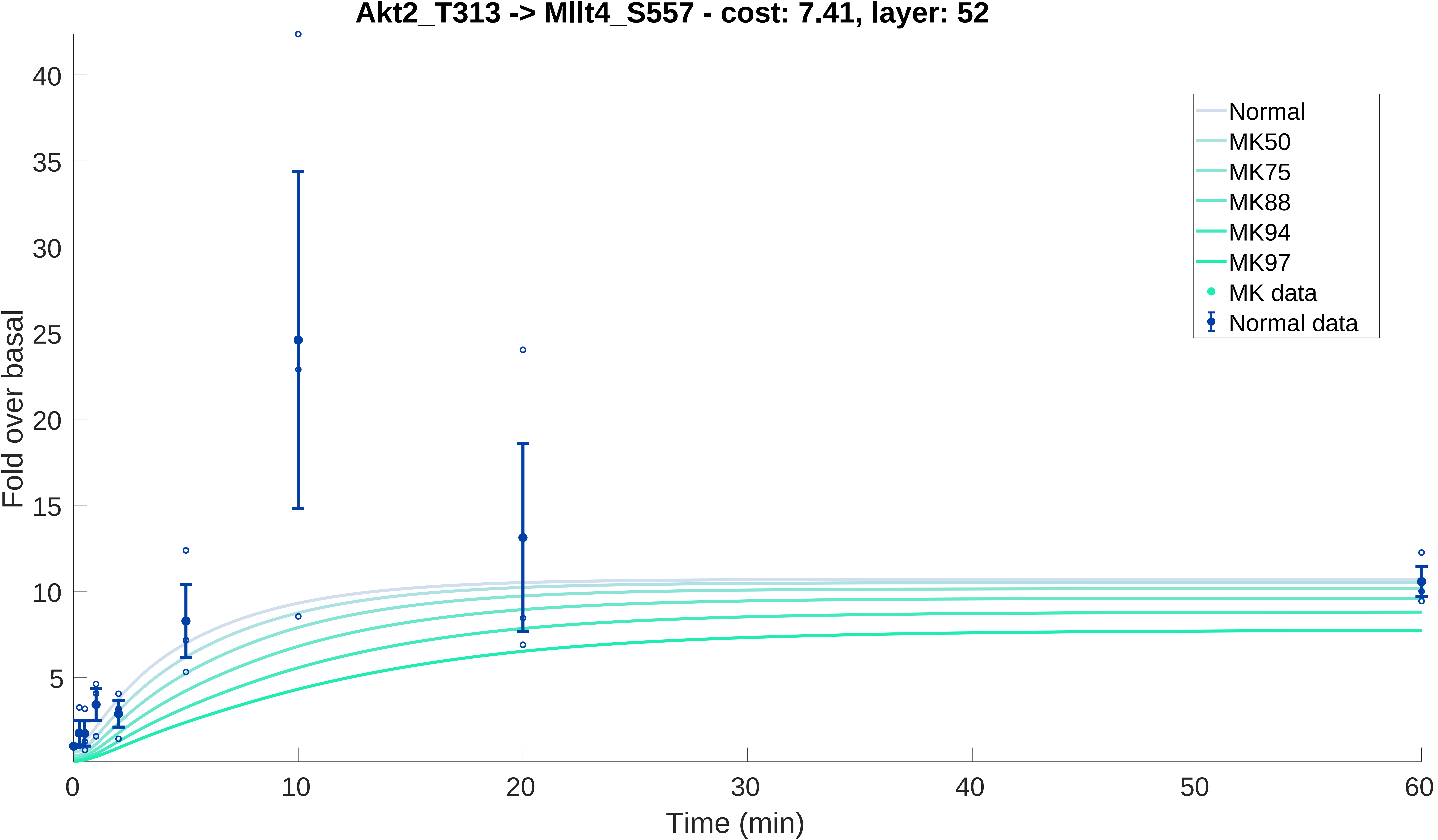

Akt2_T313 -> Mllt4_S557 - cost: 7.41, layer: 52
Fold over basal
Time (min)
0
10
20
30
40
50
60
5
10
15
20
25
30
35
40
Normal
MK50
MK75
MK88
MK94
MK97
MK data
Normal data

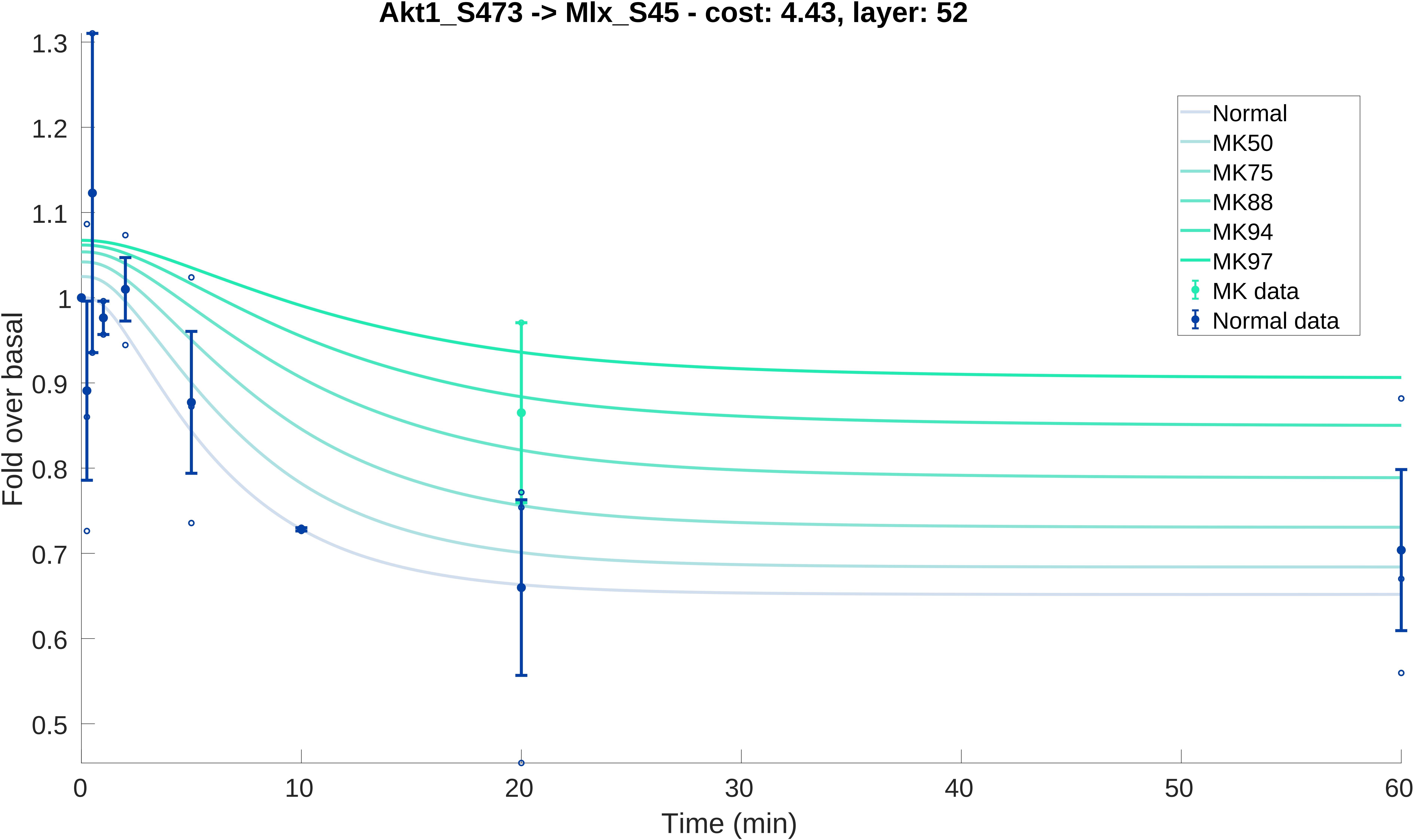
Akt1_S473 -> Mlx_S45 - cost: 4.43, layer: 52
Fold over basal
1.3
1.2
1.1
1
0.9
0.8
0.7
0.6
0.5
0
10
20
30
40
50
60
Time (min)
Normal
MK50
MK75
MK88
MK94
MK97
MK data
Normal data

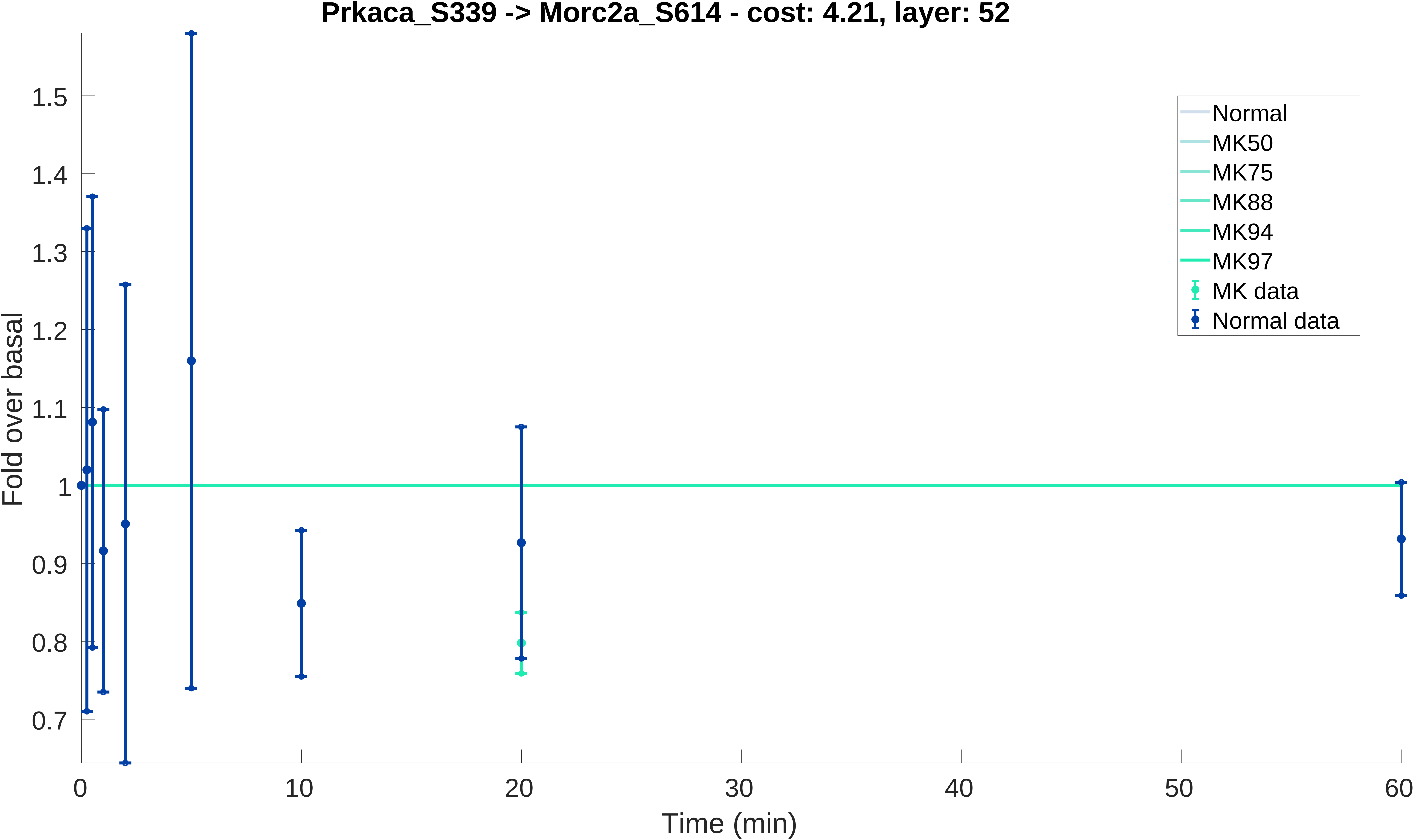

Prkaca_S339 -> Morc2a_S614 - cost: 4.21, layer: 52
Fold over basal
1.5
1.4
1.3
1.2
1.1
1
0.9
0.8
0.7
0
10
20
30
40
50
60
Time (min)
Normal
MK50
MK75
MK88
MK94
MK97
MK data
Normal data

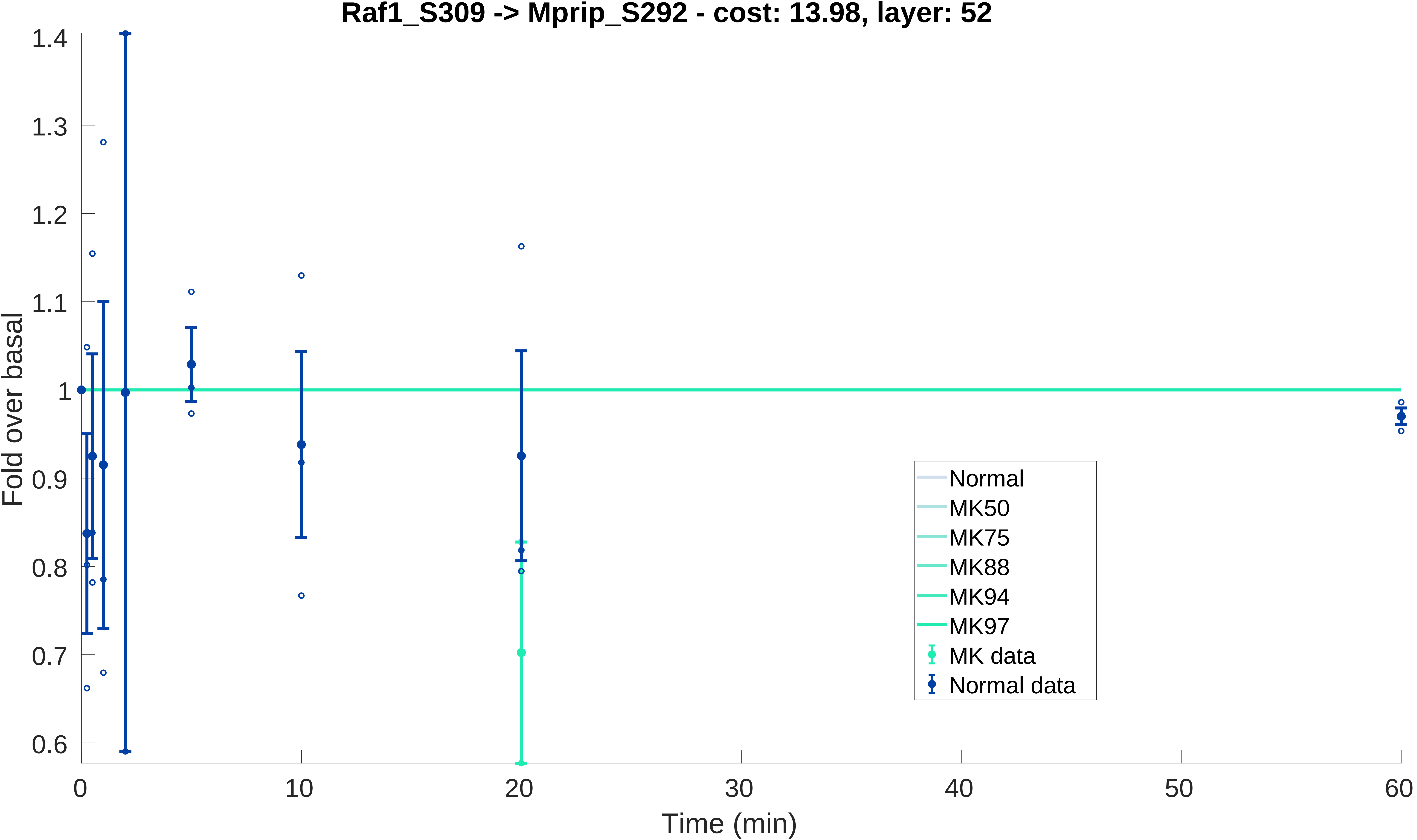
Raf1_S309 -> Mprip_S292 - cost: 13.98, layer: 52
Fold over basal
1.4
1.3
1.2
1.1
1
0.9
0.8
0.7
0.6
0
10
20
30
40
50
60
Time (min)
Normal
MK50
MK75
MK88
MK94
MK97
MK data
Normal data

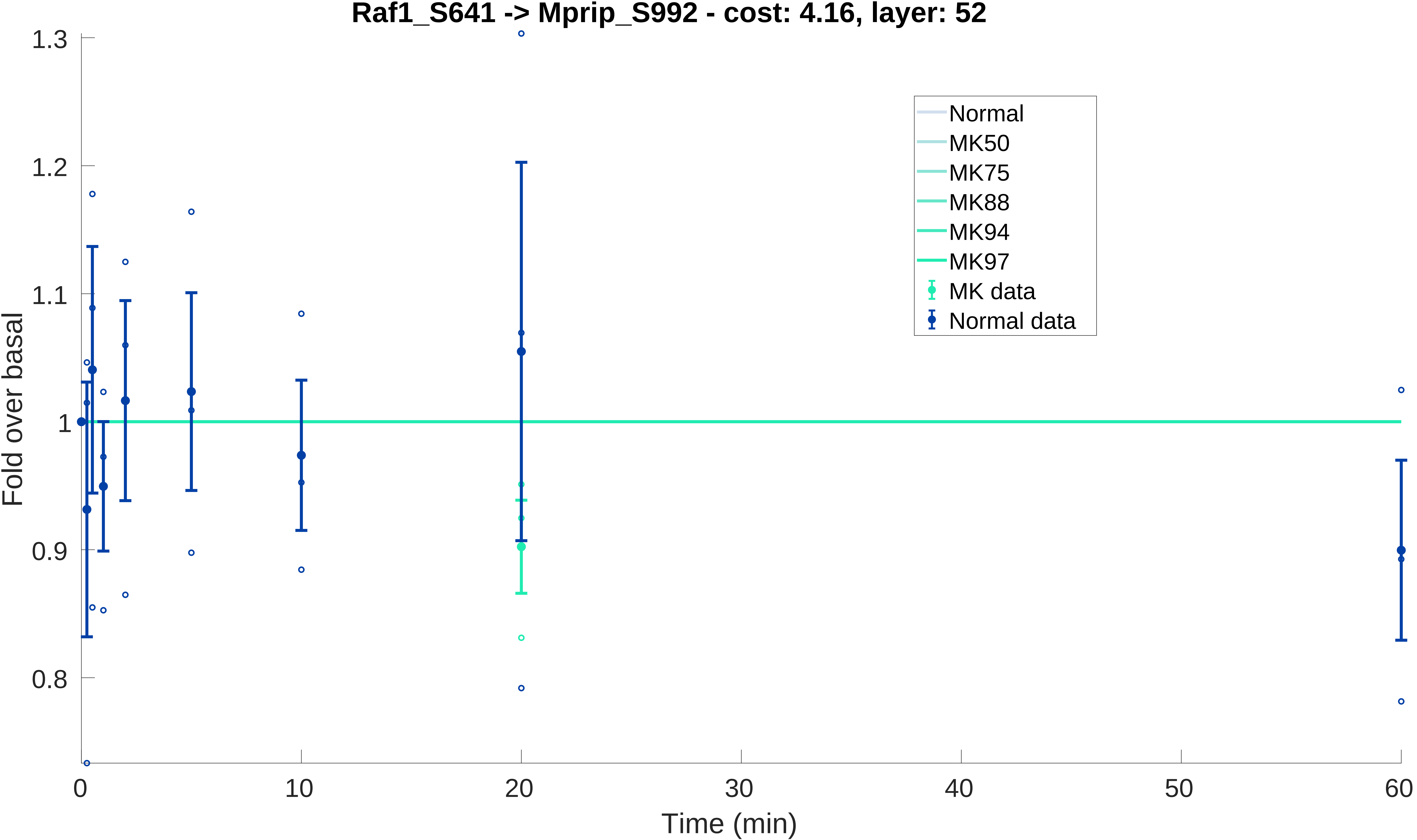

Raf1_S641 -> Mprip_S992 - cost: 4.16, layer: 52
1.3
1.2
1.1
1
0.9
0.8
Fold over basal
0
10
20
30
40
50
60
Time (min)
Normal
MK50
MK75
MK88
MK94
MK97
MK data
Normal data

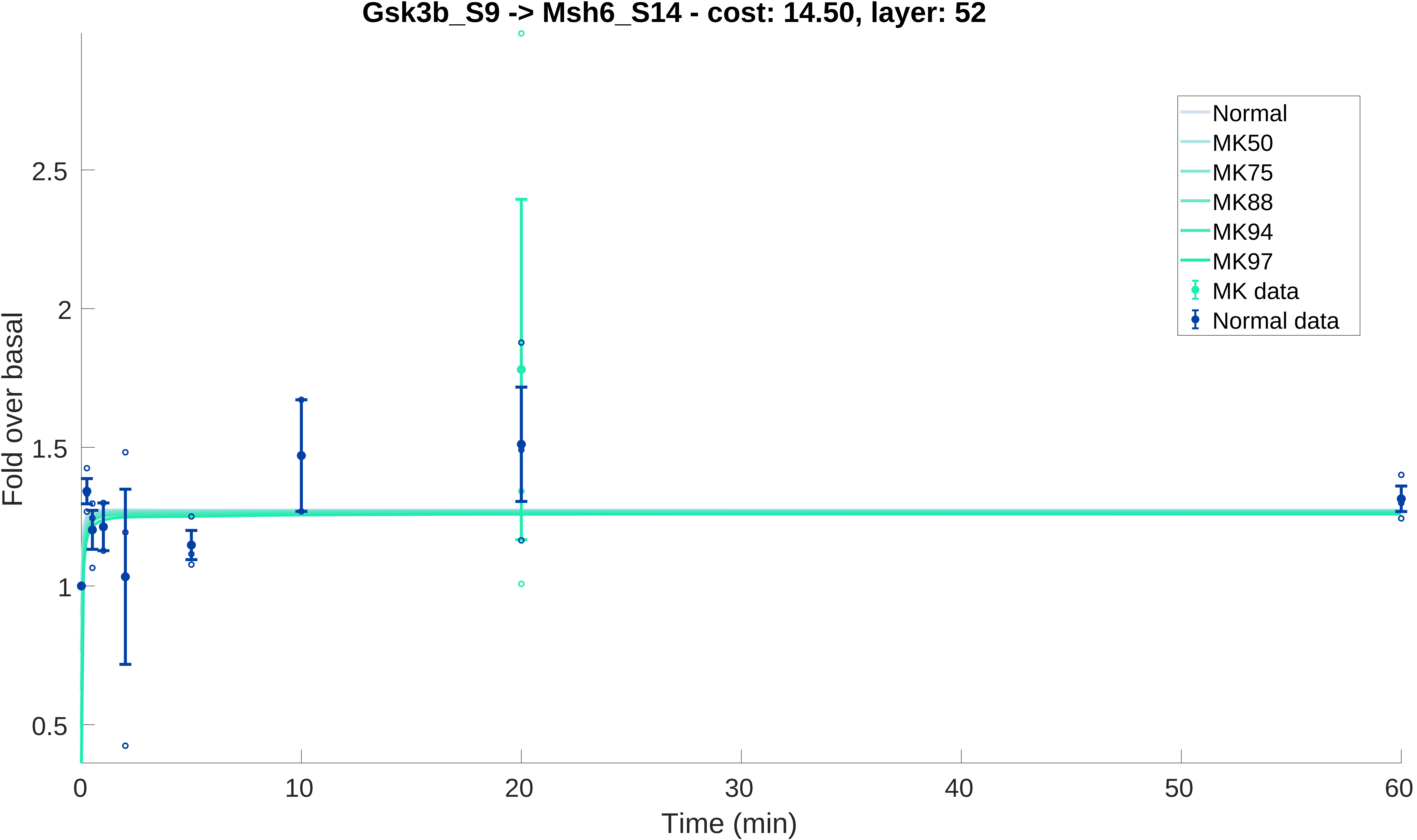

Gsk3b_S9 -> Msh6_S14 - cost: 14.50, layer: 52
Fold over basal
Time (min)
0.5
1
1.5
2
2.5
0
10
20
30
40
50
60
Normal
MK50
MK75
MK88
MK94
MK97
MK data
Normal data

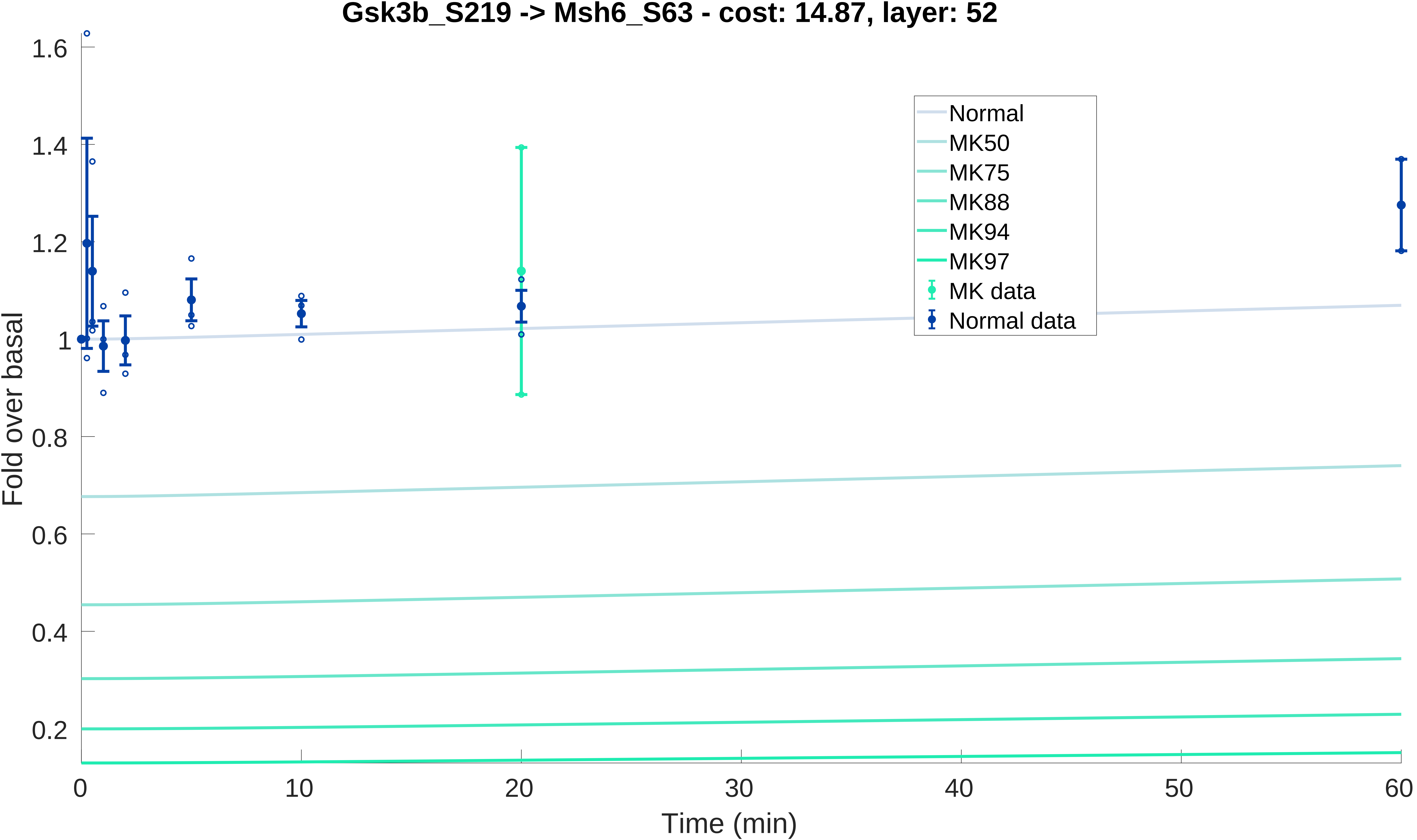

Gsk3b_S219 -> Msh6_S63 - cost: 14.87, layer: 52
Fold over basal
Time (min)
1.6
1.4
1.2
1
0.8
0.6
0.4
0.2
0
10
20
30
40
50
60
Normal
MK50
MK75
MK88
MK94
MK97
MK data
Normal data

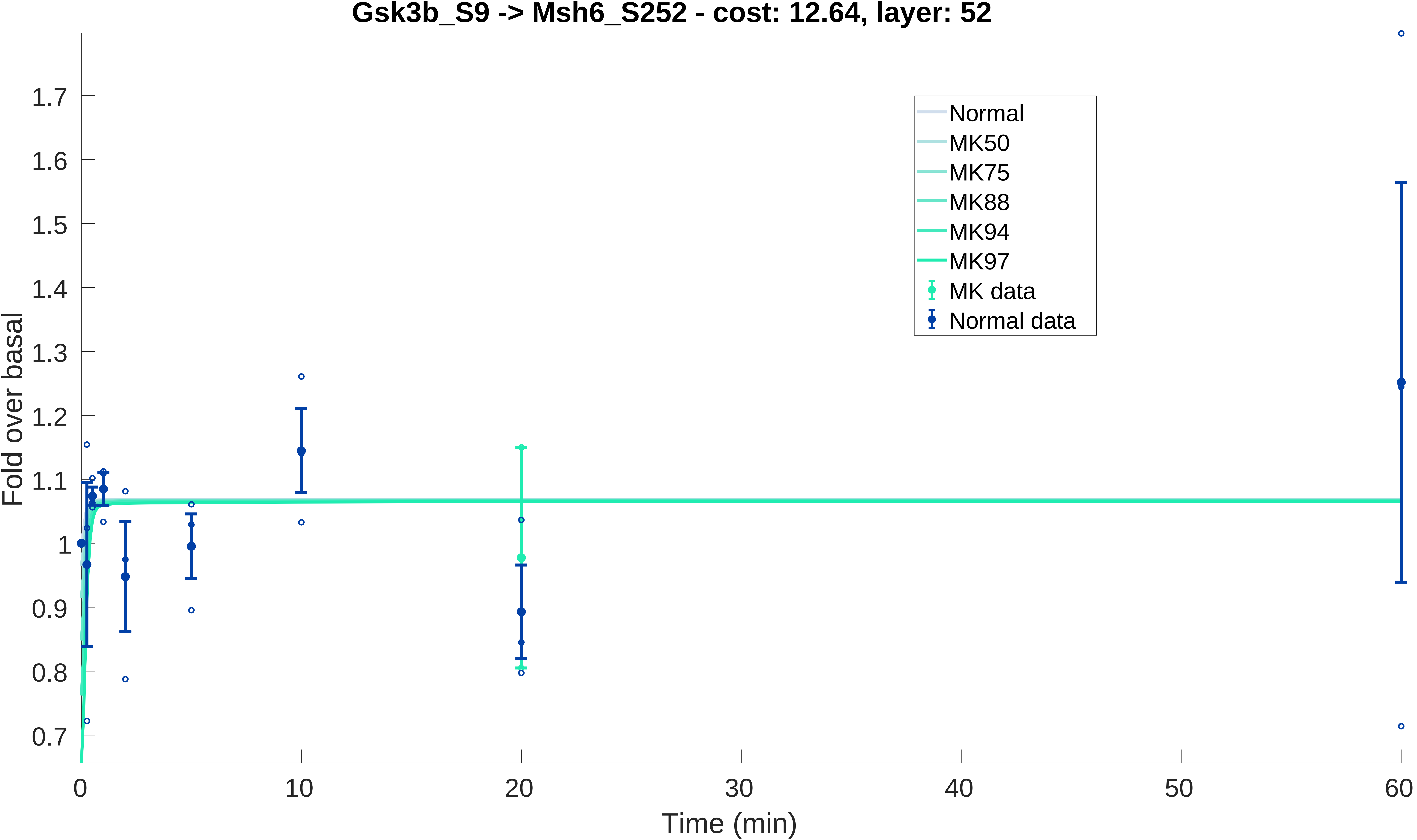

Gsk3b_S9 -> Msh6_S252 - cost: 12.64, layer: 52
Fold over basal
Time (min)
1.7
1.6
1.5
1.4
1.3
1.2
1.1
1
0.9
0.8
0.7
0
10
20
30
40
50
60
Normal
MK50
MK75
MK88
MK94
MK97
MK data
Normal data

Gsk3b_S9 -> Msh6_S254 - cost: 8.22, layer: 52

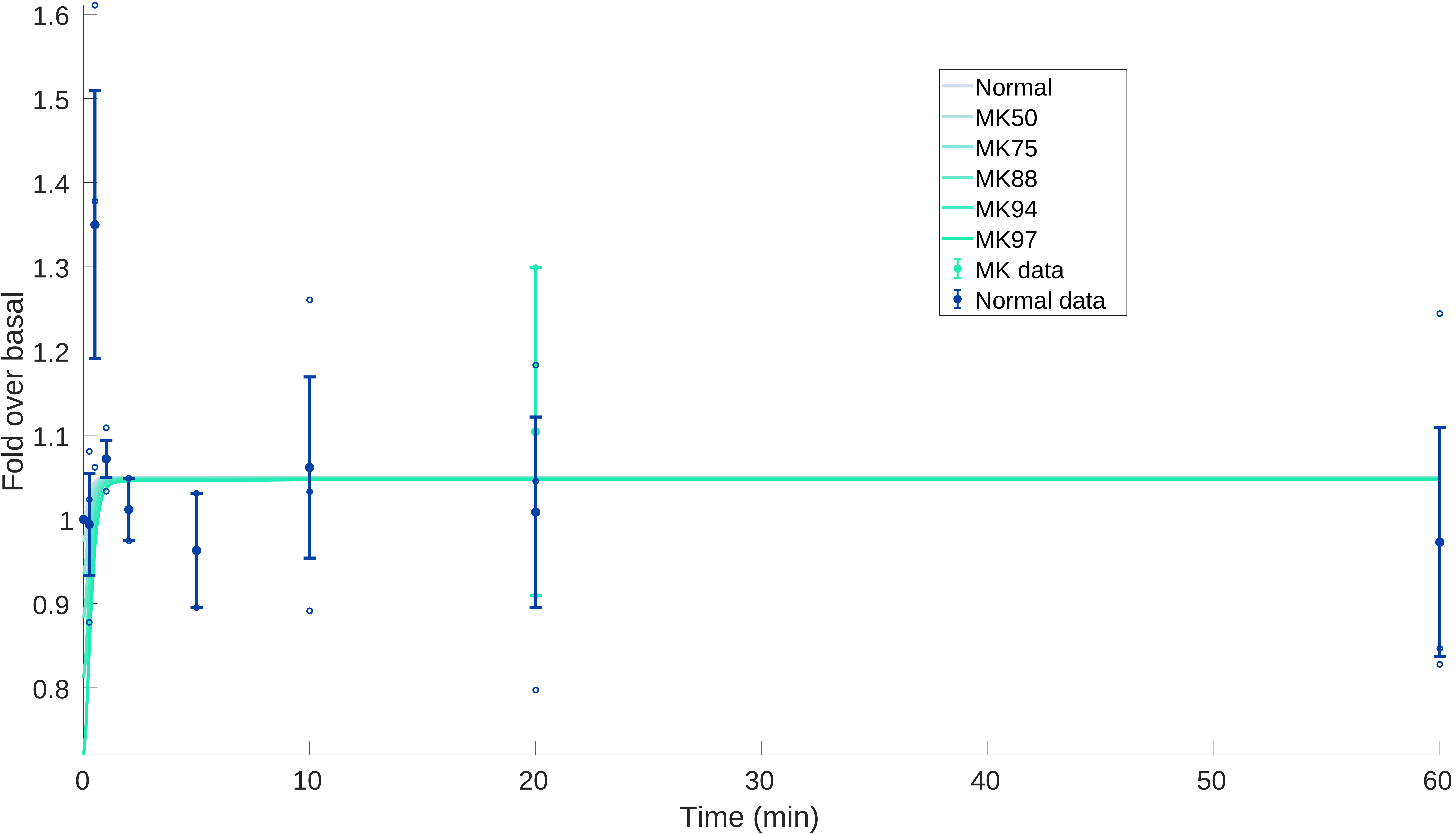

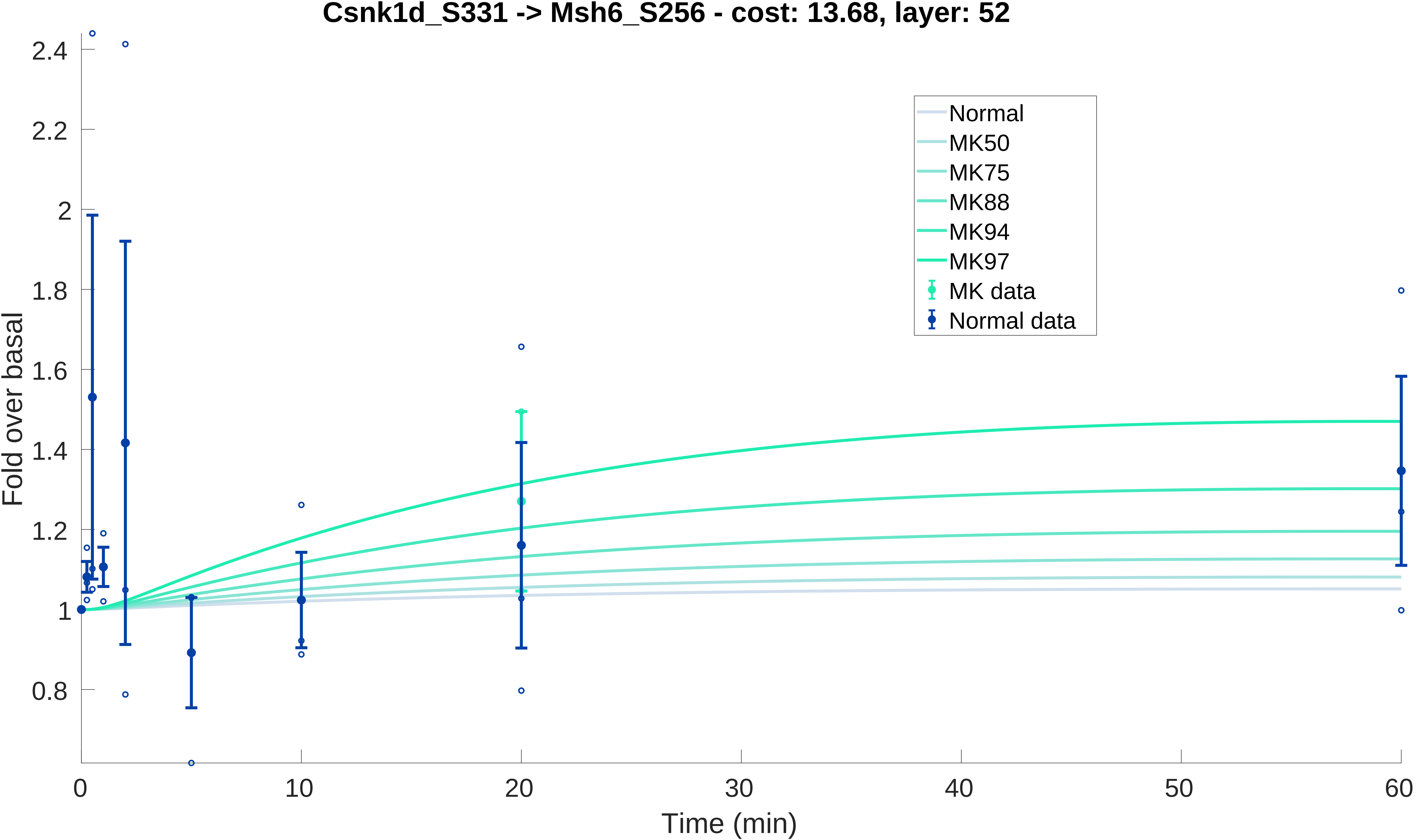
Csnk1d_S331 -> Msh6_S256 - cost: 13.68, layer: 52
Fold over basal
Time (min)
Normal
MK50
MK75
MK88
MK94
MK97
MK data
Normal data
2.4
2.2
2
1.8
1.6
1.4
1.2
1
0.8
0
10
20
30
40
50
60

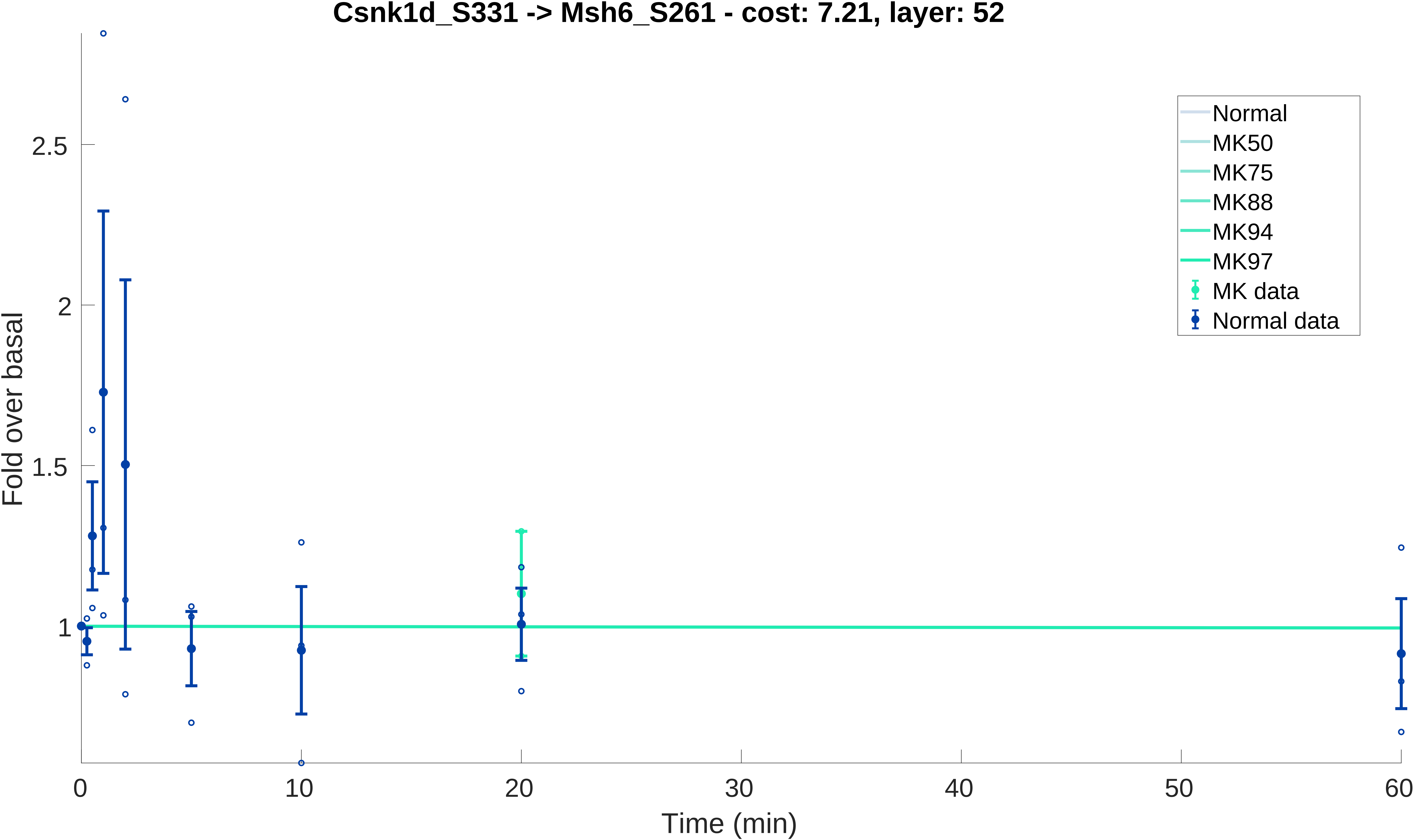

Csnk1d_S331 -> Msh6_S261 - cost: 7.21, layer: 52
Fold over basal
2.5
2
1.5
1
0
10
20
30
40
50
60
Time (min)
Normal
MK50
MK75
MK88
MK94
MK97
MK data
Normal data

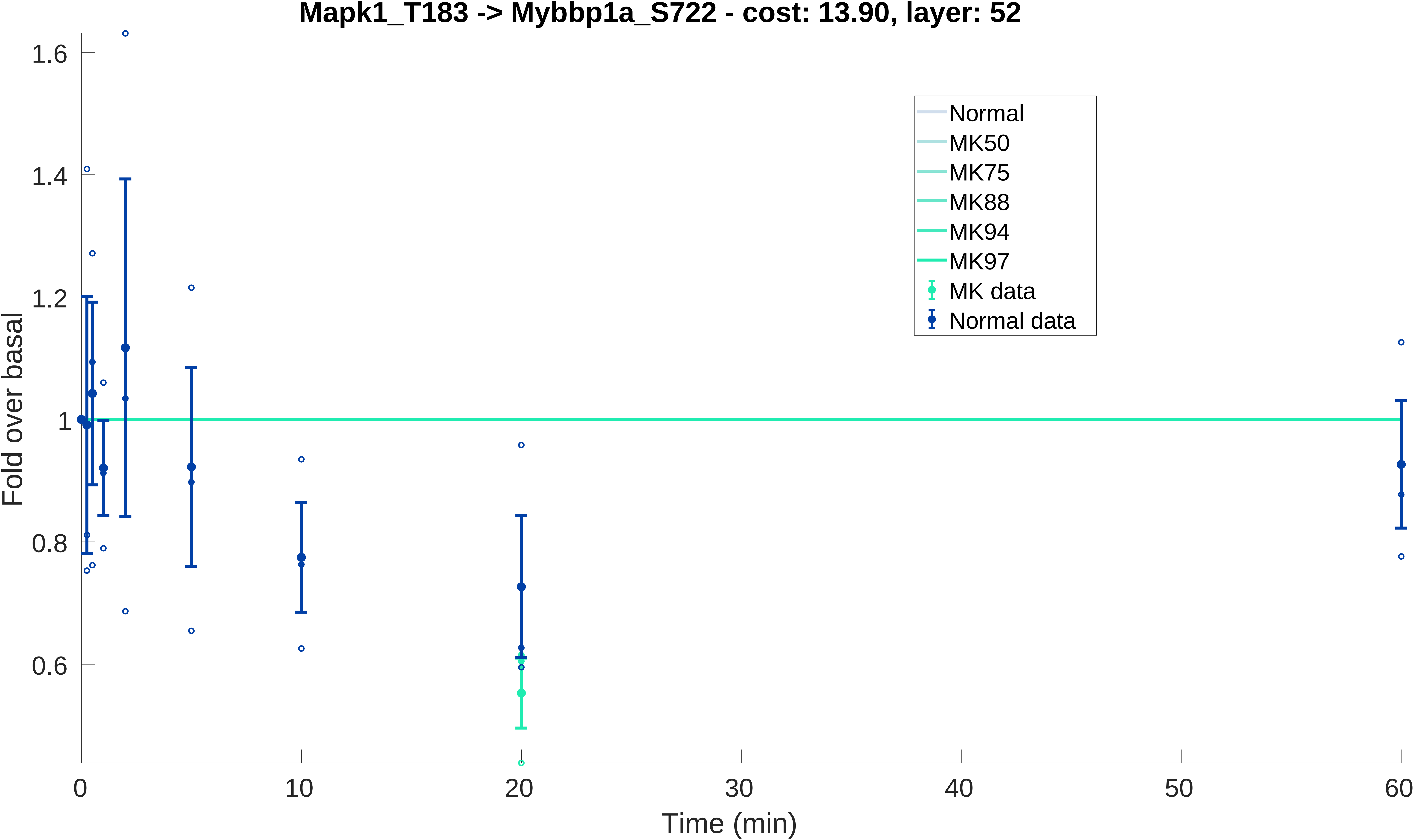

Mapk1_T183 -> Mybbp1a_S722 - cost: 13.90, layer: 52
Fold over basal
1.6
1.4
1.2
1
0.8
0.6
0
10
20
30
40
50
60
Time (min)
Normal
MK50
MK75
MK88
MK94
MK97
MK data
Normal data

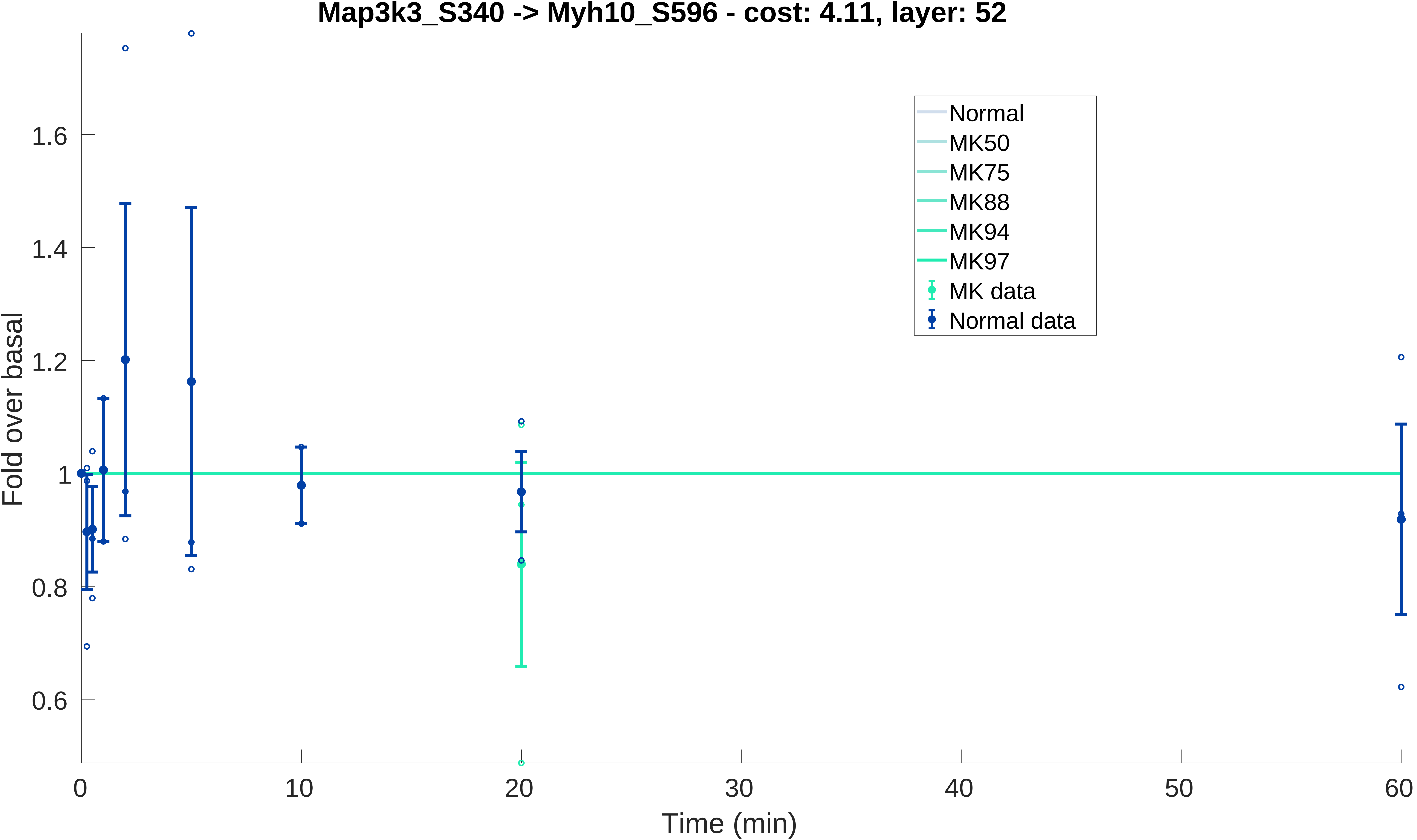

Map3k3_S340 -> Myh10_S596 - cost: 4.11, layer: 52
Normal
MK50
MK75
MK88
MK94
MK97
MK data
Normal data
Fold over basal
1.6
1.4
1.2
1
0.8
0.6
0
10
20
30
40
50
60
Time (min)

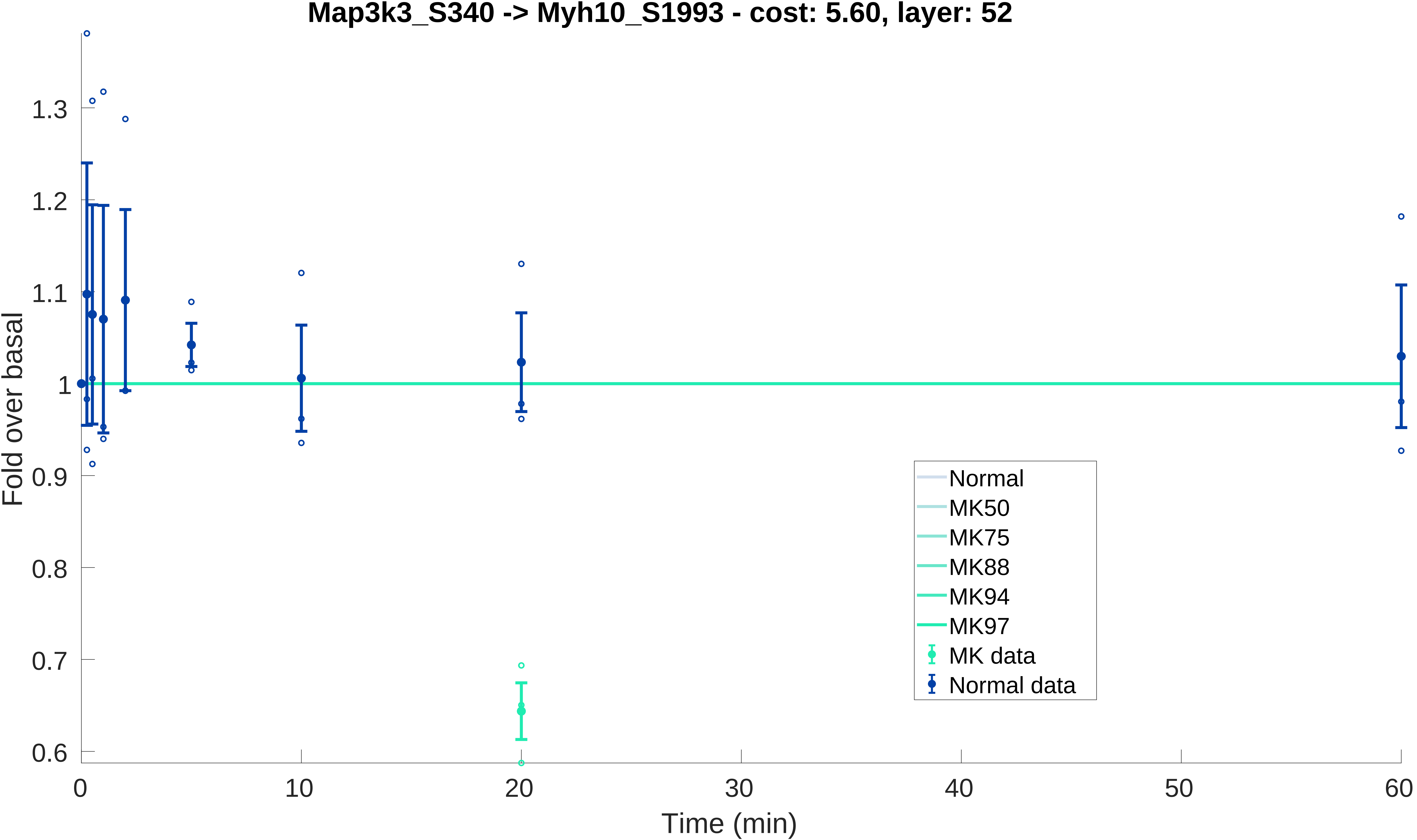

Map3k3_S340 -> Myh10_S1993 - cost: 5.60, layer: 52
Fold over basal
Time (min)
0.6
0.7
0.8
0.9
1
1.1
1.2
1.3
0
10
20
30
40
50
60
Normal
MK50
MK75
MK88
MK94
MK97
MK data
Normal data

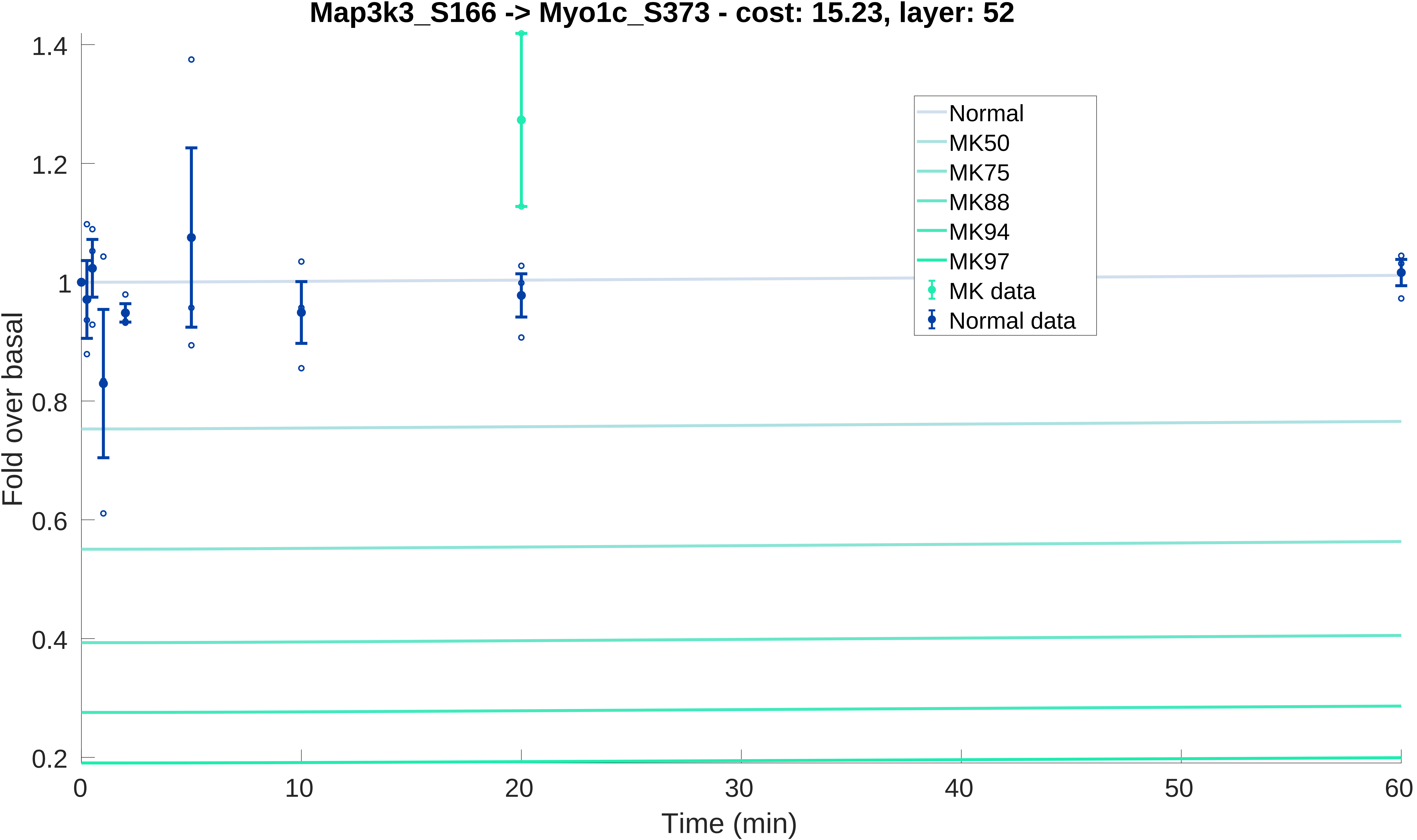
Map3k3_S166 -> Myo1c_S373 - cost: 15.23, layer: 52
Fold over basal
Time (min)
Normal
MK50
MK75
MK88
MK94
MK97
MK data
Normal data
1.4
1.2
1
0.8
0.6
0.4
0.2
0
10
20
30
40
50
60

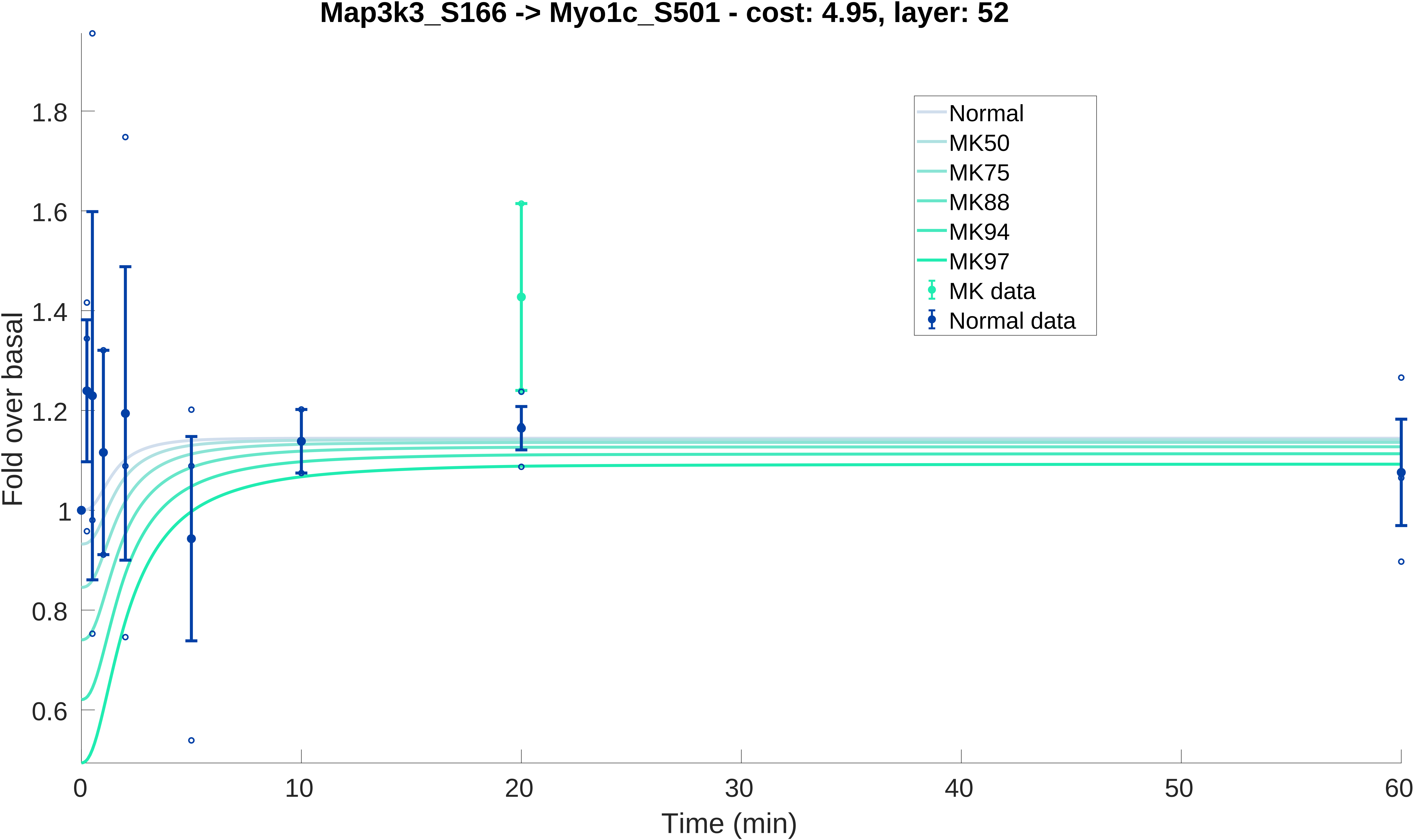

Map3k3_S166 -> Myo1c_S501 - cost: 4.95, layer: 52
Fold over basal
0.6
0.8
1
1.2
1.4
1.6
1.8
Time (min)
0
10
20
30
40
50
60
Normal
MK50
MK75
MK88
MK94
MK97
MK data
Normal data

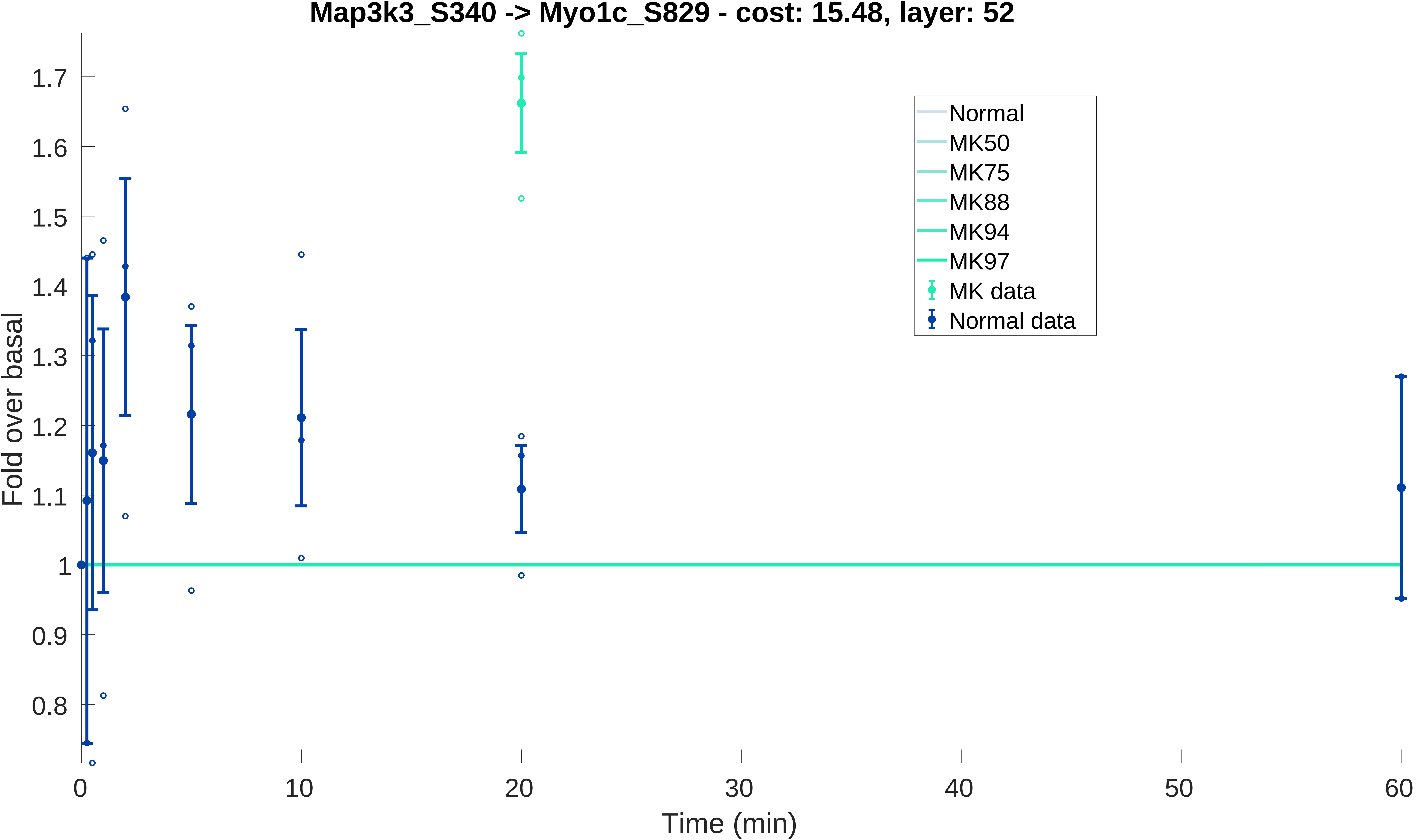

Map3k3_S340 -> Myo1c_S829 - cost: 15.48, layer: 52
Normal
MK50
MK75
MK88
MK94
MK97
MK data
Normal data
Fold over basal
Time (min)
1.7
1.6
1.5
1.4
1.3
1.2
1.1
1
0.9
0.8
0
10
20
30
40
50
60

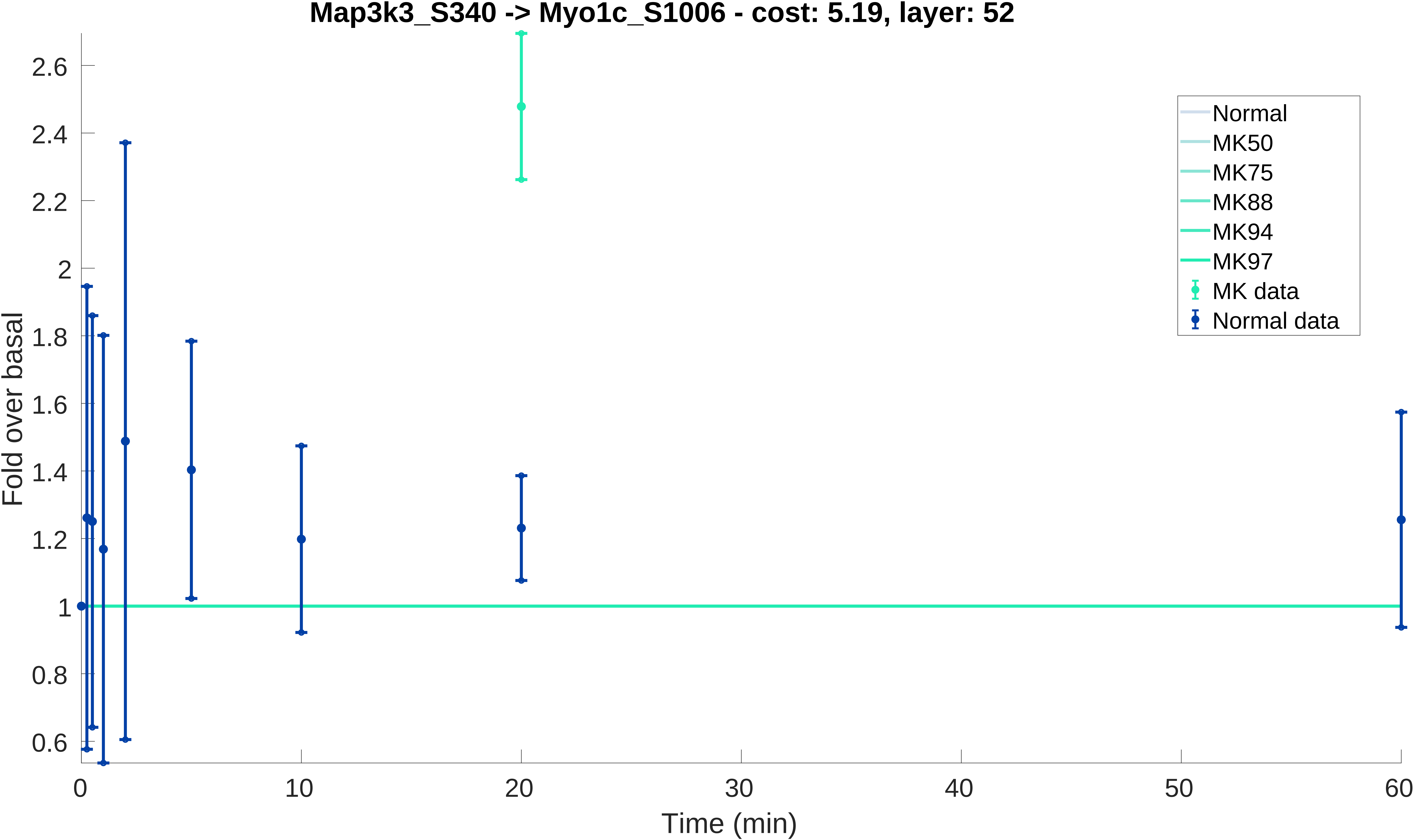

Map3k3_S340 -> Myo1c_S1006 - cost: 5.19, layer: 52
Fold over basal
Time (min)
2.6
2.4
2.2
2
1.8
1.6
1.4
1.2
1
0.8
0.6
0
10
20
30
40
50
60
Normal
MK50
MK75
MK88
MK94
MK97
MK data
Normal data

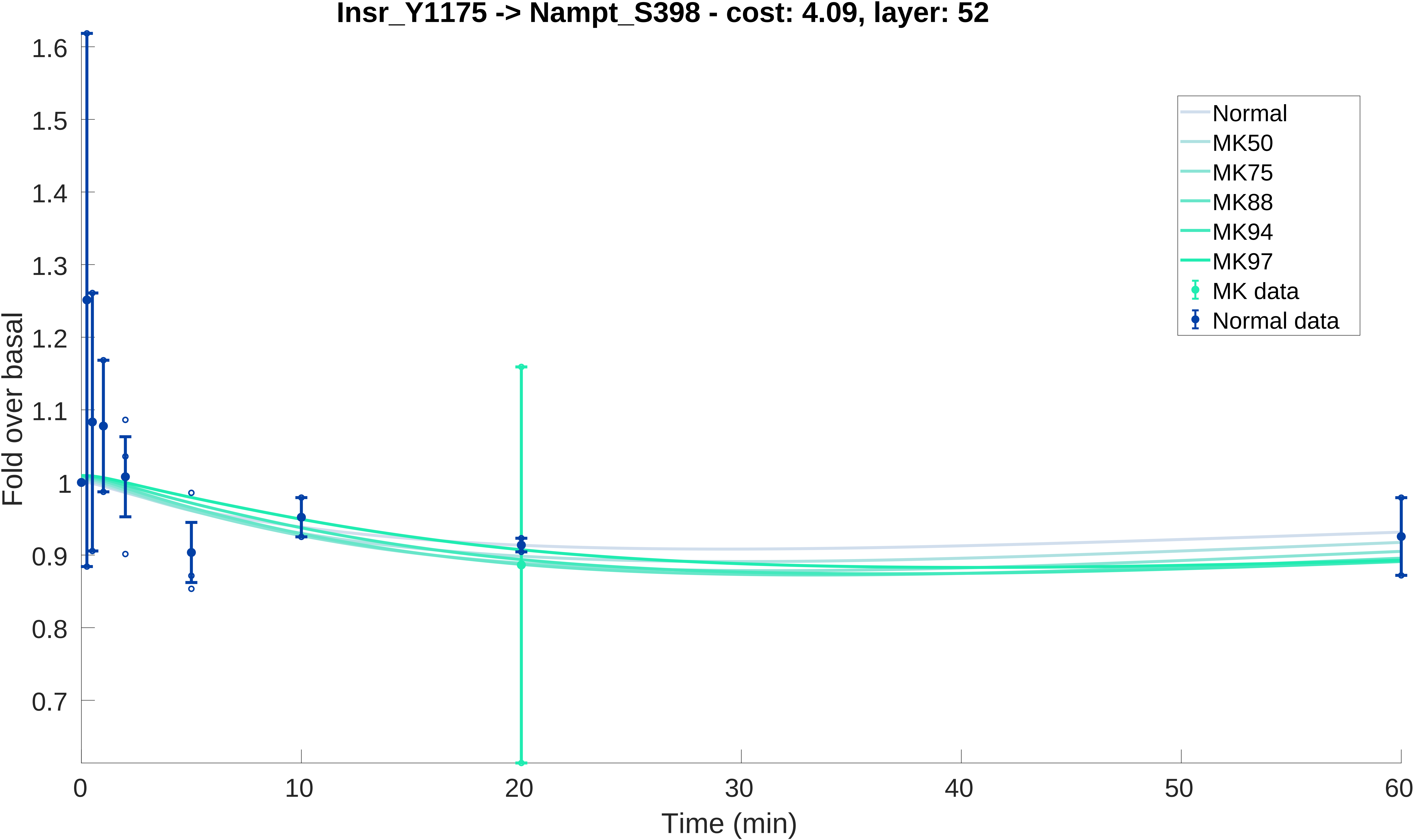
Insr_Y1175 -> Nampt_S398 - cost: 4.09, layer: 52
Fold over basal
1.6
1.5
1.4
1.3
1.2
1.1
1
0.9
0.8
0.7
0
10
20
30
40
50
60
Time (min)
Normal
MK50
MK75
MK88
MK94
MK97
MK data
Normal data

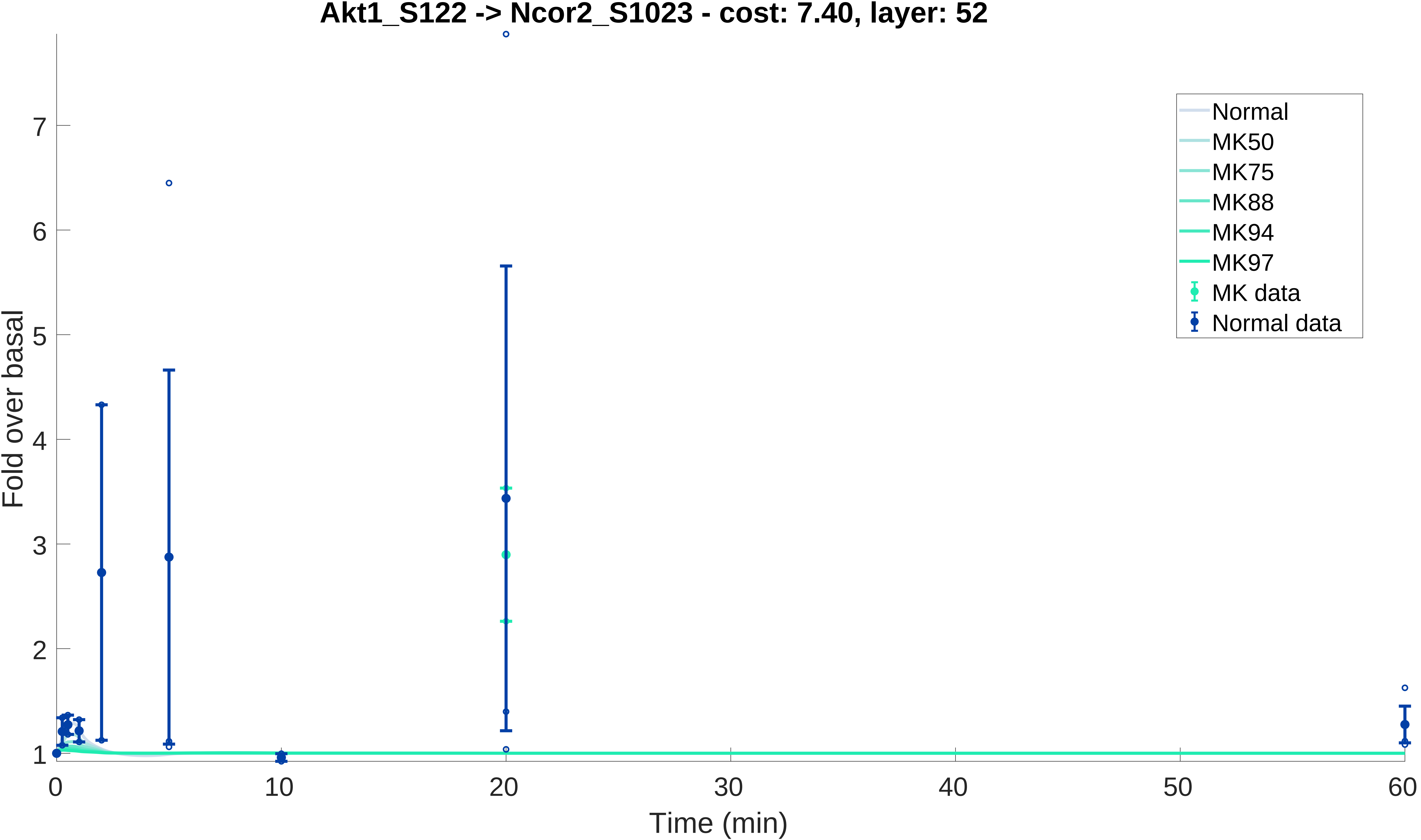
Akt1_S122 -> Ncor2_S1023 - cost: 7.40, layer: 52
Fold over basal
Time (min)
1
2
3
4
5
6
7
0
10
20
30
40
50
60
Normal
MK50
MK75
MK88
MK94
MK97
MK data
Normal data

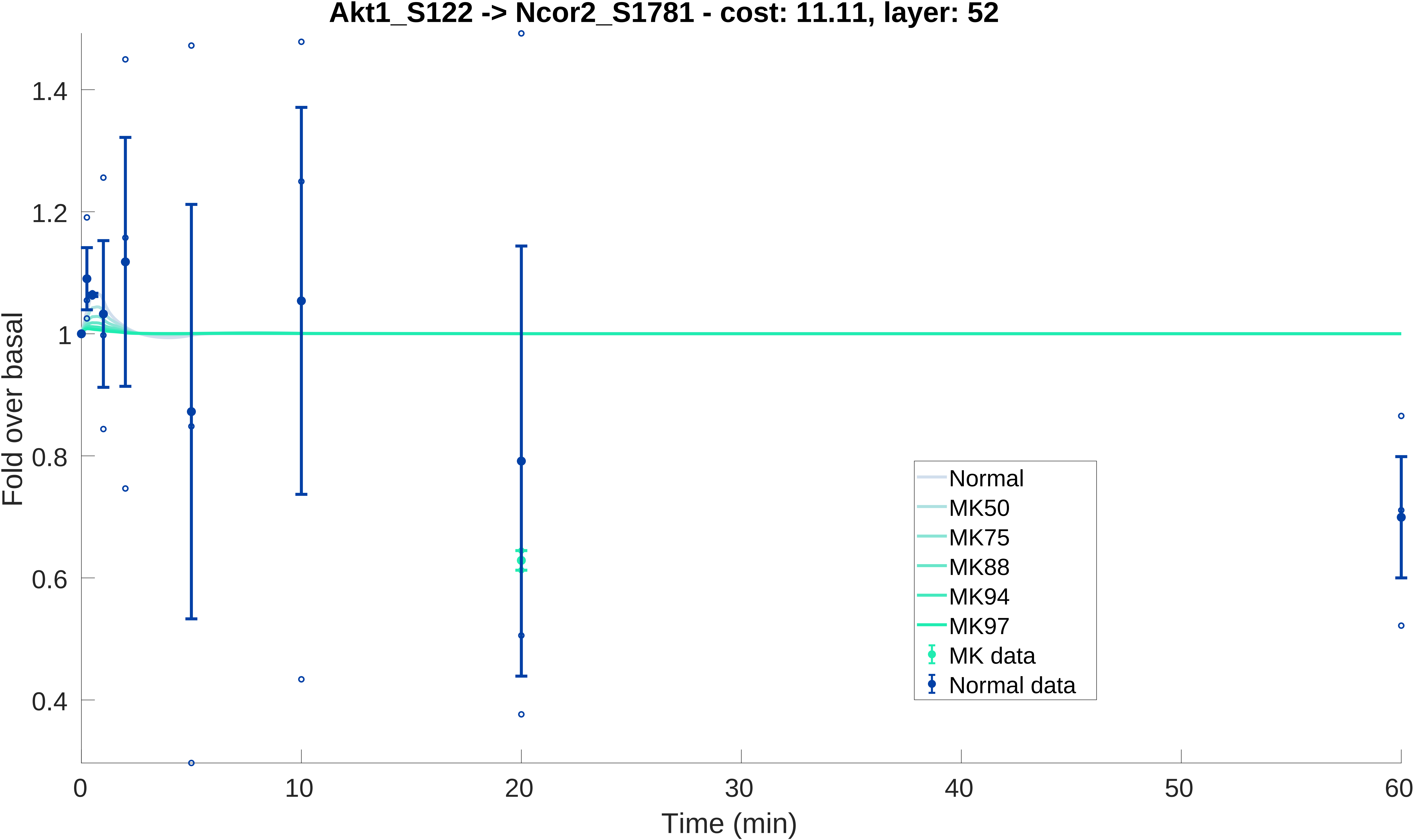
Akt1_S122 -> Ncor2_S1781 - cost: 11.11, layer: 52
Fold over basal
1.4
1.2
1
0.8
0.6
0.4
0
10
20
30
40
50
60
Time (min)
Normal
MK50
MK75
MK88
MK94
MK97
MK data
Normal data

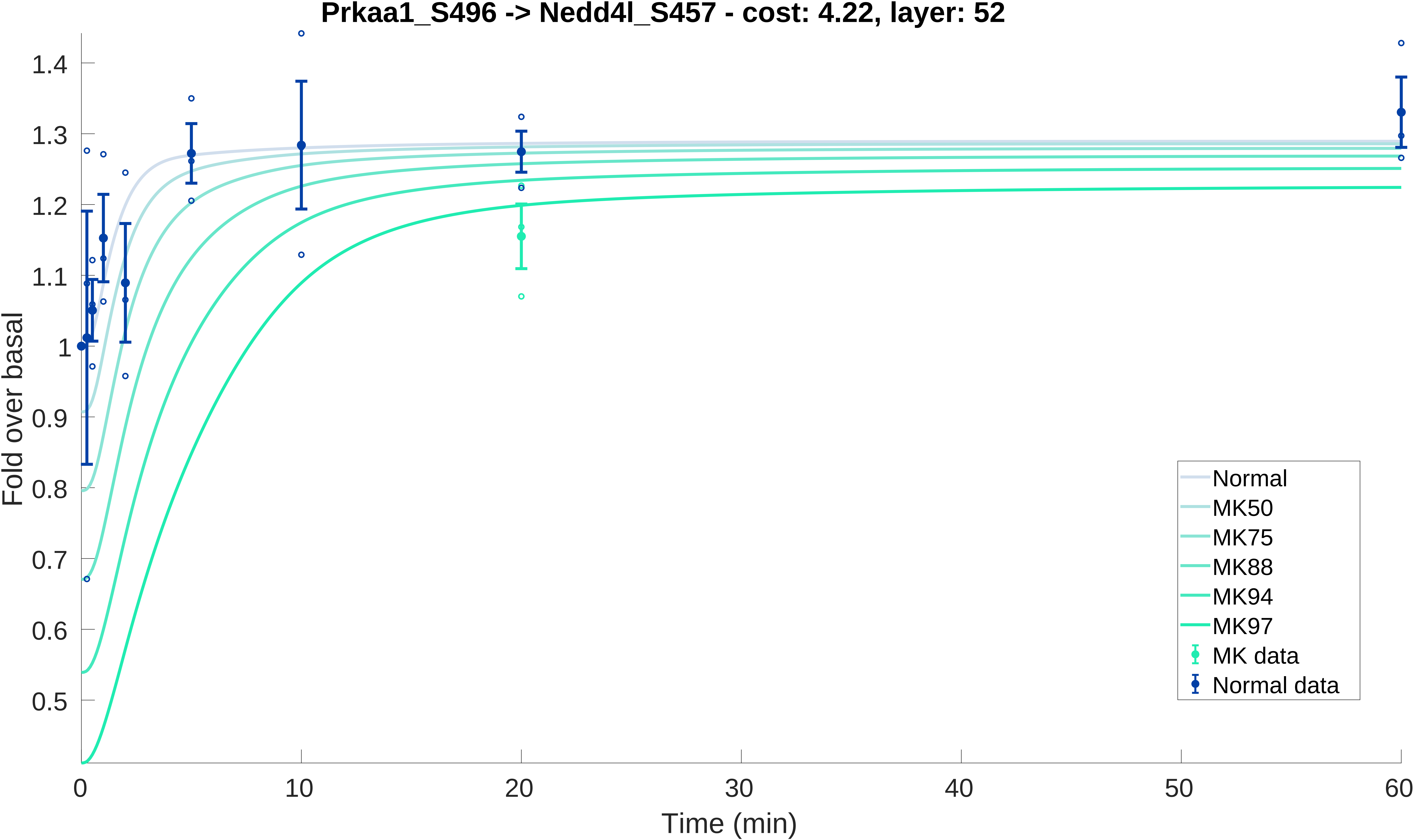

Prkaa1_S496 -> Nedd4l_S457 - cost: 4.22, layer: 52
Fold over basal
Time (min)
1.4
1.3
1.2
1.1
1
0.9
0.8
0.7
0.6
0.5
0
10
20
30
40
50
60
Normal
MK50
MK75
MK88
MK94
MK97
MK data
Normal data

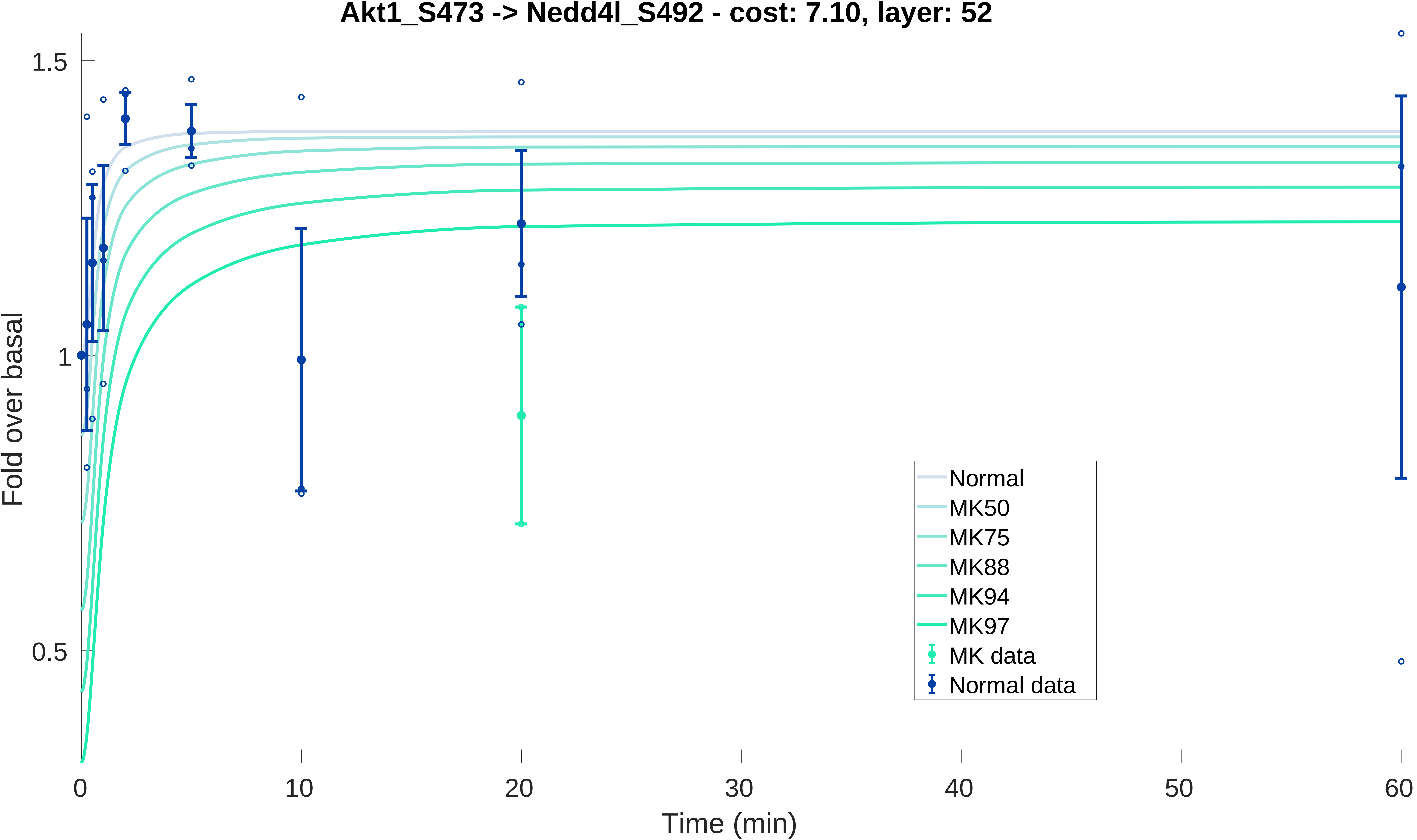

Akt1_S473 -> Nedd4l_S492 - cost: 7.10, layer: 52
Fold over basal
Time (min)
1.5
1
0.5
0
10
20
30
40
50
60
Normal
MK50
MK75
MK88
MK94
MK97
MK data
Normal data

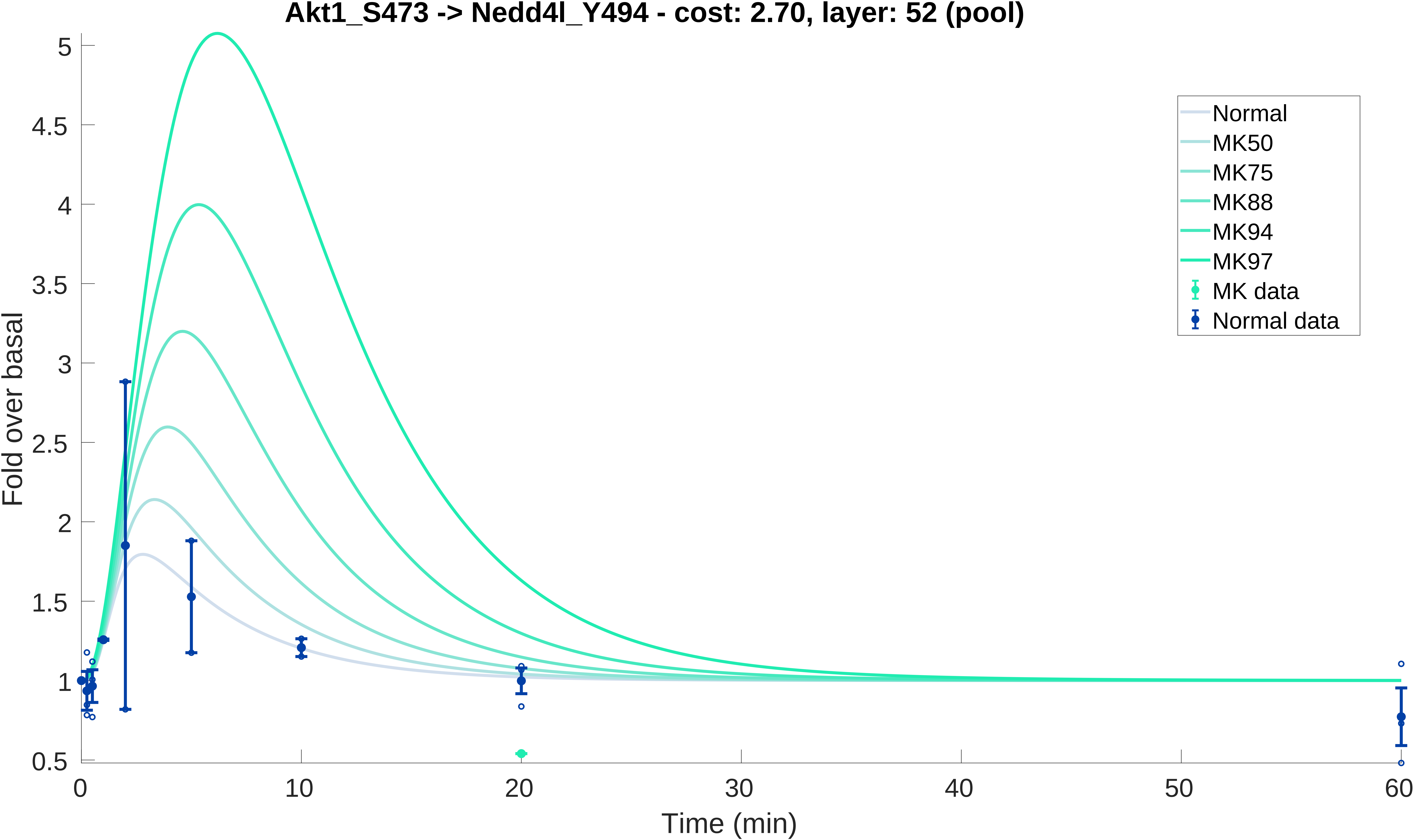

Akt1_S473 -> Nedd4l_Y494 - cost: 2.70, layer: 52 (pool)
Fold over basal
Time (min)
5
4.5
4
3.5
3
2.5
2
1.5
1
0.5
0
10
20
30
40
50
60
Normal
MK50
MK75
MK88
MK94
MK97
MK data
Normal data

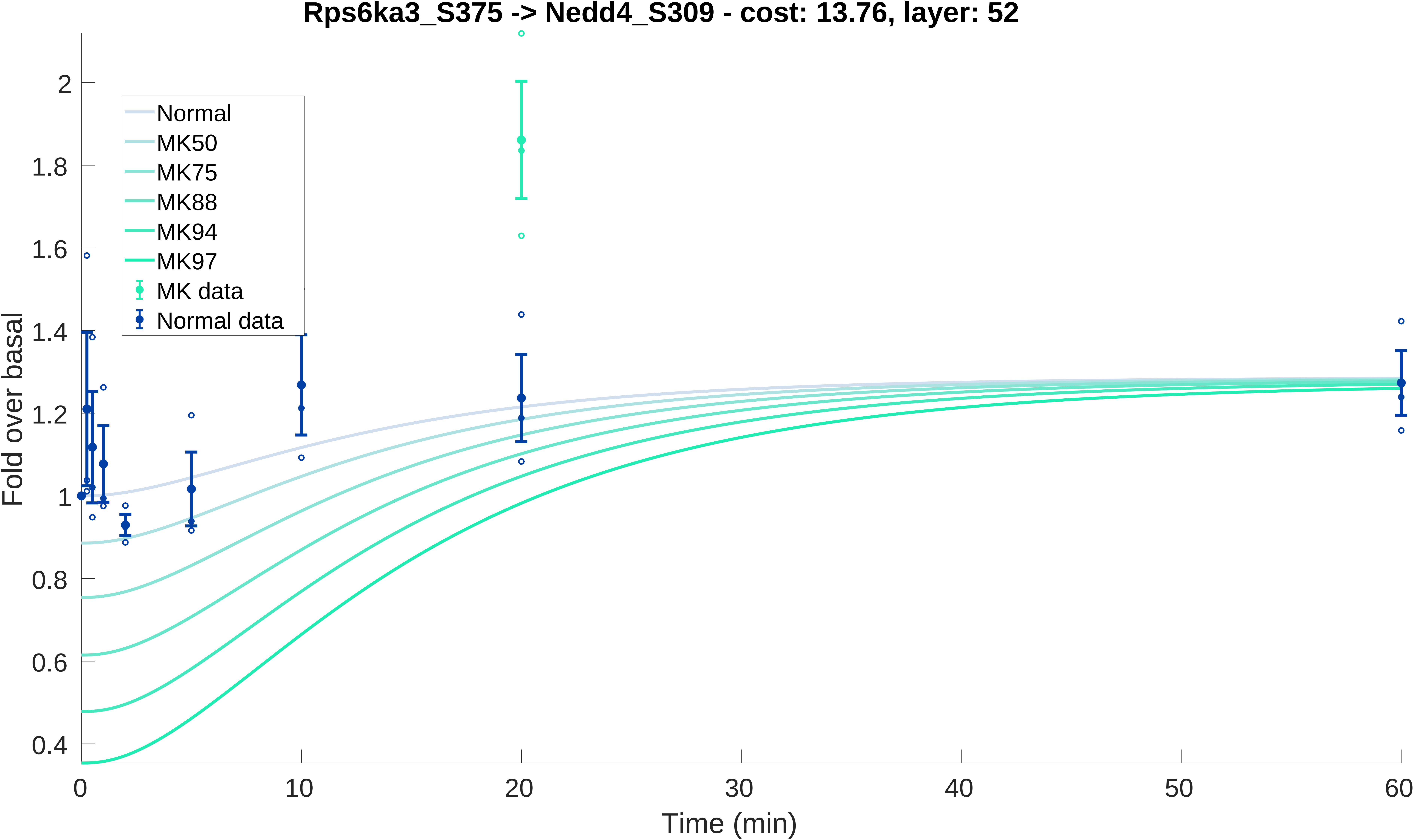

Rps6ka3_S375 -> Nedd4_S309 - cost: 13.76, layer: 52
Fold over basal
Time (min)
0.4
0.6
0.8
1
1.2
1.4
1.6
1.8
2
0
10
20
30
40
50
60
Normal
MK50
MK75
MK88
MK94
MK97
MK data
Normal data

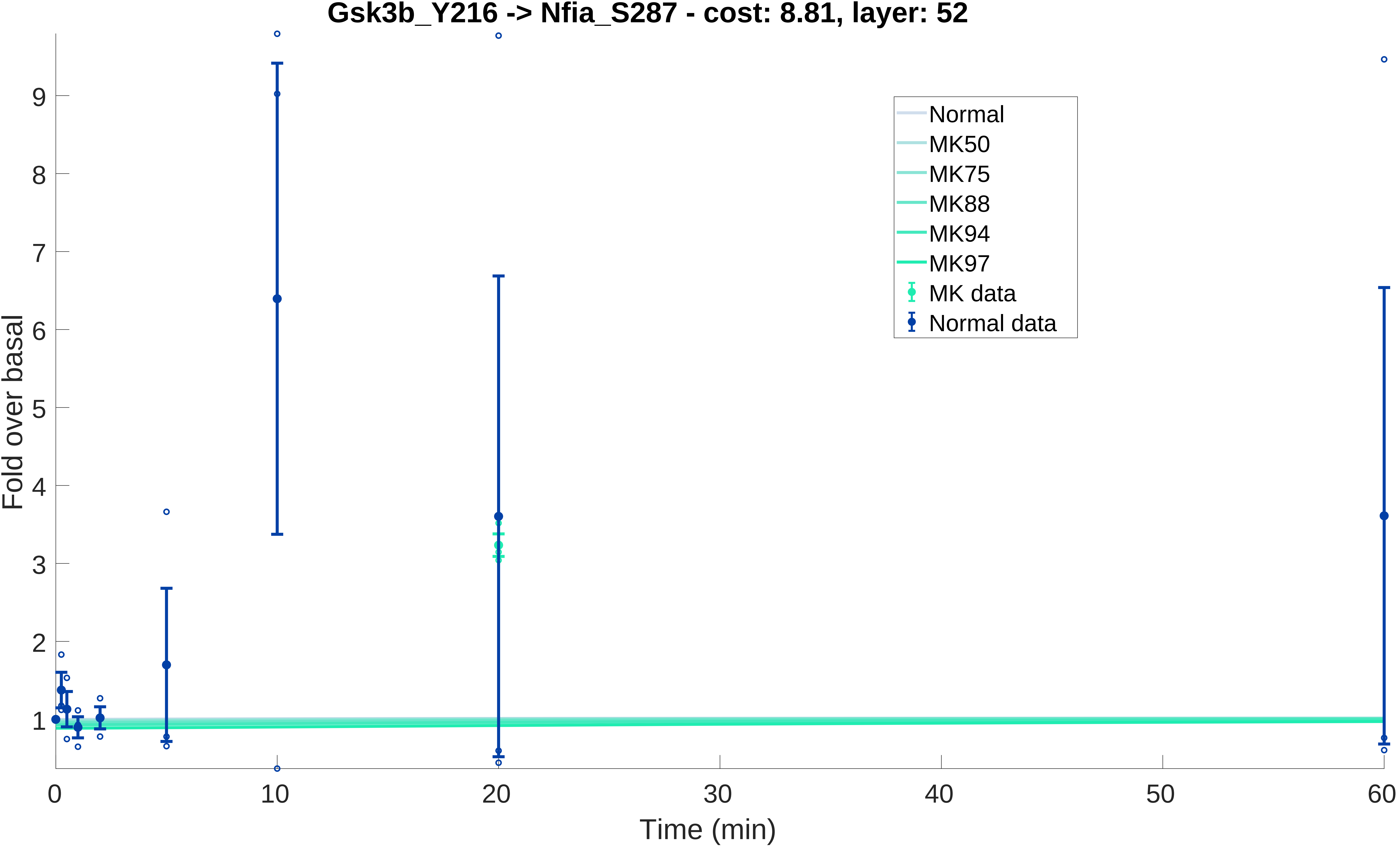

Gsk3b_Y216 -> Nfia_S287 - cost: 8.81, layer: 52
Fold over basal
Time (min)
0
10
20
30
40
50
60
1
2
3
4
5
6
7
8
9
Normal
MK50
MK75
MK88
MK94
MK97
MK data
Normal data

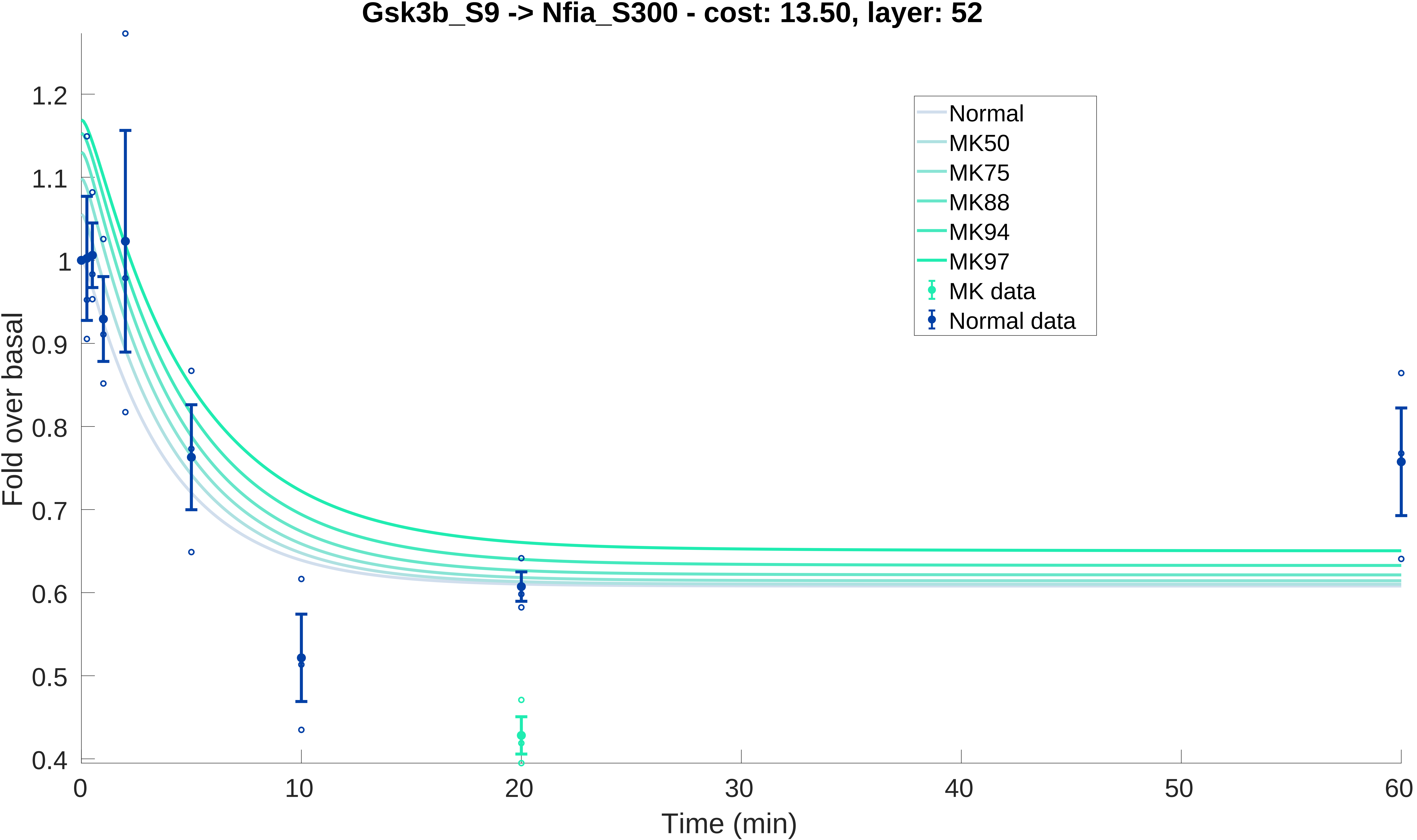
Gsk3b_S9 -> Nfia_S300 - cost: 13.50, layer: 52
Normal
MK50
MK75
MK88
MK94
MK97
MK data
Normal data
Fold over basal
Time (min)
0.4
0.5
0.6
0.7
0.8
0.9
1
1.1
1.2
0
10
20
30
40
50
60

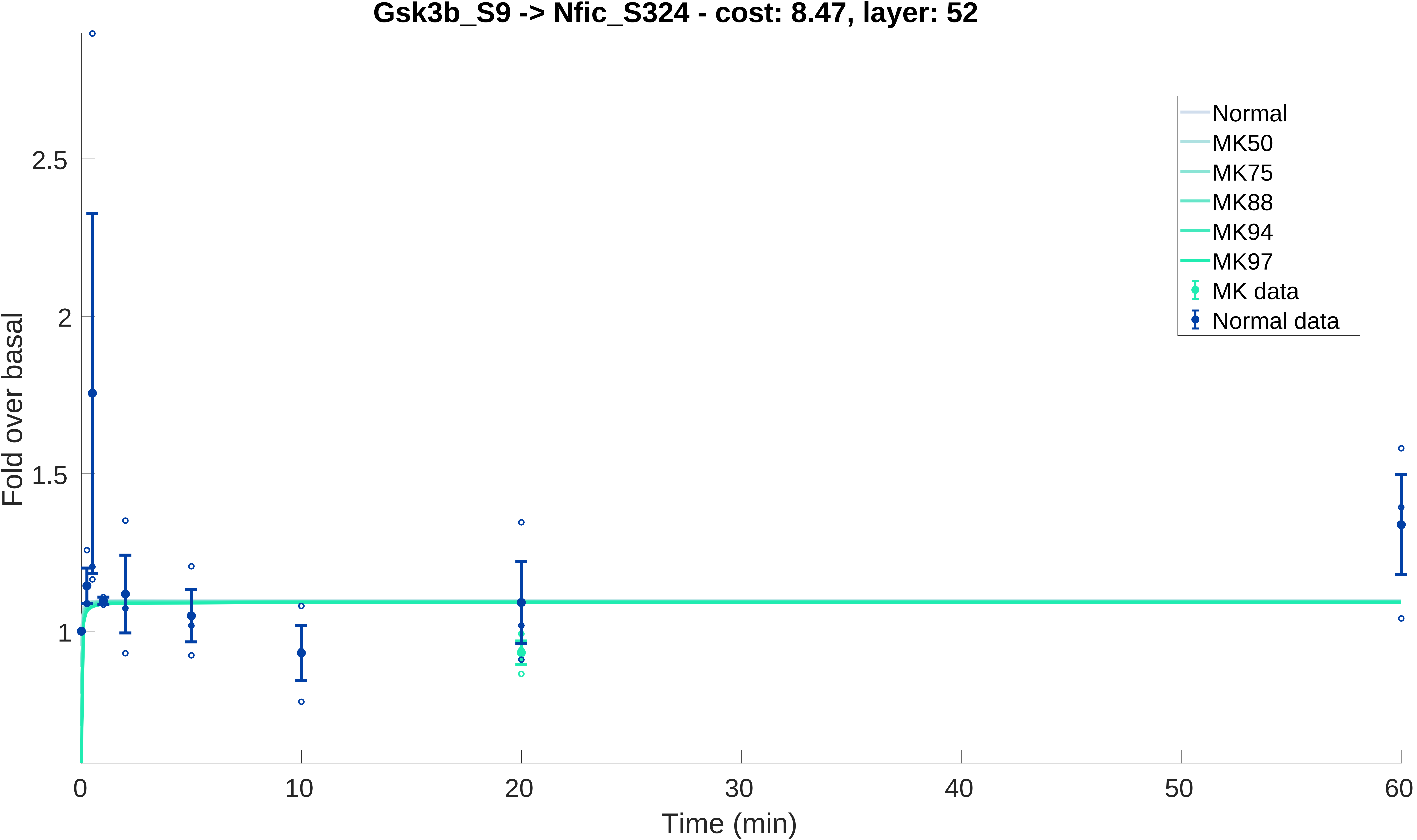

Gsk3b_S9 -> Nfic_S324 - cost: 8.47, layer: 52
Fold over basal
2.5
2
1.5
1
0
10
20
30
40
50
60
Time (min)
Normal
MK50
MK75
MK88
MK94
MK97
MK data
Normal data

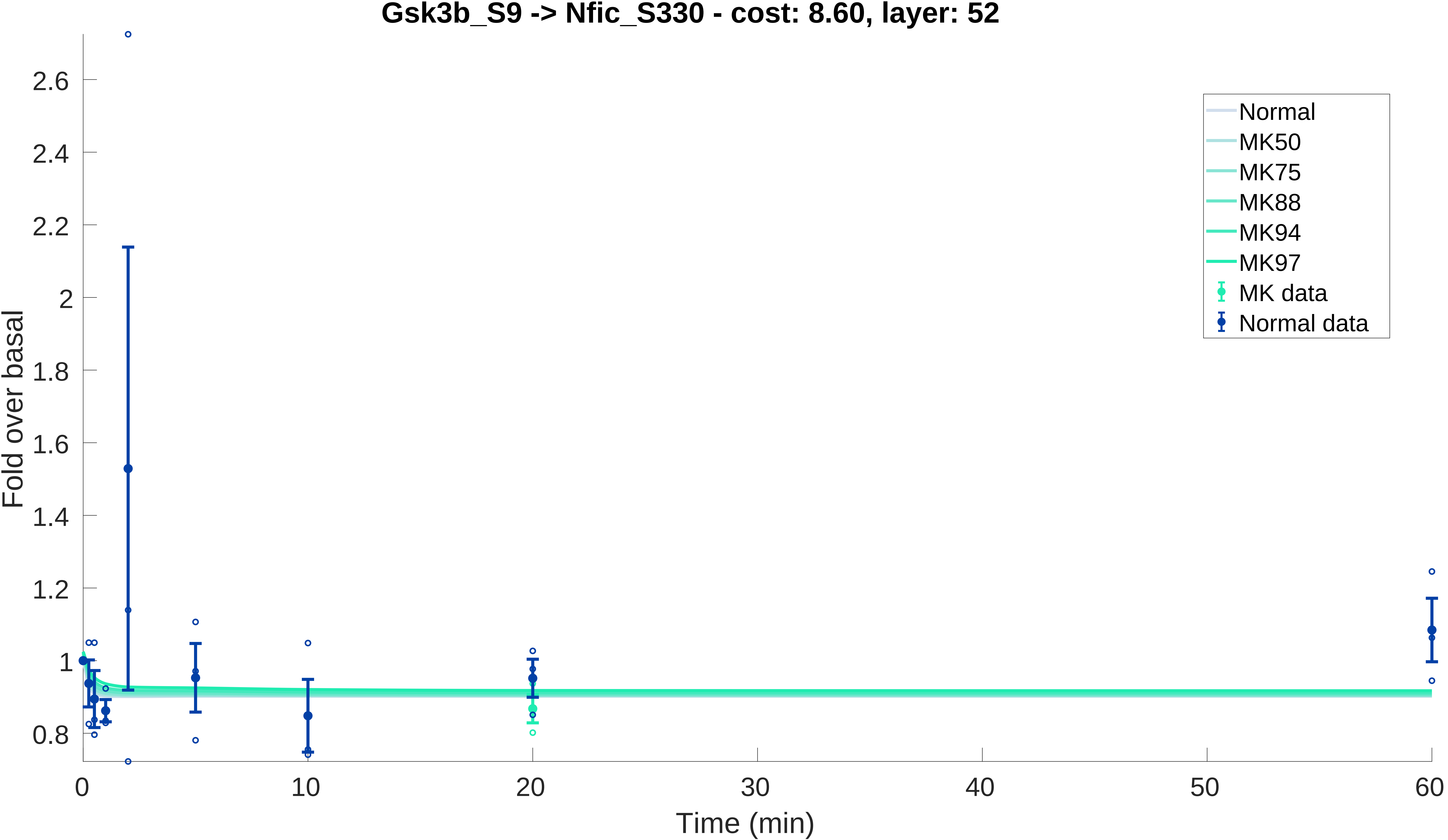

Gsk3b_S9 -> Nfic_S330 - cost: 8.60, layer: 52
Fold over basal
Time (min)
0
10
20
30
40
50
60
0.8
1
1.2
1.4
1.6
1.8
2
2.2
2.4
2.6
Normal
MK50
MK75
MK88
MK94
MK97
MK data
Normal data

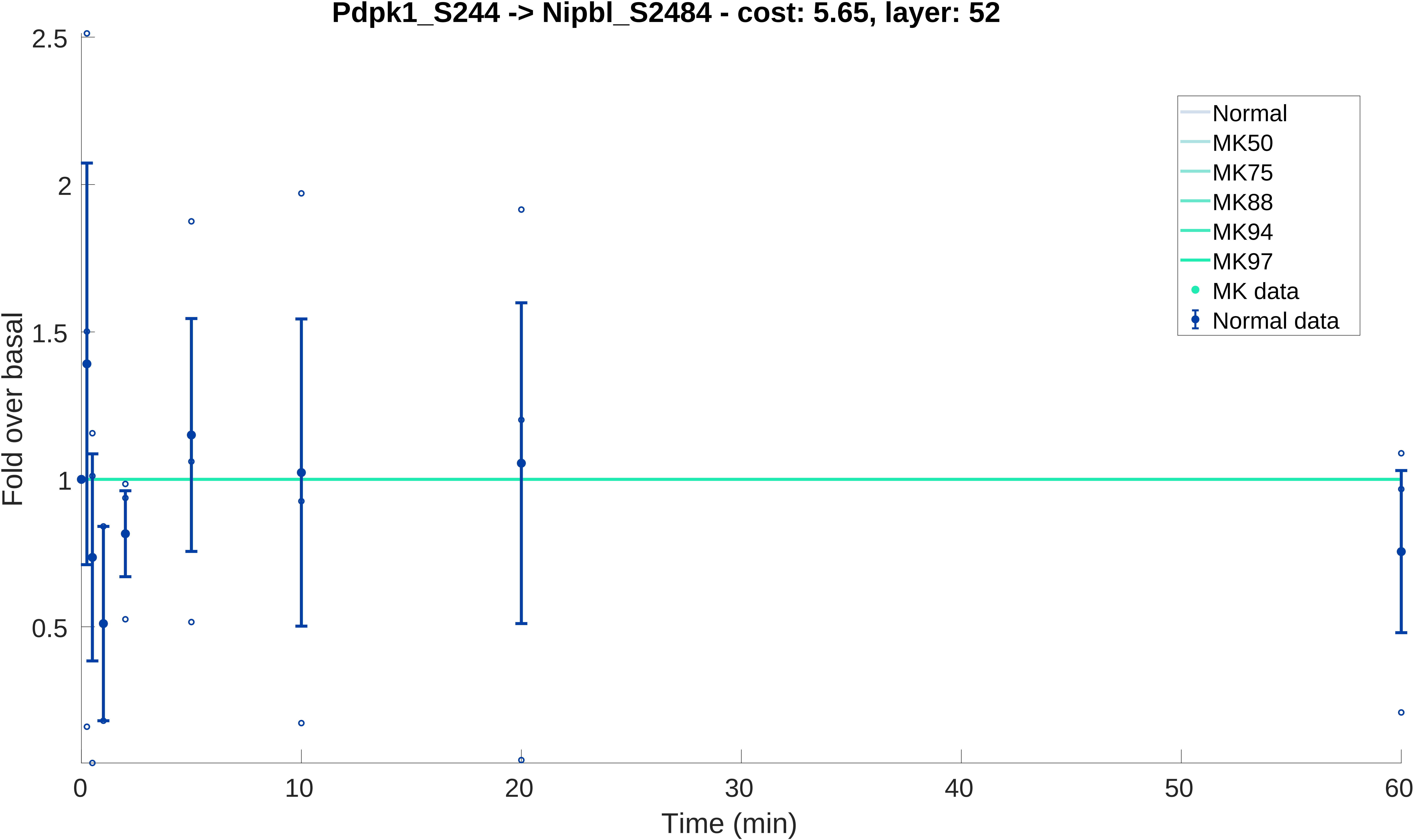

Pdpk1_S244 -> Nipbl_S2484 - cost: 5.65, layer: 52
Fold over basal
Time (min)
0.5
1
1.5
2
2.5
0
10
20
30
40
50
60
Normal
MK50
MK75
MK88
MK94
MK97
MK data
Normal data

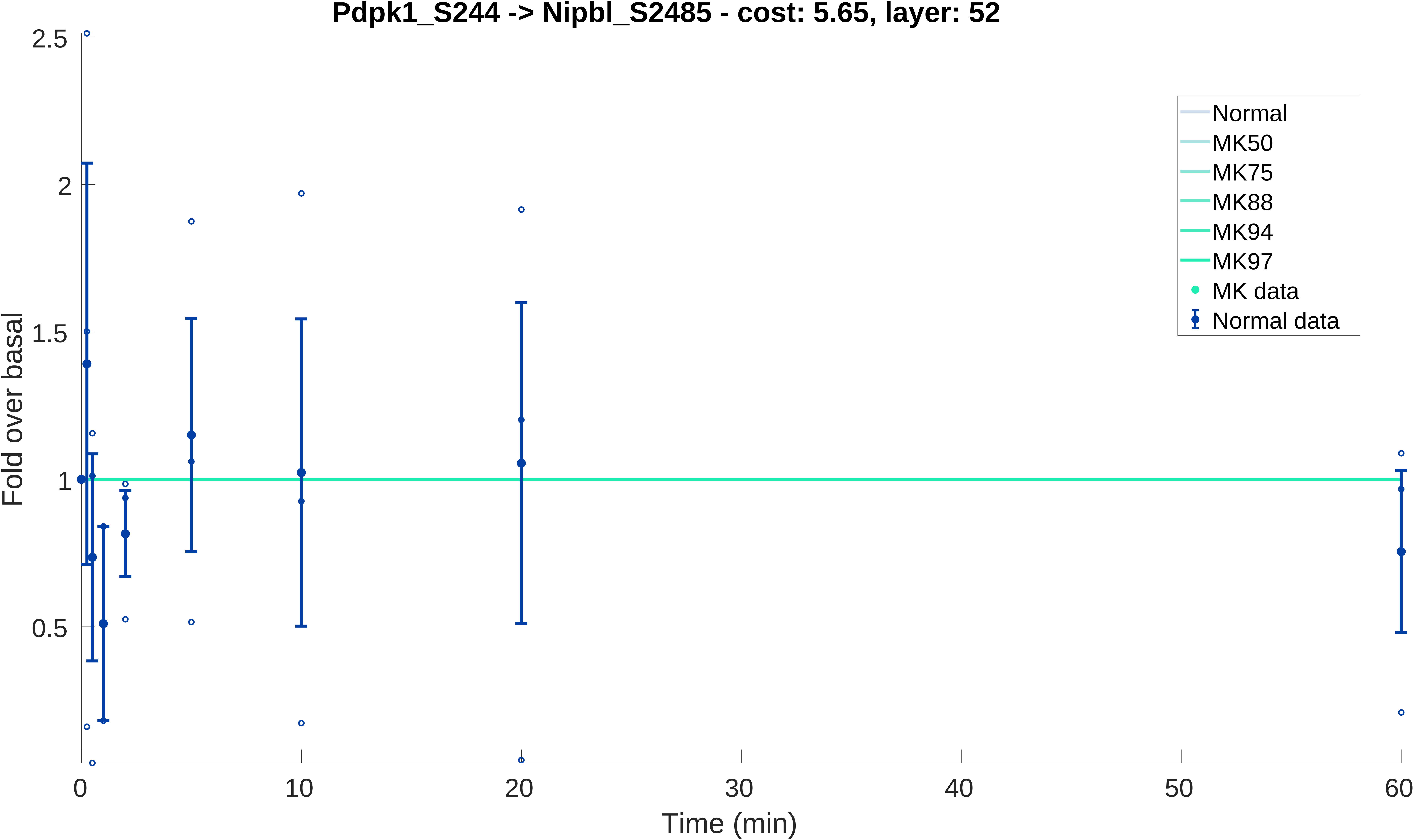

Pdpk1_S244 -> Nipbl_S2485 - cost: 5.65, layer: 52
Fold over basal
Time (min)
0
10
20
30
40
50
60
0.5
1
1.5
2
2.5
Normal
MK50
MK75
MK88
MK94
MK97
MK data
Normal data

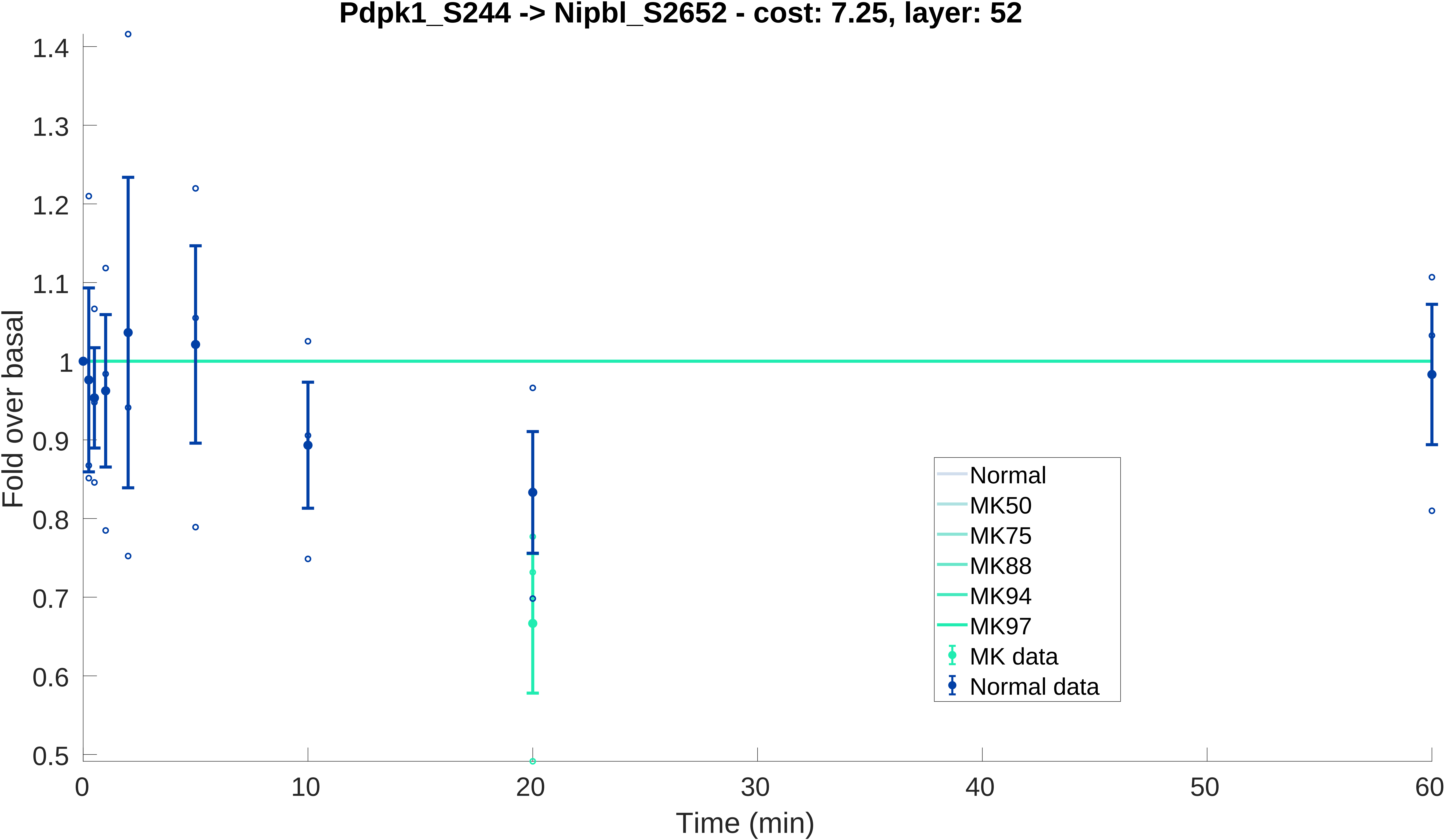
Pdpk1_S244 -> Nipbl_S2652 - cost: 7.25, layer: 52
Fold over basal
Time (min)
1.4
1.3
1.2
1.1
1
0.9
0.8
0.7
0.6
0.5
0
10
20
30
40
50
60
Normal
MK50
MK75
MK88
MK94
MK97
MK data
Normal data

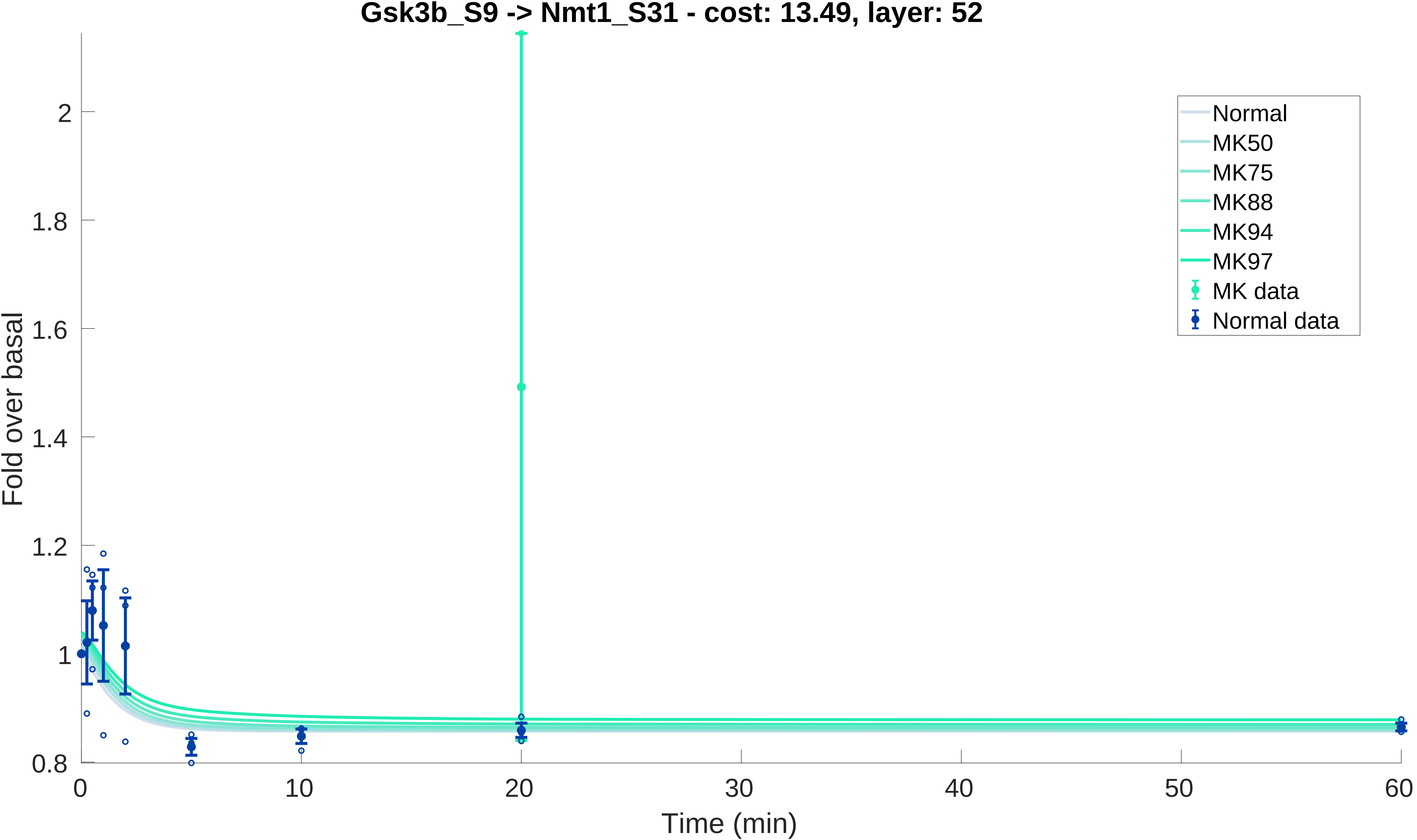

Gsk3b_S9 -> Nmt1_S31 - cost: 13.49, layer: 52
Fold over basal
Time (min)
0.8
1
1.2
1.4
1.6
1.8
2
0
10
20
30
40
50
60
Normal
MK50
MK75
MK88
MK94
MK97
MK data
Normal data

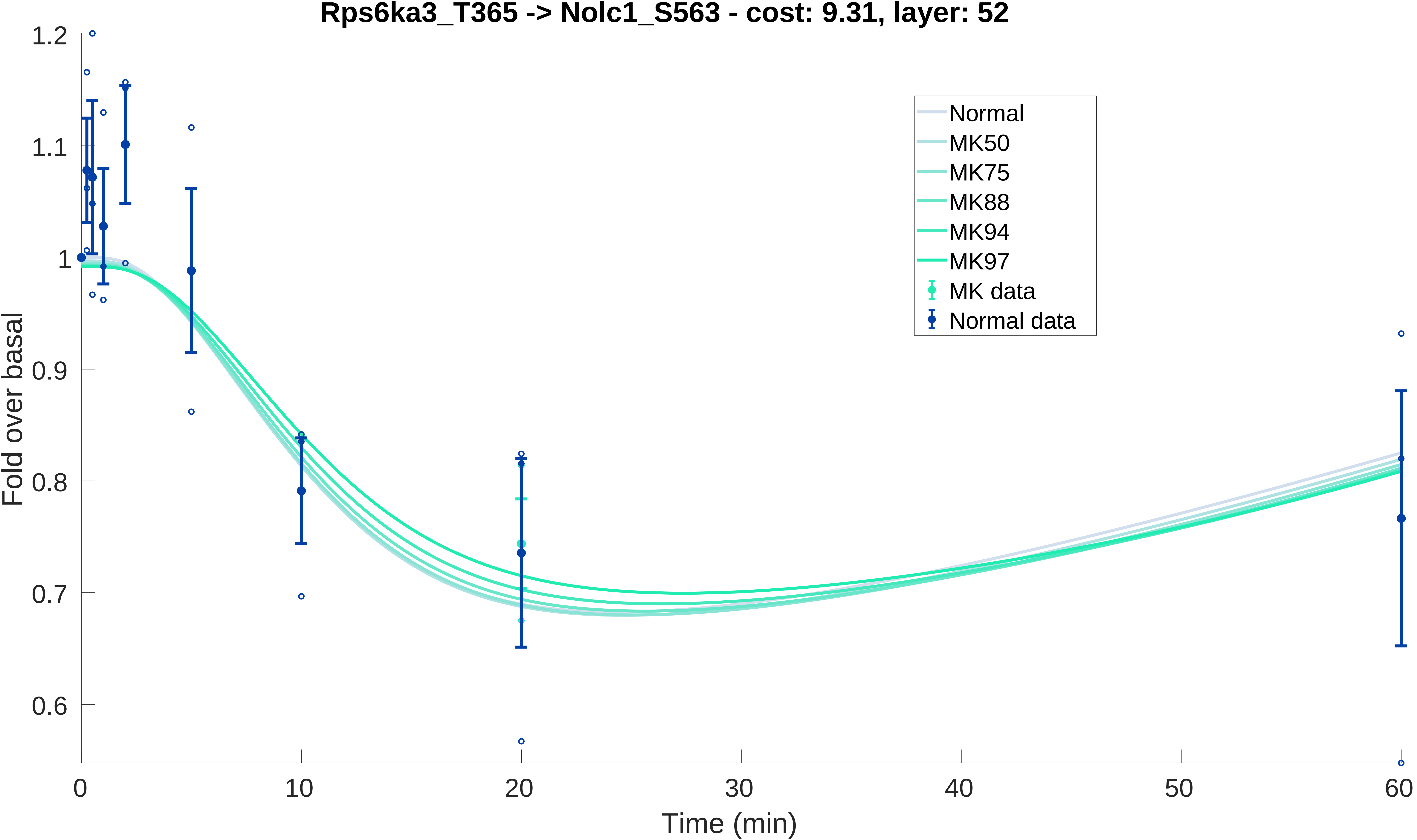

Rps6ka3_T365 -> Nolc1_S563 - cost: 9.31, layer: 52
Fold over basal
1.2
1.1
1
0.9
0.8
0.7
0.6
0
10
20
30
40
50
60
Time (min)
Normal
MK50
MK75
MK88
MK94
MK97
MK data
Normal data

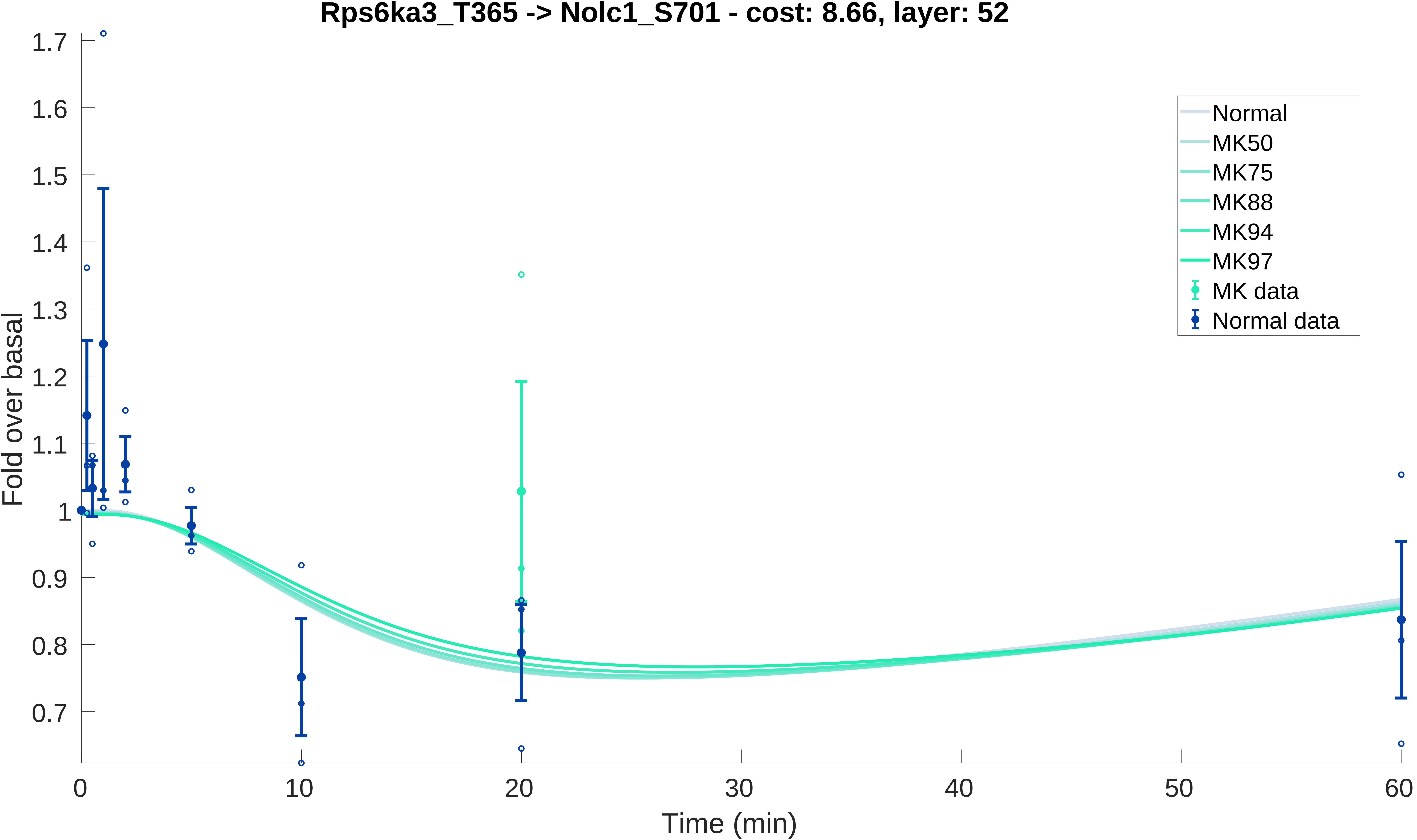
Rps6ka3_T365 -> Nolc1_S701 - cost: 8.66, layer: 52
Fold over basal
Time (min)
Normal
MK50
MK75
MK88
MK94
MK97
MK data
Normal data

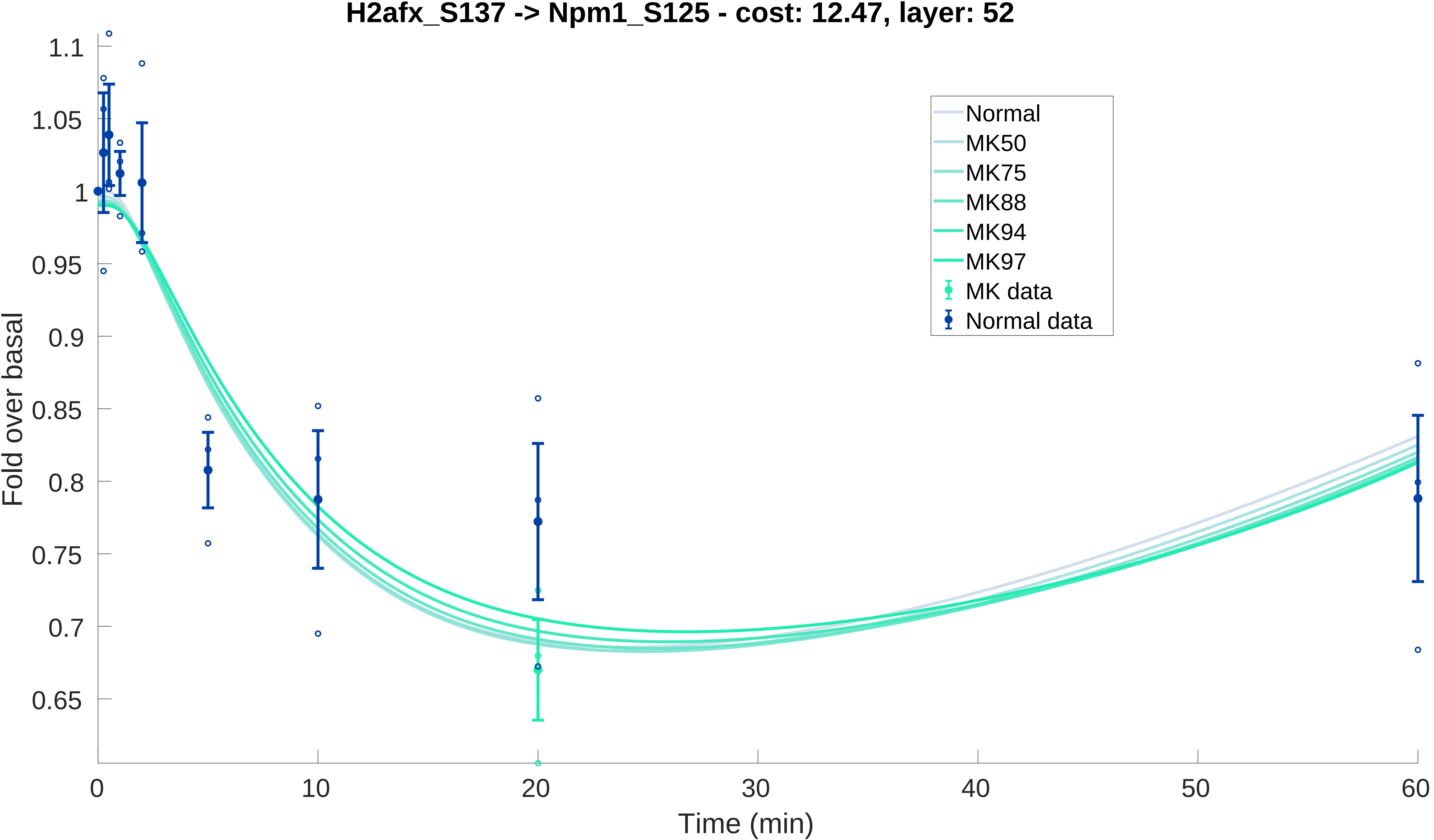

H2afx_S137 -> Npm1_S125 - cost: 12.47, layer: 52
Fold over basal
Time (min)
0
10
20
30
40
50
60
1.1
1.05
1
0.95
0.9
0.85
0.8
0.75
0.7
0.65
Normal
MK50
MK75
MK88
MK94
MK97
MK data
Normal data

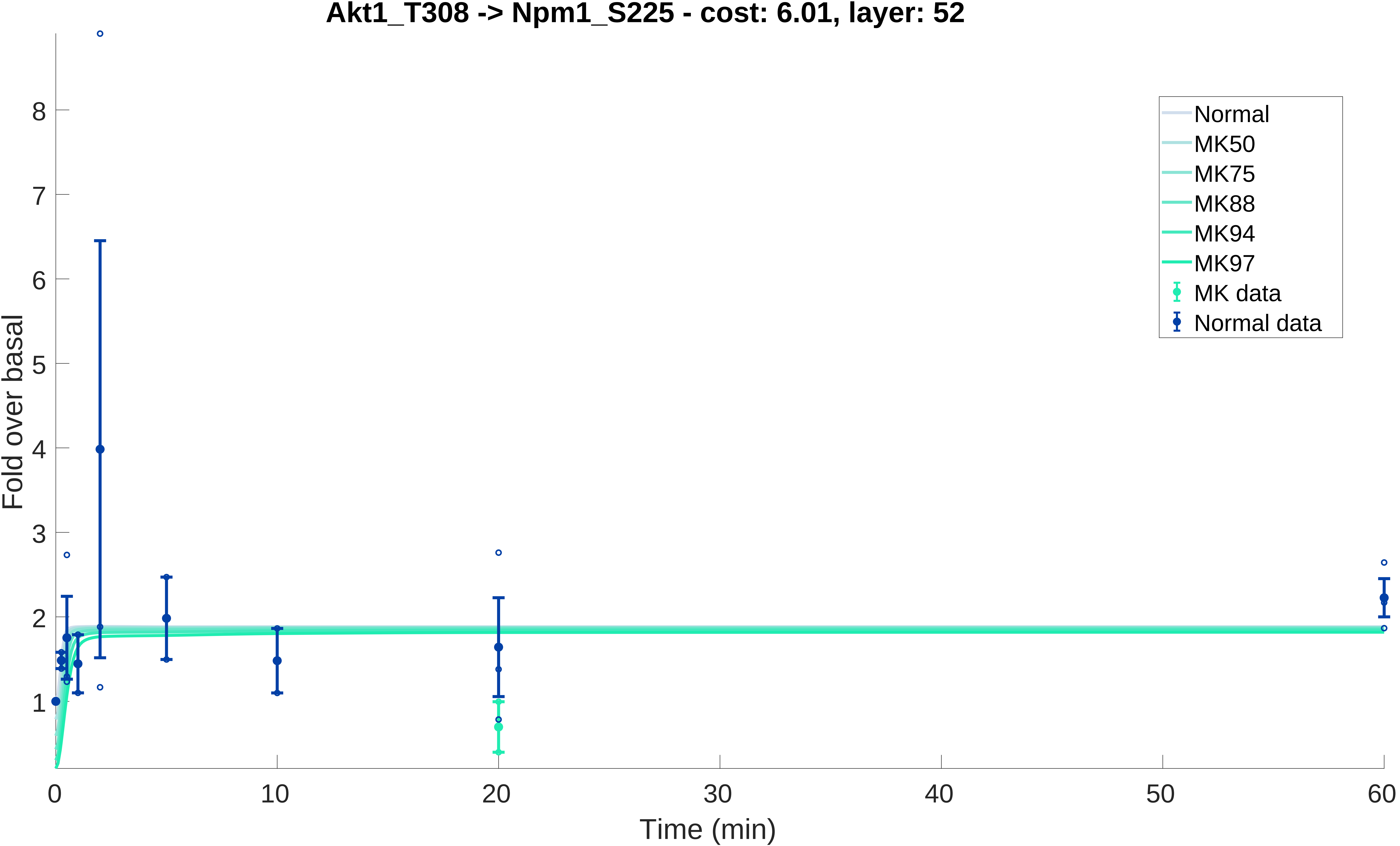

Akt1_T308 -> Npm1_S225 - cost: 6.01, layer: 52
Fold over basal
Time (min)
0
10
20
30
40
50
60
1
2
3
4
5
6
7
8
Normal
MK50
MK75
MK88
MK94
MK97
MK data
Normal data

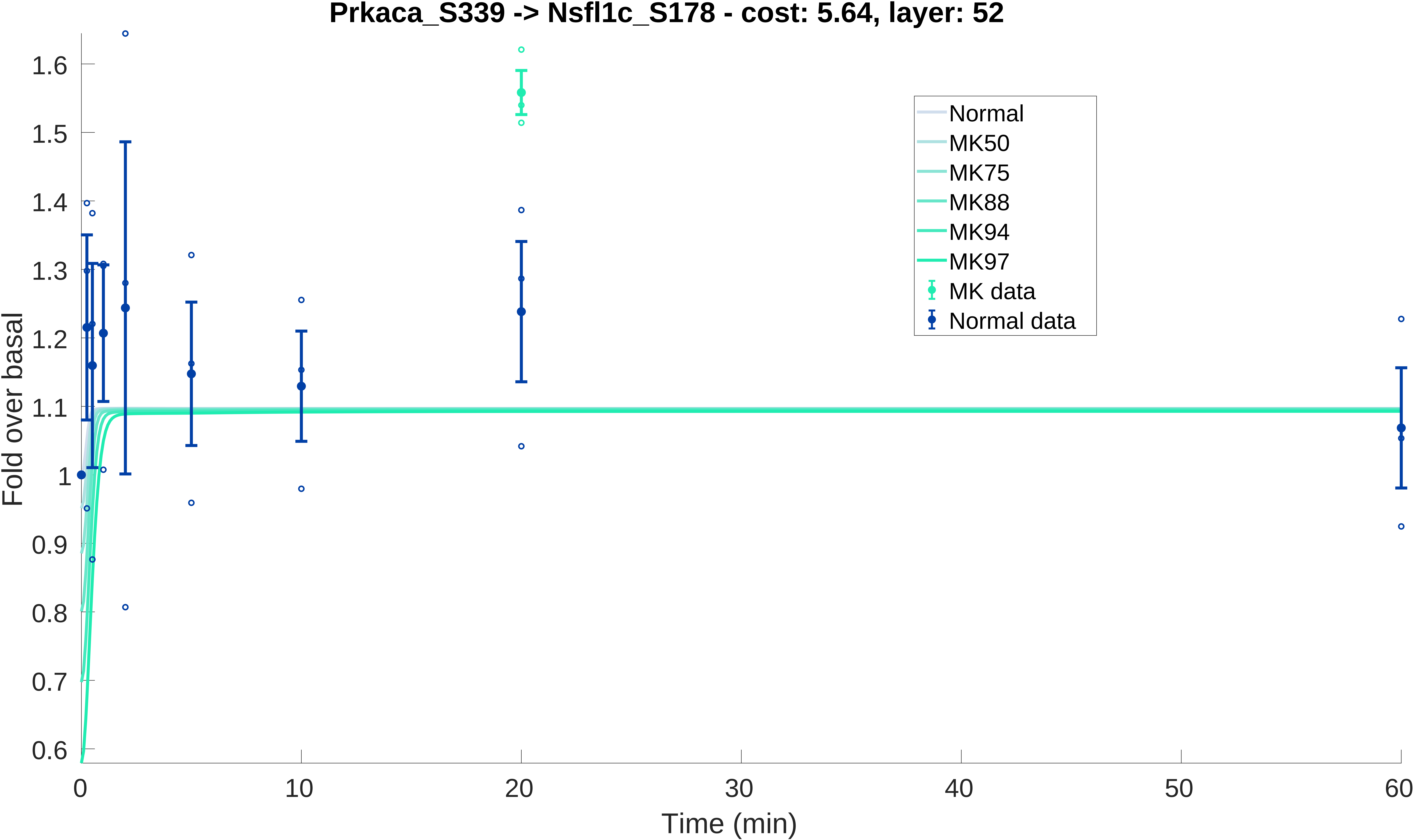

Prkaca_S339 -> Nsfl1c_S178 - cost: 5.64, layer: 52
Fold over basal
Time (min)
Normal
MK50
MK75
MK88
MK94
MK97
MK data
Normal data
1.6
1.5
1.4
1.3
1.2
1.1
1
0.9
0.8
0.7
0.6
0
10
20
30
40
50
60

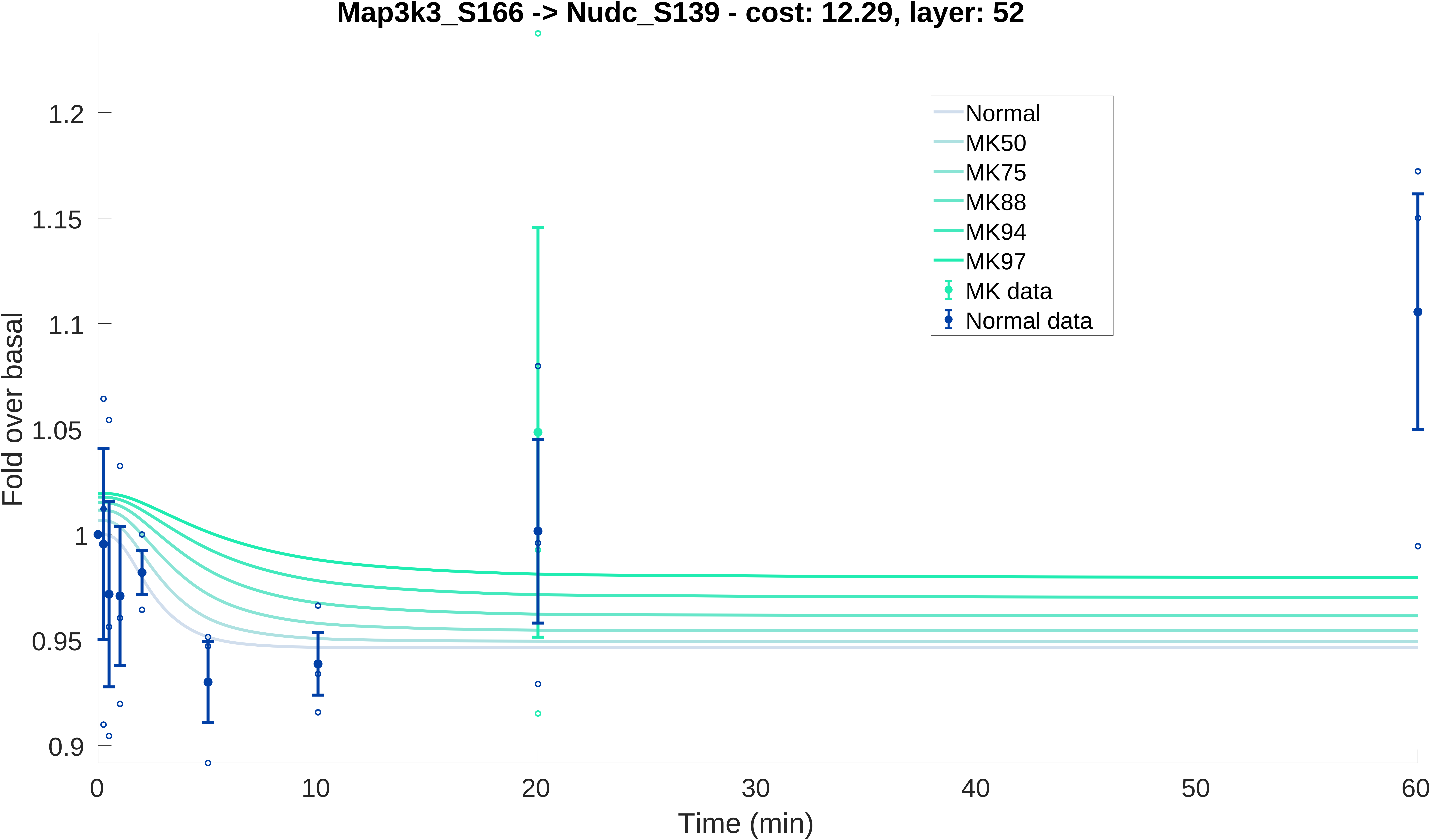
Map3k3_S166 -> Nudc_S139 - cost: 12.29, layer: 52
Fold over basal
Time (min)
1.2
1.15
1.1
1.05
1
0.95
0.9
0
10
20
30
40
50
60
Normal
MK50
MK75
MK88
MK94
MK97
MK data
Normal data

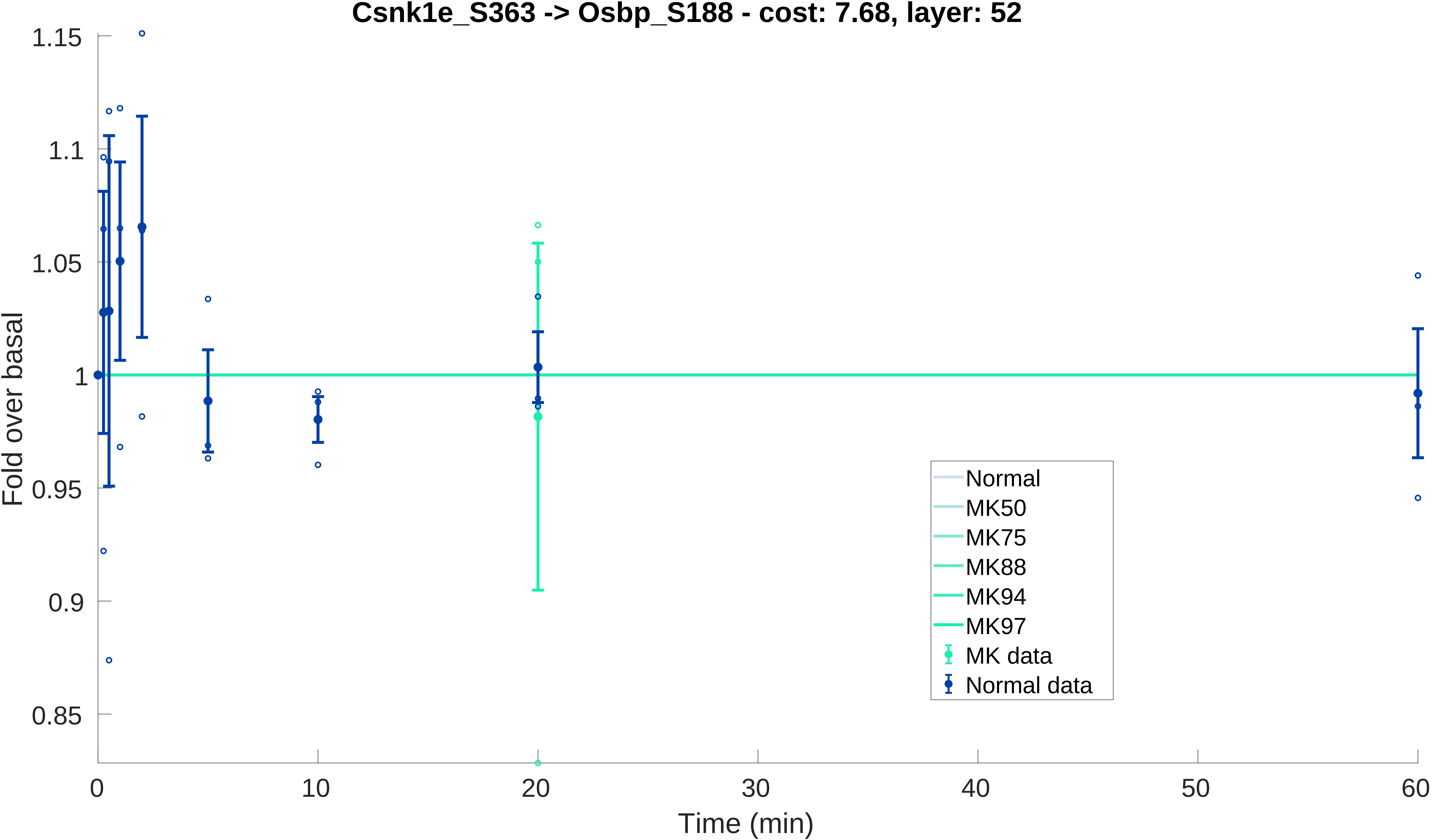

Csnk1e_S363 -> Osbp_S188 - cost: 7.68, layer: 52
Fold over basal
Time (min)
1.15
1.1
1.05
1
0.95
0.9
0.85
0
10
20
30
40
50
60
Normal
MK50
MK75
MK88
MK94
MK97
MK data
Normal data

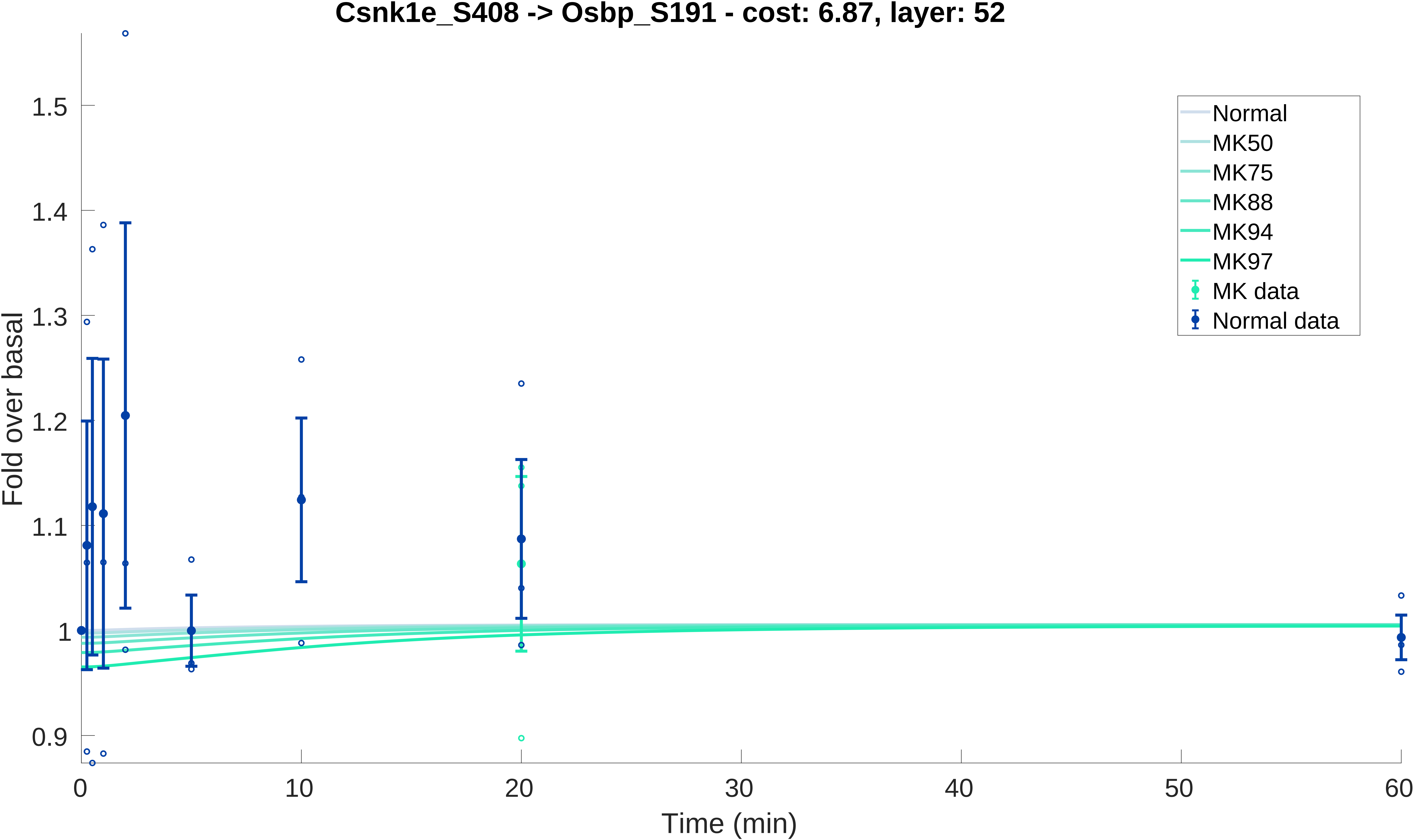

Csnk1e_S408 -> Osbp_S191 - cost: 6.87, layer: 52
Fold over basal
Time (min)
0.9
1
1.1
1.2
1.3
1.4
1.5
0
10
20
30
40
50
60
Normal
MK50
MK75
MK88
MK94
MK97
MK data
Normal data

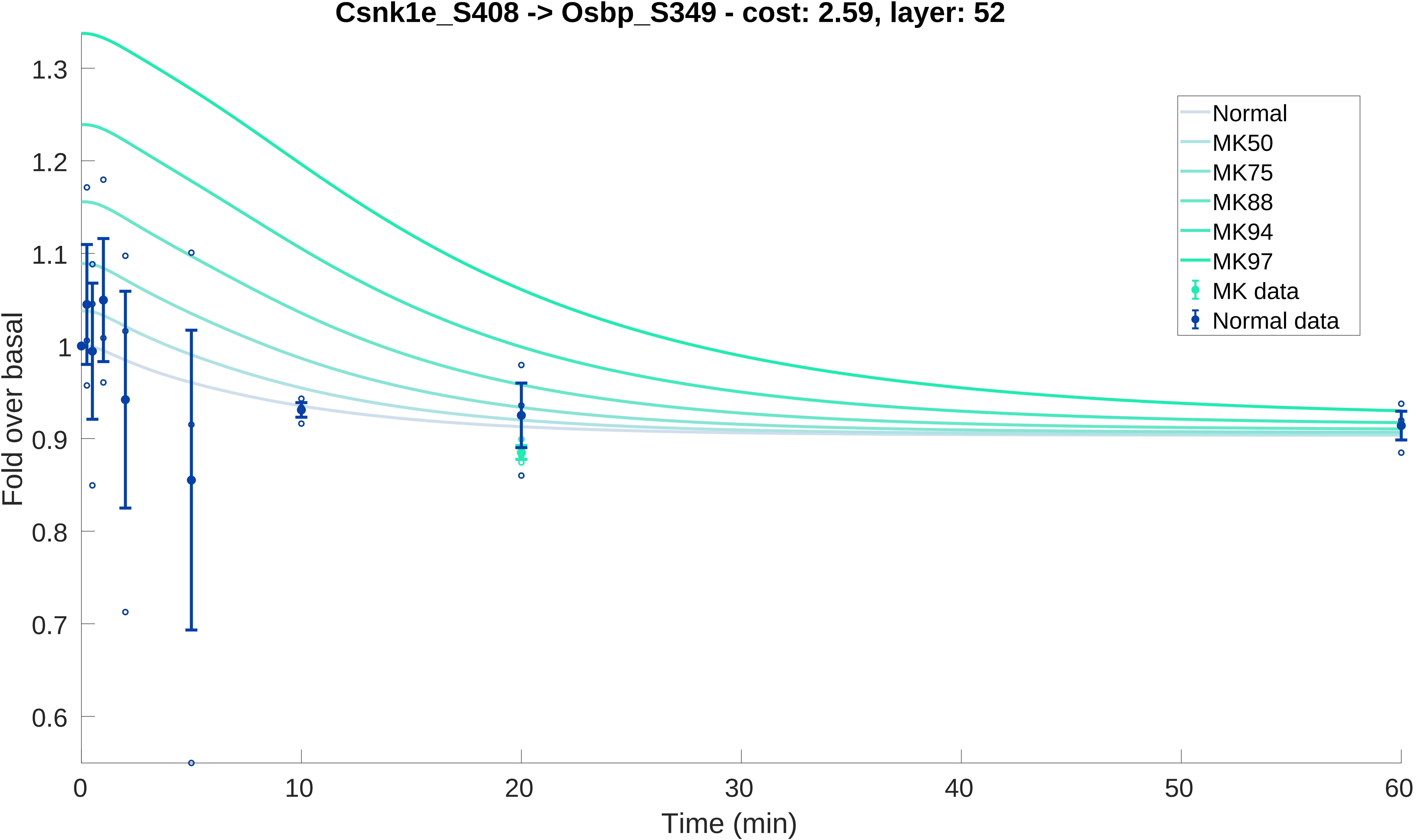

Csnk1e_S408 -> Osbp_S349 - cost: 2.59, layer: 52
Fold over basal
Time (min)
Normal
MK50
MK75
MK88
MK94
MK97
MK data
Normal data
0.6
0.7
0.8
0.9
1
1.1
1.2
1.3
0
10
20
30
40
50
60

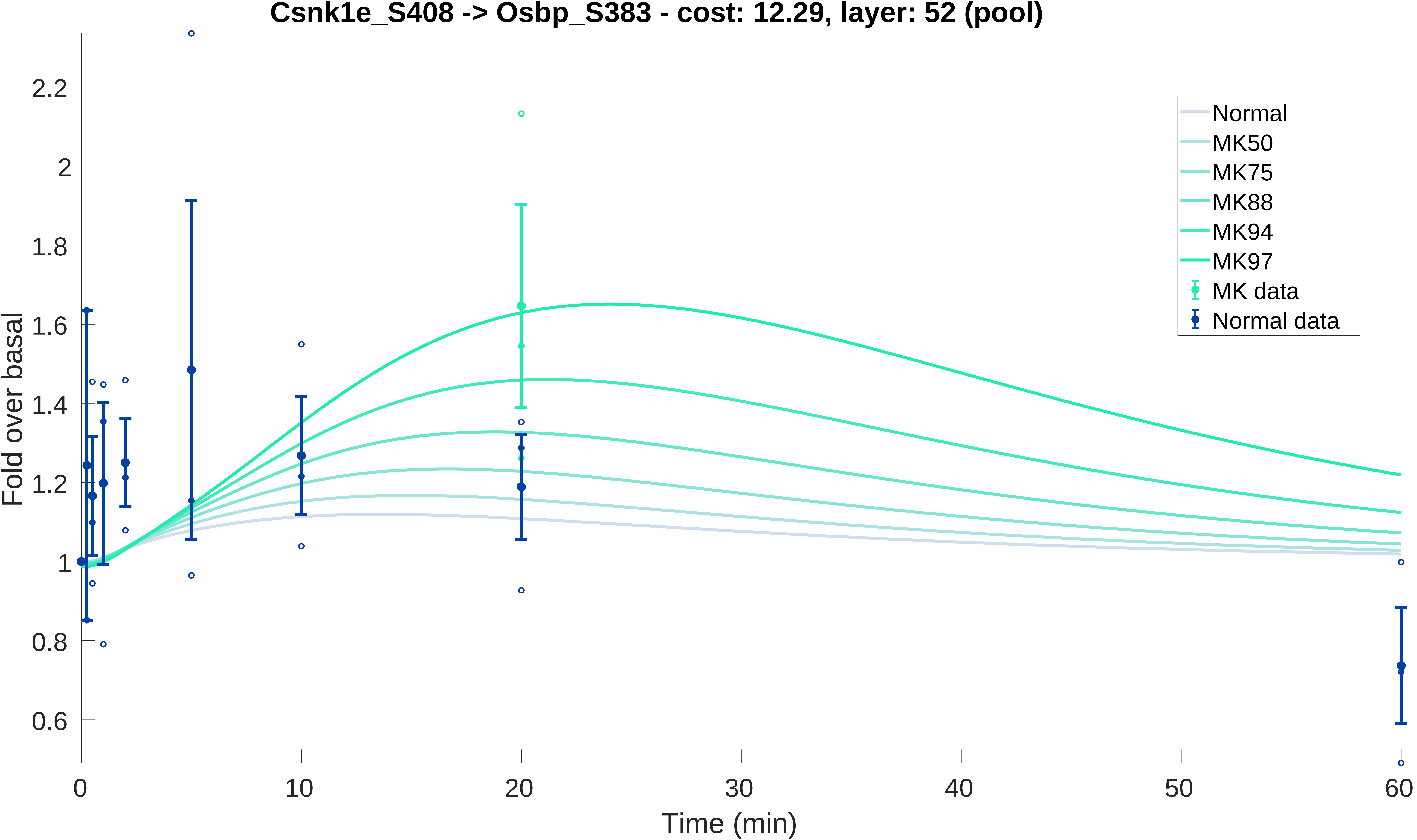
Csnk1e_S408 -> Osbp_S383 - cost: 12.29, layer: 52 (pool)
Fold over basal
Time (min)
0
10
20
30
40
50
60
0.6
0.8
1
1.2
1.4
1.6
1.8
2
2.2
Normal
MK50
MK75
MK88
MK94
MK97
MK data
Normal data

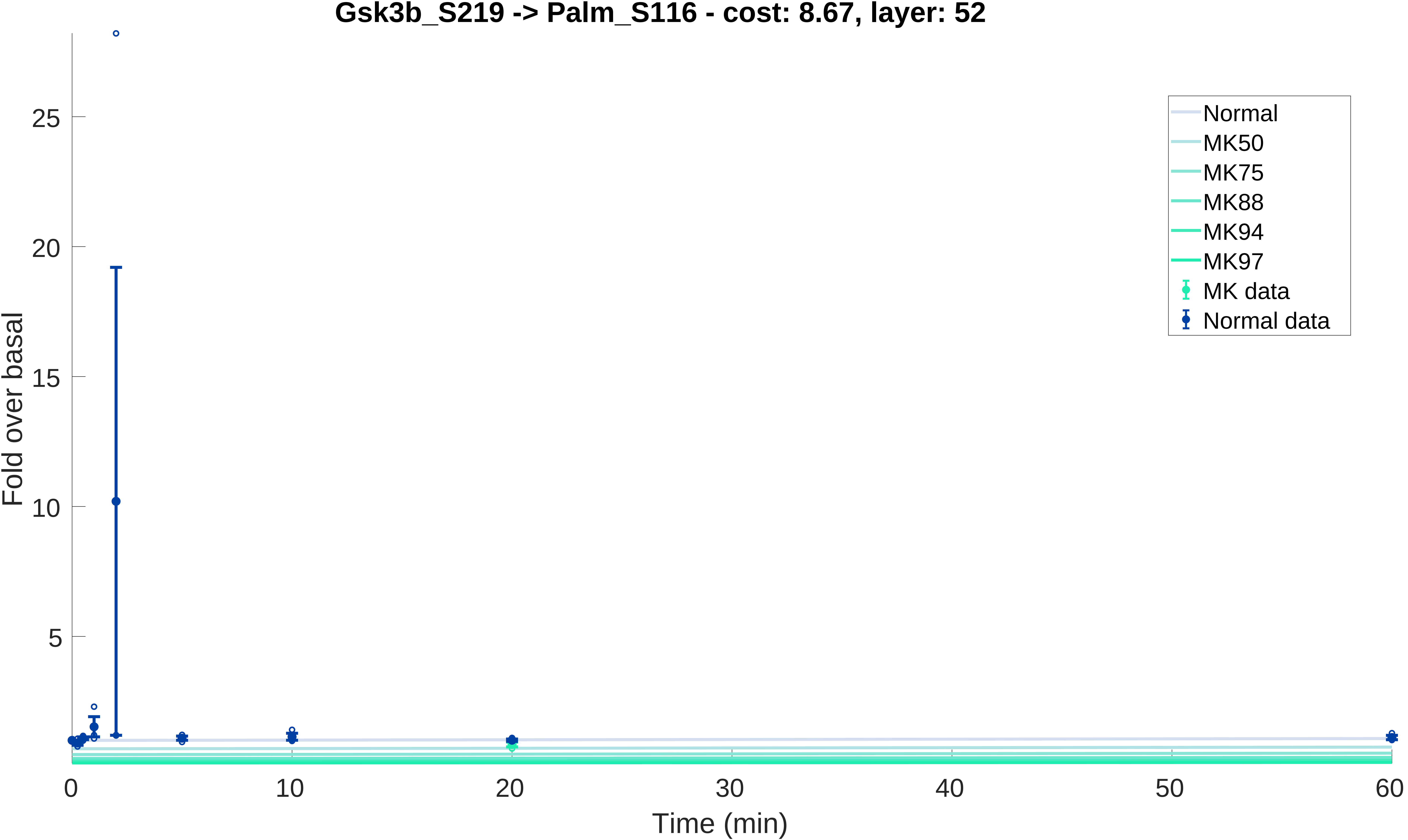
Gsk3b_S219 -> Palm_S116 - cost: 8.67, layer: 52
Fold over basal
Time (min)
0
5
10
15
20
25
0
10
20
30
40
50
60
Normal
MK50
MK75
MK88
MK94
MK97
MK data
Normal data

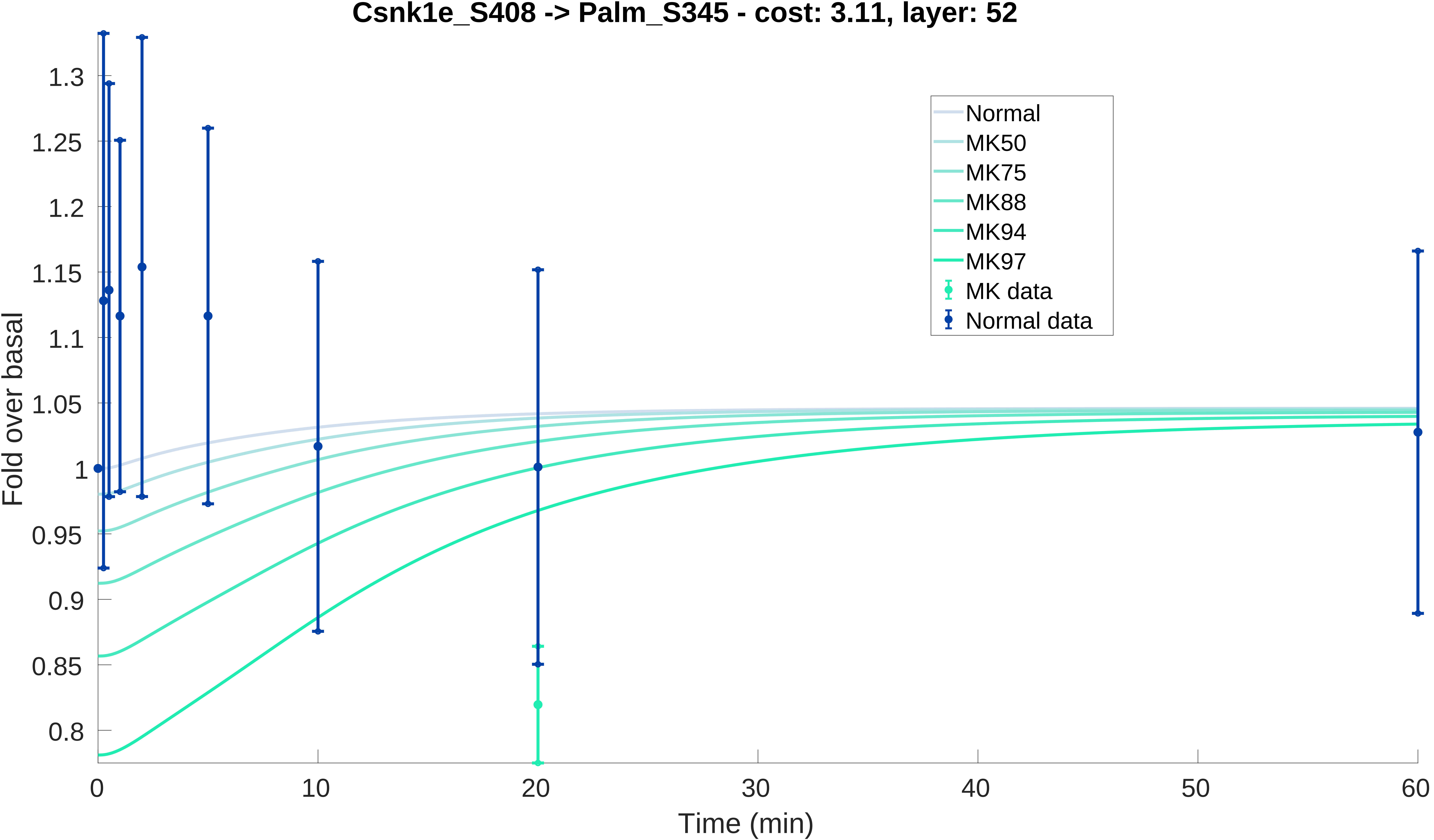
Csnk1e_S408 -> Palm_S345 - cost: 3.11, layer: 52
Fold over basal
Time (min)
1.3
1.25
1.2
1.15
1.1
1.05
1
0.95
0.9
0.85
0.8
0
10
20
30
40
50
60
Normal
MK50
MK75
MK88
MK94
MK97
MK data
Normal data

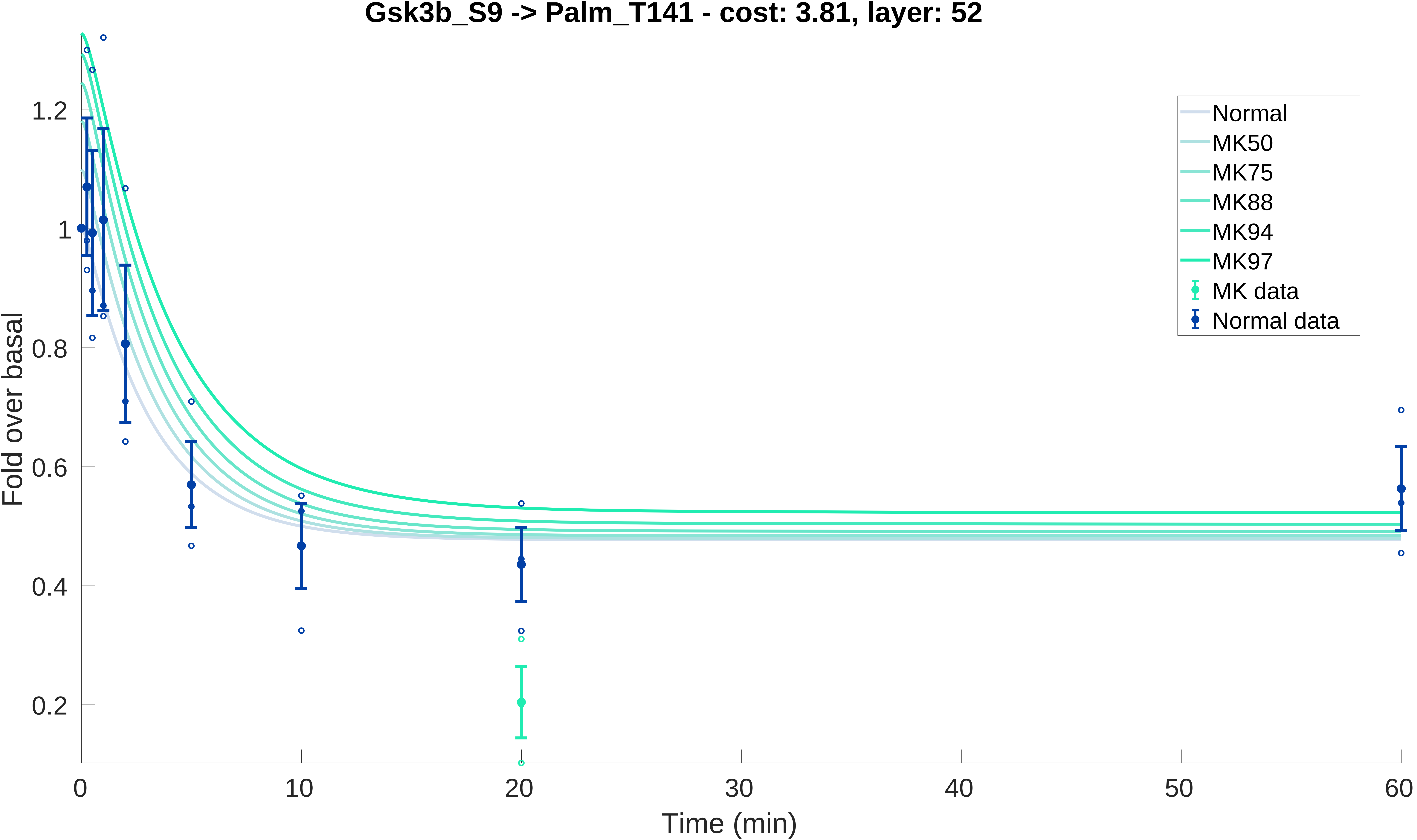
Gsk3b_S9 -> Palm_T141 - cost: 3.81, layer: 52
Fold over basal
Time (min)
1.2
1
0.8
0.6
0.4
0.2
0
10
20
30
40
50
60
Normal
MK50
MK75
MK88
MK94
MK97
MK data
Normal data

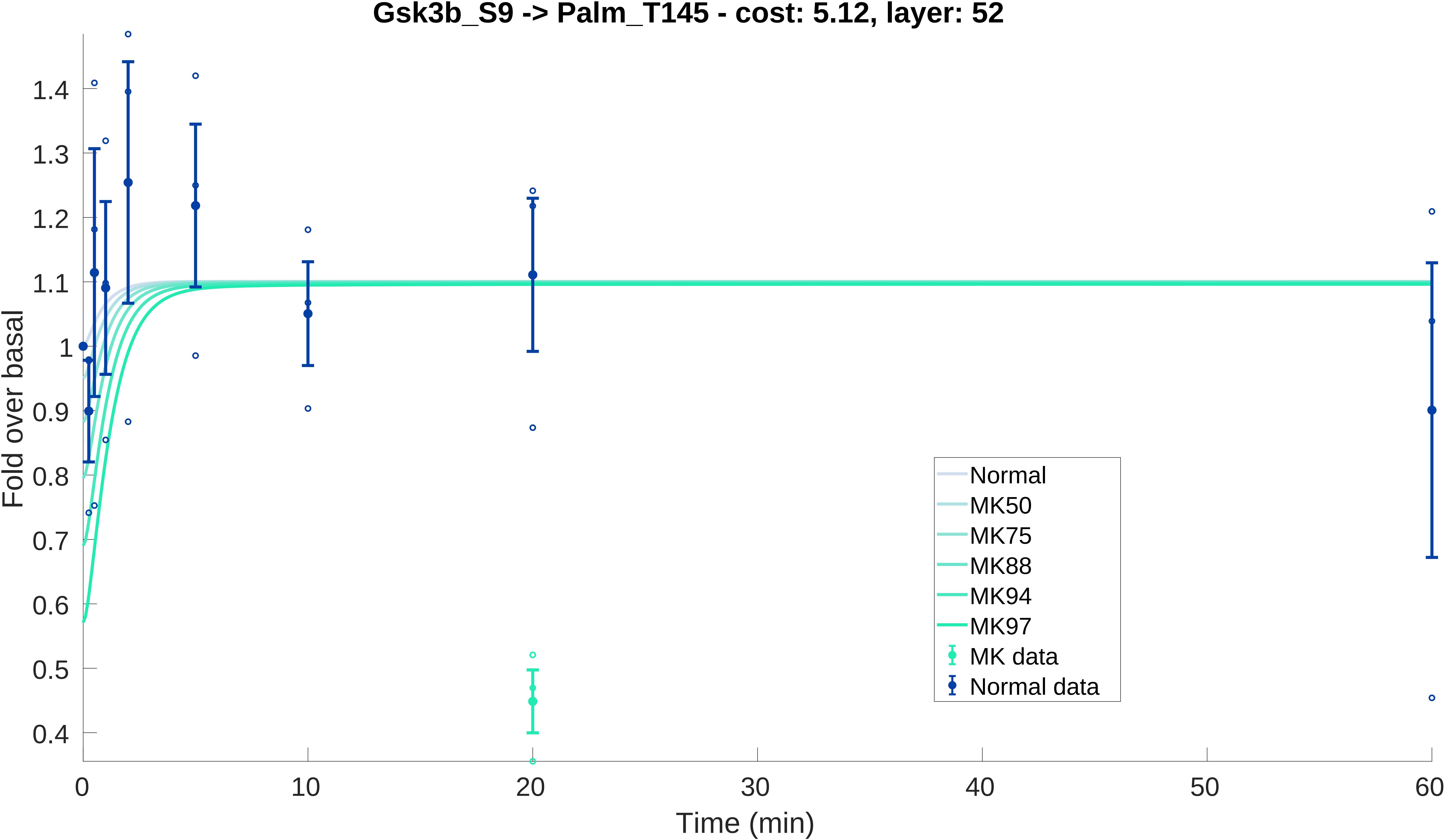

Gsk3b_S9 -> Palm_T145 - cost: 5.12, layer: 52
Fold over basal
1.4
1.3
1.2
1.1
1
0.9
0.8
0.7
0.6
0.5
0.4
0
10
20
30
40
50
60
Time (min)
Normal
MK50
MK75
MK88
MK94
MK97
MK data
Normal data

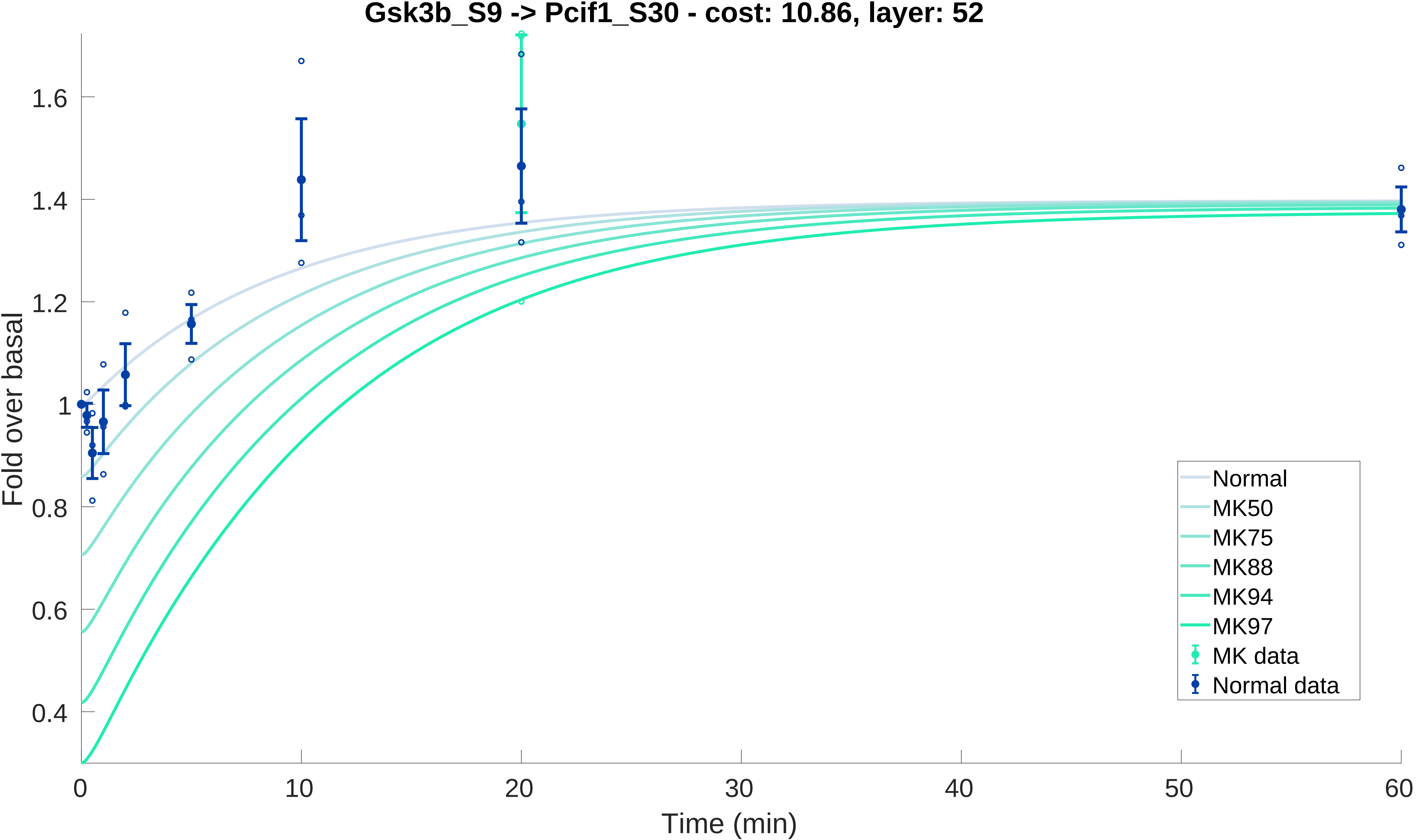
Gsk3b_S9 -> Pcif1_S30 - cost: 10.86, layer: 52
Fold over basal
Time (min)
0
10
20
30
40
50
60
0.4
0.6
0.8
1
1.2
1.4
1.6
Normal
MK50
MK75
MK88
MK94
MK97
MK data
Normal data

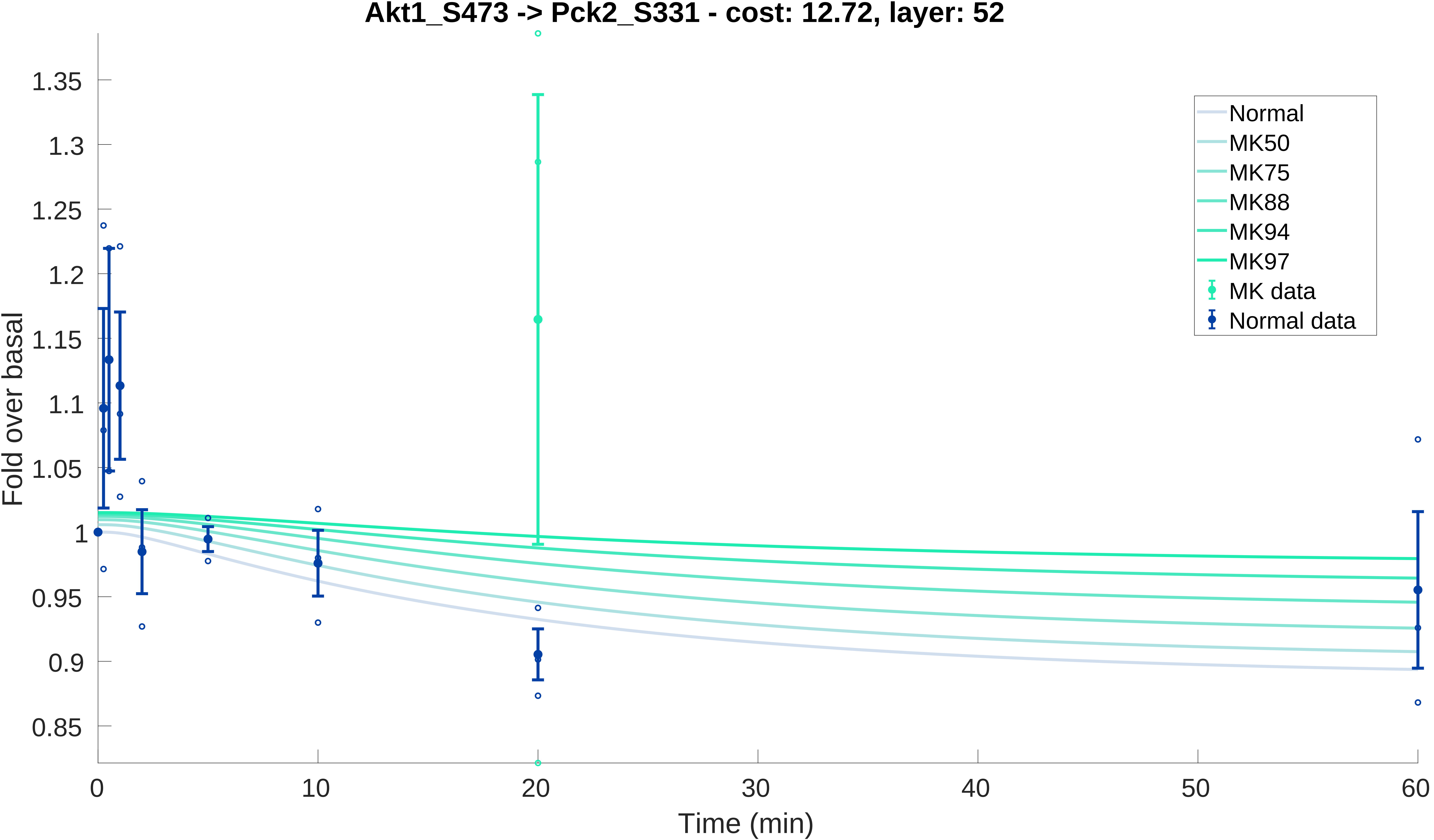

Akt1_S473 -> Pck2_S331 - cost: 12.72, layer: 52
Fold over basal
Time (min)
1.35
1.3
1.25
1.2
1.15
1.1
1.05
1
0.95
0.9
0.85
0
10
20
30
40
50
60
Normal
MK50
MK75
MK88
MK94
MK97
MK data
Normal data

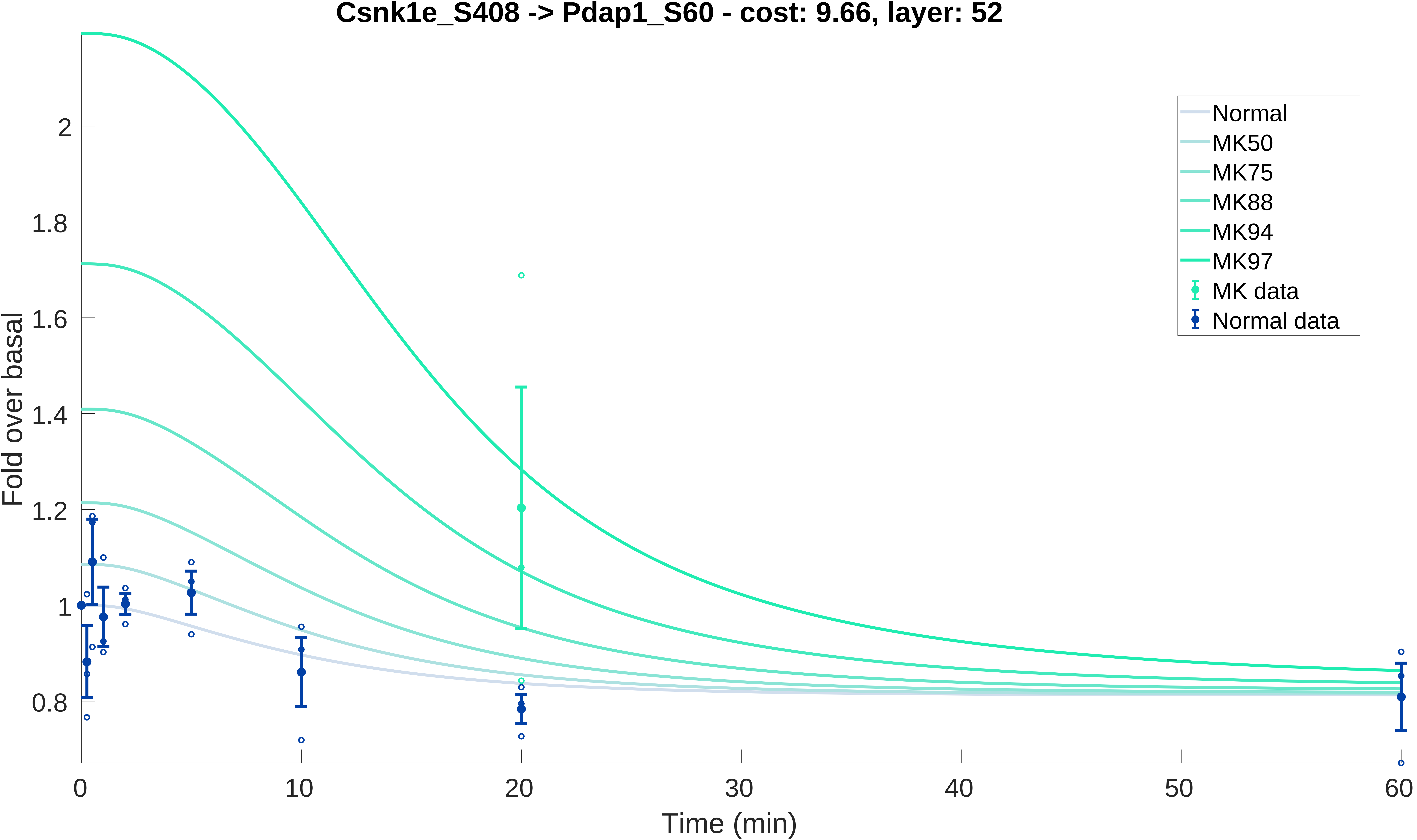

Csnk1e_S408 -> Pdap1_S60 - cost: 9.66, layer: 52
Fold over basal
Time (min)
0
10
20
30
40
50
60
0.8
1
1.2
1.4
1.6
1.8
2
Normal
MK50
MK75
MK88
MK94
MK97
MK data
Normal data

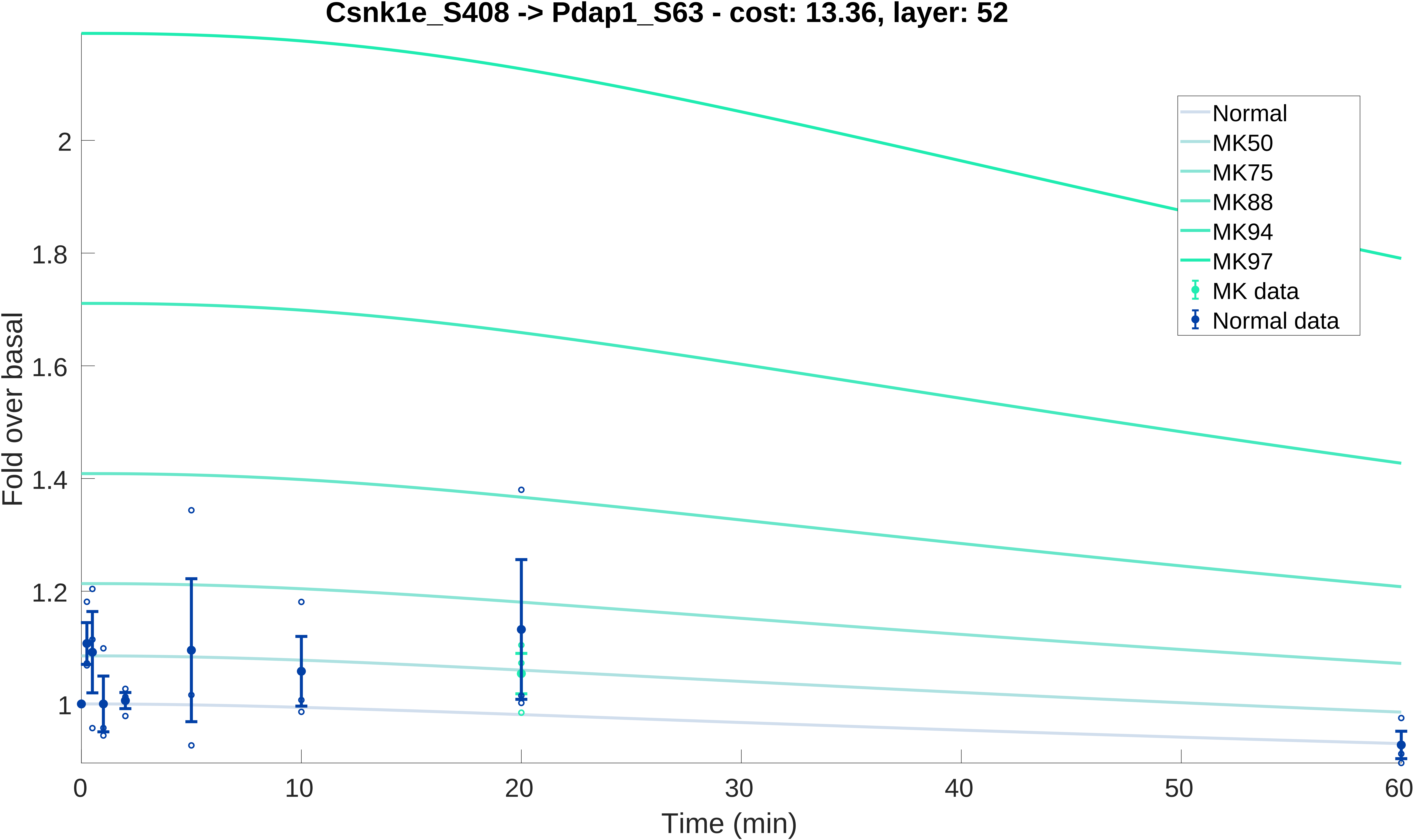

Csnk1e_S408 -> Pdap1_S63 - cost: 13.36, layer: 52
Fold over basal
2
1.8
1.6
1.4
1.2
1
0
10
20
30
40
50
60
Time (min)
Normal
MK50
MK75
MK88
MK94
MK97
MK data
Normal data

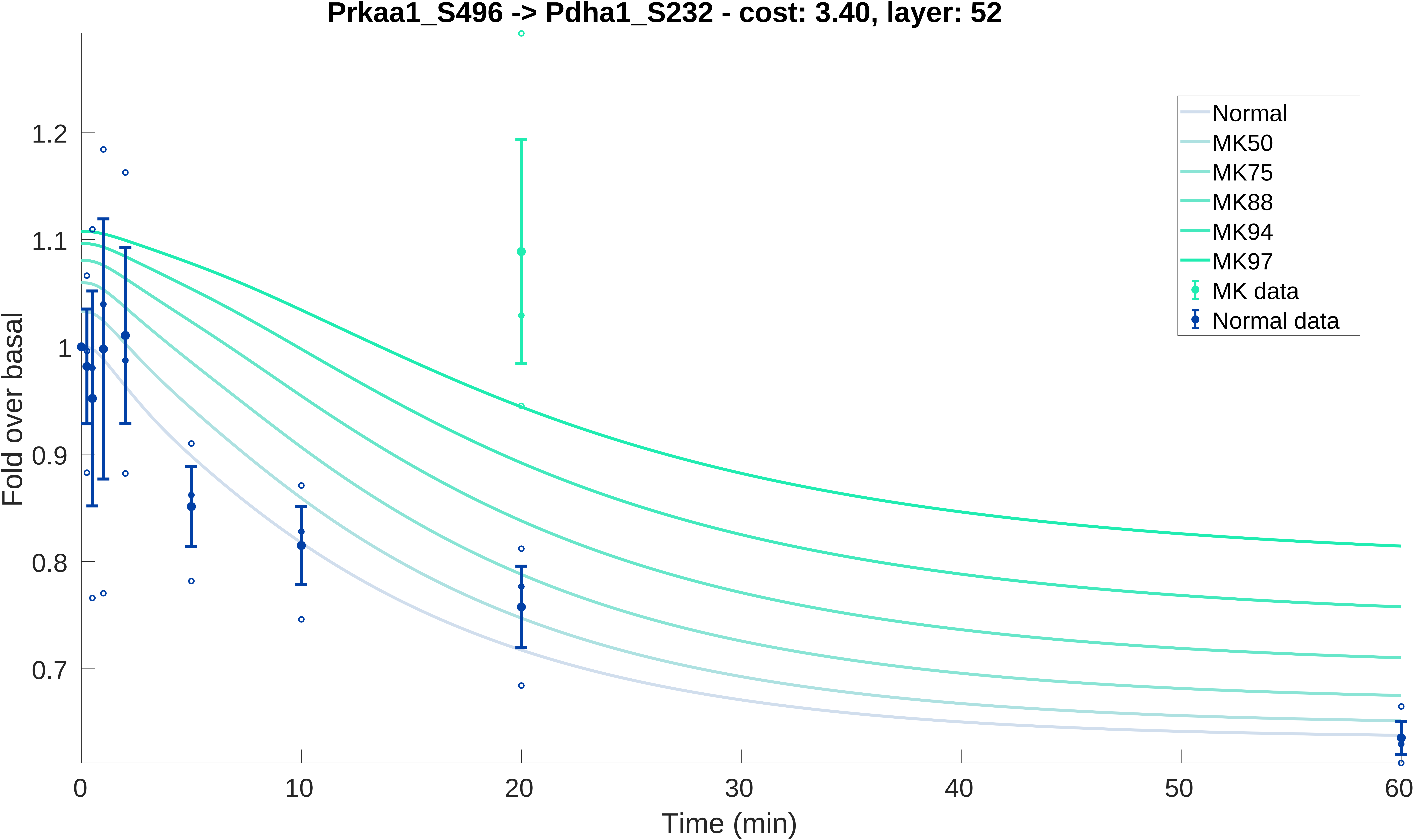

Prrkaa1_S496 -> Pdha1_S232 - cost: 3.40, layer: 52
Fold over basal
Time (min)
1.2
1.1
1
0.9
0.8
0.7
0
10
20
30
40
50
60
Normal
MK50
MK75
MK88
MK94
MK97
MK data
Normal data

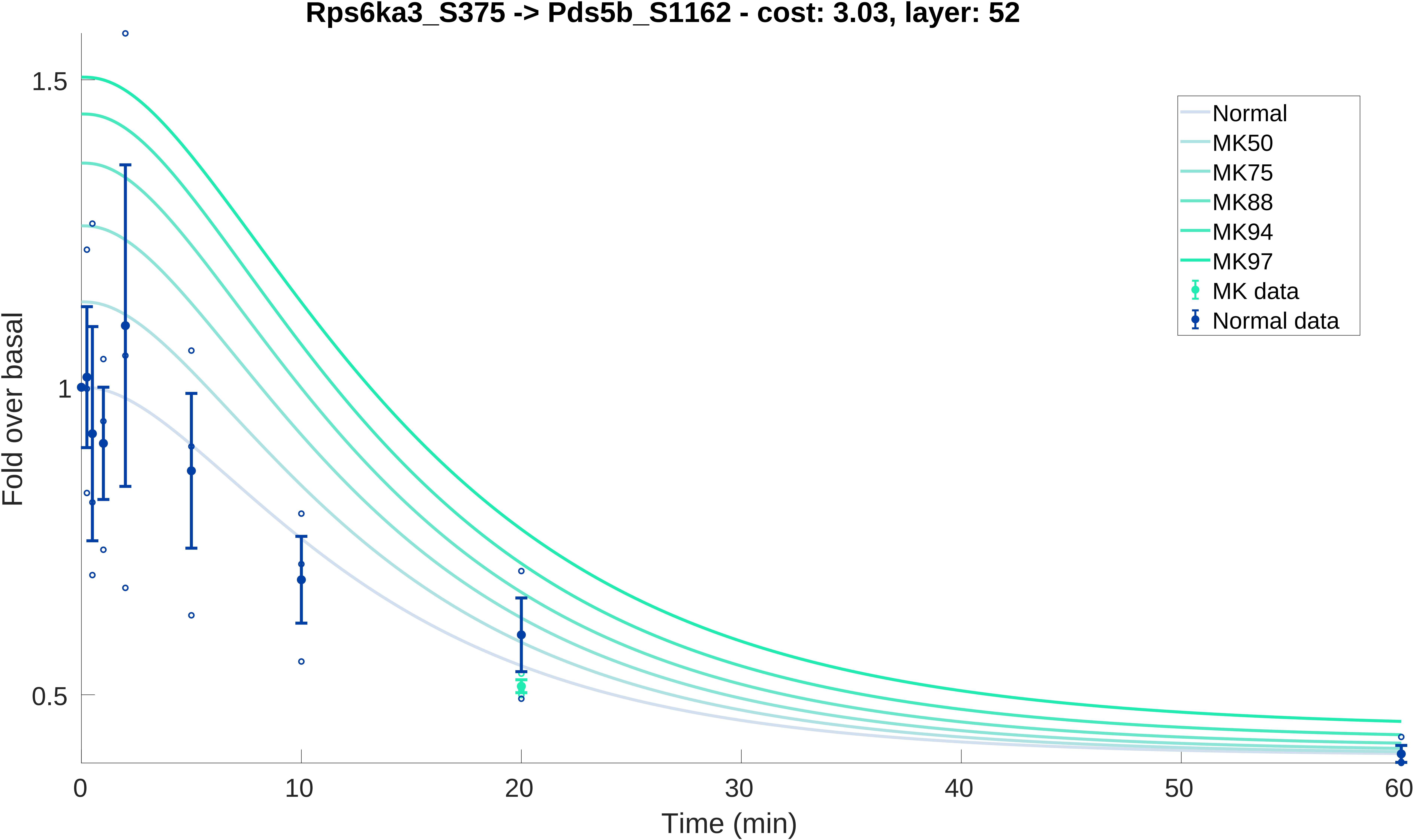

Rps6ka3_S375 -> Pds5b_S1162 - cost: 3.03, layer: 52
Fold over basal
1.5
1
0.5
0
10
20
30
40
50
60
Time (min)
Normal
MK50
MK75
MK88
MK94
MK97
MK data
Normal data

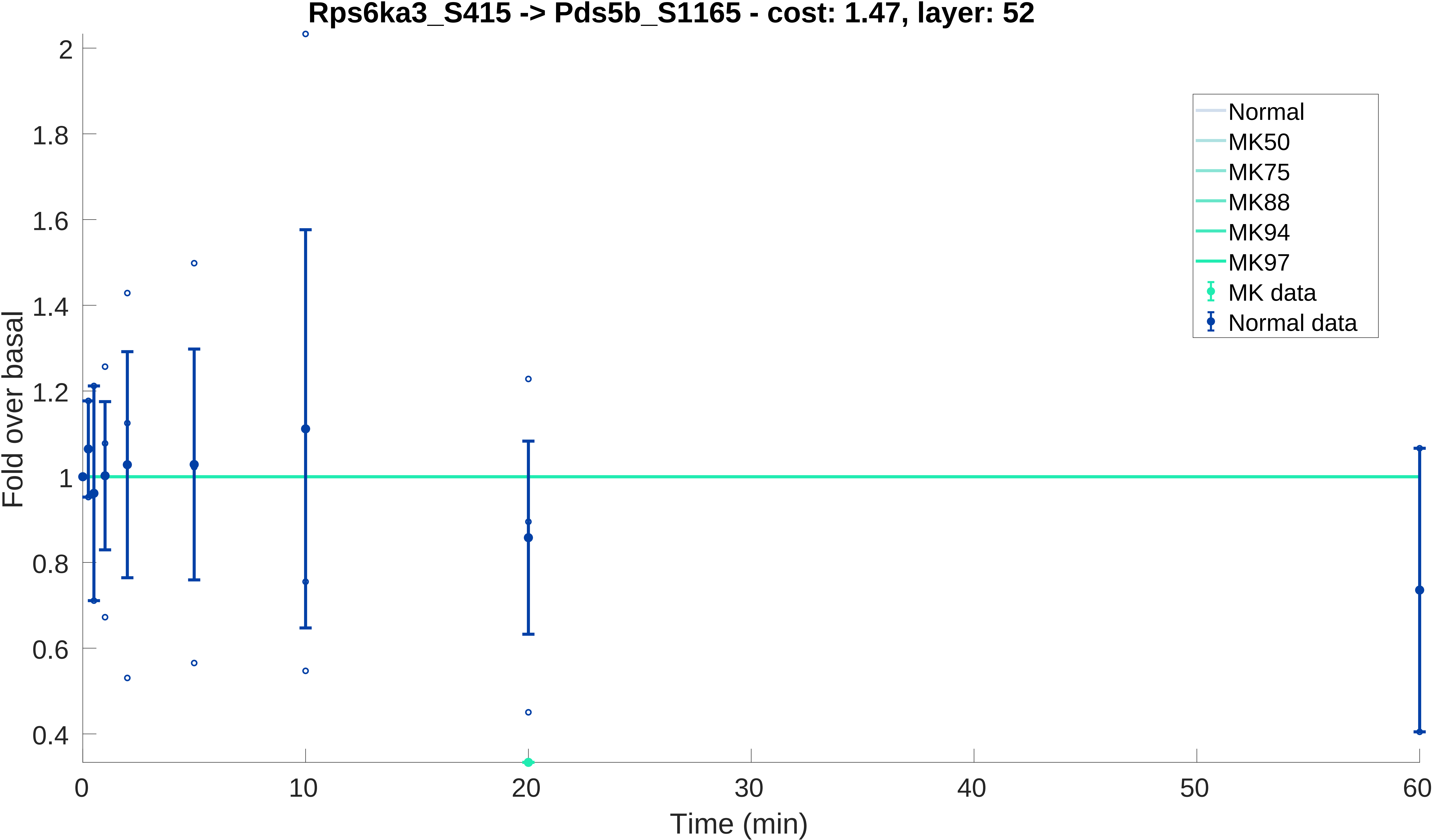

Rps6ka3_S415 -> Pds5b_S1165 - cost: 1.47, layer: 52
Fold over basal
Time (min)
0.4
0.6
0.8
1
1.2
1.4
1.6
1.8
2
0
10
20
30
40
50
60
Normal
MK50
MK75
MK88
MK94
MK97
MK data
Normal data

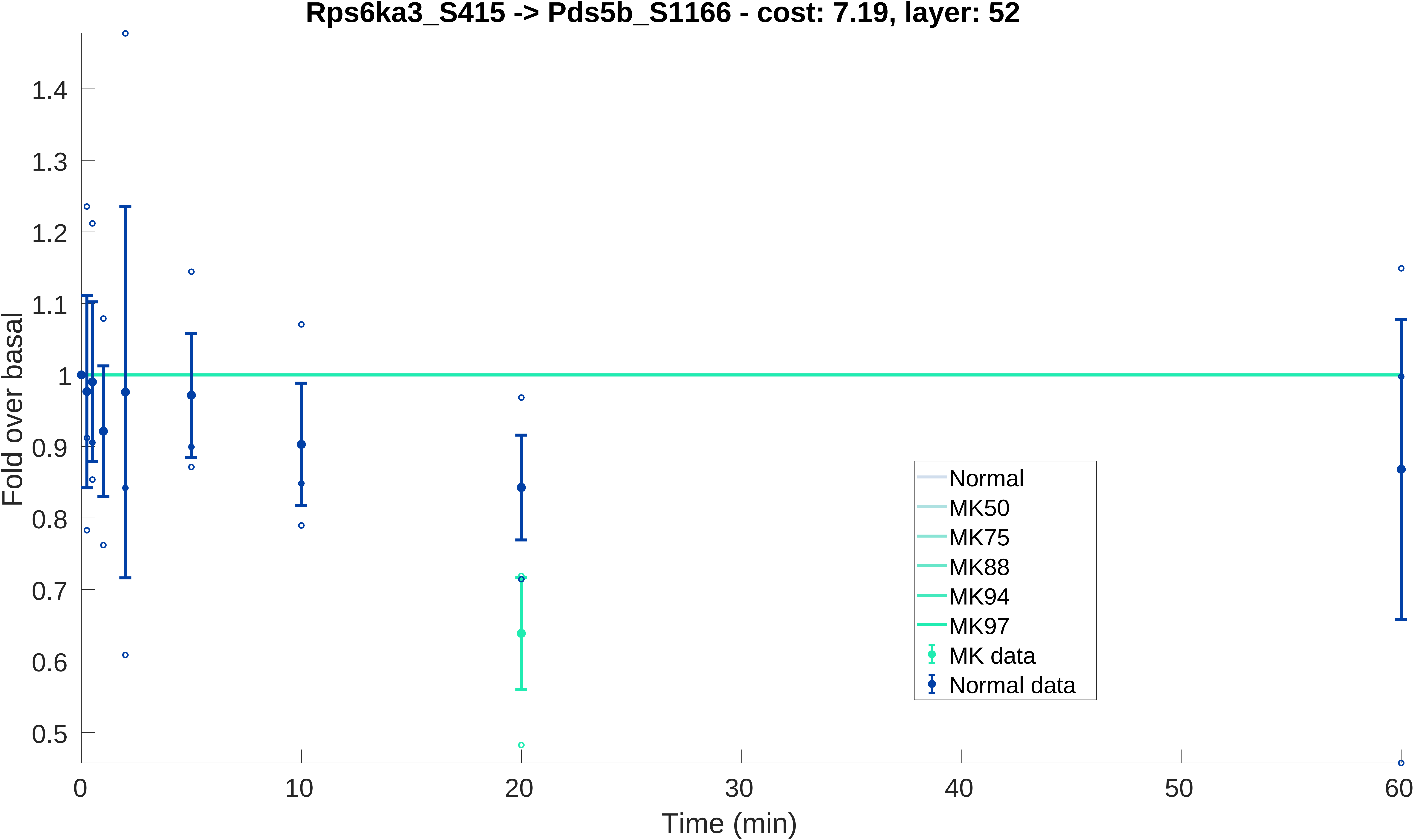

Rps6ka3_S415 -> Pds5b_S1166 - cost: 7.19, layer: 52
Fold over basal
1.4
1.3
1.2
1.1
1
0.9
0.8
0.7
0.6
0.5
0
10
20
30
40
50
60
Time (min)
Normal
MK50
MK75
MK88
MK94
MK97
MK data
Normal data

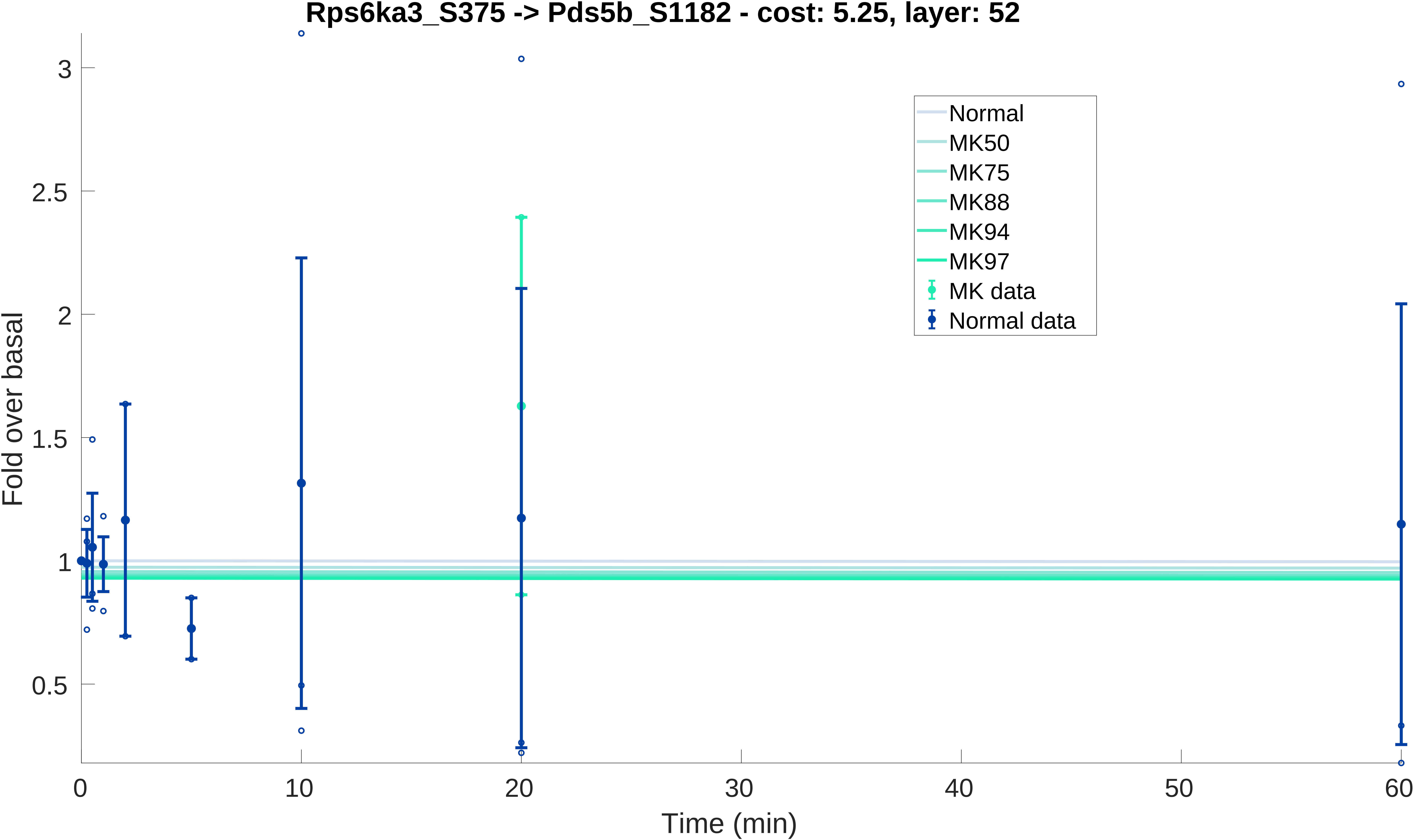

Rps6ka3_S375 -> Pds5b_S1182 - cost: 5.25, layer: 52
Fold over basal
Time (min)
0.5
1
1.5
2
2.5
3
0
10
20
30
40
50
60
Normal
MK50
MK75
MK88
MK94
MK97
MK data
Normal data

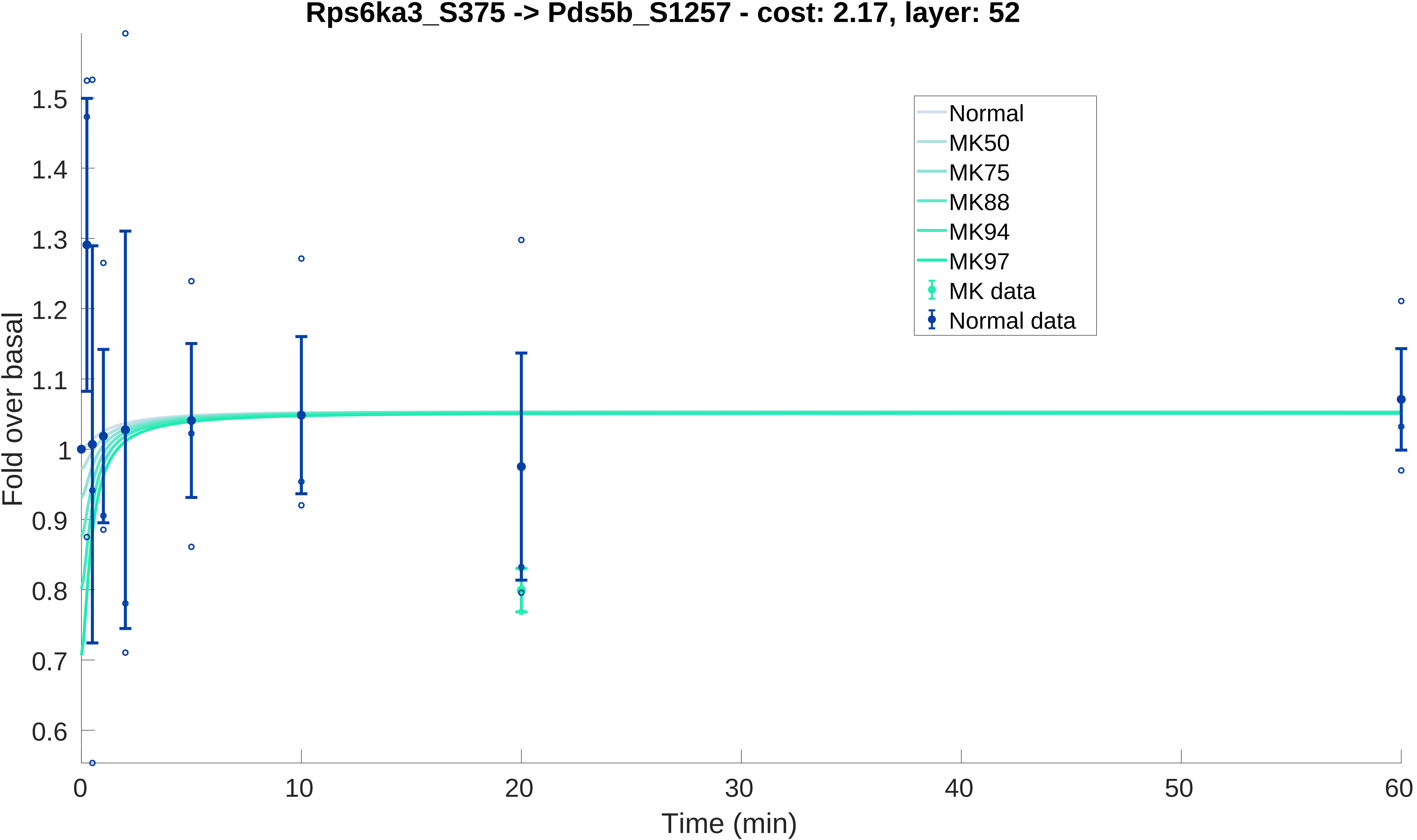

Rps6ka3_S375 -> Pds5b_S1257 - cost: 2.17, layer: 52
Fold over basal
1.5
1.4
1.3
1.2
1.1
1
0.9
0.8
0.7
0.6
0
10
20
30
40
50
60
Time (min)
Normal
MK50
MK75
MK88
MK94
MK97
MK data
Normal data

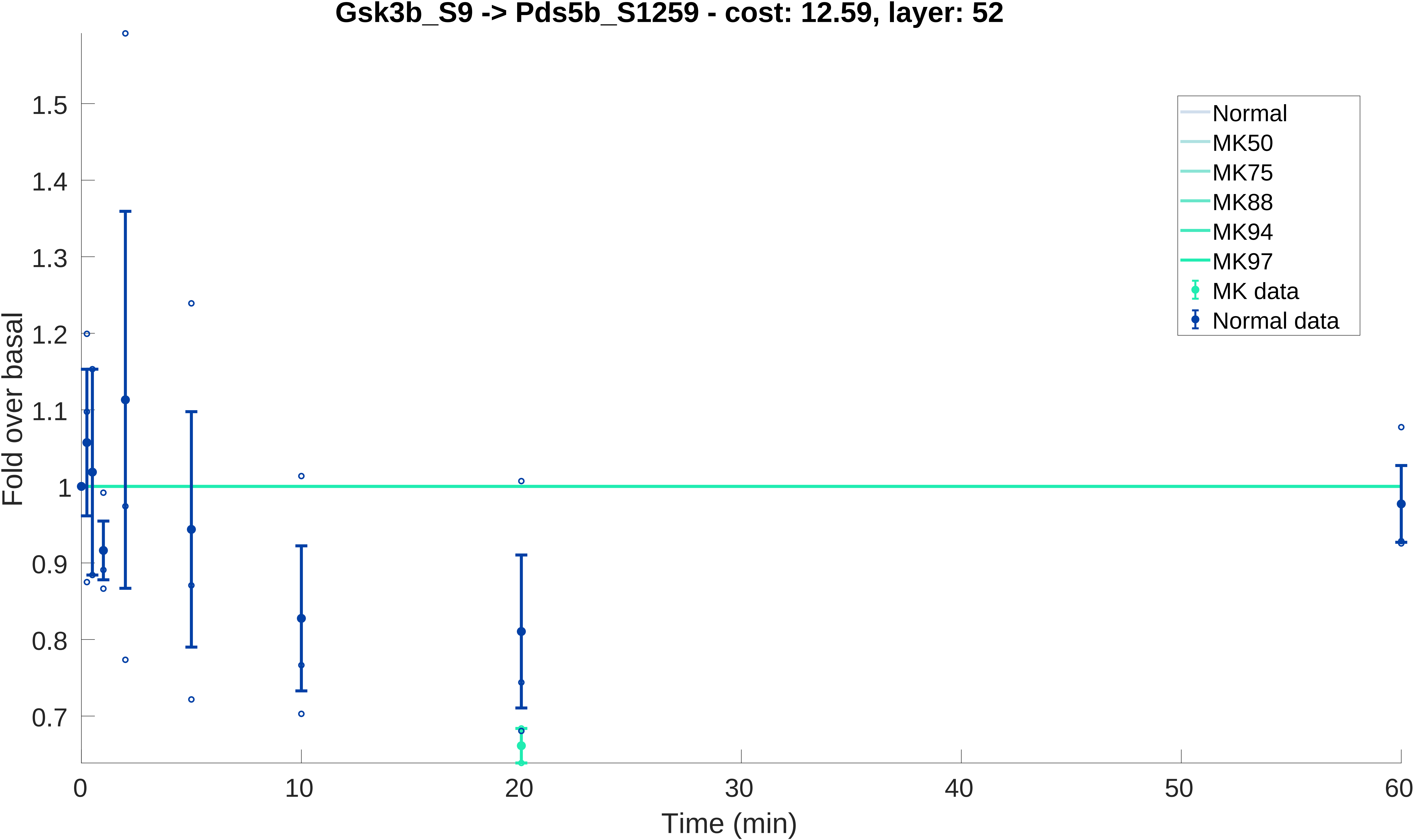

Gsk3b_S9 -> Pds5b_S1259 - cost: 12.59, layer: 52
Fold over basal
Time (min)
0.7
0.8
0.9
1
1.1
1.2
1.3
1.4
1.5
0
10
20
30
40
50
60
Normal
MK50
MK75
MK88
MK94
MK97
MK data
Normal data

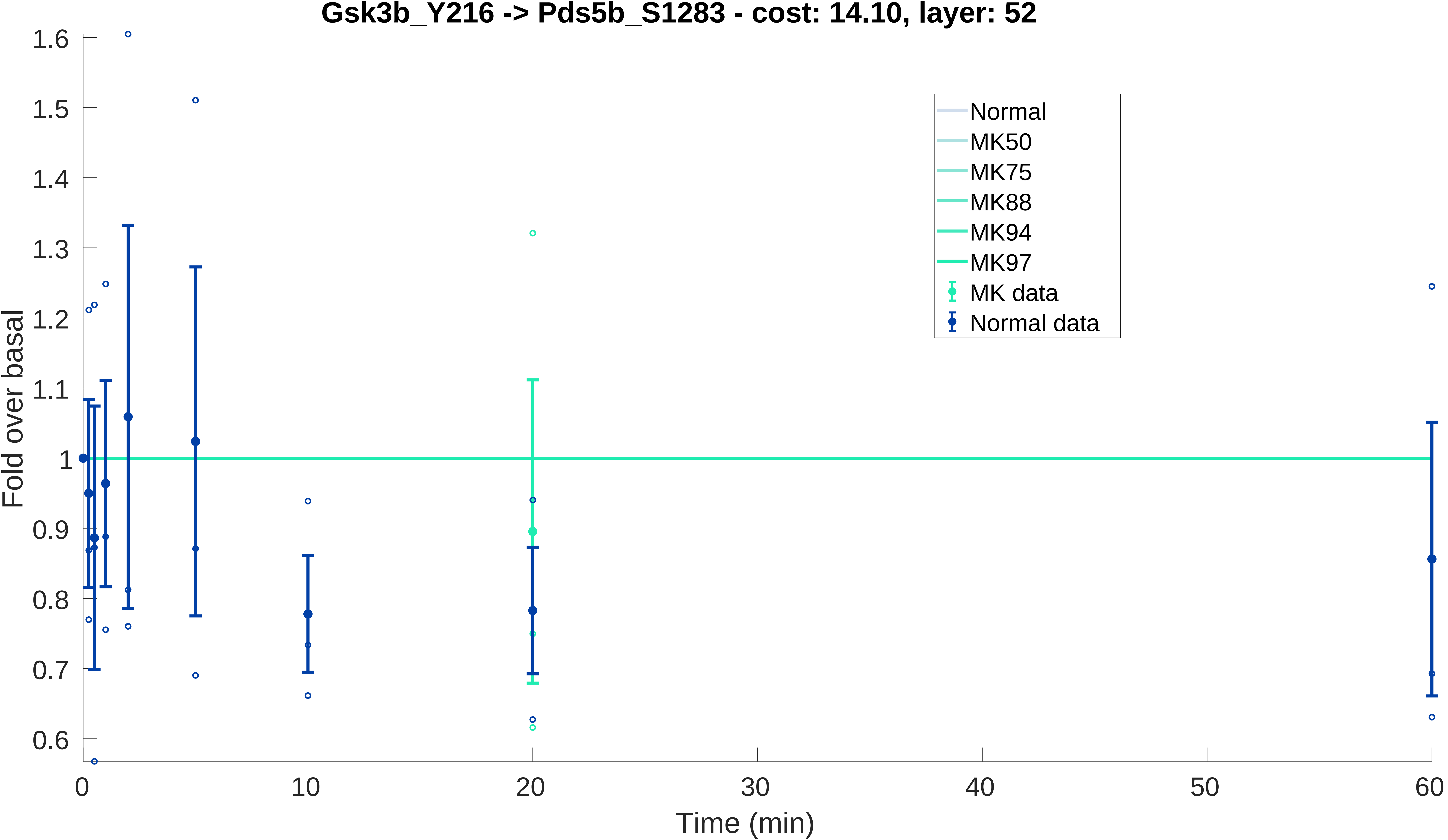

Gsk3b_Y216 -> Pds5b_S1283 - cost: 14.10, layer: 52
Fold over basal
Time (min)
1.6
1.5
1.4
1.3
1.2
1.1
1
0.9
0.8
0.7
0.6
0
10
20
30
40
50
60
Normal
MK50
MK75
MK88
MK94
MK97
MK data
Normal data

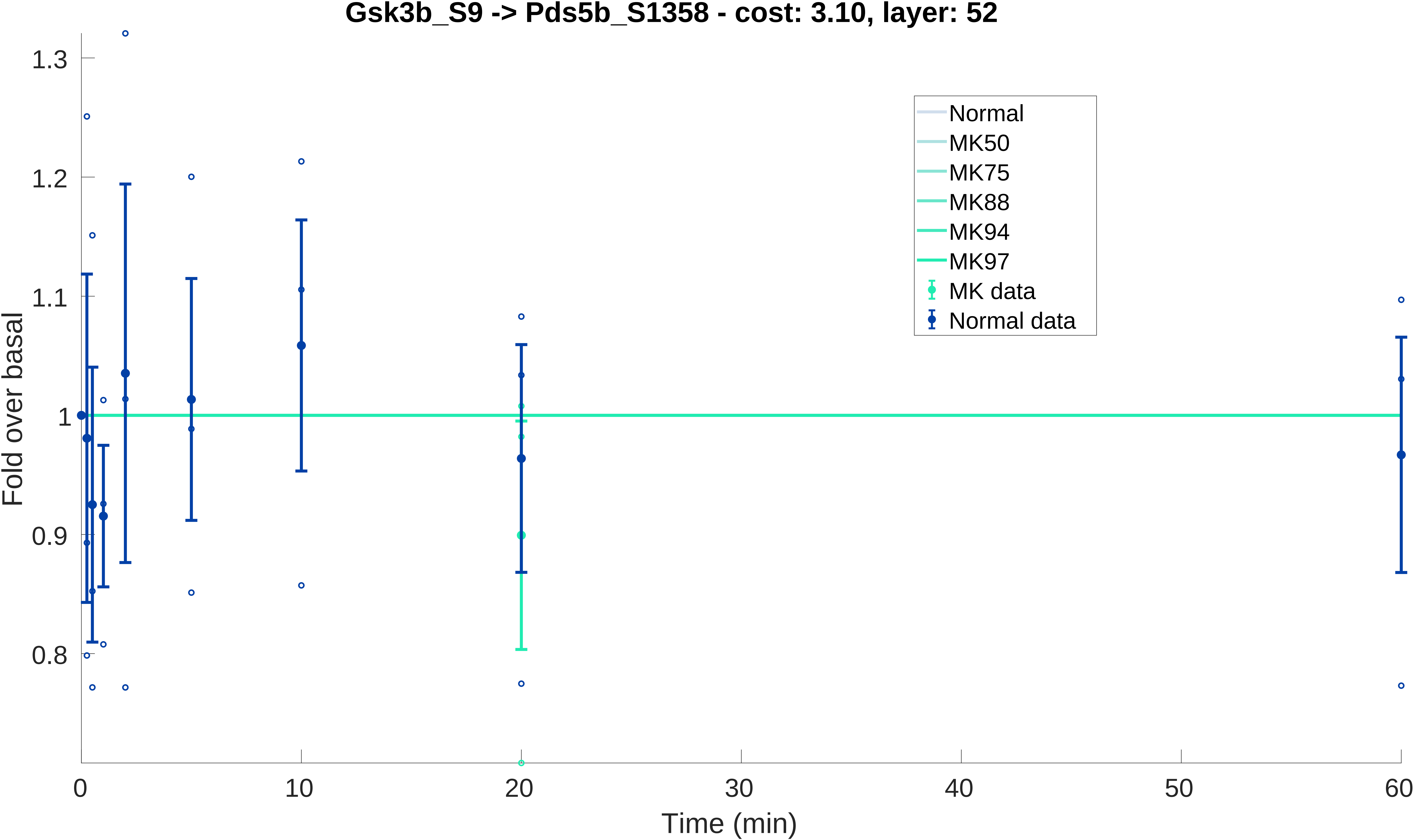
Gsk3b_S9 -> Pds5b_S1358 - cost: 3.10, layer: 52
Fold over basal
Time (min)
1.3
1.2
1.1
1
0.9
0.8
0
10
20
30
40
50
60
Normal
MK50
MK75
MK88
MK94
MK97
MK data
Normal data

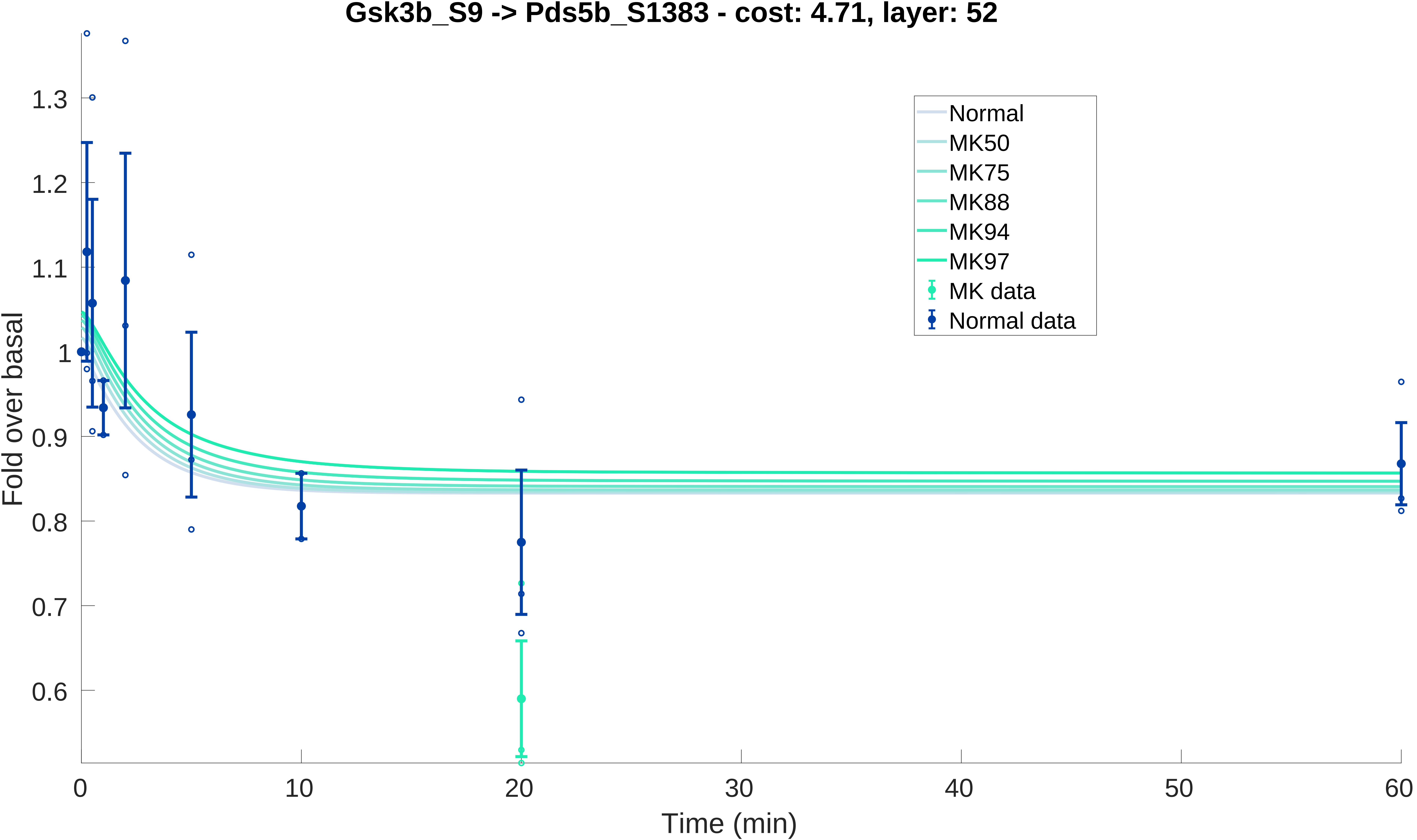

Gsk3b_S9 -> Pds5b_S1383 - cost: 4.71, layer: 52
Fold over basal
Time (min)
1.3
1.2
1.1
1
0.9
0.8
0.7
0.6
0
10
20
30
40
50
60
Normal
MK50
MK75
MK88
MK94
MK97
MK data
Normal data

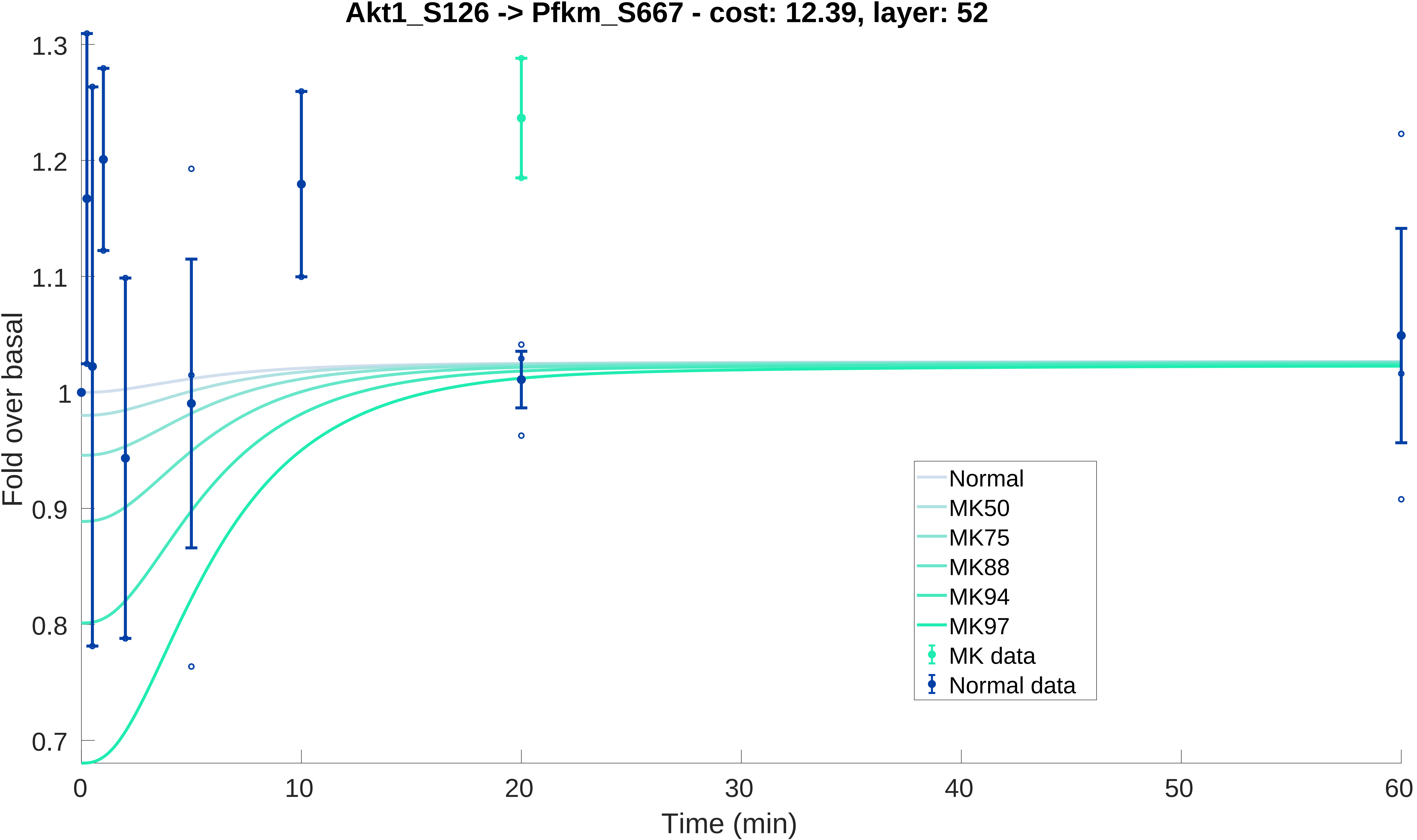

Akt1_S126 -> Pfkm_S667 - cost: 12.39, layer: 52
Fold over basal
Time (min)
1.3
1.2
1.1
1
0.9
0.8
0.7
0
10
20
30
40
50
60
Normal
MK50
MK75
MK88
MK94
MK97
MK data
Normal data

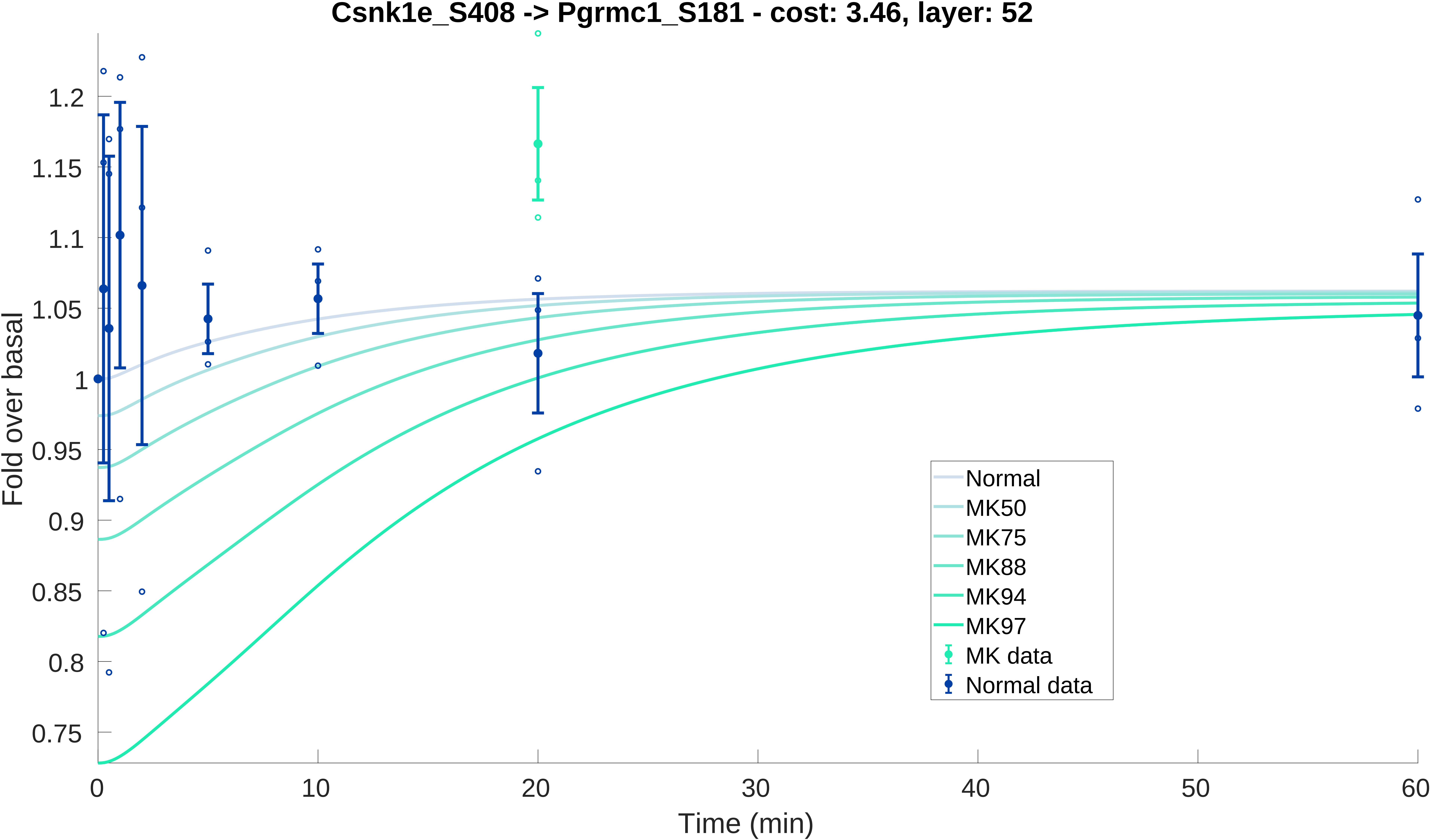

Csnk1e_S408 -> Pgrmc1_S181 - cost: 3.46, layer: 52
Fold over basal
1.2
1.15
1.1
1.05
1
0.95
0.9
0.85
0.8
0.75
0
10
20
30
40
50
60
Time (min)
Normal
MK50
MK75
MK88
MK94
MK97
MK data
Normal data

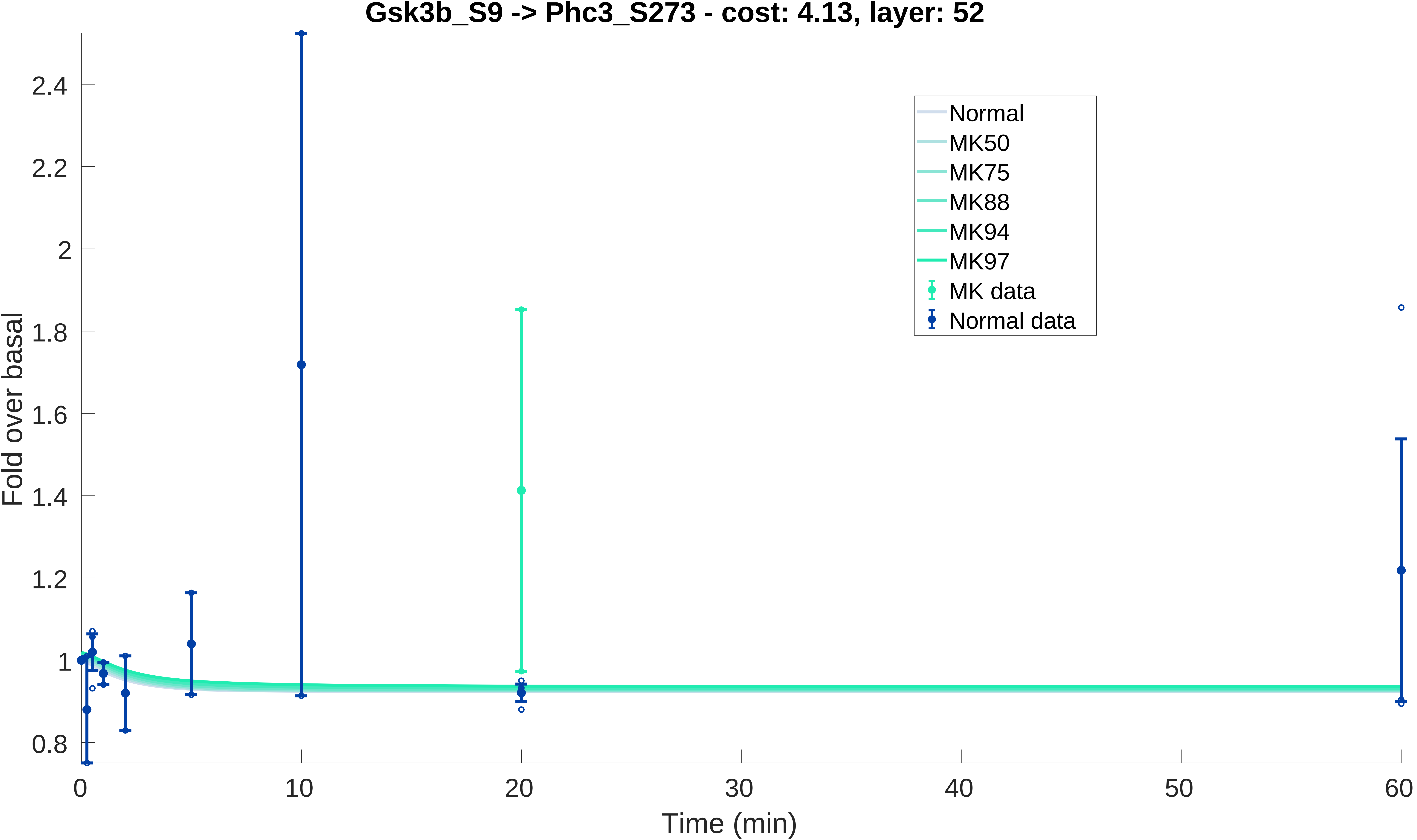
Gsk3b_S9 -> Phc3_S273 - cost: 4.13, layer: 52
Fold over basal
2.4
2.2
2
1.8
1.6
1.4
1.2
1
0.8
0
10
20
30
40
50
60
Time (min)
Normal
MK50
MK75
MK88
MK94
MK97
MK data
Normal data

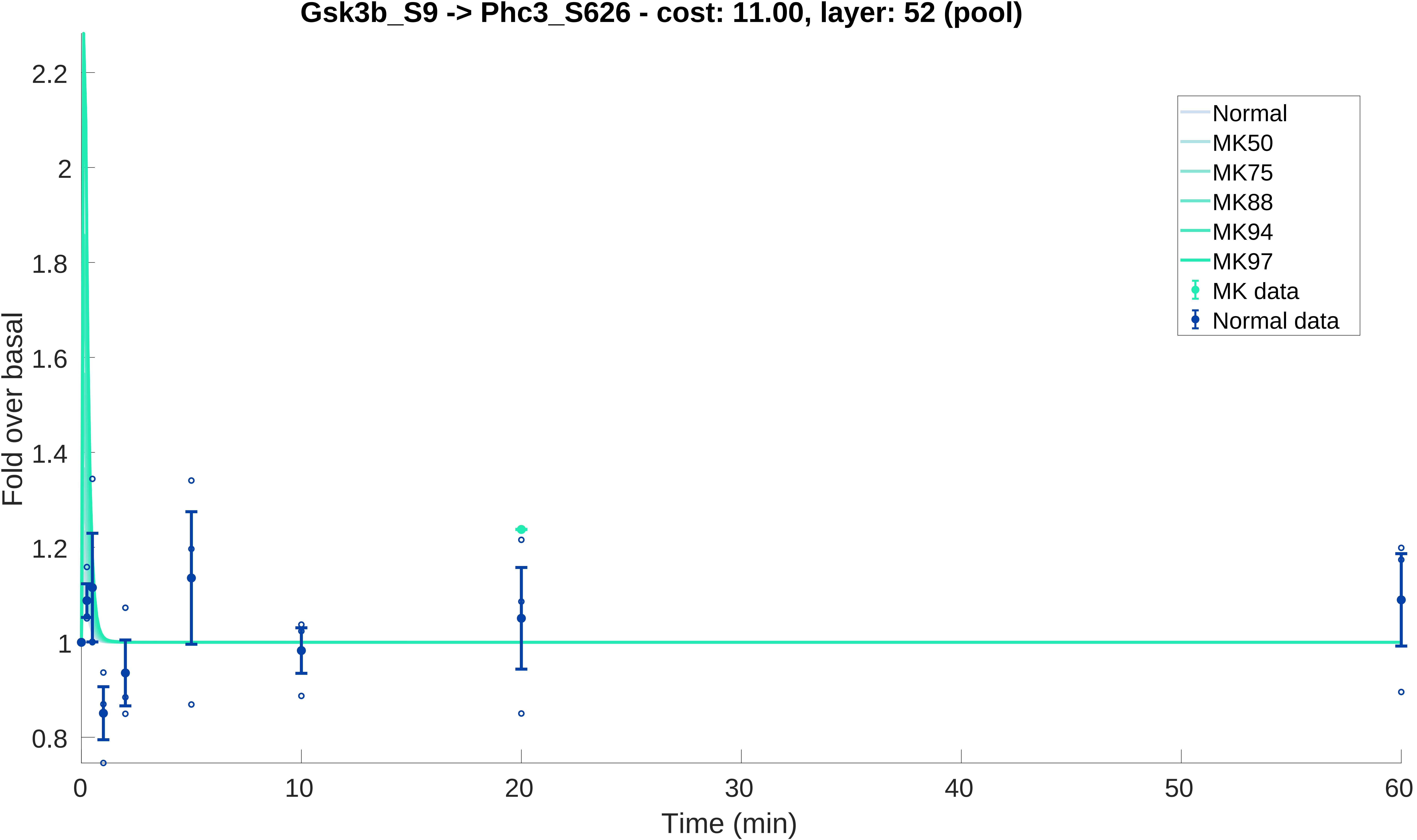

Gsk3b_S9 -> Phc3_S626 - cost: 11.00, layer: 52 (pool)
Fold over basal
Time (min)
0.8
1
1.2
1.4
1.6
1.8
2
2.2
0
10
20
30
40
50
60
Normal
MK50
MK75
MK88
MK94
MK97
MK data
Normal data

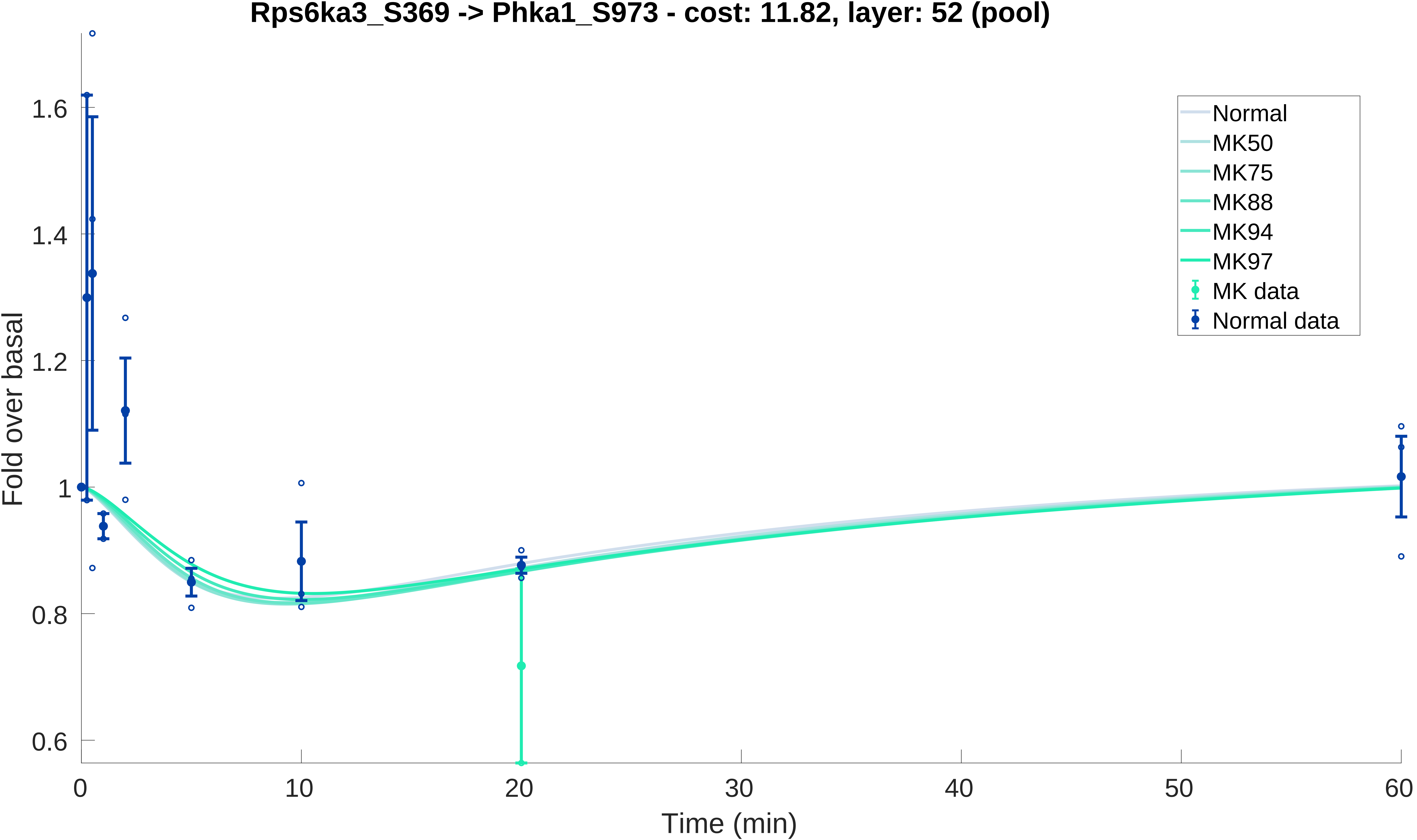

Rps6ka3_S369 -> Phka1_S973 - cost: 11.82, layer: 52 (pool)
Fold over basal
0.6
0.8
1
1.2
1.4
1.6
0
10
20
30
40
50
60
Time (min)
Normal
MK50
MK75
MK88
MK94
MK97
MK data
Normal data

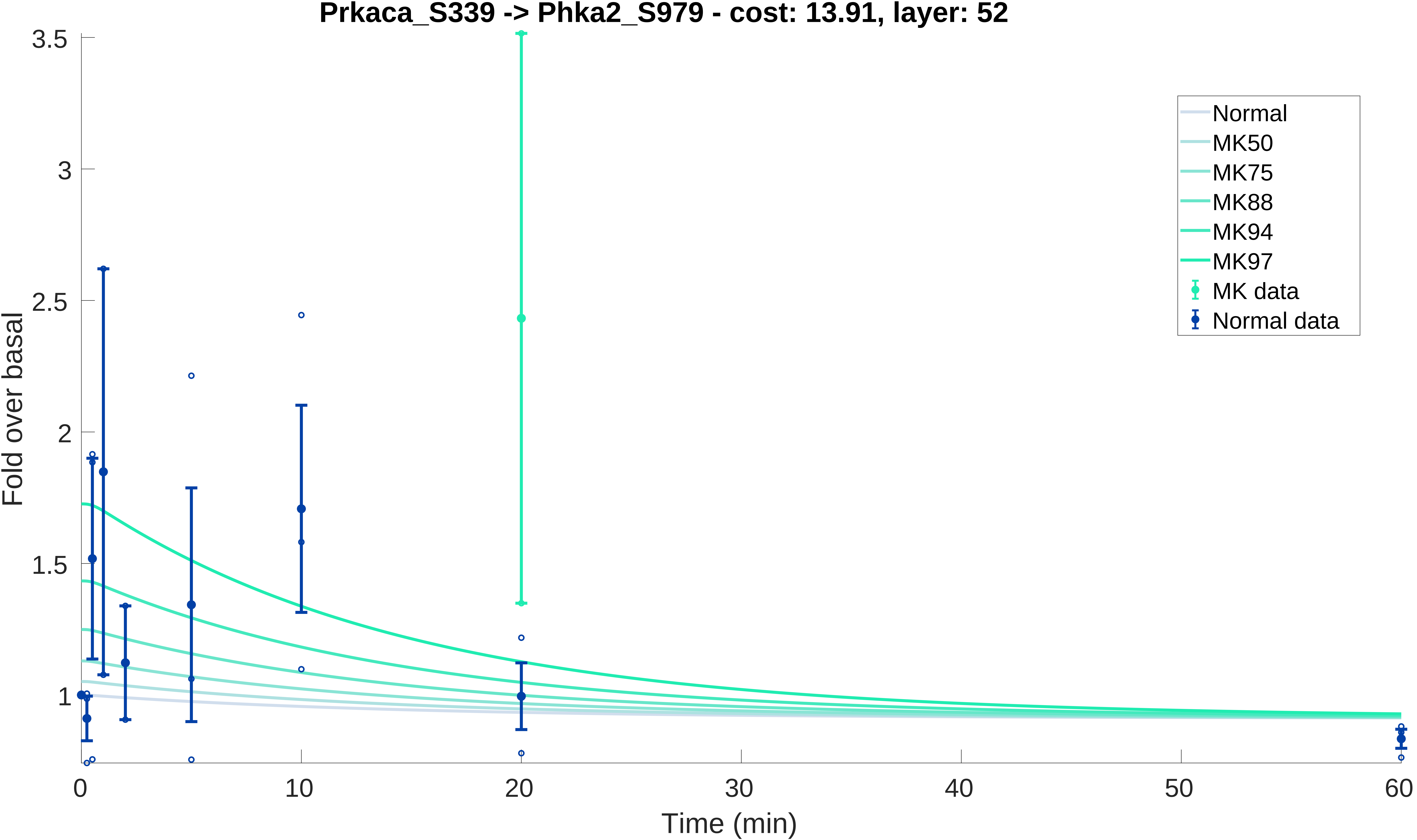

Prkaca_S339 -> Phka2_S979 - cost: 13.91, layer: 52
Fold over basal
Time (min)
3.5
3
2.5
2
1.5
1
0
10
20
30
40
50
60
Normal
MK50
MK75
MK88
MK94
MK97
MK data
Normal data

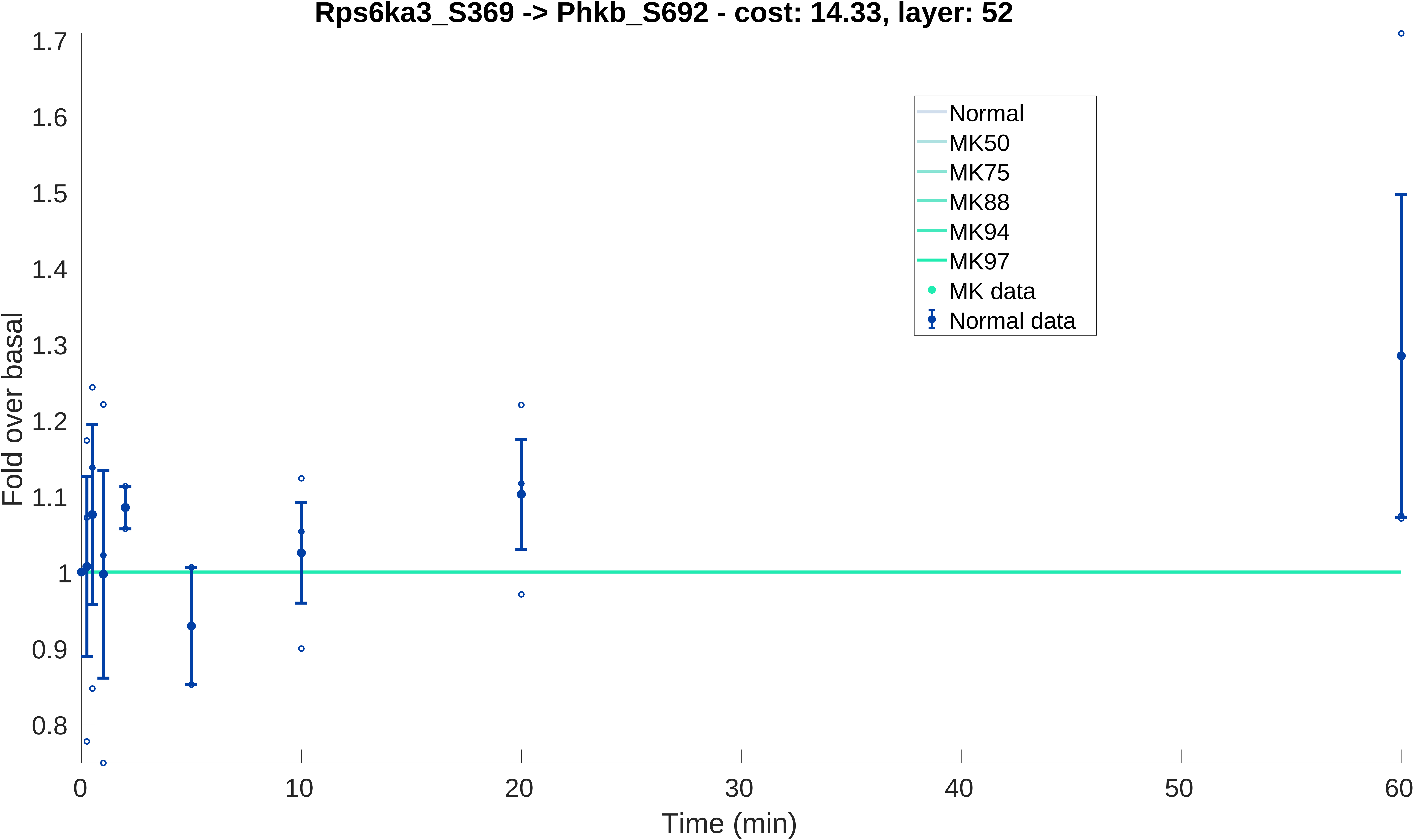

Rps6ka3_S369 -> Phkb_S692 - cost: 14.33, layer: 52
Fold over basal
Time (min)
Normal
MK50
MK75
MK88
MK94
MK97
MK data
Normal data
1.7
1.6
1.5
1.4
1.3
1.2
1.1
1
0.9
0.8
0
10
20
30
40
50
60

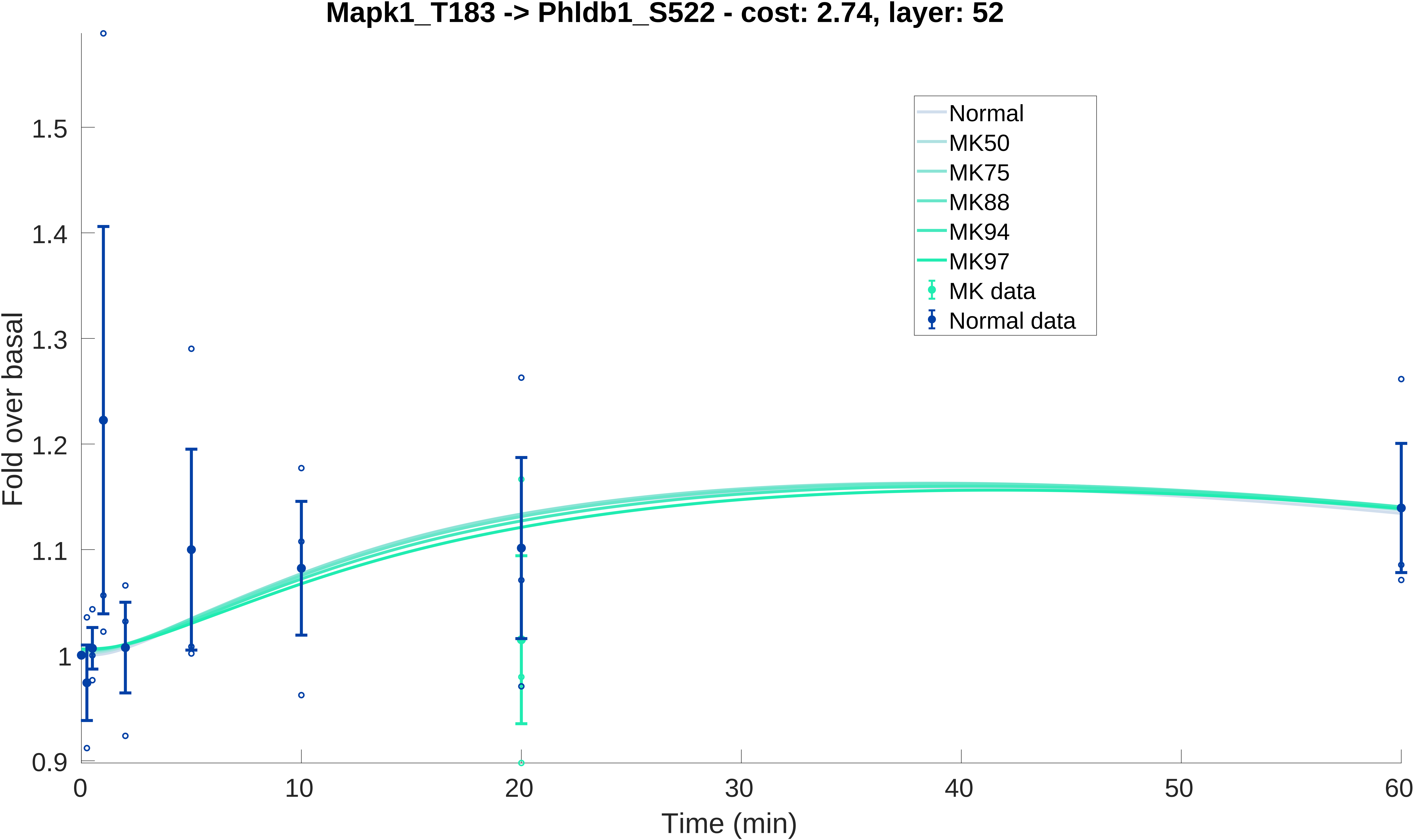

Mapk1_T183 -> Phldb1_S522 - cost: 2.74, layer: 52
Fold over basal
Time (min)
0.9
1
1.1
1.2
1.3
1.4
1.5
0
10
20
30
40
50
60
Normal
MK50
MK75
MK88
MK94
MK97
MK data
Normal data

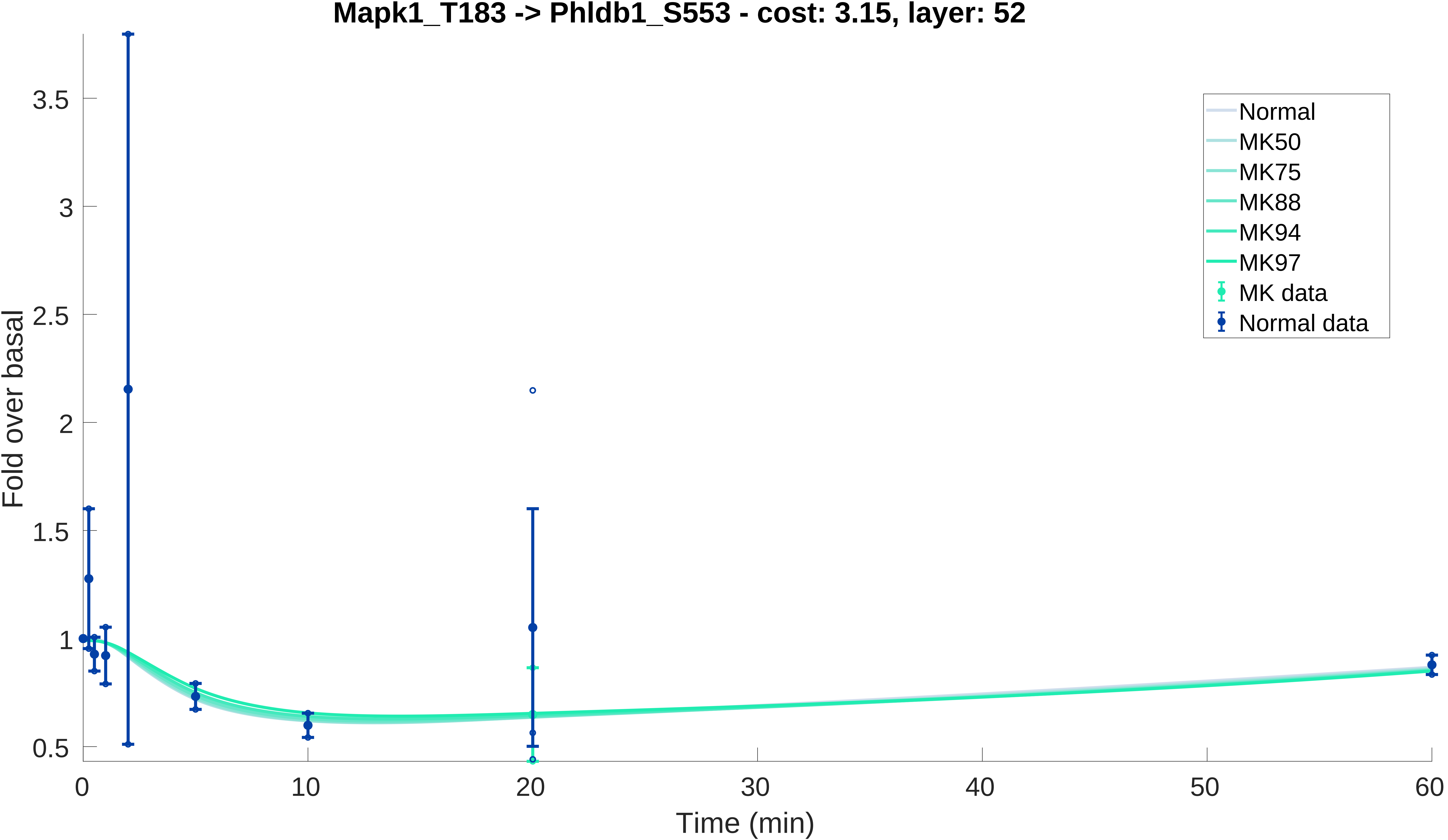

Mapk1_T183 -> Phldb1_S553 - cost: 3.15, layer: 52
Fold over basal
Time (min)
0.5
1
1.5
2
2.5
3
3.5
0
10
20
30
40
50
60
Normal
MK50
MK75
MK88
MK94
MK97
MK data
Normal data

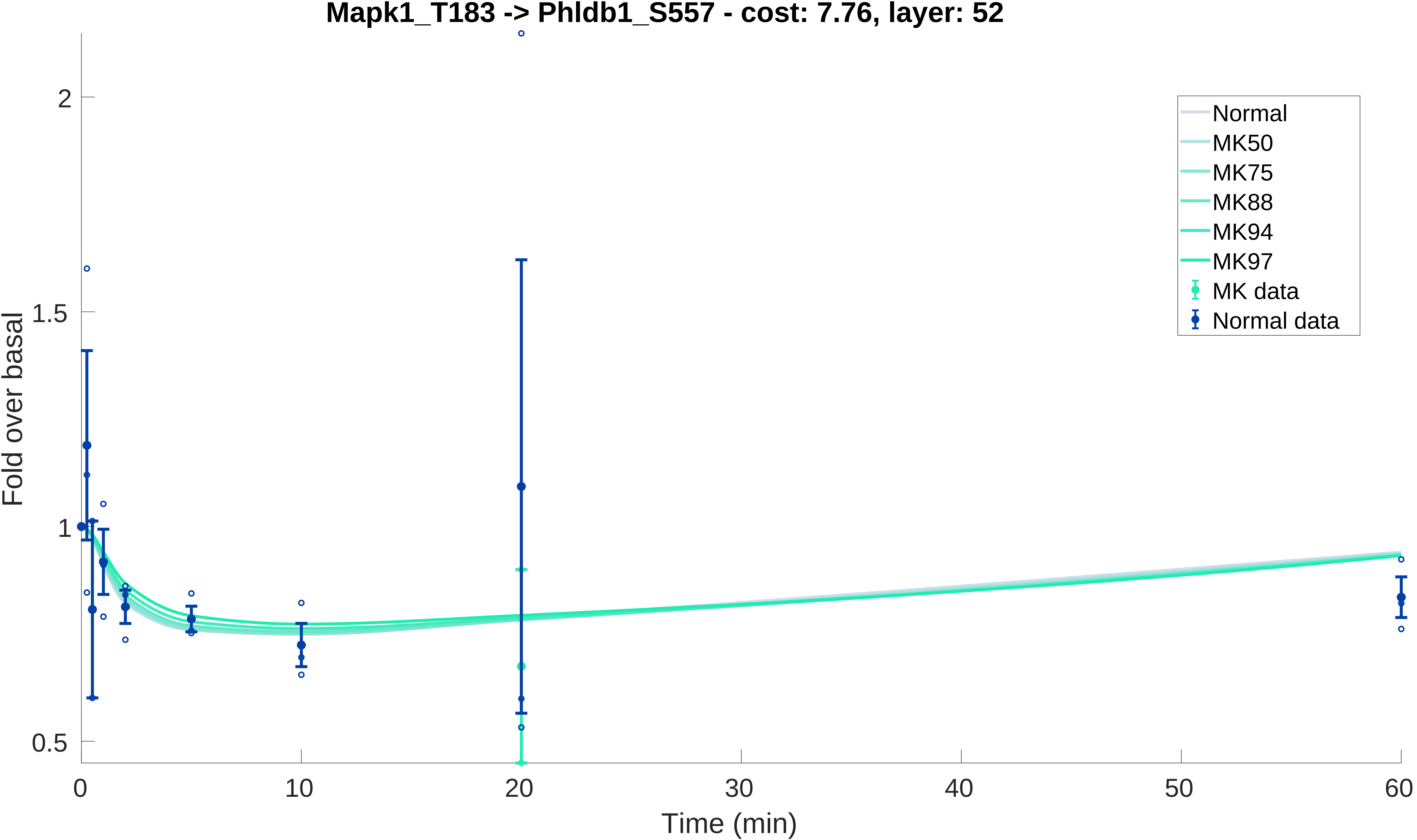

Mapk1_T183 -> Phldb1_S557 - cost: 7.76, layer: 52
Fold over basal
Time (min)
0.5
1
1.5
2
0
10
20
30
40
50
60
Normal
MK50
MK75
MK88
MK94
MK97
MK data
Normal data

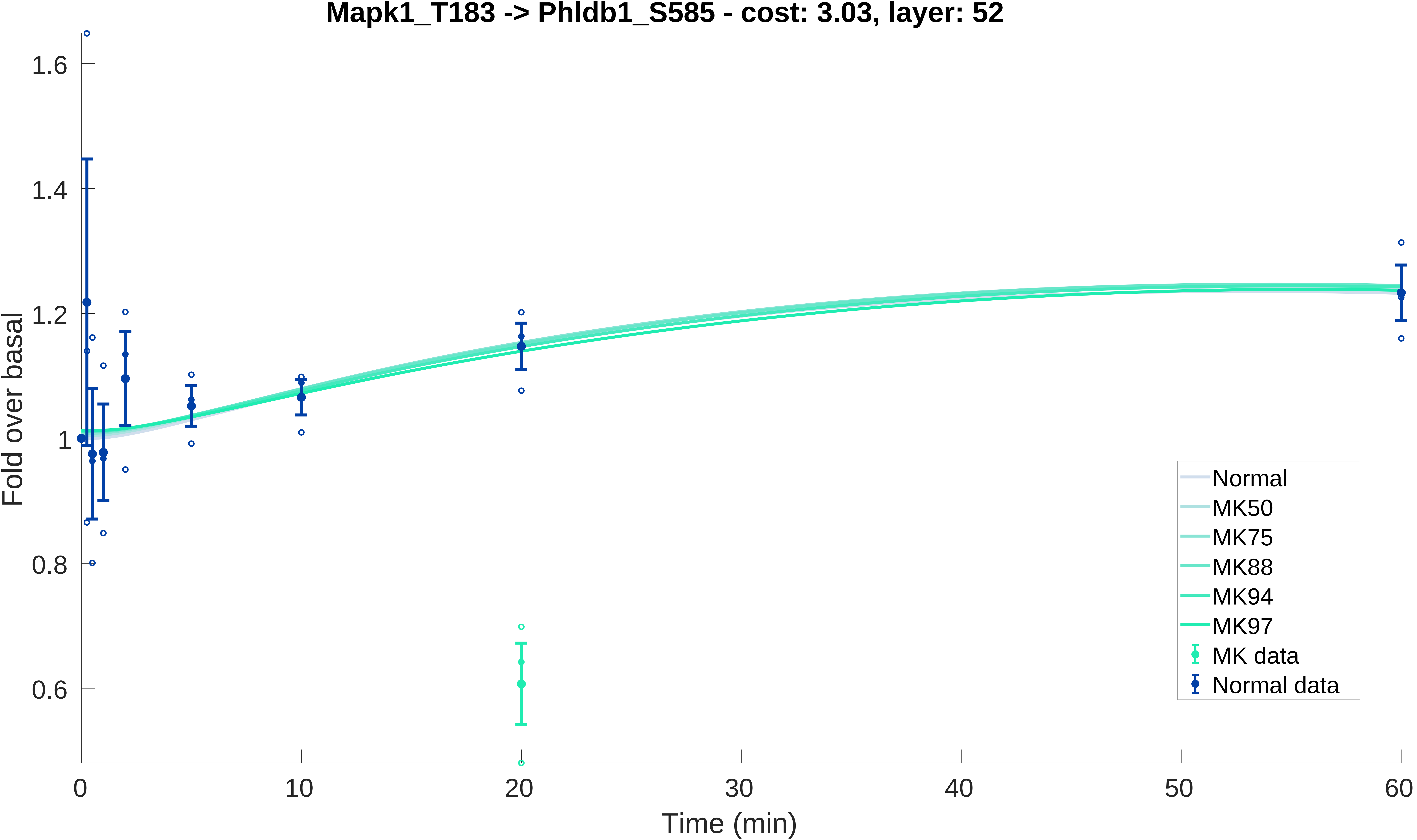
Mapk1_T183 -> Phldb1_S585 - cost: 3.03, layer: 52
Fold over basal
1.6
1.4
1.2
1
0.8
0.6
0
10
20
30
40
50
60
Time (min)
Normal
MK50
MK75
MK88
MK94
MK97
MK data
Normal data

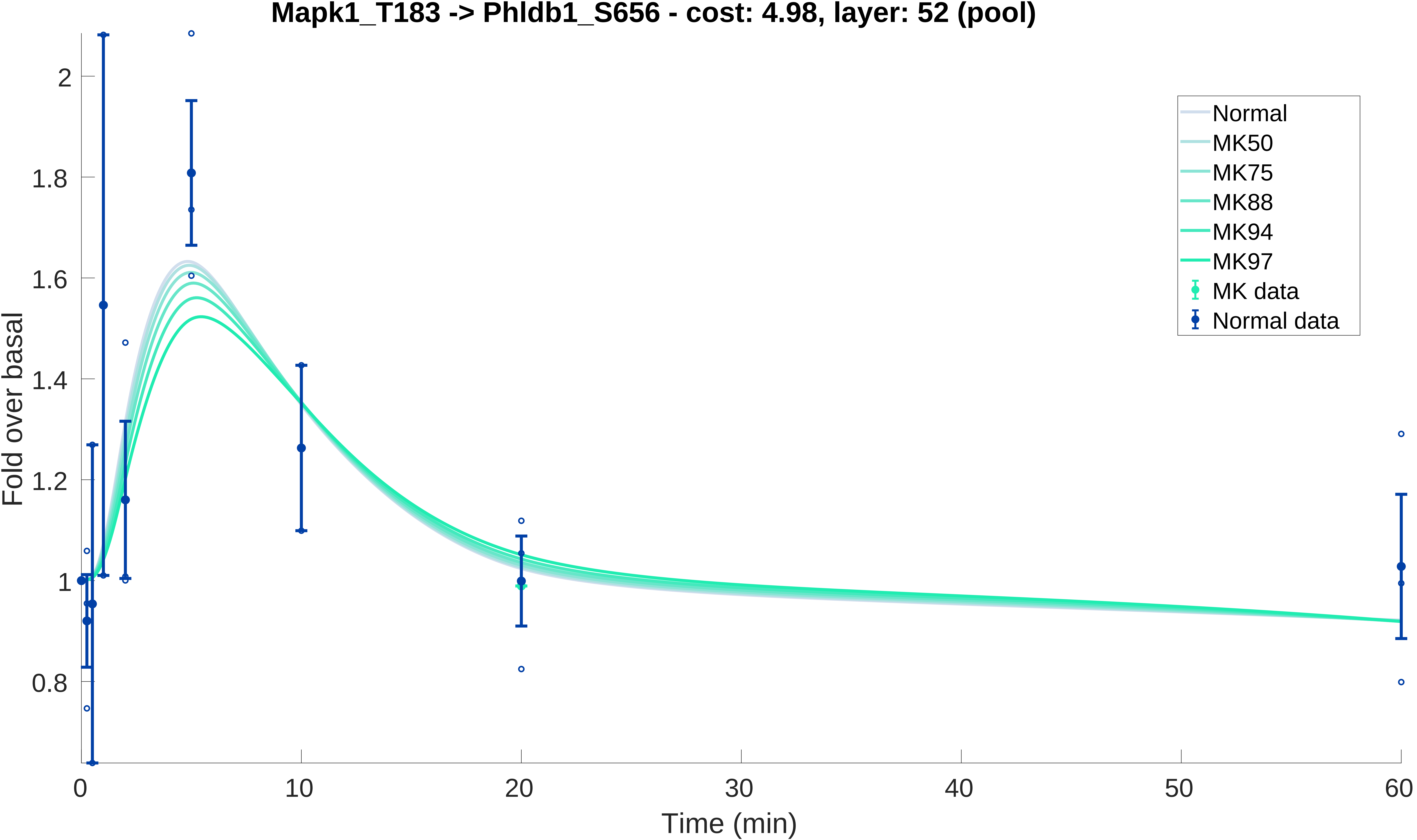

Mapk1_T183 -> Phldb1_S656 - cost: 4.98, layer: 52 (pool)
Fold over basal
Time (min)
0.8
1
1.2
1.4
1.6
1.8
2
0
10
20
30
40
50
60
Normal
MK50
MK75
MK88
MK94
MK97
MK data
Normal data

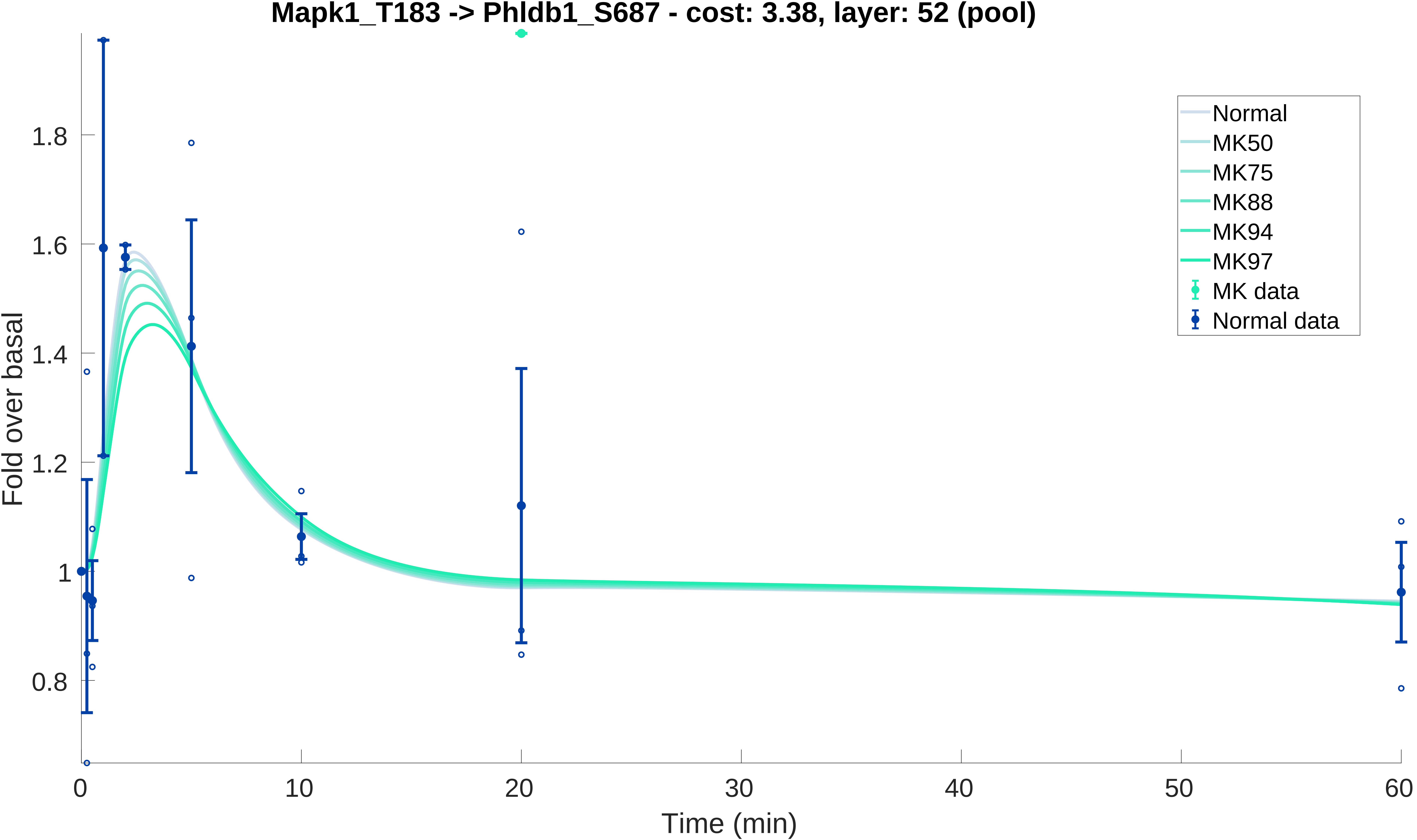
Mapk1_T183 -> Phldb1_S687 - cost: 3.38, layer: 52 (pool)
Fold over basal
1.8
1.6
1.4
1.2
1
0.8
0
10
20
30
40
50
60
Time (min)
Normal
MK50
MK75
MK88
MK94
MK97
MK data
Normal data

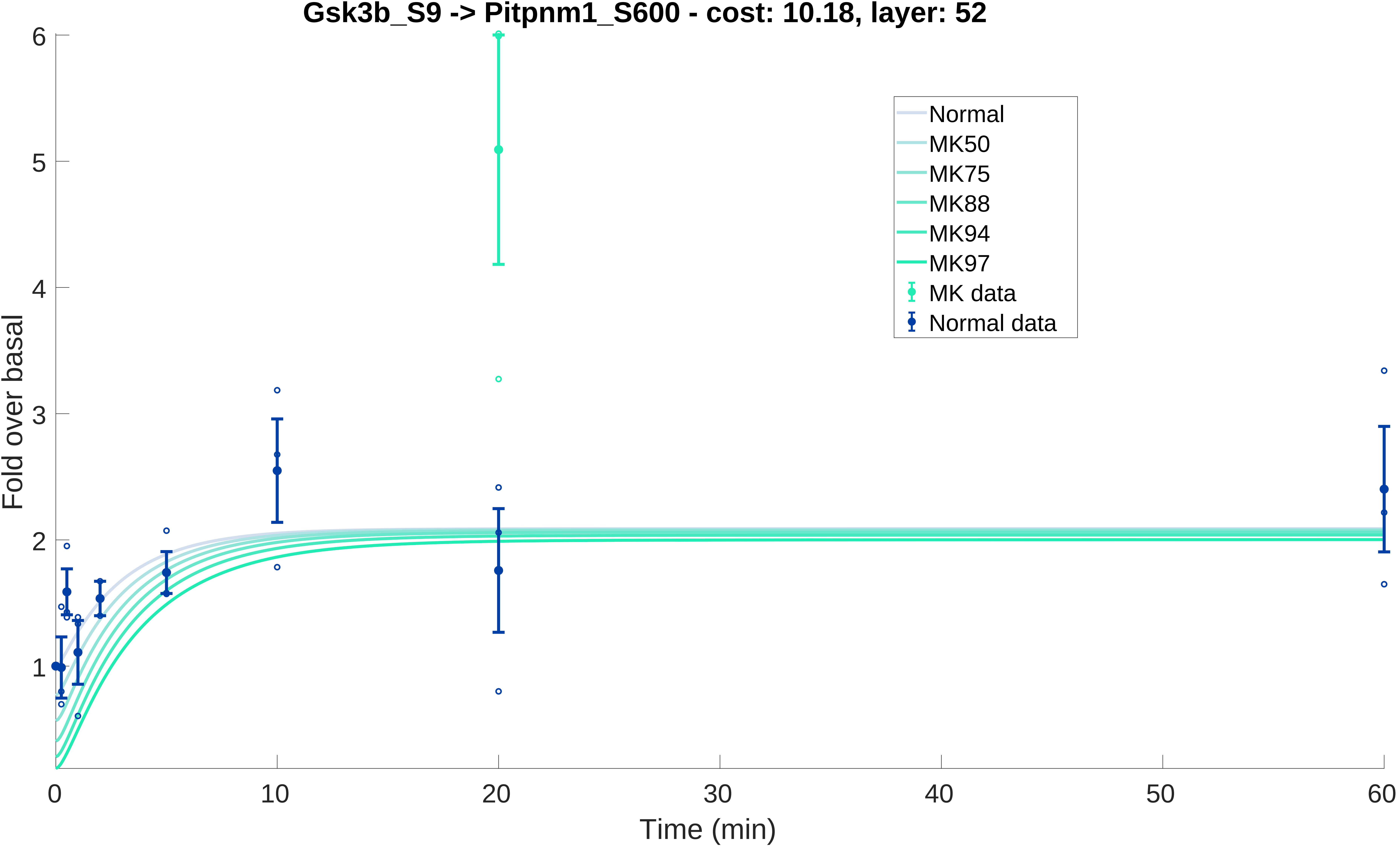

Gsk3b_S9 -> Pitpnm1_S600 - cost: 10.18, layer: 52
Fold over basal
Time (min)
0
10
20
30
40
50
60
1
2
3
4
5
6
Normal
MK50
MK75
MK88
MK94
MK97
MK data
Normal data

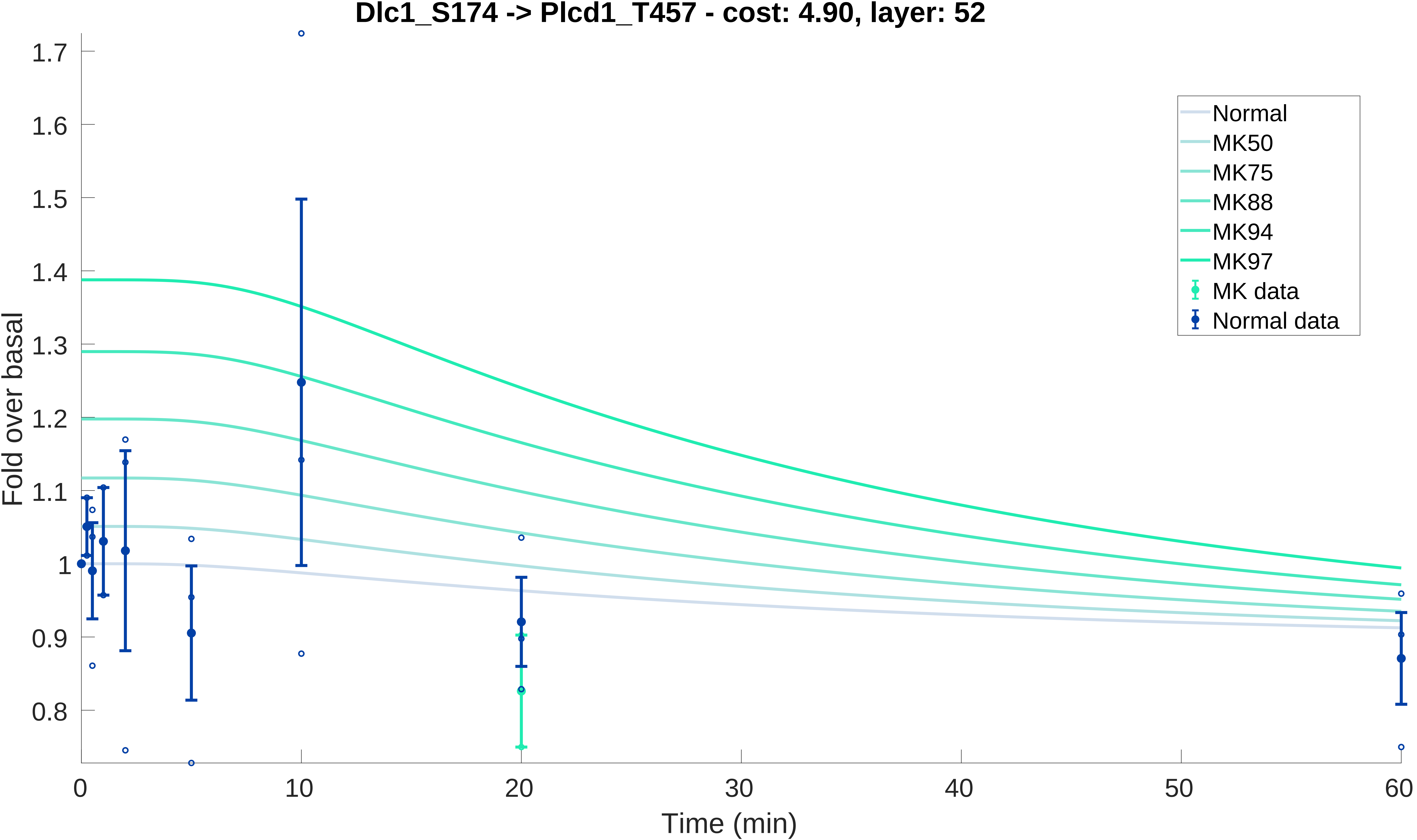

Dlc1_S174 -> Plcd1_T457 - cost: 4.90, layer: 52
Fold over basal
Time (min)
0.8
0.9
1
1.1
1.2
1.3
1.4
1.5
1.6
1.7
0
10
20
30
40
50
60
Normal
MK50
MK75
MK88
MK94
MK97
MK data
Normal data

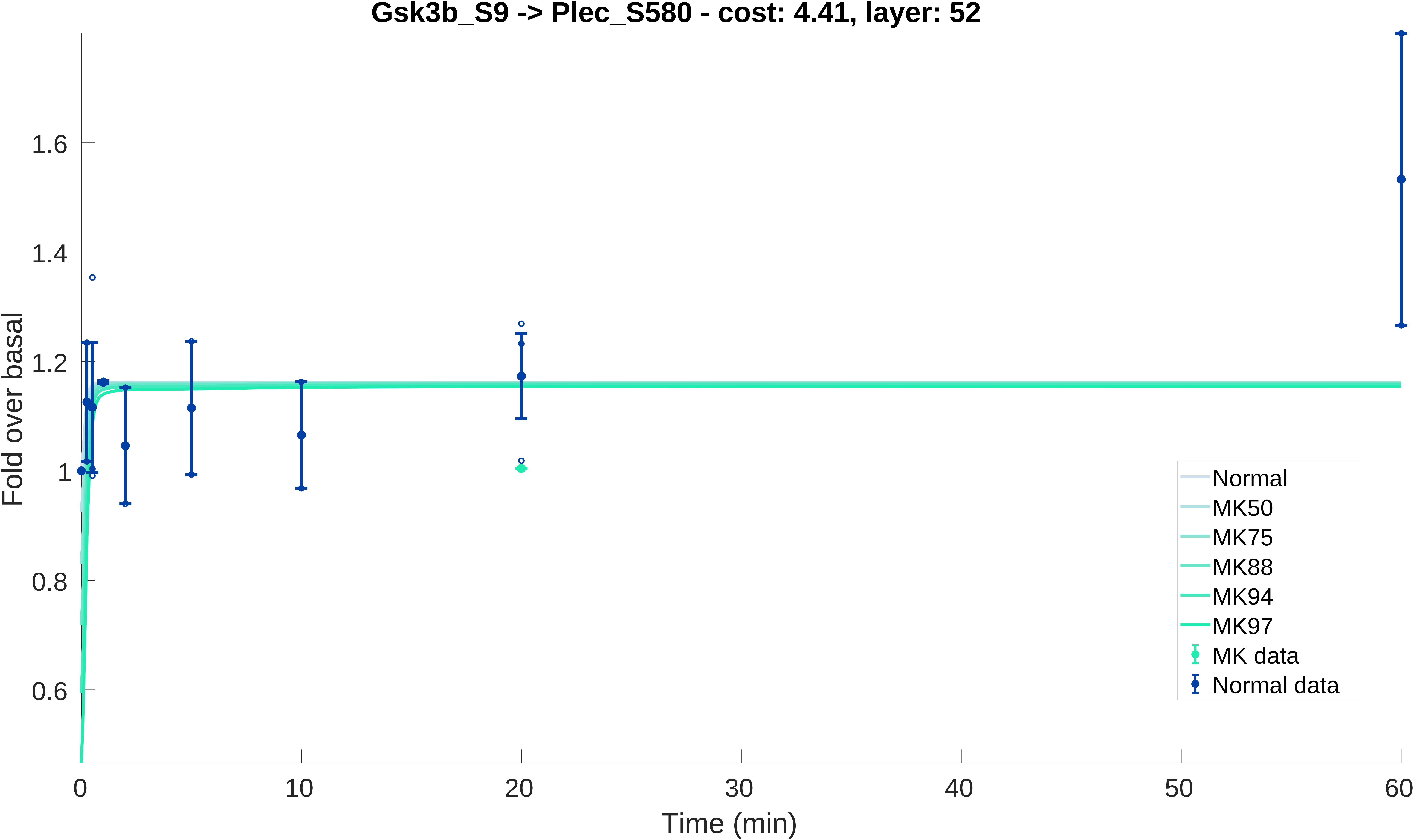
Gsk3b_S9 -> Plec_S580 - cost: 4.41, layer: 52
Fold over basal
Time (min)
1.6
1.4
1.2
1
0.8
0.6
0
10
20
30
40
50
60
Normal
MK50
MK75
MK88
MK94
MK97
MK data
Normal data

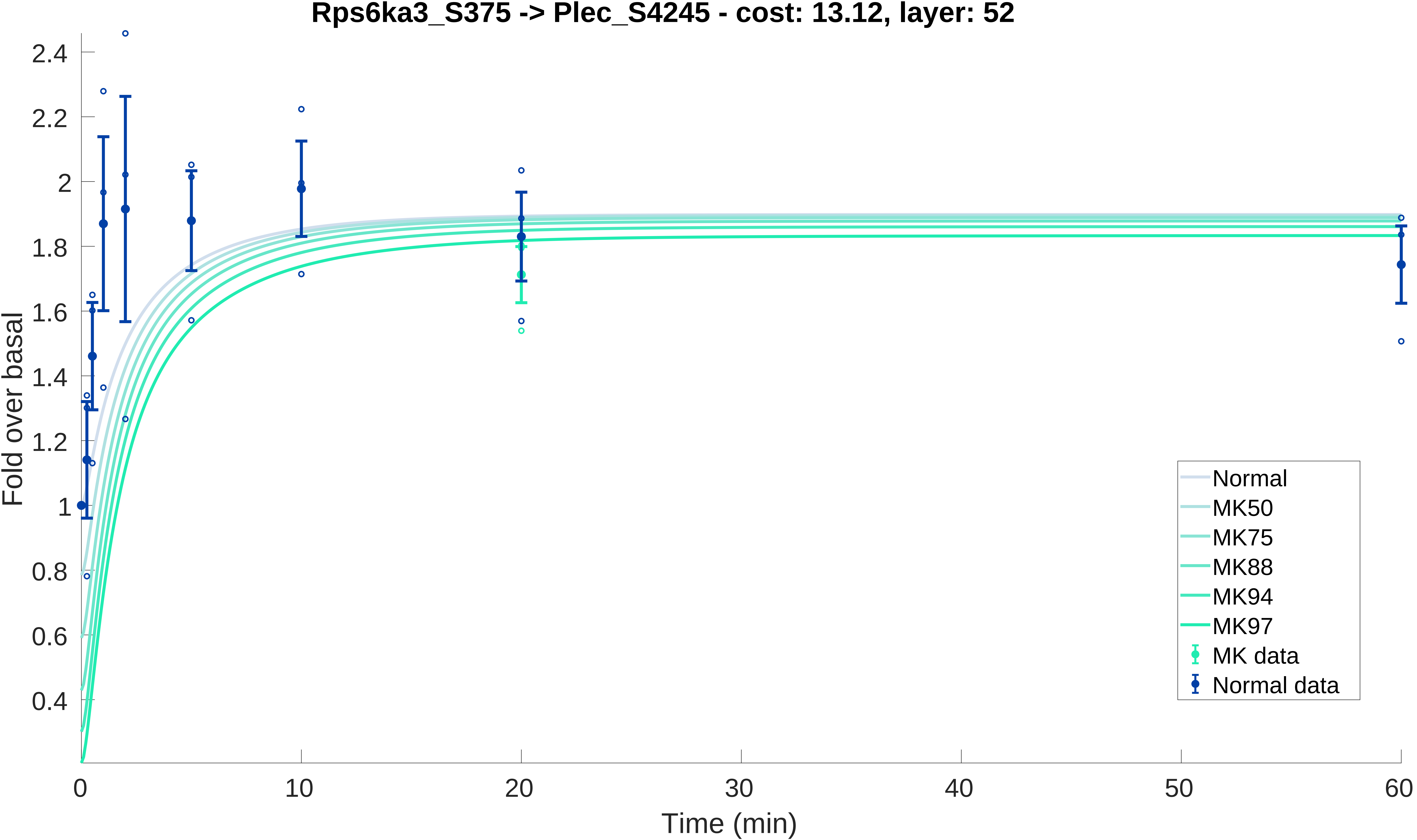

Rps6ka3_S375 -> Plec_S4245 - cost: 13.12, layer: 52
Fold over basal
Time (min)
2.4
2.2
2
1.8
1.6
1.4
1.2
1
0.8
0.6
0.4
0
10
20
30
40
50
60
Normal
MK50
MK75
MK88
MK94
MK97
MK data
Normal data

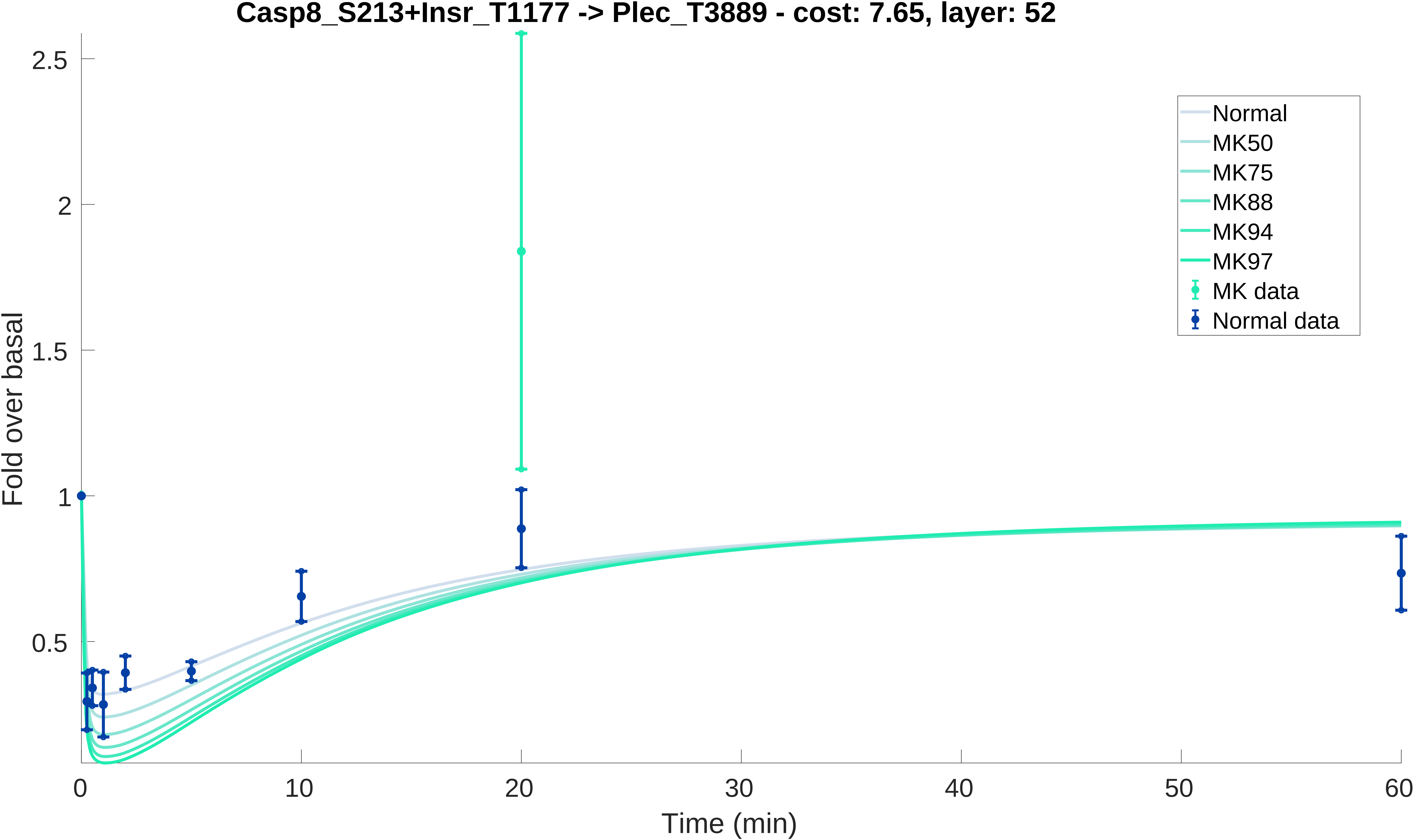

Casp8_S213+Insr_T1177 -> Plec_T3889 - cost: 7.65, layer: 52
Fold over basal
Time (min)
2.5
2
1.5
1
0.5
0
10
20
30
40
50
60
Normal
MK50
MK75
MK88
MK94
MK97
MK data
Normal data

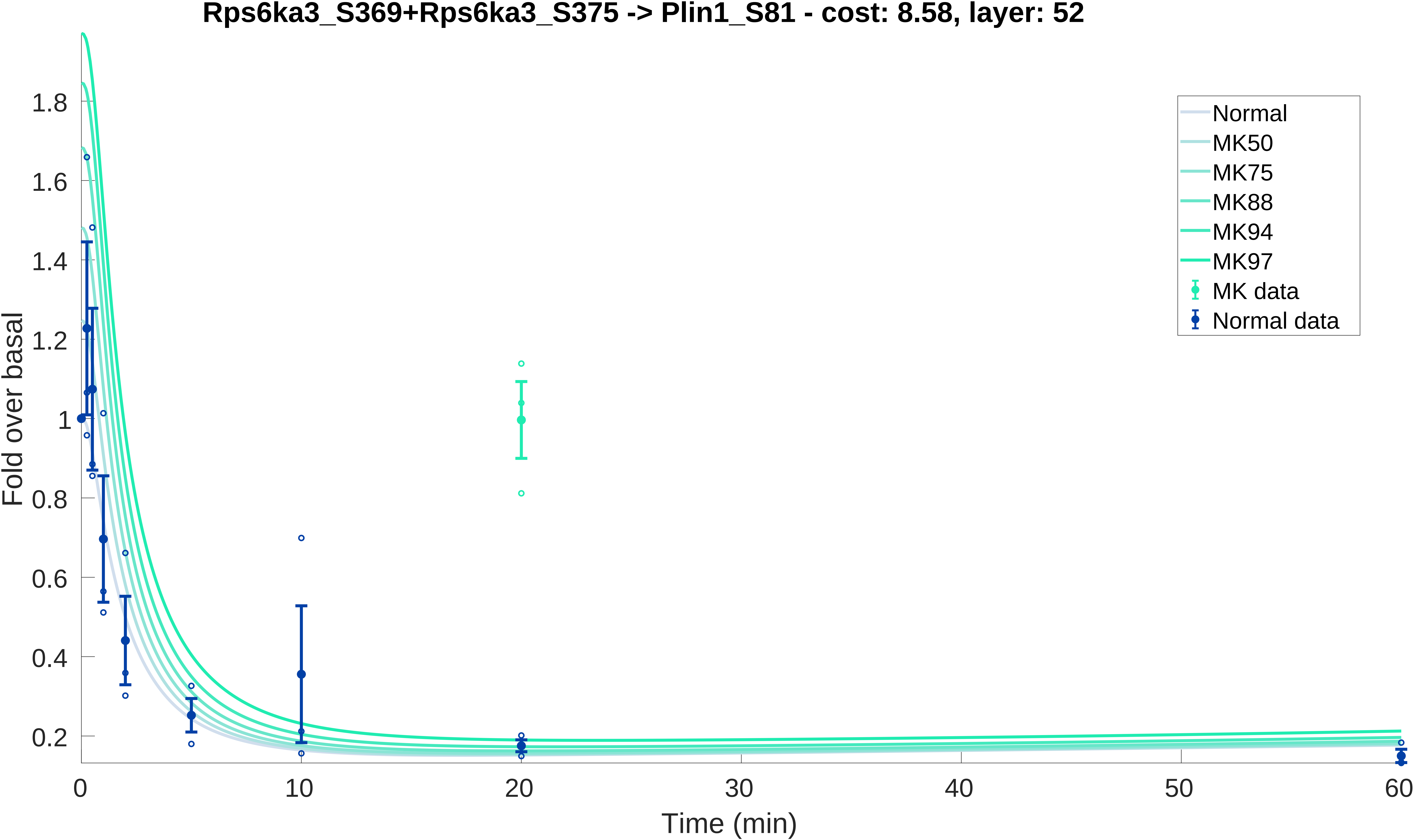
Rps6ka3_S369+Rps6ka3_S375 -> Plin1_S81 - cost: 8.58, layer: 52
Fold over basal
Time (min)
0
10
20
30
40
50
60
0.2
0.4
0.6
0.8
1
1.2
1.4
1.6
1.8
Normal
MK50
MK75
MK88
MK94
MK97
MK data
Normal data

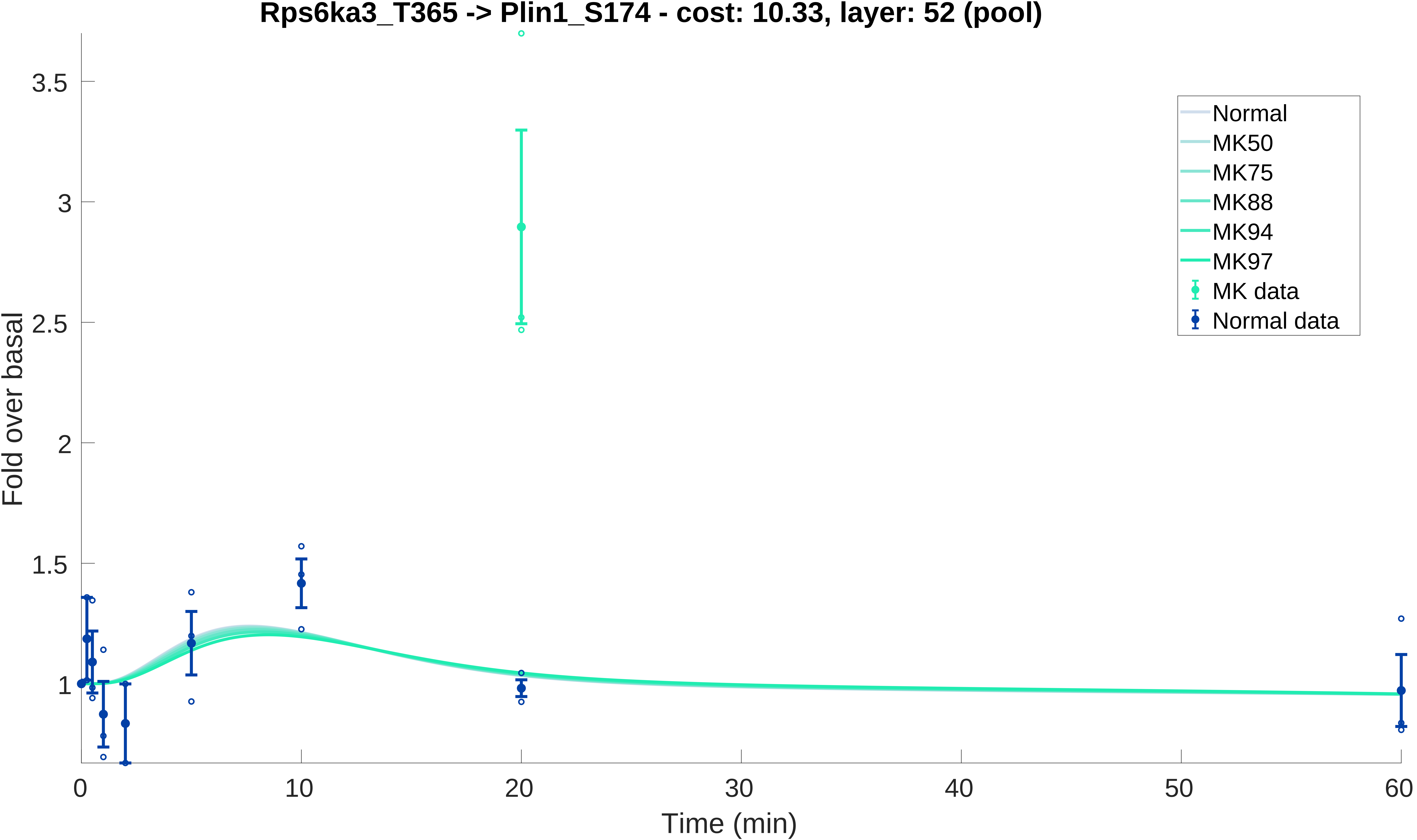

Rps6ka3_T365 -> Plin1_S174 - cost: 10.33, layer: 52 (pool)
Fold over basal
Time (min)
3.5
3
2.5
2
1.5
1
0
10
20
30
40
50
60
Normal
MK50
MK75
MK88
MK94
MK97
MK data
Normal data

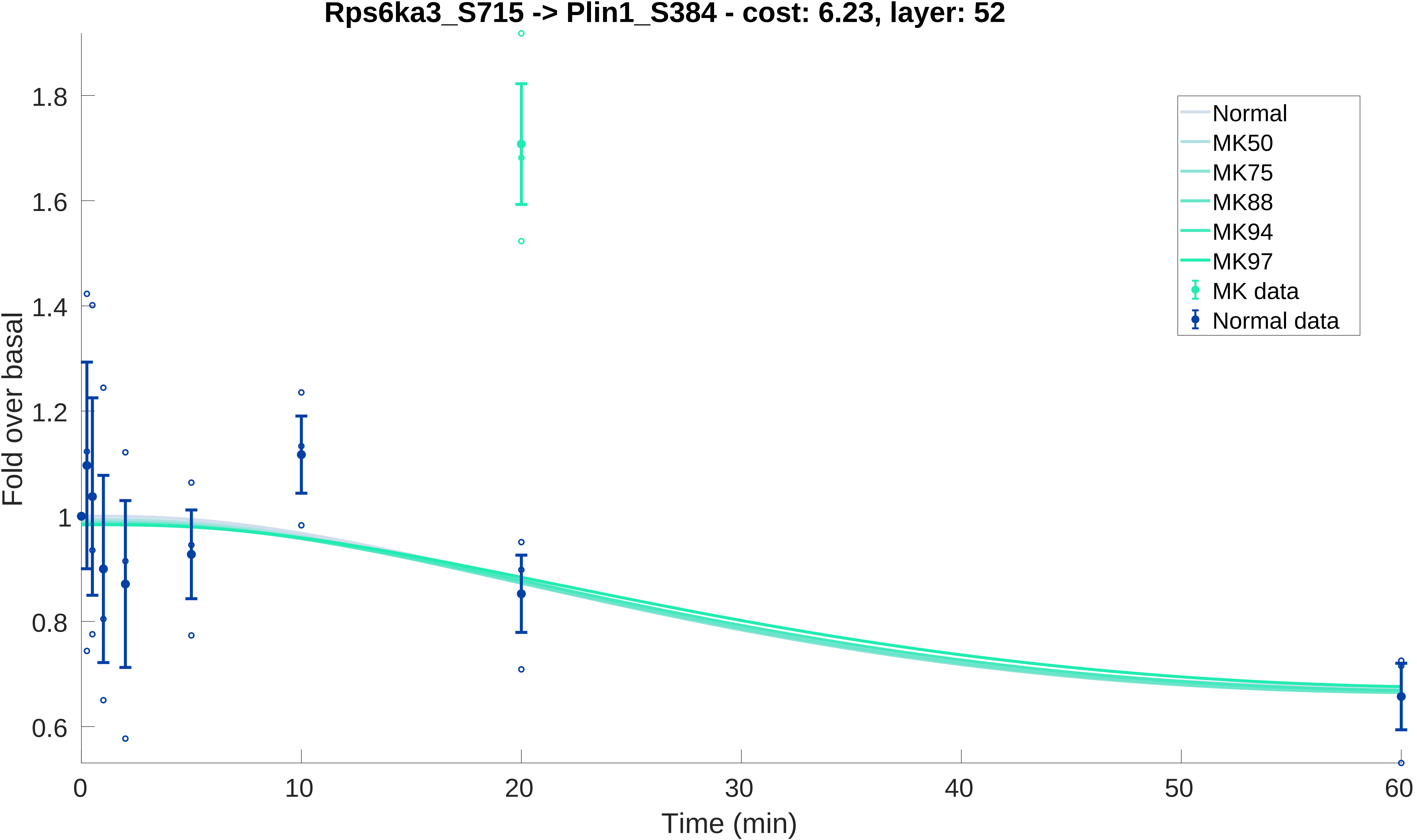
Rps6ka3_S715 -> Plin1_S384 - cost: 6.23, layer: 52
Fold over basal
Time (min)
0
10
20
30
40
50
60
0.6
0.8
1
1.2
1.4
1.6
1.8
Normal
MK50
MK75
MK88
MK94
MK97
MK data
Normal data

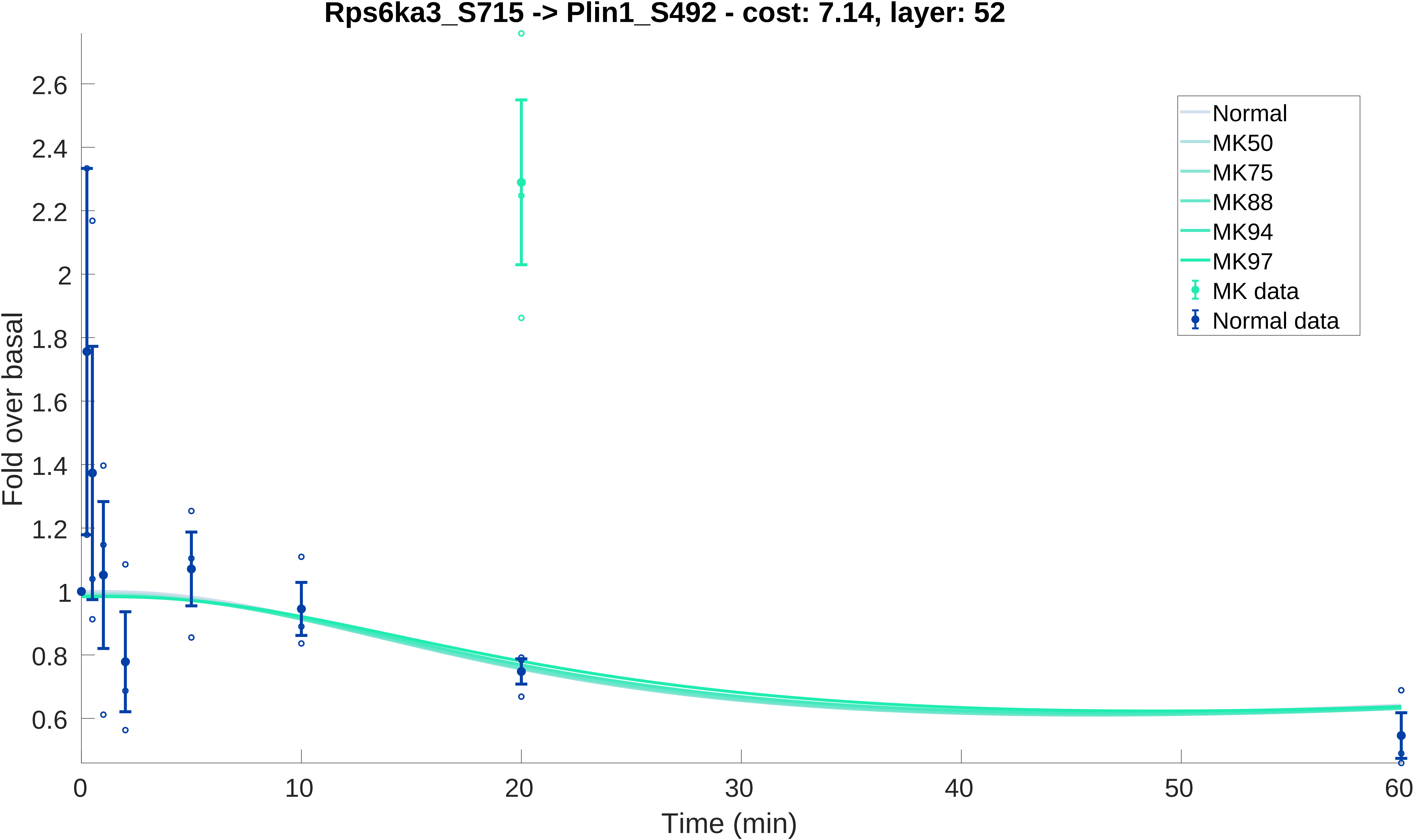

Rps6ka3_S715 -> Plin1_S492 - cost: 7.14, layer: 52
Fold over basal
Time (min)
2.6
2.4
2.2
2
1.8
1.6
1.4
1.2
1
0.8
0.6
0
10
20
30
40
50
60
Normal
MK50
MK75
MK88
MK94
MK97
MK data
Normal data

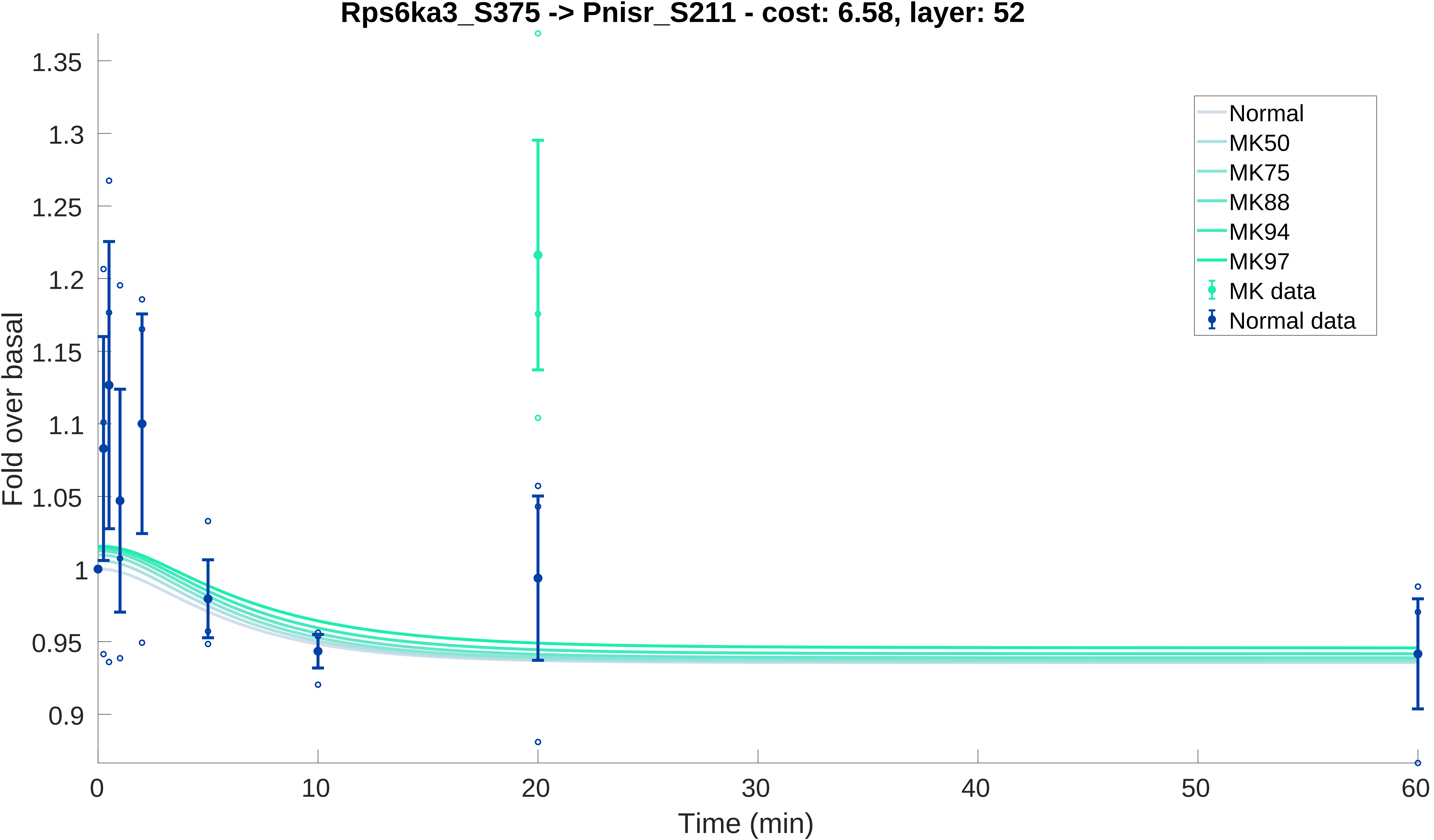

Rps6ka3_S375 -> Pnisr_S211 - cost: 6.58, layer: 52
Fold over basal
Time (min)
1.35
1.3
1.25
1.2
1.15
1.1
1.05
1
0.95
0.9
0
10
20
30
40
50
60
Normal
MK50
MK75
MK88
MK94
MK97
MK data
Normal data

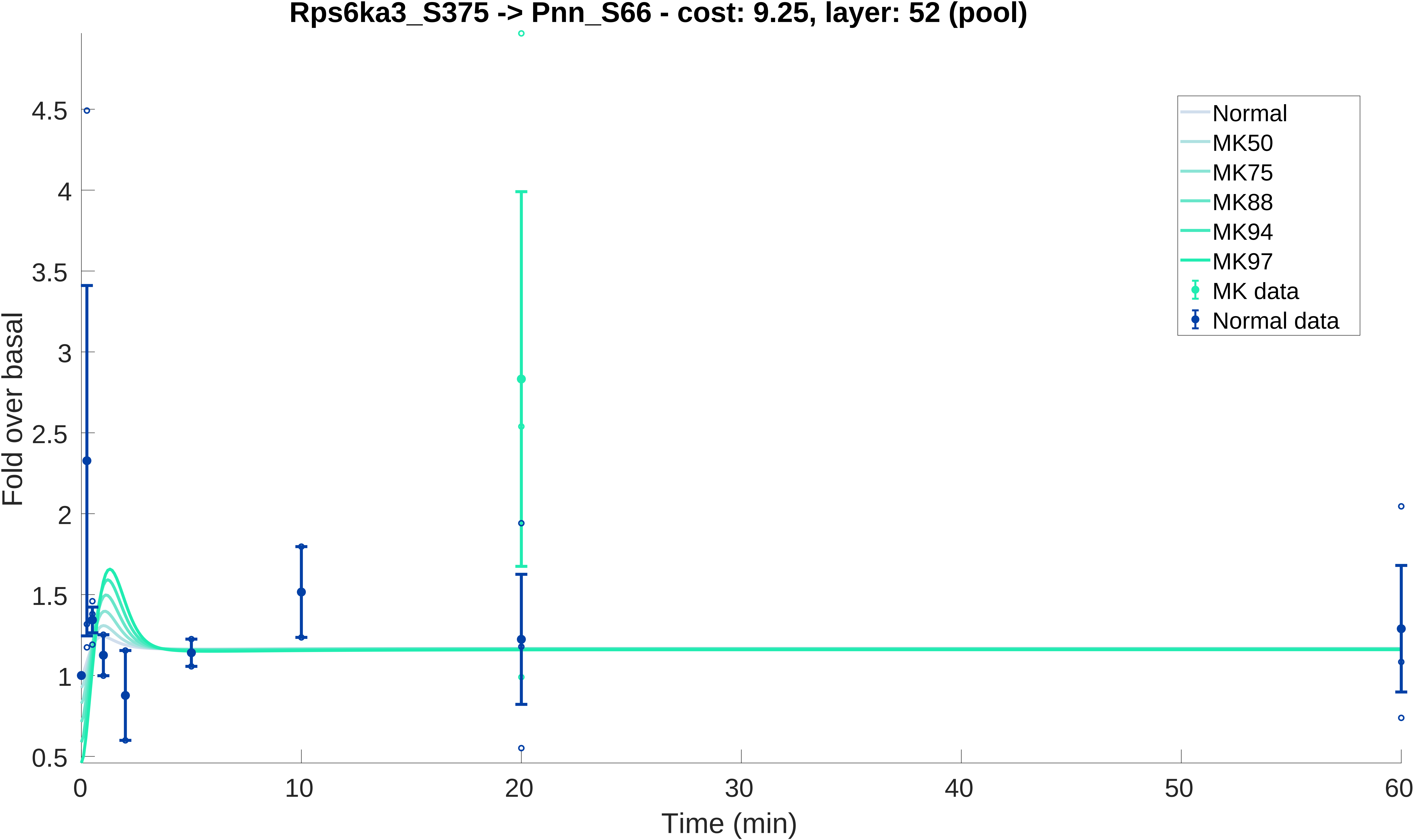

Rps6ka3_S375 -> Pnn_S66 - cost: 9.25, layer: 52 (pool)
Fold over basal
Time (min)
0.5
1
1.5
2
2.5
3
3.5
4
4.5
0
10
20
30
40
50
60
Normal
MK50
MK75
MK88
MK94
MK97
MK data
Normal data

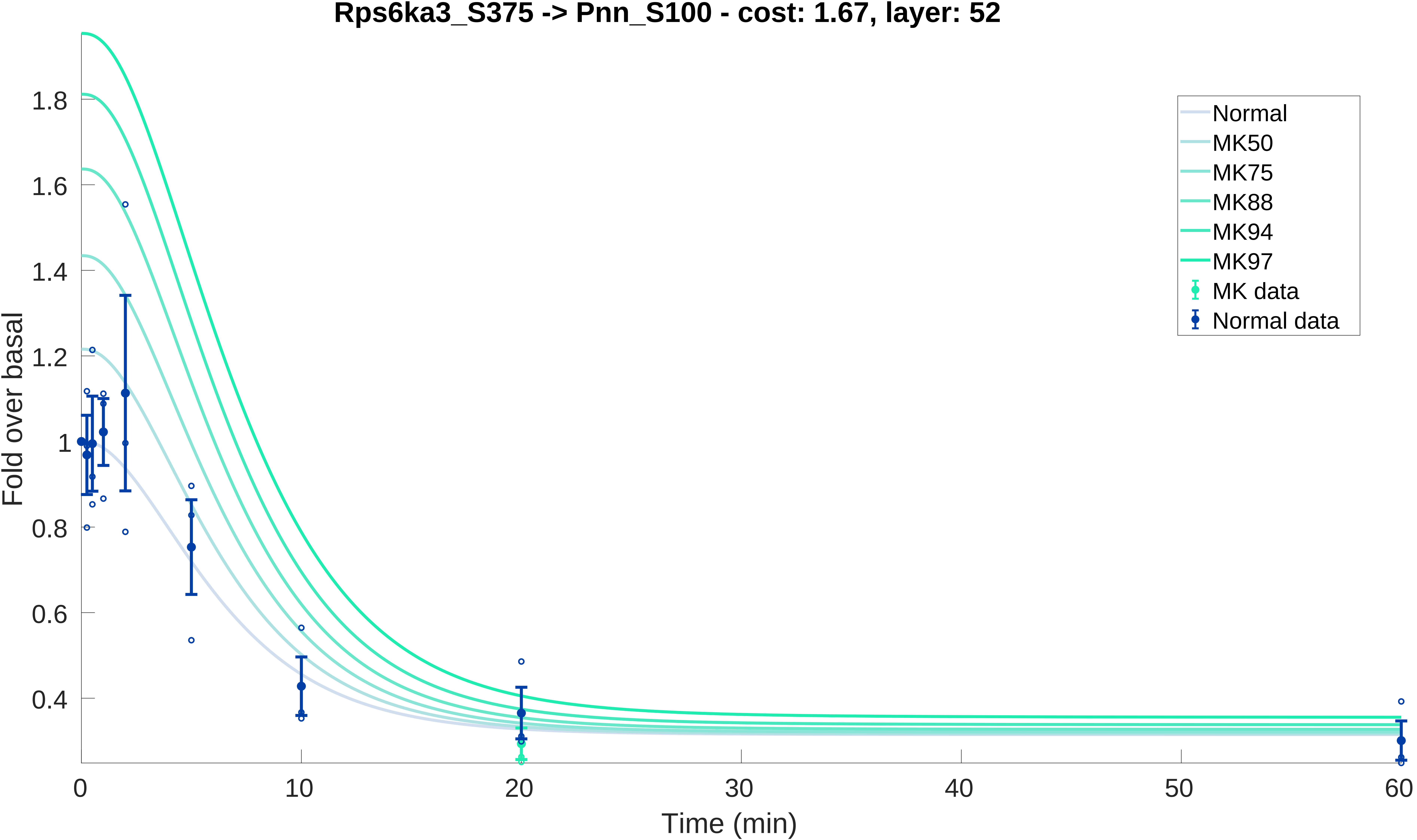

Rps6ka3_S375 -> Pnn_S100 - cost: 1.67, layer: 52
Fold over basal
Time (min)
0.4
0.6
0.8
1
1.2
1.4
1.6
1.8
0
10
20
30
40
50
60
Normal
MK50
MK75
MK88
MK94
MK97
MK data
Normal data

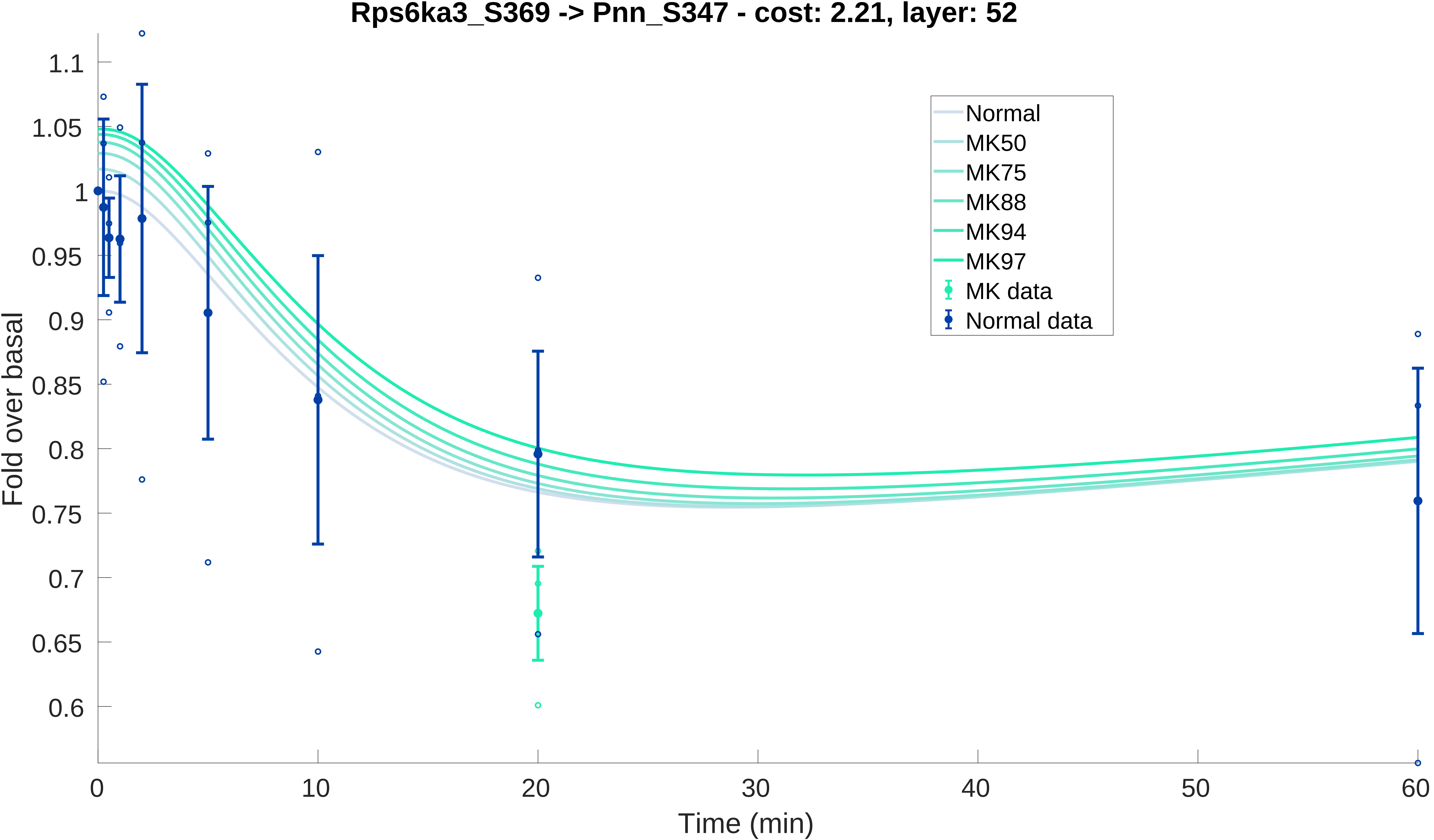

Rps6ka3_S369 -> Pnn_S347 - cost: 2.21, layer: 52
Fold over basal
Time (min)
1.1
1.05
1
0.95
0.9
0.85
0.8
0.75
0.7
0.65
0.6
0
10
20
30
40
50
60
Normal
MK50
MK75
MK88
MK94
MK97
MK data
Normal data

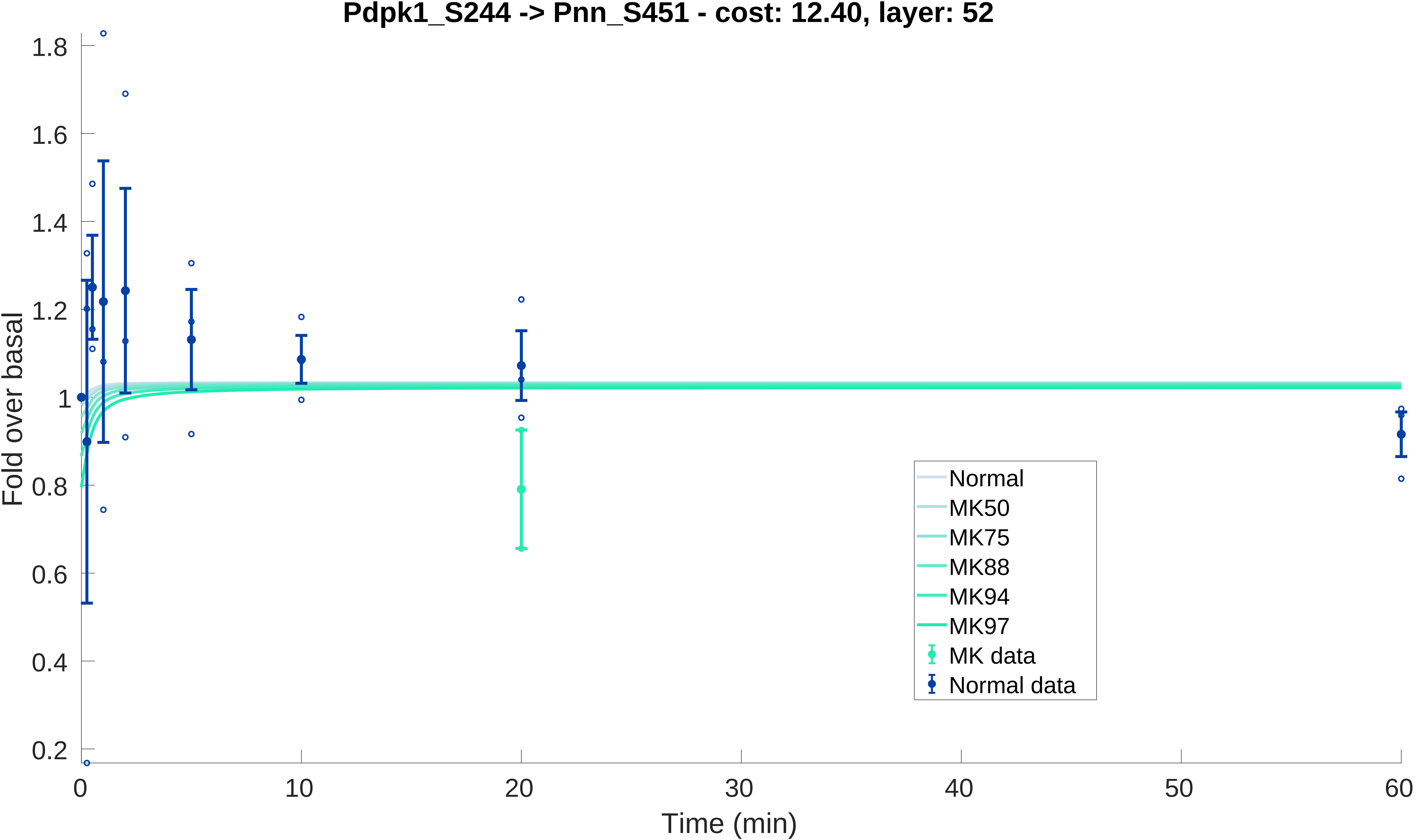
Pdpk1_S244 -> Pnn_S451 - cost: 12.40, layer: 52
Fold over basal
Time (min)
1.8
1.6
1.4
1.2
1
0.8
0.6
0.4
0.2
0
10
20
30
40
50
60
Normal
MK50
MK75
MK88
MK94
MK97
MK data
Normal data

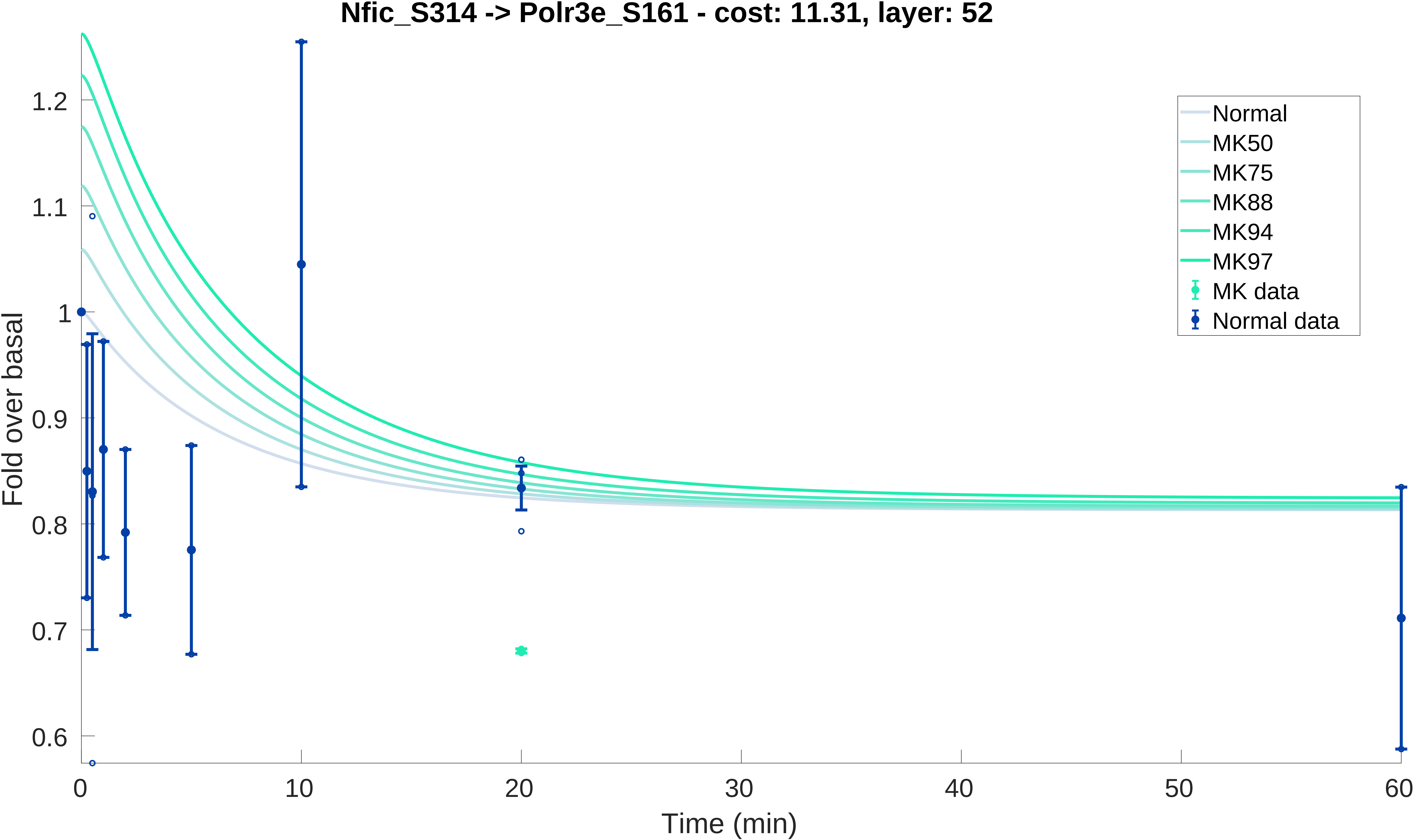

Nfic_S314 -> Polr3e_S161 - cost: 11.31, layer: 52
Fold over basal
Time (min)
0.6
0.7
0.8
0.9
1
1.1
1.2
0
10
20
30
40
50
60
Normal
MK50
MK75
MK88
MK94
MK97
MK data
Normal data

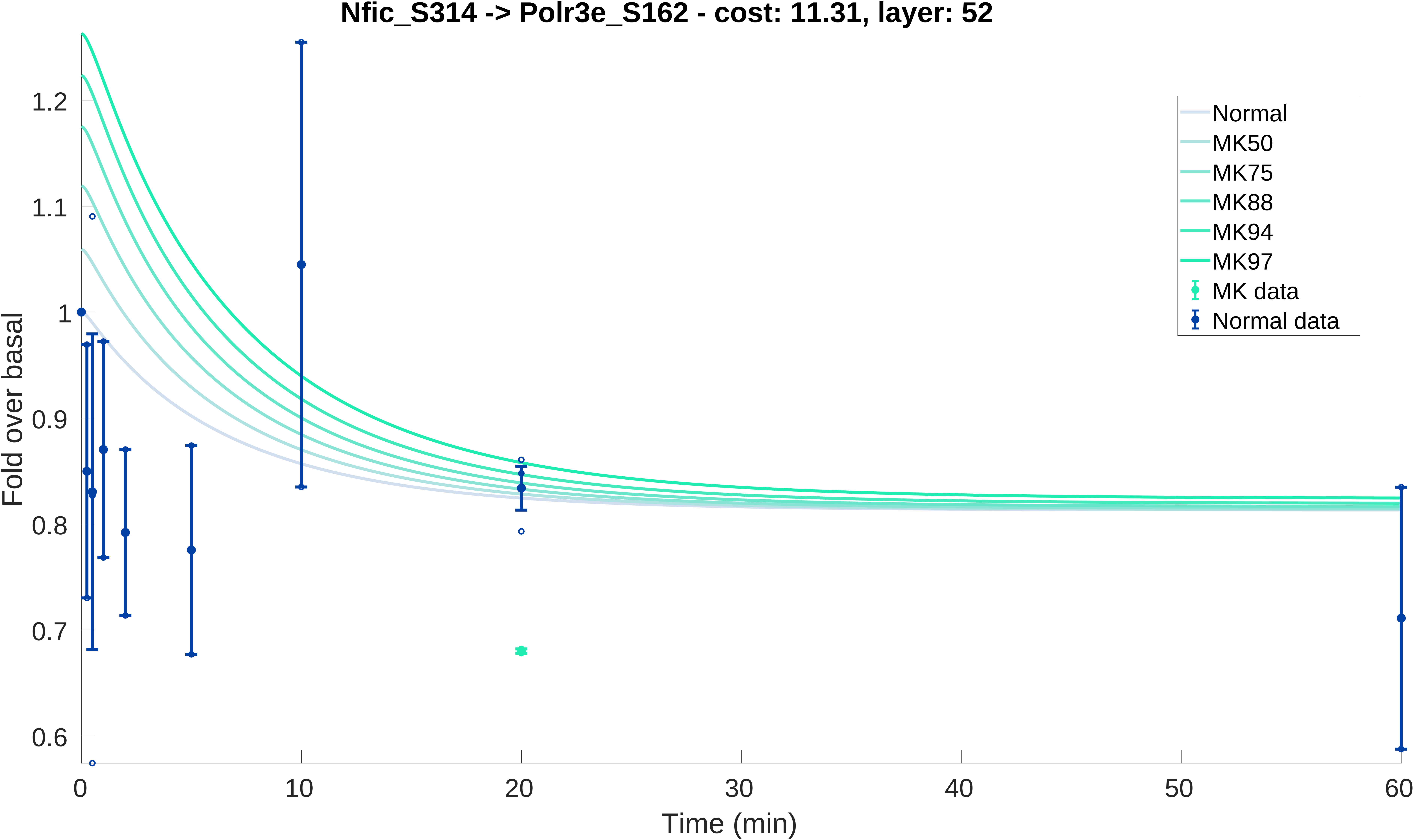

Nfic_S314 -> Polr3e_S162 - cost: 11.31, layer: 52
Fold over basal
Time (min)
0
10
20
30
40
50
60
0.6
0.7
0.8
0.9
1
1.1
1.2
Normal
MK50
MK75
MK88
MK94
MK97
MK data
Normal data

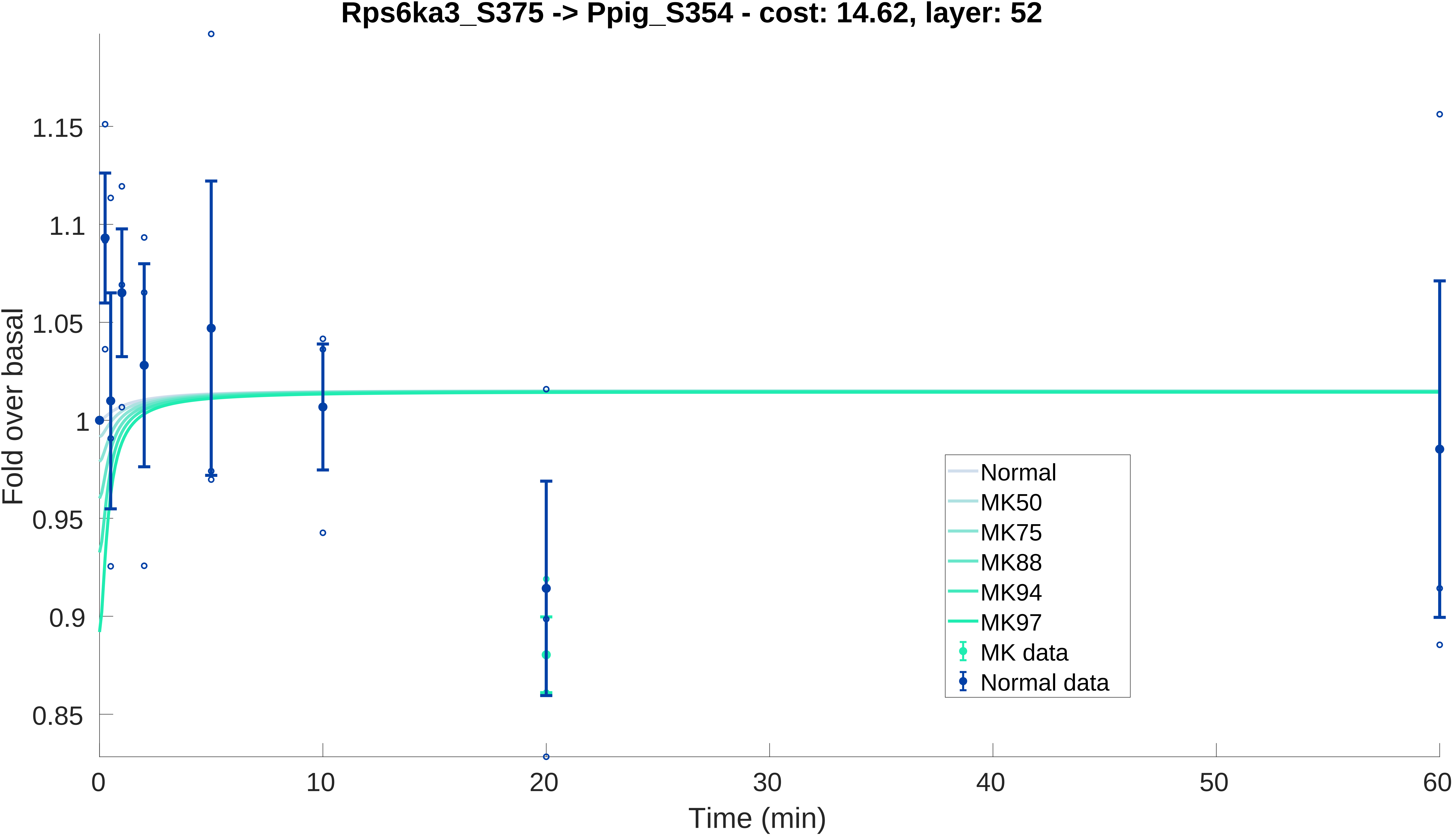
Rps6ka3_S375 -> Ppig_S354 - cost: 14.62, layer: 52
Fold over basal
Time (min)
1.15
1.1
1.05
1
0.95
0.9
0.85
0
10
20
30
40
50
60
Normal
MK50
MK75
MK88
MK94
MK97
MK data
Normal data

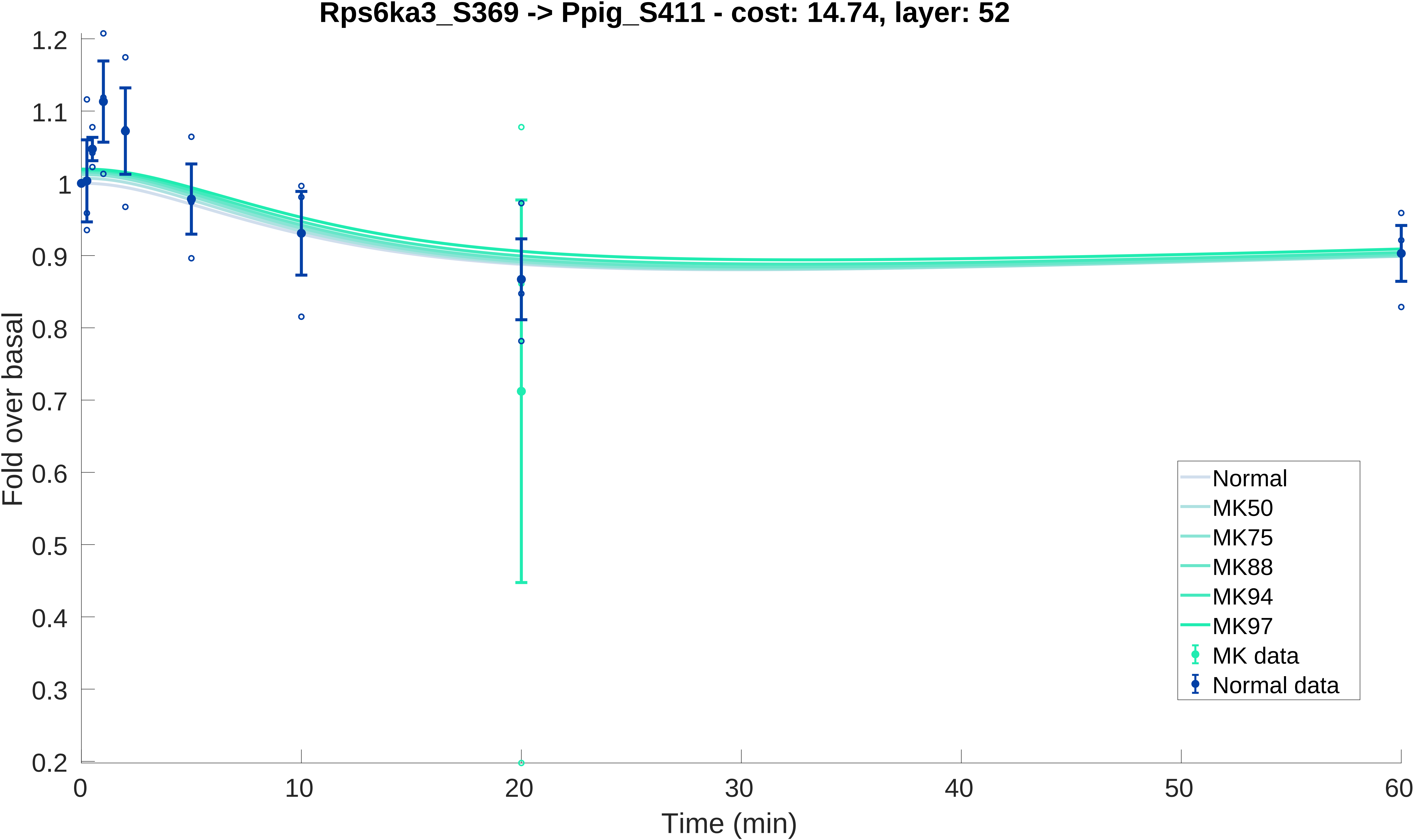
Rps6ka3_S369 -> Ppig_S411 - cost: 14.74, layer: 52
Fold over basal
Time (min)
0
10
20
30
40
50
60
0.2
0.3
0.4
0.5
0.6
0.7
0.8
0.9
1
1.1
1.2
Normal
MK50
MK75
MK88
MK94
MK97
MK data
Normal data

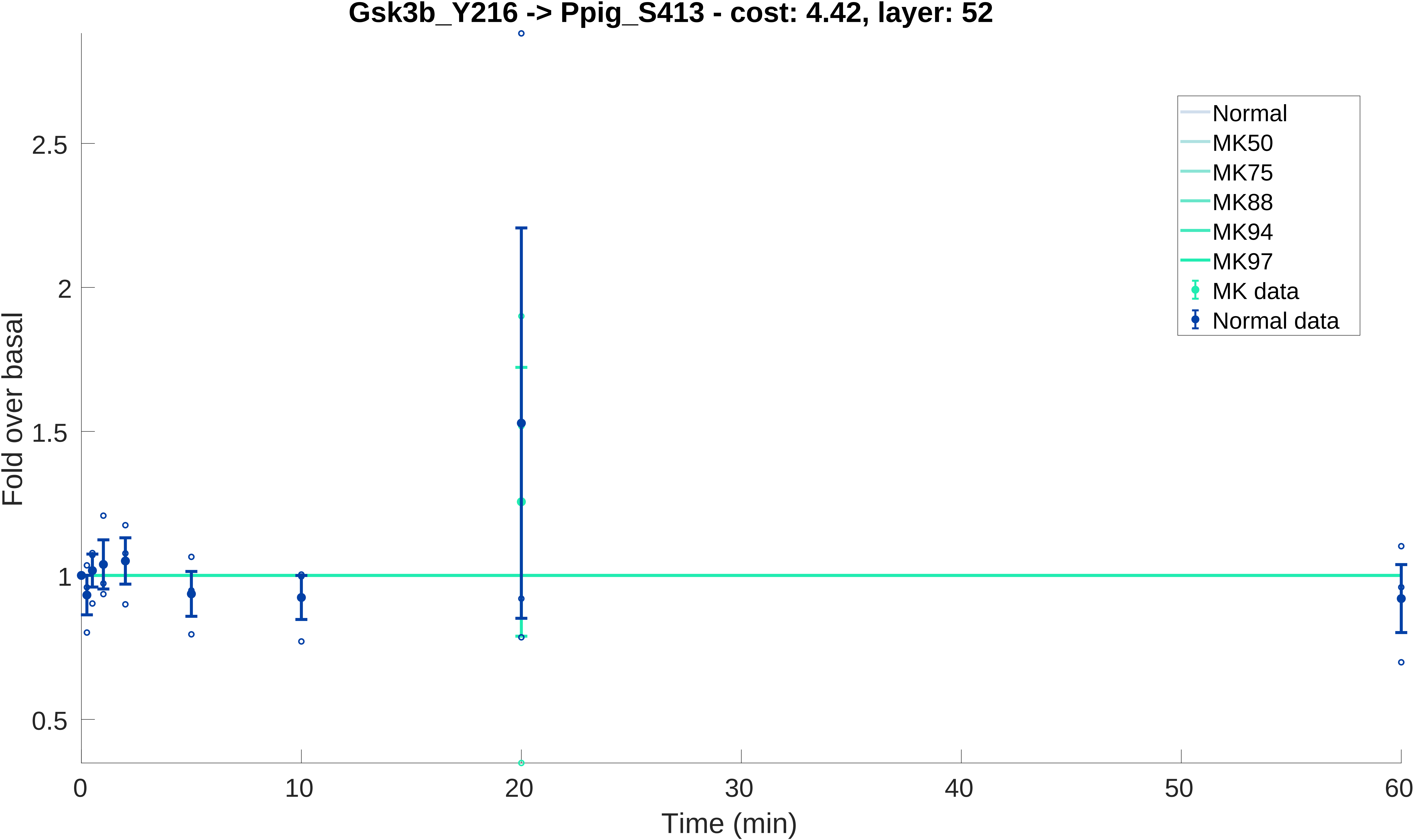

Gsk3b_Y216 -> Ppig_S413 - cost: 4.42, layer: 52
Fold over basal
Time (min)
0
10
20
30
40
50
60
0.5
1
1.5
2
2.5
Normal
MK50
MK75
MK88
MK94
MK97
MK data
Normal data

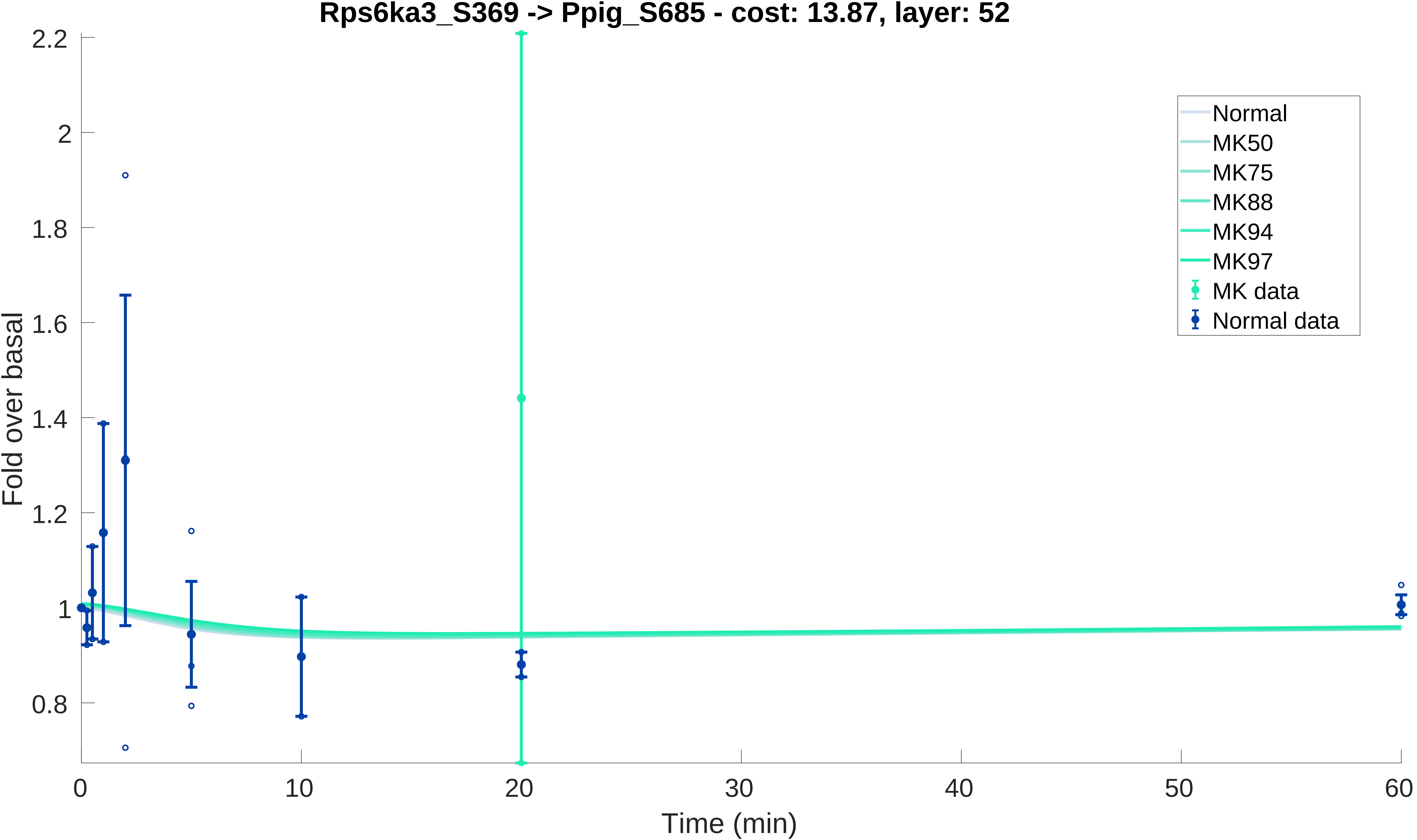

Rps6ka3_S369 -> Ppig_S685 - cost: 13.87, layer: 52
Fold over basal
Time (min)
0.8
1
1.2
1.4
1.6
1.8
2
2.2
0
10
20
30
40
50
60
Normal
MK50
MK75
MK88
MK94
MK97
MK data
Normal data

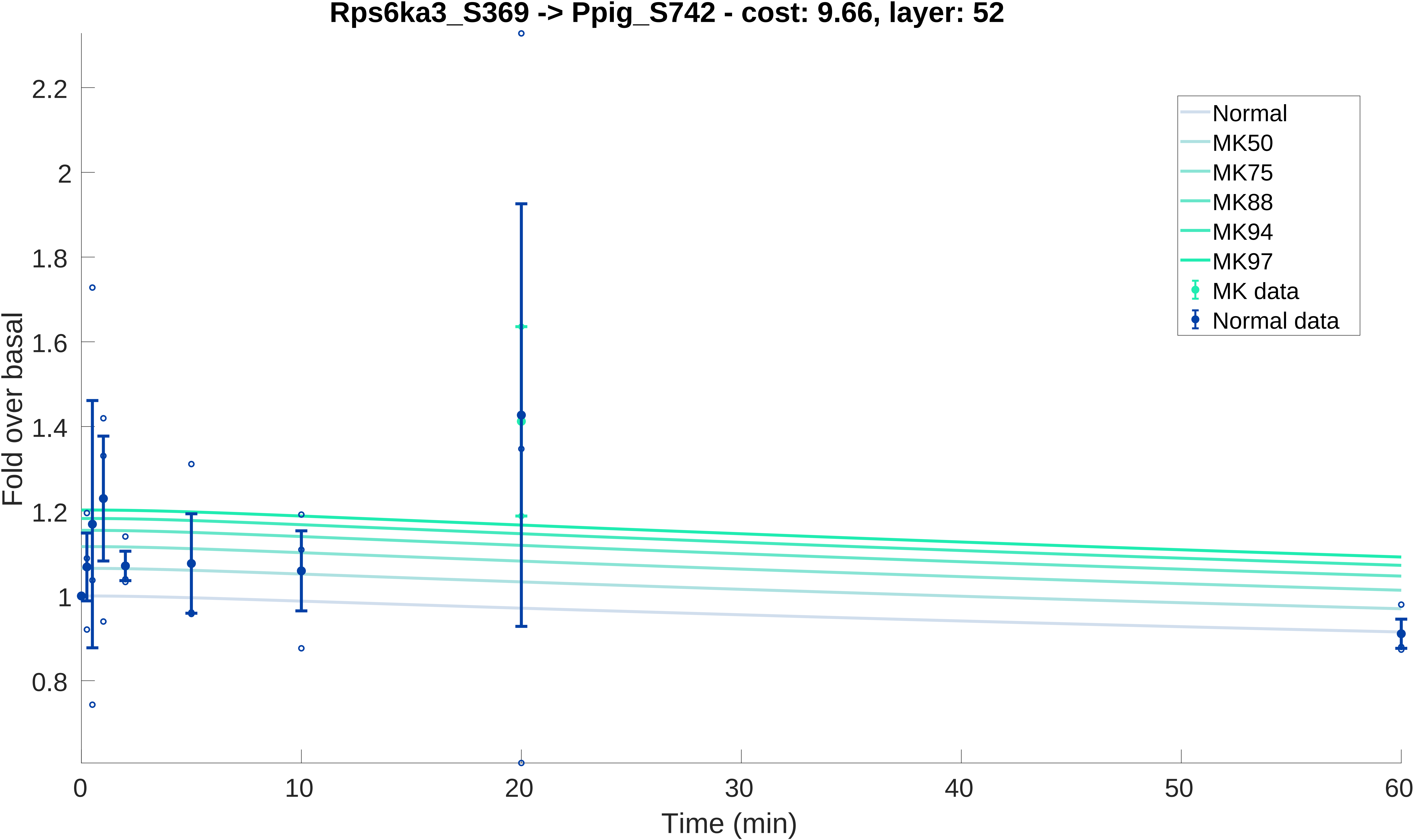

Rps6ka3_S369 -> Ppig_S742 - cost: 9.66, layer: 52
Fold over basal
Time (min)
0.8
1
1.2
1.4
1.6
1.8
2
2.2
0
10
20
30
40
50
60
Normal
MK50
MK75
MK88
MK94
MK97
MK data
Normal data

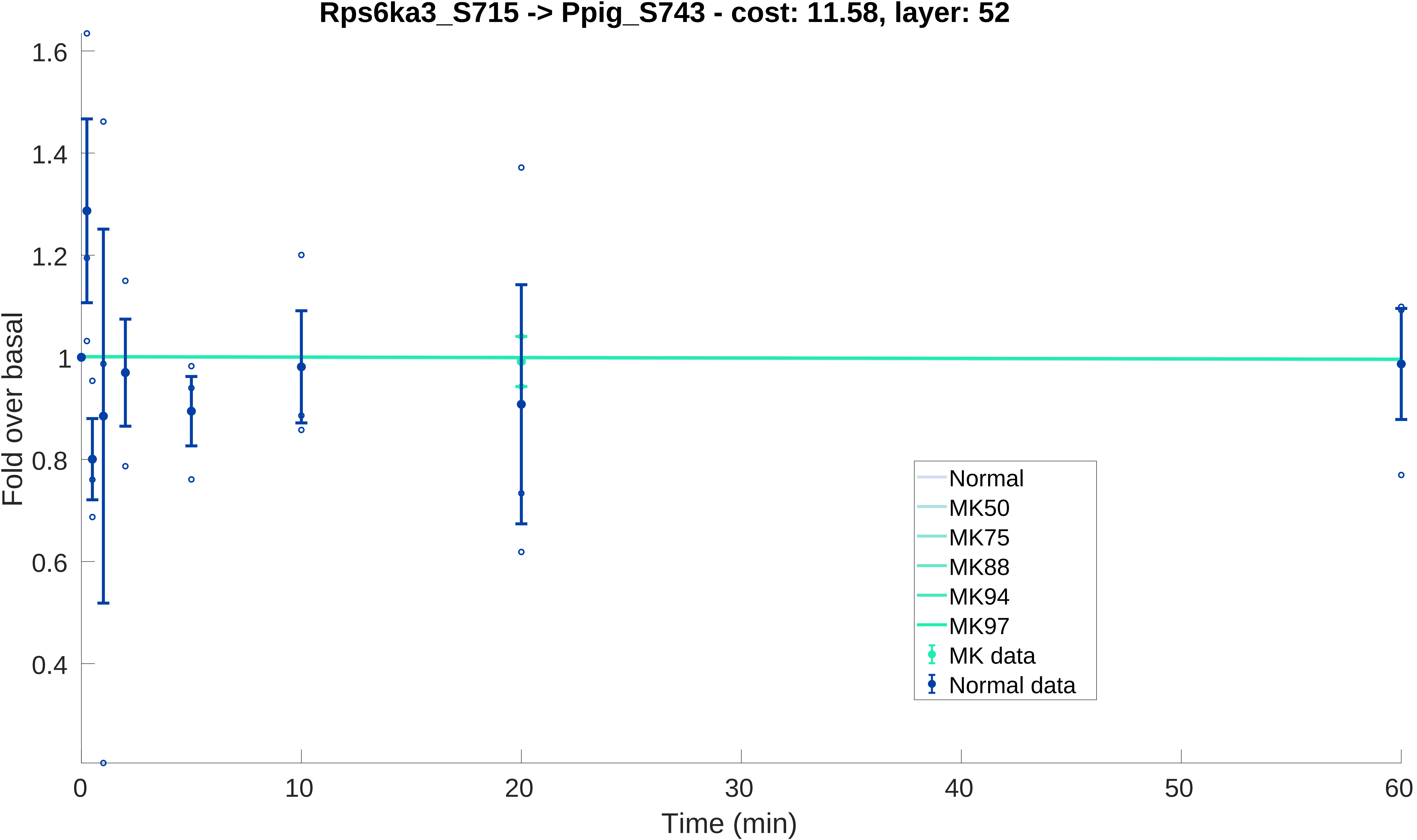

Rps6ka3_S715 -> Ppig_S743 - cost: 11.58, layer: 52
Fold over basal
Time (min)
1.6
1.4
1.2
1
0.8
0.6
0.4
0
10
20
30
40
50
60
Normal
MK50
MK75
MK88
MK94
MK97
MK data
Normal data

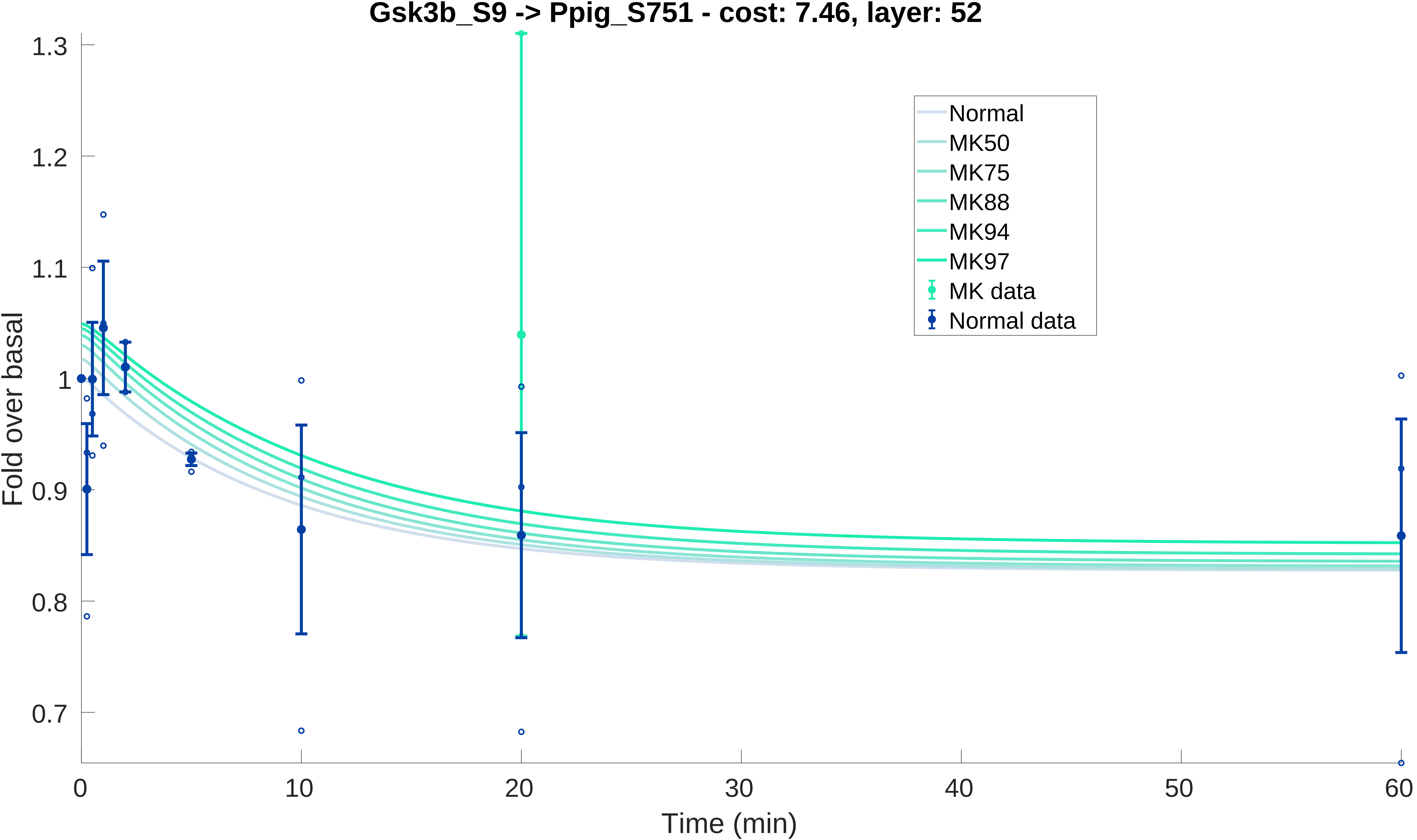

Gsk3b_S9 -> Ppig_S751 - cost: 7.46, layer: 52
Fold over basal
Time (min)
Normal
MK50
MK75
MK88
MK94
MK97
MK data
Normal data
1.3
1.2
1.1
1
0.9
0.8
0.7
0
10
20
30
40
50
60

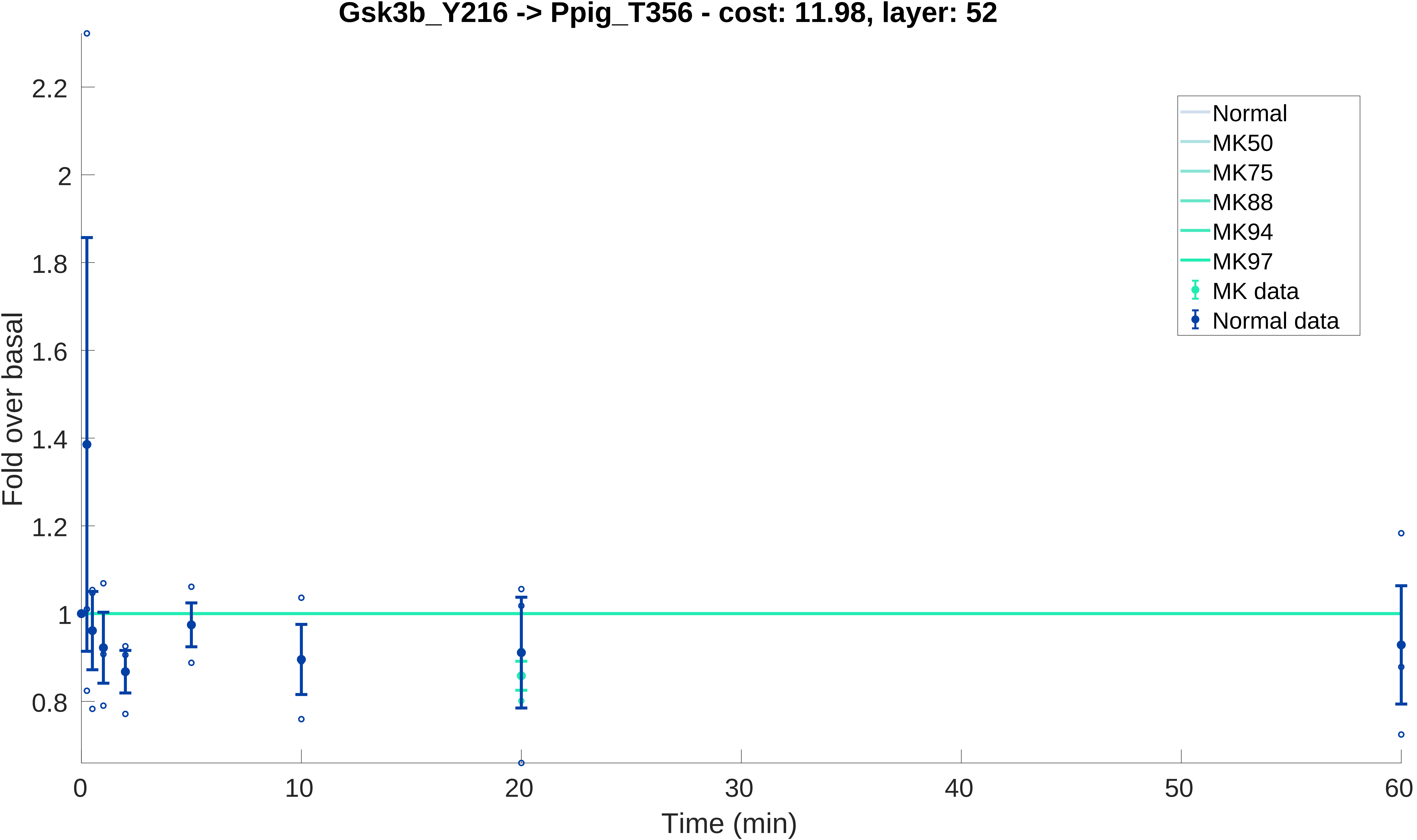

Gsk3b_Y216 -> Ppig_T356 - cost: 11.98, layer: 52
Fold over basal
Time (min)
2.2
2
1.8
1.6
1.4
1.2
1
0.8
0
10
20
30
40
50
60
Normal
MK50
MK75
MK88
MK94
MK97
MK data
Normal data

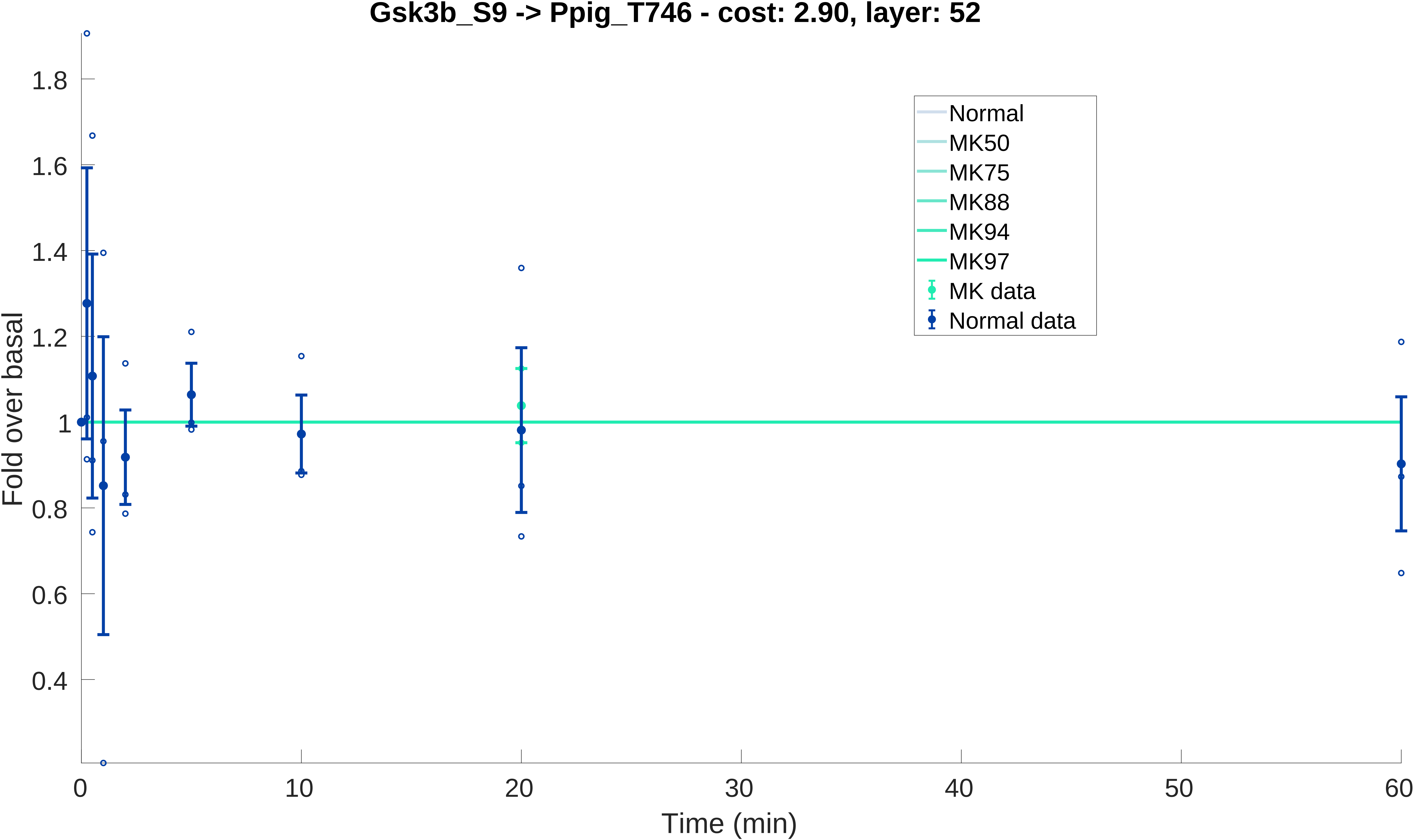
Gsk3b_S9 -> Ppig_T746 - cost: 2.90, layer: 52
Fold over basal
Time (min)
1.8
1.6
1.4
1.2
1
0.8
0.6
0.4
0
10
20
30
40
50
60
Normal
MK50
MK75
MK88
MK94
MK97
MK data
Normal data

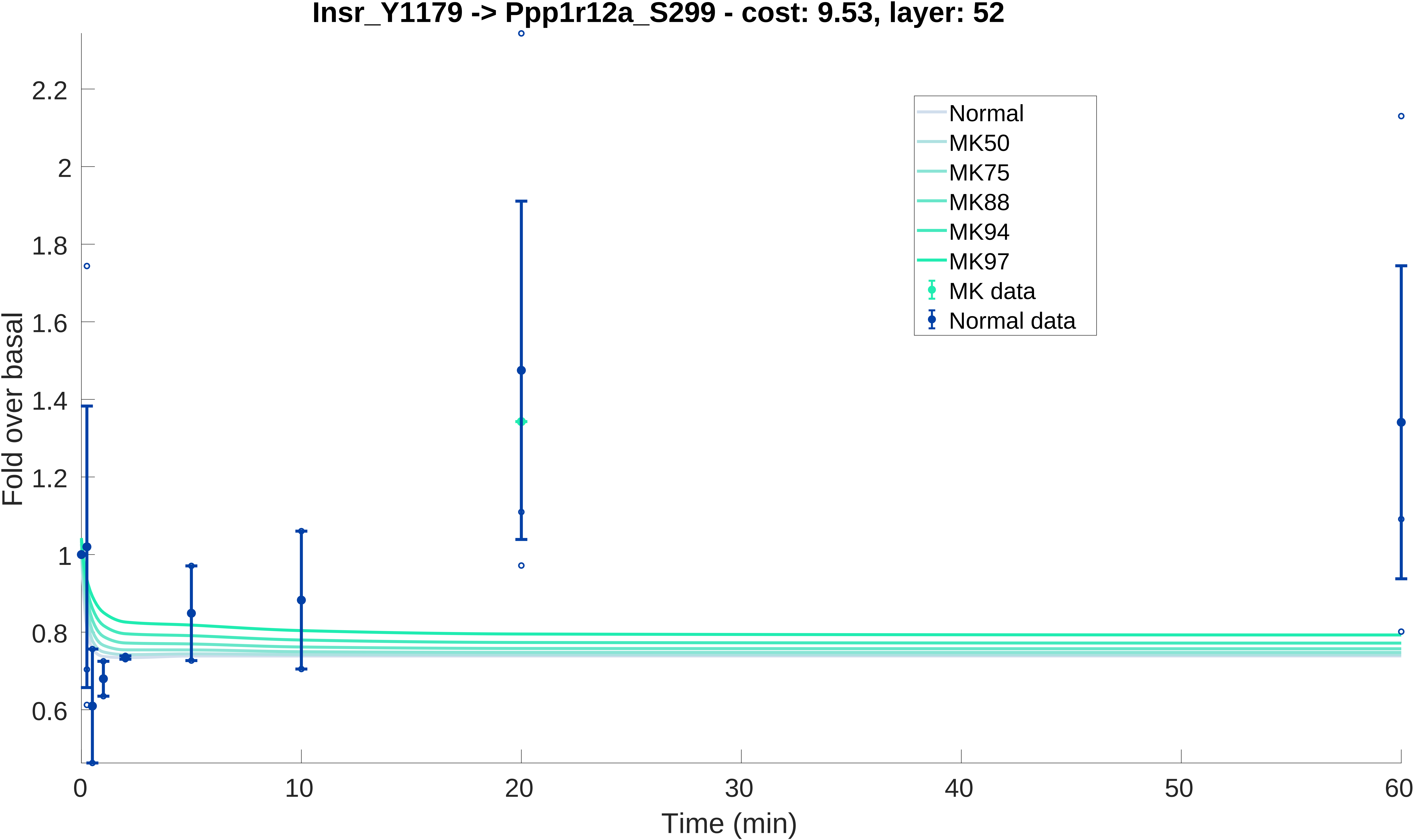

Insr_Y1179 -> Ppp1r12a_S299 - cost: 9.53, layer: 52
Fold over basal
Time (min)
Normal
MK50
MK75
MK88
MK94
MK97
MK data
Normal data
0.6
0.8
1
1.2
1.4
1.6
1.8
2
2.2
0
10
20
30
40
50
60

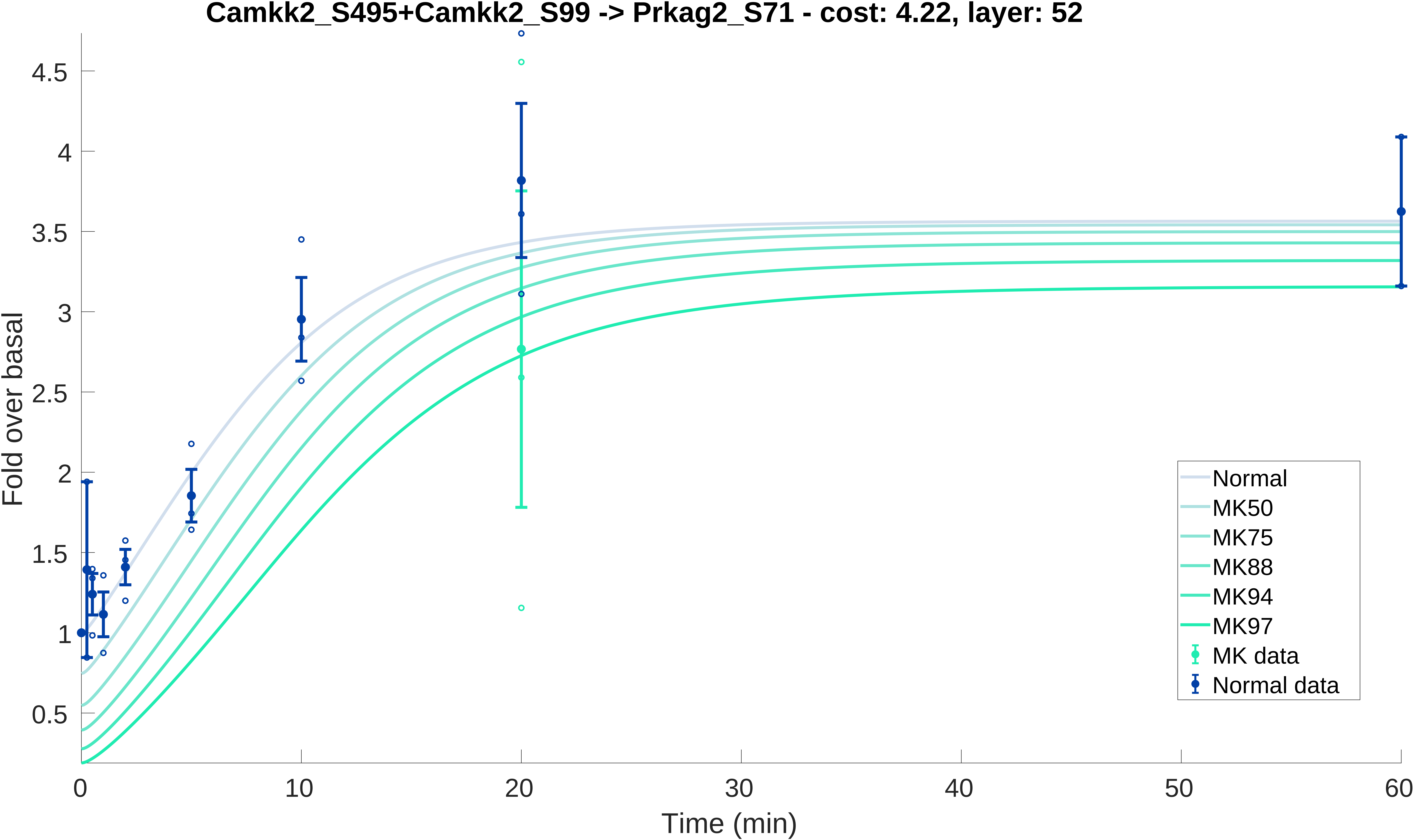

Camkk2_S495+Camkk2_S99 -> Prkag2_S71 - cost: 4.22, layer: 52
Fold over basal
Time (min)
0.5
1
1.5
2
2.5
3
3.5
4
4.5
0
10
20
30
40
50
60
Normal
MK50
MK75
MK88
MK94
MK97
MK data
Normal data

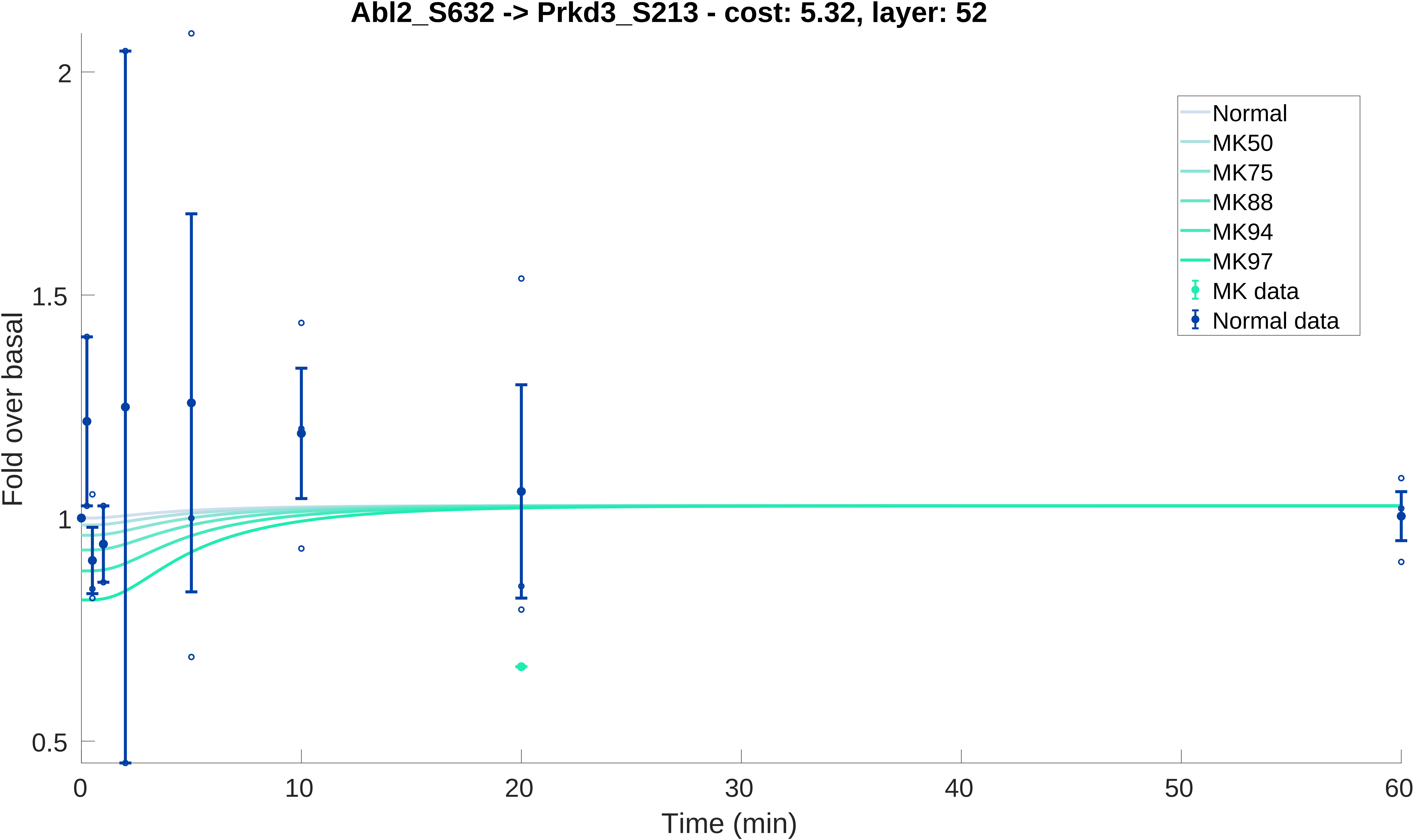

Abl2_S632 -> Prkd3_S213 - cost: 5.32, layer: 52
Fold over basal
Time (min)
0.5
1
1.5
2
0
10
20
30
40
50
60
Normal
MK50
MK75
MK88
MK94
MK97
MK data
Normal data

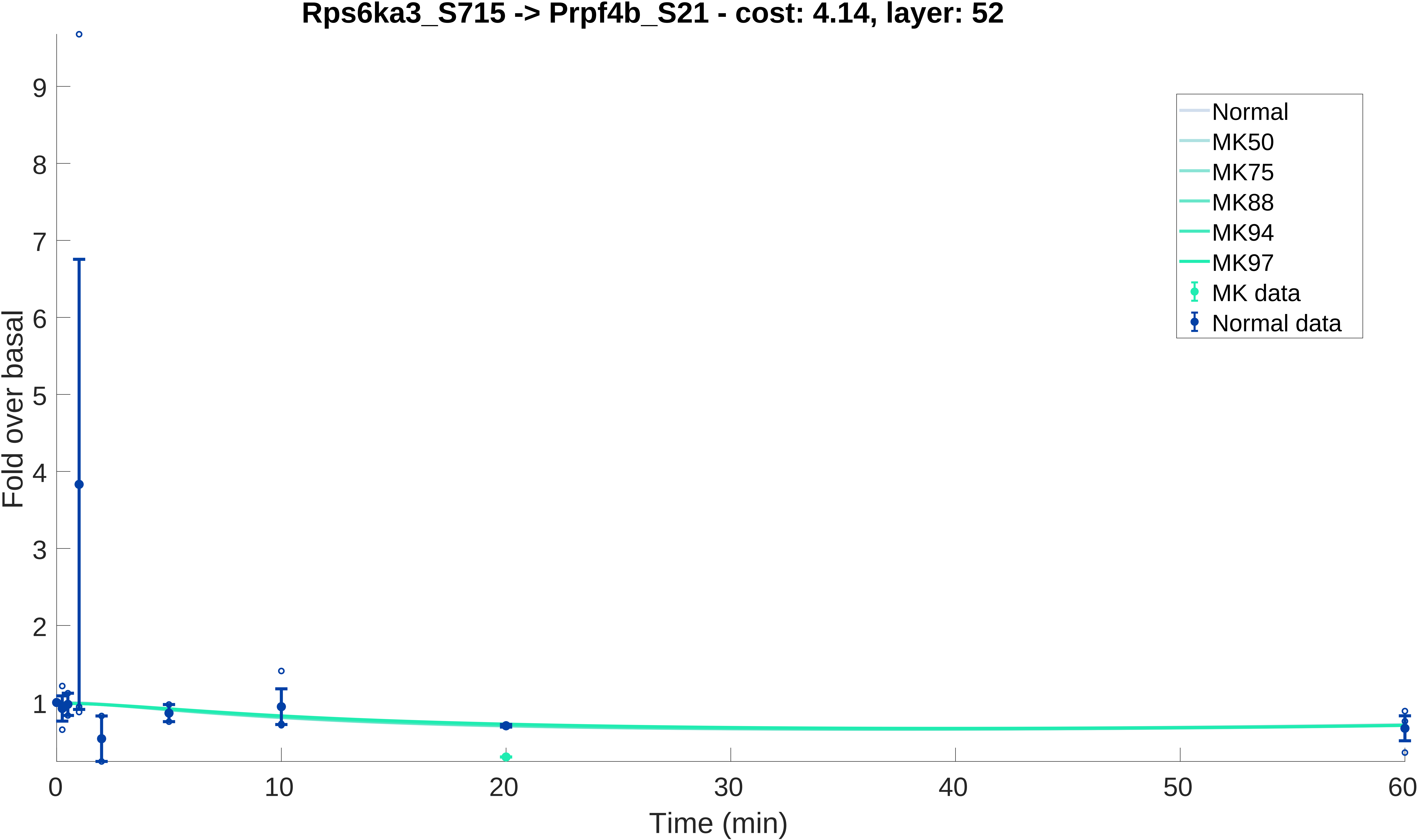

Rps6ka3_S715 -> Prpf4b_S21 - cost: 4.14, layer: 52
Fold over basal
Time (min)
0
10
20
30
40
50
60
1
2
3
4
5
6
7
8
9
Normal
MK50
MK75
MK88
MK94
MK97
MK data
Normal data

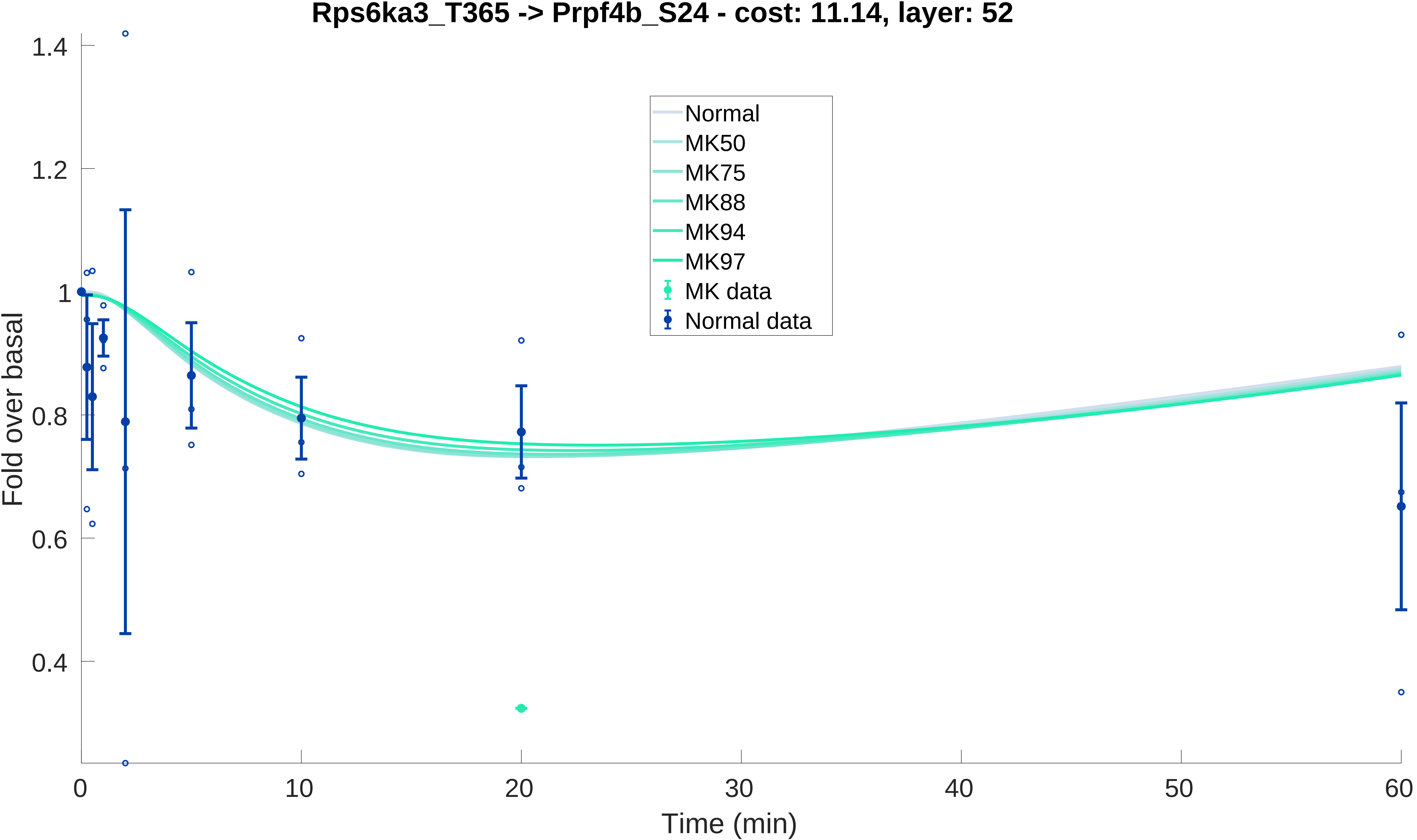

Rps6ka3_T365 -> Prpf4b_S24 - cost: 11.14, layer: 52
Fold over basal
1.4
1.2
1
0.8
0.6
0.4
Normal
MK50
MK75
MK88
MK94
MK97
MK data
Normal data
0
10
20
30
40
50
60
Time (min)

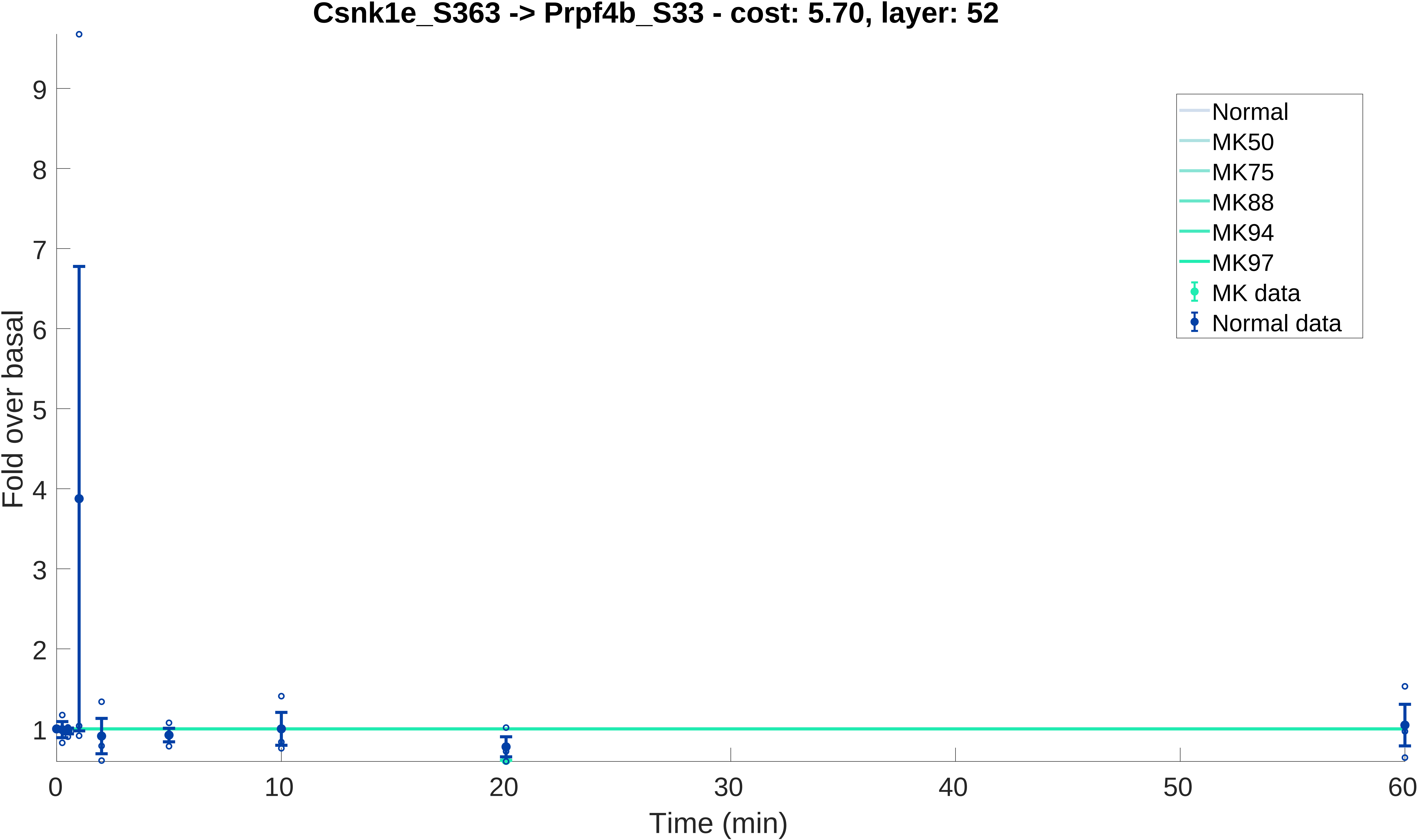
Csnk1e_S363 -> Prpf4b_S33 - cost: 5.70, layer: 52
Fold over basal
Time (min)
0
10
20
30
40
50
60
1
2
3
4
5
6
7
8
9
Normal
MK50
MK75
MK88
MK94
MK97
MK data
Normal data

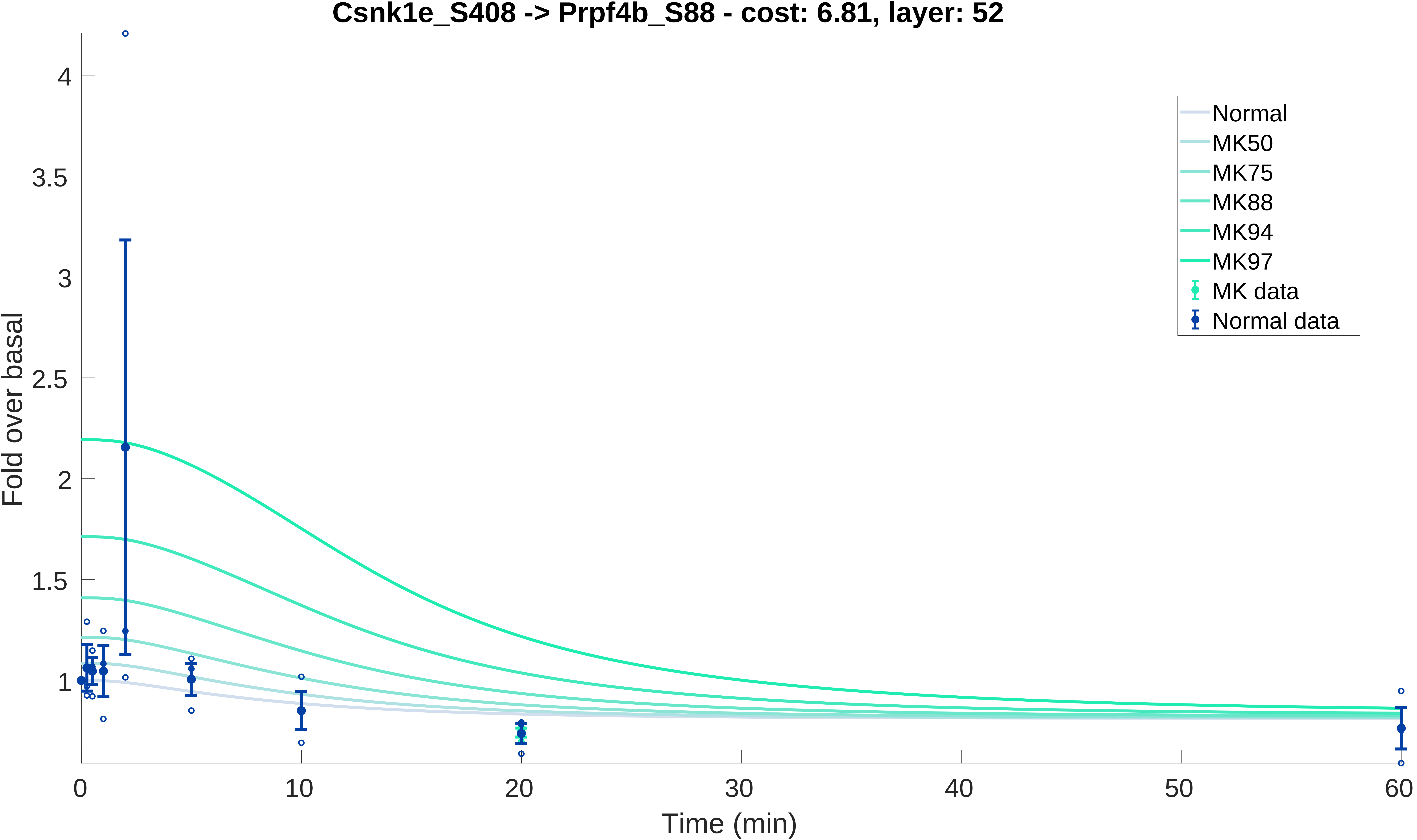

Csnk1e_S408 -> Prpf4b_S88 - cost: 6.81, layer: 52
Fold over basal
4
3.5
3
2.5
2
1.5
1
0
10
20
30
40
50
60
Time (min)
Normal
MK50
MK75
MK88
MK94
MK97
MK data
Normal data

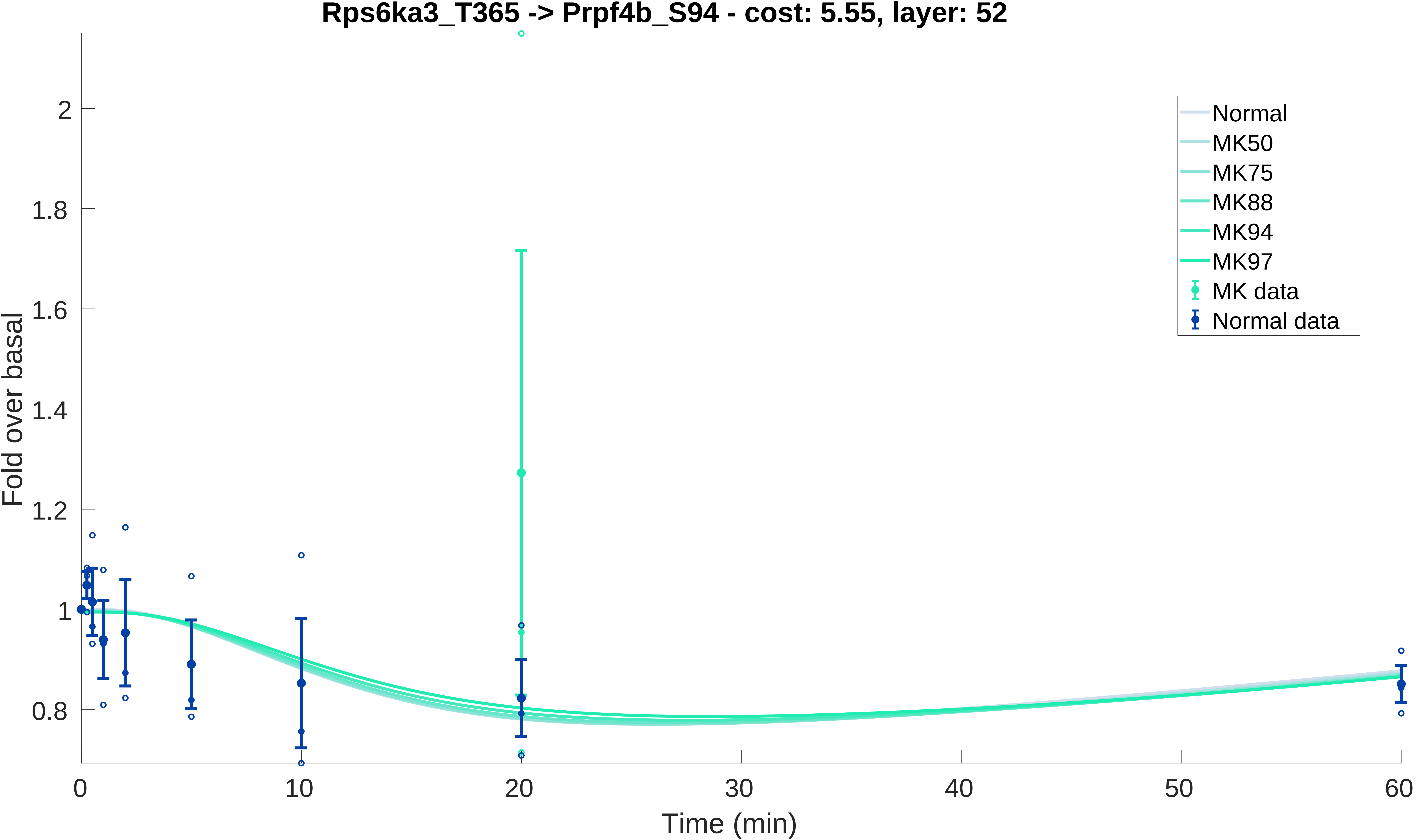

Rps6ka3_T365 -> Prpf4b_S94 - cost: 5.55, layer: 52
Fold over basal
Time (min)
0.8
1
1.2
1.4
1.6
1.8
2
0
10
20
30
40
50
60
Normal
MK50
MK75
MK88
MK94
MK97
MK data
Normal data

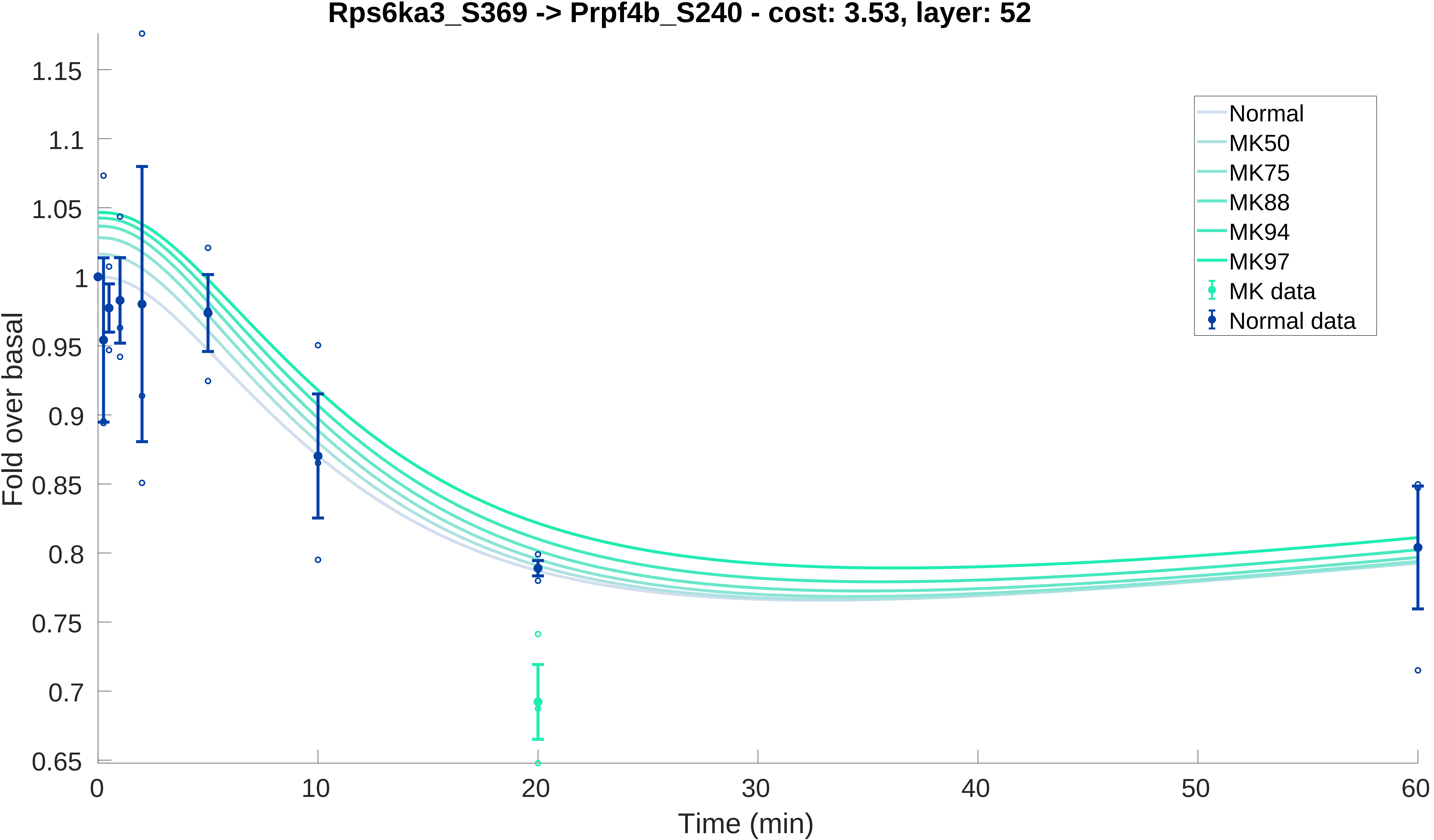

Rps6ka3_S369 -> Prpf4b_S240 - cost: 3.53, layer: 52
Fold over basal
1.15
1.1
1.05
1
0.95
0.9
0.85
0.8
0.75
0.7
0.65
0
10
20
30
40
50
60
Time (min)
Normal
MK50
MK75
MK88
MK94
MK97
MK data
Normal data

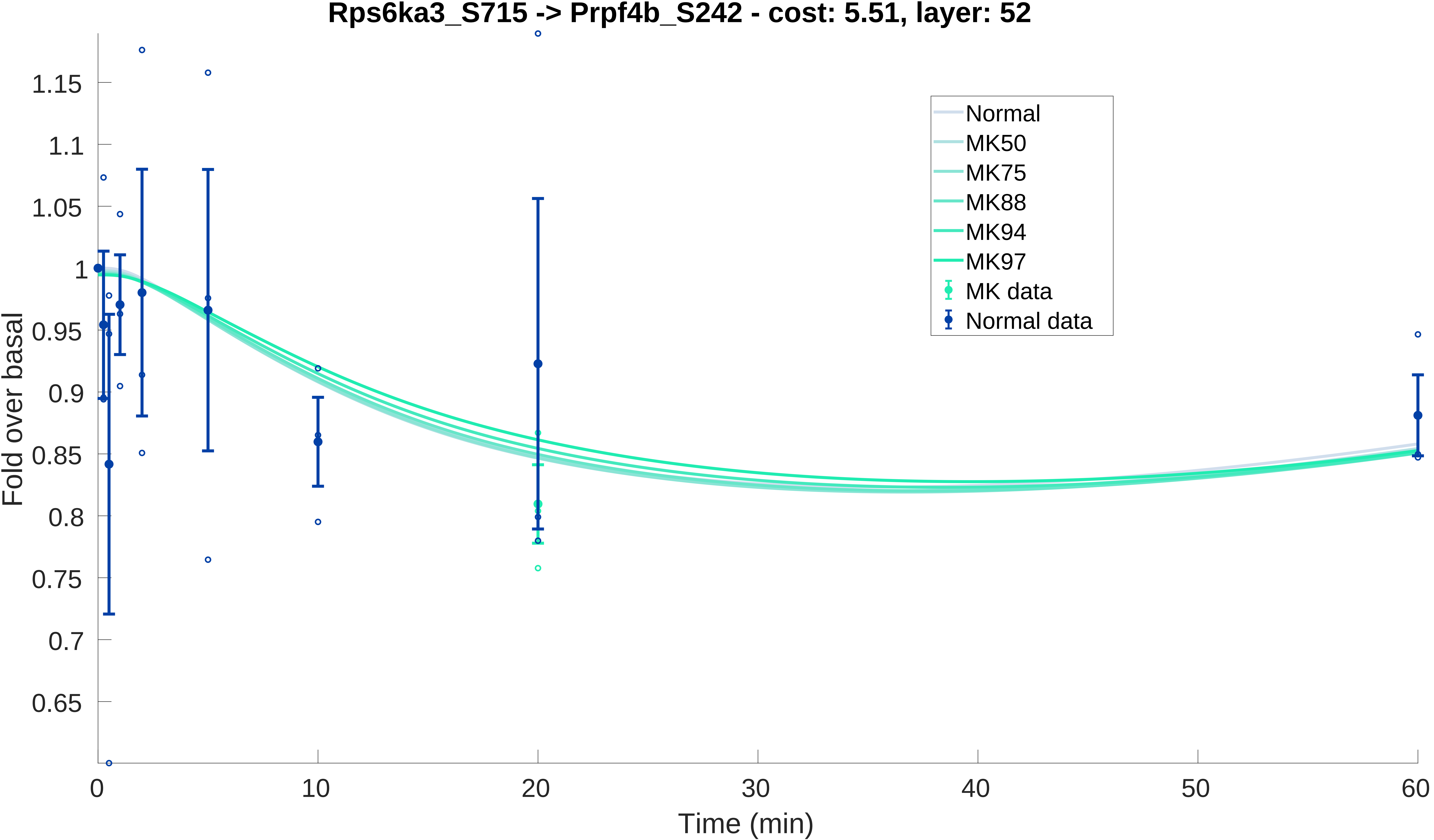
Rps6ka3_S715 -> Prpf4b_S242 - cost: 5.51, layer: 52
Fold over basal
Time (min)
Normal
MK50
MK75
MK88
MK94
MK97
MK data
Normal data
1.15
1.1
1.05
1
0.95
0.9
0.85
0.8
0.75
0.7
0.65
0
10
20
30
40
50
60

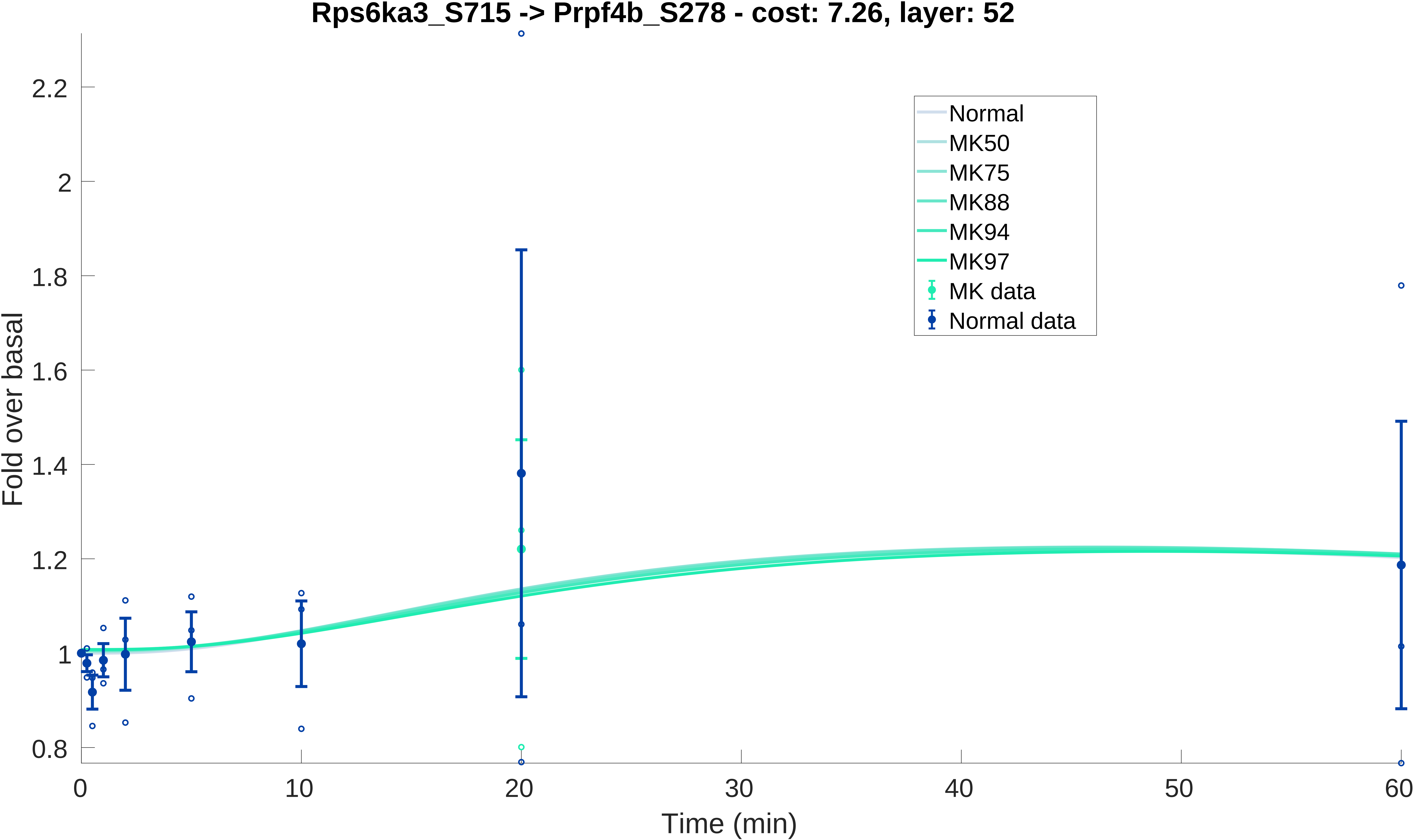
Rps6ka3_S715 -> Prpf4b_S278 - cost: 7.26, layer: 52
Fold over basal
Time (min)
Normal
MK50
MK75
MK88
MK94
MK97
MK data
Normal data
0.8
1
1.2
1.4
1.6
1.8
2
2.2
0
10
20
30
40
50
60

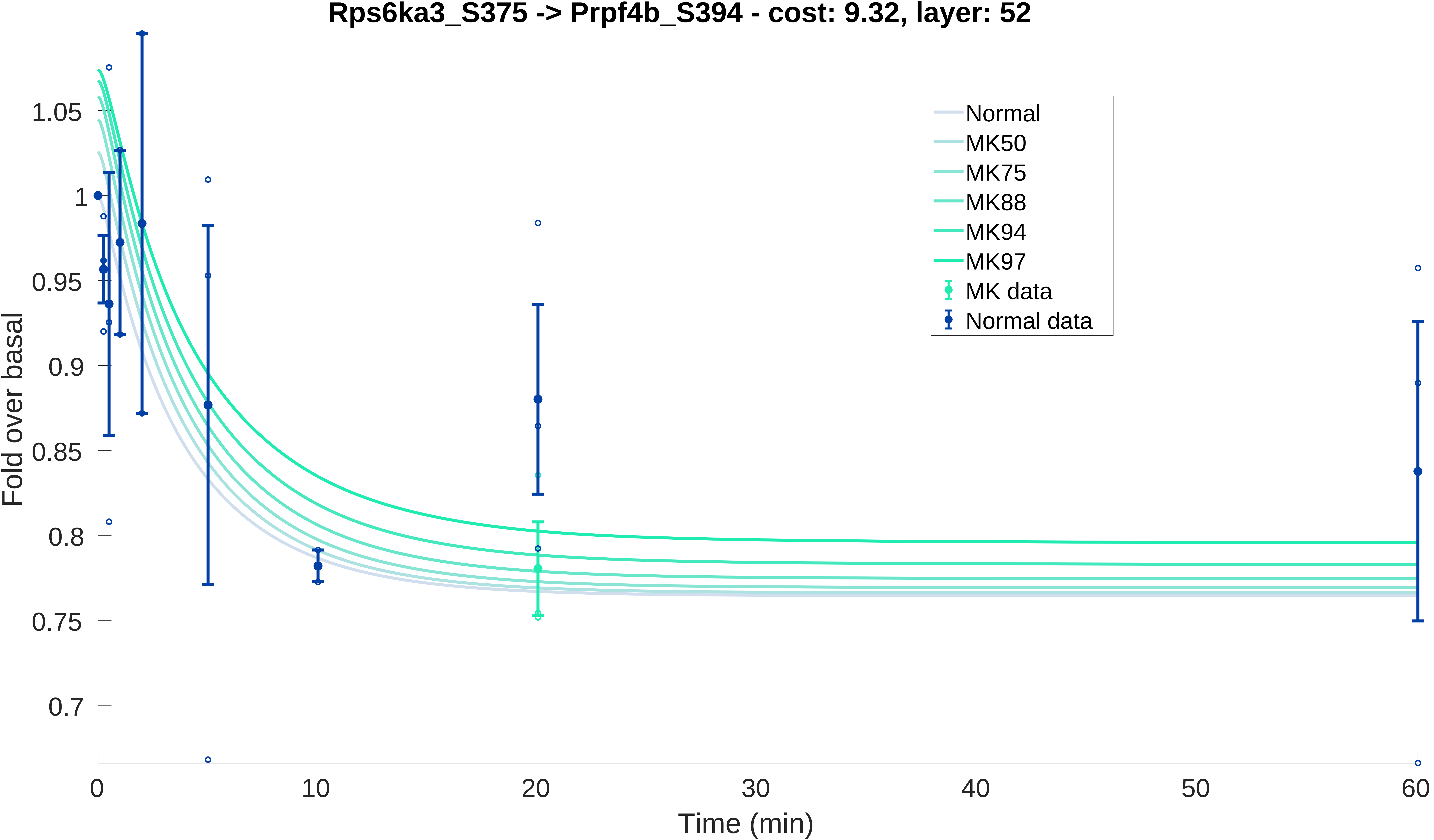

Rps6ka3_S375 -> Prpf4b_S394 - cost: 9.32, layer: 52
Fold over basal
Time (min)
Normal
MK50
MK75
MK88
MK94
MK97
MK data
Normal data
1.05
1
0.95
0.9
0.85
0.8
0.75
0.7
0
10
20
30
40
50
60

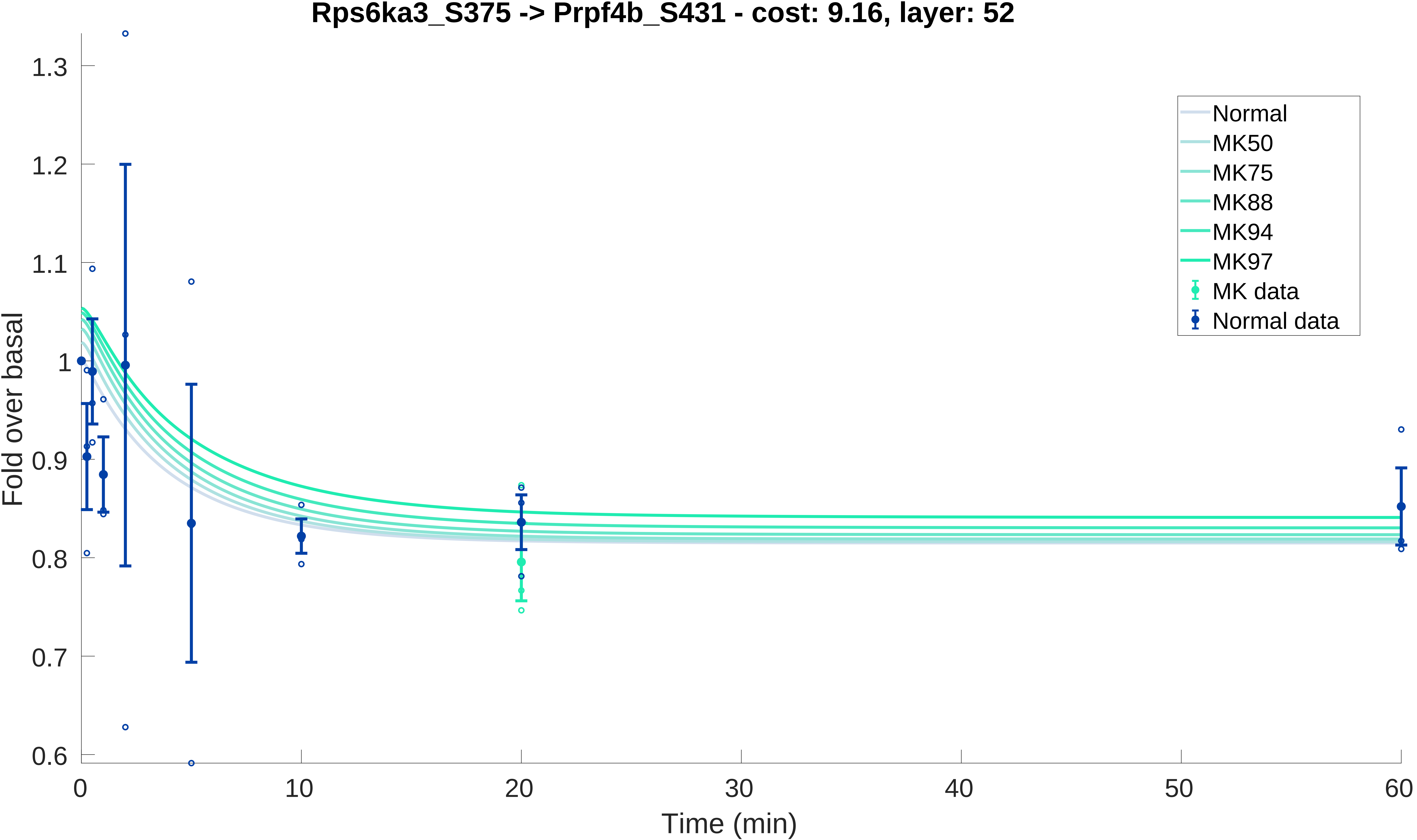
Rps6ka3_S375 -> Prpf4b_S431 - cost: 9.16, layer: 52
Fold over basal
1.3
1.2
1.1
1
0.9
0.8
0.7
0.6
0
10
20
30
40
50
60
Time (min)
Normal
MK50
MK75
MK88
MK94
MK97
MK data
Normal data

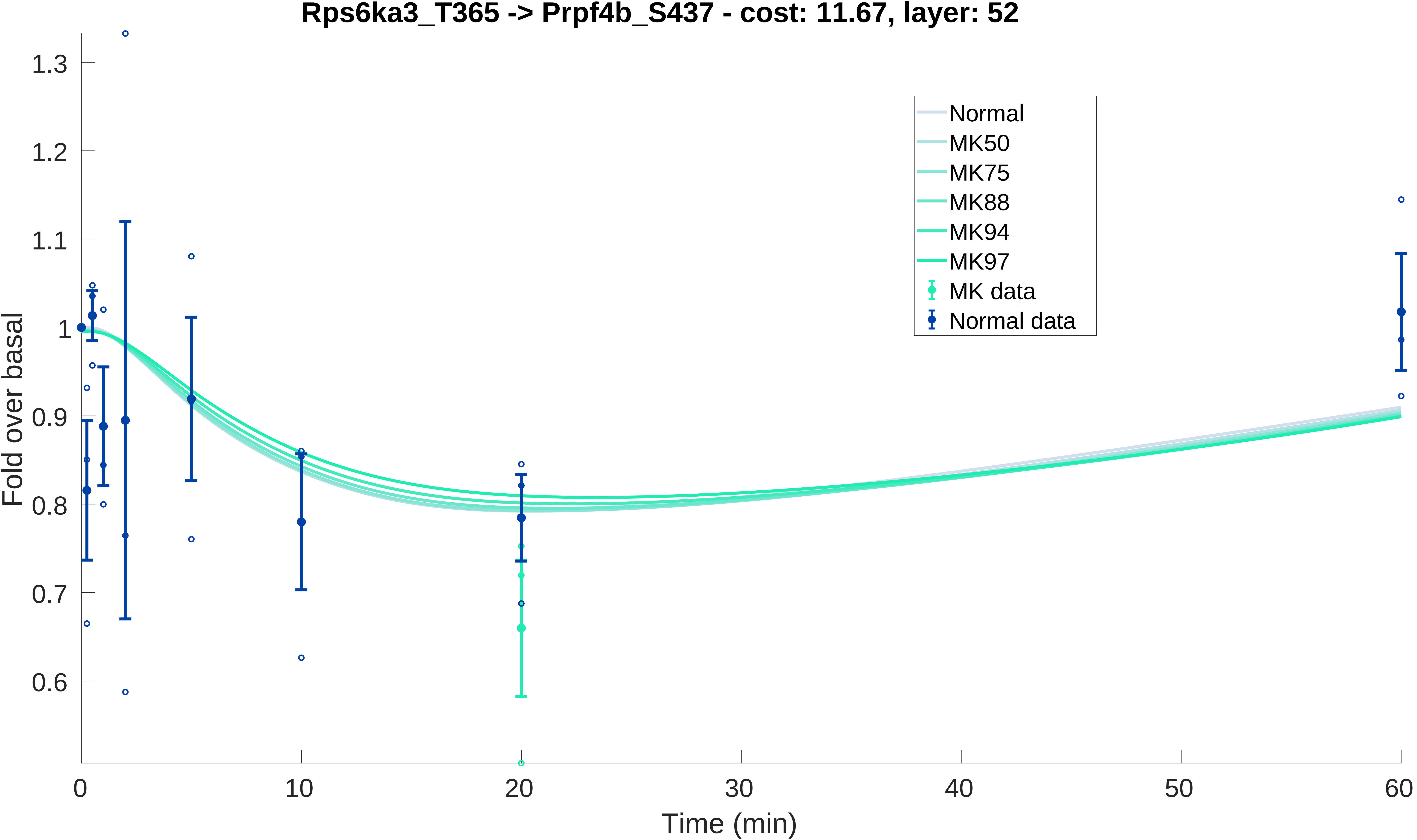

Rps6ka3_T365 -> Prpf4b_S437 - cost: 11.67, layer: 52
Fold over basal
Time (min)
1.3
1.2
1.1
1
0.9
0.8
0.7
0.6
0
10
20
30
40
50
60
Normal
MK50
MK75
MK88
MK94
MK97
MK data
Normal data

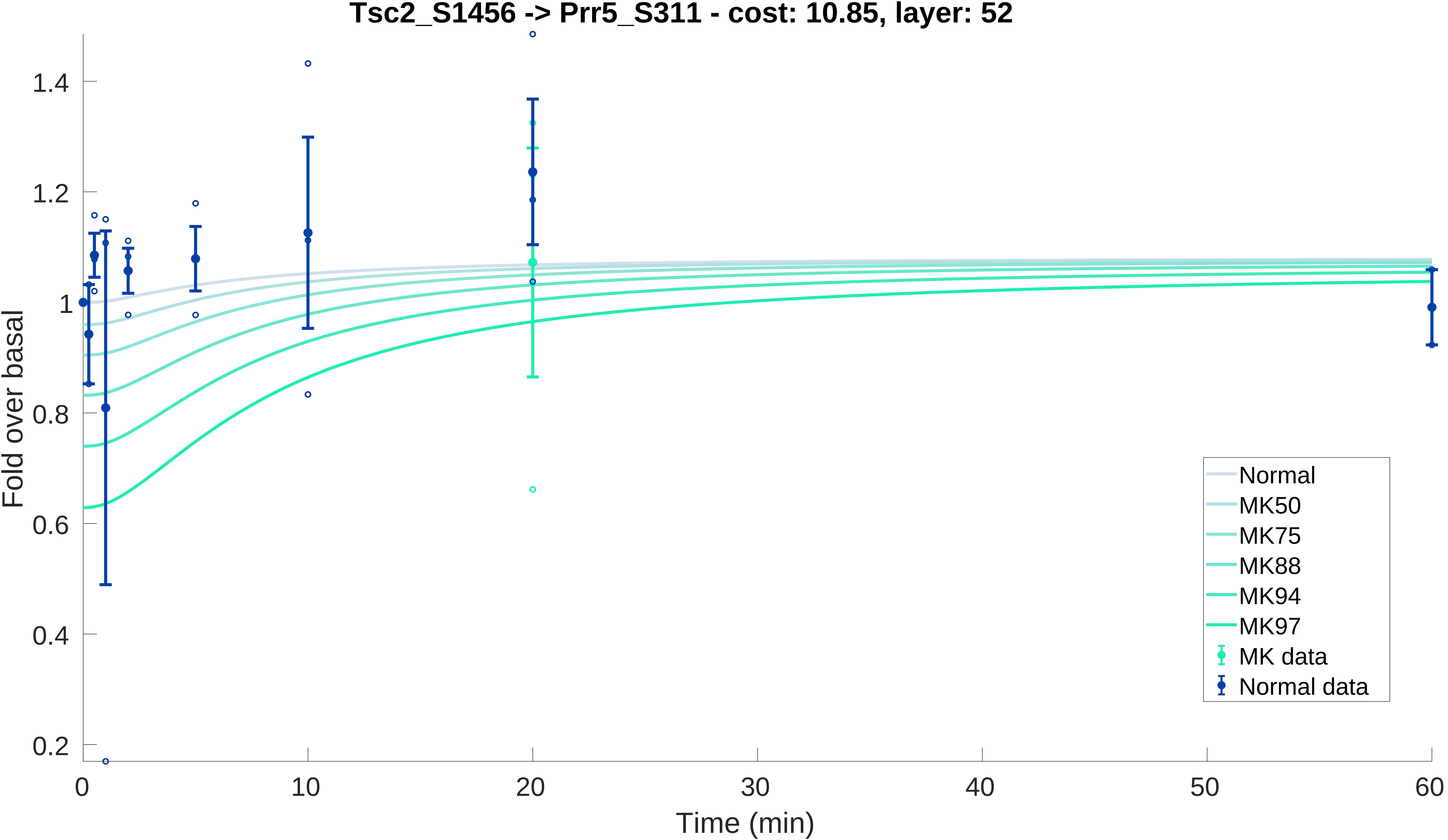

Tsc2_S1456 -> Prr5_S311 - cost: 10.85, layer: 52
Fold over basal
Time (min)
0.2
0.4
0.6
0.8
1
1.2
1.4
0
10
20
30
40
50
60
Normal
MK50
MK75
MK88
MK94
MK97
MK data
Normal data

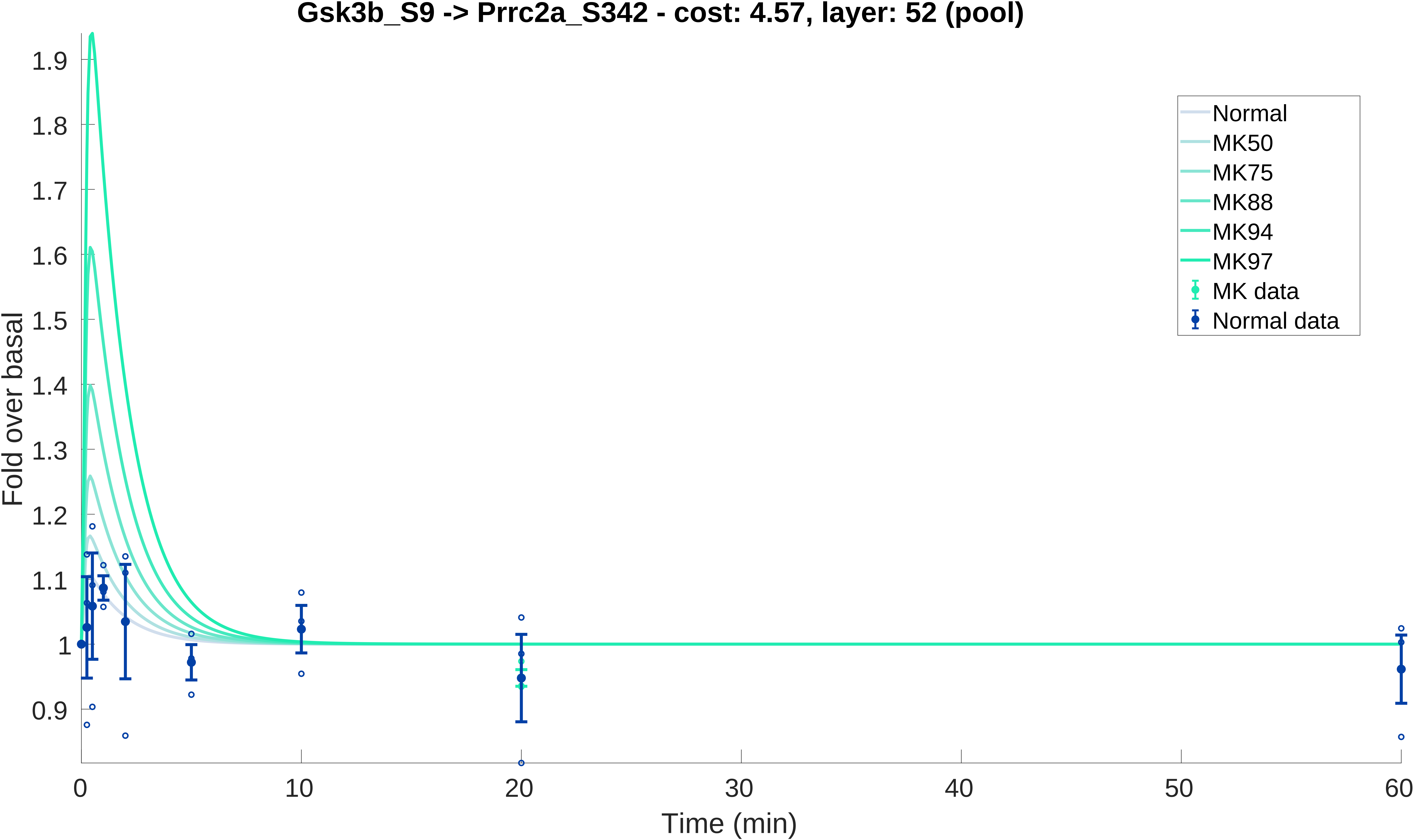

Gsk3b_S9 -> Prrc2a_S342 - cost: 4.57, layer: 52 (pool)
Fold over basal
1.9
1.8
1.7
1.6
1.5
1.4
1.3
1.2
1.1
1
0.9
0
10
20
30
40
50
60
Time (min)
Normal
MK50
MK75
MK88
MK94
MK97
MK data
Normal data

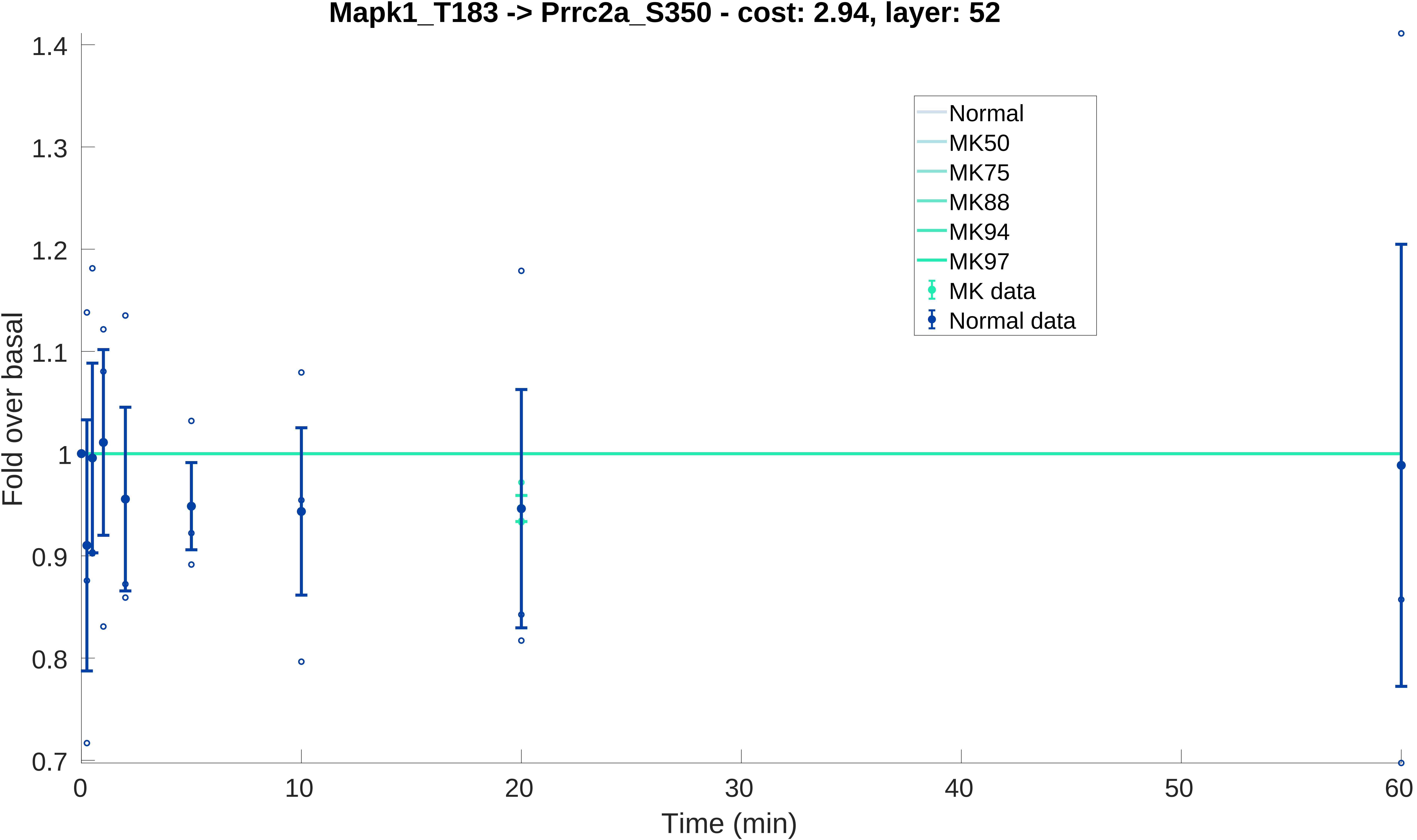

Mapk1_T183 -> Prrc2a_S350 - cost: 2.94, layer: 52
Fold over basal
Time (min)
0.7
0.8
0.9
1
1.1
1.2
1.3
1.4
0
10
20
30
40
50
60
Normal
MK50
MK75
MK88
MK94
MK97
MK data
Normal data

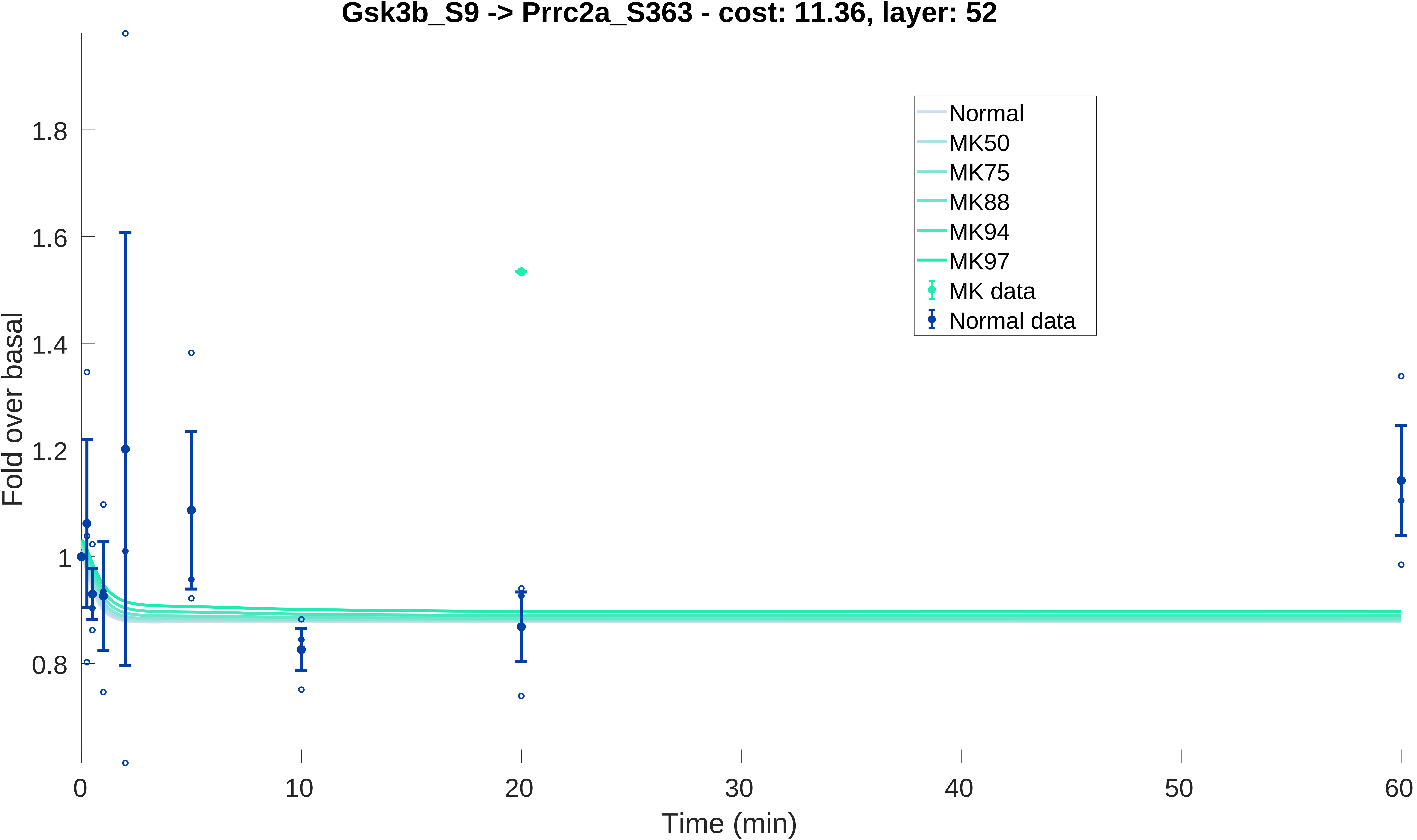

Gsk3b_S9 -> Prrc2a_S363 - cost: 11.36, layer: 52
1.8
1.6
1.4
1.2
1
0.8
Fold over basal
0
10
20
30
40
50
60
Time (min)
Normal
MK50
MK75
MK88
MK94
MK97
MK data
Normal data

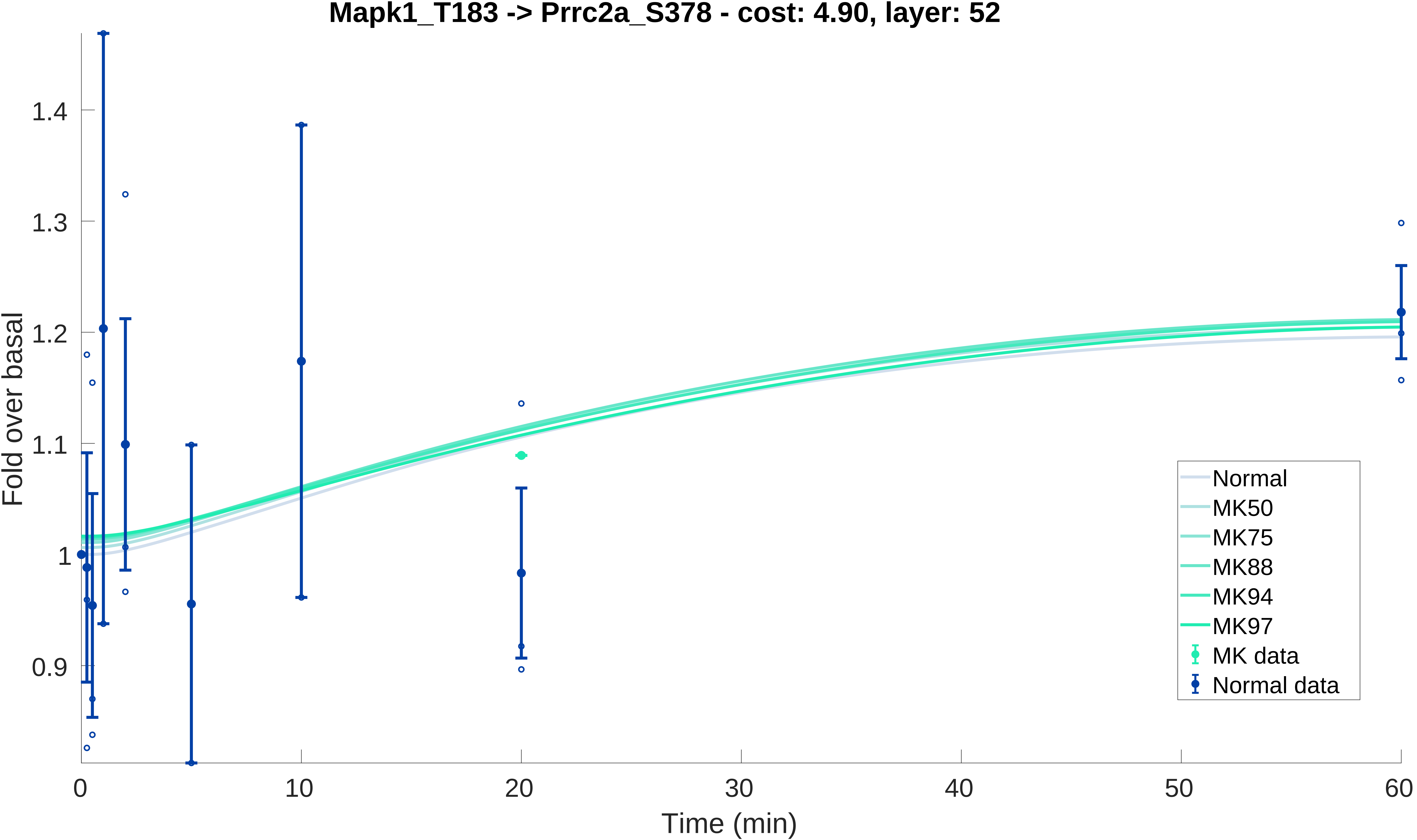

Mapk1_T183 -> Prrc2a_S378 - cost: 4.90, layer: 52
Fold over basal
1.4
1.3
1.2
1.1
1
0.9
0
10
20
30
40
50
60
Time (min)
Normal
MK50
MK75
MK88
MK94
MK97
MK data
Normal data

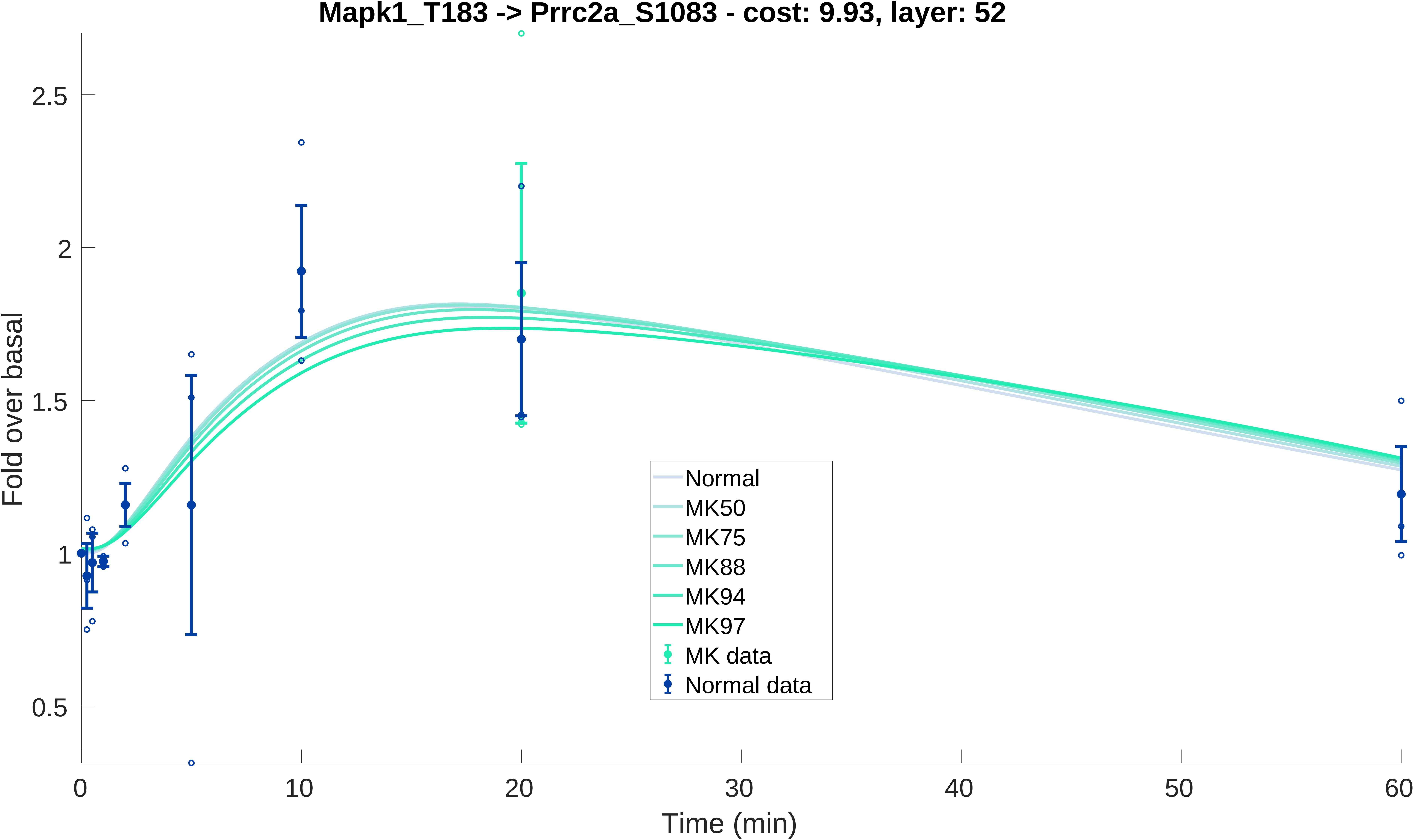

Mapk1_T183 -> Prrc2a_S1083 - cost: 9.93, layer: 52
Fold over basal
Time (min)
0.5
1
1.5
2
2.5
0
10
20
30
40
50
60
Normal
MK50
MK75
MK88
MK94
MK97
MK data
Normal data

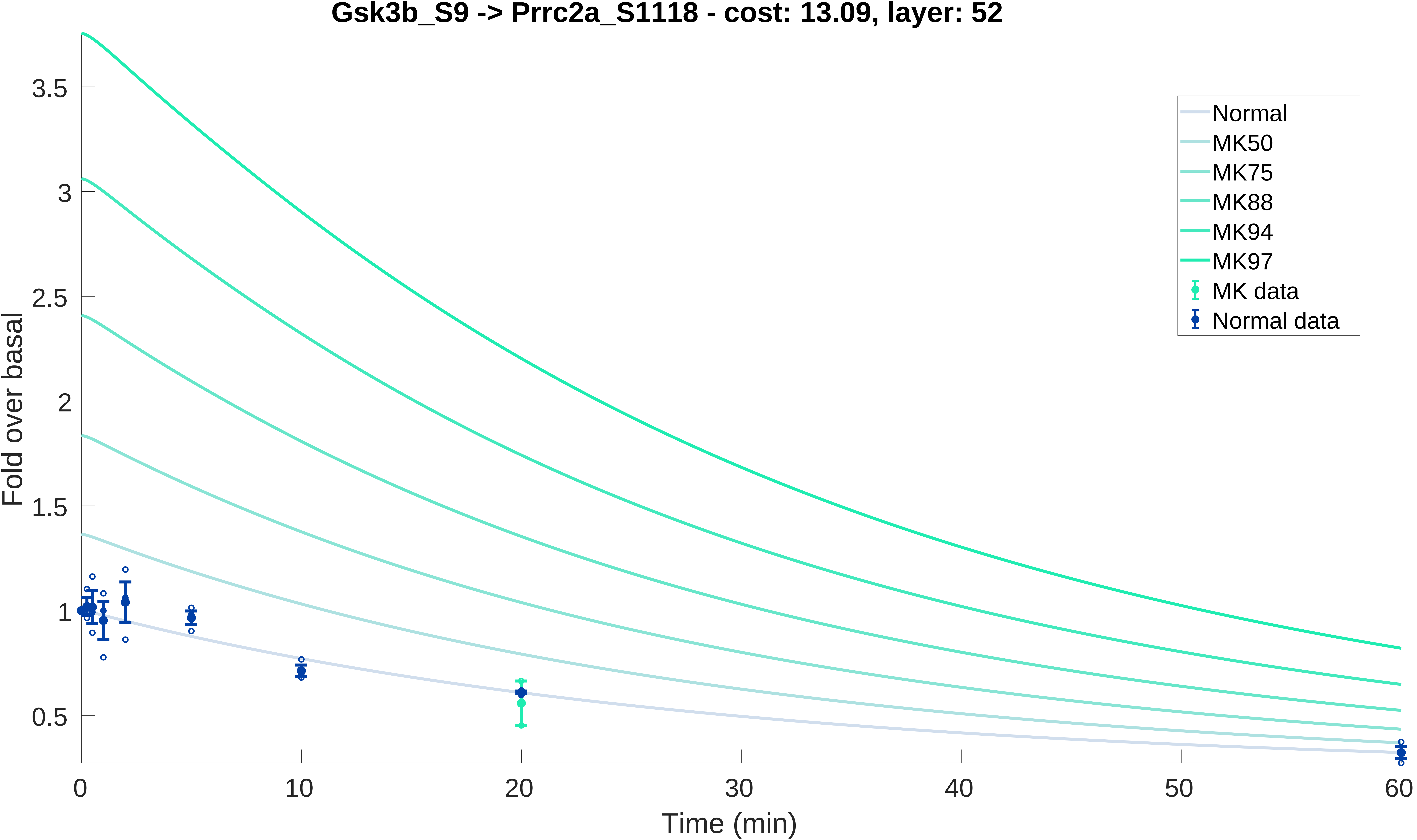
Gsk3b_S9 -> Prrc2a_S1118 - cost: 13.09, layer: 52
Fold over basal
Time (min)
3.5
3
2.5
2
1.5
1
0.5
0
10
20
30
40
50
60
Normal
MK50
MK75
MK88
MK94
MK97
MK data
Normal data

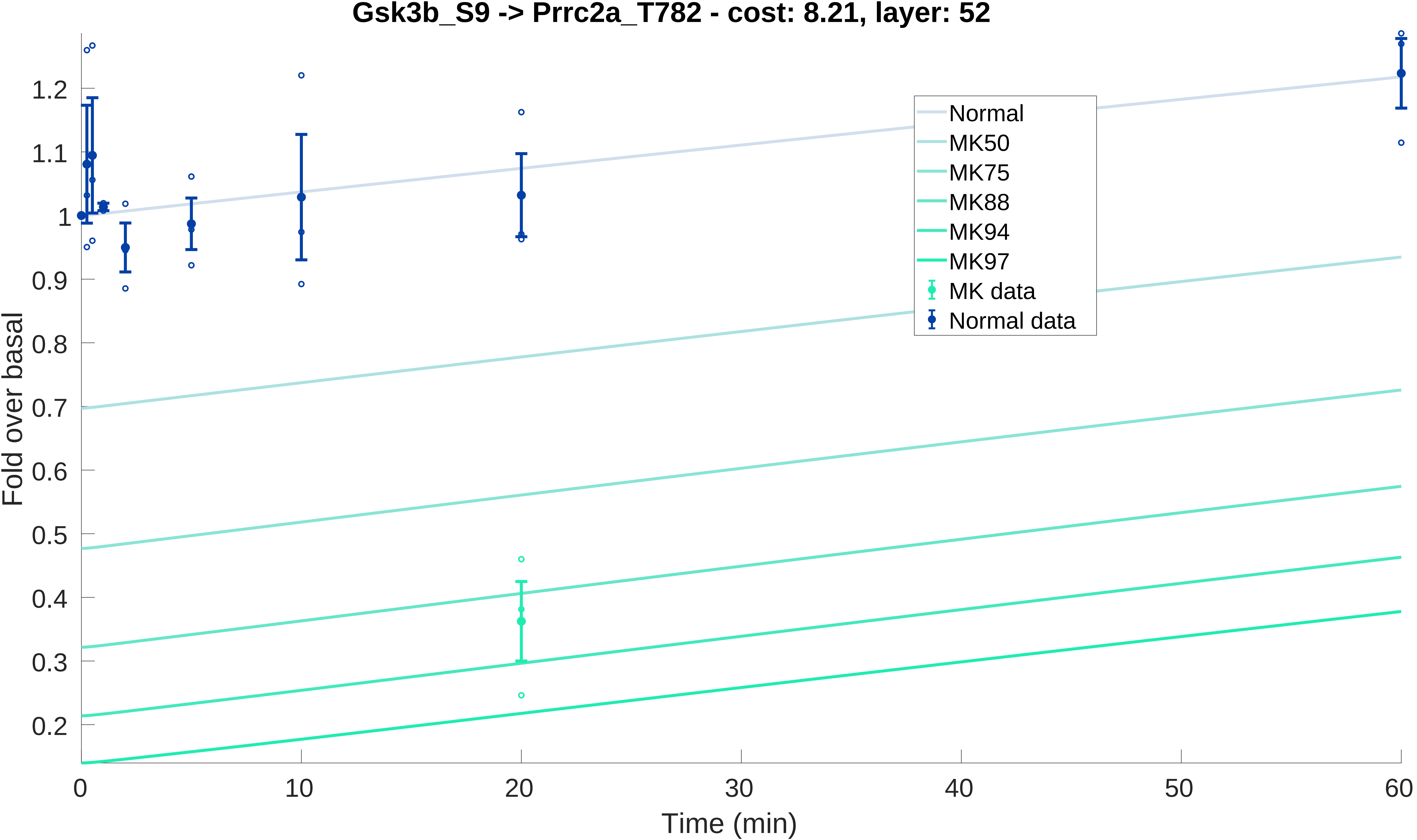
Gsk3b_S9 -> Prrc2a_T782 - cost: 8.21, layer: 52
Fold over basal
Time (min)
1.2
1.1
1
0.9
0.8
0.7
0.6
0.5
0.4
0.3
0.2
0
10
20
30
40
50
60
Normal
MK50
MK75
MK88
MK94
MK97
MK data
Normal data

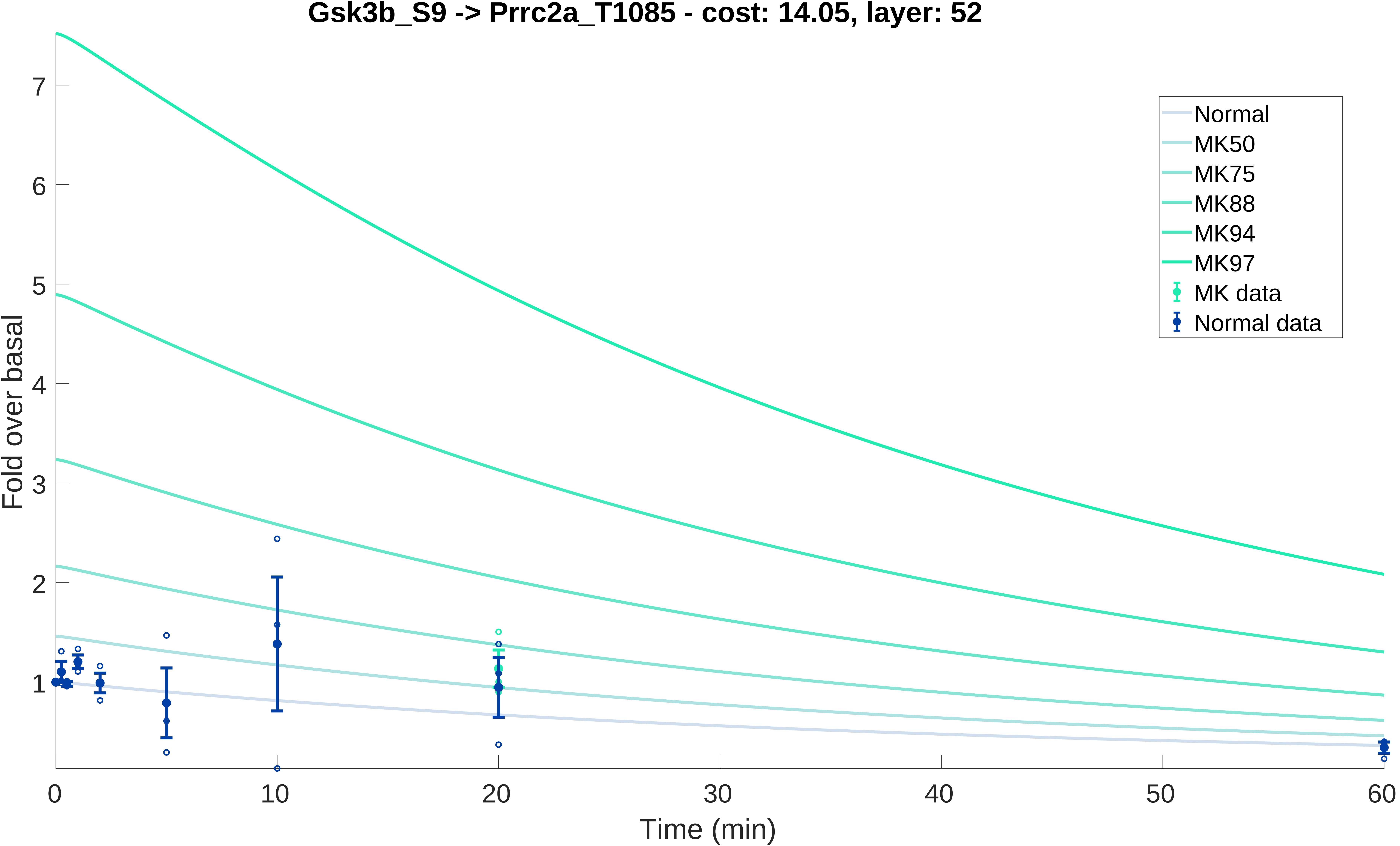

Gsk3b_S9 -> Prrc2a_T1085 - cost: 14.05, layer: 52
Fold over basal
Time (min)
0
10
20
30
40
50
60
1
2
3
4
5
6
7
Normal
MK50
MK75
MK88
MK94
MK97
MK data
Normal data

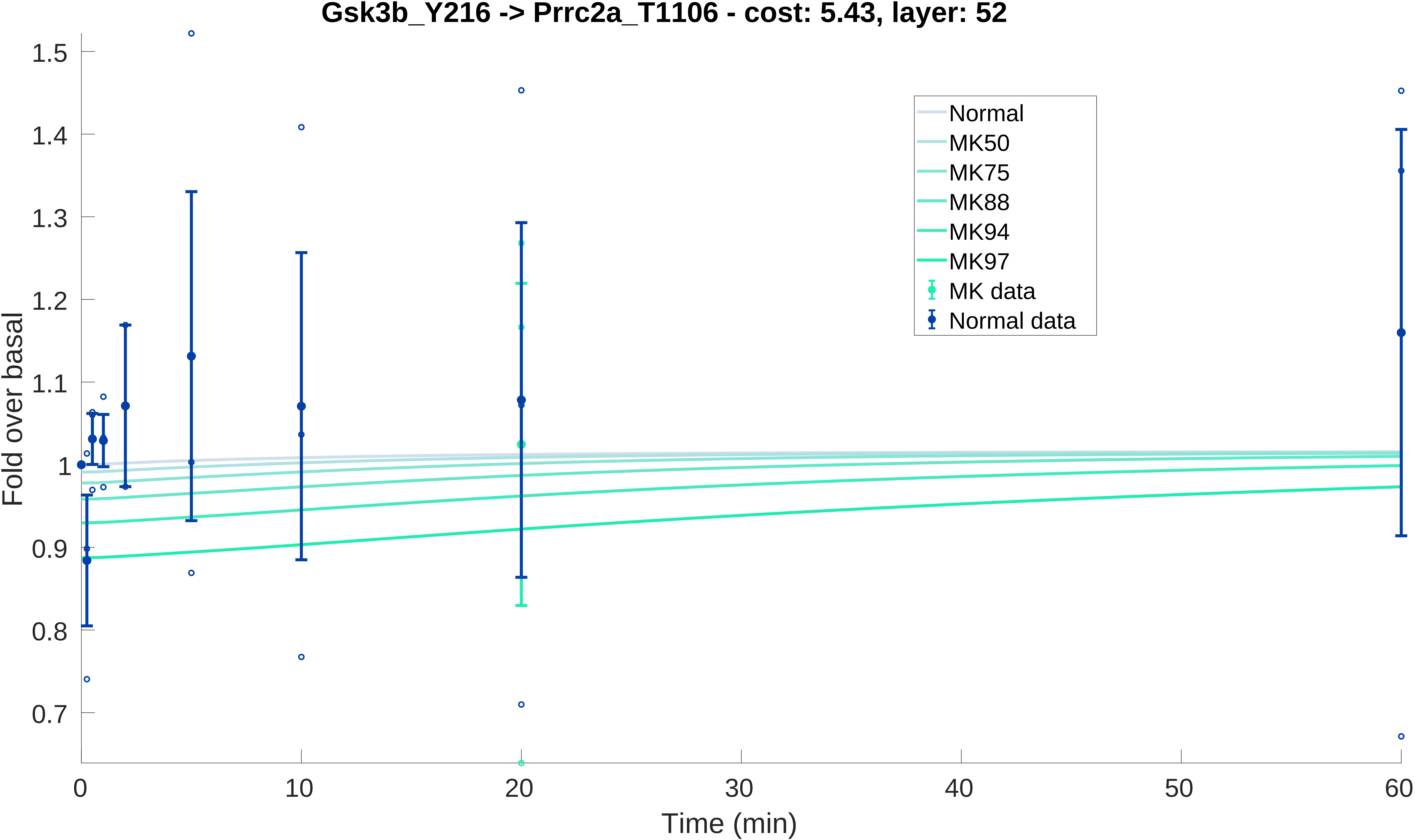

Gsk3b_Y216 -> Prrc2a_T1106 - cost: 5.43, layer: 52
Fold over basal
Time (min)
Normal
MK50
MK75
MK88
MK94
MK97
MK data
Normal data
1.5
1.4
1.3
1.2
1.1
1
0.9
0.8
0.7
0
10
20
30
40
50
60

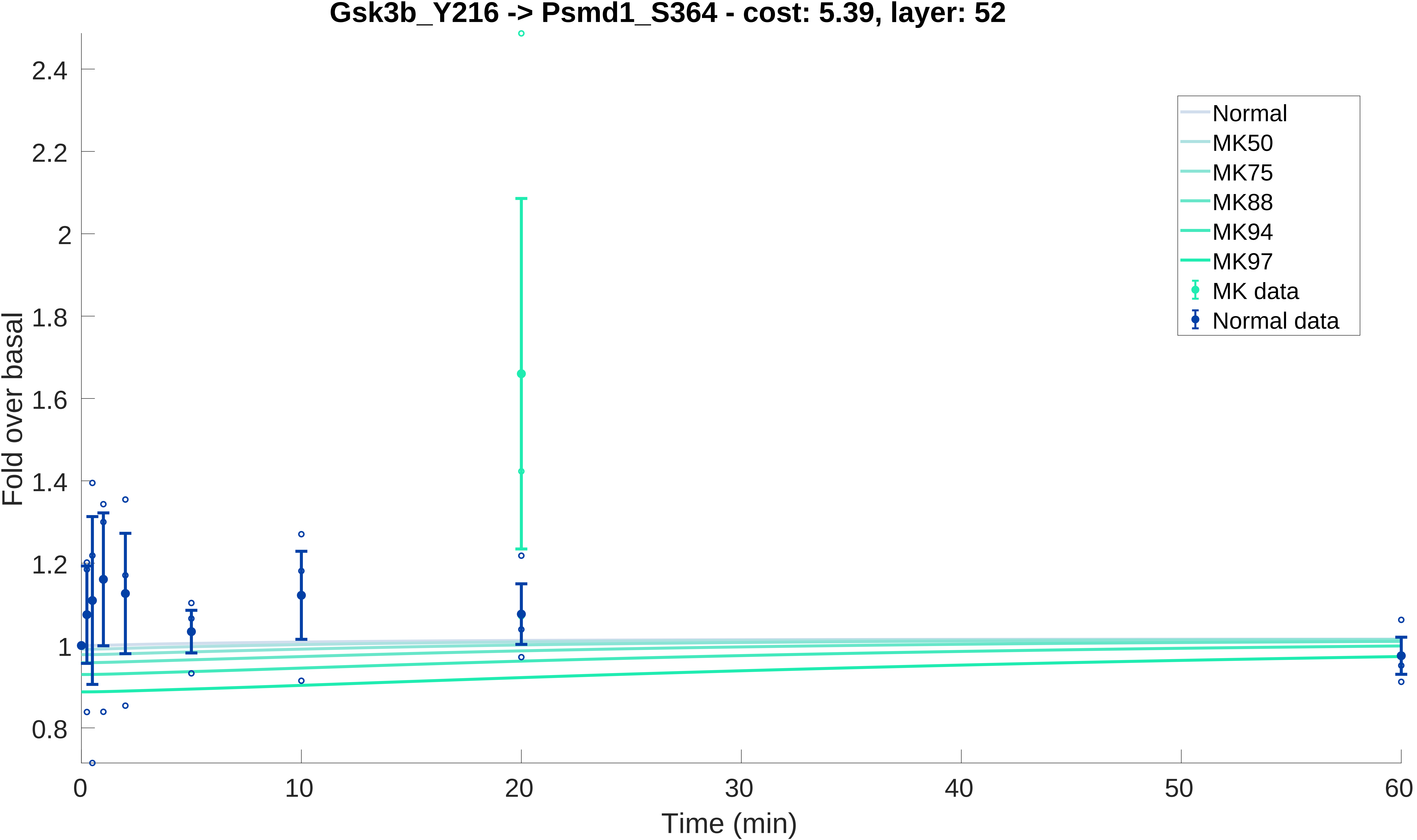

Gsk3b_Y216 -> Psmd1_S364 - cost: 5.39, layer: 52
Fold over basal
Time (min)
0.8
1
1.2
1.4
1.6
1.8
2
2.2
2.4
0
10
20
30
40
50
60
Normal
MK50
MK75
MK88
MK94
MK97
MK data
Normal data

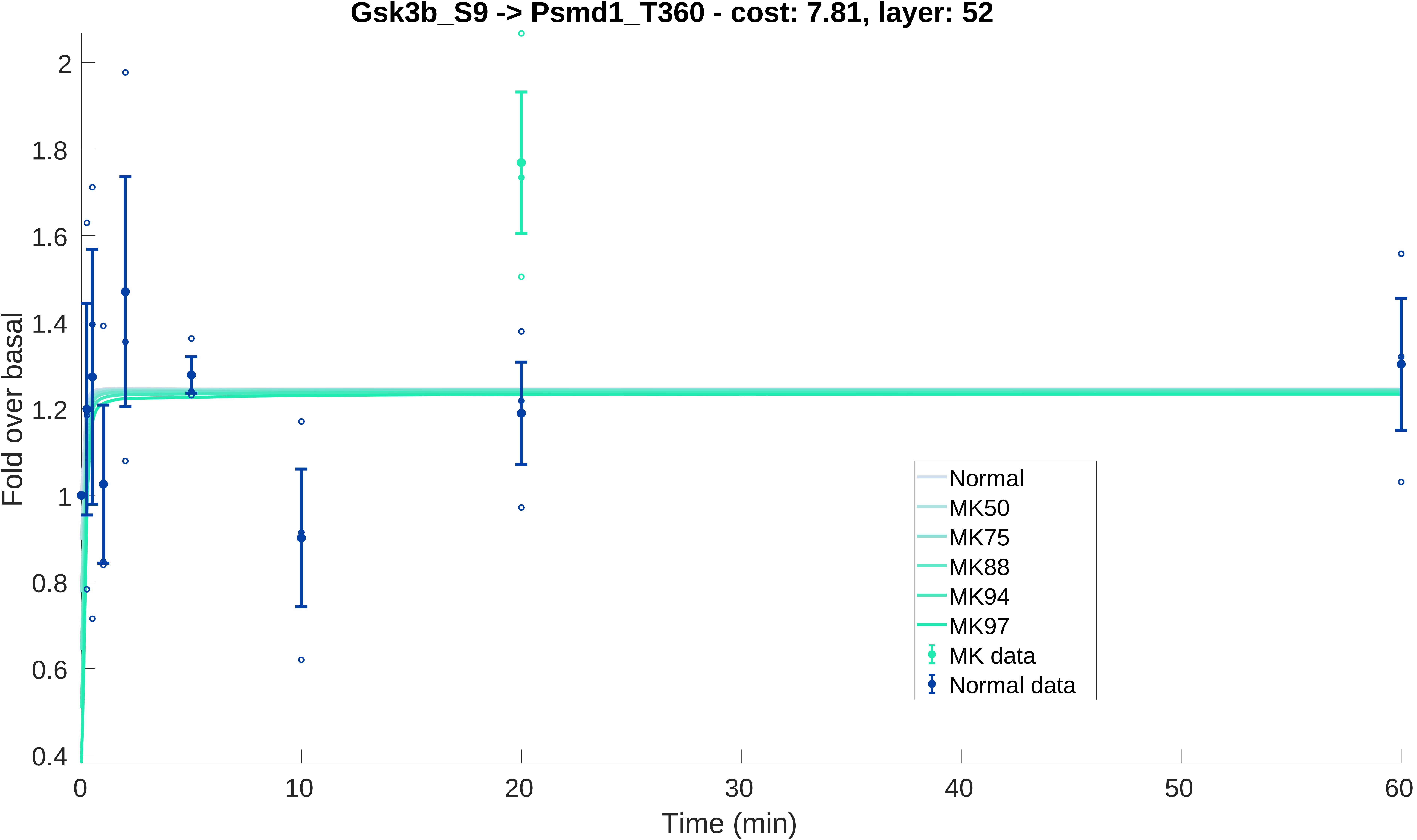

Gsk3b_S9 -> Psmd1_T360 - cost: 7.81, layer: 52
Fold over basal
Time (min)
0.4
0.6
0.8
1
1.2
1.4
1.6
1.8
2
0
10
20
30
40
50
60
Normal
MK50
MK75
MK88
MK94
MK97
MK data
Normal data

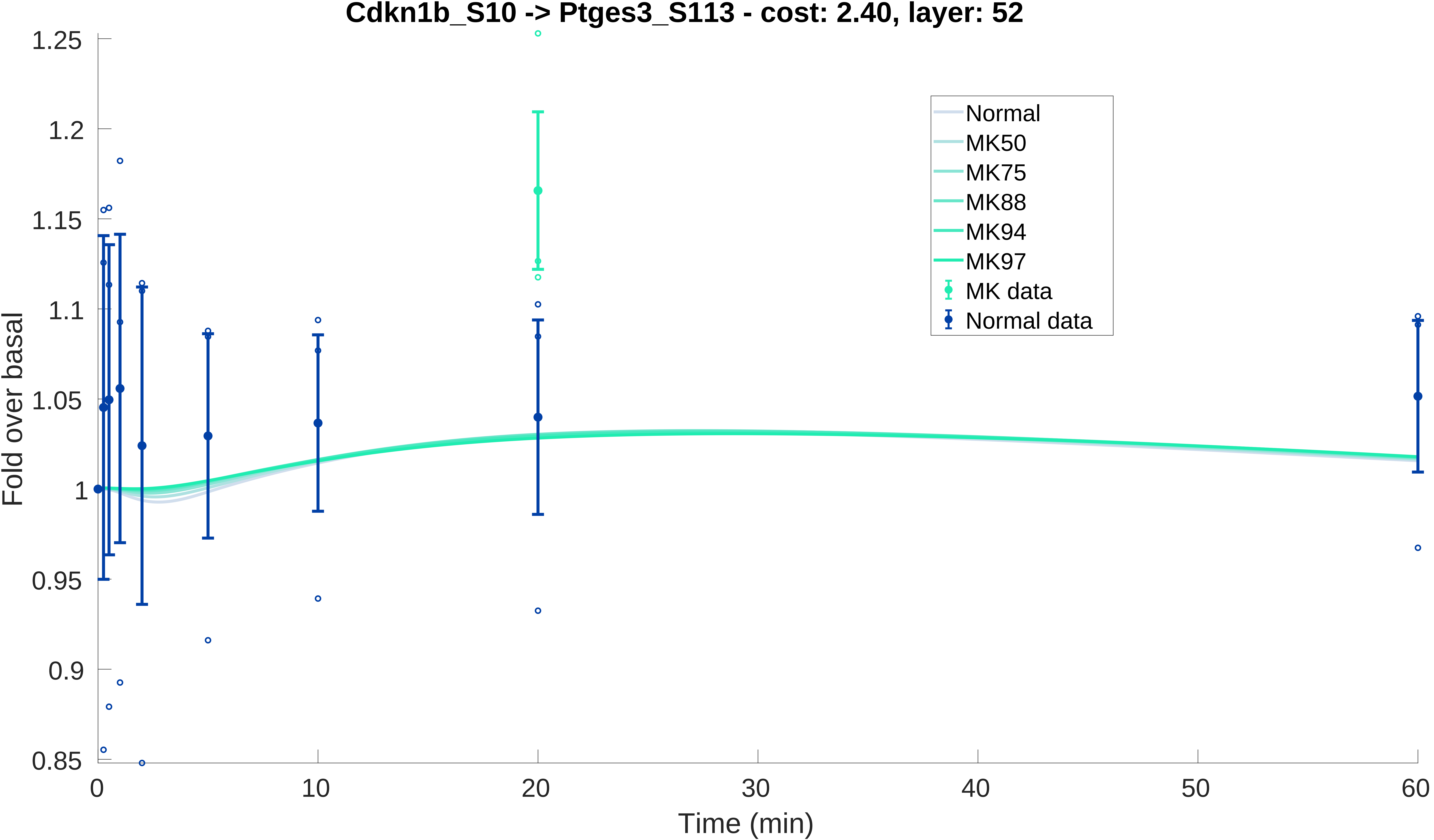

Cdkn1b_S10 -> Ptges3_S113 - cost: 2.40, layer: 52
Fold over basal
Time (min)
1.25
1.2
1.15
1.1
1.05
1
0.95
0.9
0.85
0
10
20
30
40
50
60
Normal
MK50
MK75
MK88
MK94
MK97
MK data
Normal data

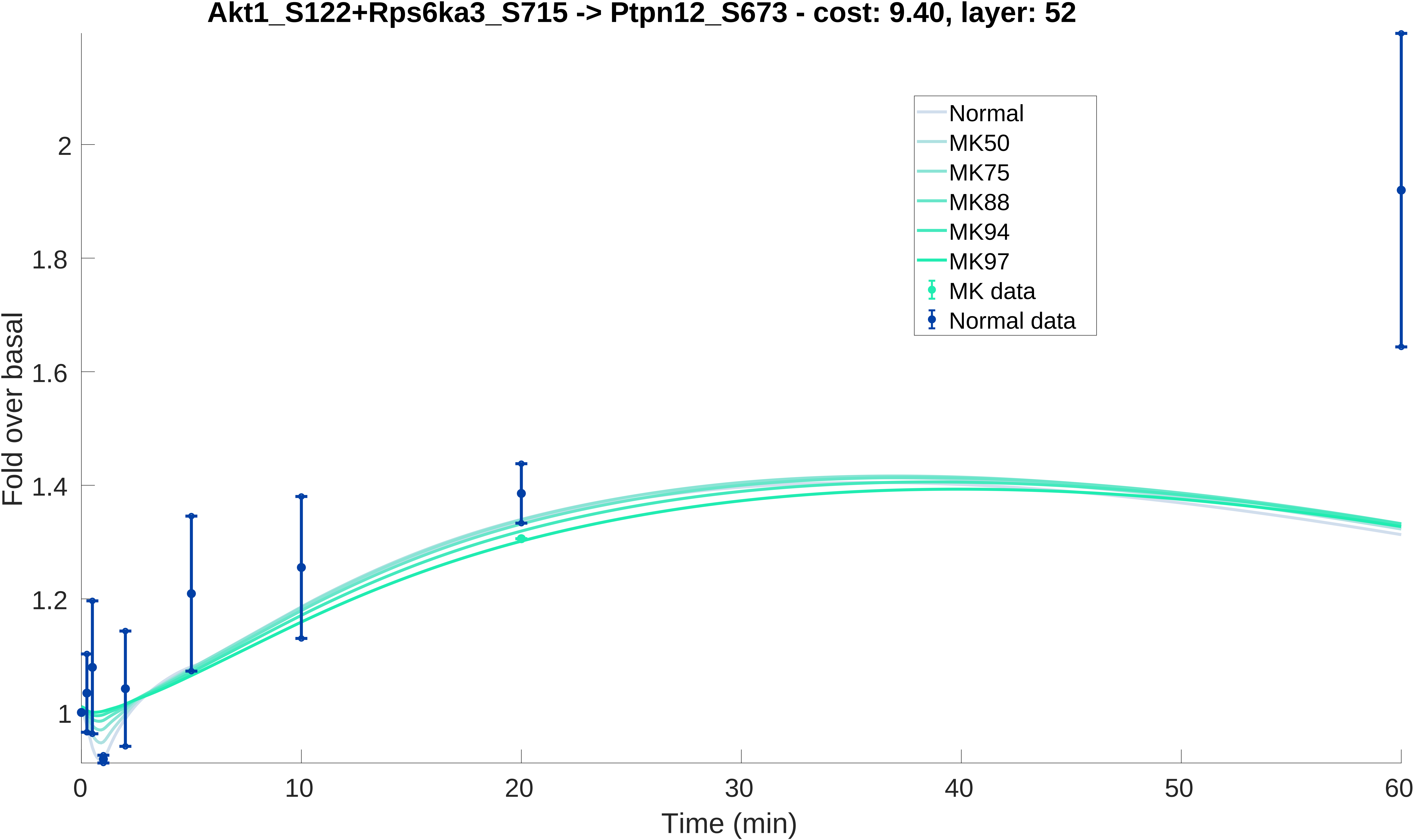
Akt1_S122+Rps6ka3_S715 -> Ptpn12_S673 - cost: 9.40, layer: 52
Fold over basal
Time (min)
2
1.8
1.6
1.4
1.2
1
0
10
20
30
40
50
60
Normal
MK50
MK75
MK88
MK94
MK97
MK data
Normal data

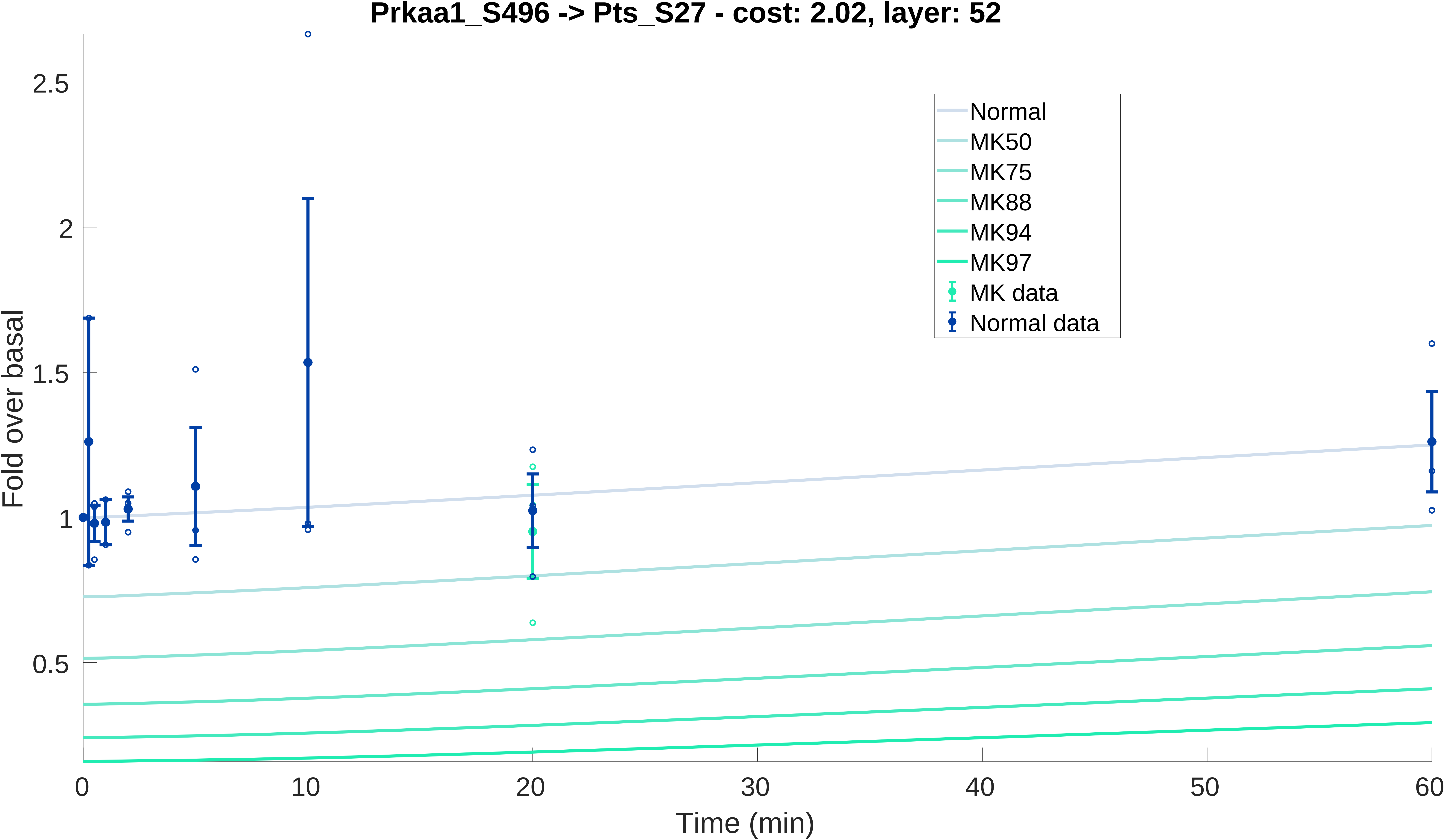

Prkaa1_S496 -> Pts_S27 - cost: 2.02, layer: 52
Fold over basal
Time (min)
0
10
20
30
40
50
60
0.5
1
1.5
2
2.5
Normal
MK50
MK75
MK88
MK94
MK97
MK data
Normal data

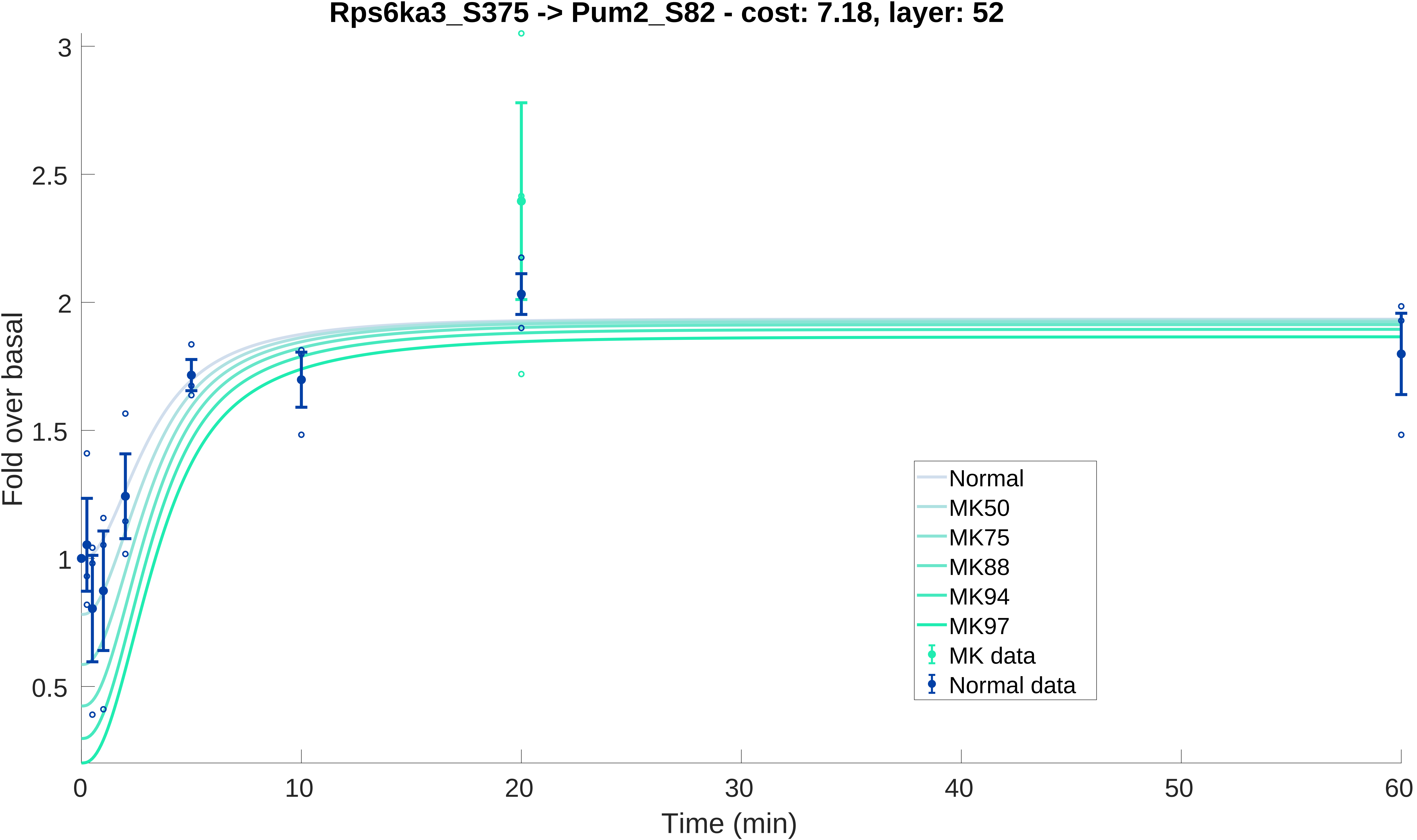
Rps6ka3_S375 -> Pum2_S82 - cost: 7.18, layer: 52
Fold over basal
Time (min)
0
10
20
30
40
50
60
0.5
1
1.5
2
2.5
3
Normal
MK50
MK75
MK88
MK94
MK97
MK data
Normal data

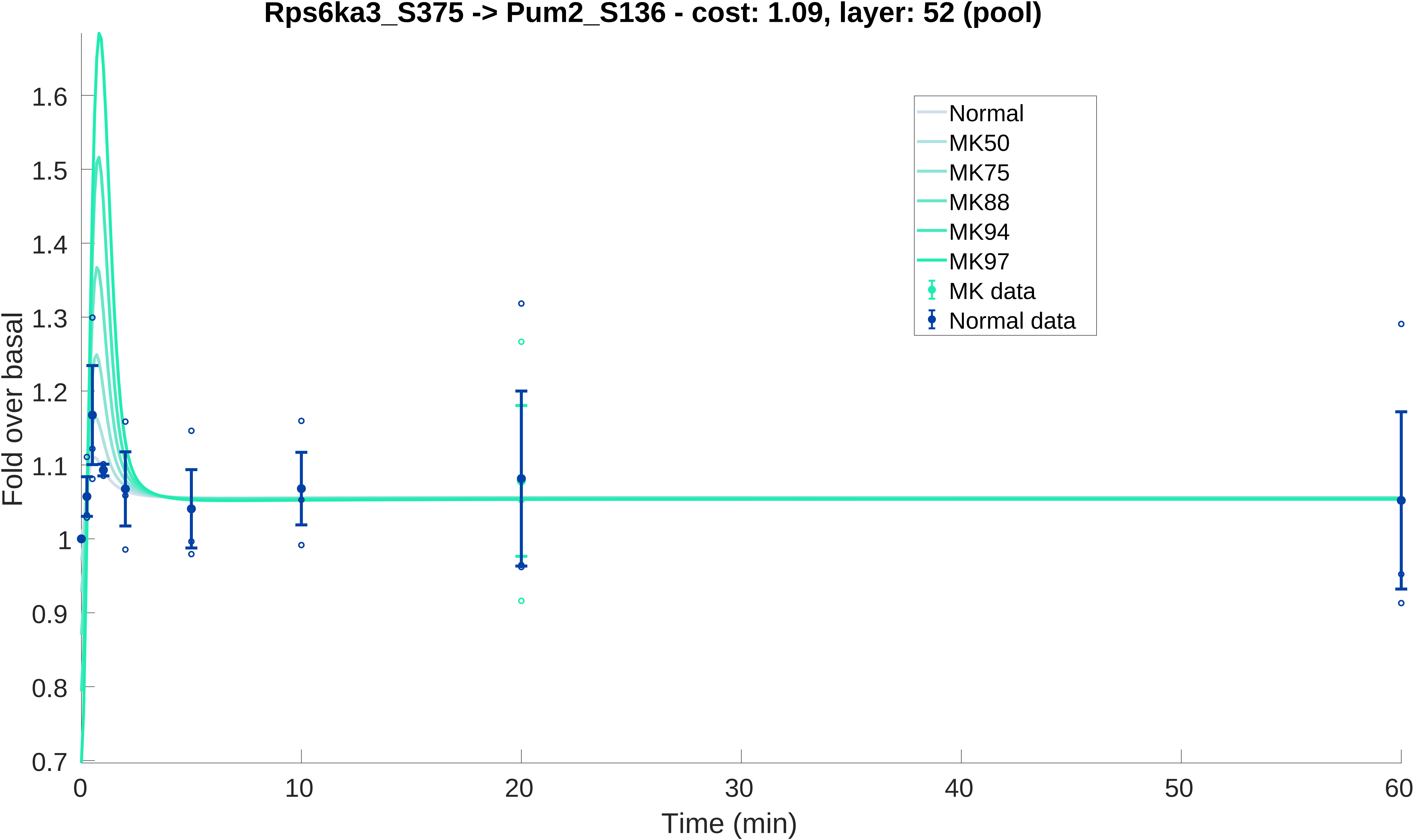
Rps6ka3_S375 -> Pum2_S136 - cost: 1.09, layer: 52 (pool)
Fold over basal
Time (min)
Normal
MK50
MK75
MK88
MK94
MK97
MK data
Normal data
1.6
1.5
1.4
1.3
1.2
1.1
1
0.9
0.8
0.7
0
10
20
30
40
50
60

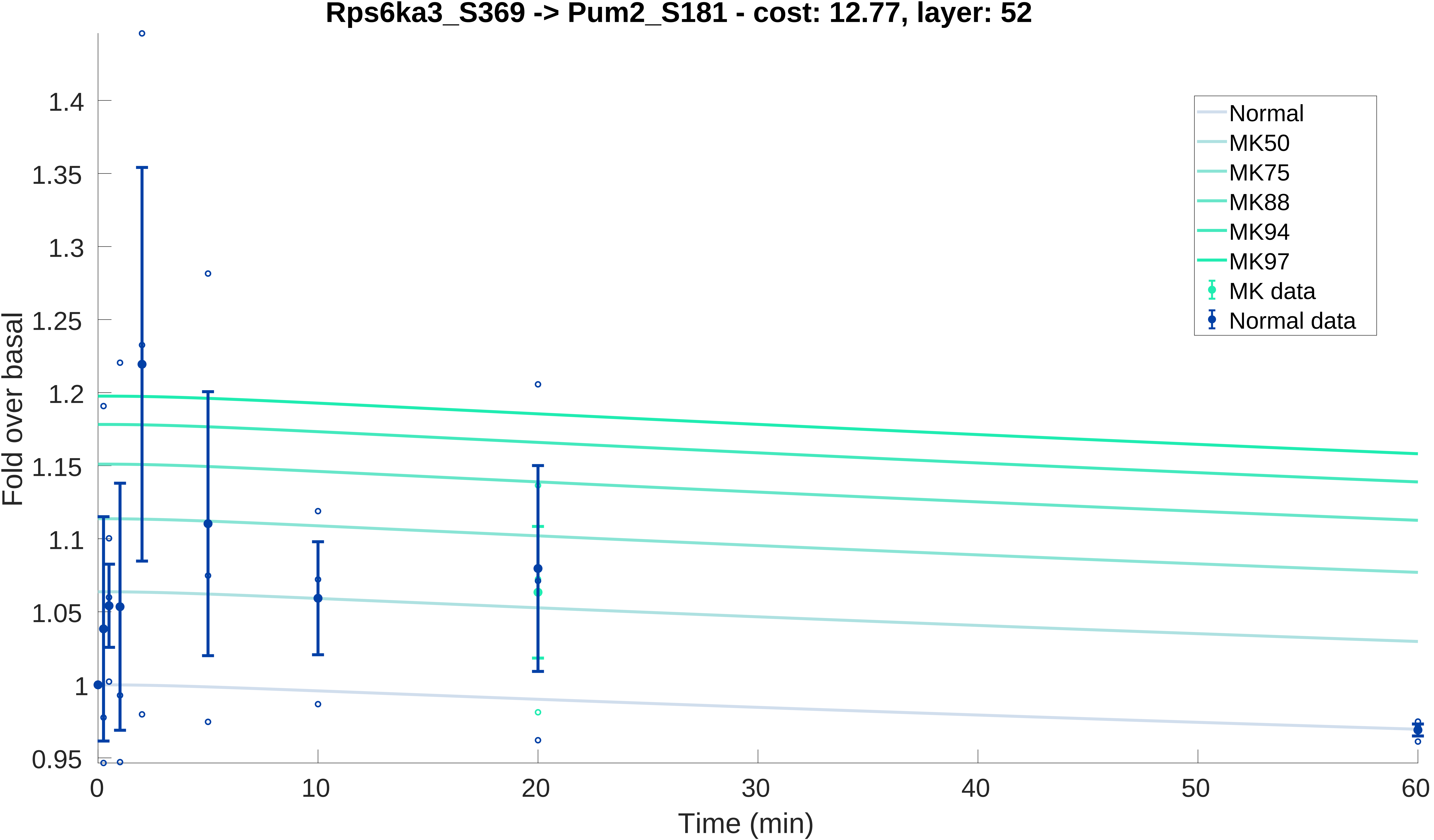

Rps6ka3_S369 -> Pum2_S181 - cost: 12.77, layer: 52
Fold over basal
Time (min)
Normal
MK50
MK75
MK88
MK94
MK97
MK data
Normal data
1.4
1.35
1.3
1.25
1.2
1.15
1.1
1.05
1
0.95
0
10
20
30
40
50
60

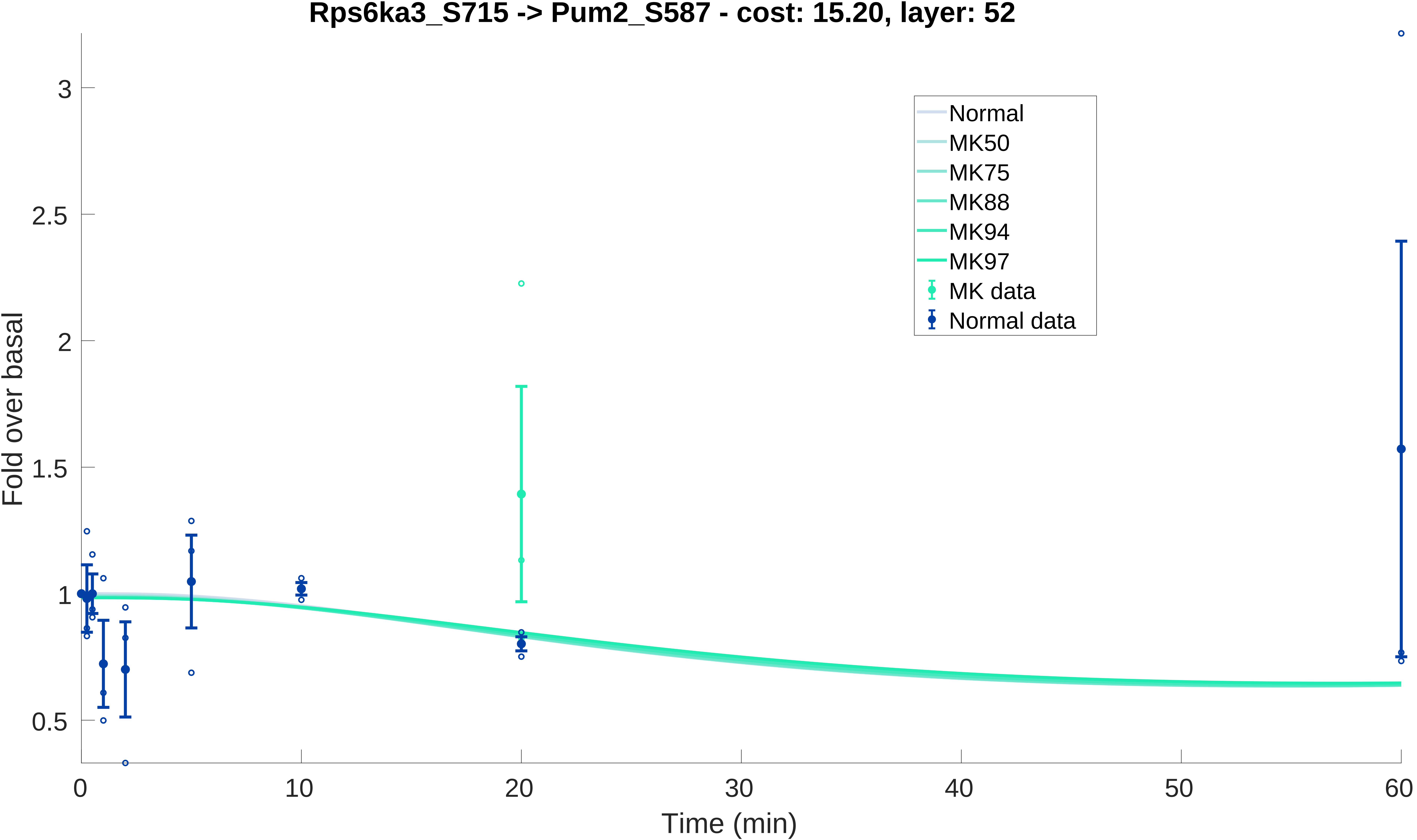
Rps6ka3_S715 -> Pum2_S587 - cost: 15.20, layer: 52
Fold over basal
Time (min)
0.5
1
1.5
2
2.5
3
0
10
20
30
40
50
60
Normal
MK50
MK75
MK88
MK94
MK97
MK data
Normal data

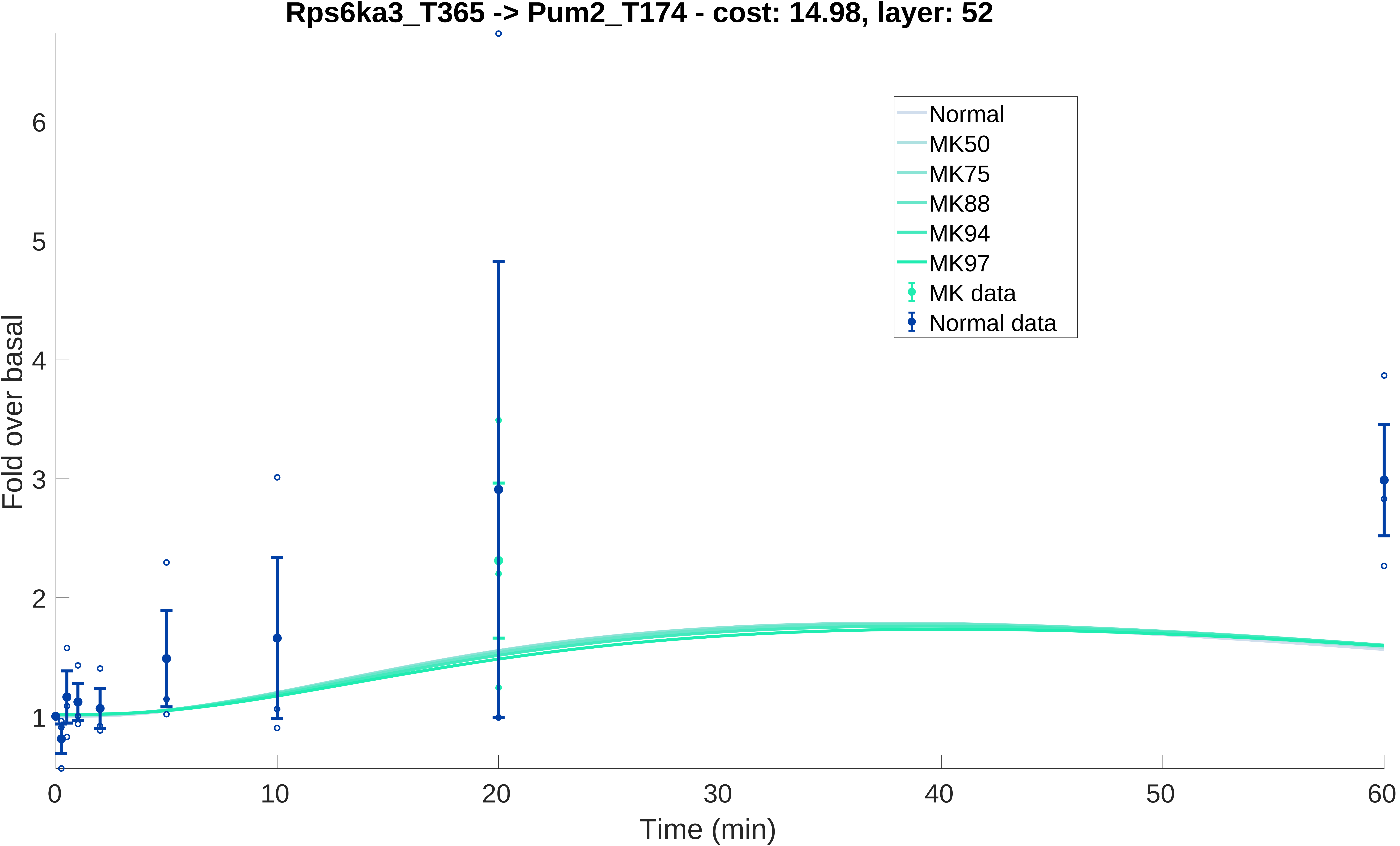

Rps6ka3_T365 -> Pum2_T174 - cost: 14.98, layer: 52
Fold over basal
Time (min)
Normal
MK50
MK75
MK88
MK94
MK97
MK data
Normal data
1
2
3
4
5
6
0
10
20
30
40
50
60

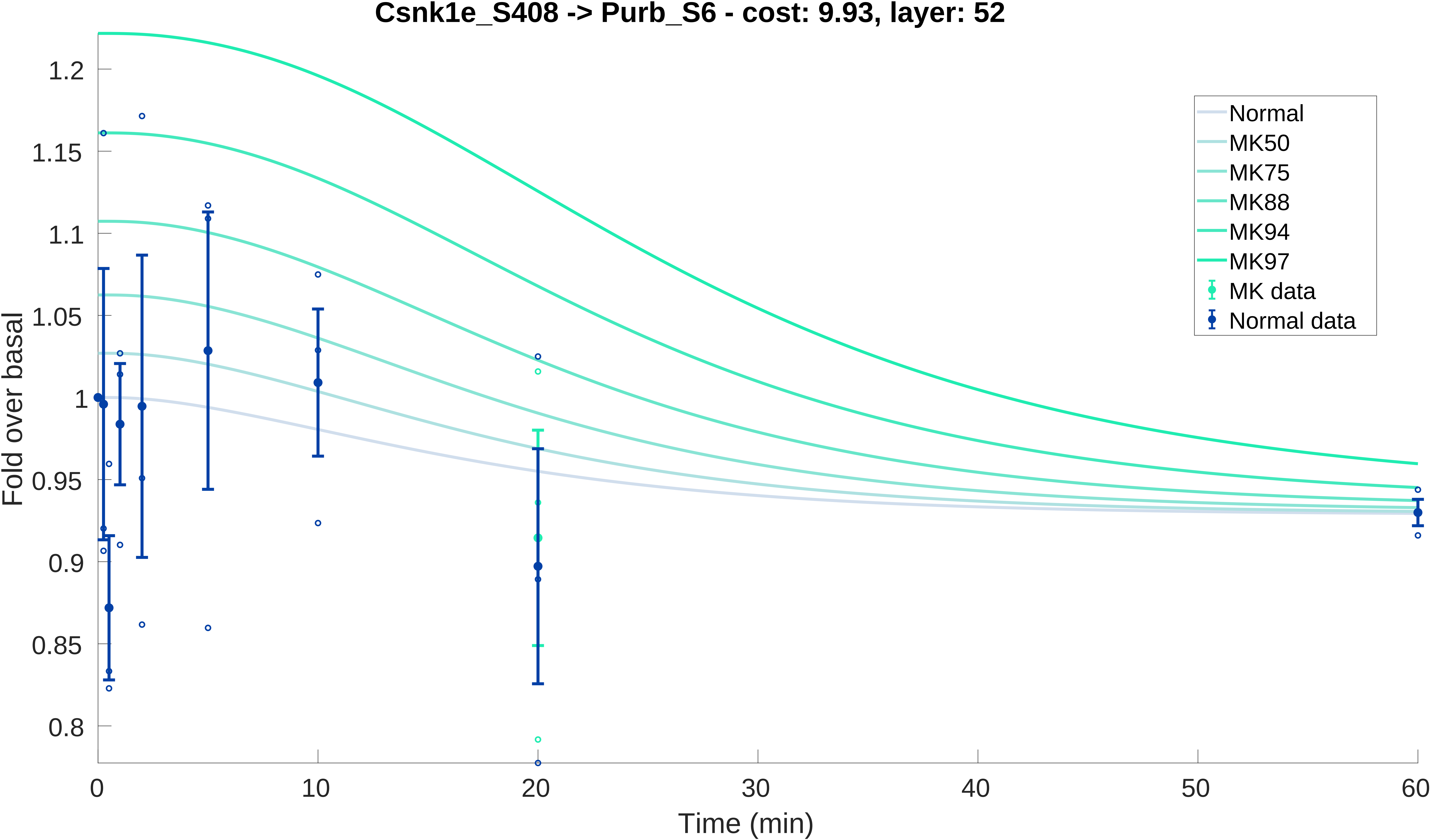

Csnk1e_S408 -> Purb_S6 - cost: 9.93, layer: 52
Fold over basal
Time (min)
1.2
1.15
1.1
1.05
1
0.95
0.9
0.85
0.8
0
10
20
30
40
50
60
Normal
MK50
MK75
MK88
MK94
MK97
MK data
Normal data

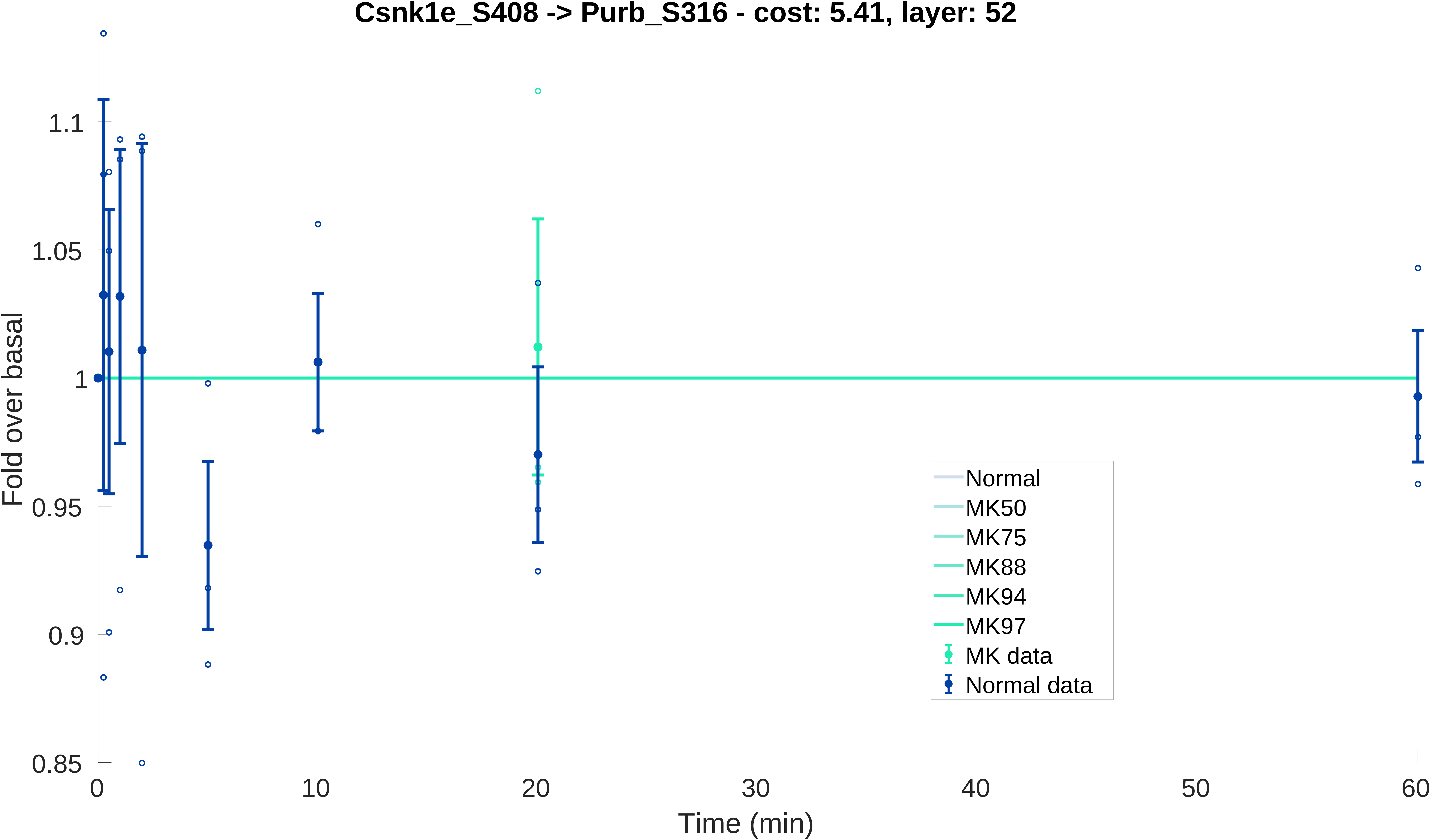

Csnk1e_S408 -> Purb_S316 - cost: 5.41, layer: 52
Fold over basal
1.1
1.05
1
0.95
0.9
0.85
0
10
20
30
40
50
60
Time (min)
Normal
MK50
MK75
MK88
MK94
MK97
MK data
Normal data

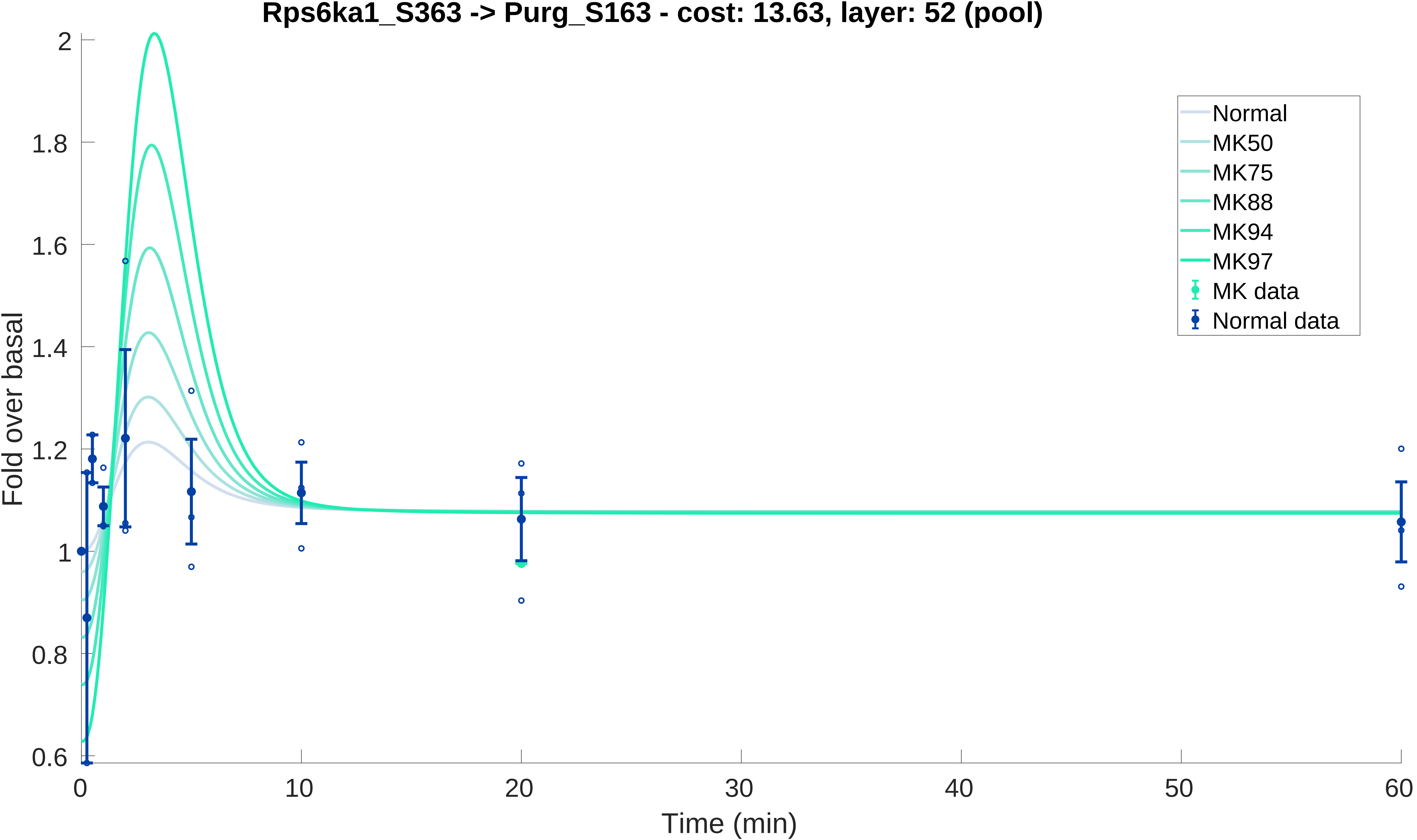

Rps6ka1_S363 -> Purg_S163 - cost: 13.63, layer: 52 (pool)
Fold over basal
Time (min)
0.6
0.8
1
1.2
1.4
1.6
1.8
2
0
10
20
30
40
50
60
Normal
MK50
MK75
MK88
MK94
MK97
MK data
Normal data

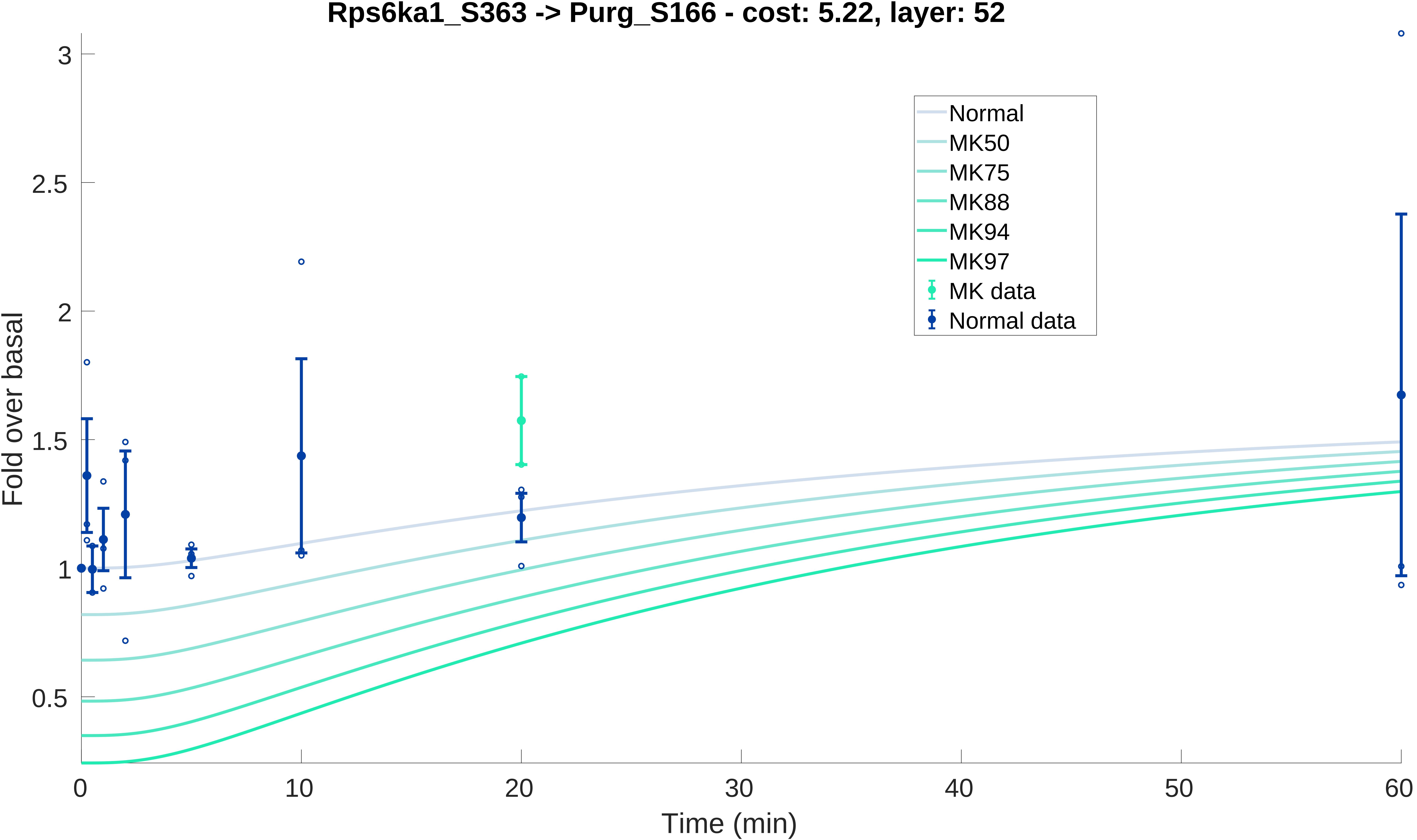
Rps6ka1_S363 -> Purg_S166 - cost: 5.22, layer: 52
Fold over basal
Time (min)
0.5
1
1.5
2
2.5
3
0
10
20
30
40
50
60
Normal
MK50
MK75
MK88
MK94
MK97
MK data
Normal data

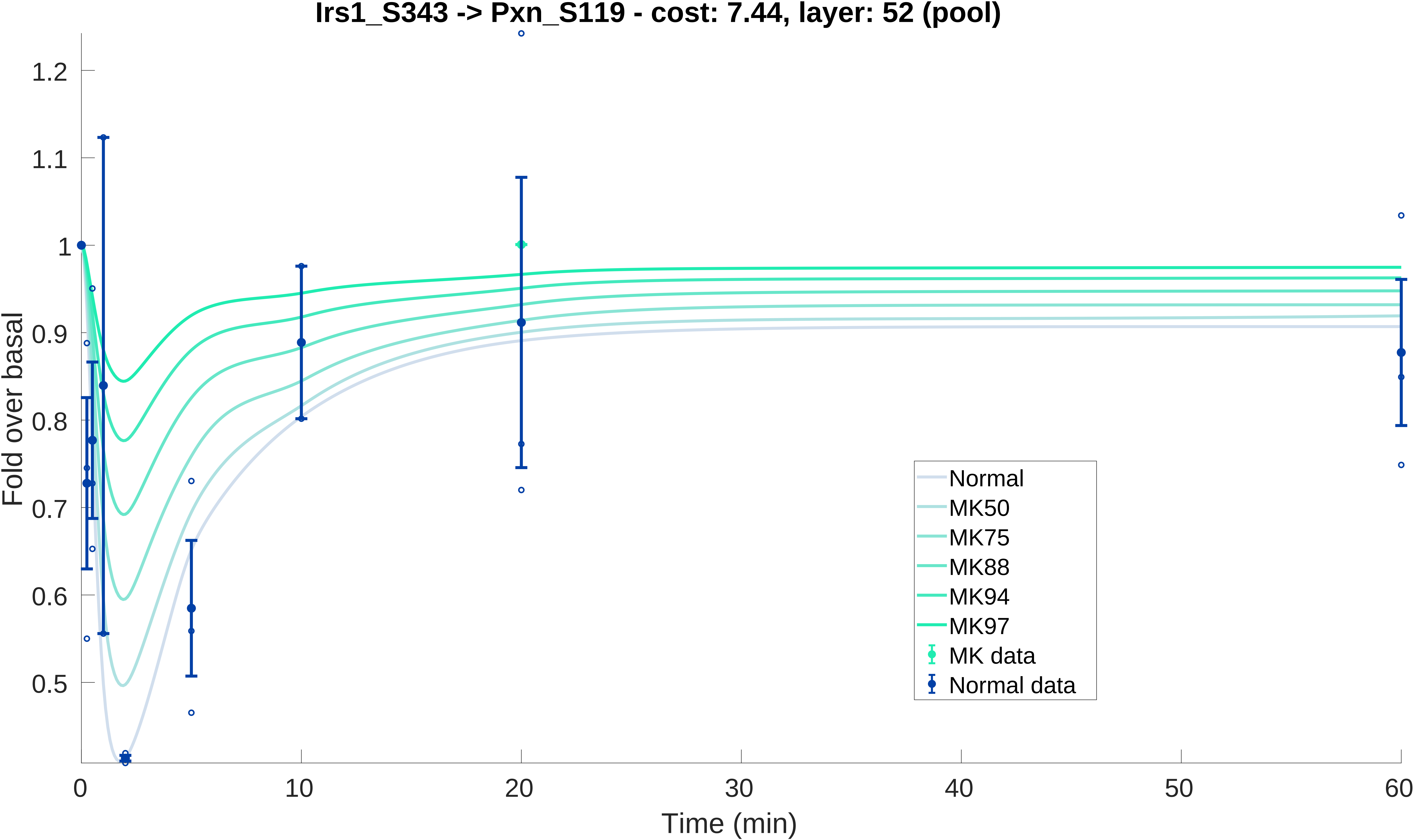

Irs1_S343 -> Pxn_S119 - cost: 7.44, layer: 52 (pool)
Fold over basal
1.2
1.1
1
0.9
0.8
0.7
0.6
0.5
0
10
20
30
40
50
60
Time (min)
Normal
MK50
MK75
MK88
MK94
MK97
MK data
Normal data

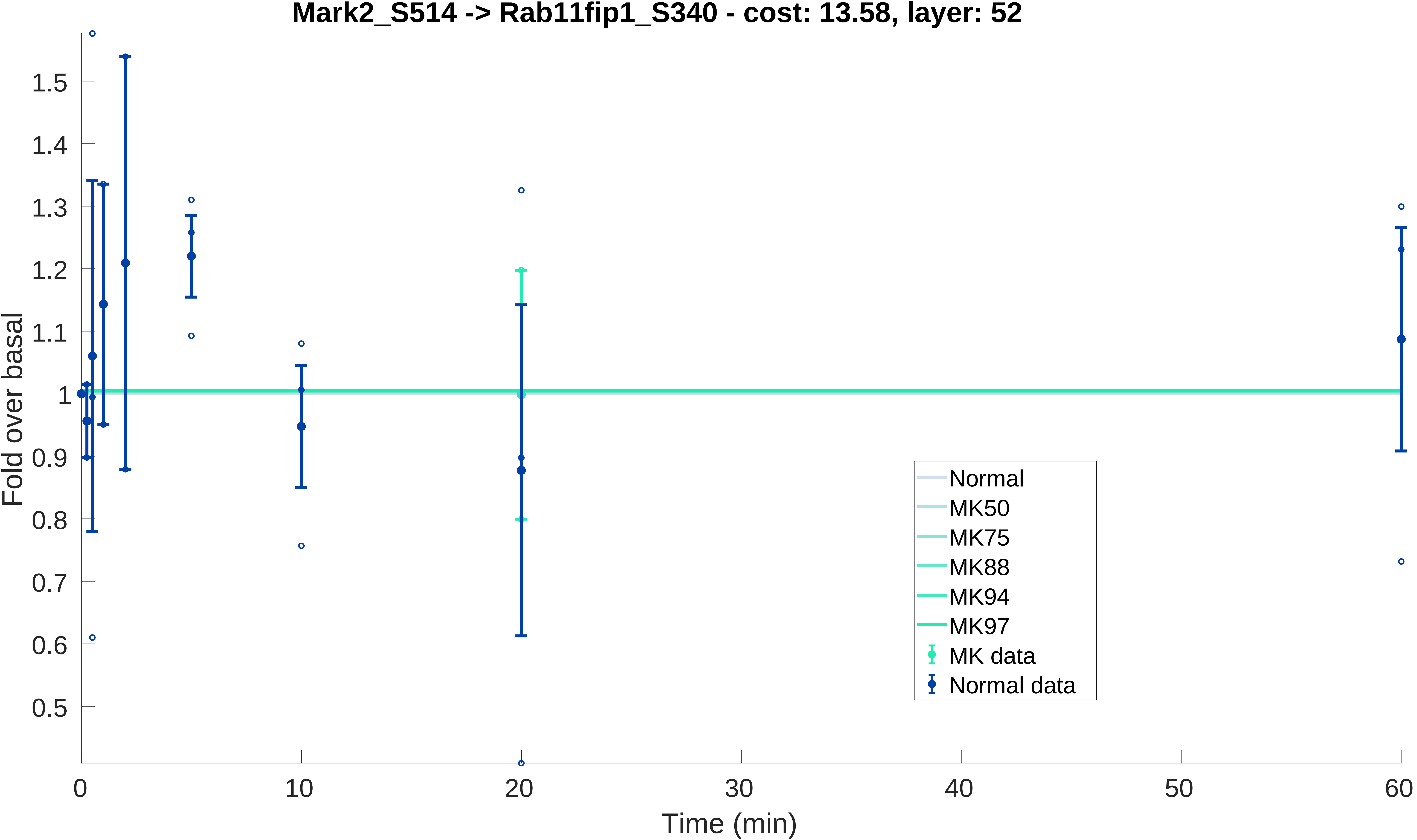
Mark2_S514 -> Rab11fip1_S340 - cost: 13.58, layer: 52
Fold over basal
1.5
1.4
1.3
1.2
1.1
1
0.9
0.8
0.7
0.6
0.5
0
10
20
30
40
50
60
Time (min)
Normal
MK50
MK75
MK88
MK94
MK97
MK data
Normal data

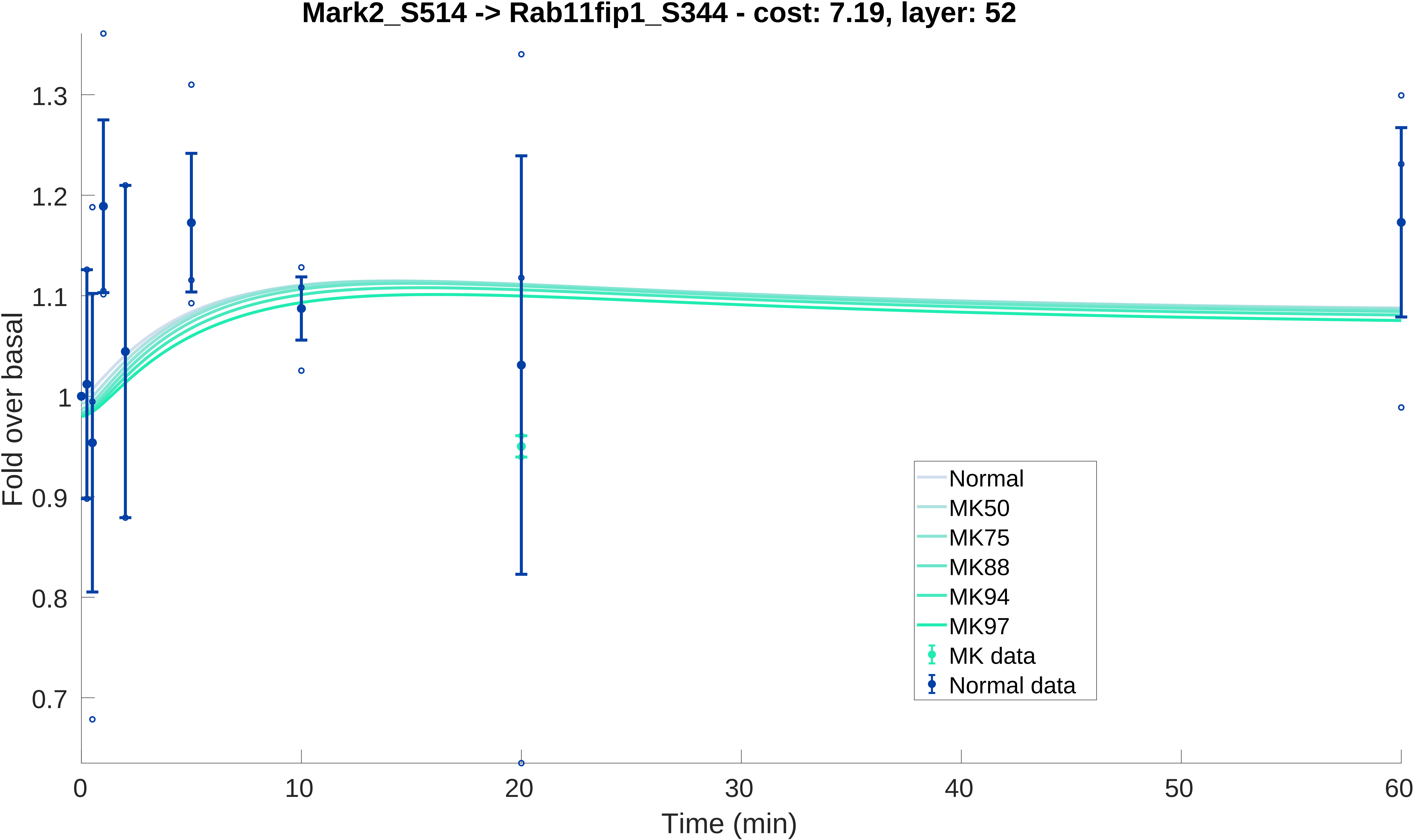

Mark2_S514 -> Rab11fip1_S344 - cost: 7.19, layer: 52
Fold over basal
1.3
1.2
1.1
1
0.9
0.8
0.7
0
10
20
30
40
50
60
Time (min)
Normal
MK50
MK75
MK88
MK94
MK97
MK data
Normal data

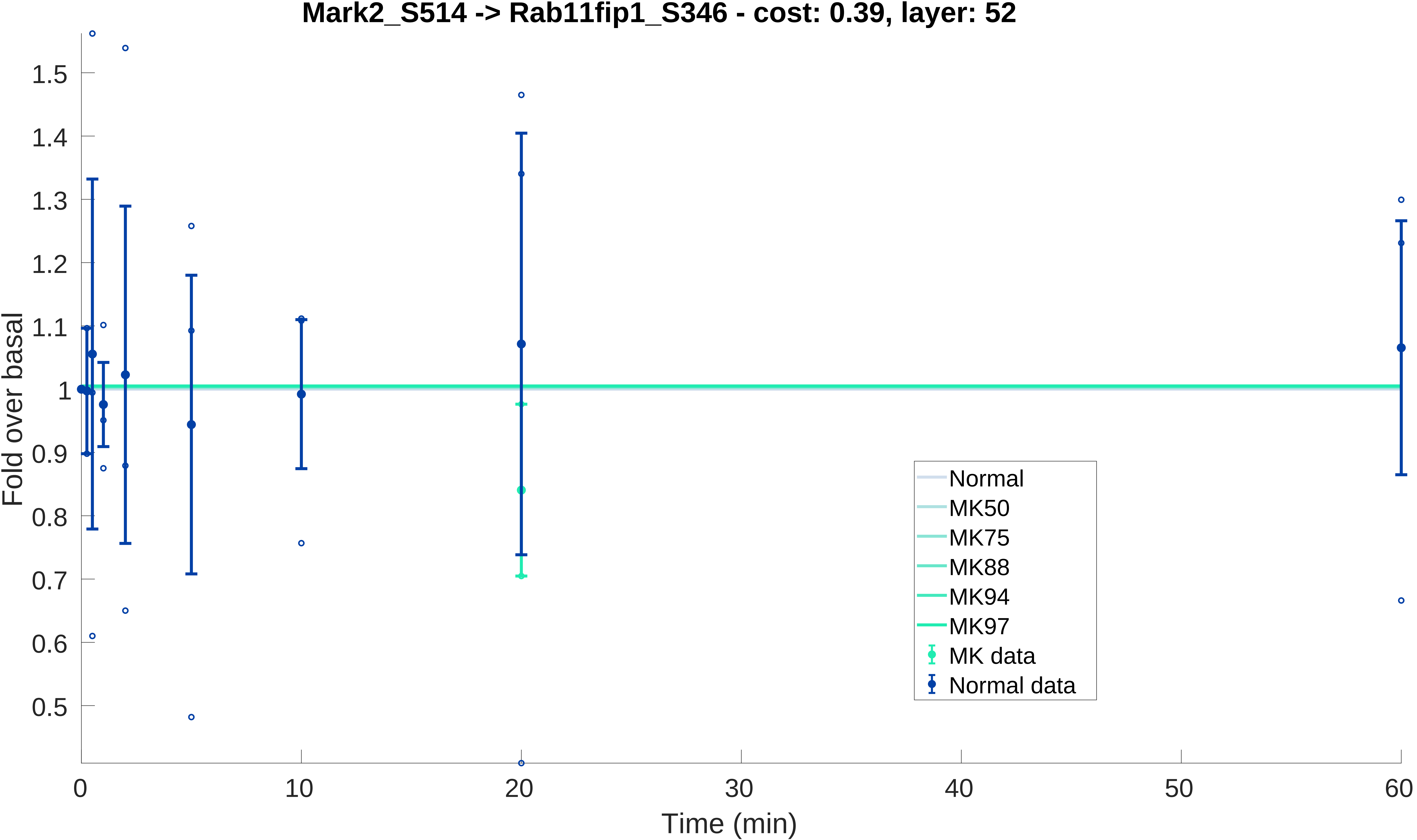

Mark2_S514 -> Rab11fip1_S346 - cost: 0.39, layer: 52
Fold over basal
Time (min)
0.5
0.6
0.7
0.8
0.9
1
1.1
1.2
1.3
1.4
1.5
0
10
20
30
40
50
60
Normal
MK50
MK75
MK88
MK94
MK97
MK data
Normal data

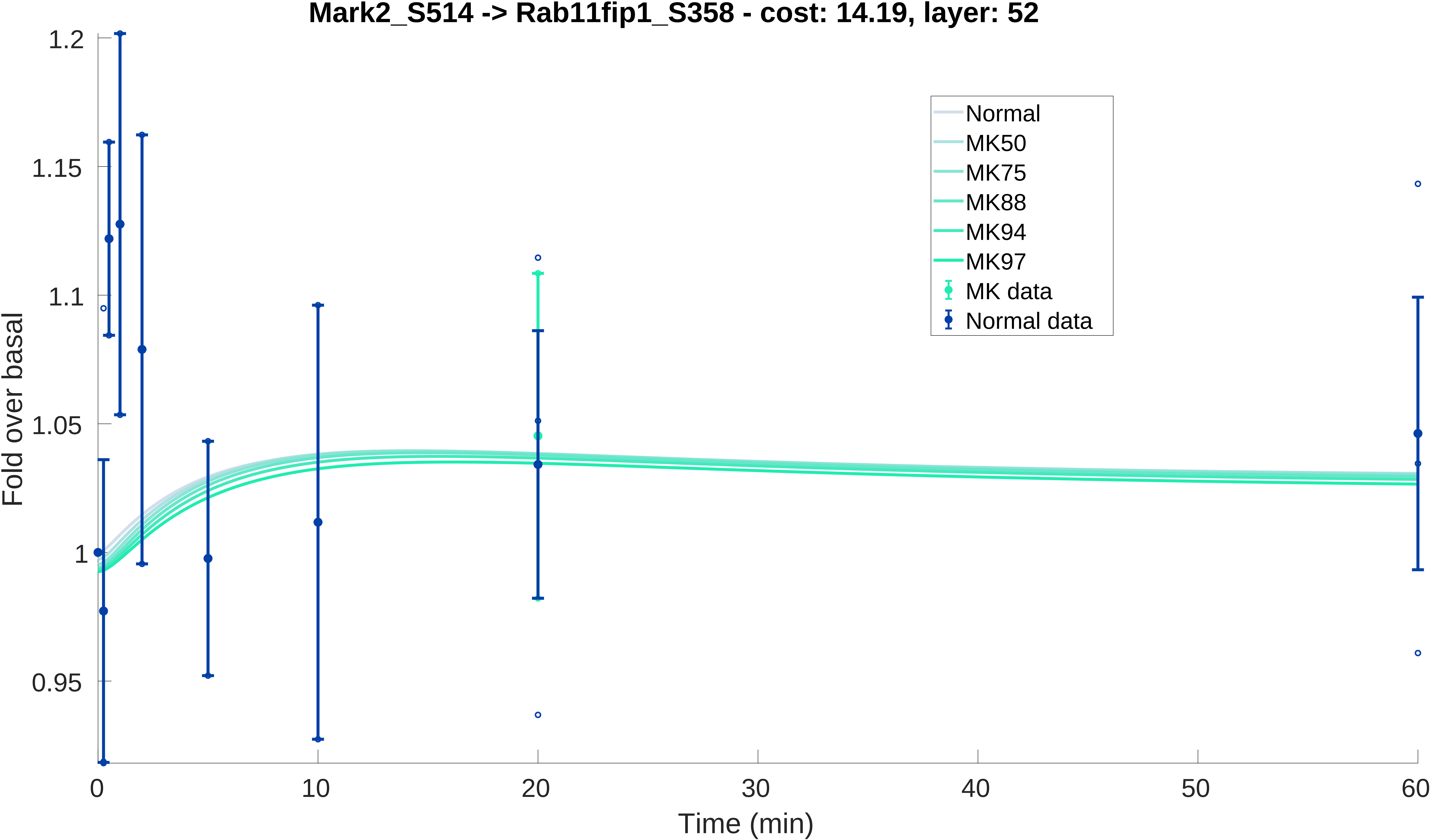

Mark2_S514 -> Rab11fip1_S358 - cost: 14.19, layer: 52
Fold over basal
Time (min)
1.2
1.15
1.1
1.05
1
0.95
0
10
20
30
40
50
60
Normal
MK50
MK75
MK88
MK94
MK97
MK data
Normal data

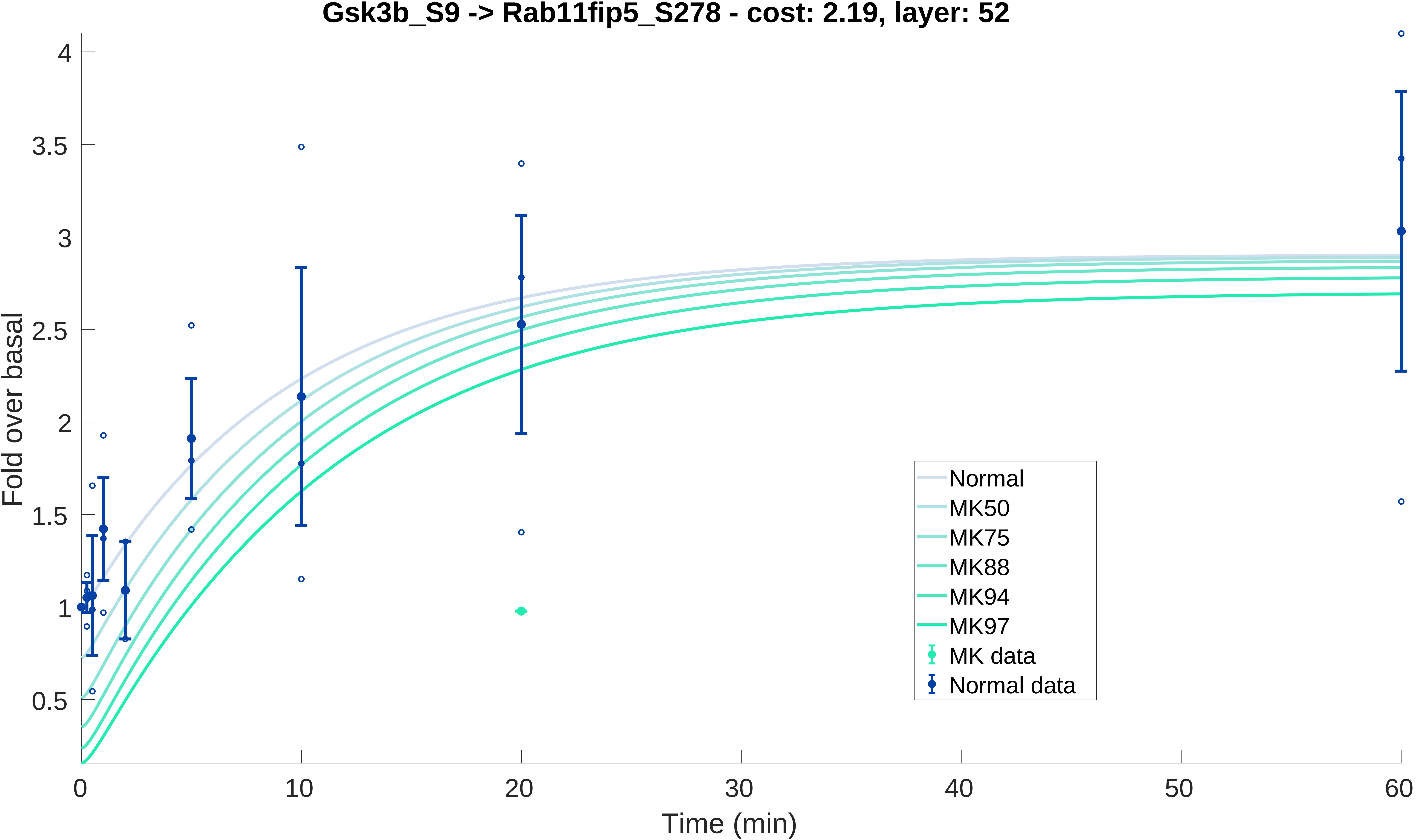
Gsk3b_S9 -> Rab11fip5_S278 - cost: 2.19, layer: 52
Fold over basal
Time (min)
0
10
20
30
40
50
60
0.5
1
1.5
2
2.5
3
3.5
4
Normal
MK50
MK75
MK88
MK94
MK97
MK data
Normal data

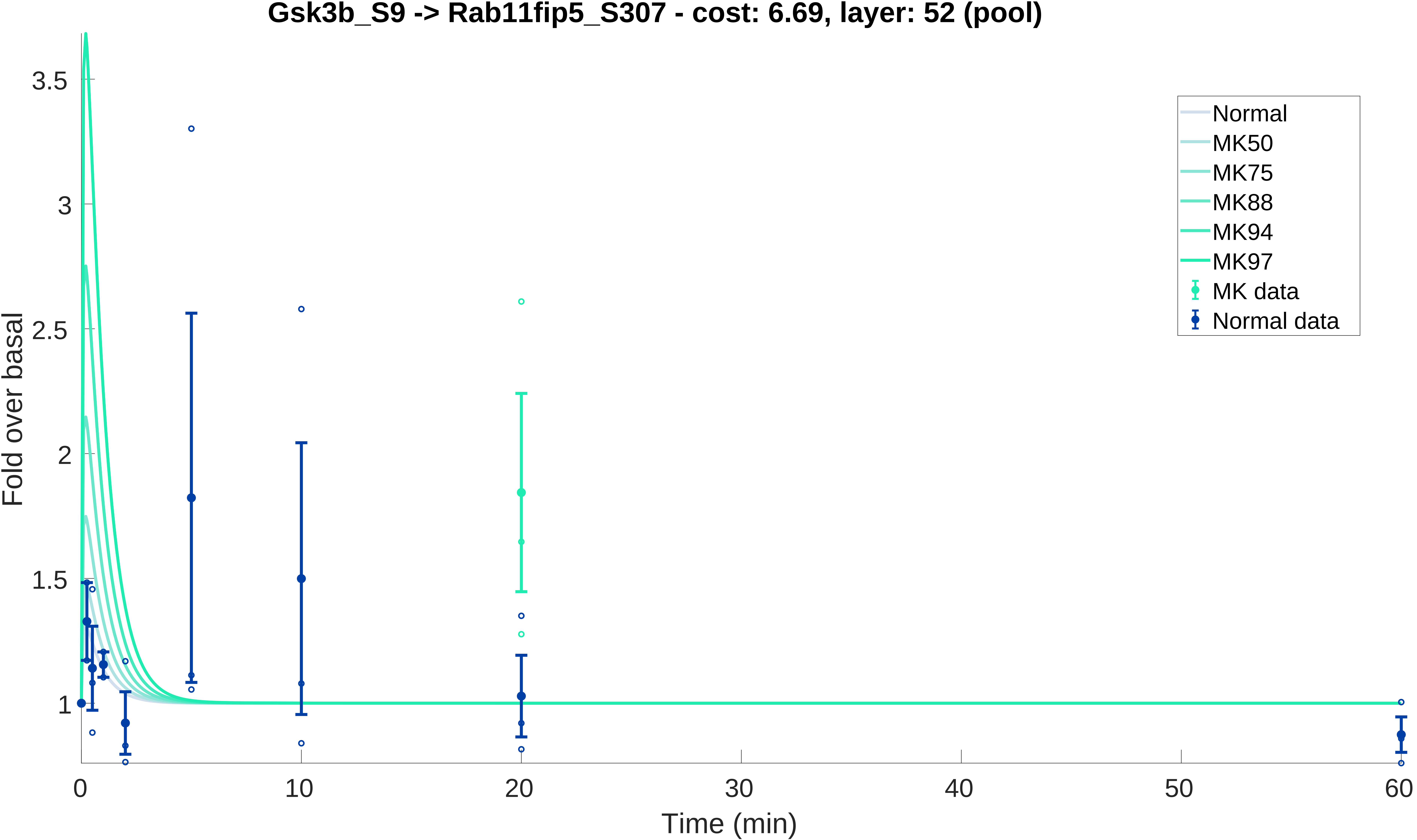

Gsk3b_S9 -> Rab11fip5_S307 - cost: 6.69, layer: 52 (pool)
Fold over basal
Time (min)
Normal
MK50
MK75
MK88
MK94
MK97
MK data
Normal data
0
10
20
30
40
50
60
1
1.5
2
2.5
3
3.5

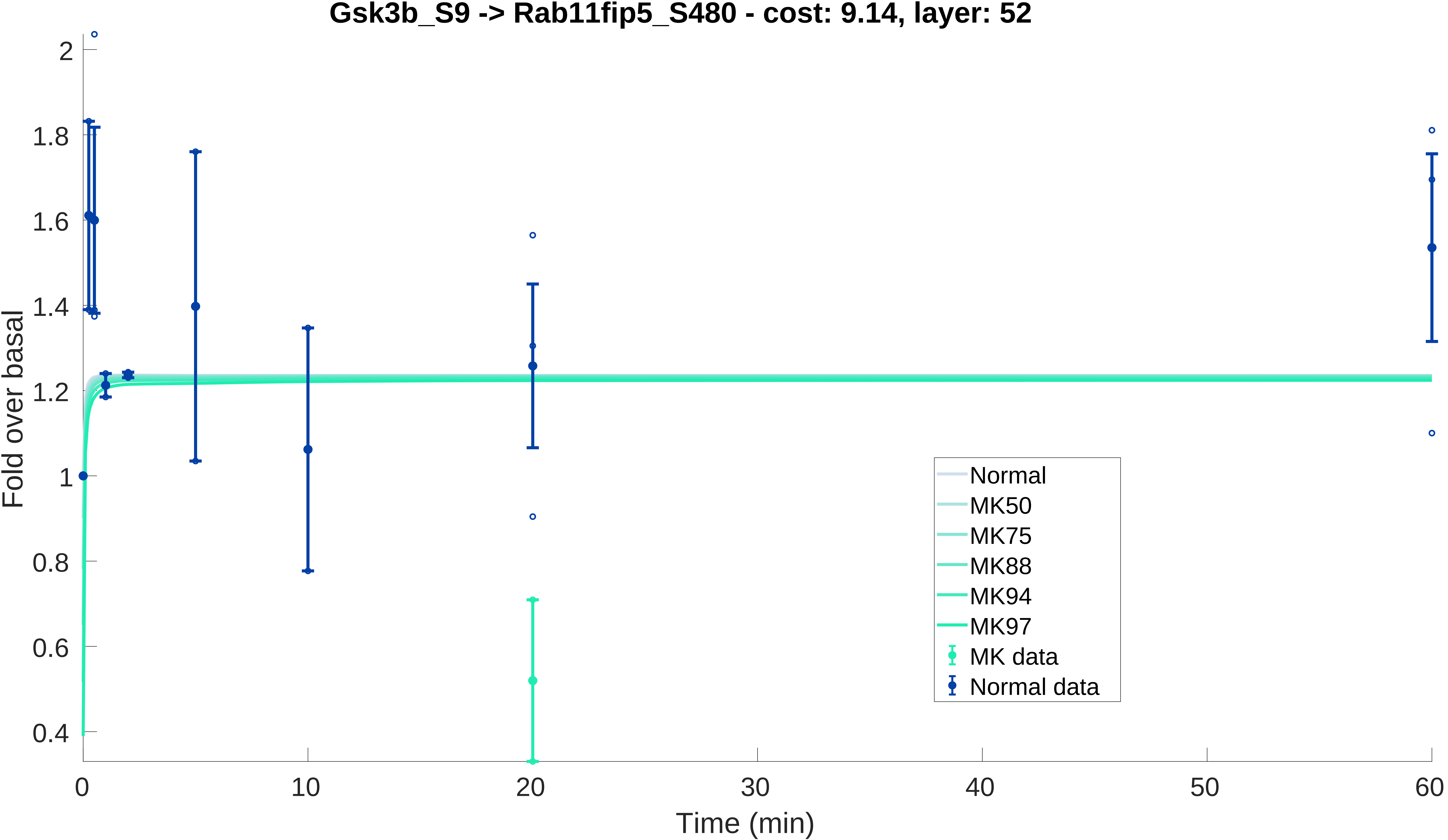

Gsk3b_S9 -> Rab11fip5_S480 - cost: 9.14, layer: 52
Fold over basal
Time (min)
2
1.8
1.6
1.4
1.2
1
0.8
0.6
0.4
0
10
20
30
40
50
60
Normal
MK50
MK75
MK88
MK94
MK97
MK data
Normal data

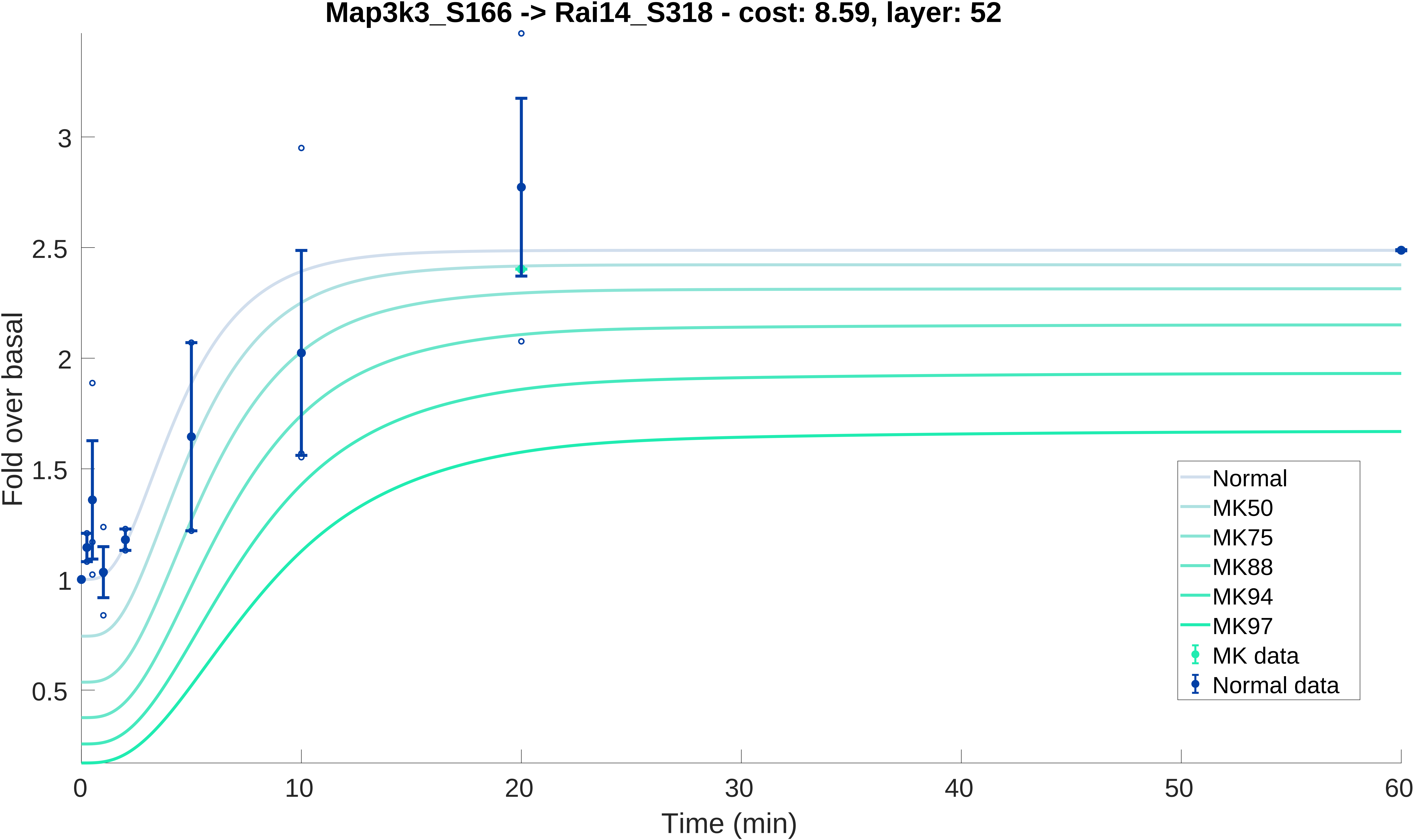
Map3k3_S166 -> Rai14_S318 - cost: 8.59, layer: 52
Fold over basal
0.5
1
1.5
2
2.5
3
Time (min)
0
10
20
30
40
50
60
Normal
MK50
MK75
MK88
MK94
MK97
MK data
Normal data

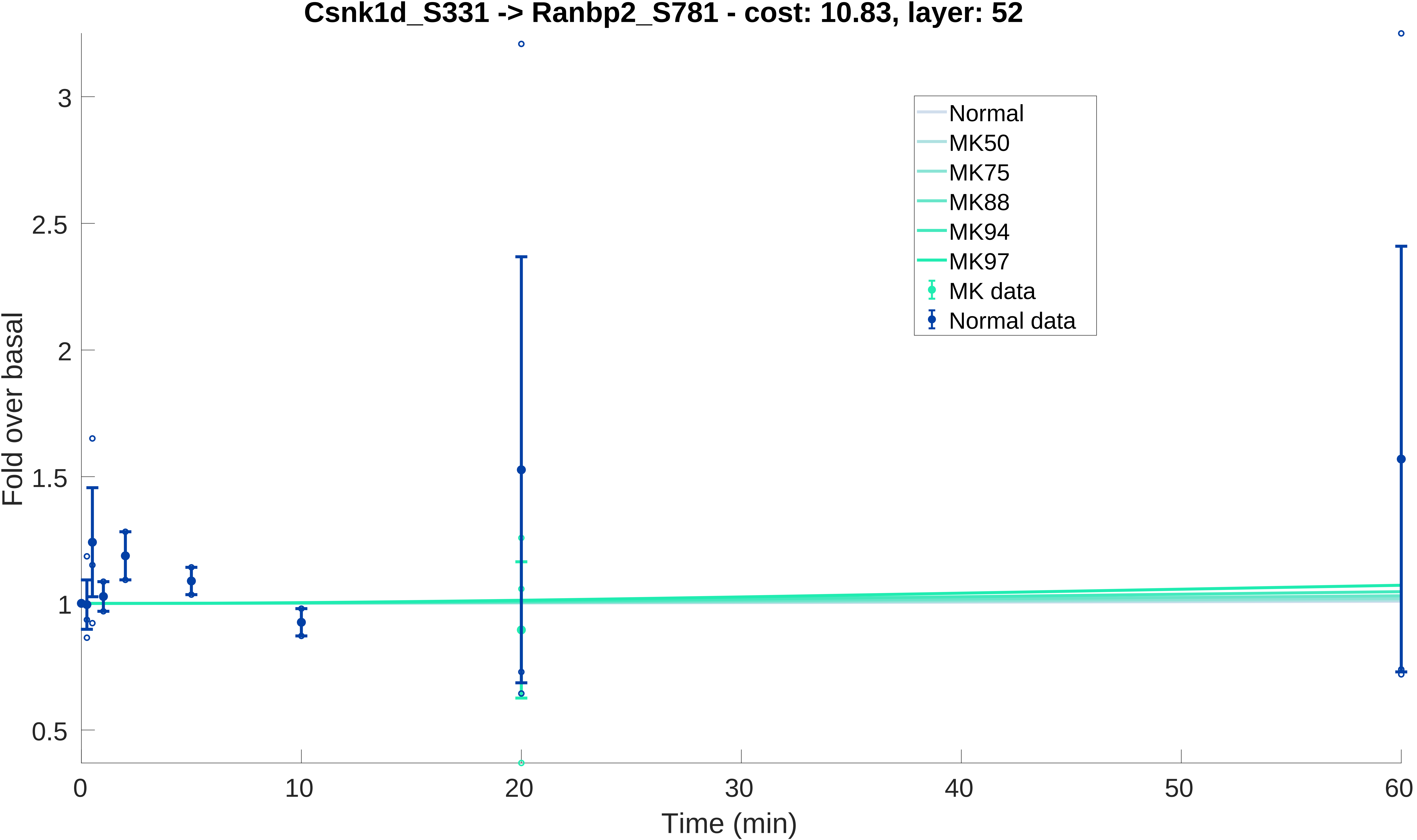

Csnk1d_S331 -> Ranbp2_S781 - cost: 10.83, layer: 52
Fold over basal
Time (min)
0.5
1
1.5
2
2.5
3
0
10
20
30
40
50
60
Normal
MK50
MK75
MK88
MK94
MK97
MK data
Normal data

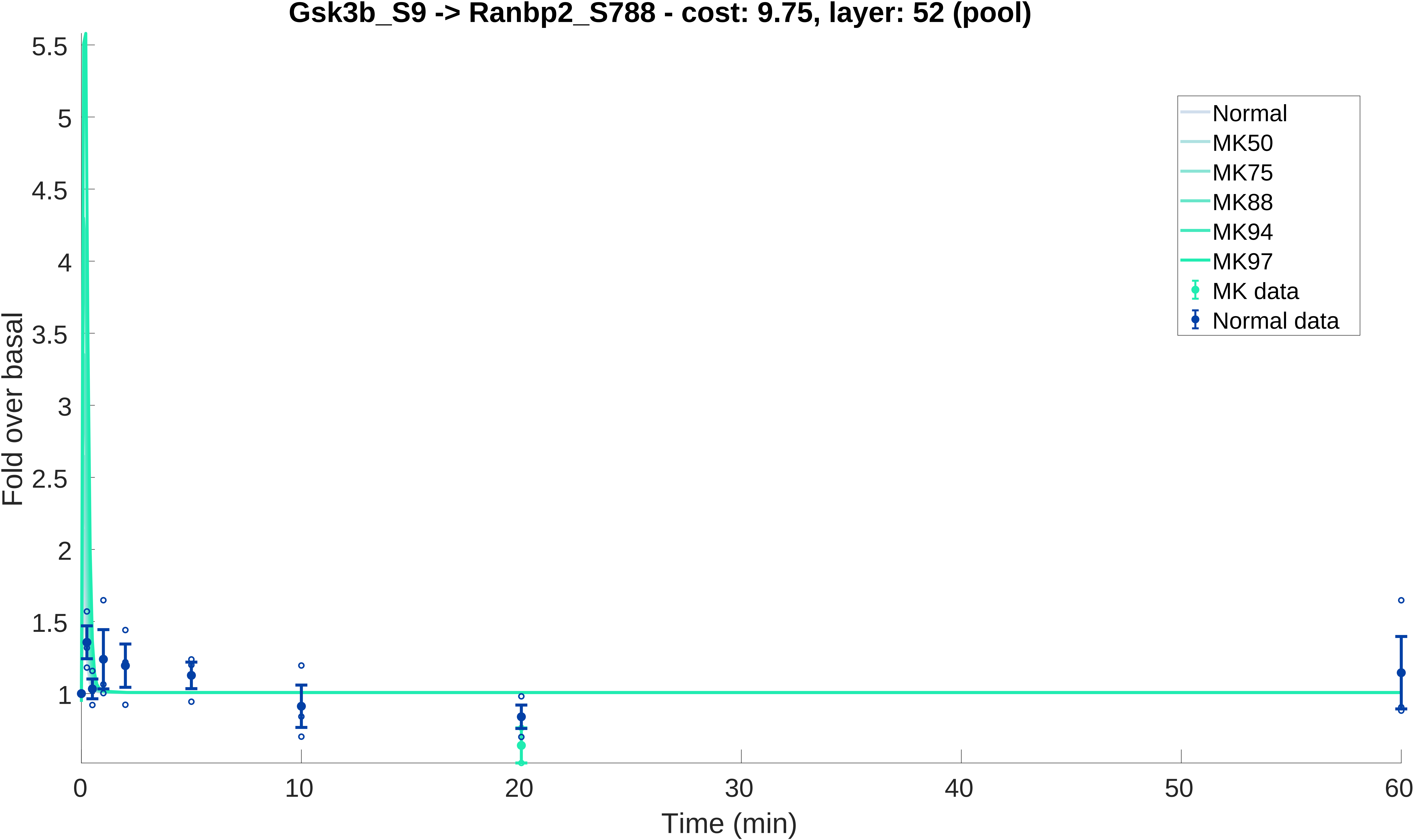

Gsk3b_S9 -> Ranbp2_S788 - cost: 9.75, layer: 52 (pool)
Fold over basal
5.5
5
4.5
4
3.5
3
2.5
2
1.5
1
0
10
20
30
40
50
60
Time (min)
Normal
MK50
MK75
MK88
MK94
MK97
MK data
Normal data

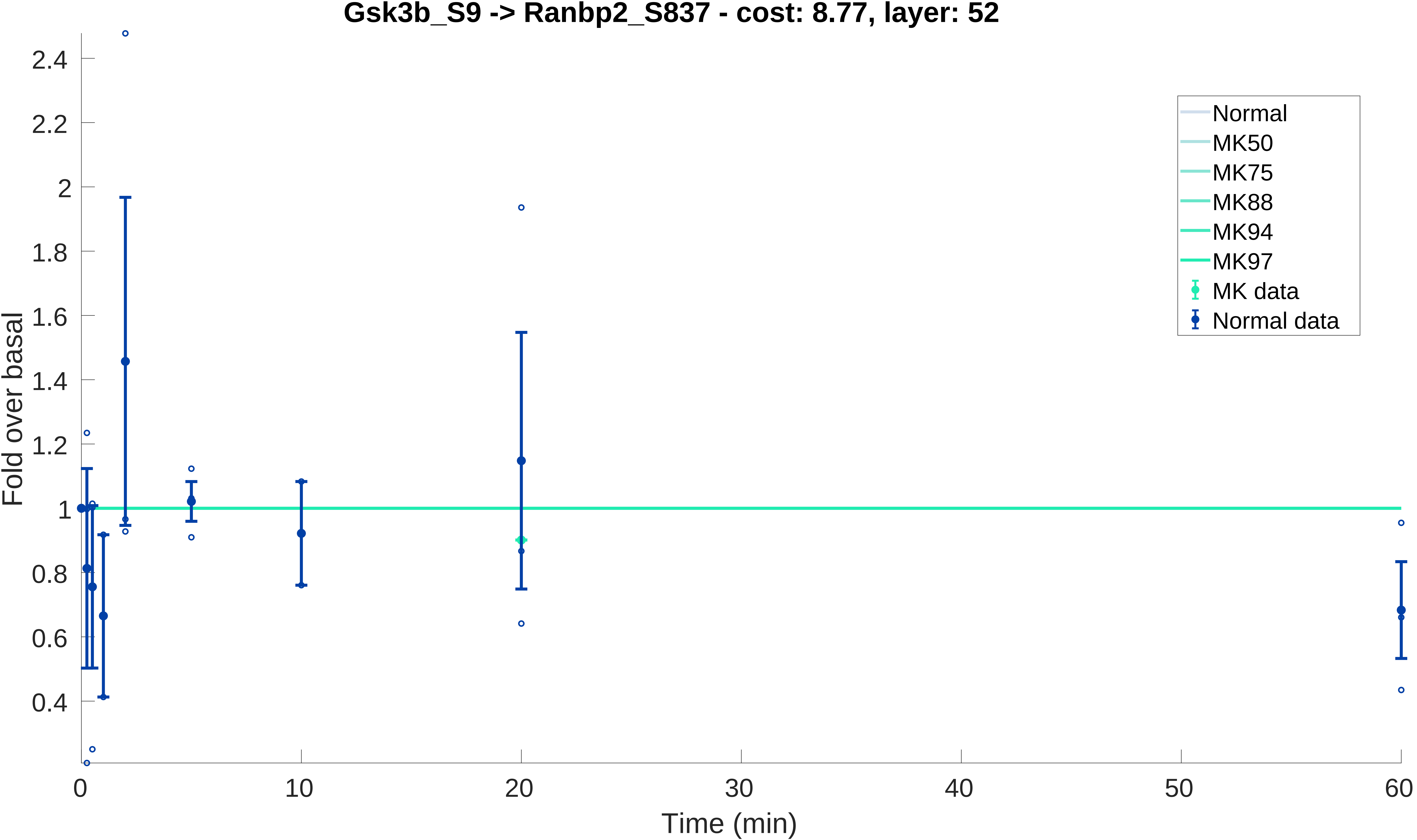

Gsk3b_S9 -> Ranbp2_S837 - cost: 8.77, layer: 52
Fold over basal
Time (min)
0.4
0.6
0.8
1
1.2
1.4
1.6
1.8
2
2.2
2.4
0
10
20
30
40
50
60
Normal
MK50
MK75
MK88
MK94
MK97
MK data
Normal data

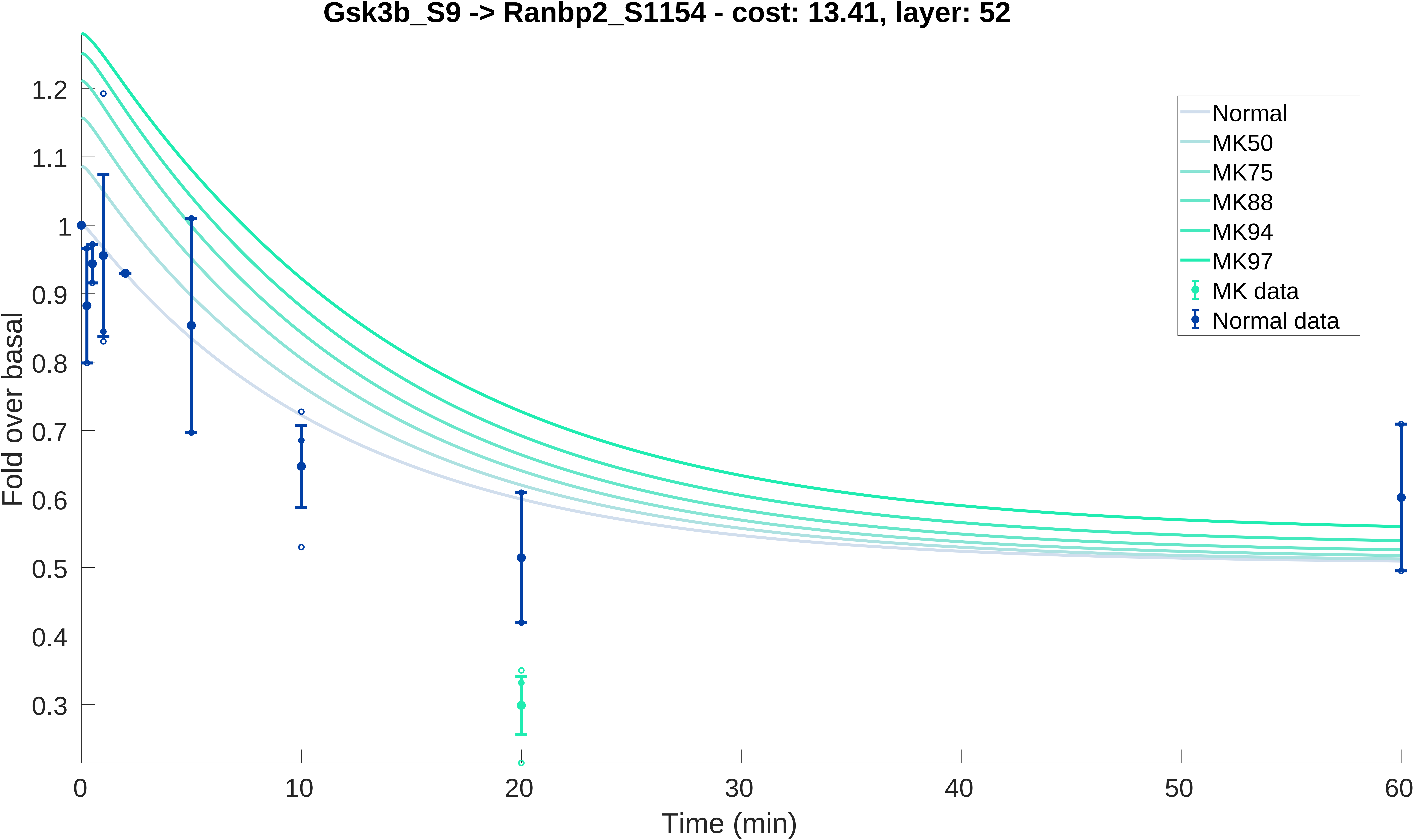
Gsk3b_S9 -> Ranbp2_S1154 - cost: 13.41, layer: 52
Fold over basal
Time (min)
1.2
1.1
1
0.9
0.8
0.7
0.6
0.5
0.4
0.3
0
10
20
30
40
50
60
Normal
MK50
MK75
MK88
MK94
MK97
MK data
Normal data

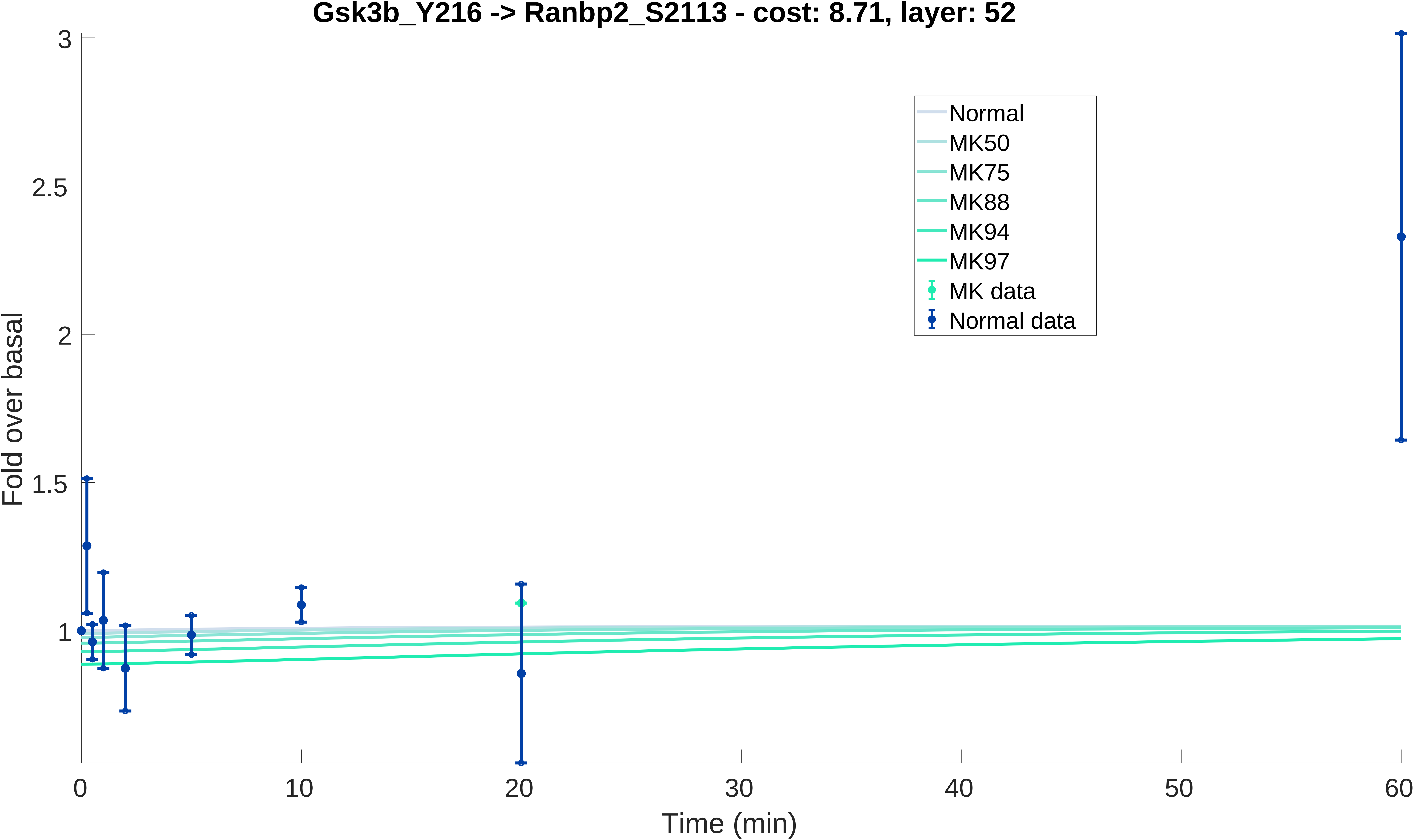

Gsk3b_Y216 -> Ranbp2_S2113 - cost: 8.71, layer: 52
Fold over basal
Time (min)
Normal
MK50
MK75
MK88
MK94
MK97
MK data
Normal data
3
2.5
2
1.5
1
0
10
20
30
40
50
60

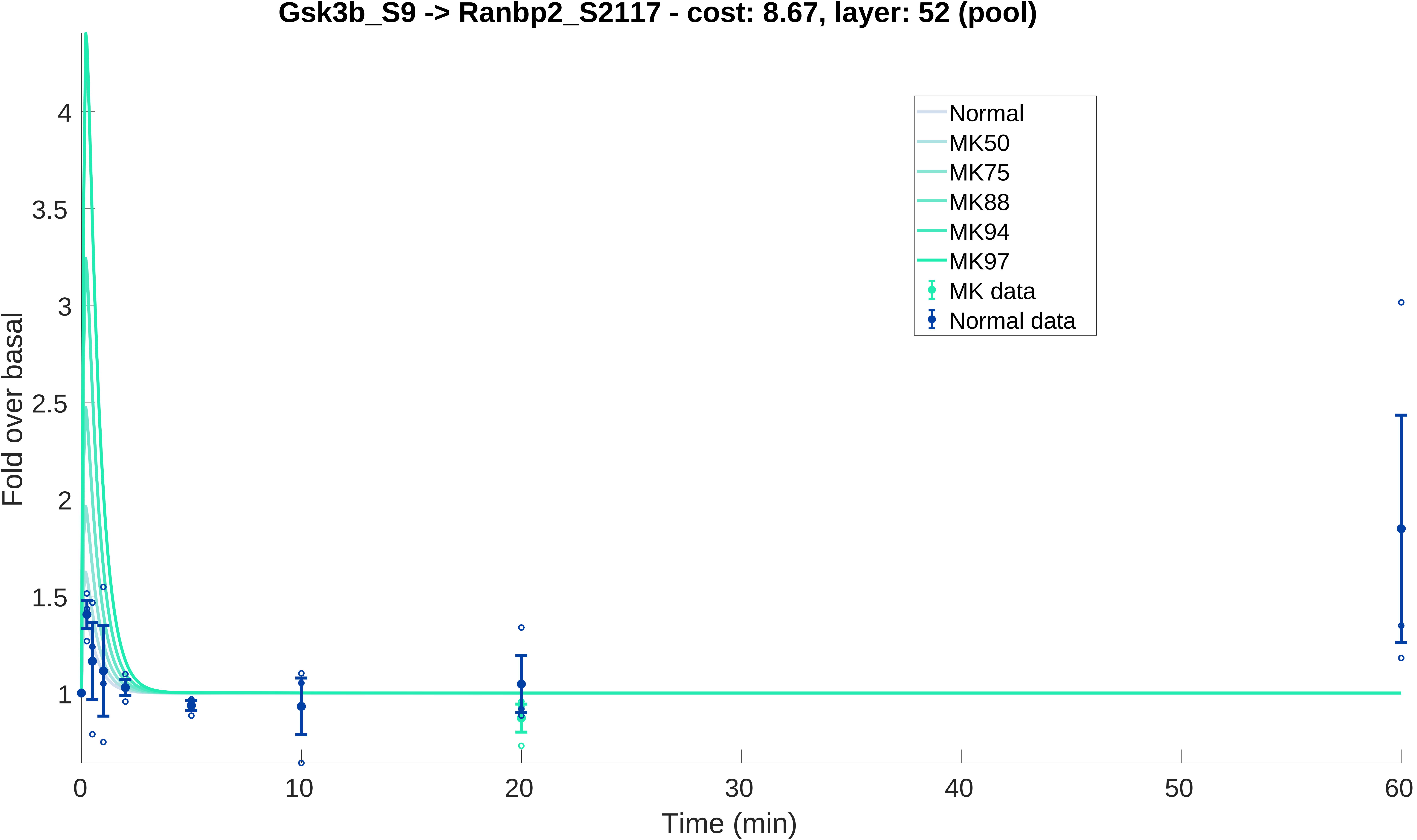

Gsk3b_S9 -> Ranbp2_S2117 - cost: 8.67, layer: 52 (pool)
Fold over basal
Time (min)
4
3.5
3
2.5
2
1.5
1
0
10
20
30
40
50
60
Normal
MK50
MK75
MK88
MK94
MK97
MK data
Normal data

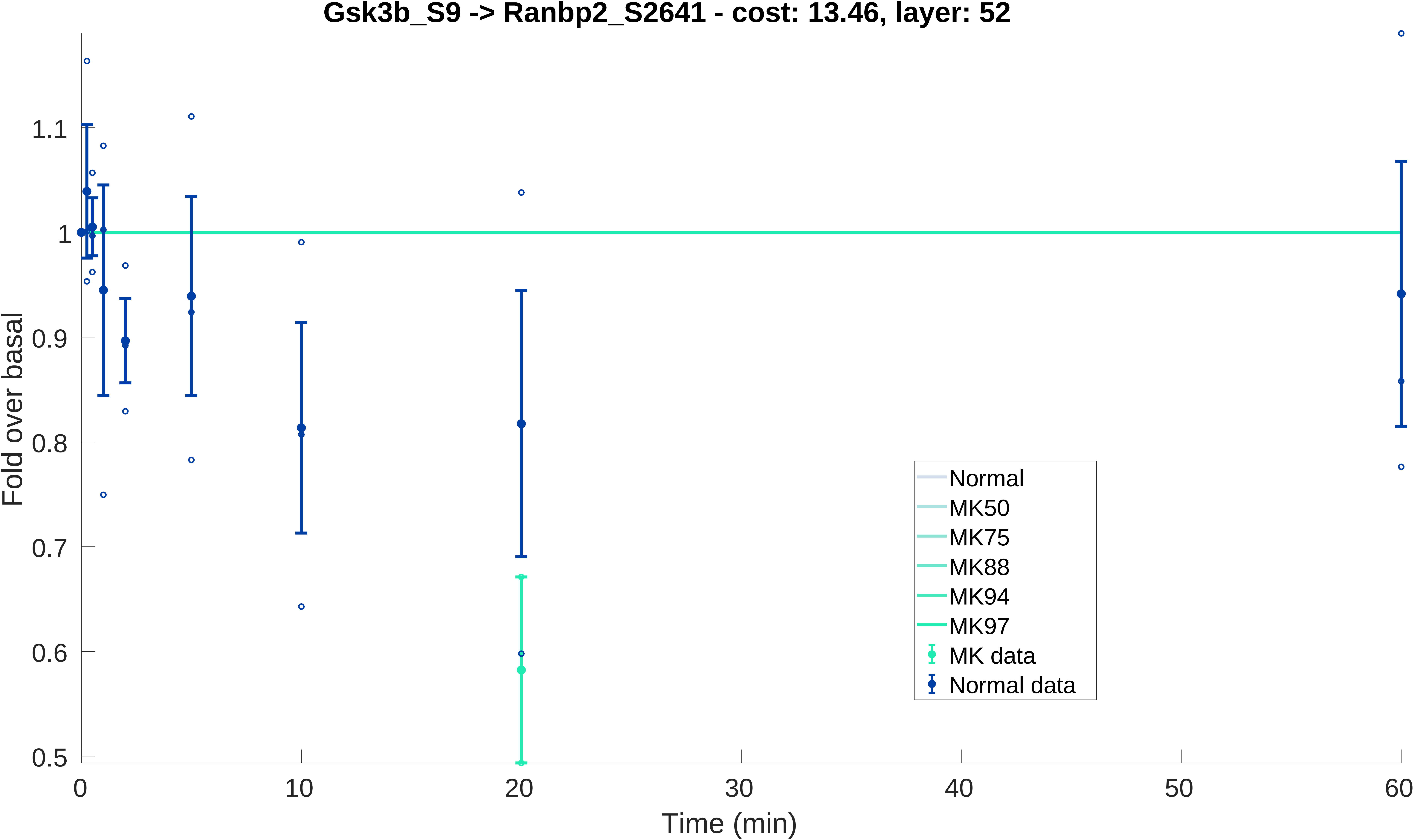
Gsk3b_S9 -> Ranbp2_S2641 - cost: 13.46, layer: 52
Fold over basal
1.1
1
0.9
0.8
0.7
0.6
0.5
0
10
20
30
40
50
60
Time (min)
Normal
MK50
MK75
MK88
MK94
MK97
MK data
Normal data

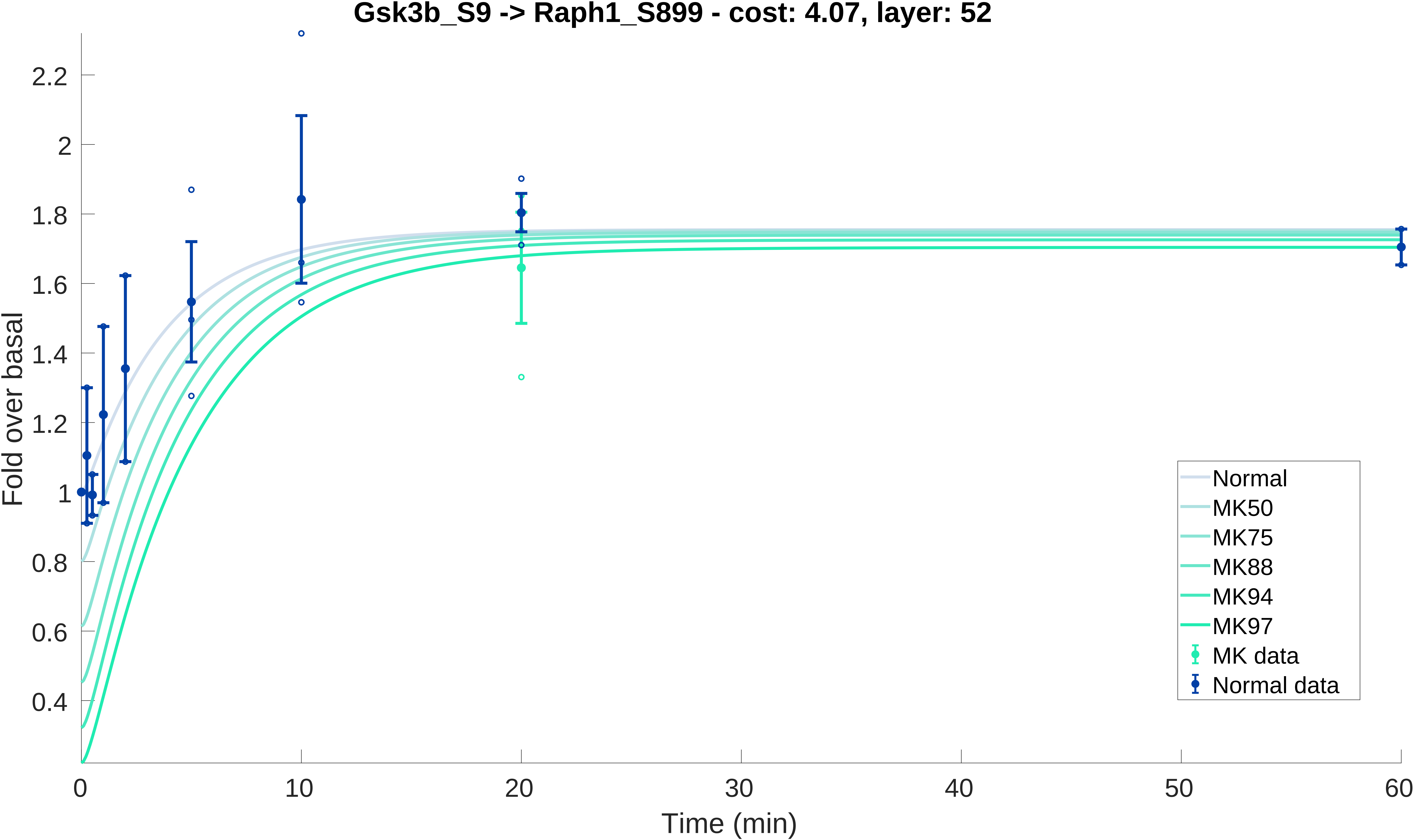

Gsk3b_S9 -> Raph1_S899 - cost: 4.07, layer: 52
Fold over basal
2.2
2
1.8
1.6
1.4
1.2
1
0.8
0.6
0.4
0
10
20
30
40
50
60
Time (min)
Normal
MK50
MK75
MK88
MK94
MK97
MK data
Normal data

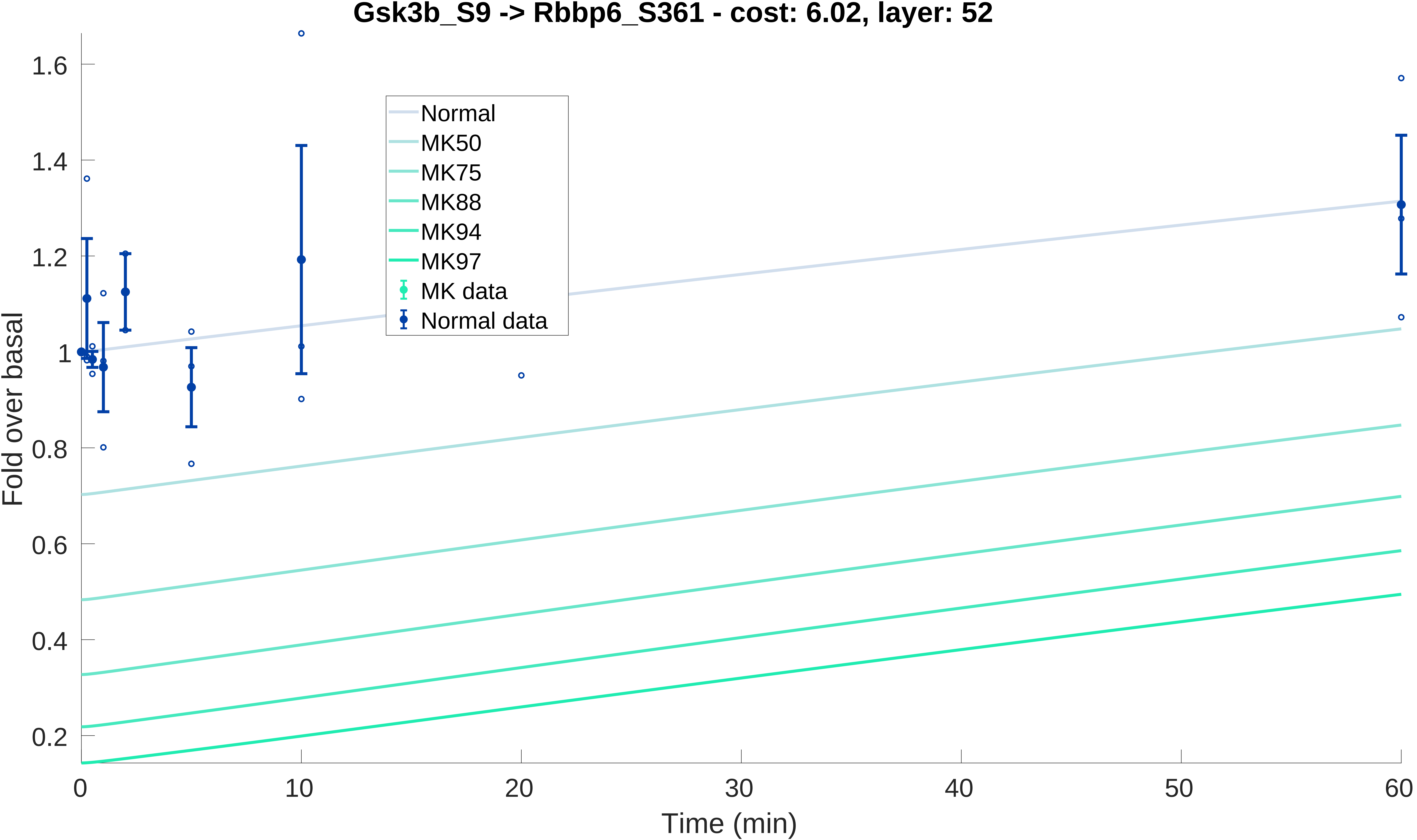

Gsk3b_S9 -> Rbbp6_S361 - cost: 6.02, layer: 52
Fold over basal
Time (min)
1.6
1.4
1.2
1
0.8
0.6
0.4
0.2
0
10
20
30
40
50
60
Normal
MK50
MK75
MK88
MK94
MK97
MK data
Normal data

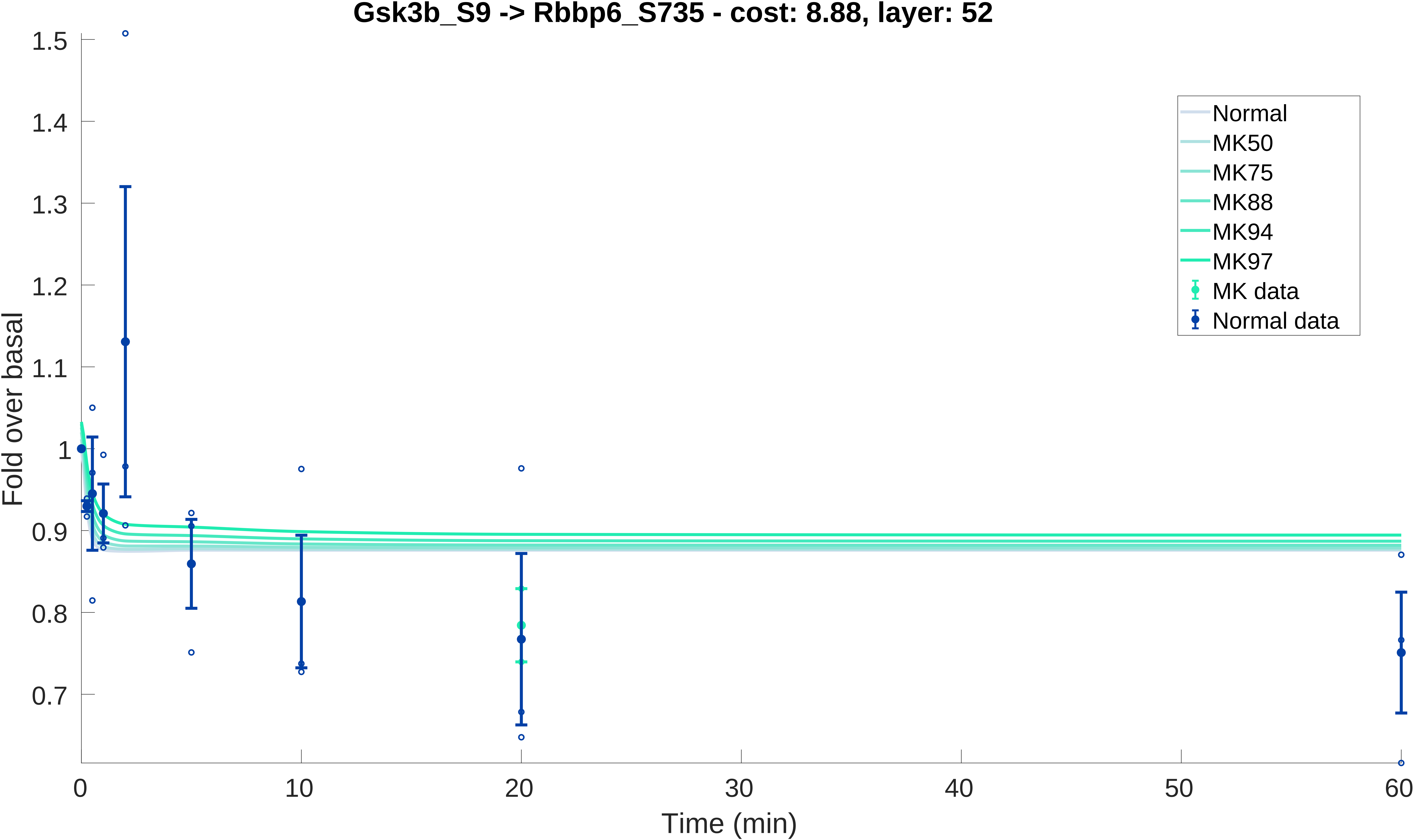

Gsk3b_S9 -> Rbbp6_S735 - cost: 8.88, layer: 52
Fold over basal
Time (min)
1.5
1.4
1.3
1.2
1.1
1
0.9
0.8
0.7
0
10
20
30
40
50
60
Normal
MK50
MK75
MK88
MK94
MK97
MK data
Normal data

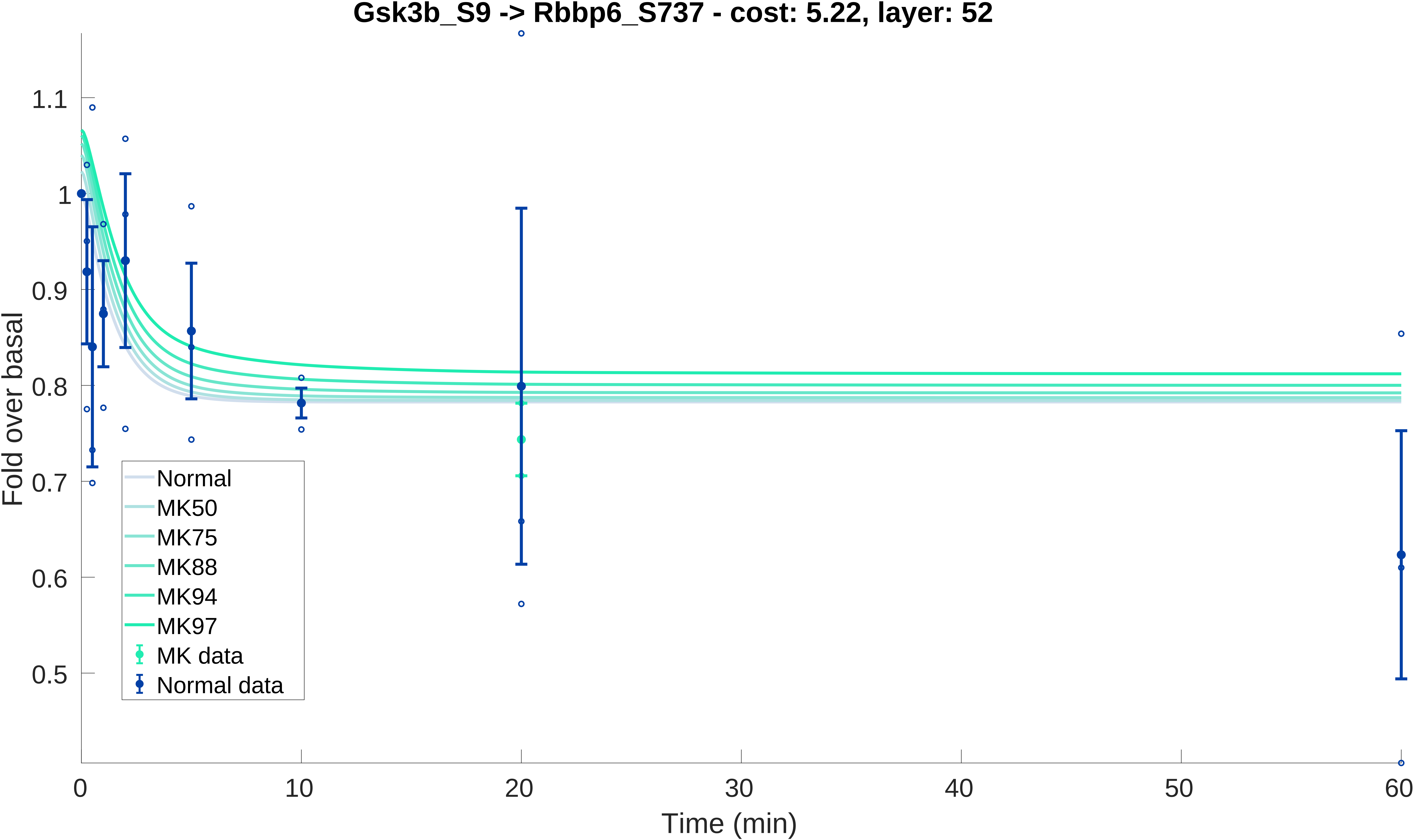
Gsk3b_S9 -> Rbbp6_S737 - cost: 5.22, layer: 52
Fold over basal
1.1
1
0.9
0.8
0.7
0.6
0.5
0
10
20
30
40
50
60
Time (min)
Normal
MK50
MK75
MK88
MK94
MK97
MK data
Normal data

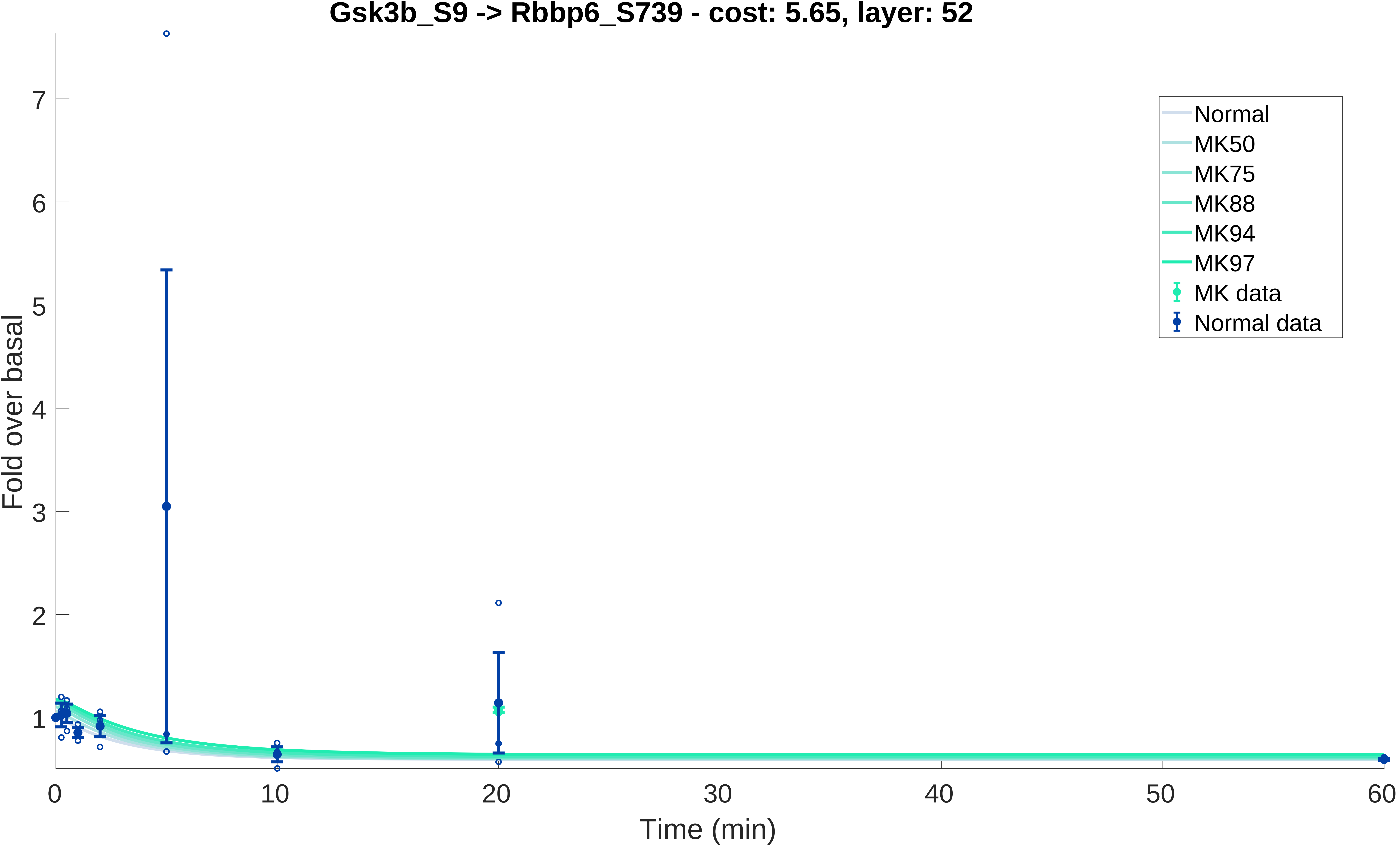

Gsk3b_S9 -> Rbbp6_S739 - cost: 5.65, layer: 52
Fold over basal
Time (min)
0
10
20
30
40
50
60
1
2
3
4
5
6
7
Normal
MK50
MK75
MK88
MK94
MK97
MK data
Normal data

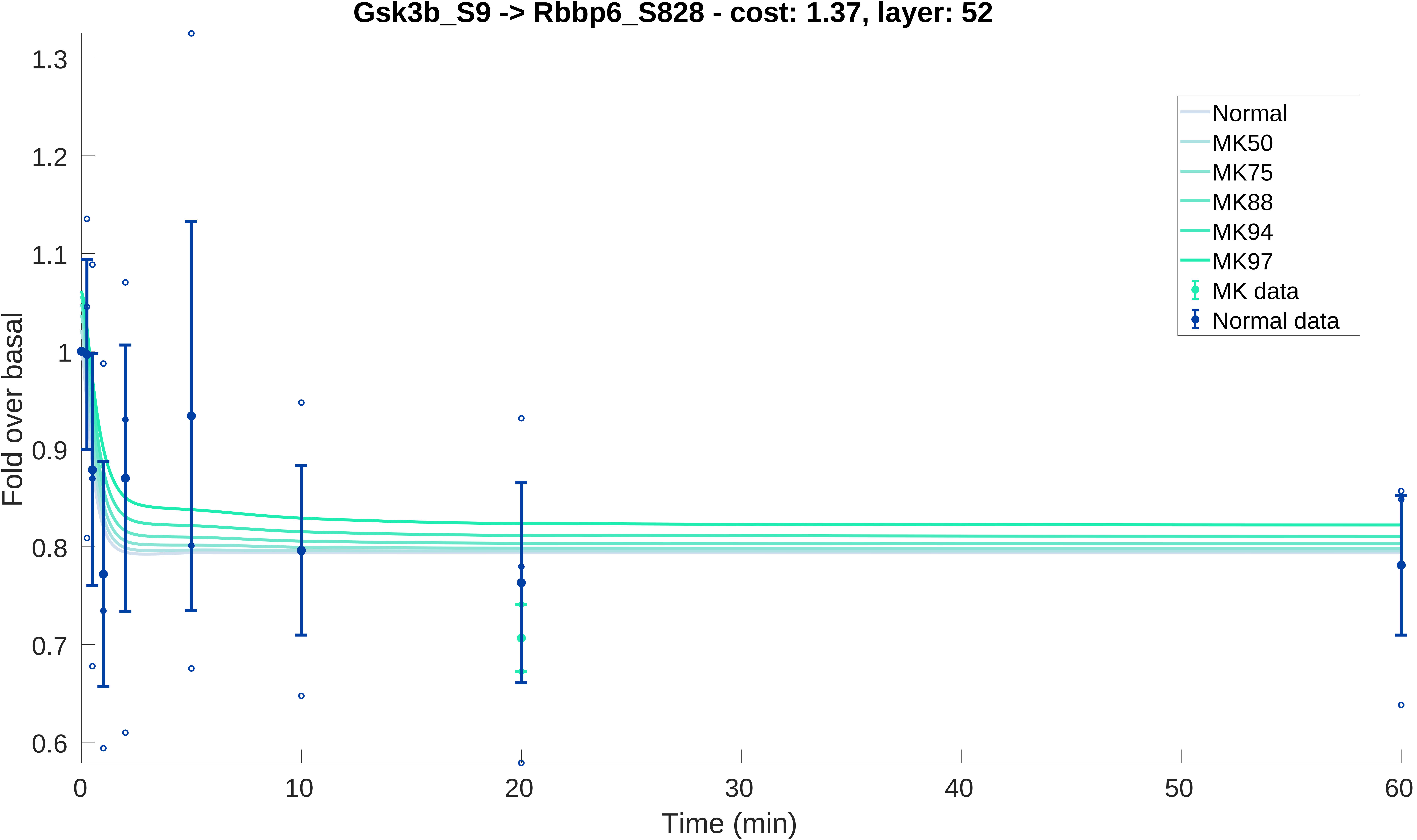

Gsk3b_S9 -> Rbbp6_S828 - cost: 1.37, layer: 52
Fold over basal
Time (min)
1.3
1.2
1.1
1
0.9
0.8
0.7
0.6
0
10
20
30
40
50
60
Normal
MK50
MK75
MK88
MK94
MK97
MK data
Normal data

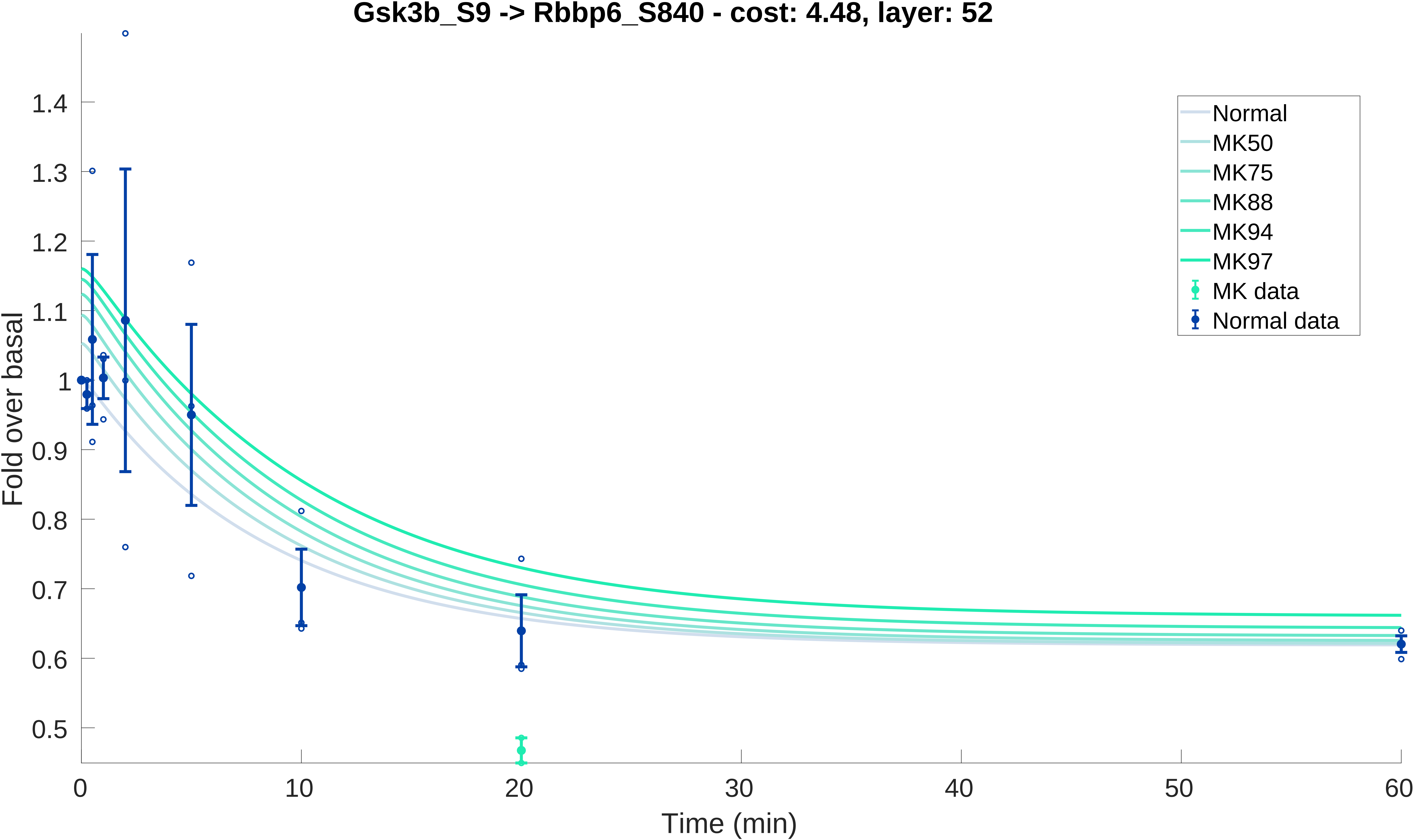

Gsk3b_S9 -> Rbbp6_S840 - cost: 4.48, layer: 52
Fold over basal
1.4
1.3
1.2
1.1
1
0.9
0.8
0.7
0.6
0.5
0
10
20
30
40
50
60
Time (min)
Normal
MK50
MK75
MK88
MK94
MK97
MK data
Normal data

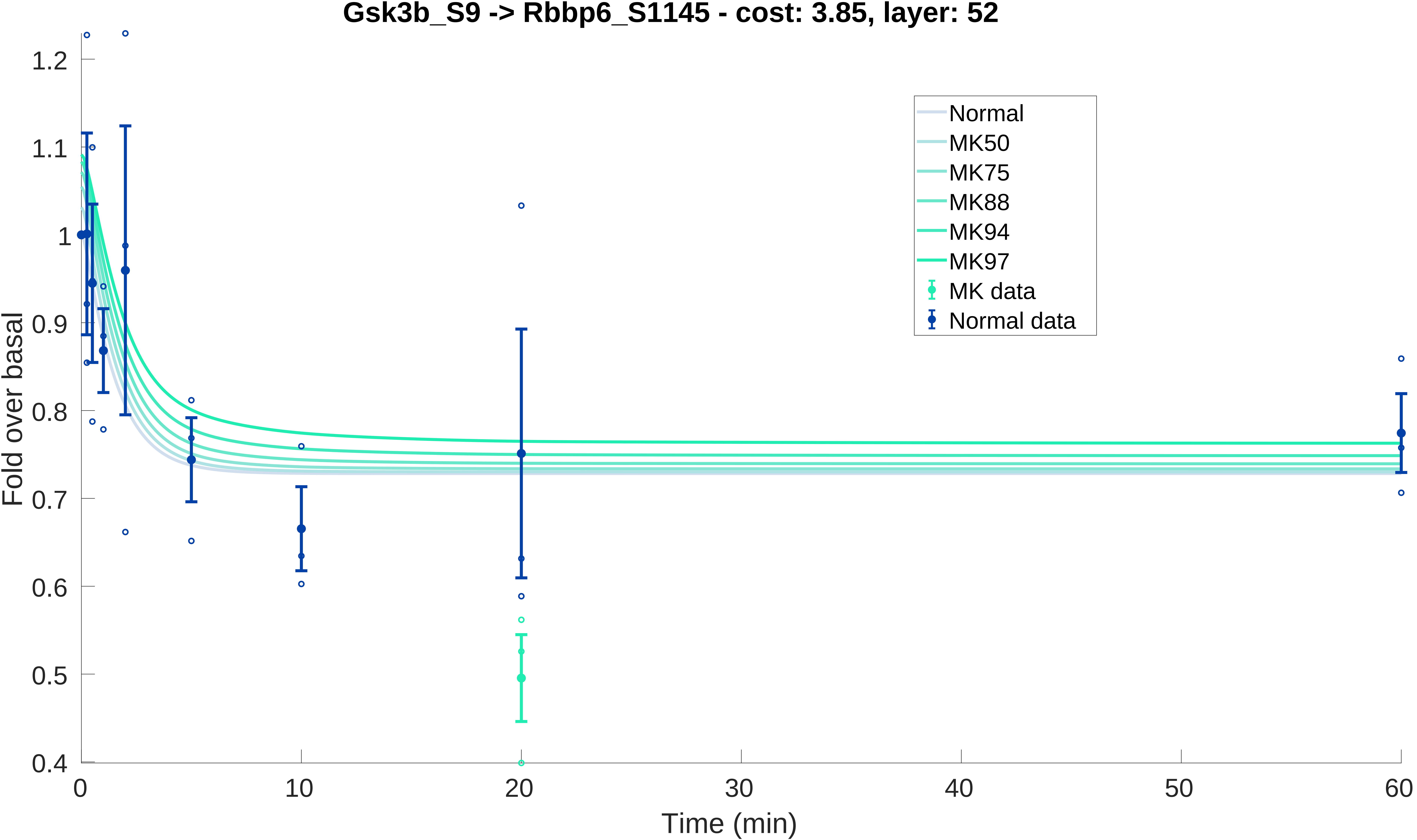

Gsk3b_S9 -> Rbbp6_S1145 - cost: 3.85, layer: 52
Fold over basal
Time (min)
0.4
0.5
0.6
0.7
0.8
0.9
1
1.1
1.2
0
10
20
30
40
50
60
Normal
MK50
MK75
MK88
MK94
MK97
MK data
Normal data

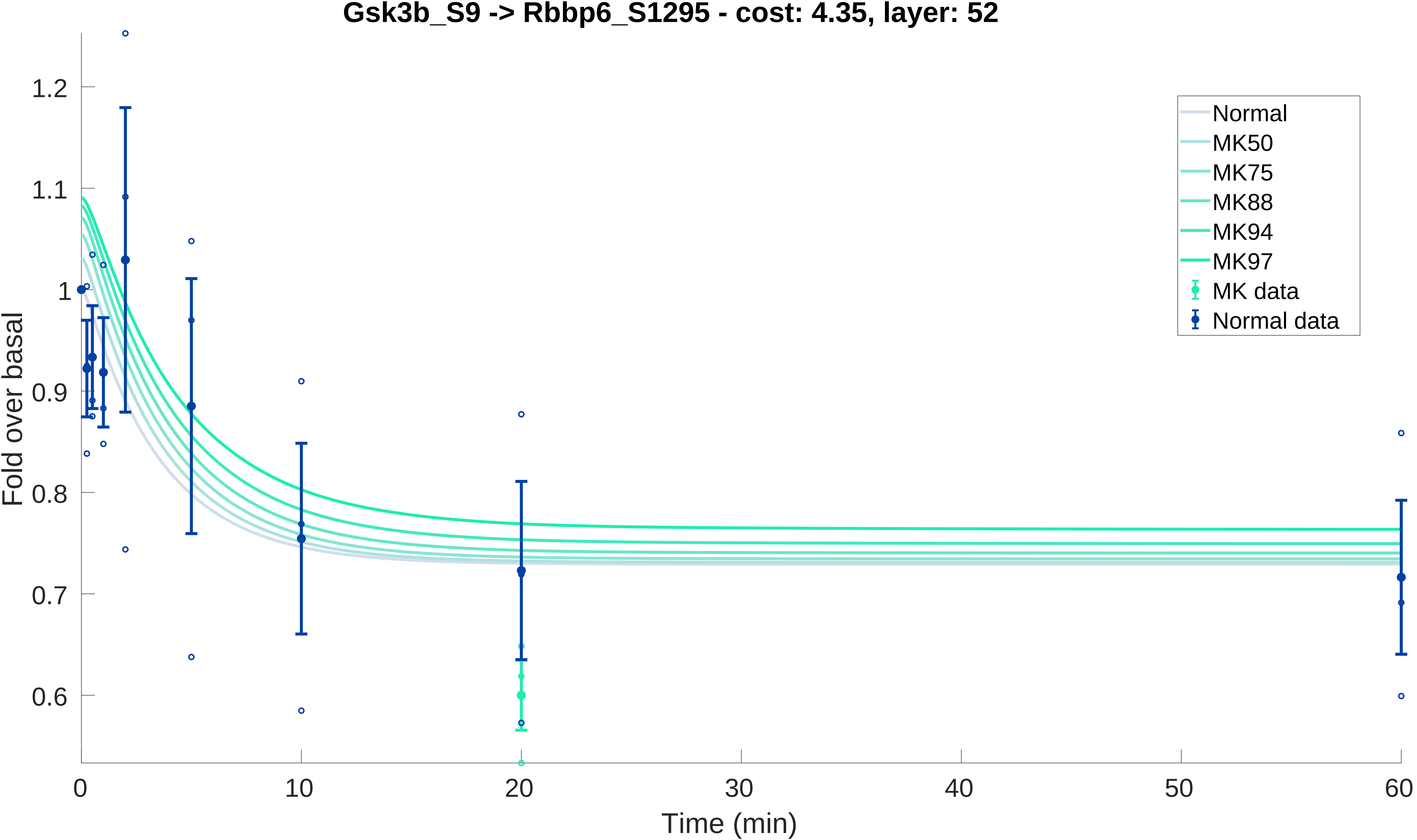

Gsk3b_S9 -> Rbbp6_S1295 - cost: 4.35, layer: 52
Fold over basal
Time (min)
Normal
MK50
MK75
MK88
MK94
MK97
MK data
Normal data
1.2
1.1
1
0.9
0.8
0.7
0.6
0
10
20
30
40
50
60

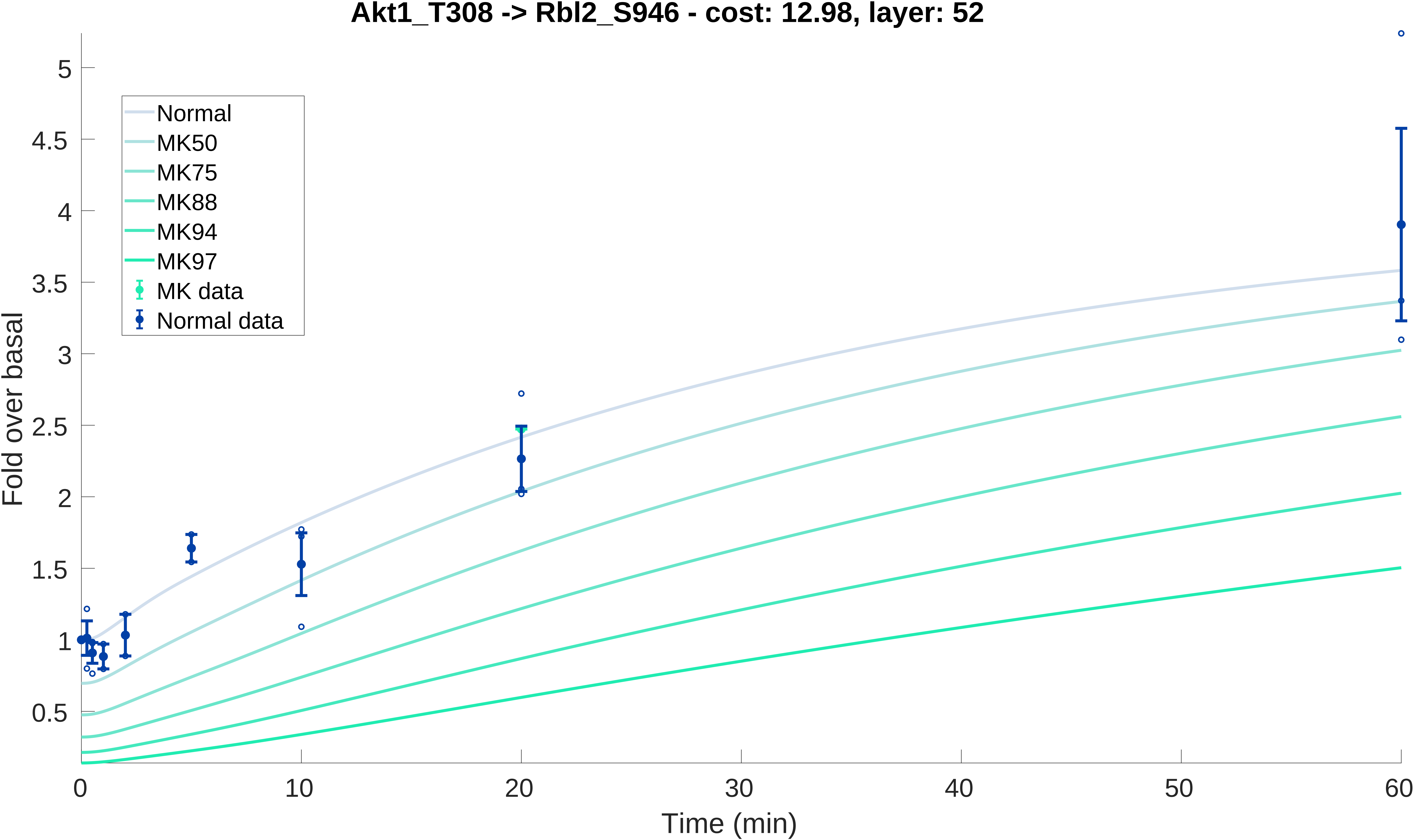

Akt1_T308 -> Rbl2_S946 - cost: 12.98, layer: 52
Normal
MK50
MK75
MK88
MK94
MK97
MK data
Normal data
Fold over basal
Time (min)
0.5
1
1.5
2
2.5
3
3.5
4
4.5
5
0
10
20
30
40
50
60

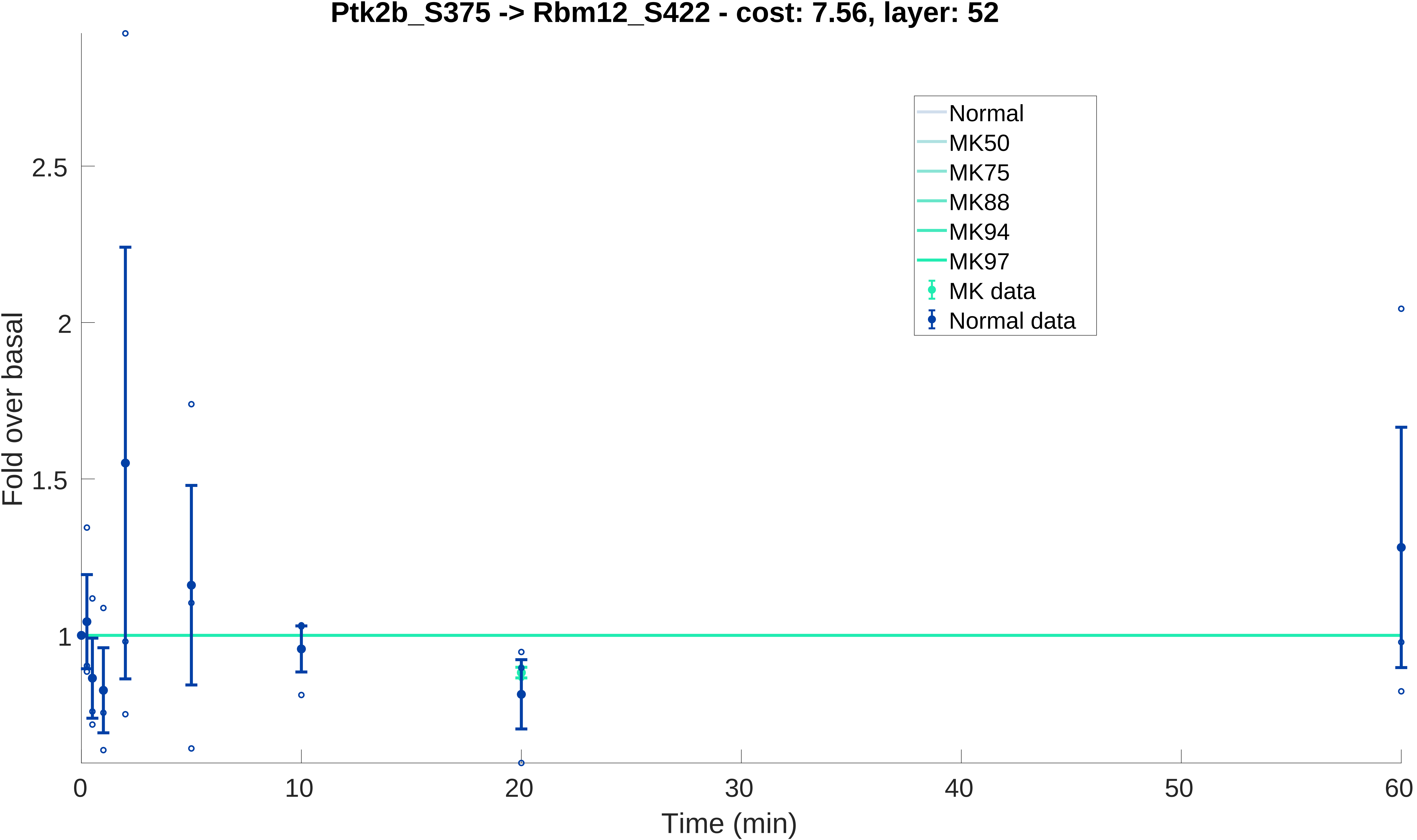
Ptk2b_S375 -> Rbm12_S422 - cost: 7.56, layer: 52
Normal
MK50
MK75
MK88
MK94
MK97
MK data
Normal data
Fold over basal
Time (min)
2.5
2
1.5
1
0
10
20
30
40
50
60

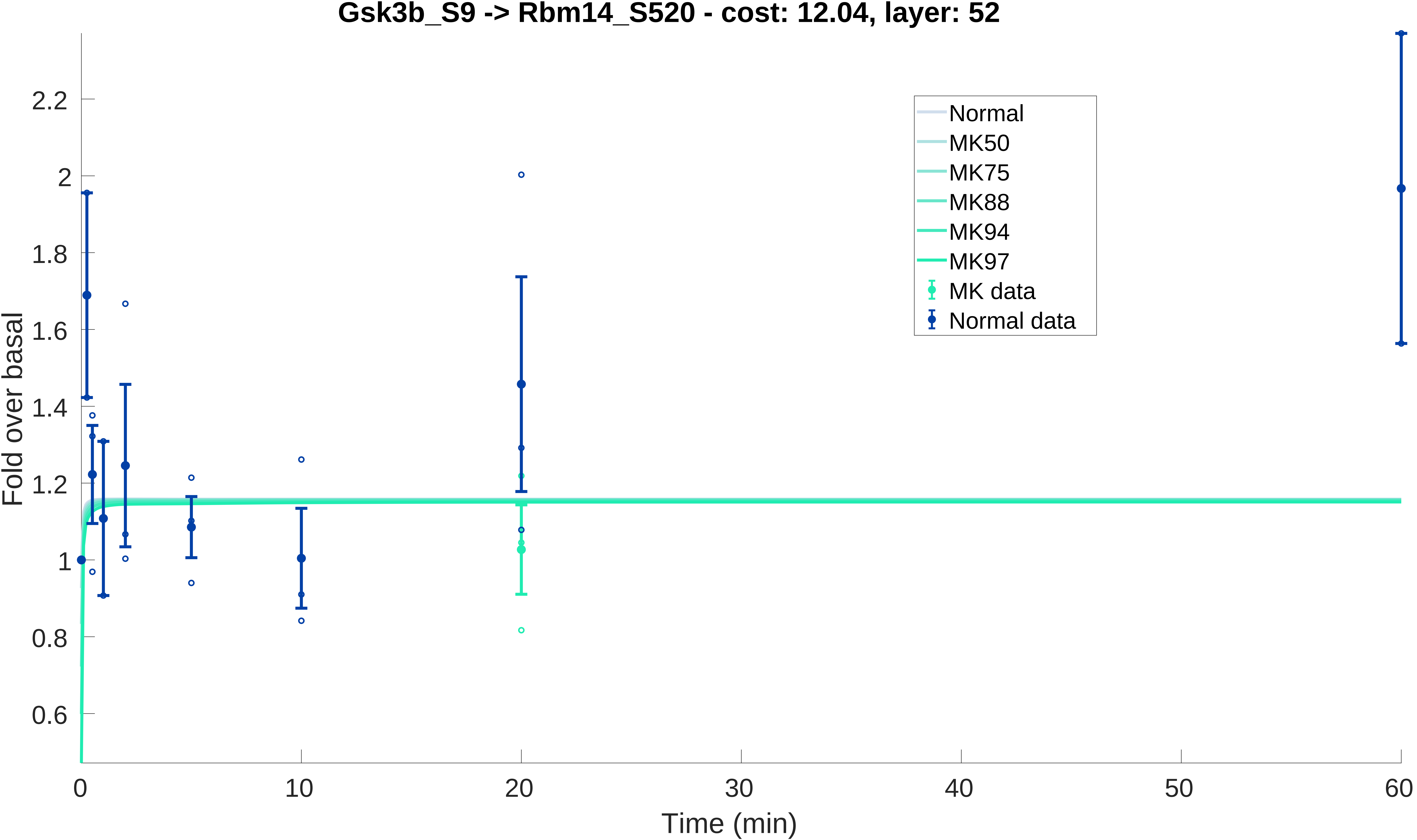
Gsk3b_S9 -> Rbm14_S520 - cost: 12.04, layer: 52
Fold over basal
Time (min)
Normal
MK50
MK75
MK88
MK94
MK97
MK data
Normal data
0.6
0.8
1
1.2
1.4
1.6
1.8
2
2.2
0
10
20
30
40
50
60

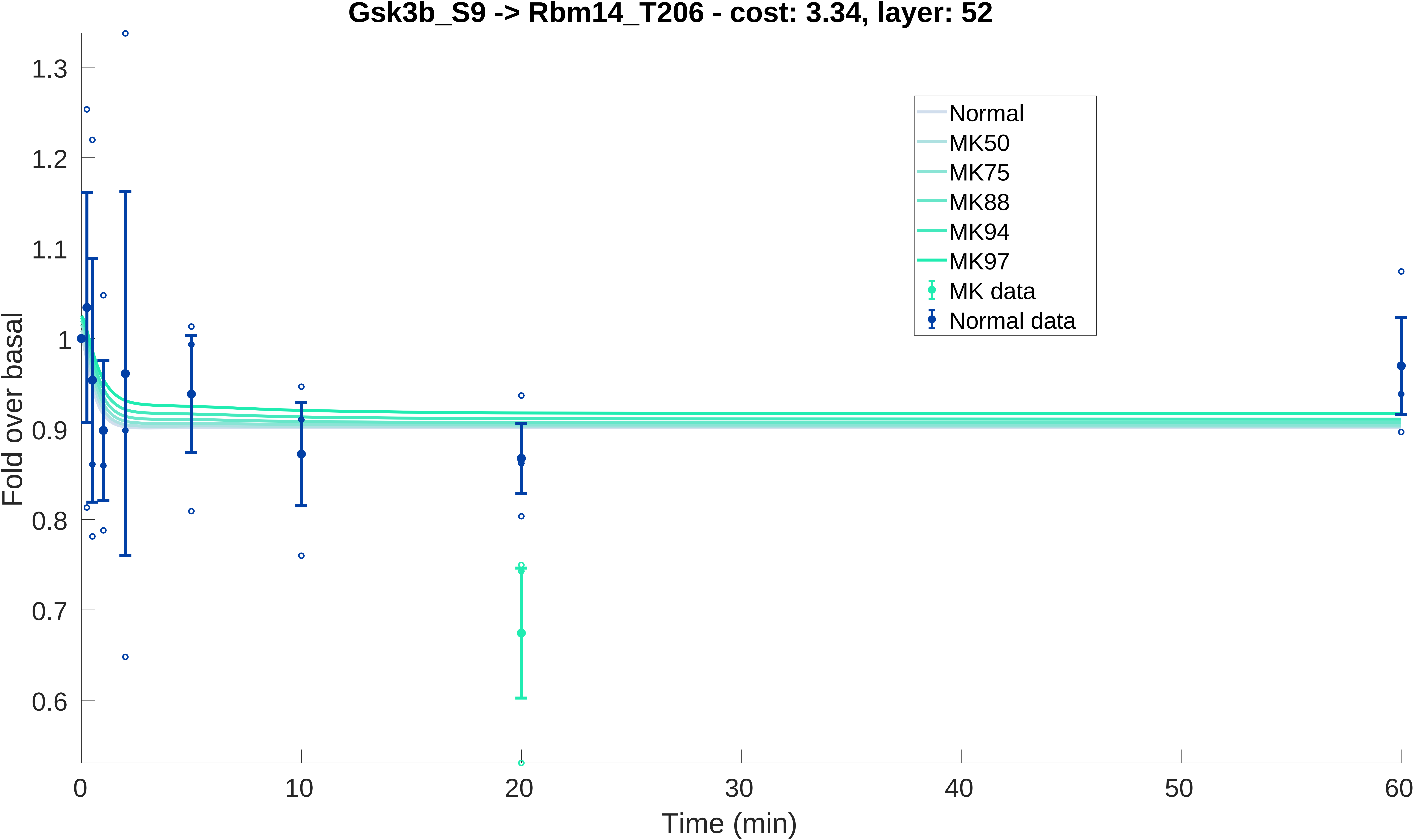

Gsk3b_S9 -> Rbm14_T206 - cost: 3.34, layer: 52
Fold over basal
Time (min)
1.3
1.2
1.1
1
0.9
0.8
0.7
0.6
0
10
20
30
40
50
60
Normal
MK50
MK75
MK88
MK94
MK97
MK data
Normal data

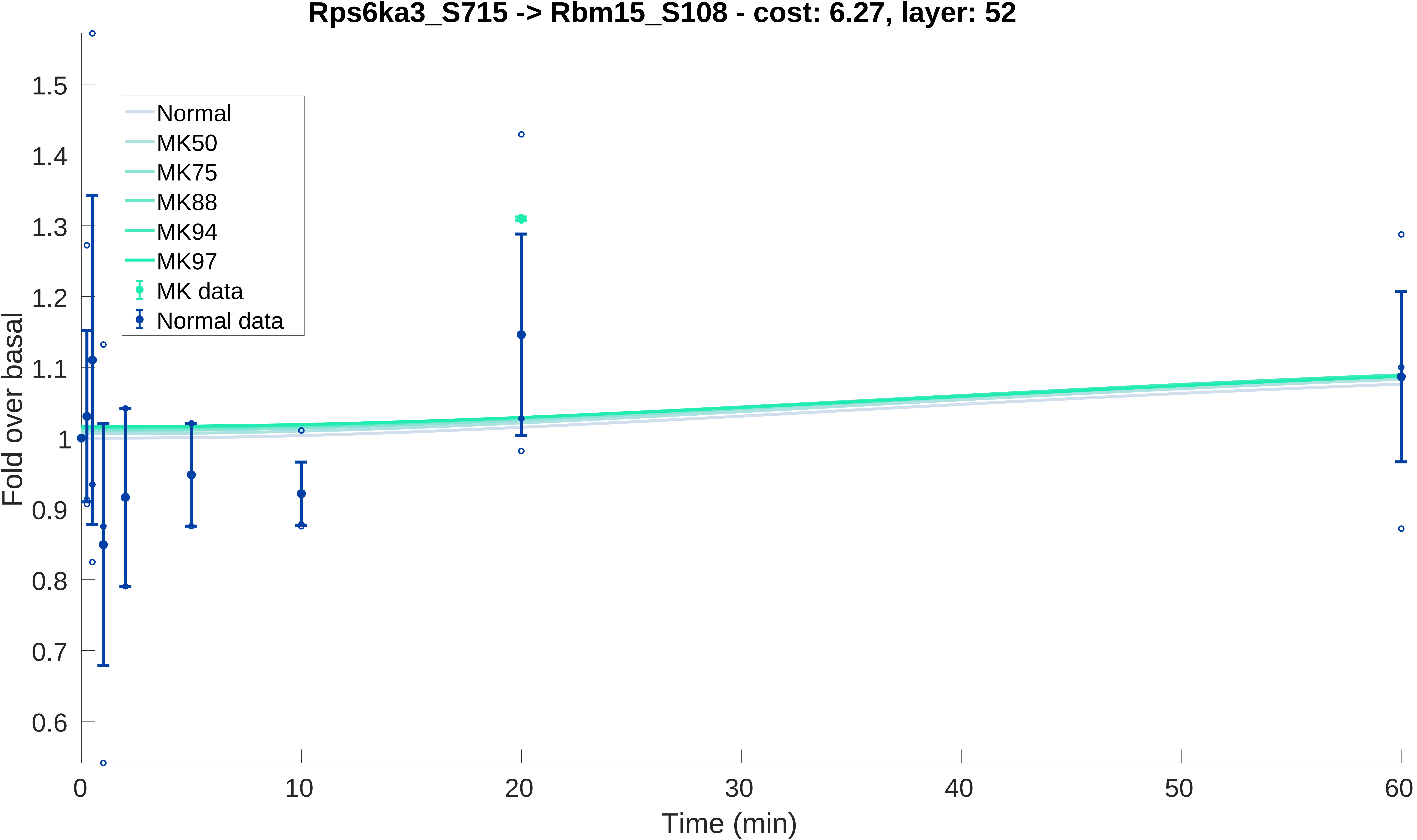

Rps6ka3_S715 -> Rbm15_S108 - cost: 6.27, layer: 52
Normal
MK50
MK75
MK88
MK94
MK97
MK data
Normal data
Fold over basal
Time (min)
1.5
1.4
1.3
1.2
1.1
1
0.9
0.8
0.7
0.6
0
10
20
30
40
50
60

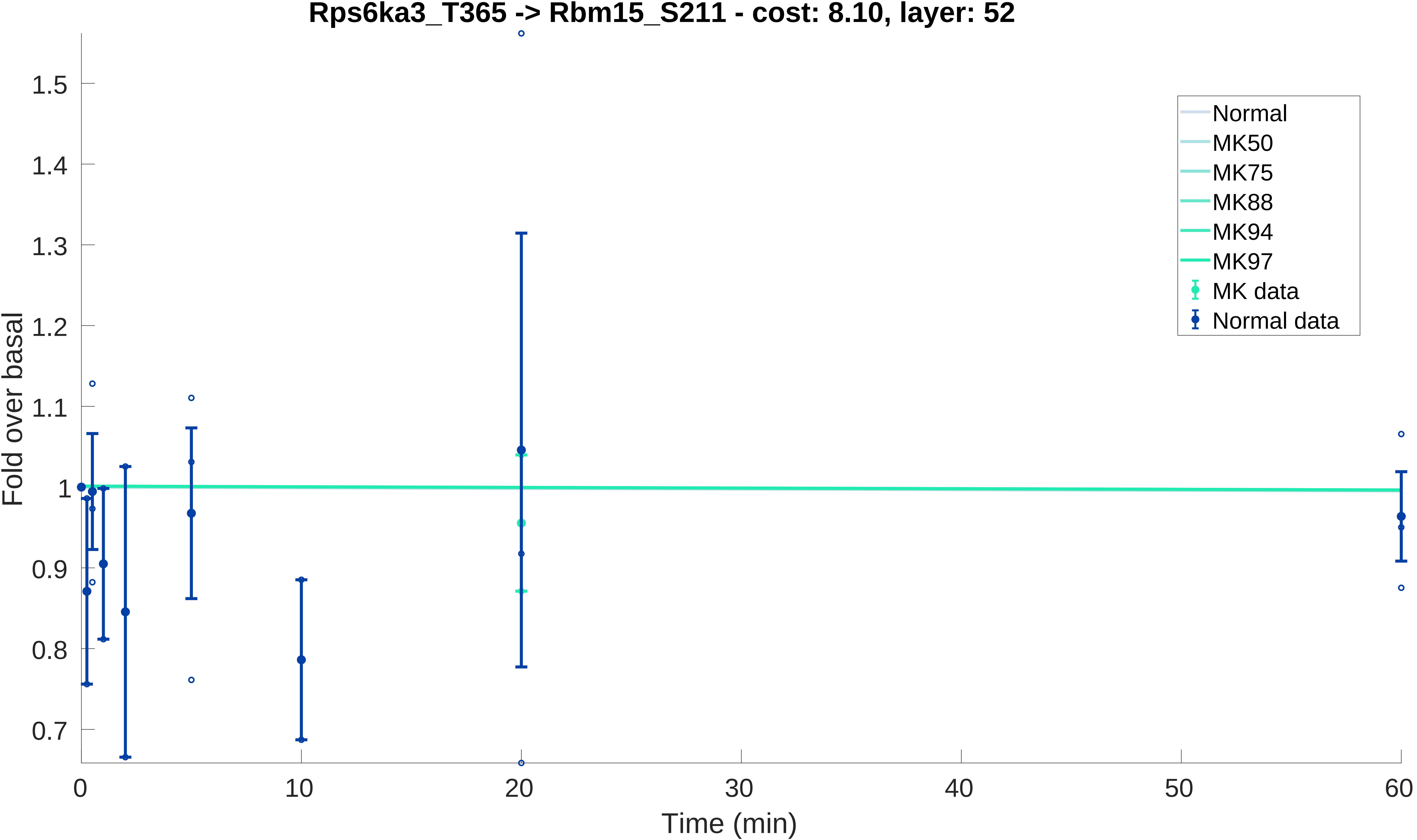
Rps6ka3_T365 -> Rbm15_S211 - cost: 8.10, layer: 52
Fold over basal
Time (min)
1.5
1.4
1.3
1.2
1.1
1
0.9
0.8
0.7
0
10
20
30
40
50
60
Normal
MK50
MK75
MK88
MK94
MK97
MK data
Normal data

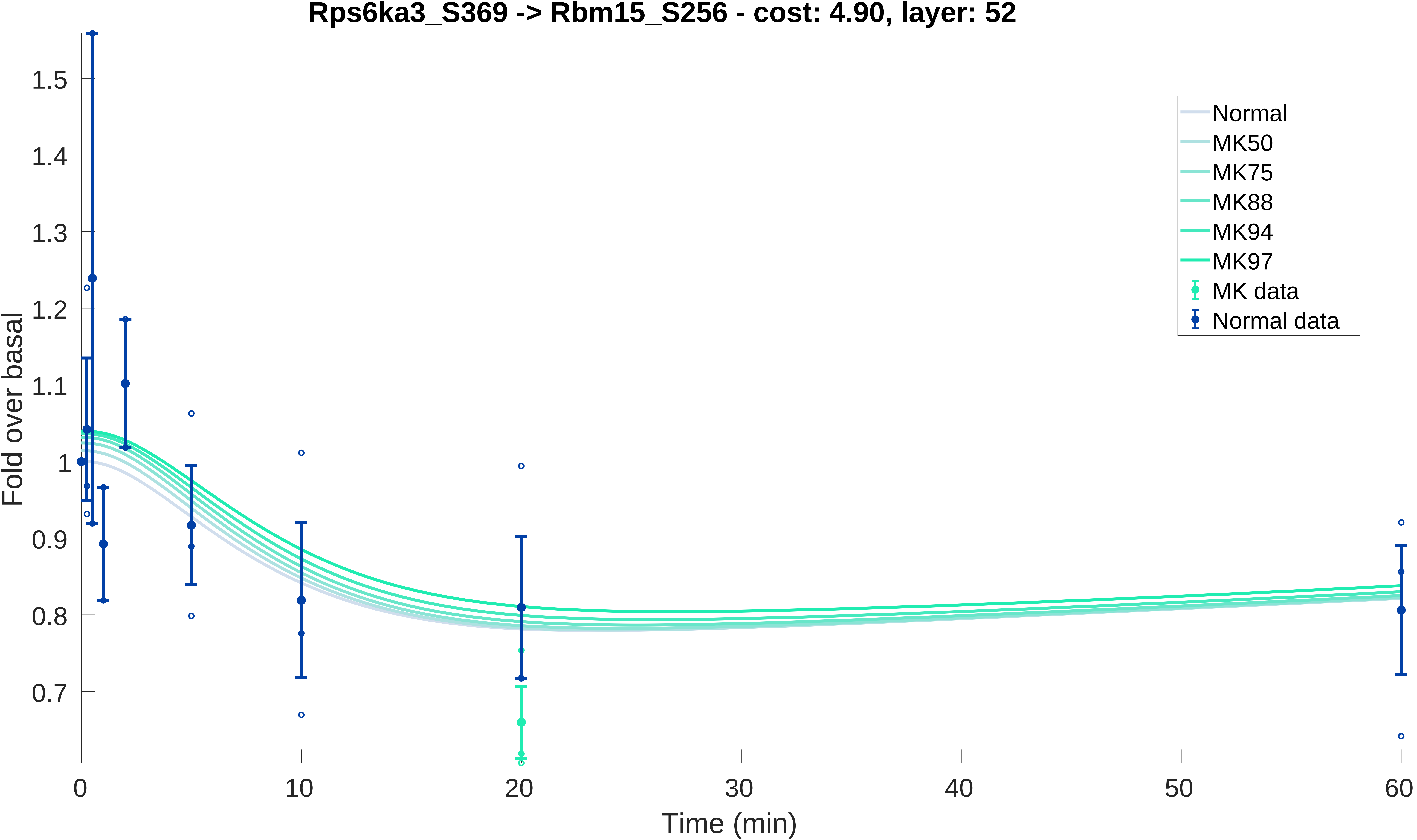

Rps6ka3_S369 -> Rbm15_S256 - cost: 4.90, layer: 52
Fold over basal
Time (min)
0
10
20
30
40
50
60
0.7
0.8
0.9
1
1.1
1.2
1.3
1.4
1.5
Normal
MK50
MK75
MK88
MK94
MK97
MK data
Normal data

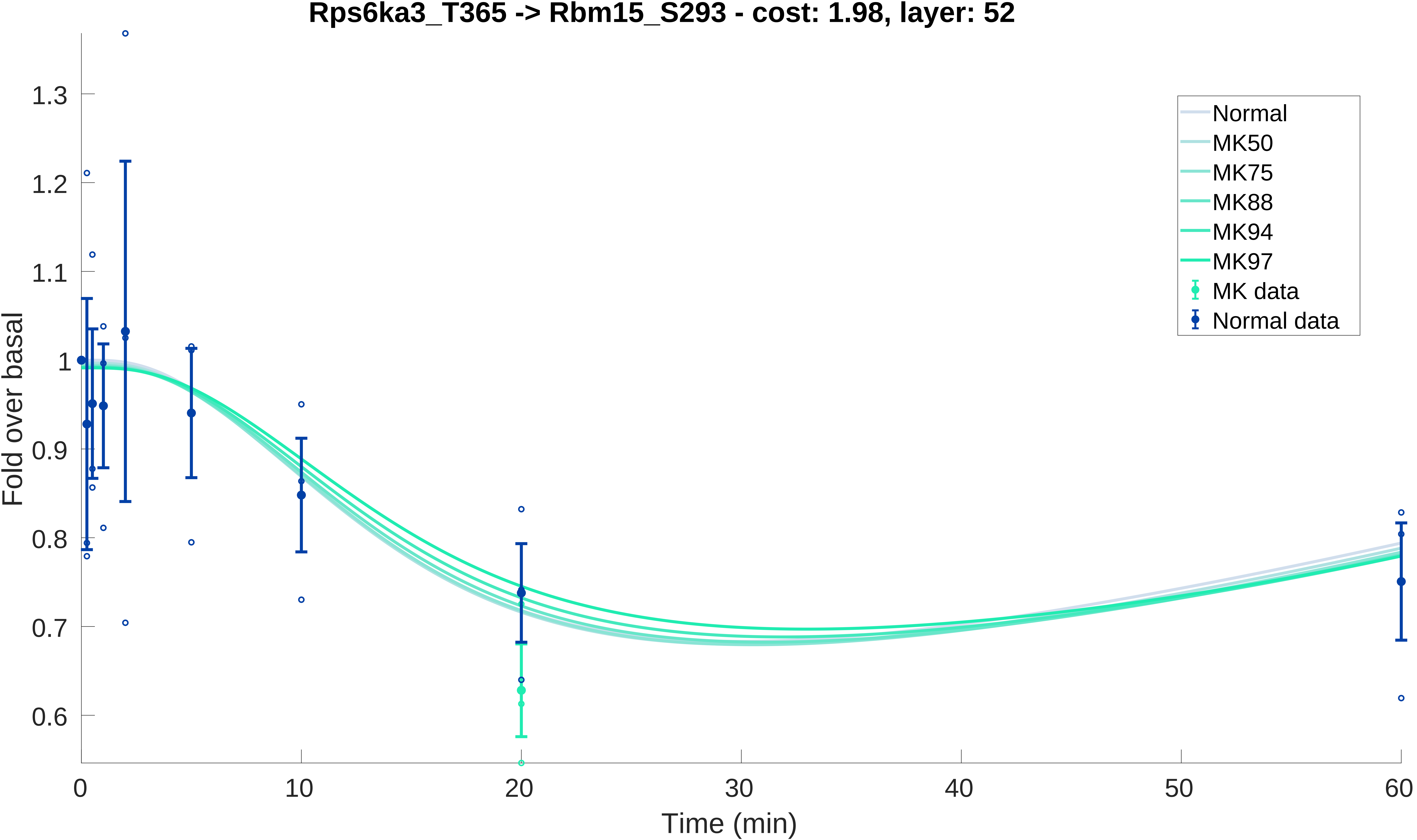

Rps6ka3_T365 -> Rbm15_S293 - cost: 1.98, layer: 52
Fold over basal
1.3
1.2
1.1
1
0.9
0.8
0.7
0.6
0
10
20
30
40
50
60
Time (min)
Normal
MK50
MK75
MK88
MK94
MK97
MK data
Normal data

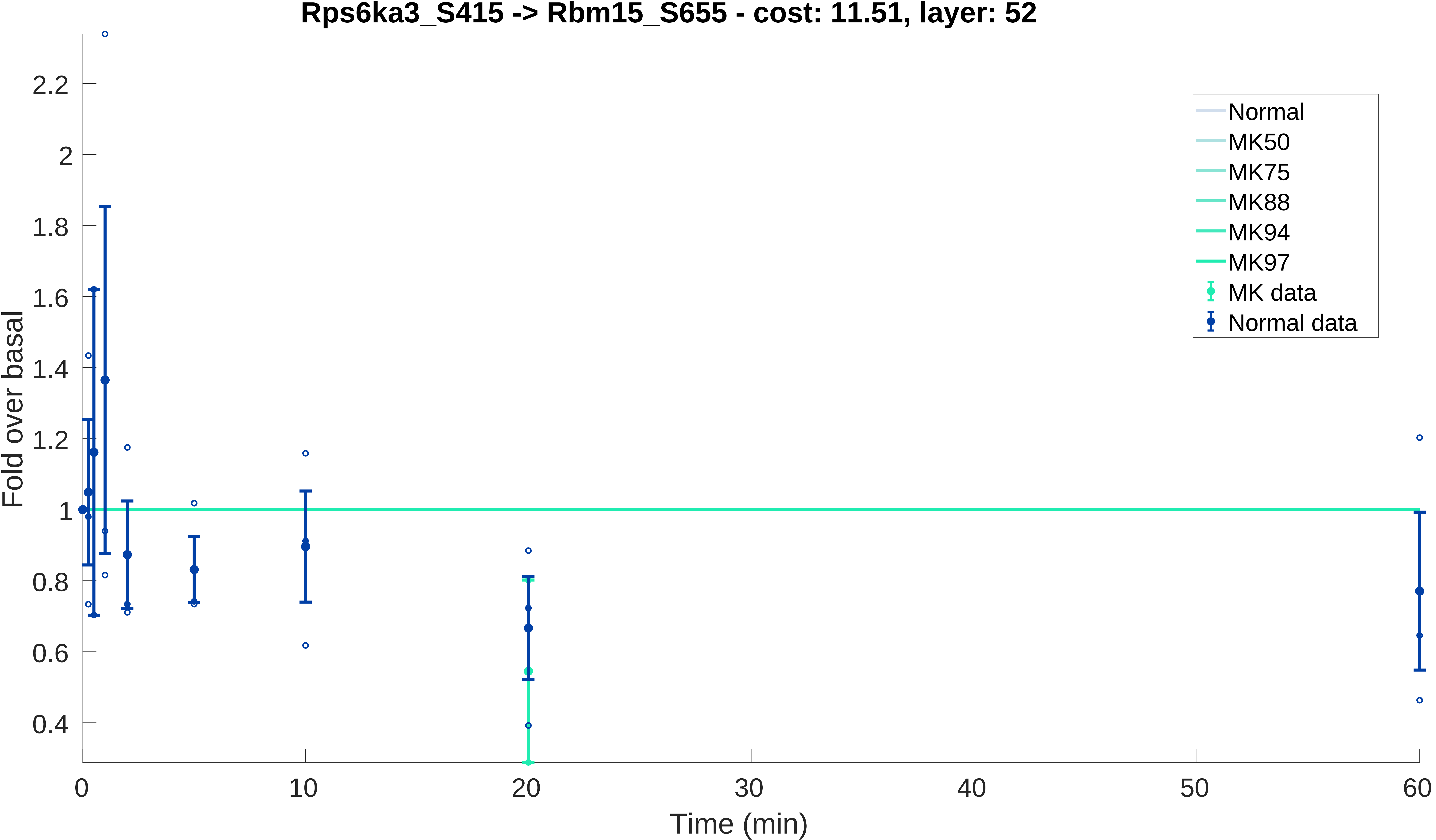
Rps6ka3_S415 -> Rbm15_S655 - cost: 11.51, layer: 52
Fold over basal
Time (min)
0
10
20
30
40
50
60
0.4
0.6
0.8
1
1.2
1.4
1.6
1.8
2
2.2
Normal
MK50
MK75
MK88
MK94
MK97
MK data
Normal data

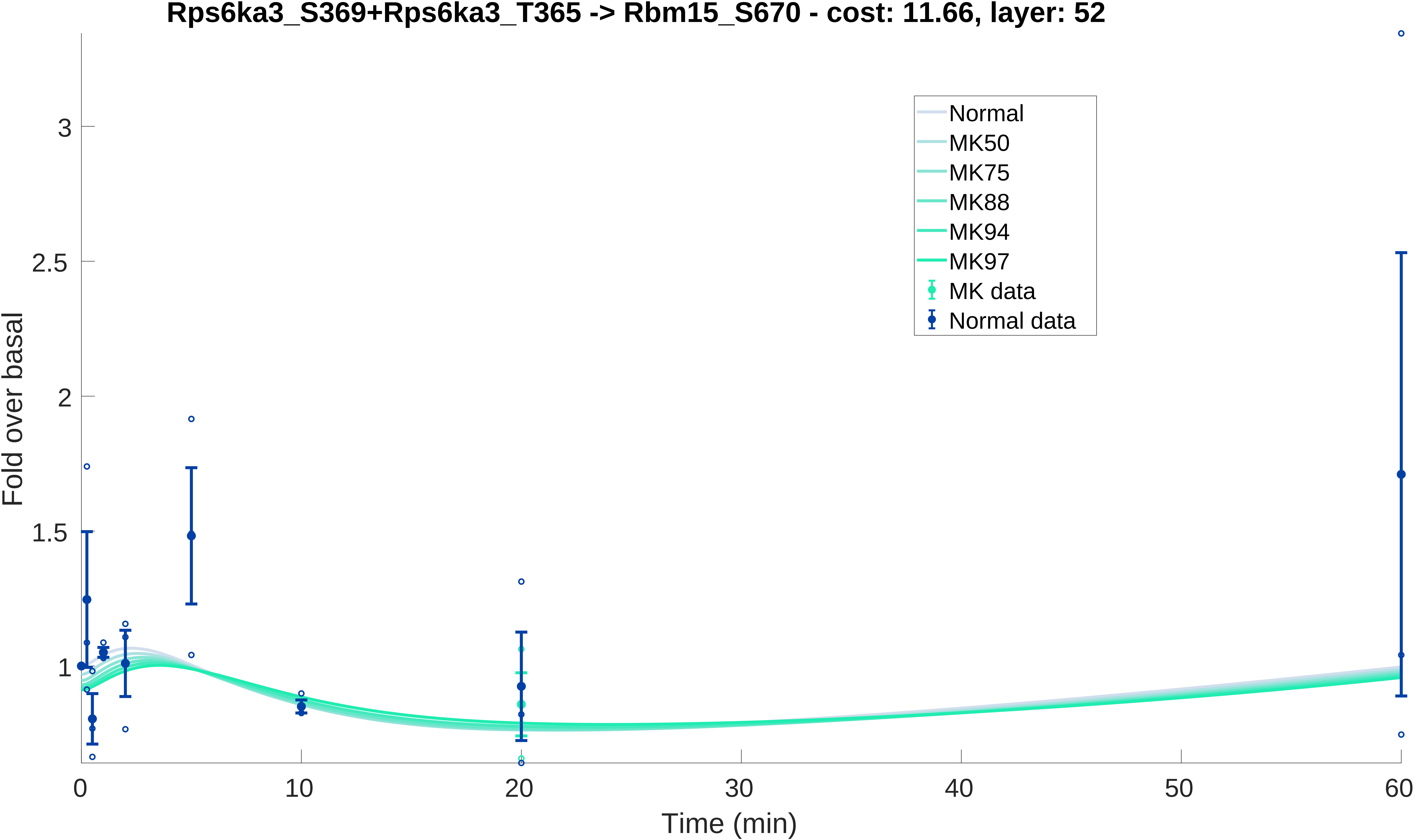

Rps6ka3_S369+Rps6ka3_T365 -> Rbm15_S670 - cost: 11.66, layer: 52
Fold over basal
Time (min)
0
10
20
30
40
50
60
1
1.5
2
2.5
3
Normal
MK50
MK75
MK88
MK94
MK97
MK data
Normal data

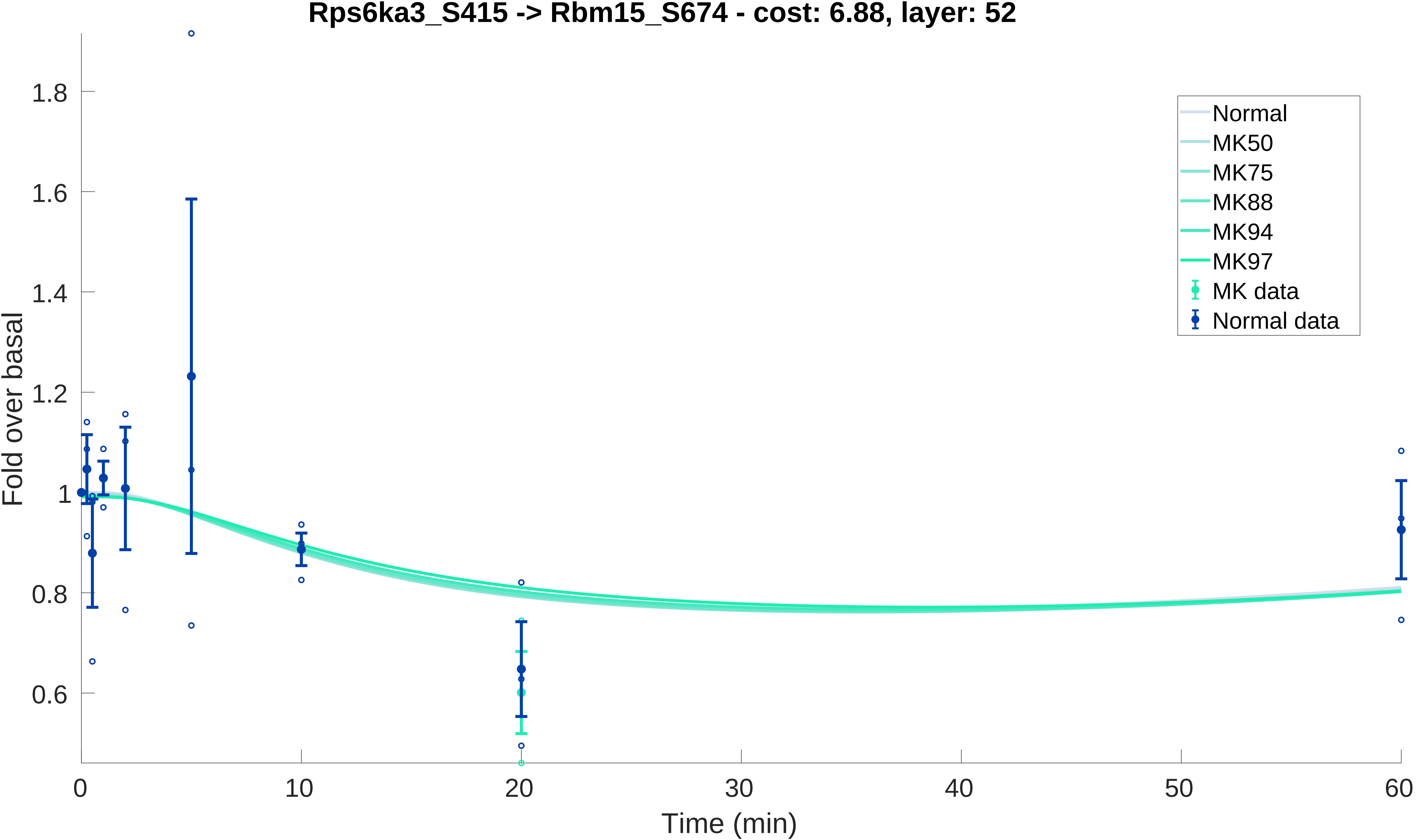

Rps6ka3_S415 -> Rbm15_S674 - cost: 6.88, layer: 52
Fold over basal
Time (min)
0.6
0.8
1
1.2
1.4
1.6
1.8
0
10
20
30
40
50
60
Normal
MK50
MK75
MK88
MK94
MK97
MK data
Normal data

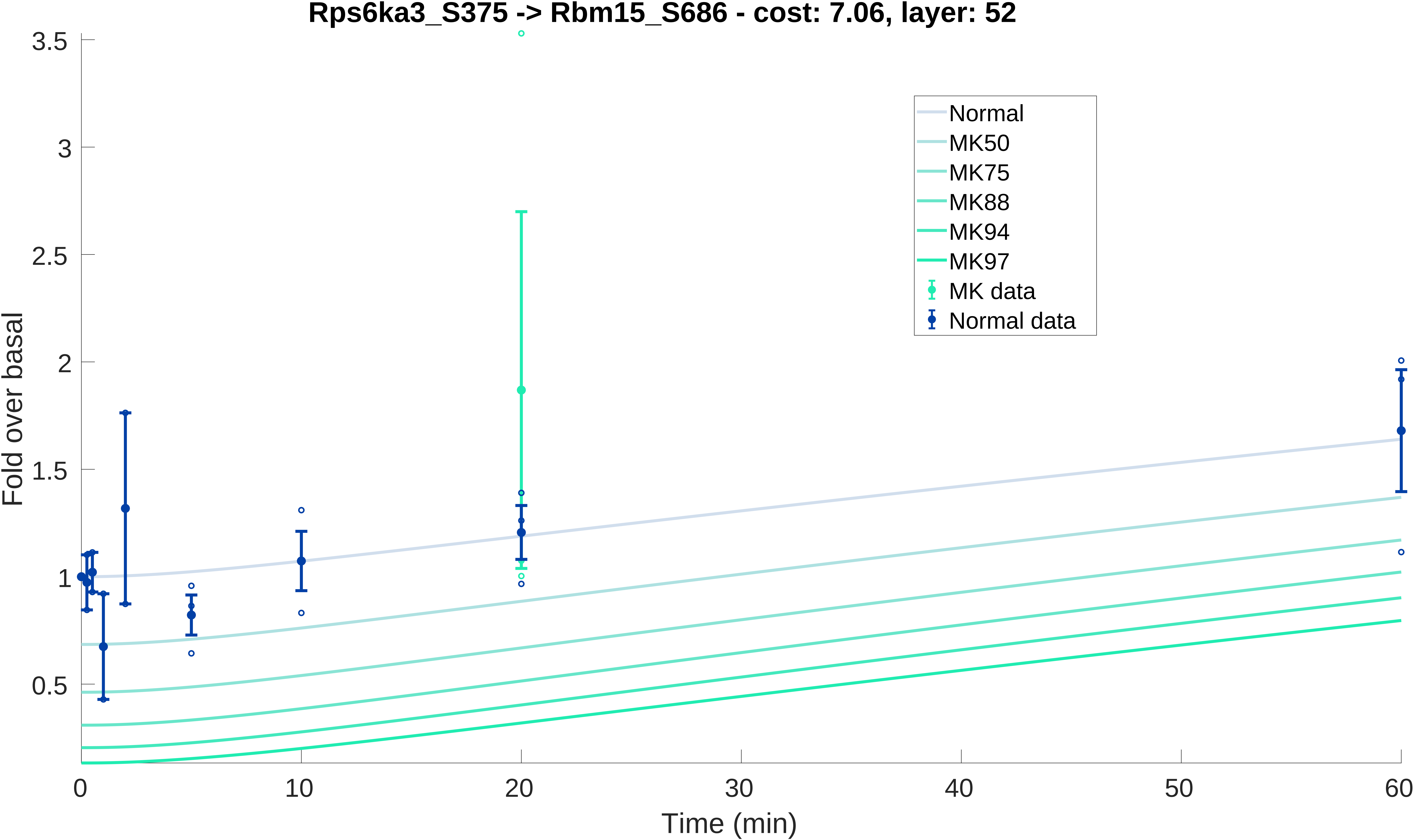

Rps6ka3_S375 -> Rbm15_S686 - cost: 7.06, layer: 52
Normal
MK50
MK75
MK88
MK94
MK97
MK data
Normal data
Fold over basal
Time (min)
0
10
20
30
40
50
60
0.5
1
1.5
2
2.5
3
3.5

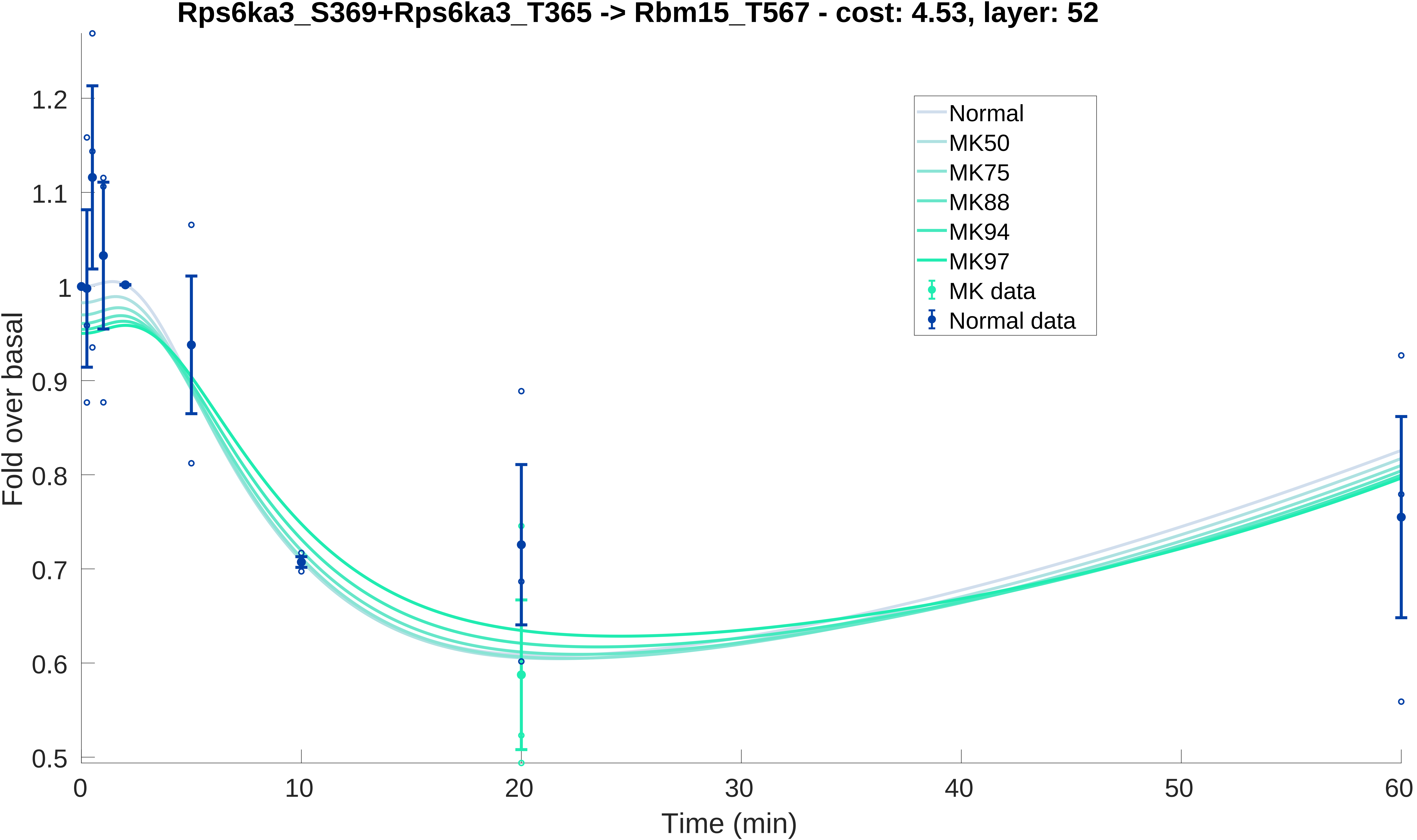

Rps6ka3_S369+Rps6ka3_T365 -> Rbm15_T567 - cost: 4.53, layer: 52
Fold over basal
Time (min)
Normal
MK50
MK75
MK88
MK94
MK97
MK data
Normal data
0.5
0.6
0.7
0.8
0.9
1
1.1
1.2
0
10
20
30
40
50
60

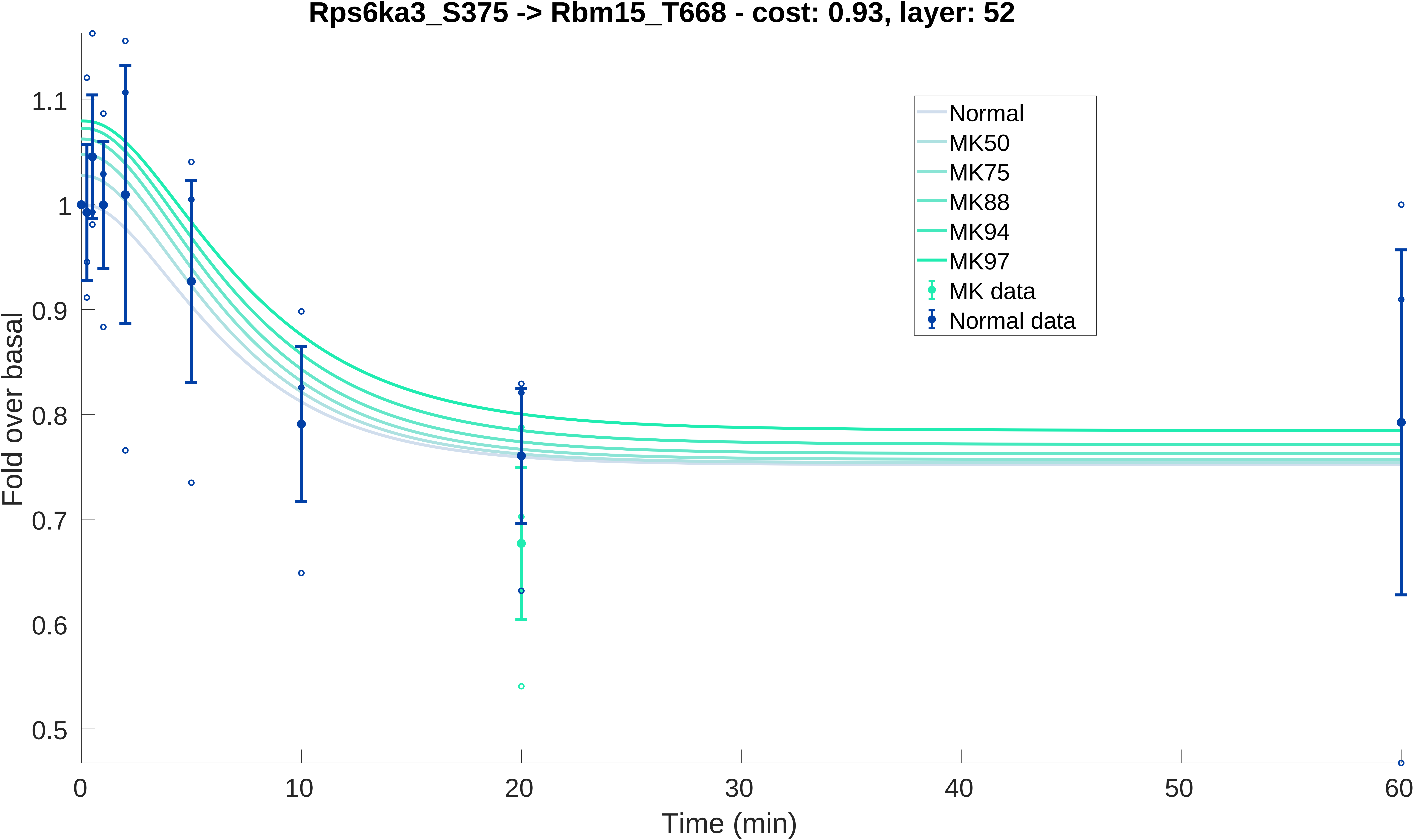

Rps6ka3_S375 -> Rbm15_T668 - cost: 0.93, layer: 52
Normal
MK50
MK75
MK88
MK94
MK97
MK data
Normal data
Fold over basal
Time (min)
0.5
0.6
0.7
0.8
0.9
1
1.1
0
10
20
30
40
50
60

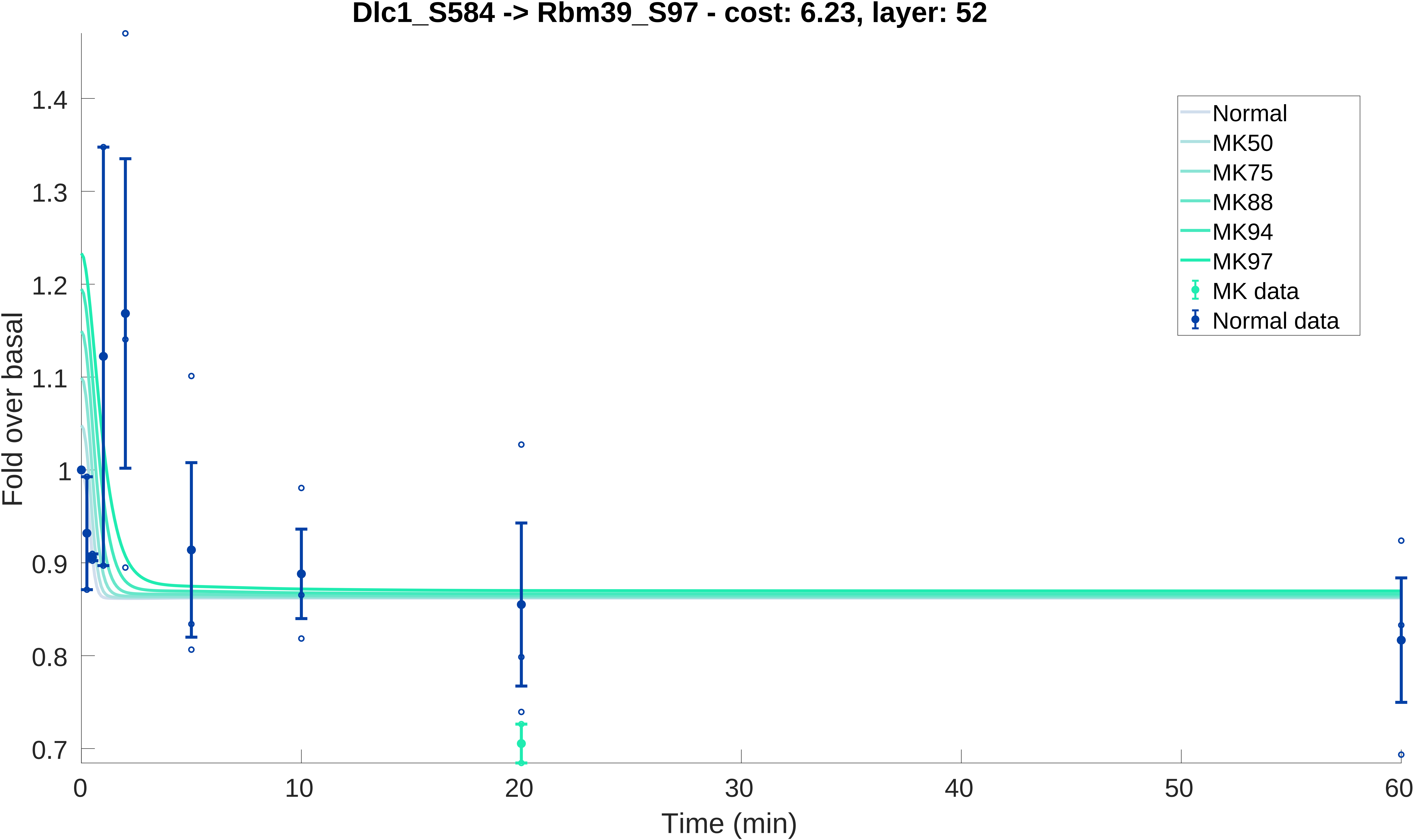

Dlc1_S584 -> Rbm39_S97 - cost: 6.23, layer: 52
Fold over basal
Time (min)
0.7
0.8
0.9
1
1.1
1.2
1.3
1.4
0
10
20
30
40
50
60
Normal
MK50
MK75
MK88
MK94
MK97
MK data
Normal data

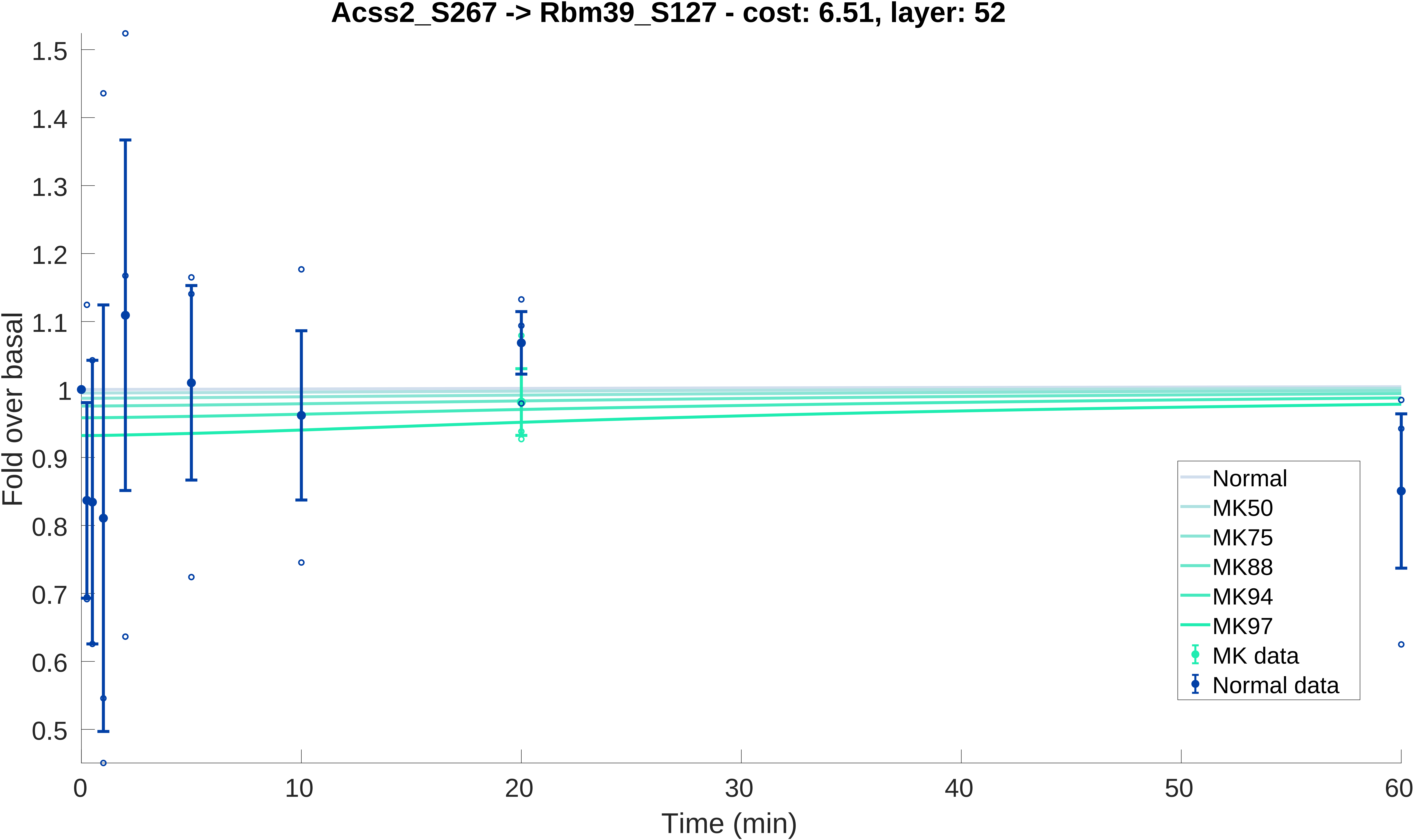

Acss2_S267 -> Rbm39_S127 - cost: 6.51, layer: 52
Fold over basal
Time (min)
1.5
1.4
1.3
1.2
1.1
1
0.9
0.8
0.7
0.6
0.5
0
10
20
30
40
50
60
Normal
MK50
MK75
MK88
MK94
MK97
MK data
Normal data

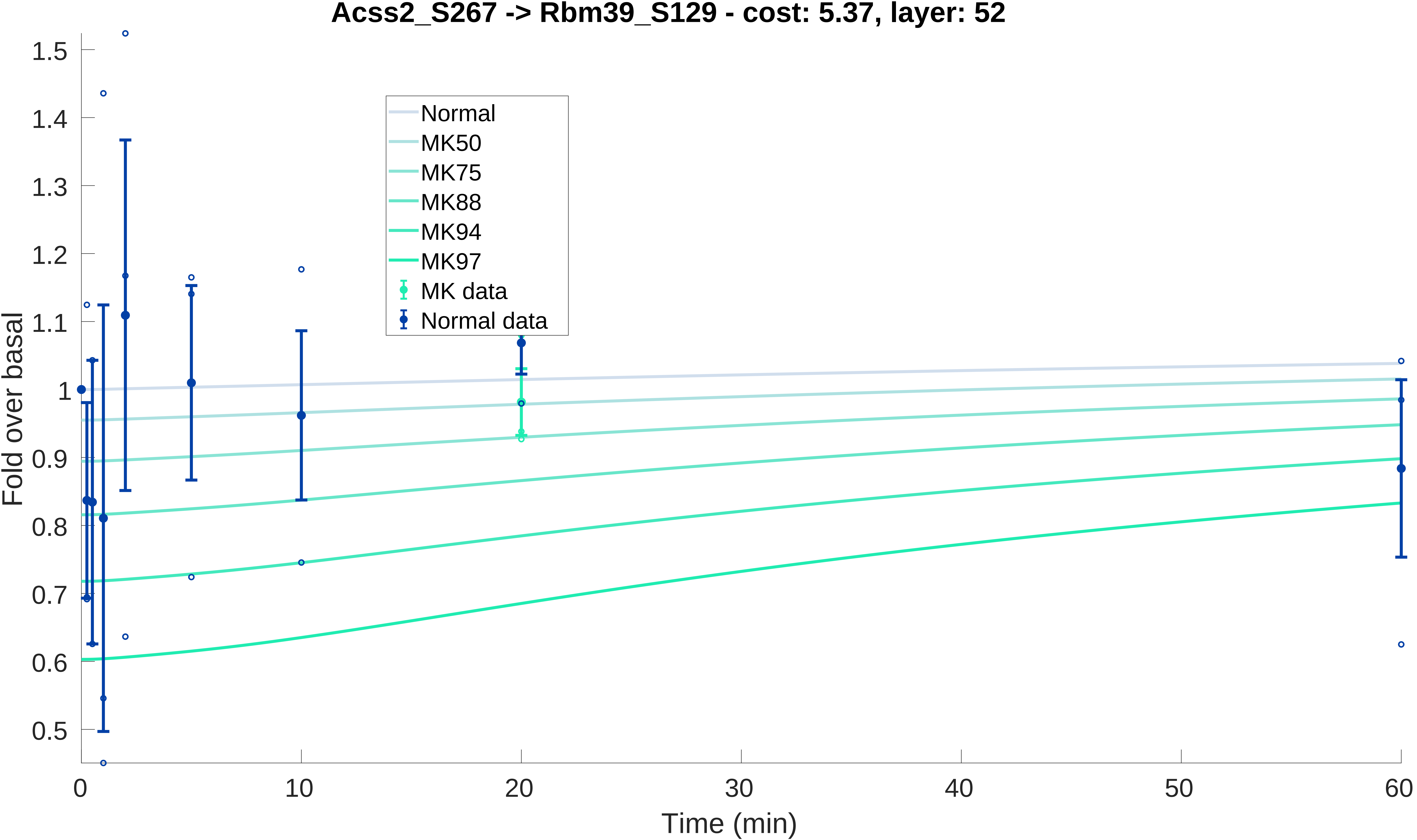

Acss2_S267 -> Rbm39_S129 - cost: 5.37, layer: 52
Normal
MK50
MK75
MK88
MK94
MK97
MK data
Normal data
Fold over basal
Time (min)
0
10
20
30
40
50
60
0.5
0.6
0.7
0.8
0.9
1
1.1
1.2
1.3
1.4
1.5

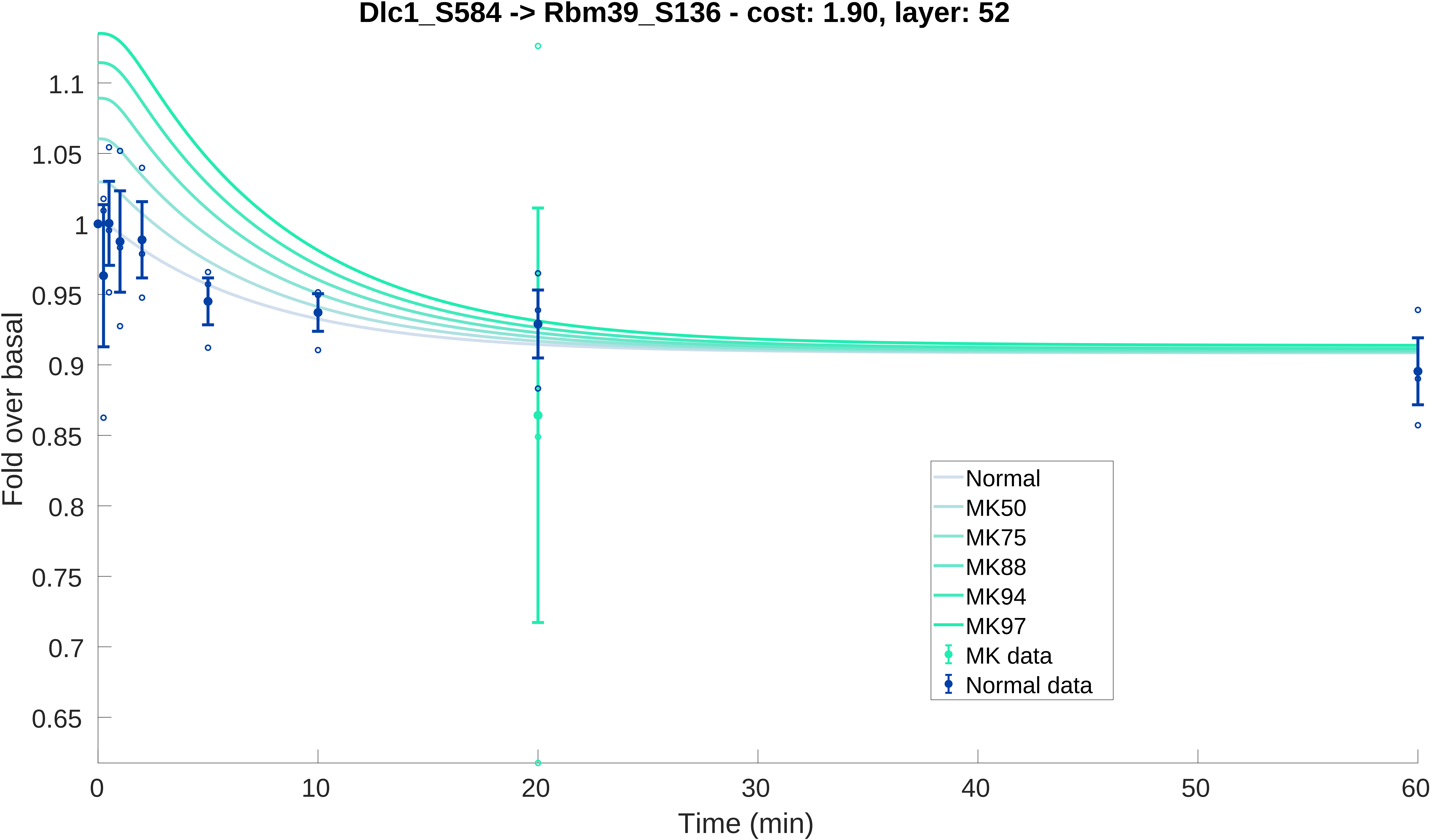

Dlc1_S584 -> Rbm39_S136 - cost: 1.90, layer: 52
Fold over basal
Time (min)
1.1
1.05
1
0.95
0.9
0.85
0.8
0.75
0.7
0.65
0
10
20
30
40
50
60
Normal
MK50
MK75
MK88
MK94
MK97
MK data
Normal data

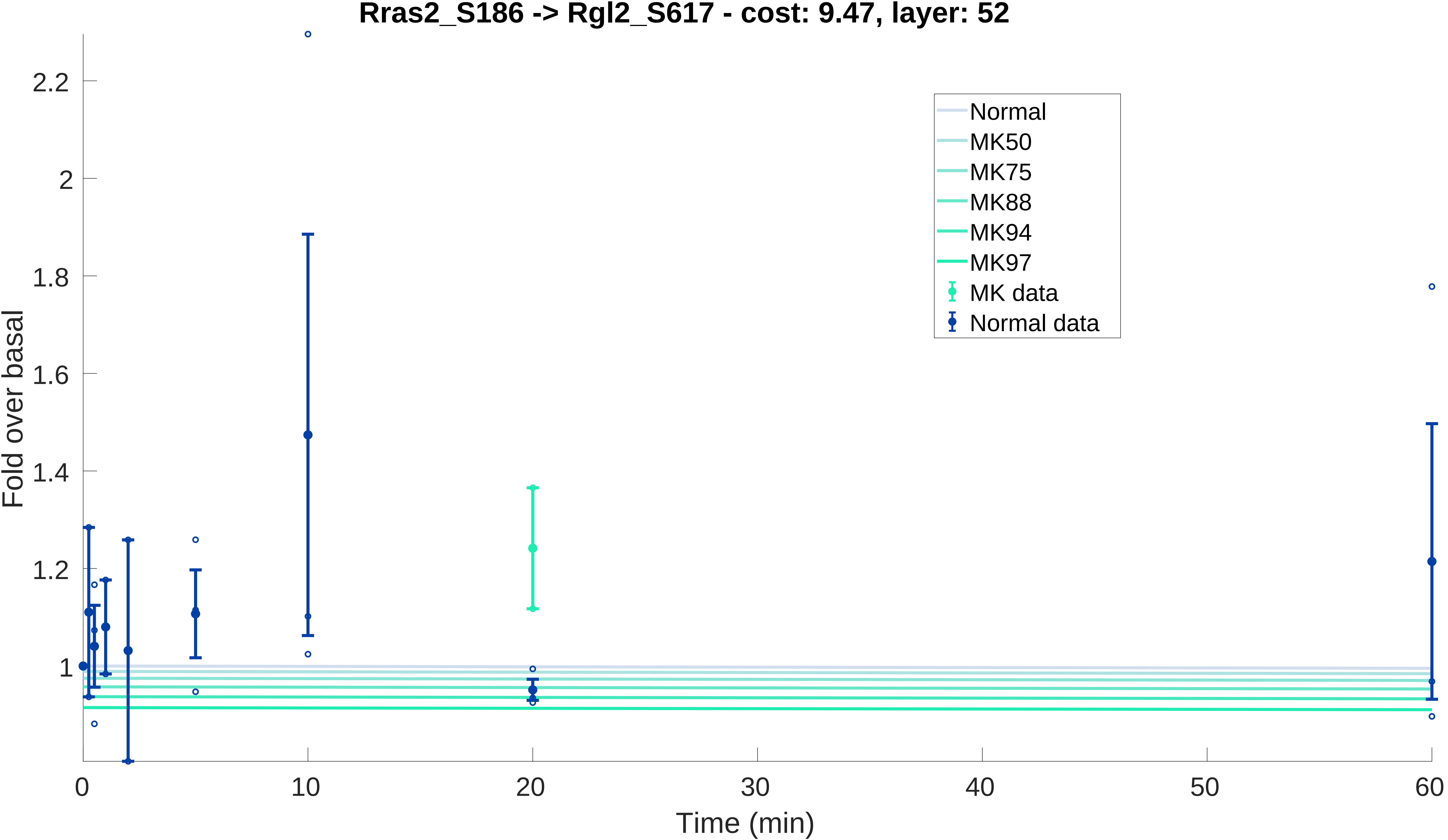

Rras2_S186 -> Rgl2_S617 - cost: 9.47, layer: 52
Fold over basal
Time (min)
2.2
2
1.8
1.6
1.4
1.2
1
0
10
20
30
40
50
60
Normal
MK50
MK75
MK88
MK94
MK97
MK data
Normal data

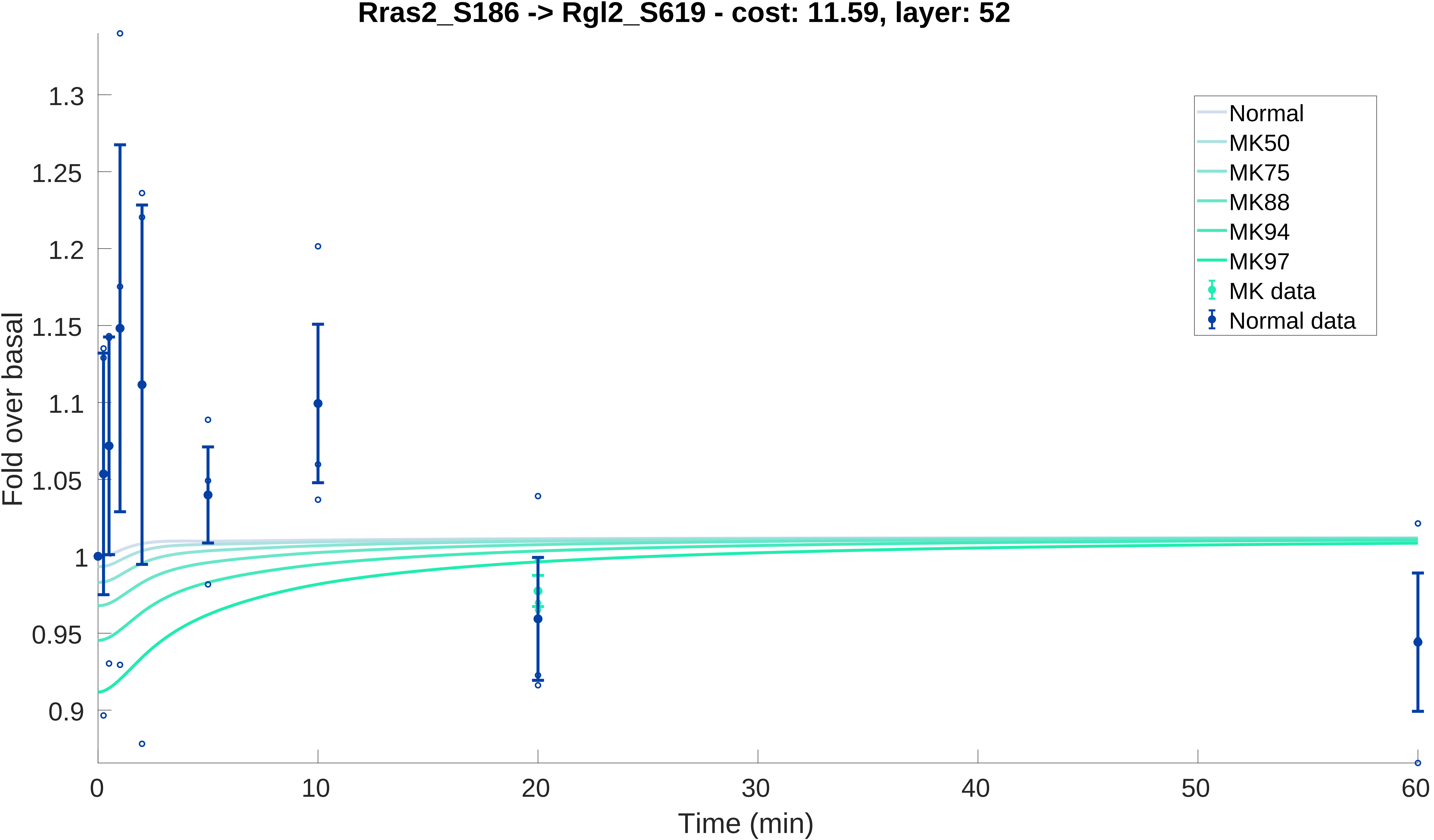

Rras2_S186 -> Rgl2_S619 - cost: 11.59, layer: 52
Fold over basal
Time (min)
0.9
0.95
1
1.05
1.1
1.15
1.2
1.25
1.3
0
10
20
30
40
50
60
Normal
MK50
MK75
MK88
MK94
MK97
MK data
Normal data

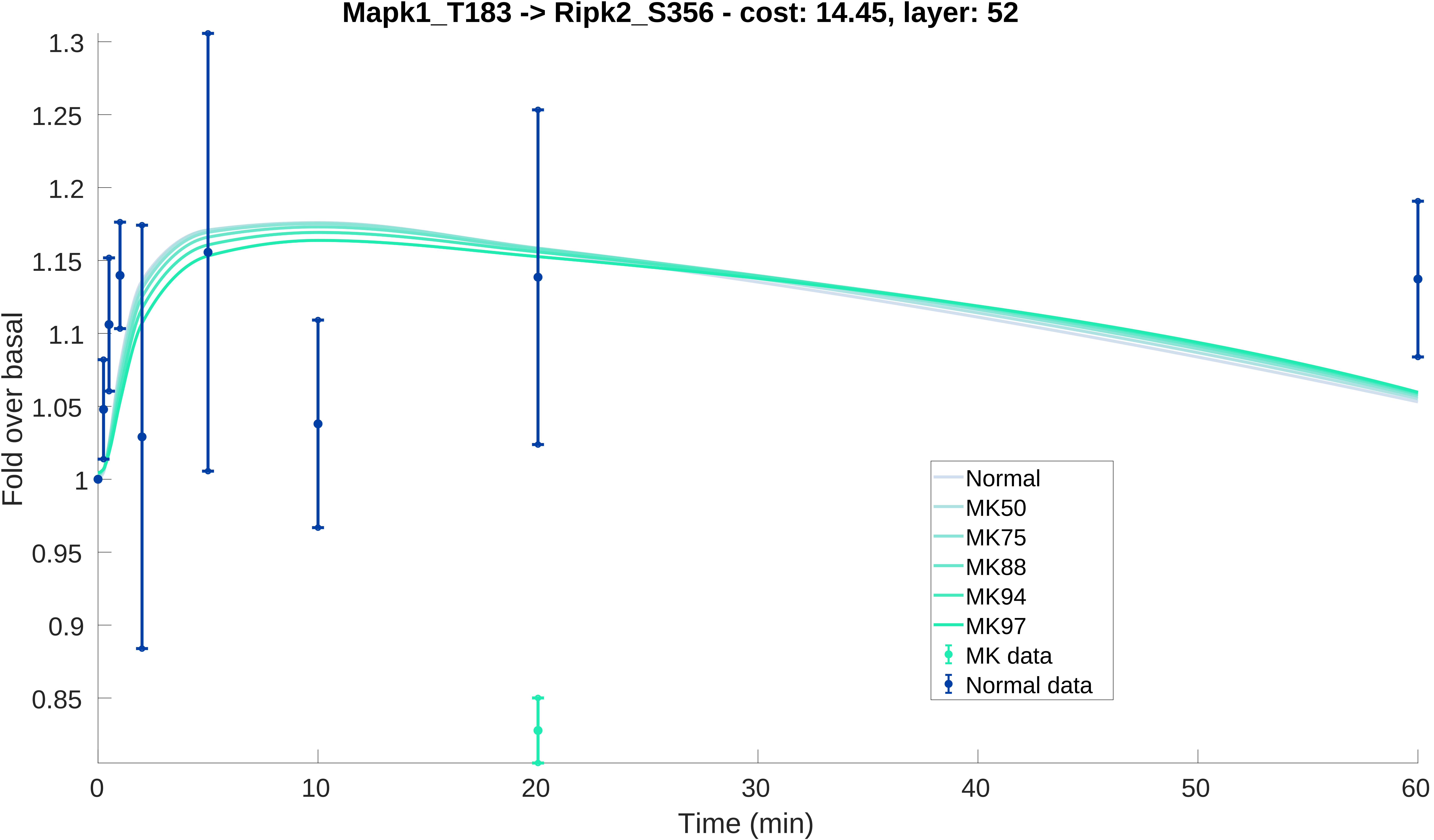

Mapk1_T183 -> Ripk2_S356 - cost: 14.45, layer: 52
Fold over basal
Time (min)
1.3
1.25
1.2
1.15
1.1
1.05
1
0.95
0.9
0.85
0
10
20
30
40
50
60
Normal
MK50
MK75
MK88
MK94
MK97
MK data
Normal data

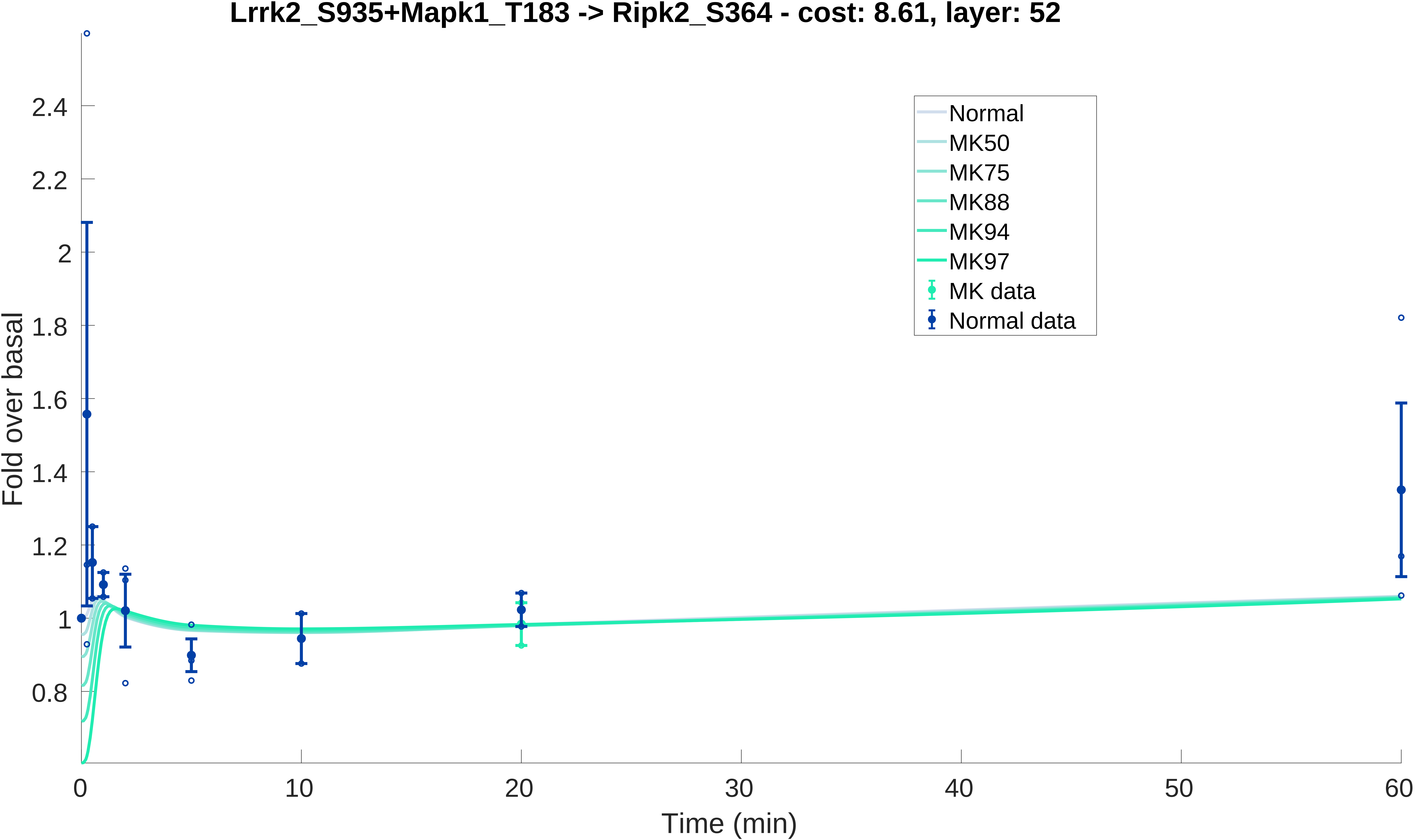

Lrrk2_S935+Mapk1_T183 -> Ripk2_S364 - cost: 8.61, layer: 52
Fold over basal
Time (min)
0.8
1
1.2
1.4
1.6
1.8
2
2.2
2.4
0
10
20
30
40
50
60
Normal
MK50
MK75
MK88
MK94
MK97
MK data
Normal data

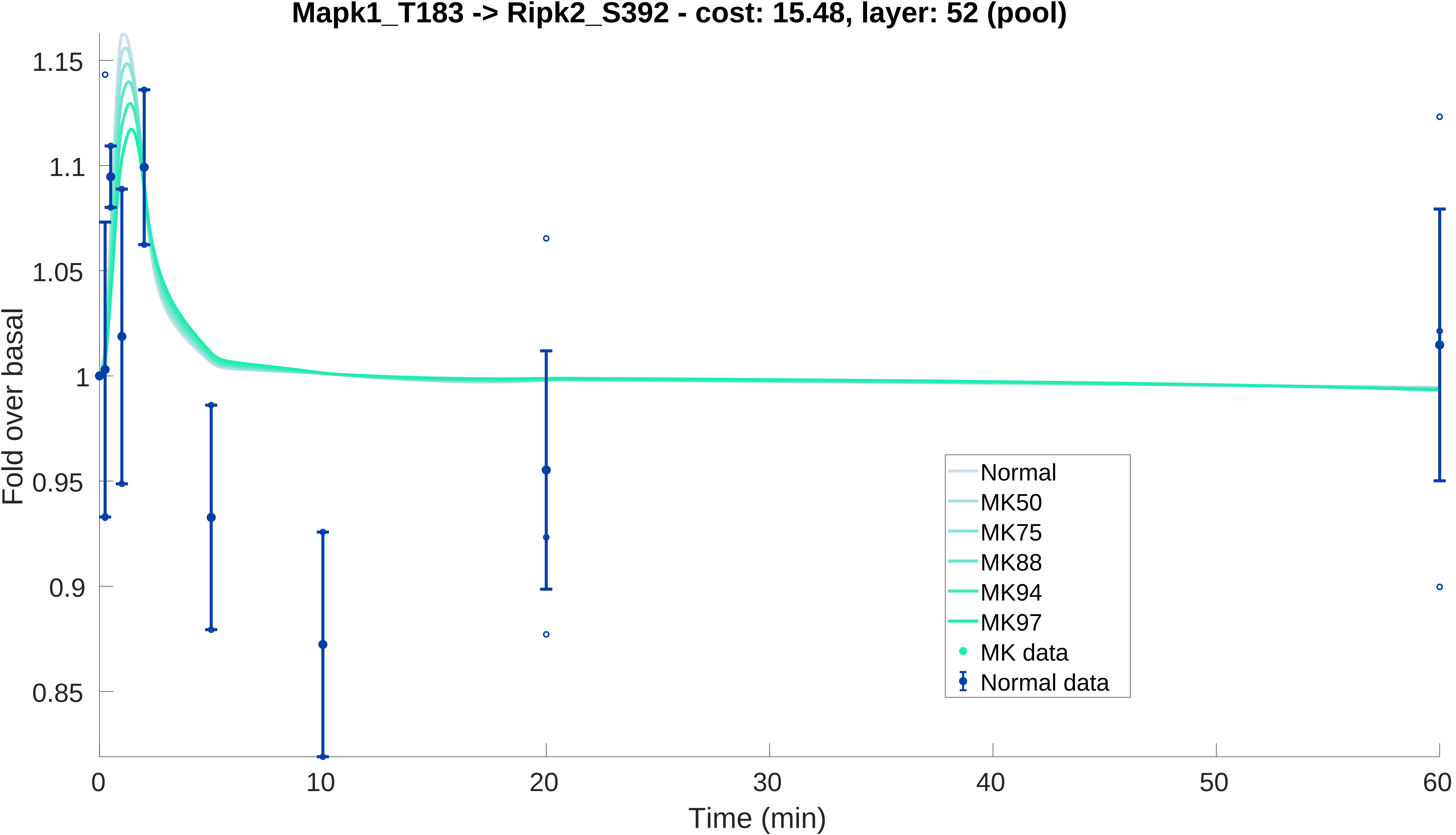

Mapk1_T183 -> Ripk2_S392 - cost: 15.48, layer: 52 (pool)
Fold over basal
1.15
1.1
1.05
1
0.95
0.9
0.85
0
10
20
30
40
50
60
Time (min)
Normal
MK50
MK75
MK88
MK94
MK97
MK data
Normal data

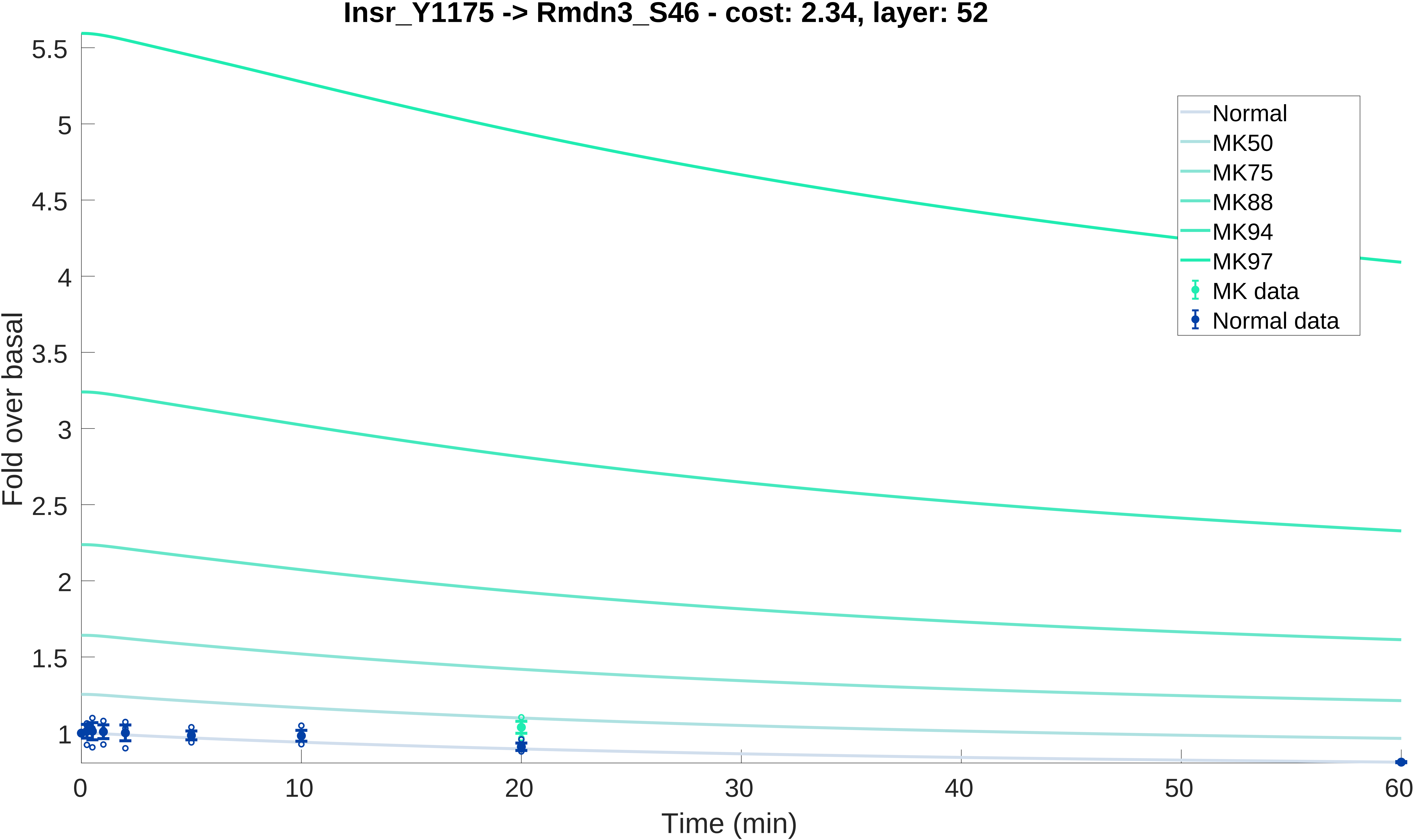

Insr_Y1175 -> Rmdn3_S46 - cost: 2.34, layer: 52
Fold over basal
Time (min)
Normal
MK50
MK75
MK88
MK94
MK97
MK data
Normal data
5.5
5
4.5
4
3.5
3
2.5
2
1.5
1
0
10
20
30
40
50
60

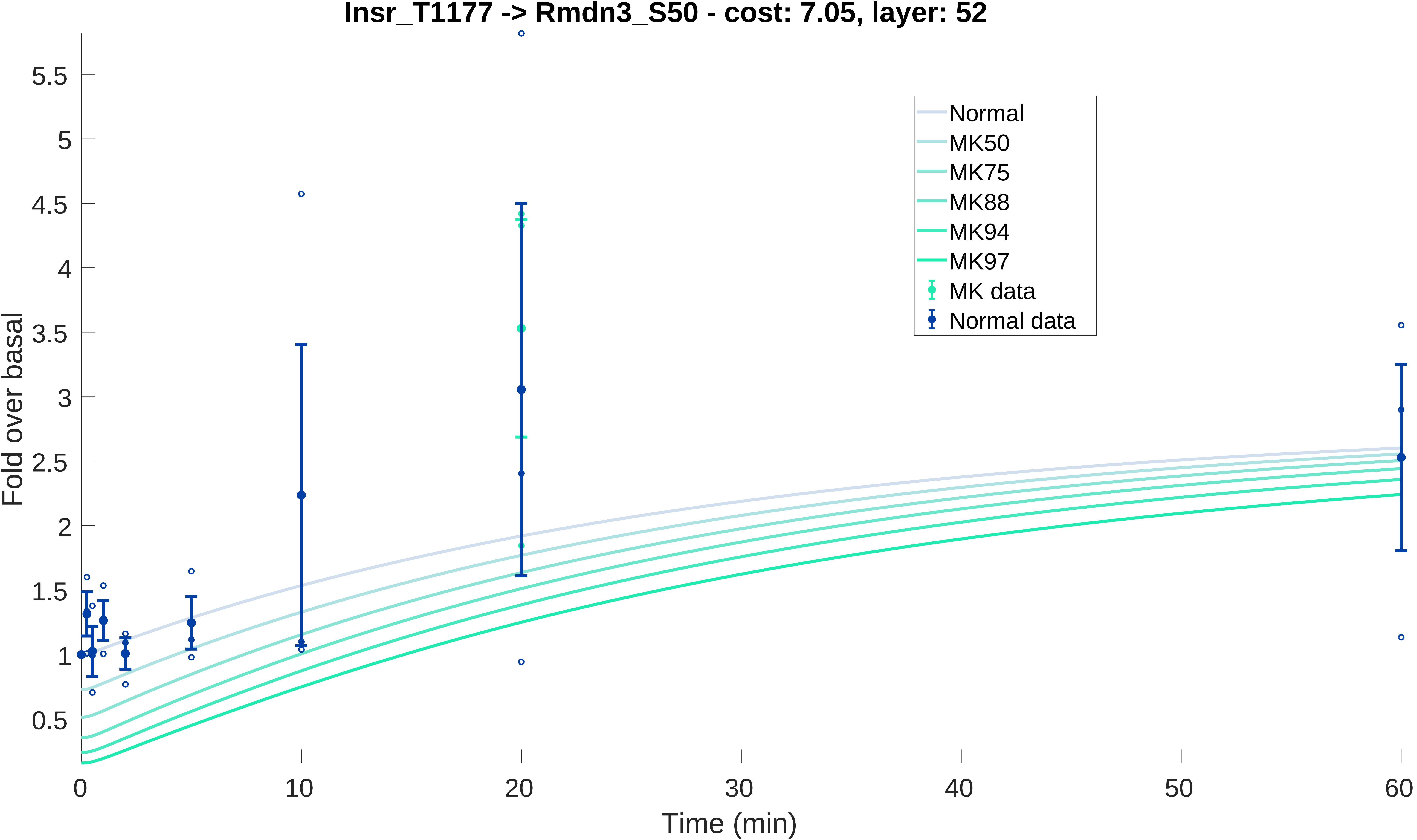
Insr_T1177 -> Rmdn3_S50 - cost: 7.05, layer: 52
Fold over basal
Time (min)
Normal
MK50
MK75
MK88
MK94
MK97
MK data
Normal data
0
10
20
30
40
50
60
0.5
1
1.5
2
2.5
3
3.5
4
4.5
5
5.5

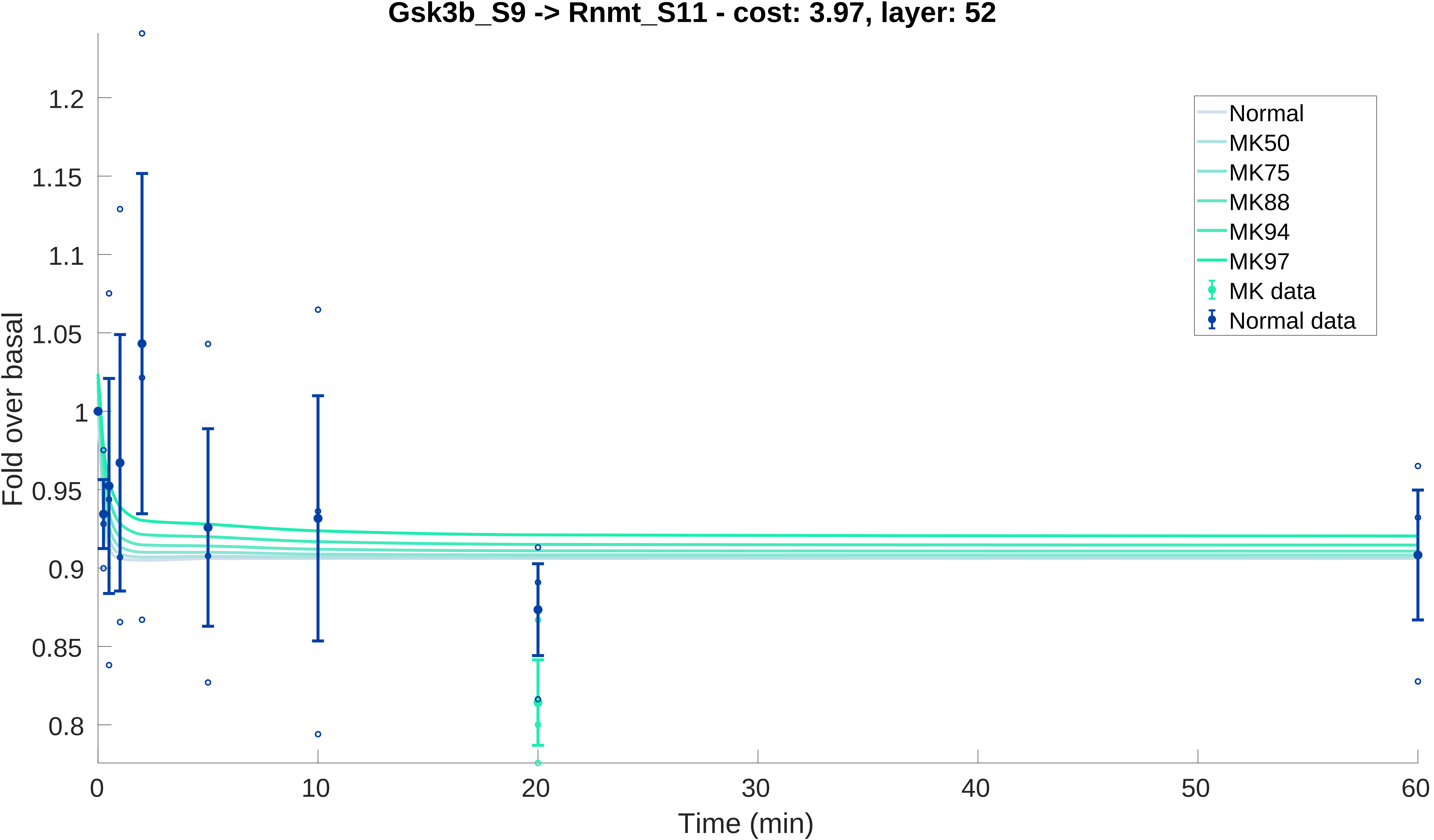

Gsk3b_S9 -> Rnmt_S11 - cost: 3.97, layer: 52
Fold over basal
Time (min)
1.2
1.15
1.1
1.05
1
0.95
0.9
0.85
0.8
0
10
20
30
40
50
60
Normal
MK50
MK75
MK88
MK94
MK97
MK data
Normal data

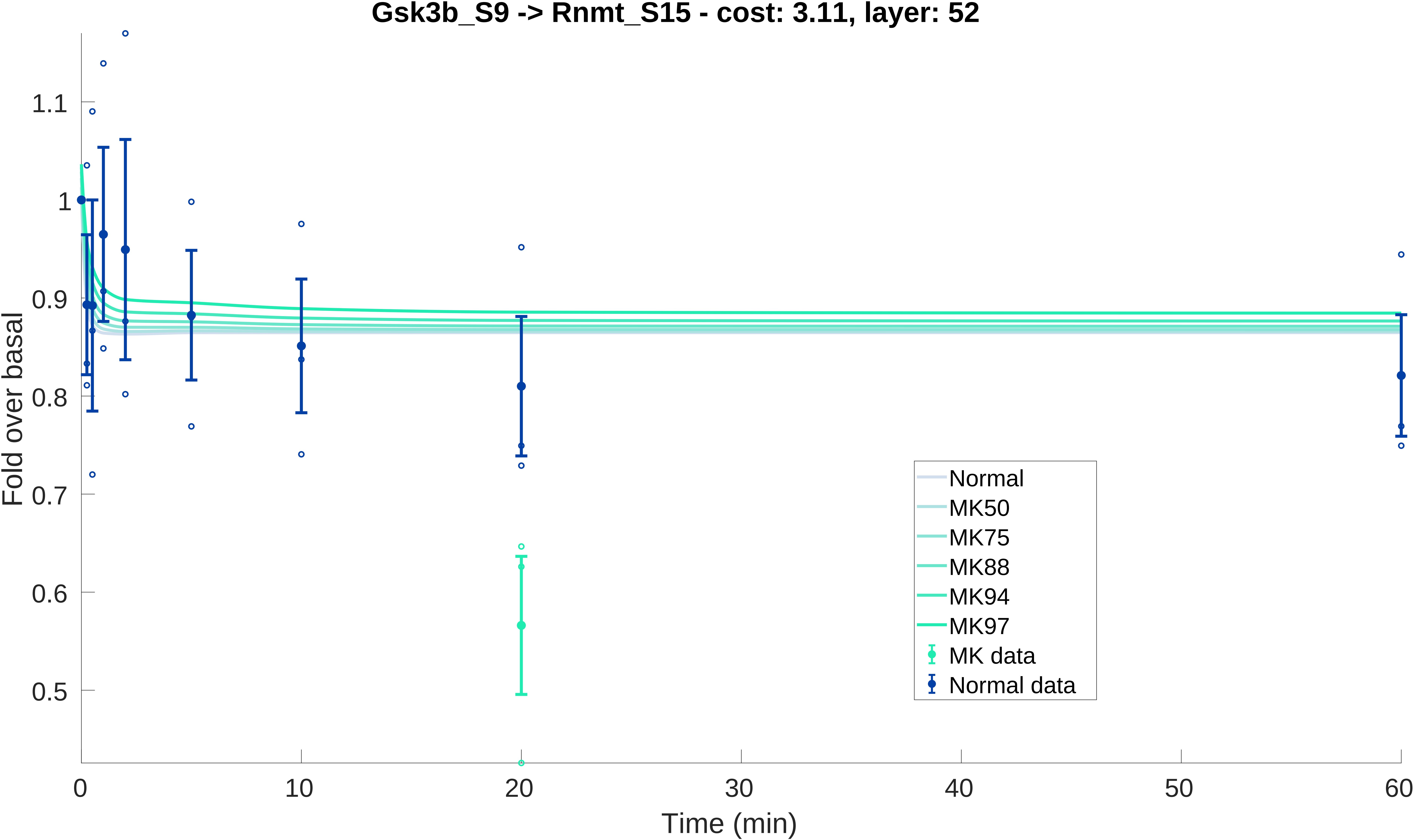

Gsk3b_S9 -> Rnmt_S15 - cost: 3.11, layer: 52
Fold over basal
1.1
1
0.9
0.8
0.7
0.6
0.5
0
10
20
30
40
50
60
Time (min)
Normal
MK50
MK75
MK88
MK94
MK97
MK data
Normal data

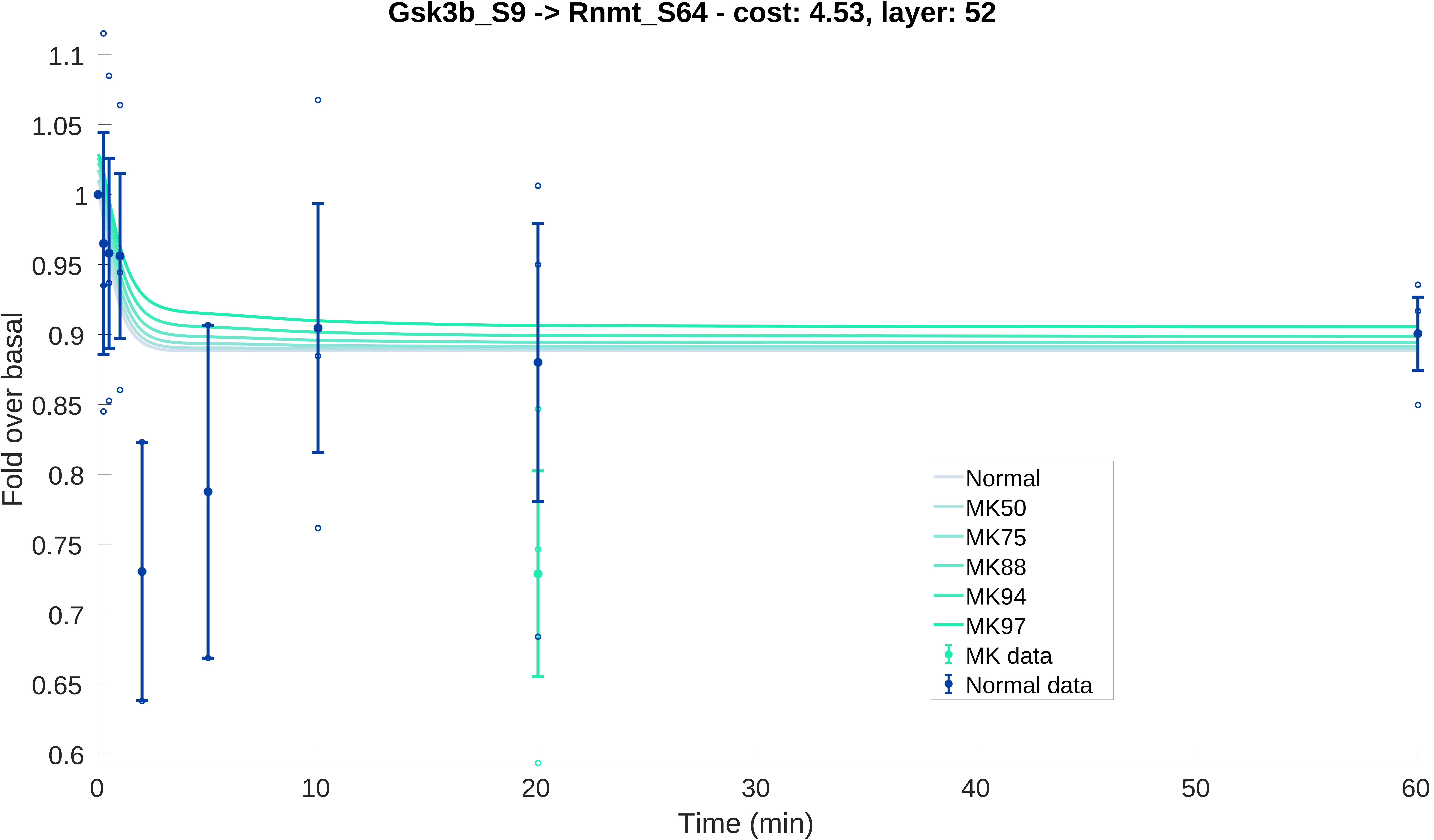

Gsk3b_S9 -> Rnmt_S64 - cost: 4.53, layer: 52
Fold over basal
Time (min)
1.1
1.05
1
0.95
0.9
0.85
0.8
0.75
0.7
0.65
0.6
0
10
20
30
40
50
60
Normal
MK50
MK75
MK88
MK94
MK97
MK data
Normal data

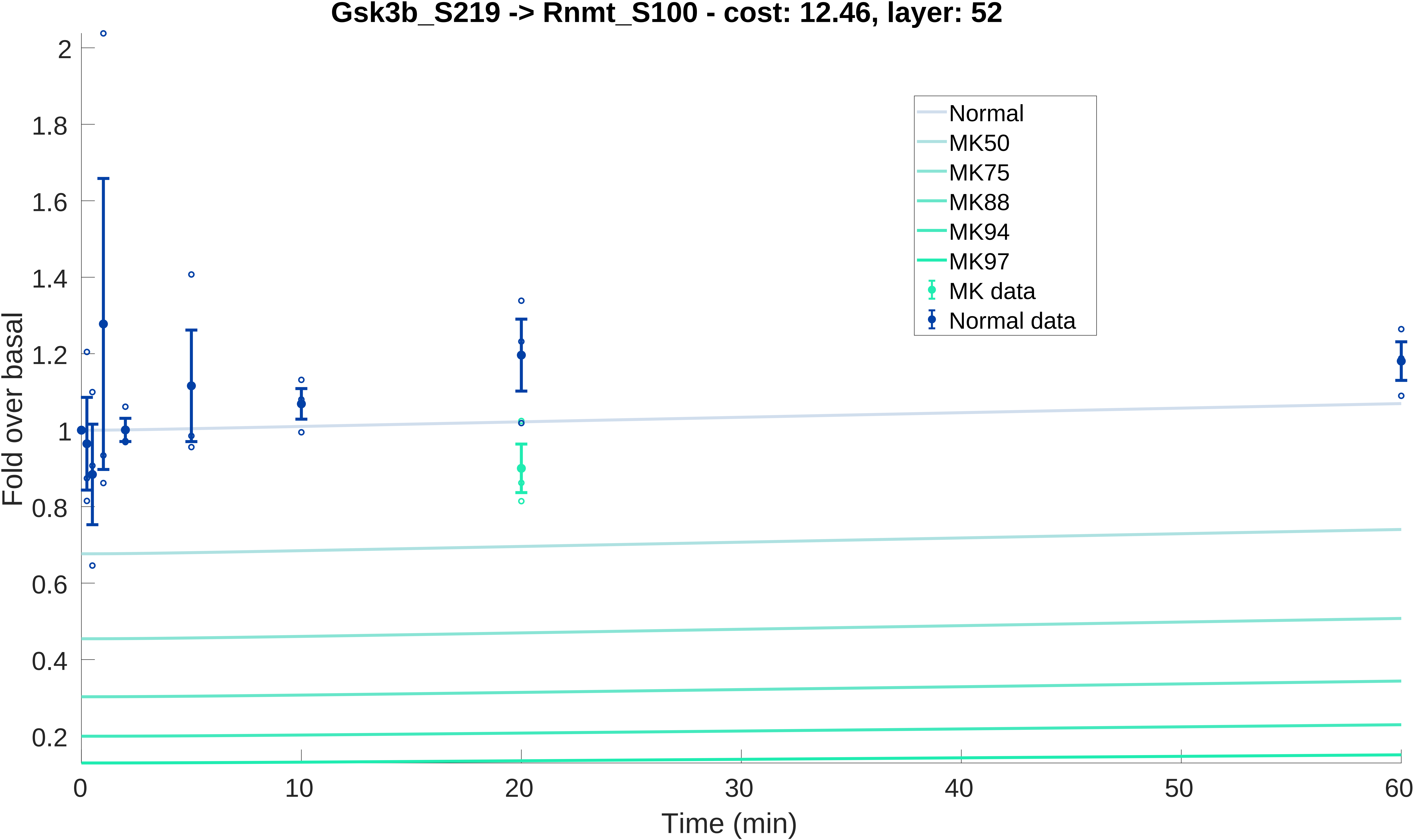
Gsk3b_S219 -> Rnmt_S100 - cost: 12.46, layer: 52
Fold over basal
Time (min)
0
10
20
30
40
50
60
0.2
0.4
0.6
0.8
1
1.2
1.4
1.6
1.8
2
Normal
MK50
MK75
MK88
MK94
MK97
MK data
Normal data

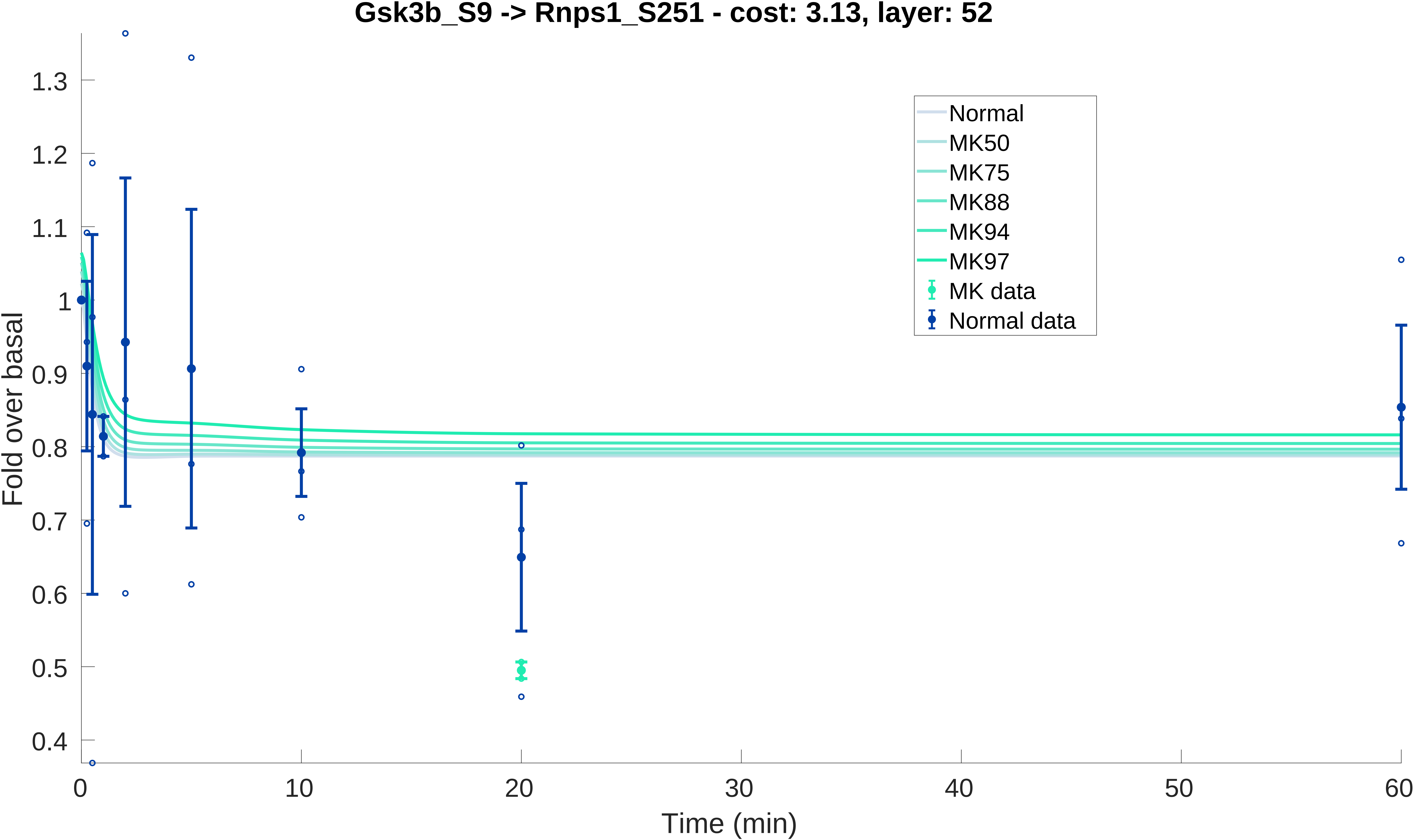
Gsk3b_S9 -> Rnps1_S251 - cost: 3.13, layer: 52
Fold over basal
Time (min)
1.3
1.2
1.1
1
0.9
0.8
0.7
0.6
0.5
0.4
0
10
20
30
40
50
60
Normal
MK50
MK75
MK88
MK94
MK97
MK data
Normal data

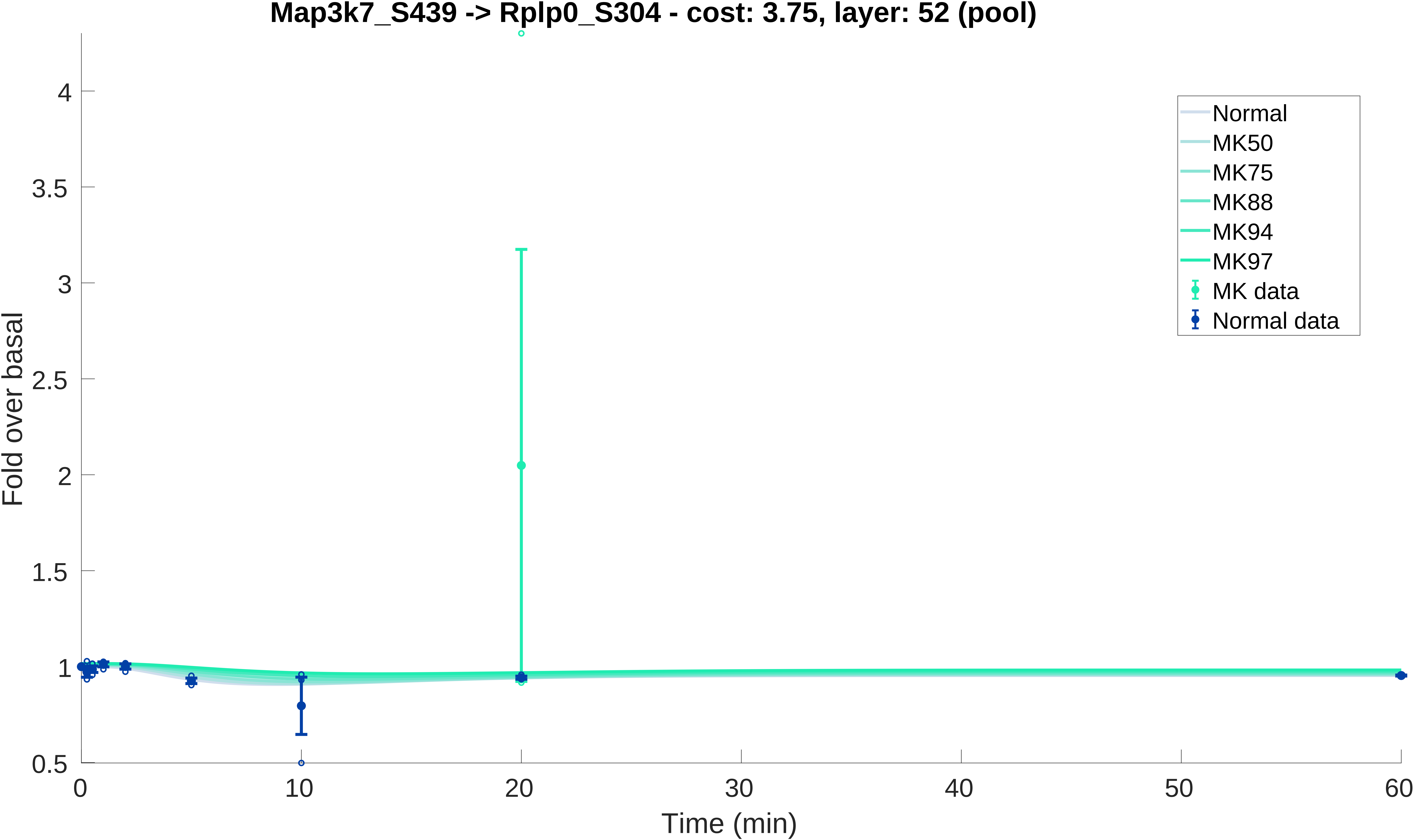
Map3k7_S439 -> Rplp0_S304 - cost: 3.75, layer: 52 (pool)
Fold over basal
Time (min)
0
10
20
30
40
50
60
0.5
1
1.5
2
2.5
3
3.5
4
Normal
MK50
MK75
MK88
MK94
MK97
MK data
Normal data

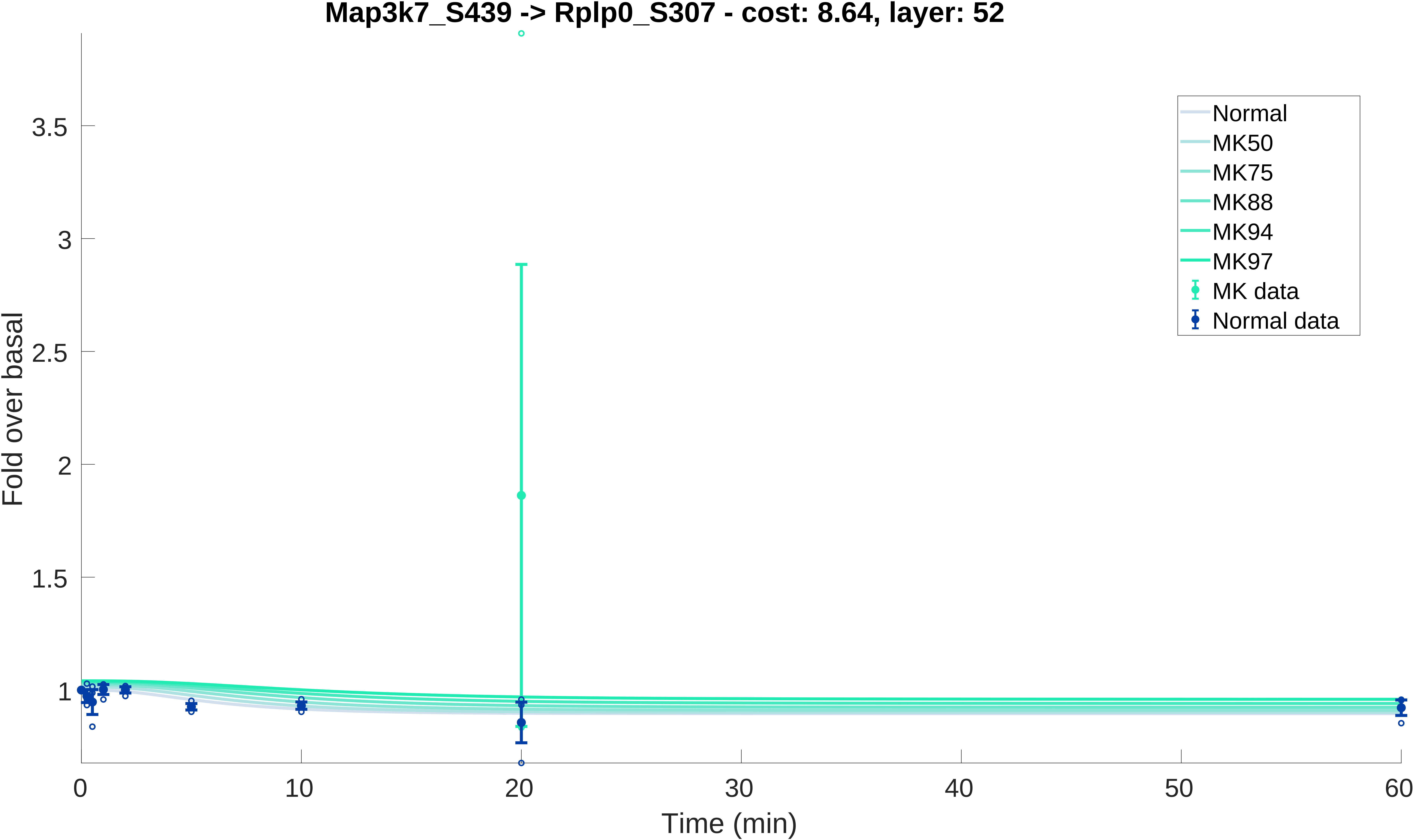

Map3k7_S439 -> Rplp0_S307 - cost: 8.64, layer: 52
Fold over basal
Time (min)
0
10
20
30
40
50
60
1
1.5
2
2.5
3
3.5
Normal
MK50
MK75
MK88
MK94
MK97
MK data
Normal data

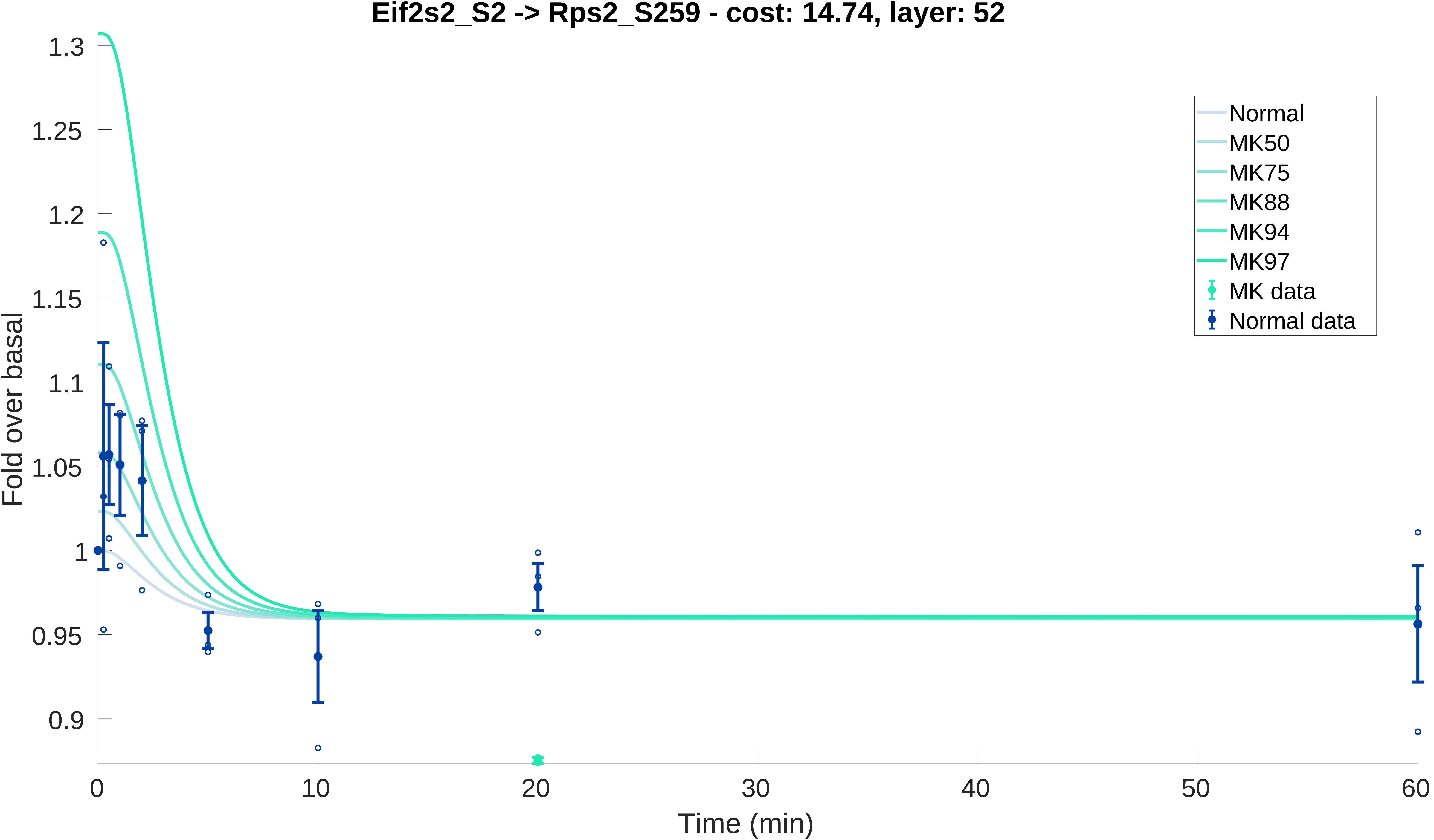

Eif2s2_S2 -> Rps2_S259 - cost: 14.74, layer: 52
Fold over basal
Time (min)
1.3
1.25
1.2
1.15
1.1
1.05
1
0.95
0.9
0
10
20
30
40
50
60
Normal
MK50
MK75
MK88
MK94
MK97
MK data
Normal data

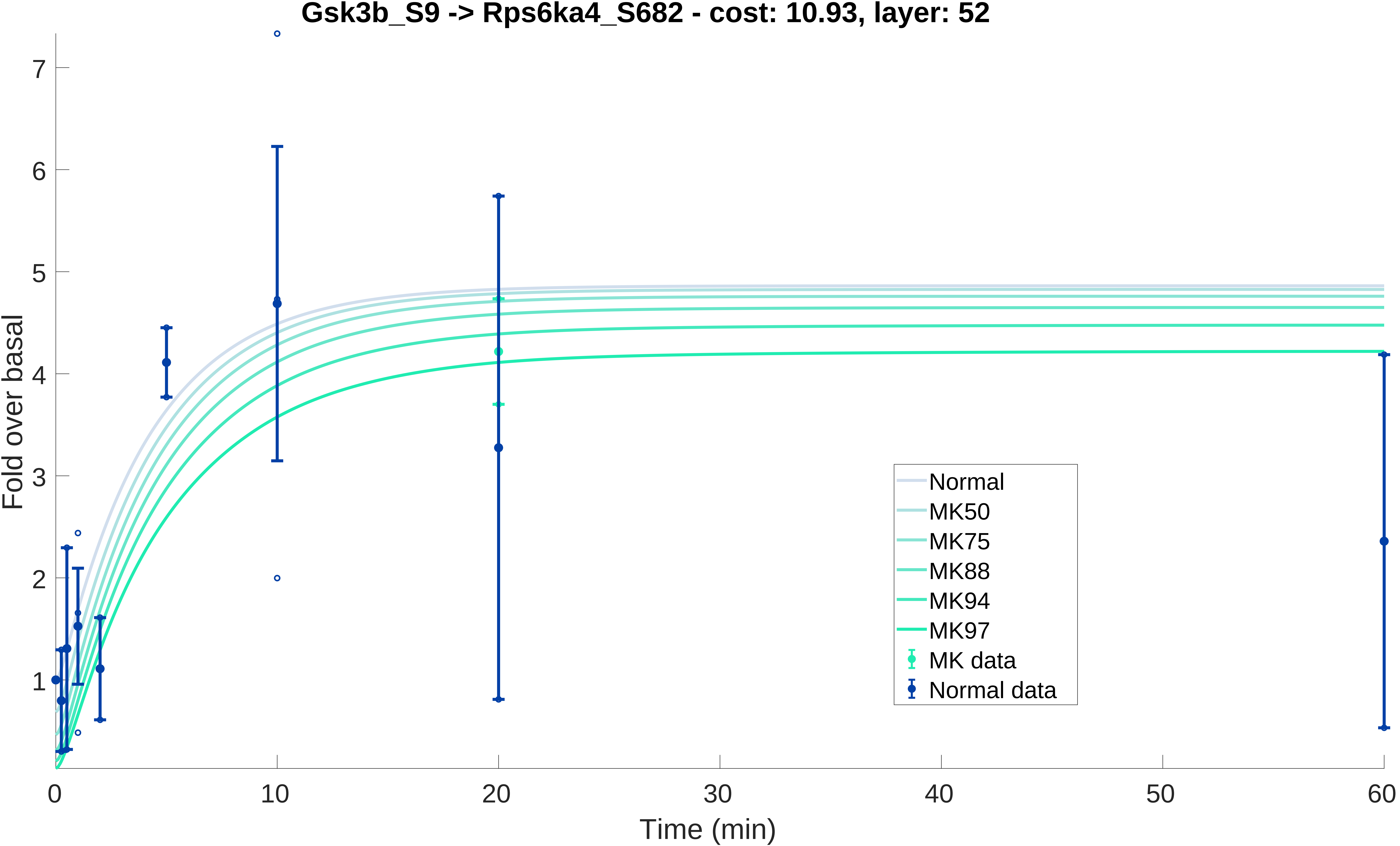
Gsk3b_S9 -> Rps6ka4_S682 - cost: 10.93, layer: 52
Fold over basal
Time (min)
0
10
20
30
40
50
60
1
2
3
4
5
6
7
Normal
MK50
MK75
MK88
MK94
MK97
MK data
Normal data

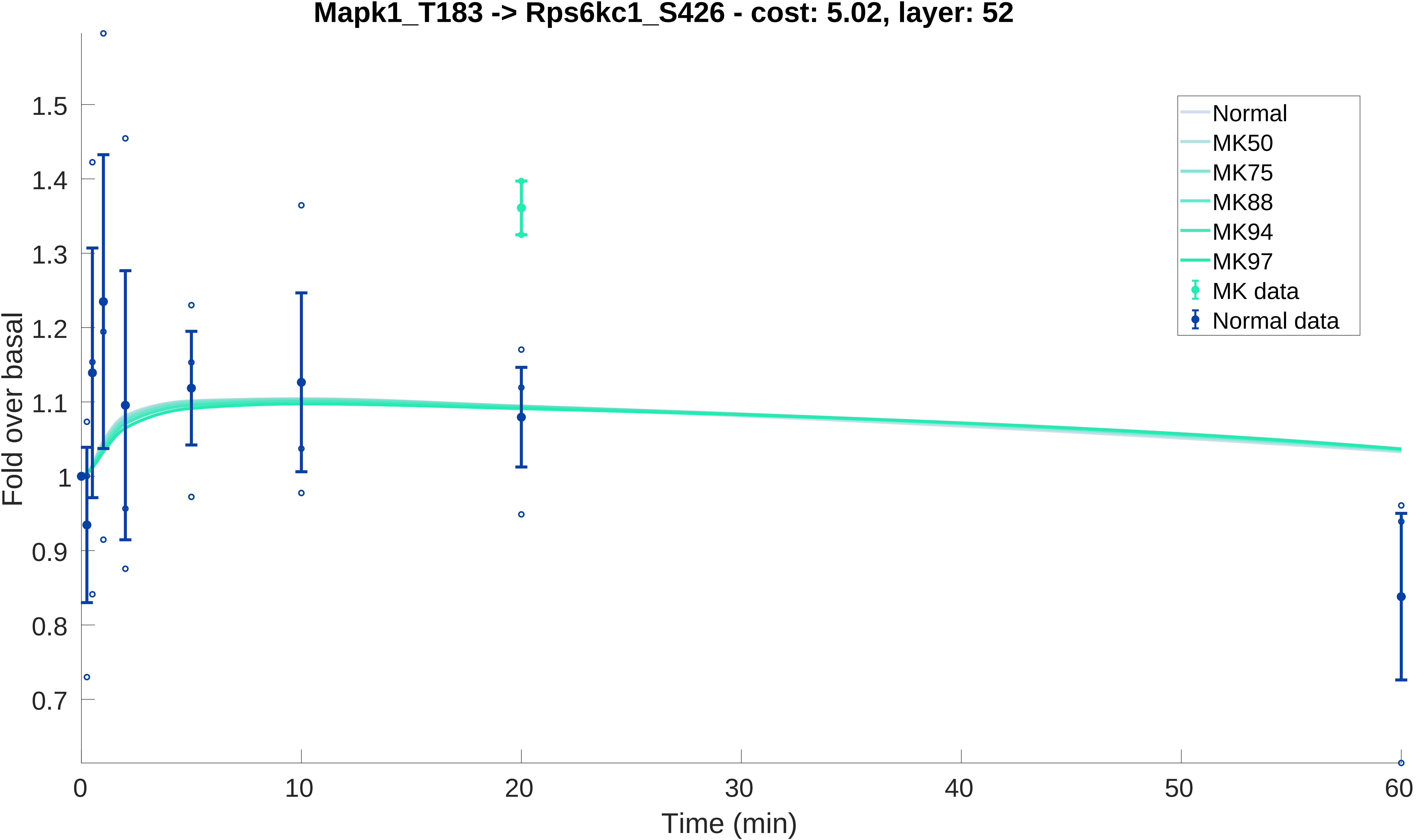
Mapk1_T183 -> Rps6kc1_S426 - cost: 5.02, layer: 52
Fold over basal
1.5
1.4
1.3
1.2
1.1
1
0.9
0.8
0.7
0
10
20
30
40
50
60
Time (min)
Normal
MK50
MK75
MK88
MK94
MK97
MK data
Normal data

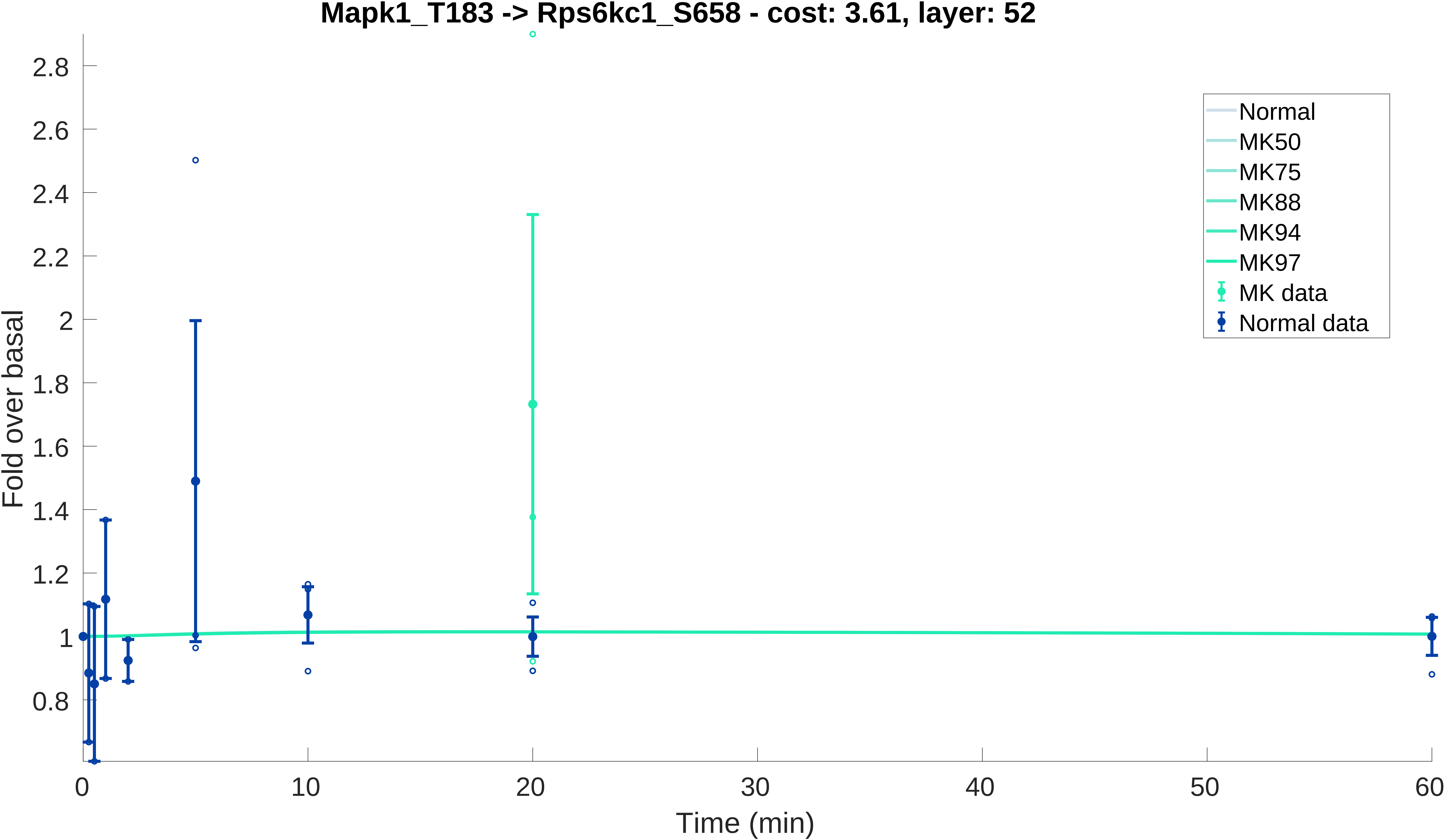

Mapk1_T183 -> Rps6kc1_S658 - cost: 3.61, layer: 52
Fold over basal
Time (min)
Normal
MK50
MK75
MK88
MK94
MK97
MK data
Normal data
0.8
1
1.2
1.4
1.6
1.8
2
2.2
2.4
2.6
2.8
0
10
20
30
40
50
60

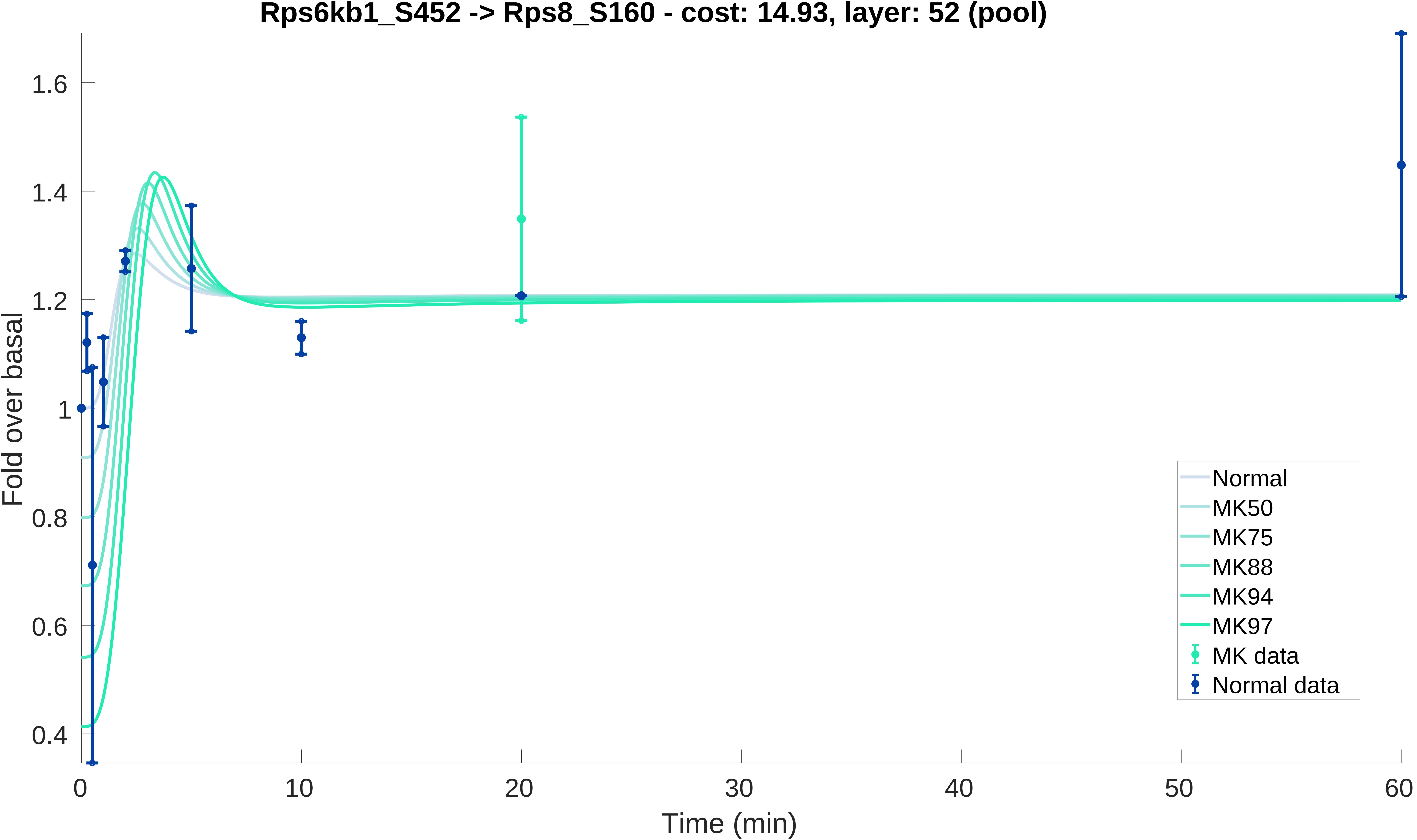
Rps6kb1_S452 -> Rps8_S160 - cost: 14.93, layer: 52 (pool)
Fold over basal
1.6
1.4
1.2
1
0.8
0.6
0.4
0
10
20
30
40
50
60
Time (min)
Normal
MK50
MK75
MK88
MK94
MK97
MK data
Normal data

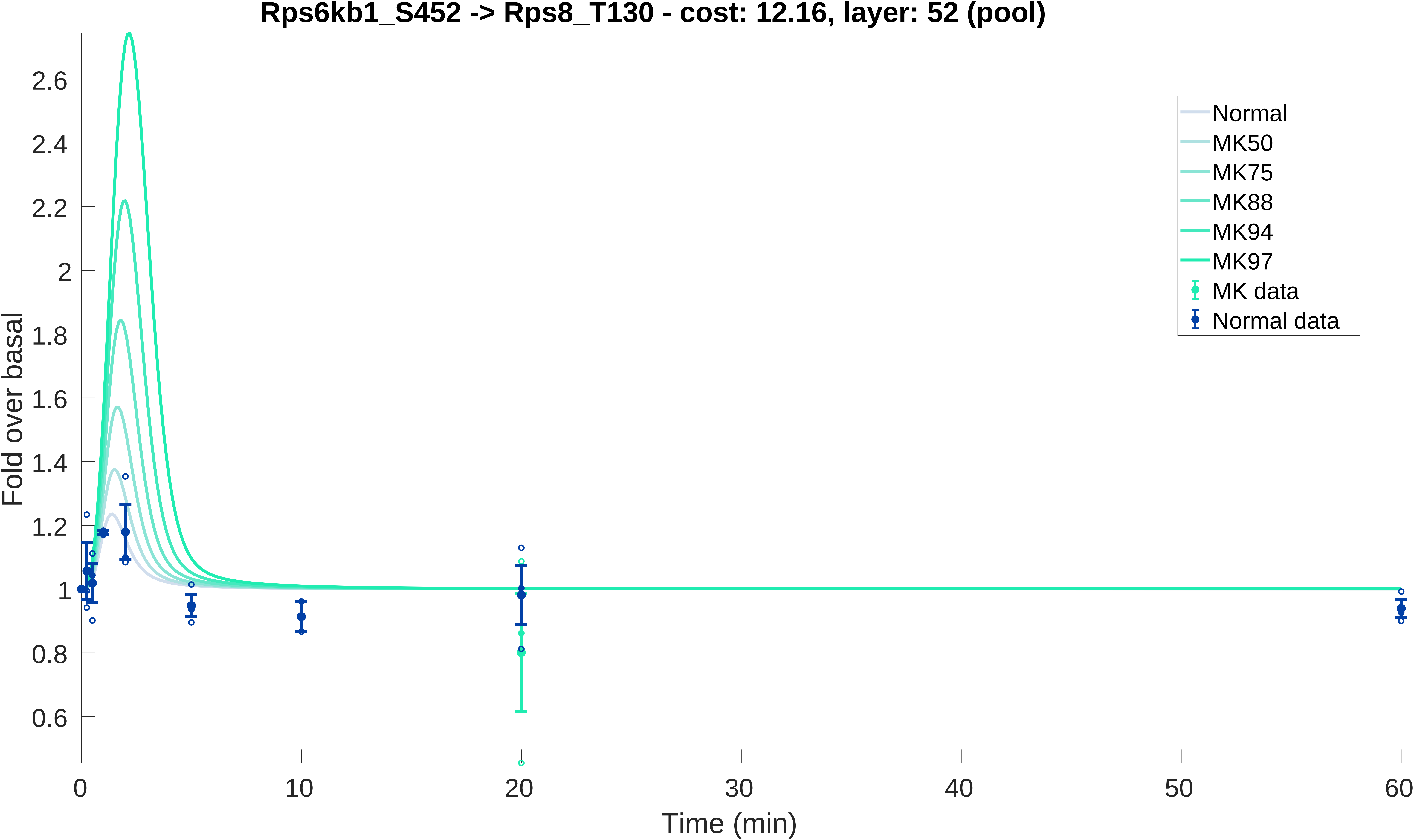

Rps6kb1_S452 -> Rps8_T130 - cost: 12.16, layer: 52 (pool)
Fold over basal
Time (min)
0.6
0.8
1
1.2
1.4
1.6
1.8
2
2.2
2.4
2.6
0
10
20
30
40
50
60
Normal
MK50
MK75
MK88
MK94
MK97
MK data
Normal data

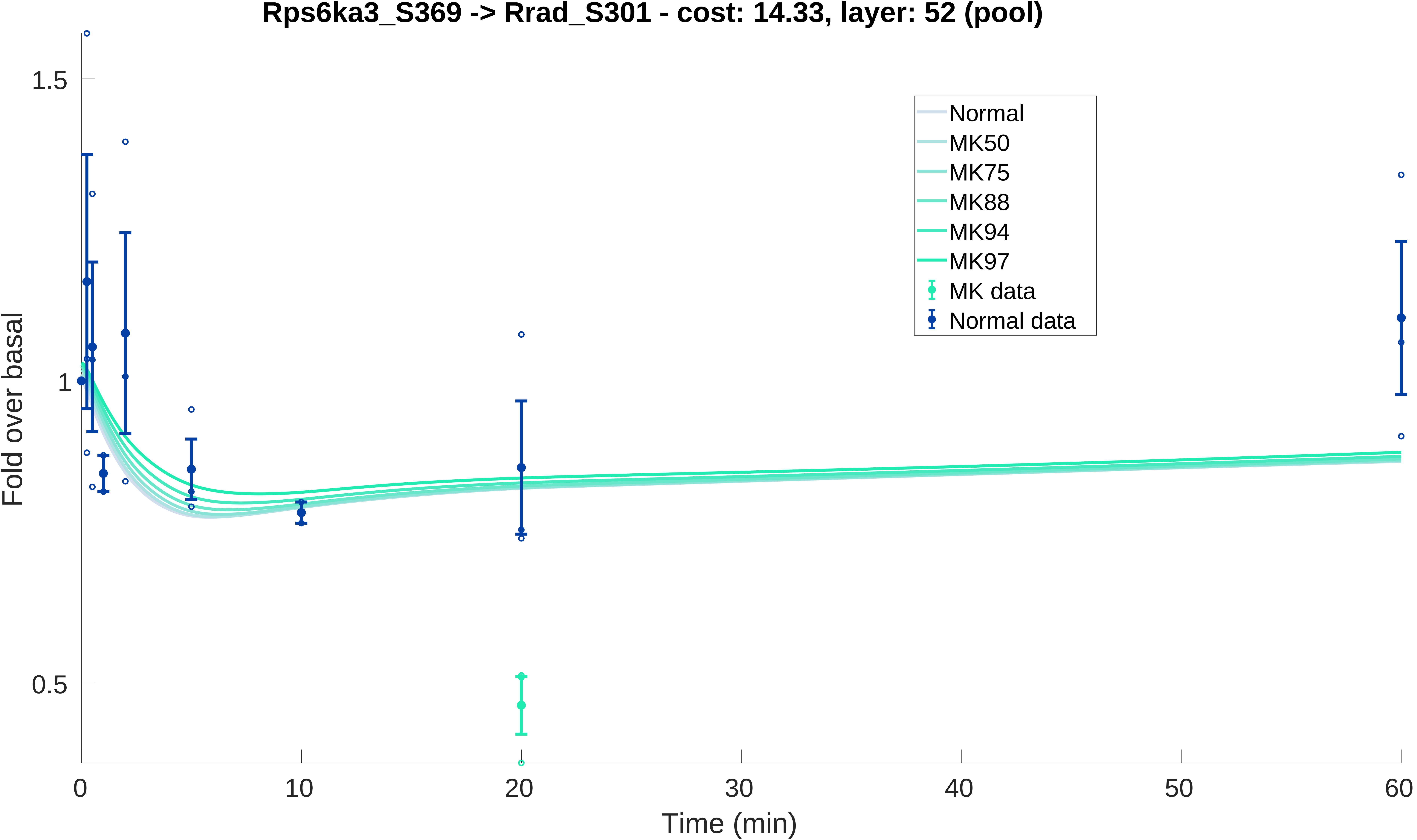

Rps6ka3_S369 -> Rrad_S301 - cost: 14.33, layer: 52 (pool)
Fold over basal
1.5
1
0.5
Time (min)
0
10
20
30
40
50
60
Normal
MK50
MK75
MK88
MK94
MK97
MK data
Normal data

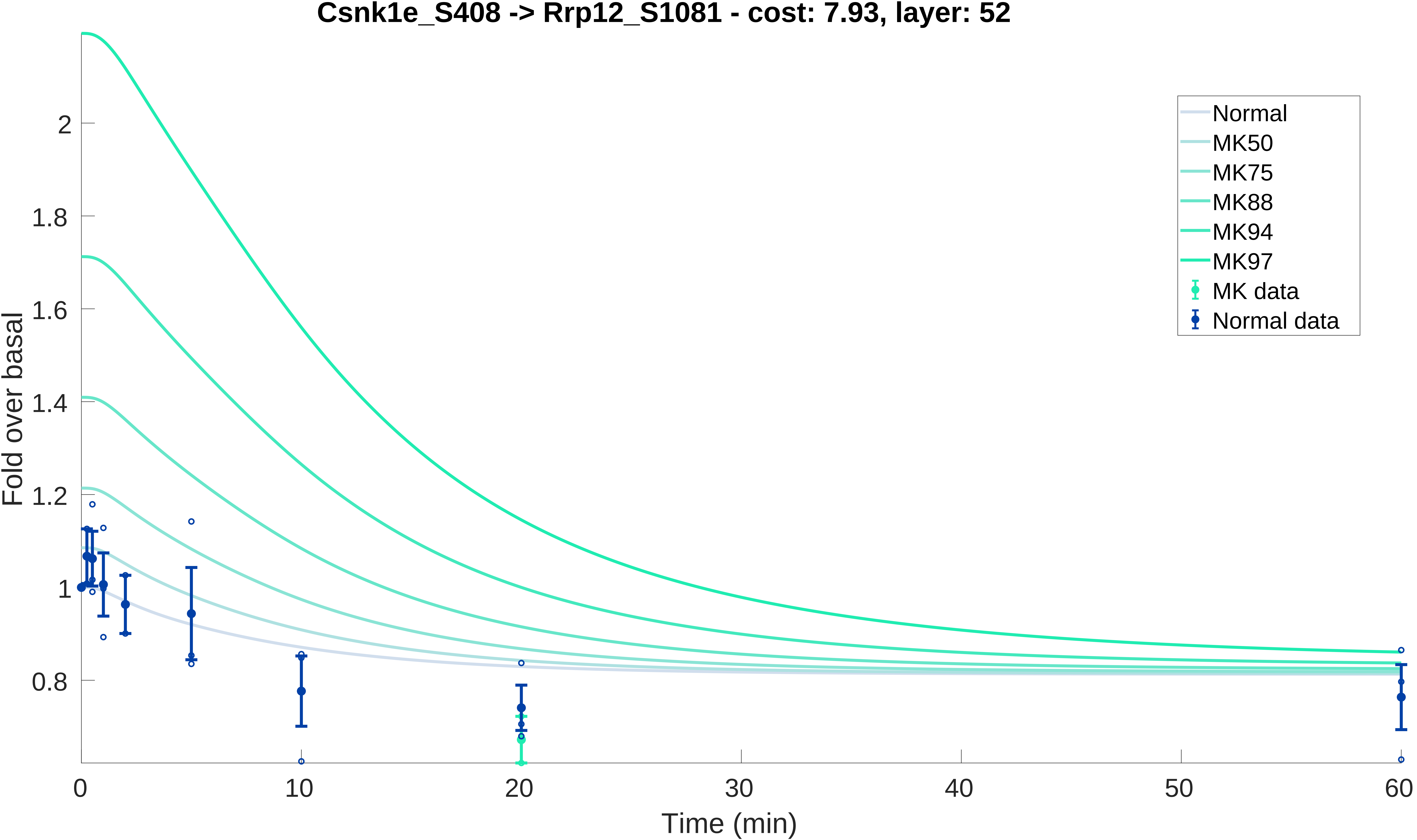
Csnk1e_S408 -> Rrp12_S1081 - cost: 7.93, layer: 52
Fold over basal
Time (min)
Normal
MK50
MK75
MK88
MK94
MK97
MK data
Normal data
0.8
1
1.2
1.4
1.6
1.8
2
0
10
20
30
40
50
60

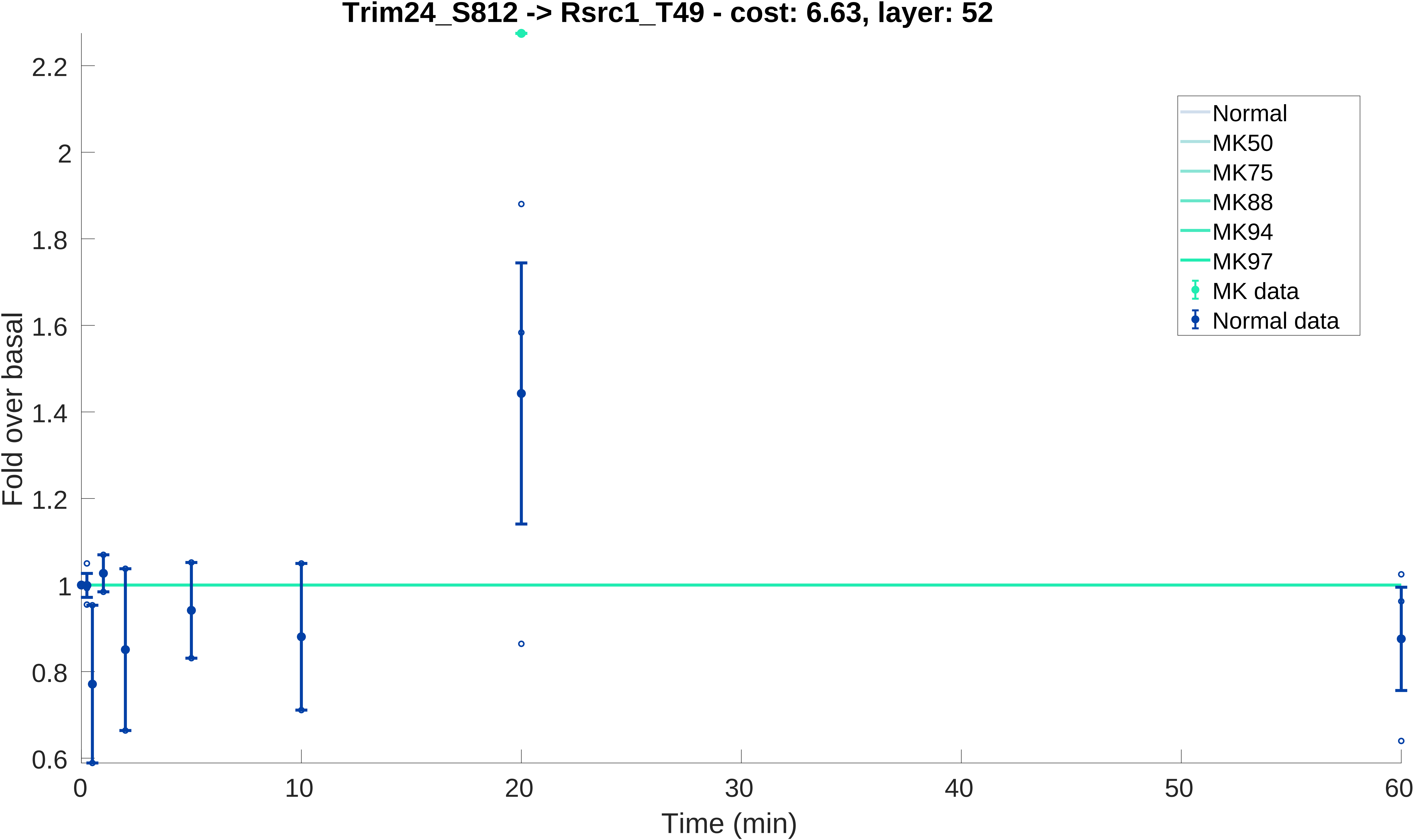
Trim24_S812 -> Rsrc1_T49 - cost: 6.63, layer: 52
Fold over basal
Time (min)
0.6
0.8
1
1.2
1.4
1.6
1.8
2
2.2
0
10
20
30
40
50
60
Normal
MK50
MK75
MK88
MK94
MK97
MK data
Normal data

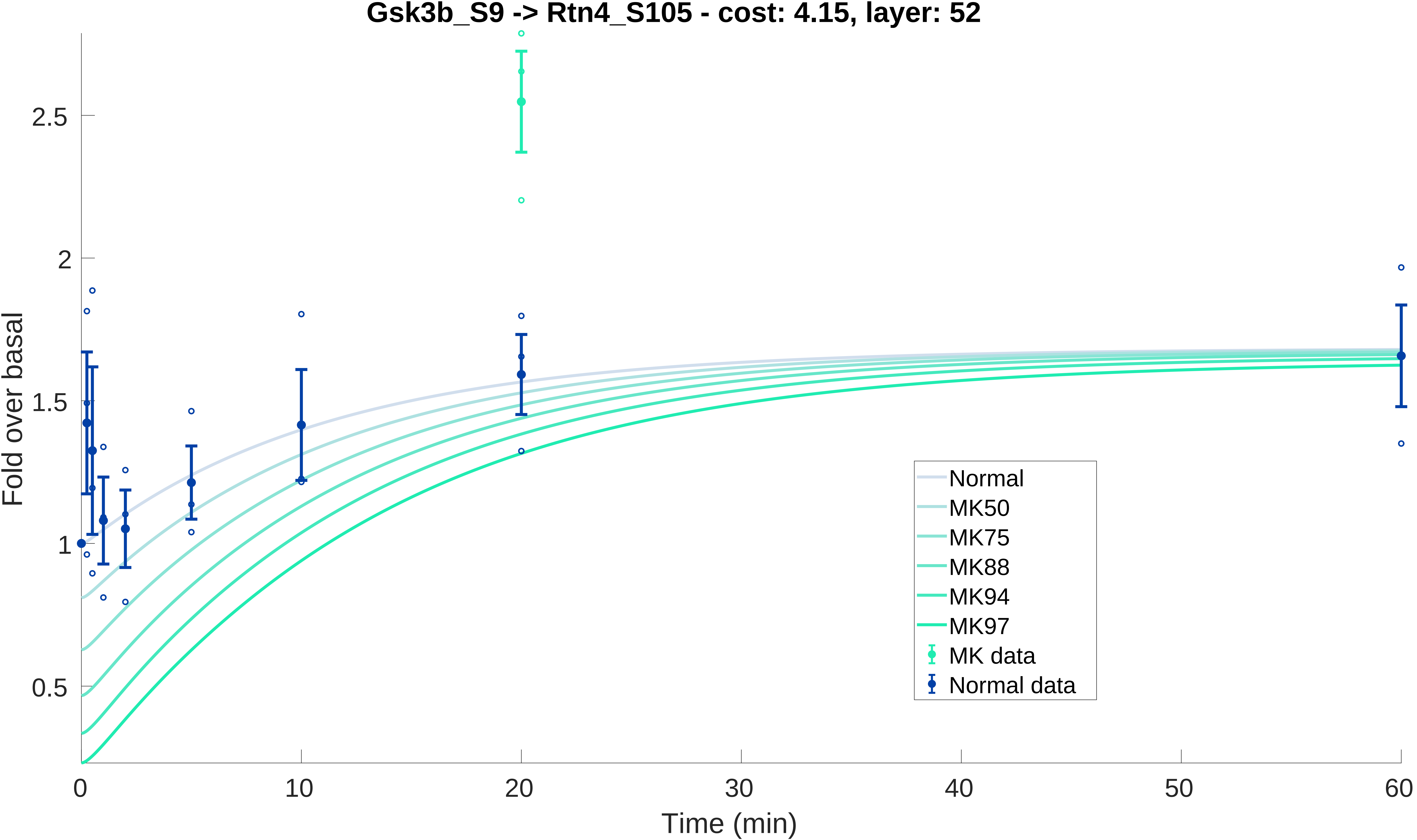
Gsk3b_S9 -> Rtn4_S105 - cost: 4.15, layer: 52
Fold over basal
2.5
2
1.5
1
0.5
0
10
20
30
40
50
60
Time (min)
Normal
MK50
MK75
MK88
MK94
MK97
MK data
Normal data

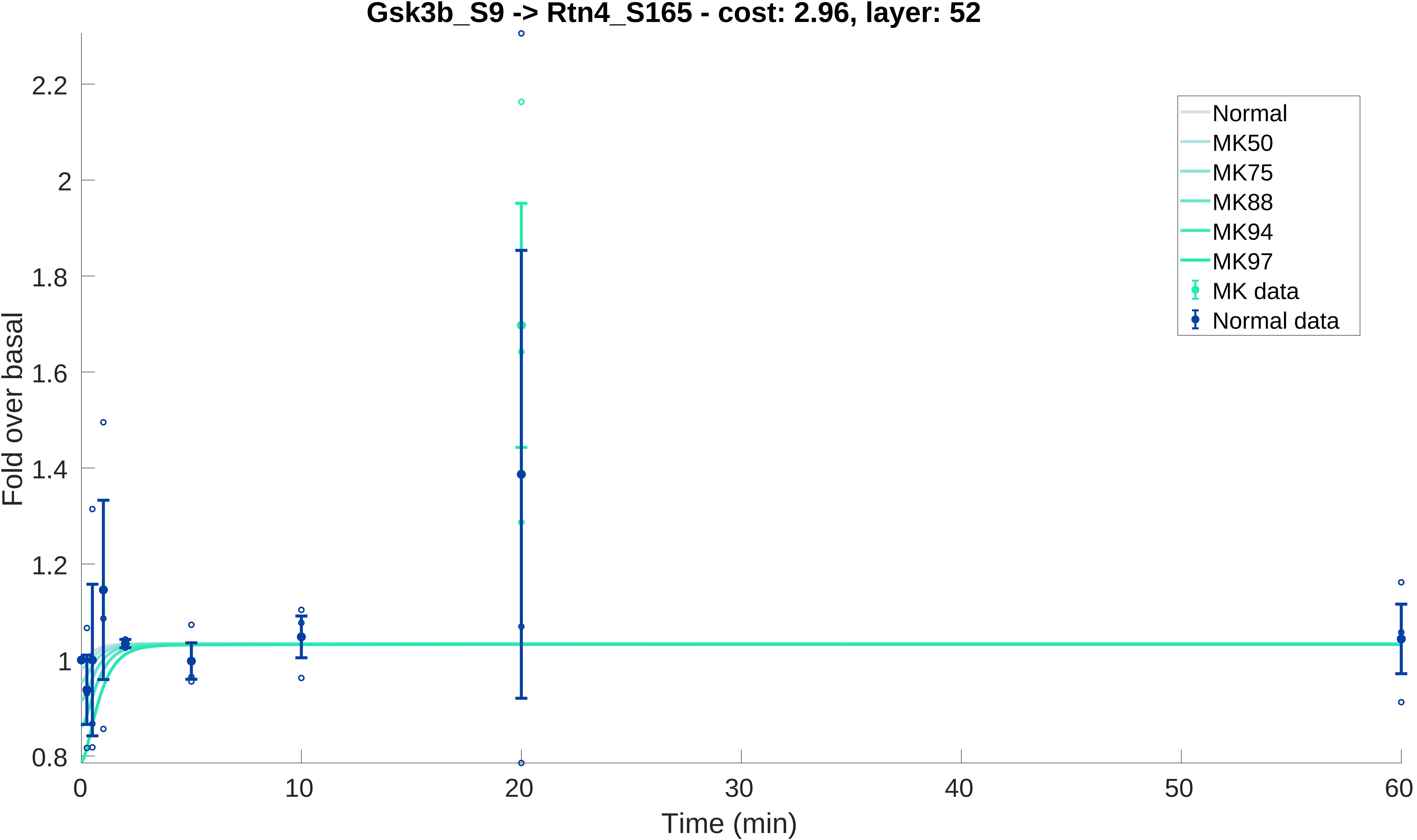
Gsk3b_S9 -> Rtn4_S165 - cost: 2.96, layer: 52
Fold over basal
Time (min)
0.8
1
1.2
1.4
1.6
1.8
2
2.2
0
10
20
30
40
50
60
Normal
MK50
MK75
MK88
MK94
MK97
MK data
Normal data

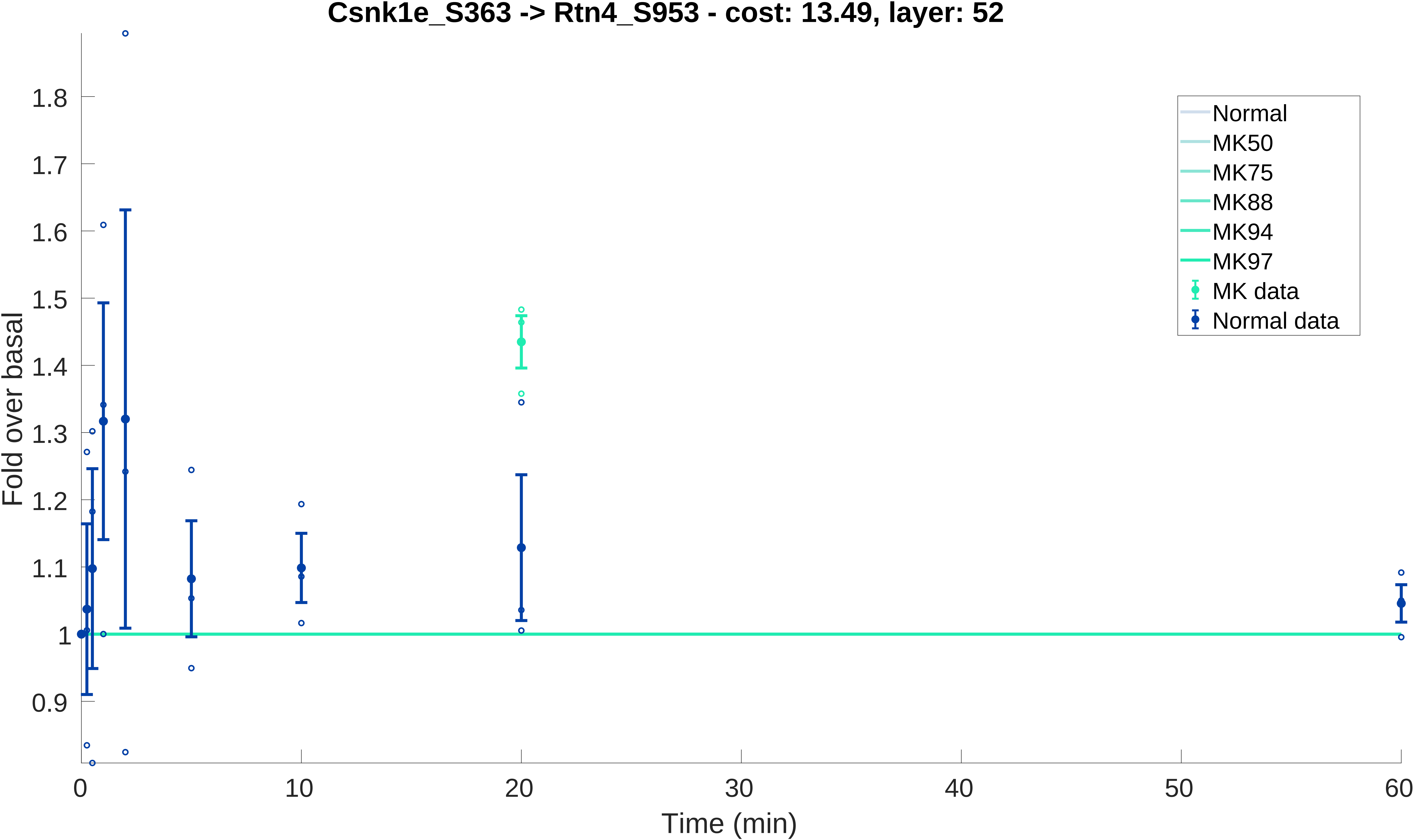
Csnk1e_S363 -> Rtn4_S953 - cost: 13.49, layer: 52
Fold over basal
Time (min)
0.9
1
1.1
1.2
1.3
1.4
1.5
1.6
1.7
1.8
0
10
20
30
40
50
60
Normal
MK50
MK75
MK88
MK94
MK97
MK data
Normal data

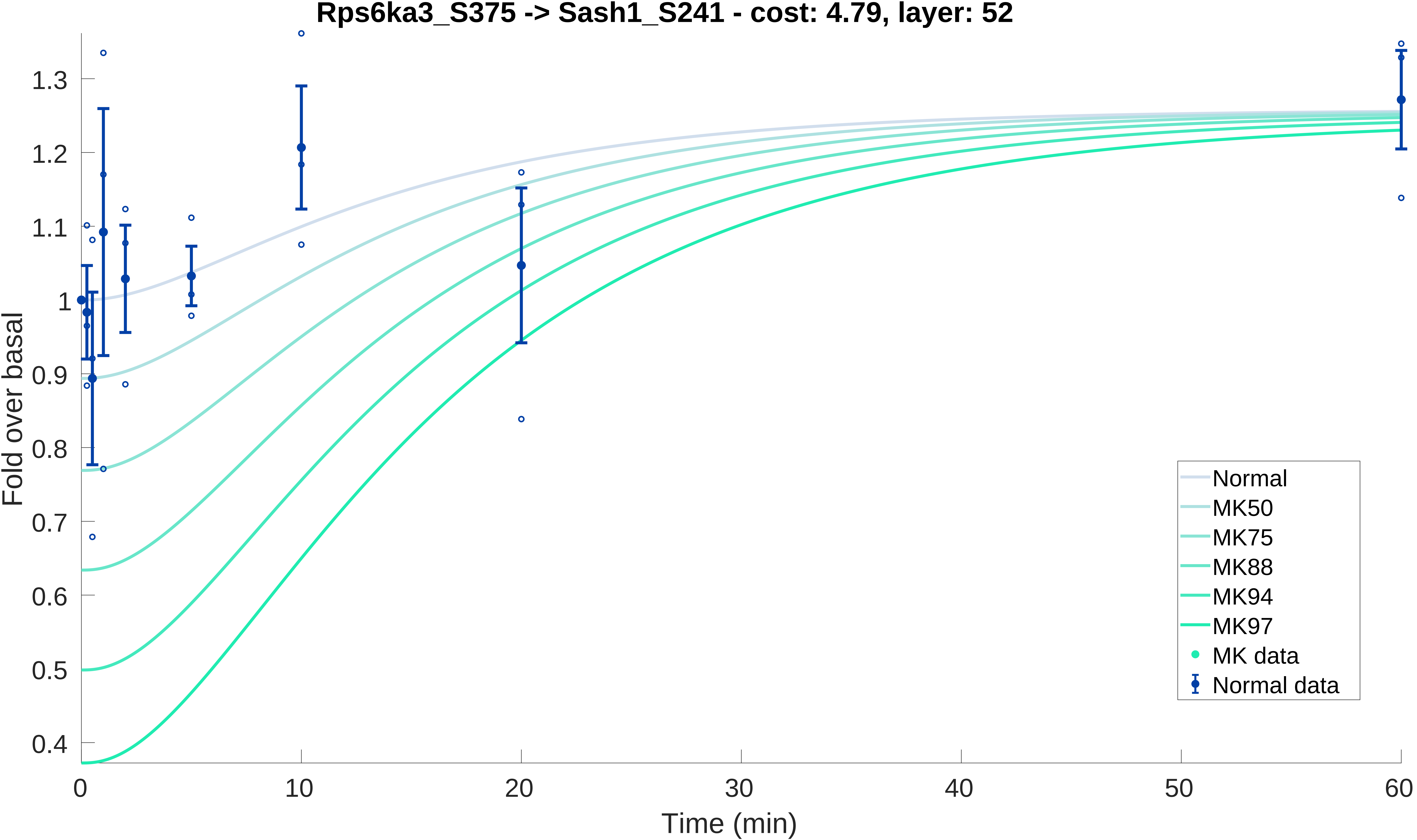

Rps6ka3_S375 -> Sash1_S241 - cost: 4.79, layer: 52
Fold over basal
Time (min)
0.4
0.5
0.6
0.7
0.8
0.9
1
1.1
1.2
1.3
0
10
20
30
40
50
60
Normal
MK50
MK75
MK88
MK94
MK97
MK data
Normal data

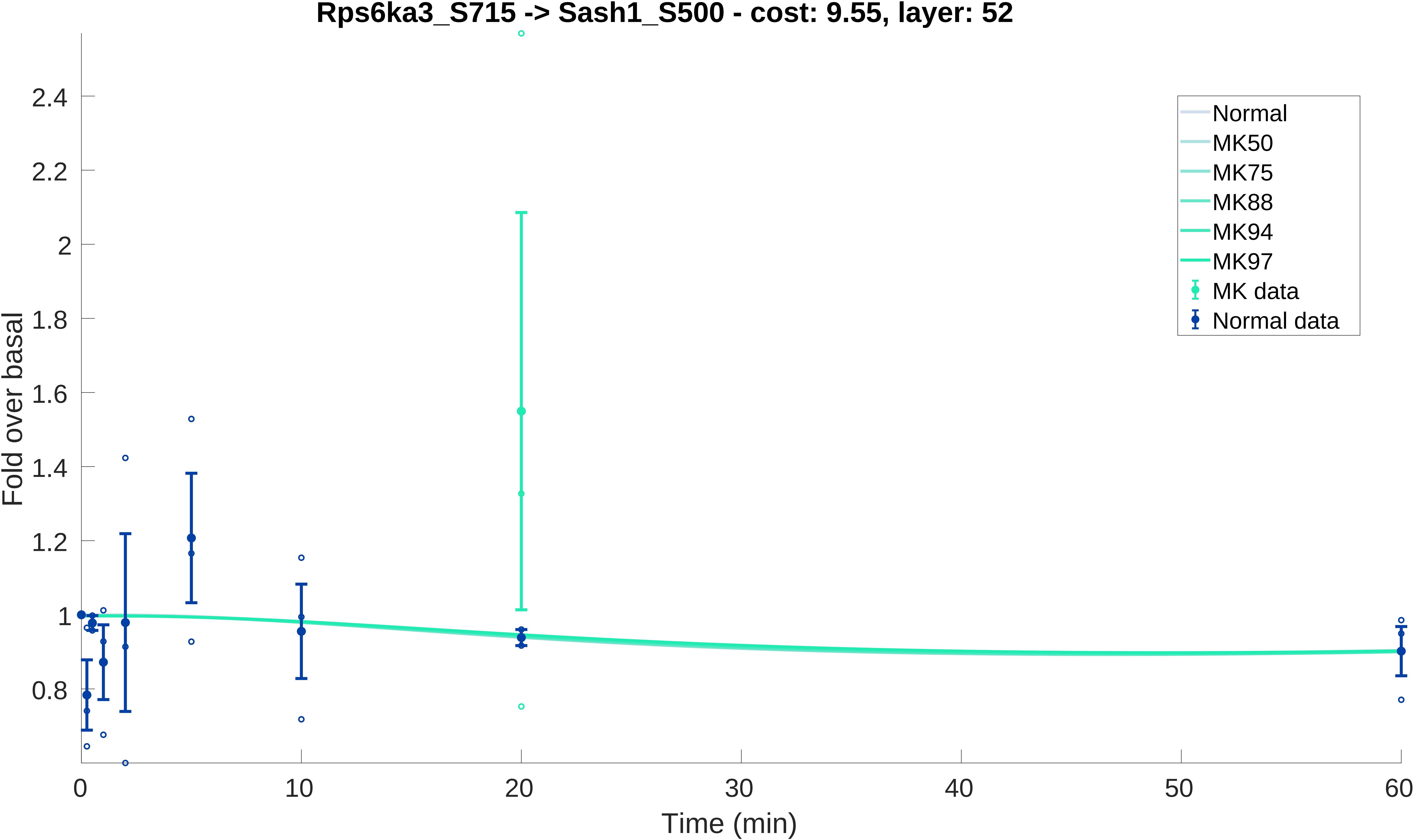
Rps6ka3_S715 -> Sash1_S500 - cost: 9.55, layer: 52
Fold over basal
2.4
2.2
2
1.8
1.6
1.4
1.2
1
0.8
0
10
20
30
40
50
60
Time (min)
Normal
MK50
MK75
MK88
MK94
MK97
MK data
Normal data

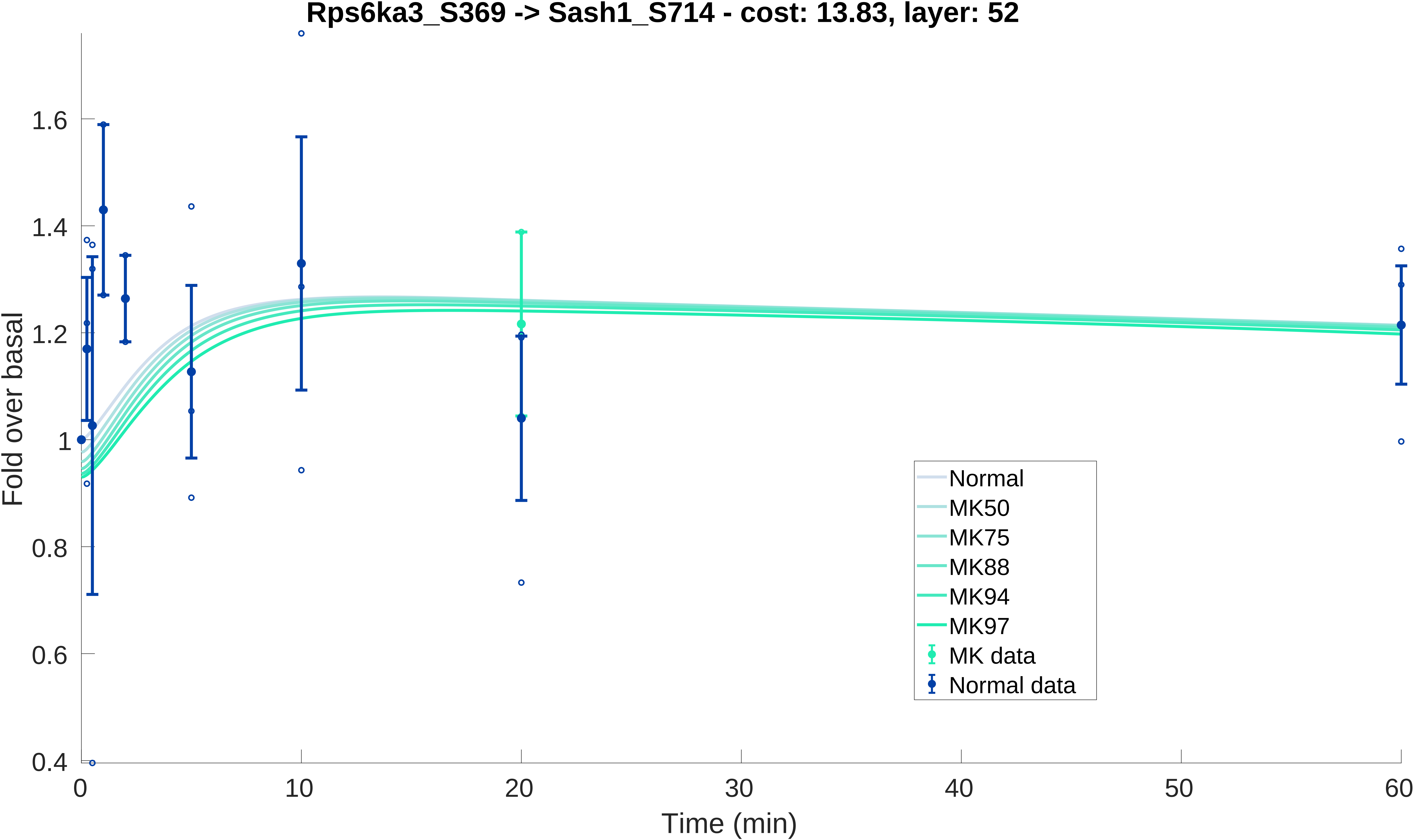

Rps6ka3_S369 -> Sash1_S714 - cost: 13.83, layer: 52
Fold over basal
Time (min)
0.4
0.6
0.8
1
1.2
1.4
1.6
0
10
20
30
40
50
60
Normal
MK50
MK75
MK88
MK94
MK97
MK data
Normal data

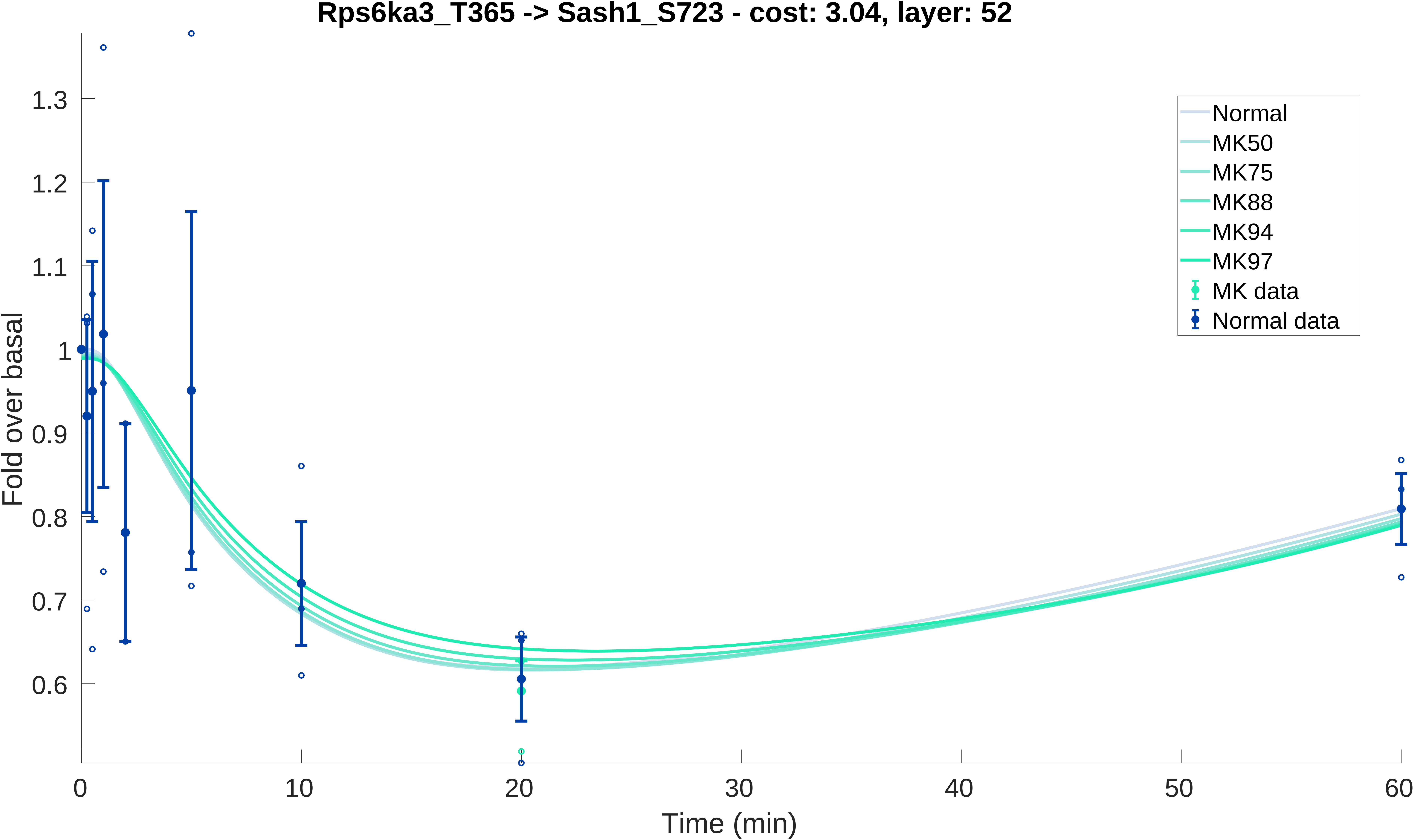
Rps6ka3_T365 -> Sash1_S723 - cost: 3.04, layer: 52
Fold over basal
Time (min)
0
10
20
30
40
50
60
0.6
0.7
0.8
0.9
1
1.1
1.2
1.3
Normal
MK50
MK75
MK88
MK94
MK97
MK data
Normal data

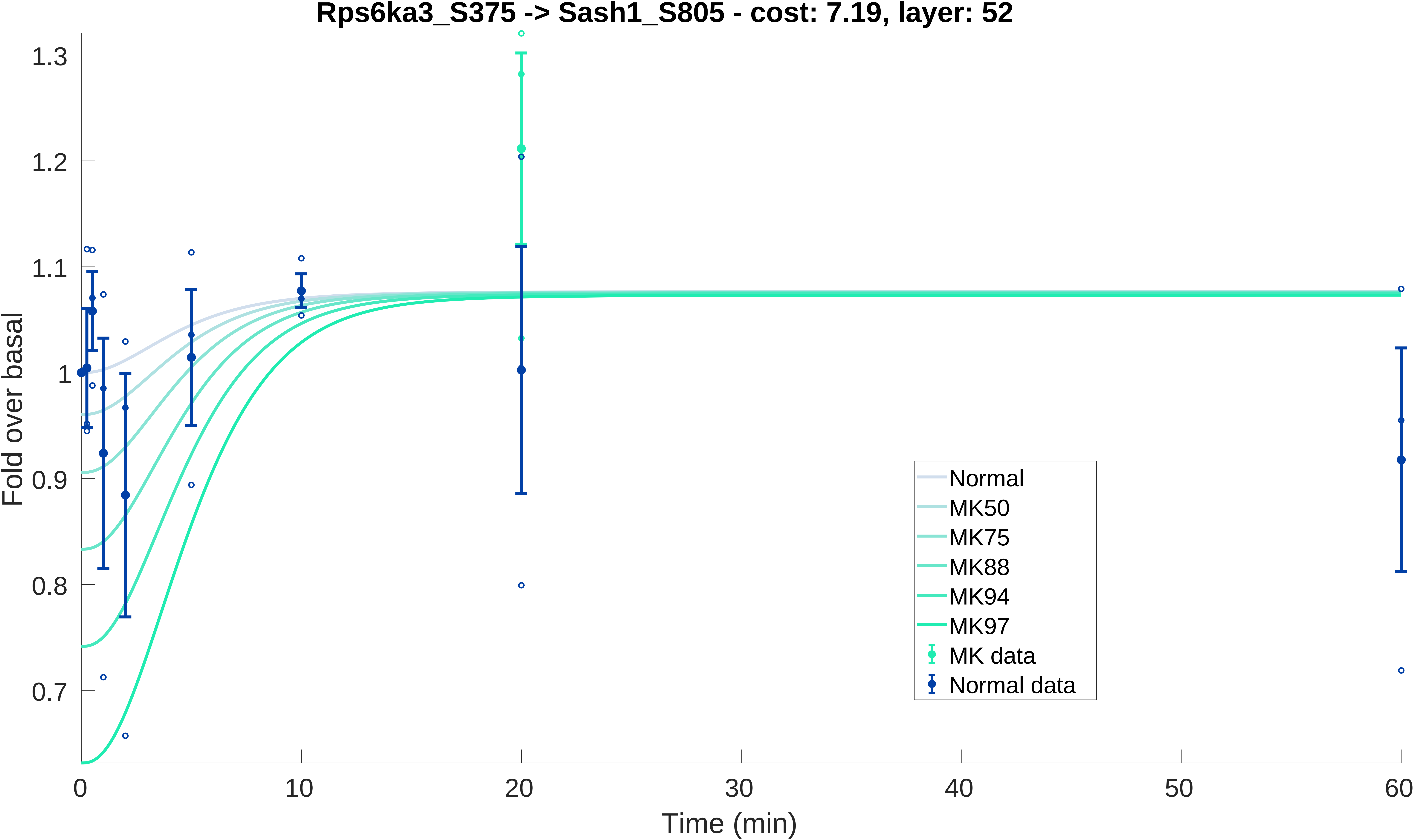

Rps6ka3_S375 -> Sash1_S805 - cost: 7.19, layer: 52
Fold over basal
Time (min)
1.3
1.2
1.1
1
0.9
0.8
0.7
0
10
20
30
40
50
60
Normal
MK50
MK75
MK88
MK94
MK97
MK data
Normal data

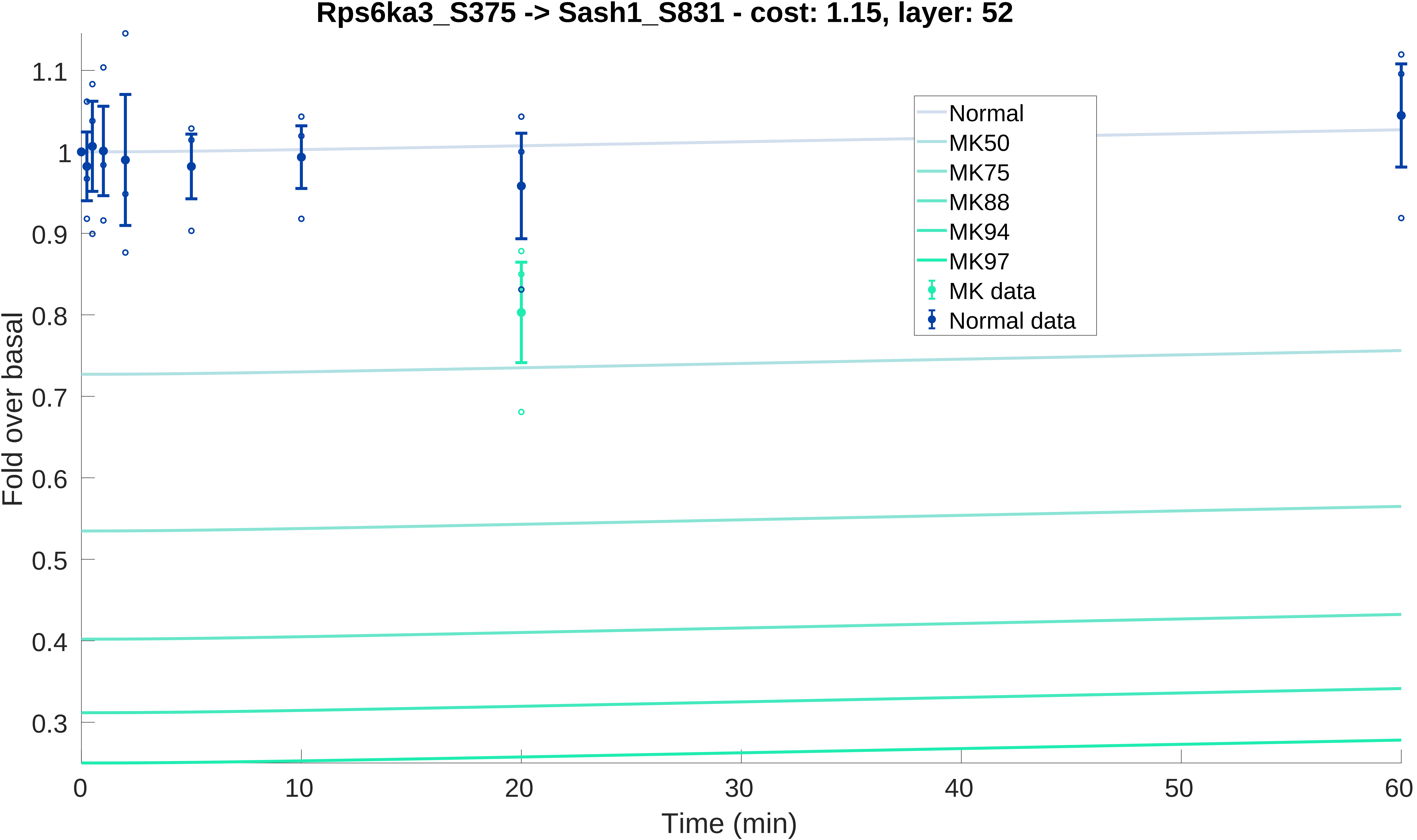

Rps6ka3_S375 -> Sash1_S831 - cost: 1.15, layer: 52
Fold over basal
Time (min)
1.1
1
0.9
0.8
0.7
0.6
0.5
0.4
0.3
0
10
20
30
40
50
60
Normal
MK50
MK75
MK88
MK94
MK97
MK data
Normal data

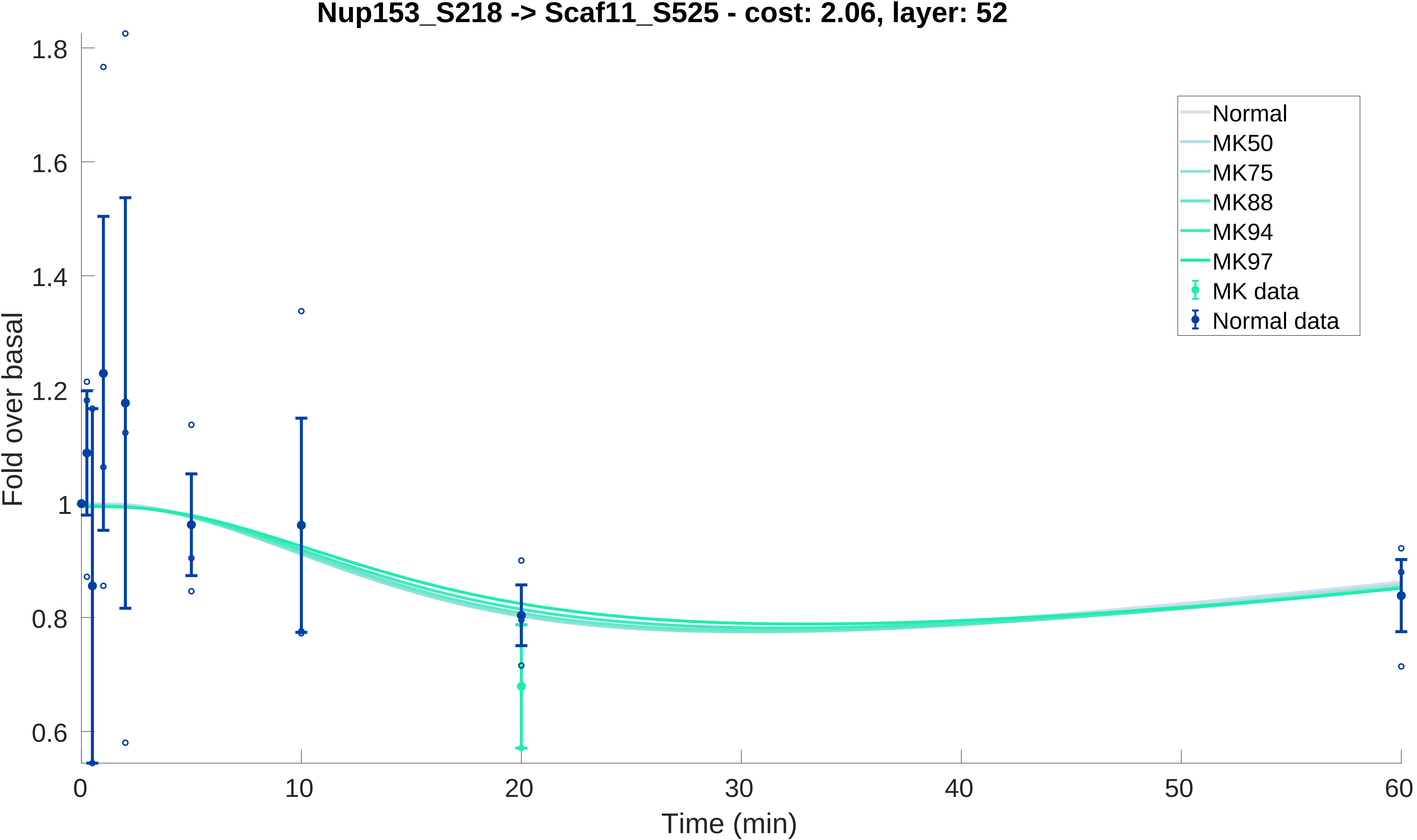

Nup153_S218 -> Scaf11_S525 - cost: 2.06, layer: 52
Fold over basal
Time (min)
Normal
MK50
MK75
MK88
MK94
MK97
MK data
Normal data
1.8
1.6
1.4
1.2
1
0.8
0.6
0
10
20
30
40
50
60

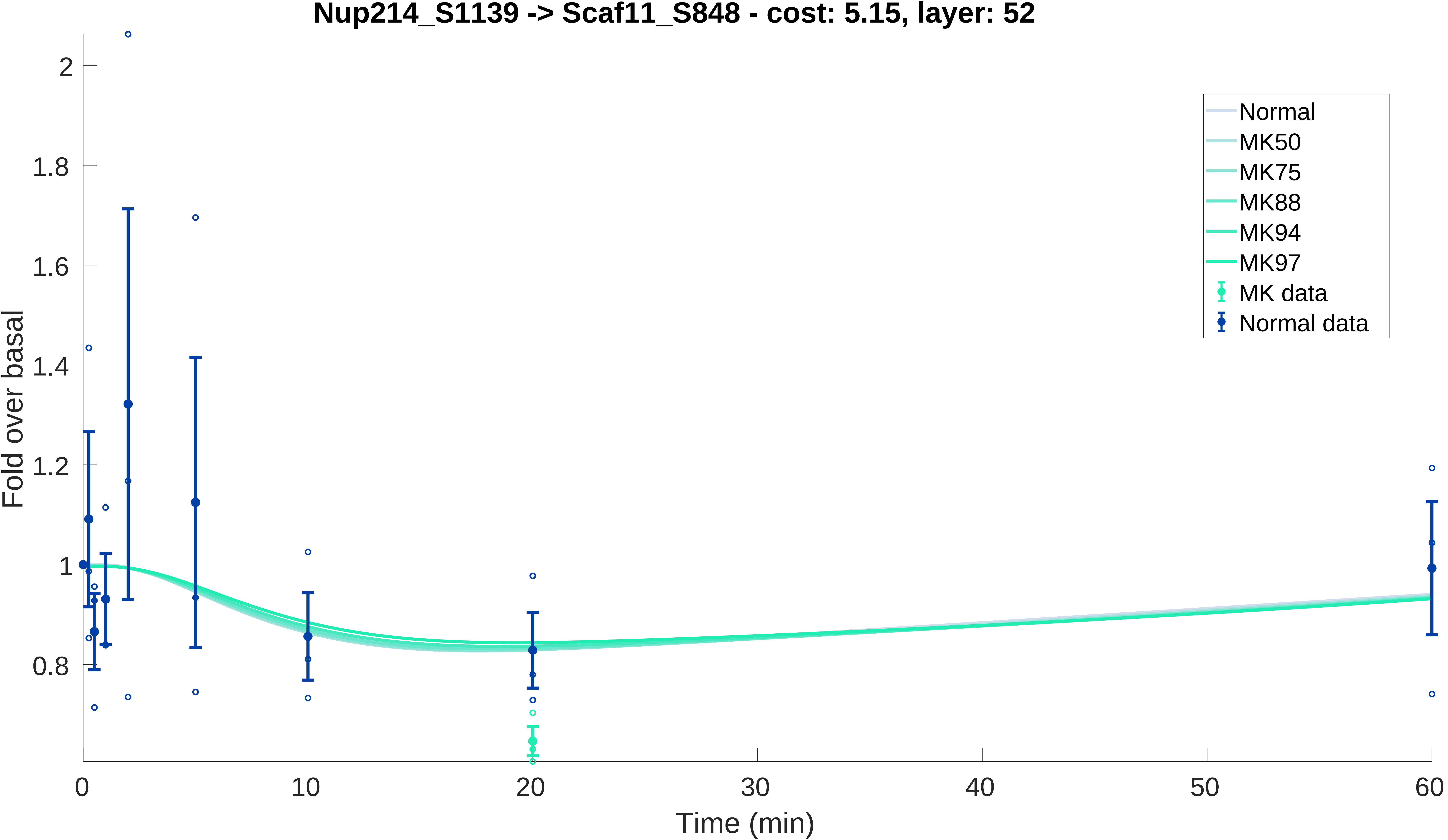

Nup214_S1139 -> Scaf11_S848 - cost: 5.15, layer: 52
Fold over basal
2
1.8
1.6
1.4
1.2
1
0.8
0
10
20
30
40
50
60
Time (min)
Normal
MK50
MK75
MK88
MK94
MK97
MK data
Normal data

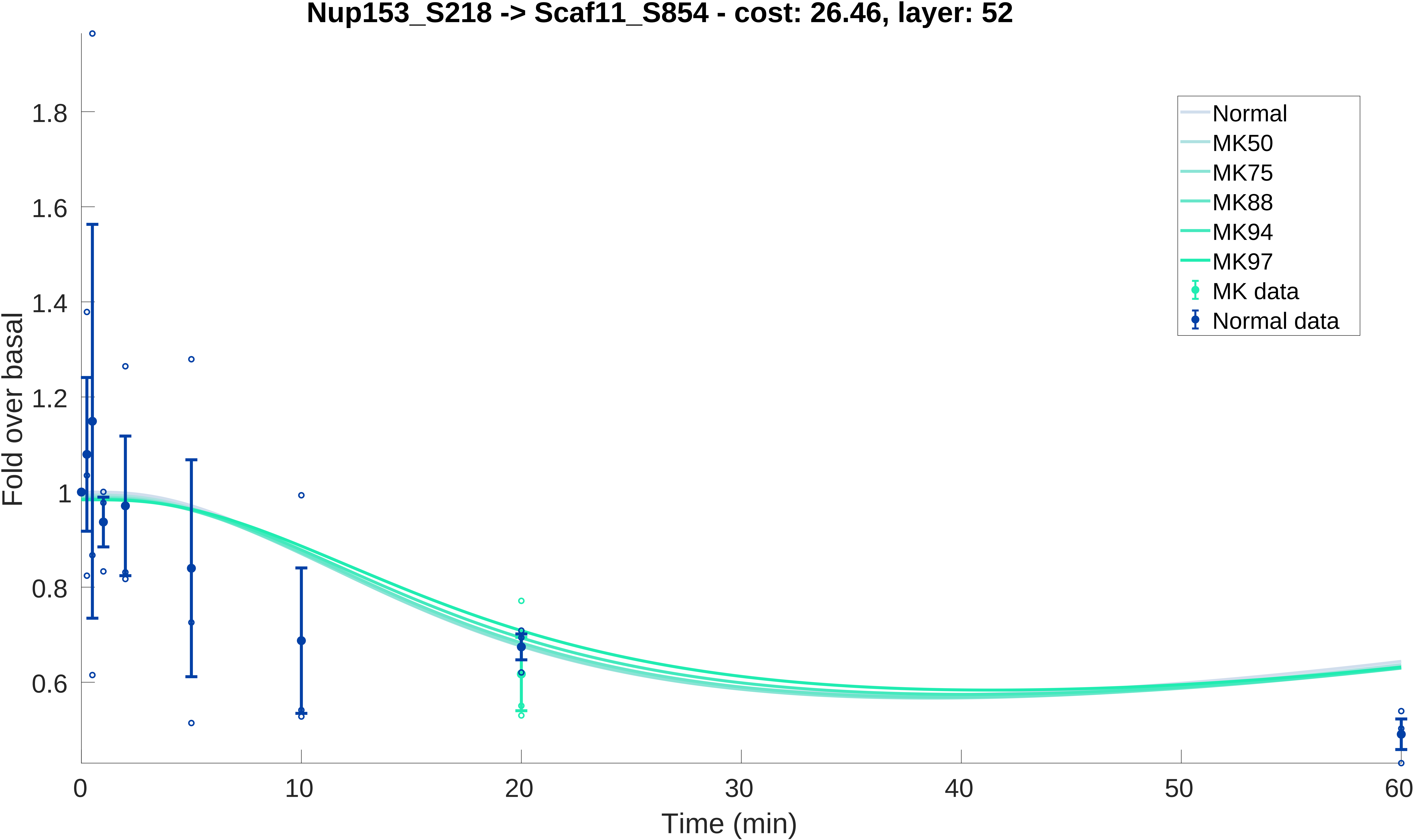

Nup153_S218 -> Scaf11_S854 - cost: 26.46, layer: 52
Fold over basal
1.8
1.6
1.4
1.2
1
0.8
0.6
0
10
20
30
40
50
60
Time (min)
Normal
MK50
MK75
MK88
MK94
MK97
MK data
Normal data

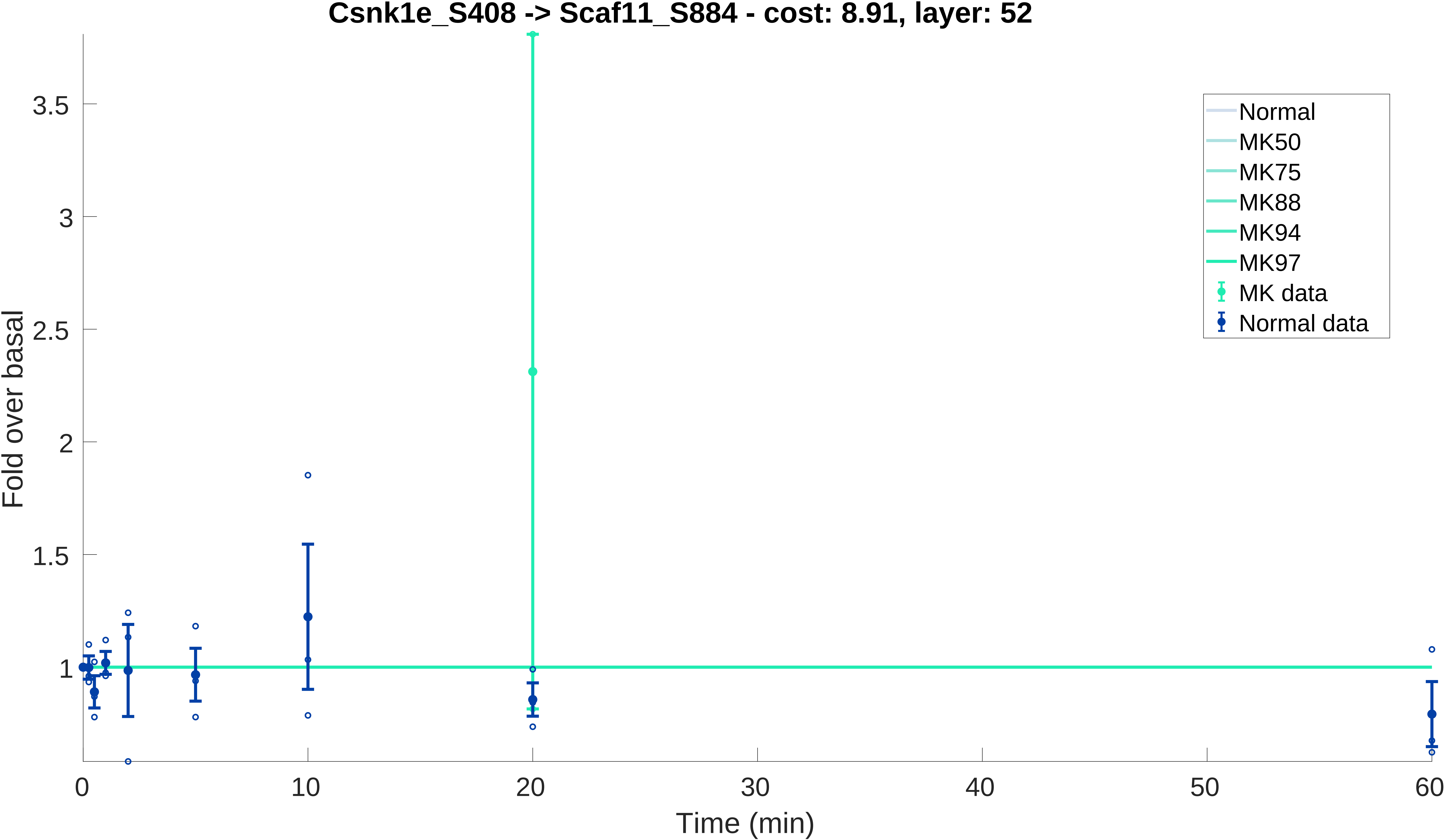

Csnk1e_S408 -> Scaf11_S884 - cost: 8.91, layer: 52
Fold over basal
3.5
3
2.5
2
1.5
1
Time (min)
0
10
20
30
40
50
60
Normal
MK50
MK75
MK88
MK94
MK97
MK data
Normal data

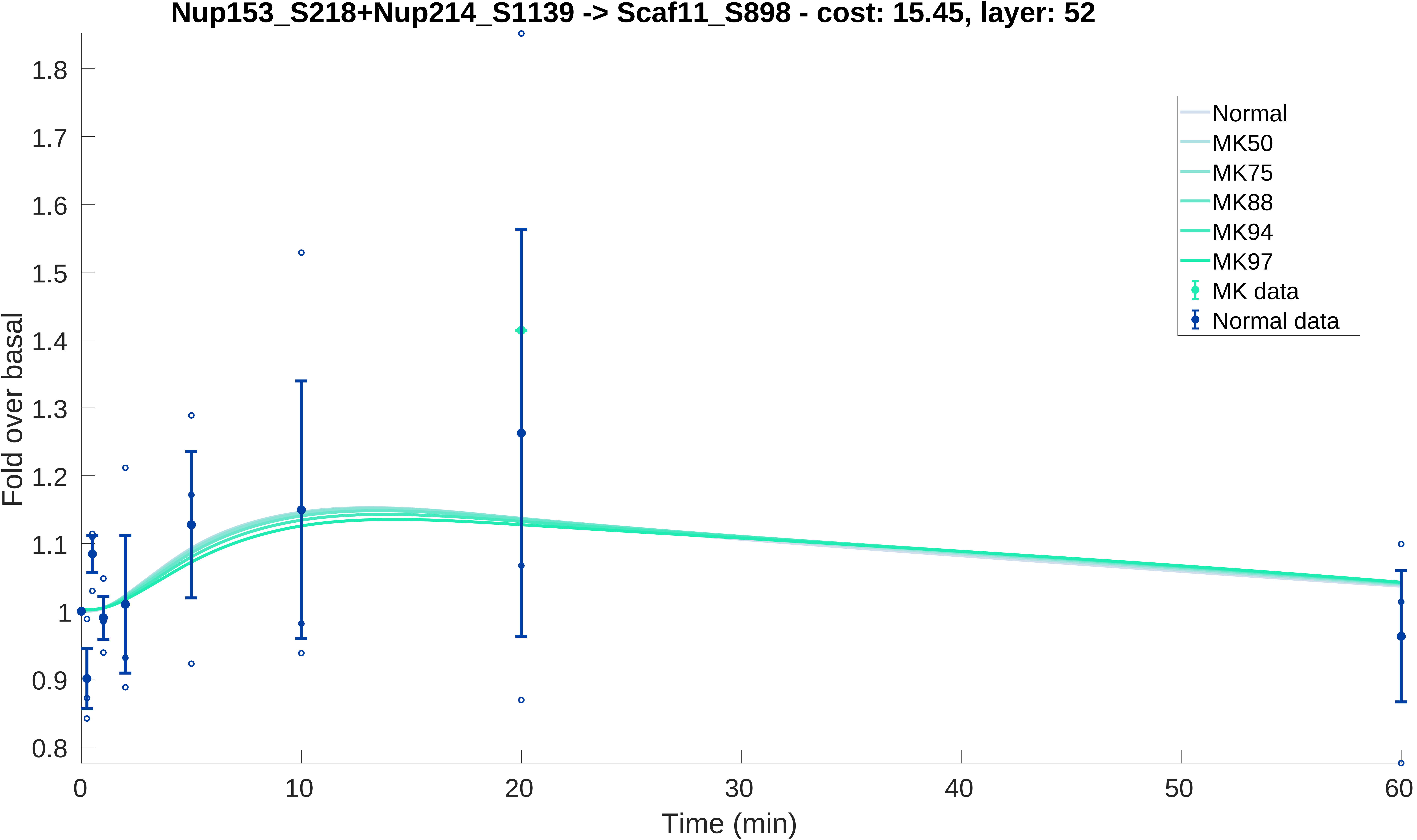

Nup153_S218+Nup214_S1139 -> Scaf11_S898 - cost: 15.45, layer: 52
Fold over basal
1.8
1.7
1.6
1.5
1.4
1.3
1.2
1.1
1
0.9
0.8
0
10
20
30
40
50
60
Time (min)
Normal
MK50
MK75
MK88
MK94
MK97
MK data
Normal data

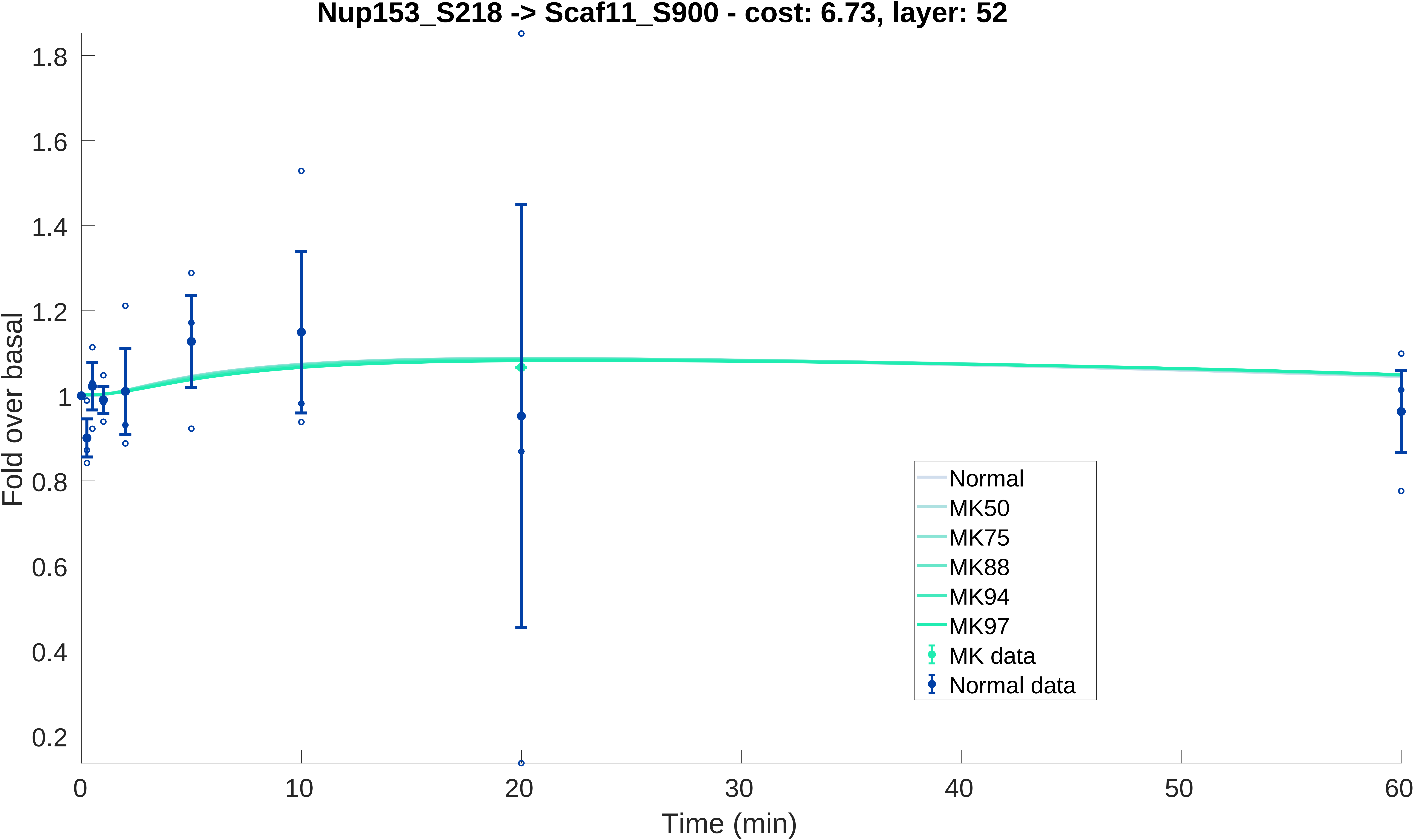

Nup153_S218 -> Scaf11_S900 - cost: 6.73, layer: 52
Fold over basal
Time (min)
0.2
0.4
0.6
0.8
1
1.2
1.4
1.6
1.8
0
10
20
30
40
50
60
Normal
MK50
MK75
MK88
MK94
MK97
MK data
Normal data

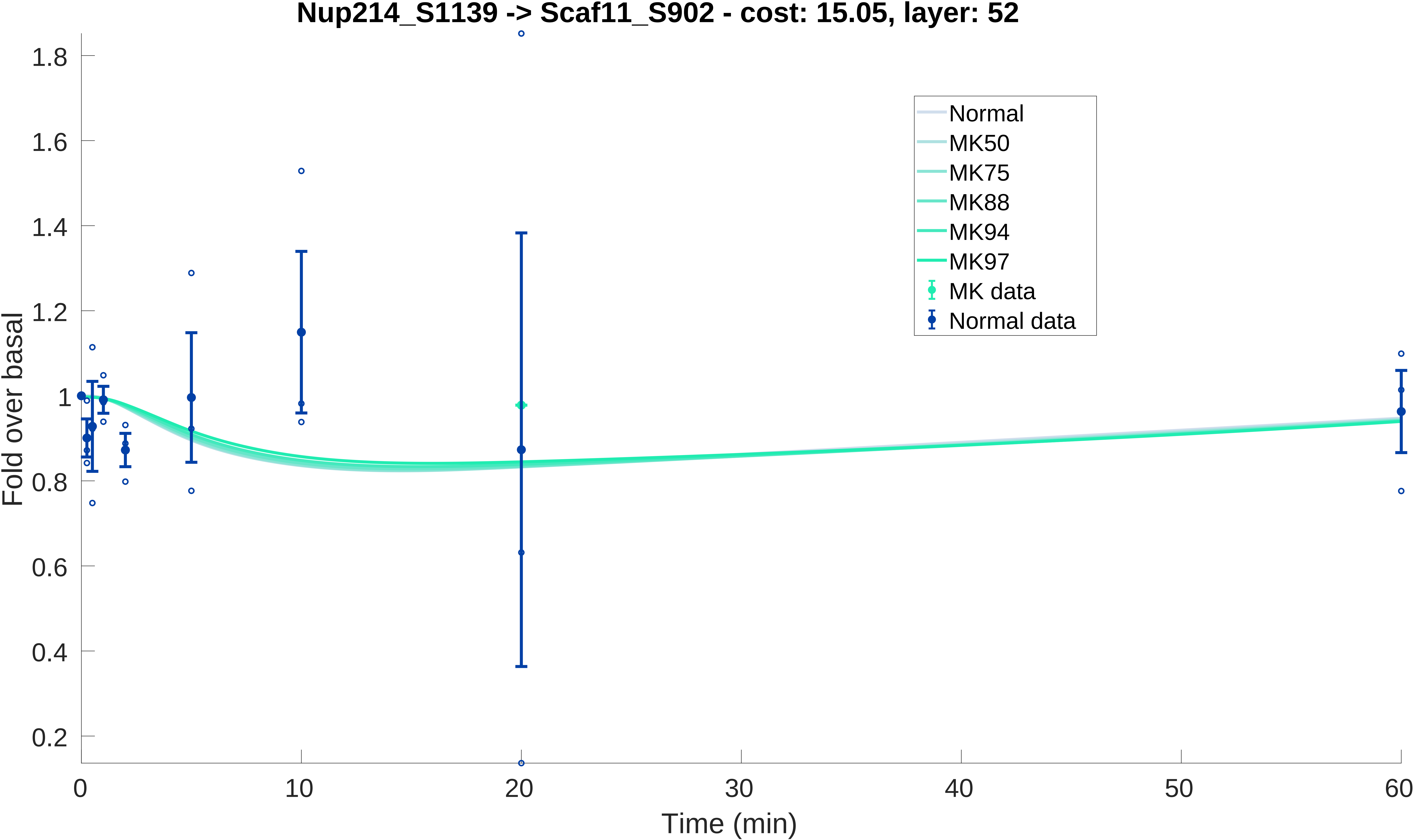

Nup214_S1139 -> Scaf11_S902 - cost: 15.05, layer: 52
Fold over basal
1.8
1.6
1.4
1.2
1
0.8
0.6
0.4
0.2
0
10
20
30
40
50
60
Time (min)
Normal
MK50
MK75
MK88
MK94
MK97
MK data
Normal data

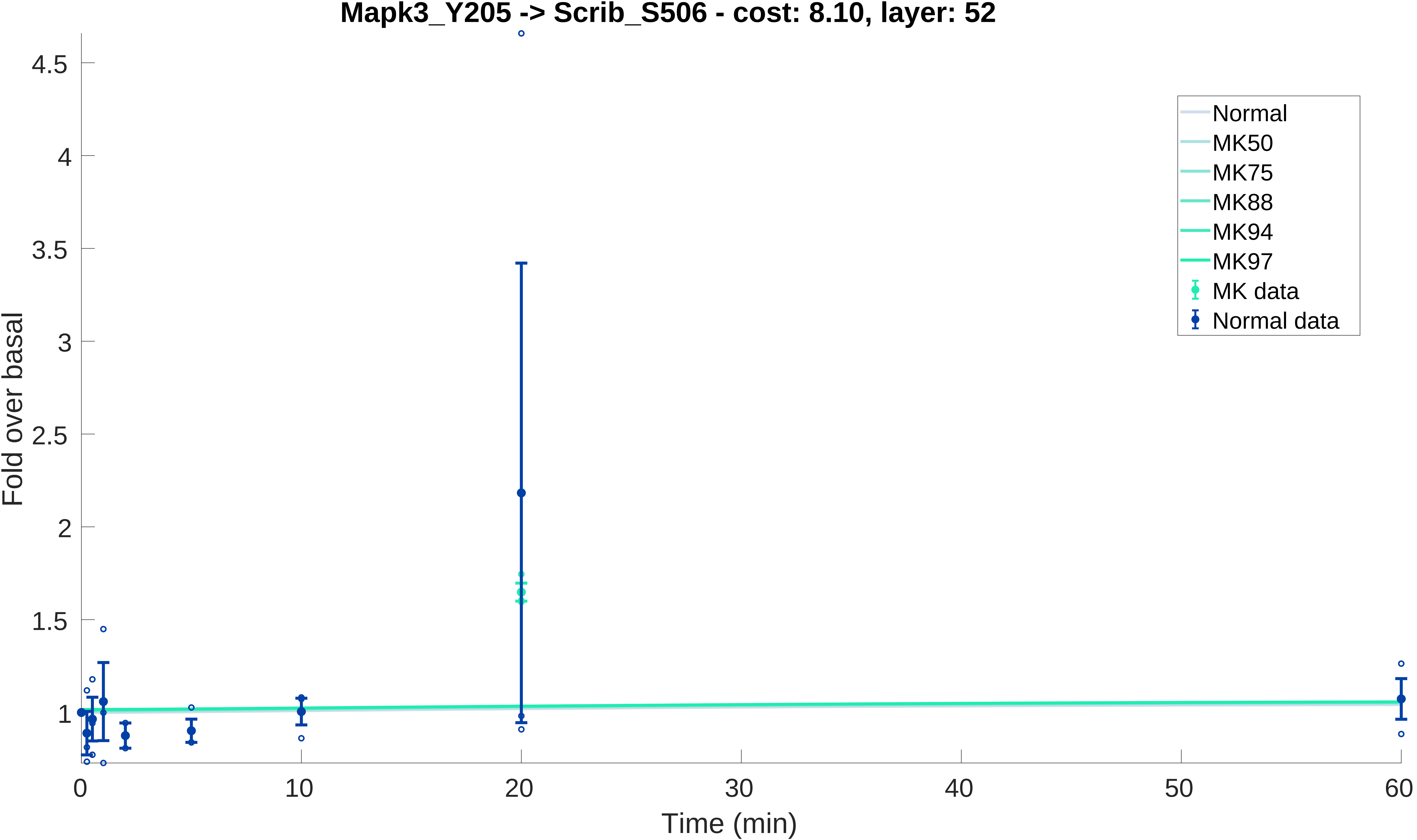
Mapk3_Y205 -> Scrib_S506 - cost: 8.10, layer: 52
Fold over basal
Time (min)
4.5
4
3.5
3
2.5
2
1.5
1
0
10
20
30
40
50
60
Normal
MK50
MK75
MK88
MK94
MK97
MK data
Normal data

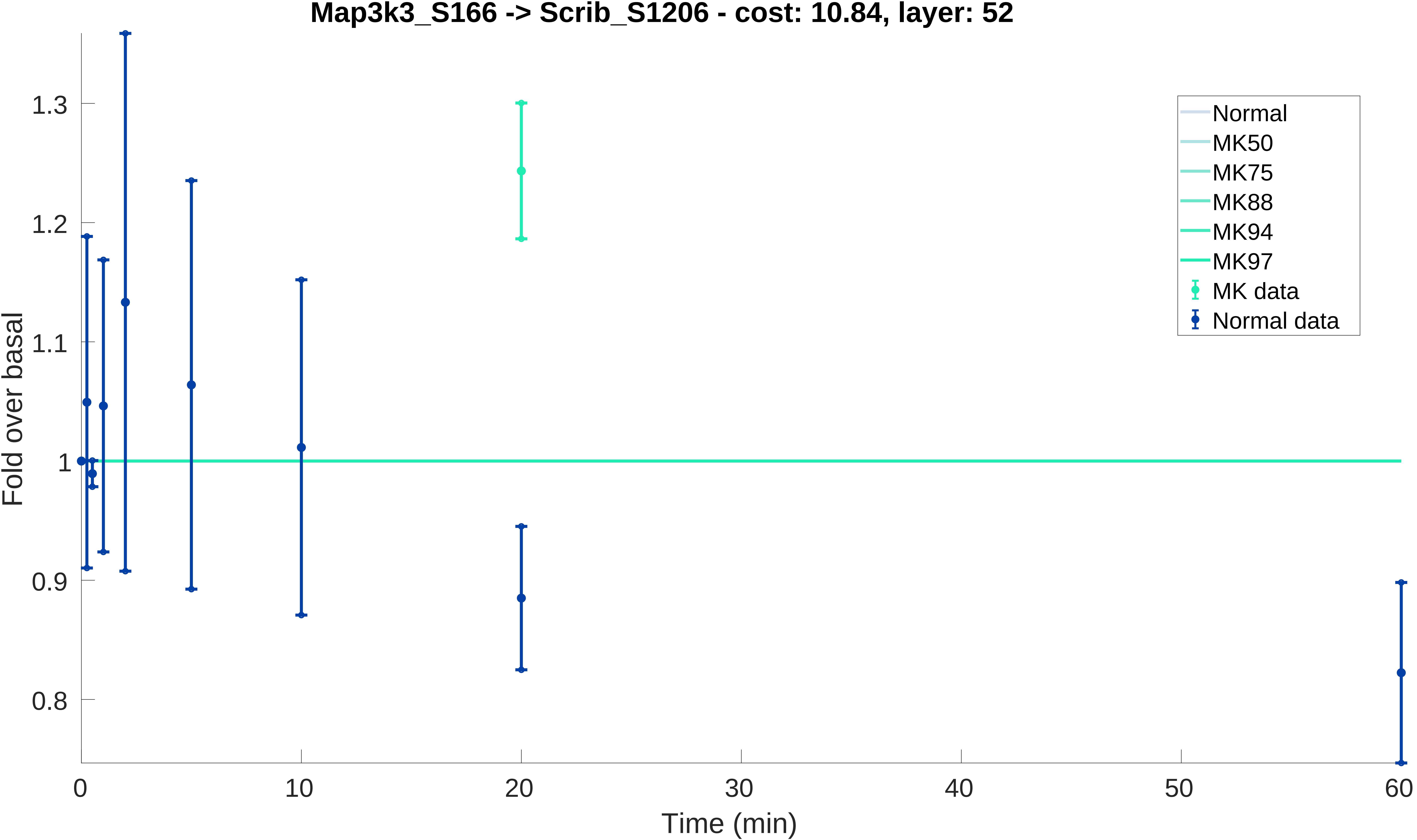

Map3k3_S166 -> Scrib_S1206 - cost: 10.84, layer: 52
Fold over basal
Time (min)
0
10
20
30
40
50
60
0.8
0.9
1
1.1
1.2
1.3
Normal
MK50
MK75
MK88
MK94
MK97
MK data
Normal data

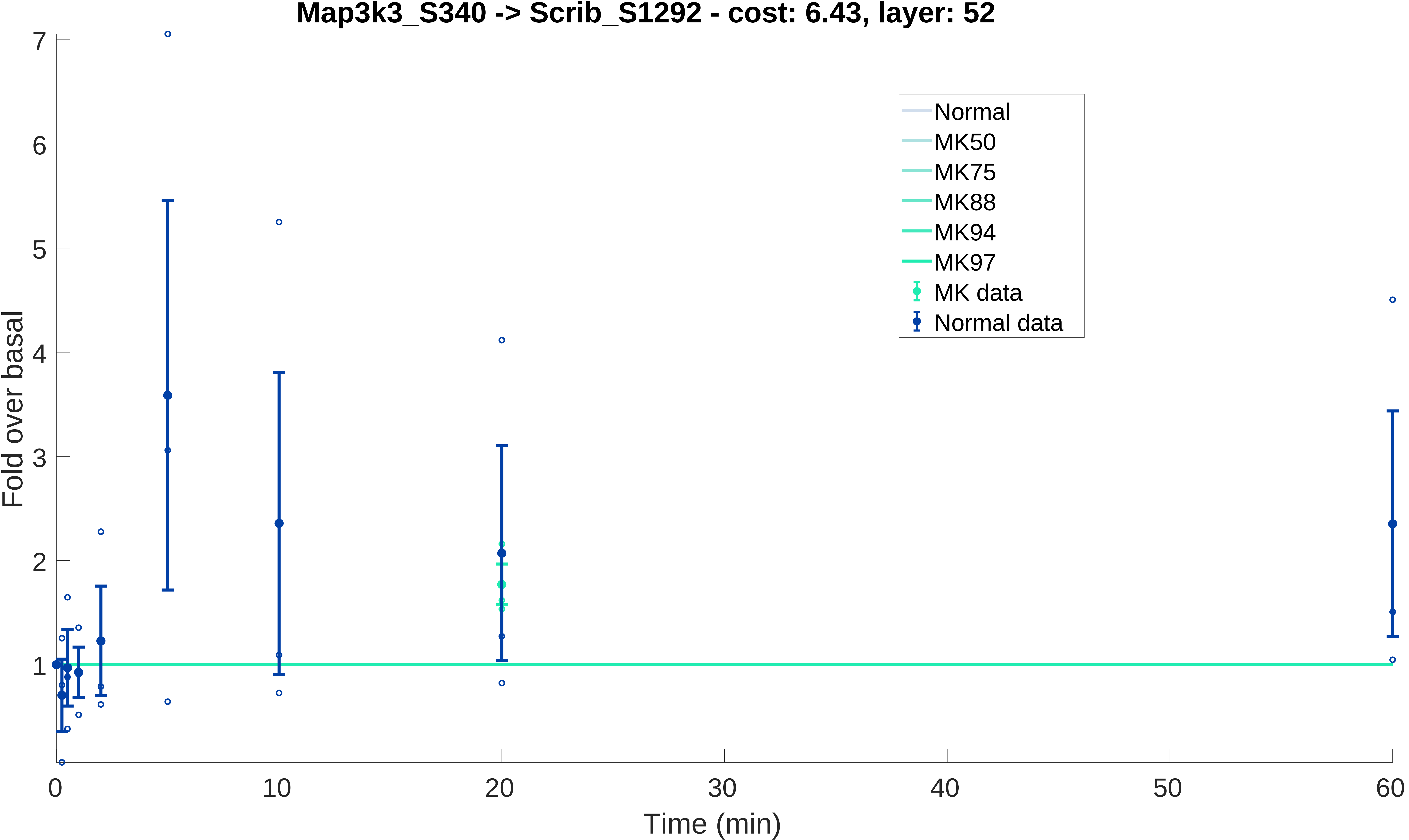

Map3k3_S340 -> Scrib_S1292 - cost: 6.43, layer: 52
Fold over basal
Time (min)
Normal
MK50
MK75
MK88
MK94
MK97
MK data
Normal data
0
10
20
30
40
50
60
1
2
3
4
5
6
7

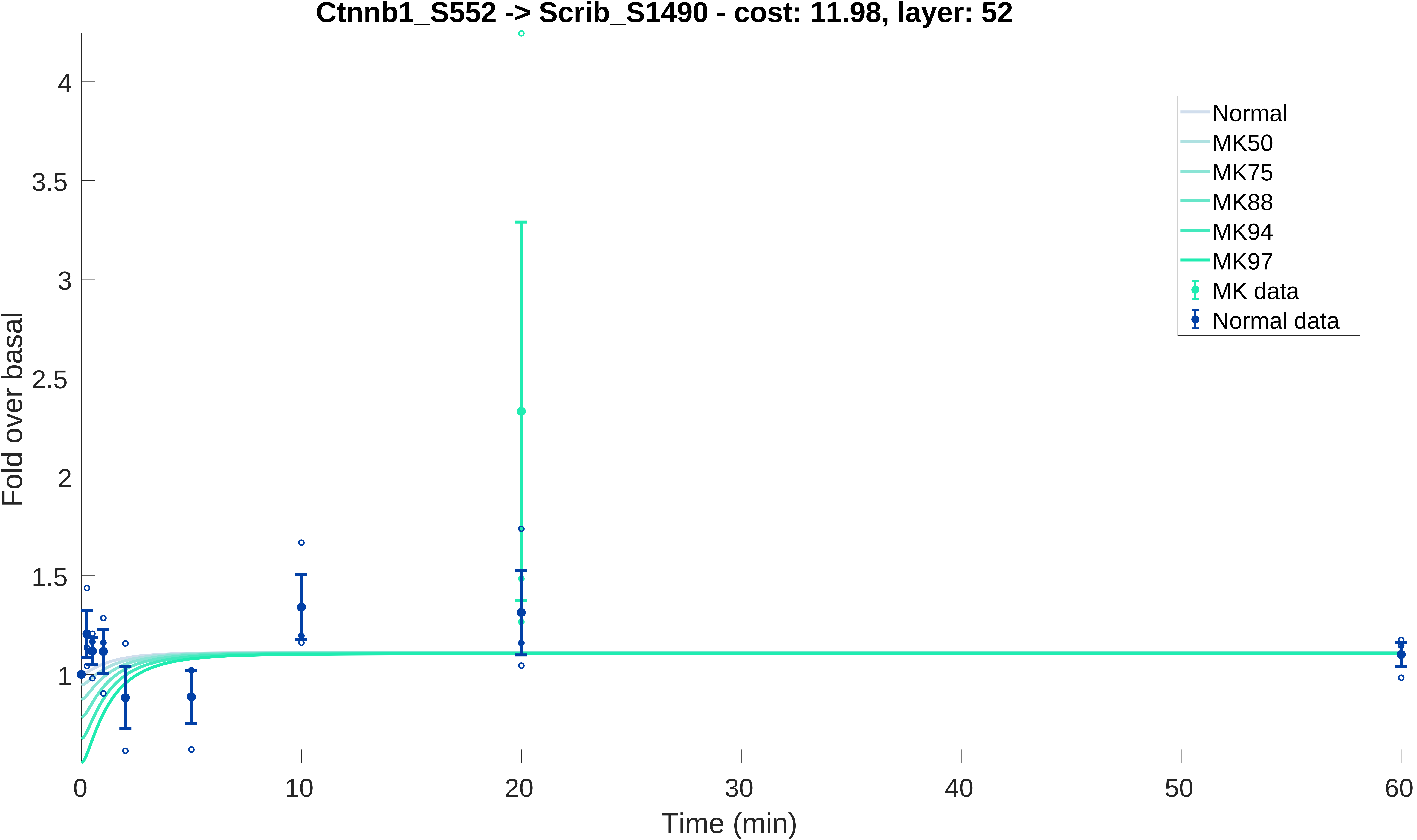

Ctnnb1_S552 -> Scrib_S1490 - cost: 11.98, layer: 52
Fold over basal
Time (min)
Normal
MK50
MK75
MK88
MK94
MK97
MK data
Normal data
0
10
20
30
40
50
60
1
1.5
2
2.5
3
3.5
4

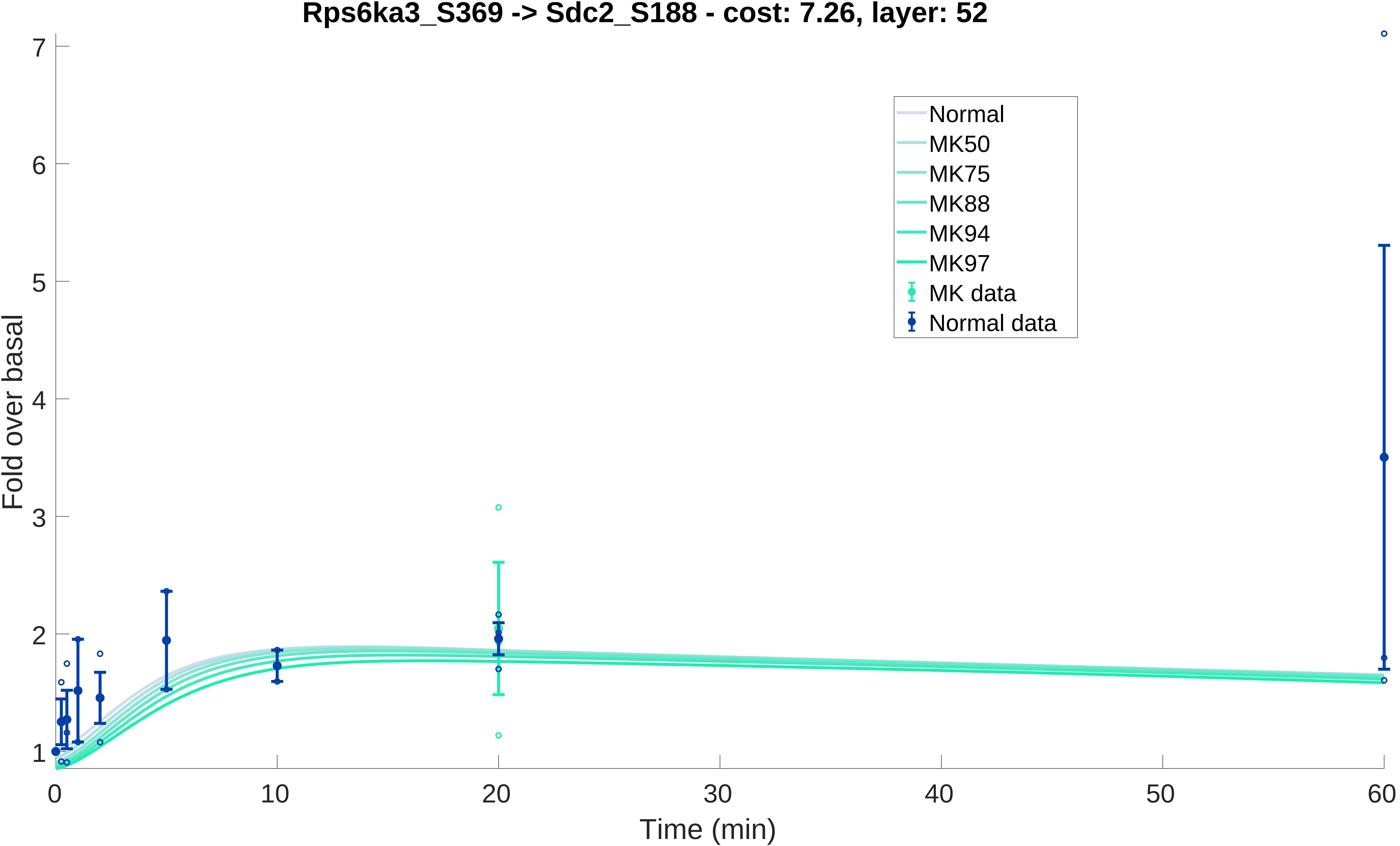

Rps6ka3_S369 -> Sdc2_S188 - cost: 7.26, layer: 52
Fold over basal
Time (min)
Normal
MK50
MK75
MK88
MK94
MK97
MK data
Normal data
0
10
20
30
40
50
60
1
2
3
4
5
6
7

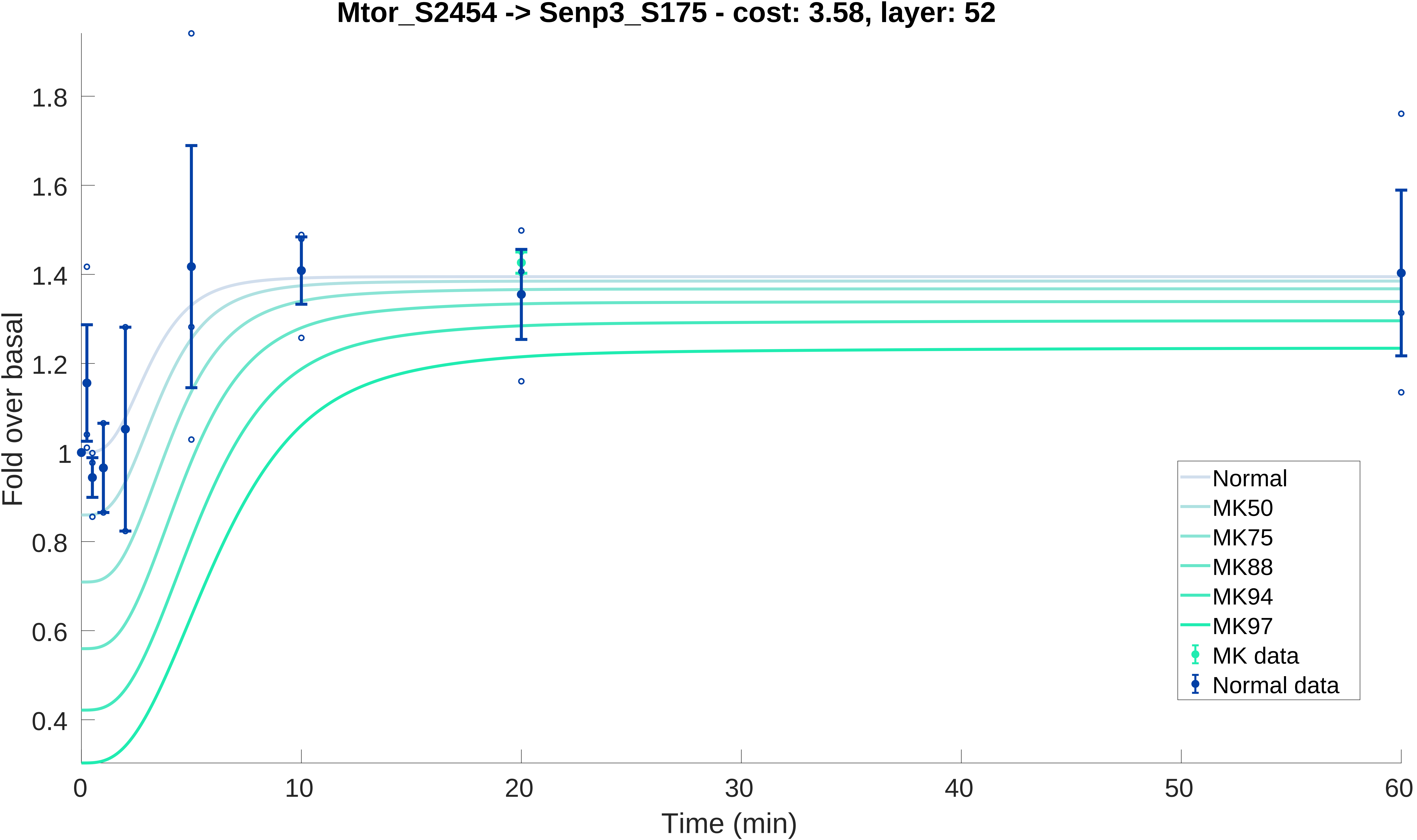

Mtor_S2454 -> Senp3_S175 - cost: 3.58, layer: 52
Fold over basal
1.8
1.6
1.4
1.2
1
0.8
0.6
0.4
0
10
20
30
40
50
60
Time (min)
Normal
MK50
MK75
MK88
MK94
MK97
MK data
Normal data

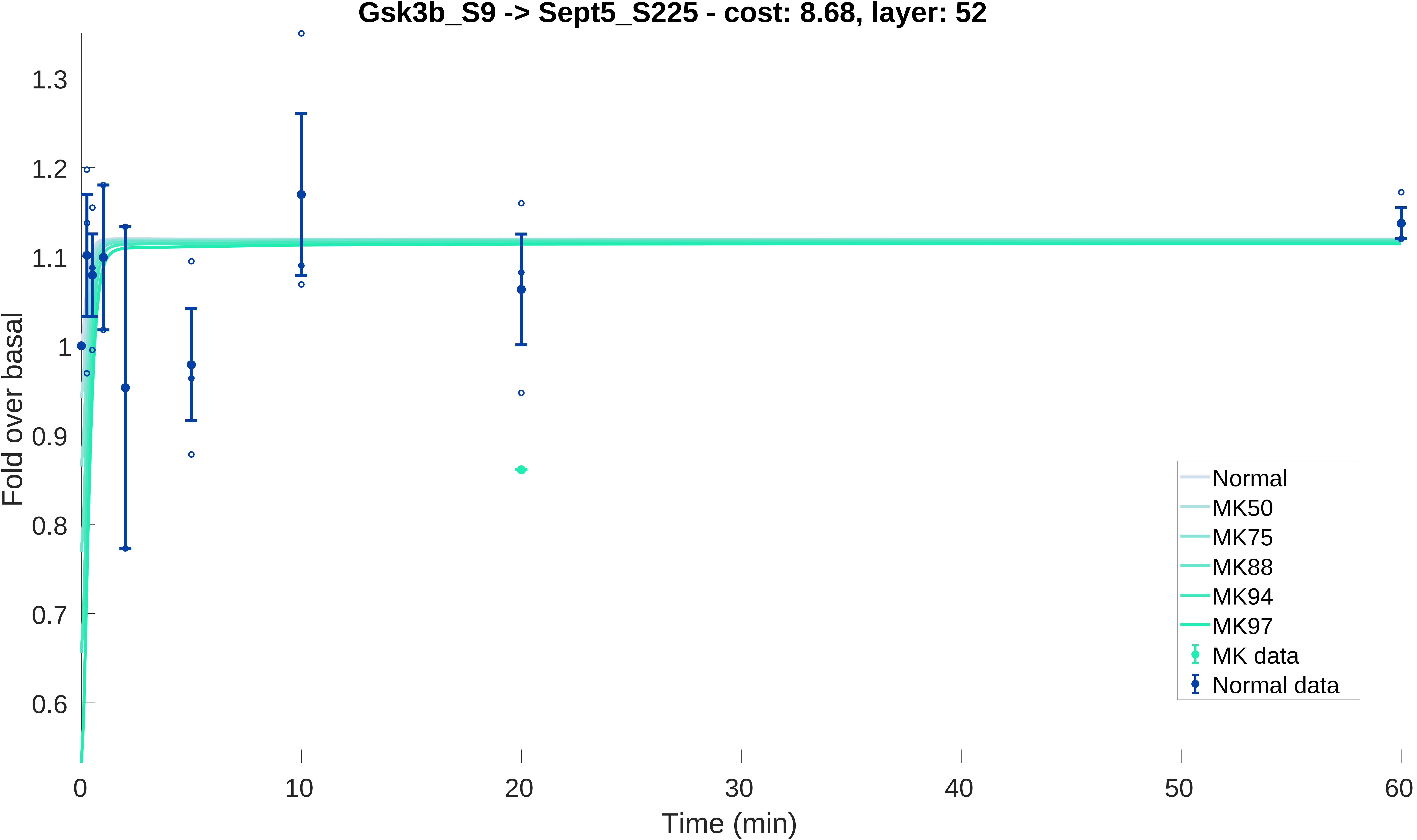

Gsk3b_S9 -> Sept5_S225 - cost: 8.68, layer: 52
Fold over basal
Time (min)
1.3
1.2
1.1
1
0.9
0.8
0.7
0.6
0
10
20
30
40
50
60
Normal
MK50
MK75
MK88
MK94
MK97
MK data
Normal data

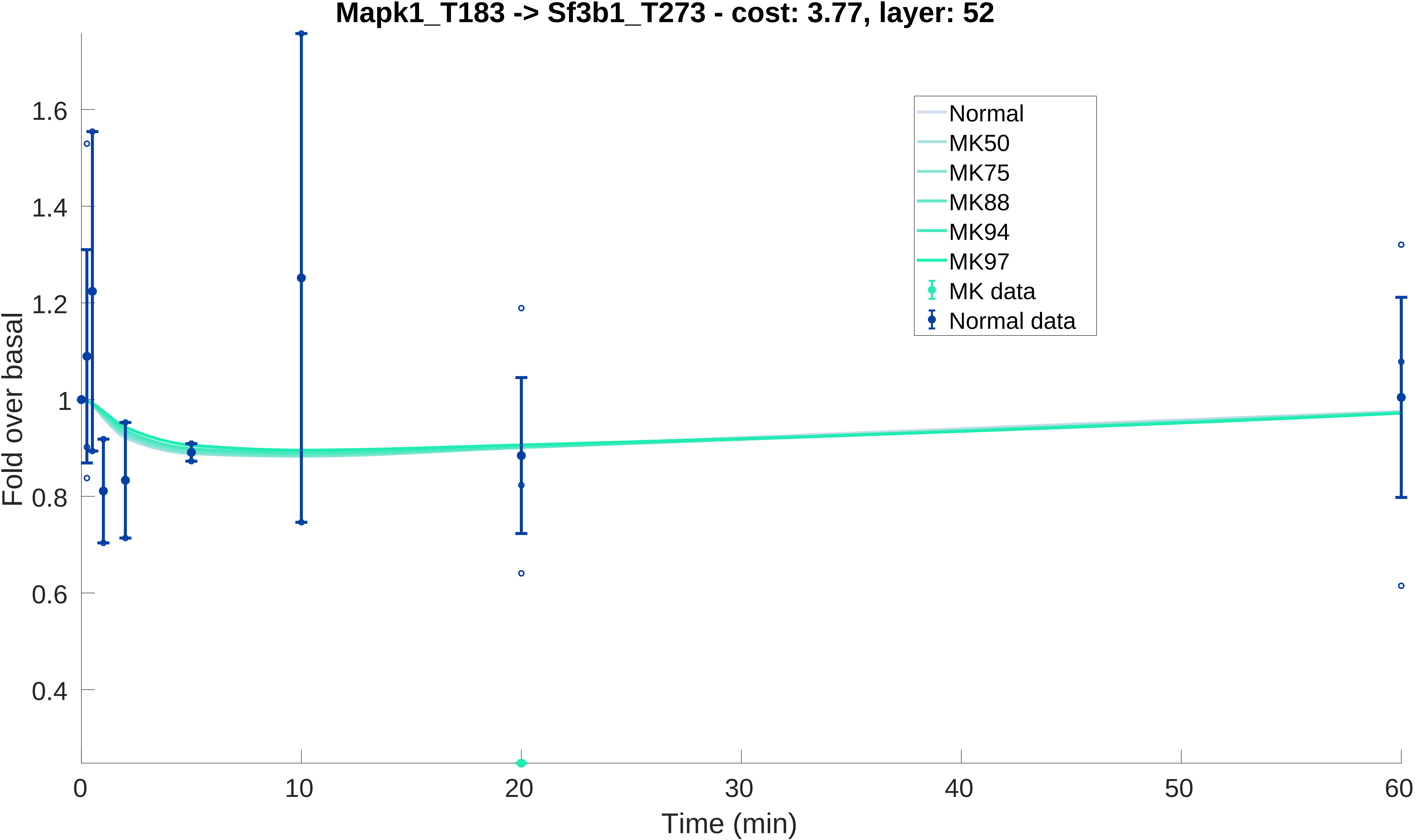
Mapk1_T183 -> Sf3b1_T273 - cost: 3.77, layer: 52
Fold over basal
1.6
1.4
1.2
1
0.8
0.6
0.4
0
10
20
30
40
50
60
Time (min)
Normal
MK50
MK75
MK88
MK94
MK97
MK data
Normal data

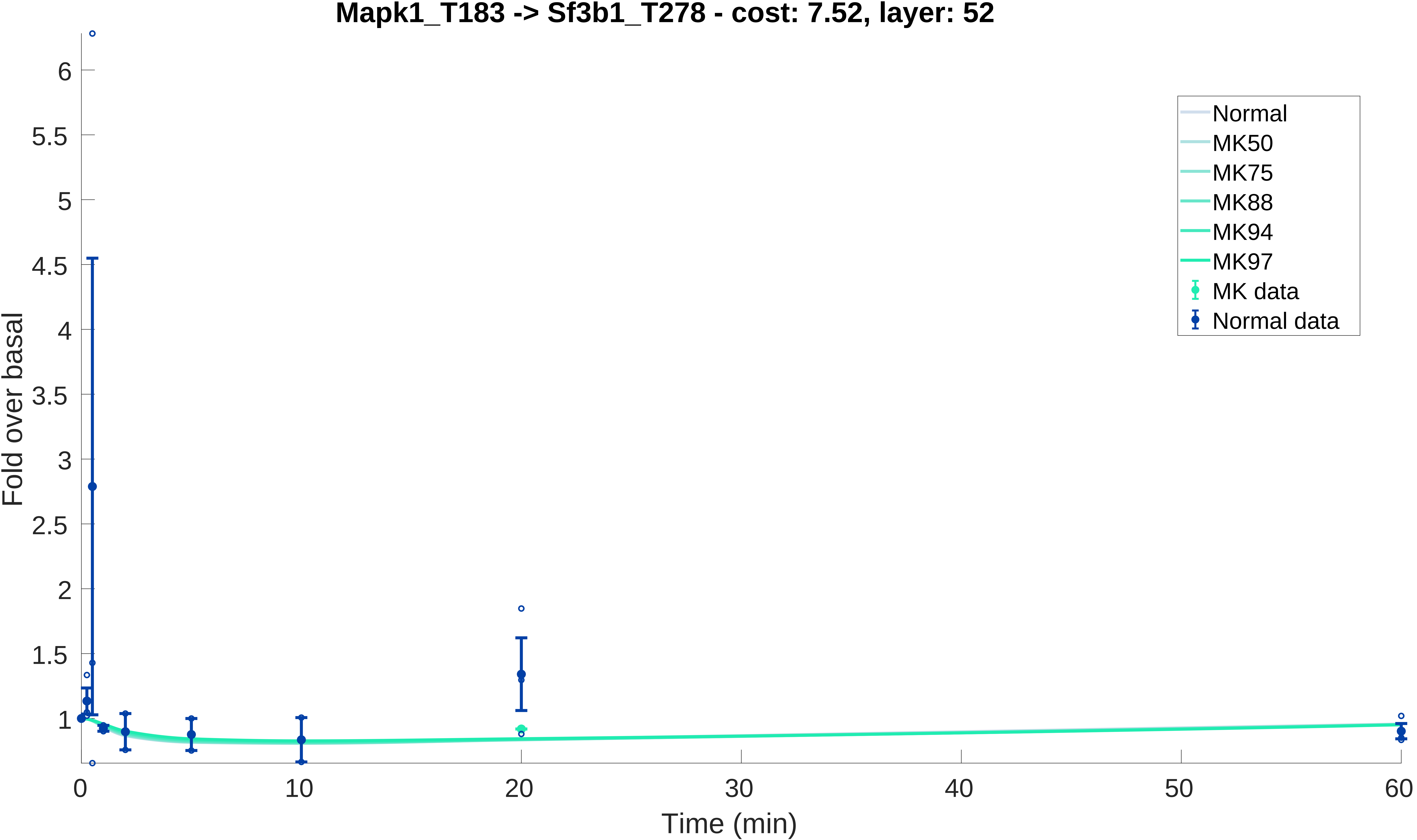

Mapk1_T183 -> Sf3b1_T278 - cost: 7.52, layer: 52
Fold over basal
Time (min)
1
1.5
2
2.5
3
3.5
4
4.5
5
5.5
6
0
10
20
30
40
50
60
Normal
MK50
MK75
MK88
MK94
MK97
MK data
Normal data

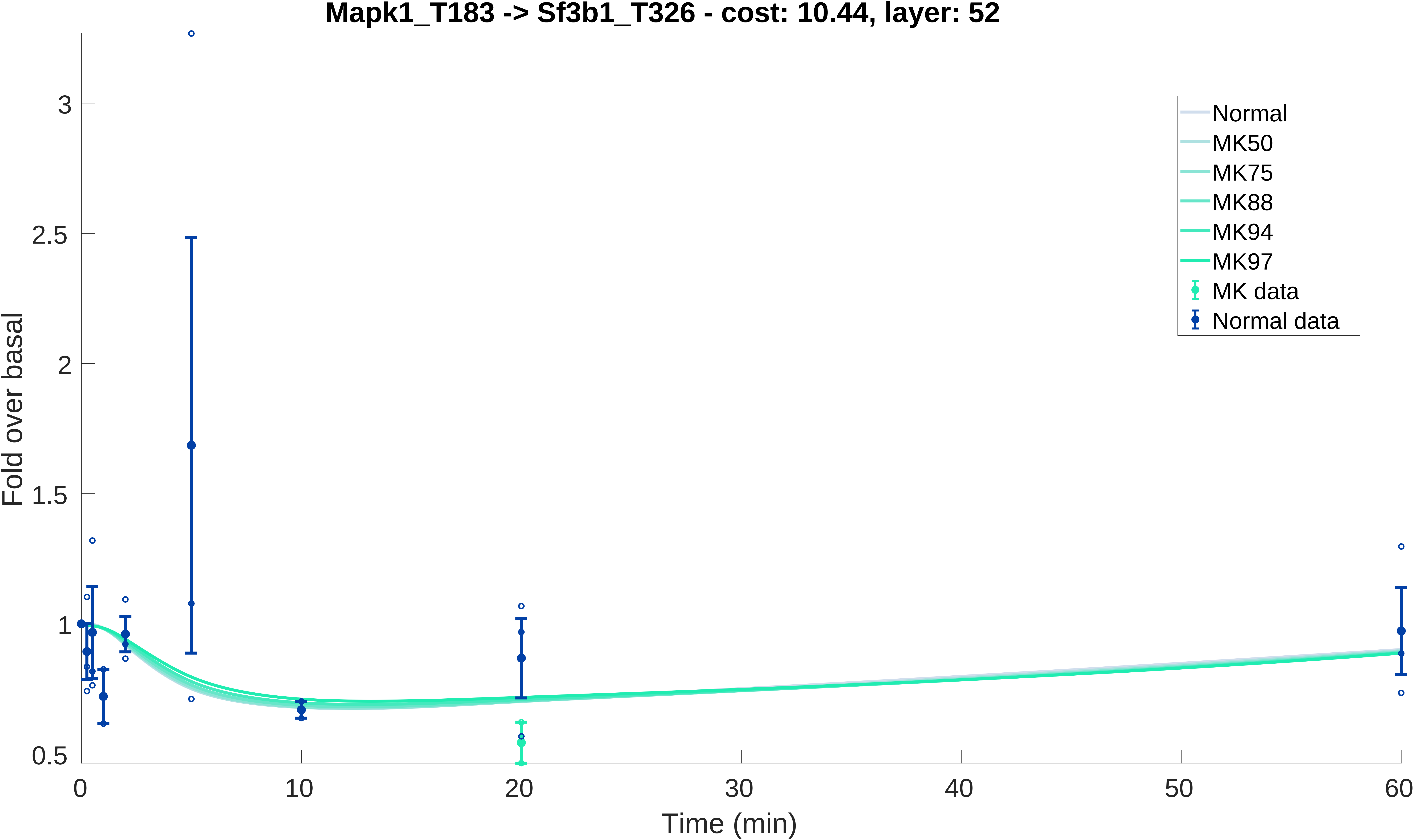
Mapk1_T183 -> Sf3b1_T326 - cost: 10.44, layer: 52
Fold over basal
Time (min)
0.5
1
1.5
2
2.5
3
0
10
20
30
40
50
60
Normal
MK50
MK75
MK88
MK94
MK97
MK data
Normal data

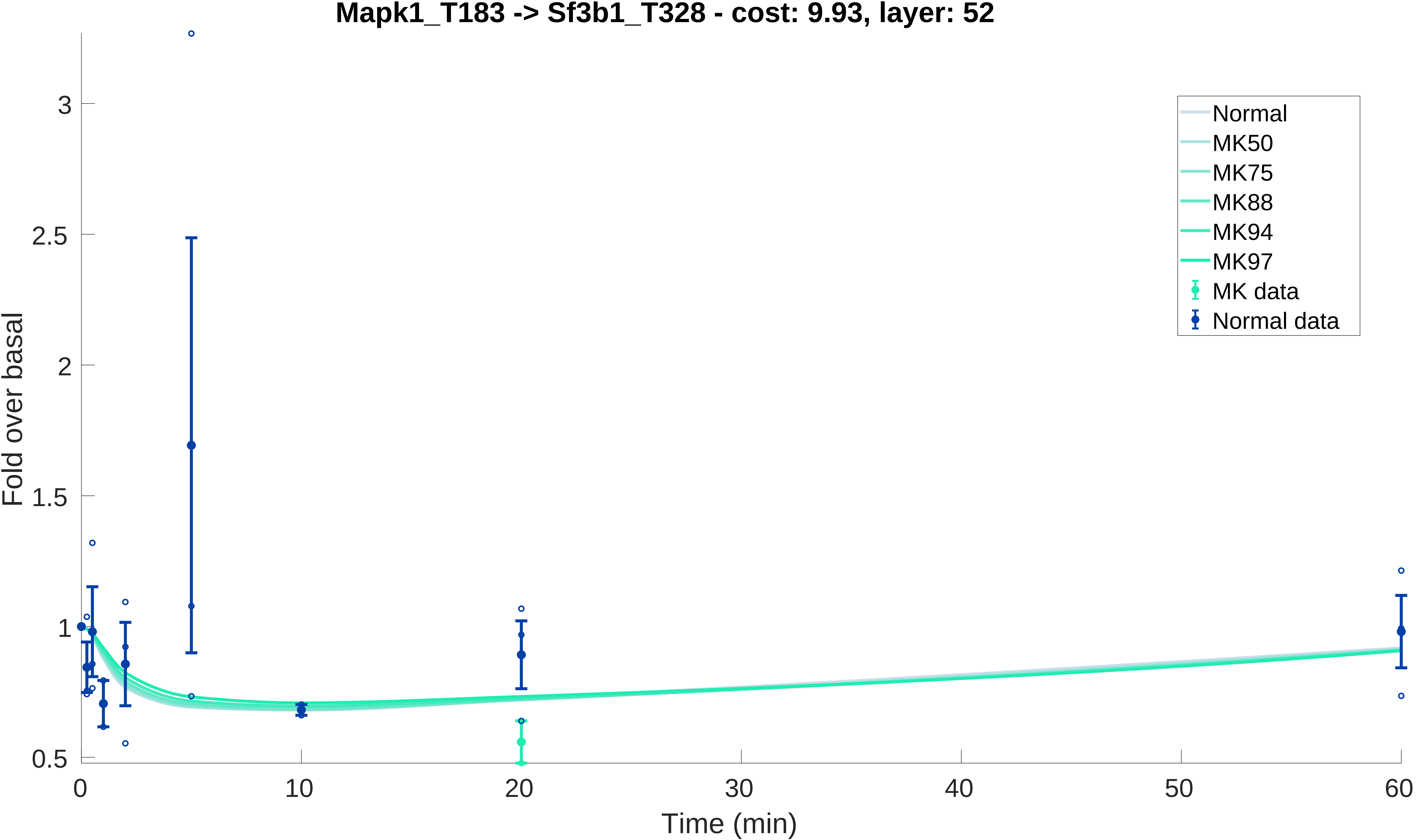

Mapk1_T183 -> Sf3b1_T328 - cost: 9.93, layer: 52
Fold over basal
Time (min)
0.5
1
1.5
2
2.5
3
0
10
20
30
40
50
60
Normal
MK50
MK75
MK88
MK94
MK97
MK data
Normal data

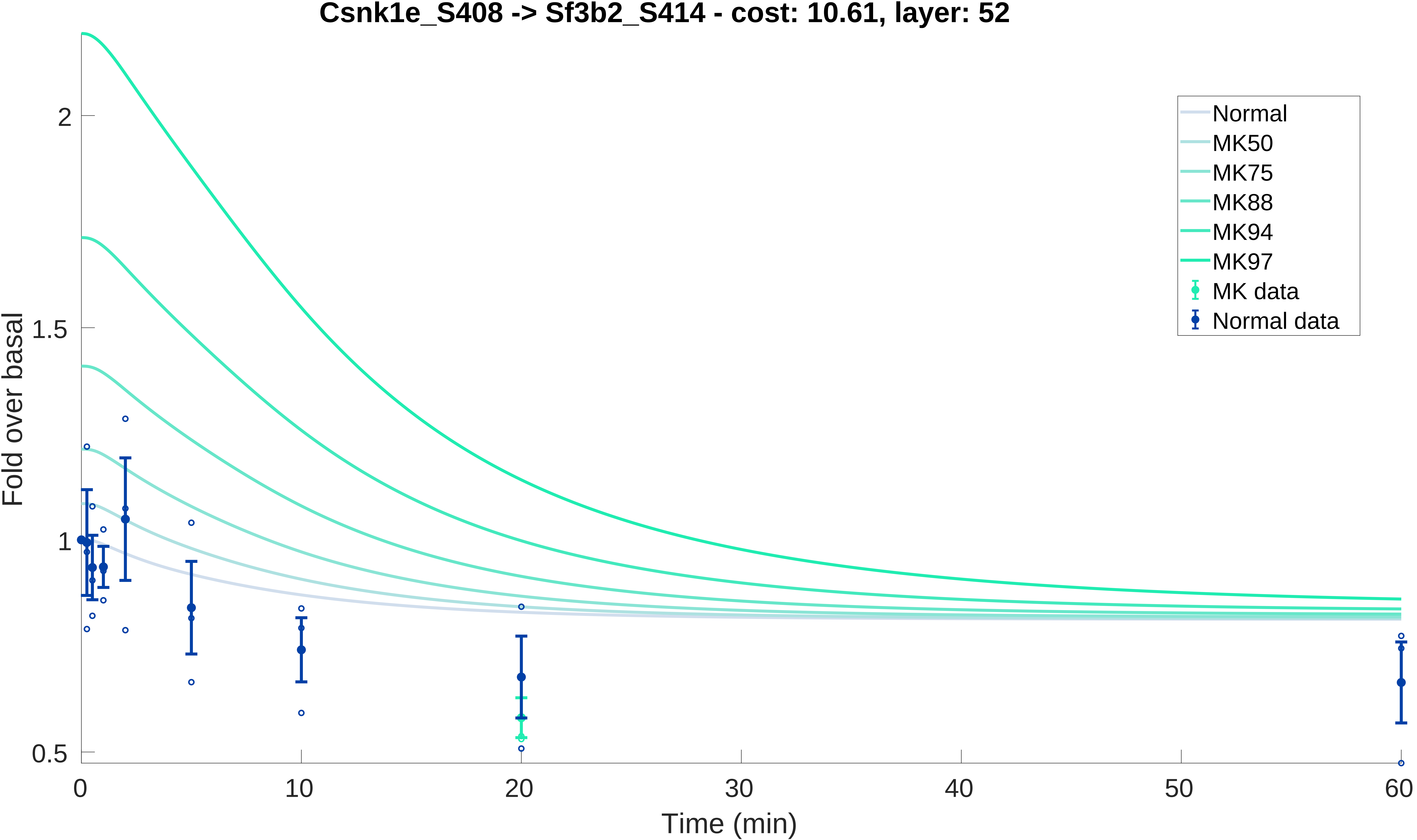

Csnk1e_S408 -> Sf3b2_S414 - cost: 10.61, layer: 52
Fold over basal
Time (min)
0.5
1
1.5
2
0
10
20
30
40
50
60
Normal
MK50
MK75
MK88
MK94
MK97
MK data
Normal data

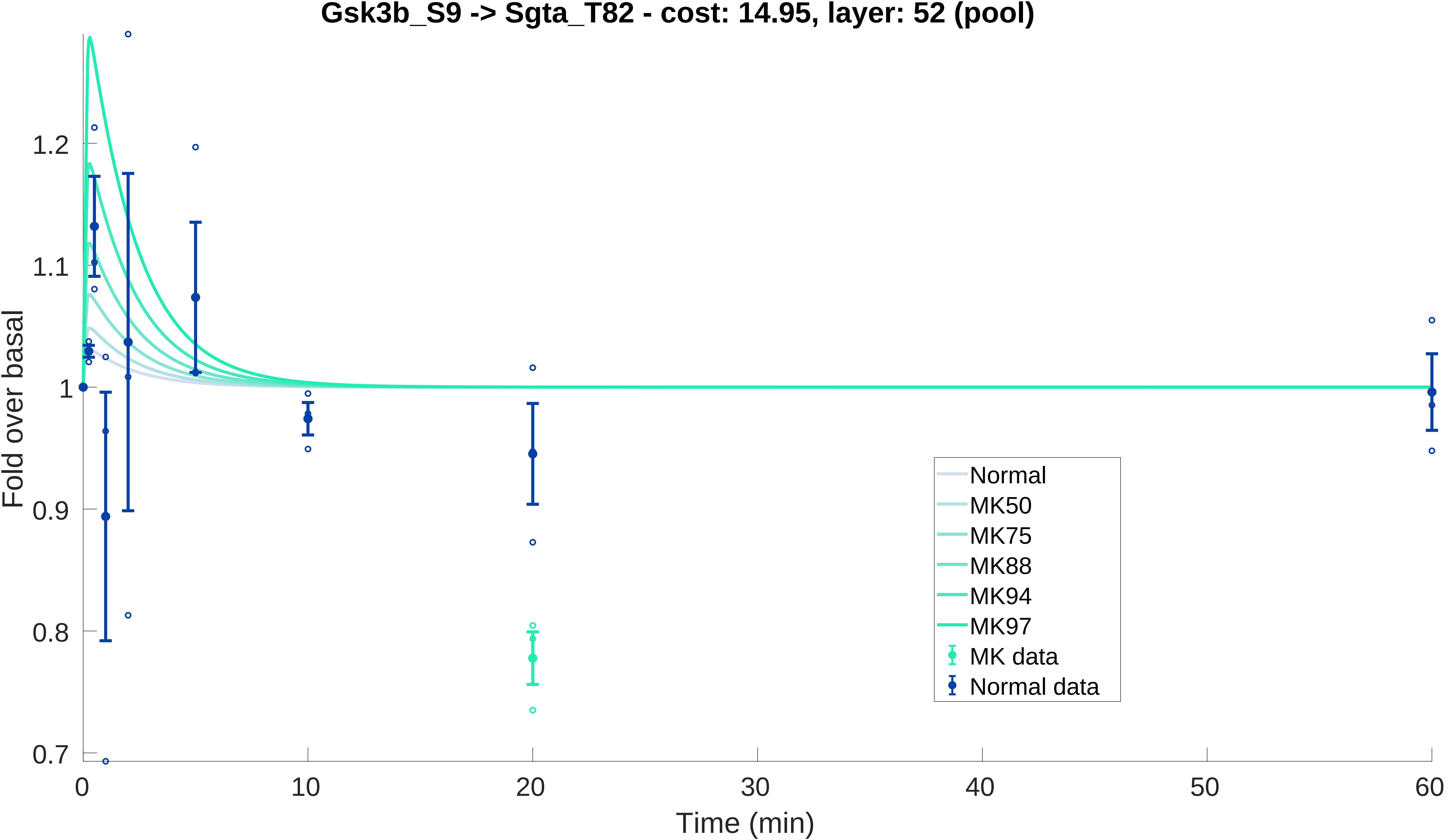

Gsk3b_S9 -> Sgta_T82 - cost: 14.95, layer: 52 (pool)
Fold over basal
Time (min)
1.2
1.1
1
0.9
0.8
0.7
0
10
20
30
40
50
60
Normal
MK50
MK75
MK88
MK94
MK97
MK data
Normal data

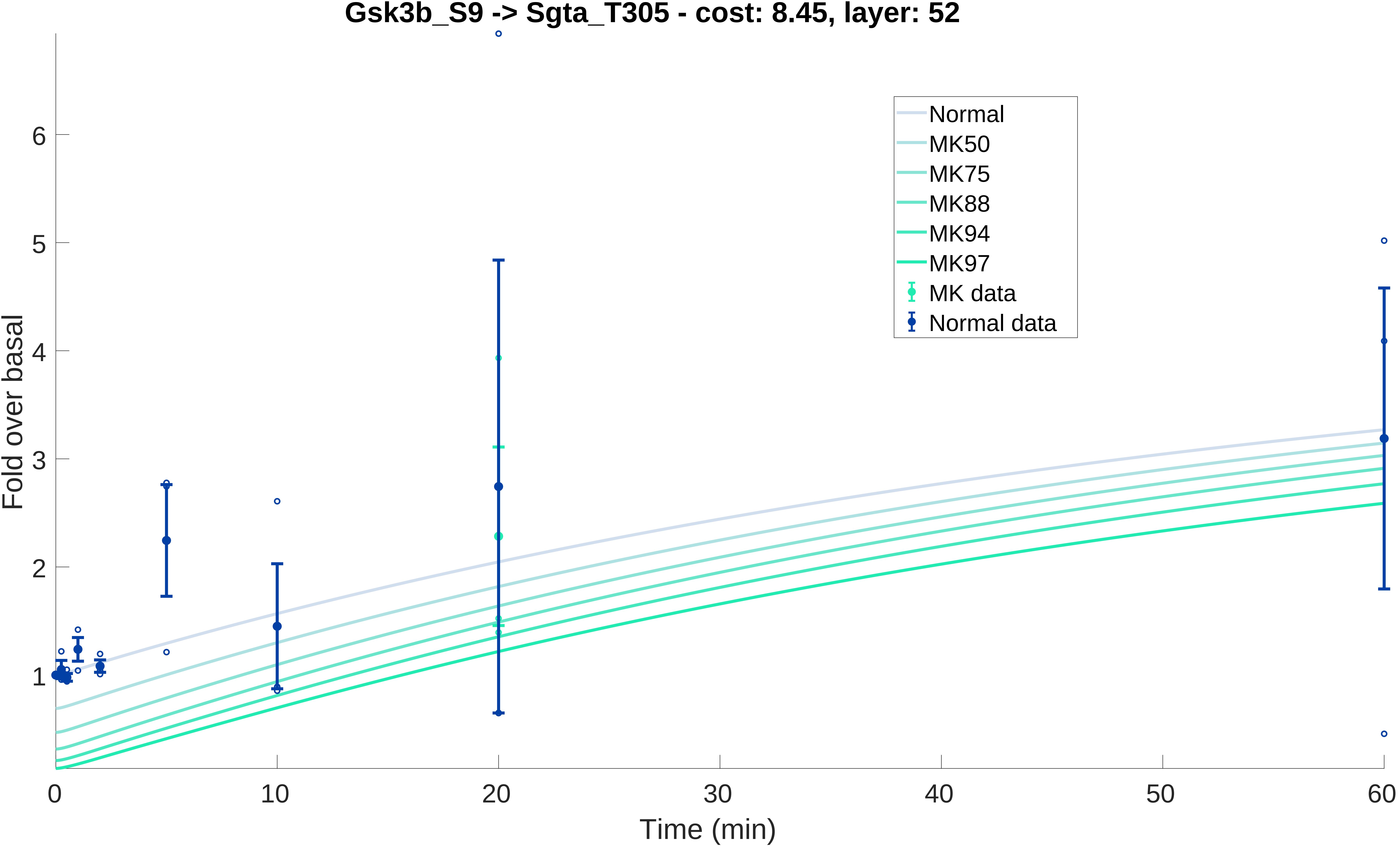
Gsk3b_S9 -> Sgta_T305 - cost: 8.45, layer: 52
Fold over basal
Time (min)
0
10
20
30
40
50
60
1
2
3
4
5
6
Normal
MK50
MK75
MK88
MK94
MK97
MK data
Normal data

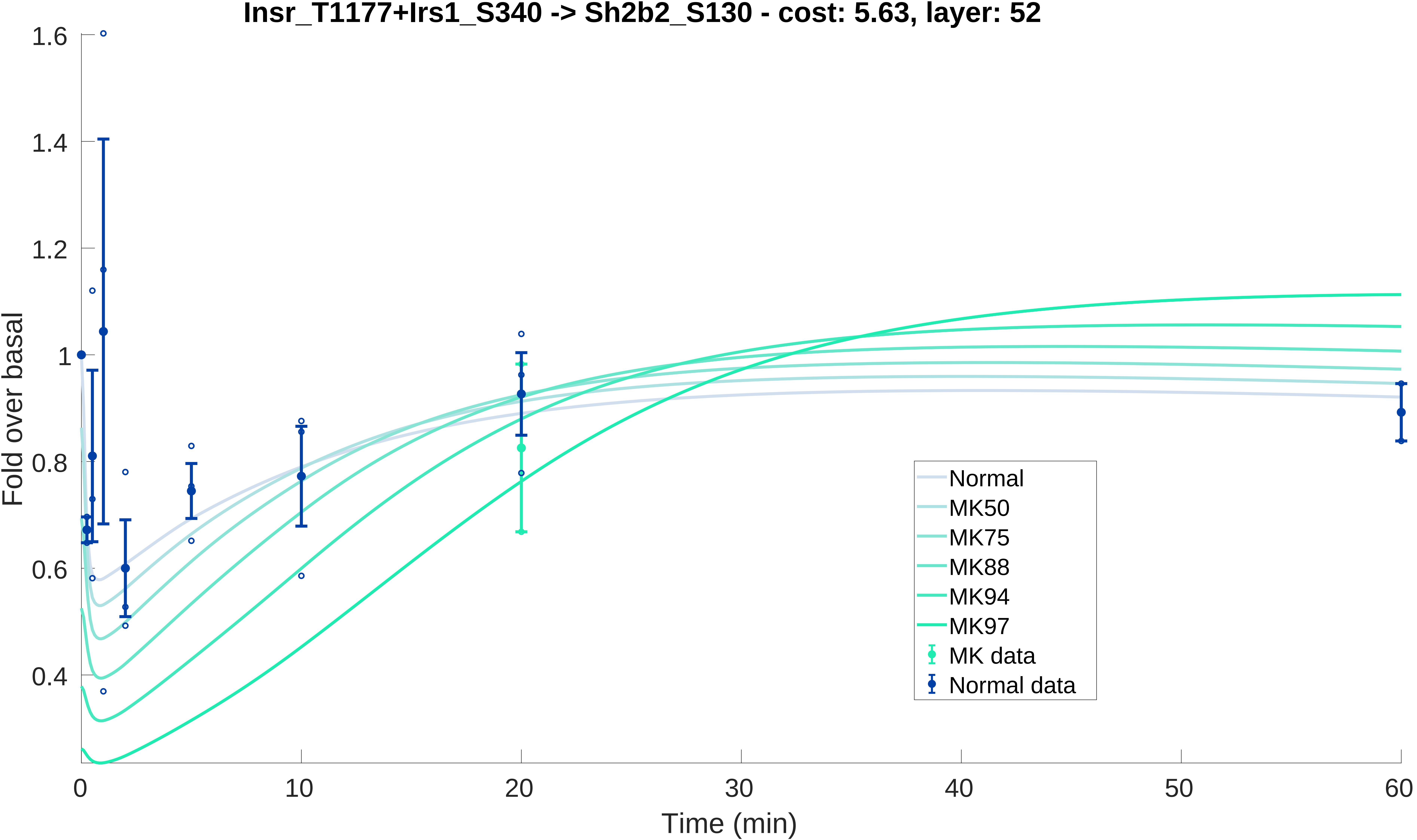

Insr_T1177+Irs1_S340 -> Sh2b2_S130 - cost: 5.63, layer: 52
Fold over basal
Time (min)
1.6
1.4
1.2
1
0.8
0.6
0.4
0
10
20
30
40
50
60
Normal
MK50
MK75
MK88
MK94
MK97
MK data
Normal data

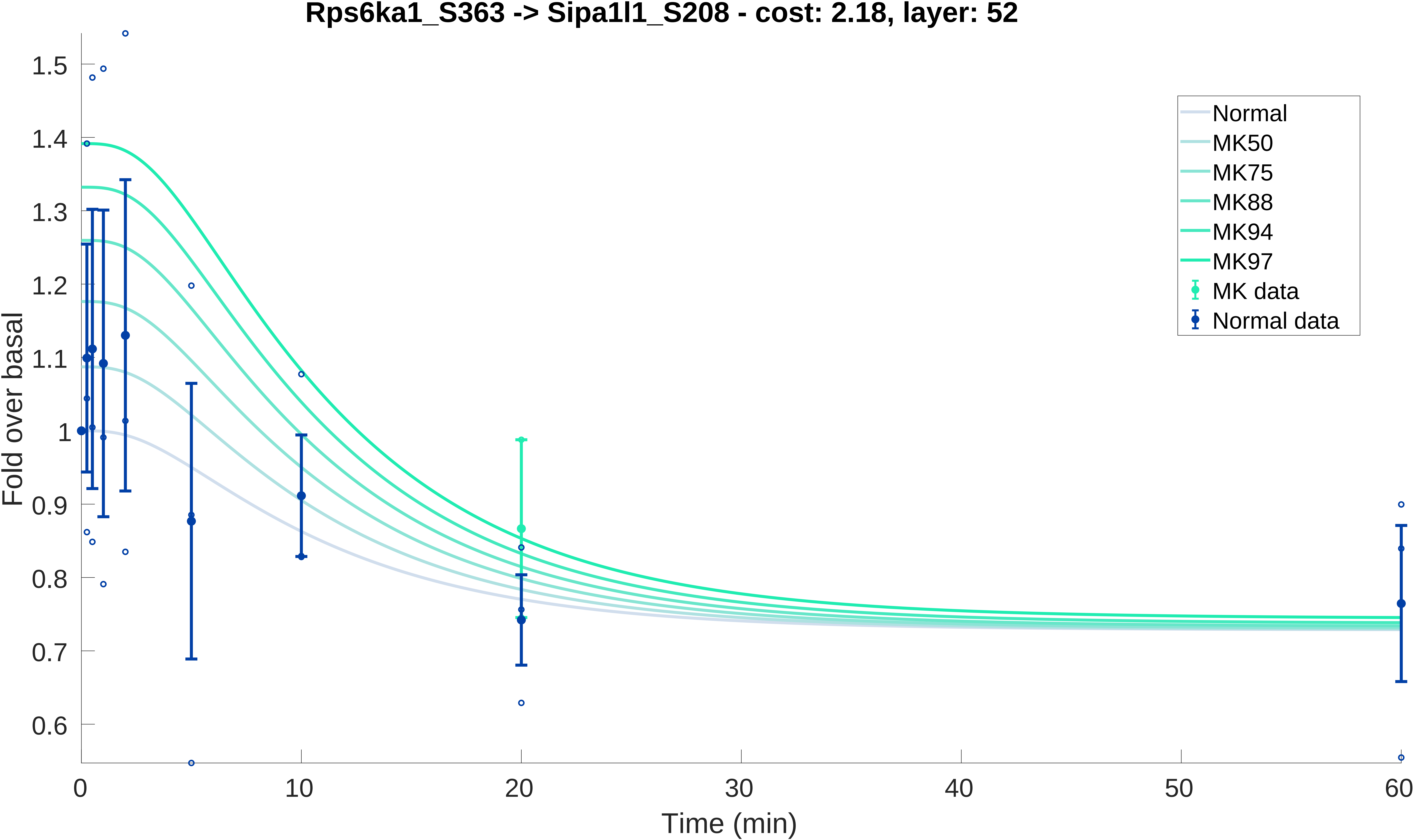
Rps6ka1_S363 -> Sipa1l1_S208 - cost: 2.18, layer: 52
Fold over basal
Time (min)
Normal
MK50
MK75
MK88
MK94
MK97
MK data
Normal data
1.5
1.4
1.3
1.2
1.1
1
0.9
0.8
0.7
0.6
0
10
20
30
40
50
60

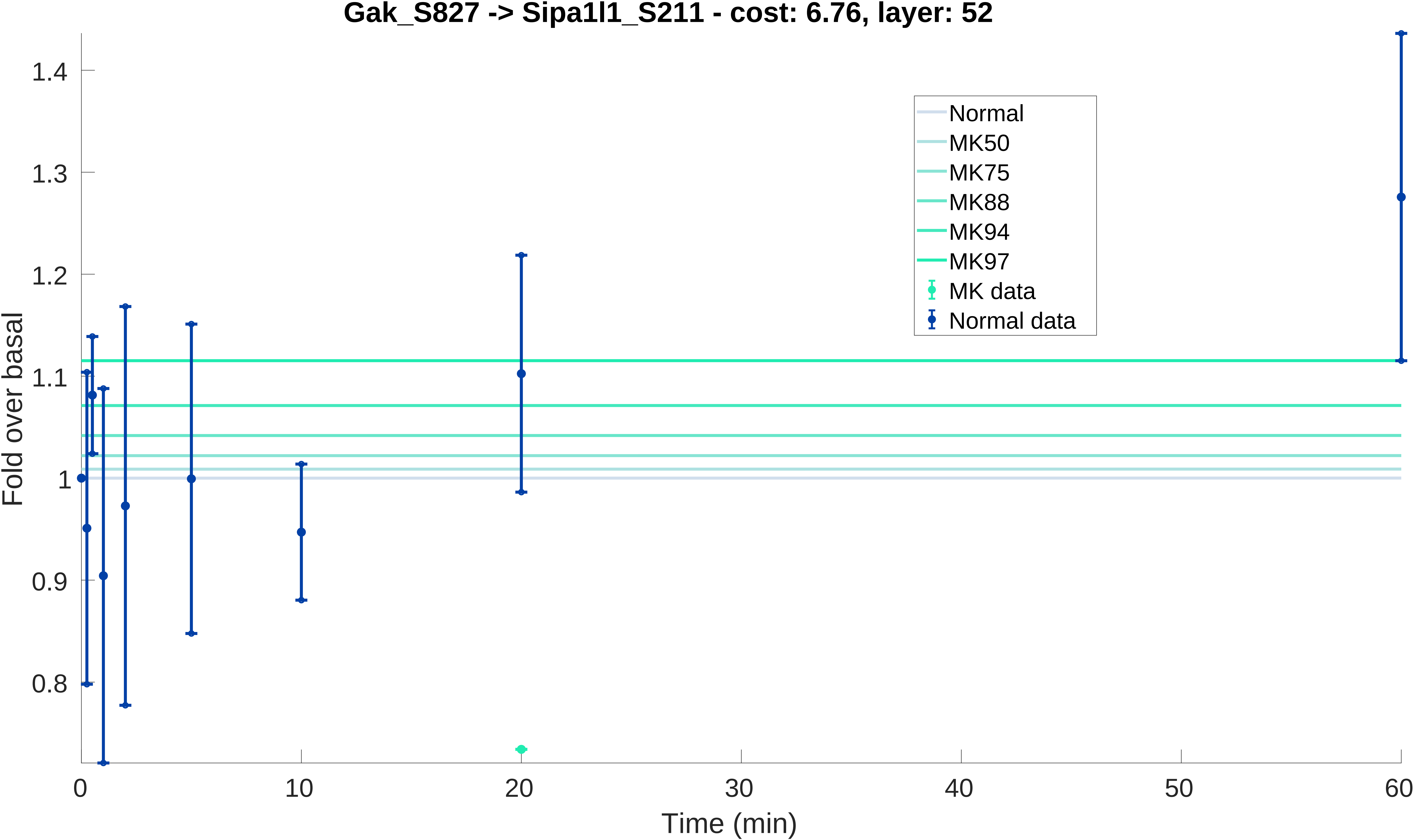

Gak_S827 -> Sipa1l1_S211 - cost: 6.76, layer: 52
Fold over basal
1.4
1.3
1.2
1.1
1
0.9
0.8
Time (min)
0
10
20
30
40
50
60
Normal
MK50
MK75
MK88
MK94
MK97
MK data
Normal data

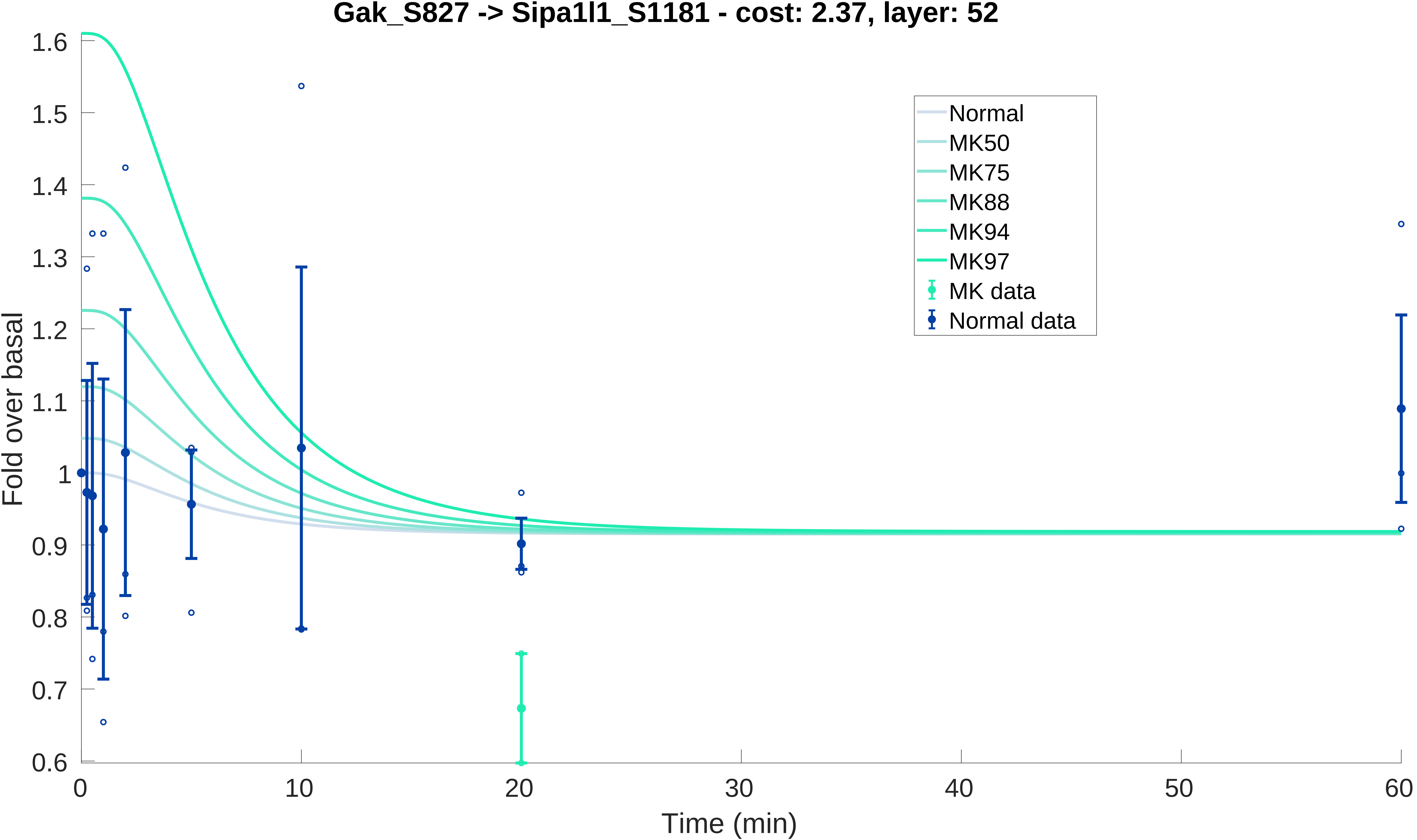

Gak_S827 -> Sipa1l1_S1181 - cost: 2.37, layer: 52
Fold over basal
Time (min)
Normal
MK50
MK75
MK88
MK94
MK97
MK data
Normal data
1.6
1.5
1.4
1.3
1.2
1.1
1
0.9
0.8
0.7
0.6
0
10
20
30
40
50
60

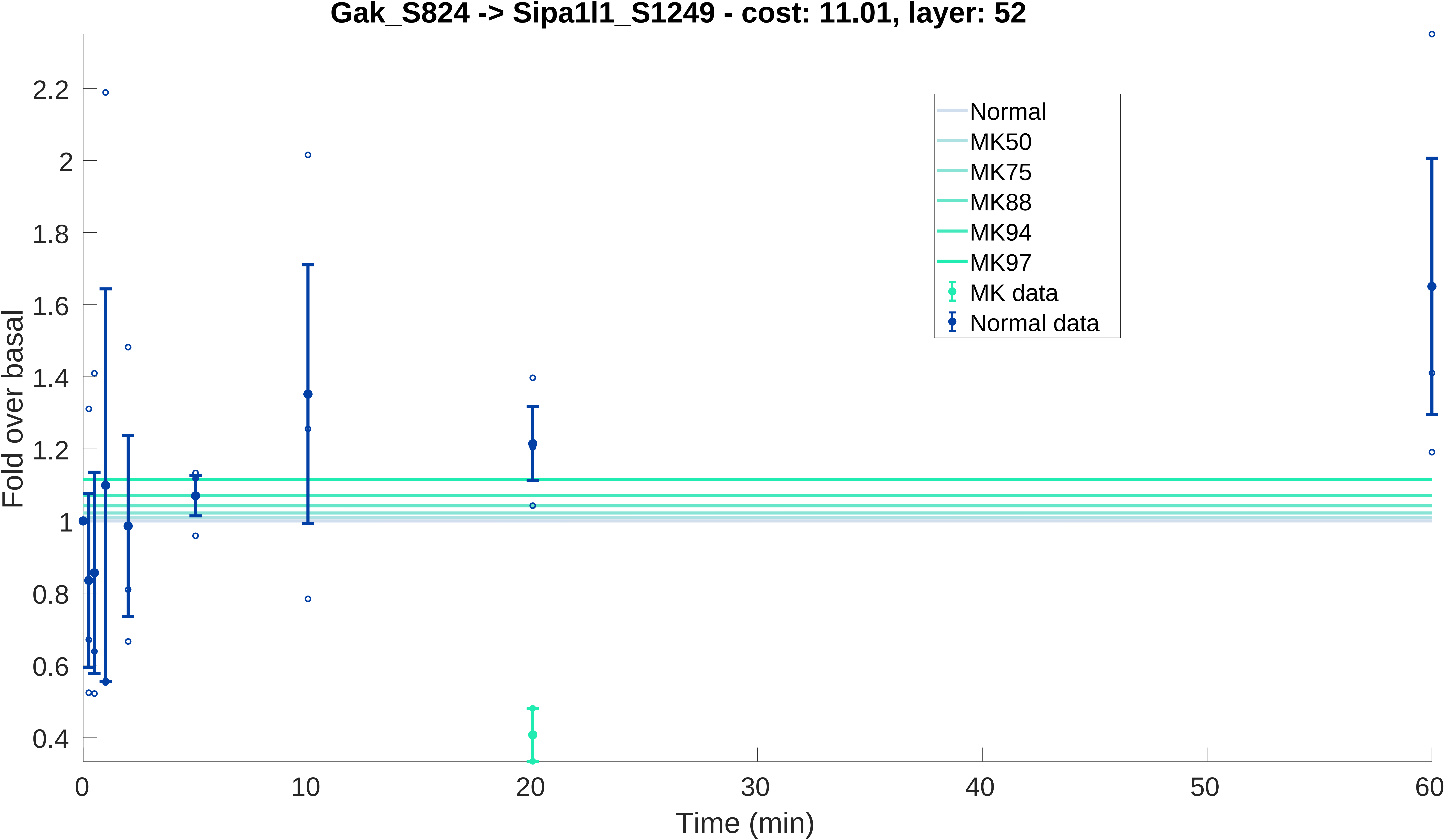

Gak_S824 -> Sipa1l1_S1249 - cost: 11.01, layer: 52
Fold over basal
Time (min)
2.2
2
1.8
1.6
1.4
1.2
1
0.8
0.6
0.4
0
10
20
30
40
50
60
Normal
MK50
MK75
MK88
MK94
MK97
MK data
Normal data

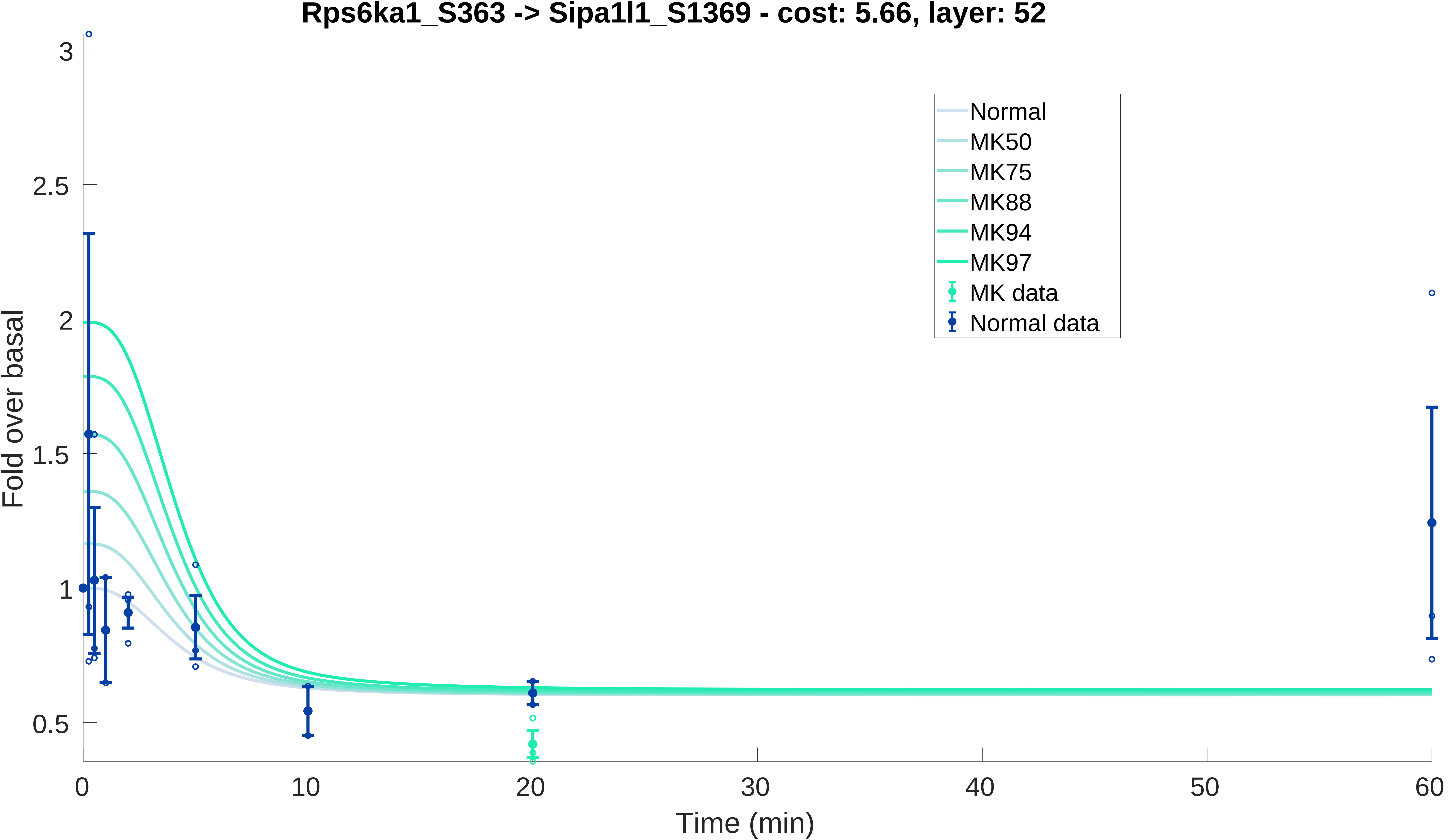

Rps6ka1_S363 -> Sipa1l1_S1369 - cost: 5.66, layer: 52
Fold over basal
Time (min)
Normal
MK50
MK75
MK88
MK94
MK97
MK data
Normal data
0.5
1
1.5
2
2.5
3
0
10
20
30
40
50
60

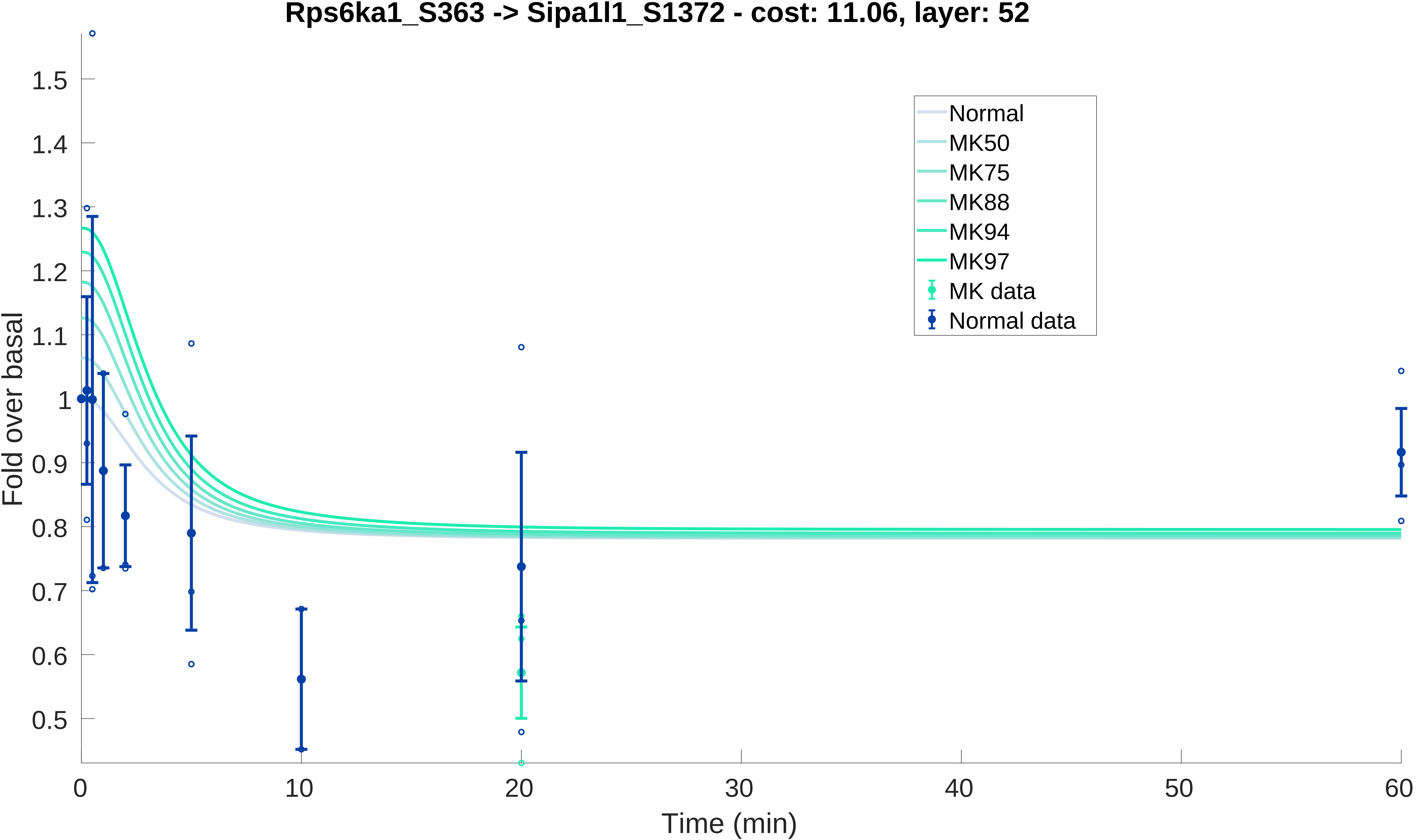

Rps6ka1_S363 -> Sipa1l1_S1372 - cost: 11.06, layer: 52
Fold over basal
1.5
1.4
1.3
1.2
1.1
1
0.9
0.8
0.7
0.6
0.5
0
10
20
30
40
50
60
Time (min)
Normal
MK50
MK75
MK88
MK94
MK97
MK data
Normal data

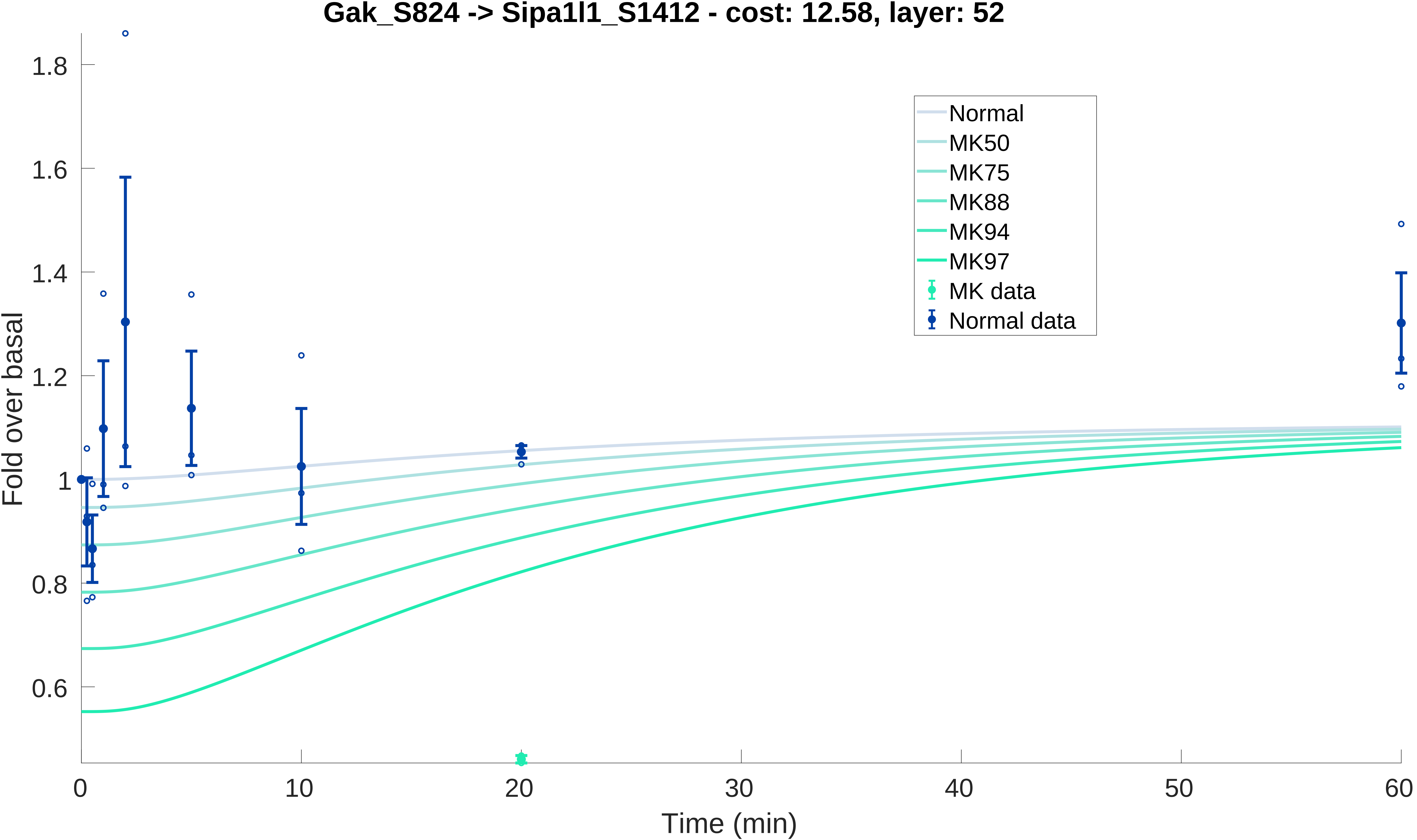

Gak_S824 -> Sipa1l1_S1412 - cost: 12.58, layer: 52
Fold over basal
Time (min)
0.6
0.8
1
1.2
1.4
1.6
1.8
0
10
20
30
40
50
60
Normal
MK50
MK75
MK88
MK94
MK97
MK data
Normal data

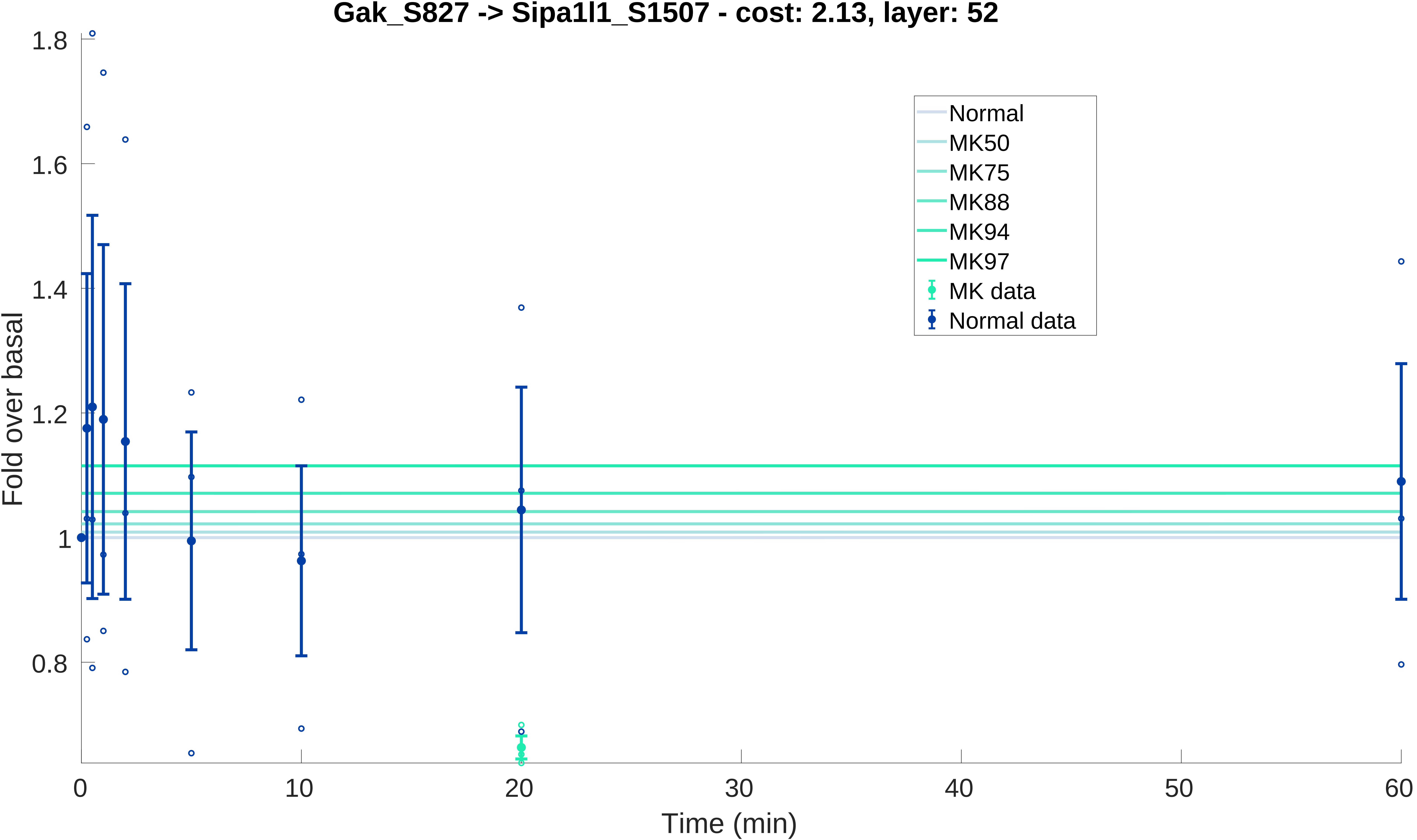
Gak_S827 -> Sipa1l1_S1507 - cost: 2.13, layer: 52
Fold over basal
Time (min)
Normal
MK50
MK75
MK88
MK94
MK97
MK data
Normal data

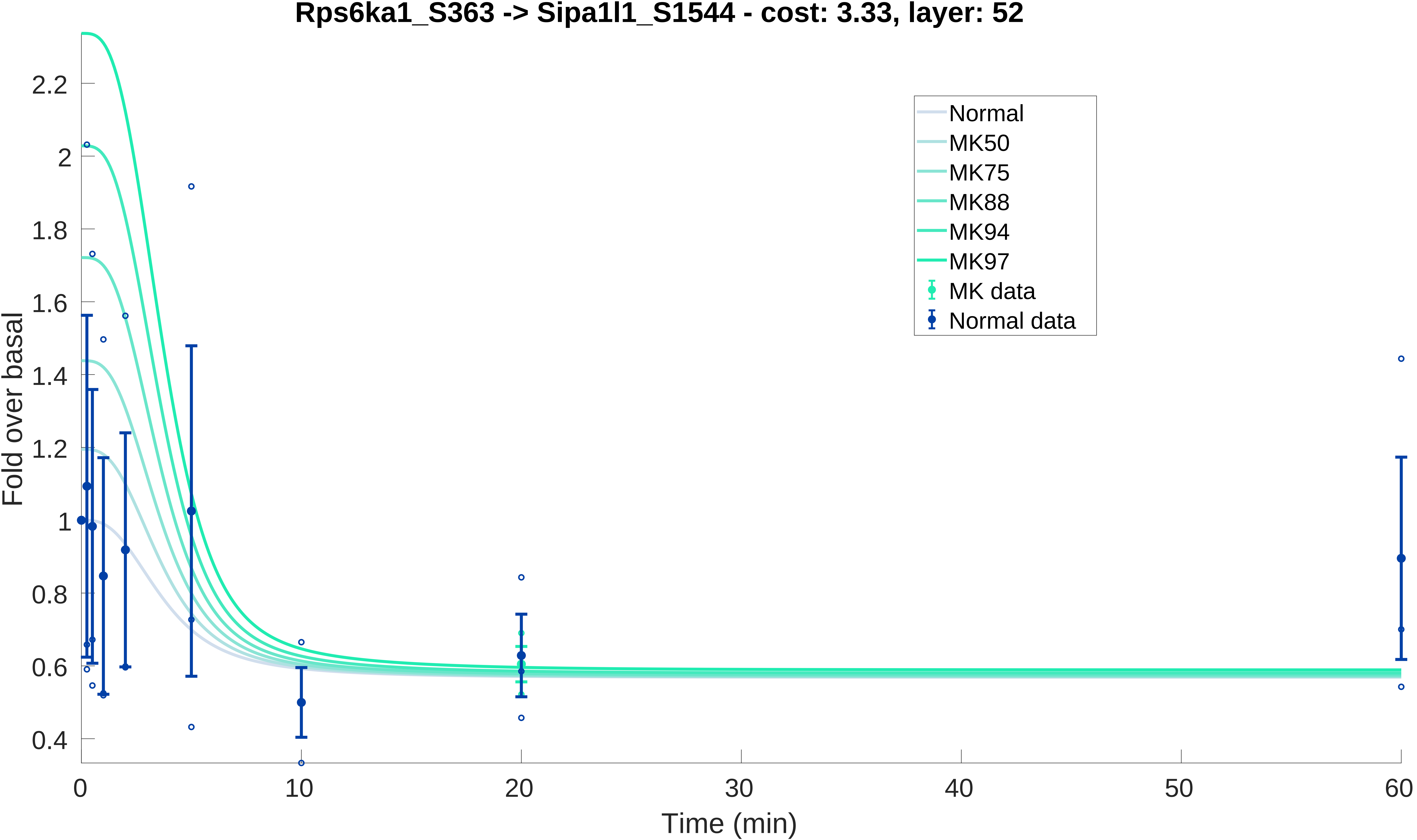

Rps6ka1_S363 -> Sipa1l1_S1544 - cost: 3.33, layer: 52
Fold over basal
Time (min)
0.4
0.6
0.8
1
1.2
1.4
1.6
1.8
2
2.2
0
10
20
30
40
50
60
Normal
MK50
MK75
MK88
MK94
MK97
MK data
Normal data

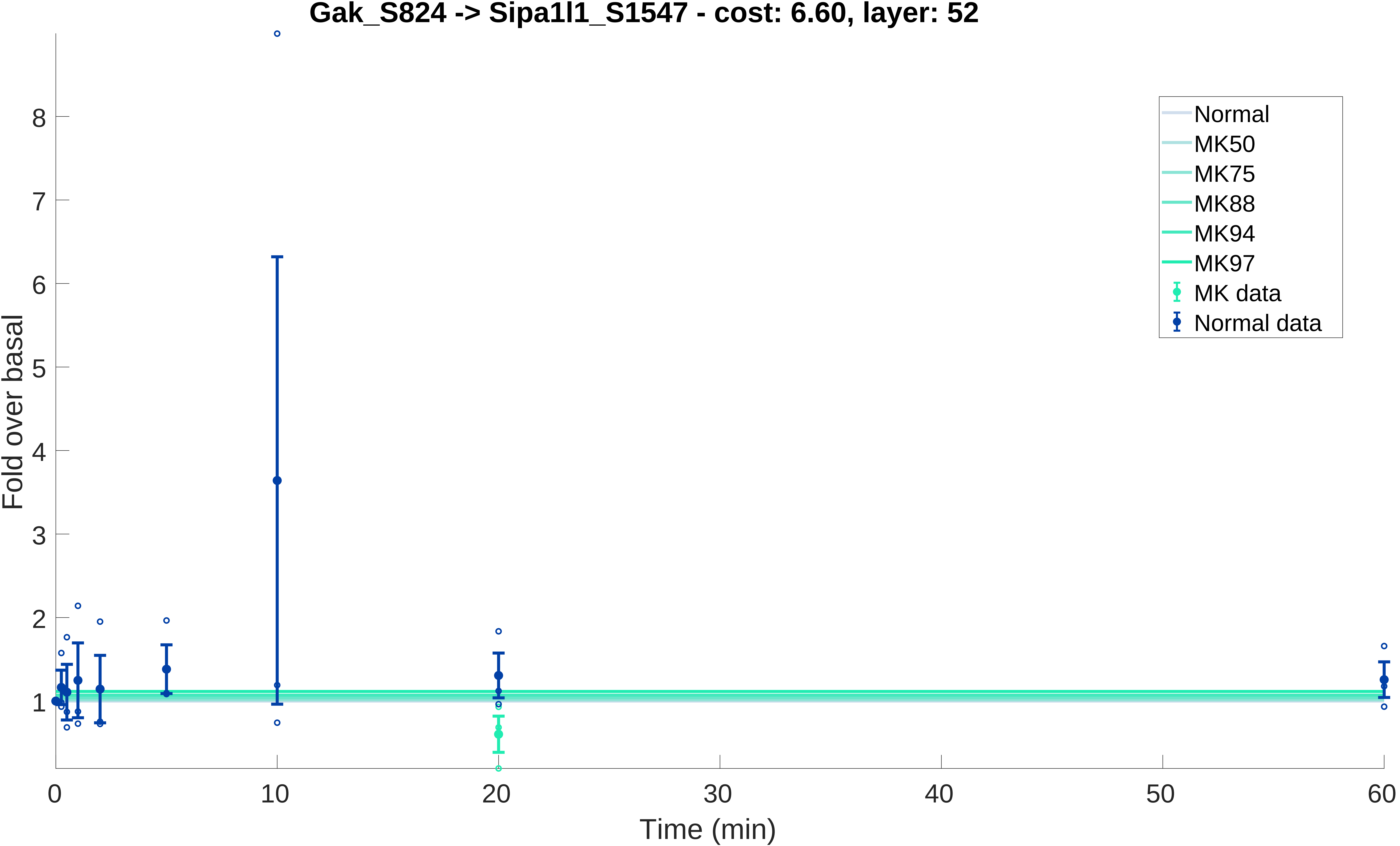
Gak_S824 -> Sipa1l1_S1547 - cost: 6.60, layer: 52
Fold over basal
Time (min)
Normal
MK50
MK75
MK88
MK94
MK97
MK data
Normal data
0
10
20
30
40
50
60
1
2
3
4
5
6
7
8

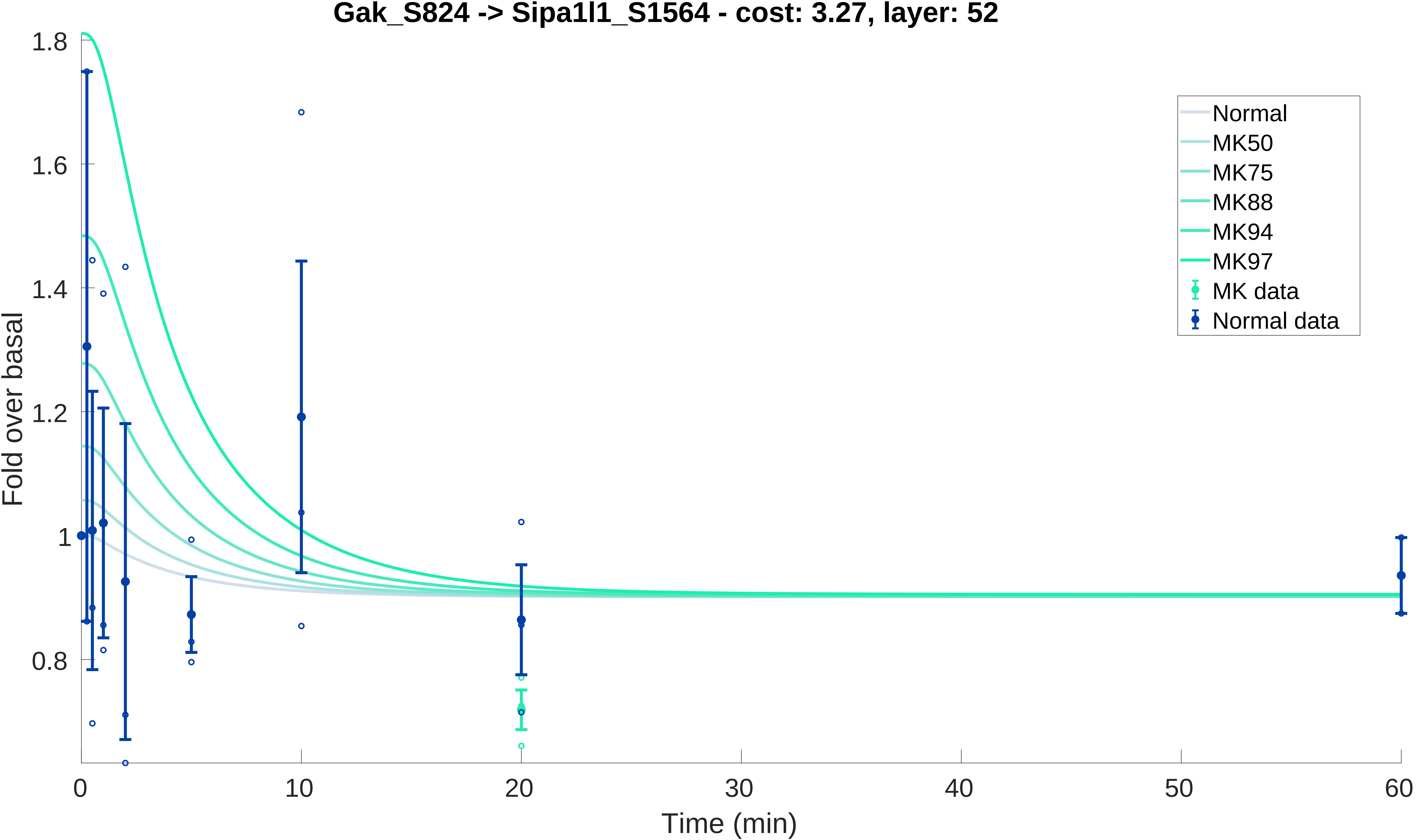

Gak_S824 -> Sipa1l1_S1564 - cost: 3.27, layer: 52
Fold over basal
Time (min)
1.8
1.6
1.4
1.2
1
0.8
0
10
20
30
40
50
60
Normal
MK50
MK75
MK88
MK94
MK97
MK data
Normal data

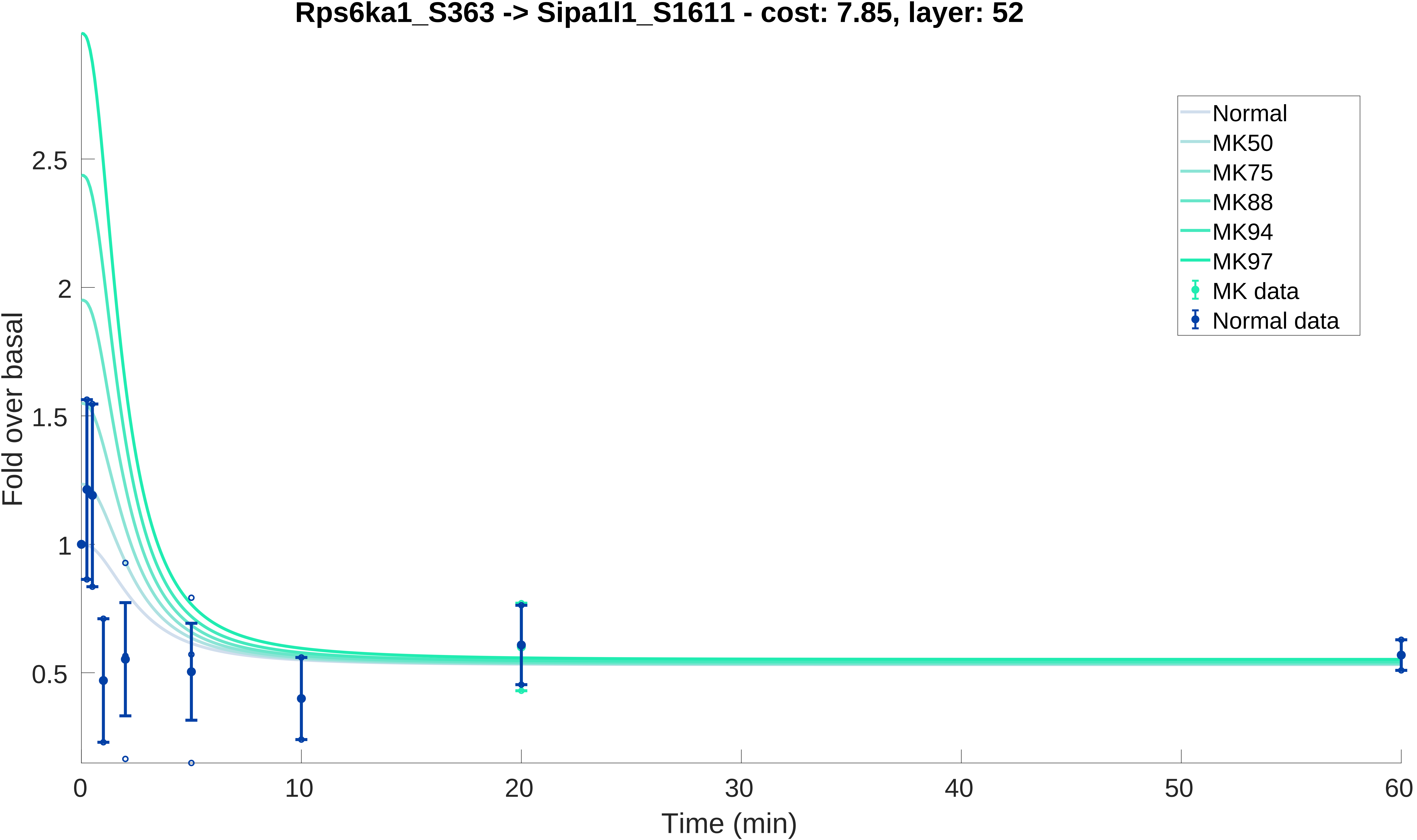

Rps6ka1_S363 -> Sipa1l1_S1611 - cost: 7.85, layer: 52
Fold over basal
Time (min)
0.5
1
1.5
2
2.5
0
10
20
30
40
50
60
Normal
MK50
MK75
MK88
MK94
MK97
MK data
Normal data

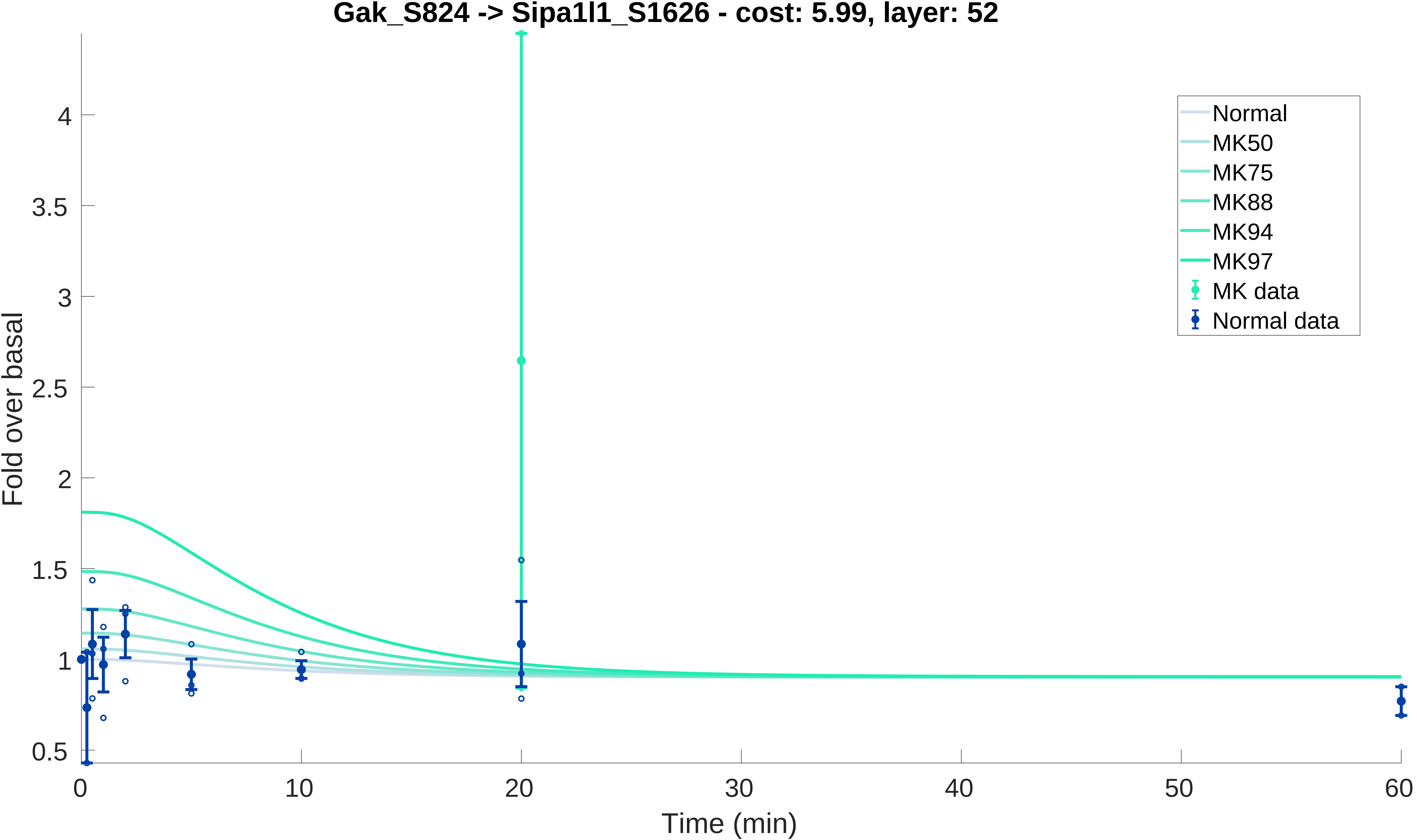

Gak_S824 -> Sipa1l1_S1626 - cost: 5.99, layer: 52
Fold over basal
Time (min)
Normal
MK50
MK75
MK88
MK94
MK97
MK data
Normal data
0.5
1
1.5
2
2.5
3
3.5
4
0
10
20
30
40
50
60

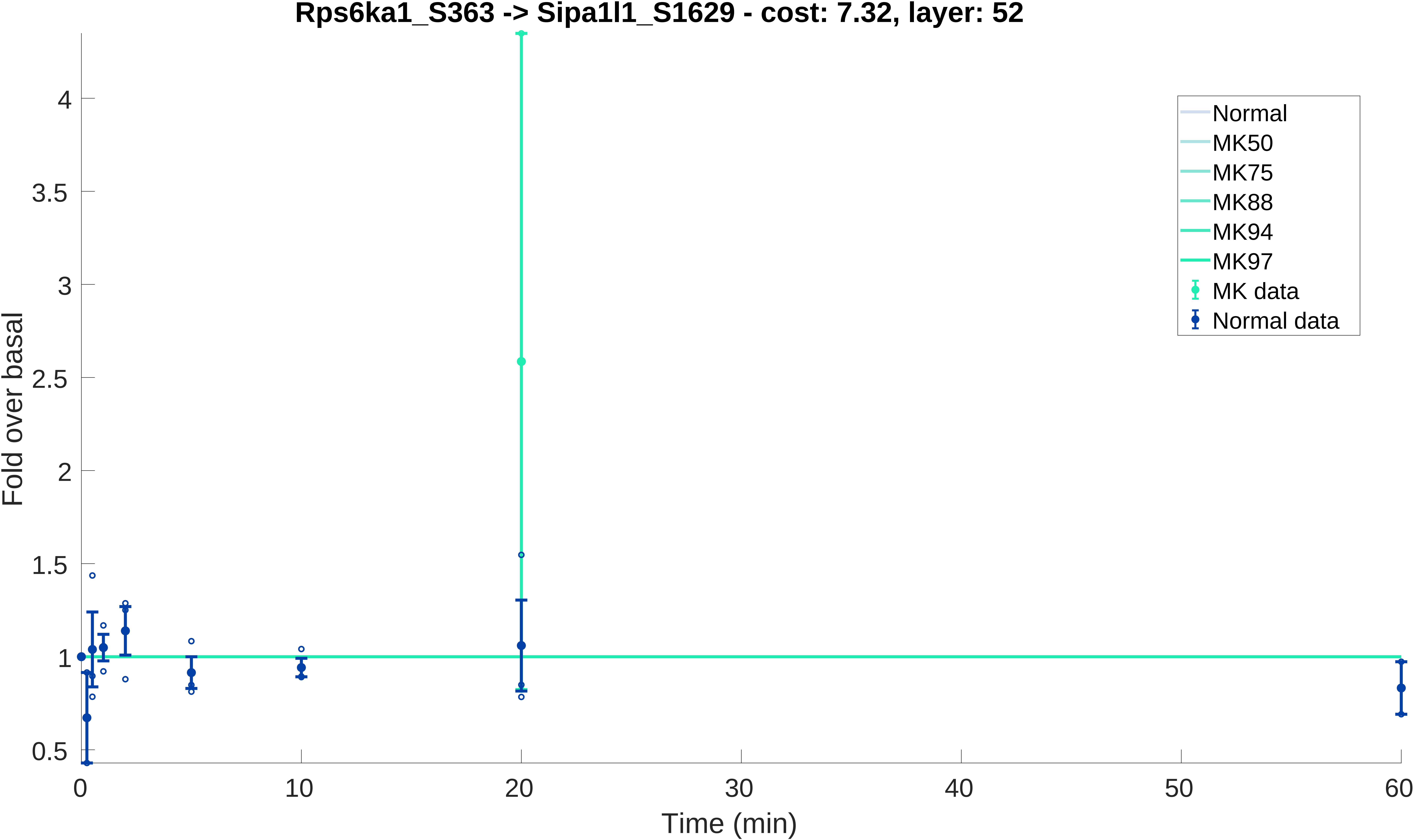

Rps6ka1_S363 -> Sipa1l1_S1629 - cost: 7.32, layer: 52
Fold over basal
Time (min)
0.5
1
1.5
2
2.5
3
3.5
4
0
10
20
30
40
50
60
Normal
MK50
MK75
MK88
MK94
MK97
MK data
Normal data

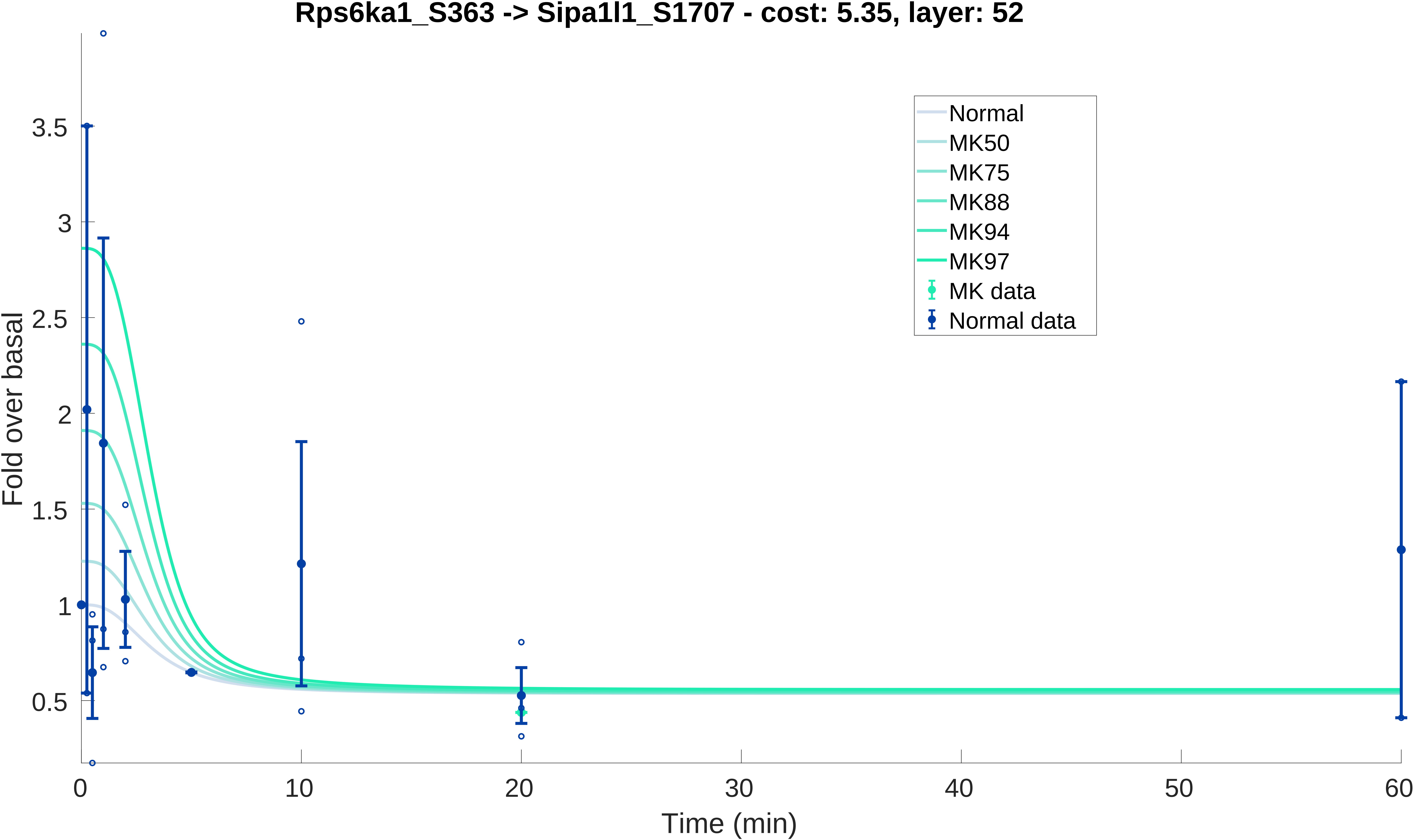

Rps6ka1_S363 -> Sipa1l1_S1707 - cost: 5.35, layer: 52
Fold over basal
Time (min)
0
10
20
30
40
50
60
0.5
1
1.5
2
2.5
3
3.5
Normal
MK50
MK75
MK88
MK94
MK97
MK data
Normal data

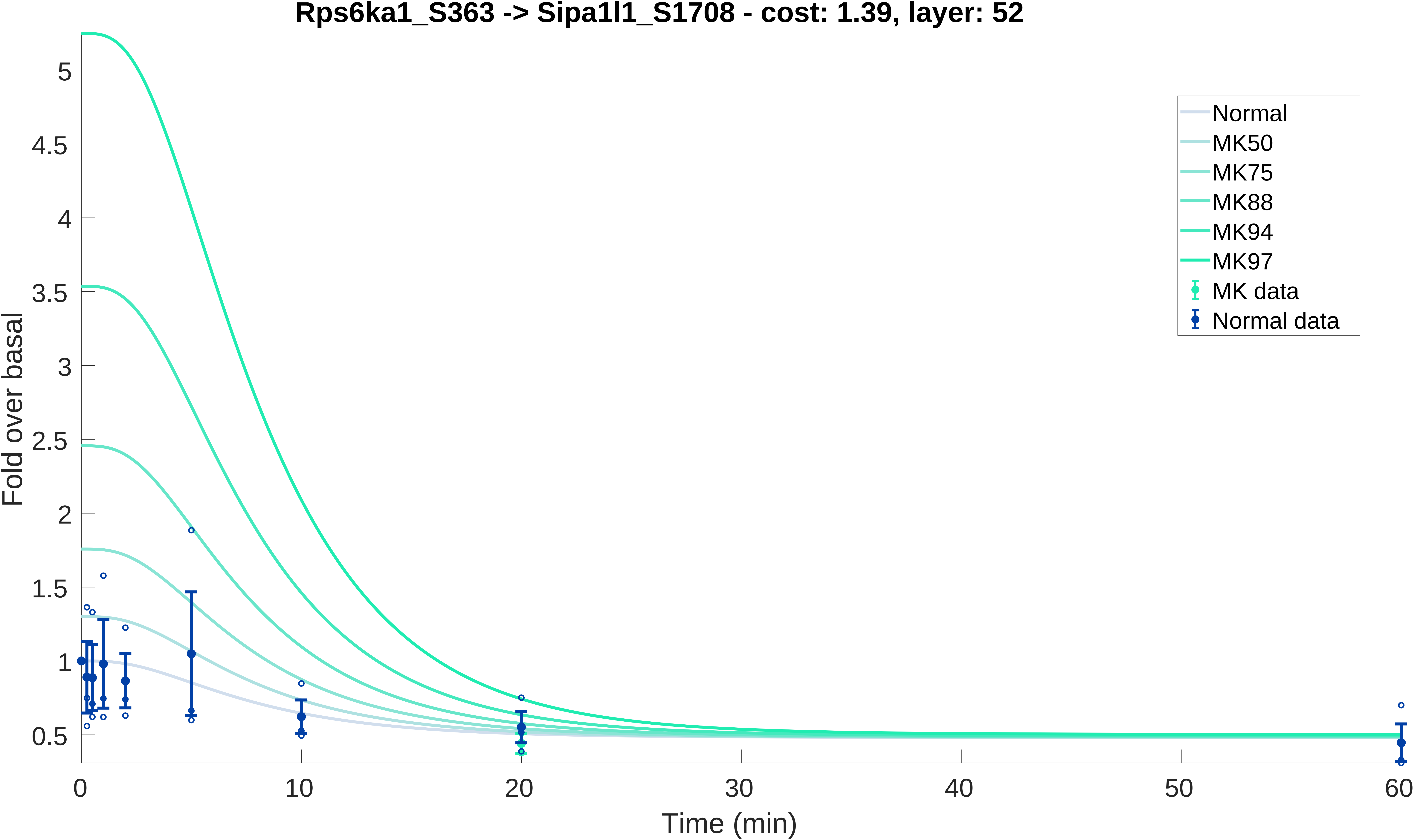

Rps6ka1_S363 -> Sipa1l1_S1708 - cost: 1.39, layer: 52
Fold over basal
Time (min)
Normal
MK50
MK75
MK88
MK94
MK97
MK data
Normal data
0.5
1
1.5
2
2.5
3
3.5
4
4.5
5
0
10
20
30
40
50
60

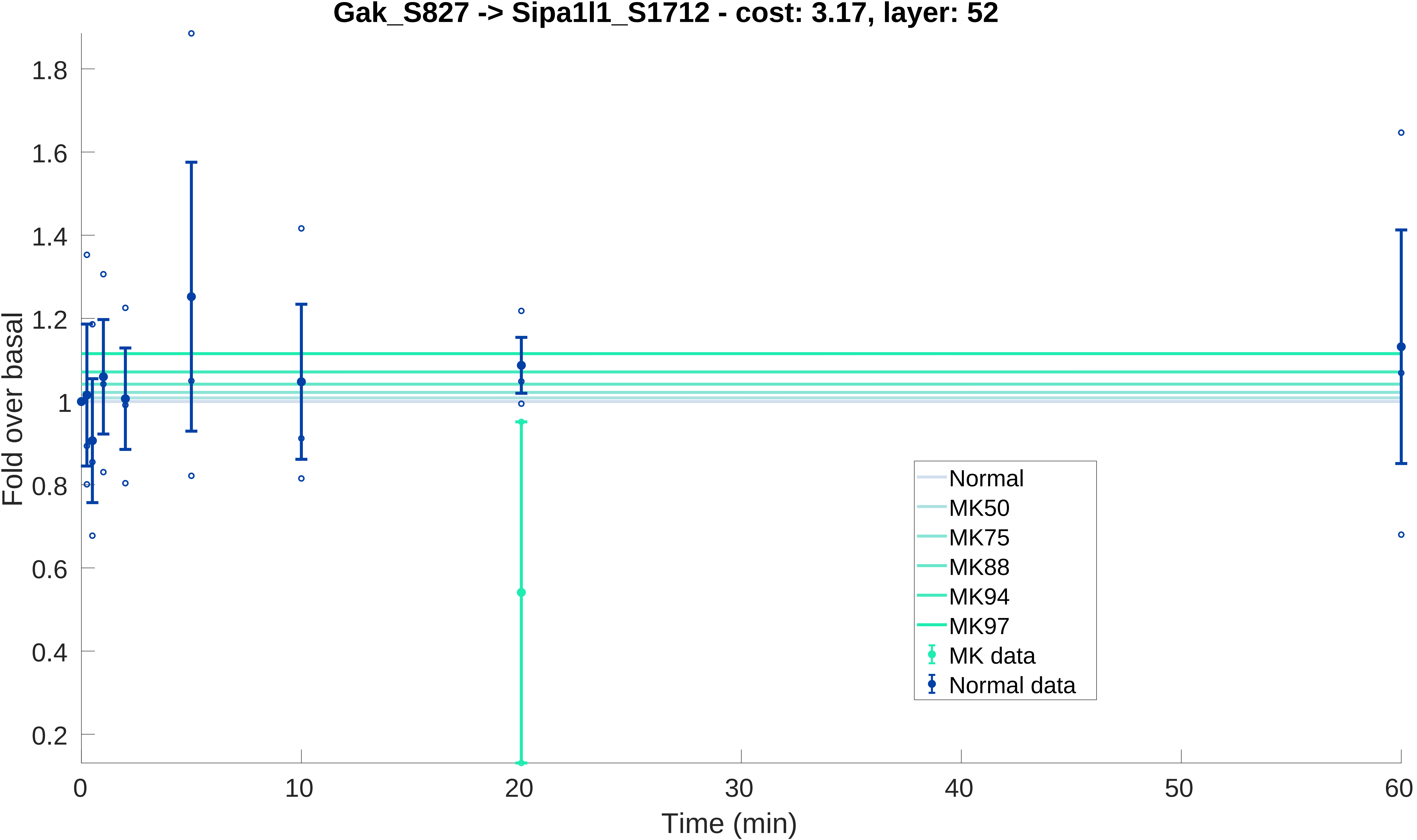
Gak_S827 -> Sipa1l1_S1712 - cost: 3.17, layer: 52
Fold over basal
Time (min)
0
10
20
30
40
50
60
0.2
0.4
0.6
0.8
1
1.2
1.4
1.6
1.8
Normal
MK50
MK75
MK88
MK94
MK97
MK data
Normal data

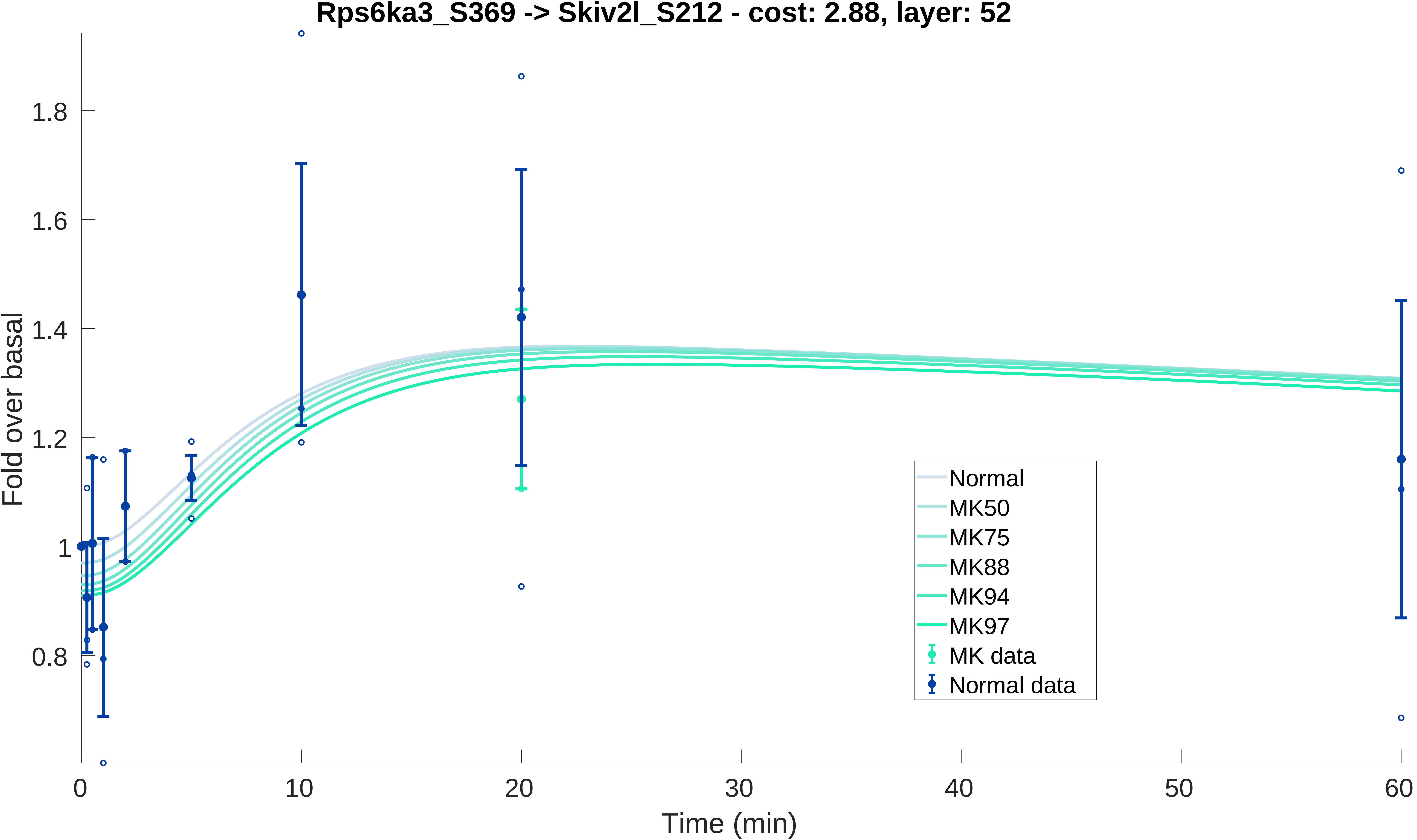
Rps6ka3_S369 -> Skiv2l_S212 - cost: 2.88, layer: 52
Fold over basal
1.8
1.6
1.4
1.2
1
0.8
0
10
20
30
40
50
60
Time (min)
Normal
MK50
MK75
MK88
MK94
MK97
MK data
Normal data

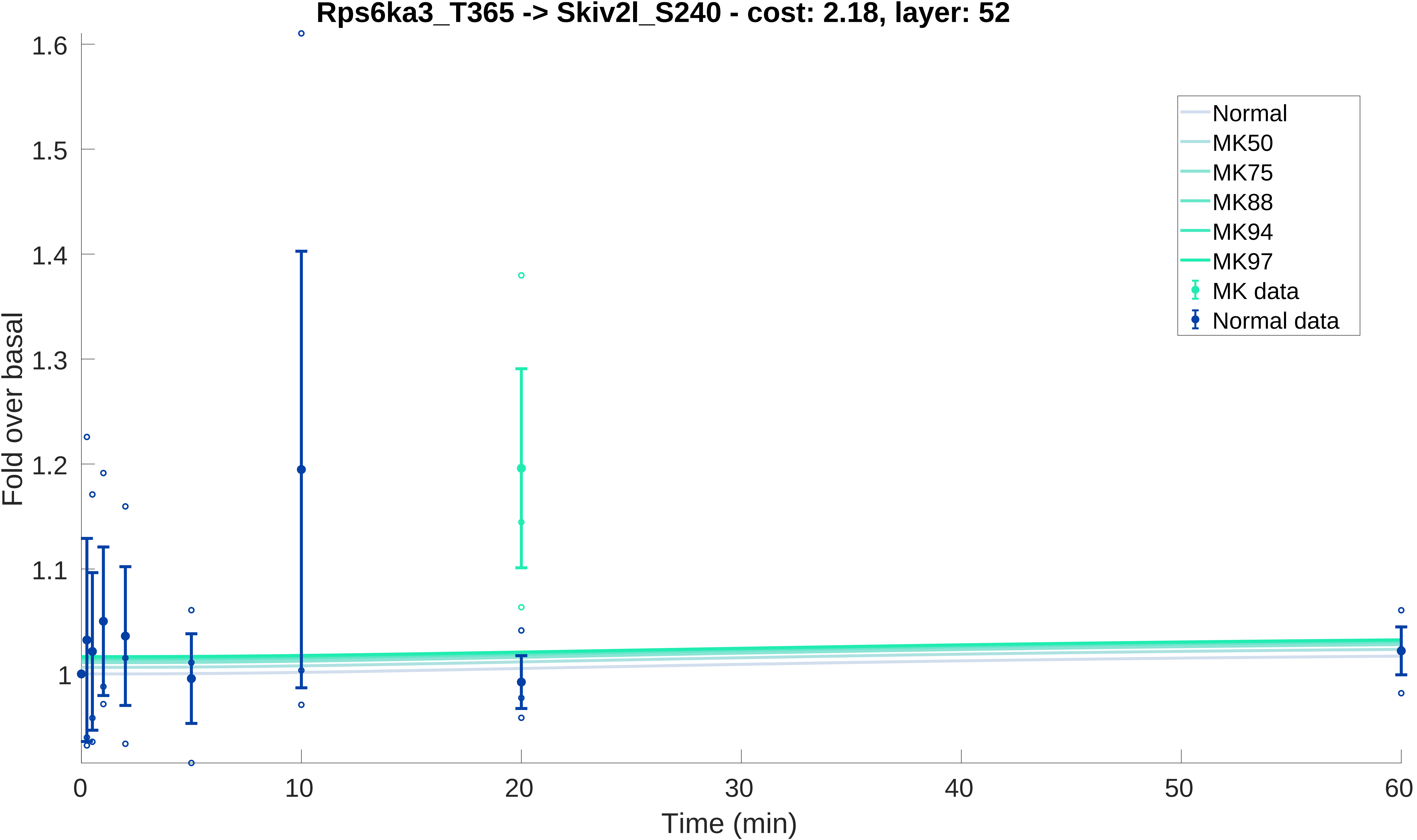

Rps6ka3_T365 -> Skiv2l_S240 - cost: 2.18, layer: 52
Fold over basal
1.6
1.5
1.4
1.3
1.2
1.1
1
0
10
20
30
40
50
60
Time (min)
Normal
MK50
MK75
MK88
MK94
MK97
MK data
Normal data

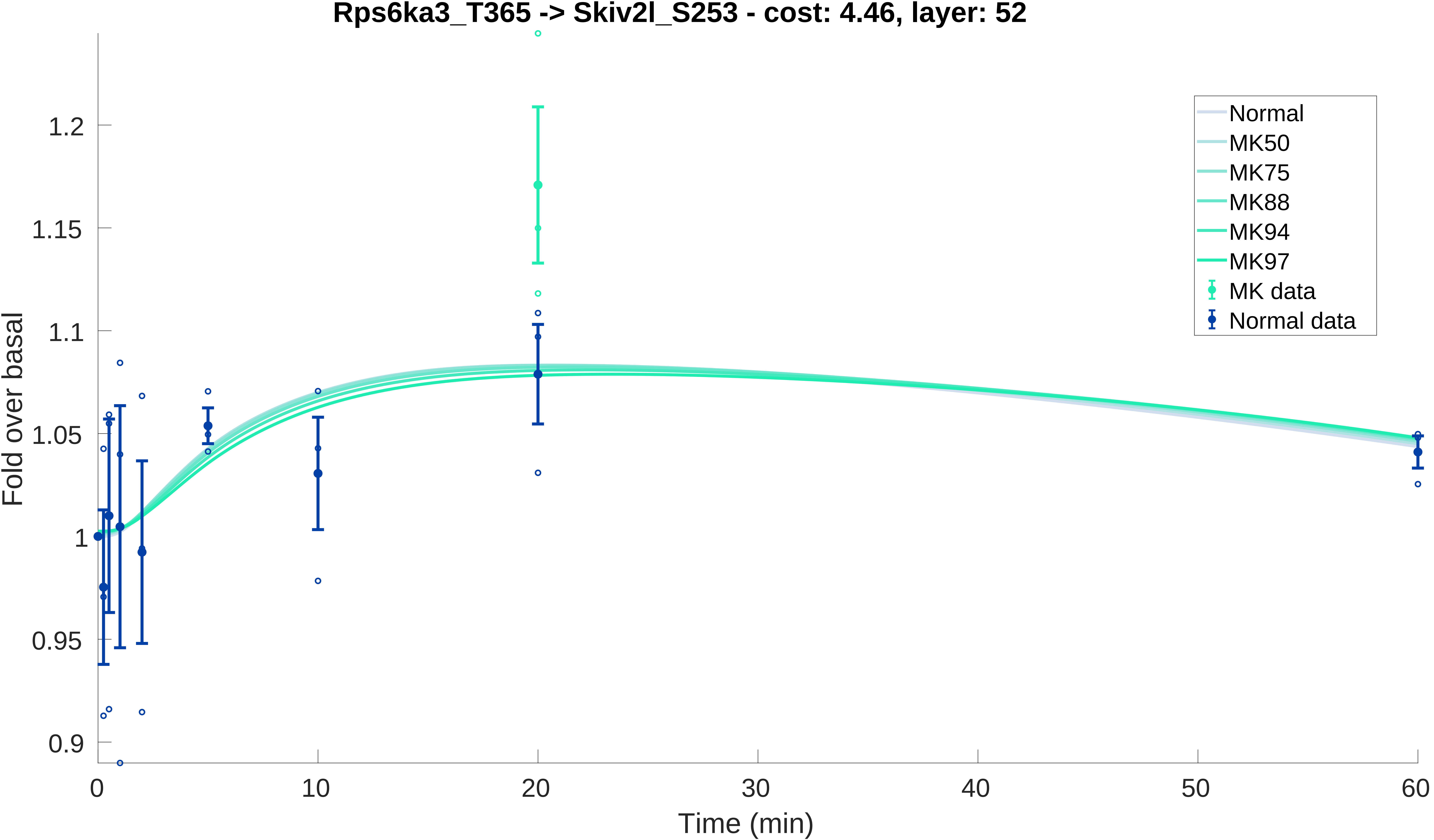

Rps6ka3_T365 -> Skiv2l_S253 - cost: 4.46, layer: 52
Fold over basal
Time (min)
Normal
MK50
MK75
MK88
MK94
MK97
MK data
Normal data
1.2
1.15
1.1
1.05
1
0.95
0.9
0
10
20
30
40
50
60

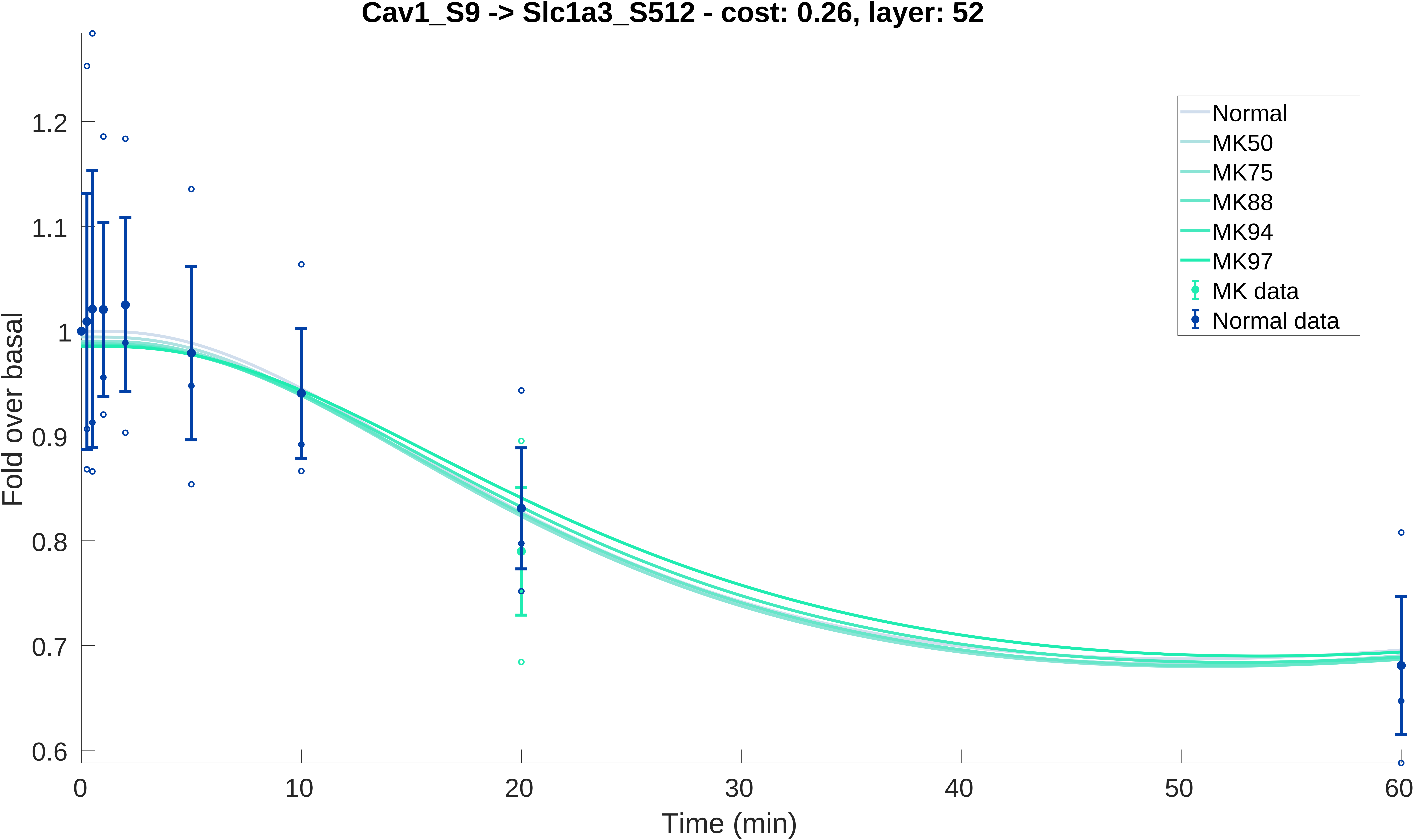

Cav1_S9 -> Slc1a3_S512 - cost: 0.26, layer: 52
Fold over basal
Time (min)
Normal
MK50
MK75
MK88
MK94
MK97
MK data
Normal data
1.2
1.1
1
0.9
0.8
0.7
0.6
0
10
20
30
40
50
60

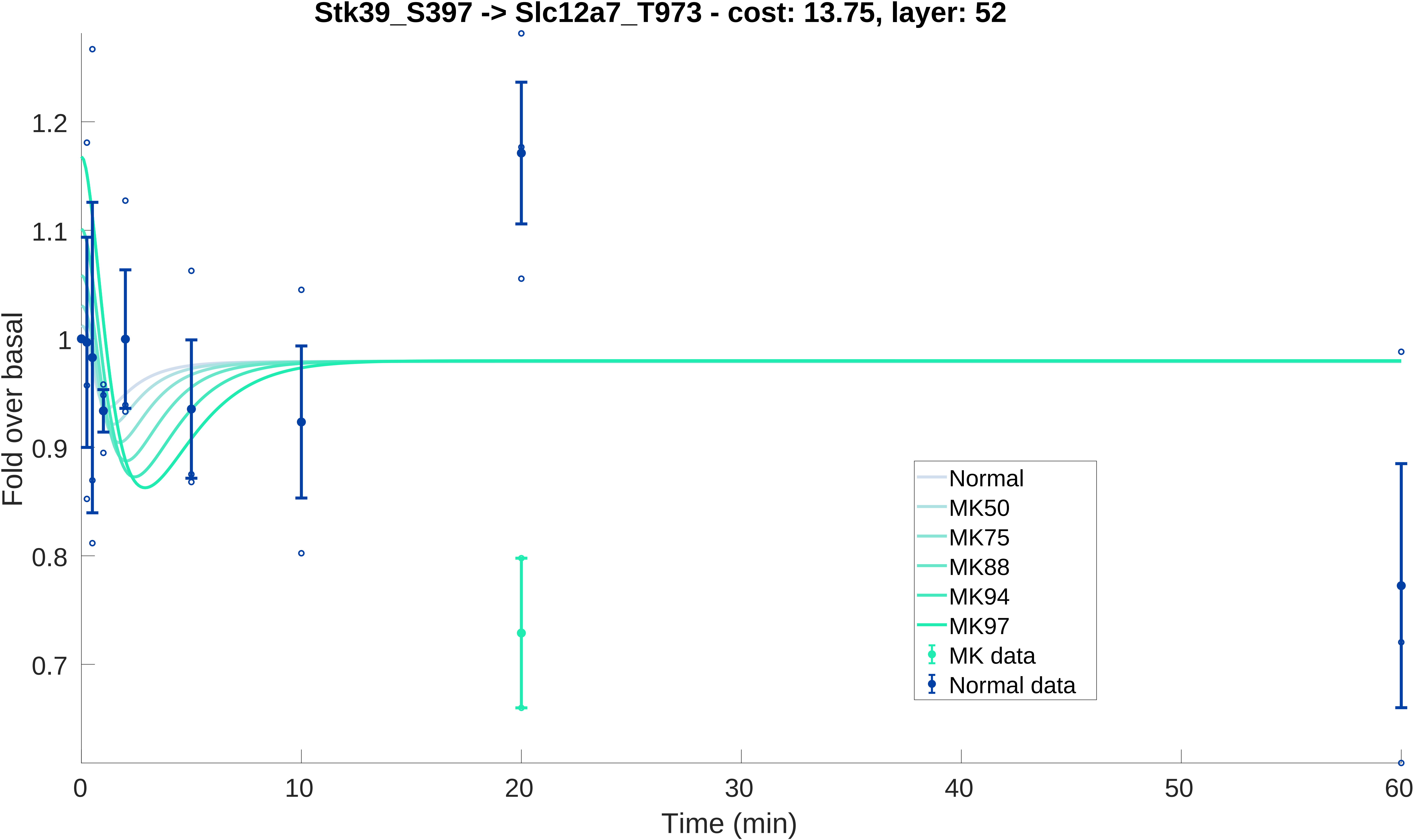

Stk39_S397 -> Slc12a7_T973 - cost: 13.75, layer: 52
Fold over basal
1.2
1.1
1
0.9
0.8
0.7
0
10
20
30
40
50
60
Time (min)
Normal
MK50
MK75
MK88
MK94
MK97
MK data
Normal data

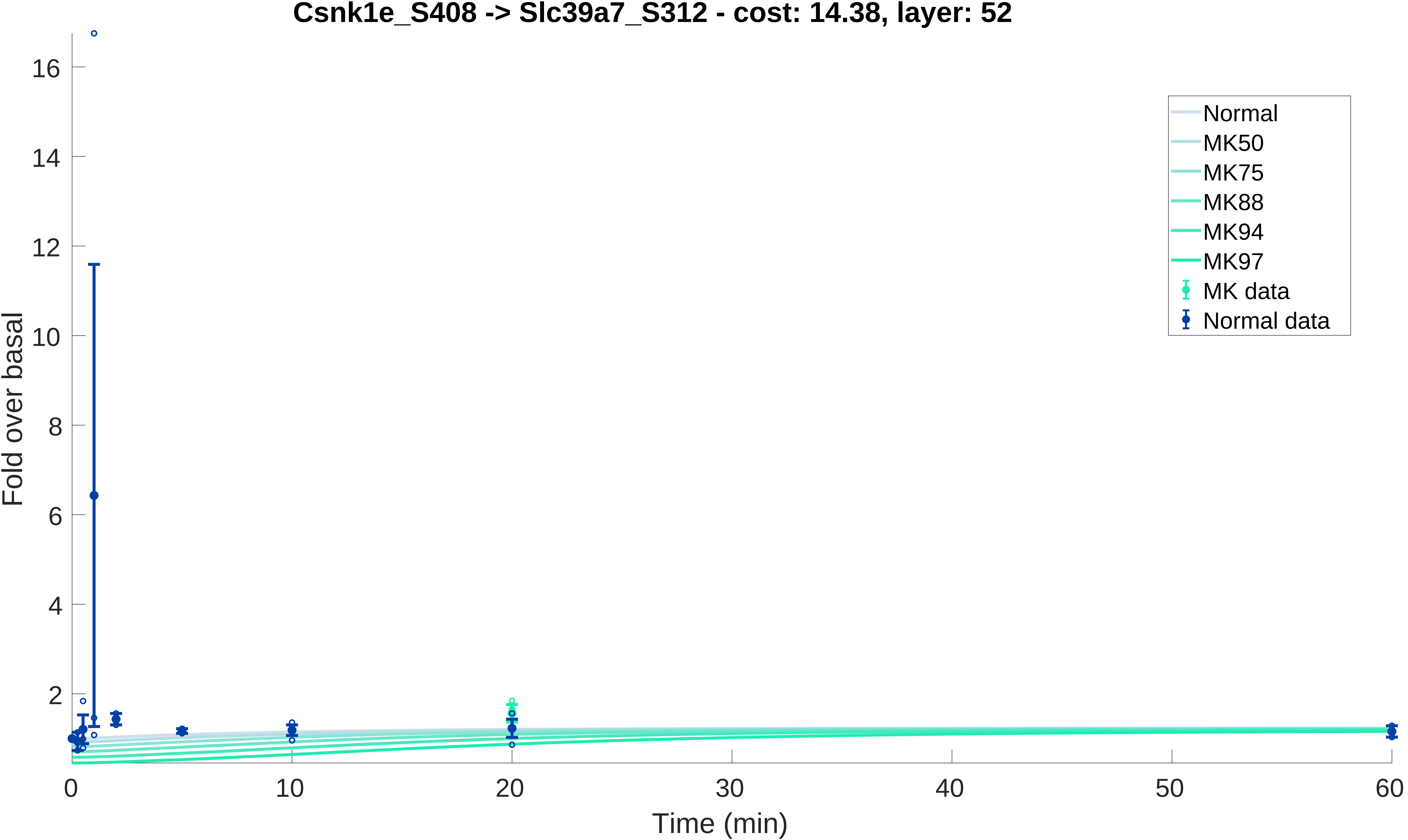

Csnk1e_S408 -> Slc39a7_S312 - cost: 14.38, layer: 52
Fold over basal
Time (min)
Normal
MK50
MK75
MK88
MK94
MK97
MK data
Normal data
16
14
12
10
8
6
4
2
0
10
20
30
40
50
60

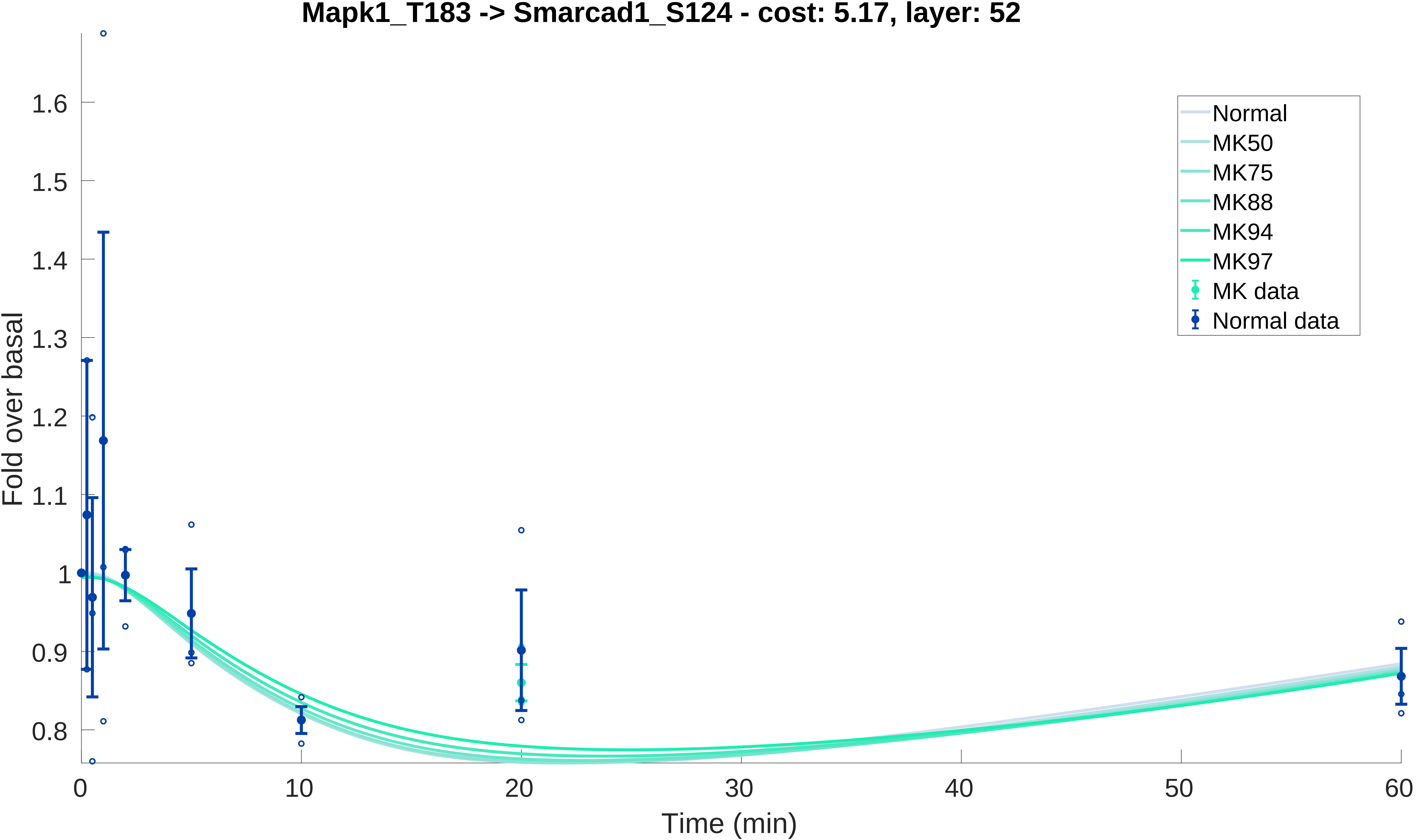
Mapk1_T183 -> Smarcad1_S124 - cost: 5.17, layer: 52
Fold over basal
1.6
1.5
1.4
1.3
1.2
1.1
1
0.9
0.8
0
10
20
30
40
50
60
Time (min)
Normal
MK50
MK75
MK88
MK94
MK97
MK data
Normal data

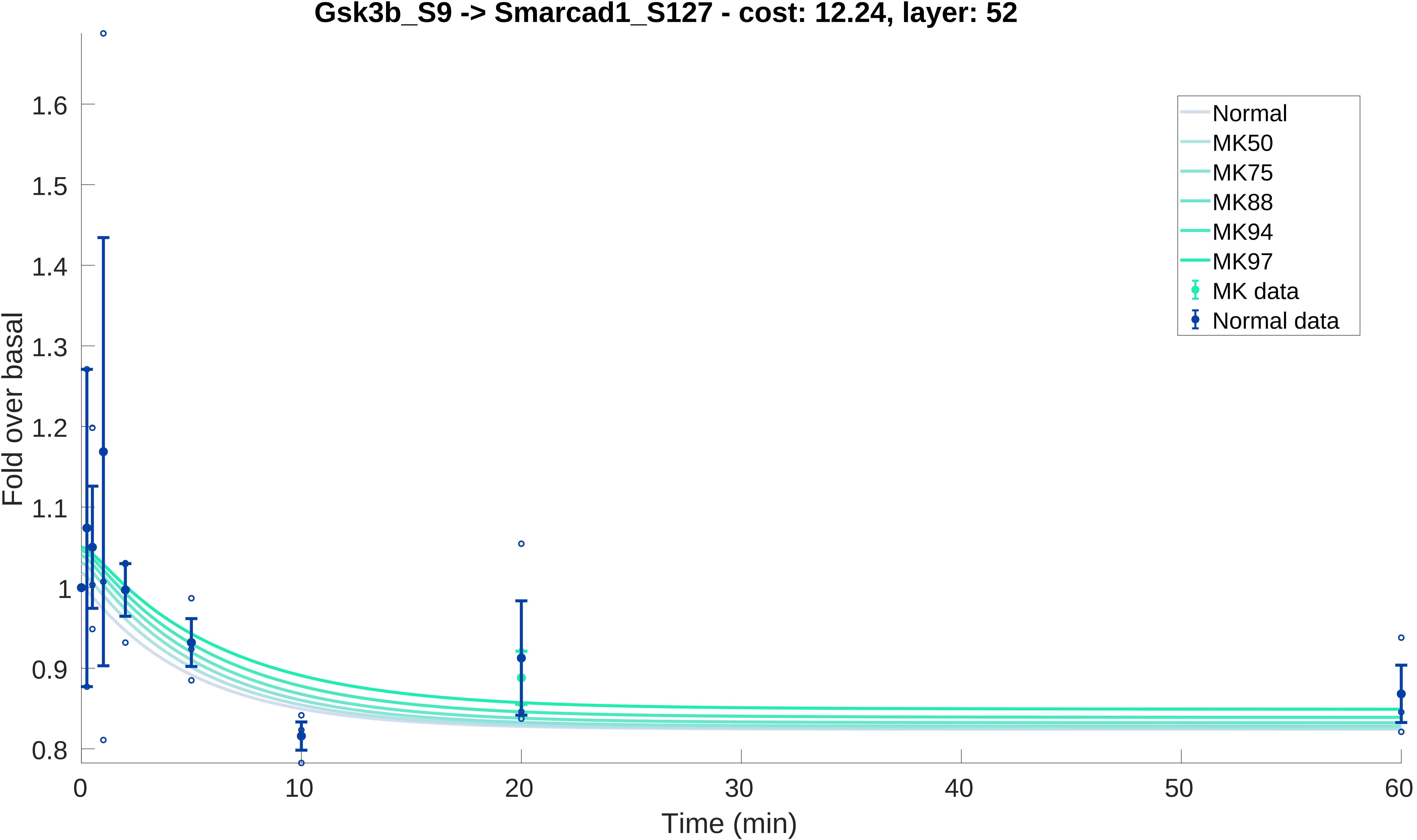

Gsk3b_S9 -> Smarcad1_S127 - cost: 12.24, layer: 52
Fold over basal
1.6
1.5
1.4
1.3
1.2
1.1
1
0.9
0.8
0
10
20
30
40
50
60
Time (min)
Normal
MK50
MK75
MK88
MK94
MK97
MK data
Normal data

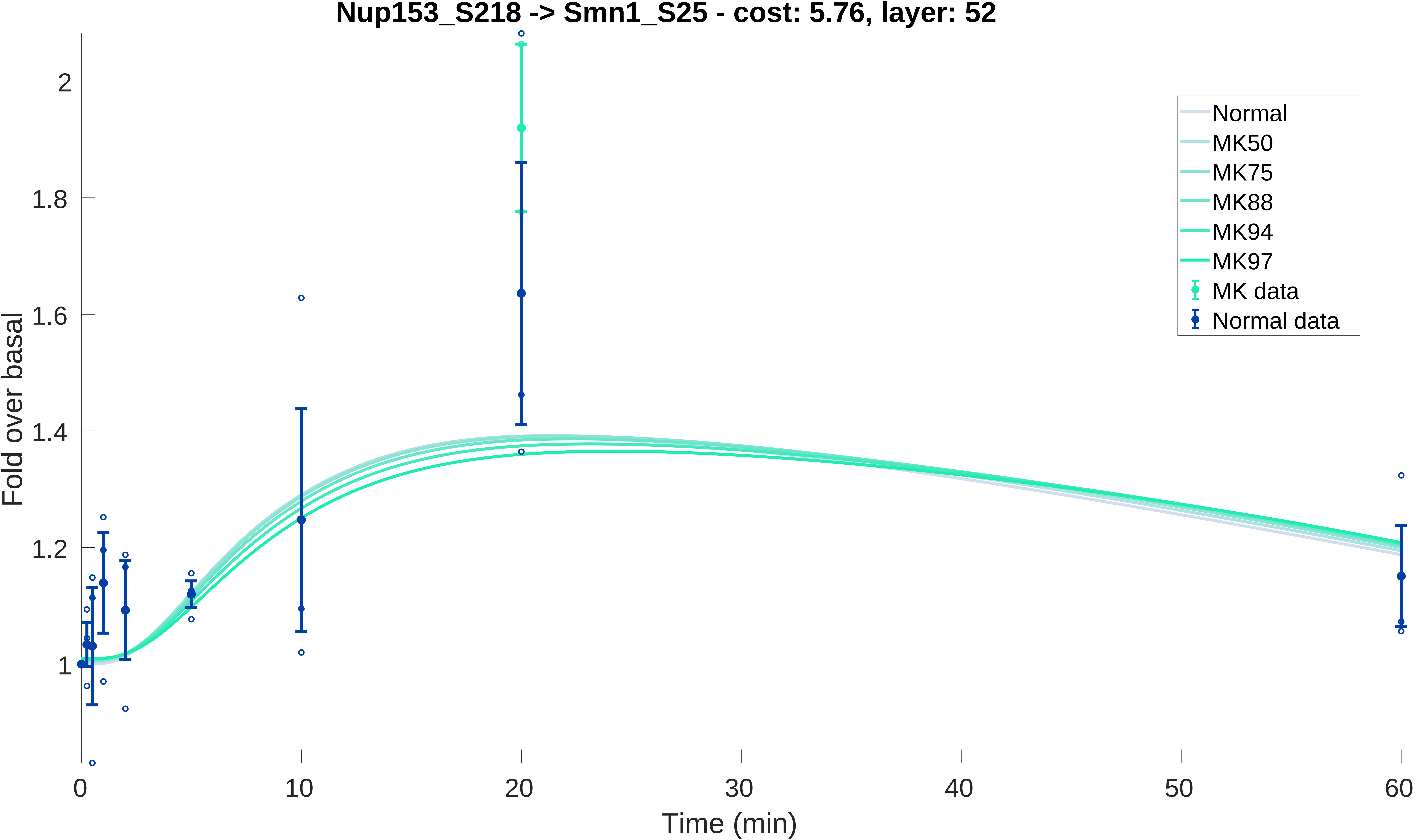

Nup153_S218 -> Smn1_S25 - cost: 5.76, layer: 52
Fold over basal
Time (min)
Normal
MK50
MK75
MK88
MK94
MK97
MK data
Normal data
0
10
20
30
40
50
60
1
1.2
1.4
1.6
1.8
2

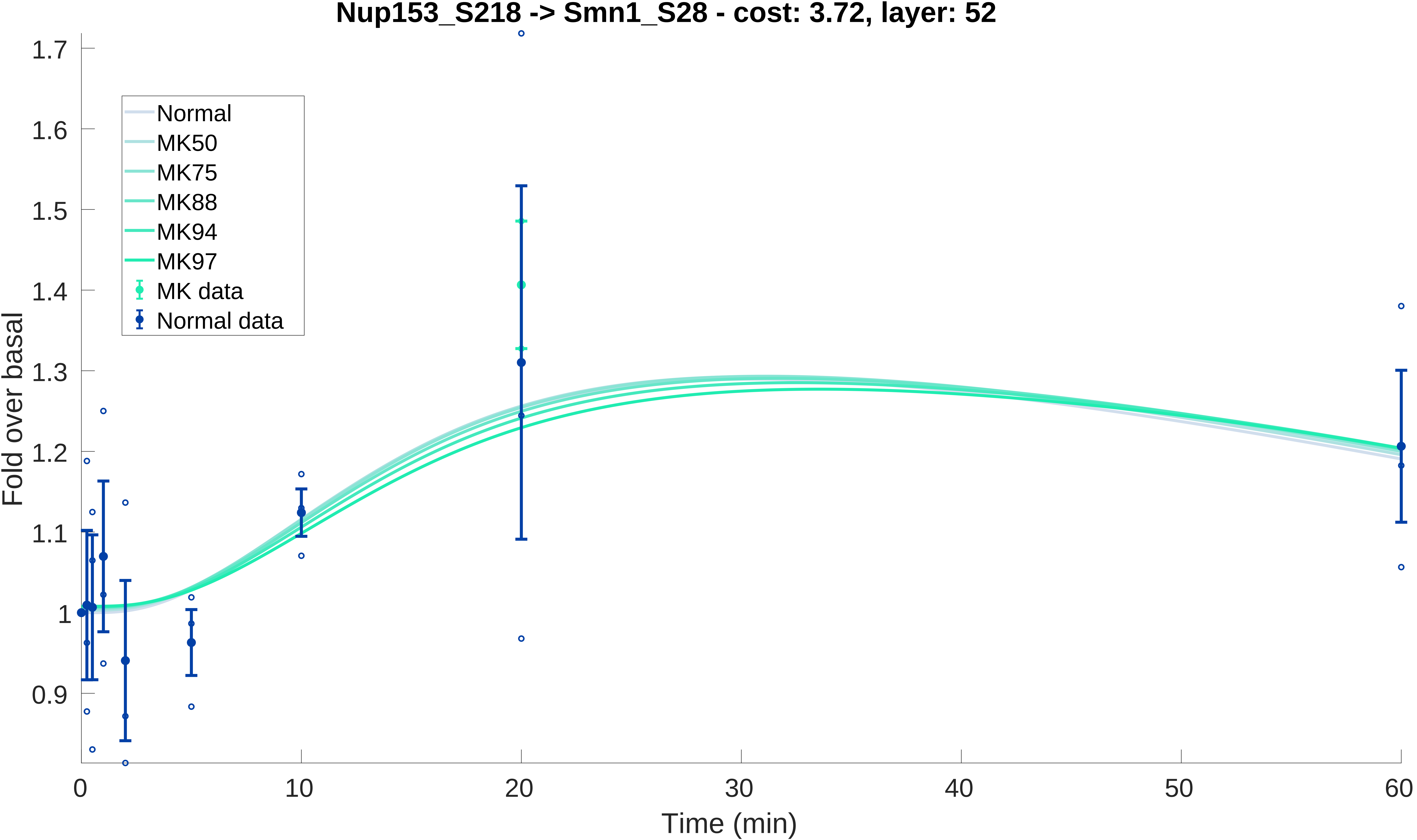

Nup153_S218 -> Smn1_S28 - cost: 3.72, layer: 52
Fold over basal
Time (min)
0.9
1
1.1
1.2
1.3
1.4
1.5
1.6
1.7
0
10
20
30
40
50
60
Normal
MK50
MK75
MK88
MK94
MK97
MK data
Normal data

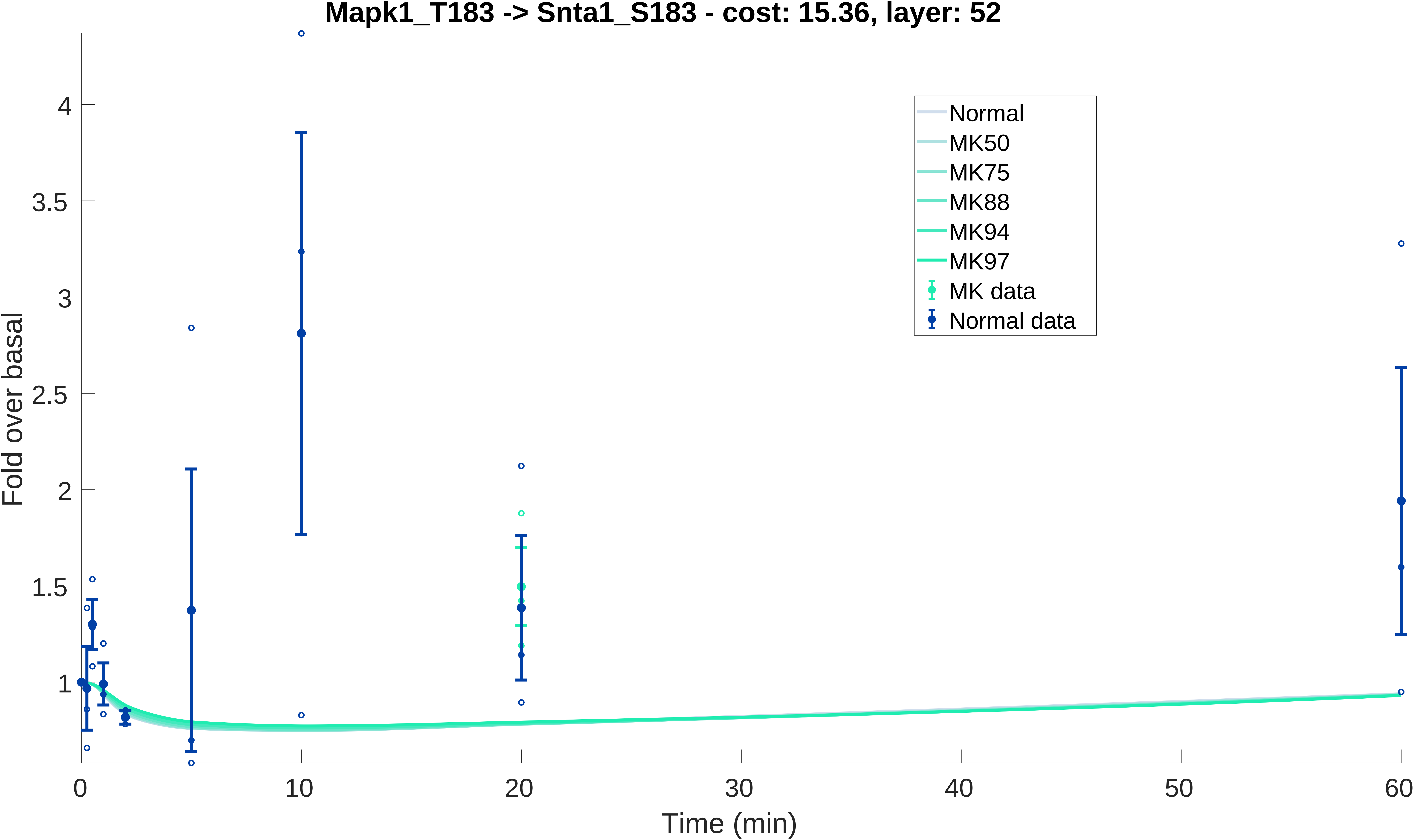
Mapk1_T183 -> Snta1_S183 - cost: 15.36, layer: 52
Fold over basal
Time (min)
0
10
20
30
40
50
60
1
1.5
2
2.5
3
3.5
4
Normal
MK50
MK75
MK88
MK94
MK97
MK data
Normal data

Sqstm1_T269 -> Sod1_S108 - cost: 5.51, layer: 52

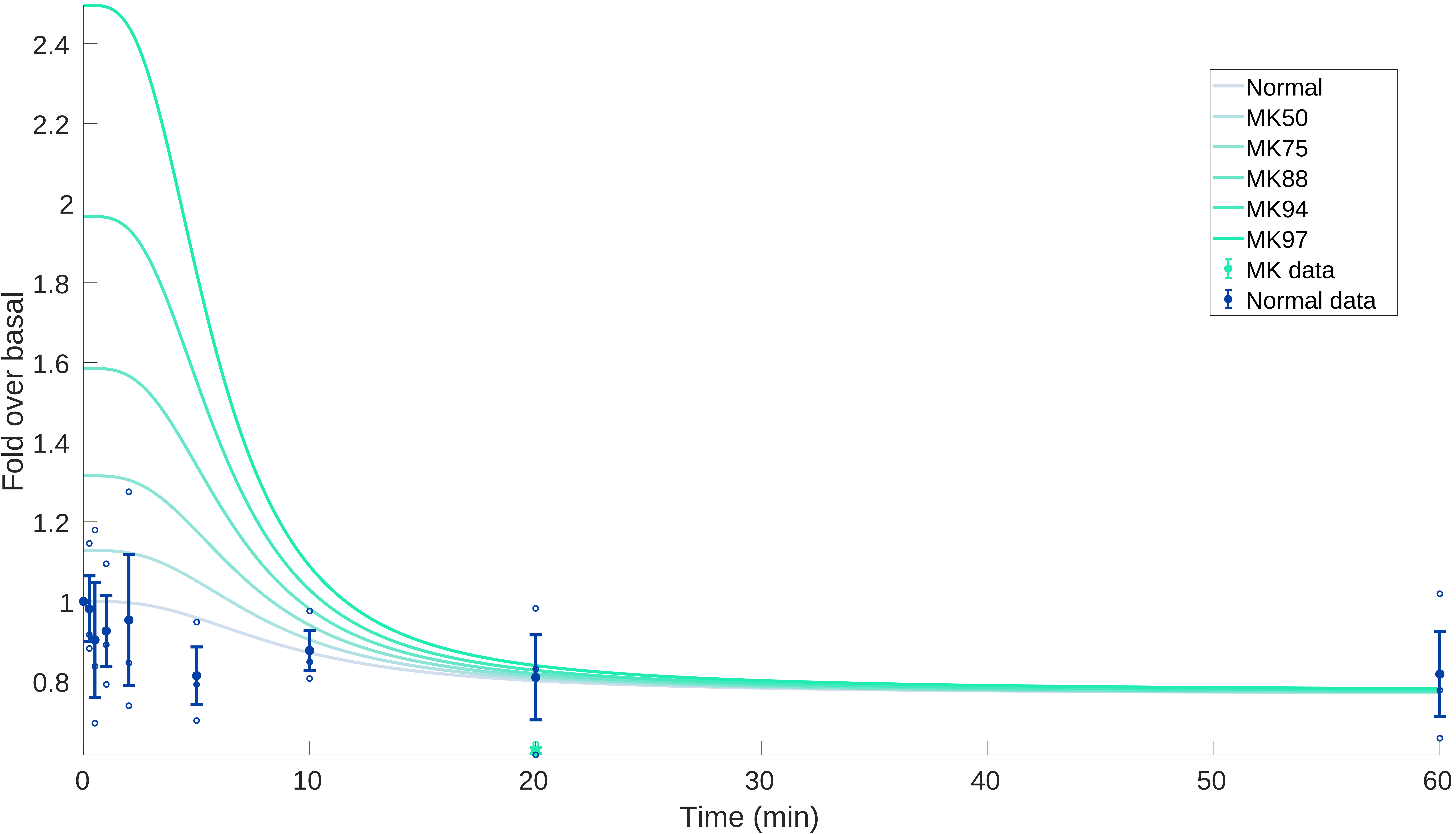

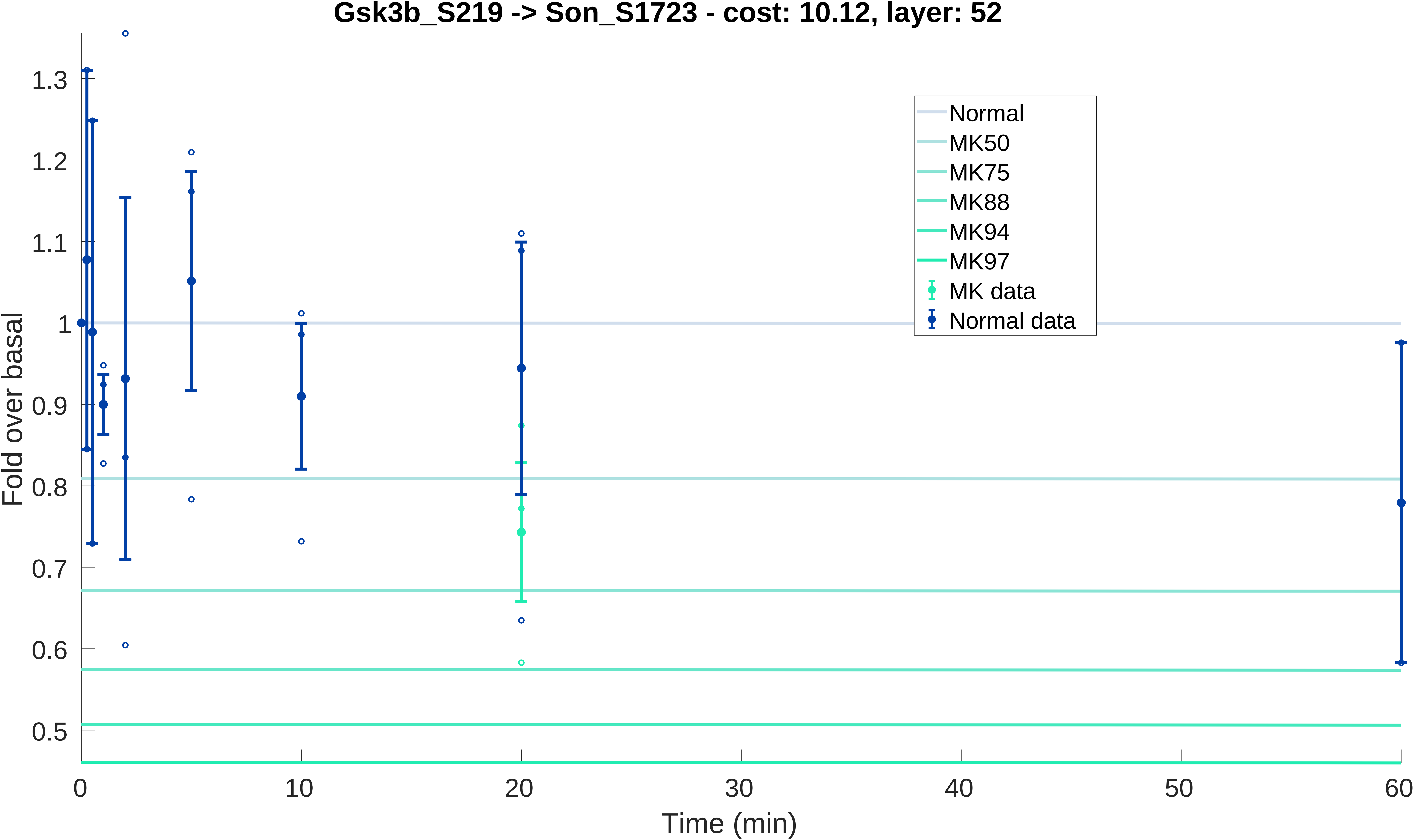

Gsk3b_S219 -> Son_S1723 - cost: 10.12, layer: 52
Fold over basal
Time (min)
0
10
20
30
40
50
60
0.5
0.6
0.7
0.8
0.9
1
1.1
1.2
1.3
Normal
MK50
MK75
MK88
MK94
MK97
MK data
Normal data

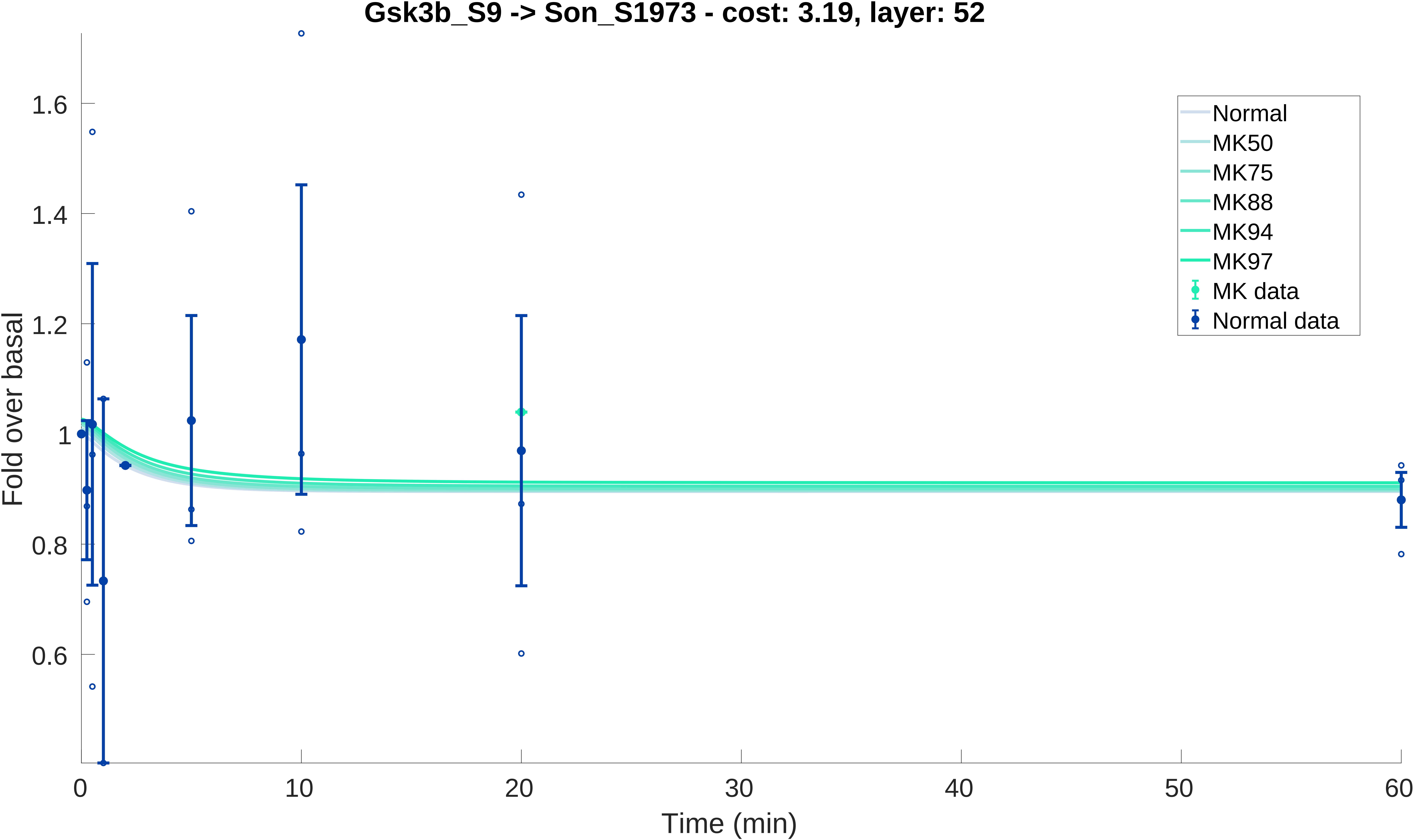

Gsk3b_S9 -> Son_S1973 - cost: 3.19, layer: 52
Fold over basal
1.6
1.4
1.2
1
0.8
0.6
0
10
20
30
40
50
60
Time (min)
Normal
MK50
MK75
MK88
MK94
MK97
MK data
Normal data

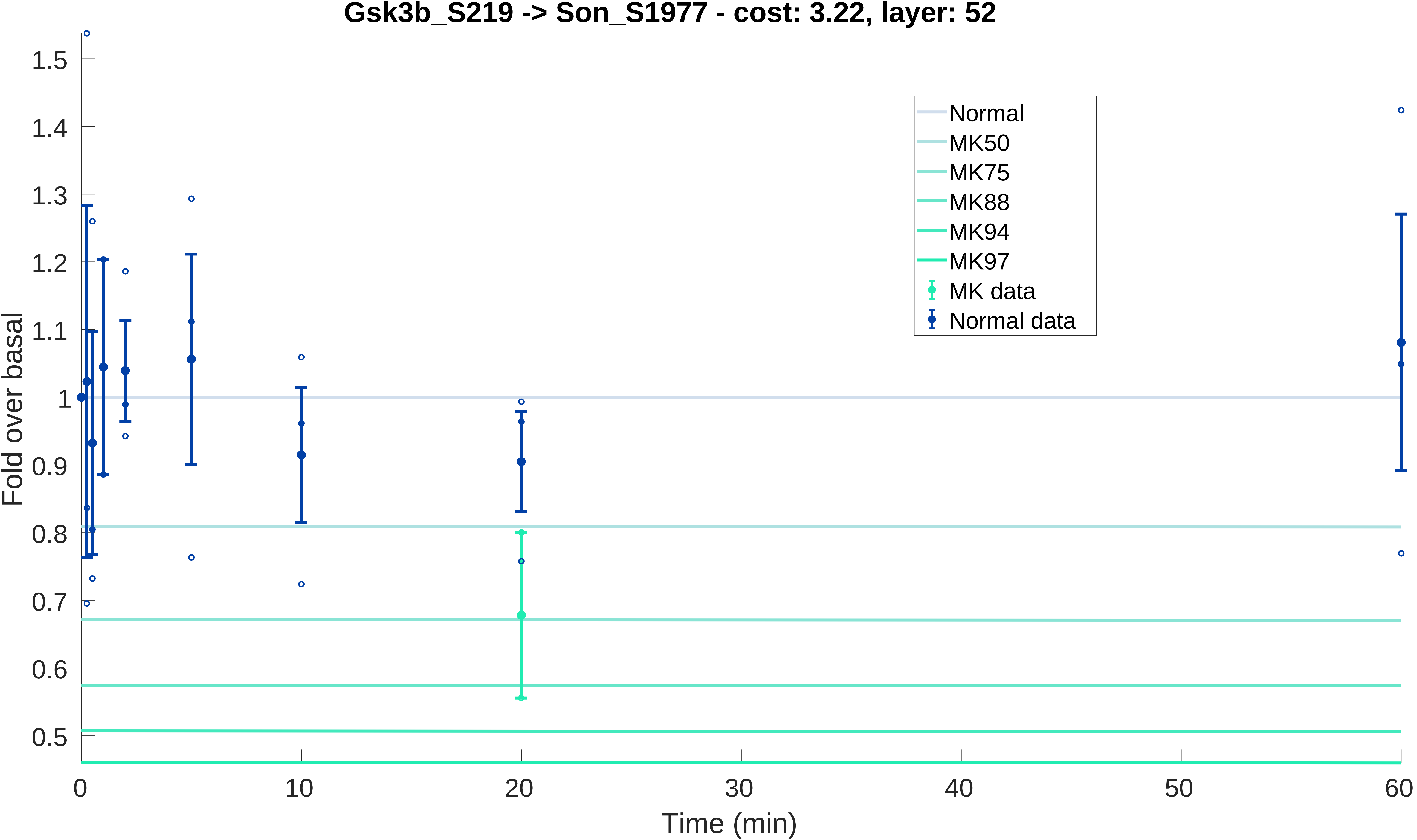

Gsk3b_S219 -> Son_S1977 - cost: 3.22, layer: 52
Fold over basal
Time (min)
1.5
1.4
1.3
1.2
1.1
1
0.9
0.8
0.7
0.6
0.5
0
10
20
30
40
50
60
Normal
MK50
MK75
MK88
MK94
MK97
MK data
Normal data

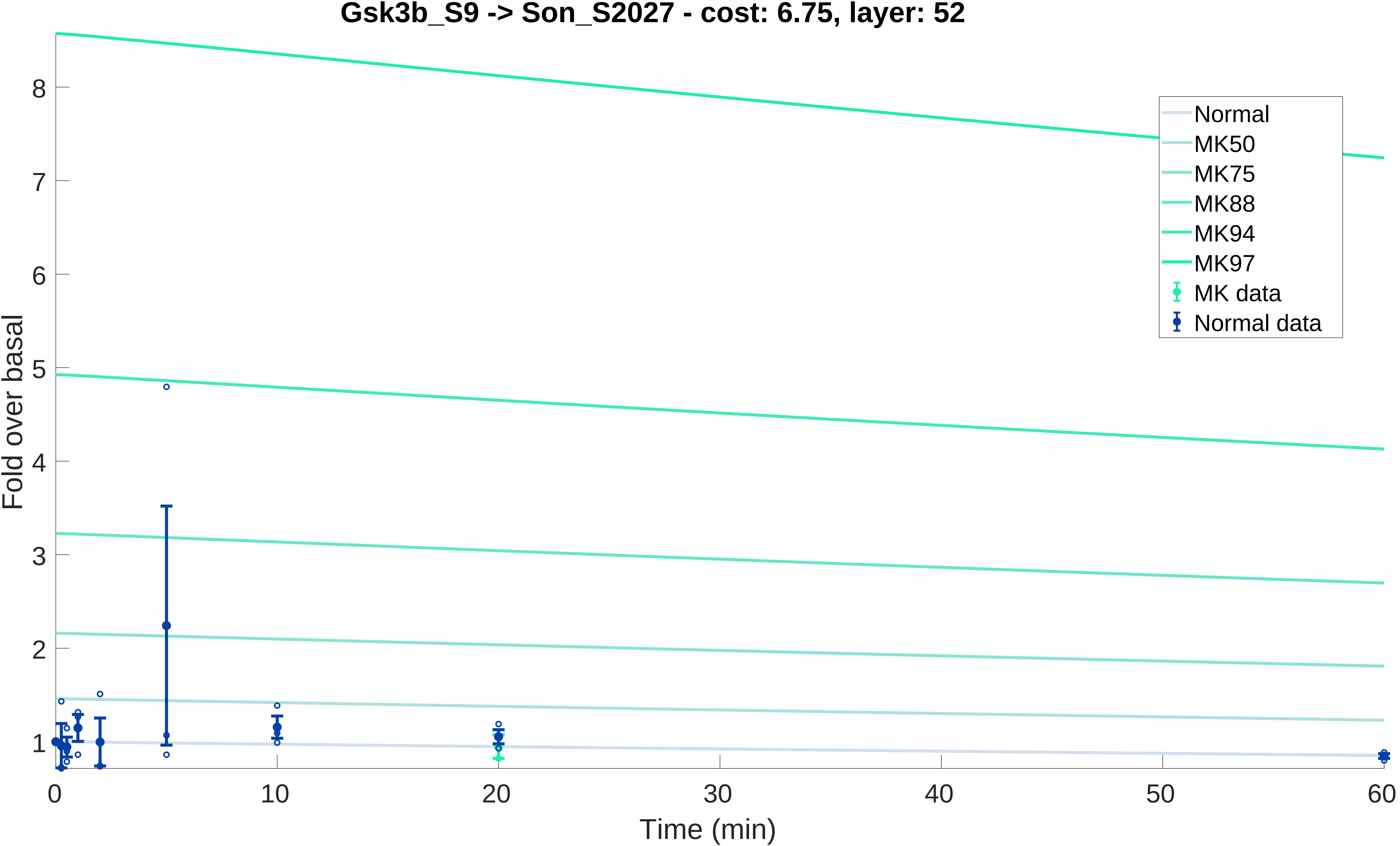

Gsk3b_S9 -> Son_S2027 - cost: 6.75, layer: 52
Fold over basal
Time (min)
Normal
MK50
MK75
MK88
MK94
MK97
MK data
Normal data
0
10
20
30
40
50
60
1
2
3
4
5
6
7
8

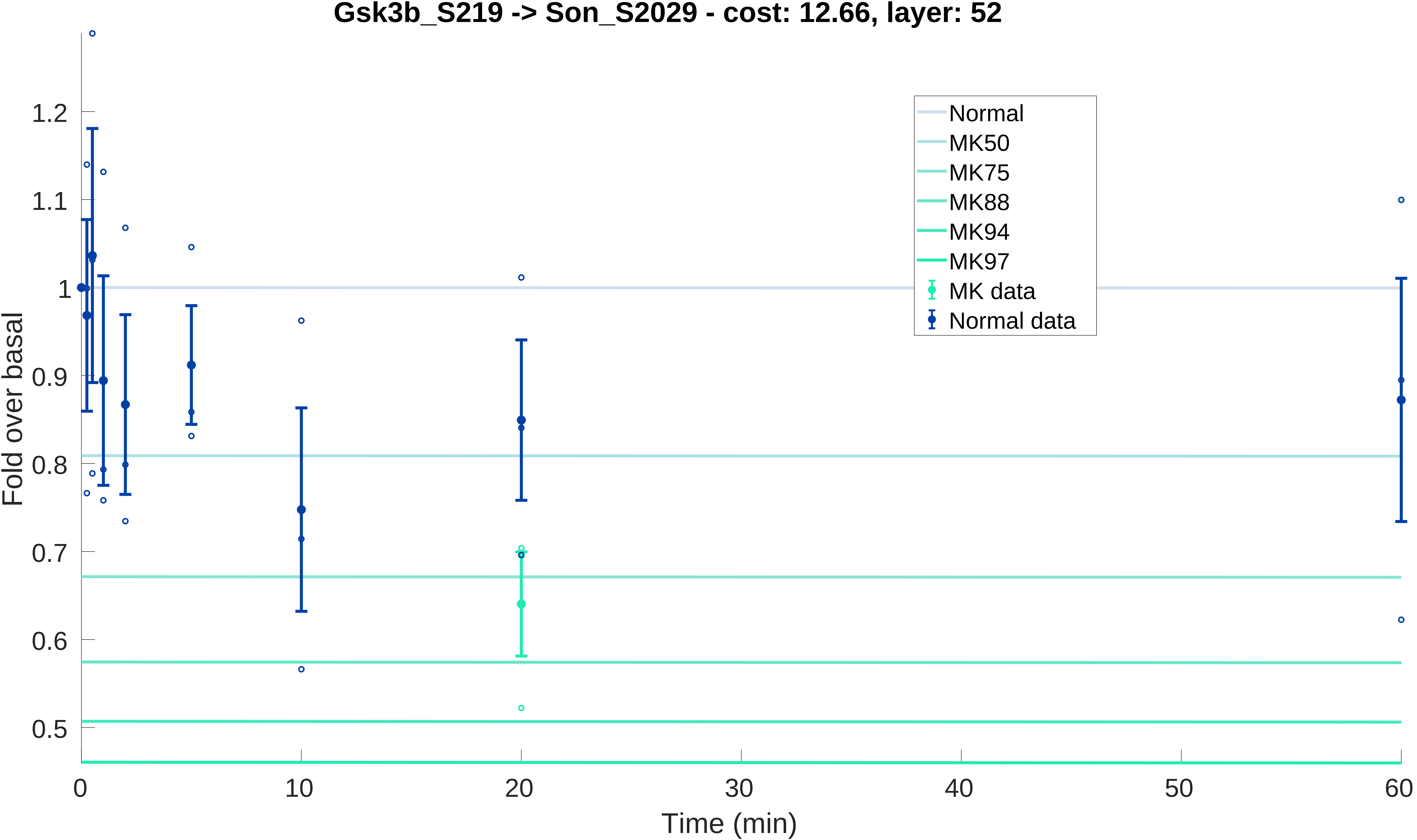
Gsk3b_S219 -> Son_S2029 - cost: 12.66, layer: 52
Fold over basal
Time (min)
0.5
0.6
0.7
0.8
0.9
1
1.1
1.2
0
10
20
30
40
50
60
Normal
MK50
MK75
MK88
MK94
MK97
MK data
Normal data

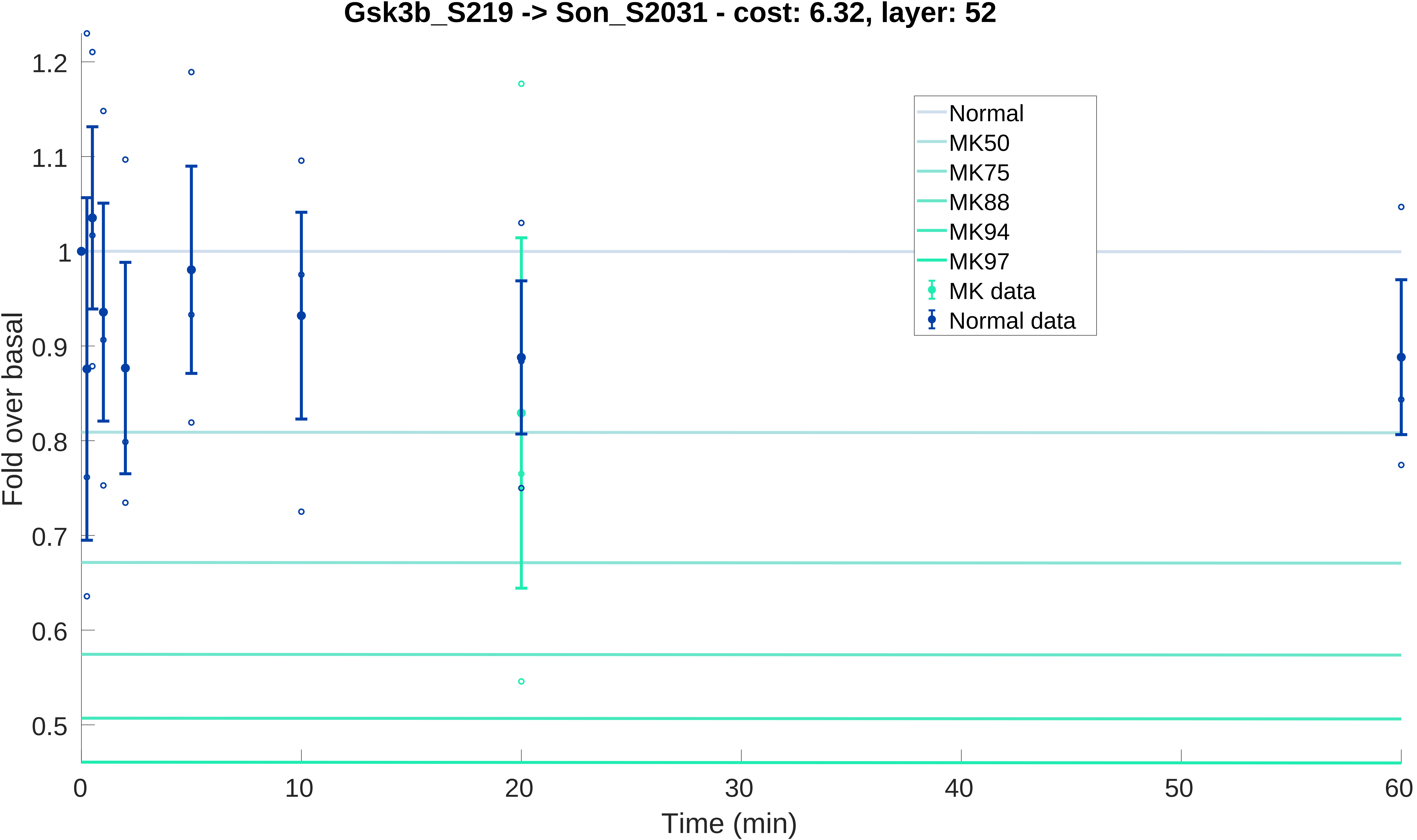
Gsk3b_S219 -> Son_S2031 - cost: 6.32, layer: 52
Fold over basal
Time (min)
Normal
MK50
MK75
MK88
MK94
MK97
MK data
Normal data
1.2
1.1
1
0.9
0.8
0.7
0.6
0.5
0
10
20
30
40
50
60

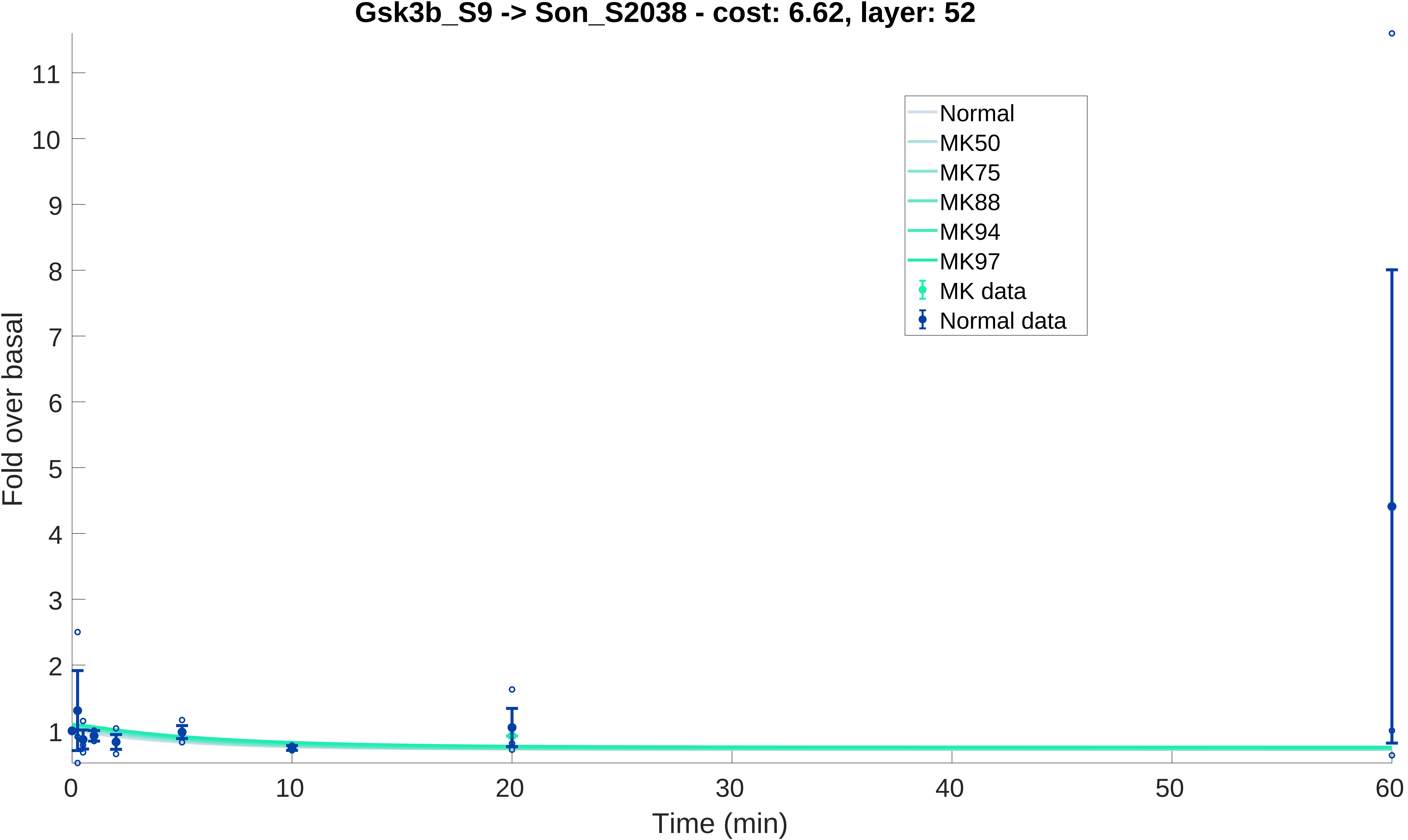

Gsk3b_S9 -> Son_S2038 - cost: 6.62, layer: 52
Fold over basal
Time (min)
Normal
MK50
MK75
MK88
MK94
MK97
MK data
Normal data
0
10
20
30
40
50
60
1
2
3
4
5
6
7
8
9
10
11

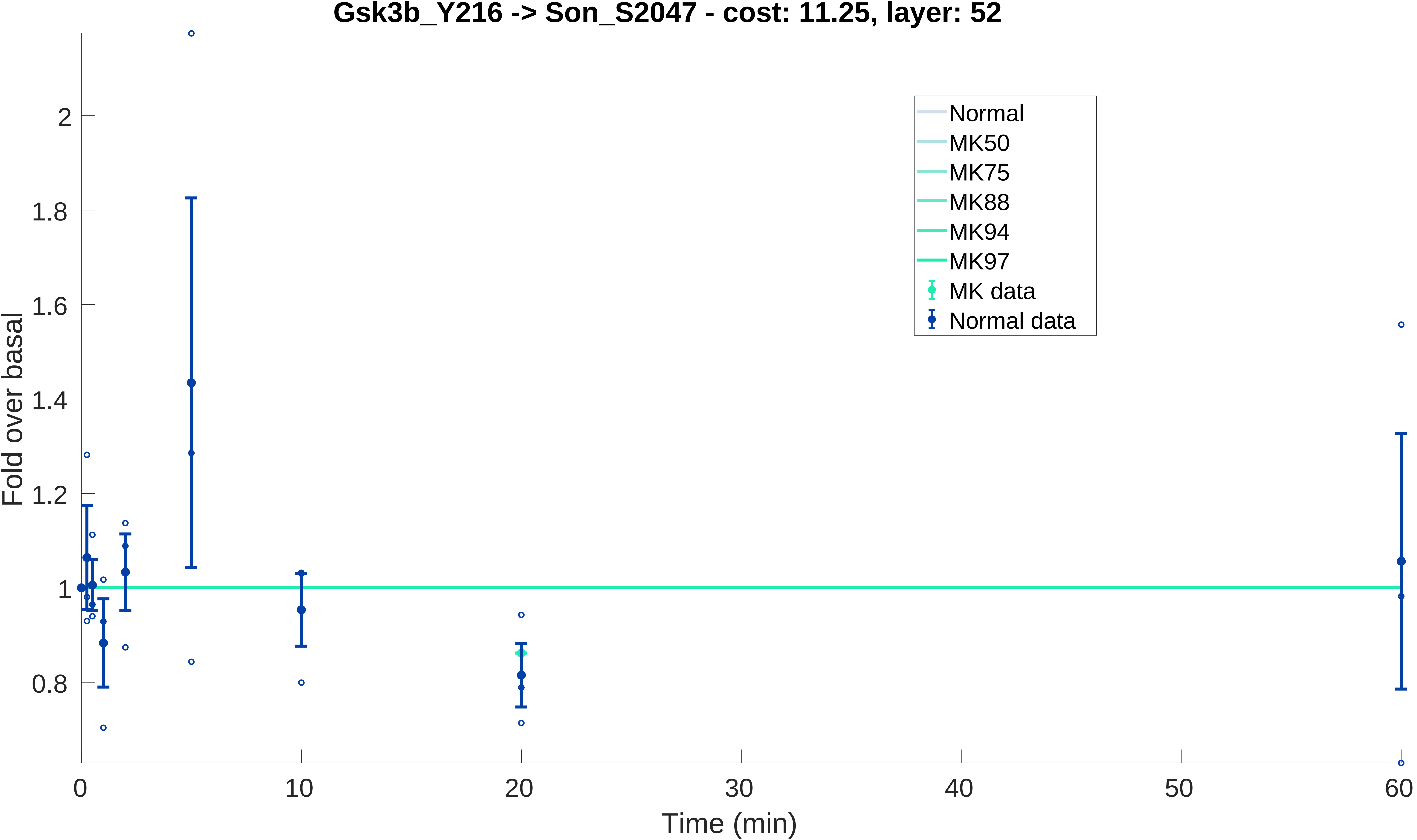

Gsk3b_Y216 -> Son_S2047 - cost: 11.25, layer: 52
Normal
MK50
MK75
MK88
MK94
MK97
MK data
Normal data
Fold over basal
Time (min)
0.8
1
1.2
1.4
1.6
1.8
2
0
10
20
30
40
50
60

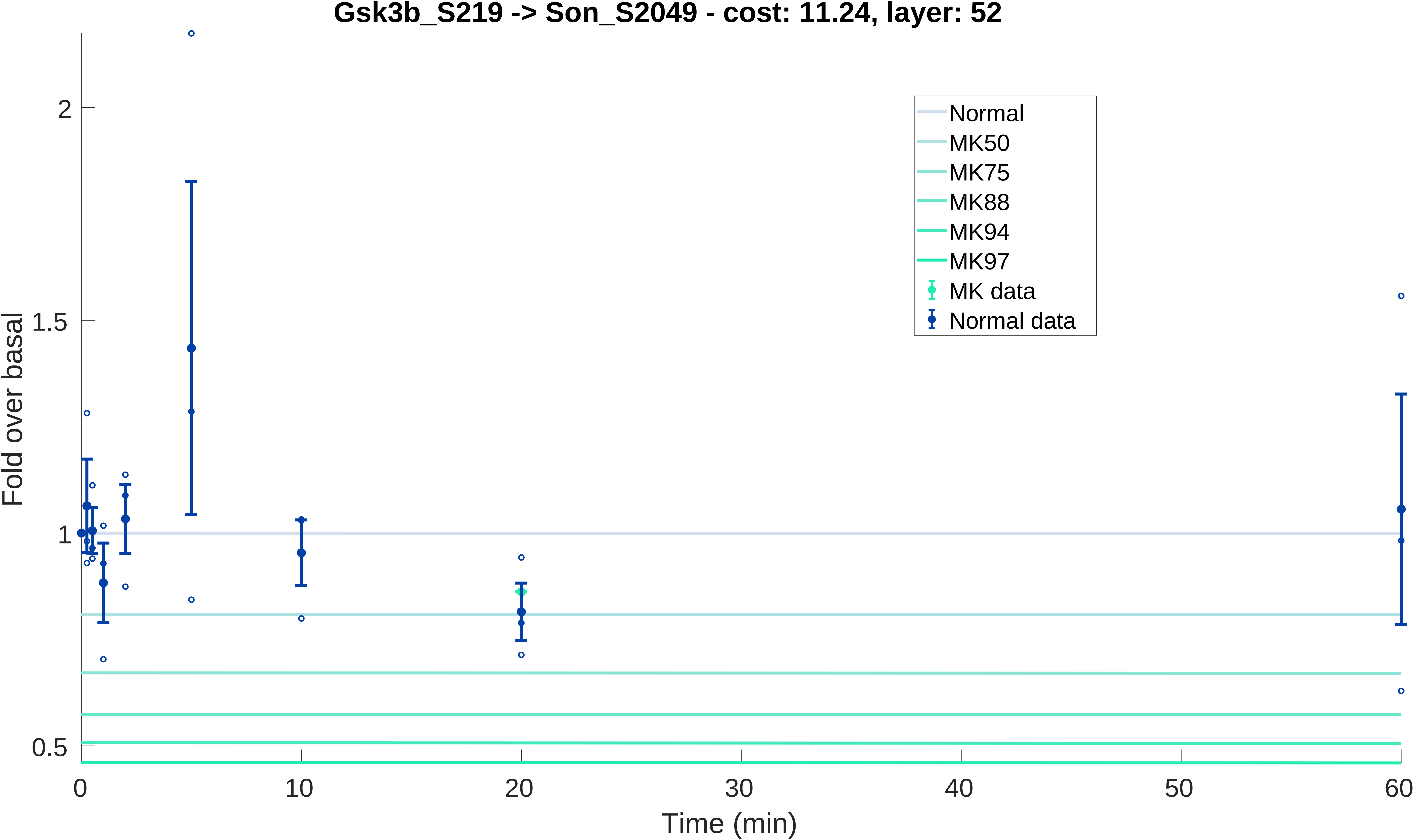

Gsk3b_S219 -> Son_S2049 - cost: 11.24, layer: 52
Fold over basal
Time (min)
0.5
1
1.5
2
0
10
20
30
40
50
60
Normal
MK50
MK75
MK88
MK94
MK97
MK data
Normal data

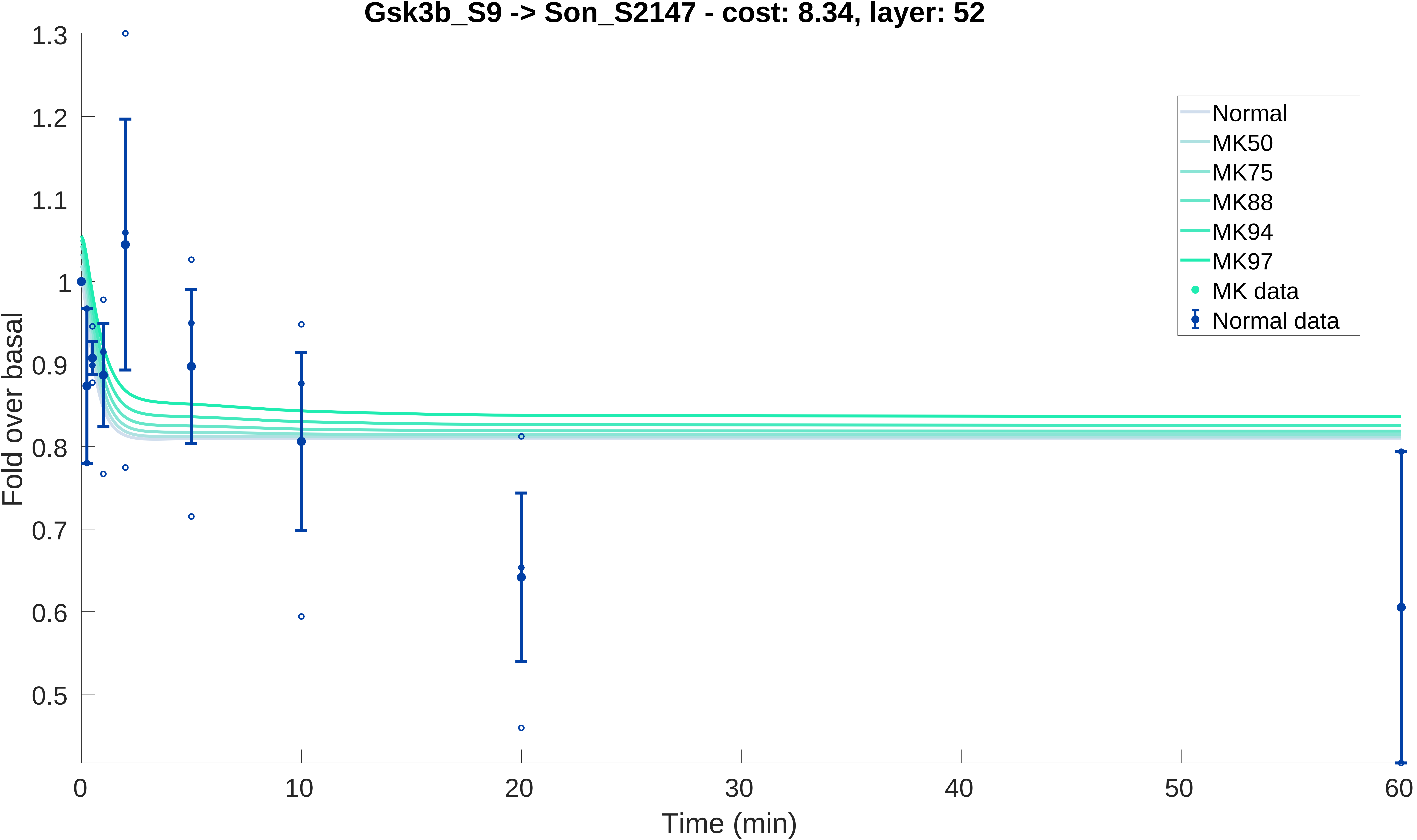
Gsk3b_S9 -> Son_S2147 - cost: 8.34, layer: 52
Fold over basal
Time (min)
Normal
MK50
MK75
MK88
MK94
MK97
MK data
Normal data
1.3
1.2
1.1
1
0.9
0.8
0.7
0.6
0.5
0
10
20
30
40
50
60

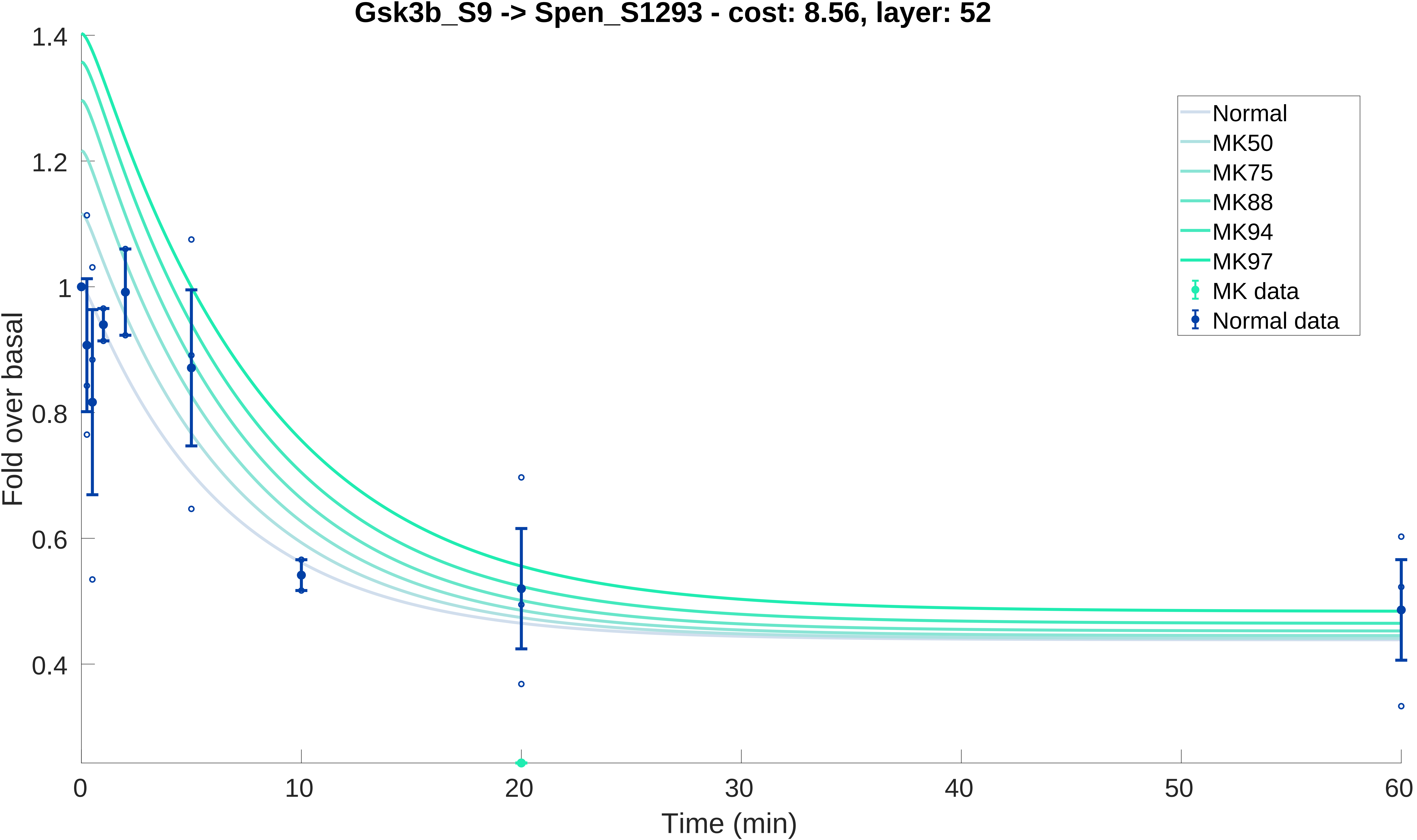

Gsk3b_S9 -> Spen_S1293 - cost: 8.56, layer: 52
Fold over basal
Time (min)
1.4
1.2
1
0.8
0.6
0.4
0
10
20
30
40
50
60
Normal
MK50
MK75
MK88
MK94
MK97
MK data
Normal data

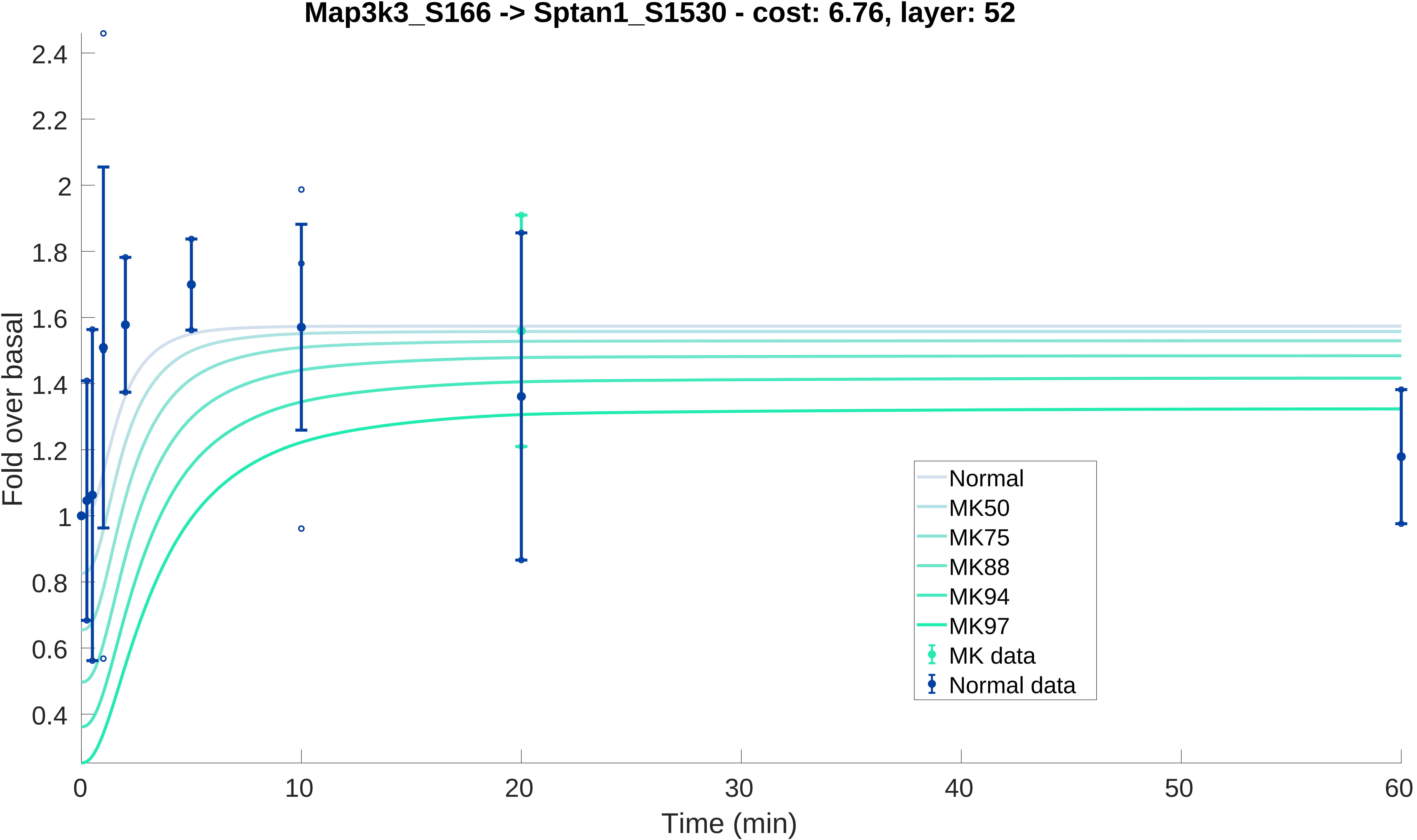

Map3k3_S166 -> Sptan1_S1530 - cost: 6.76, layer: 52
Fold over basal
2.4
2.2
2
1.8
1.6
1.4
1.2
1
0.8
0.6
0.4
0
10
20
30
40
50
60
Time (min)
Normal
MK50
MK75
MK88
MK94
MK97
MK data
Normal data

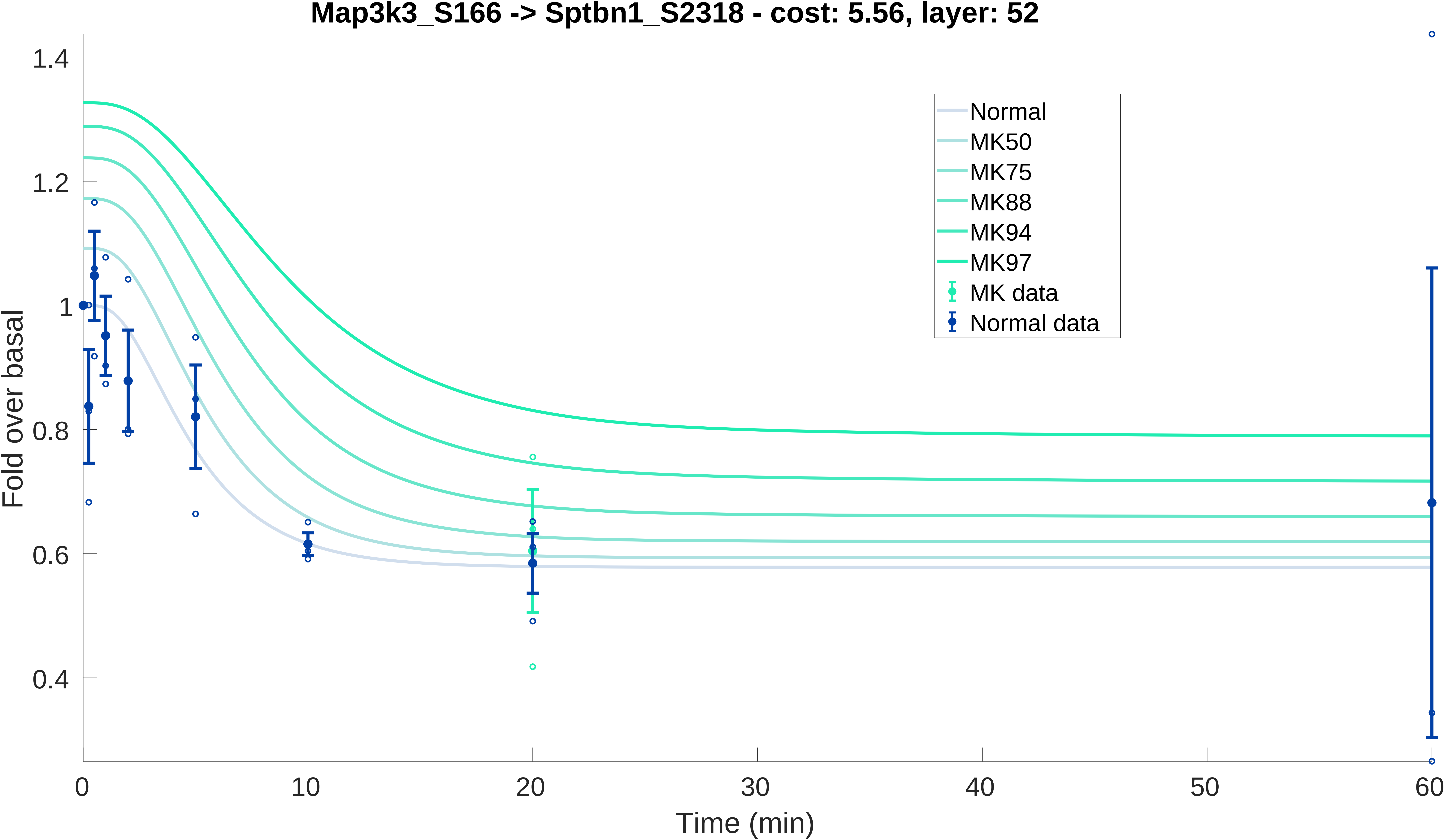
Map3k3_S166 -> Sptbn1_S2318 - cost: 5.56, layer: 52
1.4
1.2
1
0.8
0.6
0.4
Fold over basal
0
10
20
30
40
50
60
Time (min)
Normal
MK50
MK75
MK88
MK94
MK97
MK data
Normal data

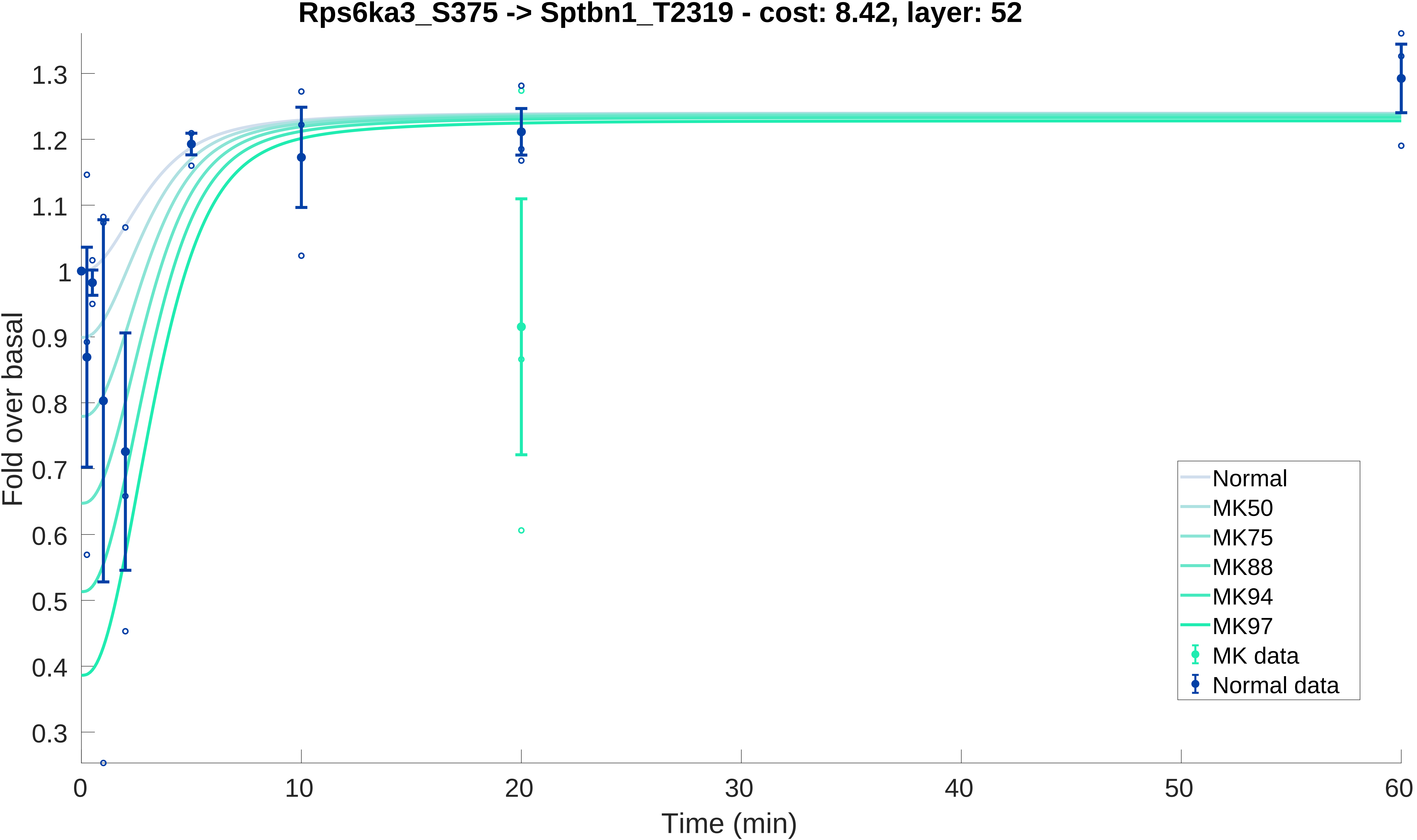

Rps6ka3_S375 -> Sptbn1_T2319 - cost: 8.42, layer: 52
Fold over basal
Time (min)
0
10
20
30
40
50
60
0.3
0.4
0.5
0.6
0.7
0.8
0.9
1
1.1
1.2
1.3
Normal
MK50
MK75
MK88
MK94
MK97
MK data
Normal data

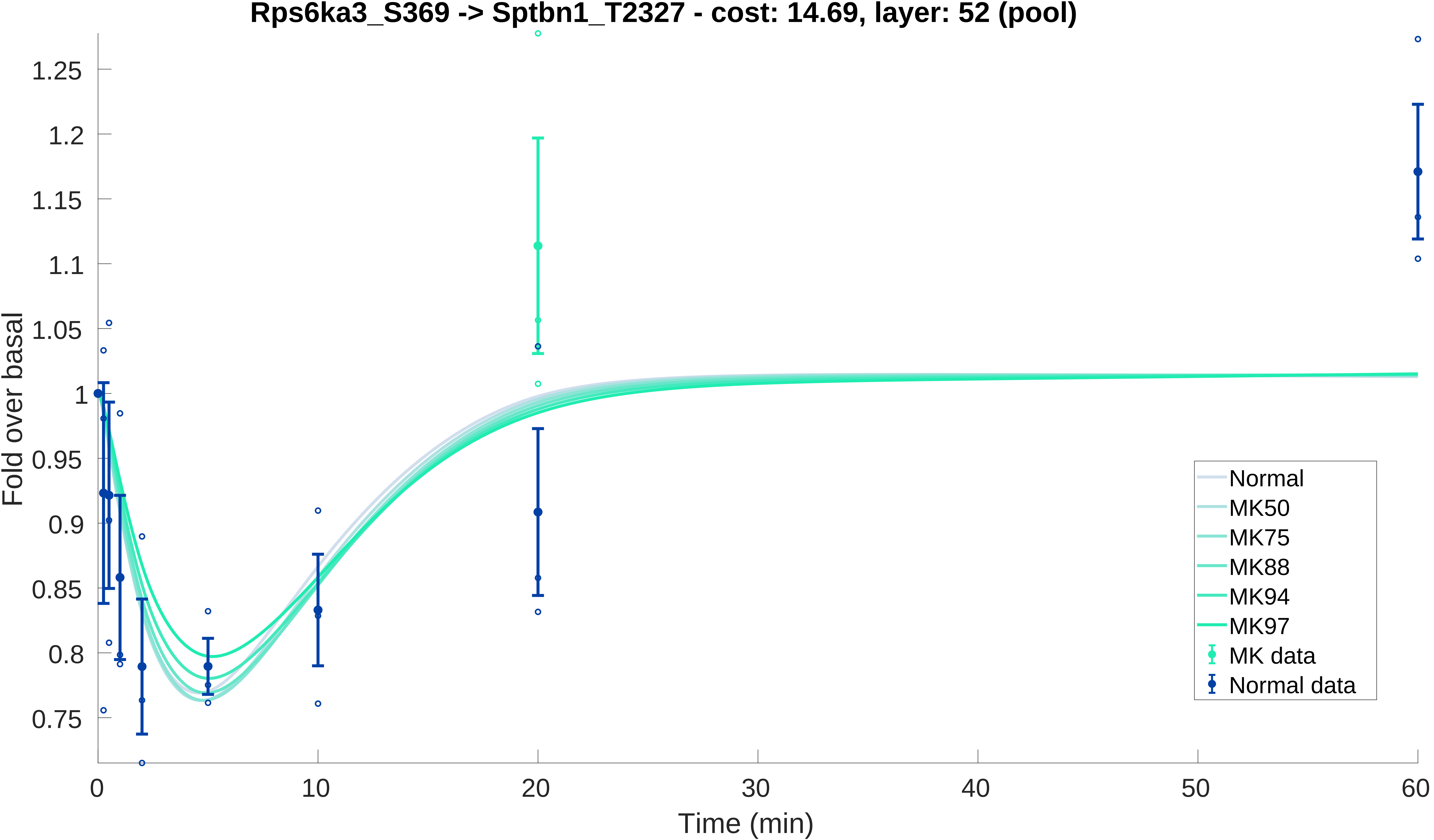

Rps6ka3_S369 -> Sptbn1_T2327 - cost: 14.69, layer: 52 (pool)
Fold over basal
Time (min)
1.25
1.2
1.15
1.1
1.05
1
0.95
0.9
0.85
0.8
0.75
0
10
20
30
40
50
60
Normal
MK50
MK75
MK88
MK94
MK97
MK data
Normal data

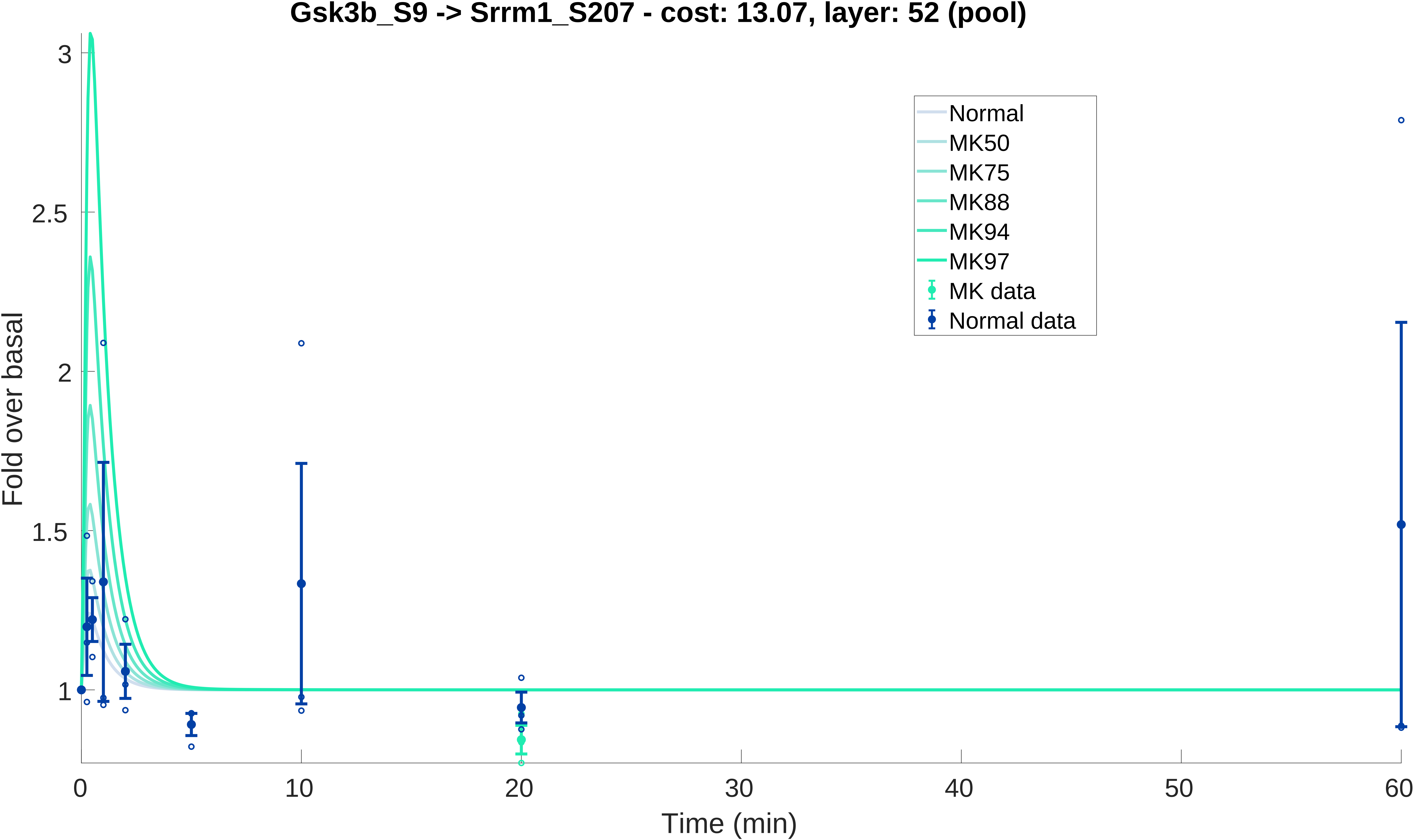
Gsk3b_S9 -> Srrm1_S207 - cost: 13.07, layer: 52 (pool)
Fold over basal
Time (min)
Normal
MK50
MK75
MK88
MK94
MK97
MK data
Normal data
0
10
20
30
40
50
60
1
1.5
2
2.5
3

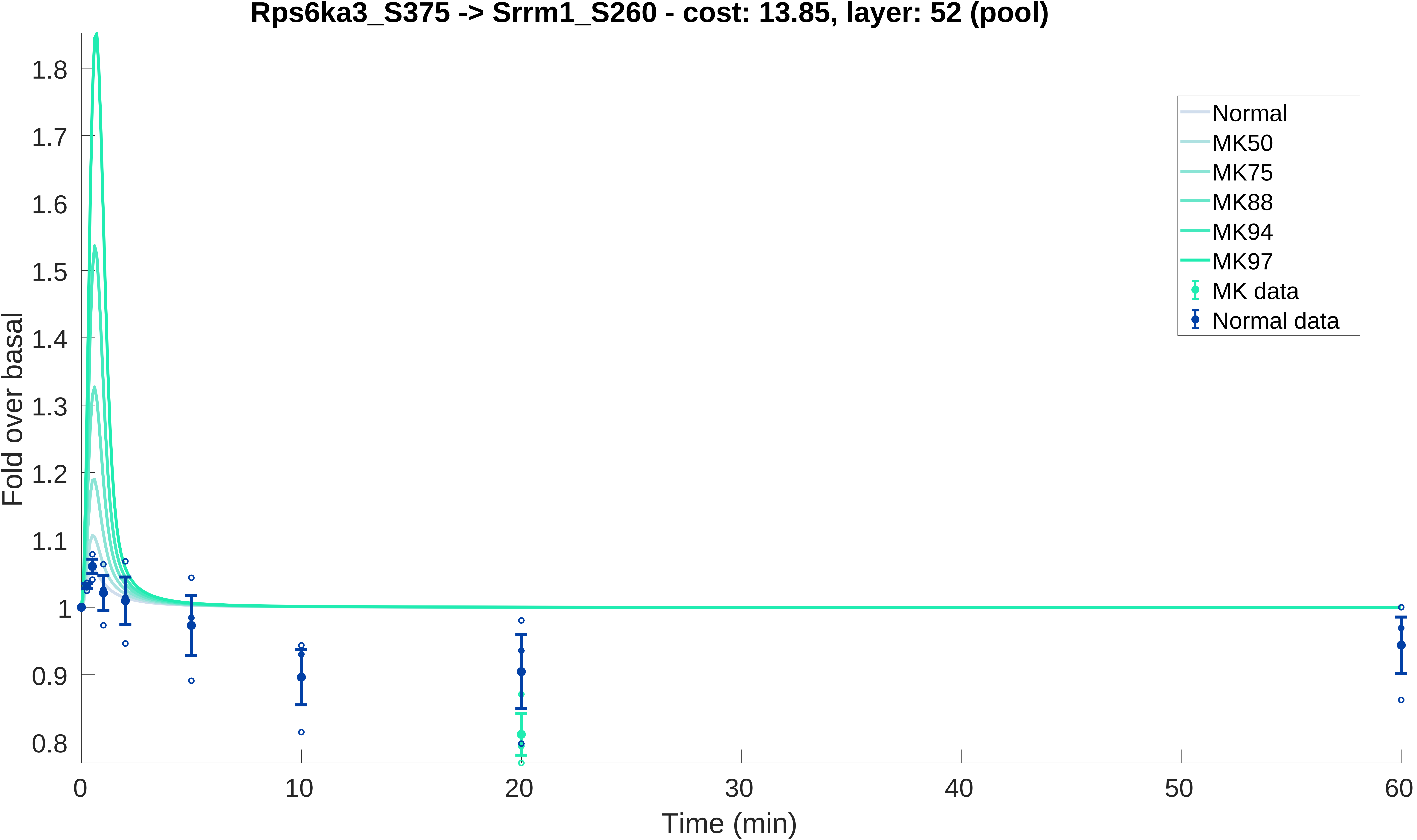
Rps6ka3_S375 -> Srrm1_S260 - cost: 13.85, layer: 52 (pool)
Fold over basal
Time (min)
1.8
1.7
1.6
1.5
1.4
1.3
1.2
1.1
1
0.9
0.8
0
10
20
30
40
50
60
Normal
MK50
MK75
MK88
MK94
MK97
MK data
Normal data

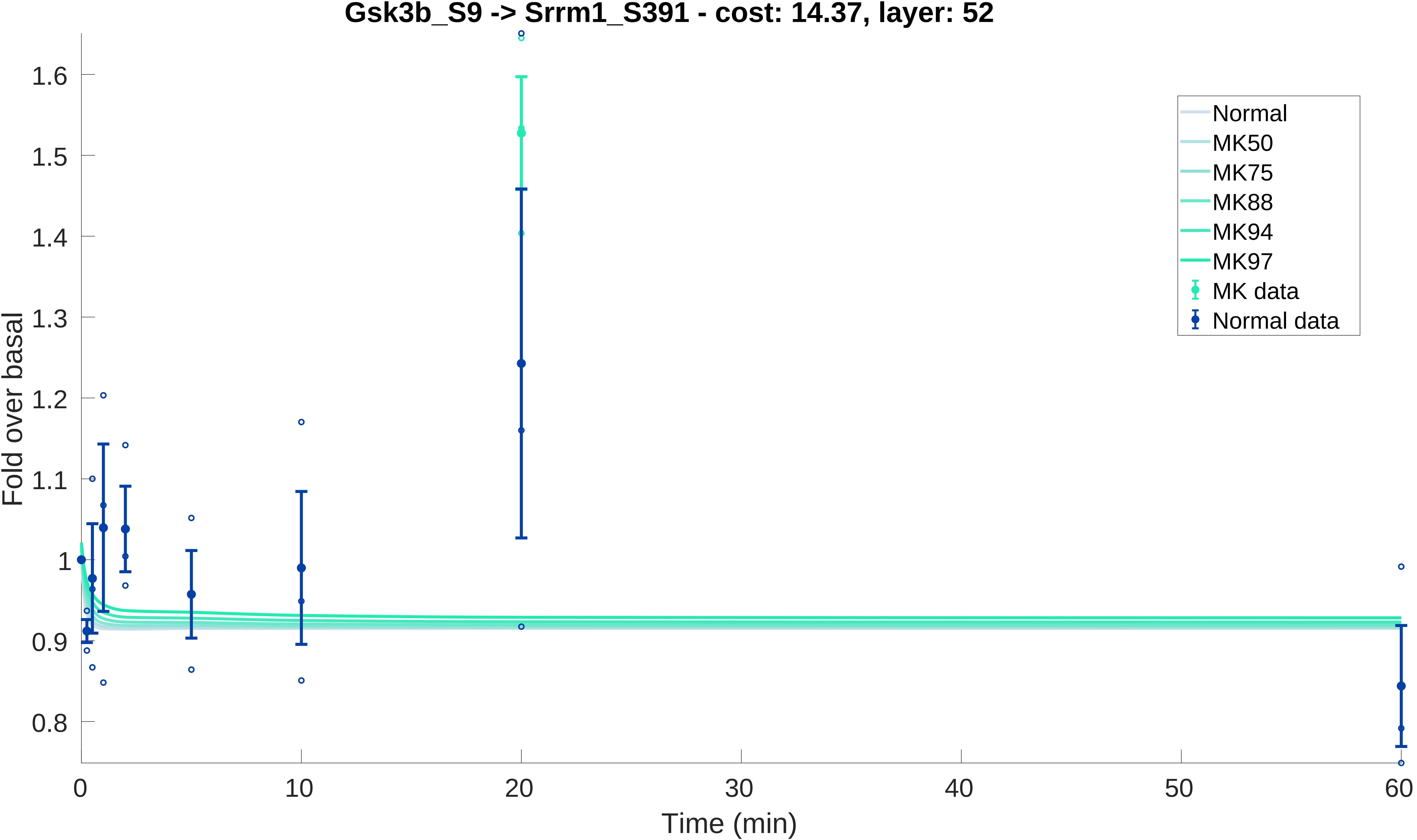

Gsk3b_S9 -> Srrm1_S391 - cost: 14.37, layer: 52
Fold over basal
Time (min)
Normal
MK50
MK75
MK88
MK94
MK97
MK data
Normal data
1.6
1.5
1.4
1.3
1.2
1.1
1
0.9
0.8
0
10
20
30
40
50
60

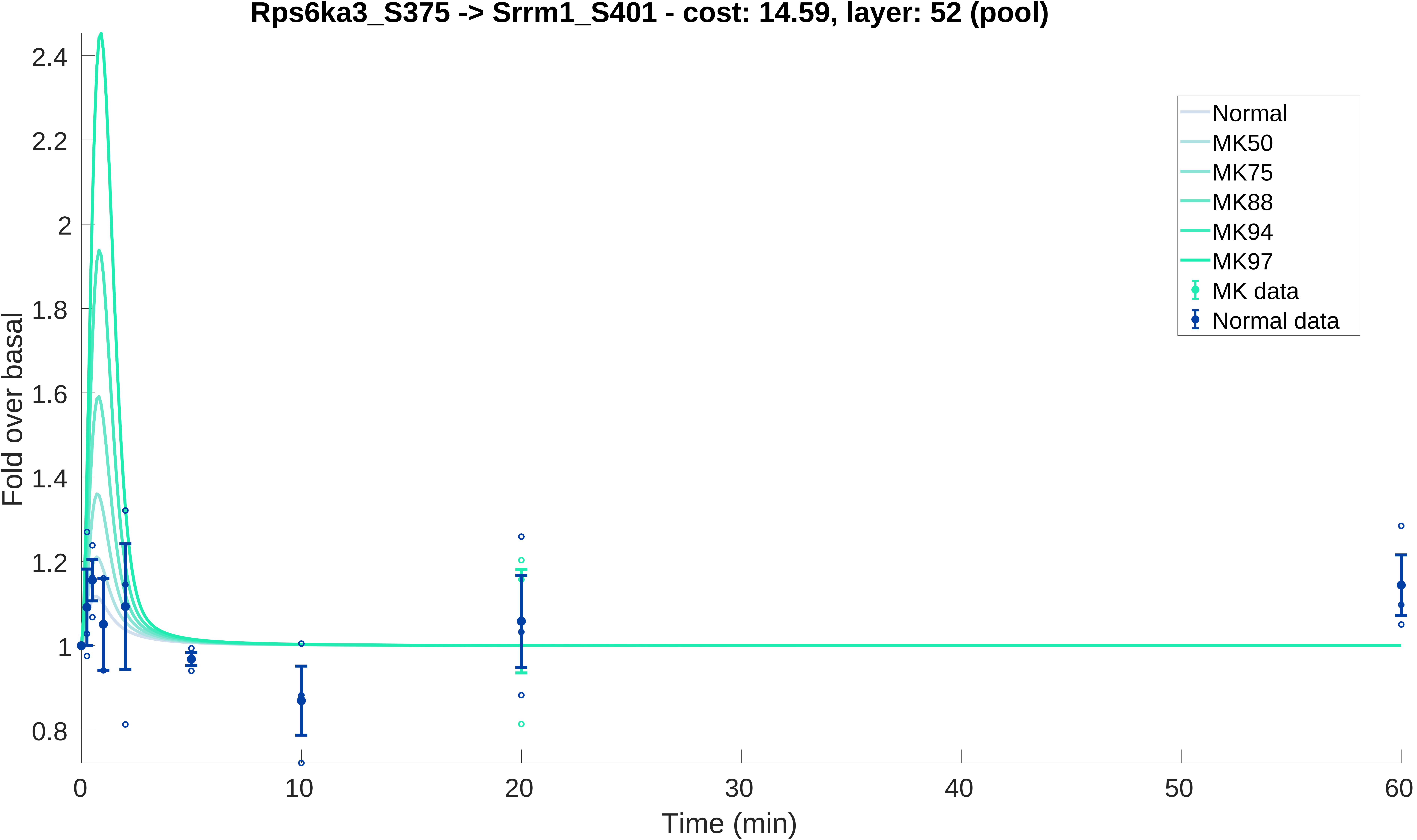

Rps6ka3_S375 -> Srrm1_S401 - cost: 14.59, layer: 52 (pool)
Fold over basal
Time (min)
0.8
1
1.2
1.4
1.6
1.8
2
2.2
2.4
0
10
20
30
40
50
60
Normal
MK50
MK75
MK88
MK94
MK97
MK data
Normal data

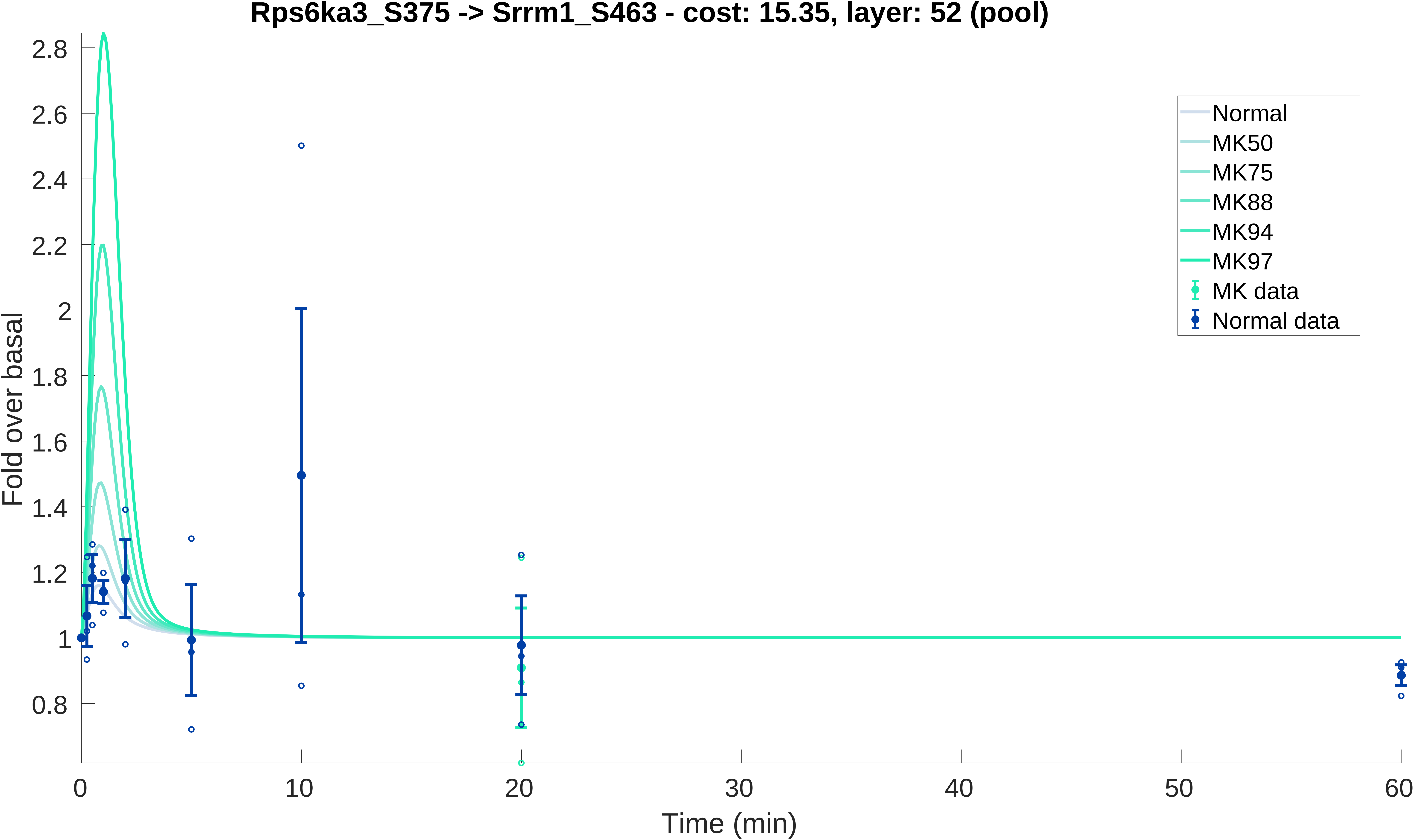

Rps6ka3_S375 -> Srrm1_S463 - cost: 15.35, layer: 52 (pool)
Fold over basal
Time (min)
Normal
MK50
MK75
MK88
MK94
MK97
MK data
Normal data
2.8
2.6
2.4
2.2
2
1.8
1.6
1.4
1.2
1
0.8
0
10
20
30
40
50
60

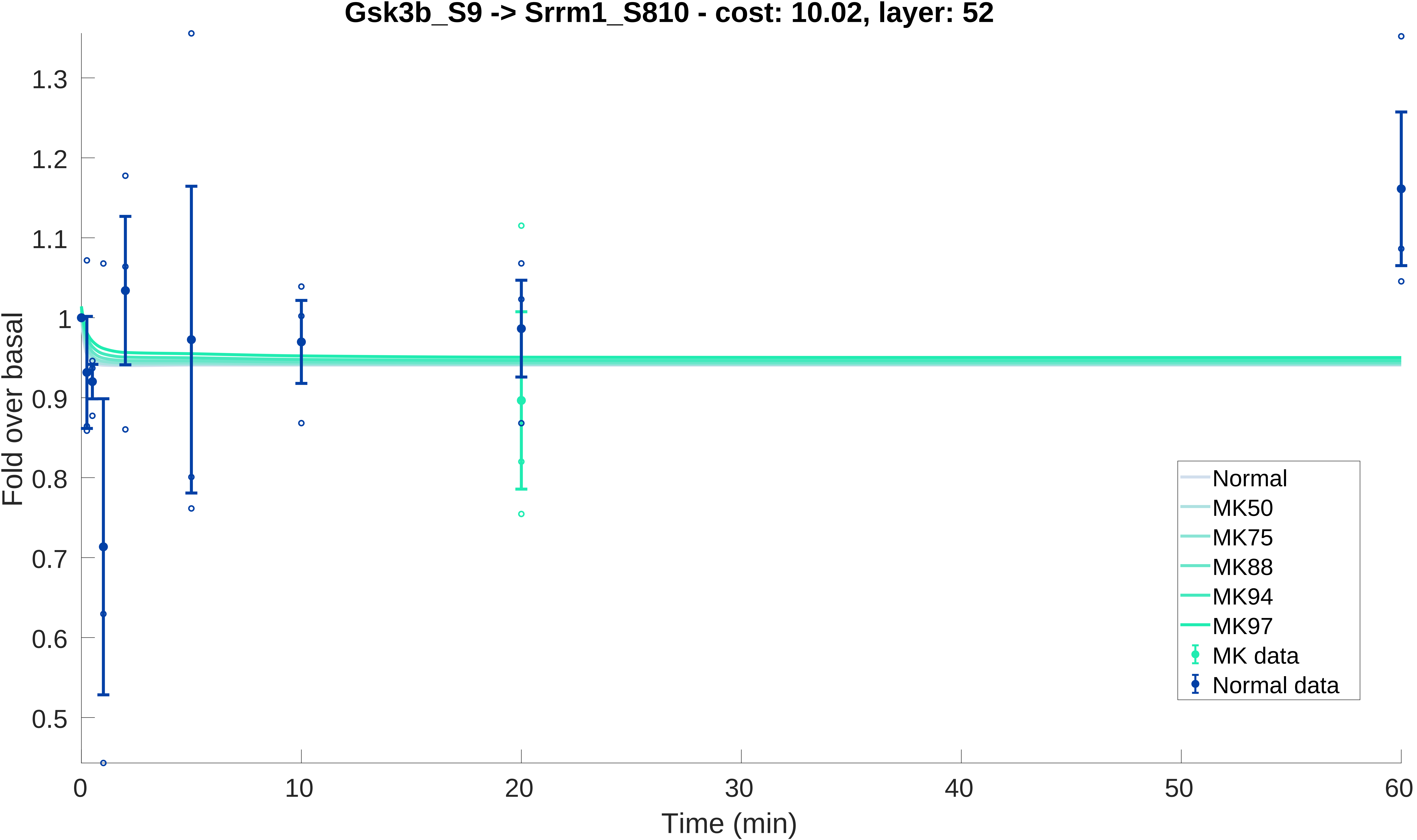

Gsk3b_S9 -> Srrm1_S810 - cost: 10.02, layer: 52
Fold over basal
1.3
1.2
1.1
1
0.9
0.8
0.7
0.6
0.5
0
10
20
30
40
50
60
Time (min)
Normal
MK50
MK75
MK88
MK94
MK97
MK data
Normal data

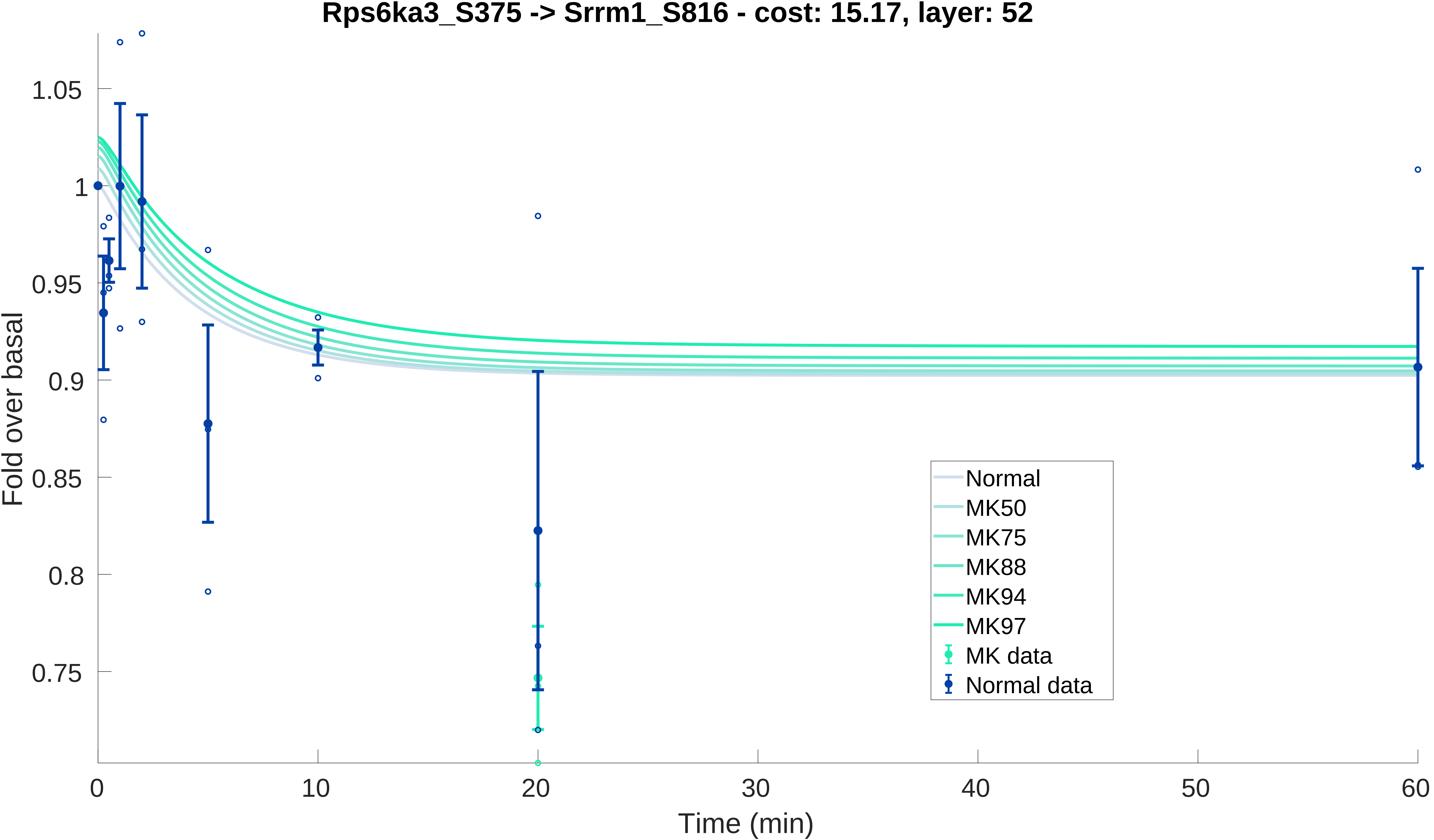

Rps6ka3_S375 -> Srrm1_S816 - cost: 15.17, layer: 52
Fold over basal
1.05
1
0.95
0.9
0.85
0.8
0.75
0
10
20
30
40
50
60
Time (min)
Normal
MK50
MK75
MK88
MK94
MK97
MK data
Normal data

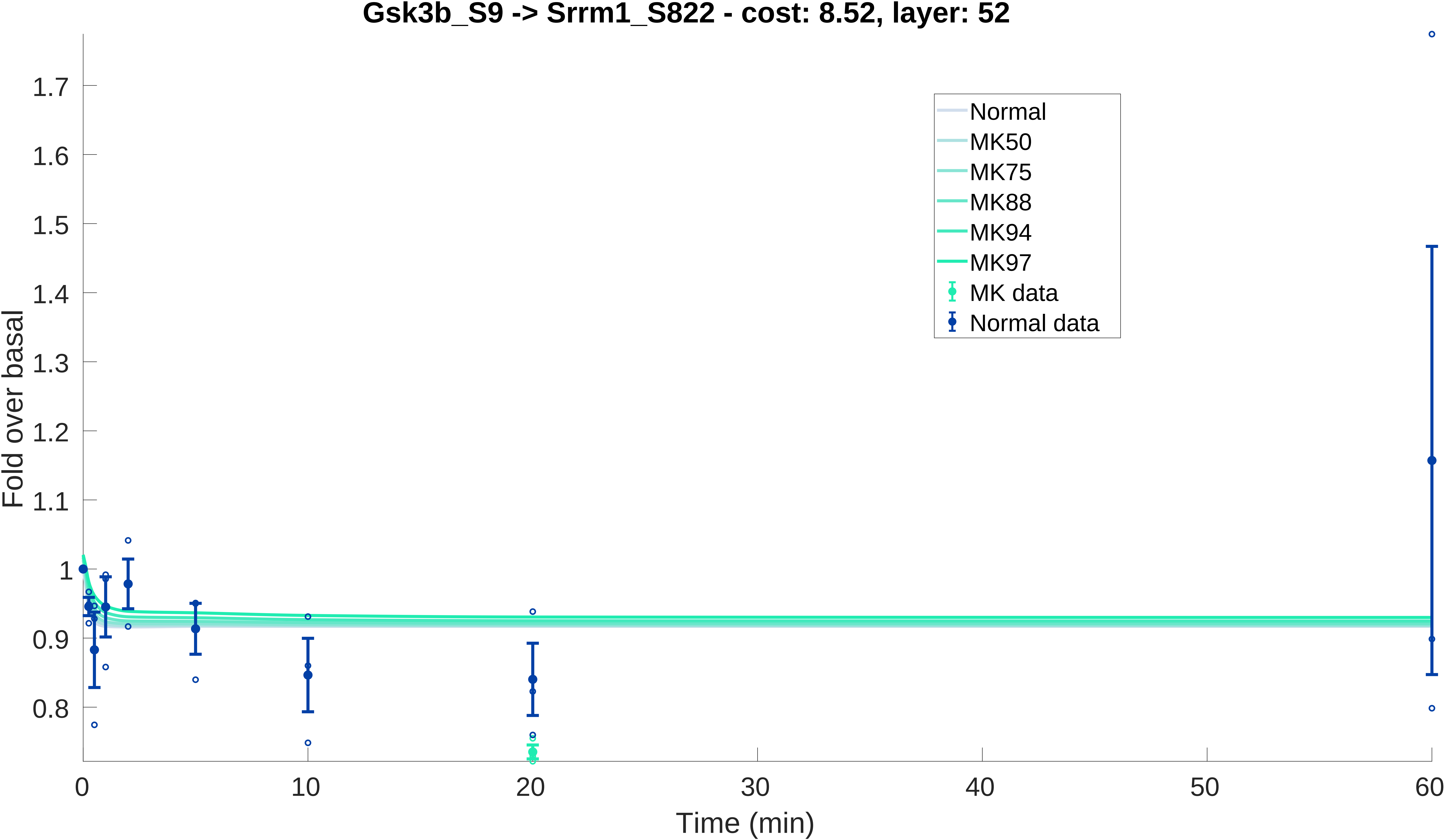

Gsk3b_S9 -> Srrm1_S822 - cost: 8.52, layer: 52
Fold over basal
Time (min)
Normal
MK50
MK75
MK88
MK94
MK97
MK data
Normal data
0.8
0.9
1
1.1
1.2
1.3
1.4
1.5
1.6
1.7
0
10
20
30
40
50
60

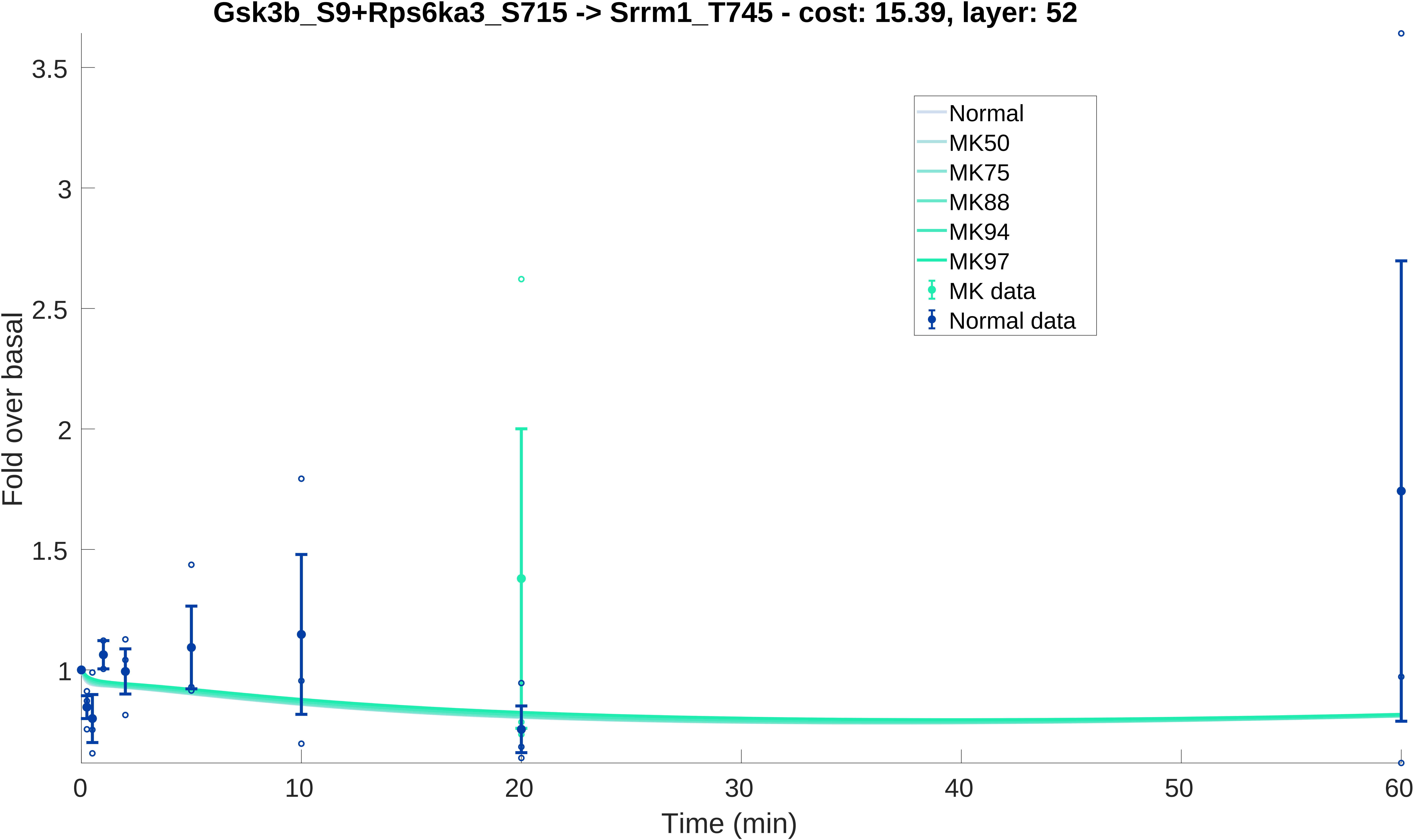
Gsk3b_S9+Rps6ka3_S715 -> Srrm1_T745 - cost: 15.39, layer: 52
Fold over basal
3.5
3
2.5
2
1.5
1
0
10
20
30
40
50
60
Time (min)
Normal
MK50
MK75
MK88
MK94
MK97
MK data
Normal data

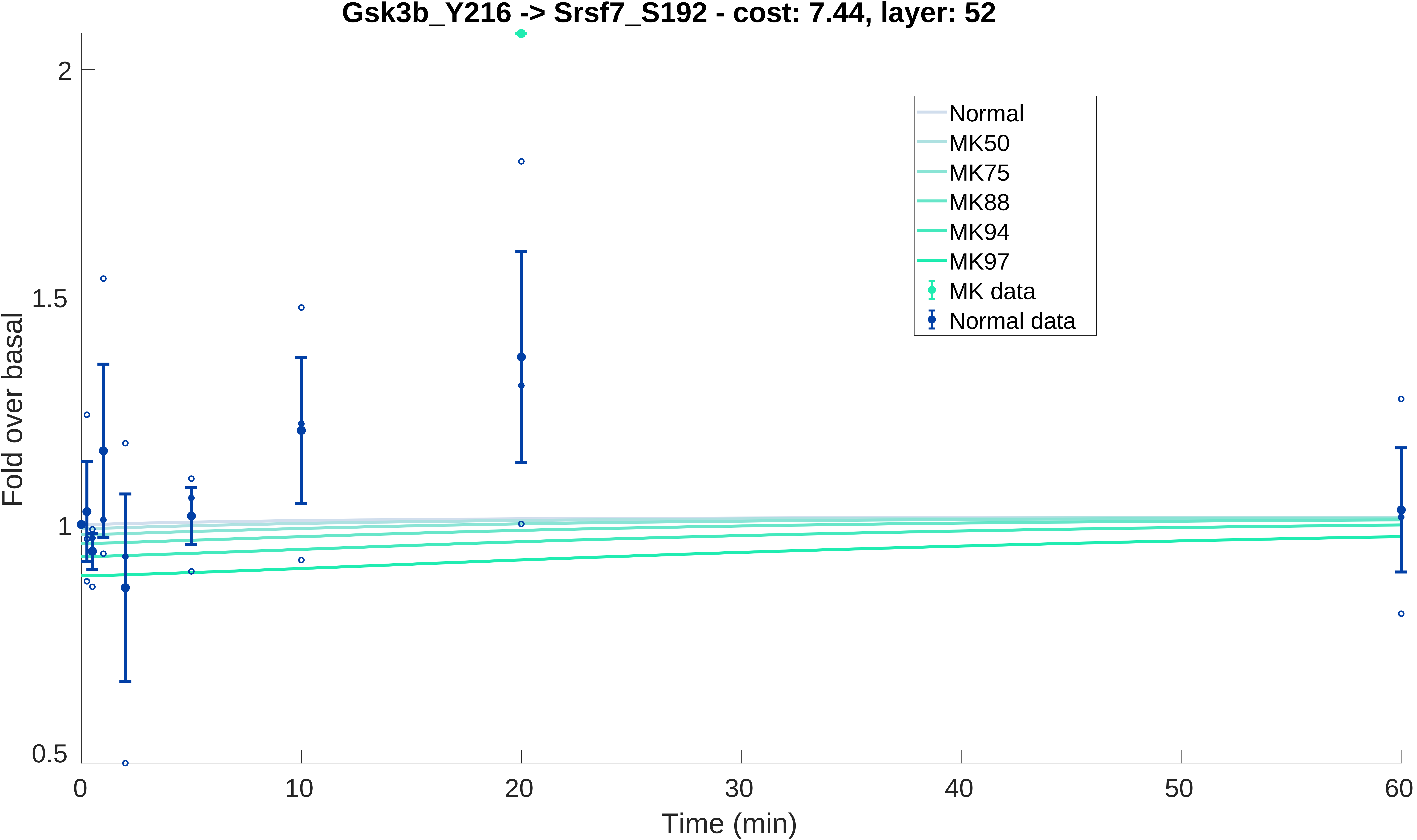
Gsk3b_Y216 -> Srsf7_S192 - cost: 7.44, layer: 52
Fold over basal
Time (min)
Normal
MK50
MK75
MK88
MK94
MK97
MK data
Normal data
2
1.5
1
0.5
0
10
20
30
40
50
60

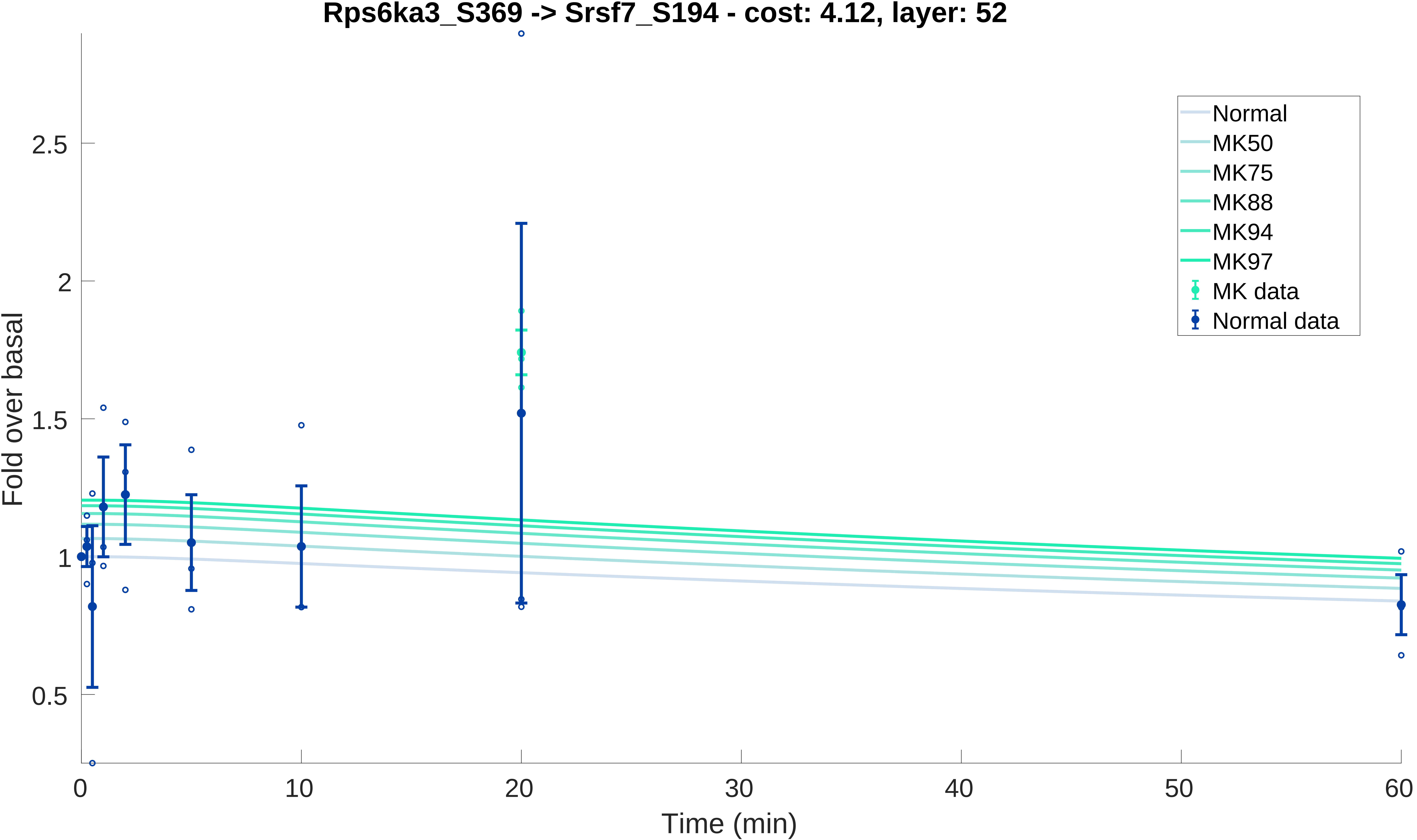

Rps6ka3_S369 -> Srsf7_S194 - cost: 4.12, layer: 52
Fold over basal
2.5
2
1.5
1
0.5
0
10
20
30
40
50
60
Time (min)
Normal
MK50
MK75
MK88
MK94
MK97
MK data
Normal data

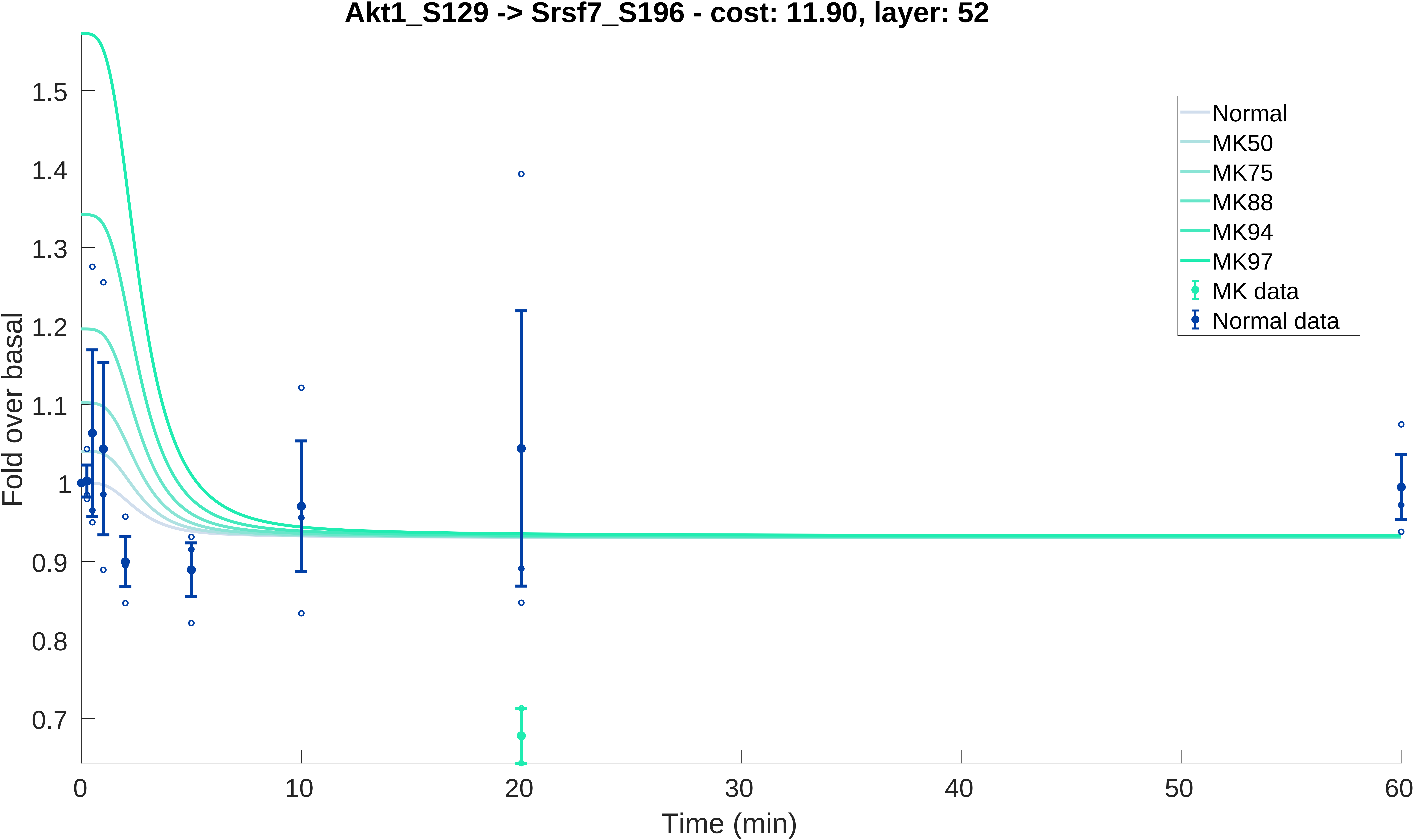

Akt1_S129 -> Srsf7_S196 - cost: 11.90, layer: 52
Fold over basal
Time (min)
0
10
20
30
40
50
60
0.7
0.8
0.9
1
1.1
1.2
1.3
1.4
1.5
Normal
MK50
MK75
MK88
MK94
MK97
MK data
Normal data

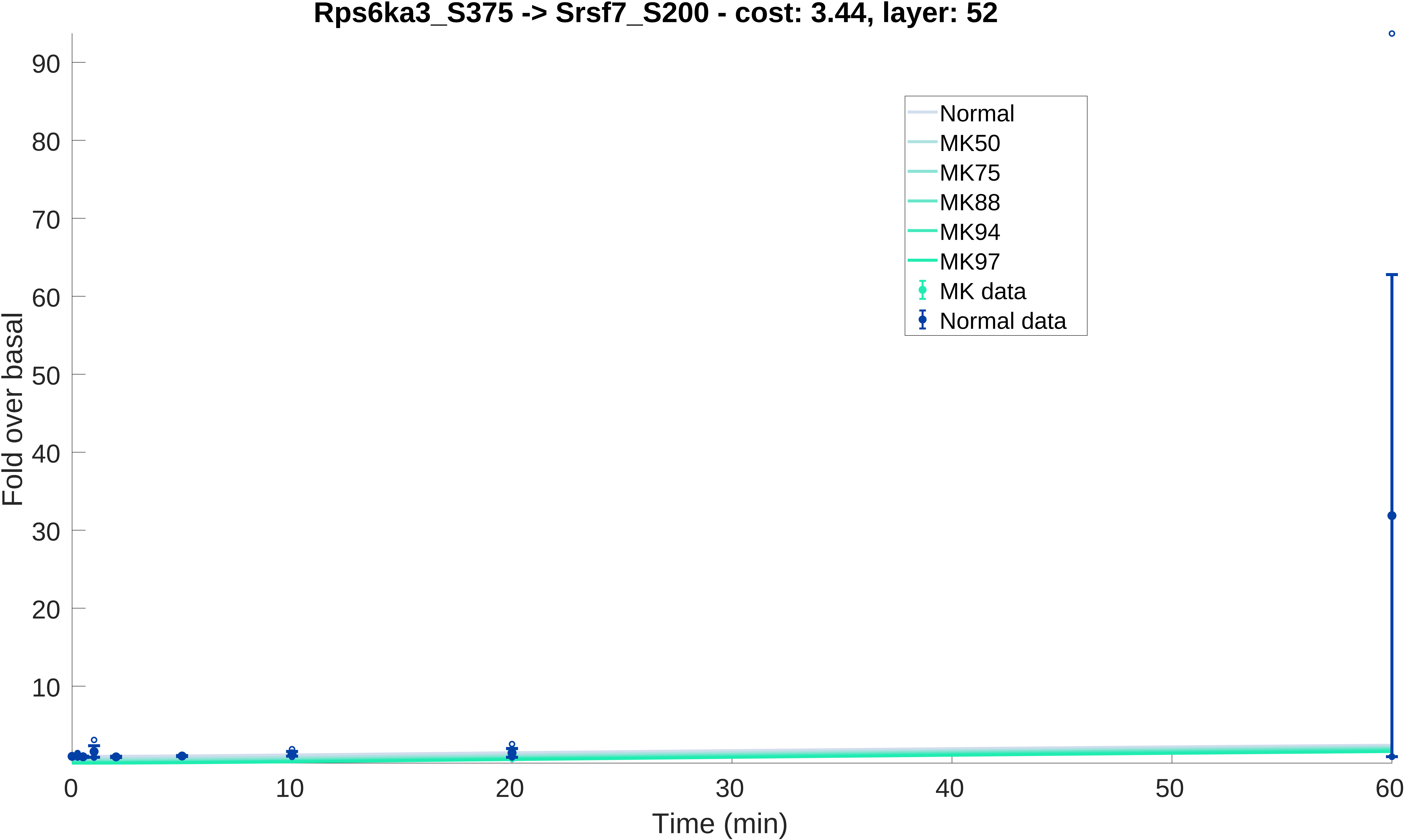

Rps6ka3_S375 -> Srsf7_S200 - cost: 3.44, layer: 52
Fold over basal
Time (min)
Normal
MK50
MK75
MK88
MK94
MK97
MK data
Normal data
0
10
20
30
40
50
60
70
80
90

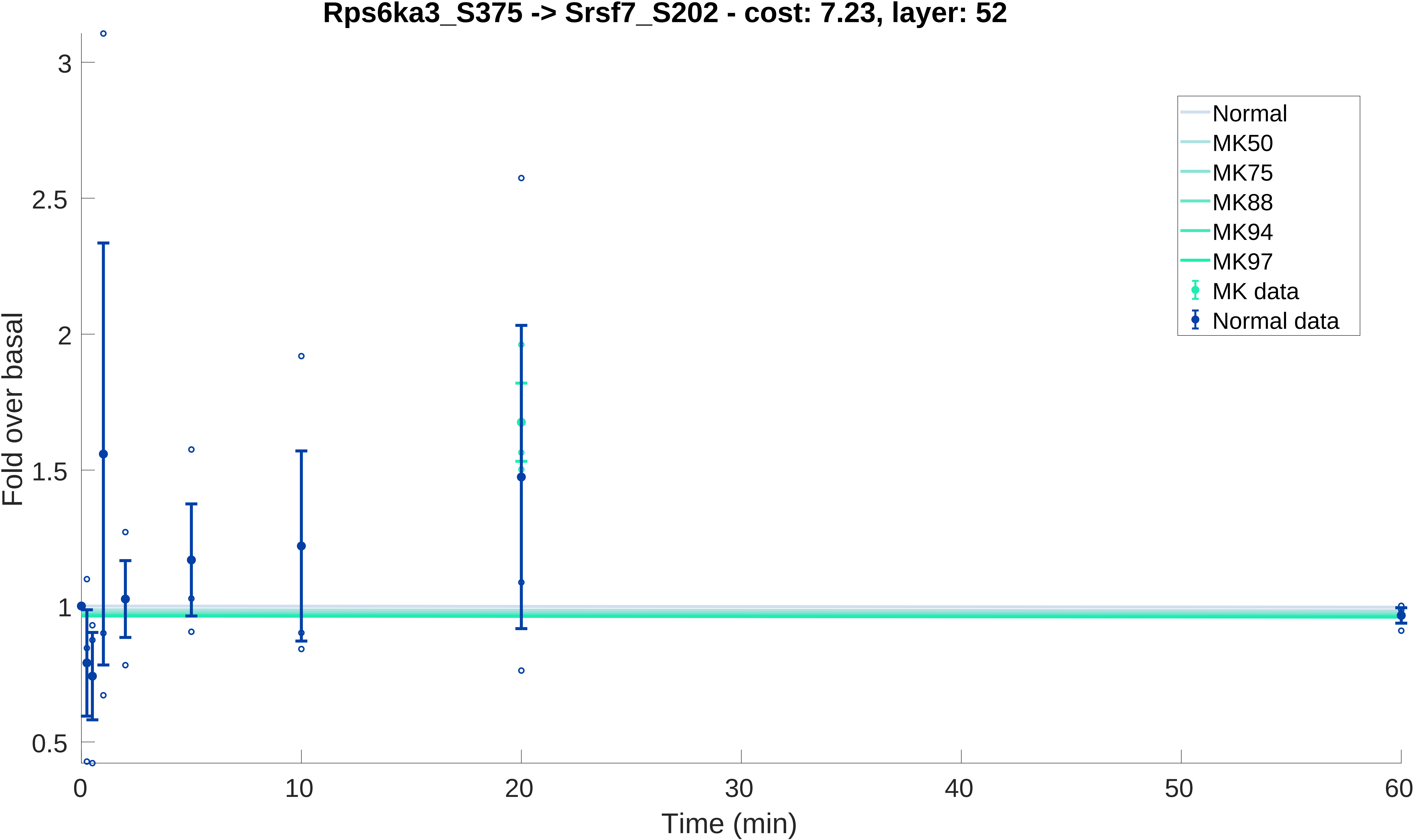

Rps6ka3_S375 -> Srsf7_S202 - cost: 7.23, layer: 52
Fold over basal
Time (min)
0.5
1
1.5
2
2.5
3
0
10
20
30
40
50
60
Normal
MK50
MK75
MK88
MK94
MK97
MK data
Normal data

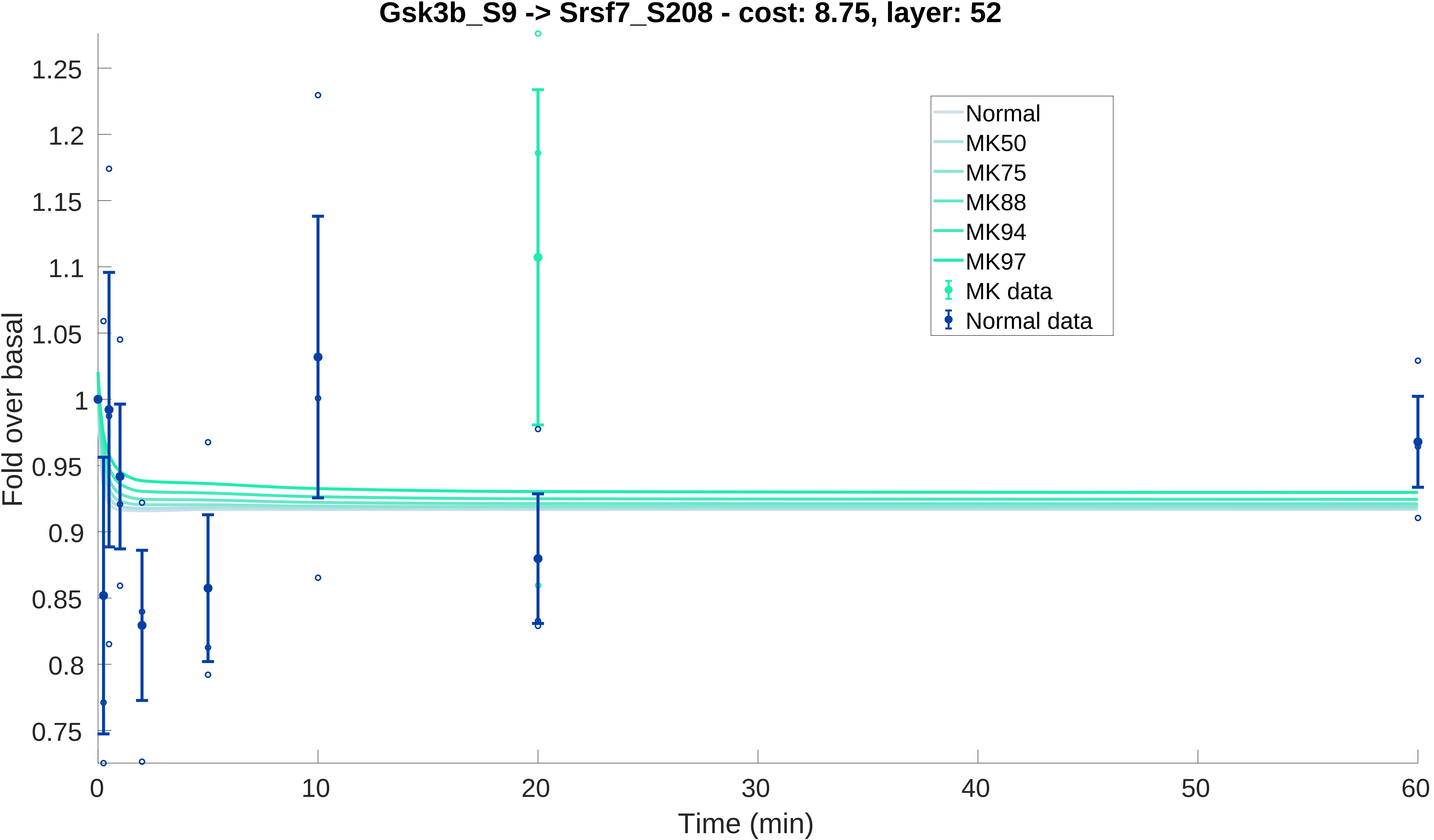

Gsk3b_S9 -> Srsf7_S208 - cost: 8.75, layer: 52
1.25
1.2
1.15
1.1
1.05
1
0.95
0.9
0.85
0.8
0.75
Fold over basal
0
10
20
30
40
50
60
Time (min)
Normal
MK50
MK75
MK88
MK94
MK97
MK data
Normal data

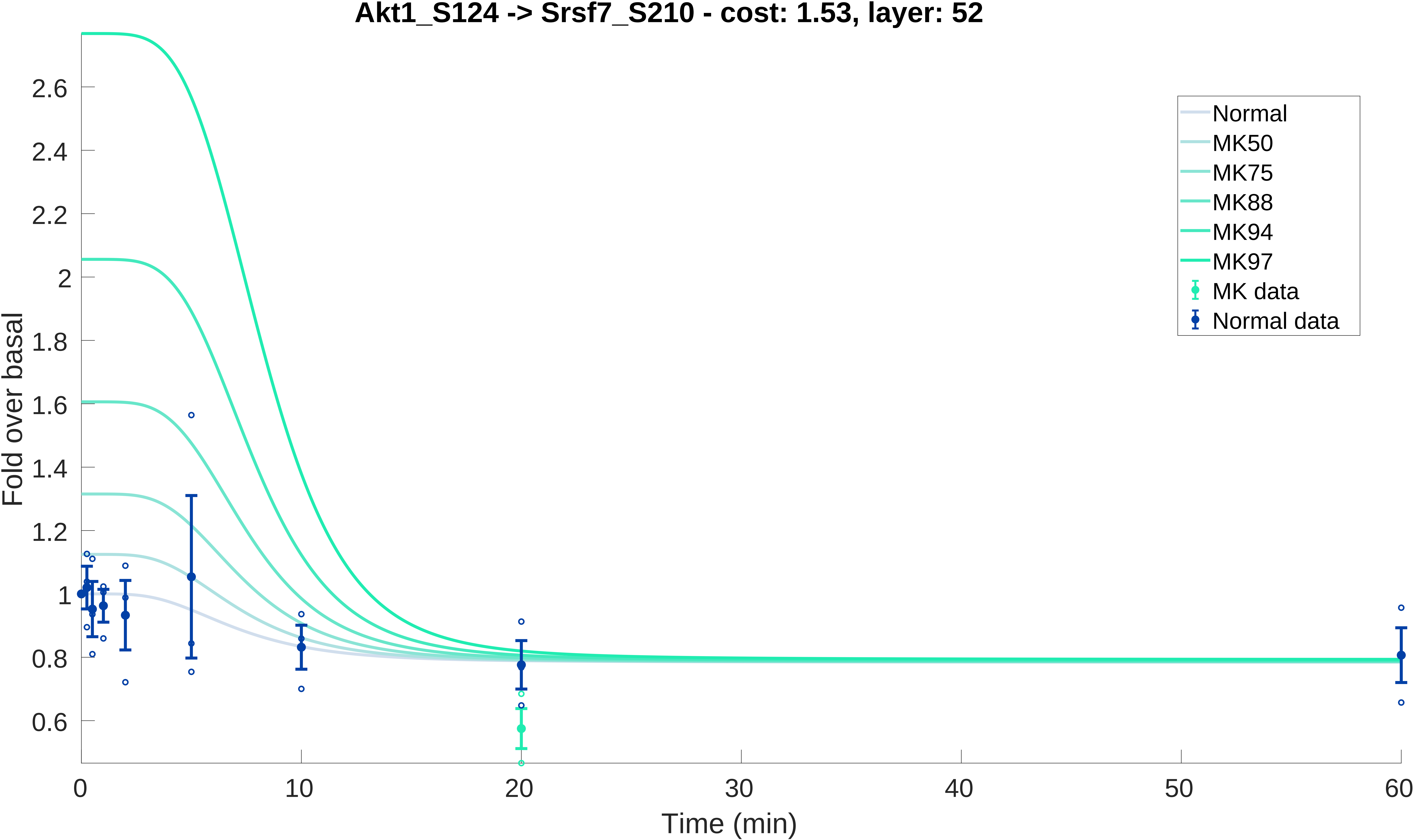

Akt1_S124 -> Srsf7_S210 - cost: 1.53, layer: 52
Fold over basal
Time (min)
2.6
2.4
2.2
2
1.8
1.6
1.4
1.2
1
0.8
0.6
0
10
20
30
40
50
60
Normal
MK50
MK75
MK88
MK94
MK97
MK data
Normal data

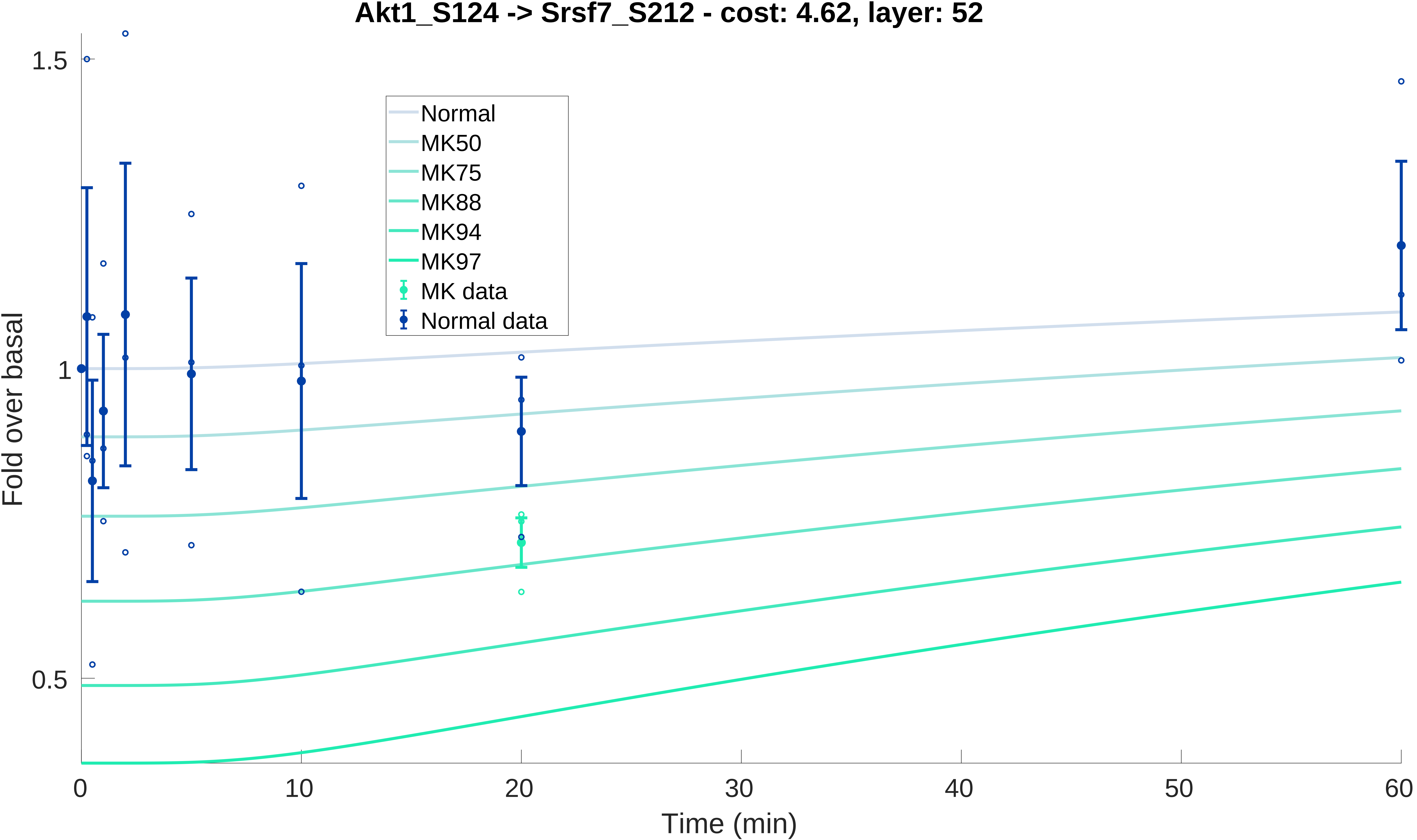
Akt1_S124 -> Srsf7_S212 - cost: 4.62, layer: 52
Fold over basal
1.5
1
0.5
Time (min)
0
10
20
30
40
50
60
Normal
MK50
MK75
MK88
MK94
MK97
MK data
Normal data

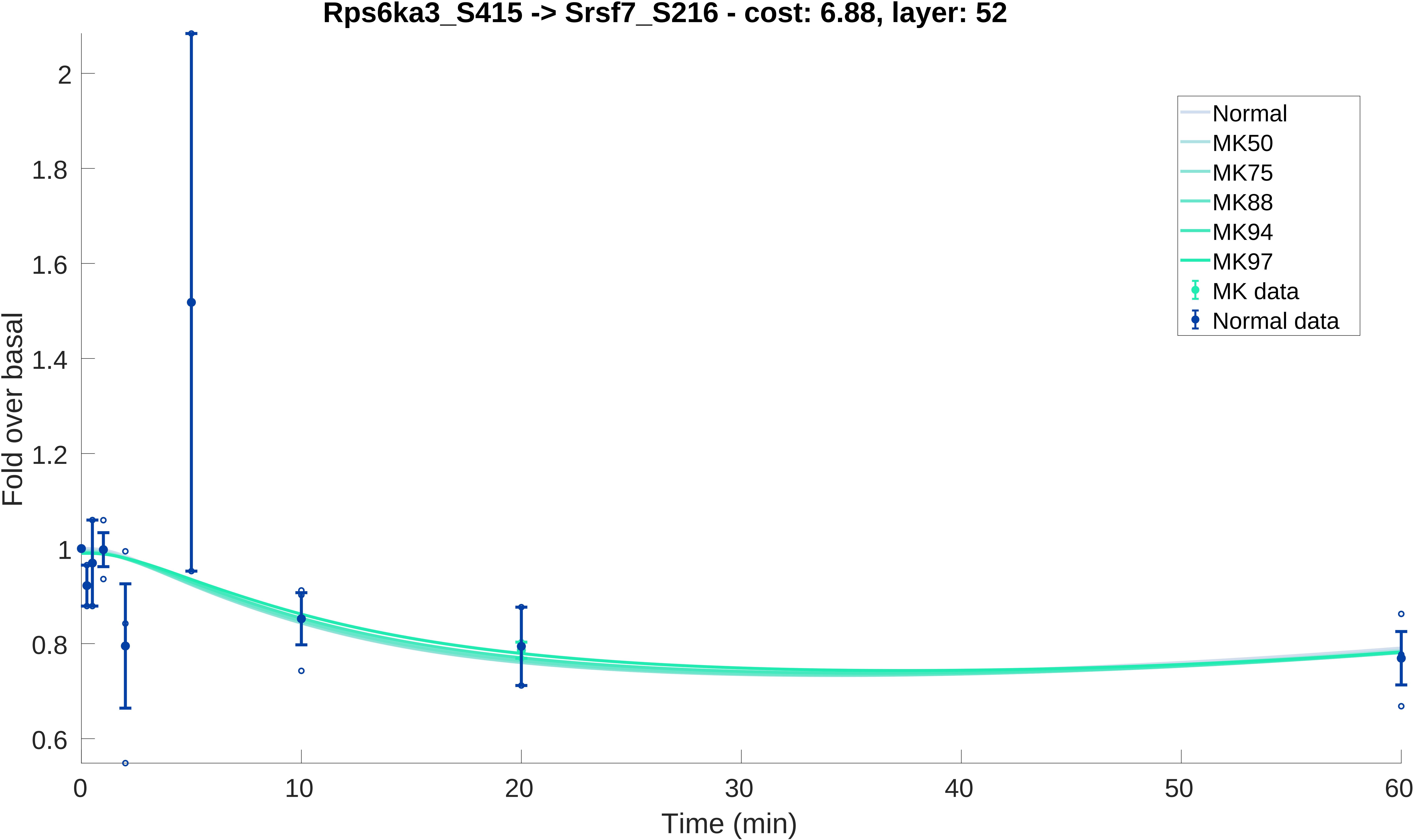

Rps6ka3_S415 -> Srsf7_S216 - cost: 6.88, layer: 52
Fold over basal
Time (min)
0
10
20
30
40
50
60
0.6
0.8
1
1.2
1.4
1.6
1.8
2
Normal
MK50
MK75
MK88
MK94
MK97
MK data
Normal data

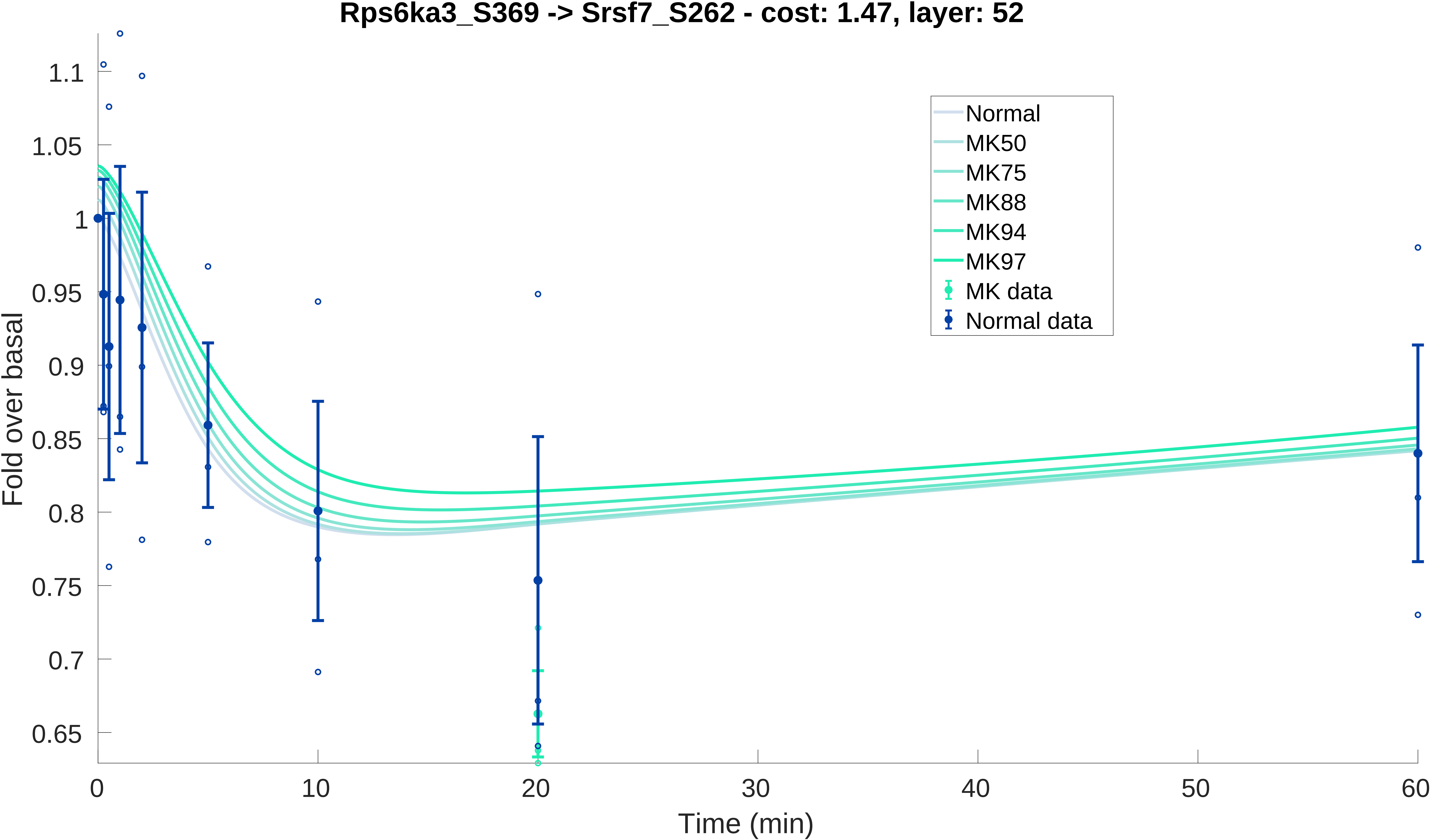

Rps6ka3_S369 -> Srsf7_S262 - cost: 1.47, layer: 52
Fold over basal
1.1
1.05
1
0.95
0.9
0.85
0.8
0.75
0.7
0.65
0
10
20
30
40
50
60
Time (min)
Normal
MK50
MK75
MK88
MK94
MK97
MK data
Normal data

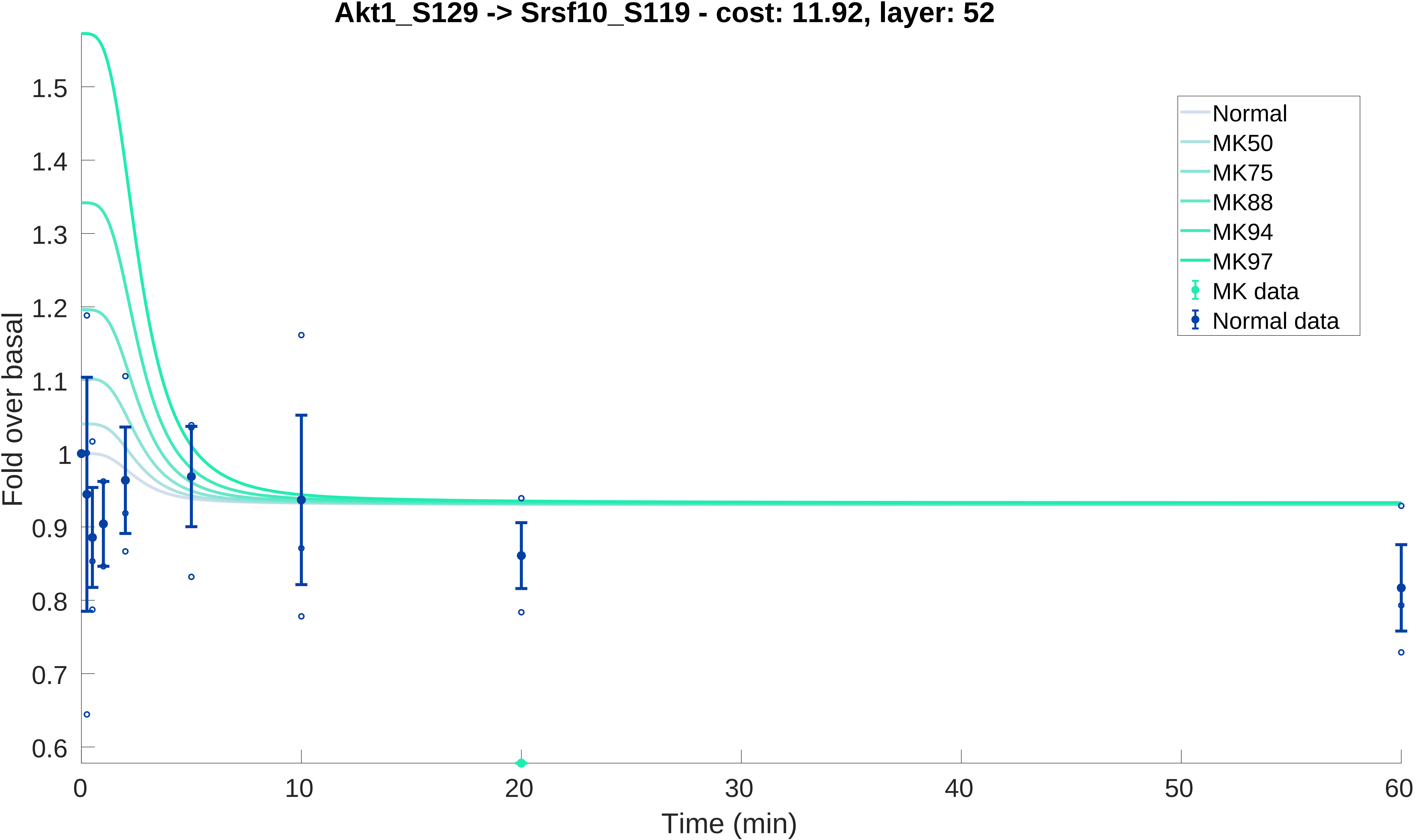
Akt1_S129 -> Srsf10_S119 - cost: 11.92, layer: 52
Fold over basal
1.5
1.4
1.3
1.2
1.1
1
0.9
0.8
0.7
0.6
0
10
20
30
40
50
60
Time (min)
Normal
MK50
MK75
MK88
MK94
MK97
MK data
Normal data

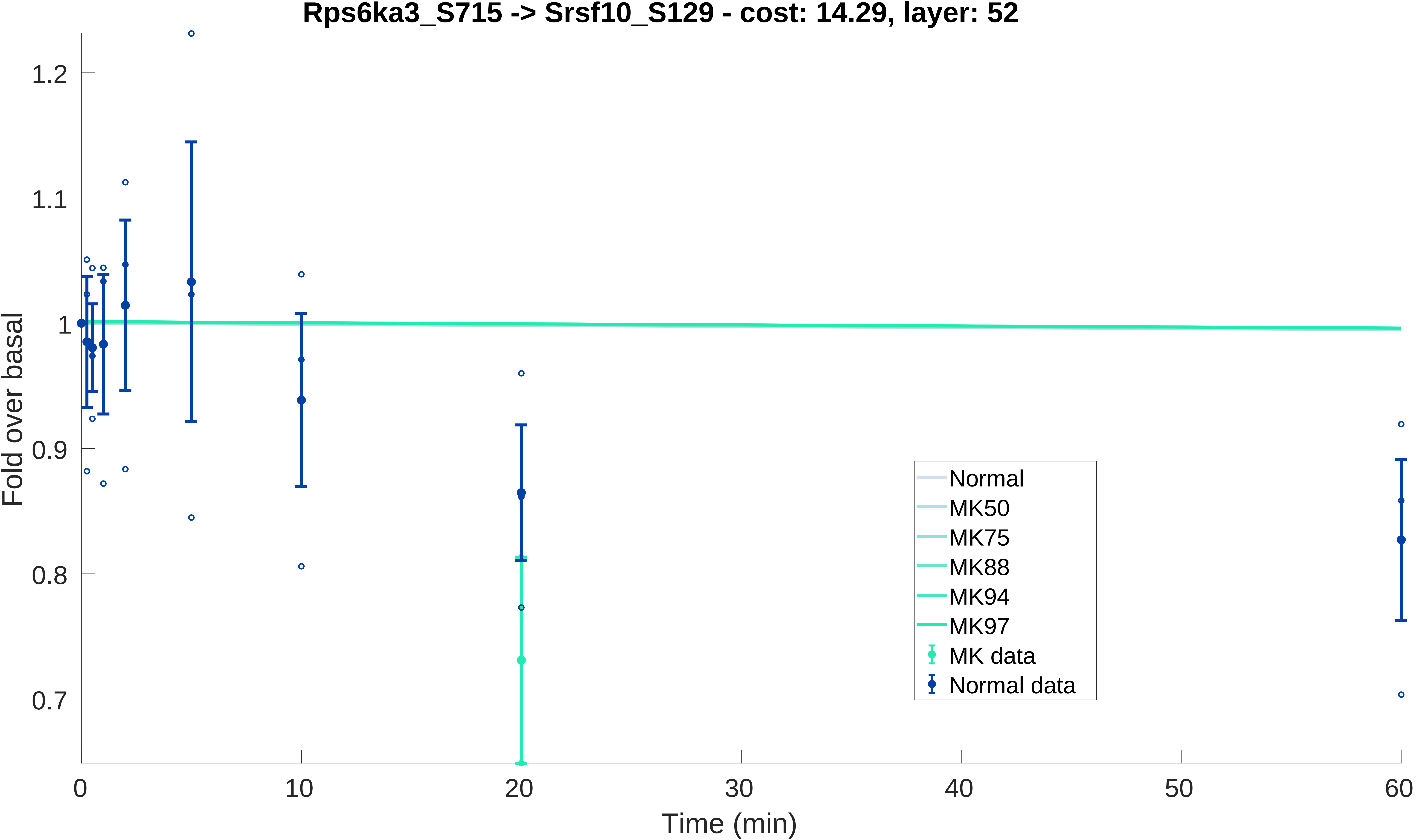

Rps6ka3_S715 -> Srsf10_S129 - cost: 14.29, layer: 52
Fold over basal
Time (min)
1.2
1.1
1
0.9
0.8
0.7
0
10
20
30
40
50
60
Normal
MK50
MK75
MK88
MK94
MK97
MK data
Normal data

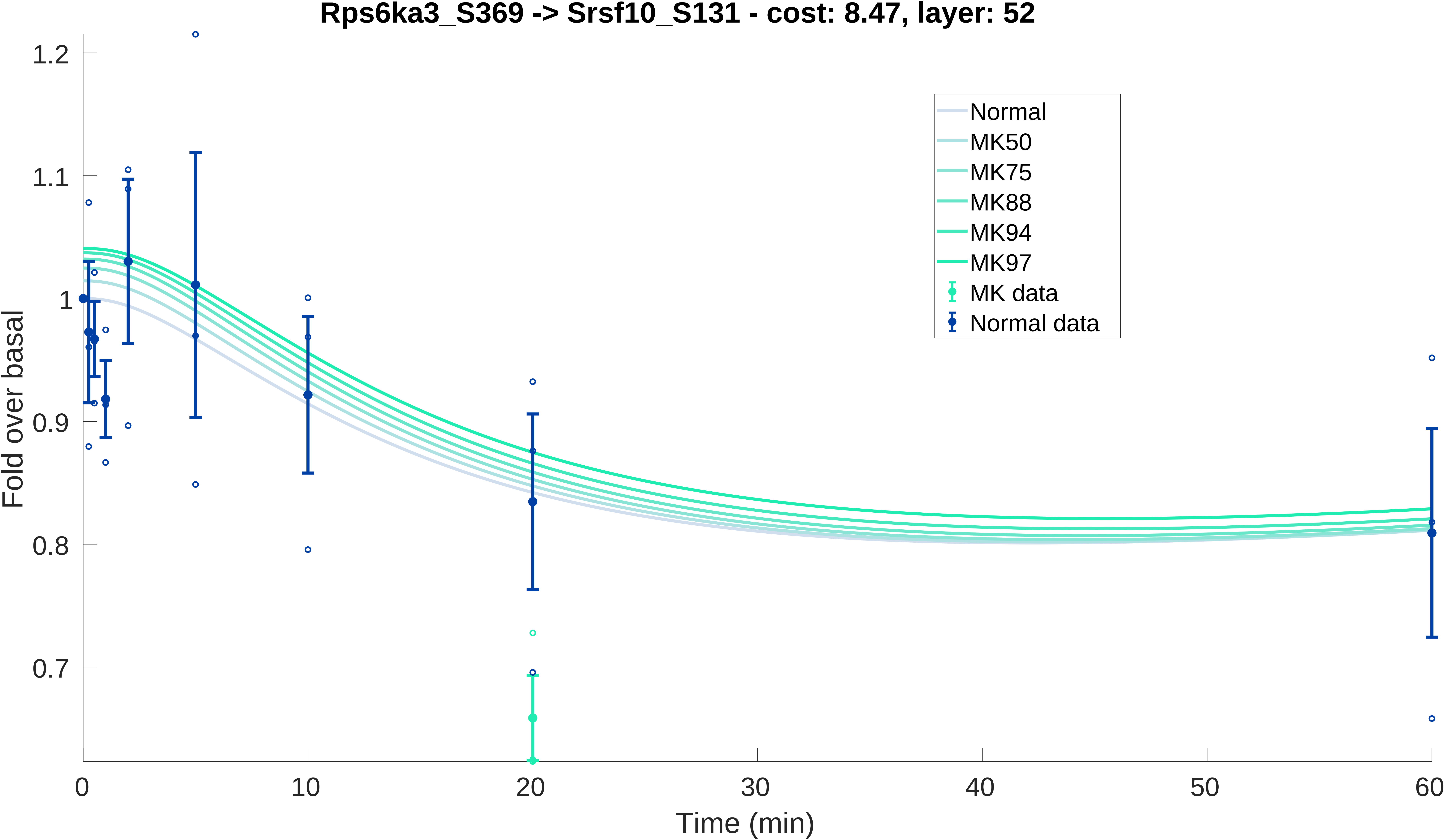
Rps6ka3_S369 -> Srsf10_S131 - cost: 8.47, layer: 52
Fold over basal
1.2
1.1
1
0.9
0.8
0.7
0
10
20
30
40
50
60
Time (min)
Normal
MK50
MK75
MK88
MK94
MK97
MK data
Normal data

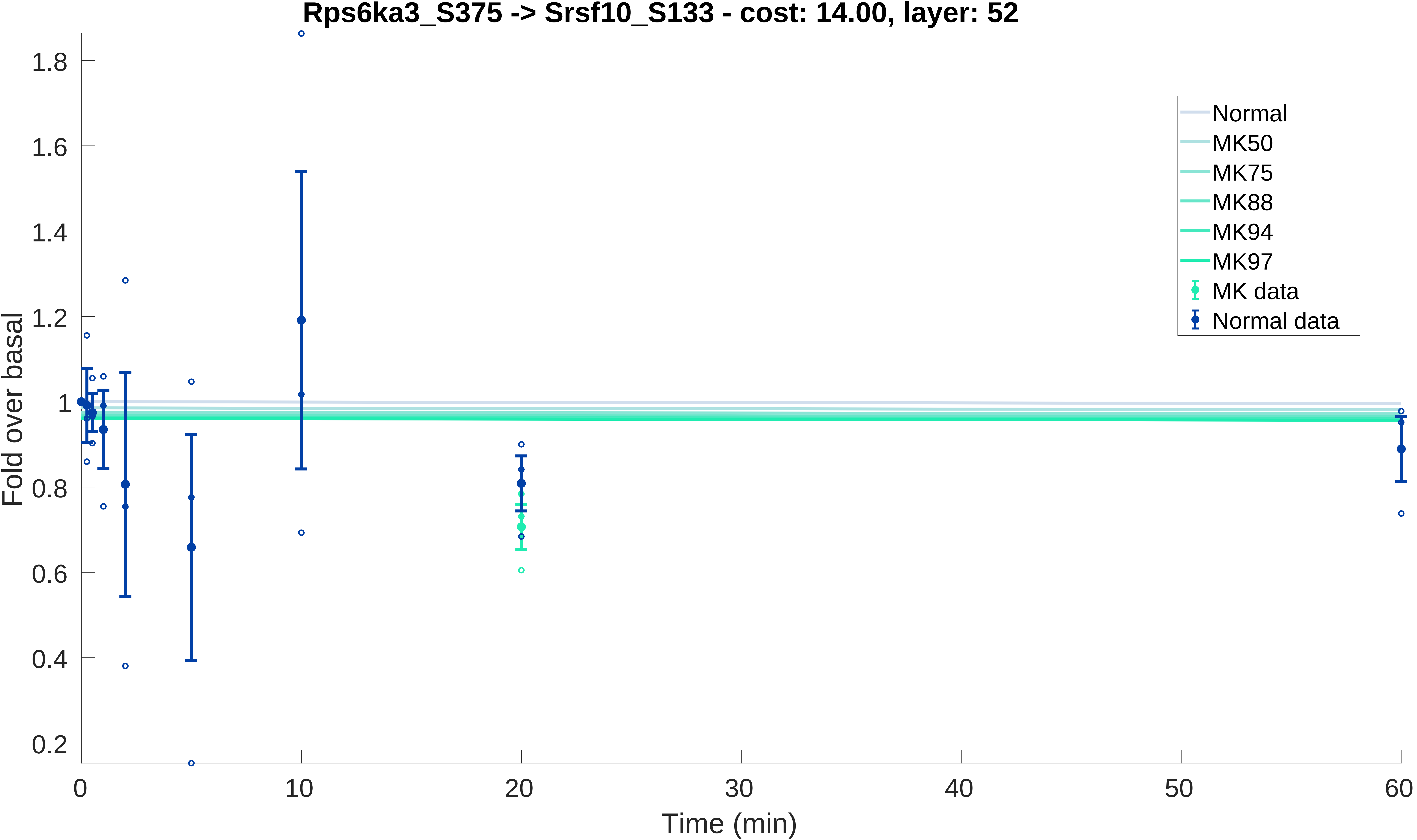

Rps6ka3_S375 -> Srsf10_S133 - cost: 14.00, layer: 52
Fold over basal
Time (min)
1.8
1.6
1.4
1.2
1
0.8
0.6
0.4
0.2
0
10
20
30
40
50
60
Normal
MK50
MK75
MK88
MK94
MK97
MK data
Normal data

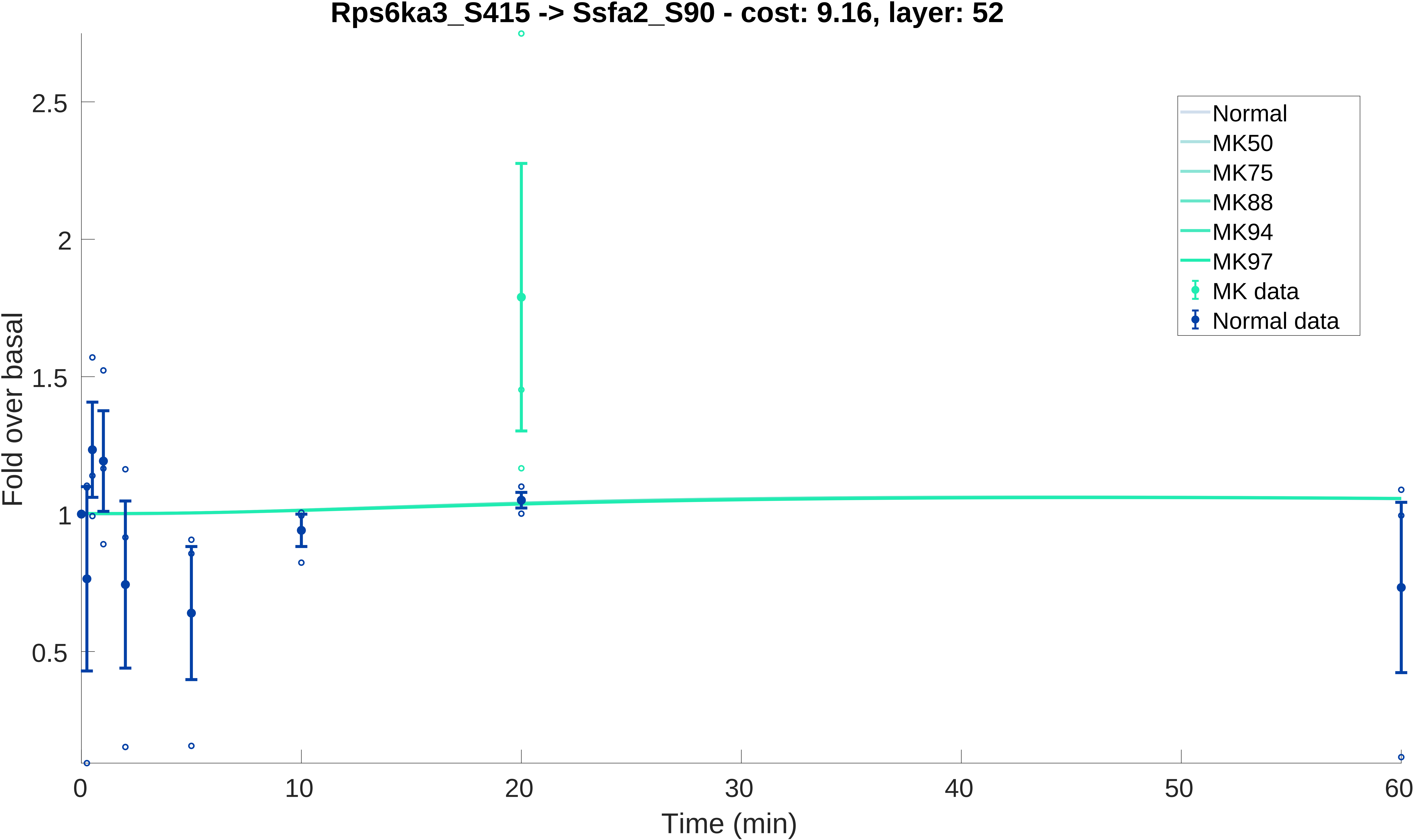

Rps6ka3_S415 -> Ssfa2_S90 - cost: 9.16, layer: 52
Fold over basal
Time (min)
0.5
1
1.5
2
2.5
0
10
20
30
40
50
60
Normal
MK50
MK75
MK88
MK94
MK97
MK data
Normal data

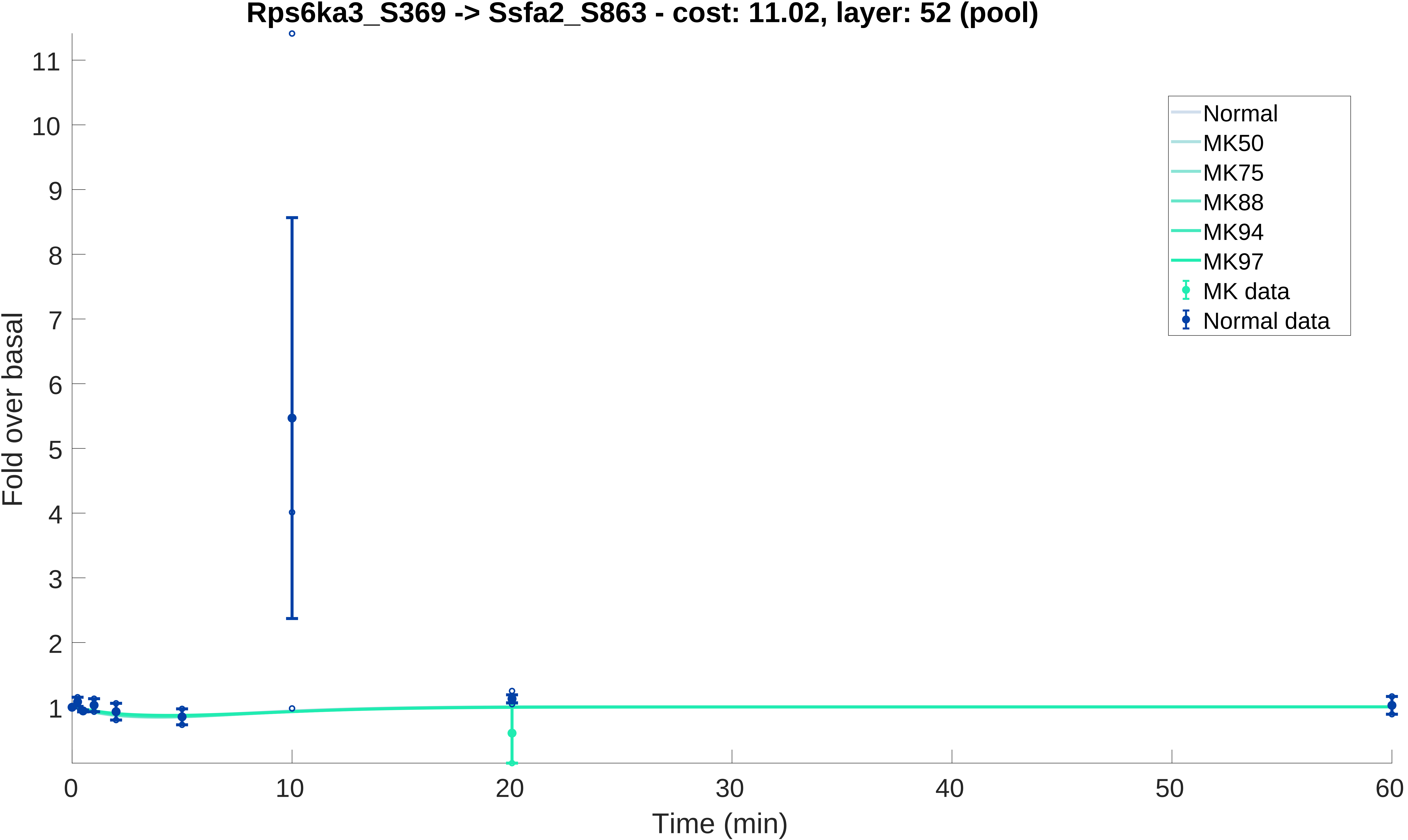

Rps6ka3_S369 -> Ssfa2_S863 - cost: 11.02, layer: 52 (pool)
Fold over basal
Time (min)
Normal
MK50
MK75
MK88
MK94
MK97
MK data
Normal data

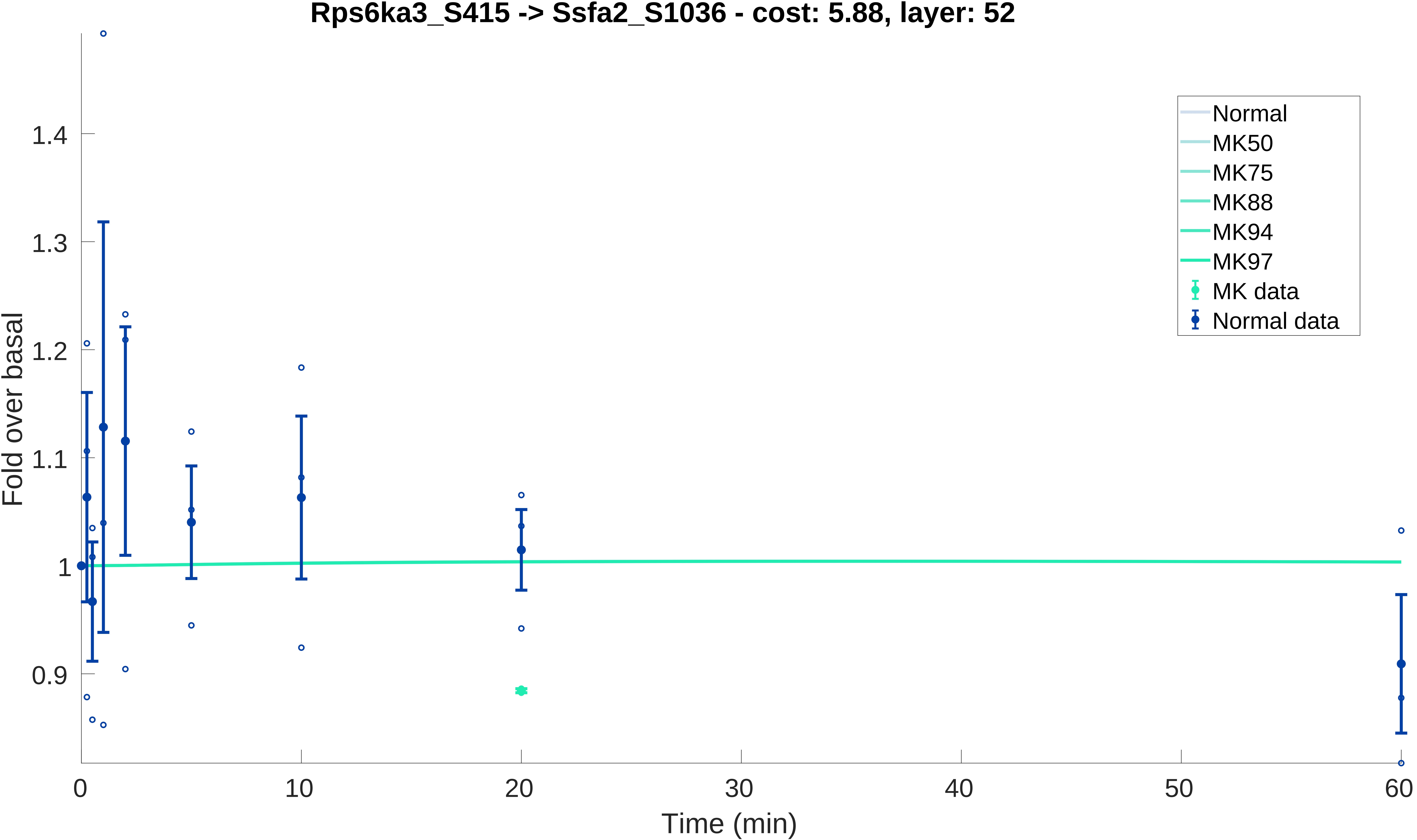

Rps6ka3_S415 -> Ssfa2_S1036 - cost: 5.88, layer: 52
Fold over basal
Time (min)
1.4
1.3
1.2
1.1
1
0.9
0
10
20
30
40
50
60
Normal
MK50
MK75
MK88
MK94
MK97
MK data
Normal data

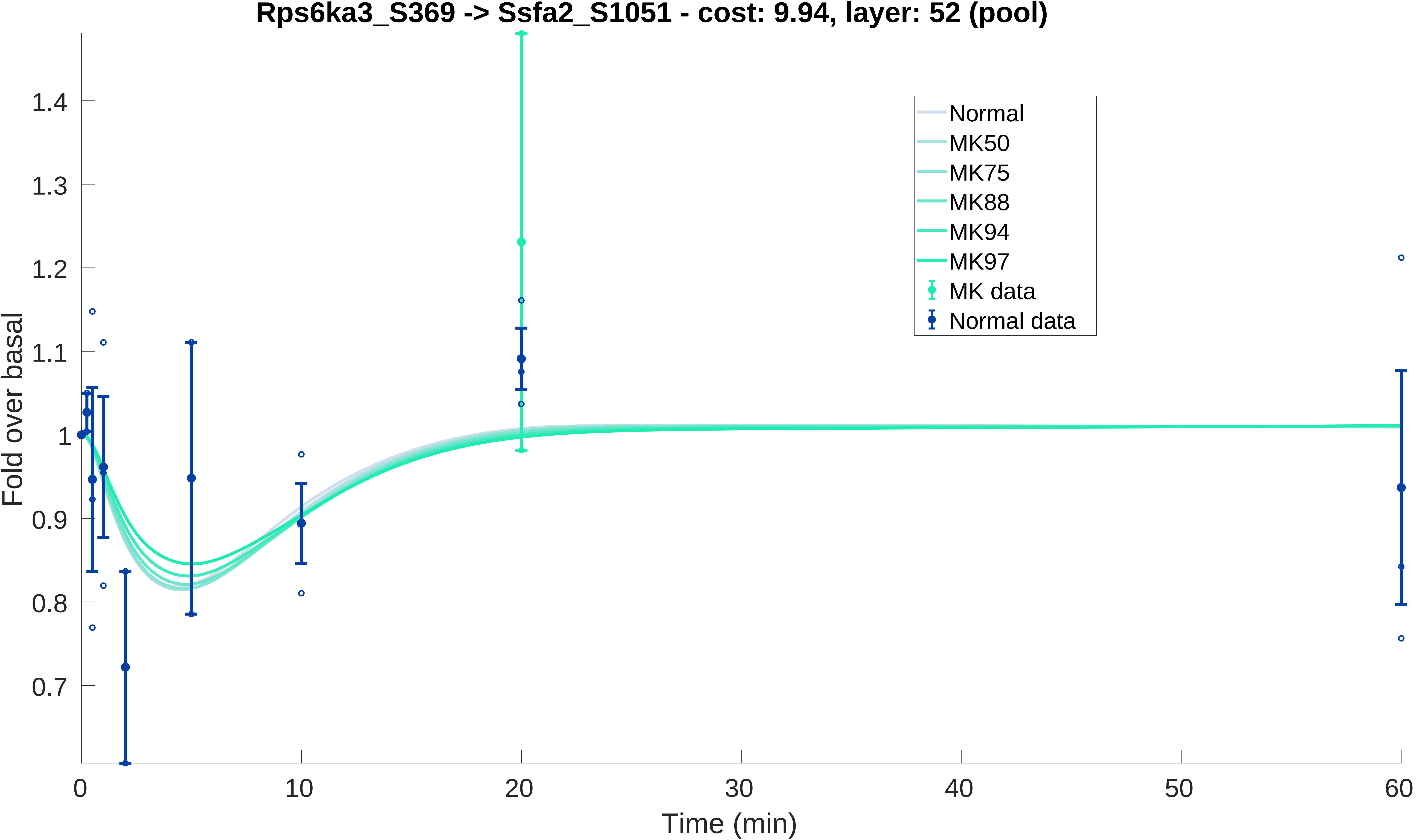

Rps6ka3_S369 -> Ssfa2_S1051 - cost: 9.94, layer: 52 (pool)
Normal
MK50
MK75
MK88
MK94
MK97
MK data
Normal data
Fold over basal
1.4
1.3
1.2
1.1
1
0.9
0.8
0.7
0
10
20
30
40
50
60
Time (min)

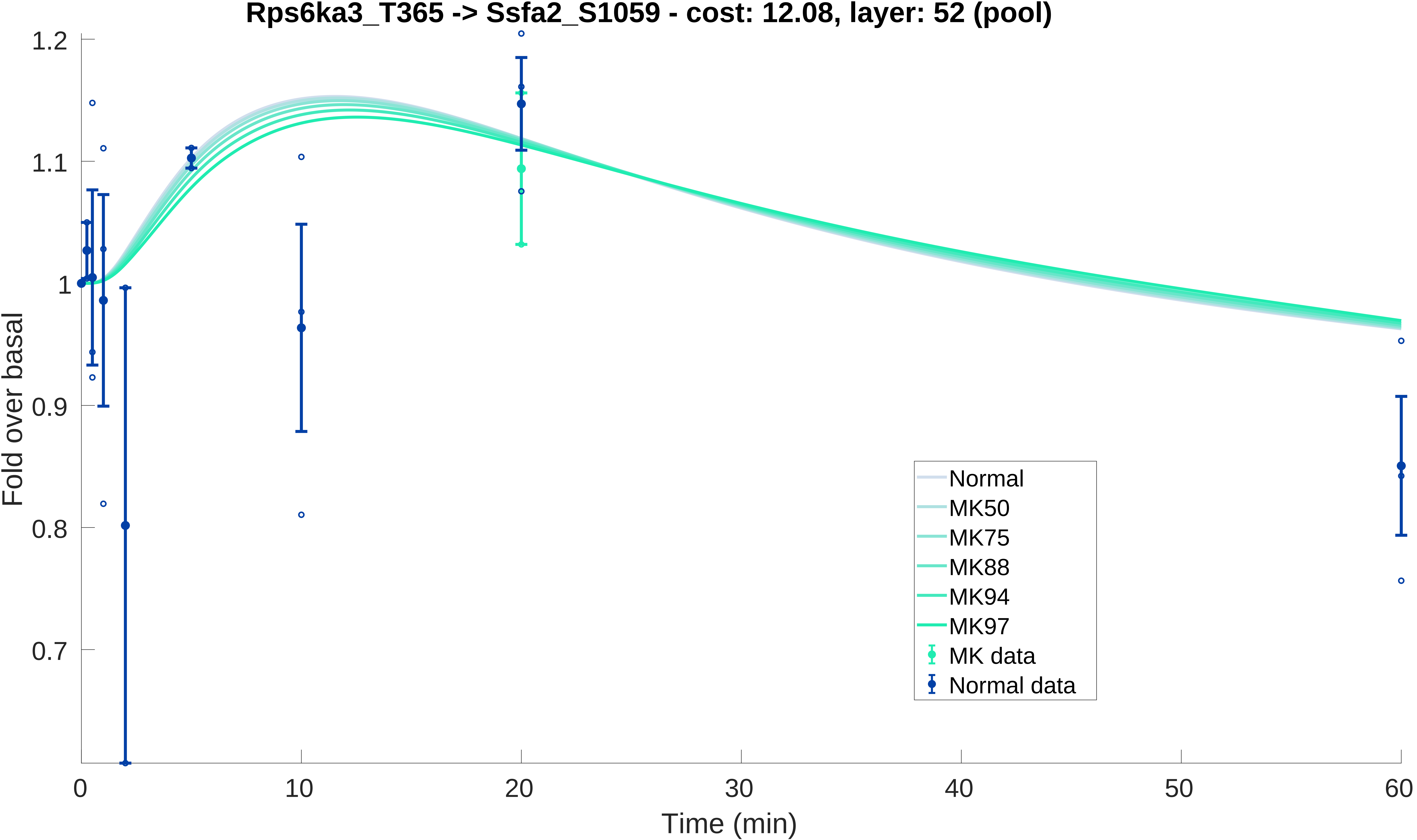

Rps6ka3_T365 -> Ssfa2_S1059 - cost: 12.08, layer: 52 (pool)
Fold over basal
Time (min)
1.2
1.1
1
0.9
0.8
0.7
0
10
20
30
40
50
60
Normal
MK50
MK75
MK88
MK94
MK97
MK data
Normal data

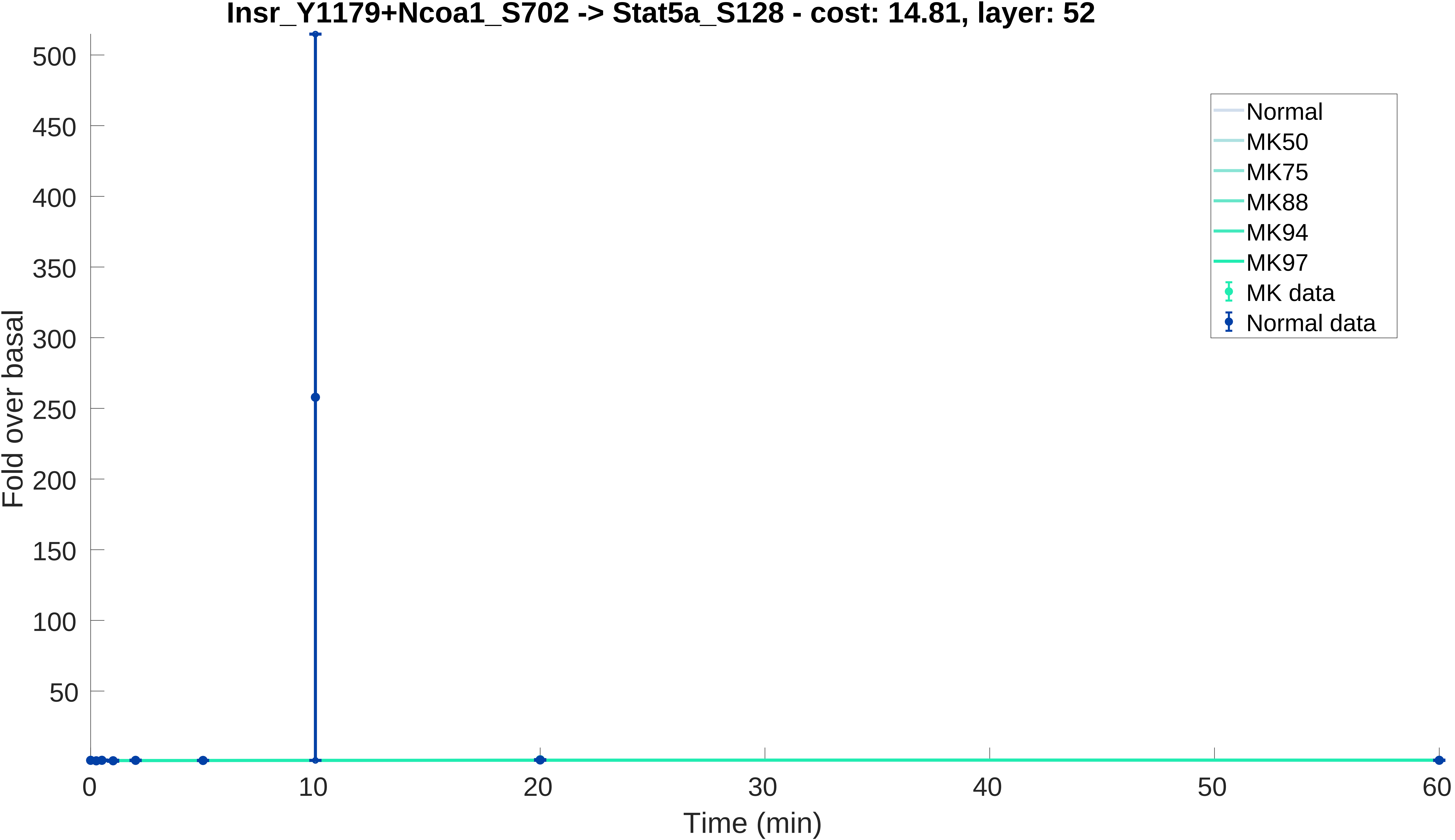
Insr_Y1179+Ncoa1_S702 -> Stat5a_S128 - cost: 14.81, layer: 52
Fold over basal
Time (min)
500
450
400
350
300
250
200
150
100
50
0
10
20
30
40
50
60
Normal
MK50
MK75
MK88
MK94
MK97
MK data
Normal data

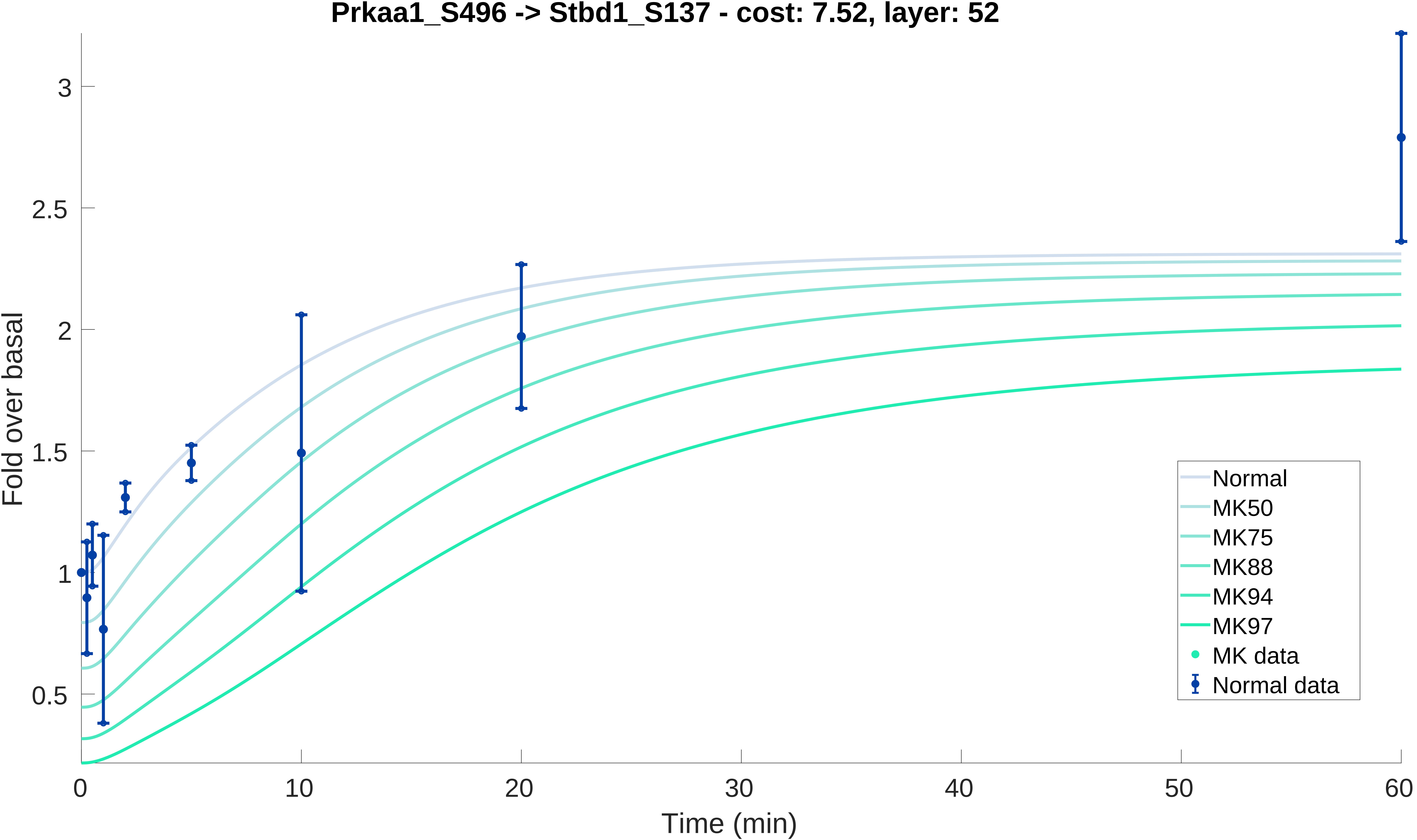

Prkaa1_S496 -> Stbd1_S137 - cost: 7.52, layer: 52
Fold over basal
Time (min)
0.5
1
1.5
2
2.5
3
0
10
20
30
40
50
60
Normal
MK50
MK75
MK88
MK94
MK97
MK data
Normal data

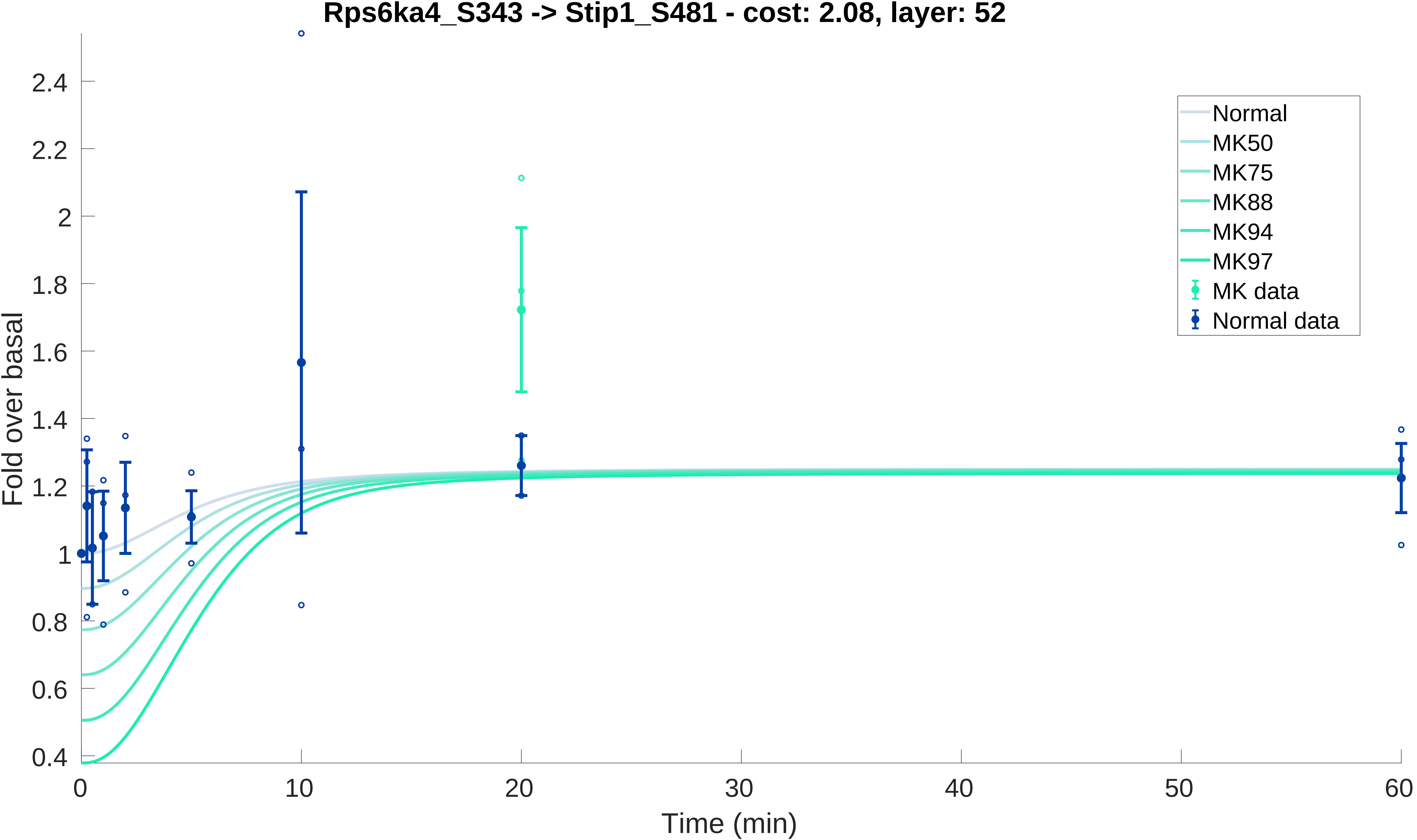
Rps6ka4_S343 -> Stip1_S481 - cost: 2.08, layer: 52
Fold over basal
Time (min)
Normal
MK50
MK75
MK88
MK94
MK97
MK data
Normal data
0.4
0.6
0.8
1
1.2
1.4
1.6
1.8
2
2.2
2.4
0
10
20
30
40
50
60

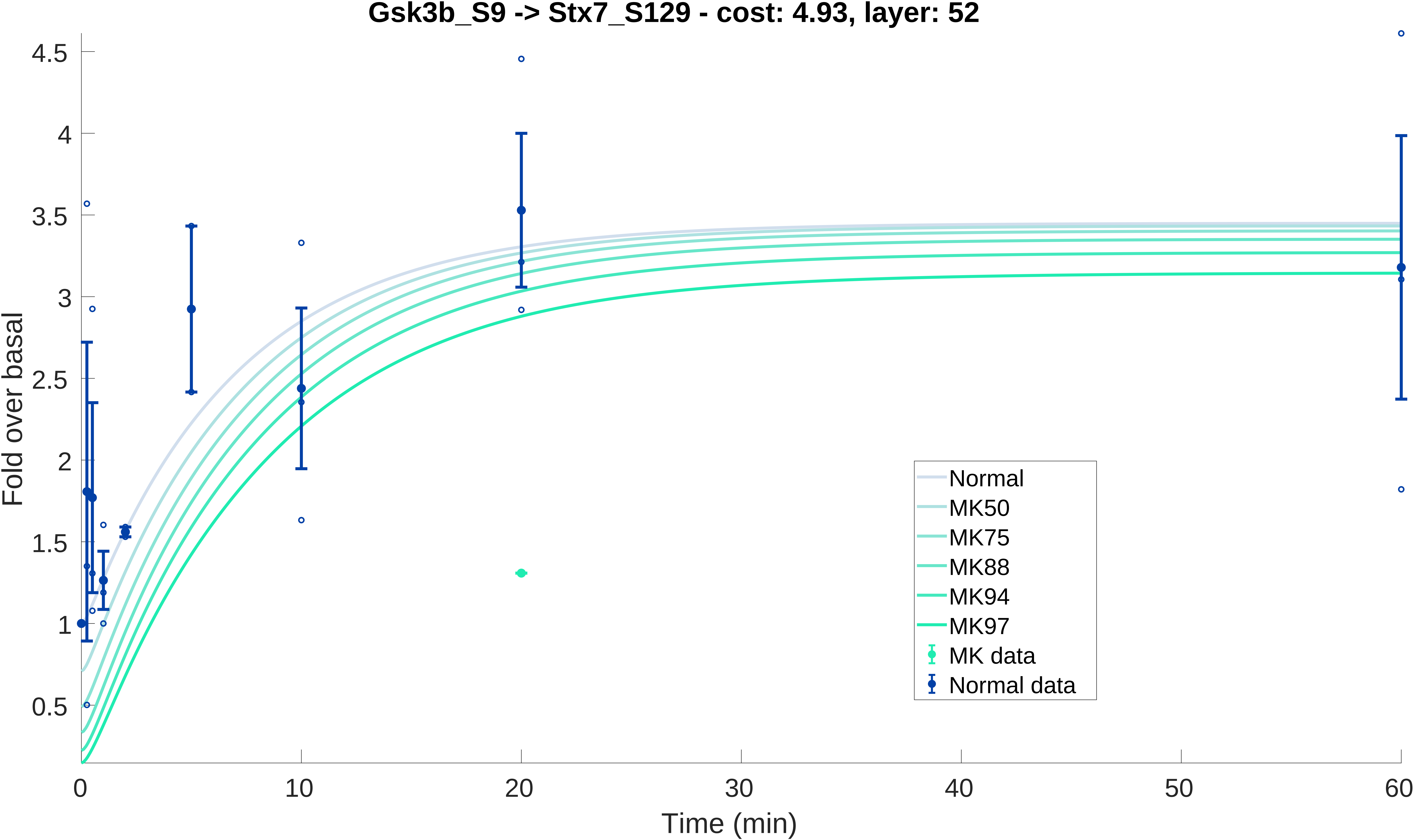

Gsk3b_S9 -> Stx7_S129 - cost: 4.93, layer: 52
Fold over basal
Time (min)
0.5
1
1.5
2
2.5
3
3.5
4
4.5
0
10
20
30
40
50
60
Normal
MK50
MK75
MK88
MK94
MK97
MK data
Normal data

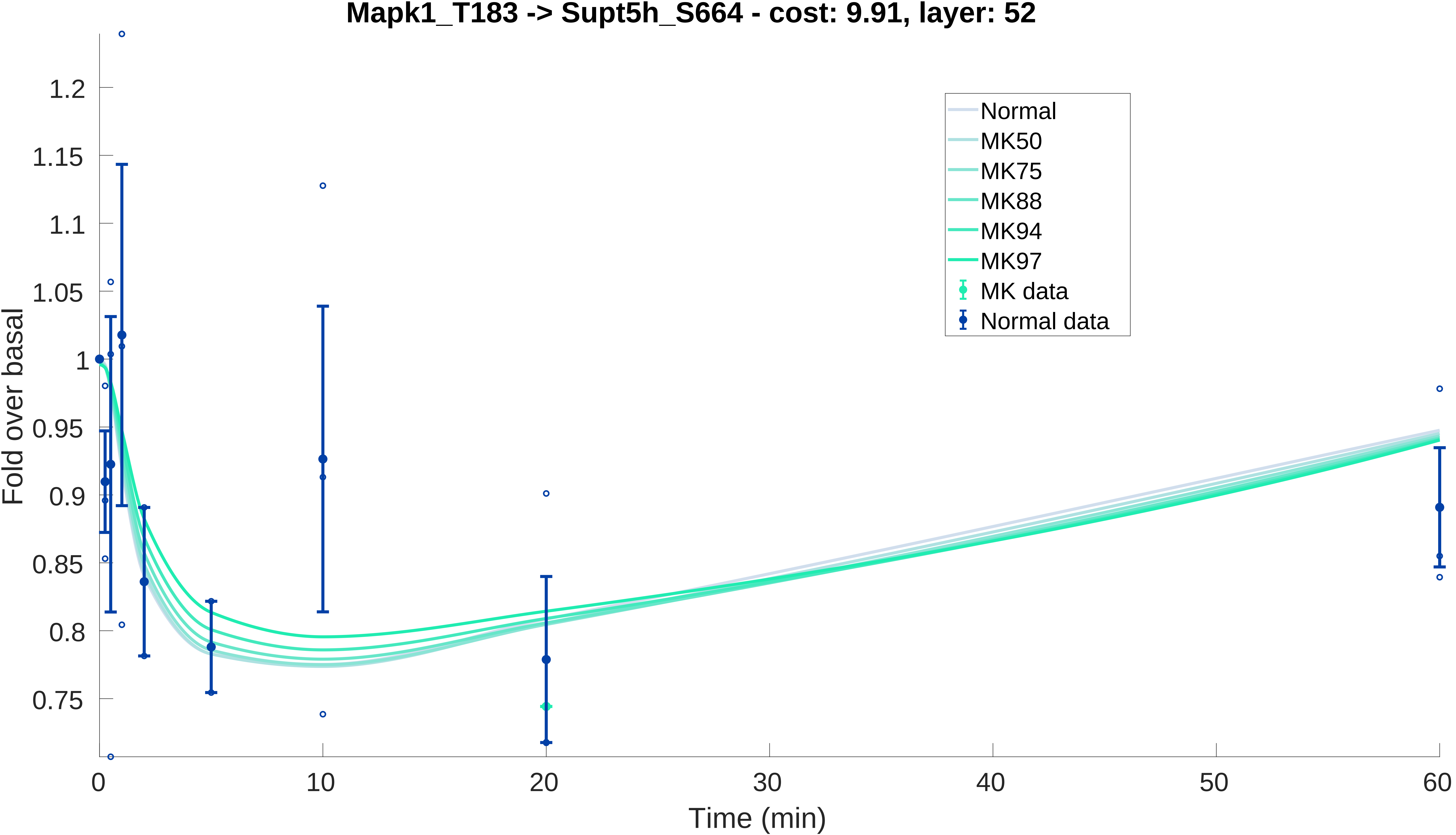

Mapk1_T183 -> Supt5h_S664 - cost: 9.91, layer: 52
Fold over basal
Time (min)
1.2
1.15
1.1
1.05
1
0.95
0.9
0.85
0.8
0.75
0
10
20
30
40
50
60
Normal
MK50
MK75
MK88
MK94
MK97
MK data
Normal data

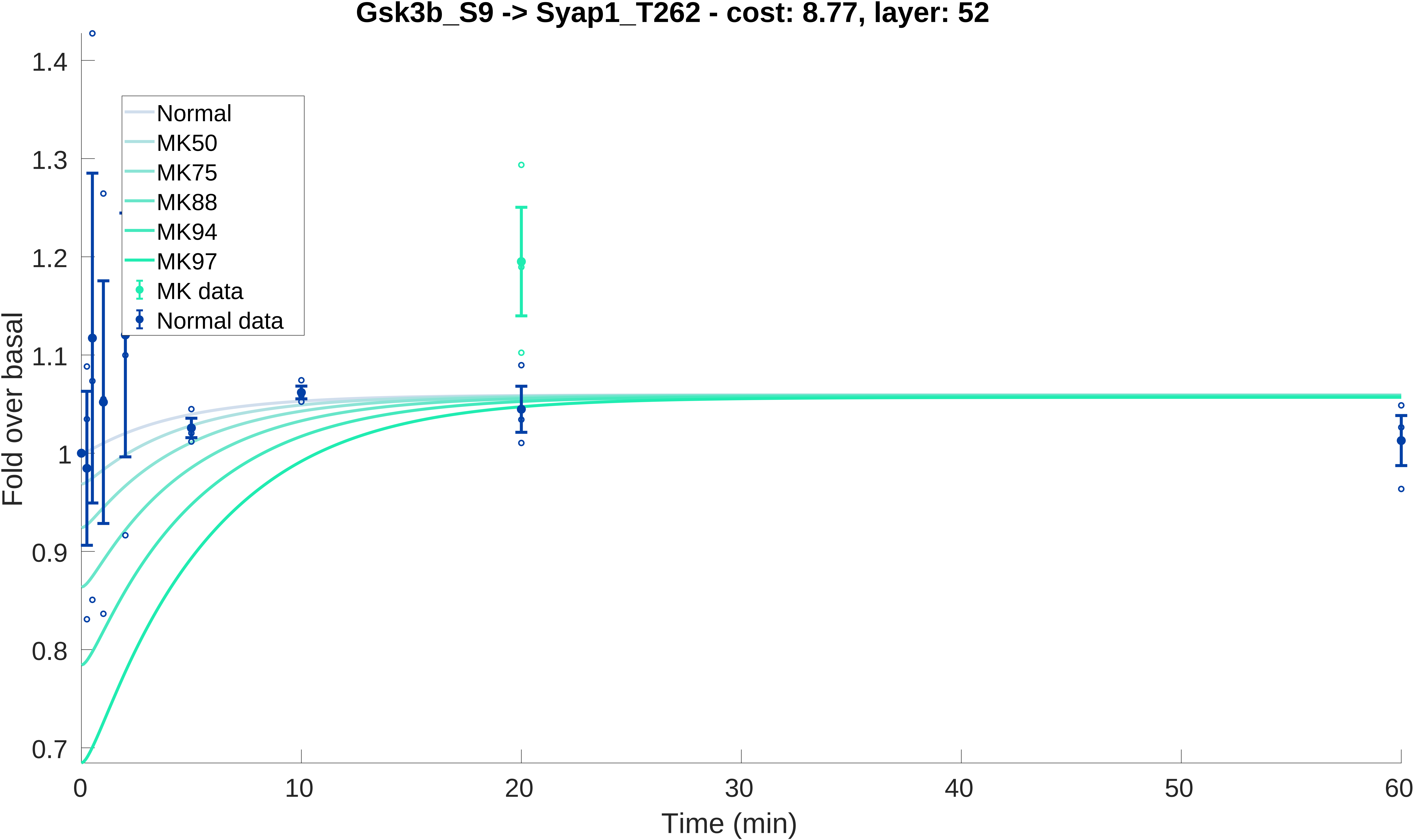

Gsk3b_S9 -> Syap1_T262 - cost: 8.77, layer: 52
Fold over basal
Time (min)
1.4
1.3
1.2
1.1
1
0.9
0.8
0.7
0
10
20
30
40
50
60
Normal
MK50
MK75
MK88
MK94
MK97
MK data
Normal data

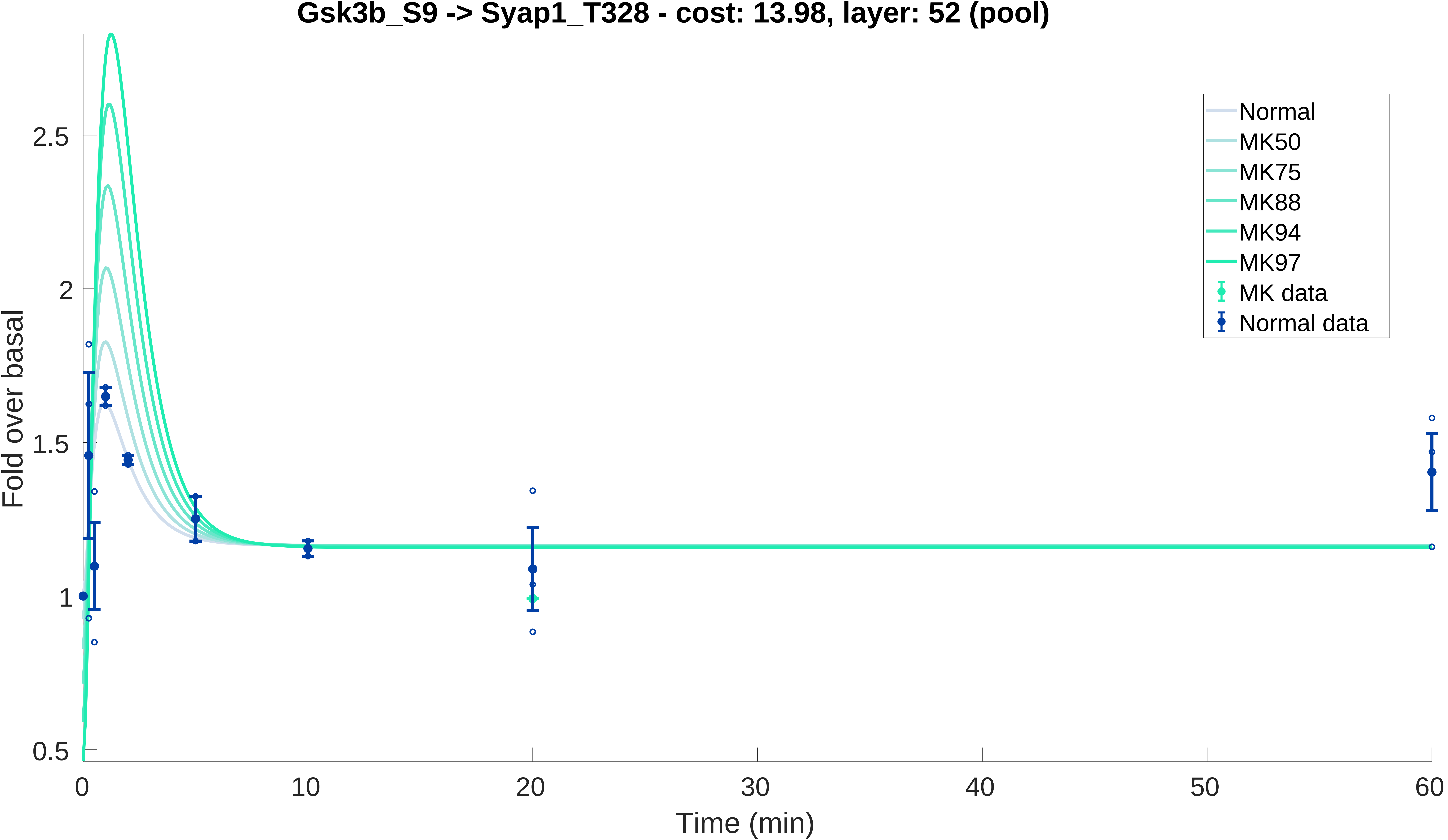
Gsk3b_S9 -> Syap1_T328 - cost: 13.98, layer: 52 (pool)
Normal
MK50
MK75
MK88
MK94
MK97
MK data
Normal data
Fold over basal
Time (min)
0.5
1
1.5
2
2.5
0
10
20
30
40
50
60

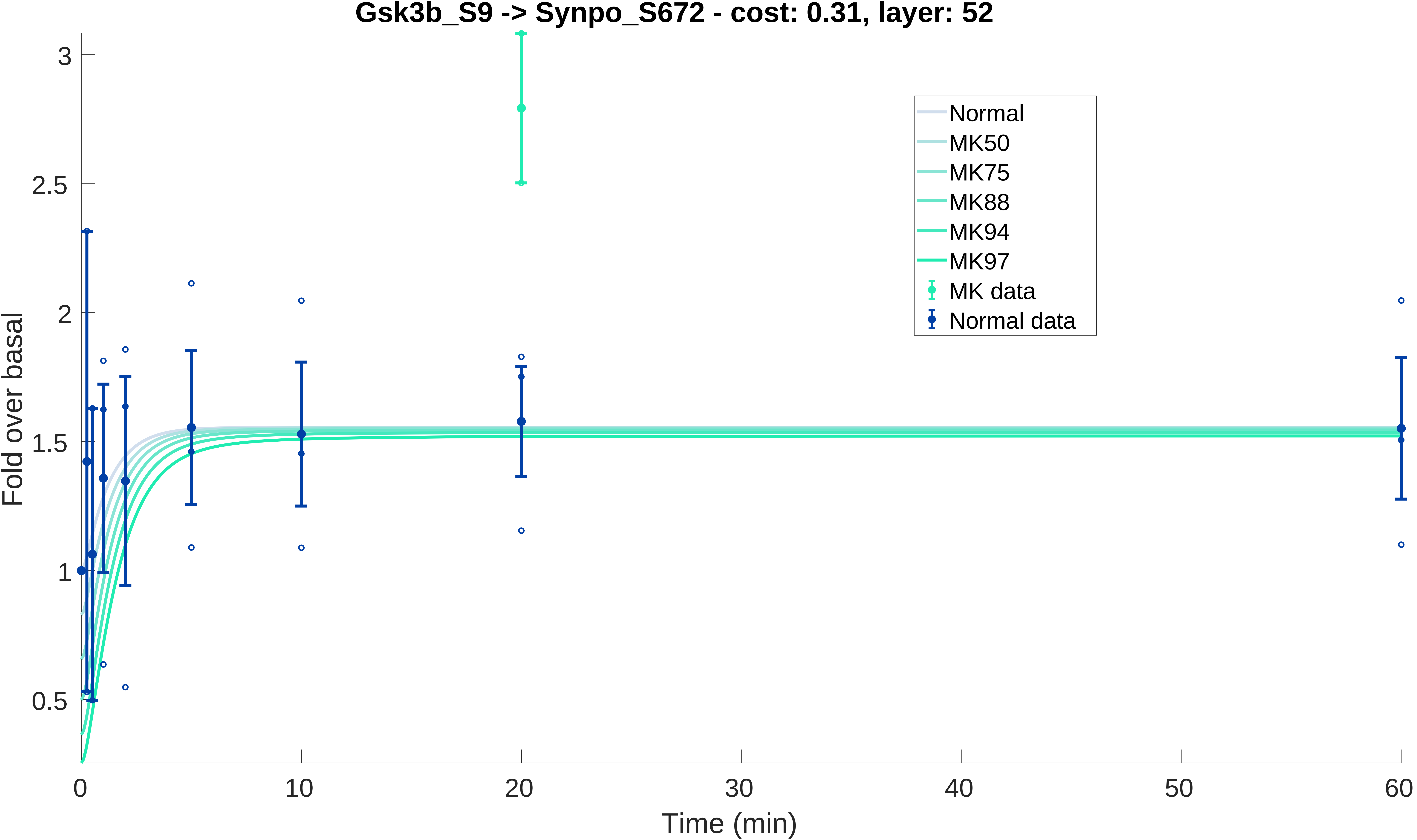
Gsk3b_S9 -> Synpo_S672 - cost: 0.31, layer: 52
Fold over basal
Time (min)
Normal
MK50
MK75
MK88
MK94
MK97
MK data
Normal data
0.5
1
1.5
2
2.5
3
0
10
20
30
40
50
60

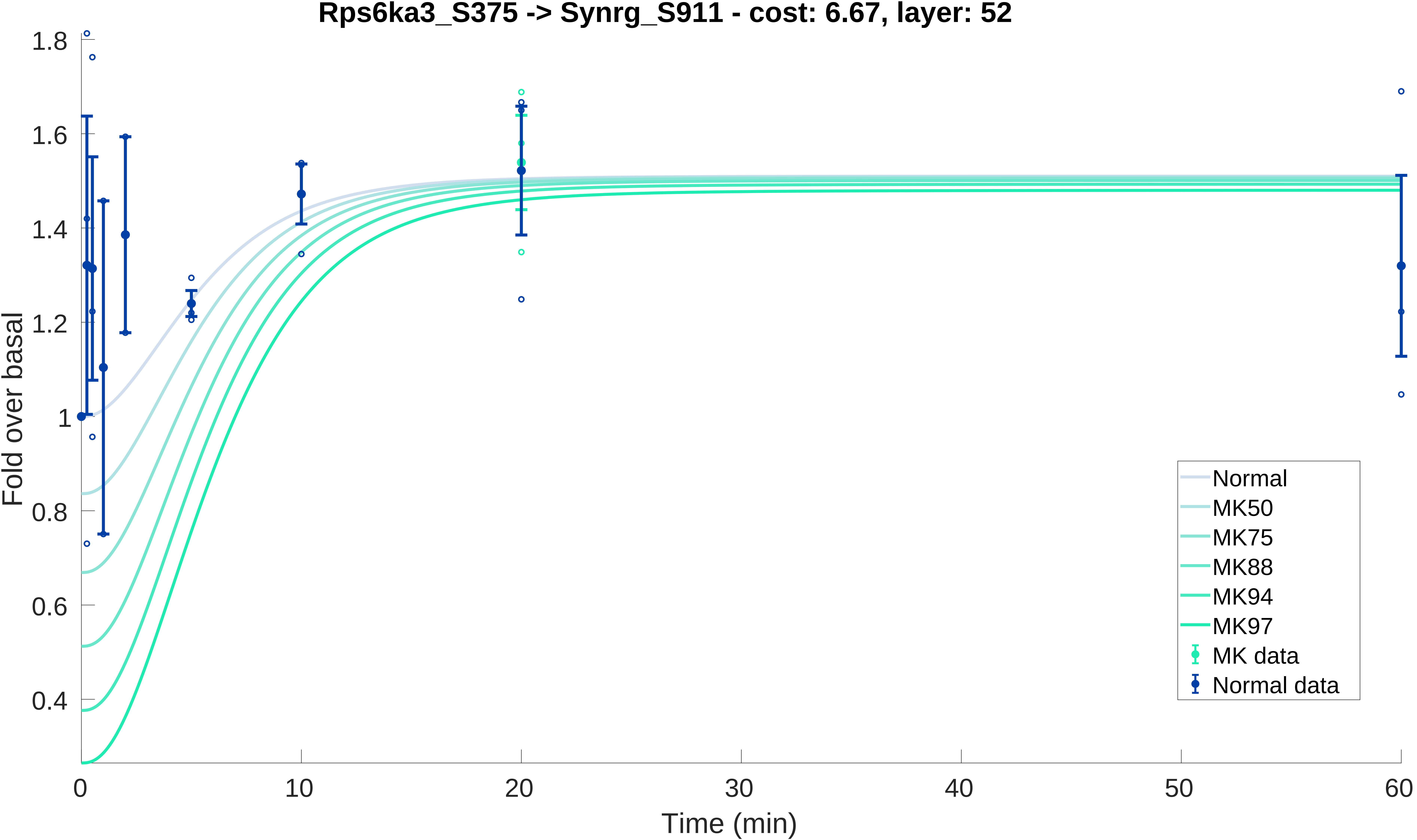

Rps6ka3_S375 -> Synrg_S911 - cost: 6.67, layer: 52
1.8
1.6
1.4
1.2
1
0.8
0.6
0.4
Fold over basal
0
10
20
30
40
50
60
Time (min)
Normal
MK50
MK75
MK88
MK94
MK97
MK data
Normal data

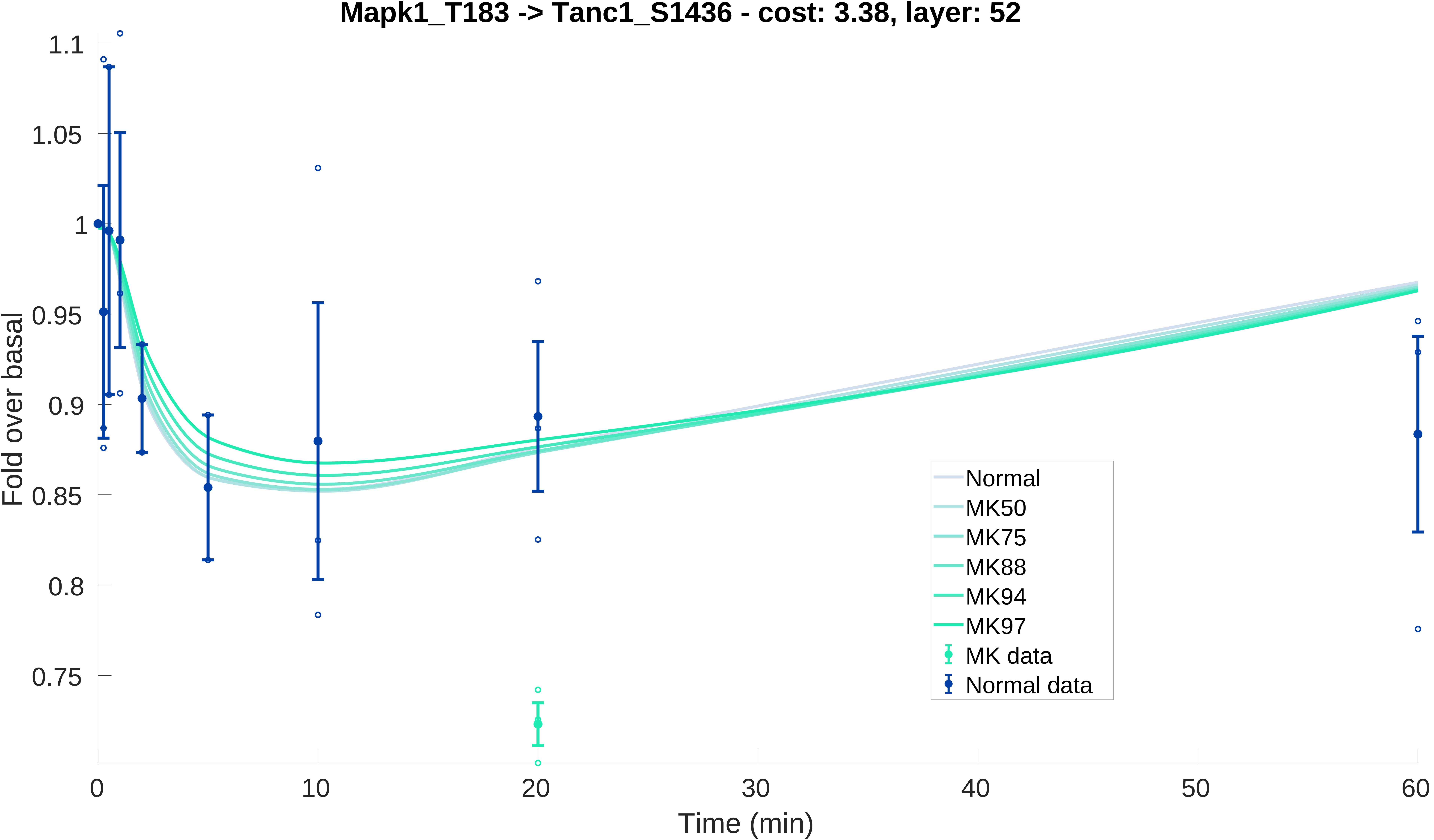

Mapk1_T183 -> Tanc1_S1436 - cost: 3.38, layer: 52
Fold over basal
1.1
1.05
1
0.95
0.9
0.85
0.8
0.75
0
10
20
30
40
50
60
Time (min)
Normal
MK50
MK75
MK88
MK94
MK97
MK data
Normal data

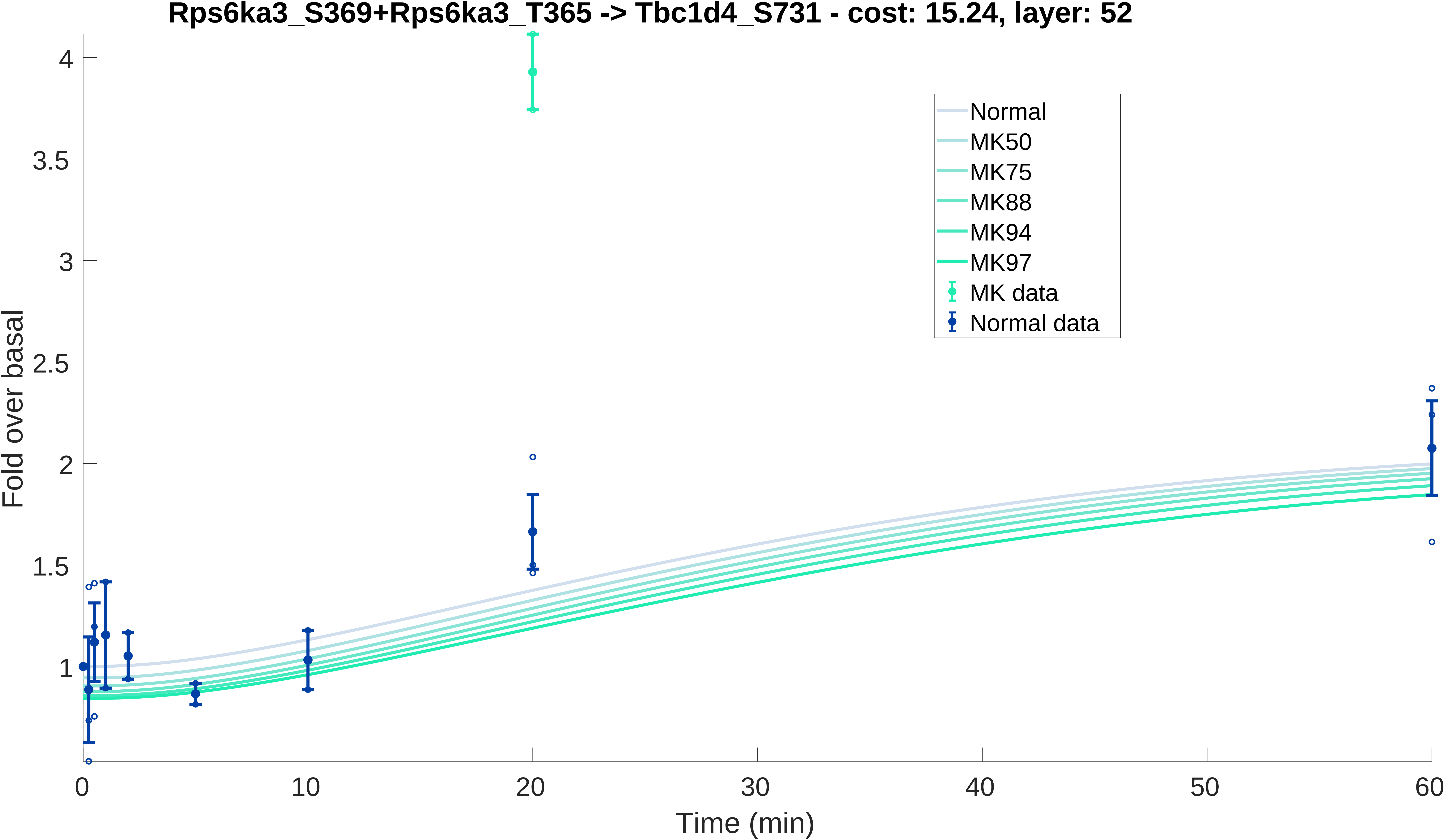
Rps6ka3_S369+Rps6ka3_T365 -> Tbc1d4_S731 - cost: 15.24, layer: 52
Fold over basal
4
3.5
3
2.5
2
1.5
1
0
10
20
30
40
50
60
Time (min)
Normal
MK50
MK75
MK88
MK94
MK97
MK data
Normal data

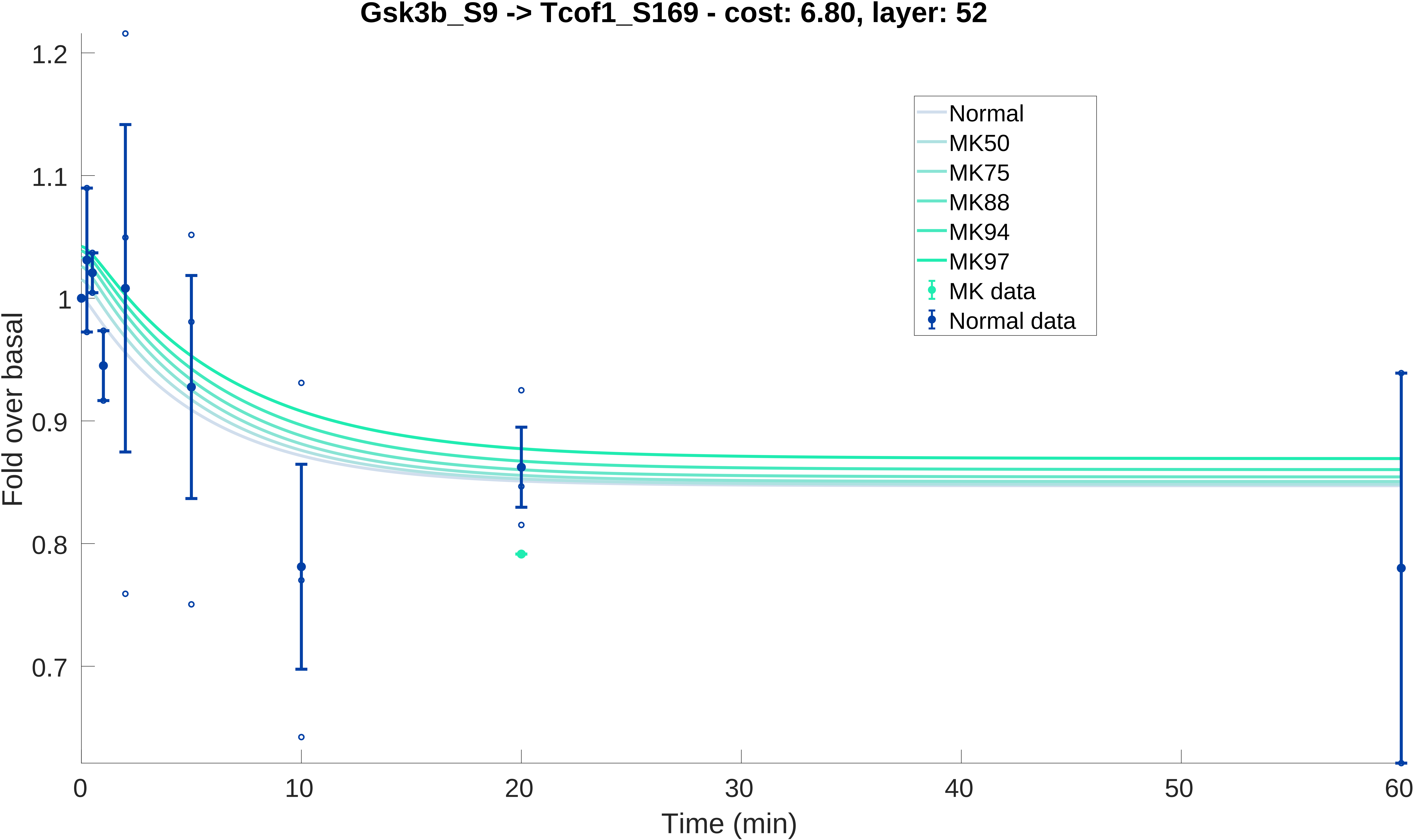

Gsk3b_S9 -> Tcof1_S169 - cost: 6.80, layer: 52
Fold over basal
Time (min)
1.2
1.1
1
0.9
0.8
0.7
0
10
20
30
40
50
60
Normal
MK50
MK75
MK88
MK94
MK97
MK data
Normal data

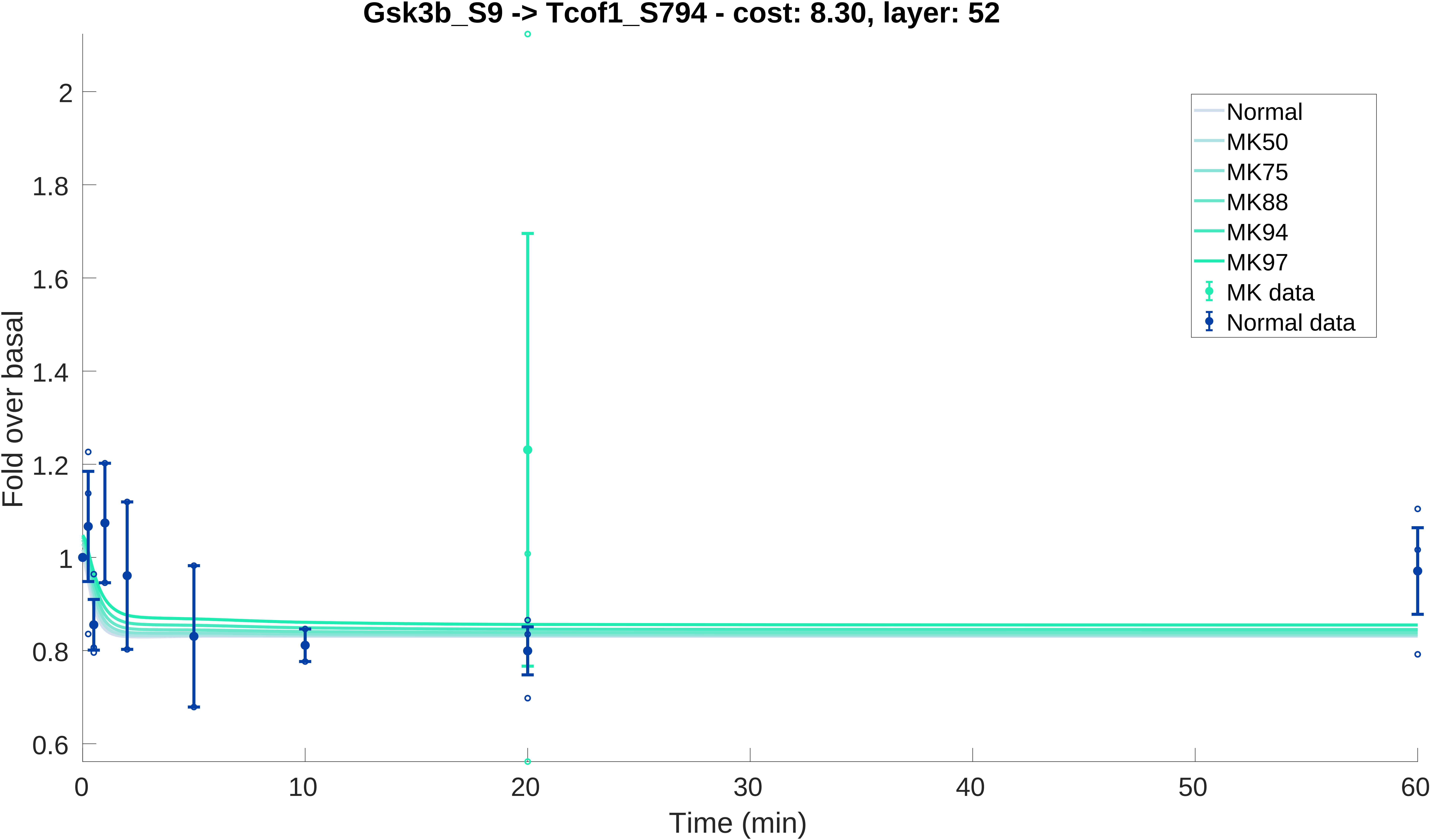
Gsk3b_S9 -> Tcof1_S794 - cost: 8.30, layer: 52
Fold over basal
Time (min)
0.6
0.8
1
1.2
1.4
1.6
1.8
2
0
10
20
30
40
50
60
Normal
MK50
MK75
MK88
MK94
MK97
MK data
Normal data

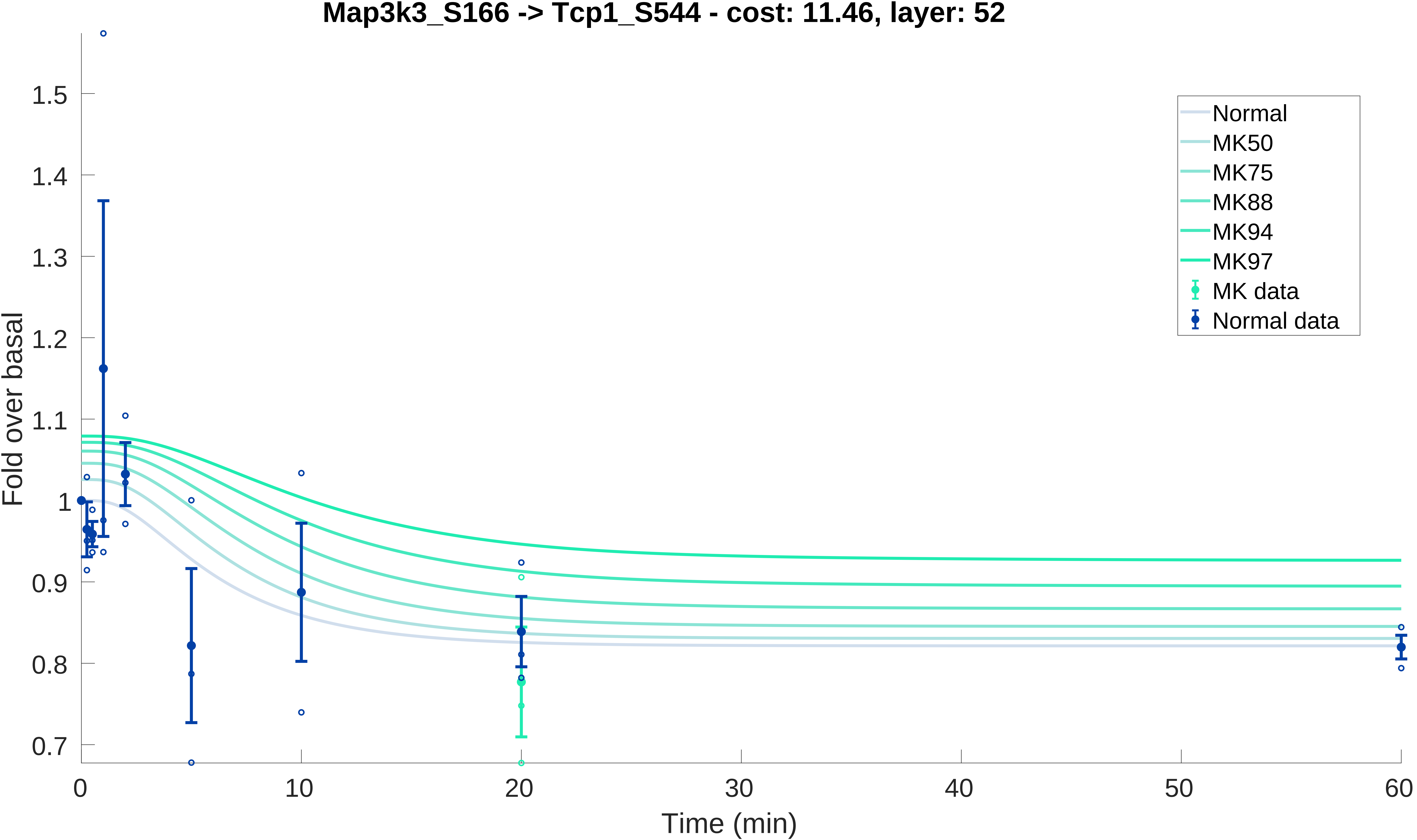

Map3k3_S166 -> Tcp1_S544 - cost: 11.46, layer: 52
Fold over basal
Time (min)
0.7
0.8
0.9
1
1.1
1.2
1.3
1.4
1.5
0
10
20
30
40
50
60
Normal
MK50
MK75
MK88
MK94
MK97
MK data
Normal data

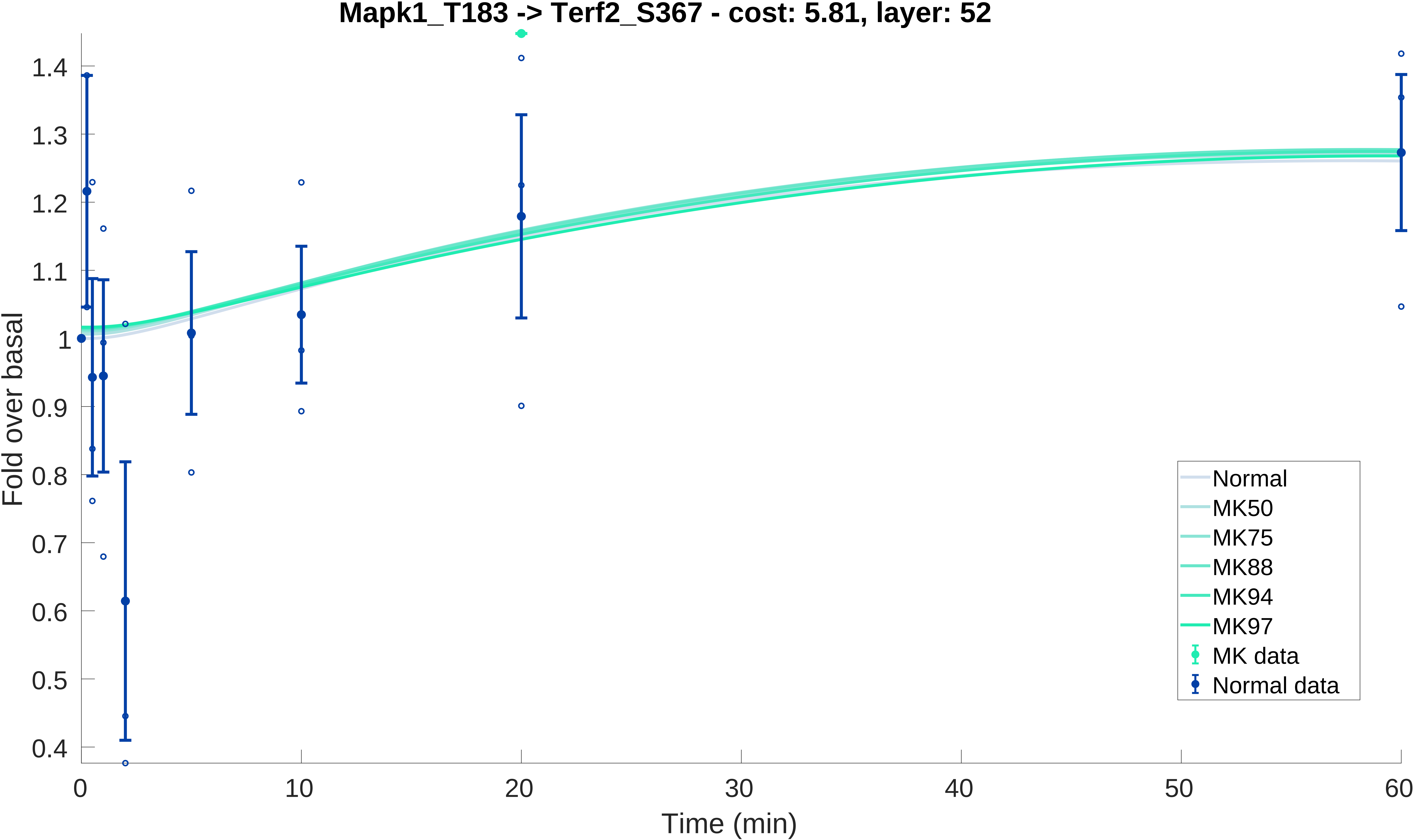
Mapk1_T183 -> Terf2_S367 - cost: 5.81, layer: 52
Fold over basal
Time (min)
0
10
20
30
40
50
60
0.4
0.5
0.6
0.7
0.8
0.9
1
1.1
1.2
1.3
1.4
Normal
MK50
MK75
MK88
MK94
MK97
MK data
Normal data

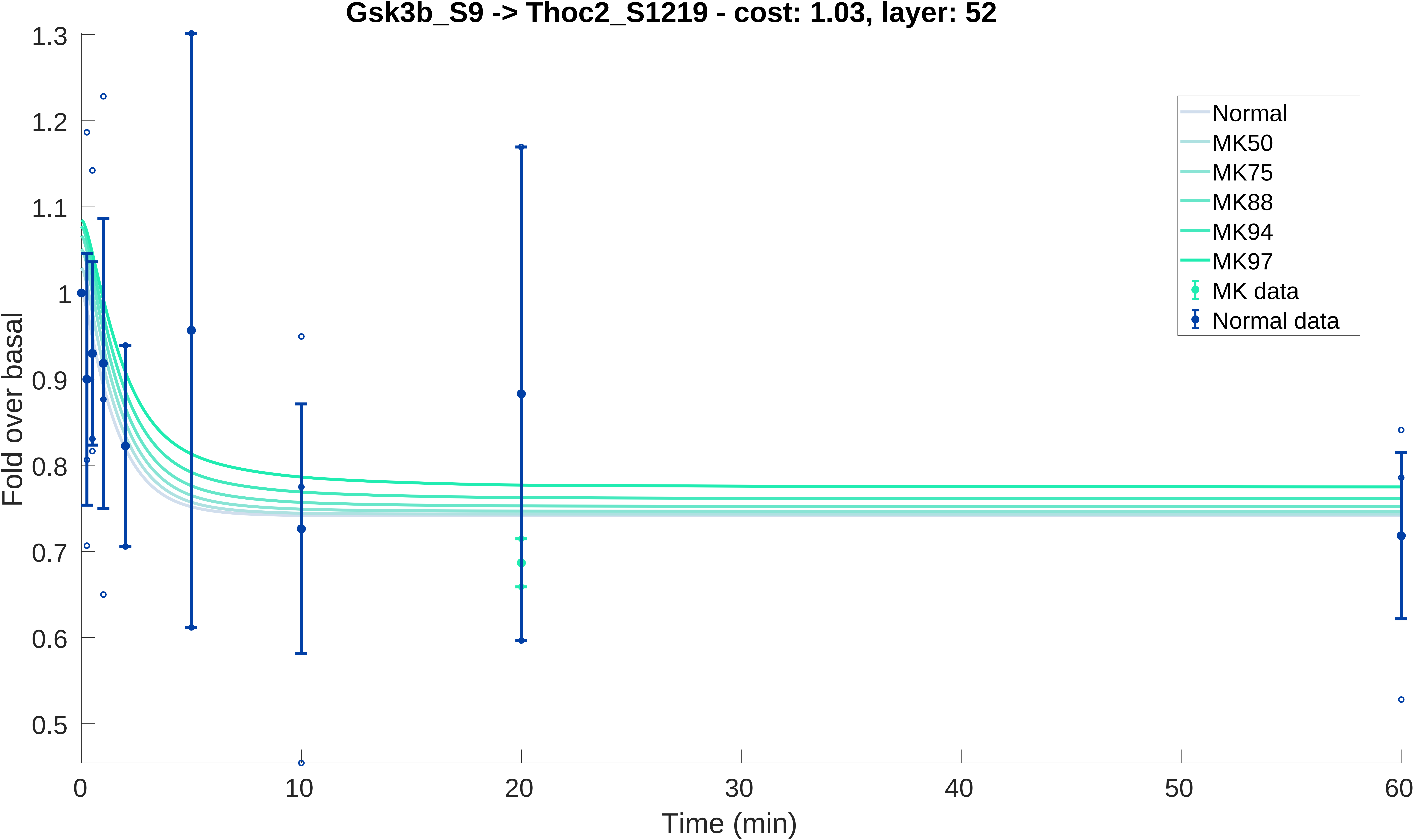

Gsk3b_S9 -> Thoc2_S1219 - cost: 1.03, layer: 52
Fold over basal
Time (min)
1.3
1.2
1.1
1
0.9
0.8
0.7
0.6
0.5
0
10
20
30
40
50
60
Normal
MK50
MK75
MK88
MK94
MK97
MK data
Normal data

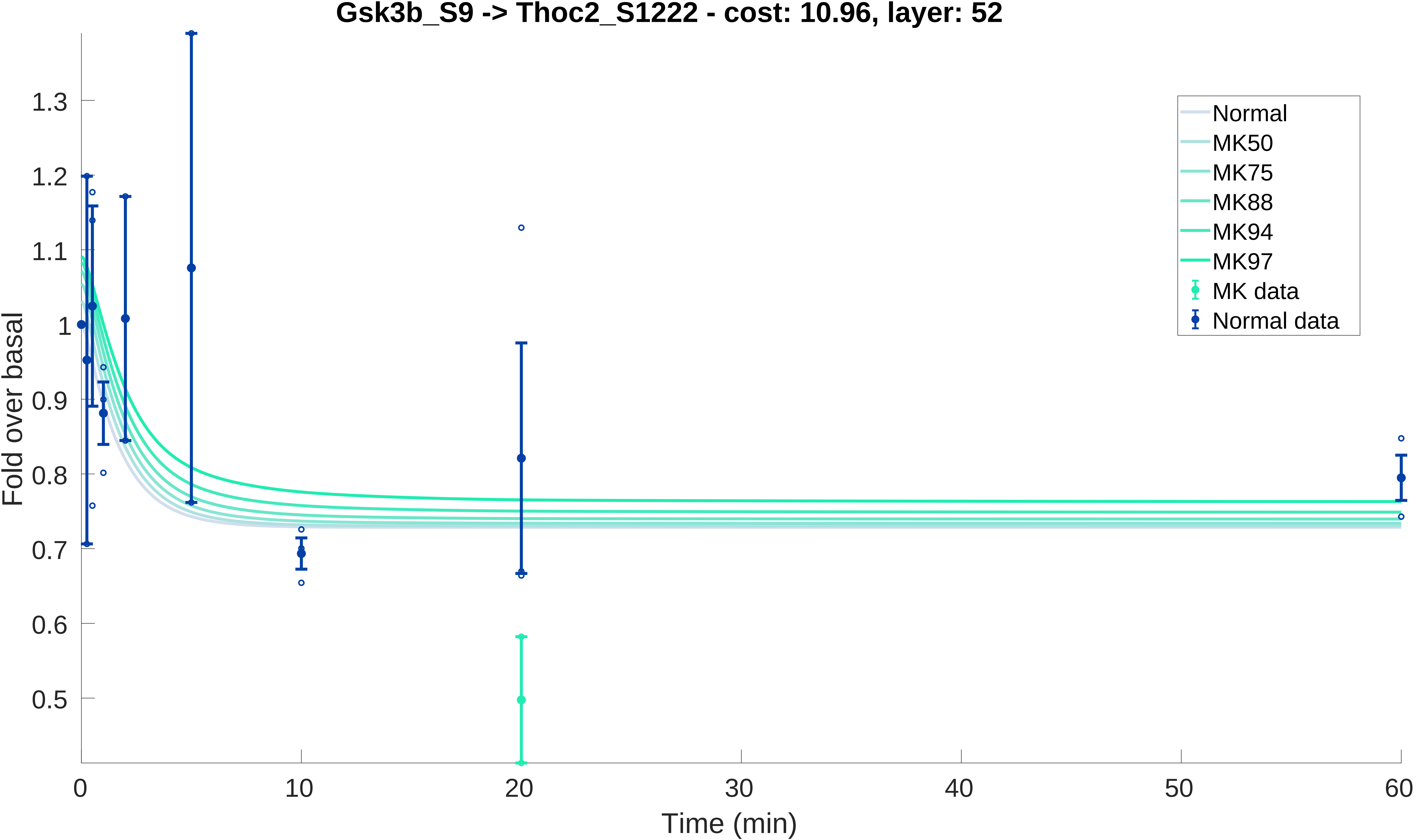

Gsk3b_S9 -> Thoc2_S1222 - cost: 10.96, layer: 52
Fold over basal
Time (min)
1.3
1.2
1.1
1
0.9
0.8
0.7
0.6
0.5
0
10
20
30
40
50
60
Normal
MK50
MK75
MK88
MK94
MK97
MK data
Normal data

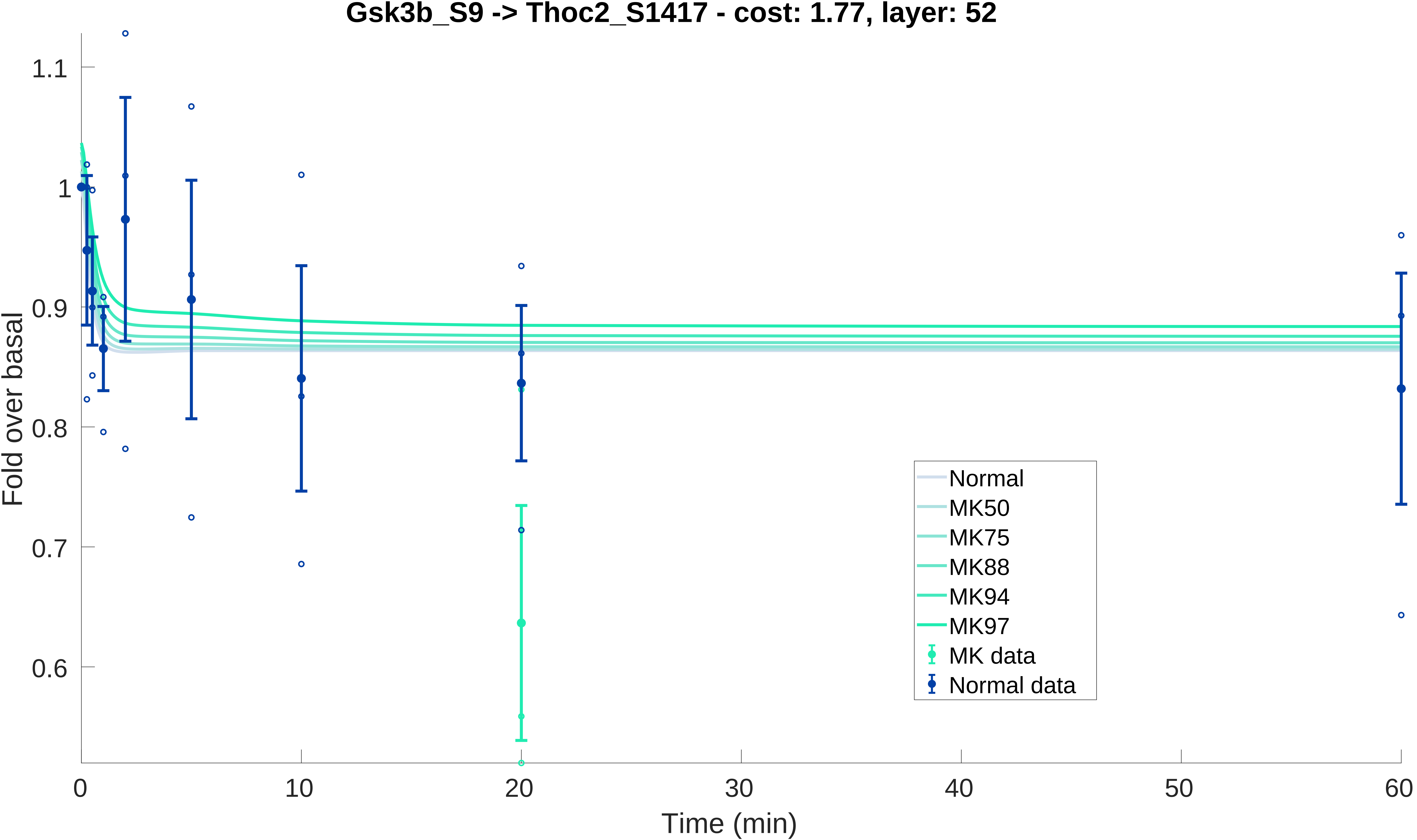
Gsk3b_S9 -> Thoc2_S1417 - cost: 1.77, layer: 52
Fold over basal
Time (min)
1.1
1
0.9
0.8
0.7
0.6
0
10
20
30
40
50
60
Normal
MK50
MK75
MK88
MK94
MK97
MK data
Normal data

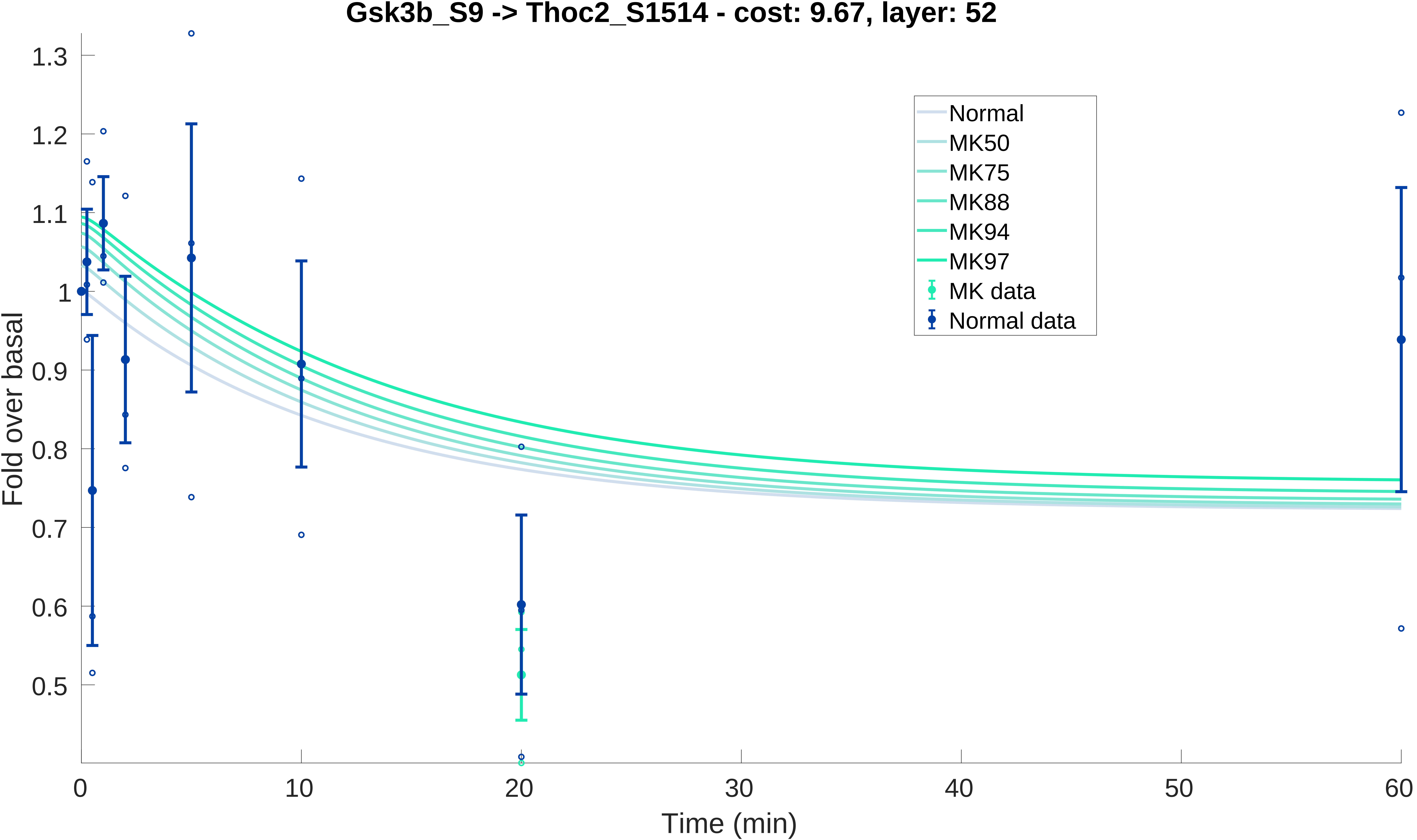

Gsk3b_S9 -> Thoc2_S1514 - cost: 9.67, layer: 52
Fold over basal
Time (min)
1.3
1.2
1.1
1
0.9
0.8
0.7
0.6
0.5
0
10
20
30
40
50
60
Normal
MK50
MK75
MK88
MK94
MK97
MK data
Normal data

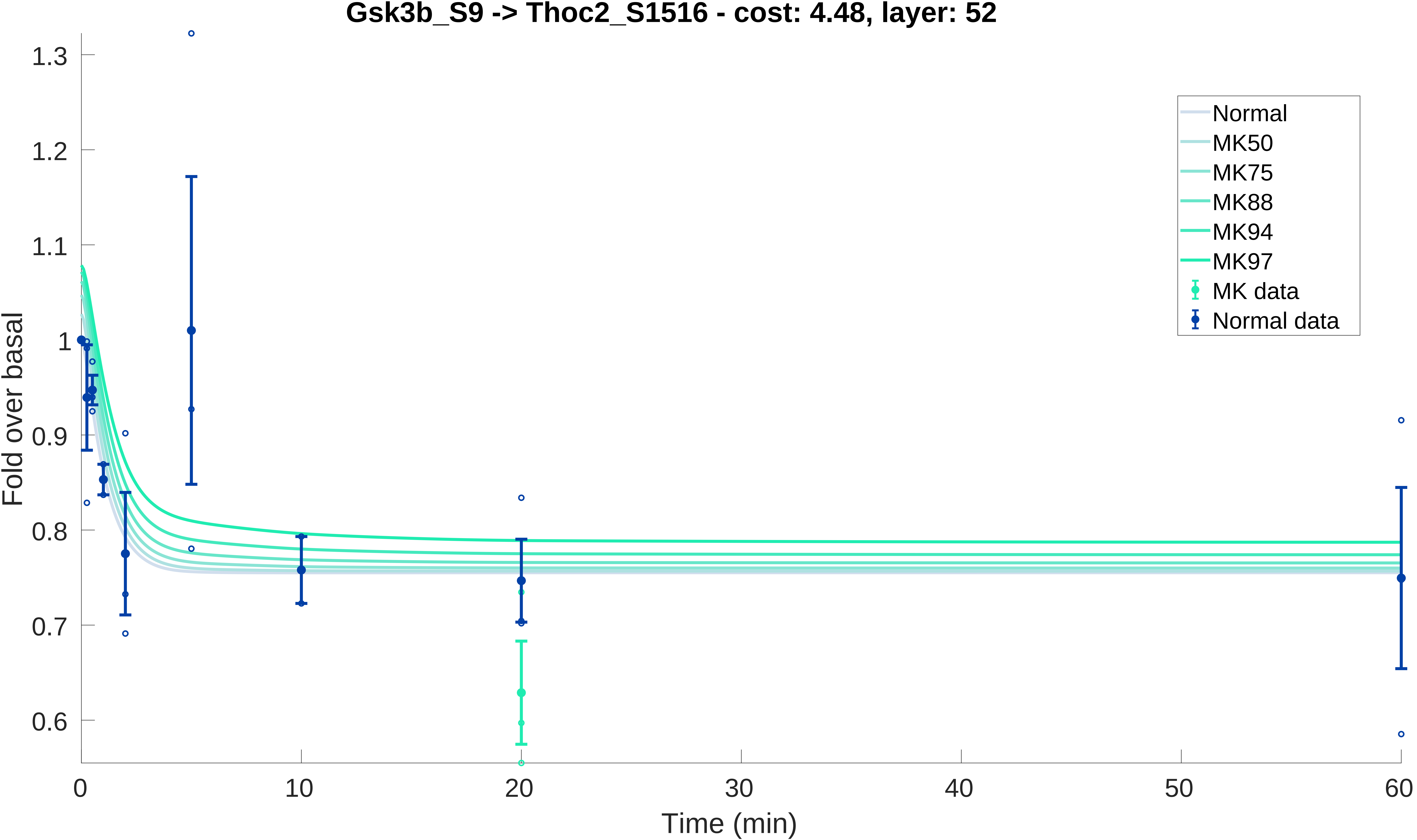

Gsk3b_S9 -> Thoc2_S1516 - cost: 4.48, layer: 52
Fold over basal
1.3
1.2
1.1
1
0.9
0.8
0.7
0.6
0
10
20
30
40
50
60
Time (min)
Normal
MK50
MK75
MK88
MK94
MK97
MK data
Normal data

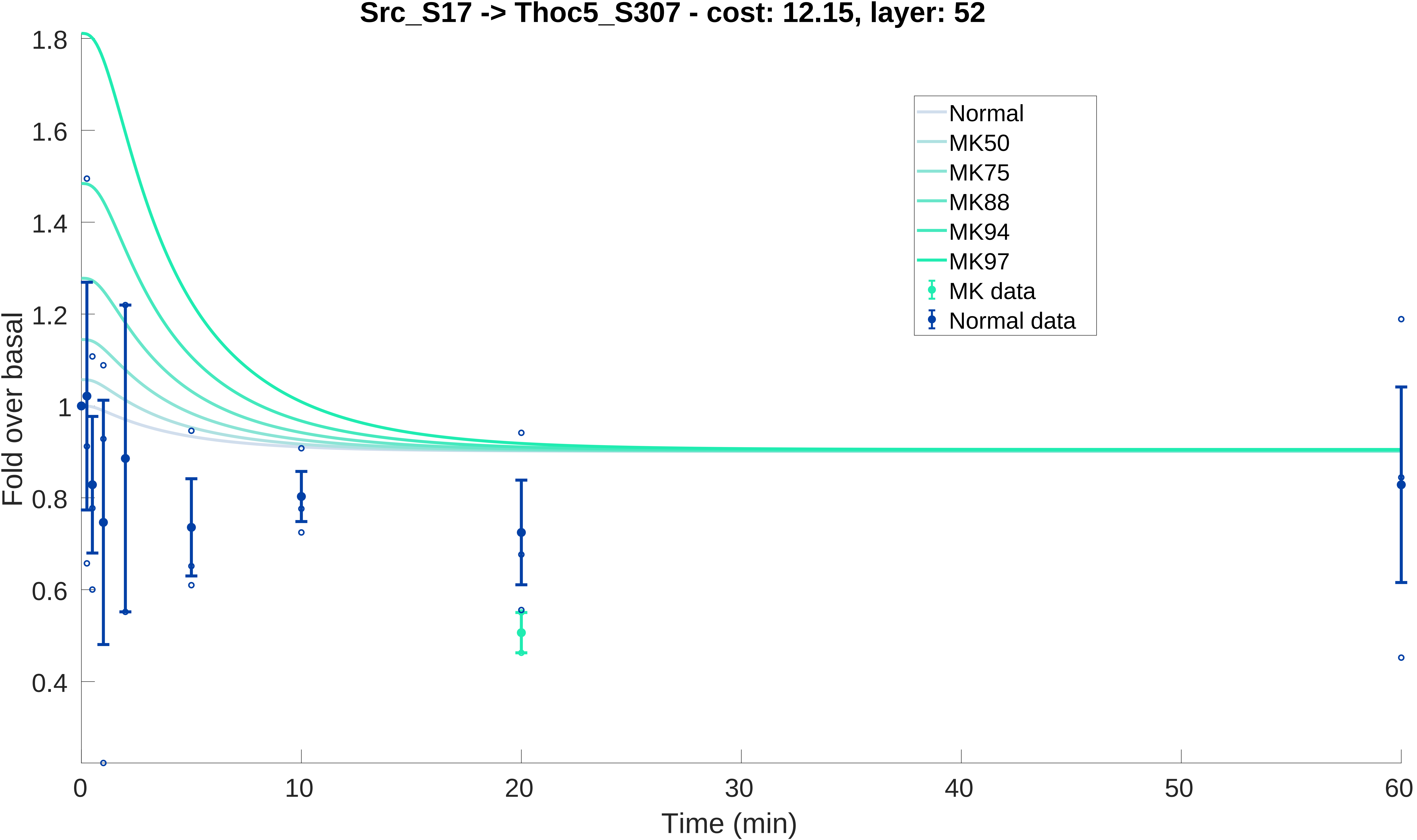
Src_S17 -> Thoc5_S307 - cost: 12.15, layer: 52
Fold over basal
Time (min)
1.8
1.6
1.4
1.2
1
0.8
0.6
0.4
0
10
20
30
40
50
60
Normal
MK50
MK75
MK88
MK94
MK97
MK data
Normal data

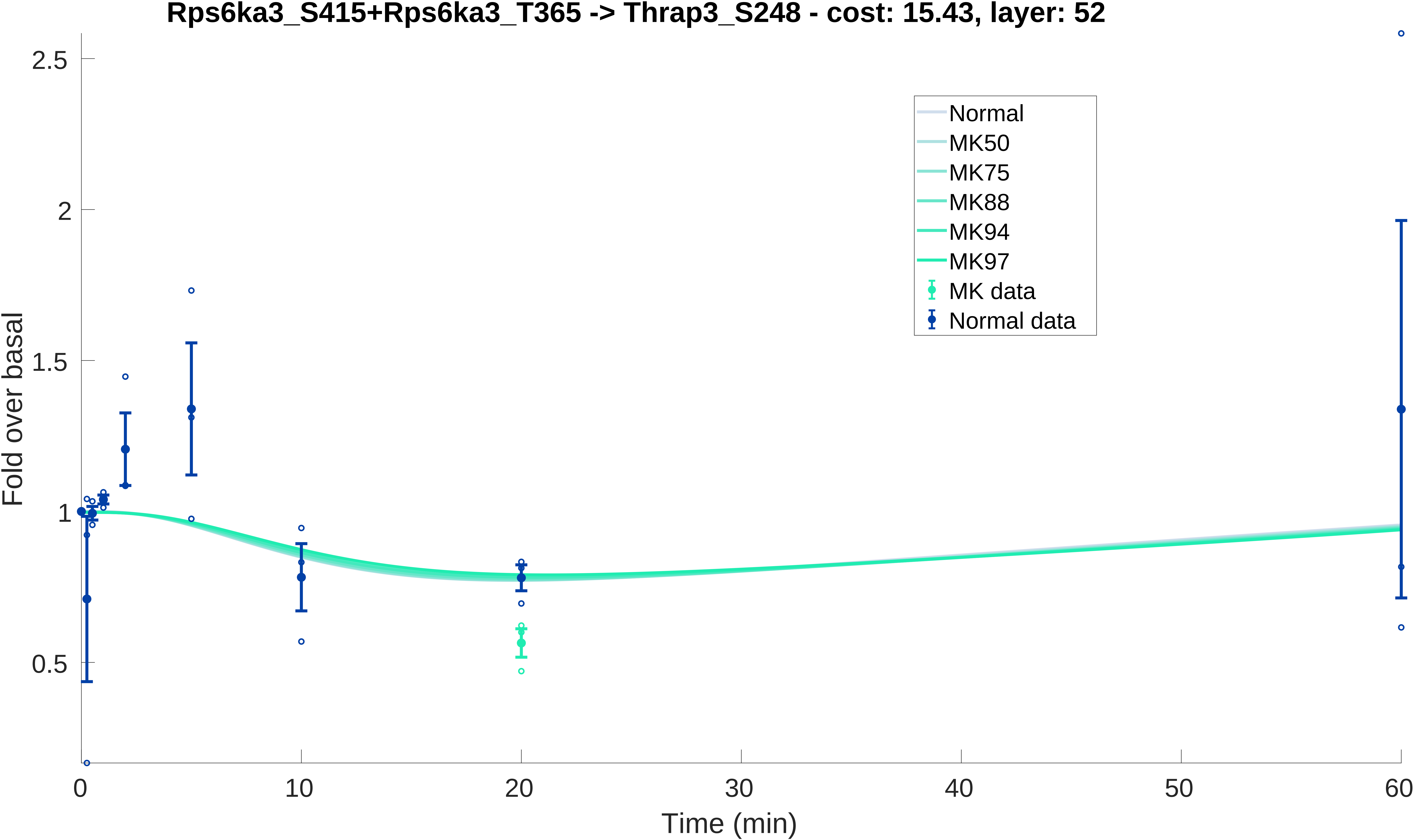

Rps6ka3_S415+Rps6ka3_T365 -> Thrap3_S248 - cost: 15.43, layer: 52
Fold over basal
Time (min)
0
10
20
30
40
50
60
0.5
1
1.5
2
2.5
Normal
MK50
MK75
MK88
MK94
MK97
MK data
Normal data

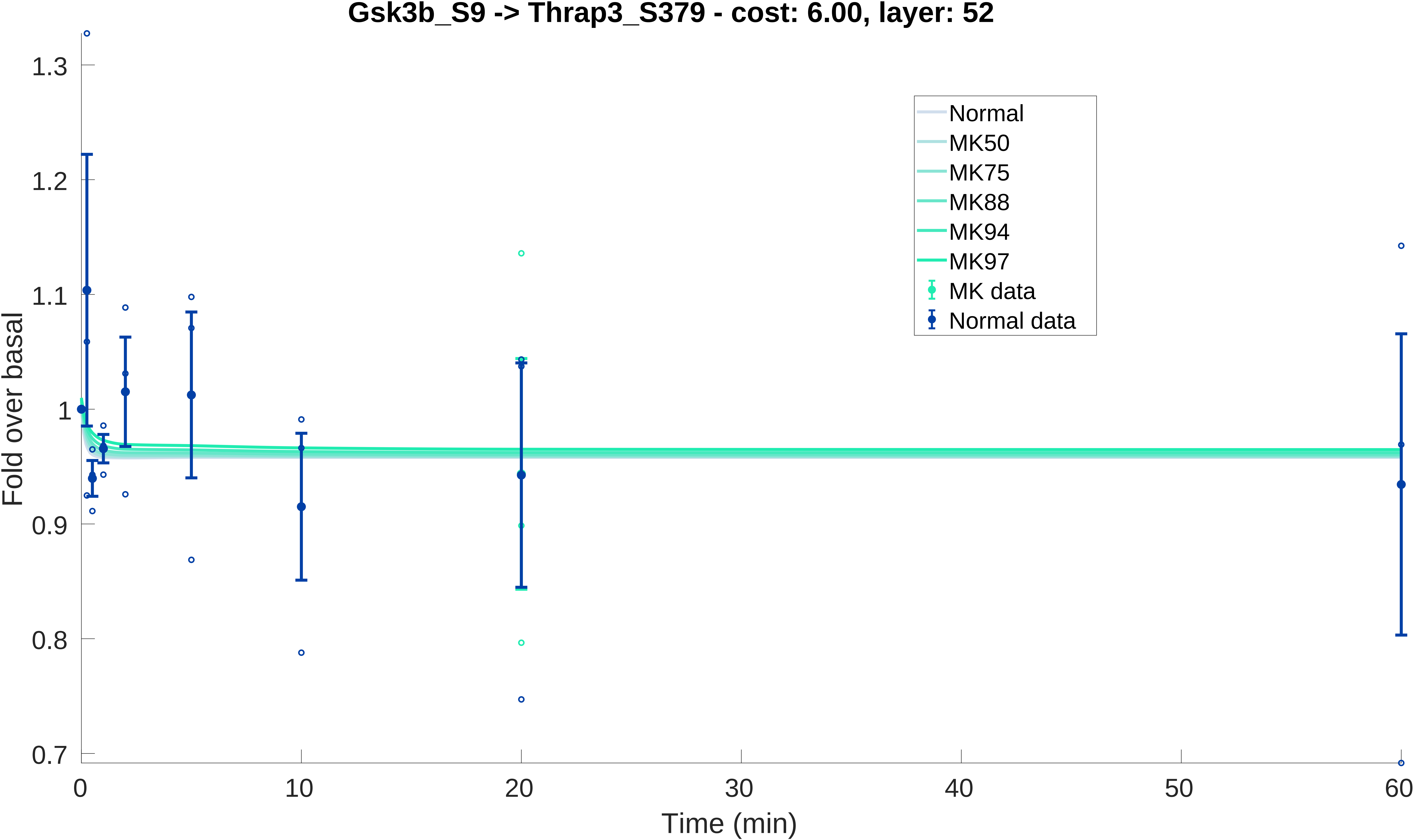
Gsk3b_S9 -> Thrap3_S379 - cost: 6.00, layer: 52
Fold over basal
Time (min)
1.3
1.2
1.1
1
0.9
0.8
0.7
0
10
20
30
40
50
60
Normal
MK50
MK75
MK88
MK94
MK97
MK data
Normal data

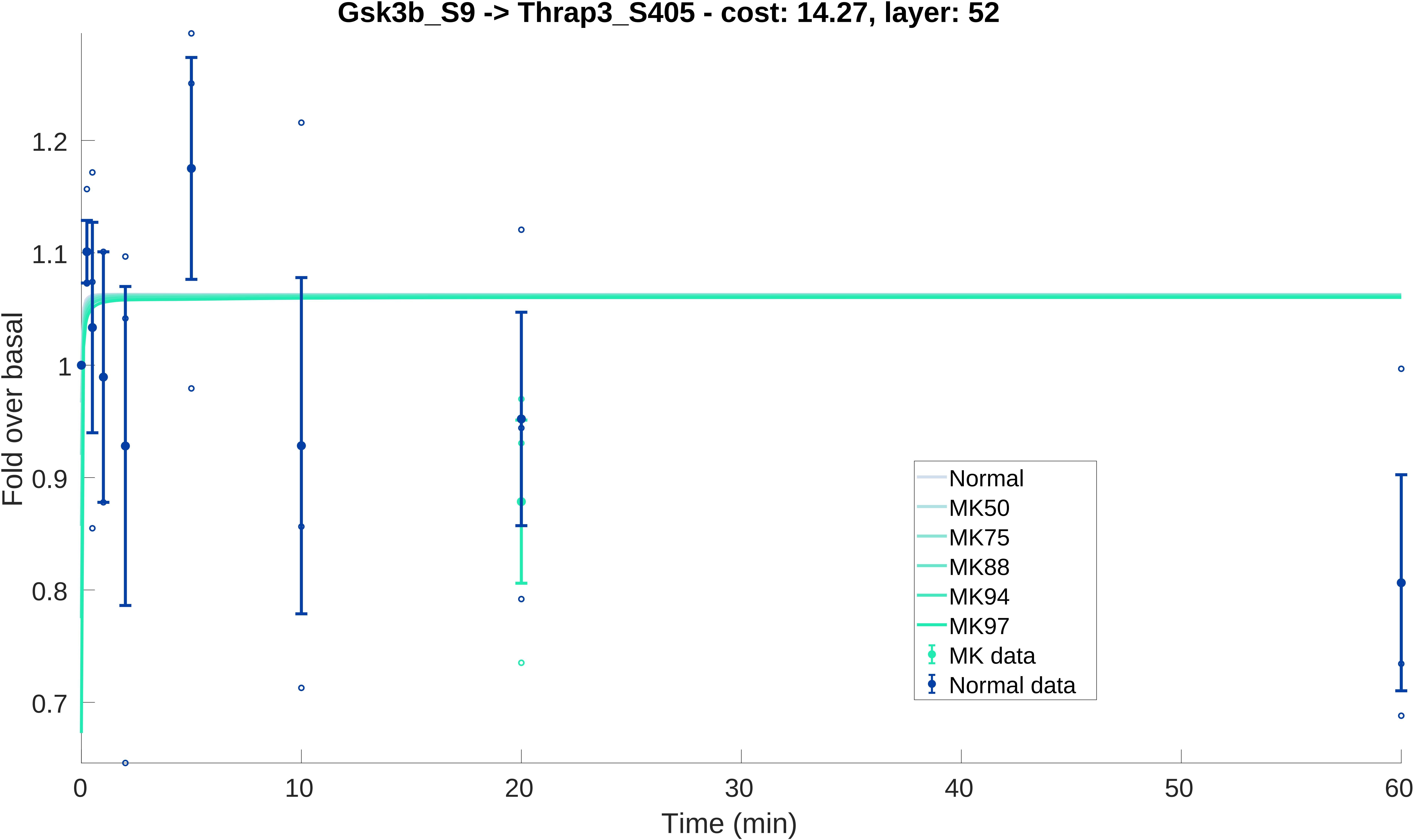
Gsk3b_S9 -> Thrap3_S405 - cost: 14.27, layer: 52
Fold over basal
Time (min)
0
10
20
30
40
50
60
0.7
0.8
0.9
1
1.1
1.2
Normal
MK50
MK75
MK88
MK94
MK97
MK data
Normal data

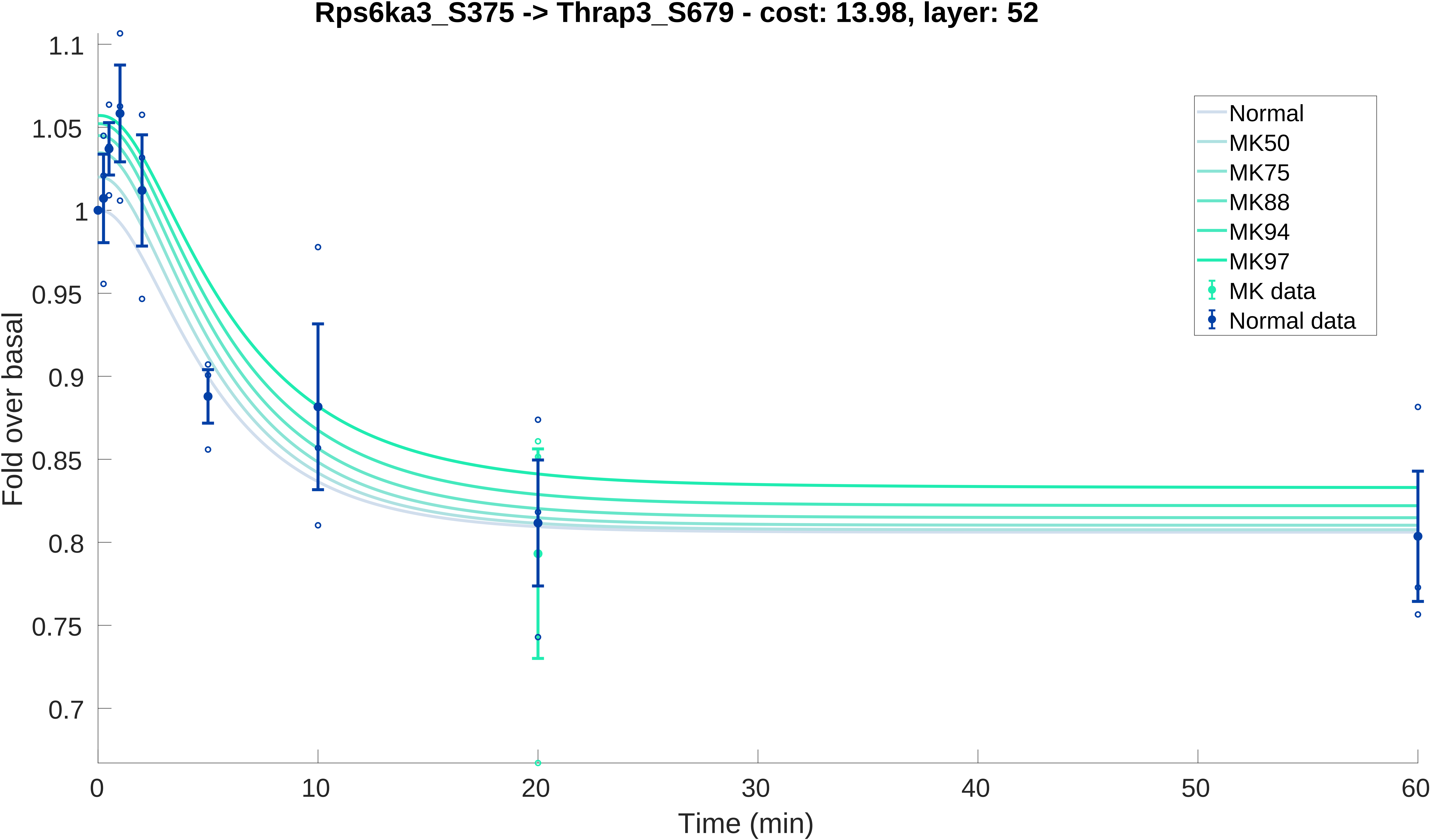

Rps6ka3_S375 -> Thrap3_S679 - cost: 13.98, layer: 52
Fold over basal
Time (min)
1.1
1.05
1
0.95
0.9
0.85
0.8
0.75
0.7
0
10
20
30
40
50
60
Normal
MK50
MK75
MK88
MK94
MK97
MK data
Normal data

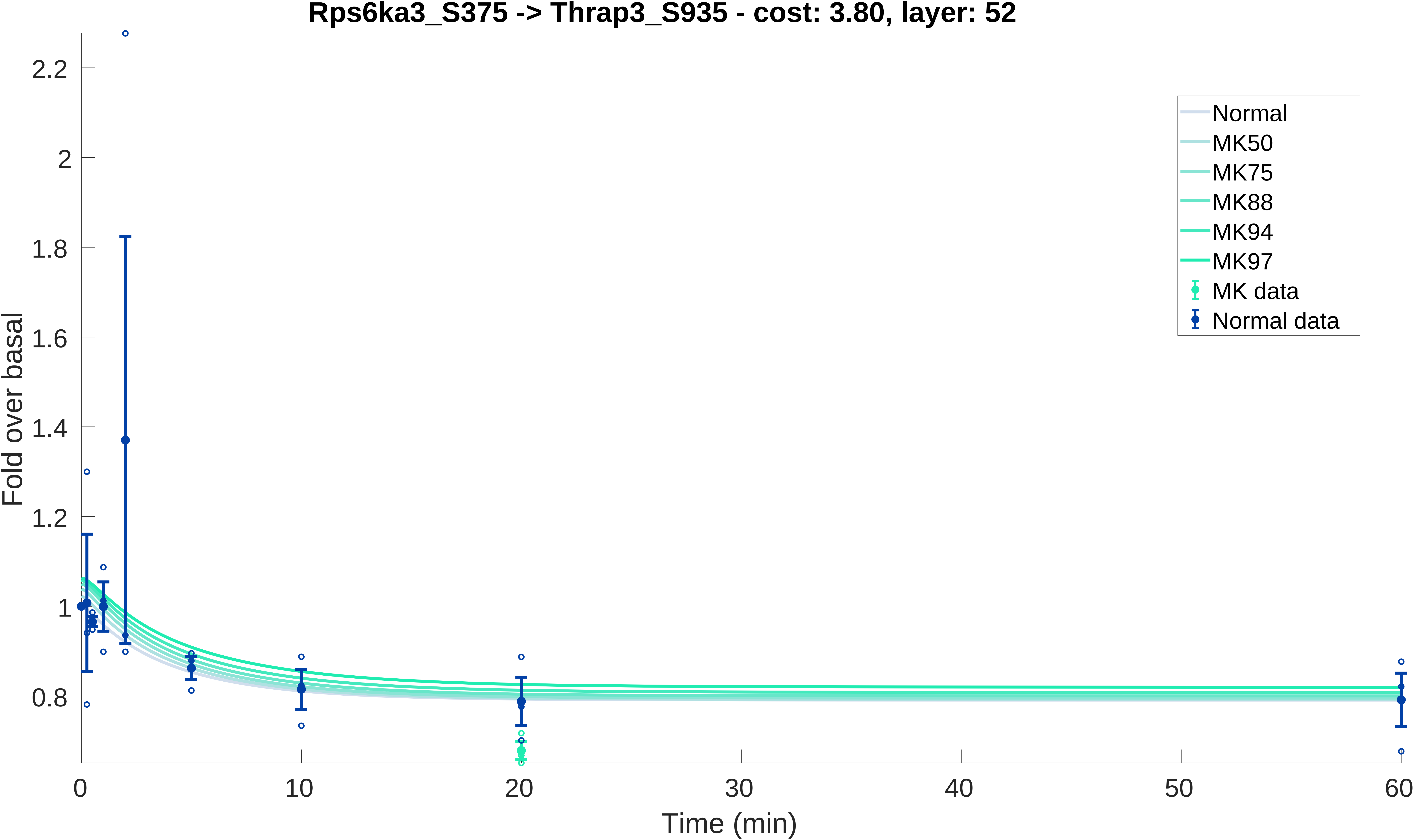
Rps6ka3_S375 -> Thrap3_S935 - cost: 3.80, layer: 52
Fold over basal
Time (min)
Normal
MK50
MK75
MK88
MK94
MK97
MK data
Normal data
0.8
1
1.2
1.4
1.6
1.8
2
2.2
0
10
20
30
40
50
60

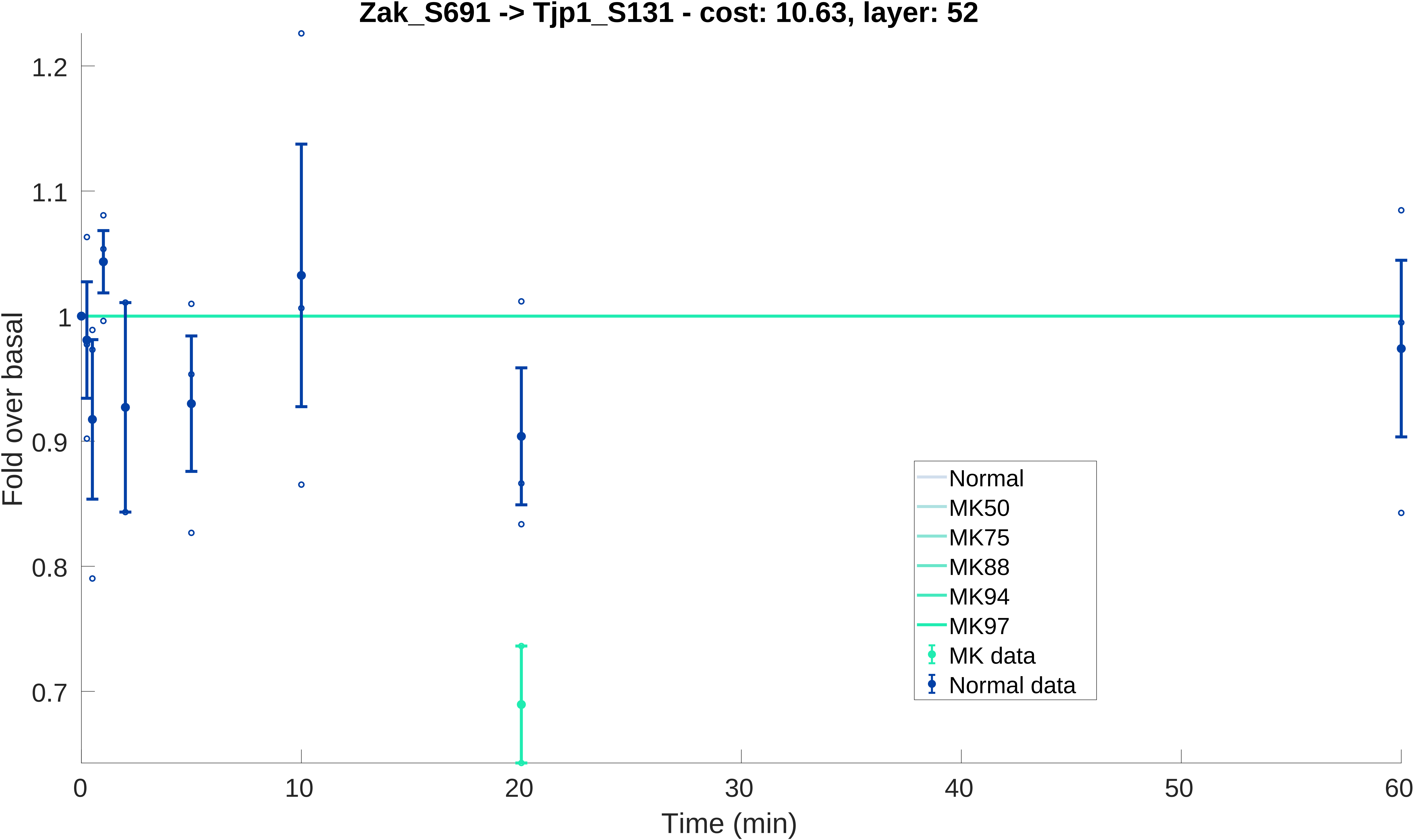
Zak_S691 -> Tjp1_S131 - cost: 10.63, layer: 52
Fold over basal
1.2
1.1
1
0.9
0.8
0.7
0
10
20
30
40
50
60
Time (min)
Normal
MK50
MK75
MK88
MK94
MK97
MK data
Normal data

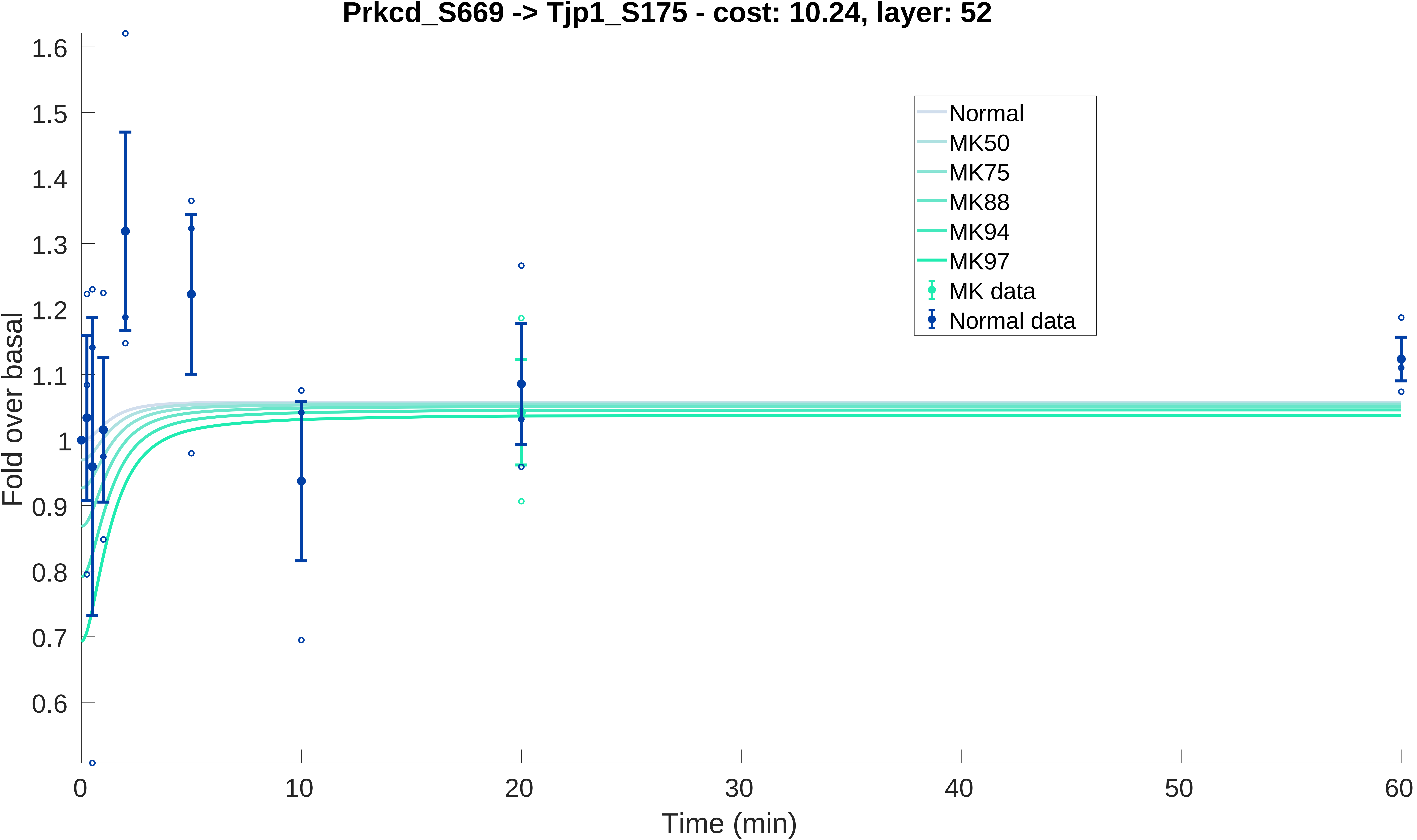

Prkcd_S669 -> Tjp1_S175 - cost: 10.24, layer: 52
Fold over basal
1.6
1.5
1.4
1.3
1.2
1.1
1
0.9
0.8
0.7
0.6
0
10
20
30
40
50
60
Time (min)
Normal
MK50
MK75
MK88
MK94
MK97
MK data
Normal data

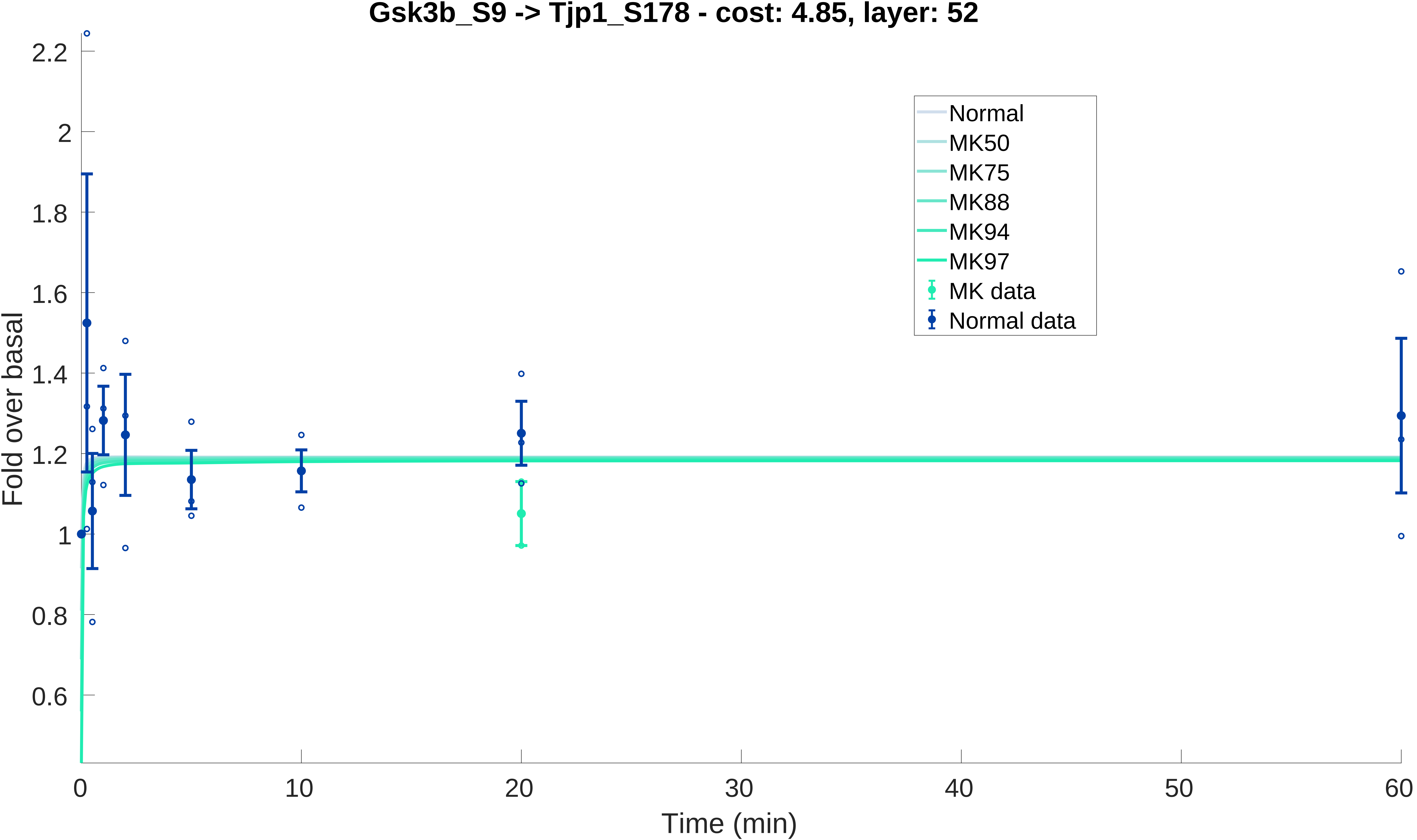

Gsk3b_S9 -> Tjp1_S178 - cost: 4.85, layer: 52
Normal
MK50
MK75
MK88
MK94
MK97
MK data
Normal data
Fold over basal
Time (min)
2.2
2
1.8
1.6
1.4
1.2
1
0.8
0.6
0
10
20
30
40
50
60

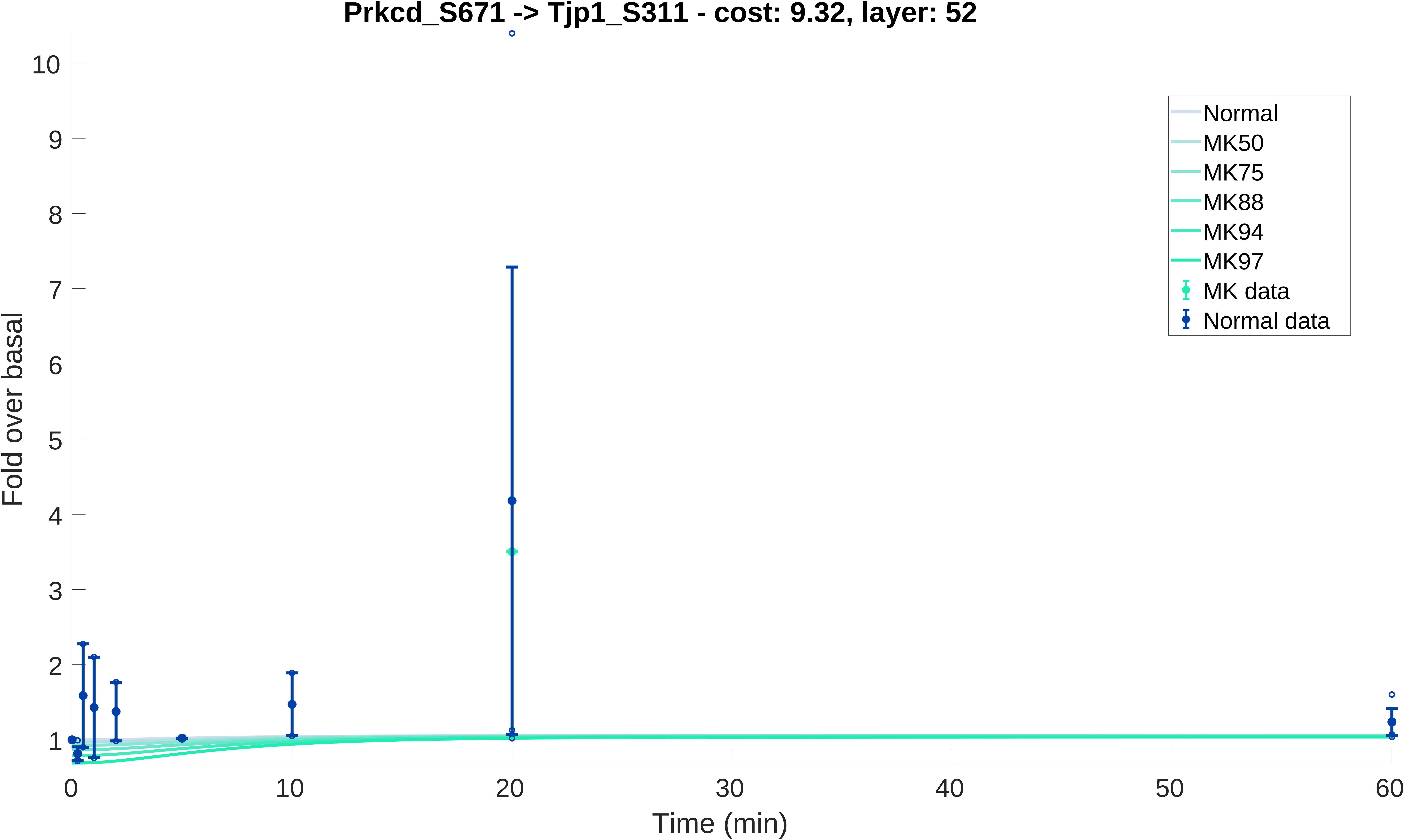
Prkcd_S671 -> Tjp1_S311 - cost: 9.32, layer: 52
Fold over basal
Time (min)
0
10
20
30
40
50
60
1
2
3
4
5
6
7
8
9
10
Normal
MK50
MK75
MK88
MK94
MK97
MK data
Normal data

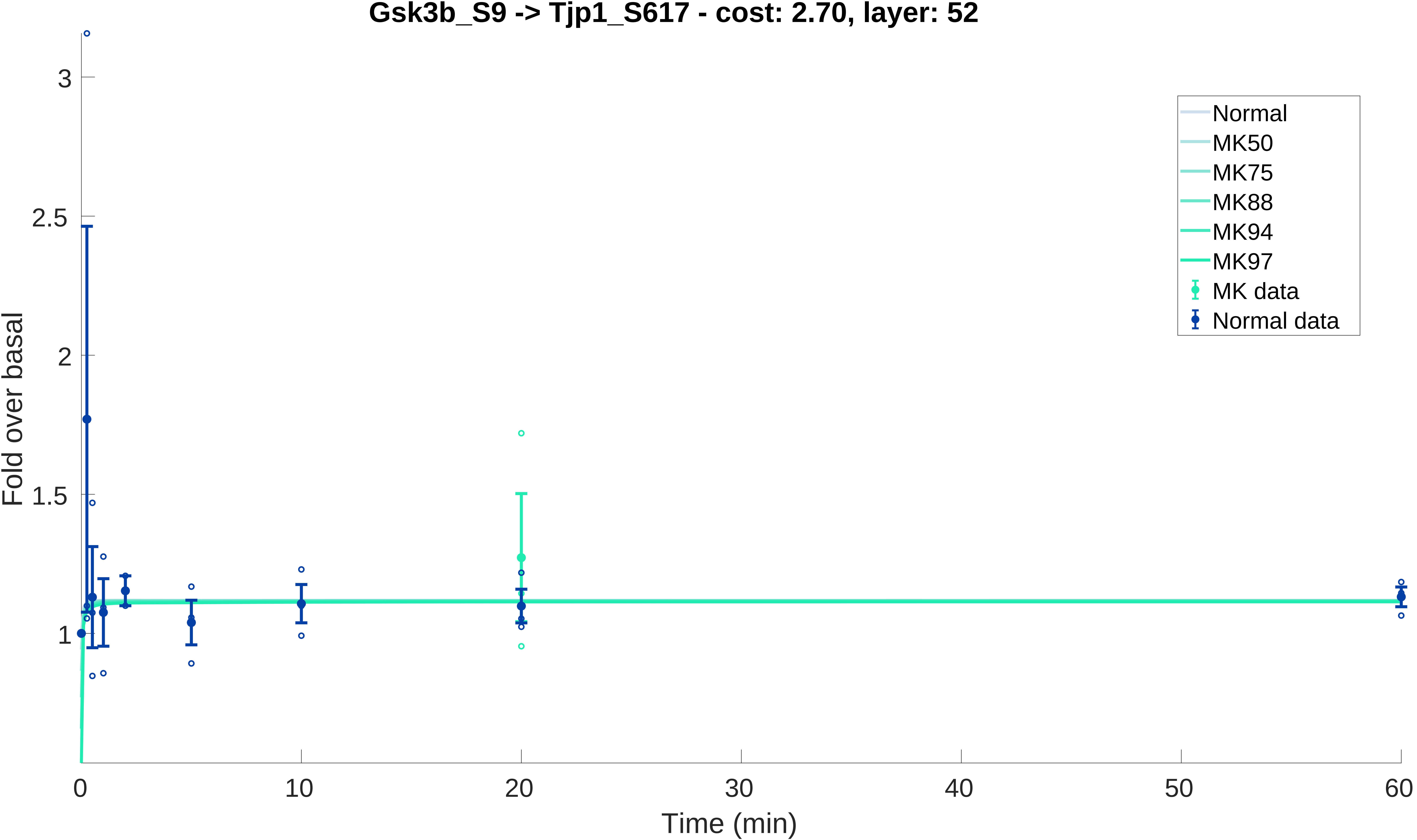

Gsk3b_S9 -> Tjp1_S617 - cost: 2.70, layer: 52
Fold over basal
3
2.5
2
1.5
1
0
10
20
30
40
50
60
Time (min)
Normal
MK50
MK75
MK88
MK94
MK97
MK data
Normal data

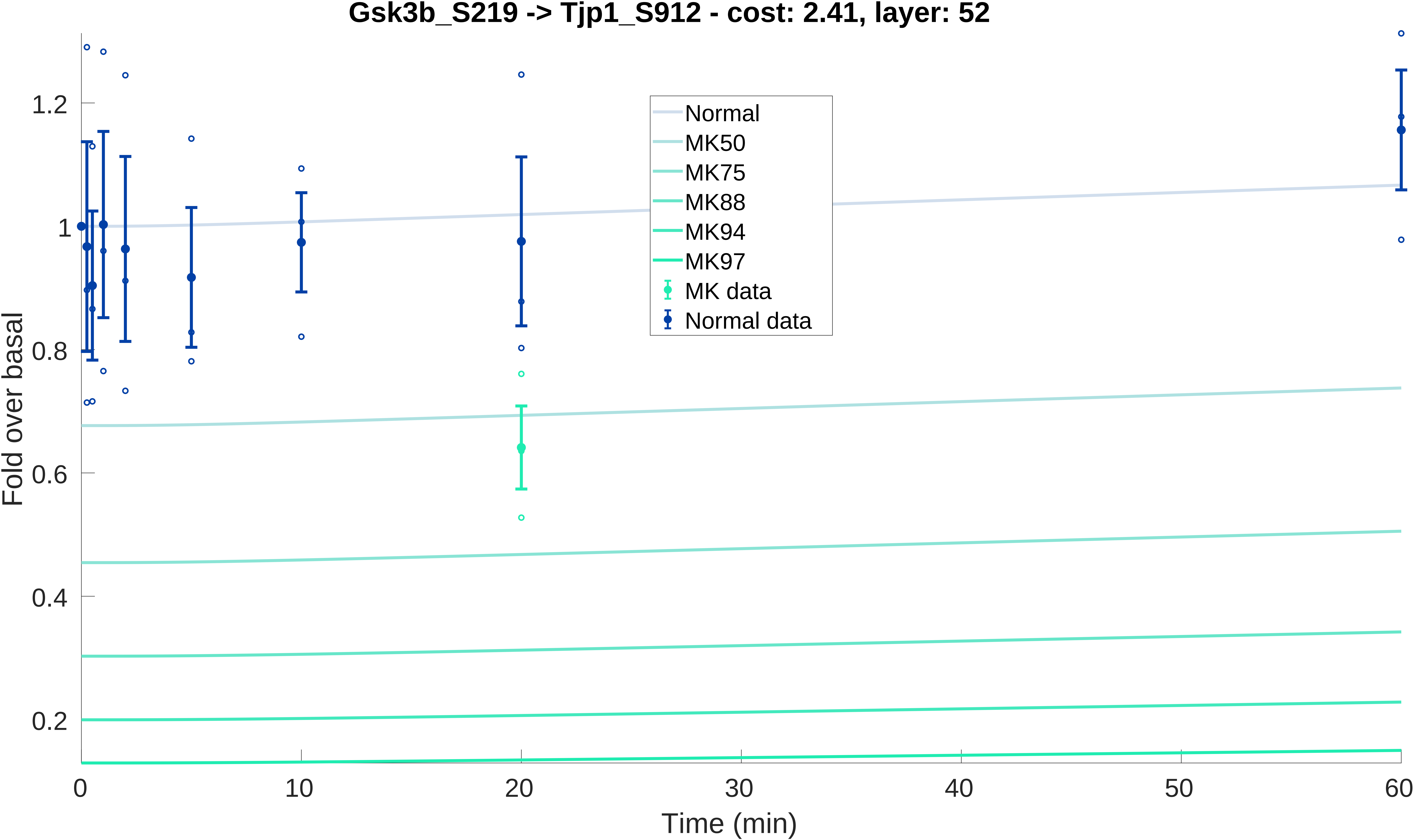

Gsk3b_S219 -> Tjp1_S912 - cost: 2.41, layer: 52
Normal
MK50
MK75
MK88
MK94
MK97
MK data
Normal data
Fold over basal
0.2
0.4
0.6
0.8
1
1.2
0
10
20
30
40
50
60
Time (min)

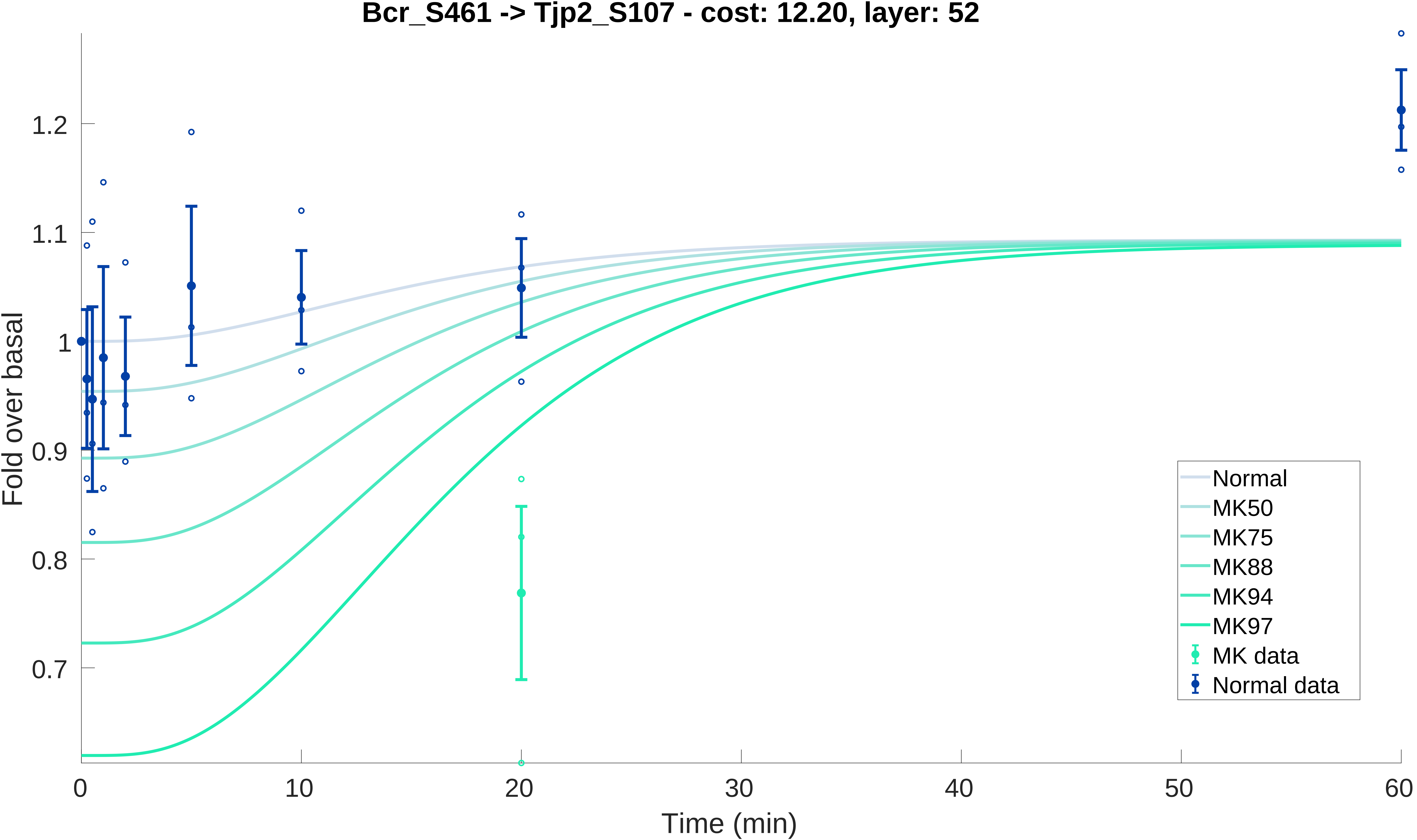

Bcr_S461 -> Tjp2_S107 - cost: 12.20, layer: 52
Fold over basal
Time (min)
0
10
20
30
40
50
60
0.7
0.8
0.9
1
1.1
1.2
Normal
MK50
MK75
MK88
MK94
MK97
MK data
Normal data

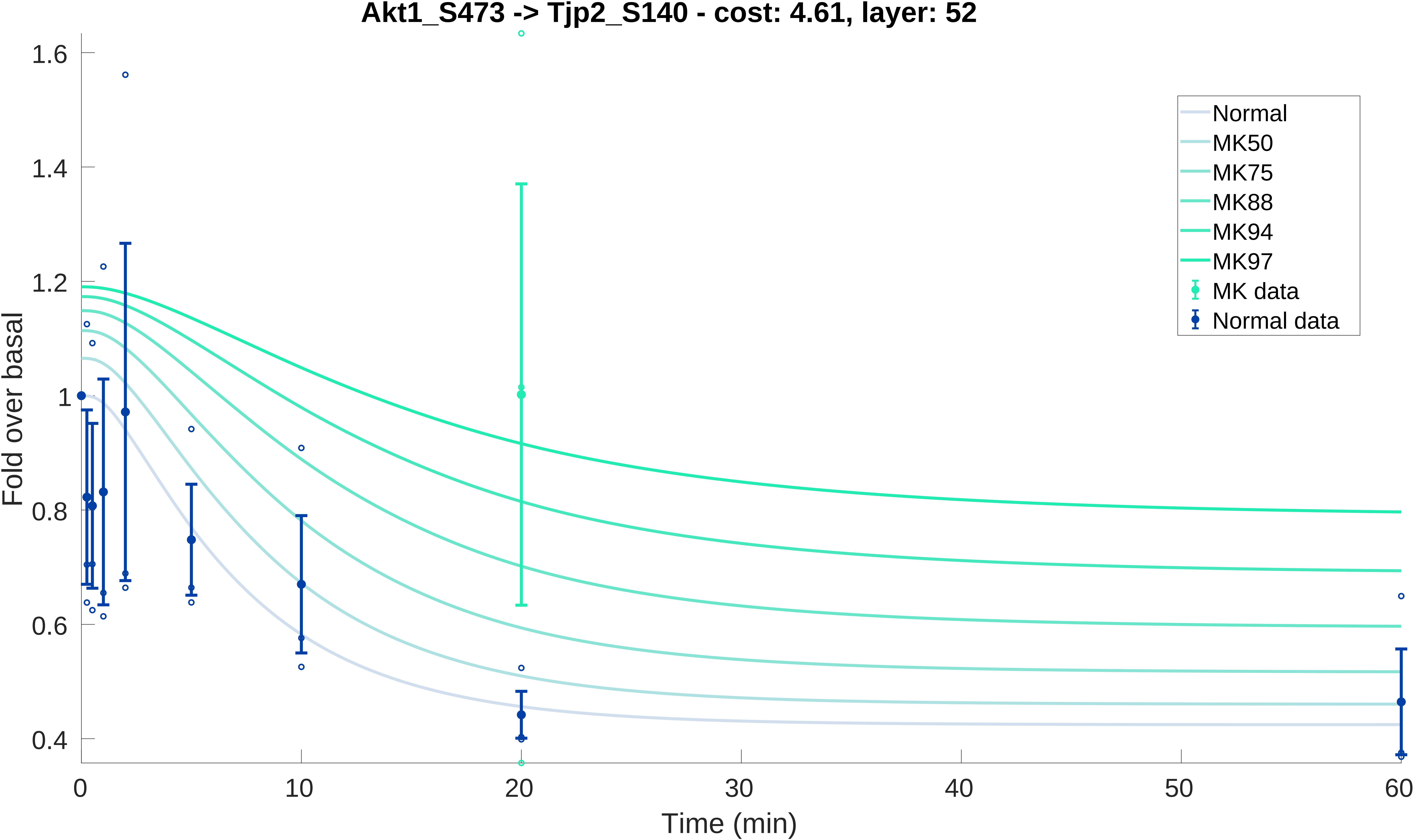
Akt1_S473 -> Tjp2_S140 - cost: 4.61, layer: 52
Fold over basal
Time (min)
1.6
1.4
1.2
1
0.8
0.6
0.4
0
10
20
30
40
50
60
Normal
MK50
MK75
MK88
MK94
MK97
MK data
Normal data

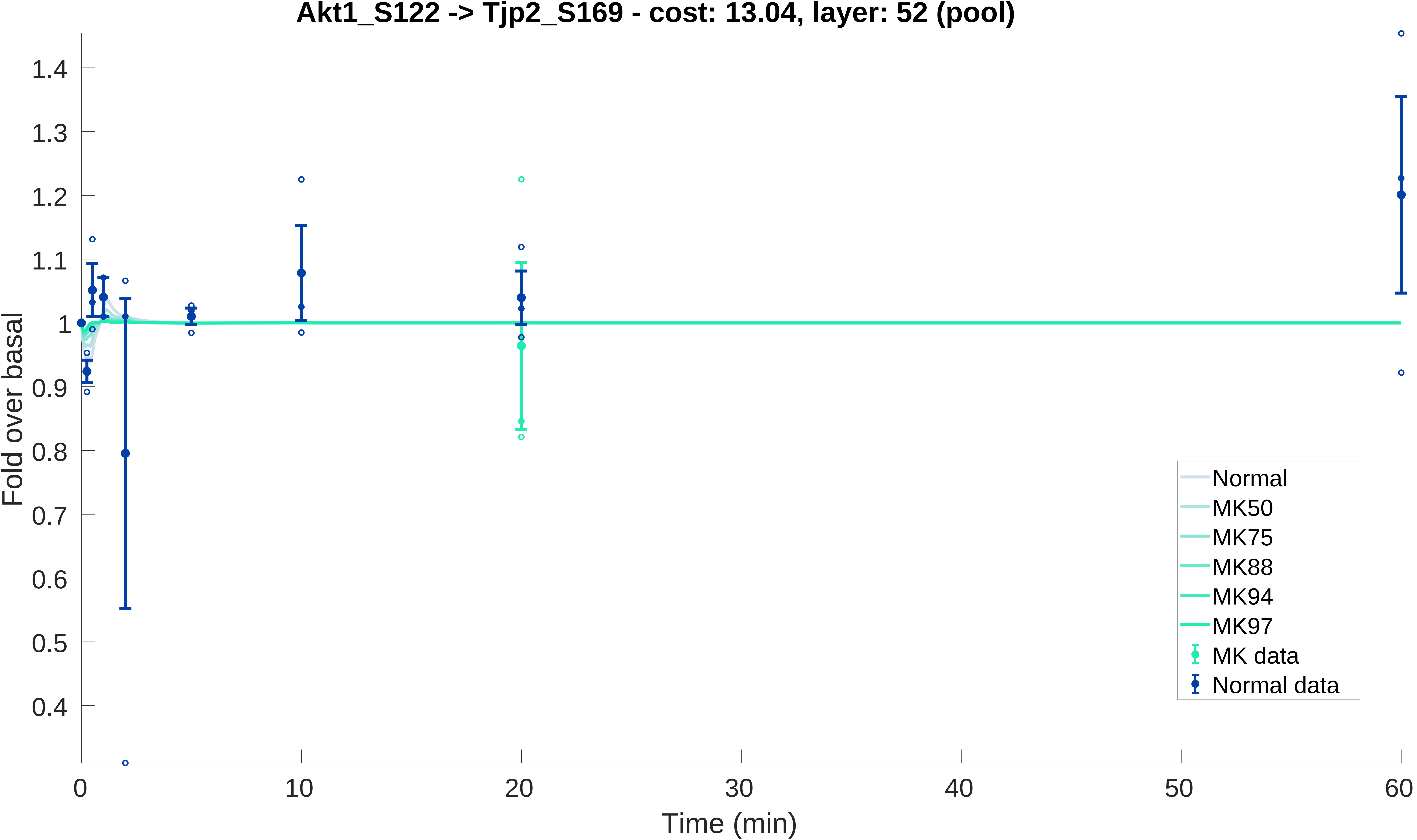
Akt1_S122 -> Tjp2_S169 - cost: 13.04, layer: 52 (pool)
Fold over basal
Time (min)
0.4
0.5
0.6
0.7
0.8
0.9
1
1.1
1.2
1.3
1.4
0
10
20
30
40
50
60
Normal
MK50
MK75
MK88
MK94
MK97
MK data
Normal data

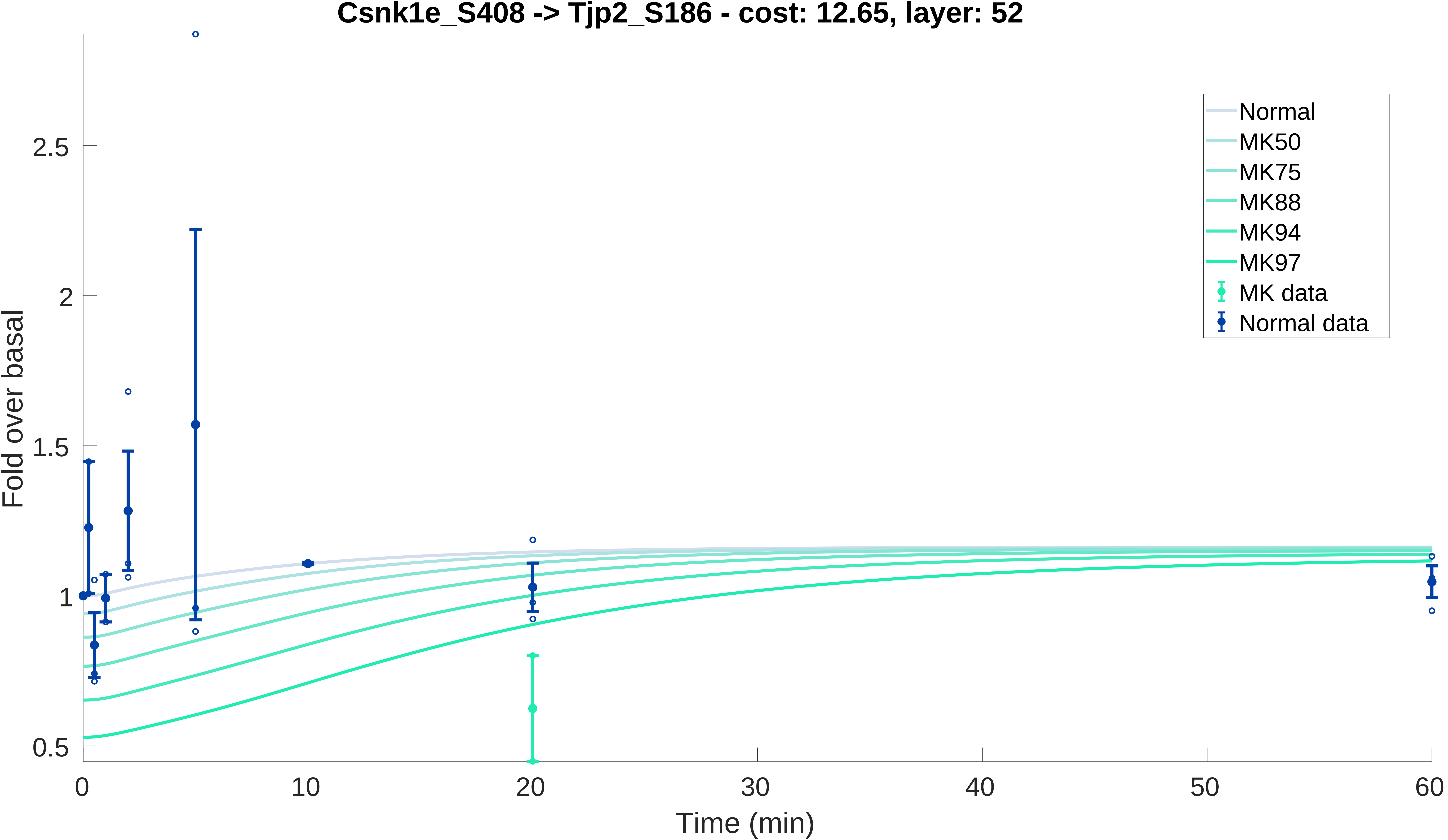

Csnk1e_S408 -> Tjp2_S186 - cost: 12.65, layer: 52
Fold over basal
Time (min)
0
10
20
30
40
50
60
0.5
1
1.5
2
2.5
Normal
MK50
MK75
MK88
MK94
MK97
MK data
Normal data

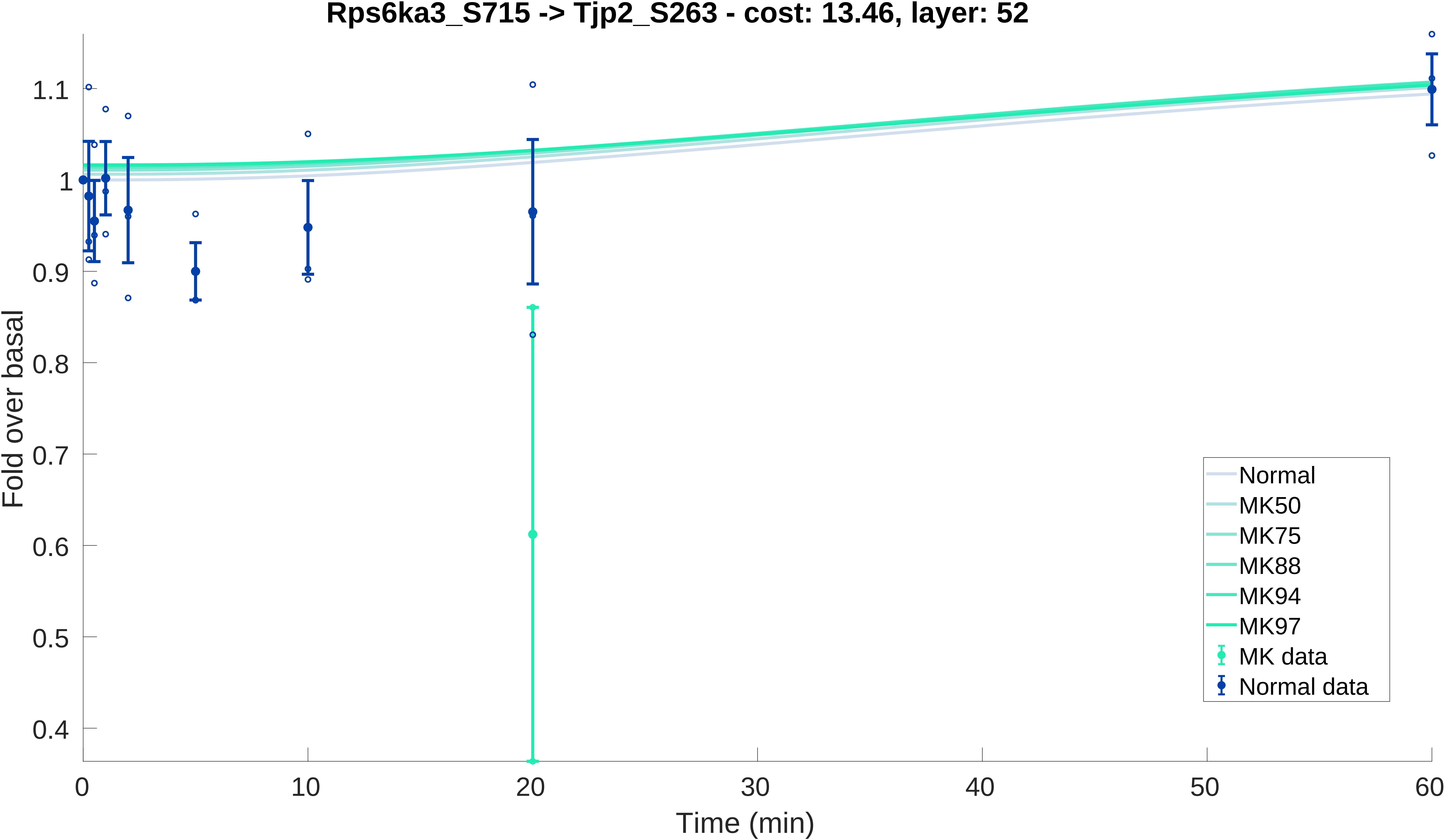

Rps6ka3_S715 -> Tjp2_S263 - cost: 13.46, layer: 52
Fold over basal
1.1
1
0.9
0.8
0.7
0.6
0.5
0.4
0
10
20
30
40
50
60
Time (min)
Normal
MK50
MK75
MK88
MK94
MK97
MK data
Normal data

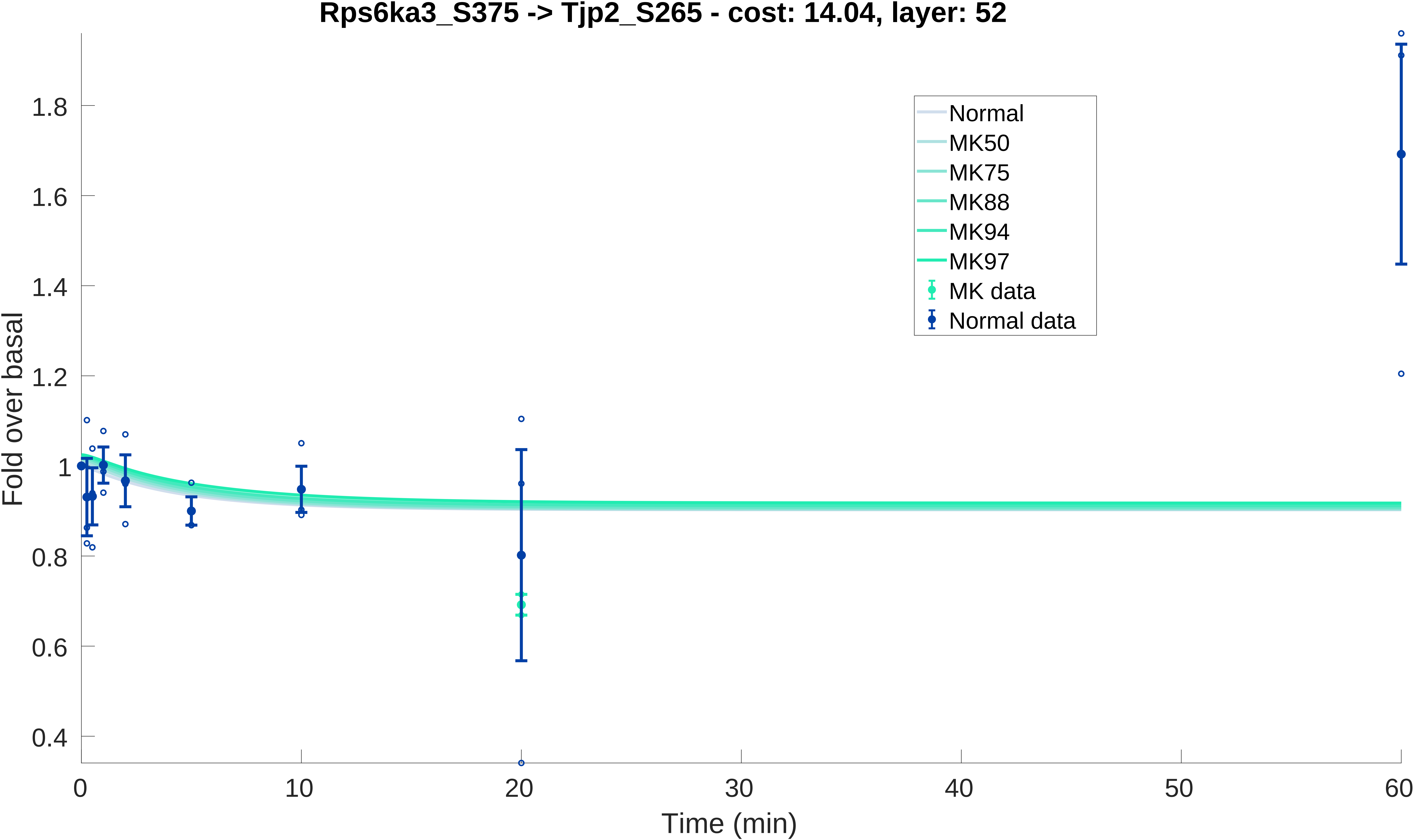

Rps6ka3_S375 -> Tjp2_S265 - cost: 14.04, layer: 52
Fold over basal
Time (min)
0
10
20
30
40
50
60
0.4
0.6
0.8
1
1.2
1.4
1.6
1.8
Normal
MK50
MK75
MK88
MK94
MK97
MK data
Normal data

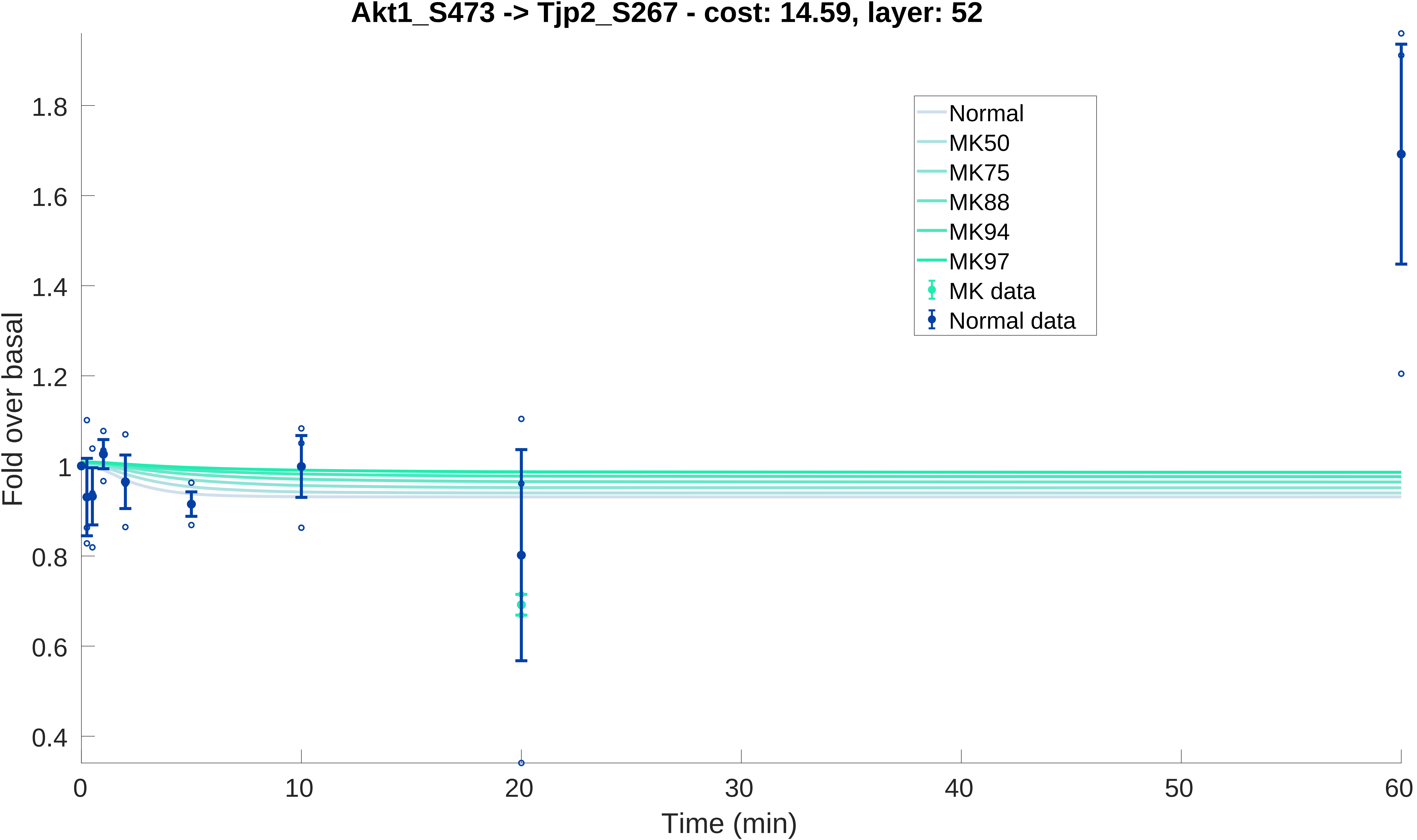
Akt1_S473 -> Tjp2_S267 - cost: 14.59, layer: 52
Normal
MK50
MK75
MK88
MK94
MK97
MK data
Normal data
Fold over basal
Time (min)
0.4
0.6
0.8
1
1.2
1.4
1.6
1.8
0
10
20
30
40
50
60

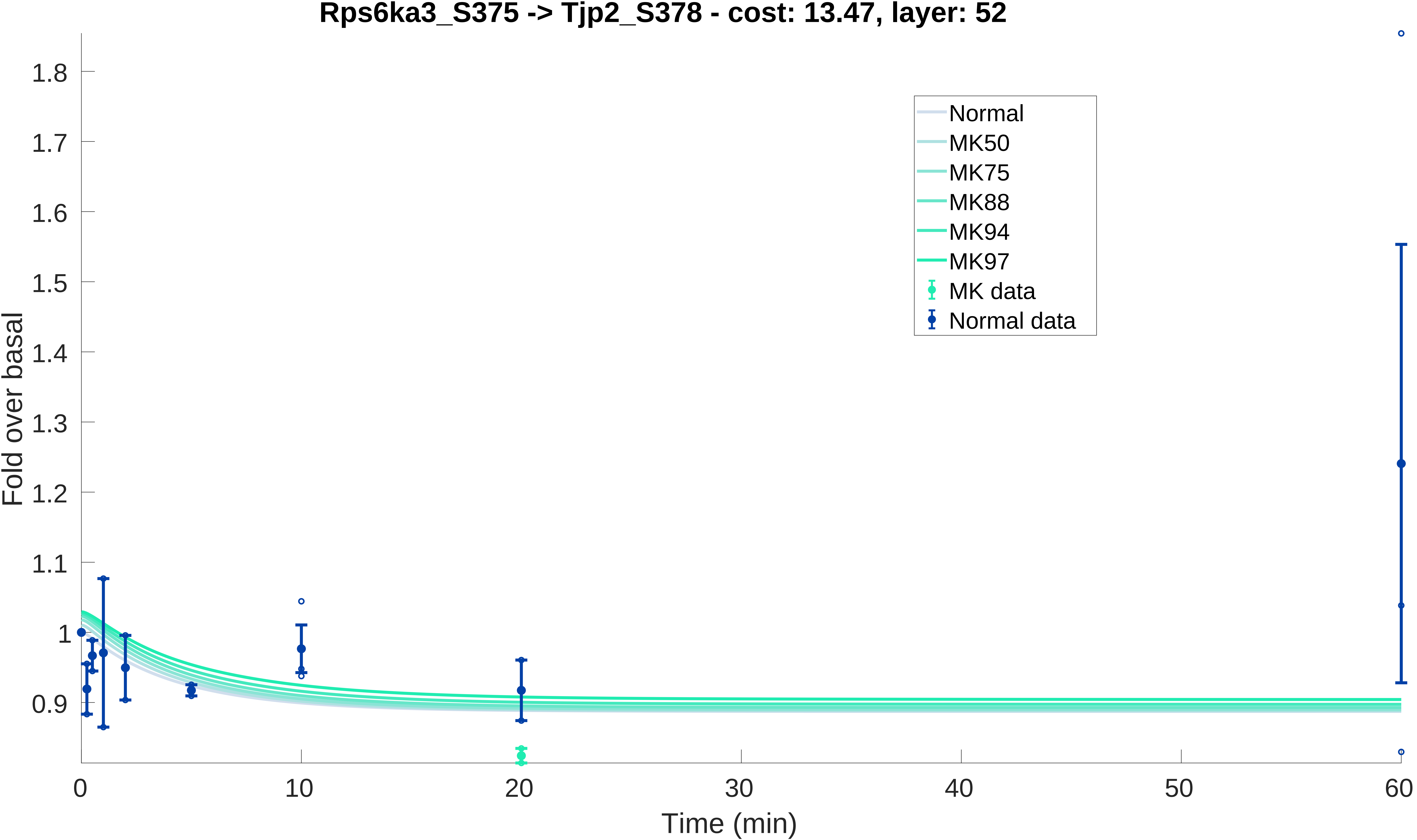
Rps6ka3_S375 -> Tjp2_S378 - cost: 13.47, layer: 52
Fold over basal
Time (min)
1.8
1.7
1.6
1.5
1.4
1.3
1.2
1.1
1
0.9
0
10
20
30
40
50
60
Normal
MK50
MK75
MK88
MK94
MK97
MK data
Normal data

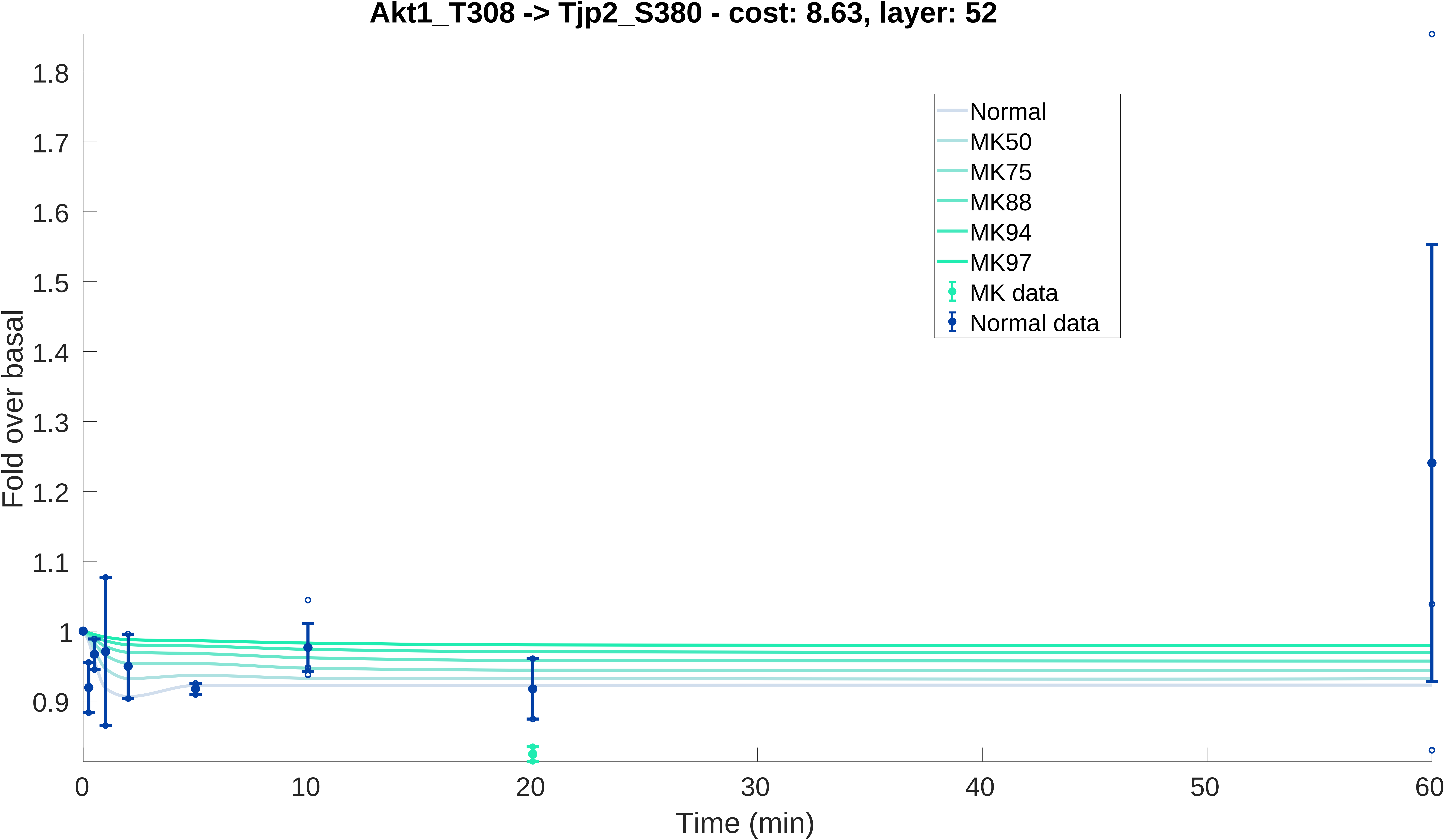
Akt1_T308 -> Tjp2_S380 - cost: 8.63, layer: 52
Fold over basal
Time (min)
0.9
1
1.1
1.2
1.3
1.4
1.5
1.6
1.7
1.8
0
10
20
30
40
50
60
Normal
MK50
MK75
MK88
MK94
MK97
MK data
Normal data

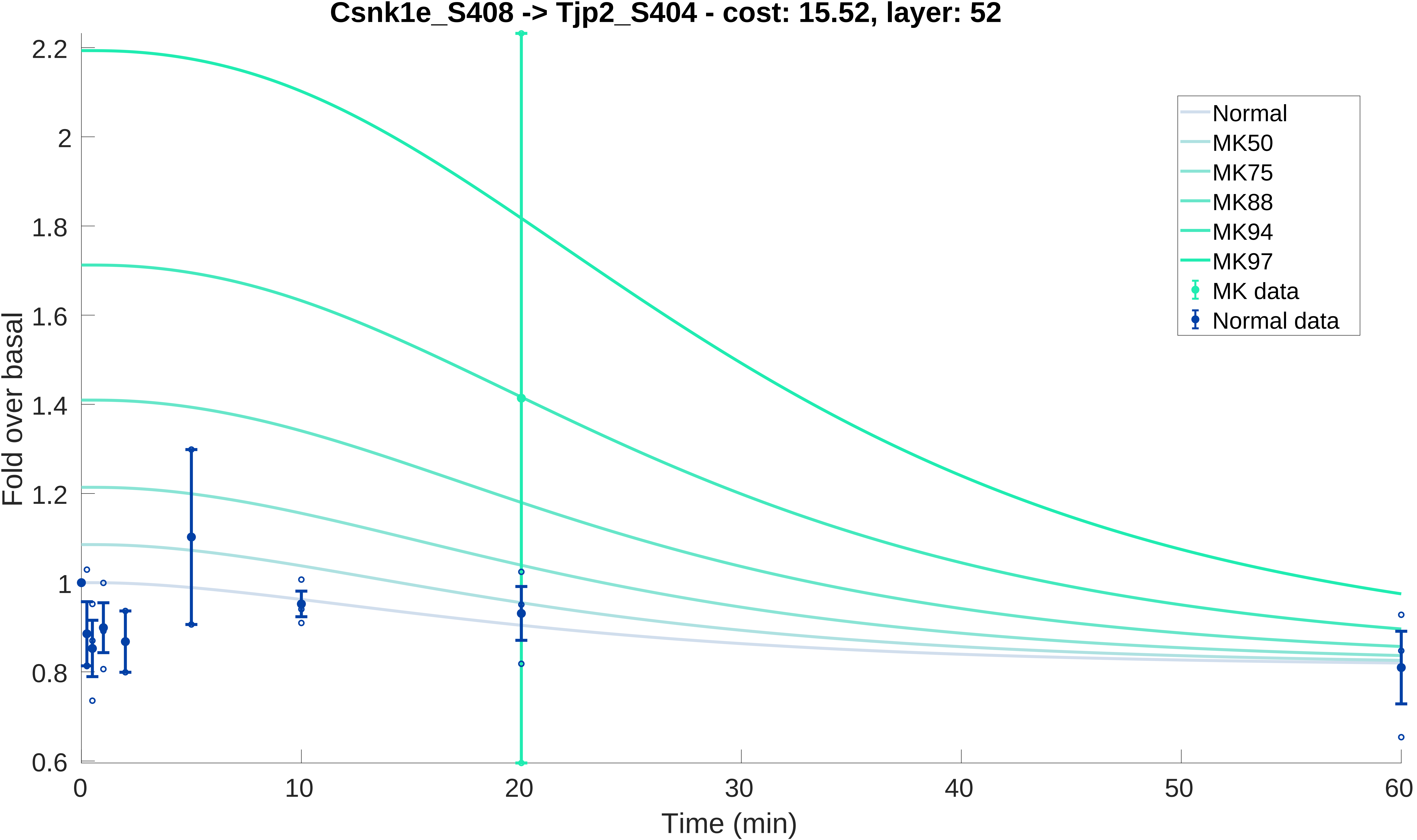

Csnk1e_S408 -> Tjp2_S404 - cost: 15.52, layer: 52
Fold over basal
Time (min)
Normal
MK50
MK75
MK88
MK94
MK97
MK data
Normal data
0.6
0.8
1
1.2
1.4
1.6
1.8
2
2.2
0
10
20
30
40
50
60

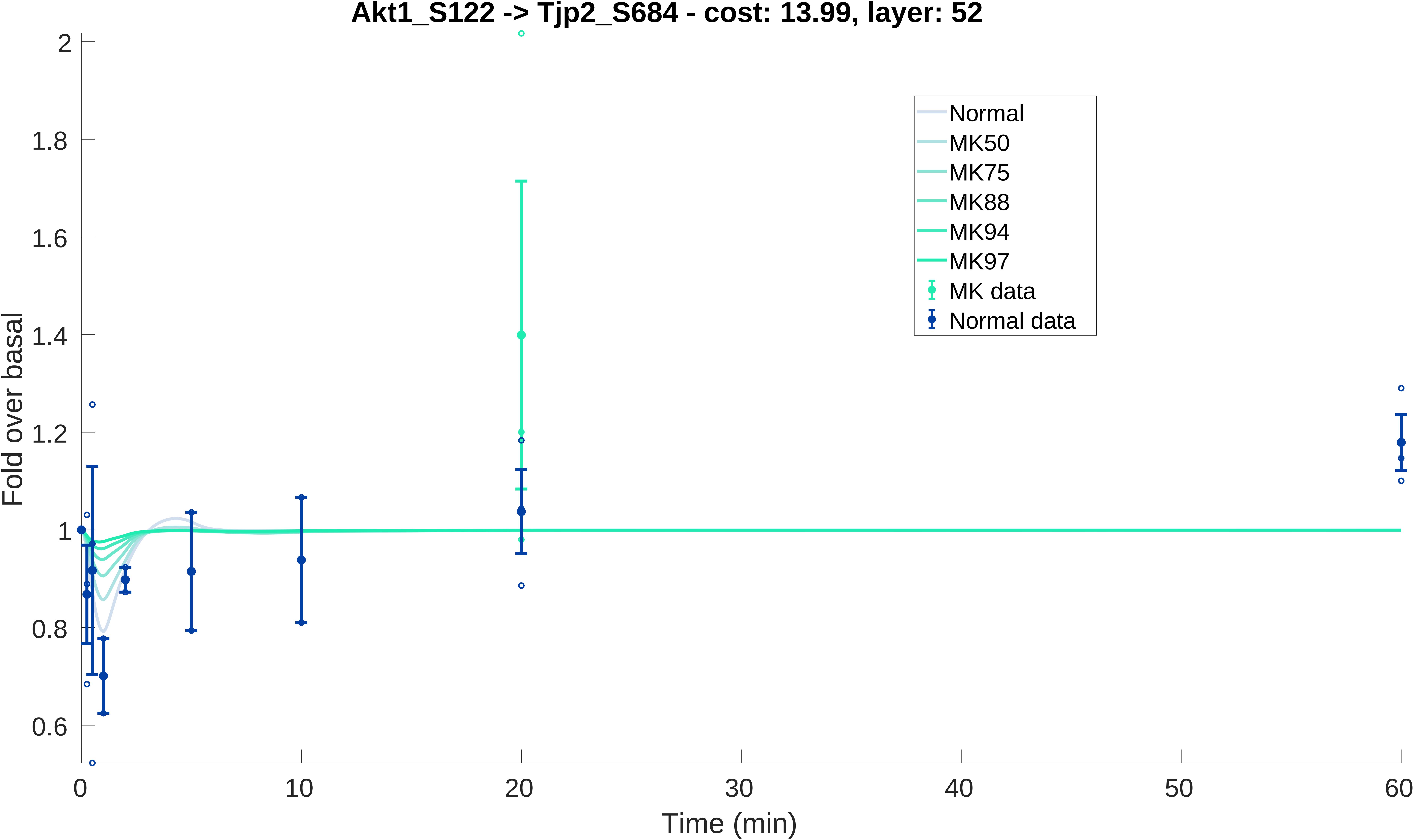

Akt1_S122 -> Tjp2_S684 - cost: 13.99, layer: 52
Fold over basal
Time (min)
2
1.8
1.6
1.4
1.2
1
0.8
0.6
0
10
20
30
40
50
60
Normal
MK50
MK75
MK88
MK94
MK97
MK data
Normal data

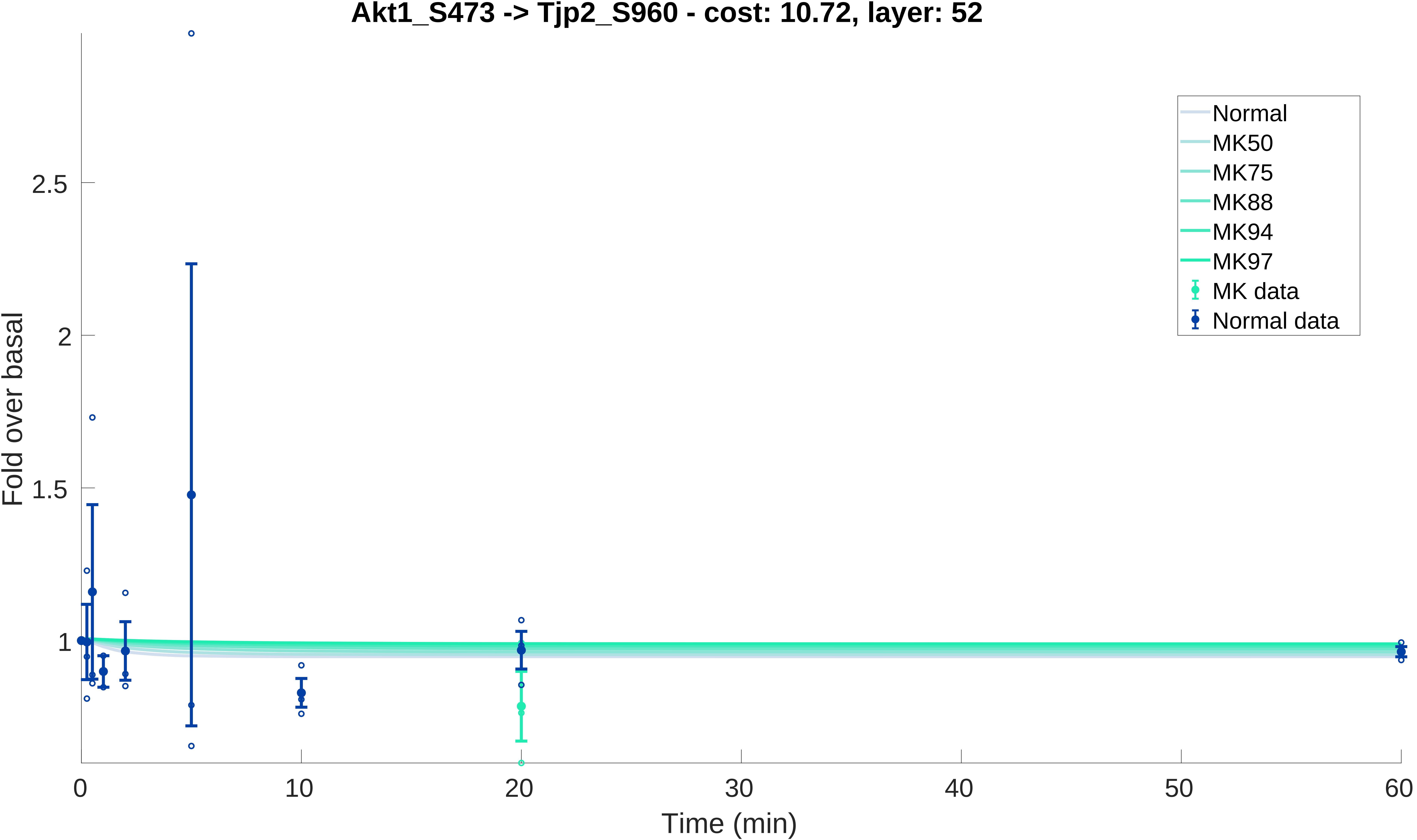

Akt1_S473 -> Tjp2_S960 - cost: 10.72, layer: 52
Fold over basal
Time (min)
2.5
2
1.5
1
0
10
20
30
40
50
60
Normal
MK50
MK75
MK88
MK94
MK97
MK data
Normal data

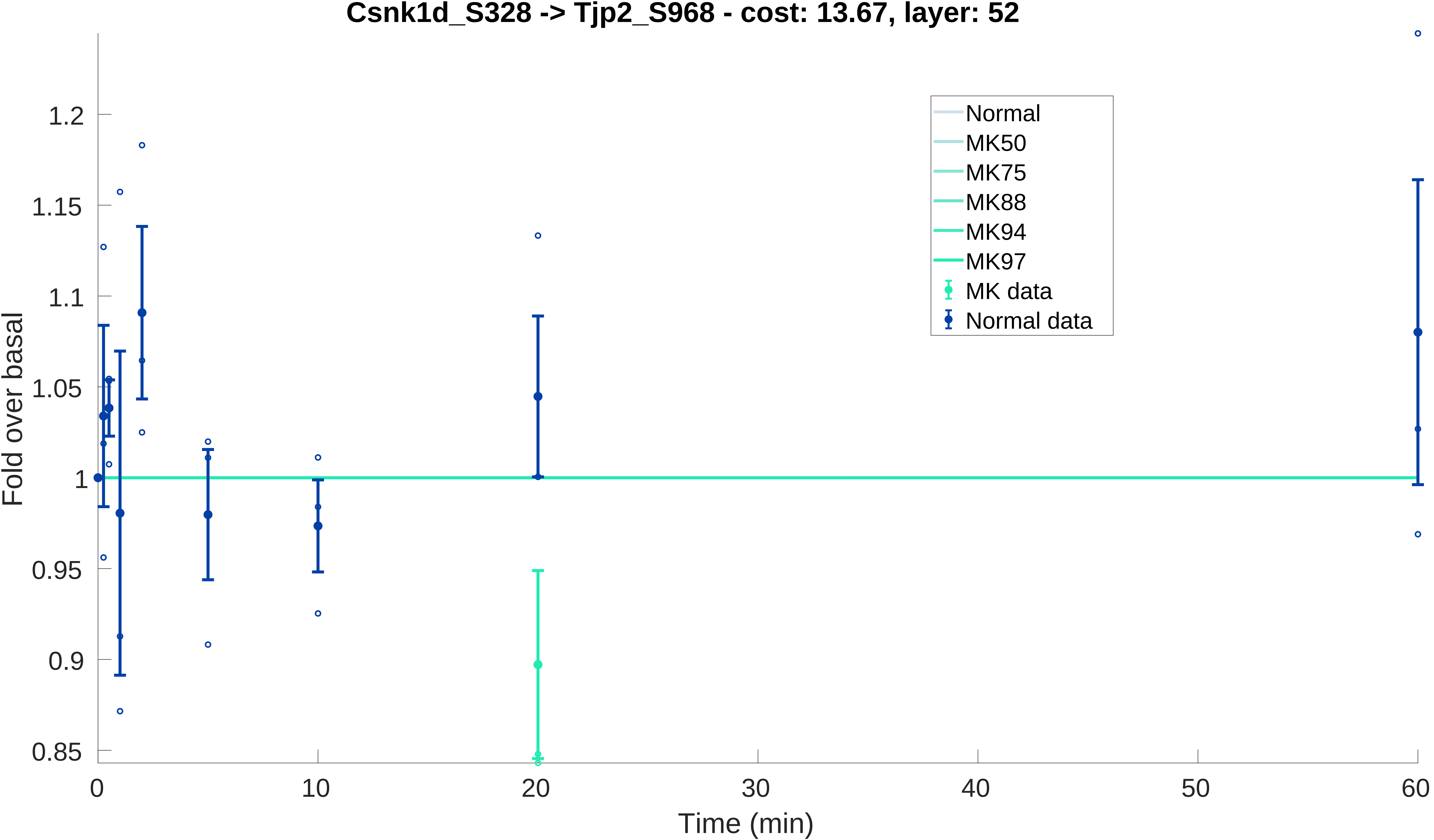

Csnk1d_S328 -> Tjp2_S968 - cost: 13.67, layer: 52
Fold over basal
Time (min)
1.2
1.15
1.1
1.05
1
0.95
0.9
0.85
0
10
20
30
40
50
60
Normal
MK50
MK75
MK88
MK94
MK97
MK data
Normal data

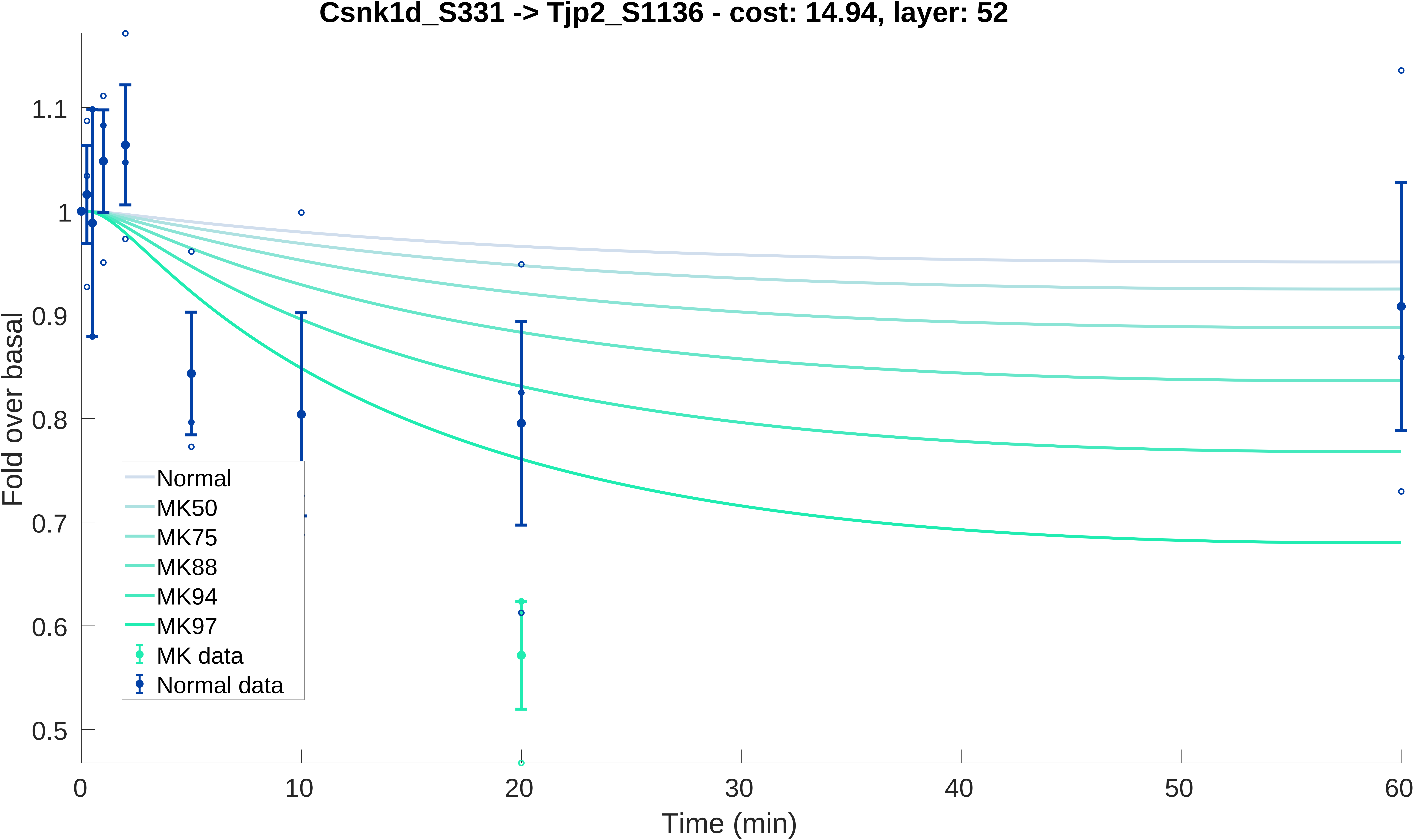

Csnk1d_S331 -> Tjp2_S1136 - cost: 14.94, layer: 52
Fold over basal
Time (min)
0.5
0.6
0.7
0.8
0.9
1
1.1
0
10
20
30
40
50
60
Normal
MK50
MK75
MK88
MK94
MK97
MK data
Normal data

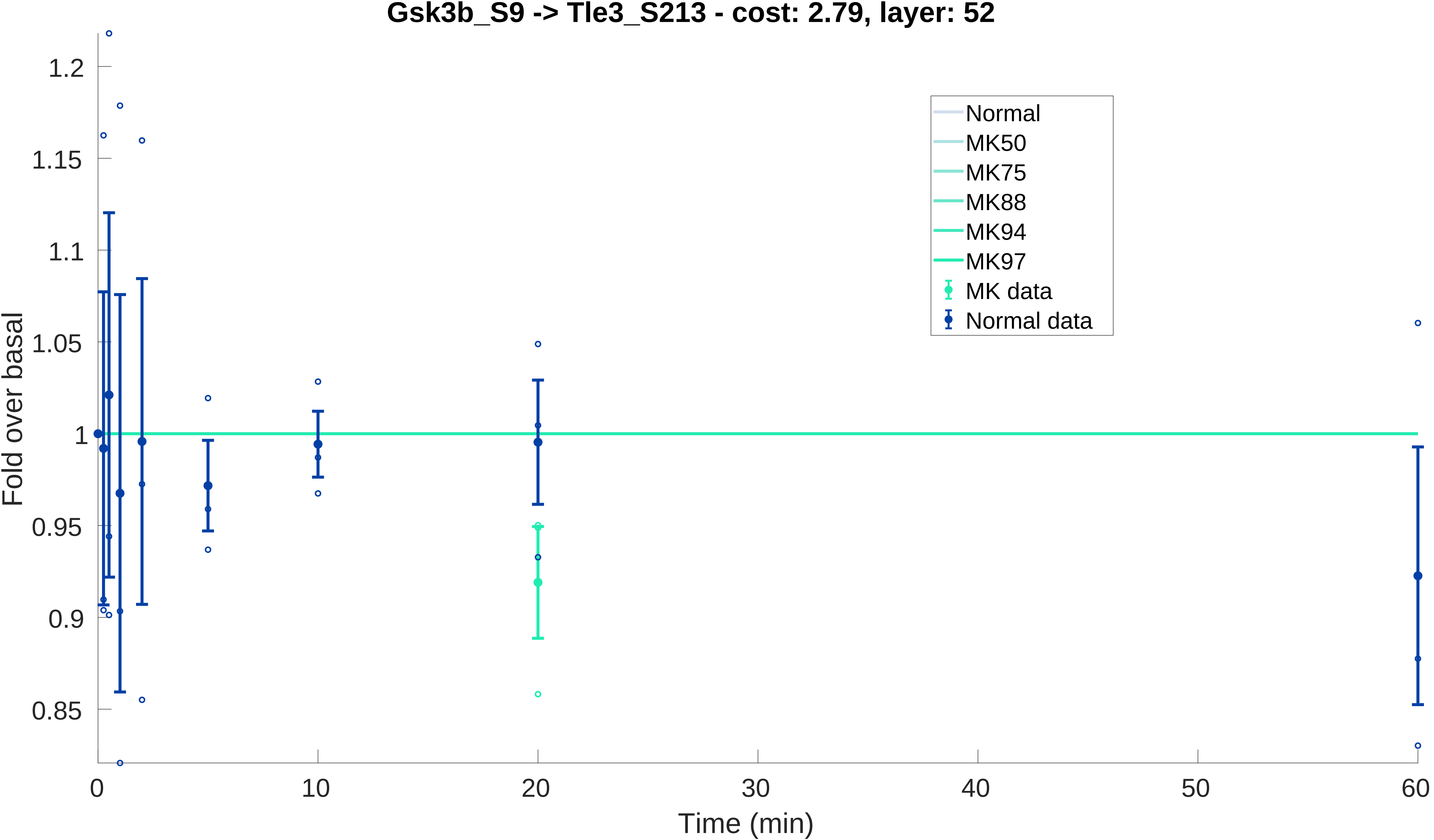

Gsk3b_S9 -> Tle3_S213 - cost: 2.79, layer: 52
Fold over basal
Time (min)
1.2
1.15
1.1
1.05
1
0.95
0.9
0.85
0
10
20
30
40
50
60
Normal
MK50
MK75
MK88
MK94
MK97
MK data
Normal data

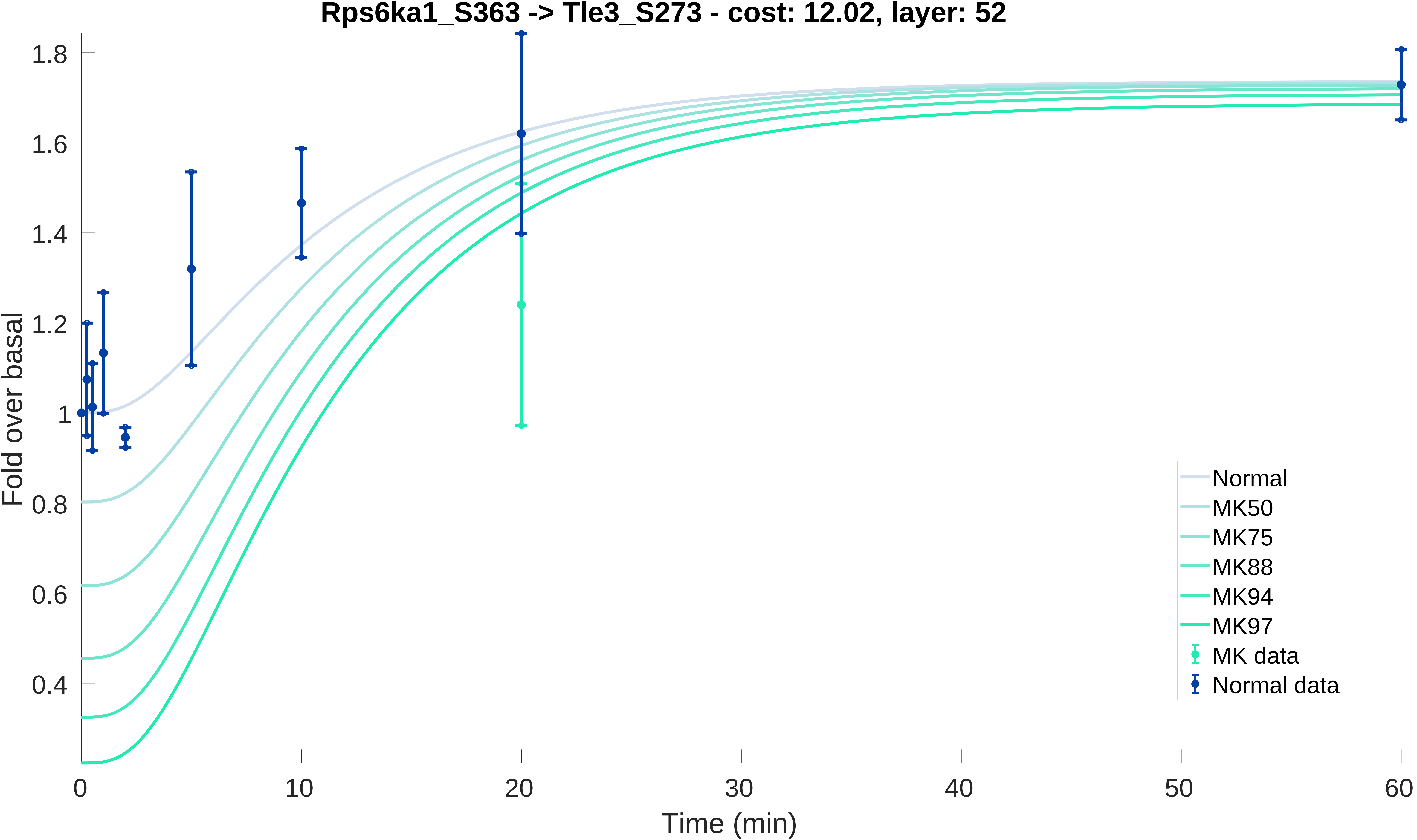

Rps6ka1_S363 -> Tle3_S273 - cost: 12.02, layer: 52
Fold over basal
1.8
1.6
1.4
1.2
1
0.8
0.6
0.4
0
10
20
30
40
50
60
Time (min)
Normal
MK50
MK75
MK88
MK94
MK97
MK data
Normal data

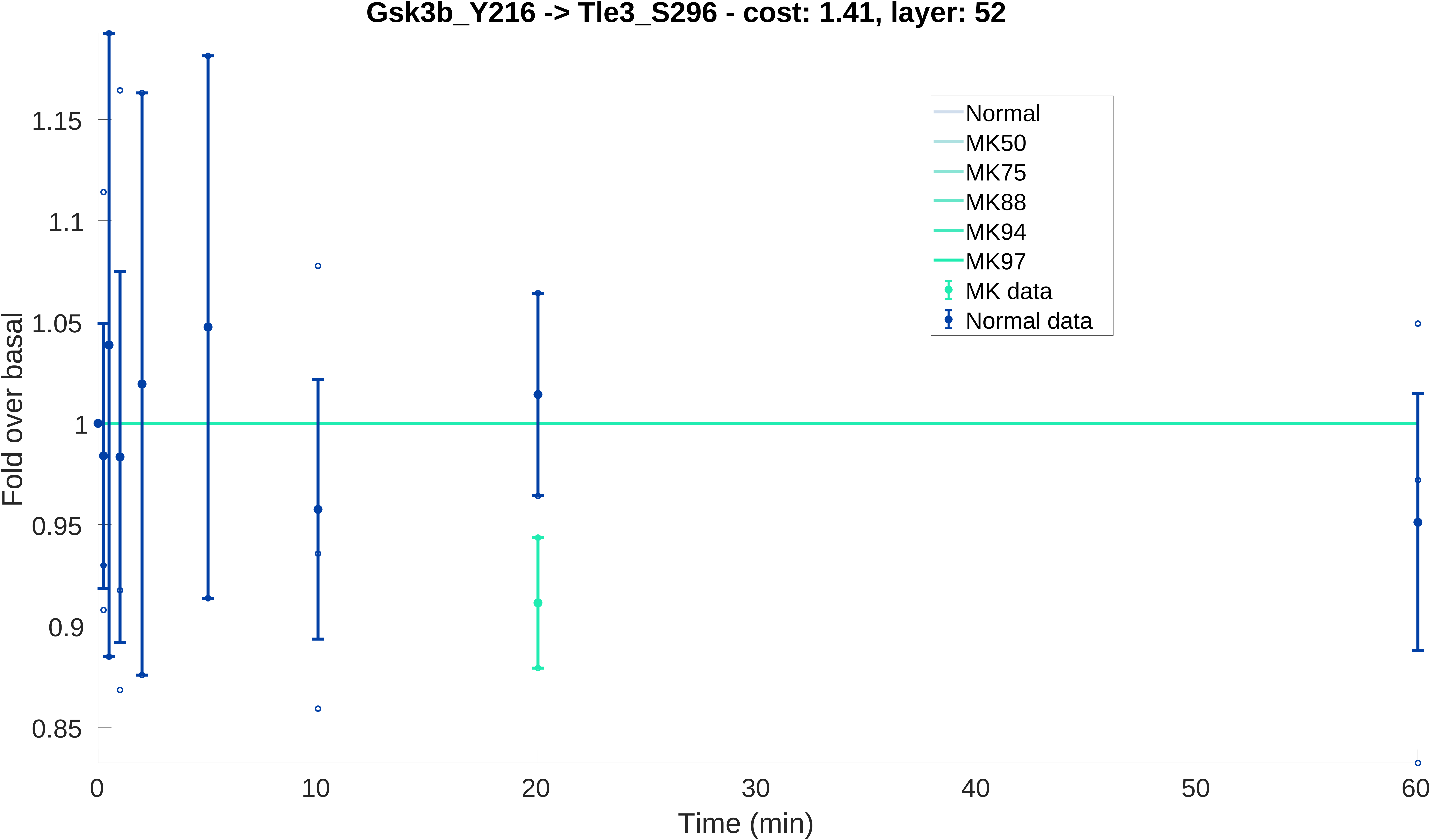

Gsk3b_Y216 -> Tle3_S296 - cost: 1.41, layer: 52
Fold over basal
Time (min)
1.15
1.1
1.05
1
0.95
0.9
0.85
0
10
20
30
40
50
60
Normal
MK50
MK75
MK88
MK94
MK97
MK data
Normal data

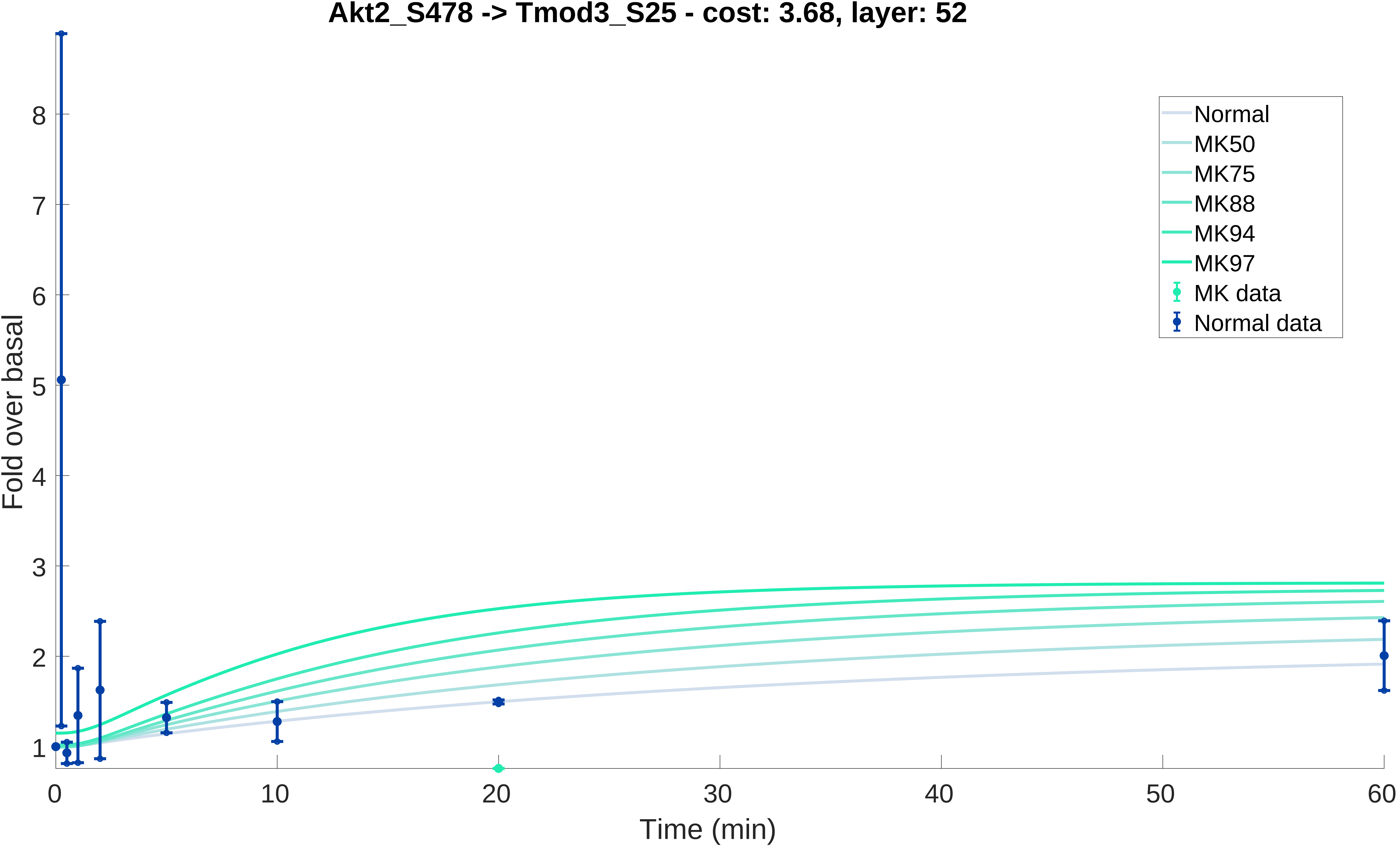

Akt2_S478 -> Tmod3_S25 - cost: 3.68, layer: 52
Fold over basal
Time (min)
0
10
20
30
40
50
60
1
2
3
4
5
6
7
8
Normal
MK50
MK75
MK88
MK94
MK97
MK data
Normal data

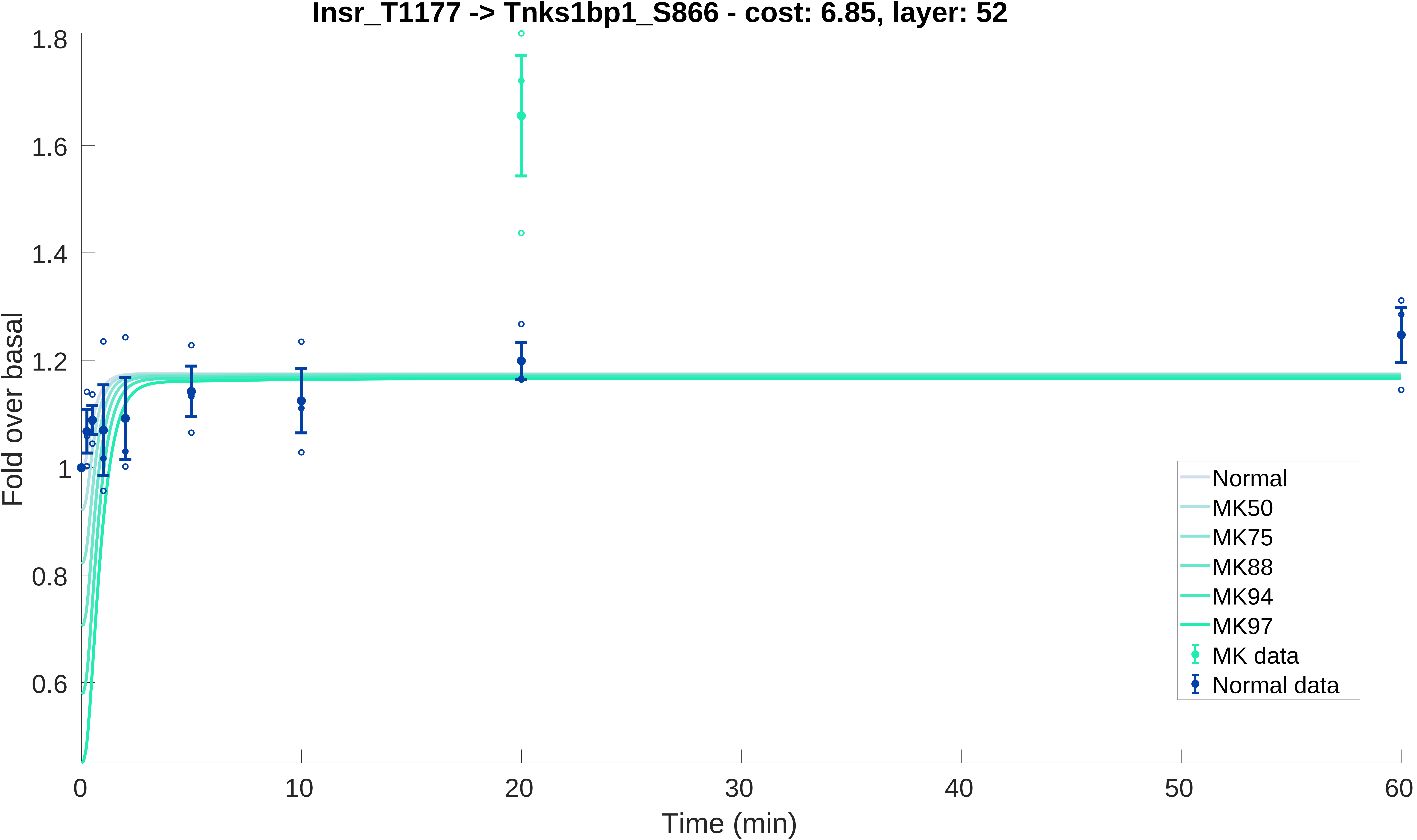

Insr_T1177 -> Tnks1bp1_S866 - cost: 6.85, layer: 52
Fold over basal
1.8
1.6
1.4
1.2
1
0.8
0.6
0
10
20
30
40
50
60
Time (min)
Normal
MK50
MK75
MK88
MK94
MK97
MK data
Normal data

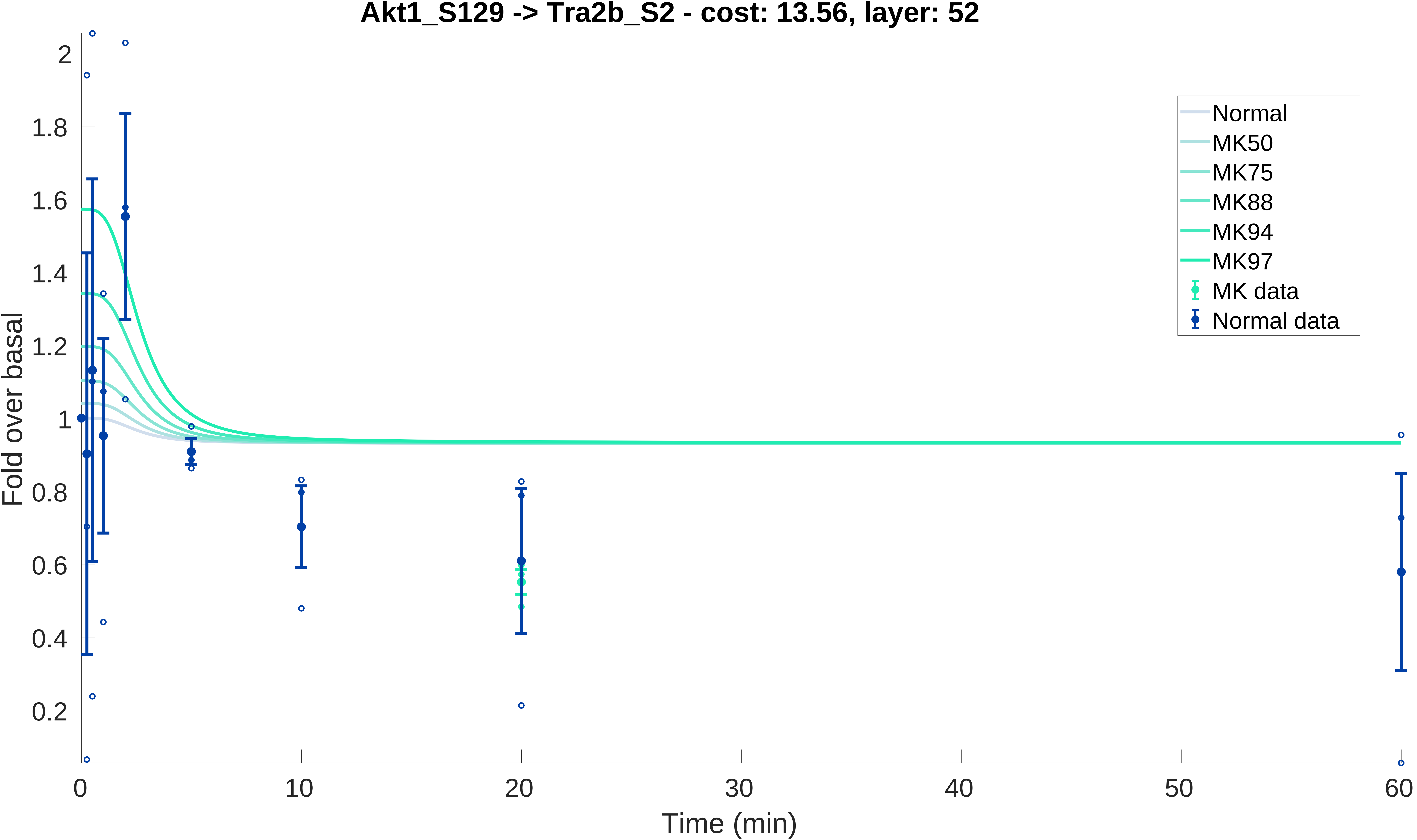

Akt1_S129 -> Tra2b_S2 - cost: 13.56, layer: 52
Fold over basal
Time (min)
2
1.8
1.6
1.4
1.2
1
0.8
0.6
0.4
0.2
0
10
20
30
40
50
60
Normal
MK50
MK75
MK88
MK94
MK97
MK data
Normal data

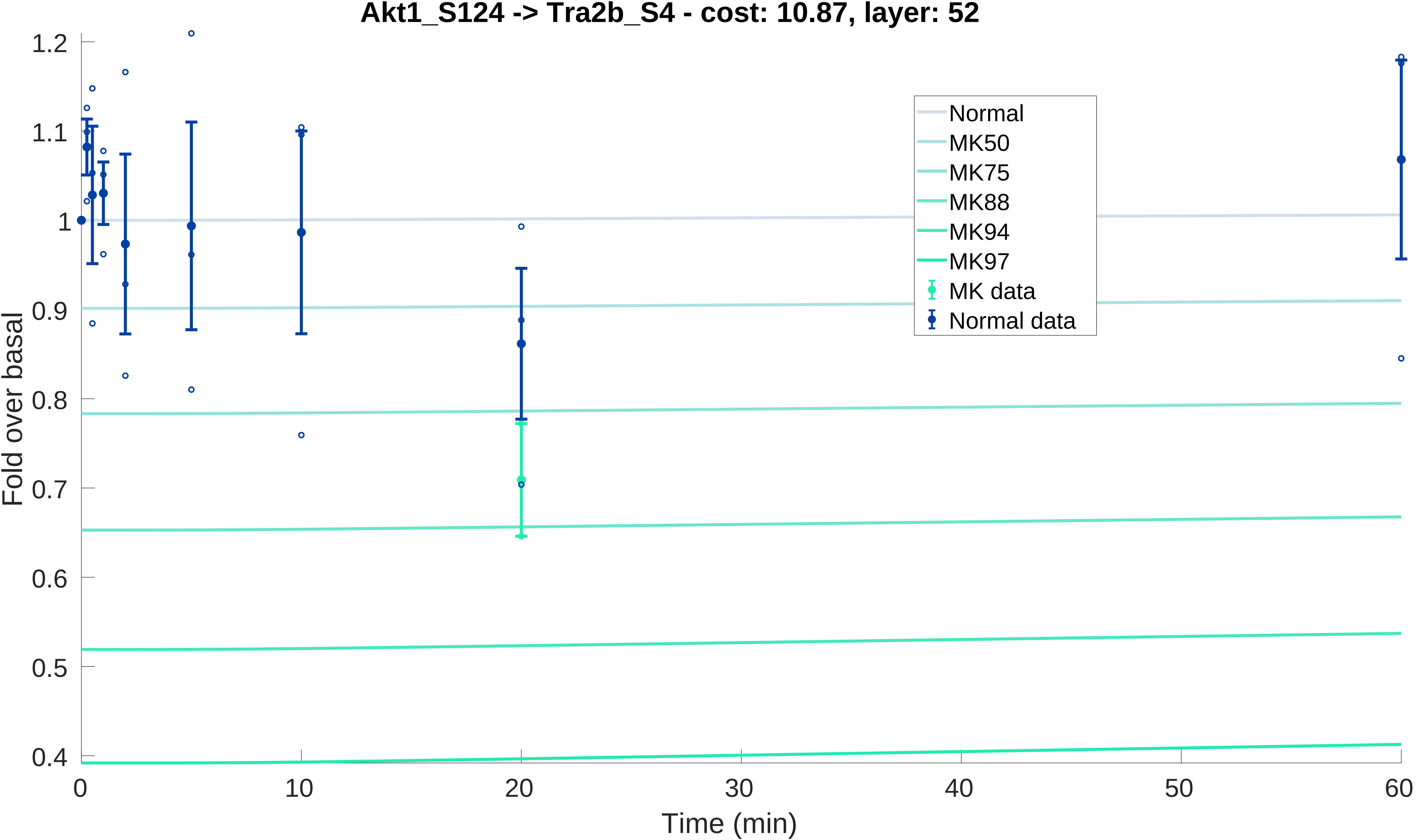

Akt1_S124 -> Tra2b_S4 - cost: 10.87, layer: 52
Fold over basal
Time (min)
0
10
20
30
40
50
60
0.4
0.5
0.6
0.7
0.8
0.9
1
1.1
1.2
Normal
MK50
MK75
MK88
MK94
MK97
MK data
Normal data

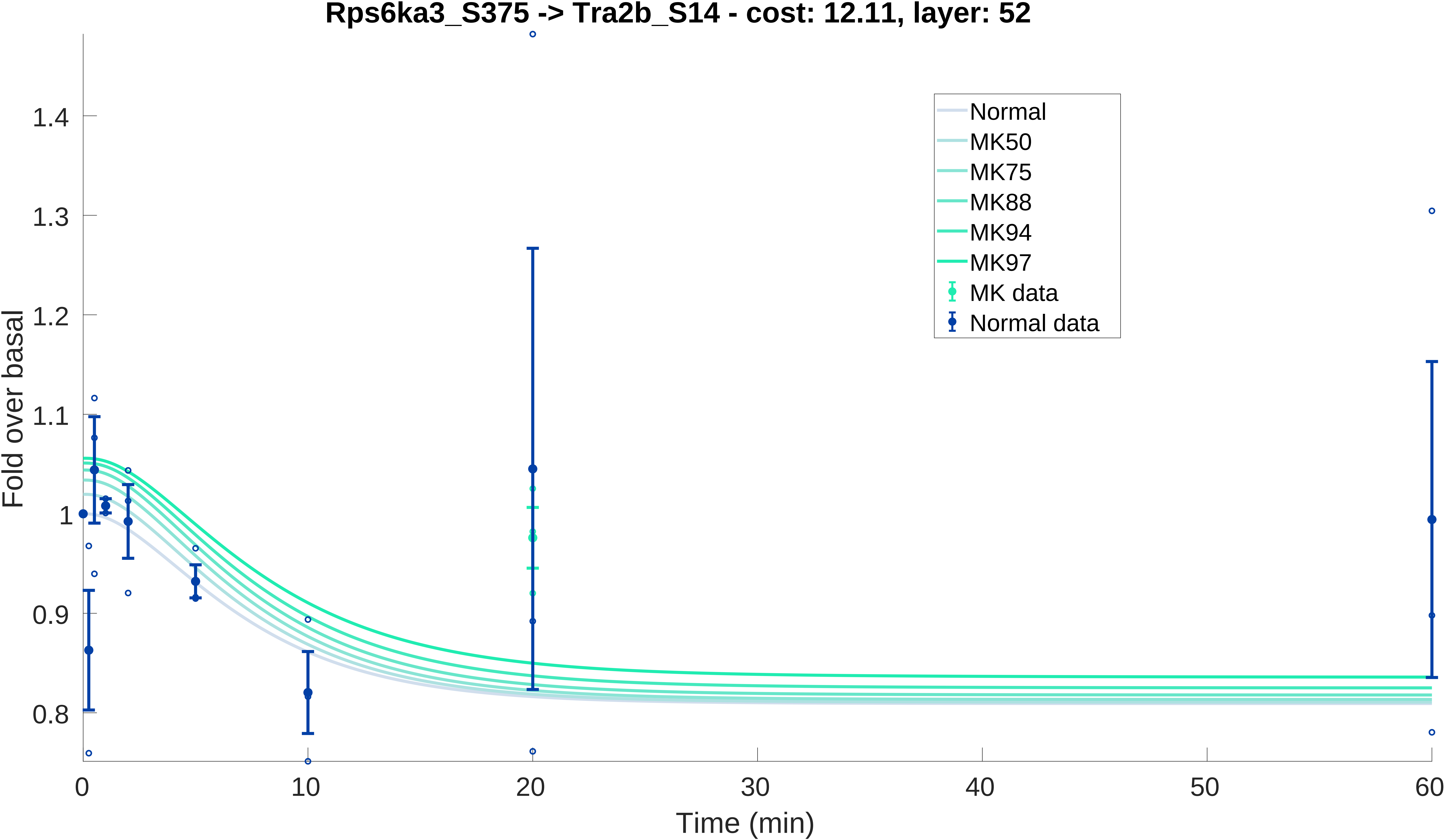

Rps6ka3_S375 -> Tra2b_S14 - cost: 12.11, layer: 52
Fold over basal
1.4
1.3
1.2
1.1
1
0.9
0.8
0
10
20
30
40
50
60
Time (min)
Normal
MK50
MK75
MK88
MK94
MK97
MK data
Normal data

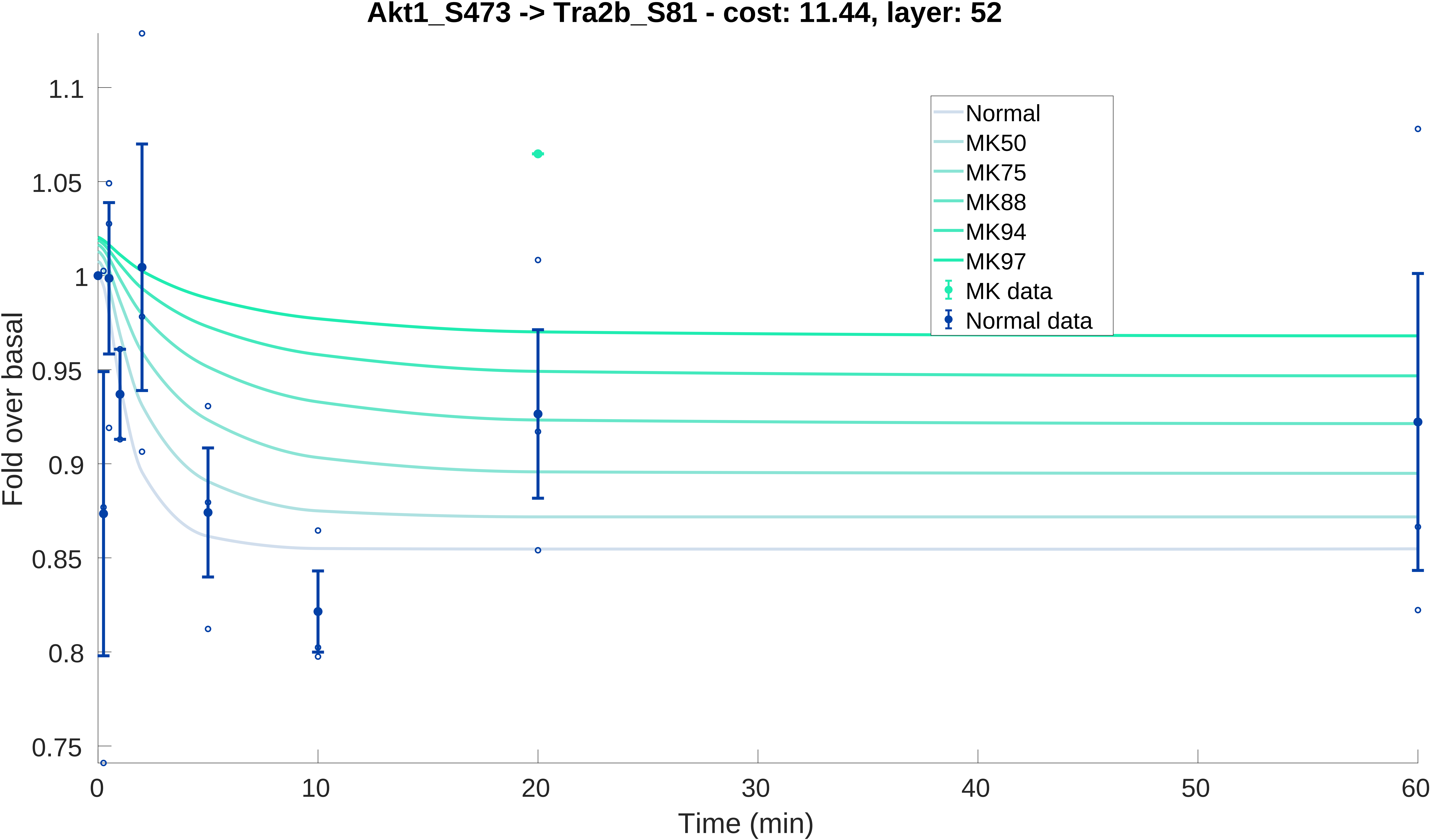

Akt1_S473 -> Tra2b_S81 - cost: 11.44, layer: 52
Fold over basal
Time (min)
1.1
1.05
1
0.95
0.9
0.85
0.8
0.75
0
10
20
30
40
50
60
Normal
MK50
MK75
MK88
MK94
MK97
MK data
Normal data

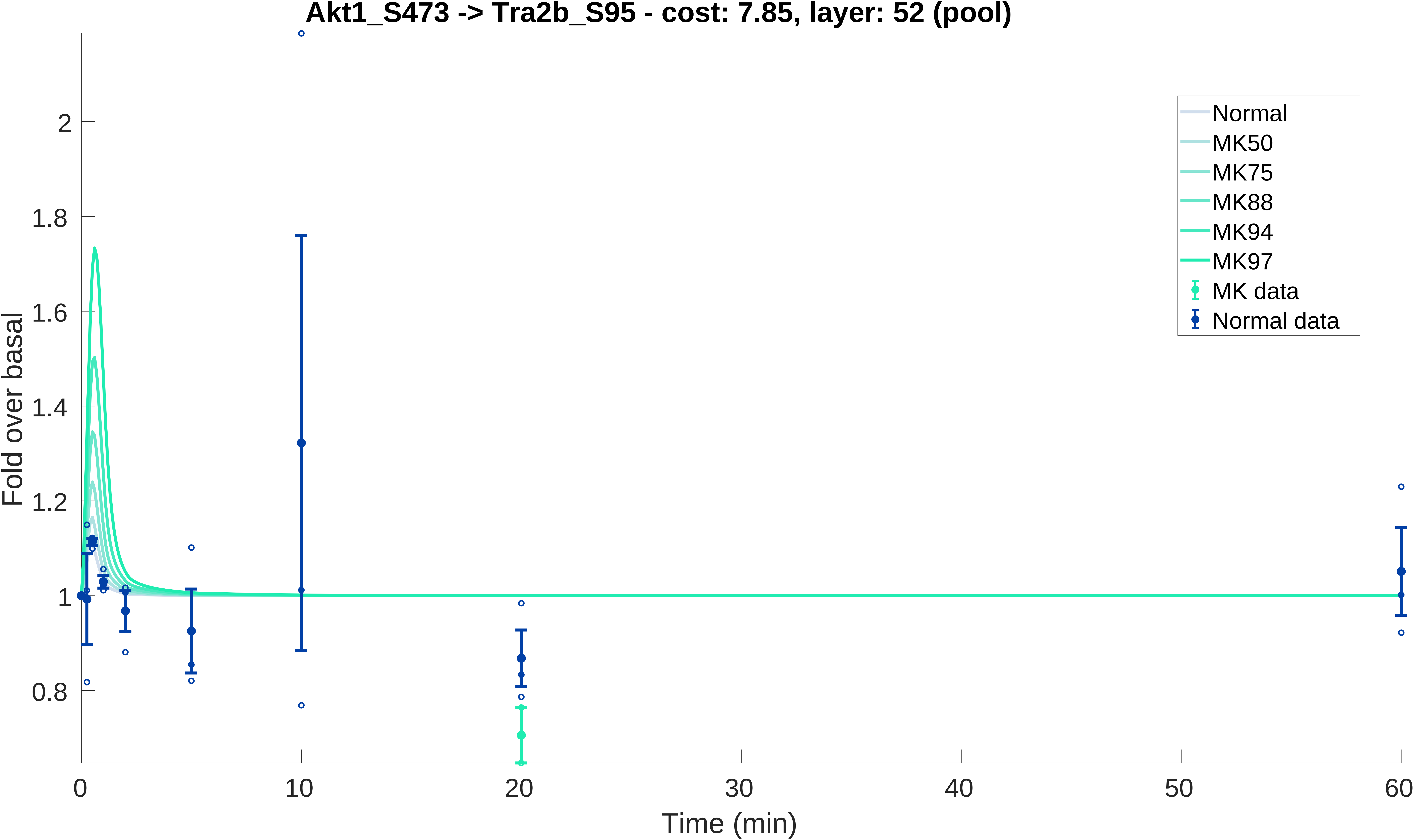

Akt1_S473 -> Tra2b_S95 - cost: 7.85, layer: 52 (pool)
Fold over basal
Time (min)
0.8
1
1.2
1.4
1.6
1.8
2
0
10
20
30
40
50
60
Normal
MK50
MK75
MK88
MK94
MK97
MK data
Normal data

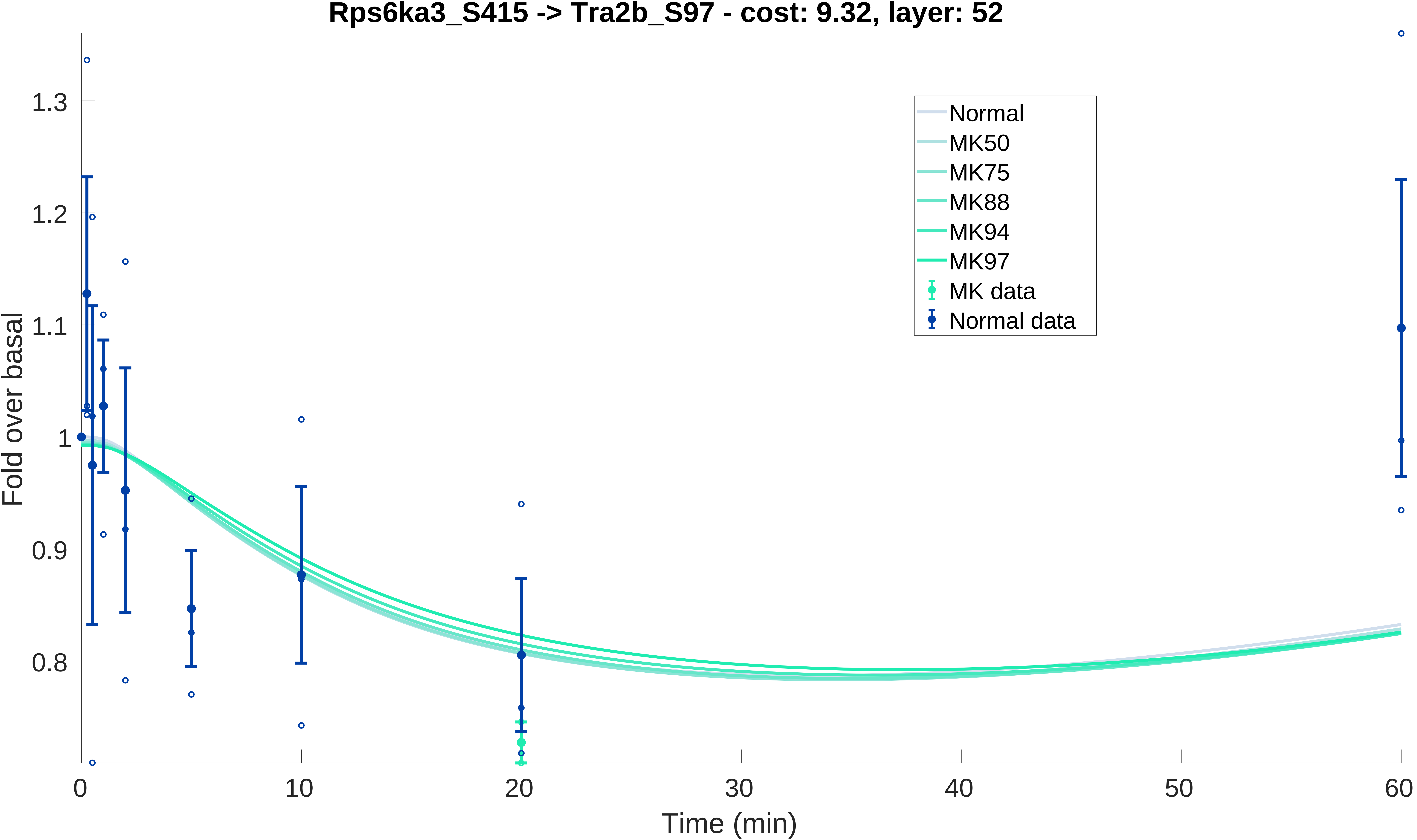

Rps6ka3_S415 -> Tra2b_S97 - cost: 9.32, layer: 52
Fold over basal
Time (min)
Normal
MK50
MK75
MK88
MK94
MK97
MK data
Normal data
1.3
1.2
1.1
1
0.9
0.8
0
10
20
30
40
50
60

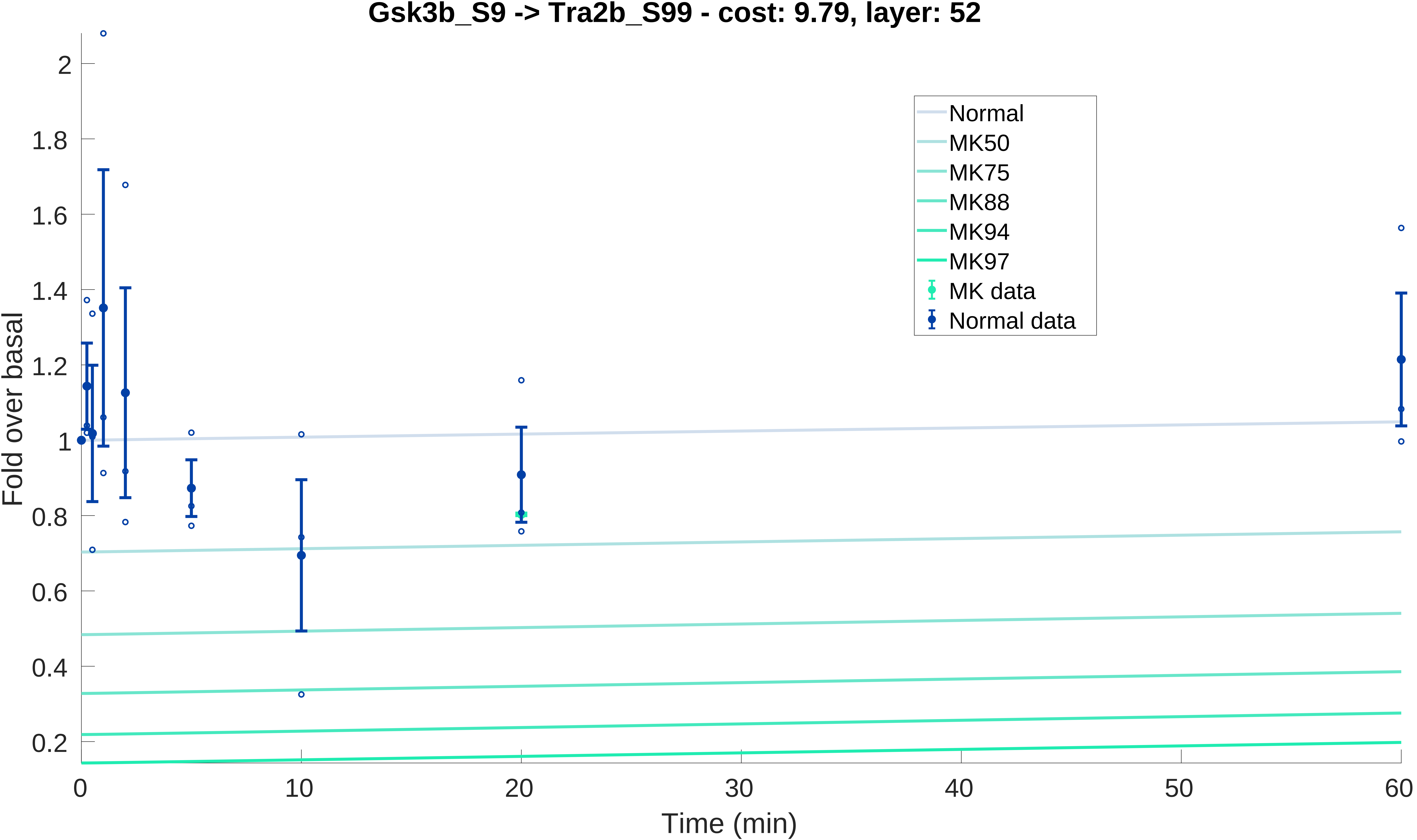

Gsk3b_S9 -> Tra2b_S99 - cost: 9.79, layer: 52
Fold over basal
Time (min)
0
10
20
30
40
50
60
0.2
0.4
0.6
0.8
1
1.2
1.4
1.6
1.8
2
Normal
MK50
MK75
MK88
MK94
MK97
MK data
Normal data

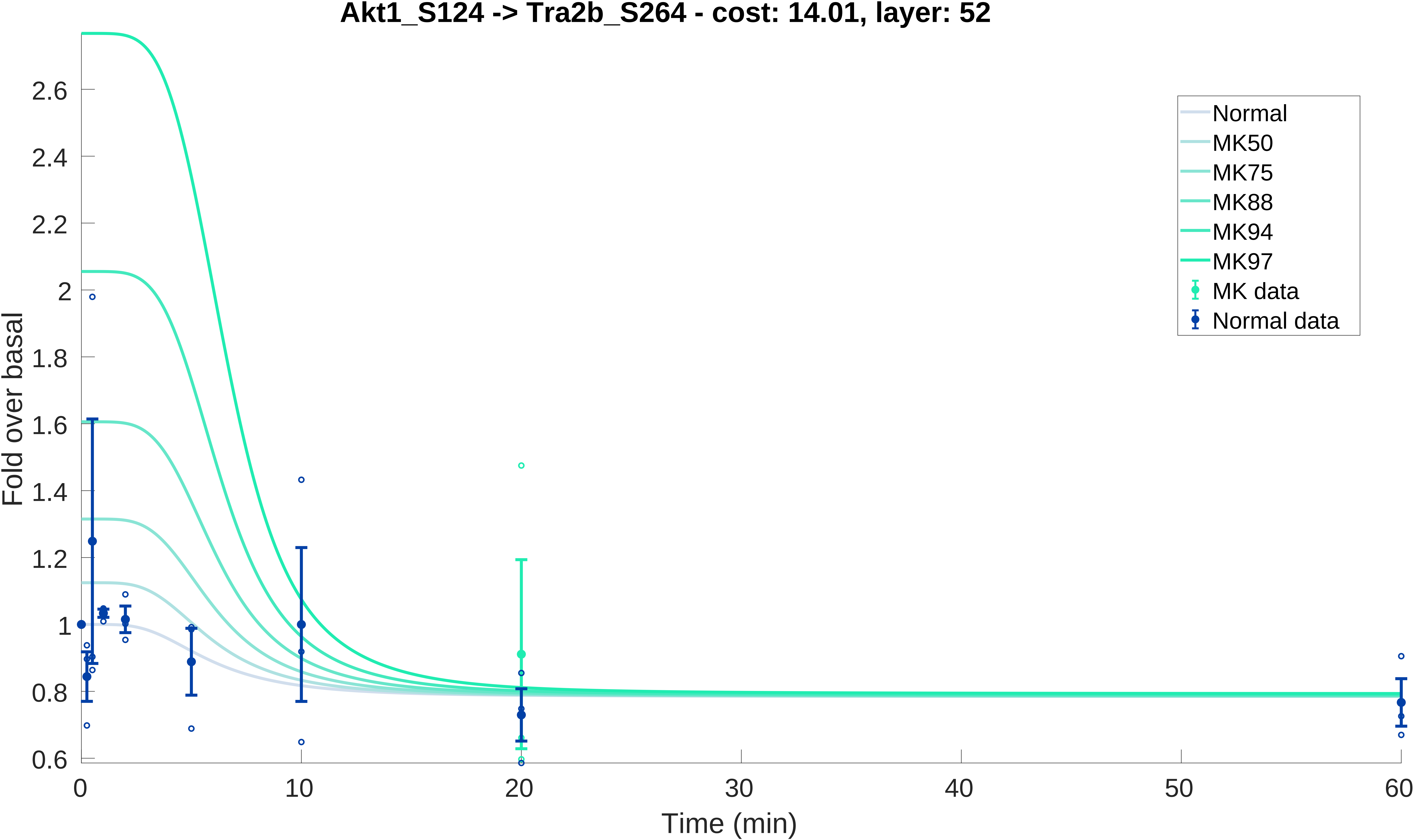

Akt1_S124 -> Tra2b_S264 - cost: 14.01, layer: 52
Fold over basal
Time (min)
0.6
0.8
1
1.2
1.4
1.6
1.8
2
2.2
2.4
2.6
0
10
20
30
40
50
60
Normal
MK50
MK75
MK88
MK94
MK97
MK data
Normal data

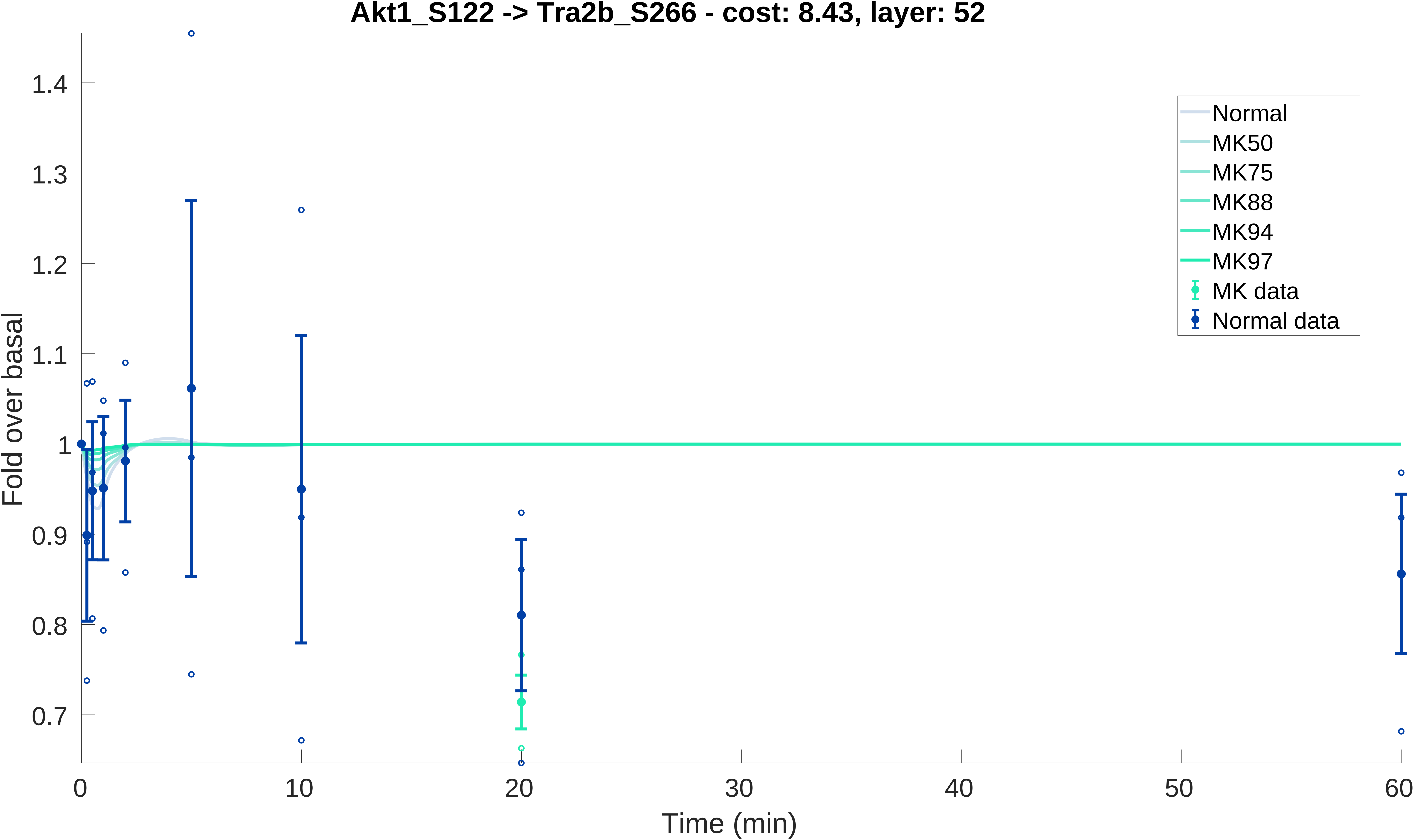
Akt1_S122 -> Tra2b_S266 - cost: 8.43, layer: 52
Fold over basal
1.4
1.3
1.2
1.1
1
0.9
0.8
0.7
0
10
20
30
40
50
60
Time (min)
Normal
MK50
MK75
MK88
MK94
MK97
MK data
Normal data

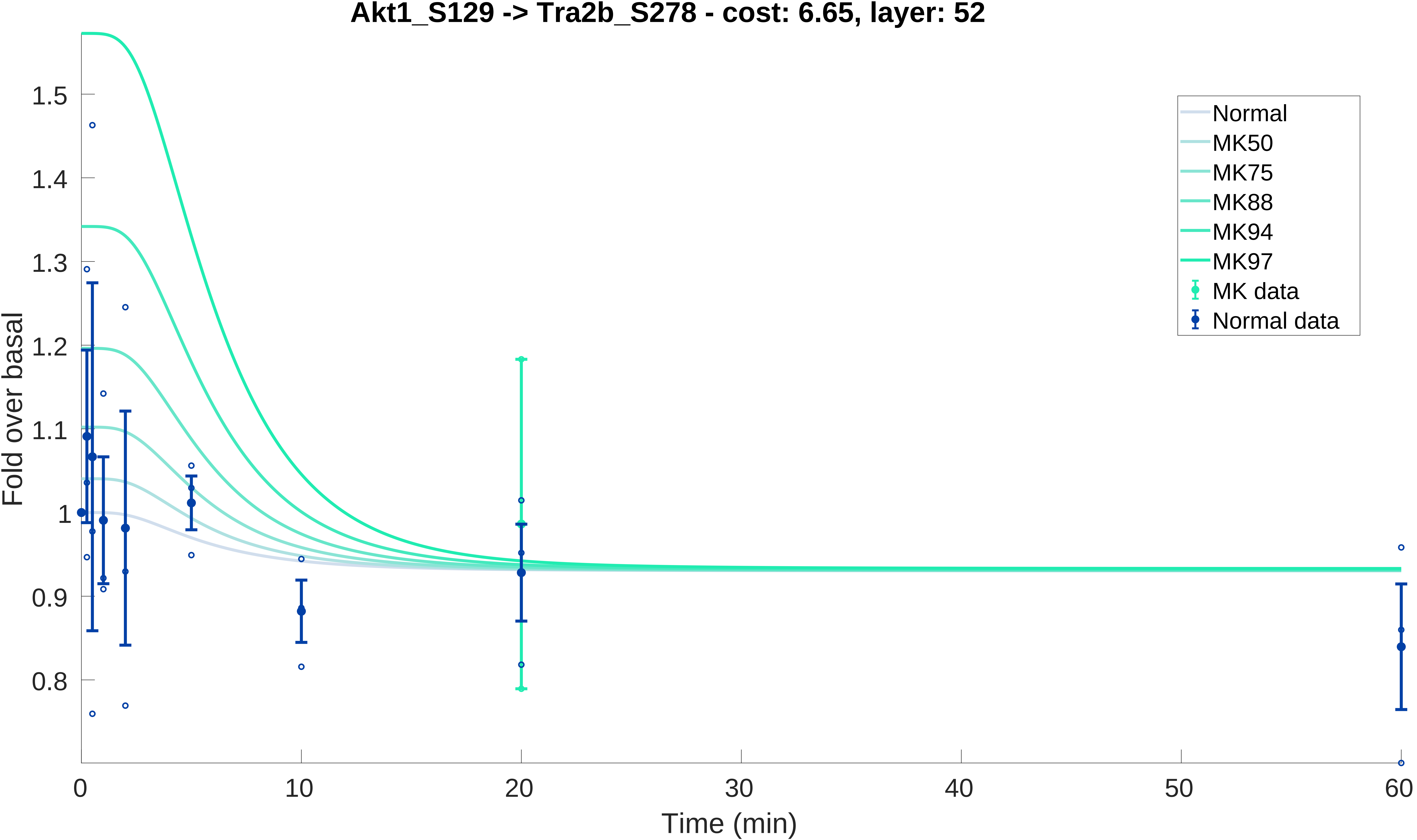

Akt1_S129 -> Tra2b_S278 - cost: 6.65, layer: 52
Fold over basal
Time (min)
1.5
1.4
1.3
1.2
1.1
1
0.9
0.8
0
10
20
30
40
50
60
Normal
MK50
MK75
MK88
MK94
MK97
MK data
Normal data

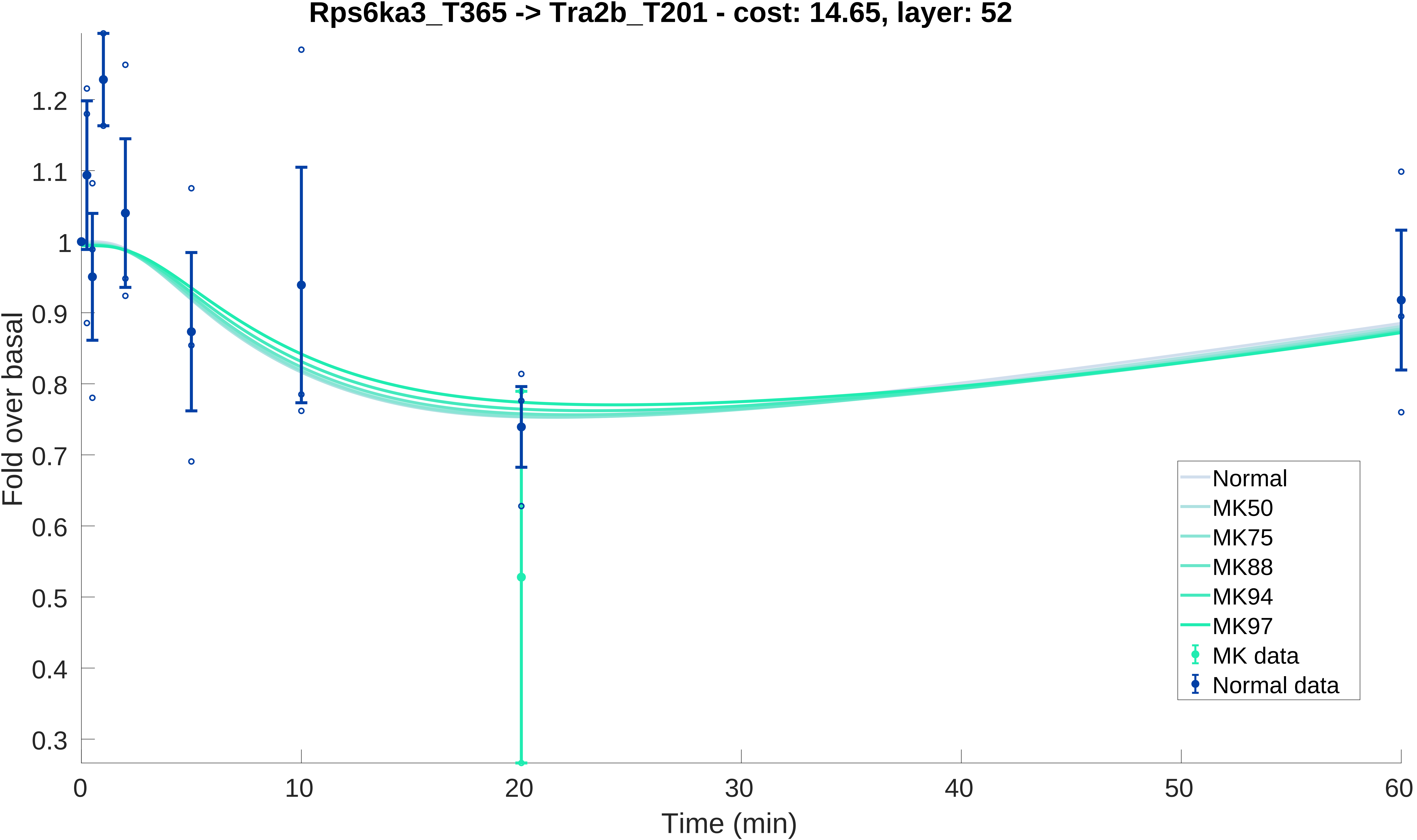

Rps6ka3_T365 -> Tra2b_T201 - cost: 14.65, layer: 52
Fold over basal
Time (min)
0.3
0.4
0.5
0.6
0.7
0.8
0.9
1
1.1
1.2
0
10
20
30
40
50
60
Normal
MK50
MK75
MK88
MK94
MK97
MK data
Normal data

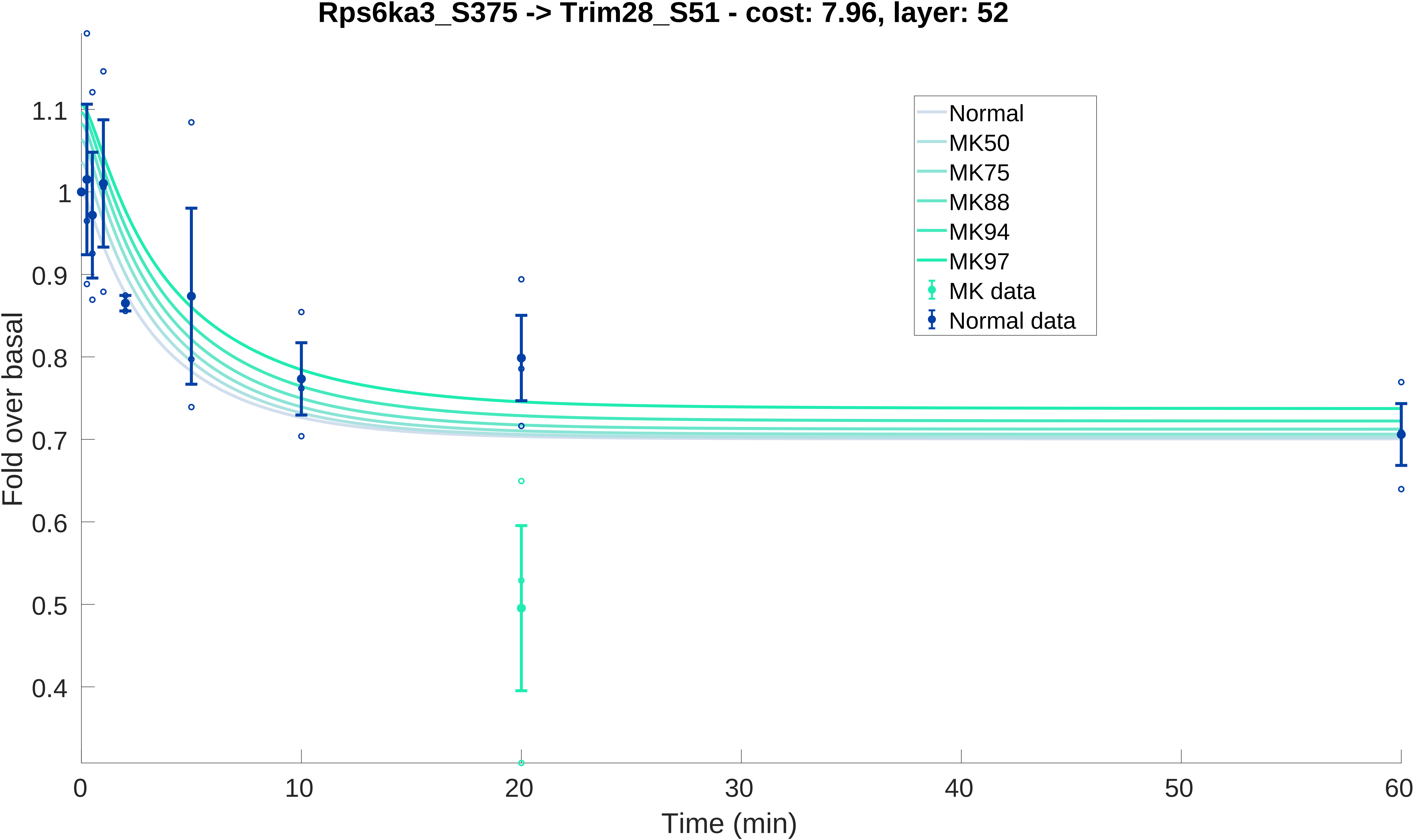

Rps6ka3_S375 -> Trim28_S51 - cost: 7.96, layer: 52
Fold over basal
Time (min)
0.4
0.5
0.6
0.7
0.8
0.9
1
1.1
0
10
20
30
40
50
60
Normal
MK50
MK75
MK88
MK94
MK97
MK data
Normal data

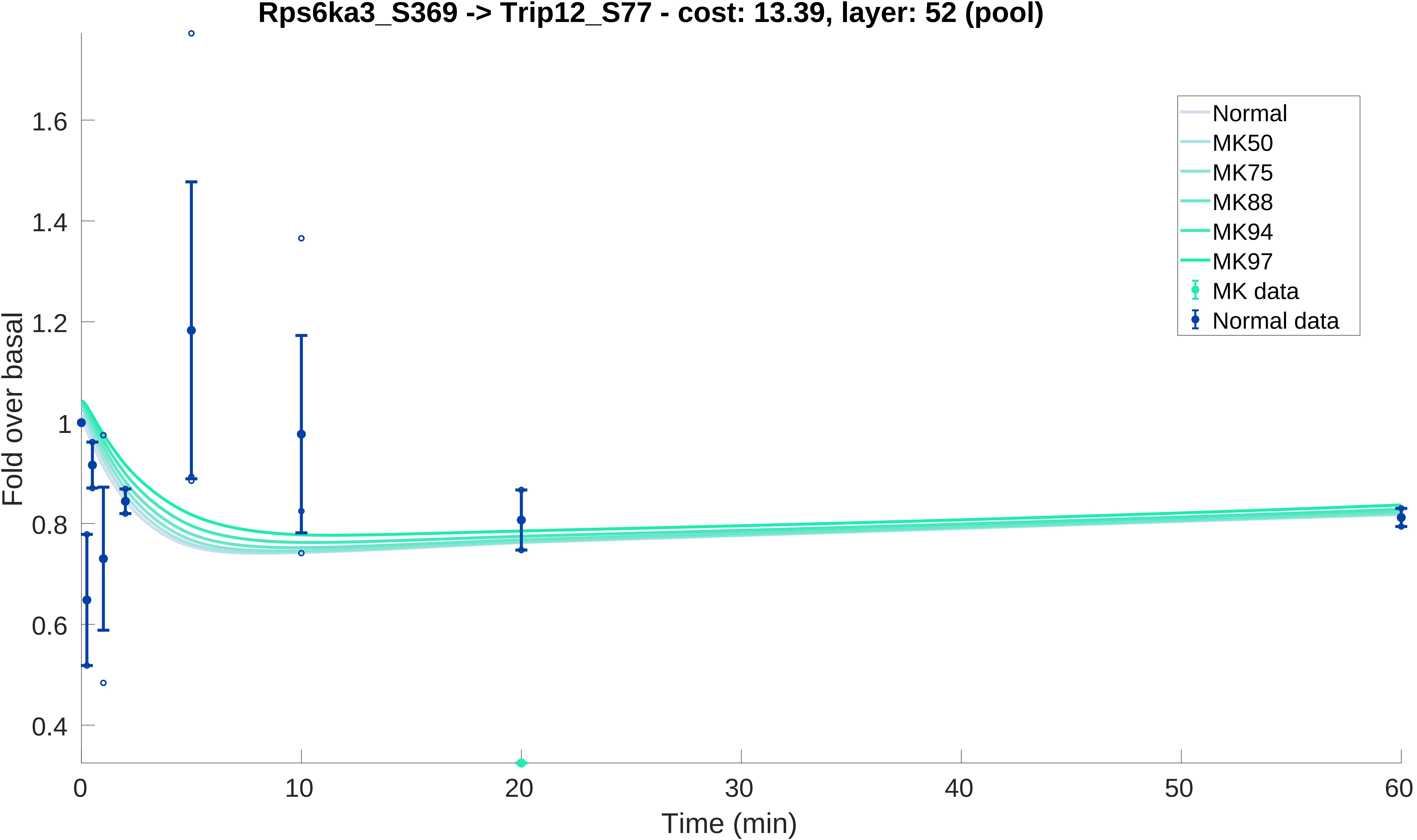

Rps6ka3_S369 -> Trip12_S77 - cost: 13.39, layer: 52 (pool)
Fold over basal
Time (min)
0.4
0.6
0.8
1
1.2
1.4
1.6
0
10
20
30
40
50
60
Normal
MK50
MK75
MK88
MK94
MK97
MK data
Normal data

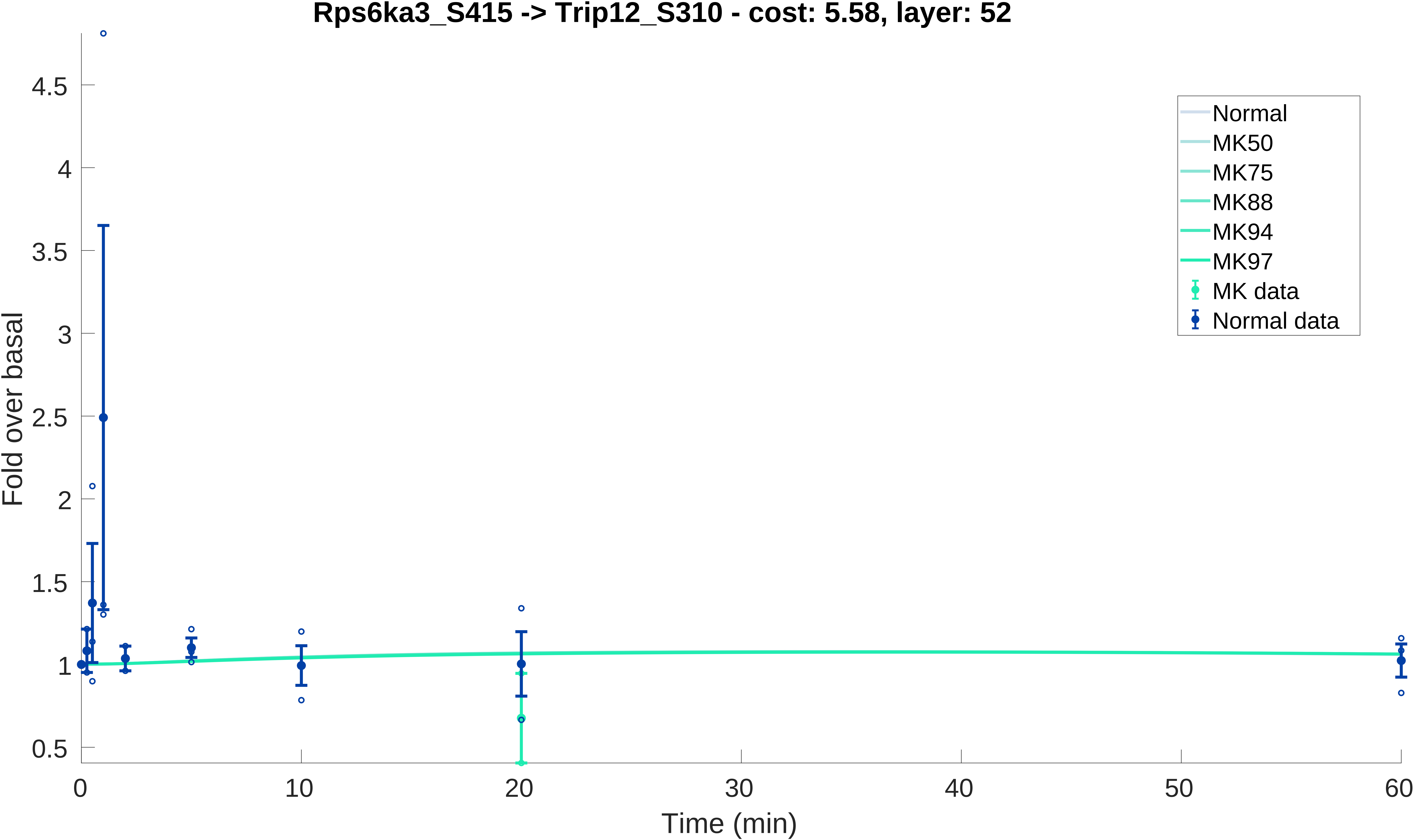

Rps6ka3_S415 -> Trip12_S310 - cost: 5.58, layer: 52
Fold over basal
Time (min)
0.5
1
1.5
2
2.5
3
3.5
4
4.5
0
10
20
30
40
50
60
Normal
MK50
MK75
MK88
MK94
MK97
MK data
Normal data

Rps6ka3_T365 -> Trip12_S312 - cost: 7.78, layer: 52

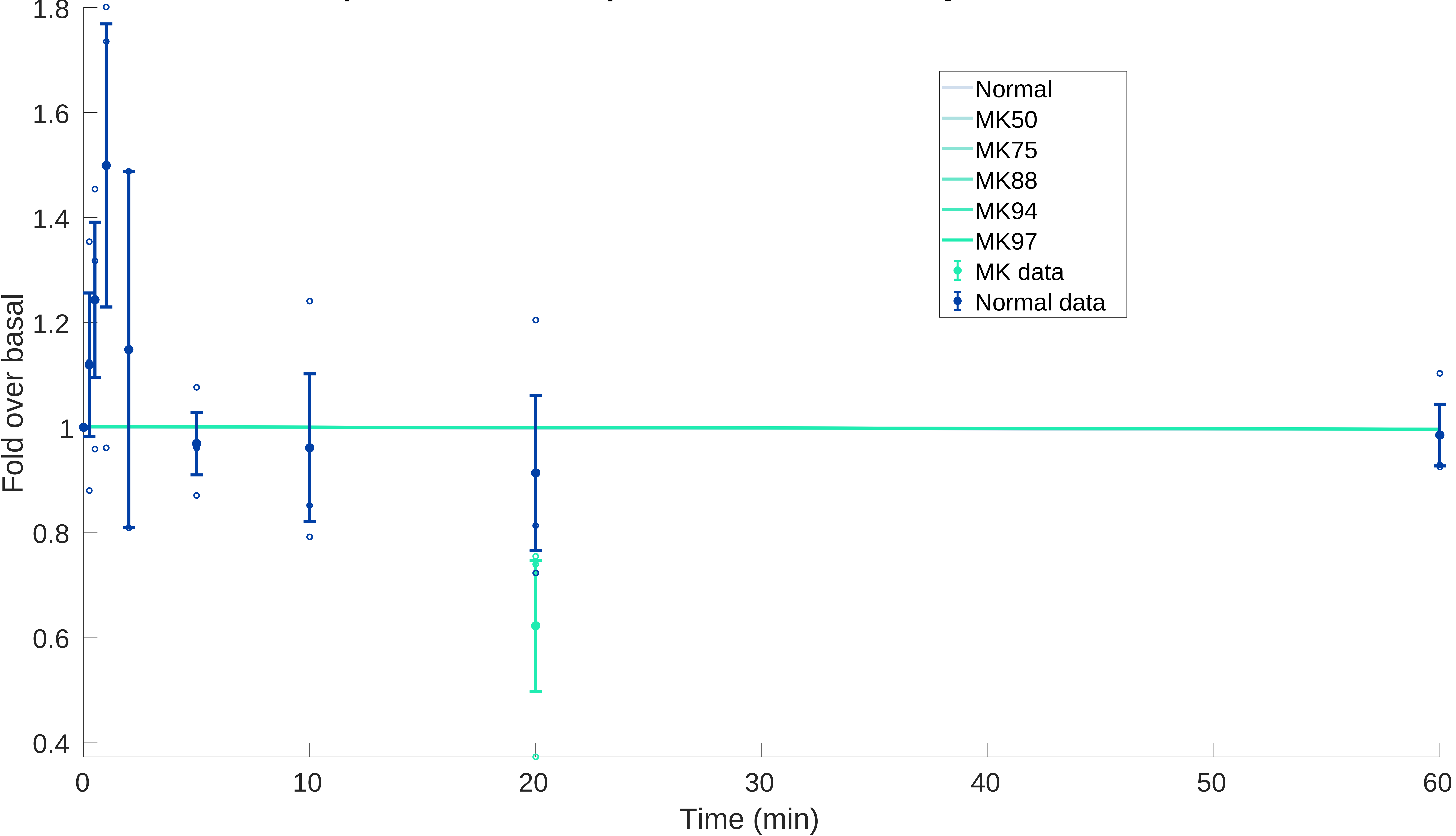

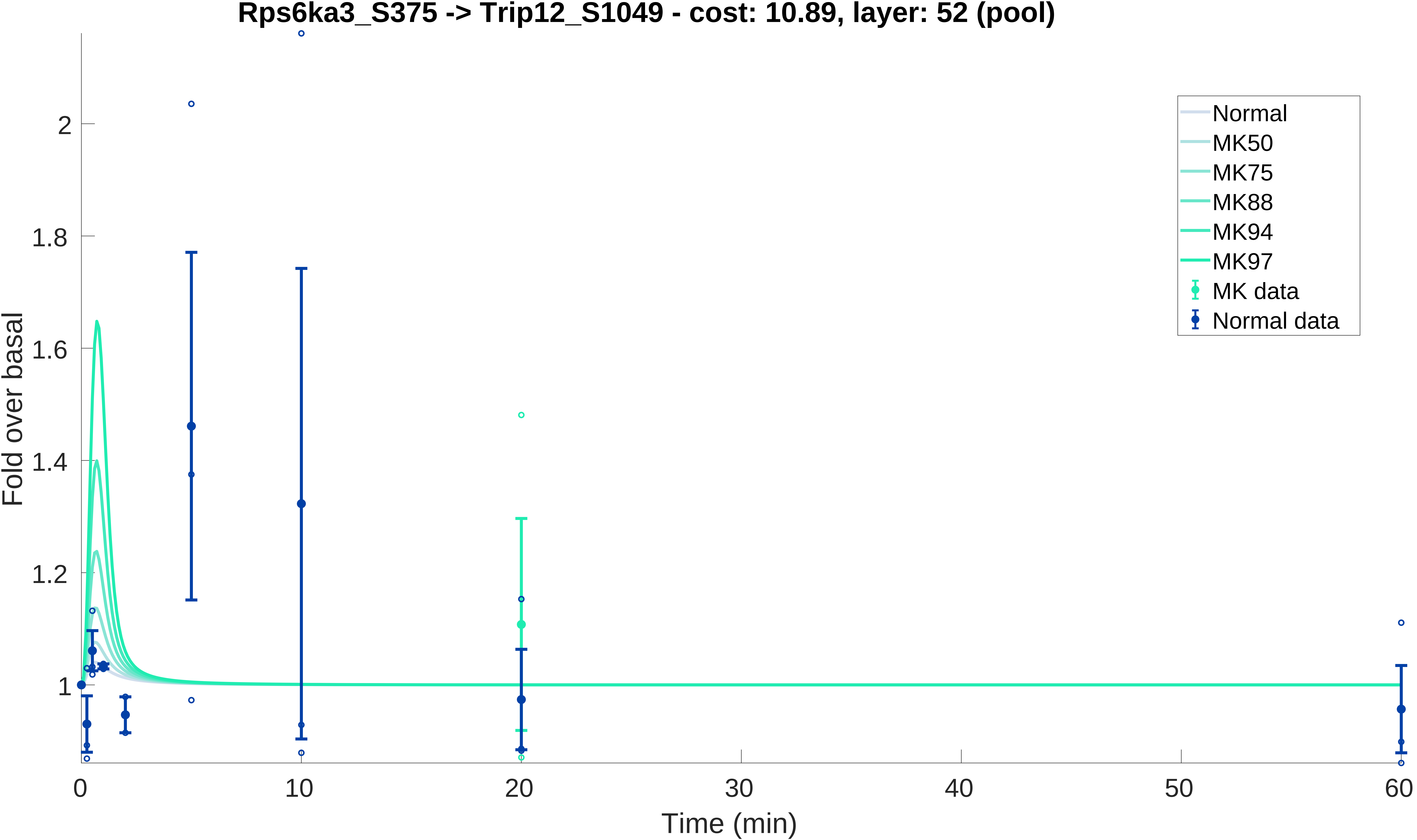

Rps6ka3_S375 -> Trip12_S1049 - cost: 10.89, layer: 52 (pool)
Fold over basal
Time (min)
0
10
20
30
40
50
60
1
1.2
1.4
1.6
1.8
2
Normal
MK50
MK75
MK88
MK94
MK97
MK data
Normal data

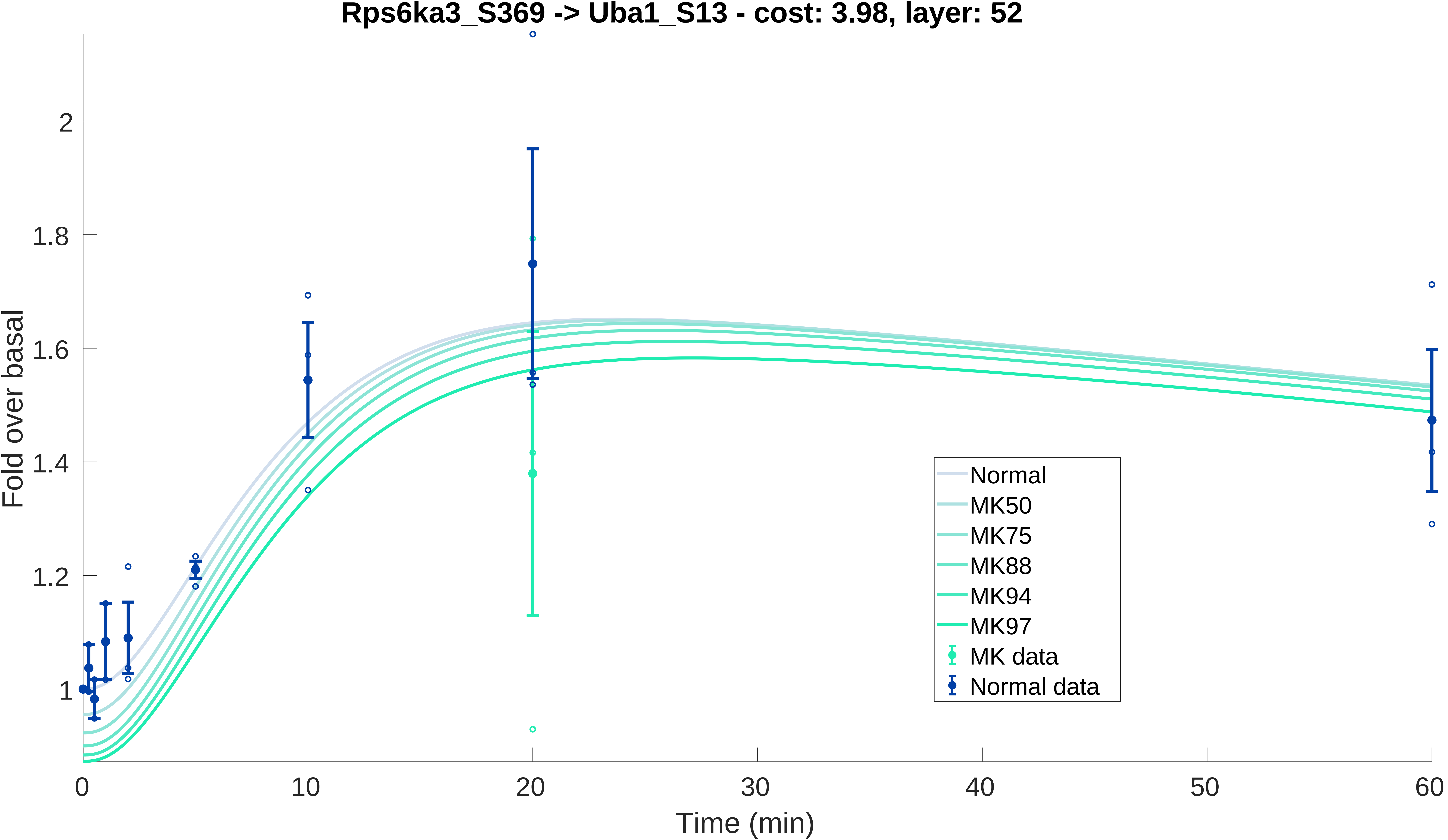

Rps6ka3_S369 -> Uba1_S13 - cost: 3.98, layer: 52
Fold over basal
Time (min)
2
1.8
1.6
1.4
1.2
1
0
10
20
30
40
50
60
Normal
MK50
MK75
MK88
MK94
MK97
MK data
Normal data

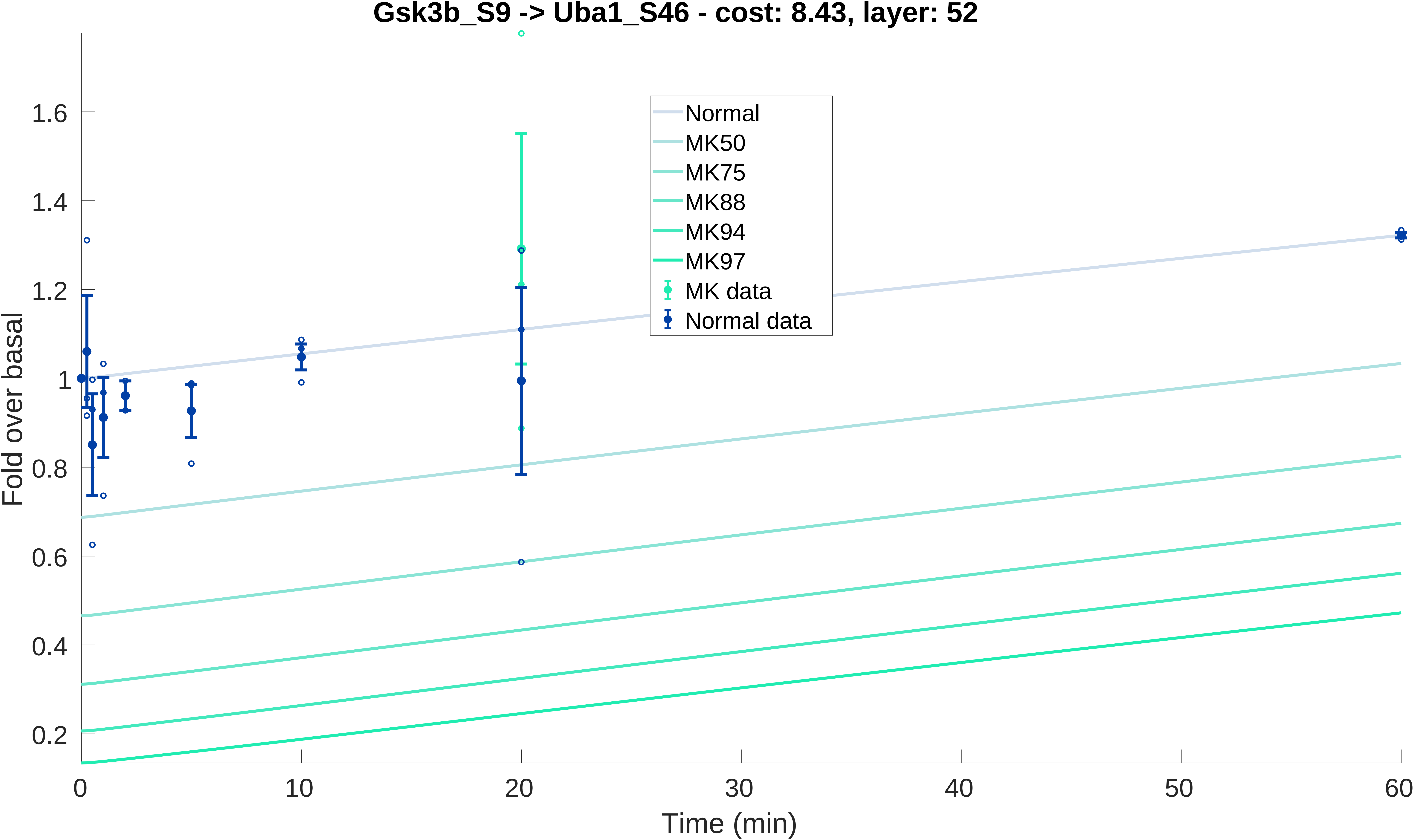

Gsk3b_S9 -> Uba1_S46 - cost: 8.43, layer: 52
1.6
1.4
1.2
1
0.8
0.6
0.4
0.2
Fold over basal
0
10
20
30
40
50
60
Time (min)
Normal
MK50
MK75
MK88
MK94
MK97
MK data
Normal data

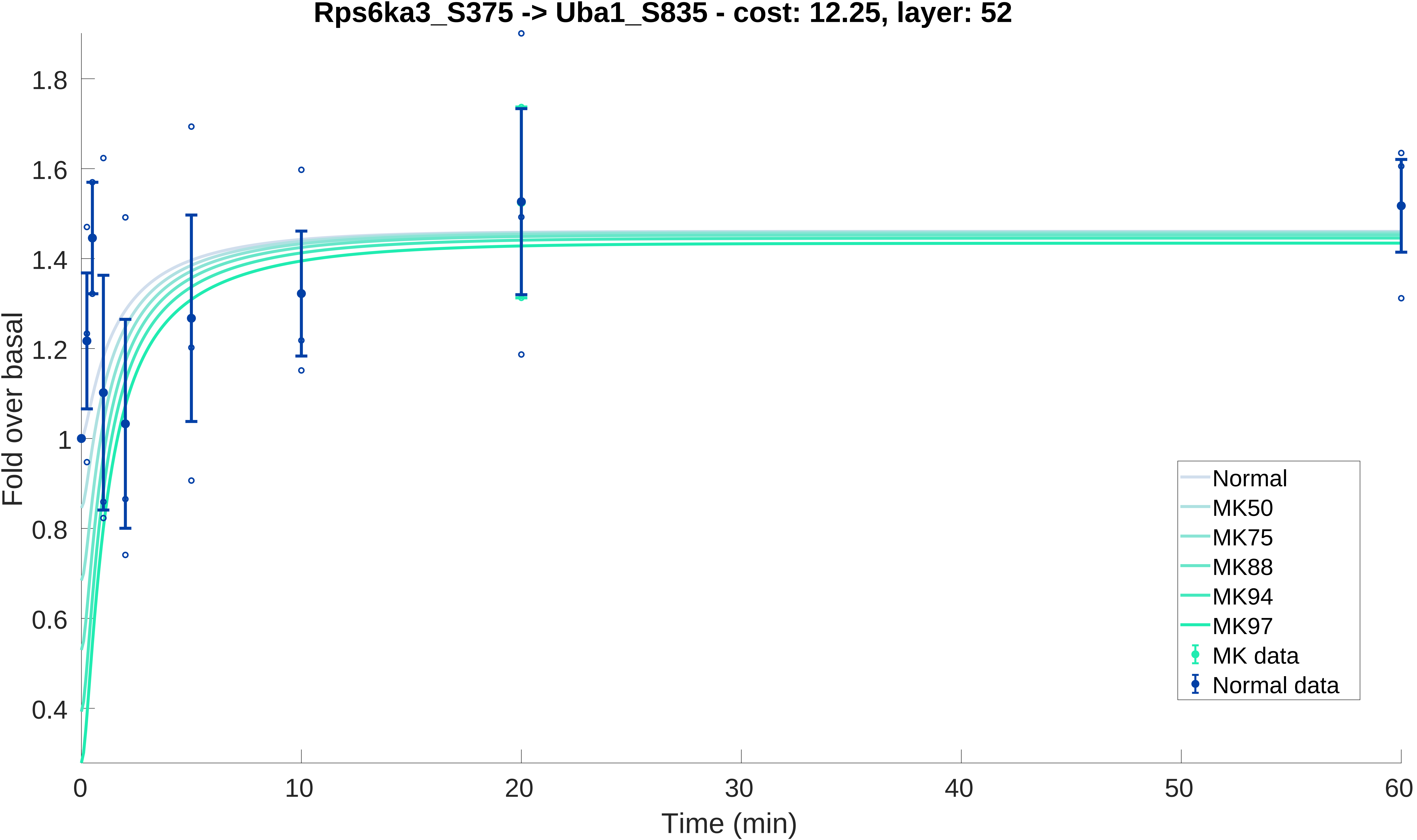

Rps6ka3_S375 -> Uba1_S835 - cost: 12.25, layer: 52
Fold over basal
Time (min)
1.8
1.6
1.4
1.2
1
0.8
0.6
0.4
0
10
20
30
40
50
60
Normal
MK50
MK75
MK88
MK94
MK97
MK data
Normal data

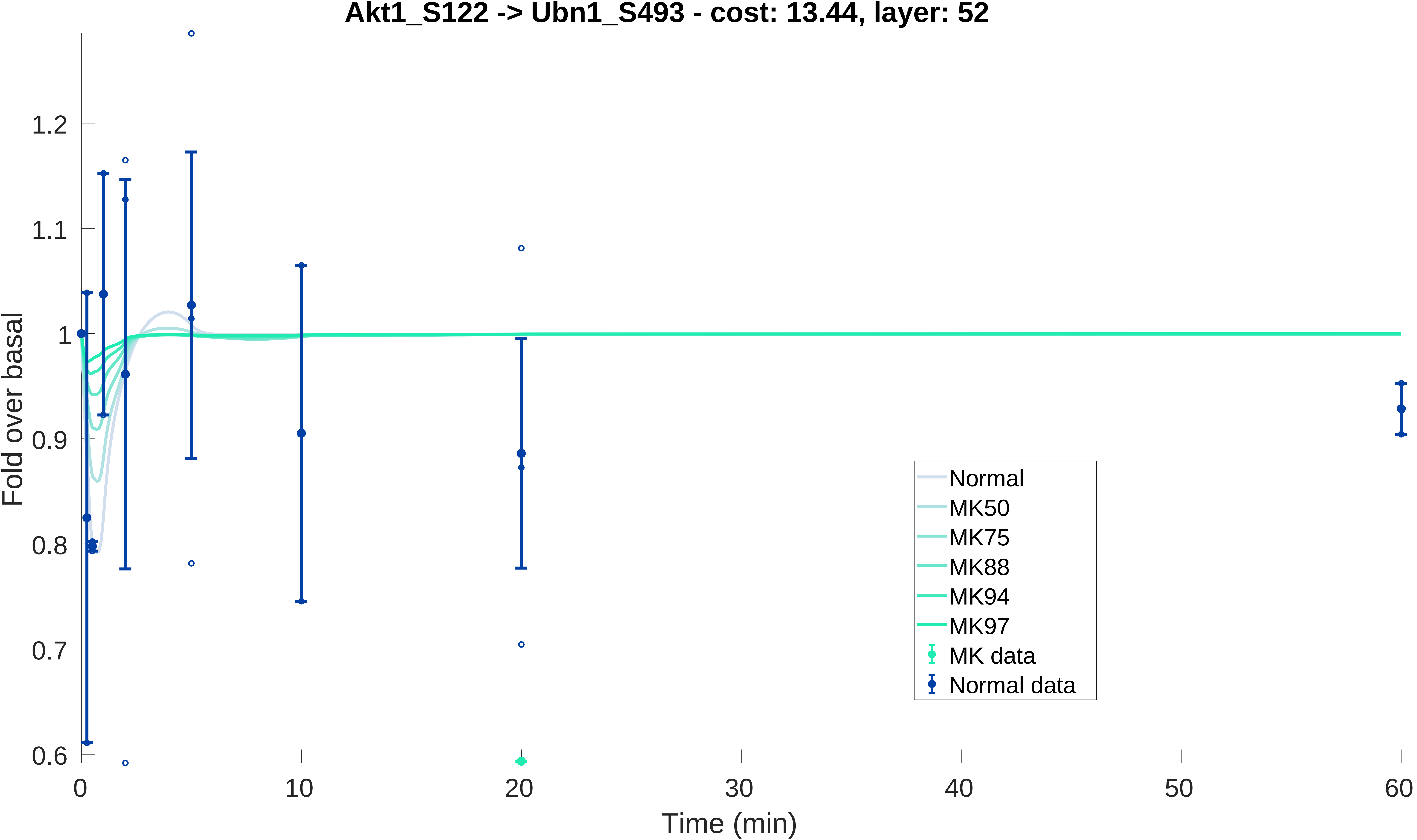

Akt1_S122 -> Ubn1_S493 - cost: 13.44, layer: 52
Fold over basal
Time (min)
1.2
1.1
1
0.9
0.8
0.7
0.6
0
10
20
30
40
50
60
Normal
MK50
MK75
MK88
MK94
MK97
MK data
Normal data

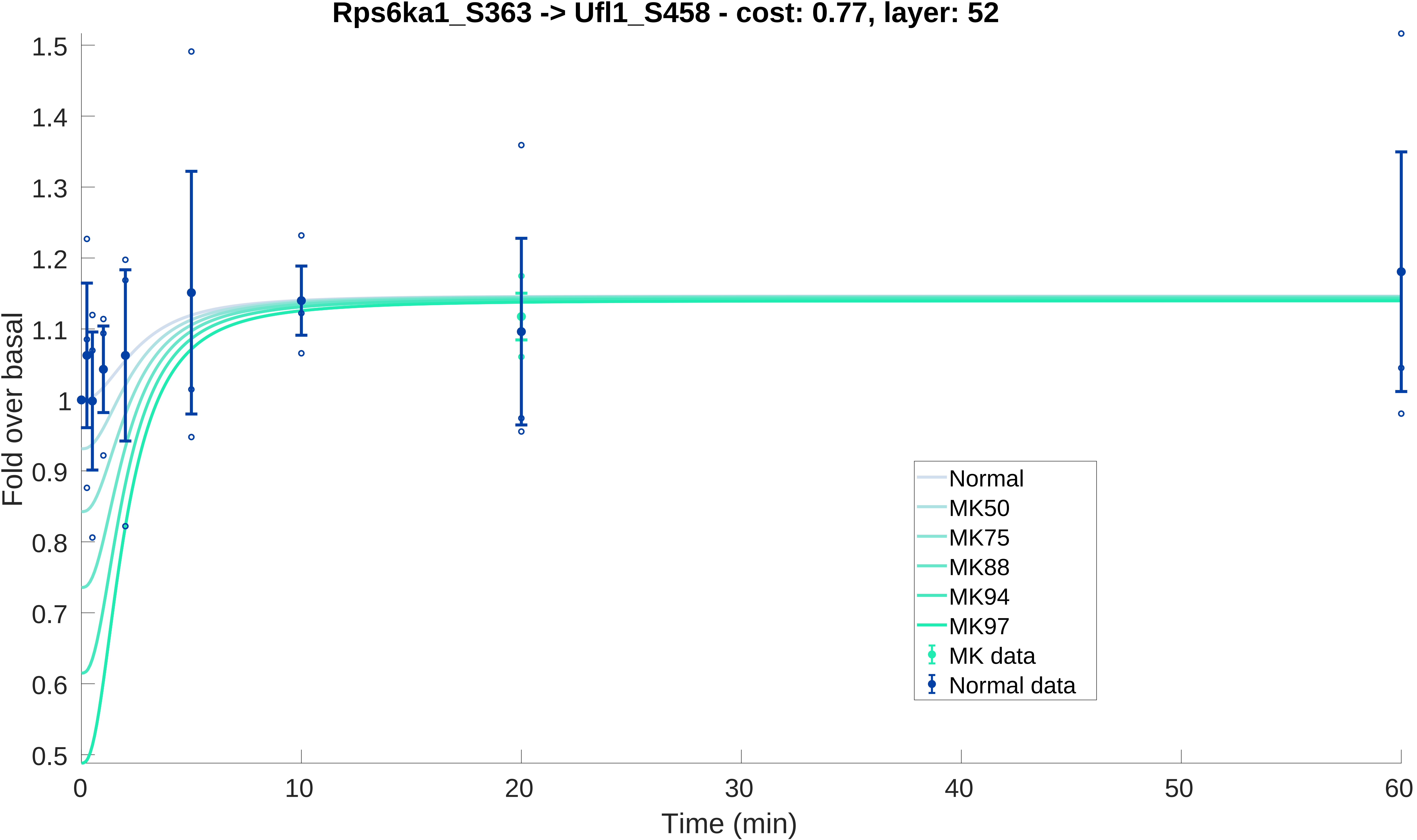
Rps6ka1_S363 -> Ufl1_S458 - cost: 0.77, layer: 52
Fold over basal
Time (min)
1.5
1.4
1.3
1.2
1.1
1
0.9
0.8
0.7
0.6
0.5
0
10
20
30
40
50
60
Normal
MK50
MK75
MK88
MK94
MK97
MK data
Normal data

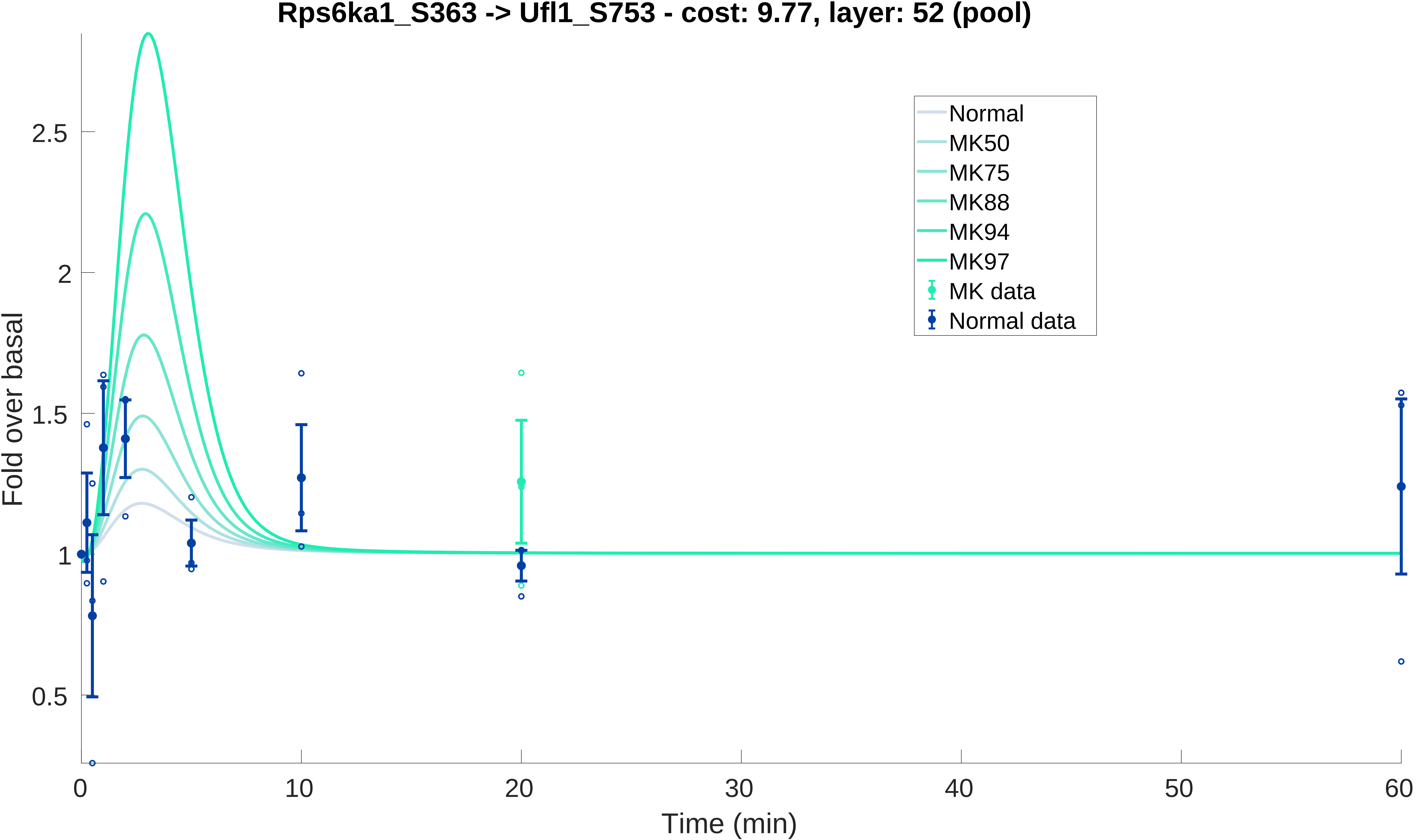

Rps6ka1_S363 -> Ufl1_S753 - cost: 9.77, layer: 52 (pool)
Fold over basal
Time (min)
2.5
2
1.5
1
0.5
0
10
20
30
40
50
60
Normal
MK50
MK75
MK88
MK94
MK97
MK data
Normal data

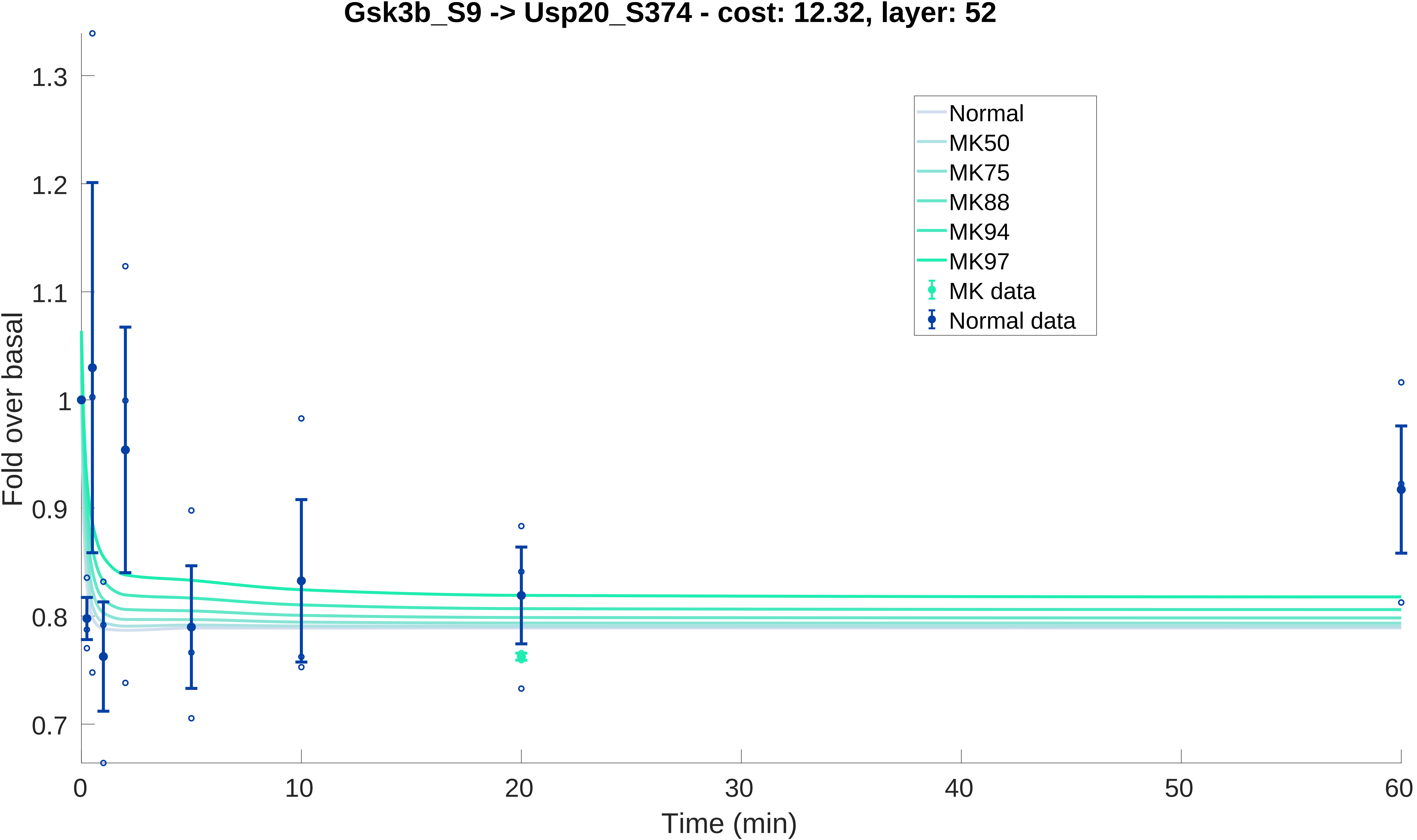

Gsk3b_S9 -> Usp20_S374 - cost: 12.32, layer: 52
Fold over basal
Time (min)
1.3
1.2
1.1
1
0.9
0.8
0.7
0
10
20
30
40
50
60
Normal
MK50
MK75
MK88
MK94
MK97
MK data
Normal data

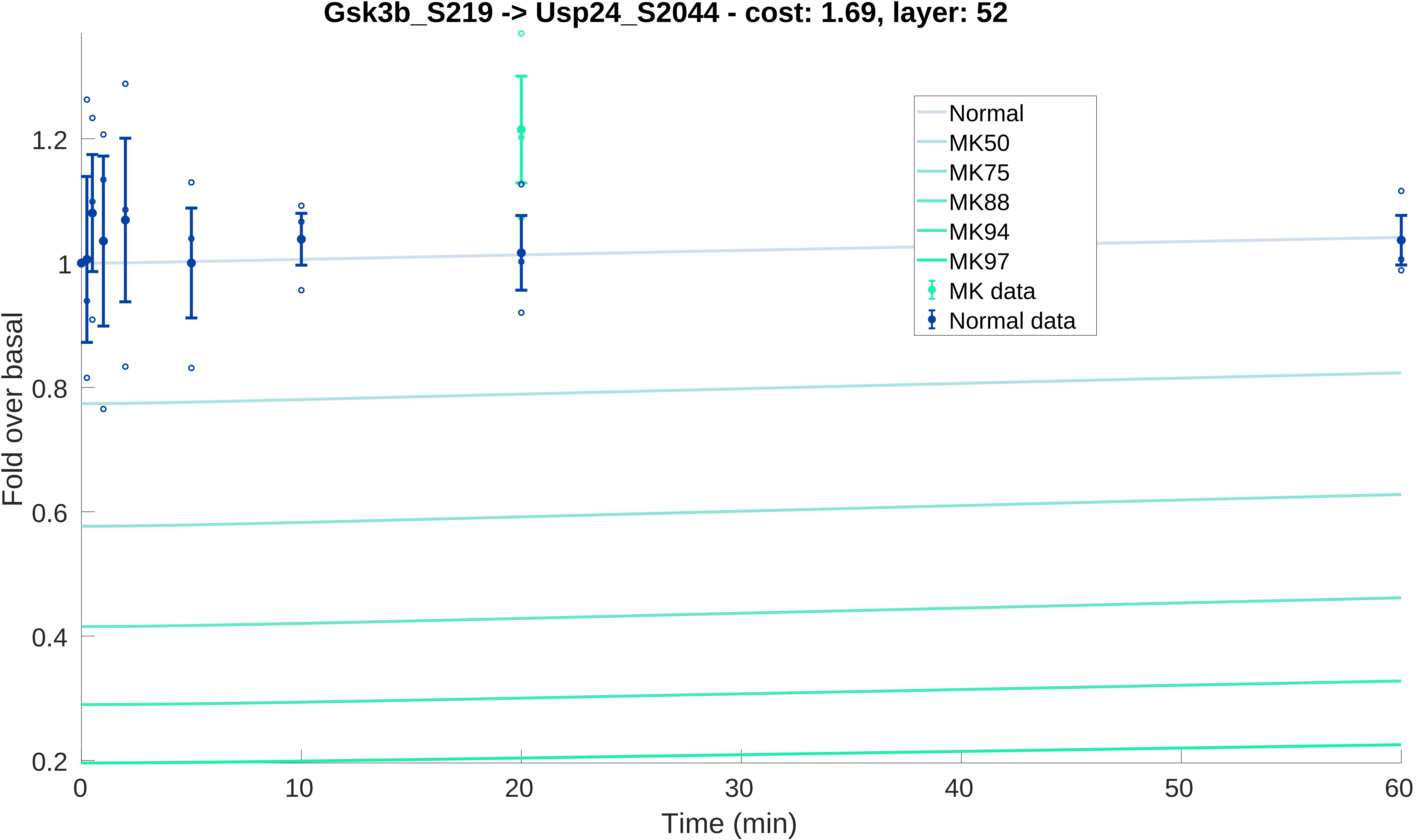
Gsk3b_S219 -> Usp24_S2044 - cost: 1.69, layer: 52
Fold over basal
Time (min)
0.2
0.4
0.6
0.8
1
1.2
0
10
20
30
40
50
60
Normal
MK50
MK75
MK88
MK94
MK97
MK data
Normal data

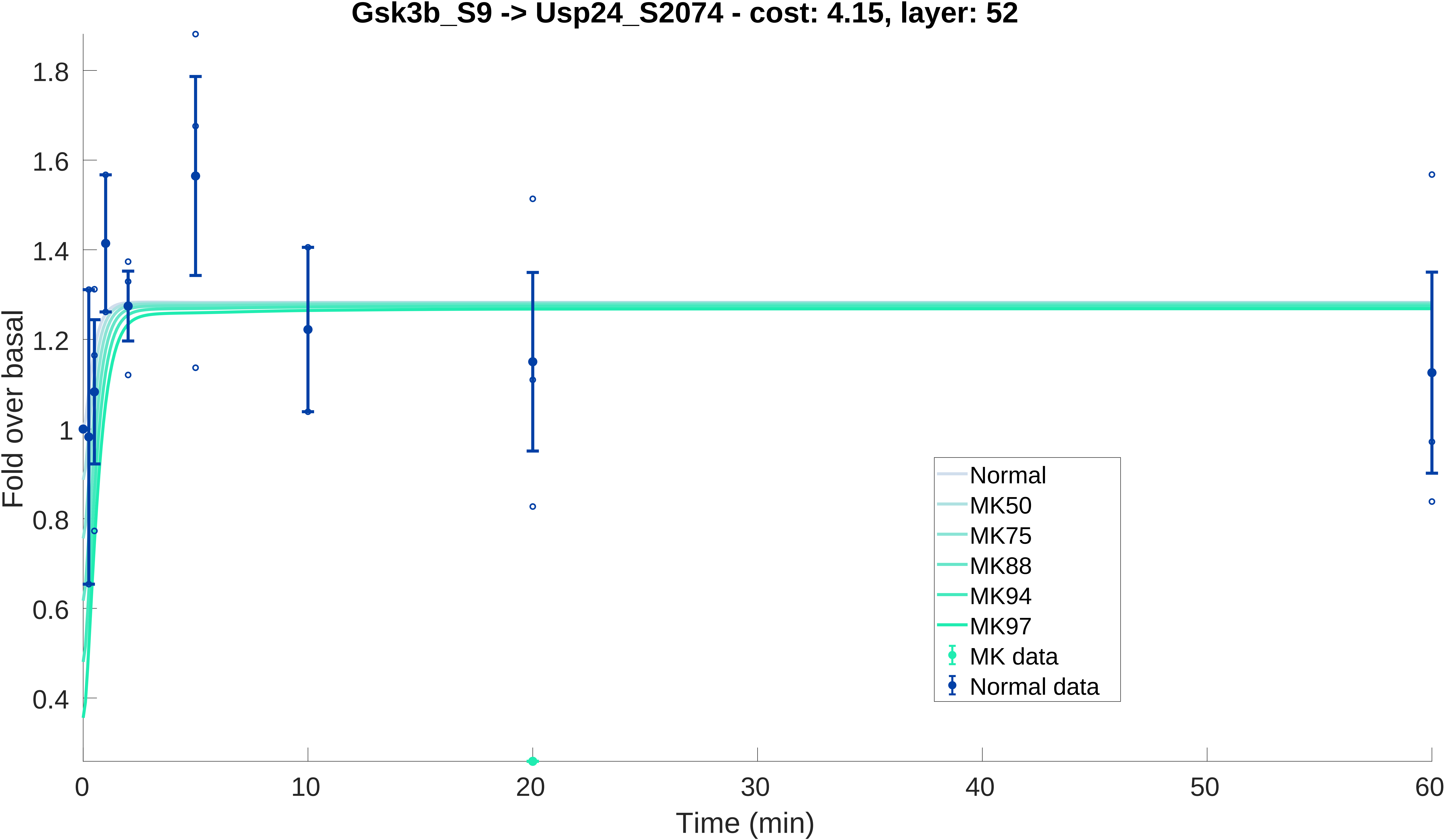

Gsk3b_S9 -> Usp24_S2074 - cost: 4.15, layer: 52
Fold over basal
0.4
0.6
0.8
1
1.2
1.4
1.6
1.8
0
10
20
30
40
50
60
Time (min)
Normal
MK50
MK75
MK88
MK94
MK97
MK data
Normal data

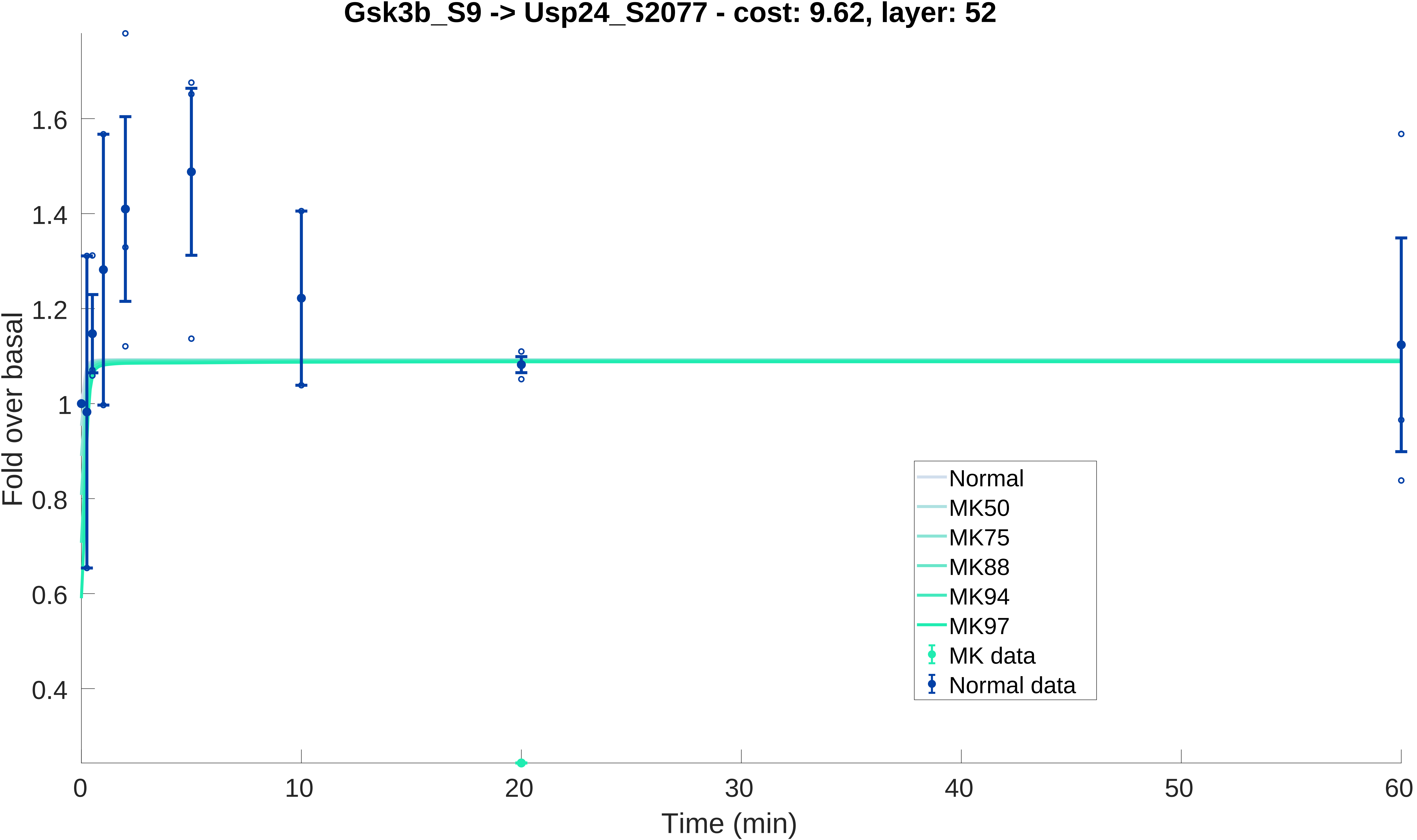

Gsk3b_S9 -> Usp24_S2077 - cost: 9.62, layer: 52
Fold over basal
Time (min)
1.6
1.4
1.2
1
0.8
0.6
0.4
0
10
20
30
40
50
60
Normal
MK50
MK75
MK88
MK94
MK97
MK data
Normal data

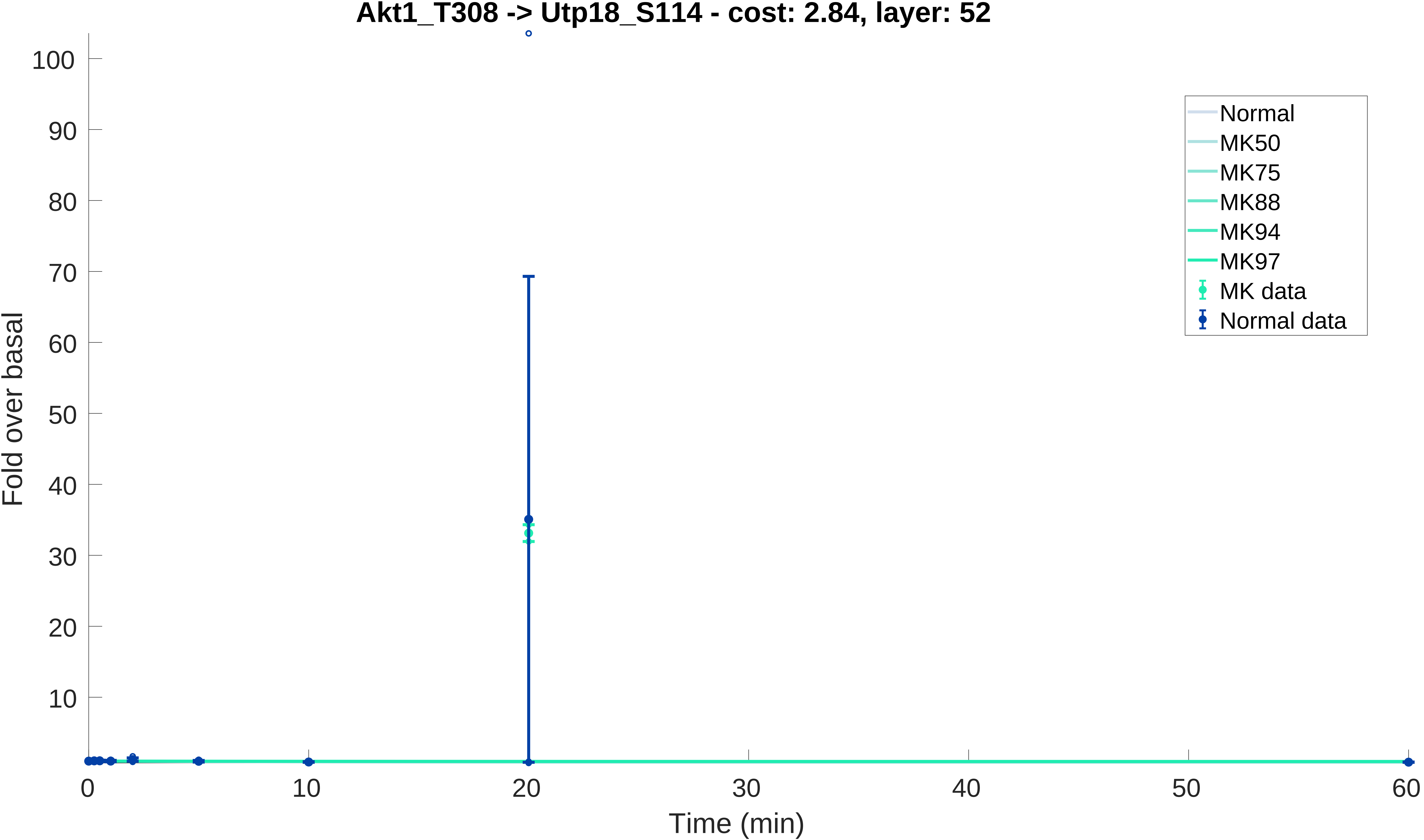

Akt1_T308 -> Utp18_S114 - cost: 2.84, layer: 52
Fold over basal
100
90
80
70
60
50
40
30
20
10
0
10
20
30
40
50
60
Time (min)
Normal
MK50
MK75
MK88
MK94
MK97
MK data
Normal data

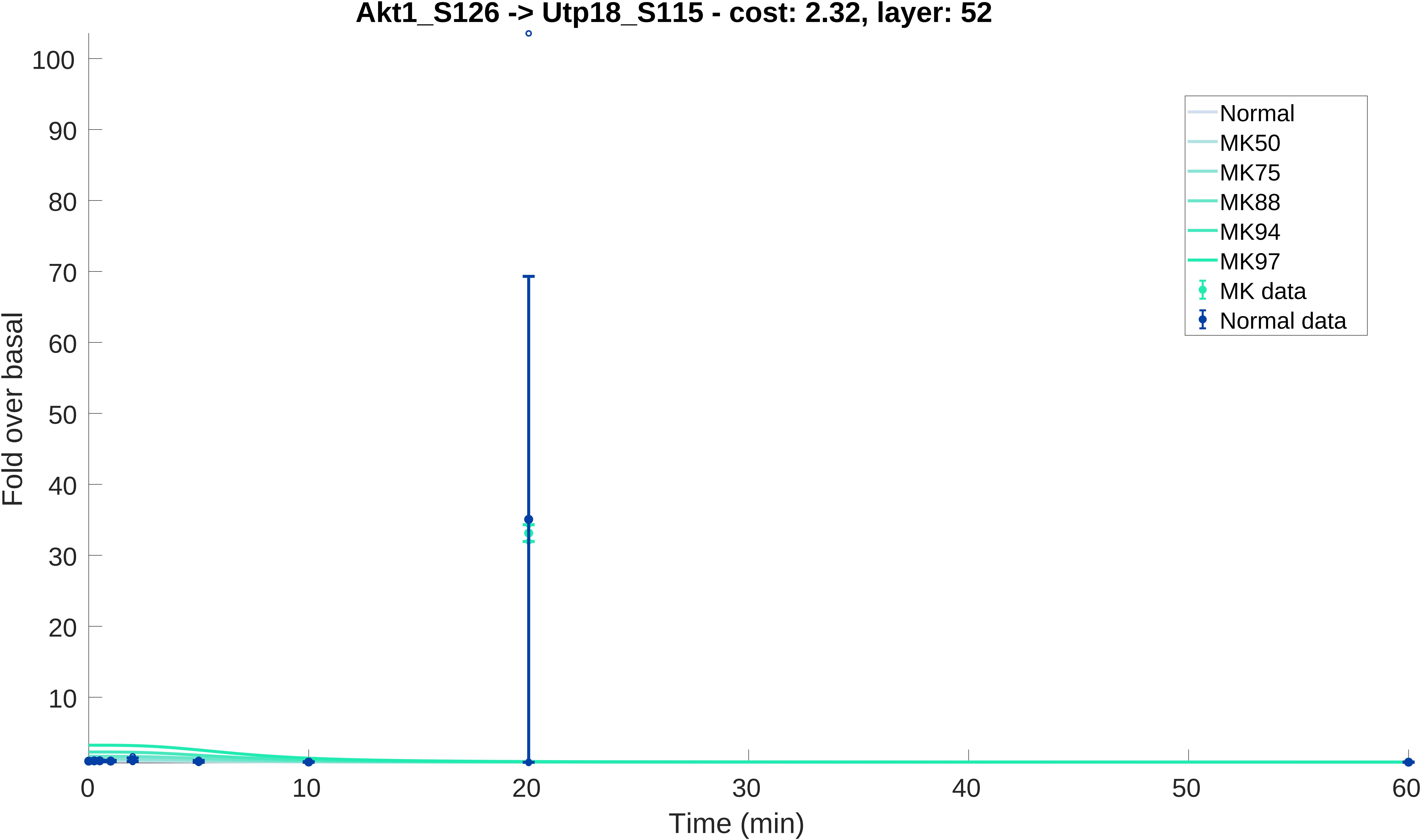

Akt1_S126 -> Utp18_S115 - cost: 2.32, layer: 52
Fold over basal
Time (min)
Normal
MK50
MK75
MK88
MK94
MK97
MK data
Normal data
0
10
20
30
40
50
60
70
80
90
100

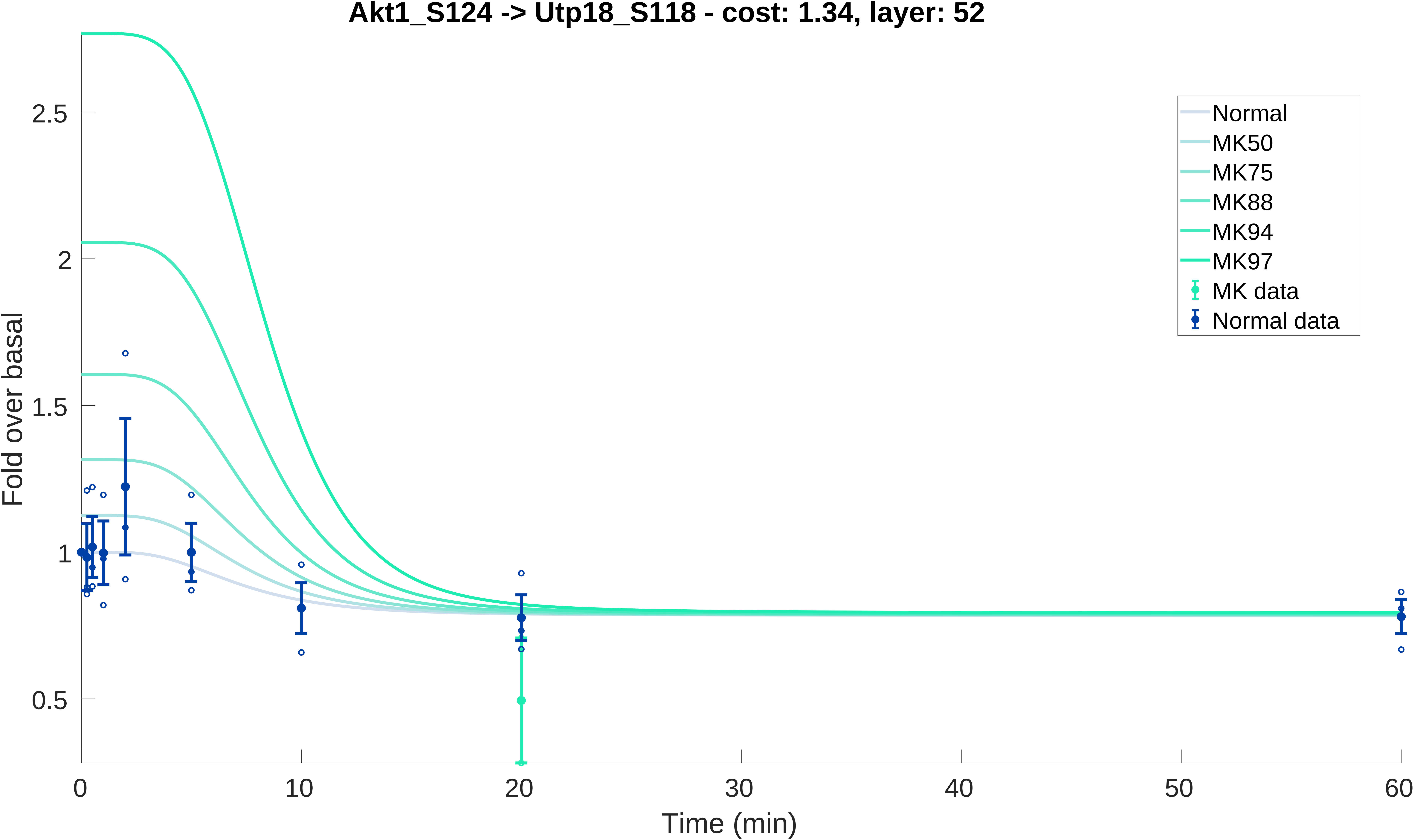

Akt1_S124 -> Utp18_S118 - cost: 1.34, layer: 52
Fold over basal
2.5
2
1.5
1
0.5
0
10
20
30
40
50
60
Time (min)
Normal
MK50
MK75
MK88
MK94
MK97
MK data
Normal data

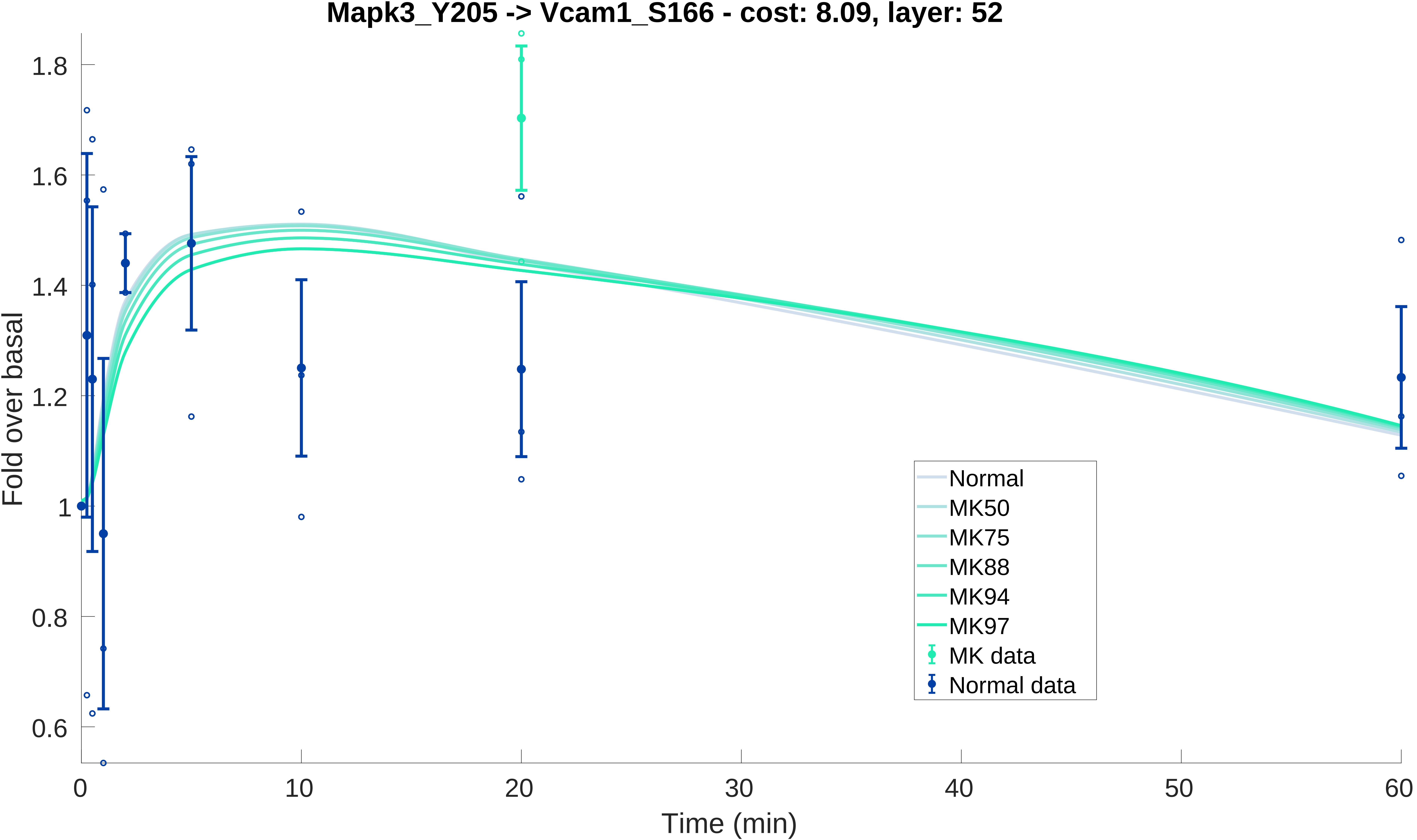

Mapk3_Y205 -> Vcam1_S166 - cost: 8.09, layer: 52
Fold over basal
1.8
1.6
1.4
1.2
1
0.8
0.6
0
10
20
30
40
50
60
Time (min)
Normal
MK50
MK75
MK88
MK94
MK97
MK data
Normal data

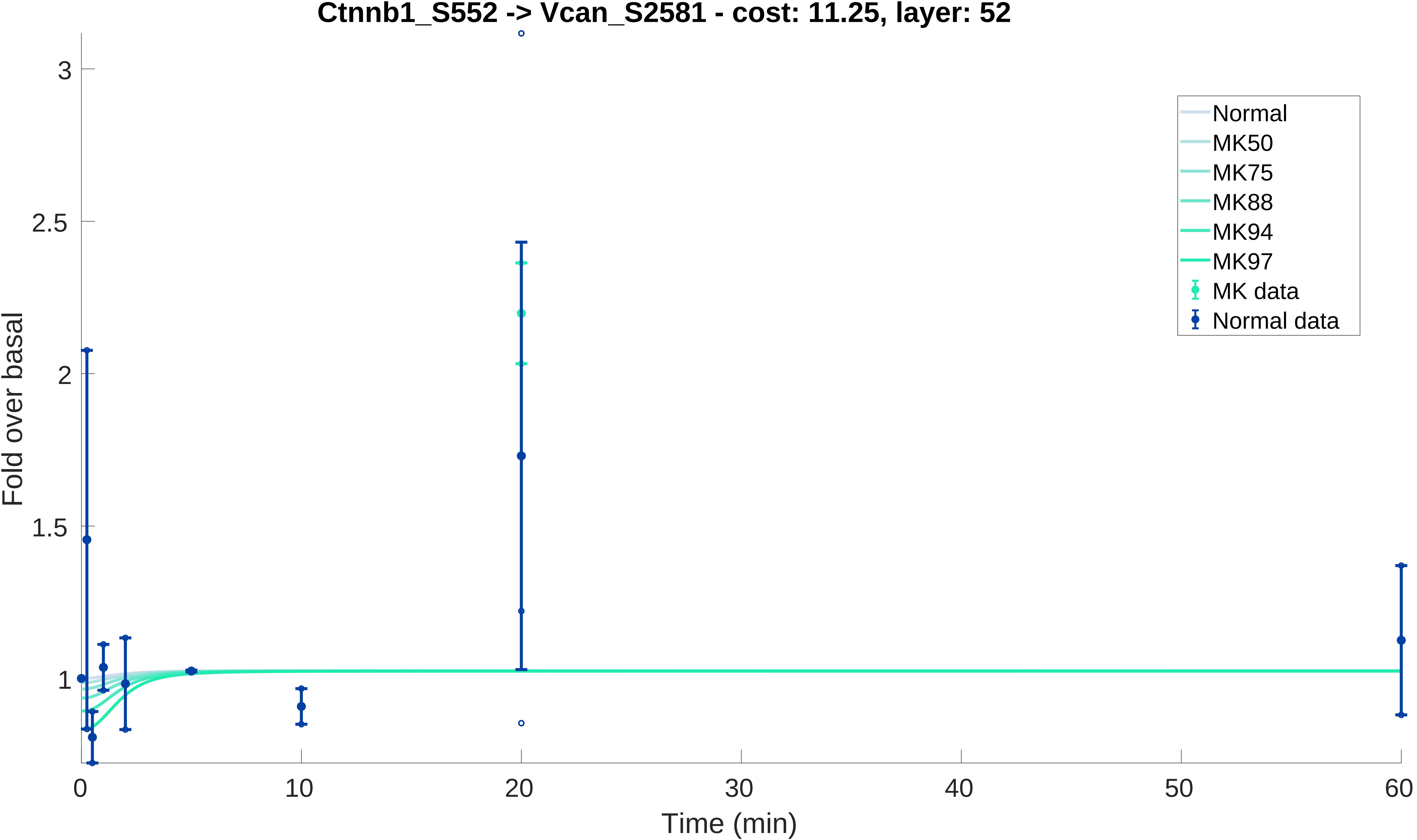

Ctnnb1_S552 -> Vcan_S2581 - cost: 11.25, layer: 52
Fold over basal
Time (min)
1
1.5
2
2.5
3
0
10
20
30
40
50
60
Normal
MK50
MK75
MK88
MK94
MK97
MK data
Normal data

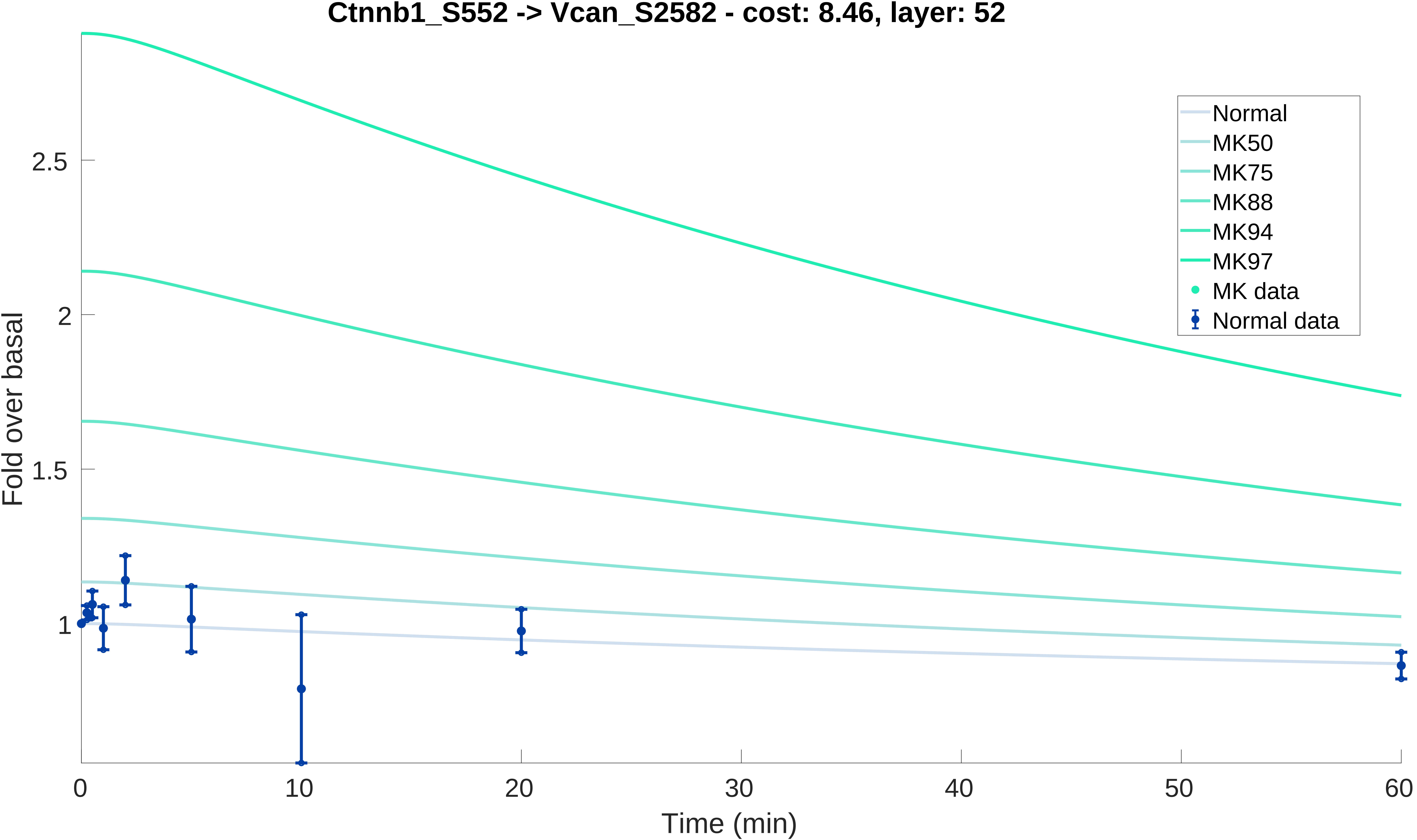

Ctnnb1_S552 -> Vcan_S2582 - cost: 8.46, layer: 52
Fold over basal
Time (min)
2.5
2
1.5
1
0
10
20
30
40
50
60
Normal
MK50
MK75
MK88
MK94
MK97
MK data
Normal data

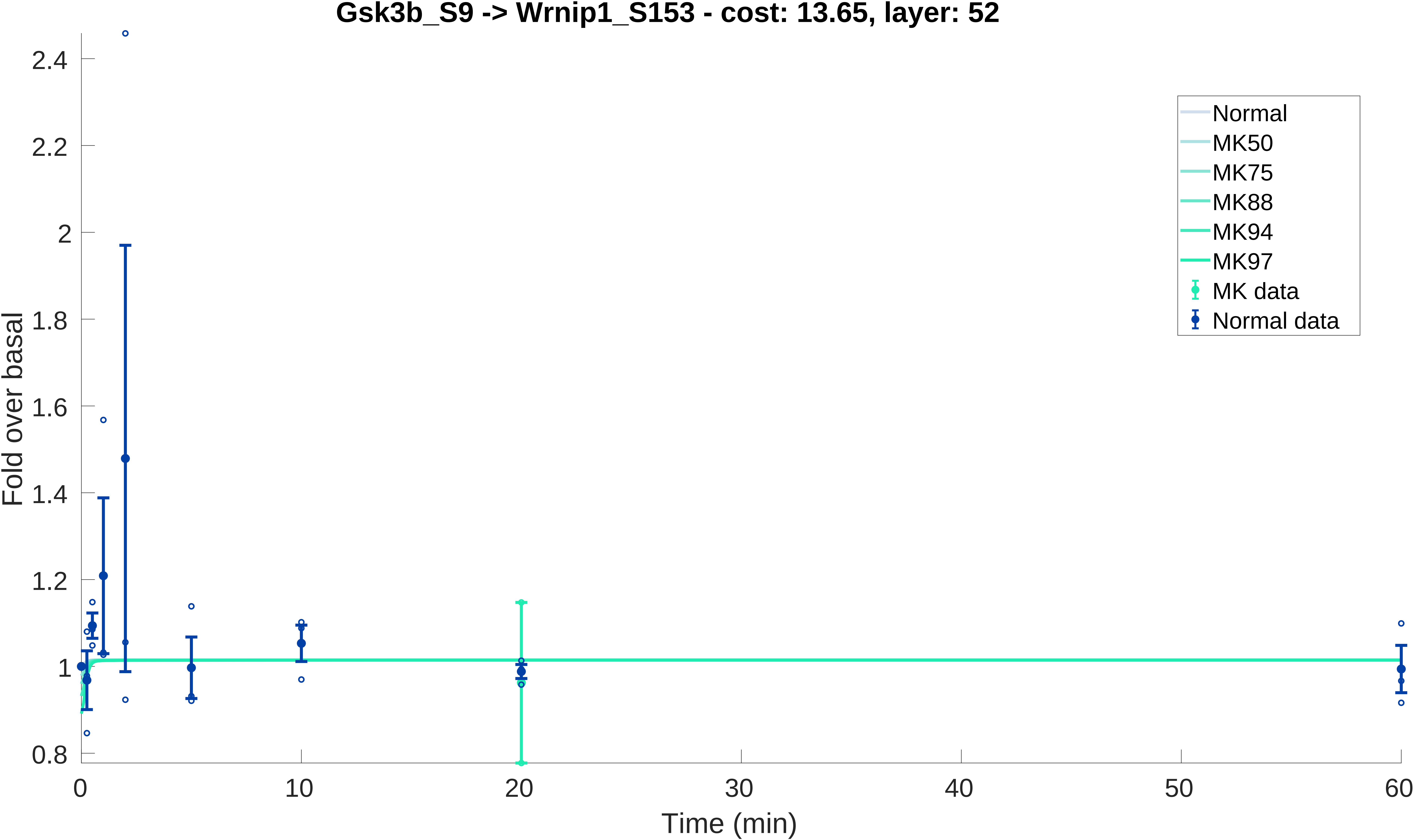
Gsk3b_S9 -> Wrnip1_S153 - cost: 13.65, layer: 52
Fold over basal
Time (min)
Normal
MK50
MK75
MK88
MK94
MK97
MK data
Normal data
0.8
1
1.2
1.4
1.6
1.8
2
2.2
2.4
0
10
20
30
40
50
60

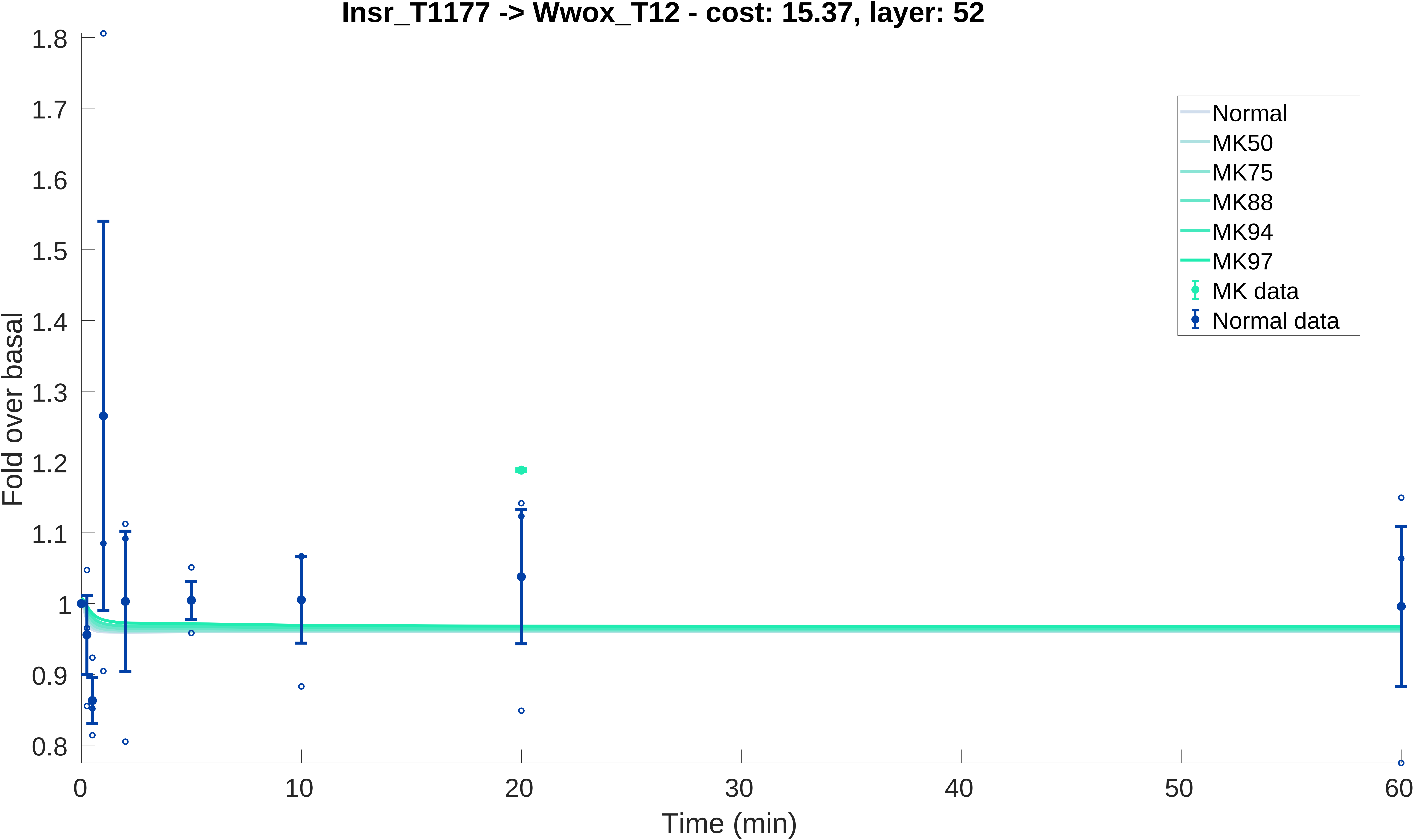

Insr_T1177 -> Wwox_T12 - cost: 15.37, layer: 52
Fold over basal
Time (min)
1.8
1.7
1.6
1.5
1.4
1.3
1.2
1.1
1
0.9
0.8
0
10
20
30
40
50
60
Normal
MK50
MK75
MK88
MK94
MK97
MK data
Normal data

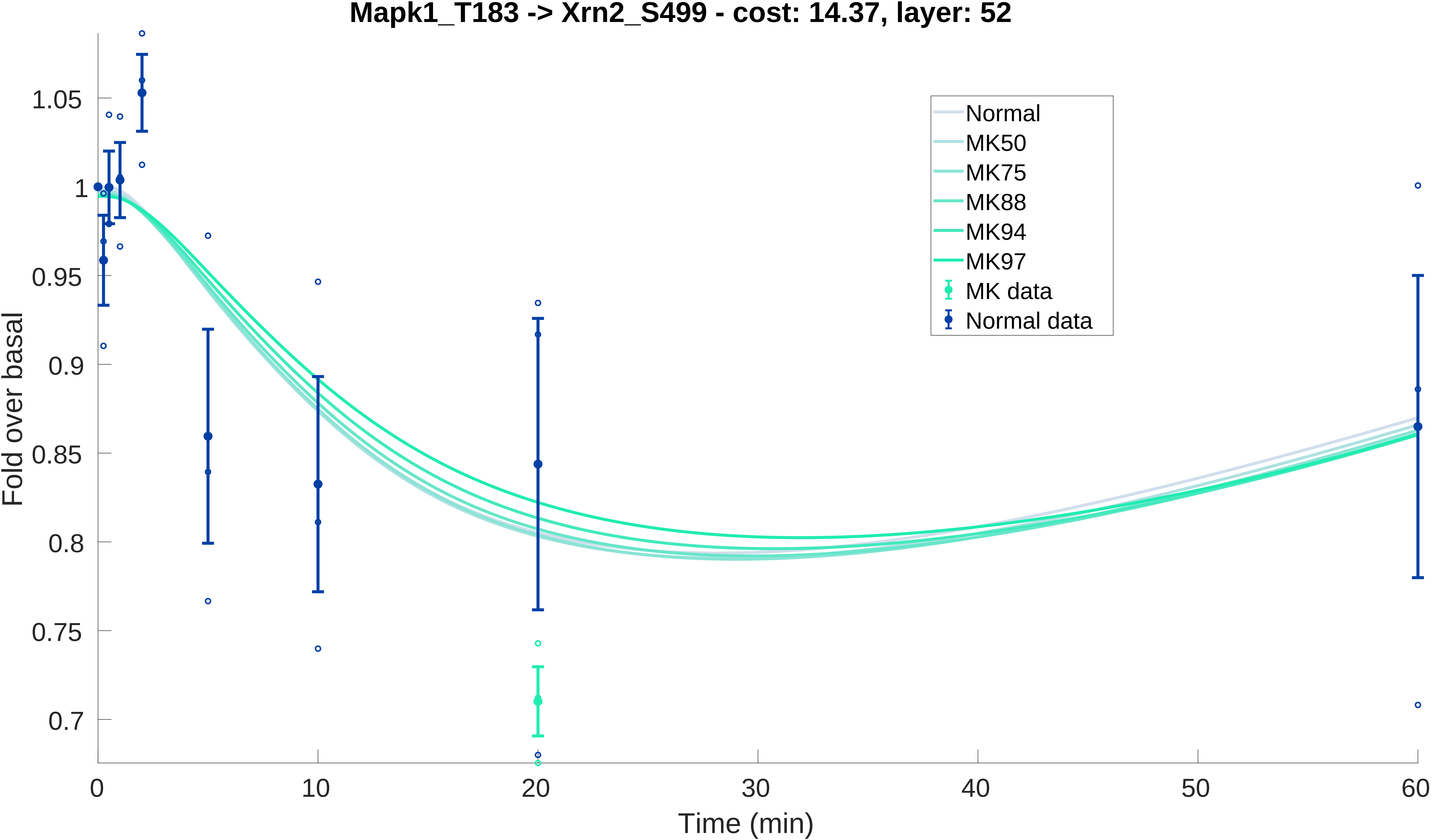

Mapk1_T183 -> Xrn2_S499 - cost: 14.37, layer: 52
Fold over basal
1.05
1
0.95
0.9
0.85
0.8
0.75
0.7
0
10
20
30
40
50
60
Time (min)
Normal
MK50
MK75
MK88
MK94
MK97
MK data
Normal data

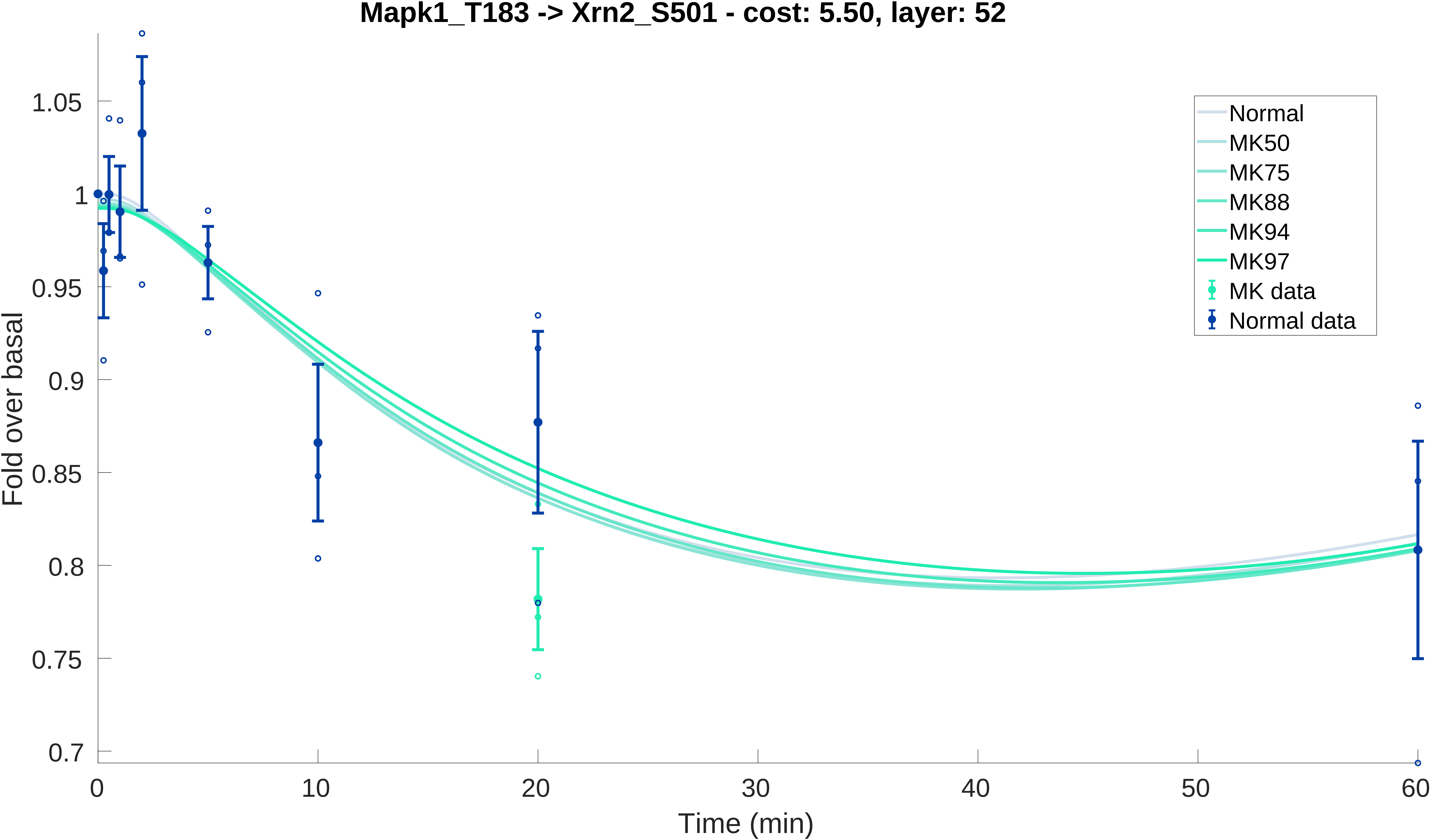
Mapk1_T183 -> Xrn2_S501 - cost: 5.50, layer: 52
Fold over basal
1.05
1
0.95
0.9
0.85
0.8
0.75
0.7
0
10
20
30
40
50
60
Time (min)
Normal
MK50
MK75
MK88
MK94
MK97
MK data
Normal data

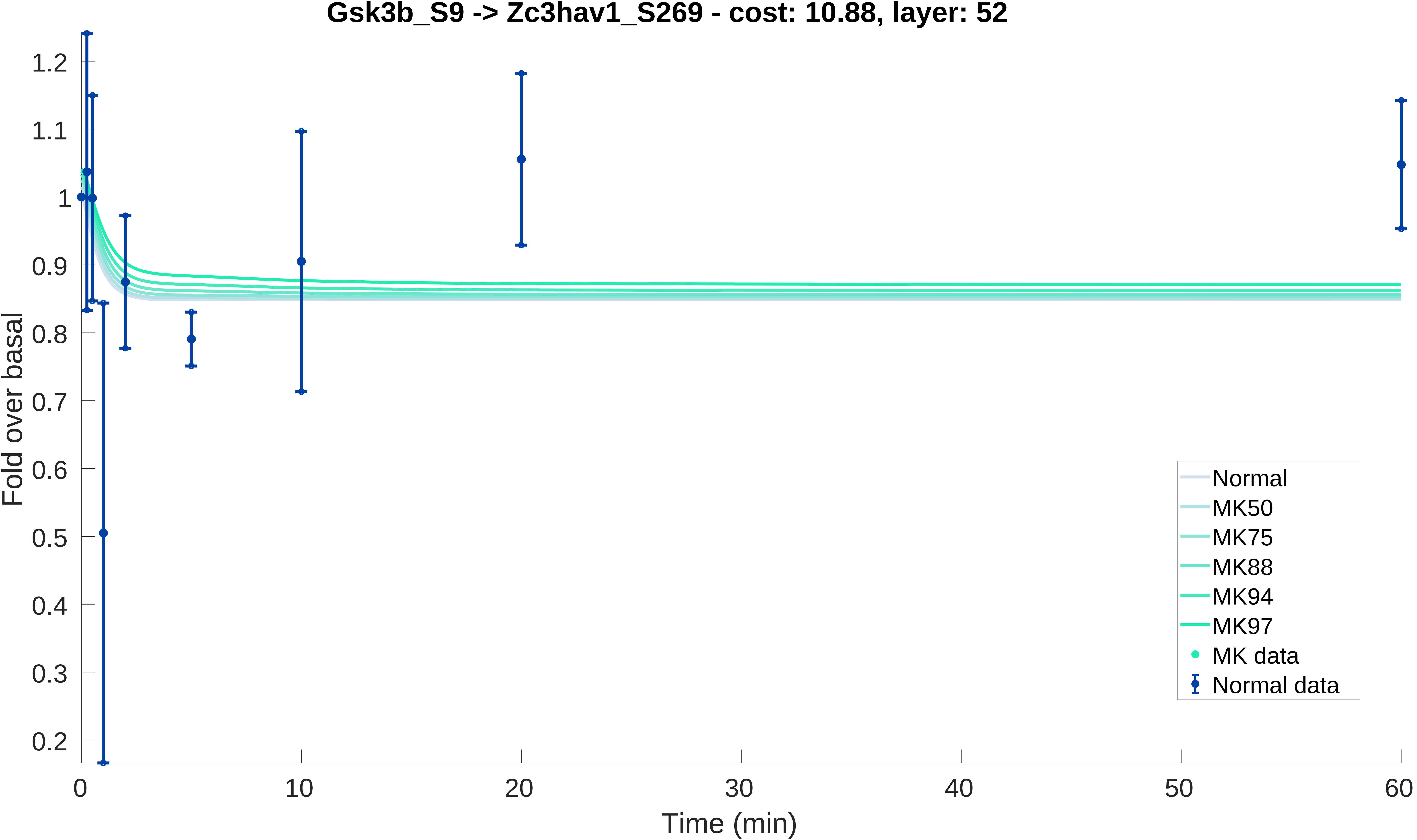

Gsk3b_S9 -> Zc3hav1_S269 - cost: 10.88, layer: 52
Fold over basal
1.2
1.1
1
0.9
0.8
0.7
0.6
0.5
0.4
0.3
0.2
0
10
20
30
40
50
60
Time (min)
Normal
MK50
MK75
MK88
MK94
MK97
MK data
Normal data

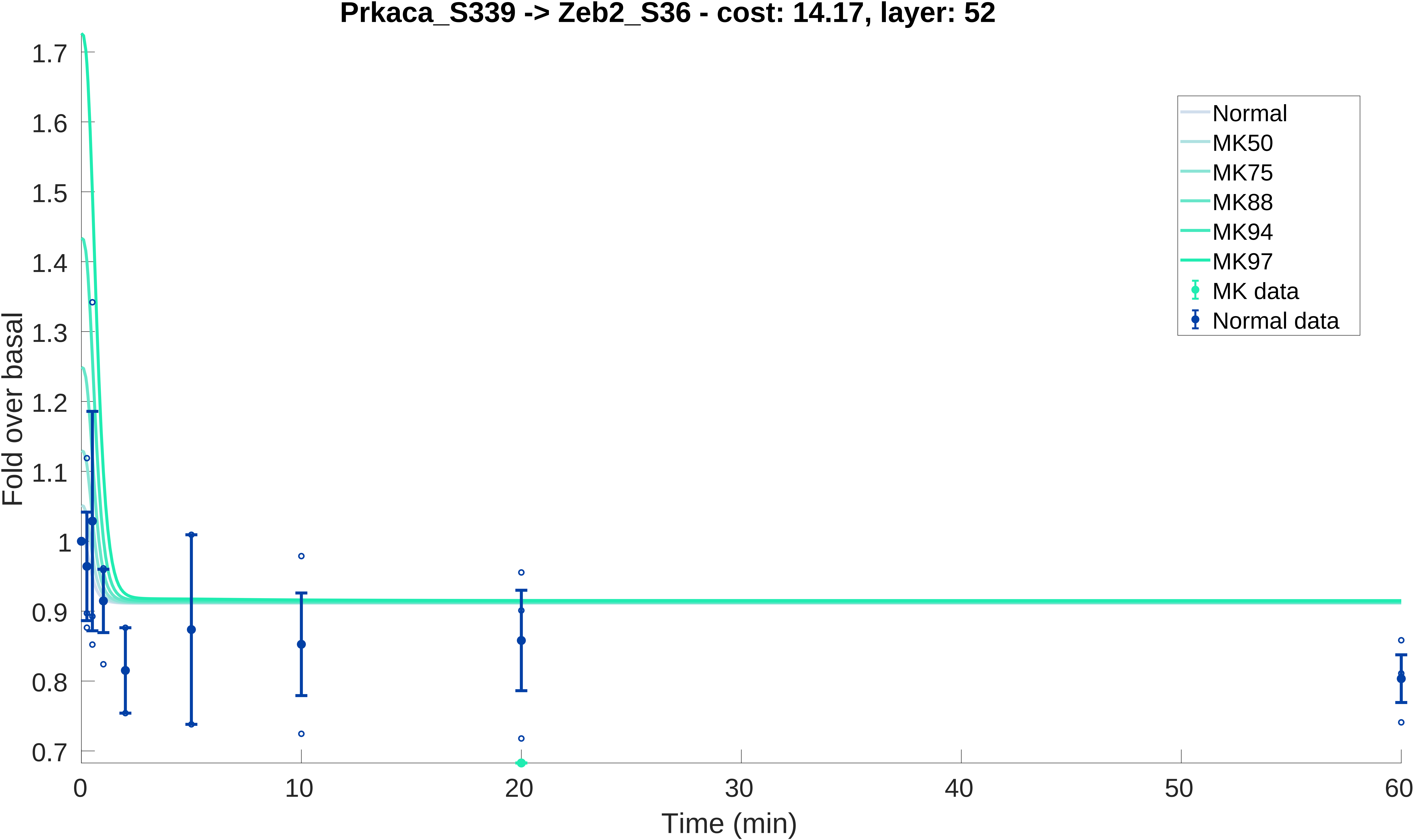

Prrkaca_S339 -> Zeb2_S36 - cost: 14.17, layer: 52
Fold over basal
Time (min)
Normal
MK50
MK75
MK88
MK94
MK97
MK data
Normal data

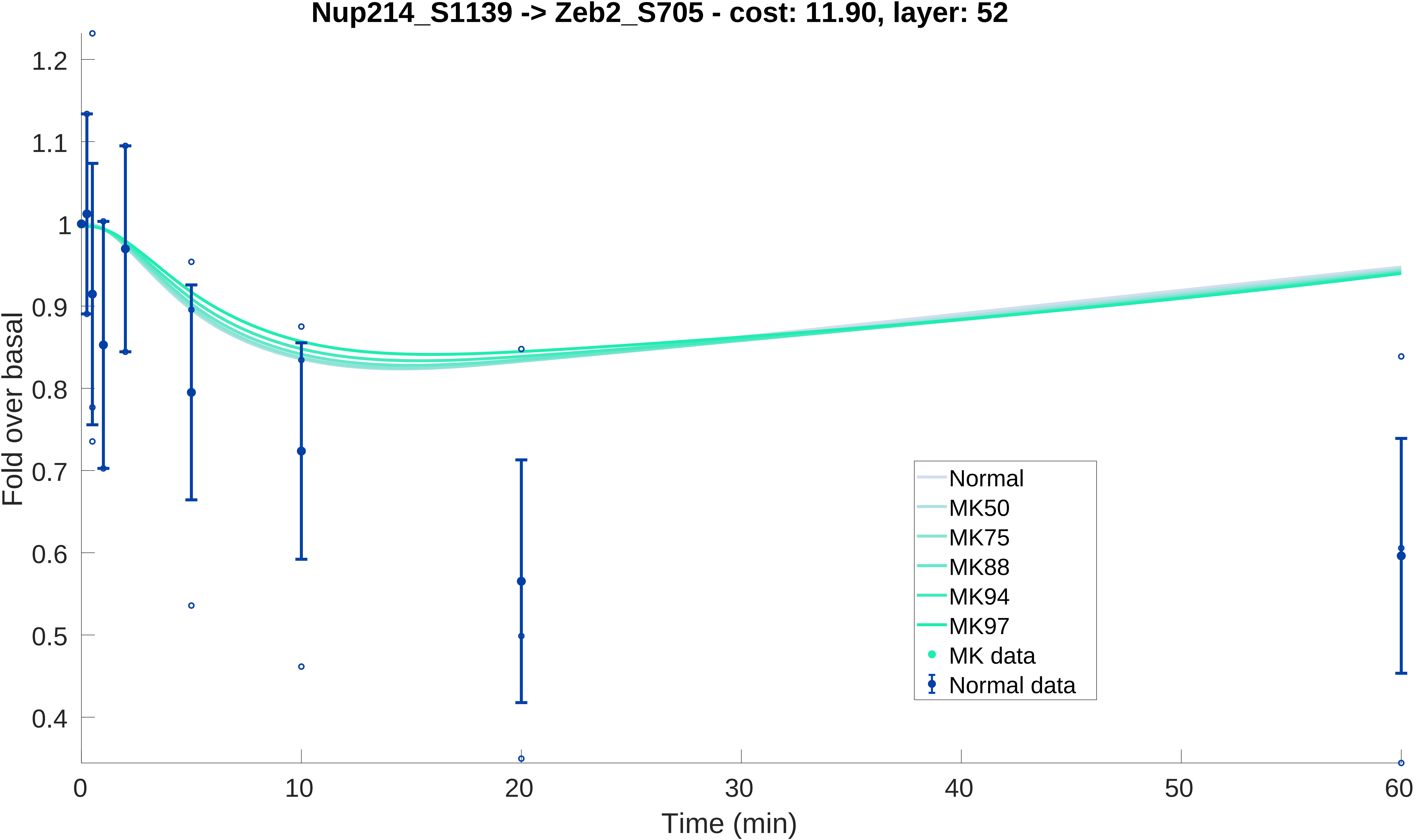

Nup214_S1139 -> Zeb2_S705 - cost: 11.90, layer: 52
Fold over basal
Time (min)
1.2
1.1
1
0.9
0.8
0.7
0.6
0.5
0.4
0
10
20
30
40
50
60
Normal
MK50
MK75
MK88
MK94
MK97
MK data
Normal data

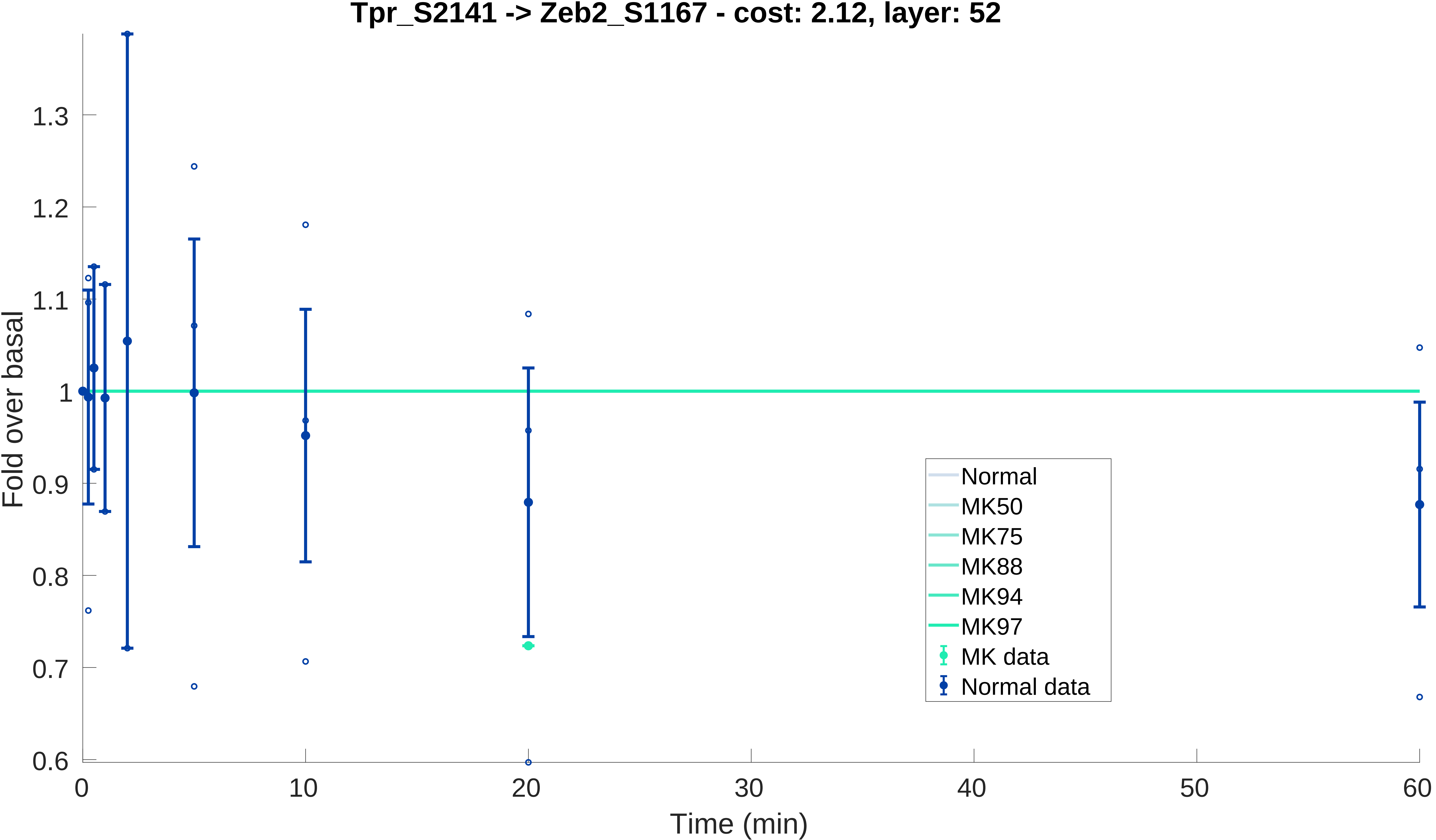

Tpr_S2141 -> Zeb2_S1167 - cost: 2.12, layer: 52
Fold over basal
1.3
1.2
1.1
1
0.9
0.8
0.7
0.6
0
10
20
30
40
50
60
Time (min)
Normal
MK50
MK75
MK88
MK94
MK97
MK data
Normal data

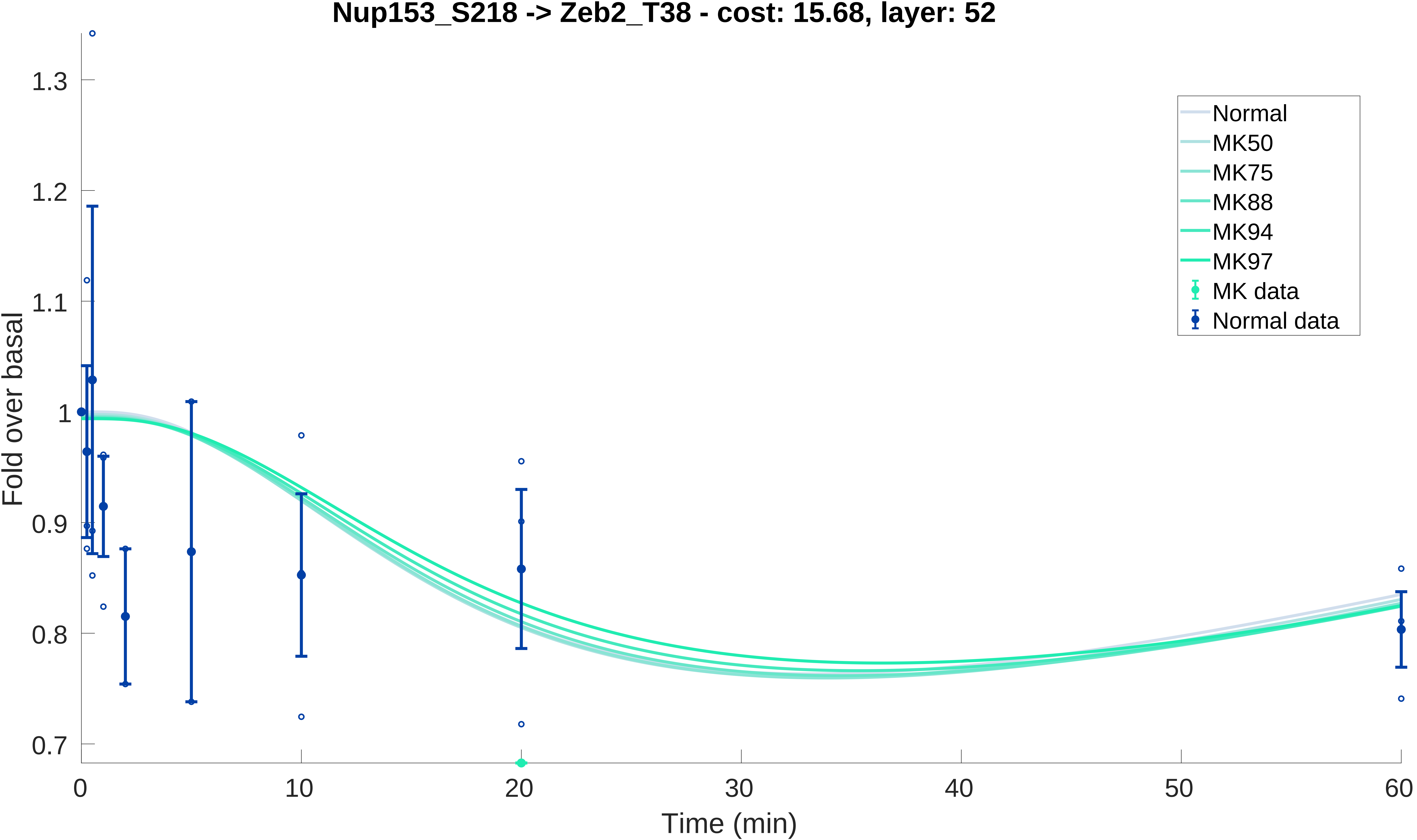

Nup153_S218 -> Zeb2_T38 - cost: 15.68, layer: 52
Fold over basal
1.3
1.2
1.1
1
0.9
0.8
0.7
0
10
20
30
40
50
60
Time (min)
Normal
MK50
MK75
MK88
MK94
MK97
MK data
Normal data

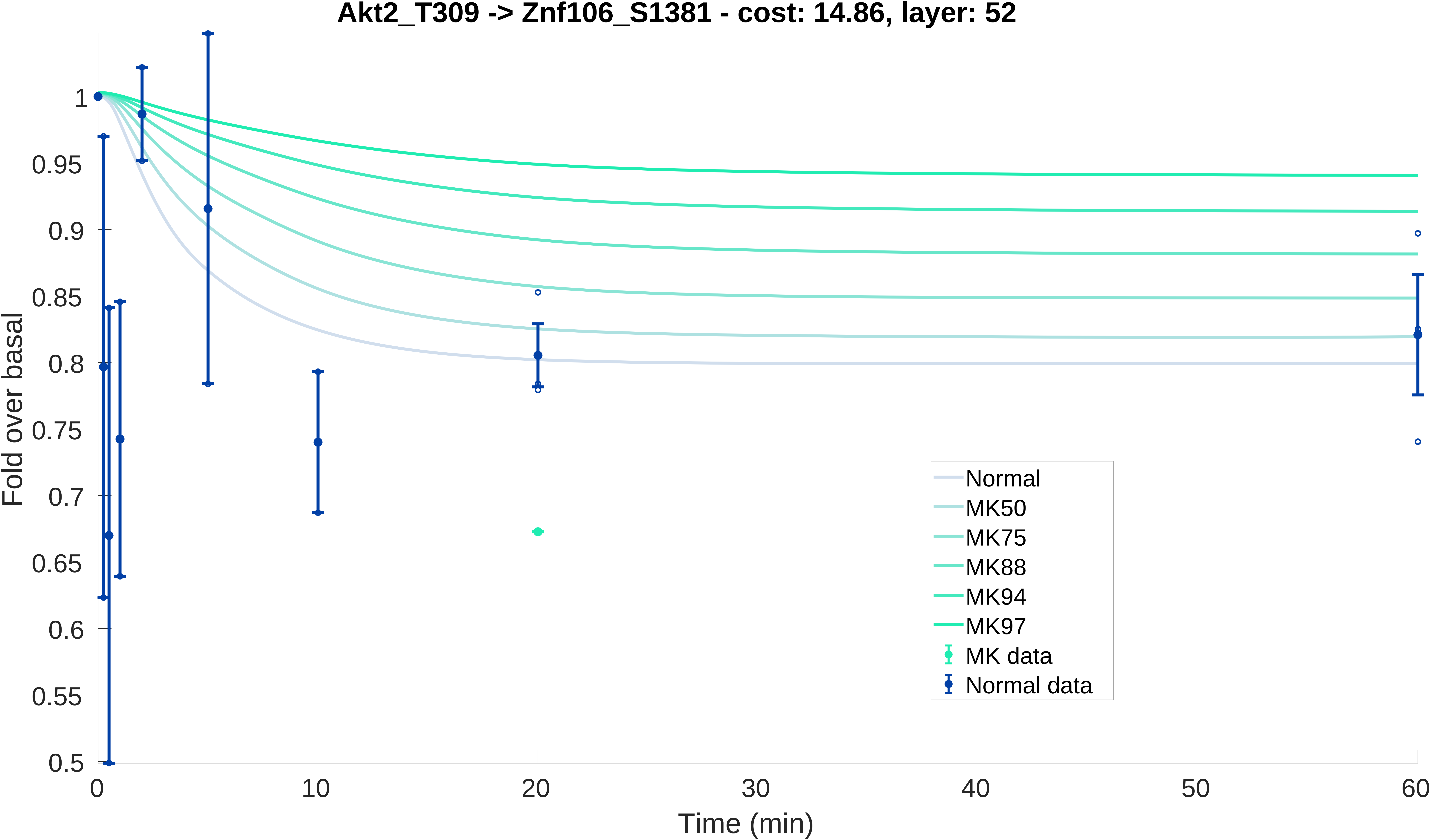

Akt2_T309 -> Znf106_S1381 - cost: 14.86, layer: 52
Fold over basal
1
0.95
0.9
0.85
0.8
0.75
0.7
0.65
0.6
0.55
0.5
0
10
20
30
40
50
60
Time (min)
Normal
MK50
MK75
MK88
MK94
MK97
MK data
Normal data

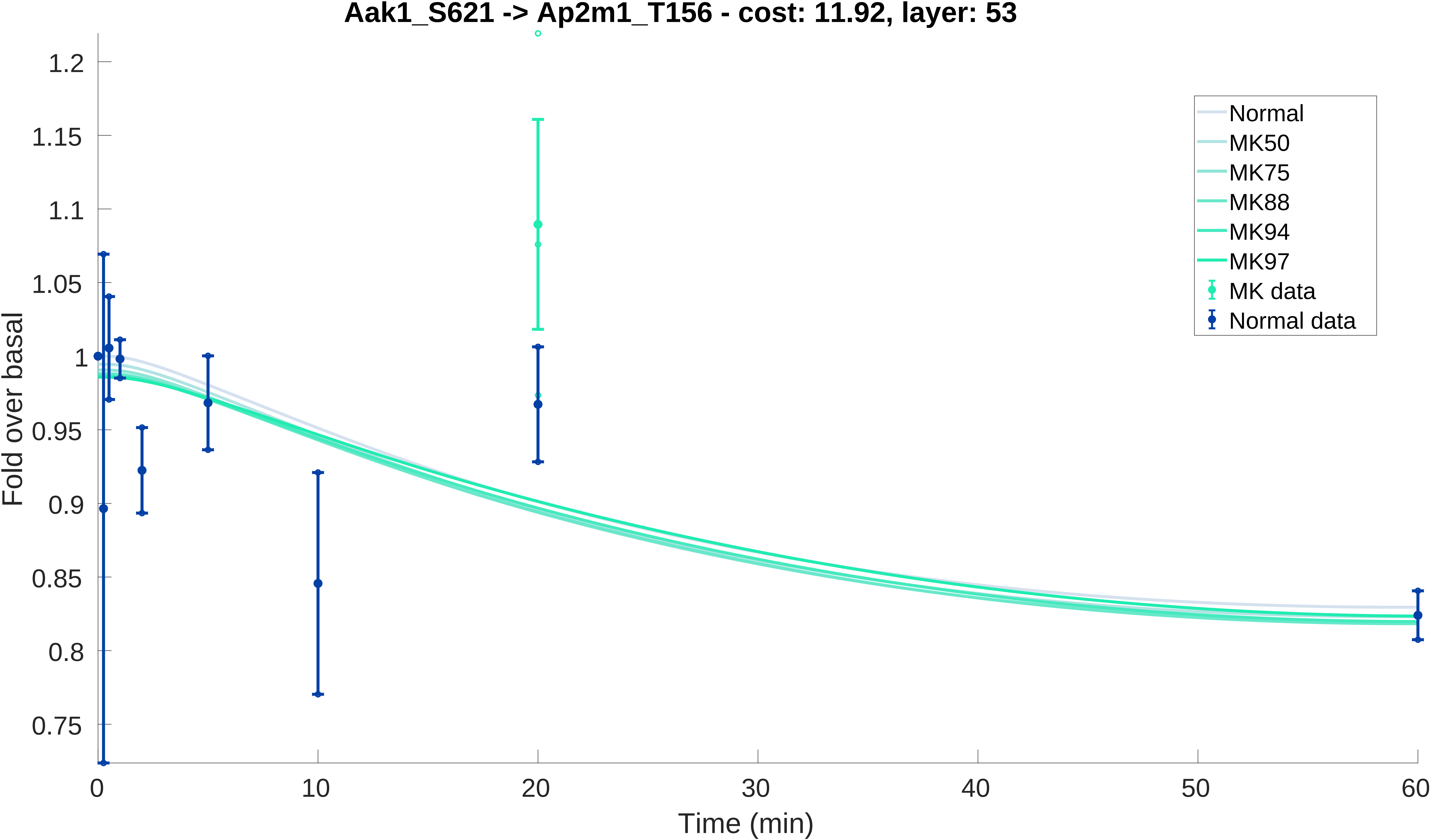
Aak1_S621 -> Ap2m1_T156 - cost: 11.92, layer: 53
Fold over basal
1.2
1.15
1.1
1.05
1
0.95
0.9
0.85
0.8
0.75
0
10
20
30
40
50
60
Time (min)
Normal
MK50
MK75
MK88
MK94
MK97
MK data
Normal data

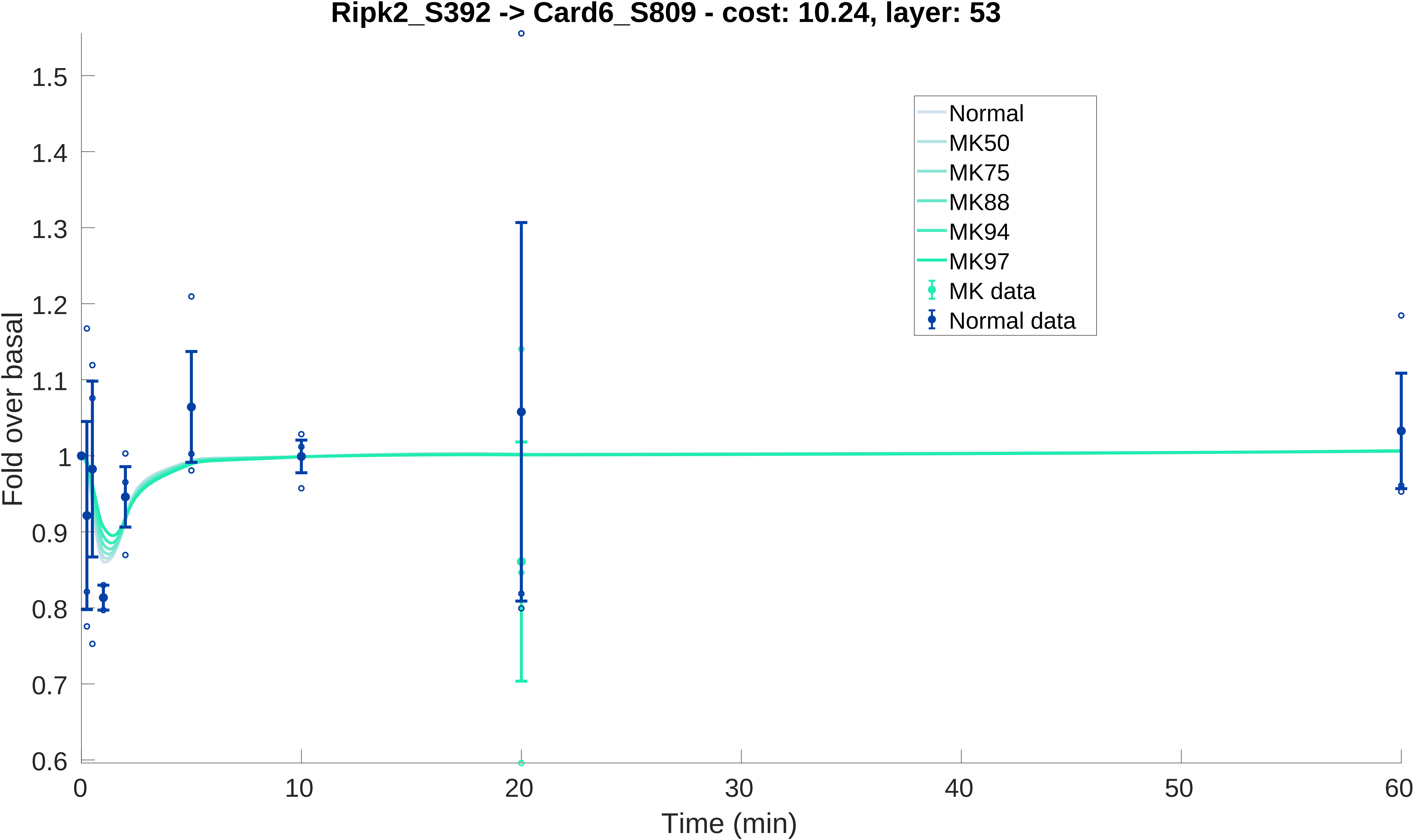

Ripk2_S392 -> Card6_S809 - cost: 10.24, layer: 53
Fold over basal
Time (min)
0.6
0.7
0.8
0.9
1
1.1
1.2
1.3
1.4
1.5
0
10
20
30
40
50
60
Normal
MK50
MK75
MK88
MK94
MK97
MK data
Normal data

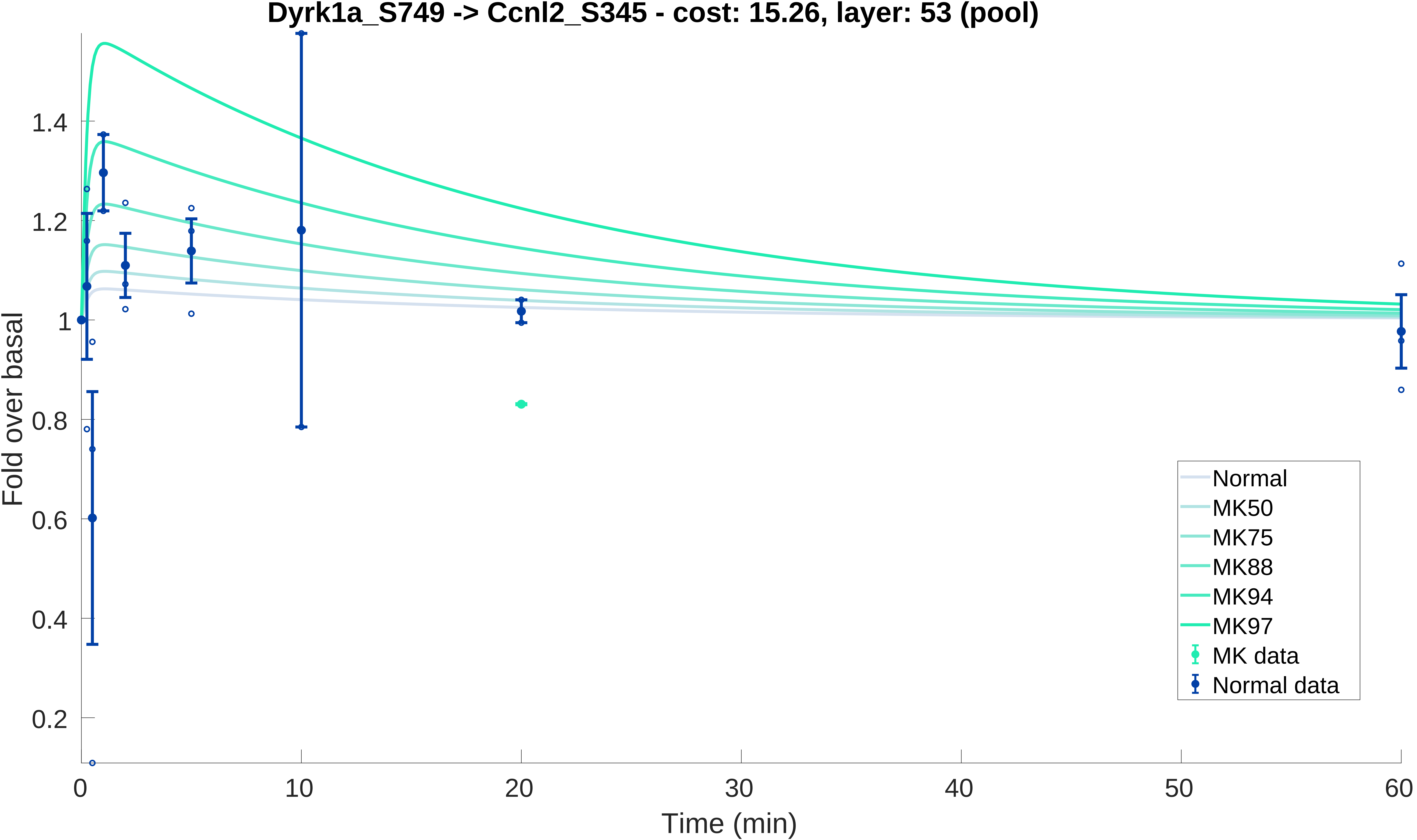

Dyrk1a_S749 -> Ccnl2_S345 - cost: 15.26, layer: 53 (pool)
Fold over basal
Time (min)
0
10
20
30
40
50
60
0.2
0.4
0.6
0.8
1
1.2
1.4
Normal
MK50
MK75
MK88
MK94
MK97
MK data
Normal data

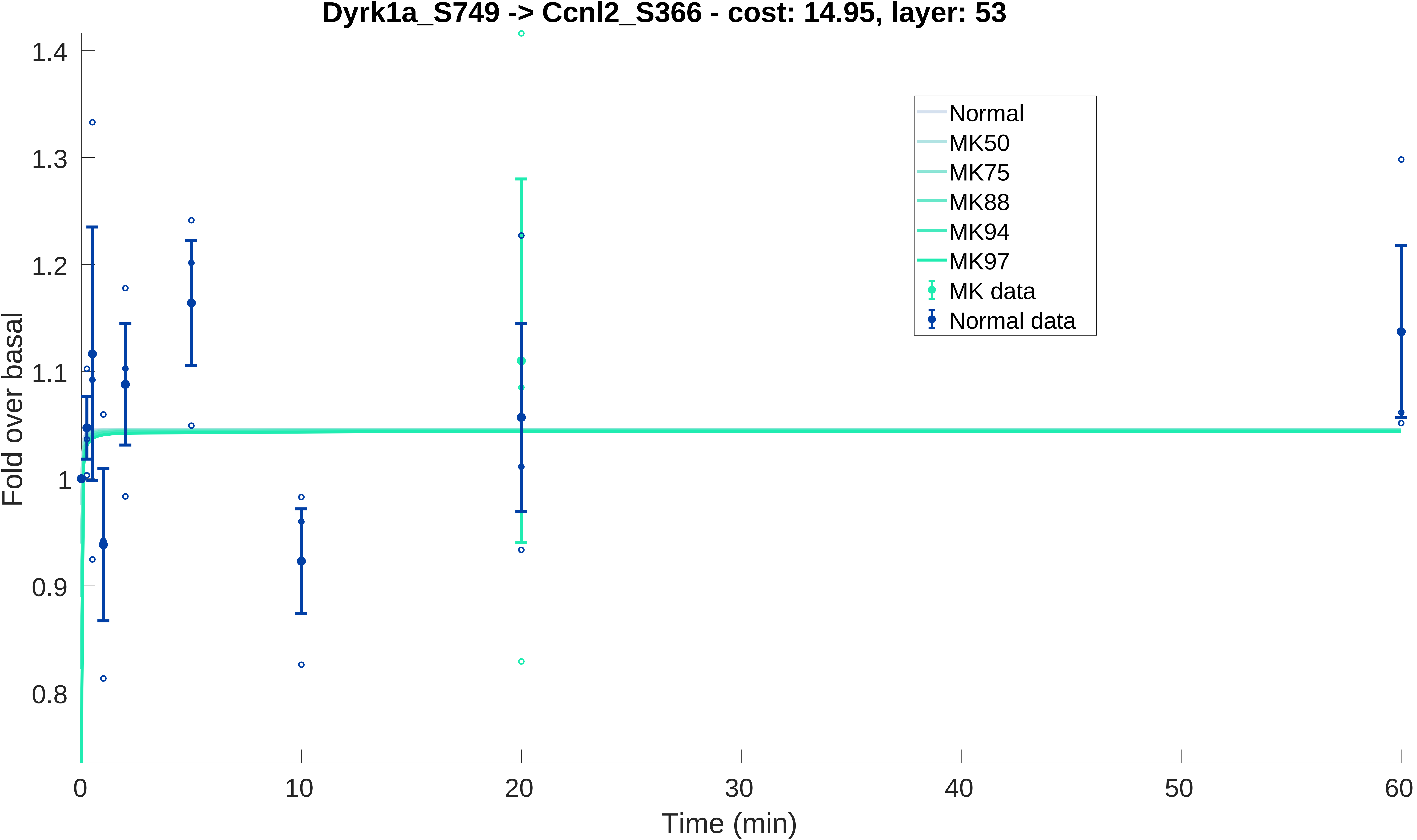

Dyrk1a_S749 -> Ccnl2_S366 - cost: 14.95, layer: 53
Fold over basal
Time (min)
Normal
MK50
MK75
MK88
MK94
MK97
MK data
Normal data
1.4
1.3
1.2
1.1
1
0.9
0.8
0
10
20
30
40
50
60

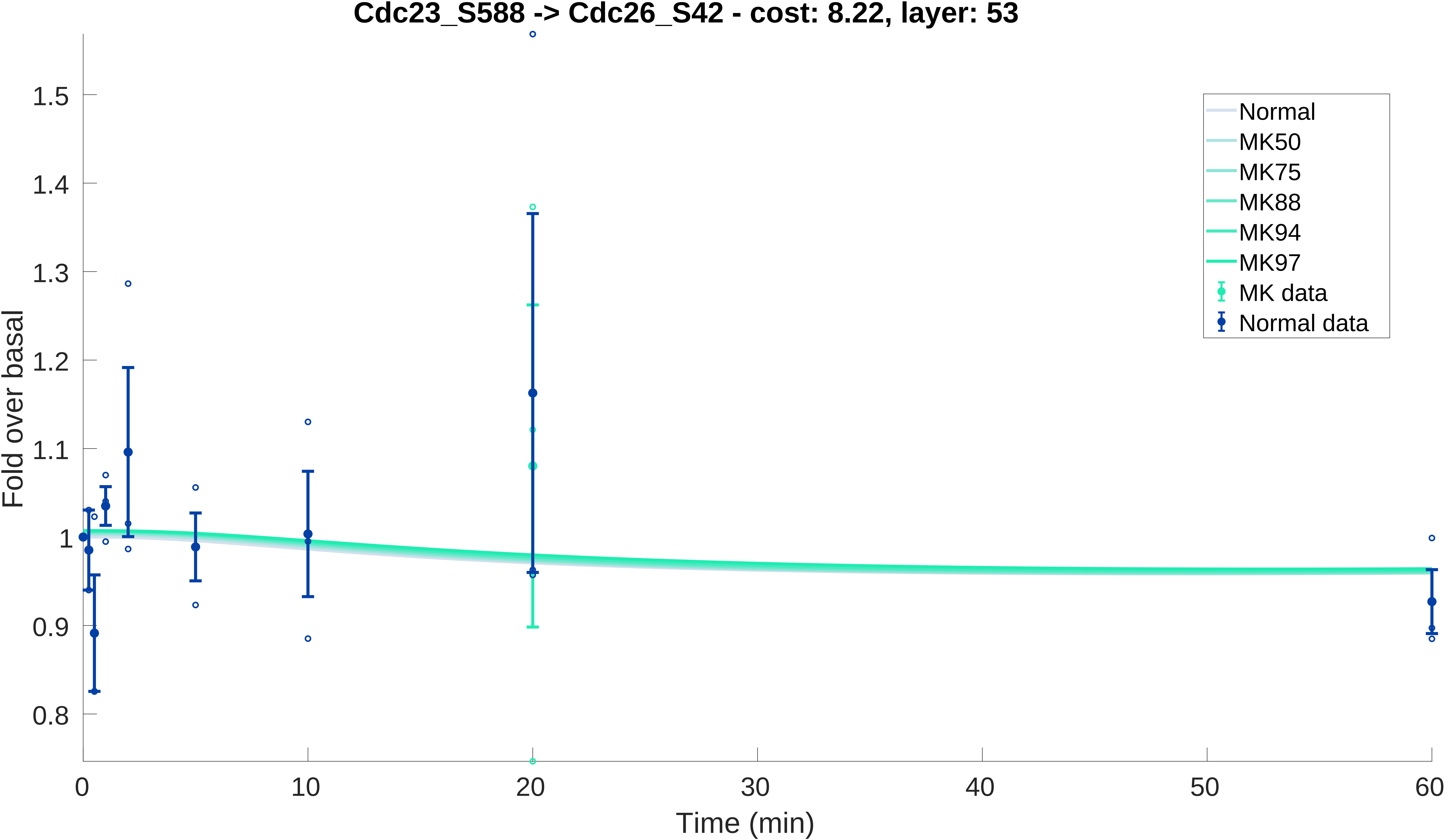

Cdc23_S588 -> Cdc26_S42 - cost: 8.22, layer: 53
Fold over basal
Time (min)
0.8
0.9
1
1.1
1.2
1.3
1.4
1.5
0
10
20
30
40
50
60
Normal
MK50
MK75
MK88
MK94
MK97
MK data
Normal data

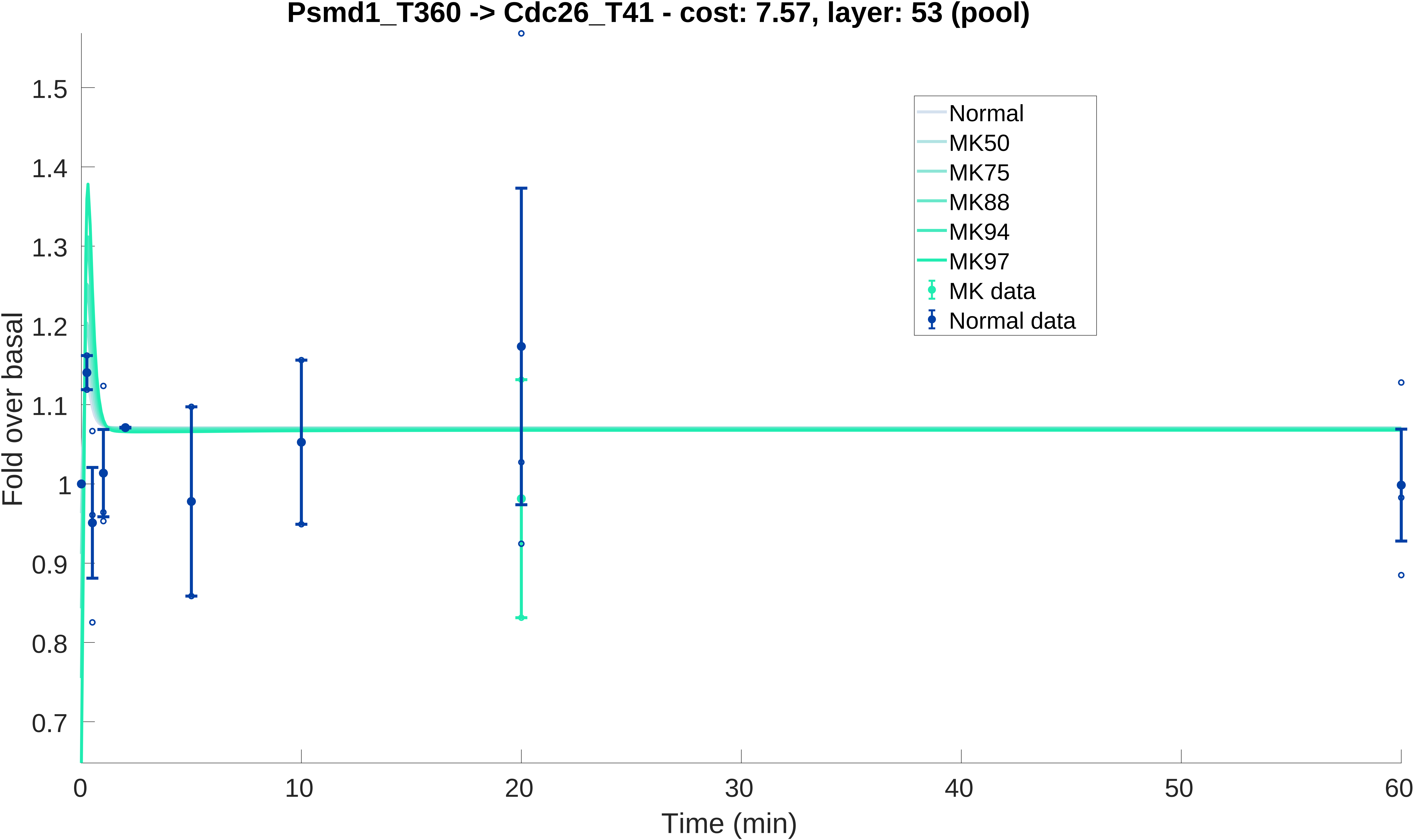
Psmd1_T360 -> Cdc26_T41 - cost: 7.57, layer: 53 (pool)
Fold over basal
Time (min)
1.5
1.4
1.3
1.2
1.1
1
0.9
0.8
0.7
0
10
20
30
40
50
60
Normal
MK50
MK75
MK88
MK94
MK97
MK data
Normal data

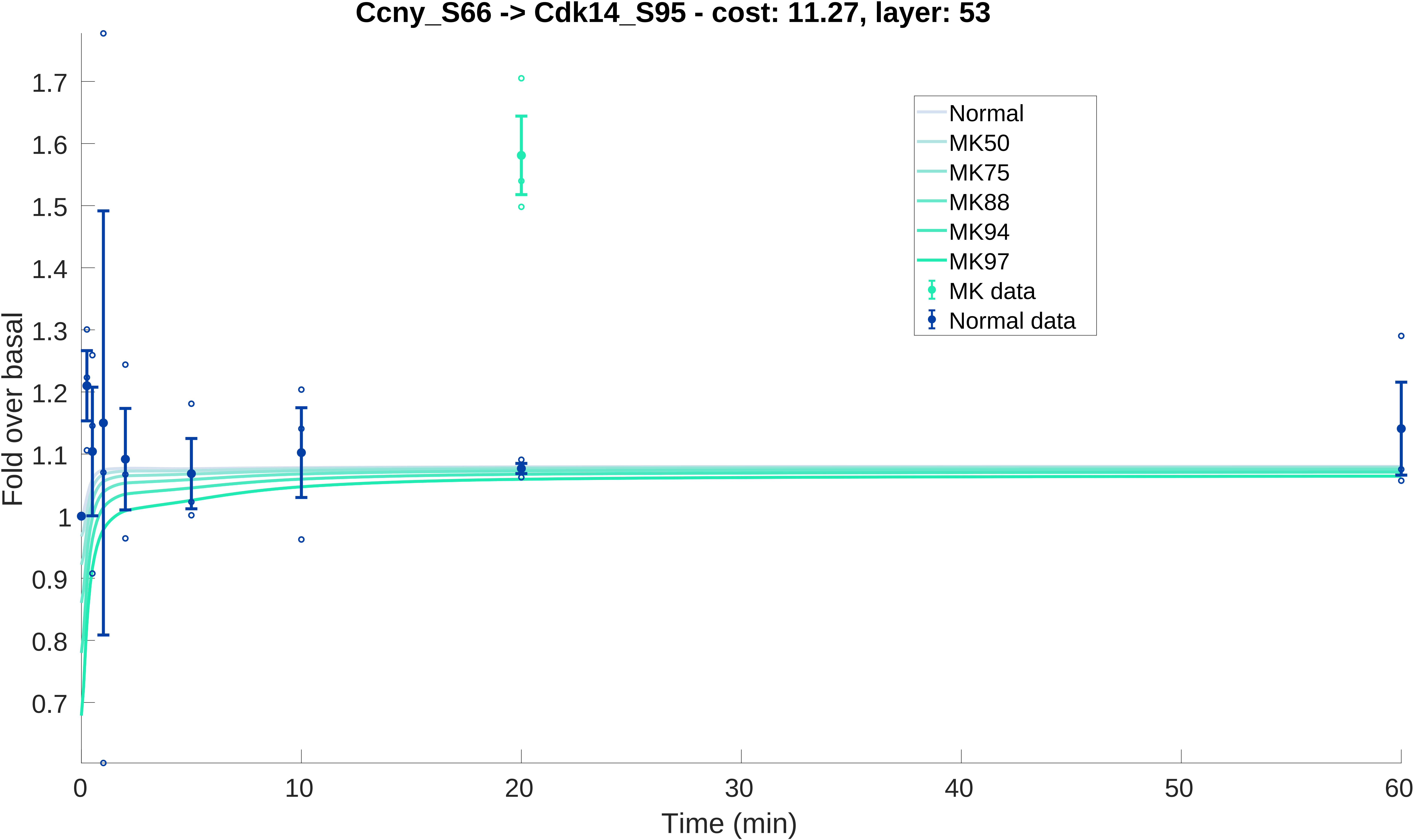

Ccny_S66 -> Cdk14_S95 - cost: 11.27, layer: 53
Fold over basal
Time (min)
1.7
1.6
1.5
1.4
1.3
1.2
1.1
1
0.9
0.8
0.7
0
10
20
30
40
50
60
Normal
MK50
MK75
MK88
MK94
MK97
MK data
Normal data

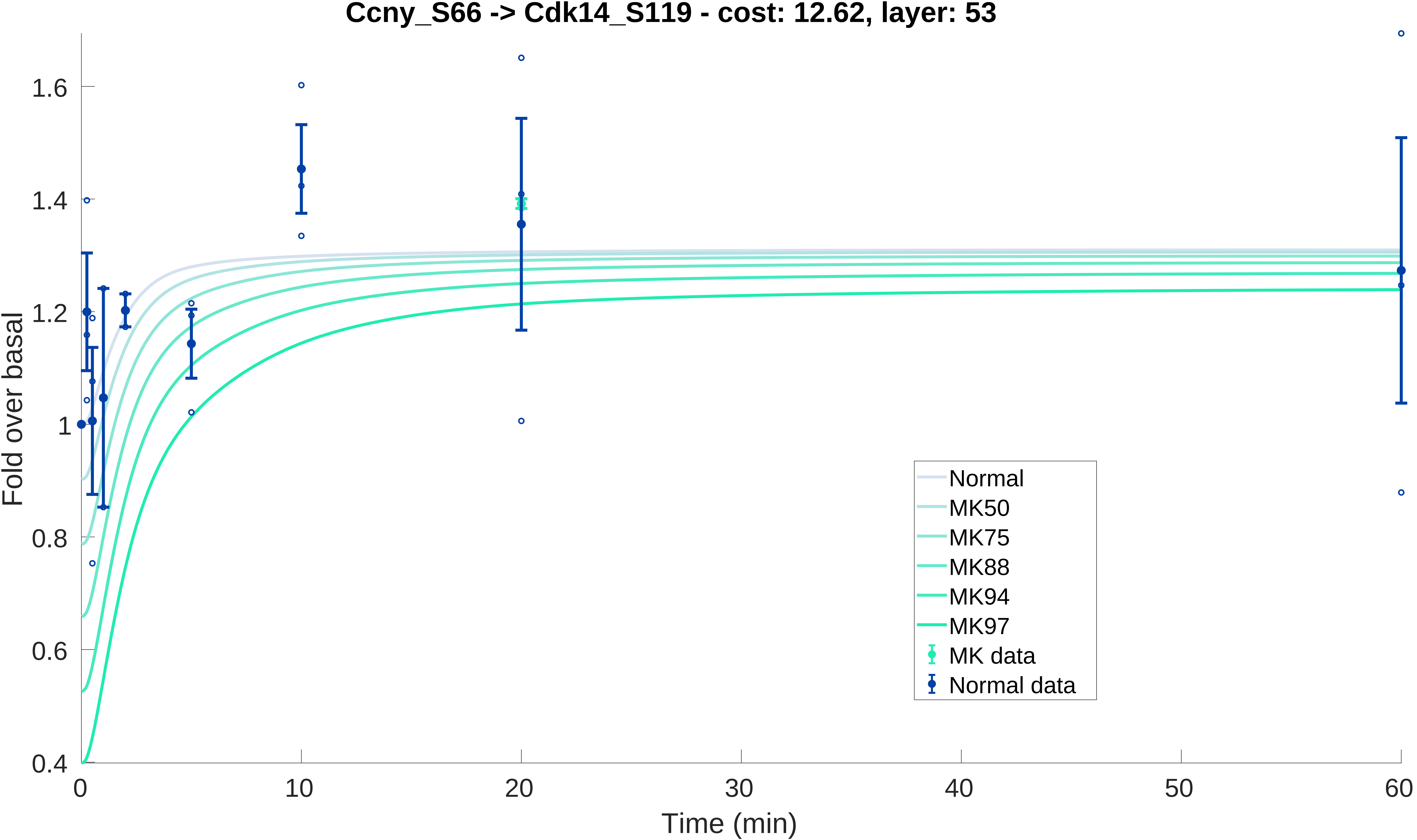

Ccny_S66 -> Cdk14_S119 - cost: 12.62, layer: 53
Fold over basal
Time (min)
0
10
20
30
40
50
60
0.4
0.6
0.8
1
1.2
1.4
1.6
Normal
MK50
MK75
MK88
MK94
MK97
MK data
Normal data

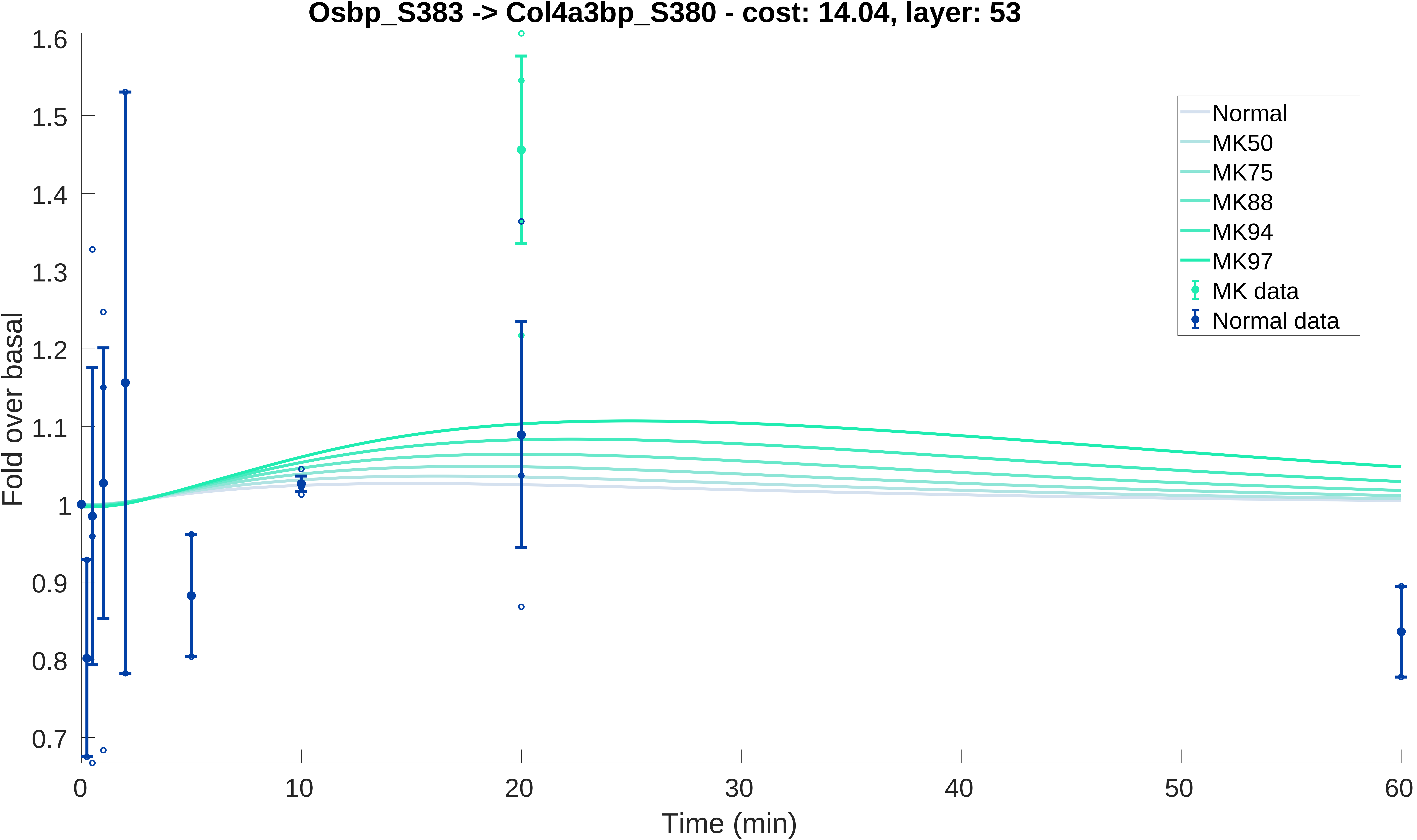

Osbp_S383 -> Col4a3bp_S380 - cost: 14.04, layer: 53
Fold over basal
Time (min)
Normal
MK50
MK75
MK88
MK94
MK97
MK data
Normal data
0.7
0.8
0.9
1
1.1
1.2
1.3
1.4
1.5
1.6
0
10
20
30
40
50
60

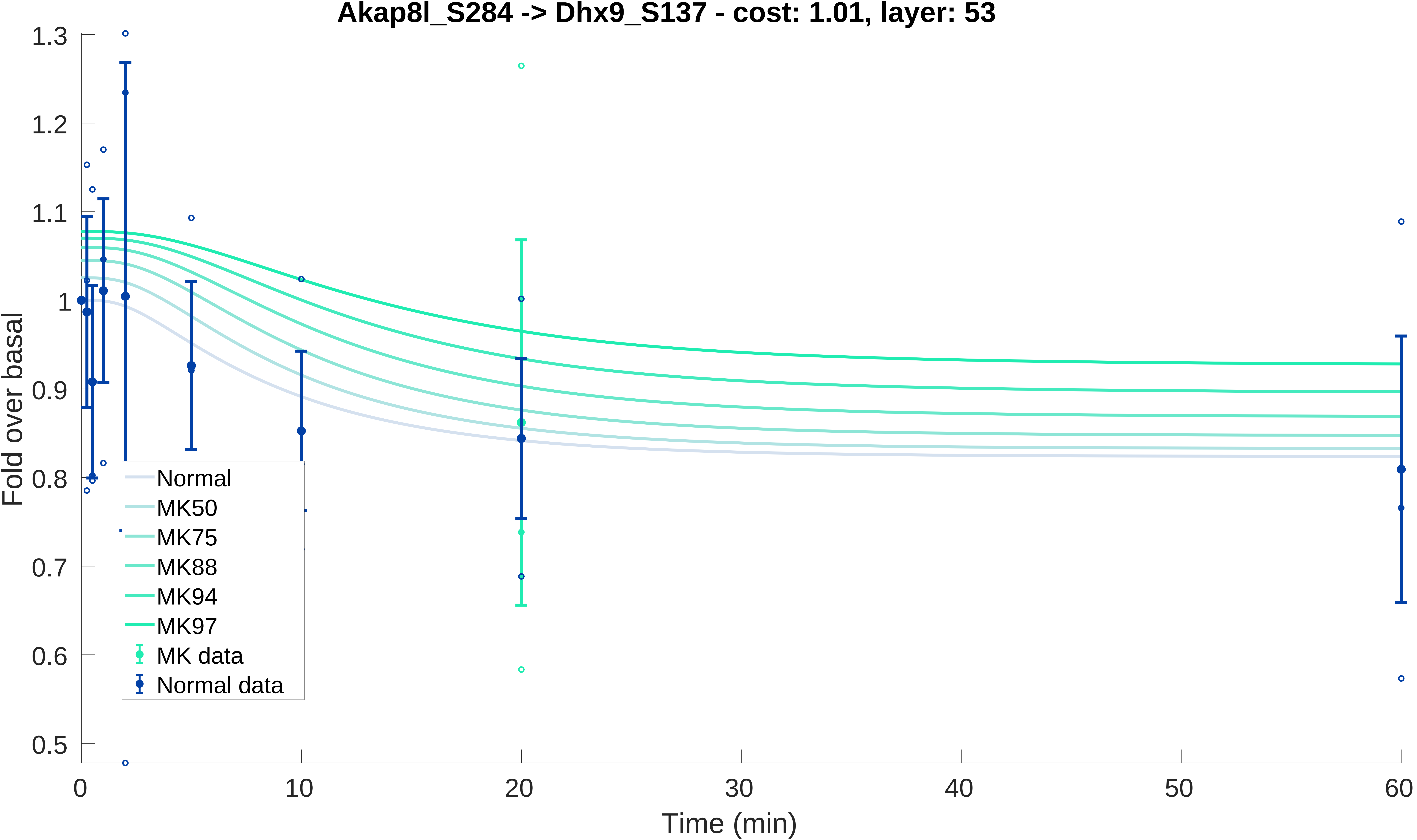

Akap8l_S284 -> Dhx9_S137 - cost: 1.01, layer: 53
1.3
1.2
1.1
1
0.9
0.8
0.7
0.6
0.5
Fold over basal
0
10
20
30
40
50
60
Time (min)
Normal
MK50
MK75
MK88
MK94
MK97
MK data
Normal data

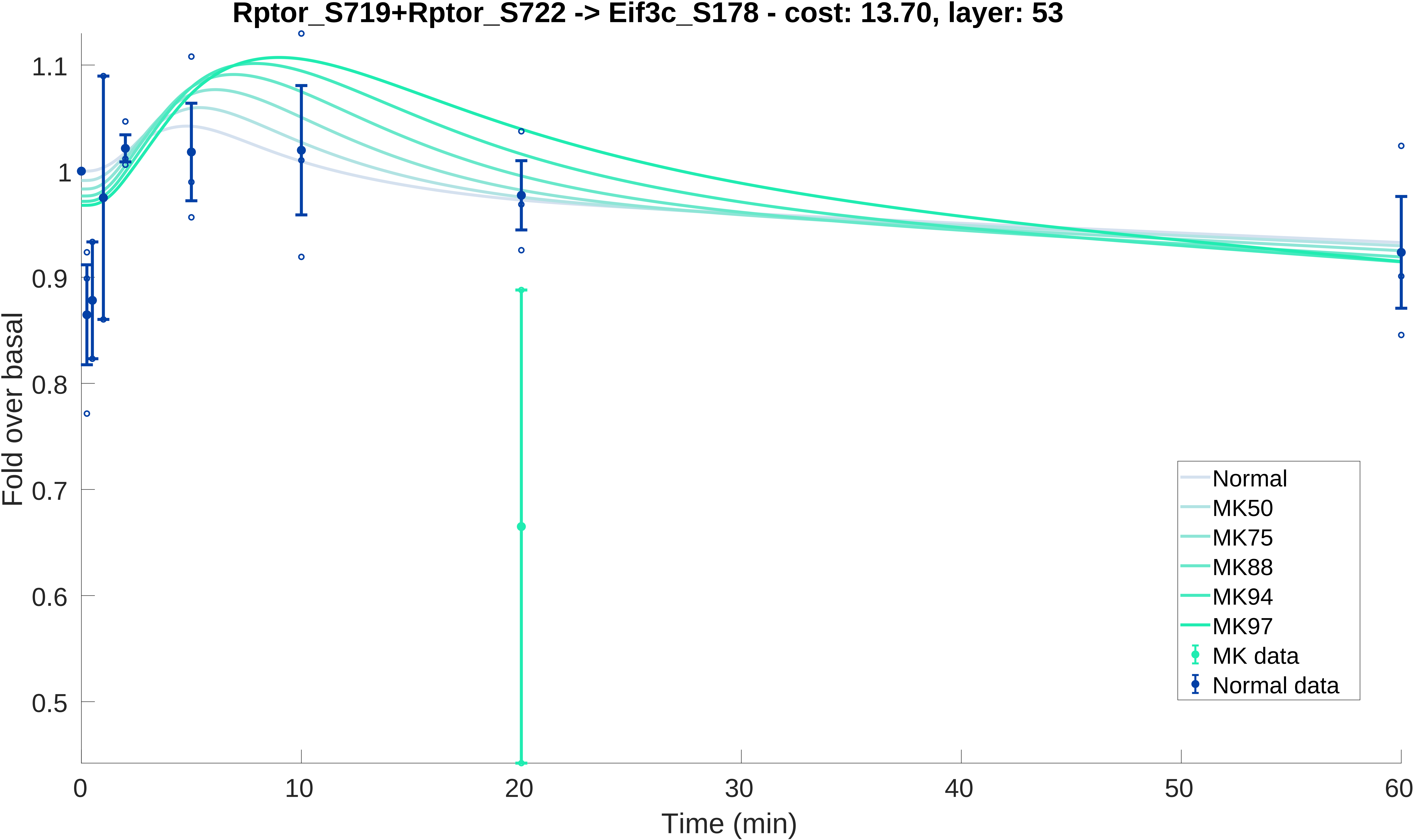

Rptor_S719+Rptor_S722 -> Eif3c_S178 - cost: 13.70, layer: 53
Fold over basal
Time (min)
1.1
1
0.9
0.8
0.7
0.6
0.5
0
10
20
30
40
50
60
Normal
MK50
MK75
MK88
MK94
MK97
MK data
Normal data

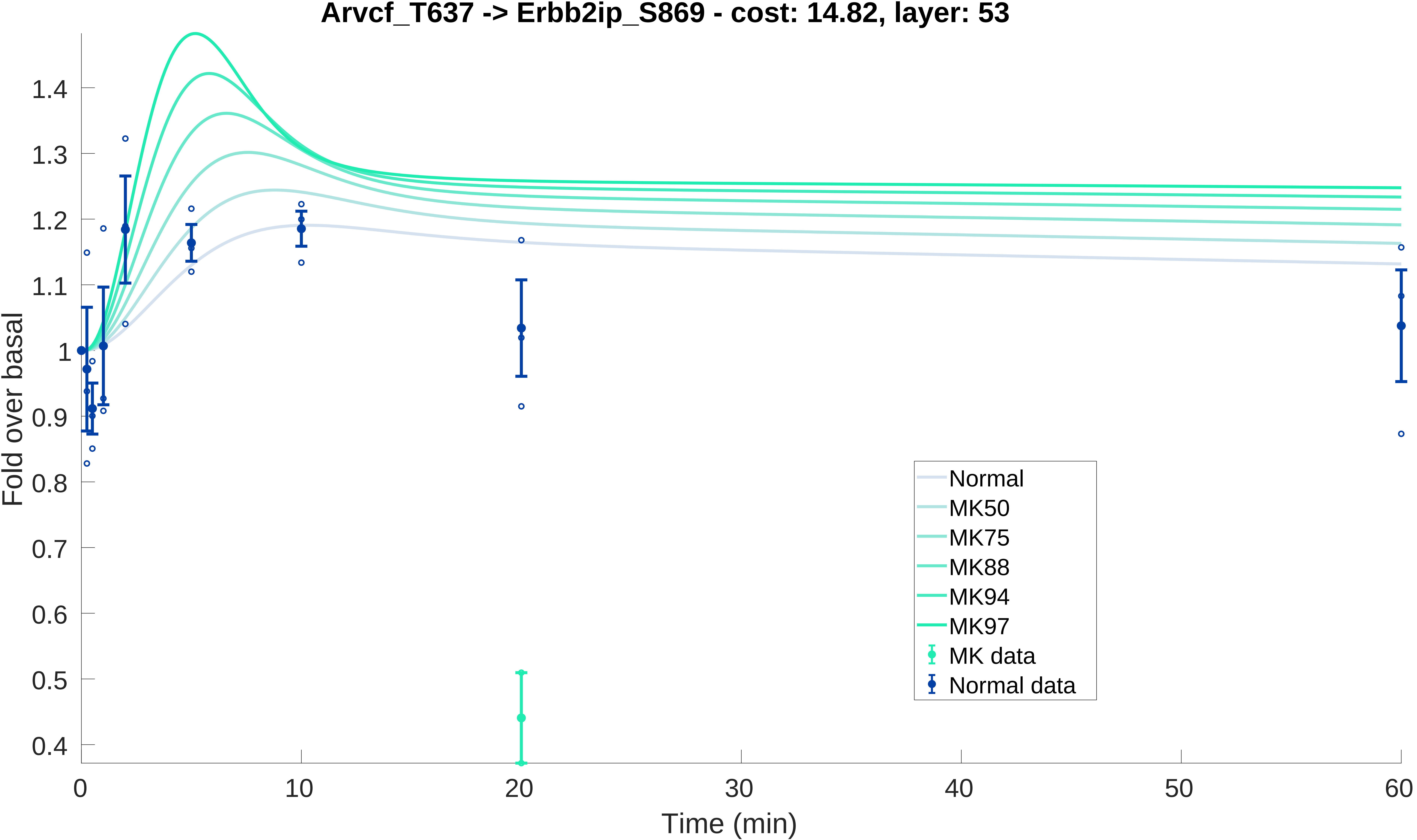

Arvcf_T637 -> Erbb2ip_S869 - cost: 14.82, layer: 53
Fold over basal
Time (min)
0.4
0.5
0.6
0.7
0.8
0.9
1
1.1
1.2
1.3
1.4
0
10
20
30
40
50
60
Normal
MK50
MK75
MK88
MK94
MK97
MK data
Normal data

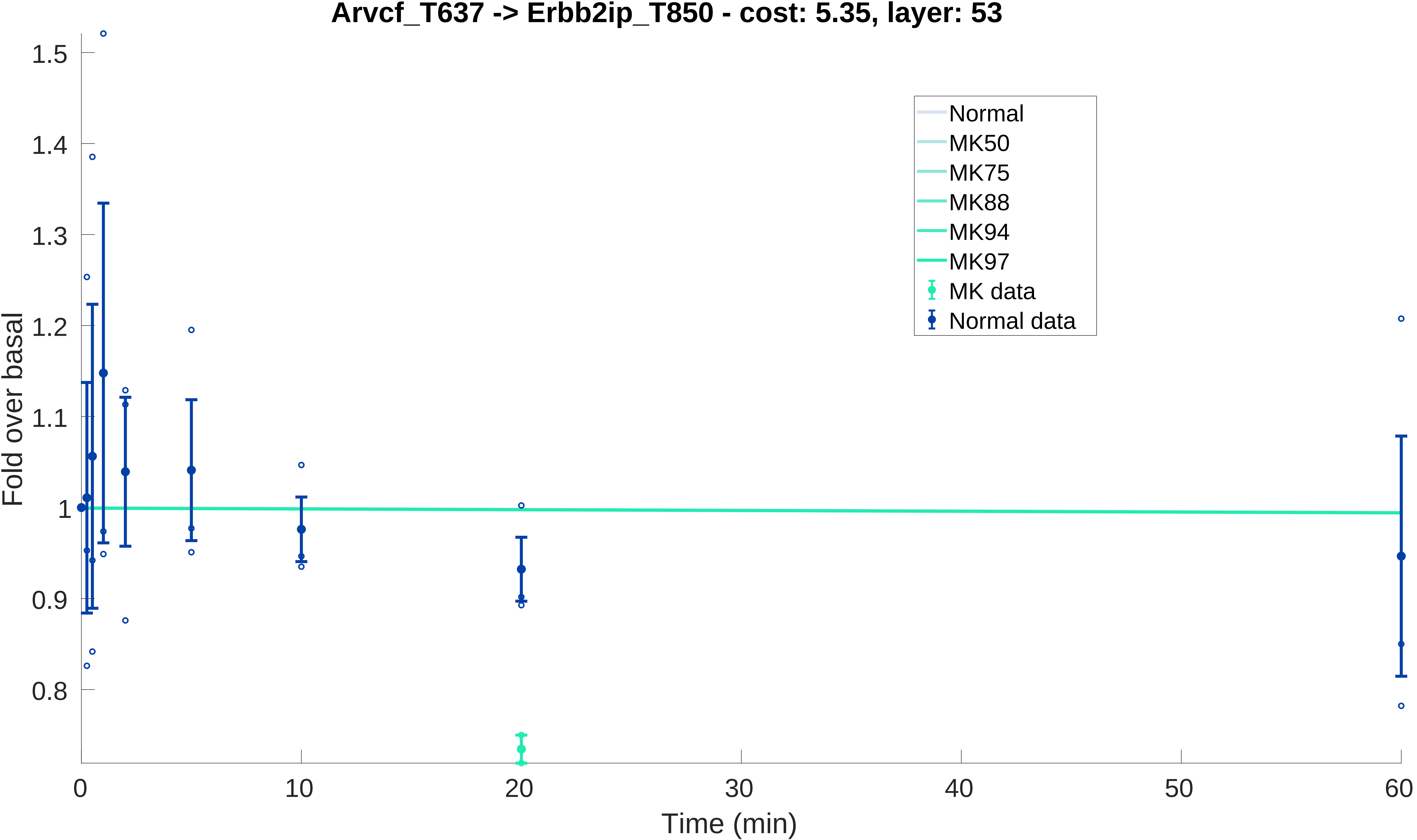

Arvcf_T637 -> Erbb2ip_T850 - cost: 5.35, layer: 53
Fold over basal
Time (min)
1.5
1.4
1.3
1.2
1.1
1
0.9
0.8
0
10
20
30
40
50
60
Normal
MK50
MK75
MK88
MK94
MK97
MK data
Normal data

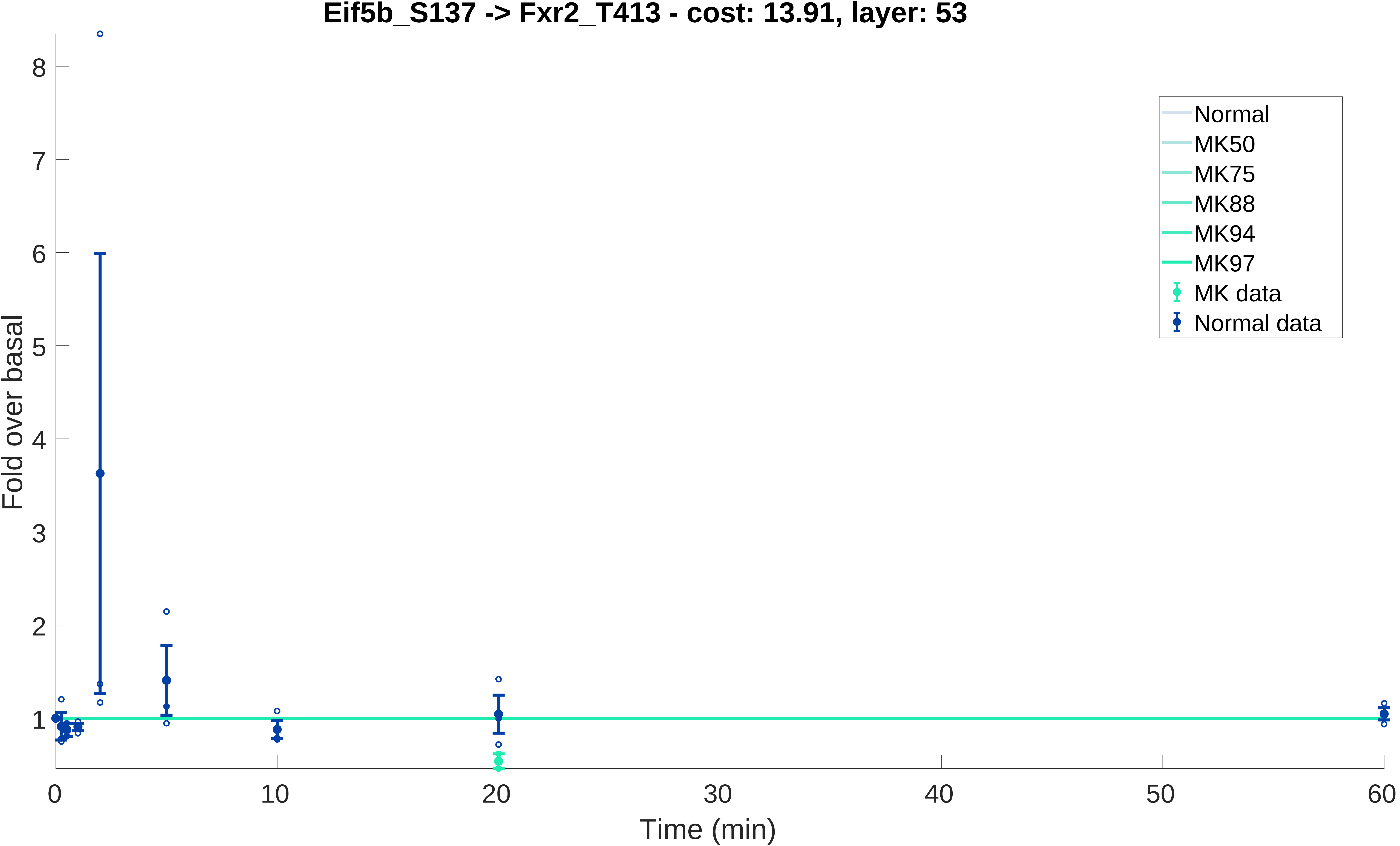

Eif5b_S137 -> Fxr2_T413 - cost: 13.91, layer: 53
Fold over basal
Time (min)
8
7
6
5
4
3
2
1
0
10
20
30
40
50
60
Normal
MK50
MK75
MK88
MK94
MK97
MK data
Normal data

Cblb_S525+Cblb_S529 -> Ifngr1_T373 - cost: 12.34, layer: 53

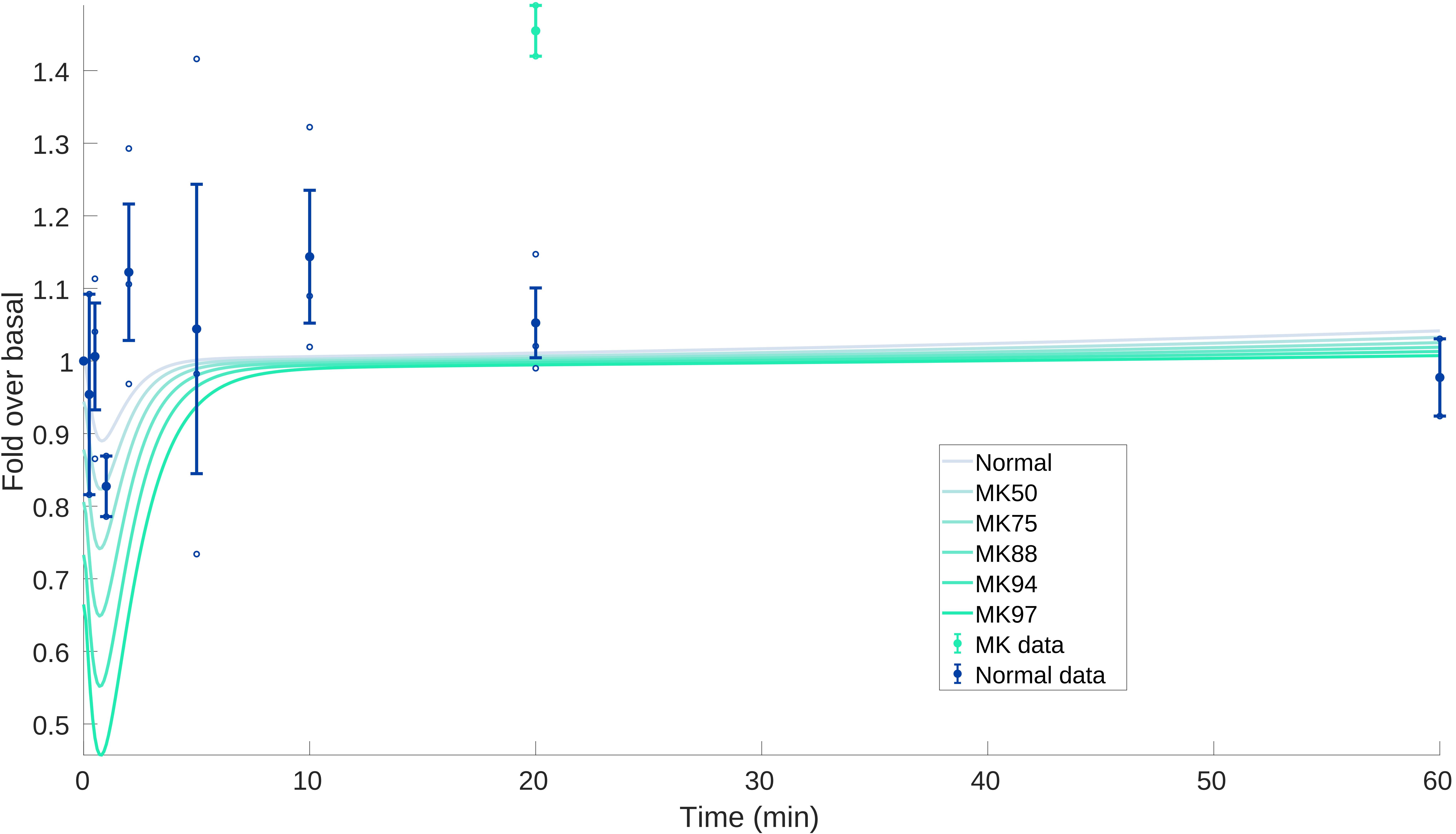

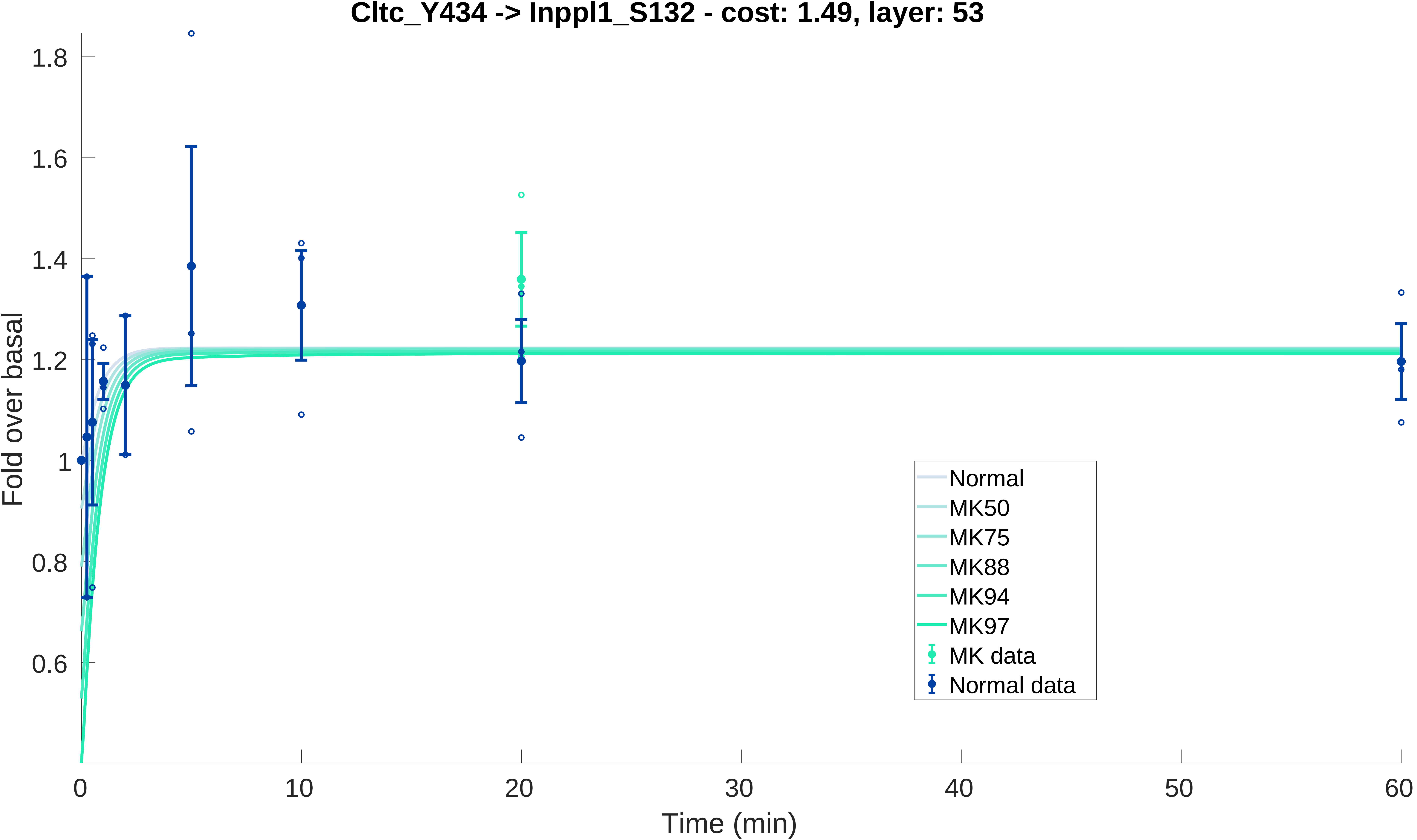
Cltc_Y434 -> Inppl1_S132 - cost: 1.49, layer: 53
Fold over basal
Time (min)
1.8
1.6
1.4
1.2
1
0.8
0.6
0
10
20
30
40
50
60
Normal
MK50
MK75
MK88
MK94
MK97
MK data
Normal data

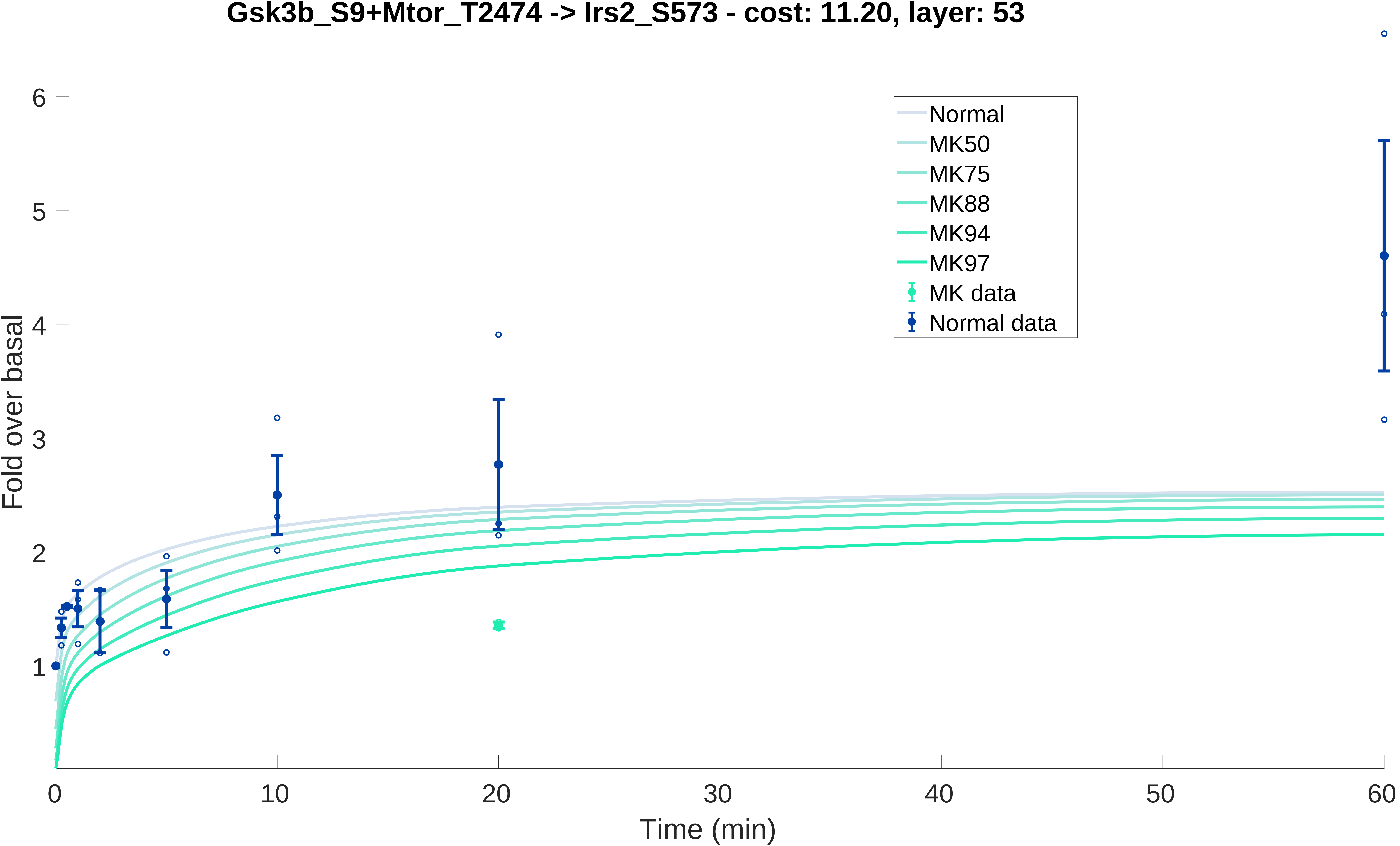
Gsk3b_S9+Mtor_T2474 -> Irs2_S573 - cost: 11.20, layer: 53
Fold over basal
Time (min)
Normal
MK50
MK75
MK88
MK94
MK97
MK data
Normal data
0
10
20
30
40
50
60
1
2
3
4
5
6

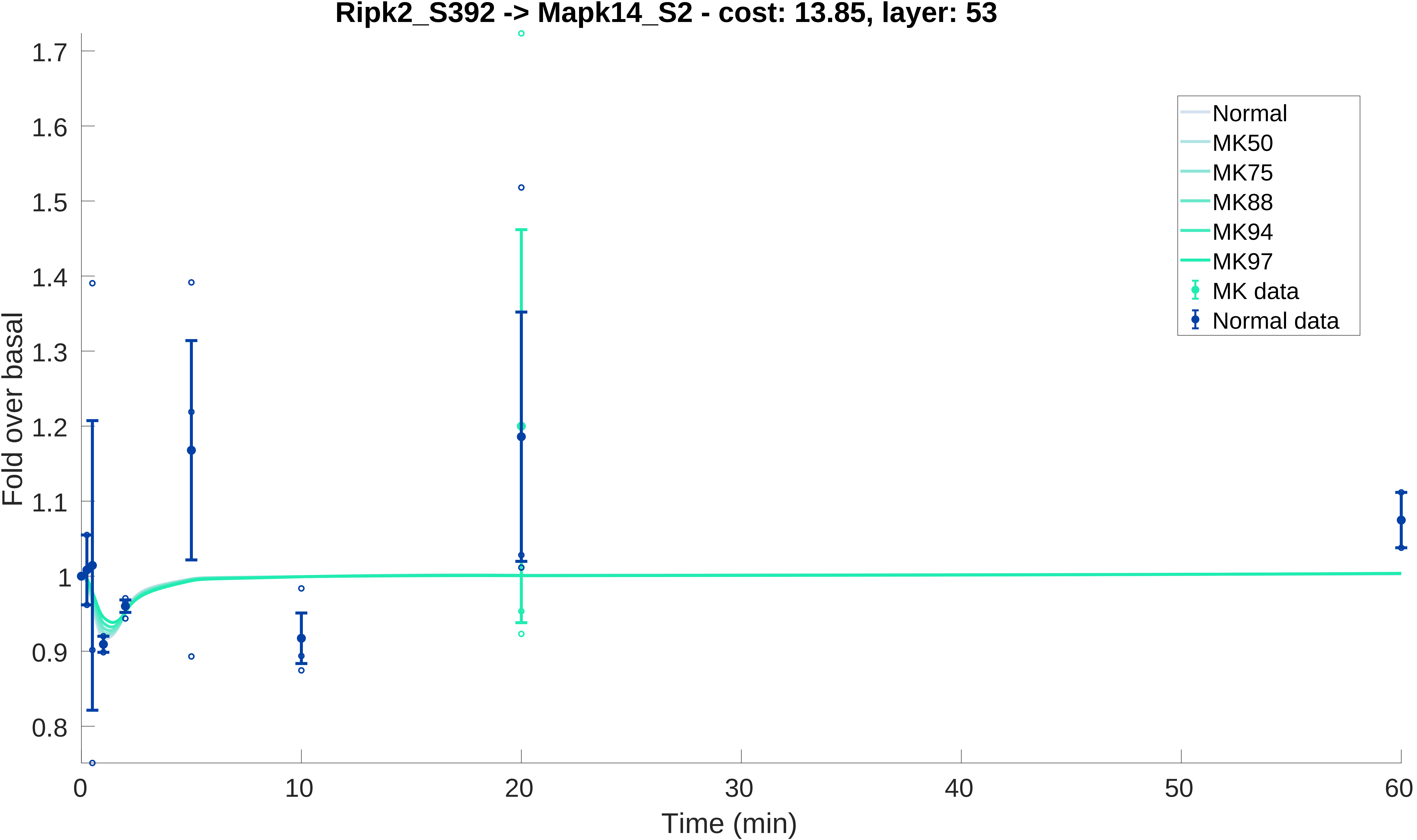

Ripk2_S392 -> Mapk14_S2 - cost: 13.85, layer: 53
Fold over basal
Time (min)
1.7
1.6
1.5
1.4
1.3
1.2
1.1
1
0.9
0.8
0
10
20
30
40
50
60
Normal
MK50
MK75
MK88
MK94
MK97
MK data
Normal data

Akt2_S126+Rras2_S186 -> Mllt4_S1282 - cost: 9.54, layer: 53

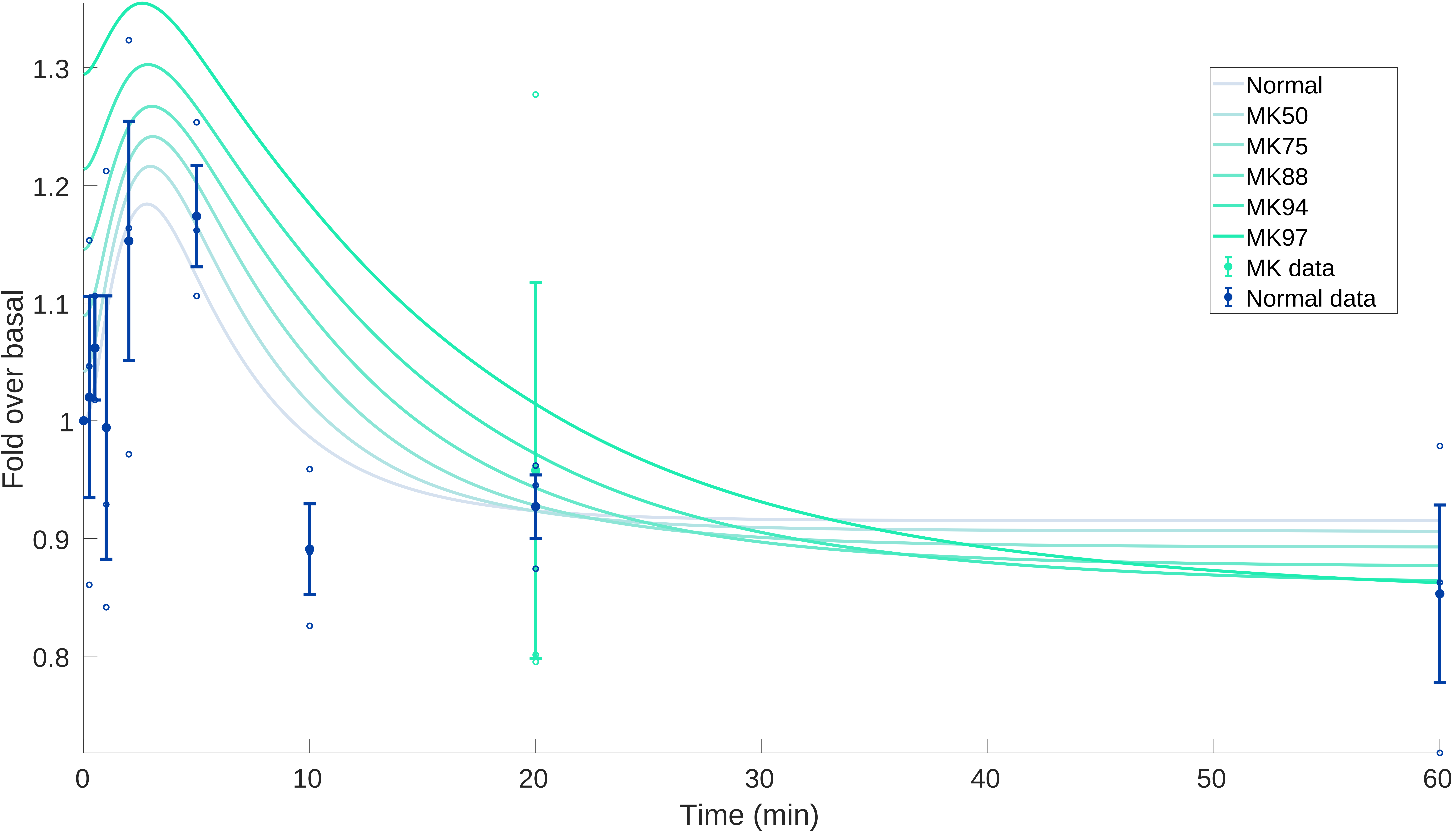

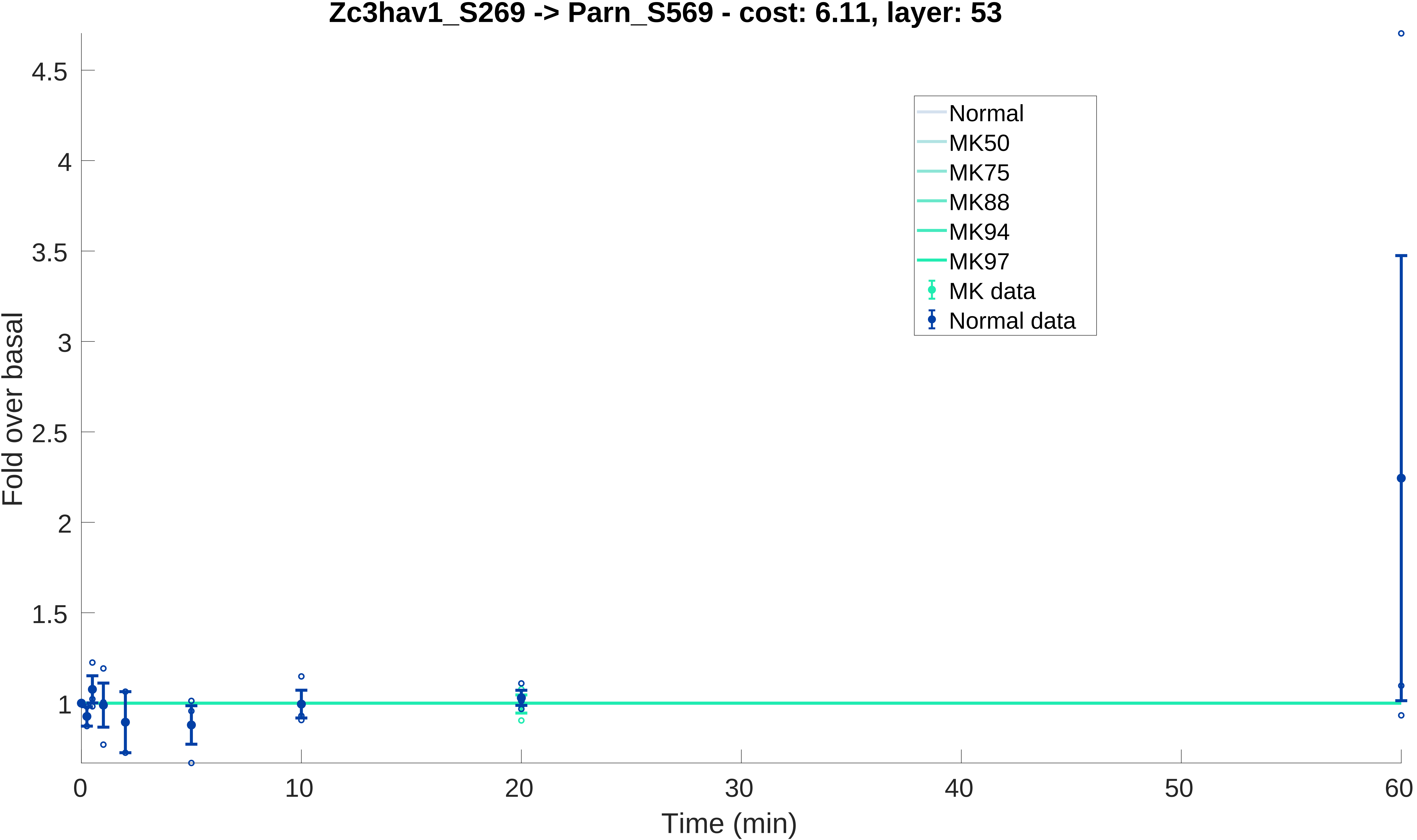

Zc3hav1_S269 -> Parn_S569 - cost: 6.11, layer: 53
Fold over basal
4.5
4
3.5
3
2.5
2
1.5
1
0
10
20
30
40
50
60
Time (min)
Normal
MK50
MK75
MK88
MK94
MK97
MK data
Normal data

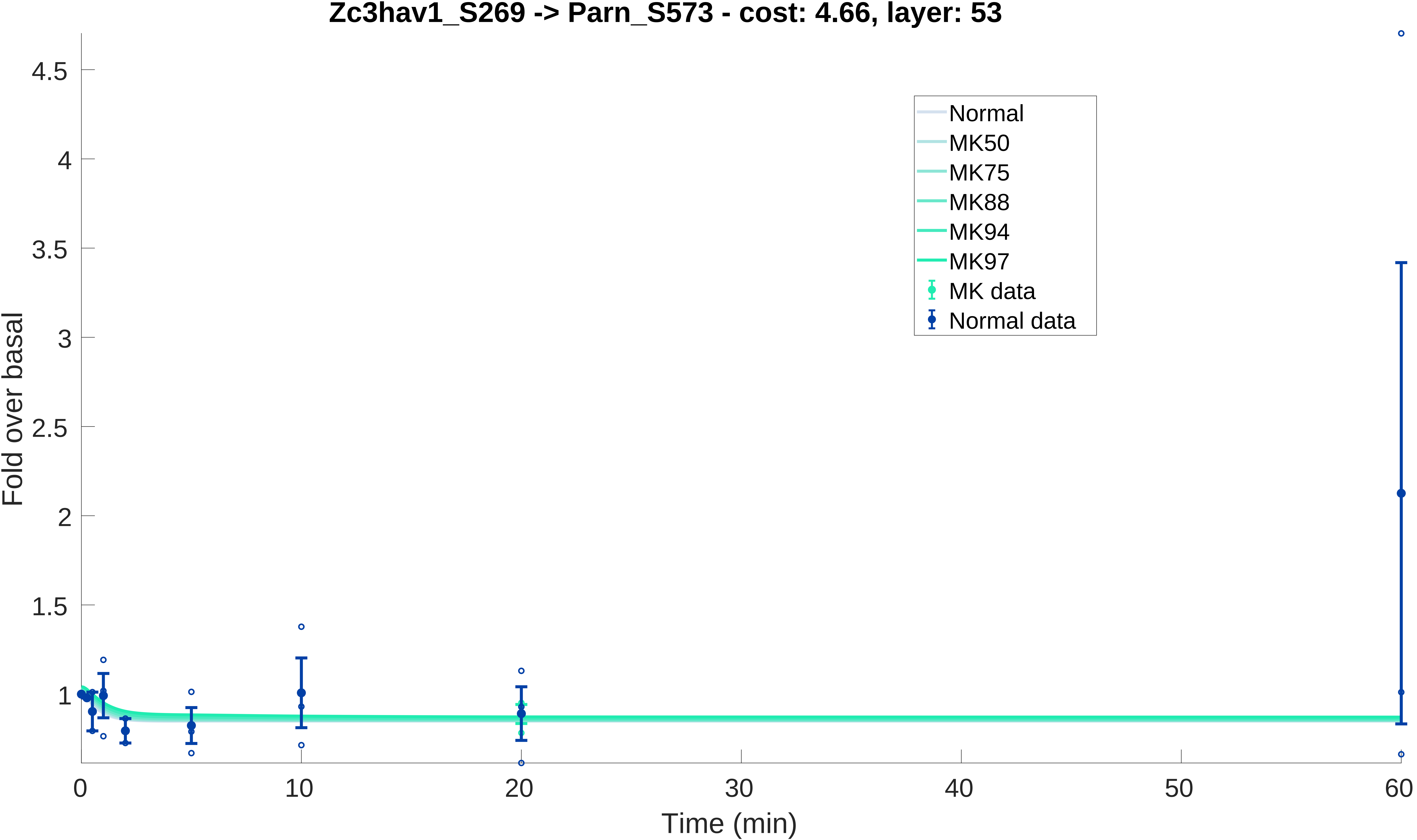

Zc3hav1_S269 -> Parn_S573 - cost: 4.66, layer: 53
Fold over basal
Time (min)
Normal
MK50
MK75
MK88
MK94
MK97
MK data
Normal data
4.5
4
3.5
3
2.5
2
1.5
1
0
10
20
30
40
50
60

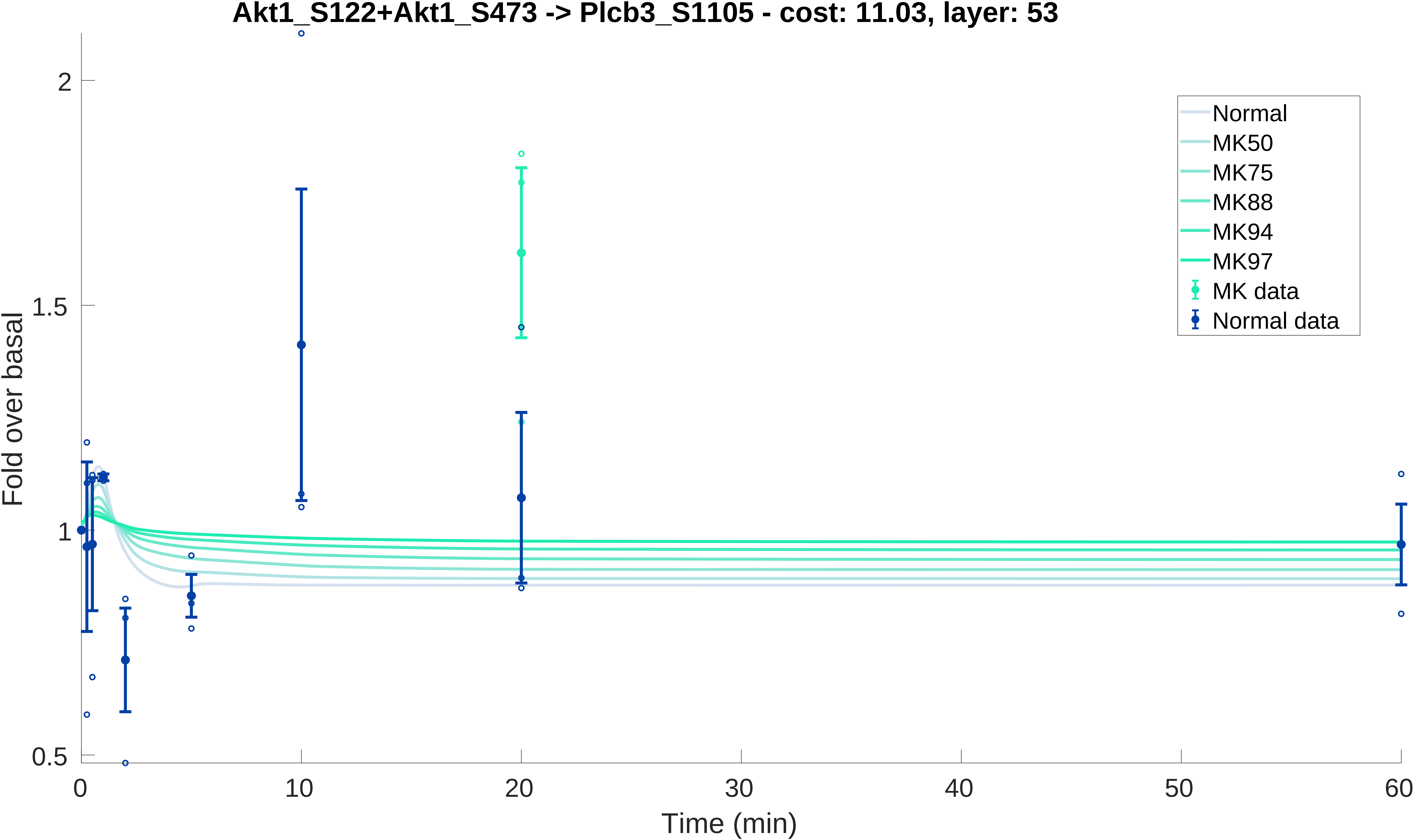
Akt1_S122+Akt1_S473 -> Plcb3_S1105 - cost: 11.03, layer: 53
Fold over basal
Time (min)
0.5
1
1.5
2
0
10
20
30
40
50
60
Normal
MK50
MK75
MK88
MK94
MK97
MK data
Normal data

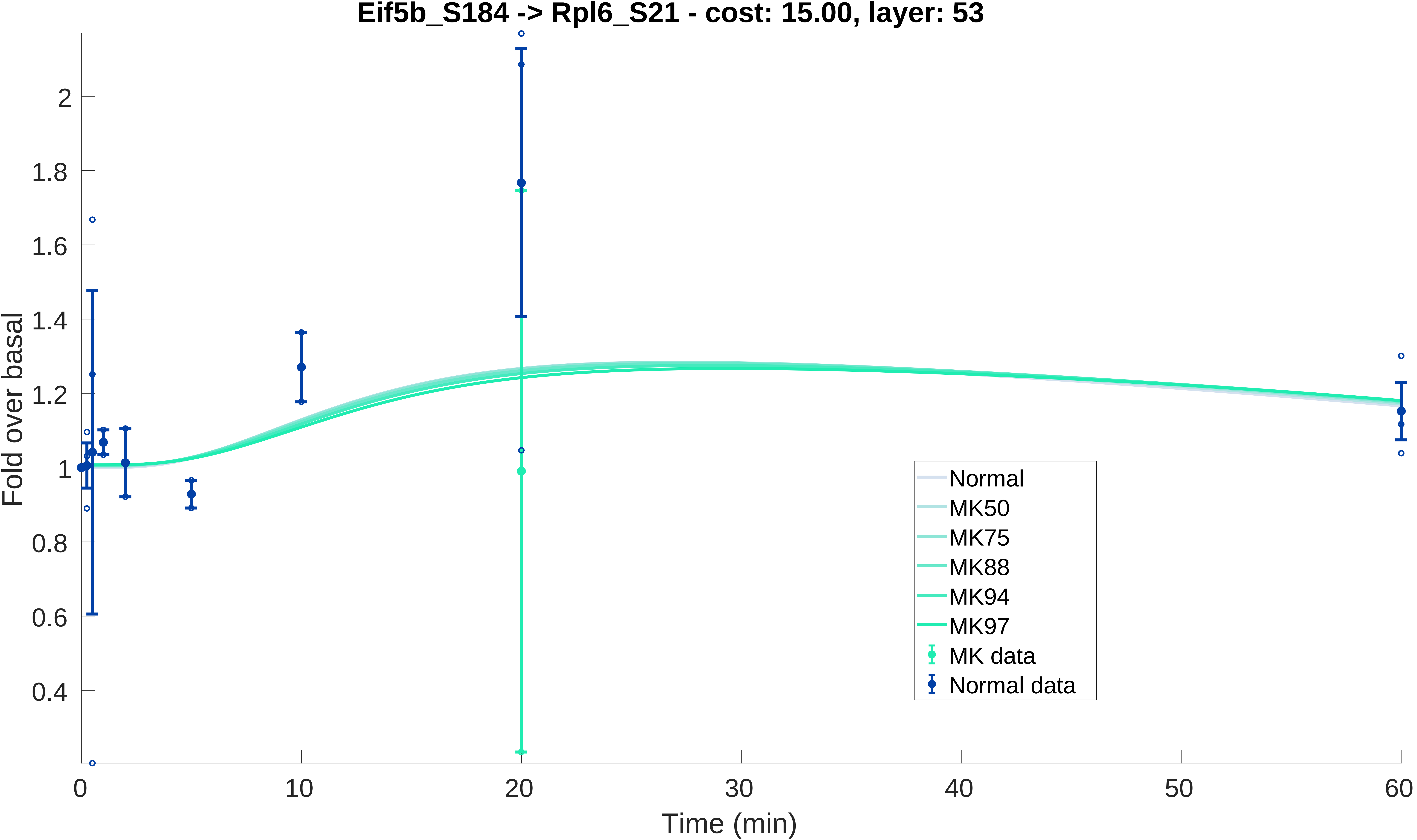

Eif5b_S184 -> Rpl6_S21 - cost: 15.00, layer: 53
Fold over basal
2
1.8
1.6
1.4
1.2
1
0.8
0.6
0.4
0
10
20
30
40
50
60
Time (min)
Normal
MK50
MK75
MK88
MK94
MK97
MK data
Normal data

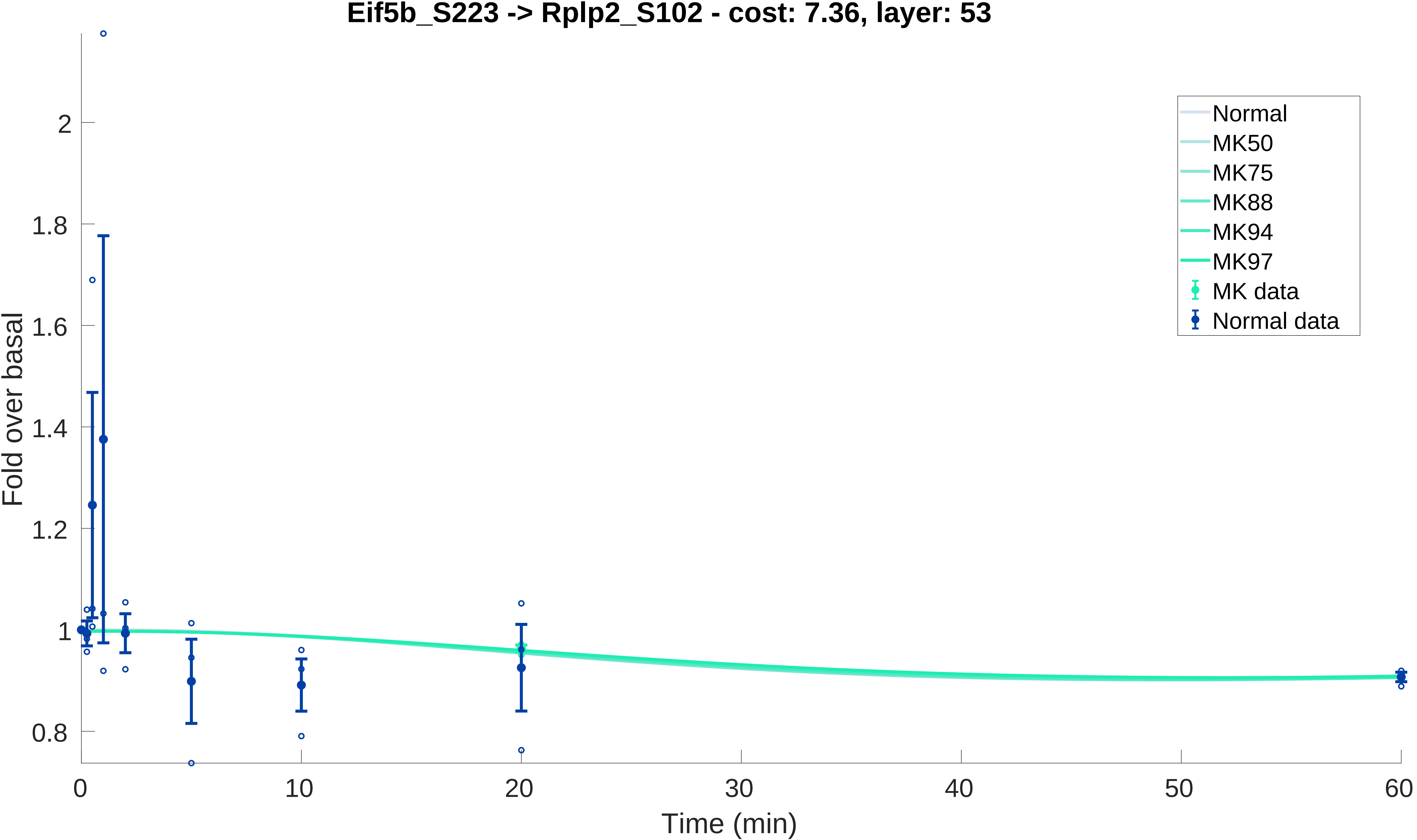

Eif5b_S223 -> Rplp2_S102 - cost: 7.36, layer: 53
Fold over basal
Time (min)
0.8
1
1.2
1.4
1.6
1.8
2
0
10
20
30
40
50
60
Normal
MK50
MK75
MK88
MK94
MK97
MK data
Normal data

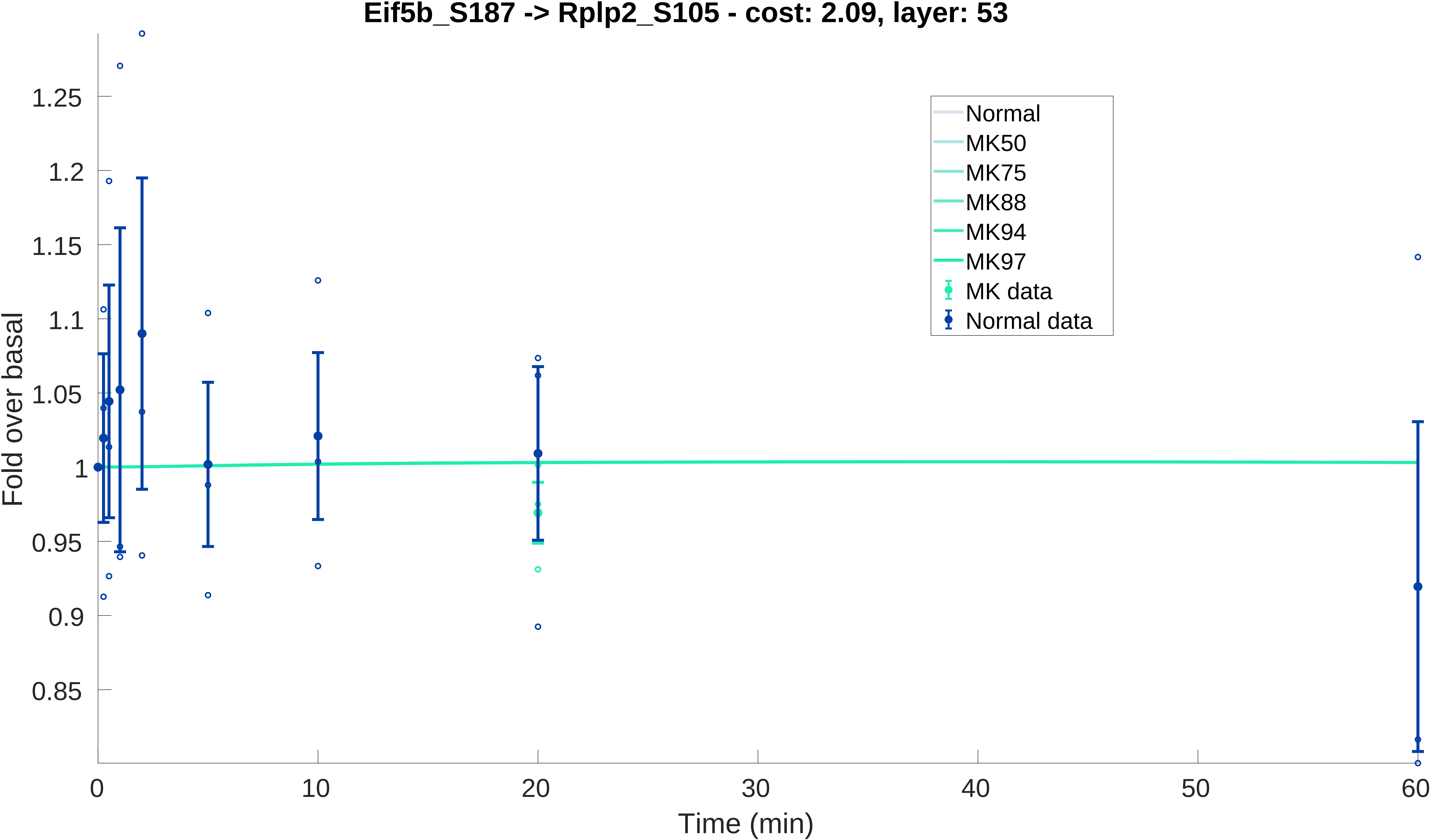

Eif5b_S187 -> Rplp2_S105 - cost: 2.09, layer: 53
Normal
MK50
MK75
MK88
MK94
MK97
MK data
Normal data
Fold over basal
1.25
1.2
1.15
1.1
1.05
1
0.95
0.9
0.85
0
10
20
30
40
50
60
Time (min)

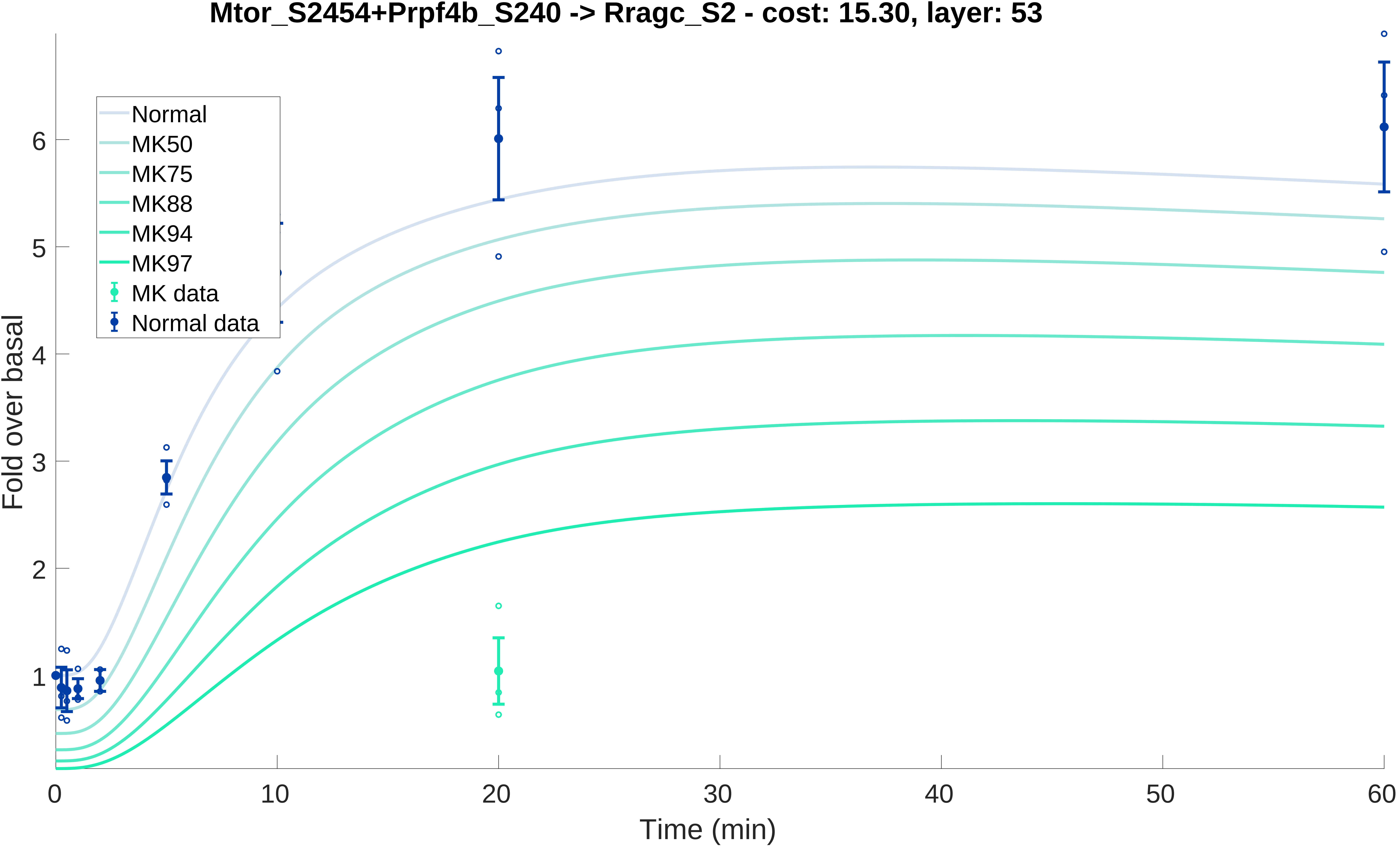

Mtor_S2454+Prpf4b_S240 -> Rragc_S2 - cost: 15.30, layer: 53
Normal
MK50
MK75
MK88
MK94
MK97
MK data
Normal data
Fold over basal
Time (min)
0
10
20
30
40
50
60
1
2
3
4
5
6

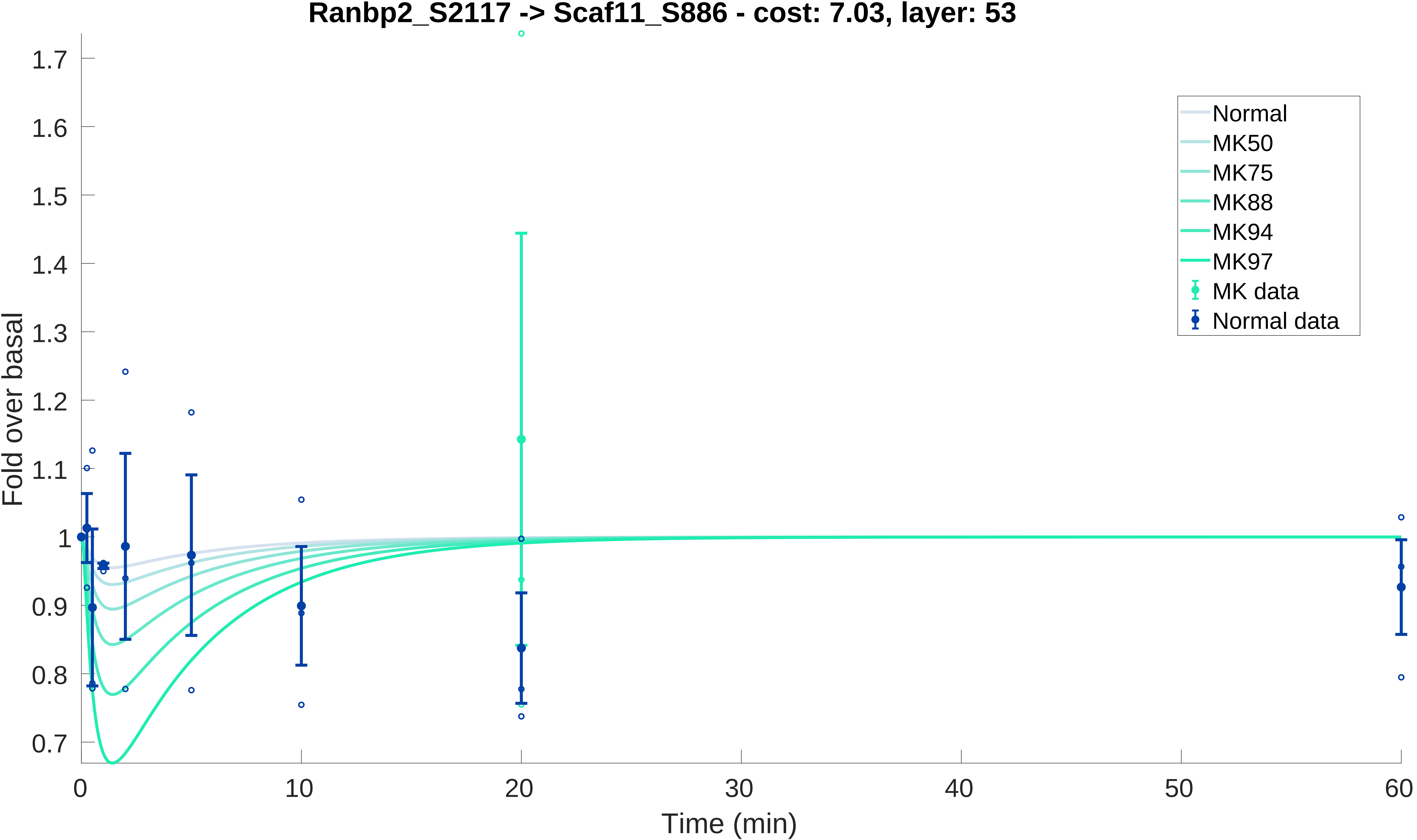

Ranbp2_S2117 -> Scaf11_S886 - cost: 7.03, layer: 53
Fold over basal
Time (min)
1.7
1.6
1.5
1.4
1.3
1.2
1.1
1
0.9
0.8
0.7
0
10
20
30
40
50
60
Normal
MK50
MK75
MK88
MK94
MK97
MK data
Normal data

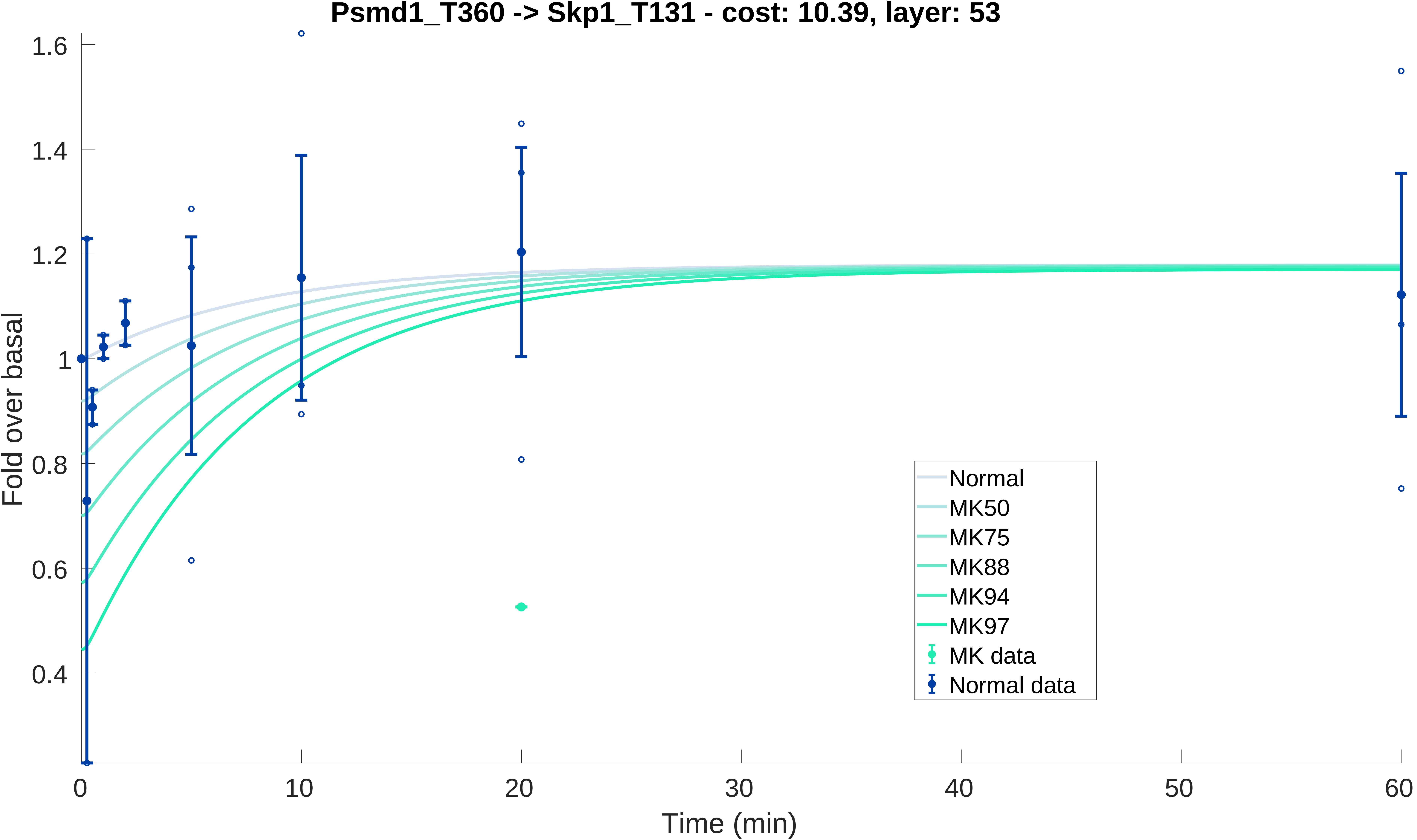

Psmd1_T360 -> Skp1_T131 - cost: 10.39, layer: 53
Fold over basal
Time (min)
1.6
1.4
1.2
1
0.8
0.6
0.4
0
10
20
30
40
50
60
Normal
MK50
MK75
MK88
MK94
MK97
MK data
Normal data

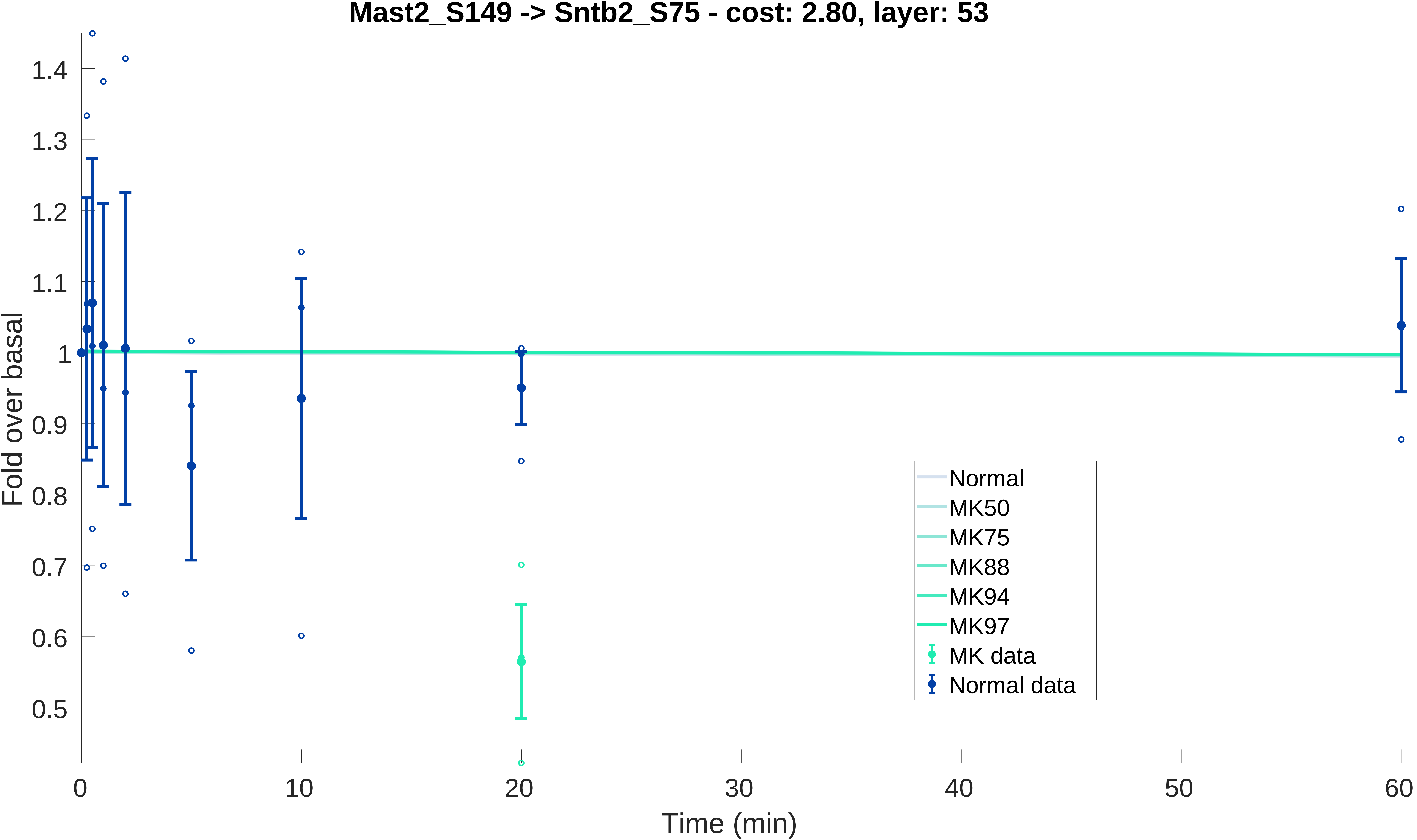

Mast2_S149 -> Sntb2_S75 - cost: 2.80, layer: 53
Fold over basal
Time (min)
0.5
0.6
0.7
0.8
0.9
1
1.1
1.2
1.3
1.4
0
10
20
30
40
50
60
Normal
MK50
MK75
MK88
MK94
MK97
MK data
Normal data

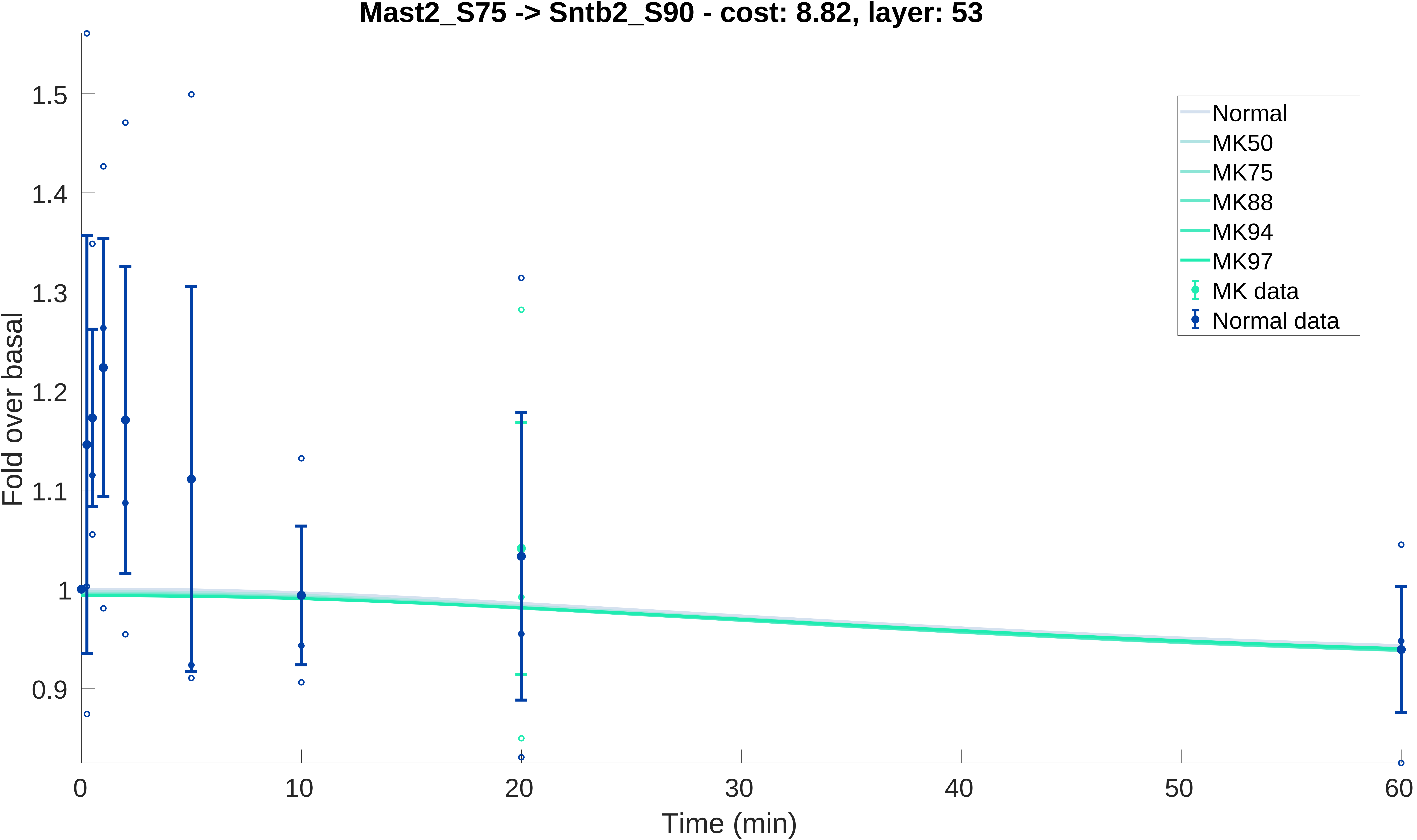
Mast2_S75 -> Sntb2_S90 - cost: 8.82, layer: 53
Fold over basal
1.5
1.4
1.3
1.2
1.1
1
0.9
0
10
20
30
40
50
60
Time (min)
Normal
MK50
MK75
MK88
MK94
MK97
MK data
Normal data

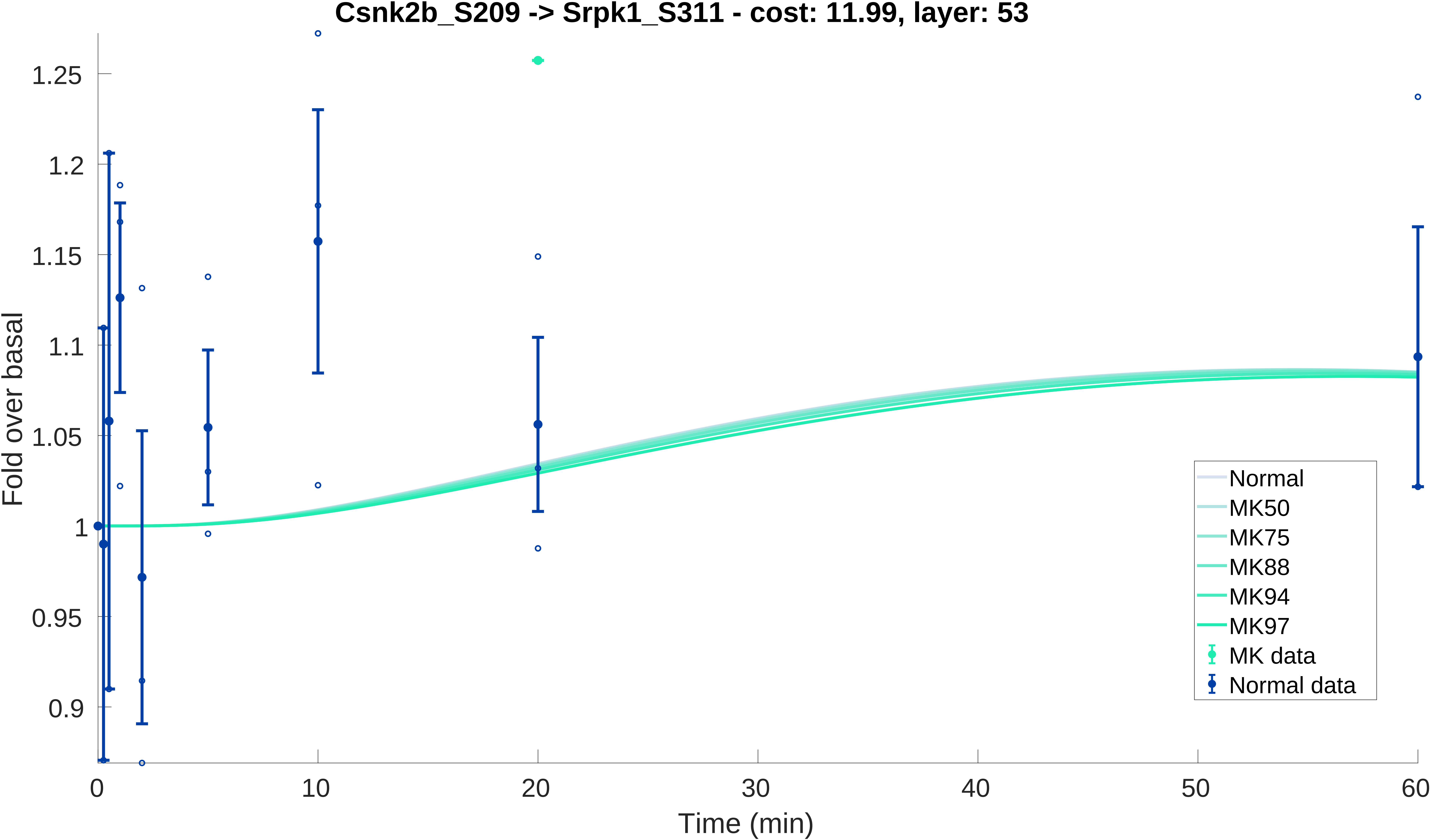
Csnk2b_S209 -> Srpk1_S311 - cost: 11.99, layer: 53
Fold over basal
1.25
1.2
1.15
1.1
1.05
1
0.95
0.9
0
10
20
30
40
50
60
Time (min)
Normal
MK50
MK75
MK88
MK94
MK97
MK data
Normal data

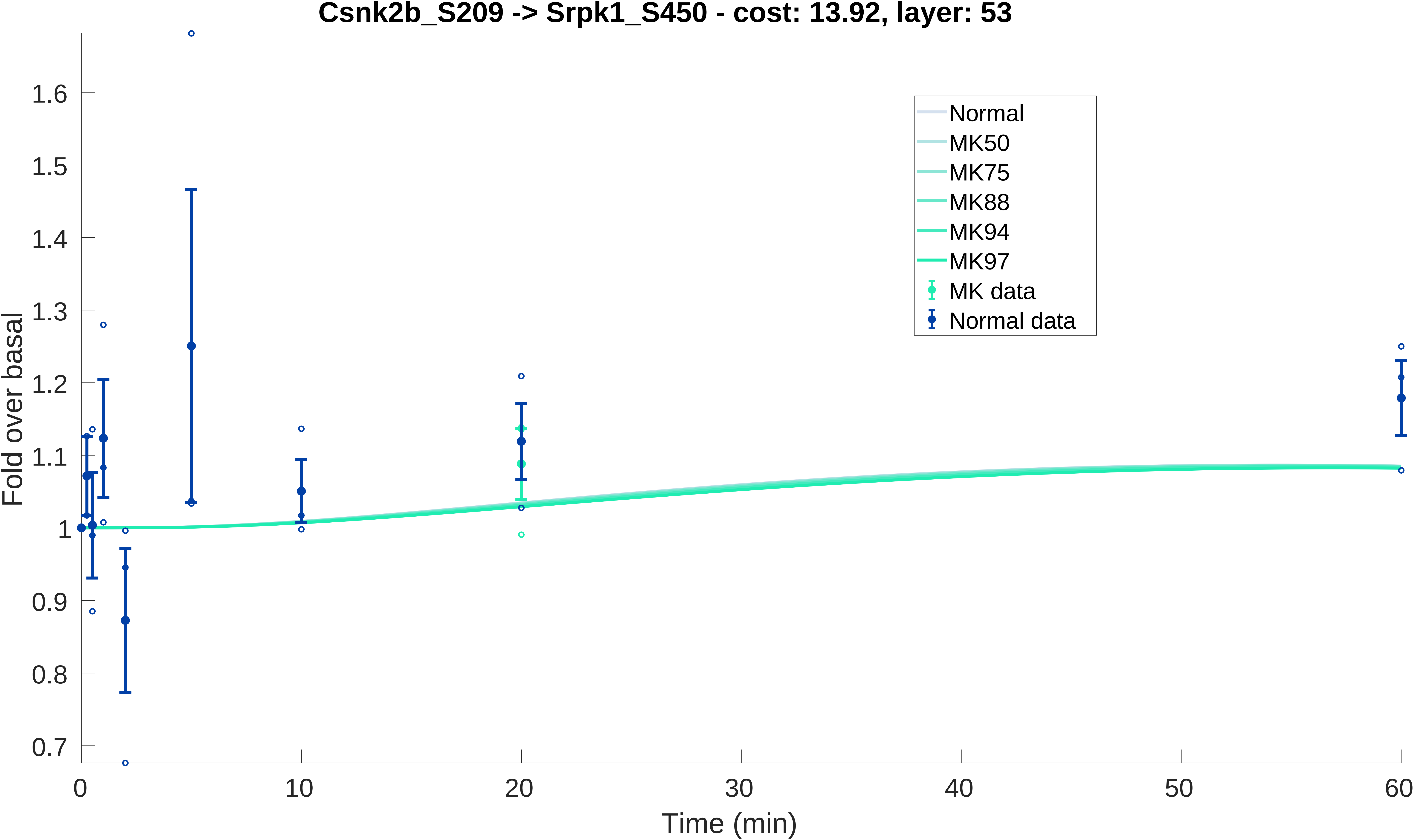

Csnk2b_S209 -> Srpk1_S450 - cost: 13.92, layer: 53
Fold over basal
Time (min)
1.6
1.5
1.4
1.3
1.2
1.1
1
0.9
0.8
0.7
0
10
20
30
40
50
60
Normal
MK50
MK75
MK88
MK94
MK97
MK data
Normal data

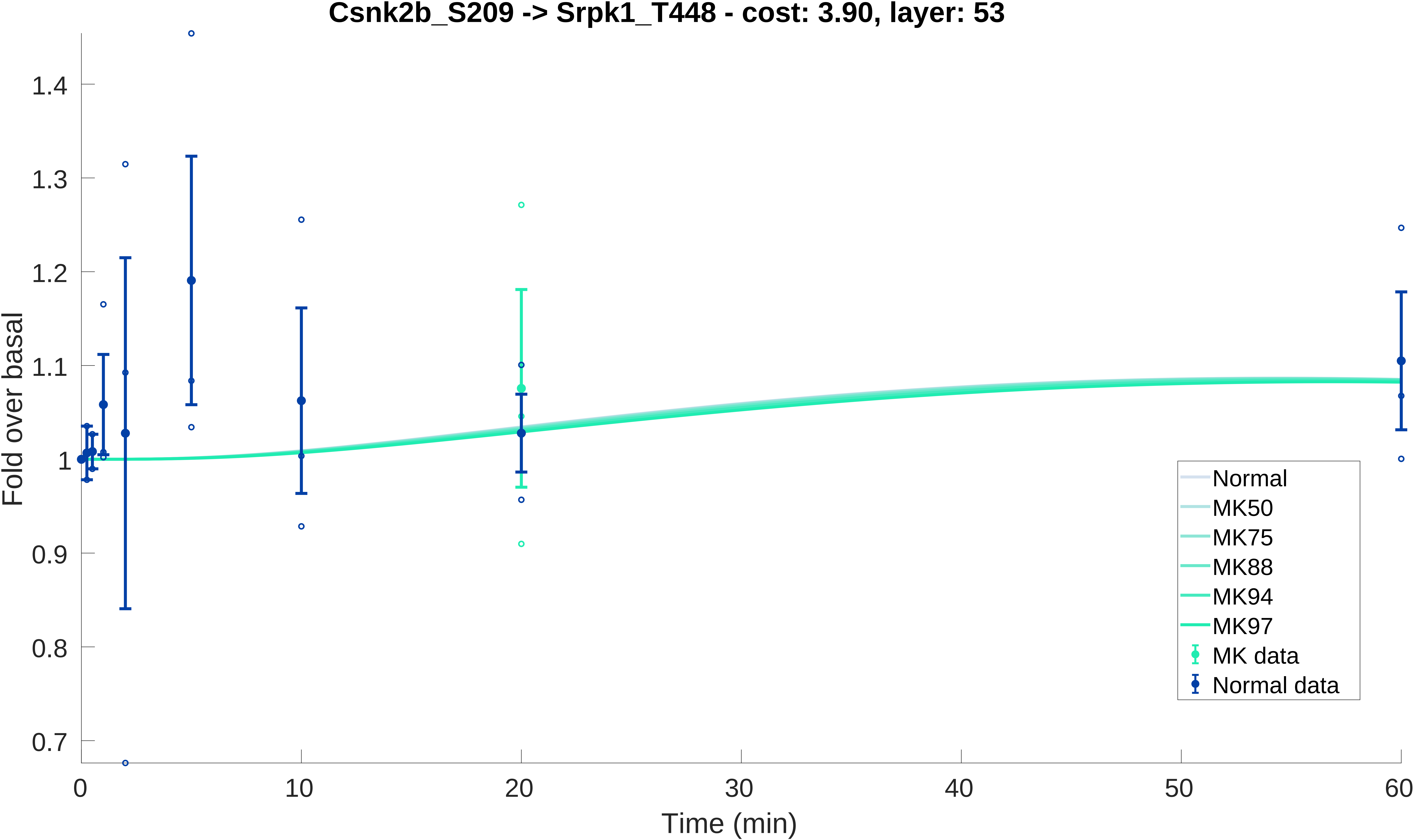

Csnk2b_S209 -> Srpk1_T448 - cost: 3.90, layer: 53
Fold over basal
Time (min)
1.4
1.3
1.2
1.1
1
0.9
0.8
0.7
0
10
20
30
40
50
60
Normal
MK50
MK75
MK88
MK94
MK97
MK data
Normal data

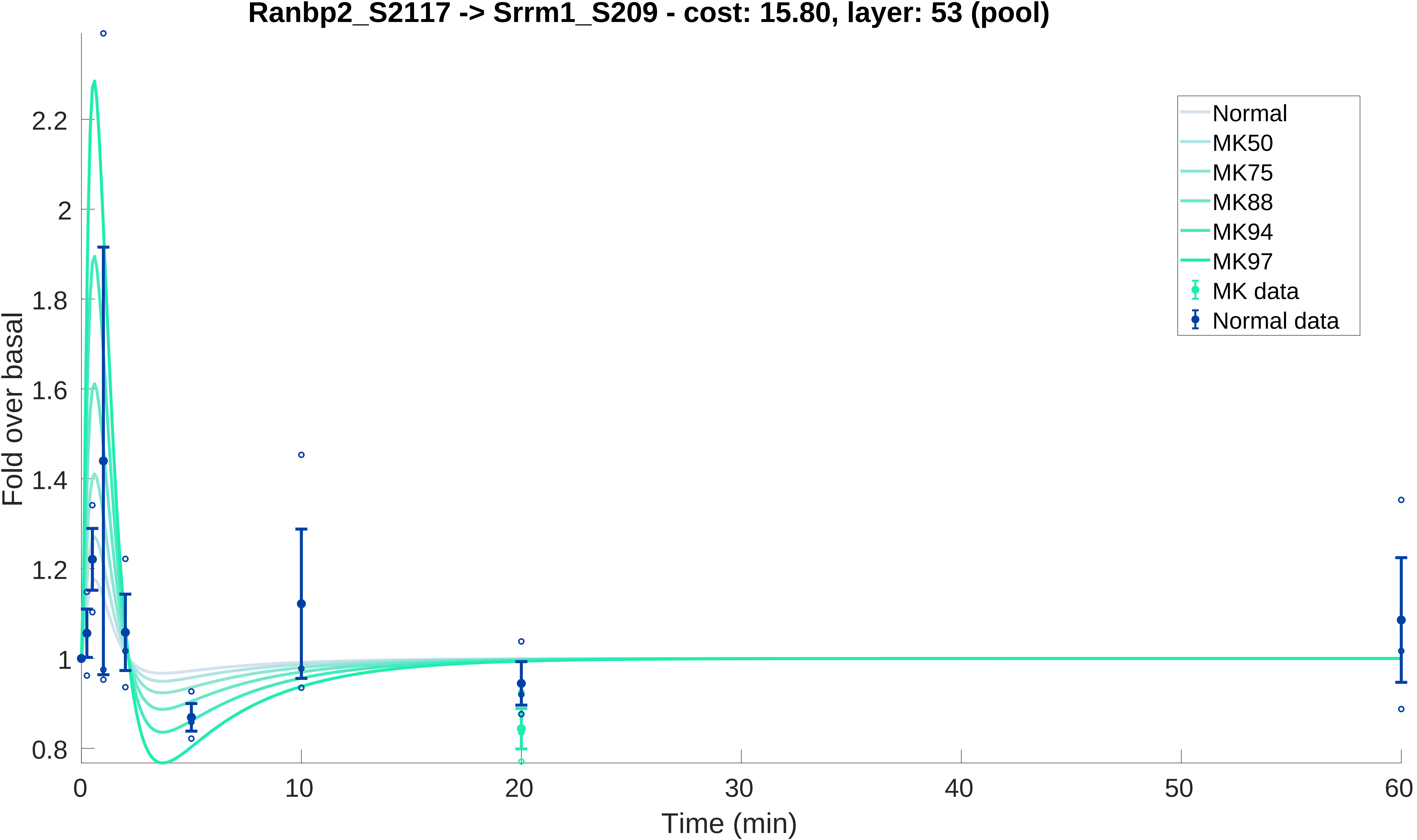

Ranbp2_S2117 -> Srrm1_S209 - cost: 15.80, layer: 53 (pool)
Normal
MK50
MK75
MK88
MK94
MK97
MK data
Normal data
Fold over basal
Time (min)
0.8
1
1.2
1.4
1.6
1.8
2
2.2
0
10
20
30
40
50
60

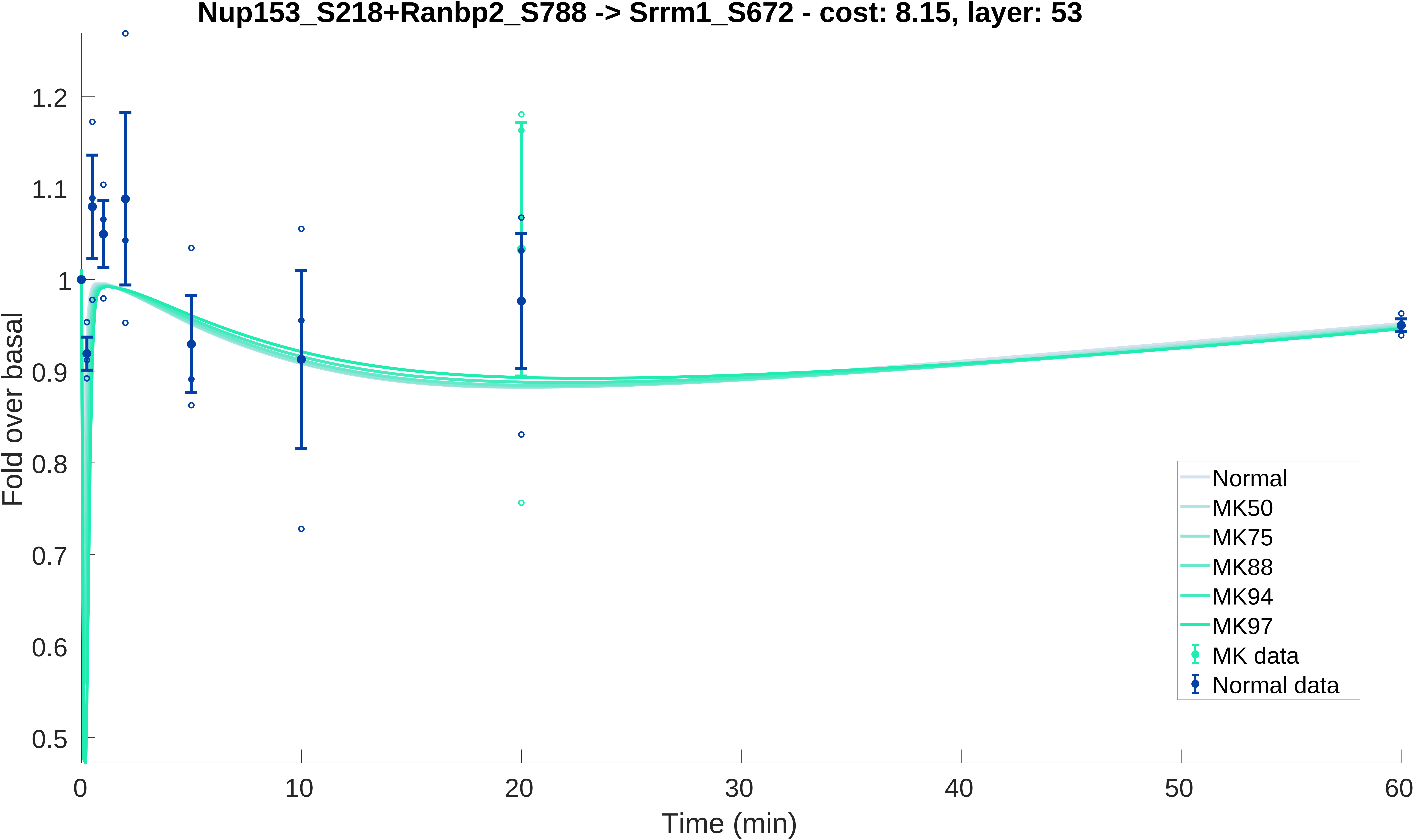

Nup153_S218+Ranbp2_S788 -> Srrm1_S672 - cost: 8.15, layer: 53
Fold over basal
1.2
1.1
1
0.9
0.8
0.7
0.6
0.5
0
10
20
30
40
50
60
Time (min)
Normal
MK50
MK75
MK88
MK94
MK97
MK data
Normal data

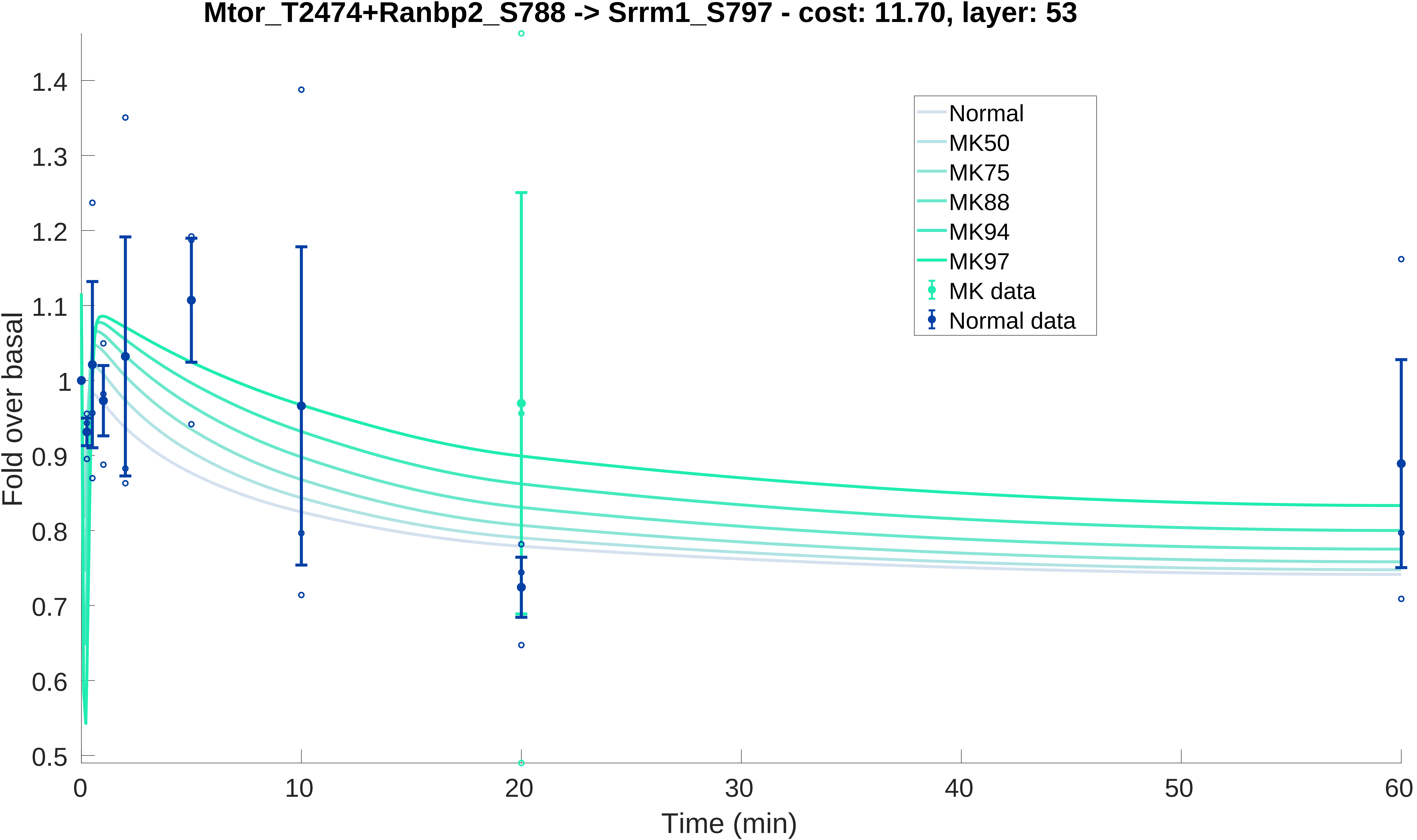

Mtor_T2474+Ranbp2_S788 -> Srrm1_S797 - cost: 11.70, layer: 53
Fold over basal
Time (min)
0.5
0.6
0.7
0.8
0.9
1
1.1
1.2
1.3
1.4
0
10
20
30
40
50
60
Normal
MK50
MK75
MK88
MK94
MK97
MK data
Normal data

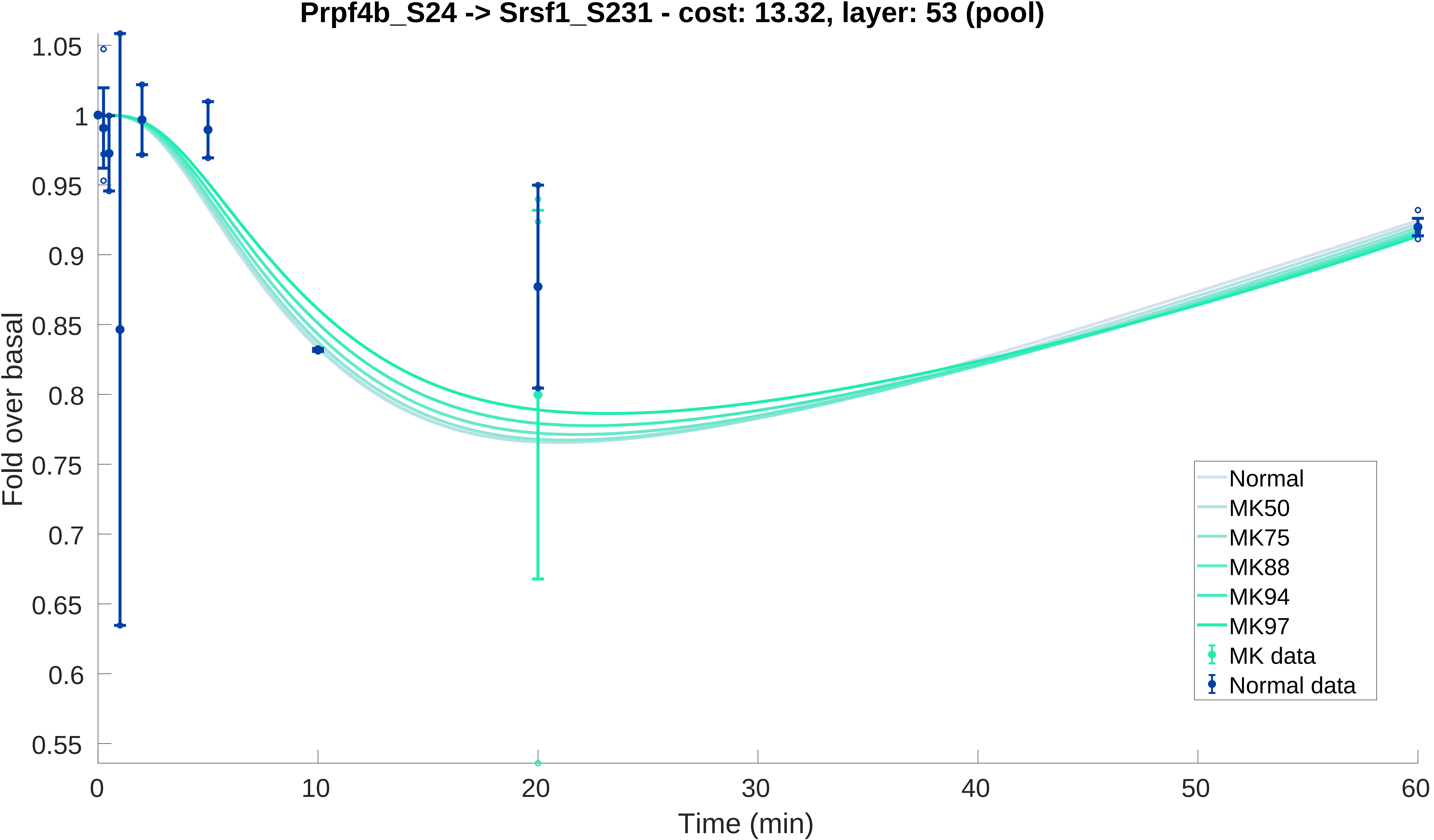
Prpf4b_S24 -> Srsf1_S231 - cost: 13.32, layer: 53 (pool)
Fold over basal
Time (min)
1.05
1
0.95
0.9
0.85
0.8
0.75
0.7
0.65
0.6
0.55
0
10
20
30
40
50
60
Normal
MK50
MK75
MK88
MK94
MK97
MK data
Normal data

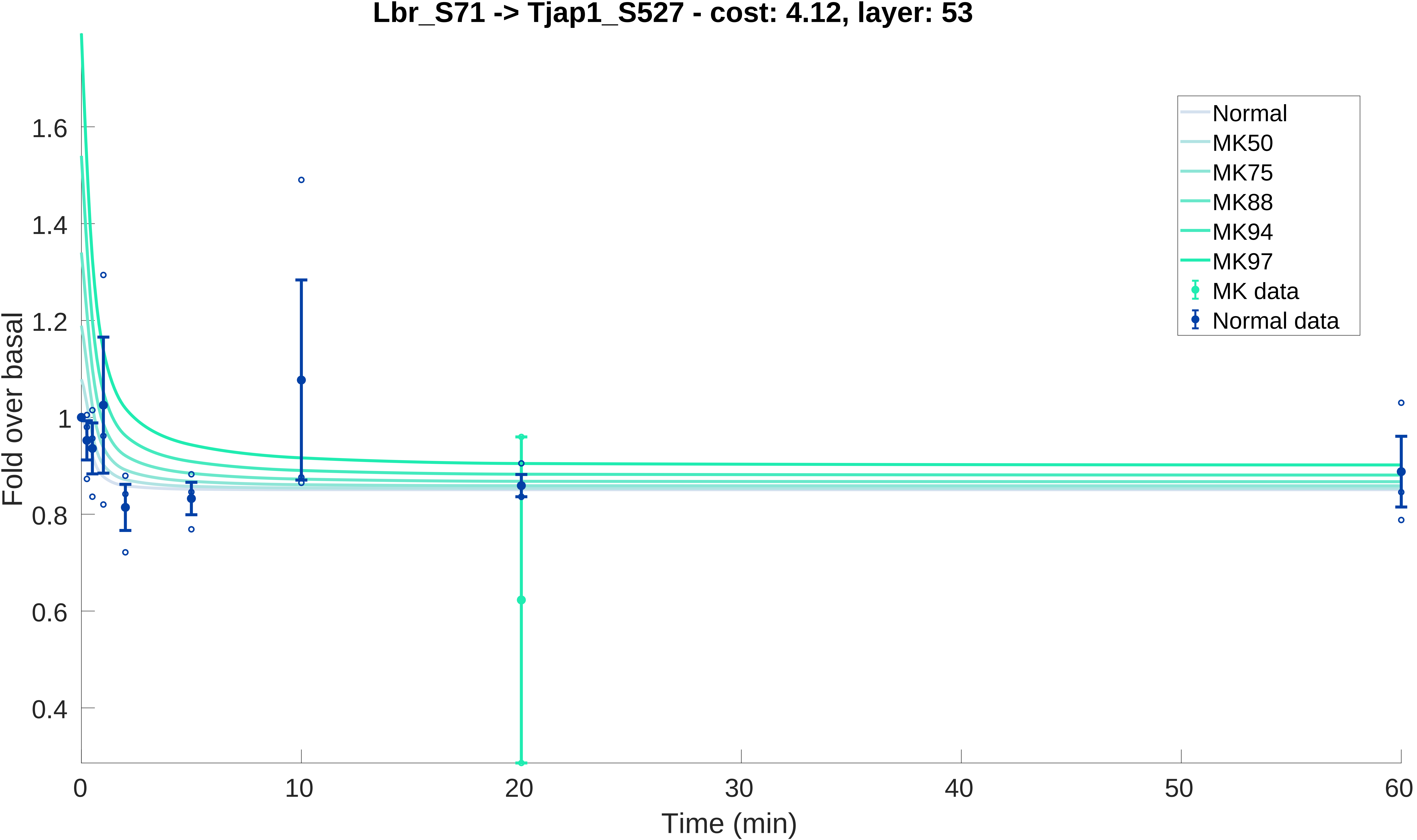
Lbr_S71 -> Tjap1_S527 - cost: 4.12, layer: 53
Fold over basal
Time (min)
0.4
0.6
0.8
1
1.2
1.4
1.6
0
10
20
30
40
50
60
Normal
MK50
MK75
MK88
MK94
MK97
MK data
Normal data

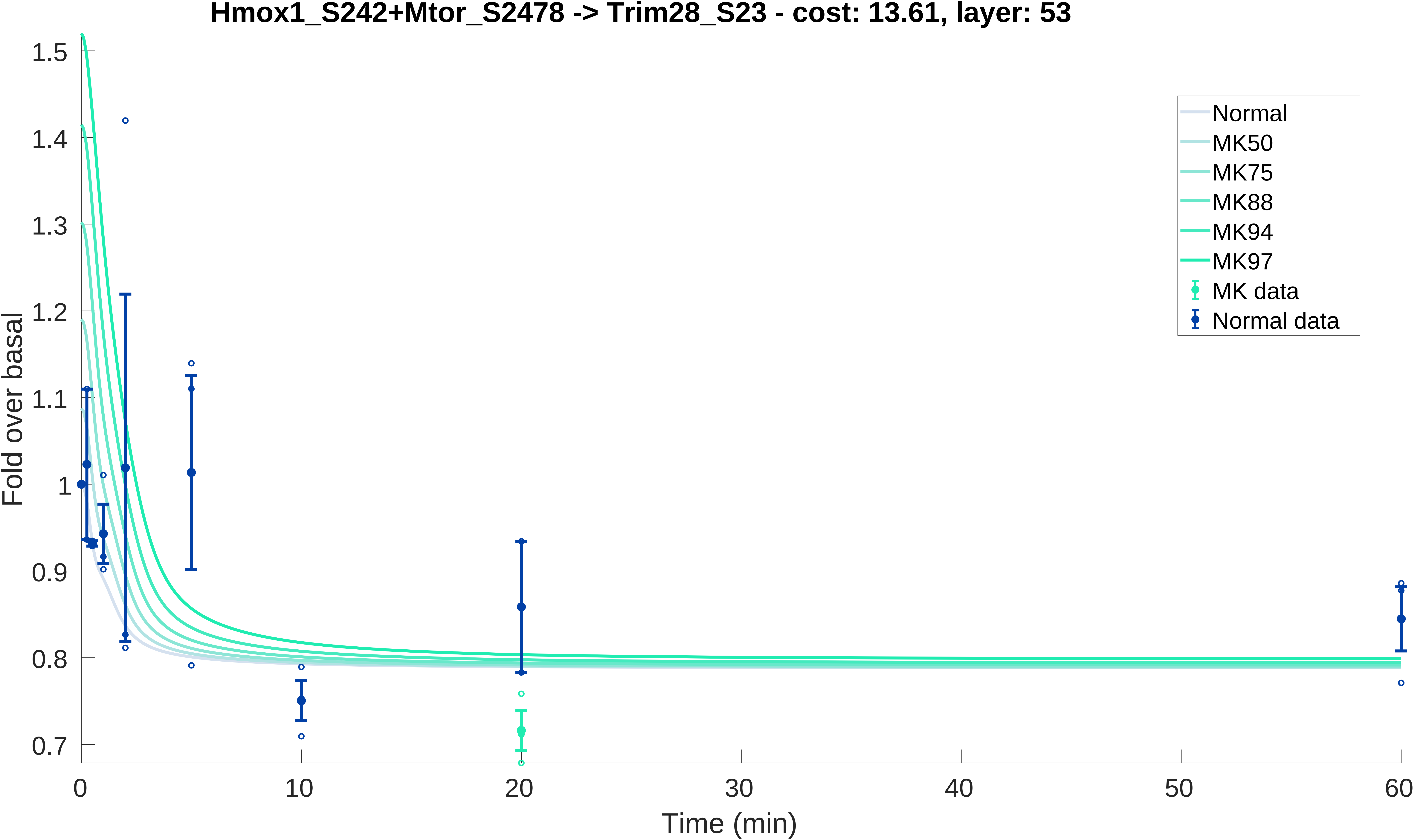

Hmox1_S242+Mtor_S2478 -> Trim28_S23 - cost: 13.61, layer: 53
Fold over basal
Time (min)
1.5
1.4
1.3
1.2
1.1
1
0.9
0.8
0.7
0
10
20
30
40
50
60
Normal
MK50
MK75
MK88
MK94
MK97
MK data
Normal data

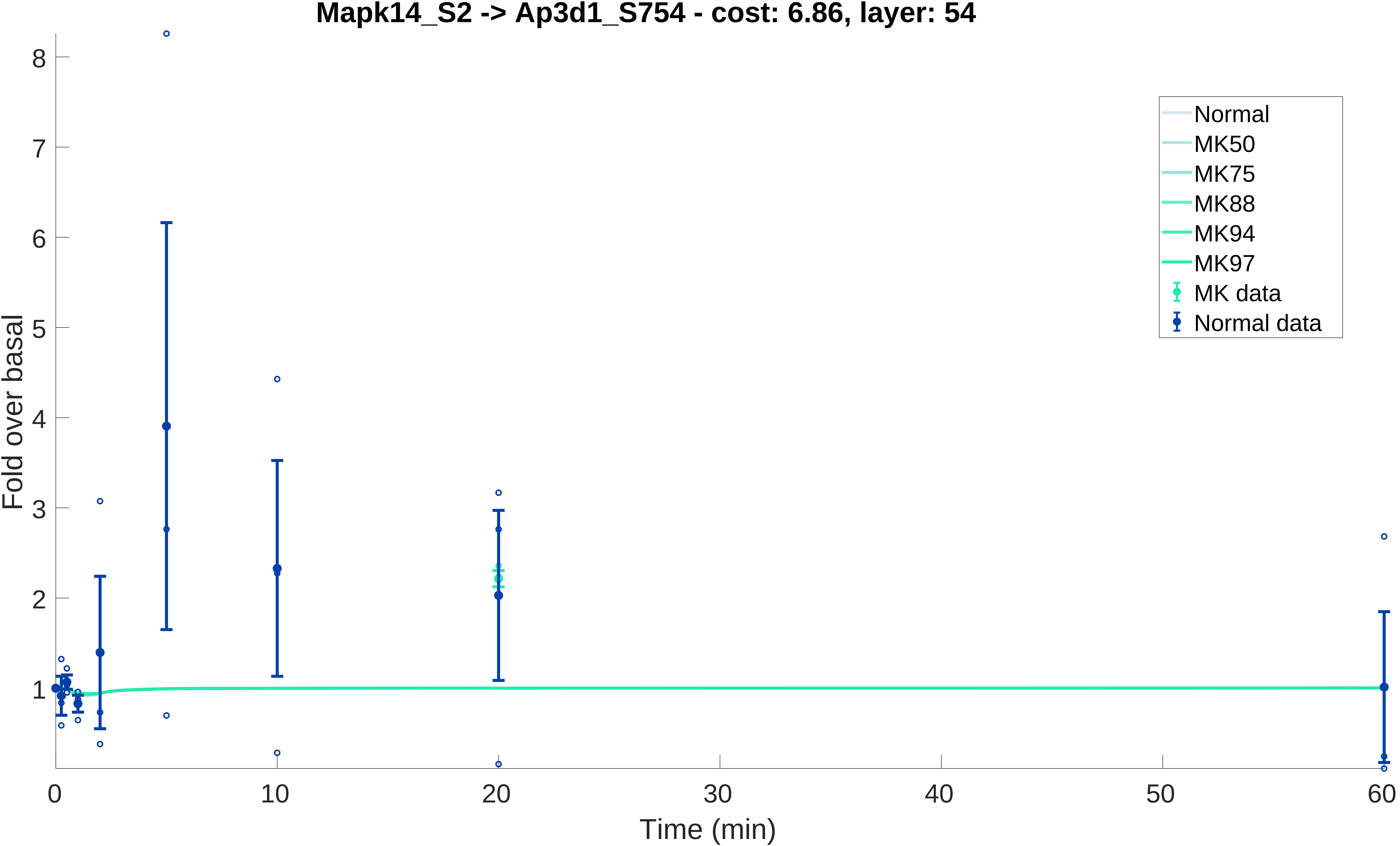

Mapk14_S2 -> Ap3d1_S754 - cost: 6.86, layer: 54
Fold over basal
Time (min)
1
2
3
4
5
6
7
8
0
10
20
30
40
50
60
Normal
MK50
MK75
MK88
MK94
MK97
MK data
Normal data

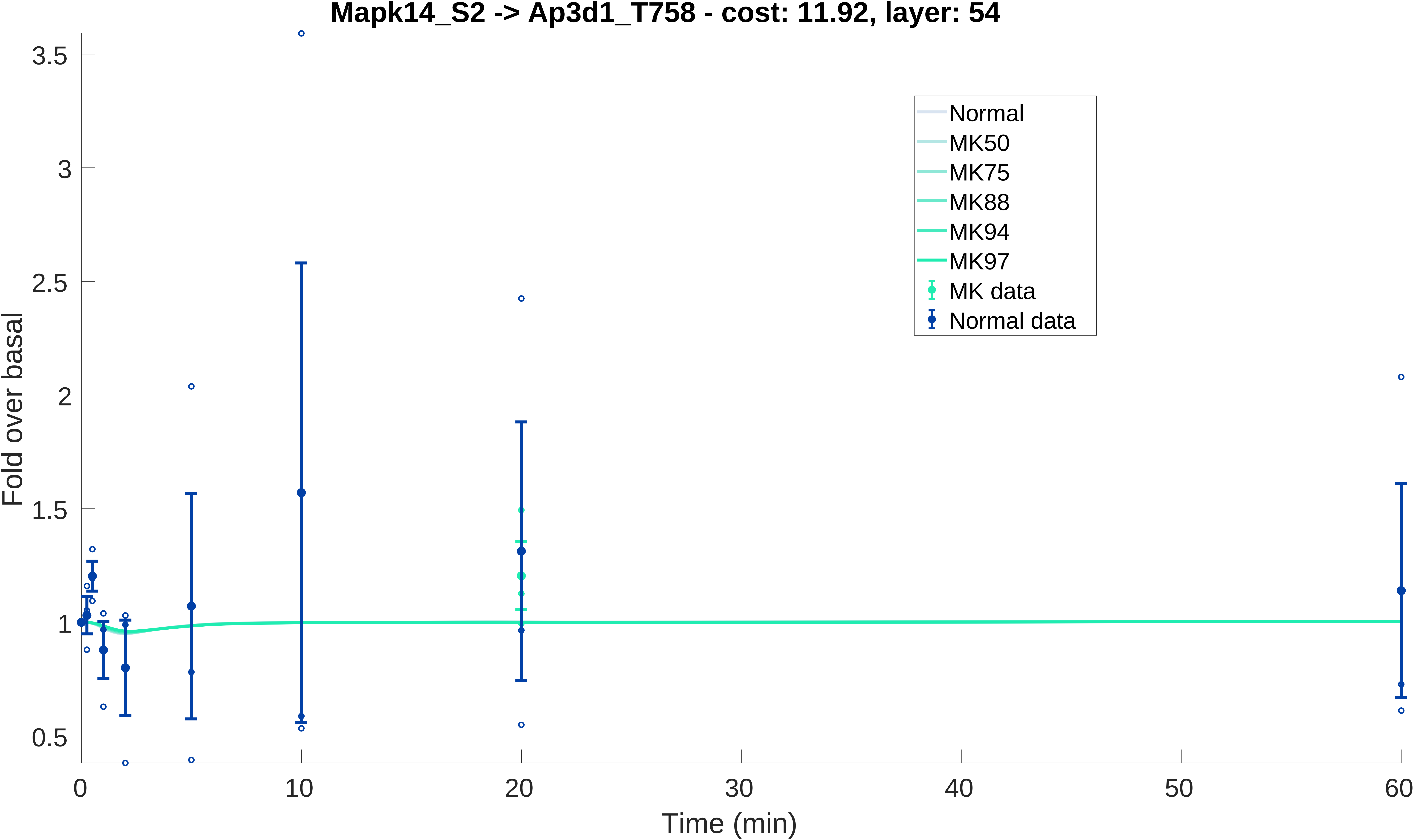

Mapk14_S2 -> Ap3d1_T758 - cost: 11.92, layer: 54
Normal
MK50
MK75
MK88
MK94
MK97
MK data
Normal data
Fold over basal
Time (min)
0.5
1
1.5
2
2.5
3
3.5
0
10
20
30
40
50
60

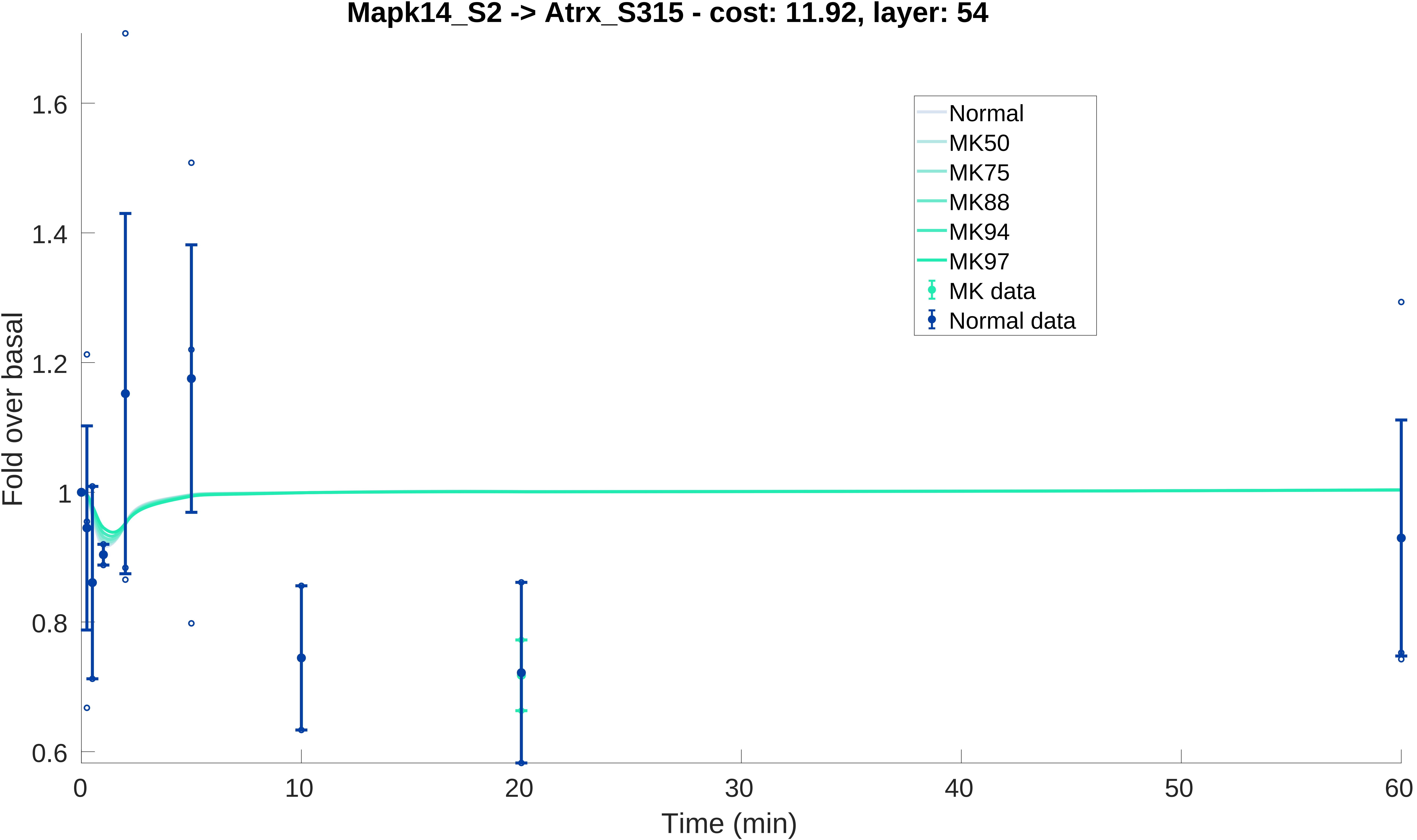

Mapk14_S2 -> Atrx_S315 - cost: 11.92, layer: 54
Fold over basal
Time (min)
0.6
0.8
1
1.2
1.4
1.6
0
10
20
30
40
50
60
Normal
MK50
MK75
MK88
MK94
MK97
MK data
Normal data

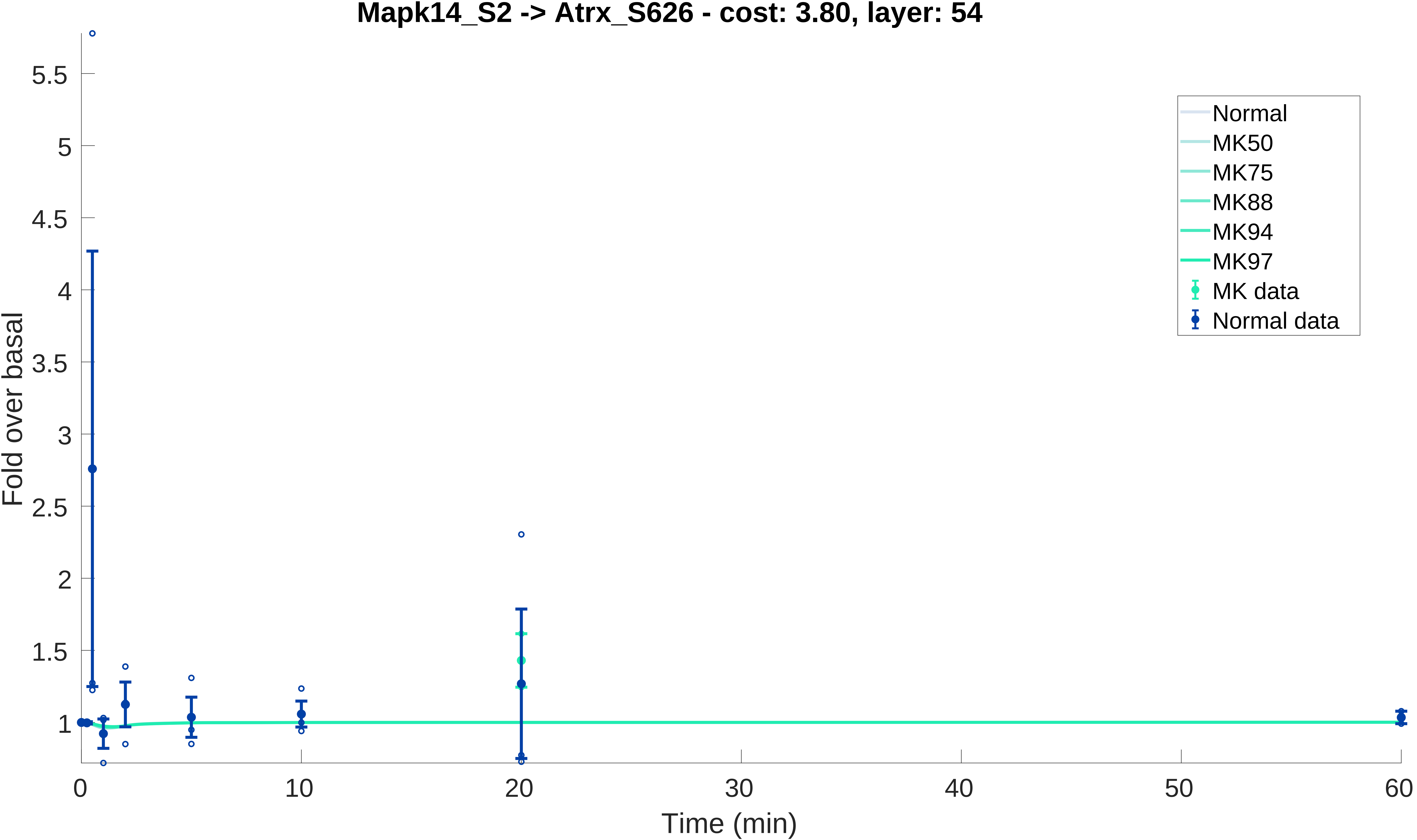
Mapk14_S2 -> Atrx_S626 - cost: 3.80, layer: 54
Fold over basal
5.5
5
4.5
4
3.5
3
2.5
2
1.5
1
Time (min)
0
10
20
30
40
50
60
Normal
MK50
MK75
MK88
MK94
MK97
MK data
Normal data

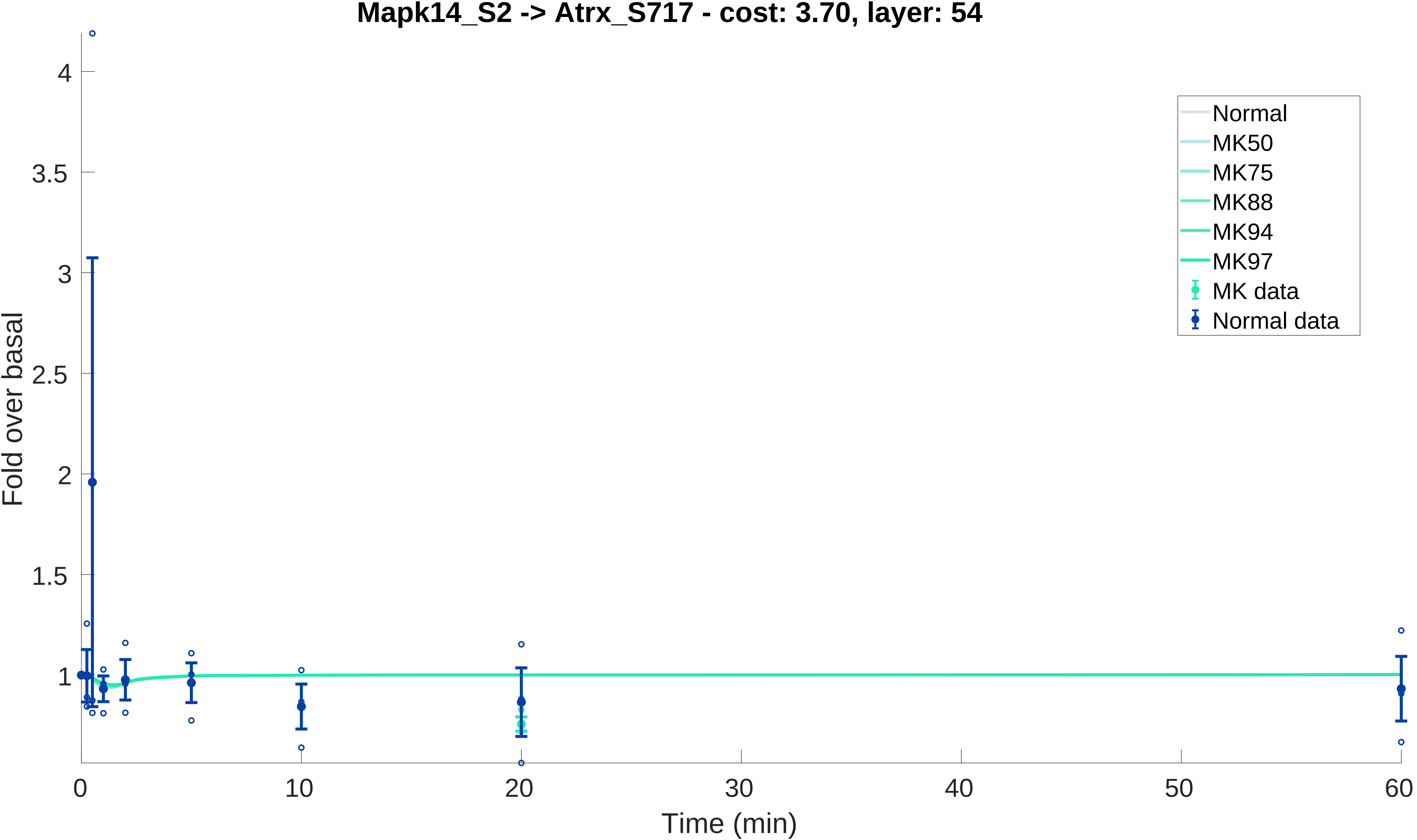

Mapk14_S2 -> Atrx_S717 - cost: 3.70, layer: 54
Fold over basal
4
3.5
3
2.5
2
1.5
1
0
10
20
30
40
50
60
Time (min)
Normal
MK50
MK75
MK88
MK94
MK97
MK data
Normal data

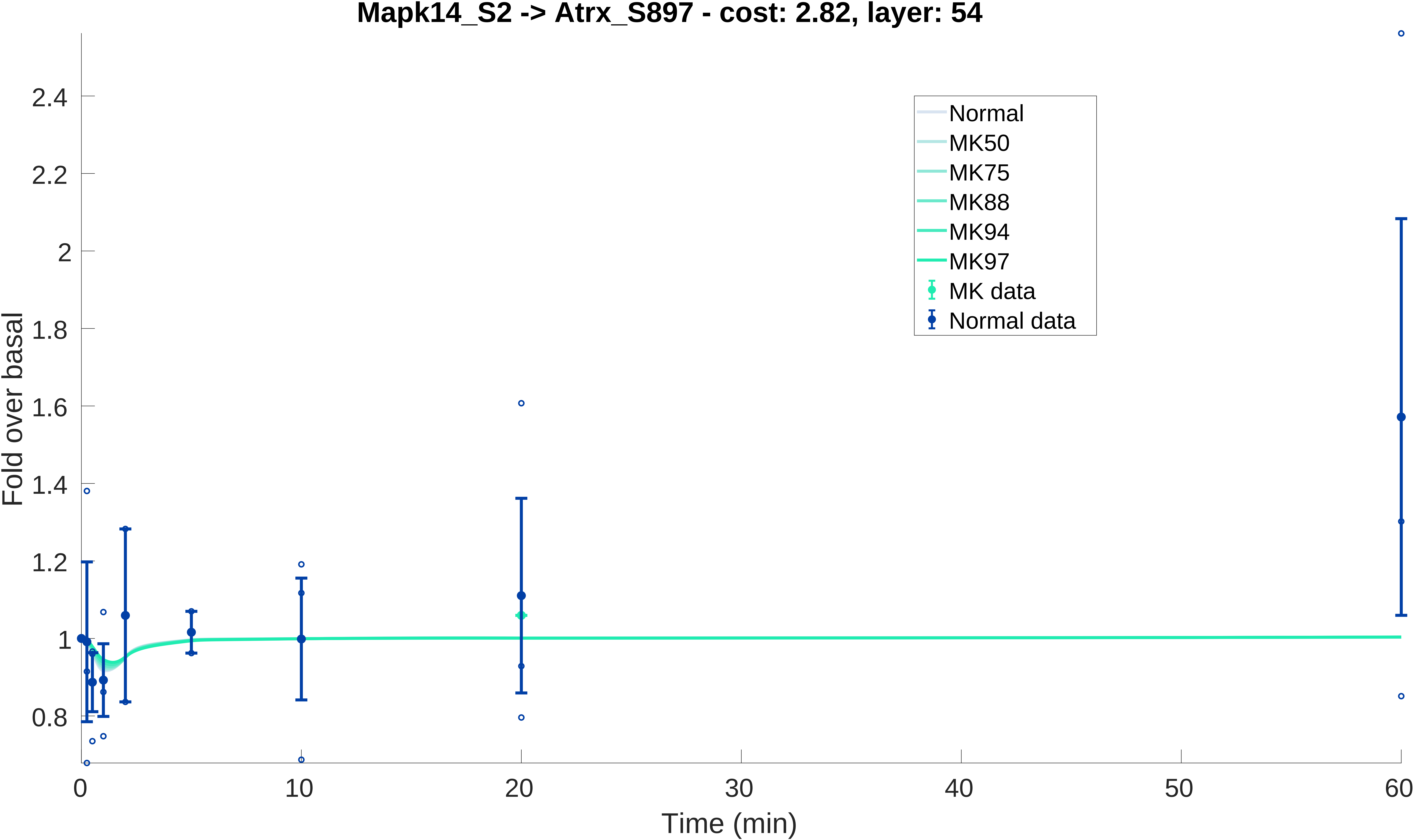
Mapk14_S2 -> Atrx_S897 - cost: 2.82, layer: 54
Normal
MK50
MK75
MK88
MK94
MK97
MK data
Normal data
Fold over basal
Time (min)
0.8
1
1.2
1.4
1.6
1.8
2
2.2
2.4
0
10
20
30
40
50
60

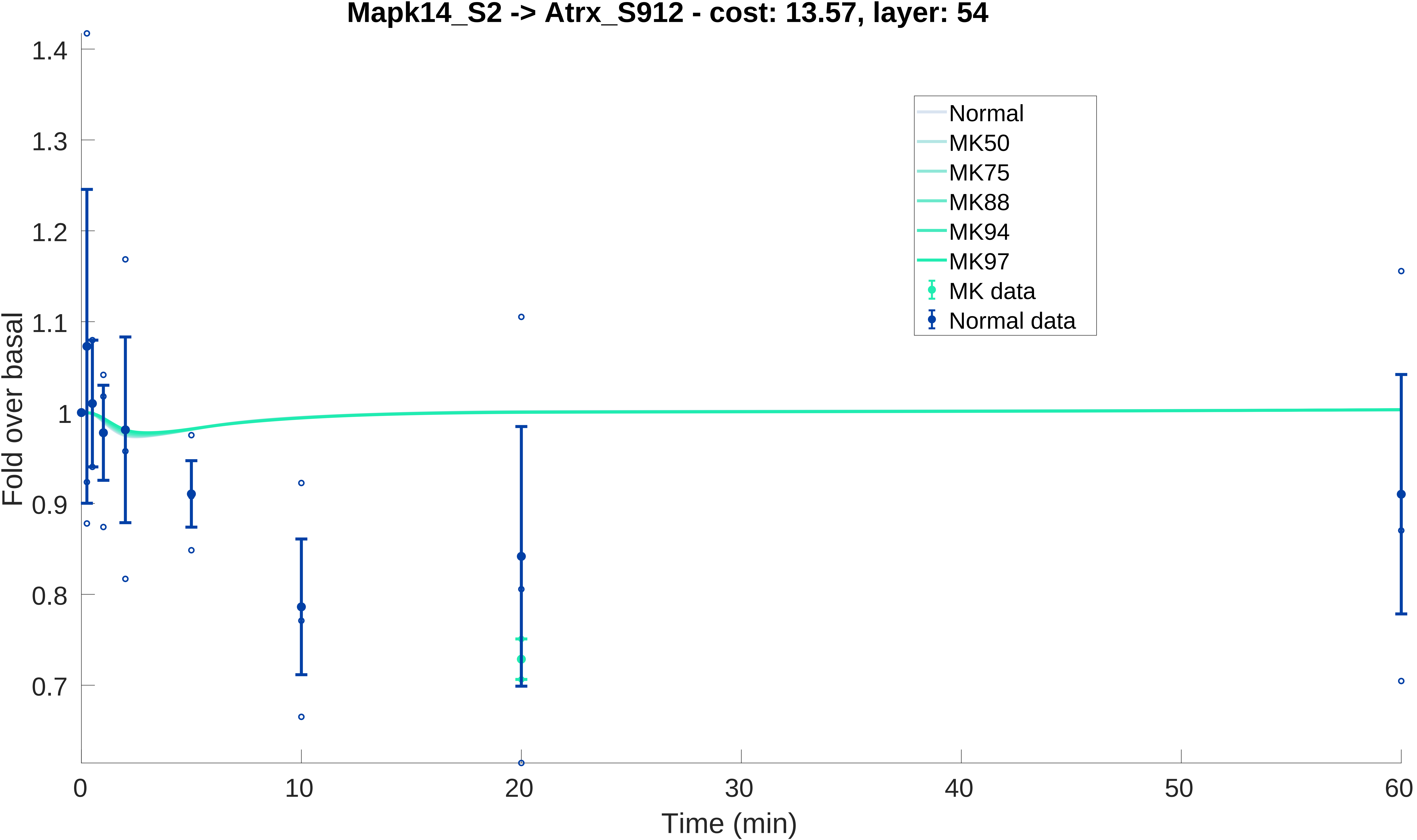

Mapk14_S2 -> Atrx_S912 - cost: 13.57, layer: 54
Fold over basal
Time (min)
1.4
1.3
1.2
1.1
1
0.9
0.8
0.7
0
10
20
30
40
50
60
Normal
MK50
MK75
MK88
MK94
MK97
MK data
Normal data

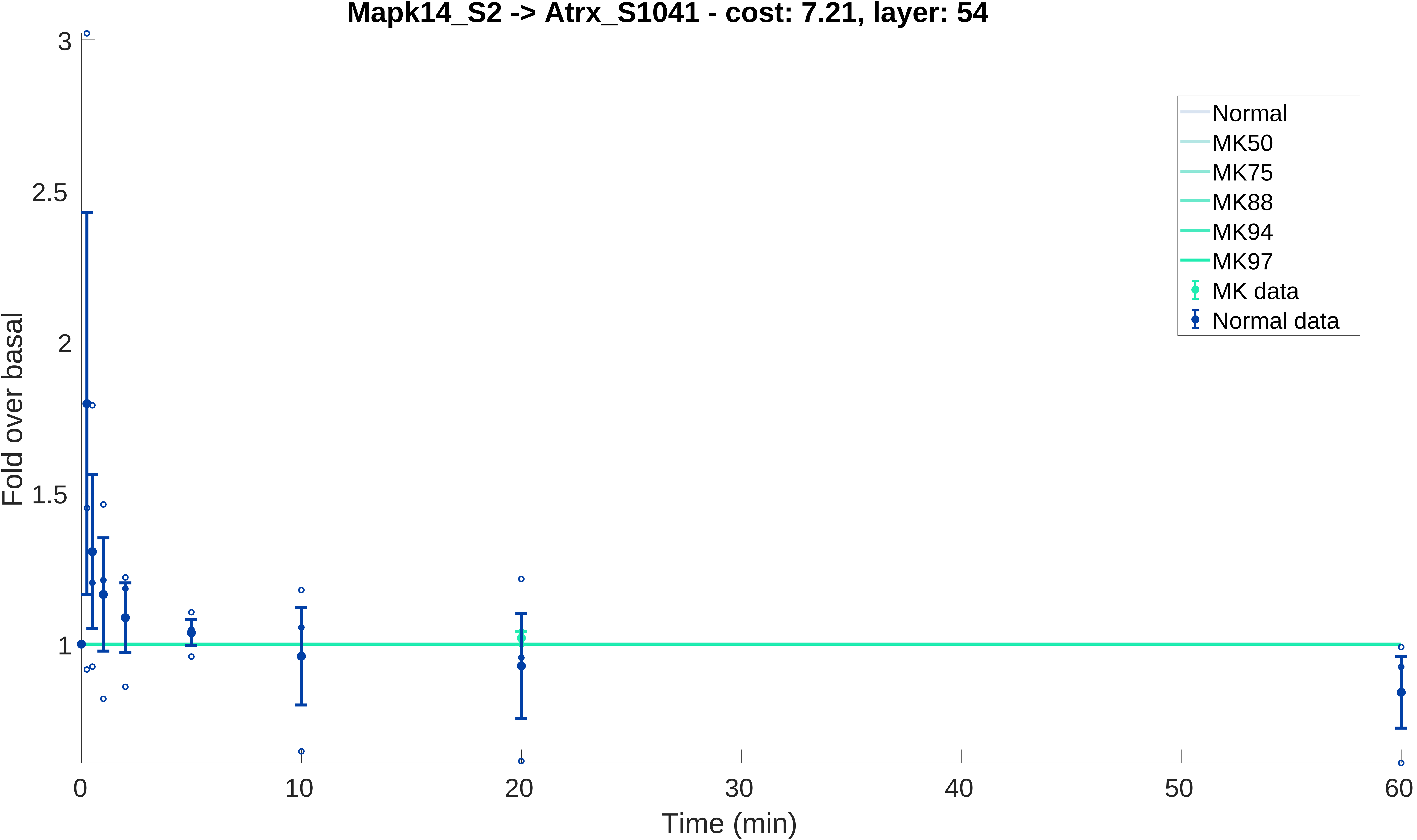

Mapk14_S2 -> Atrx_S1041 - cost: 7.21, layer: 54
Fold over basal
Time (min)
Normal
MK50
MK75
MK88
MK94
MK97
MK data
Normal data
3
2.5
2
1.5
1
0
10
20
30
40
50
60

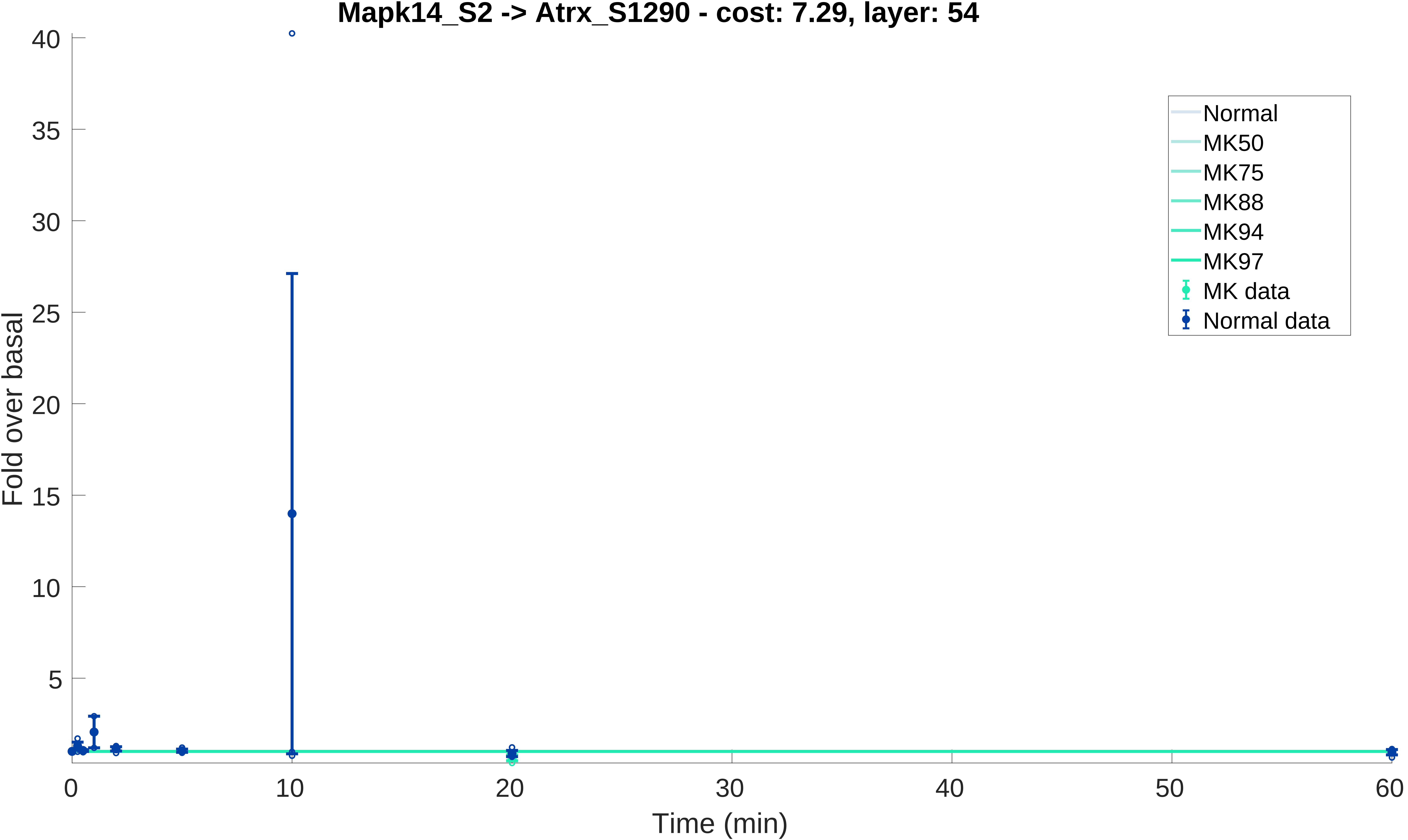

Mapk14_S2 -> Atrx_S1290 - cost: 7.29, layer: 54
Fold over basal
Time (min)
0
10
20
30
40
50
60
5
10
15
20
25
30
35
40
Normal
MK50
MK75
MK88
MK94
MK97
MK data
Normal data

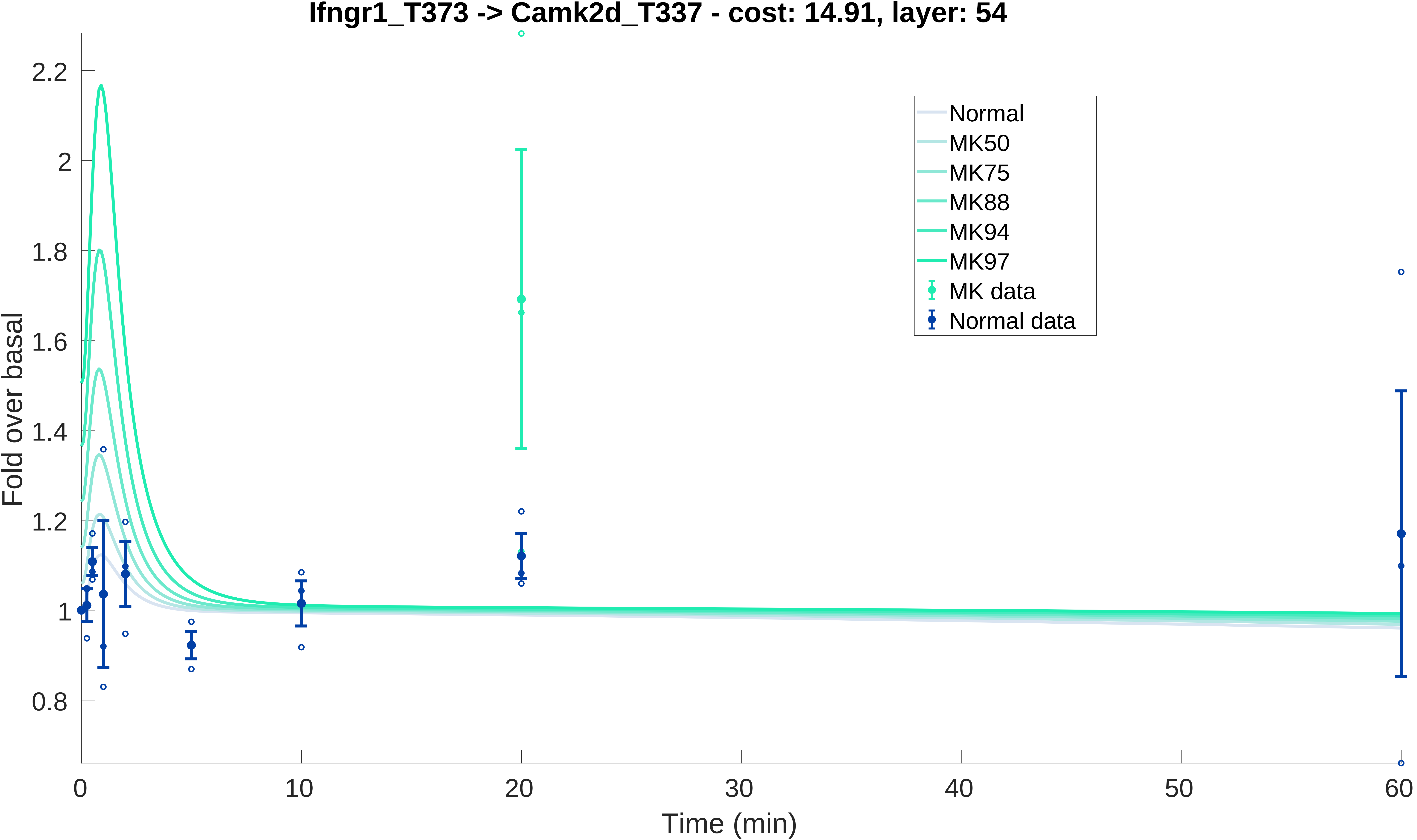

Ifngr1_T373 -> Camk2d_T337 - cost: 14.91, layer: 54
Fold over basal
Time (min)
0.8
1
1.2
1.4
1.6
1.8
2
2.2
0
10
20
30
40
50
60
Normal
MK50
MK75
MK88
MK94
MK97
MK data
Normal data

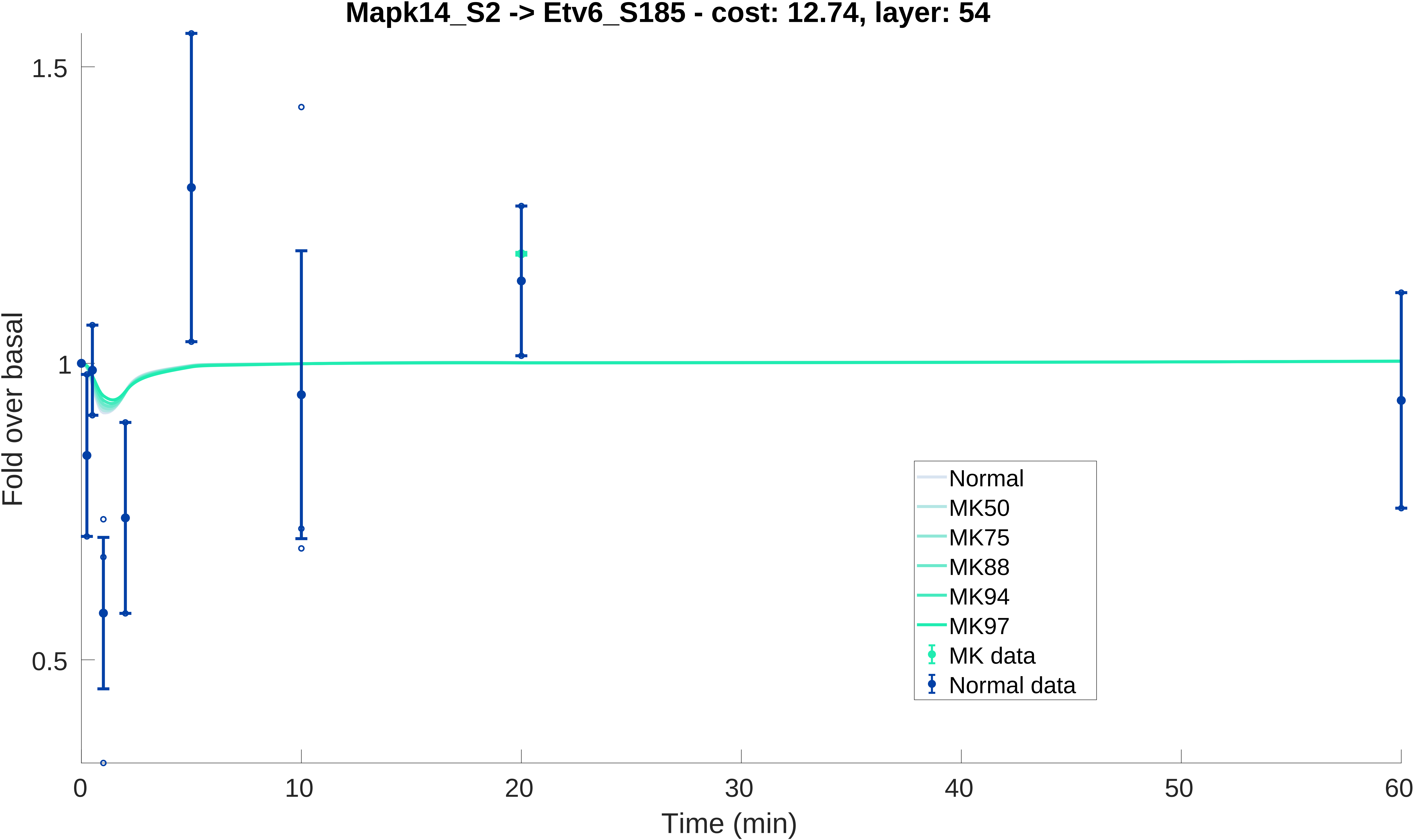

Mapk14_S2 -> Etv6_S185 - cost: 12.74, layer: 54
Fold over basal
Time (min)
1.5
1
0.5
0
10
20
30
40
50
60
Normal
MK50
MK75
MK88
MK94
MK97
MK data
Normal data

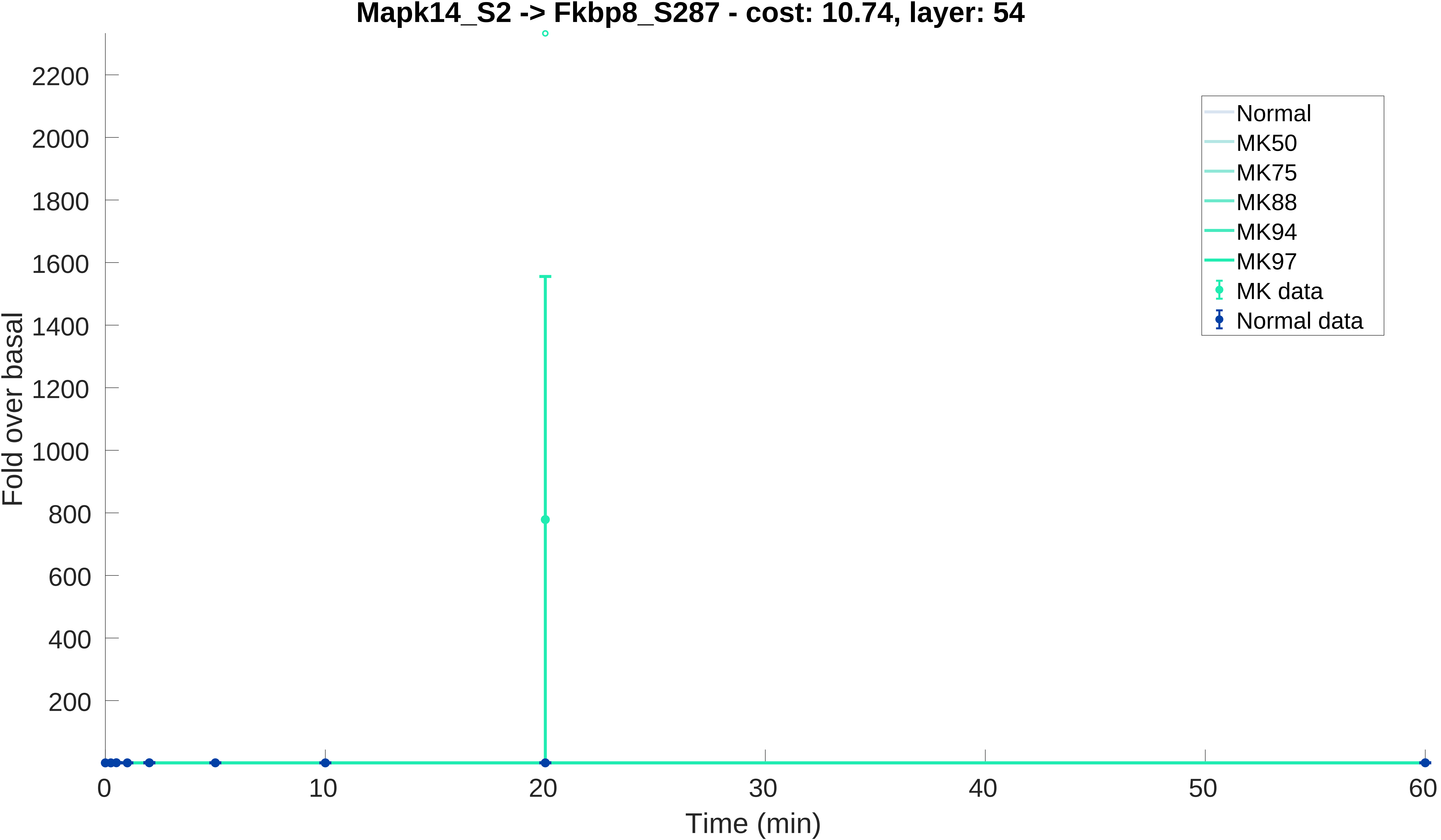

Mapk14_S2 -> Fkbp8_S287 - cost: 10.74, layer: 54
Fold over basal
Time (min)
2200
2000
1800
1600
1400
1200
1000
800
600
400
200
0
10
20
30
40
50
60
Normal
MK50
MK75
MK88
MK94
MK97
MK data
Normal data

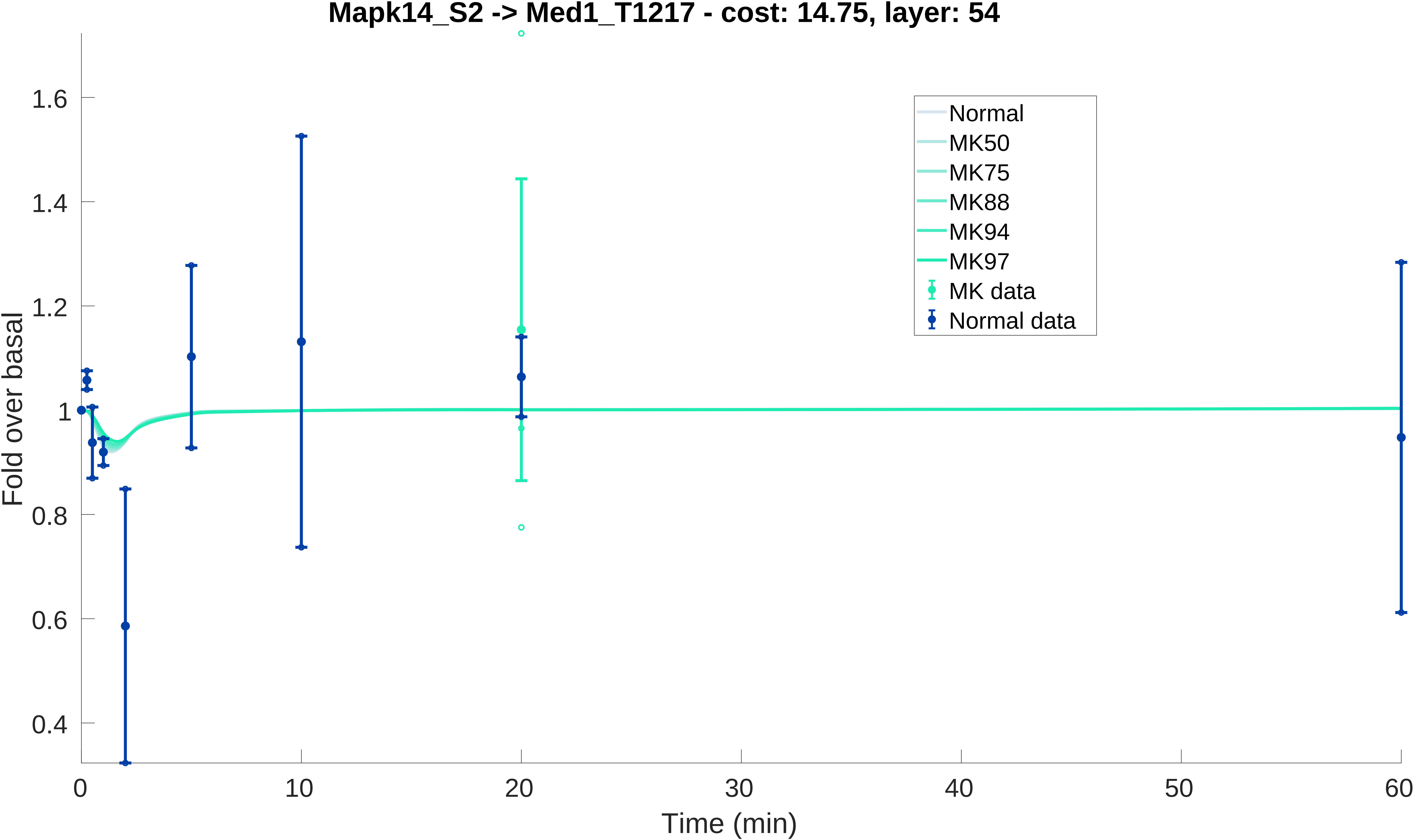
Mapk14_S2 -> Med1_T1217 - cost: 14.75, layer: 54
Fold over basal
Time (min)
Normal
MK50
MK75
MK88
MK94
MK97
MK data
Normal data
0.4
0.6
0.8
1
1.2
1.4
1.6
0
10
20
30
40
50
60

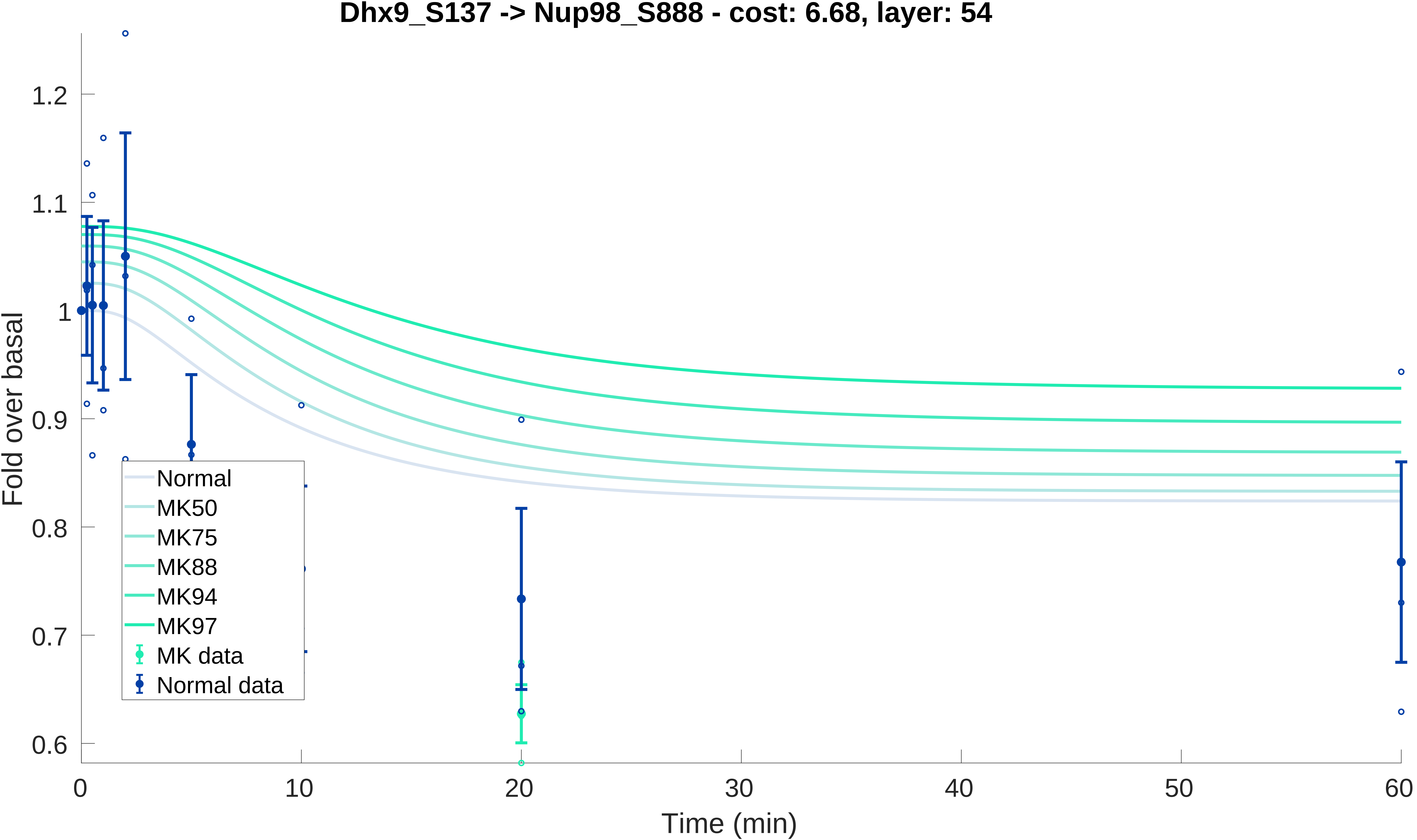

Dhx9_S137 -> Nup98_S888 - cost: 6.68, layer: 54
Fold over basal
Time (min)
0.6
0.7
0.8
0.9
1
1.1
1.2
0
10
20
30
40
50
60
Normal
MK50
MK75
MK88
MK94
MK97
MK data
Normal data

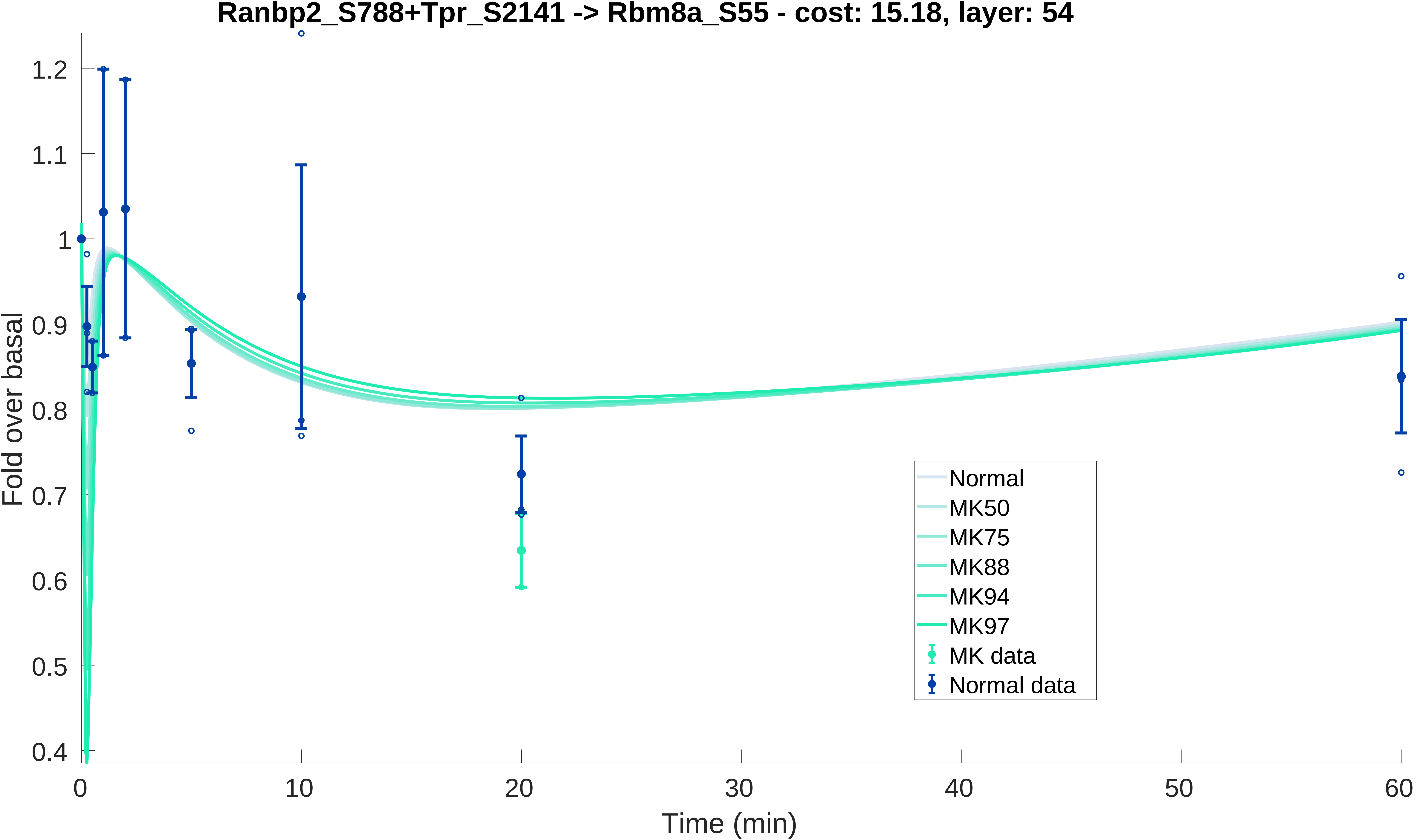

Ranbp2_S788+Tpr_S2141 -> Rbm8a_S55 - cost: 15.18, layer: 54
Fold over basal
Time (min)
1.2
1.1
1
0.9
0.8
0.7
0.6
0.5
0.4
0
10
20
30
40
50
60
Normal
MK50
MK75
MK88
MK94
MK97
MK data
Normal data

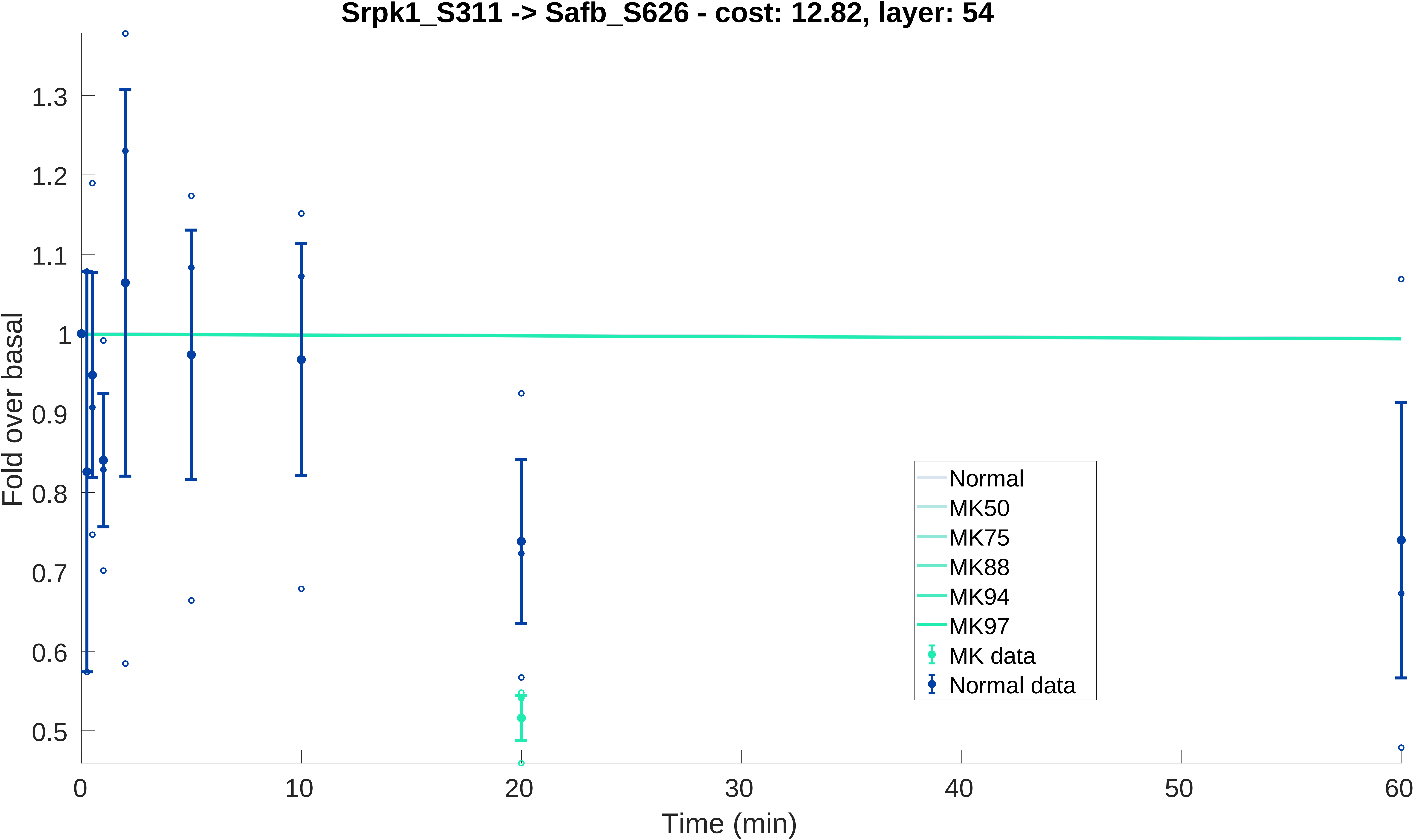

Srpk1_S311 -> Safb_S626 - cost: 12.82, layer: 54
Fold over basal
1.3
1.2
1.1
1
0.9
0.8
0.7
0.6
0.5
0
10
20
30
40
50
60
Time (min)
Normal
MK50
MK75
MK88
MK94
MK97
MK data
Normal data

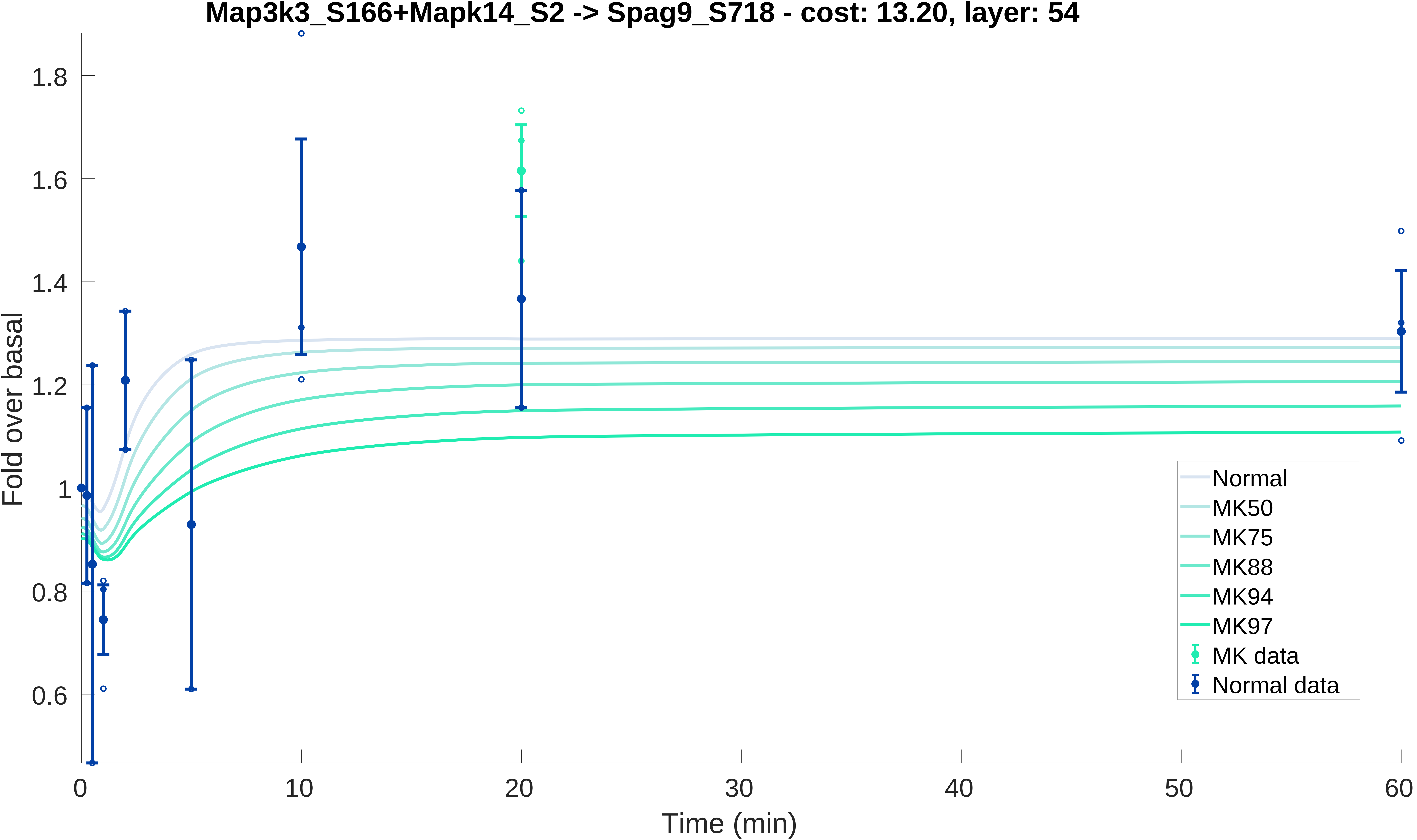

Map3k3_S166+Mapk14_S2 -> Spag9_S718 - cost: 13.20, layer: 54
Fold over basal
1.8
1.6
1.4
1.2
1
0.8
0.6
0
10
20
30
40
50
60
Time (min)
Normal
MK50
MK75
MK88
MK94
MK97
MK data
Normal data

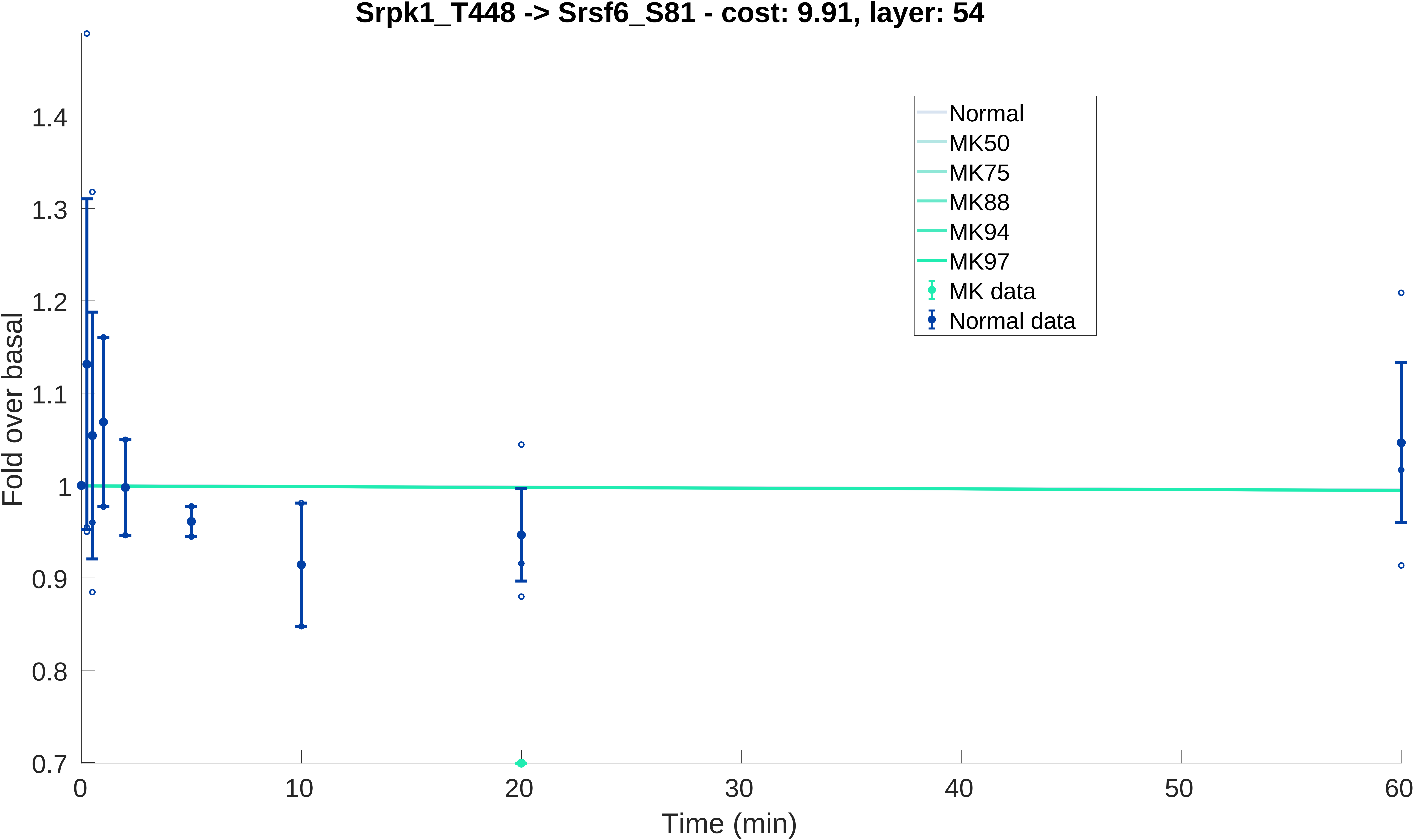

Srpk1_T448 -> Srsf6_S81 - cost: 9.91, layer: 54
Fold over basal
Time (min)
0.7
0.8
0.9
1
1.1
1.2
1.3
1.4
0
10
20
30
40
50
60
Normal
MK50
MK75
MK88
MK94
MK97
MK data
Normal data

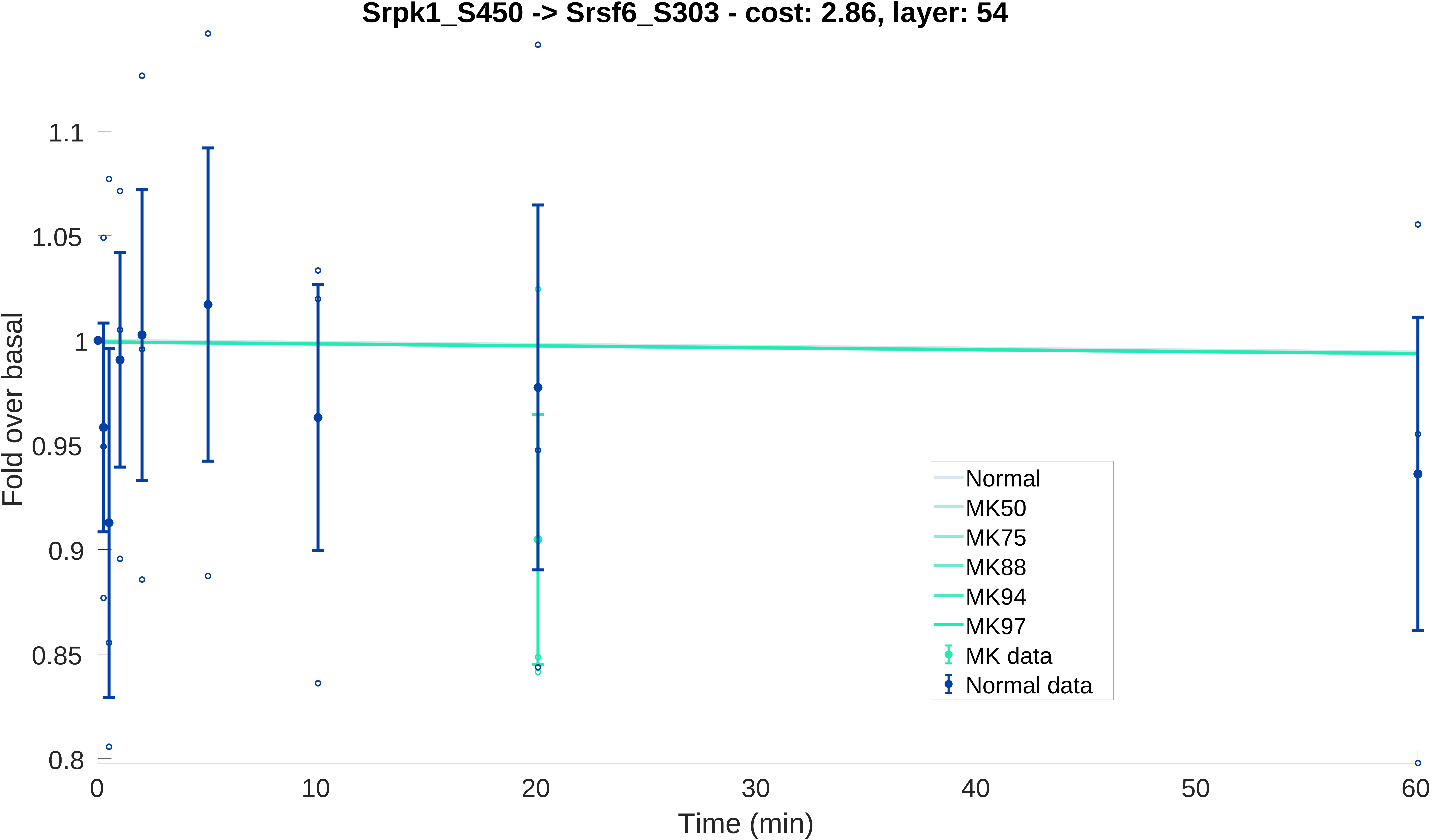

Srpk1_S450 -> Srsf6_S303 - cost: 2.86, layer: 54
Fold over basal
Time (min)
0.8
0.85
0.9
0.95
1
1.05
1.1
0
10
20
30
40
50
60
Normal
MK50
MK75
MK88
MK94
MK97
MK data
Normal data

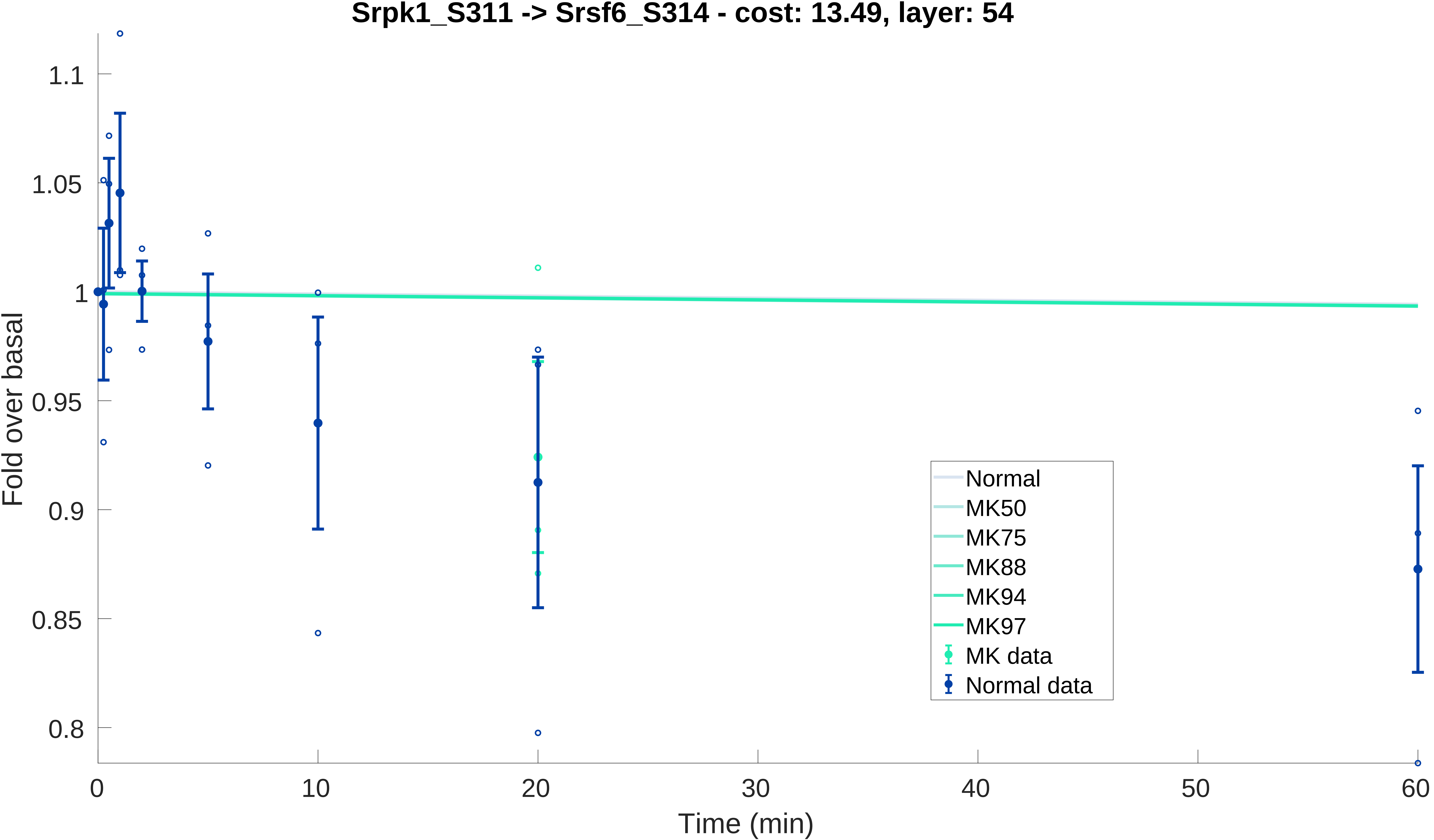
Srpk1_S311 -> Srsf6_S314 - cost: 13.49, layer: 54
Fold over basal
Time (min)
1.1
1.05
1
0.95
0.9
0.85
0.8
0
10
20
30
40
50
60
Normal
MK50
MK75
MK88
MK94
MK97
MK data
Normal data

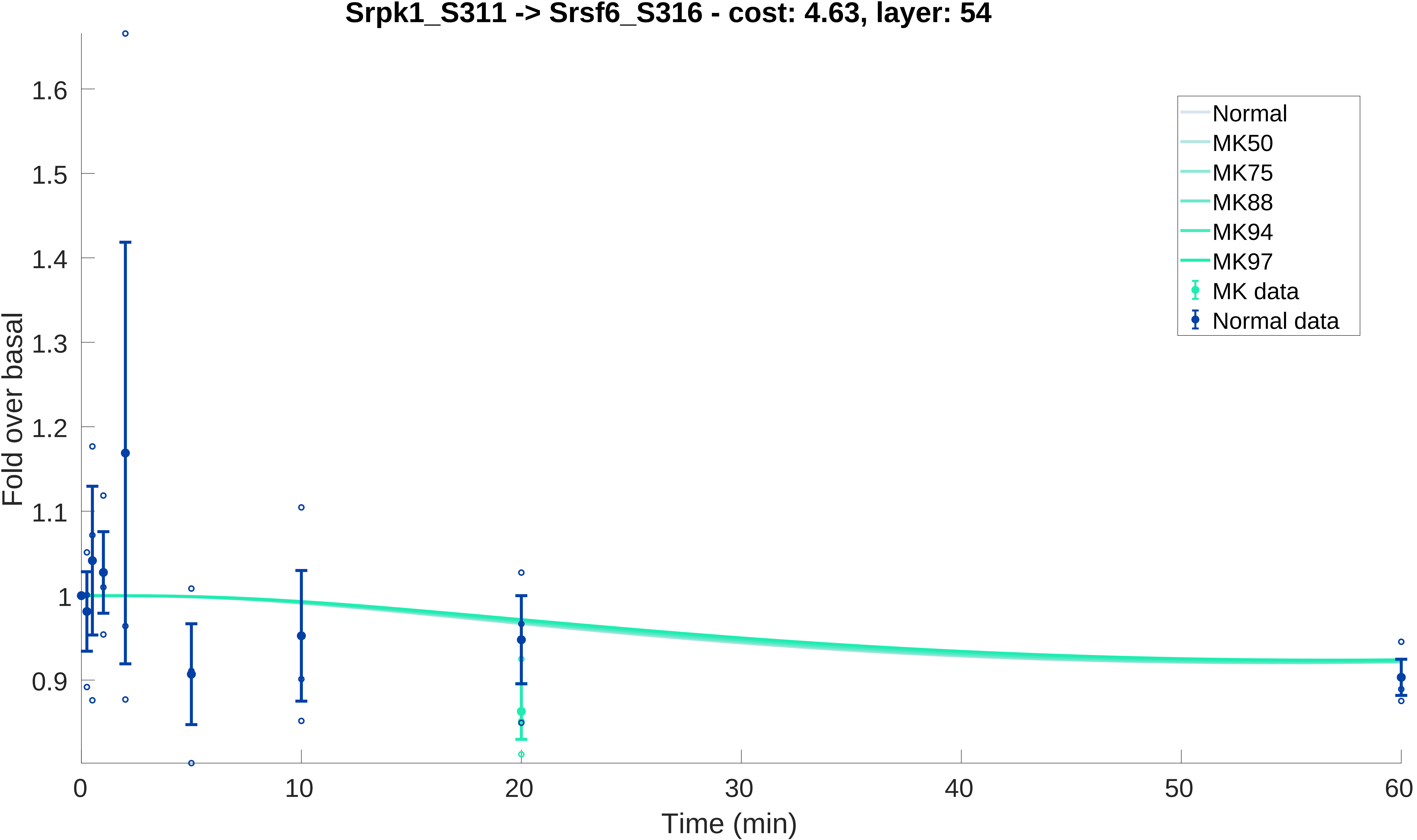
Srpk1_S311 -> Srsf6_S316 - cost: 4.63, layer: 54
Fold over basal
Time (min)
Normal
MK50
MK75
MK88
MK94
MK97
MK data
Normal data
0.9
1
1.1
1.2
1.3
1.4
1.5
1.6
0
10
20
30
40
50
60

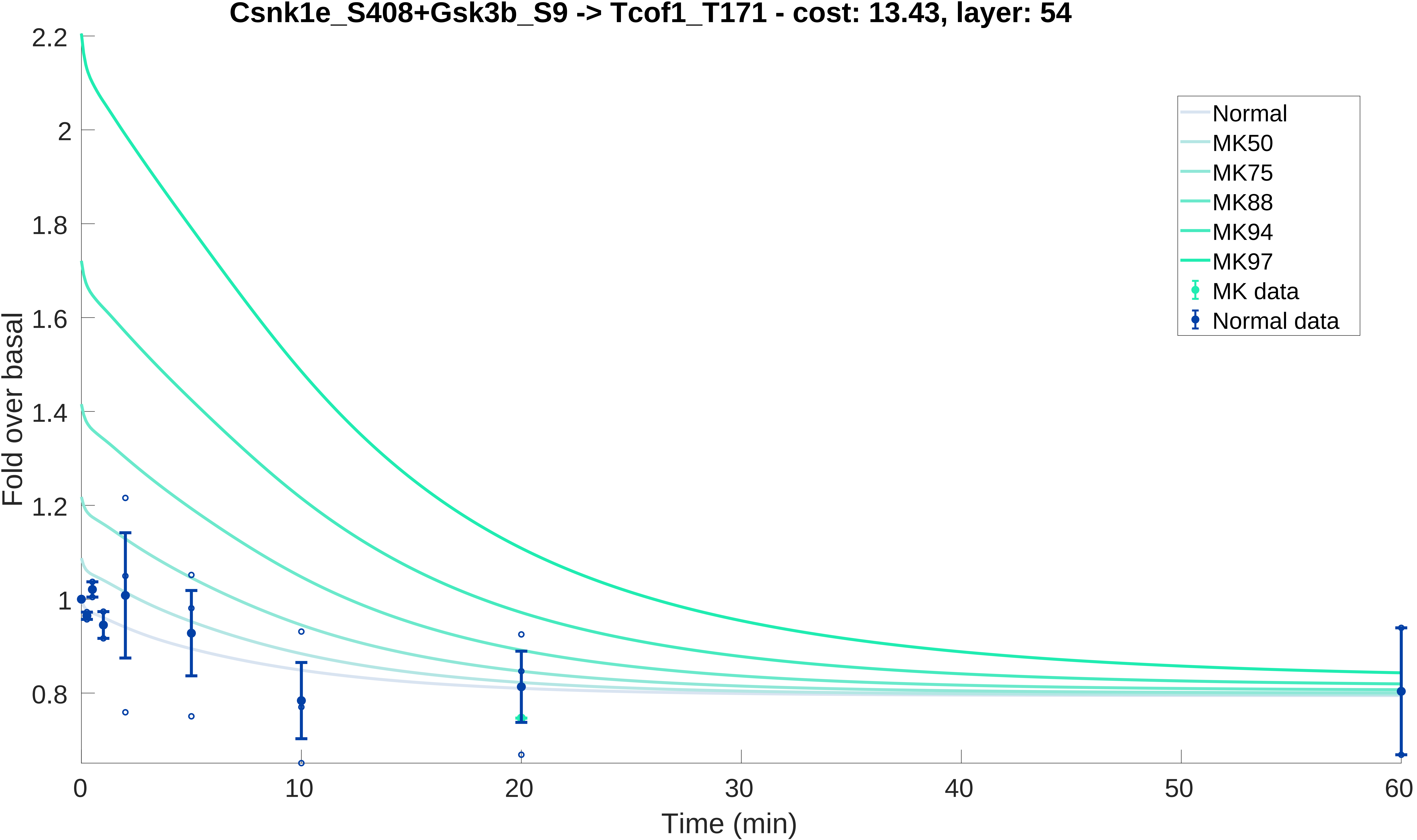

Csnk1e_S408+Gsk3b_S9 -> Tcof1_T171 - cost: 13.43, layer: 54
Fold over basal
Time (min)
2.2
2
1.8
1.6
1.4
1.2
1
0.8
0
10
20
30
40
50
60
Normal
MK50
MK75
MK88
MK94
MK97
MK data
Normal data

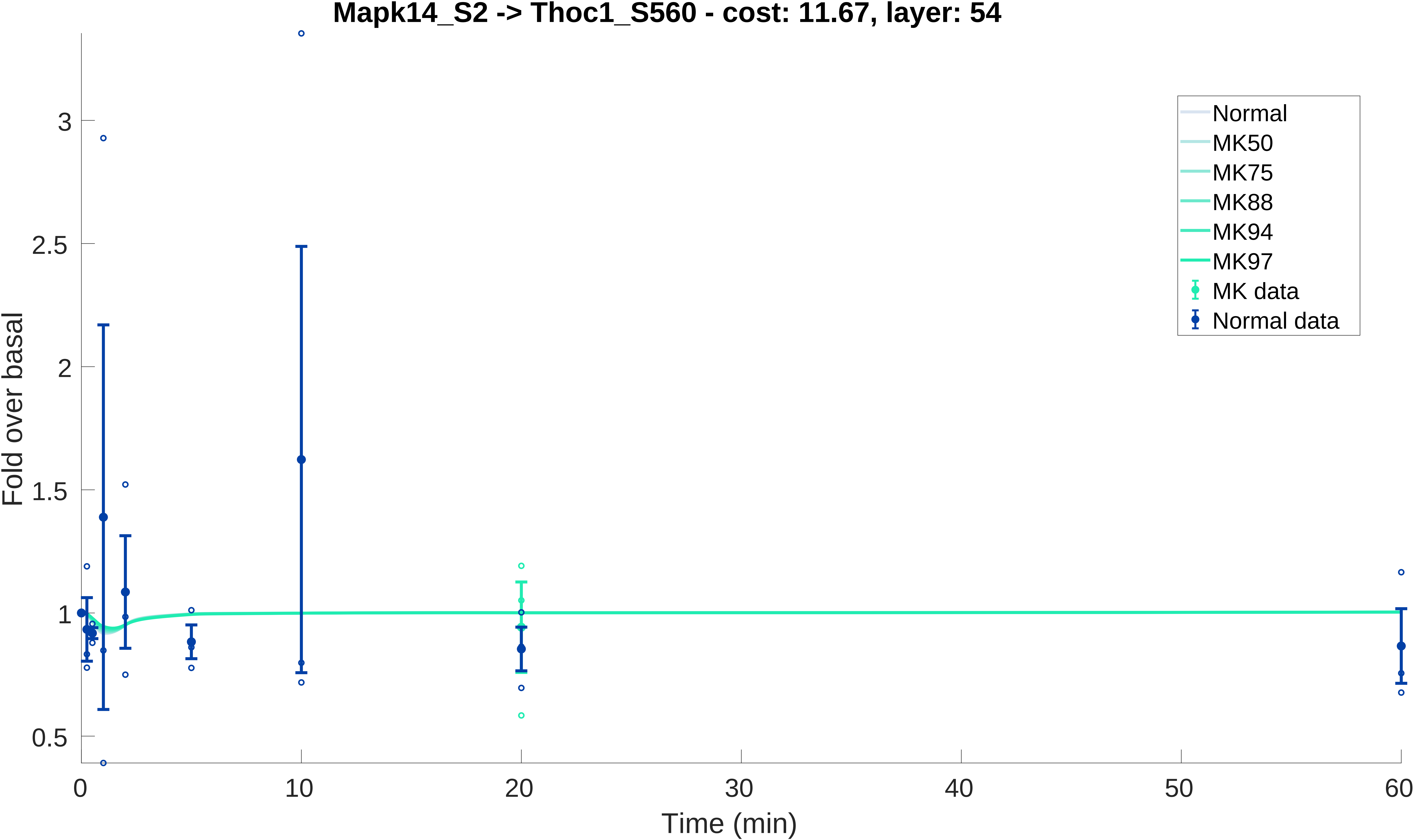

Mapk14_S2 -> Thoc1_S560 - cost: 11.67, layer: 54
Fold over basal
Time (min)
Normal
MK50
MK75
MK88
MK94
MK97
MK data
Normal data
0.5
1
1.5
2
2.5
3
0
10
20
30
40
50
60

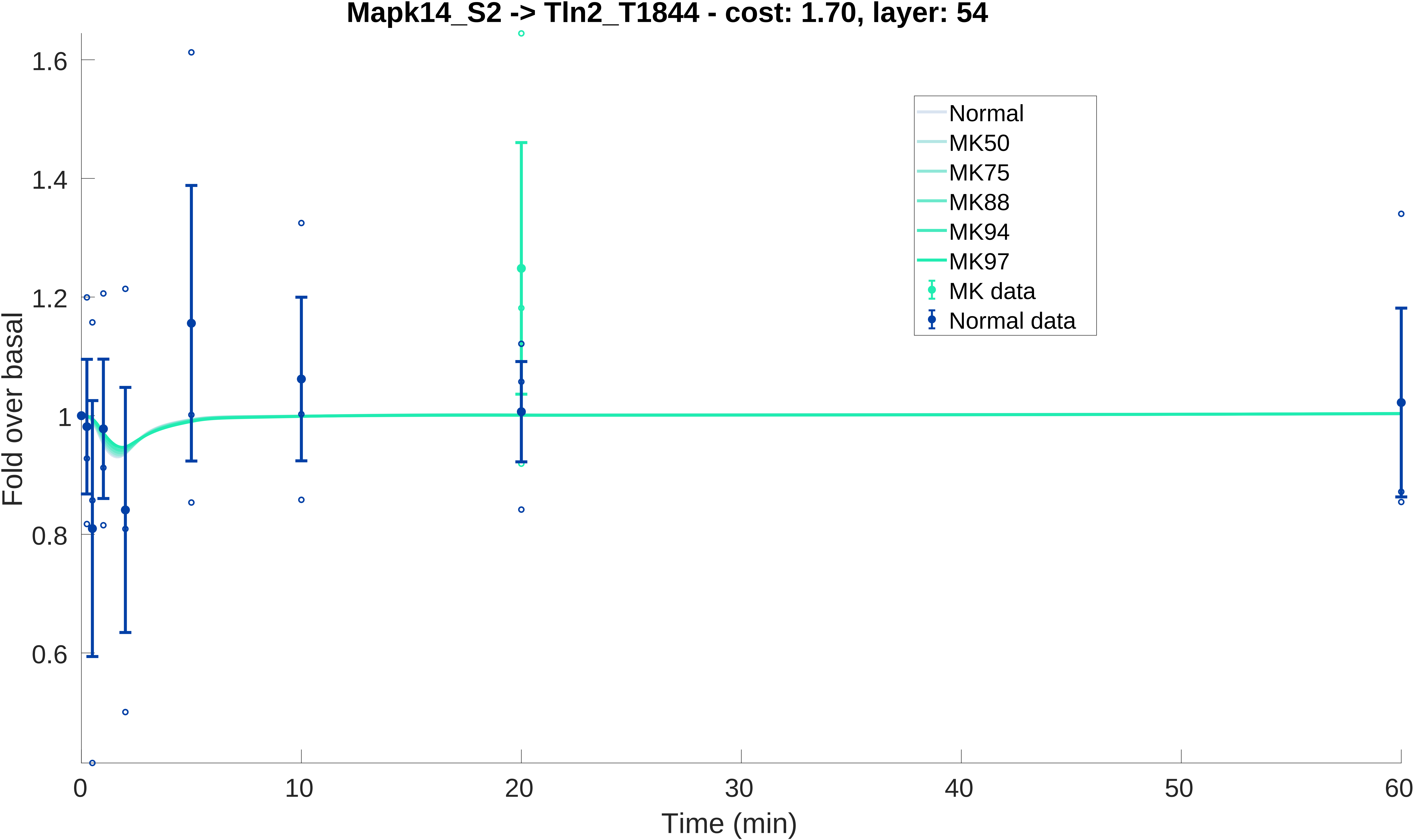

Mapk14_S2 -> Tln2_T1844 - cost: 1.70, layer: 54
Fold over basal
Time (min)
0.6
0.8
1
1.2
1.4
1.6
0
10
20
30
40
50
60
Normal
MK50
MK75
MK88
MK94
MK97
MK data
Normal data

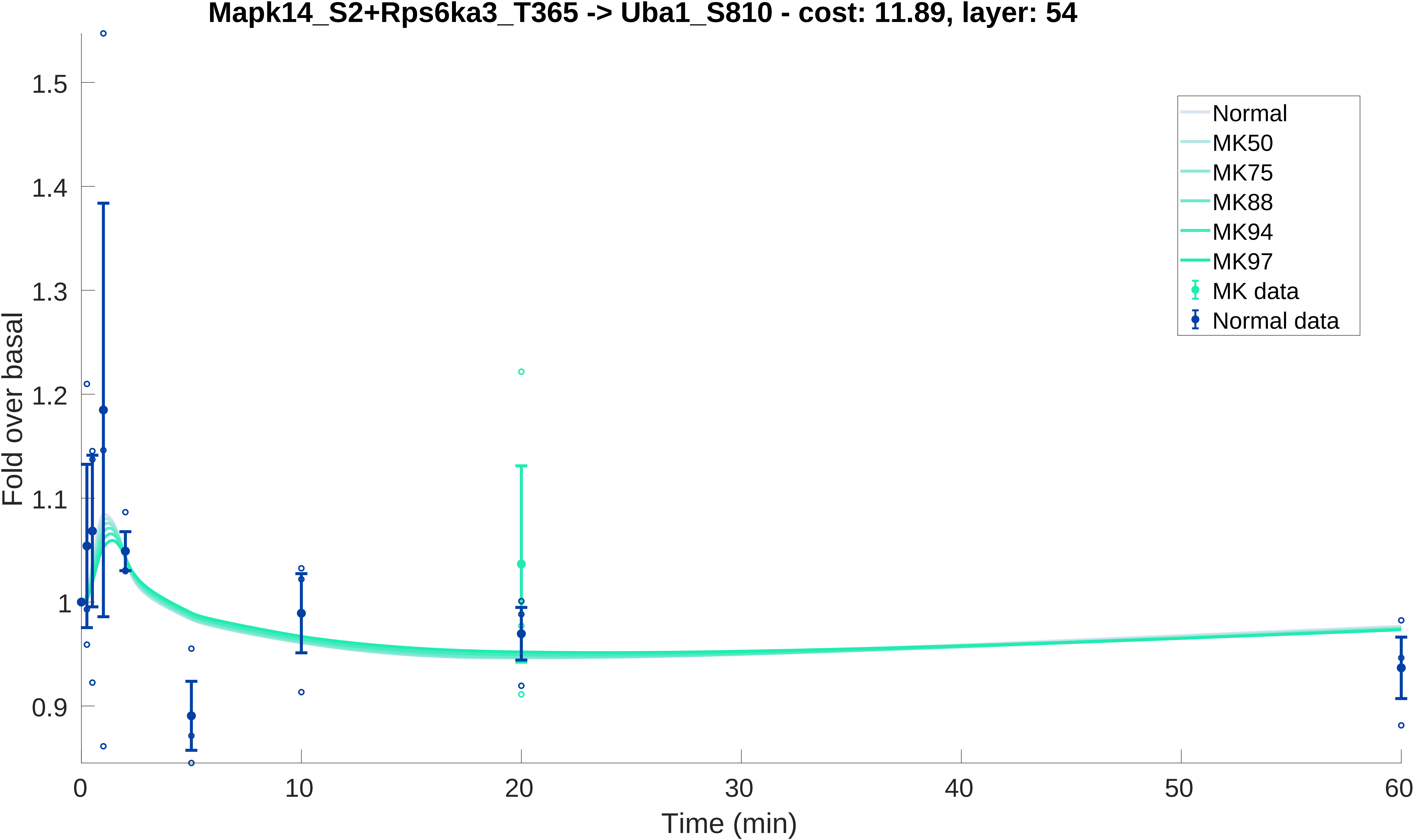

Mapk14_S2+Rps6ka3_T365 -> Uba1_S810 - cost: 11.89, layer: 54
Fold over basal
Time (min)
1.5
1.4
1.3
1.2
1.1
1
0.9
0
10
20
30
40
50
60
Normal
MK50
MK75
MK88
MK94
MK97
MK data
Normal data

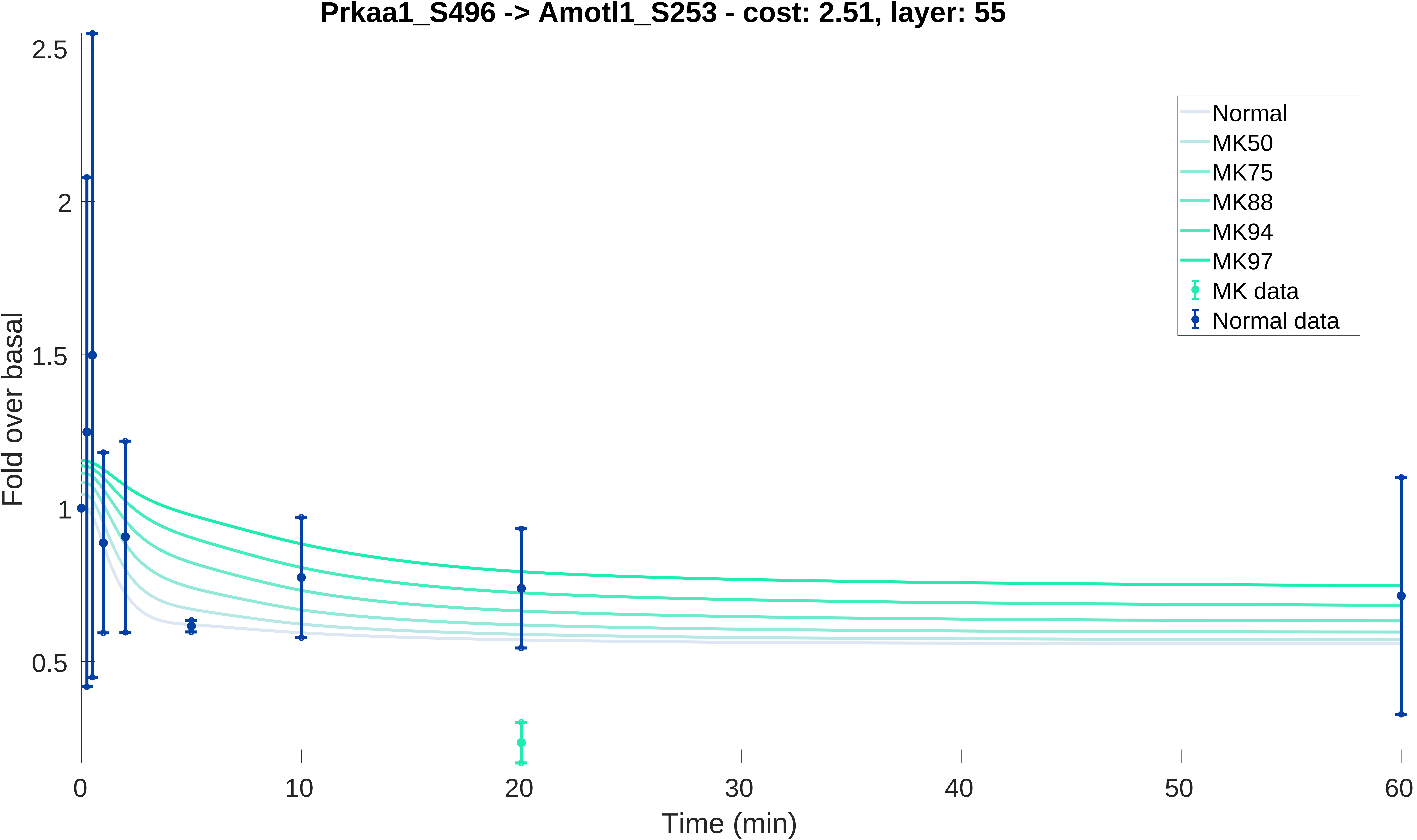

Prkaa1_S496 -> Amotl1_S253 - cost: 2.51, layer: 55
Fold over basal
Time (min)
0
10
20
30
40
50
60
0.5
1
1.5
2
2.5
Normal
MK50
MK75
MK88
MK94
MK97
MK data
Normal data

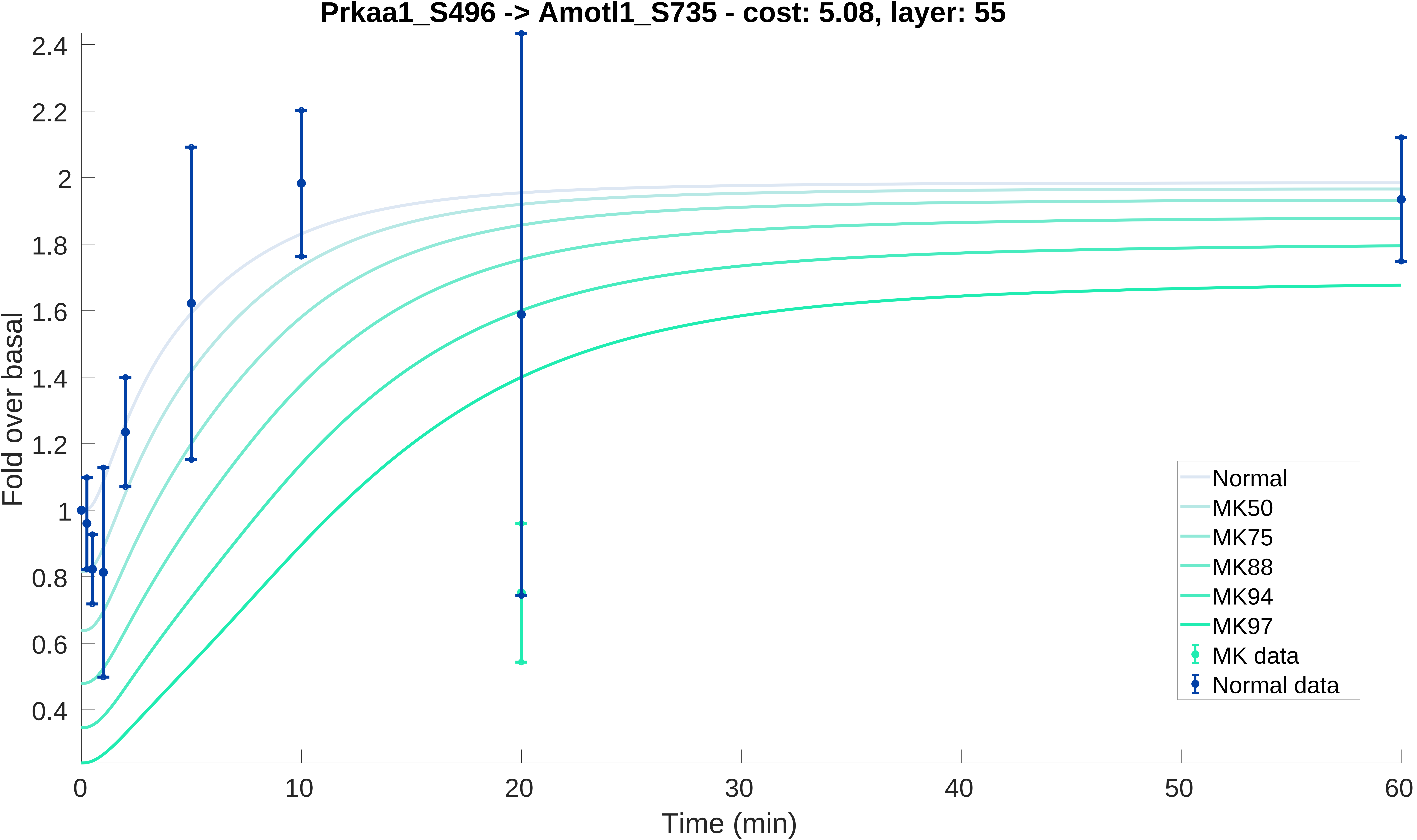

Prkaa1_S496 -> Amotl1_S735 - cost: 5.08, layer: 55
Fold over basal
Time (min)
0.4
0.6
0.8
1
1.2
1.4
1.6
1.8
2
2.2
2.4
0
10
20
30
40
50
60
Normal
MK50
MK75
MK88
MK94
MK97
MK data
Normal data

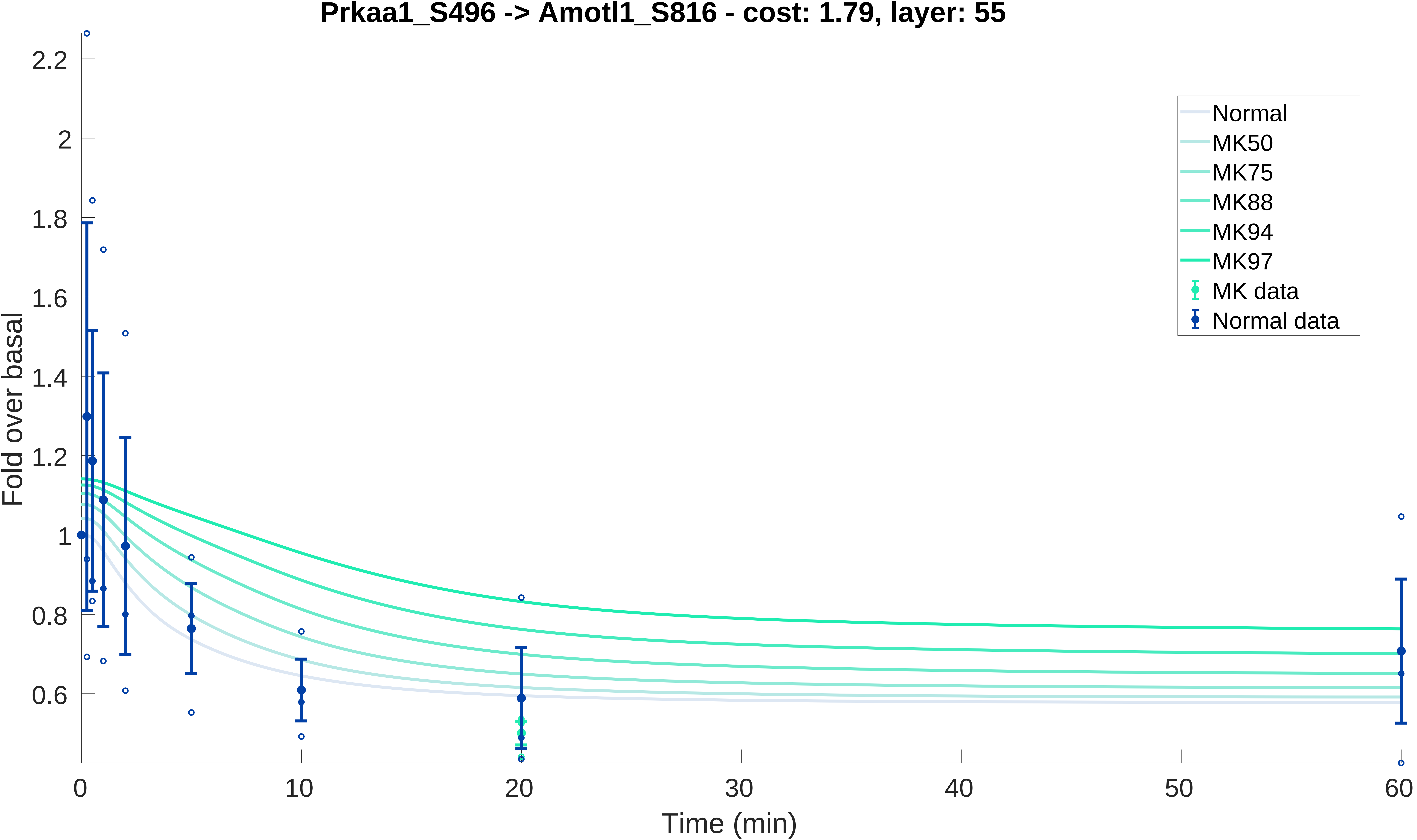
Prkaa1_S496 -> Amotl1_S816 - cost: 1.79, layer: 55
Fold over basal
Time (min)
0
10
20
30
40
50
60
0.6
0.8
1
1.2
1.4
1.6
1.8
2
2.2
Normal
MK50
MK75
MK88
MK94
MK97
MK data
Normal data

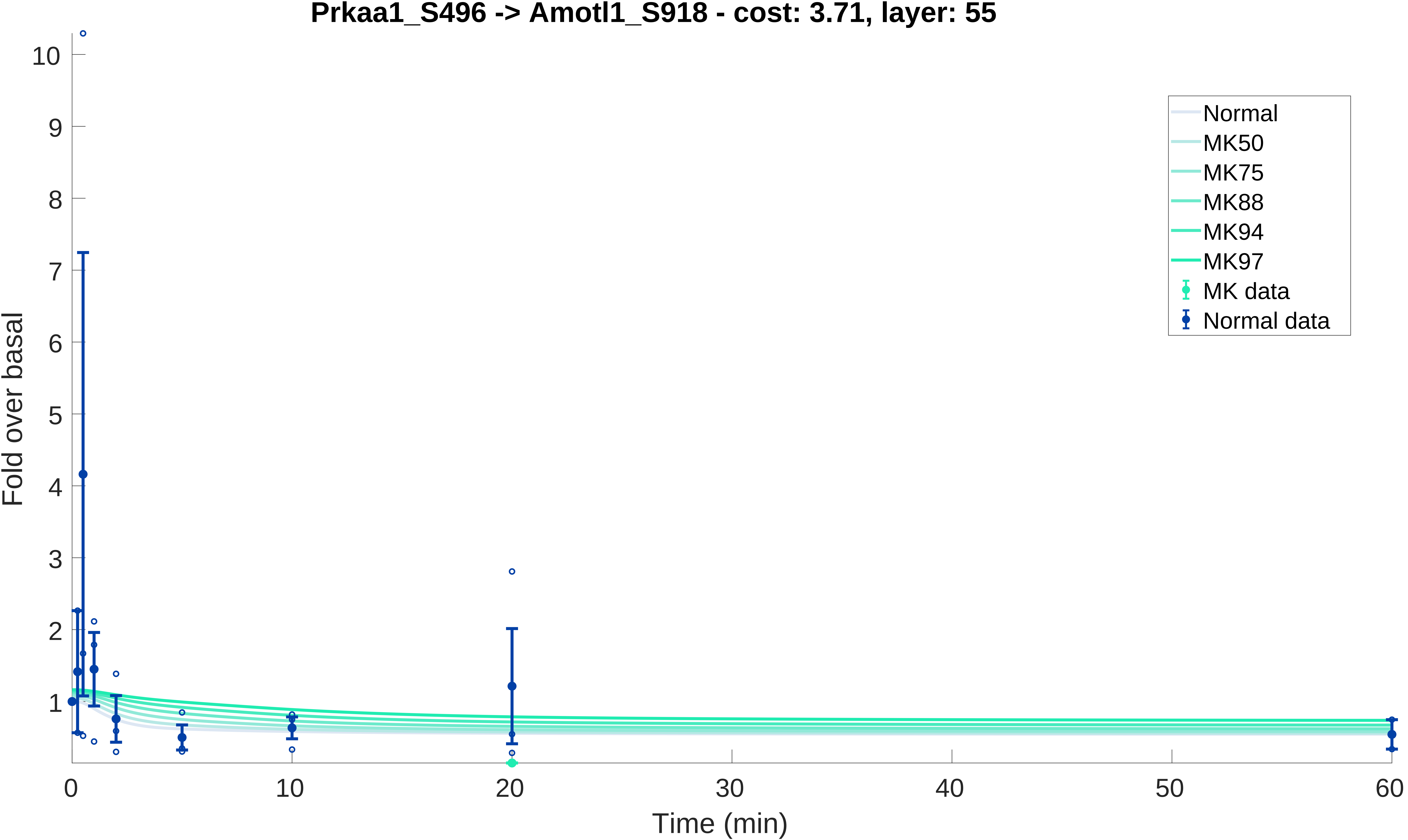

Prkaa1_S496 -> Amotl1_S918 - cost: 3.71, layer: 55
Fold over basal
Time (min)
0
10
20
30
40
50
60
1
2
3
4
5
6
7
8
9
10
Normal
MK50
MK75
MK88
MK94
MK97
MK data
Normal data

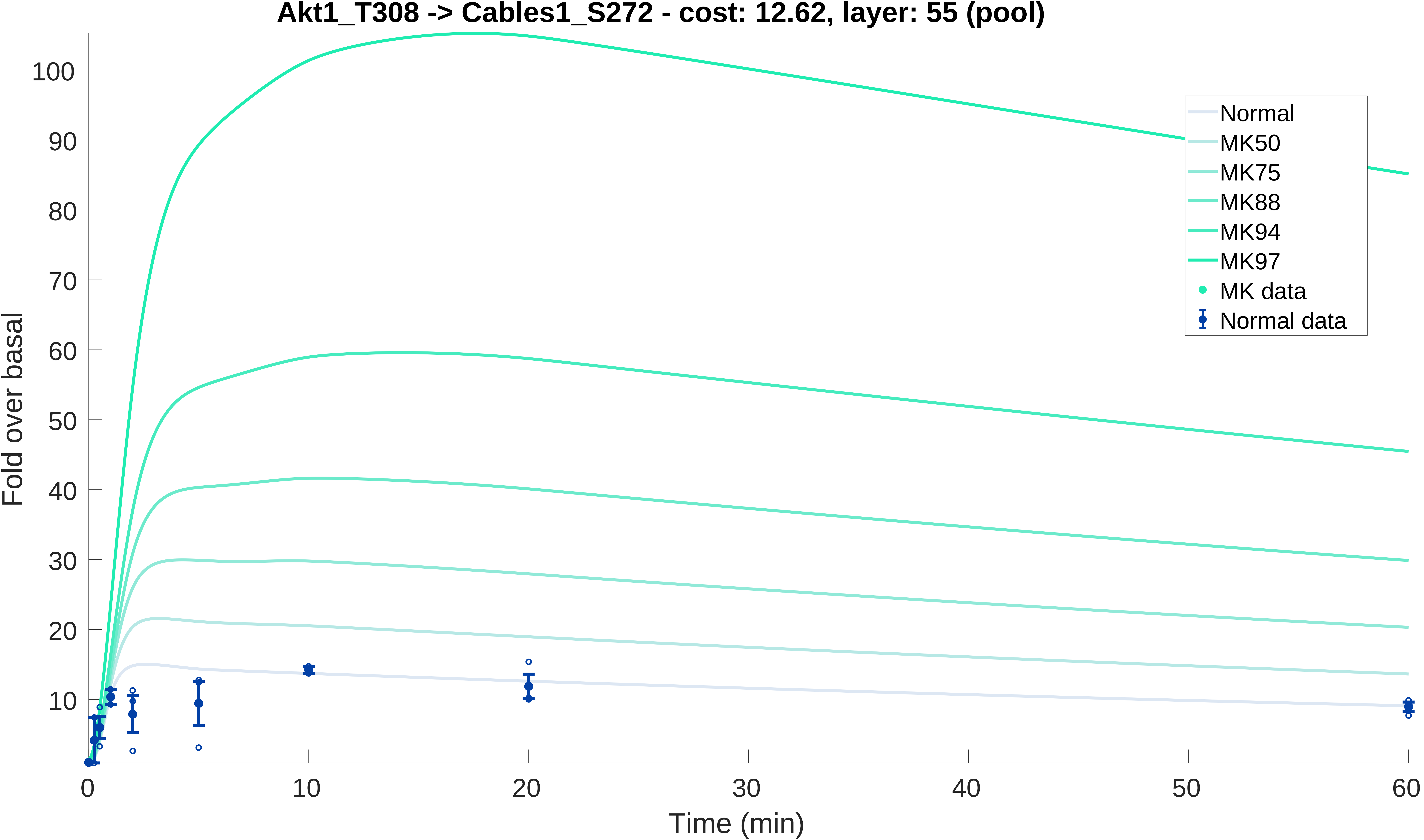
Akt1_T308 -> Cables1_S272 - cost: 12.62, layer: 55 (pool)
Fold over basal
100
90
80
70
60
50
40
30
20
10
0
10
20
30
40
50
60
Time (min)
Normal
MK50
MK75
MK88
MK94
MK97
MK data
Normal data

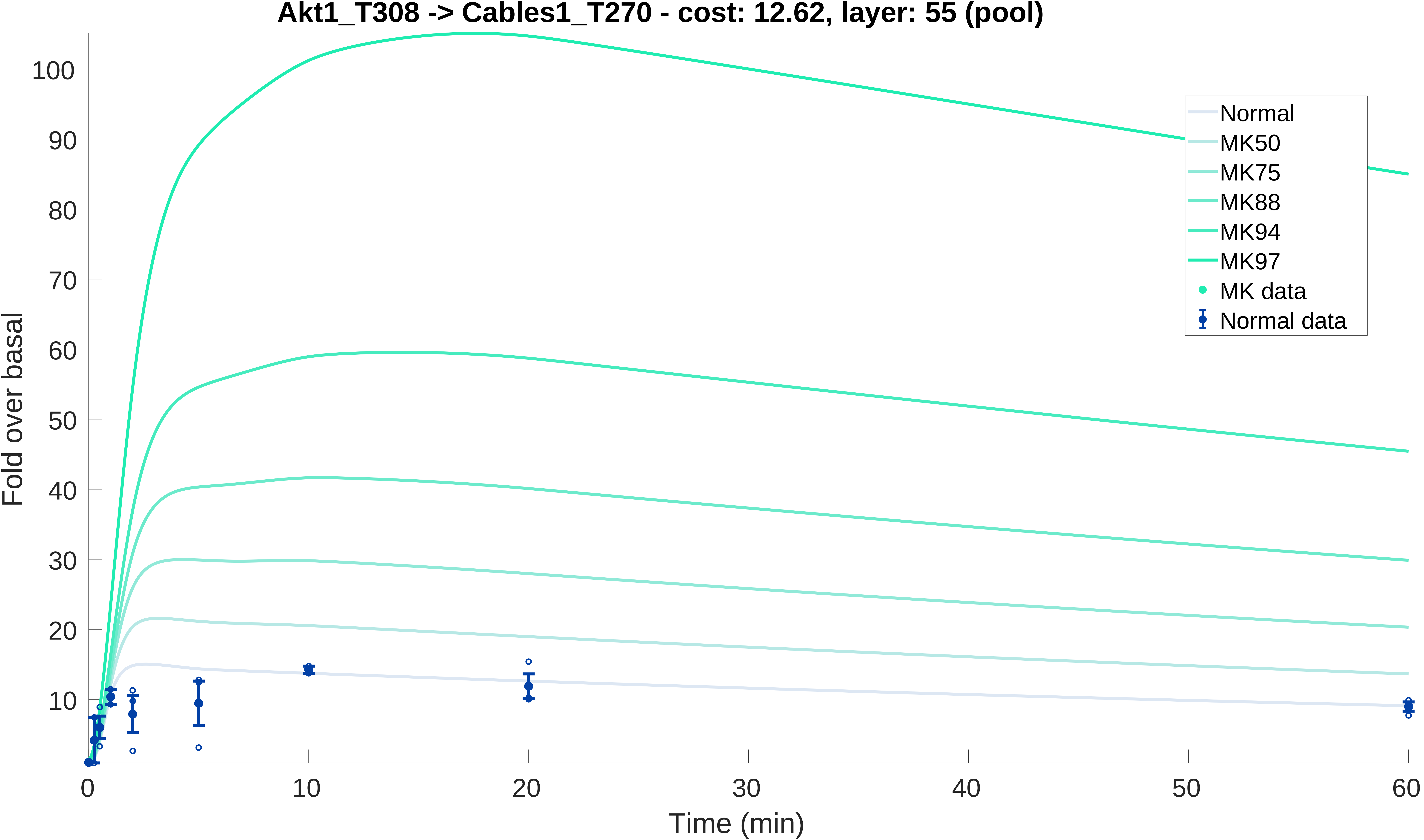
Akt1_T308 -> Cables1_T270 - cost: 12.62, layer: 55 (pool)
Fold over basal
Time (min)
0
10
20
30
40
50
60
100
90
80
70
60
50
40
30
20
10
Normal
MK50
MK75
MK88
MK94
MK97
MK data
Normal data

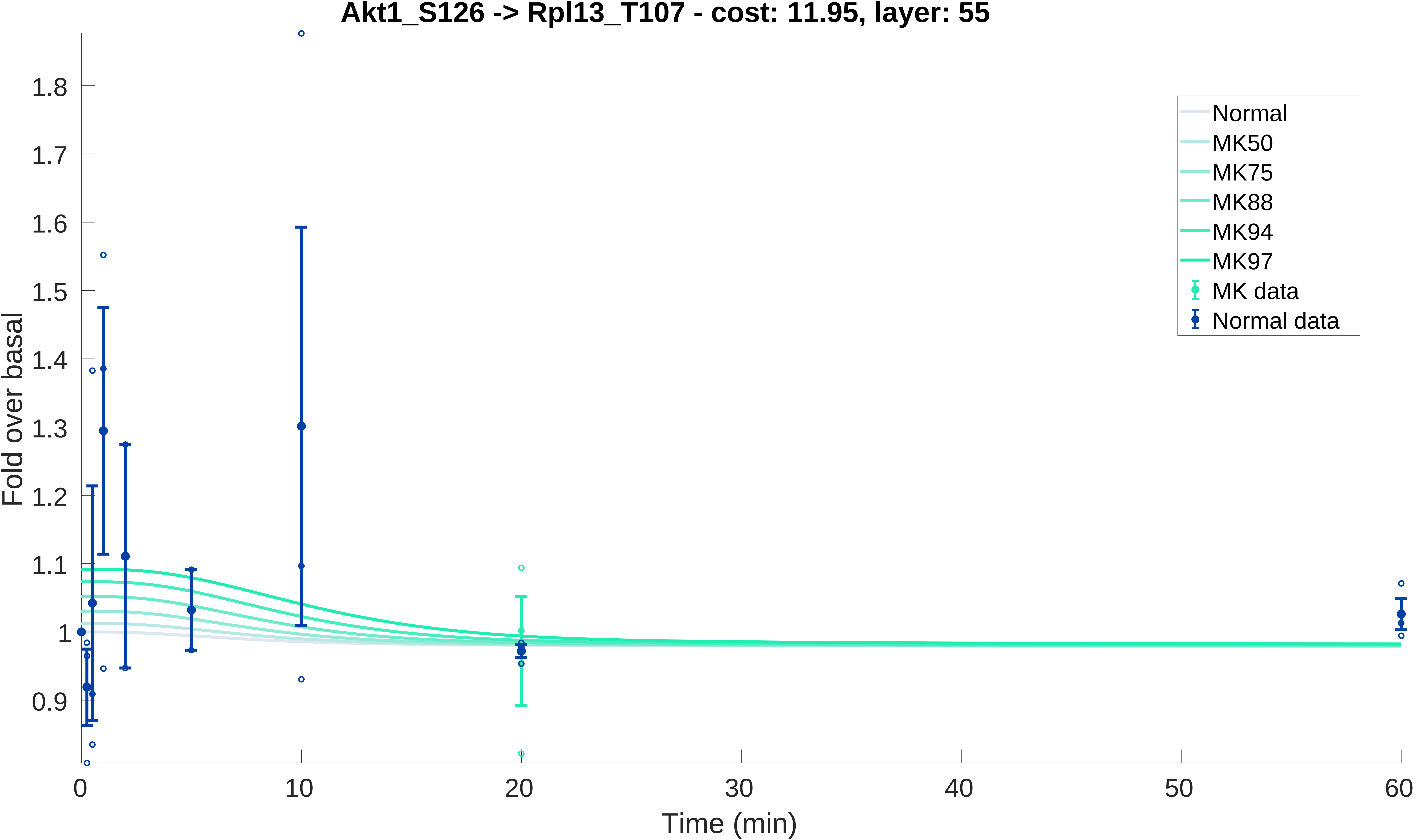

Akt1_S126 -> Rpl13_T107 - cost: 11.95, layer: 55
Fold over basal
1.8
1.7
1.6
1.5
1.4
1.3
1.2
1.1
1
0.9
0
10
20
30
40
50
60
Time (min)
Normal
MK50
MK75
MK88
MK94
MK97
MK data
Normal data

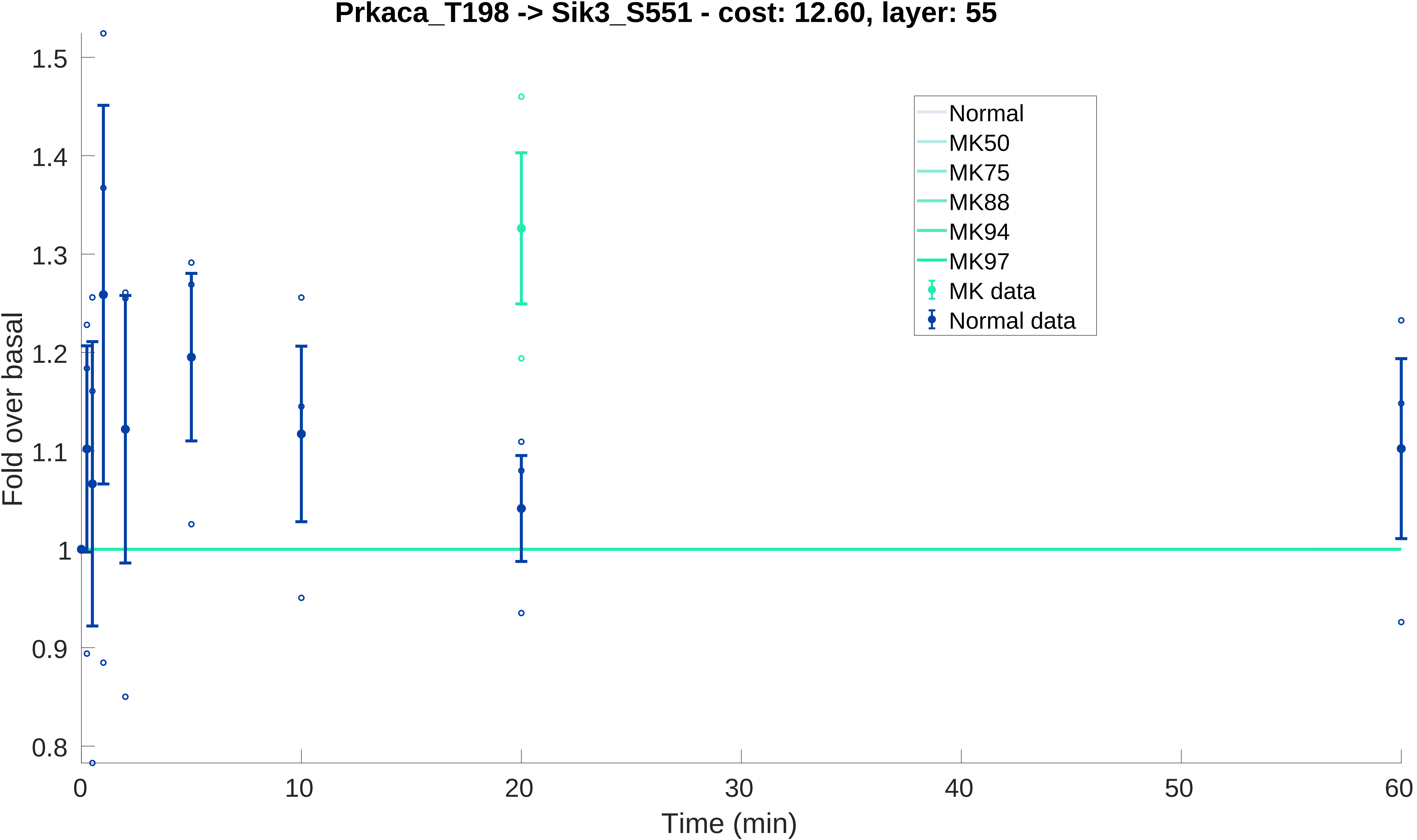

Prkaca_T198 -> Sik3_S551 - cost: 12.60, layer: 55
Fold over basal
Time (min)
0.8
0.9
1
1.1
1.2
1.3
1.4
1.5
0
10
20
30
40
50
60
Normal
MK50
MK75
MK88
MK94
MK97
MK data
Normal data

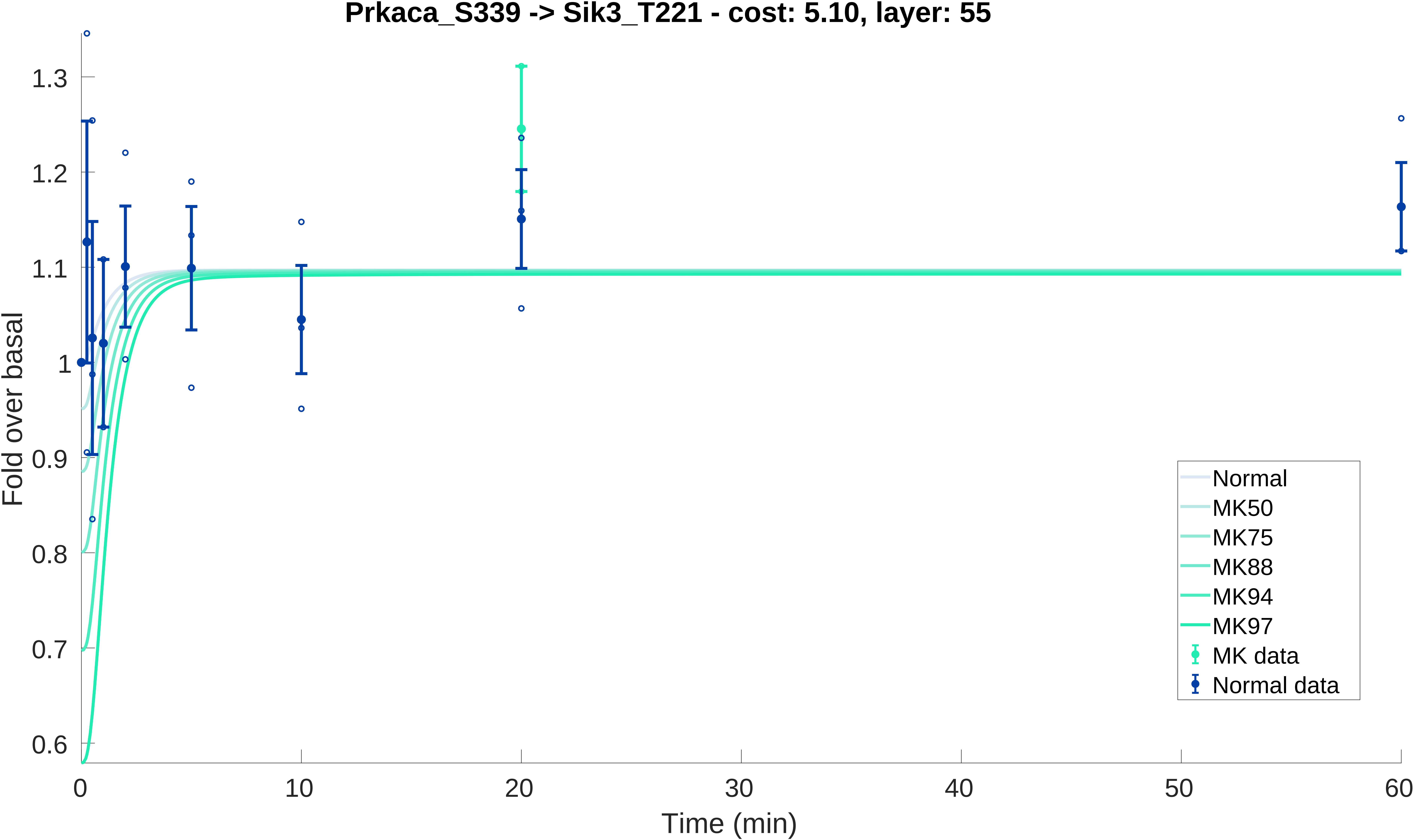

Prkaca_S339 -> Sik3_T221 - cost: 5.10, layer: 55
Fold over basal
Time (min)
0.6
0.7
0.8
0.9
1
1.1
1.2
1.3
0
10
20
30
40
50
60
Normal
MK50
MK75
MK88
MK94
MK97
MK data
Normal data

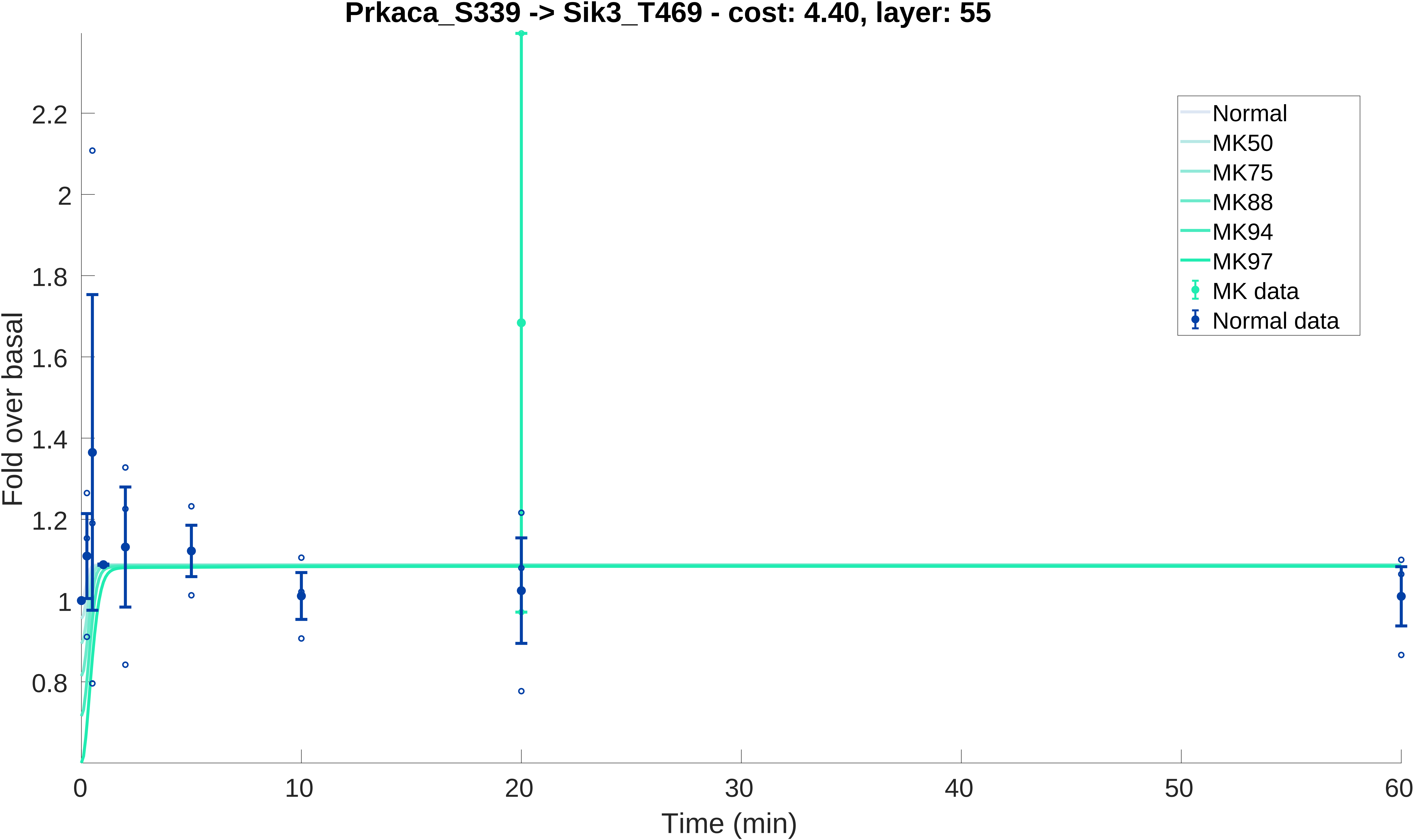

Prkaca_S339 -> Sik3_T469 - cost: 4.40, layer: 55
Fold over basal
Time (min)
0.8
1
1.2
1.4
1.6
1.8
2
2.2
0
10
20
30
40
50
60
Normal
MK50
MK75
MK88
MK94
MK97
MK data
Normal data

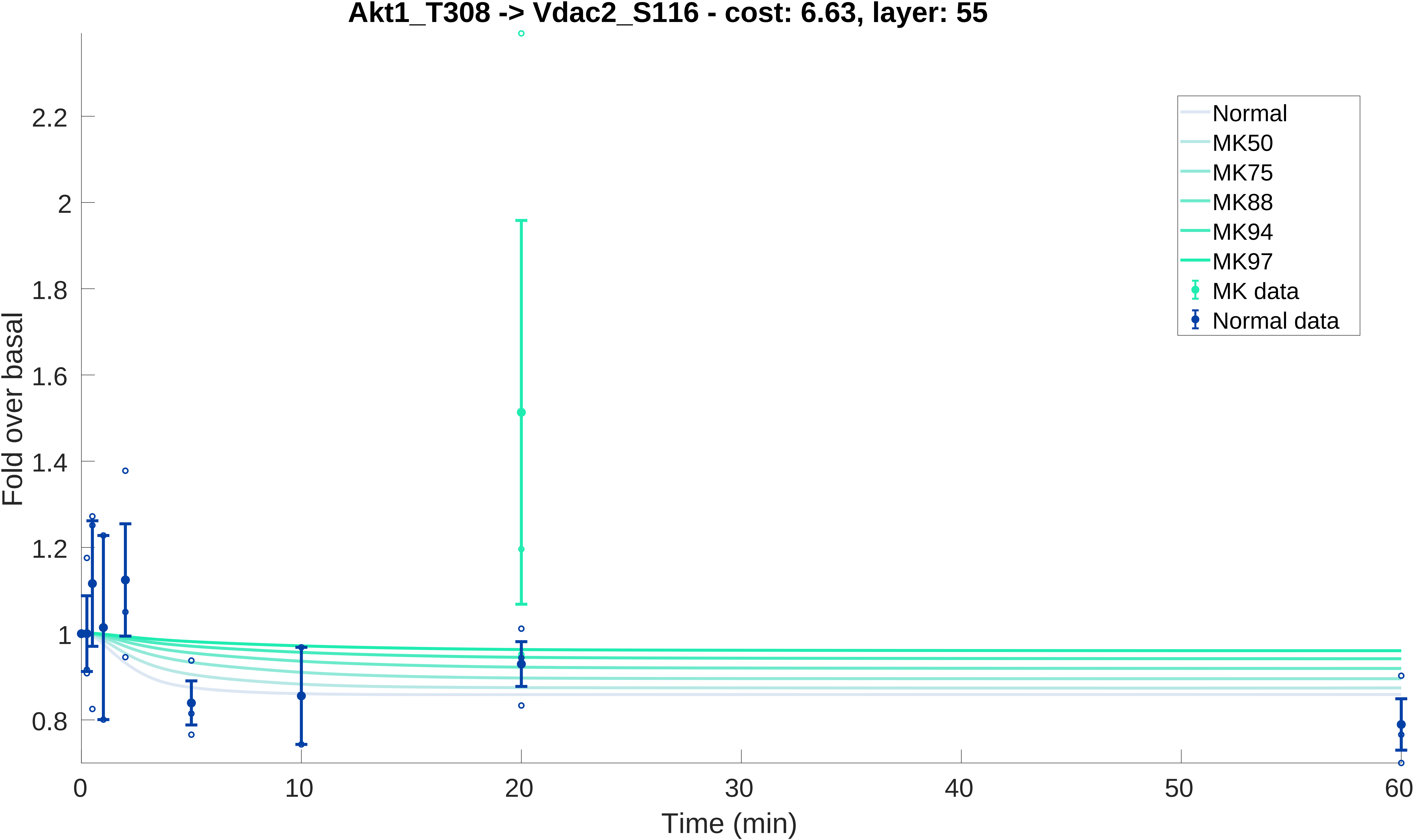
Akt1_T308 -> Vdac2_S116 - cost: 6.63, layer: 55
Fold over basal
Time (min)
Normal
MK50
MK75
MK88
MK94
MK97
MK data
Normal data
2.2
2
1.8
1.6
1.4
1.2
1
0.8
0
10
20
30
40
50
60

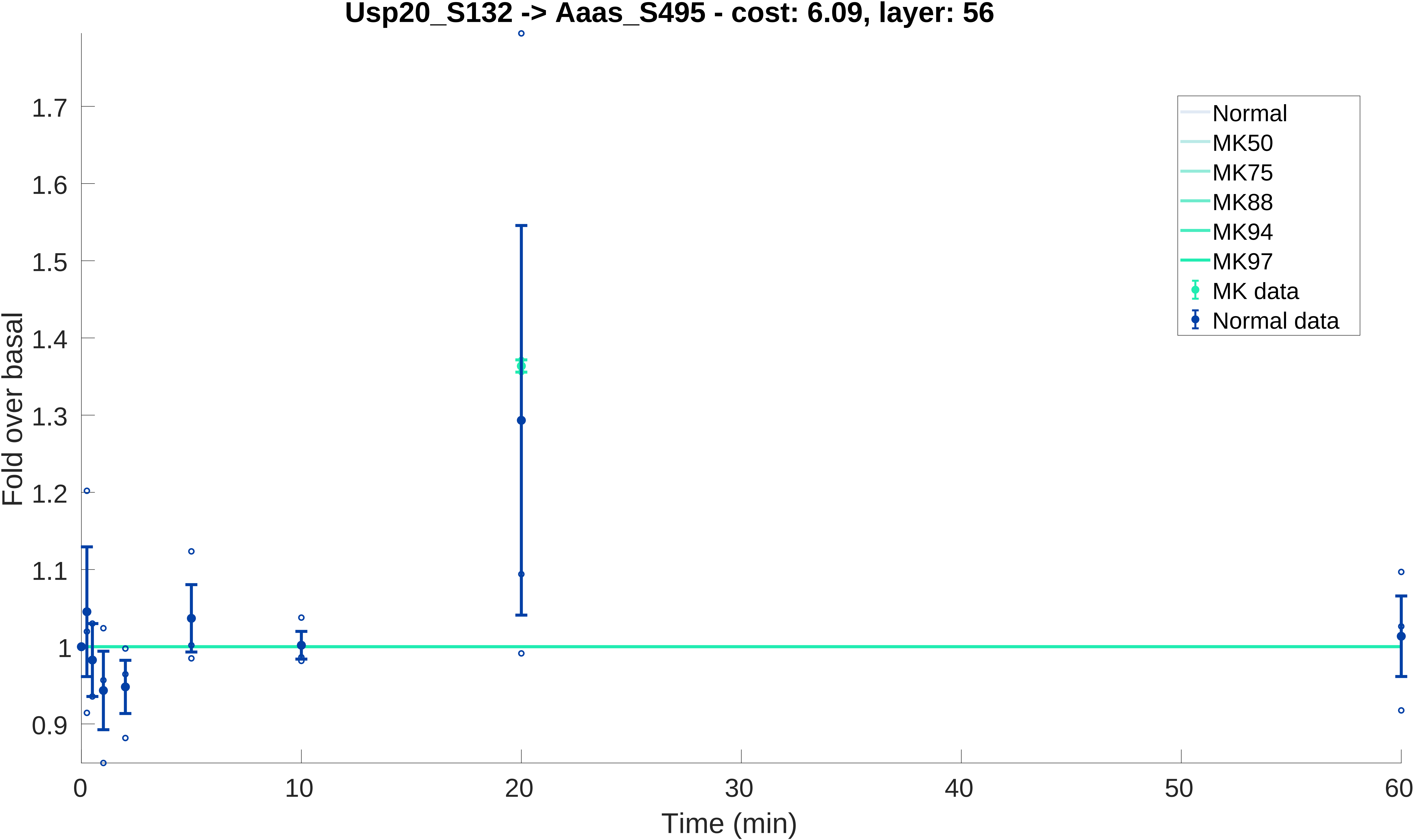

Usp20_S132 -> Aaas_S495 - cost: 6.09, layer: 56
Fold over basal
1.7
1.6
1.5
1.4
1.3
1.2
1.1
1
0.9
0
10
20
30
40
50
60
Time (min)
Normal
MK50
MK75
MK88
MK94
MK97
MK data
Normal data

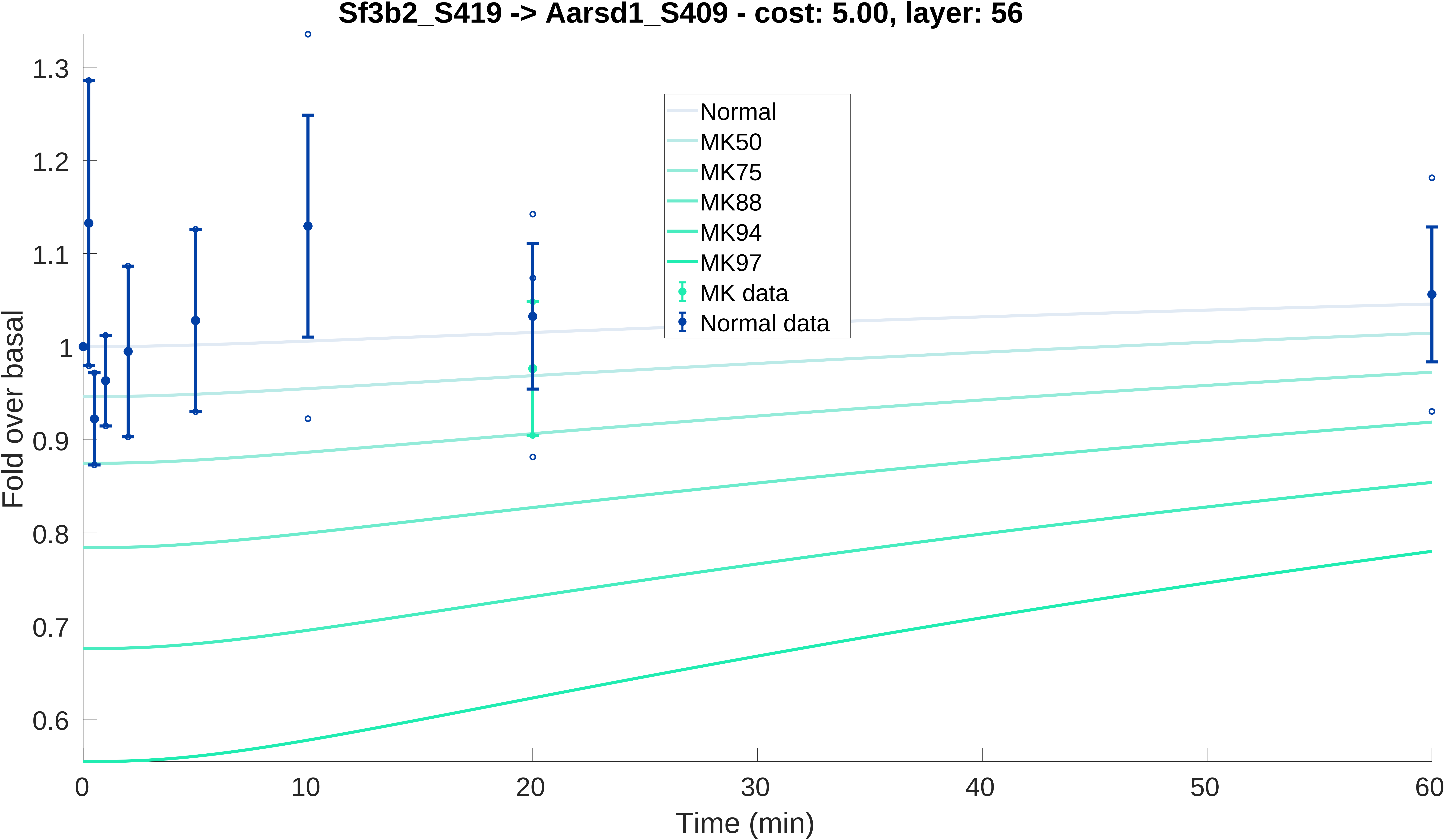

Sf3b2_S419 -> Aarsd1_S409 - cost: 5.00, layer: 56
Fold over basal
Time (min)
1.3
1.2
1.1
1
0.9
0.8
0.7
0.6
0
10
20
30
40
50
60
Normal
MK50
MK75
MK88
MK94
MK97
MK data
Normal data

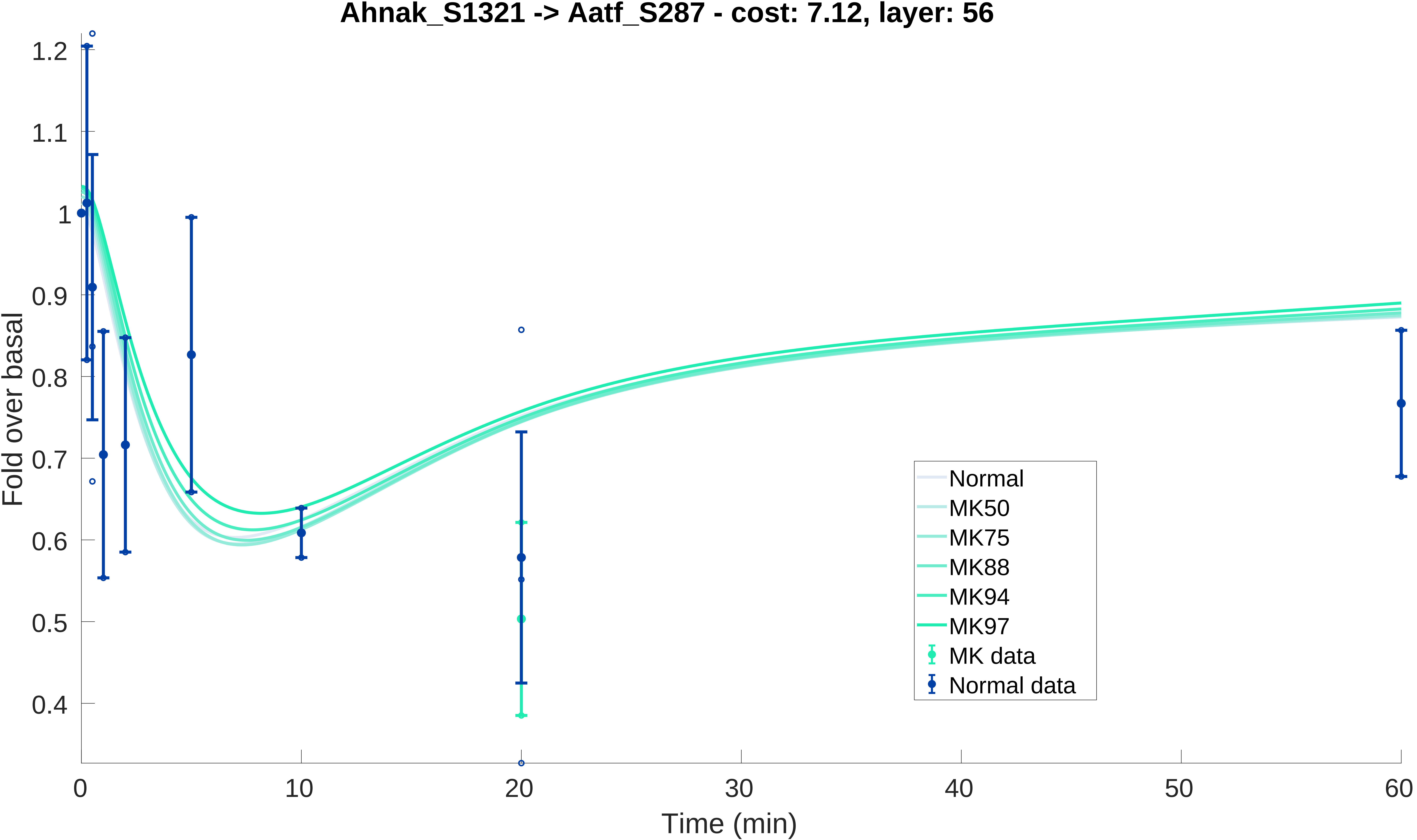

Ahnak_S1321 -> Aatf_S287 - cost: 7.12, layer: 56
Fold over basal
Time (min)
0
10
20
30
40
50
60
0.4
0.5
0.6
0.7
0.8
0.9
1
1.1
1.2
Normal
MK50
MK75
MK88
MK94
MK97
MK data
Normal data

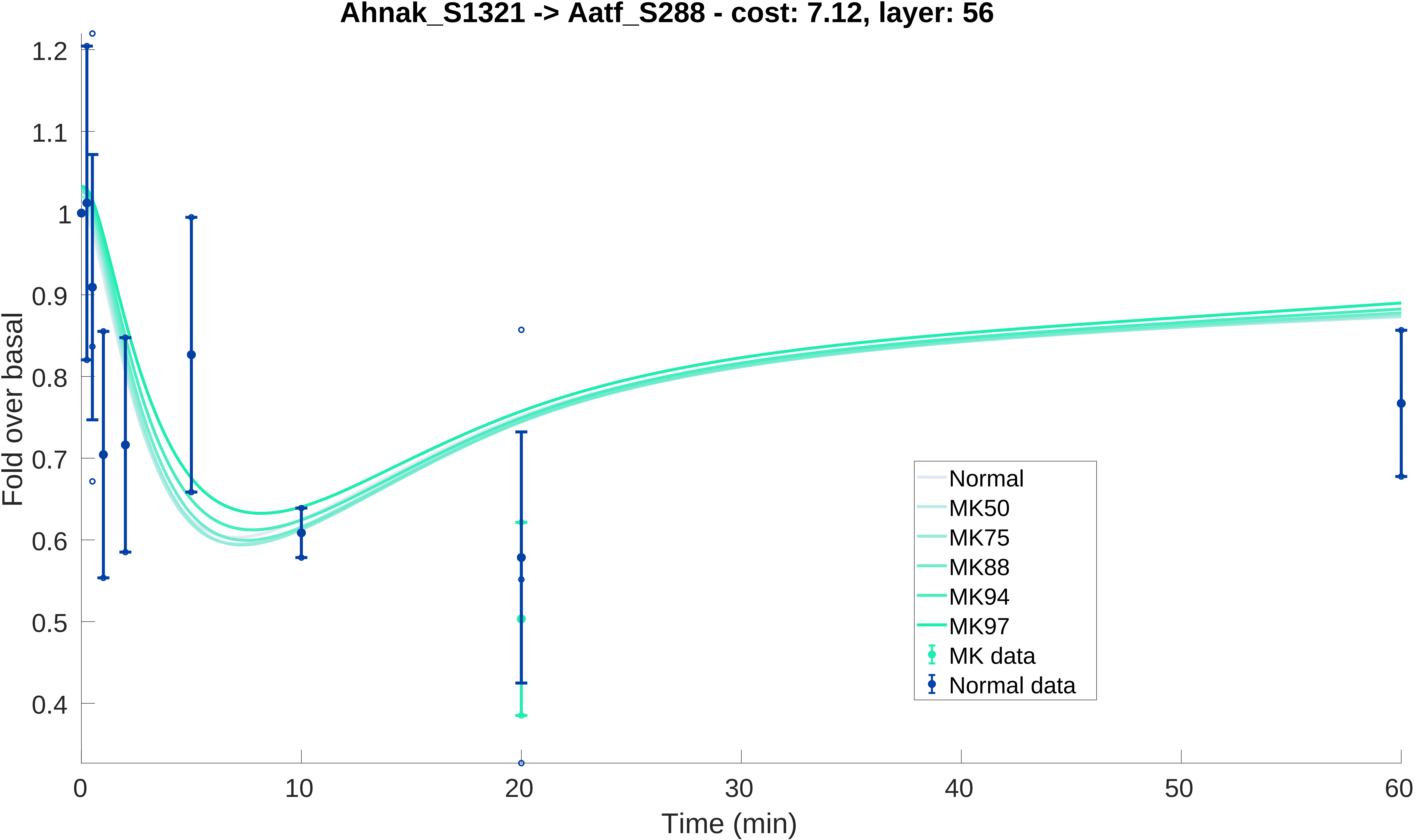
Ahnak_S1321 -> Aatf_S288 - cost: 7.12, layer: 56
Fold over basal
Time (min)
0
10
20
30
40
50
60
0.4
0.5
0.6
0.7
0.8
0.9
1
1.1
1.2
Normal
MK50
MK75
MK88
MK94
MK97
MK data
Normal data

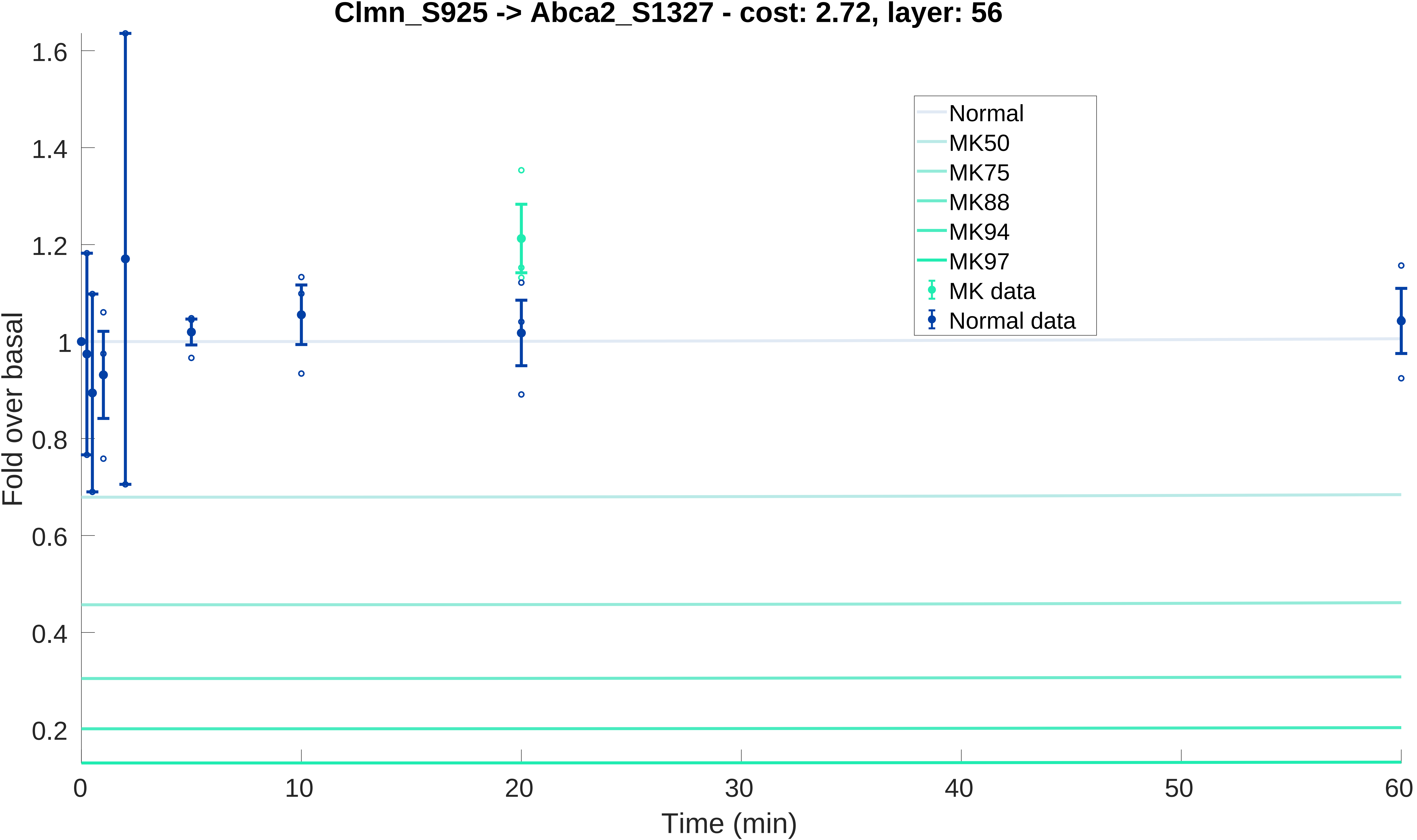

Clmn_S925 -> Abca2_S1327 - cost: 2.72, layer: 56
Fold over basal
Time (min)
0
10
20
30
40
50
60
0.2
0.4
0.6
0.8
1
1.2
1.4
1.6
Normal
MK50
MK75
MK88
MK94
MK97
MK data
Normal data

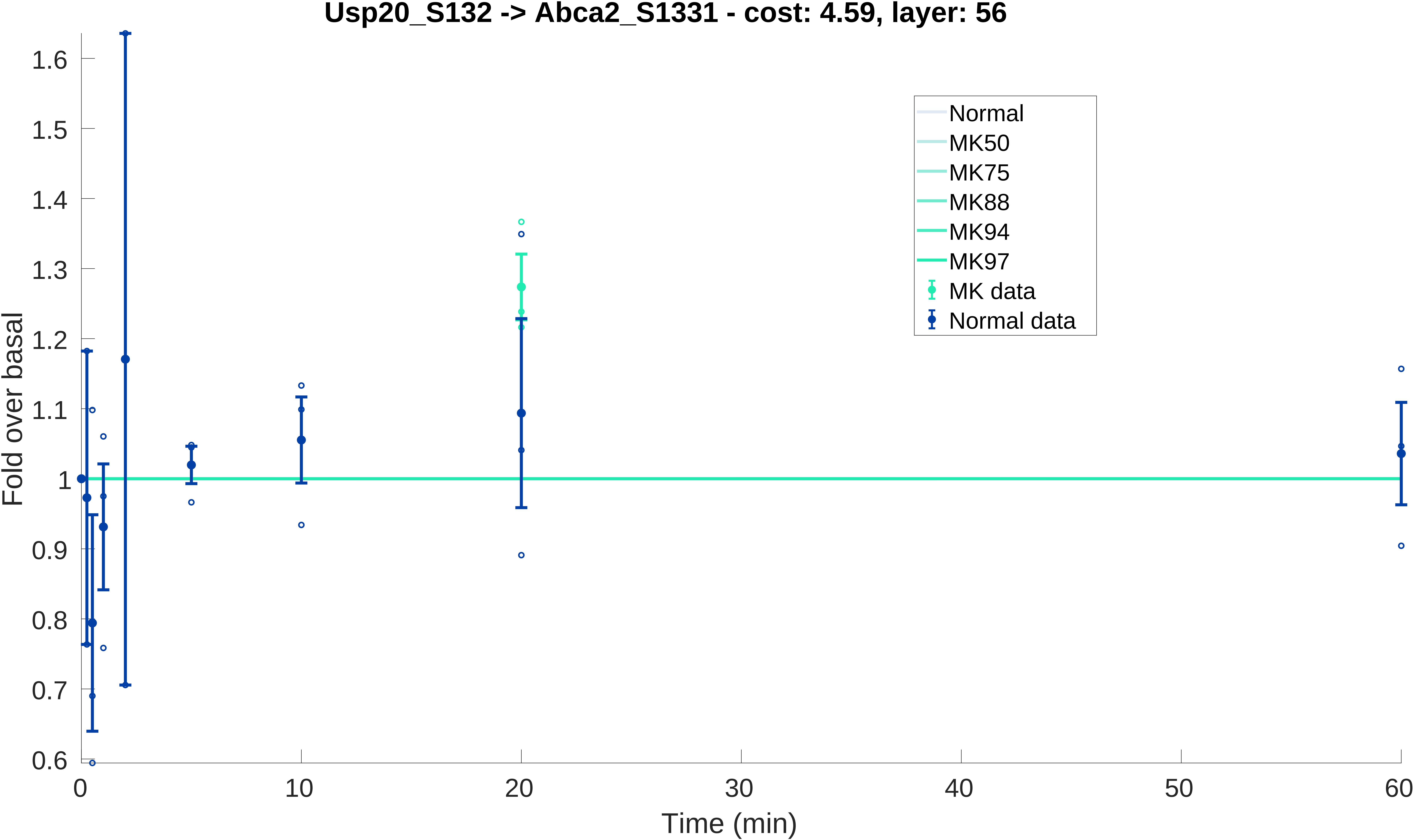
Usp20_S132 -> Abca2_S1331 - cost: 4.59, layer: 56
Fold over basal
Time (min)
1.6
1.5
1.4
1.3
1.2
1.1
1
0.9
0.8
0.7
0.6
0
10
20
30
40
50
60
Normal
MK50
MK75
MK88
MK94
MK97
MK data
Normal data

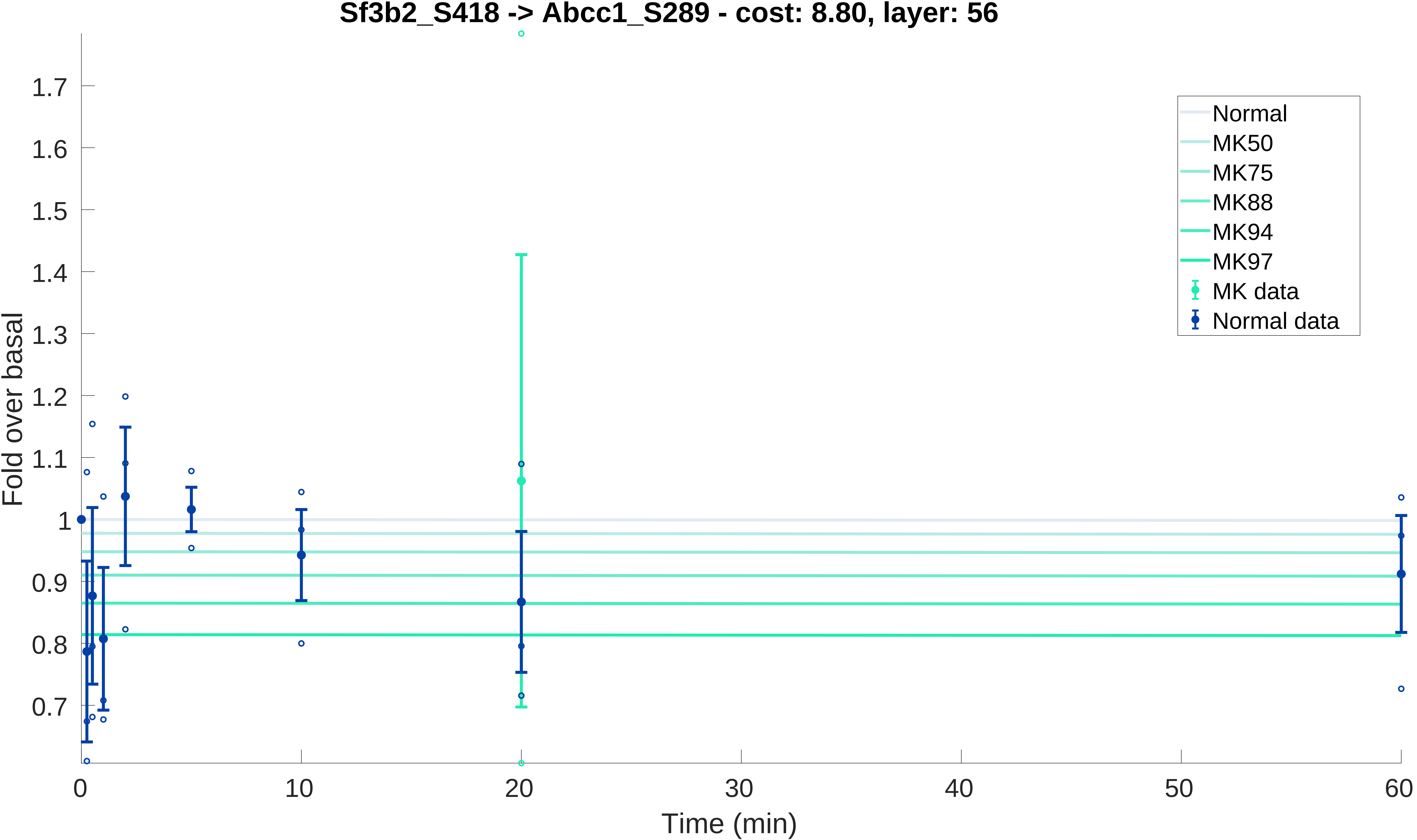

Sf3b2_S418 -> Abcc1_S289 - cost: 8.80, layer: 56
Fold over basal
Time (min)
1.7
1.6
1.5
1.4
1.3
1.2
1.1
1
0.9
0.8
0.7
0
10
20
30
40
50
60
Normal
MK50
MK75
MK88
MK94
MK97
MK data
Normal data

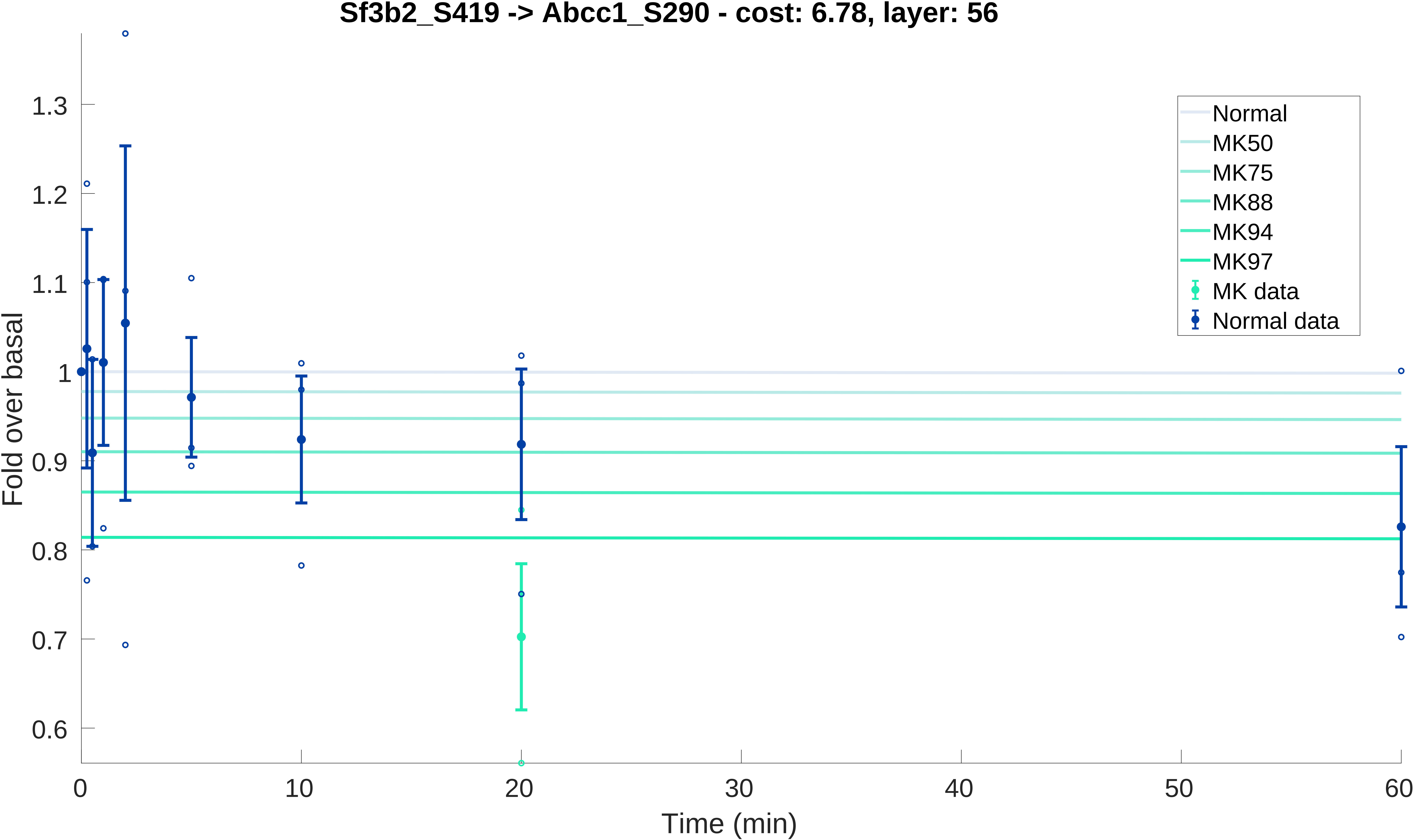
Sf3b2_S419 -> Abcc1_S290 - cost: 6.78, layer: 56
Fold over basal
Time (min)
0.6
0.7
0.8
0.9
1
1.1
1.2
1.3
0
10
20
30
40
50
60
Normal
MK50
MK75
MK88
MK94
MK97
MK data
Normal data

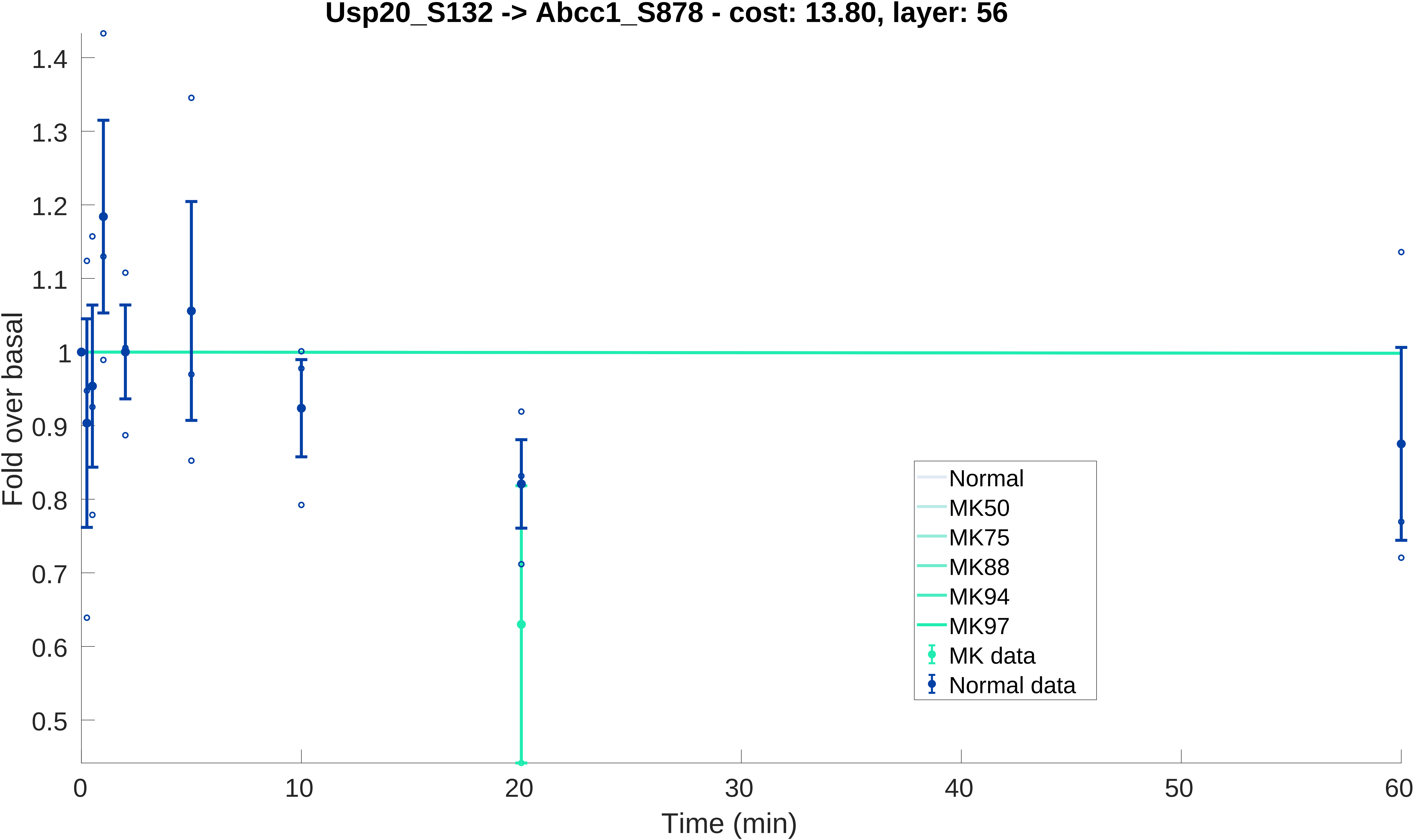

Usp20_S132 -> Abcc1_S878 - cost: 13.80, layer: 56
Fold over basal
1.4
1.3
1.2
1.1
1
0.9
0.8
0.7
0.6
0.5
0
10
20
30
40
50
60
Time (min)
Normal
MK50
MK75
MK88
MK94
MK97
MK data
Normal data

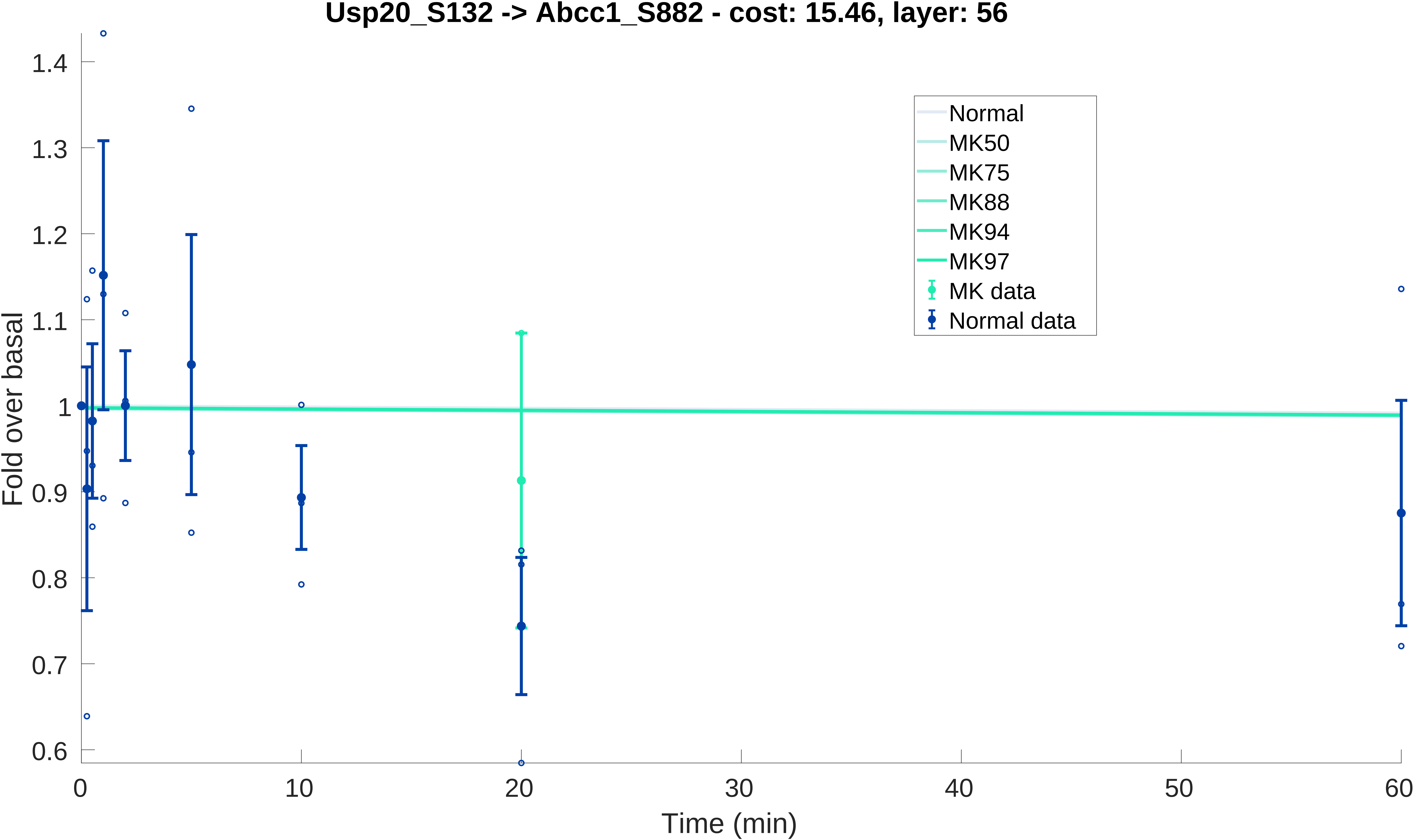

Usp20_S132 -> Abcc1_S882 - cost: 15.46, layer: 56
Fold over basal
Time (min)
Normal
MK50
MK75
MK88
MK94
MK97
MK data
Normal data
1.4
1.3
1.2
1.1
1
0.9
0.8
0.7
0.6
0
10
20
30
40
50
60

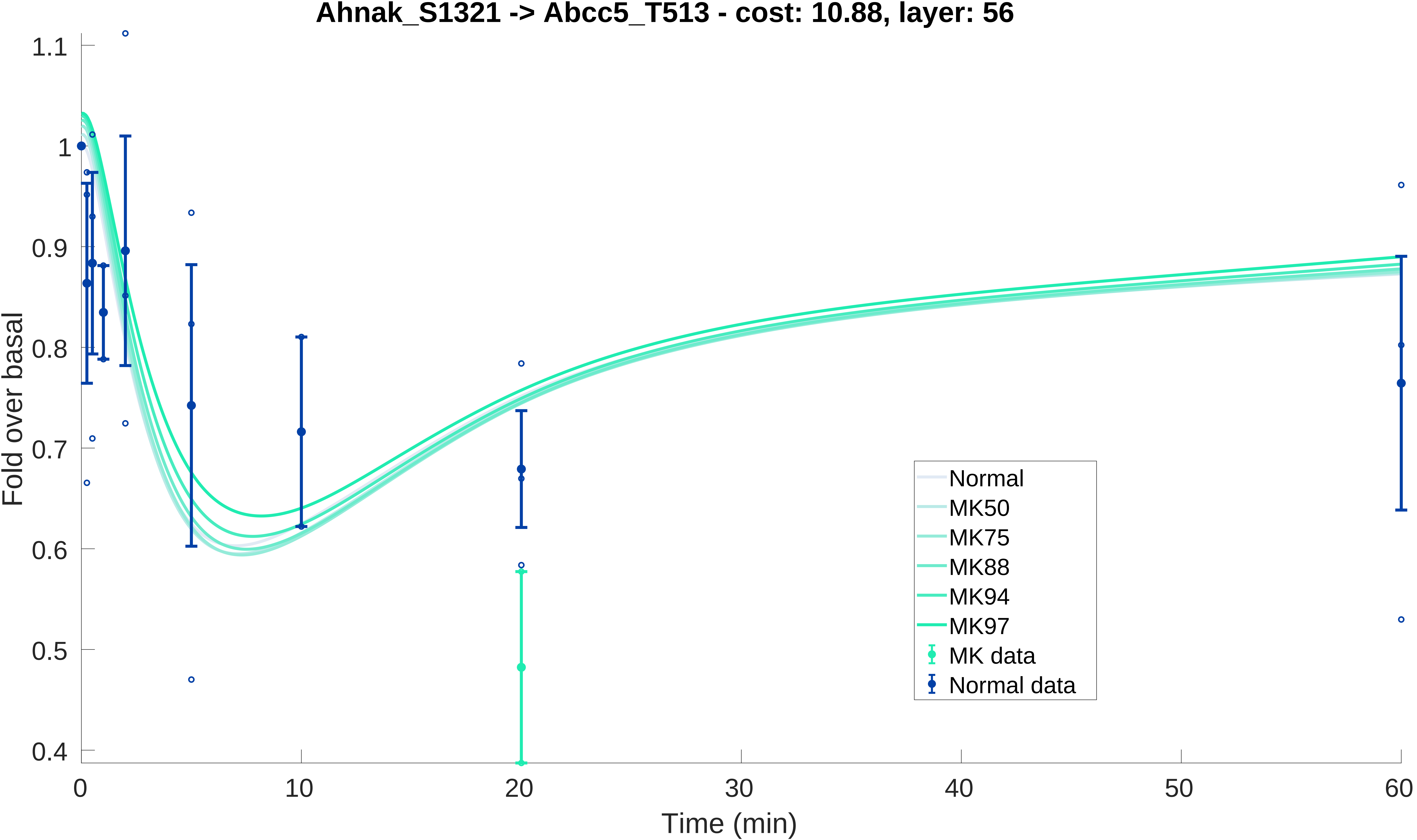

Ahnak_S1321 -> Abcc5_T513 - cost: 10.88, layer: 56
Fold over basal
Time (min)
1.1
1
0.9
0.8
0.7
0.6
0.5
0.4
0
10
20
30
40
50
60
Normal
MK50
MK75
MK88
MK94
MK97
MK data
Normal data

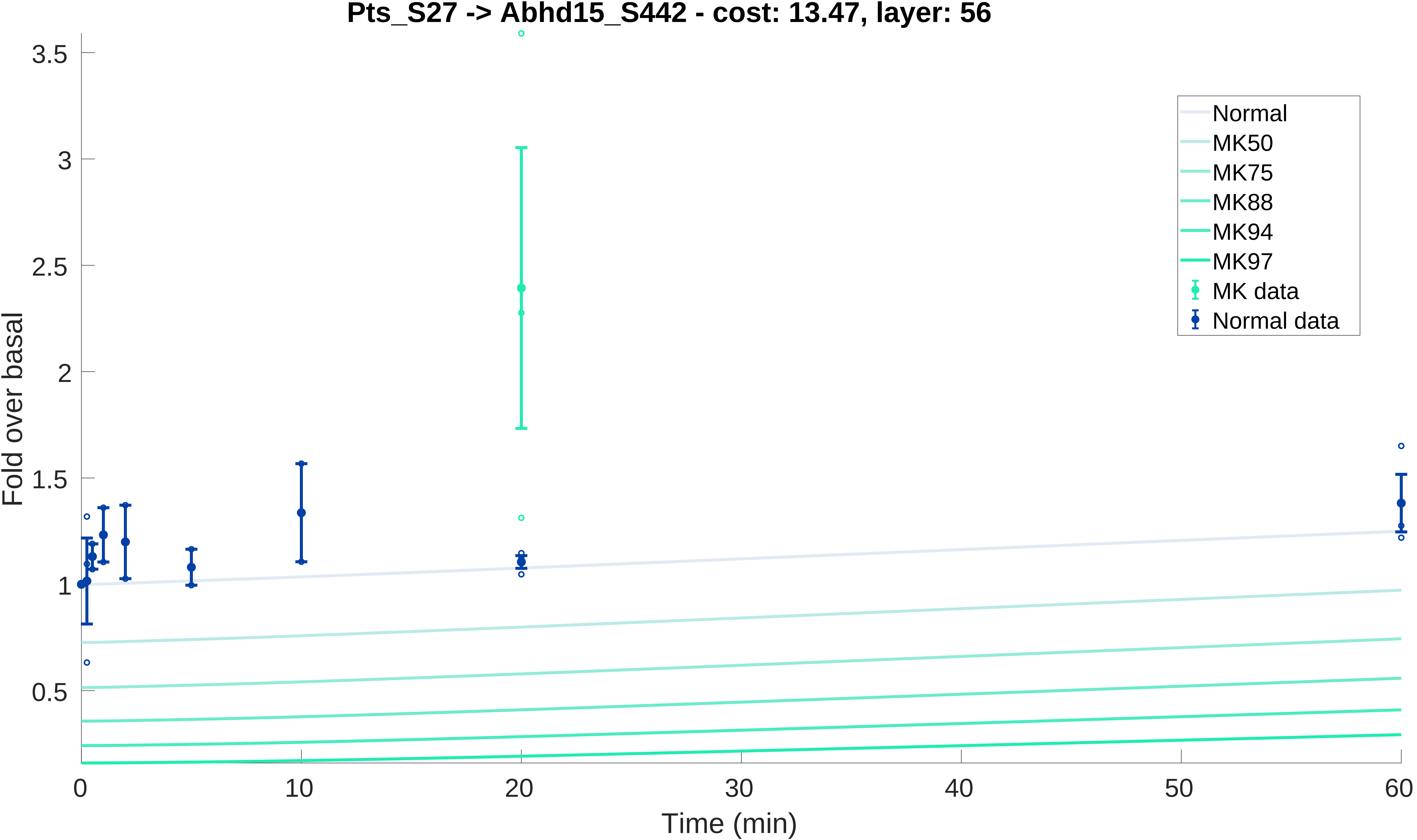

Pts_S27 -> Abhd15_S442 - cost: 13.47, layer: 56
Fold over basal
Time (min)
0
10
20
30
40
50
60
0.5
1
1.5
2
2.5
3
3.5
Normal
MK50
MK75
MK88
MK94
MK97
MK data
Normal data

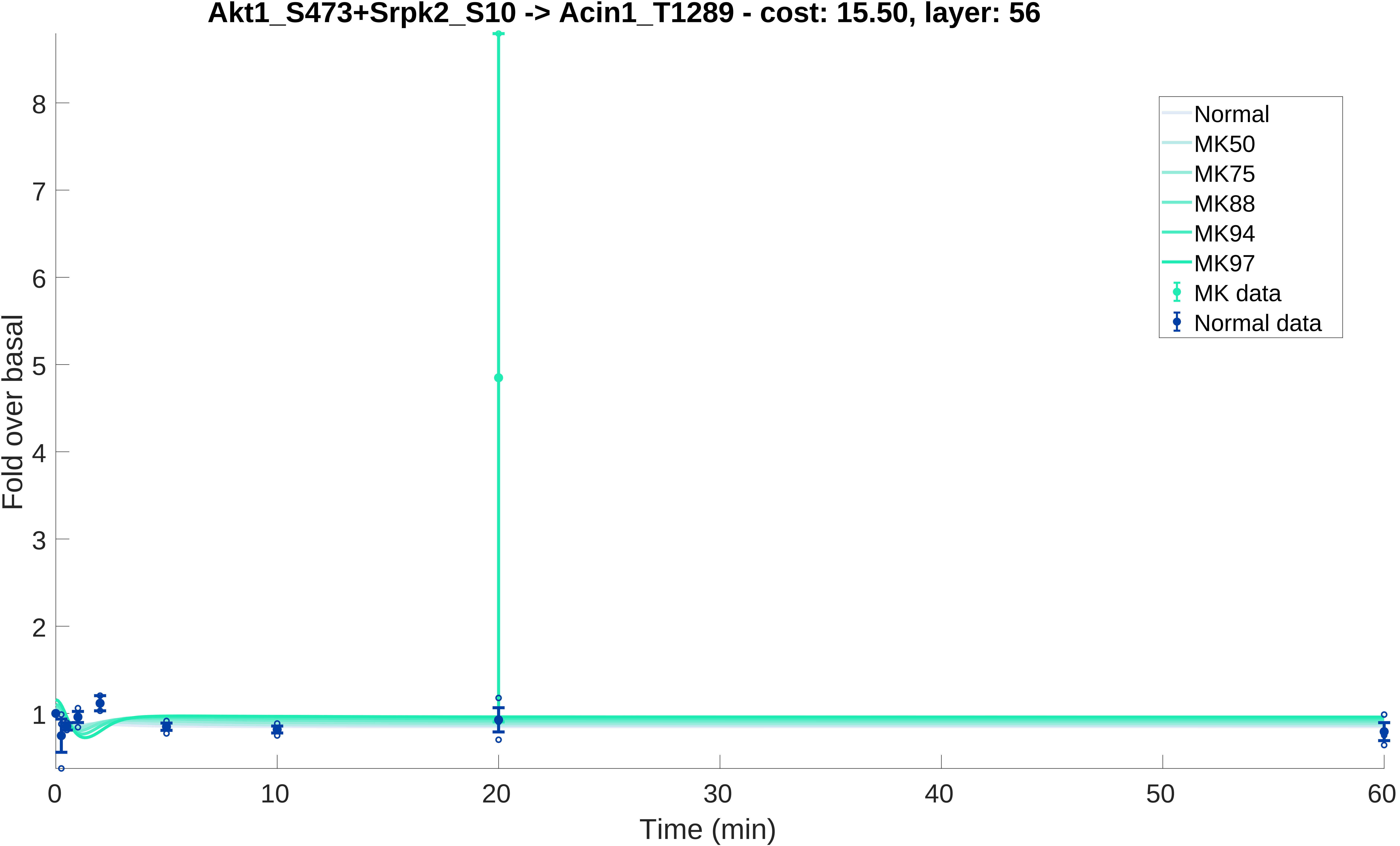
Akt1_S473+Srpk2_S10 -> Acin1_T1289 - cost: 15.50, layer: 56
Fold over basal
Time (min)
0
10
20
30
40
50
60
1
2
3
4
5
6
7
8
Normal
MK50
MK75
MK88
MK94
MK97
MK data
Normal data

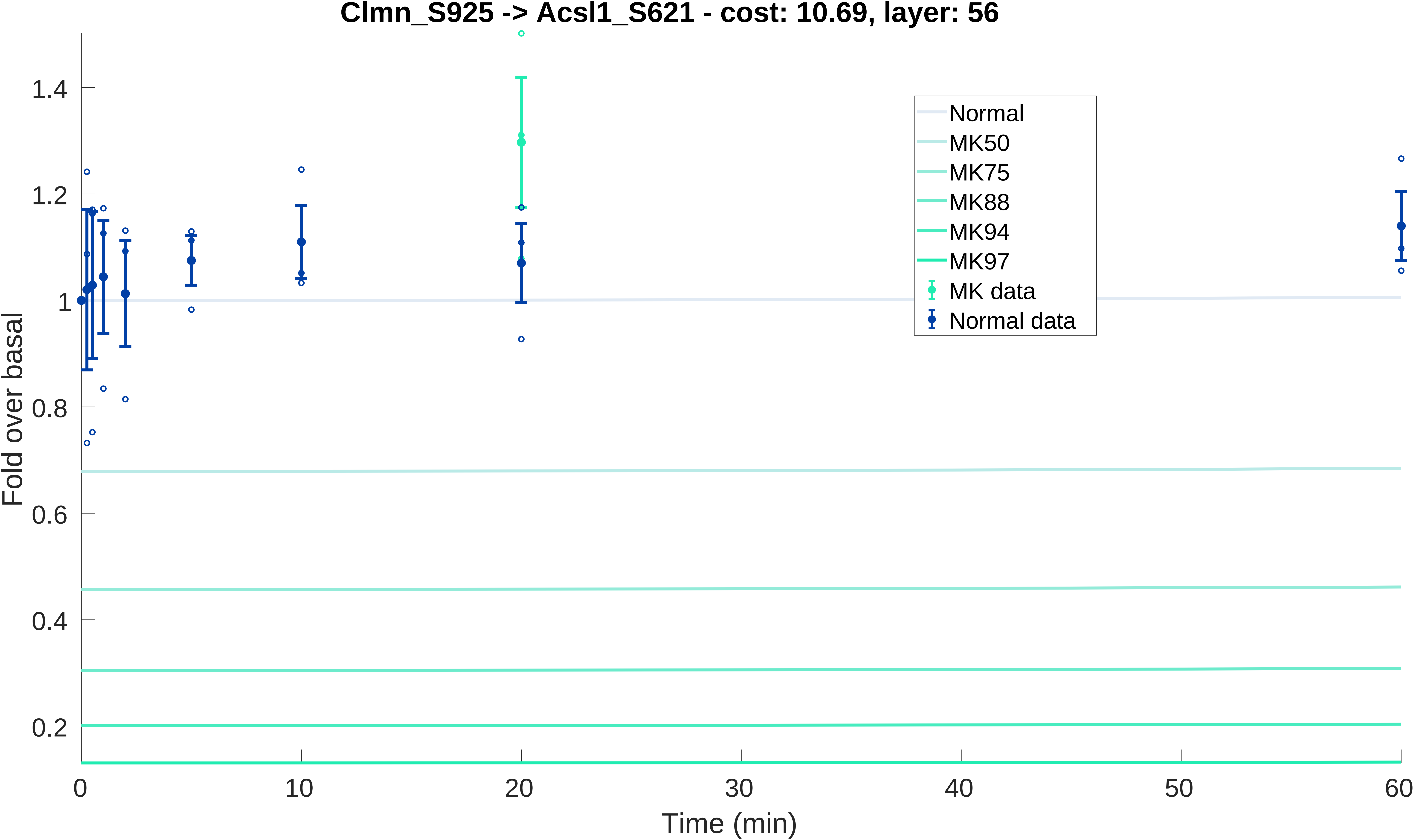
Clmn_S925 -> Acsl1_S621 - cost: 10.69, layer: 56
Fold over basal
Time (min)
0
10
20
30
40
50
60
0.2
0.4
0.6
0.8
1
1.2
1.4
Normal
MK50
MK75
MK88
MK94
MK97
MK data
Normal data

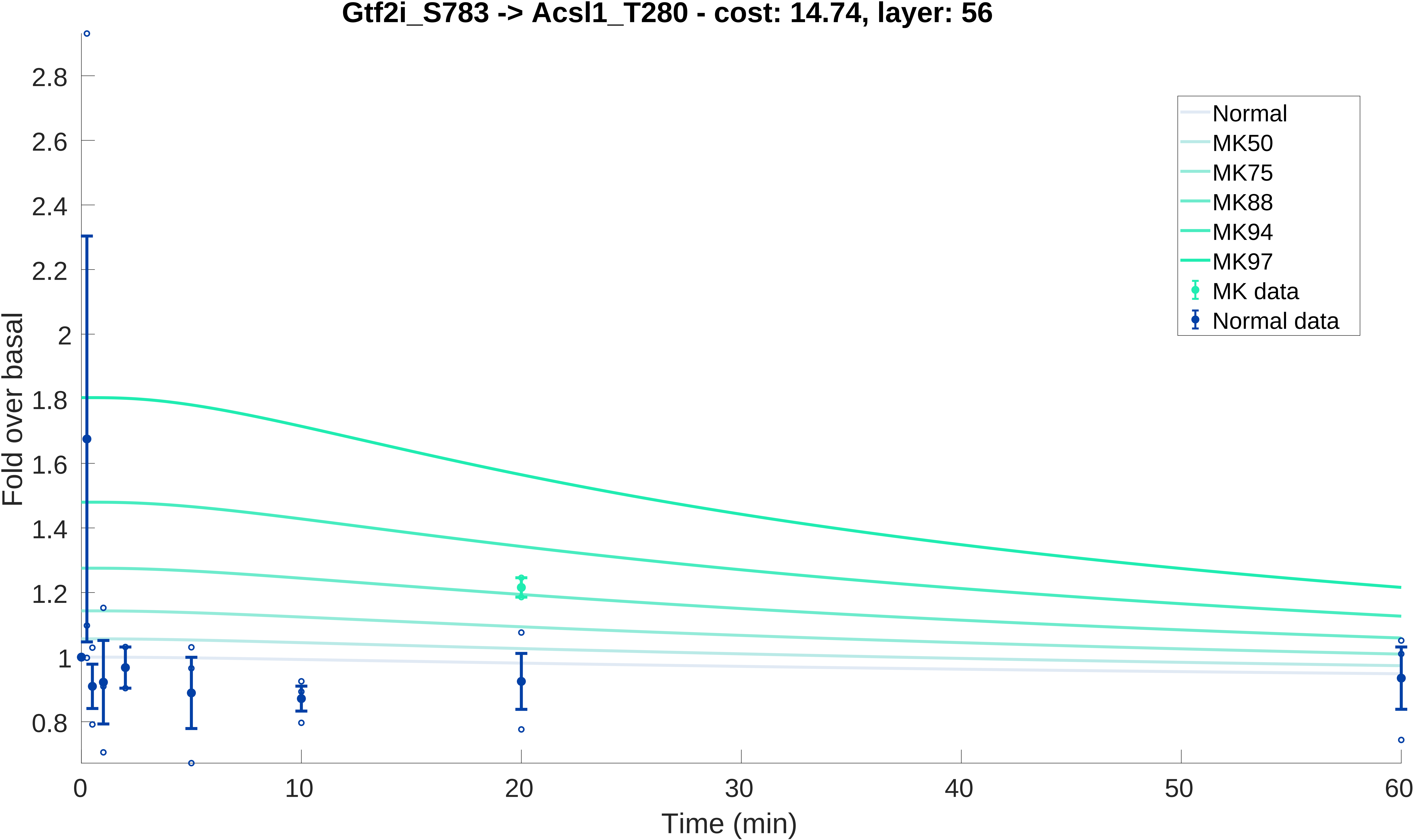

Gtf2i_S783 -> Acsl1_T280 - cost: 14.74, layer: 56
Fold over basal
Time (min)
0.8
1
1.2
1.4
1.6
1.8
2
2.2
2.4
2.6
2.8
0
10
20
30
40
50
60
Normal
MK50
MK75
MK88
MK94
MK97
MK data
Normal data

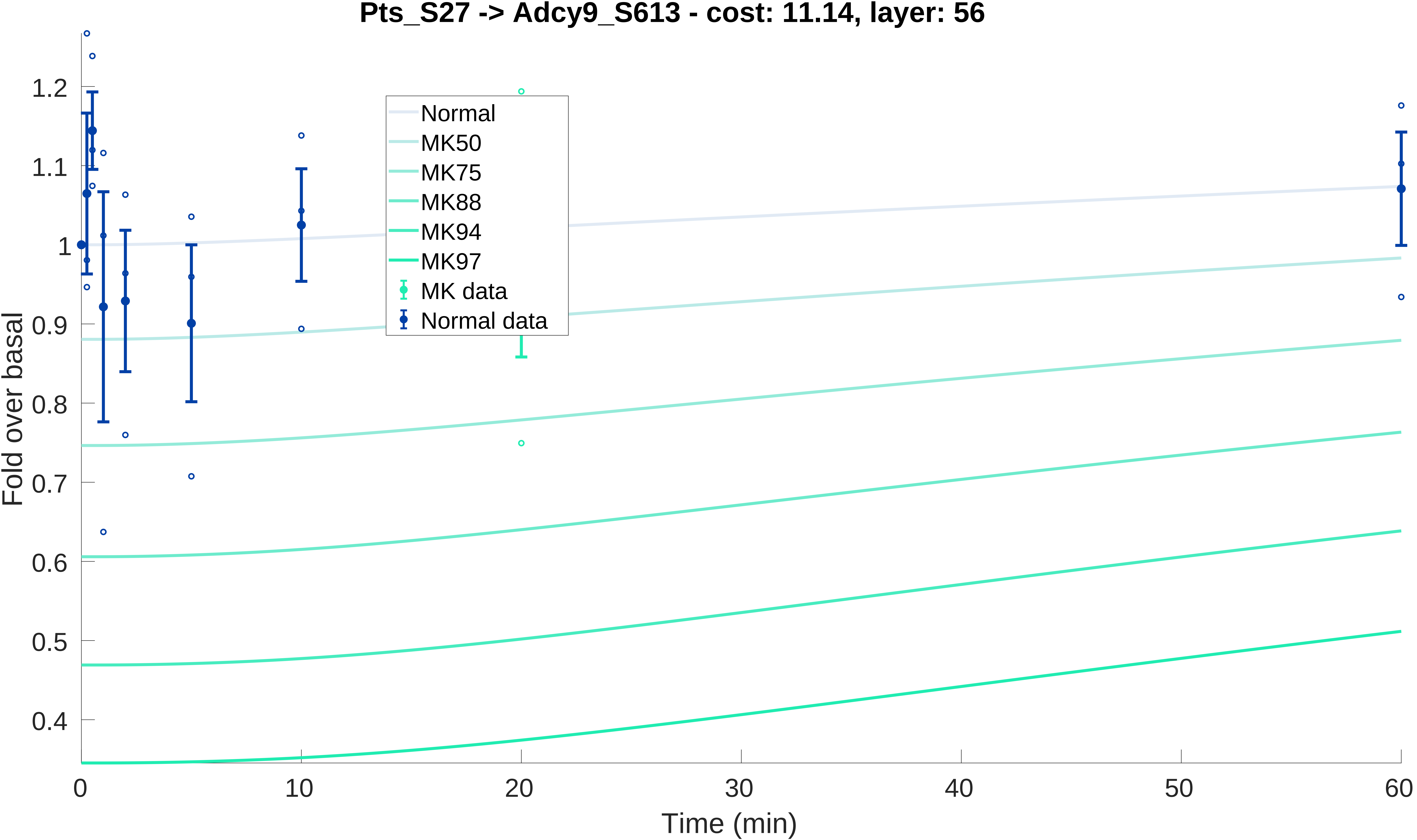

Pts_S27 -> Adcy9_S613 - cost: 11.14, layer: 56
Fold over basal
Time (min)
1.2
1.1
1
0.9
0.8
0.7
0.6
0.5
0.4
0
10
20
30
40
50
60
Normal
MK50
MK75
MK88
MK94
MK97
MK data
Normal data

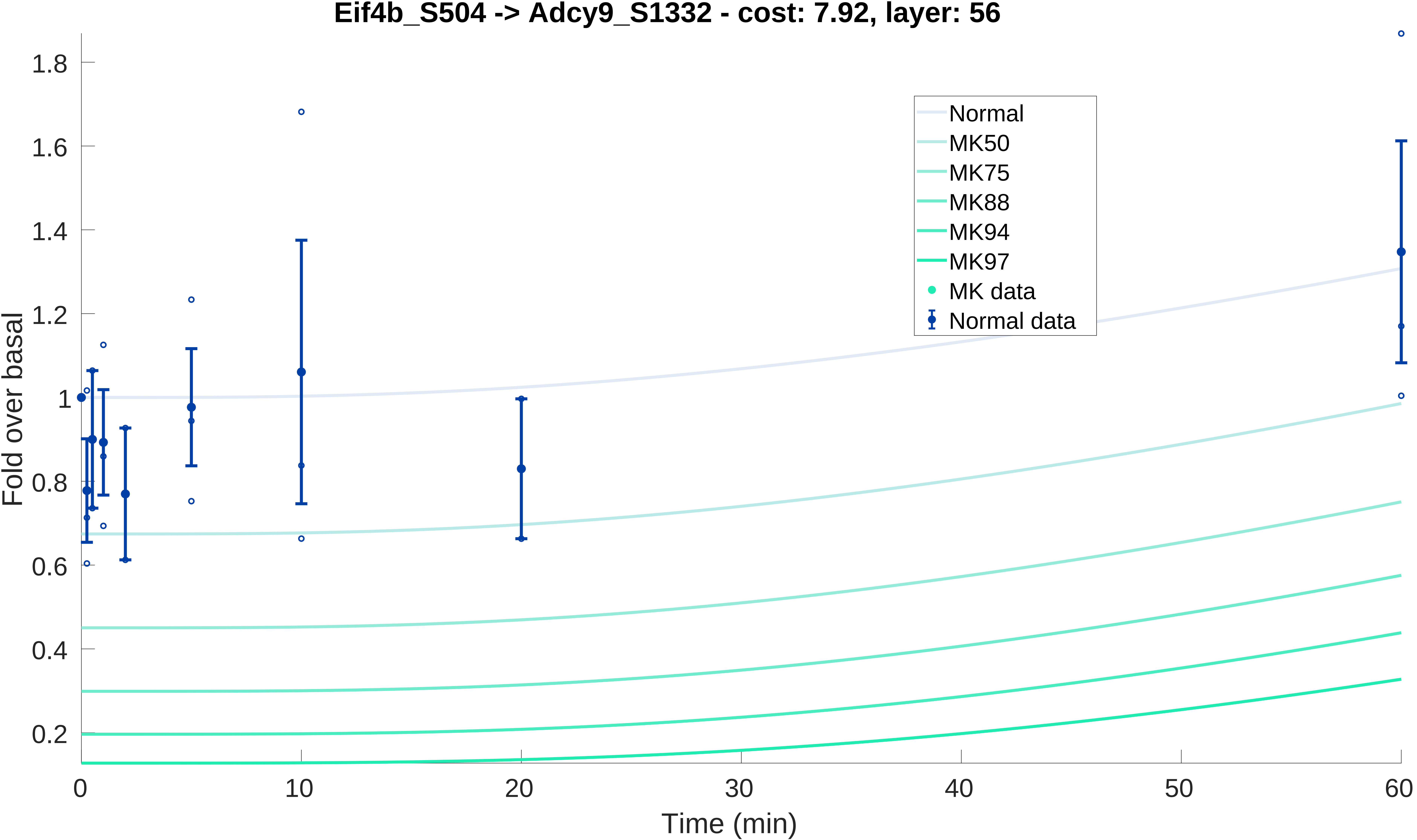

Eif4b_S504 -> Adcy9_S1332 - cost: 7.92, layer: 56
Fold over basal
1.8
1.6
1.4
1.2
1
0.8
0.6
0.4
0.2
0
10
20
30
40
50
60
Time (min)
Normal
MK50
MK75
MK88
MK94
MK97
MK data
Normal data

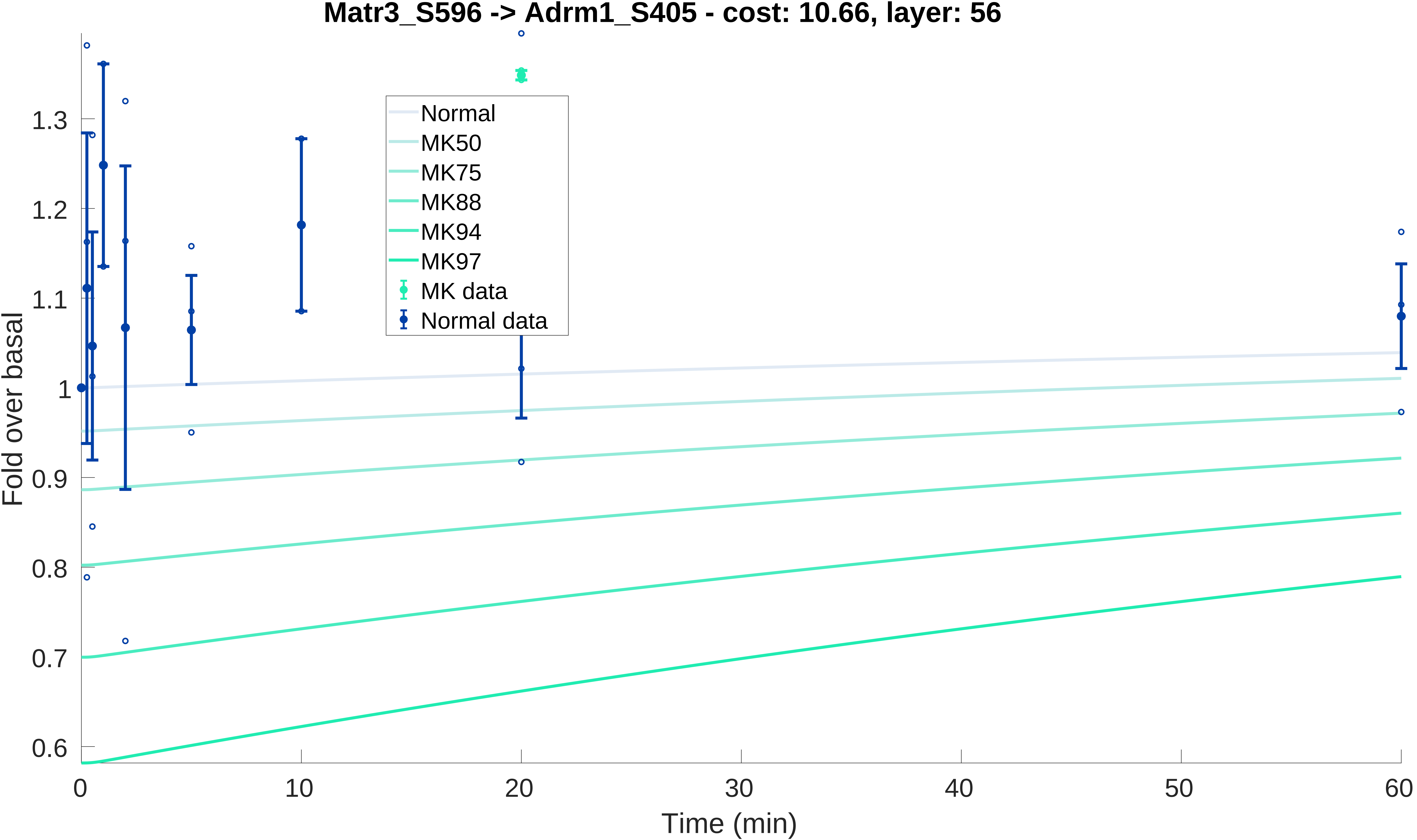

Matr3_S596 -> Adrm1_S405 - cost: 10.66, layer: 56
Normal
MK50
MK75
MK88
MK94
MK97
MK data
Normal data
Fold over basal
Time (min)
0.6
0.7
0.8
0.9
1
1.1
1.2
1.3
0
10
20
30
40
50
60

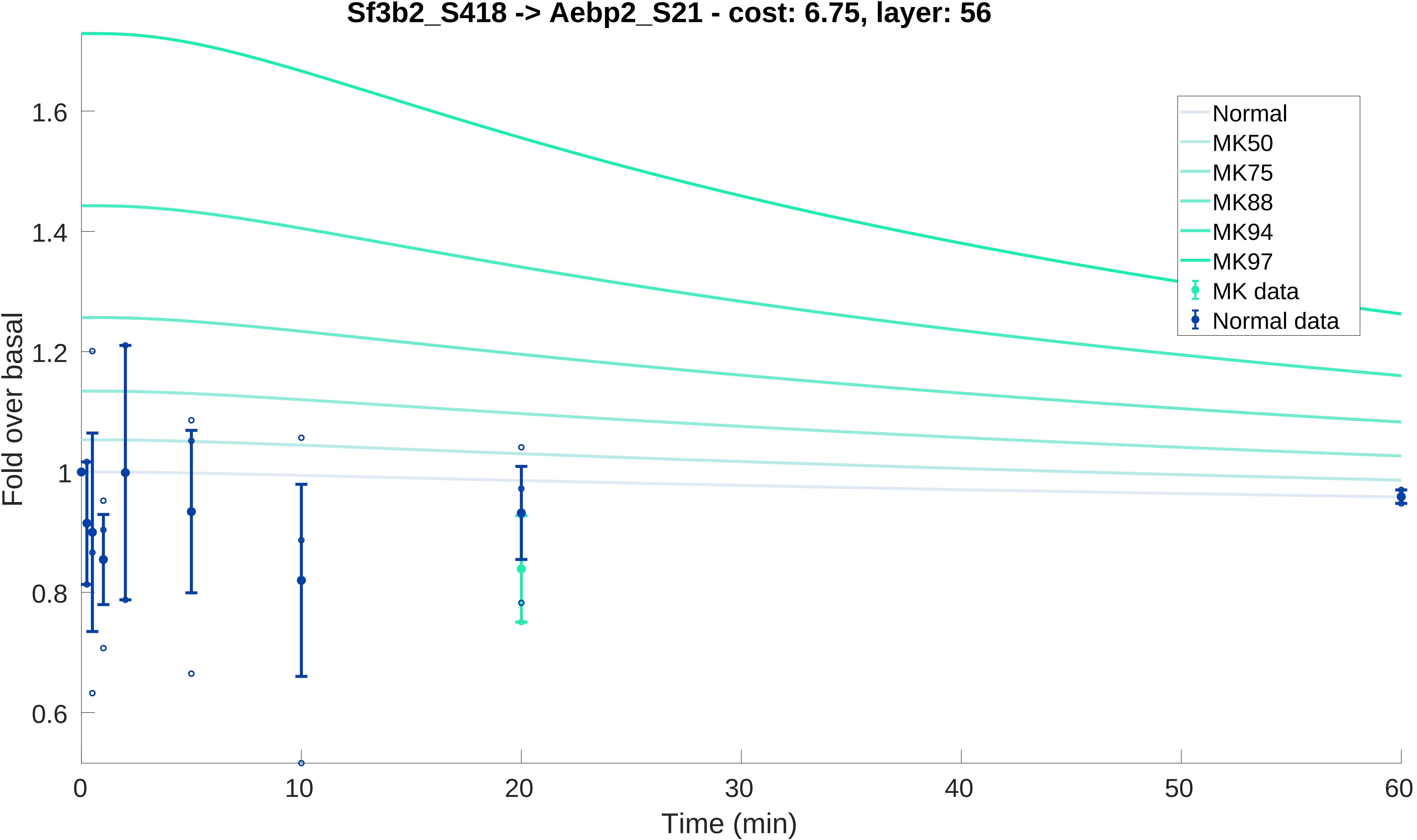

Sf3b2_S418 -> Aebp2_S21 - cost: 6.75, layer: 56
Fold over basal
Time (min)
0.6
0.8
1
1.2
1.4
1.6
0
10
20
30
40
50
60
Normal
MK50
MK75
MK88
MK94
MK97
MK data
Normal data

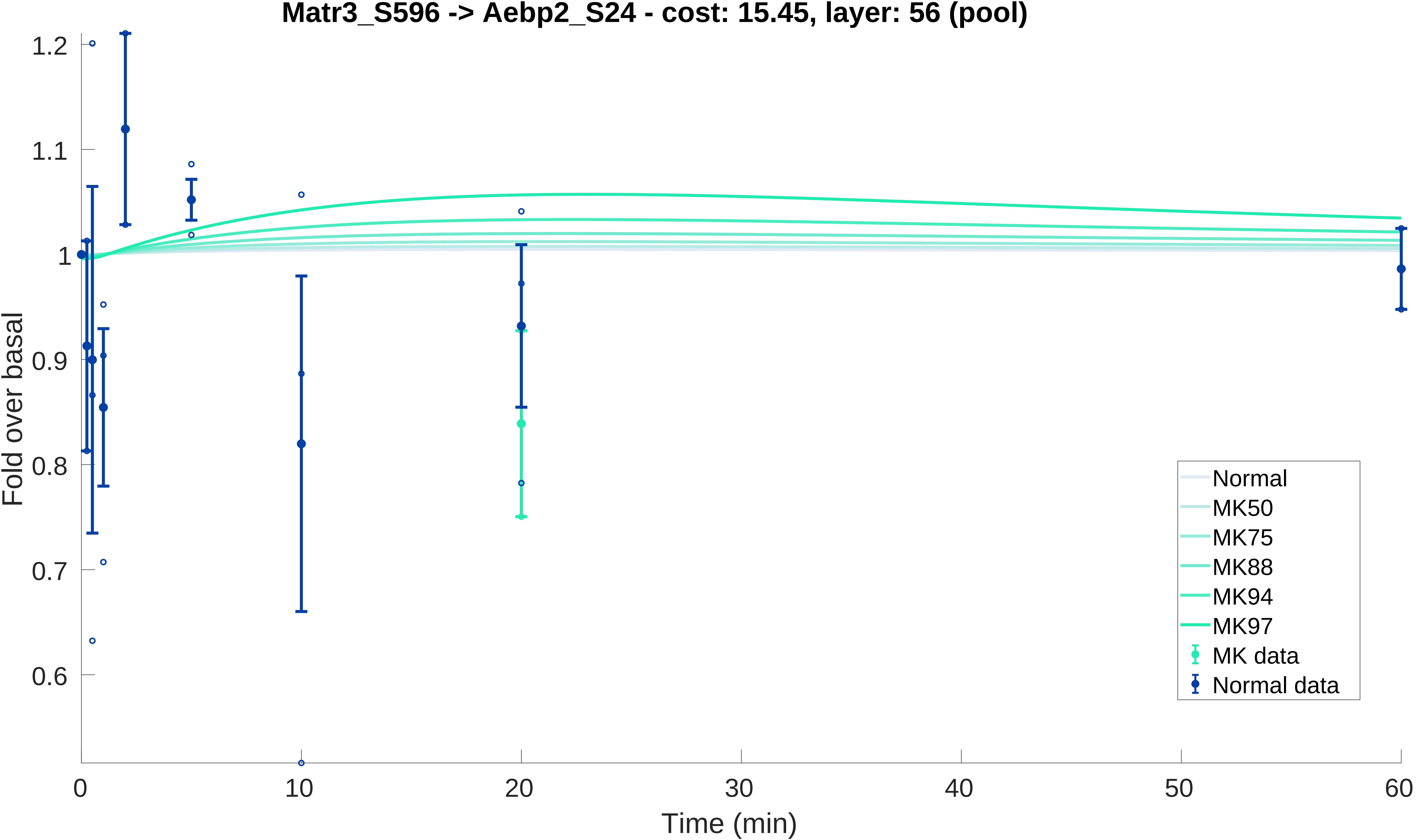
Matr3_S596 -> Aebp2_S24 - cost: 15.45, layer: 56 (pool)
Fold over basal
Time (min)
1.2
1.1
1
0.9
0.8
0.7
0.6
0
10
20
30
40
50
60
Normal
MK50
MK75
MK88
MK94
MK97
MK data
Normal data

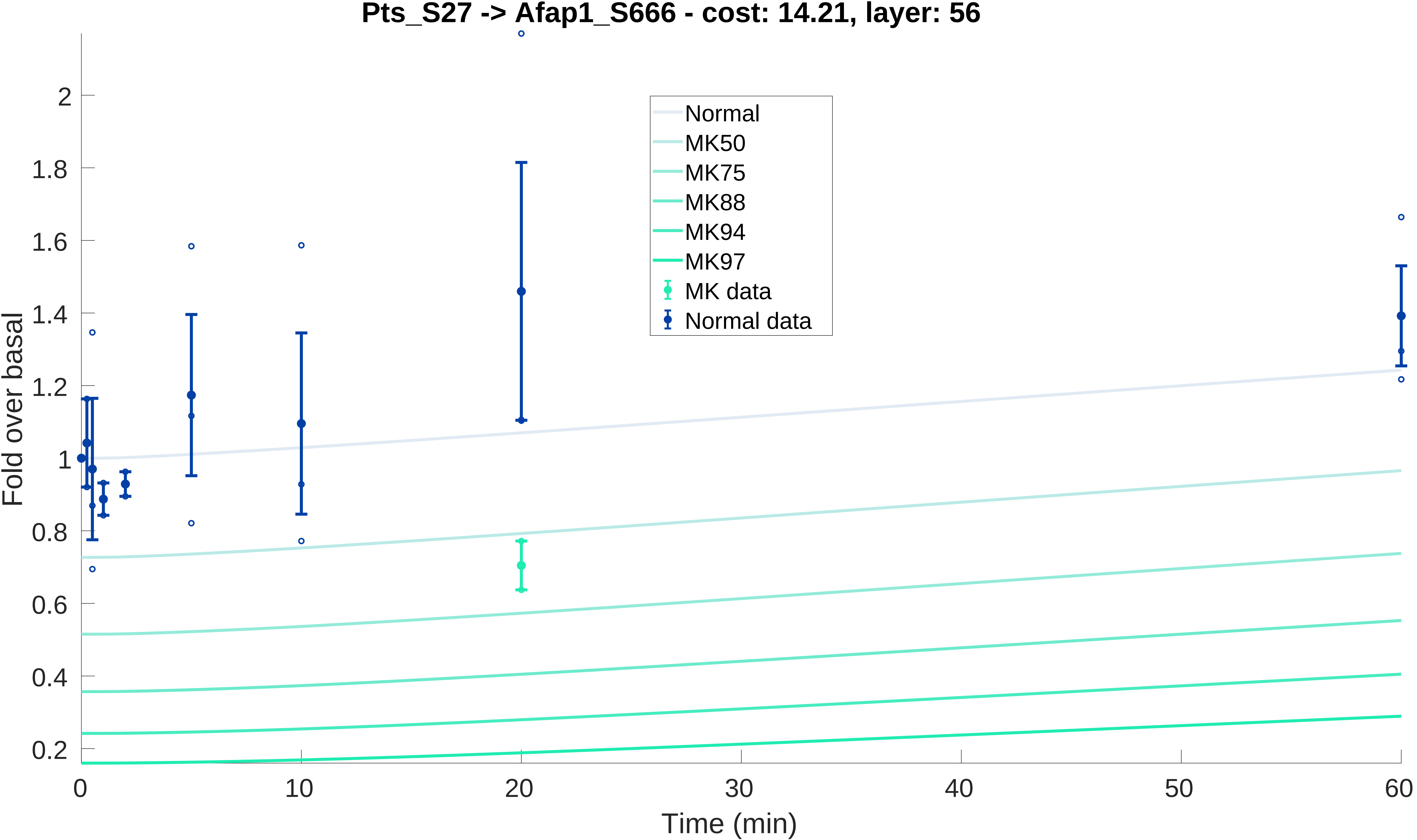

Pts_S27 -> Afap1_S666 - cost: 14.21, layer: 56
Fold over basal
Time (min)
0.2
0.4
0.6
0.8
1
1.2
1.4
1.6
1.8
2
0
10
20
30
40
50
60
Normal
MK50
MK75
MK88
MK94
MK97
MK data
Normal data

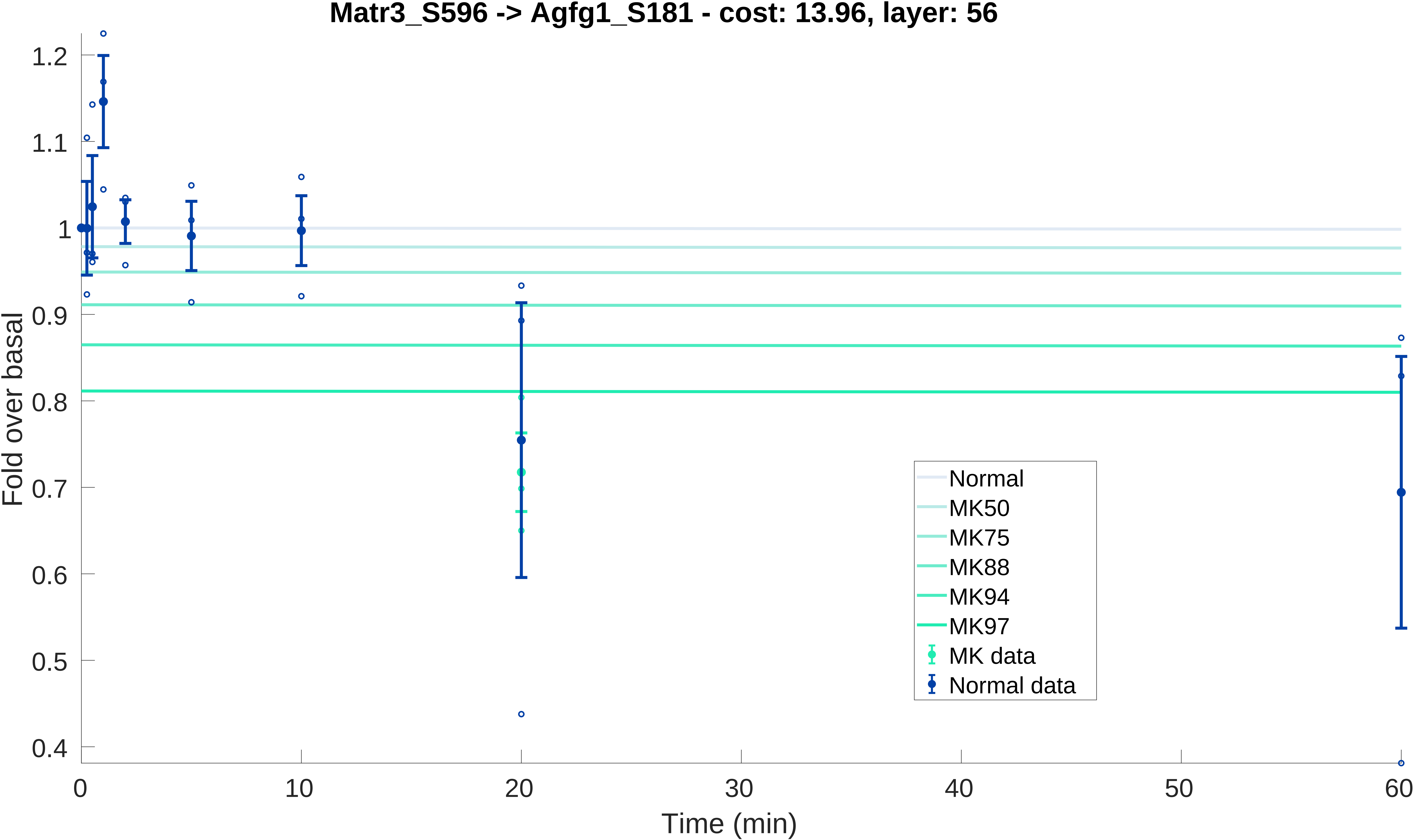

Matr3_S596 -> Agfg1_S181 - cost: 13.96, layer: 56
Fold over basal
Time (min)
1.2
1.1
1
0.9
0.8
0.7
0.6
0.5
0.4
0
10
20
30
40
50
60
Normal
MK50
MK75
MK88
MK94
MK97
MK data
Normal data

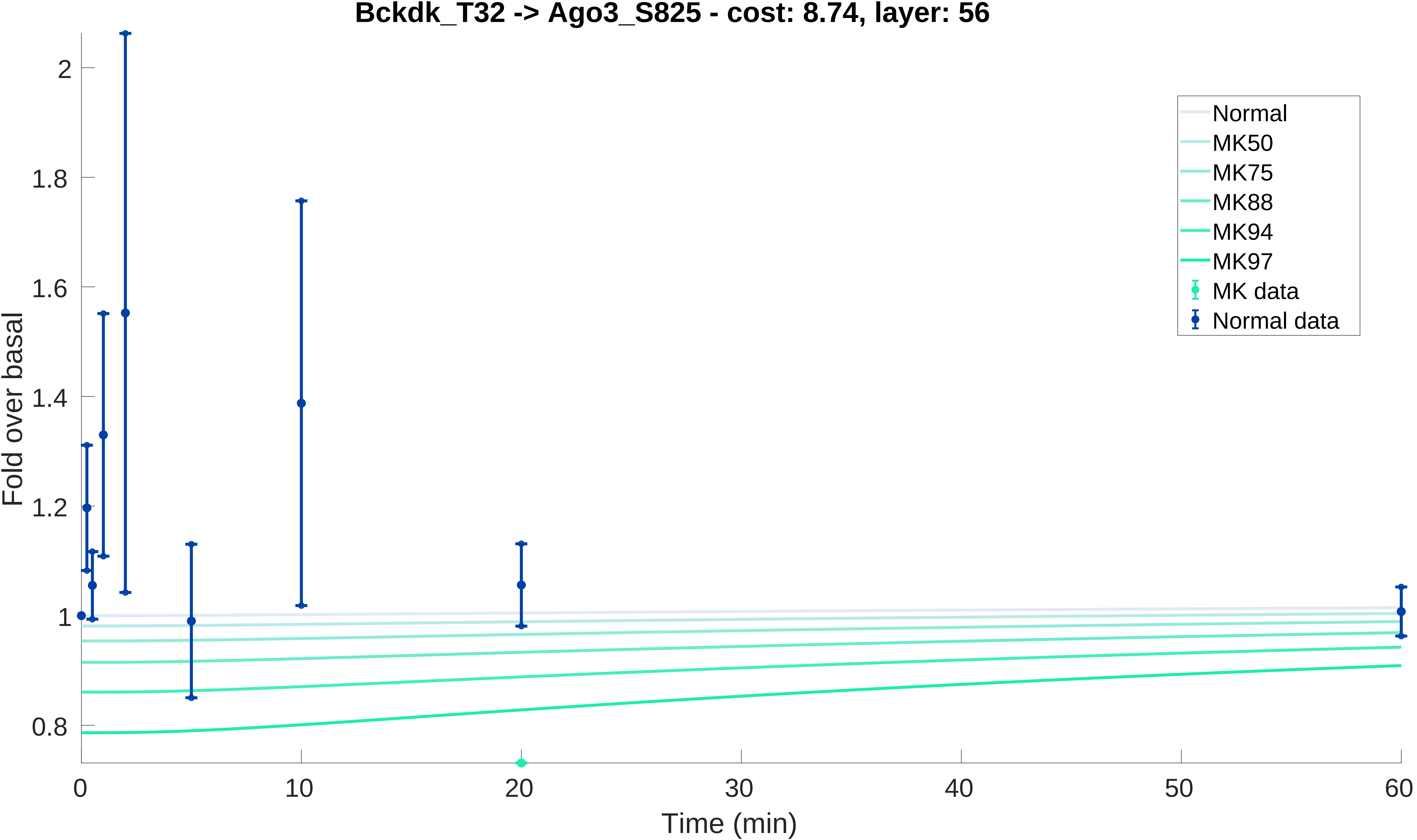

Bckdk_T32 -> Ago3_S825 - cost: 8.74, layer: 56
Fold over basal
Time (min)
0.8
1
1.2
1.4
1.6
1.8
2
0
10
20
30
40
50
60
Normal
MK50
MK75
MK88
MK94
MK97
MK data
Normal data

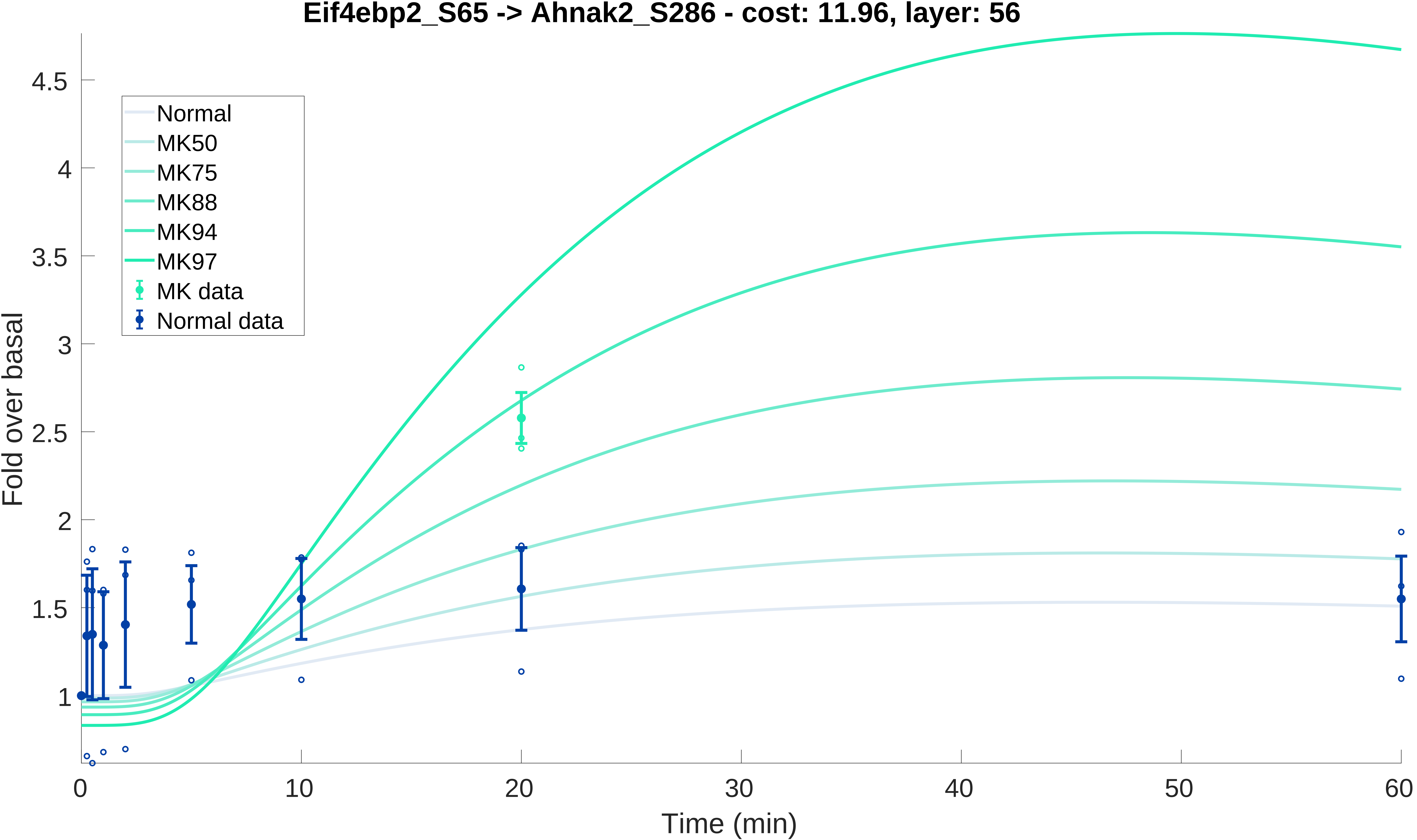

Eif4ebp2_S65 -> Ahnak2_S286 - cost: 11.96, layer: 56
Normal
MK50
MK75
MK88
MK94
MK97
MK data
Normal data
Fold over basal
Time (min)
4.5
4
3.5
3
2.5
2
1.5
1
0
10
20
30
40
50
60

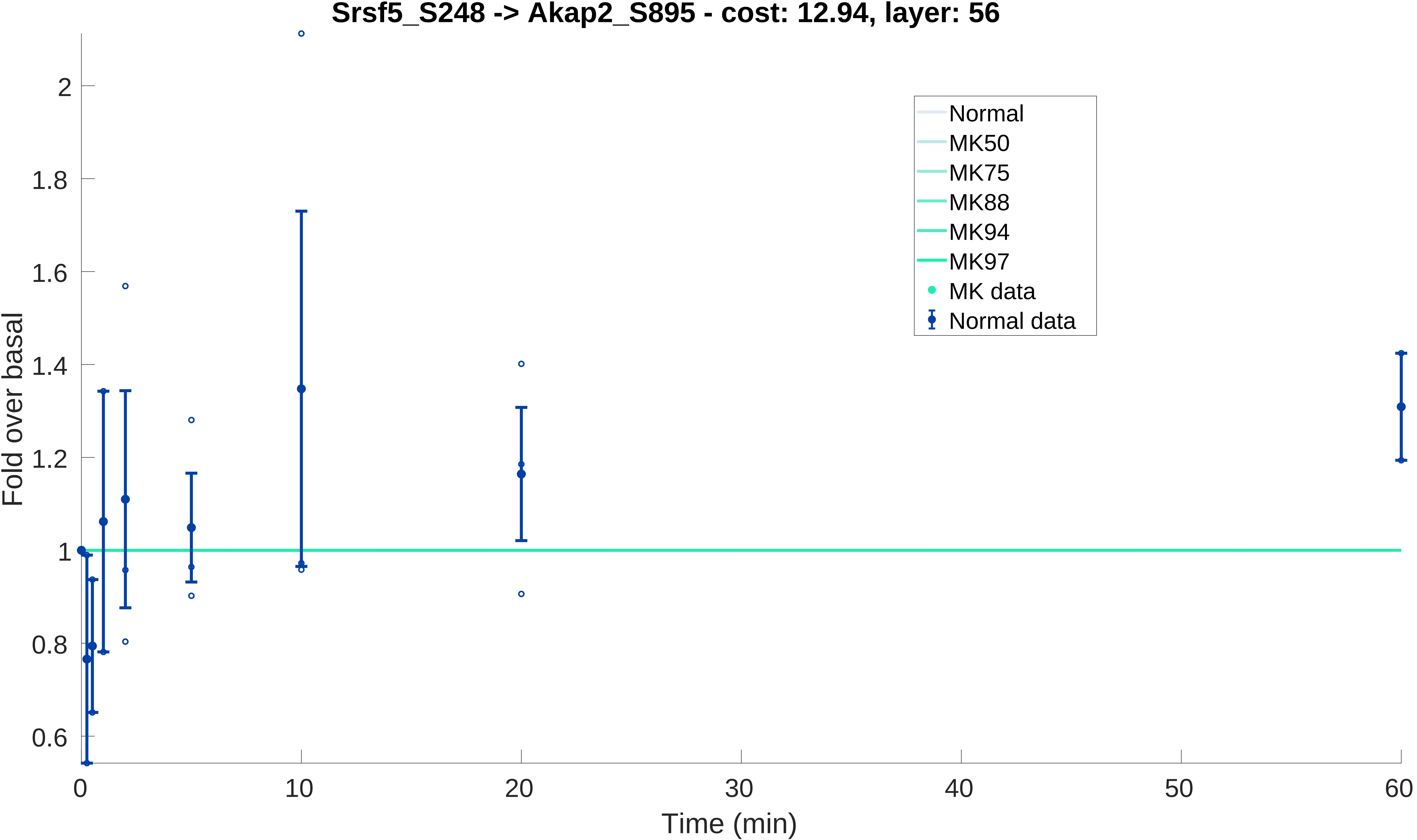

Srsf5_S248 -> Akap2_S895 - cost: 12.94, layer: 56
Fold over basal
Time (min)
0.6
0.8
1
1.2
1.4
1.6
1.8
2
0
10
20
30
40
50
60
Normal
MK50
MK75
MK88
MK94
MK97
MK data
Normal data

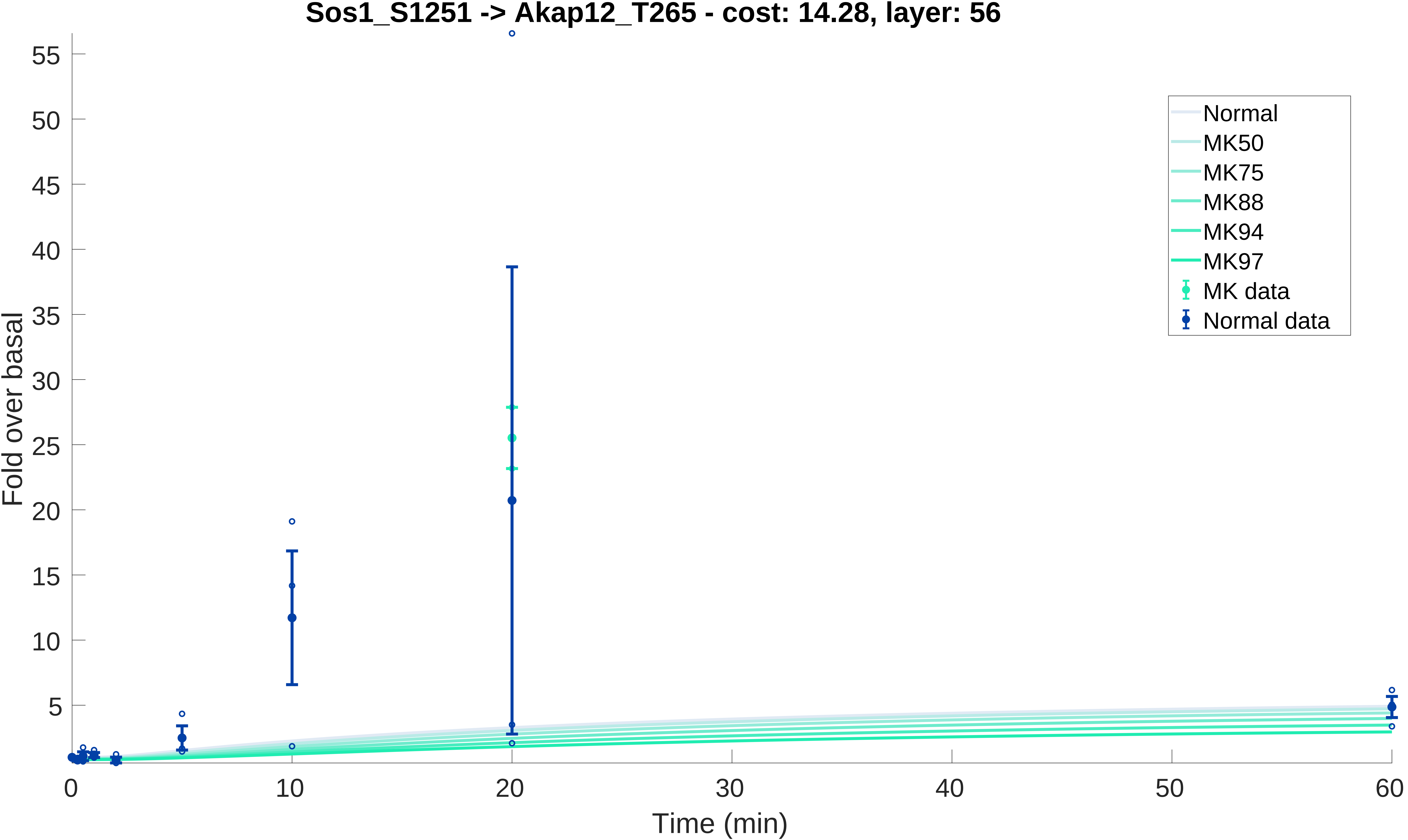

Sos1_S1251 -> Akap12_T265 - cost: 14.28, layer: 56
Fold over basal
Time (min)
0
10
20
30
40
50
60
5
10
15
20
25
30
35
40
45
50
55
Normal
MK50
MK75
MK88
MK94
MK97
MK data
Normal data

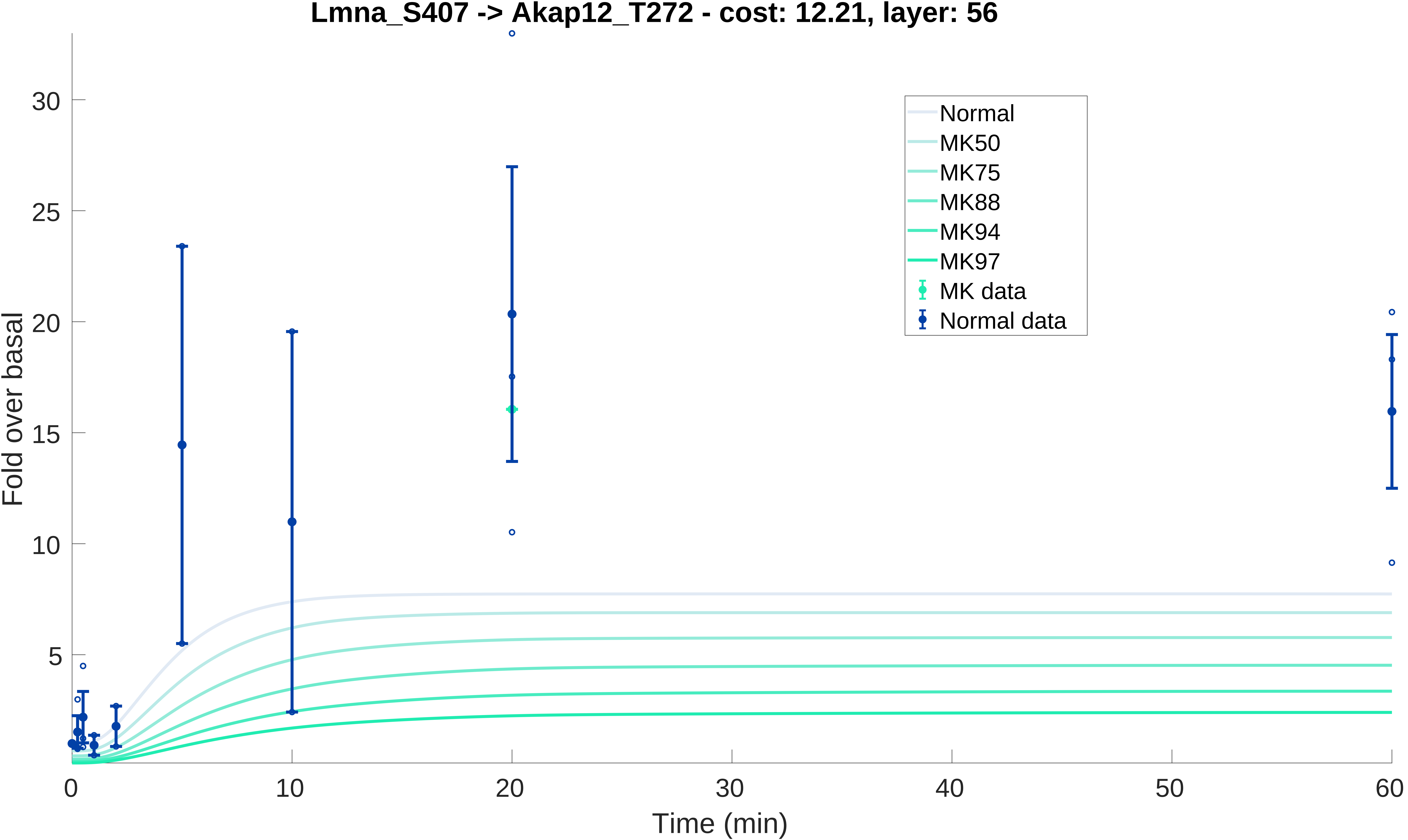

Lmna_S407 -> Akap12_T272 - cost: 12.21, layer: 56
Fold over basal
Time (min)
Normal
MK50
MK75
MK88
MK94
MK97
MK data
Normal data
0
10
20
30
40
50
60
5
10
15
20
25
30

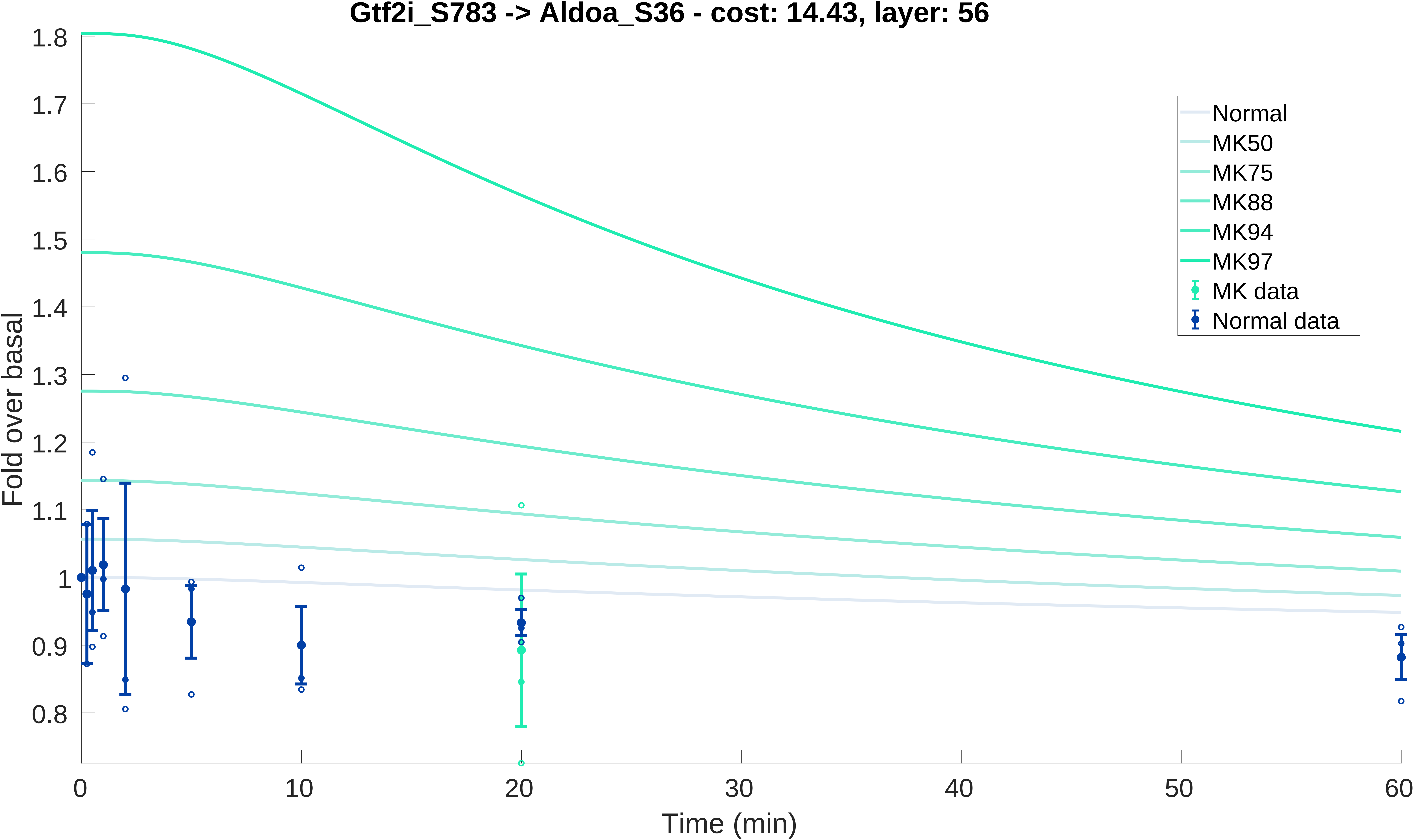

Gtf2i_S783 -> Aldoa_S36 - cost: 14.43, layer: 56
Fold over basal
Time (min)
0.8
0.9
1
1.1
1.2
1.3
1.4
1.5
1.6
1.7
1.8
0
10
20
30
40
50
60
Normal
MK50
MK75
MK88
MK94
MK97
MK data
Normal data

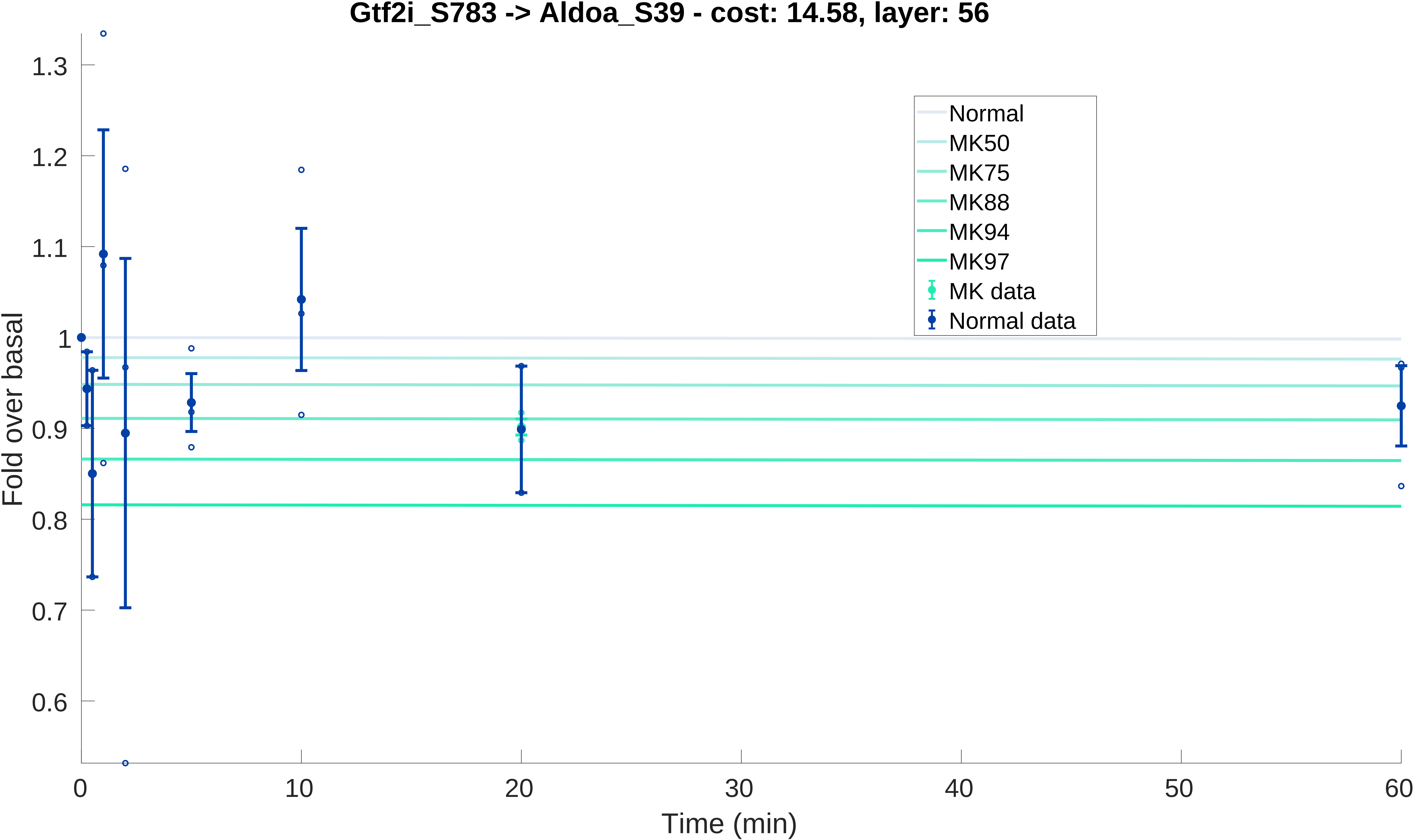

Gtf2i_S783 -> Aldoa_S39 - cost: 14.58, layer: 56
Fold over basal
Time (min)
Normal
MK50
MK75
MK88
MK94
MK97
MK data
Normal data
1.3
1.2
1.1
1
0.9
0.8
0.7
0.6
0
10
20
30
40
50
60

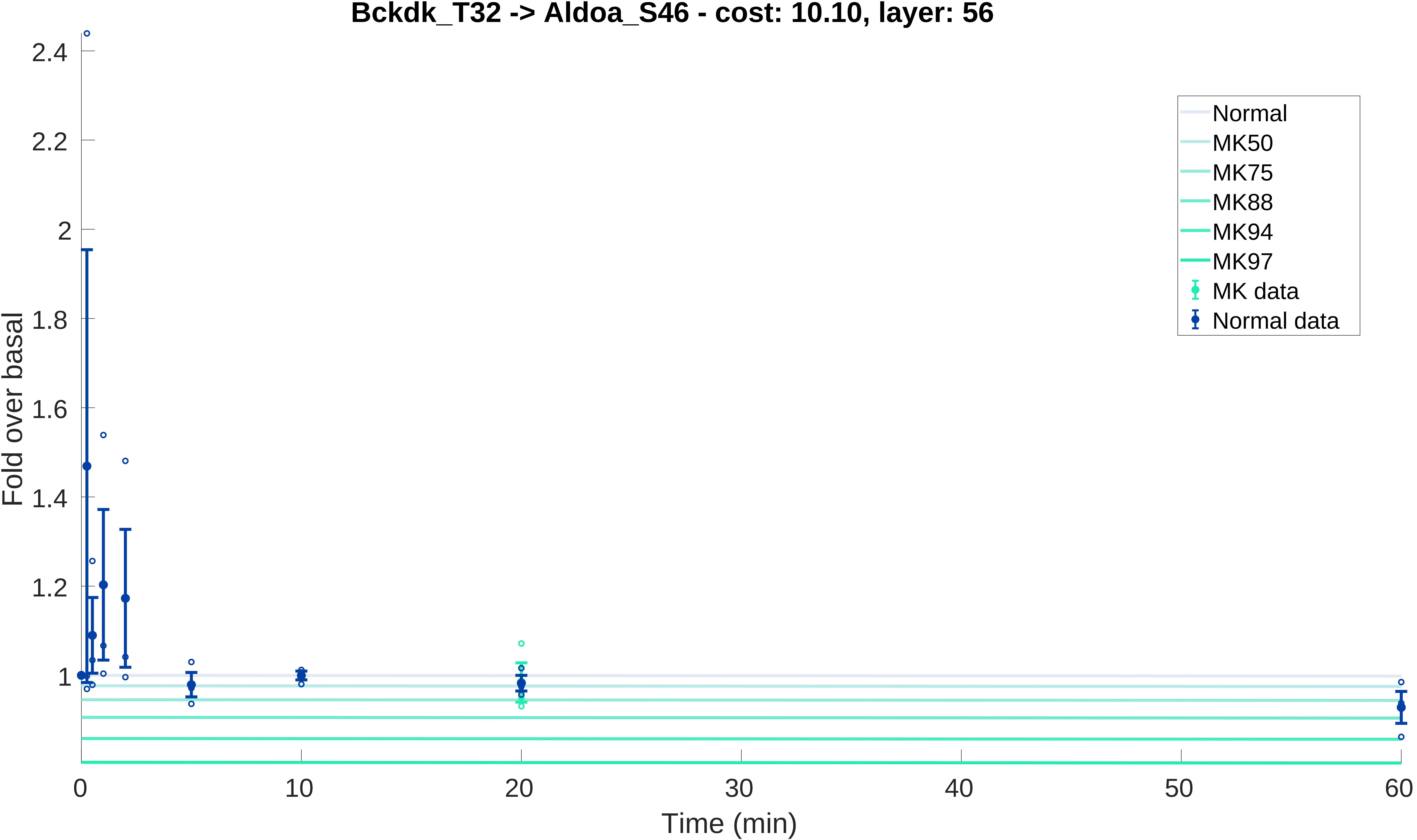
Bckdk_T32 -> Aldoa_S46 - cost: 10.10, layer: 56
Fold over basal
Time (min)
2.4
2.2
2
1.8
1.6
1.4
1.2
1
0
10
20
30
40
50
60
Normal
MK50
MK75
MK88
MK94
MK97
MK data
Normal data

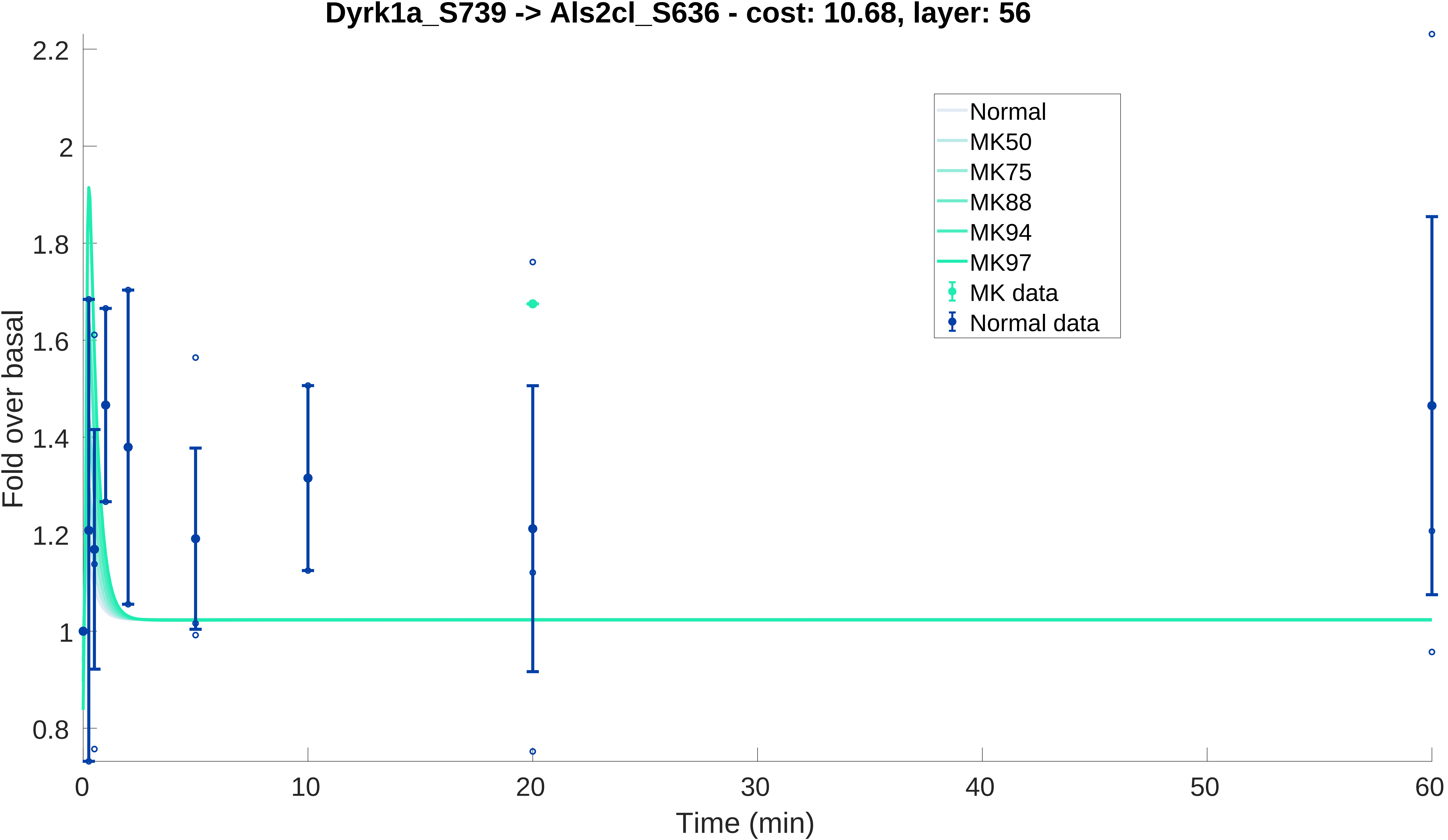

Dyrk1a_S739 -> Als2cl_S636 - cost: 10.68, layer: 56
Fold over basal
Time (min)
0.8
1
1.2
1.4
1.6
1.8
2
2.2
0
10
20
30
40
50
60
Normal
MK50
MK75
MK88
MK94
MK97
MK data
Normal data

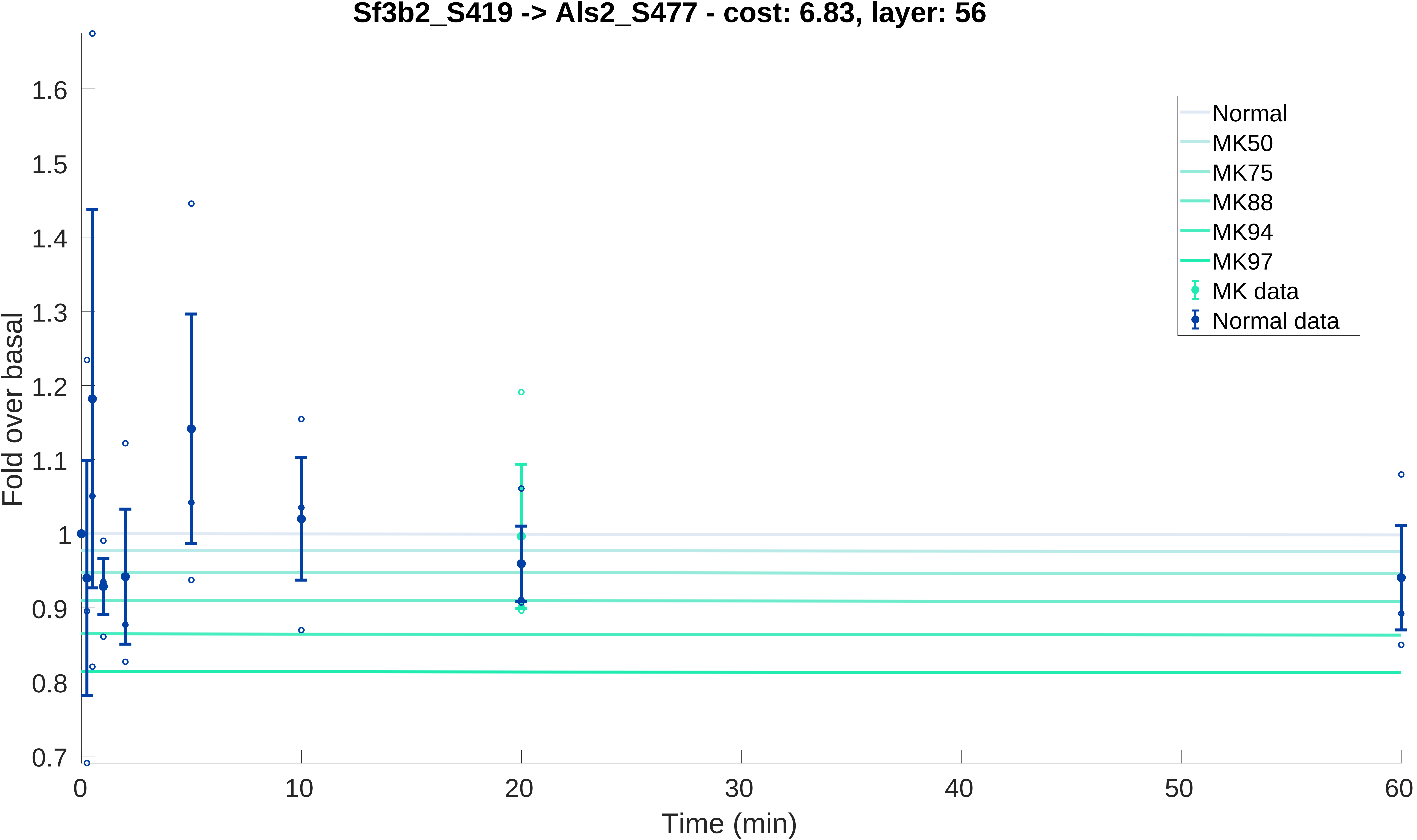

Sf3b2_S419 -> Als2_S477 - cost: 6.83, layer: 56
Fold over basal
Time (min)
0.7
0.8
0.9
1
1.1
1.2
1.3
1.4
1.5
1.6
0
10
20
30
40
50
60
Normal
MK50
MK75
MK88
MK94
MK97
MK data
Normal data

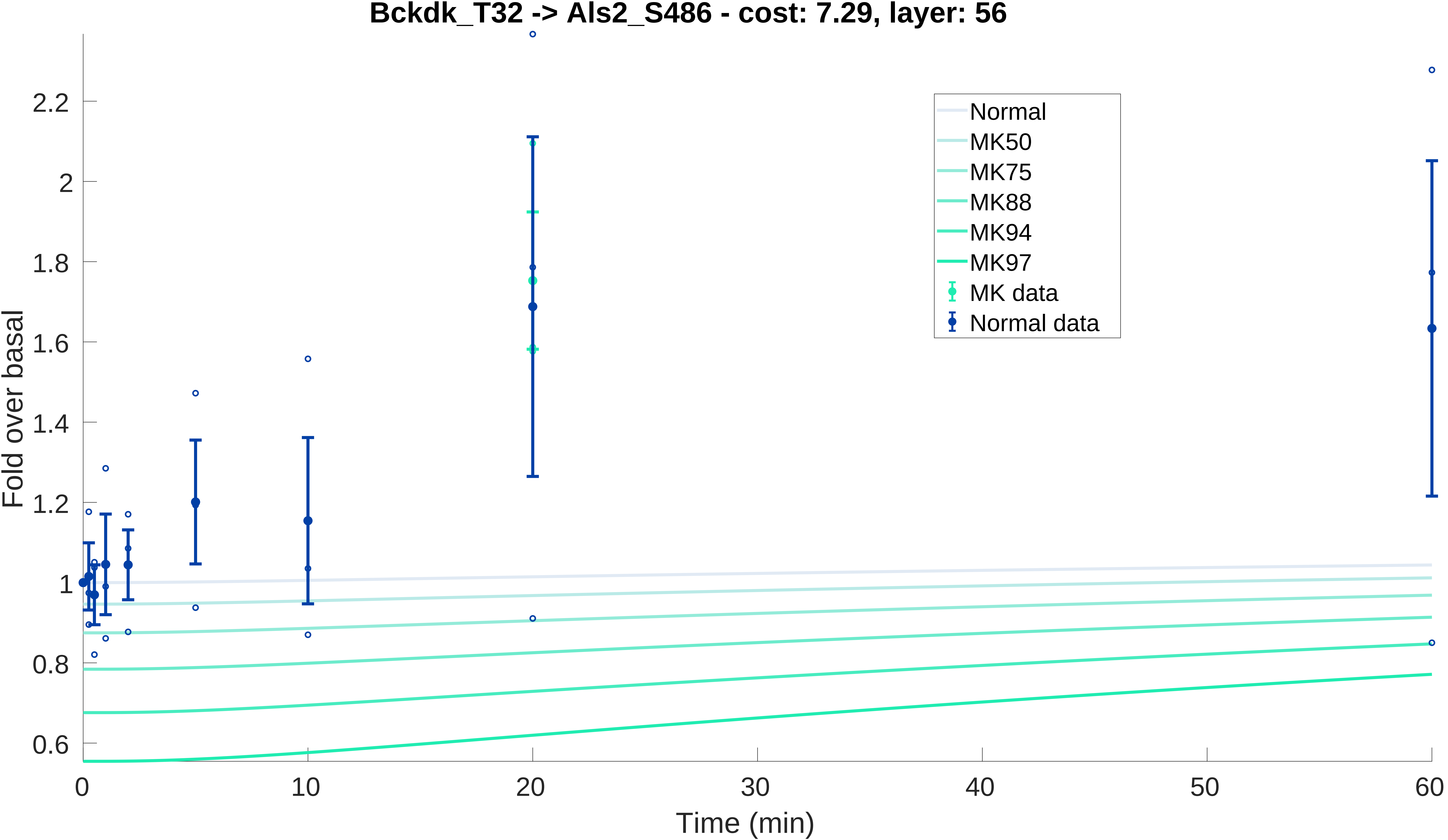
Bckdk_T32 -> Als2_S486 - cost: 7.29, layer: 56
Normal
MK50
MK75
MK88
MK94
MK97
MK data
Normal data
Fold over basal
Time (min)
0.6
0.8
1
1.2
1.4
1.6
1.8
2
2.2
0
10
20
30
40
50
60

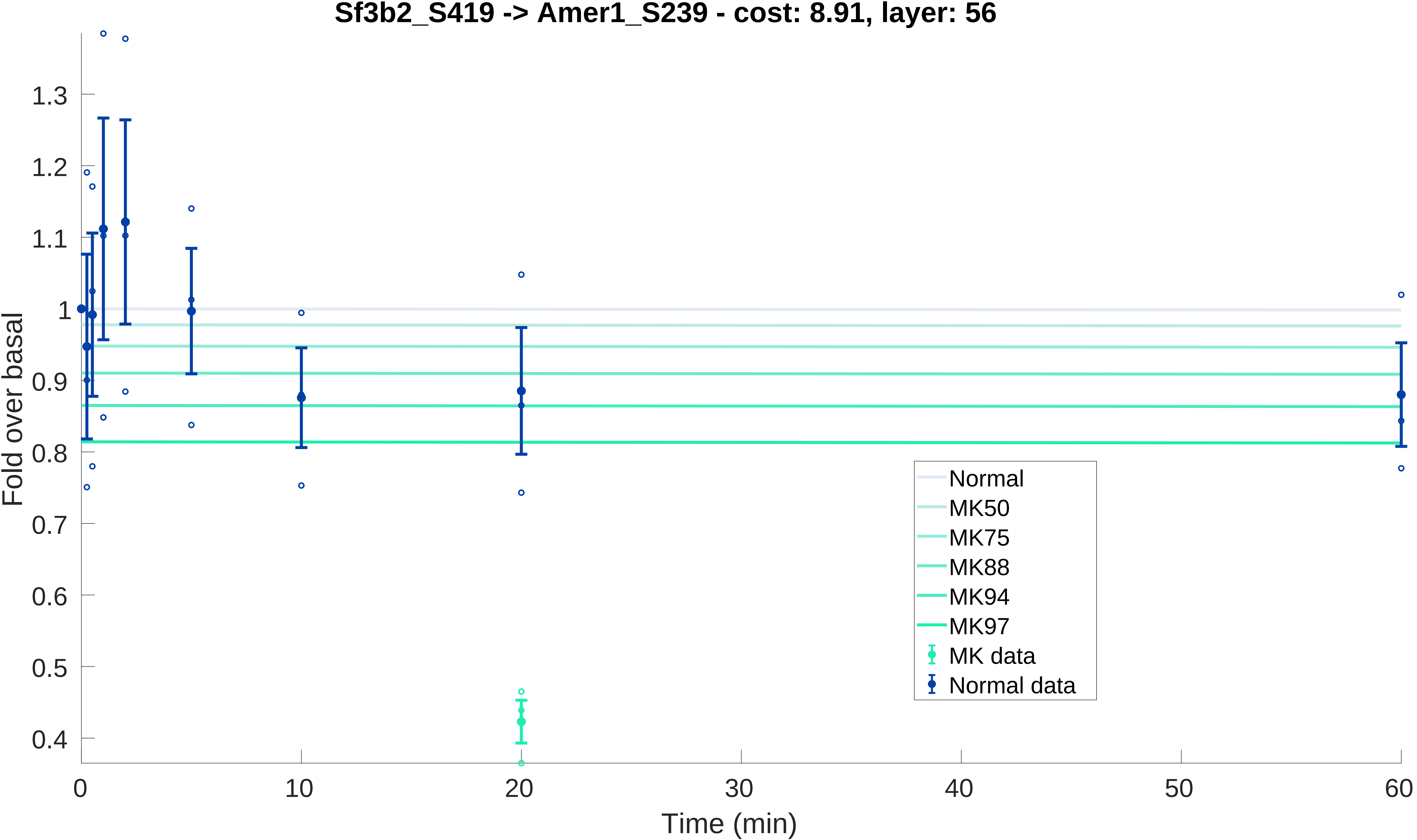

Sf3b2_S419 -> Amer1_S239 - cost: 8.91, layer: 56
Fold over basal
Time (min)
1.3
1.2
1.1
1
0.9
0.8
0.7
0.6
0.5
0.4
0
10
20
30
40
50
60
Normal
MK50
MK75
MK88
MK94
MK97
MK data
Normal data

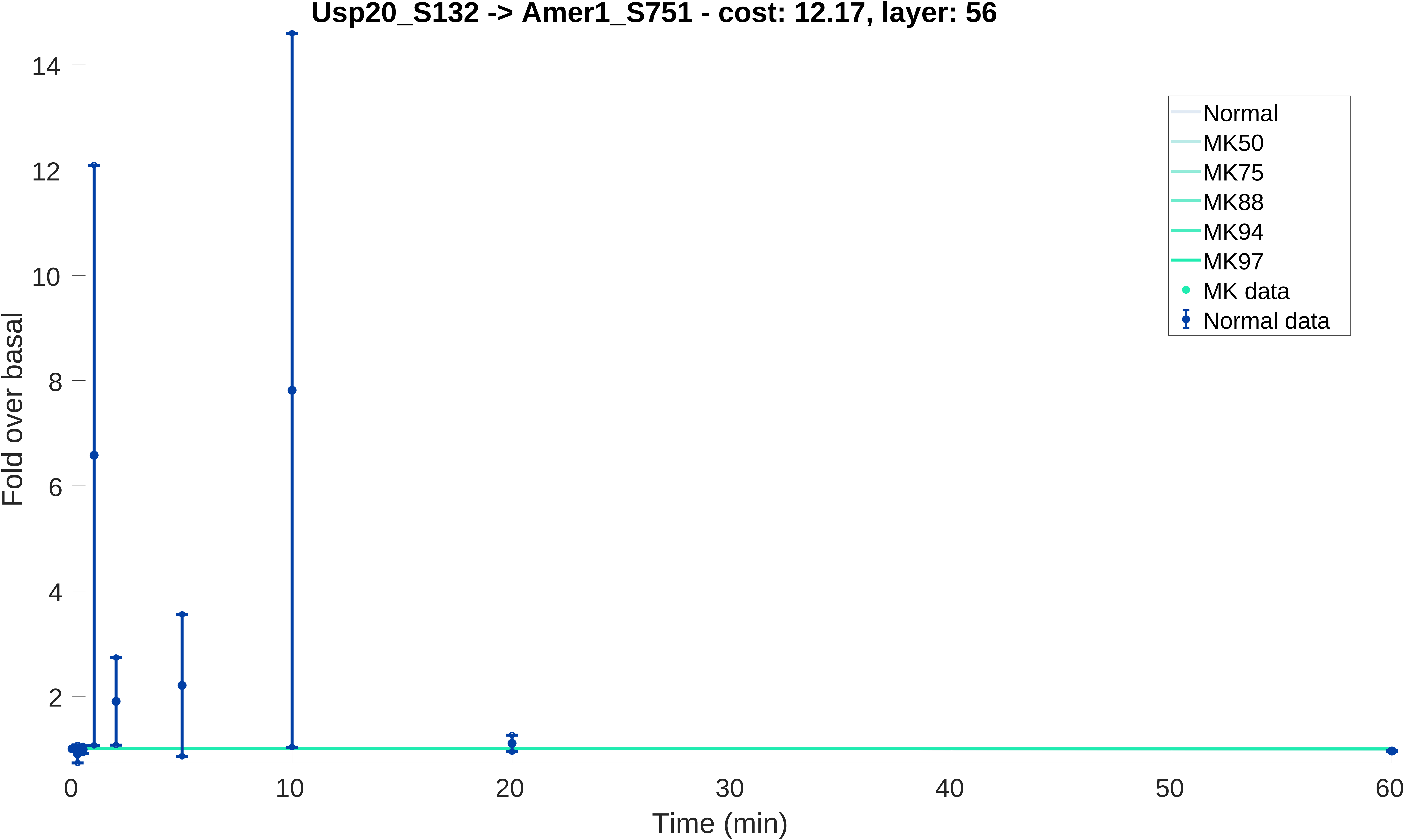

Usp20_S132 -> Amer1_S751 - cost: 12.17, layer: 56
Fold over basal
Time (min)
Normal
MK50
MK75
MK88
MK94
MK97
MK data
Normal data
0
10
20
30
40
50
60
2
4
6
8
10
12
14

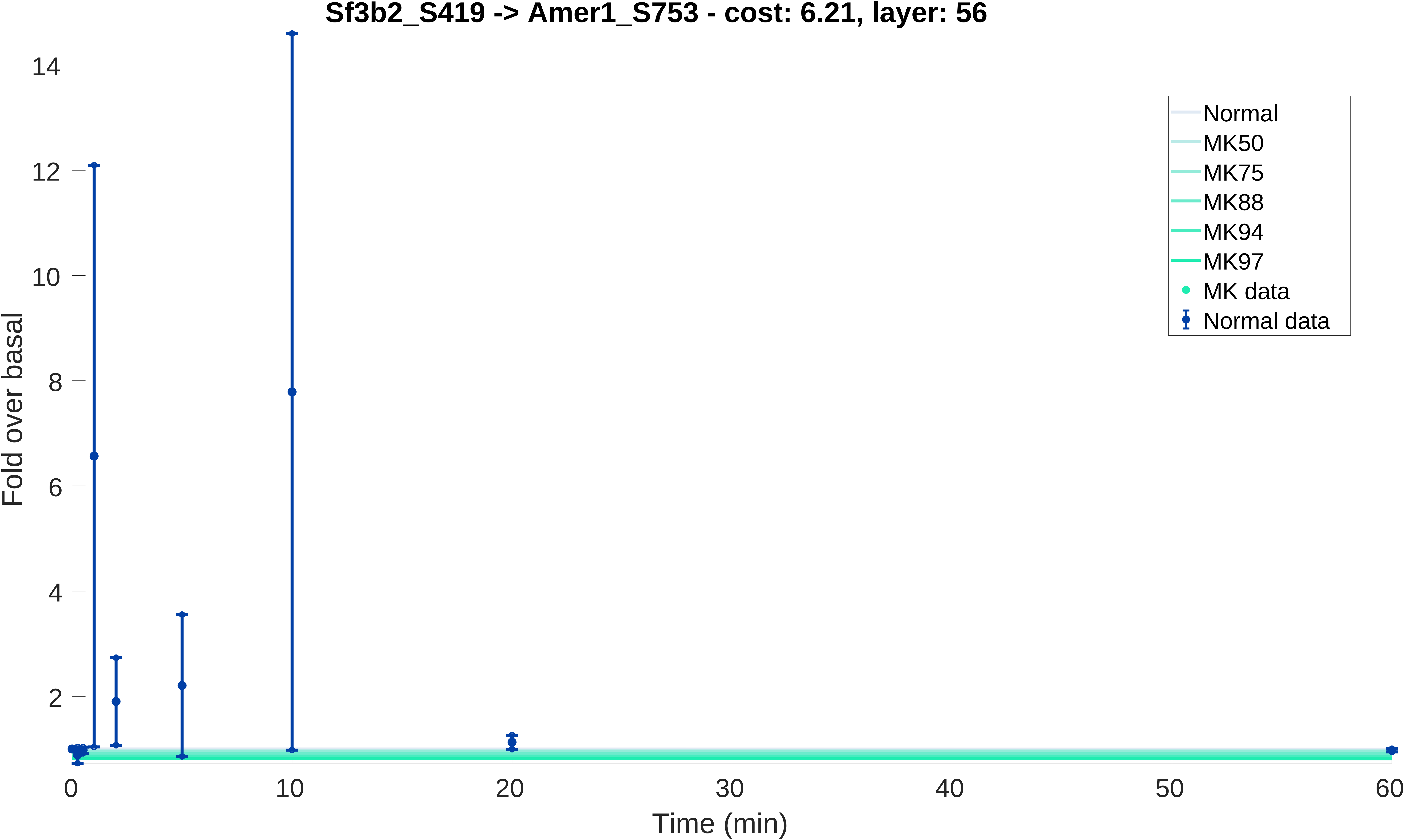

Sf3b2_S419 -> Amer1_S753 - cost: 6.21, layer: 56
Fold over basal
Time (min)
0
10
20
30
40
50
60
2
4
6
8
10
12
14
Normal
MK50
MK75
MK88
MK94
MK97
MK data
Normal data

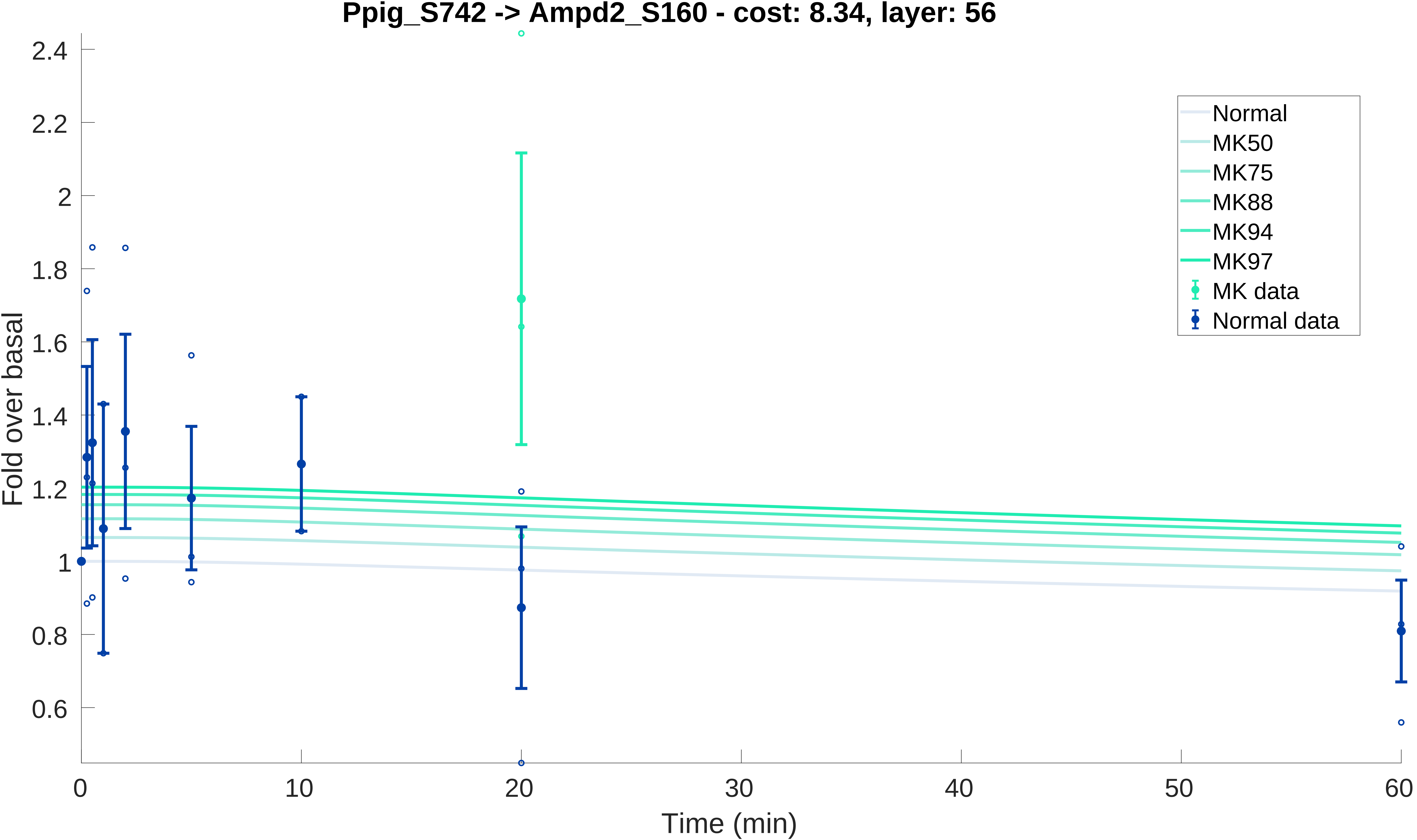

Ppig_S742 -> Ampd2_S160 - cost: 8.34, layer: 56
Fold over basal
Time (min)
Normal
MK50
MK75
MK88
MK94
MK97
MK data
Normal data
2.4
2.2
2
1.8
1.6
1.4
1.2
1
0.8
0.6
0
10
20
30
40
50
60

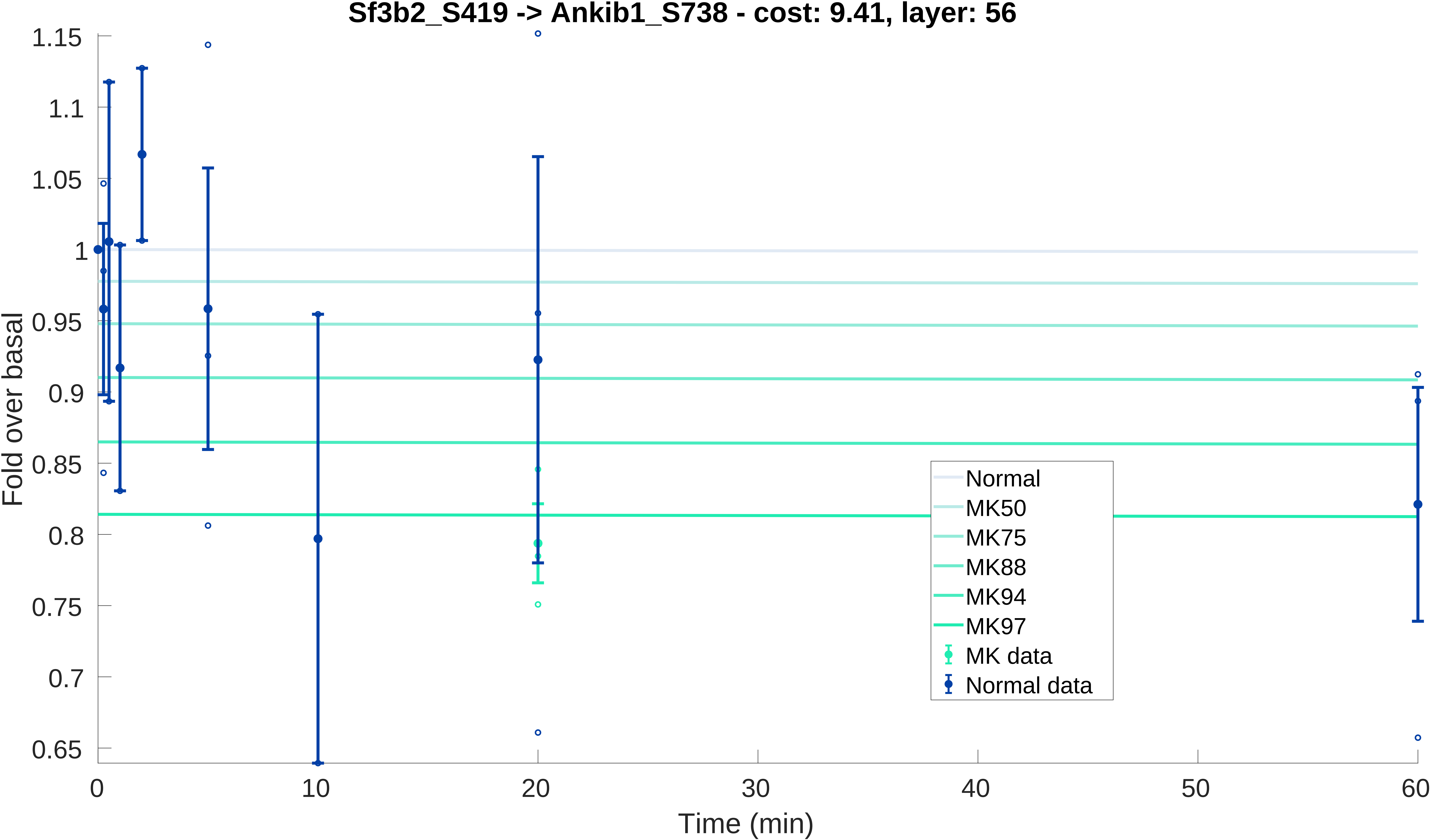

Sf3b2_S419 -> Ankib1_S738 - cost: 9.41, layer: 56
Fold over basal
1.15
1.1
1.05
1
0.95
0.9
0.85
0.8
0.75
0.7
0.65
Time (min)
0
10
20
30
40
50
60
Normal
MK50
MK75
MK88
MK94
MK97
MK data
Normal data

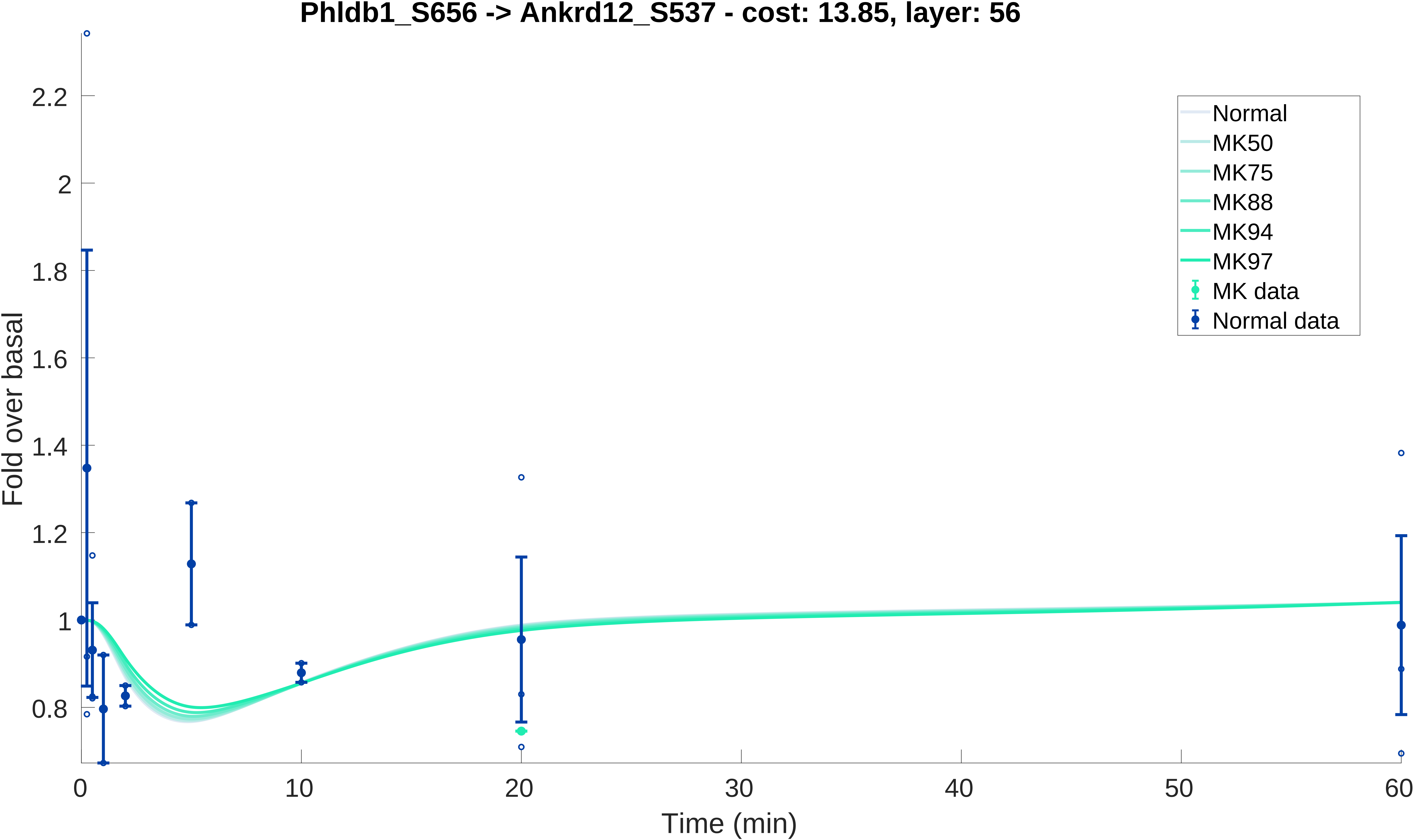

Phldb1_S656 -> Ankrd12_S537 - cost: 13.85, layer: 56
Fold over basal
Time (min)
0.8
1
1.2
1.4
1.6
1.8
2
2.2
0
10
20
30
40
50
60
Normal
MK50
MK75
MK88
MK94
MK97
MK data
Normal data

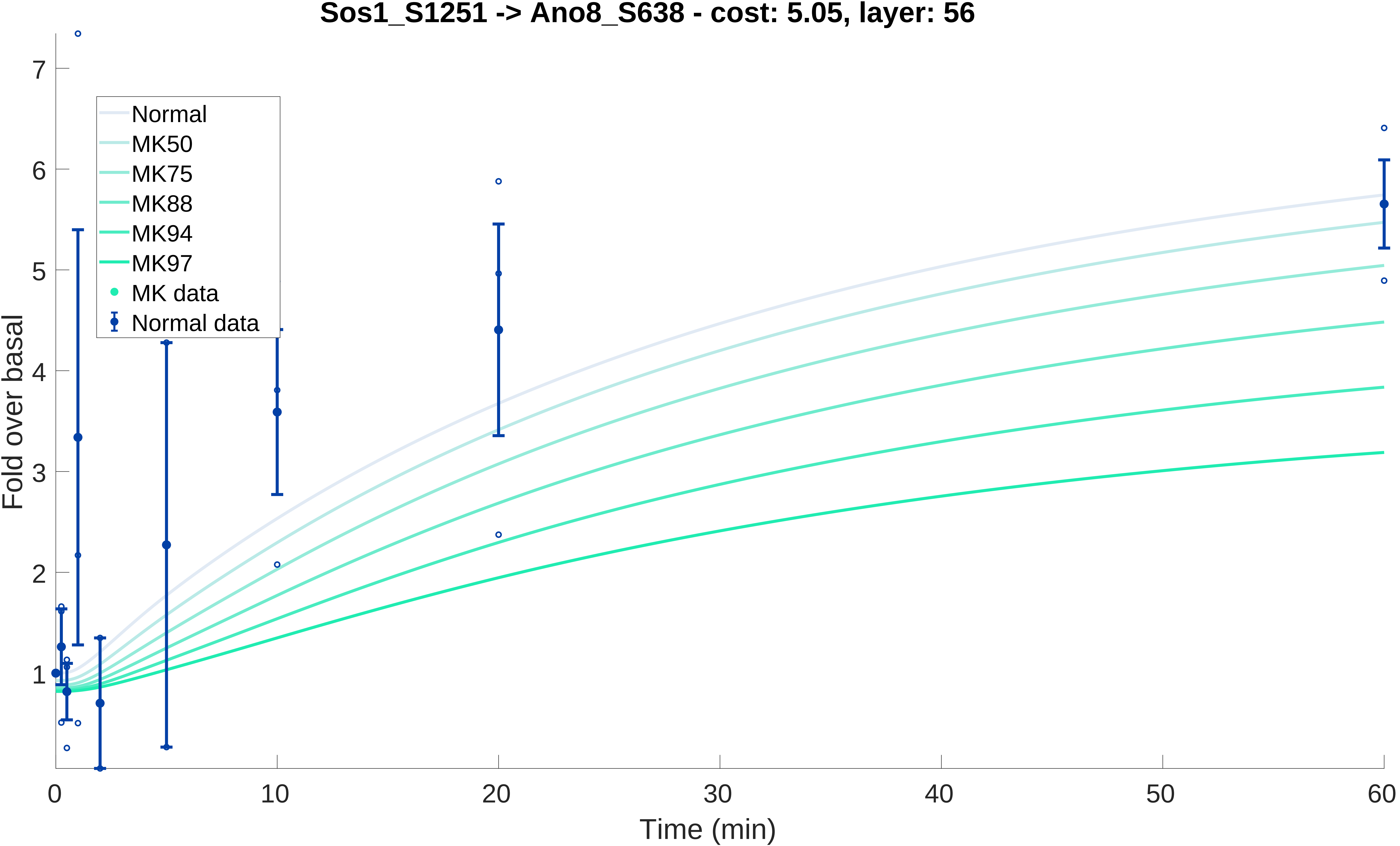

Sos1_S1251 -> Ano8_S638 - cost: 5.05, layer: 56
Normal
MK50
MK75
MK88
MK94
MK97
MK data
Normal data
Fold over basal
Time (min)
0
10
20
30
40
50
60
1
2
3
4
5
6
7

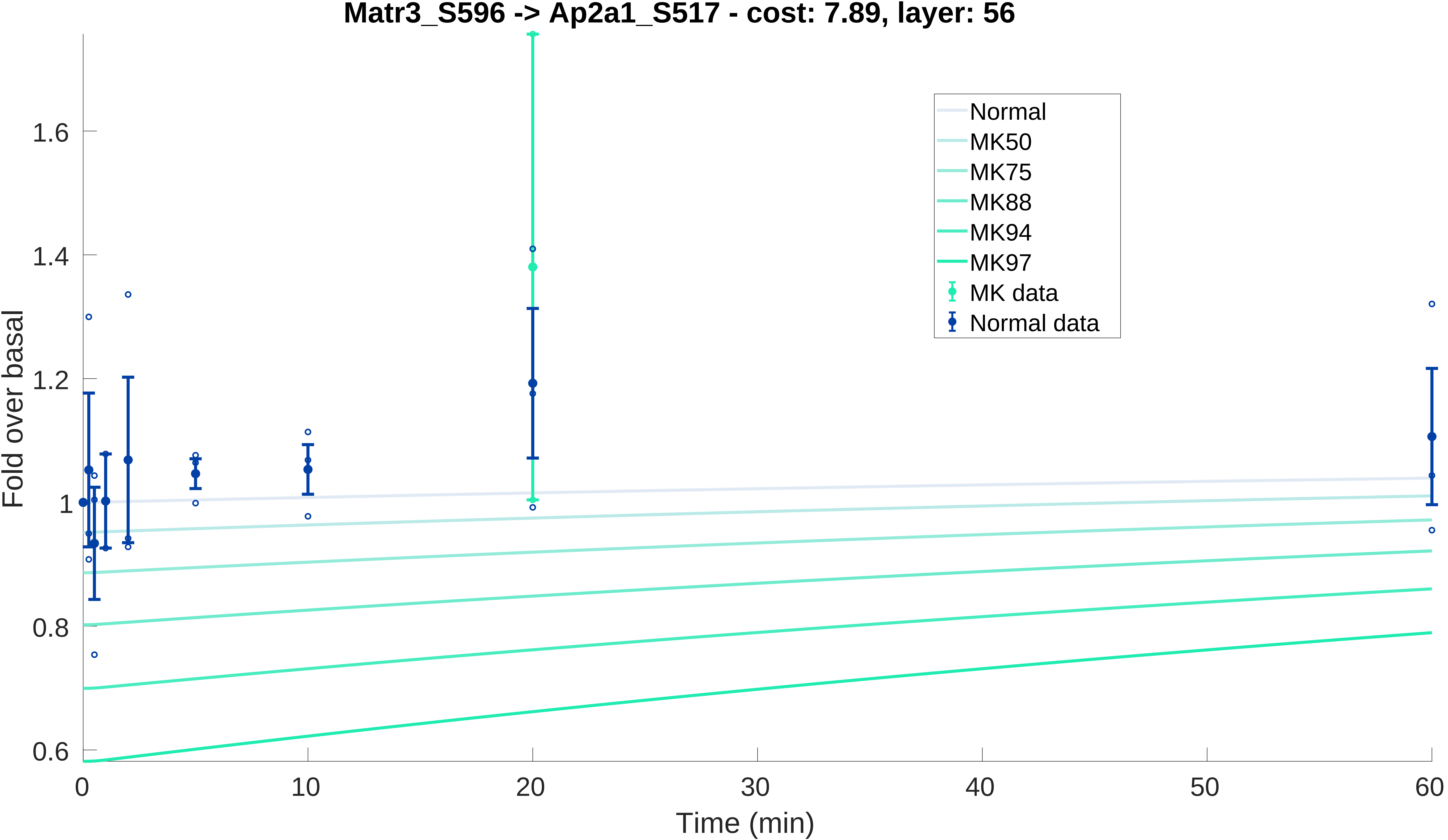

Matr3_S596 -> Ap2a1_S517 - cost: 7.89, layer: 56
Fold over basal
Time (min)
Normal
MK50
MK75
MK88
MK94
MK97
MK data
Normal data
1.6
1.4
1.2
1
0.8
0.6
0
10
20
30
40
50
60

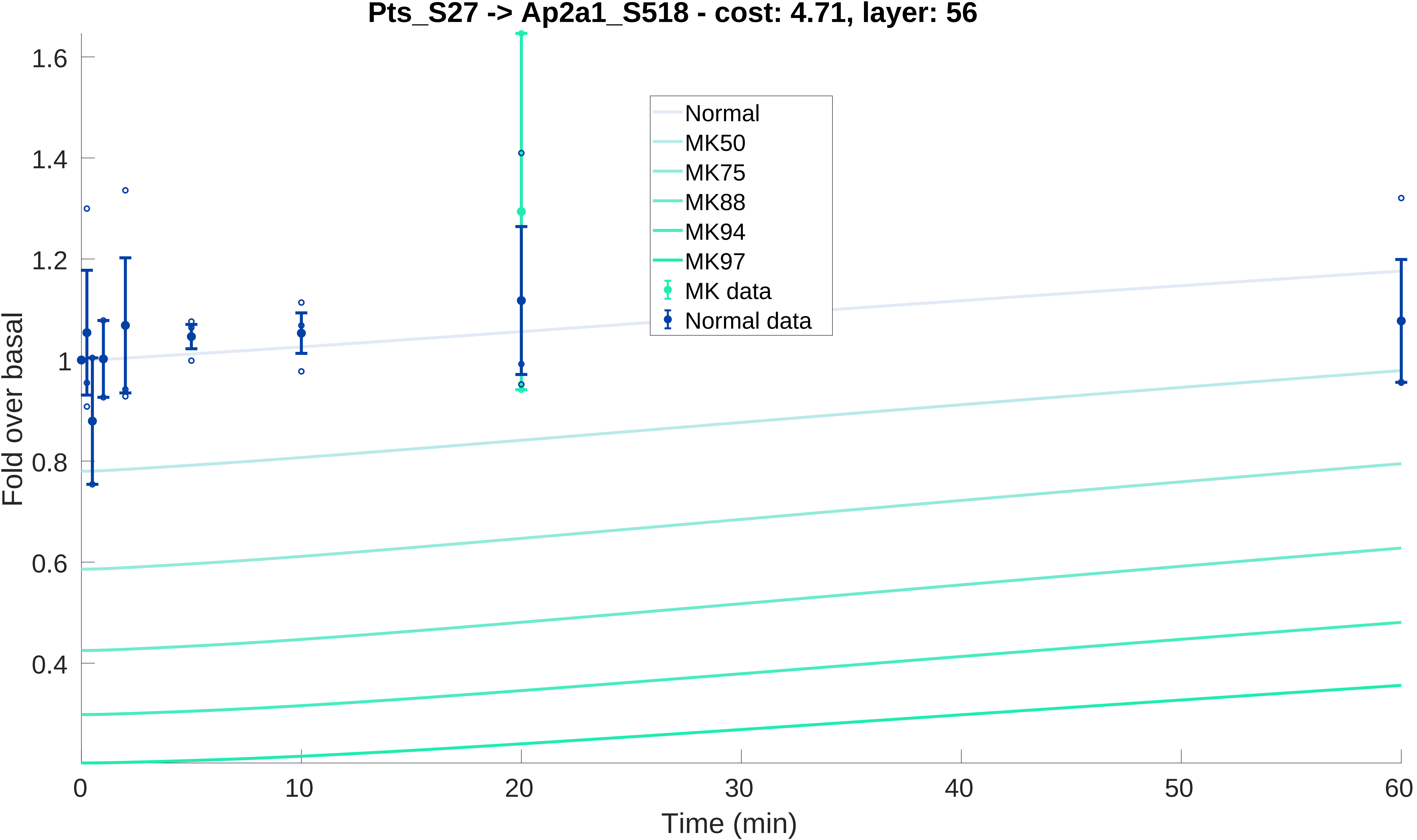
Pts_S27 -> Ap2a1_S518 - cost: 4.71, layer: 56
Fold over basal
Time (min)
0
10
20
30
40
50
60
0.4
0.6
0.8
1
1.2
1.4
1.6
Normal
MK50
MK75
MK88
MK94
MK97
MK data
Normal data

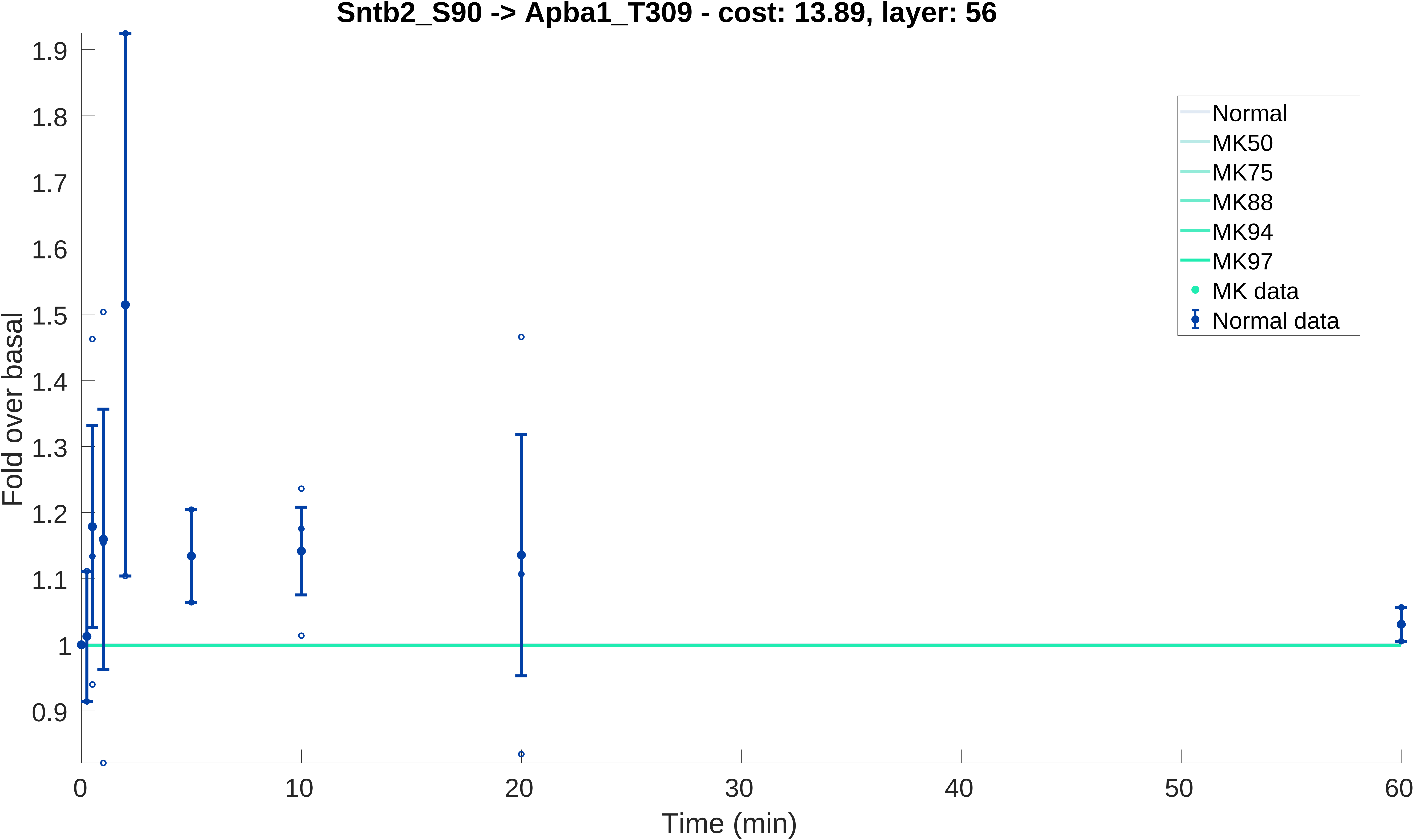

Sntb2_S90 -> Apba1_T309 - cost: 13.89, layer: 56
Fold over basal
Time (min)
0.9
1
1.1
1.2
1.3
1.4
1.5
1.6
1.7
1.8
1.9
0
10
20
30
40
50
60
Normal
MK50
MK75
MK88
MK94
MK97
MK data
Normal data

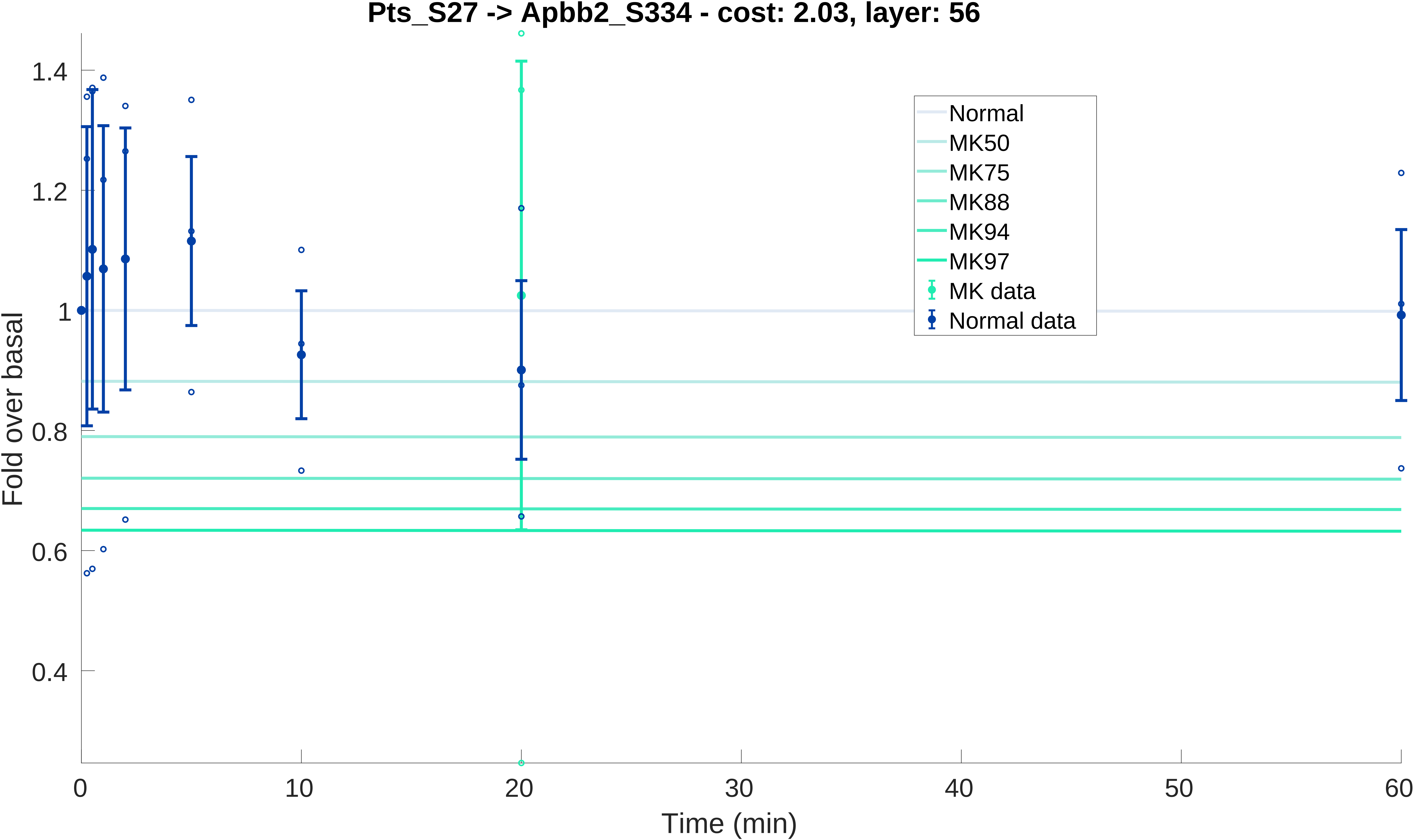

Pts_S27 -> Apbb2_S334 - cost: 2.03, layer: 56
Fold over basal
Time (min)
0.4
0.6
0.8
1
1.2
1.4
0
10
20
30
40
50
60
Normal
MK50
MK75
MK88
MK94
MK97
MK data
Normal data

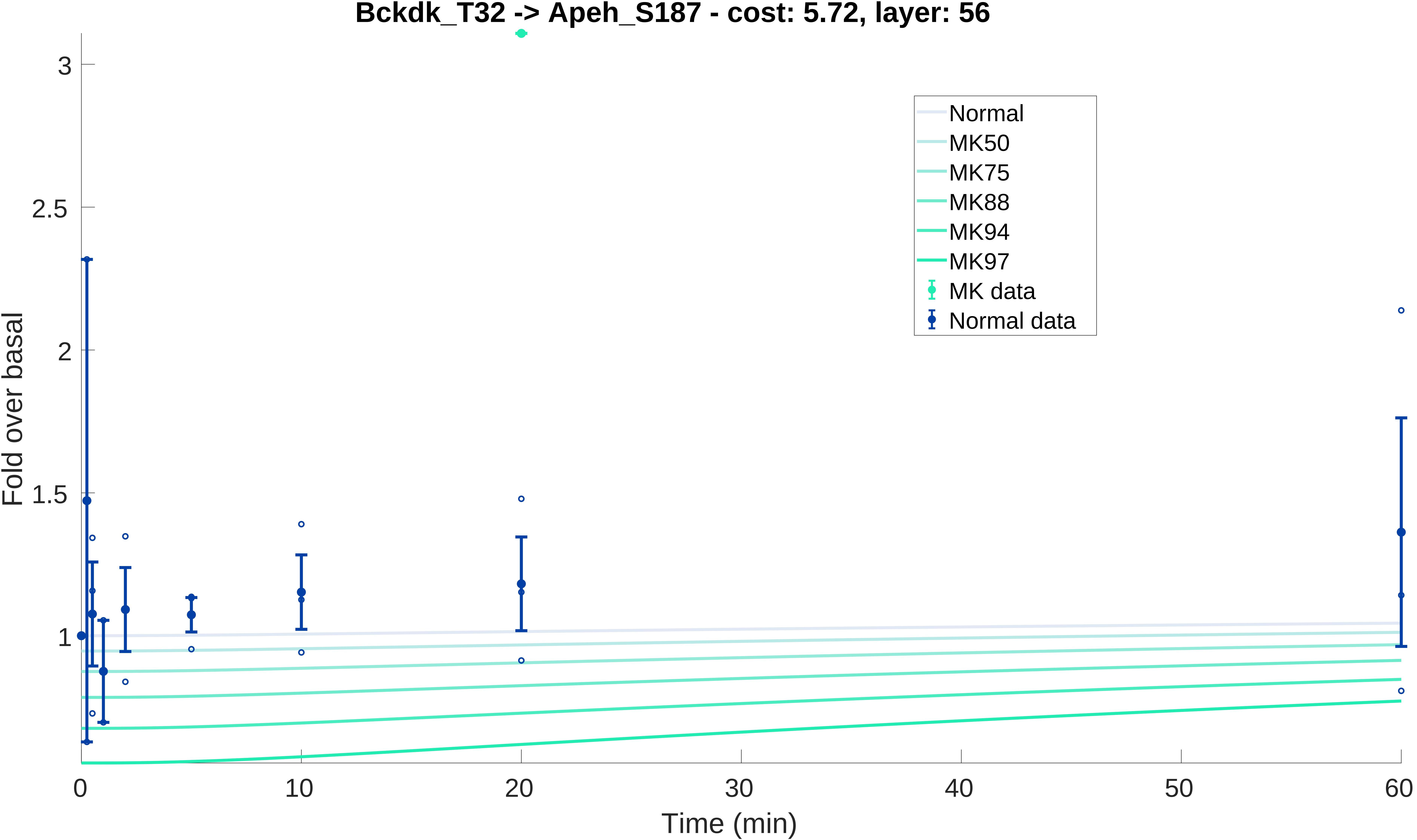

Bckdk_T32 -> Apeh_S187 - cost: 5.72, layer: 56
Fold over basal
Time (min)
0
10
20
30
40
50
60
1
1.5
2
2.5
3
Normal
MK50
MK75
MK88
MK94
MK97
MK data
Normal data

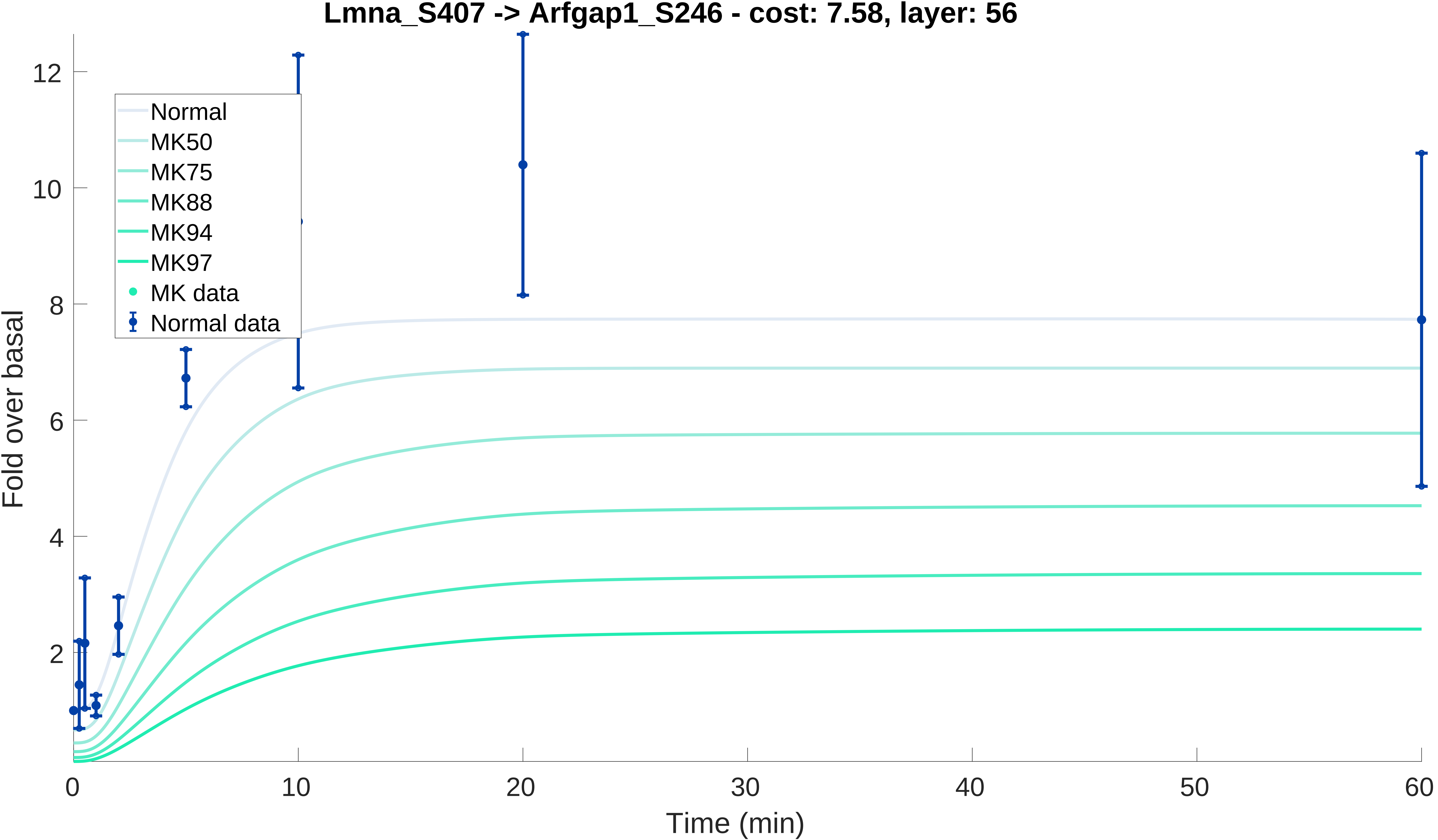

Lmna_S407 -> Arfgap1_S246 - cost: 7.58, layer: 56
Normal
MK50
MK75
MK88
MK94
MK97
MK data
Normal data
Fold over basal
Time (min)
0
10
20
30
40
50
60
2
4
6
8
10
12

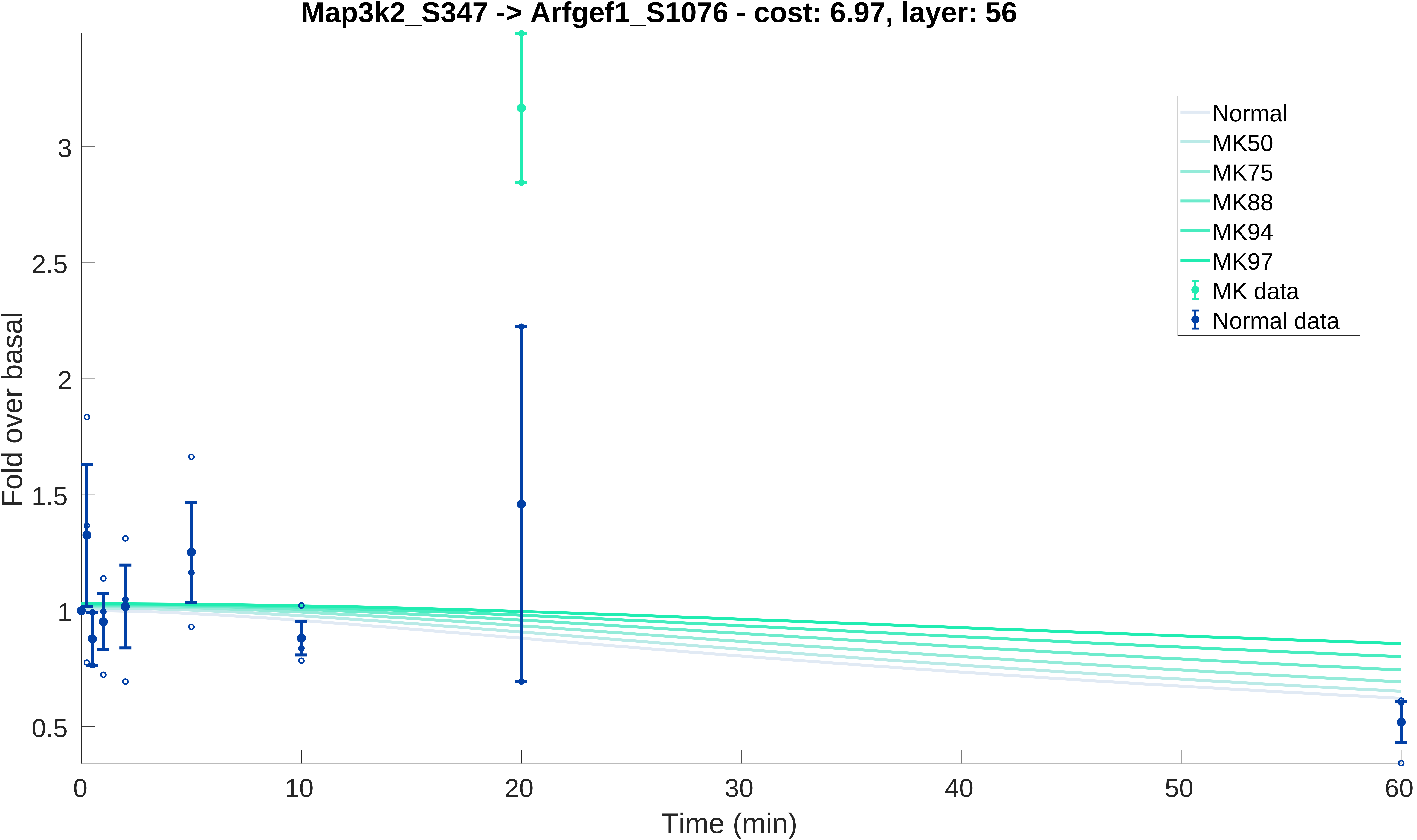

Map3k2_S347 -> Arfgef1_S1076 - cost: 6.97, layer: 56
Fold over basal
Time (min)
0.5
1
1.5
2
2.5
3
0
10
20
30
40
50
60
Normal
MK50
MK75
MK88
MK94
MK97
MK data
Normal data

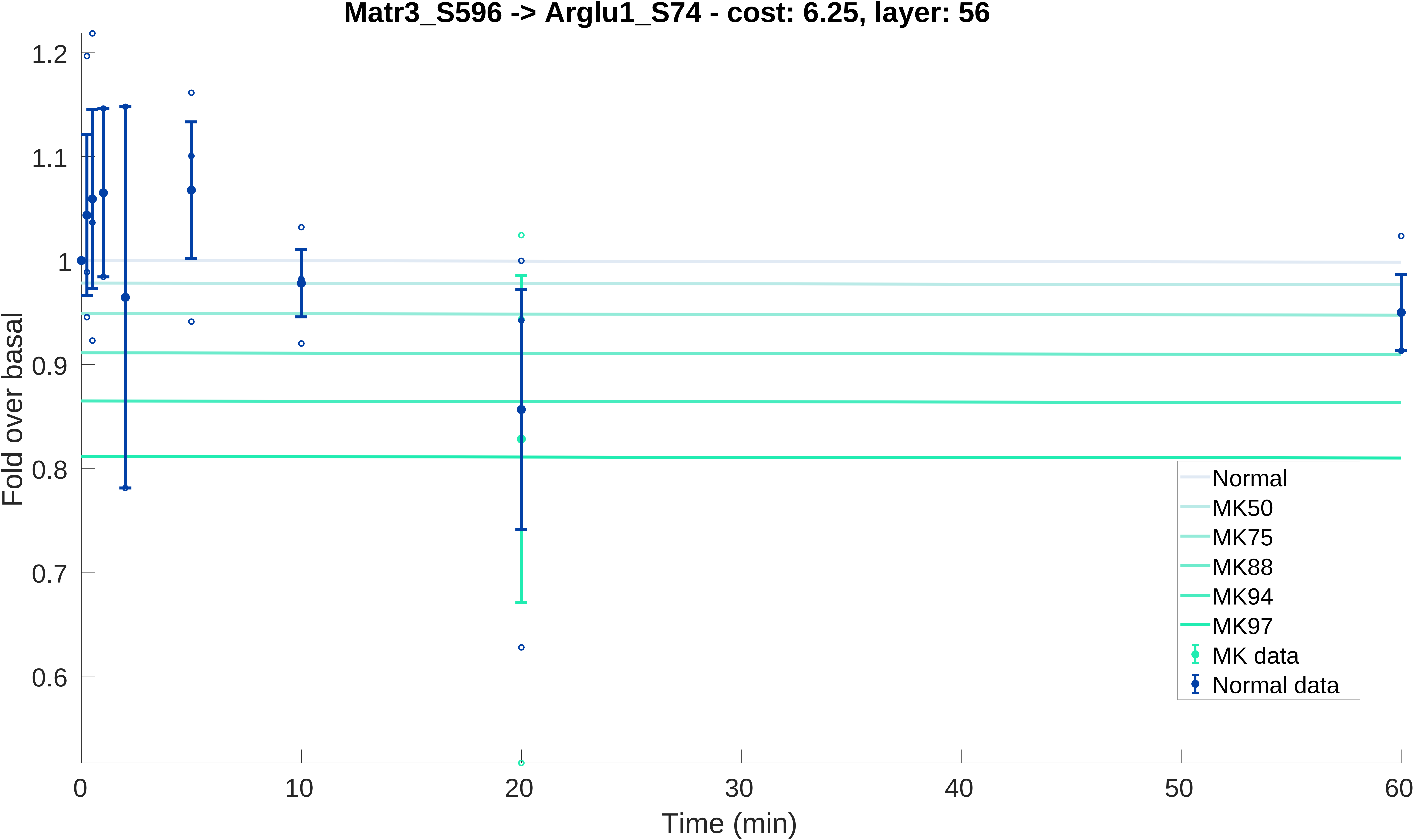

Matr3_S596 -> Arglu1_S74 - cost: 6.25, layer: 56
Fold over basal
1.2
1.1
1
0.9
0.8
0.7
0.6
0
10
20
30
40
50
60
Time (min)
Normal
MK50
MK75
MK88
MK94
MK97
MK data
Normal data

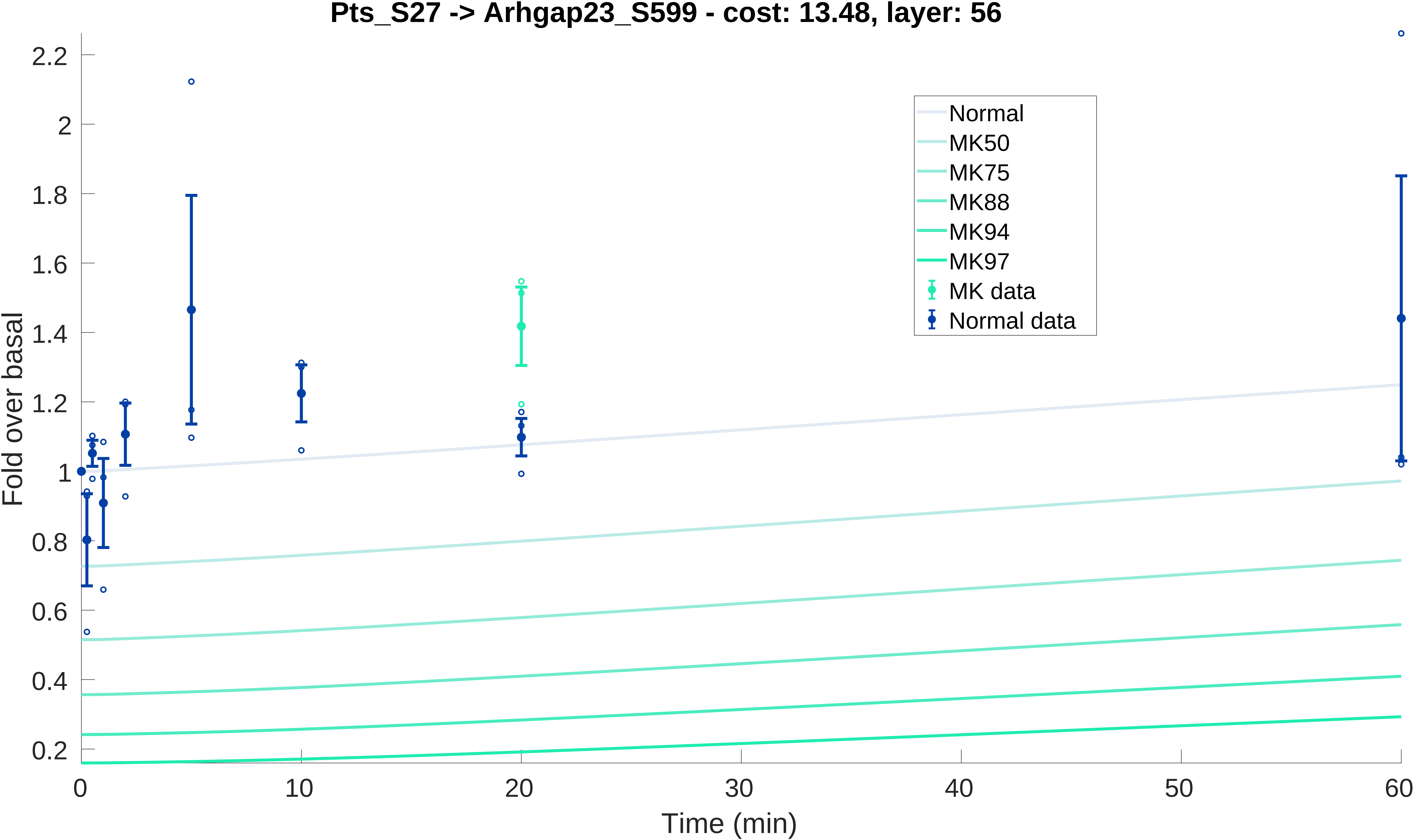
Pts_S27 -> Arhgap23_S599 - cost: 13.48, layer: 56
Fold over basal
Time (min)
0
10
20
30
40
50
60
0.2
0.4
0.6
0.8
1
1.2
1.4
1.6
1.8
2
2.2
Normal
MK50
MK75
MK88
MK94
MK97
MK data
Normal data

Clmn_S925 -> Arhgap23_S624 - cost: 8.52, layer: 56

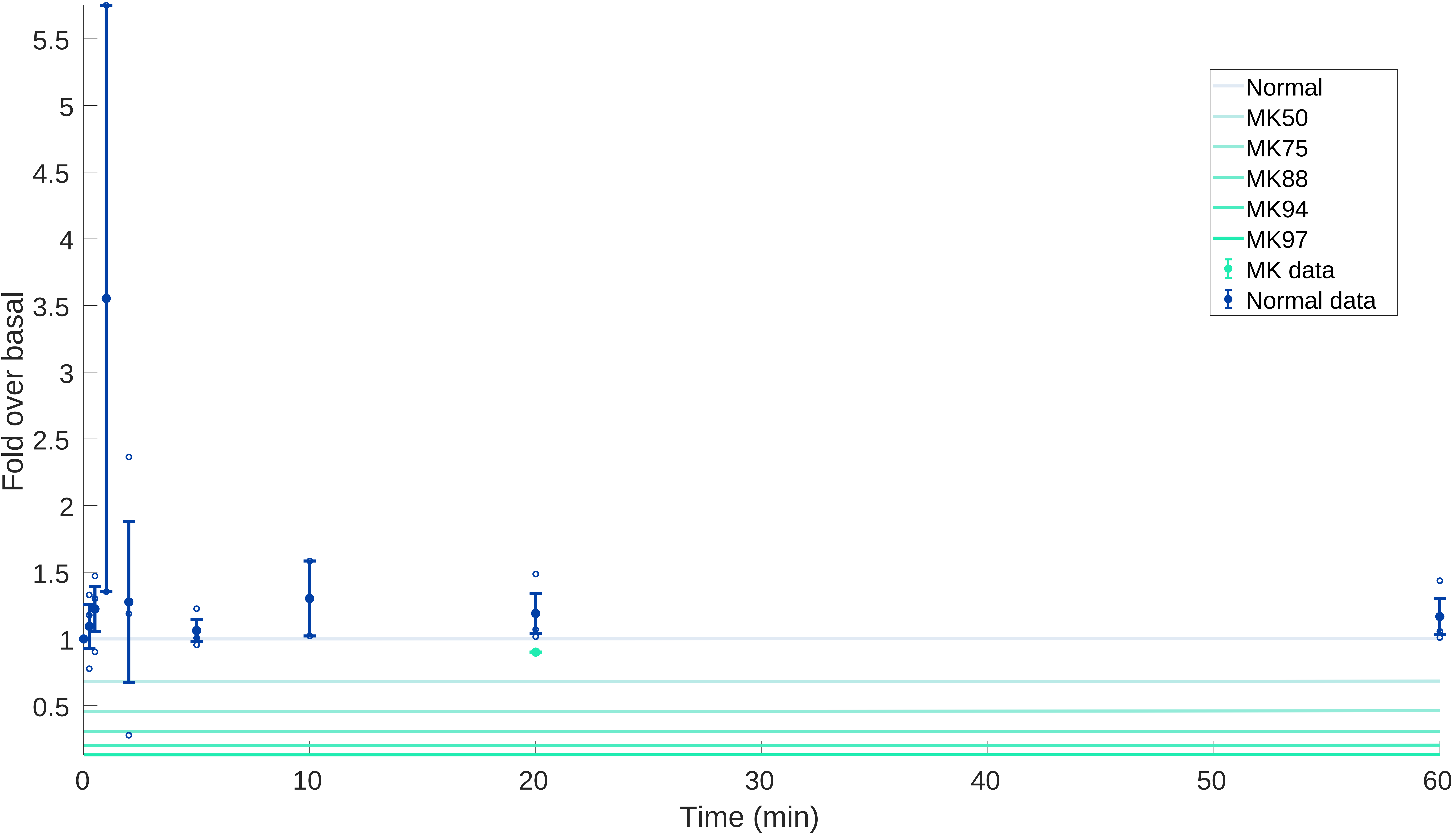

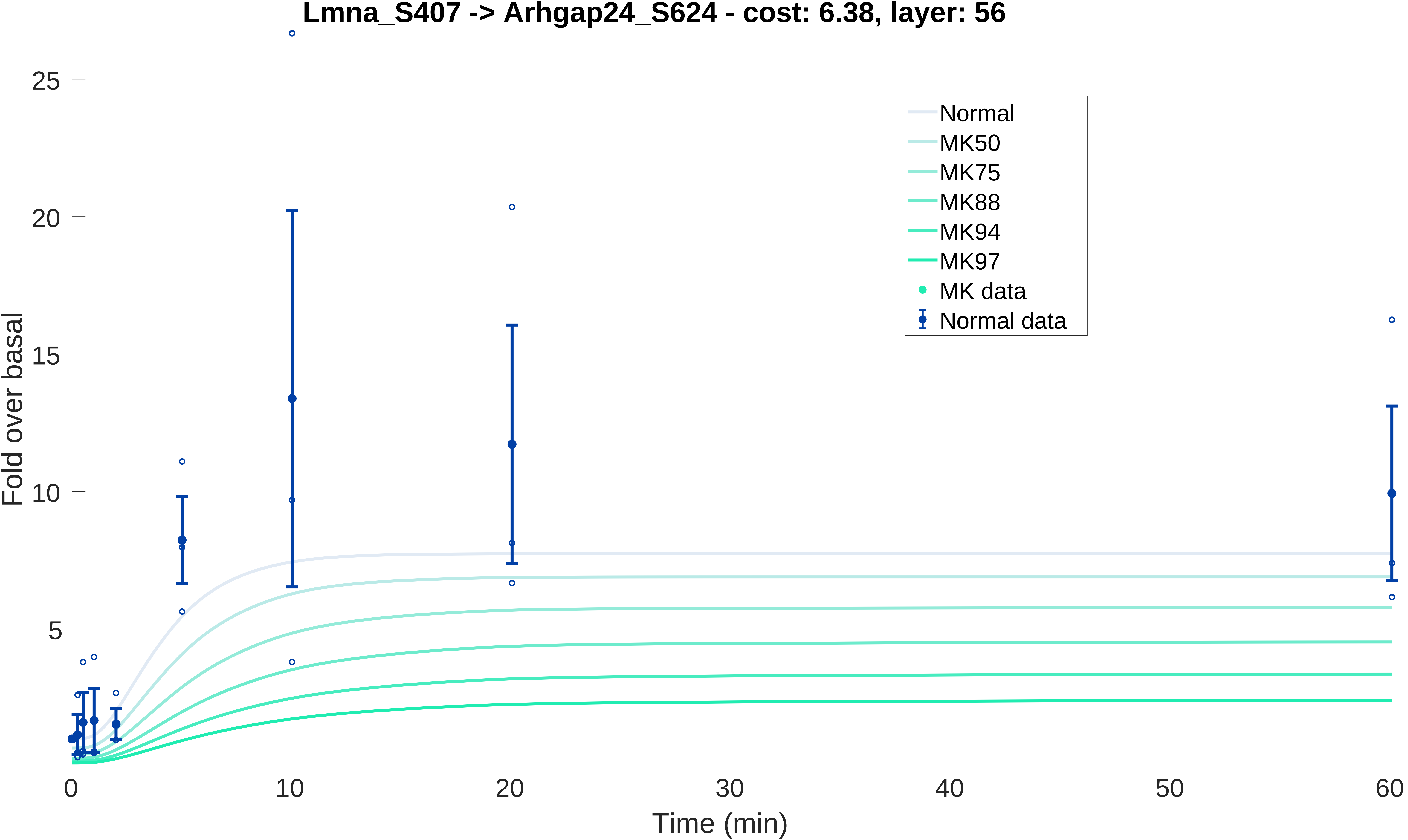

Lmna_S407 -> Arhgap24_S624 - cost: 6.38, layer: 56
Fold over basal
Time (min)
0
5
10
15
20
25
30
40
50
60
Normal
MK50
MK75
MK88
MK94
MK97
MK data
Normal data

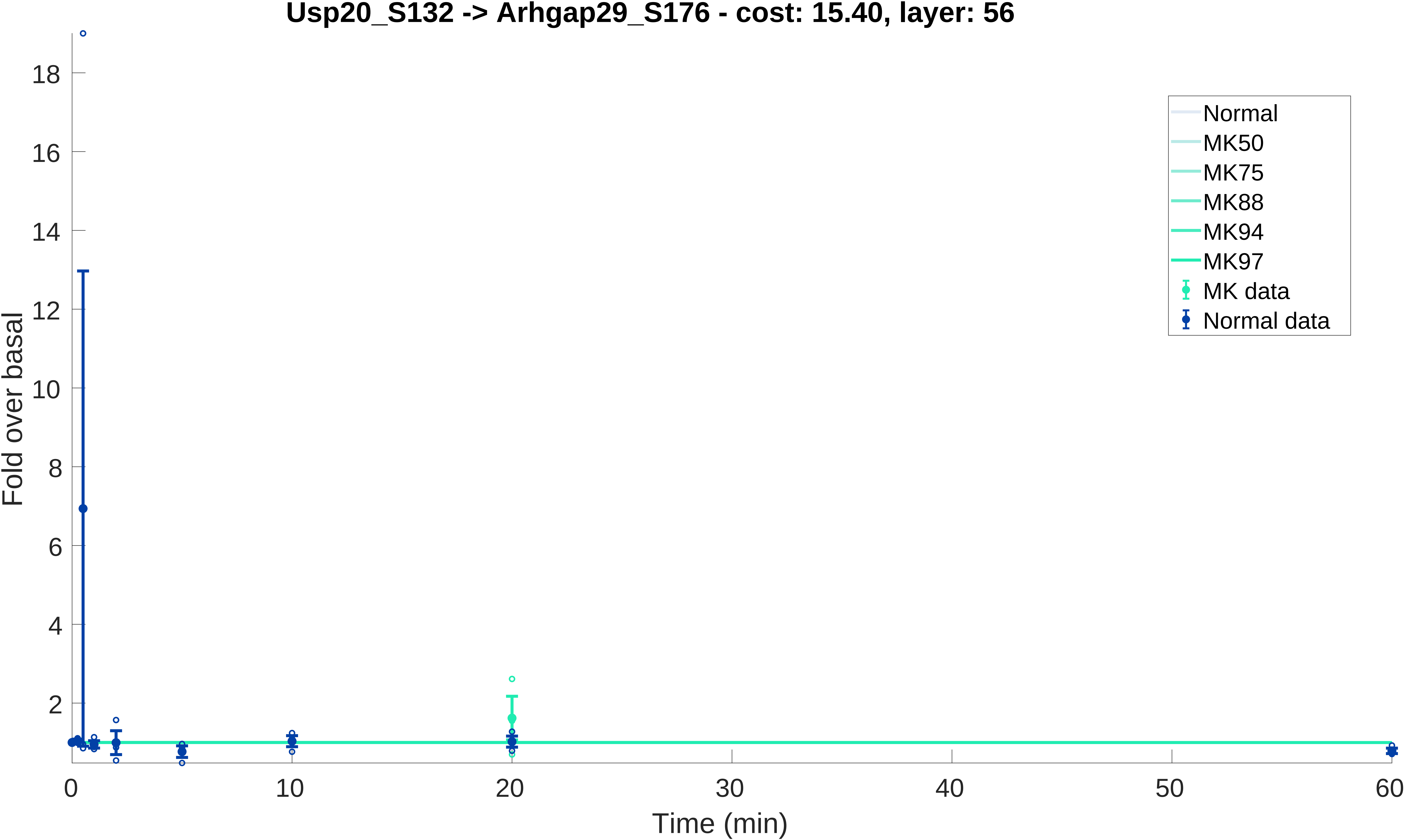

Usp20_S132 -> Arhgap29_S176 - cost: 15.40, layer: 56
Fold over basal
Time (min)
0
10
20
30
40
50
60
2
4
6
8
10
12
14
16
18
Normal
MK50
MK75
MK88
MK94
MK97
MK data
Normal data

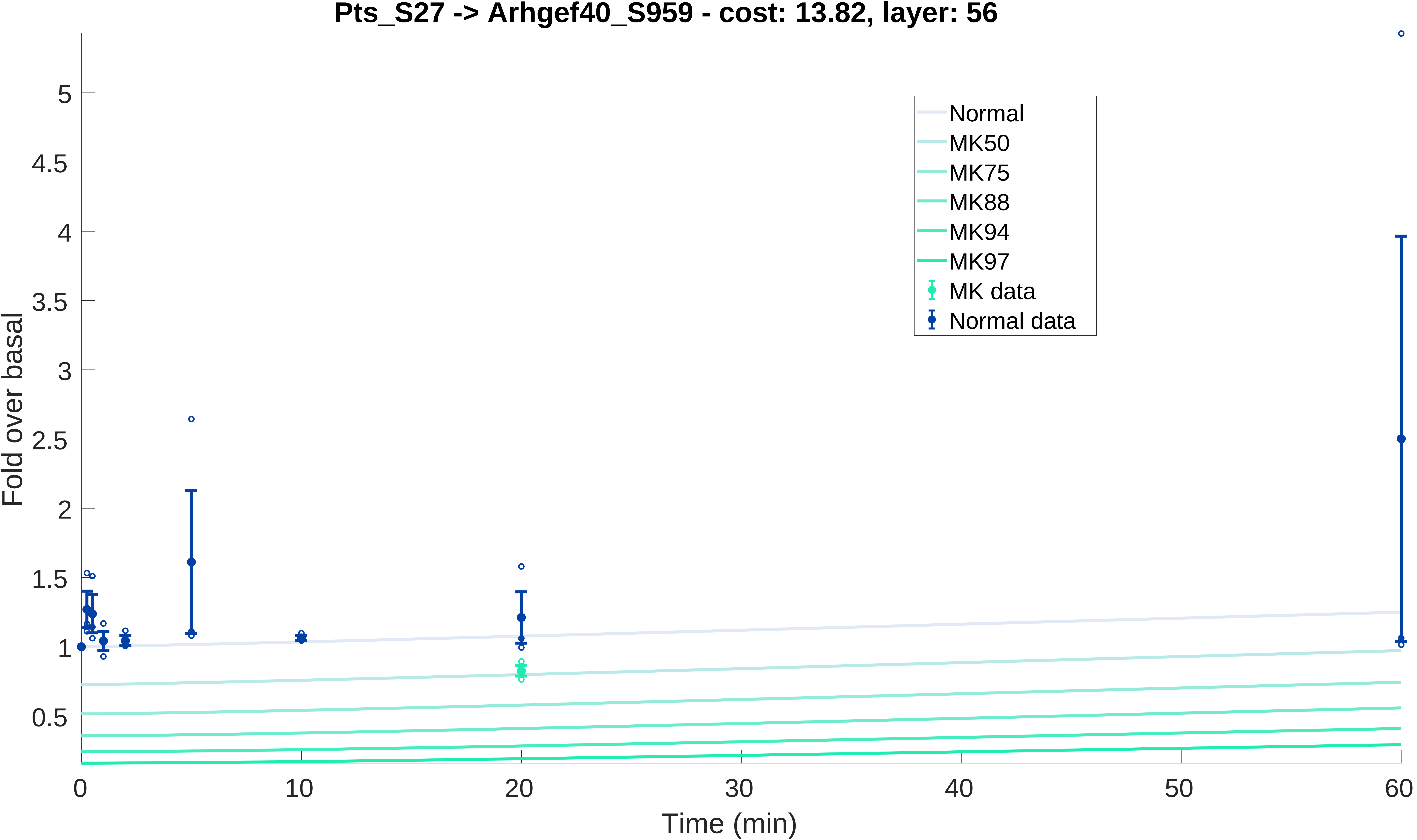

Pts_S27 -> Arhgef40_S959 - cost: 13.82, layer: 56
Fold over basal
Time (min)
0.5
1
1.5
2
2.5
3
3.5
4
4.5
5
0
10
20
30
40
50
60
Normal
MK50
MK75
MK88
MK94
MK97
MK data
Normal data

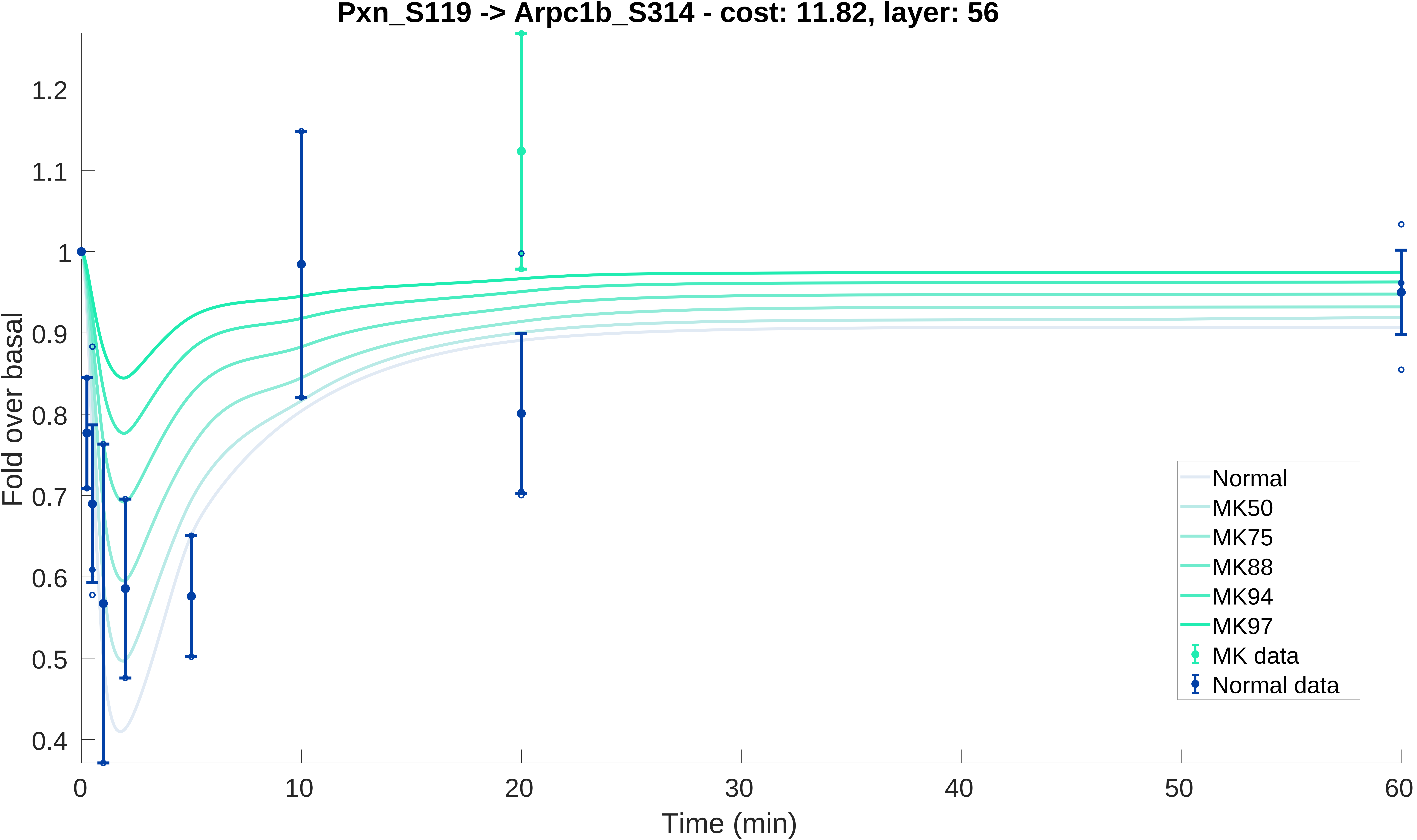

Pxn_S119 -> Arpc1b_S314 - cost: 11.82, layer: 56
Fold over basal
Time (min)
0.4
0.5
0.6
0.7
0.8
0.9
1
1.1
1.2
0
10
20
30
40
50
60
Normal
MK50
MK75
MK88
MK94
MK97
MK data
Normal data

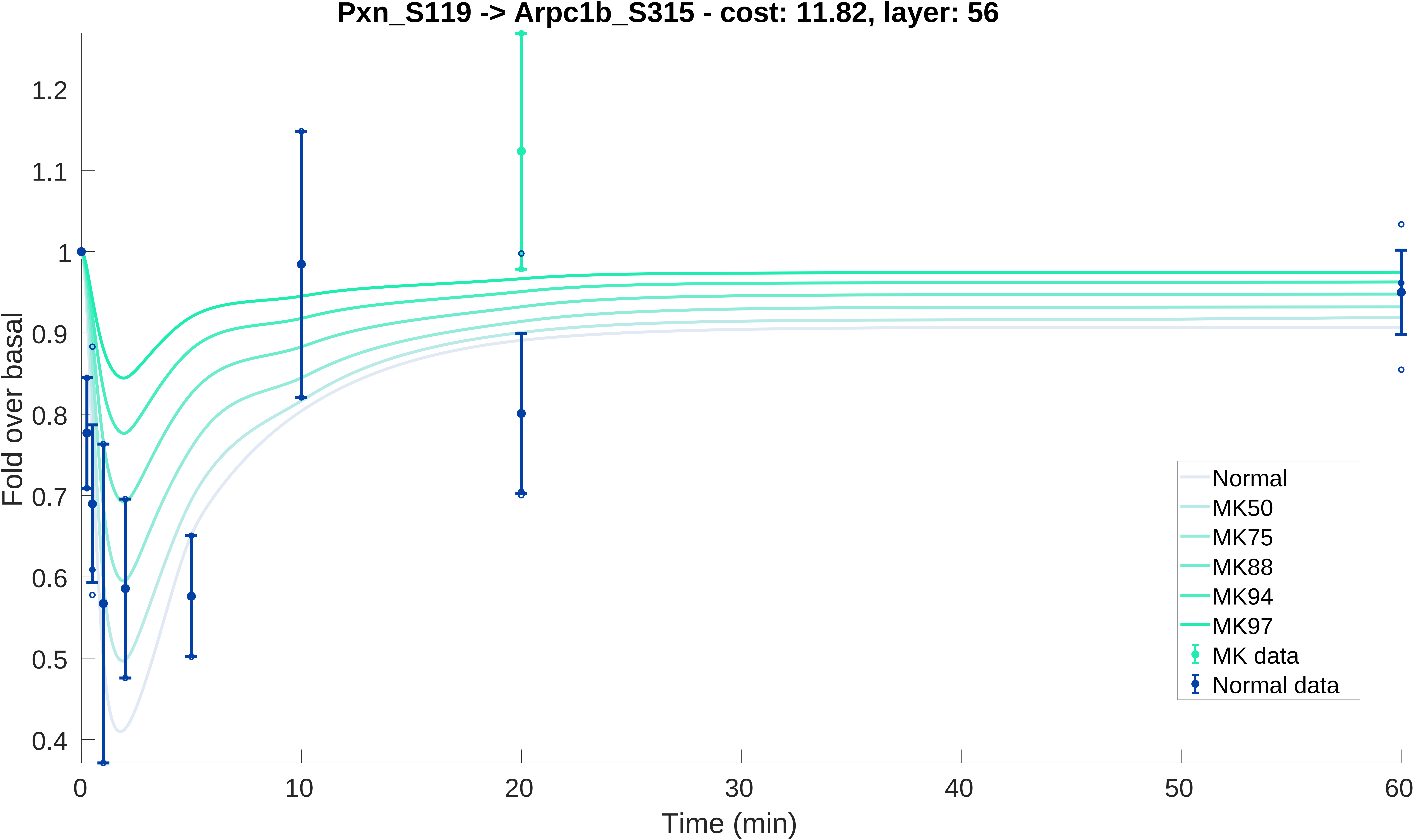

Pxn_S119 -> Arpc1b_S315 - cost: 11.82, layer: 56
Fold over basal
Time (min)
0.4
0.5
0.6
0.7
0.8
0.9
1
1.1
1.2
0
10
20
30
40
50
60
Normal
MK50
MK75
MK88
MK94
MK97
MK data
Normal data

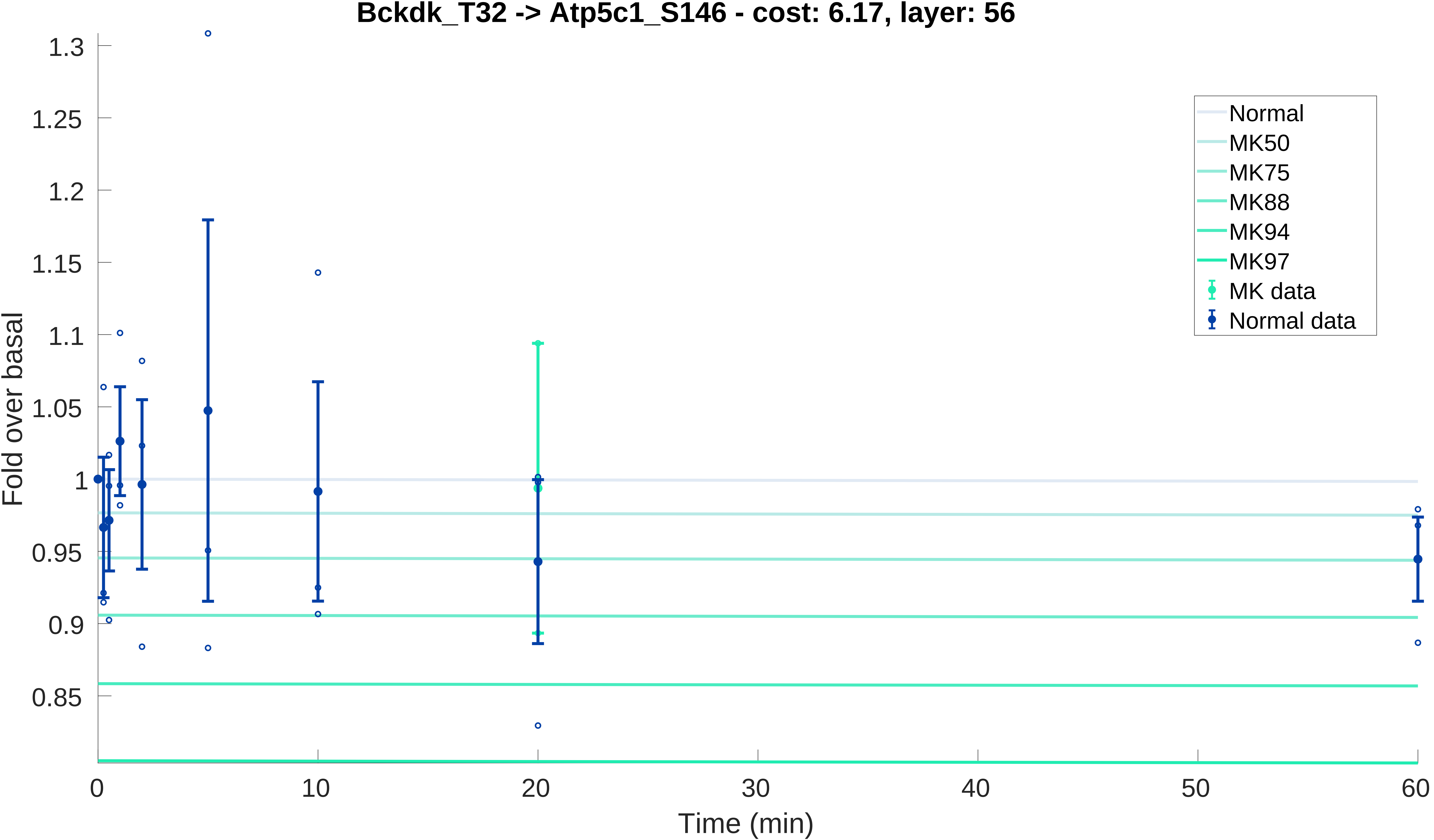
Bckdk_T32 -> Atp5c1_S146 - cost: 6.17, layer: 56
Fold over basal
Time (min)
1.3
1.25
1.2
1.15
1.1
1.05
1
0.95
0.9
0.85
0
10
20
30
40
50
60
Normal
MK50
MK75
MK88
MK94
MK97
MK data
Normal data

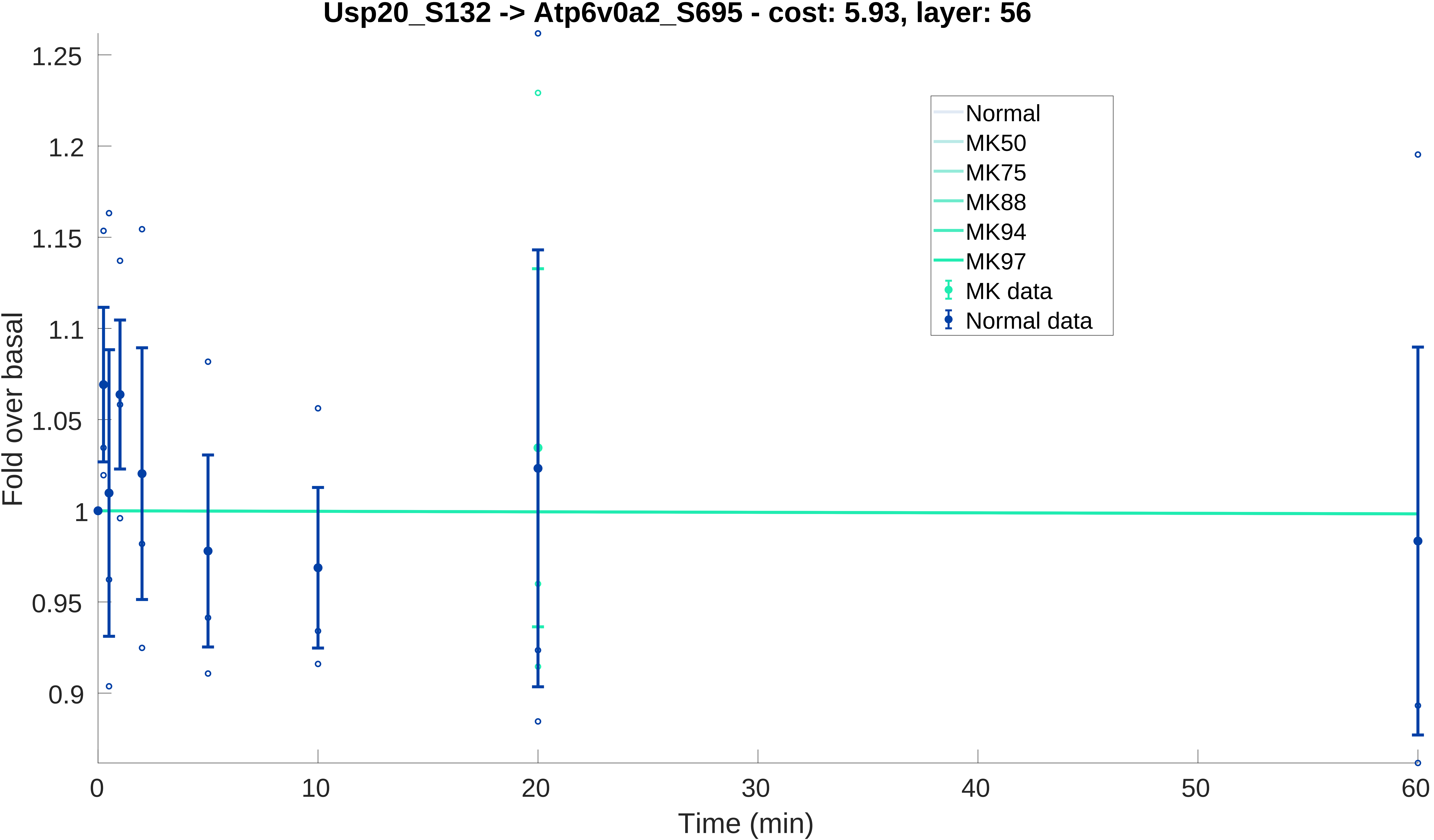

Usp20_S132 -> Atp6v0a2_S695 - cost: 5.93, layer: 56
Fold over basal
Time (min)
1.25
1.2
1.15
1.1
1.05
1
0.95
0.9
0
10
20
30
40
50
60
Normal
MK50
MK75
MK88
MK94
MK97
MK data
Normal data

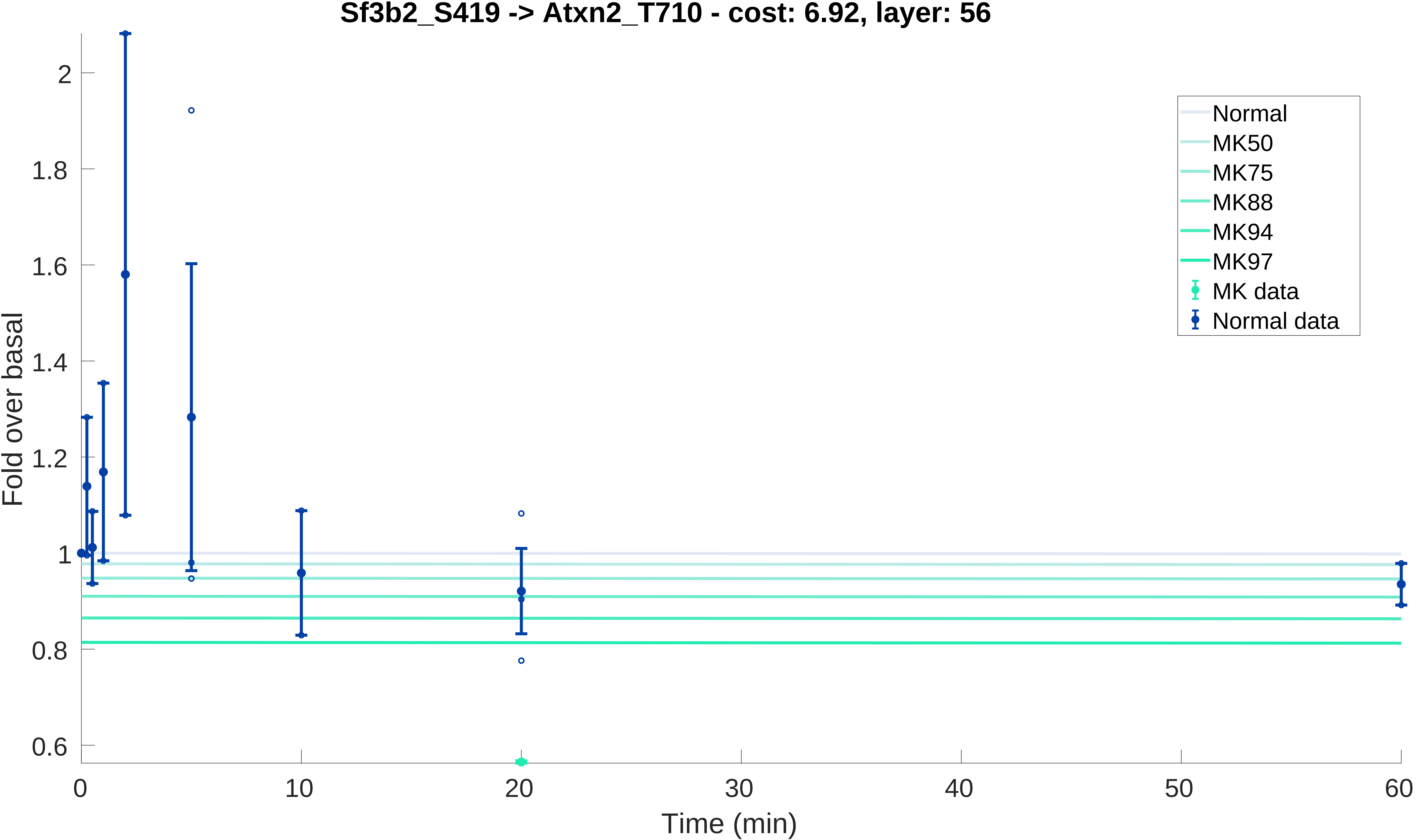
Sf3b2_S419 -> Atxn2_T710 - cost: 6.92, layer: 56
Fold over basal
Time (min)
0.6
0.8
1
1.2
1.4
1.6
1.8
2
0
10
20
30
40
50
60
Normal
MK50
MK75
MK88
MK94
MK97
MK data
Normal data

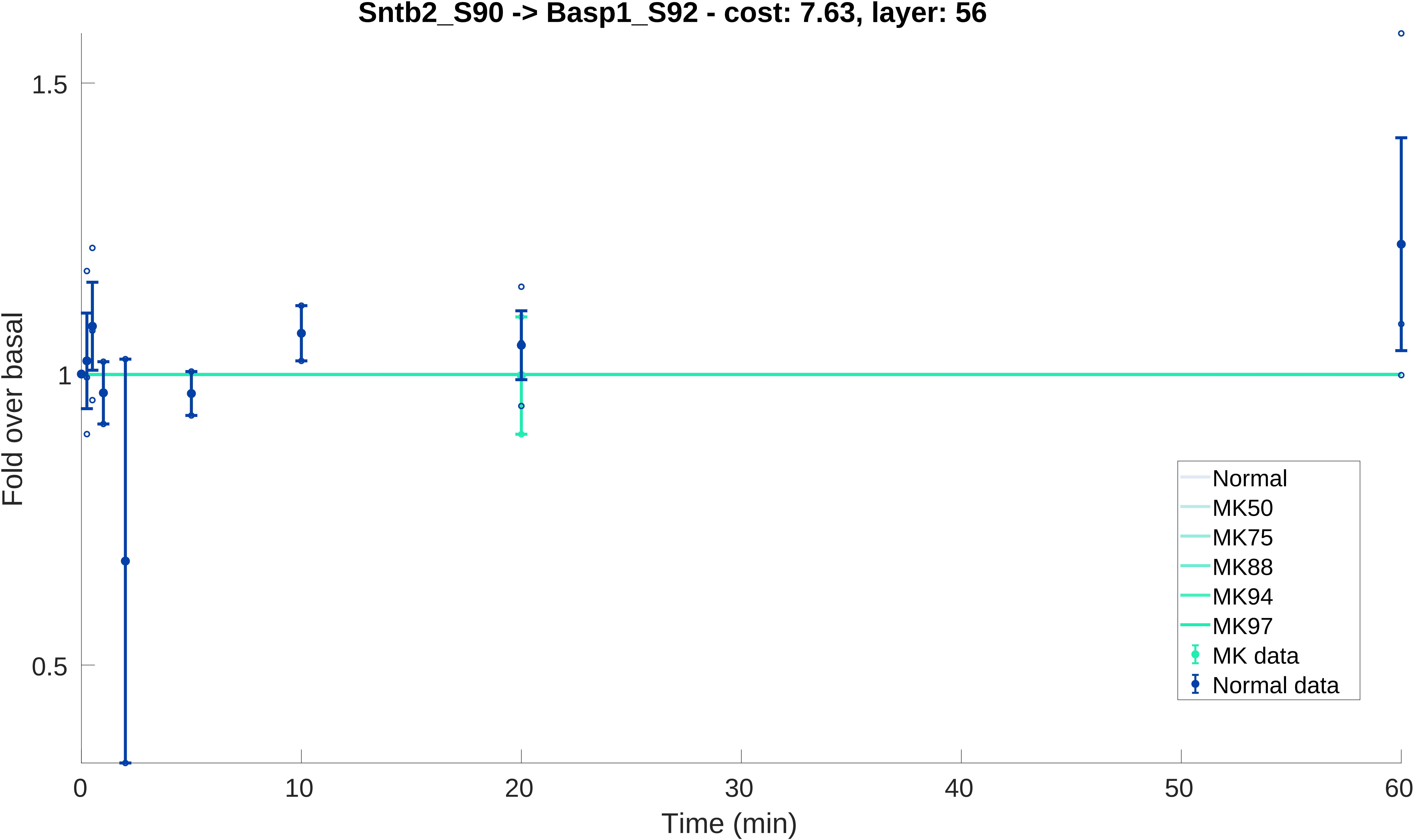

Sntb2_S90 -> Basp1_S92 - cost: 7.63, layer: 56
Fold over basal
1.5
1
0.5
0
10
20
30
40
50
60
Time (min)
Normal
MK50
MK75
MK88
MK94
MK97
MK data
Normal data

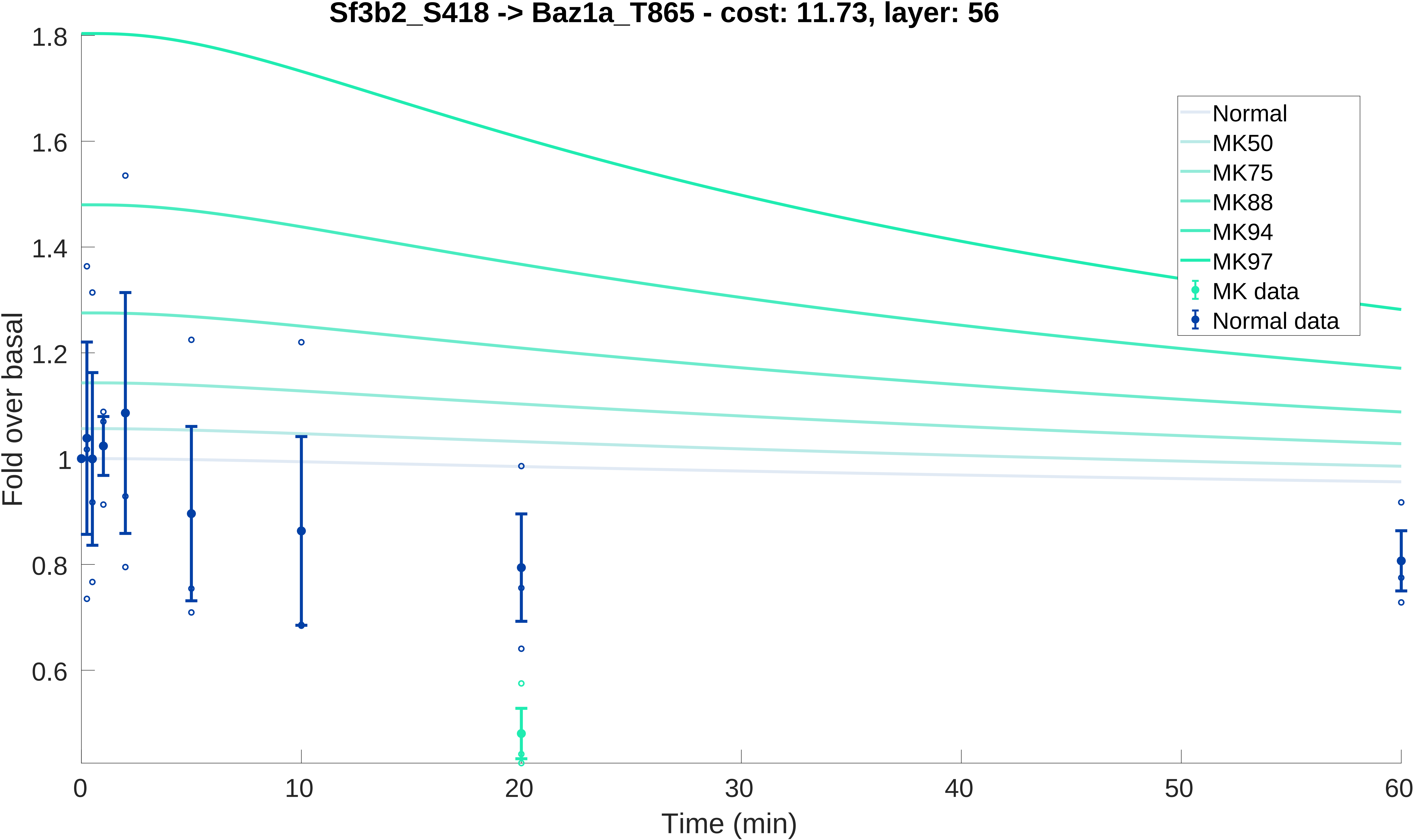

Sf3b2_S418 -> Baz1a_T865 - cost: 11.73, layer: 56
Fold over basal
1.8
1.6
1.4
1.2
1
0.8
0.6
Time (min)
0
10
20
30
40
50
60
Normal
MK50
MK75
MK88
MK94
MK97
MK data
Normal data

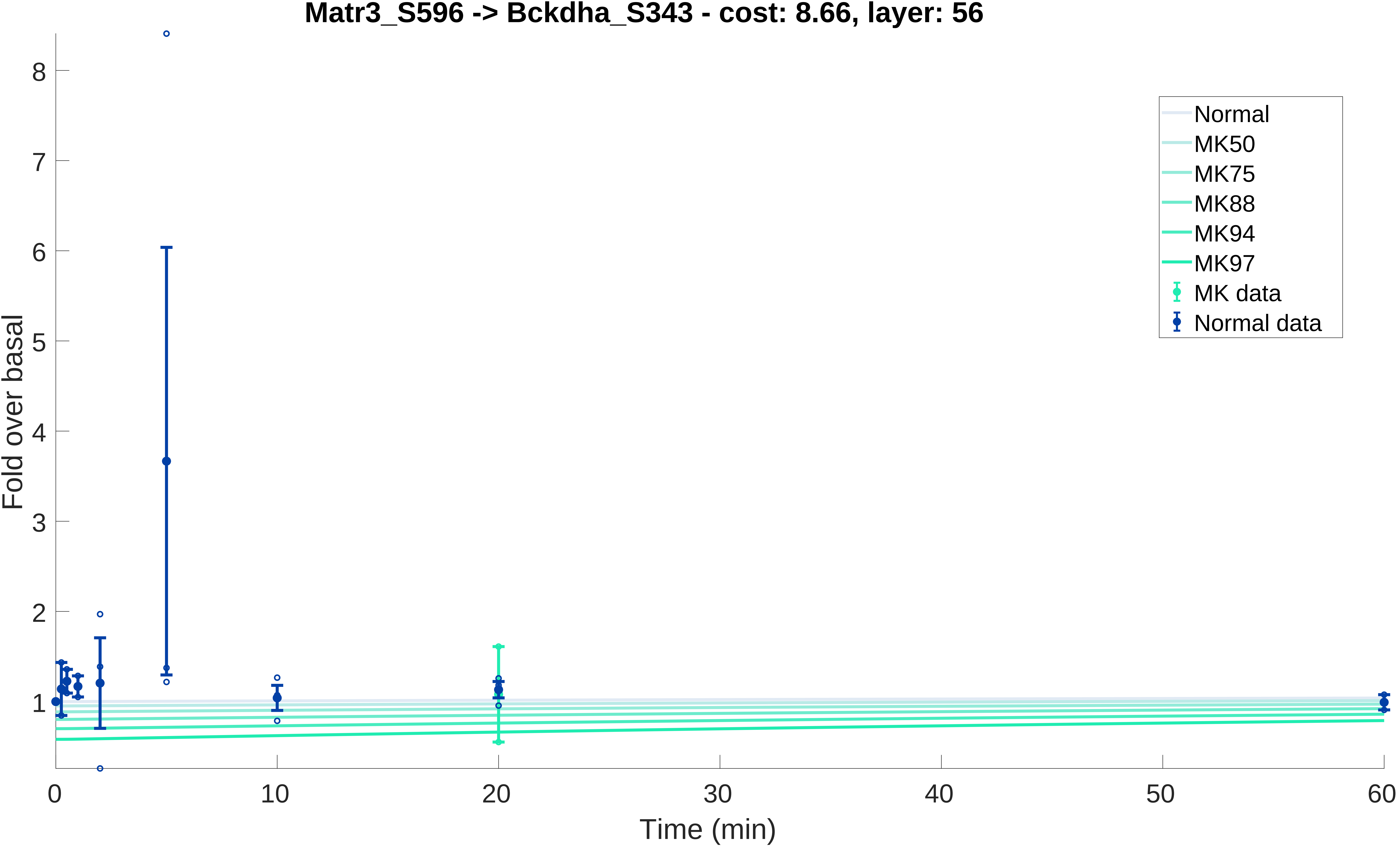

Matr3_S596 -> Bckdha_S343 - cost: 8.66, layer: 56
Fold over basal
Time (min)
0
10
20
30
40
50
60
1
2
3
4
5
6
7
8
Normal
MK50
MK75
MK88
MK94
MK97
MK data
Normal data

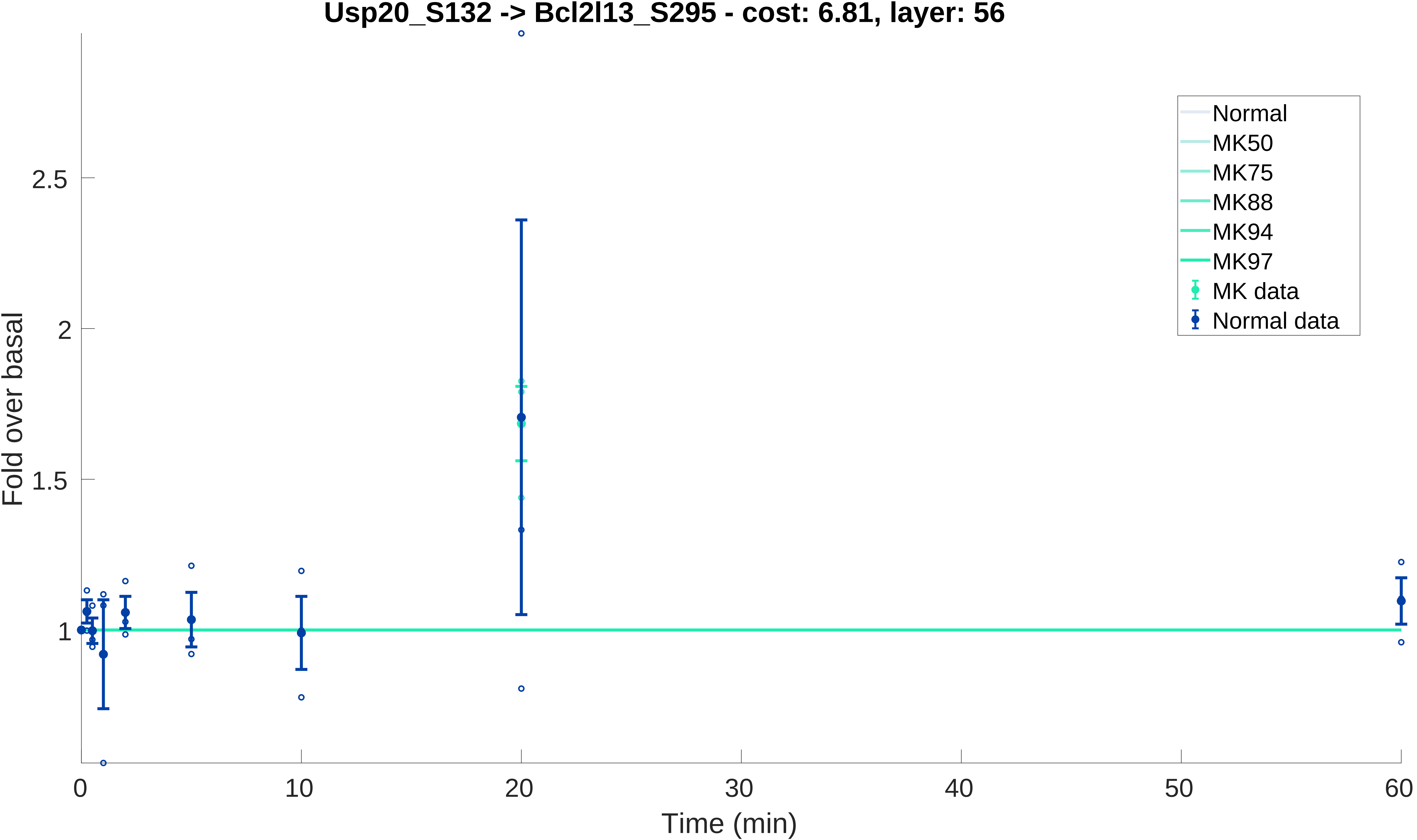

Usp20_S132 -> Bcl2l13_S295 - cost: 6.81, layer: 56
Fold over basal
Time (min)
2.5
2
1.5
1
0
10
20
30
40
50
60
Normal
MK50
MK75
MK88
MK94
MK97
MK data
Normal data

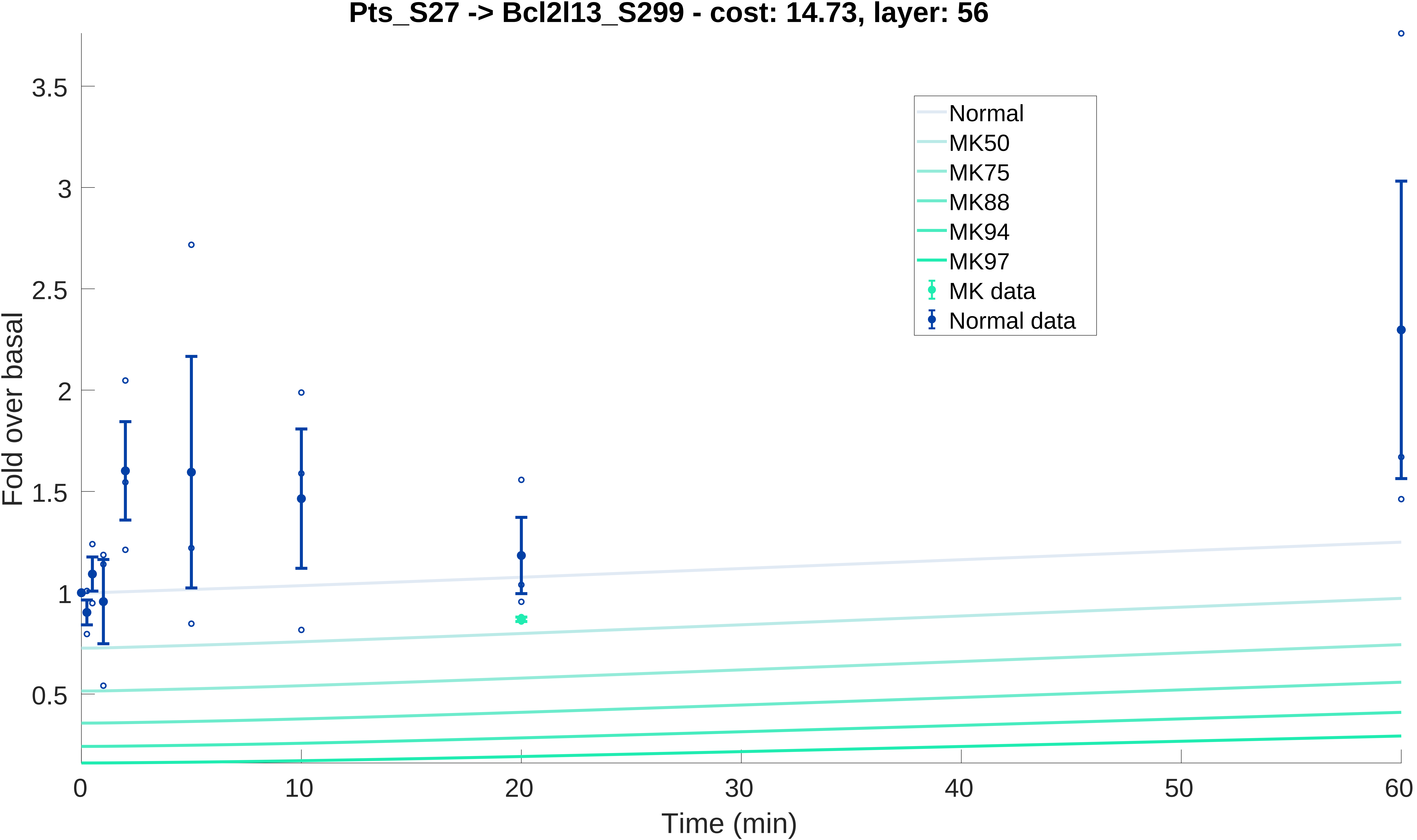
Pts_S27 -> Bcl2l13_S299 - cost: 14.73, layer: 56
Fold over basal
Time (min)
0.5
1
1.5
2
2.5
3
3.5
0
10
20
30
40
50
60
Normal
MK50
MK75
MK88
MK94
MK97
MK data
Normal data

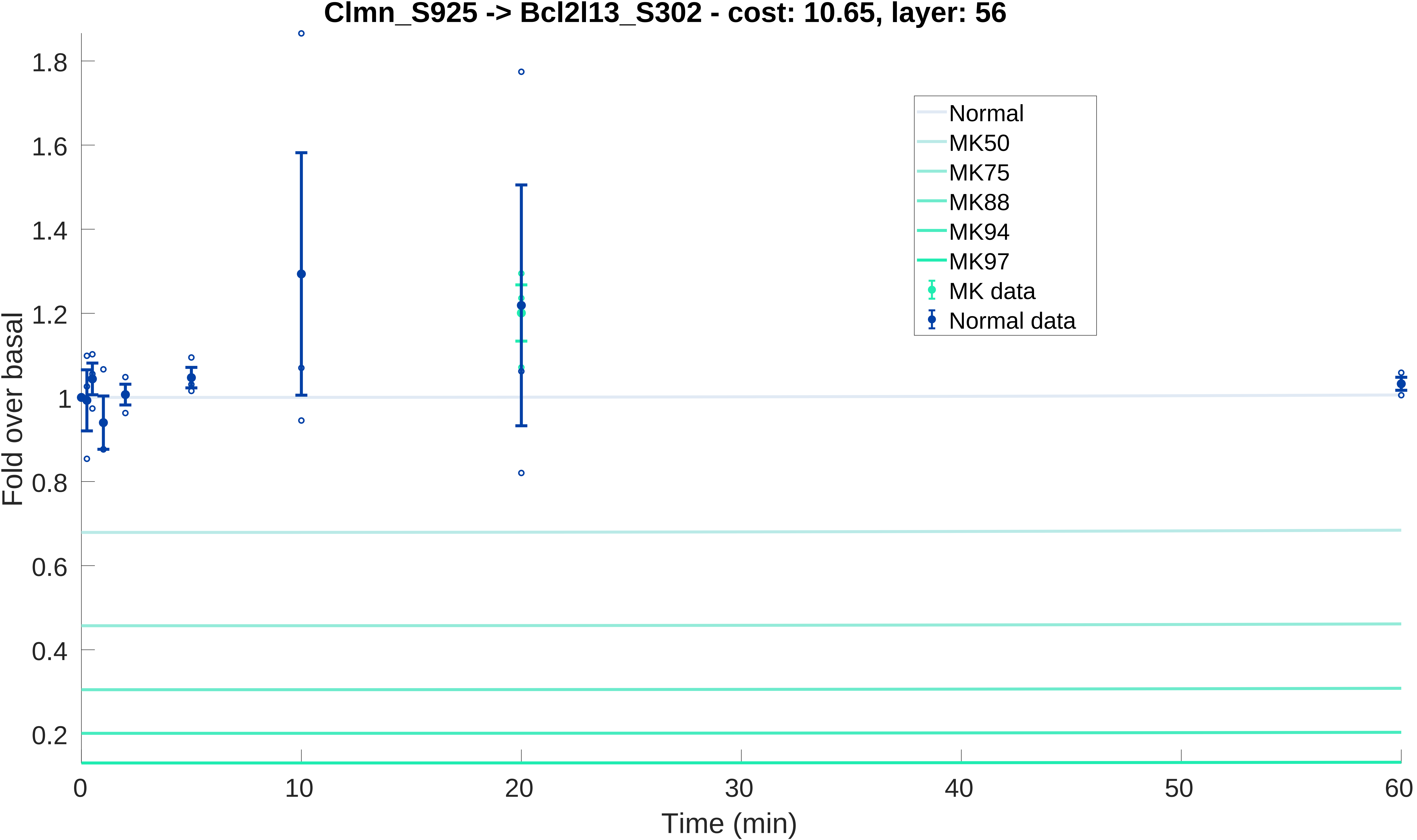

Clmn_S925 -> Bcl2l13_S302 - cost: 10.65, layer: 56
Fold over basal
Time (min)
0.2
0.4
0.6
0.8
1
1.2
1.4
1.6
1.8
0
10
20
30
40
50
60
Normal
MK50
MK75
MK88
MK94
MK97
MK data
Normal data

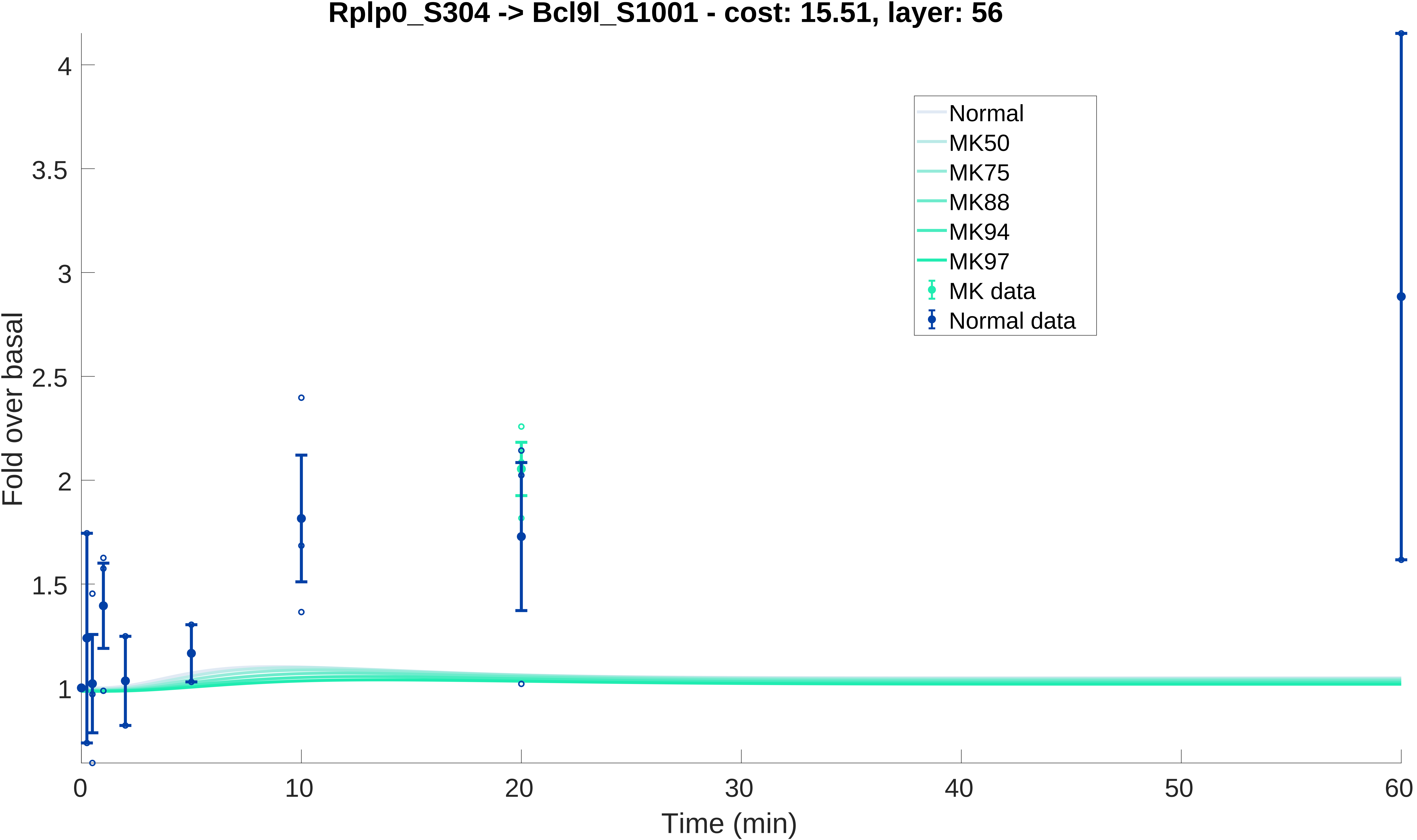

Rplp0_S304 -> Bcl9l_S1001 - cost: 15.51, layer: 56
Fold over basal
Time (min)
Normal
MK50
MK75
MK88
MK94
MK97
MK data
Normal data
0
10
20
30
40
50
60
1
1.5
2
2.5
3
3.5
4

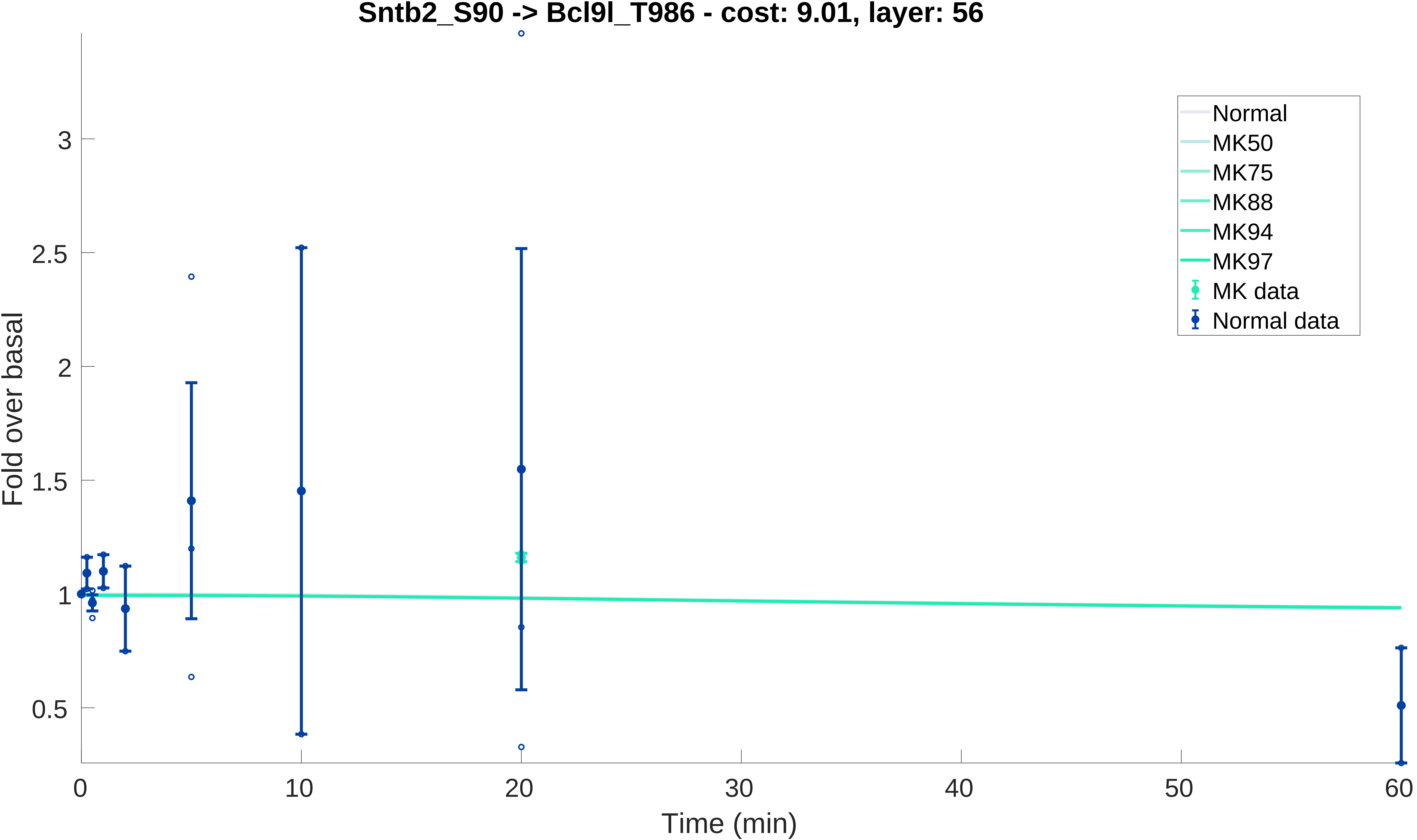

Sntb2_S90 -> Bcl9l_T986 - cost: 9.01, layer: 56
Fold over basal
3
2.5
2
1.5
1
0.5
0
10
20
30
40
50
60
Time (min)
Normal
MK50
MK75
MK88
MK94
MK97
MK data
Normal data

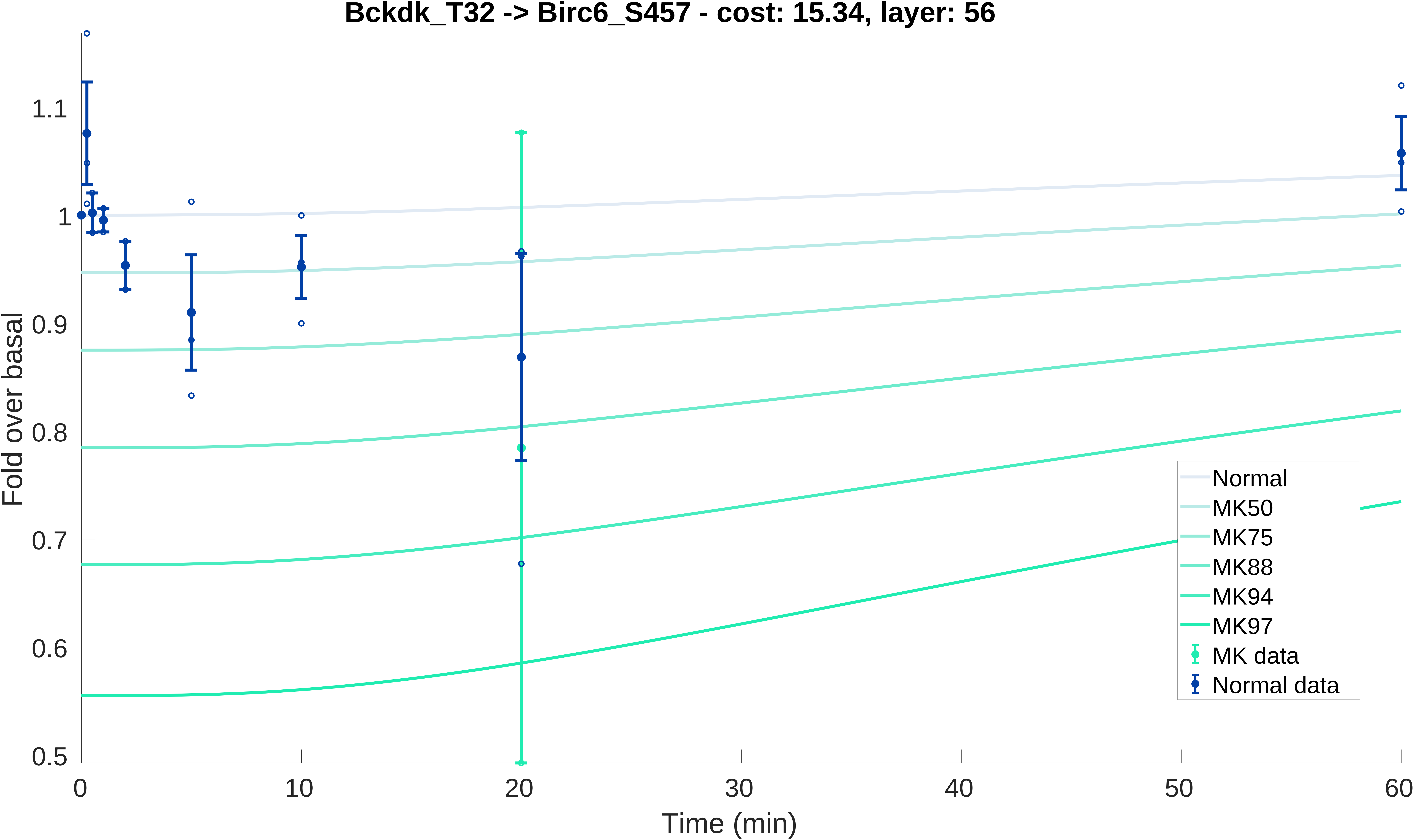

Bckdk_T32 -> Birc6_S457 - cost: 15.34, layer: 56
Fold over basal
Time (min)
1.1
1
0.9
0.8
0.7
0.6
0.5
0
10
20
30
40
50
60
Normal
MK50
MK75
MK88
MK94
MK97
MK data
Normal data

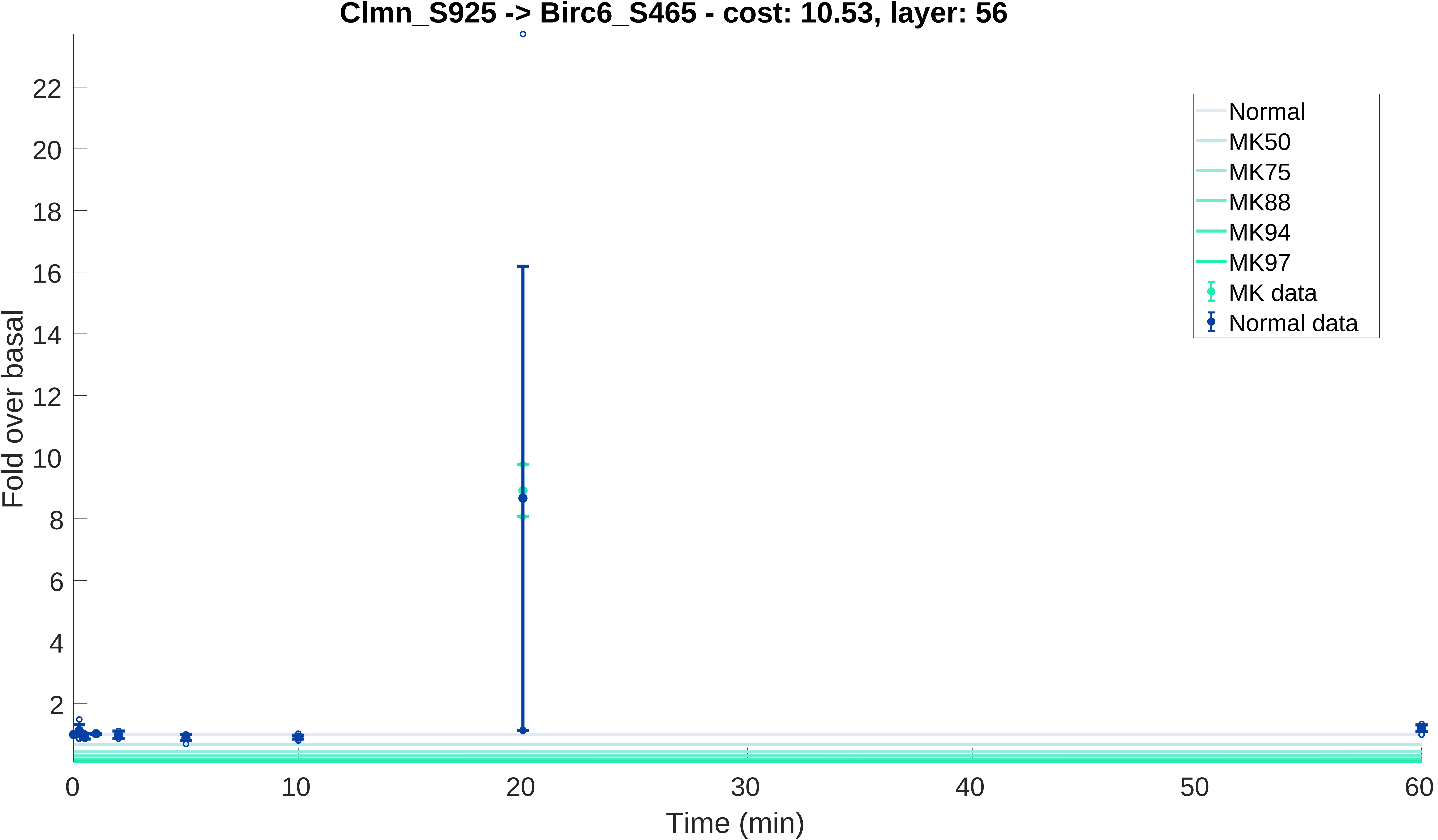

Clmn_S925 -> Birc6_S465 - cost: 10.53, layer: 56
Fold over basal
Time (min)
0
10
20
30
40
50
60
2
4
6
8
10
12
14
16
18
20
22
Normal
MK50
MK75
MK88
MK94
MK97
MK data
Normal data

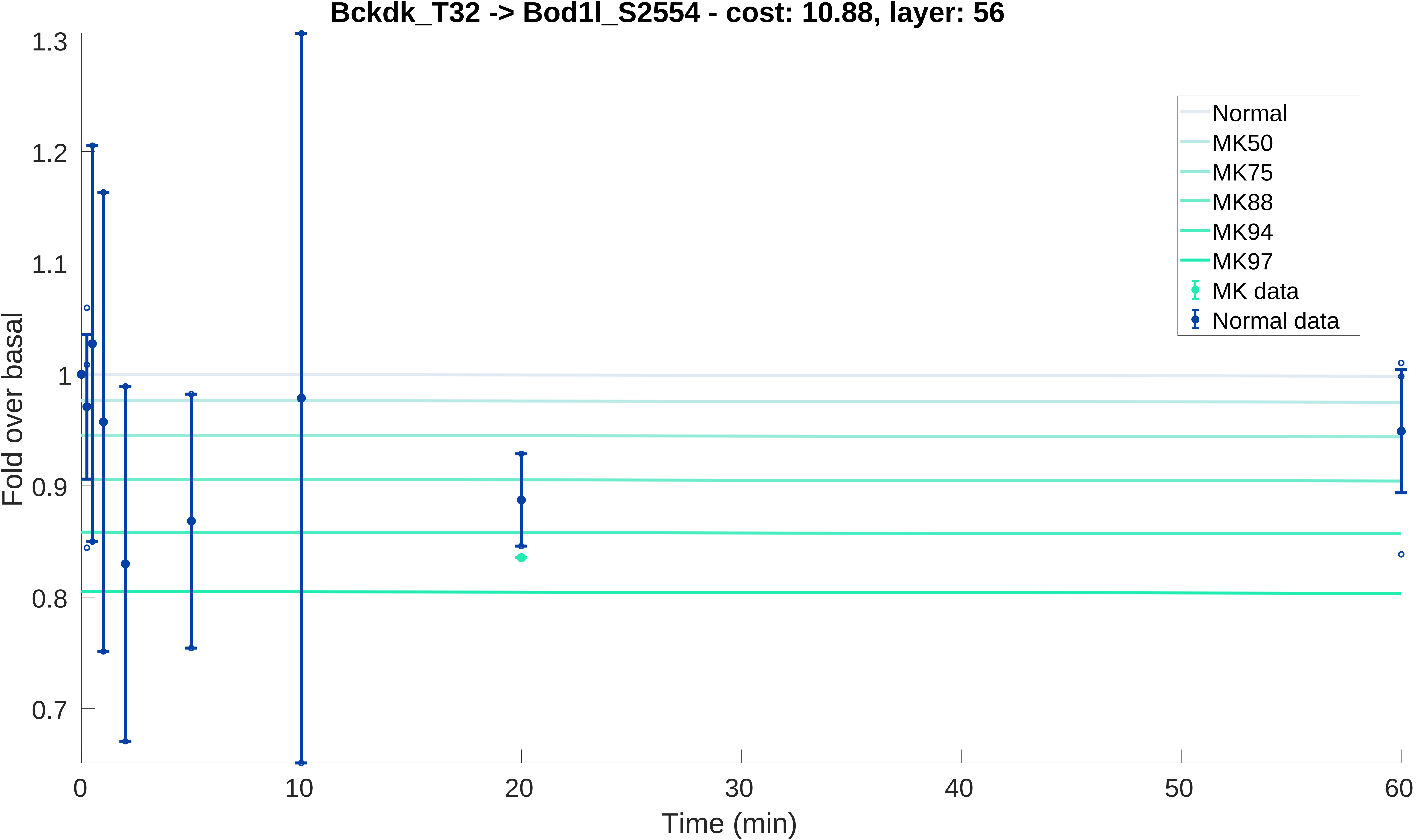

Bckdk_T32 -> Bod1l_S2554 - cost: 10.88, layer: 56
Fold over basal
Time (min)
1.3
1.2
1.1
1
0.9
0.8
0.7
0
10
20
30
40
50
60
Normal
MK50
MK75
MK88
MK94
MK97
MK data
Normal data

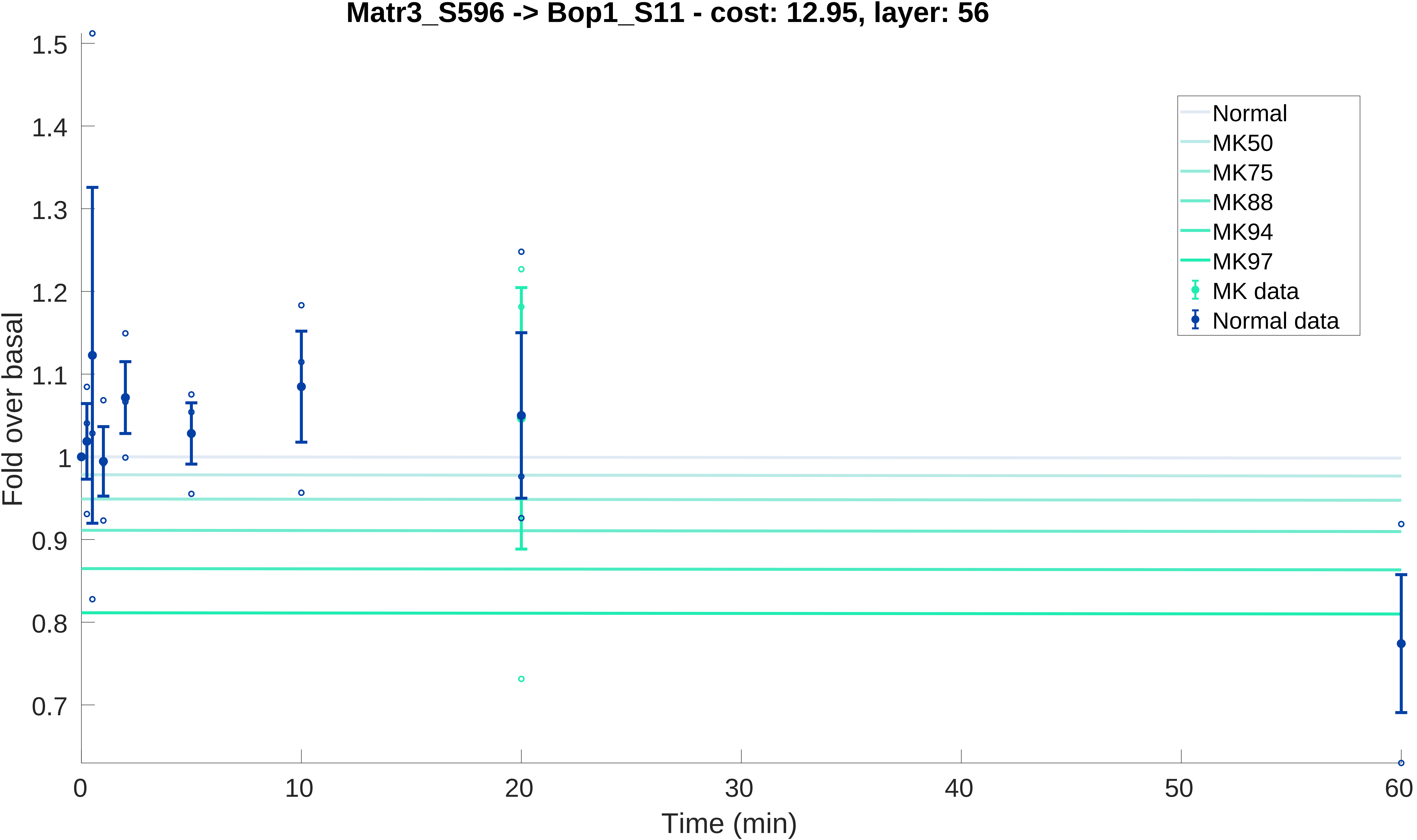

Matr3_S596 -> Bop1_S11 - cost: 12.95, layer: 56
Fold over basal
Time (min)
Normal
MK50
MK75
MK88
MK94
MK97
MK data
Normal data
1.5
1.4
1.3
1.2
1.1
1
0.9
0.8
0.7
0
10
20
30
40
50
60

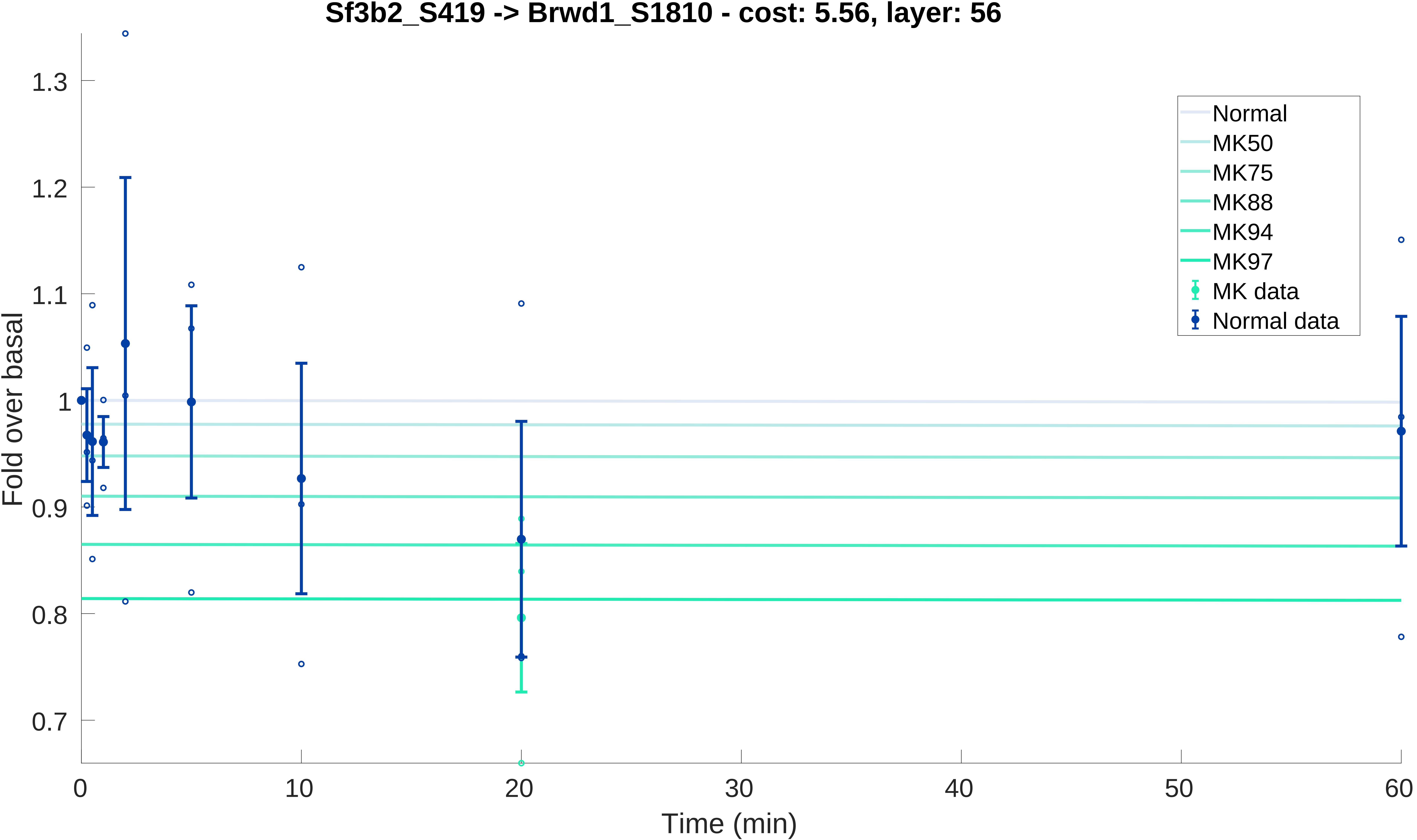

Sf3b2_S419 -> Brwd1_S1810 - cost: 5.56, layer: 56
Fold over basal
Time (min)
1.3
1.2
1.1
1
0.9
0.8
0.7
0
10
20
30
40
50
60
Normal
MK50
MK75
MK88
MK94
MK97
MK data
Normal data

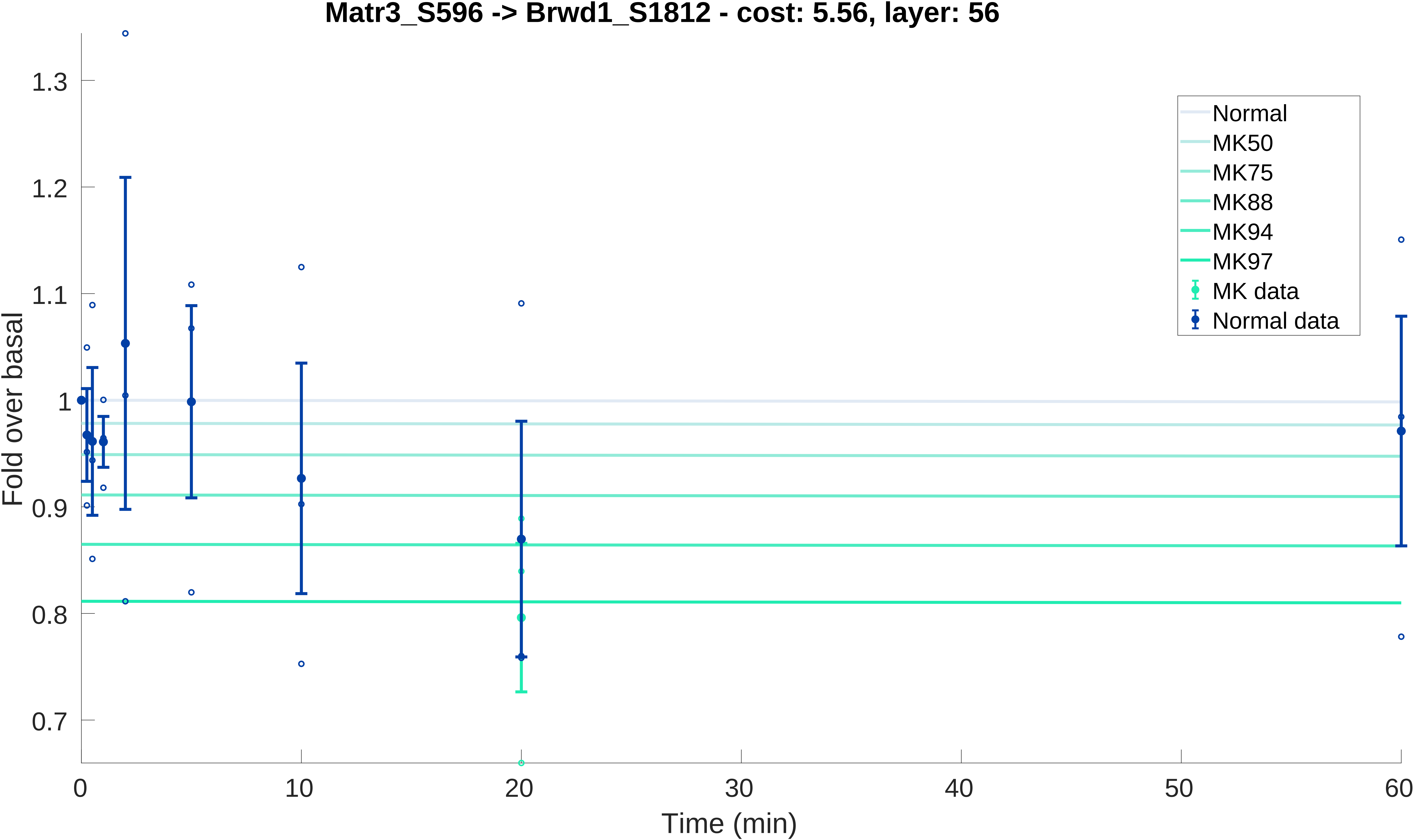

Matr3_S596 -> Brwd1_S1812 - cost: 5.56, layer: 56
Fold over basal
Time (min)
0.7
0.8
0.9
1
1.1
1.2
1.3
0
10
20
30
40
50
60
Normal
MK50
MK75
MK88
MK94
MK97
MK data
Normal data

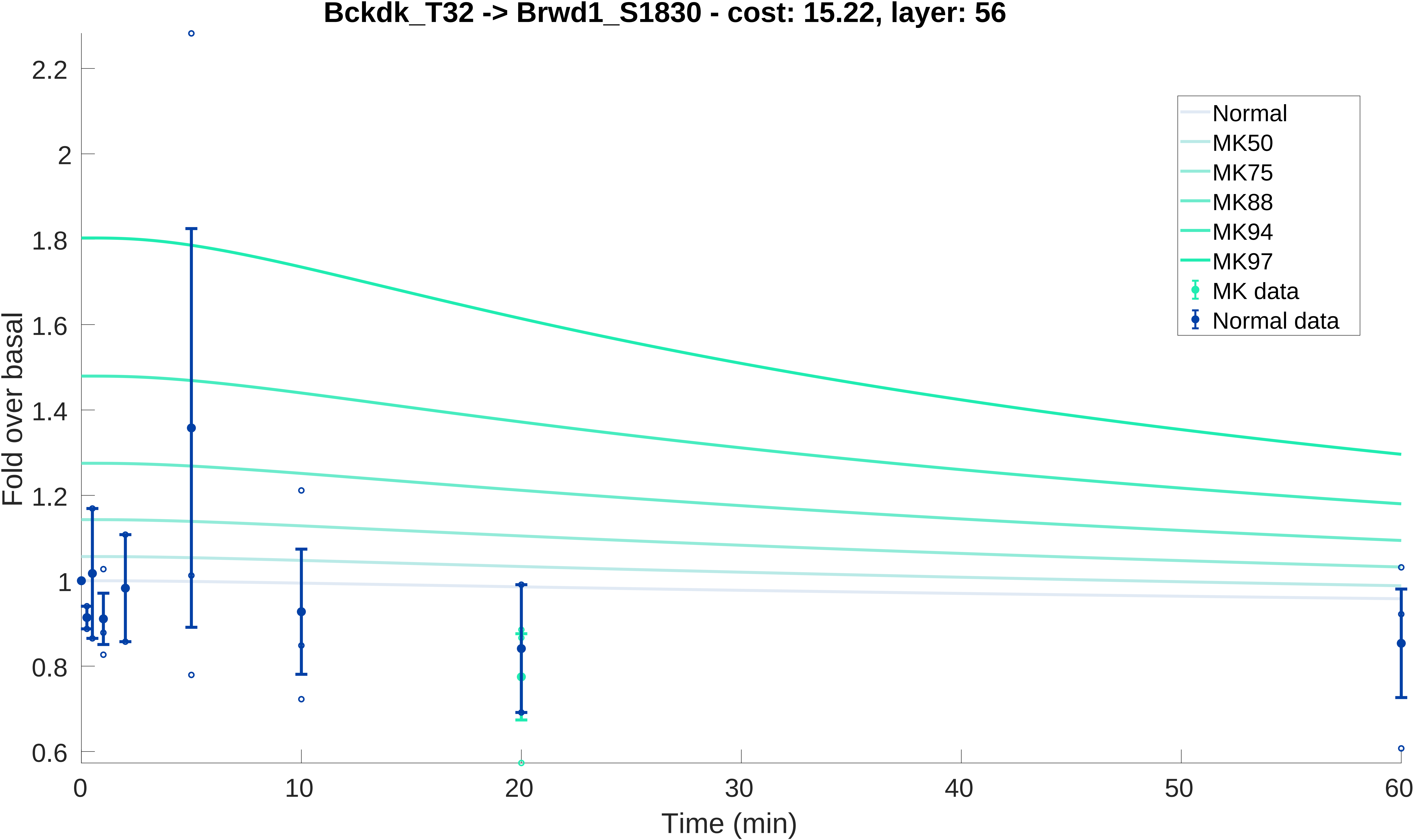

Bckdk_T32 -> Brwd1_S1830 - cost: 15.22, layer: 56
Fold over basal
Time (min)
Normal
MK50
MK75
MK88
MK94
MK97
MK data
Normal data
0.6
0.8
1
1.2
1.4
1.6
1.8
2
2.2
0
10
20
30
40
50
60

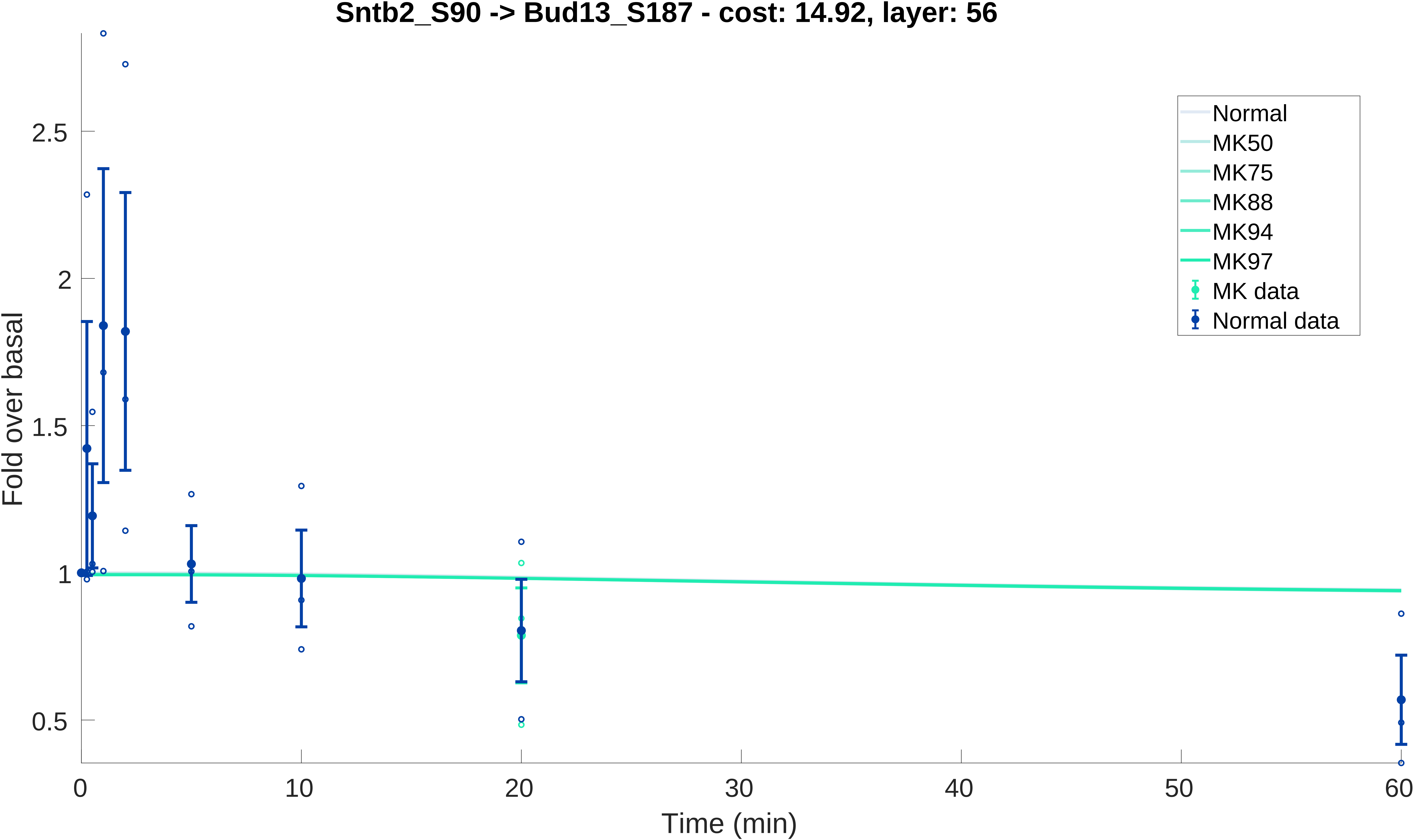

Sntb2_S90 -> Bud13_S187 - cost: 14.92, layer: 56
Fold over basal
Time (min)
0.5
1
1.5
2
2.5
0
10
20
30
40
50
60
Normal
MK50
MK75
MK88
MK94
MK97
MK data
Normal data

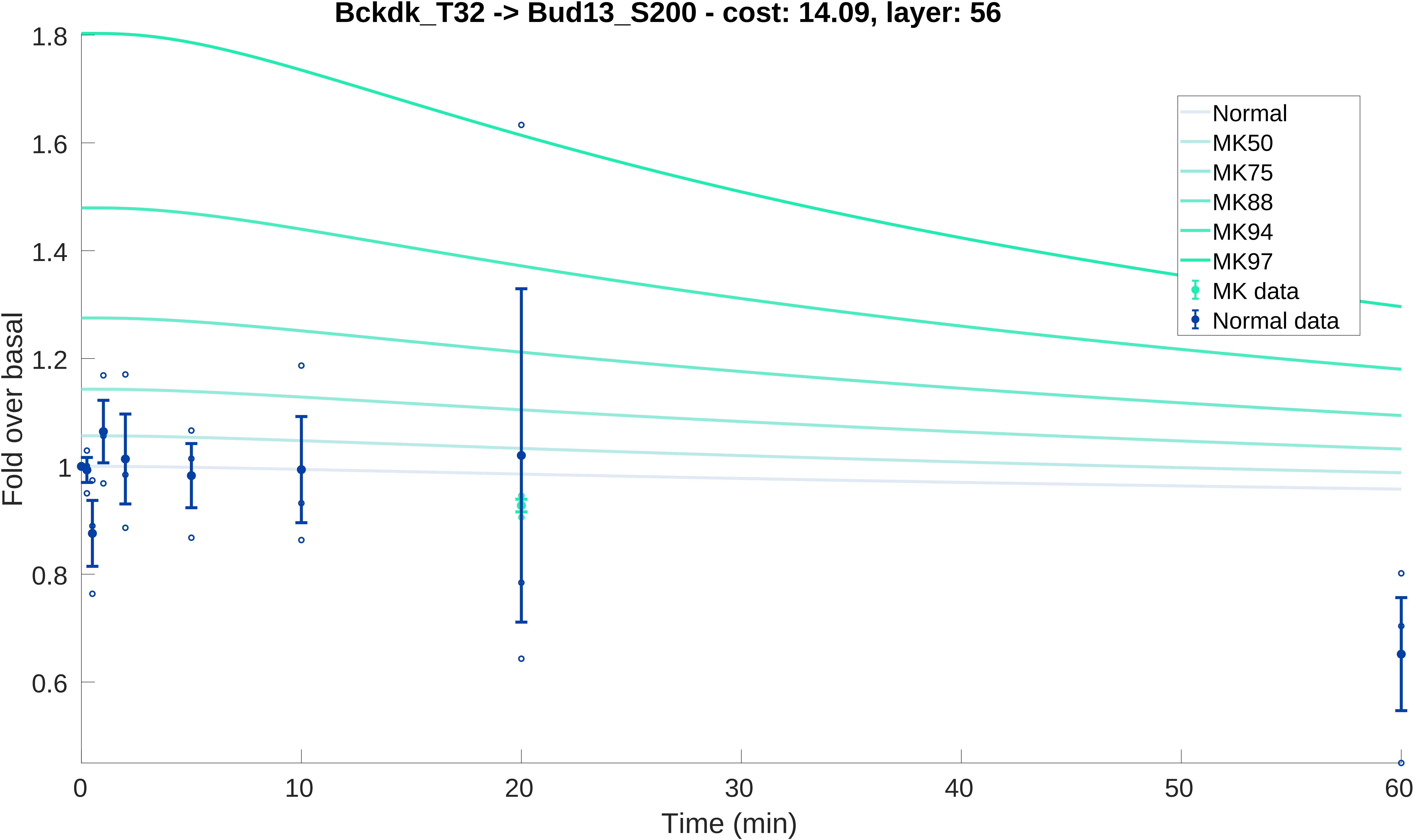

Bckdk_T32 -> Bud13_S200 - cost: 14.09, layer: 56
Fold over basal
Time (min)
Normal
MK50
MK75
MK88
MK94
MK97
MK data
Normal data
1.8
1.6
1.4
1.2
1
0.8
0.6
0
10
20
30
40
50
60

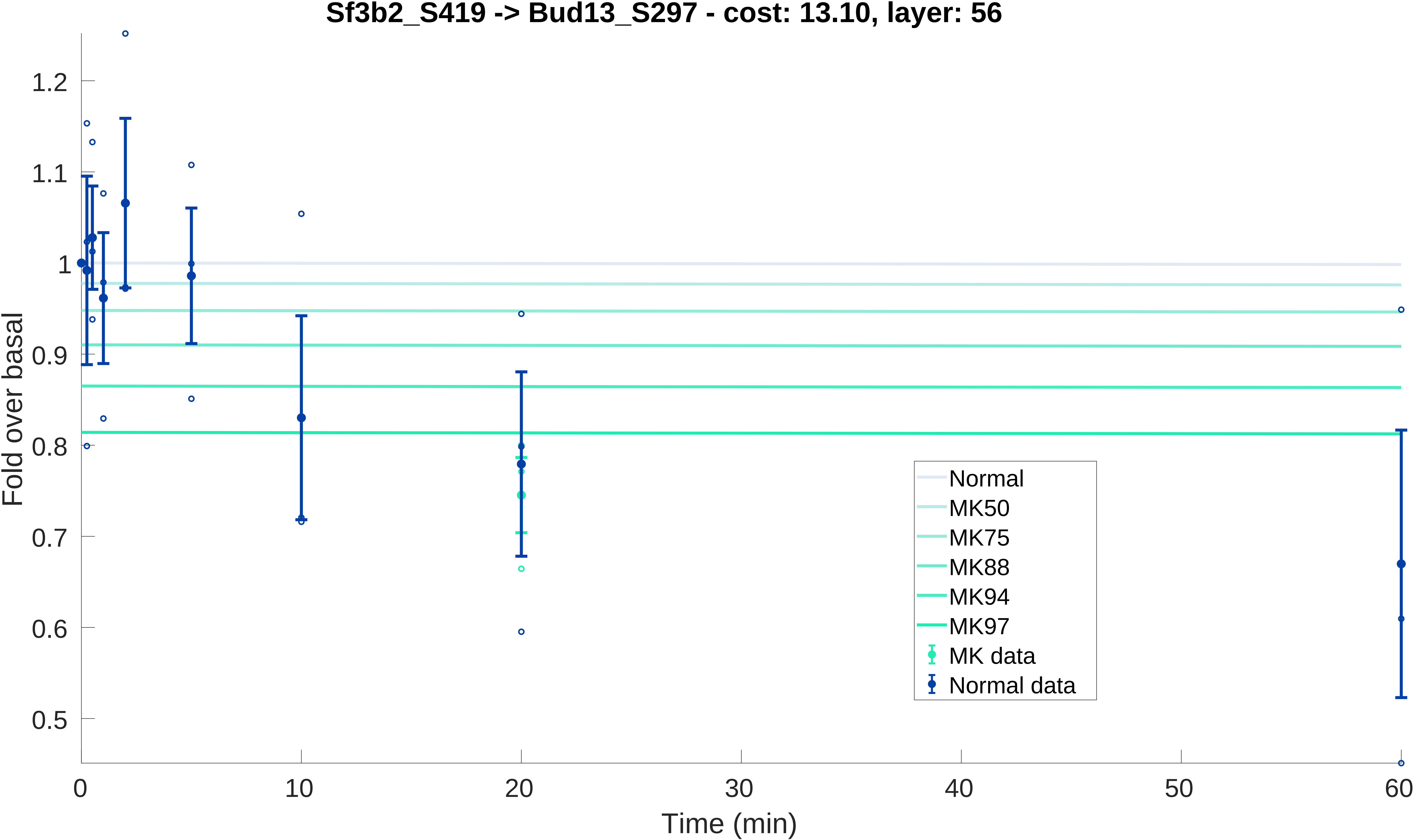

Sf3b2_S419 -> Bud13_S297 - cost: 13.10, layer: 56
Fold over basal
Time (min)
1.2
1.1
1
0.9
0.8
0.7
0.6
0.5
0
10
20
30
40
50
60
Normal
MK50
MK75
MK88
MK94
MK97
MK data
Normal data

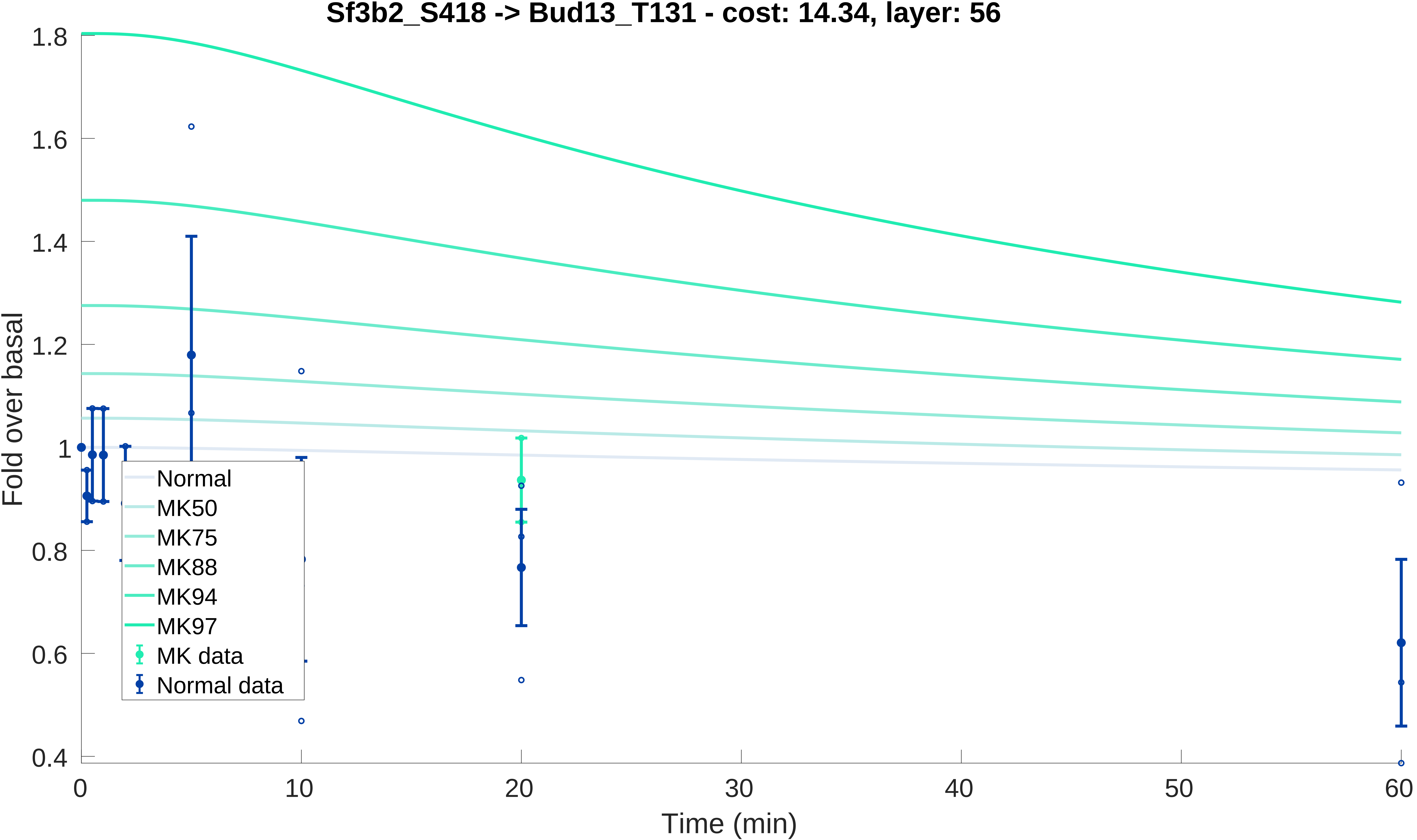

Sf3b2_S418 -> Bud13_T131 - cost: 14.34, layer: 56
Fold over basal
Time (min)
1.8
1.6
1.4
1.2
1
0.8
0.6
0.4
0
10
20
30
40
50
60
Normal
MK50
MK75
MK88
MK94
MK97
MK data
Normal data

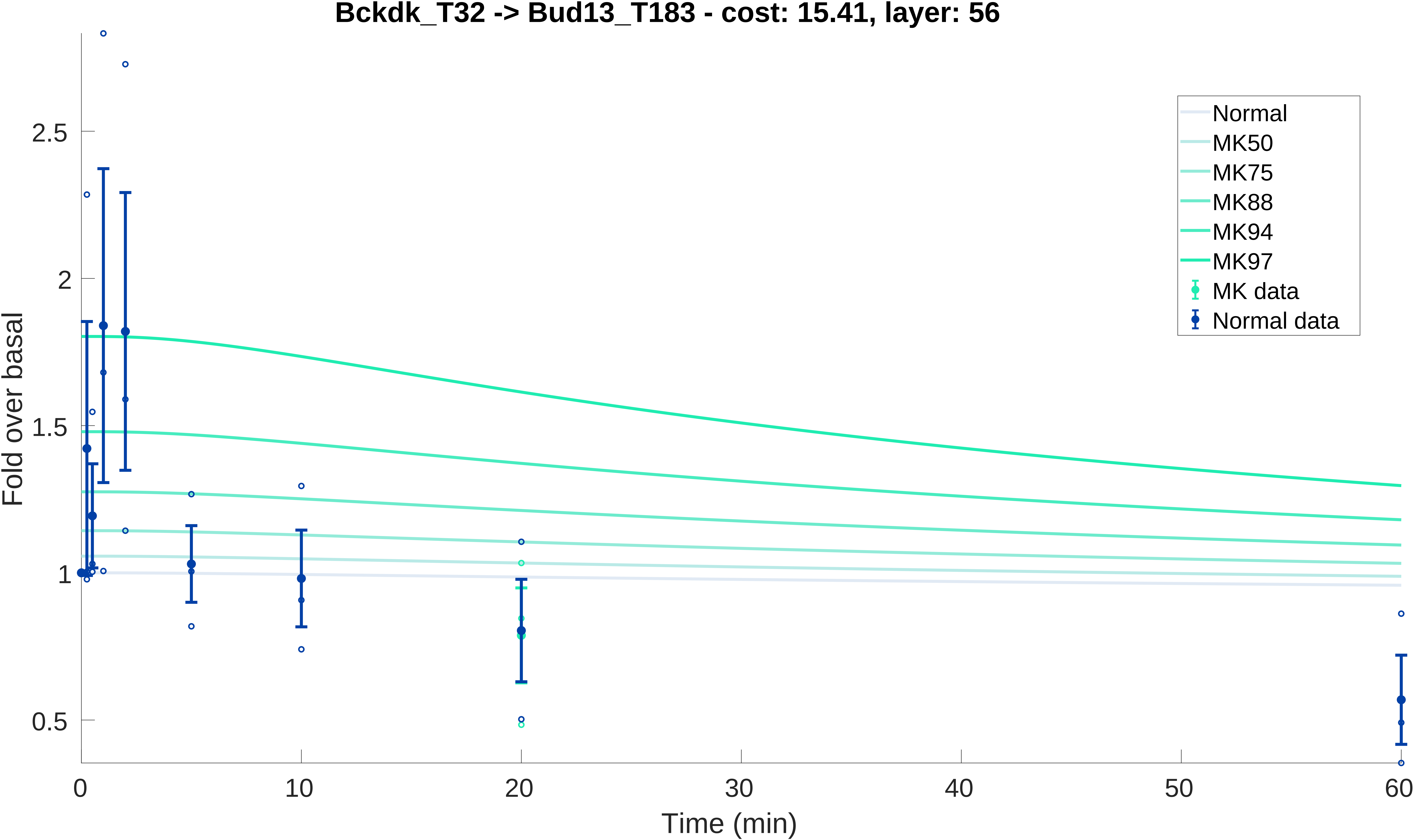

Bckdk_T32 -> Bud13_T183 - cost: 15.41, layer: 56
Fold over basal
Time (min)
0.5
1
1.5
2
2.5
0
10
20
30
40
50
60
Normal
MK50
MK75
MK88
MK94
MK97
MK data
Normal data

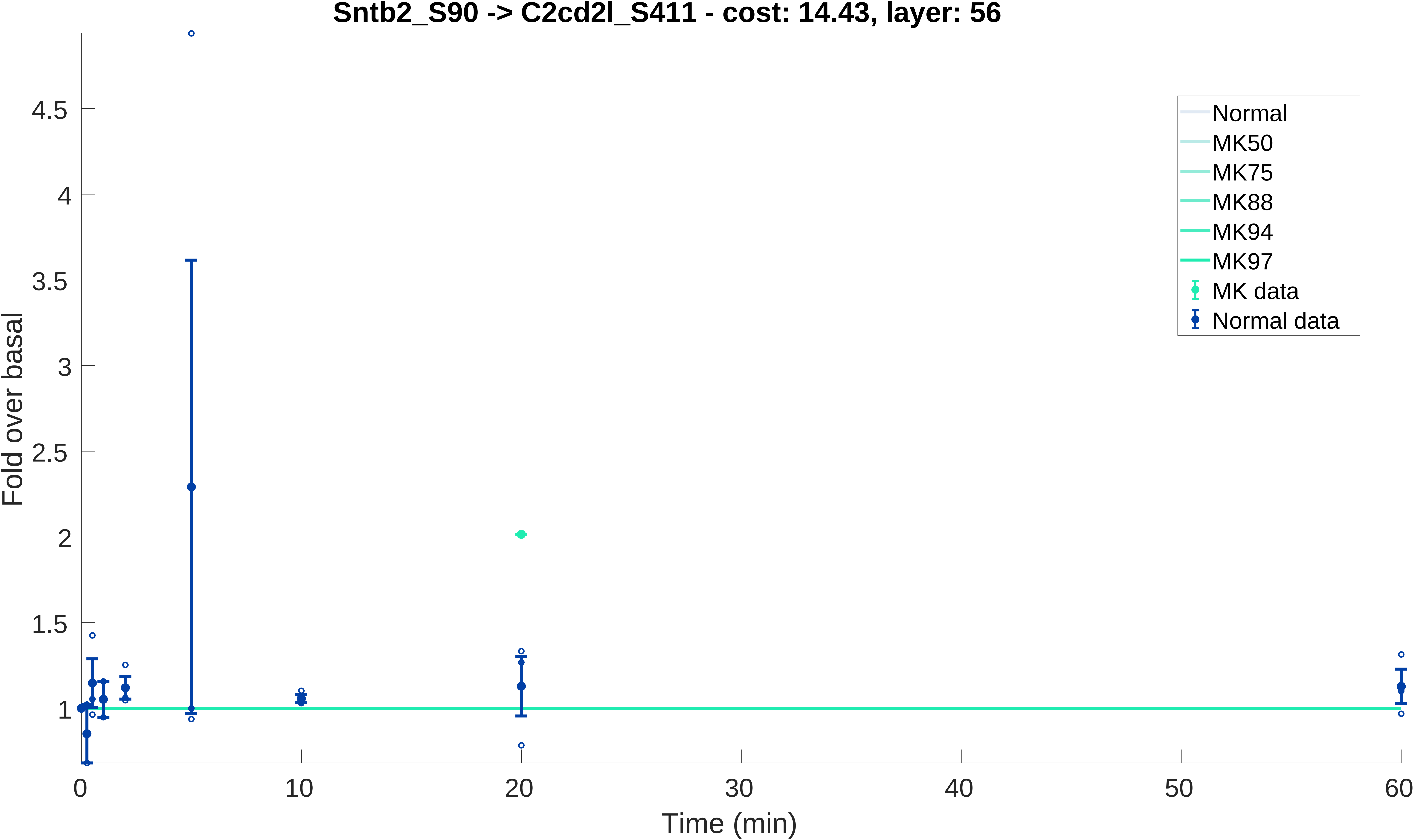

Sntb2_S90 -> C2cd2l_S411 - cost: 14.43, layer: 56
Fold over basal
Time (min)
4.5
4
3.5
3
2.5
2
1.5
1
0
10
20
30
40
50
60
Normal
MK50
MK75
MK88
MK94
MK97
MK data
Normal data

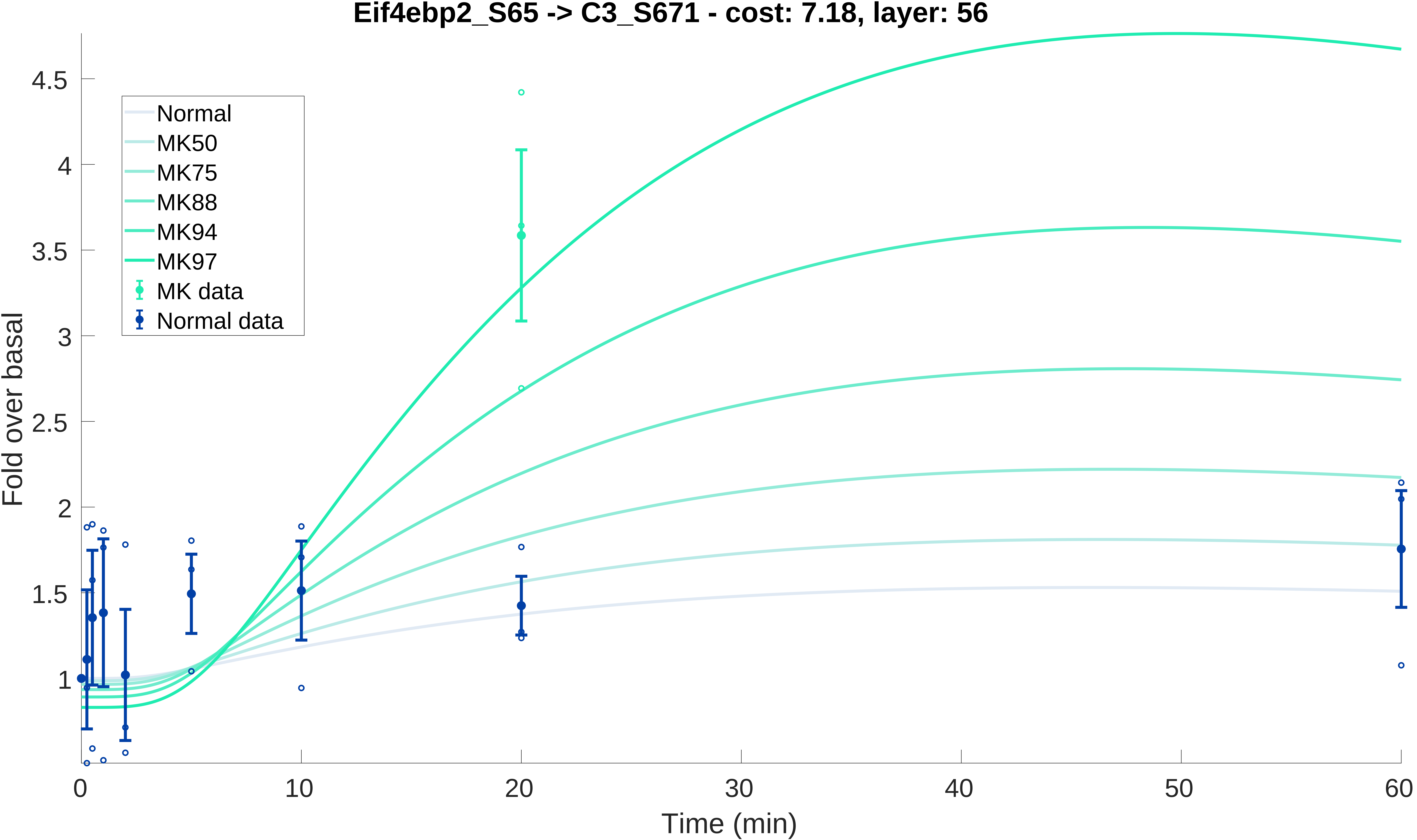

Eif4ebp2_S65 -> C3_S671 - cost: 7.18, layer: 56
Normal
MK50
MK75
MK88
MK94
MK97
MK data
Normal data
Fold over basal
Time (min)
4.5
4
3.5
3
2.5
2
1.5
1
0
10
20
30
40
50
60

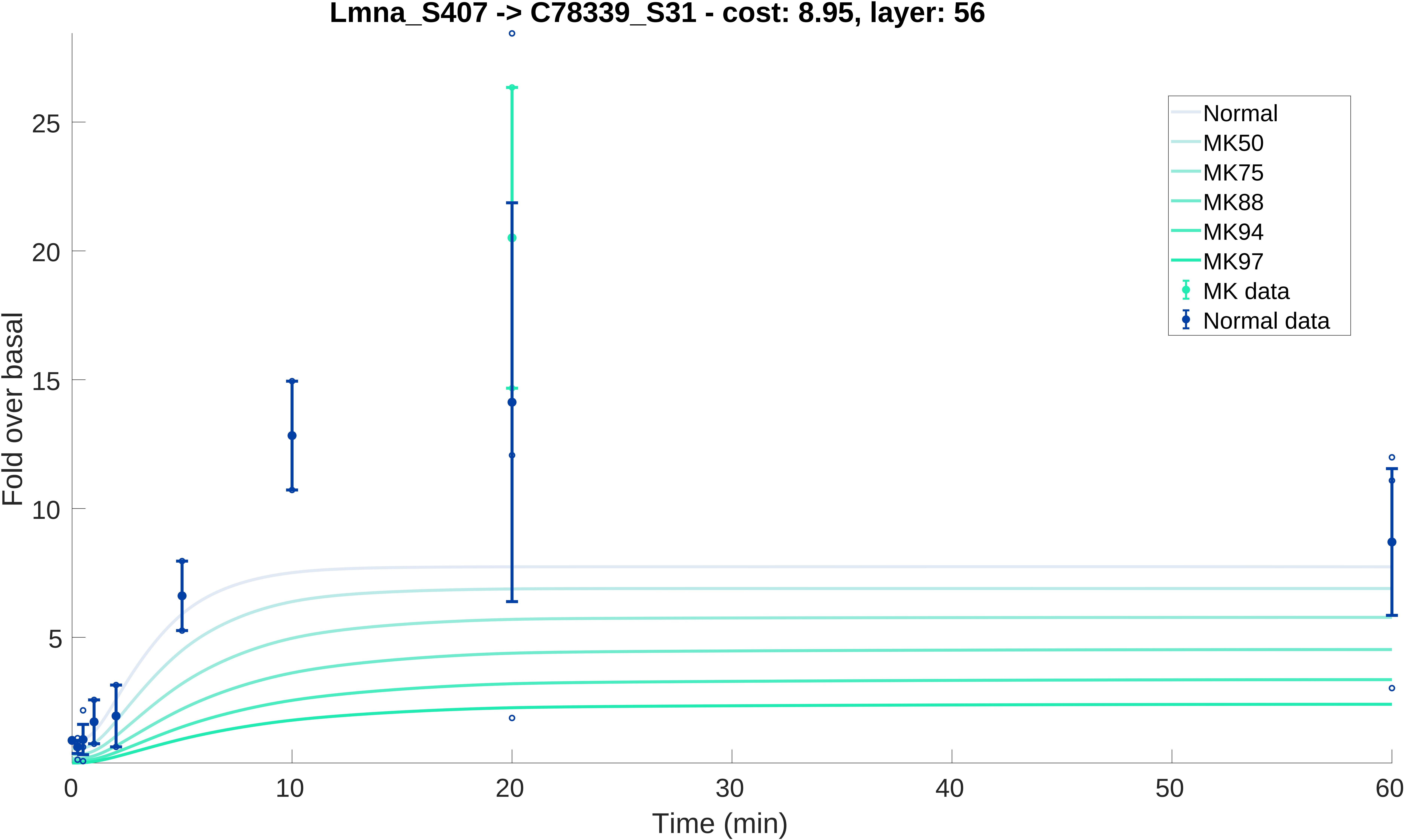
Lmna_S407 -> C78339_S31 - cost: 8.95, layer: 56
Fold over basal
Time (min)
0
10
20
30
40
50
60
5
10
15
20
25
Normal
MK50
MK75
MK88
MK94
MK97
MK data
Normal data

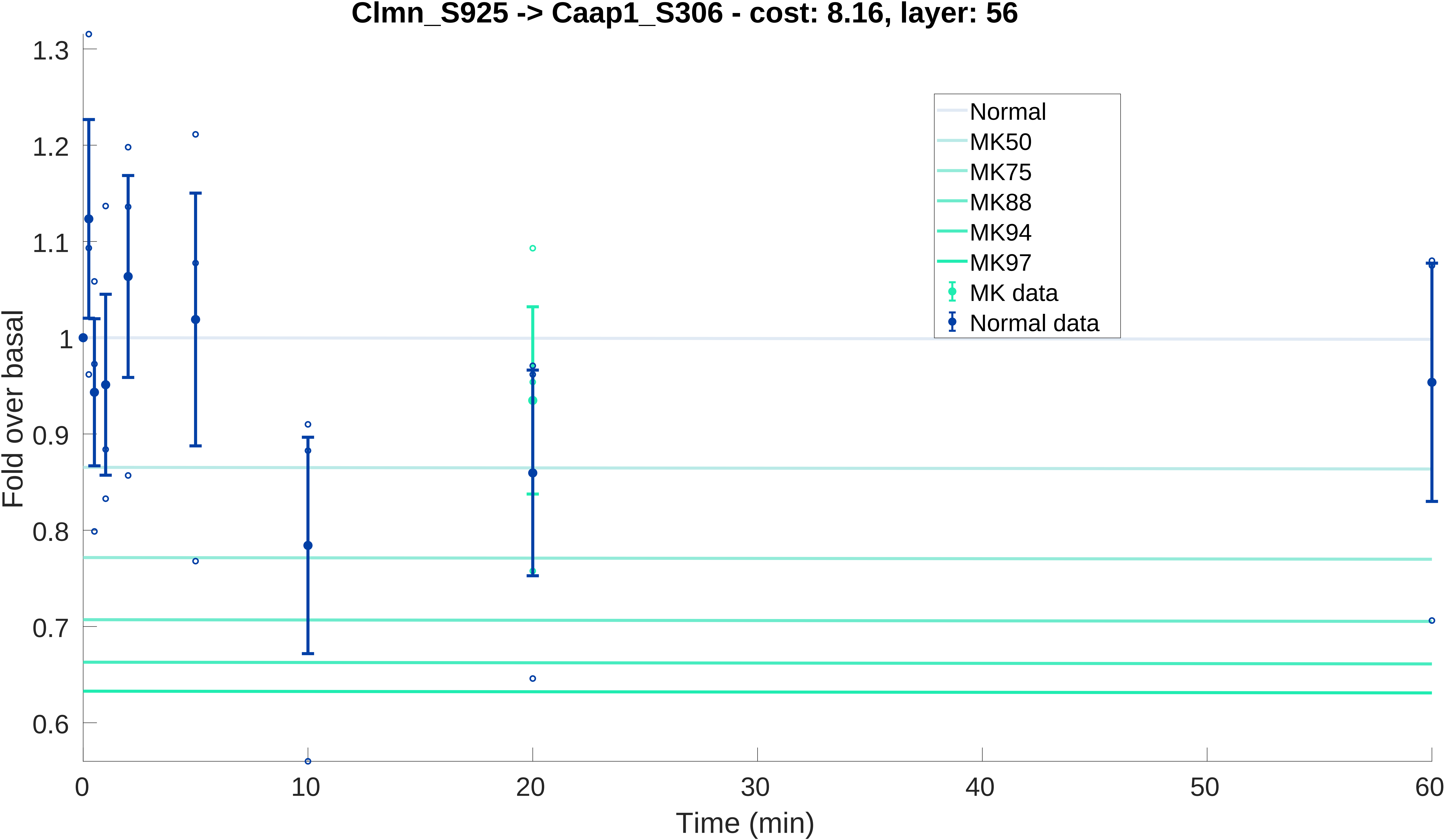

Clmn_S925 -> Caap1_S306 - cost: 8.16, layer: 56
Fold over basal
Time (min)
0
10
20
30
40
50
60
0.6
0.7
0.8
0.9
1
1.1
1.2
1.3
Normal
MK50
MK75
MK88
MK94
MK97
MK data
Normal data

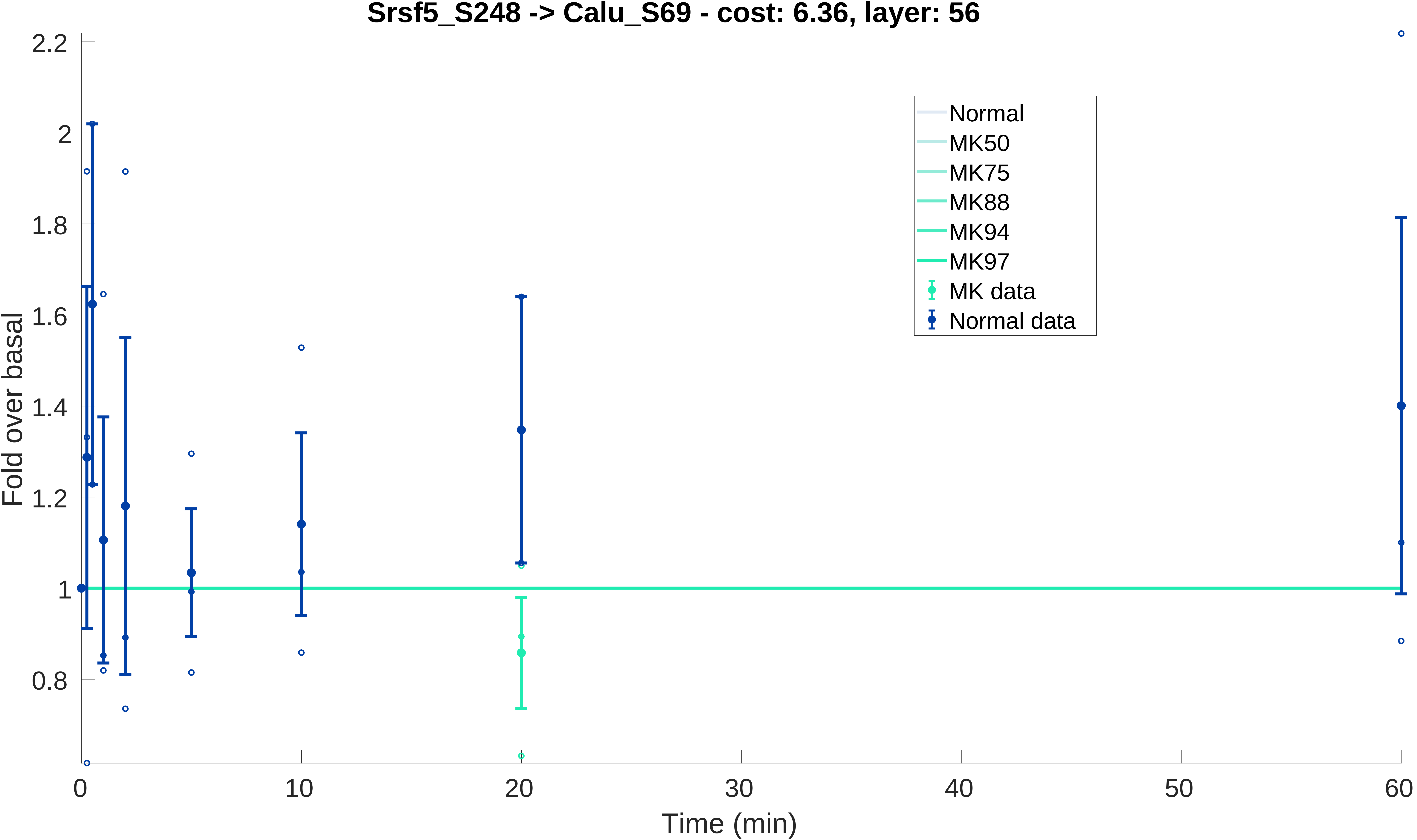

Srsf5_S248 -> Calu_S69 - cost: 6.36, layer: 56
Fold over basal
Time (min)
0
10
20
30
40
50
60
0.8
1
1.2
1.4
1.6
1.8
2
2.2
Normal
MK50
MK75
MK88
MK94
MK97
MK data
Normal data

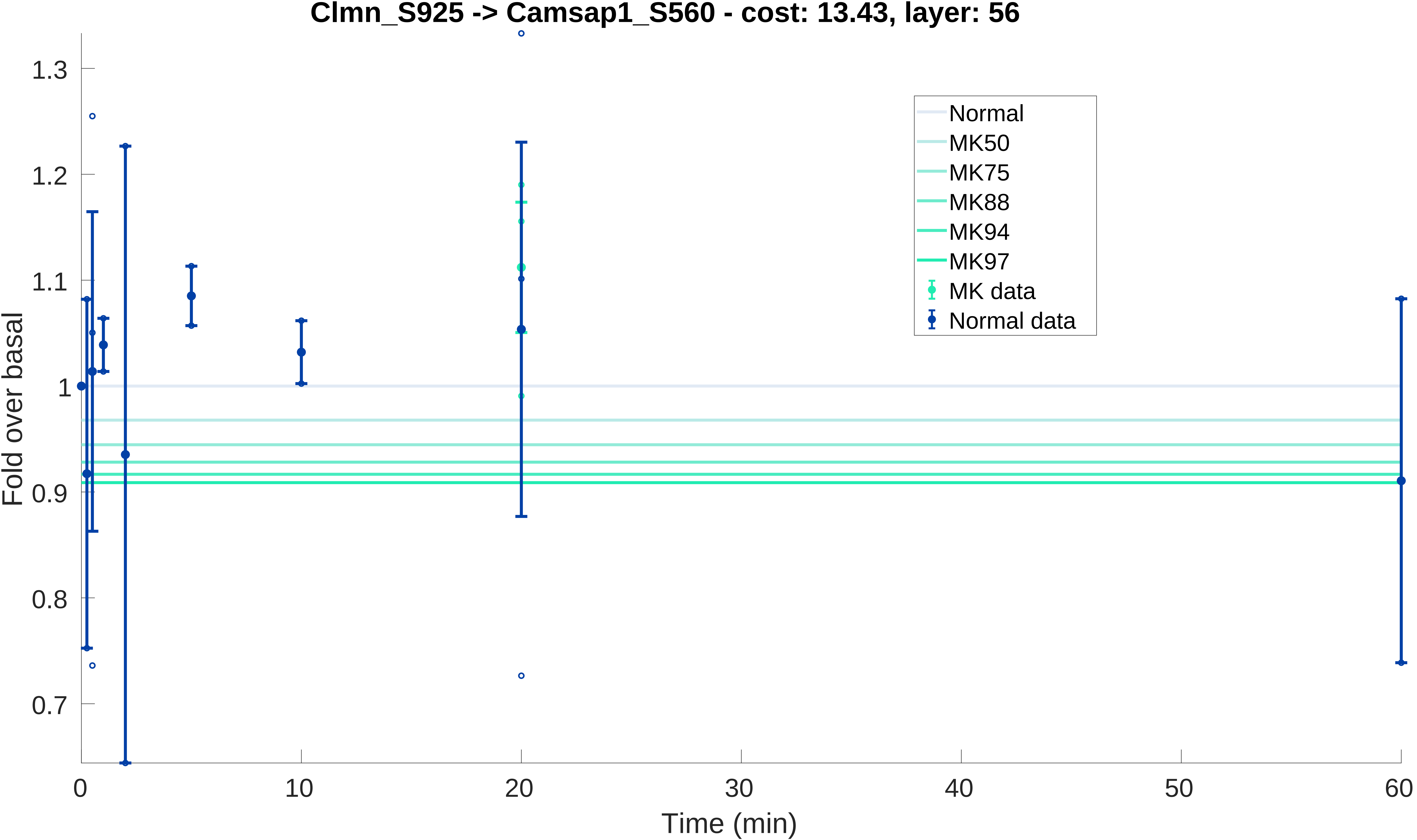

Clmn_S925 -> Camsap1_S560 - cost: 13.43, layer: 56
Fold over basal
Time (min)
1.3
1.2
1.1
1
0.9
0.8
0.7
0
10
20
30
40
50
60
Normal
MK50
MK75
MK88
MK94
MK97
MK data
Normal data

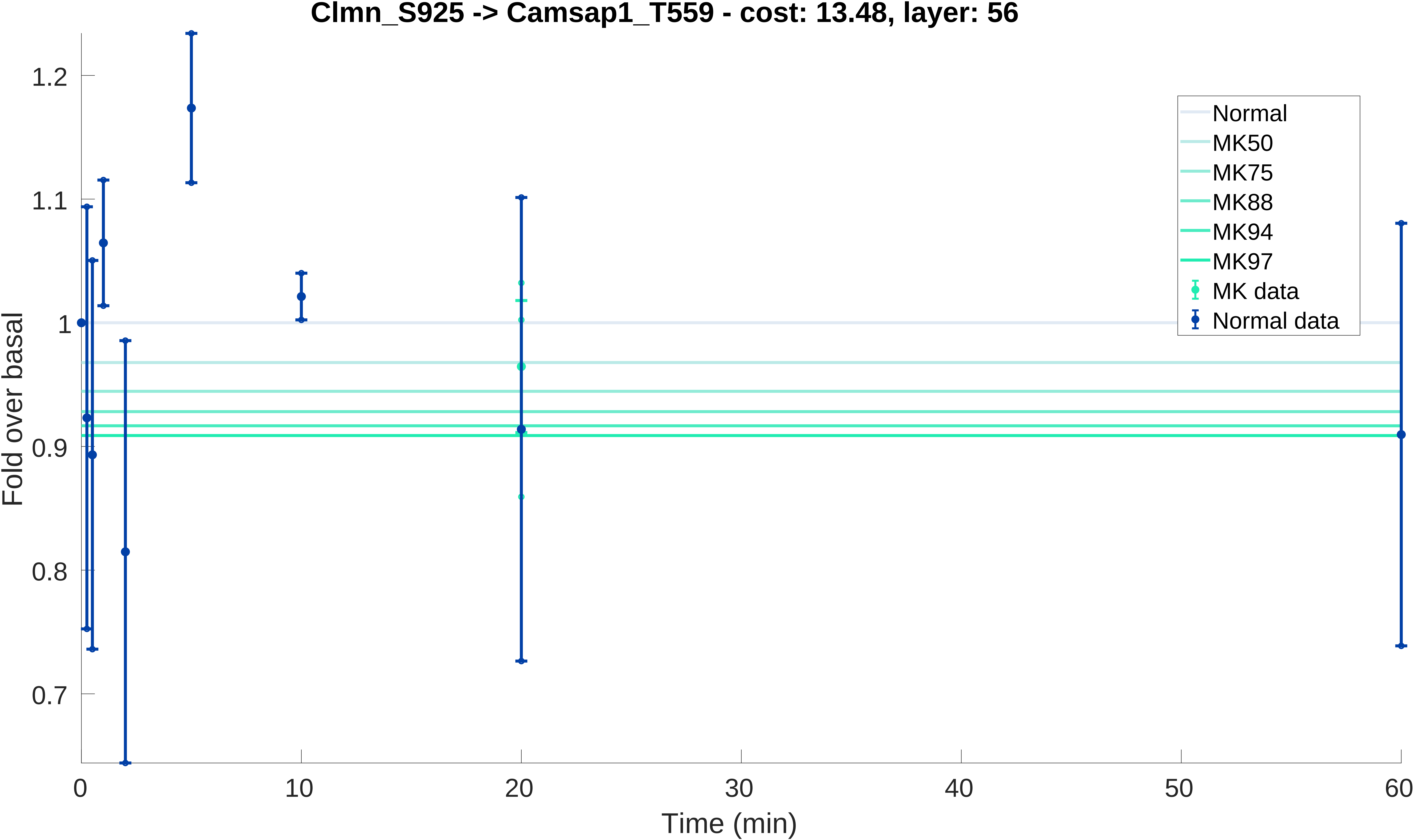
Clmn_S925 -> Camsap1_T559 - cost: 13.48, layer: 56
Fold over basal
1.2
1.1
1
0.9
0.8
0.7
0
10
20
30
40
50
60
Time (min)
Normal
MK50
MK75
MK88
MK94
MK97
MK data
Normal data

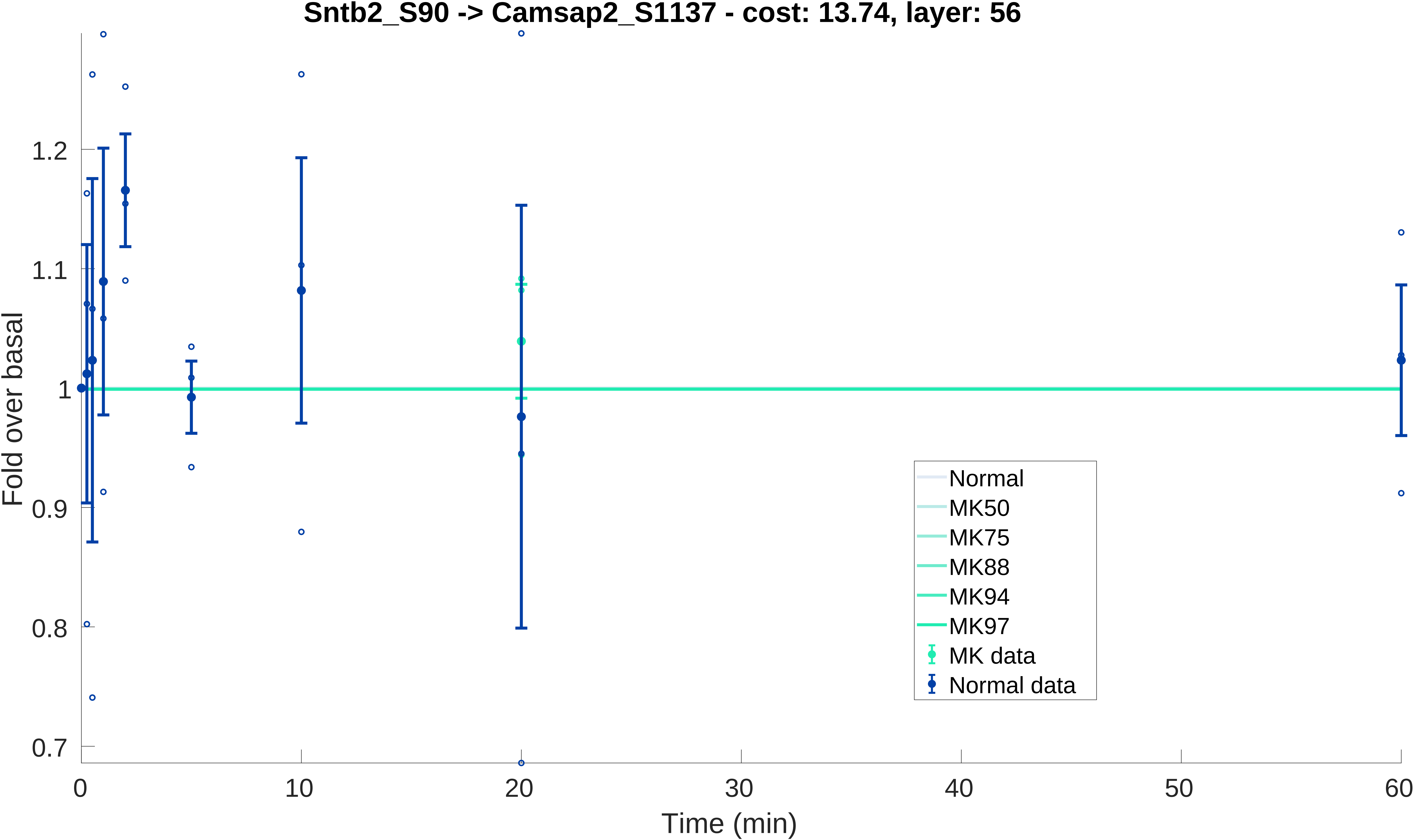

Sntb2_S90 -> Camsap2_S1137 - cost: 13.74, layer: 56
Fold over basal
Time (min)
0.7
0.8
0.9
1
1.1
1.2
0
10
20
30
40
50
60
Normal
MK50
MK75
MK88
MK94
MK97
MK data
Normal data

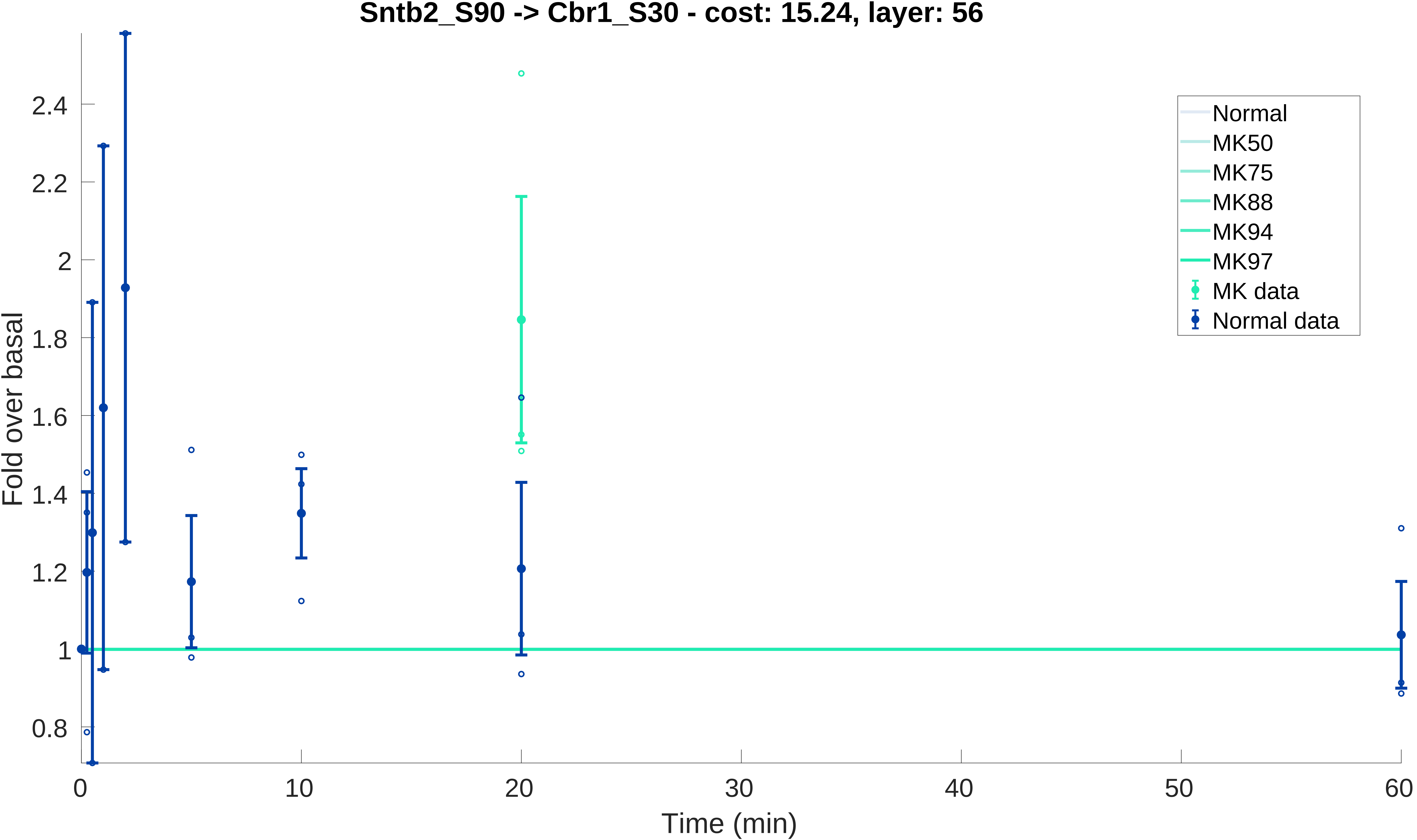

Sntb2_S90 -> Cbr1_S30 - cost: 15.24, layer: 56
Fold over basal
Time (min)
0.8
1
1.2
1.4
1.6
1.8
2
2.2
2.4
0
10
20
30
40
50
60
Normal
MK50
MK75
MK88
MK94
MK97
MK data
Normal data

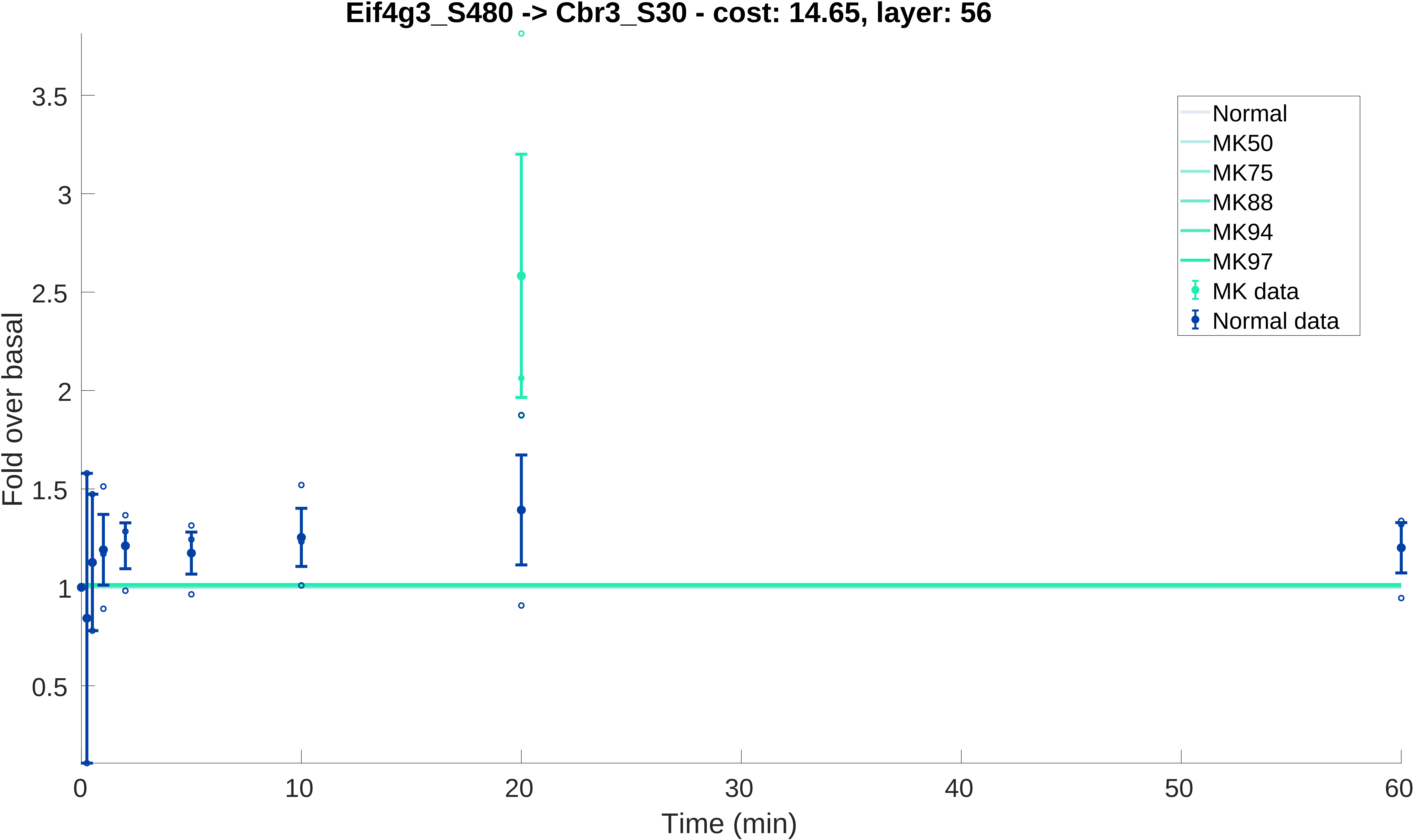
Eif4g3_S480 -> Cbr3_S30 - cost: 14.65, layer: 56
Fold over basal
3.5
3
2.5
2
1.5
1
0.5
0
10
20
30
40
50
60
Time (min)
Normal
MK50
MK75
MK88
MK94
MK97
MK data
Normal data

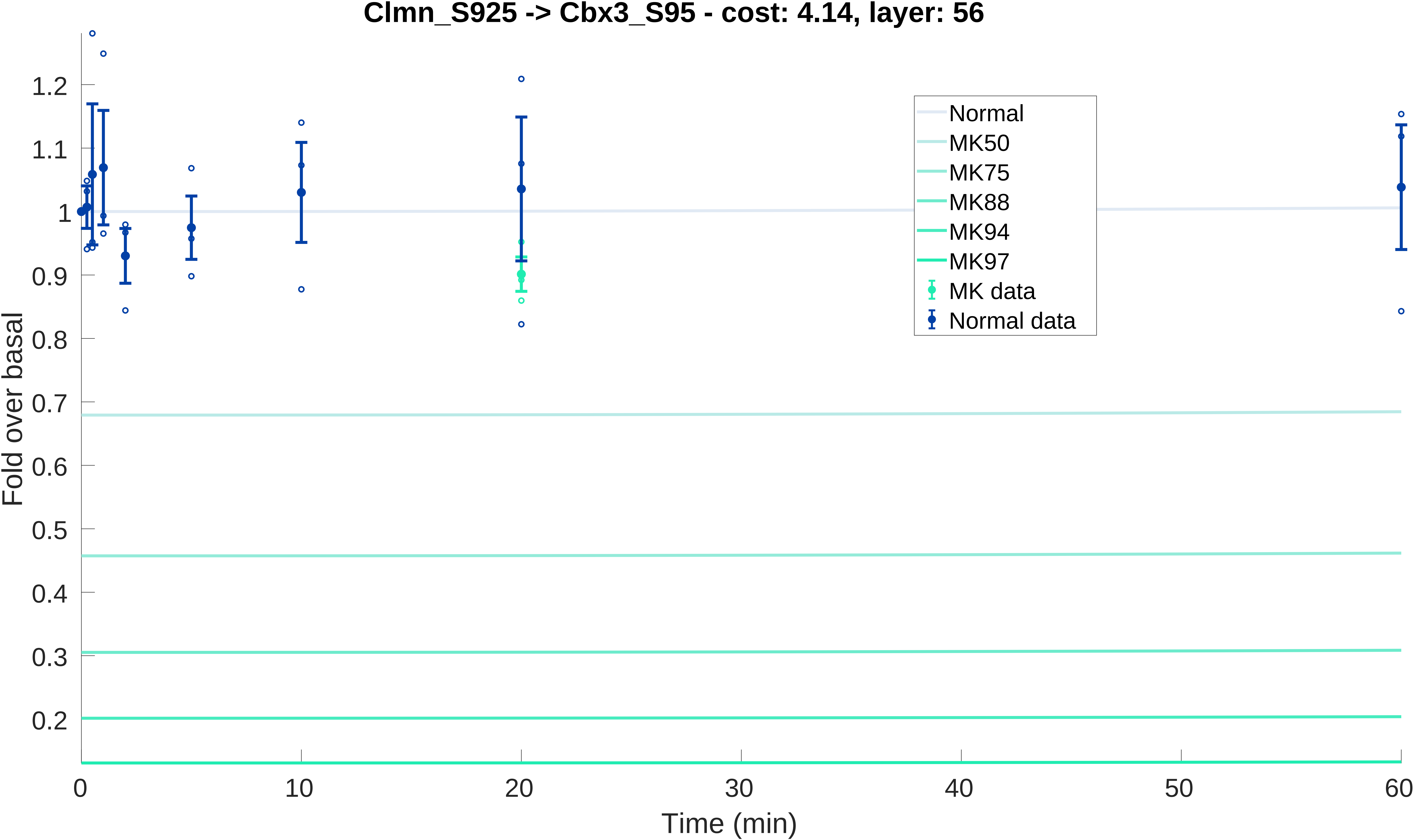

Clmn_S925 -> Cbx3_S95 - cost: 4.14, layer: 56
Fold over basal
Time (min)
1.2
1.1
1
0.9
0.8
0.7
0.6
0.5
0.4
0.3
0.2
0
10
20
30
40
50
60
Normal
MK50
MK75
MK88
MK94
MK97
MK data
Normal data

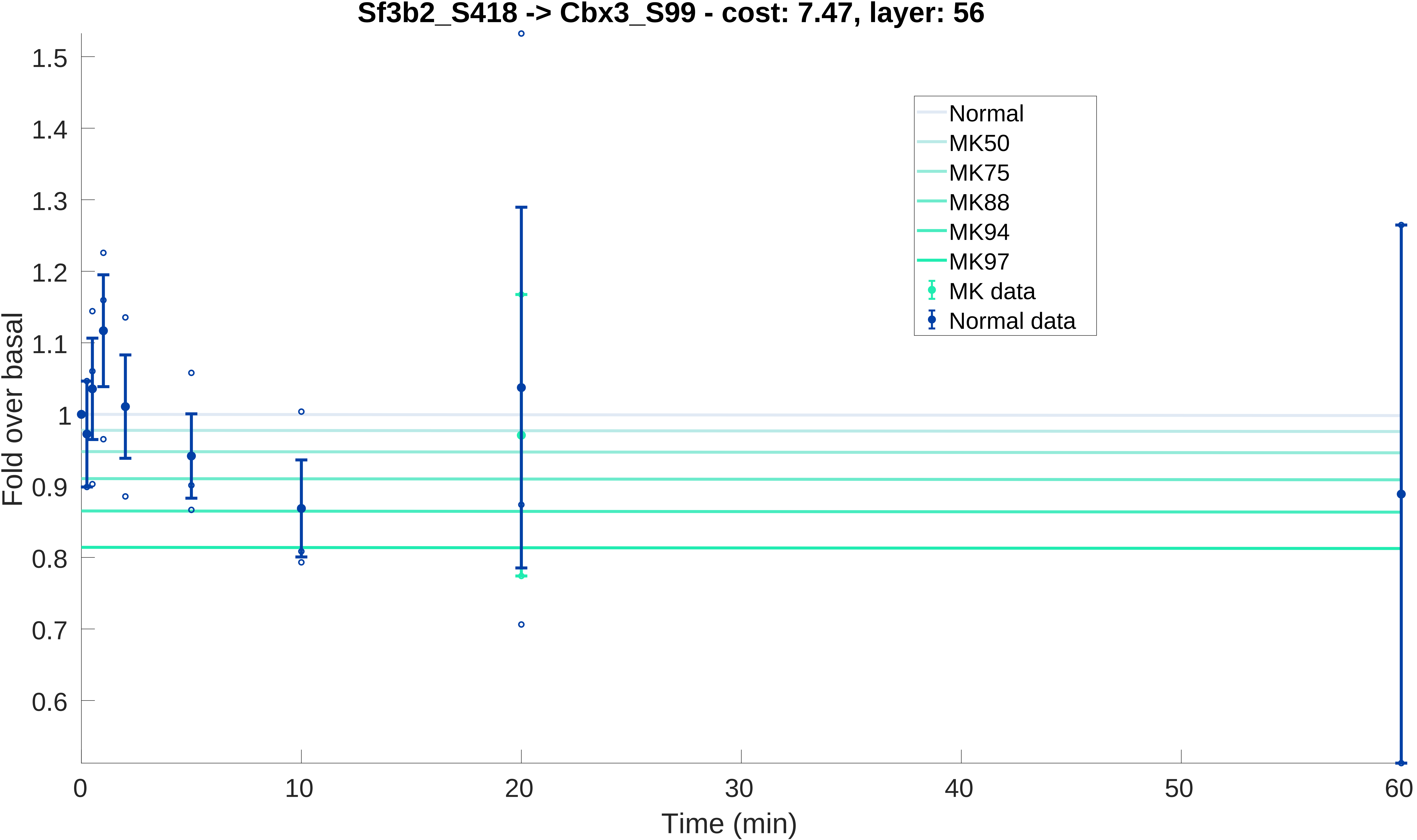
Sf3b2_S418 -> Cbx3_S99 - cost: 7.47, layer: 56
Fold over basal
Time (min)
1.5
1.4
1.3
1.2
1.1
1
0.9
0.8
0.7
0.6
0
10
20
30
40
50
60
Normal
MK50
MK75
MK88
MK94
MK97
MK data
Normal data

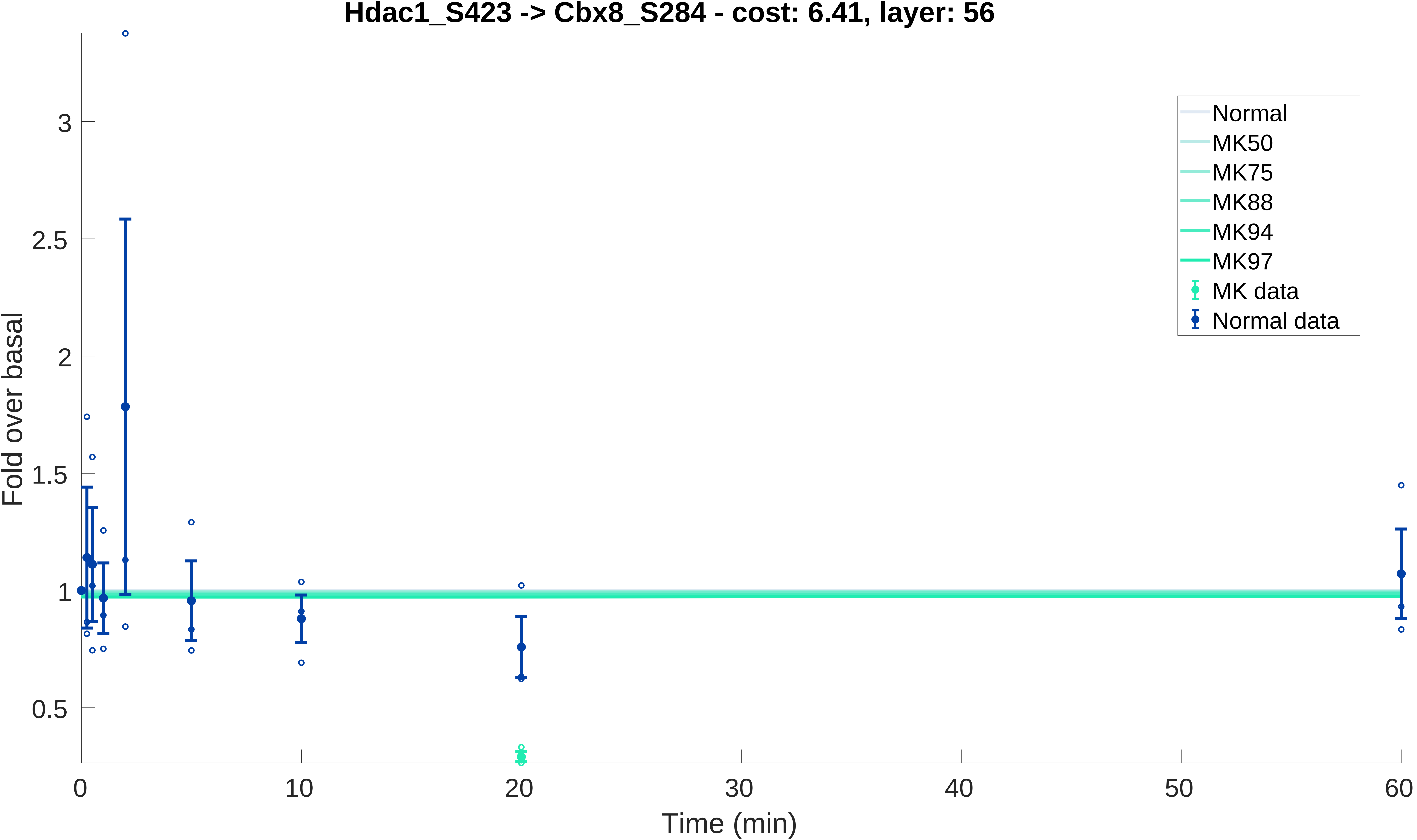

Hdac1_S423 -> Cbx8_S284 - cost: 6.41, layer: 56
Fold over basal
Time (min)
0
10
20
30
40
50
60
0.5
1
1.5
2
2.5
3
Normal
MK50
MK75
MK88
MK94
MK97
MK data
Normal data

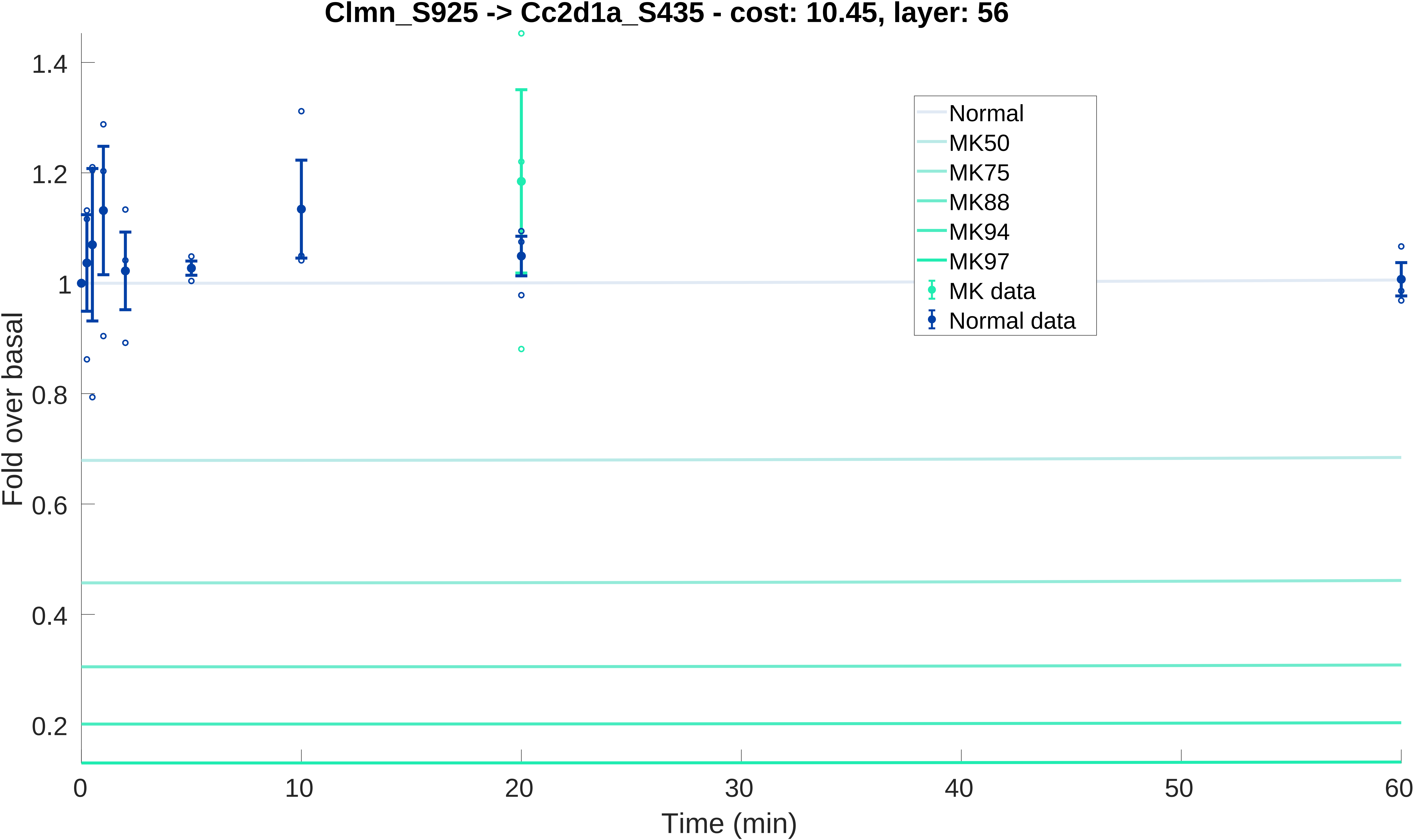

Clmn_S925 -> Cc2d1a_S435 - cost: 10.45, layer: 56
Fold over basal
Time (min)
Normal
MK50
MK75
MK88
MK94
MK97
MK data
Normal data
0
10
20
30
40
50
60
0.2
0.4
0.6
0.8
1
1.2
1.4

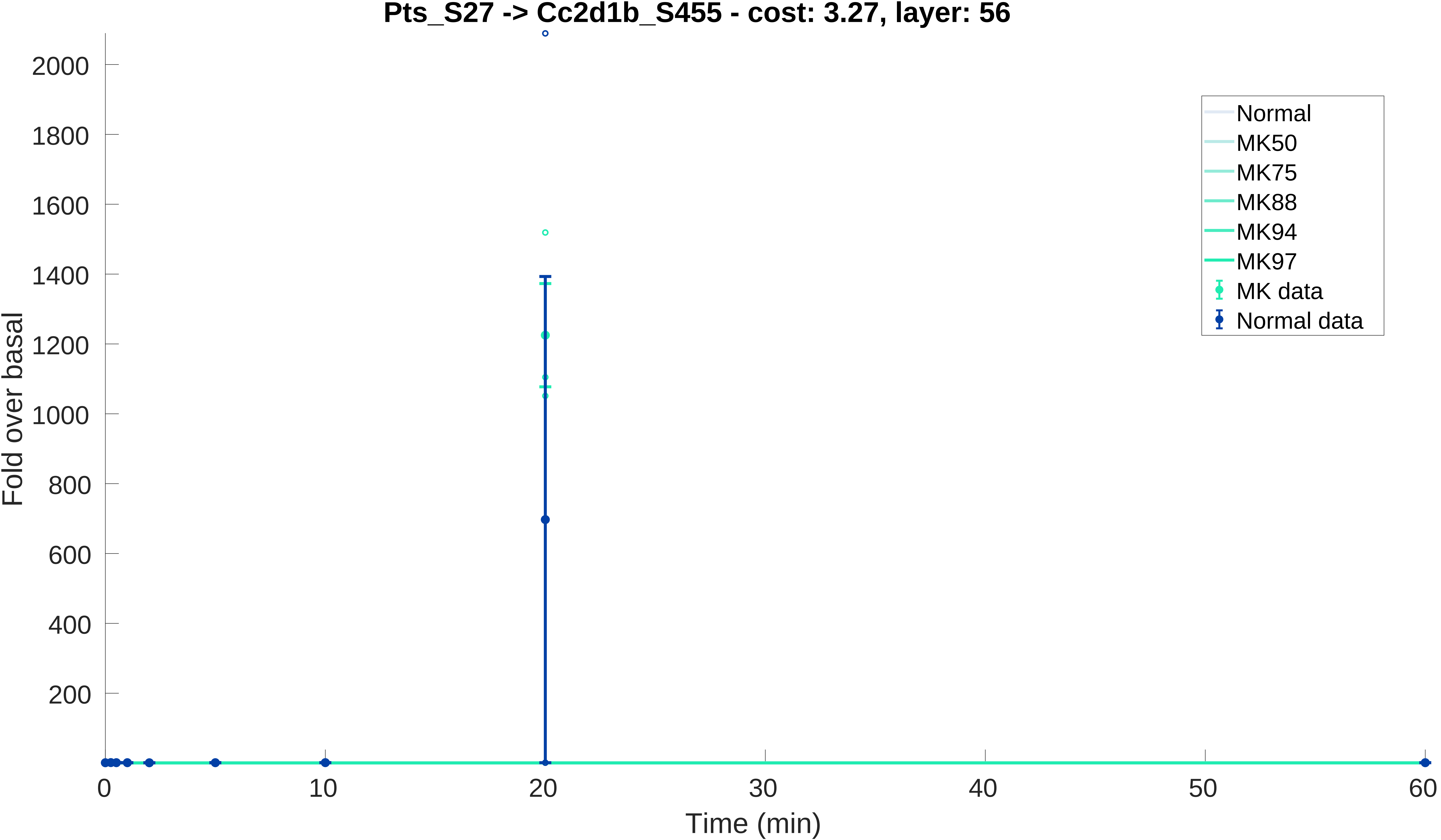

Pts_S27 -> Cc2d1b_S455 - cost: 3.27, layer: 56
Fold over basal
2000
1800
1600
1400
1200
1000
800
600
400
200
0
10
20
30
40
50
60
Time (min)
Normal
MK50
MK75
MK88
MK94
MK97
MK data
Normal data

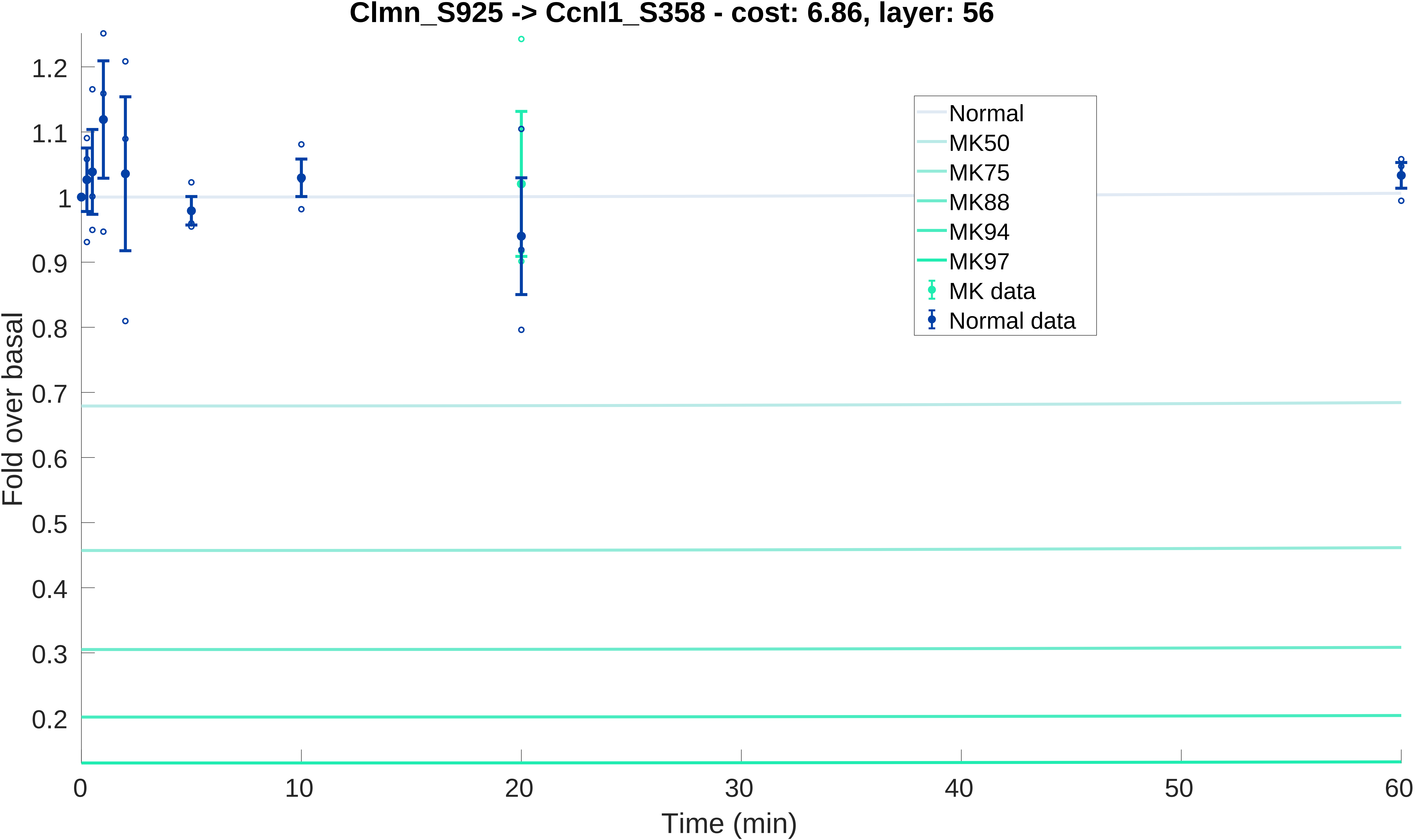

Clmn_S925 -> Ccnl1_S358 - cost: 6.86, layer: 56
Fold over basal
Time (min)
Normal
MK50
MK75
MK88
MK94
MK97
MK data
Normal data
1.2
1.1
1
0.9
0.8
0.7
0.6
0.5
0.4
0.3
0.2
0
10
20
30
40
50
60

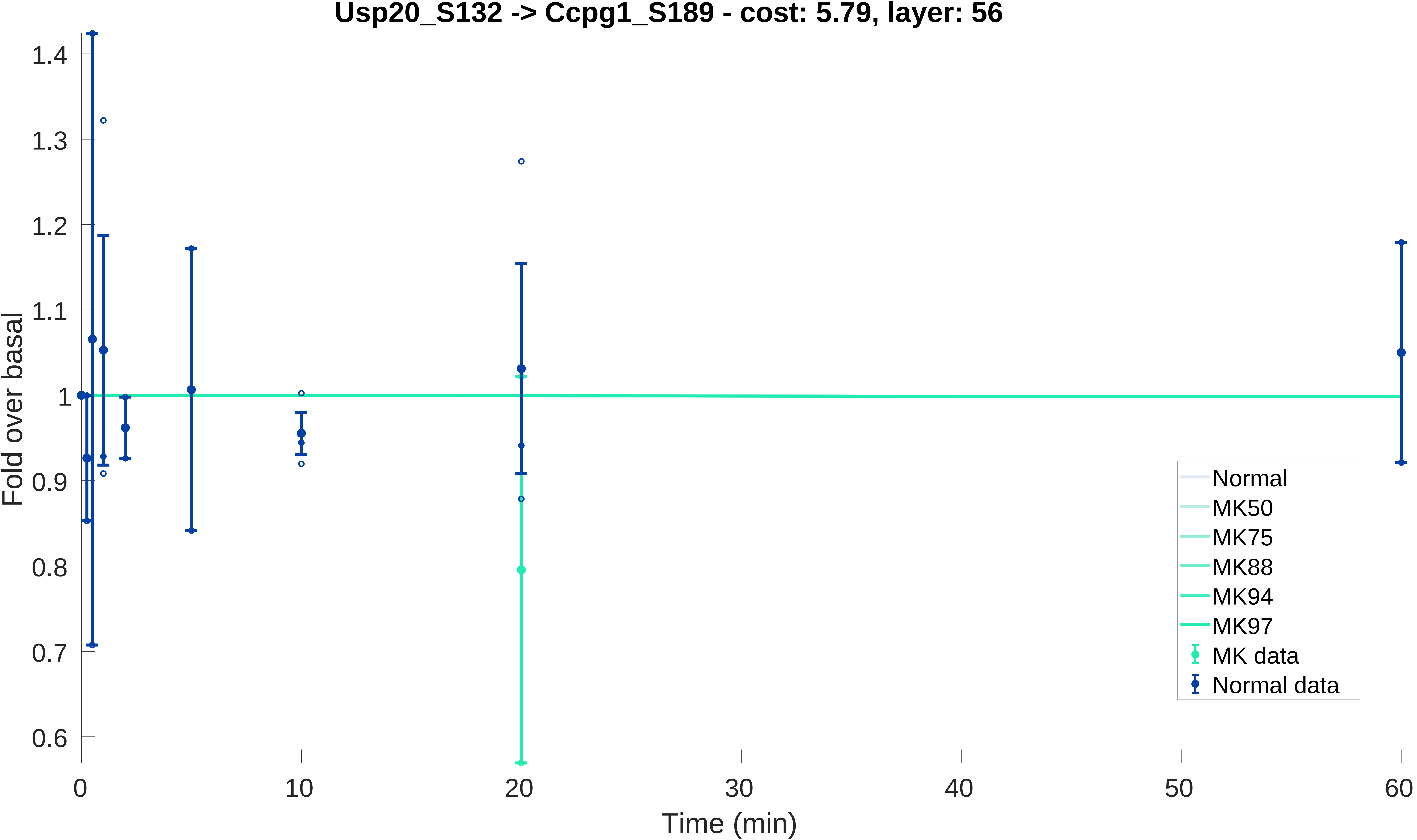

Usp20_S132 -> Ccpg1_S189 - cost: 5.79, layer: 56
Fold over basal
1.4
1.3
1.2
1.1
1
0.9
0.8
0.7
0.6
0
10
20
30
40
50
60
Time (min)
Normal
MK50
MK75
MK88
MK94
MK97
MK data
Normal data

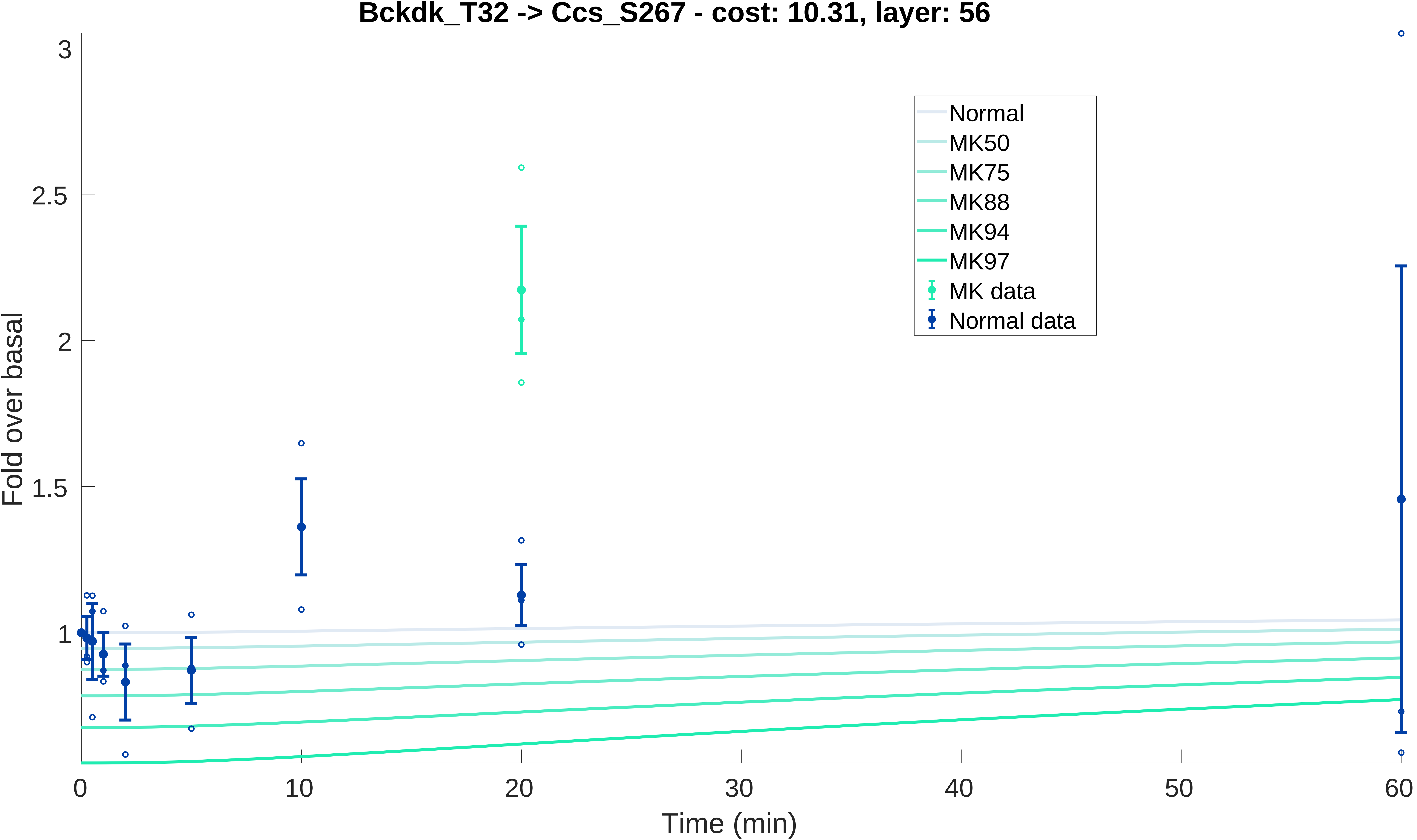

Bckdk_T32 -> Ccs_S267 - cost: 10.31, layer: 56
Fold over basal
Time (min)
3
2.5
2
1.5
1
0
10
20
30
40
50
60
Normal
MK50
MK75
MK88
MK94
MK97
MK data
Normal data

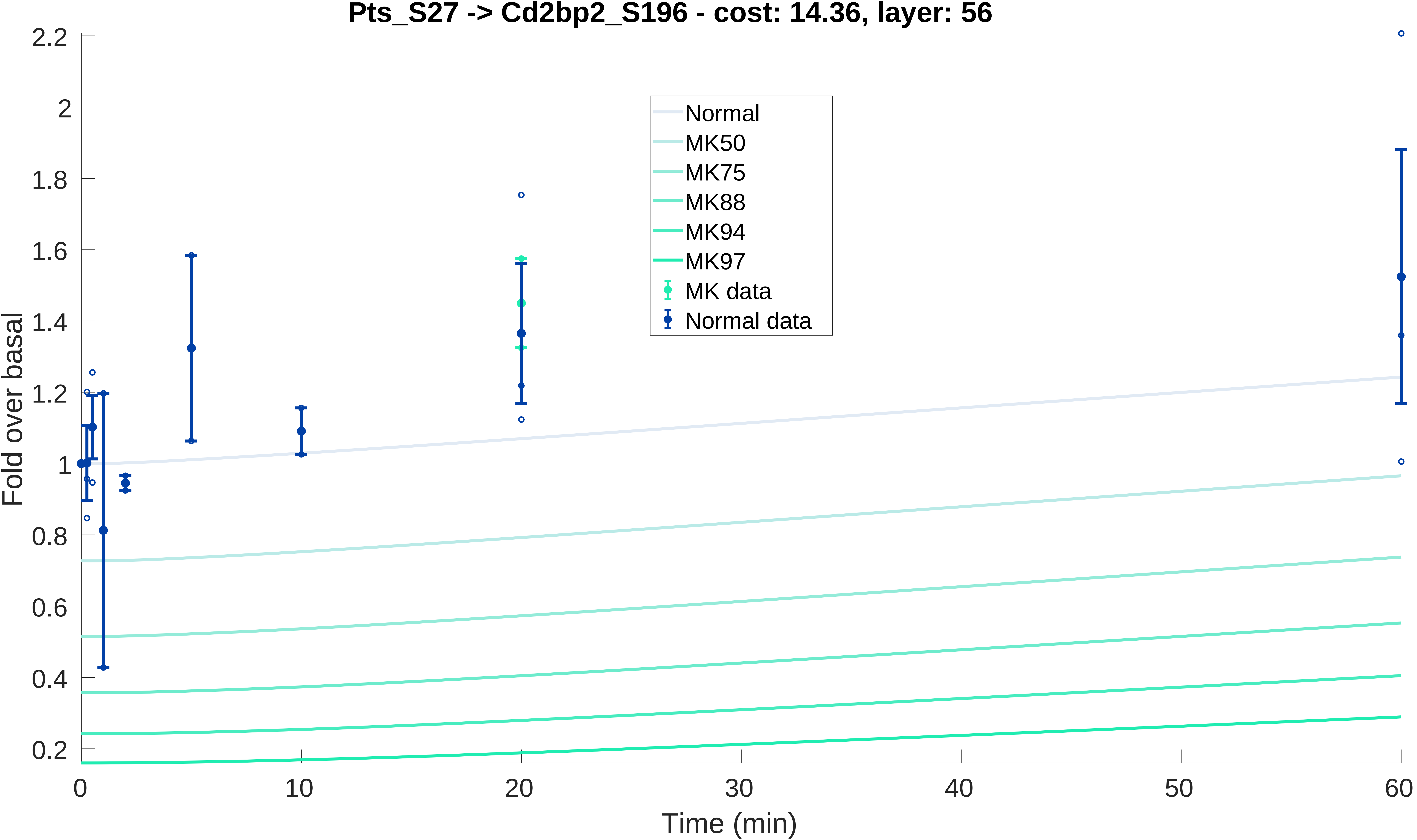

Pts_S27 -> Cd2bp2_S196 - cost: 14.36, layer: 56
Normal
MK50
MK75
MK88
MK94
MK97
MK data
Normal data
Fold over basal
Time (min)
2.2
2
1.8
1.6
1.4
1.2
1
0.8
0.6
0.4
0.2
0
10
20
30
40
50
60

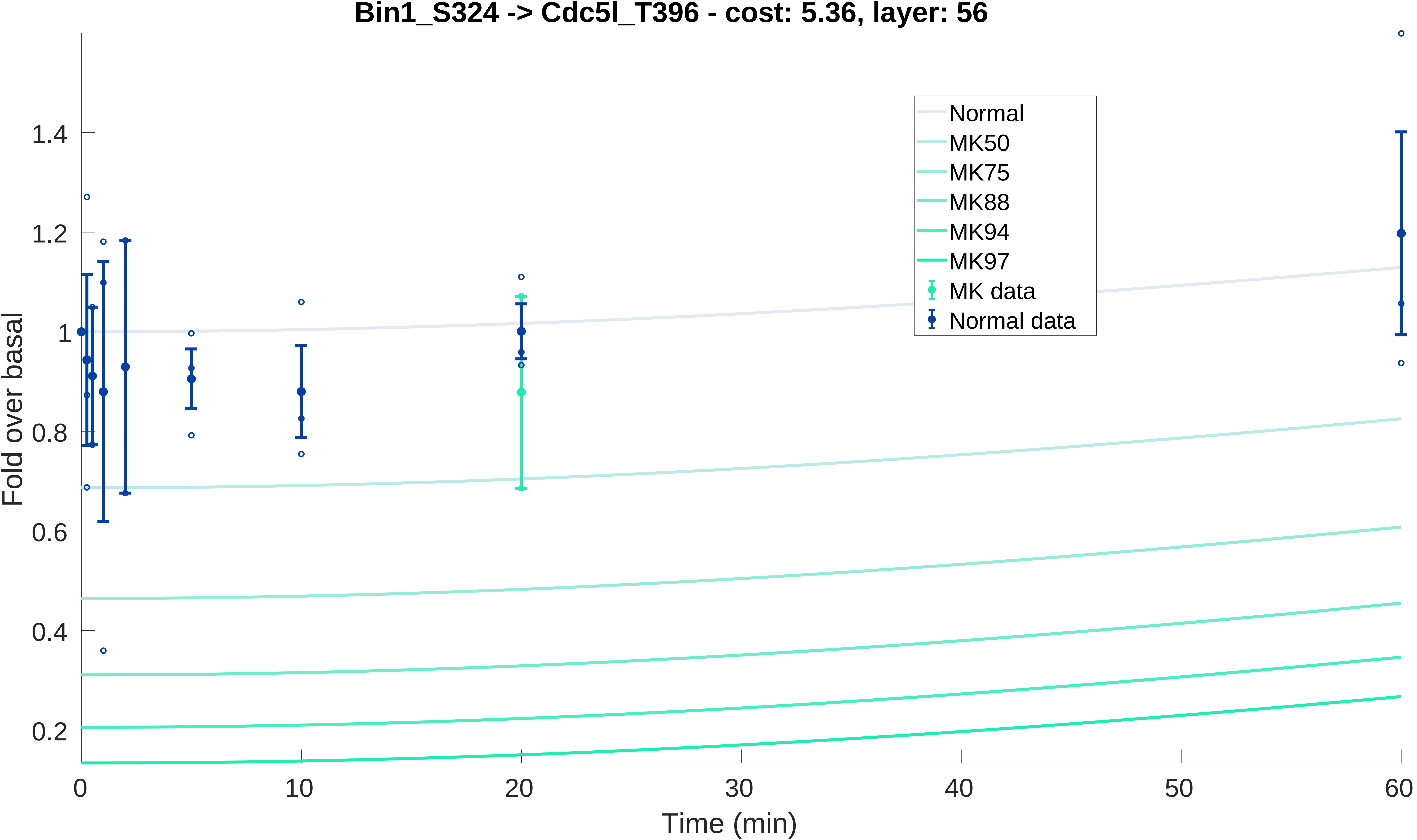

Bin1_S324 -> Cdc5l_T396 - cost: 5.36, layer: 56
Fold over basal
Time (min)
0.2
0.4
0.6
0.8
1
1.2
1.4
0
10
20
30
40
50
60
Normal
MK50
MK75
MK88
MK94
MK97
MK data
Normal data

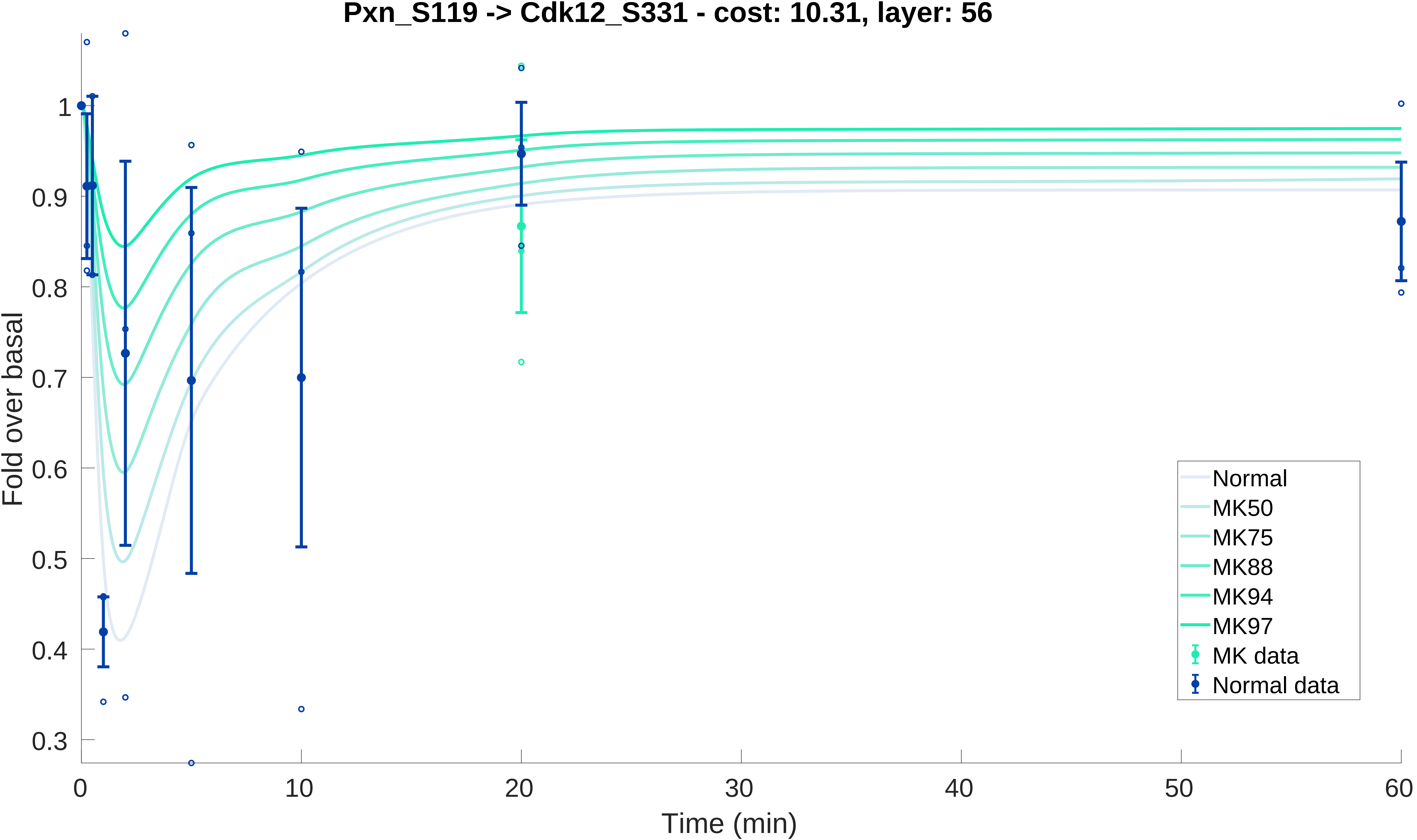

Pxn_S119 -> Cdk12_S331 - cost: 10.31, layer: 56
Fold over basal
Time (min)
0.3
0.4
0.5
0.6
0.7
0.8
0.9
1
0
10
20
30
40
50
60
Normal
MK50
MK75
MK88
MK94
MK97
MK data
Normal data

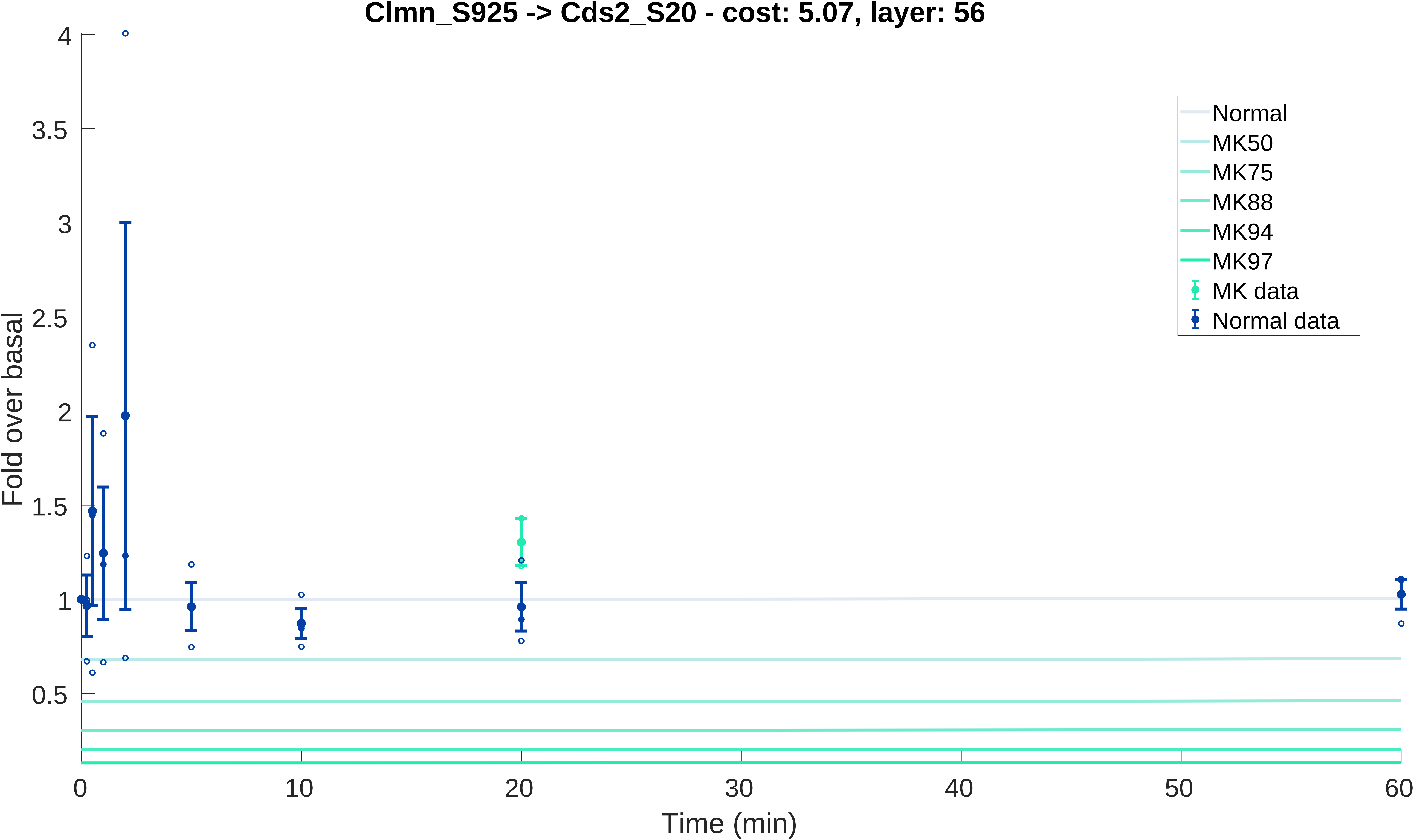

Clmn_S925 -> Cds2_S20 - cost: 5.07, layer: 56
Fold over basal
4
3.5
3
2.5
2
1.5
1
0.5
0
10
20
30
40
50
60
Time (min)
Normal
MK50
MK75
MK88
MK94
MK97
MK data
Normal data

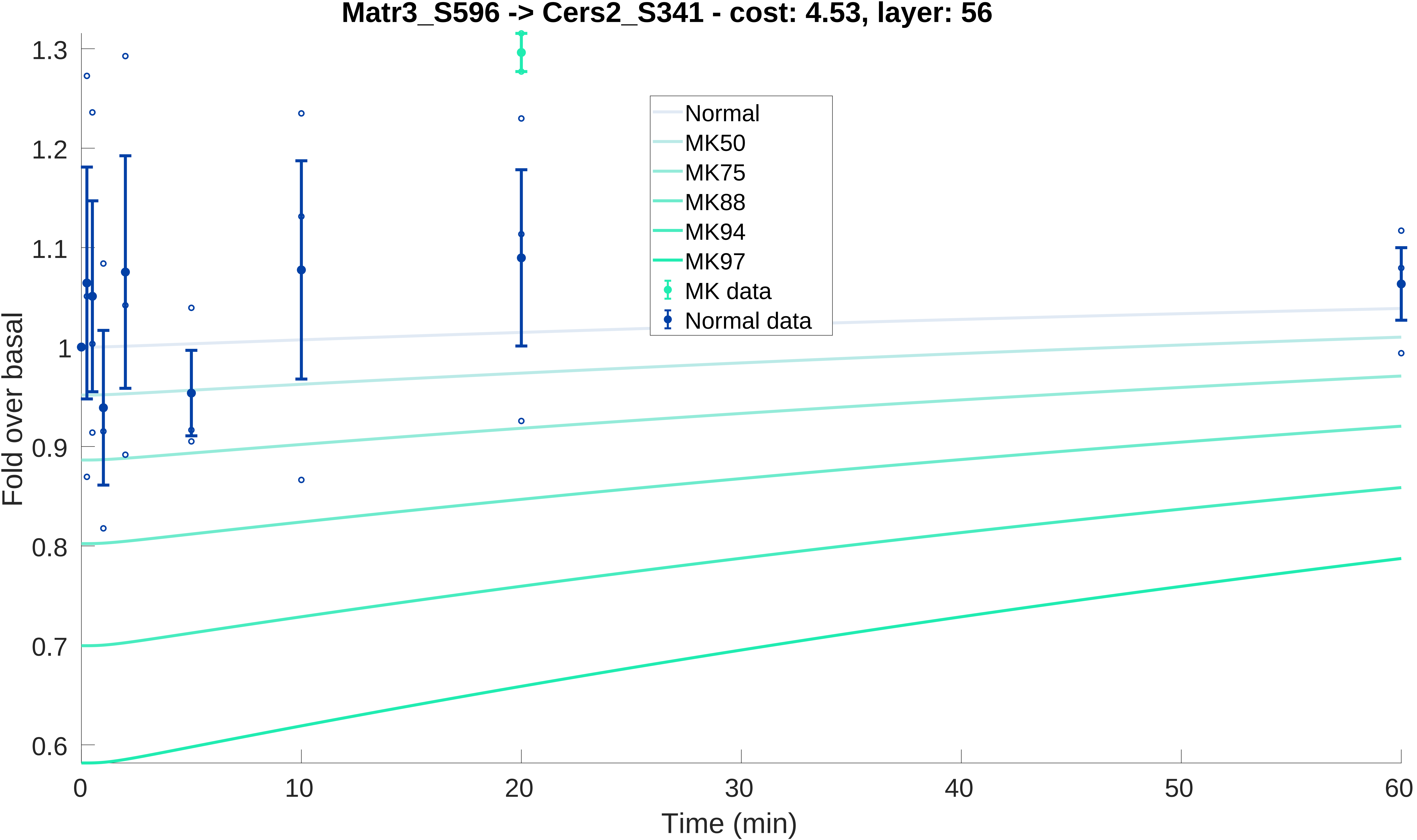

Matr3_S596 -> Cers2_S341 - cost: 4.53, layer: 56
Normal
MK50
MK75
MK88
MK94
MK97
MK data
Normal data
Fold over basal
Time (min)
0
10
20
30
40
50
60
0.6
0.7
0.8
0.9
1
1.1
1.2
1.3

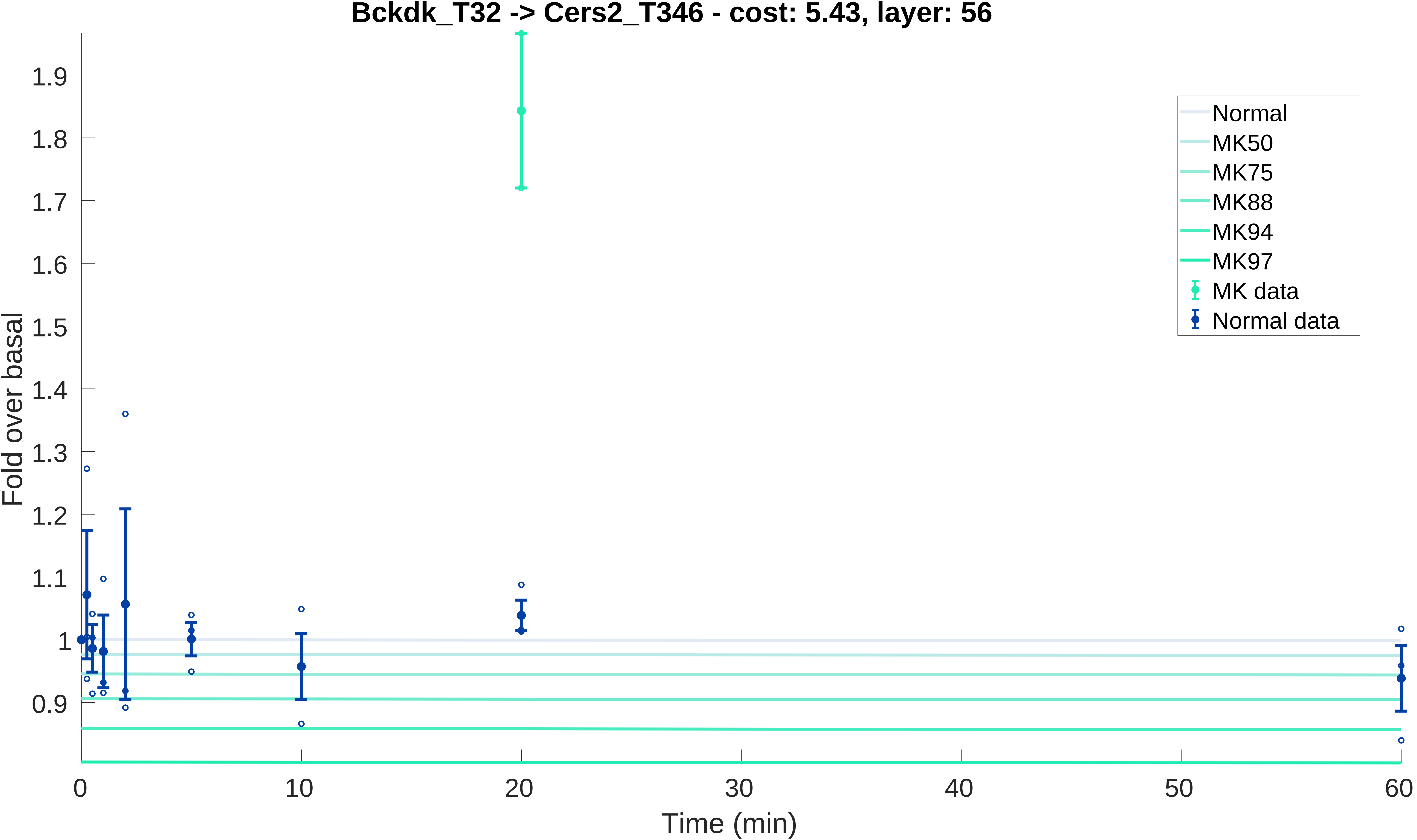

Bckdk_T32 -> Cers2_T346 - cost: 5.43, layer: 56
Fold over basal
Time (min)
Normal
MK50
MK75
MK88
MK94
MK97
MK data
Normal data
0
10
20
30
40
50
60
0.9
1
1.1
1.2
1.3
1.4
1.5
1.6
1.7
1.8
1.9

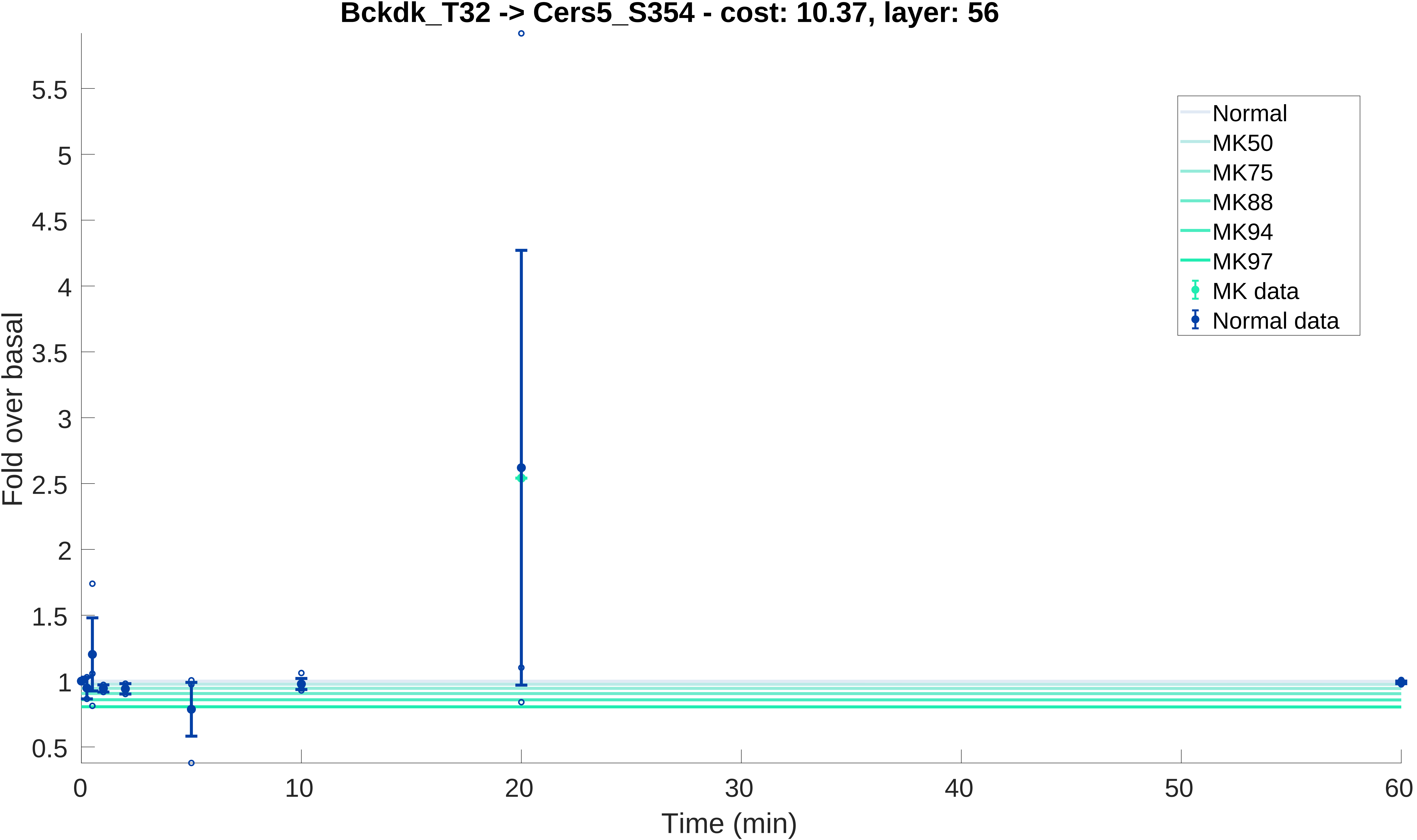

Bckdk_T32 -> Cers5_S354 - cost: 10.37, layer: 56
Fold over basal
Time (min)
0.5
1
1.5
2
2.5
3
3.5
4
4.5
5
5.5
0
10
20
30
40
50
60
Normal
MK50
MK75
MK88
MK94
MK97
MK data
Normal data

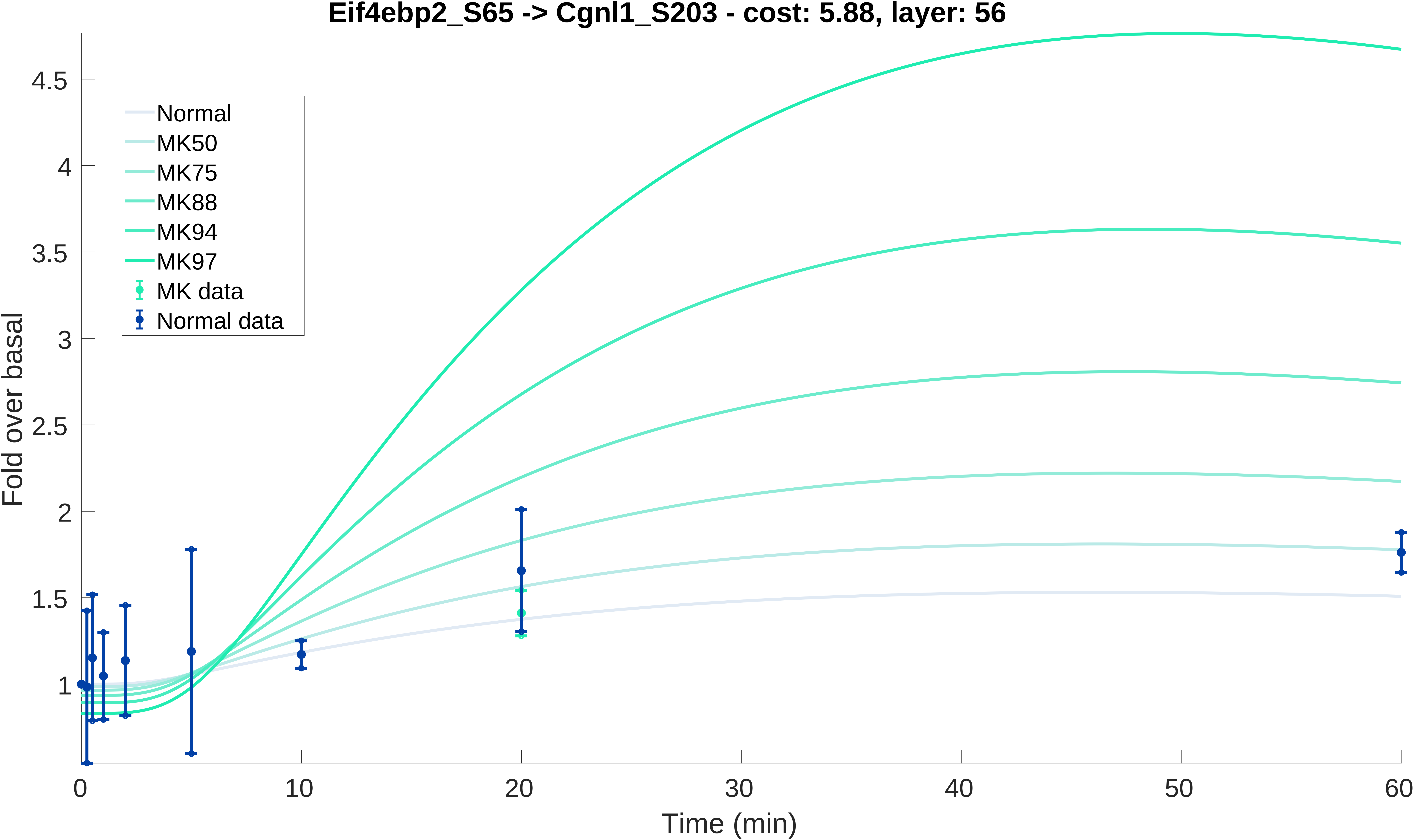

Eif4ebp2_S65 -> Cgnl1_S203 - cost: 5.88, layer: 56
Fold over basal
Time (min)
Normal
MK50
MK75
MK88
MK94
MK97
MK data
Normal data
4.5
4
3.5
3
2.5
2
1.5
1
0
10
20
30
40
50
60

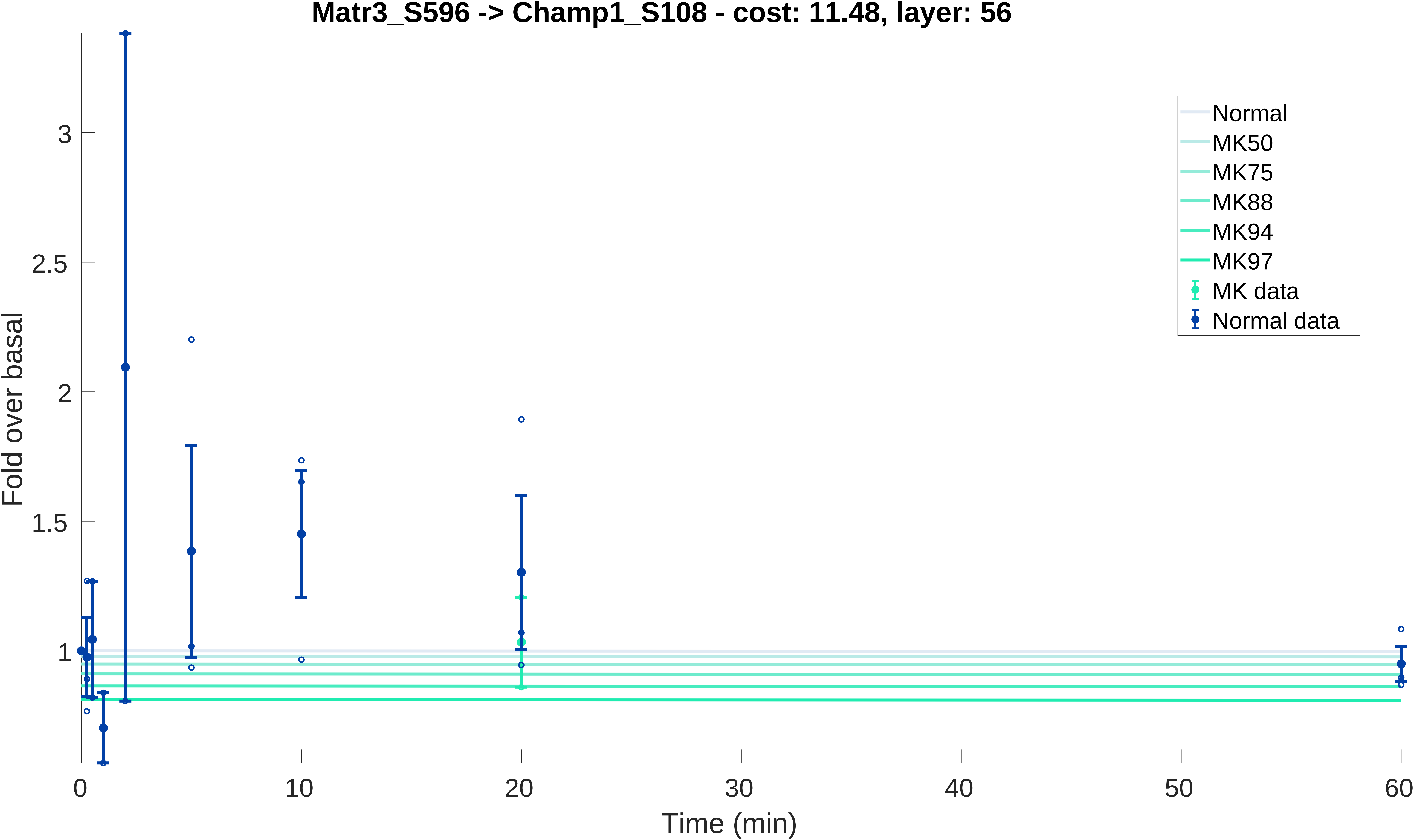

Matr3_S596 -> Champ1_S108 - cost: 11.48, layer: 56
Fold over basal
Time (min)
3
2.5
2
1.5
1
0
10
20
30
40
50
60
Normal
MK50
MK75
MK88
MK94
MK97
MK data
Normal data

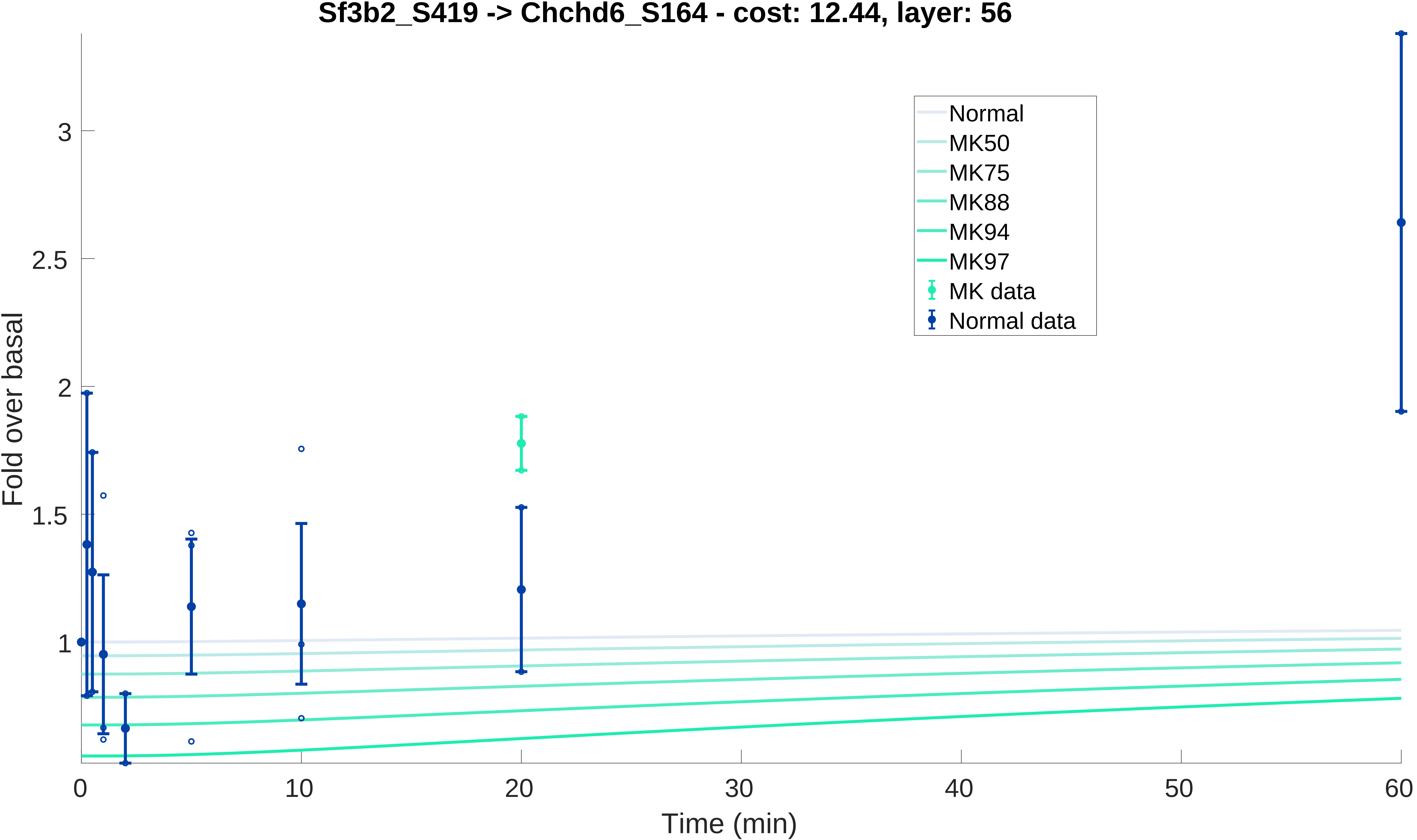

Sf3b2_S419 -> Chchd6_S164 - cost: 12.44, layer: 56
Fold over basal
Time (min)
Normal
MK50
MK75
MK88
MK94
MK97
MK data
Normal data
3
2.5
2
1.5
1
0
10
20
30
40
50
60

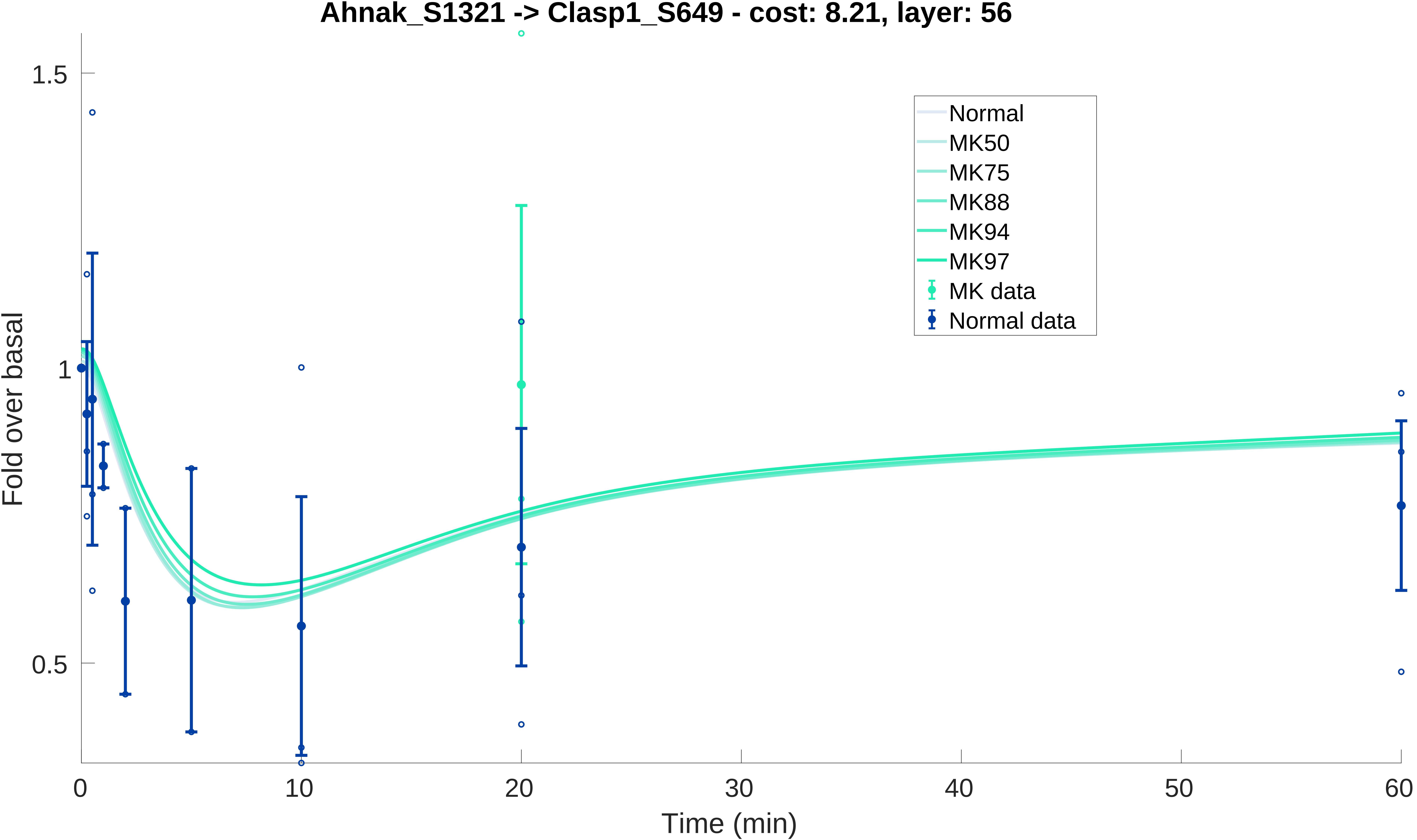

Ahnak_S1321 -> Clasp1_S649 - cost: 8.21, layer: 56
Fold over basal
Time (min)
1.5
1
0.5
0
10
20
30
40
50
60
Normal
MK50
MK75
MK88
MK94
MK97
MK data
Normal data

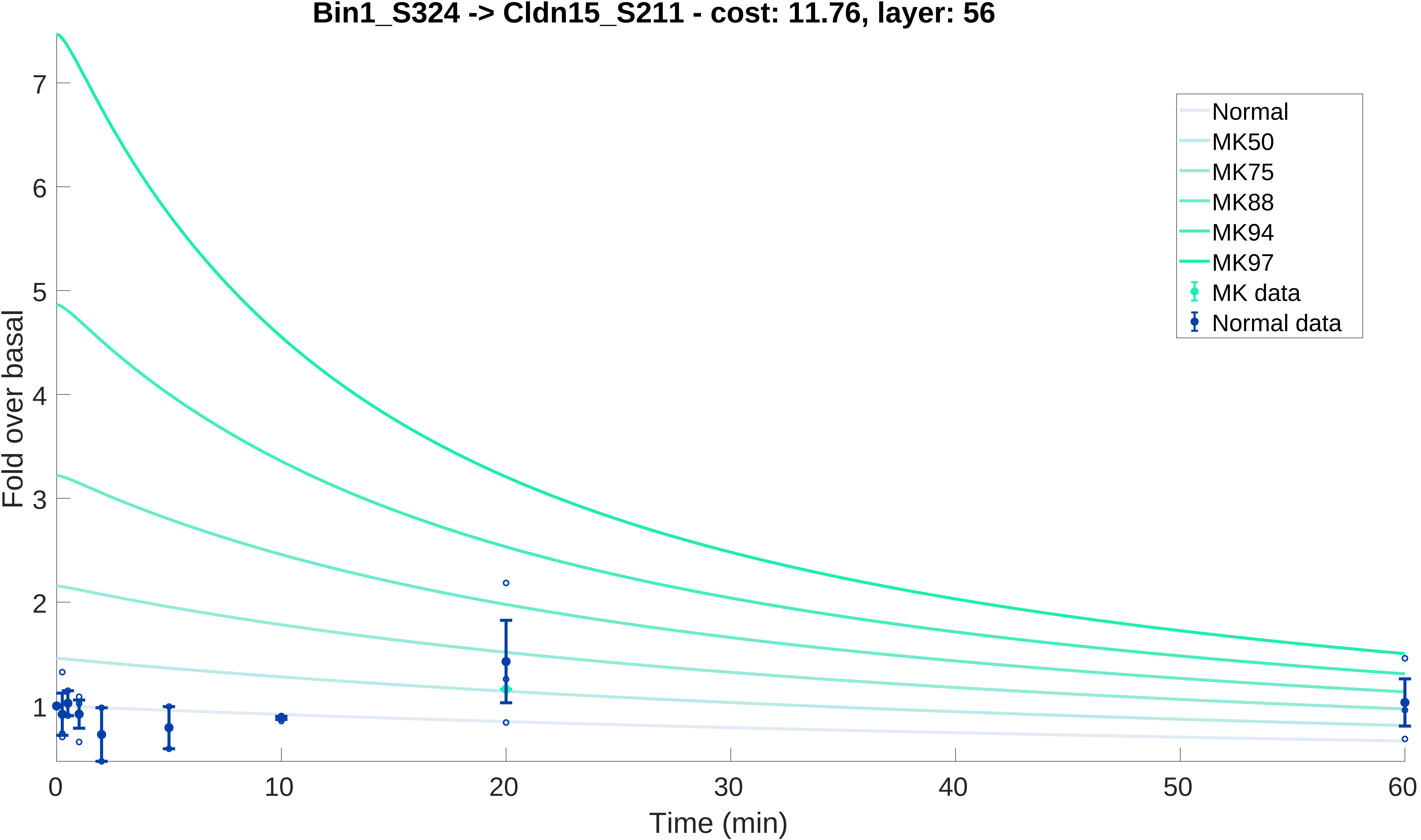
Bin1_S324 -> Cldn15_S211 - cost: 11.76, layer: 56
Fold over basal
Time (min)
Normal
MK50
MK75
MK88
MK94
MK97
MK data
Normal data
1
2
3
4
5
6
7
0
10
20
30
40
50
60

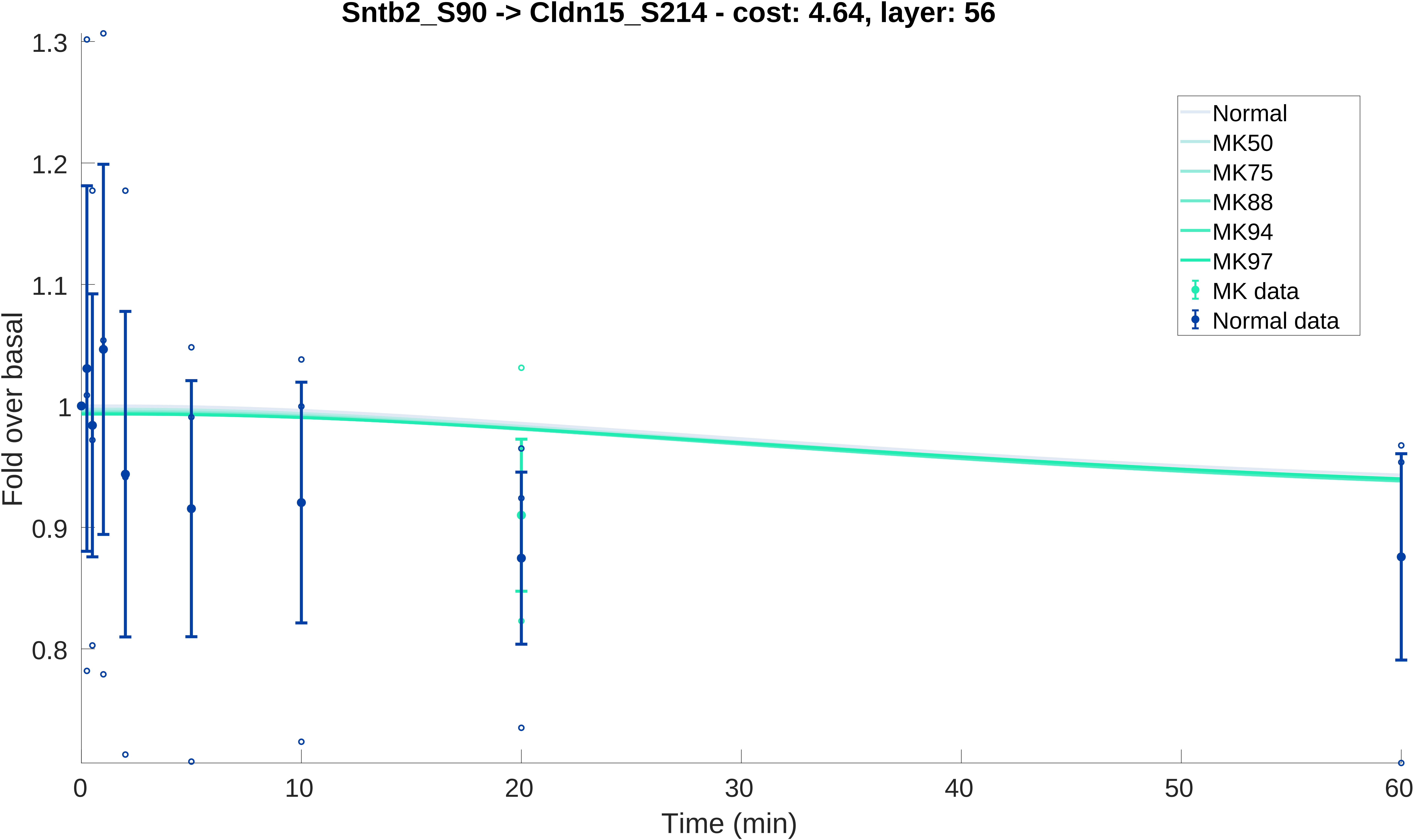
Sntb2_S90 -> Cldn15_S214 - cost: 4.64, layer: 56
Normal
MK50
MK75
MK88
MK94
MK97
MK data
Normal data
Fold over basal
Time (min)
1.3
1.2
1.1
1
0.9
0.8
0
10
20
30
40
50
60

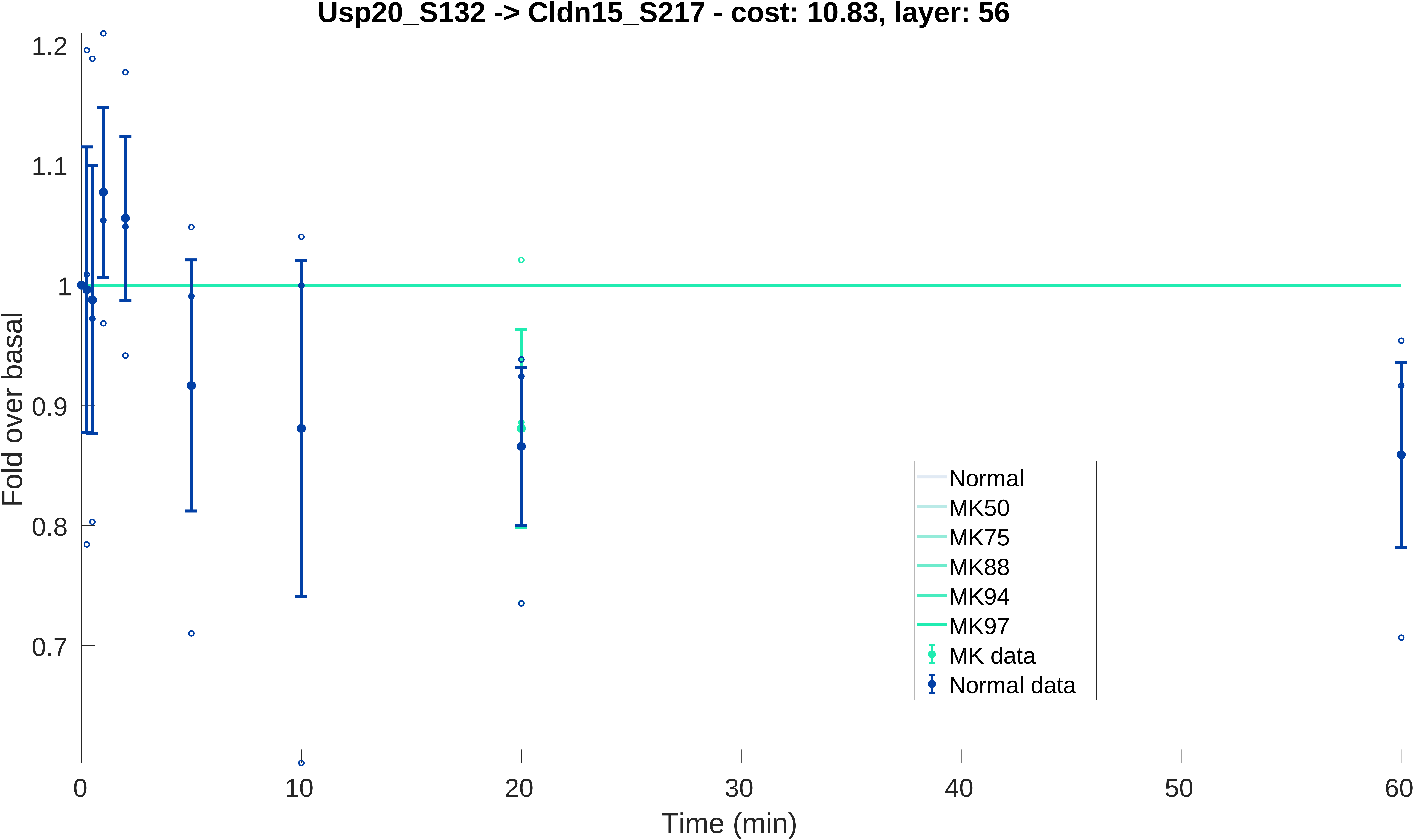
Usp20_S132 -> Cldn15_S217 - cost: 10.83, layer: 56
Fold over basal
Time (min)
1.2
1.1
1
0.9
0.8
0.7
0
10
20
30
40
50
60
Normal
MK50
MK75
MK88
MK94
MK97
MK data
Normal data

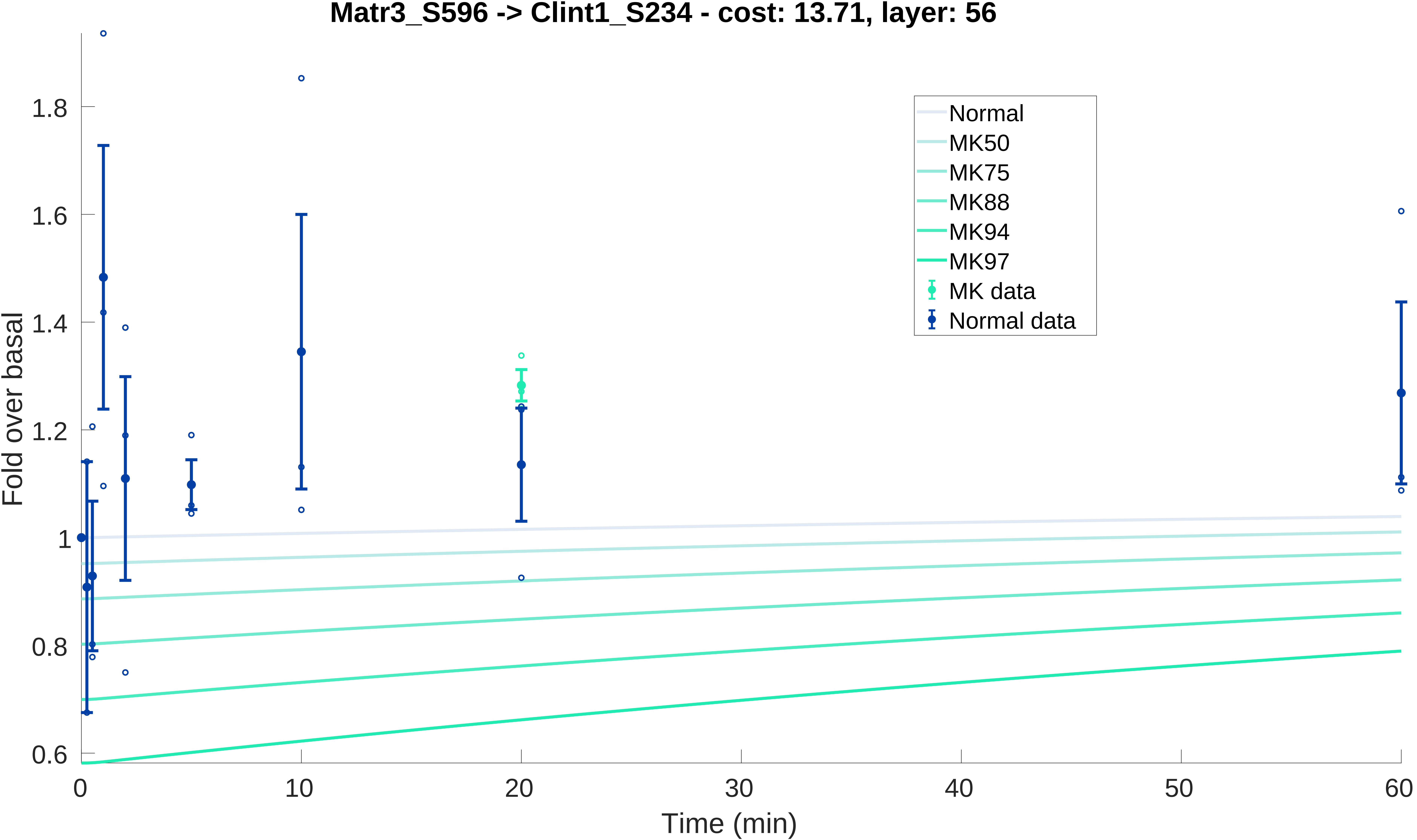
Matr3_S596 -> Clint1_S234 - cost: 13.71, layer: 56
Fold over basal
Time (min)
0.6
0.8
1
1.2
1.4
1.6
1.8
0
10
20
30
40
50
60
Normal
MK50
MK75
MK88
MK94
MK97
MK data
Normal data

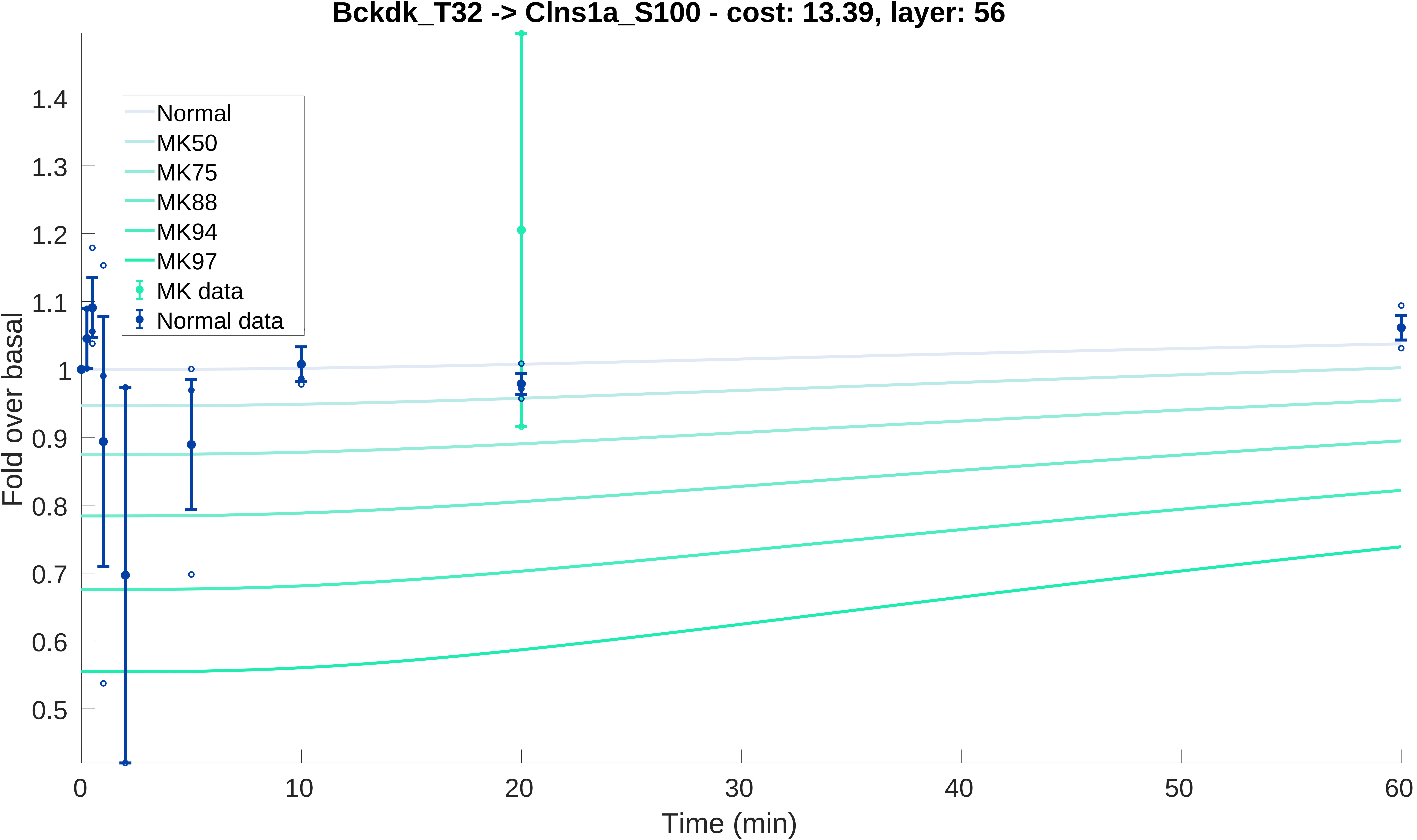

Bckdk_T32 -> Clns1a_S100 - cost: 13.39, layer: 56
Normal
MK50
MK75
MK88
MK94
MK97
MK data
Normal data
Fold over basal
Time (min)
1.4
1.3
1.2
1.1
1
0.9
0.8
0.7
0.6
0.5
0
10
20
30
40
50
60

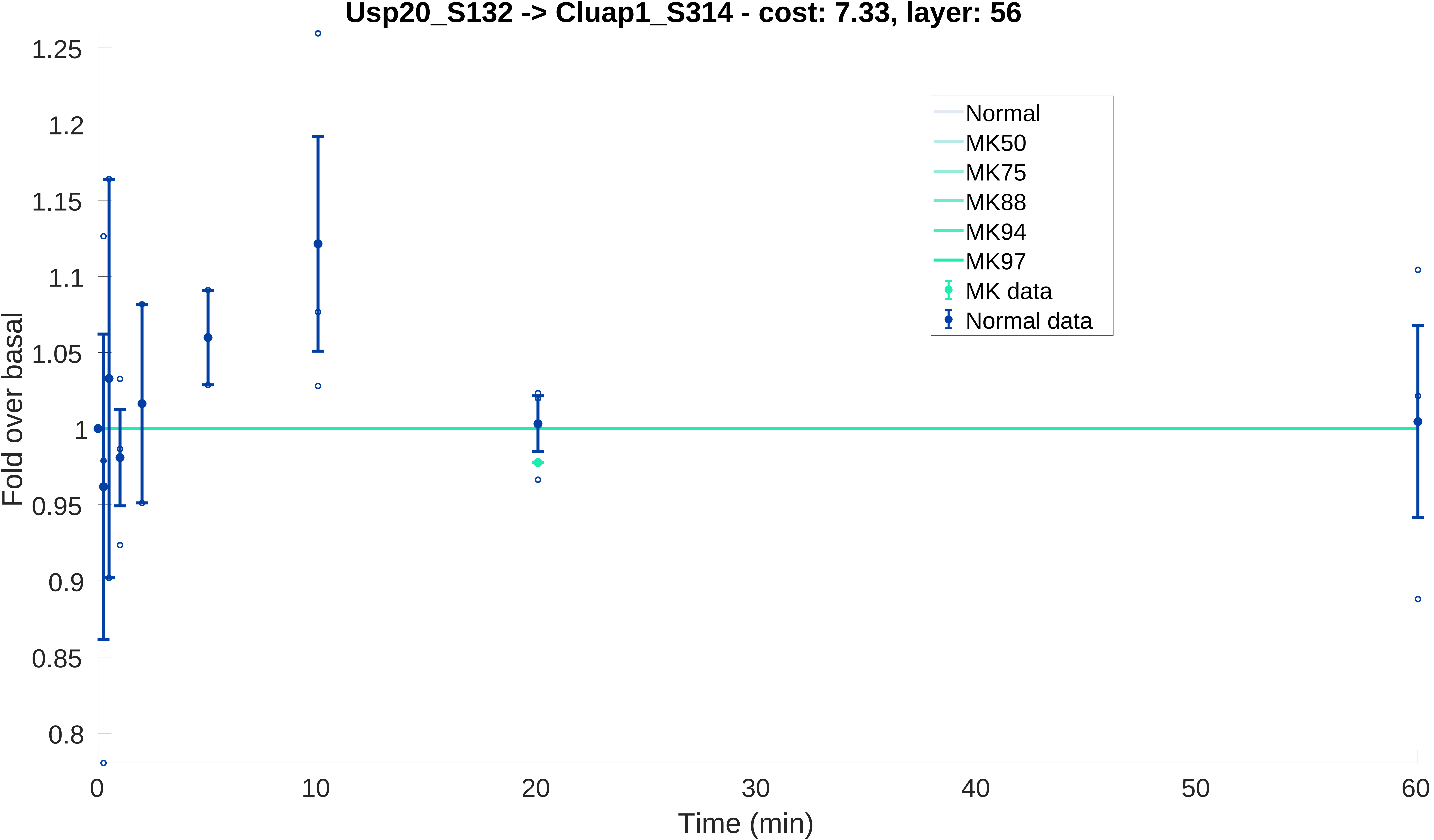

Usp20_S132 -> Cluap1_S314 - cost: 7.33, layer: 56
Fold over basal
Time (min)
Normal
MK50
MK75
MK88
MK94
MK97
MK data
Normal data
1.25
1.2
1.15
1.1
1.05
1
0.95
0.9
0.85
0.8
0
10
20
30
40
50
60

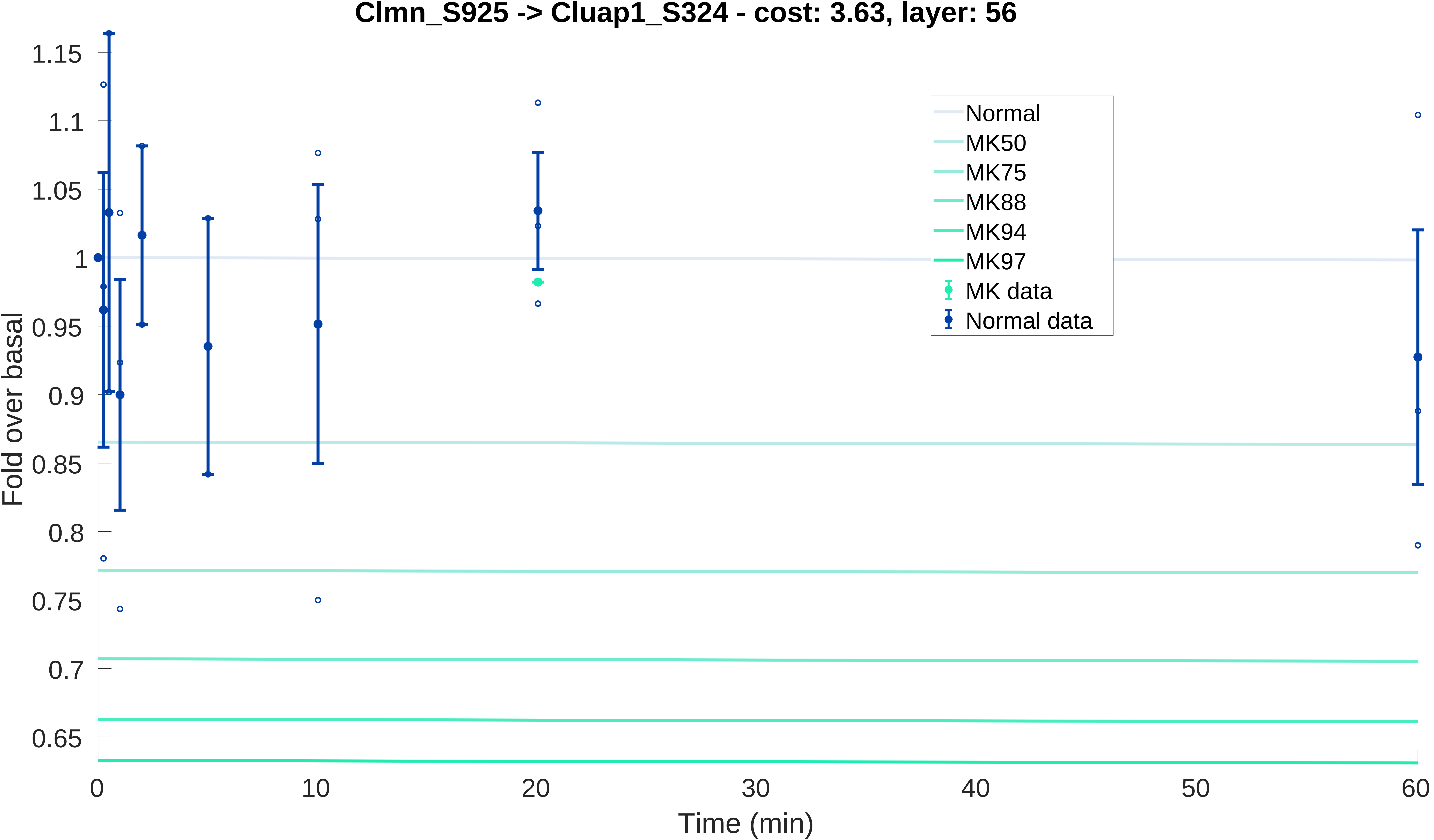
Clmn_S925 -> Cluap1_S324 - cost: 3.63, layer: 56
Fold over basal
Time (min)
1.15
1.1
1.05
1
0.95
0.9
0.85
0.8
0.75
0.7
0.65
0
10
20
30
40
50
60
Normal
MK50
MK75
MK88
MK94
MK97
MK data
Normal data

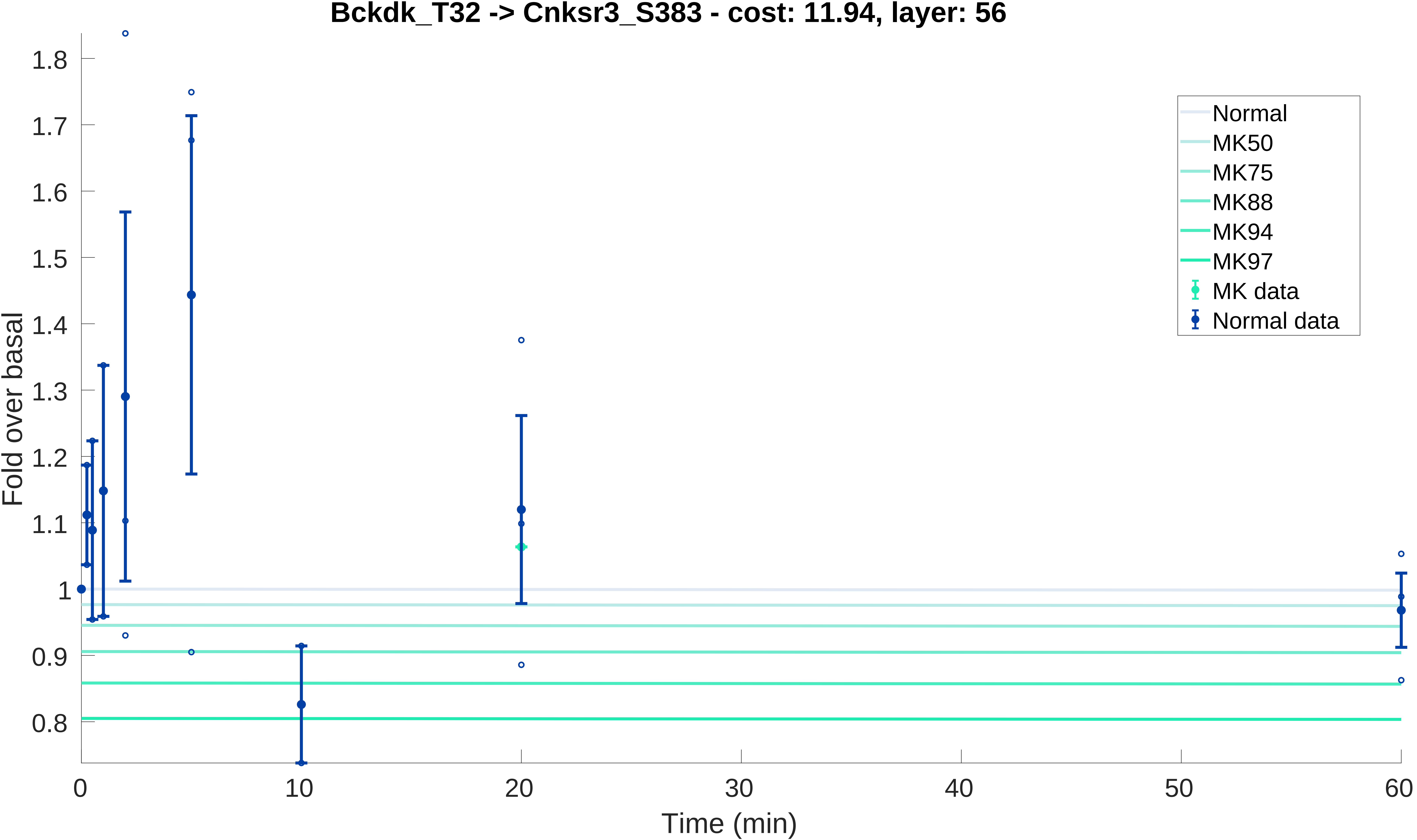

Bckdk_T32 -> Cnksr3_S383 - cost: 11.94, layer: 56
Fold over basal
1.8
1.7
1.6
1.5
1.4
1.3
1.2
1.1
1
0.9
0.8
Time (min)
0
10
20
30
40
50
60
Normal
MK50
MK75
MK88
MK94
MK97
MK data
Normal data

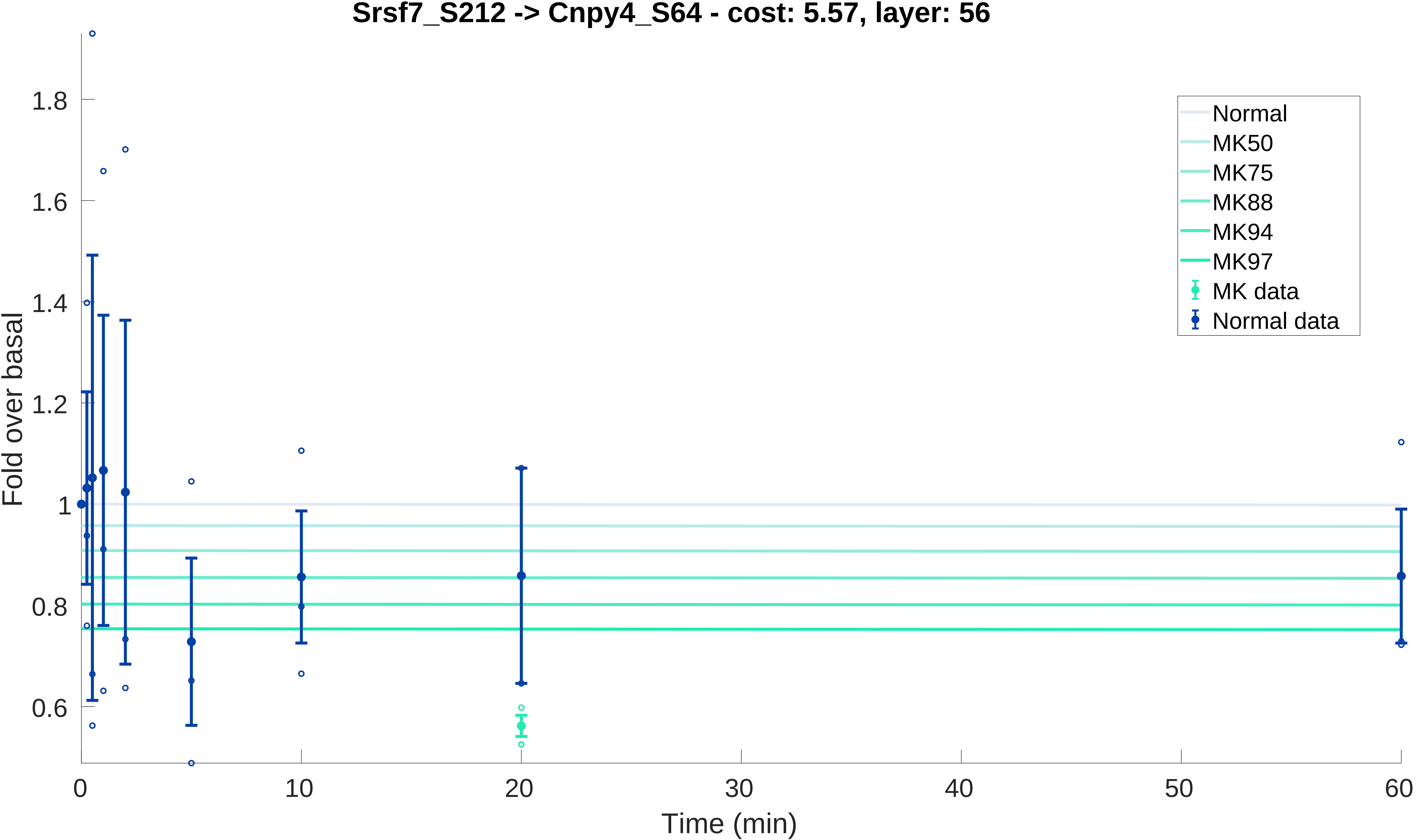
Srsf7_S212 -> Cnpy4_S64 - cost: 5.57, layer: 56
Fold over basal
Time (min)
Normal
MK50
MK75
MK88
MK94
MK97
MK data
Normal data
0.6
0.8
1
1.2
1.4
1.6
1.8
0
10
20
30
40
50
60

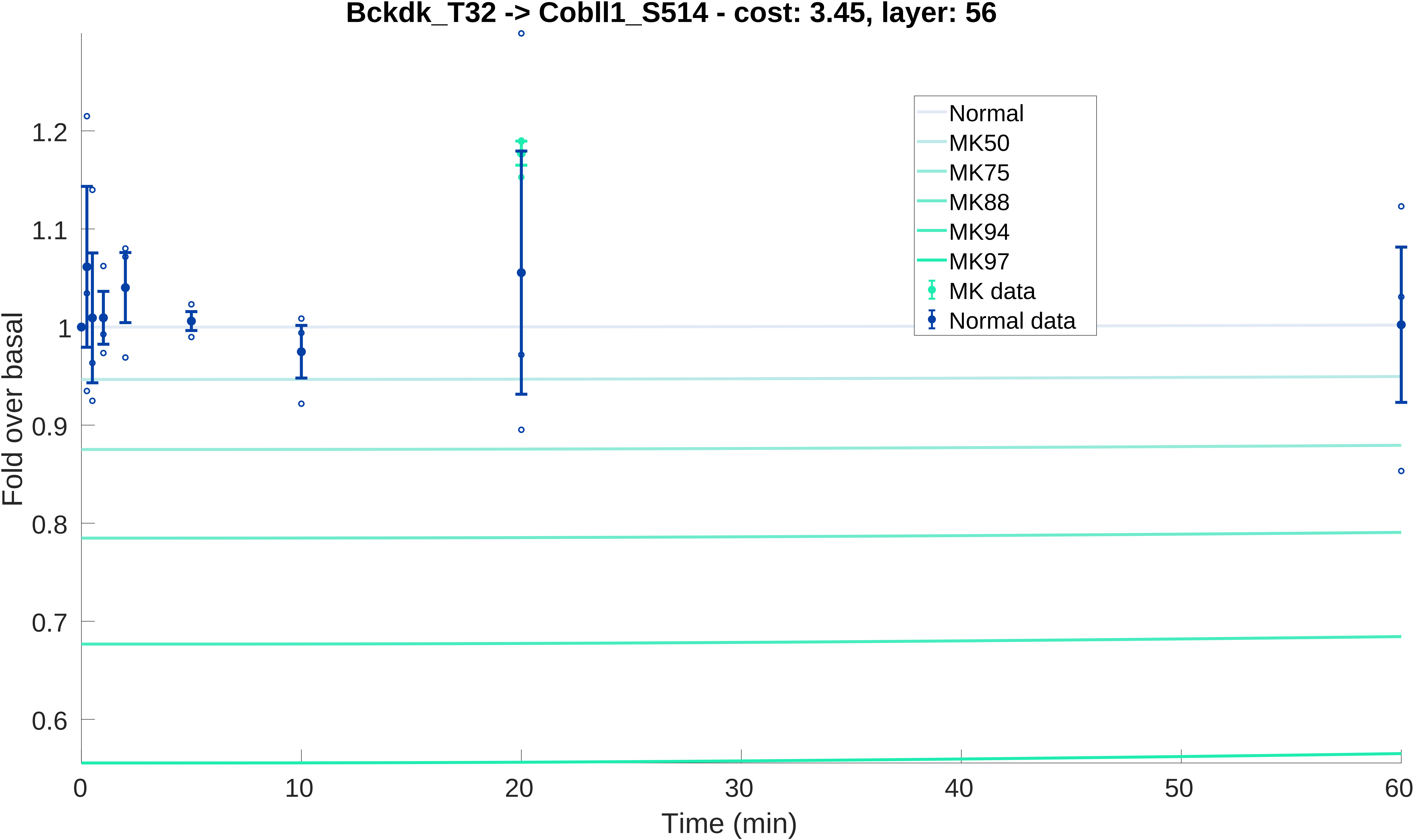

Bckdk_T32 -> Cobll1_S514 - cost: 3.45, layer: 56
Fold over basal
1.2
1.1
1
0.9
0.8
0.7
0.6
0
10
20
30
40
50
60
Time (min)
Normal
MK50
MK75
MK88
MK94
MK97
MK data
Normal data

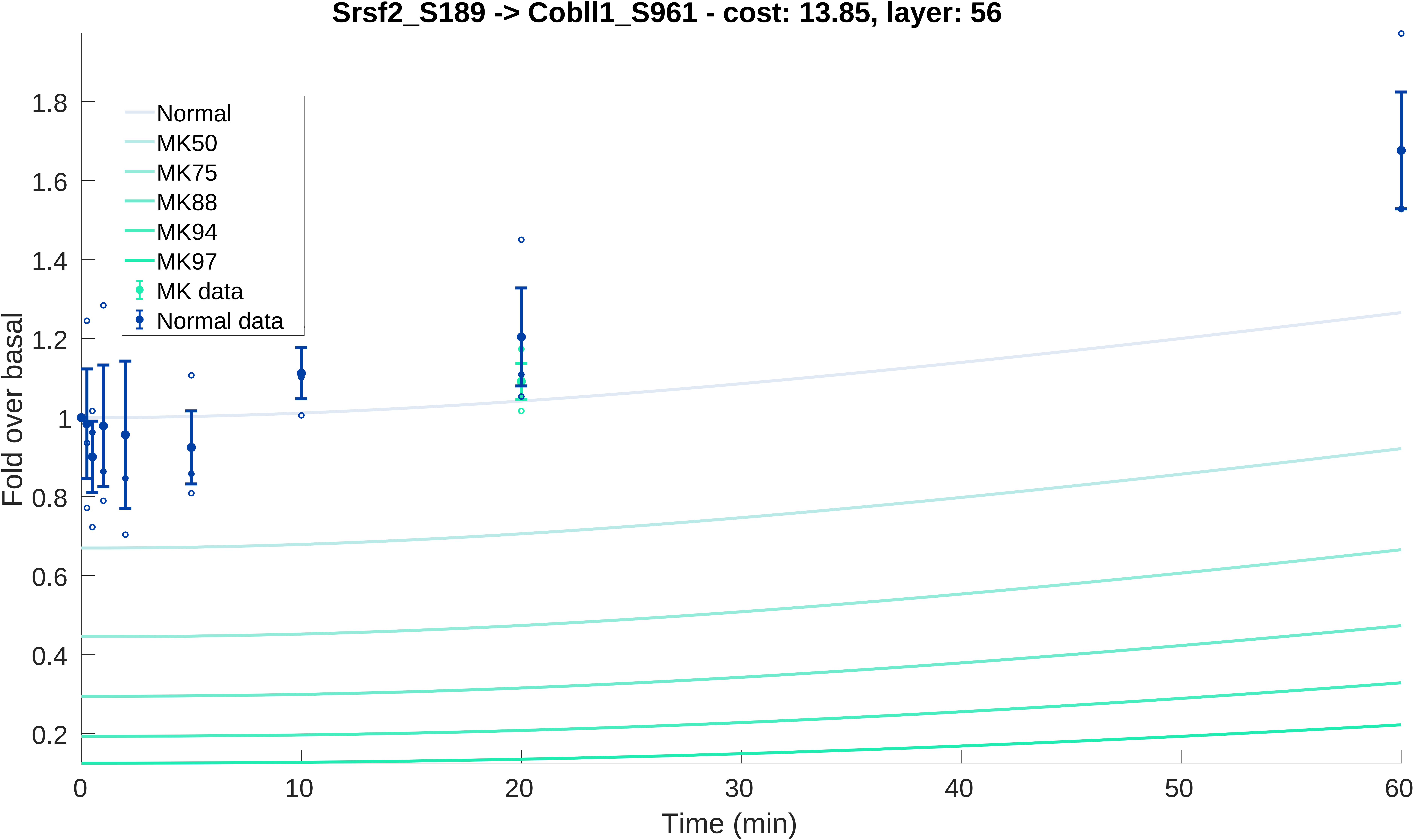

Srsf2_S189 -> Cobll1_S961 - cost: 13.85, layer: 56
Normal
MK50
MK75
MK88
MK94
MK97
MK data
Normal data
Fold over basal
0.2
0.4
0.6
0.8
1
1.2
1.4
1.6
1.8
Time (min)
0
10
20
30
40
50
60

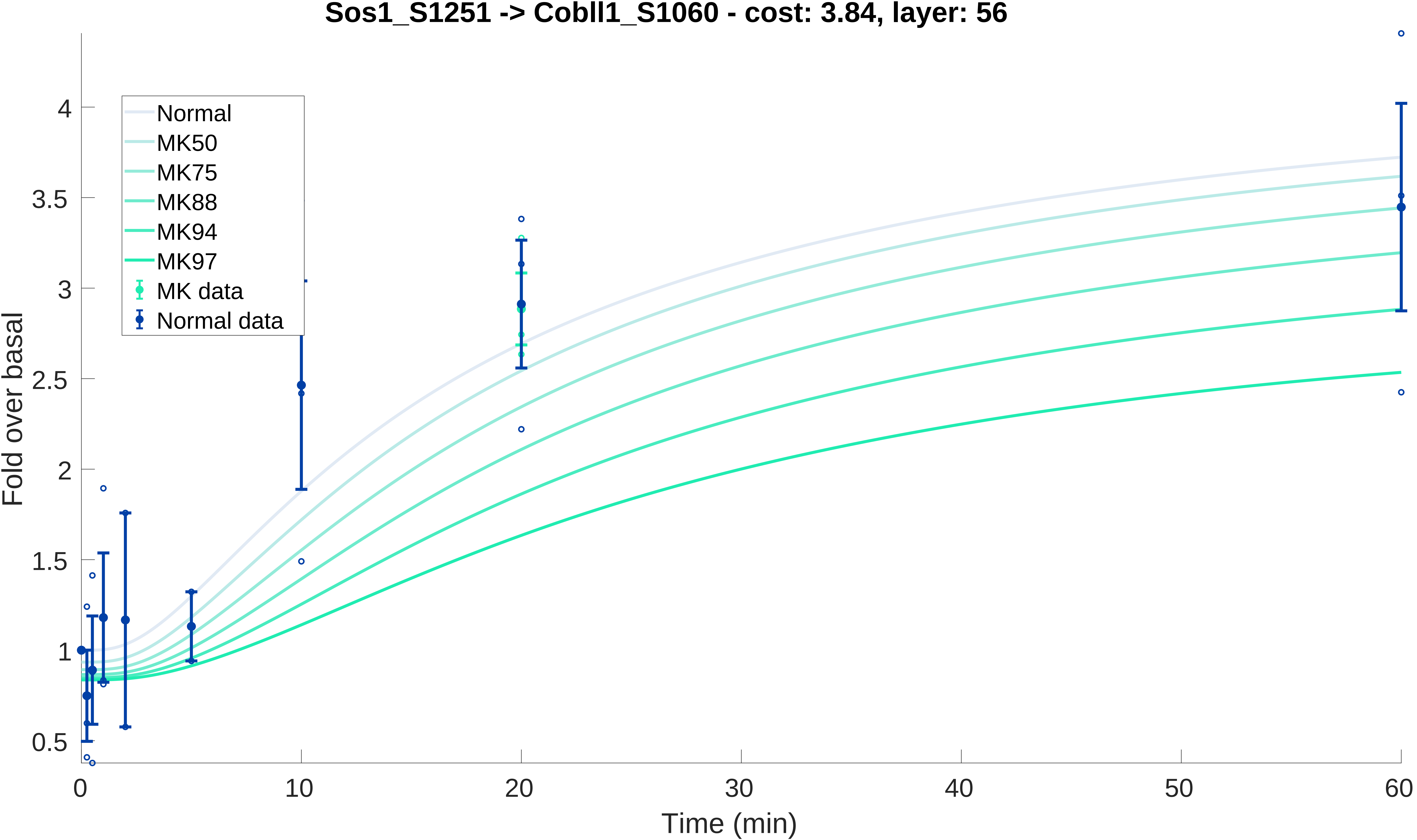

Sos1_S1251 -> Cobll1_S1060 - cost: 3.84, layer: 56
Normal
MK50
MK75
MK88
MK94
MK97
MK data
Normal data
Fold over basal
Time (min)
0.5
1
1.5
2
2.5
3
3.5
4
0
10
20
30
40
50
60

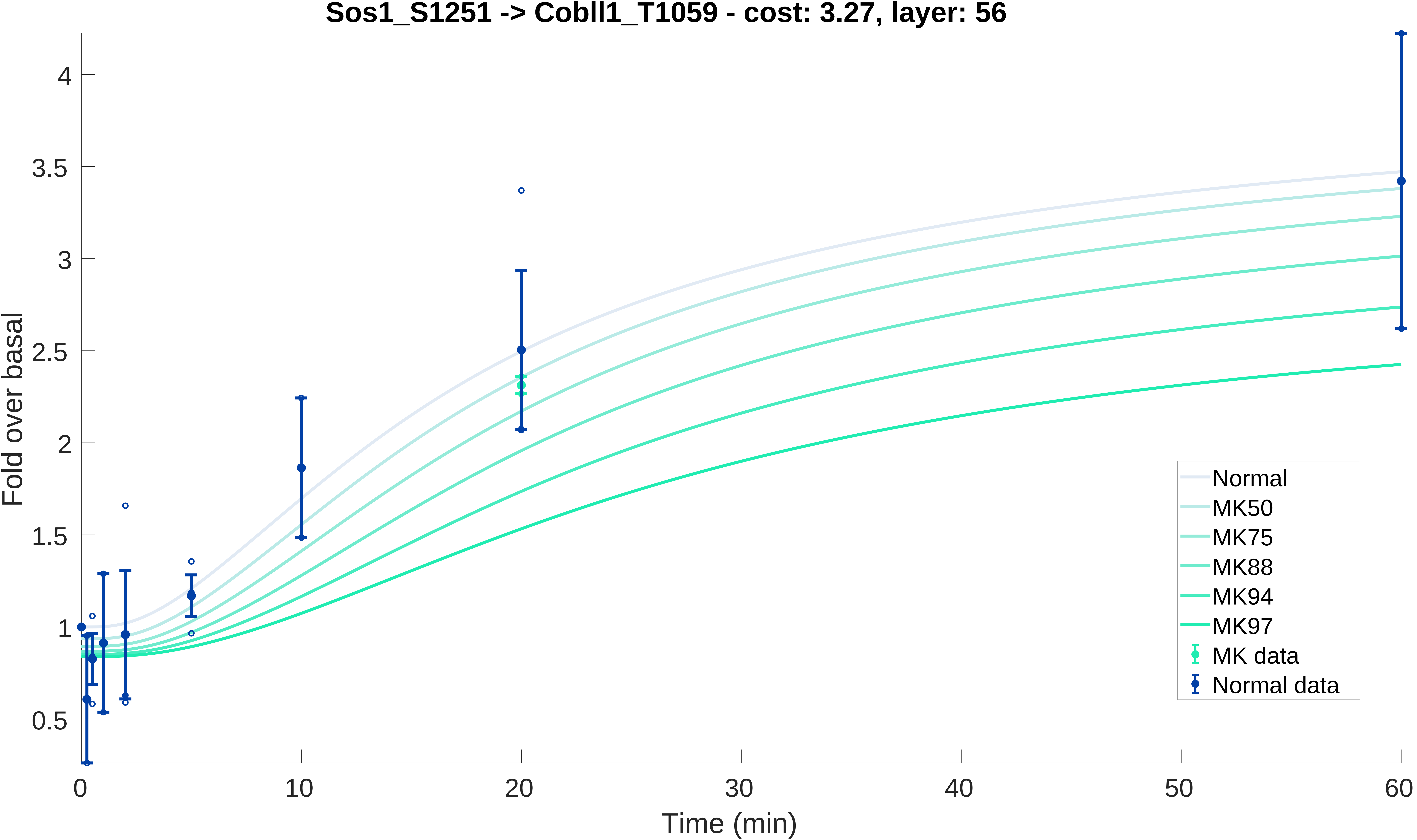

Sos1_S1251 -> Cobll1_T1059 - cost: 3.27, layer: 56
Fold over basal
Time (min)
0.5
1
1.5
2
2.5
3
3.5
4
0
10
20
30
40
50
60
Normal
MK50
MK75
MK88
MK94
MK97
MK data
Normal data

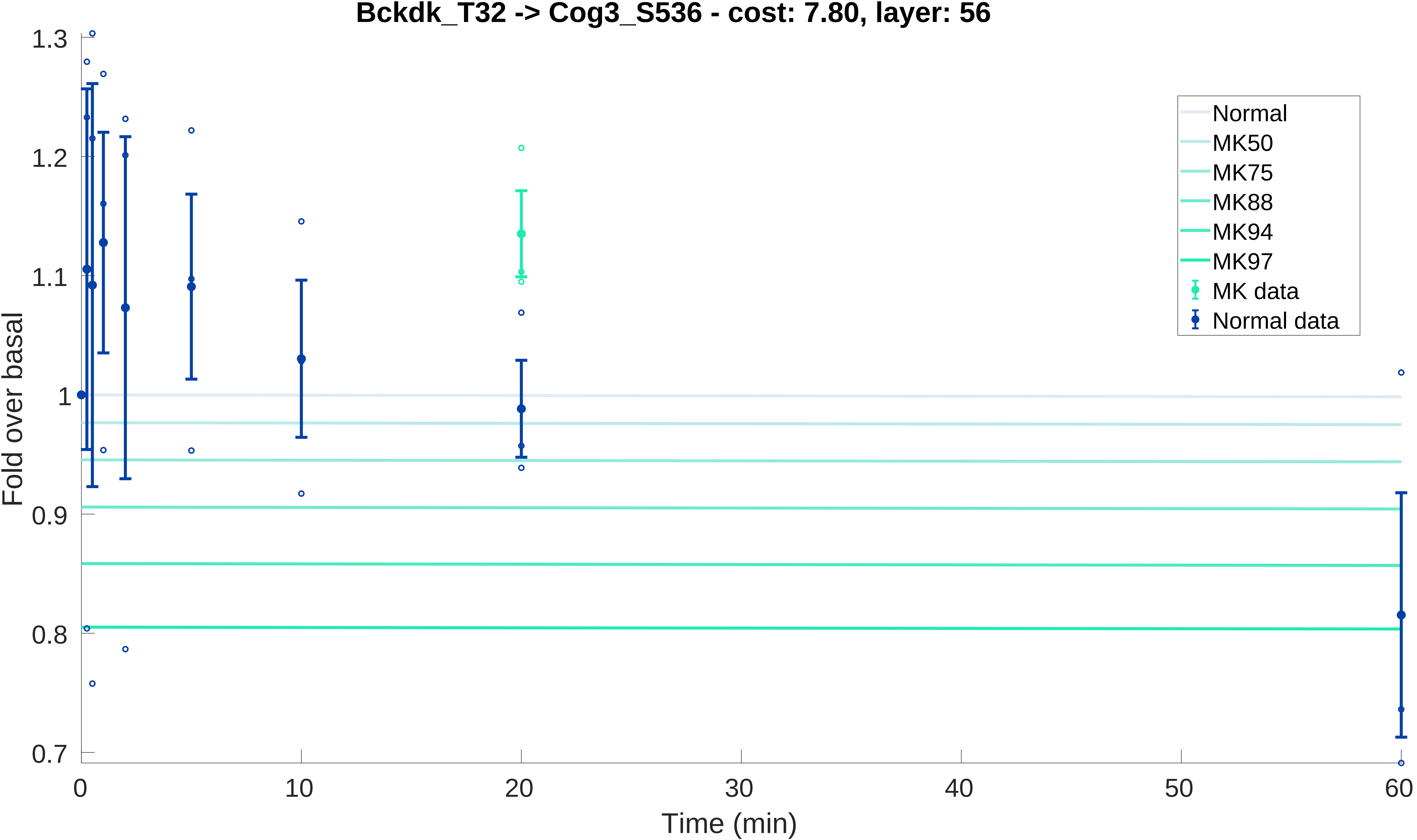

Bckdk_T32 -> Cog3_S536 - cost: 7.80, layer: 56
Fold over basal
Time (min)
1.3
1.2
1.1
1
0.9
0.8
0.7
0
10
20
30
40
50
60
Normal
MK50
MK75
MK88
MK94
MK97
MK data
Normal data

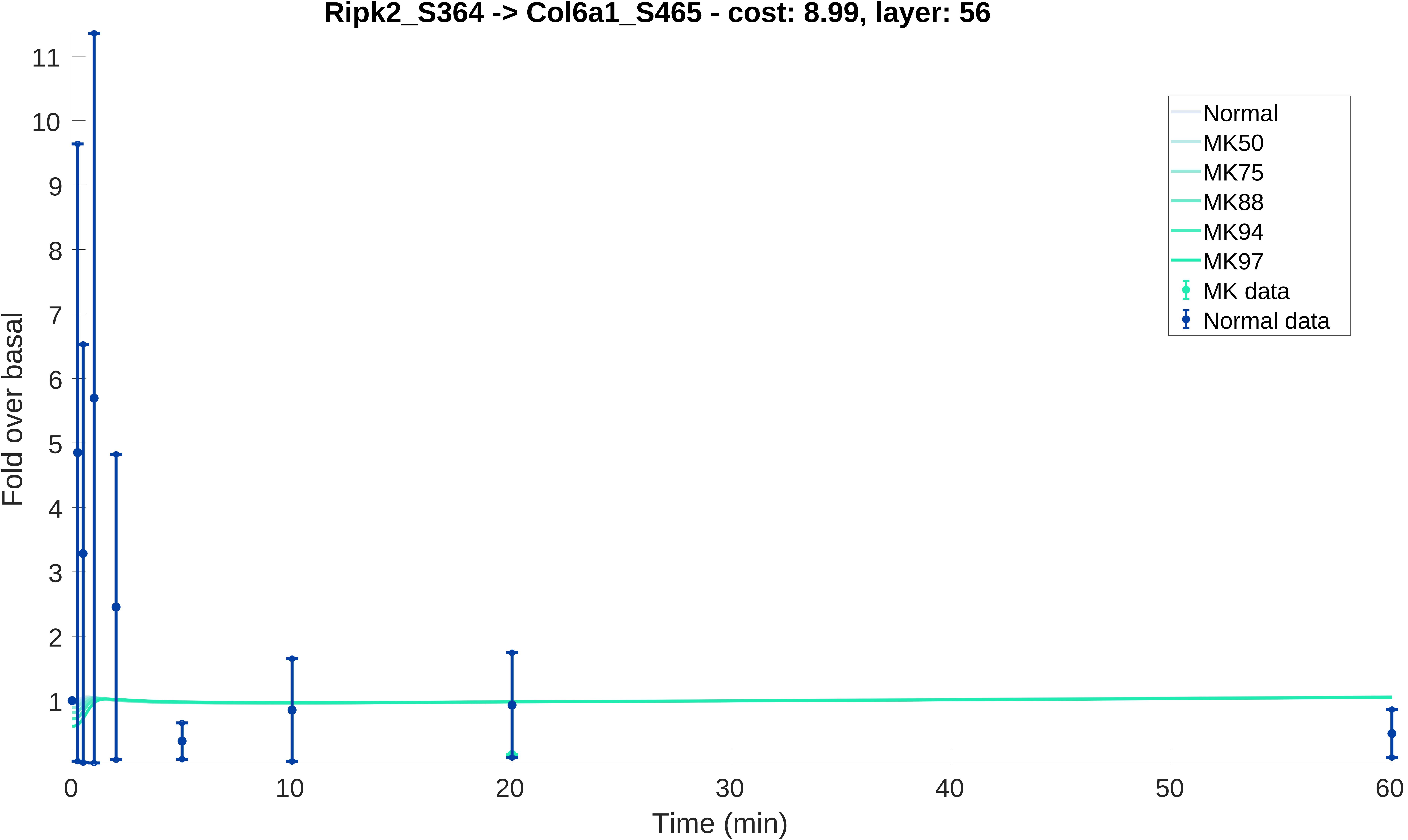

Ripk2_S364 -> Col6a1_S465 - cost: 8.99, layer: 56
Fold over basal
Time (min)
0
10
20
30
40
50
60
1
2
3
4
5
6
7
8
9
10
11
Normal
MK50
MK75
MK88
MK94
MK97
MK data
Normal data

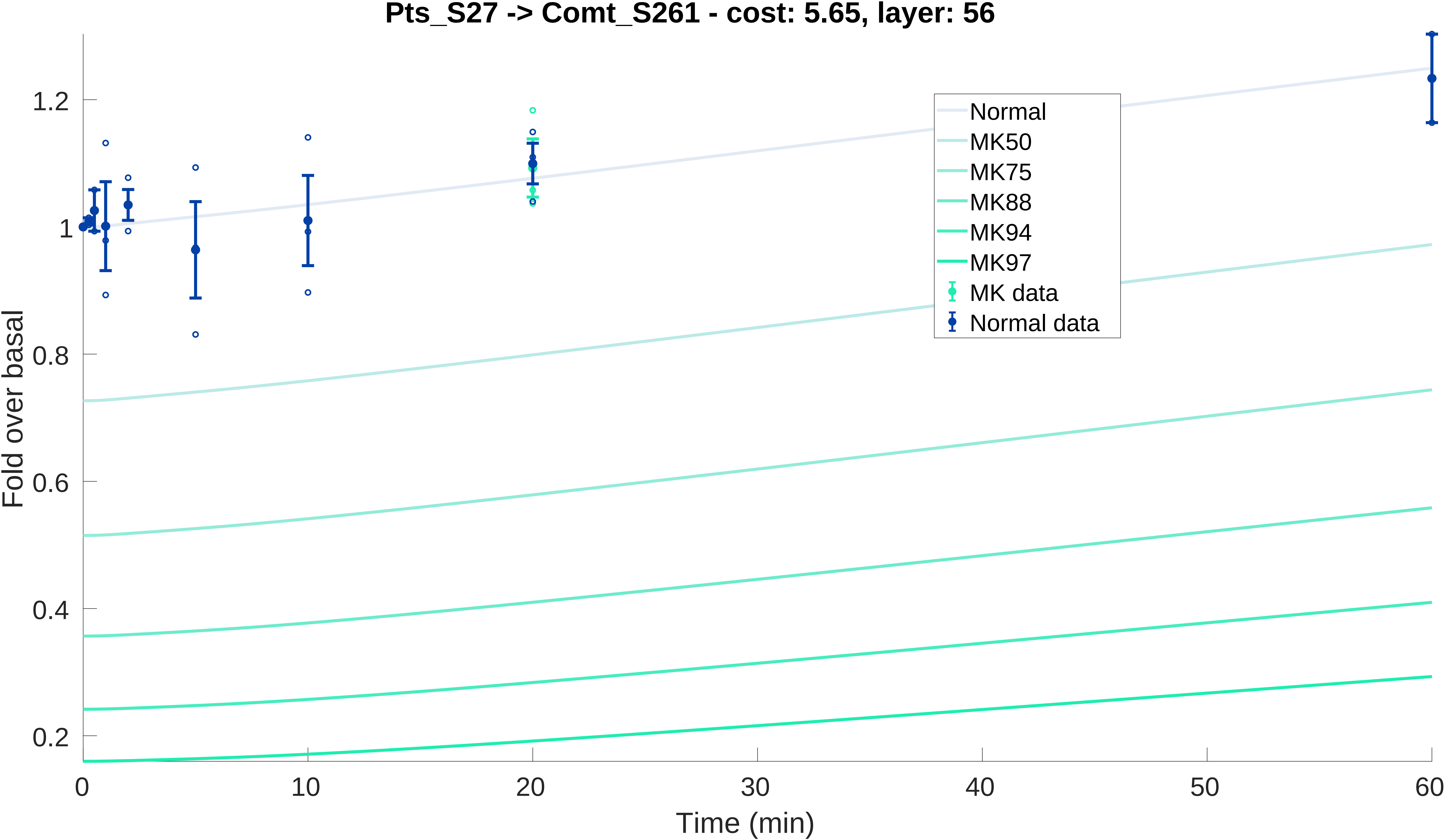

Pts_S27 -> Comt_S261 - cost: 5.65, layer: 56
Fold over basal
Time (min)
Normal
MK50
MK75
MK88
MK94
MK97
MK data
Normal data
0.2
0.4
0.6
0.8
1
1.2
0
10
20
30
40
50
60

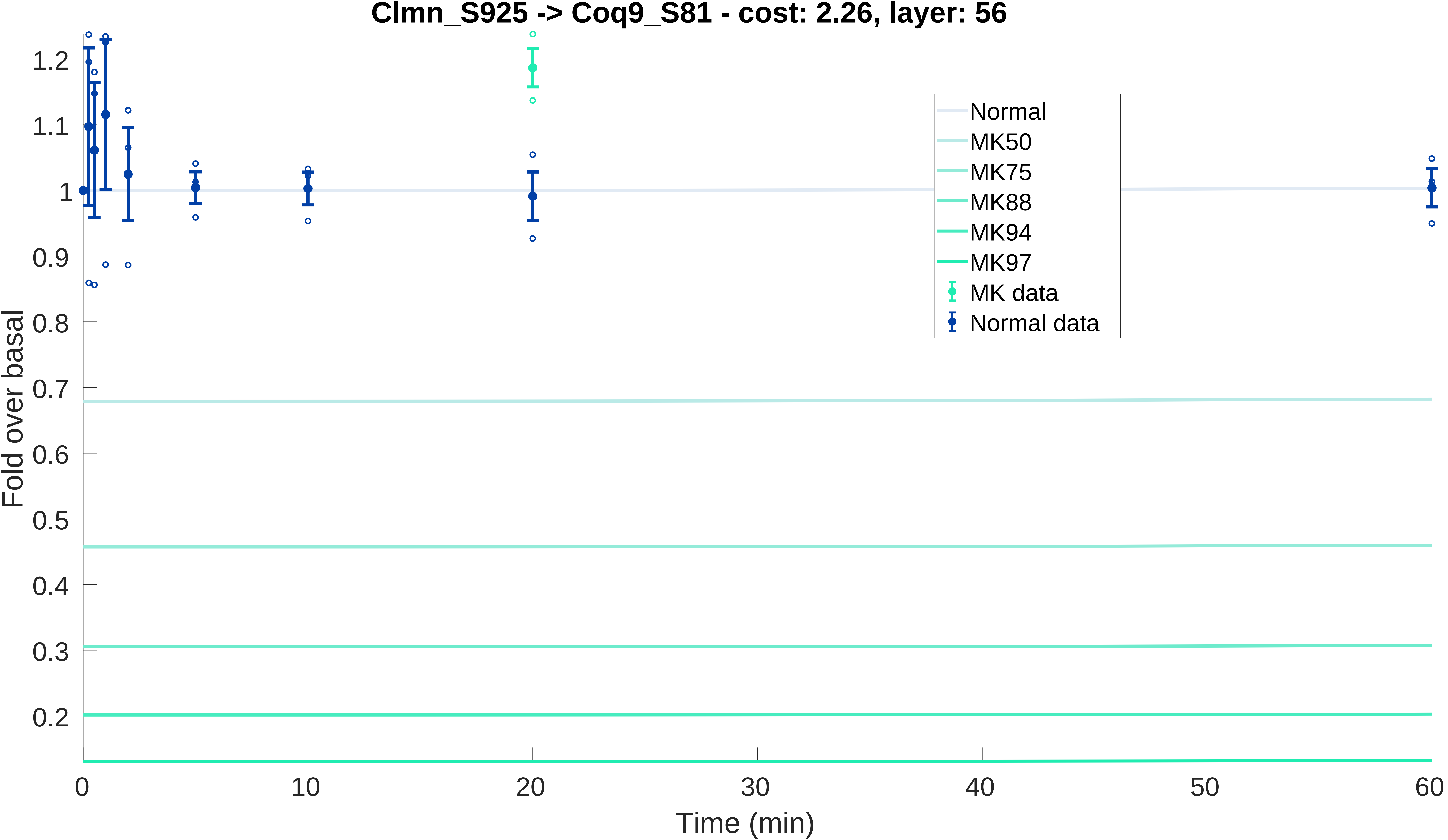

Clmn_S925 -> Coq9_S81 - cost: 2.26, layer: 56
Fold over basal
1.2
1.1
1
0.9
0.8
0.7
0.6
0.5
0.4
0.3
0.2
0
10
20
30
40
50
60
Time (min)
Normal
MK50
MK75
MK88
MK94
MK97
MK data
Normal data

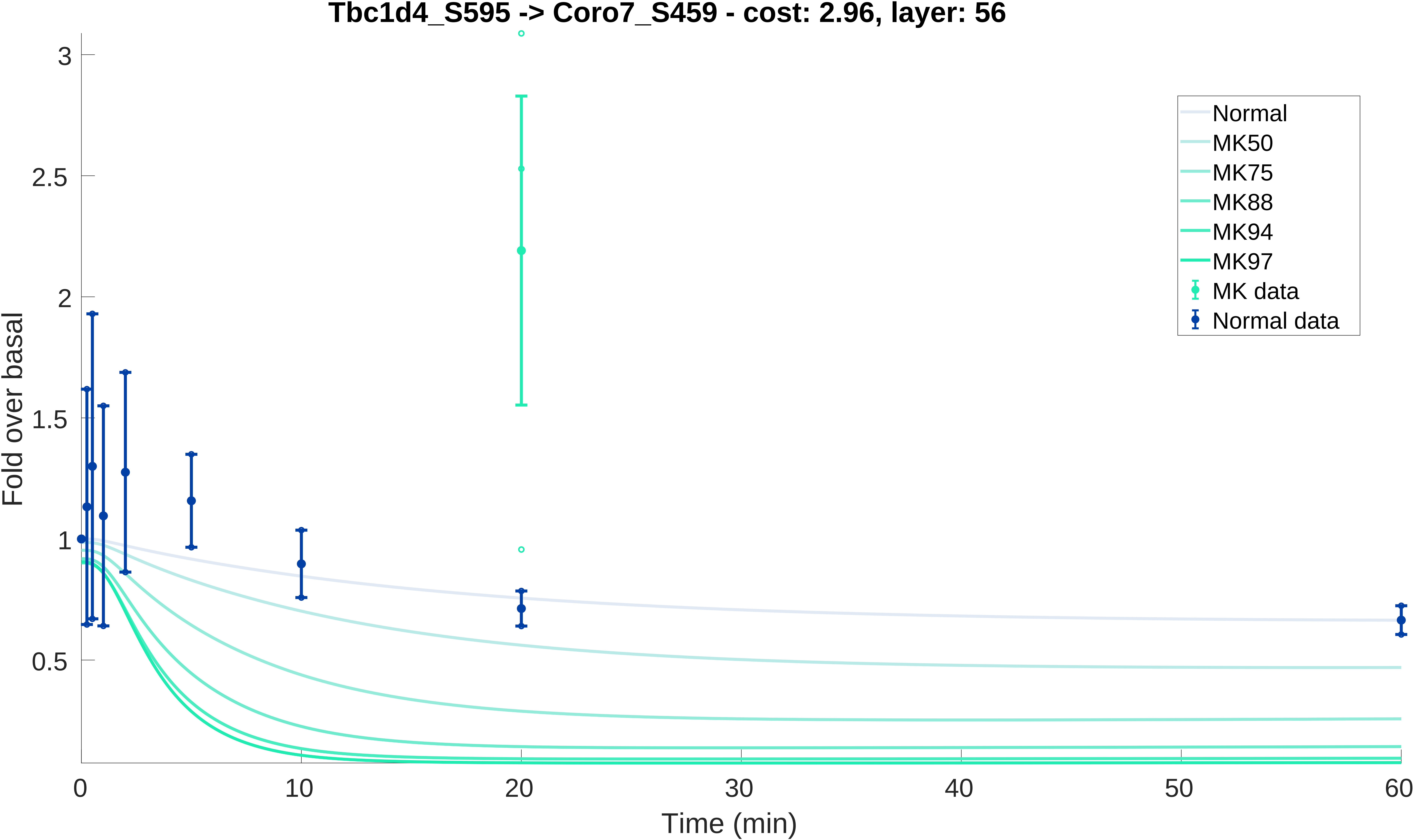
Tbc1d4_S595 -> Coro7_S459 - cost: 2.96, layer: 56
Fold over basal
Time (min)
0
10
20
30
40
50
60
0.5
1
1.5
2
2.5
3
Normal
MK50
MK75
MK88
MK94
MK97
MK data
Normal data

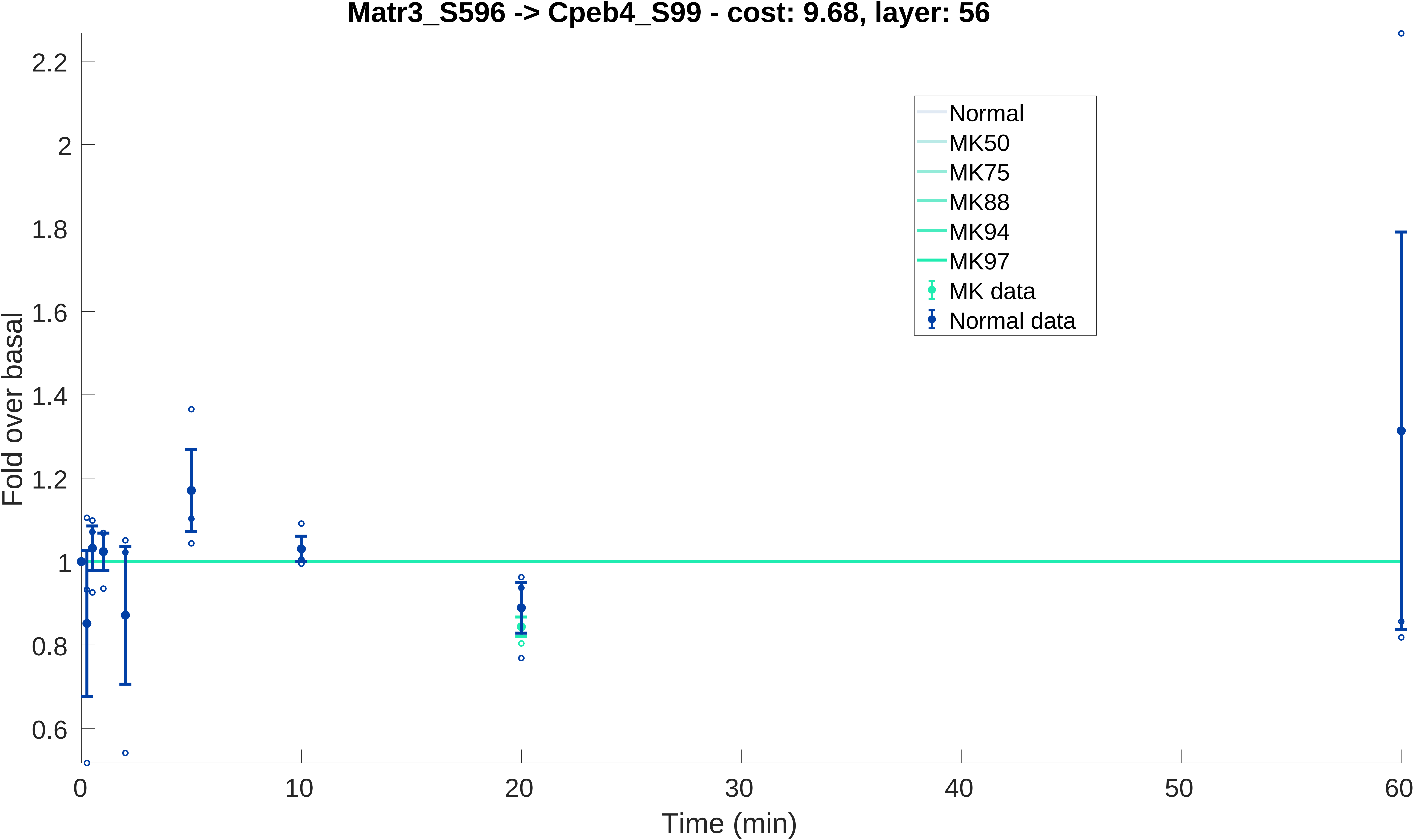

Matr3_S596 -> Cpeb4_S99 - cost: 9.68, layer: 56
Normal
MK50
MK75
MK88
MK94
MK97
MK data
Normal data
Fold over basal
Time (min)
0.6
0.8
1
1.2
1.4
1.6
1.8
2
2.2
0
10
20
30
40
50
60

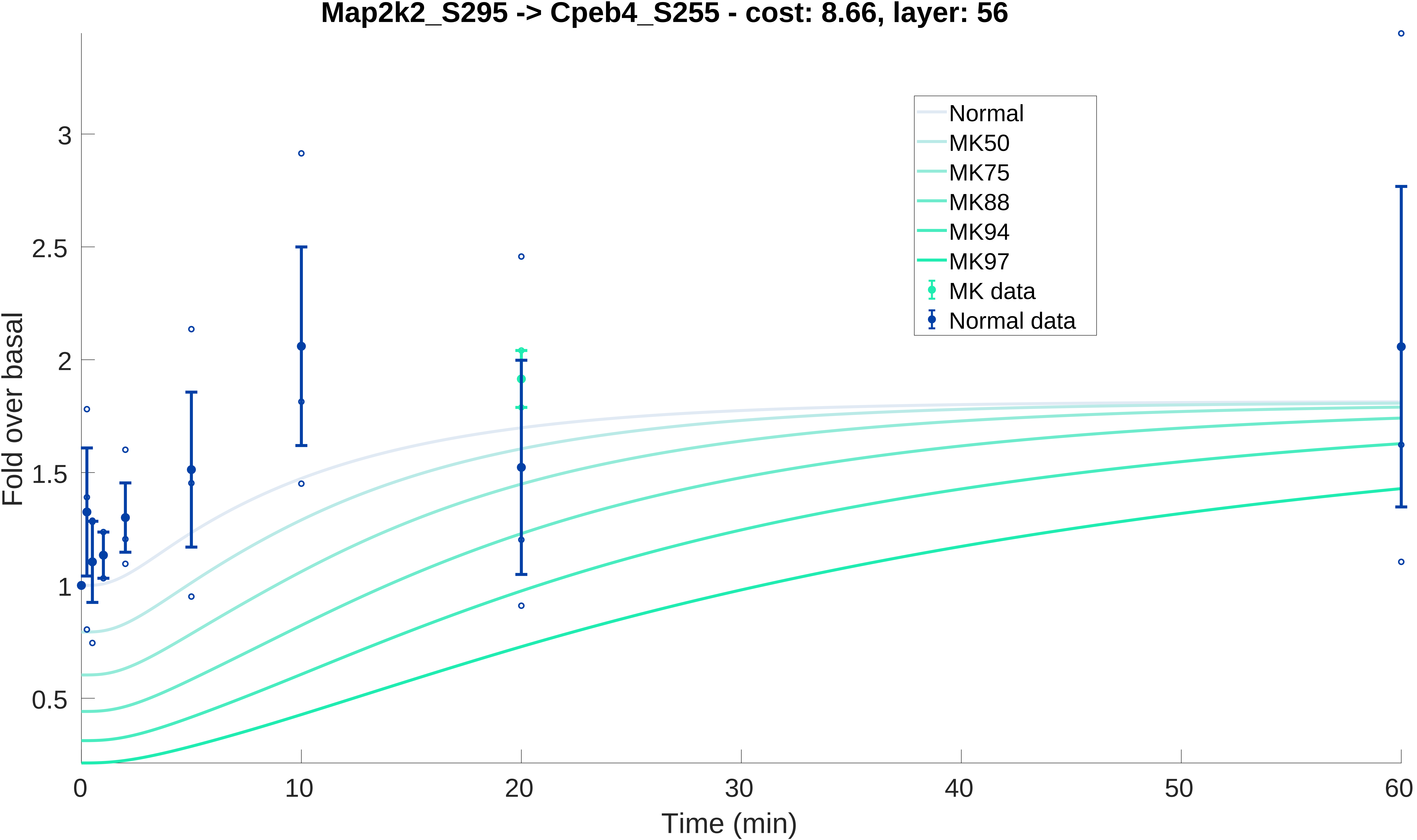

Map2k2_S295 -> Cpeb4_S255 - cost: 8.66, layer: 56
Normal
MK50
MK75
MK88
MK94
MK97
MK data
Normal data
Fold over basal
Time (min)
0.5
1
1.5
2
2.5
3
0
10
20
30
40
50
60

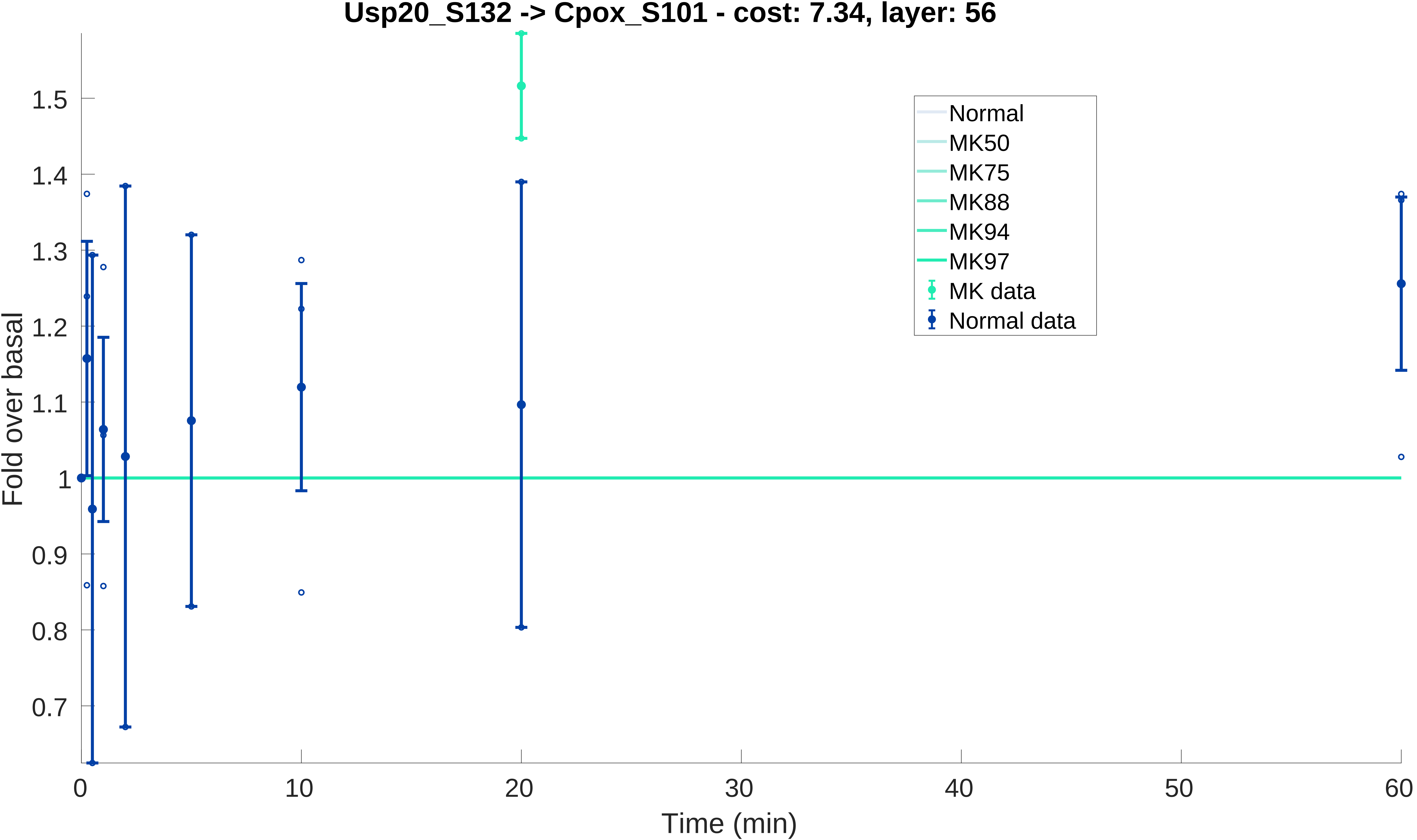
Usp20_S132 -> Cpox_S101 - cost: 7.34, layer: 56
Fold over basal
Time (min)
Normal
MK50
MK75
MK88
MK94
MK97
MK data
Normal data
0.7
0.8
0.9
1
1.1
1.2
1.3
1.4
1.5
0
10
20
30
40
50
60

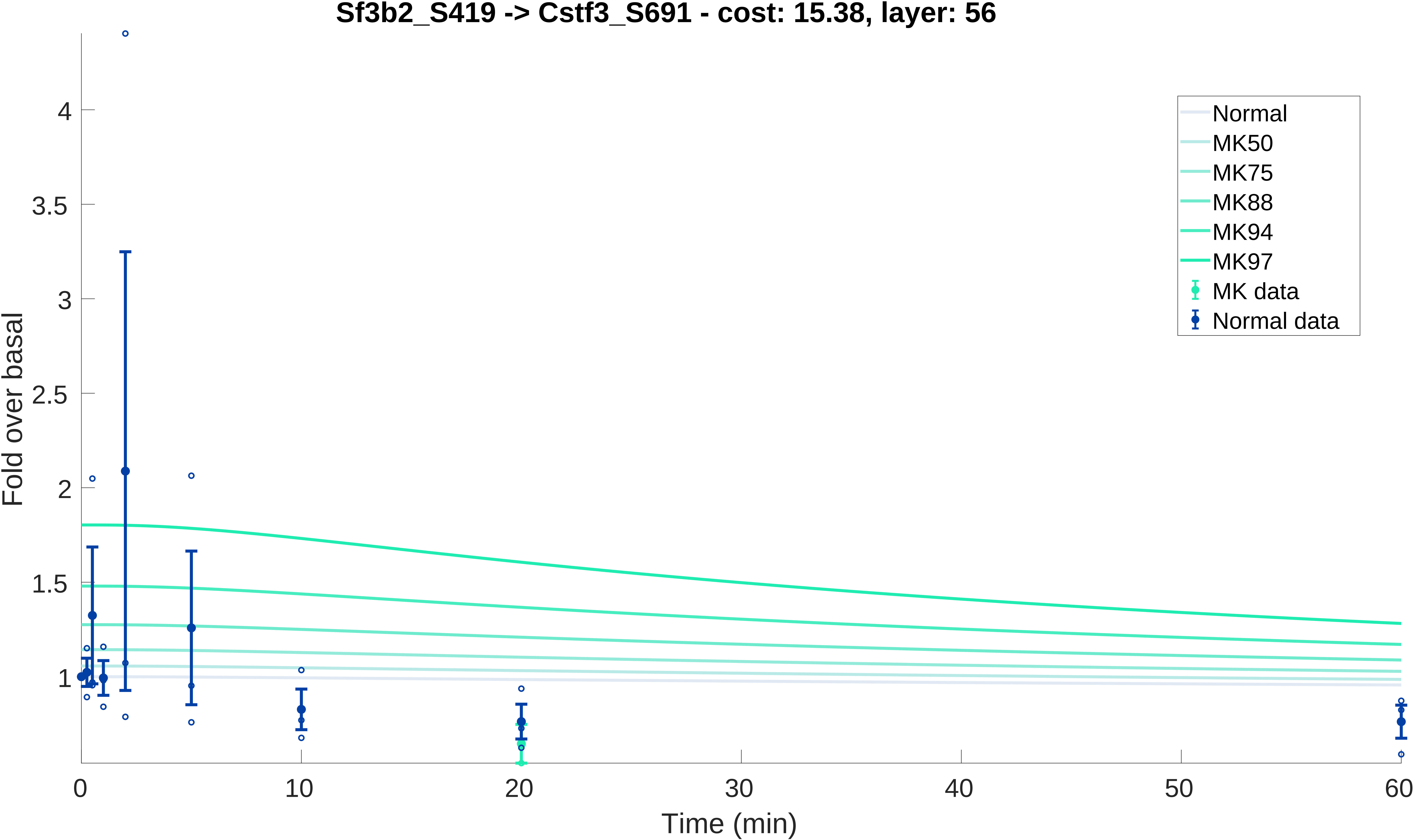

Sf3b2_S419 -> Cstf3_S691 - cost: 15.38, layer: 56
Fold over basal
Time (min)
0
10
20
30
40
50
60
1
1.5
2
2.5
3
3.5
4
Normal
MK50
MK75
MK88
MK94
MK97
MK data
Normal data

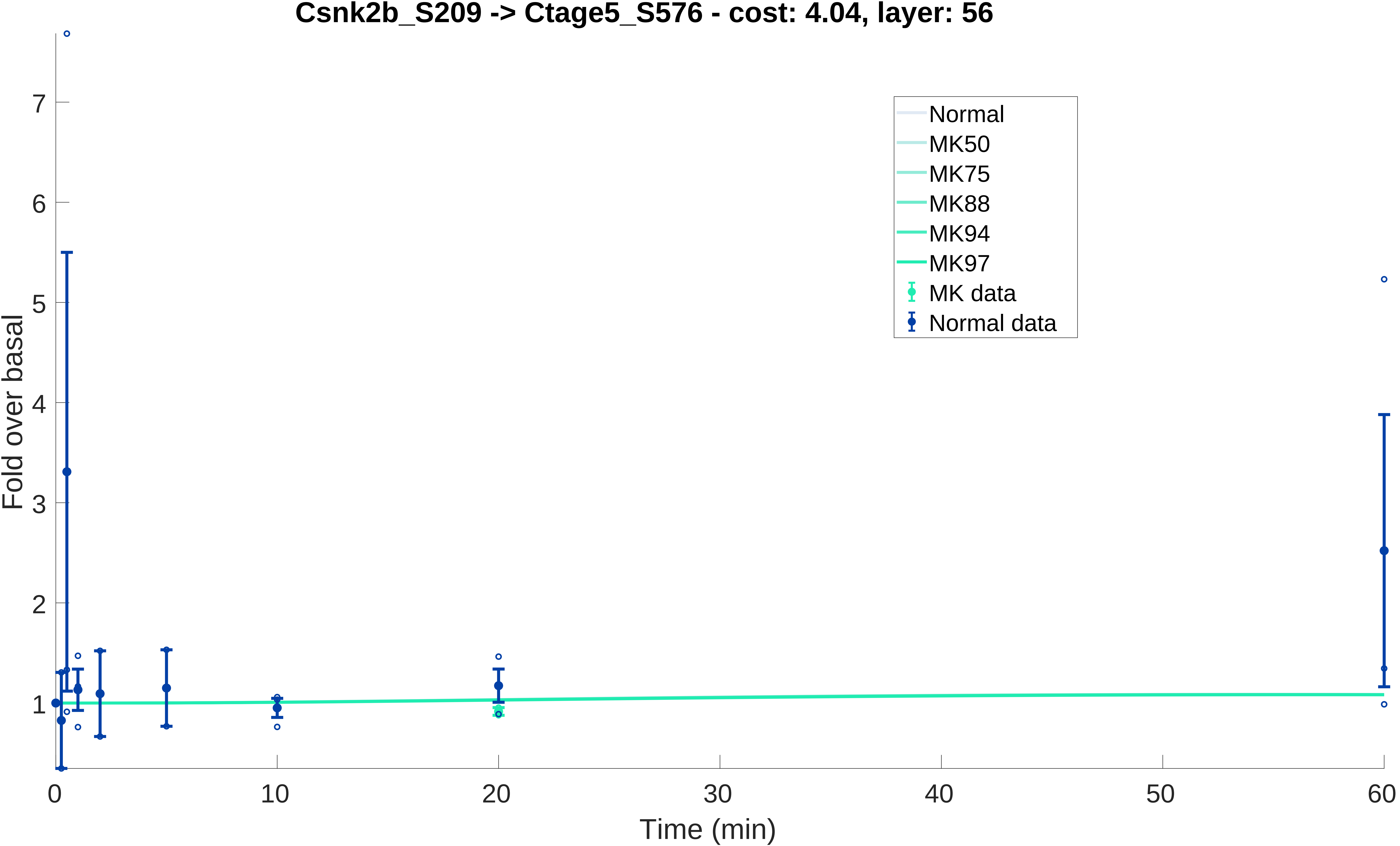

Csnk2b_S209 -> Ctage5_S576 - cost: 4.04, layer: 56
Fold over basal
Time (min)
0
10
20
30
40
50
60
1
2
3
4
5
6
7
Normal
MK50
MK75
MK88
MK94
MK97
MK data
Normal data

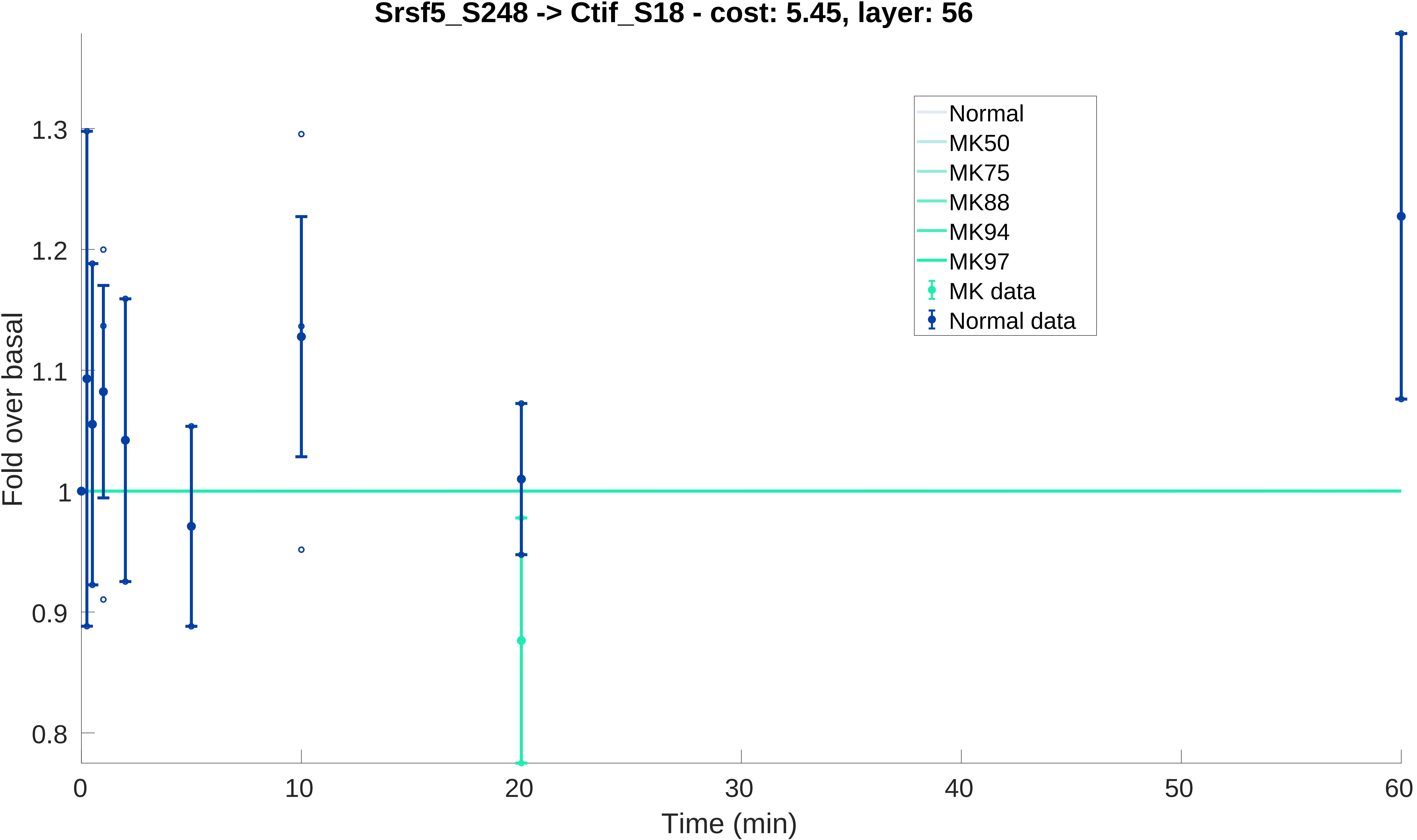

Srsf5_S248 -> Ctif_S18 - cost: 5.45, layer: 56
Fold over basal
Time (min)
1.3
1.2
1.1
1
0.9
0.8
0
10
20
30
40
50
60
Normal
MK50
MK75
MK88
MK94
MK97
MK data
Normal data

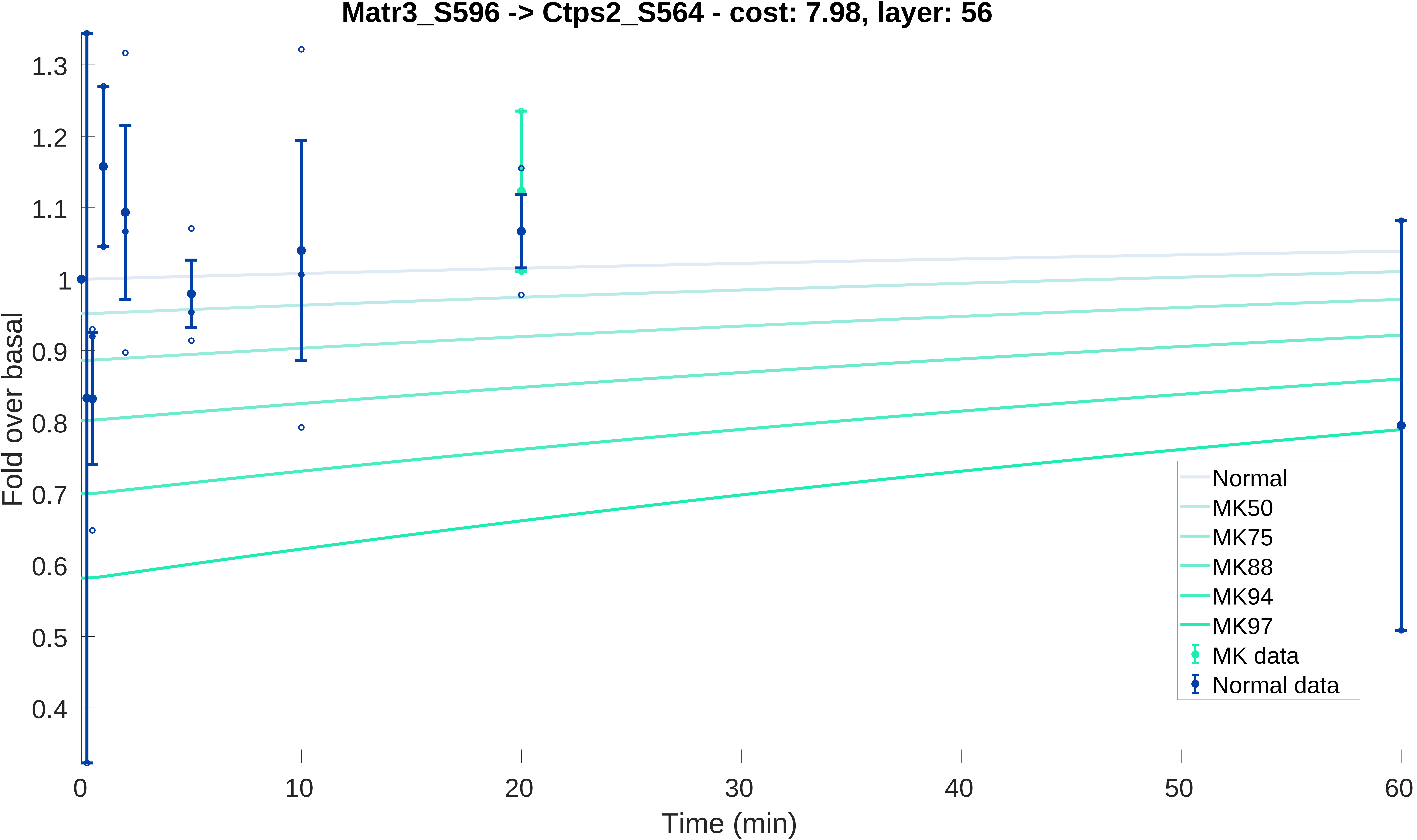
Matr3_S596 -> Ctps2_S564 - cost: 7.98, layer: 56
Fold over basal
1.3
1.2
1.1
1
0.9
0.8
0.7
0.6
0.5
0.4
0
10
20
30
40
50
60
Time (min)
Normal
MK50
MK75
MK88
MK94
MK97
MK data
Normal data

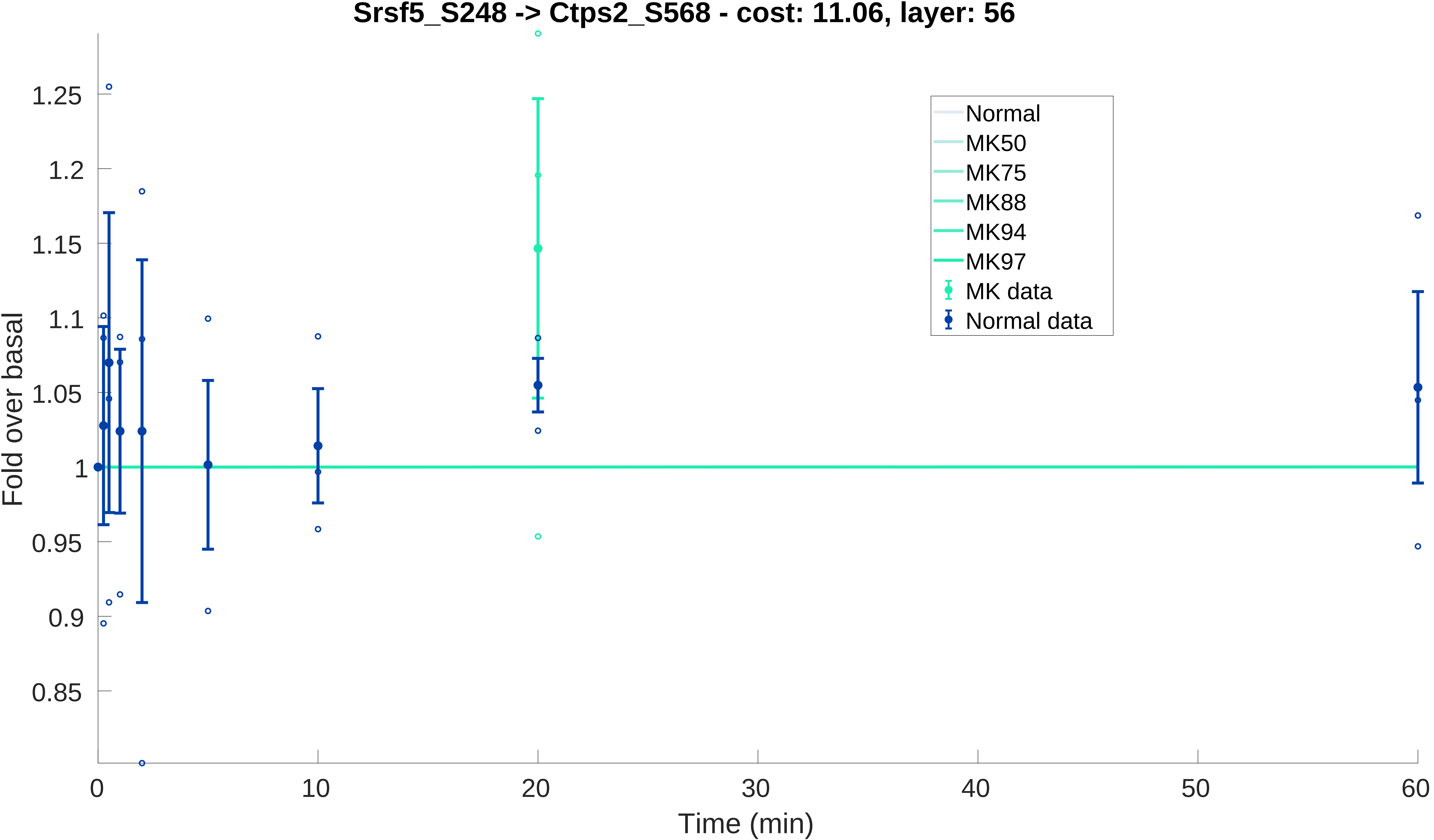
Srsf5_S248 -> Ctps2_S568 - cost: 11.06, layer: 56
Fold over basal
Time (min)
1.25
1.2
1.15
1.1
1.05
1
0.95
0.9
0.85
0
10
20
30
40
50
60
Normal
MK50
MK75
MK88
MK94
MK97
MK data
Normal data

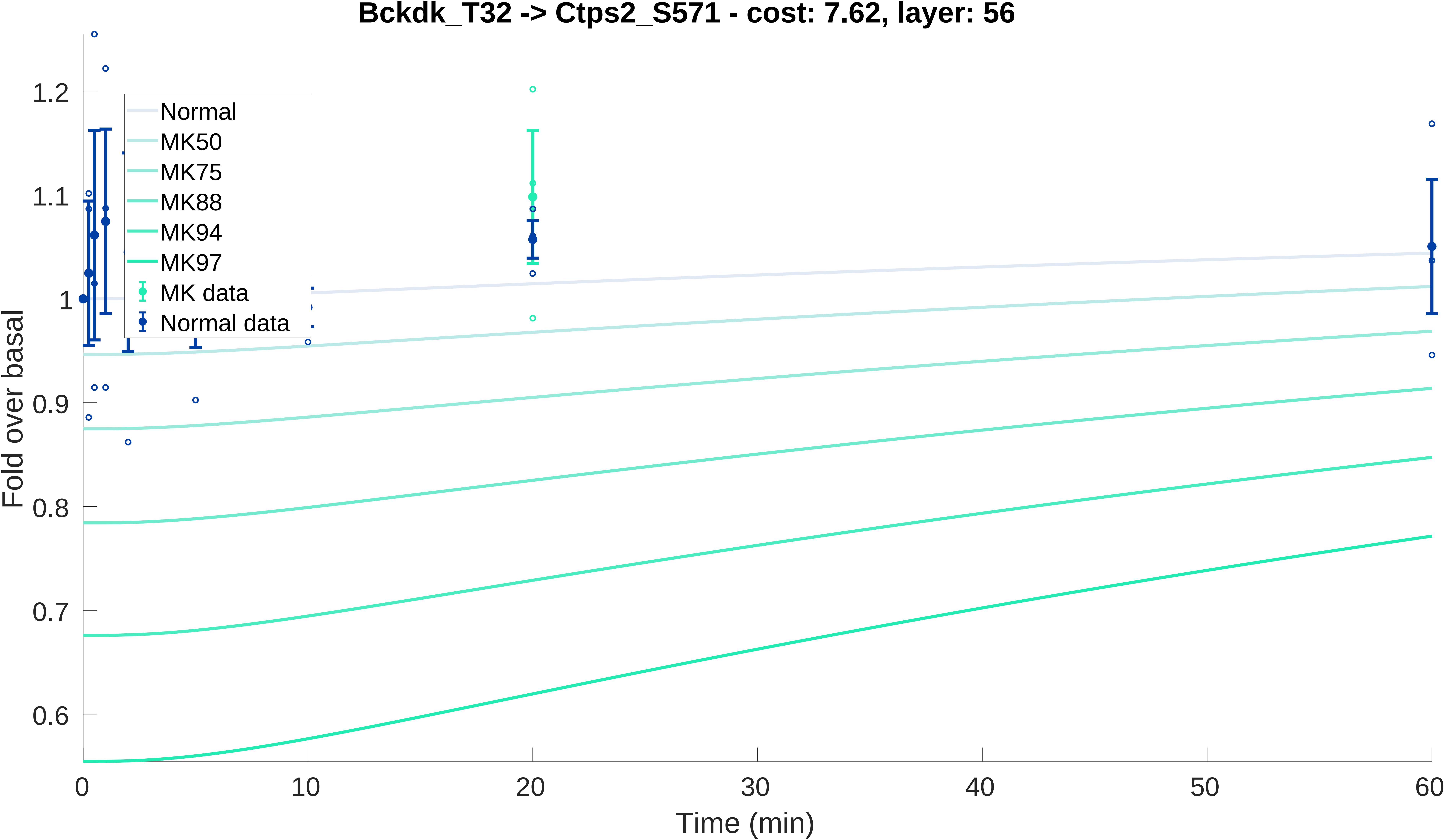

Bckdk_T32 -> Ctps2_S571 - cost: 7.62, layer: 56
Fold over basal
1.2
1.1
1
0.9
0.8
0.7
0.6
0
10
20
30
40
50
60
Time (min)
Normal
MK50
MK75
MK88
MK94
MK97
MK data
Normal data

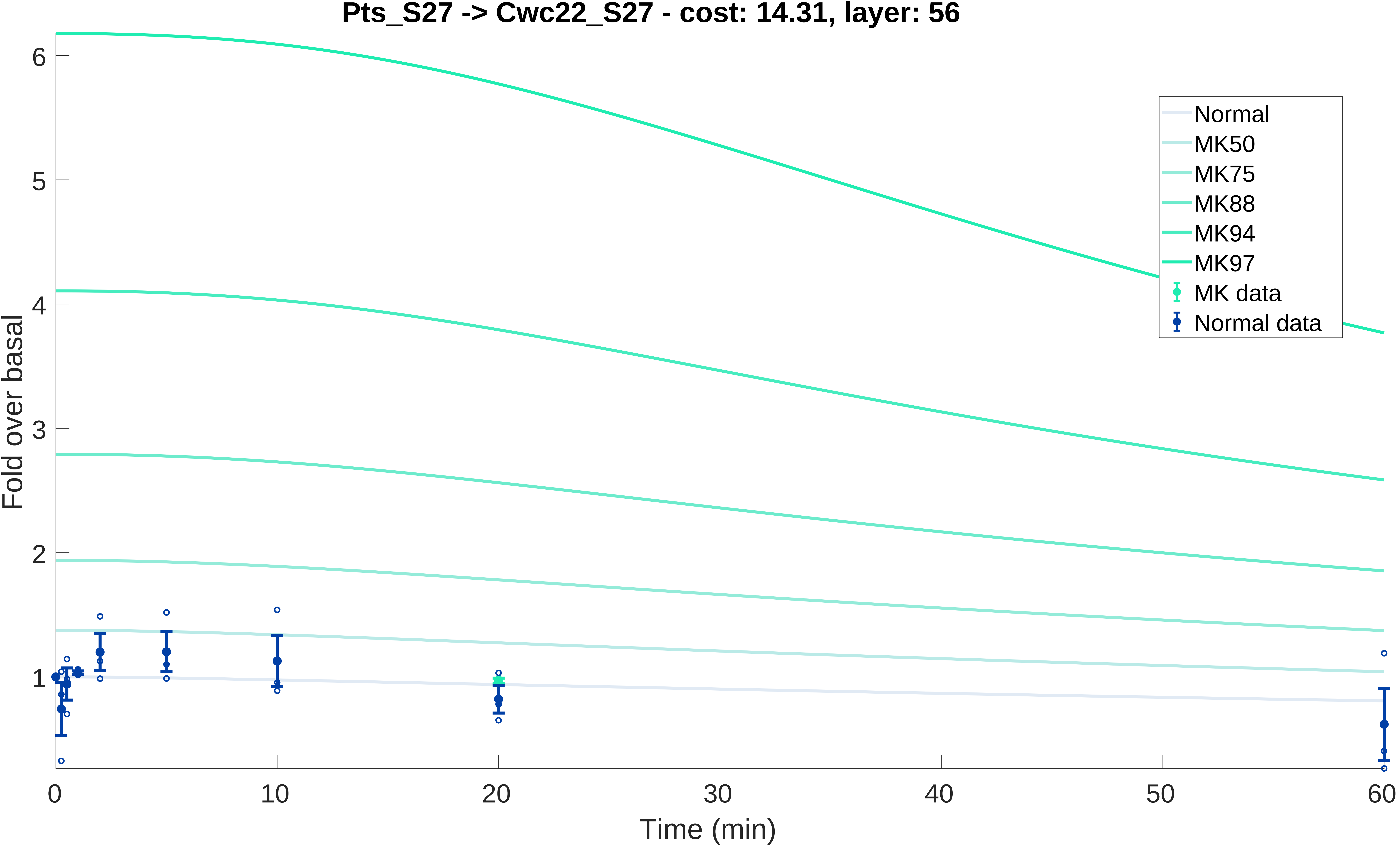

Pts_S27 -> Cwc22_S27 - cost: 14.31, layer: 56
Fold over basal
Time (min)
Normal
MK50
MK75
MK88
MK94
MK97
MK data
Normal data
0
10
20
30
40
50
60
1
2
3
4
5
6

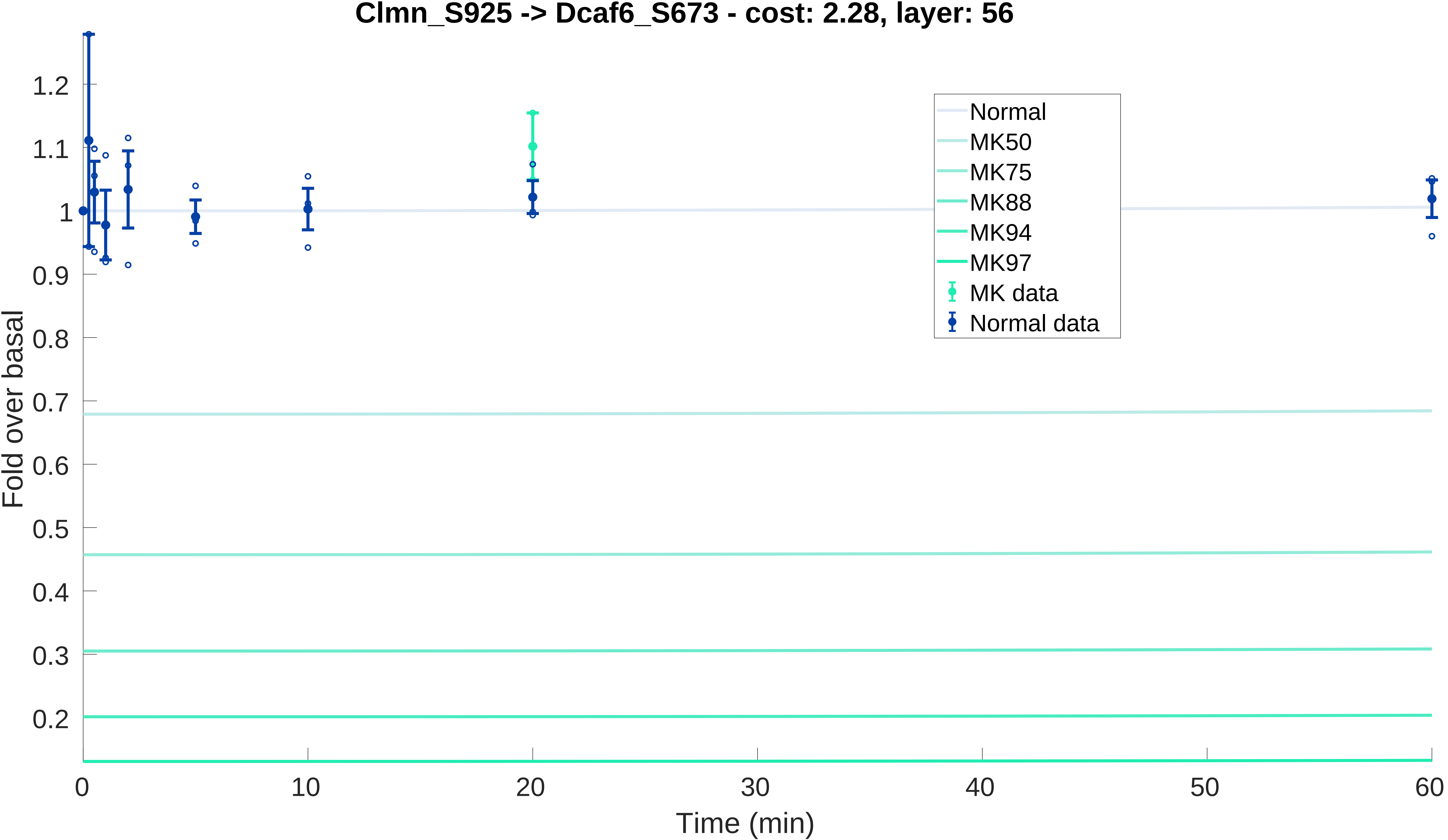

Clmn_S925 -> Dcaf6_S673 - cost: 2.28, layer: 56
Fold over basal
Time (min)
0
10
20
30
40
50
60
1.2
1.1
1
0.9
0.8
0.7
0.6
0.5
0.4
0.3
0.2
Normal
MK50
MK75
MK88
MK94
MK97
MK data
Normal data

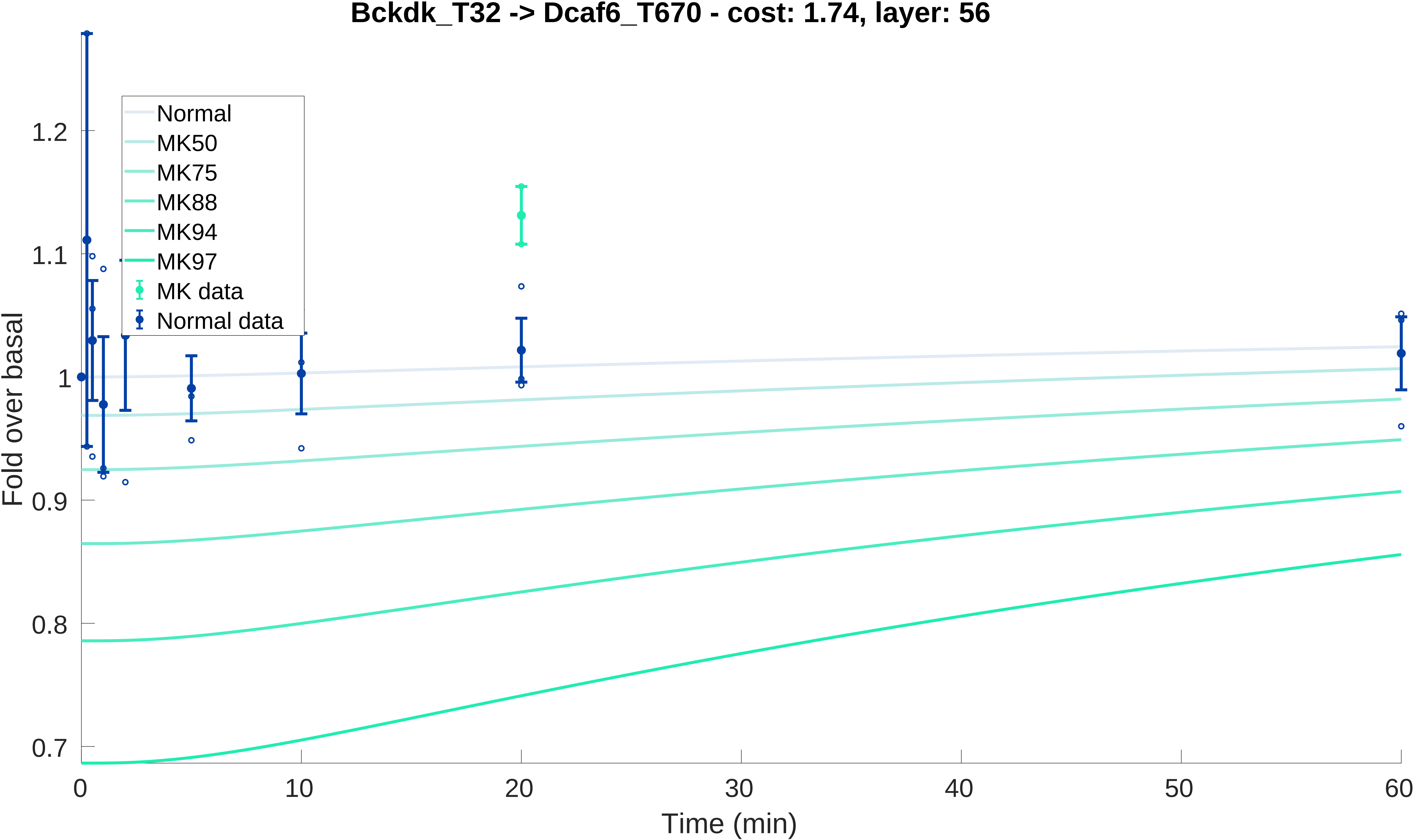

Bckdk_T32 -> Dcaf6_T670 - cost: 1.74, layer: 56
Fold over basal
Time (min)
Normal
MK50
MK75
MK88
MK94
MK97
MK data
Normal data
1.2
1.1
1
0.9
0.8
0.7
0
10
20
30
40
50
60

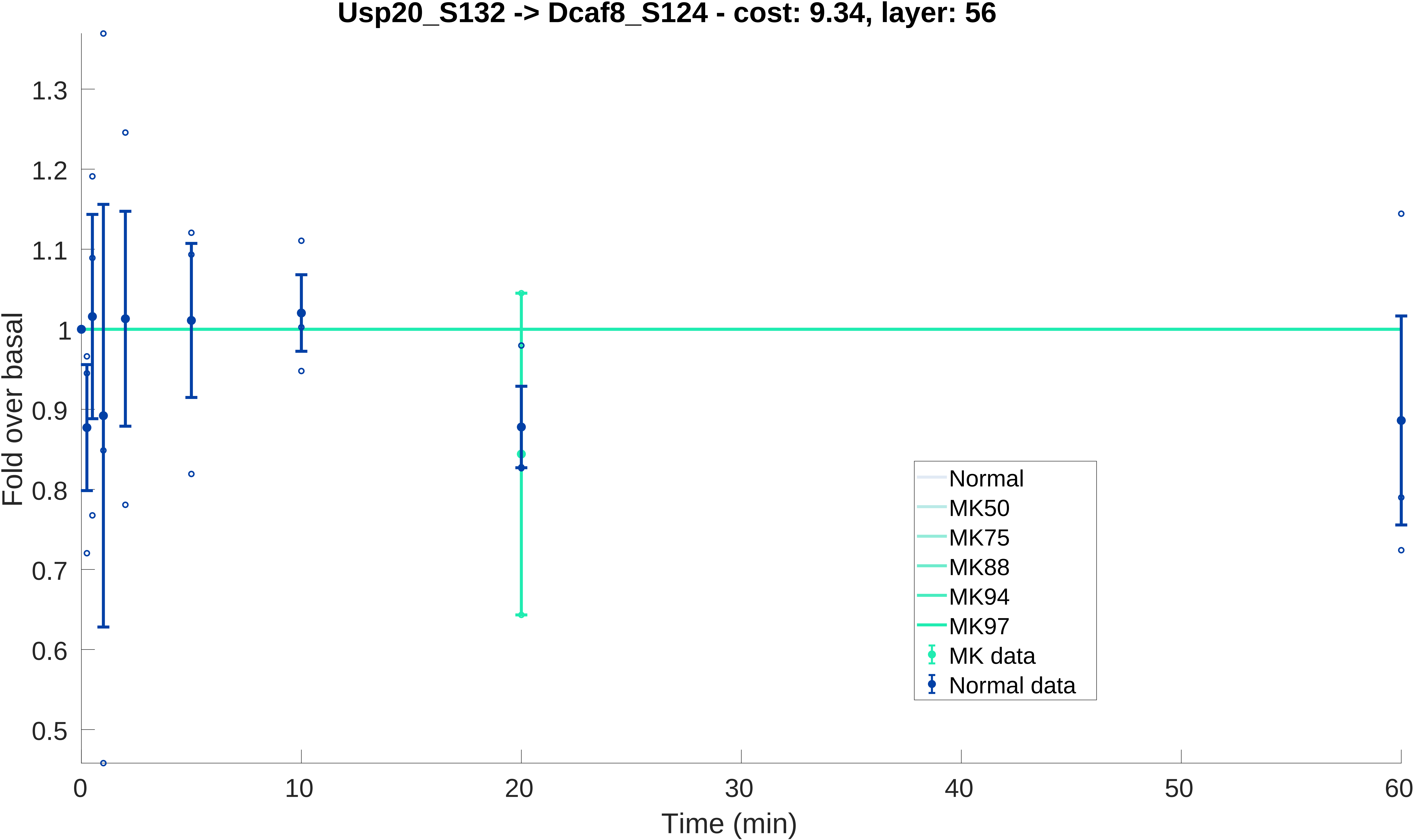
Usp20_S132 -> Dcaf8_S124 - cost: 9.34, layer: 56
Fold over basal
Time (min)
1.3
1.2
1.1
1
0.9
0.8
0.7
0.6
0.5
0
10
20
30
40
50
60
Normal
MK50
MK75
MK88
MK94
MK97
MK data
Normal data

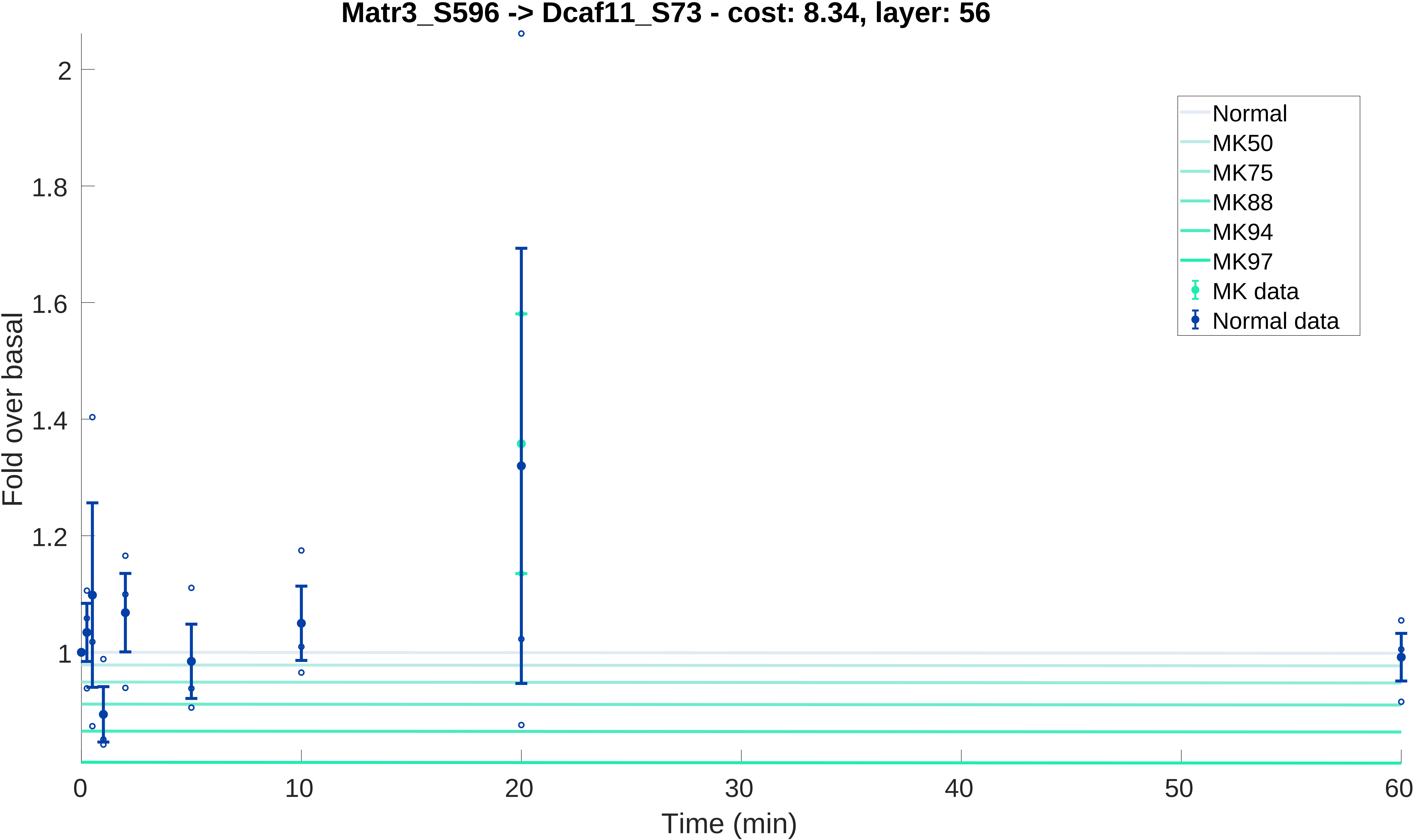

Matr3_S596 -> Dcaf11_S73 - cost: 8.34, layer: 56
Fold over basal
Time (min)
Normal
MK50
MK75
MK88
MK94
MK97
MK data
Normal data
2
1.8
1.6
1.4
1.2
1
0
10
20
30
40
50
60

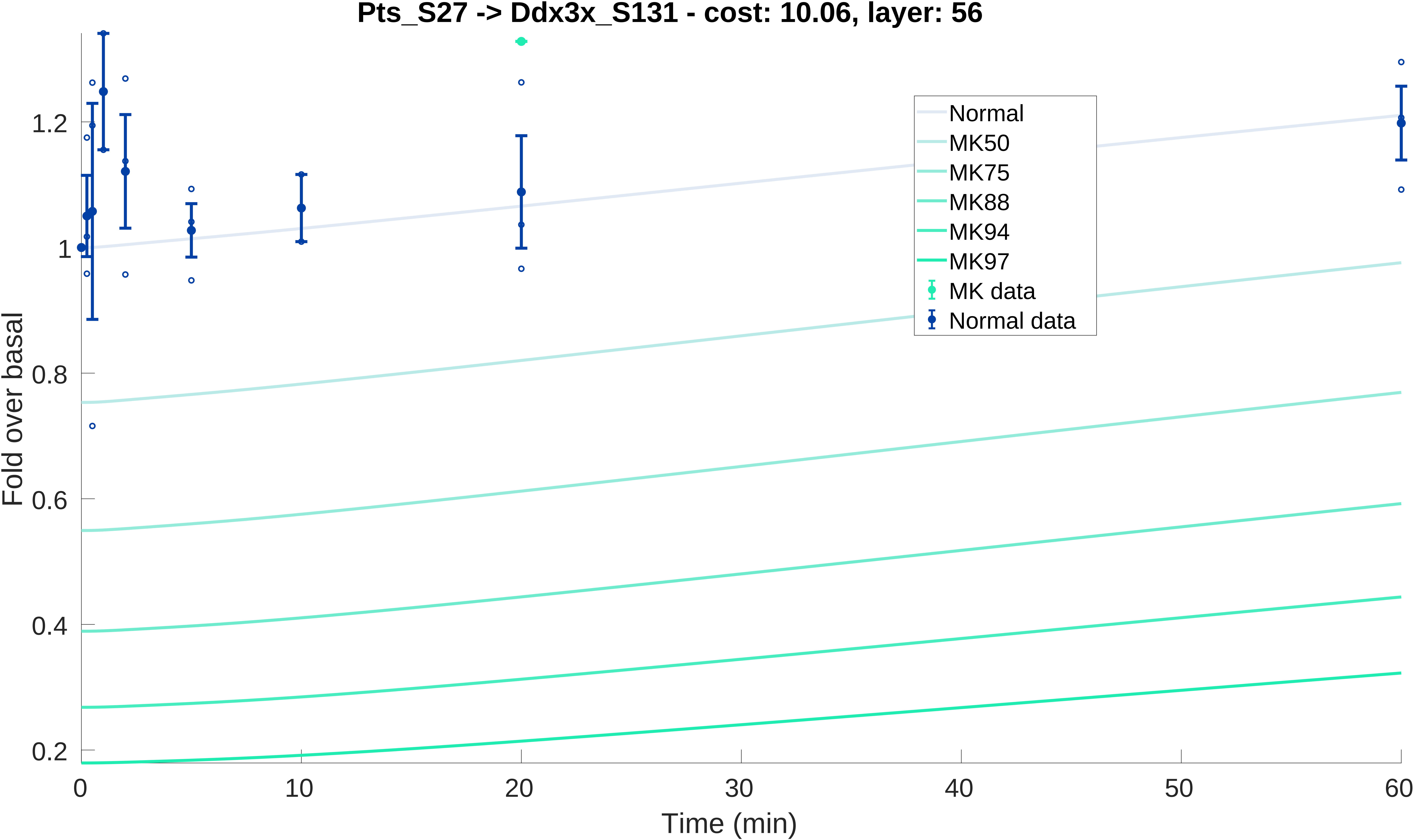

Pts_S27 -> Ddx3x_S131 - cost: 10.06, layer: 56
Fold over basal
Time (min)
0.2
0.4
0.6
0.8
1
1.2
0
10
20
30
40
50
60
Normal
MK50
MK75
MK88
MK94
MK97
MK data
Normal data

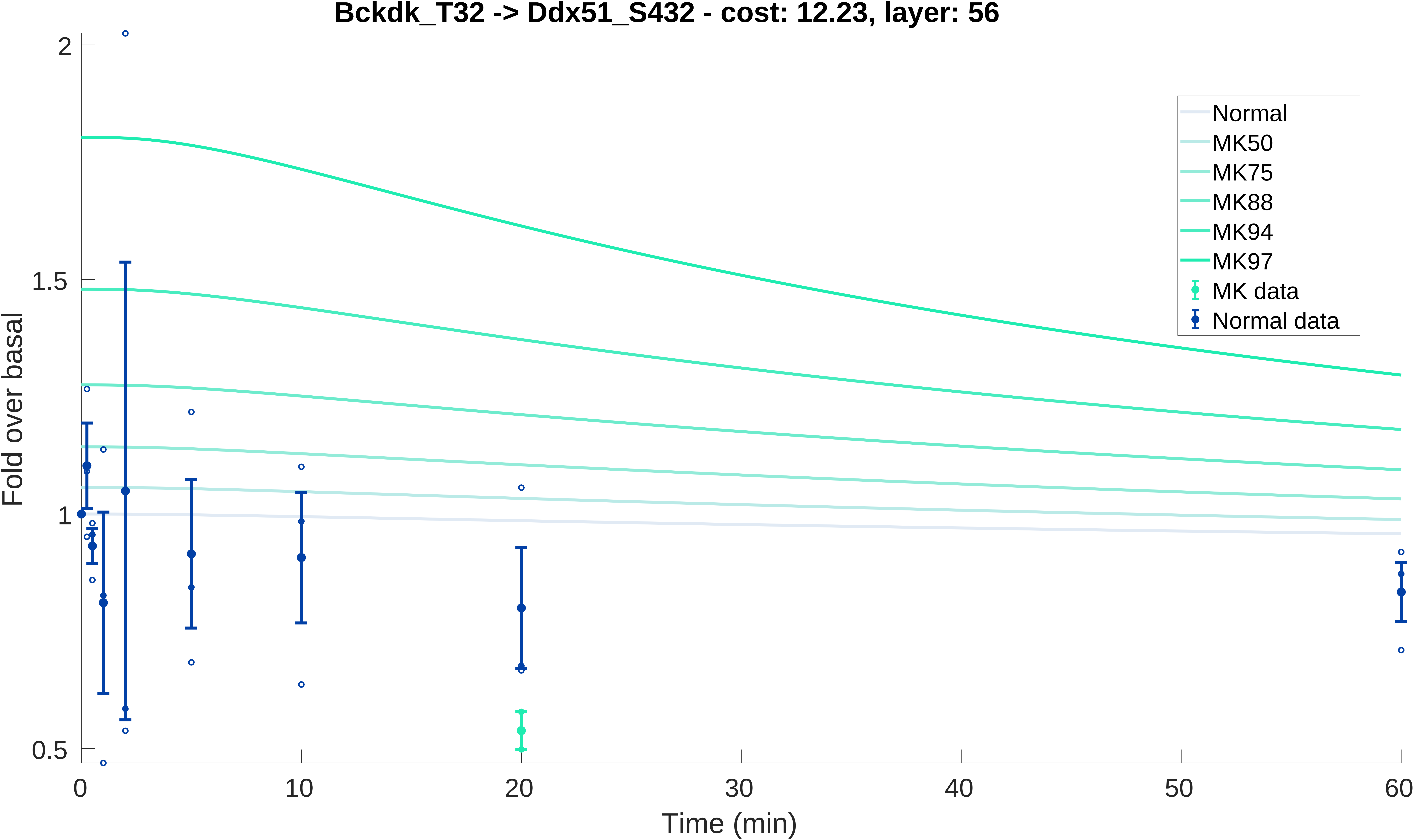
Bckdk_T32 -> Ddx51_S432 - cost: 12.23, layer: 56
Fold over basal
Time (min)
0.5
1
1.5
2
0
10
20
30
40
50
60
Normal
MK50
MK75
MK88
MK94
MK97
MK data
Normal data

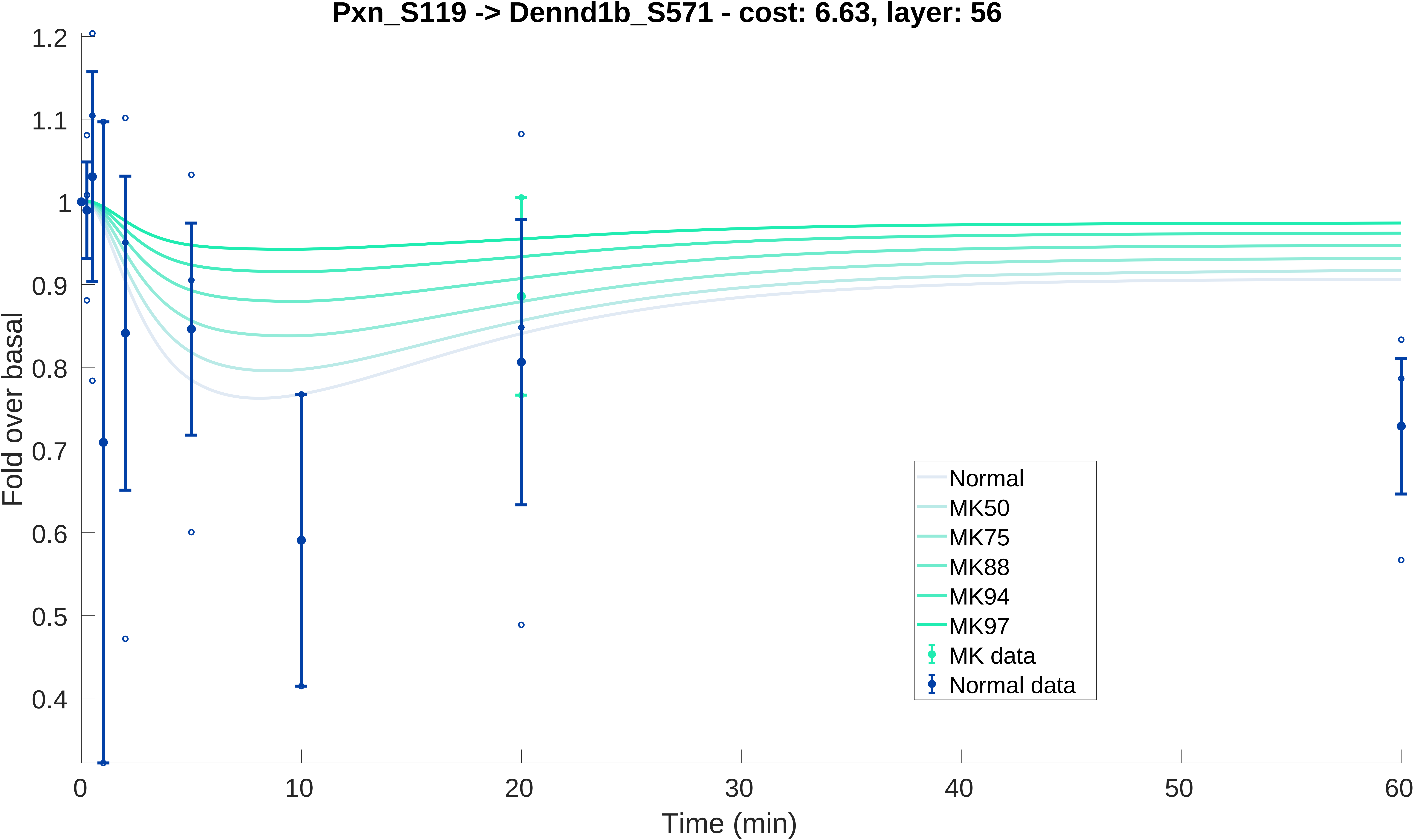
Pxn_S119 -> Dennd1b_S571 - cost: 6.63, layer: 56
Fold over basal
1.2
1.1
1
0.9
0.8
0.7
0.6
0.5
0.4
0
10
20
30
40
50
60
Time (min)
Normal
MK50
MK75
MK88
MK94
MK97
MK data
Normal data

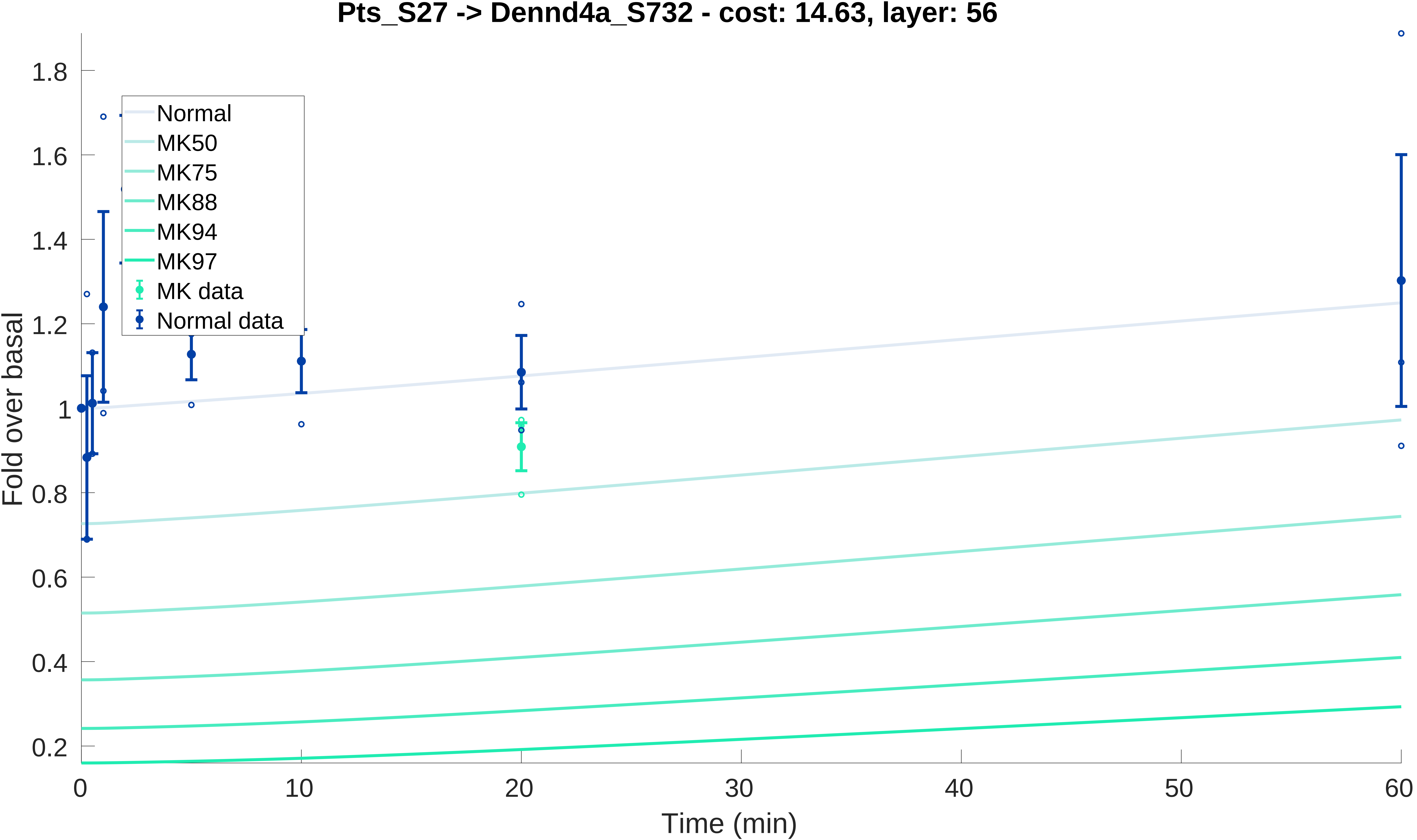

Pts_S27 -> Dennd4a_S732 - cost: 14.63, layer: 56
Fold over basal
Time (min)
Normal
MK50
MK75
MK88
MK94
MK97
MK data
Normal data
0
10
20
30
40
50
60
0.2
0.4
0.6
0.8
1
1.2
1.4
1.6
1.8

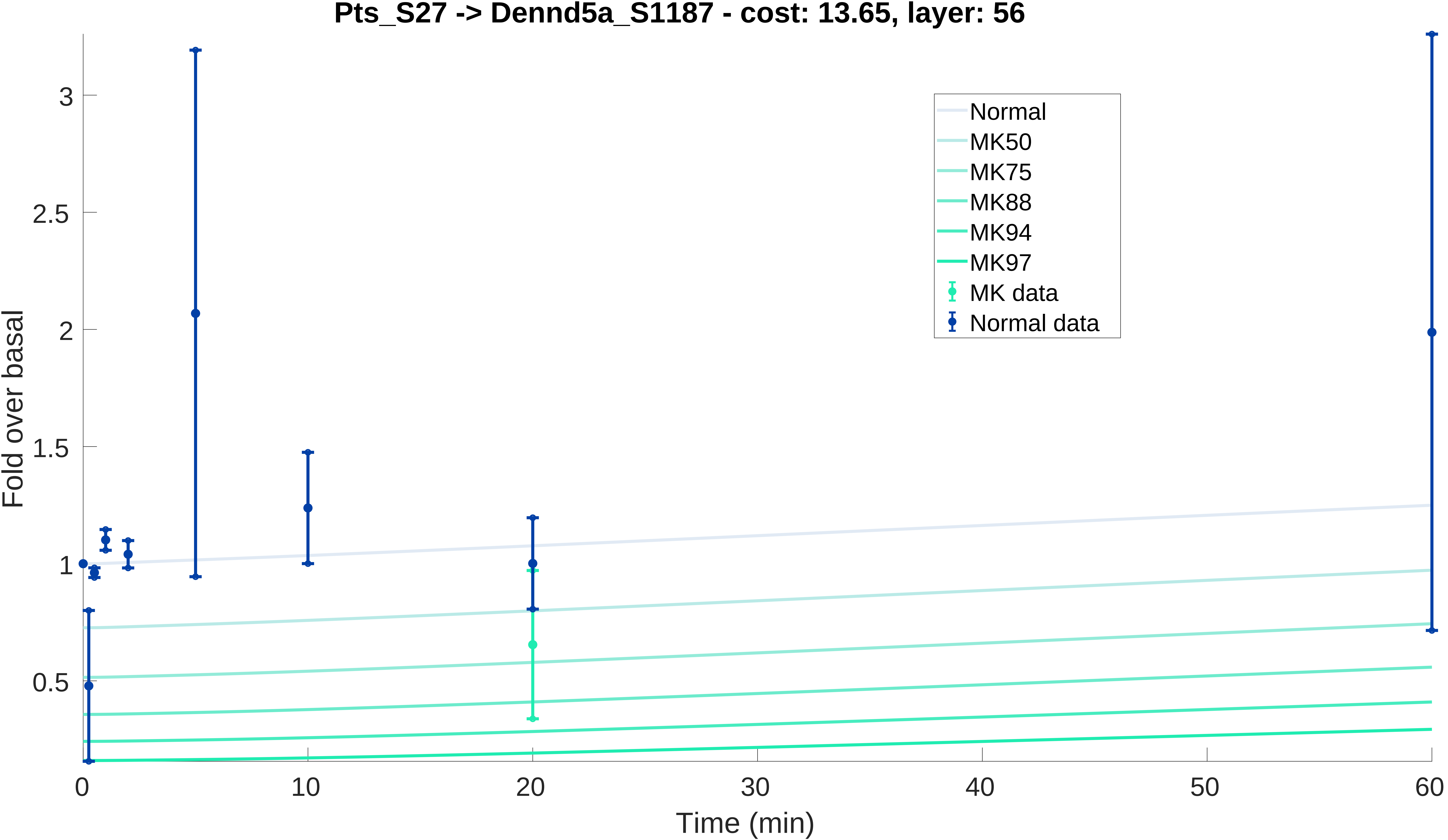

Pts_S27 -> Dennd5a_S1187 - cost: 13.65, layer: 56
Fold over basal
Time (min)
0
10
20
30
40
50
60
0.5
1
1.5
2
2.5
3
Normal
MK50
MK75
MK88
MK94
MK97
MK data
Normal data

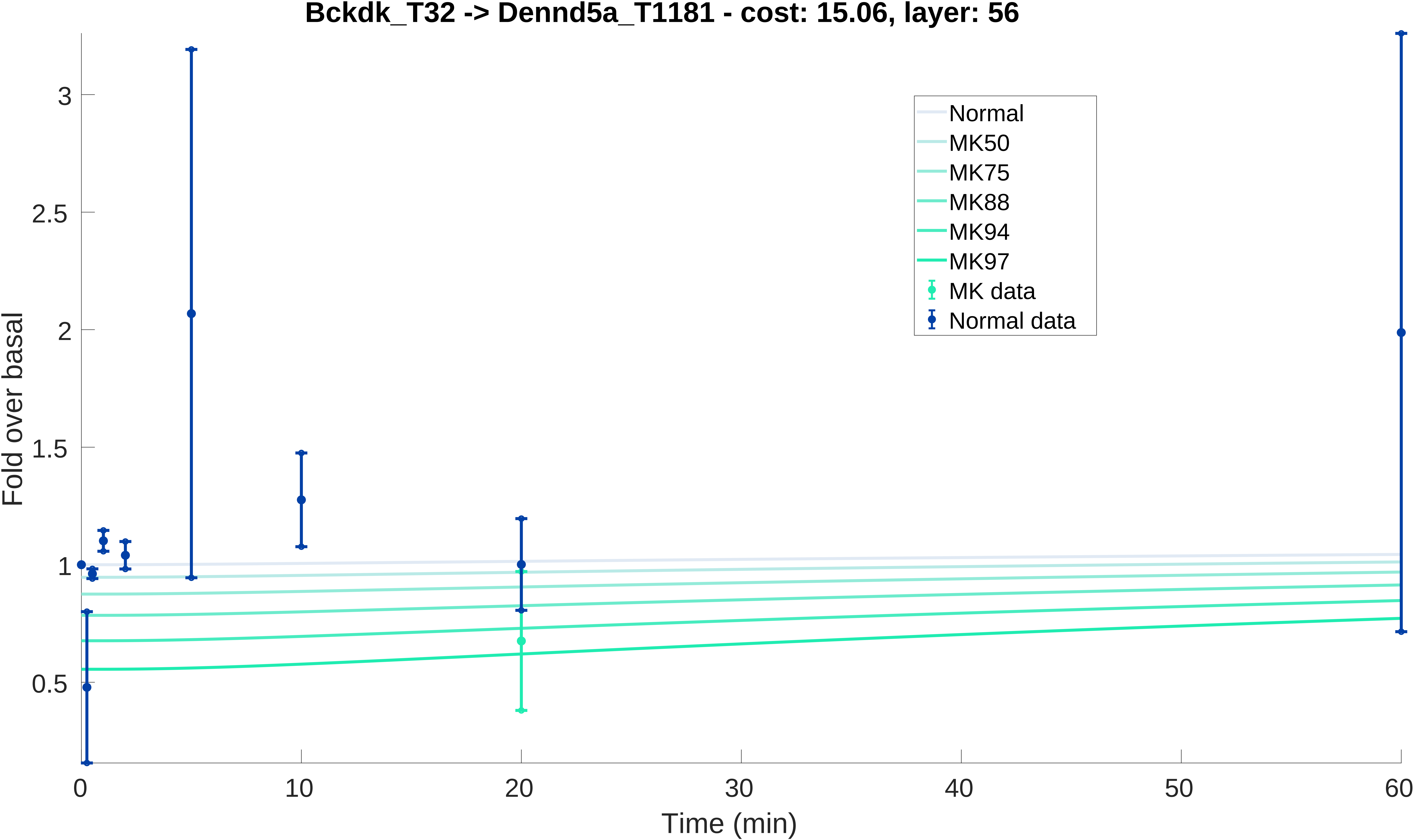

Bckdk_T32 -> Dennd5a_T1181 - cost: 15.06, layer: 56
Fold over basal
Time (min)
0.5
1
1.5
2
2.5
3
0
10
20
30
40
50
60
Normal
MK50
MK75
MK88
MK94
MK97
MK data
Normal data

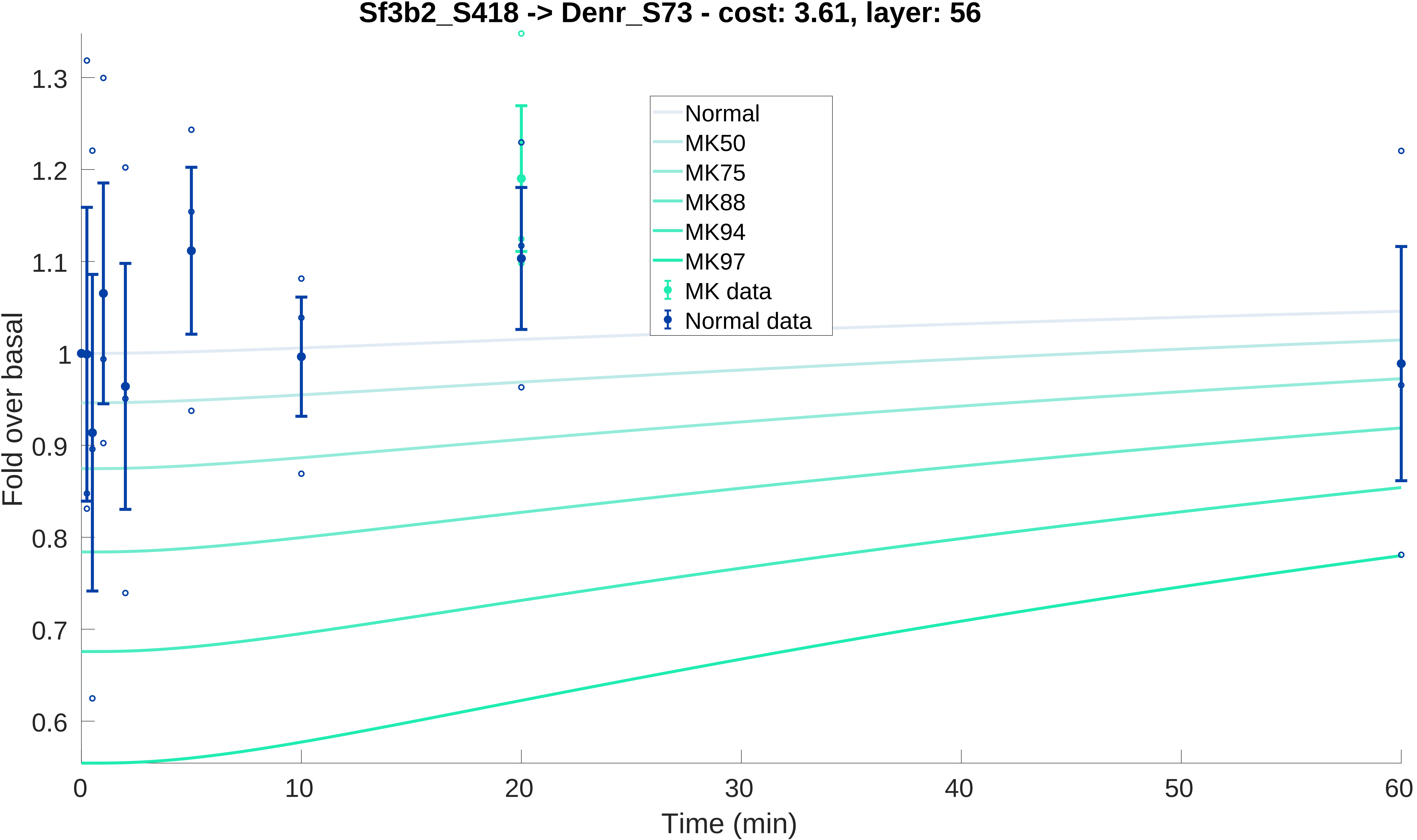

Sf3b2_S418 -> Denr_S73 - cost: 3.61, layer: 56
Normal
MK50
MK75
MK88
MK94
MK97
MK data
Normal data
Fold over basal
1.3
1.2
1.1
1
0.9
0.8
0.7
0.6
0
10
20
30
40
50
60
Time (min)

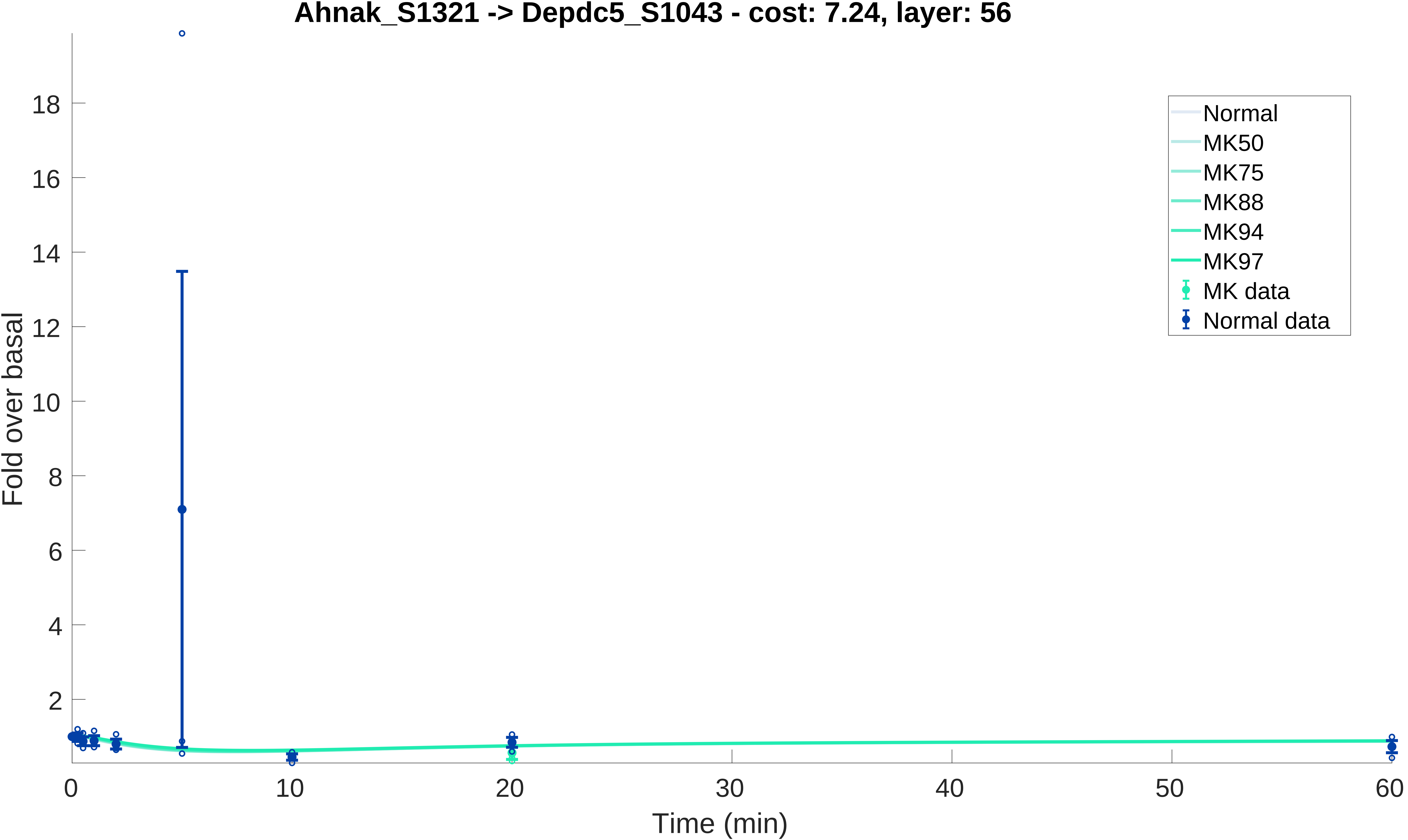

Ahnak_S1321 -> Depdc5_S1043 - cost: 7.24, layer: 56
Fold over basal
18
16
14
12
10
8
6
4
2
0
10
20
30
40
50
60
Time (min)
Normal
MK50
MK75
MK88
MK94
MK97
MK data
Normal data

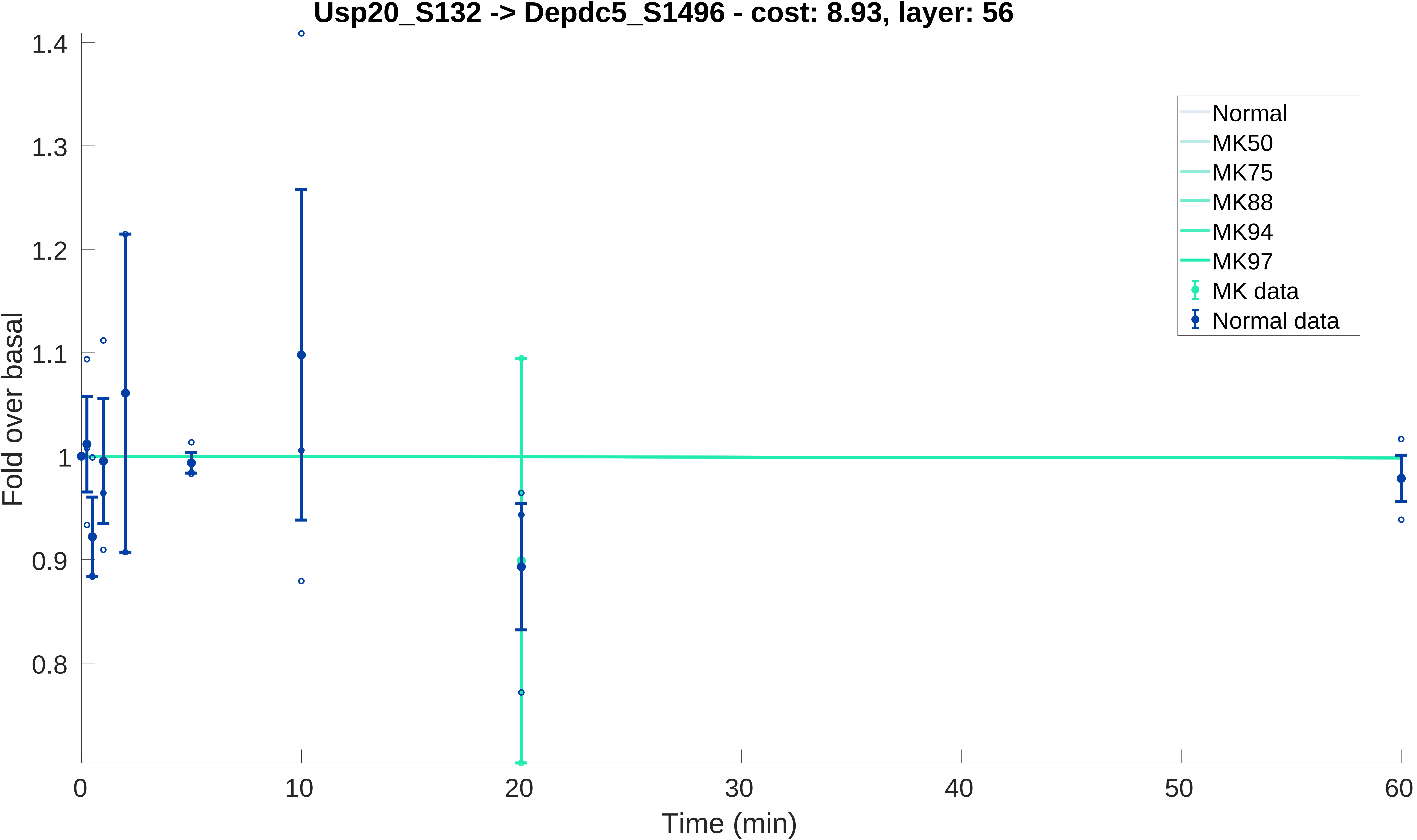

Usp20_S132 -> Depdc5_S1496 - cost: 8.93, layer: 56
Fold over basal
Time (min)
1.4
1.3
1.2
1.1
1
0.9
0.8
0
10
20
30
40
50
60
Normal
MK50
MK75
MK88
MK94
MK97
MK data
Normal data

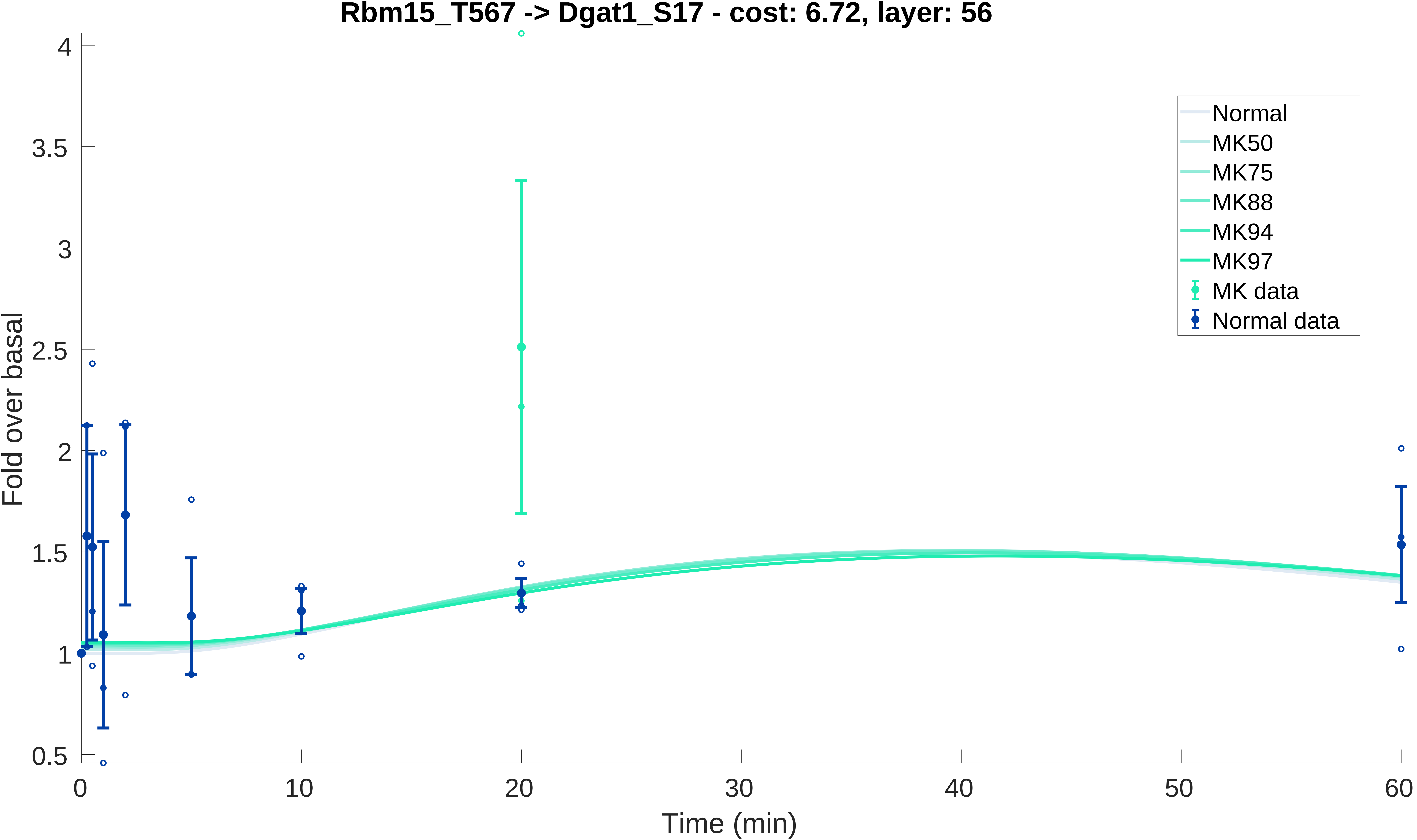

Rbm15_T567 -> Dgat1_S17 - cost: 6.72, layer: 56
Fold over basal
Time (min)
Normal
MK50
MK75
MK88
MK94
MK97
MK data
Normal data
0.5
1
1.5
2
2.5
3
3.5
4
0
10
20
30
40
50
60

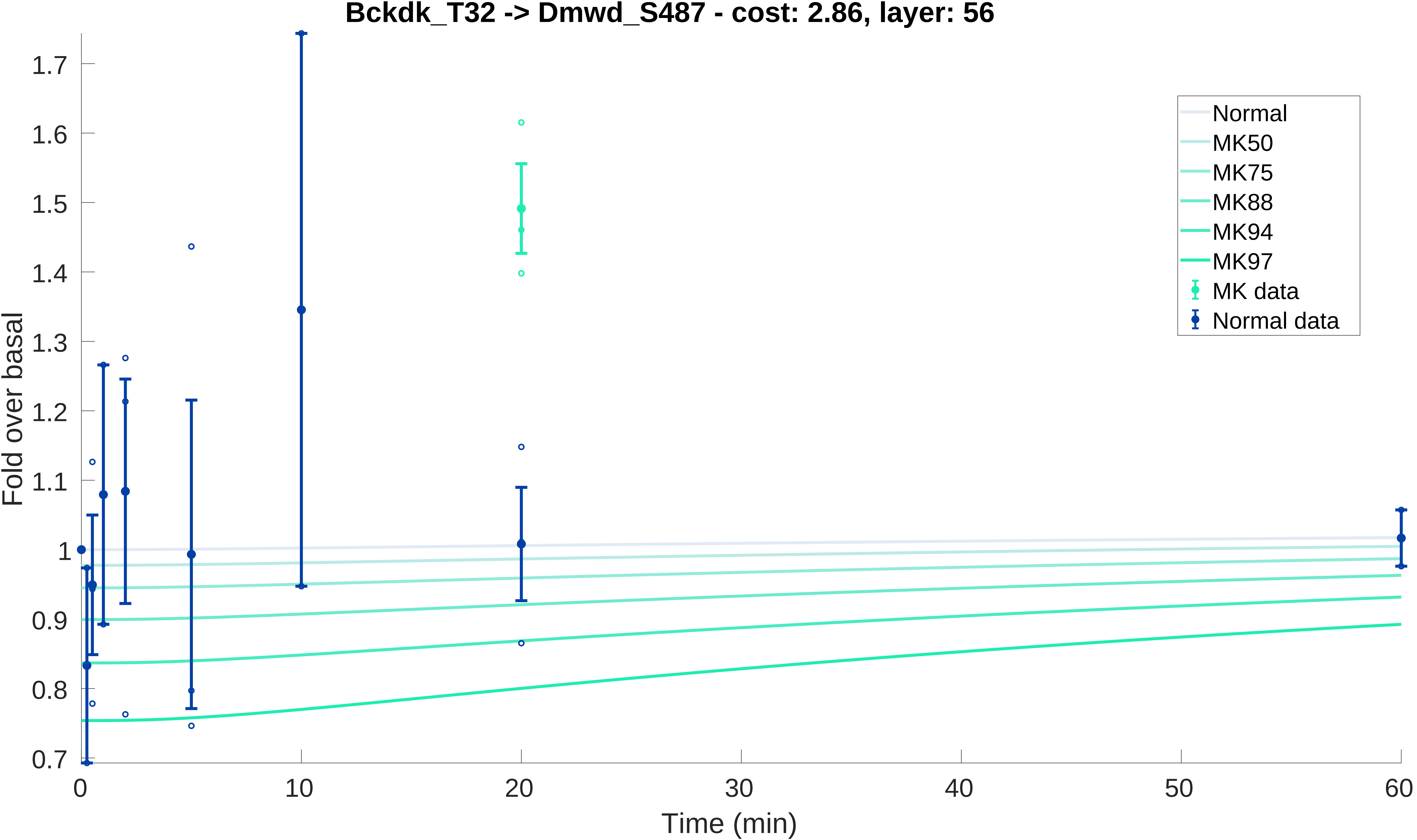

Bckdk_T32 -> Dmwd_S487 - cost: 2.86, layer: 56
Fold over basal
Time (min)
0.7
0.8
0.9
1
1.1
1.2
1.3
1.4
1.5
1.6
1.7
0
10
20
30
40
50
60
Normal
MK50
MK75
MK88
MK94
MK97
MK data
Normal data

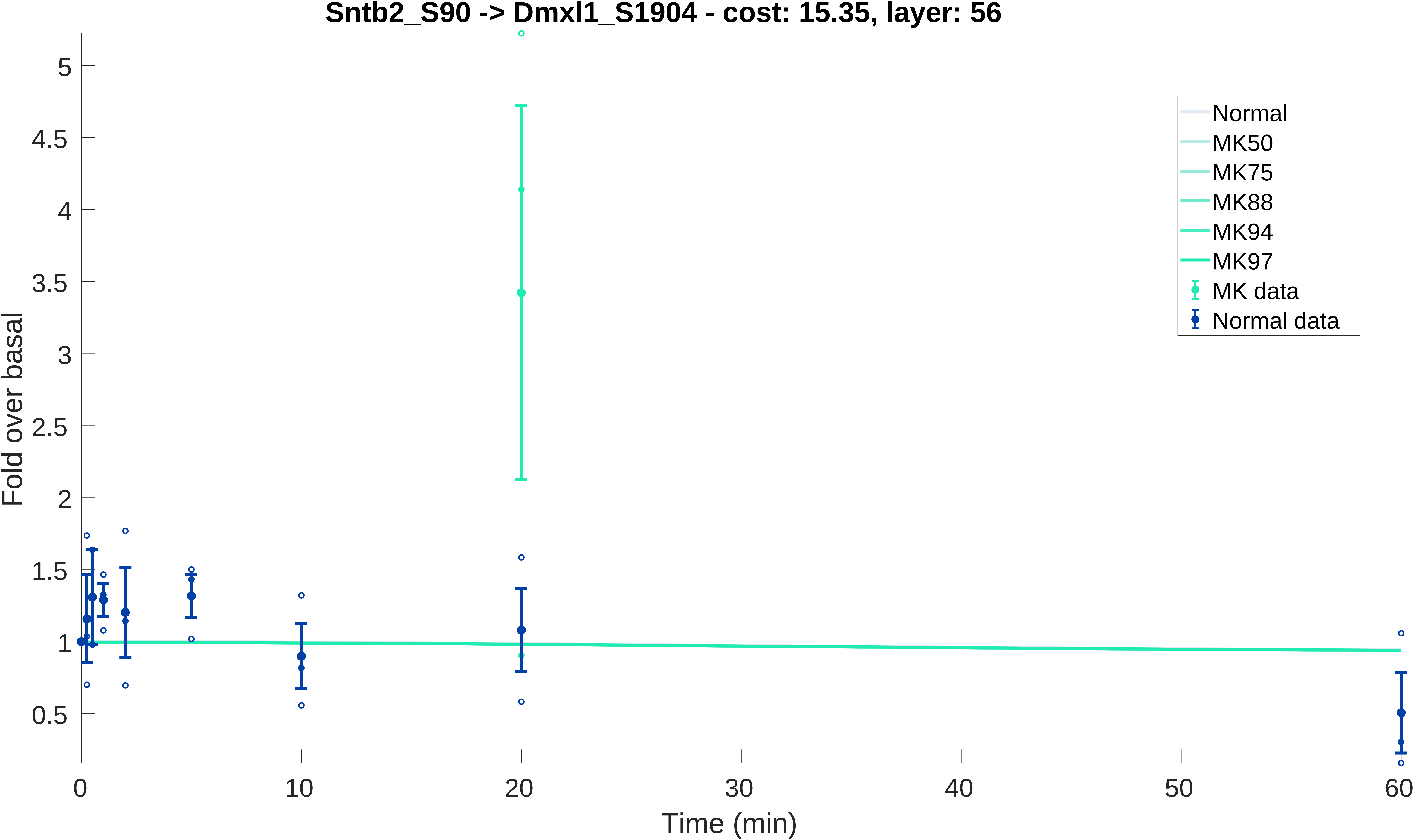

Sntb2_S90 -> Dmxl1_S1904 - cost: 15.35, layer: 56
Fold over basal
Time (min)
0
10
20
30
40
50
60
0.5
1
1.5
2
2.5
3
3.5
4
4.5
5
Normal
MK50
MK75
MK88
MK94
MK97
MK data
Normal data

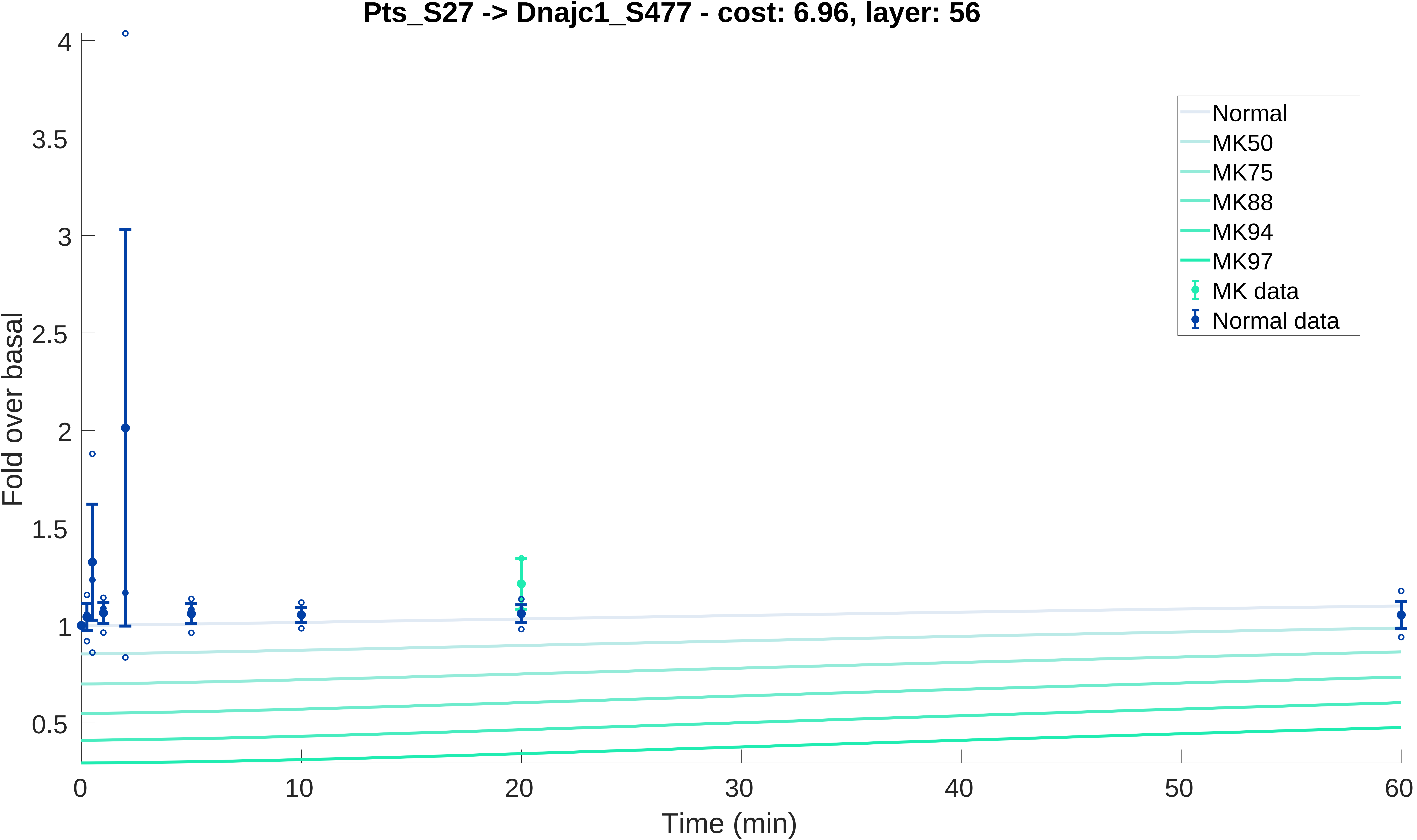
Pts_S27 -> Dnajc1_S477 - cost: 6.96, layer: 56
Fold over basal
Time (min)
0.5
1
1.5
2
2.5
3
3.5
4
0
10
20
30
40
50
60
Normal
MK50
MK75
MK88
MK94
MK97
MK data
Normal data

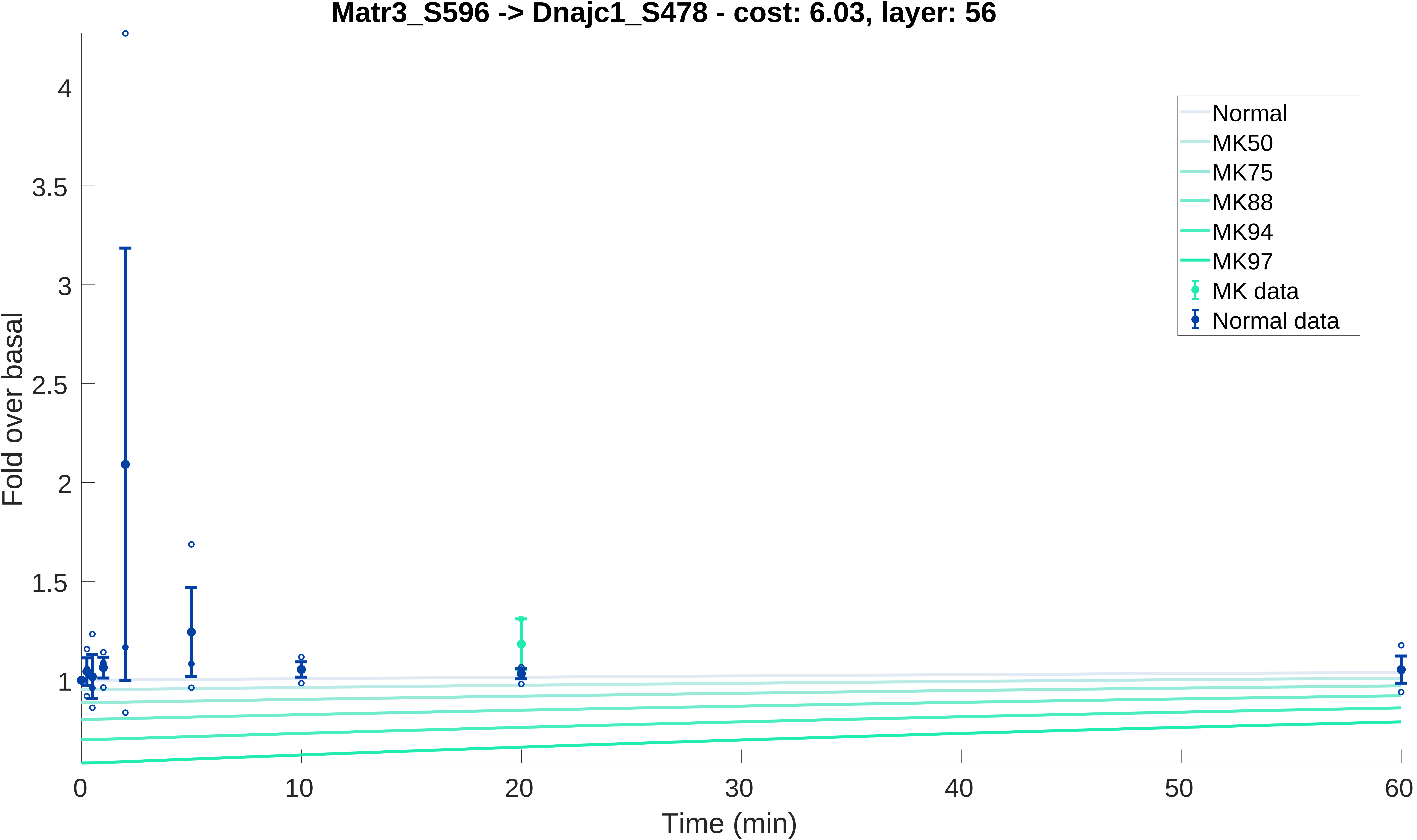

Matr3_S596 -> Dnajc1_S478 - cost: 6.03, layer: 56
Fold over basal
Time (min)
Normal
MK50
MK75
MK88
MK94
MK97
MK data
Normal data

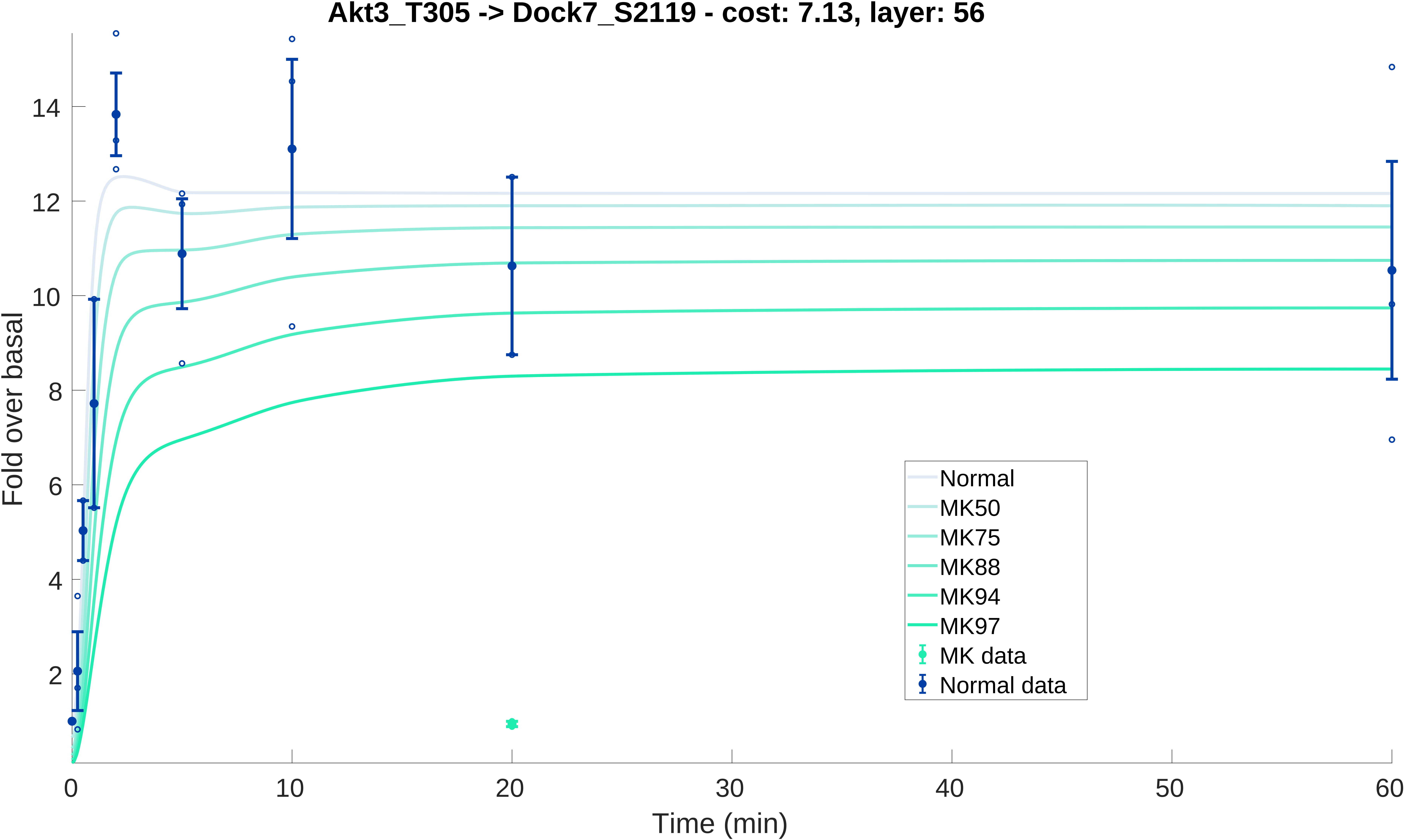

Akt3_T305 -> Dock7_S2119 - cost: 7.13, layer: 56
Fold over basal
Time (min)
0
10
20
30
40
50
60
2
4
6
8
10
12
14
Normal
MK50
MK75
MK88
MK94
MK97
MK data
Normal data

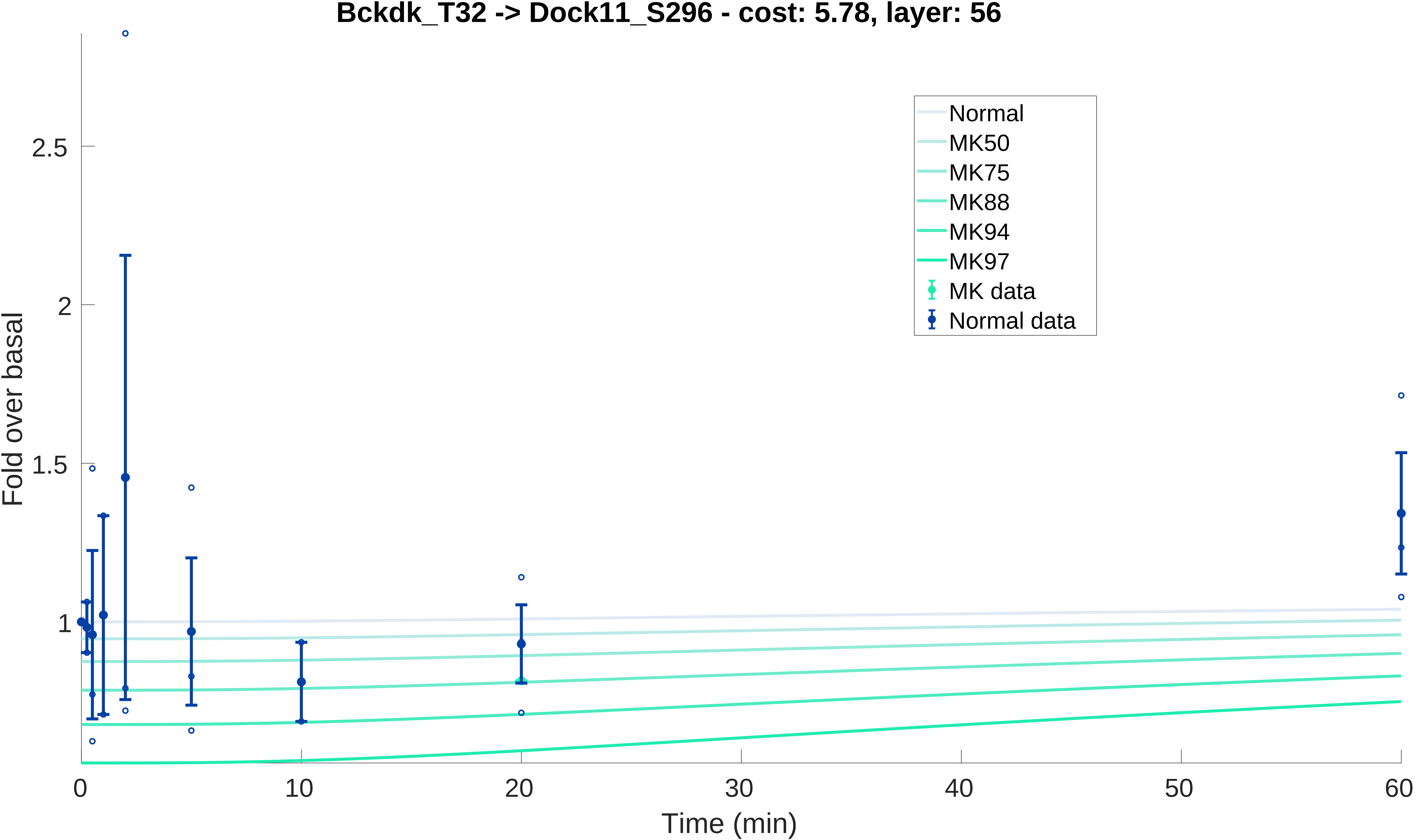

Bckdk_T32 -> Dock11_S296 - cost: 5.78, layer: 56
Normal
MK50
MK75
MK88
MK94
MK97
MK data
Normal data
Fold over basal
2.5
2
1.5
1
0
10
20
30
40
50
60
Time (min)

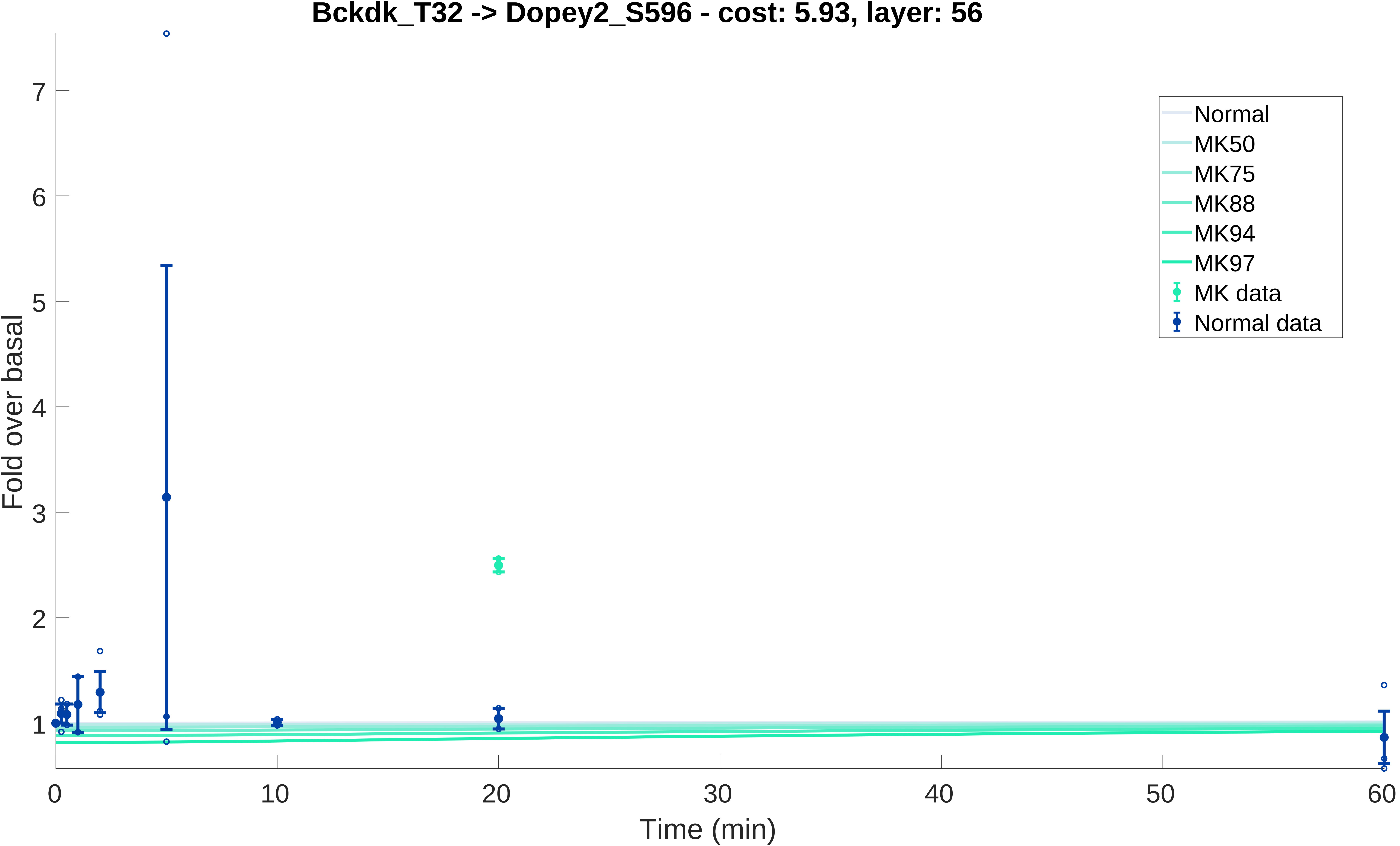

Bckdk_T32 -> Dopey2_S596 - cost: 5.93, layer: 56
Fold over basal
Time (min)
1
2
3
4
5
6
7
0
10
20
30
40
50
60
Normal
MK50
MK75
MK88
MK94
MK97
MK data
Normal data

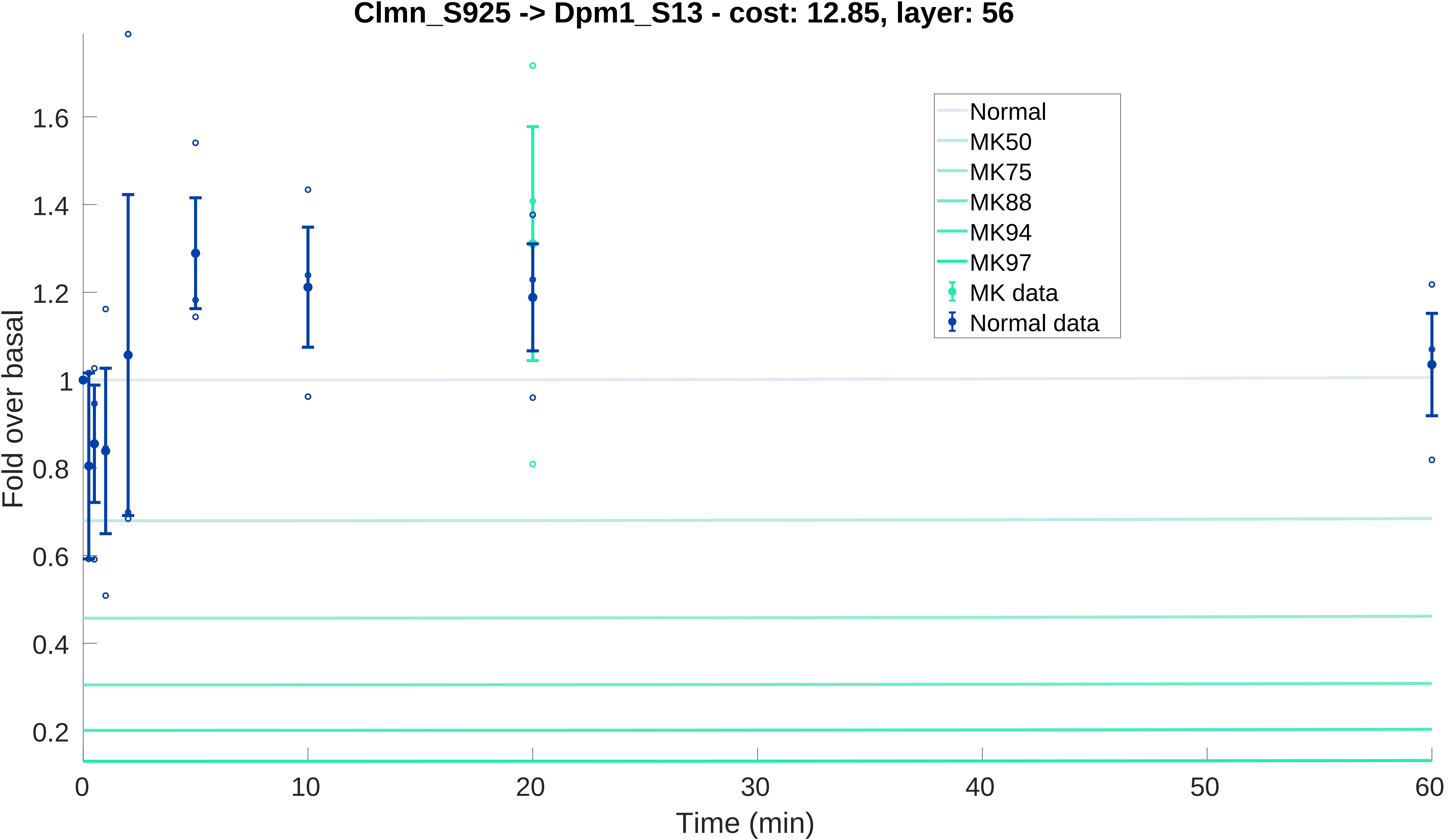

Clmn_S925 -> Dpm1_S13 - cost: 12.85, layer: 56
Fold over basal
Time (min)
0
10
20
30
40
50
60
0.2
0.4
0.6
0.8
1
1.2
1.4
1.6
Normal
MK50
MK75
MK88
MK94
MK97
MK data
Normal data

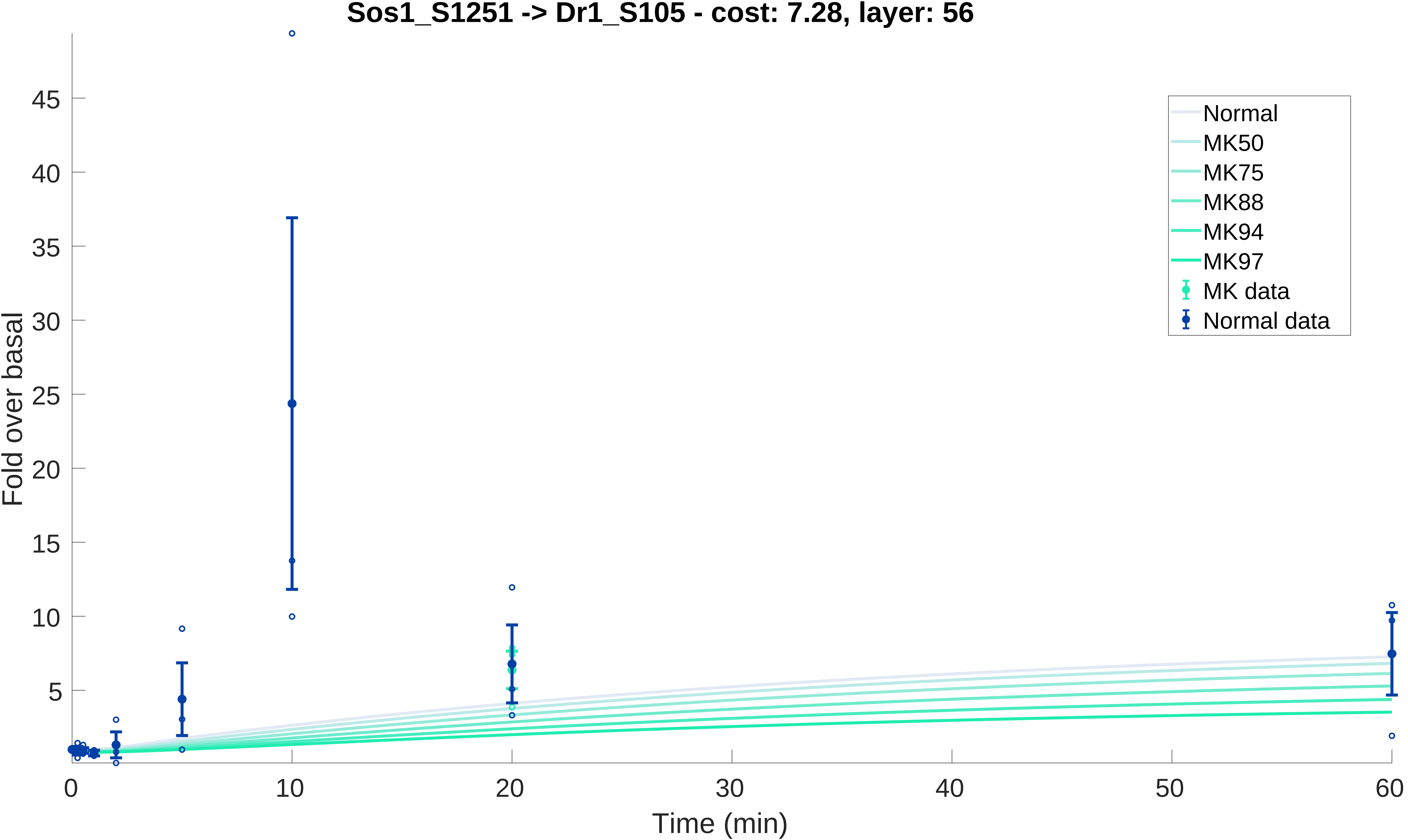
Sos1_S1251 -> Dr1_S105 - cost: 7.28, layer: 56
Fold over basal
Time (min)
Normal
MK50
MK75
MK88
MK94
MK97
MK data
Normal data
0
10
20
30
40
50
60
5
10
15
20
25
30
35
40
45

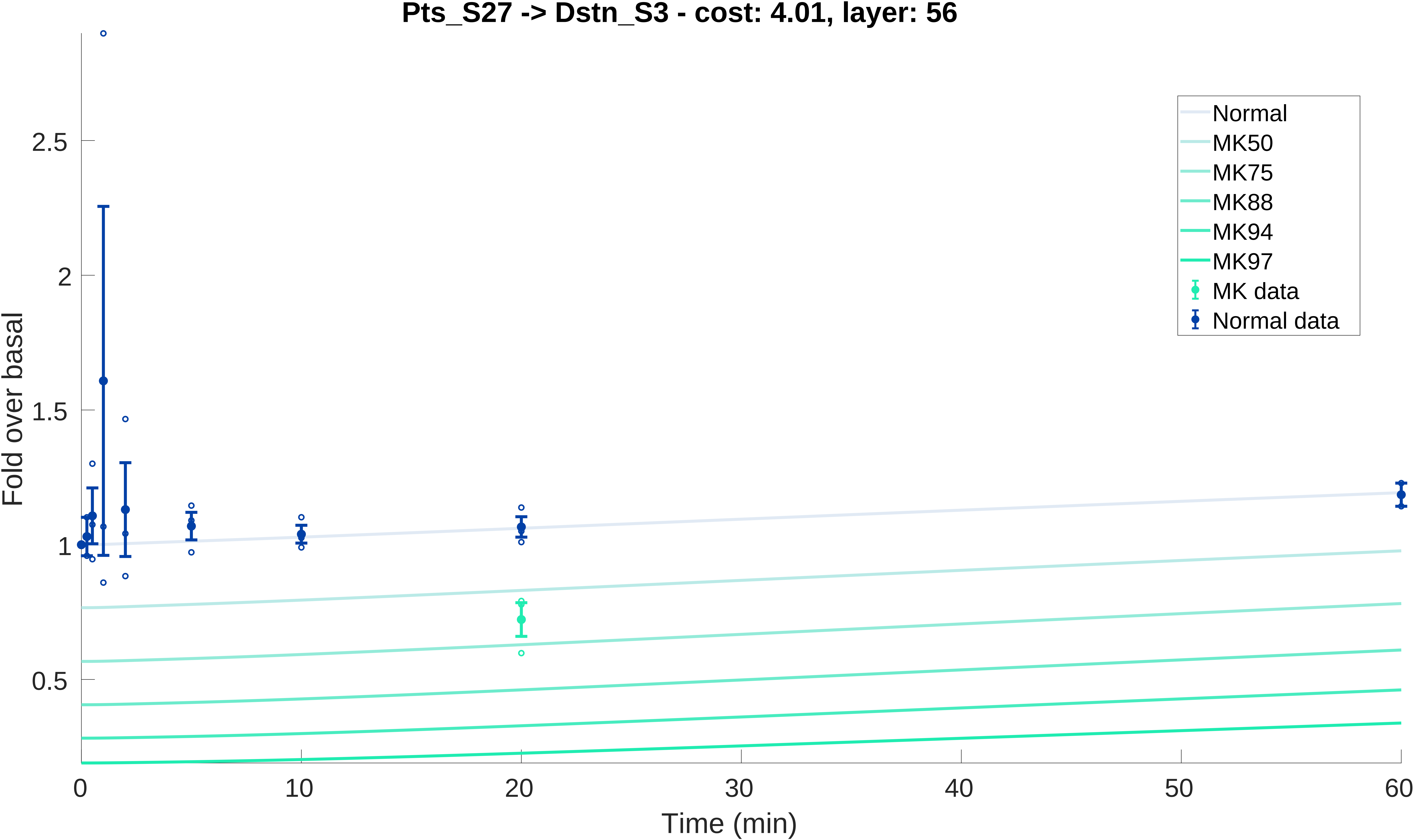
Pts_S27 -> Dstn_S3 - cost: 4.01, layer: 56
Fold over basal
2.5
2
1.5
1
0.5
0
10
20
30
40
50
60
Time (min)
Normal
MK50
MK75
MK88
MK94
MK97
MK data
Normal data

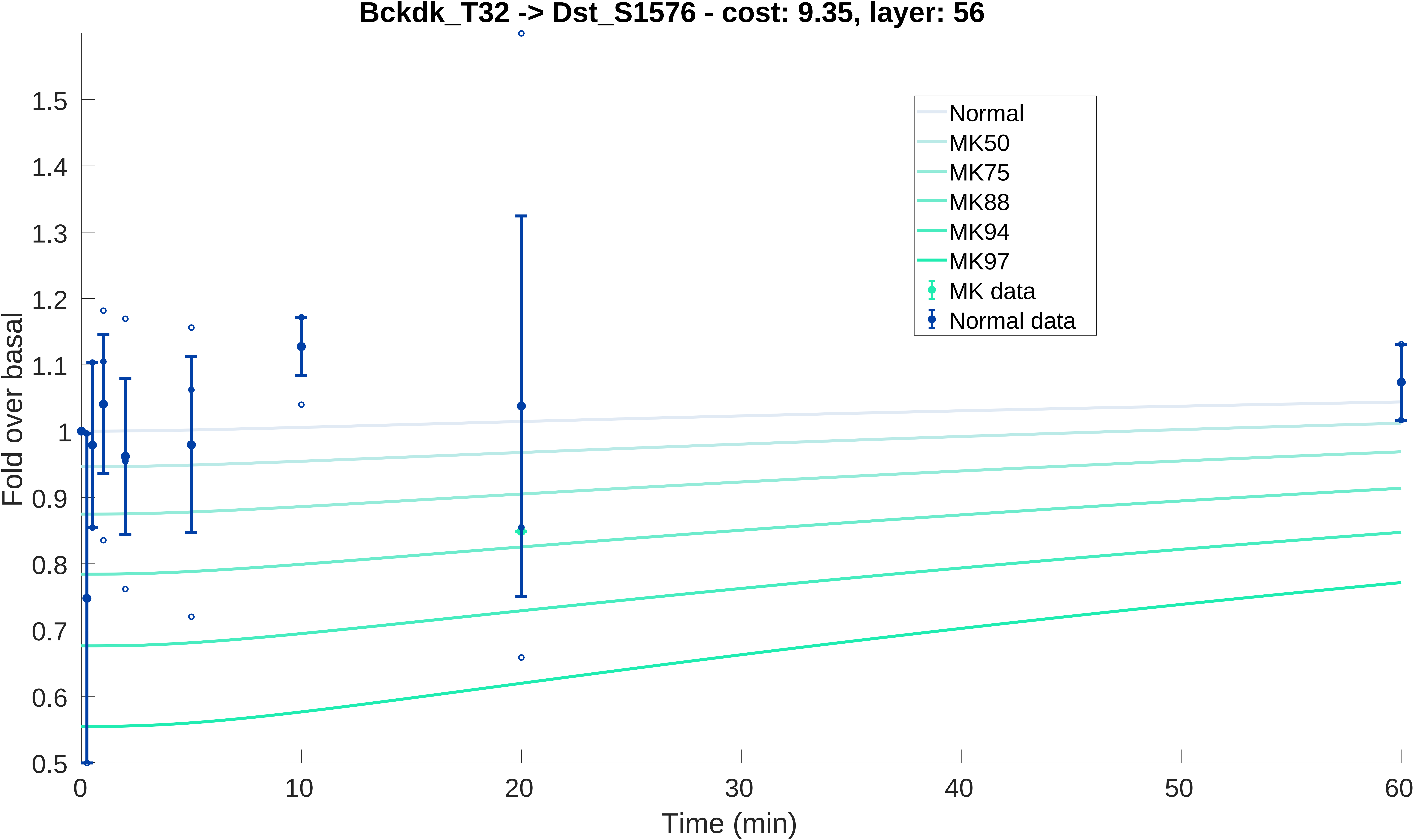

Bckdk_T32 -> Dst_S1576 - cost: 9.35, layer: 56
Normal
MK50
MK75
MK88
MK94
MK97
MK data
Normal data
Fold over basal
Time (min)
1.5
1.4
1.3
1.2
1.1
1
0.9
0.8
0.7
0.6
0.5
0
10
20
30
40
50
60

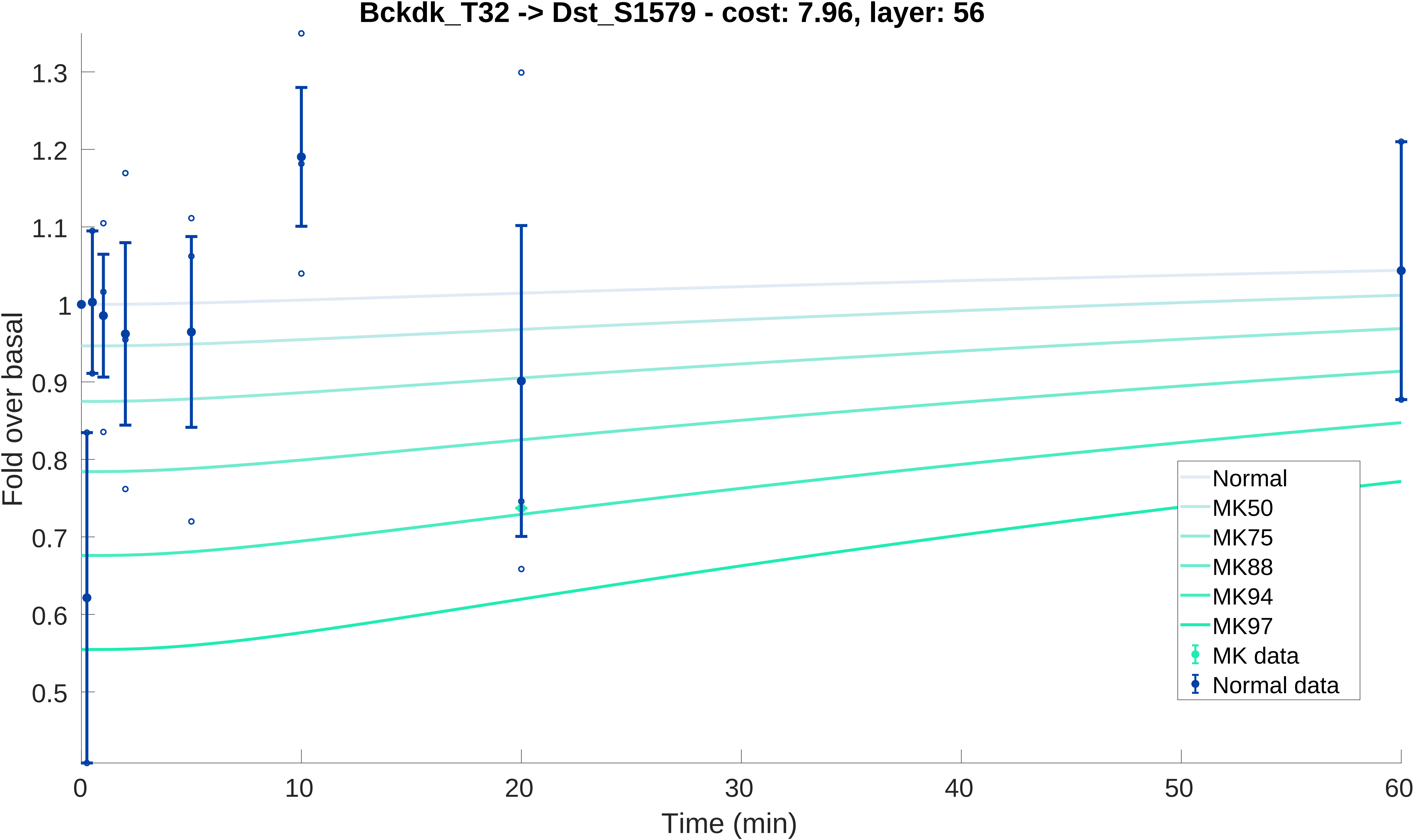

Bckdk_T32 -> Dst_S1579 - cost: 7.96, layer: 56
Fold over basal
Time (min)
1.3
1.2
1.1
1
0.9
0.8
0.7
0.6
0.5
0
10
20
30
40
50
60
Normal
MK50
MK75
MK88
MK94
MK97
MK data
Normal data

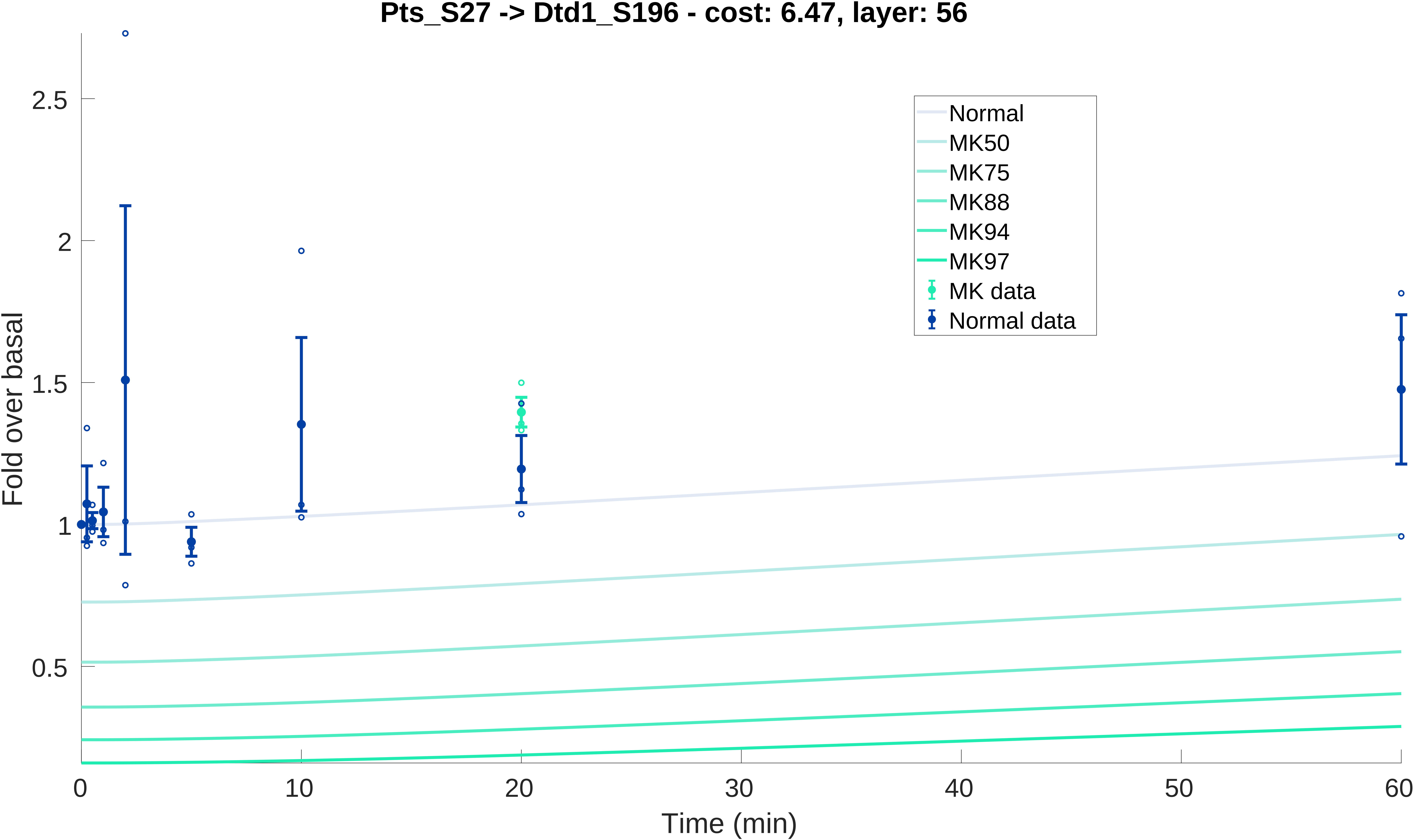

Pts_S27 -> Dtd1_S196 - cost: 6.47, layer: 56
Fold over basal
Time (min)
0.5
1
1.5
2
2.5
0
10
20
30
40
50
60
Normal
MK50
MK75
MK88
MK94
MK97
MK data
Normal data

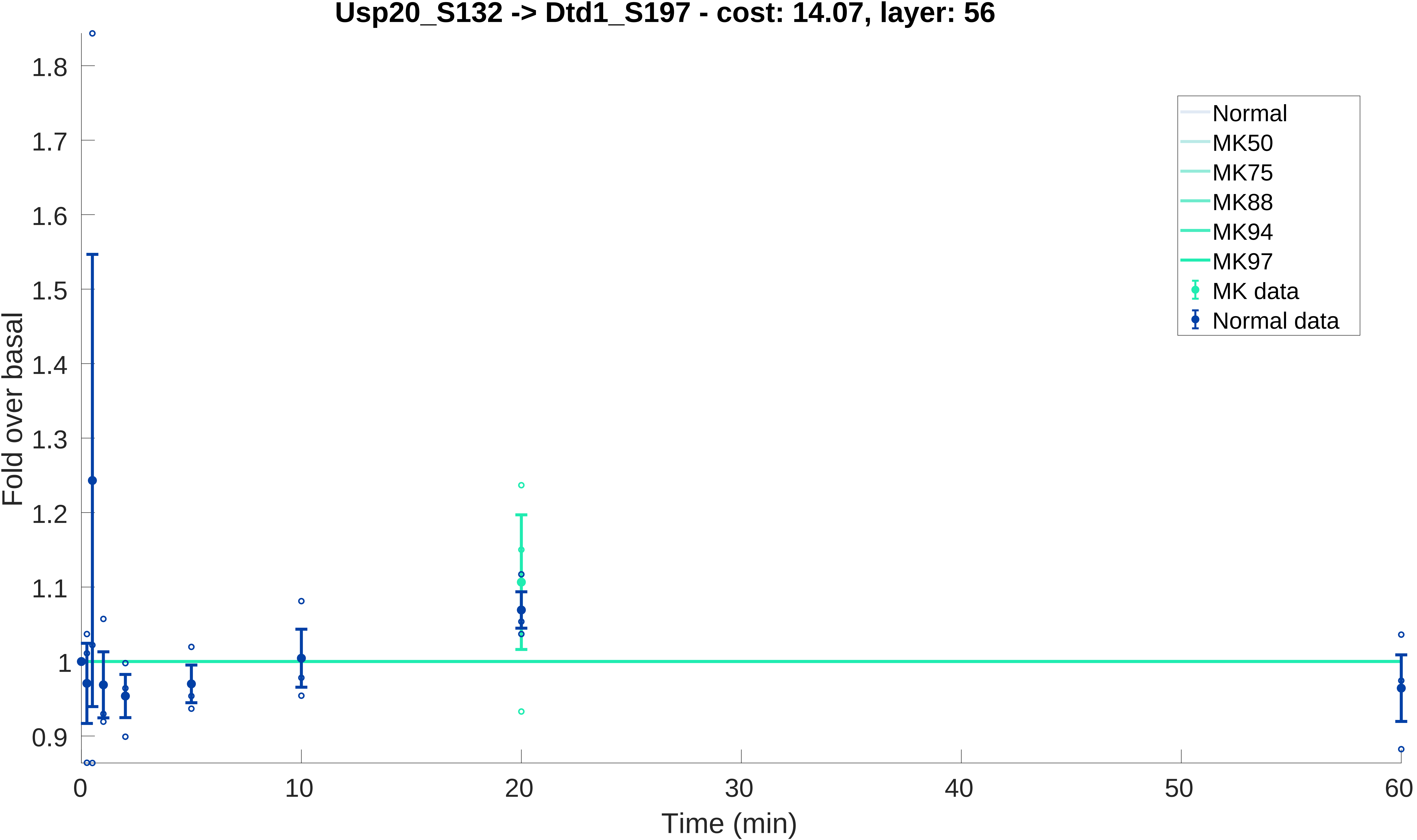
Usp20_S132 -> Dtd1_S197 - cost: 14.07, layer: 56
Fold over basal
1.8
1.7
1.6
1.5
1.4
1.3
1.2
1.1
1
0.9
0
10
20
30
40
50
60
Time (min)
Normal
MK50
MK75
MK88
MK94
MK97
MK data
Normal data

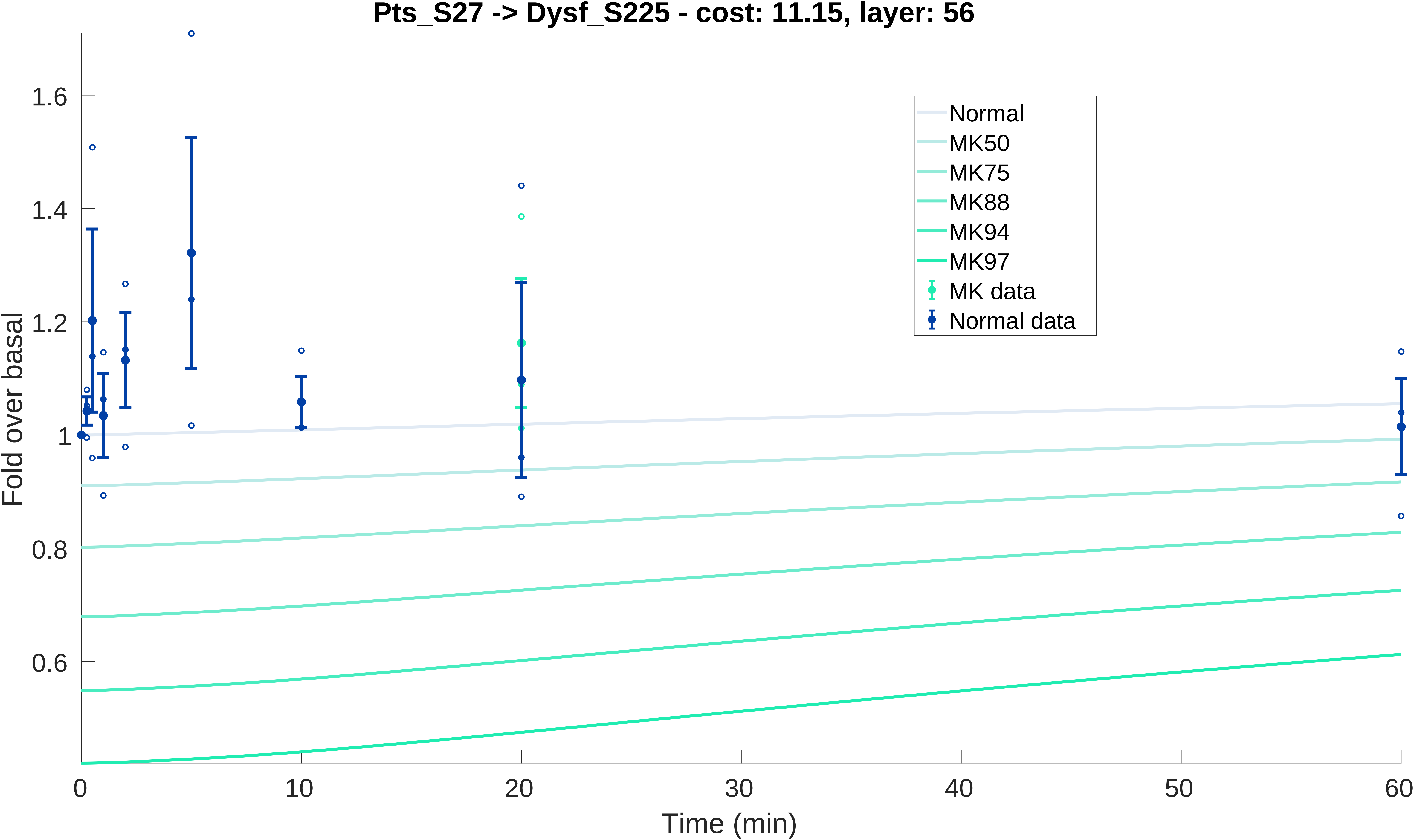

Pts_S27 -> Dysf_S225 - cost: 11.15, layer: 56
Fold over basal
1.6
1.4
1.2
1
0.8
0.6
Time (min)
0
10
20
30
40
50
60
Normal
MK50
MK75
MK88
MK94
MK97
MK data
Normal data

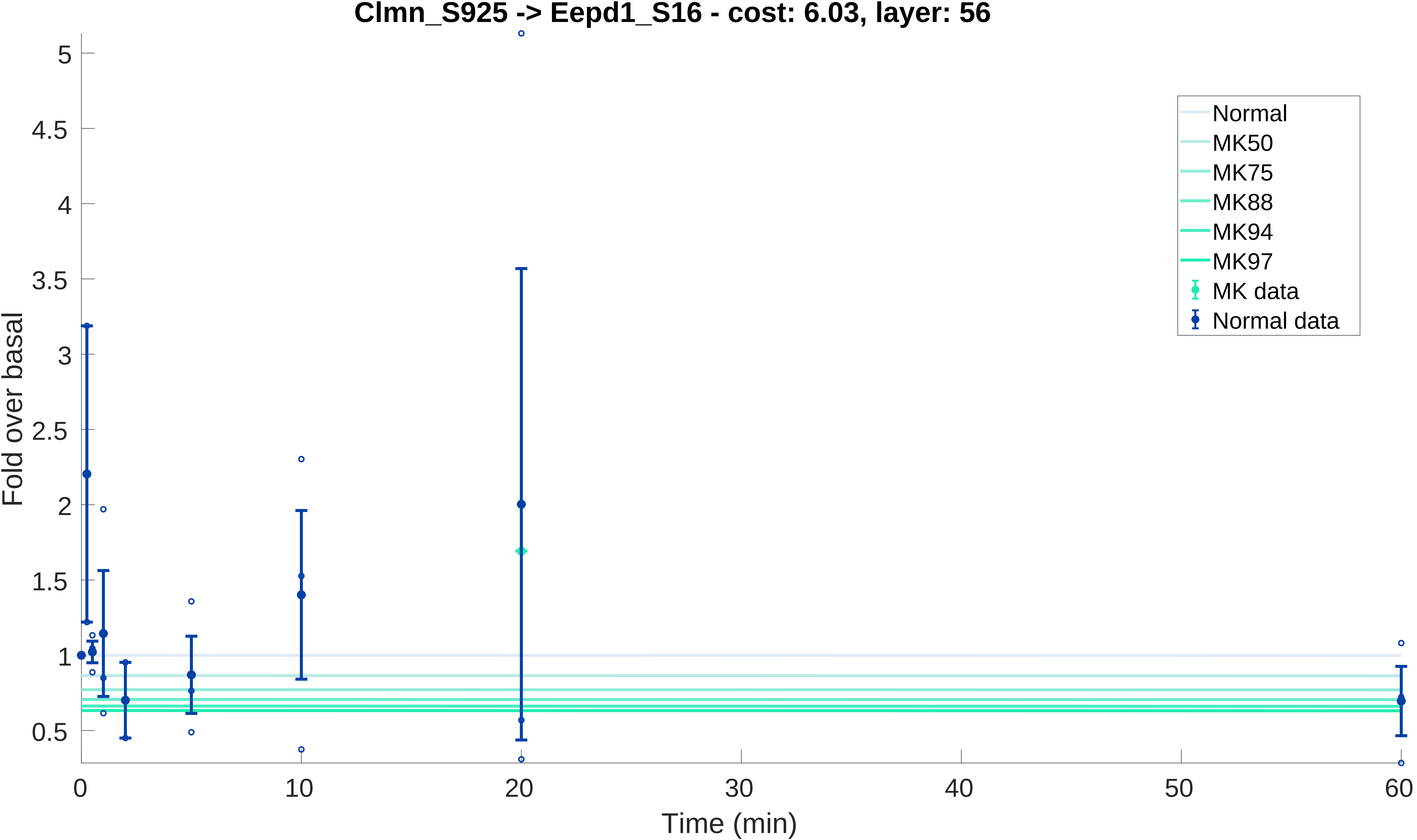
Clmn_S925 -> Eepd1_S16 - cost: 6.03, layer: 56
Fold over basal
Time (min)
0.5
1
1.5
2
2.5
3
3.5
4
4.5
5
0
10
20
30
40
50
60
Normal
MK50
MK75
MK88
MK94
MK97
MK data
Normal data

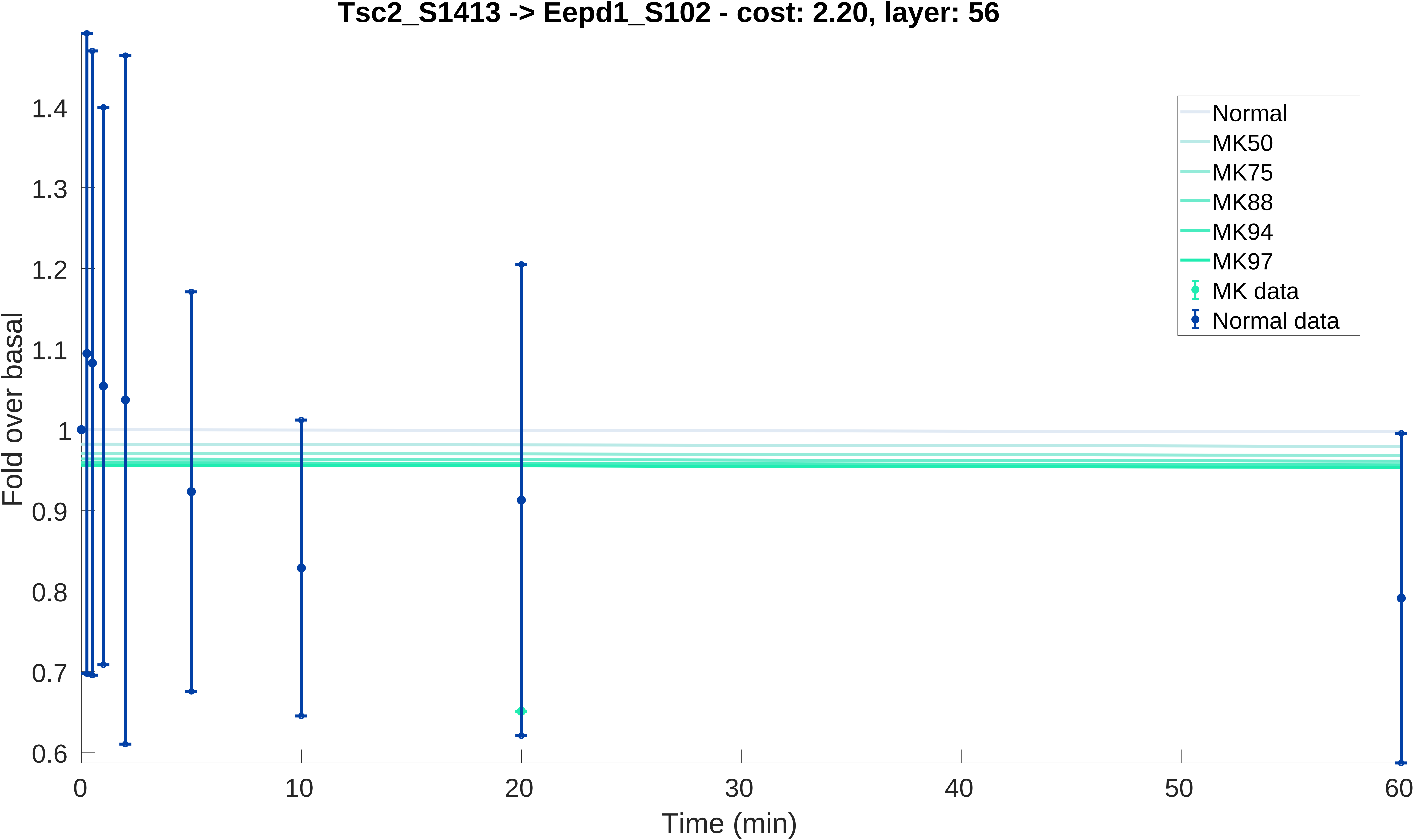

Tsc2_S1413 -> Eepd1_S102 - cost: 2.20, layer: 56
Fold over basal
1.4
1.3
1.2
1.1
1
0.9
0.8
0.7
0.6
0
10
20
30
40
50
60
Time (min)
Normal
MK50
MK75
MK88
MK94
MK97
MK data
Normal data

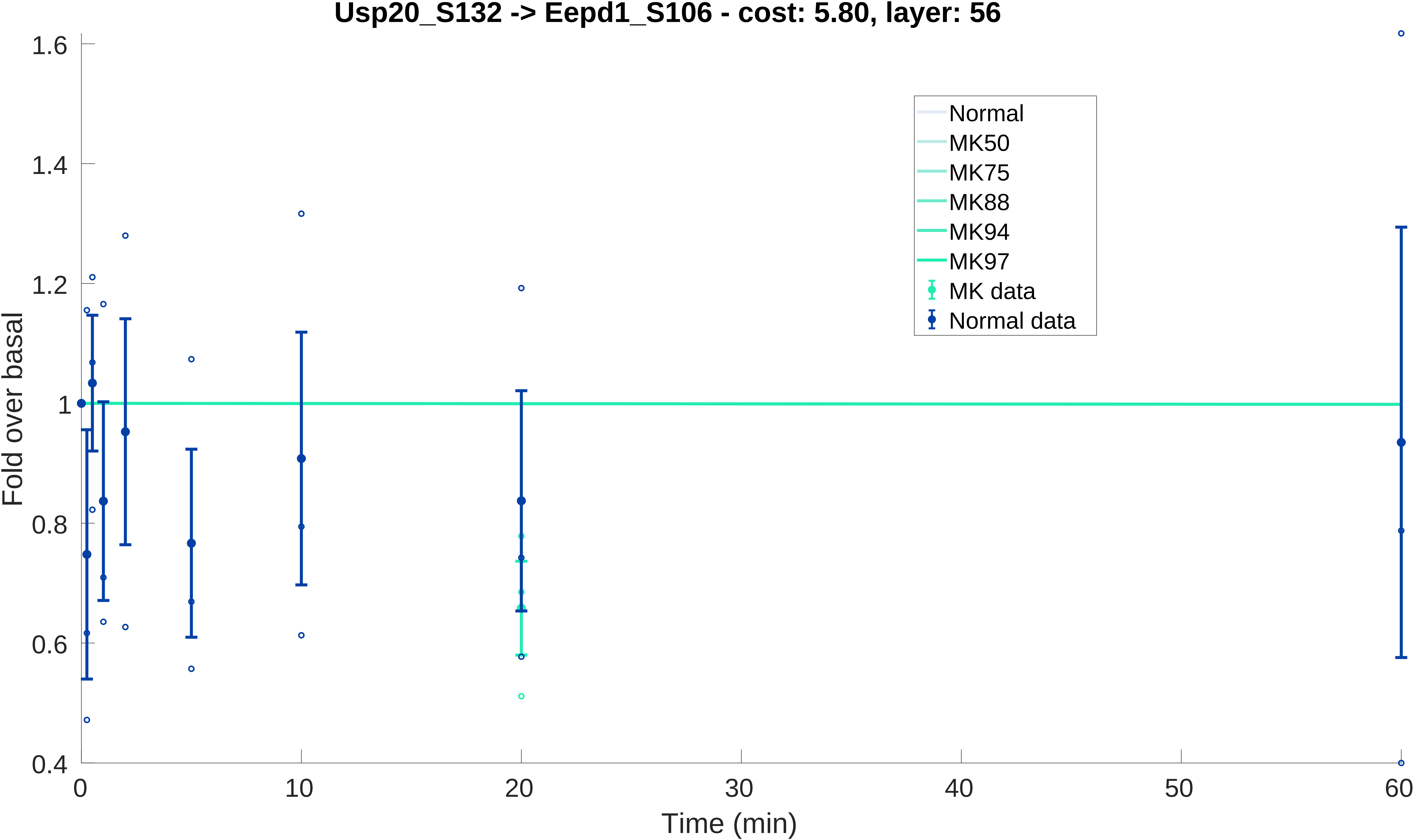
Usp20_S132 -> Eepd1_S106 - cost: 5.80, layer: 56
Fold over basal
Time (min)
0
10
20
30
40
50
60
0.4
0.6
0.8
1
1.2
1.4
1.6
Normal
MK50
MK75
MK88
MK94
MK97
MK data
Normal data

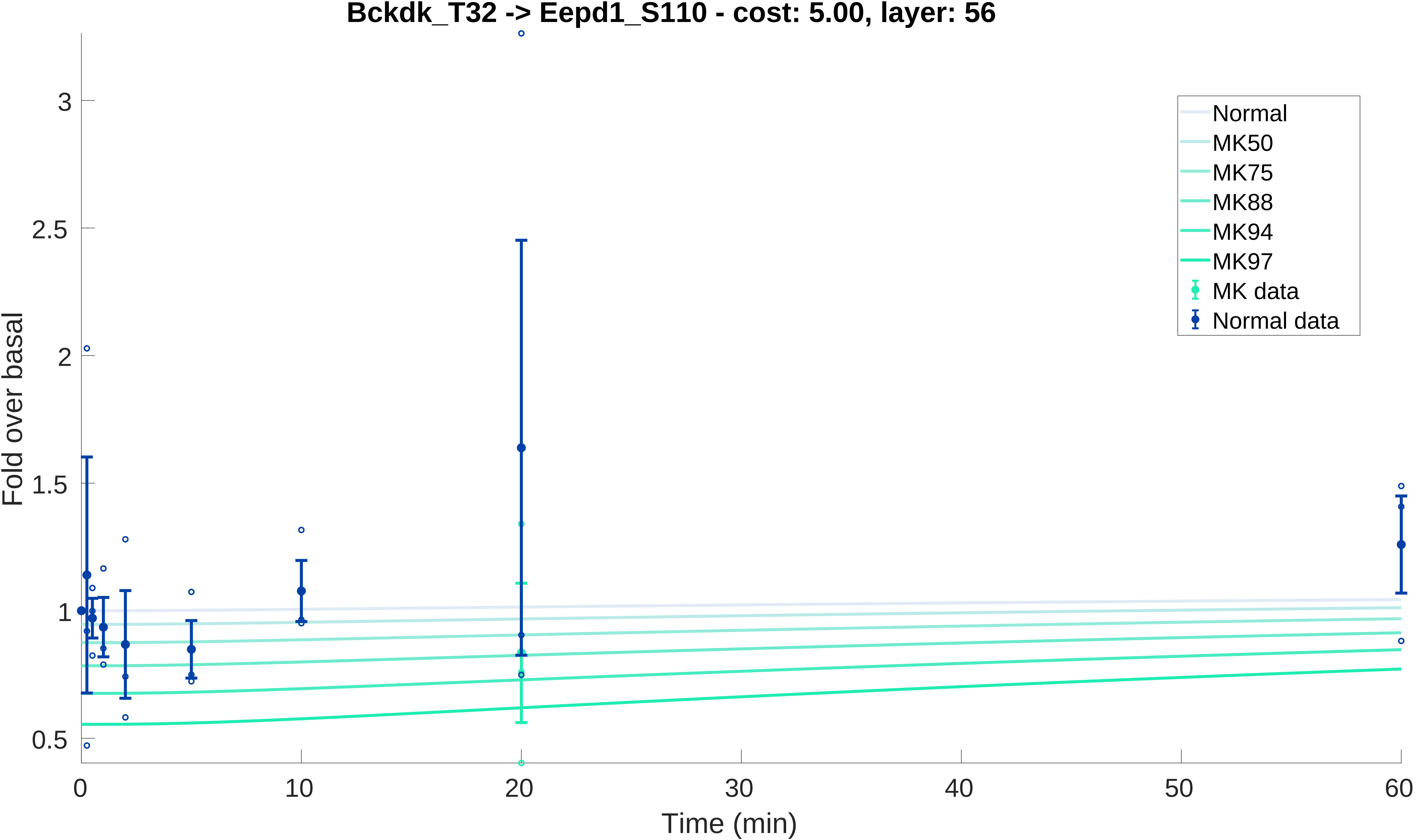

Bckdk_T32 -> Eepd1_S110 - cost: 5.00, layer: 56
Fold over basal
Time (min)
0.5
1
1.5
2
2.5
3
0
10
20
30
40
50
60
Normal
MK50
MK75
MK88
MK94
MK97
MK data
Normal data

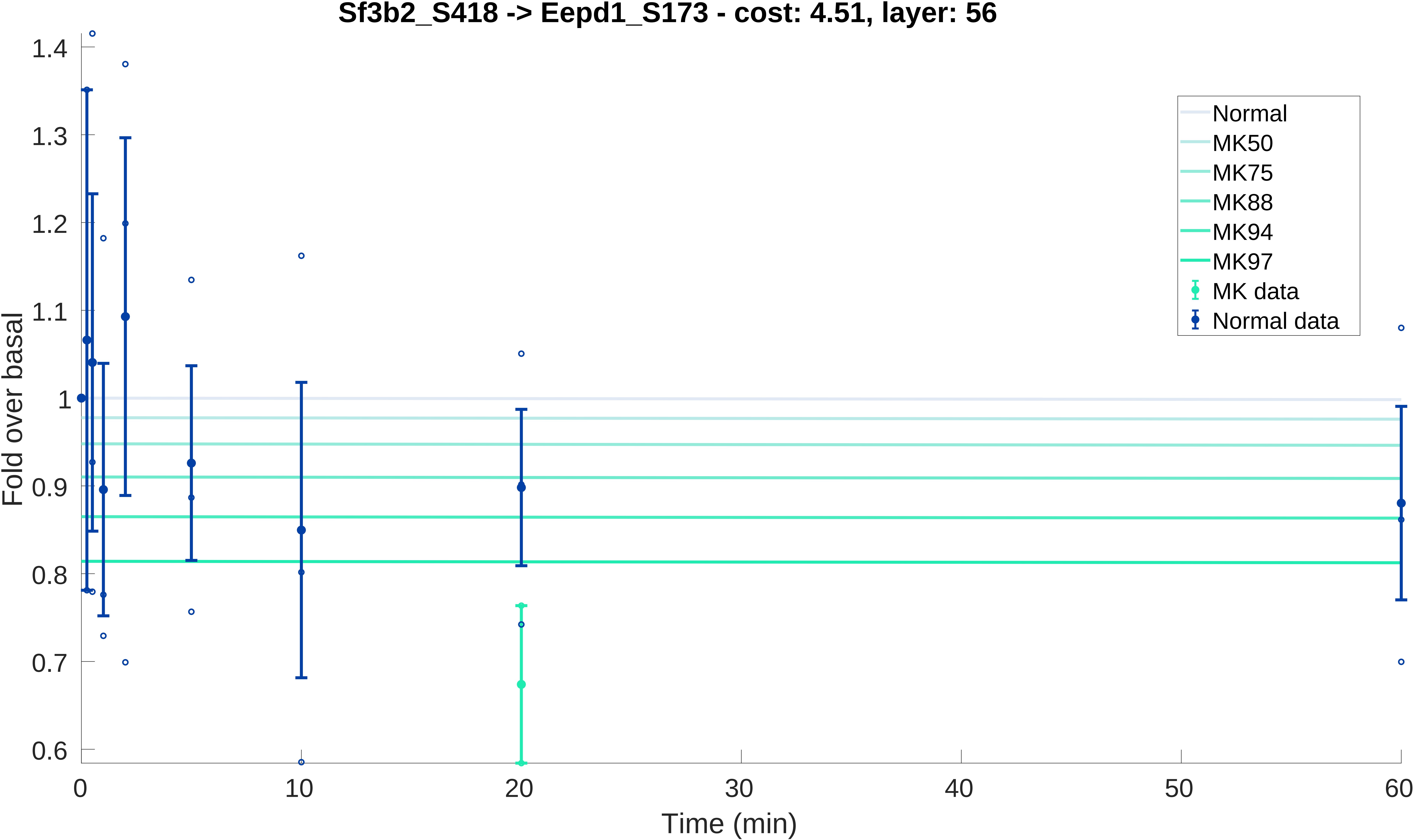

Sf3b2_S418 -> Eepd1_S173 - cost: 4.51, layer: 56
Fold over basal
Time (min)
Normal
MK50
MK75
MK88
MK94
MK97
MK data
Normal data
1.4
1.3
1.2
1.1
1
0.9
0.8
0.7
0.6
0
10
20
30
40
50
60

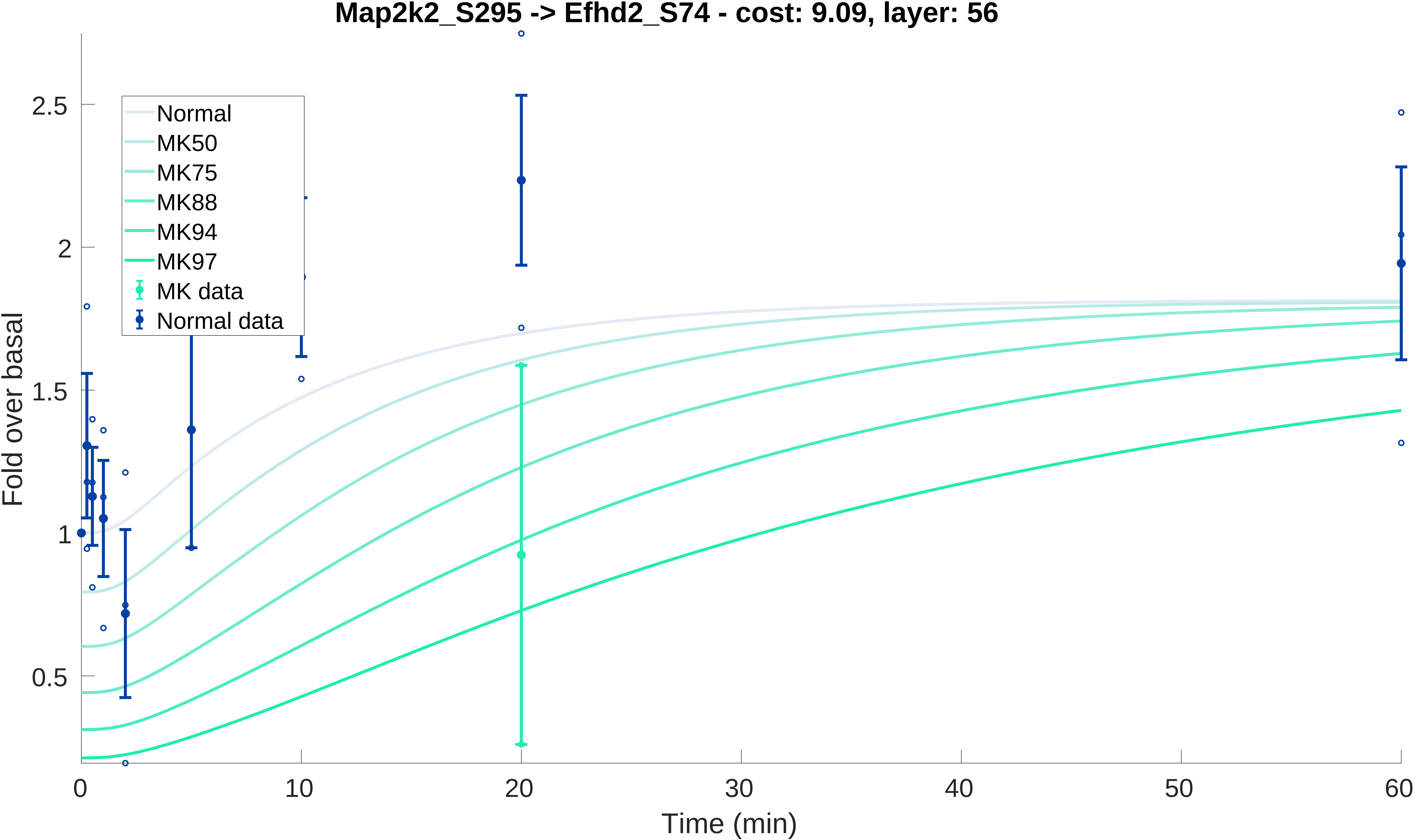
Map2k2_S295 -> Efhd2_S74 - cost: 9.09, layer: 56
Normal
MK50
MK75
MK88
MK94
MK97
MK data
Normal data
Fold over basal
Time (min)
0.5
1
1.5
2
2.5
0
10
20
30
40
50
60

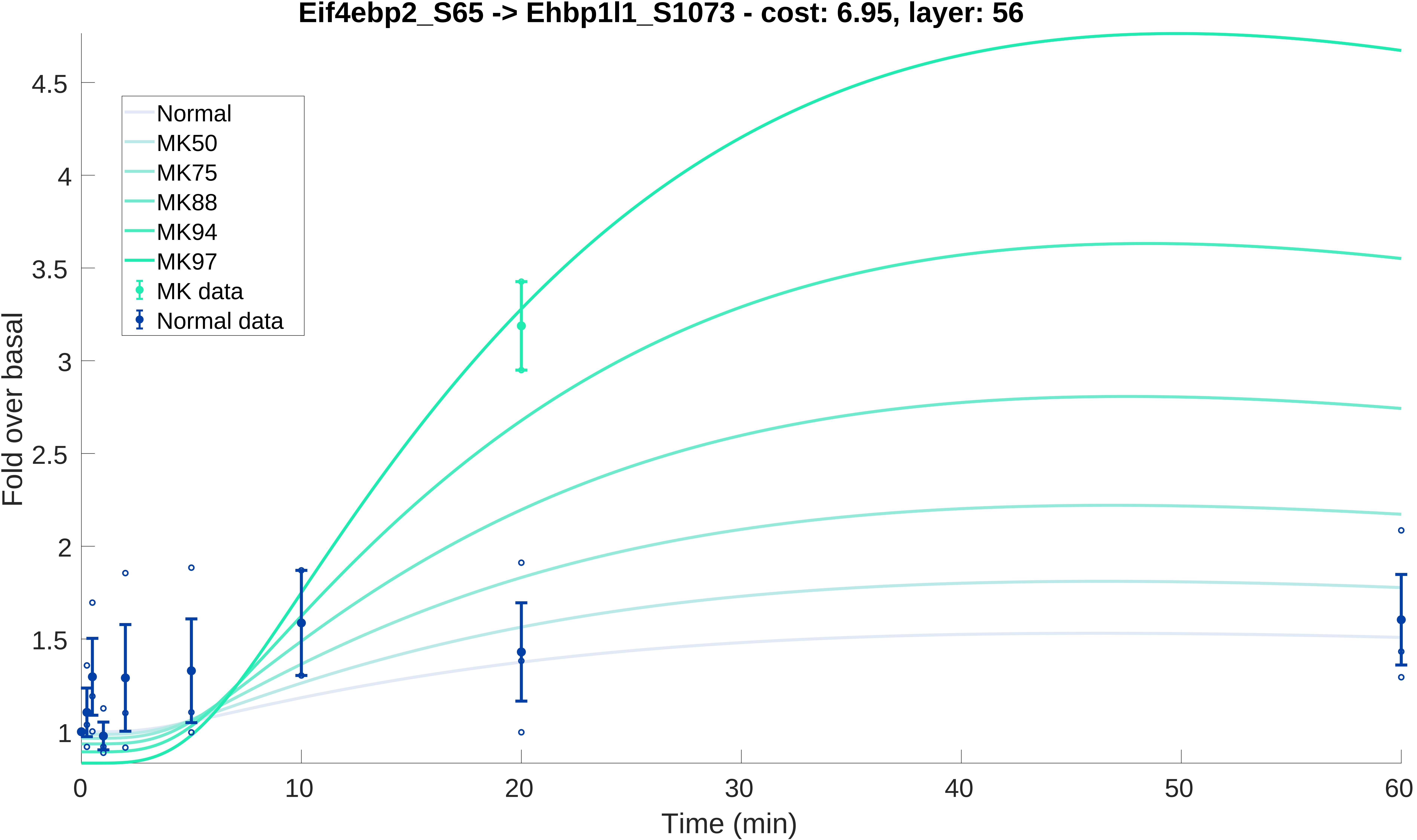

Eif4ebp2_S65 -> Ehbp1l1_S1073 - cost: 6.95, layer: 56
Normal
MK50
MK75
MK88
MK94
MK97
MK data
Normal data
Fold over basal
Time (min)
4.5
4
3.5
3
2.5
2
1.5
1
0
10
20
30
40
50
60

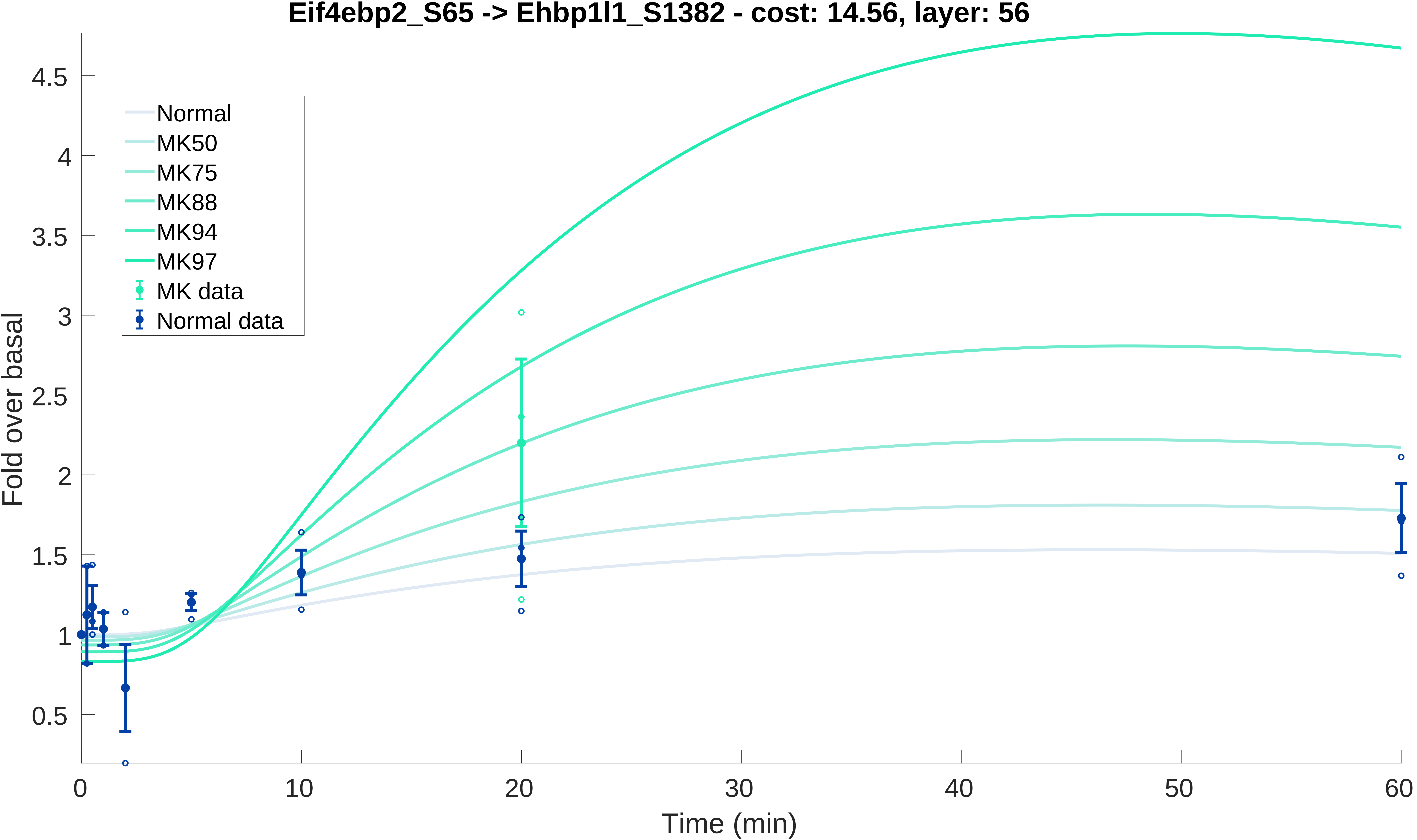

Eif4ebp2_S65 -> Ehbp1l1_S1382 - cost: 14.56, layer: 56
Normal
MK50
MK75
MK88
MK94
MK97
MK data
Normal data
Fold over basal
4.5
4
3.5
3
2.5
2
1.5
1
0.5
0
10
20
30
40
50
60
Time (min)

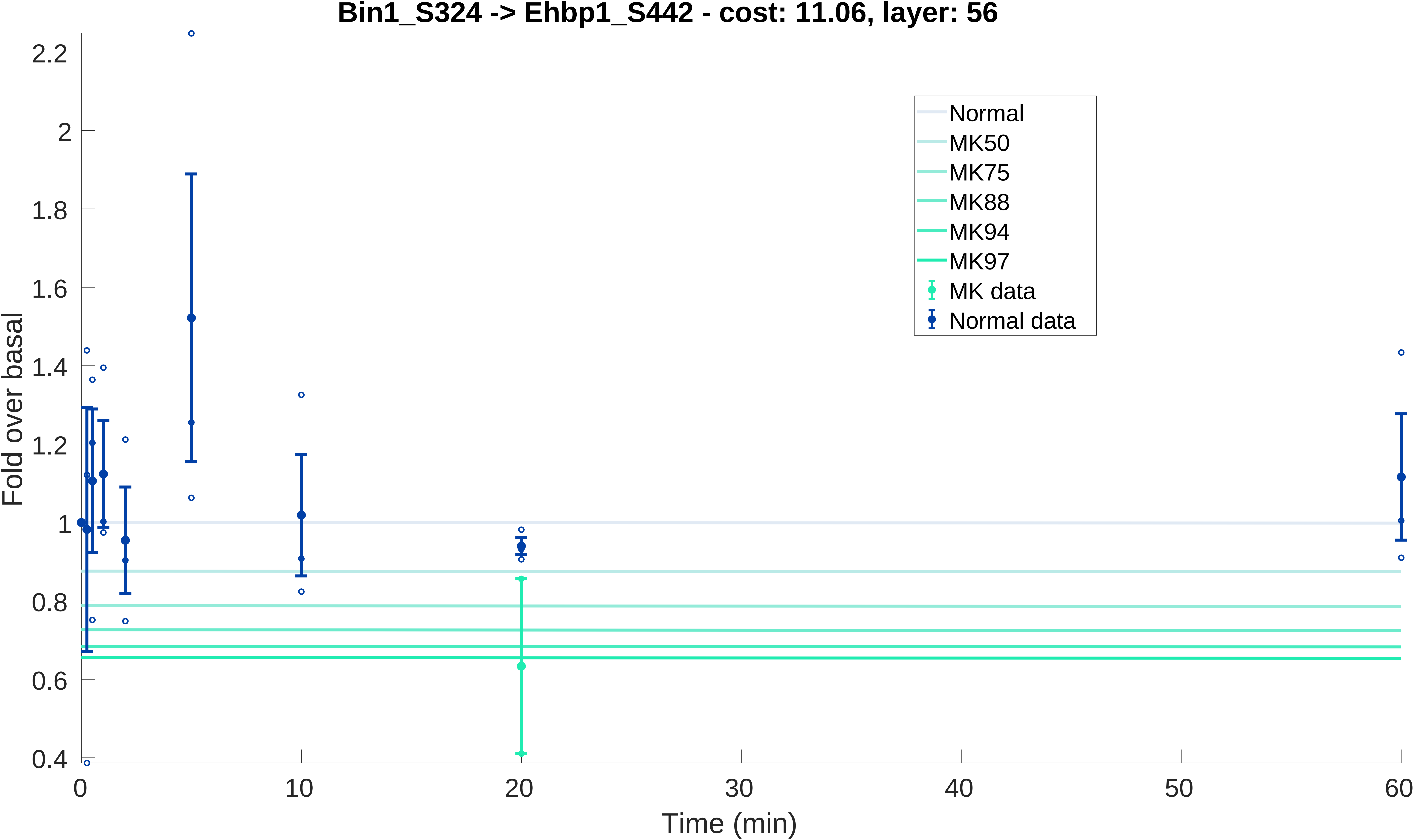

Bin1_S324 -> Ehbp1_S442 - cost: 11.06, layer: 56
Fold over basal
2.2
2
1.8
1.6
1.4
1.2
1
0.8
0.6
0.4
0
10
20
30
40
50
60
Time (min)
Normal
MK50
MK75
MK88
MK94
MK97
MK data
Normal data

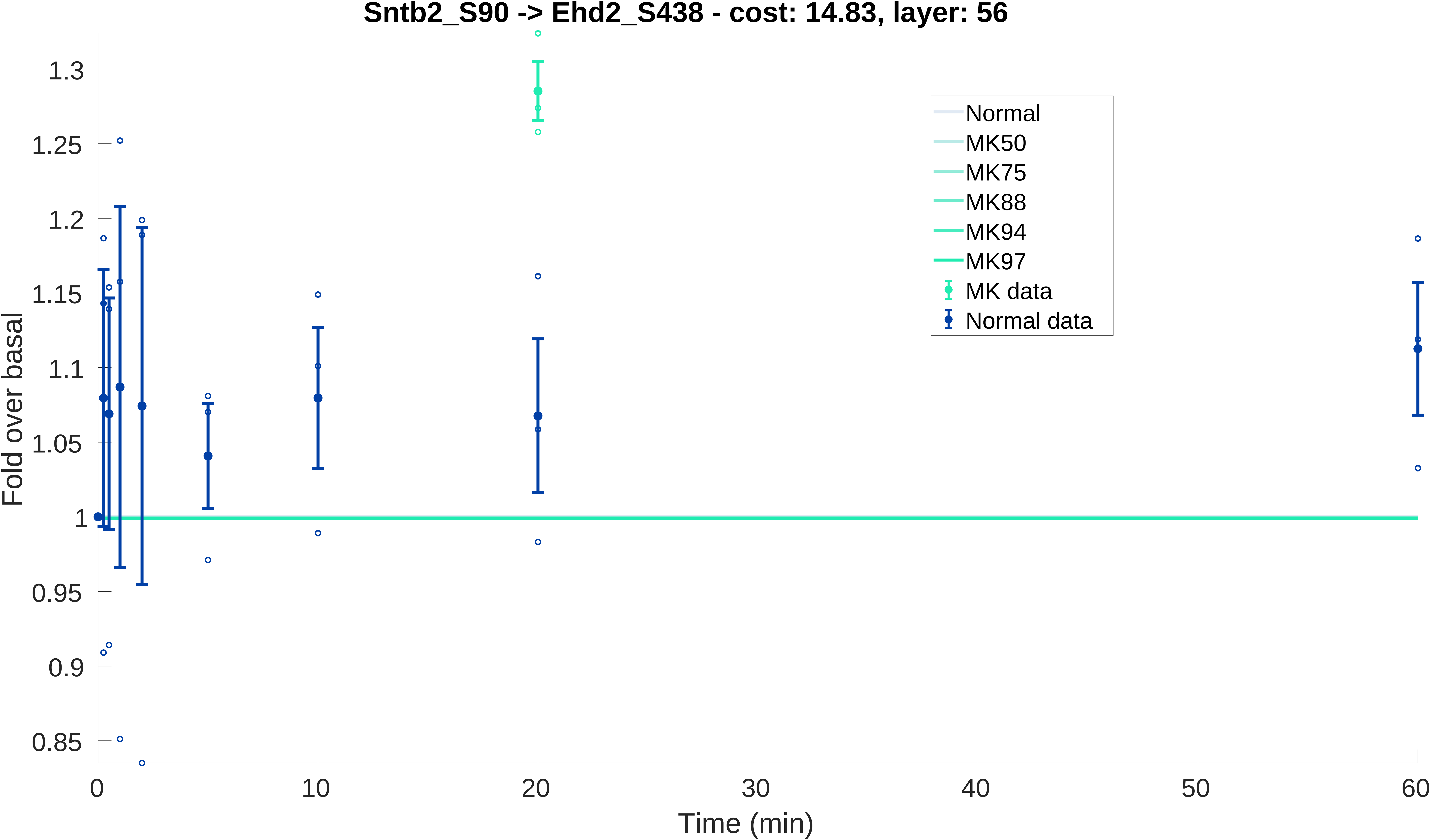

Sntb2_S90 -> Ehd2_S438 - cost: 14.83, layer: 56
Fold over basal
Time (min)
0.85
0.9
0.95
1
1.05
1.1
1.15
1.2
1.25
1.3
0
10
20
30
40
50
60
Normal
MK50
MK75
MK88
MK94
MK97
MK data
Normal data

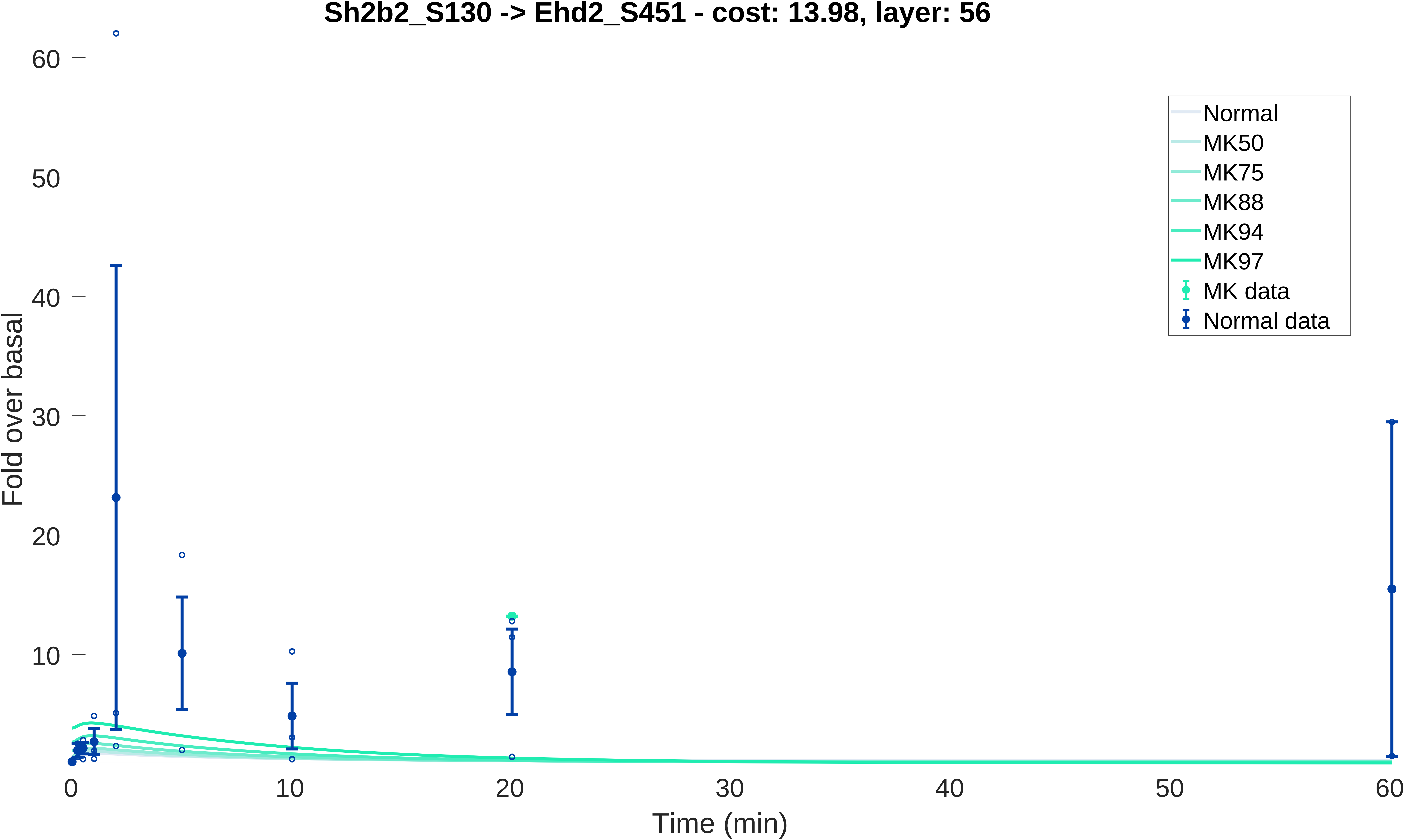

Sh2b2_S130 -> Ehd2_S451 - cost: 13.98, layer: 56
Fold over basal
Time (min)
0
10
20
30
40
50
60
Normal
MK50
MK75
MK88
MK94
MK97
MK data
Normal data

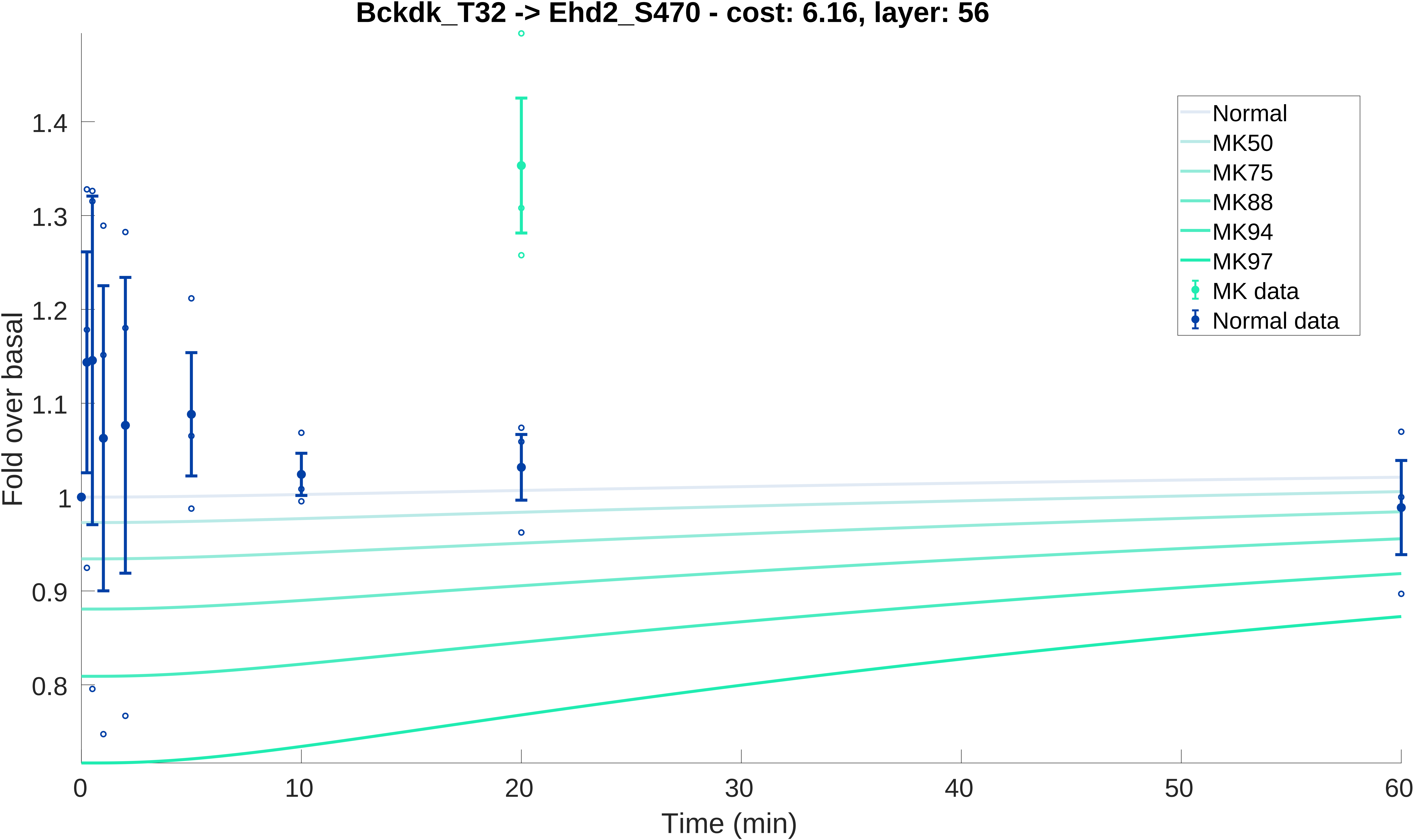

Bckdk_T32 -> Ehd2_S470 - cost: 6.16, layer: 56
Fold over basal
Time (min)
0.8
0.9
1
1.1
1.2
1.3
1.4
0
10
20
30
40
50
60
Normal
MK50
MK75
MK88
MK94
MK97
MK data
Normal data

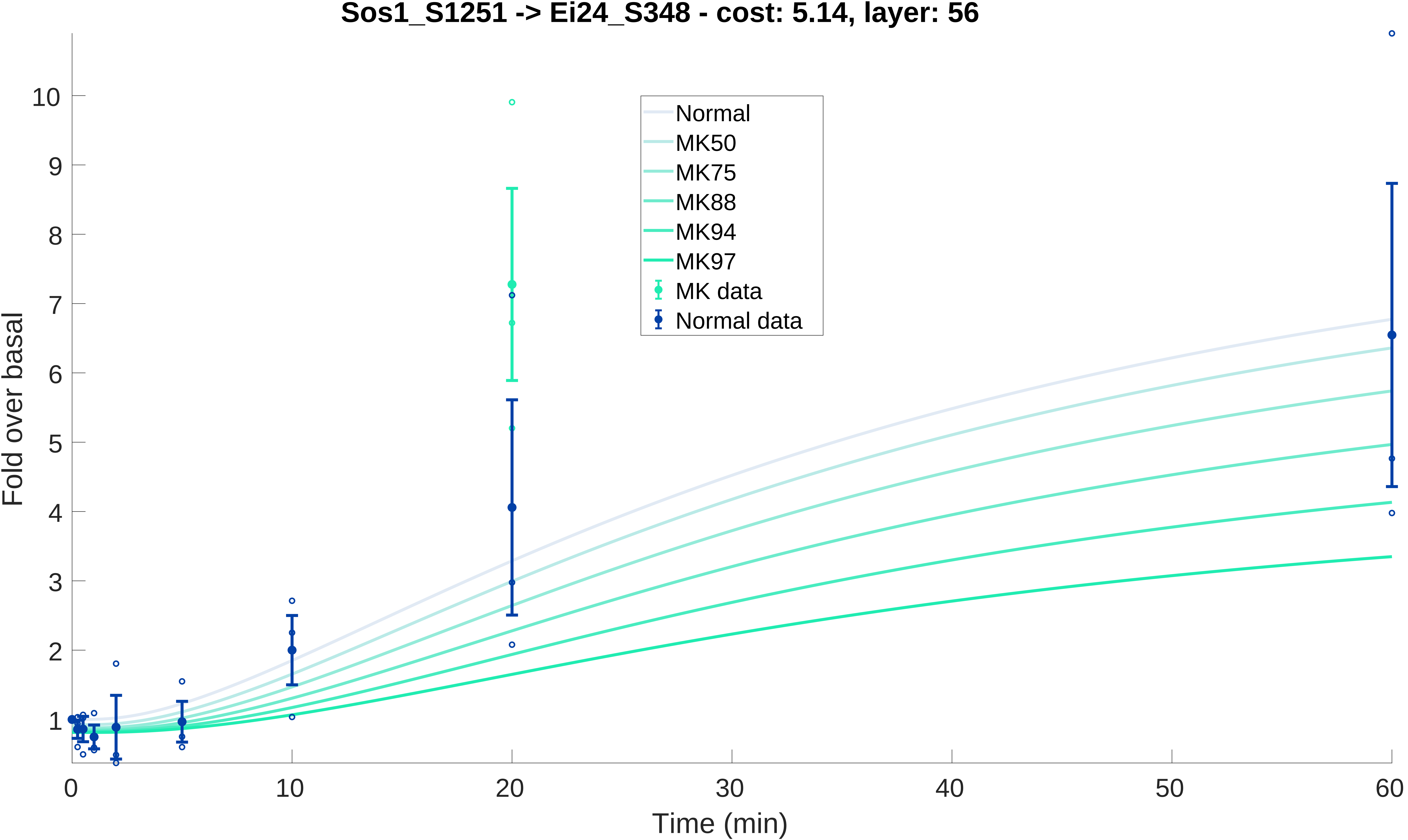

Sos1_S1251 -> Ei24_S348 - cost: 5.14, layer: 56
Fold over basal
Time (min)
Normal
MK50
MK75
MK88
MK94
MK97
MK data
Normal data
0
10
20
30
40
50
60
1
2
3
4
5
6
7
8
9
10

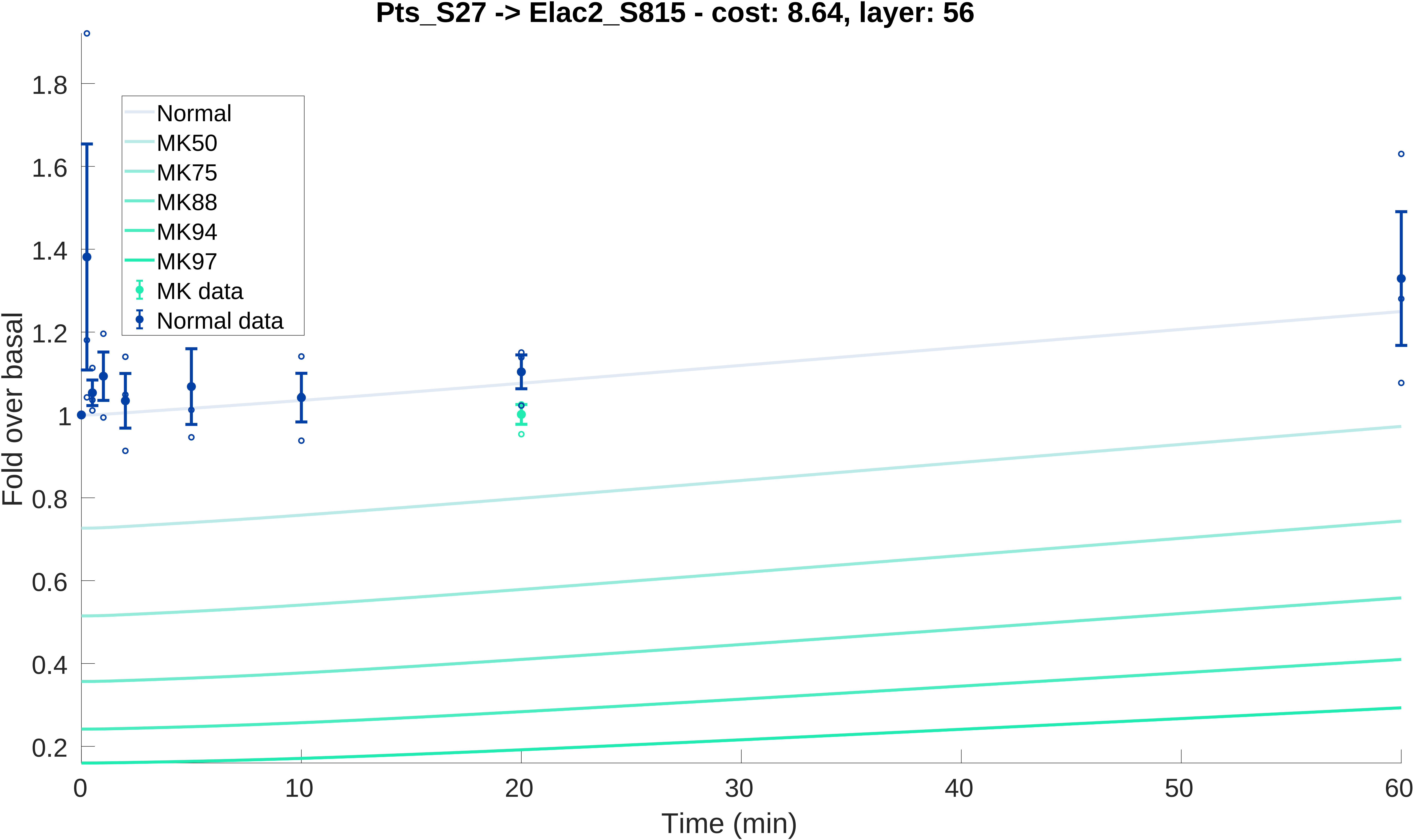

Pts_S27 -> Elac2_S815 - cost: 8.64, layer: 56
Fold over basal
Time (min)
Normal
MK50
MK75
MK88
MK94
MK97
MK data
Normal data
0
10
20
30
40
50
60
0.2
0.4
0.6
0.8
1
1.2
1.4
1.6
1.8

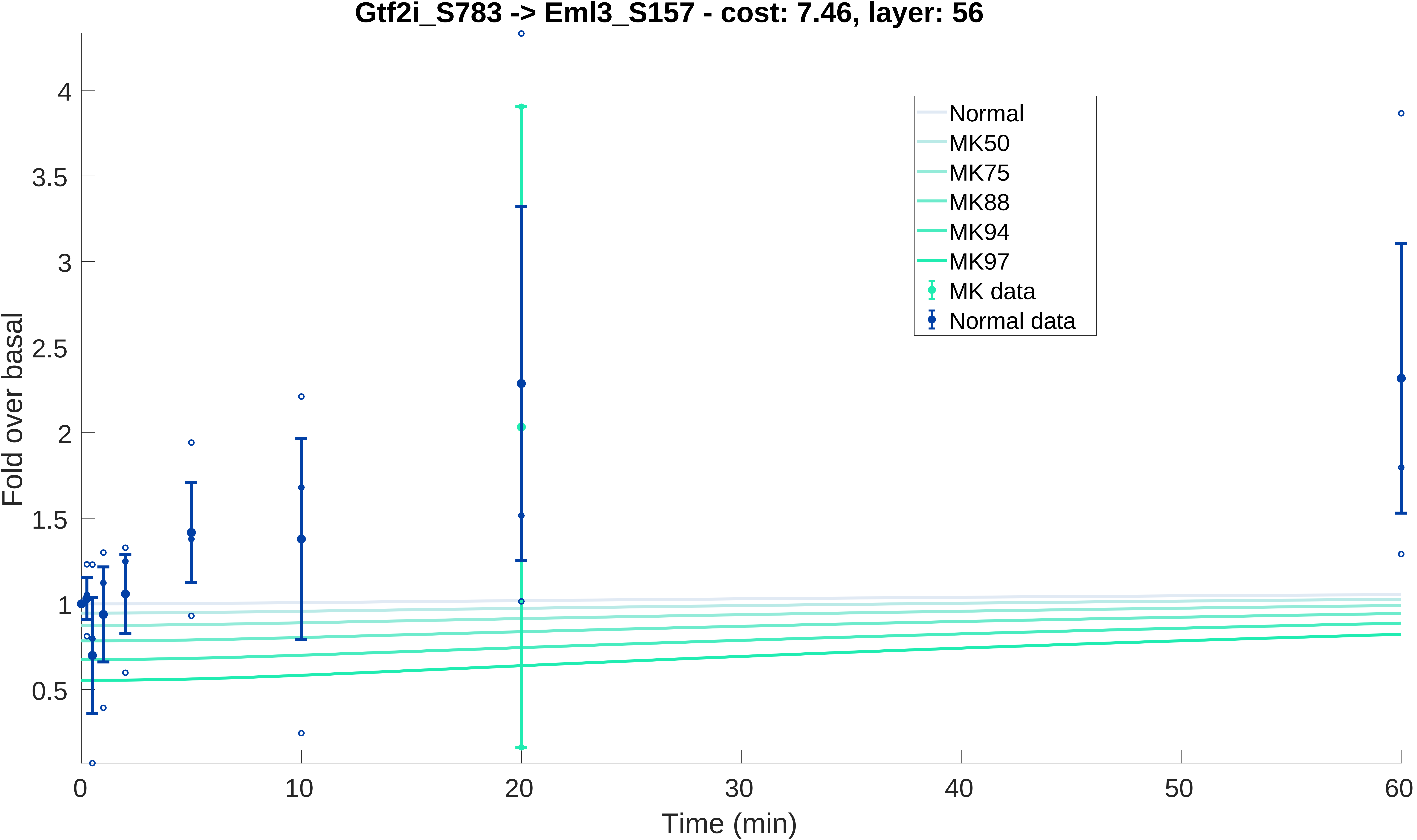
Gtf2i_S783 -> Eml3_S157 - cost: 7.46, layer: 56
Fold over basal
Time (min)
0
10
20
30
40
50
60
0.5
1
1.5
2
2.5
3
3.5
4
Normal
MK50
MK75
MK88
MK94
MK97
MK data
Normal data

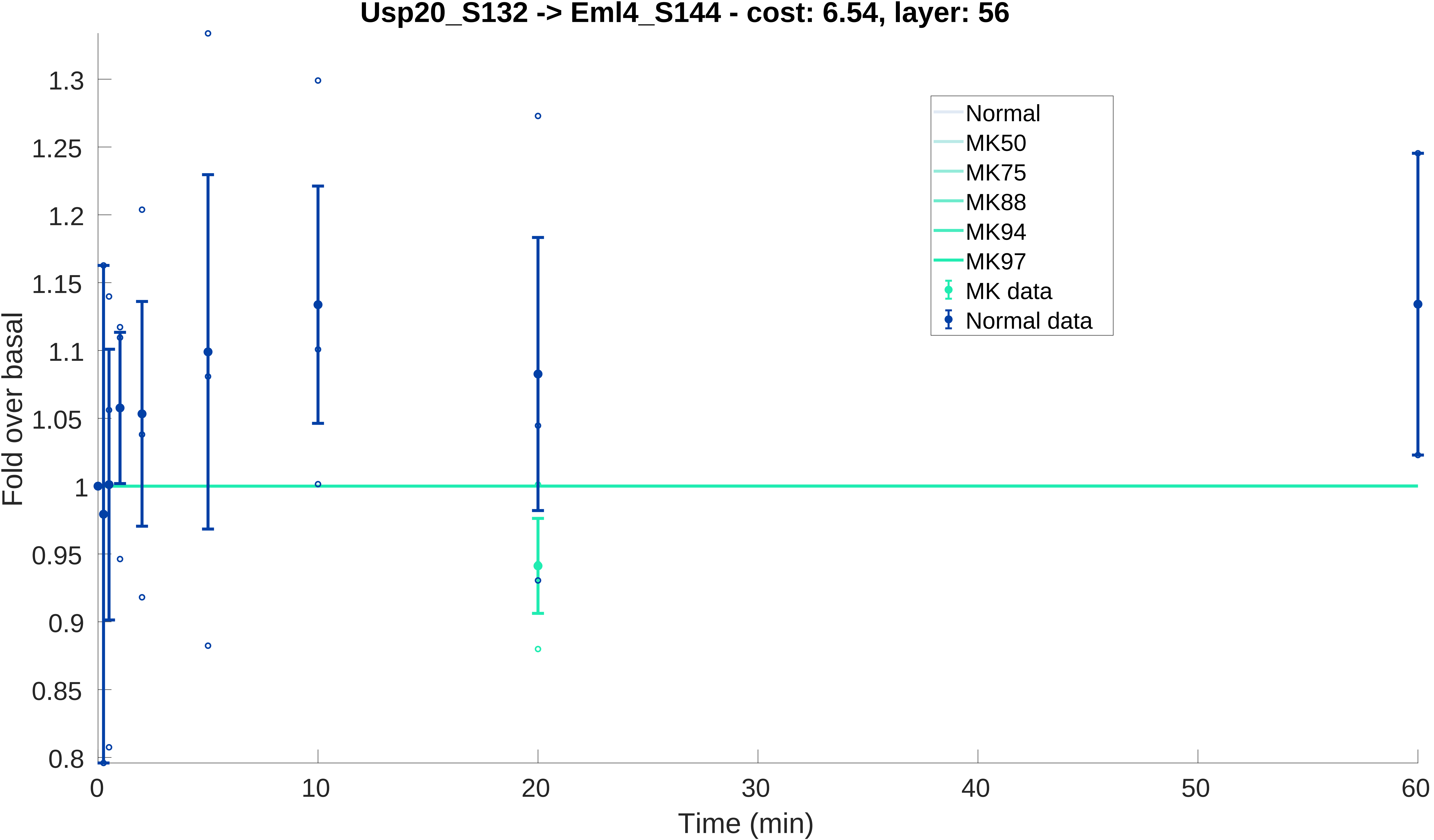

Usp20_S132 -> Eml4_S144 - cost: 6.54, layer: 56
Fold over basal
1.3
1.25
1.2
1.15
1.1
1.05
1
0.95
0.9
0.85
0.8
0
10
20
30
40
50
60
Time (min)
Normal
MK50
MK75
MK88
MK94
MK97
MK data
Normal data

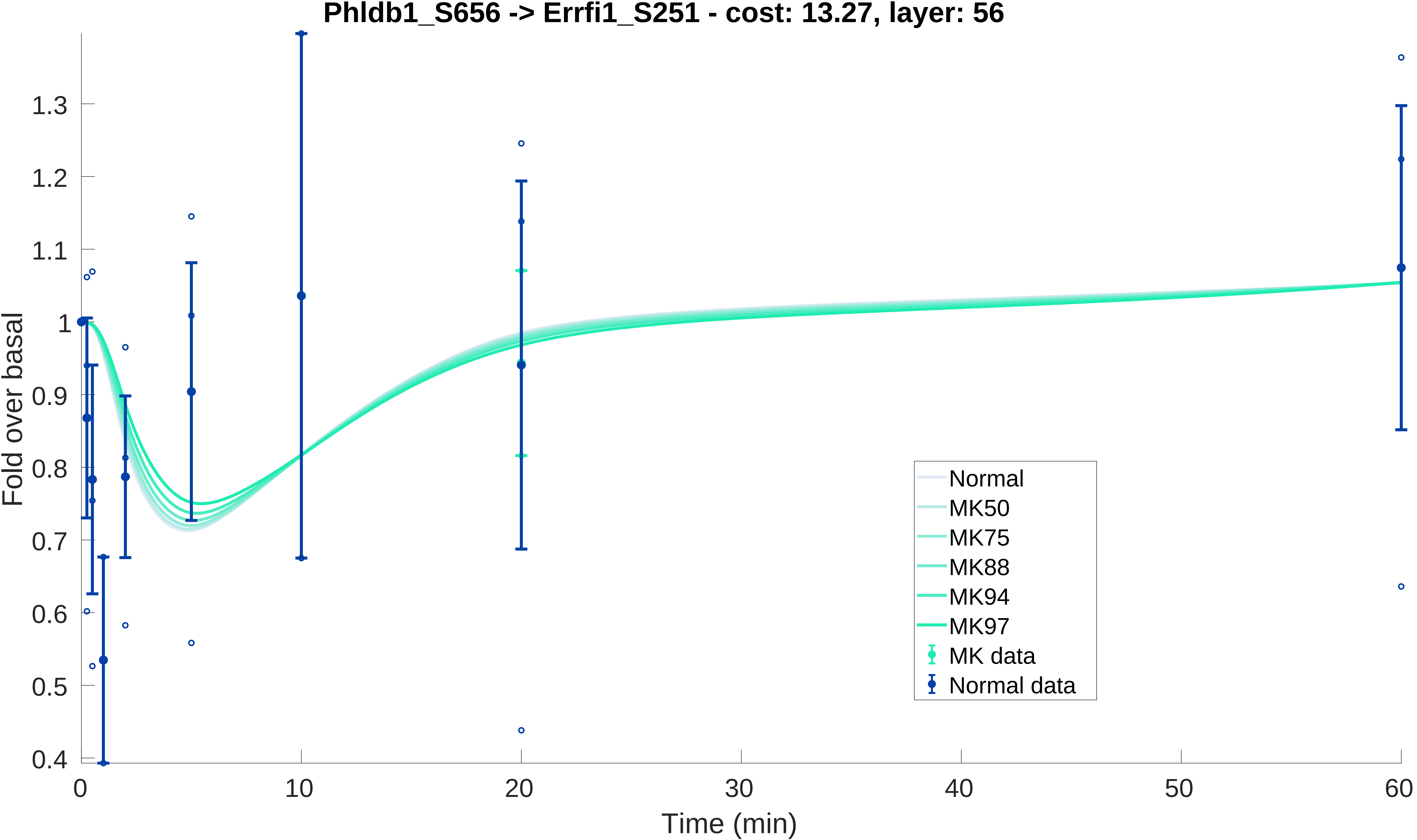
Phldb1_S656 -> Errfi1_S251 - cost: 13.27, layer: 56
Fold over basal
Time (min)
1.3
1.2
1.1
1
0.9
0.8
0.7
0.6
0.5
0.4
0
10
20
30
40
50
60
Normal
MK50
MK75
MK88
MK94
MK97
MK data
Normal data

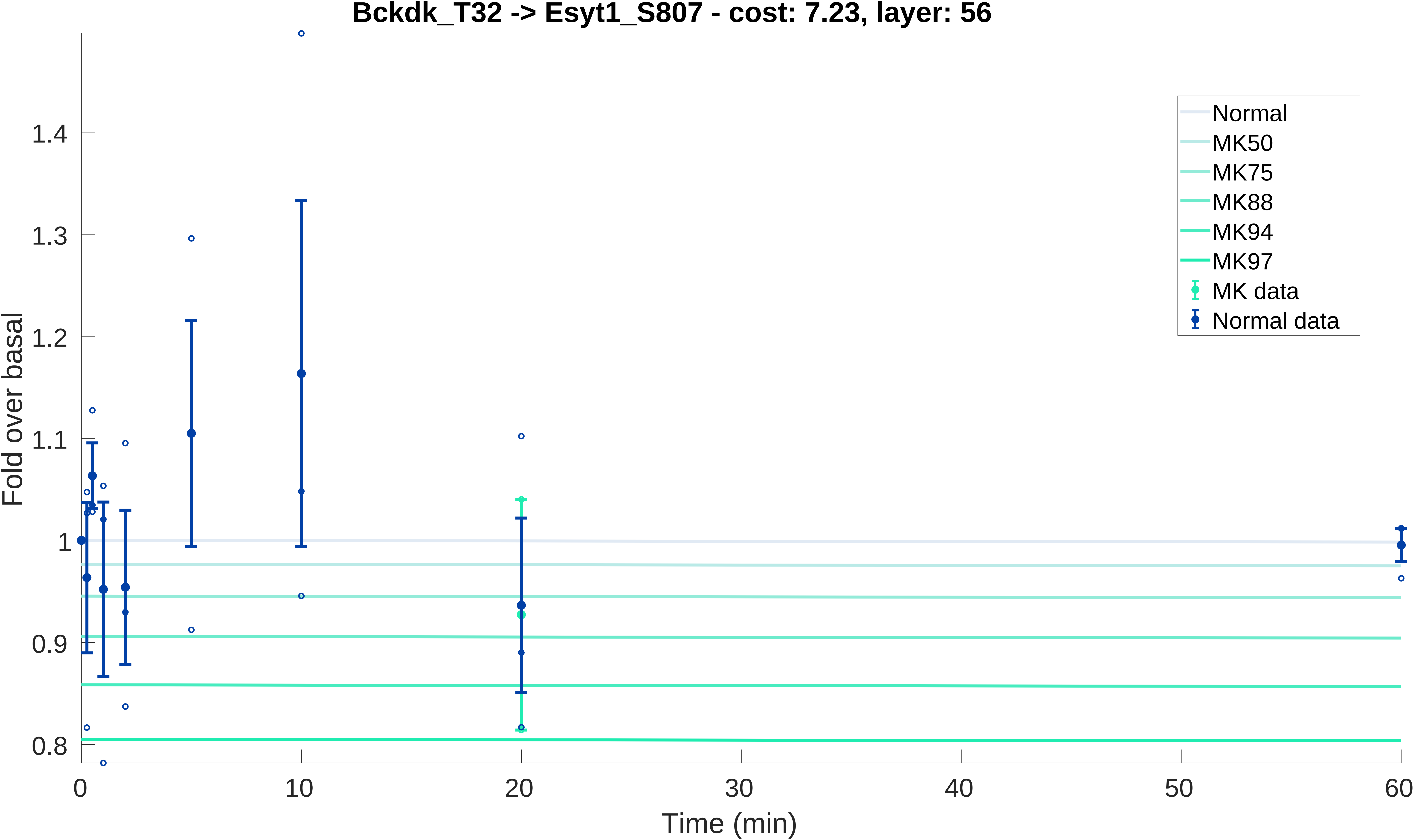

Bckdk_T32 -> Esyt1_S807 - cost: 7.23, layer: 56
Fold over basal
1.4
1.3
1.2
1.1
1
0.9
0.8
0
10
20
30
40
50
60
Time (min)
Normal
MK50
MK75
MK88
MK94
MK97
MK data
Normal data

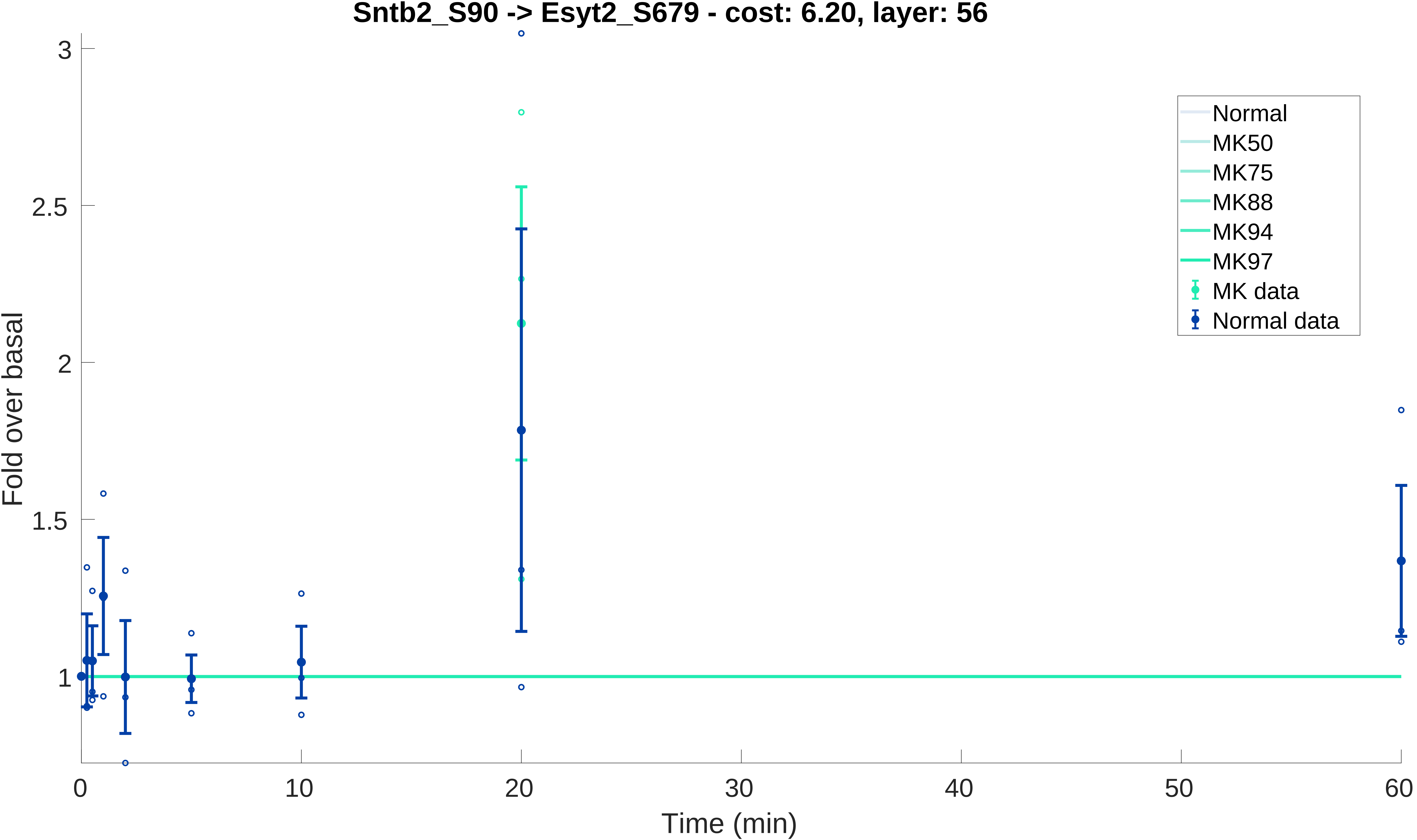

Sntb2_S90 -> Esyt2_S679 - cost: 6.20, layer: 56
Fold over basal
Time (min)
3
2.5
2
1.5
1
0
10
20
30
40
50
60
Normal
MK50
MK75
MK88
MK94
MK97
MK data
Normal data

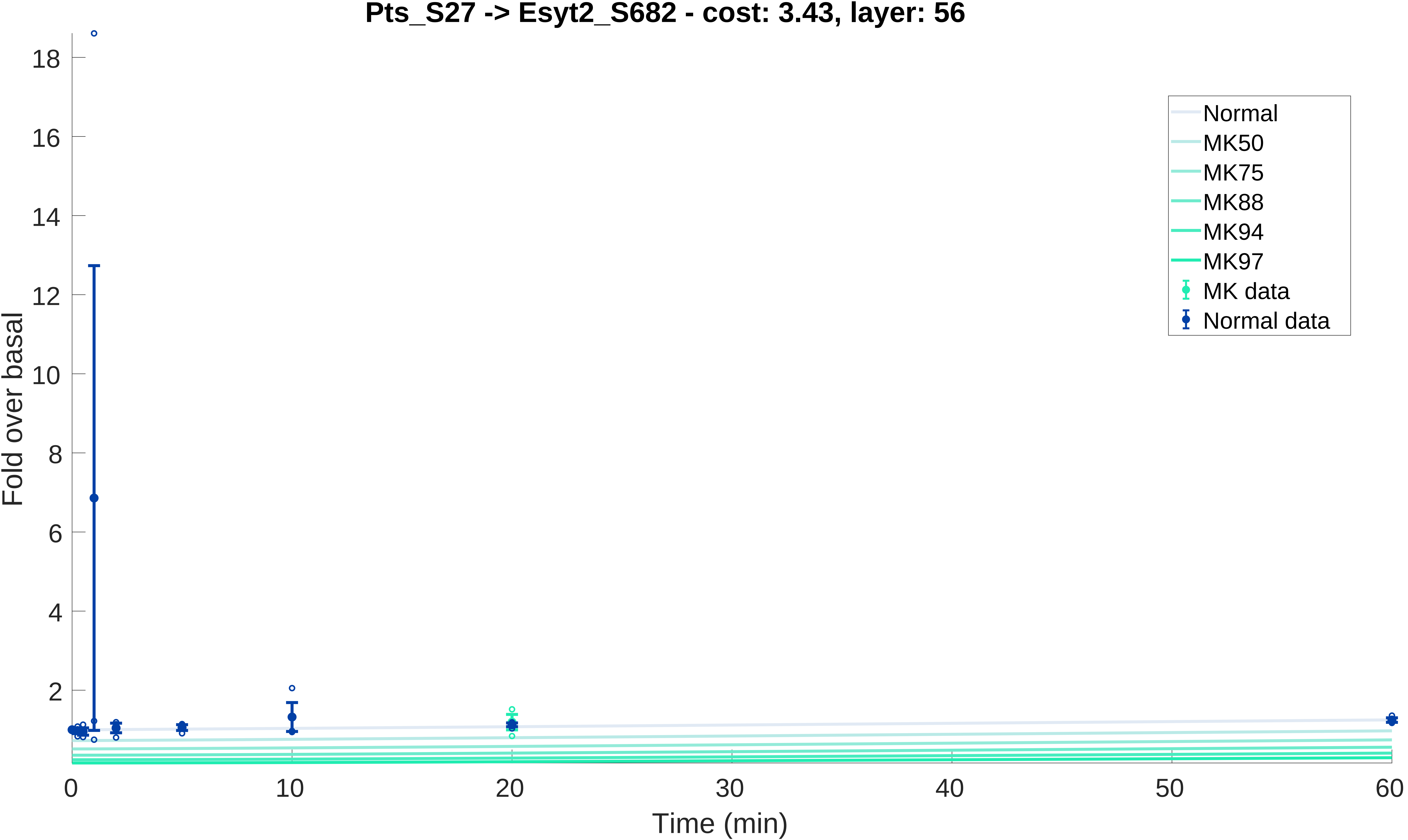

Pts_S27 -> Esyt2_S682 - cost: 3.43, layer: 56
Fold over basal
Time (min)
Normal
MK50
MK75
MK88
MK94
MK97
MK data
Normal data

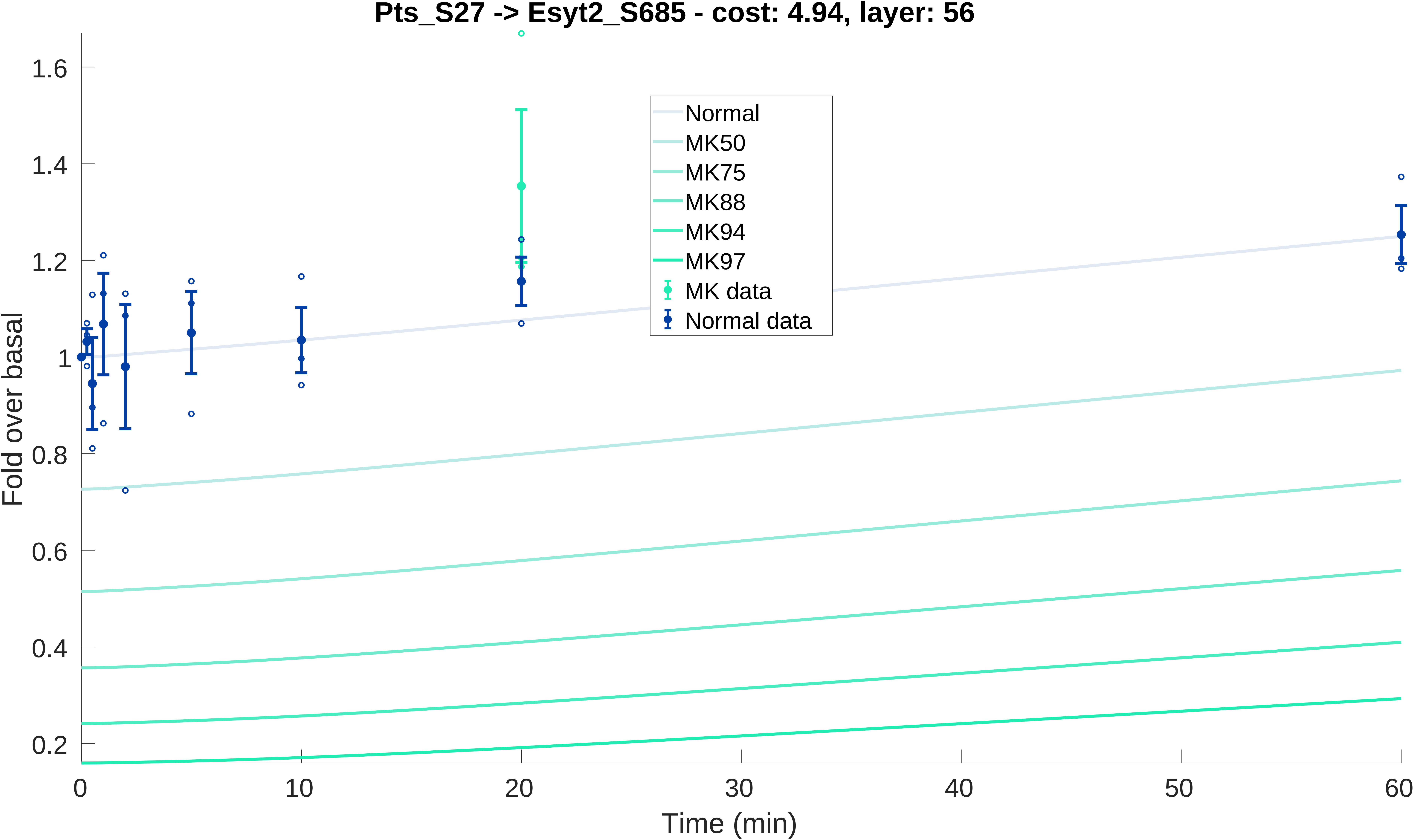
Pts_S27 -> Esyt2_S685 - cost: 4.94, layer: 56
Fold over basal
Time (min)
Normal
MK50
MK75
MK88
MK94
MK97
MK data
Normal data
0.2
0.4
0.6
0.8
1
1.2
1.4
1.6
0
10
20
30
40
50
60

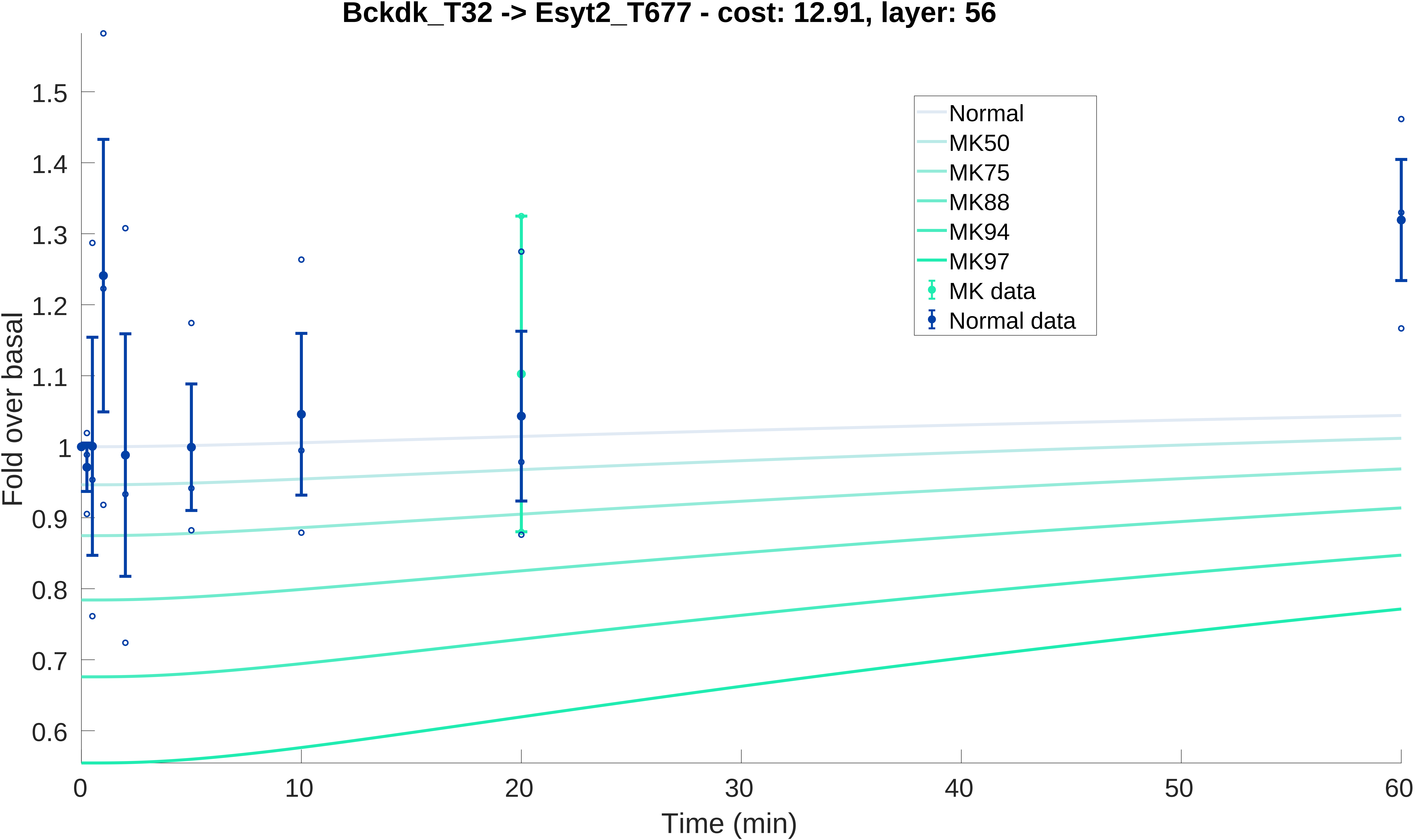

Bckdk_T32 -> Esyt2_T677 - cost: 12.91, layer: 56
Fold over basal
1.5
1.4
1.3
1.2
1.1
1
0.9
0.8
0.7
0.6
0
10
20
30
40
50
60
Time (min)
Normal
MK50
MK75
MK88
MK94
MK97
MK data
Normal data

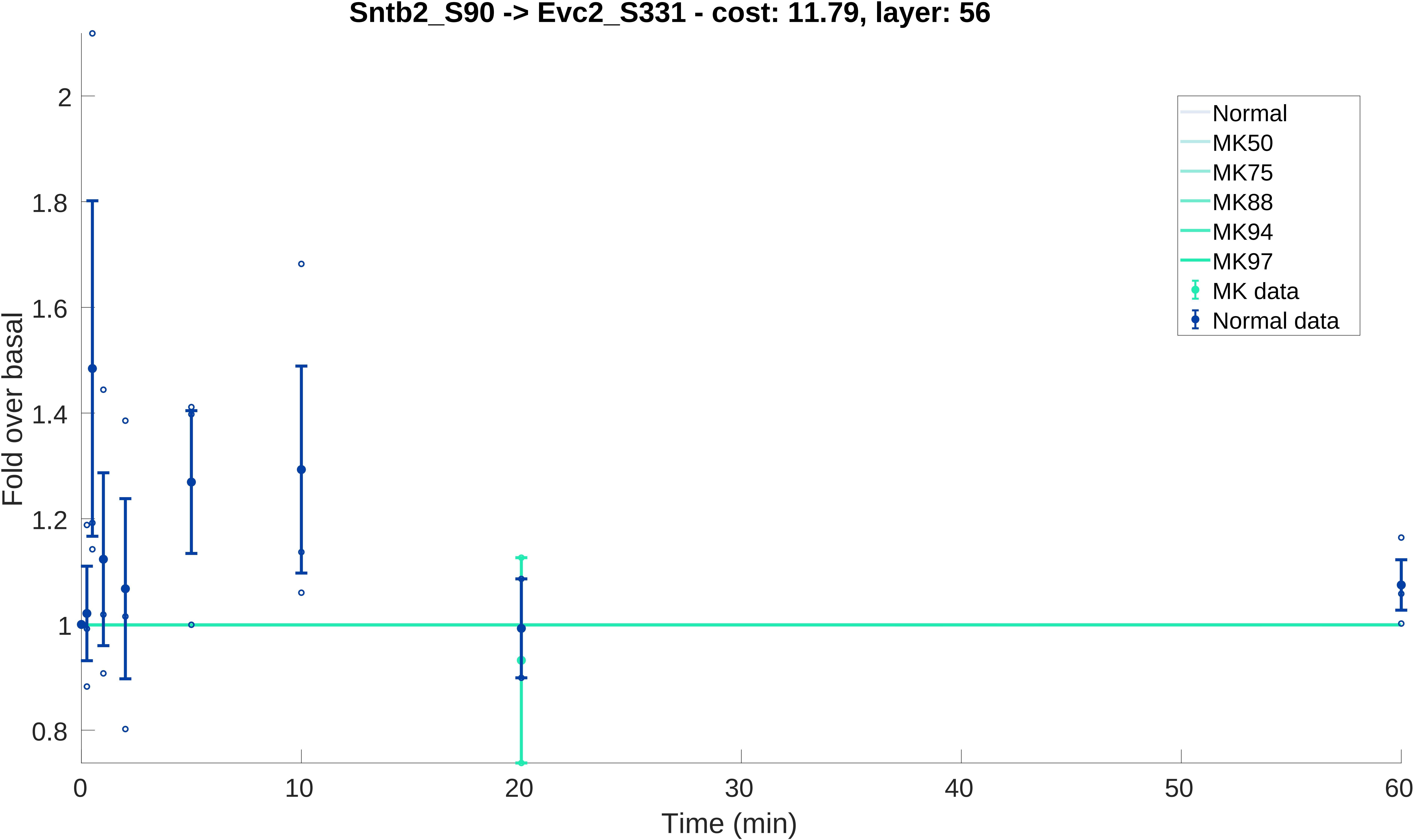
Sntb2_S90 -> Evc2_S331 - cost: 11.79, layer: 56
Fold over basal
Time (min)
0.8
1
1.2
1.4
1.6
1.8
2
0
10
20
30
40
50
60
Normal
MK50
MK75
MK88
MK94
MK97
MK data
Normal data

Pts_S27 -> Exoc1_S458 - cost: 12.37, layer: 56

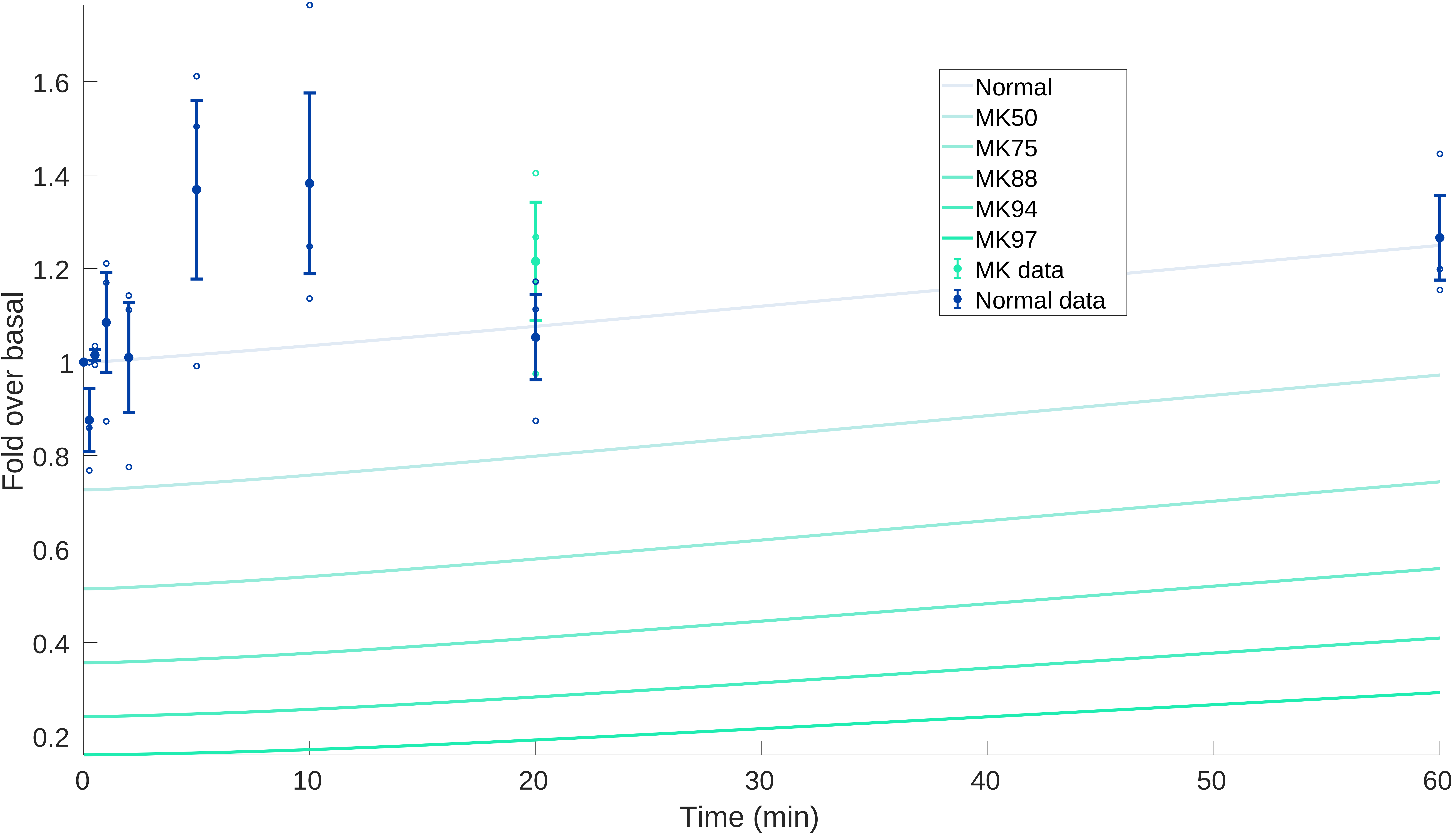

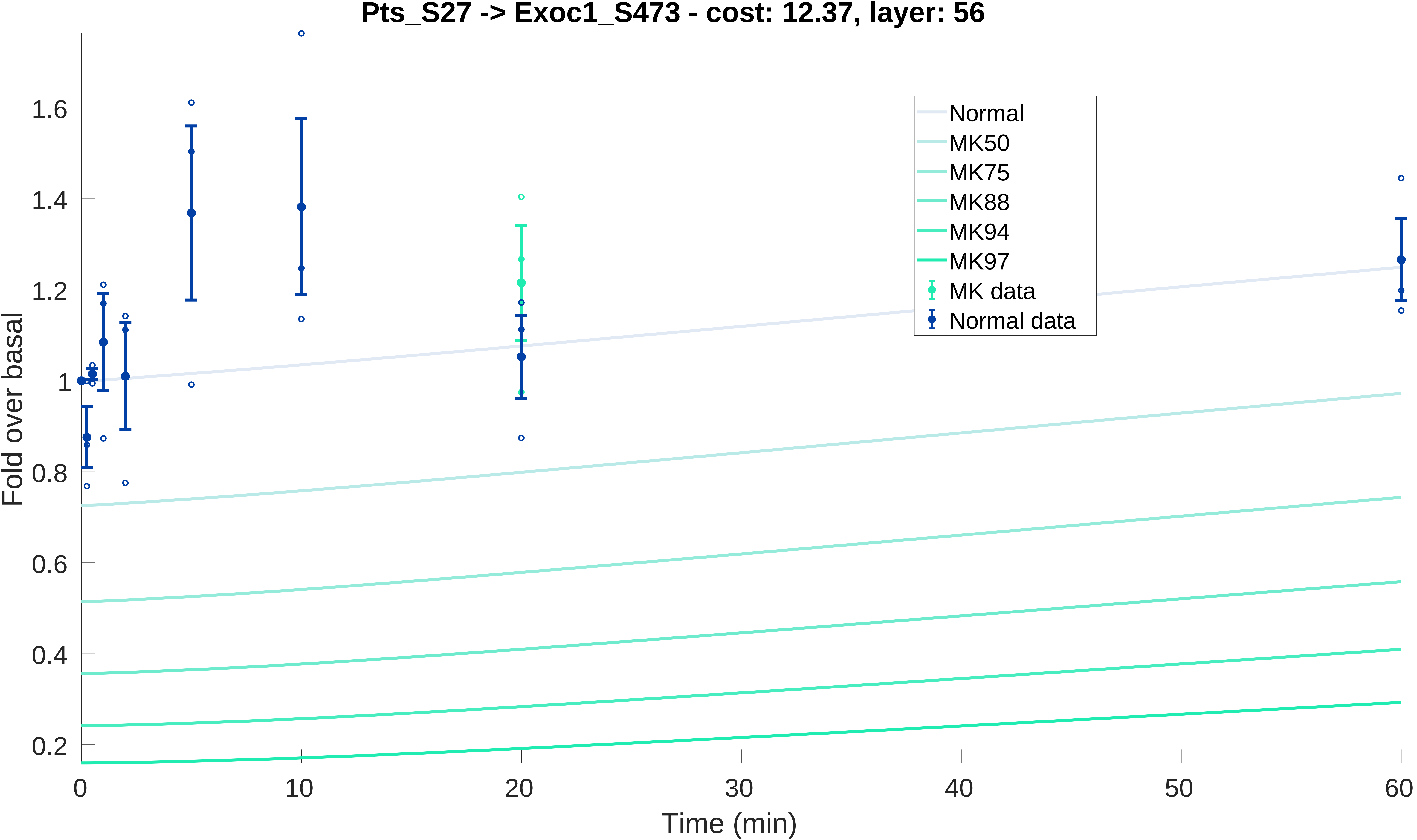

Pts_S27 -> Exoc1_S473 - cost: 12.37, layer: 56
Fold over basal
Time (min)
Normal
MK50
MK75
MK88
MK94
MK97
MK data
Normal data
0.2
0.4
0.6
0.8
1
1.2
1.4
1.6
0
10
20
30
40
50
60

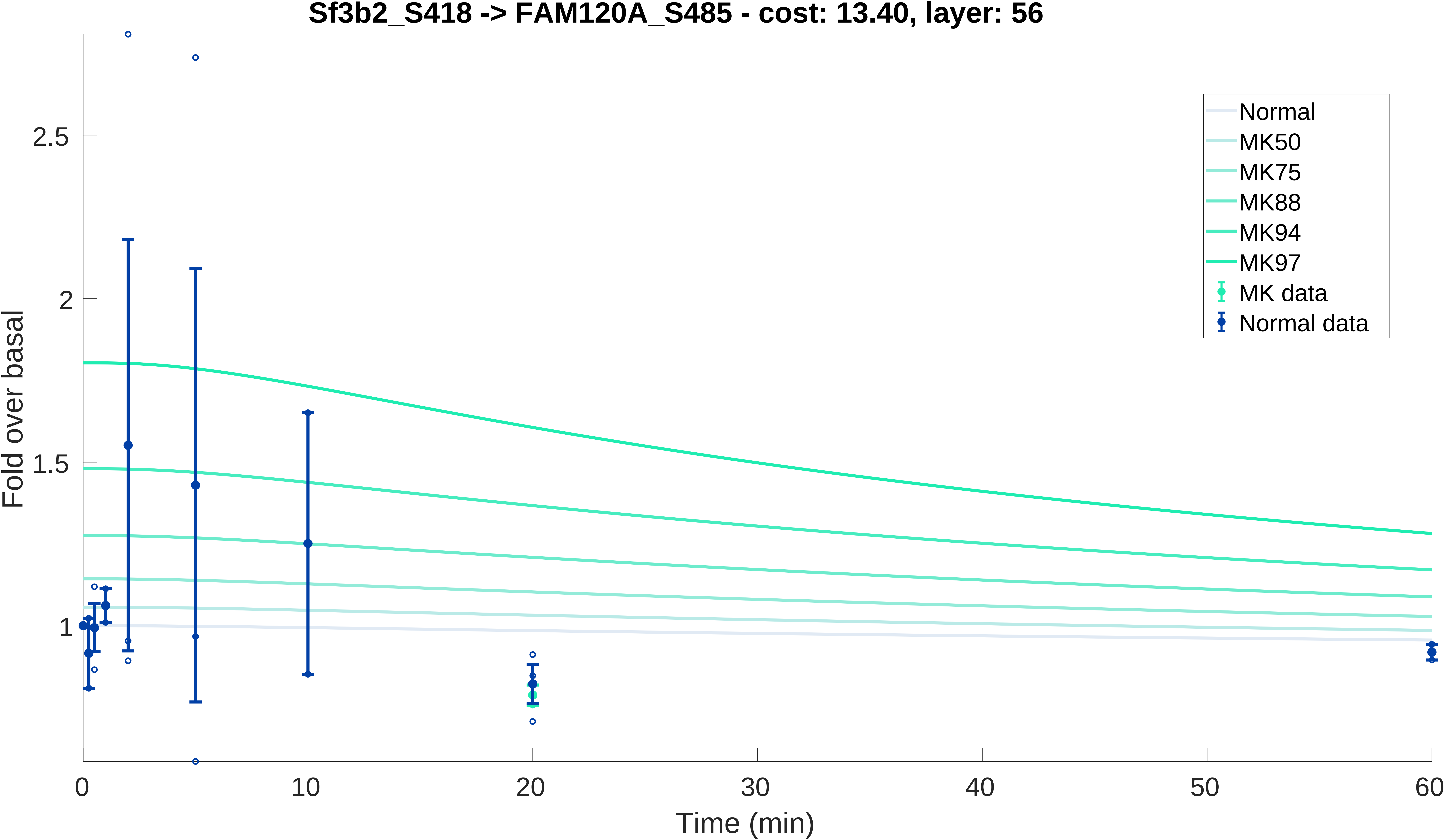

Sf3b2_S418 -> FAM120A_S485 - cost: 13.40, layer: 56
Fold over basal
2.5
2
1.5
1
0
10
20
30
40
50
60
Time (min)
Normal
MK50
MK75
MK88
MK94
MK97
MK data
Normal data

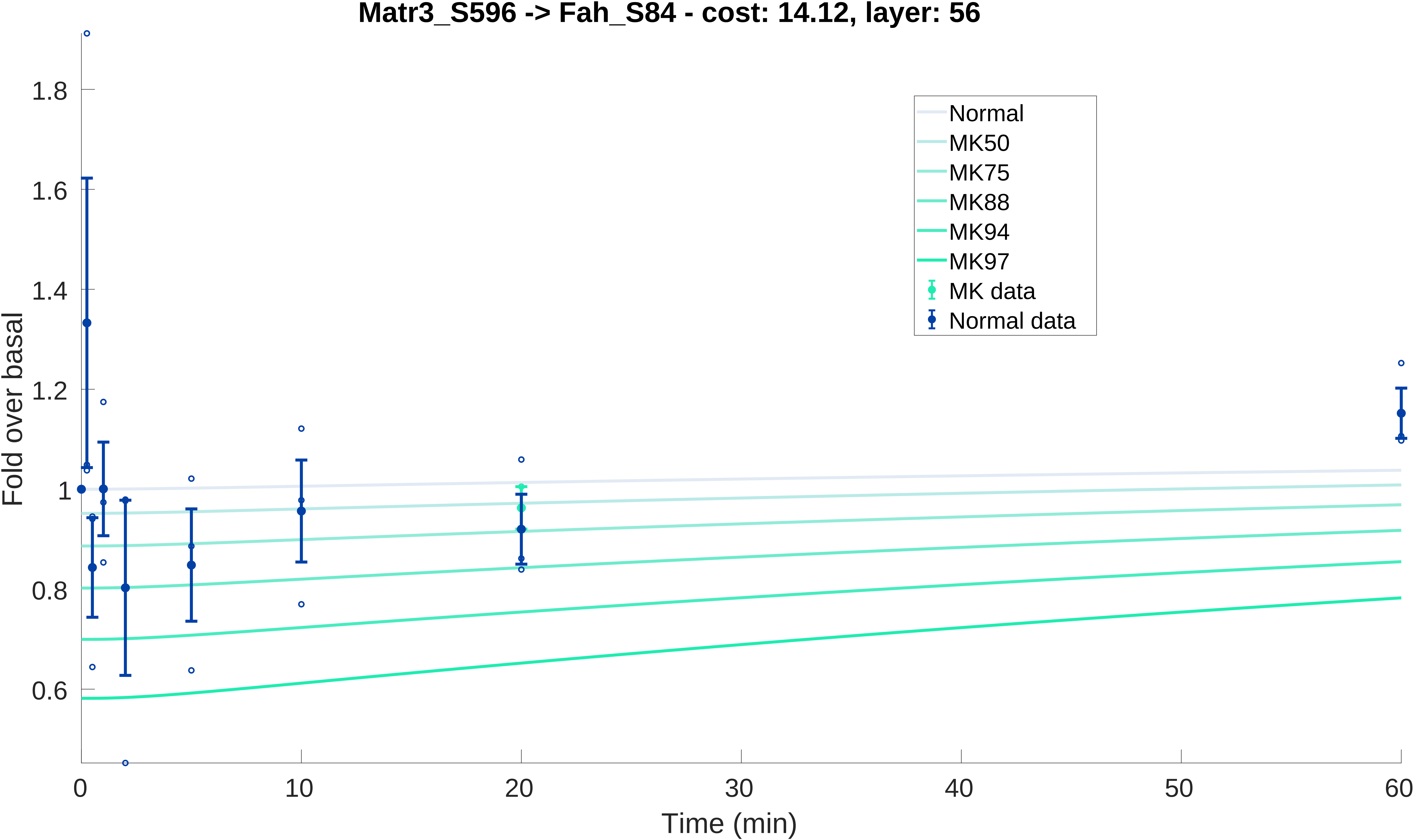

Matr3_S596 -> Fah_S84 - cost: 14.12, layer: 56
Fold over basal
Time (min)
1.8
1.6
1.4
1.2
1
0.8
0.6
0
10
20
30
40
50
60
Normal
MK50
MK75
MK88
MK94
MK97
MK data
Normal data

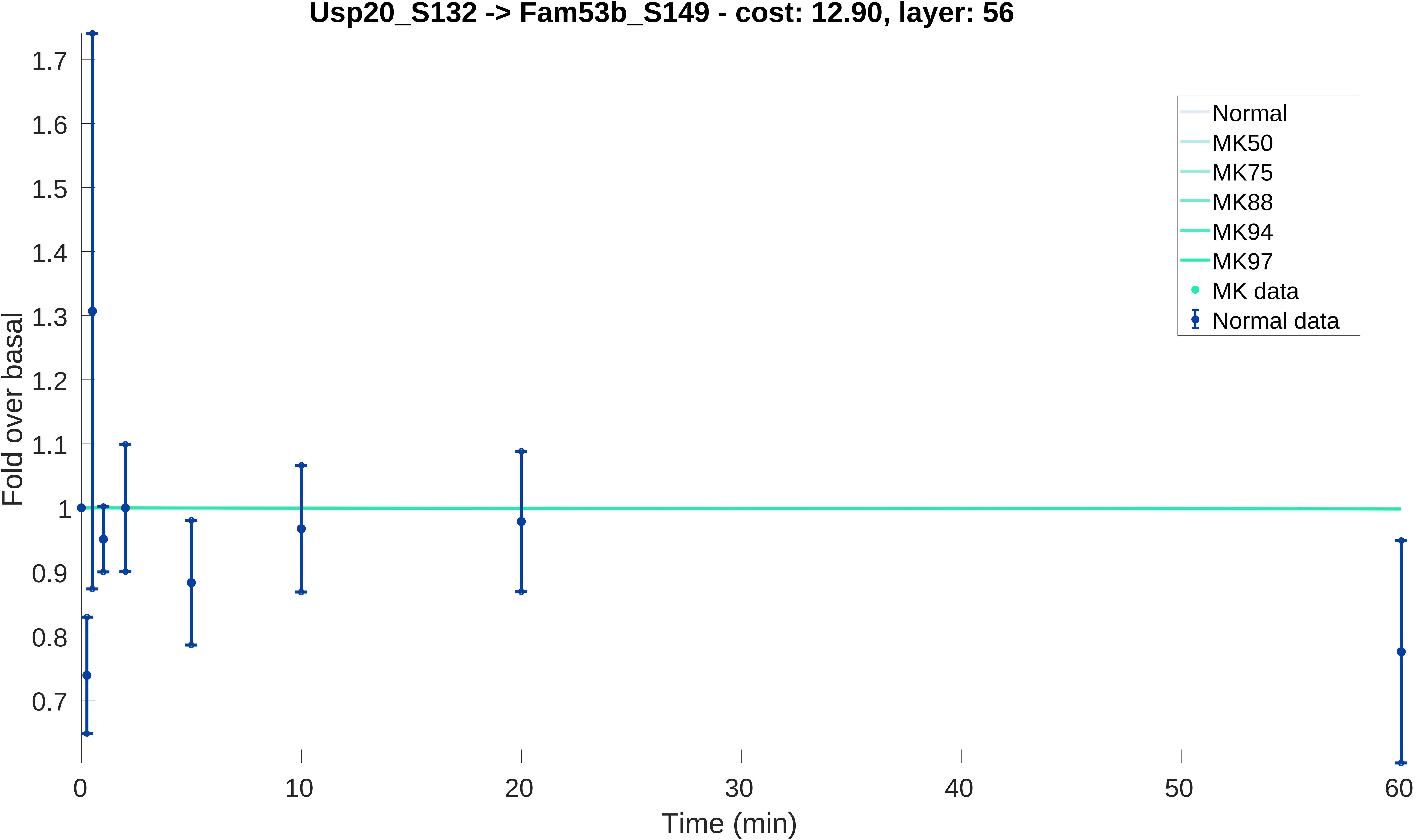
Usp20_S132 -> Fam53b_S149 - cost: 12.90, layer: 56
Fold over basal
Time (min)
1.7
1.6
1.5
1.4
1.3
1.2
1.1
1
0.9
0.8
0.7
0
10
20
30
40
50
60
Normal
MK50
MK75
MK88
MK94
MK97
MK data
Normal data

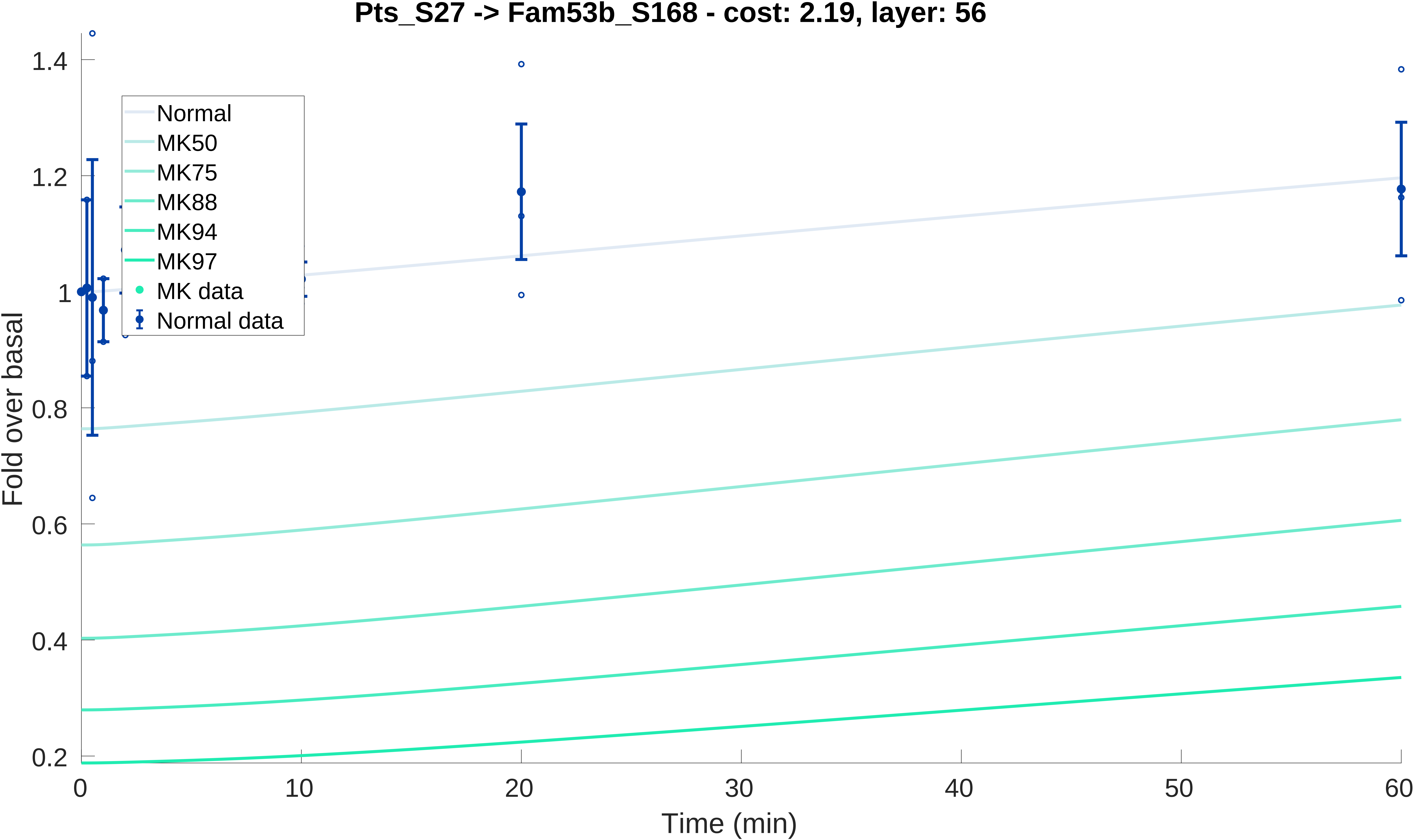

Pts_S27 -> Fam53b_S168 - cost: 2.19, layer: 56
Fold over basal
Time (min)
Normal
MK50
MK75
MK88
MK94
MK97
MK data
Normal data
0.2
0.4
0.6
0.8
1
1.2
1.4
0
10
20
30
40
50
60

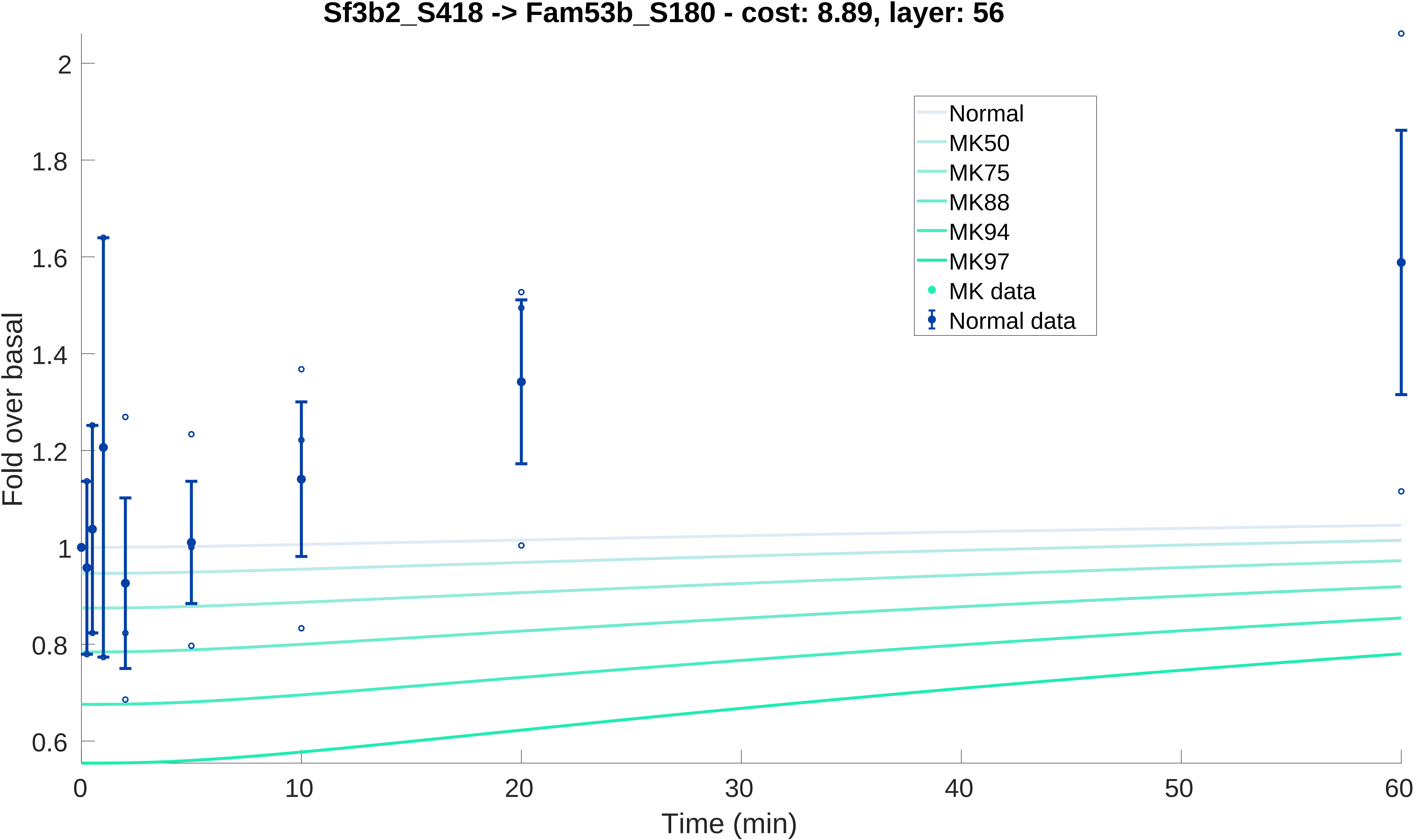

Sf3b2_S418 -> Fam53b_S180 - cost: 8.89, layer: 56
Fold over basal
Time (min)
0
10
20
30
40
50
60
0.6
0.8
1
1.2
1.4
1.6
1.8
2
Normal
MK50
MK75
MK88
MK94
MK97
MK data
Normal data

Pts_S27 -> Fam53b_S269 - cost: 4.63, layer: 56

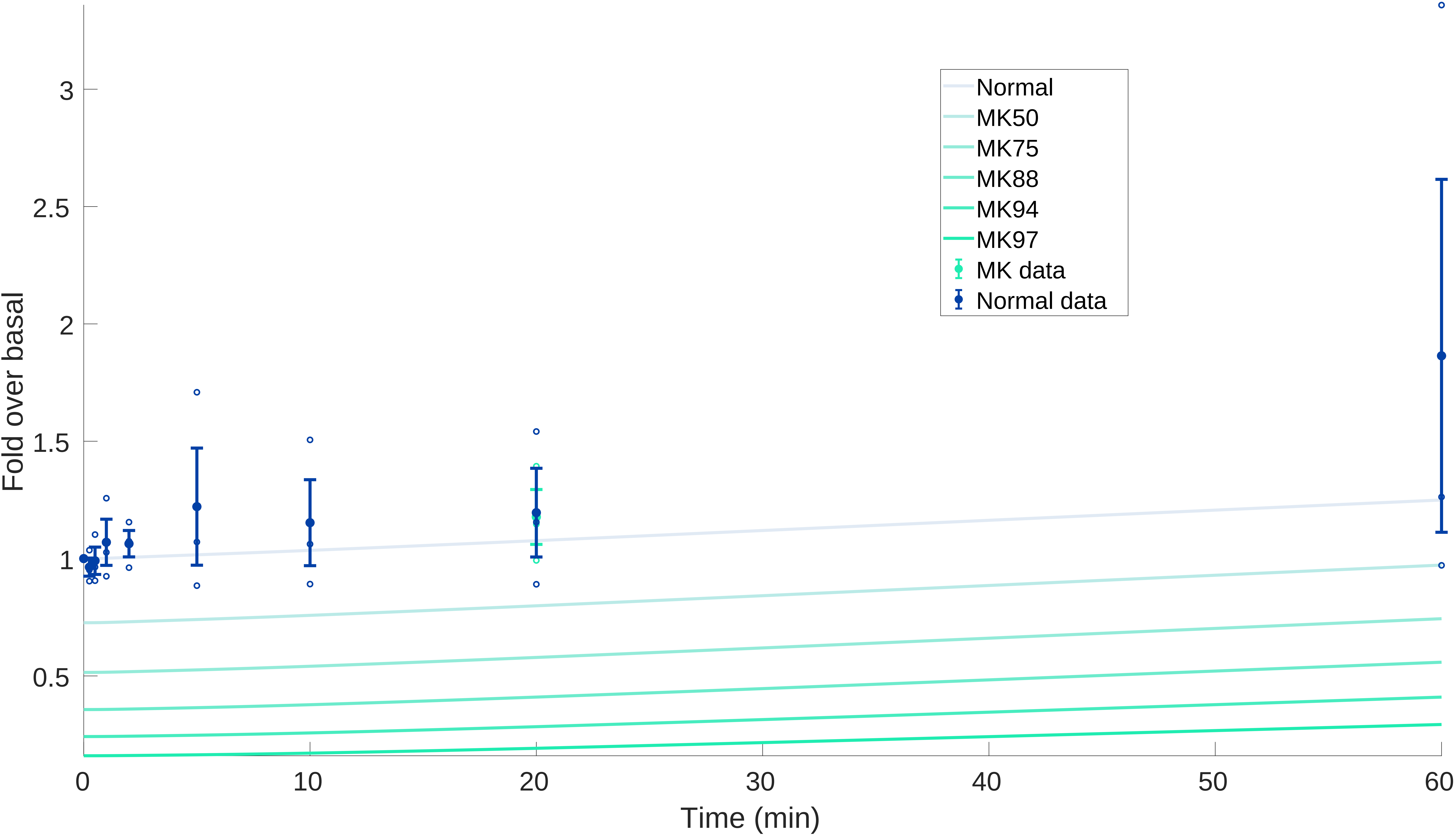

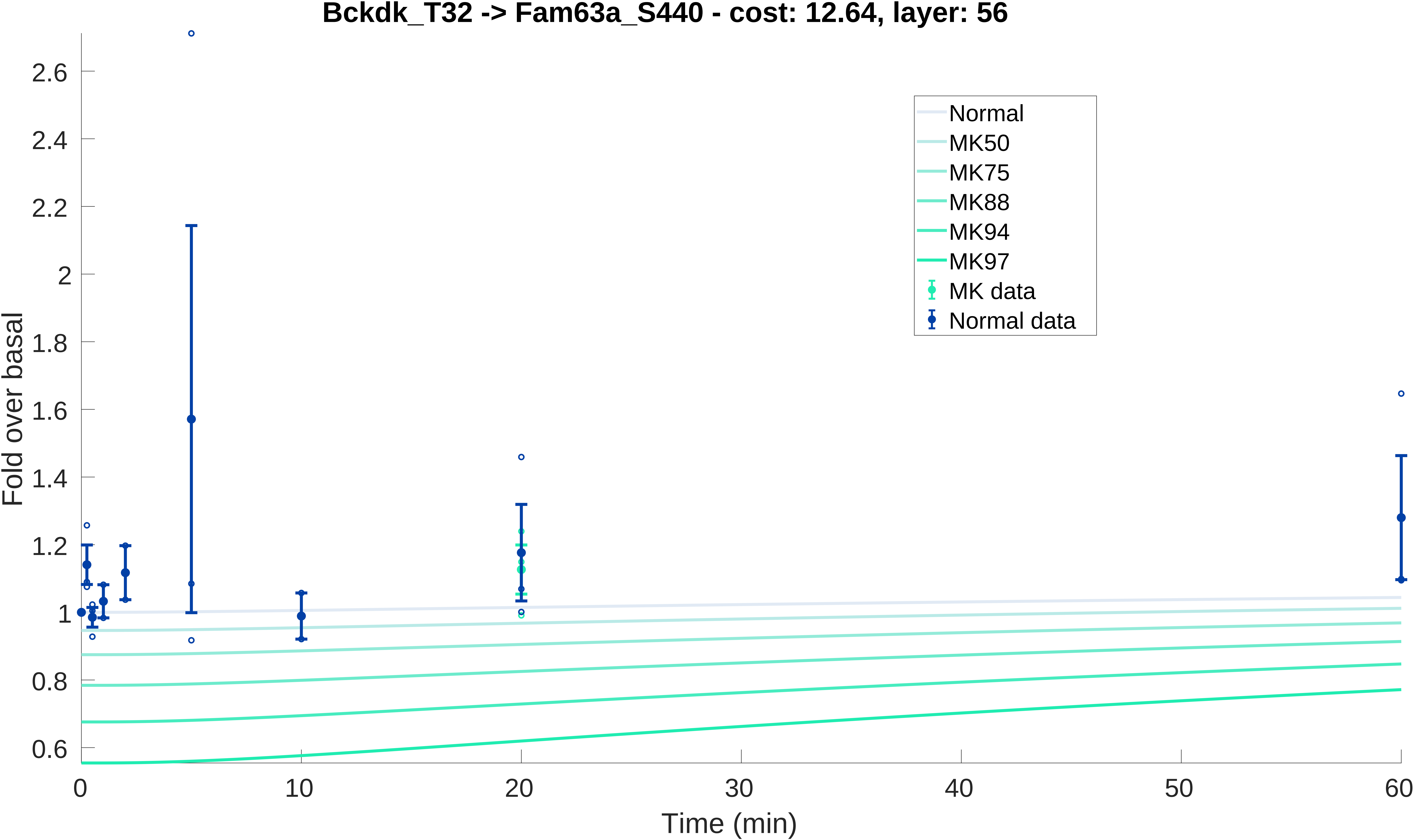

Bckdk_T32 -> Fam63a_S440 - cost: 12.64, layer: 56
Fold over basal
Time (min)
0.6
0.8
1
1.2
1.4
1.6
1.8
2
2.2
2.4
2.6
0
10
20
30
40
50
60
Normal
MK50
MK75
MK88
MK94
MK97
MK data
Normal data

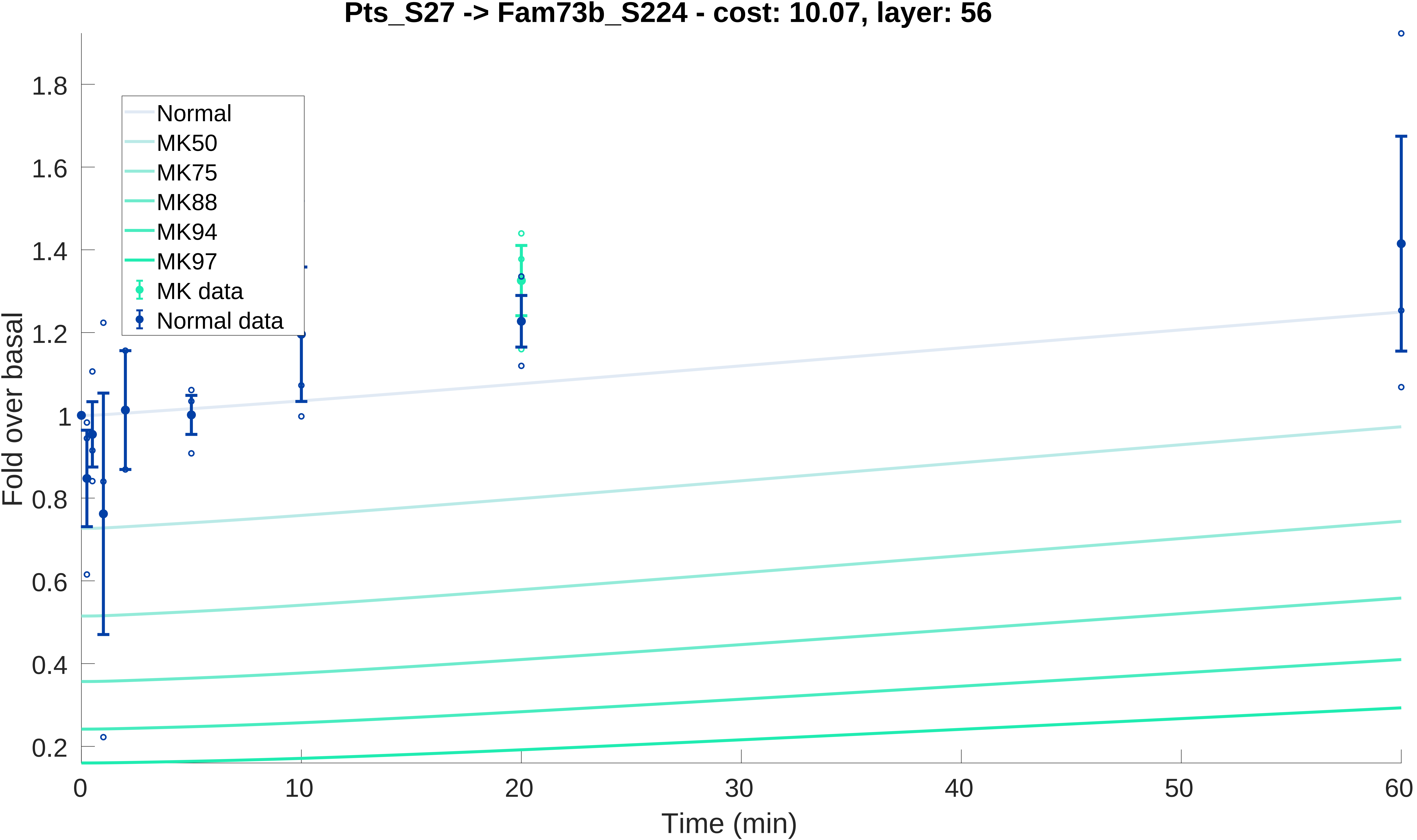

Pts_S27 -> Fam73b_S224 - cost: 10.07, layer: 56
Normal
MK50
MK75
MK88
MK94
MK97
MK data
Normal data
Fold over basal
Time (min)
0.2
0.4
0.6
0.8
1
1.2
1.4
1.6
1.8
0
10
20
30
40
50
60

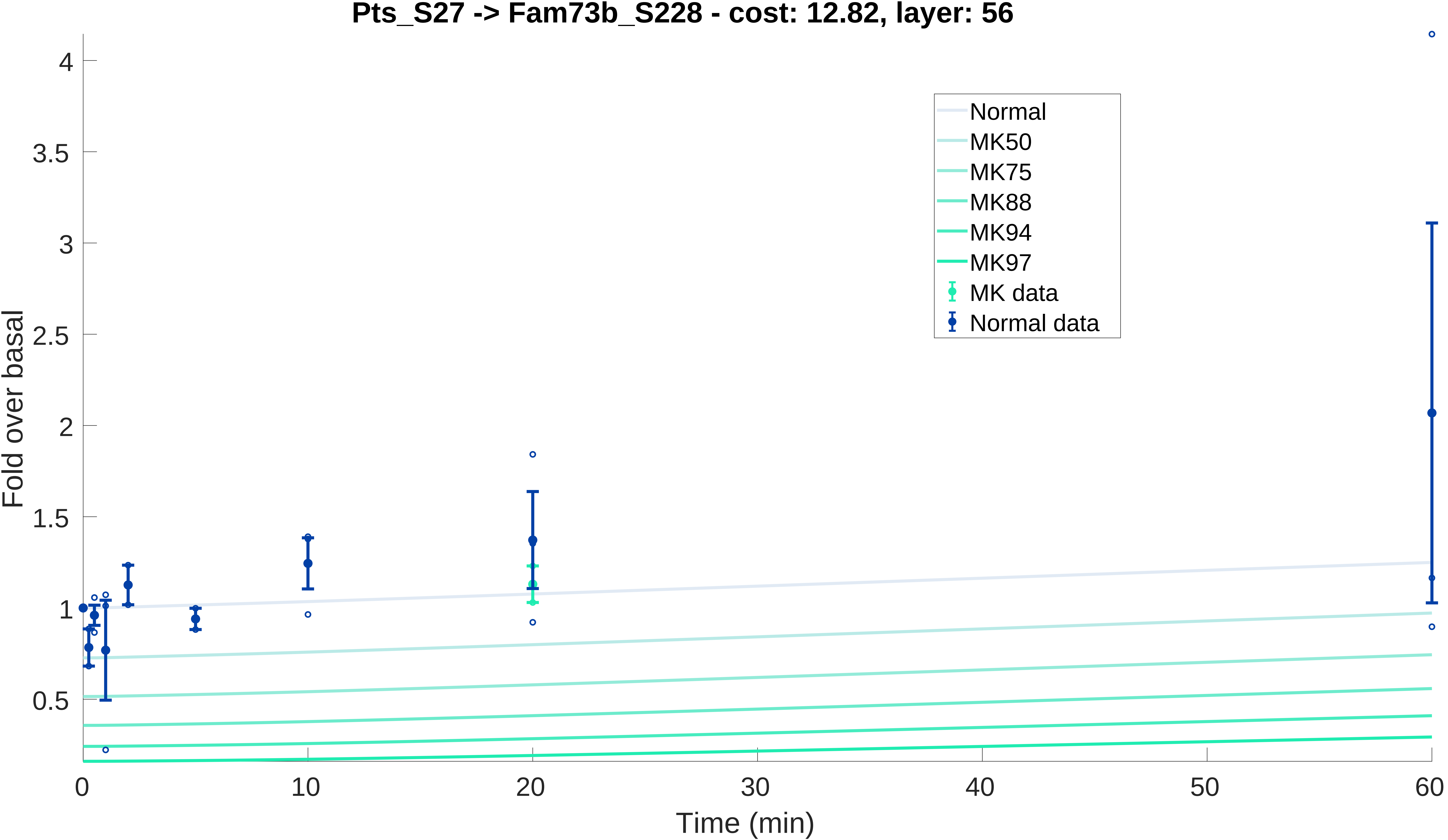
Pts_S27 -> Fam73b_S228 - cost: 12.82, layer: 56
Fold over basal
Time (min)
0
10
20
30
40
50
60
0.5
1
1.5
2
2.5
3
3.5
4
Normal
MK50
MK75
MK88
MK94
MK97
MK data
Normal data

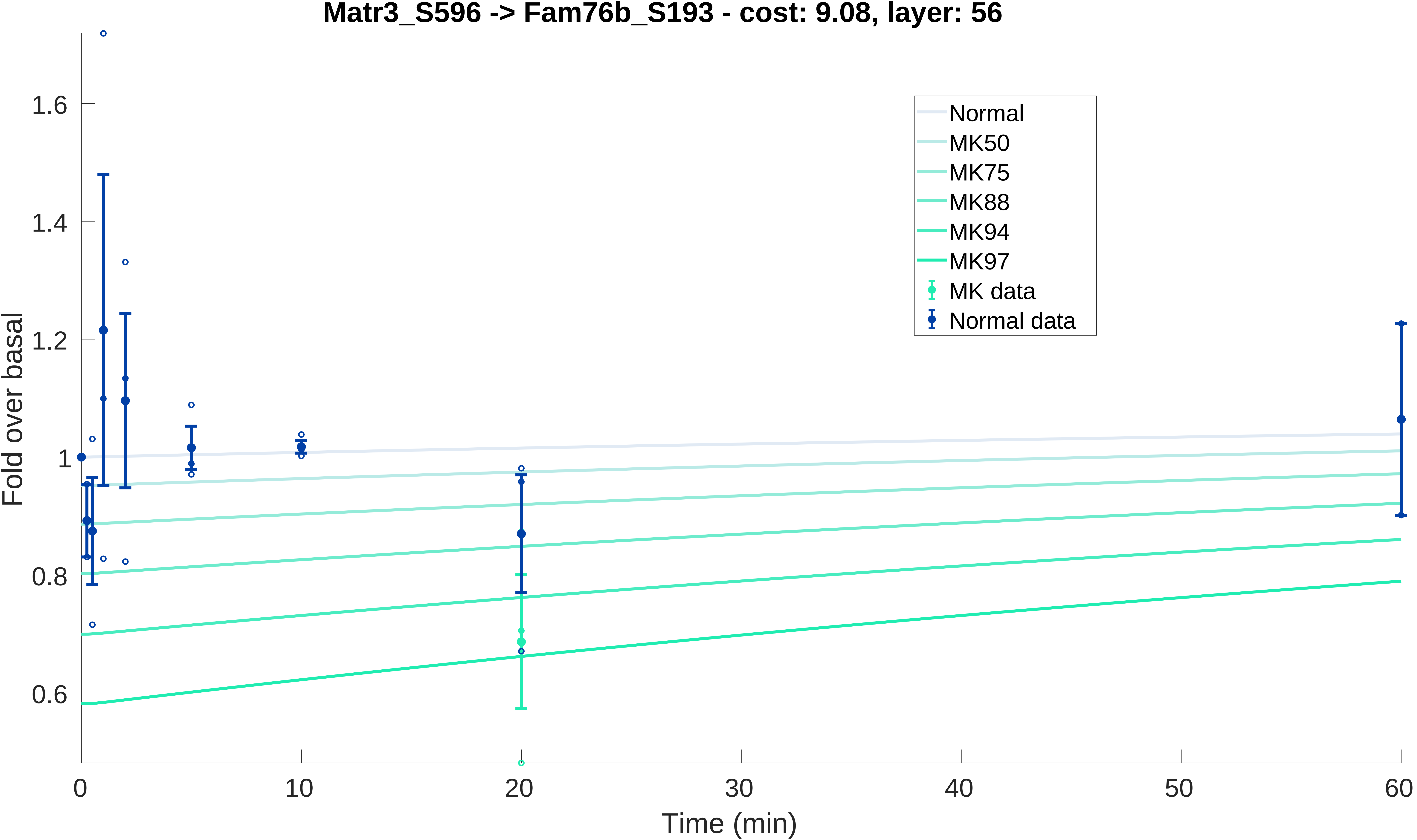

Matr3_S596 -> Fam76b_S193 - cost: 9.08, layer: 56
Fold over basal
Time (min)
0.6
0.8
1
1.2
1.4
1.6
0
10
20
30
40
50
60
Normal
MK50
MK75
MK88
MK94
MK97
MK data
Normal data

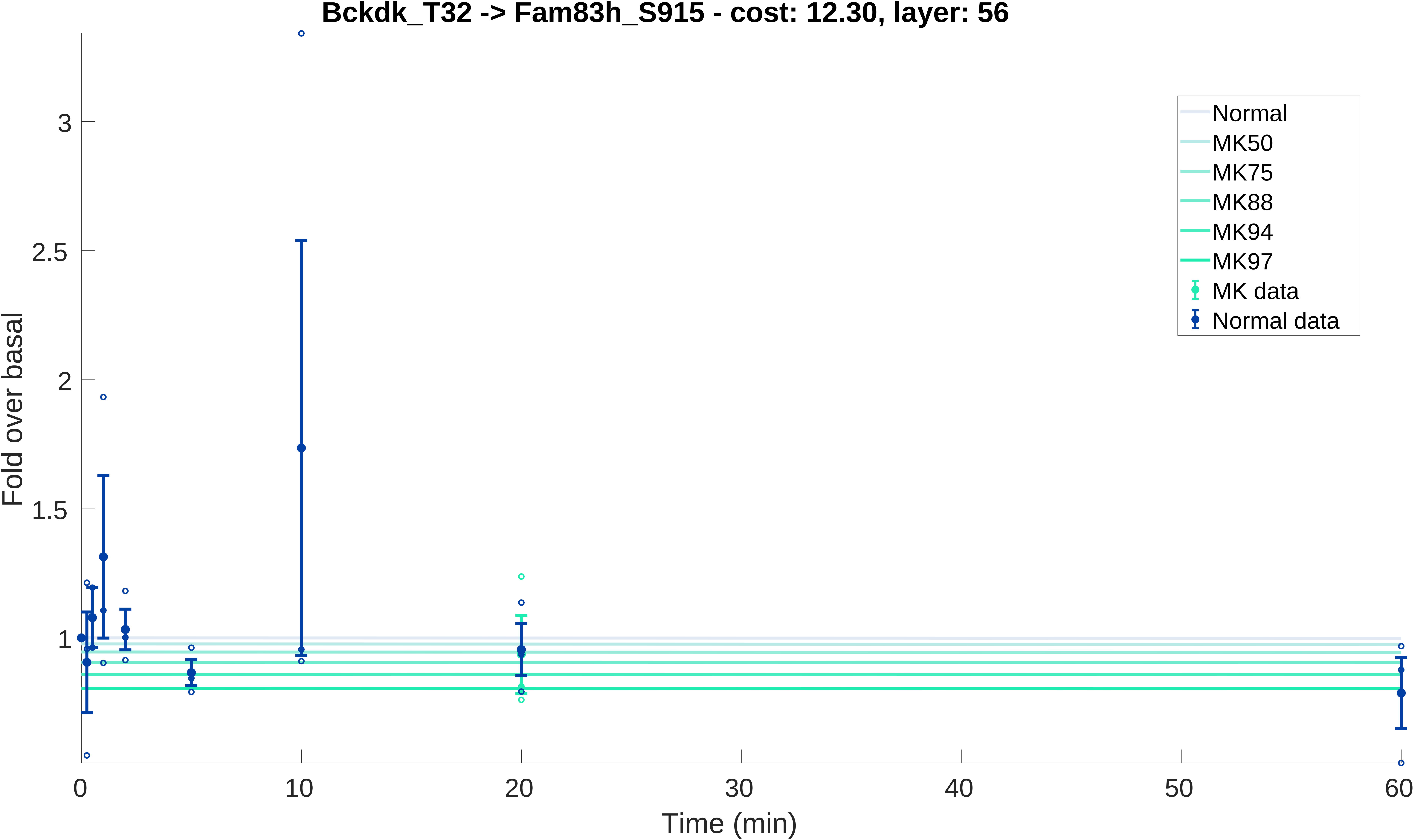

Bckdk_T32 -> Fam83h_S915 - cost: 12.30, layer: 56
Fold over basal
3
2.5
2
1.5
1
Time (min)
0
10
20
30
40
50
60
Normal
MK50
MK75
MK88
MK94
MK97
MK data
Normal data

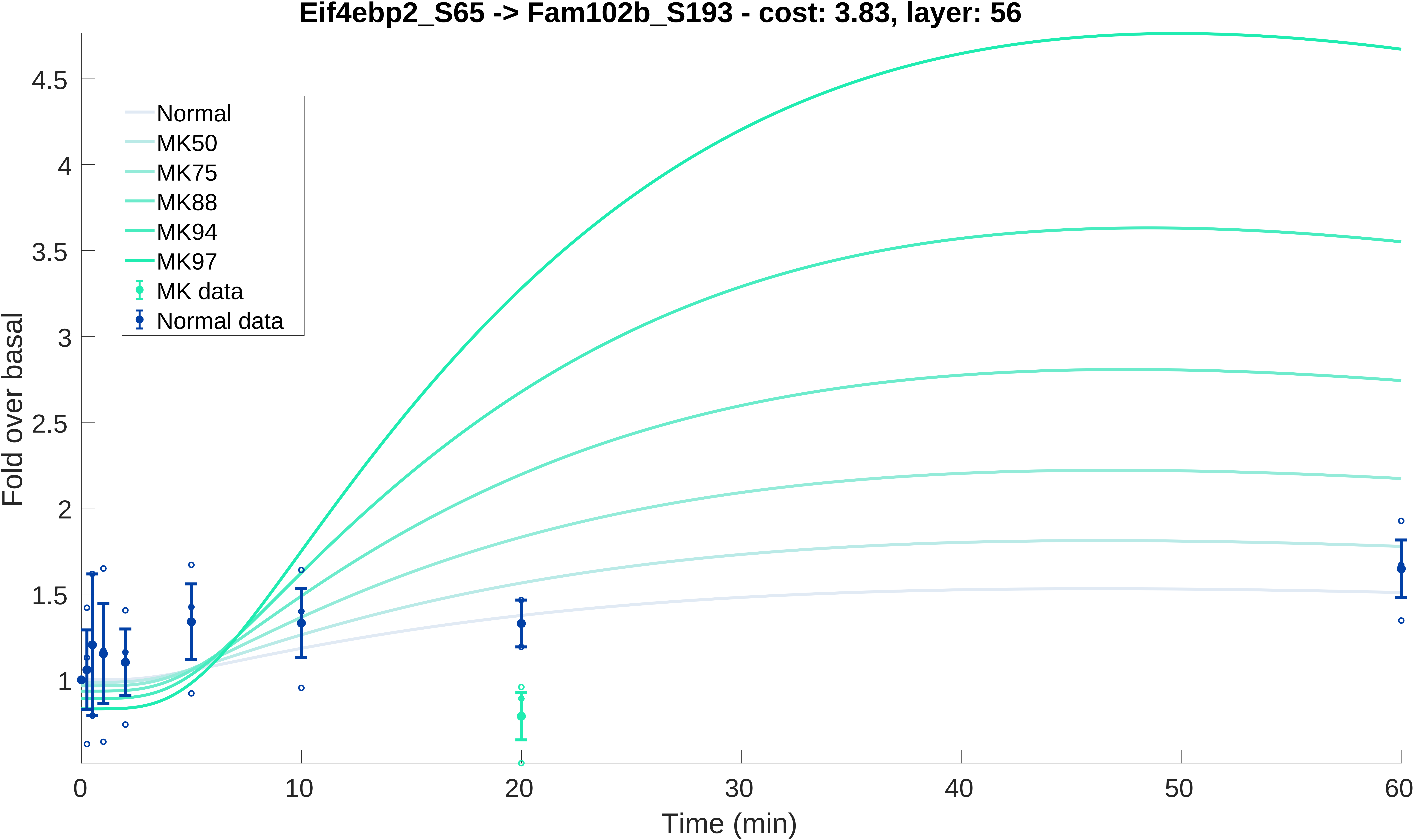

Eif4ebp2_S65 -> Fam102b_S193 - cost: 3.83, layer: 56
Normal
MK50
MK75
MK88
MK94
MK97
MK data
Normal data
Fold over basal
Time (min)
4.5
4
3.5
3
2.5
2
1.5
1
0
10
20
30
40
50
60

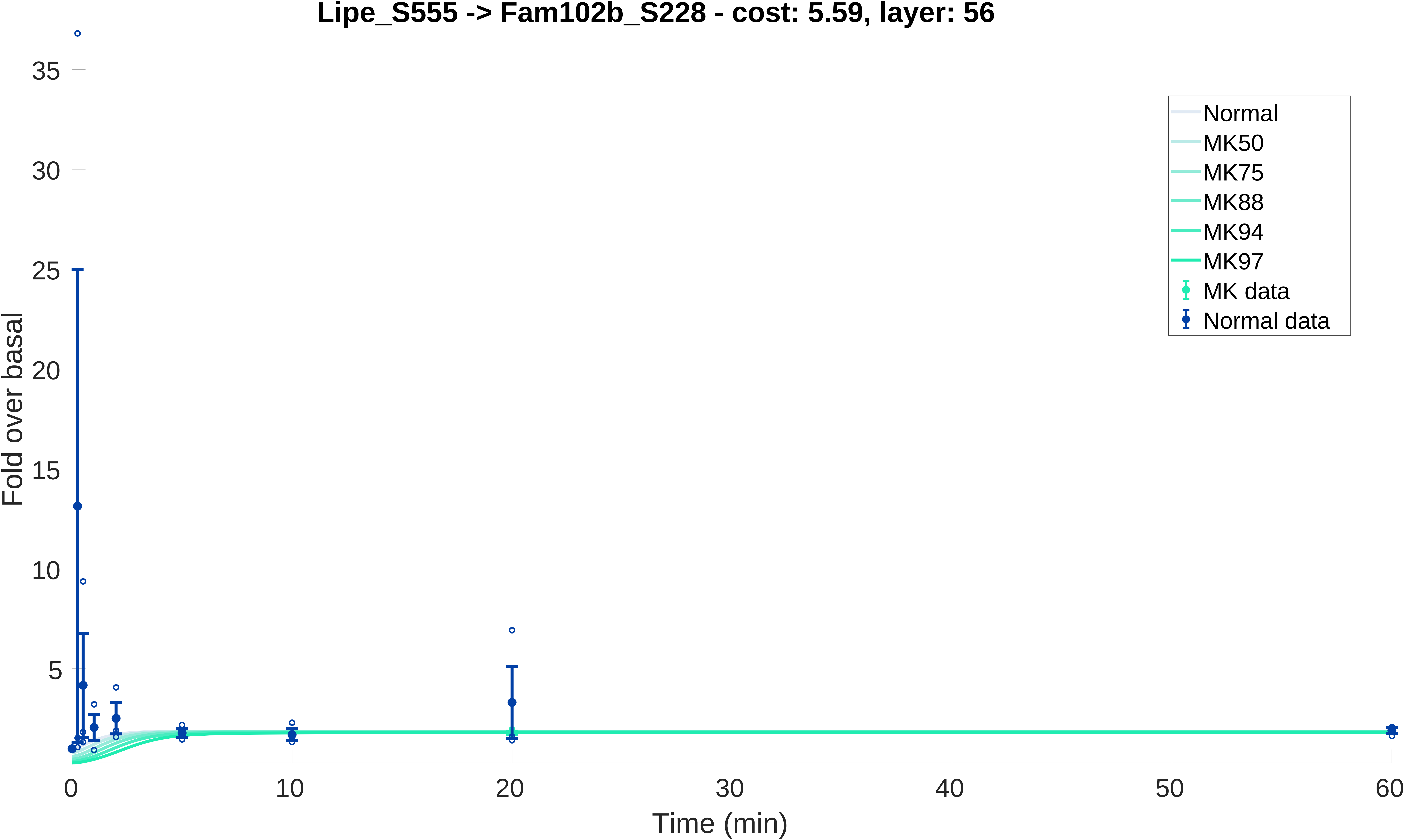

Lipe_S555 -> Fam102b_S228 - cost: 5.59, layer: 56
Fold over basal
Time (min)
0
10
20
30
40
50
60
5
10
15
20
25
30
35
Normal
MK50
MK75
MK88
MK94
MK97
MK data
Normal data

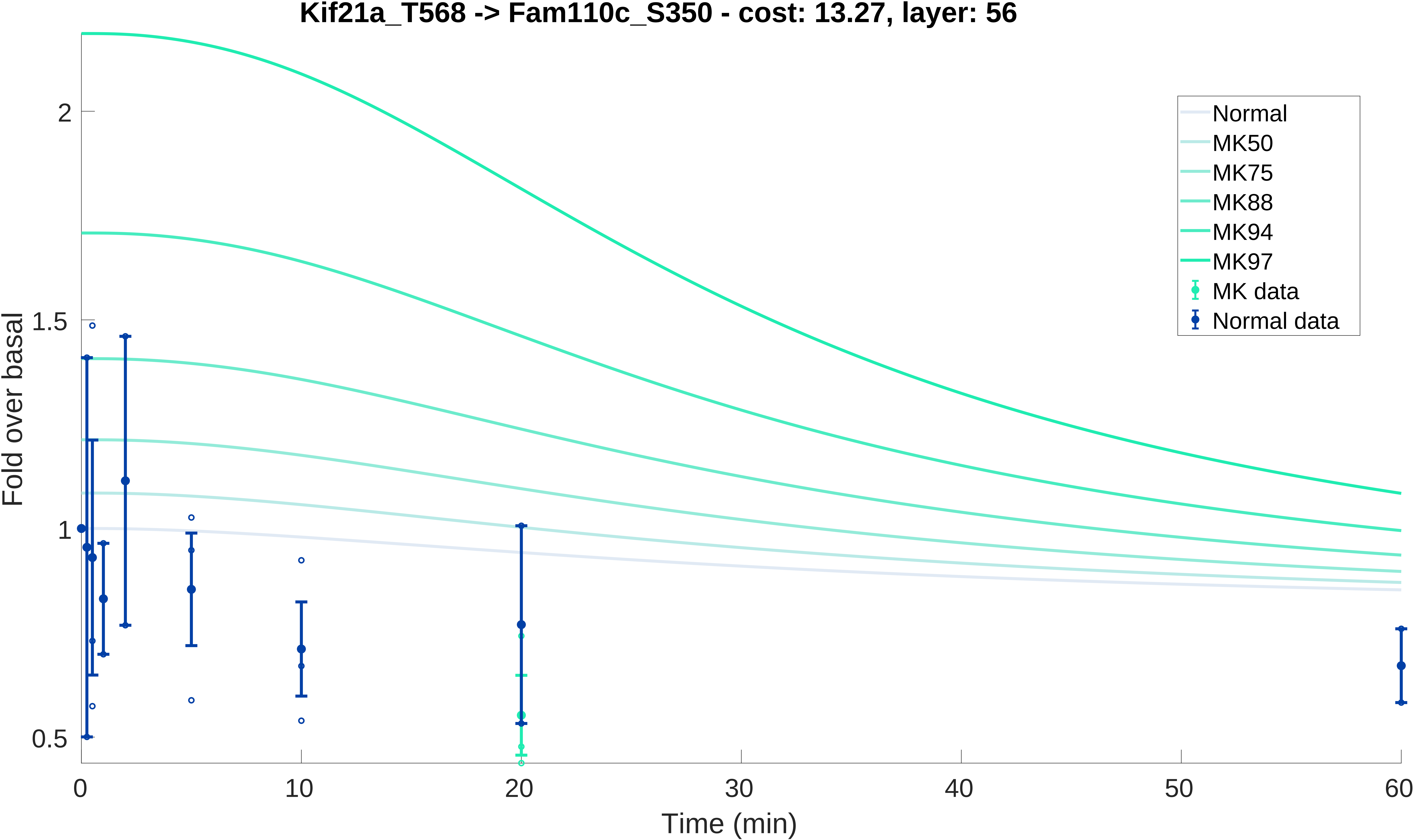

Kif21a_T568 -> Fam110c_S350 - cost: 13.27, layer: 56
Fold over basal
Time (min)
0.5
1
1.5
2
0
10
20
30
40
50
60
Normal
MK50
MK75
MK88
MK94
MK97
MK data
Normal data

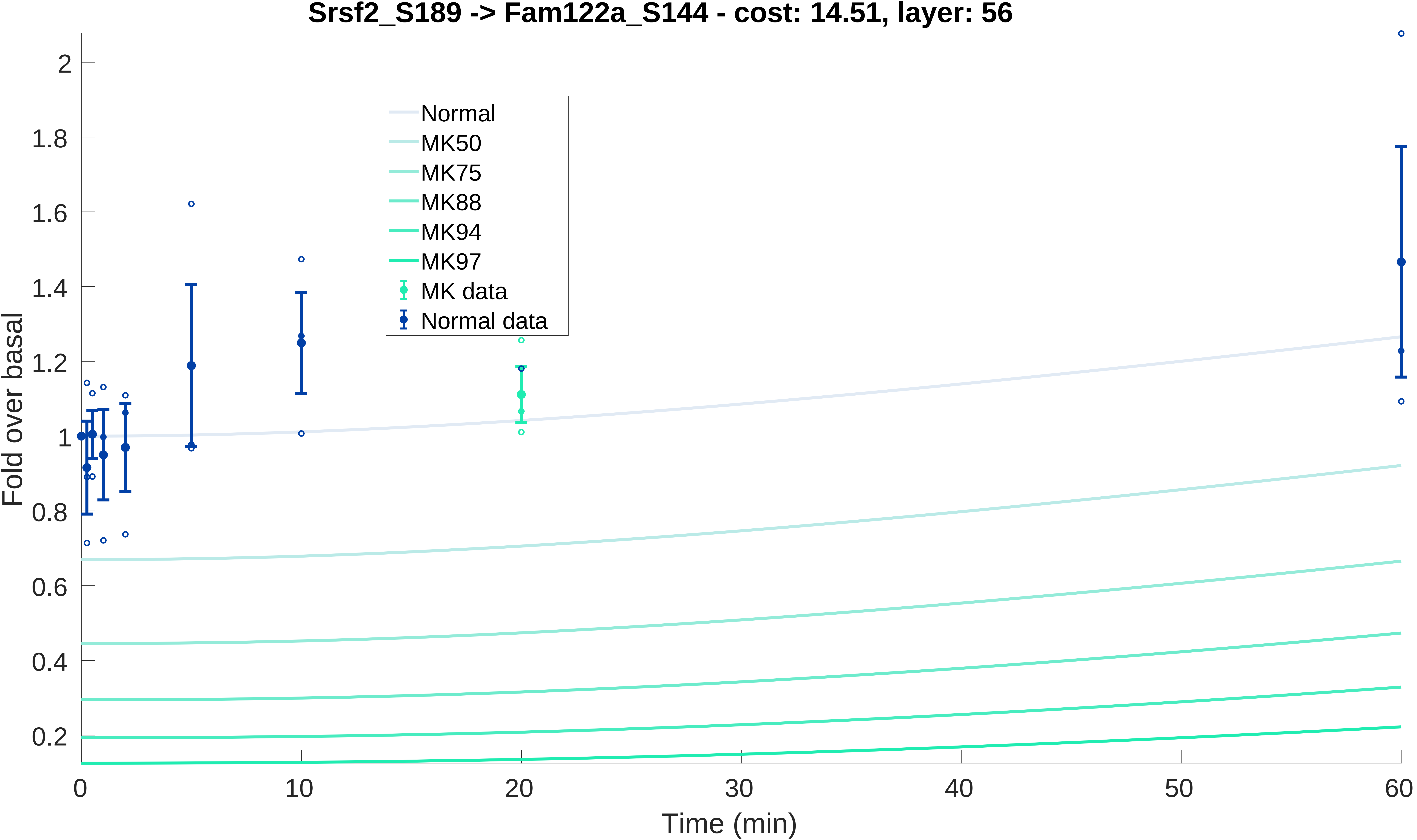
Srsf2_S189 -> Fam122a_S144 - cost: 14.51, layer: 56
Fold over basal
Time (min)
Normal
MK50
MK75
MK88
MK94
MK97
MK data
Normal data
2
1.8
1.6
1.4
1.2
1
0.8
0.6
0.4
0.2
0
10
20
30
40
50
60

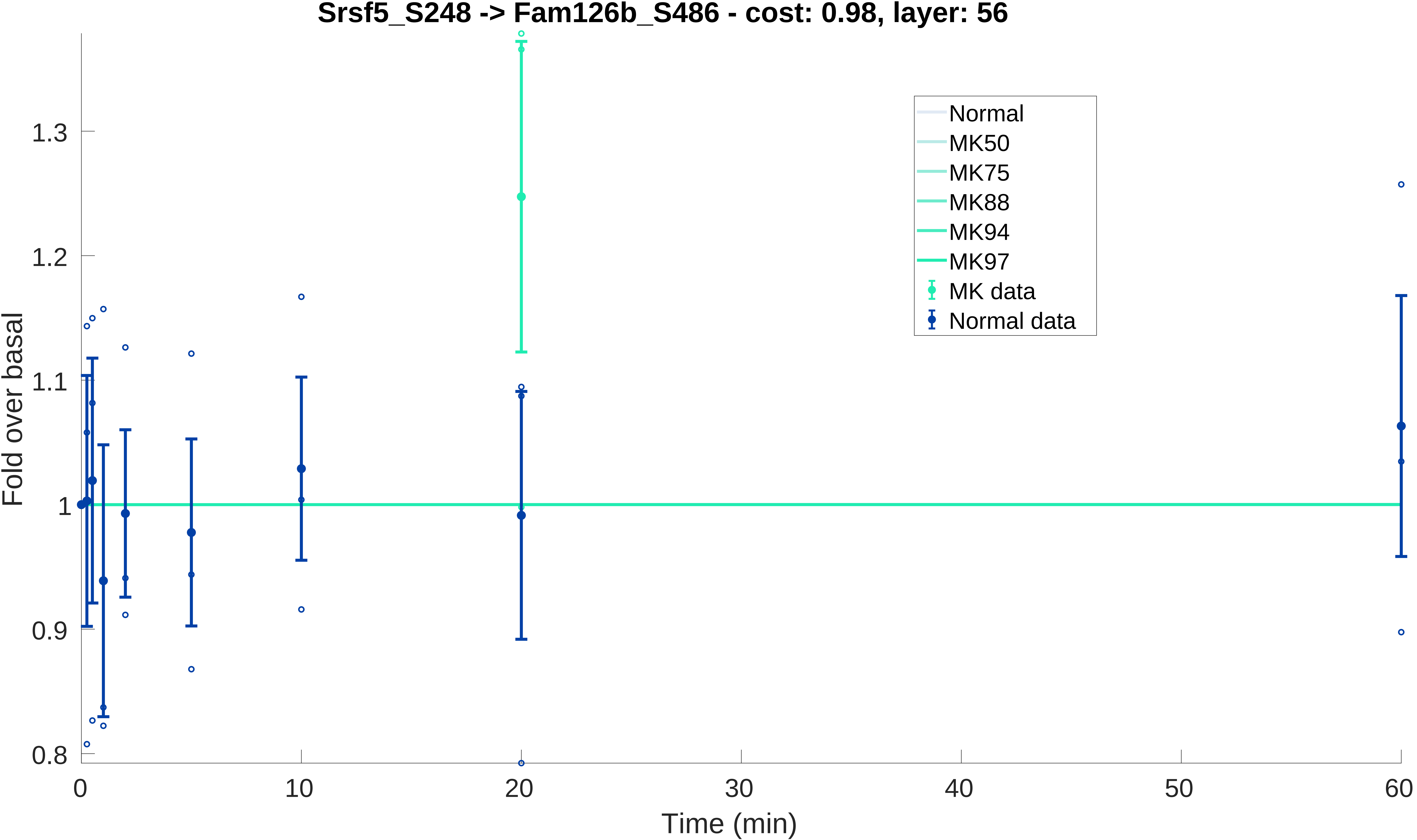

Srsf5_S248 -> Fam126b_S486 - cost: 0.98, layer: 56
Normal
MK50
MK75
MK88
MK94
MK97
MK data
Normal data
Fold over basal
Time (min)
0.8
0.9
1
1.1
1.2
1.3
0
10
20
30
40
50
60

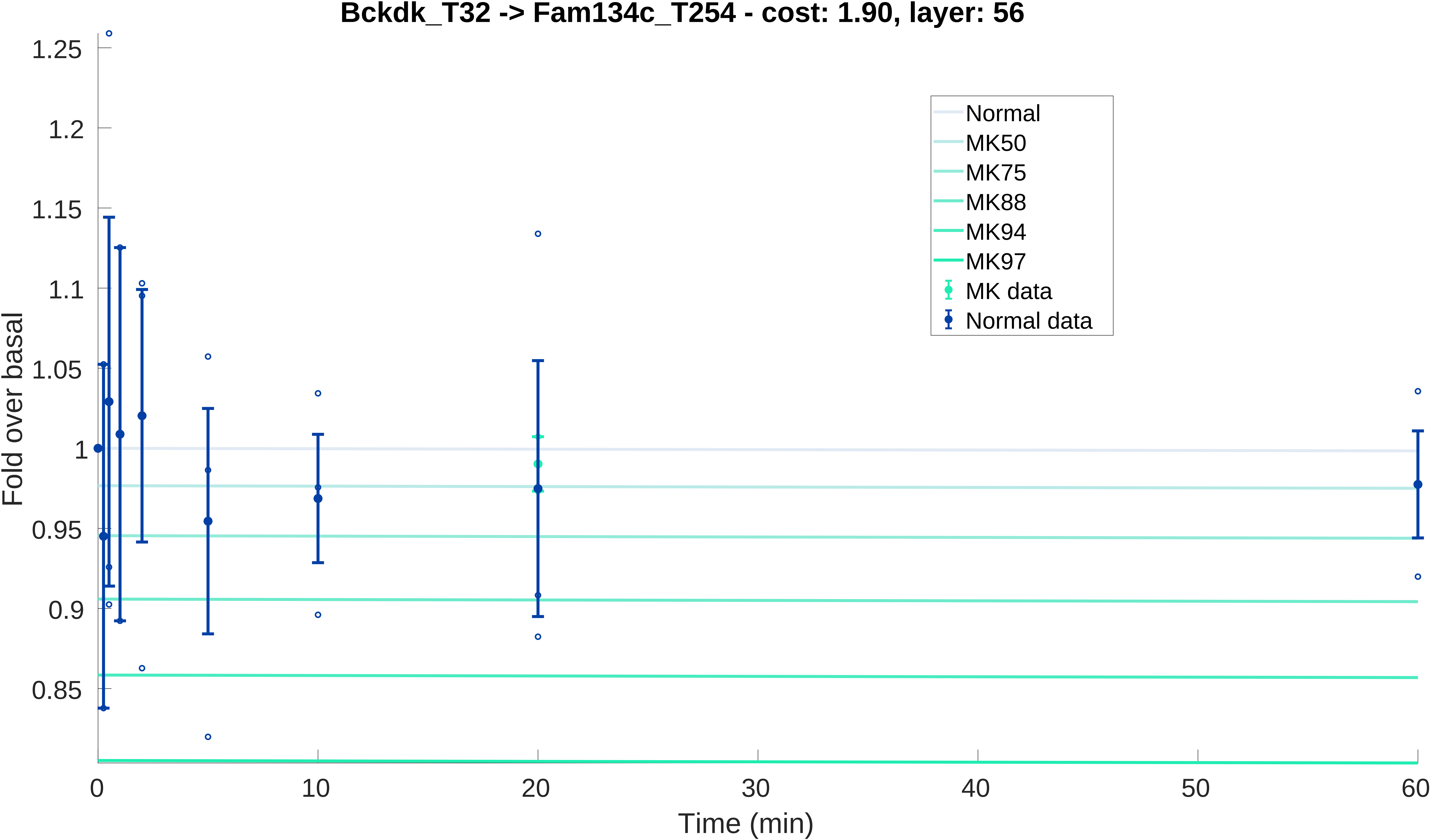

Bckdk_T32 -> Fam134c_T254 - cost: 1.90, layer: 56
Fold over basal
1.25
1.2
1.15
1.1
1.05
1
0.95
0.9
0.85
0
10
20
30
40
50
60
Time (min)
Normal
MK50
MK75
MK88
MK94
MK97
MK data
Normal data

Sntb2_S90 -> Fam134c_T307 - cost: 4.36, layer: 56

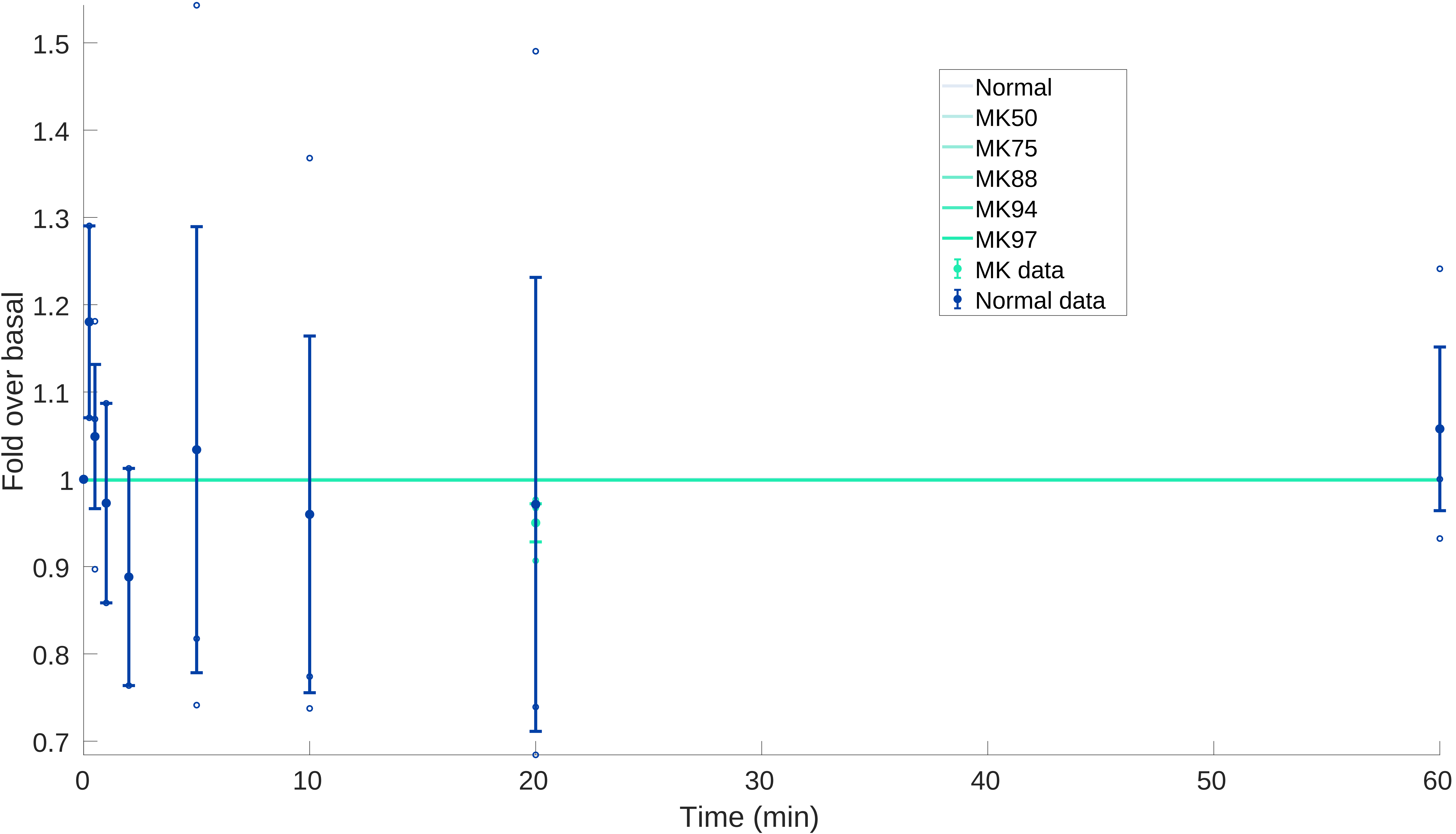

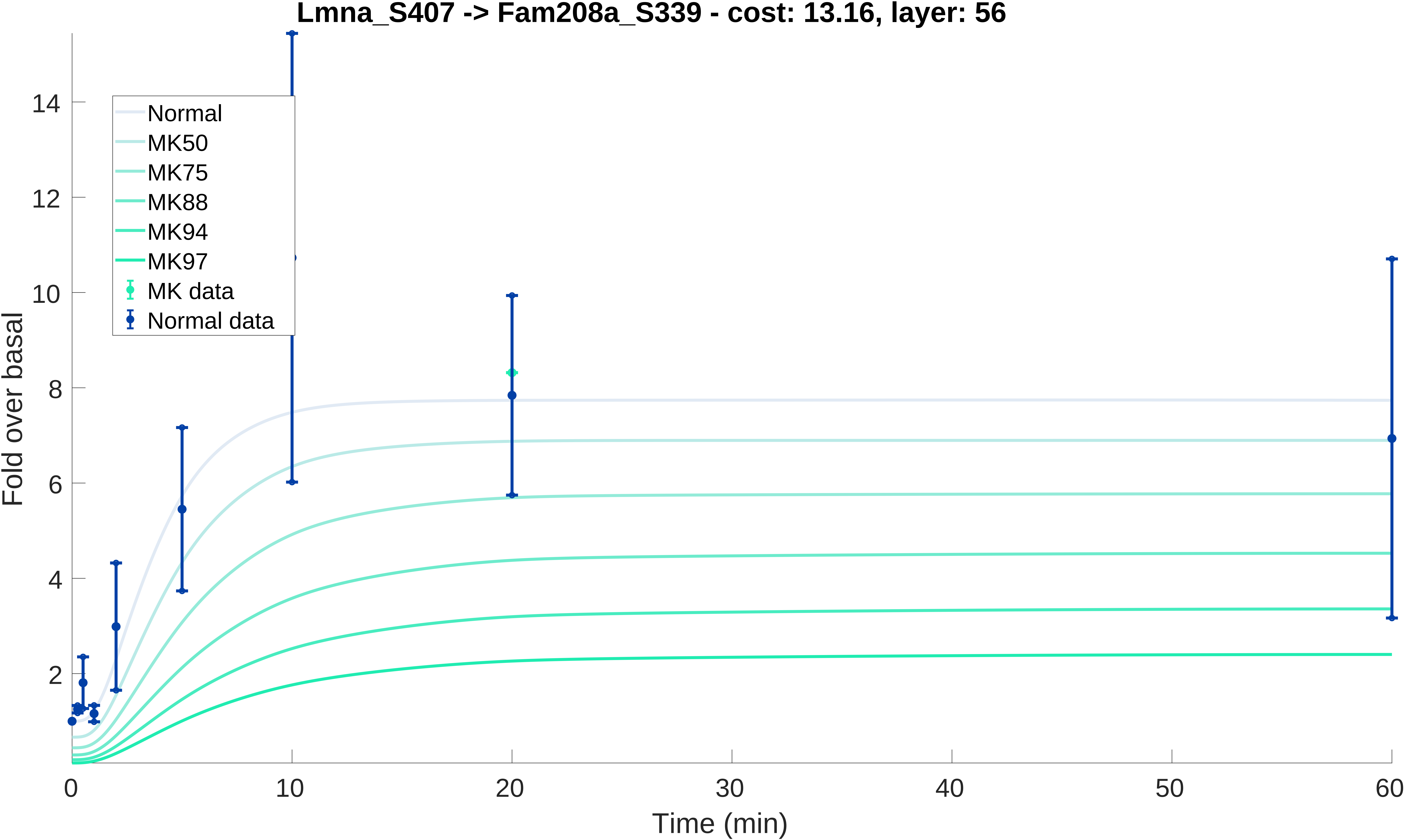

Lmna_S407 -> Fam208a_S339 - cost: 13.16, layer: 56
Normal
MK50
MK75
MK88
MK94
MK97
MK data
Normal data
Fold over basal
Time (min)
0
10
20
30
40
50
60
2
4
6
8
10
12
14

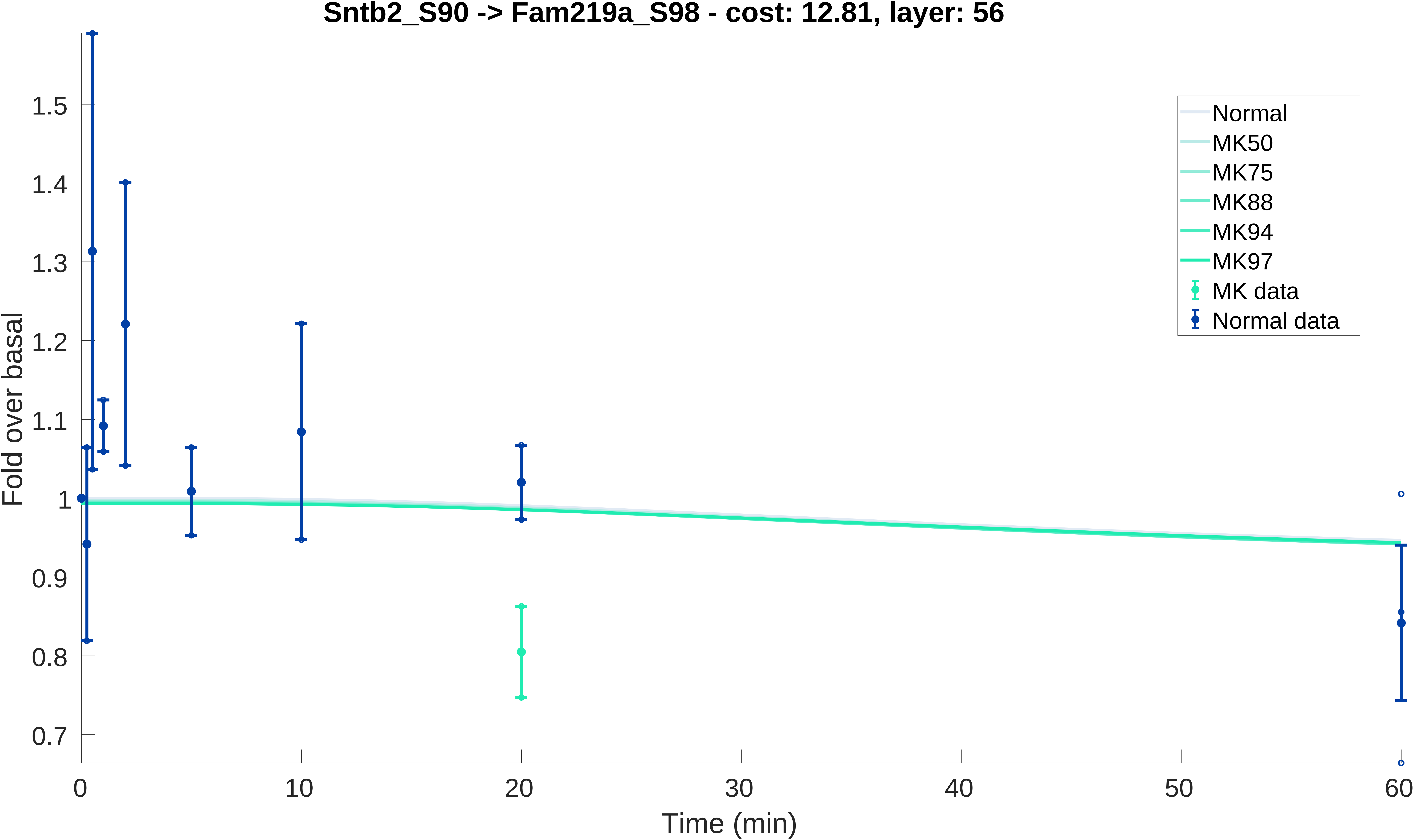

Sntb2_S90 -> Fam219a_S98 - cost: 12.81, layer: 56
Fold over basal
1.5
1.4
1.3
1.2
1.1
1
0.9
0.8
0.7
0
10
20
30
40
50
60
Time (min)
Normal
MK50
MK75
MK88
MK94
MK97
MK data
Normal data

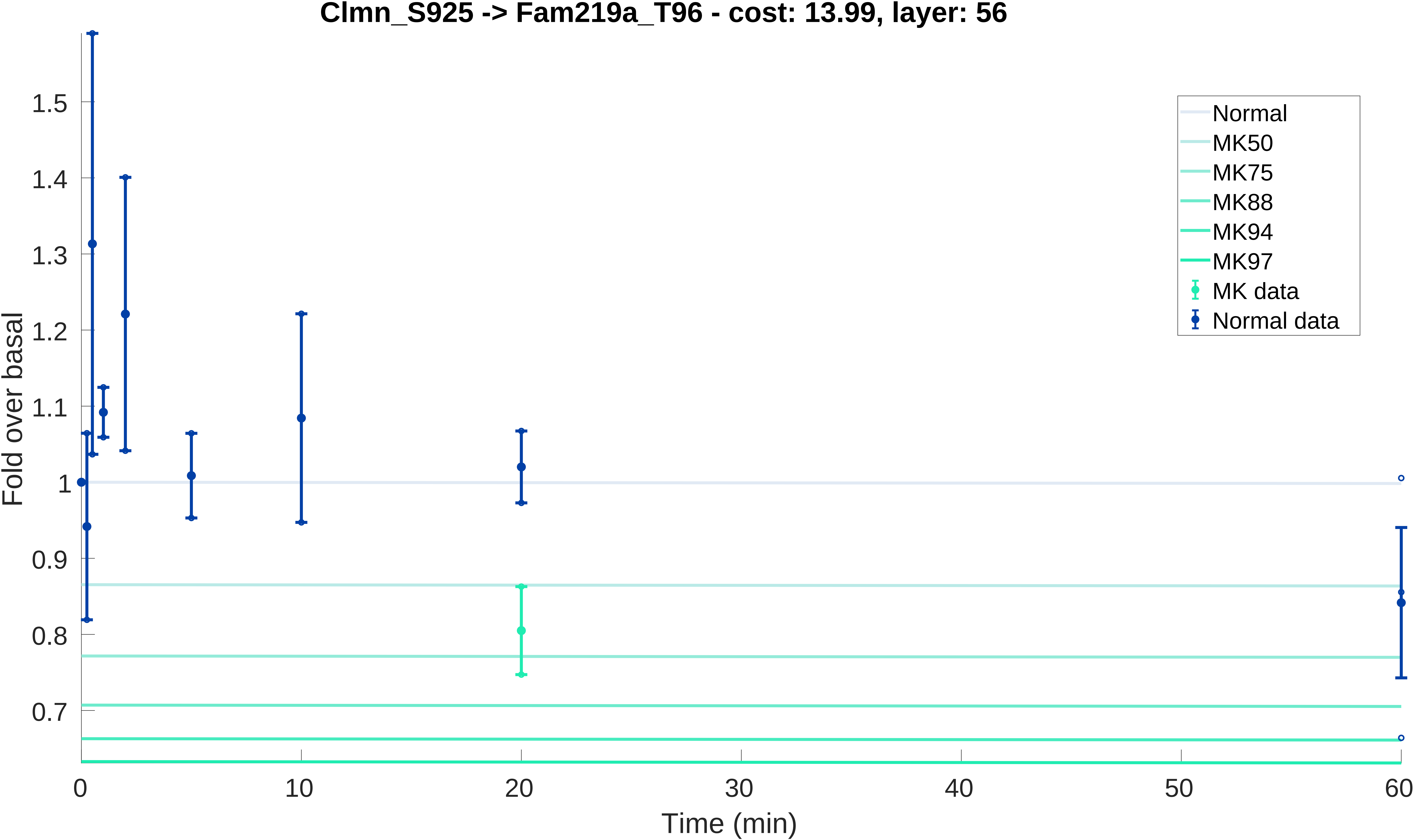

Clmn_S925 -> Fam219a_T96 - cost: 13.99, layer: 56
Fold over basal
1.5
1.4
1.3
1.2
1.1
1
0.9
0.8
0.7
0
10
20
30
40
50
60
Time (min)
Normal
MK50
MK75
MK88
MK94
MK97
MK data
Normal data

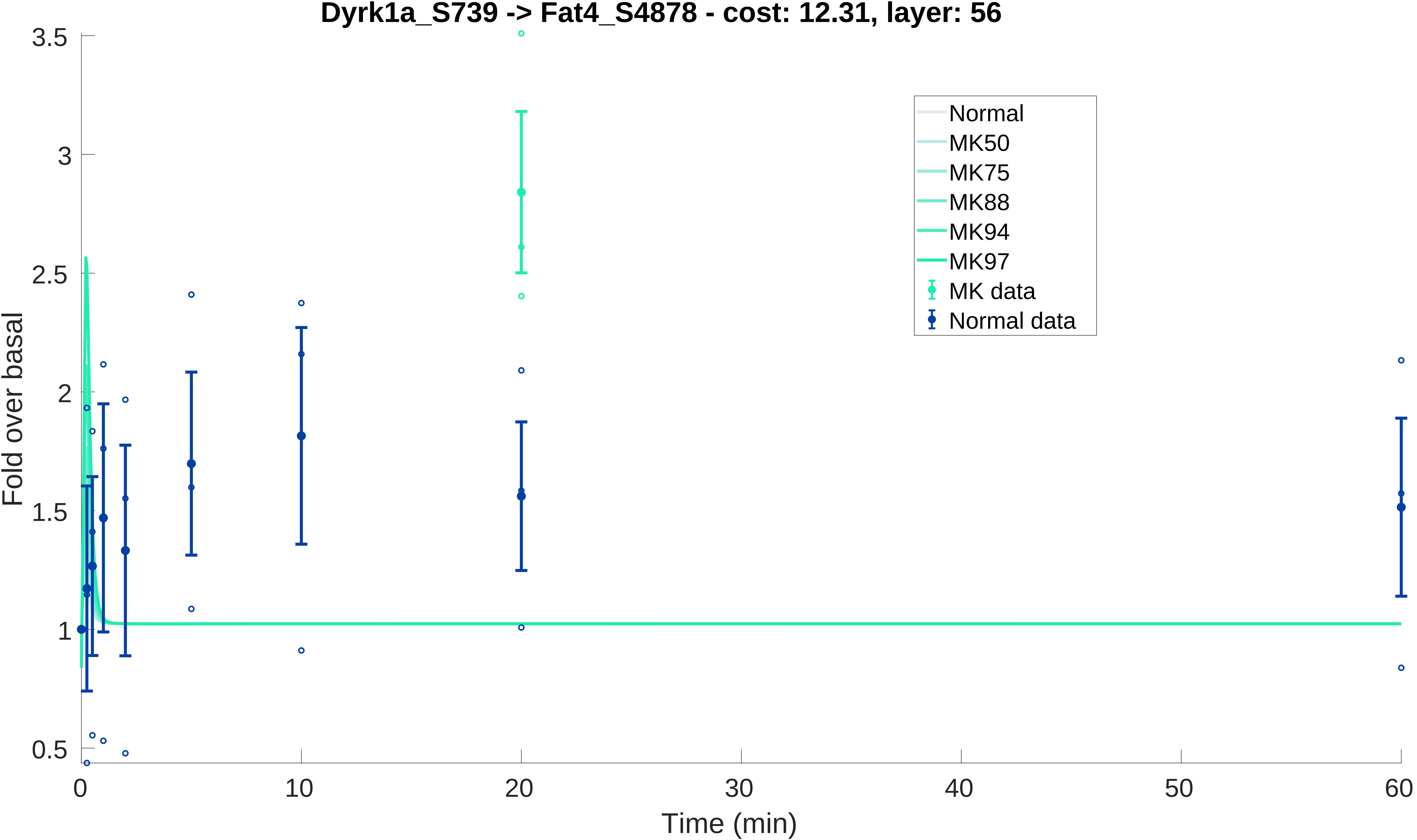

Dyrk1a_S739 -> Fat4_S4878 - cost: 12.31, layer: 56
Fold over basal
Time (min)
0.5
1
1.5
2
2.5
3
3.5
0
10
20
30
40
50
60
Normal
MK50
MK75
MK88
MK94
MK97
MK data
Normal data

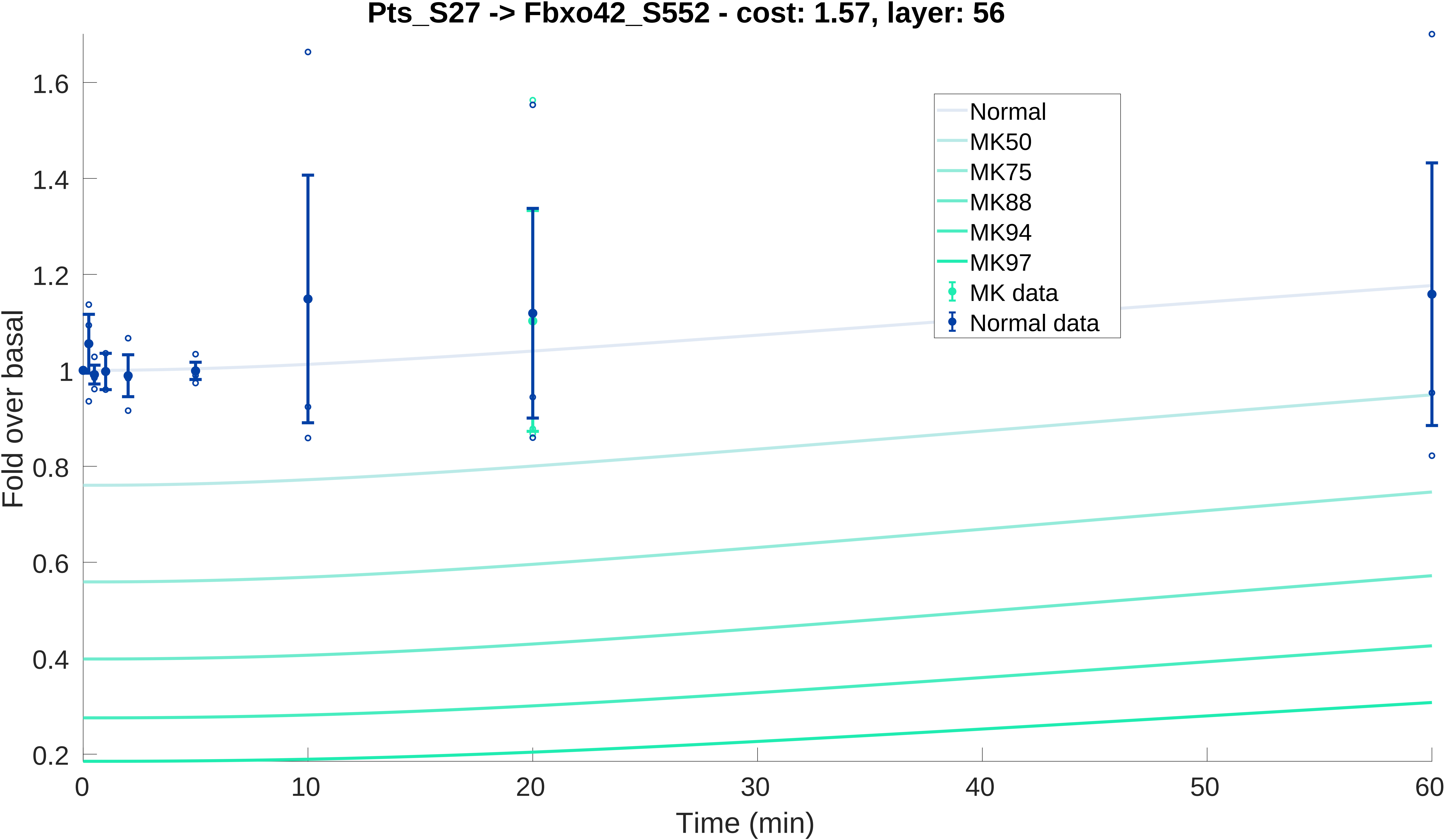
Pts_S27 -> Fbxo42_S552 - cost: 1.57, layer: 56
Fold over basal
Time (min)
1.6
1.4
1.2
1
0.8
0.6
0.4
0.2
0
10
20
30
40
50
60
Normal
MK50
MK75
MK88
MK94
MK97
MK data
Normal data

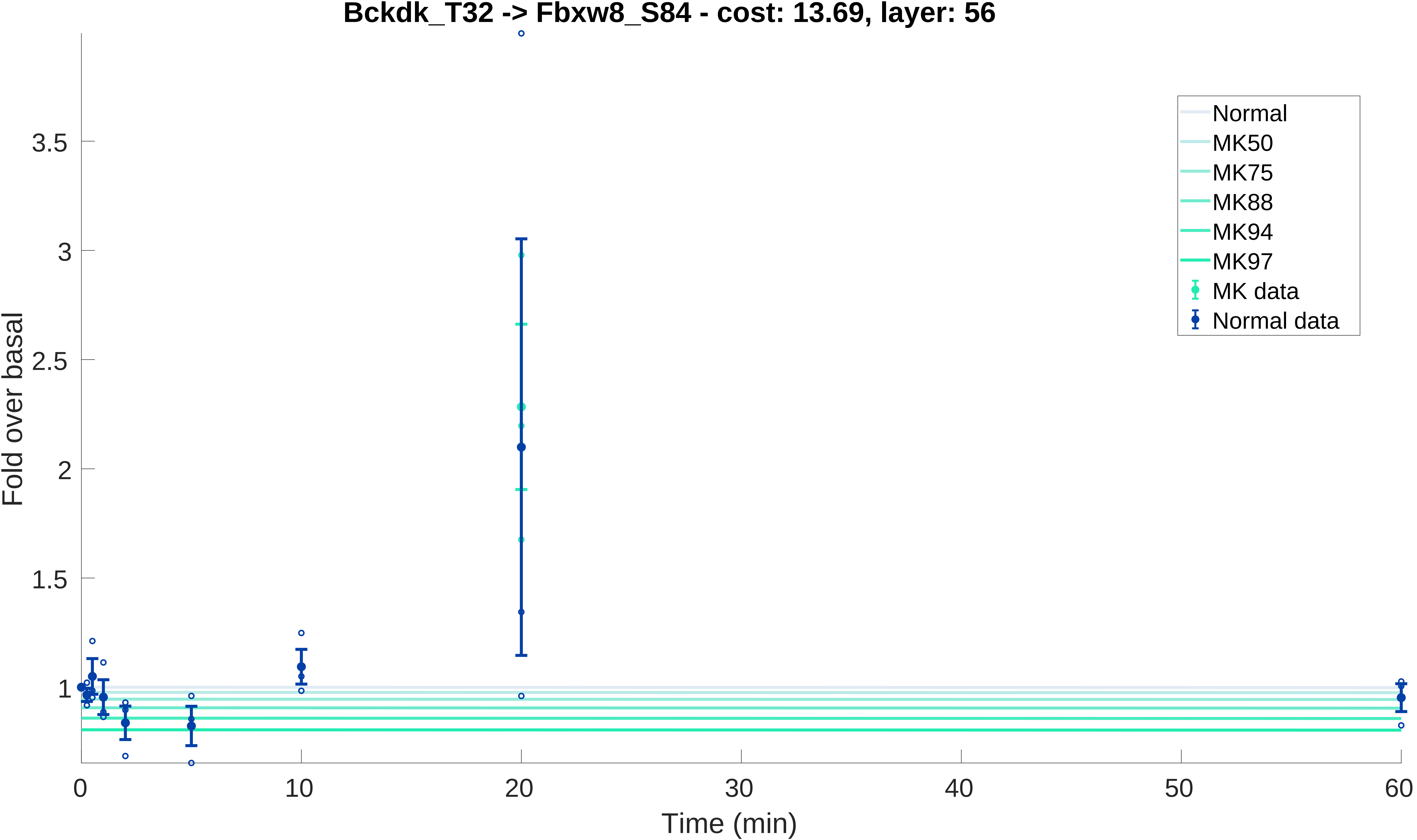

Bckdk_T32 -> Fbxw8_S84 - cost: 13.69, layer: 56
Fold over basal
Time (min)
Normal
MK50
MK75
MK88
MK94
MK97
MK data
Normal data

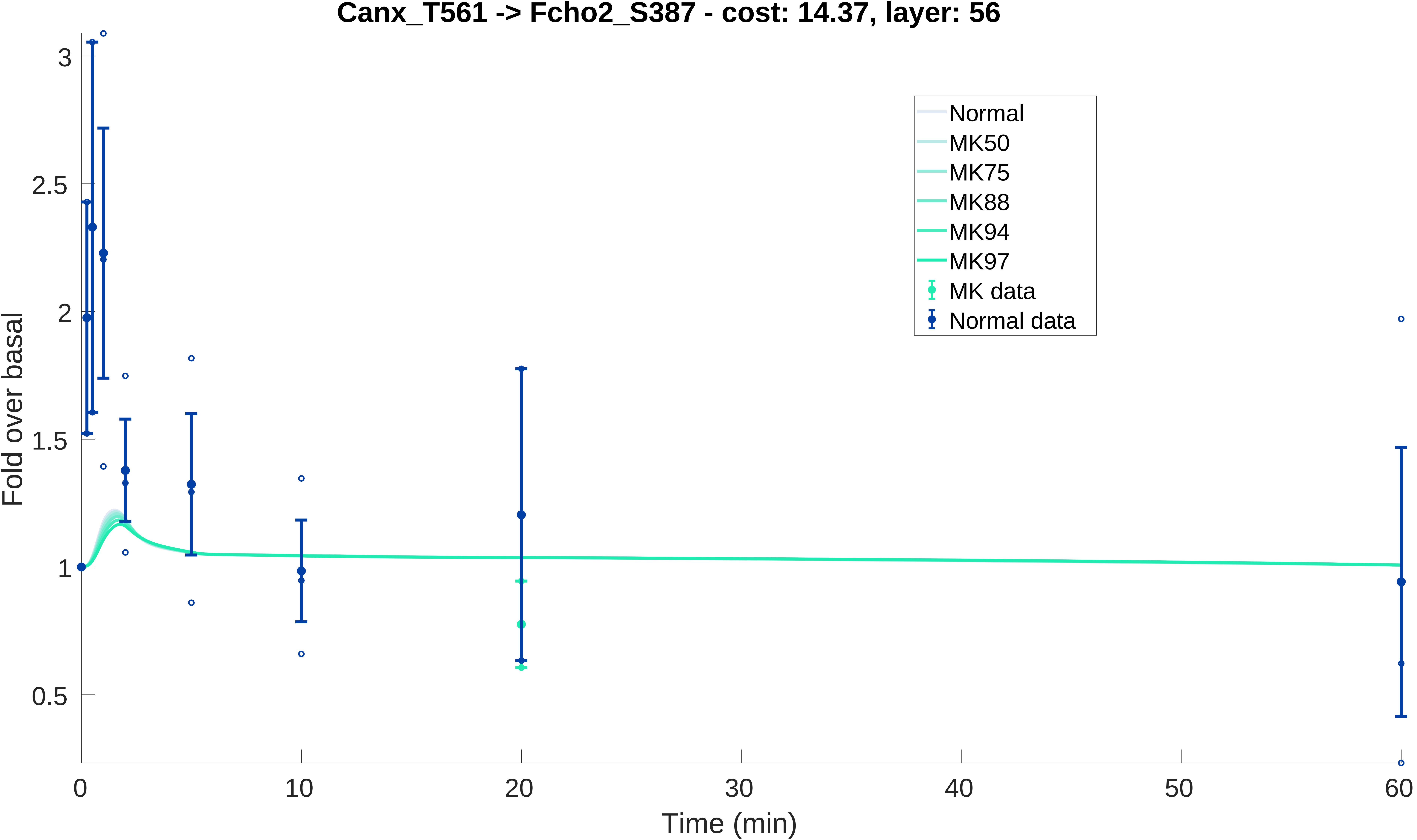
Canx_T561 -> Fcho2_S387 - cost: 14.37, layer: 56
Fold over basal
Time (min)
0
10
20
30
40
50
60
0.5
1
1.5
2
2.5
3
Normal
MK50
MK75
MK88
MK94
MK97
MK data
Normal data

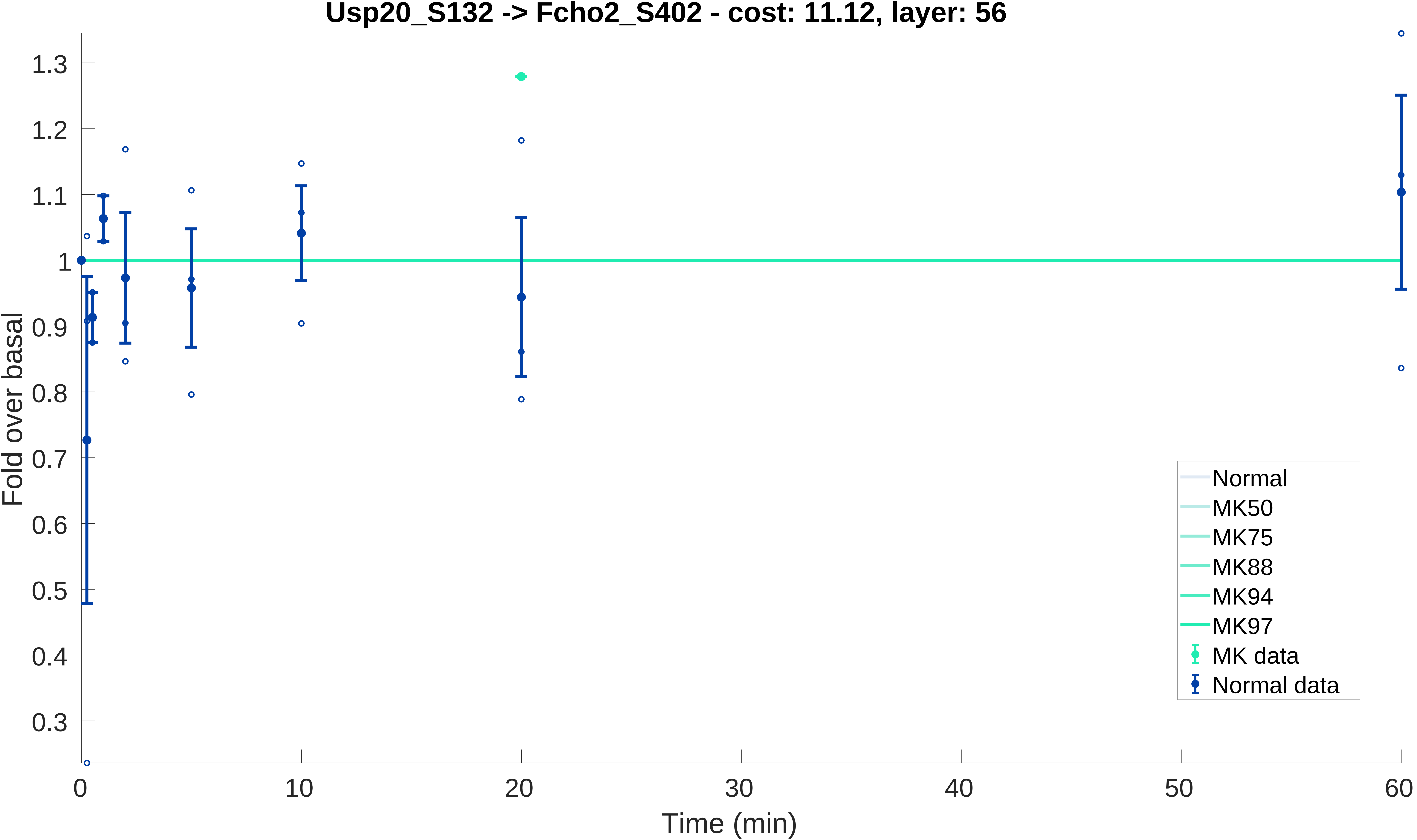
Usp20_S132 -> Fcho2_S402 - cost: 11.12, layer: 56
Fold over basal
Time (min)
1.3
1.2
1.1
1
0.9
0.8
0.7
0.6
0.5
0.4
0.3
0
10
20
30
40
50
60
Normal
MK50
MK75
MK88
MK94
MK97
MK data
Normal data

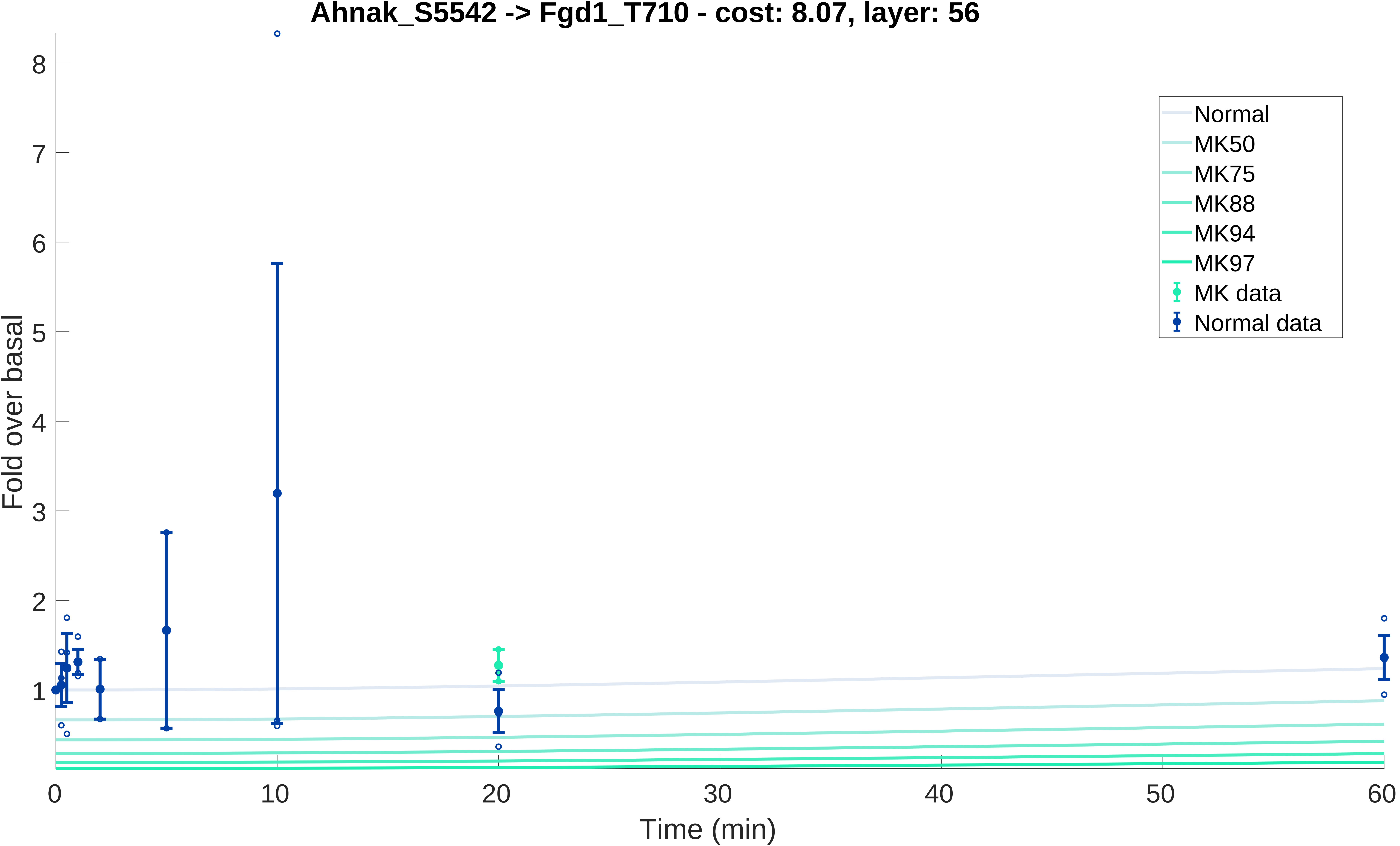

Ahnak_S5542 -> Fgd1_T710 - cost: 8.07, layer: 56
Fold over basal
Time (min)
0
10
20
30
40
50
60
1
2
3
4
5
6
7
8
Normal
MK50
MK75
MK88
MK94
MK97
MK data
Normal data

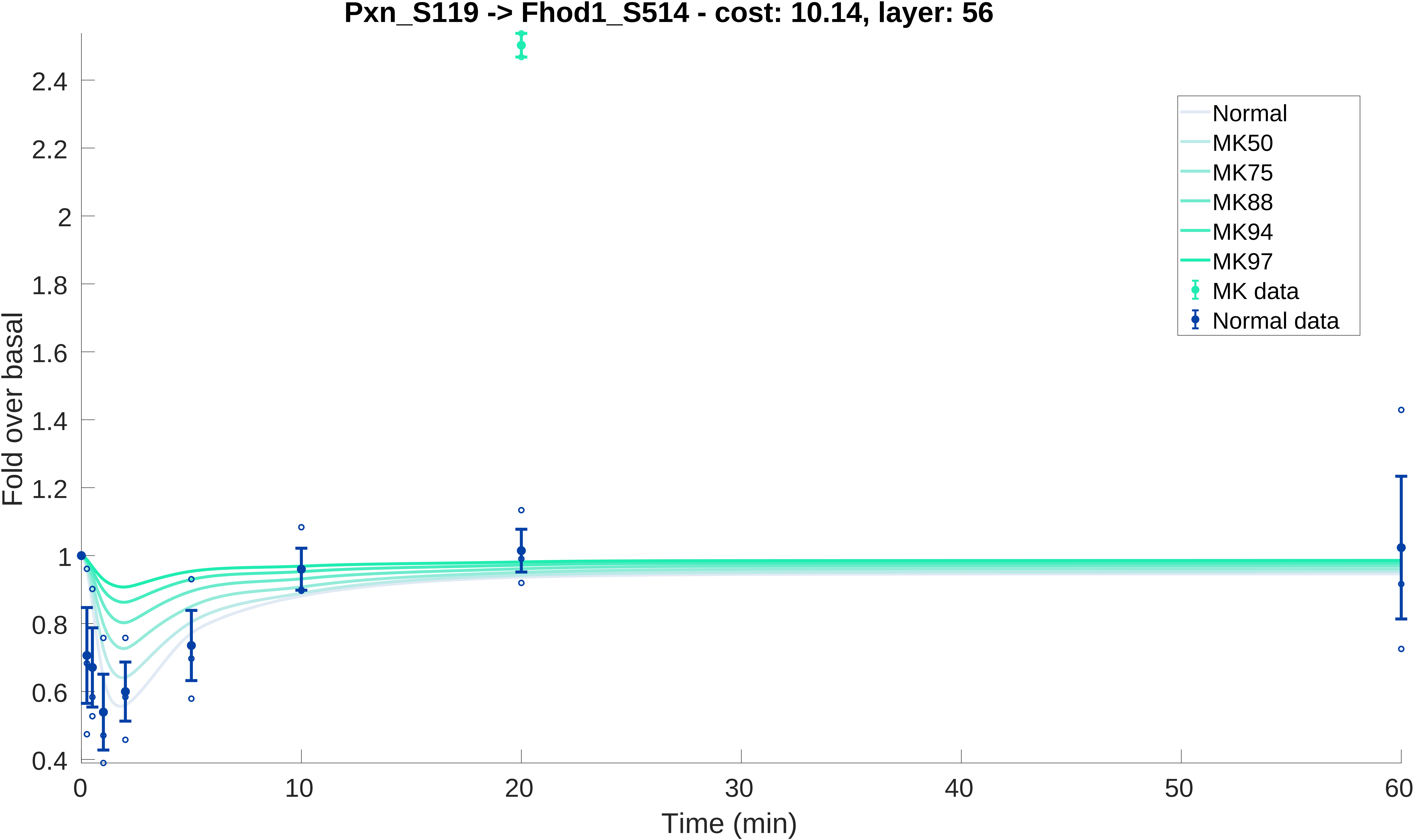

Pxn_S119 -> Fhod1_S514 - cost: 10.14, layer: 56
Fold over basal
Time (min)
0.4
0.6
0.8
1
1.2
1.4
1.6
1.8
2
2.2
2.4
0
10
20
30
40
50
60
Normal
MK50
MK75
MK88
MK94
MK97
MK data
Normal data

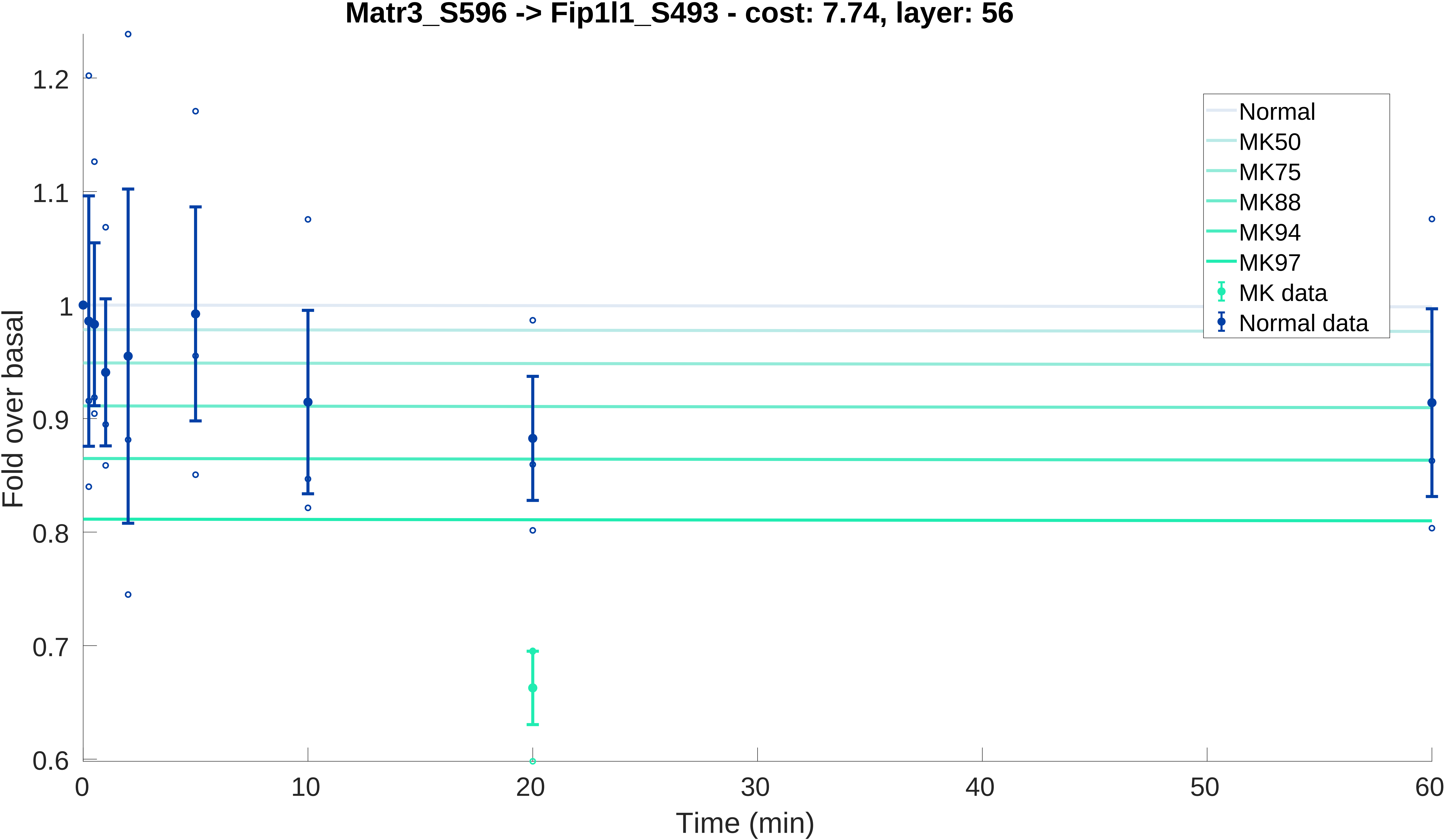

Matr3_S596 -> Fip1l1_S493 - cost: 7.74, layer: 56
Fold over basal
Time (min)
0.6
0.7
0.8
0.9
1
1.1
1.2
0
10
20
30
40
50
60
Normal
MK50
MK75
MK88
MK94
MK97
MK data
Normal data

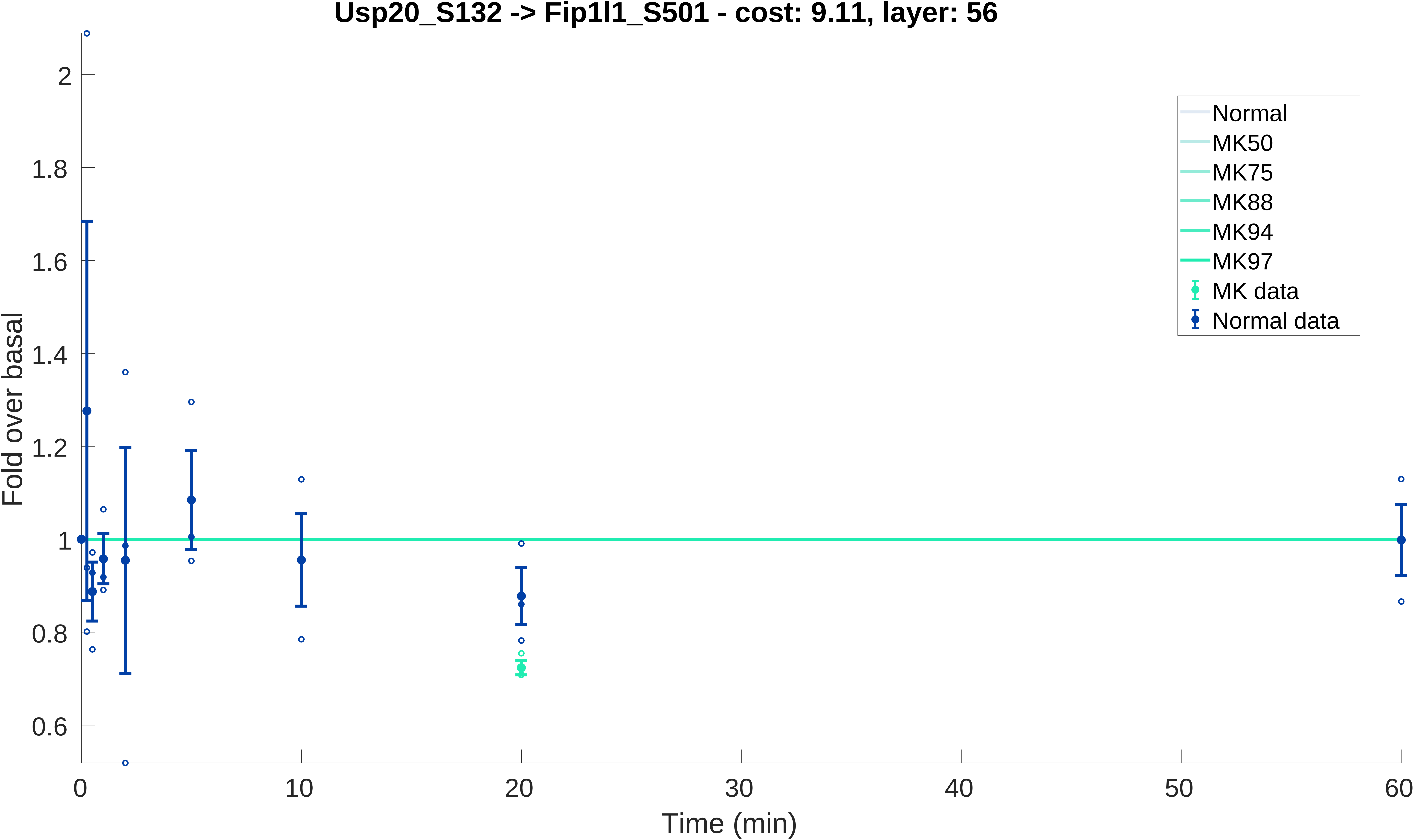
Usp20_S132 -> Fip1l1_S501 - cost: 9.11, layer: 56
Fold over basal
Time (min)
0
10
20
30
40
50
60
0.6
0.8
1
1.2
1.4
1.6
1.8
2
Normal
MK50
MK75
MK88
MK94
MK97
MK data
Normal data

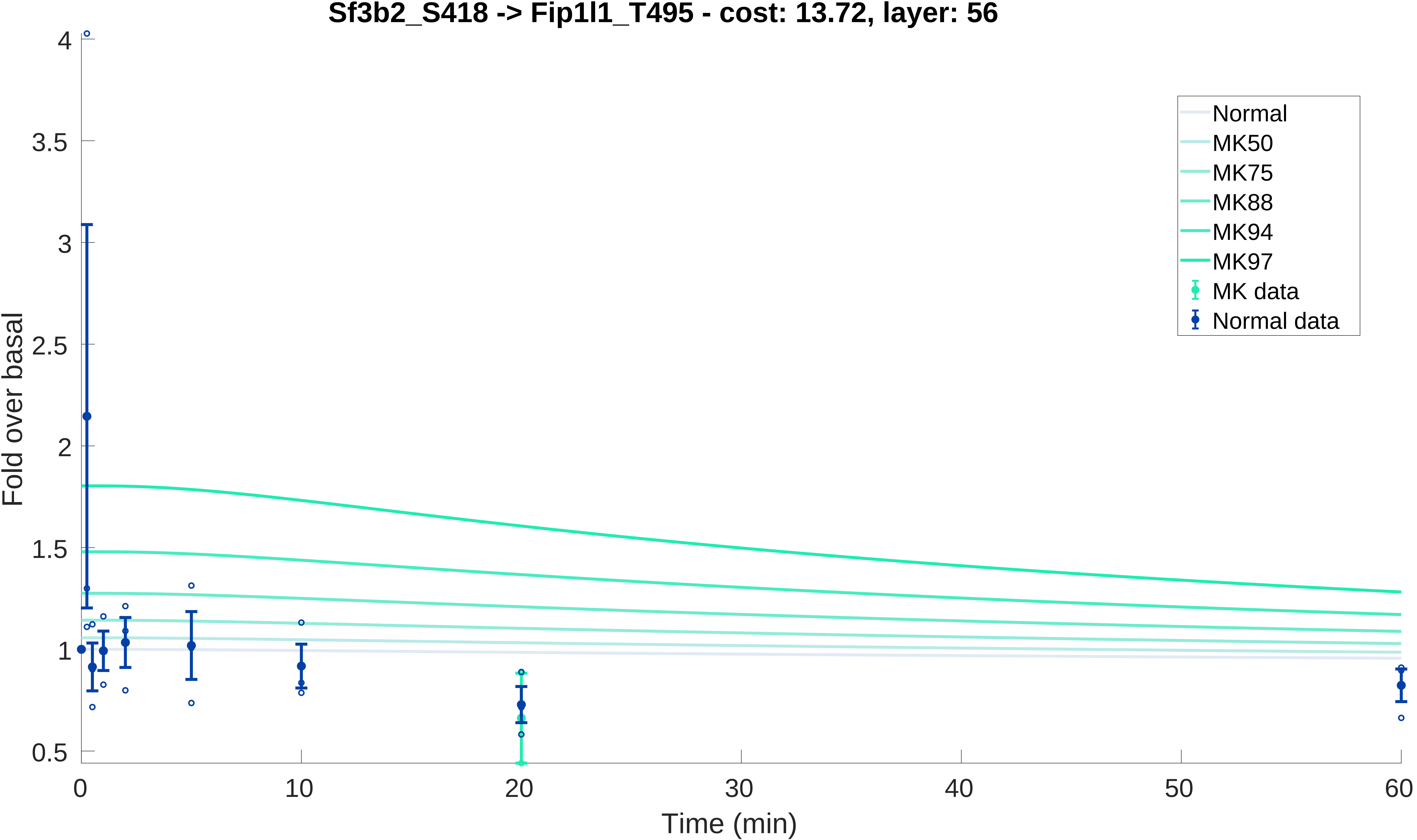

Sf3b2_S418 -> Fip1l1_T495 - cost: 13.72, layer: 56
Fold over basal
Time (min)
0.5
1
1.5
2
2.5
3
3.5
4
0
10
20
30
40
50
60
Normal
MK50
MK75
MK88
MK94
MK97
MK data
Normal data

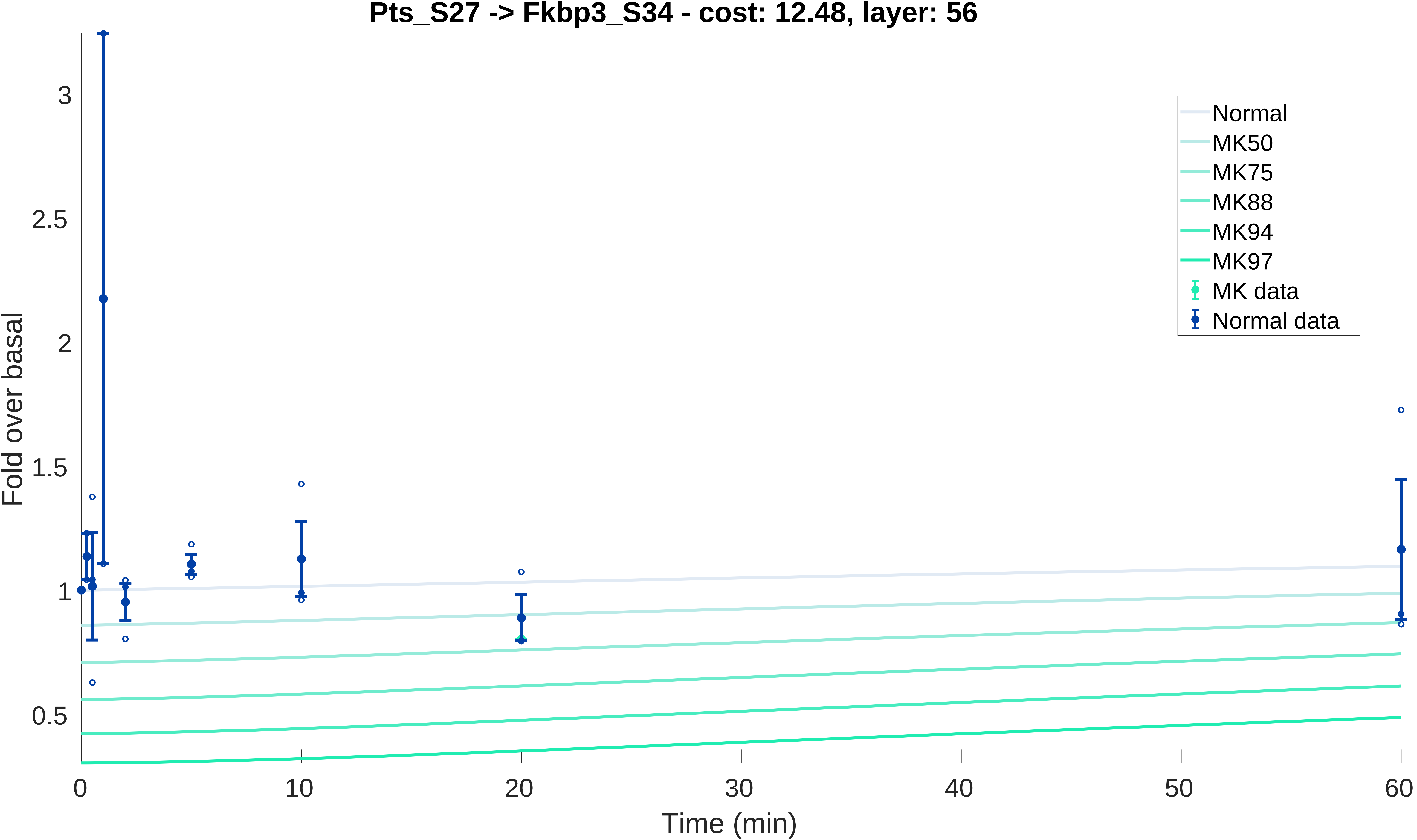
Pts_S27 -> Fkbp3_S34 - cost: 12.48, layer: 56
Fold over basal
Time (min)
0
10
20
30
40
50
60
0.5
1
1.5
2
2.5
3
Normal
MK50
MK75
MK88
MK94
MK97
MK data
Normal data

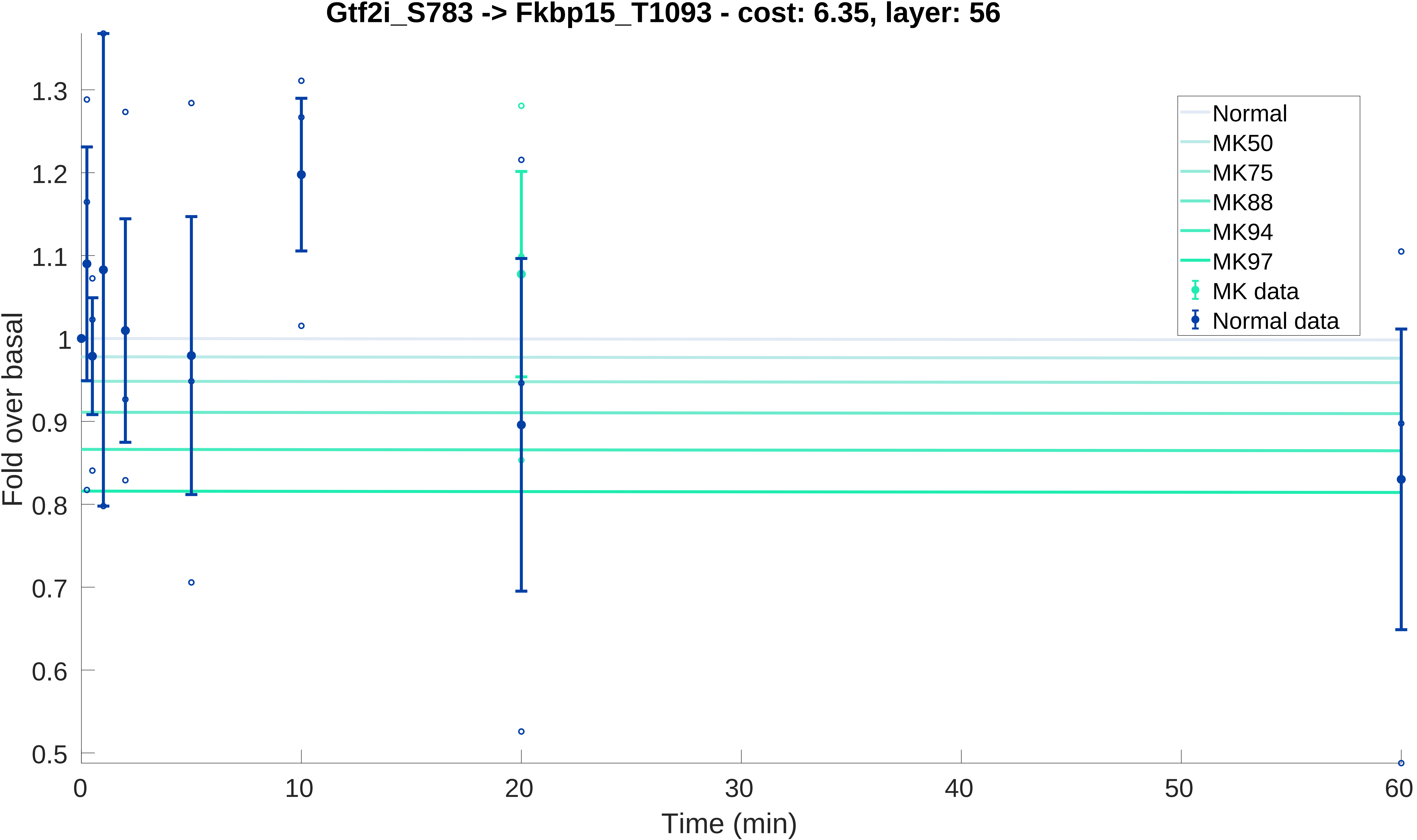

Gtf2i_S783 -> Fkbp15_T1093 - cost: 6.35, layer: 56
Fold over basal
Time (min)
Normal
MK50
MK75
MK88
MK94
MK97
MK data
Normal data
1.3
1.2
1.1
1
0.9
0.8
0.7
0.6
0.5
0
10
20
30
40
50
60

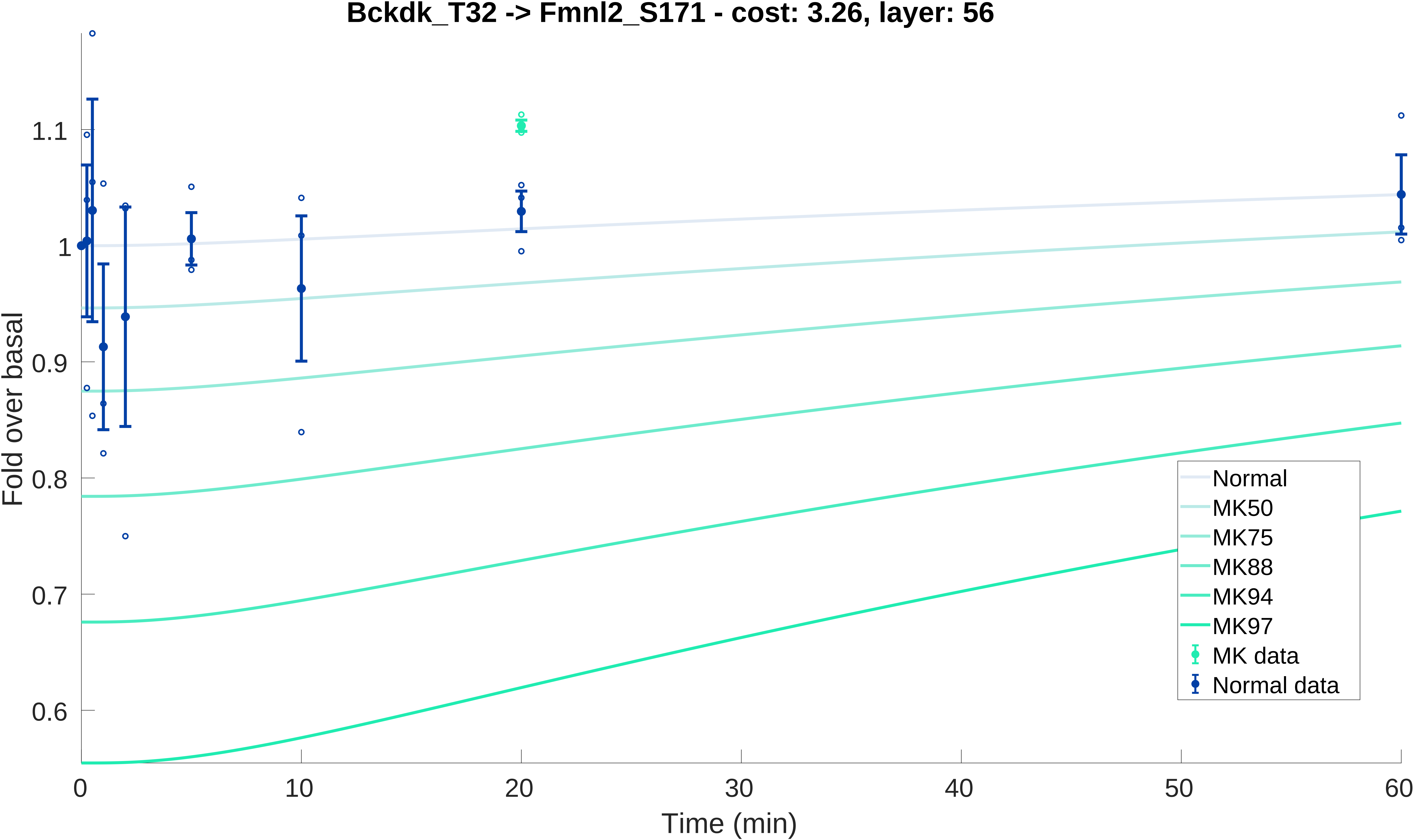

Bckdk_T32 -> Fmnl2_S171 - cost: 3.26, layer: 56
Fold over basal
Time (min)
1.1
1
0.9
0.8
0.7
0.6
0
10
20
30
40
50
60
Normal
MK50
MK75
MK88
MK94
MK97
MK data
Normal data

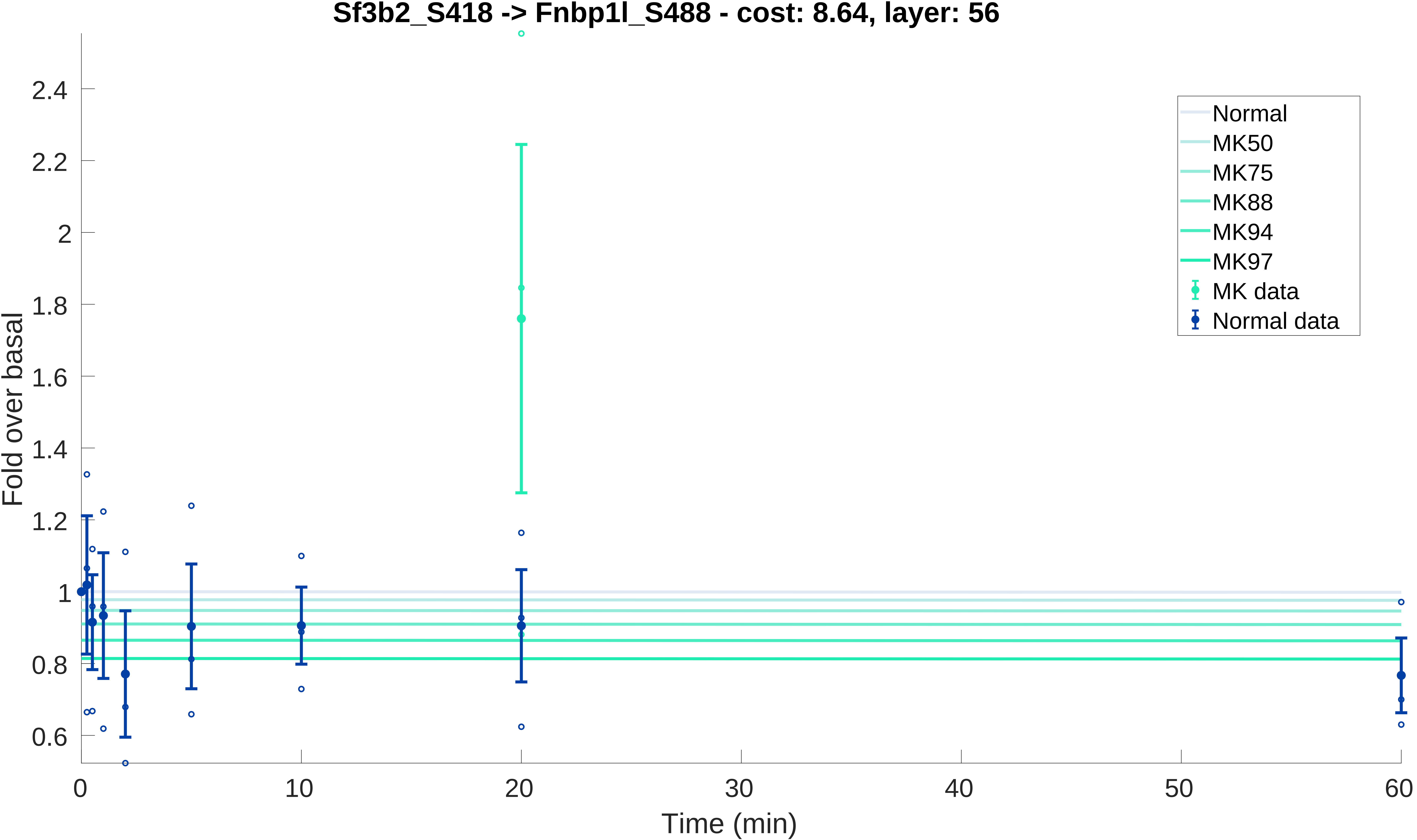

Sf3b2_S418 -> Fnbp1l_S488 - cost: 8.64, layer: 56
Fold over basal
Time (min)
0
10
20
30
40
50
60
0.6
0.8
1
1.2
1.4
1.6
1.8
2
2.2
2.4
Normal
MK50
MK75
MK88
MK94
MK97
MK data
Normal data

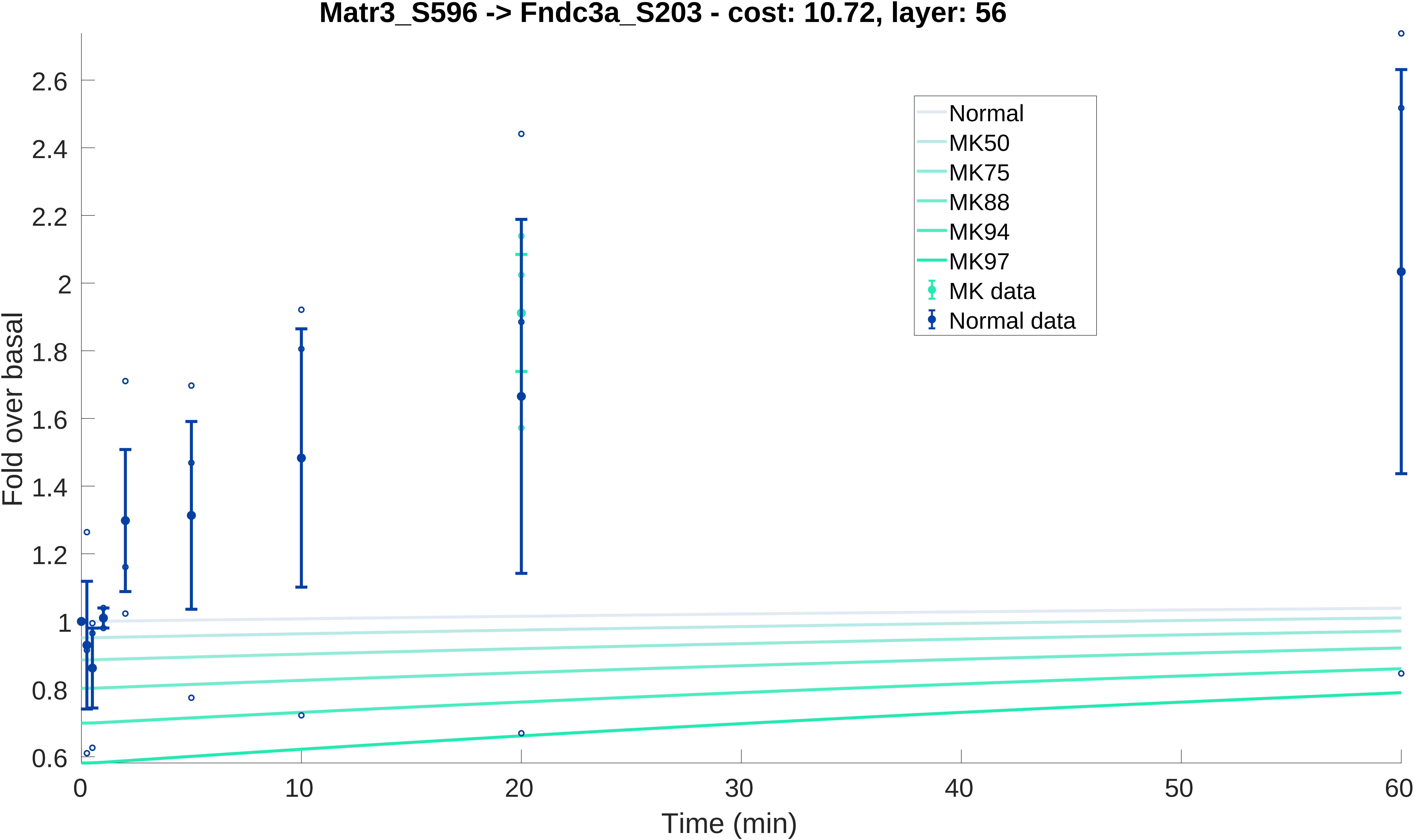

Matr3_S596 -> Fndc3a_S203 - cost: 10.72, layer: 56
Fold over basal
Time (min)
0.6
0.8
1
1.2
1.4
1.6
1.8
2
2.2
2.4
2.6
0
10
20
30
40
50
60
Normal
MK50
MK75
MK88
MK94
MK97
MK data
Normal data

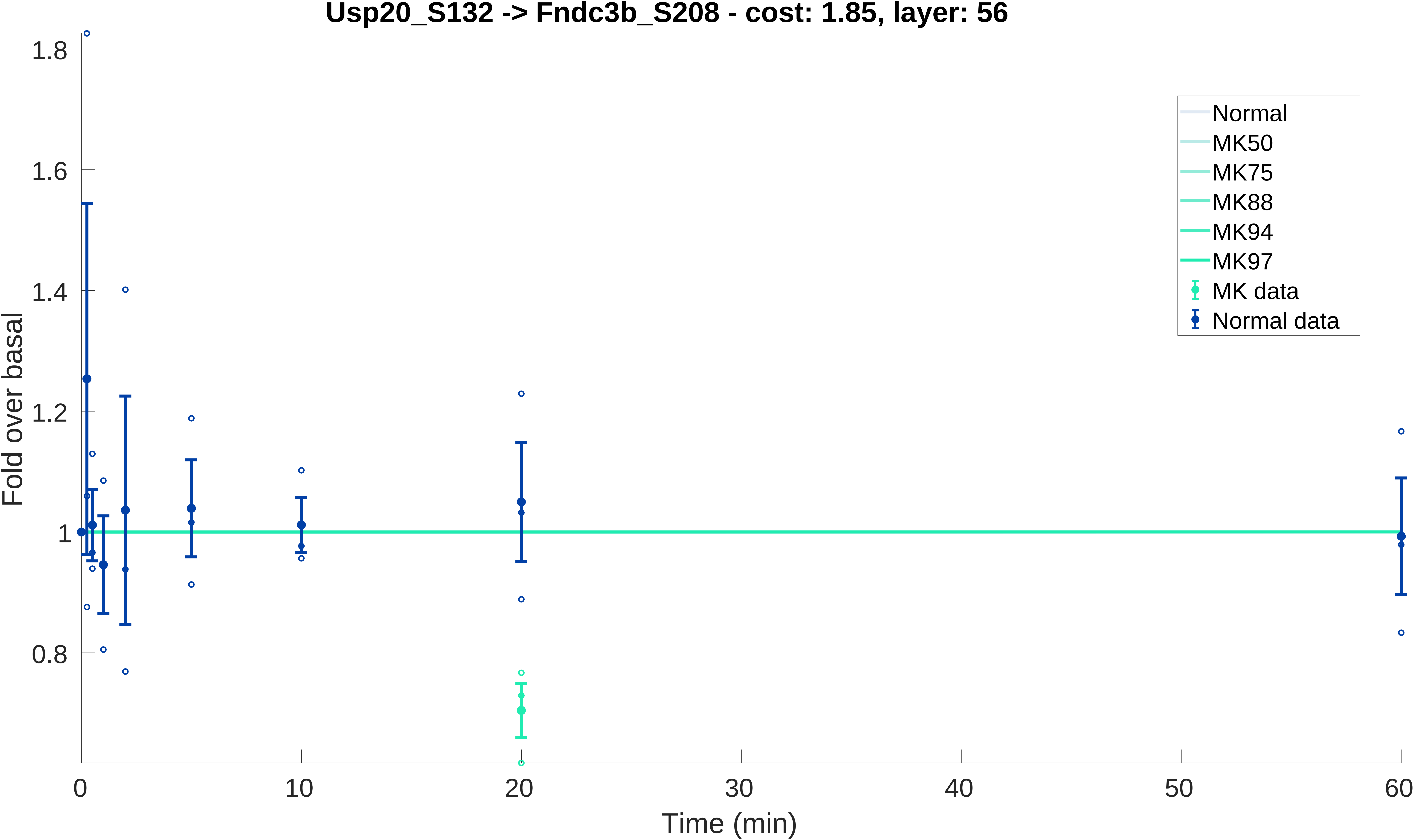

Usp20_S132 -> Fndc3b_S208 - cost: 1.85, layer: 56
Fold over basal
1.8
1.6
1.4
1.2
1
0.8
0
10
20
30
40
50
60
Time (min)
Normal
MK50
MK75
MK88
MK94
MK97
MK data
Normal data

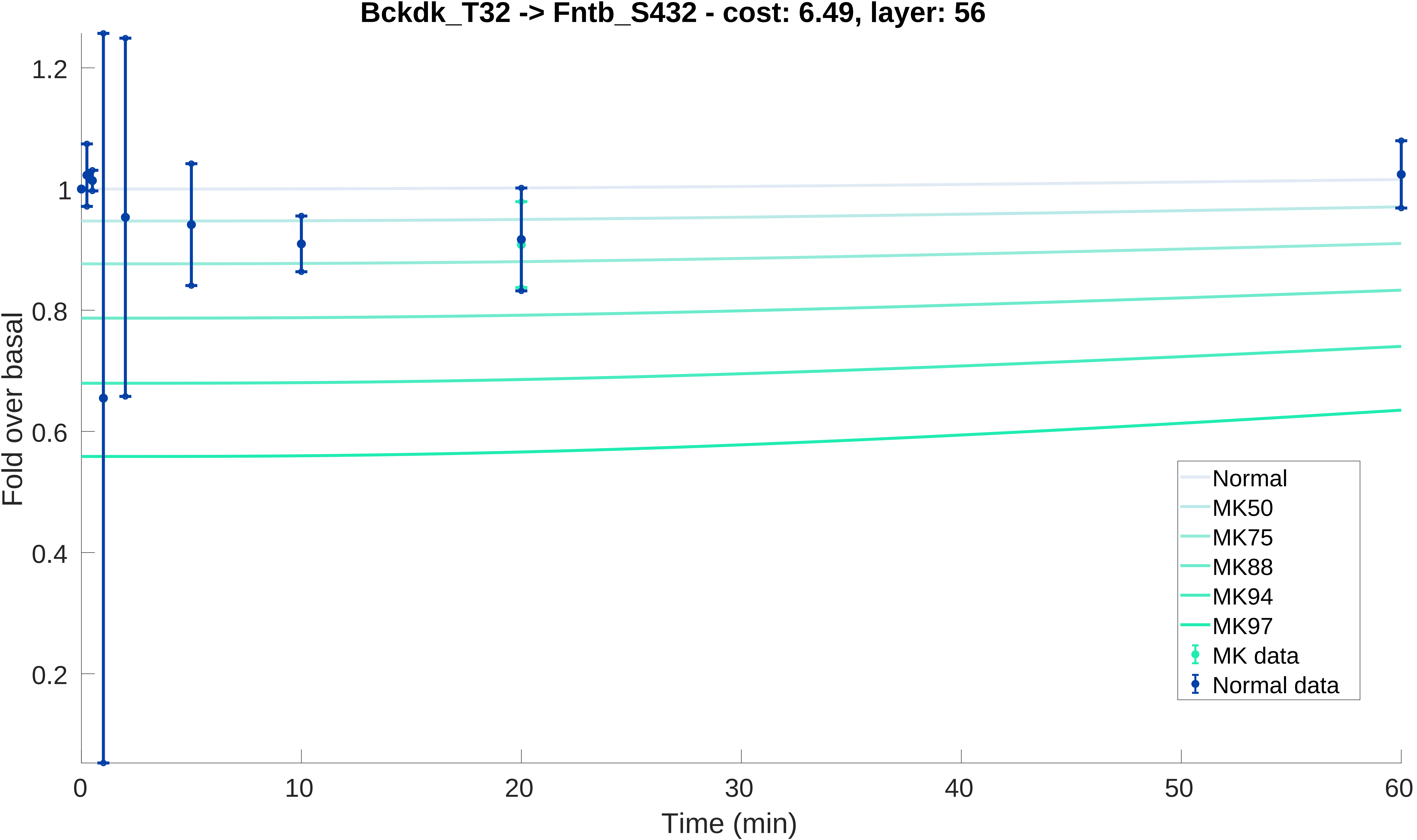
Bckdk_T32 -> Fntb_S432 - cost: 6.49, layer: 56
Fold over basal
Time (min)
0
10
20
30
40
50
60
0.2
0.4
0.6
0.8
1
1.2
Normal
MK50
MK75
MK88
MK94
MK97
MK data
Normal data

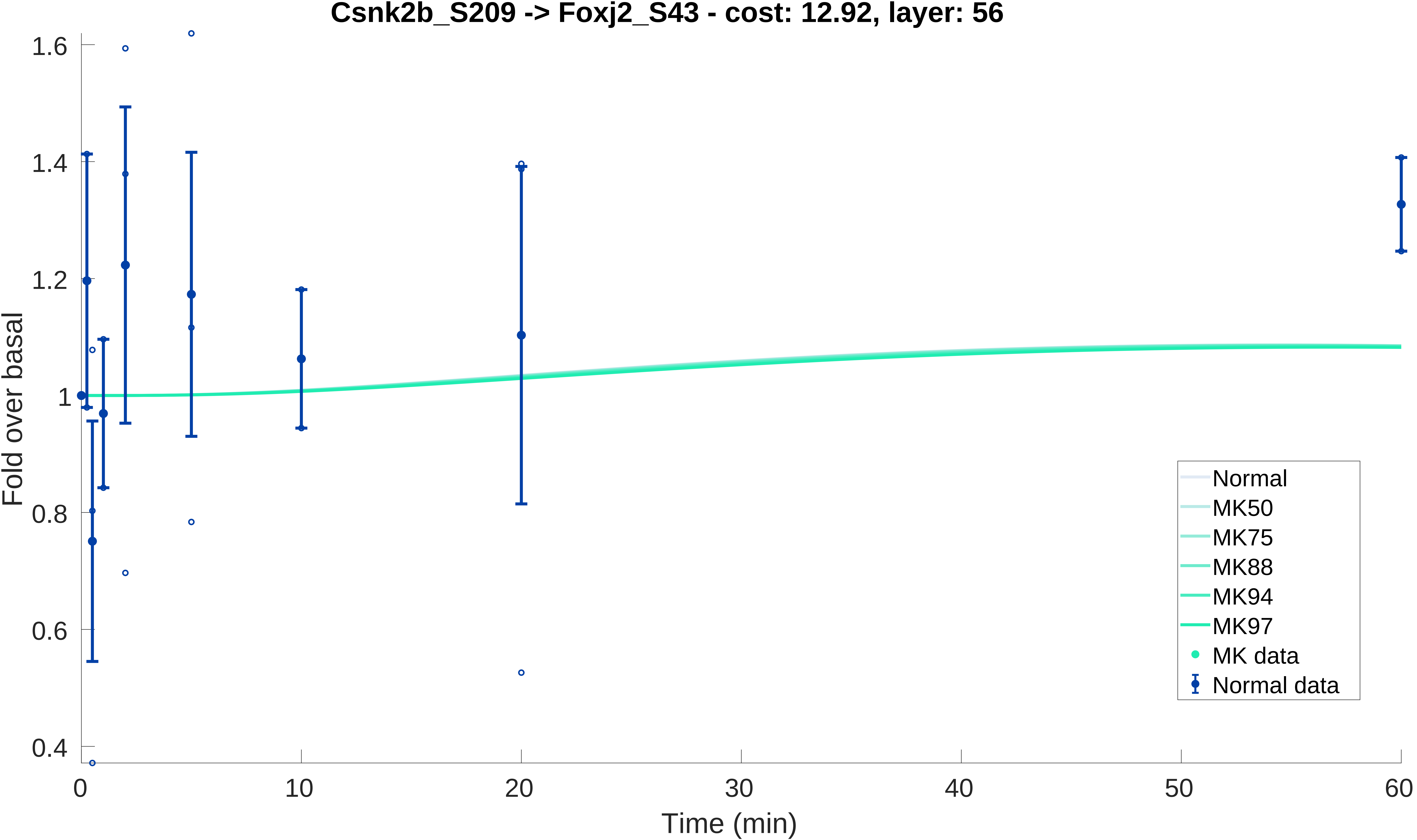

Csnk2b_S209 -> Foxj2_S43 - cost: 12.92, layer: 56
Fold over basal
Time (min)
1.6
1.4
1.2
1
0.8
0.6
0.4
0
10
20
30
40
50
60
Normal
MK50
MK75
MK88
MK94
MK97
MK data
Normal data

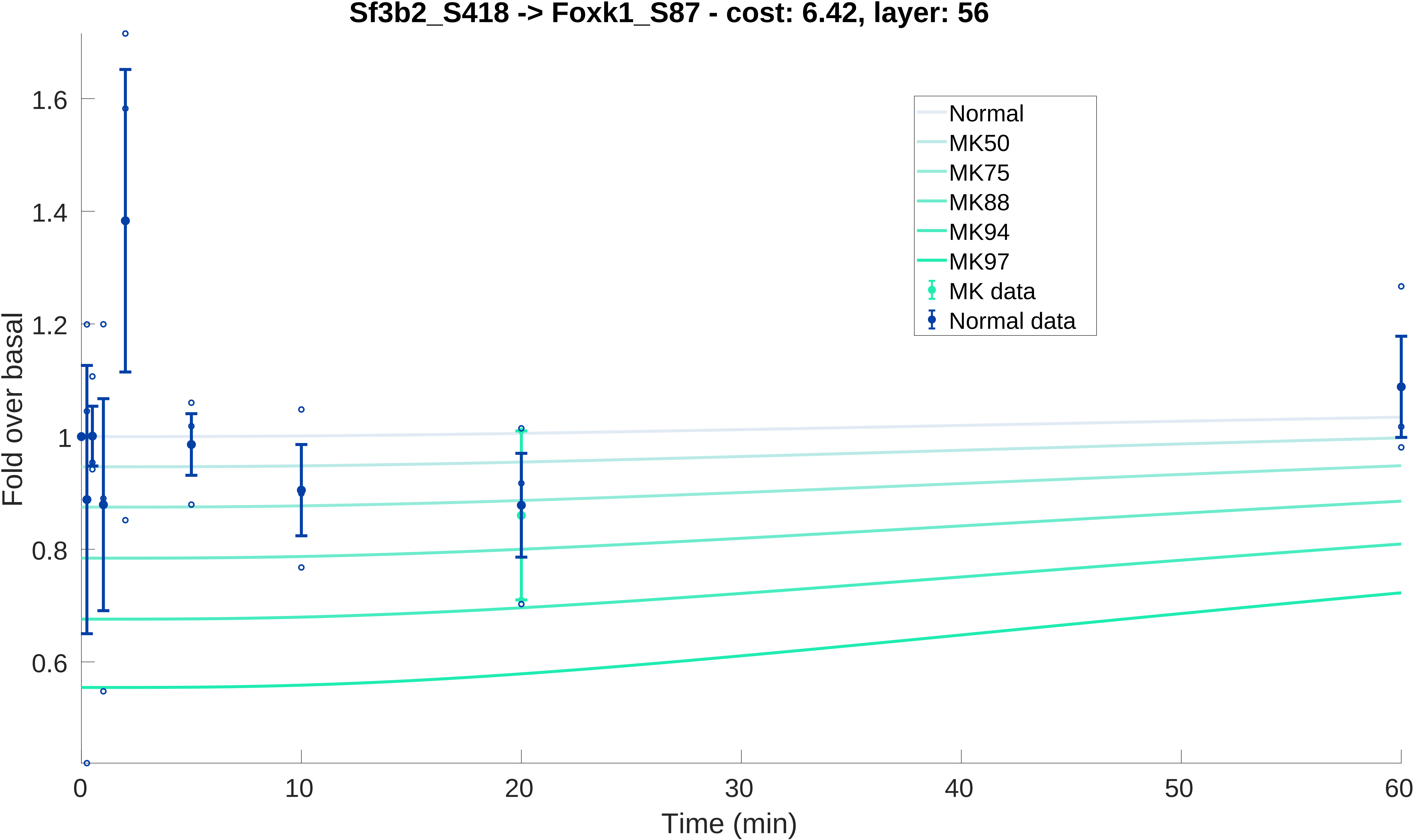

Sf3b2_S418 -> Foxk1_S87 - cost: 6.42, layer: 56
Fold over basal
1.6
1.4
1.2
1
0.8
0.6
0
10
20
30
40
50
60
Time (min)
Normal
MK50
MK75
MK88
MK94
MK97
MK data
Normal data

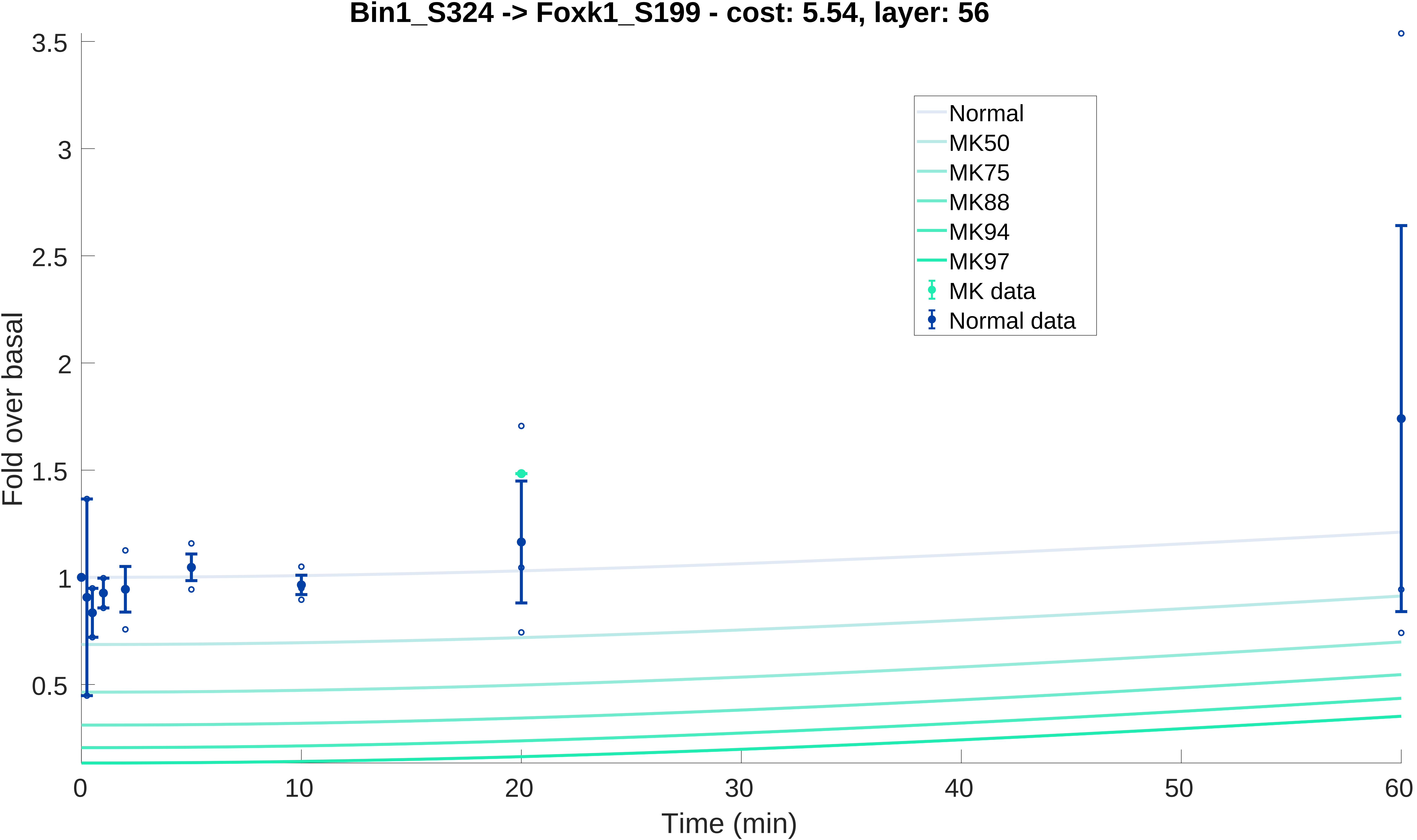

Bin1_S324 -> Foxk1_S199 - cost: 5.54, layer: 56
Fold over basal
3.5
3
2.5
2
1.5
1
0.5
0
10
20
30
40
50
60
Time (min)
Normal
MK50
MK75
MK88
MK94
MK97
MK data
Normal data

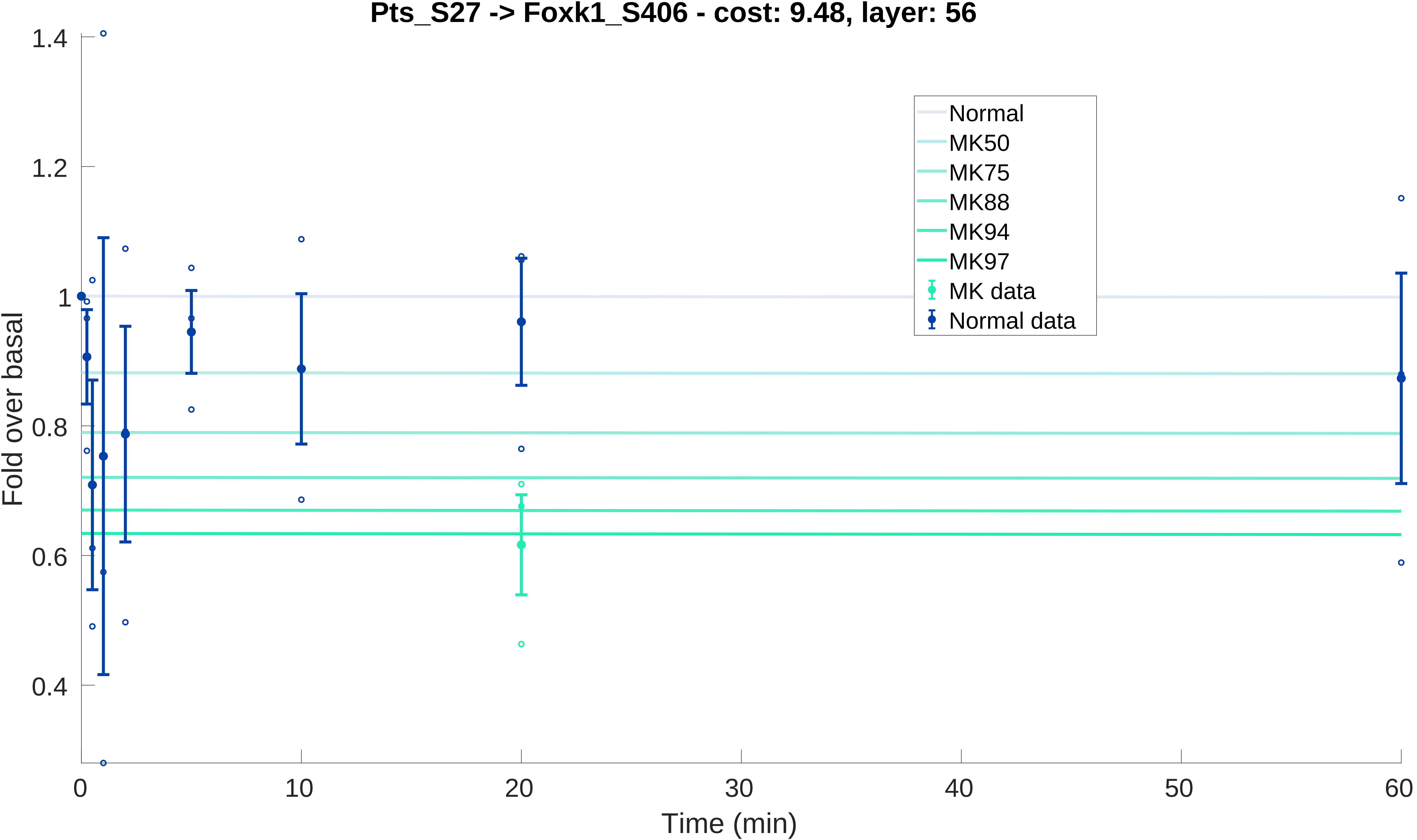

Pts_S27 -> Foxk1_S406 - cost: 9.48, layer: 56
Normal
MK50
MK75
MK88
MK94
MK97
MK data
Normal data
Fold over basal
Time (min)
1.4
1.2
1
0.8
0.6
0.4
0
10
20
30
40
50
60

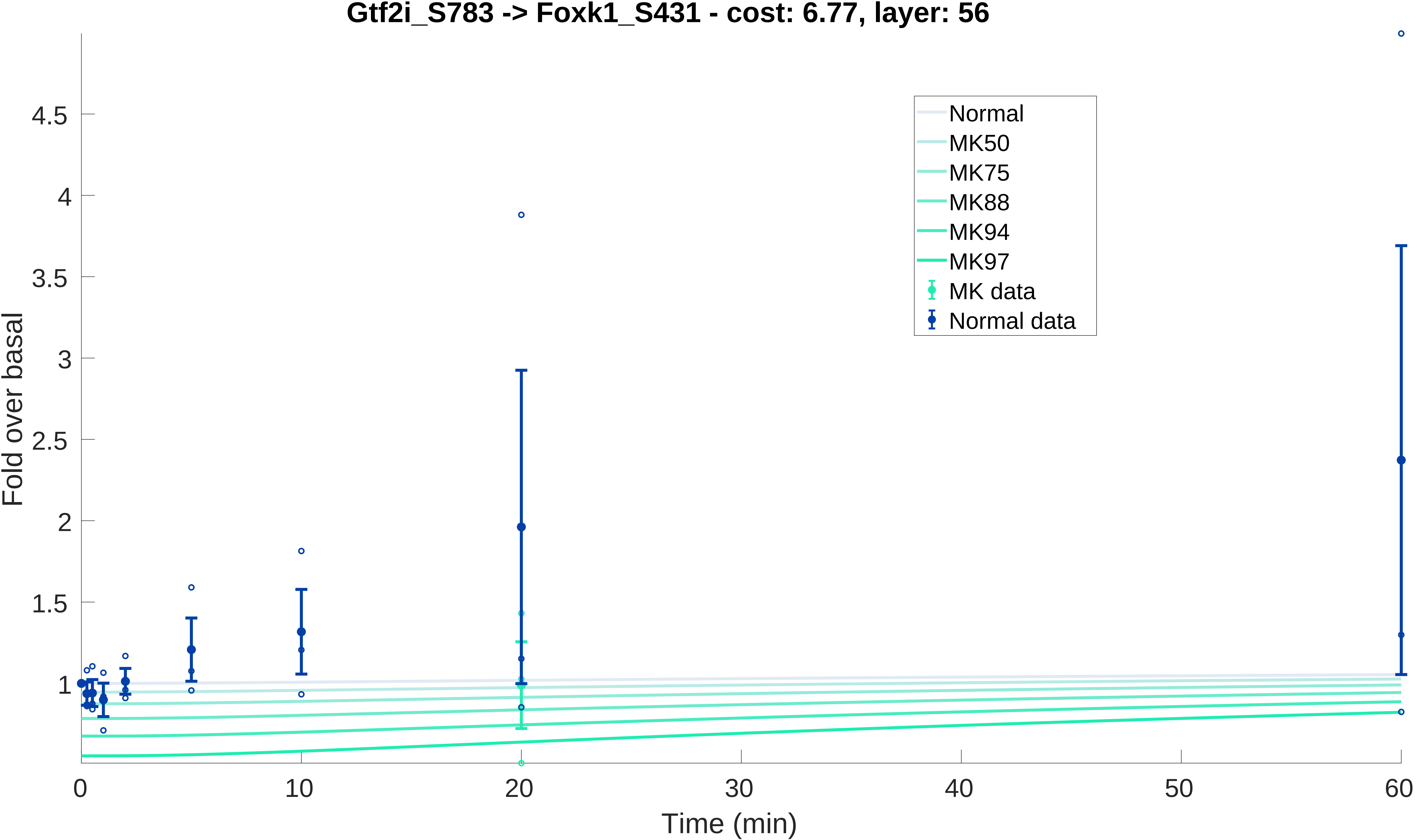

Gtf2i_S783 -> Foxk1_S431 - cost: 6.77, layer: 56
Fold over basal
Time (min)
Normal
MK50
MK75
MK88
MK94
MK97
MK data
Normal data
4.5
4
3.5
3
2.5
2
1.5
1
0
10
20
30
40
50
60

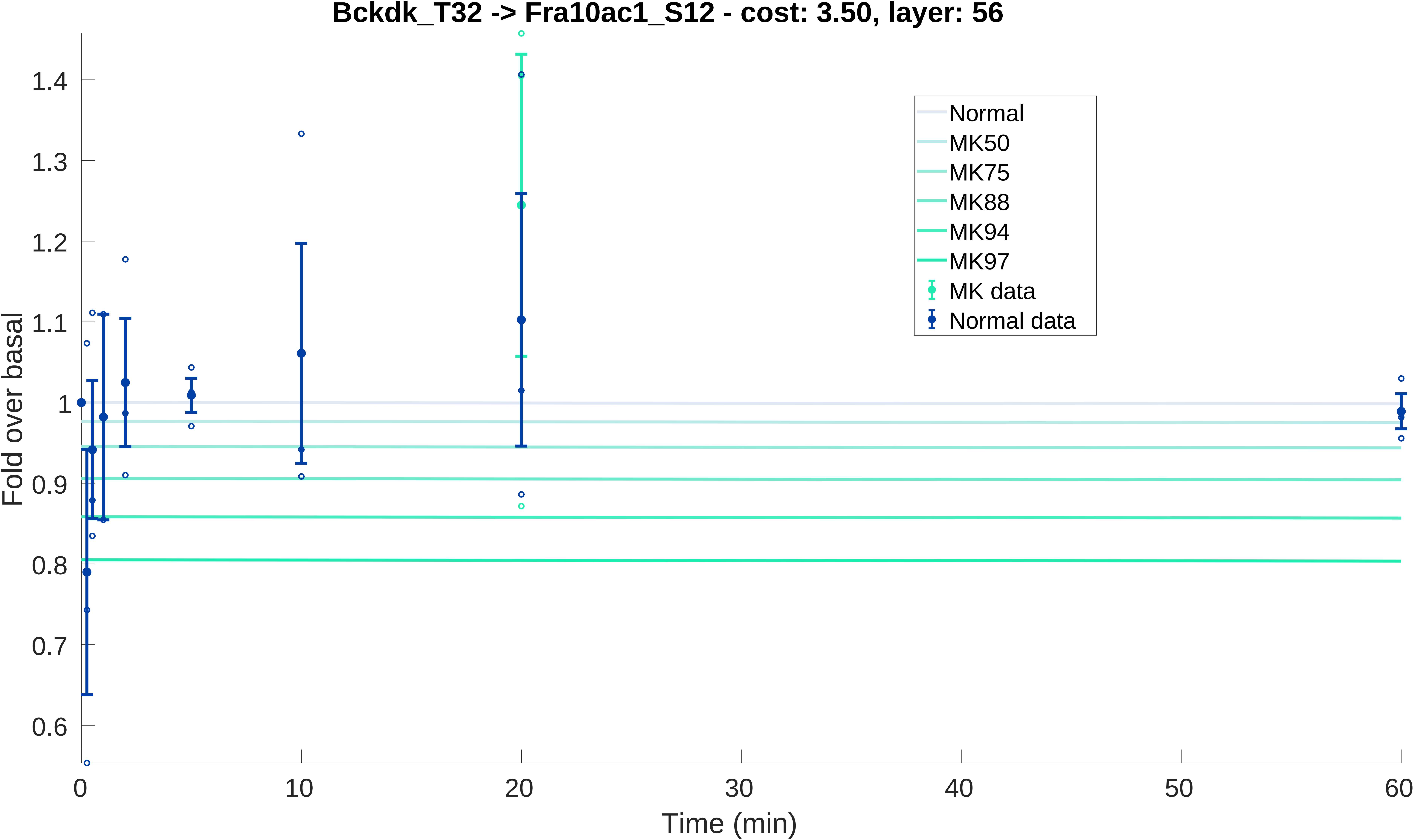

Bckdk_T32 -> Fra10ac1_S12 - cost: 3.50, layer: 56
Fold over basal
1.4
1.3
1.2
1.1
1
0.9
0.8
0.7
0.6
0
10
20
30
40
50
60
Time (min)
Normal
MK50
MK75
MK88
MK94
MK97
MK data
Normal data

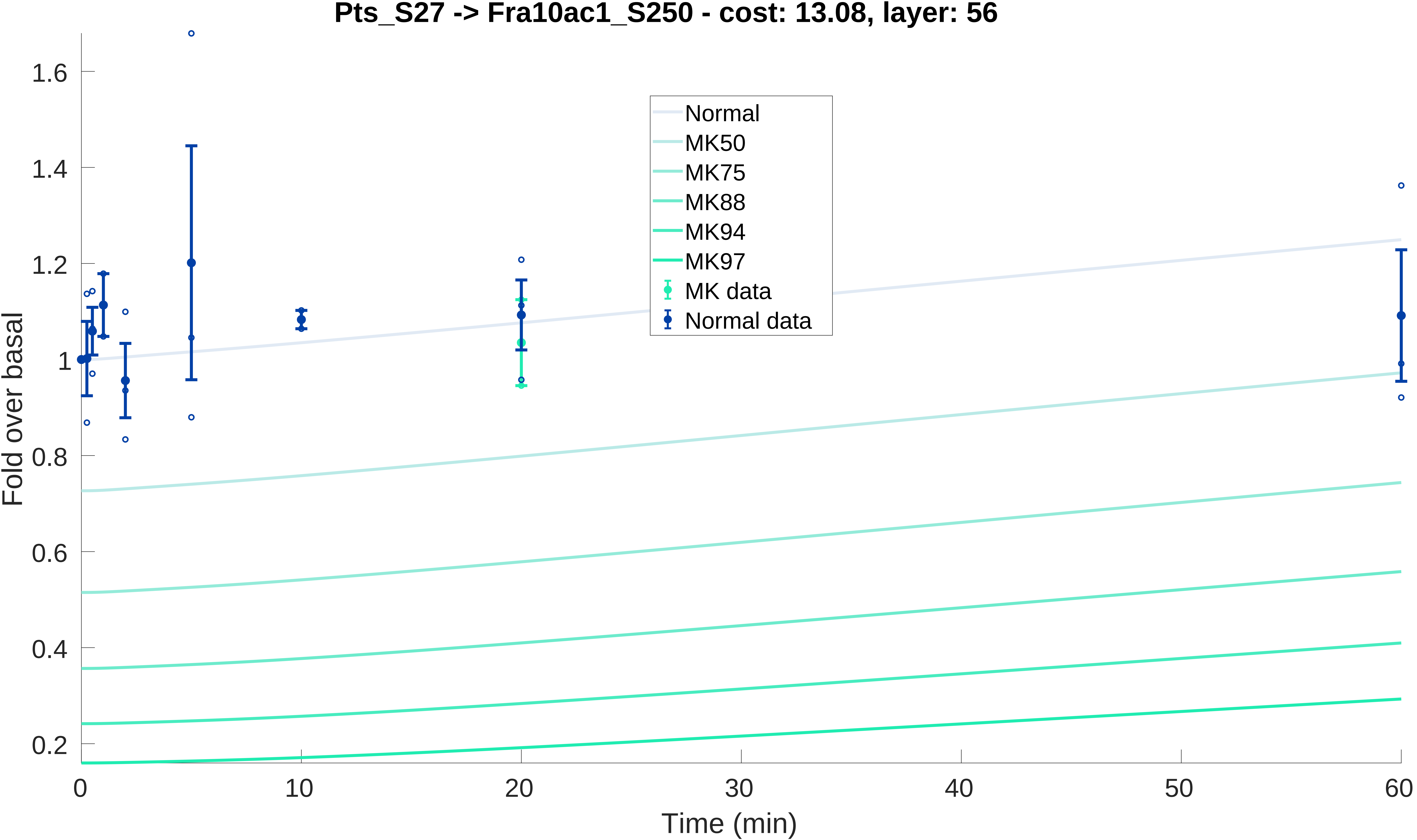
Pts_S27 -> Fra10ac1_S250 - cost: 13.08, layer: 56
Fold over basal
Time (min)
0
10
20
30
40
50
60
0.2
0.4
0.6
0.8
1
1.2
1.4
1.6
Normal
MK50
MK75
MK88
MK94
MK97
MK data
Normal data

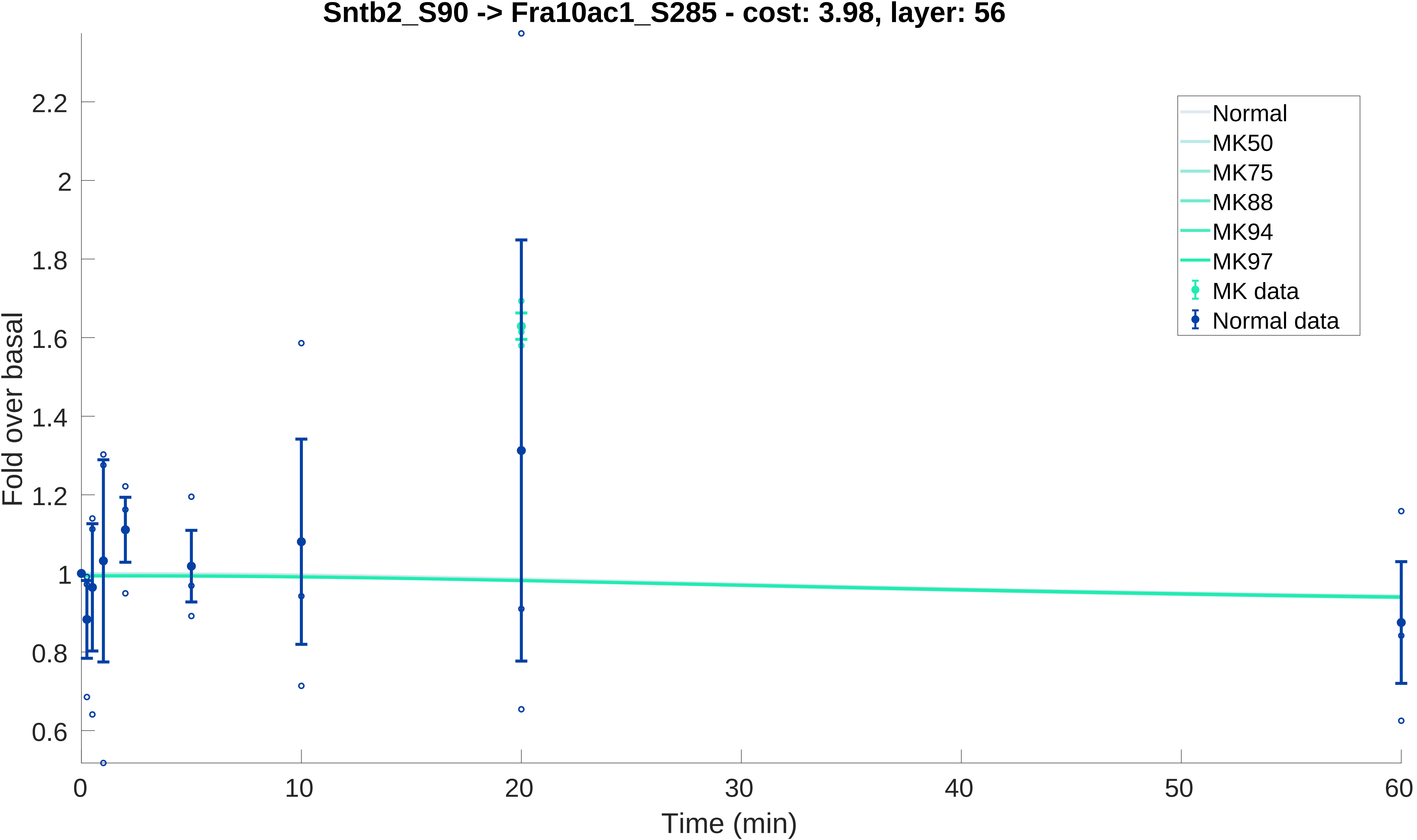

Sntb2_S90 -> Fra10ac1_S285 - cost: 3.98, layer: 56
Fold over basal
Time (min)
0.6
0.8
1
1.2
1.4
1.6
1.8
2
2.2
0
10
20
30
40
50
60
Normal
MK50
MK75
MK88
MK94
MK97
MK data
Normal data

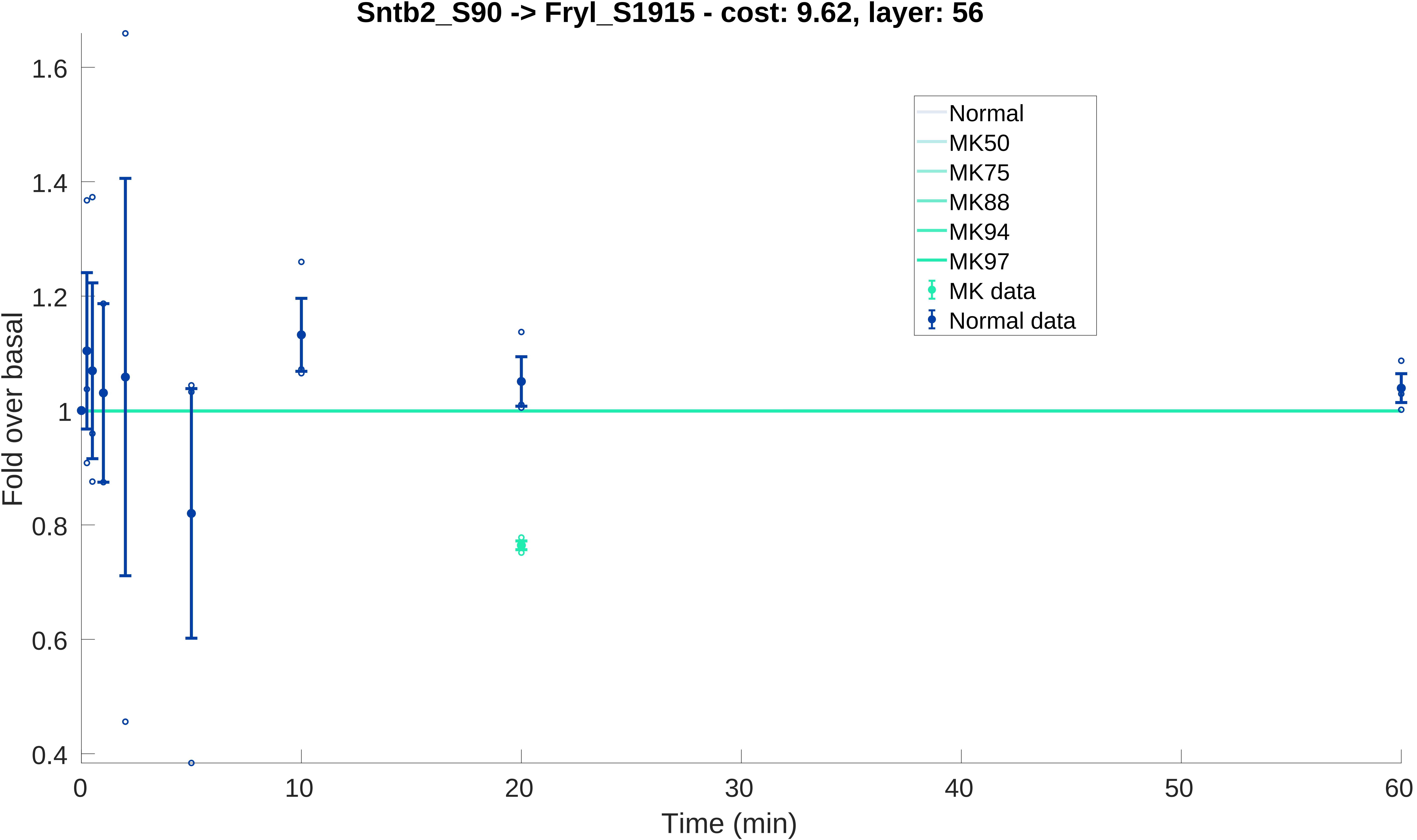

Sntb2_S90 -> Fryl_S1915 - cost: 9.62, layer: 56
Fold over basal
1.6
1.4
1.2
1
0.8
0.6
0.4
0
10
20
30
40
50
60
Time (min)
Normal
MK50
MK75
MK88
MK94
MK97
MK data
Normal data

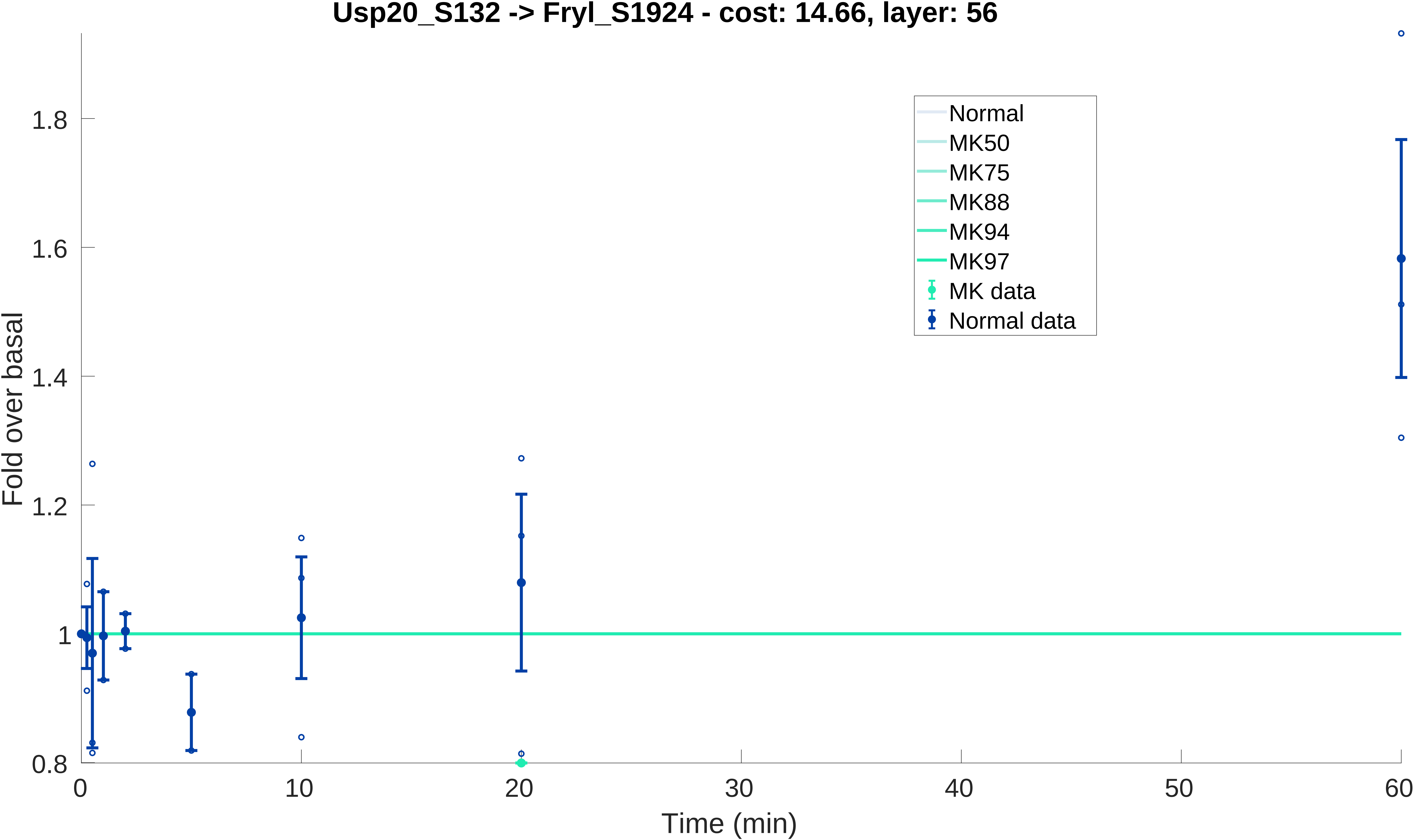

Usp20_S132 -> Fryl_S1924 - cost: 14.66, layer: 56
Fold over basal
Time (min)
0.8
1
1.2
1.4
1.6
1.8
0
10
20
30
40
50
60
Normal
MK50
MK75
MK88
MK94
MK97
MK data
Normal data

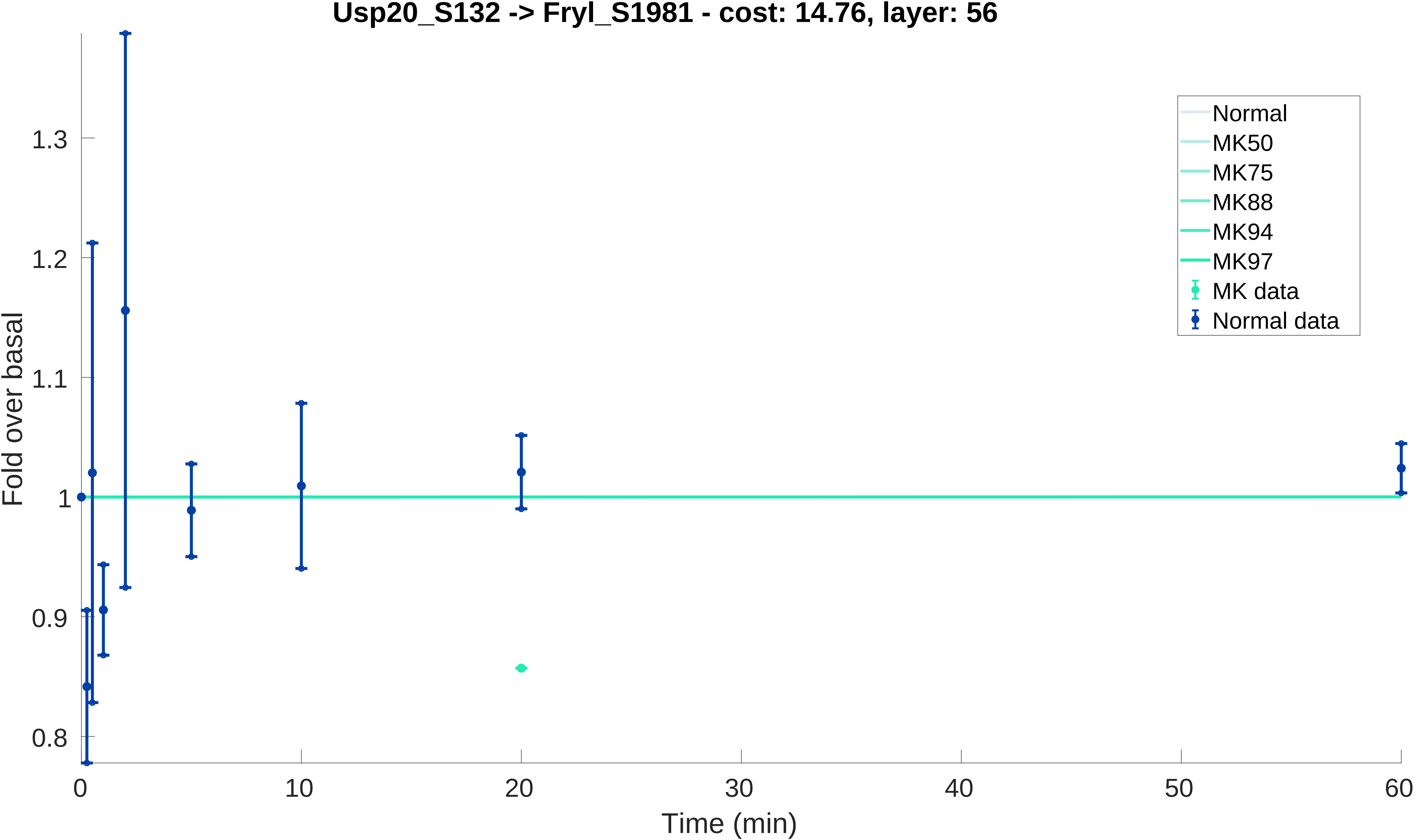

Usp20_S132 -> Fryl_S1981 - cost: 14.76, layer: 56
Fold over basal
Time (min)
1.3
1.2
1.1
1
0.9
0.8
0
10
20
30
40
50
60
Normal
MK50
MK75
MK88
MK94
MK97
MK data
Normal data

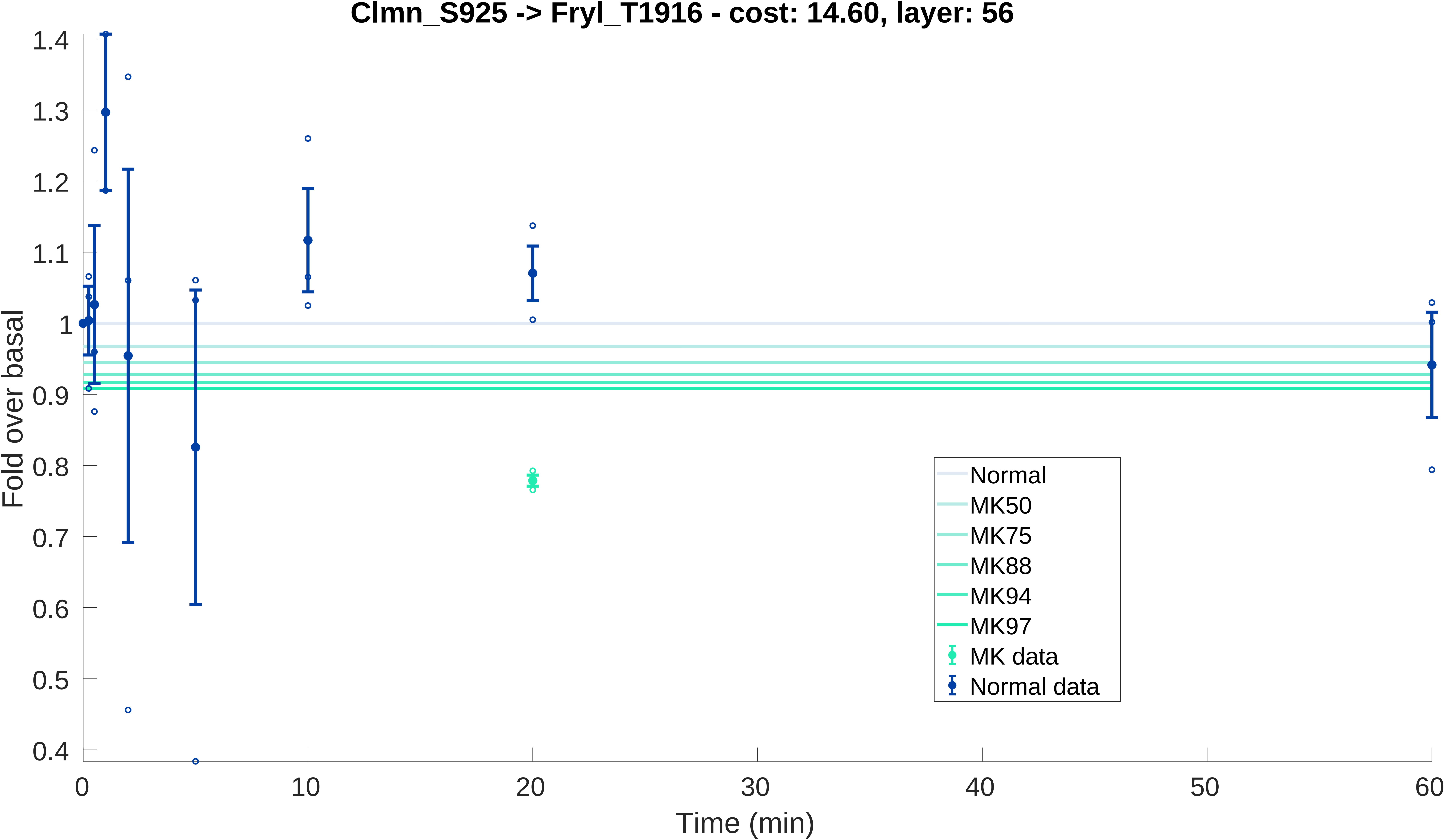

Clmn_S925 -> Fryl_T1916 - cost: 14.60, layer: 56
Fold over basal
Time (min)
0
10
20
30
40
50
60
0.4
0.5
0.6
0.7
0.8
0.9
1
1.1
1.2
1.3
1.4
Normal
MK50
MK75
MK88
MK94
MK97
MK data
Normal data

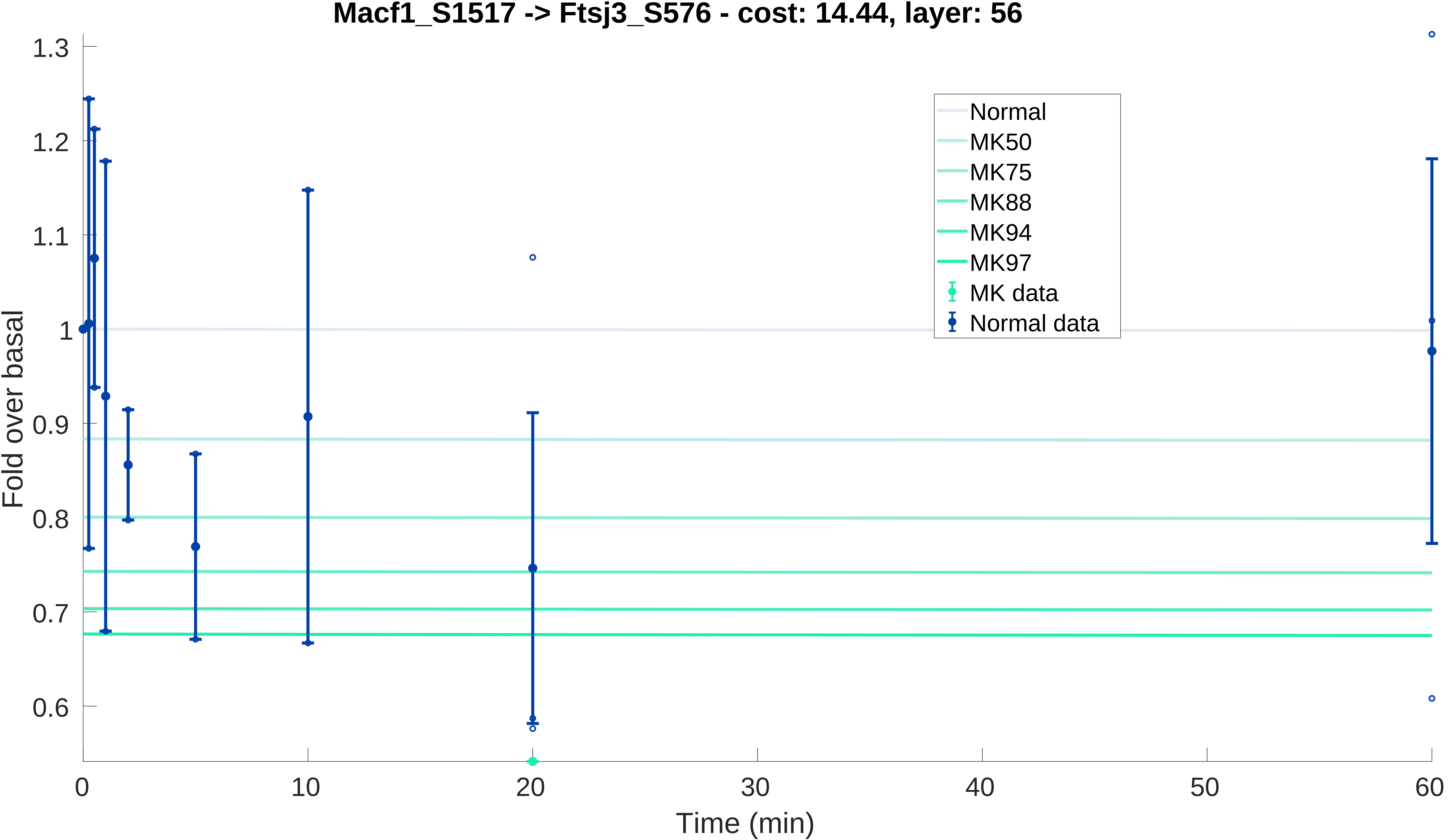

Macf1_S1517 -> Ftsj3_S576 - cost: 14.44, layer: 56
Fold over basal
Time (min)
1.3
1.2
1.1
1
0.9
0.8
0.7
0.6
0
10
20
30
40
50
60
Normal
MK50
MK75
MK88
MK94
MK97
MK data
Normal data

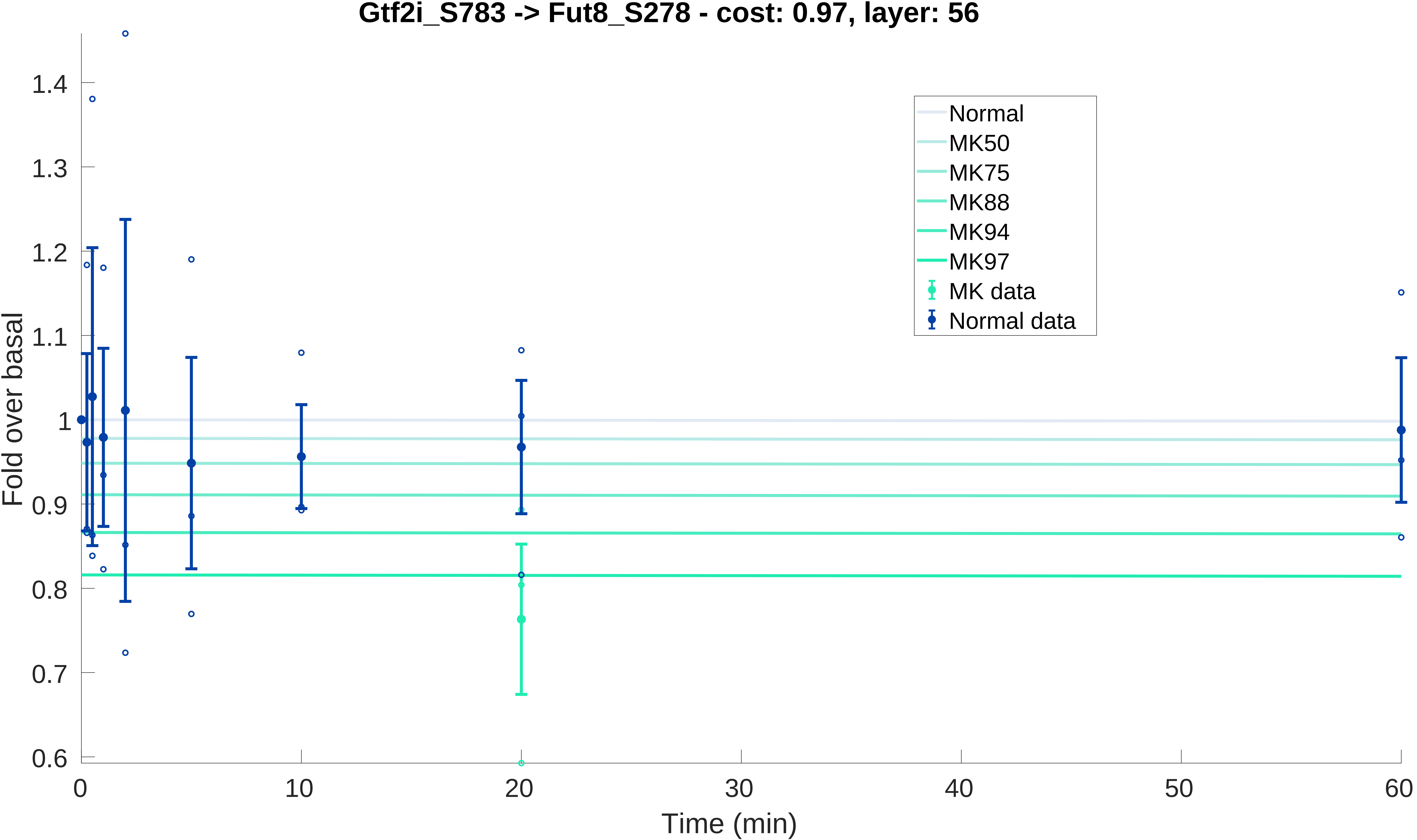

Gtf2i_S783 -> Fut8_S278 - cost: 0.97, layer: 56
Fold over basal
Time (min)
0.6
0.7
0.8
0.9
1
1.1
1.2
1.3
1.4
0
10
20
30
40
50
60
Normal
MK50
MK75
MK88
MK94
MK97
MK data
Normal data

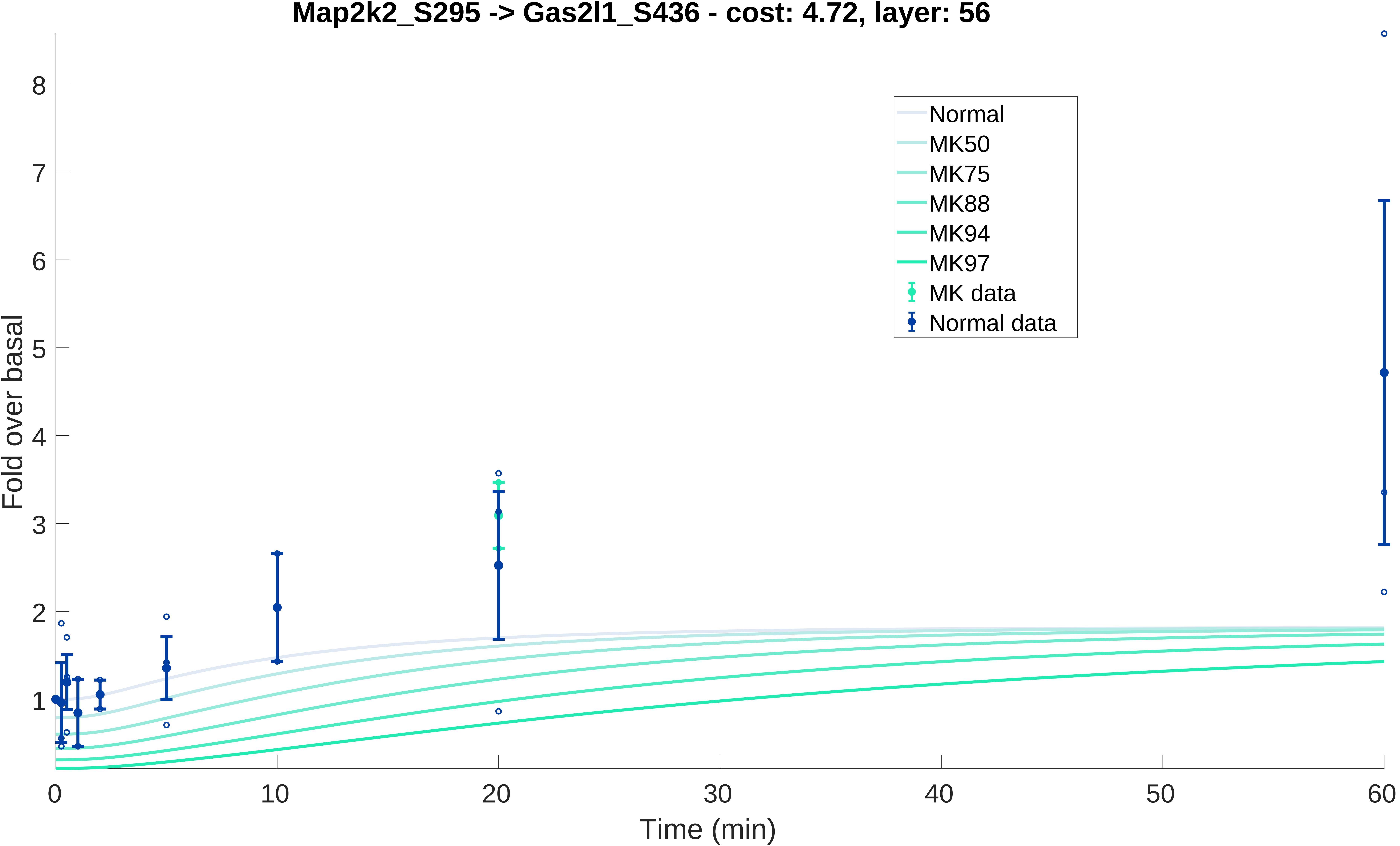
Map2k2_S295 -> Gas2l1_S436 - cost: 4.72, layer: 56
Fold over basal
1
2
3
4
5
6
7
8
0
10
20
30
40
50
60
Time (min)
Normal
MK50
MK75
MK88
MK94
MK97
MK data
Normal data

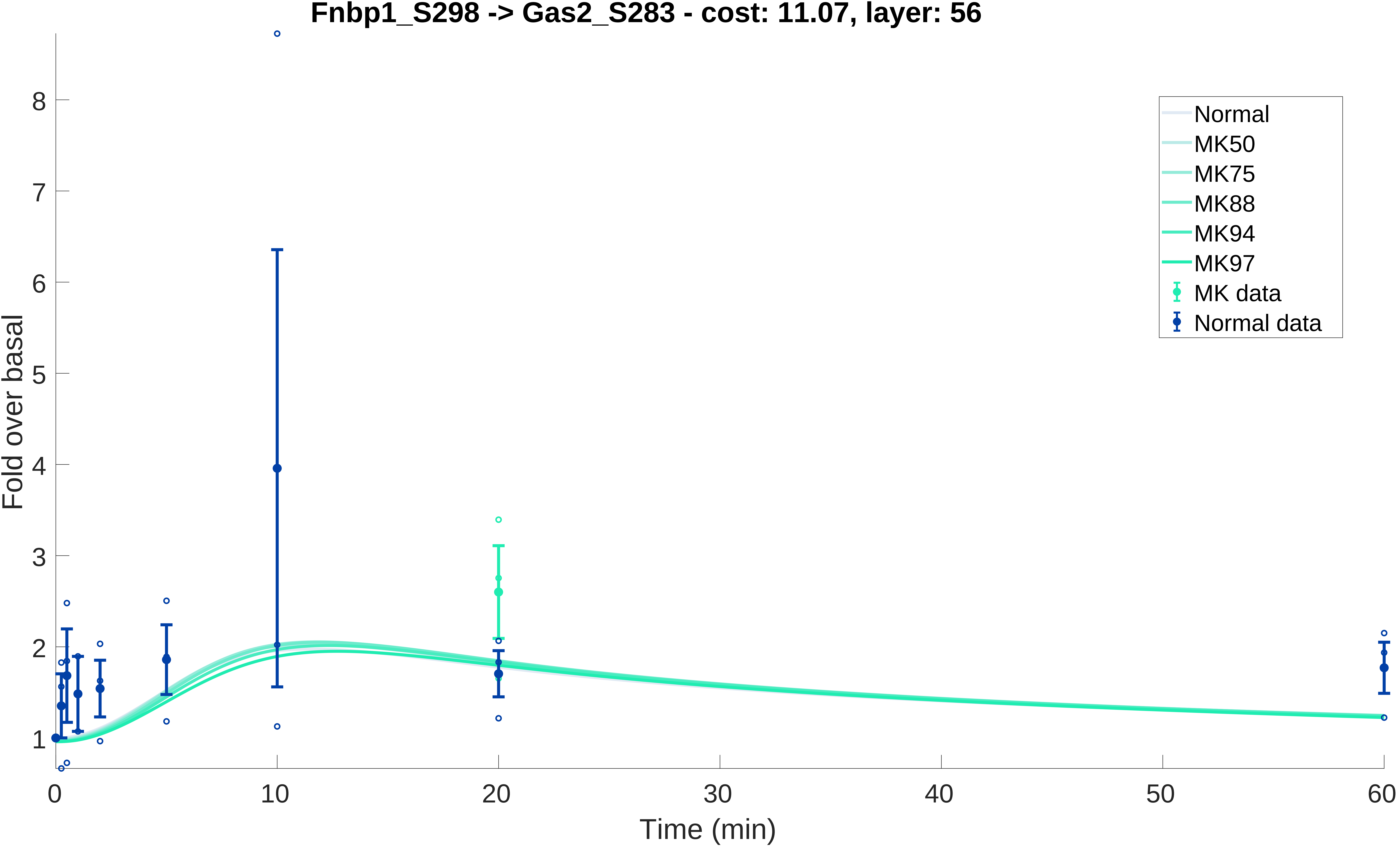

Fnbp1_S298 -> Gas2_S283 - cost: 11.07, layer: 56
Fold over basal
Time (min)
1
2
3
4
5
6
7
8
0
10
20
30
40
50
60
Normal
MK50
MK75
MK88
MK94
MK97
MK data
Normal data

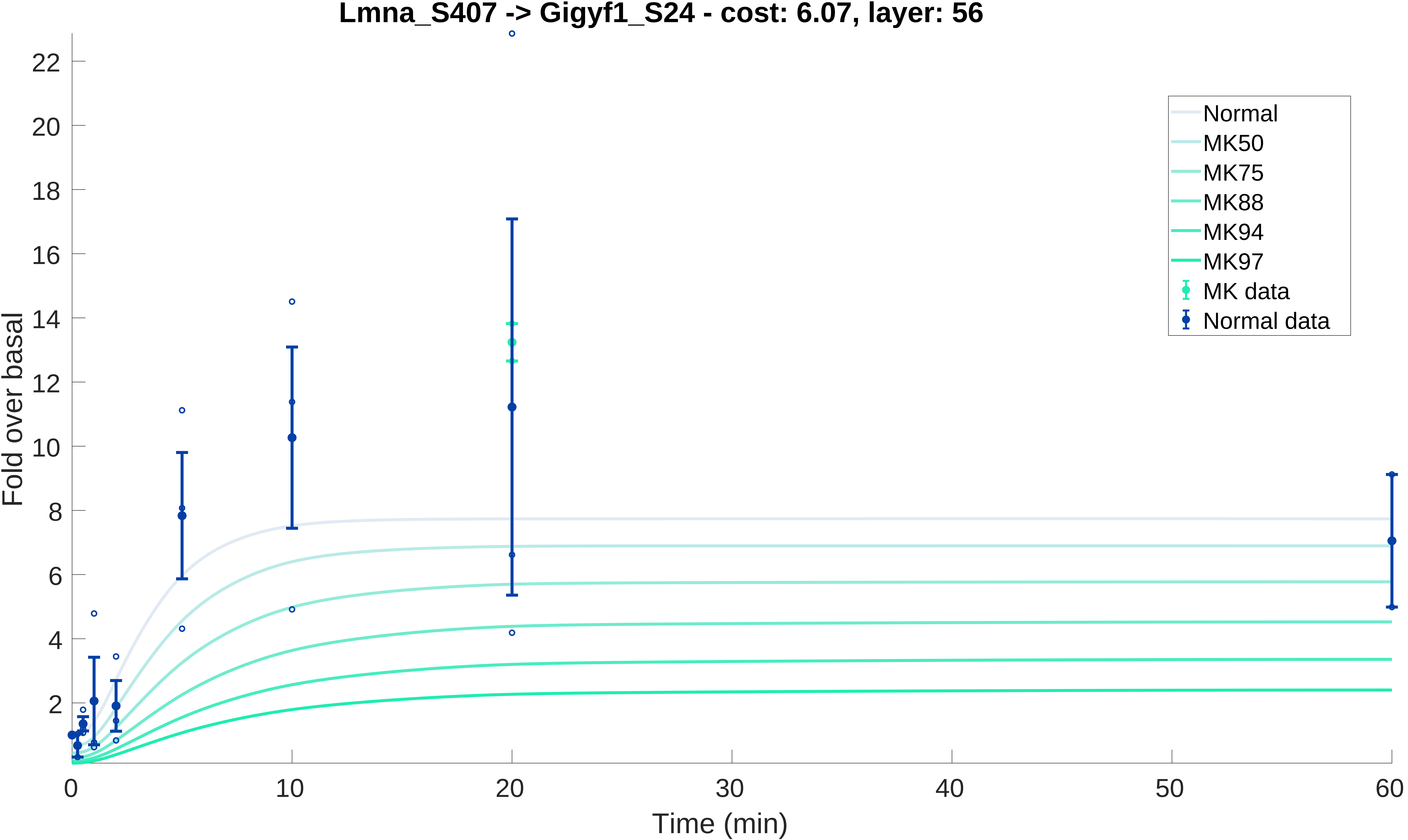

Lmna_S407 -> Gigyf1_S24 - cost: 6.07, layer: 56
Fold over basal
Time (min)
Normal
MK50
MK75
MK88
MK94
MK97
MK data
Normal data
0
10
20
30
40
50
60
2
4
6
8
10
12
14
16
18
20
22

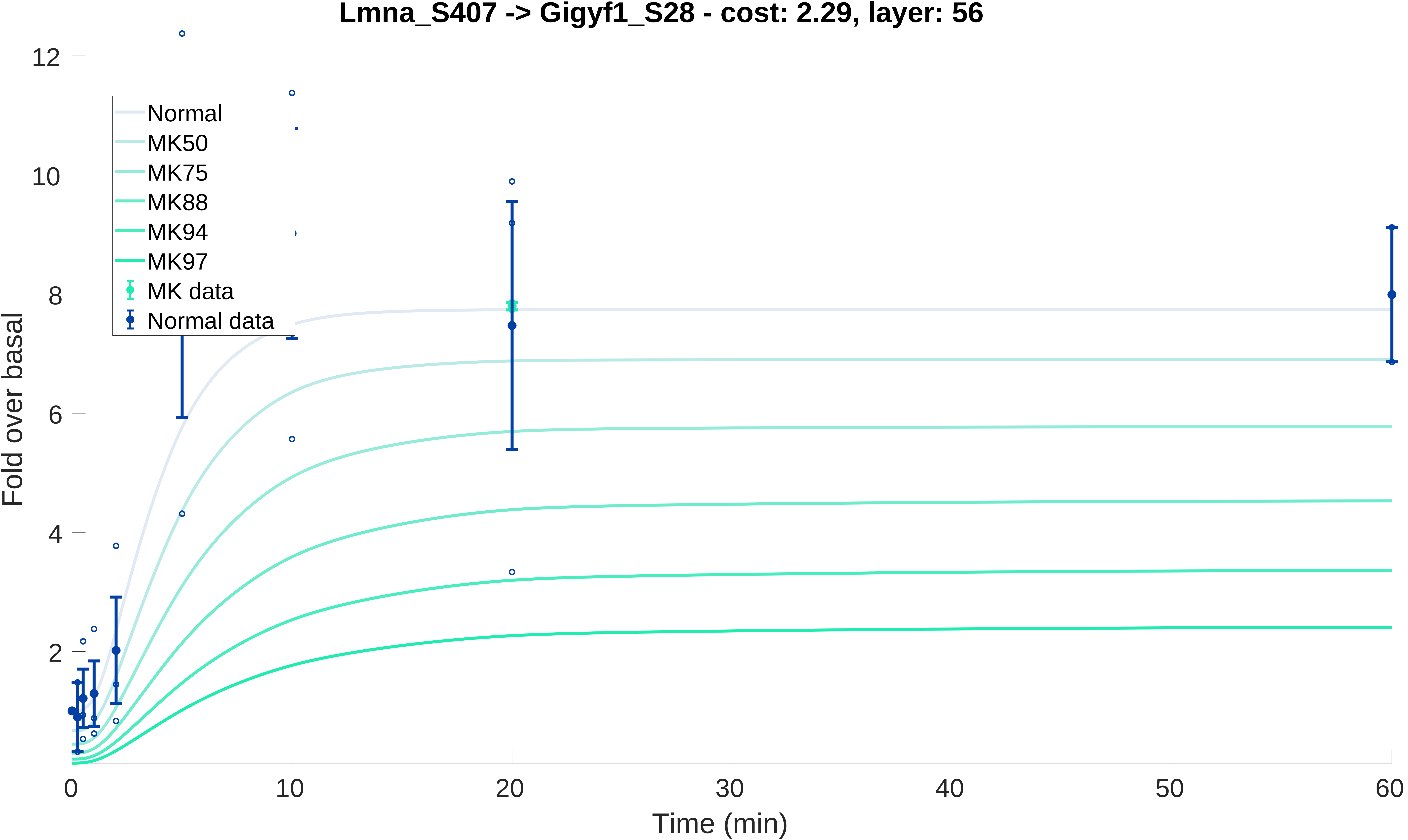

Lmna_S407 -> Gigyf1_S28 - cost: 2.29, layer: 56
Fold over basal
Time (min)
0
10
20
30
40
50
60
2
4
6
8
10
12
Normal
MK50
MK75
MK88
MK94
MK97
MK data
Normal data

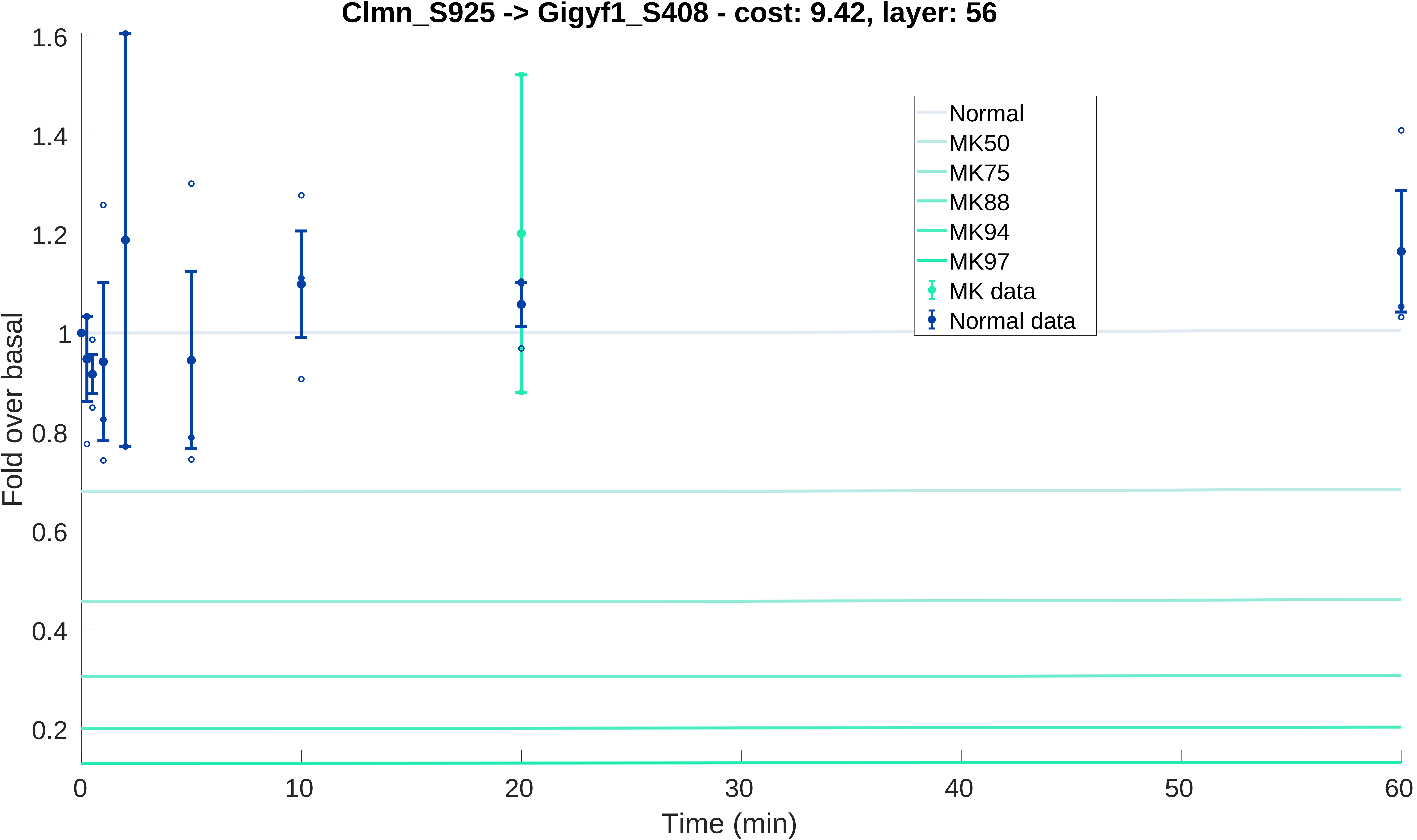

Clmn_S925 -> Gigyf1_S408 - cost: 9.42, layer: 56
Fold over basal
Time (min)
1.6
1.4
1.2
1
0.8
0.6
0.4
0.2
0
10
20
30
40
50
60
Normal
MK50
MK75
MK88
MK94
MK97
MK data
Normal data

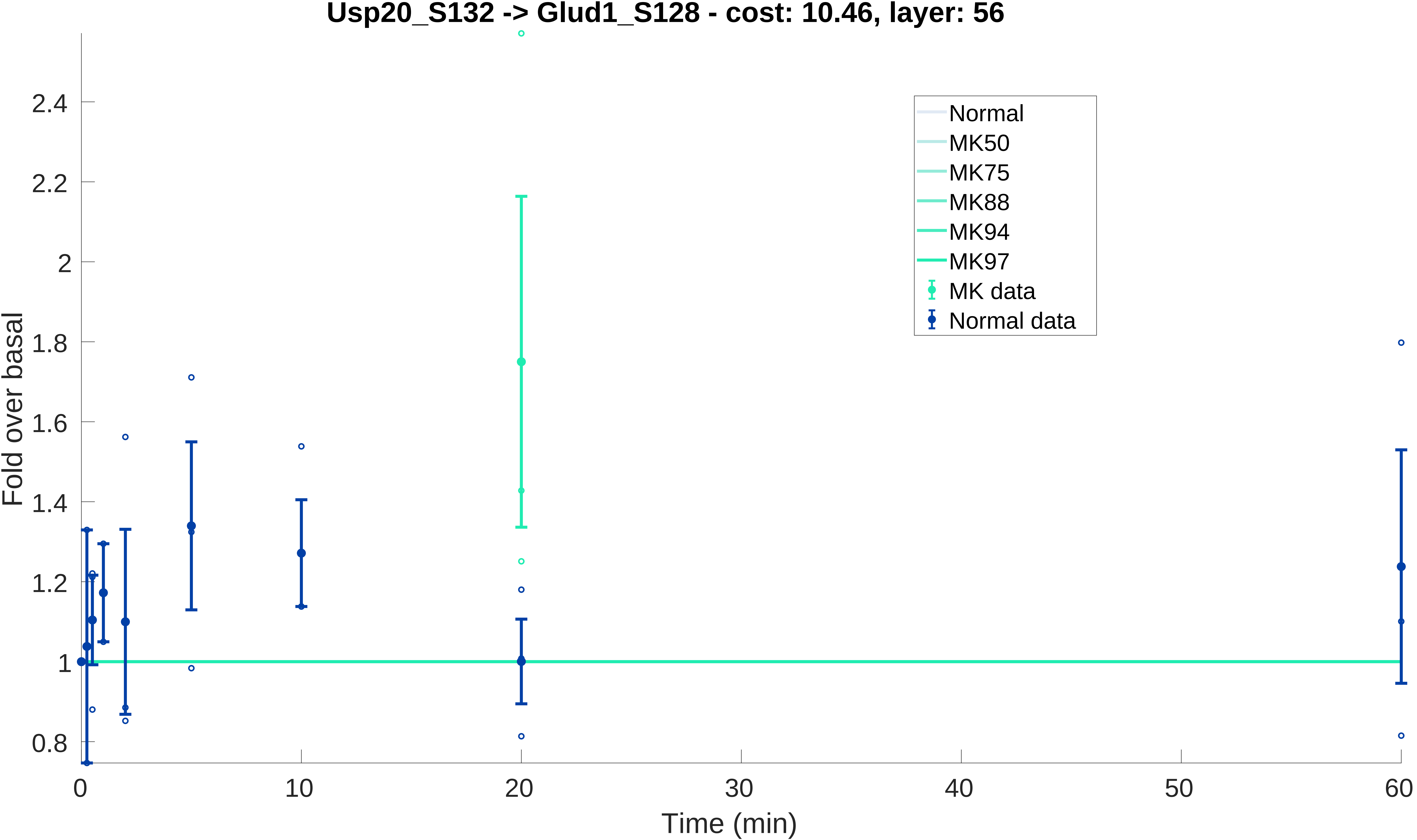

Usp20_S132 -> Glud1_S128 - cost: 10.46, layer: 56
Fold over basal
Time (min)
Normal
MK50
MK75
MK88
MK94
MK97
MK data
Normal data
2.4
2.2
2
1.8
1.6
1.4
1.2
1
0.8
0
10
20
30
40
50
60

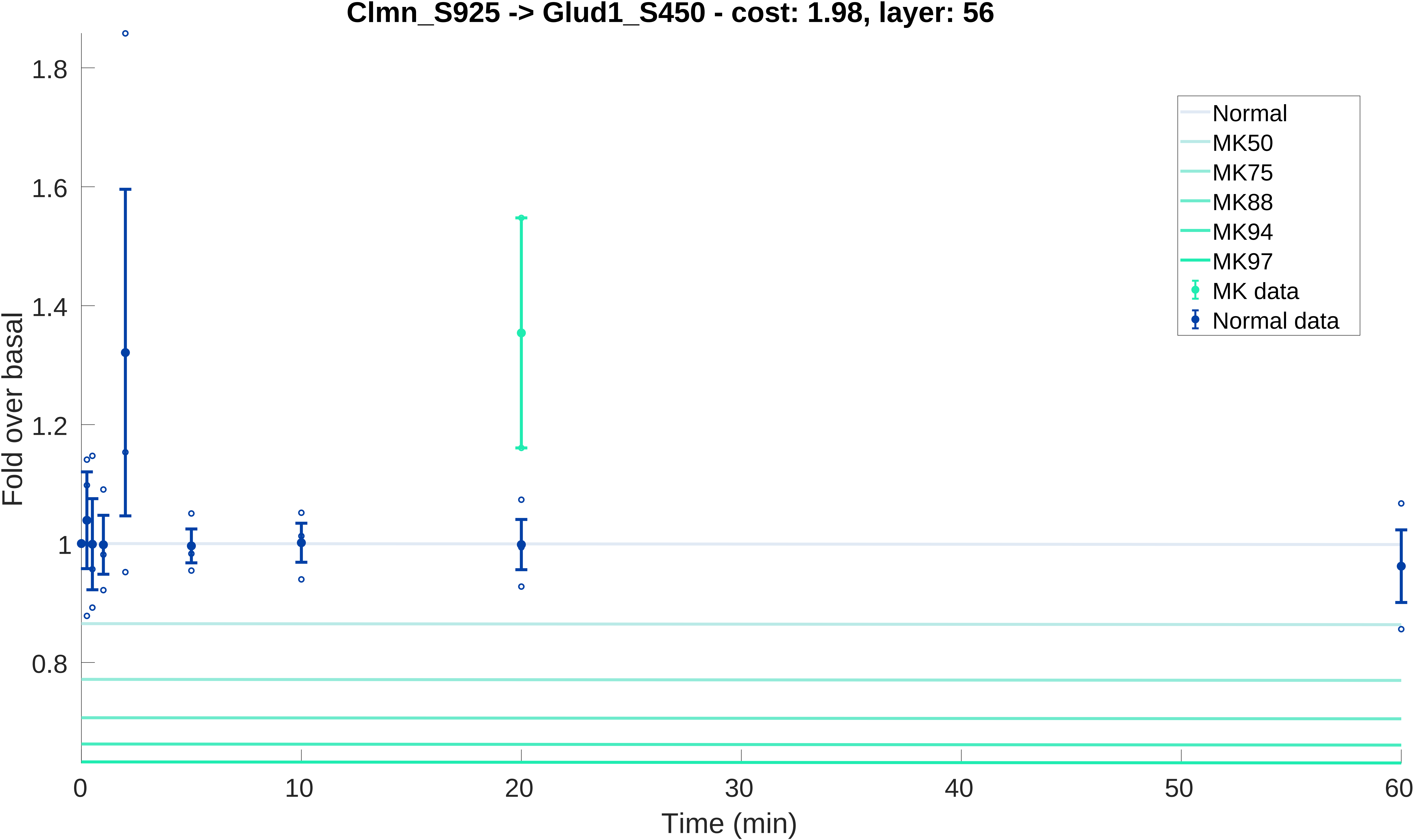

Clmn_S925 -> Glud1_S450 - cost: 1.98, layer: 56
Fold over basal
1.8
1.6
1.4
1.2
1
0.8
0
10
20
30
40
50
60
Time (min)
Normal
MK50
MK75
MK88
MK94
MK97
MK data
Normal data

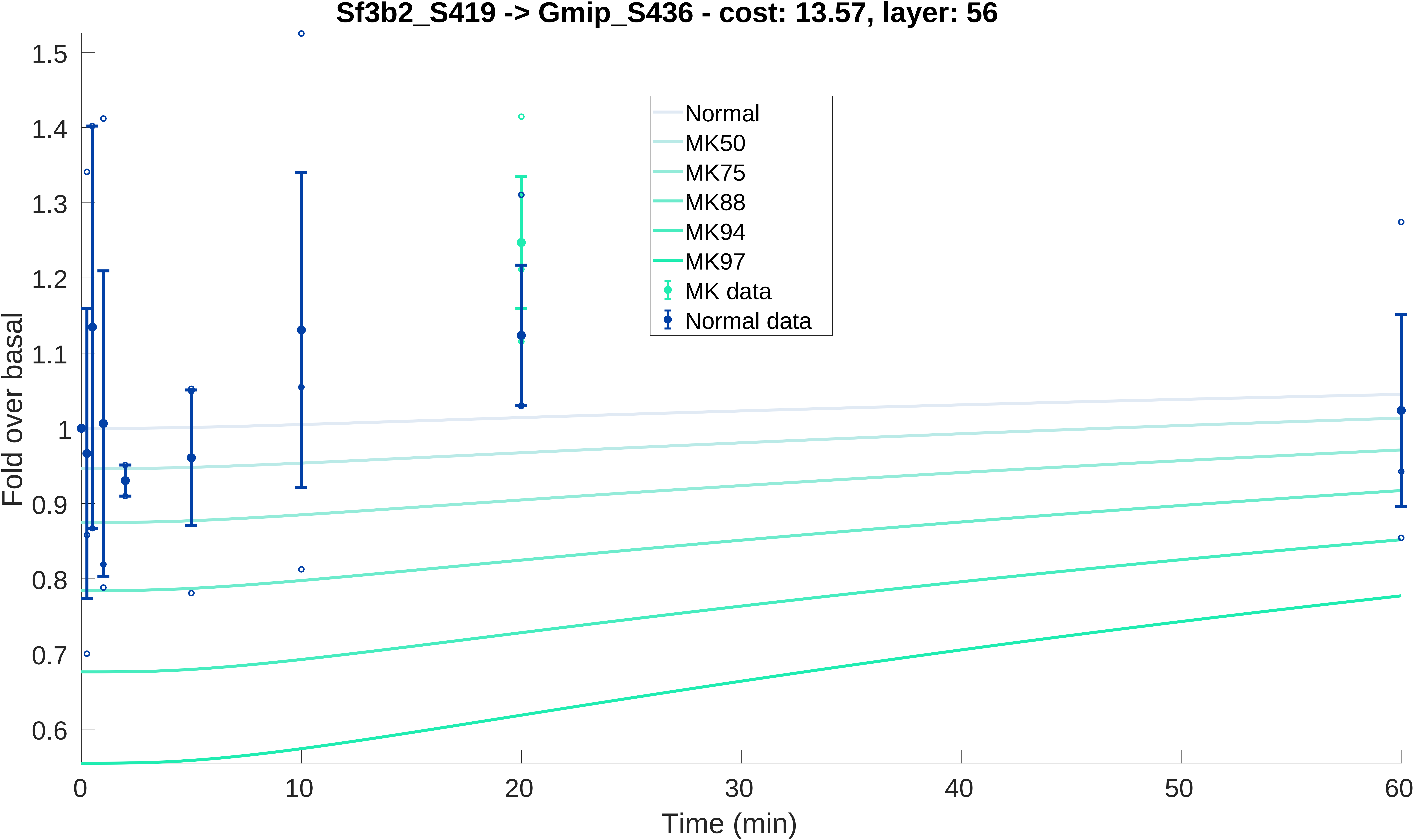
Sf3b2_S419 -> Gmip_S436 - cost: 13.57, layer: 56
Fold over basal
Time (min)
Normal
MK50
MK75
MK88
MK94
MK97
MK data
Normal data
0
10
20
30
40
50
60
0.6
0.7
0.8
0.9
1
1.1
1.2
1.3
1.4
1.5

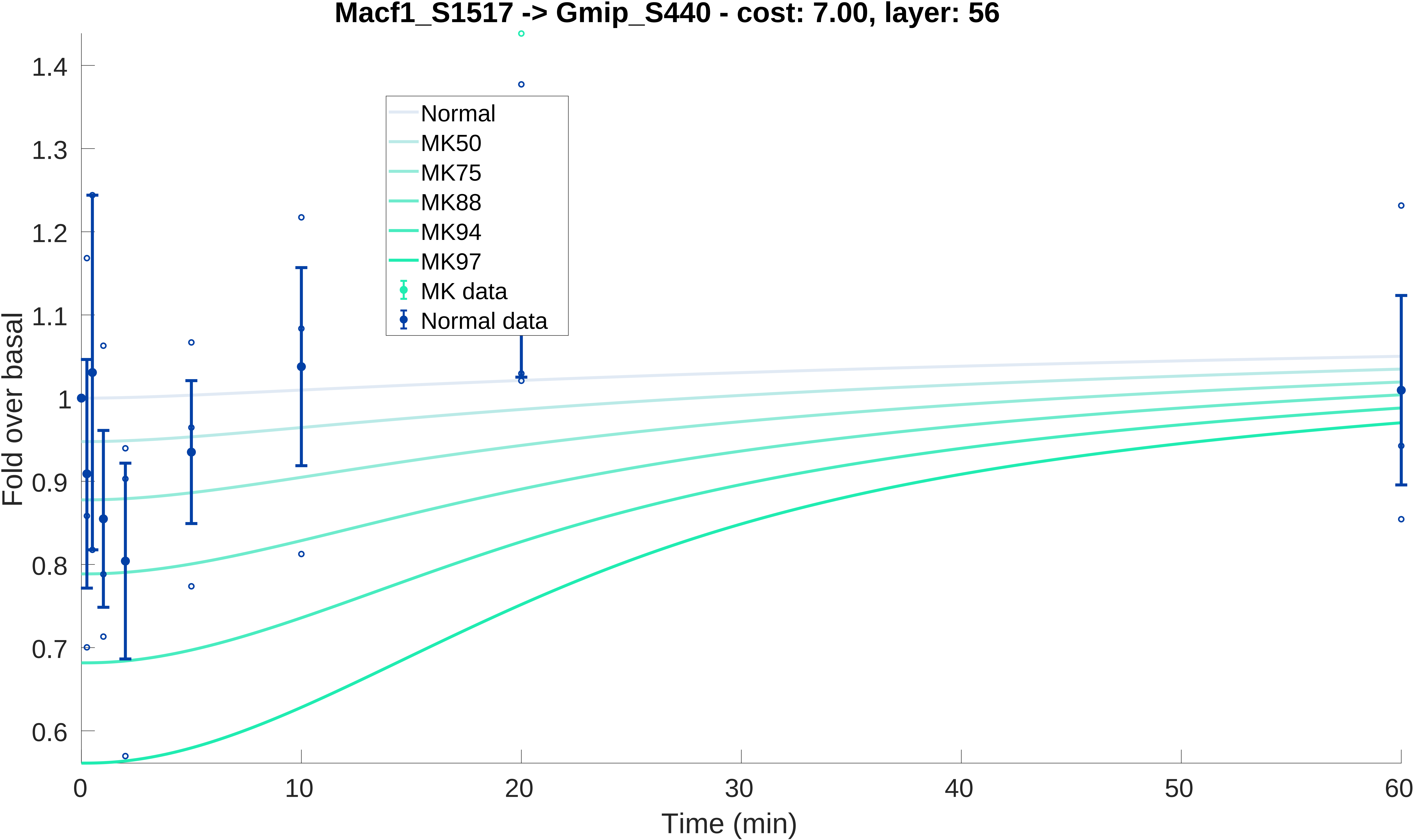

Macf1_S1517 -> Gmip_S440 - cost: 7.00, layer: 56
Normal
MK50
MK75
MK88
MK94
MK97
MK data
Normal data
Fold over basal
Time (min)
1.4
1.3
1.2
1.1
1
0.9
0.8
0.7
0.6
0
10
20
30
40
50
60

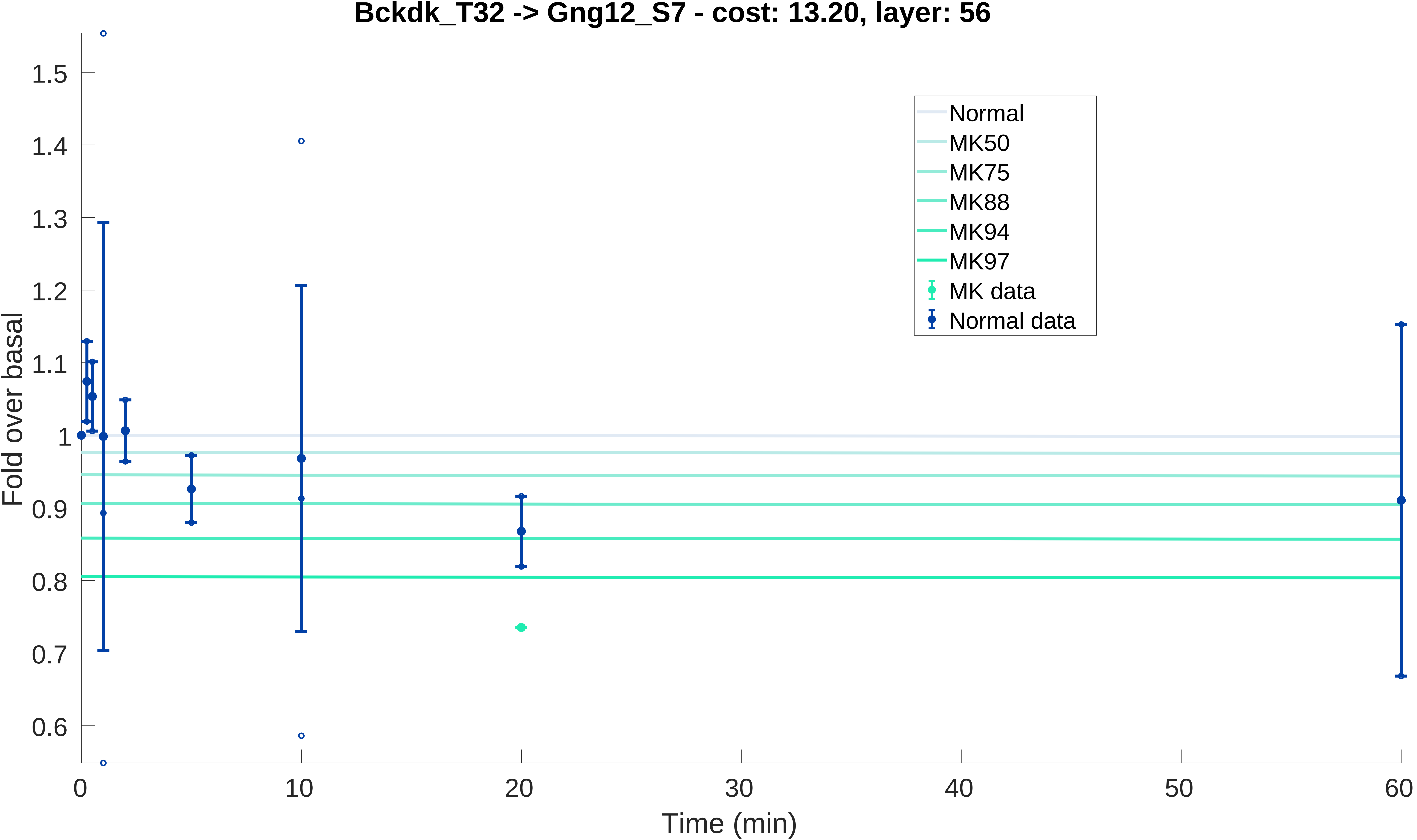

Bckdk_T32 -> Gng12_S7 - cost: 13.20, layer: 56
Fold over basal
1.5
1.4
1.3
1.2
1.1
1
0.9
0.8
0.7
0.6
0
10
20
30
40
50
60
Time (min)
Normal
MK50
MK75
MK88
MK94
MK97
MK data
Normal data

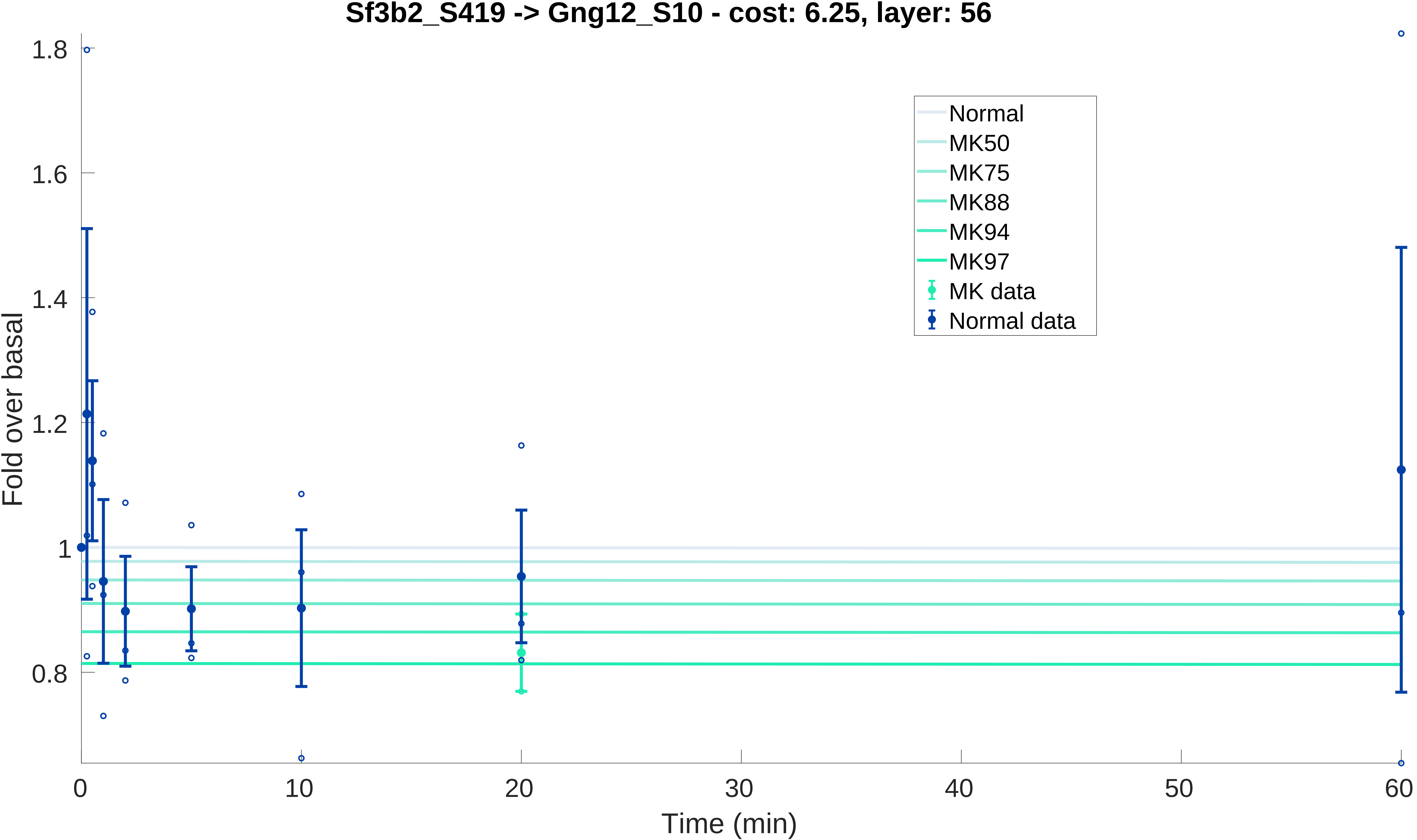

Sf3b2_S419 -> Gng12_S10 - cost: 6.25, layer: 56
Fold over basal
1.8
1.6
1.4
1.2
1
0.8
0
10
20
30
40
50
60
Time (min)
Normal
MK50
MK75
MK88
MK94
MK97
MK data
Normal data

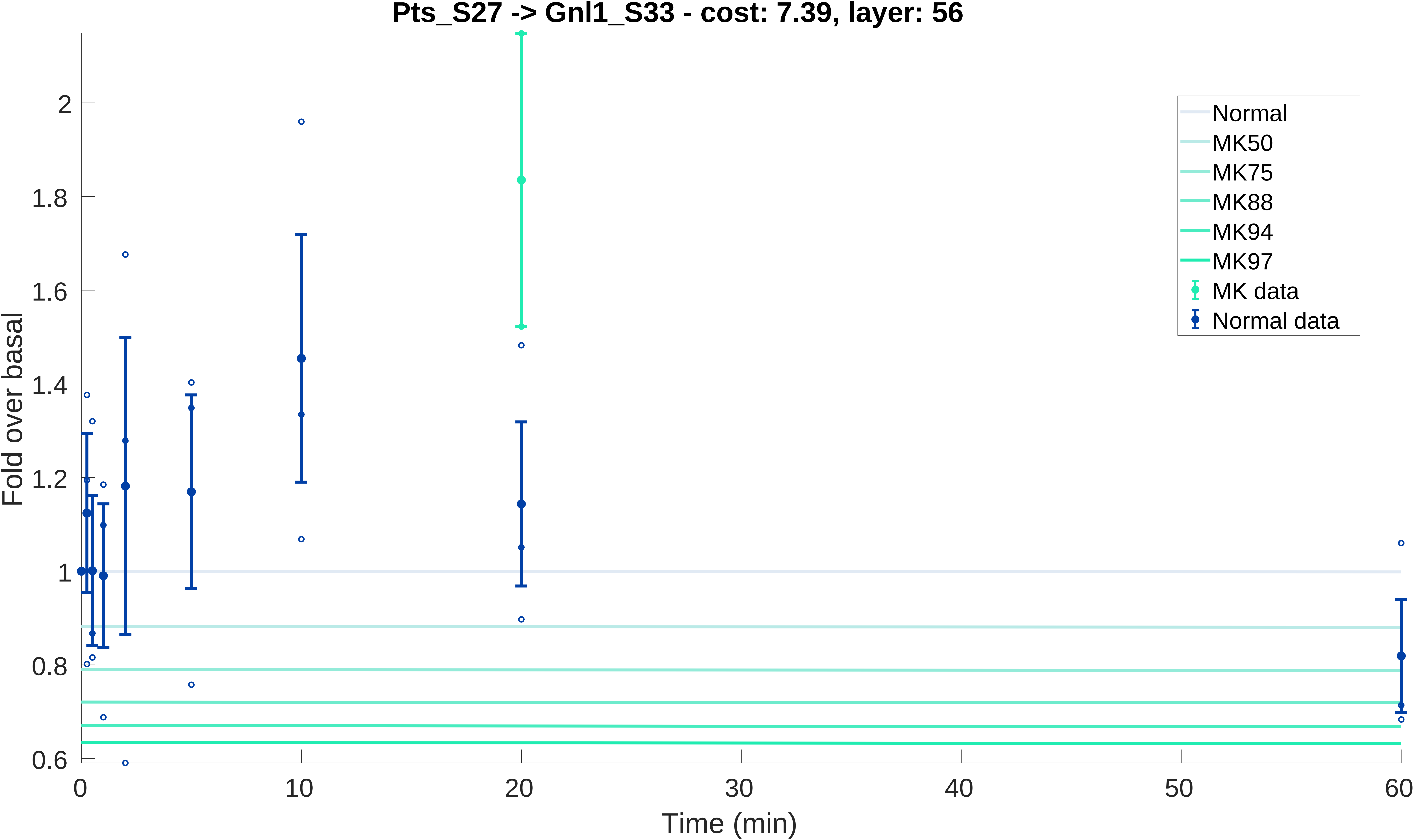
Pts_S27 -> Gnl1_S33 - cost: 7.39, layer: 56
Fold over basal
Time (min)
0.6
0.8
1
1.2
1.4
1.6
1.8
2
0
10
20
30
40
50
60
Normal
MK50
MK75
MK88
MK94
MK97
MK data
Normal data

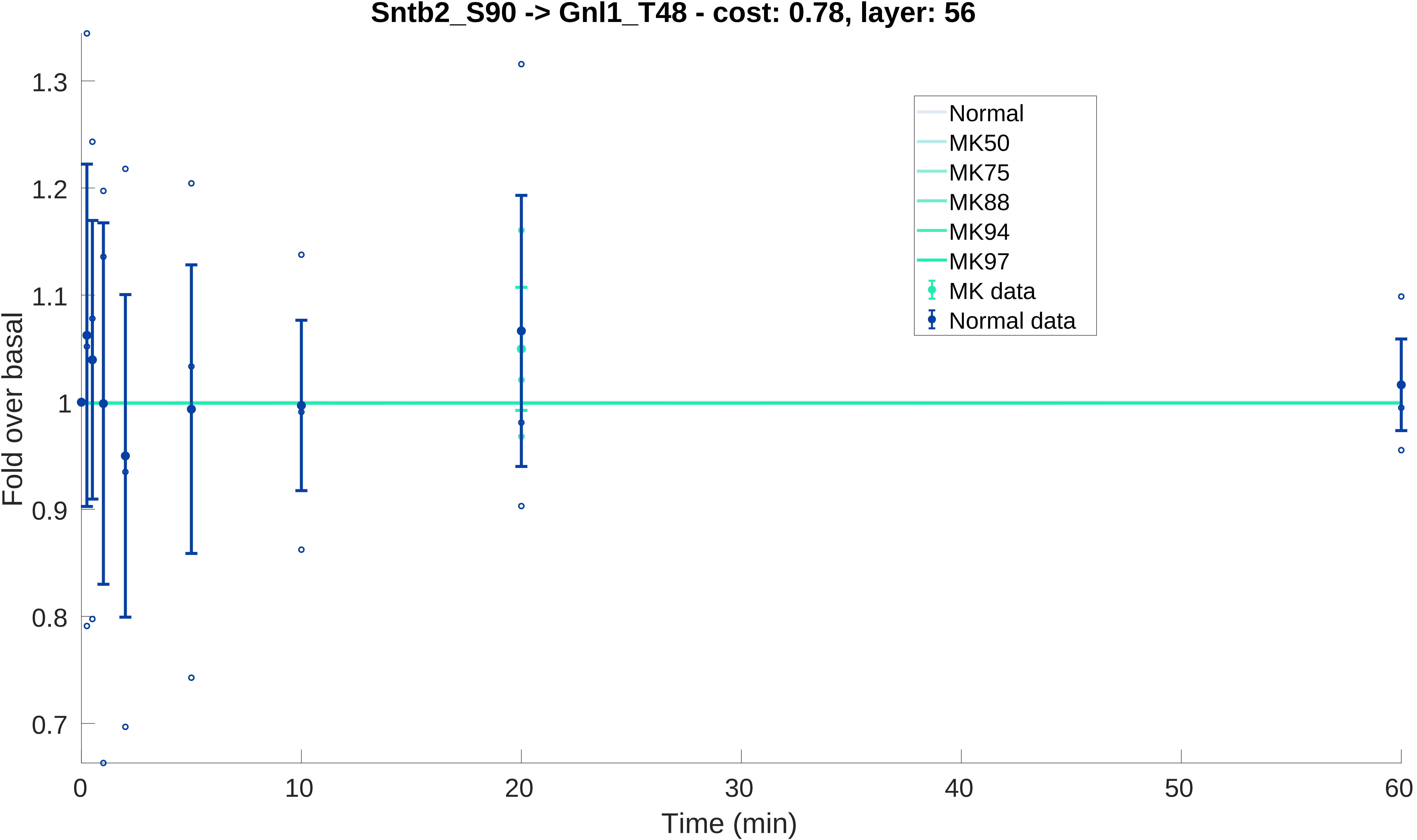

Sntb2_S90 -> Gnl1_T48 - cost: 0.78, layer: 56
Fold over basal
Time (min)
1.3
1.2
1.1
1
0.9
0.8
0.7
0
10
20
30
40
50
60
Normal
MK50
MK75
MK88
MK94
MK97
MK data
Normal data

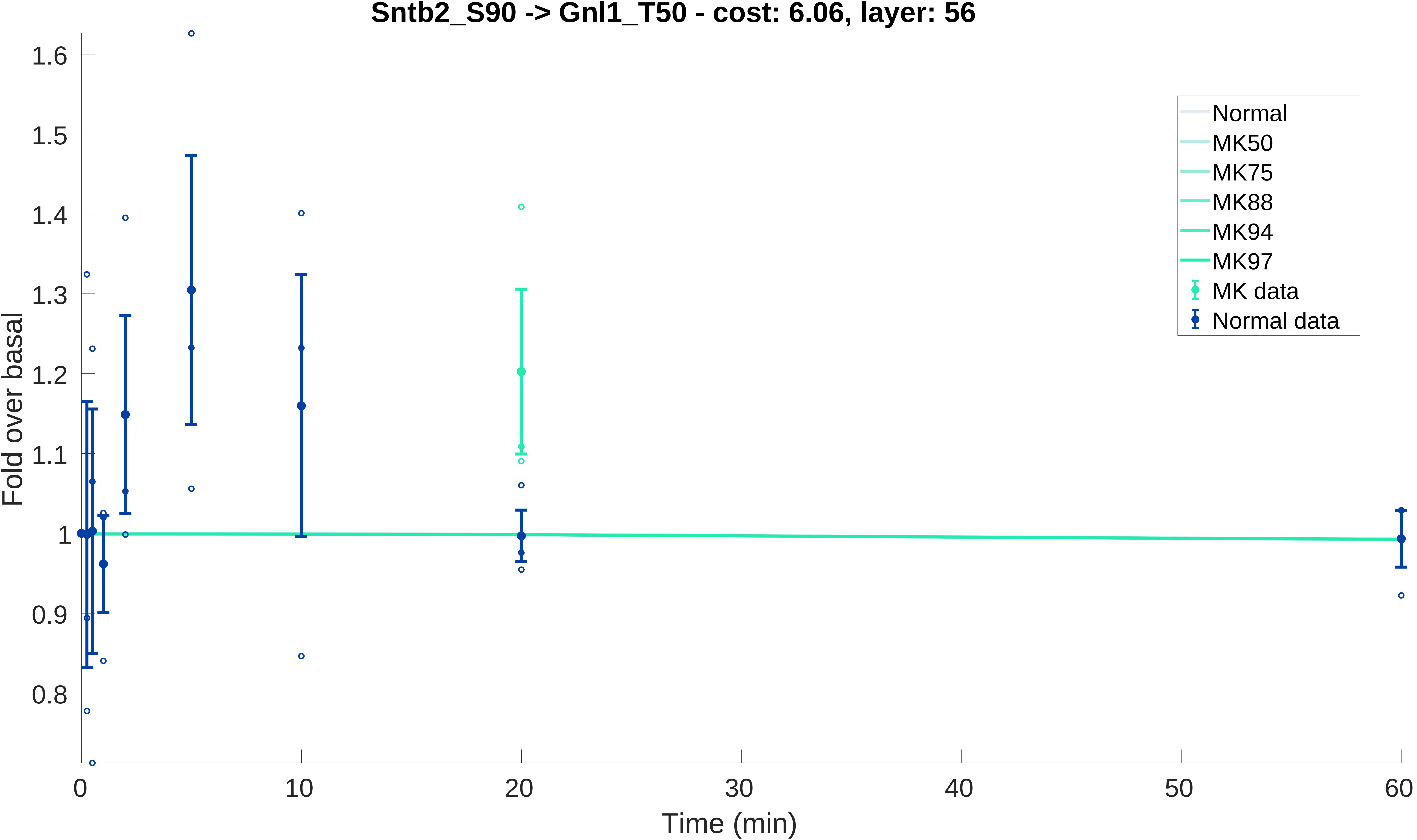

Sntb2_S90 -> Gnl1_T50 - cost: 6.06, layer: 56
Fold over basal
Time (min)
Normal
MK50
MK75
MK88
MK94
MK97
MK data
Normal data
1.6
1.5
1.4
1.3
1.2
1.1
1
0.9
0.8
0
10
20
30
40
50
60

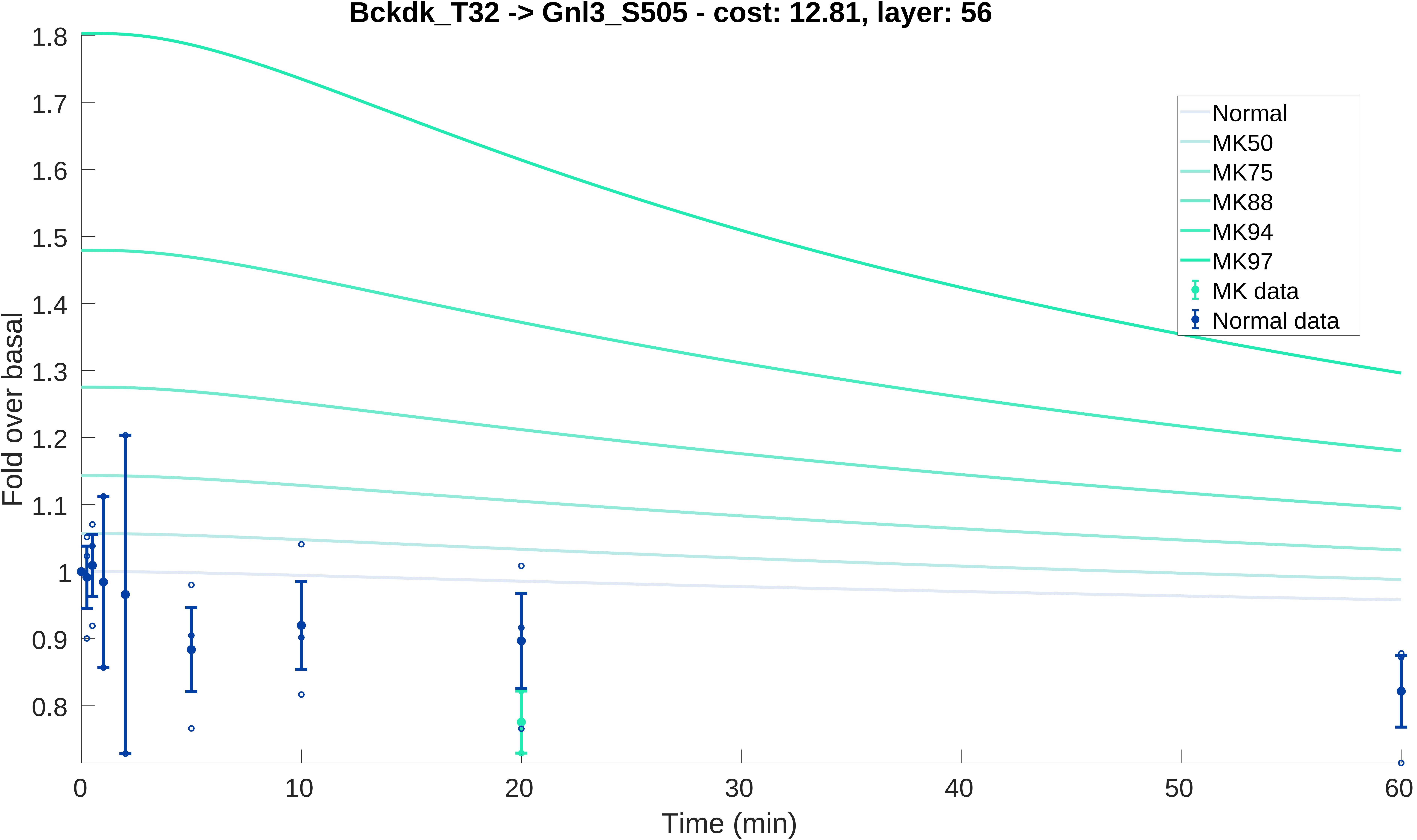

Bckdk_T32 -> Gnl3_S505 - cost: 12.81, layer: 56
Fold over basal
Time (min)
0
10
20
30
40
50
60
0.8
0.9
1
1.1
1.2
1.3
1.4
1.5
1.6
1.7
1.8
Normal
MK50
MK75
MK88
MK94
MK97
MK data
Normal data

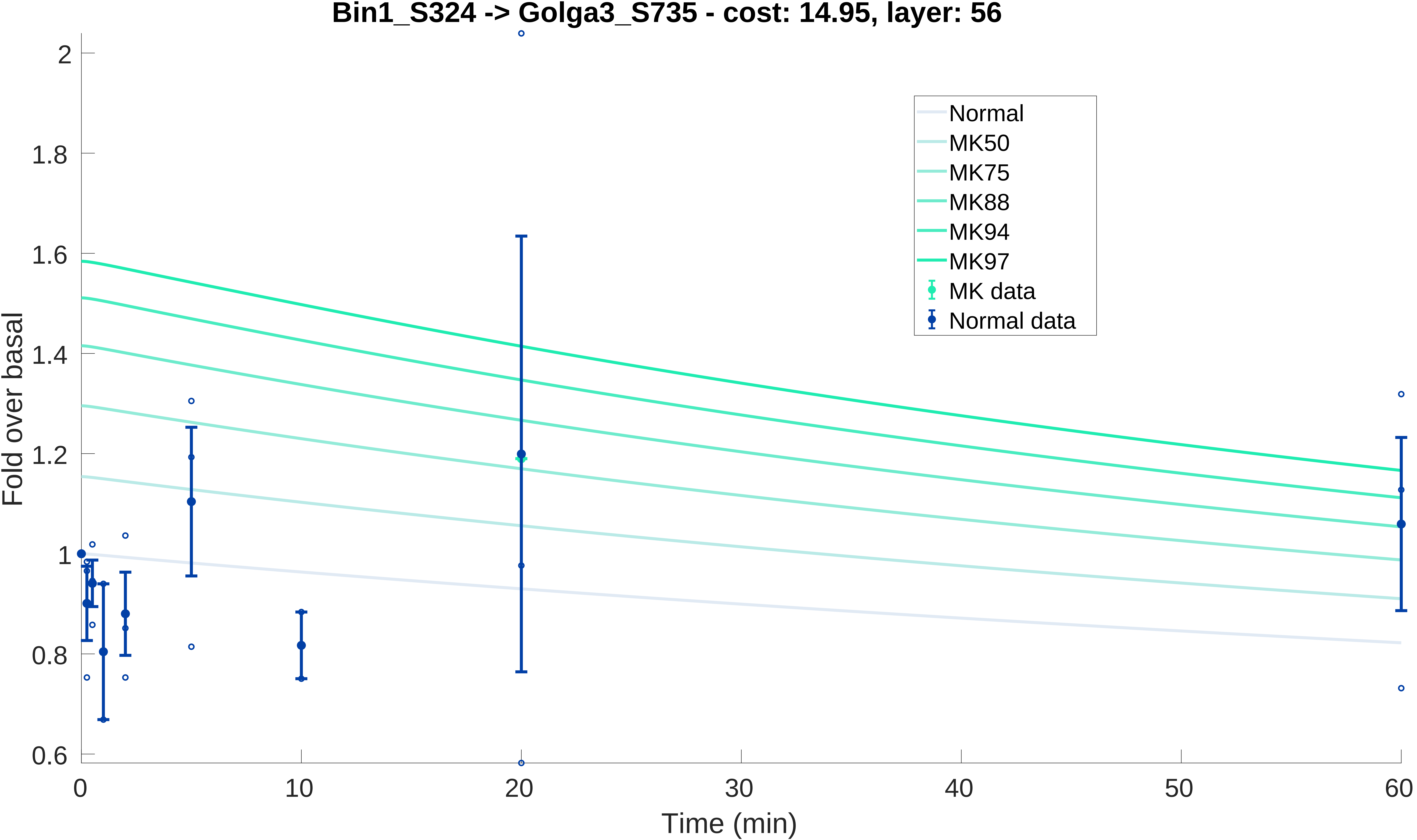

Bin1_S324 -> Golga3_S735 - cost: 14.95, layer: 56
Normal
MK50
MK75
MK88
MK94
MK97
MK data
Normal data
Fold over basal
Time (min)
0.6
0.8
1
1.2
1.4
1.6
1.8
2
0
10
20
30
40
50
60

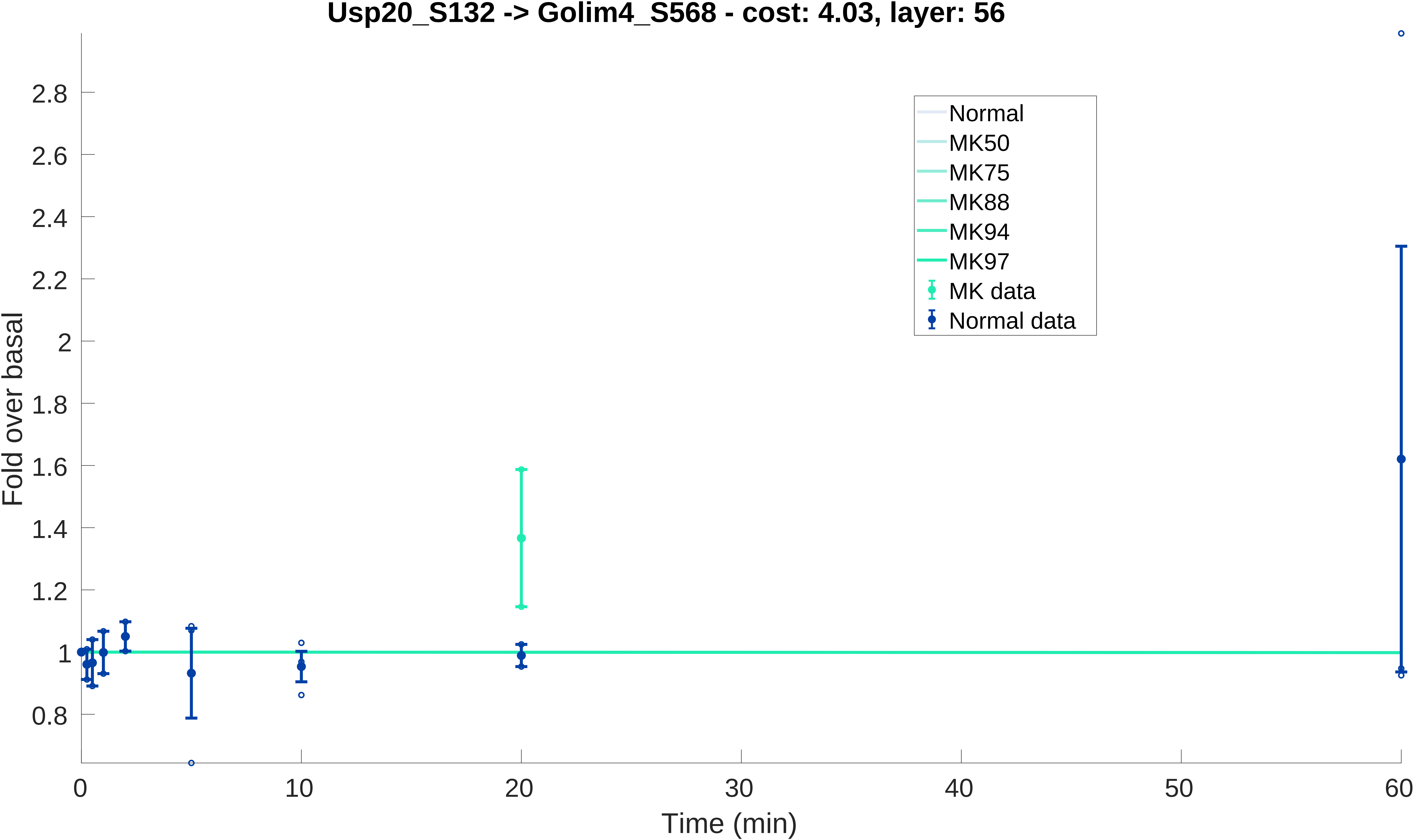
Usp20_S132 -> Golim4_S568 - cost: 4.03, layer: 56
Fold over basal
Time (min)
0
10
20
30
40
50
60
0.8
1
1.2
1.4
1.6
1.8
2
2.2
2.4
2.6
2.8
Normal
MK50
MK75
MK88
MK94
MK97
MK data
Normal data

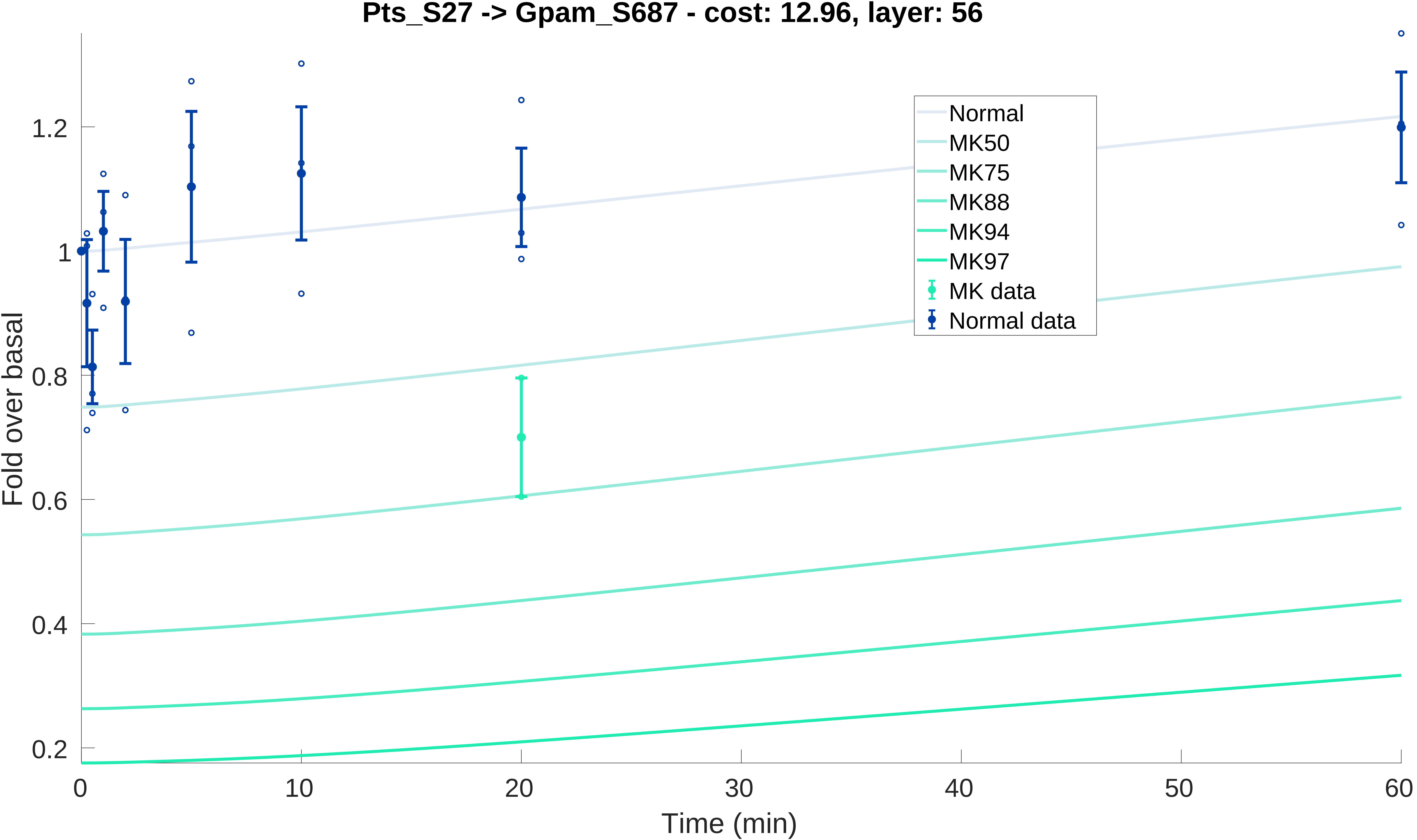
Pts_S27 -> Gpam_S687 - cost: 12.96, layer: 56
Fold over basal
Time (min)
Normal
MK50
MK75
MK88
MK94
MK97
MK data
Normal data
0.2
0.4
0.6
0.8
1
1.2
0
10
20
30
40
50
60

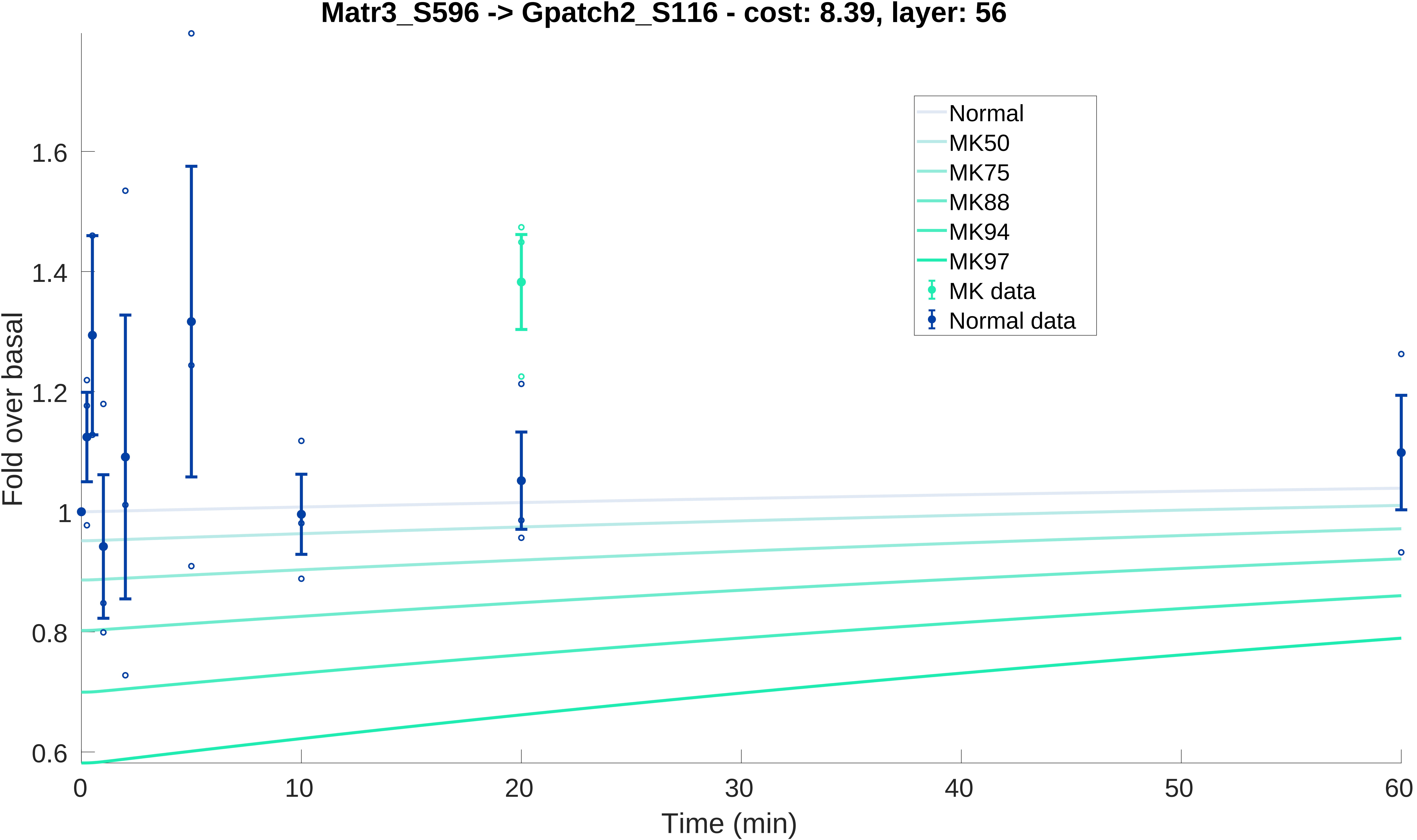

Matr3_S596 -> Gpatch2_S116 - cost: 8.39, layer: 56
Fold over basal
Time (min)
0.6
0.8
1
1.2
1.4
1.6
0
10
20
30
40
50
60
Normal
MK50
MK75
MK88
MK94
MK97
MK data
Normal data

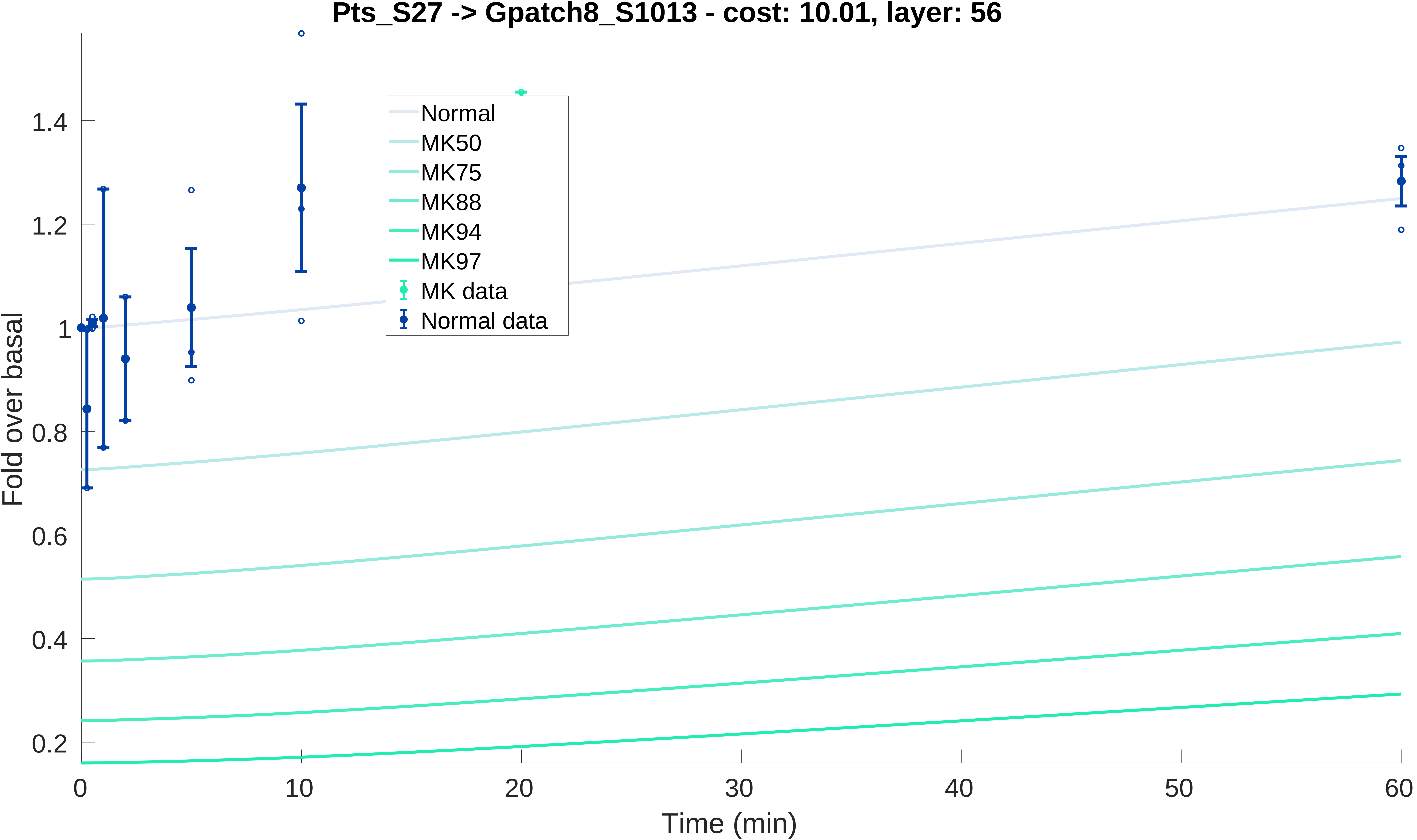

Pts_S27 -> Gpatch8_S1013 - cost: 10.01, layer: 56
Normal
MK50
MK75
MK88
MK94
MK97
MK data
Normal data
Fold over basal
Time (min)
0
10
20
30
40
50
60
0.2
0.4
0.6
0.8
1
1.2
1.4

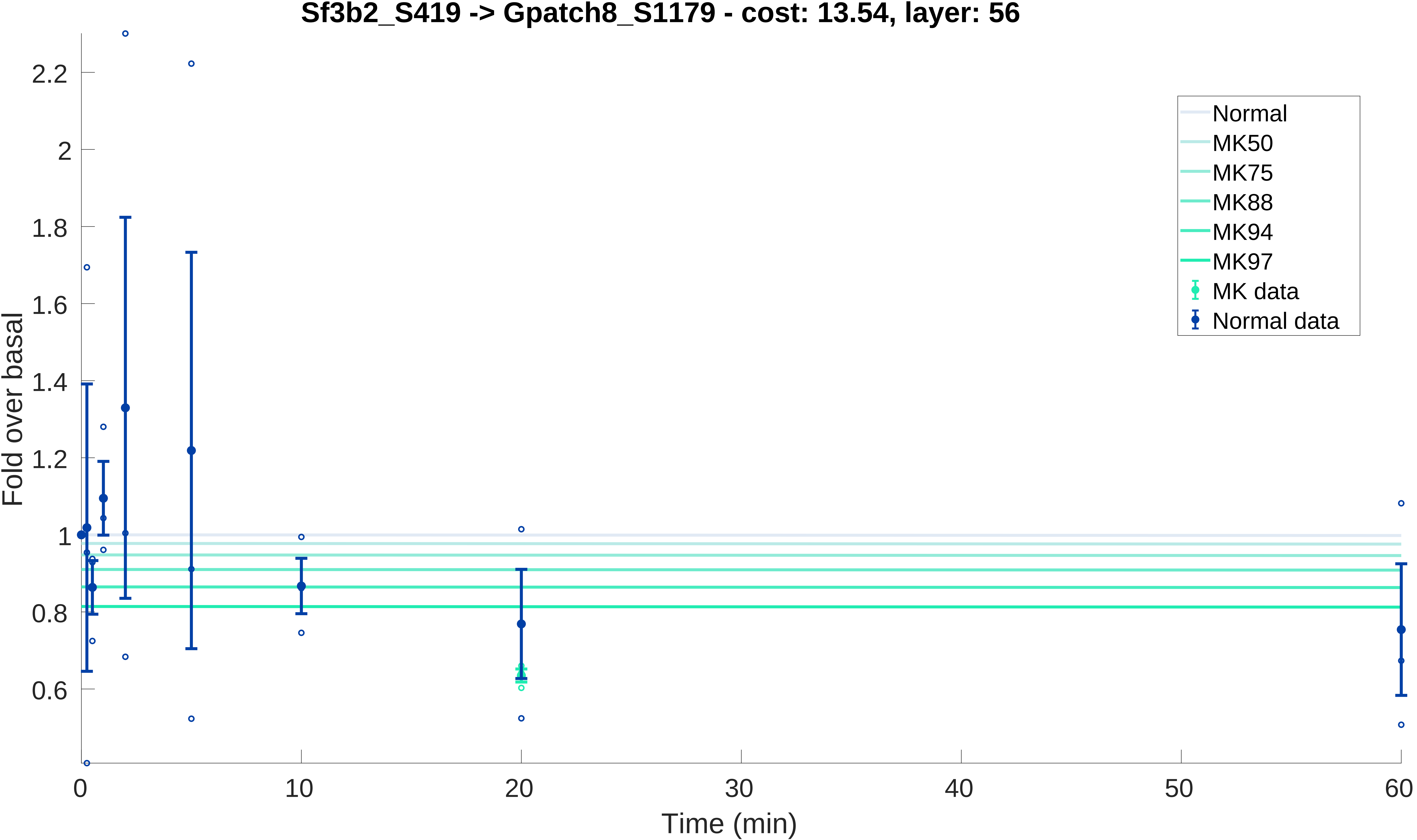
Sf3b2_S419 -> Gpatch8_S1179 - cost: 13.54, layer: 56
Fold over basal
Time (min)
0.6
0.8
1
1.2
1.4
1.6
1.8
2
2.2
0
10
20
30
40
50
60
Normal
MK50
MK75
MK88
MK94
MK97
MK data
Normal data

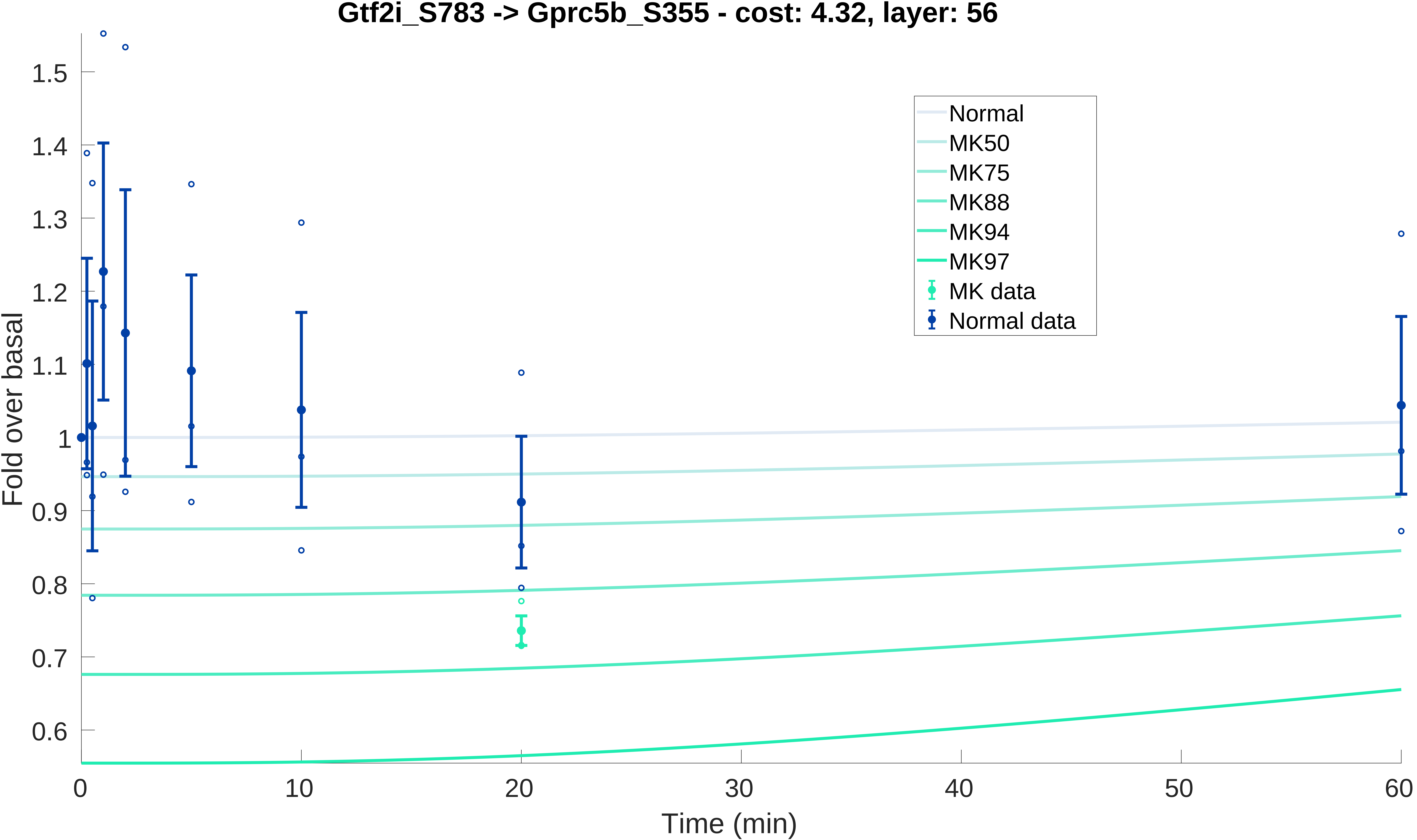
Gtf2i_S783 -> Gprc5b_S355 - cost: 4.32, layer: 56
Fold over basal
Time (min)
1.5
1.4
1.3
1.2
1.1
1
0.9
0.8
0.7
0.6
0
10
20
30
40
50
60
Normal
MK50
MK75
MK88
MK94
MK97
MK data
Normal data

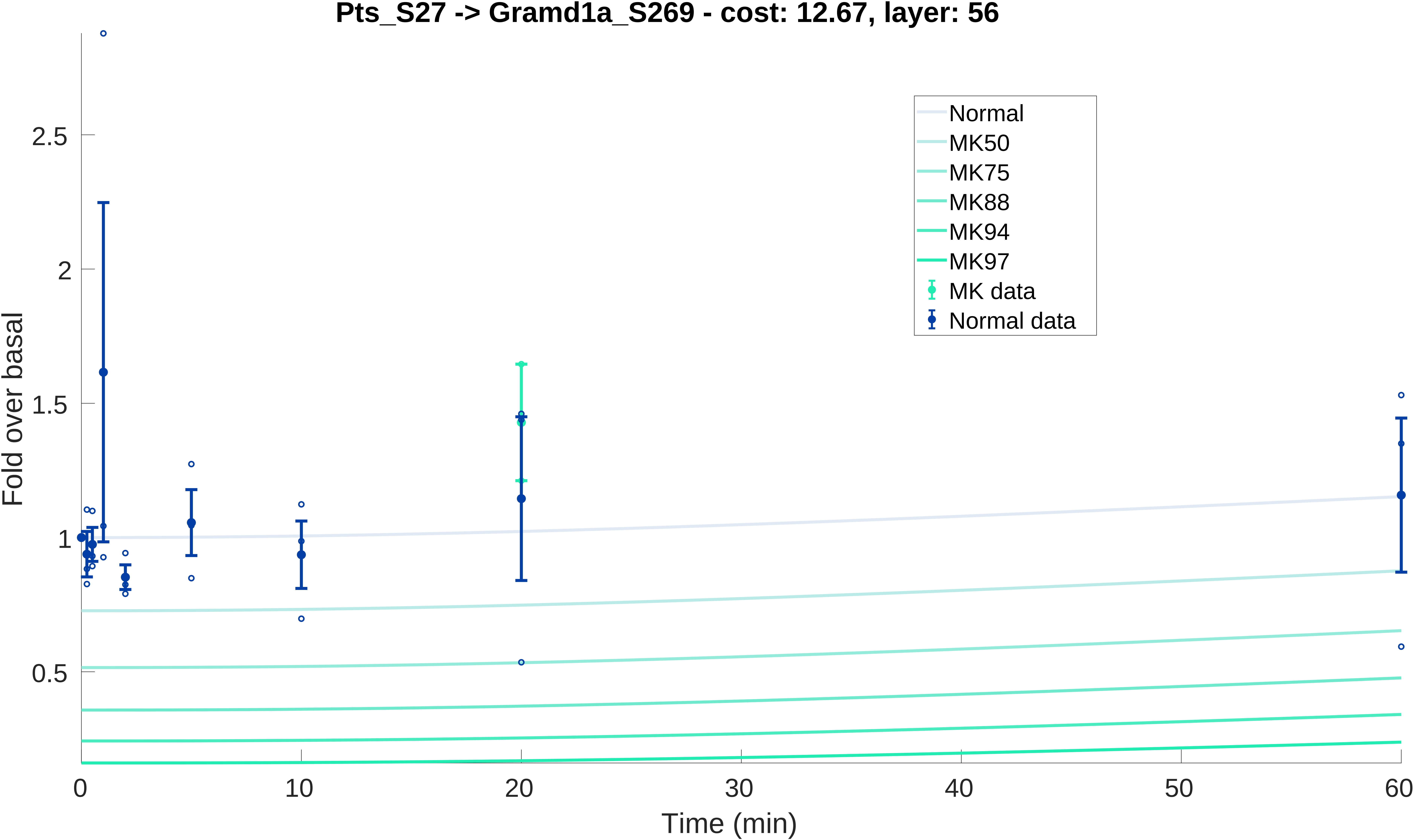

Pts_S27 -> Gramd1a_S269 - cost: 12.67, layer: 56
Fold over basal
Time (min)
0
10
20
30
40
50
60
0.5
1
1.5
2
2.5
Normal
MK50
MK75
MK88
MK94
MK97
MK data
Normal data

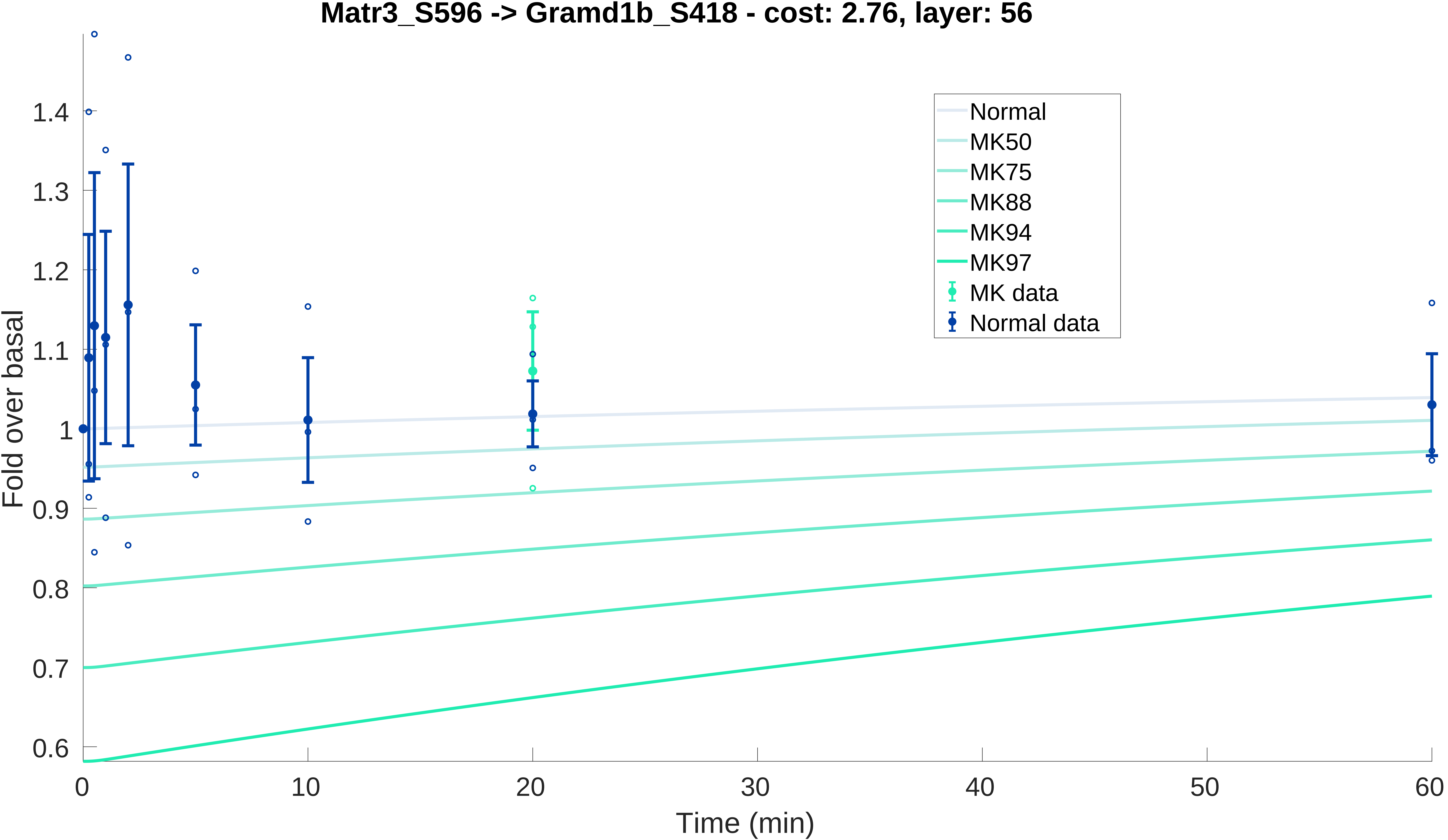
Matr3_S596 -> Gramd1b_S418 - cost: 2.76, layer: 56
Normal
MK50
MK75
MK88
MK94
MK97
MK data
Normal data
Fold over basal
Time (min)
0.6
0.7
0.8
0.9
1
1.1
1.2
1.3
1.4
0
10
20
30
40
50
60

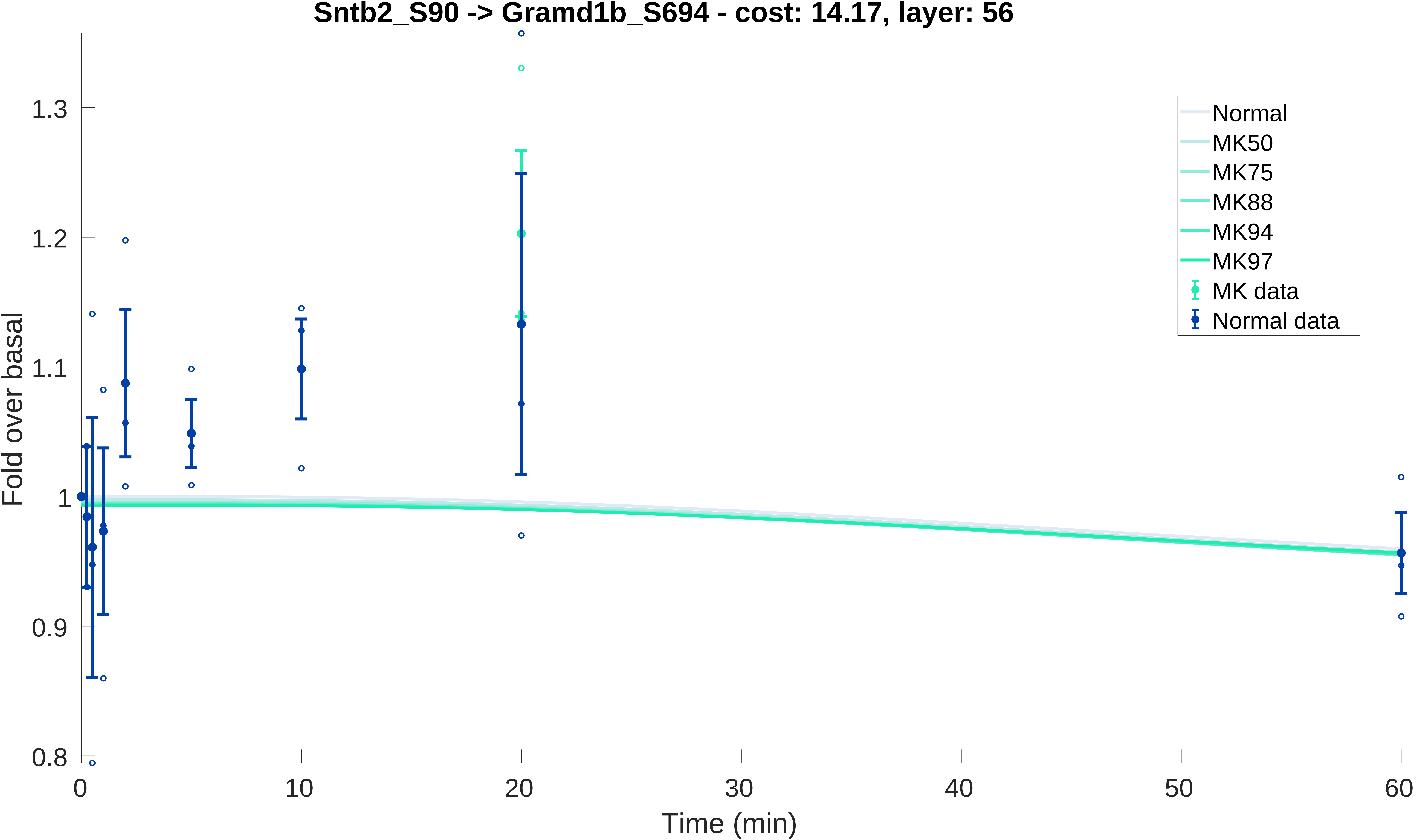

Sntb2_S90 -> Gramd1b_S694 - cost: 14.17, layer: 56
Fold over basal
Time (min)
0.8
0.9
1
1.1
1.2
1.3
0
10
20
30
40
50
60
Normal
MK50
MK75
MK88
MK94
MK97
MK data
Normal data

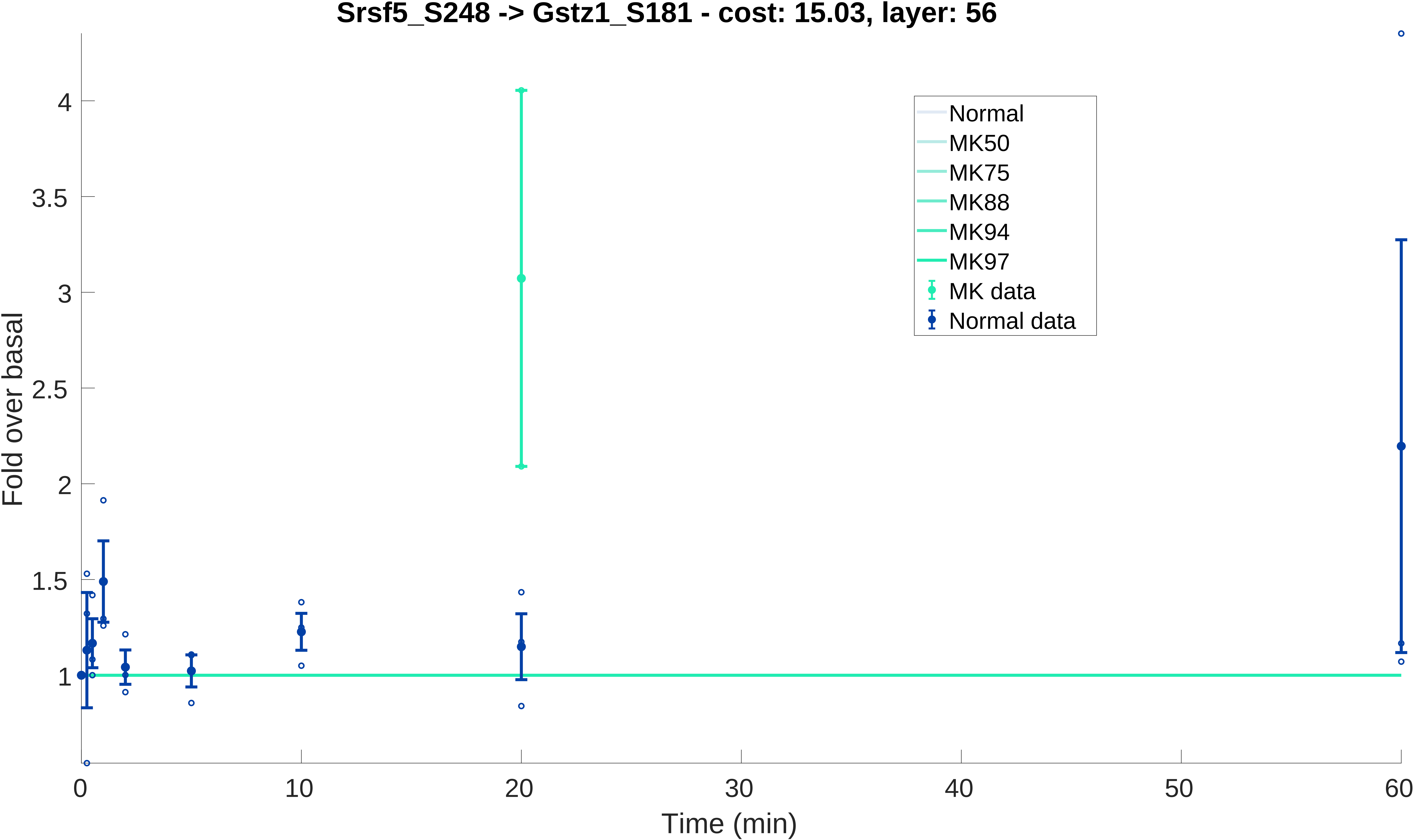

Srsf5_S248 -> Gstz1_S181 - cost: 15.03, layer: 56
Fold over basal
Time (min)
4
3.5
3
2.5
2
1.5
1
0
10
20
30
40
50
60
Normal
MK50
MK75
MK88
MK94
MK97
MK data
Normal data

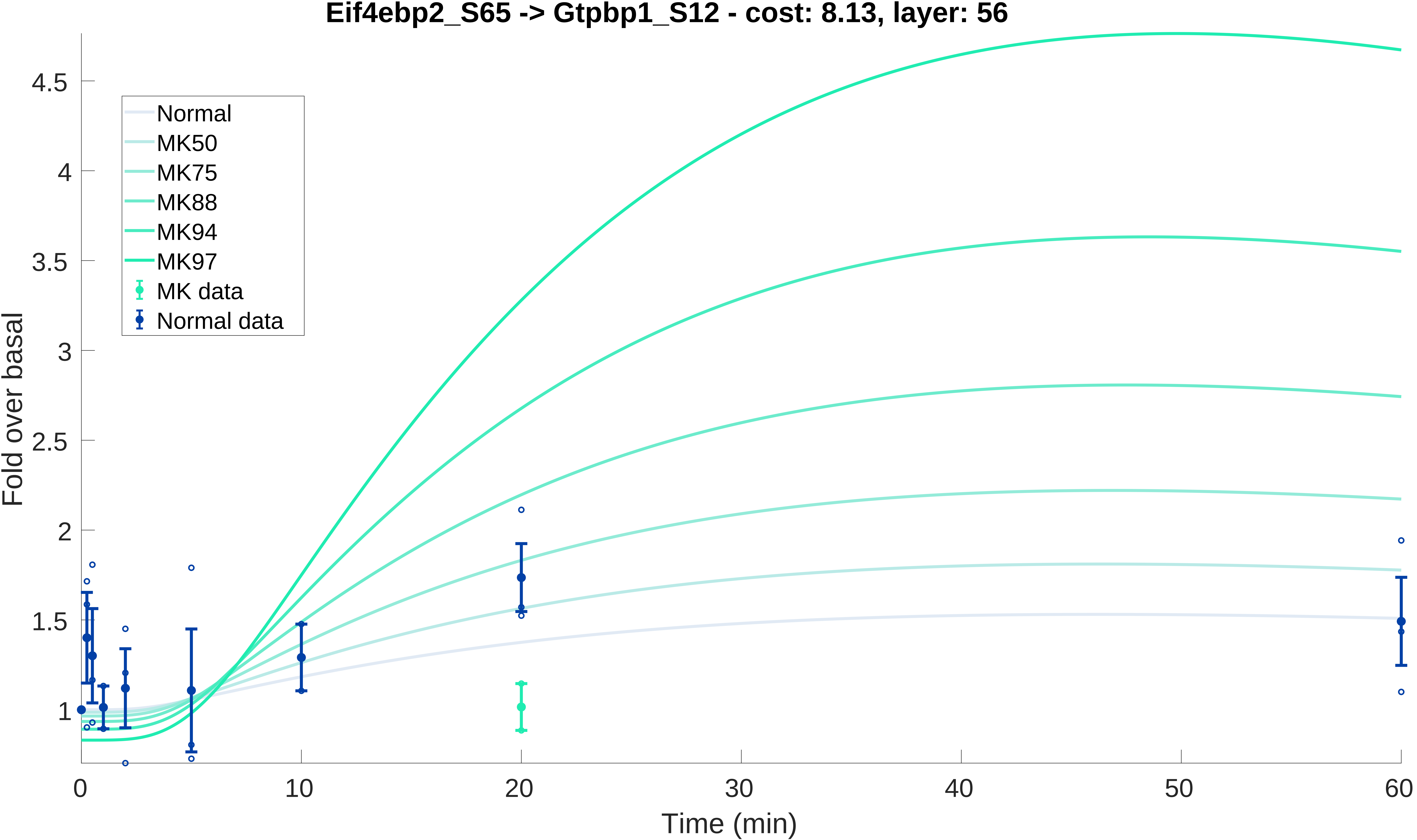

Eif4ebp2_S65 -> Gtpbp1_S12 - cost: 8.13, layer: 56
Normal
MK50
MK75
MK88
MK94
MK97
MK data
Normal data
Fold over basal
1
1.5
2
2.5
3
3.5
4
4.5
0
10
20
30
40
50
60
Time (min)

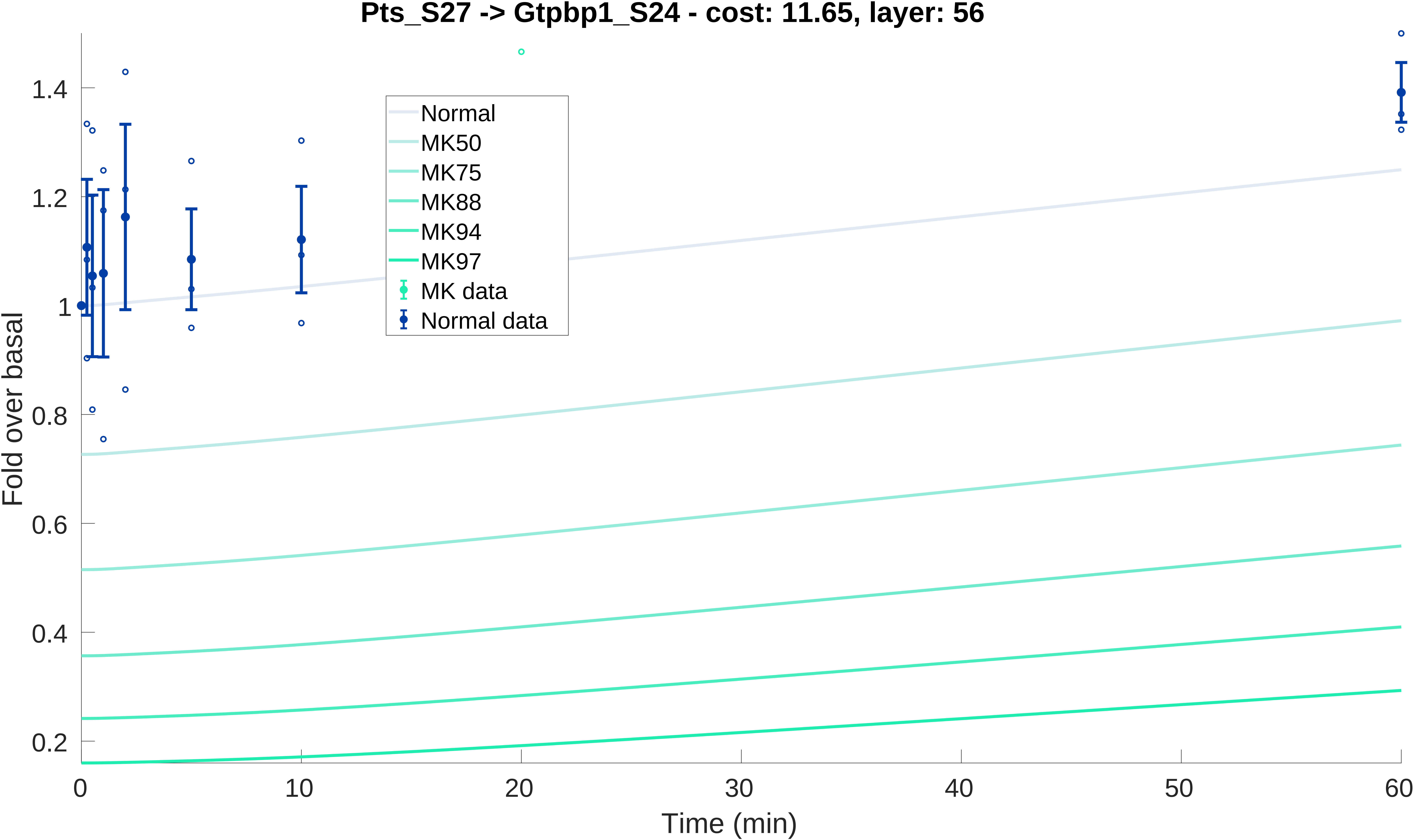
Pts_S27 -> Gtpbp1_S24 - cost: 11.65, layer: 56
Normal
MK50
MK75
MK88
MK94
MK97
MK data
Normal data
Fold over basal
Time (min)
0.2
0.4
0.6
0.8
1
1.2
1.4
0
10
20
30
40
50
60

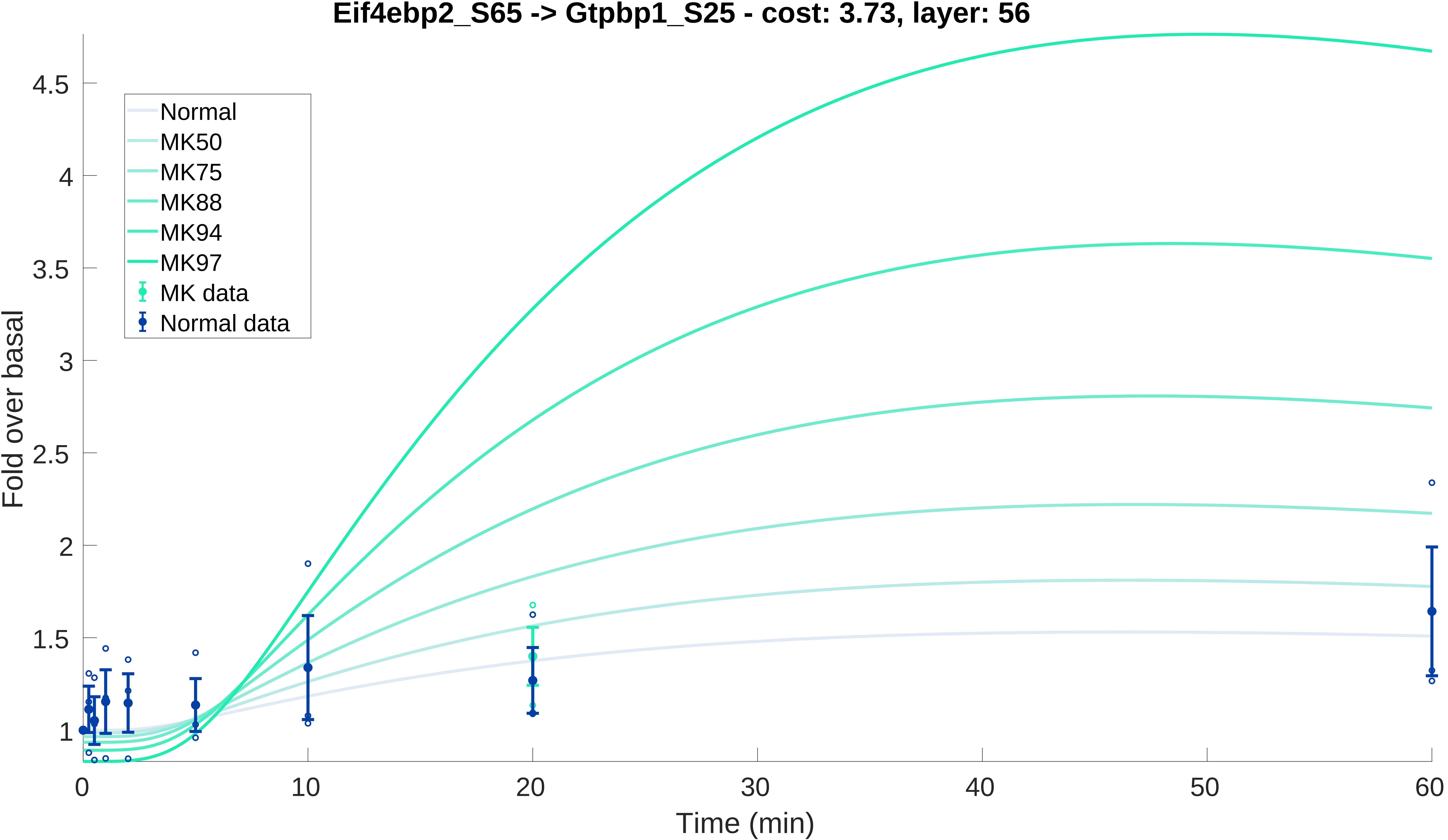

Eif4ebp2_S65 -> Gtpbp1_S25 - cost: 3.73, layer: 56
Normal
MK50
MK75
MK88
MK94
MK97
MK data
Normal data
Fold over basal
Time (min)
4.5
4
3.5
3
2.5
2
1.5
1
0
10
20
30
40
50
60

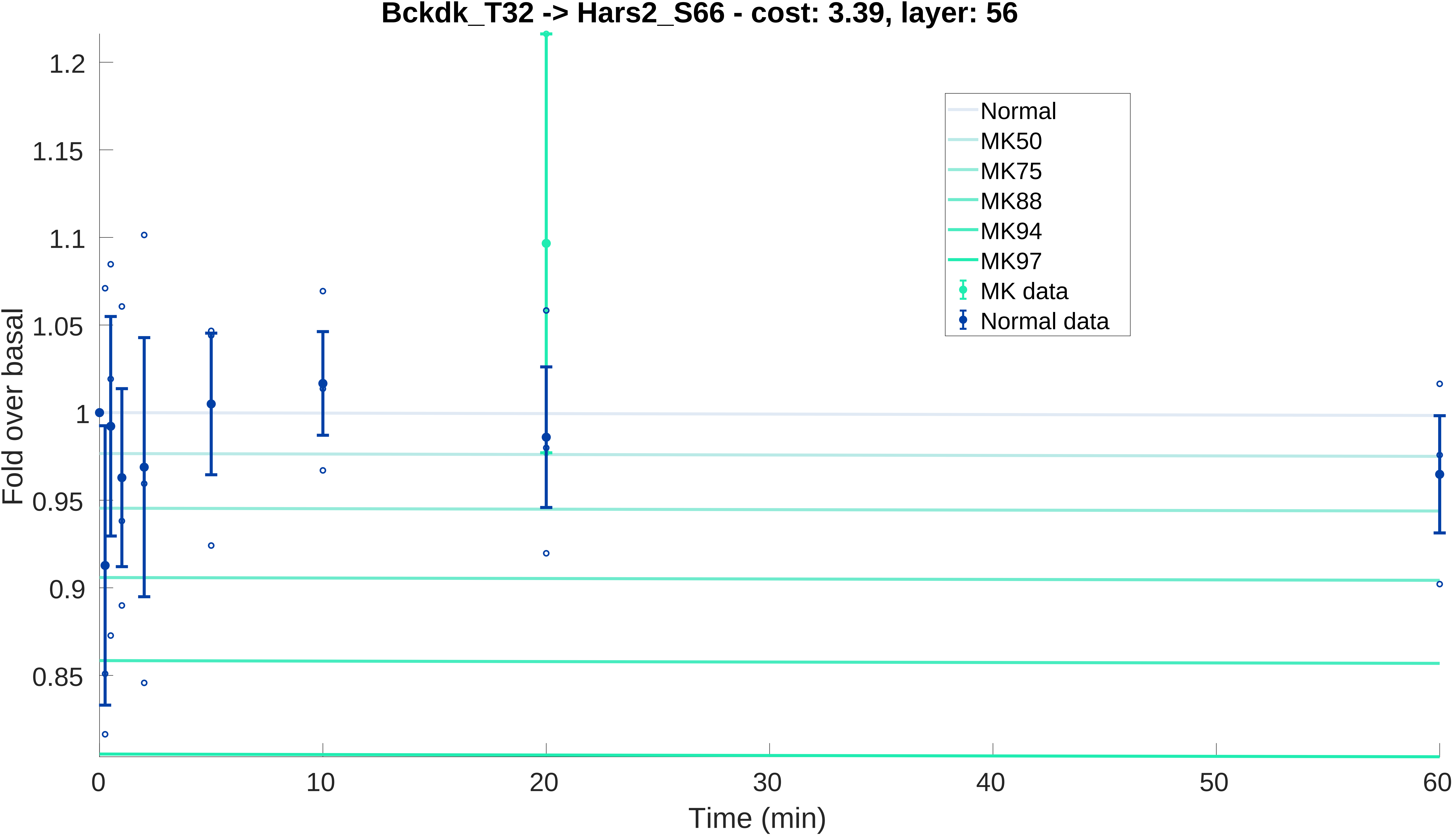
Bckdk_T32 -> Hars2_S66 - cost: 3.39, layer: 56
Fold over basal
Time (min)
Normal
MK50
MK75
MK88
MK94
MK97
MK data
Normal data
1.2
1.15
1.1
1.05
1
0.95
0.9
0.85
0
10
20
30
40
50
60

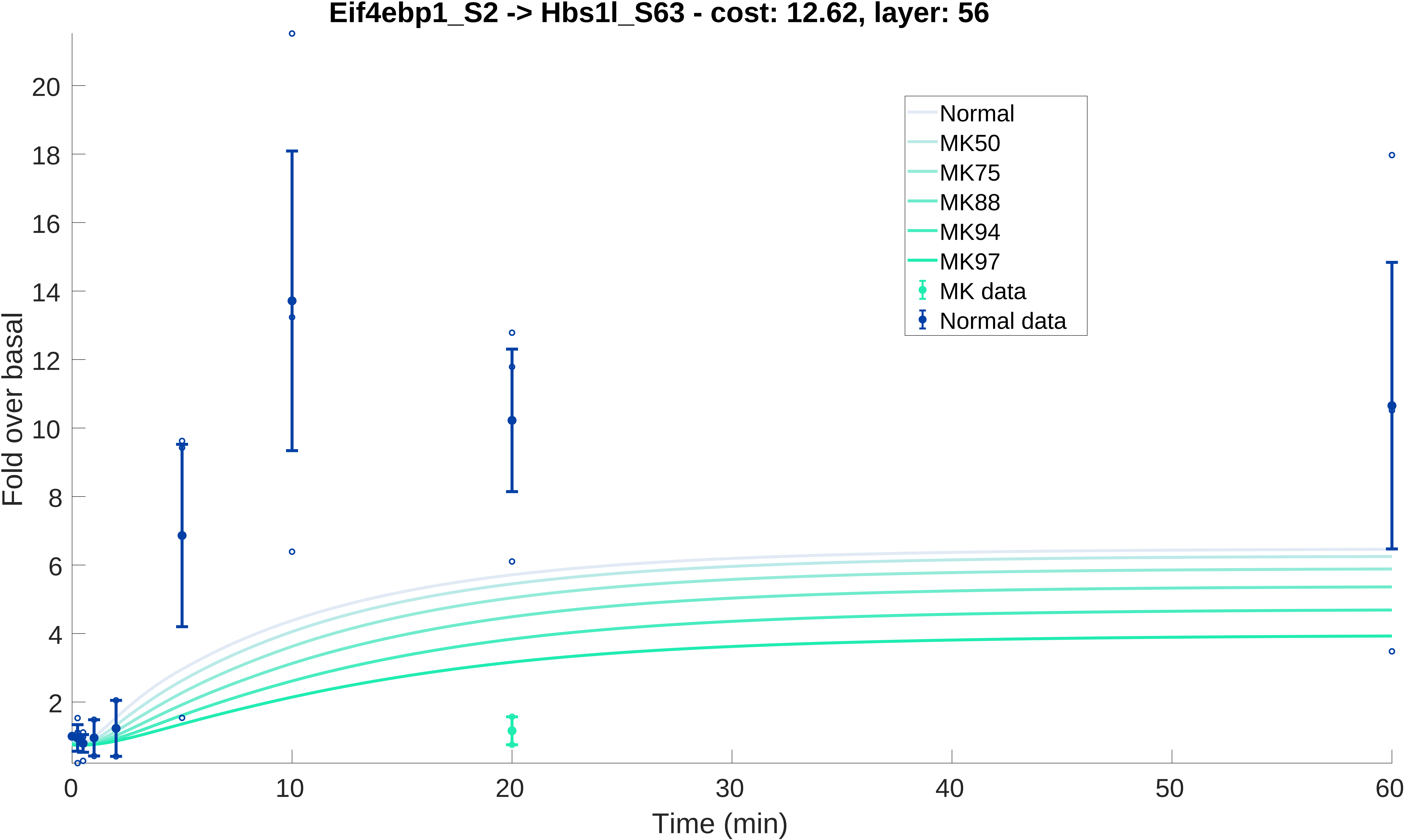

Eif4ebp1_S2 -> Hbs1l_S63 - cost: 12.62, layer: 56
Fold over basal
Time (min)
0
10
20
30
40
50
60
2
4
6
8
10
12
14
16
18
20
Normal
MK50
MK75
MK88
MK94
MK97
MK data
Normal data

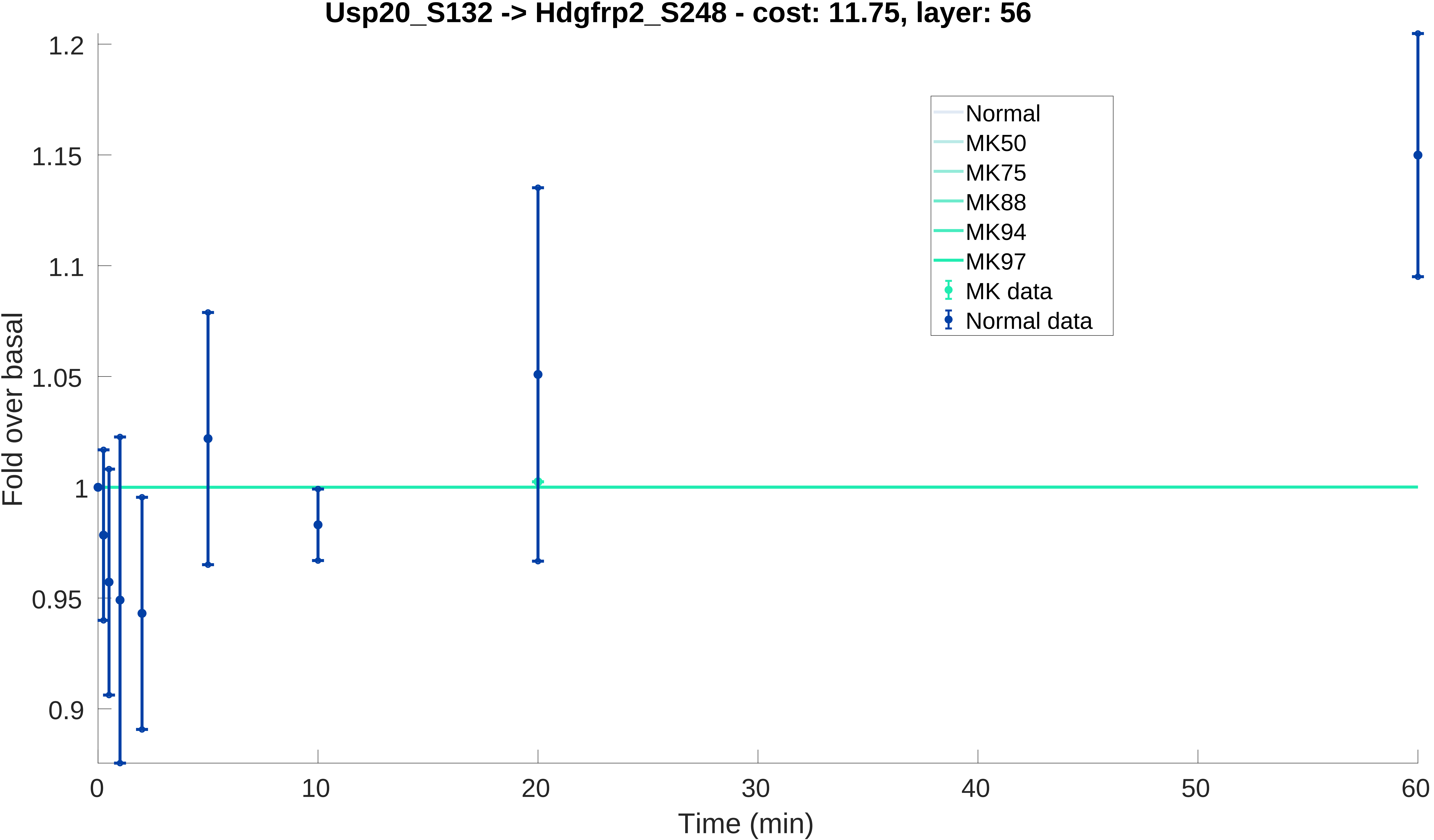

Usp20_S132 -> Hdgfrp2_S248 - cost: 11.75, layer: 56
Fold over basal
1.2
1.15
1.1
1.05
1
0.95
0.9
0
10
20
30
40
50
60
Time (min)
Normal
MK50
MK75
MK88
MK94
MK97
MK data
Normal data

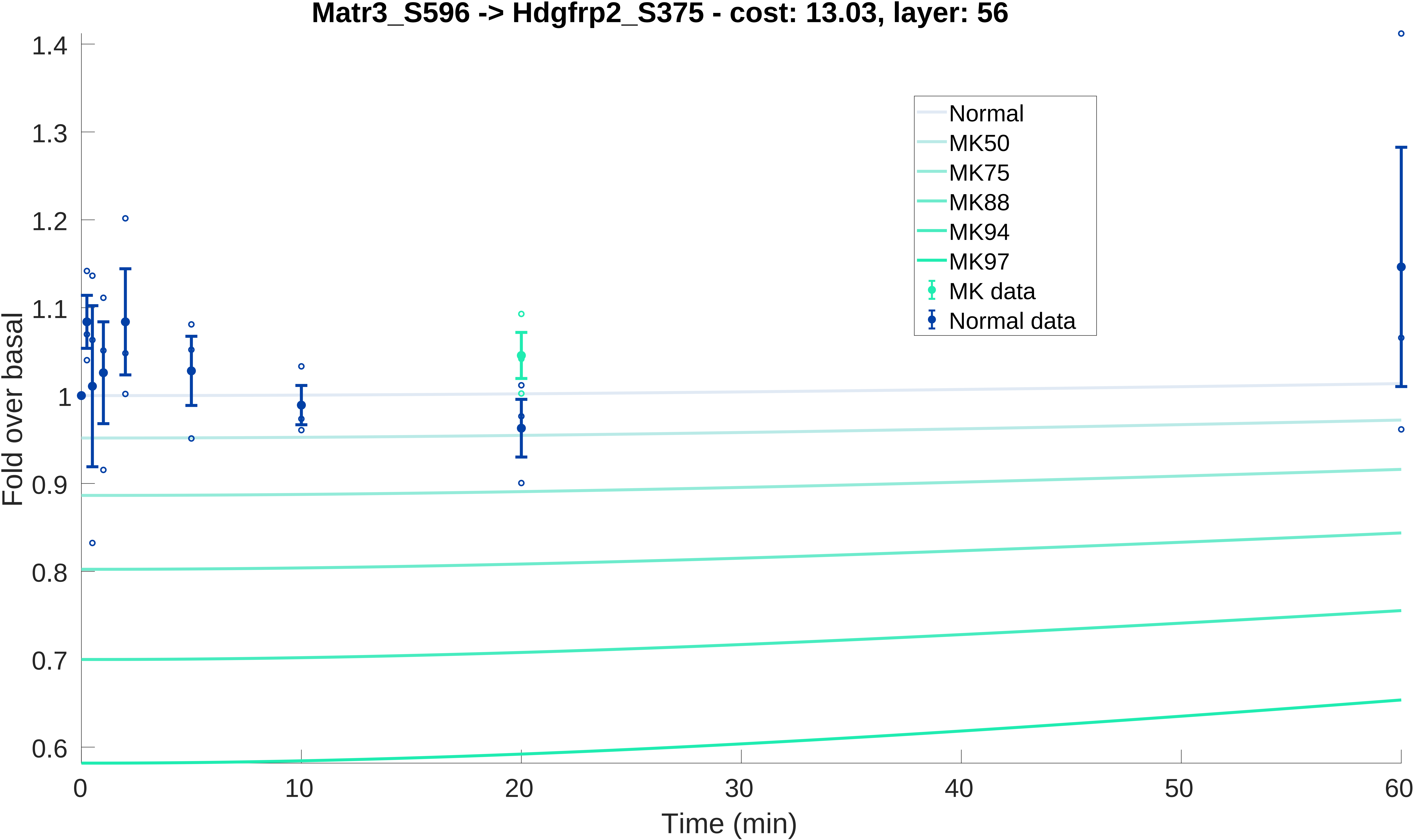

Matr3_S596 -> Hdgfrp2_S375 - cost: 13.03, layer: 56
Fold over basal
Time (min)
1.4
1.3
1.2
1.1
1
0.9
0.8
0.7
0.6
0
10
20
30
40
50
60
Normal
MK50
MK75
MK88
MK94
MK97
MK data
Normal data

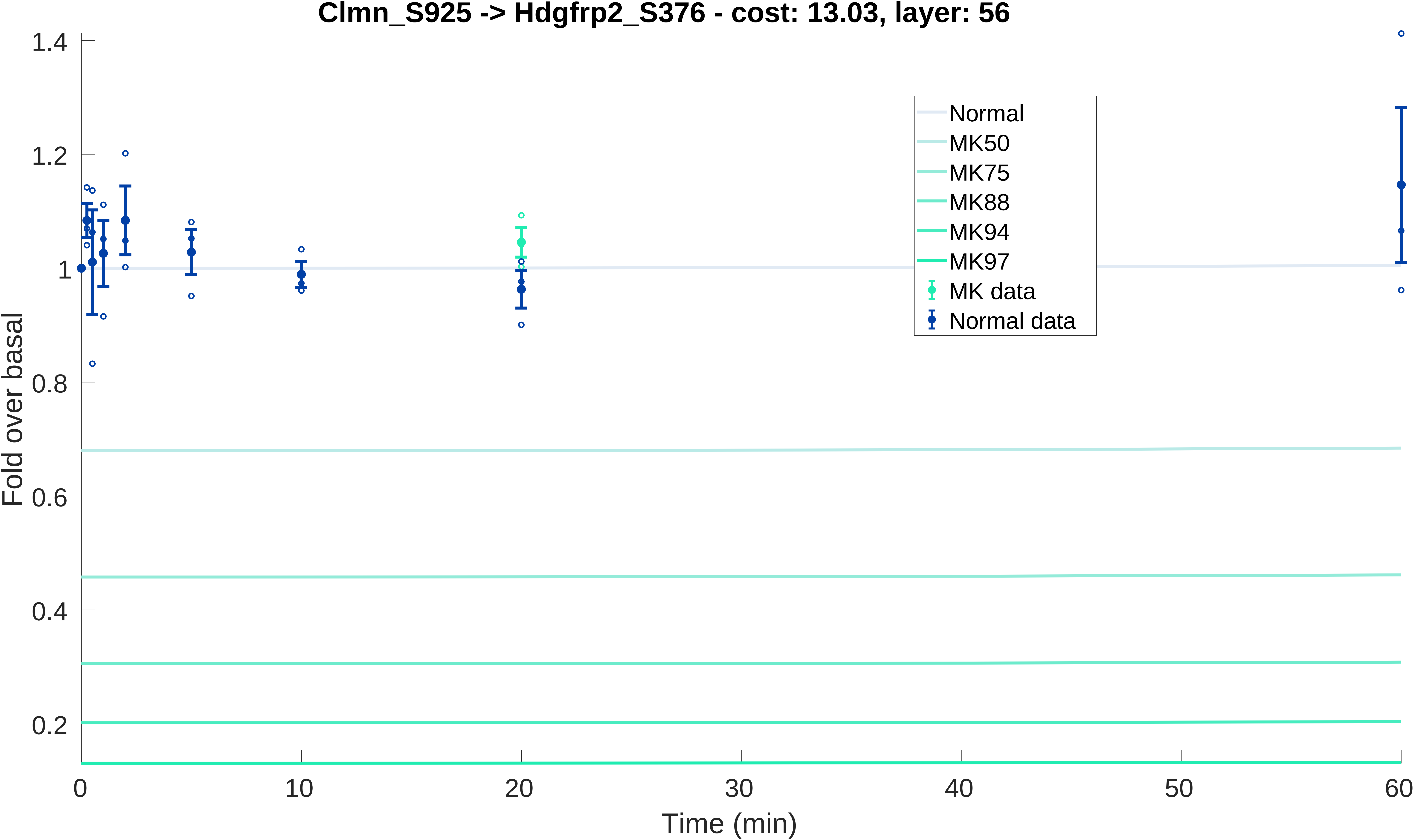

Clmn_S925 -> Hdgfrp2_S376 - cost: 13.03, layer: 56
Fold over basal
Time (min)
1.4
1.2
1
0.8
0.6
0.4
0.2
0
10
20
30
40
50
60
Normal
MK50
MK75
MK88
MK94
MK97
MK data
Normal data

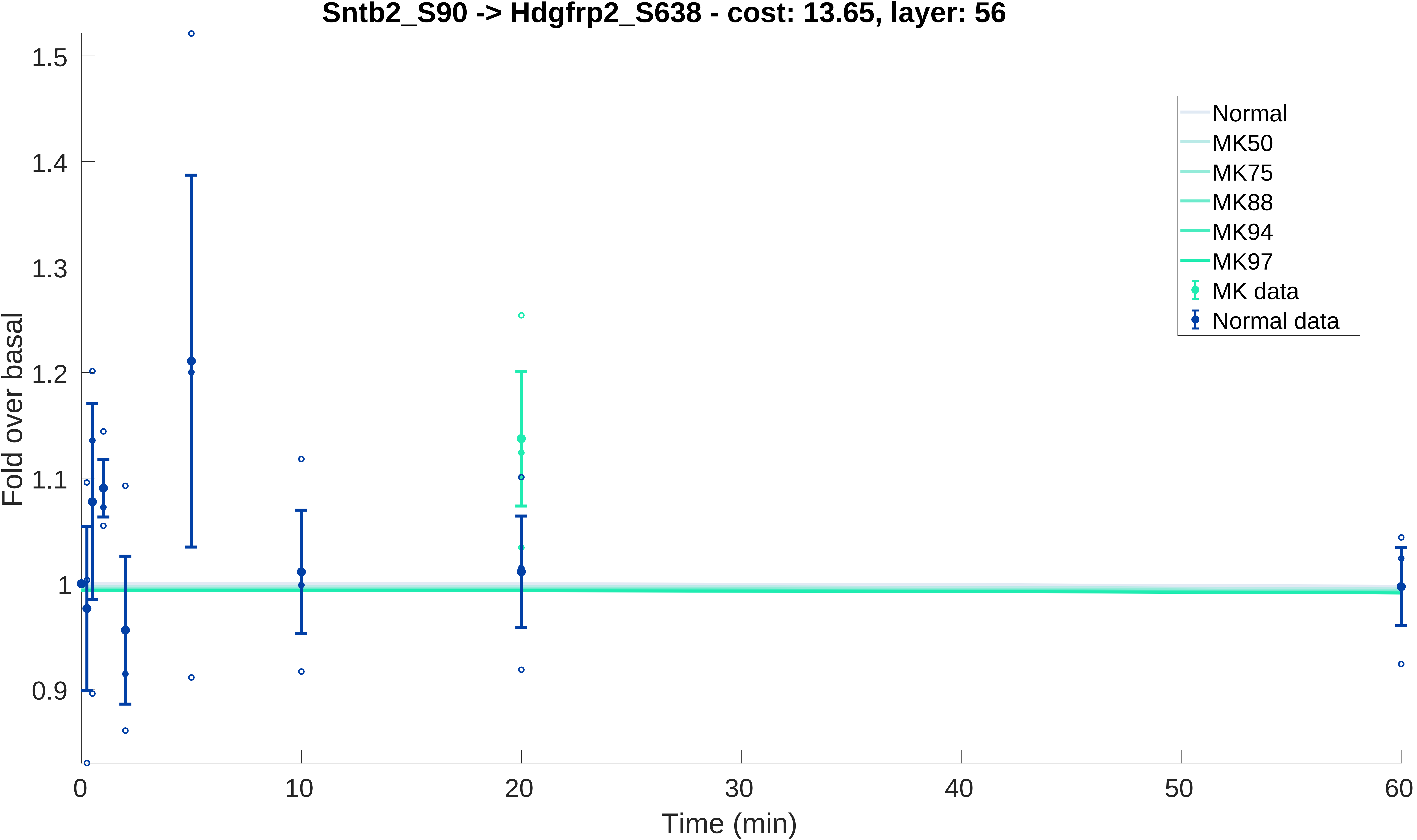

Sntb2_S90 -> Hdgfrp2_S638 - cost: 13.65, layer: 56
Fold over basal
Time (min)
1.5
1.4
1.3
1.2
1.1
1
0.9
0
10
20
30
40
50
60
Normal
MK50
MK75
MK88
MK94
MK97
MK data
Normal data

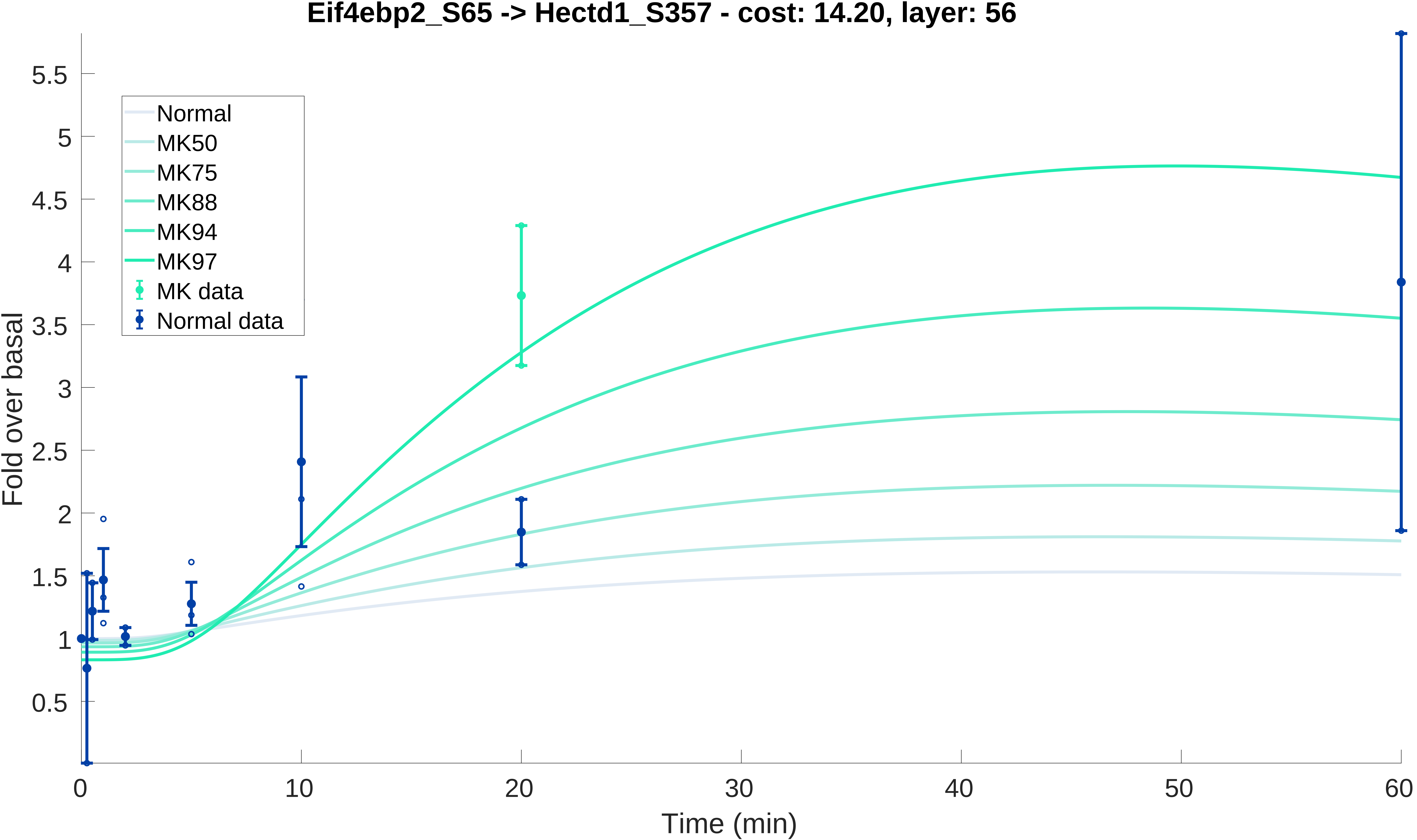

Eif4ebp2_S65 -> Hectd1_S357 - cost: 14.20, layer: 56
Normal
MK50
MK75
MK88
MK94
MK97
MK data
Normal data
Fold over basal
Time (min)
0.5
1
1.5
2
2.5
3
3.5
4
4.5
5
5.5
0
10
20
30
40
50
60

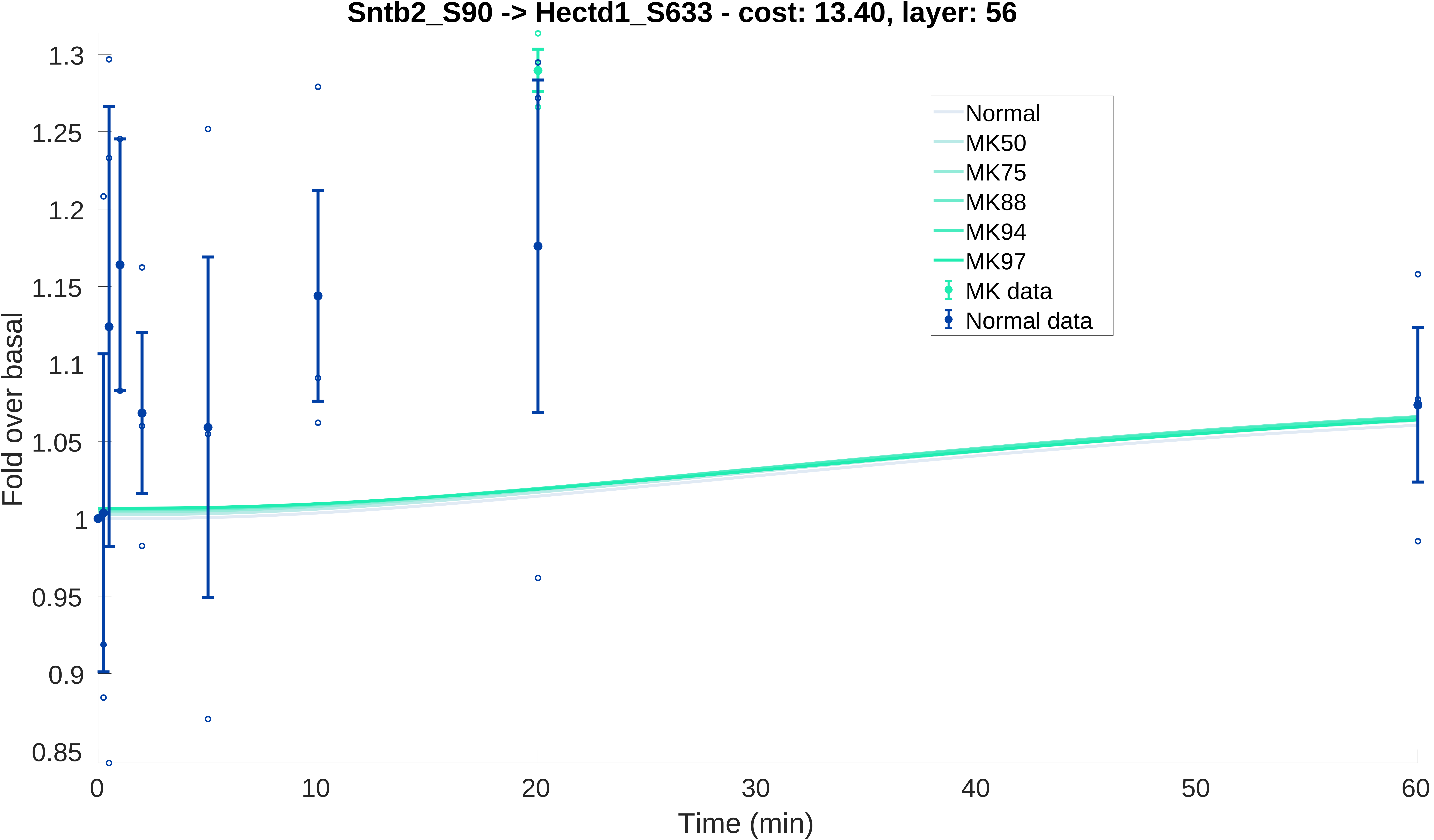

Sntb2_S90 -> Hectd1_S633 - cost: 13.40, layer: 56
Fold over basal
1.3
1.25
1.2
1.15
1.1
1.05
1
0.95
0.9
0.85
0
10
20
30
40
50
60
Time (min)
Normal
MK50
MK75
MK88
MK94
MK97
MK data
Normal data

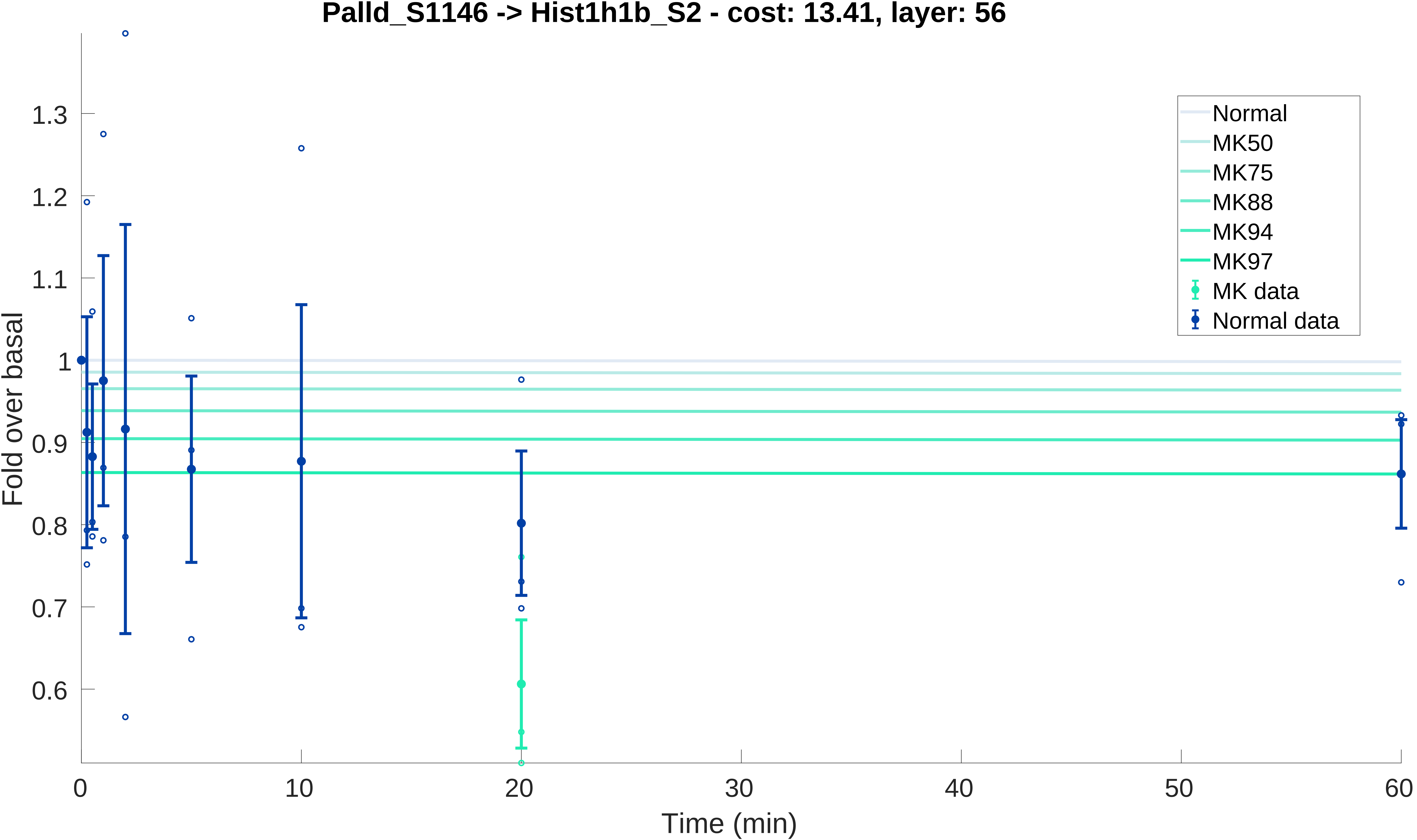

Palld_S1146 -> Hist1h1b_S2 - cost: 13.41, layer: 56
Normal
MK50
MK75
MK88
MK94
MK97
MK data
Normal data
Fold over basal
1.3
1.2
1.1
1
0.9
0.8
0.7
0.6
0
10
20
30
40
50
60
Time (min)

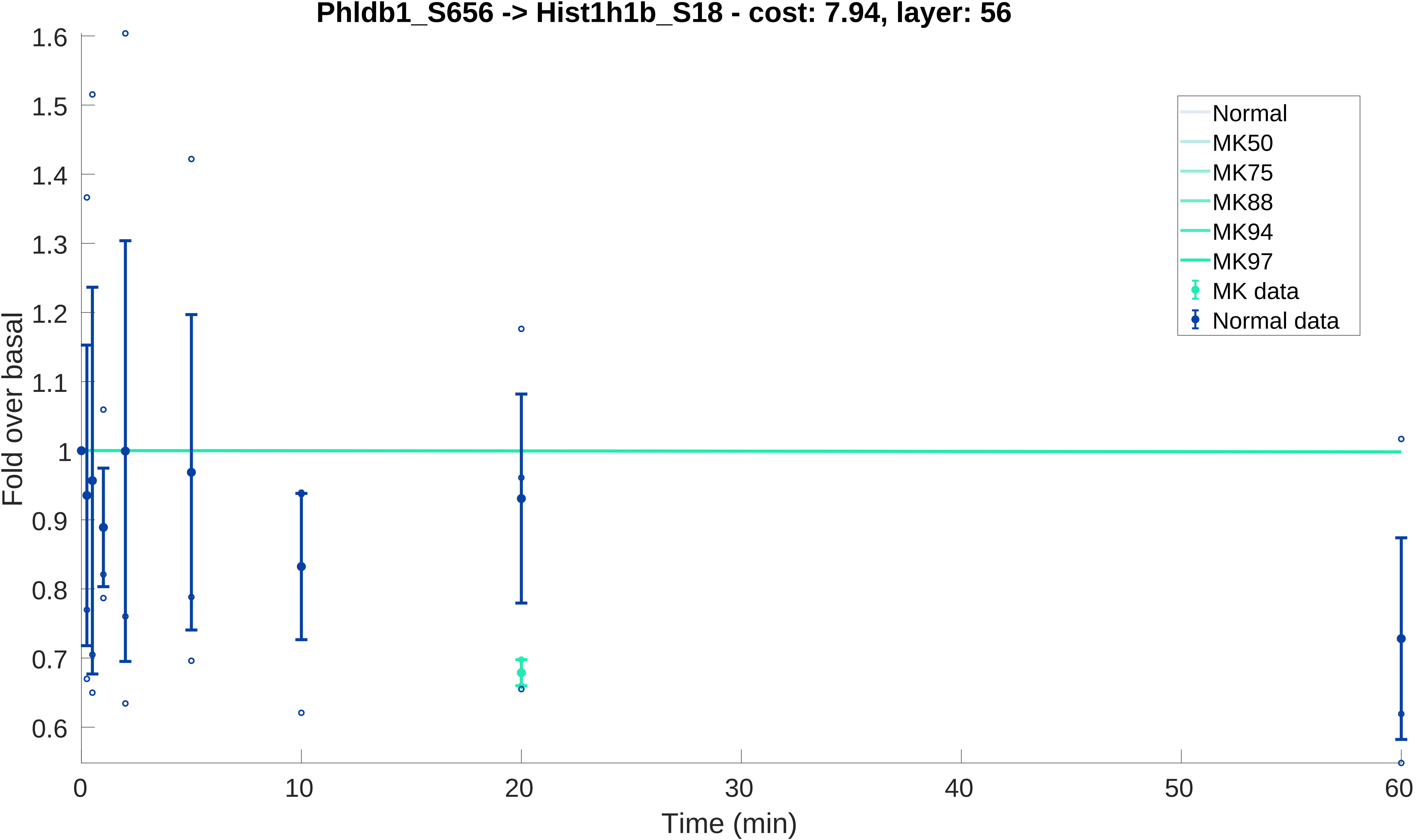

Phldb1_S656 -> Hist1h1b_S18 - cost: 7.94, layer: 56
Fold over basal
Time (min)
1.6
1.5
1.4
1.3
1.2
1.1
1
0.9
0.8
0.7
0.6
0
10
20
30
40
50
60
Normal
MK50
MK75
MK88
MK94
MK97
MK data
Normal data

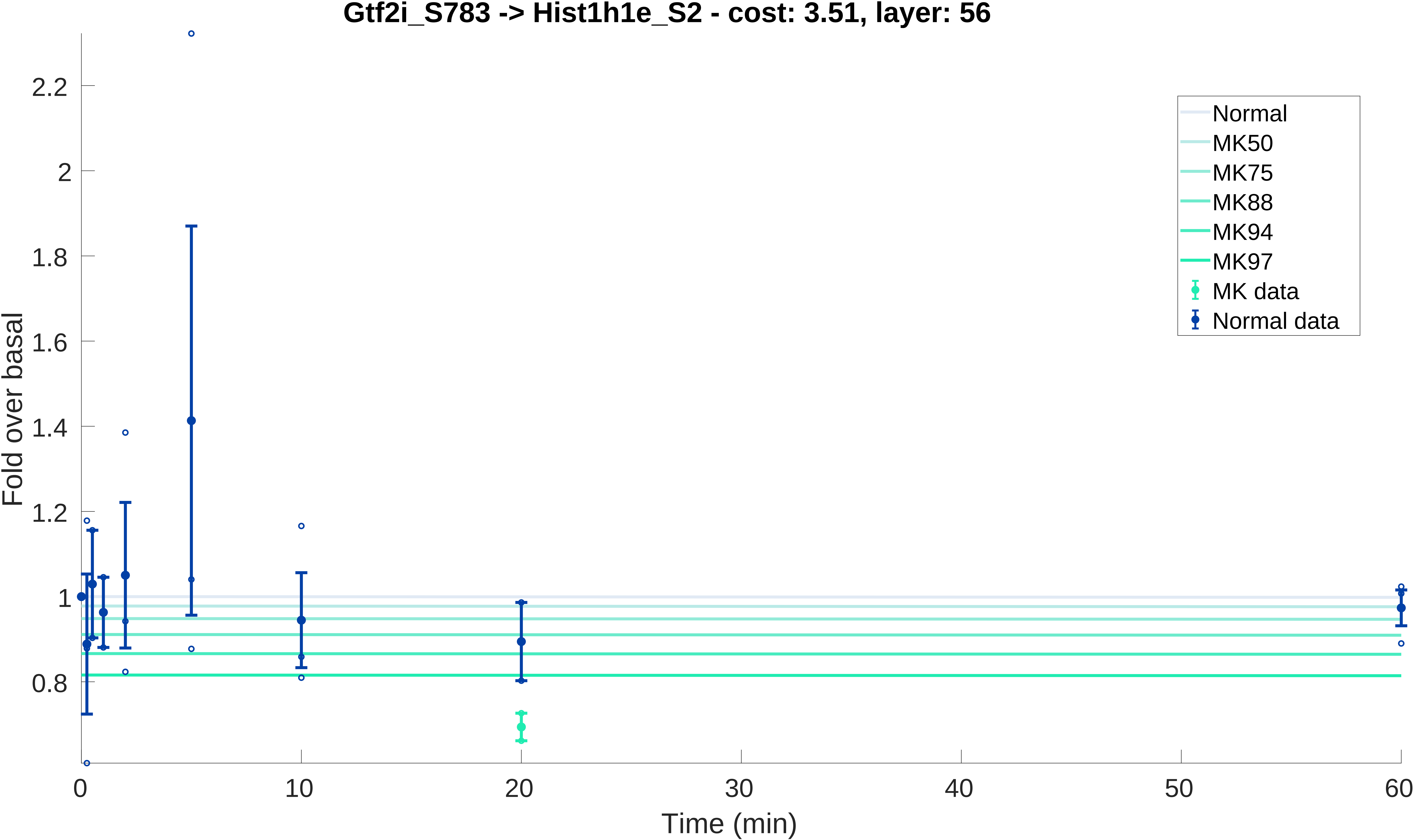

Gtf2i_S783 -> Hist1h1e_S2 - cost: 3.51, layer: 56
Fold over basal
Time (min)
0.8
1
1.2
1.4
1.6
1.8
2
2.2
0
10
20
30
40
50
60
Normal
MK50
MK75
MK88
MK94
MK97
MK data
Normal data

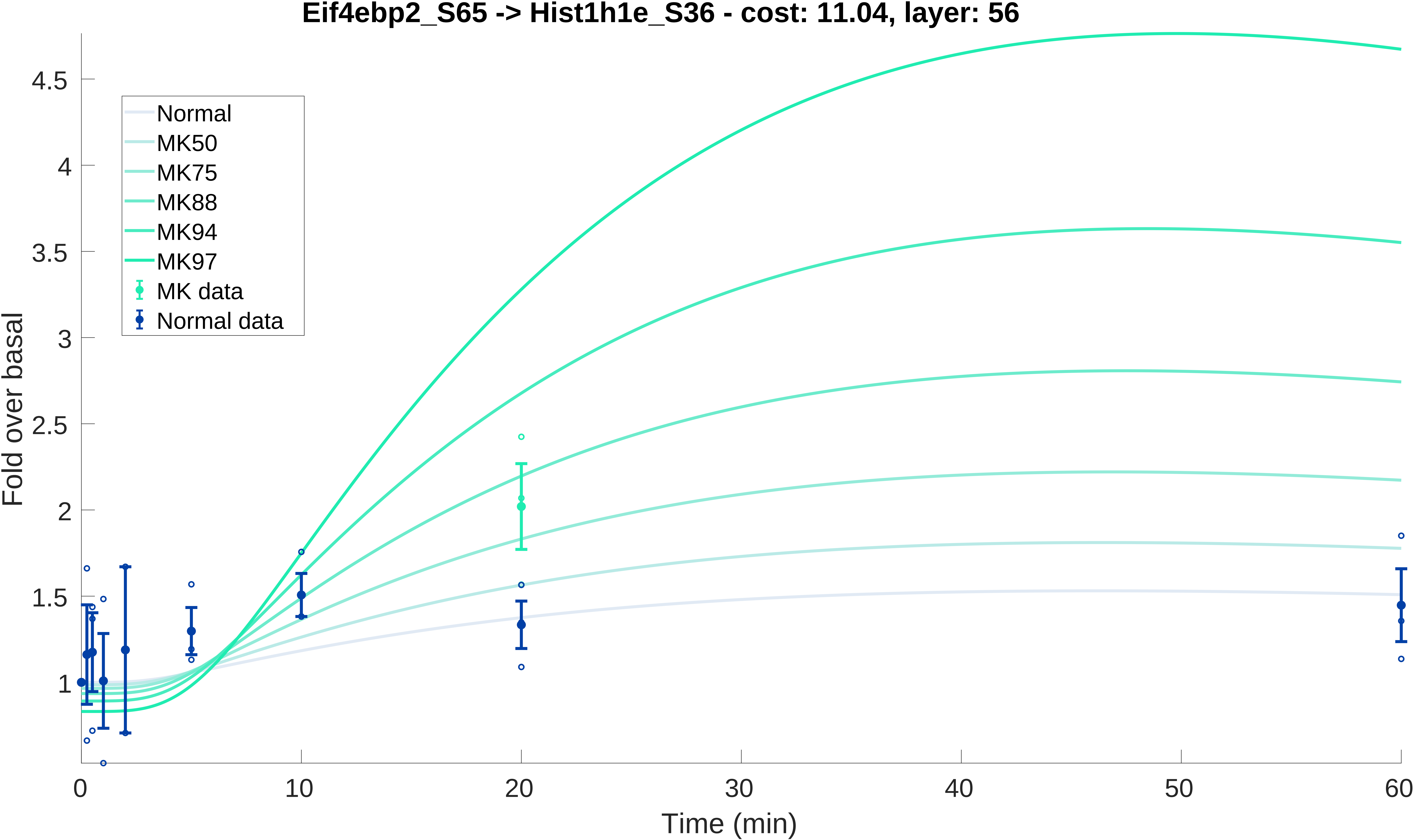

Eif4ebp2_S65 -> Hist1h1e_S36 - cost: 11.04, layer: 56
Normal
MK50
MK75
MK88
MK94
MK97
MK data
Normal data
Fold over basal
Time (min)
4.5
4
3.5
3
2.5
2
1.5
1
0
10
20
30
40
50
60

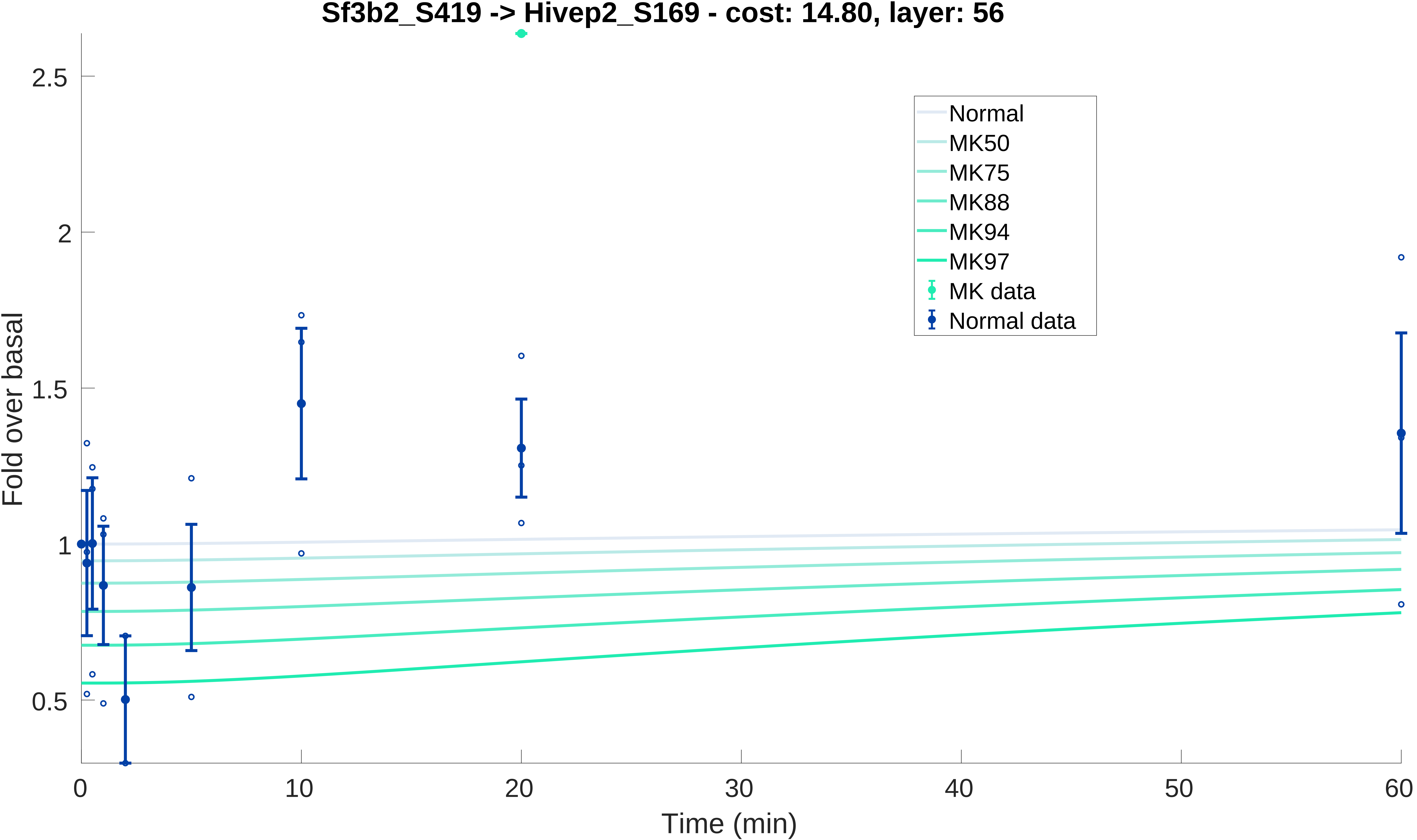

Sf3b2_S419 -> Hivep2_S169 - cost: 14.80, layer: 56
Normal
MK50
MK75
MK88
MK94
MK97
MK data
Normal data
Fold over basal
Time (min)
0
10
20
30
40
50
60
0.5
1
1.5
2
2.5

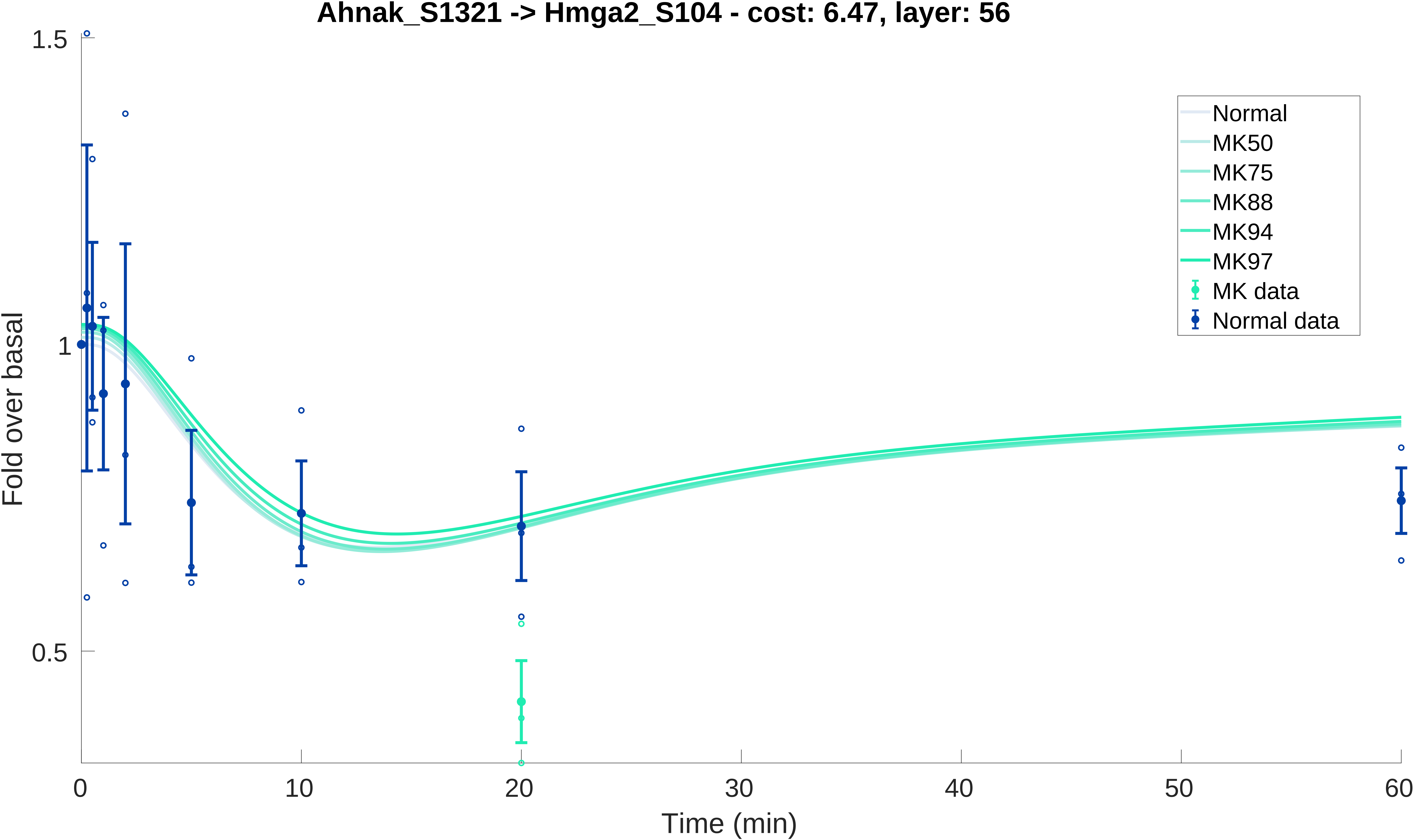
Ahnak_S1321 -> Hmga2_S104 - cost: 6.47, layer: 56
Fold over basal
1.5
1
0.5
0
10
20
30
40
50
60
Time (min)
Normal
MK50
MK75
MK88
MK94
MK97
MK data
Normal data

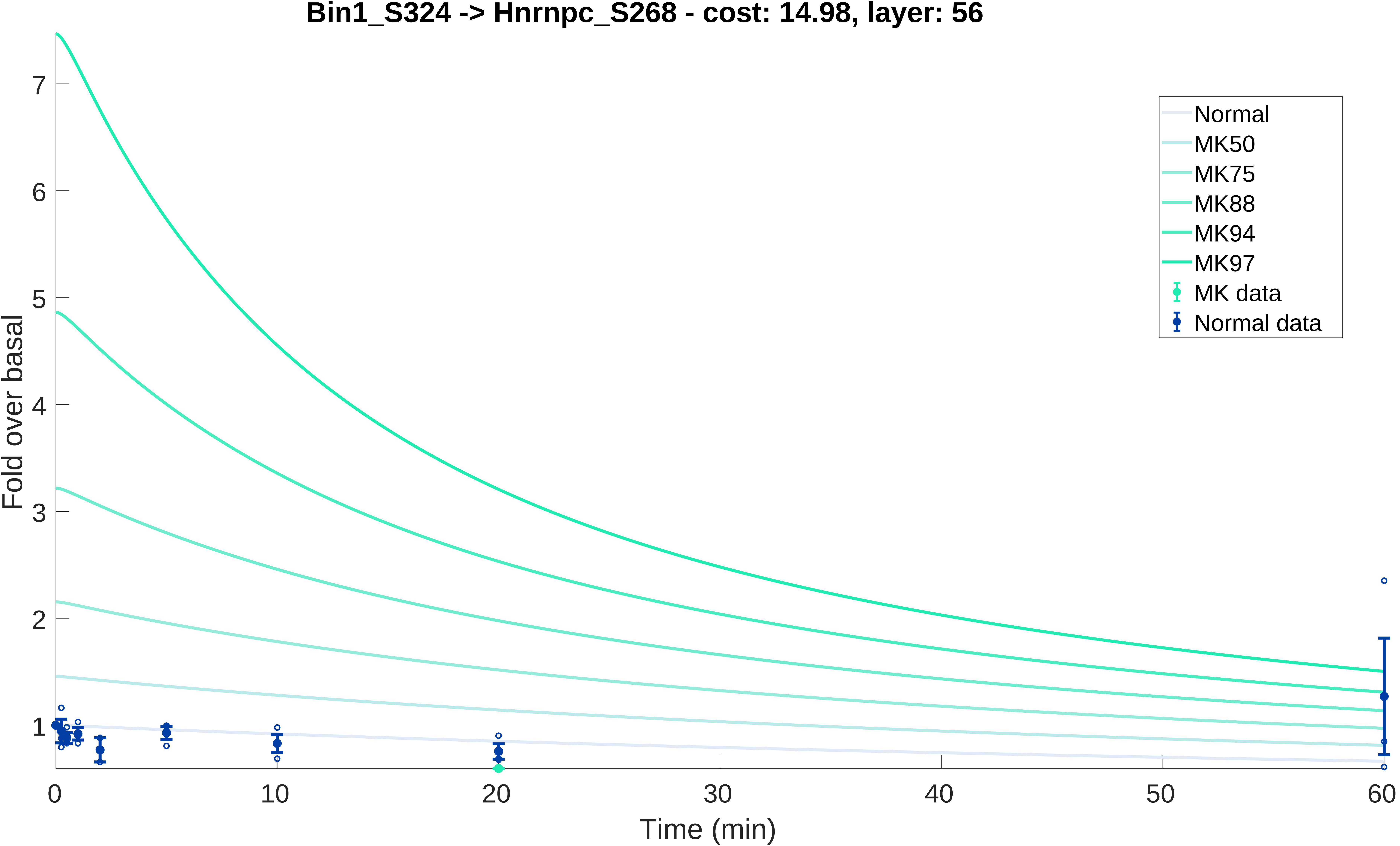

Bin1_S324 -> Hnrnpc_S268 - cost: 14.98, layer: 56
Fold over basal
Time (min)
0
10
20
30
40
50
60
1
2
3
4
5
6
7
Normal
MK50
MK75
MK88
MK94
MK97
MK data
Normal data

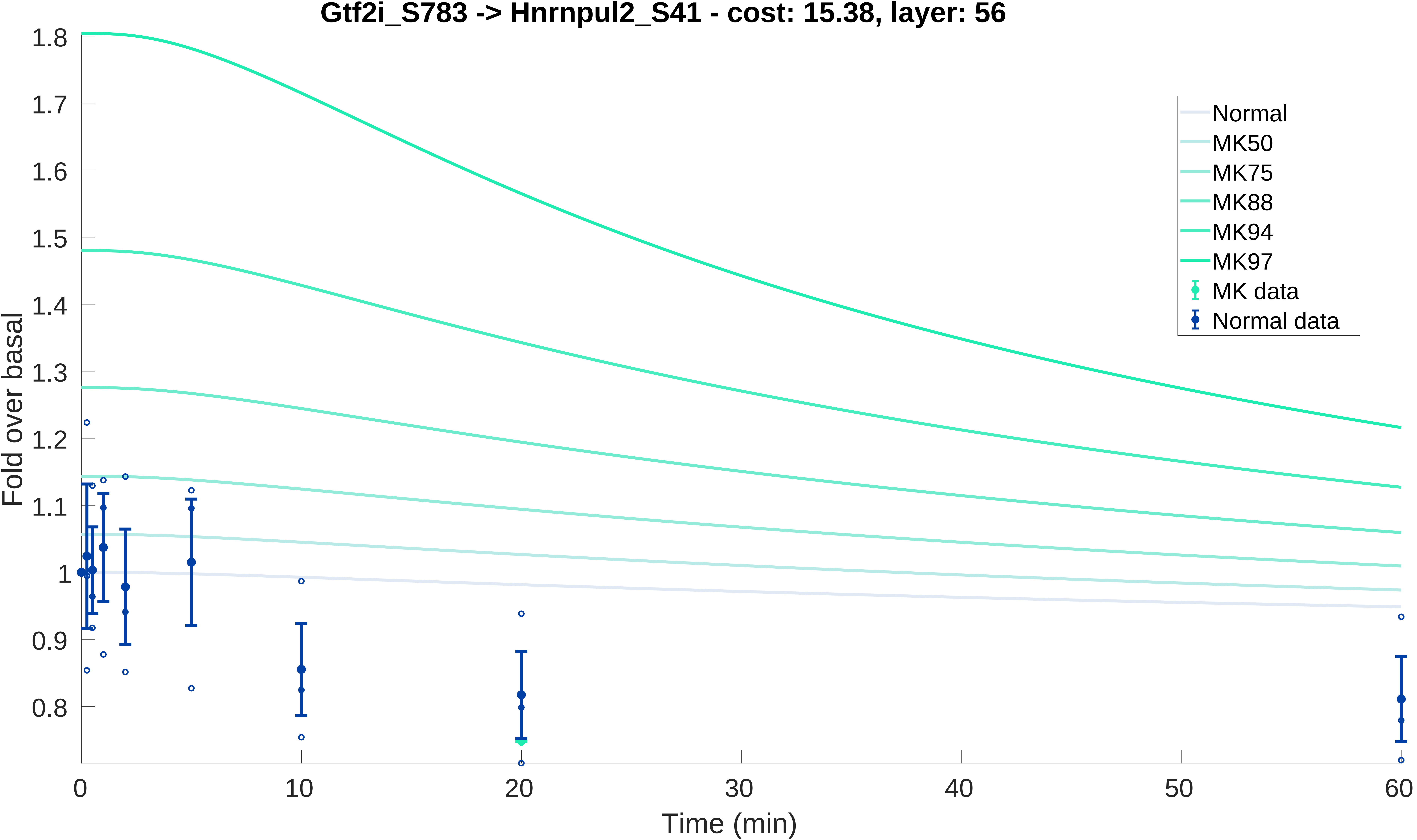

Gtf2i_S783 -> Hnrnpul2_S41 - cost: 15.38, layer: 56
Fold over basal
Time (min)
0.8
0.9
1
1.1
1.2
1.3
1.4
1.5
1.6
1.7
1.8
0
10
20
30
40
50
60
Normal
MK50
MK75
MK88
MK94
MK97
MK data
Normal data

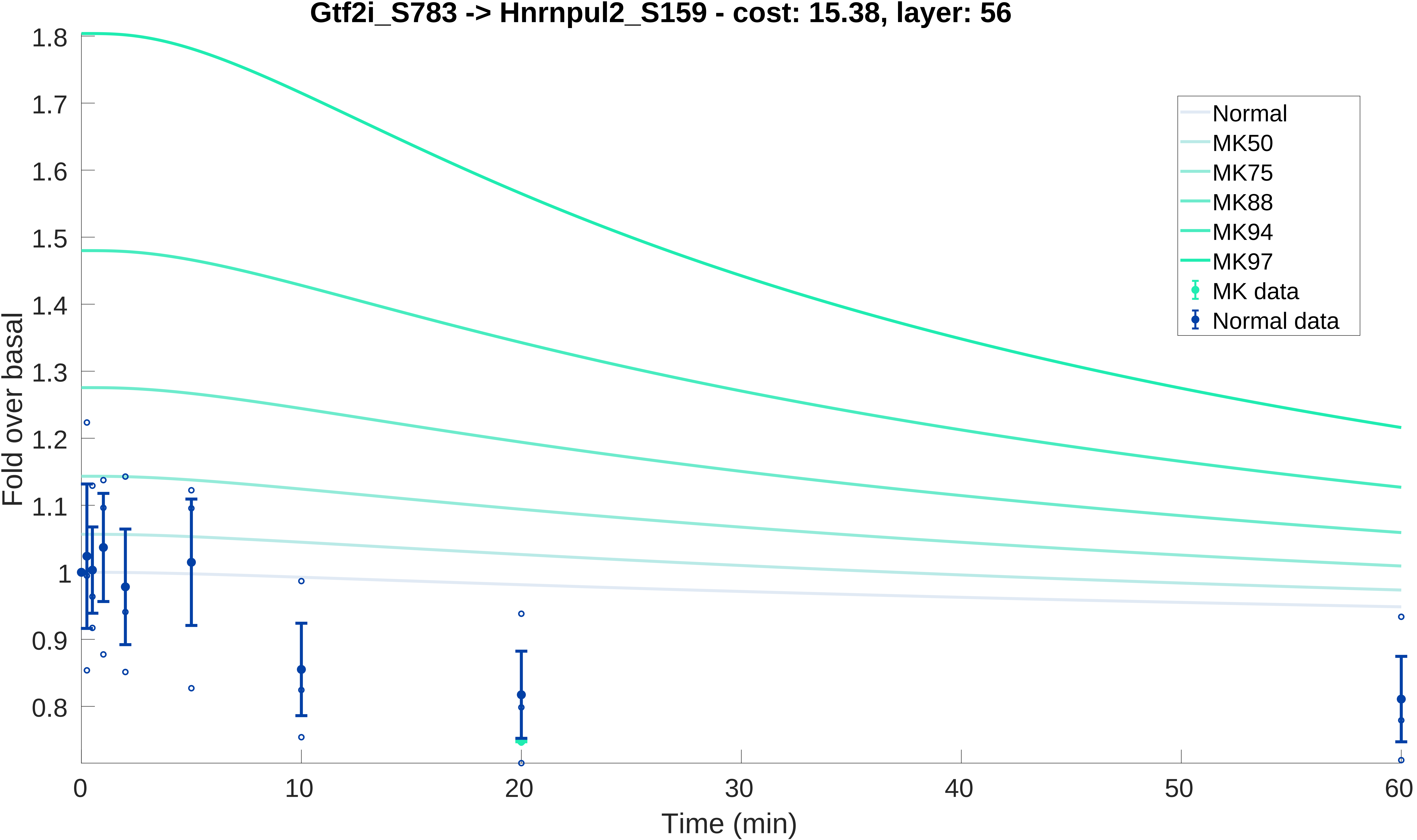

Gtf2i_S783 -> Hnrnpul2_S159 - cost: 15.38, layer: 56
1.8
1.7
1.6
1.5
1.4
1.3
1.2
1.1
1
0.9
0.8
Fold over basal
0
10
20
30
40
50
60
Time (min)
Normal
MK50
MK75
MK88
MK94
MK97
MK data
Normal data

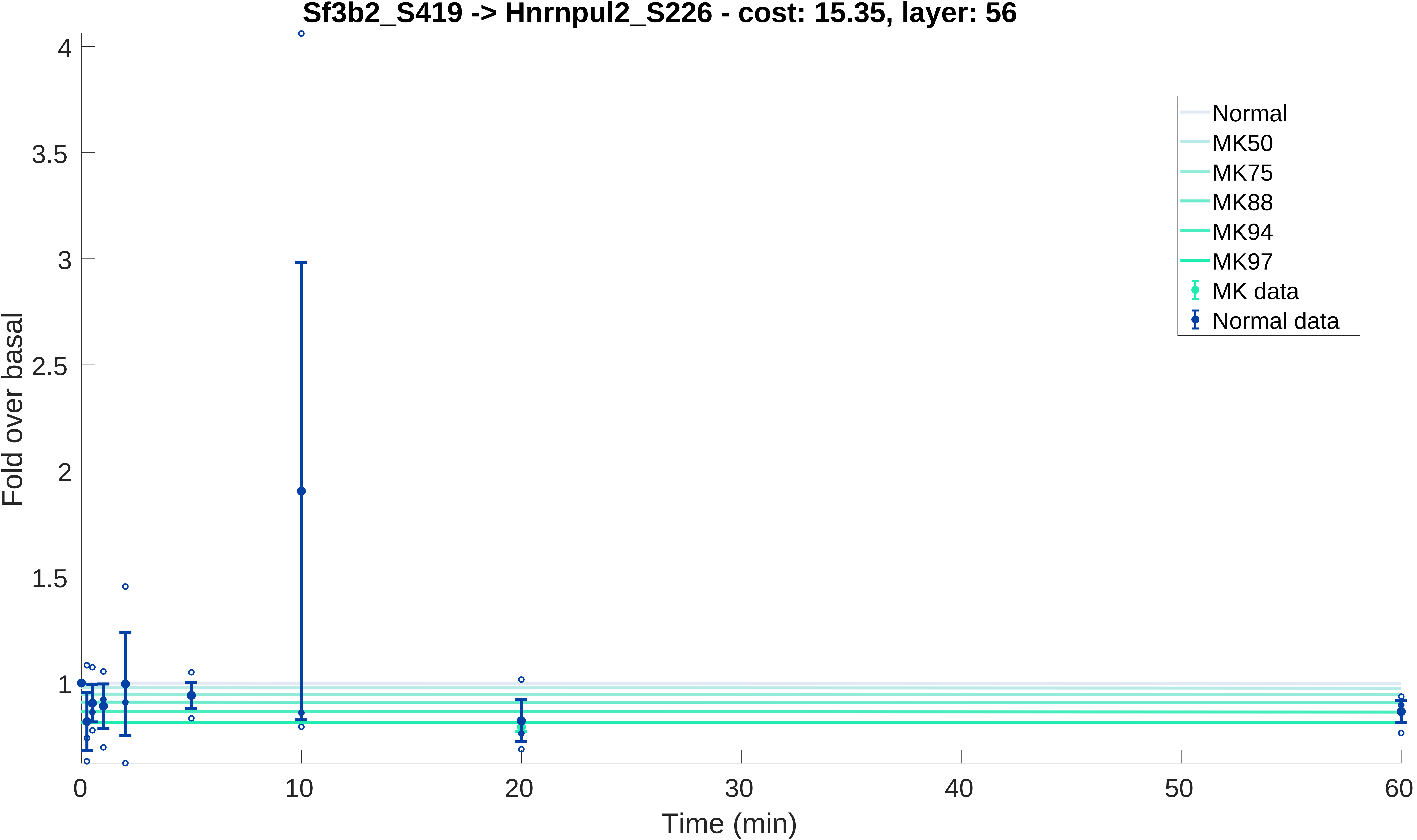

Sf3b2_S419 -> Hnrnpul2_S226 - cost: 15.35, layer: 56
Fold over basal
Time (min)
4
3.5
3
2.5
2
1.5
1
0
10
20
30
40
50
60
Normal
MK50
MK75
MK88
MK94
MK97
MK data
Normal data

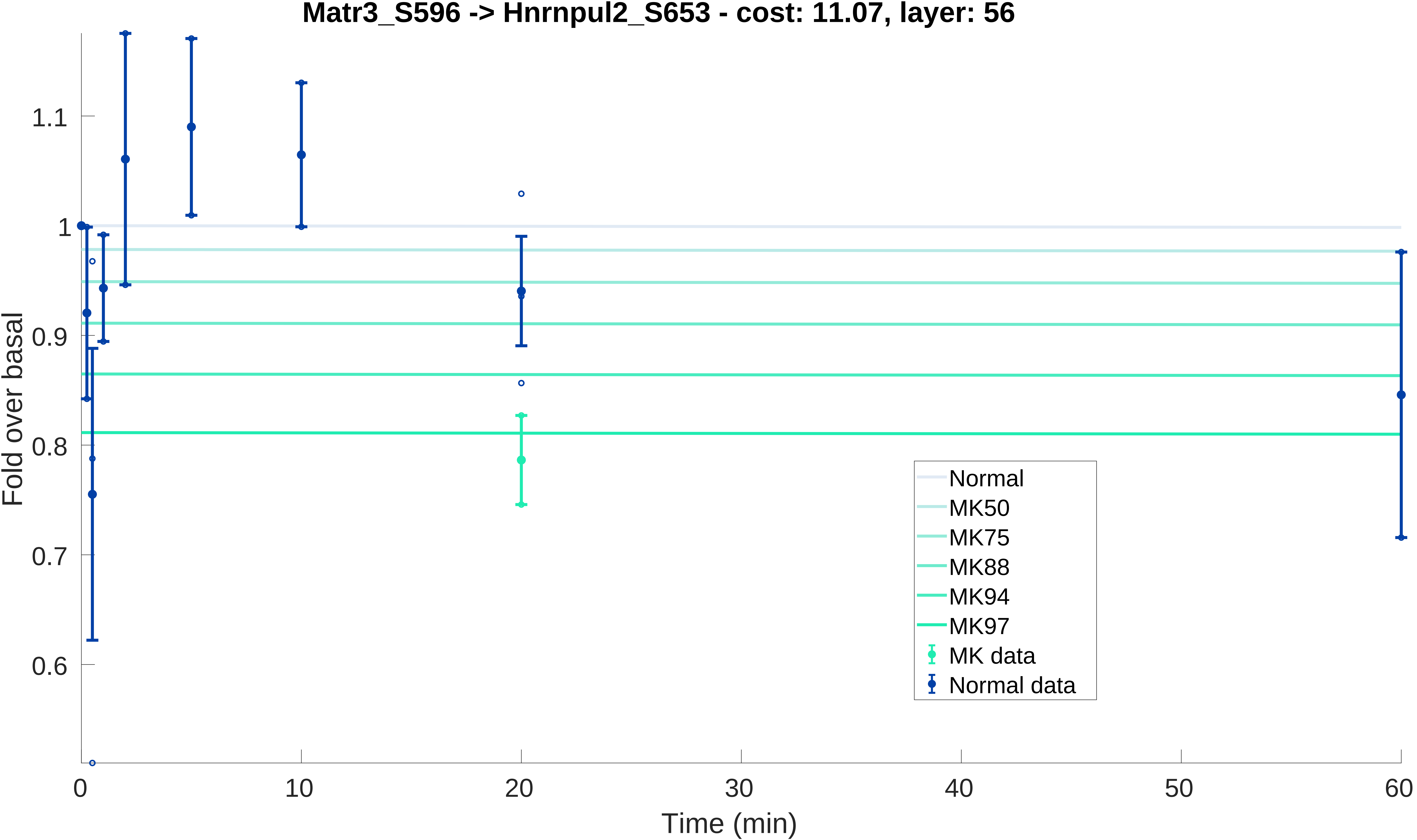

Matr3_S596 -> Hnrnpul2_S653 - cost: 11.07, layer: 56
Fold over basal
1.1
1
0.9
0.8
0.7
0.6
0
10
20
30
40
50
60
Time (min)
Normal
MK50
MK75
MK88
MK94
MK97
MK data
Normal data

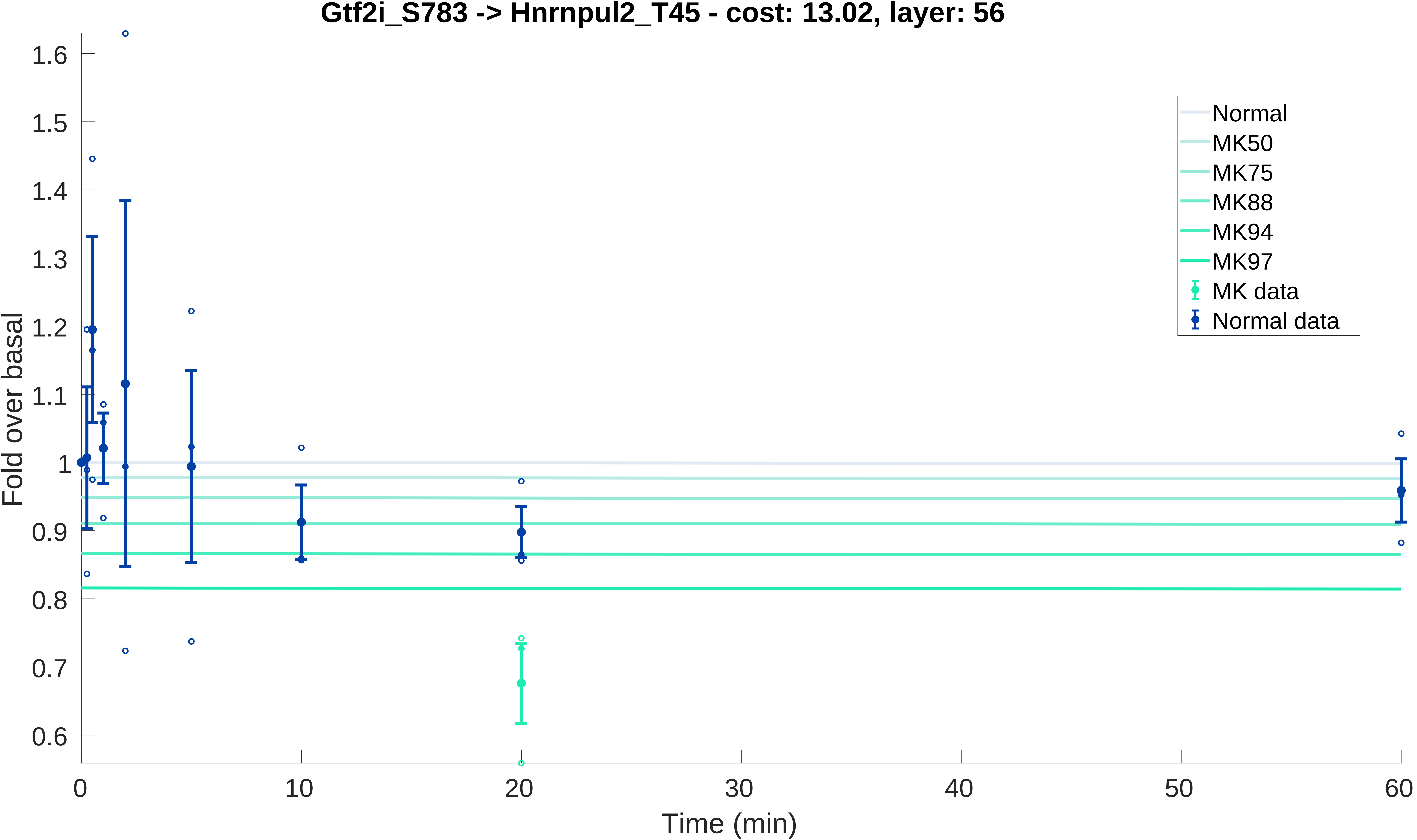

Gtf2i_S783 -> Hnrnpul2_T45 - cost: 13.02, layer: 56
Fold over basal
Time (min)
Normal
MK50
MK75
MK88
MK94
MK97
MK data
Normal data
1.6
1.5
1.4
1.3
1.2
1.1
1
0.9
0.8
0.7
0.6
0
10
20
30
40
50
60

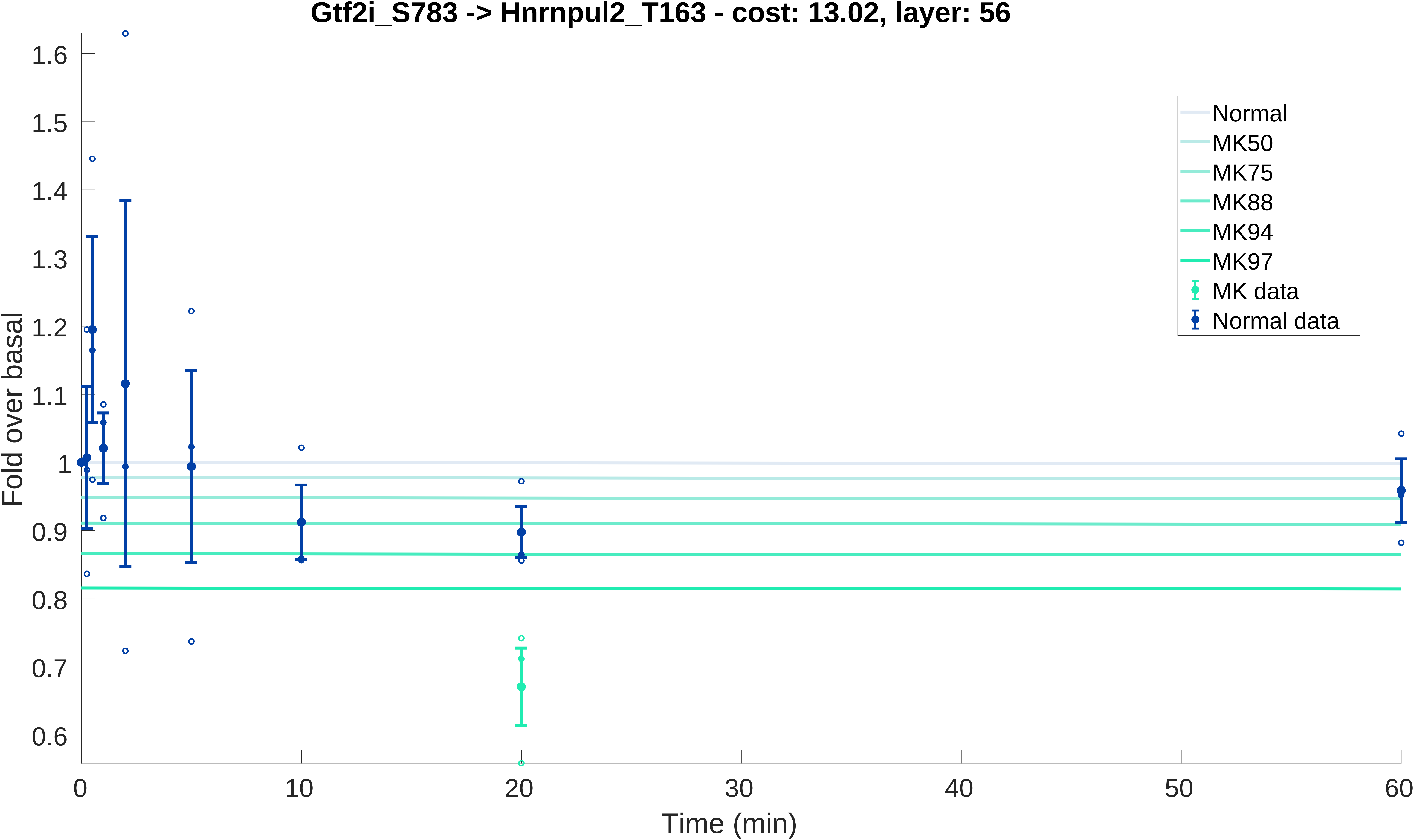

Gtf2i_S783 -> Hnrnpul2_T163 - cost: 13.02, layer: 56
Fold over basal
Time (min)
1.6
1.5
1.4
1.3
1.2
1.1
1
0.9
0.8
0.7
0.6
0
10
20
30
40
50
60
Normal
MK50
MK75
MK88
MK94
MK97
MK data
Normal data

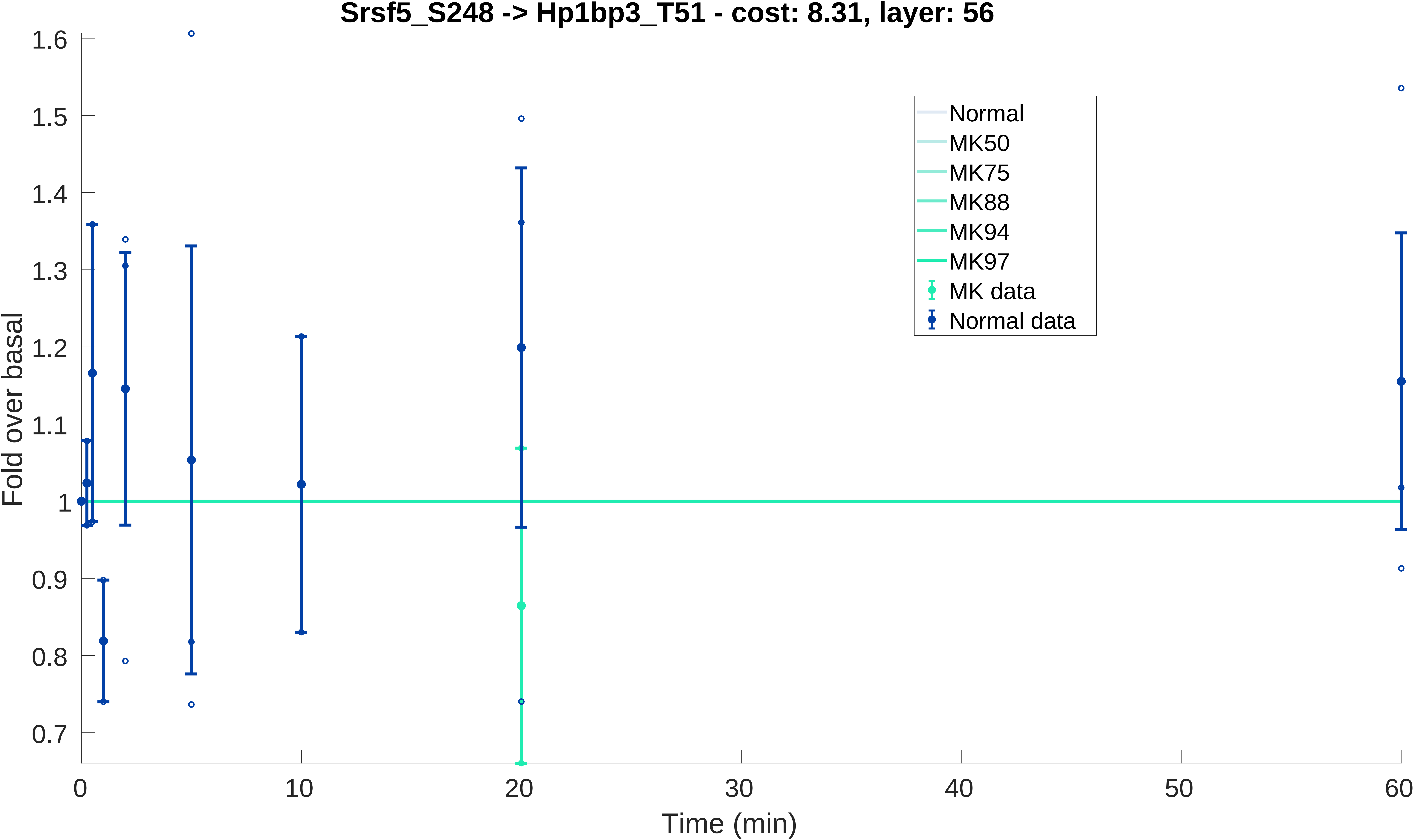

Srsf5_S248 -> Hp1bp3_T51 - cost: 8.31, layer: 56
Fold over basal
Time (min)
1.6
1.5
1.4
1.3
1.2
1.1
1
0.9
0.8
0.7
0
10
20
30
40
50
60
Normal
MK50
MK75
MK88
MK94
MK97
MK data
Normal data

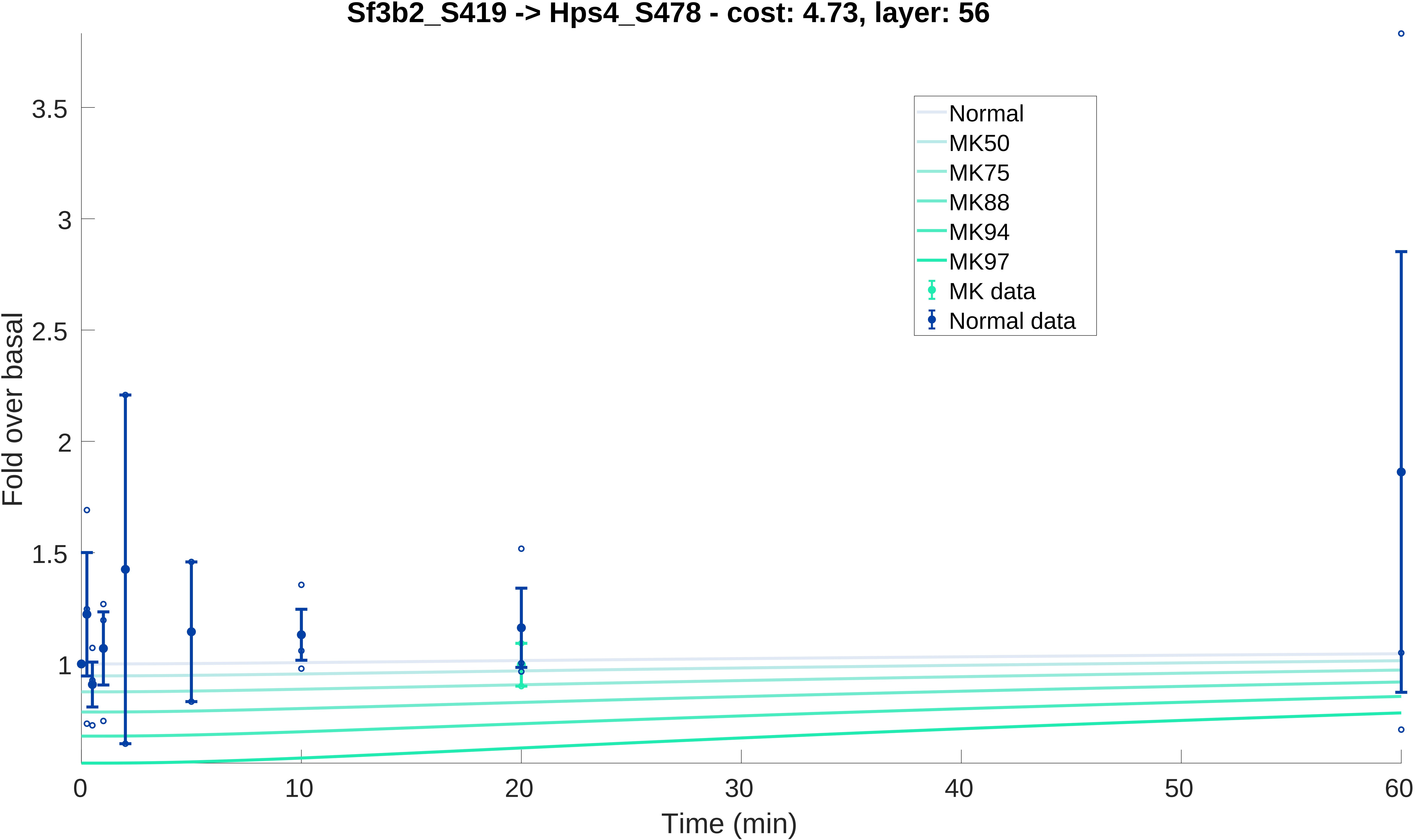

Sf3b2_S419 -> Hps4_S478 - cost: 4.73, layer: 56
Normal
MK50
MK75
MK88
MK94
MK97
MK data
Normal data
Fold over basal
Time (min)
3.5
3
2.5
2
1.5
1
0
10
20
30
40
50
60

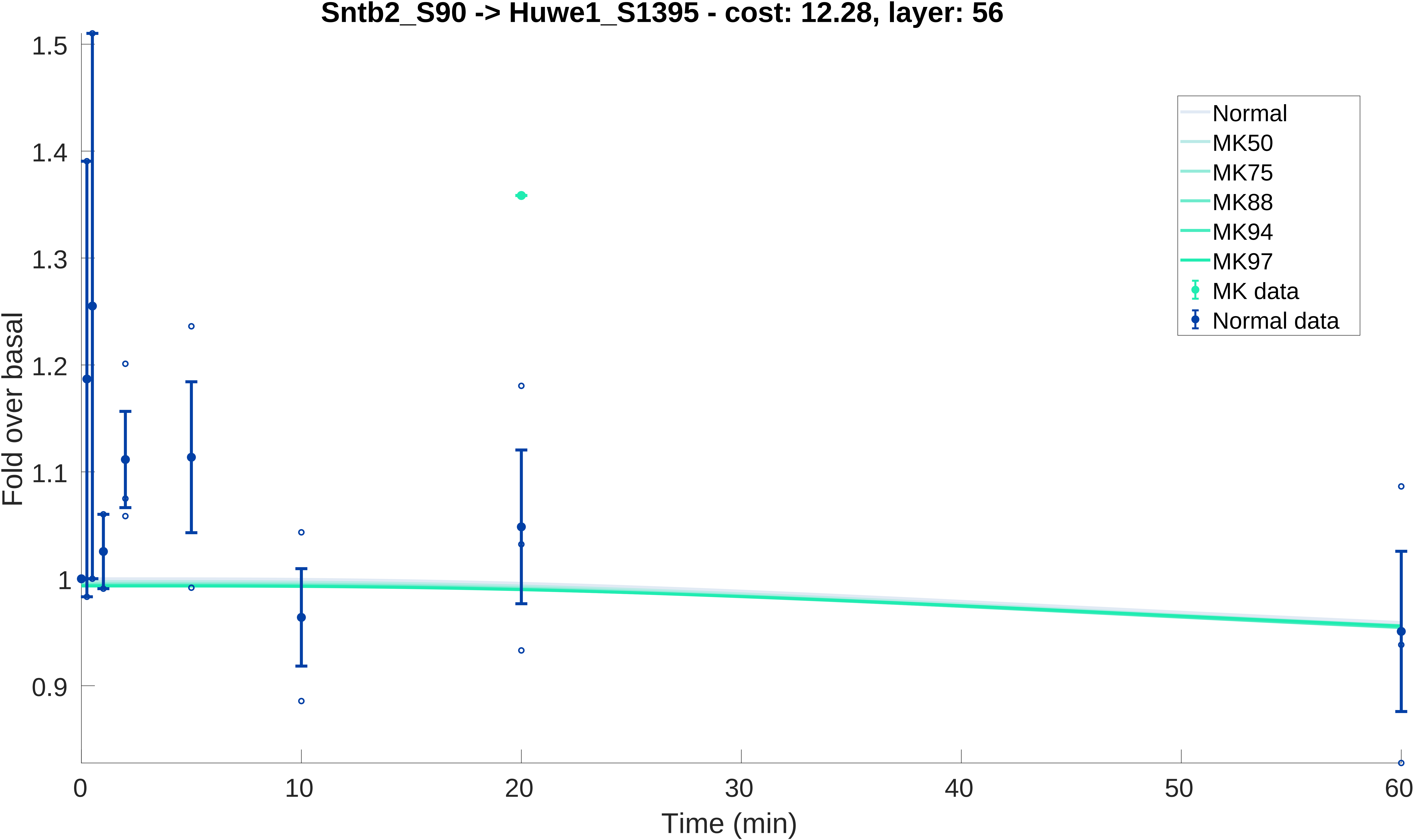

Sntb2_S90 -> Huwe1_S1395 - cost: 12.28, layer: 56
Fold over basal
Time (min)
1.5
1.4
1.3
1.2
1.1
1
0.9
0
10
20
30
40
50
60
Normal
MK50
MK75
MK88
MK94
MK97
MK data
Normal data

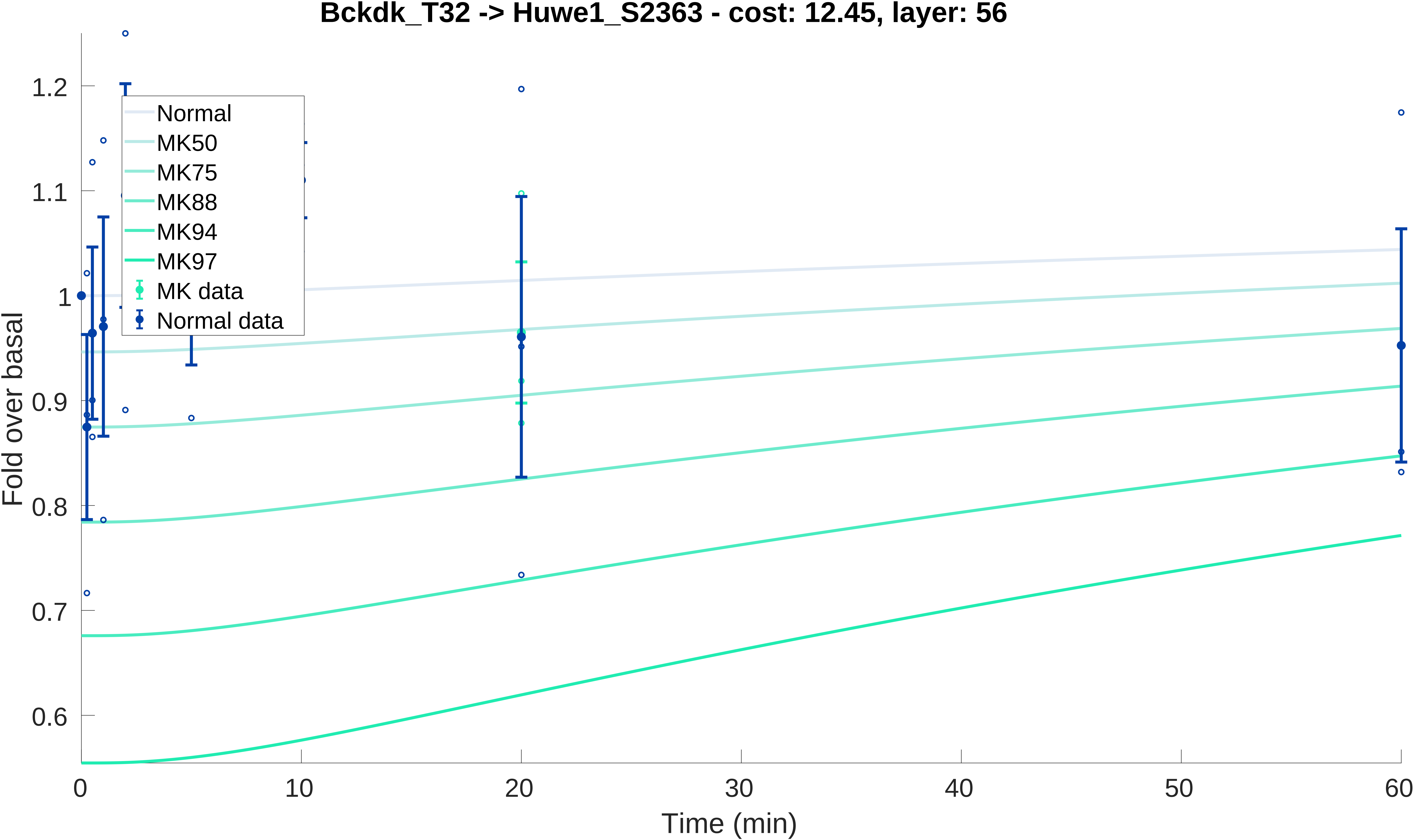

Bckdk_T32 -> Huwe1_S2363 - cost: 12.45, layer: 56
Normal
MK50
MK75
MK88
MK94
MK97
MK data
Normal data
Fold over basal
Time (min)
0.6
0.7
0.8
0.9
1
1.1
1.2
0
10
20
30
40
50
60

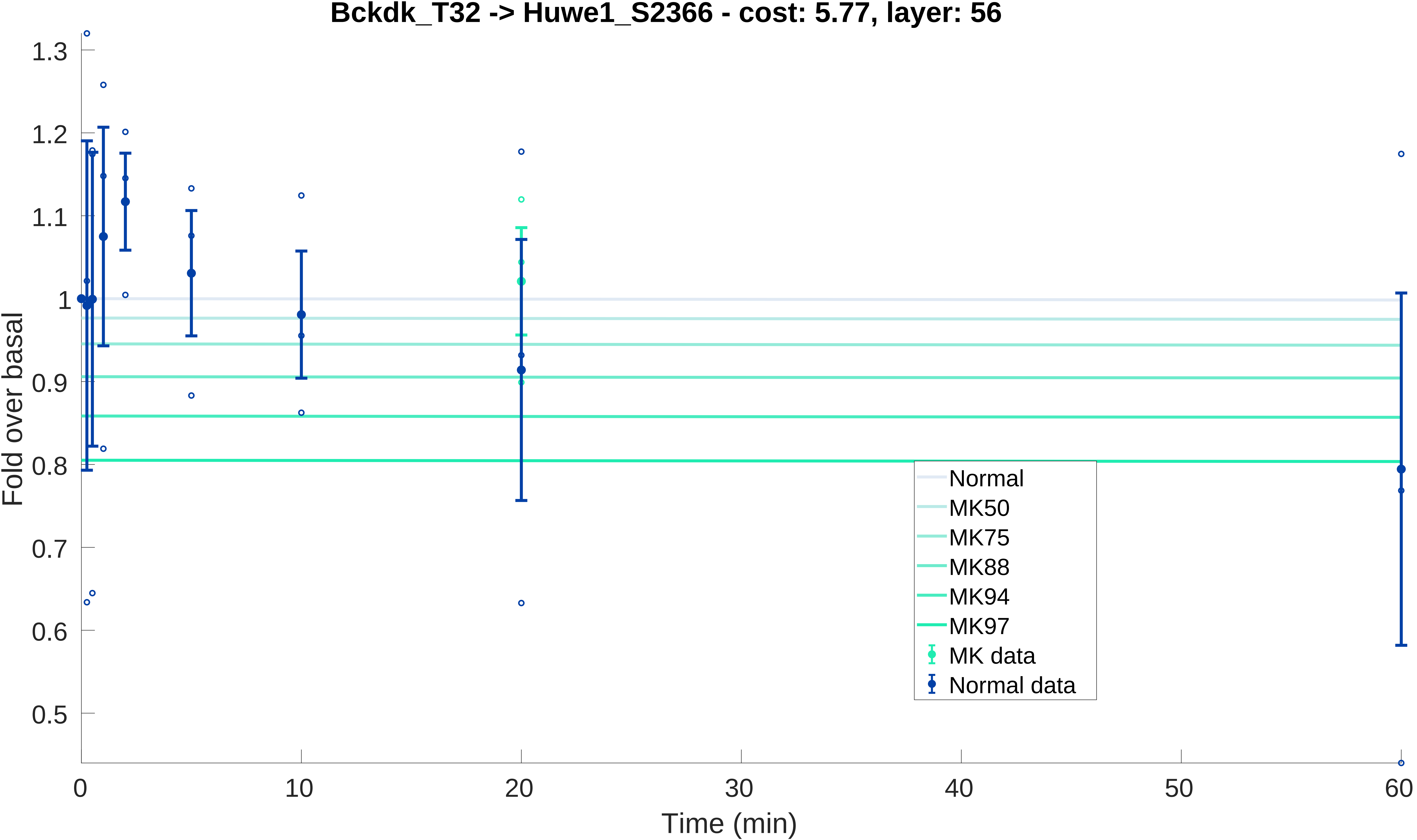

Bckdk_T32 -> Huwe1_S2366 - cost: 5.77, layer: 56
Fold over basal
Time (min)
0
10
20
30
40
50
60
0.5
0.6
0.7
0.8
0.9
1
1.1
1.2
1.3
Normal
MK50
MK75
MK88
MK94
MK97
MK data
Normal data

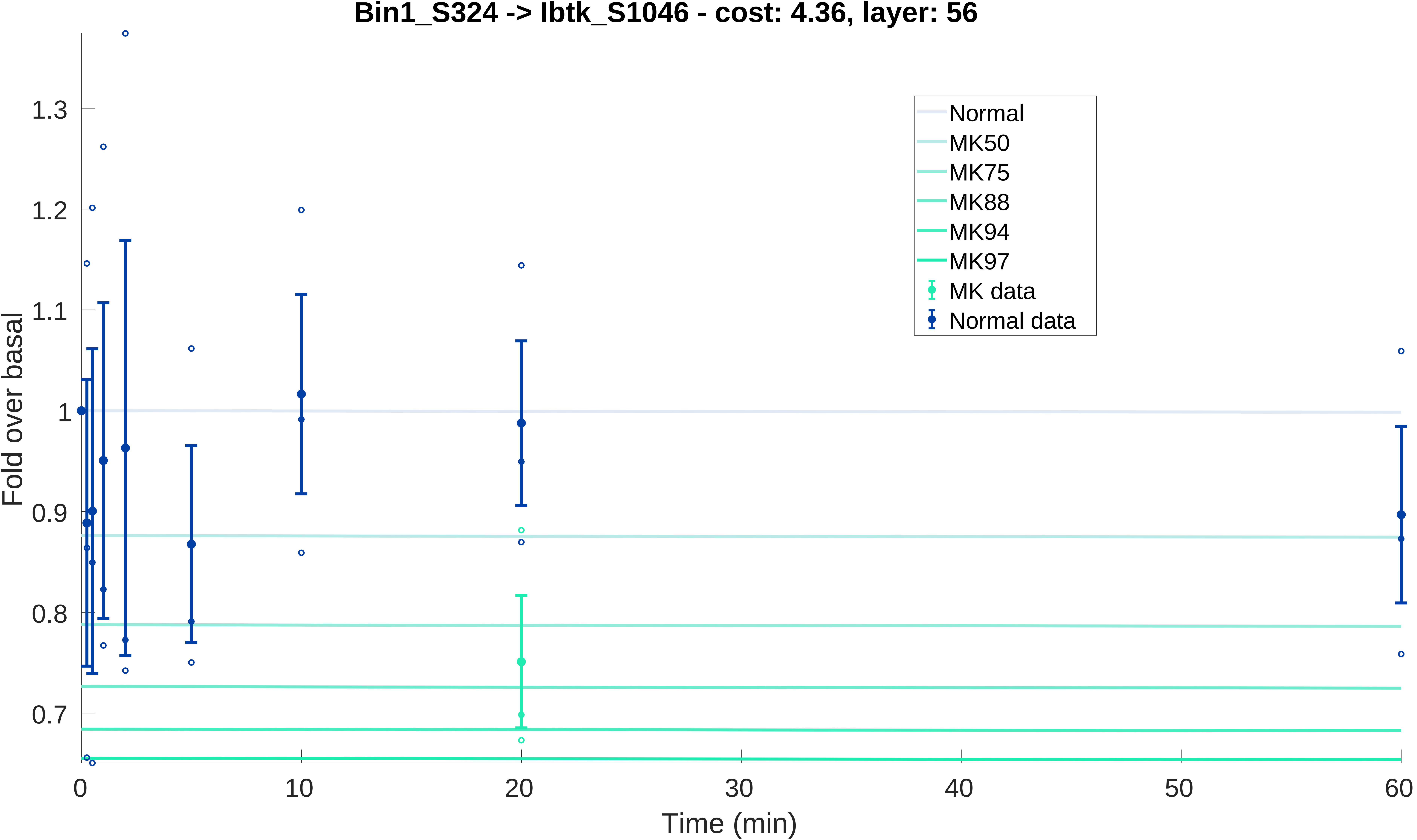
Bin1_S324 -> Ibtk_S1046 - cost: 4.36, layer: 56
Normal
MK50
MK75
MK88
MK94
MK97
MK data
Normal data
Fold over basal
Time (min)
0
10
20
30
40
50
60
0.7
0.8
0.9
1
1.1
1.2
1.3

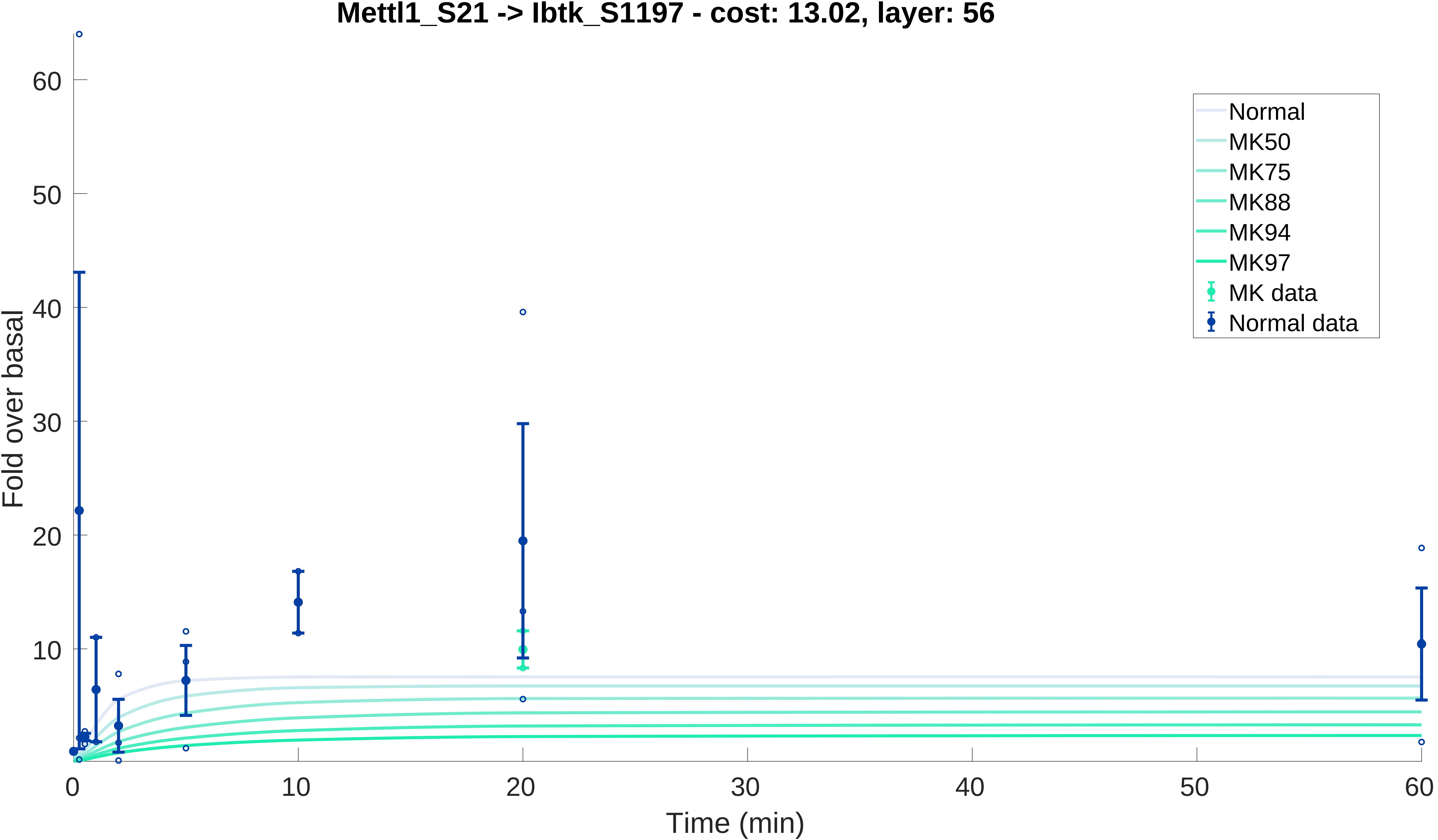

Mettl1_S21 -> Ibtk_S1197 - cost: 13.02, layer: 56
Fold over basal
Time (min)
0
10
20
30
40
50
60
Normal
MK50
MK75
MK88
MK94
MK97
MK data
Normal data

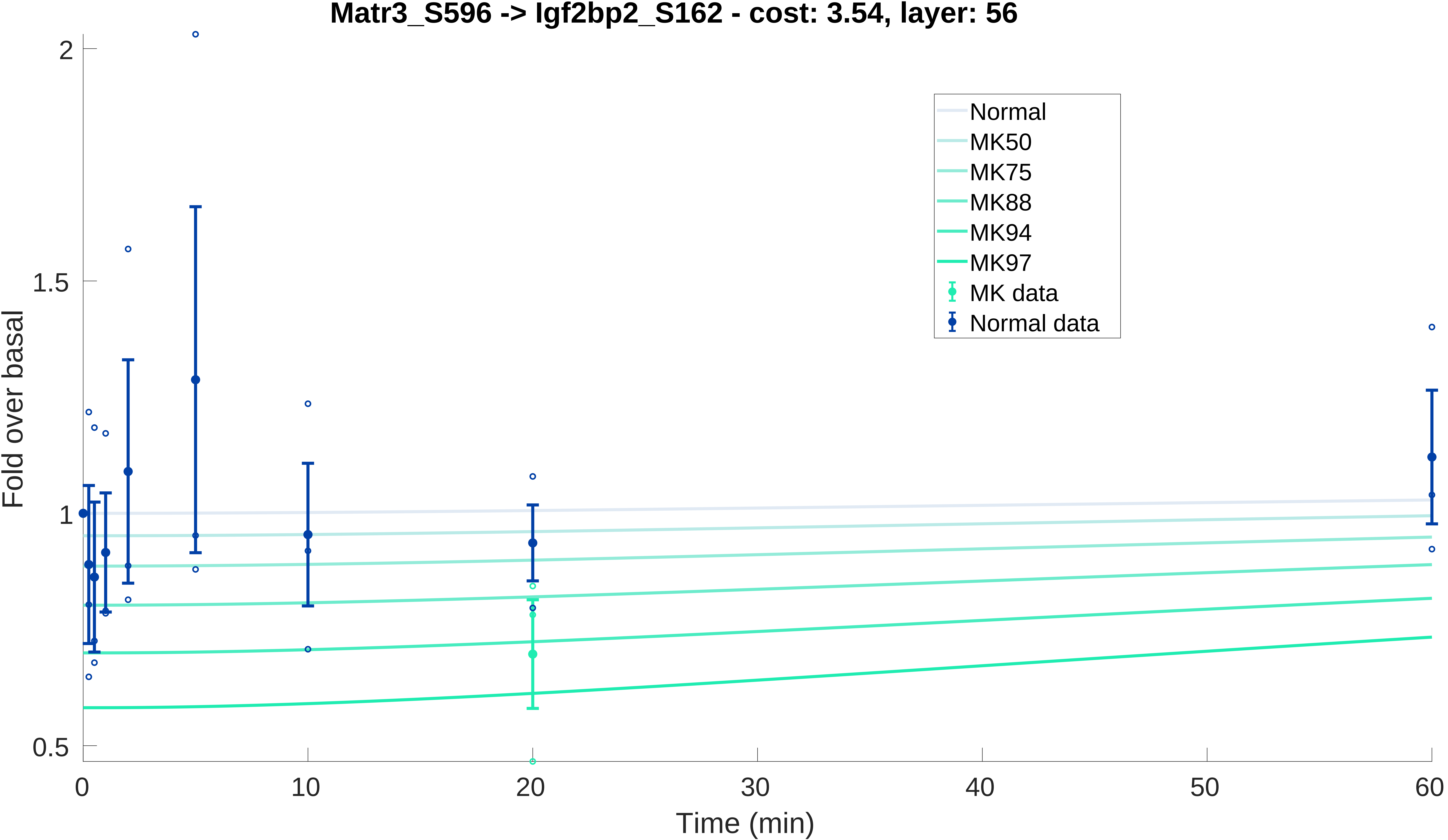

Matr3_S596 -> Igf2bp2_S162 - cost: 3.54, layer: 56
Normal
MK50
MK75
MK88
MK94
MK97
MK data
Normal data
Fold over basal
Time (min)
2
1.5
1
0.5
0
10
20
30
40
50
60

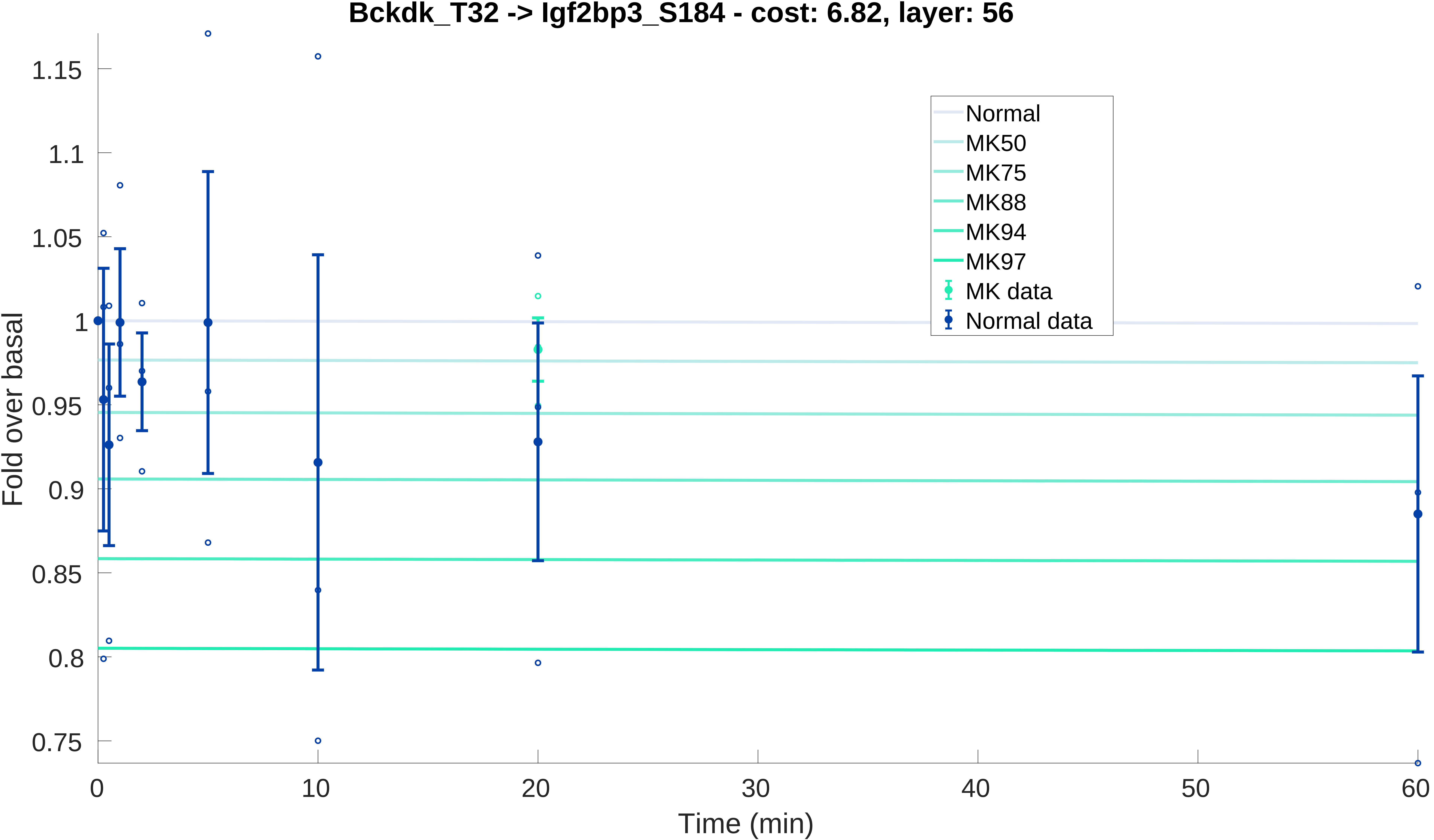

Bckdk_T32 -> Igf2bp3_S184 - cost: 6.82, layer: 56
Fold over basal
1.15
1.1
1.05
1
0.95
0.9
0.85
0.8
0.75
0
10
20
30
40
50
60
Time (min)
Normal
MK50
MK75
MK88
MK94
MK97
MK data
Normal data

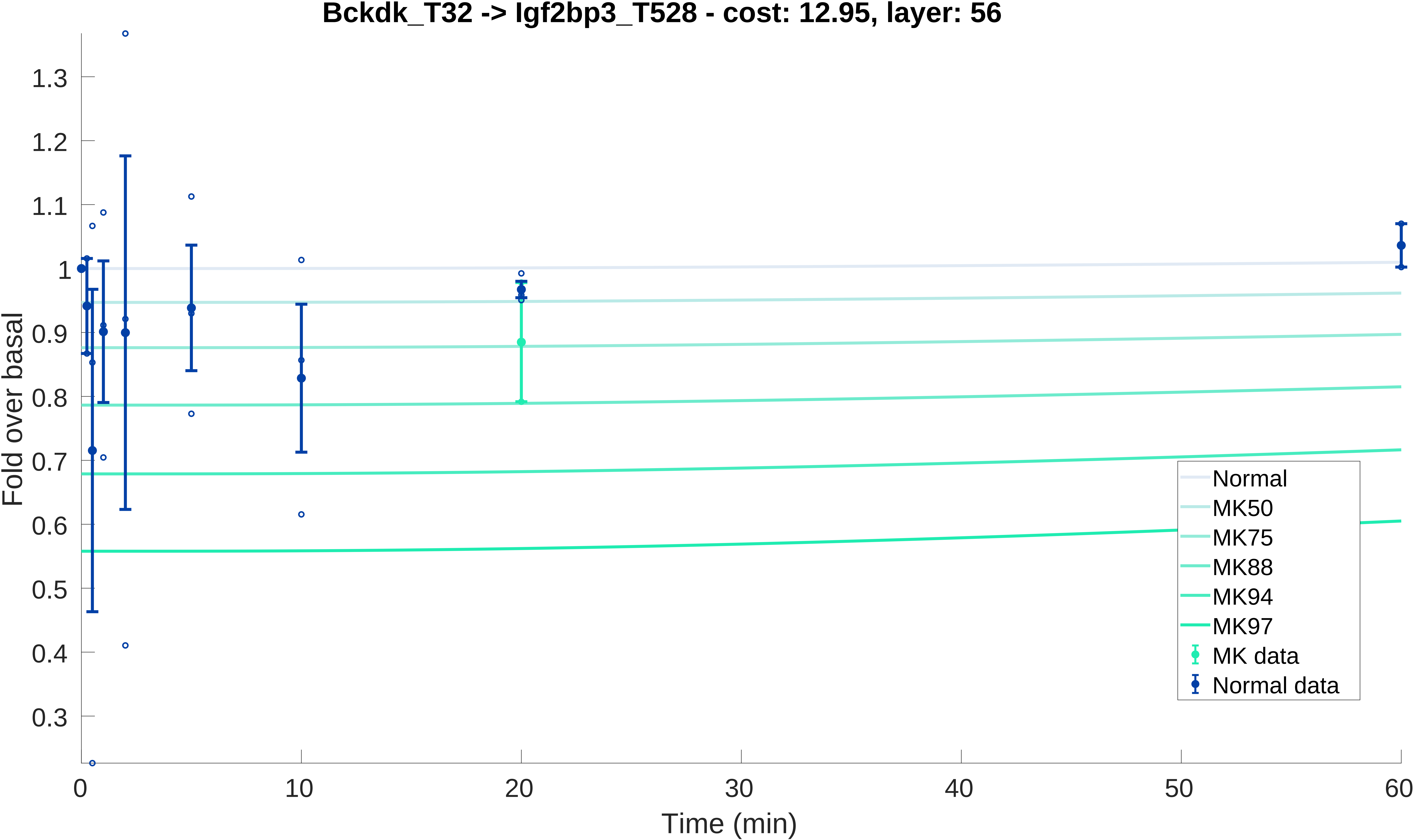
Bckdk_T32 -> Igf2bp3_T528 - cost: 12.95, layer: 56
Fold over basal
Time (min)
1.3
1.2
1.1
1
0.9
0.8
0.7
0.6
0.5
0.4
0.3
0
10
20
30
40
50
60
Normal
MK50
MK75
MK88
MK94
MK97
MK data
Normal data

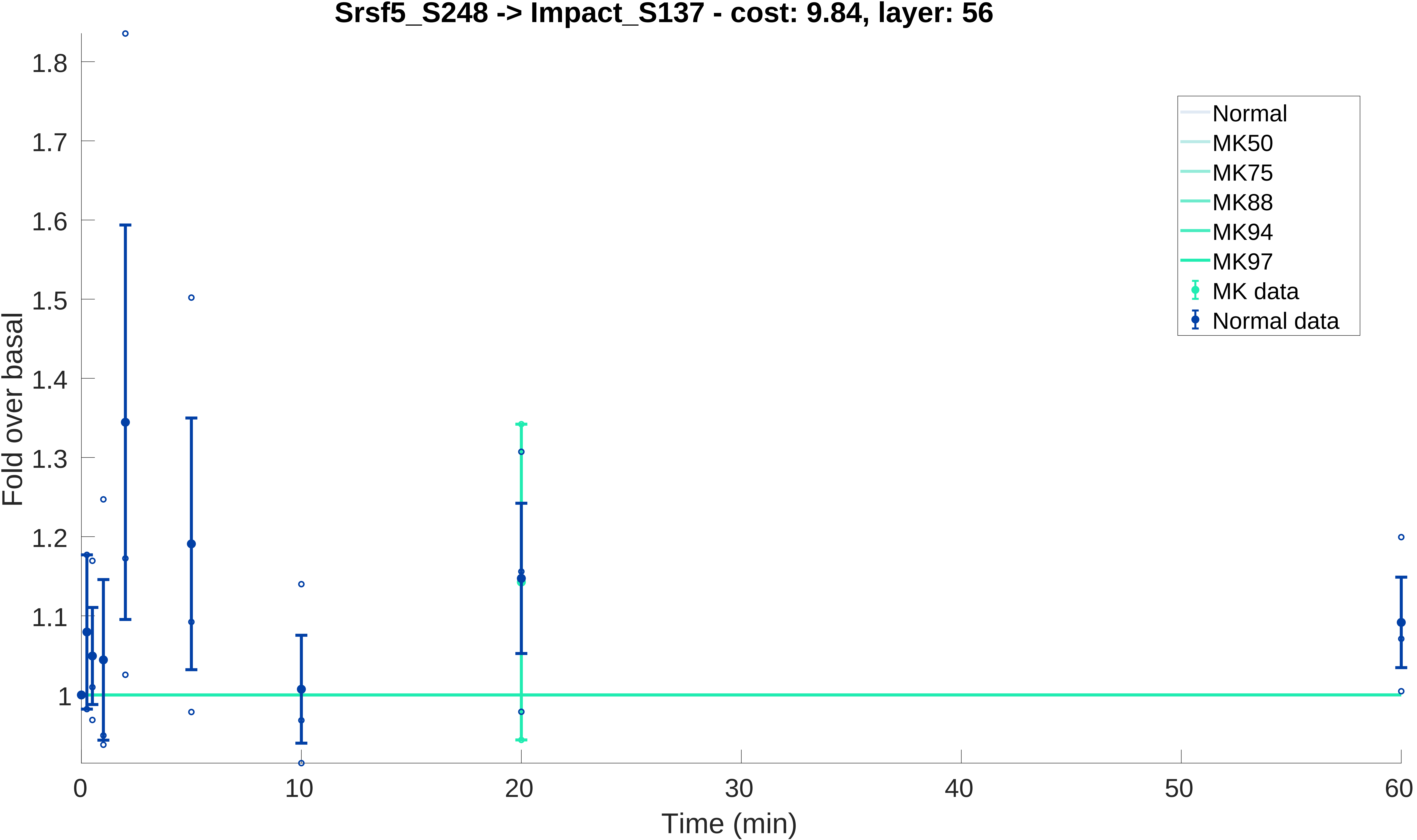

Srsf5_S248 -> Impact_S137 - cost: 9.84, layer: 56
Fold over basal
1.8
1.7
1.6
1.5
1.4
1.3
1.2
1.1
1
0
10
20
30
40
50
60
Time (min)
Normal
MK50
MK75
MK88
MK94
MK97
MK data
Normal data

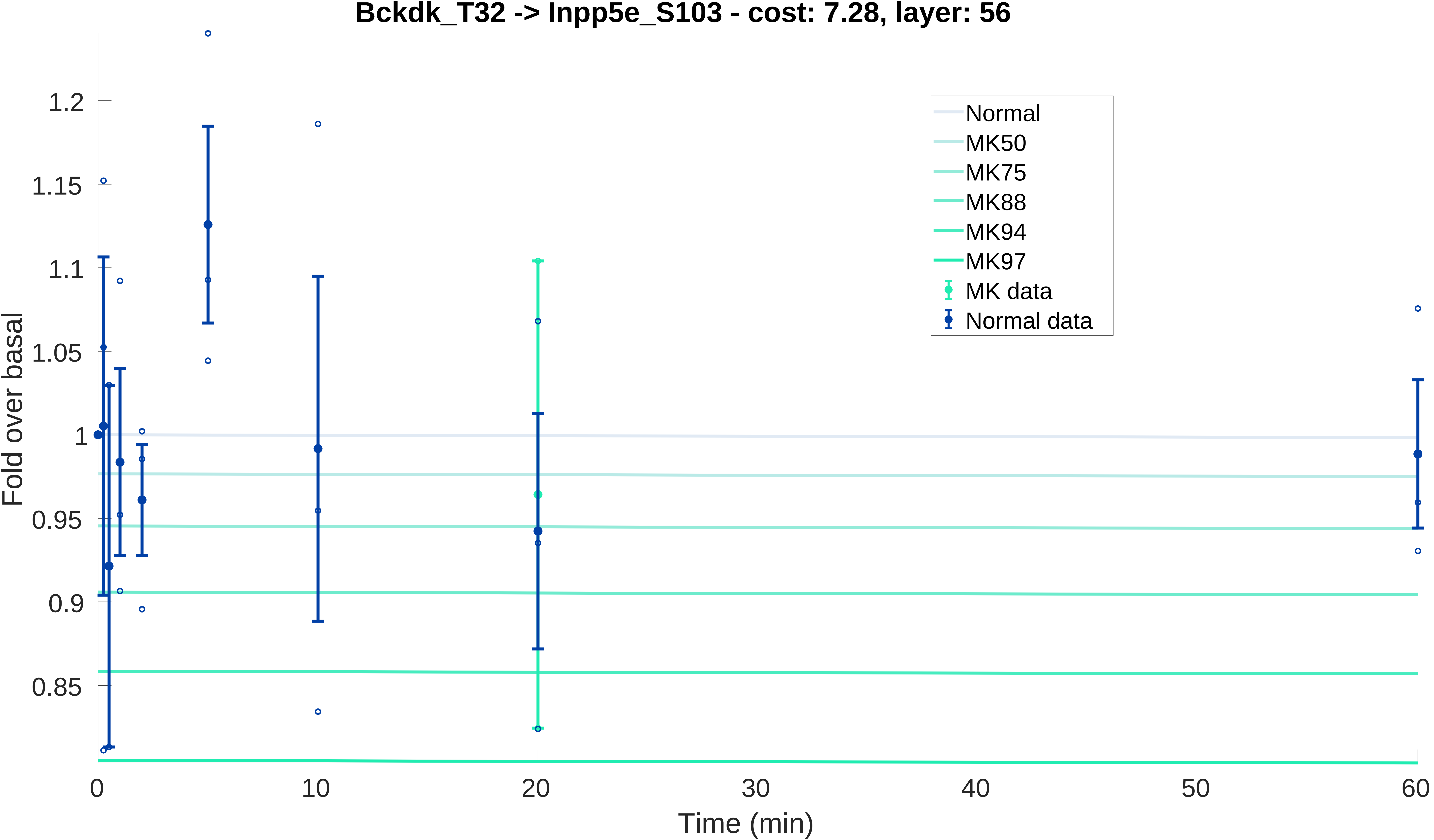

Bckdk_T32 -> Inpp5e_S103 - cost: 7.28, layer: 56
Fold over basal
Time (min)
0
10
20
30
40
50
60
0.85
0.9
0.95
1
1.05
1.1
1.15
1.2
Normal
MK50
MK75
MK88
MK94
MK97
MK data
Normal data

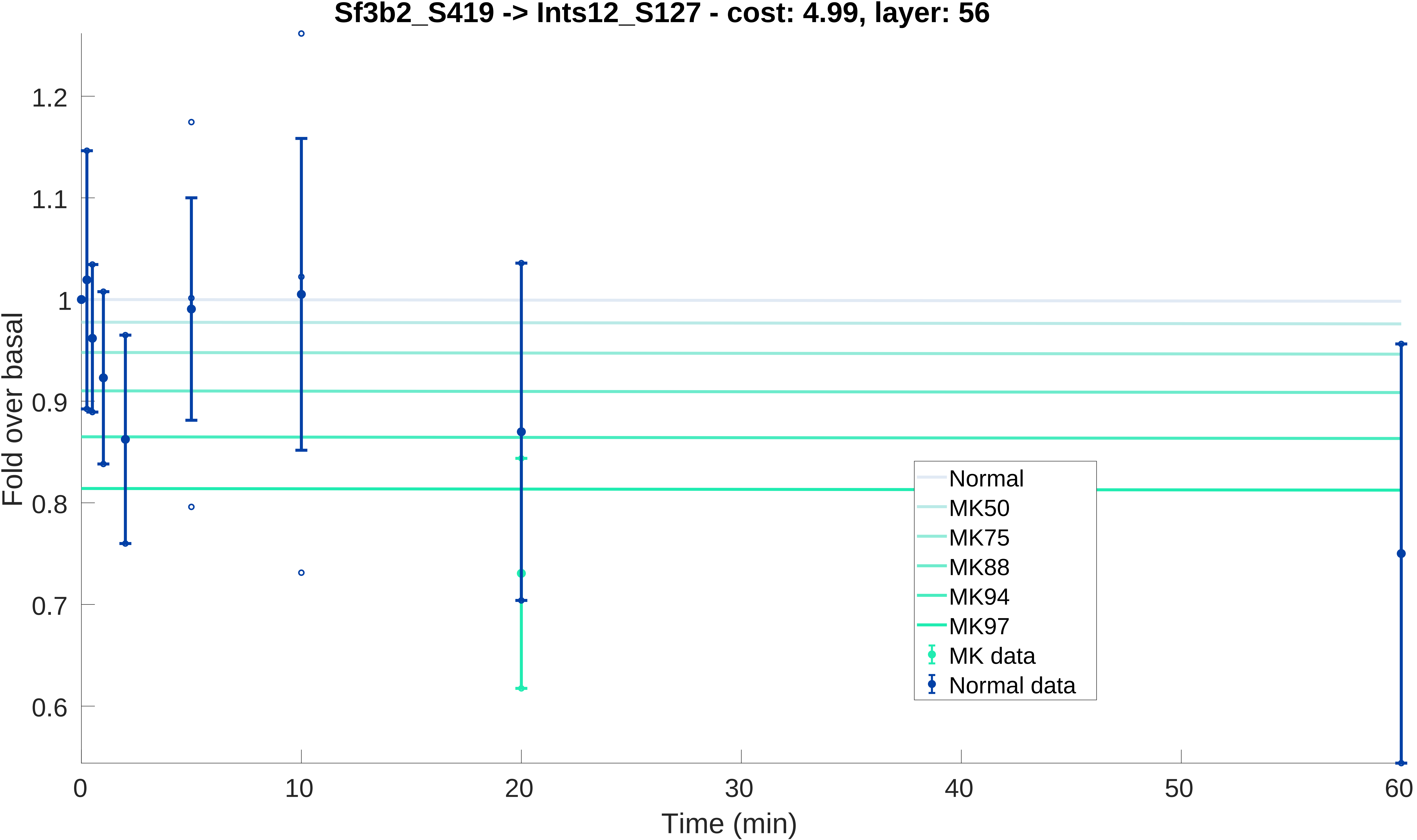

Sf3b2_S419 -> Ints12_S127 - cost: 4.99, layer: 56
Fold over basal
1.2
1.1
1
0.9
0.8
0.7
0.6
0
10
20
30
40
50
60
Time (min)
Normal
MK50
MK75
MK88
MK94
MK97
MK data
Normal data

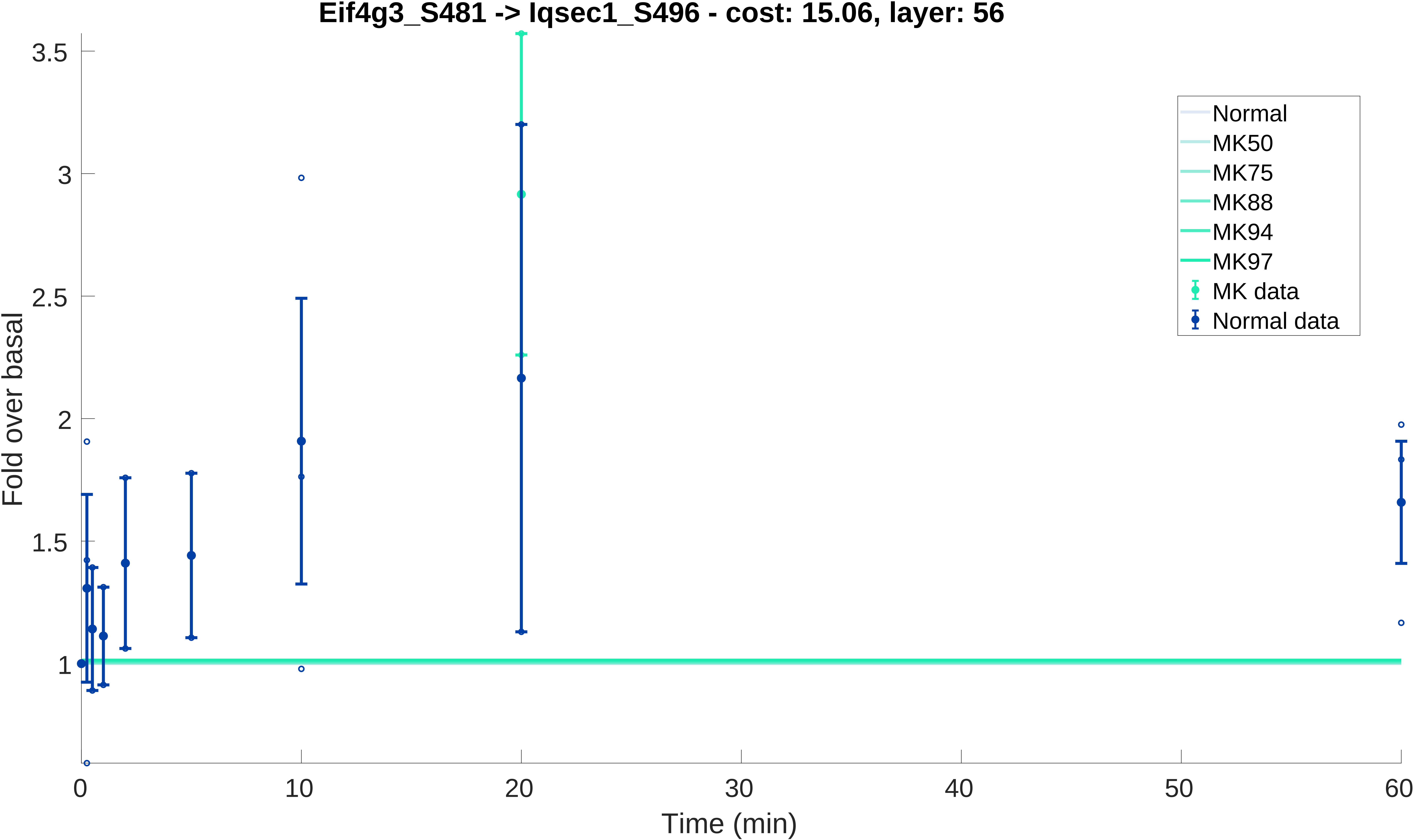

Eif4g3_S481 -> Iqsec1_S496 - cost: 15.06, layer: 56
Fold over basal
3.5
3
2.5
2
1.5
1
Time (min)
0
10
20
30
40
50
60
Normal
MK50
MK75
MK88
MK94
MK97
MK data
Normal data

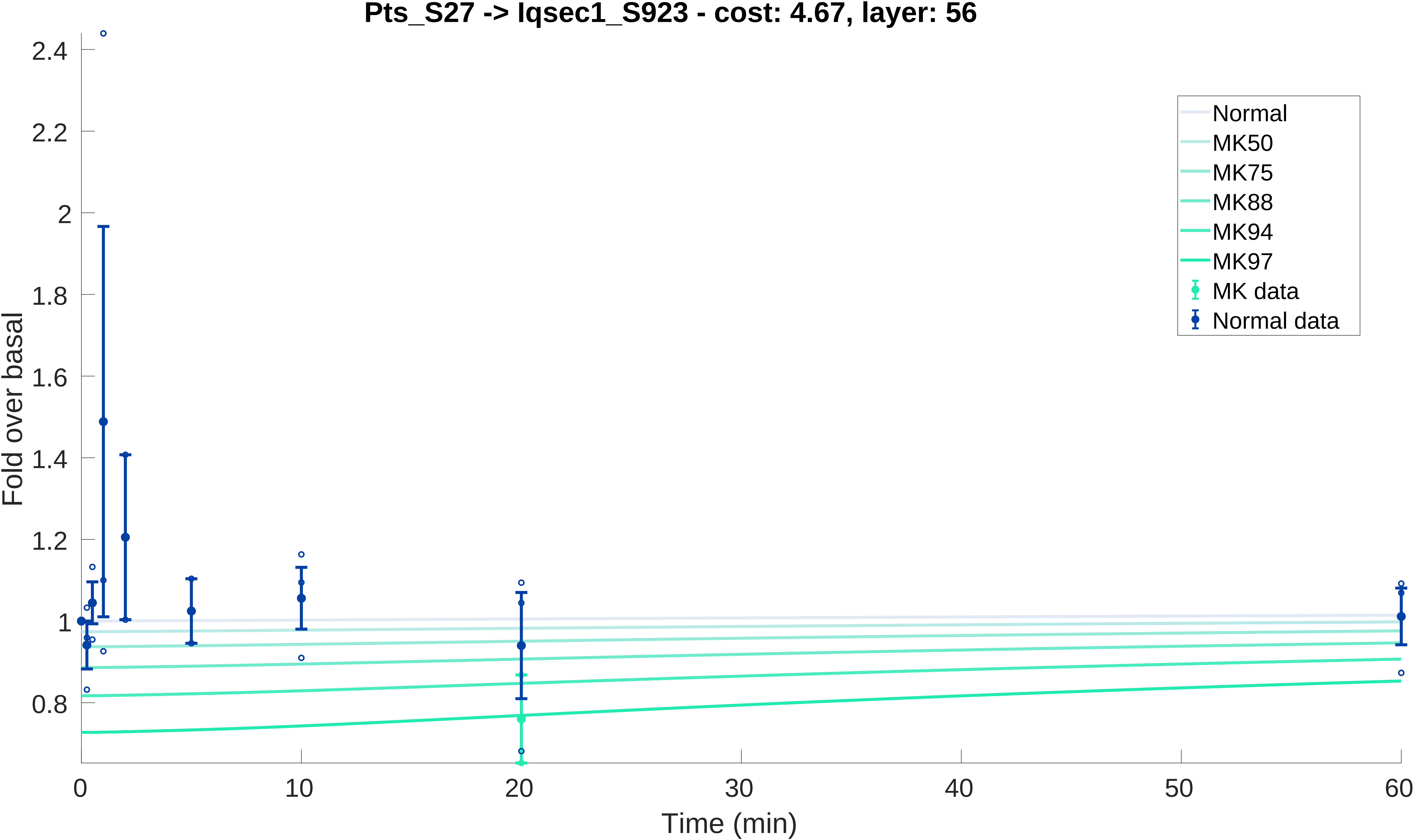

Pts_S27 -> Iqsec1_S923 - cost: 4.67, layer: 56
Fold over basal
2.4
2.2
2
1.8
1.6
1.4
1.2
1
0.8
0
10
20
30
40
50
60
Time (min)
Normal
MK50
MK75
MK88
MK94
MK97
MK data
Normal data

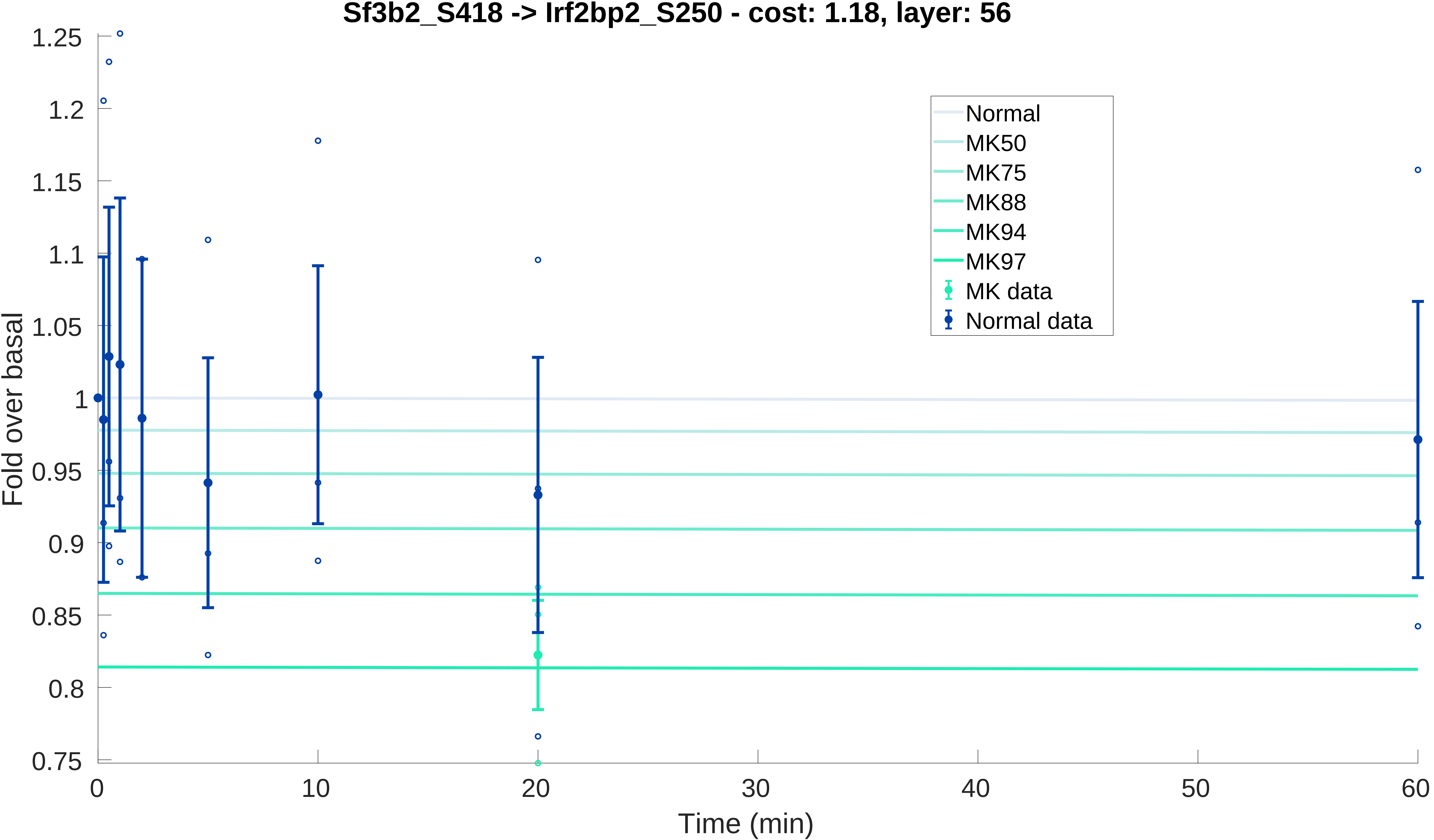
Sf3b2_S418 -> Irf2bp2_S250 - cost: 1.18, layer: 56
Fold over basal
Time (min)
Normal
MK50
MK75
MK88
MK94
MK97
MK data
Normal data
1.25
1.2
1.15
1.1
1.05
1
0.95
0.9
0.85
0.8
0.75
0
10
20
30
40
50
60

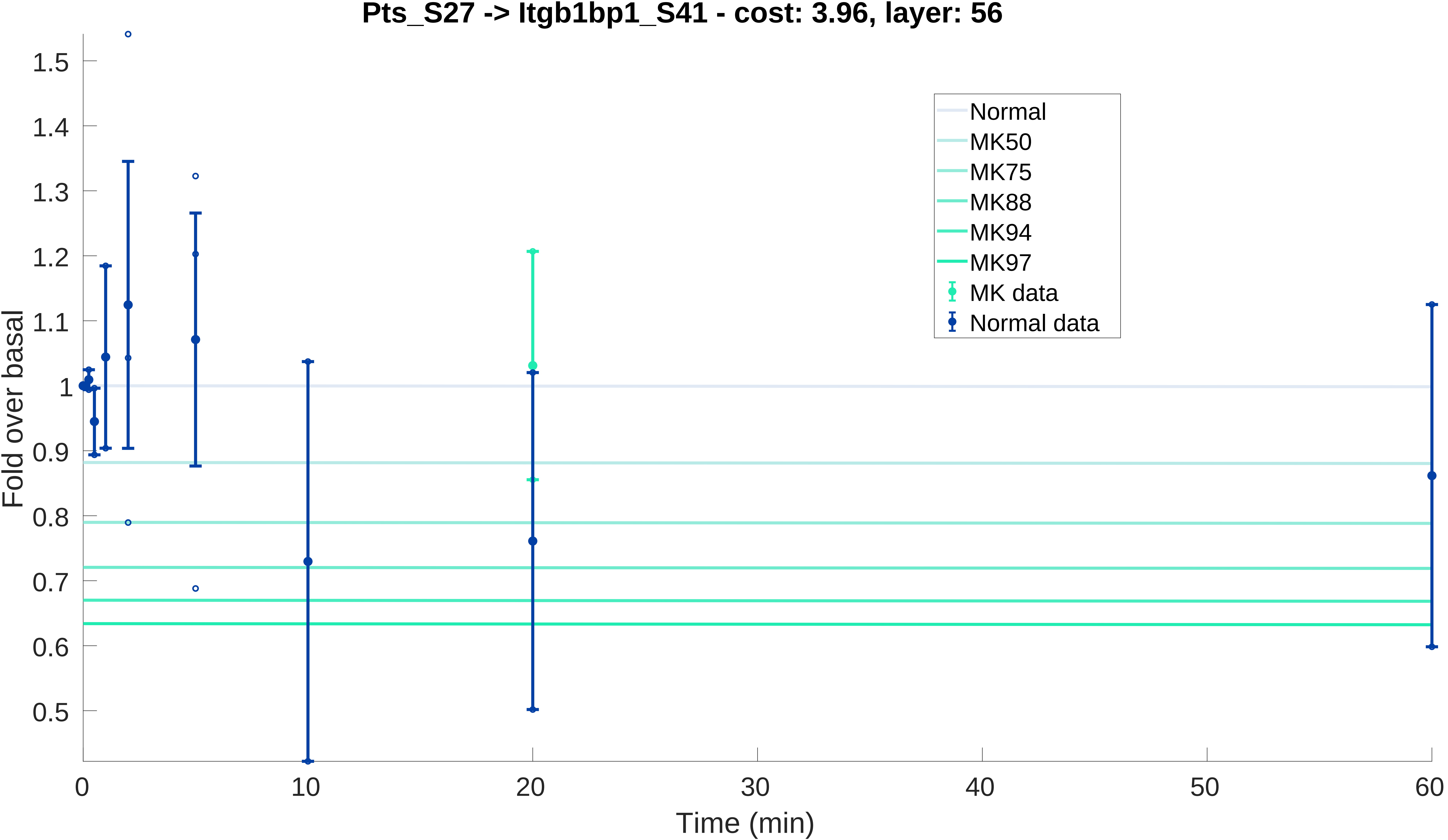

Pts_S27 -> Itgb1bp1_S41 - cost: 3.96, layer: 56
Normal
MK50
MK75
MK88
MK94
MK97
MK data
Normal data
Fold over basal
Time (min)
1.5
1.4
1.3
1.2
1.1
1
0.9
0.8
0.7
0.6
0.5
0
10
20
30
40
50
60

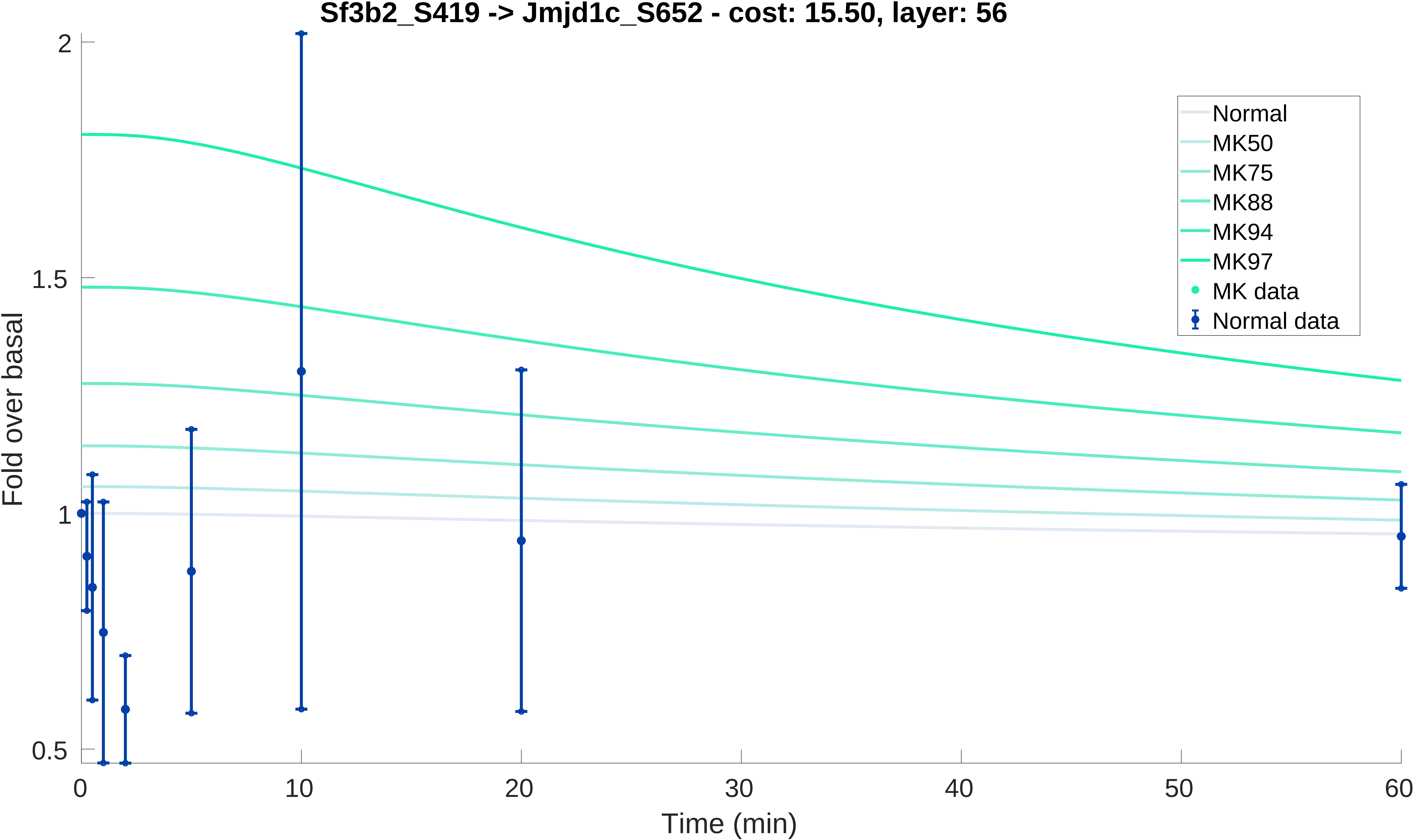

Sf3b2_S419 -> Jmjd1c_S652 - cost: 15.50, layer: 56
Fold over basal
Time (min)
0
10
20
30
40
50
60
0.5
1
1.5
2
Normal
MK50
MK75
MK88
MK94
MK97
MK data
Normal data

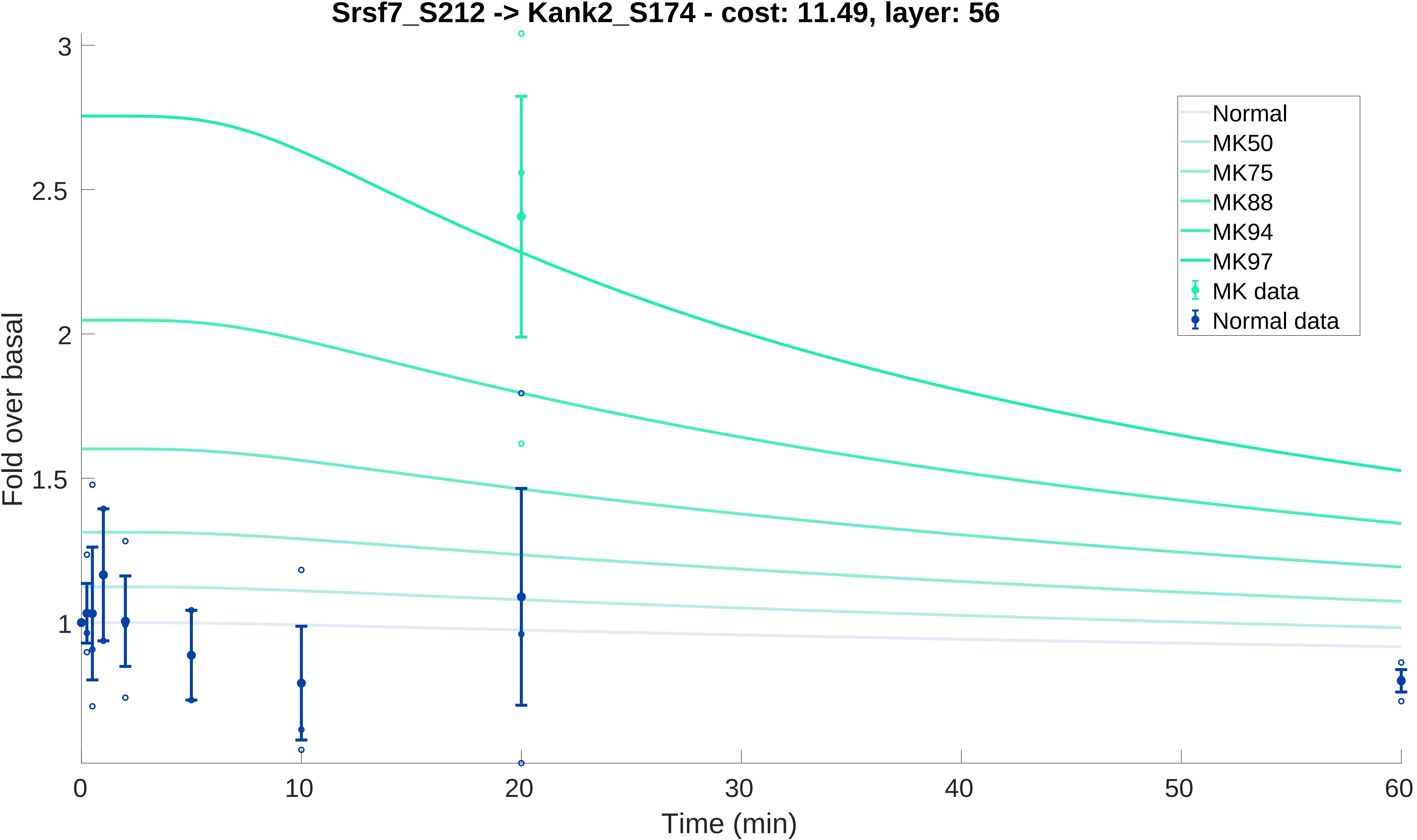

Srsf7_S212 -> Kank2_S174 - cost: 11.49, layer: 56
Fold over basal
Time (min)
3
2.5
2
1.5
1
0
10
20
30
40
50
60
Normal
MK50
MK75
MK88
MK94
MK97
MK data
Normal data

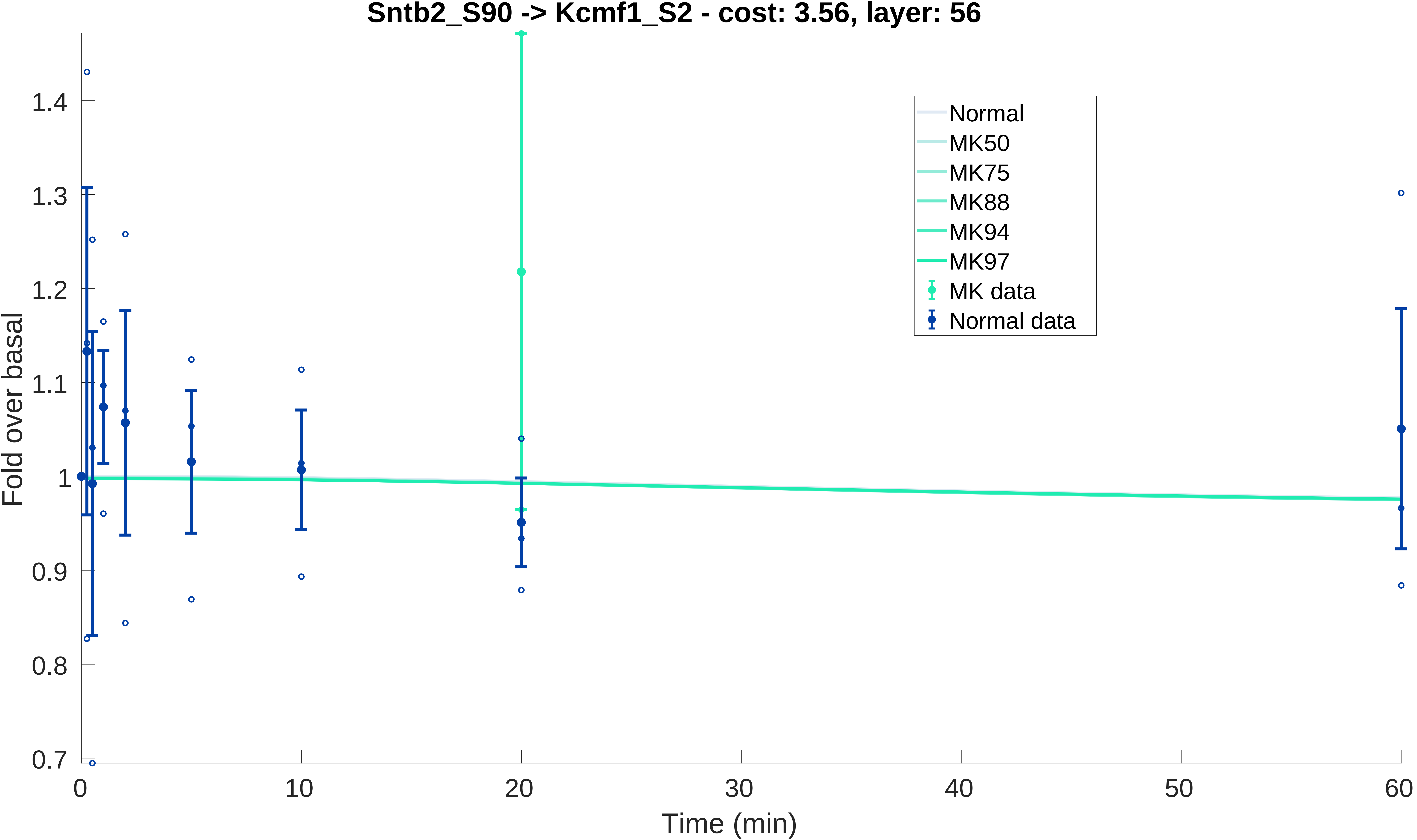
Sntb2_S90 -> Kcmf1_S2 - cost: 3.56, layer: 56
Fold over basal
Time (min)
0.7
0.8
0.9
1
1.1
1.2
1.3
1.4
0
10
20
30
40
50
60
Normal
MK50
MK75
MK88
MK94
MK97
MK data
Normal data

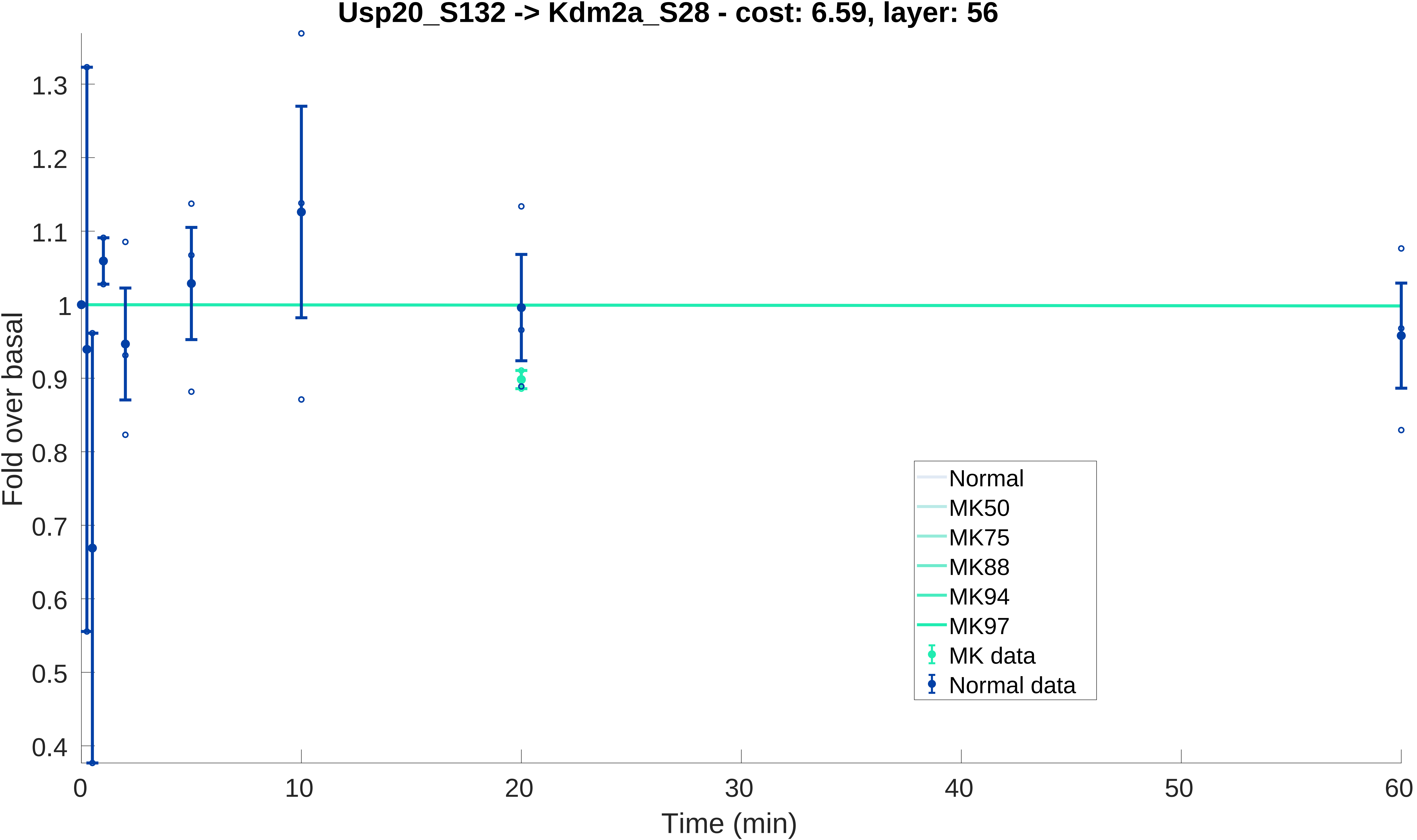

Usp20_S132 -> Kdm2a_S28 - cost: 6.59, layer: 56
Fold over basal
Time (min)
0.4
0.5
0.6
0.7
0.8
0.9
1
1.1
1.2
1.3
0
10
20
30
40
50
60
Normal
MK50
MK75
MK88
MK94
MK97
MK data
Normal data

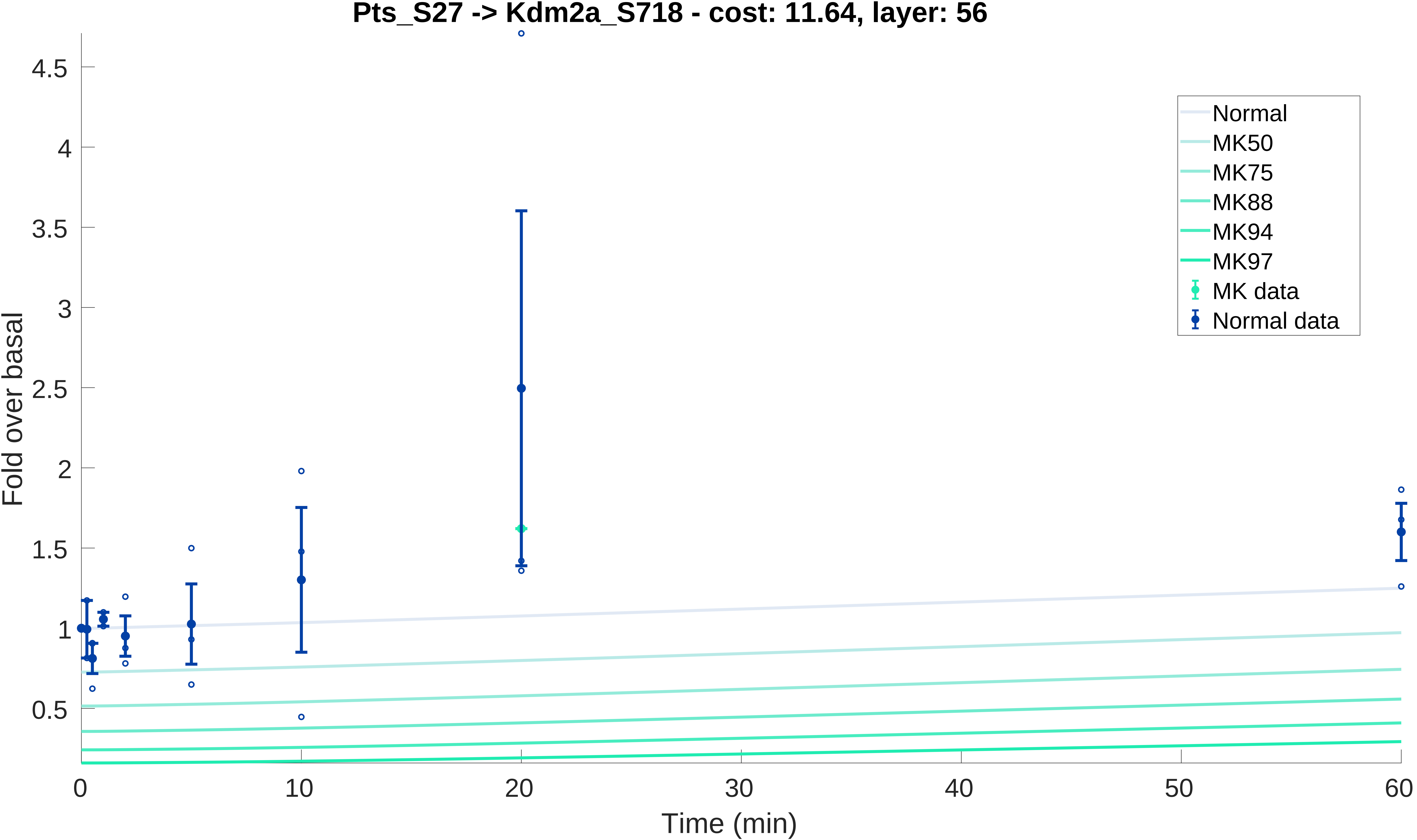

Pts_S27 -> Kdm2a_S718 - cost: 11.64, layer: 56
Fold over basal
Time (min)
0.5
1
1.5
2
2.5
3
3.5
4
4.5
0
10
20
30
40
50
60
Normal
MK50
MK75
MK88
MK94
MK97
MK data
Normal data

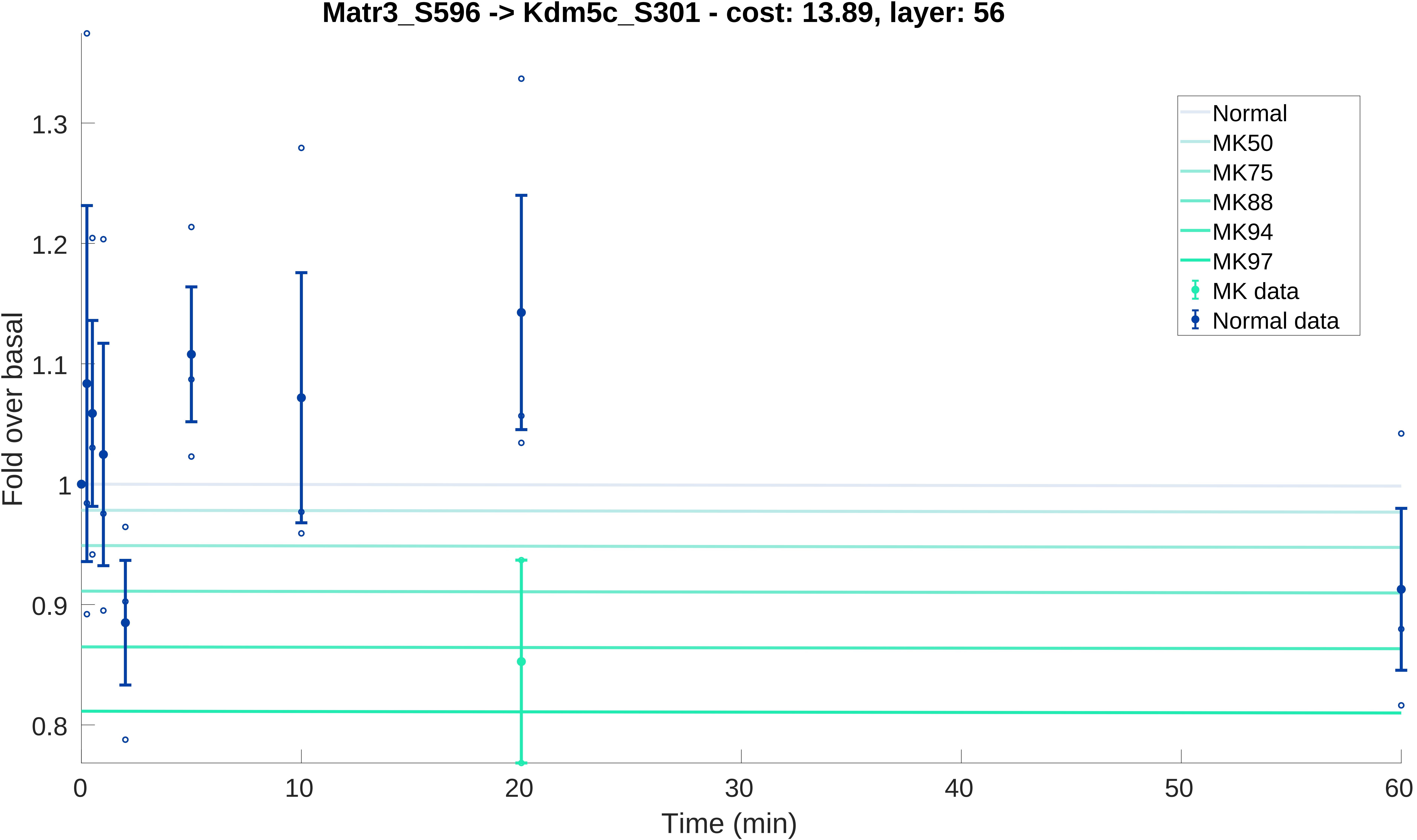

Matr3_S596 -> Kdm5c_S301 - cost: 13.89, layer: 56
Fold over basal
Time (min)
0.8
0.9
1
1.1
1.2
1.3
0
10
20
30
40
50
60
Normal
MK50
MK75
MK88
MK94
MK97
MK data
Normal data

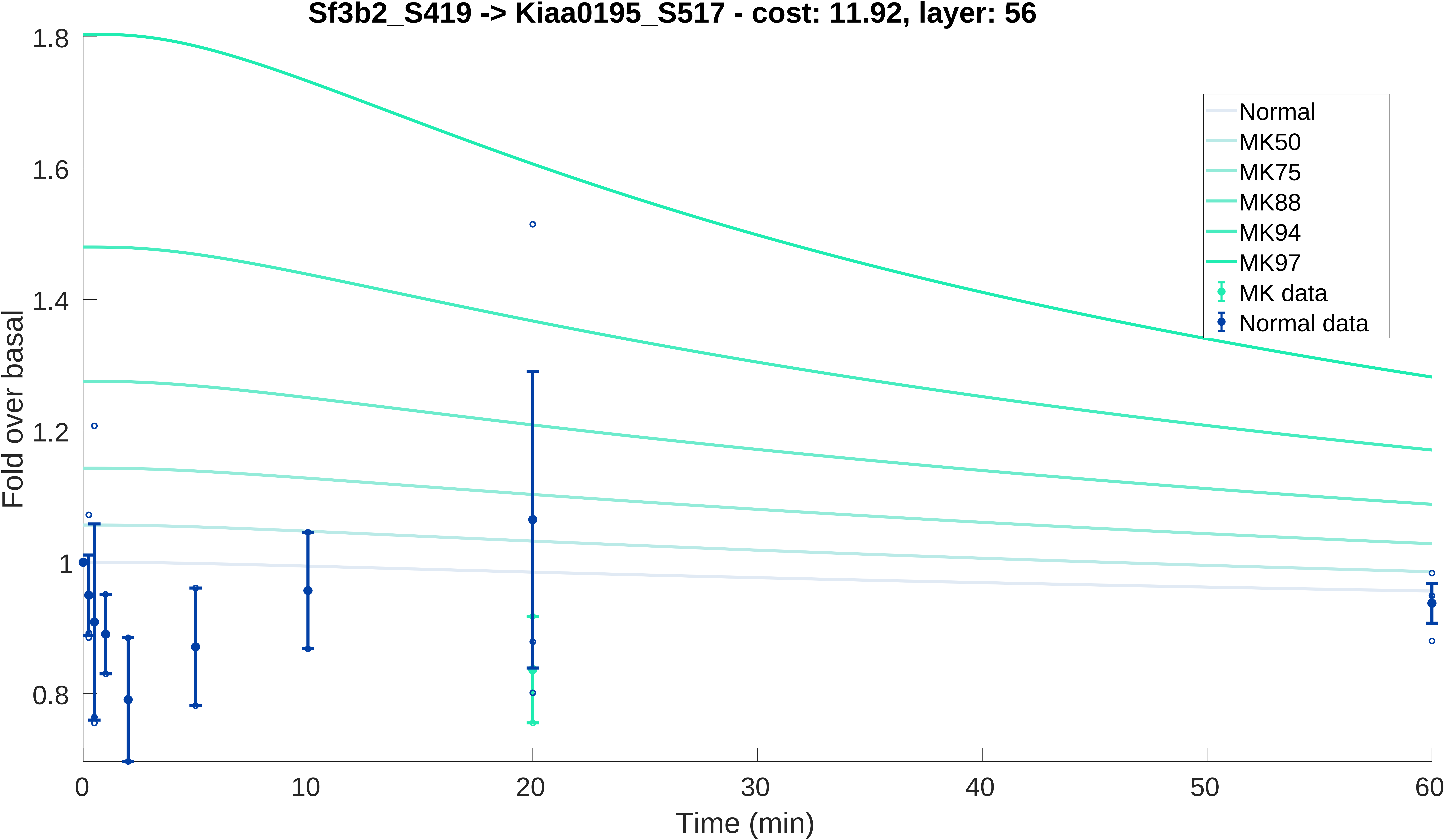
Sf3b2_S419 -> Kiaa0195_S517 - cost: 11.92, layer: 56
1.8
1.6
1.4
1.2
1
0.8
Fold over basal
0
10
20
30
40
50
60
Time (min)
Normal
MK50
MK75
MK88
MK94
MK97
MK data
Normal data

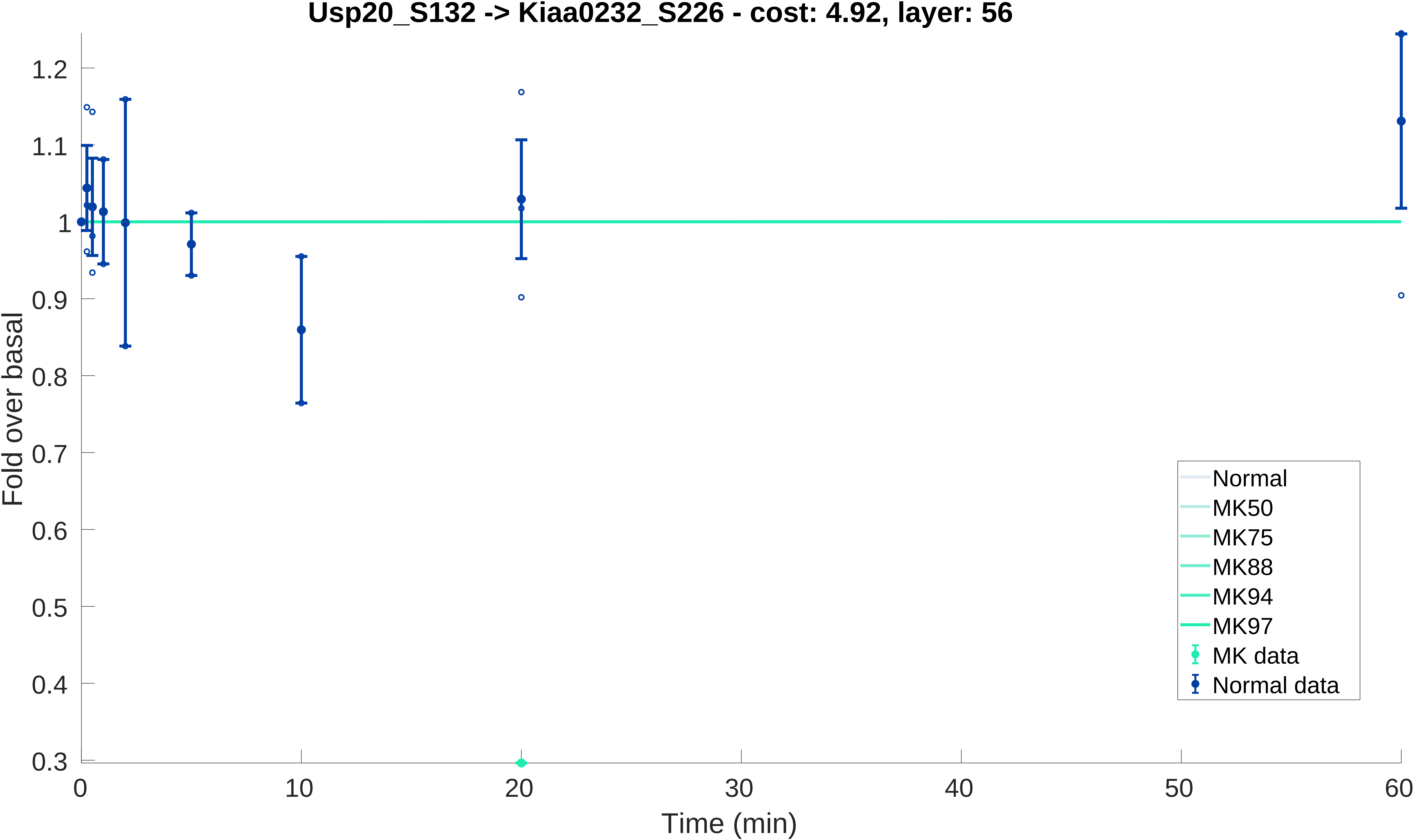

Usp20_S132 -> Kiaa0232_S226 - cost: 4.92, layer: 56
Fold over basal
Time (min)
0.3
0.4
0.5
0.6
0.7
0.8
0.9
1
1.1
1.2
0
10
20
30
40
50
60
Normal
MK50
MK75
MK88
MK94
MK97
MK data
Normal data

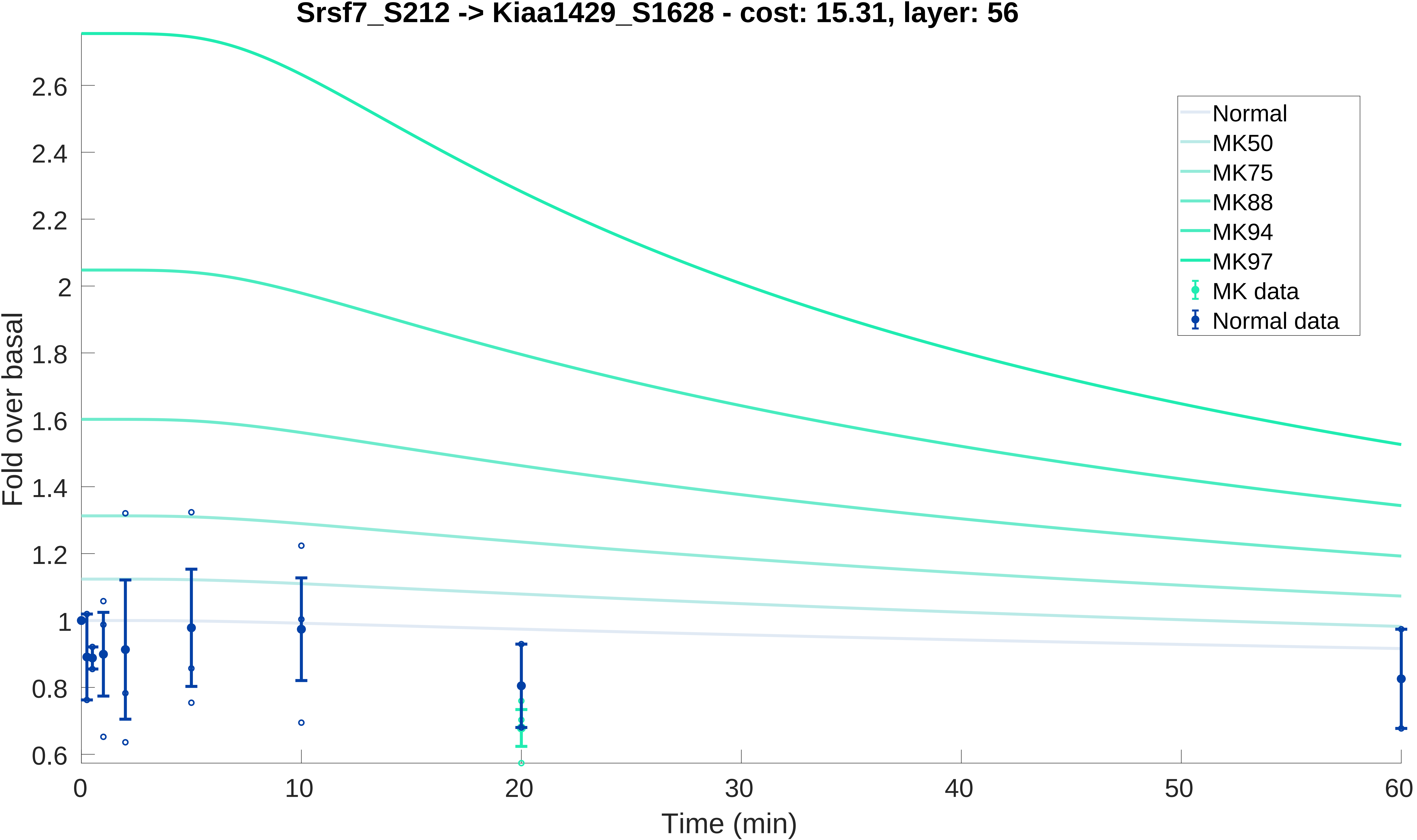

Srsf7_S212 -> Kiaa1429_S1628 - cost: 15.31, layer: 56
Fold over basal
Time (min)
2.6
2.4
2.2
2
1.8
1.6
1.4
1.2
1
0.8
0.6
0
10
20
30
40
50
60
Normal
MK50
MK75
MK88
MK94
MK97
MK data
Normal data

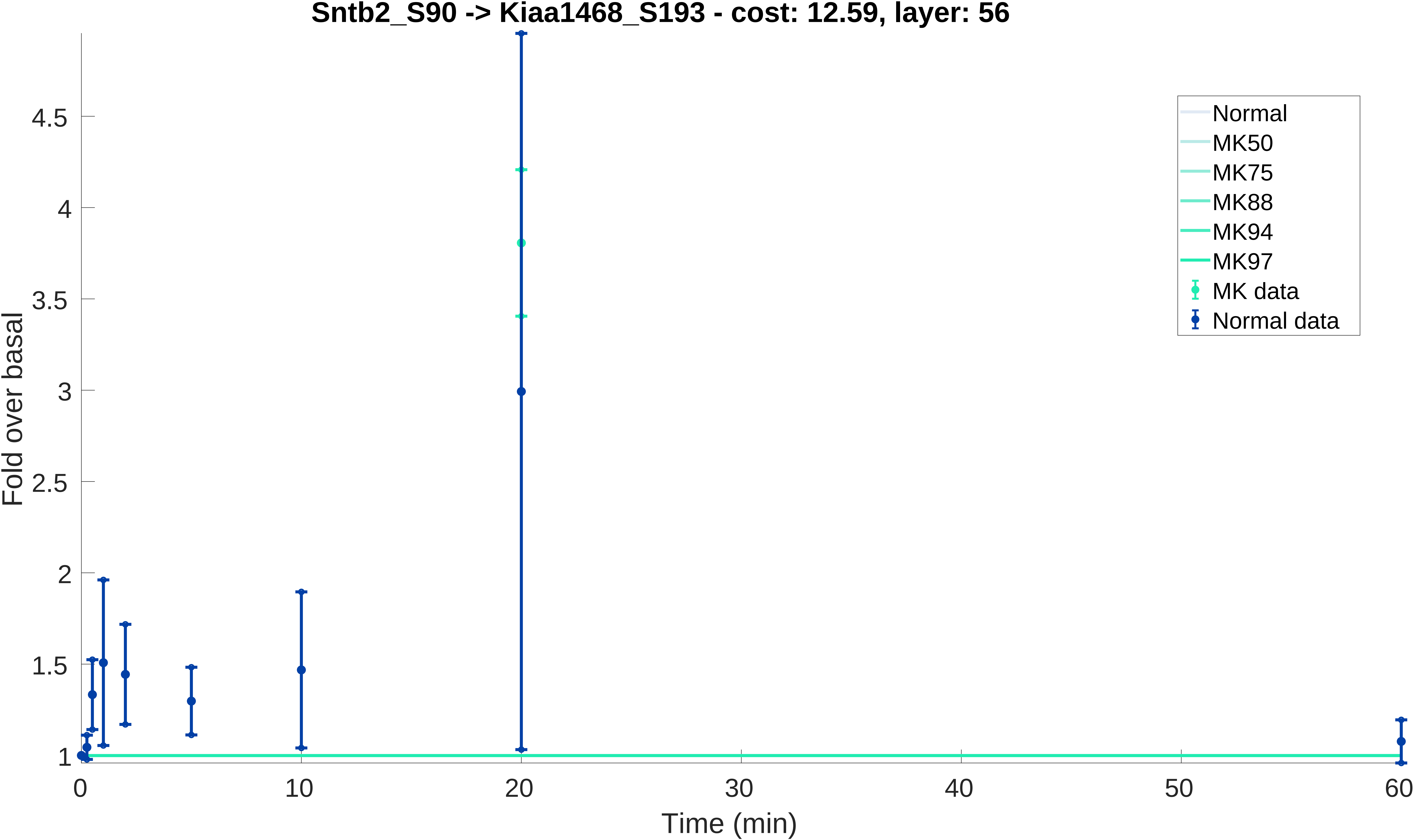
Sntb2_S90 -> Kiaa1468_S193 - cost: 12.59, layer: 56
Fold over basal
Time (min)
0
10
20
30
40
50
60
1
1.5
2
2.5
3
3.5
4
4.5
Normal
MK50
MK75
MK88
MK94
MK97
MK data
Normal data

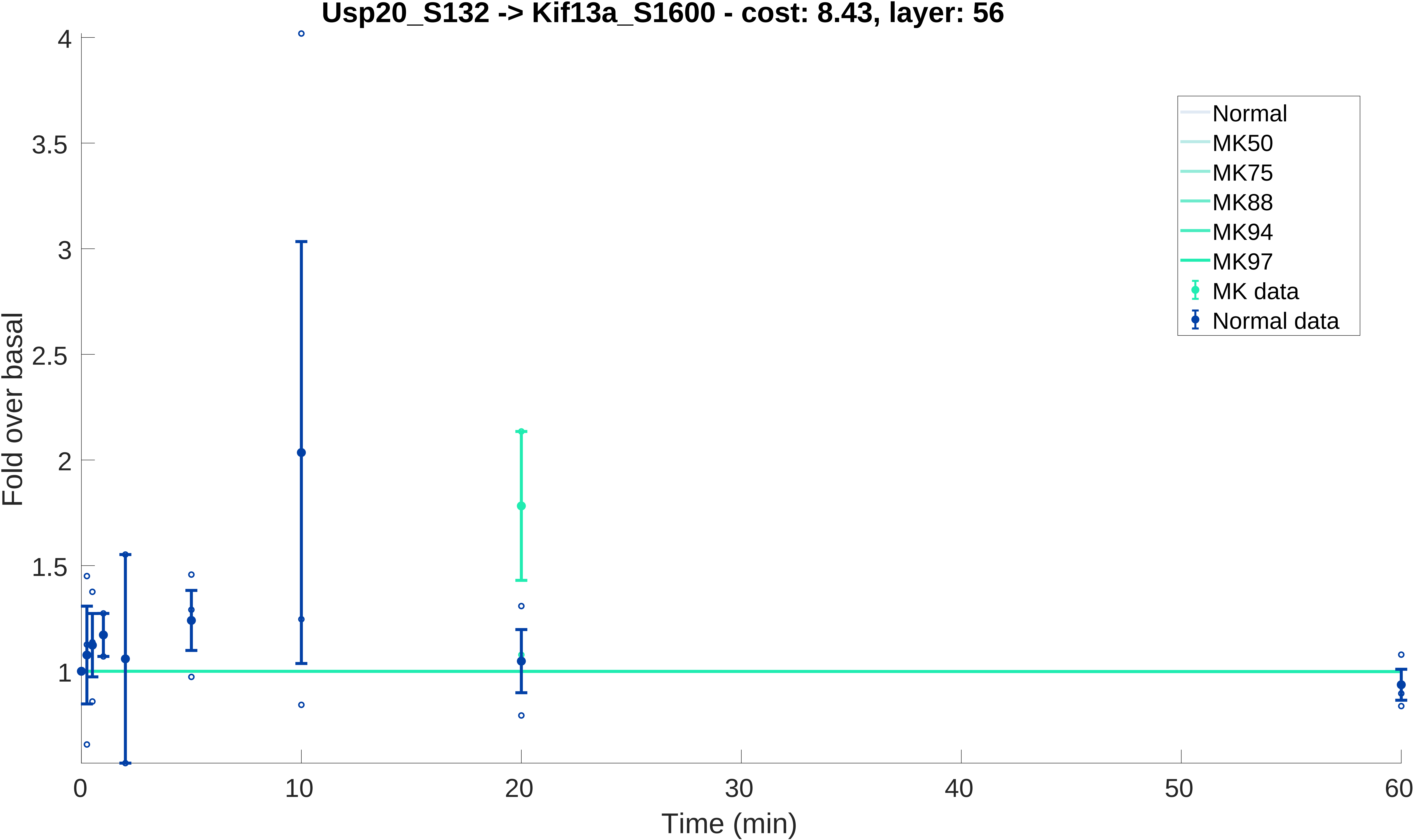

Usp20_S132 -> Kif13a_S1600 - cost: 8.43, layer: 56
Fold over basal
Time (min)
0
10
20
30
40
50
60
1
1.5
2
2.5
3
3.5
4
Normal
MK50
MK75
MK88
MK94
MK97
MK data
Normal data

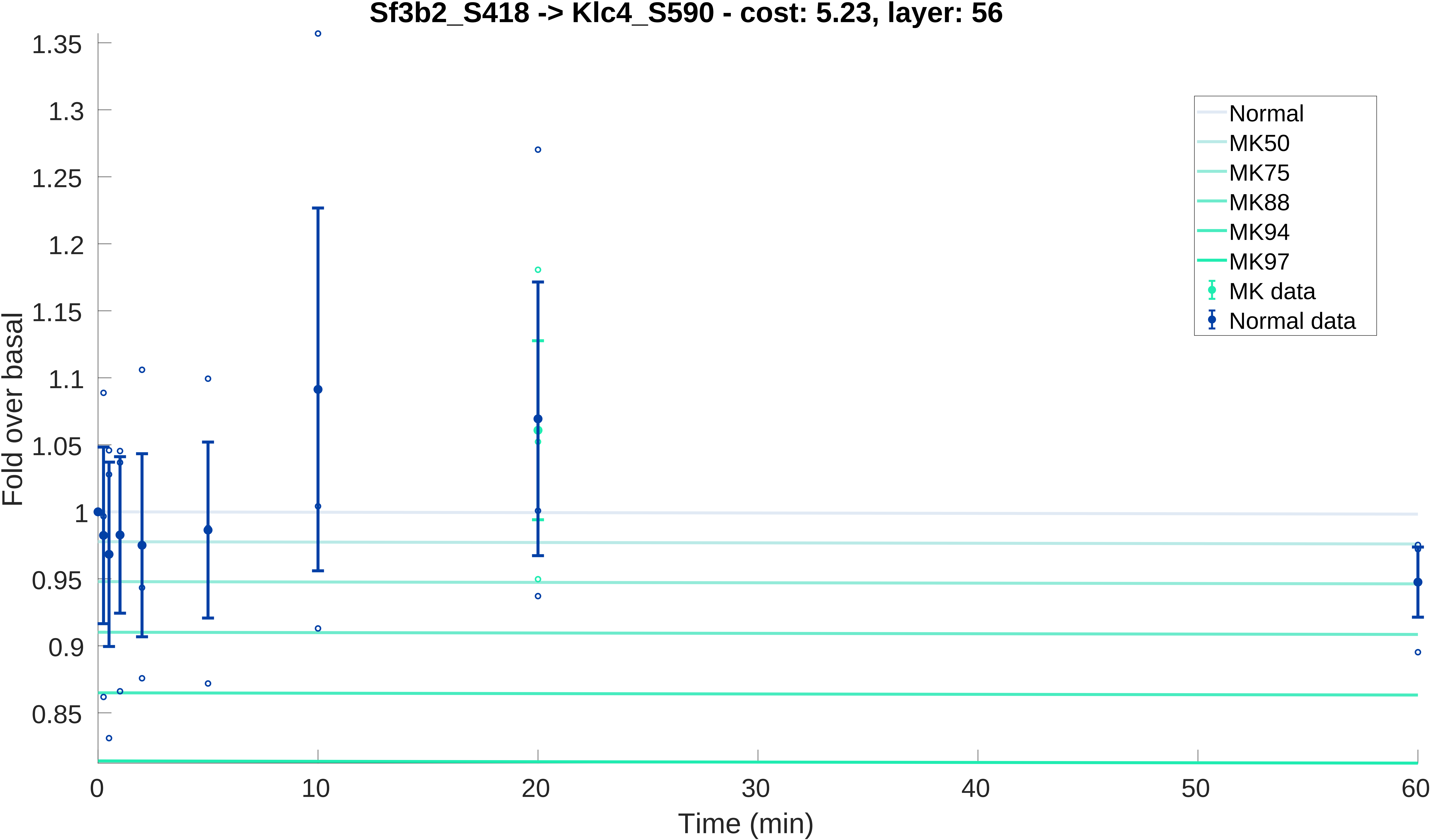

Sf3b2_S418 -> Klc4_S590 - cost: 5.23, layer: 56
Fold over basal
Time (min)
1.35
1.3
1.25
1.2
1.15
1.1
1.05
1
0.95
0.9
0.85
0
10
20
30
40
50
60
Normal
MK50
MK75
MK88
MK94
MK97
MK data
Normal data

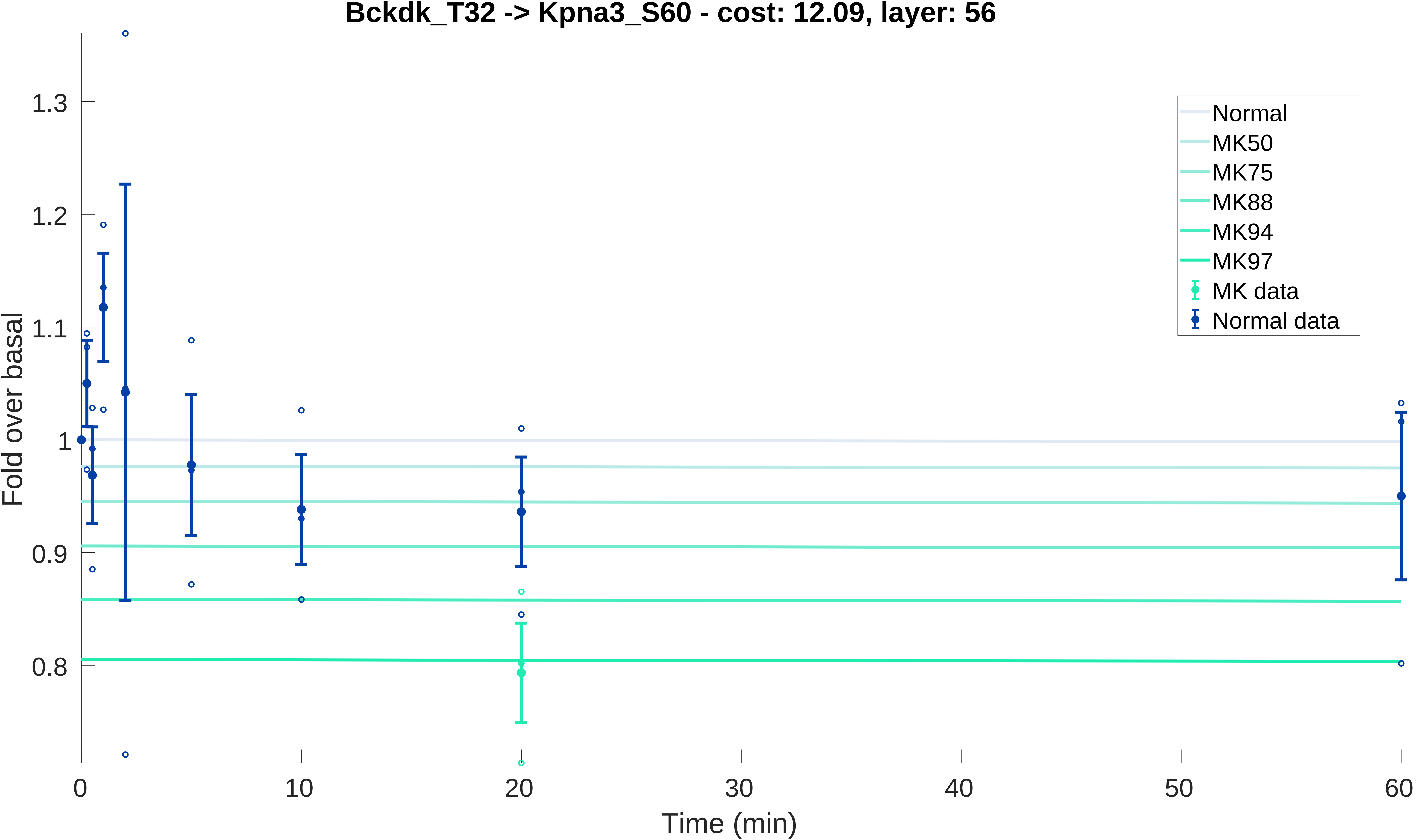

Bckdk_T32 -> Kpna3_S60 - cost: 12.09, layer: 56
Fold over basal
Time (min)
1.3
1.2
1.1
1
0.9
0.8
0
10
20
30
40
50
60
Normal
MK50
MK75
MK88
MK94
MK97
MK data
Normal data

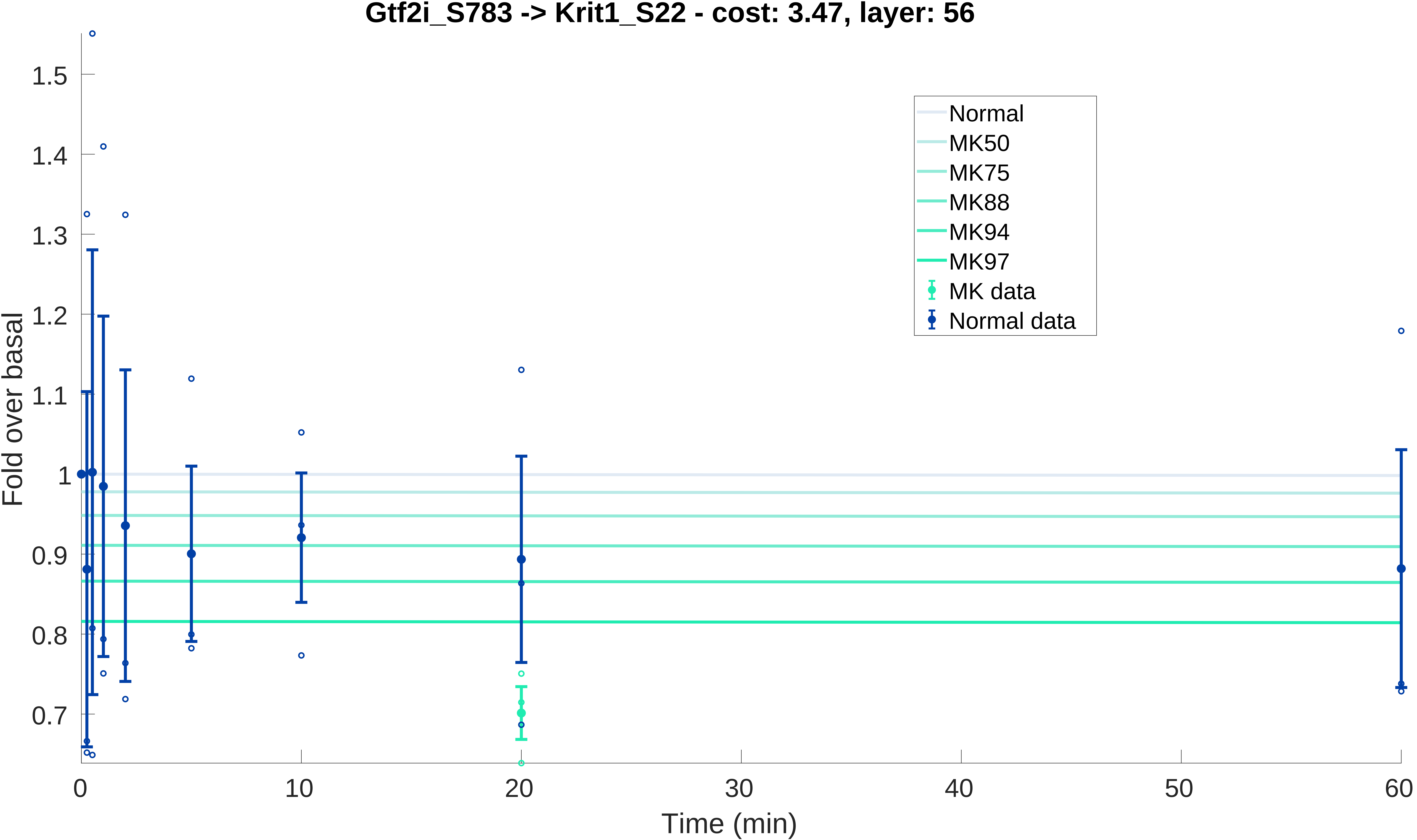

Gtf2i_S783 -> Krit1_S22 - cost: 3.47, layer: 56
Fold over basal
Time (min)
Normal
MK50
MK75
MK88
MK94
MK97
MK data
Normal data

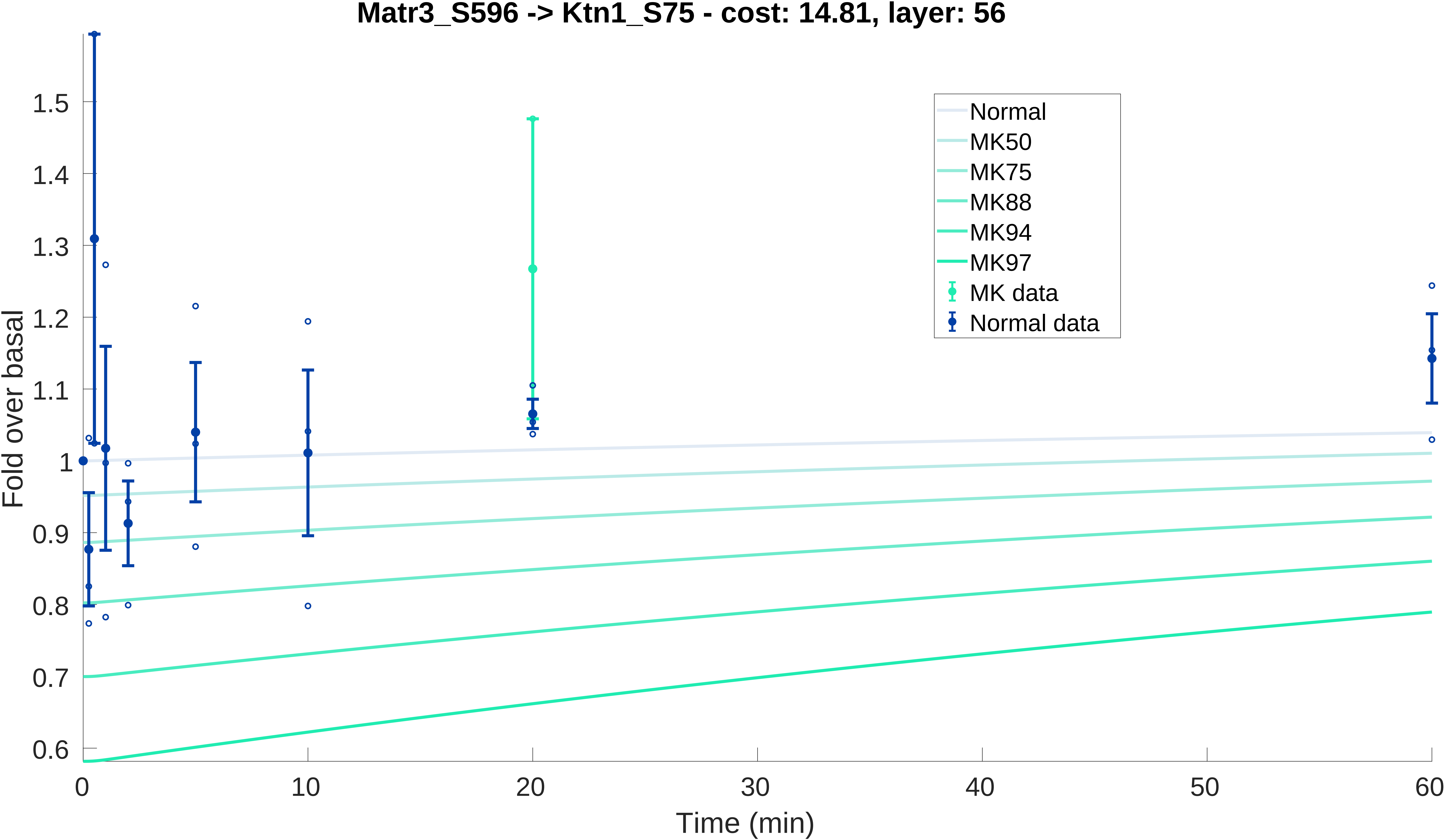

Matr3_S596 -> Ktn1_S75 - cost: 14.81, layer: 56
Fold over basal
Time (min)
1.5
1.4
1.3
1.2
1.1
1
0.9
0.8
0.7
0.6
0
10
20
30
40
50
60
Normal
MK50
MK75
MK88
MK94
MK97
MK data
Normal data

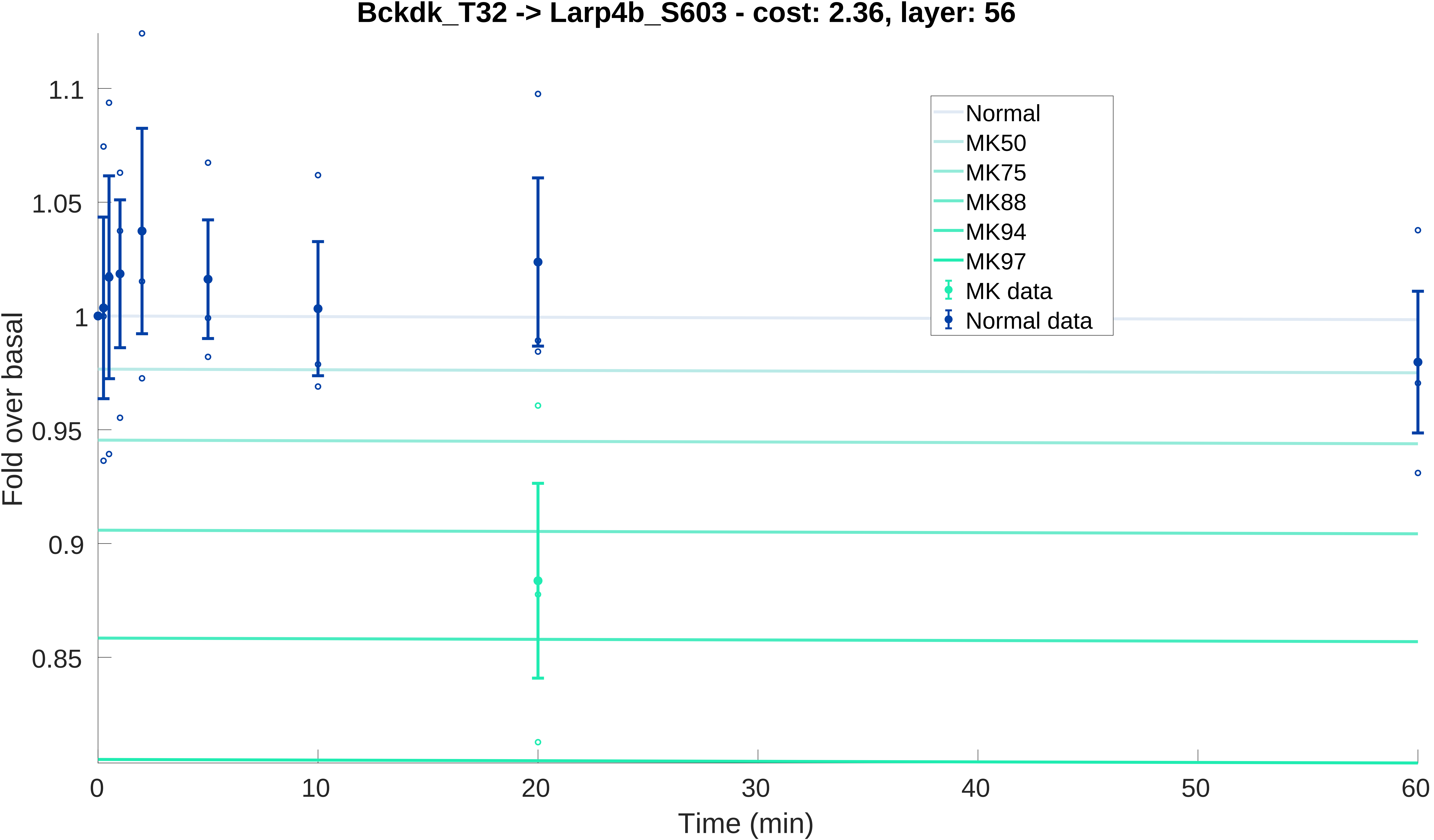

Bckdk_T32 -> Larp4b_S603 - cost: 2.36, layer: 56
Normal
MK50
MK75
MK88
MK94
MK97
MK data
Normal data
Fold over basal
1.1
1.05
1
0.95
0.9
0.85
0
10
20
30
40
50
60
Time (min)

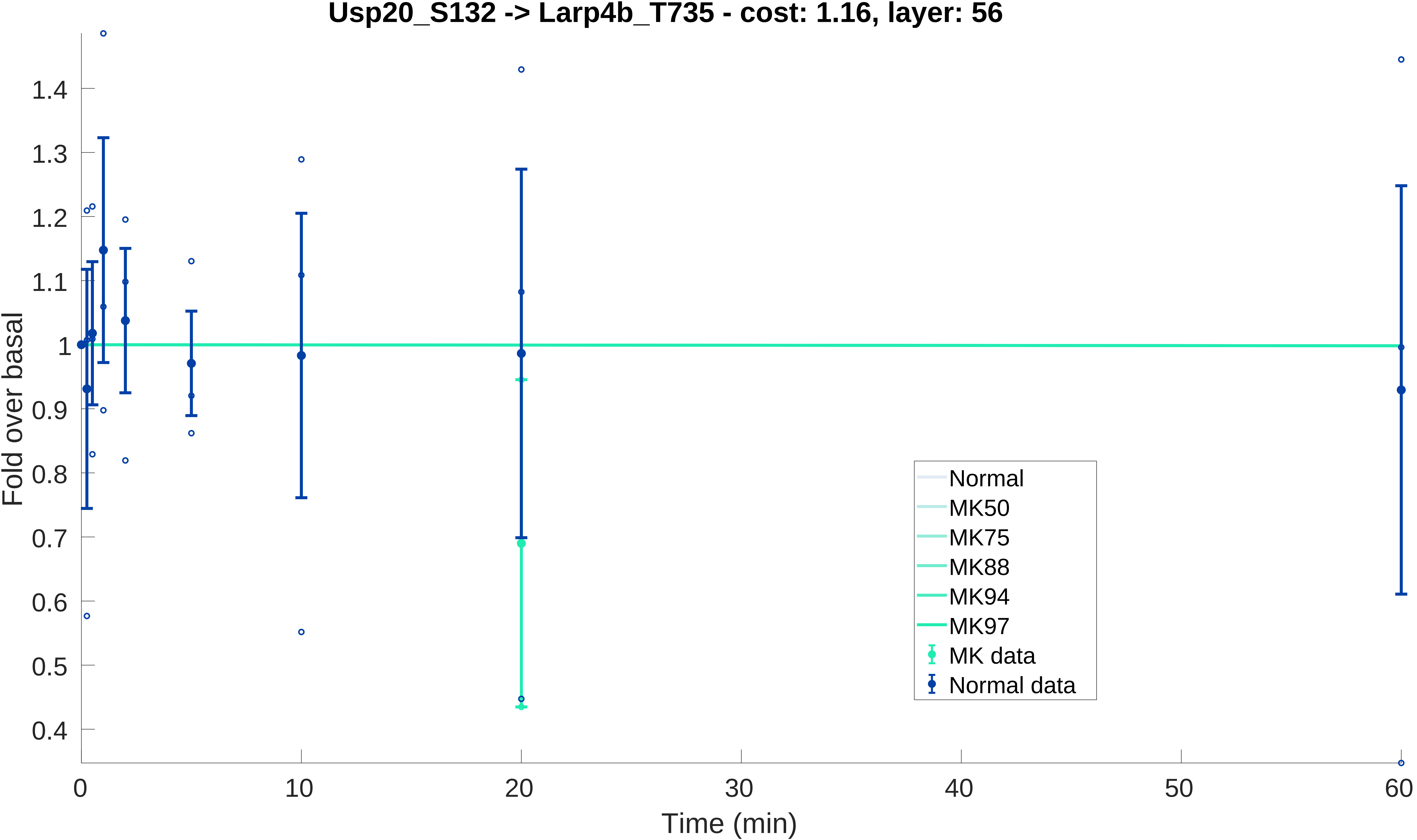

Usp20_S132 -> Larp4b_T735 - cost: 1.16, layer: 56
1.4
1.3
1.2
1.1
1
0.9
0.8
0.7
0.6
0.5
0.4
Fold over basal
0
10
20
30
40
50
60
Time (min)
Normal
MK50
MK75
MK88
MK94
MK97
MK data
Normal data

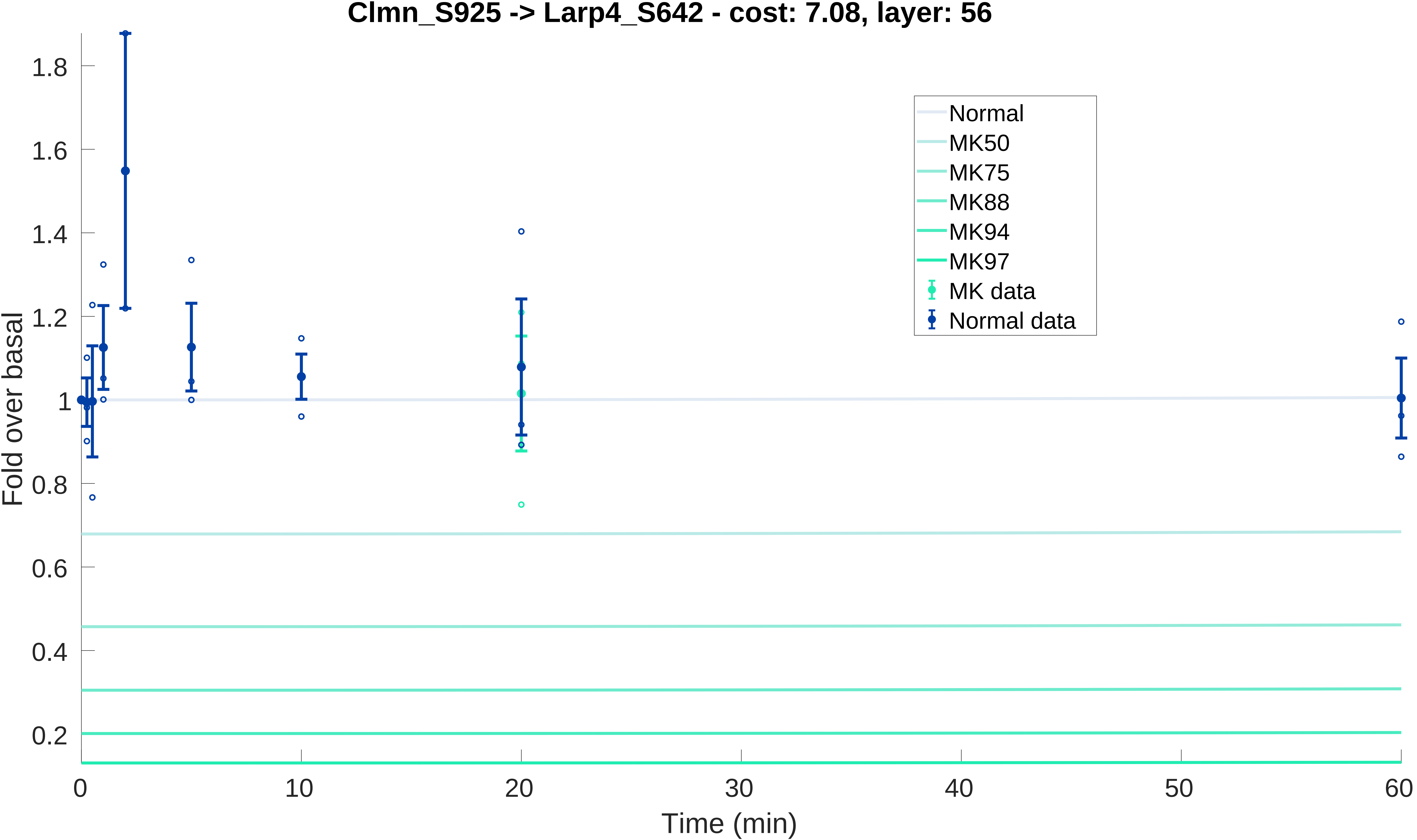
Clmn_S925 -> Larp4_S642 - cost: 7.08, layer: 56
Fold over basal
Time (min)
Normal
MK50
MK75
MK88
MK94
MK97
MK data
Normal data
1.8
1.6
1.4
1.2
1
0.8
0.6
0.4
0.2
0
10
20
30
40
50
60

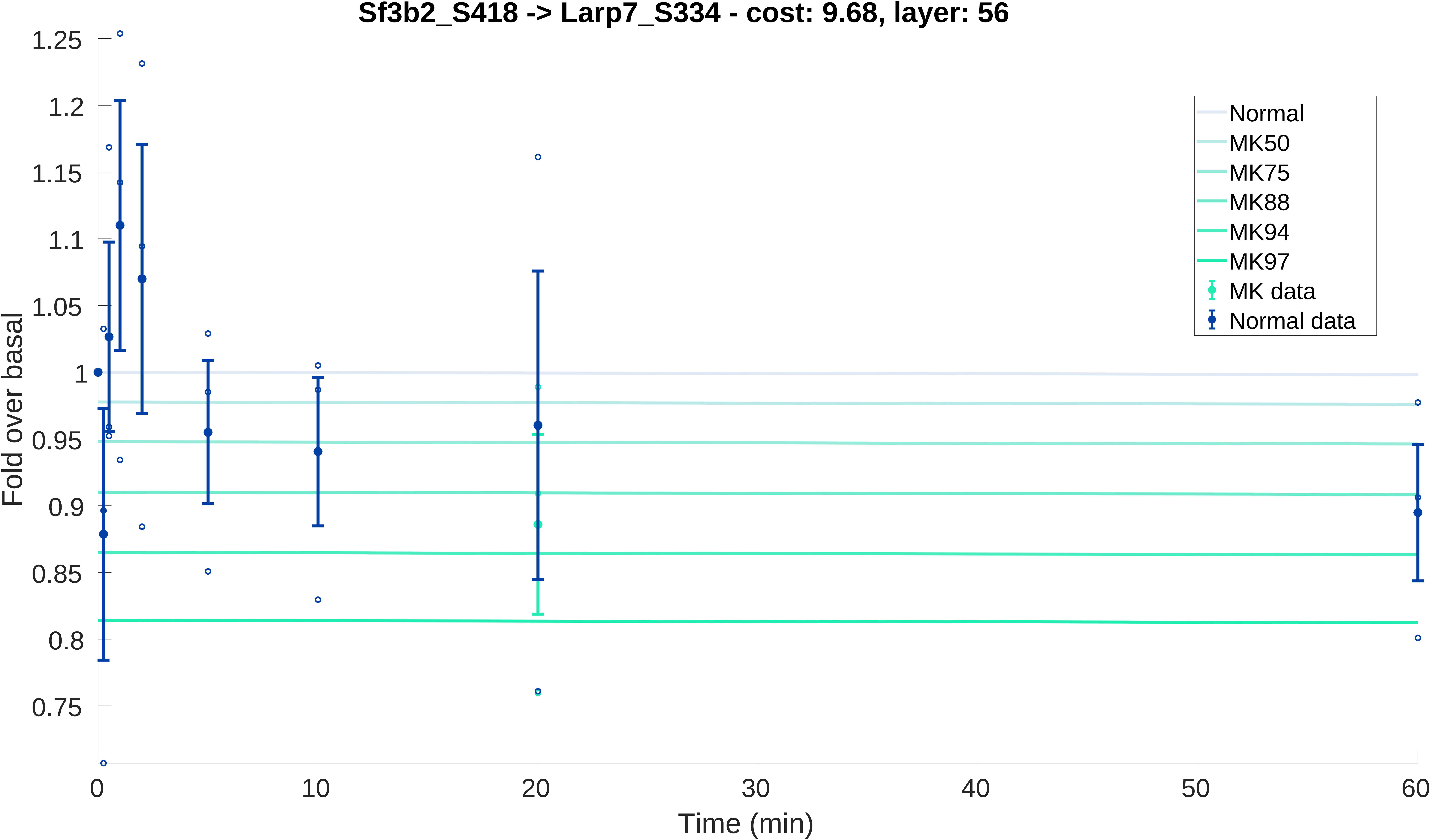

Sf3b2_S418 -> Larp7_S334 - cost: 9.68, layer: 56
Normal
MK50
MK75
MK88
MK94
MK97
MK data
Normal data
Fold over basal
Time (min)
1.25
1.2
1.15
1.1
1.05
1
0.95
0.9
0.85
0.8
0.75
0
10
20
30
40
50
60

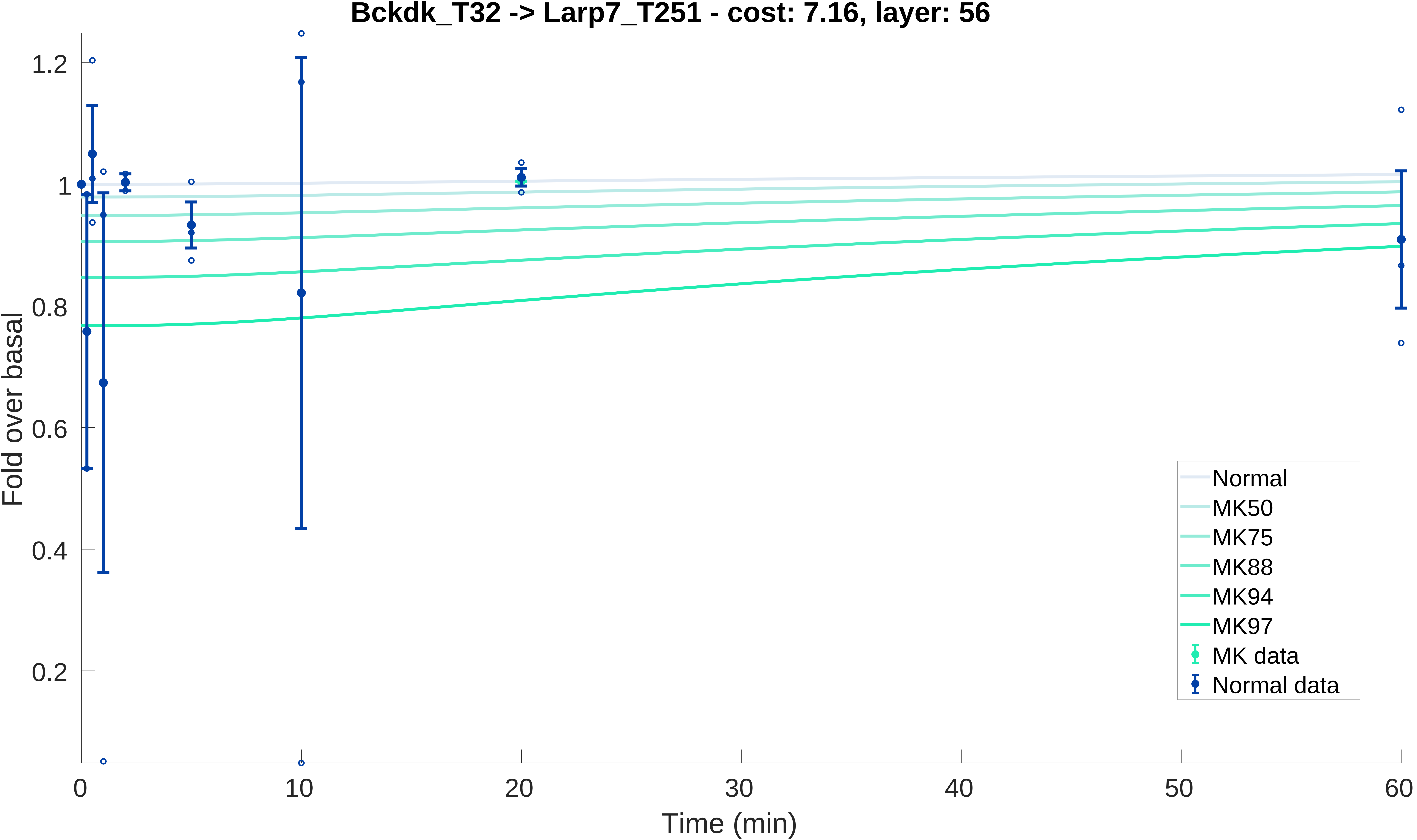
Bckdk_T32 -> Larp7_T251 - cost: 7.16, layer: 56
Fold over basal
1.2
1
0.8
0.6
0.4
0.2
0
10
20
30
40
50
60
Time (min)
Normal
MK50
MK75
MK88
MK94
MK97
MK data
Normal data

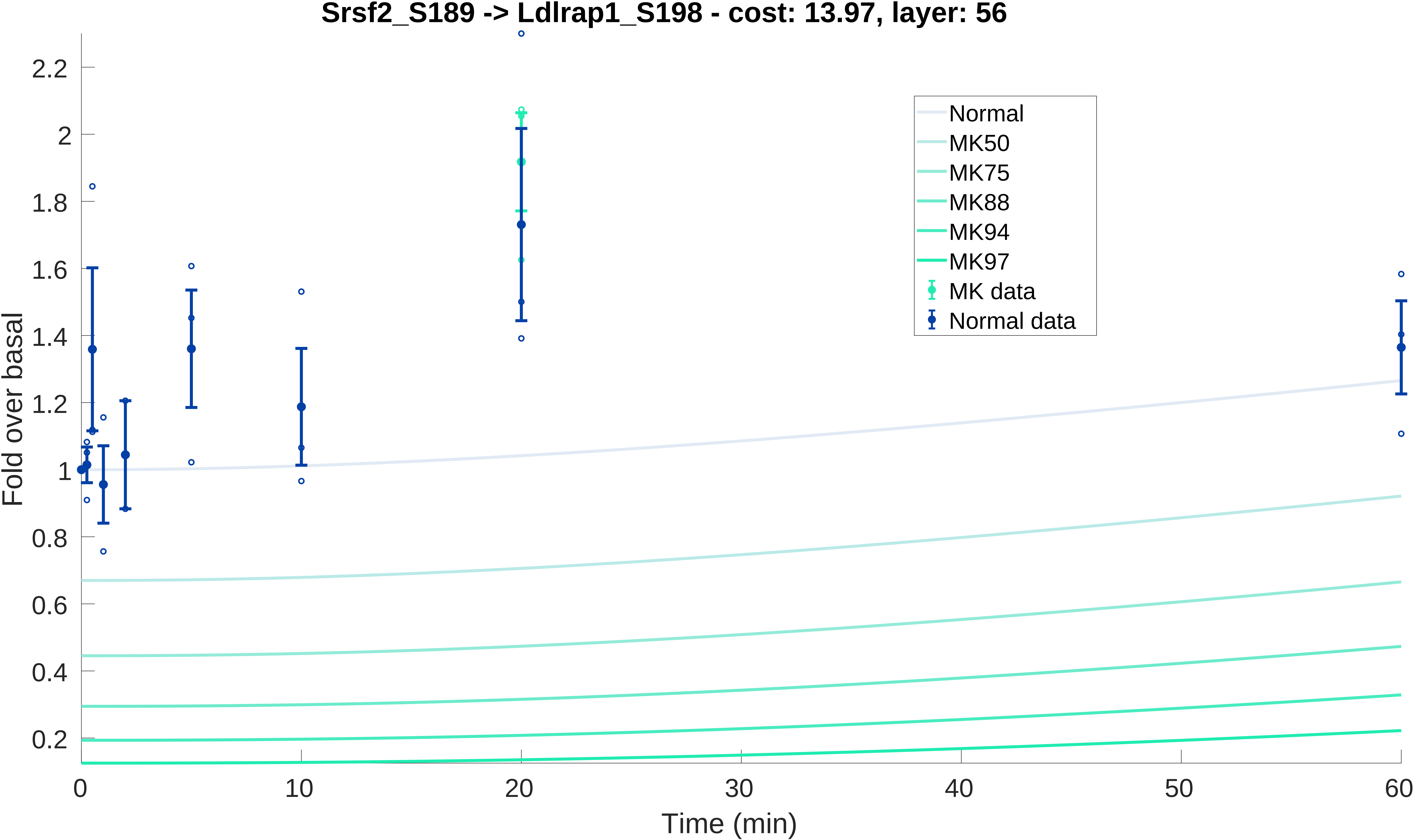
Srsf2_S189 -> Ldlrap1_S198 - cost: 13.97, layer: 56
Normal
MK50
MK75
MK88
MK94
MK97
MK data
Normal data
Fold over basal
Time (min)
0
10
20
30
40
50
60
0.2
0.4
0.6
0.8
1
1.2
1.4
1.6
1.8
2
2.2

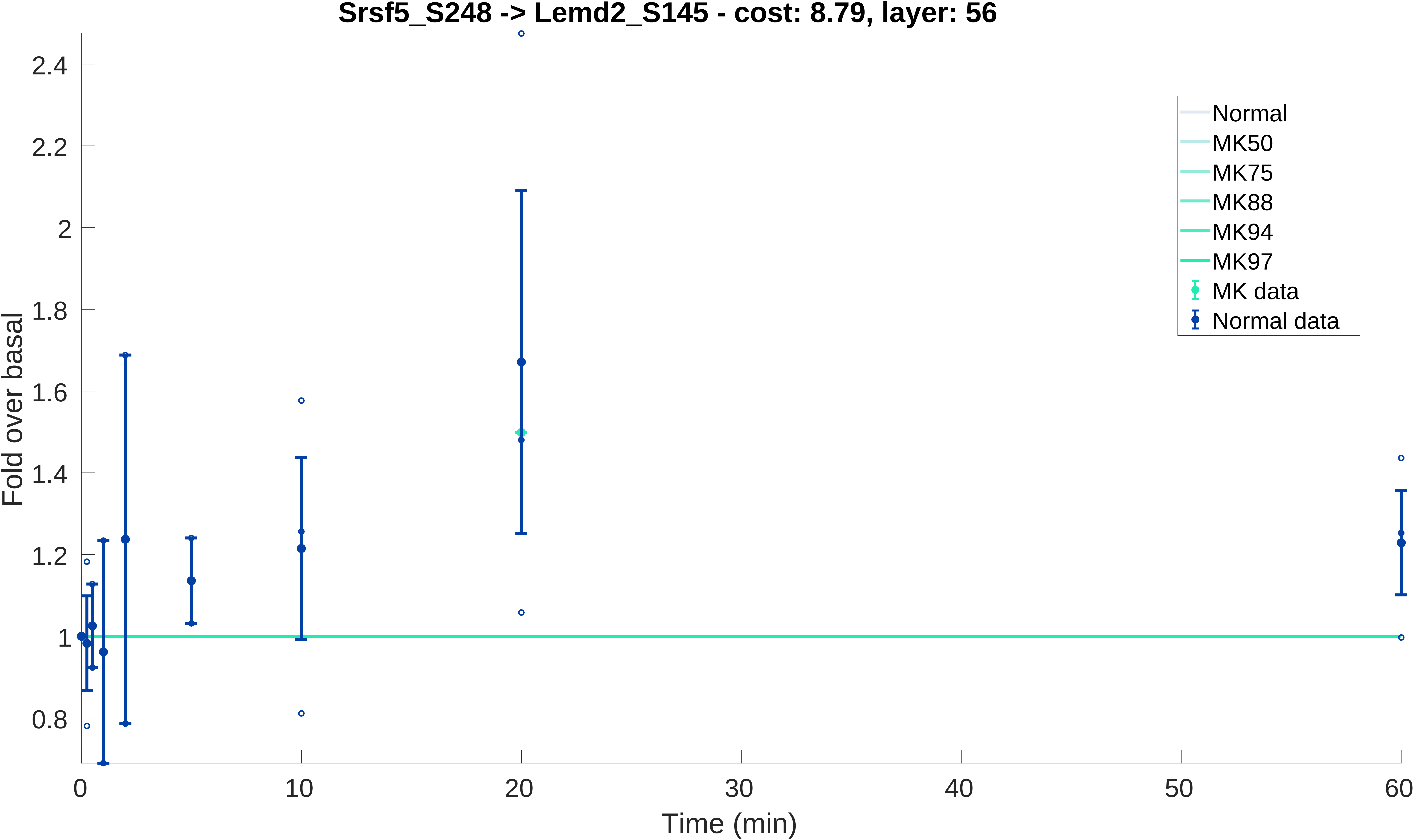
Srsf5_S248 -> Lemd2_S145 - cost: 8.79, layer: 56
Fold over basal
Time (min)
0.8
1
1.2
1.4
1.6
1.8
2
2.2
2.4
0
10
20
30
40
50
60
Normal
MK50
MK75
MK88
MK94
MK97
MK data
Normal data

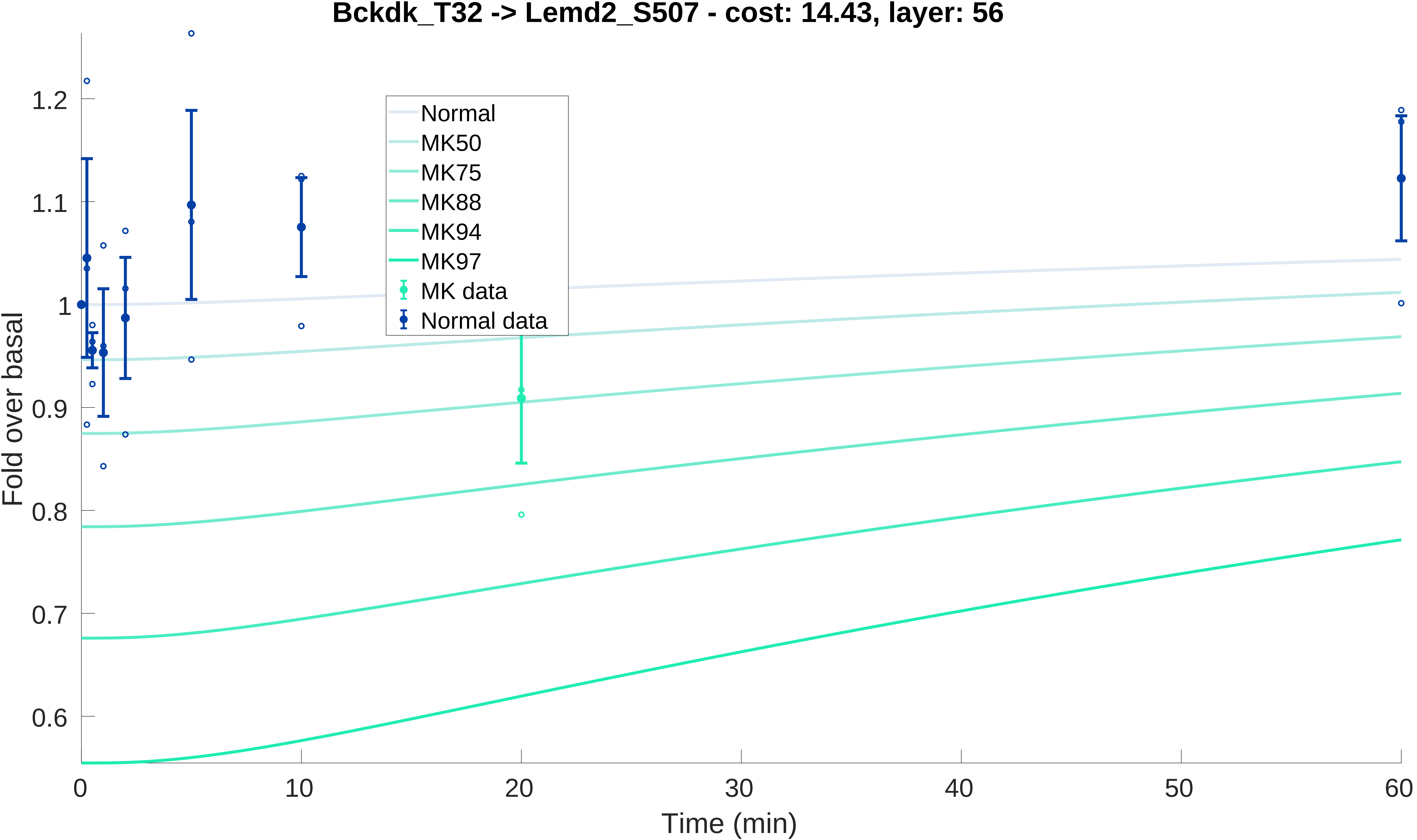

Bckdk_T32 -> Lemd2_S507 - cost: 14.43, layer: 56
Normal
MK50
MK75
MK88
MK94
MK97
MK data
Normal data
Fold over basal
Time (min)
1.2
1.1
1
0.9
0.8
0.7
0.6
0
10
20
30
40
50
60

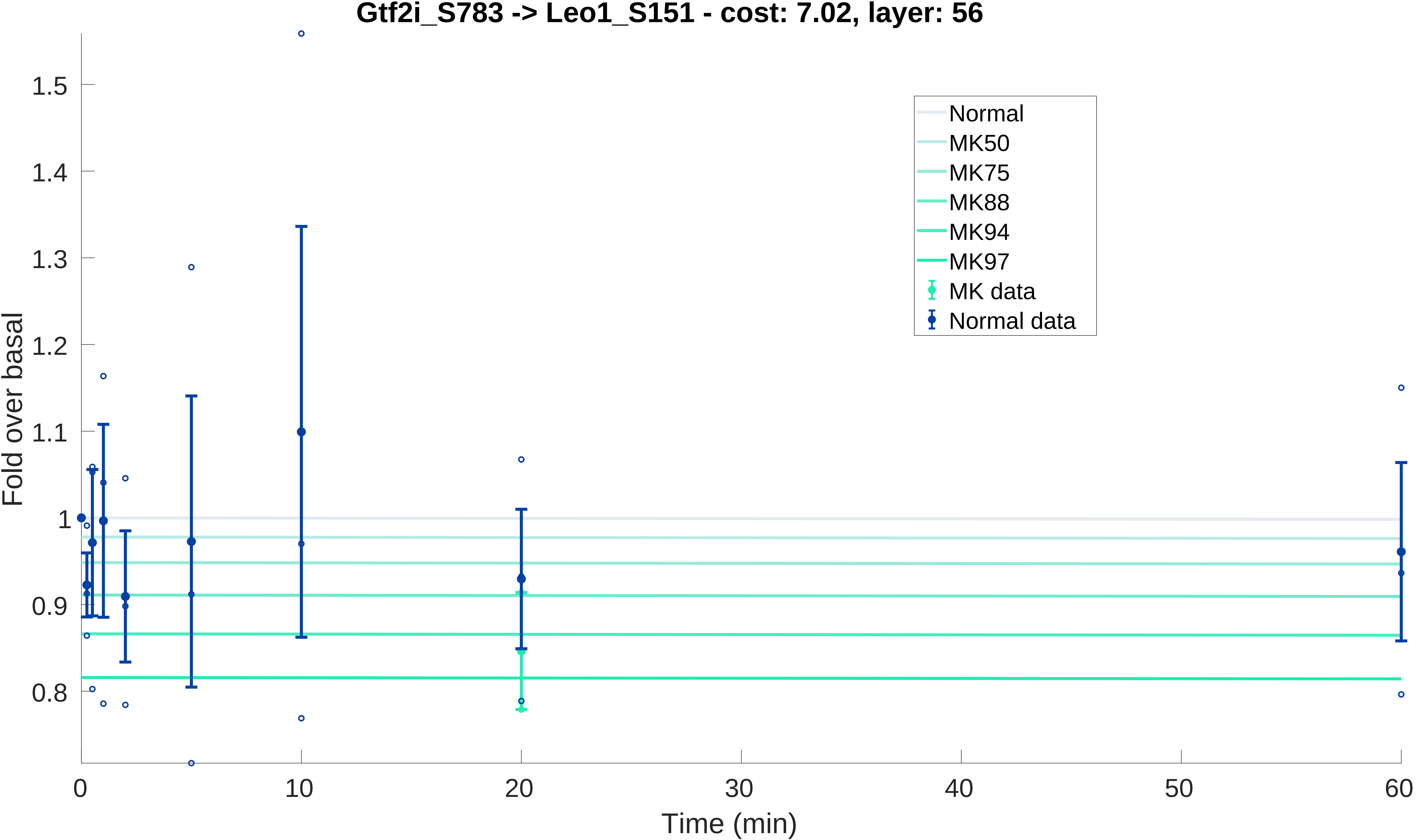
Gtf2i_S783 -> Leo1_S151 - cost: 7.02, layer: 56
Fold over basal
1.5
1.4
1.3
1.2
1.1
1
0.9
0.8
0
10
20
30
40
50
60
Time (min)
Normal
MK50
MK75
MK88
MK94
MK97
MK data
Normal data

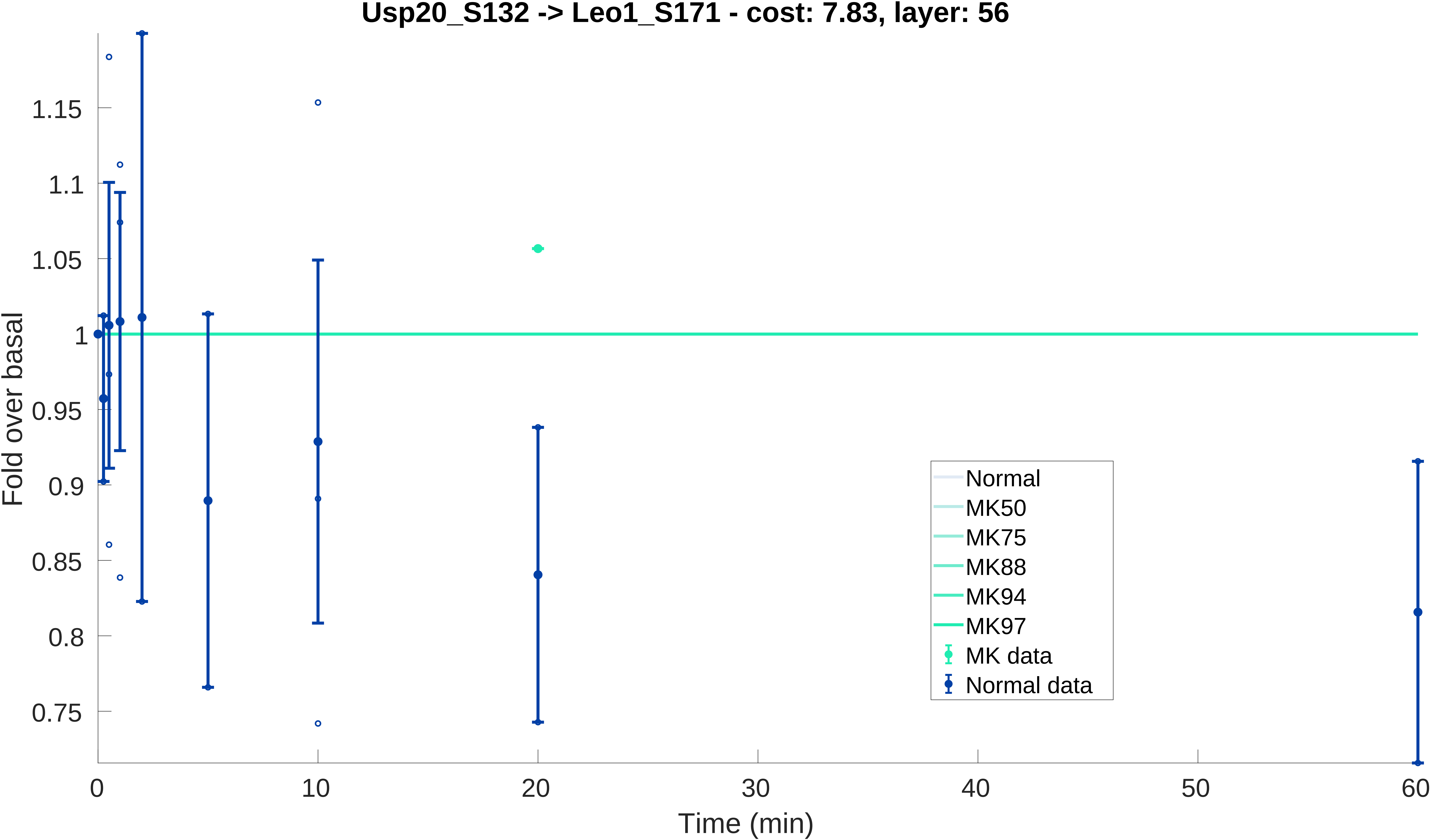

Usp20_S132 -> Leo1_S171 - cost: 7.83, layer: 56
Fold over basal
Time (min)
1.15
1.1
1.05
1
0.95
0.9
0.85
0.8
0.75
0
10
20
30
40
50
60
Normal
MK50
MK75
MK88
MK94
MK97
MK data
Normal data

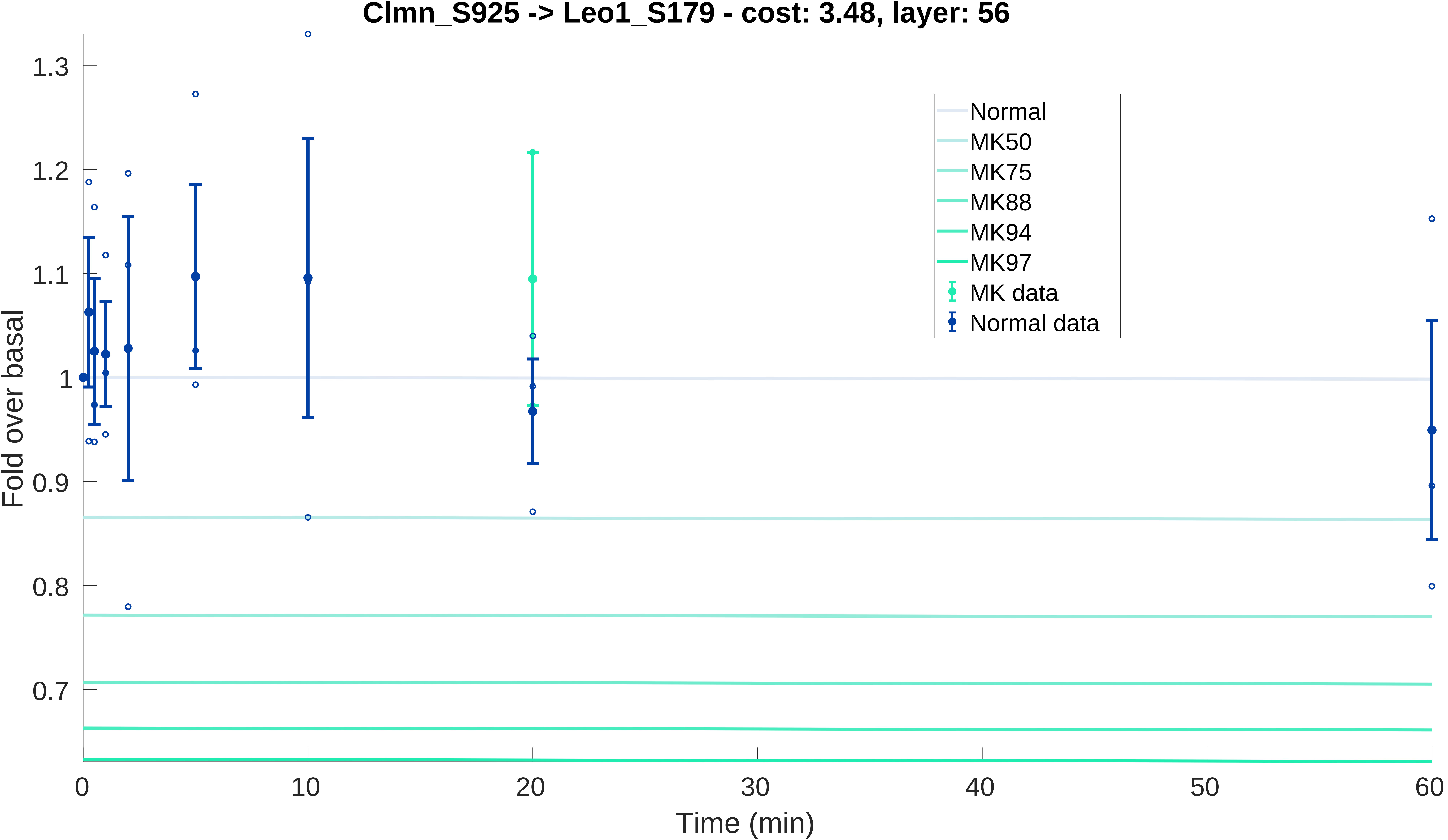

Clmn_S925 -> Leo1_S179 - cost: 3.48, layer: 56
Fold over basal
Time (min)
1.3
1.2
1.1
1
0.9
0.8
0.7
0
10
20
30
40
50
60
Normal
MK50
MK75
MK88
MK94
MK97
MK data
Normal data

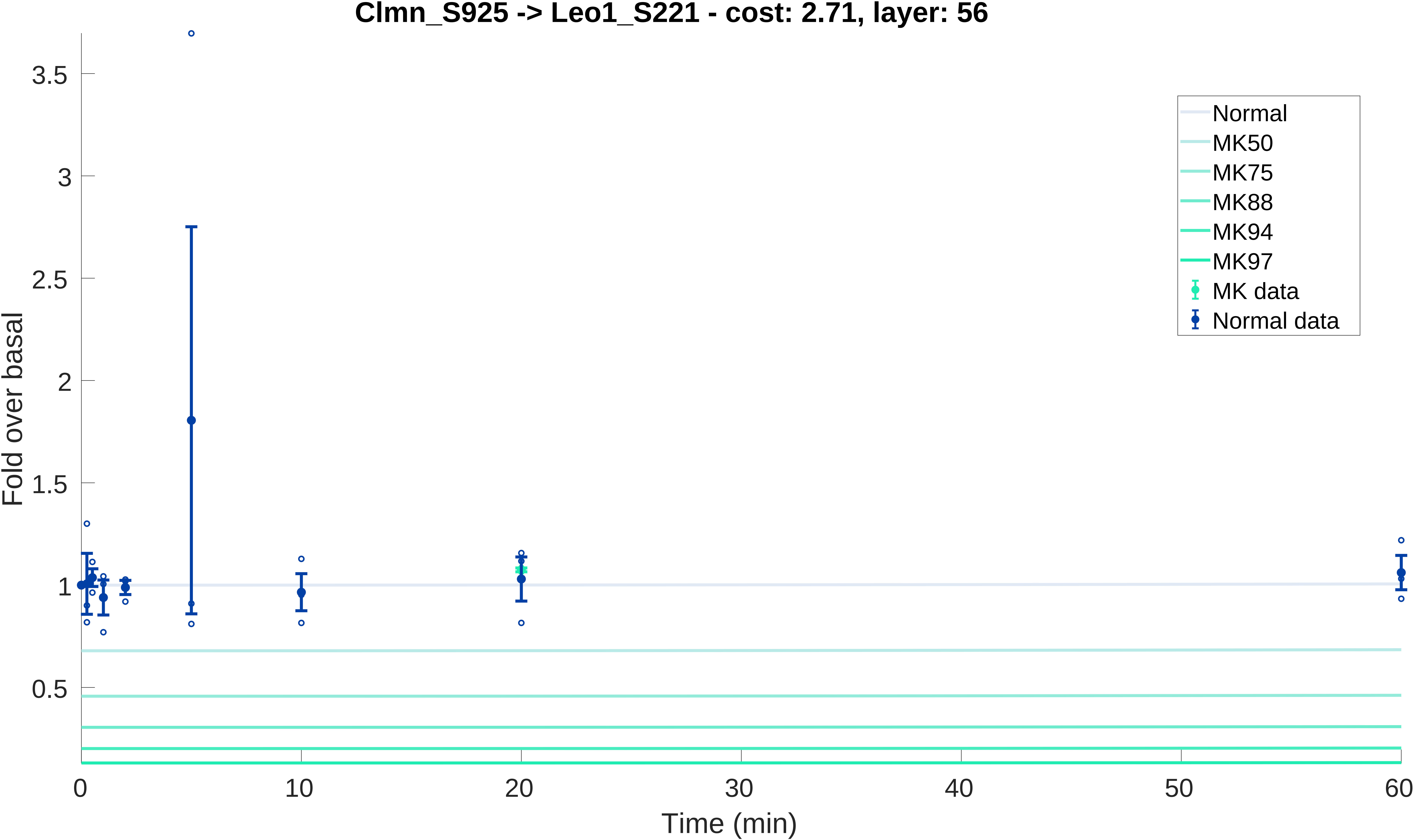
Clmn_S925 -> Leo1_S221 - cost: 2.71, layer: 56
Fold over basal
Time (min)
3.5
3
2.5
2
1.5
1
0.5
0
10
20
30
40
50
60
Normal
MK50
MK75
MK88
MK94
MK97
MK data
Normal data

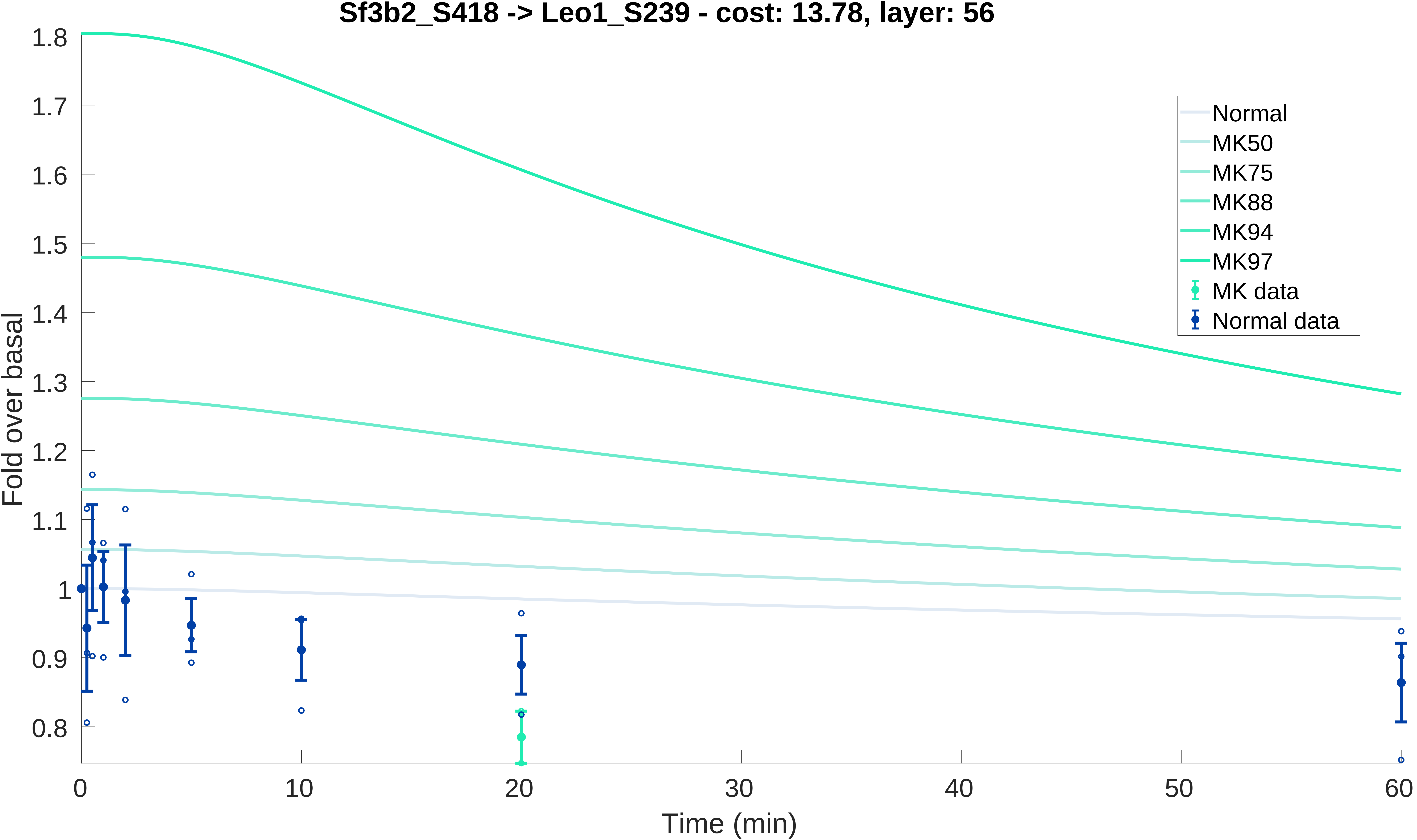

Sf3b2_S418 -> Leo1_S239 - cost: 13.78, layer: 56
Fold over basal
1.8
1.7
1.6
1.5
1.4
1.3
1.2
1.1
1
0.9
0.8
0
10
20
30
40
50
60
Time (min)
Normal
MK50
MK75
MK88
MK94
MK97
MK data
Normal data

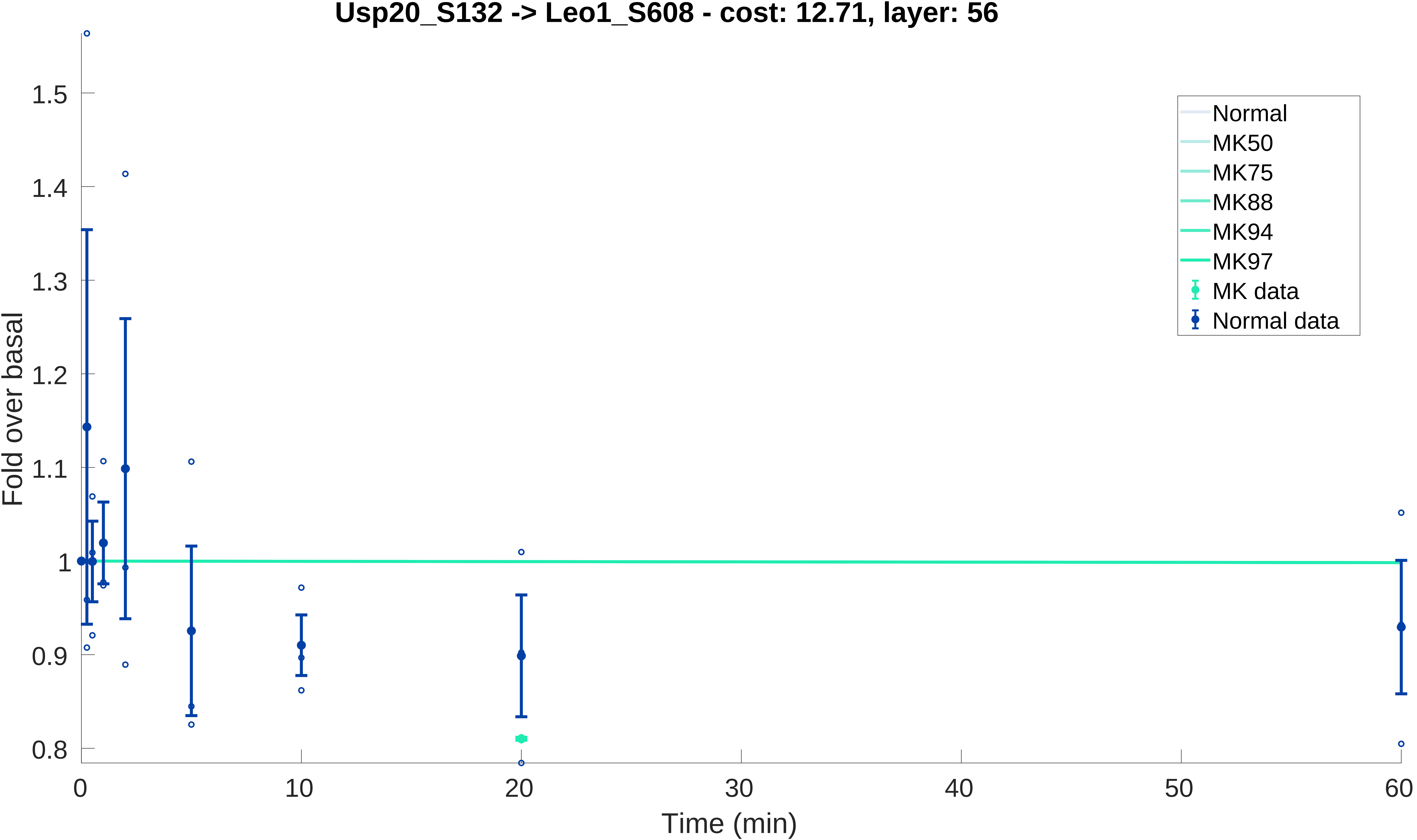

Usp20_S132 -> Leo1_S608 - cost: 12.71, layer: 56
Fold over basal
Time (min)
0.8
0.9
1
1.1
1.2
1.3
1.4
1.5
0
10
20
30
40
50
60
Normal
MK50
MK75
MK88
MK94
MK97
MK data
Normal data

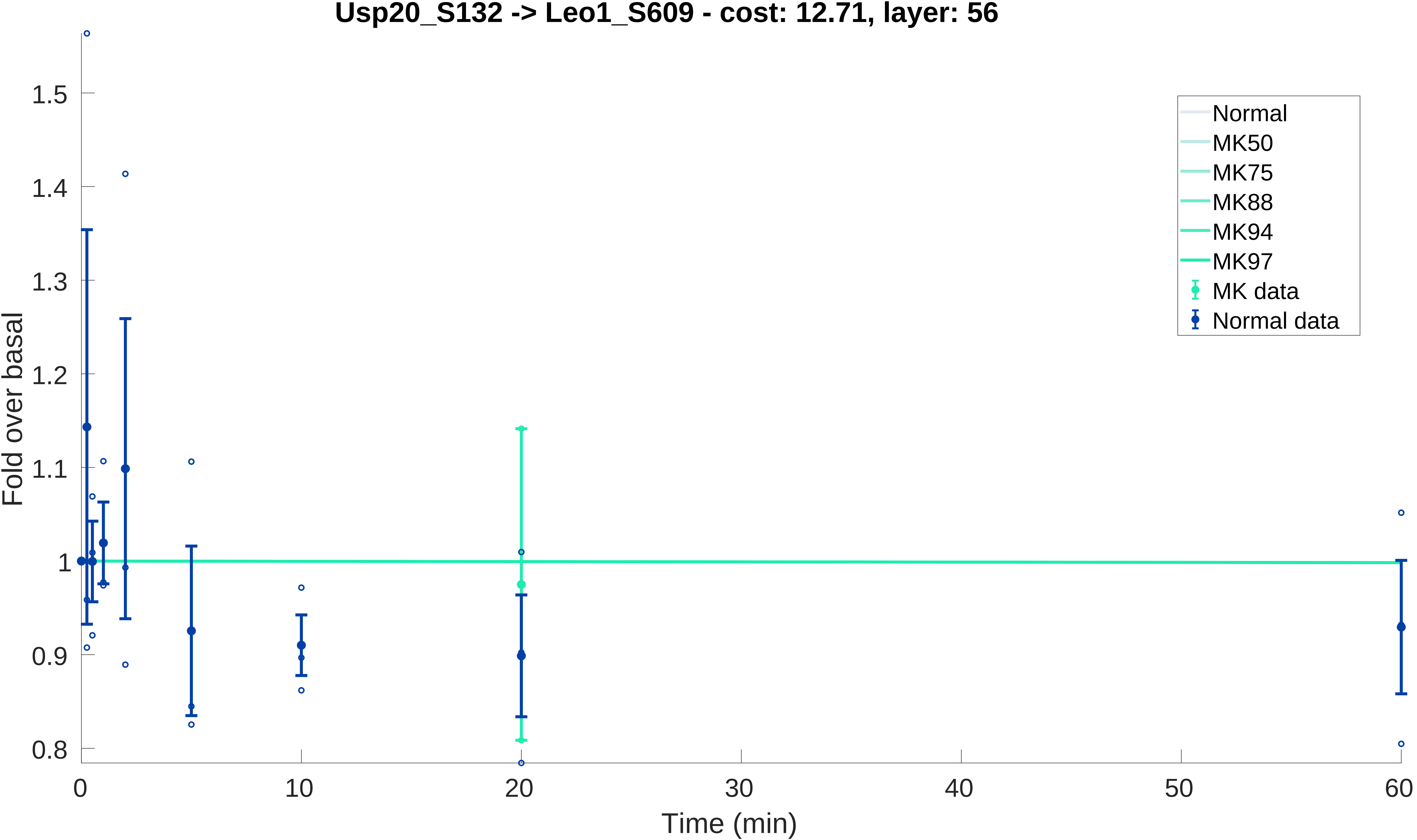

Usp20_S132 -> Leo1_S609 - cost: 12.71, layer: 56
Fold over basal
1.5
1.4
1.3
1.2
1.1
1
0.9
0.8
0
10
20
30
40
50
60
Time (min)
Normal
MK50
MK75
MK88
MK94
MK97
MK data
Normal data

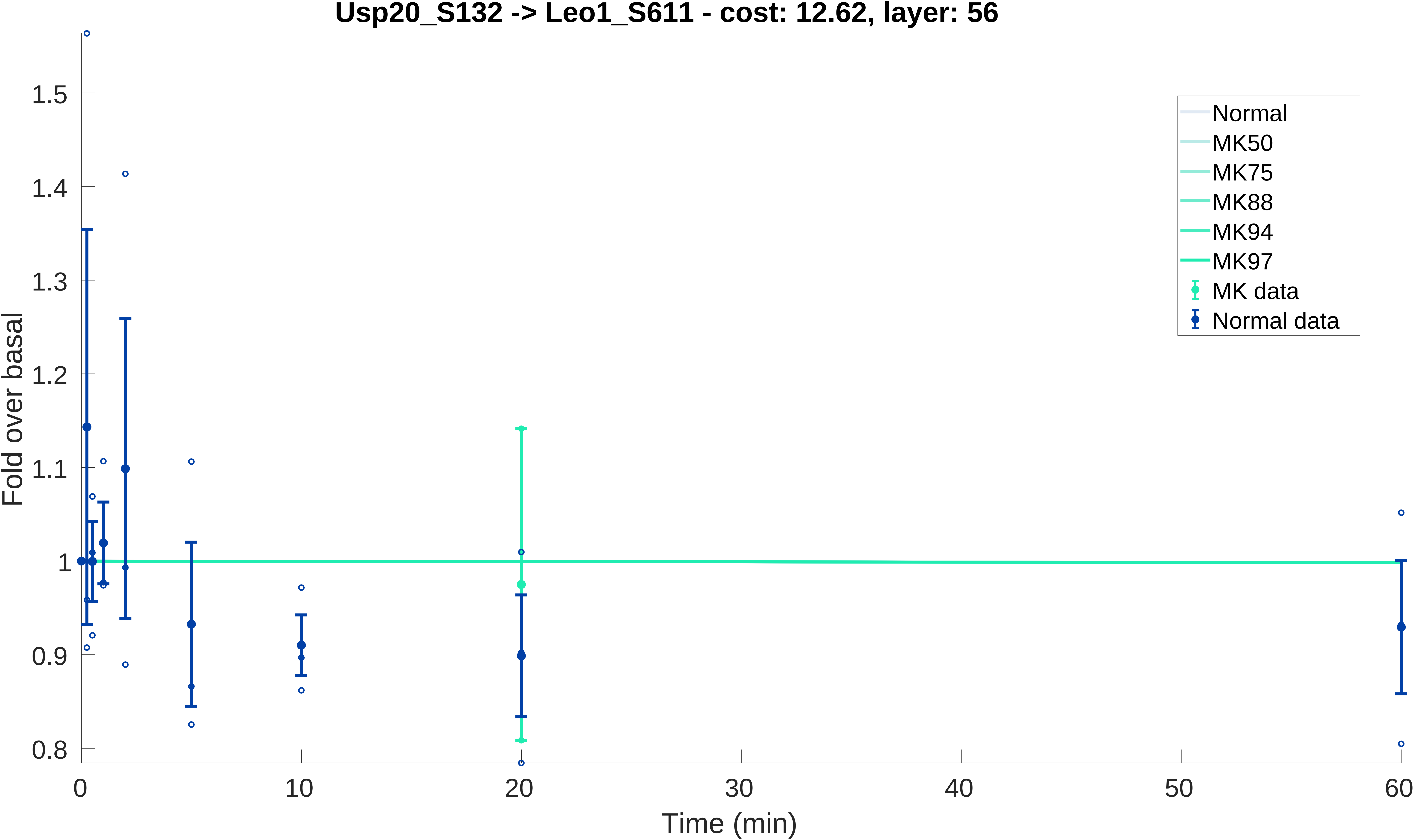

Usp20_S132 -> Leo1_S611 - cost: 12.62, layer: 56
Fold over basal
Time (min)
Normal
MK50
MK75
MK88
MK94
MK97
MK data
Normal data
0.8
0.9
1
1.1
1.2
1.3
1.4
1.5
0
10
20
30
40
50
60

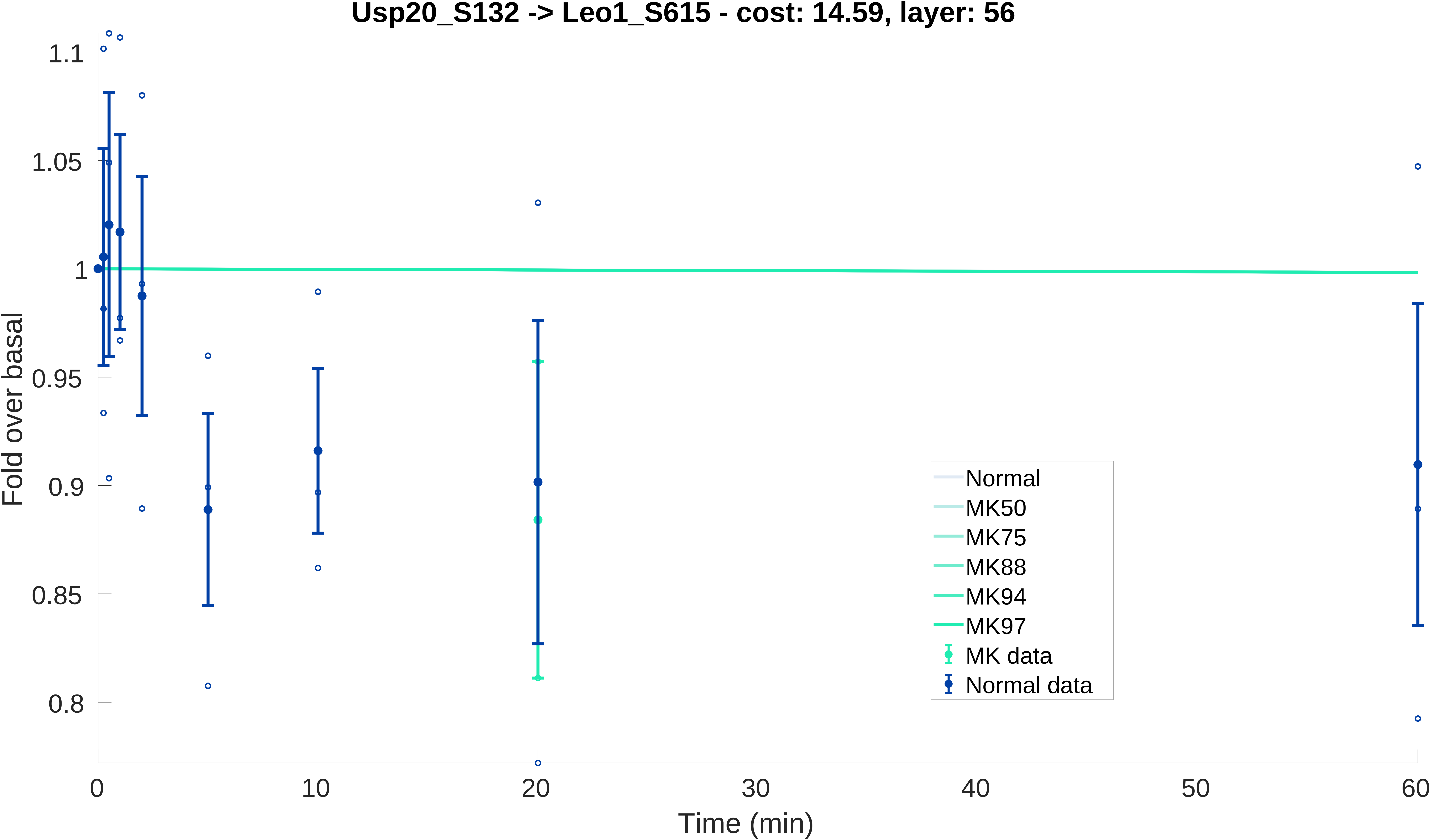
Usp20_S132 -> Leo1_S615 - cost: 14.59, layer: 56
Fold over basal
Time (min)
1.1
1.05
1
0.95
0.9
0.85
0.8
0
10
20
30
40
50
60
Normal
MK50
MK75
MK88
MK94
MK97
MK data
Normal data

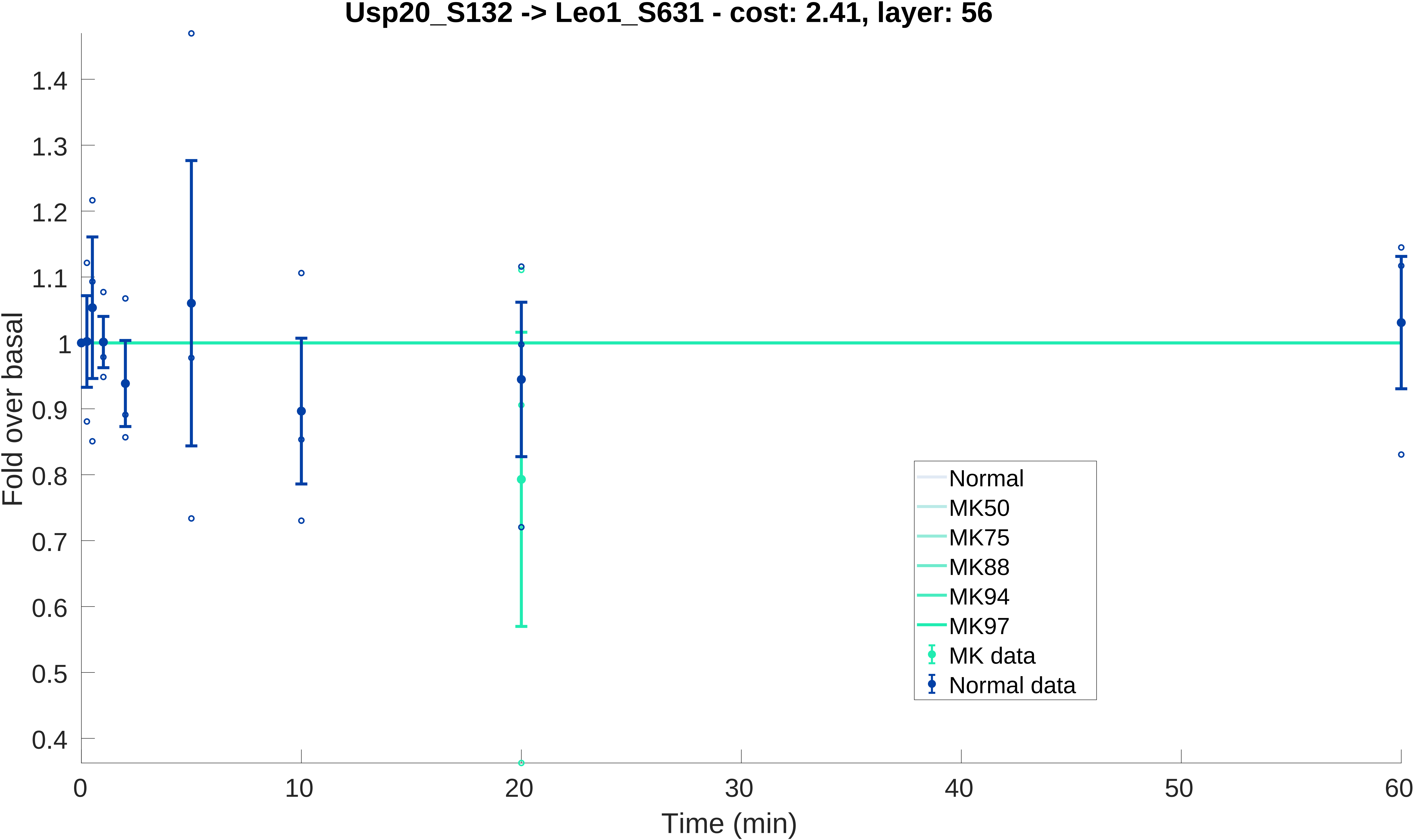

Usp20_S132 -> Leo1_S631 - cost: 2.41, layer: 56
Fold over basal
1.4
1.3
1.2
1.1
1
0.9
0.8
0.7
0.6
0.5
0.4
0
10
20
30
40
50
60
Time (min)
Normal
MK50
MK75
MK88
MK94
MK97
MK data
Normal data

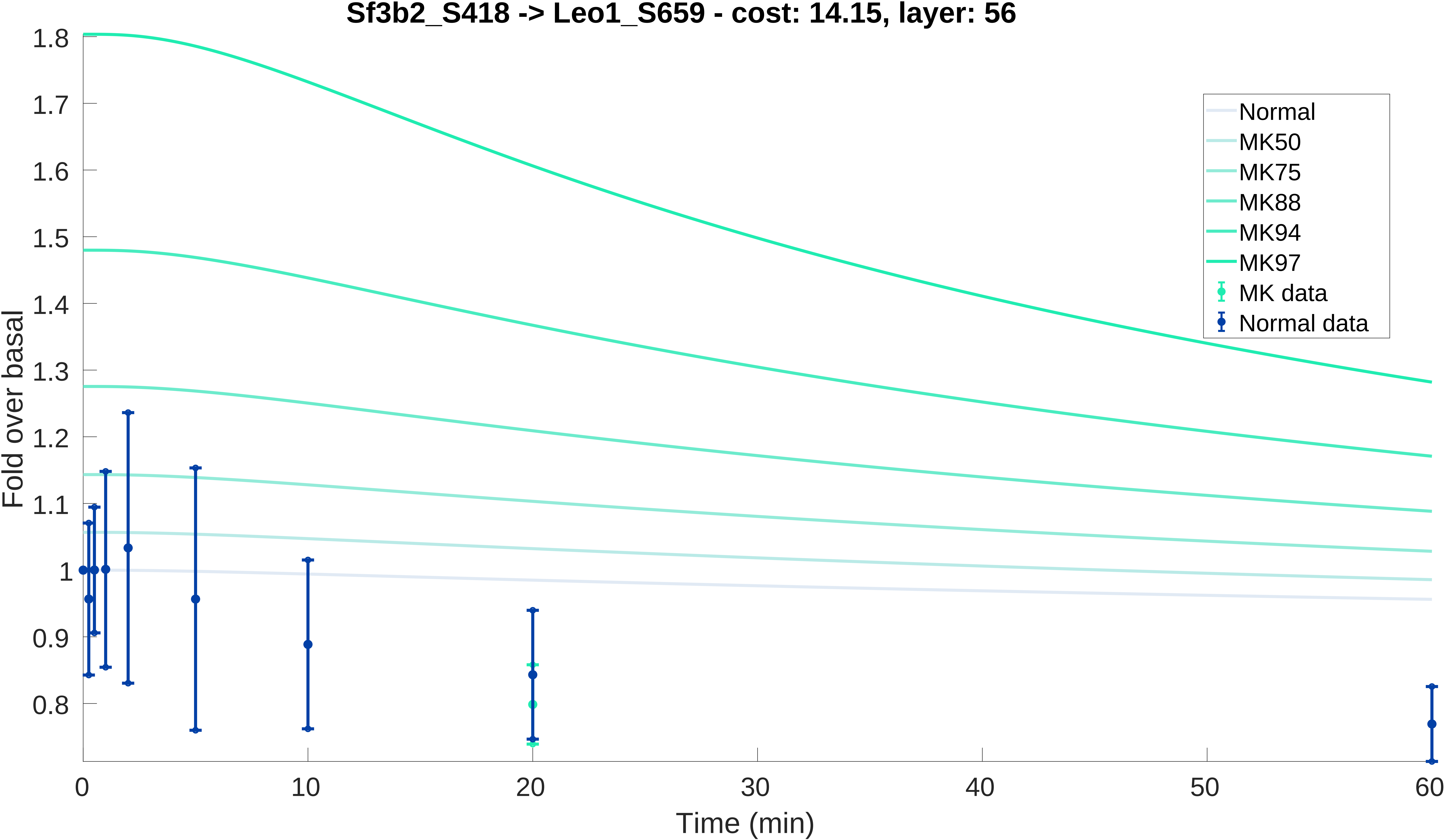
Sf3b2_S418 -> Leo1_S659 - cost: 14.15, layer: 56
Fold over basal
Time (min)
1.8
1.7
1.6
1.5
1.4
1.3
1.2
1.1
1
0.9
0.8
0
10
20
30
40
50
60
Normal
MK50
MK75
MK88
MK94
MK97
MK data
Normal data

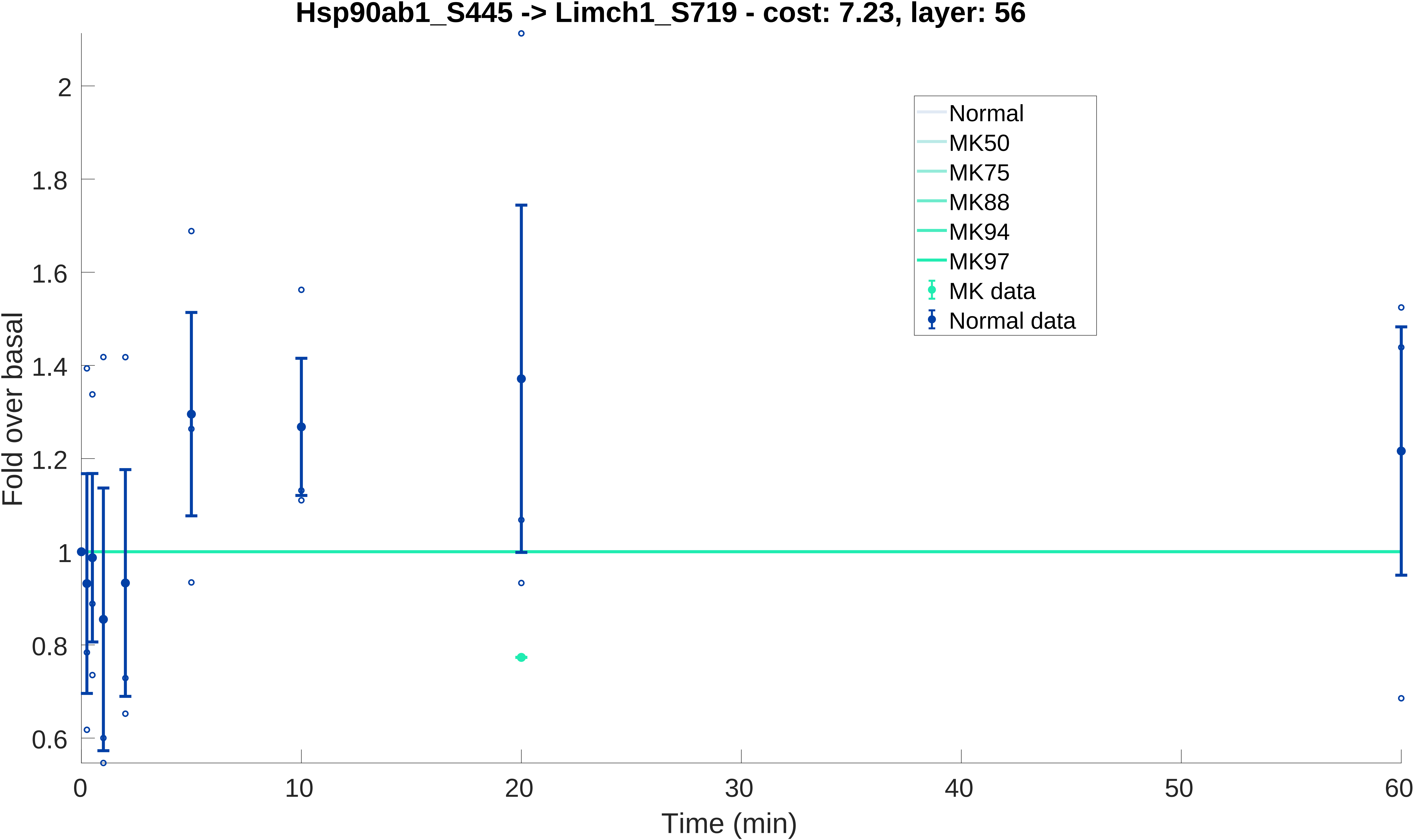

Hsp90ab1_S445 -> Limch1_S719 - cost: 7.23, layer: 56
Fold over basal
Time (min)
Normal
MK50
MK75
MK88
MK94
MK97
MK data
Normal data
2
1.8
1.6
1.4
1.2
1
0.8
0.6
0
10
20
30
40
50
60

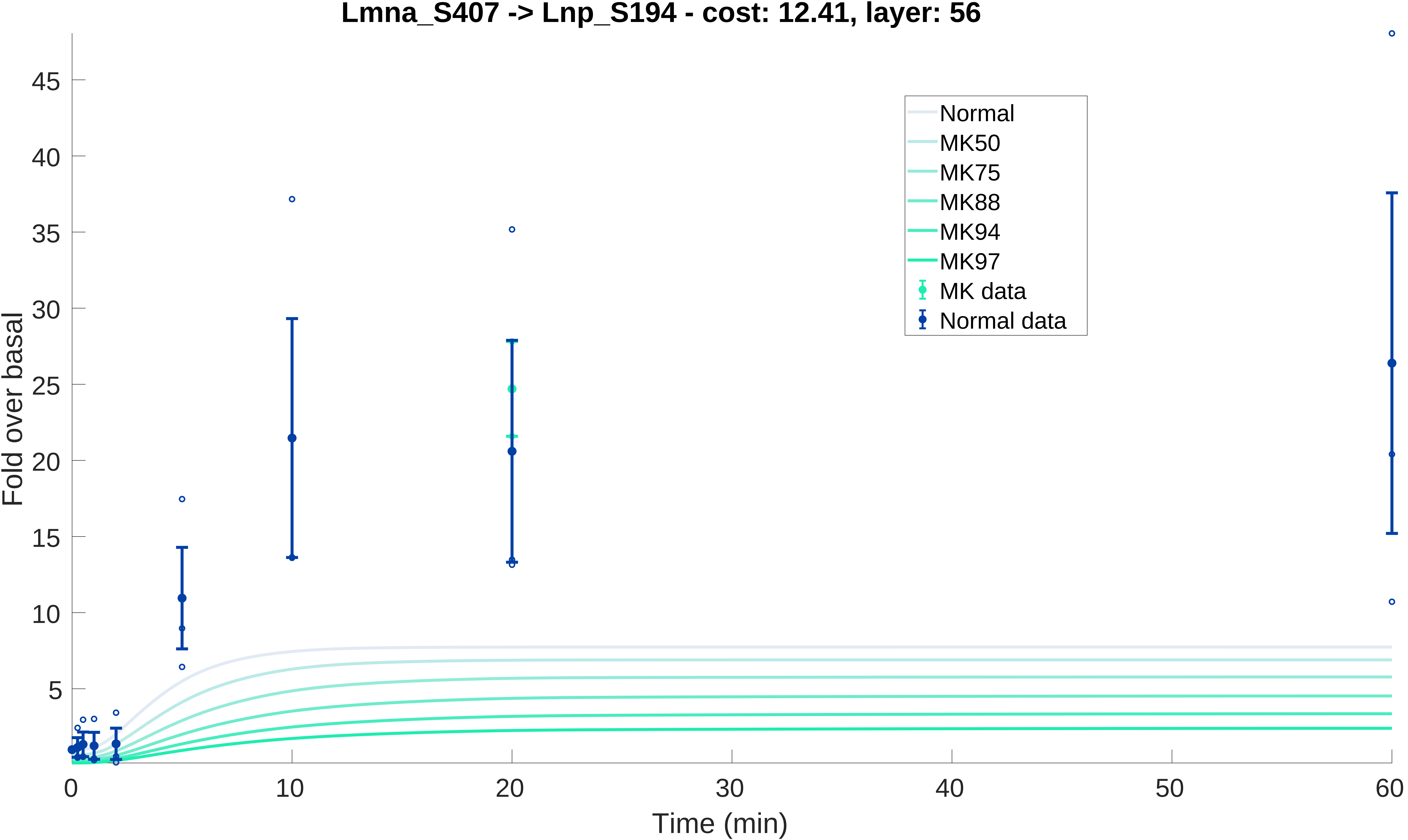

Lmna_S407 -> Lnp_S194 - cost: 12.41, layer: 56
Fold over basal
45
40
35
30
25
20
15
10
5
0
10
20
30
40
50
60
Time (min)
Normal
MK50
MK75
MK88
MK94
MK97
MK data
Normal data

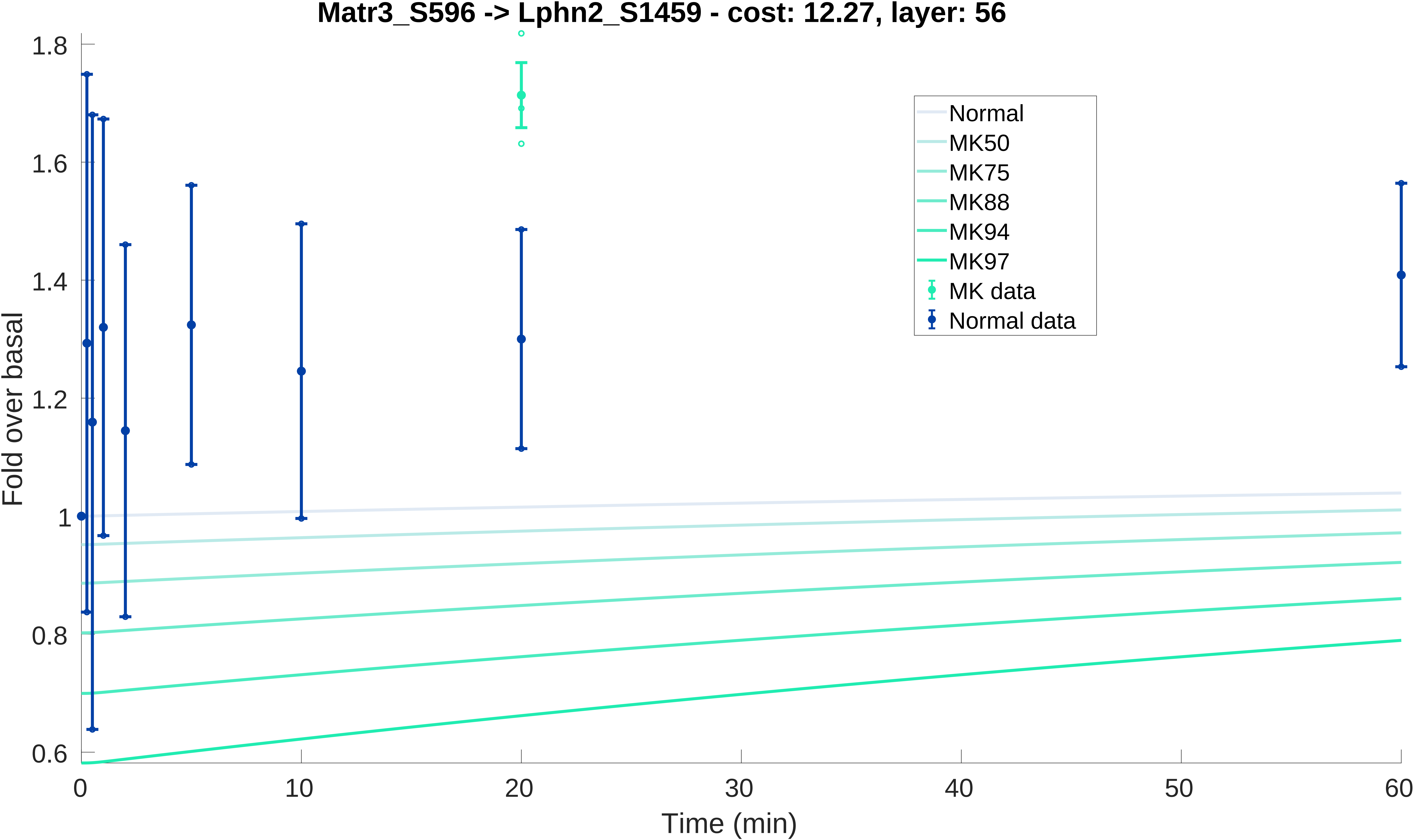

Matr3_S596 -> Lphn2_S1459 - cost: 12.27, layer: 56
Fold over basal
Time (min)
1.8
1.6
1.4
1.2
1
0.8
0.6
0
10
20
30
40
50
60
Normal
MK50
MK75
MK88
MK94
MK97
MK data
Normal data

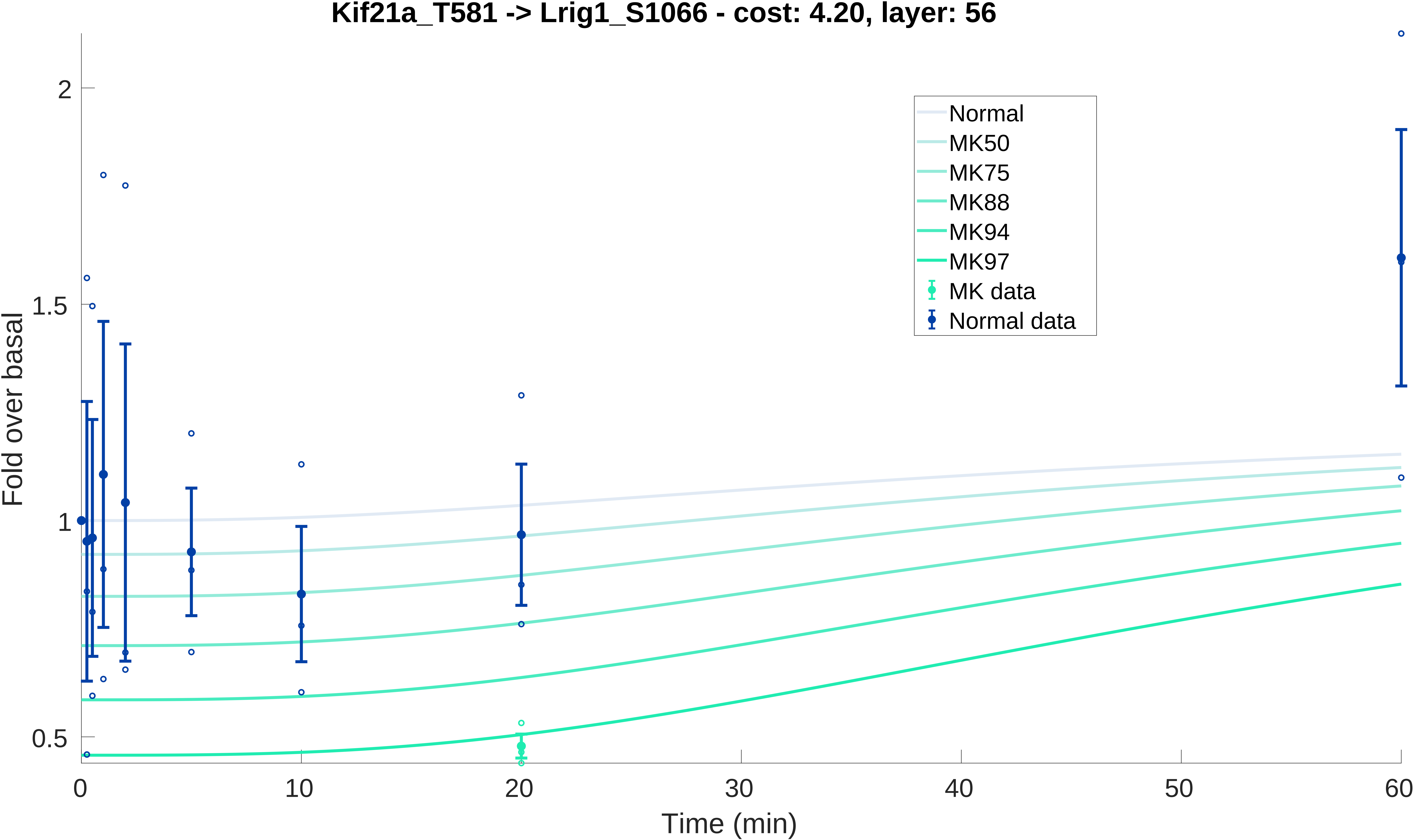
Kif21a_T581 -> Lrig1_S1066 - cost: 4.20, layer: 56
Fold over basal
Time (min)
0.5
1
1.5
2
0
10
20
30
40
50
60
Normal
MK50
MK75
MK88
MK94
MK97
MK data
Normal data

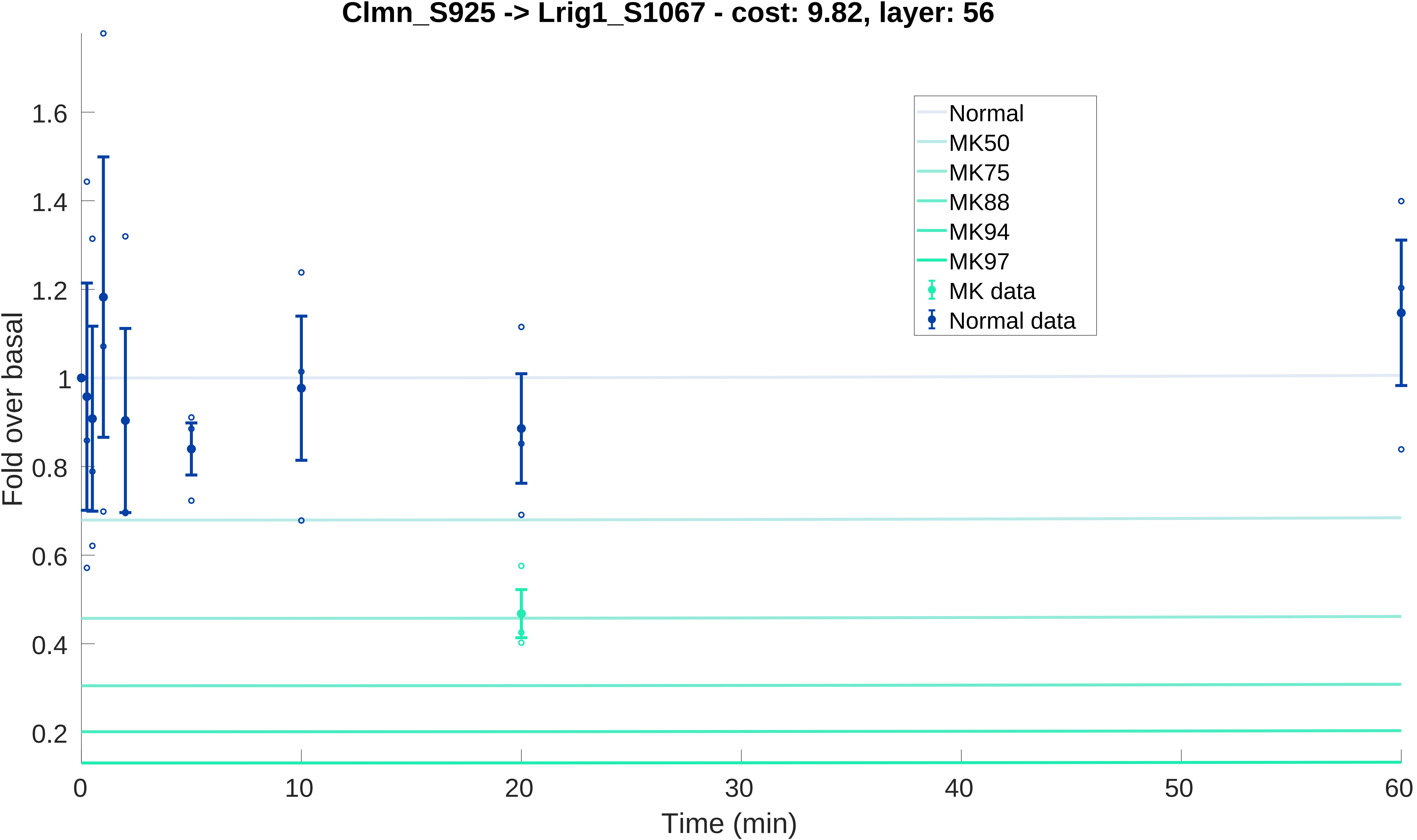

Clmn_S925 -> Lrig1_S1067 - cost: 9.82, layer: 56
Fold over basal
Time (min)
0
10
20
30
40
50
60
0.2
0.4
0.6
0.8
1
1.2
1.4
1.6
Normal
MK50
MK75
MK88
MK94
MK97
MK data
Normal data

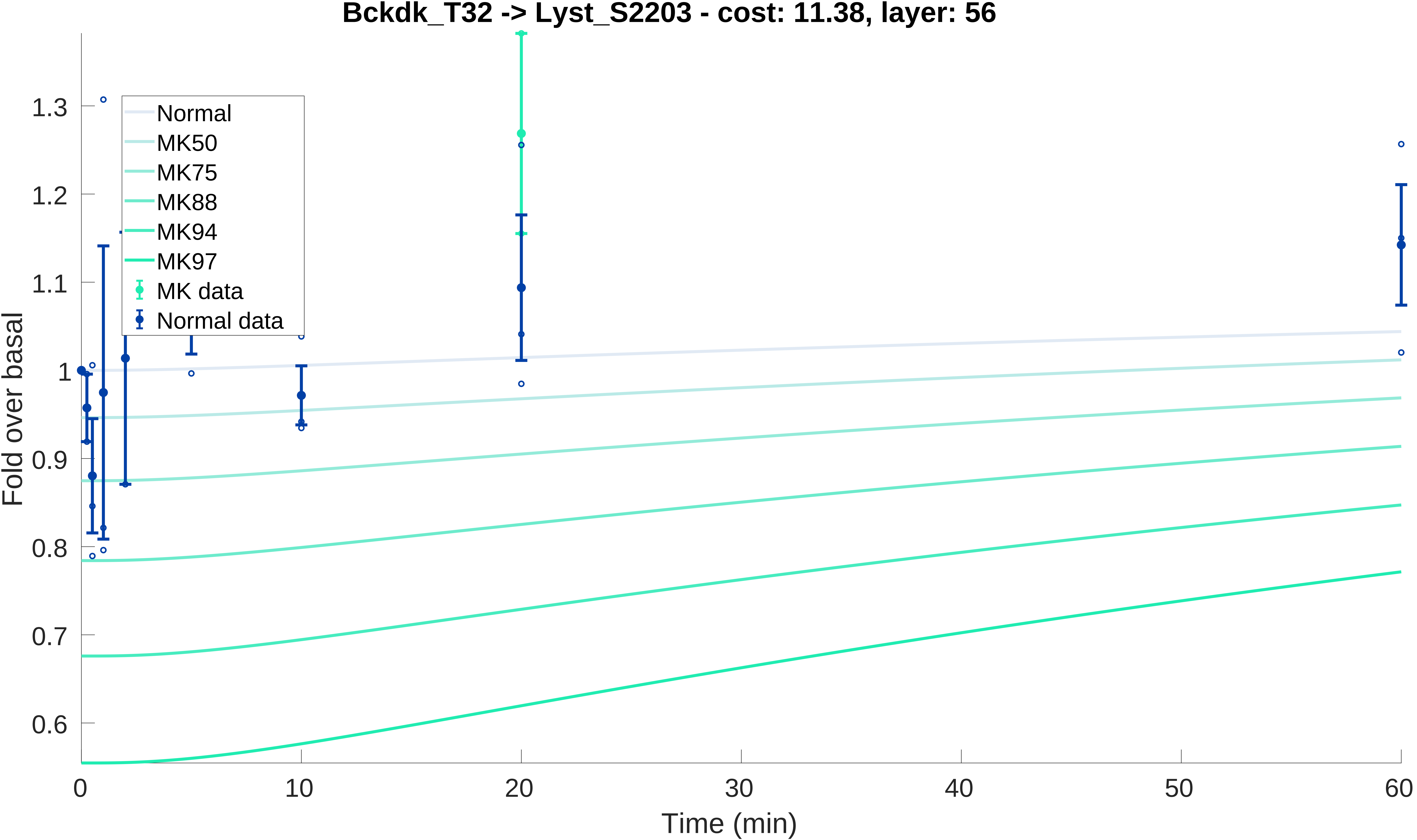

Bckdk_T32 -> Lyst_S2203 - cost: 11.38, layer: 56
Normal
MK50
MK75
MK88
MK94
MK97
MK data
Normal data
Fold over basal
Time (min)
0.6
0.7
0.8
0.9
1
1.1
1.2
1.3
0
10
20
30
40
50
60

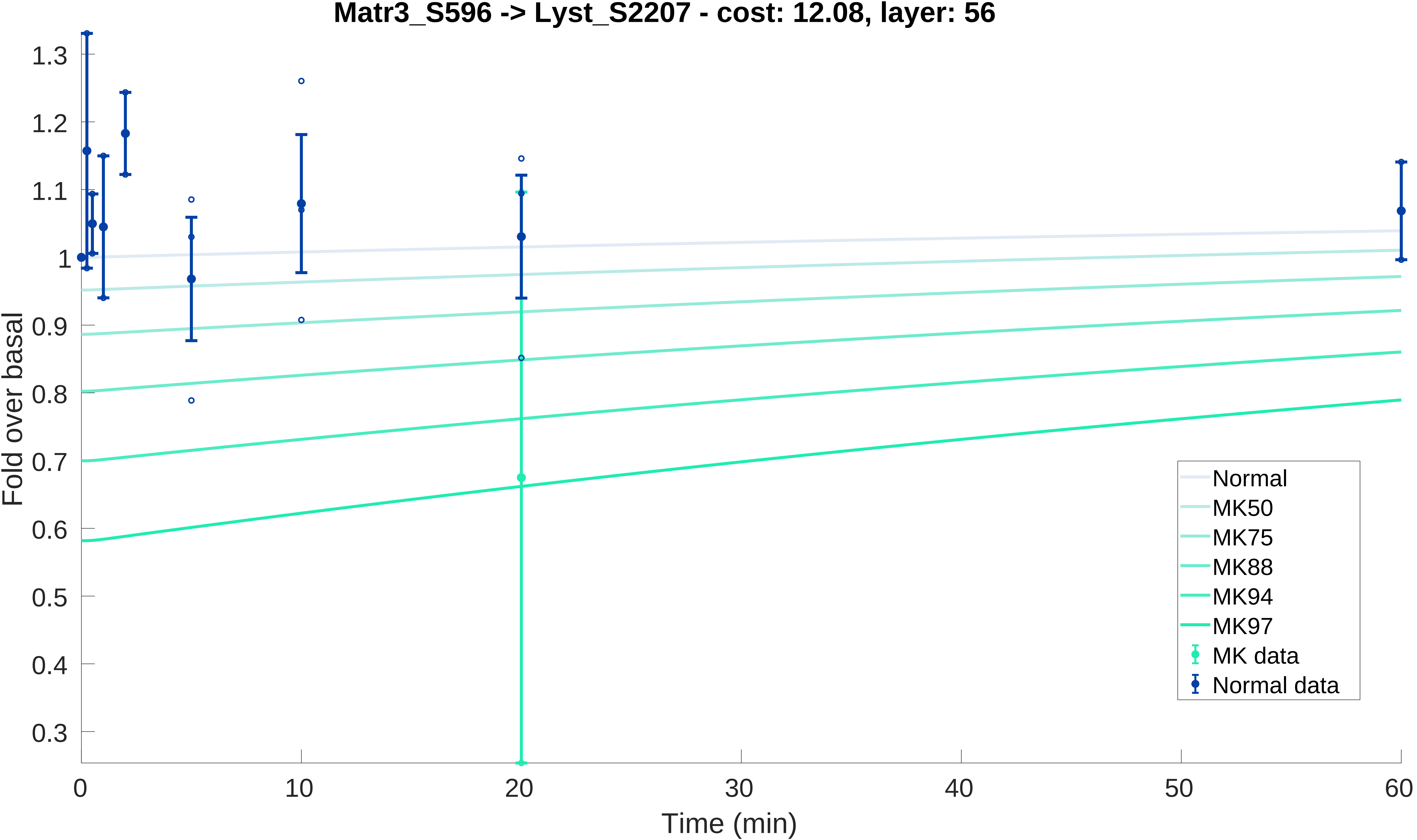

Matr3_S596 -> Lyst_S2207 - cost: 12.08, layer: 56
Fold over basal
Time (min)
0.3
0.4
0.5
0.6
0.7
0.8
0.9
1
1.1
1.2
1.3
0
10
20
30
40
50
60
Normal
MK50
MK75
MK88
MK94
MK97
MK data
Normal data

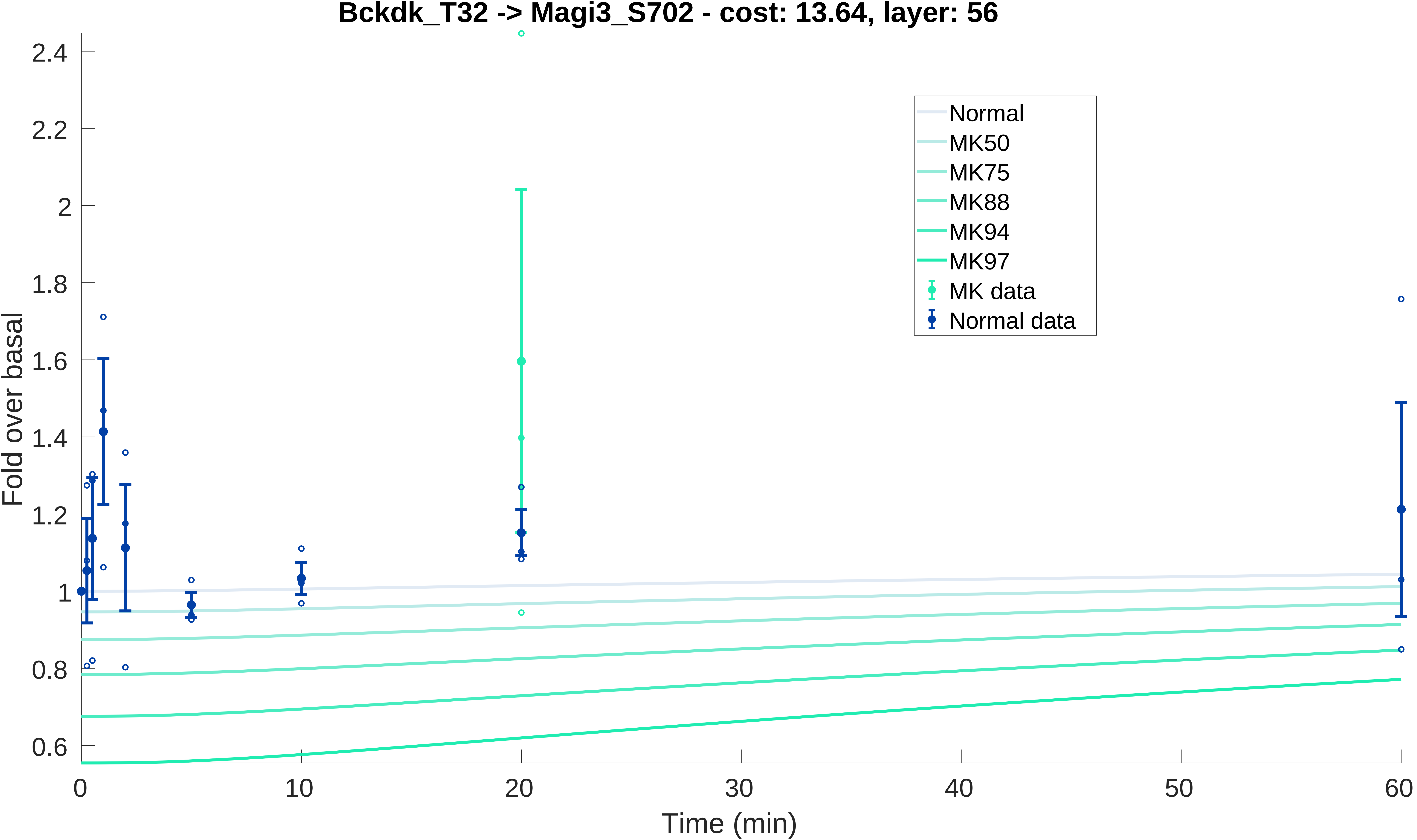
Bckdk_T32 -> Magi3_S702 - cost: 13.64, layer: 56
Fold over basal
Time (min)
Normal
MK50
MK75
MK88
MK94
MK97
MK data
Normal data
0.6
0.8
1
1.2
1.4
1.6
1.8
2
2.2
2.4
0
10
20
30
40
50
60

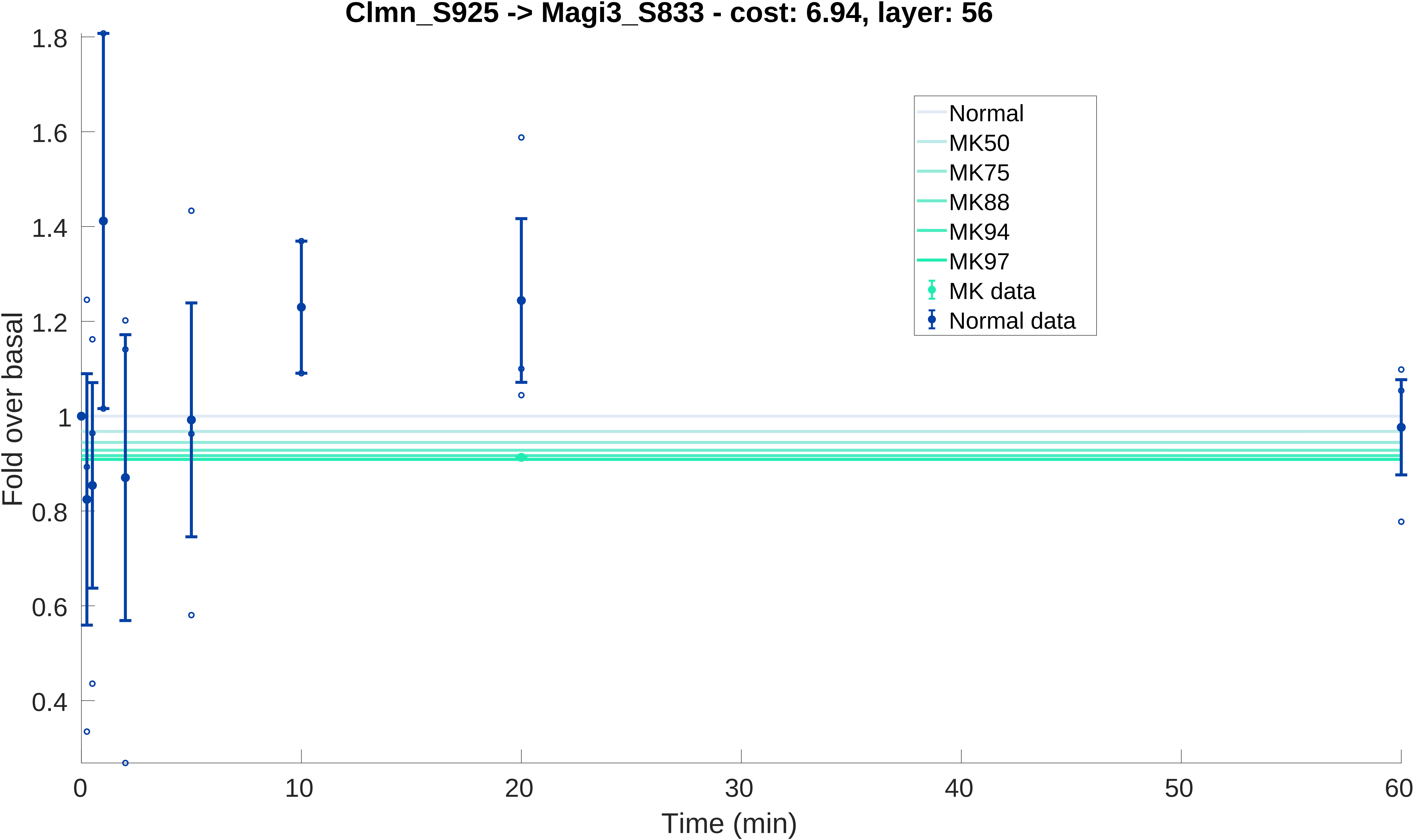
Clmn_S925 -> Magi3_S833 - cost: 6.94, layer: 56
Fold over basal
Time (min)
Normal
MK50
MK75
MK88
MK94
MK97
MK data
Normal data
1.8
1.6
1.4
1.2
1
0.8
0.6
0.4
0
10
20
30
40
50
60

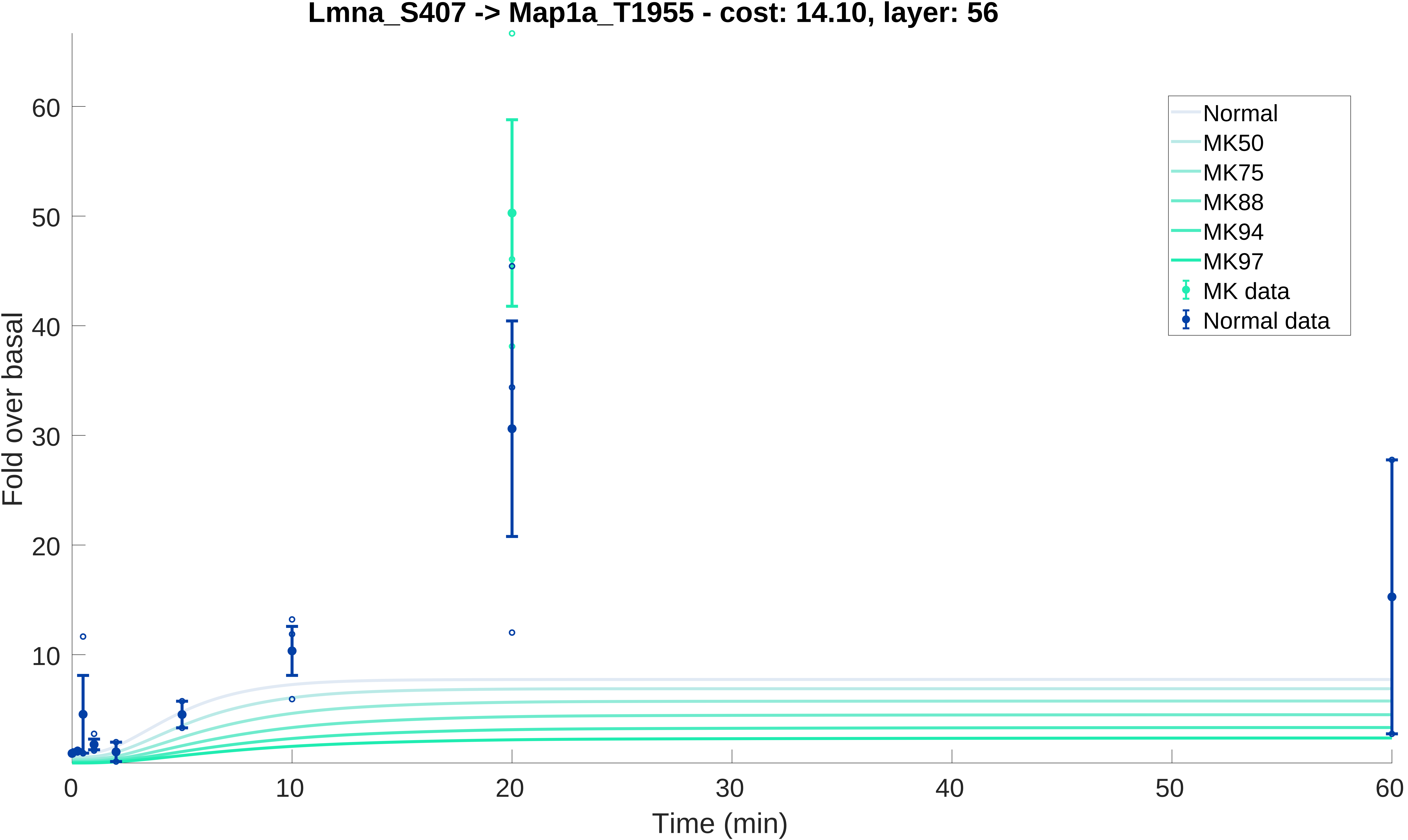
Lmna_S407 -> Map1a_T1955 - cost: 14.10, layer: 56
Fold over basal
Time (min)
0
10
20
30
40
50
60
Normal
MK50
MK75
MK88
MK94
MK97
MK data
Normal data

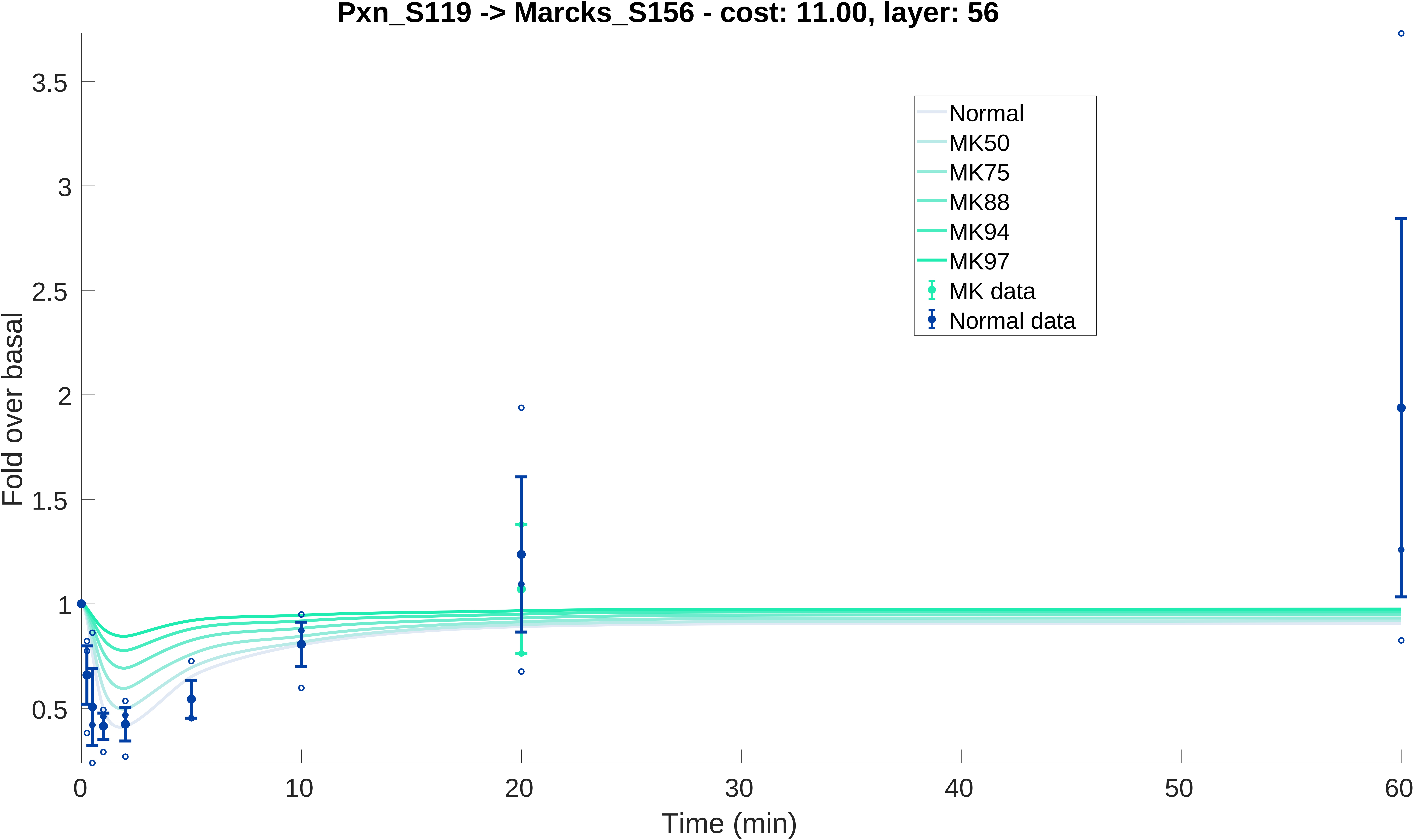

Pxn_S119 -> Marcks_S156 - cost: 11.00, layer: 56
Normal
MK50
MK75
MK88
MK94
MK97
MK data
Normal data
Fold over basal
Time (min)
0
10
20
30
40
50
60
0.5
1
1.5
2
2.5
3
3.5

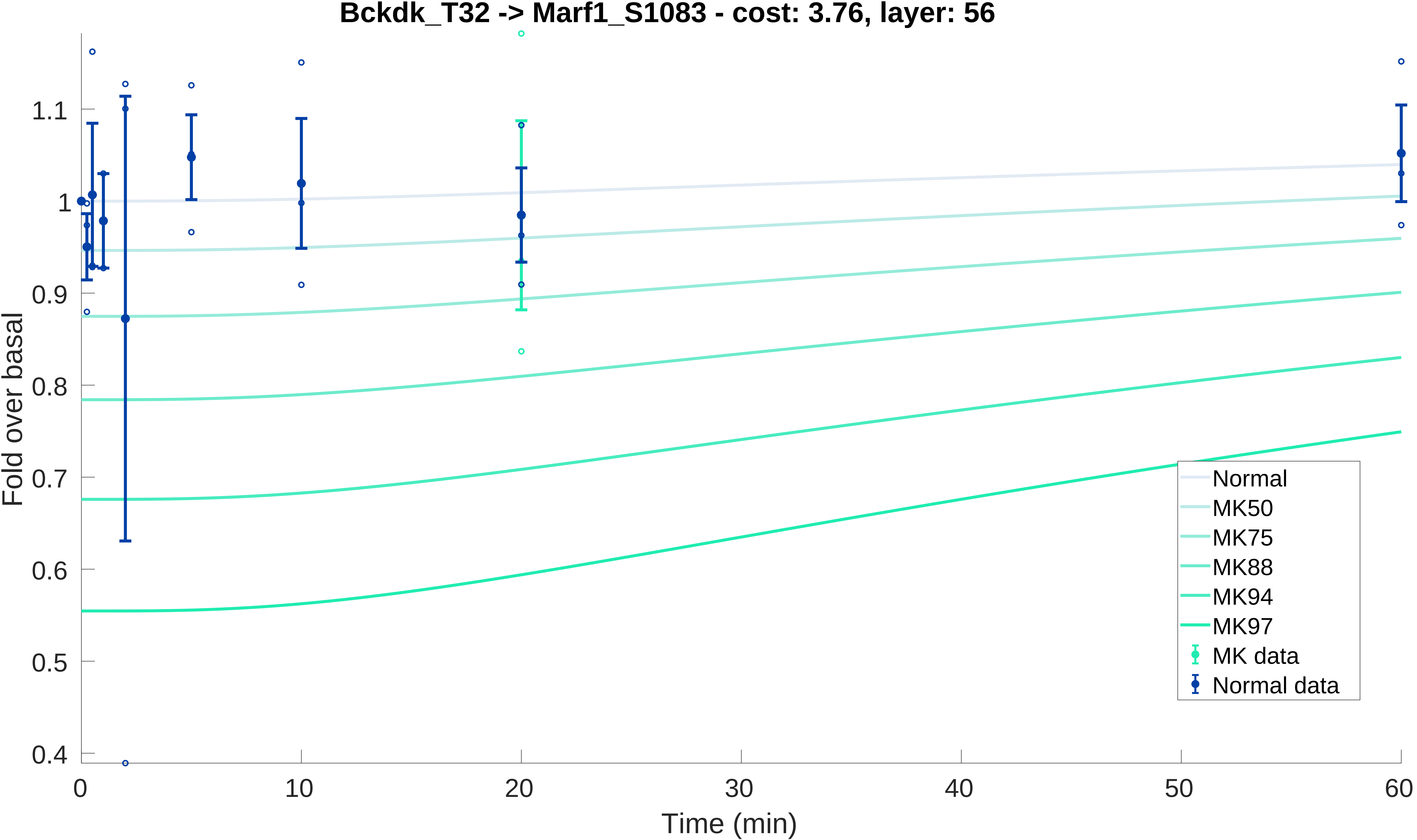

Bckdk_T32 -> Marf1_S1083 - cost: 3.76, layer: 56
Fold over basal
Time (min)
0.4
0.5
0.6
0.7
0.8
0.9
1
1.1
0
10
20
30
40
50
60
Normal
MK50
MK75
MK88
MK94
MK97
MK data
Normal data

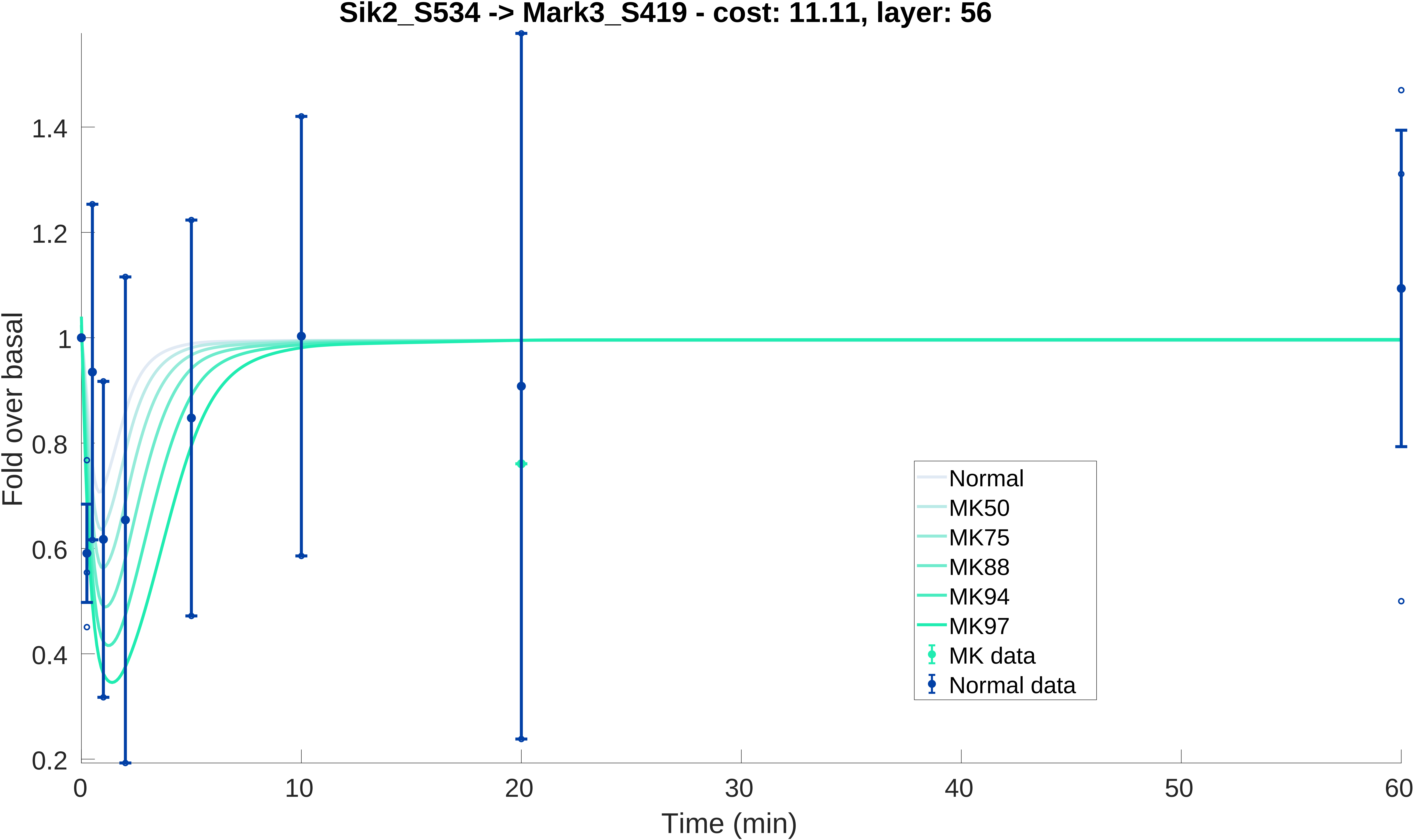

Sik2_S534 -> Mark3_S419 - cost: 11.11, layer: 56
Fold over basal
Time (min)
0.2
0.4
0.6
0.8
1
1.2
1.4
0
10
20
30
40
50
60
Normal
MK50
MK75
MK88
MK94
MK97
MK data
Normal data

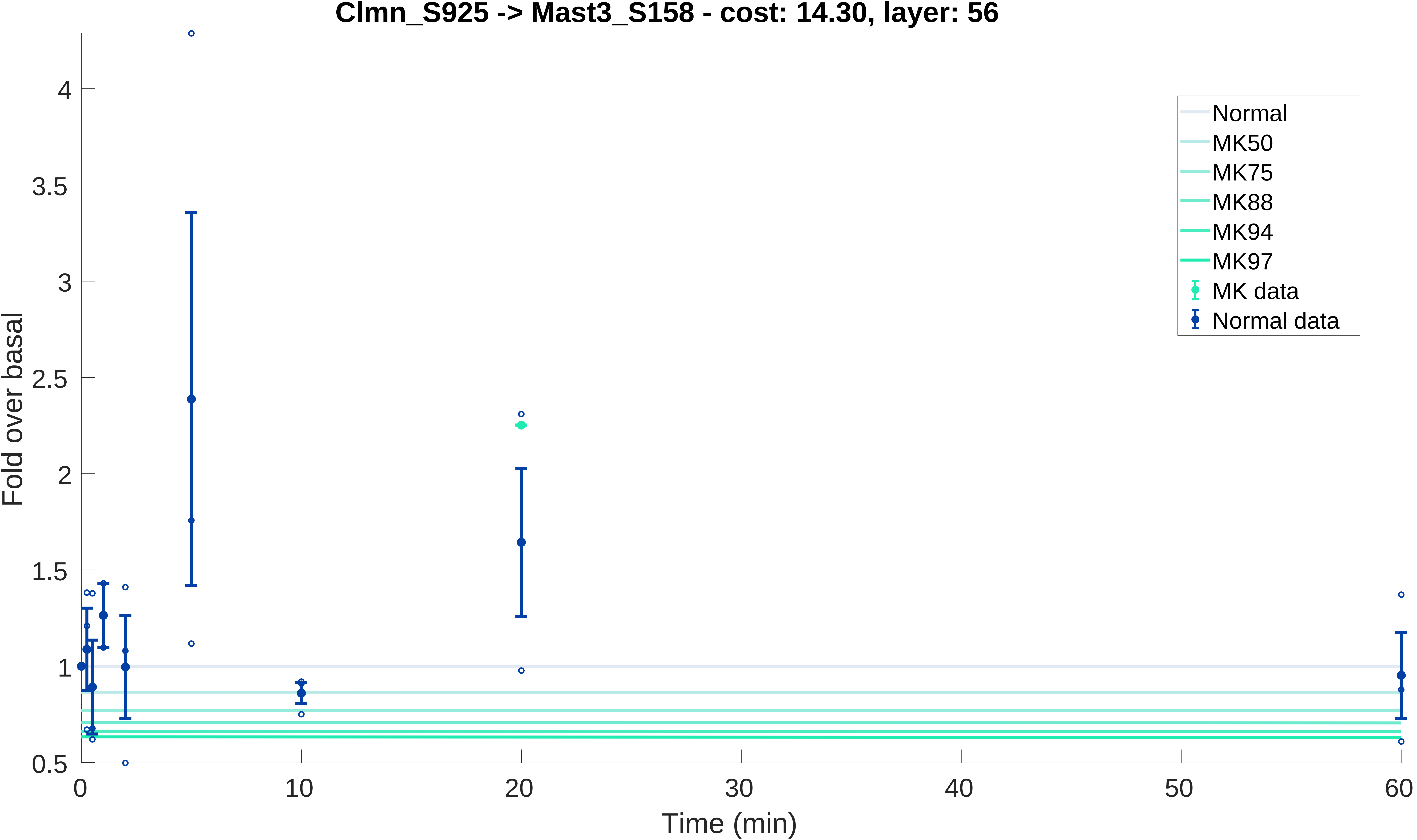
Clmn_S925 -> Mast3_S158 - cost: 14.30, layer: 56
Fold over basal
Time (min)
0
10
20
30
40
50
60
0.5
1
1.5
2
2.5
3
3.5
4
Normal
MK50
MK75
MK88
MK94
MK97
MK data
Normal data

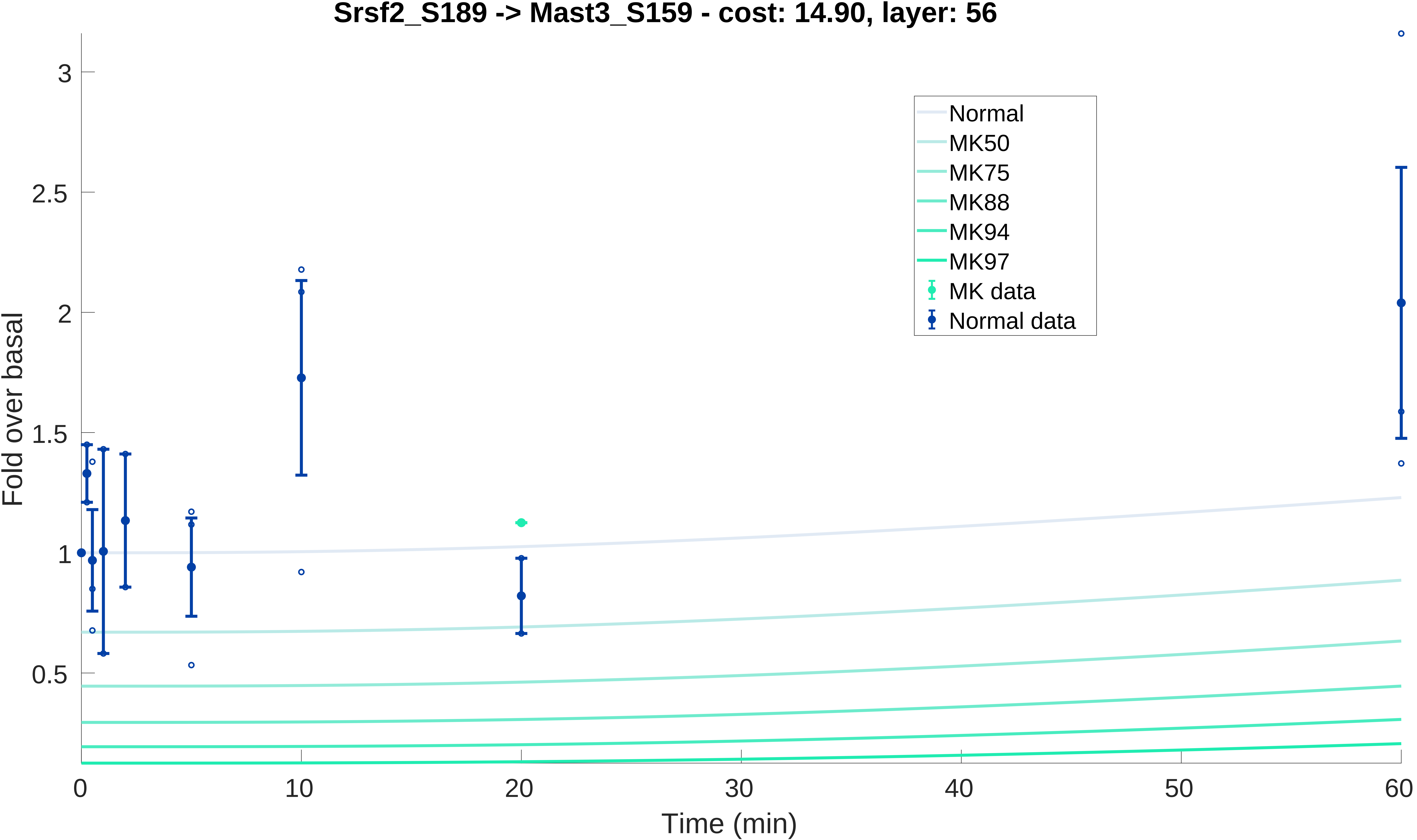
Srsf2_S189 -> Mast3_S159 - cost: 14.90, layer: 56
Fold over basal
Time (min)
0
10
20
30
40
50
60
0.5
1
1.5
2
2.5
3
Normal
MK50
MK75
MK88
MK94
MK97
MK data
Normal data

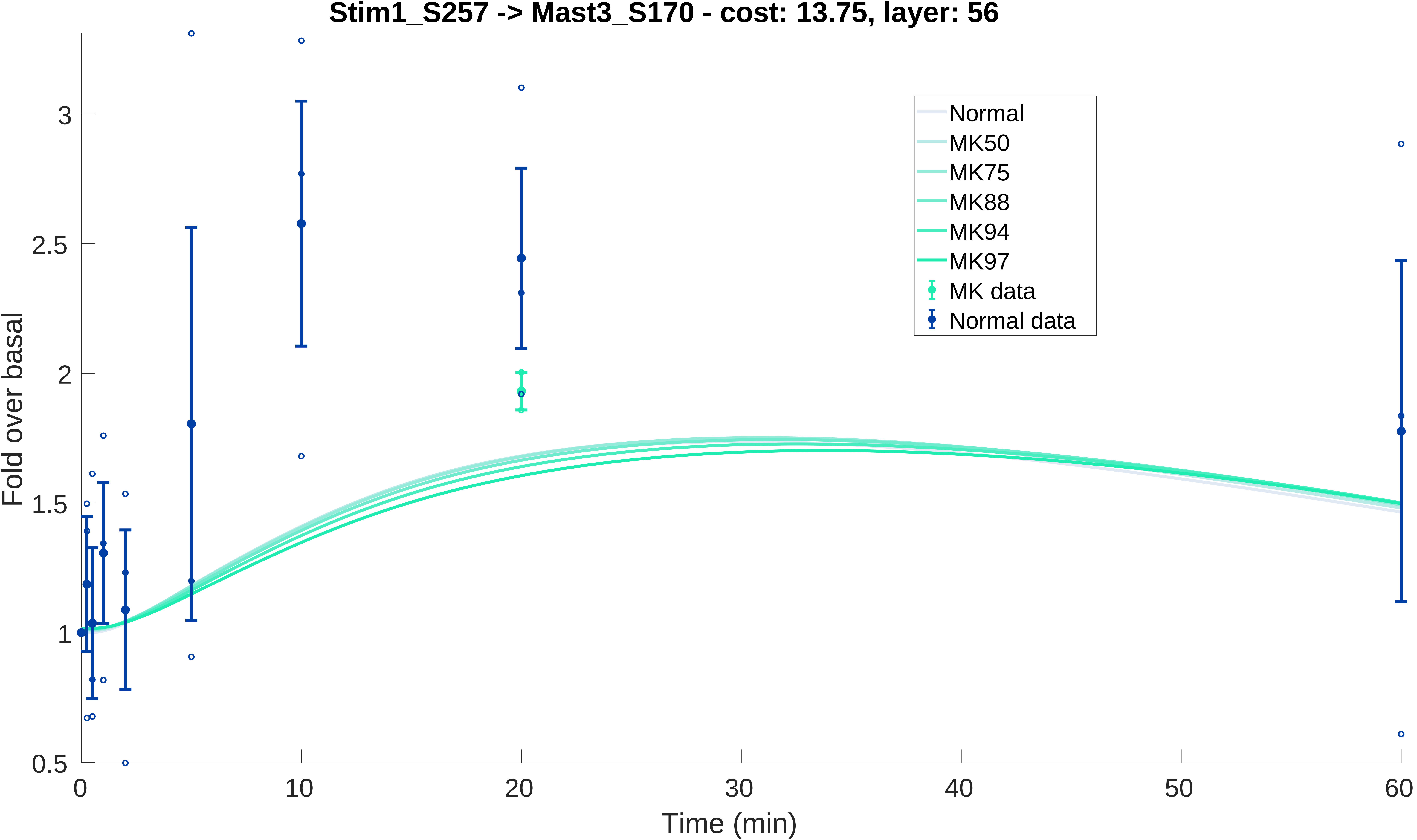

Stim1_S257 -> Mast3_S170 - cost: 13.75, layer: 56
Fold over basal
Time (min)
0.5
1
1.5
2
2.5
3
0
10
20
30
40
50
60
Normal
MK50
MK75
MK88
MK94
MK97
MK data
Normal data

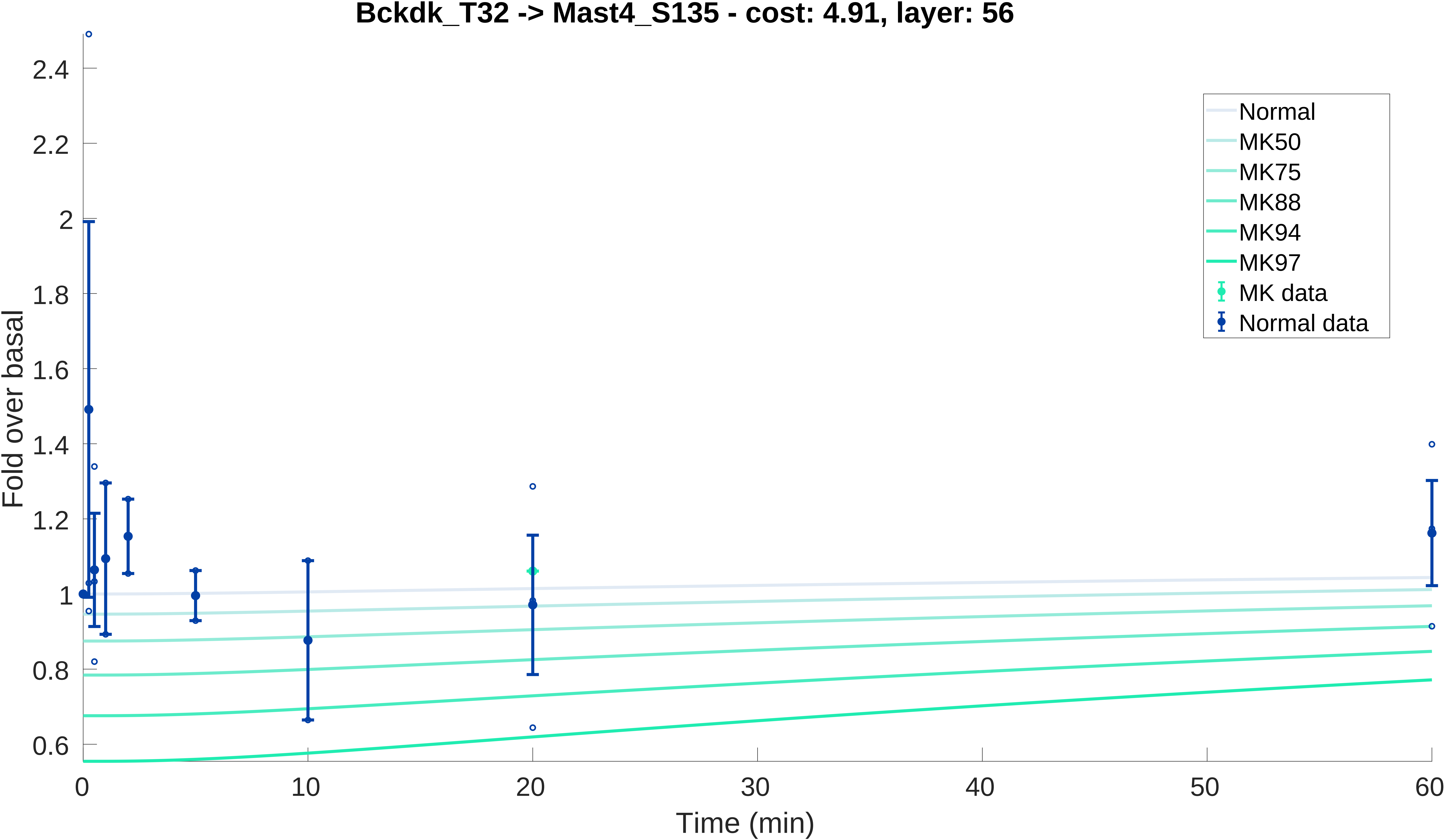

Bckdk_T32 -> Mast4_S135 - cost: 4.91, layer: 56
Fold over basal
Time (min)
0.6
0.8
1
1.2
1.4
1.6
1.8
2
2.2
2.4
0
10
20
30
40
50
60
Normal
MK50
MK75
MK88
MK94
MK97
MK data
Normal data

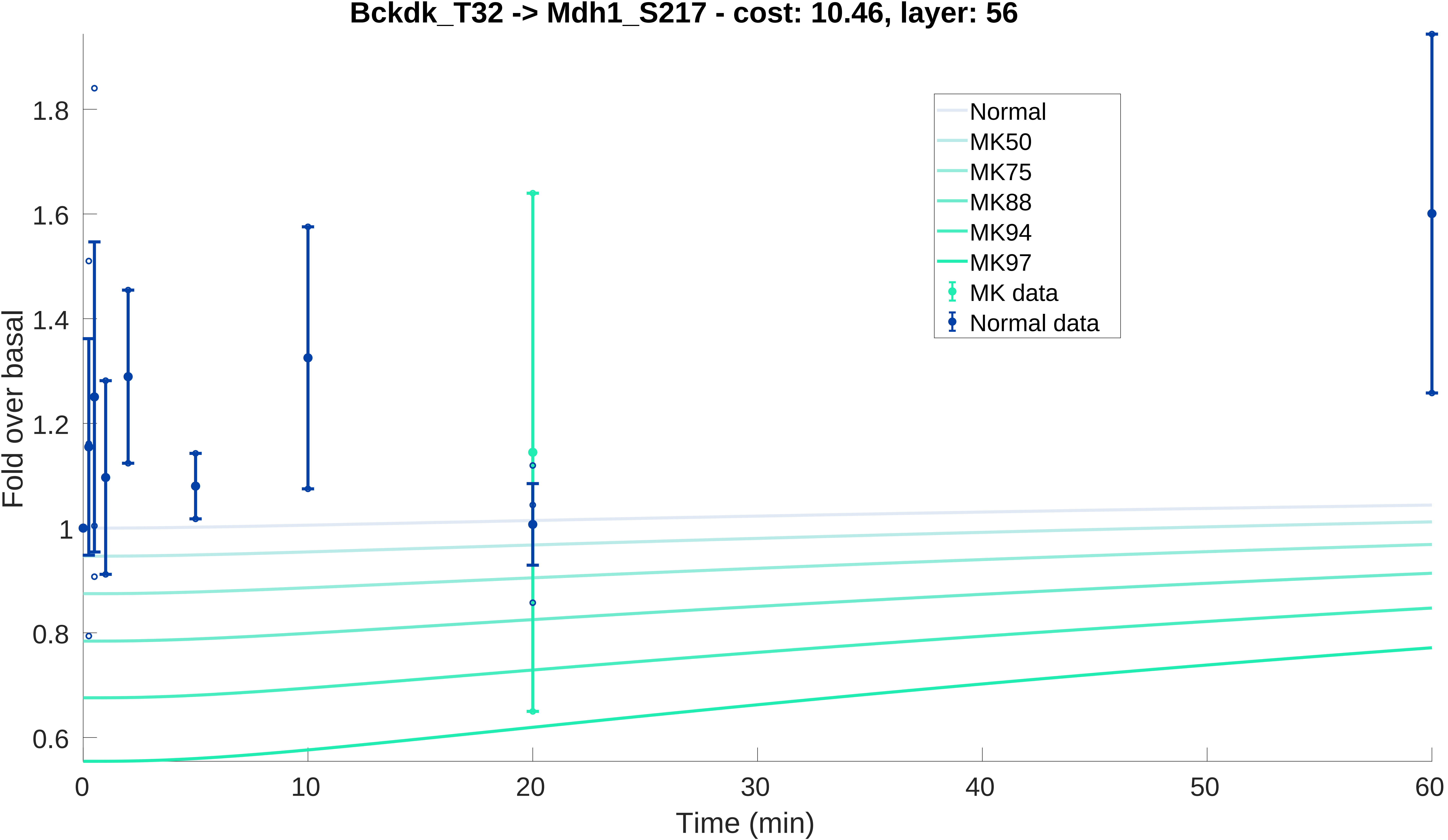

Bckdk_T32 -> Mdh1_S217 - cost: 10.46, layer: 56
Fold over basal
Time (min)
0.6
0.8
1
1.2
1.4
1.6
1.8
0
10
20
30
40
50
60
Normal
MK50
MK75
MK88
MK94
MK97
MK data
Normal data

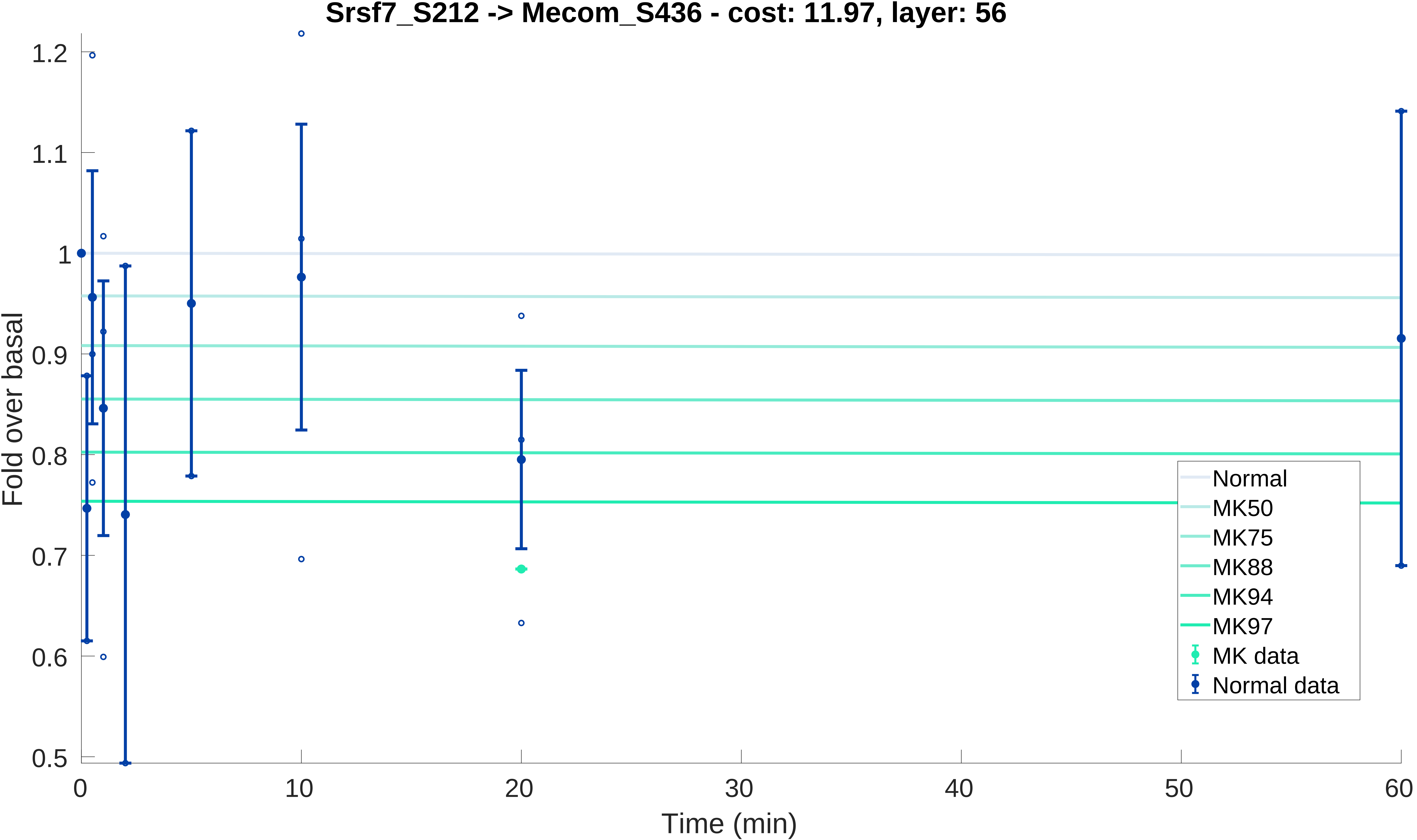

Srsf7_S212 -> Mecom_S436 - cost: 11.97, layer: 56
Fold over basal
Time (min)
1.2
1.1
1
0.9
0.8
0.7
0.6
0.5
0
10
20
30
40
50
60
Normal
MK50
MK75
MK88
MK94
MK97
MK data
Normal data

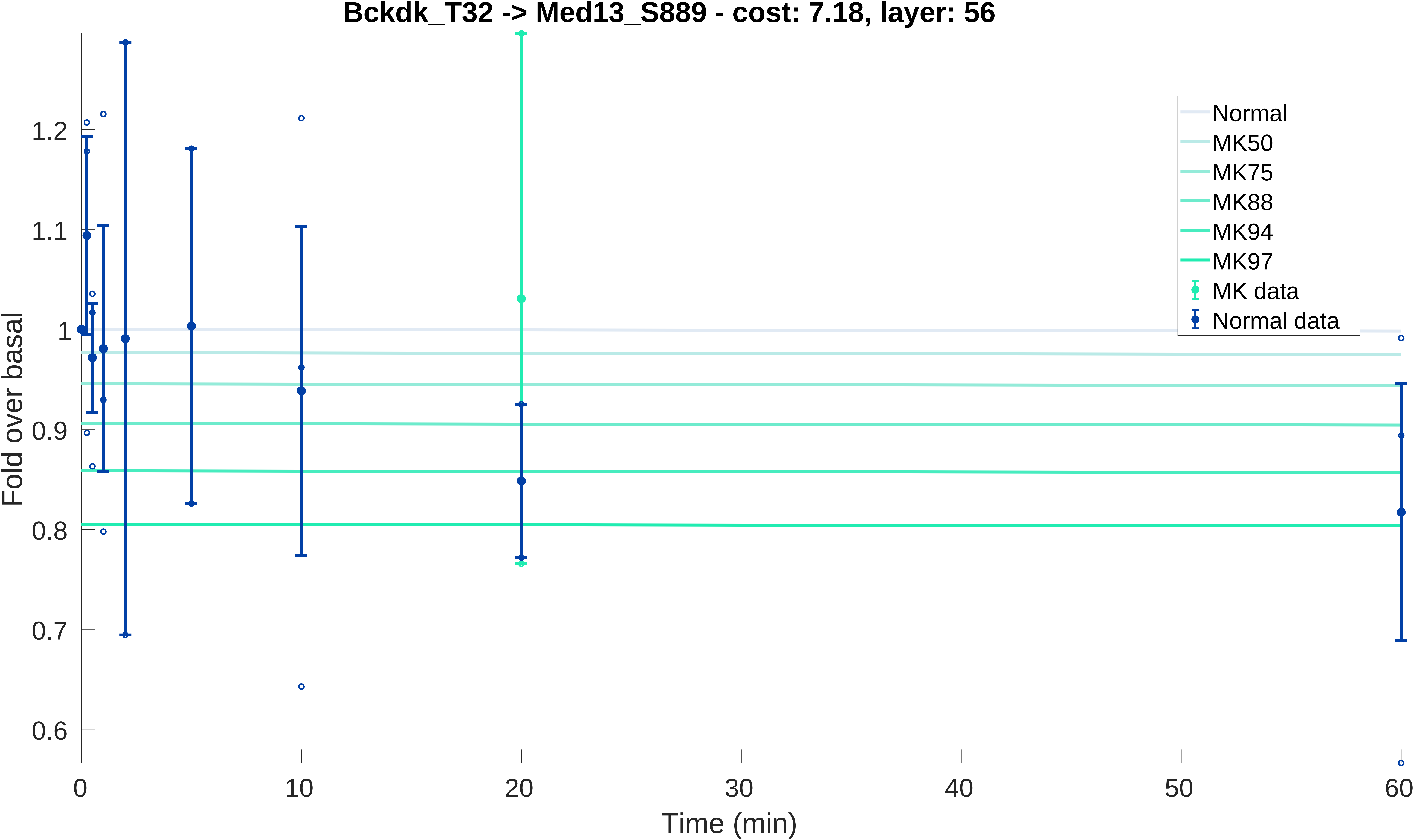
Bckdk_T32 -> Med13_S889 - cost: 7.18, layer: 56
Fold over basal
Time (min)
0.6
0.7
0.8
0.9
1
1.1
1.2
0
10
20
30
40
50
60
Normal
MK50
MK75
MK88
MK94
MK97
MK data
Normal data

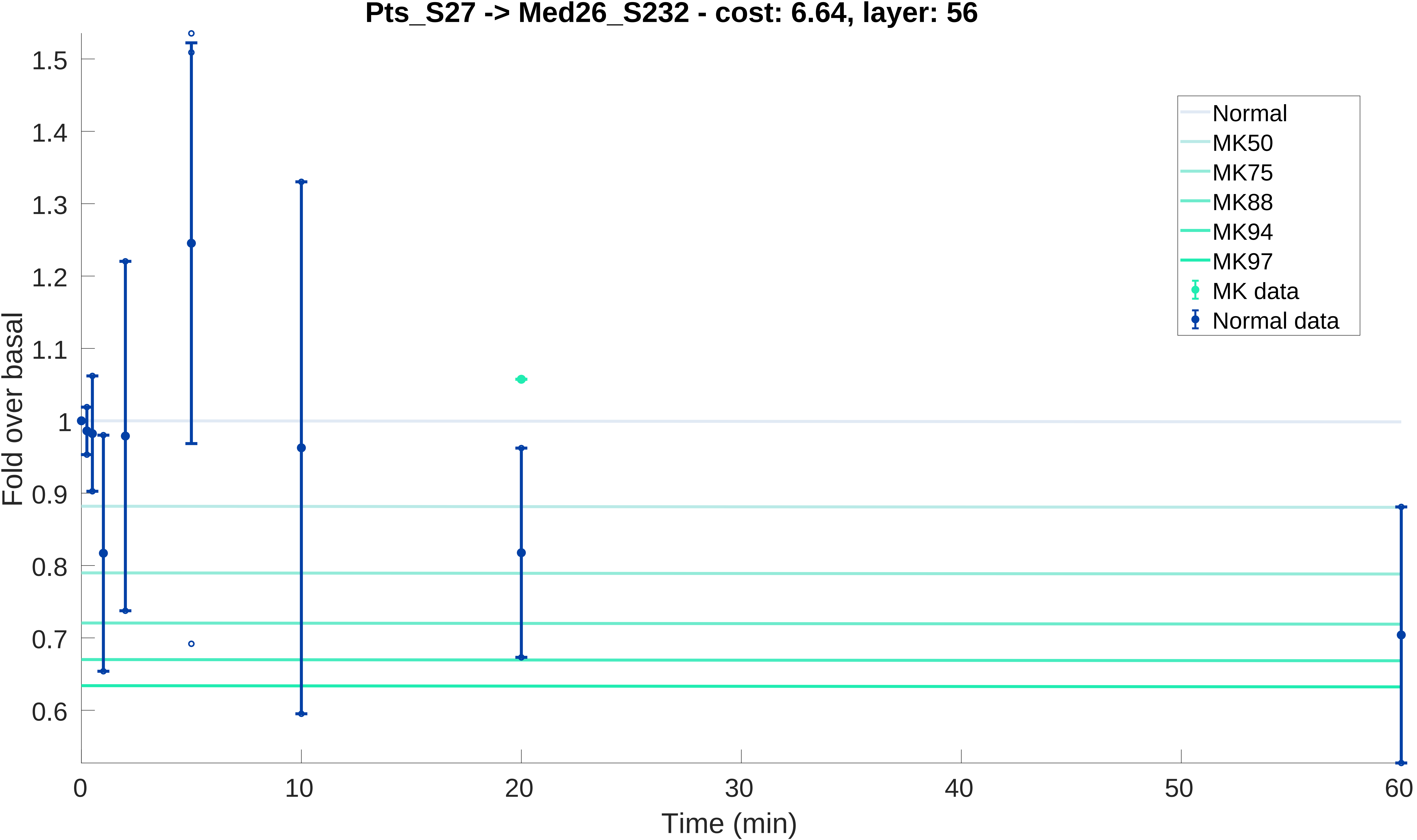

Pts_S27 -> Med26_S232 - cost: 6.64, layer: 56
Fold over basal
Time (min)
0.6
0.7
0.8
0.9
1
1.1
1.2
1.3
1.4
1.5
0
10
20
30
40
50
60
Normal
MK50
MK75
MK88
MK94
MK97
MK data
Normal data

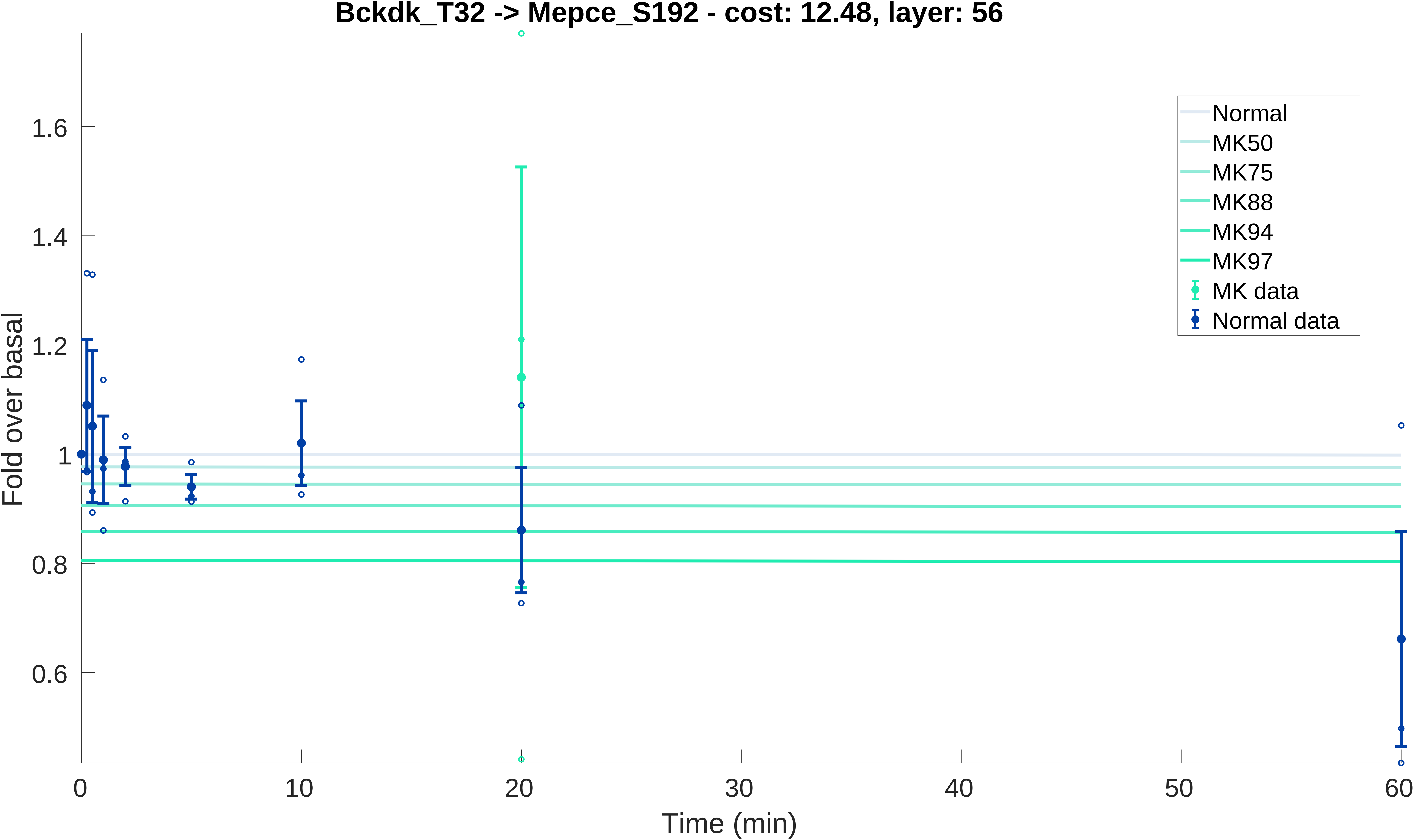

Bckdk_T32 -> Mepce_S192 - cost: 12.48, layer: 56
Fold over basal
1.6
1.4
1.2
1
0.8
0.6
0
10
20
30
40
50
60
Time (min)
Normal
MK50
MK75
MK88
MK94
MK97
MK data
Normal data

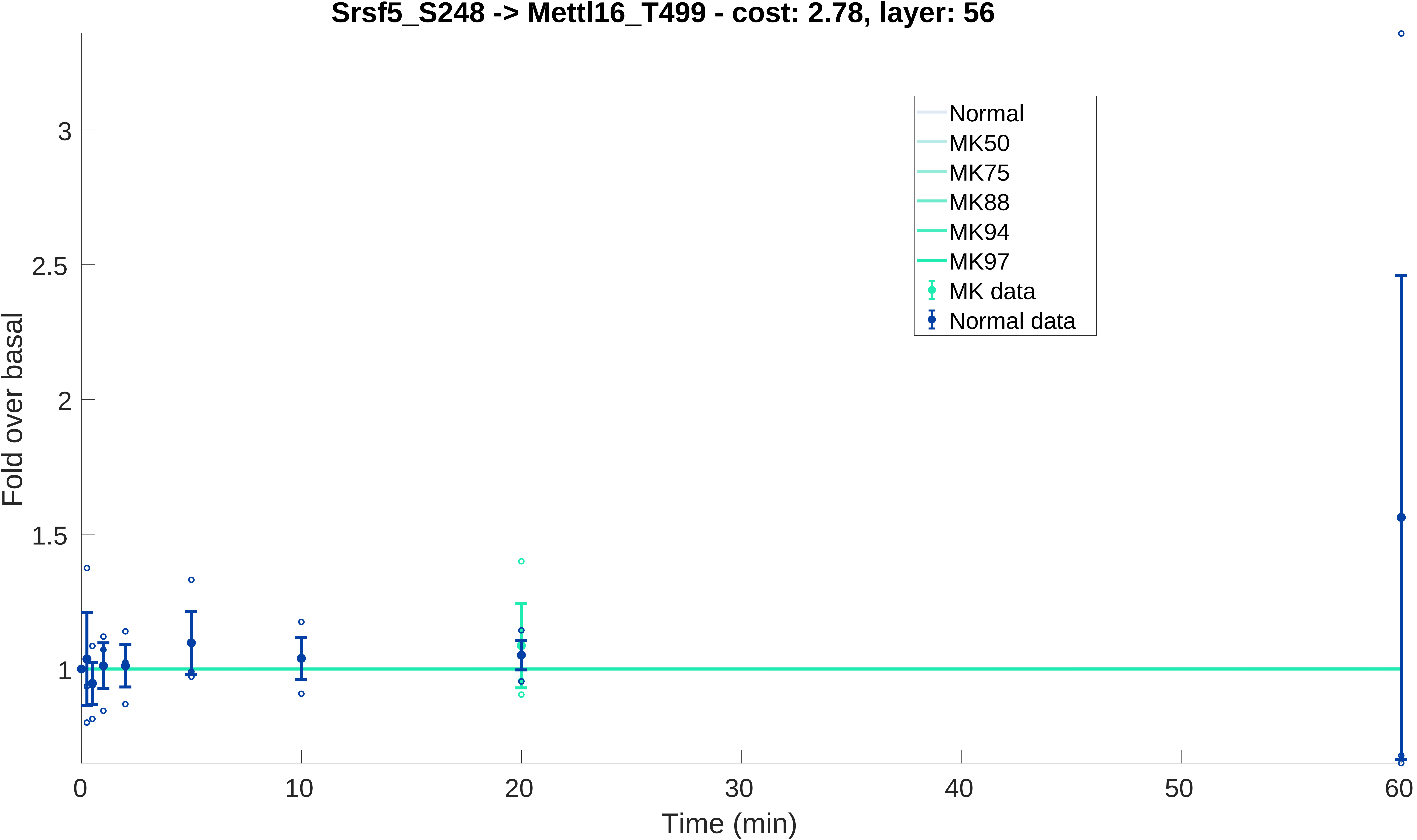

Srsf5_S248 -> Mettl16_T499 - cost: 2.78, layer: 56
Fold over basal
Time (min)
0
10
20
30
40
50
60
1
1.5
2
2.5
3
Normal
MK50
MK75
MK88
MK94
MK97
MK data
Normal data

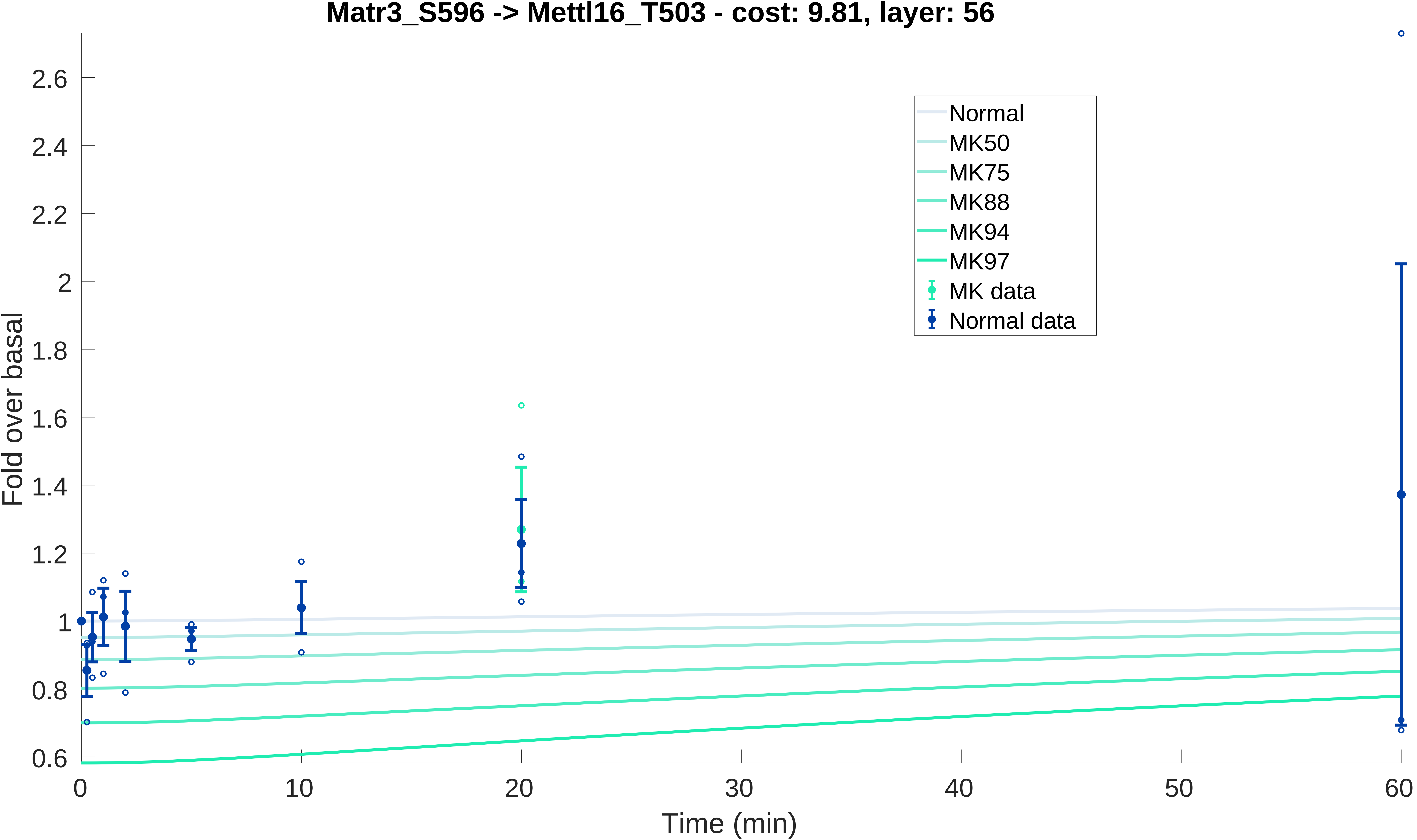
Matr3_S596 -> Mettl16_T503 - cost: 9.81, layer: 56
Normal
MK50
MK75
MK88
MK94
MK97
MK data
Normal data
Fold over basal
Time (min)
0.6
0.8
1
1.2
1.4
1.6
1.8
2
2.2
2.4
2.6
0
10
20
30
40
50
60

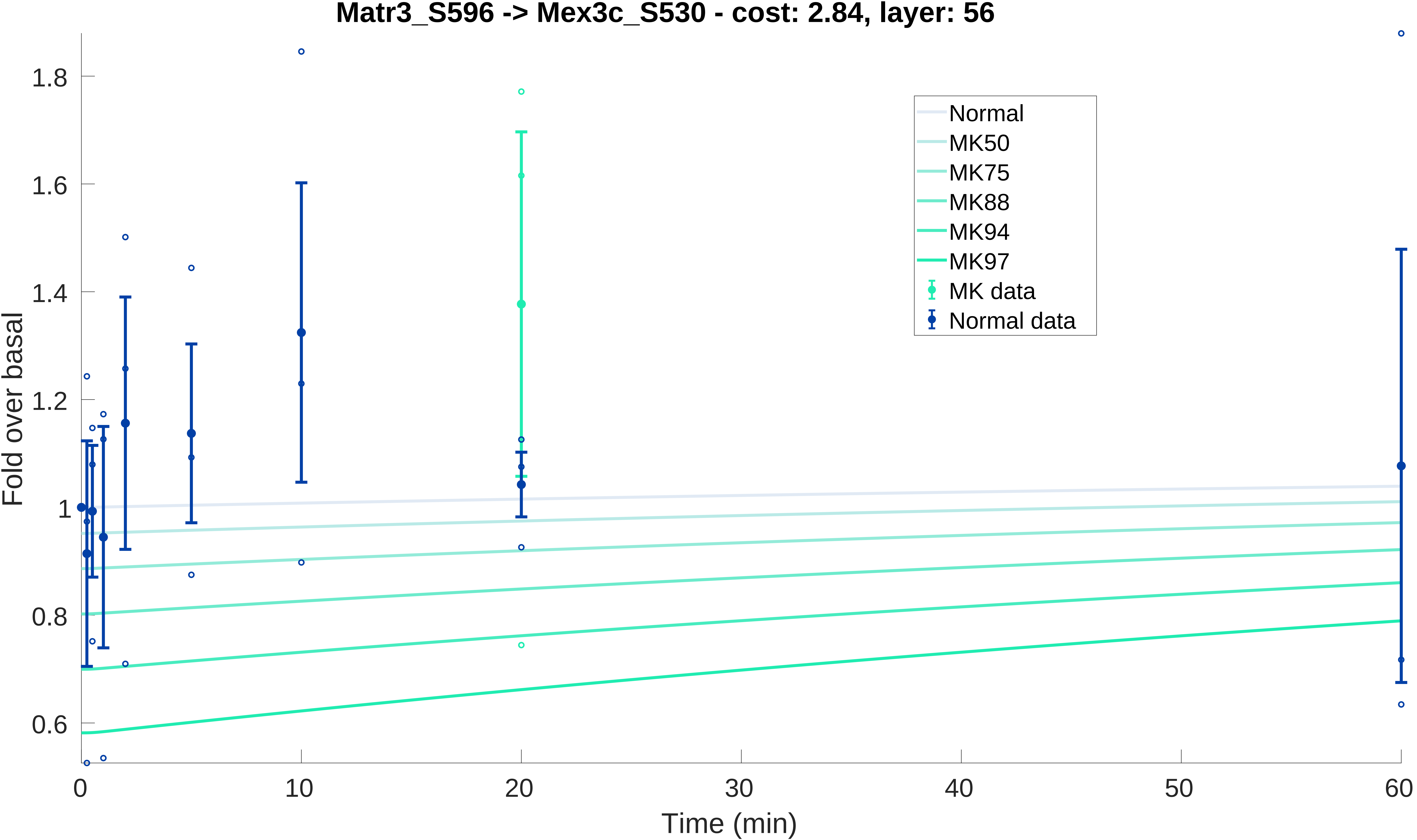

Matr3_S596 -> Mex3c_S530 - cost: 2.84, layer: 56
Fold over basal
Time (min)
0.6
0.8
1
1.2
1.4
1.6
1.8
0
10
20
30
40
50
60
Normal
MK50
MK75
MK88
MK94
MK97
MK data
Normal data

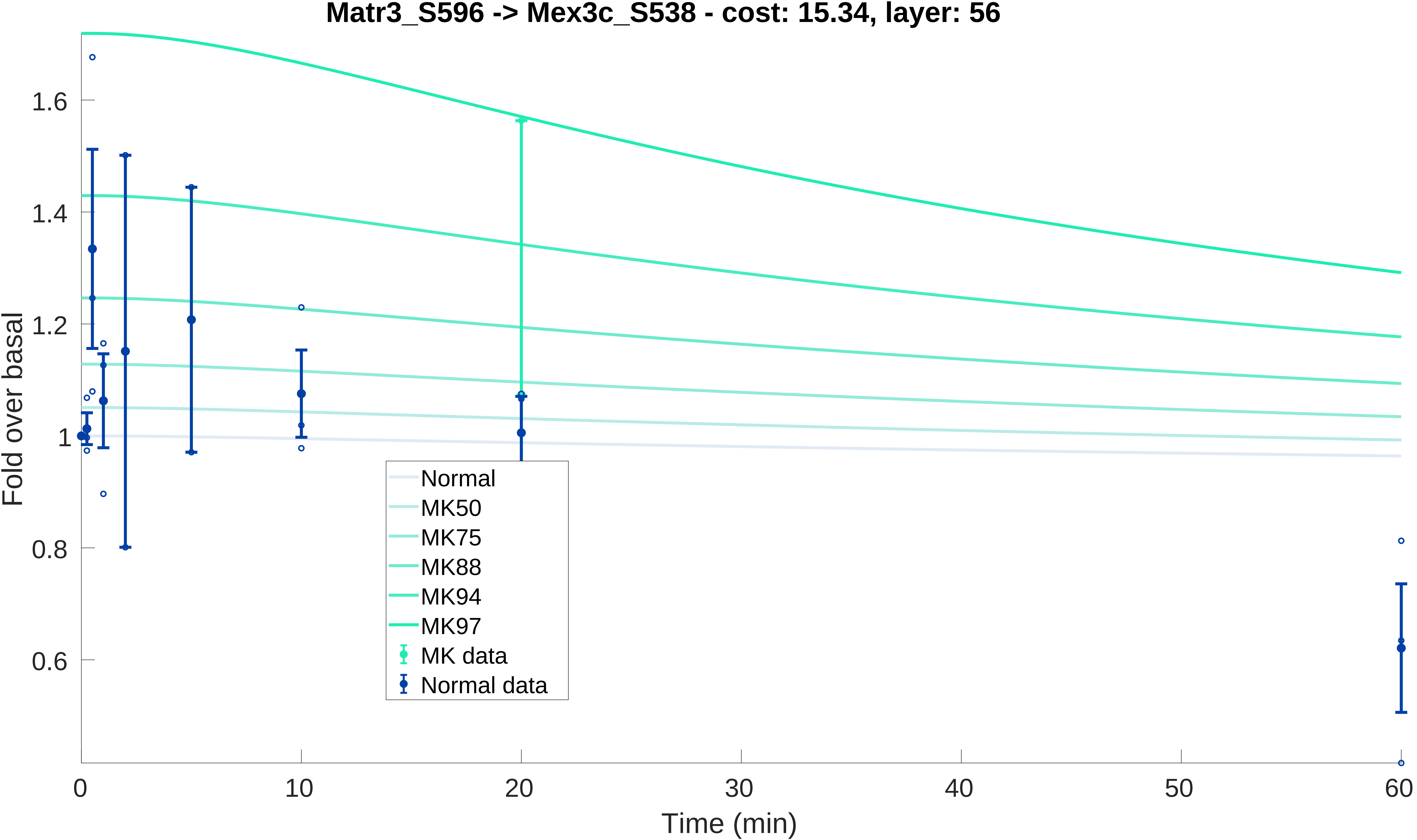

Matr3_S596 -> Mex3c_S538 - cost: 15.34, layer: 56
Fold over basal
Time (min)
0.6
0.8
1
1.2
1.4
1.6
0
10
20
30
40
50
60
Normal
MK50
MK75
MK88
MK94
MK97
MK data
Normal data

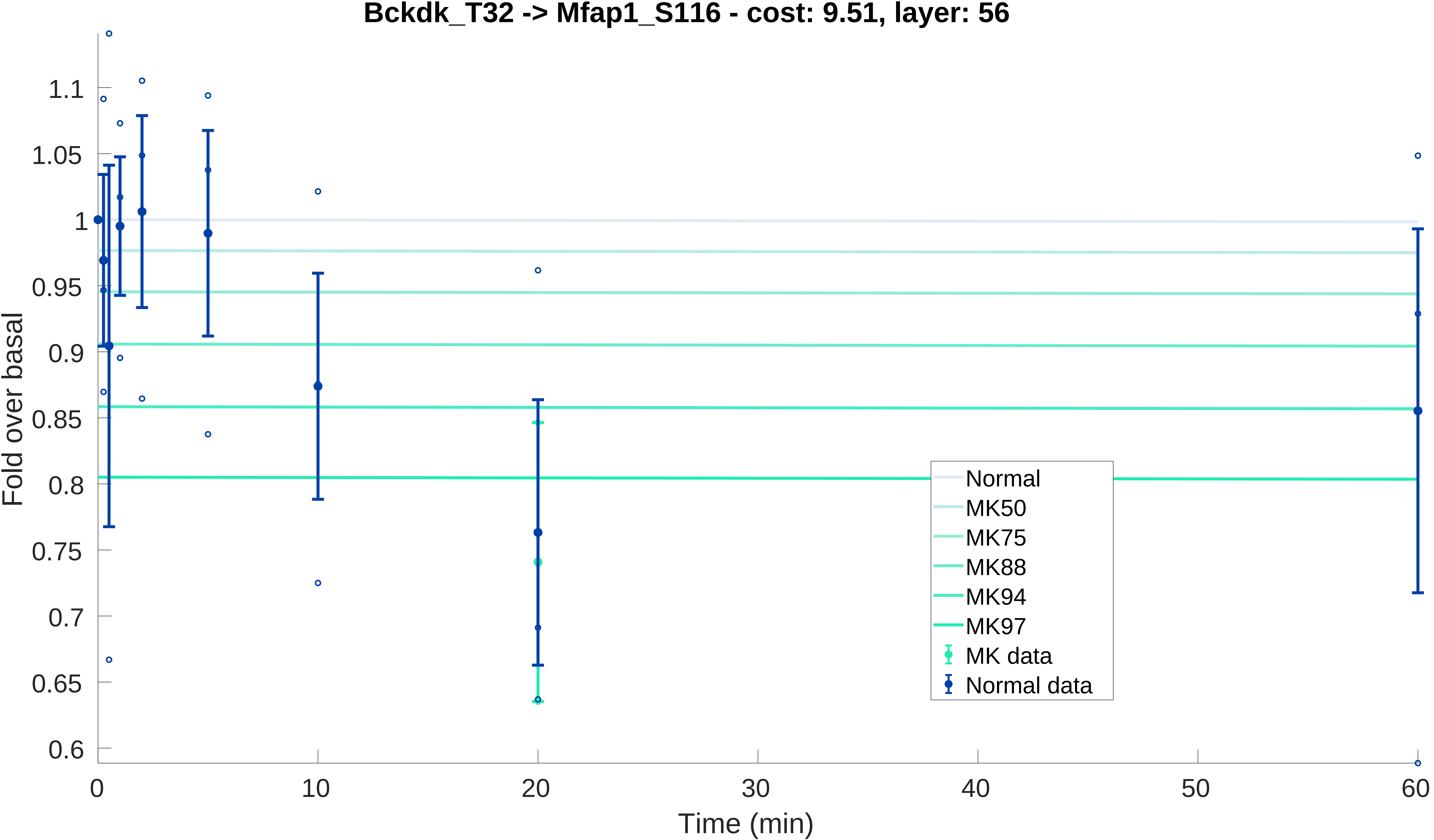
Bckdk_T32 -> Mfap1_S116 - cost: 9.51, layer: 56
Fold over basal
Time (min)
0
10
20
30
40
50
60
0.6
0.65
0.7
0.75
0.8
0.85
0.9
0.95
1
1.05
1.1
Normal
MK50
MK75
MK88
MK94
MK97
MK data
Normal data

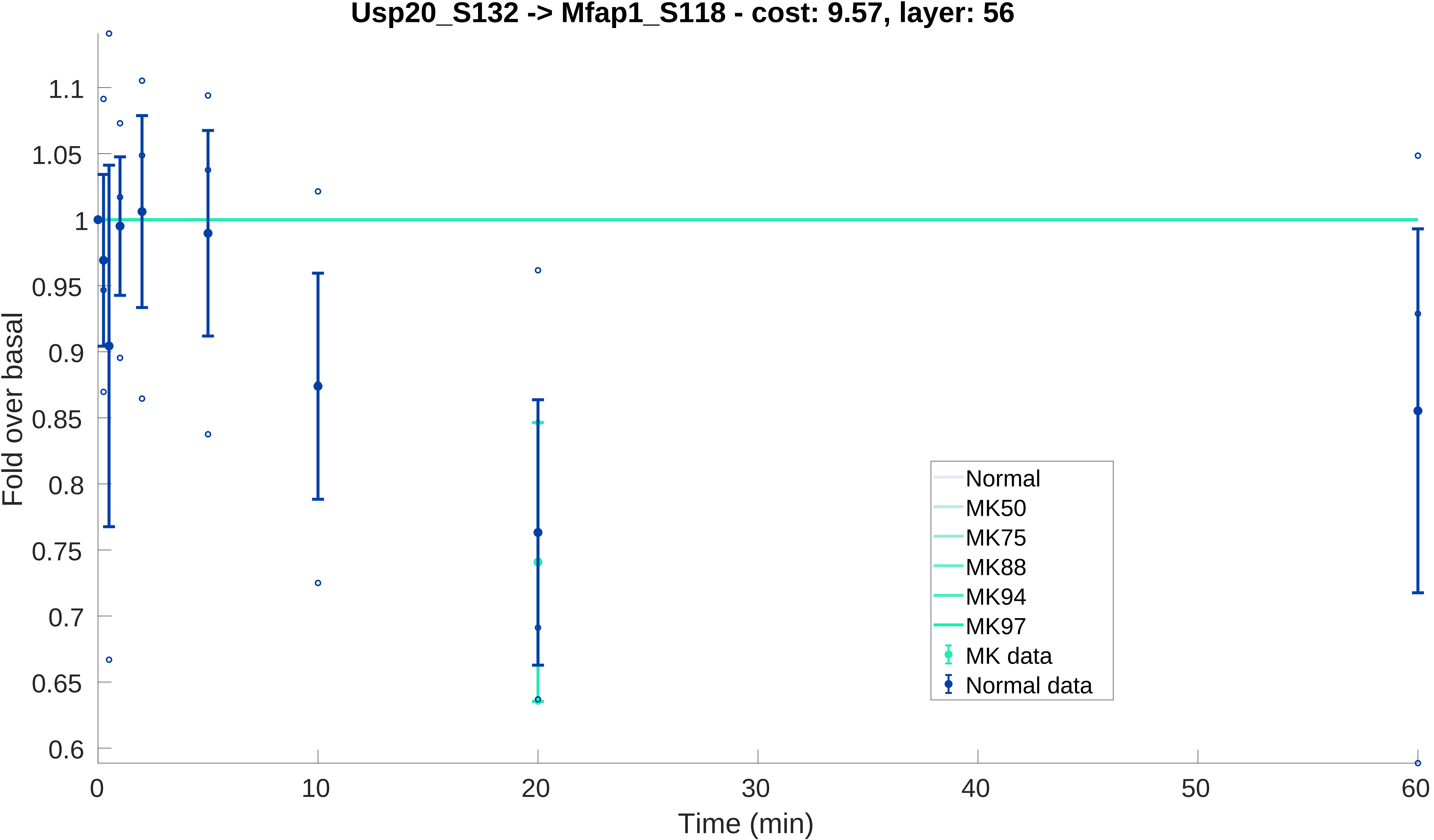
Usp20_S132 -> Mfap1_S118 - cost: 9.57, layer: 56
Fold over basal
Time (min)
1.1
1.05
1
0.95
0.9
0.85
0.8
0.75
0.7
0.65
0.6
0
10
20
30
40
50
60
Normal
MK50
MK75
MK88
MK94
MK97
MK data
Normal data

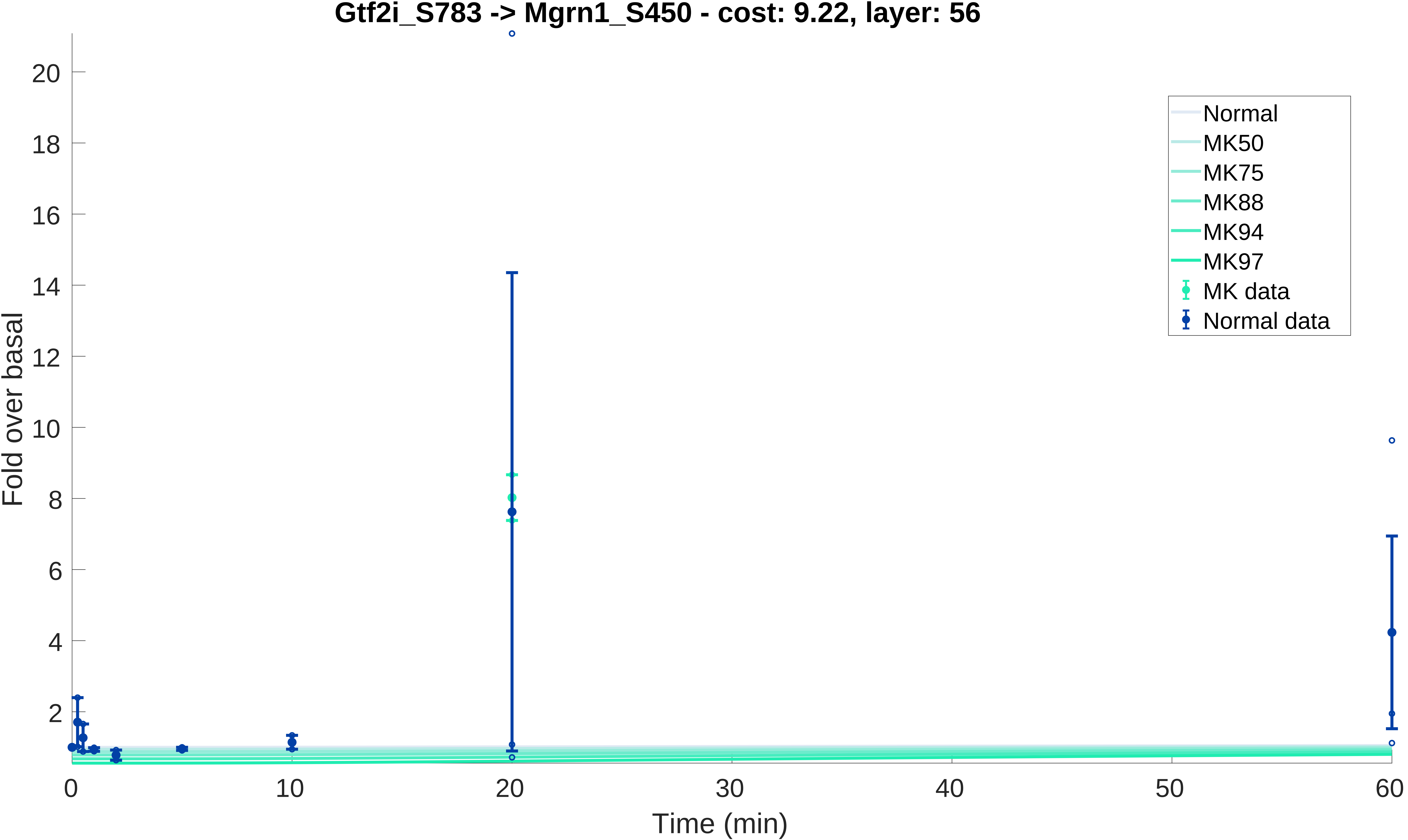

Gtf2i_S783 -> Mgrn1_S450 - cost: 9.22, layer: 56
Fold over basal
Time (min)
0
10
20
30
40
50
60
2
4
6
8
10
12
14
16
18
20
Normal
MK50
MK75
MK88
MK94
MK97
MK data
Normal data

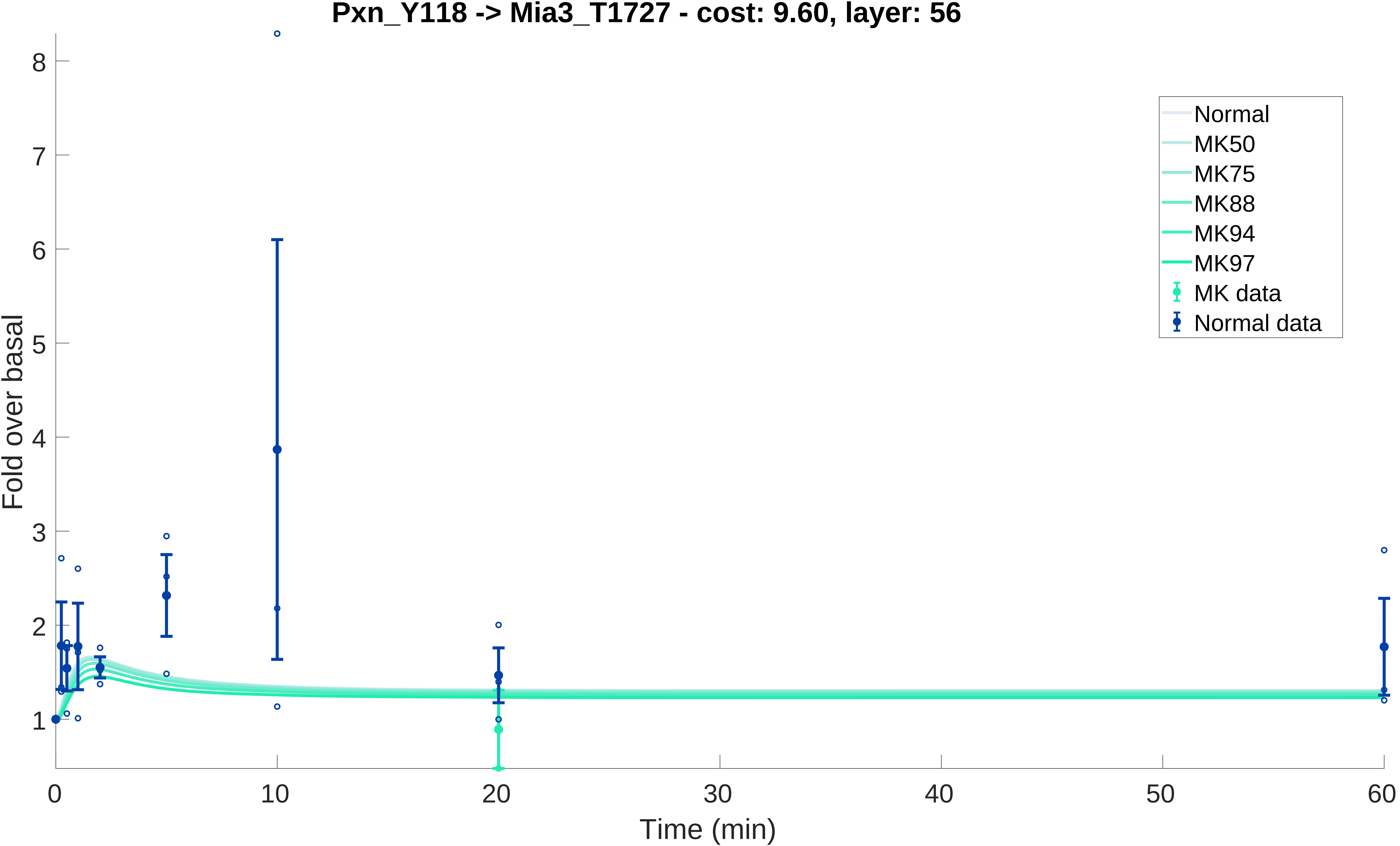

Pxn_Y118 -> Mia3_T1727 - cost: 9.60, layer: 56
Fold over basal
Time (min)
0
10
20
30
40
50
60
1
2
3
4
5
6
7
8
Normal
MK50
MK75
MK88
MK94
MK97
MK data
Normal data

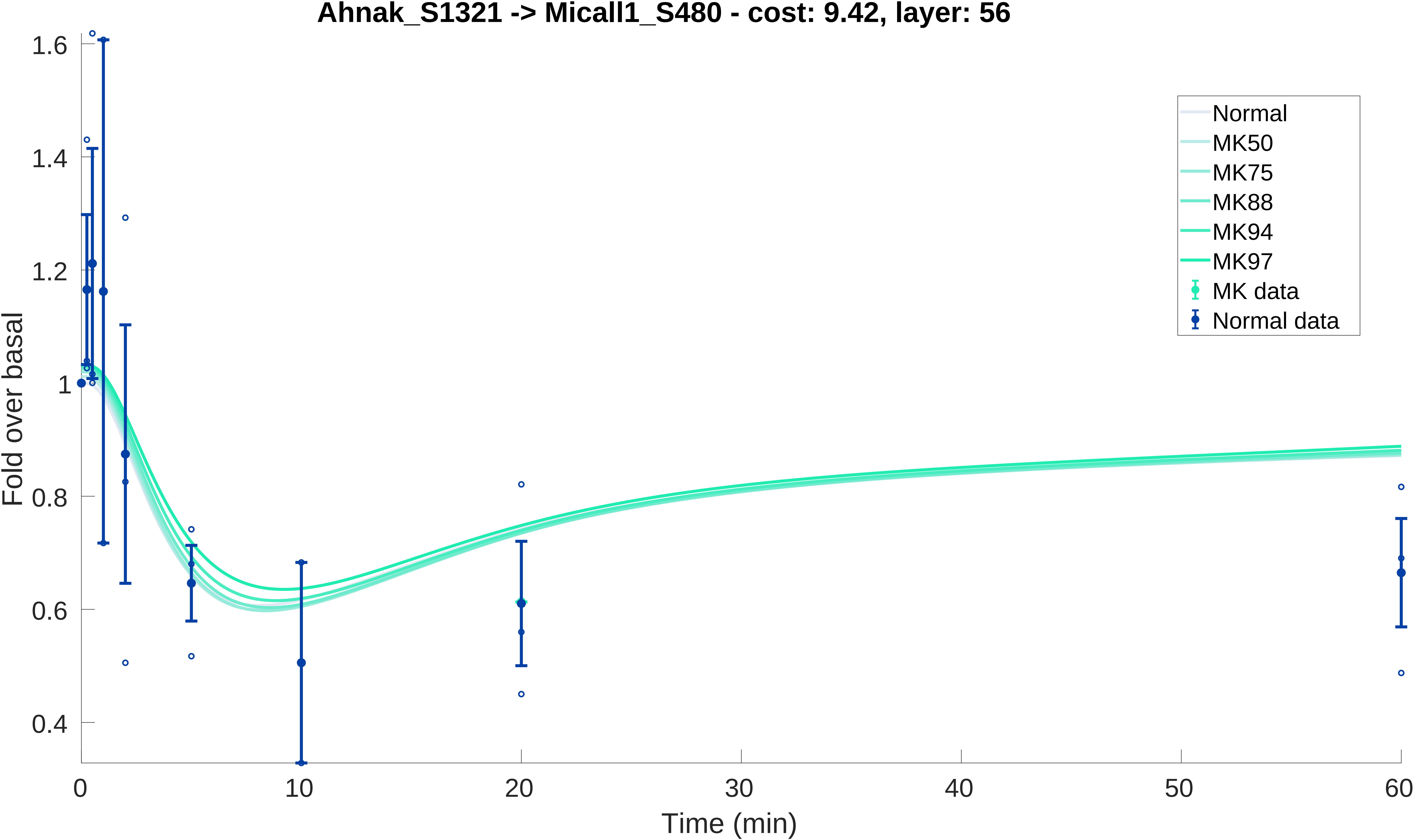

Ahnak_S1321 -> Micall1_S480 - cost: 9.42, layer: 56
Fold over basal
Time (min)
0.4
0.6
0.8
1
1.2
1.4
1.6
0
10
20
30
40
50
60
Normal
MK50
MK75
MK88
MK94
MK97
MK data
Normal data

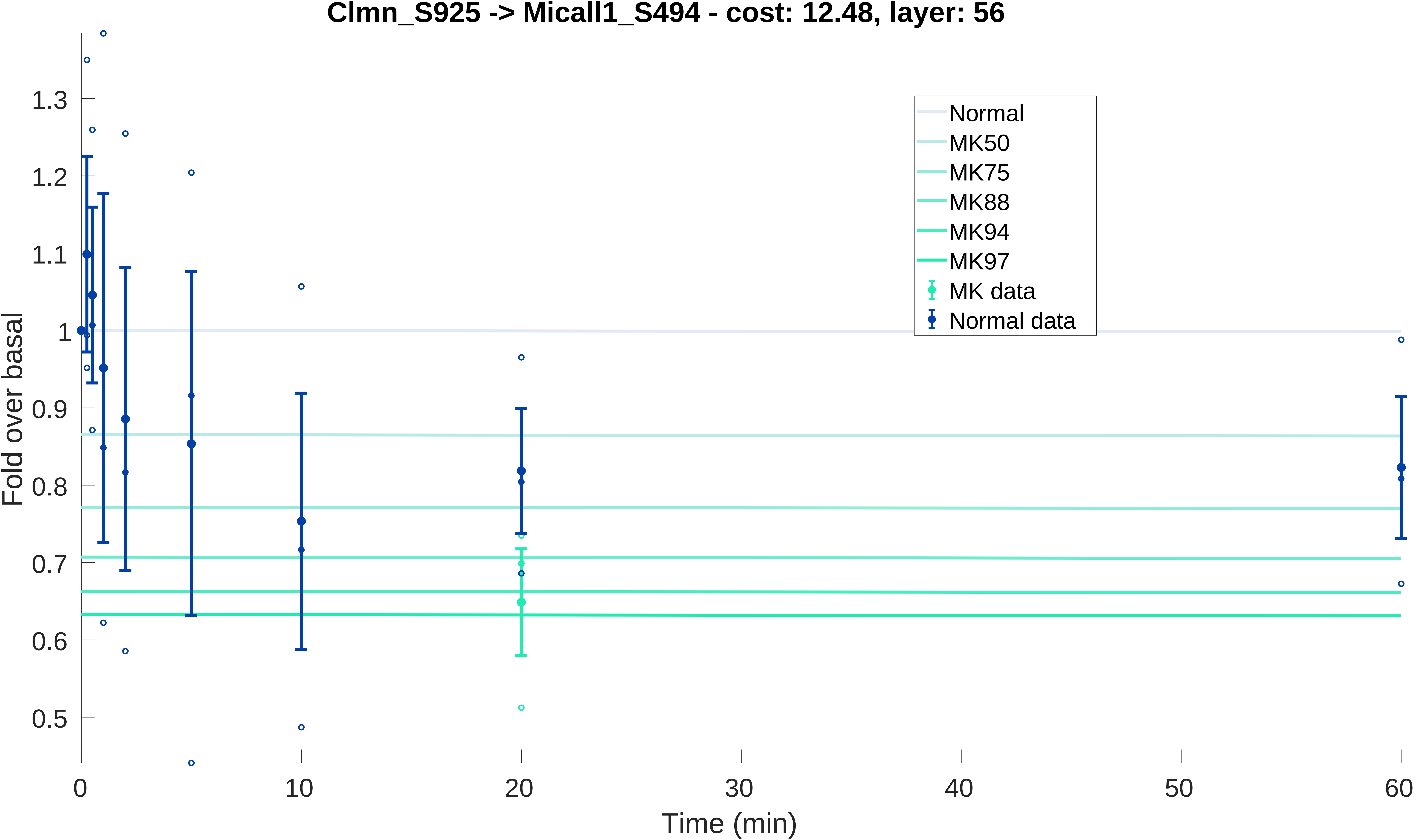

Clmn_S925 -> Micall1_S494 - cost: 12.48, layer: 56
Normal
MK50
MK75
MK88
MK94
MK97
MK data
Normal data
Fold over basal
Time (min)
1.3
1.2
1.1
1
0.9
0.8
0.7
0.6
0.5
0
10
20
30
40
50
60

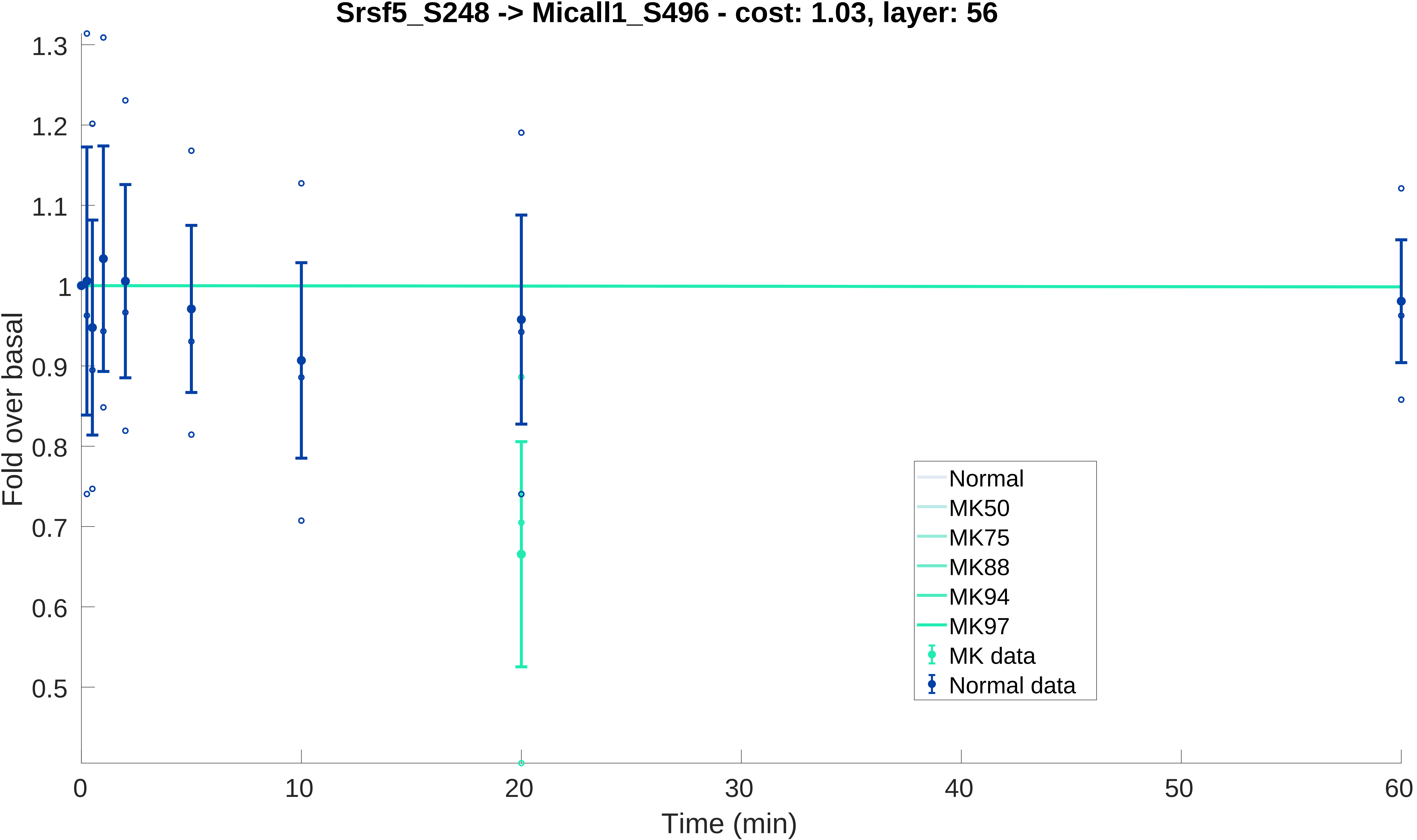
Srsf5_S248 -> Micall1_S496 - cost: 1.03, layer: 56
Fold over basal
1.3
1.2
1.1
1
0.9
0.8
0.7
0.6
0.5
0
10
20
30
40
50
60
Time (min)
Normal
MK50
MK75
MK88
MK94
MK97
MK data
Normal data

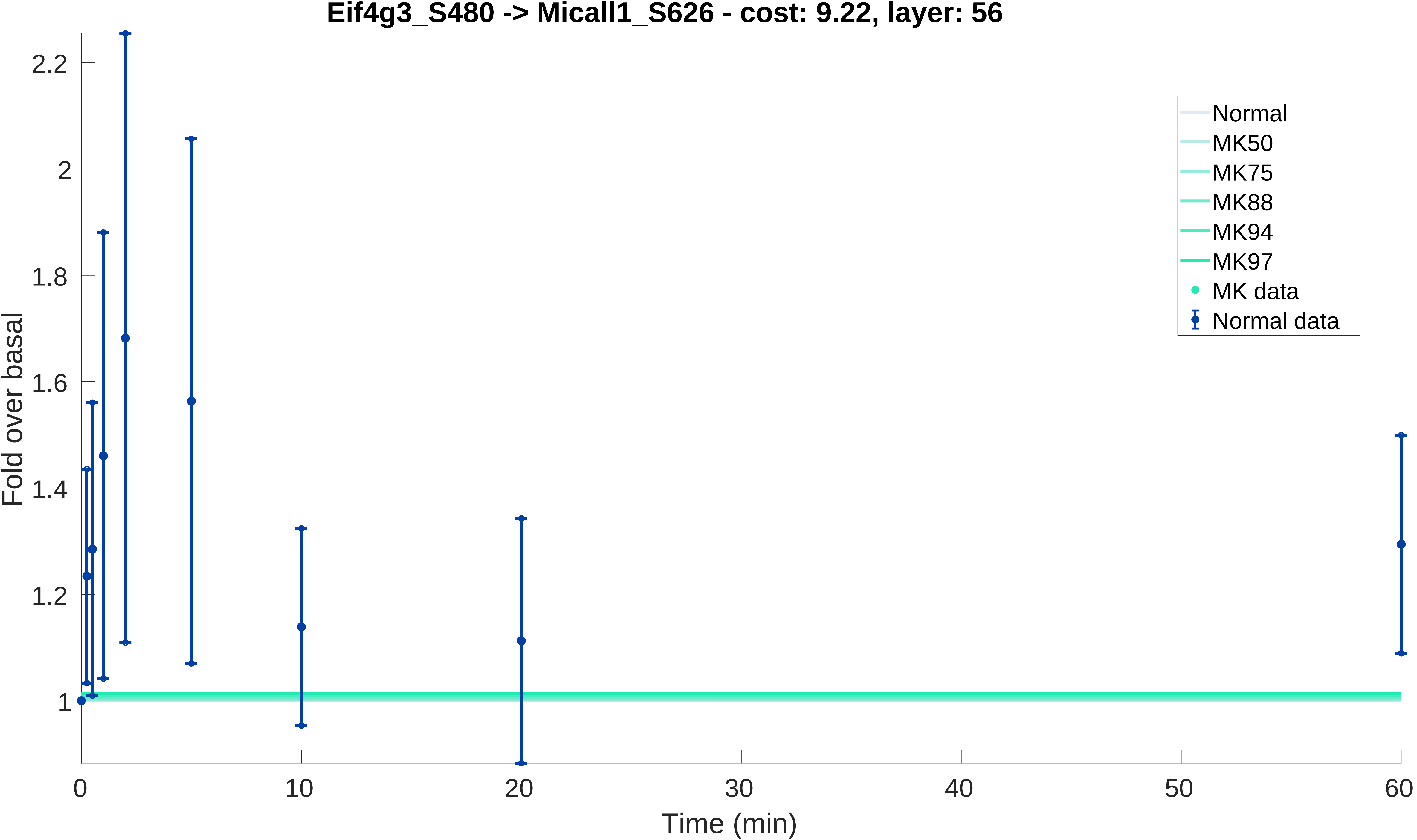
Eif4g3_S480 -> Micall1_S626 - cost: 9.22, layer: 56
Fold over basal
Time (min)
2.2
2
1.8
1.6
1.4
1.2
1
0
10
20
30
40
50
60
Normal
MK50
MK75
MK88
MK94
MK97
MK data
Normal data

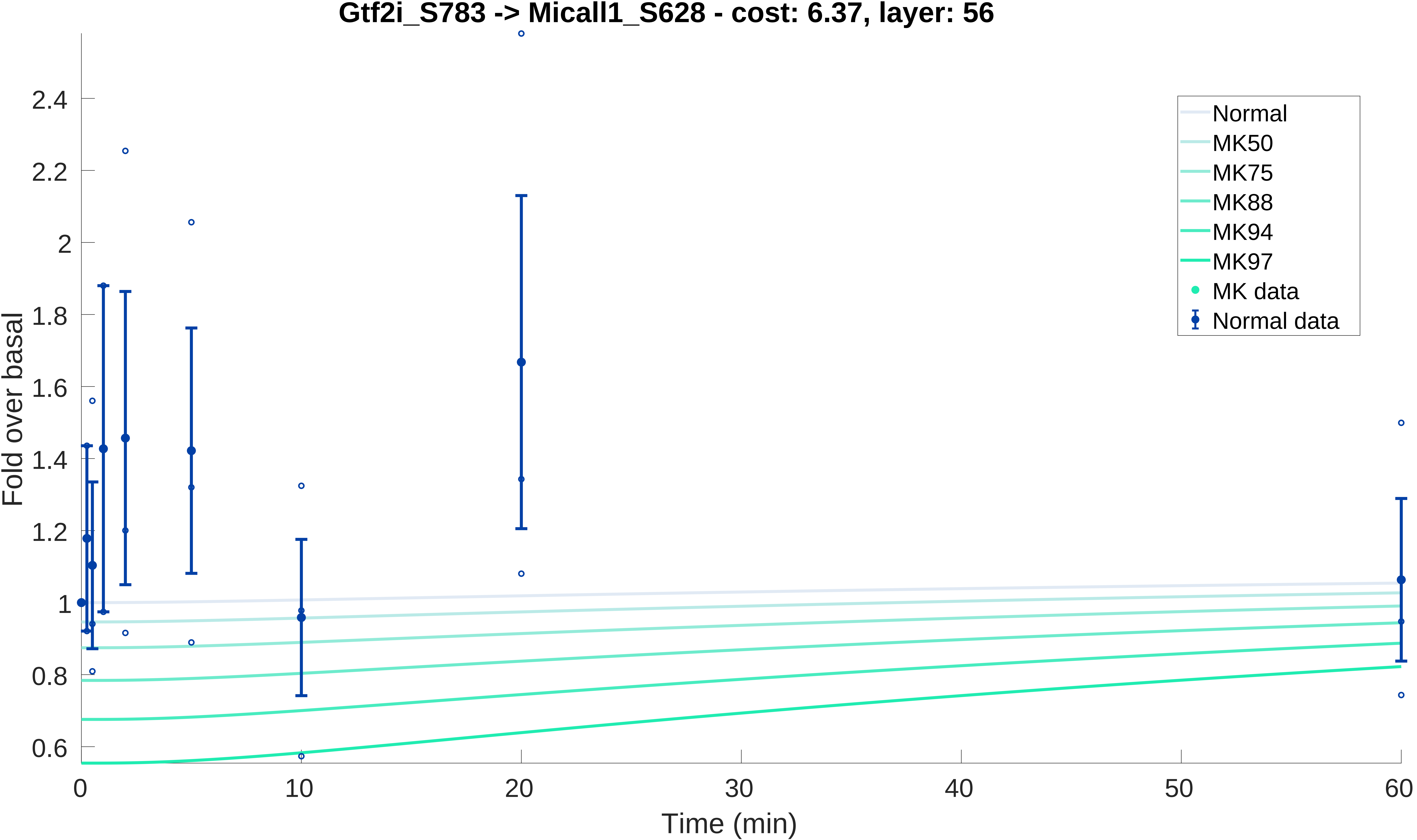

Gtf2i_S783 -> Micall1_S628 - cost: 6.37, layer: 56
Fold over basal
Time (min)
Normal
MK50
MK75
MK88
MK94
MK97
MK data
Normal data
0.6
0.8
1
1.2
1.4
1.6
1.8
2
2.2
2.4
0
10
20
30
40
50
60

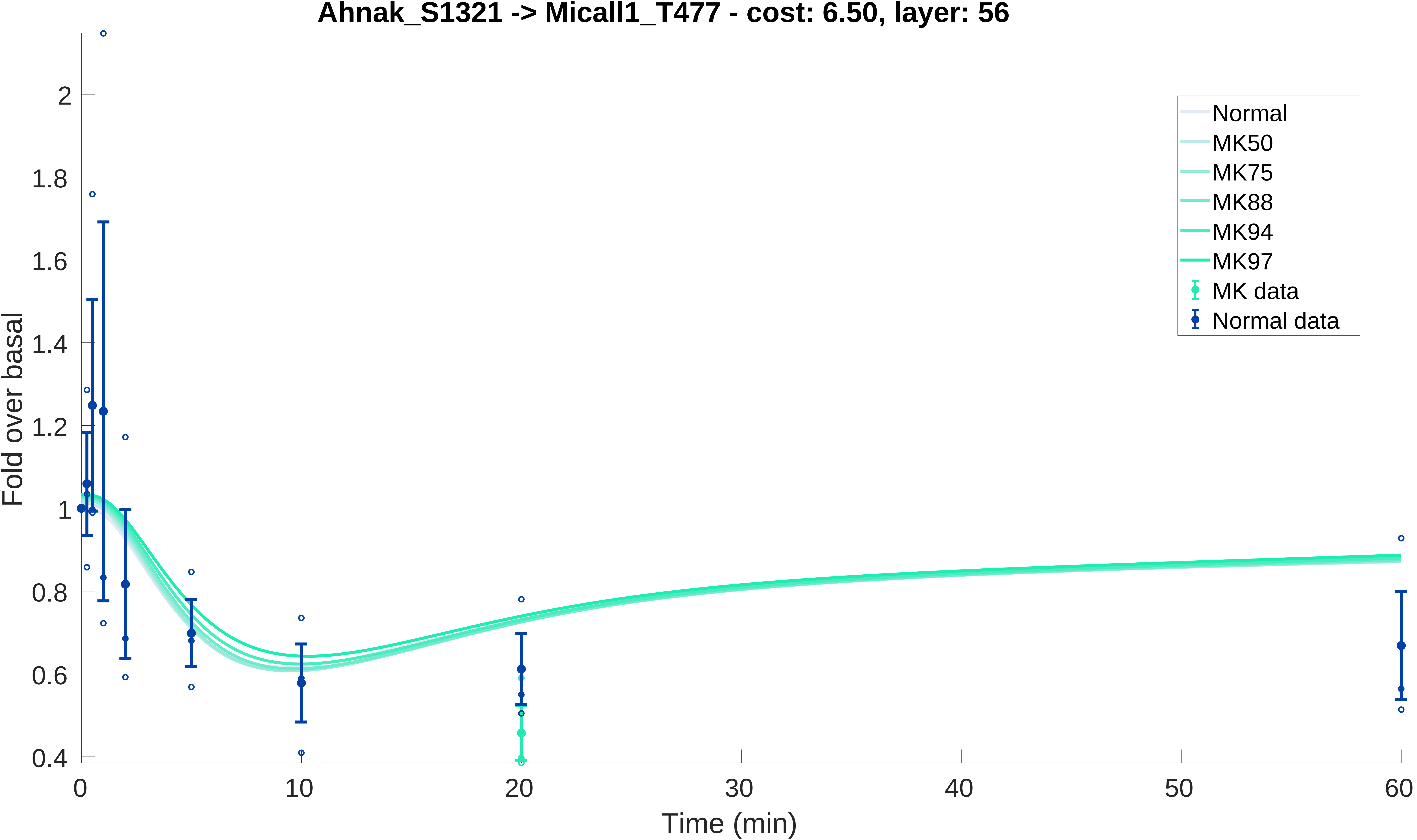

Ahnak_S1321 -> Micall1_T477 - cost: 6.50, layer: 56
Fold over basal
Time (min)
0.4
0.6
0.8
1
1.2
1.4
1.6
1.8
2
0
10
20
30
40
50
60
Normal
MK50
MK75
MK88
MK94
MK97
MK data
Normal data

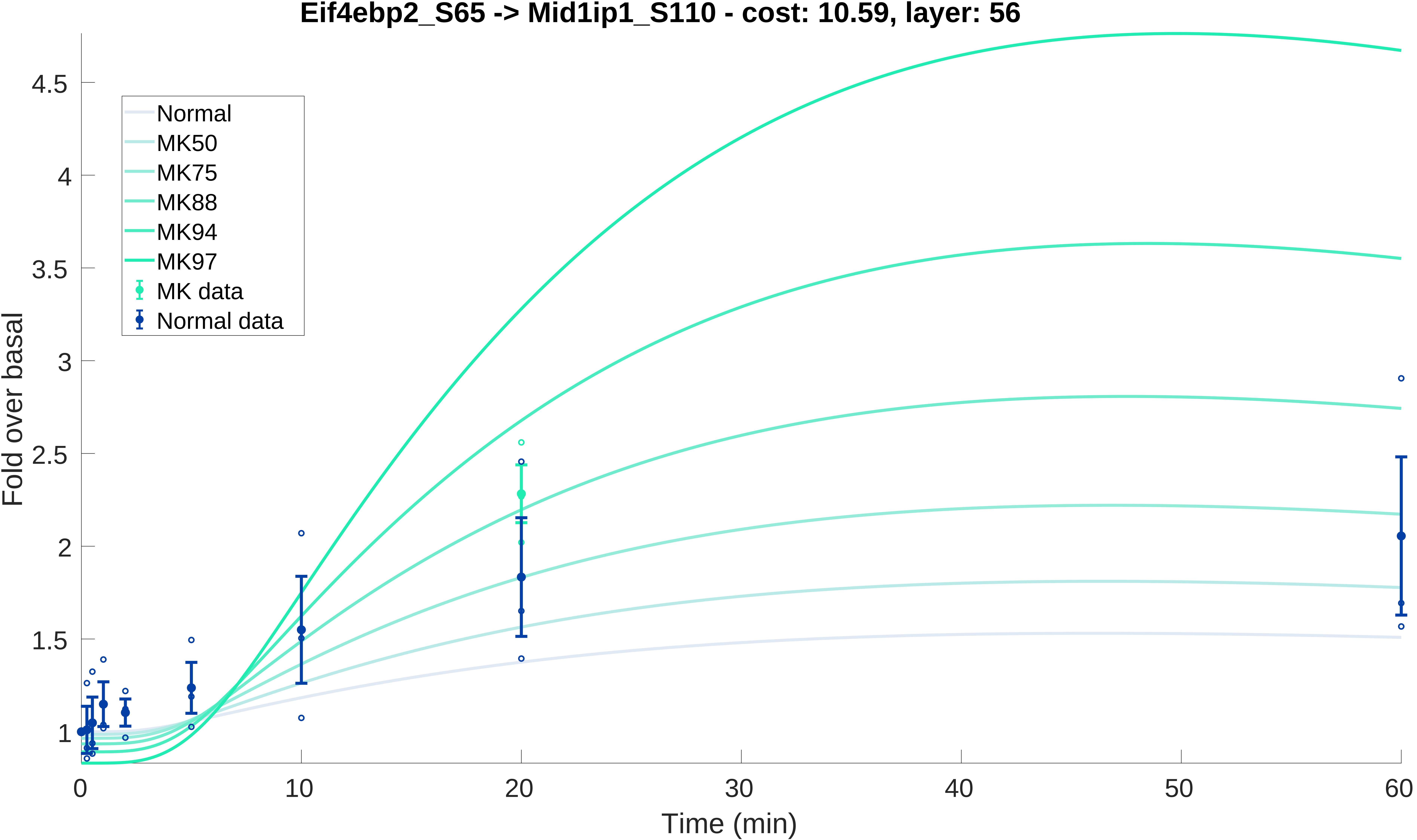

Eif4ebp2_S65 -> Mid1ip1_S110 - cost: 10.59, layer: 56
Normal
MK50
MK75
MK88
MK94
MK97
MK data
Normal data
Fold over basal
Time (min)
4.5
4
3.5
3
2.5
2
1.5
1
0
10
20
30
40
50
60

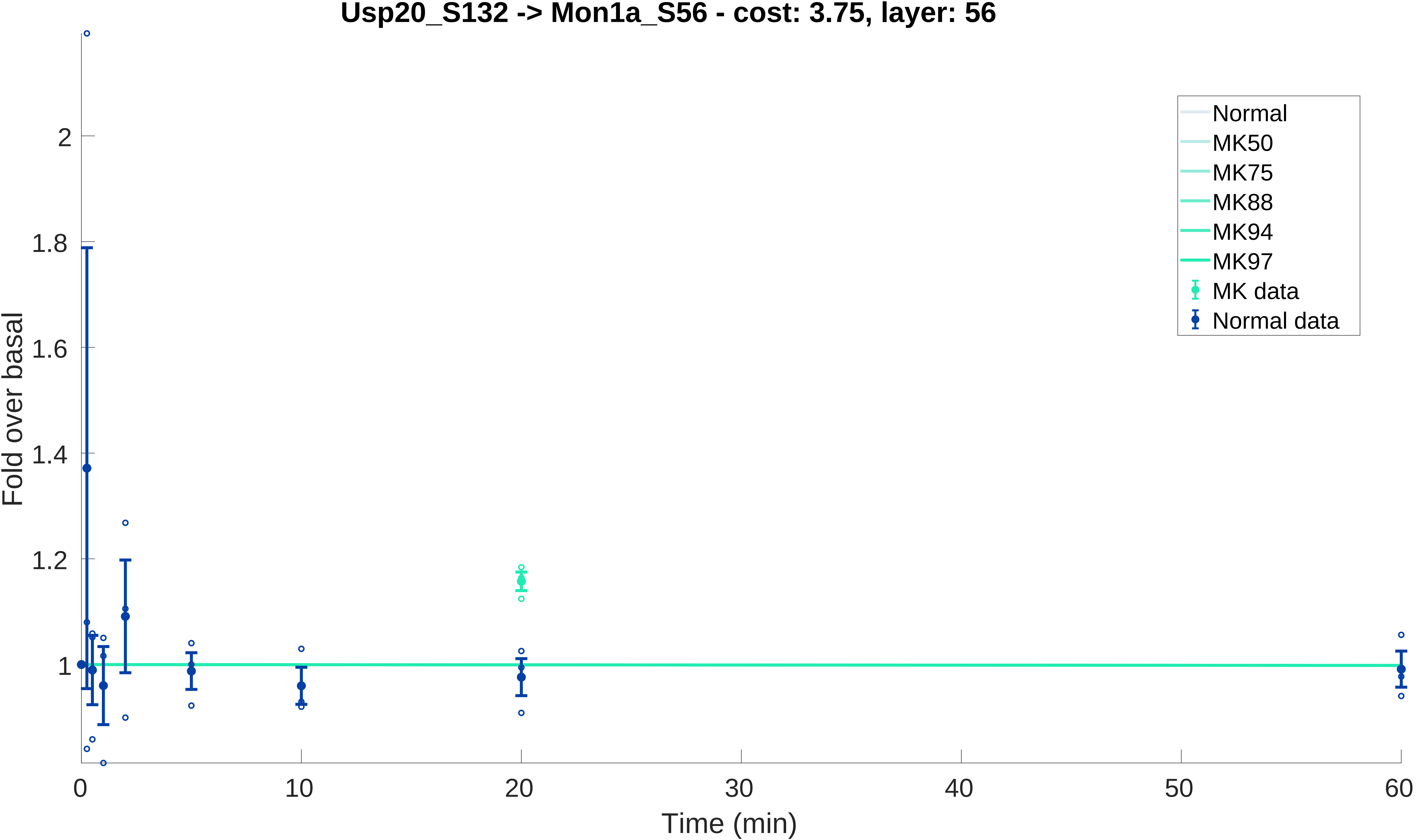
Usp20_S132 -> Mon1a_S56 - cost: 3.75, layer: 56
Fold over basal
2
1.8
1.6
1.4
1.2
1
0
10
20
30
40
50
60
Time (min)
Normal
MK50
MK75
MK88
MK94
MK97
MK data
Normal data

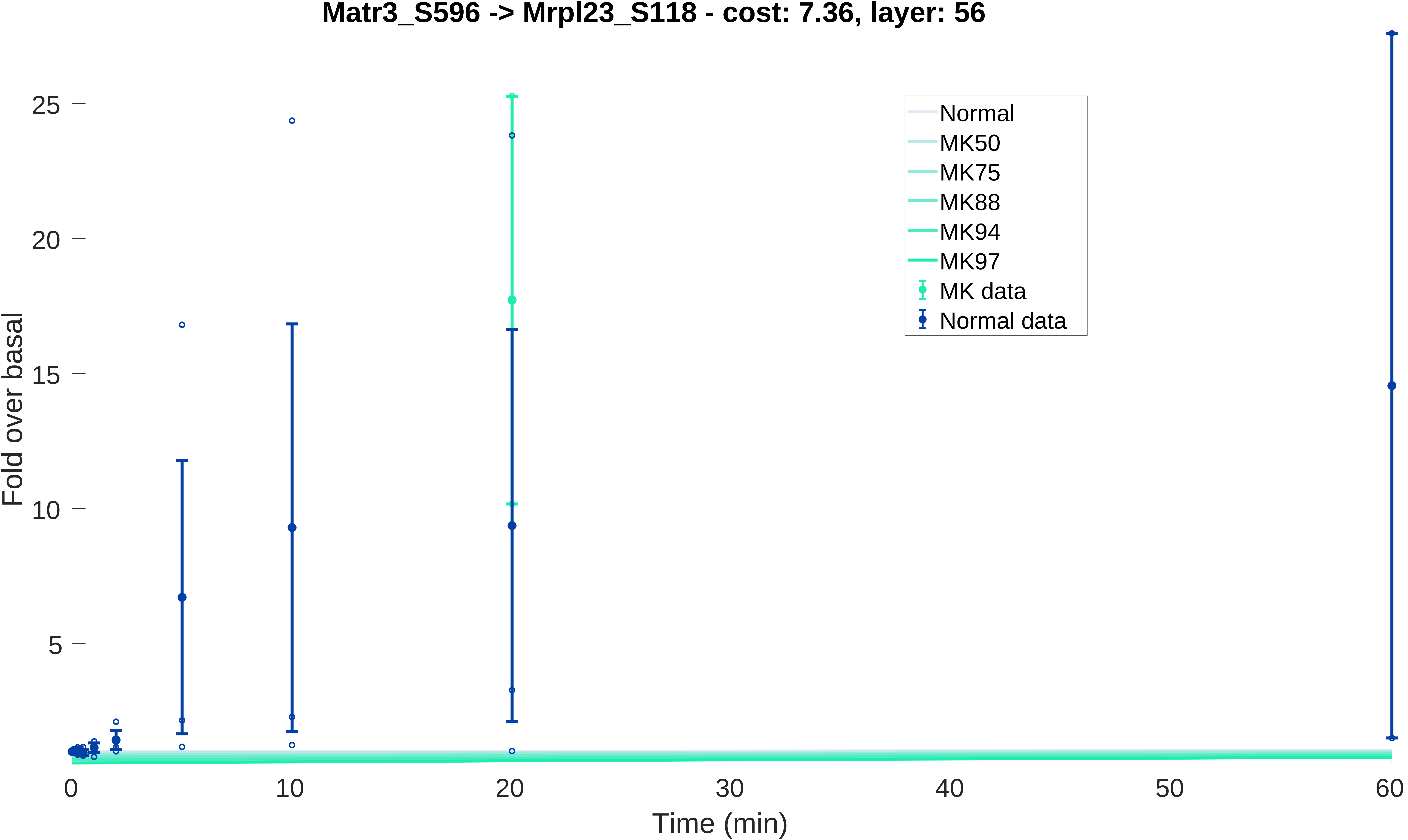

Matr3_S596 -> Mrpl23_S118 - cost: 7.36, layer: 56
Fold over basal
Time (min)
0
10
20
30
40
50
60
5
10
15
20
25
Normal
MK50
MK75
MK88
MK94
MK97
MK data
Normal data

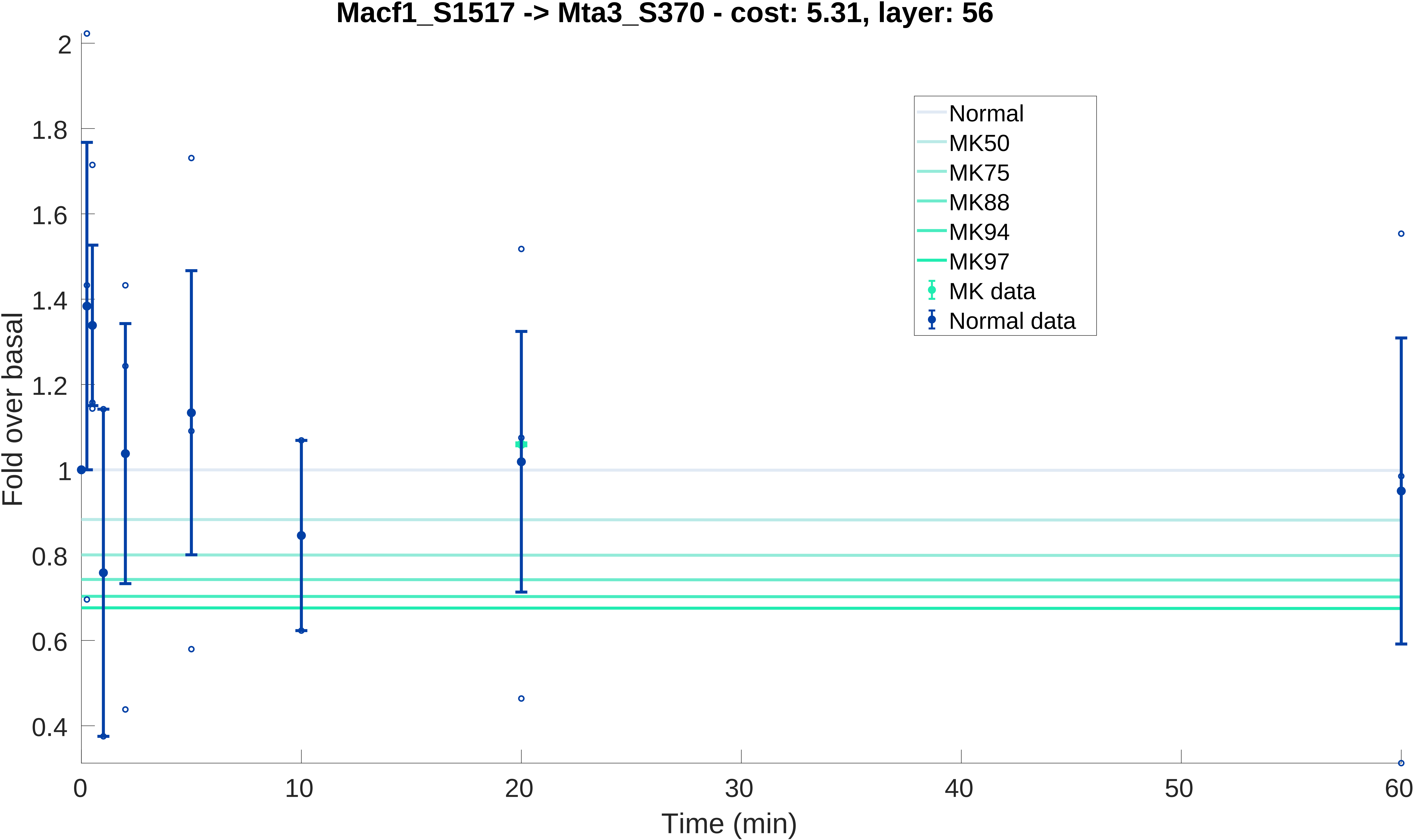

Macf1_S1517 -> Mta3_S370 - cost: 5.31, layer: 56
Fold over basal
Time (min)
0.4
0.6
0.8
1
1.2
1.4
1.6
1.8
2
0
10
20
30
40
50
60
Normal
MK50
MK75
MK88
MK94
MK97
MK data
Normal data

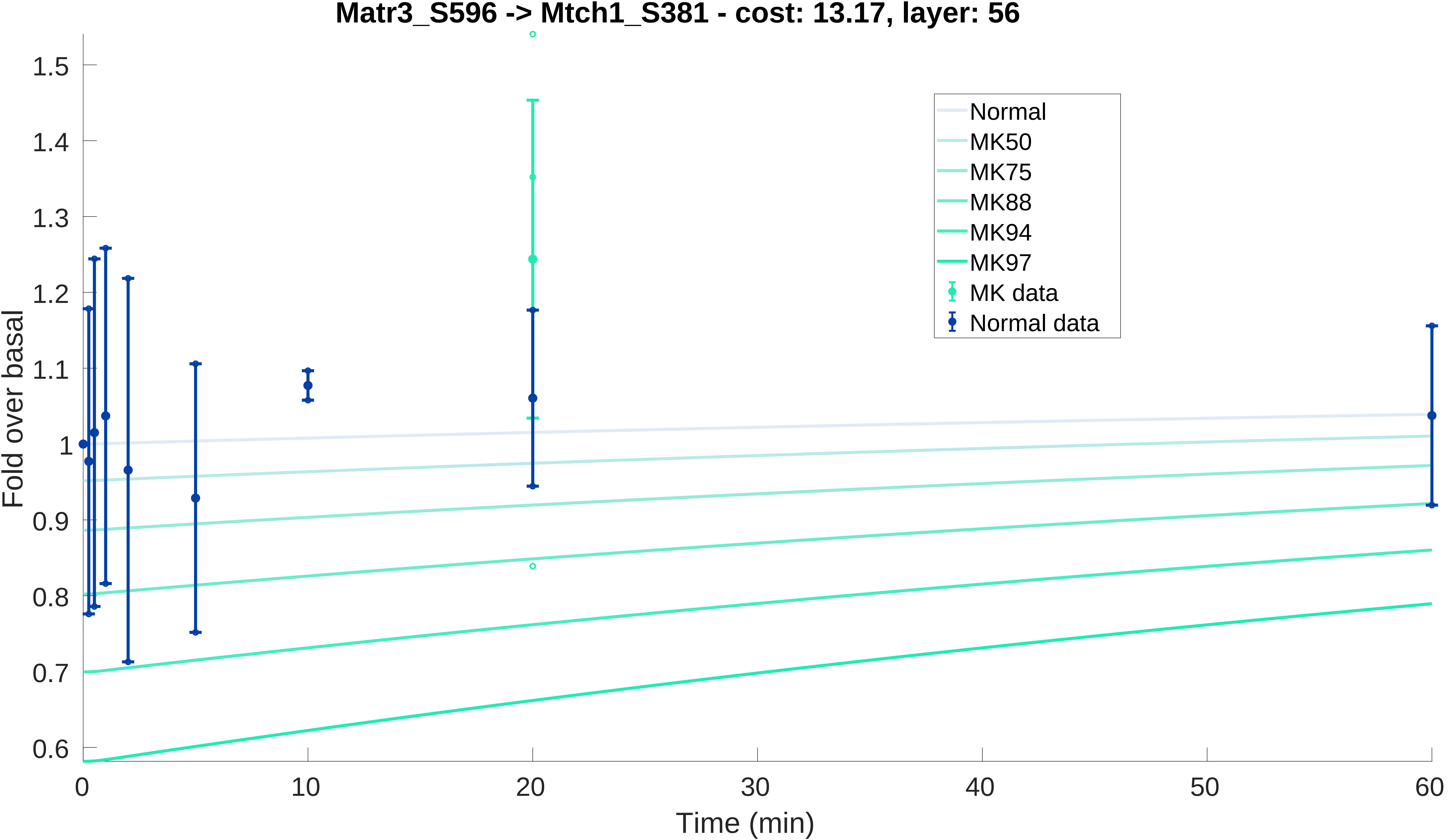

Matr3_S596 -> Mtch1_S381 - cost: 13.17, layer: 56
Fold over basal
Time (min)
Normal
MK50
MK75
MK88
MK94
MK97
MK data
Normal data
1.5
1.4
1.3
1.2
1.1
1
0.9
0.8
0.7
0.6
0
10
20
30
40
50
60

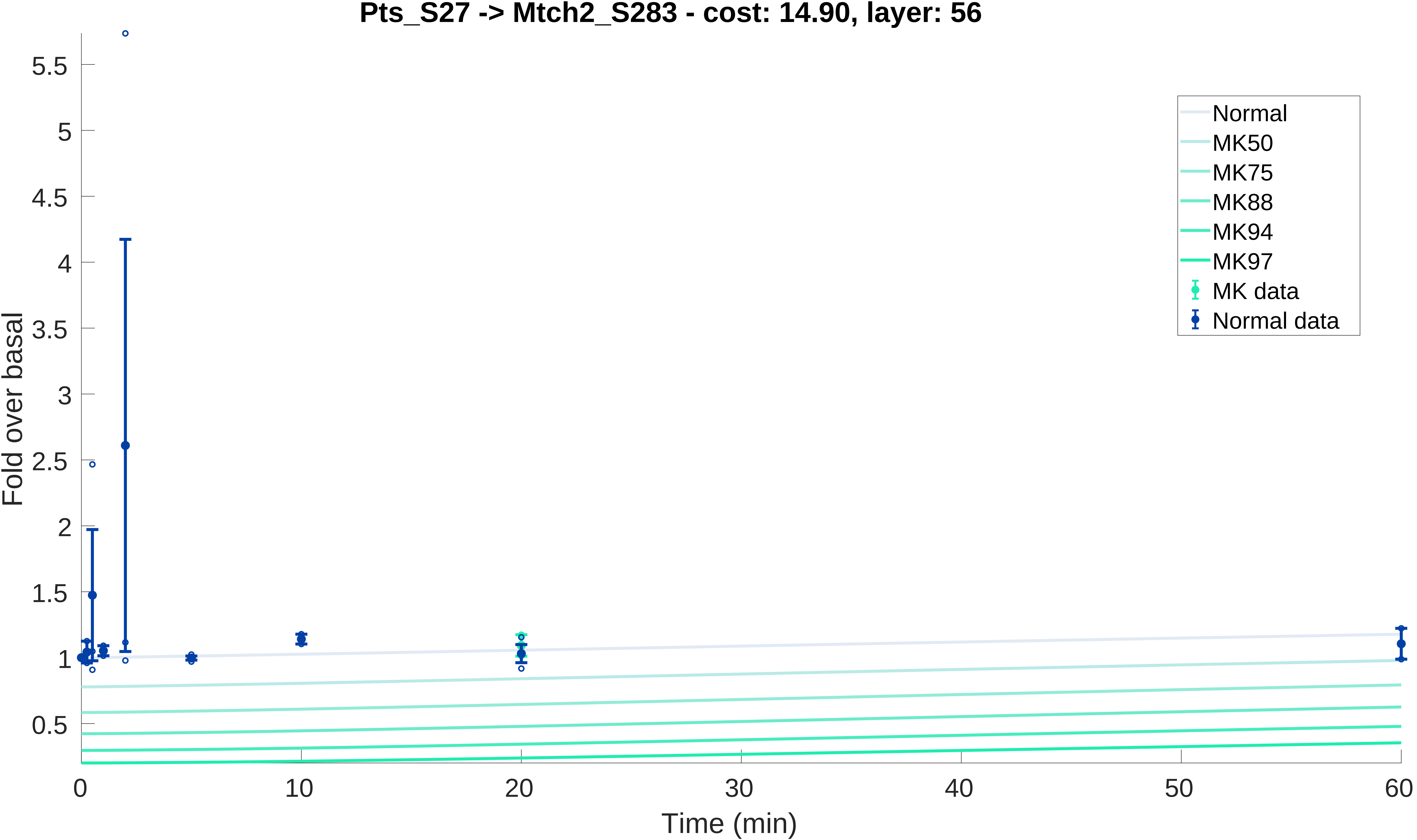
Pts_S27 -> Mtch2_S283 - cost: 14.90, layer: 56
Fold over basal
5.5
5
4.5
4
3.5
3
2.5
2
1.5
1
0.5
0
10
20
30
40
50
60
Time (min)
Normal
MK50
MK75
MK88
MK94
MK97
MK data
Normal data

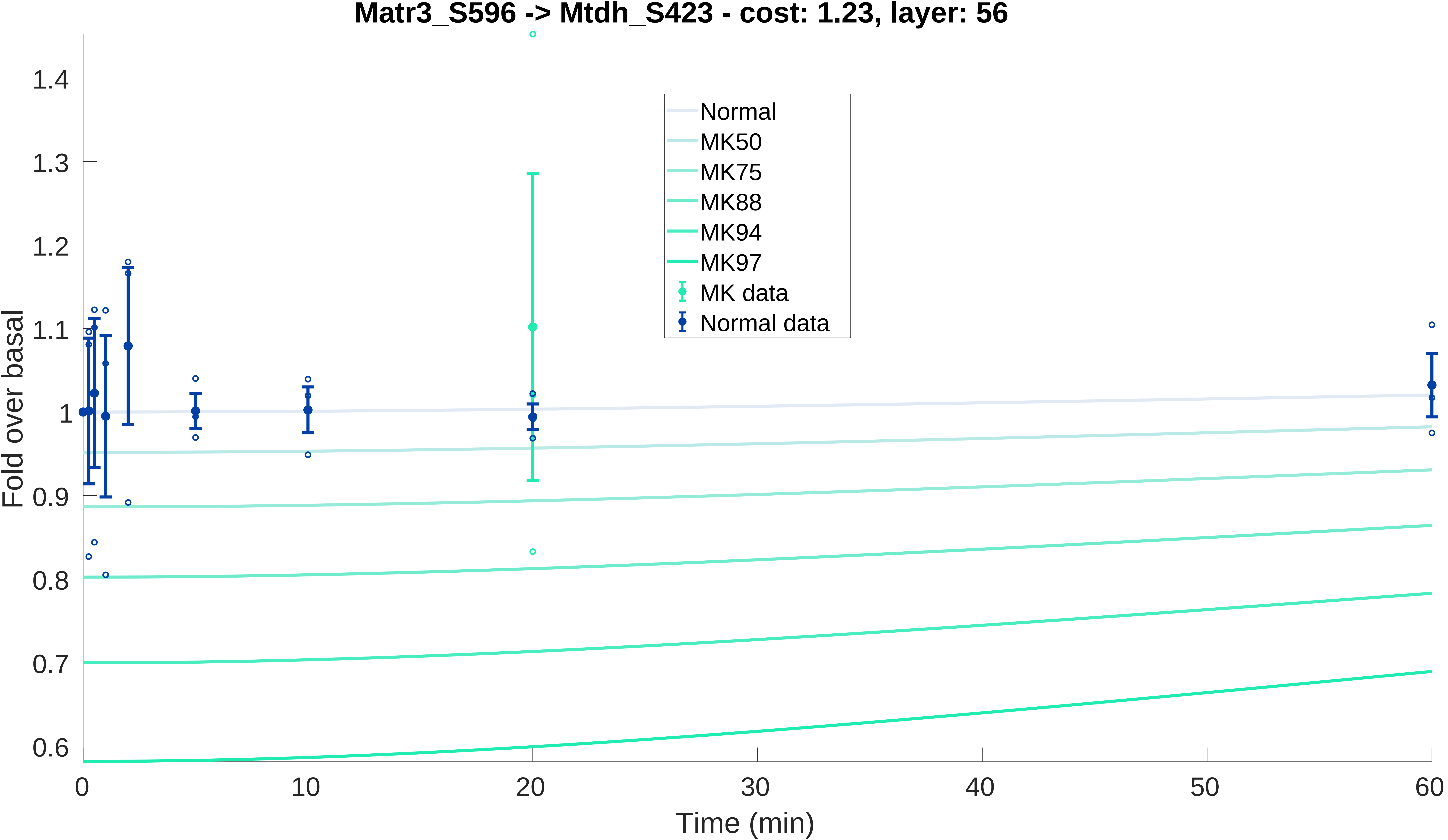

Matr3_S596 -> Mtdh_S423 - cost: 1.23, layer: 56
Fold over basal
Time (min)
1.4
1.3
1.2
1.1
1
0.9
0.8
0.7
0.6
0
10
20
30
40
50
60
Normal
MK50
MK75
MK88
MK94
MK97
MK data
Normal data

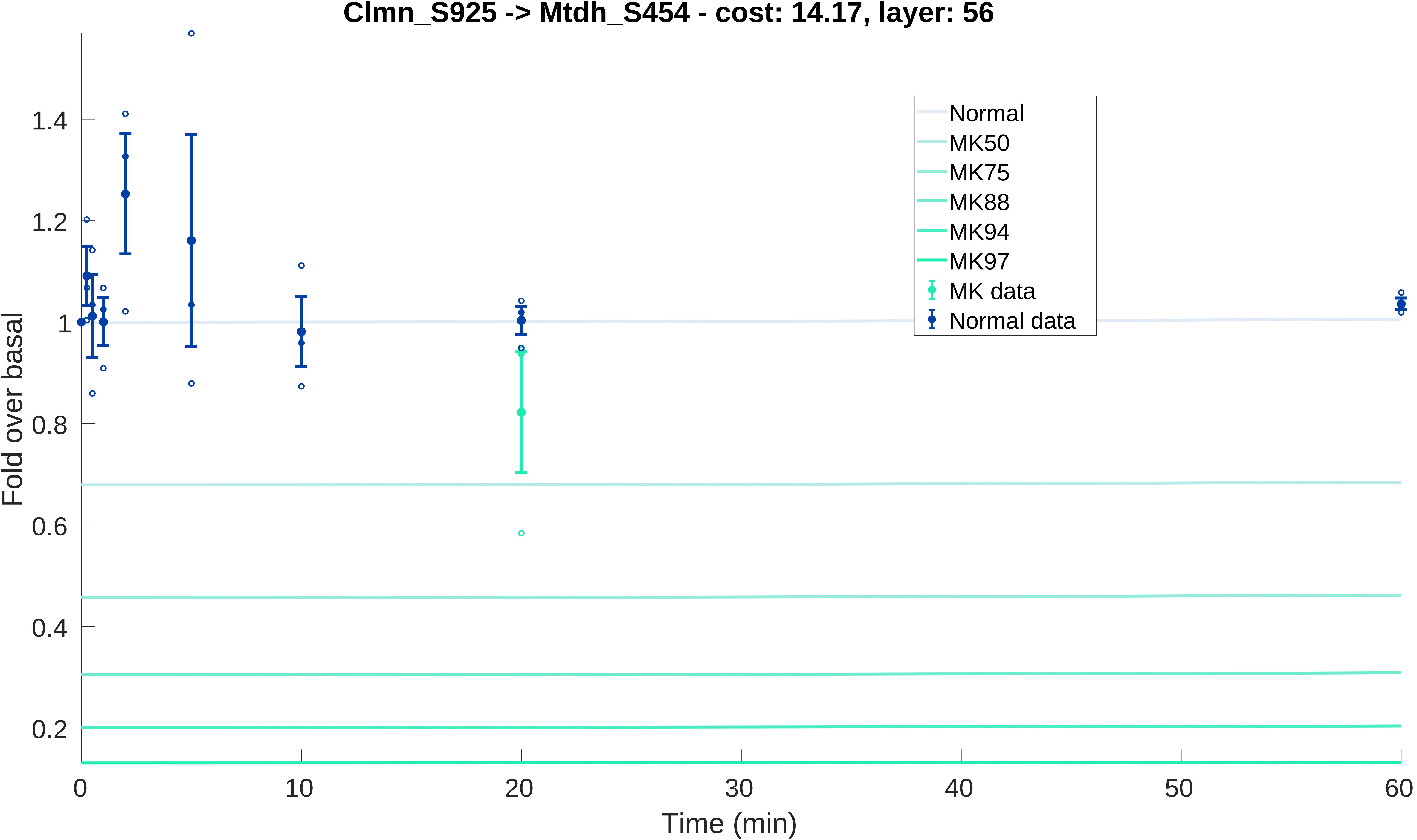

Clmn_S925 -> Mtdh_S454 - cost: 14.17, layer: 56
Fold over basal
Time (min)
0
10
20
30
40
50
60
0.2
0.4
0.6
0.8
1
1.2
1.4
Normal
MK50
MK75
MK88
MK94
MK97
MK data
Normal data

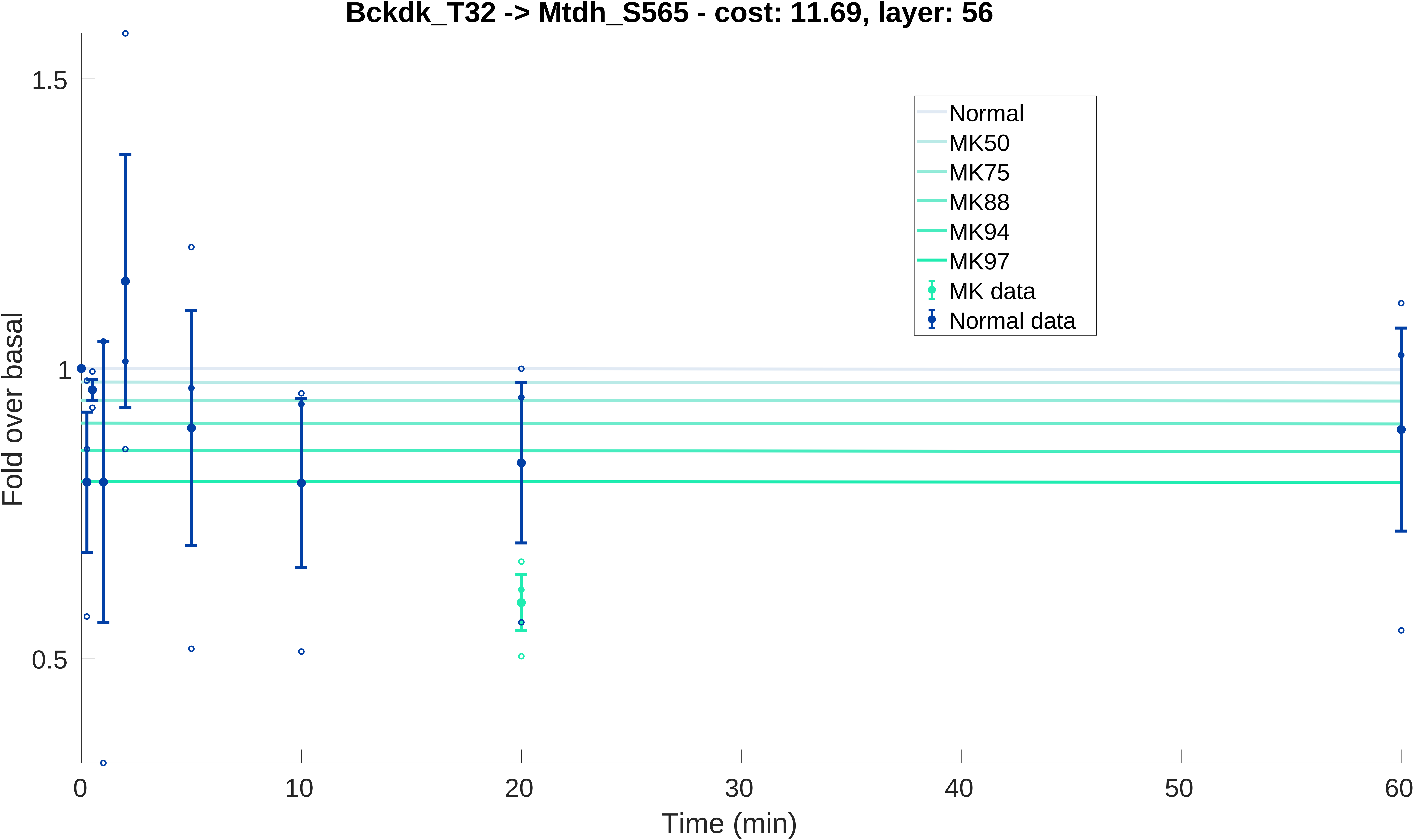
Bckdk_T32 -> Mtdh_S565 - cost: 11.69, layer: 56
Normal
MK50
MK75
MK88
MK94
MK97
MK data
Normal data
Fold over basal
1.5
1
0.5
0
10
20
30
40
50
60
Time (min)

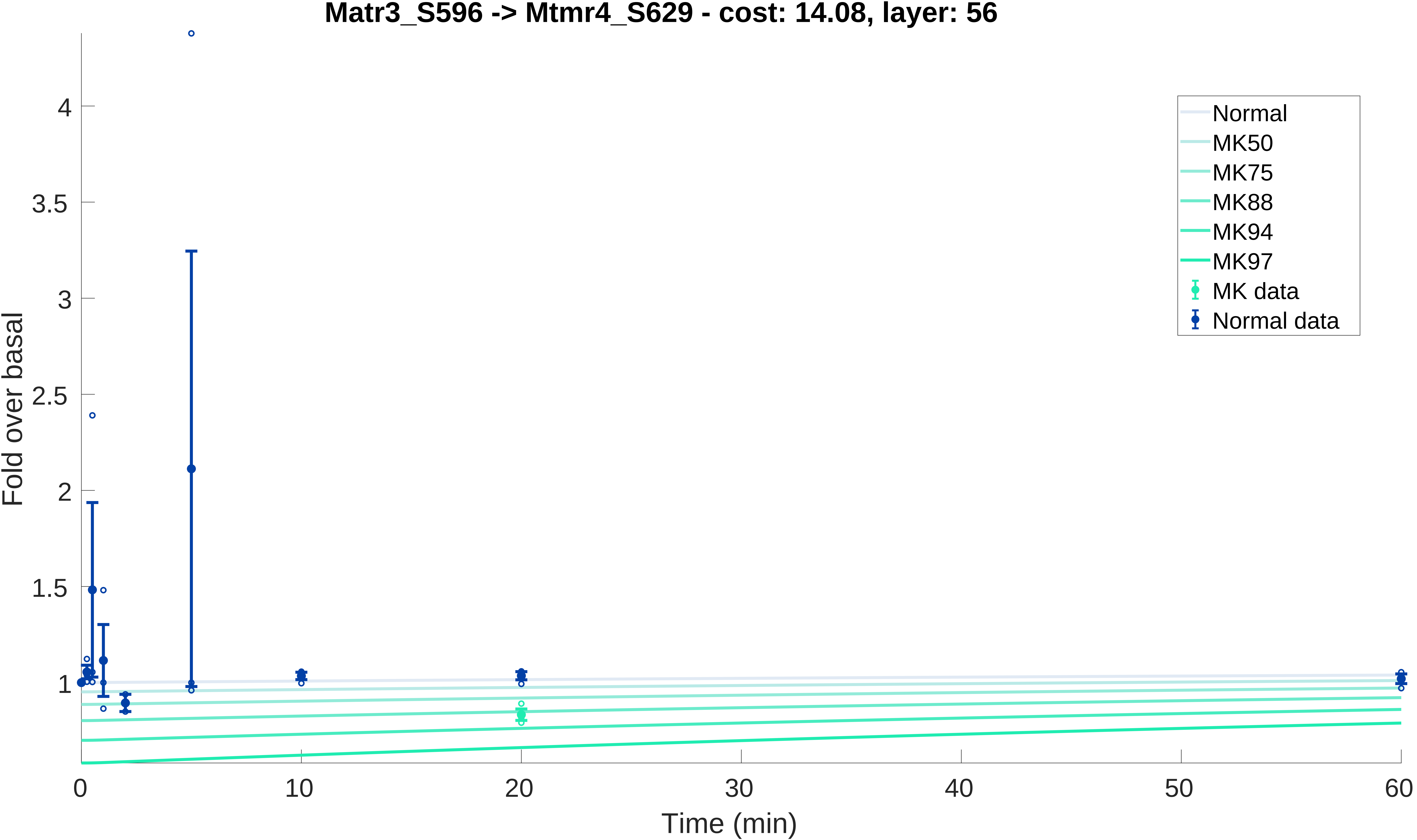

Matr3_S596 -> Mtmr4_S629 - cost: 14.08, layer: 56
Fold over basal
4
3.5
3
2.5
2
1.5
1
0
10
20
30
40
50
60
Time (min)
Normal
MK50
MK75
MK88
MK94
MK97
MK data
Normal data

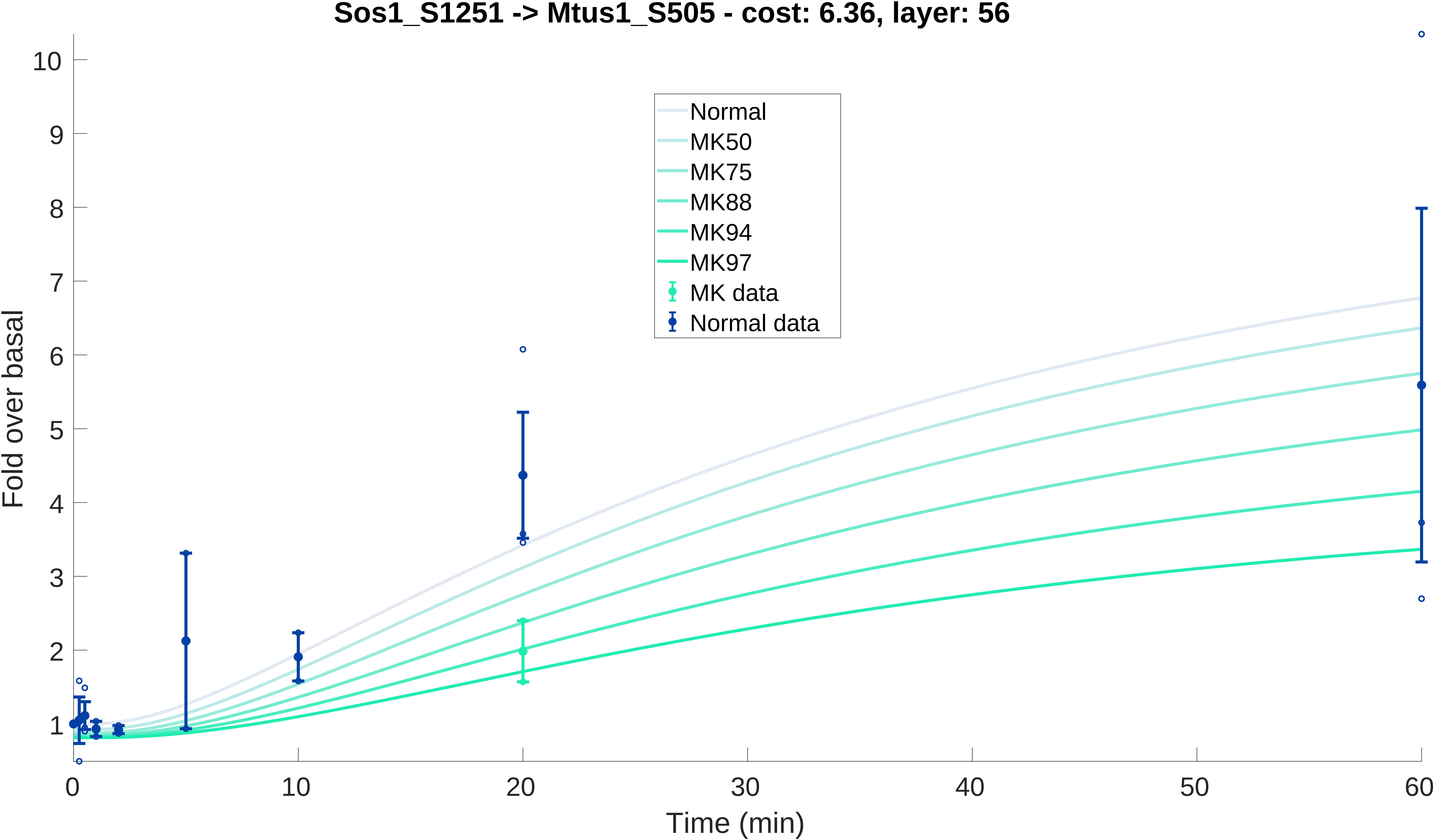

Sos1_S1251 -> Mtus1_S505 - cost: 6.36, layer: 56
Normal
MK50
MK75
MK88
MK94
MK97
MK data
Normal data
Fold over basal
Time (min)
0
10
20
30
40
50
60
1
2
3
4
5
6
7
8
9
10

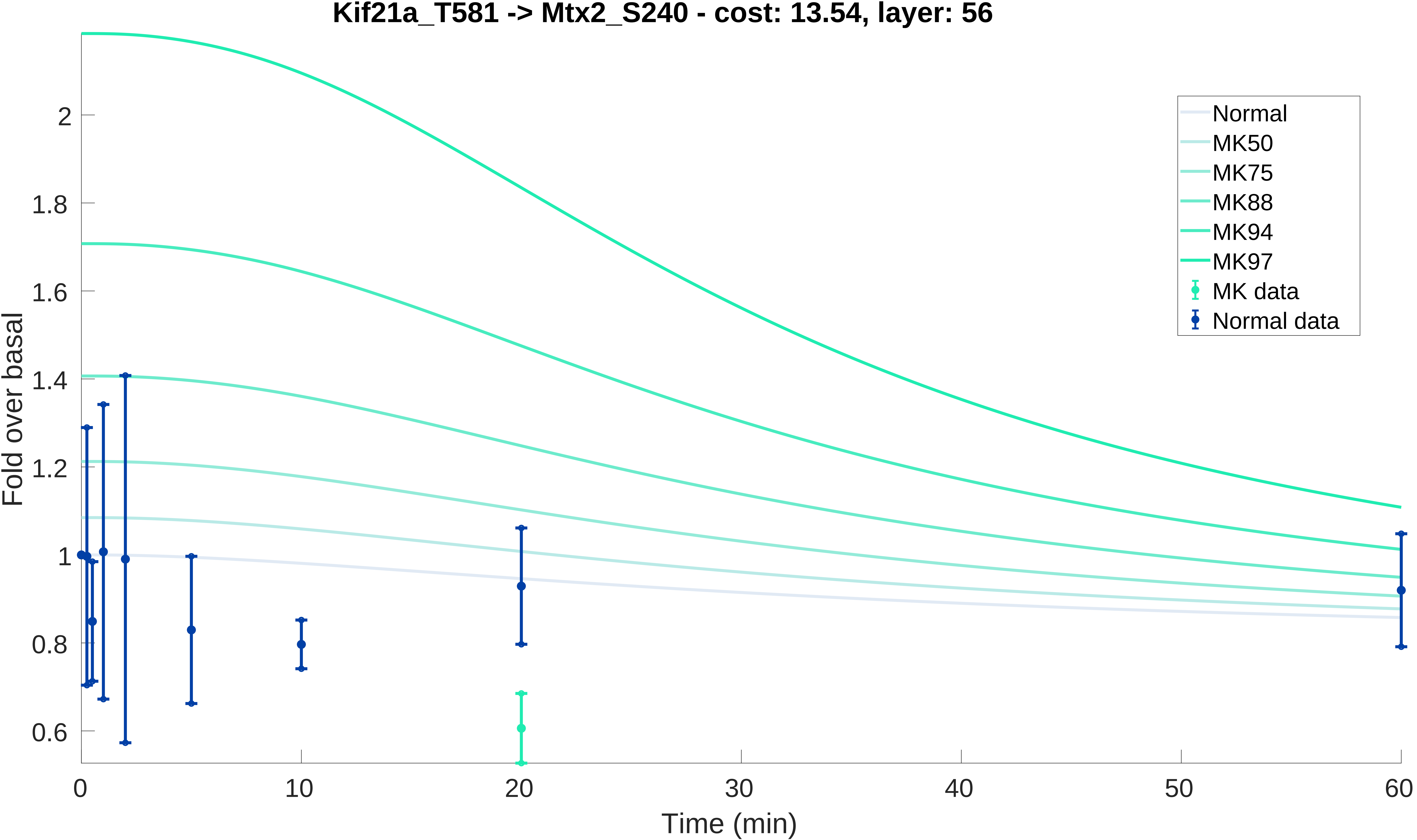
Kif21a_T581 -> Mtx2_S240 - cost: 13.54, layer: 56
Fold over basal
Time (min)
Normal
MK50
MK75
MK88
MK94
MK97
MK data
Normal data
0.6
0.8
1
1.2
1.4
1.6
1.8
2
0
10
20
30
40
50
60

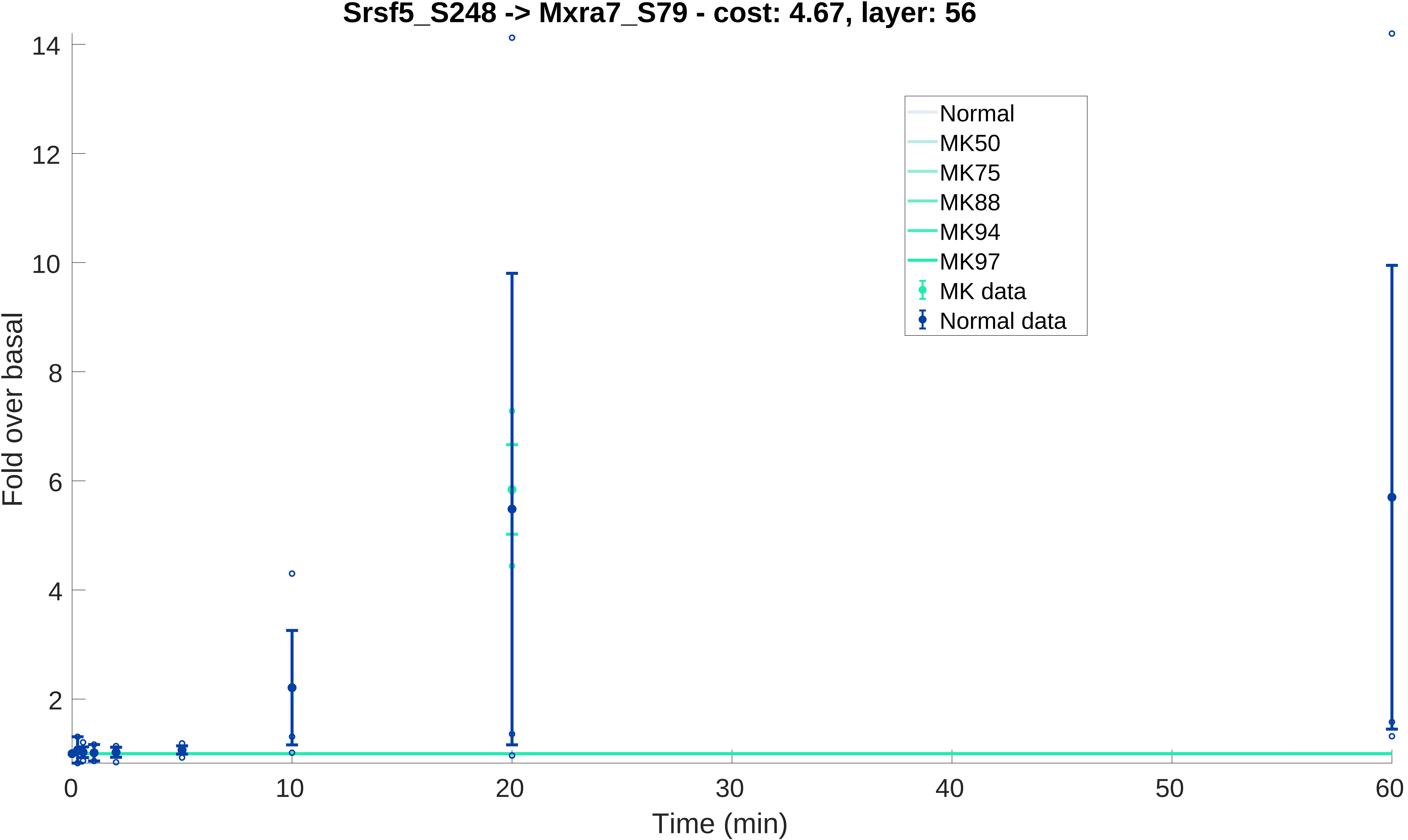

Srsf5_S248 -> Mxra7_S79 - cost: 4.67, layer: 56
Fold over basal
Time (min)
0
10
20
30
40
50
60
2
4
6
8
10
12
14
Normal
MK50
MK75
MK88
MK94
MK97
MK data
Normal data

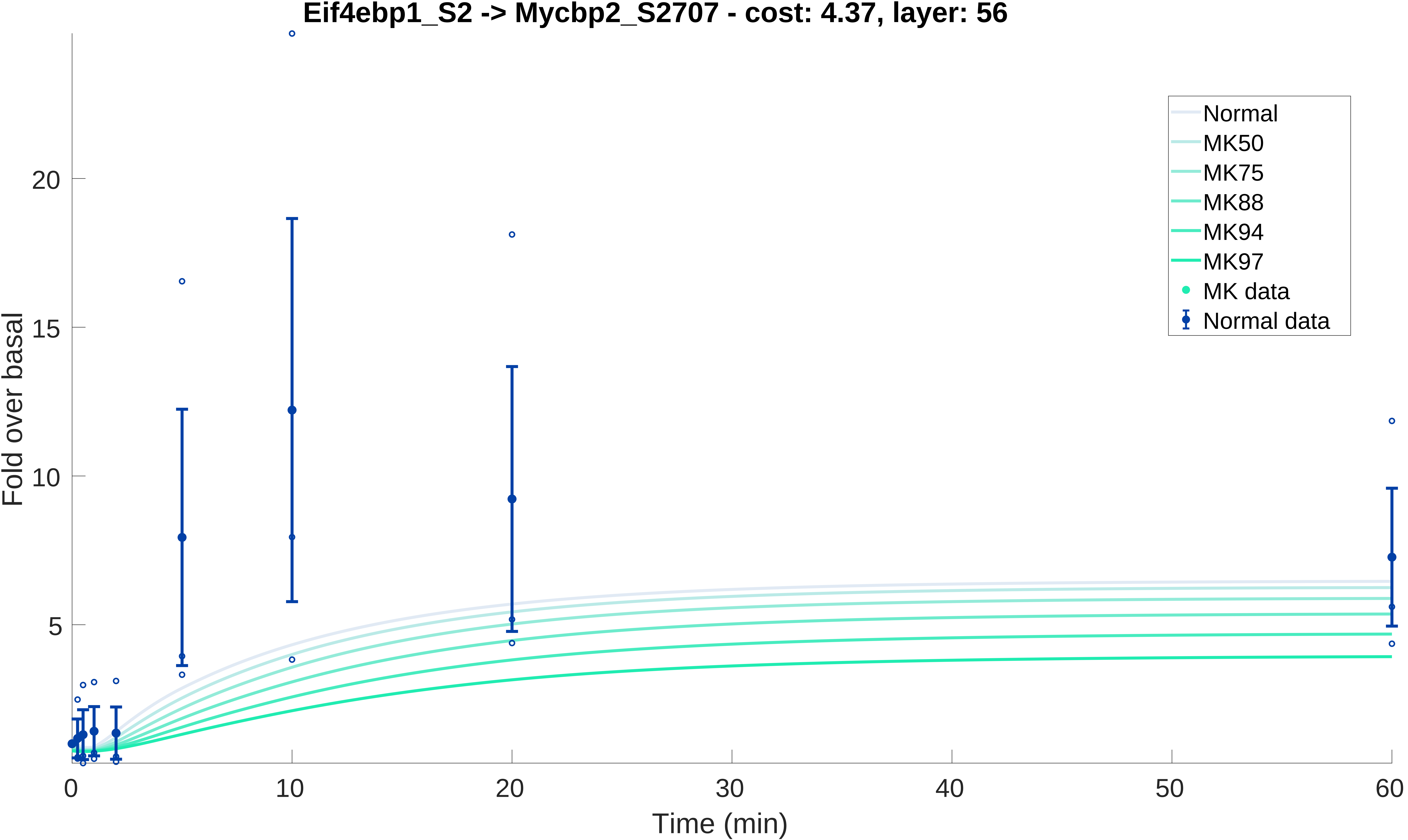
Eif4ebp1_S2 -> Mycbp2_S2707 - cost: 4.37, layer: 56
Fold over basal
Time (min)
0
10
20
30
40
50
60
5
10
15
20
Normal
MK50
MK75
MK88
MK94
MK97
MK data
Normal data

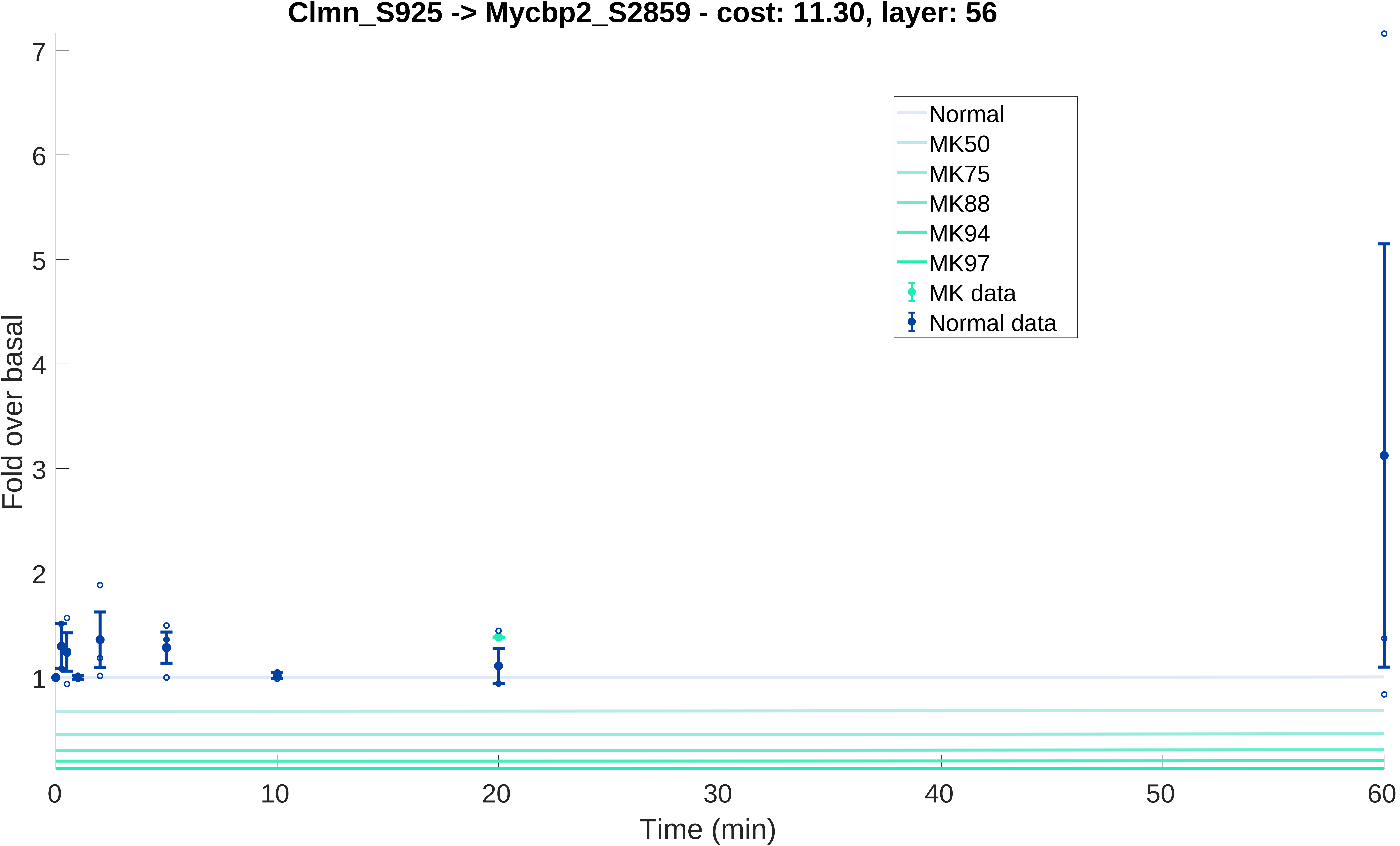

Clmn_S925 -> Mycbp2_S2859 - cost: 11.30, layer: 56
Fold over basal
Time (min)
Normal
MK50
MK75
MK88
MK94
MK97
MK data
Normal data
0
10
20
30
40
50
60
1
2
3
4
5
6
7

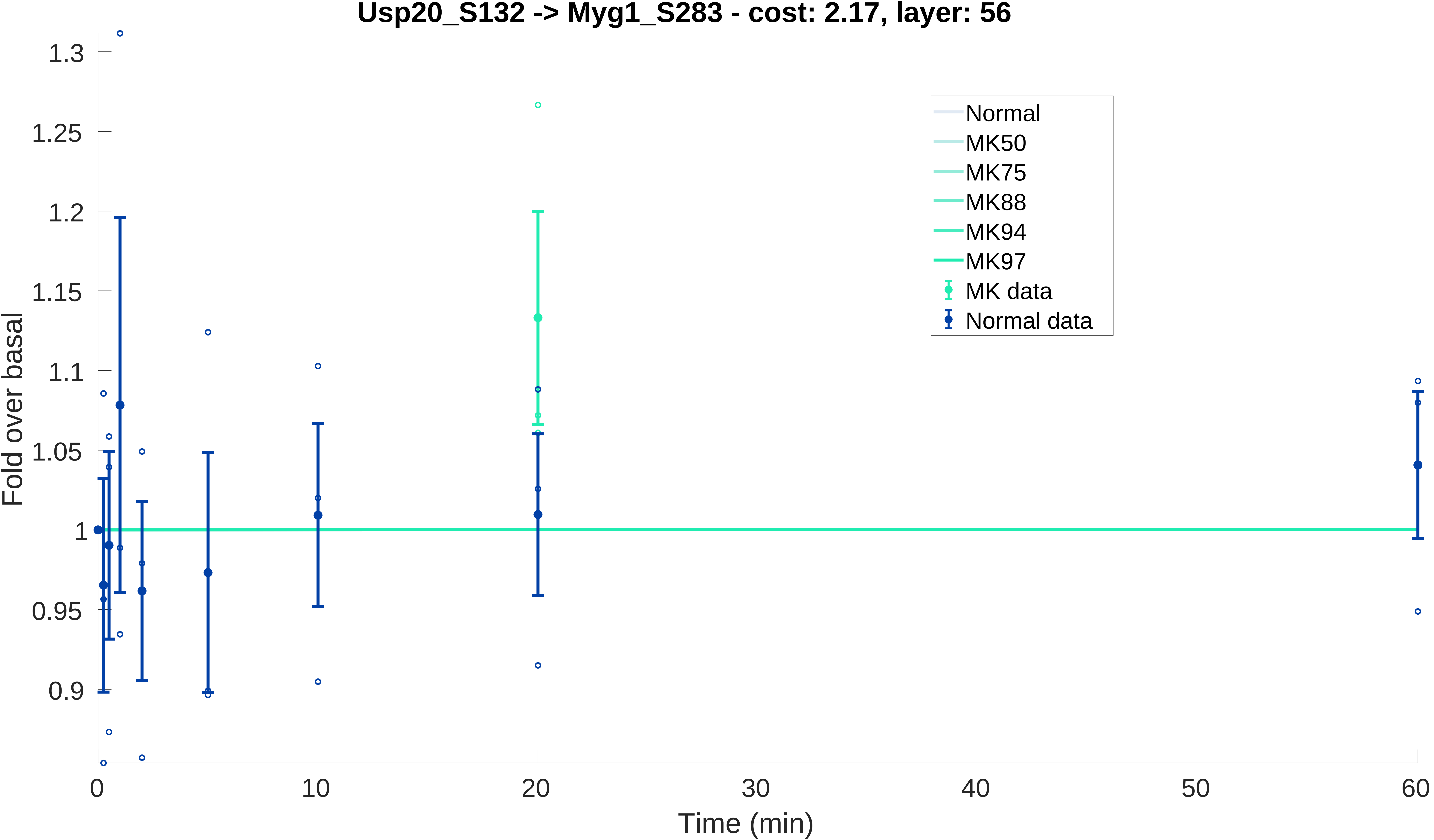
Usp20_S132 -> Myg1_S283 - cost: 2.17, layer: 56
Fold over basal
Time (min)
Normal
MK50
MK75
MK88
MK94
MK97
MK data
Normal data
1.3
1.25
1.2
1.15
1.1
1.05
1
0.95
0.9
0
10
20
30
40
50
60

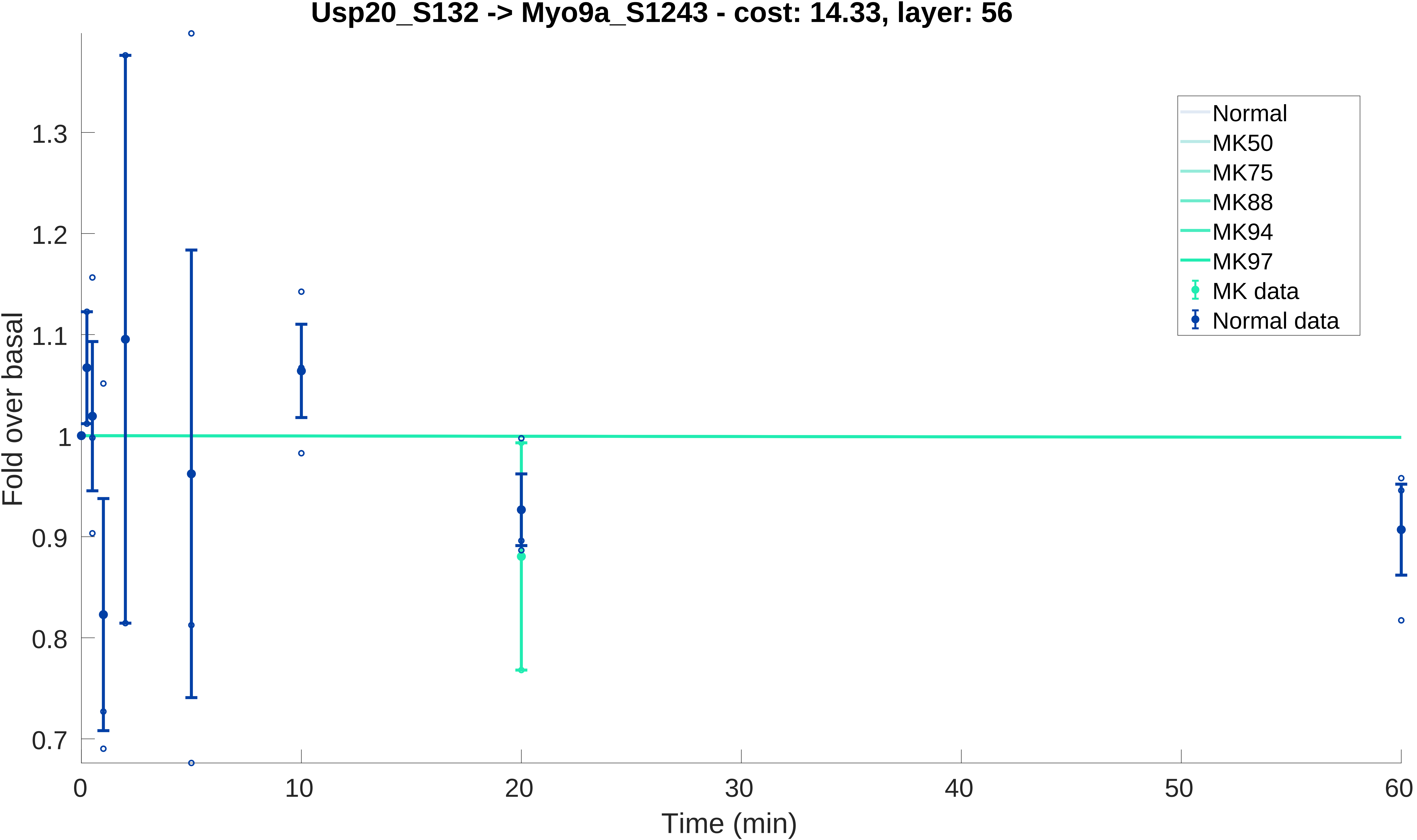

Usp20_S132 -> Myo9a_S1243 - cost: 14.33, layer: 56
Fold over basal
Time (min)
0.7
0.8
0.9
1
1.1
1.2
1.3
0
10
20
30
40
50
60
Normal
MK50
MK75
MK88
MK94
MK97
MK data
Normal data

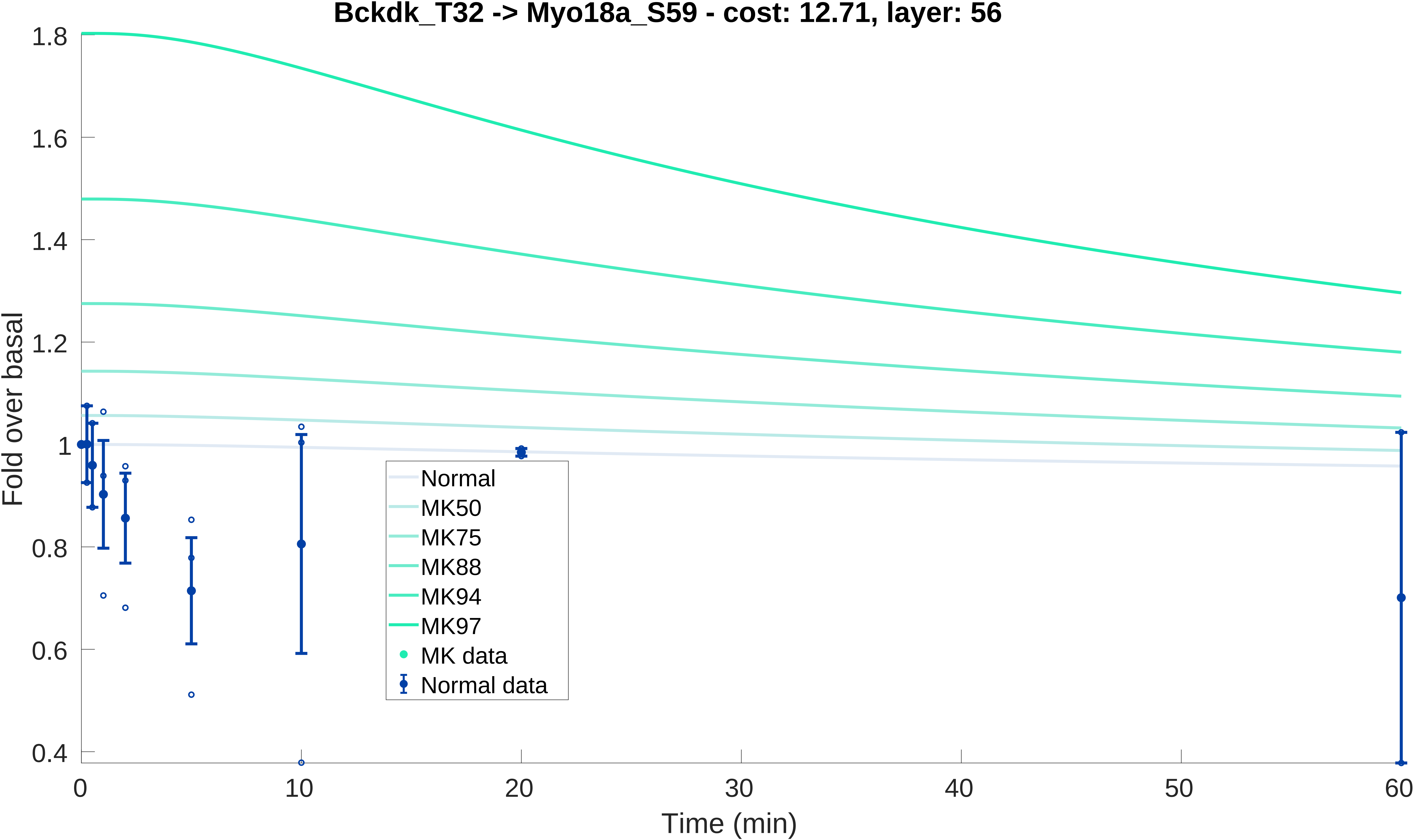
Bckdk_T32 -> Myo18a_S59 - cost: 12.71, layer: 56
Fold over basal
1.8
1.6
1.4
1.2
1
0.8
0.6
0.4
Time (min)
0
10
20
30
40
50
60
Normal
MK50
MK75
MK88
MK94
MK97
MK data
Normal data

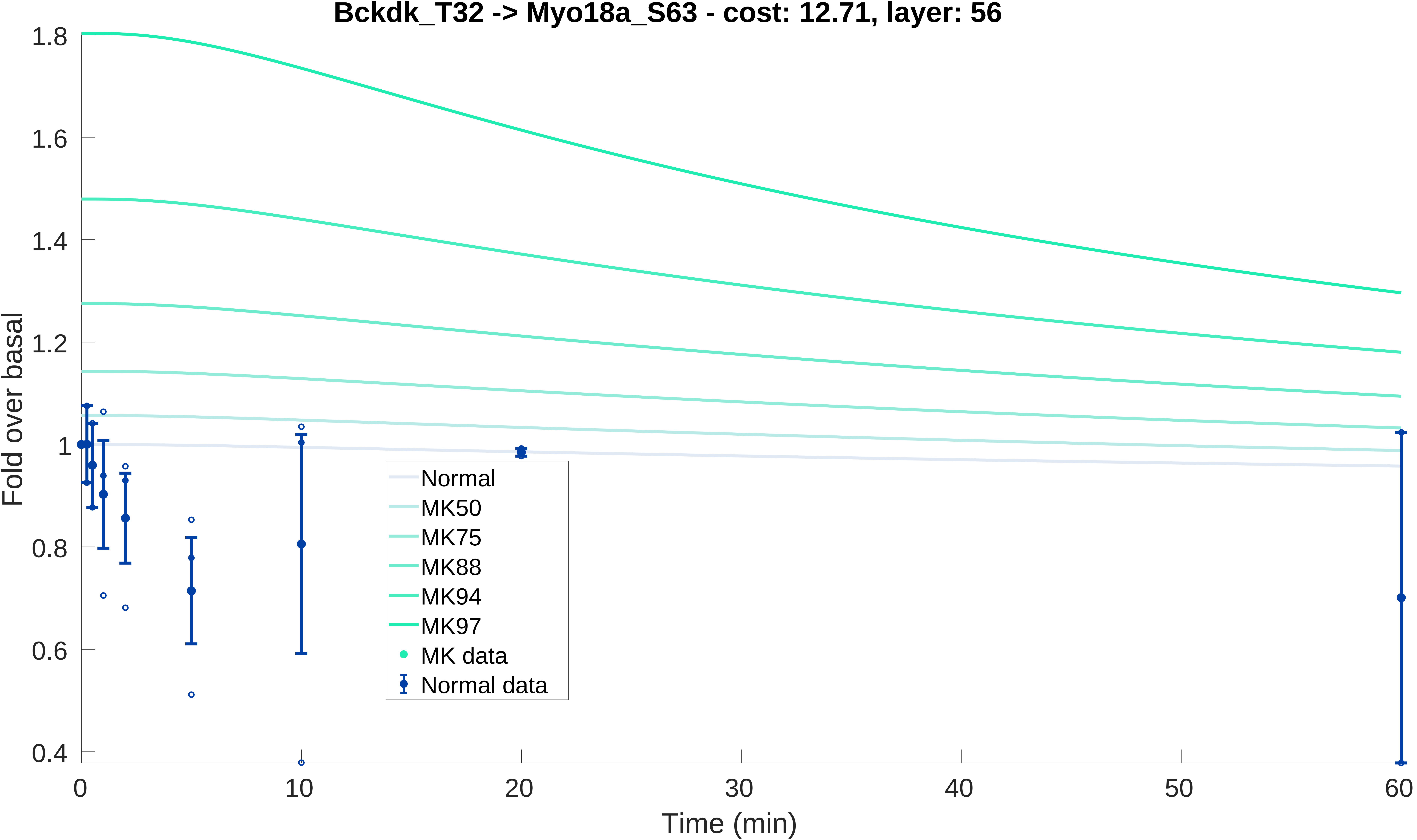

Bckdk_T32 -> Myo18a_S63 - cost: 12.71, layer: 56
Fold over basal
Time (min)
1.8
1.6
1.4
1.2
1
0.8
0.6
0.4
0
10
20
30
40
50
60
Normal
MK50
MK75
MK88
MK94
MK97
MK data
Normal data

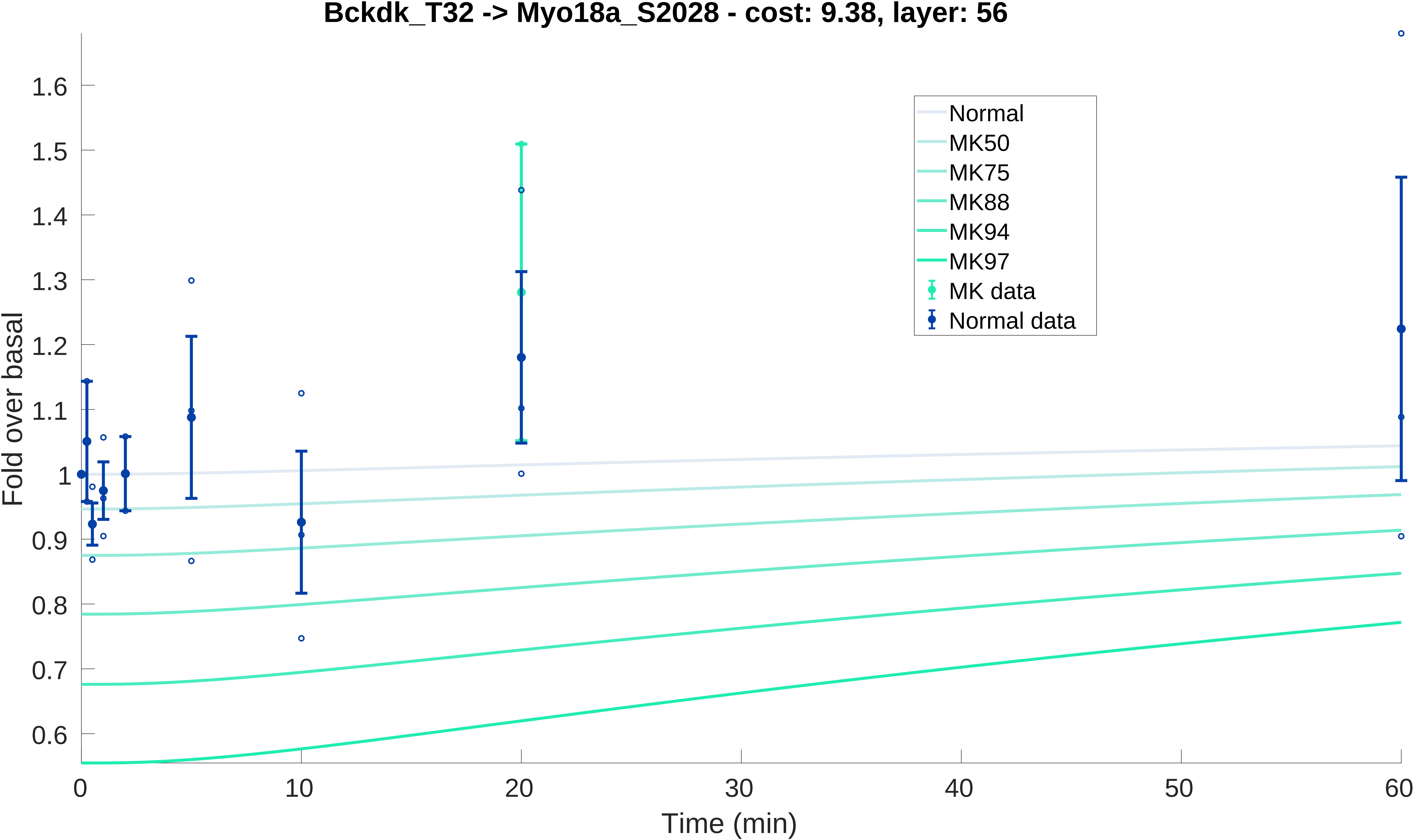

Bckdk_T32 -> Myo18a_S2028 - cost: 9.38, layer: 56
Fold over basal
1.6
1.5
1.4
1.3
1.2
1.1
1
0.9
0.8
0.7
0.6
0
10
20
30
40
50
60
Time (min)
Normal
MK50
MK75
MK88
MK94
MK97
MK data
Normal data

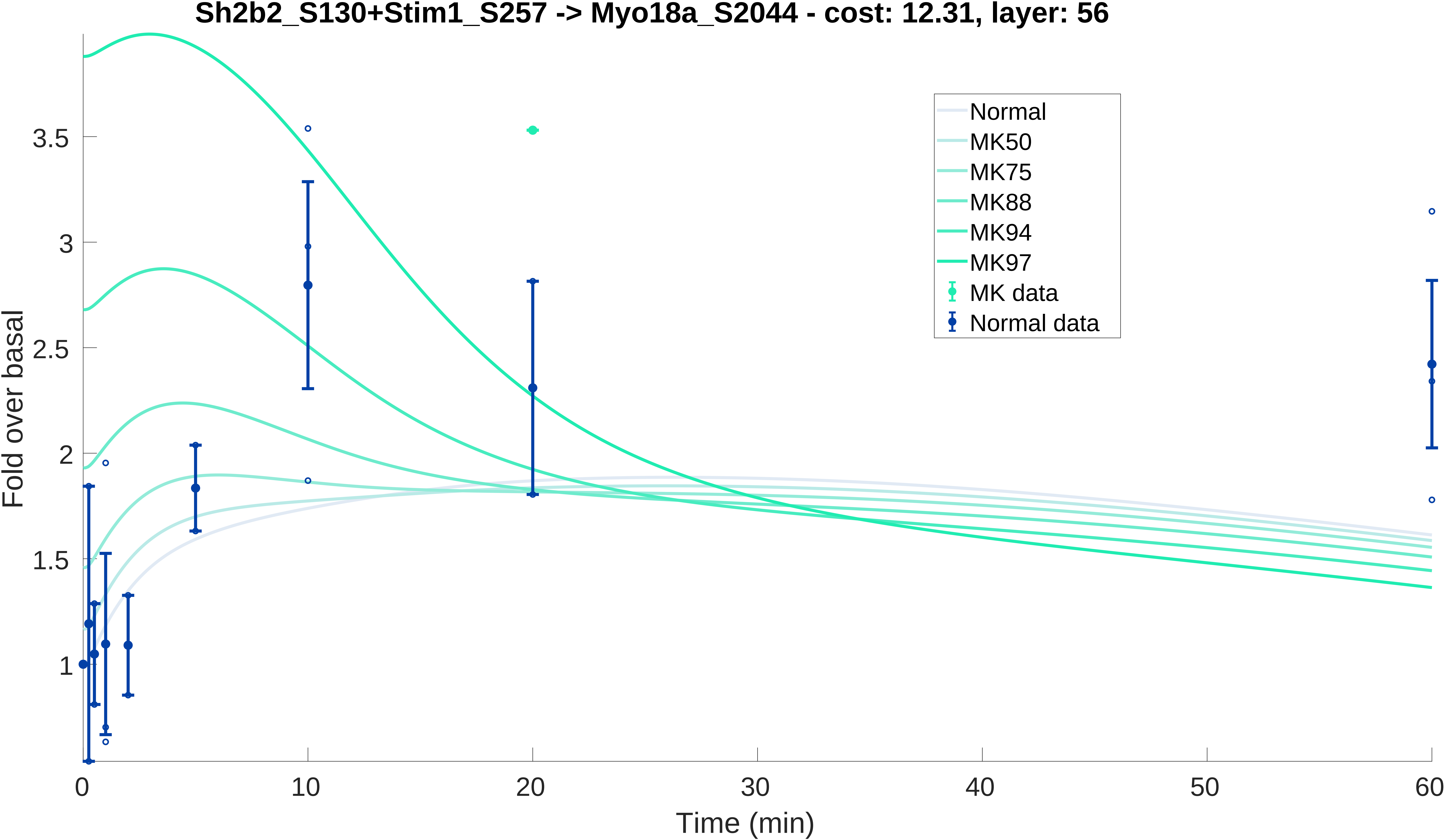

Sh2b2_S130+Stim1_S257 -> Myo18a_S2044 - cost: 12.31, layer: 56
Fold over basal
Time (min)
Normal
MK50
MK75
MK88
MK94
MK97
MK data
Normal data
3.5
3
2.5
2
1.5
1
0
10
20
30
40
50
60

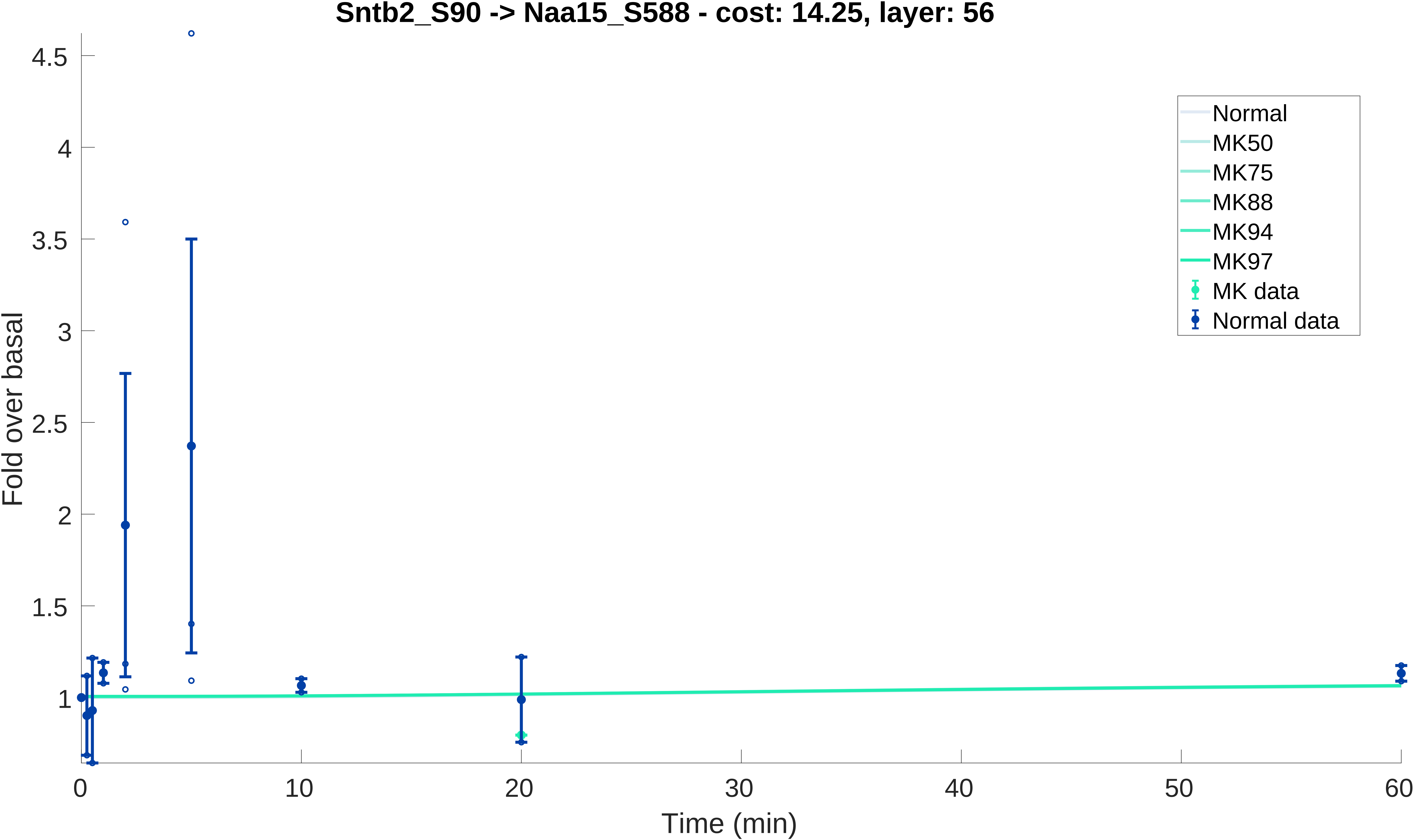
Sntb2_S90 -> Naa15_S588 - cost: 14.25, layer: 56
Fold over basal
Time (min)
4.5
4
3.5
3
2.5
2
1.5
1
0
10
20
30
40
50
60
Normal
MK50
MK75
MK88
MK94
MK97
MK data
Normal data

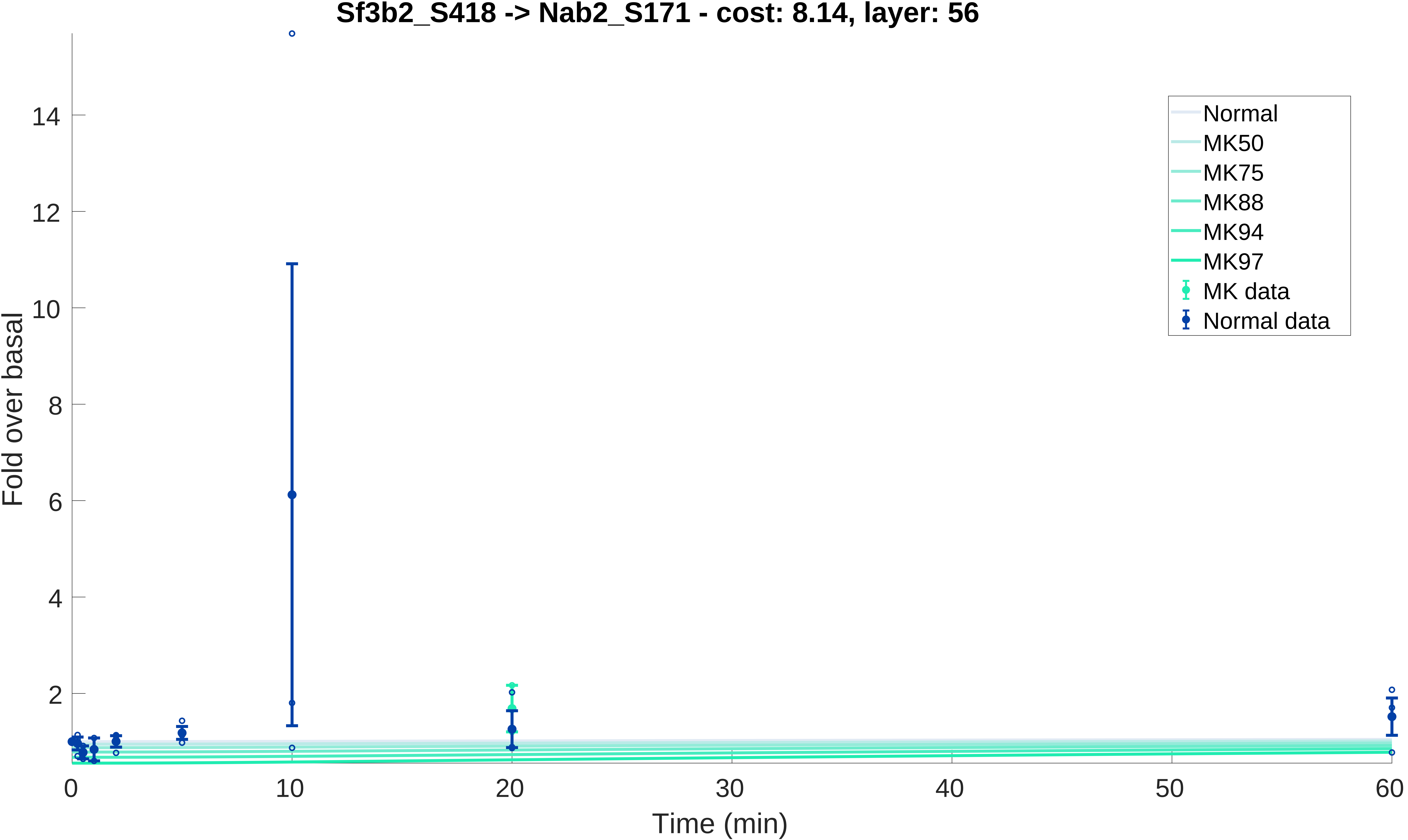

Sf3b2_S418 -> Nab2_S171 - cost: 8.14, layer: 56
Fold over basal
Time (min)
0
10
20
30
40
50
60
2
4
6
8
10
12
14
Normal
MK50
MK75
MK88
MK94
MK97
MK data
Normal data

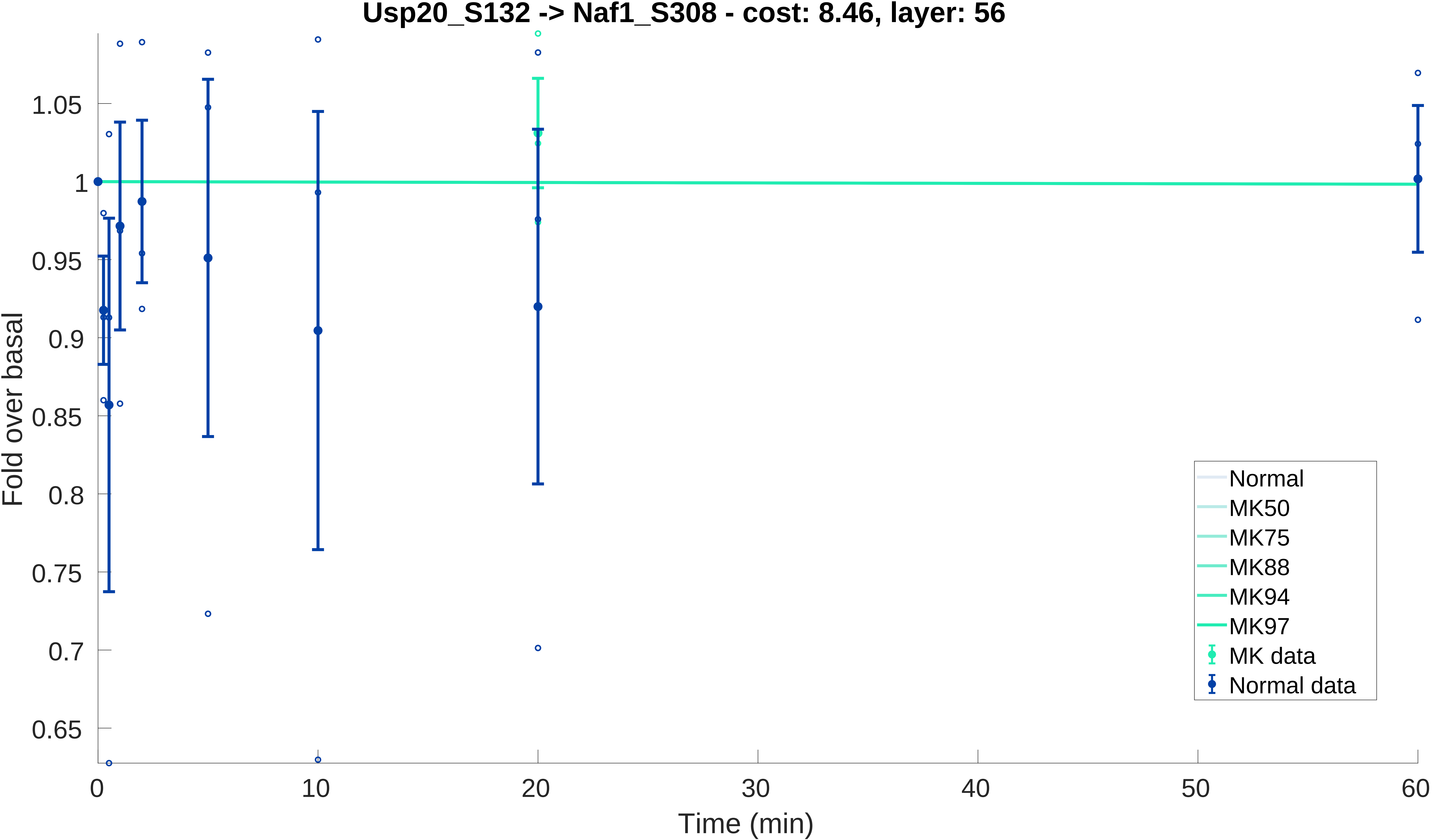

Usp20_S132 -> Naf1_S308 - cost: 8.46, layer: 56
Fold over basal
1.05
1
0.95
0.9
0.85
0.8
0.75
0.7
0.65
0
10
20
30
40
50
60
Time (min)
Normal
MK50
MK75
MK88
MK94
MK97
MK data
Normal data

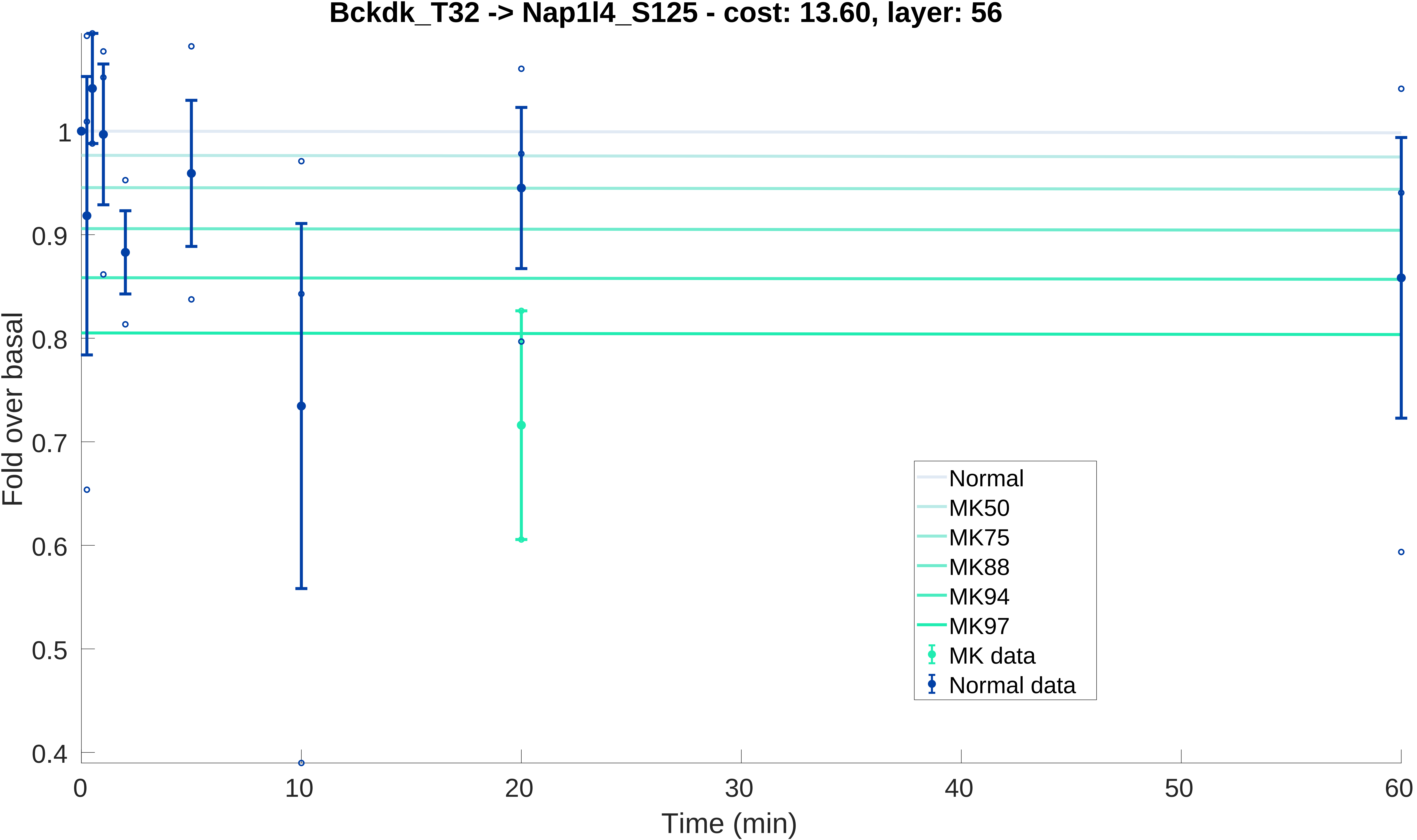

Bckdk_T32 -> Nap1l4_S125 - cost: 13.60, layer: 56
Fold over basal
Time (min)
0.4
0.5
0.6
0.7
0.8
0.9
1
0
10
20
30
40
50
60
Normal
MK50
MK75
MK88
MK94
MK97
MK data
Normal data

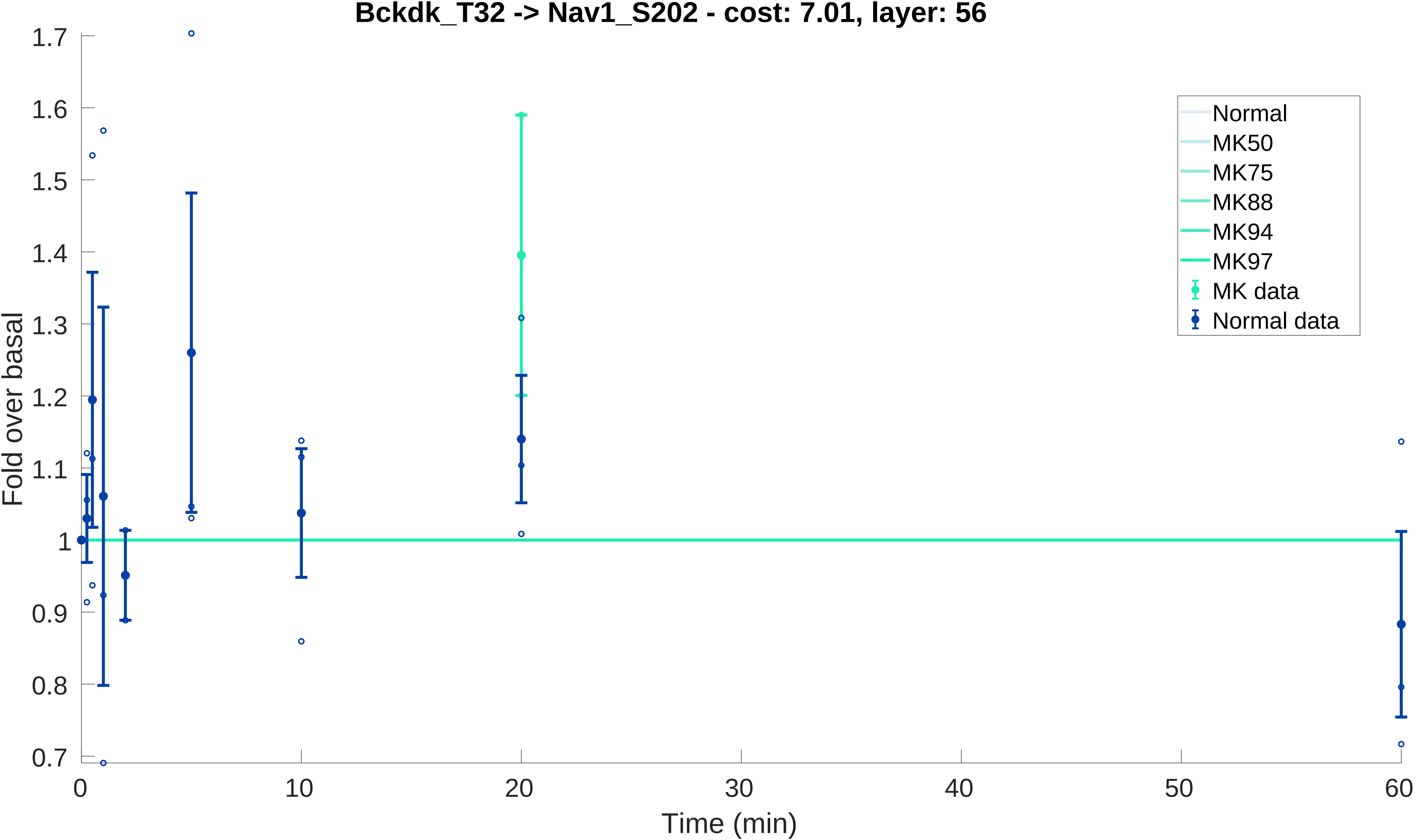

Bckdk_T32 -> Nav1_S202 - cost: 7.01, layer: 56
Fold over basal
Time (min)
1.7
1.6
1.5
1.4
1.3
1.2
1.1
1
0.9
0.8
0.7
0
10
20
30
40
50
60
Normal
MK50
MK75
MK88
MK94
MK97
MK data
Normal data

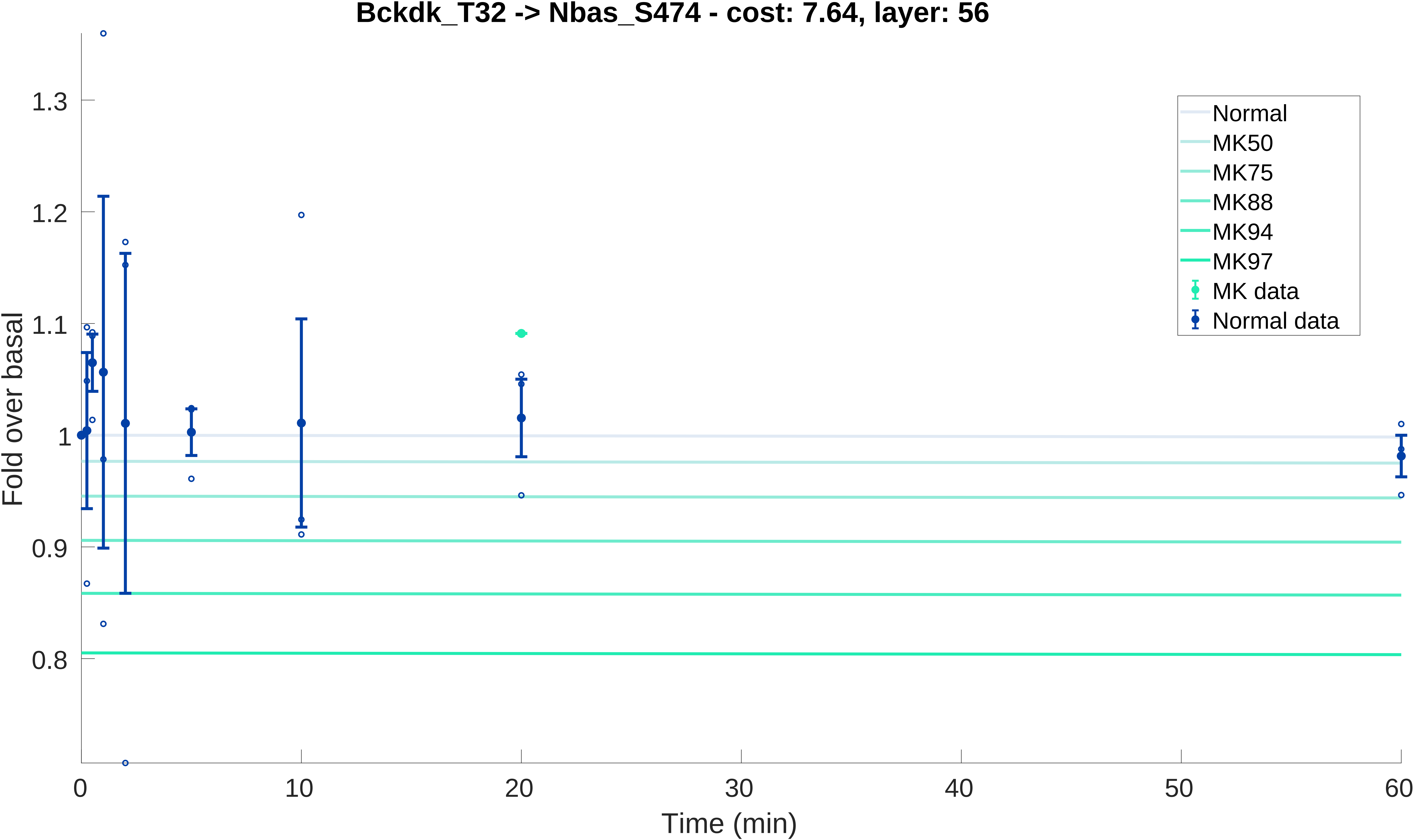

Bckdk_T32 -> Nbas_S474 - cost: 7.64, layer: 56
Fold over basal
Time (min)
1.3
1.2
1.1
1
0.9
0.8
0
10
20
30
40
50
60
Normal
MK50
MK75
MK88
MK94
MK97
MK data
Normal data

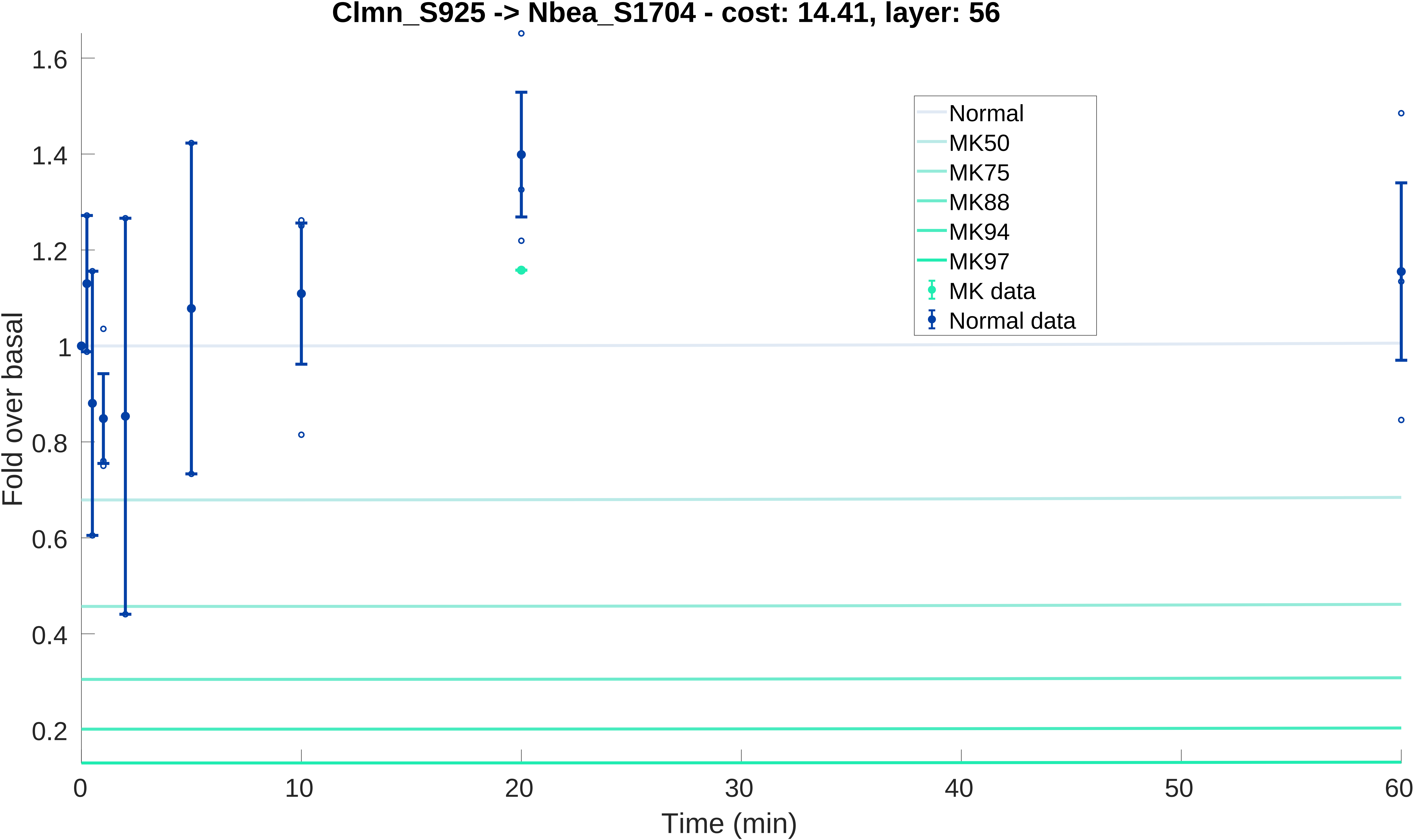
Clmn_S925 -> Nbea_S1704 - cost: 14.41, layer: 56
Fold over basal
Time (min)
1.6
1.4
1.2
1
0.8
0.6
0.4
0.2
0
10
20
30
40
50
60
Normal
MK50
MK75
MK88
MK94
MK97
MK data
Normal data

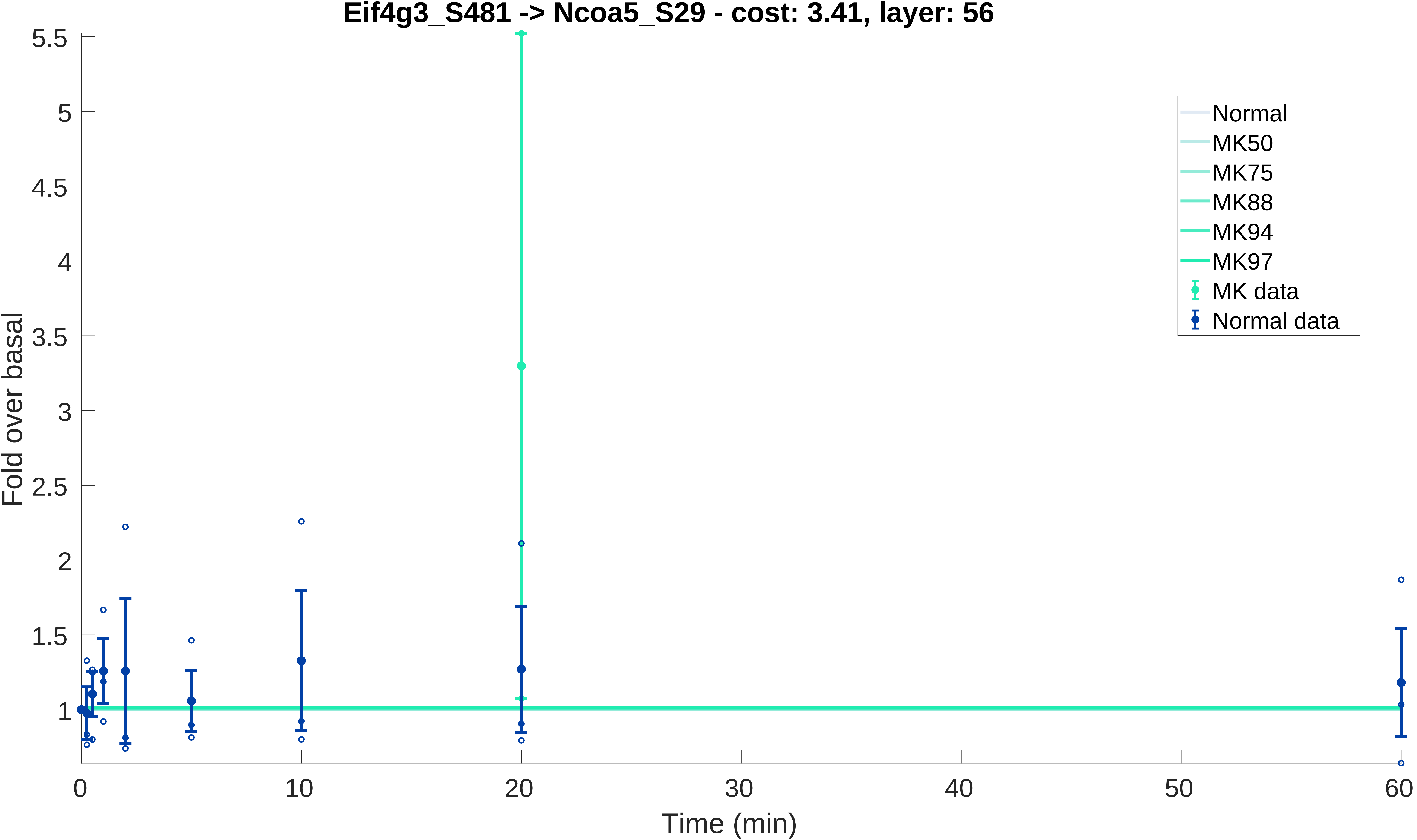

Eif4g3_S481 -> Ncoa5_S29 - cost: 3.41, layer: 56
Fold over basal
Time (min)
5.5
5
4.5
4
3.5
3
2.5
2
1.5
1
0
10
20
30
40
50
60
Normal
MK50
MK75
MK88
MK94
MK97
MK data
Normal data

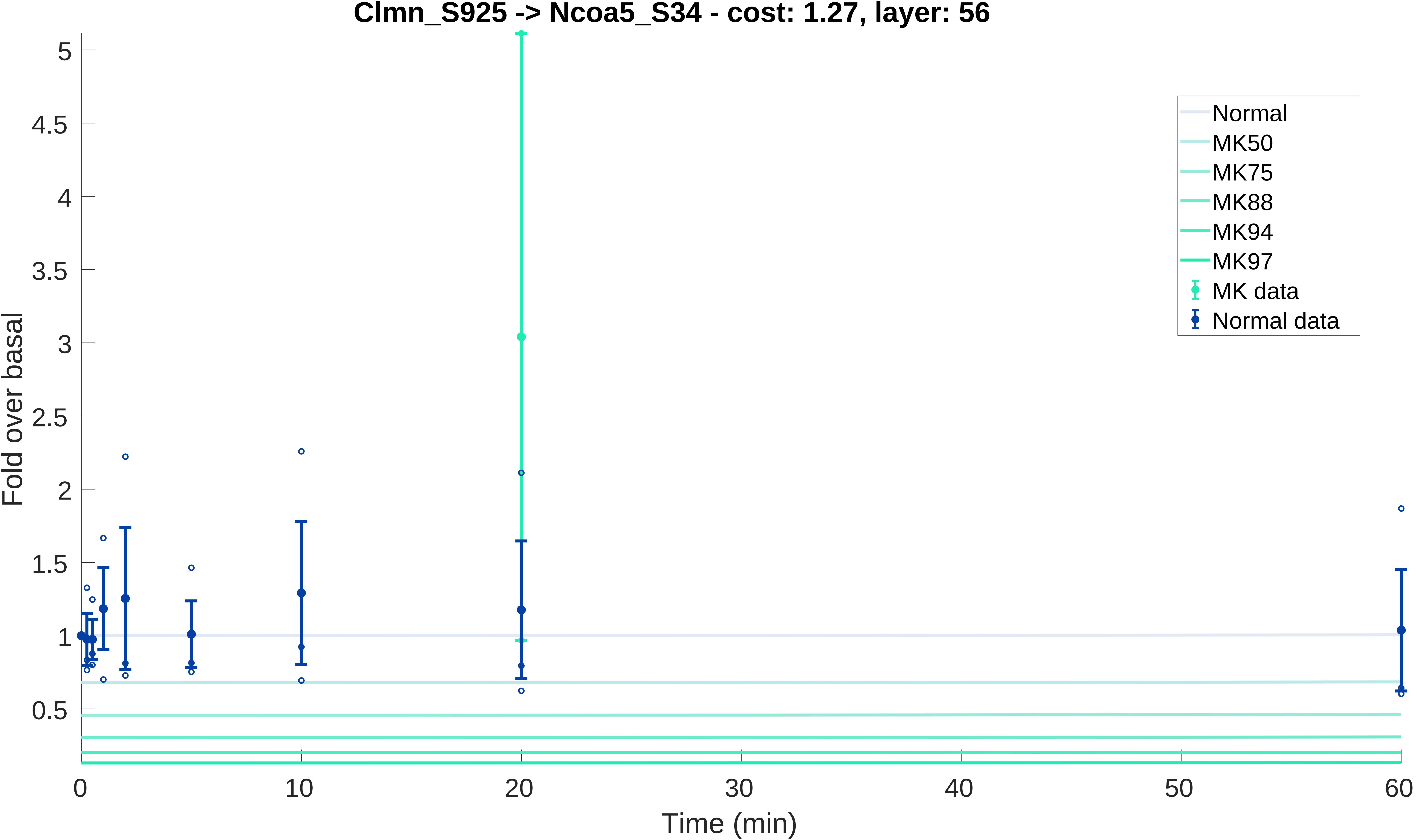

Clmn_S925 -> Ncoa5_S34 - cost: 1.27, layer: 56
Fold over basal
Time (min)
0
10
20
30
40
50
60
0.5
1
1.5
2
2.5
3
3.5
4
4.5
5
Normal
MK50
MK75
MK88
MK94
MK97
MK data
Normal data

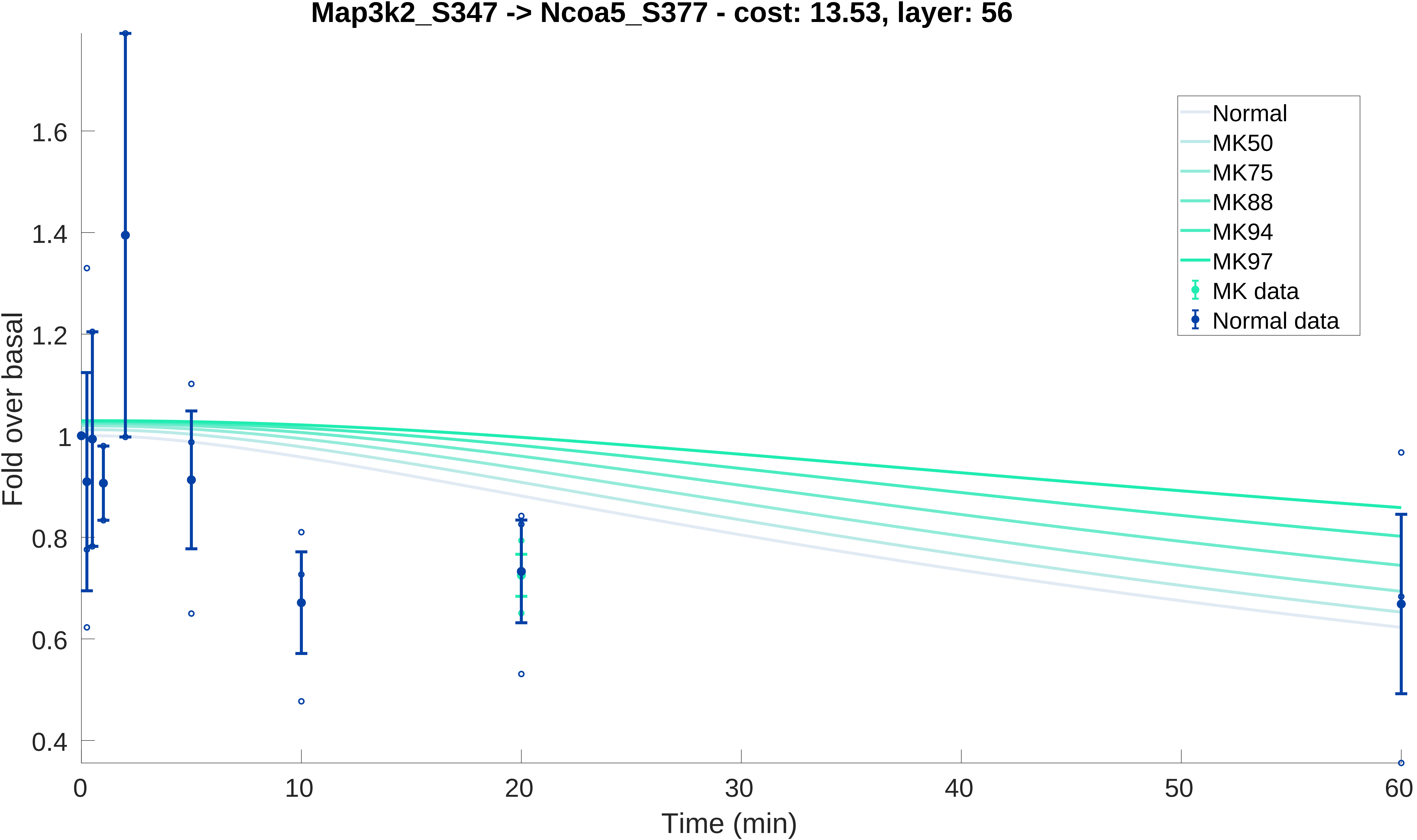
Map3k2_S347 -> Ncoa5_S377 - cost: 13.53, layer: 56
Fold over basal
Time (min)
Normal
MK50
MK75
MK88
MK94
MK97
MK data
Normal data
0.4
0.6
0.8
1
1.2
1.4
1.6
0
10
20
30
40
50
60

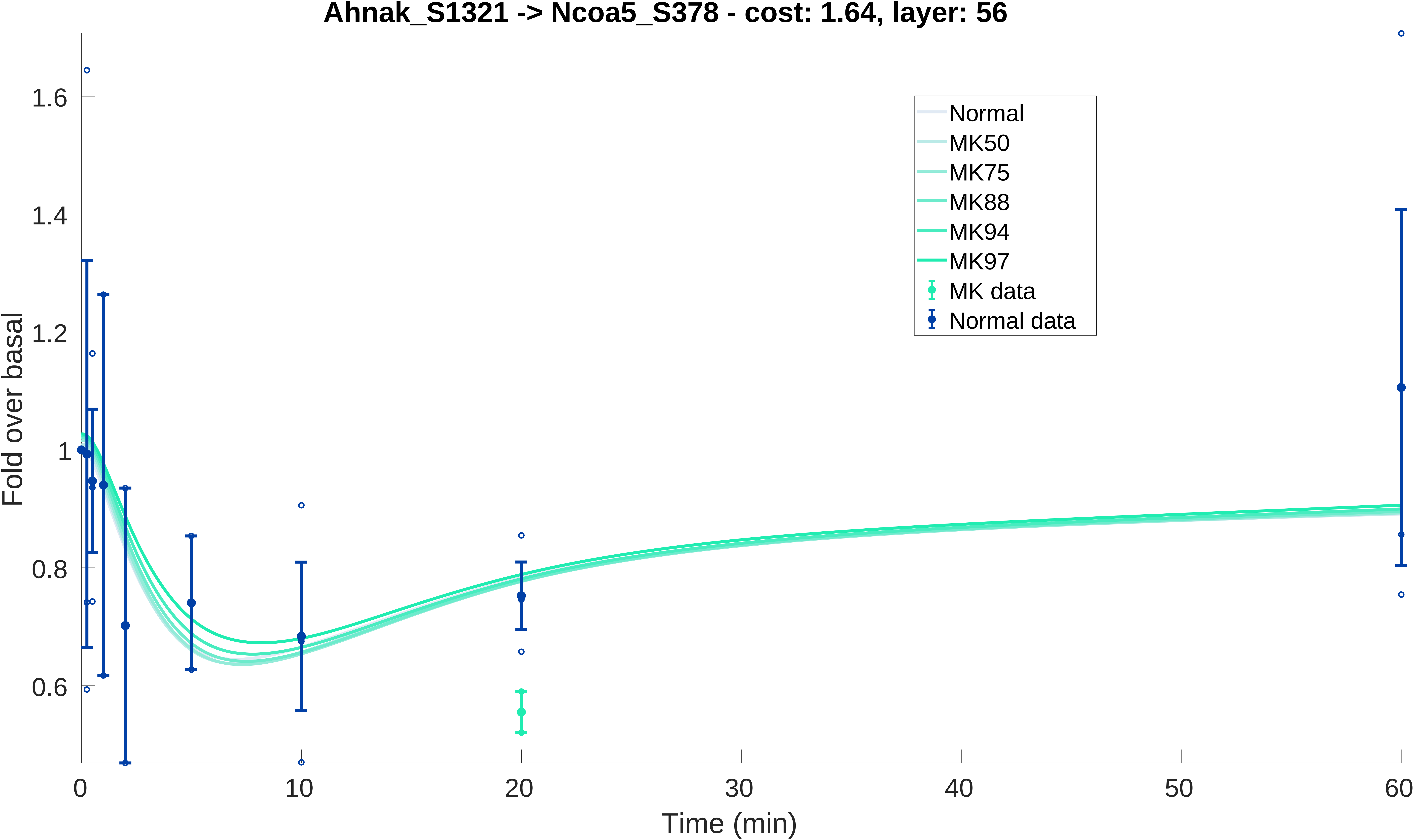
Ahnak_S1321 -> Ncoa5_S378 - cost: 1.64, layer: 56
Fold over basal
Time (min)
0.6
0.8
1
1.2
1.4
1.6
0
10
20
30
40
50
60
Normal
MK50
MK75
MK88
MK94
MK97
MK data
Normal data

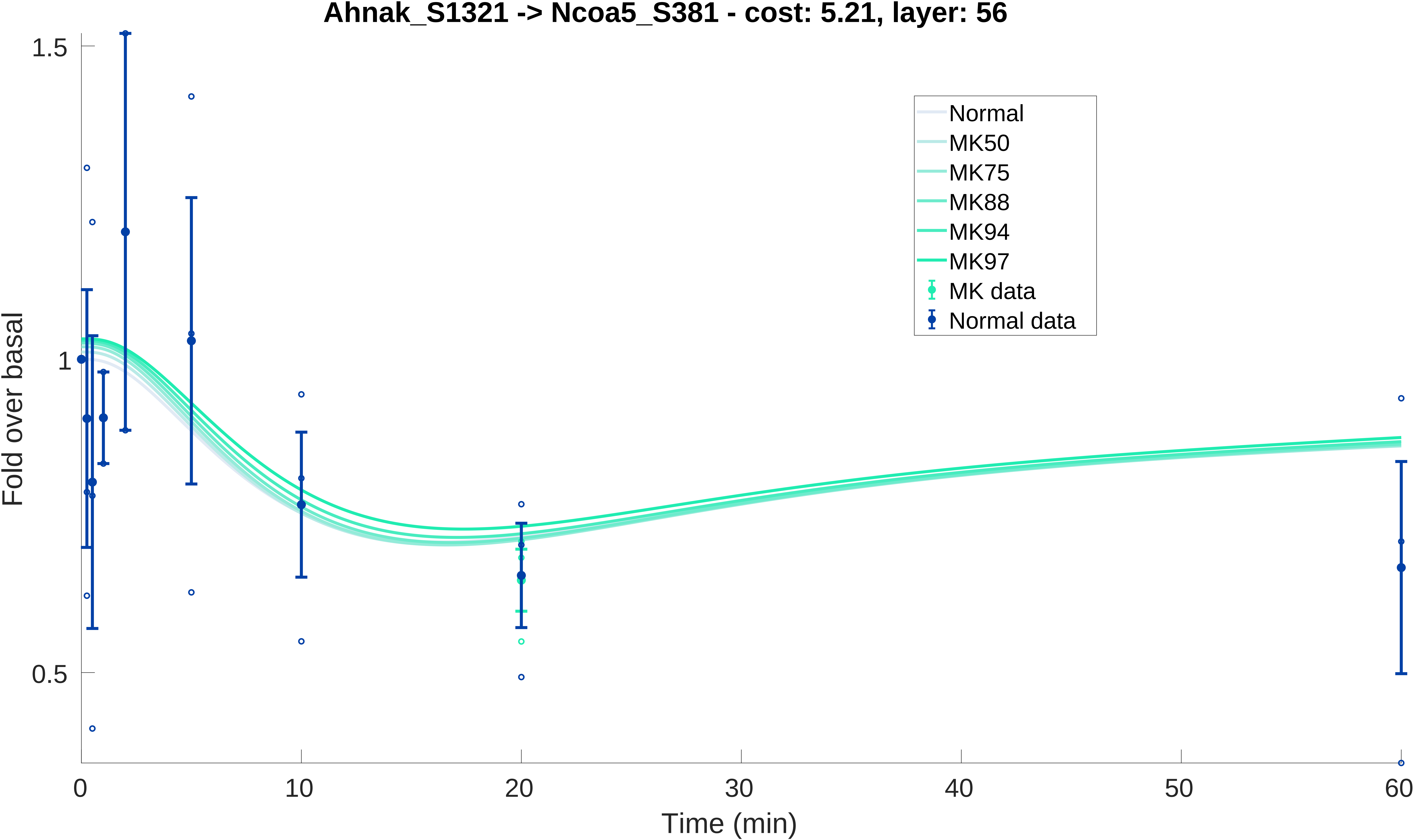
Ahnak_S1321 -> Ncoa5_S381 - cost: 5.21, layer: 56
Fold over basal
Time (min)
1.5
1
0.5
0
10
20
30
40
50
60
Normal
MK50
MK75
MK88
MK94
MK97
MK data
Normal data

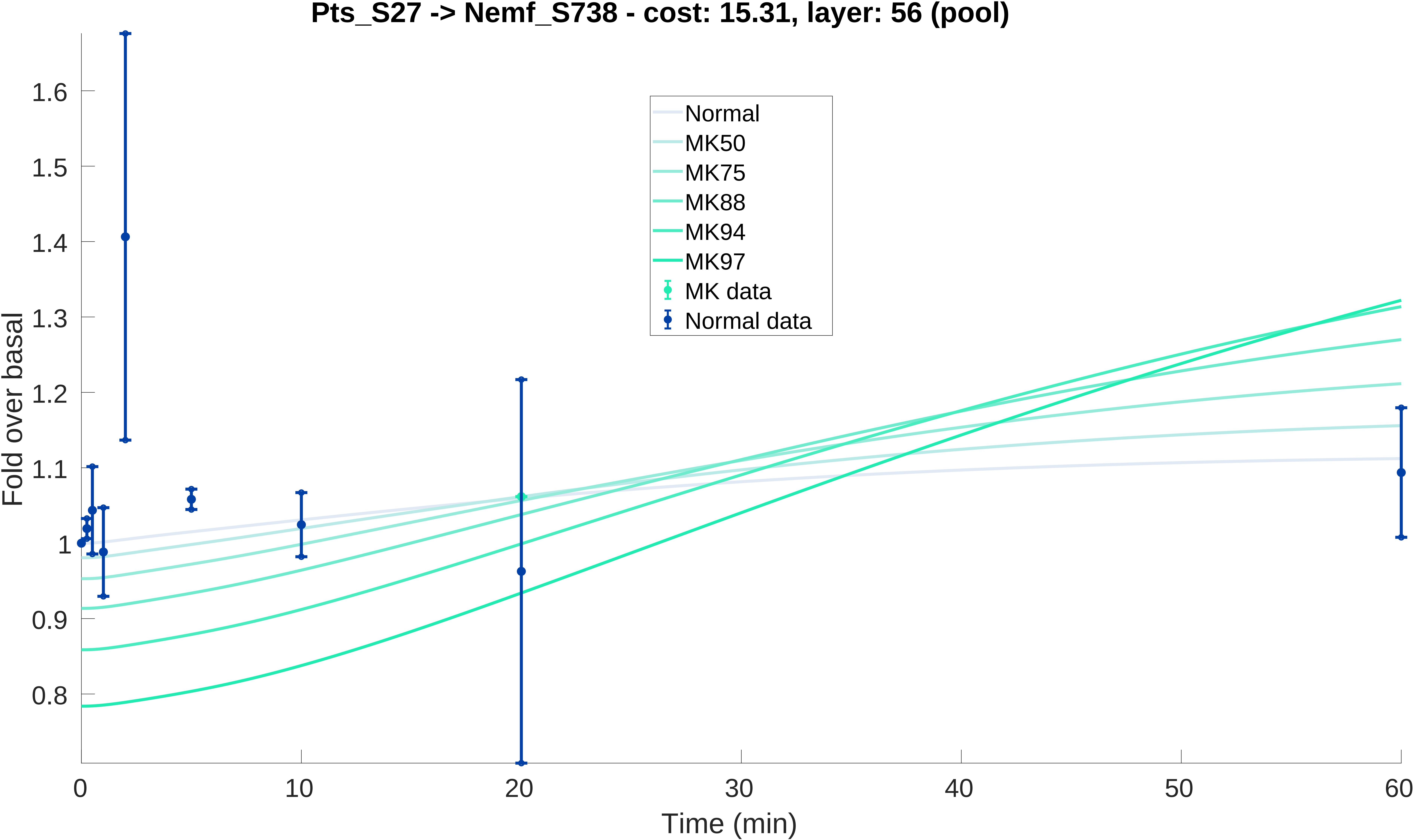

Pts_S27 -> Nemf_S738 - cost: 15.31, layer: 56 (pool)
Normal
MK50
MK75
MK88
MK94
MK97
MK data
Normal data
Fold over basal
Time (min)
1.6
1.5
1.4
1.3
1.2
1.1
1
0.9
0.8
0
10
20
30
40
50
60

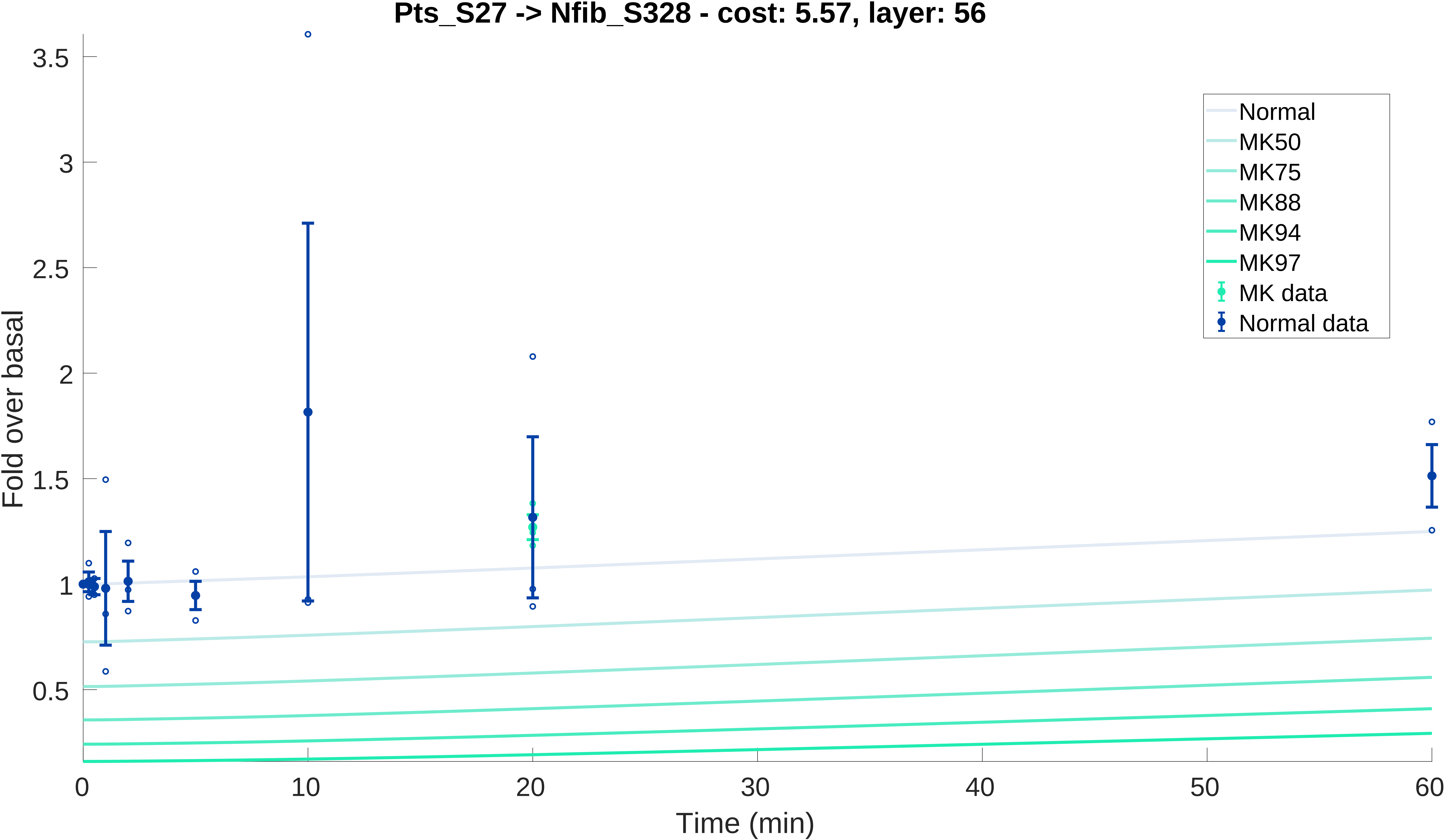

Pts_S27 -> Nfib_S328 - cost: 5.57, layer: 56
Fold over basal
3.5
3
2.5
2
1.5
1
0.5
0
10
20
30
40
50
60
Time (min)
Normal
MK50
MK75
MK88
MK94
MK97
MK data
Normal data

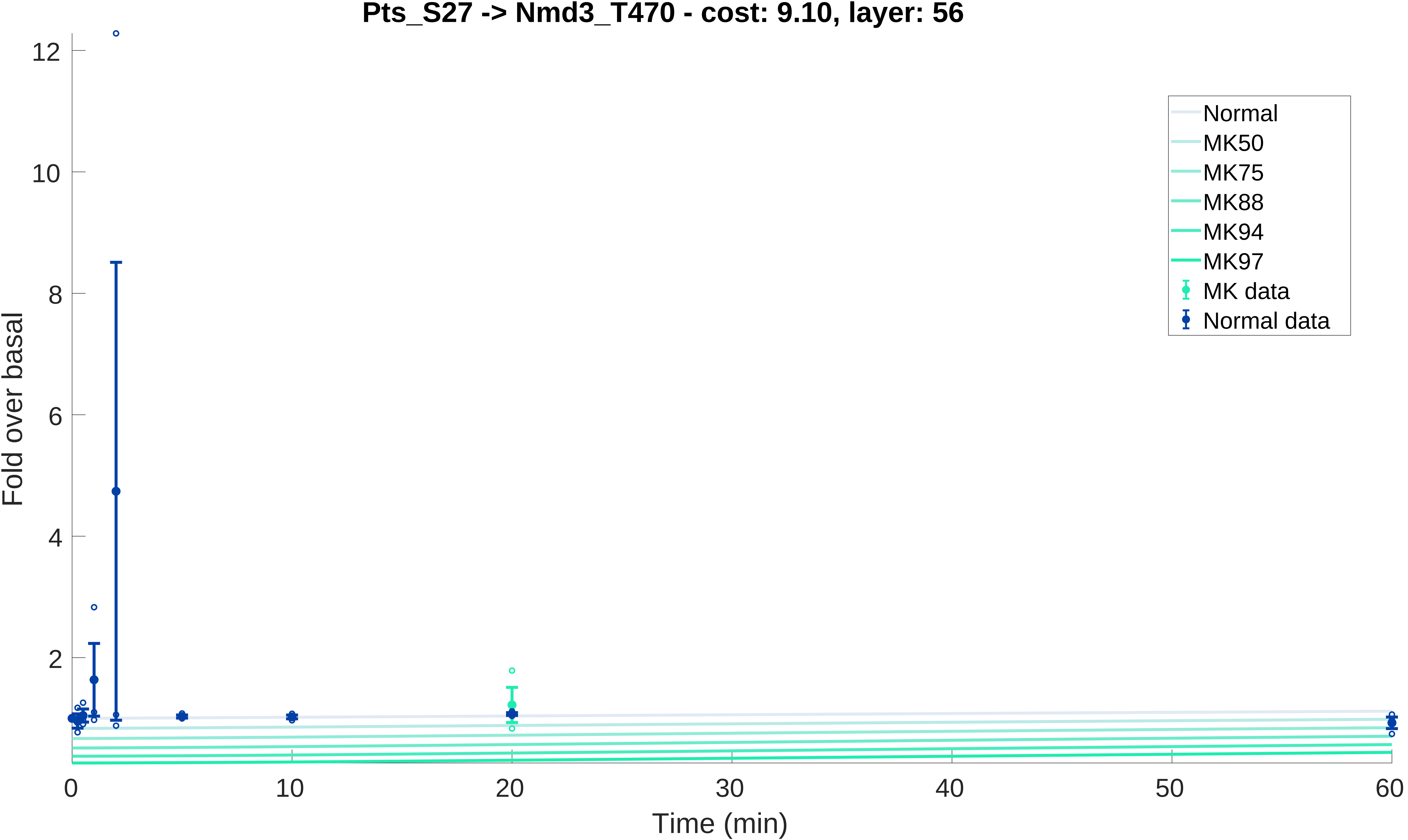

Pts_S27 -> Nmd3_T470 - cost: 9.10, layer: 56
Fold over basal
Time (min)
0
10
20
30
40
50
60
2
4
6
8
10
12
Normal
MK50
MK75
MK88
MK94
MK97
MK data
Normal data

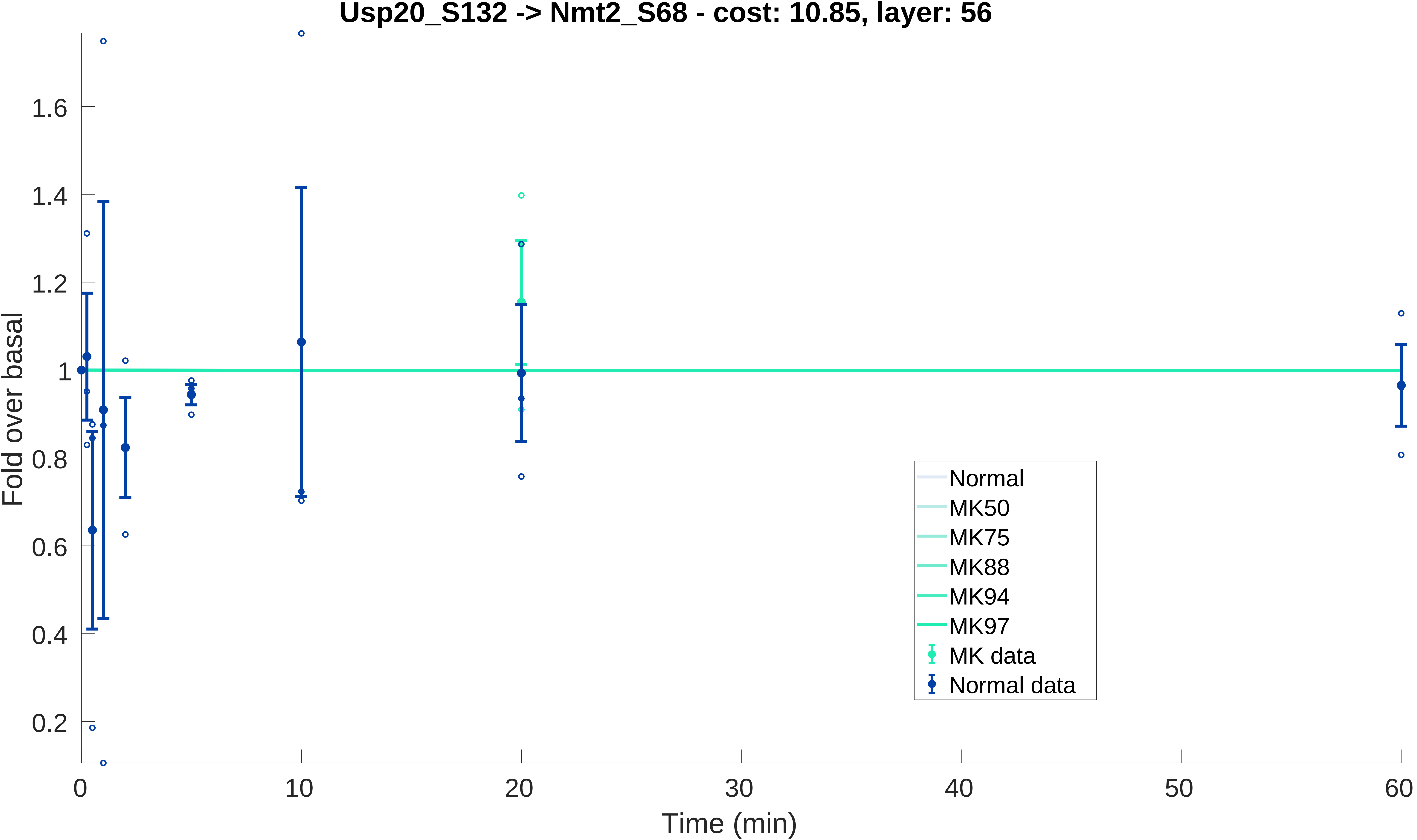

Usp20_S132 -> Nmt2_S68 - cost: 10.85, layer: 56
Fold over basal
Time (min)
1.6
1.4
1.2
1
0.8
0.6
0.4
0.2
0
10
20
30
40
50
60
Normal
MK50
MK75
MK88
MK94
MK97
MK data
Normal data

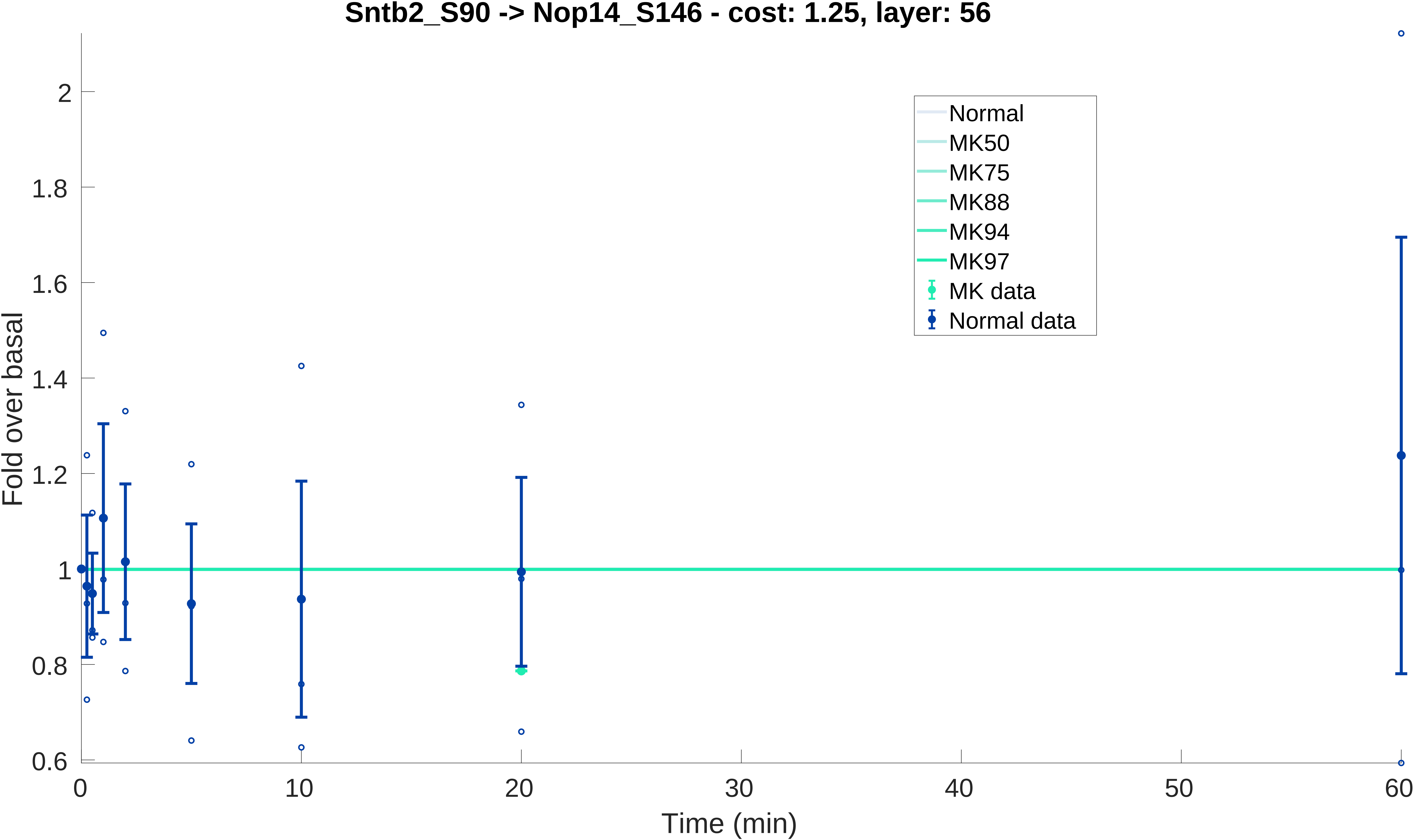

Sntb2_S90 -> Nop14_S146 - cost: 1.25, layer: 56
Normal
MK50
MK75
MK88
MK94
MK97
MK data
Normal data
Fold over basal
Time (min)
0.6
0.8
1
1.2
1.4
1.6
1.8
2
0
10
20
30
40
50
60

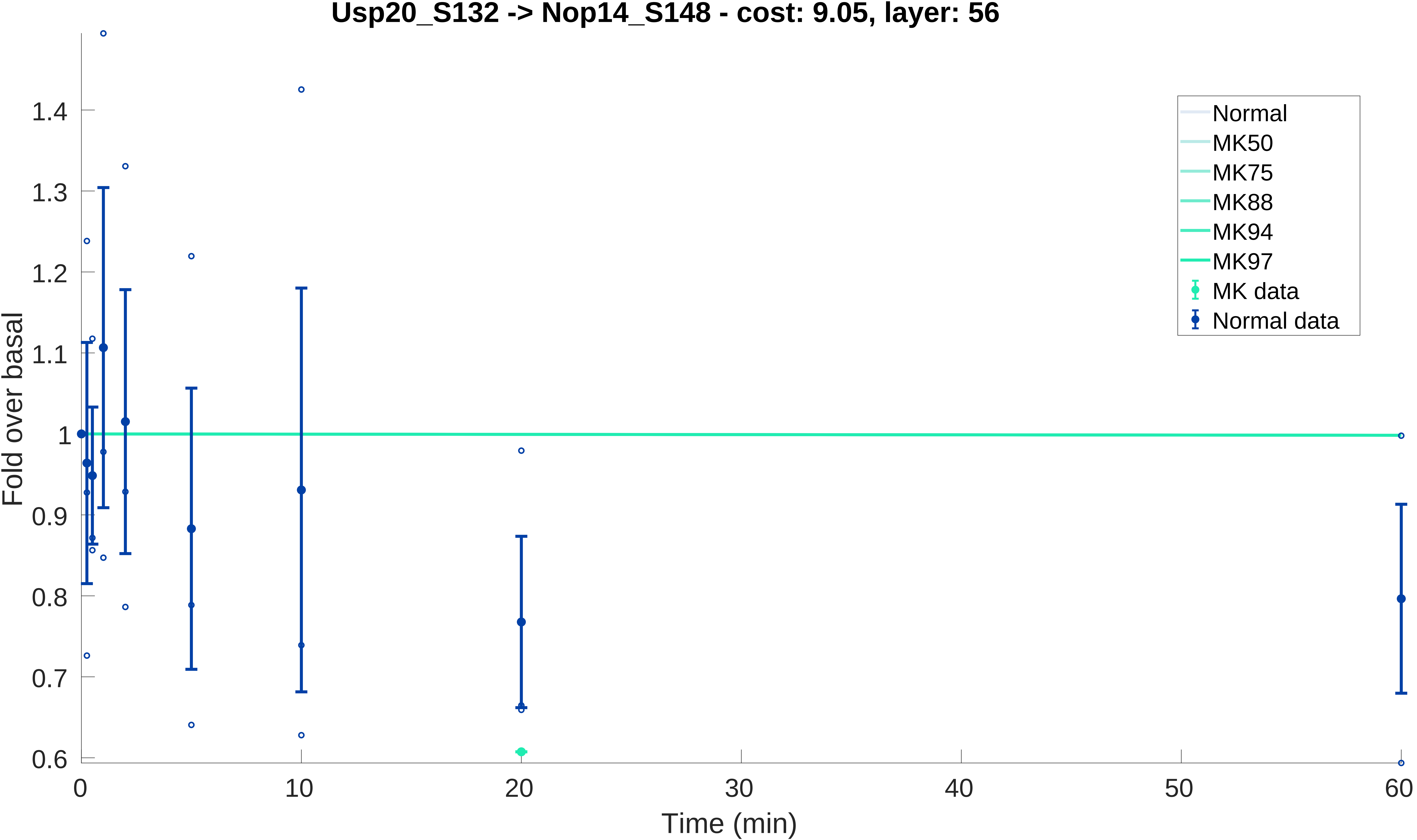

Usp20_S132 -> Nop14_S148 - cost: 9.05, layer: 56
Fold over basal
Time (min)
Normal
MK50
MK75
MK88
MK94
MK97
MK data
Normal data
0.6
0.7
0.8
0.9
1
1.1
1.2
1.3
1.4
0
10
20
30
40
50
60

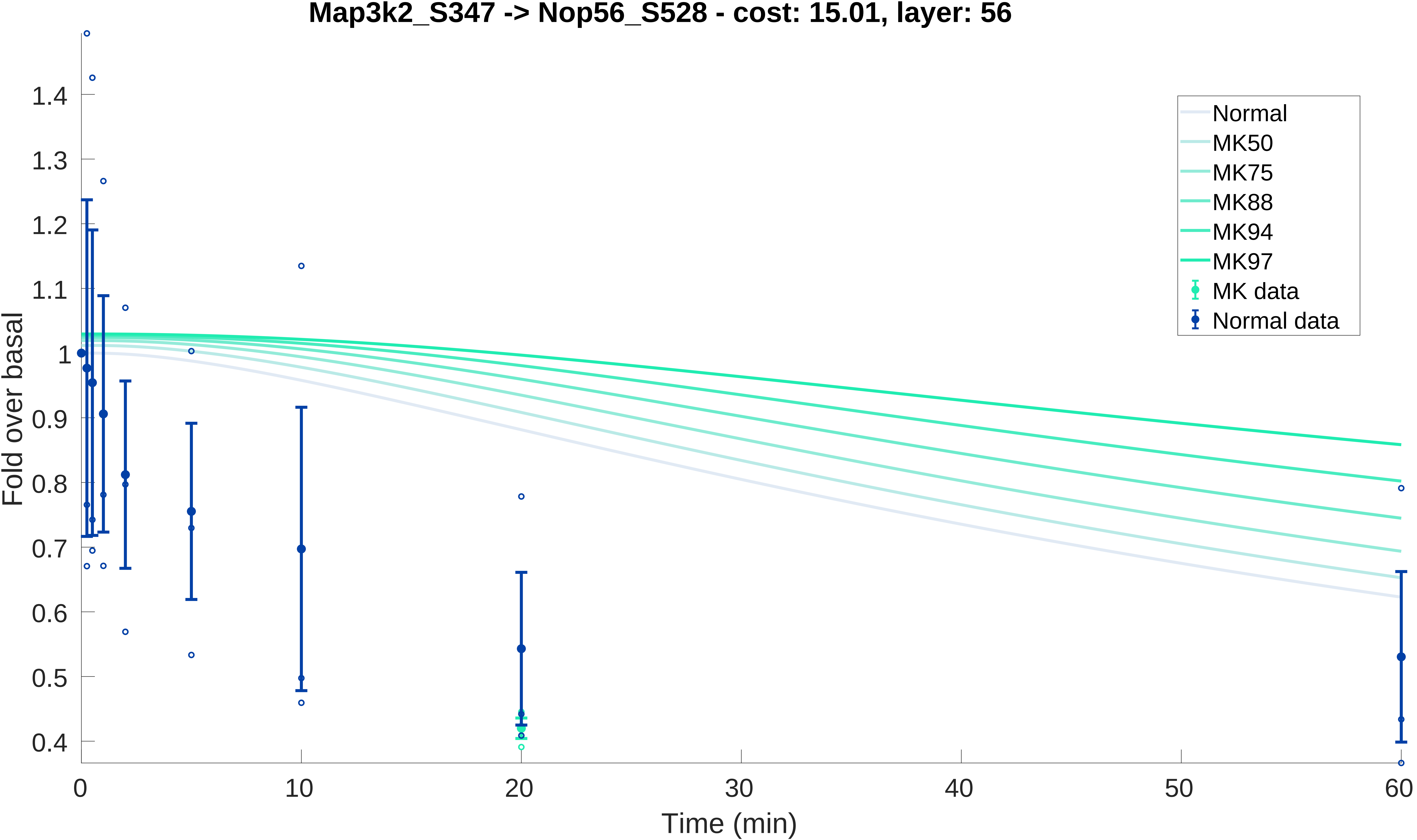

Map3k2_S347 -> Nop56_S528 - cost: 15.01, layer: 56
Fold over basal
Time (min)
0
10
20
30
40
50
60
0.4
0.5
0.6
0.7
0.8
0.9
1
1.1
1.2
1.3
1.4
Normal
MK50
MK75
MK88
MK94
MK97
MK data
Normal data

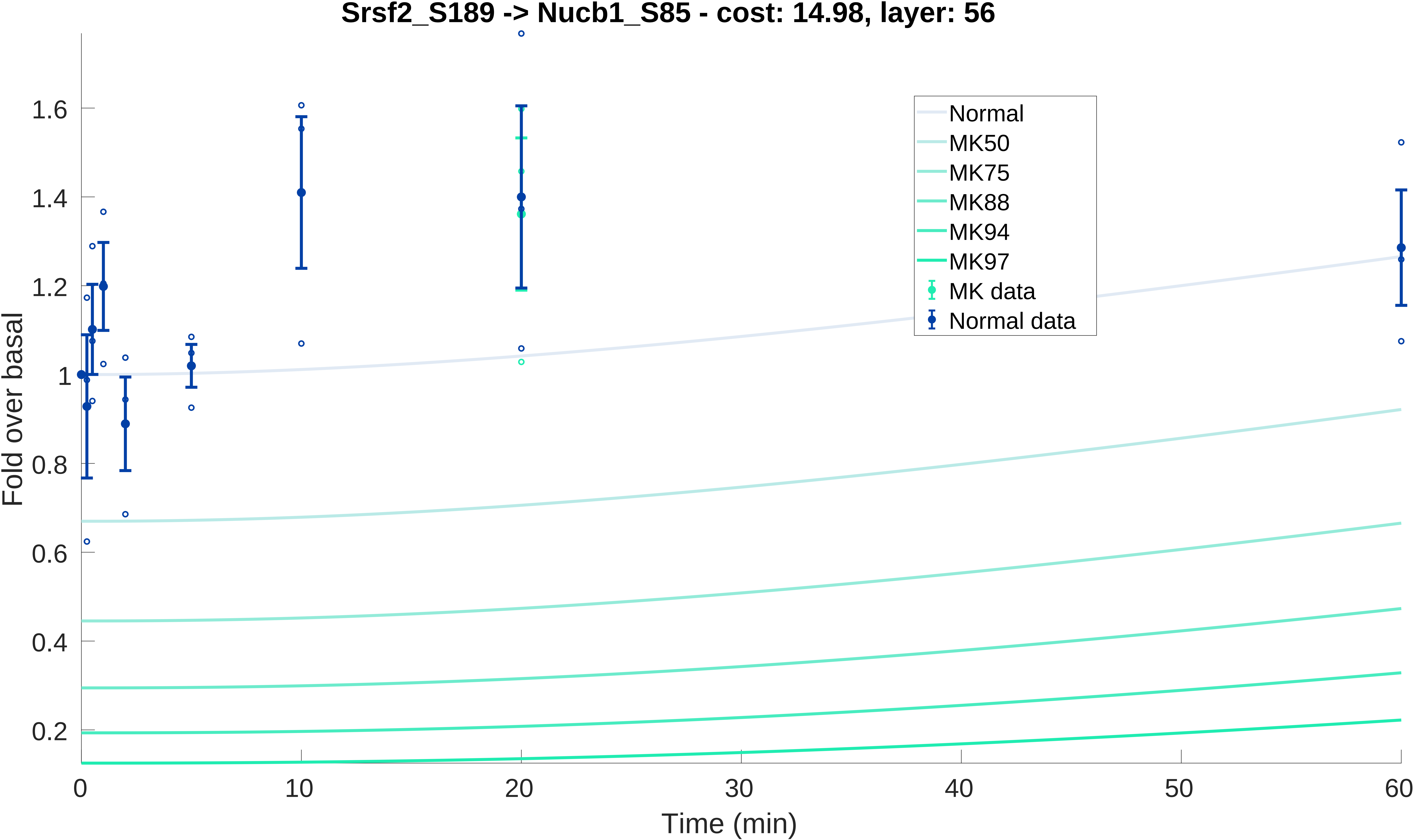

Srsf2_S189 -> Nucb1_S85 - cost: 14.98, layer: 56
Fold over basal
Time (min)
Normal
MK50
MK75
MK88
MK94
MK97
MK data
Normal data
0
10
20
30
40
50
60
0.2
0.4
0.6
0.8
1
1.2
1.4
1.6

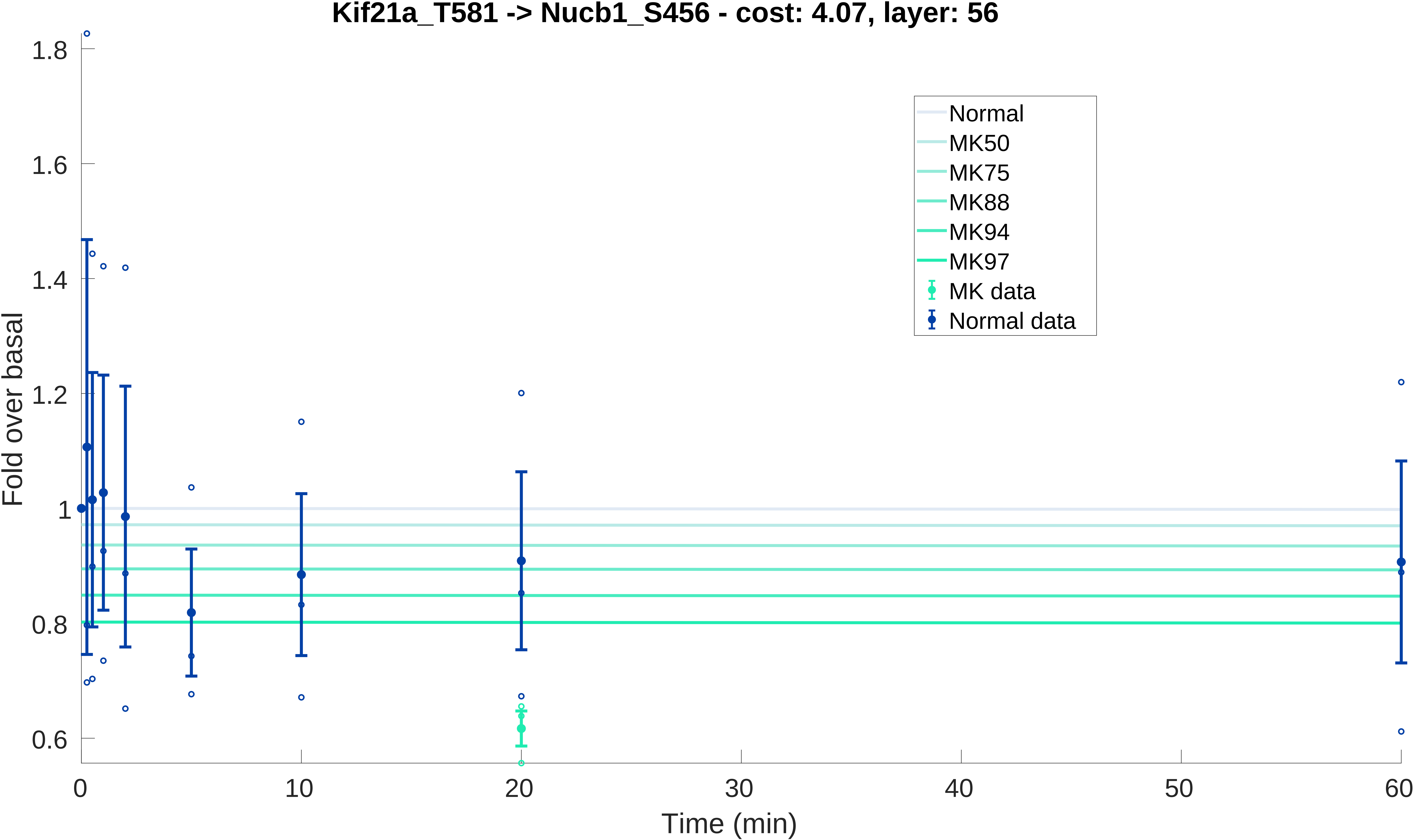

Kif21a_T581 -> Nucb1_S456 - cost: 4.07, layer: 56
Fold over basal
Time (min)
1.8
1.6
1.4
1.2
1
0.8
0.6
0
10
20
30
40
50
60
Normal
MK50
MK75
MK88
MK94
MK97
MK data
Normal data

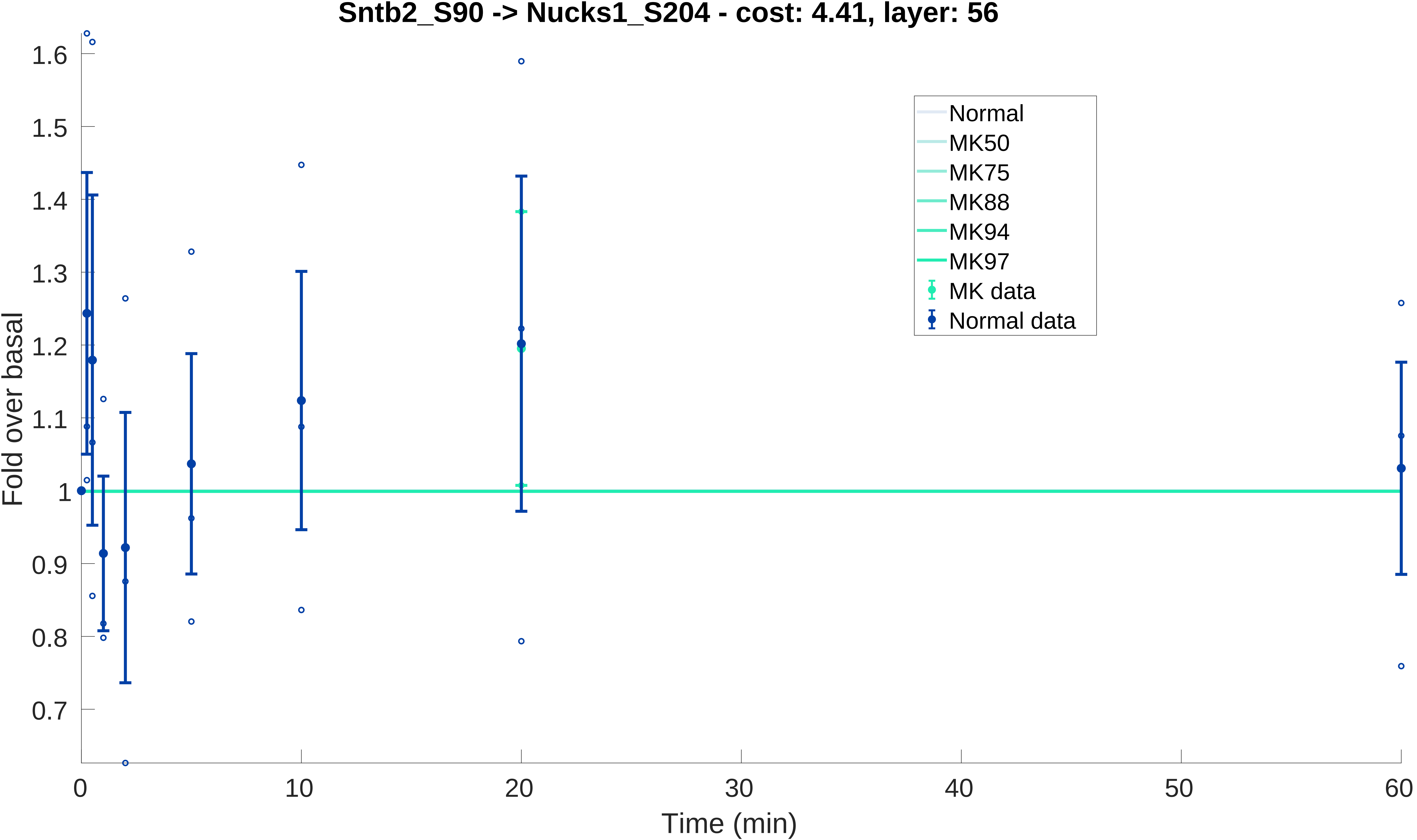

Sntb2_S90 -> Nucks1_S204 - cost: 4.41, layer: 56
Fold over basal
Time (min)
Normal
MK50
MK75
MK88
MK94
MK97
MK data
Normal data
1.6
1.5
1.4
1.3
1.2
1.1
1
0.9
0.8
0.7
0
10
20
30
40
50
60

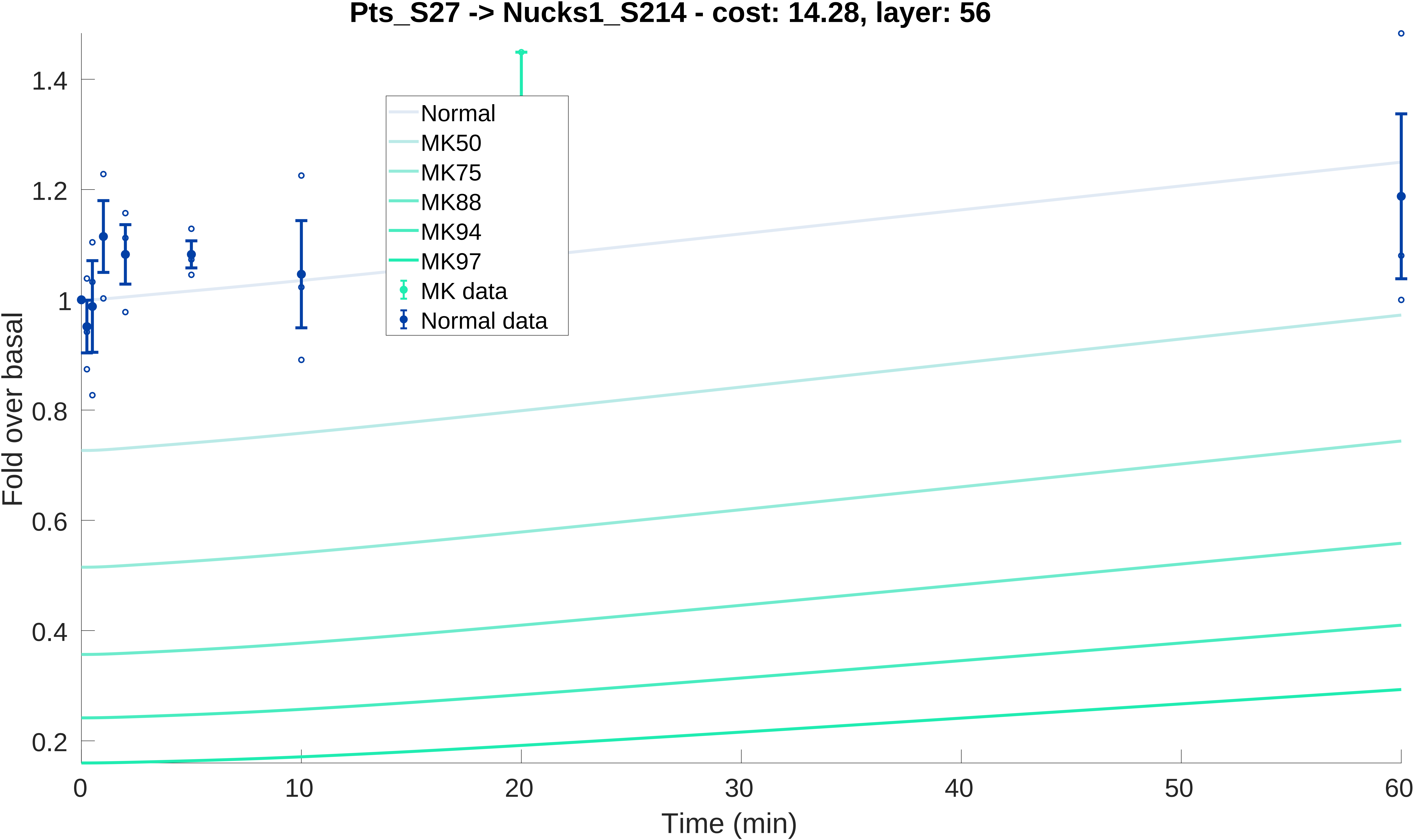

Pts_S27 -> Nucks1_S214 - cost: 14.28, layer: 56
Fold over basal
Time (min)
Normal
MK50
MK75
MK88
MK94
MK97
MK data
Normal data
0.2
0.4
0.6
0.8
1
1.2
1.4
0
10
20
30
40
50
60

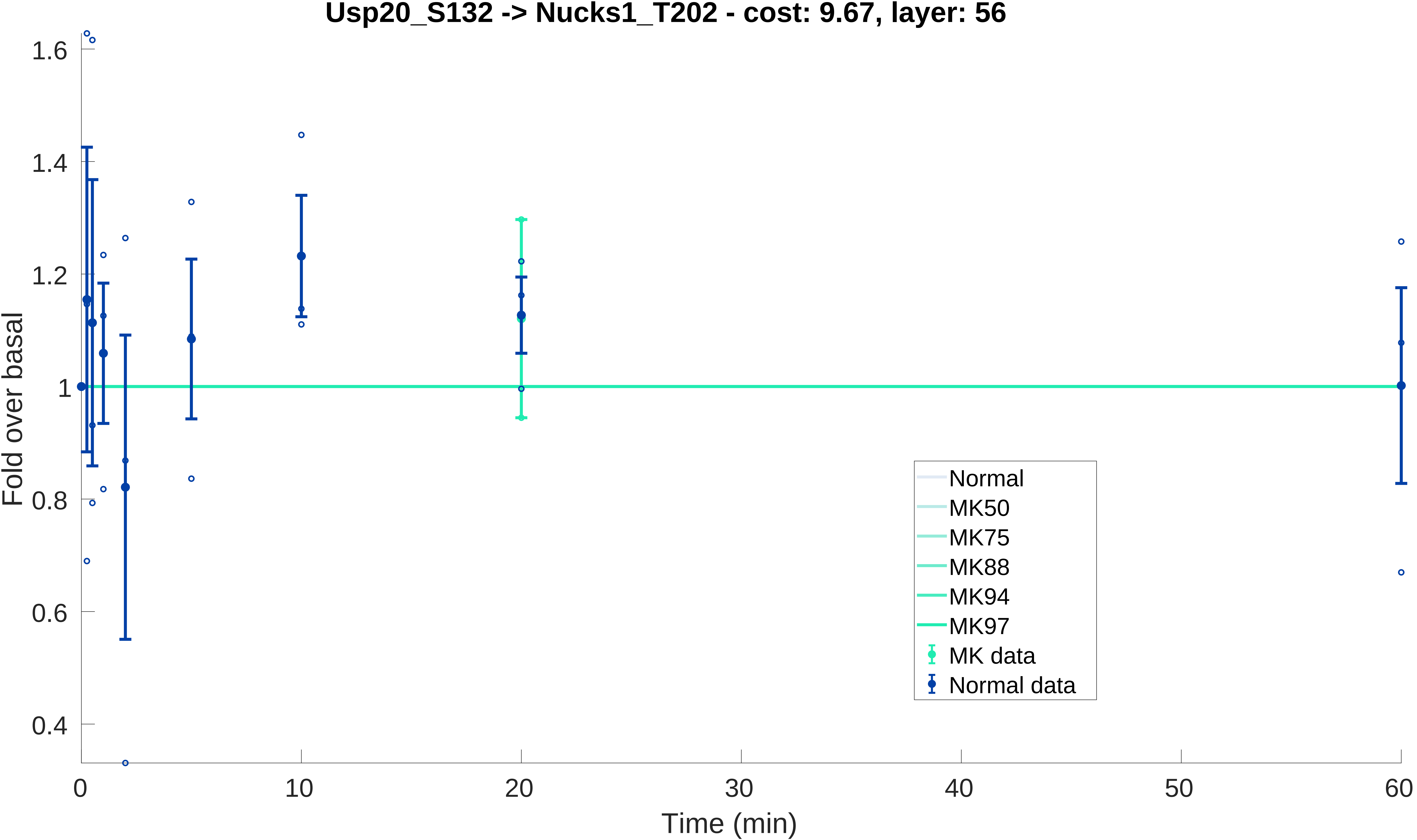

Usp20_S132 -> Nucks1_T202 - cost: 9.67, layer: 56
Fold over basal
Time (min)
0
10
20
30
40
50
60
0.4
0.6
0.8
1
1.2
1.4
1.6
Normal
MK50
MK75
MK88
MK94
MK97
MK data
Normal data

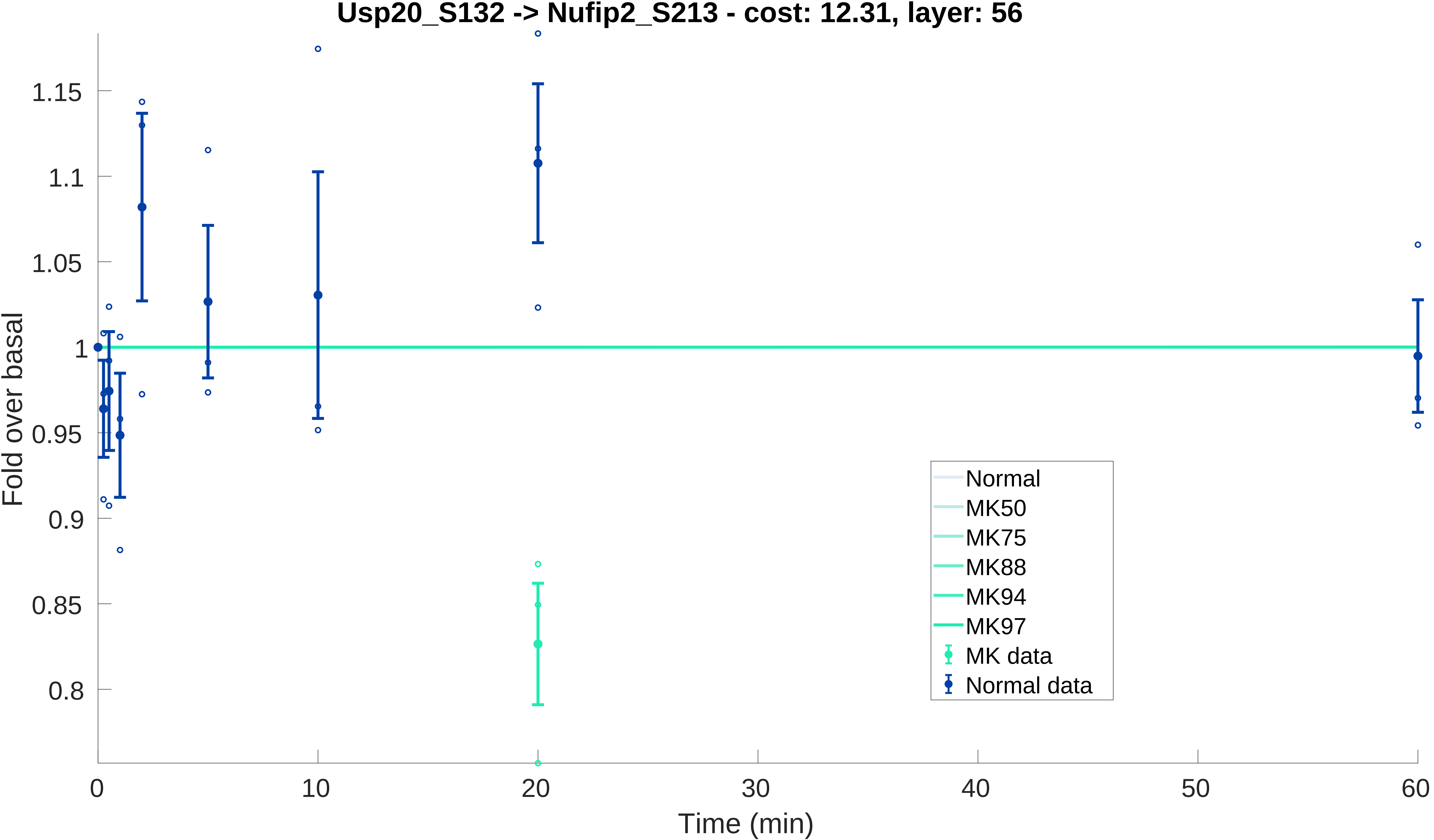
Usp20_S132 -> Nufip2_S213 - cost: 12.31, layer: 56
Fold over basal
1.15
1.1
1.05
1
0.95
0.9
0.85
0.8
0
10
20
30
40
50
60
Time (min)
Normal
MK50
MK75
MK88
MK94
MK97
MK data
Normal data

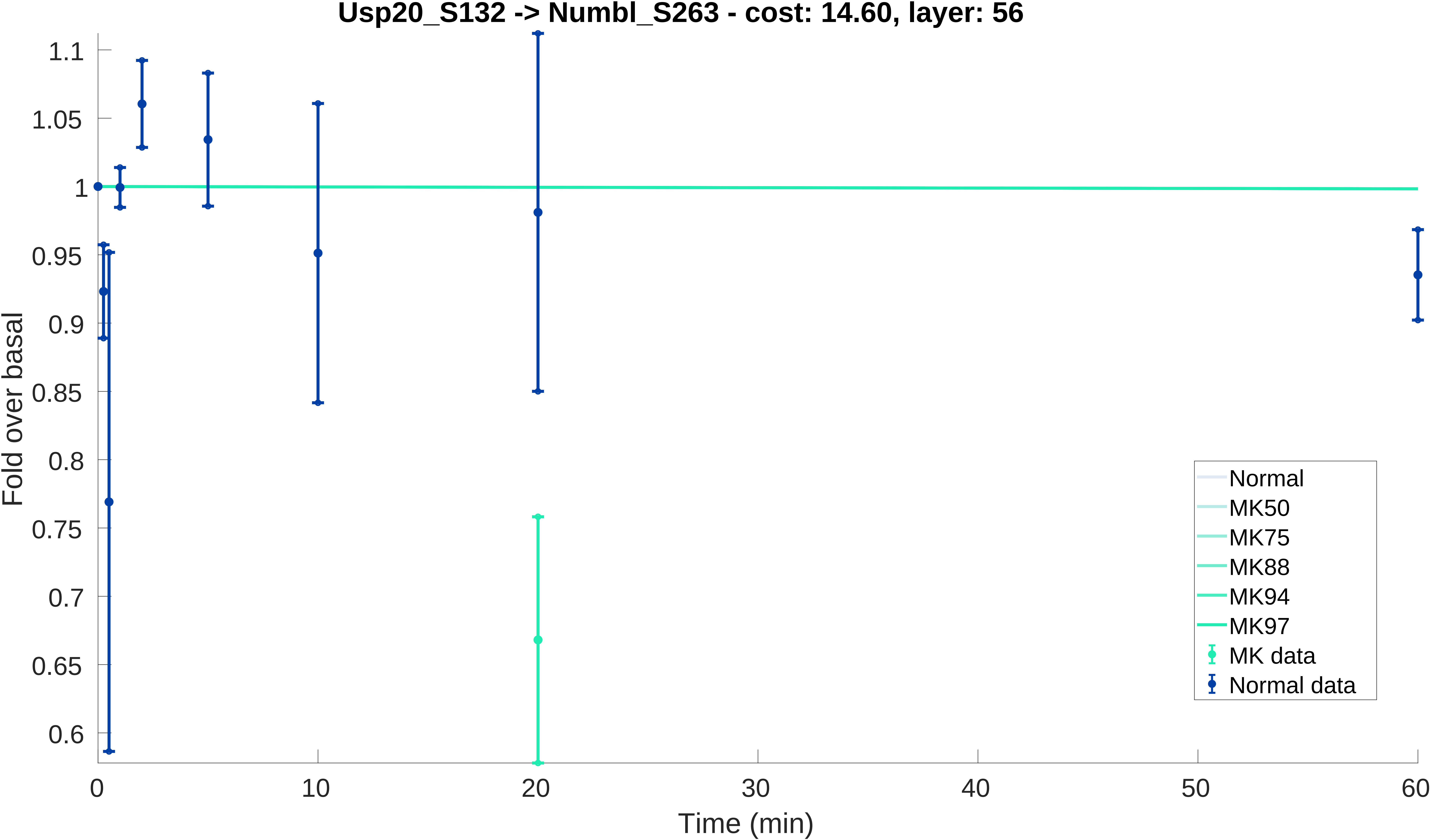

Usp20_S132 -> Numbl_S263 - cost: 14.60, layer: 56
Fold over basal
Time (min)
0
10
20
30
40
50
60
1.1
1.05
1
0.95
0.9
0.85
0.8
0.75
0.7
0.65
0.6
Normal
MK50
MK75
MK88
MK94
MK97
MK data
Normal data

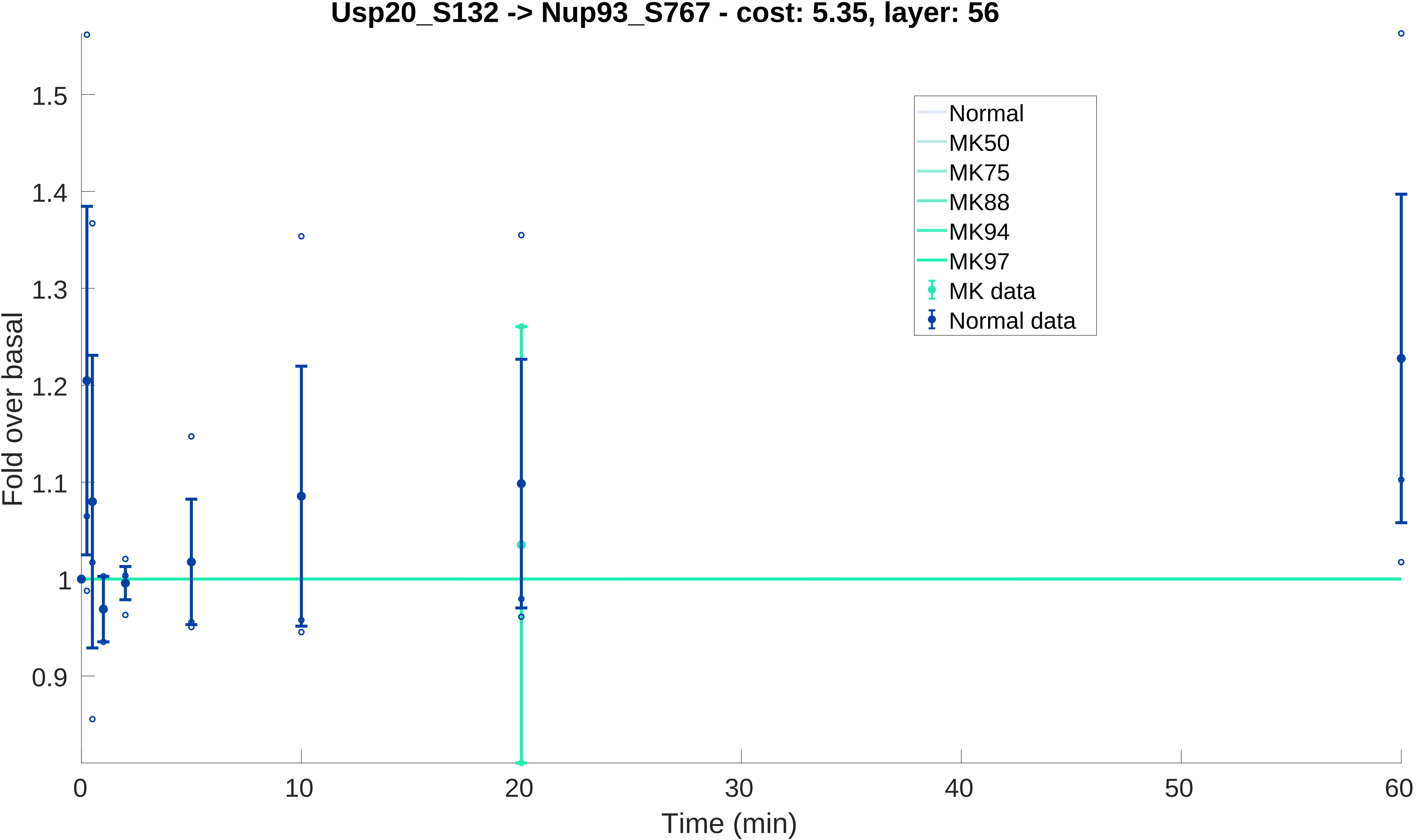

Usp20_S132 -> Nup93_S767 - cost: 5.35, layer: 56
Normal
MK50
MK75
MK88
MK94
MK97
MK data
Normal data
Fold over basal
Time (min)
1.5
1.4
1.3
1.2
1.1
1
0.9
0
10
20
30
40
50
60

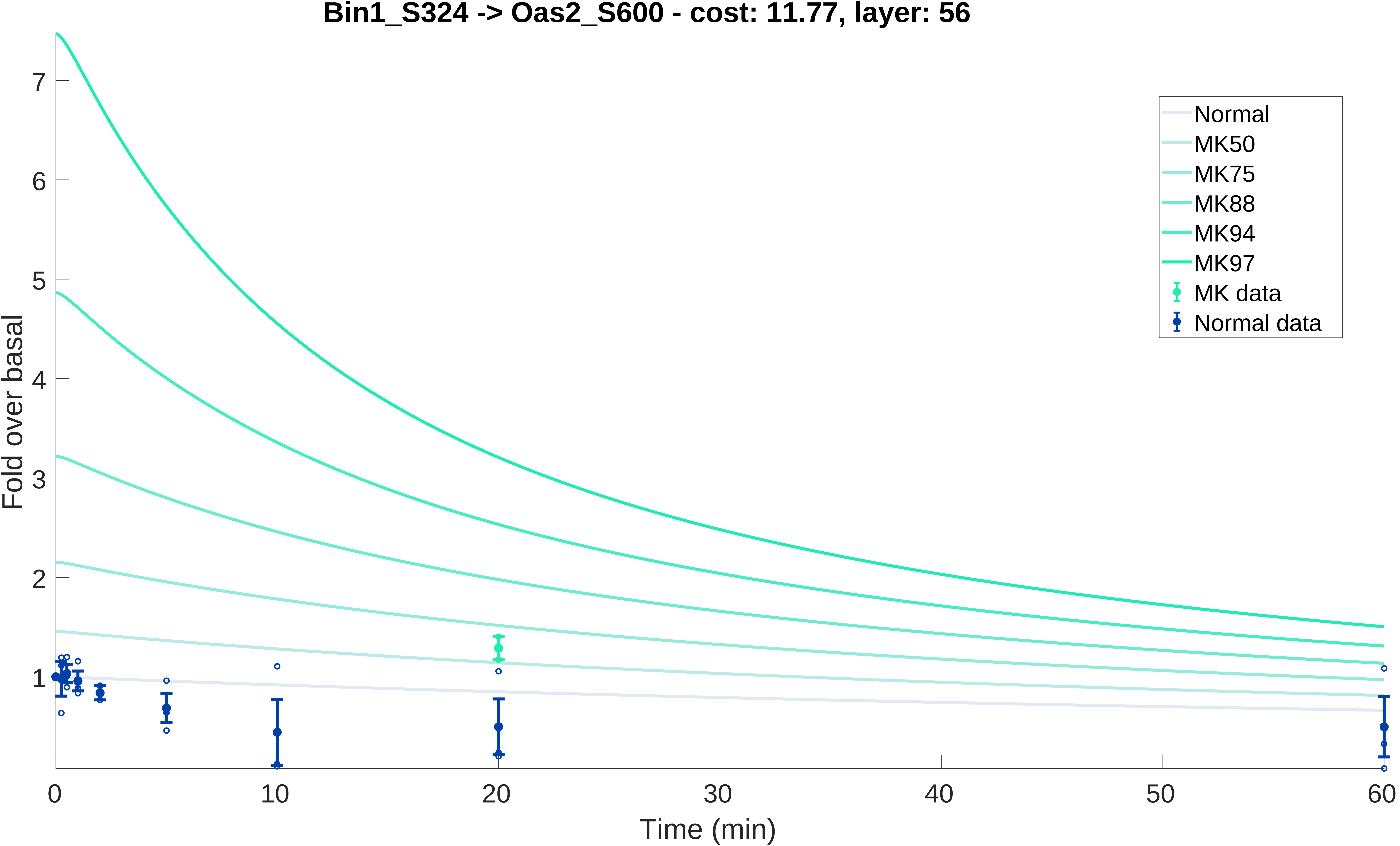
Bin1_S324 -> Oas2_S600 - cost: 11.77, layer: 56
Fold over basal
Time (min)
0
10
20
30
40
50
60
1
2
3
4
5
6
7
Normal
MK50
MK75
MK88
MK94
MK97
MK data
Normal data

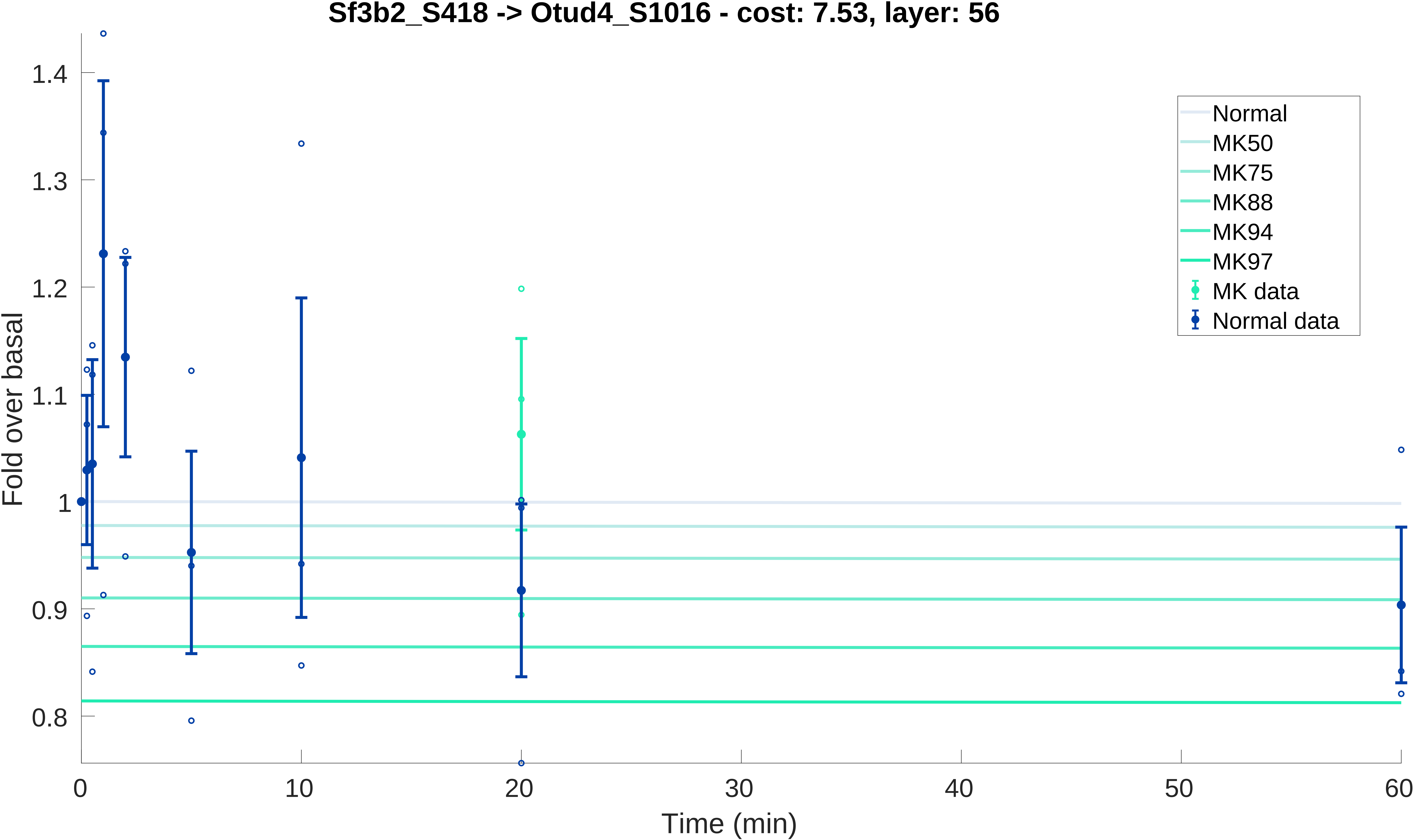

Sf3b2_S418 -> Otud4_S1016 - cost: 7.53, layer: 56
Fold over basal
Time (min)
0.8
0.9
1
1.1
1.2
1.3
1.4
0
10
20
30
40
50
60
Normal
MK50
MK75
MK88
MK94
MK97
MK data
Normal data

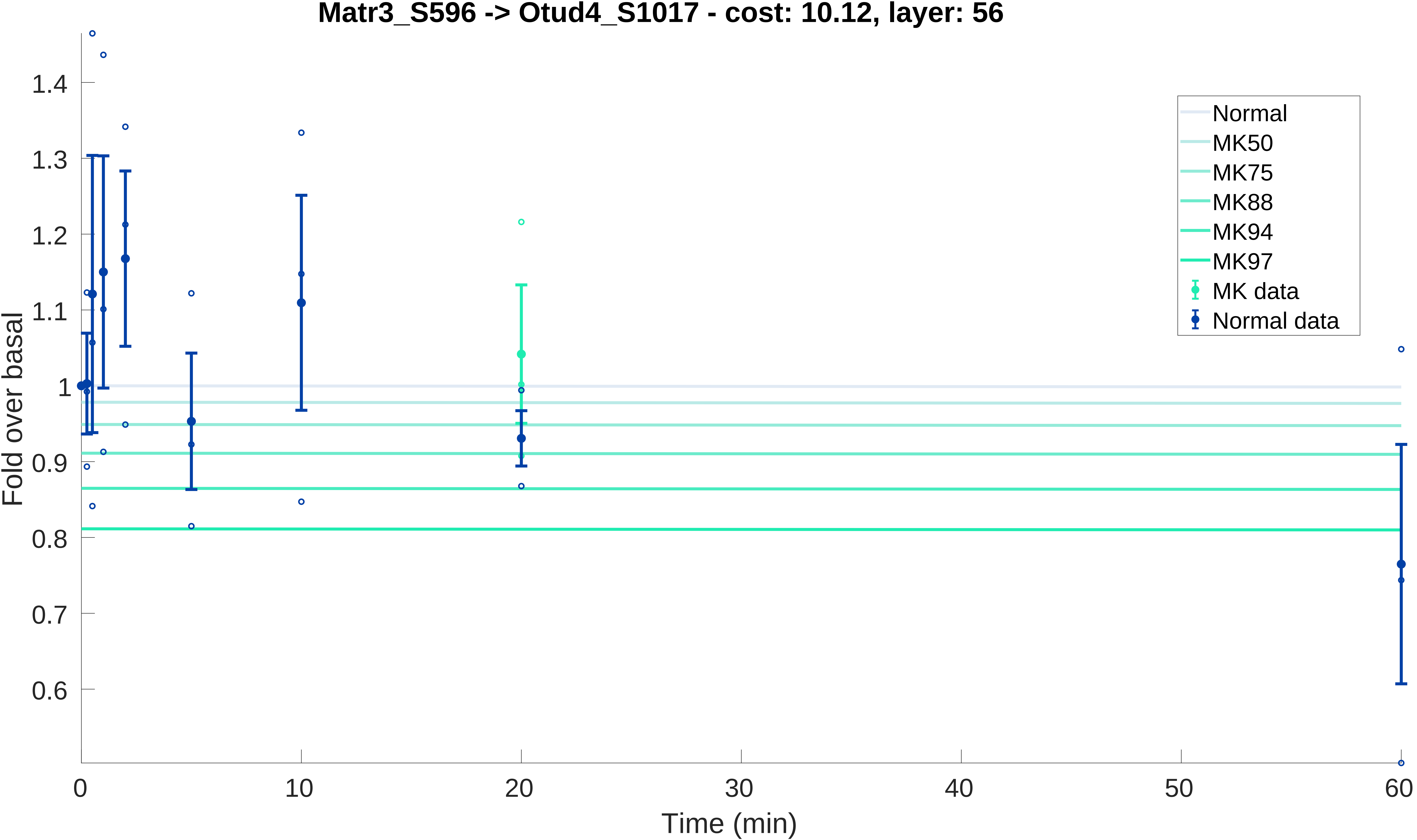

Matr3_S596 -> Otud4_S1017 - cost: 10.12, layer: 56
Fold over basal
Time (min)
1.4
1.3
1.2
1.1
1
0.9
0.8
0.7
0.6
0
10
20
30
40
50
60
Normal
MK50
MK75
MK88
MK94
MK97
MK data
Normal data

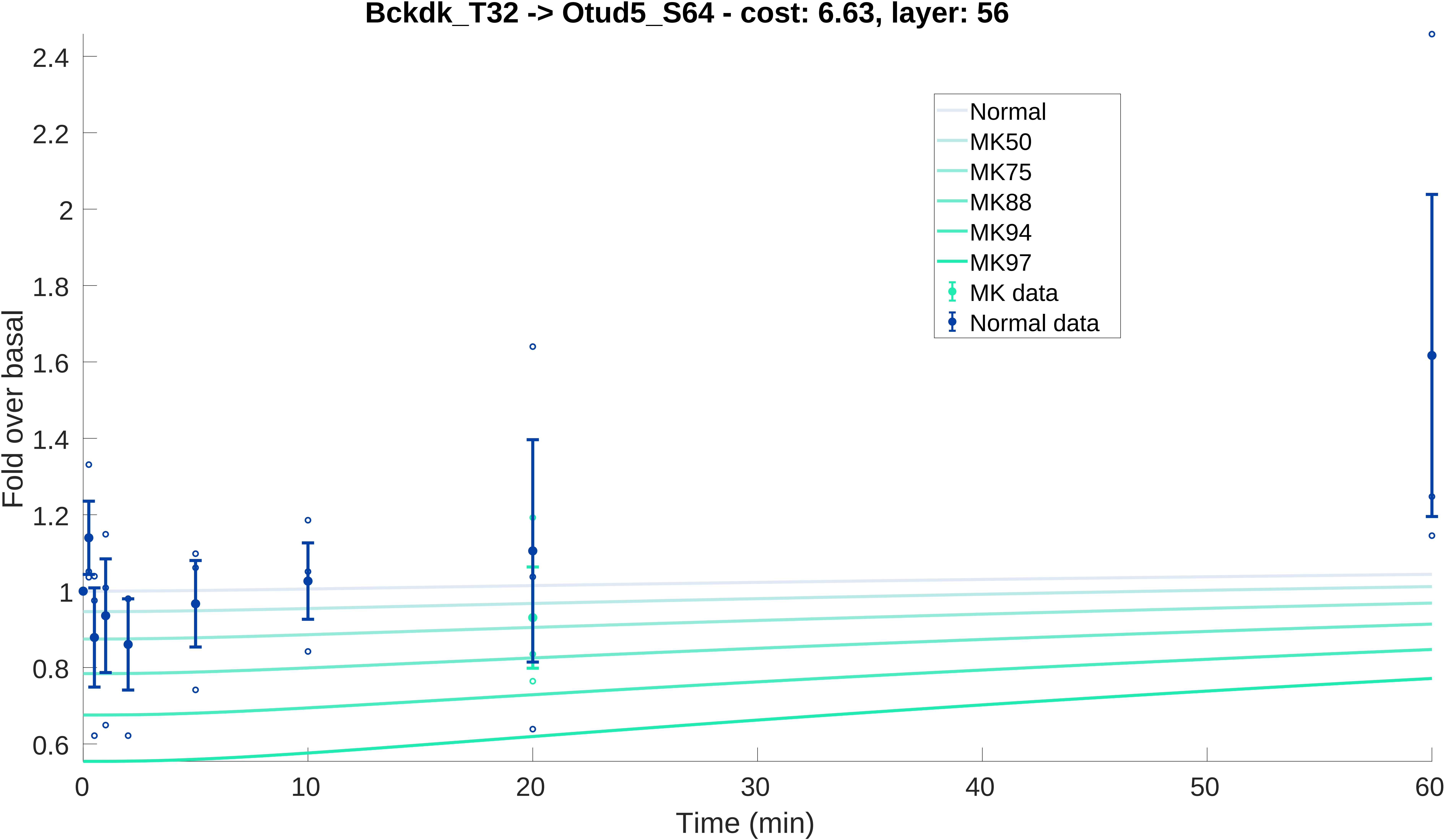
Bckdk_T32 -> Otud5_S64 - cost: 6.63, layer: 56
Fold over basal
Time (min)
0.6
0.8
1
1.2
1.4
1.6
1.8
2
2.2
2.4
0
10
20
30
40
50
60
Normal
MK50
MK75
MK88
MK94
MK97
MK data
Normal data

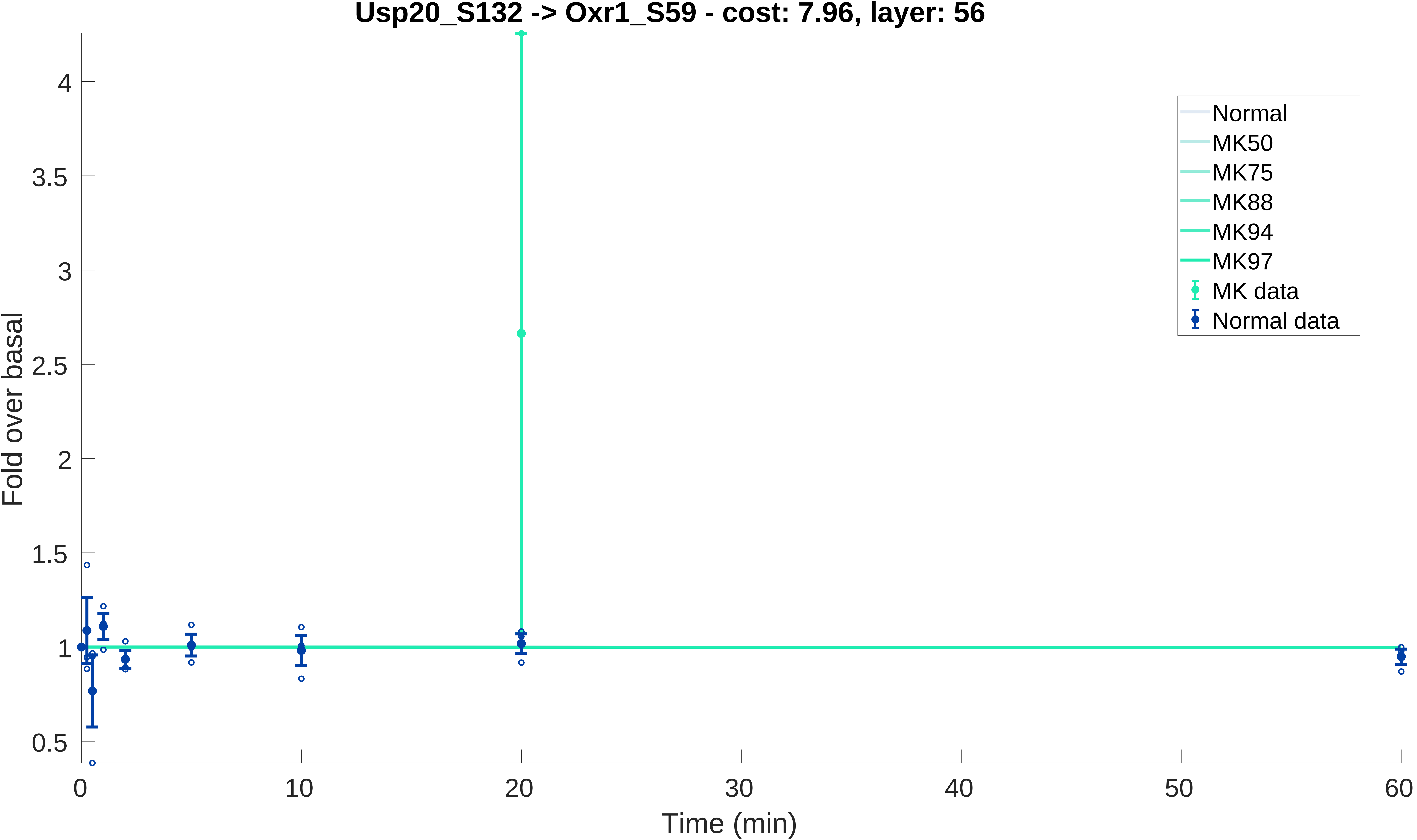

Usp20_S132 -> Oxr1_S59 - cost: 7.96, layer: 56
Fold over basal
Time (min)
0.5
1
1.5
2
2.5
3
3.5
4
0
10
20
30
40
50
60
Normal
MK50
MK75
MK88
MK94
MK97
MK data
Normal data

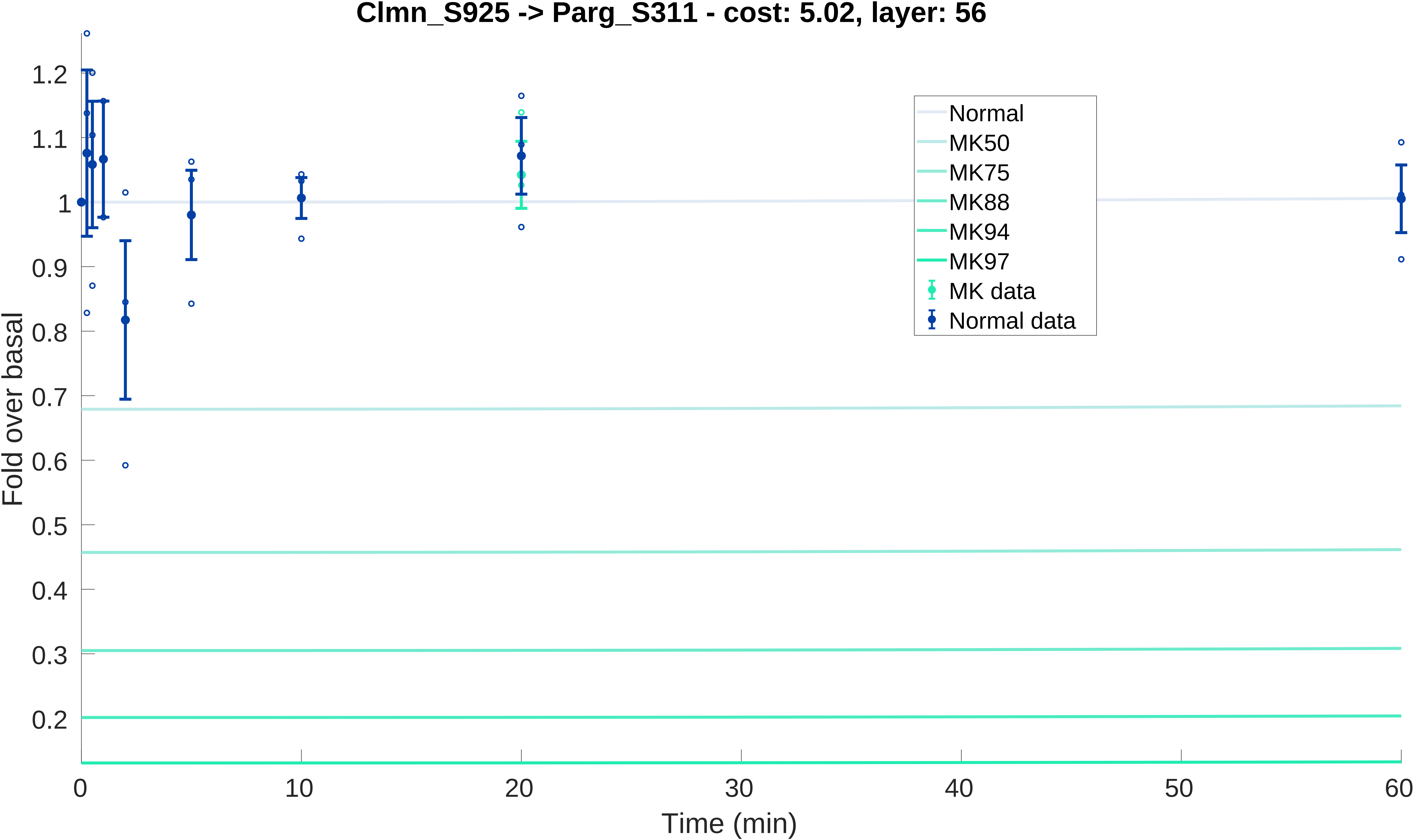
Clmn_S925 -> Parg_S311 - cost: 5.02, layer: 56
Fold over basal
Time (min)
1.2
1.1
1
0.9
0.8
0.7
0.6
0.5
0.4
0.3
0.2
0
10
20
30
40
50
60
Normal
MK50
MK75
MK88
MK94
MK97
MK data
Normal data

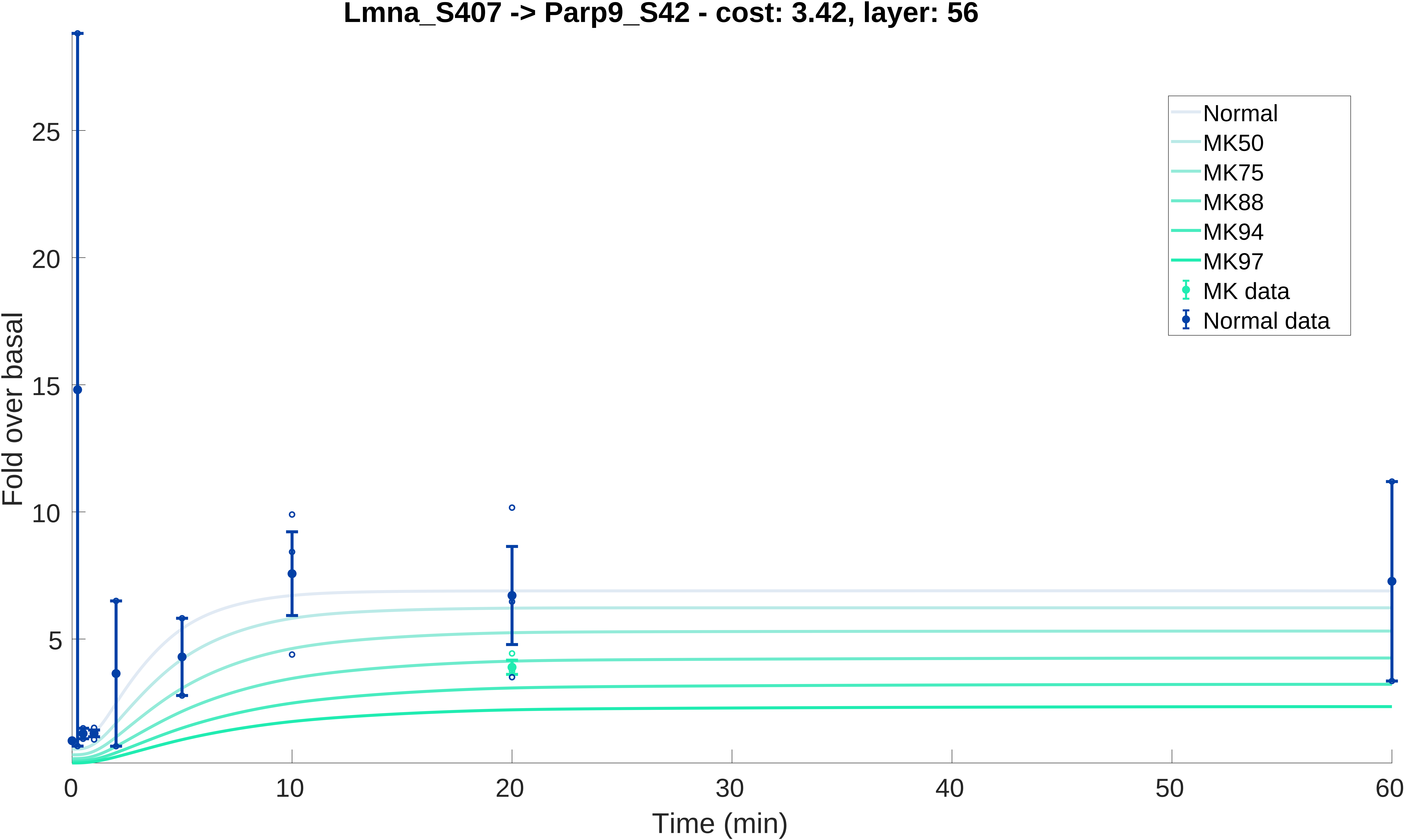

Lmna_S407 -> Parp9_S42 - cost: 3.42, layer: 56
Fold over basal
Time (min)
Normal
MK50
MK75
MK88
MK94
MK97
MK data
Normal data
0
5
10
15
20
25
0
10
20
30
40
50
60

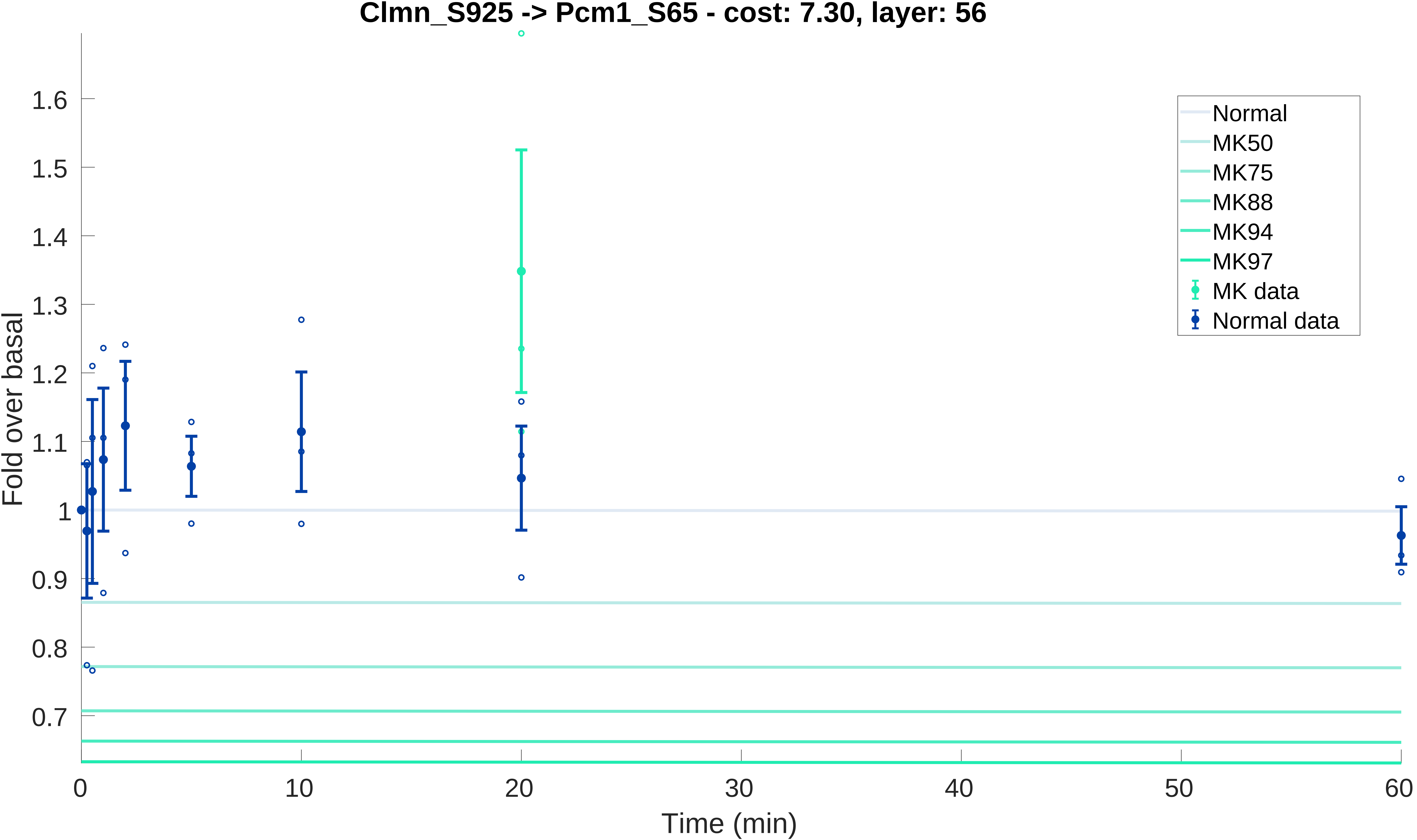
Clmn_S925 -> Pcm1_S65 - cost: 7.30, layer: 56
Fold over basal
Time (min)
0
10
20
30
40
50
60
0.7
0.8
0.9
1
1.1
1.2
1.3
1.4
1.5
1.6
Normal
MK50
MK75
MK88
MK94
MK97
MK data
Normal data

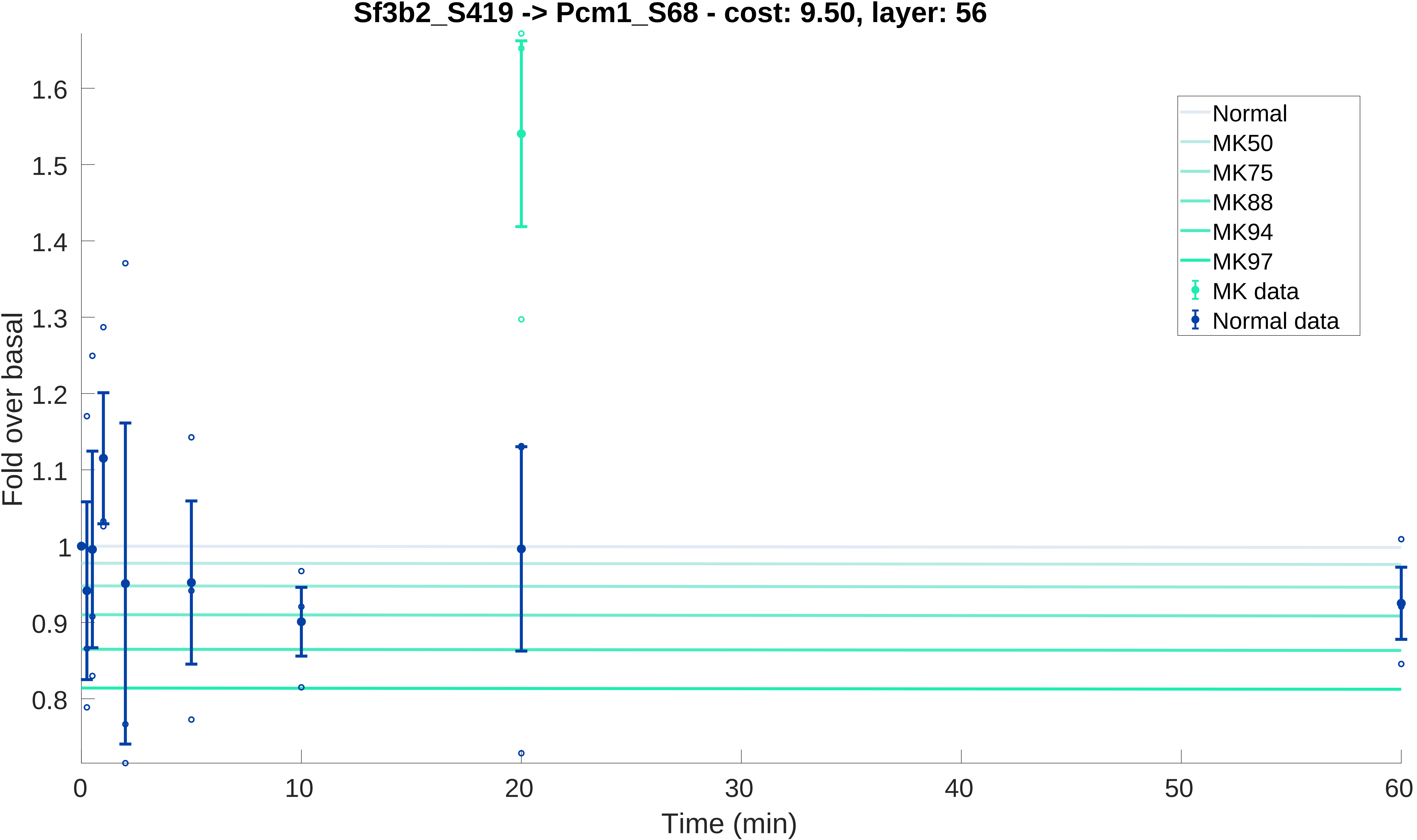
Sf3b2_S419 -> Pcm1_S68 - cost: 9.50, layer: 56
Fold over basal
Time (min)
1.6
1.5
1.4
1.3
1.2
1.1
1
0.9
0.8
0
10
20
30
40
50
60
Normal
MK50
MK75
MK88
MK94
MK97
MK data
Normal data

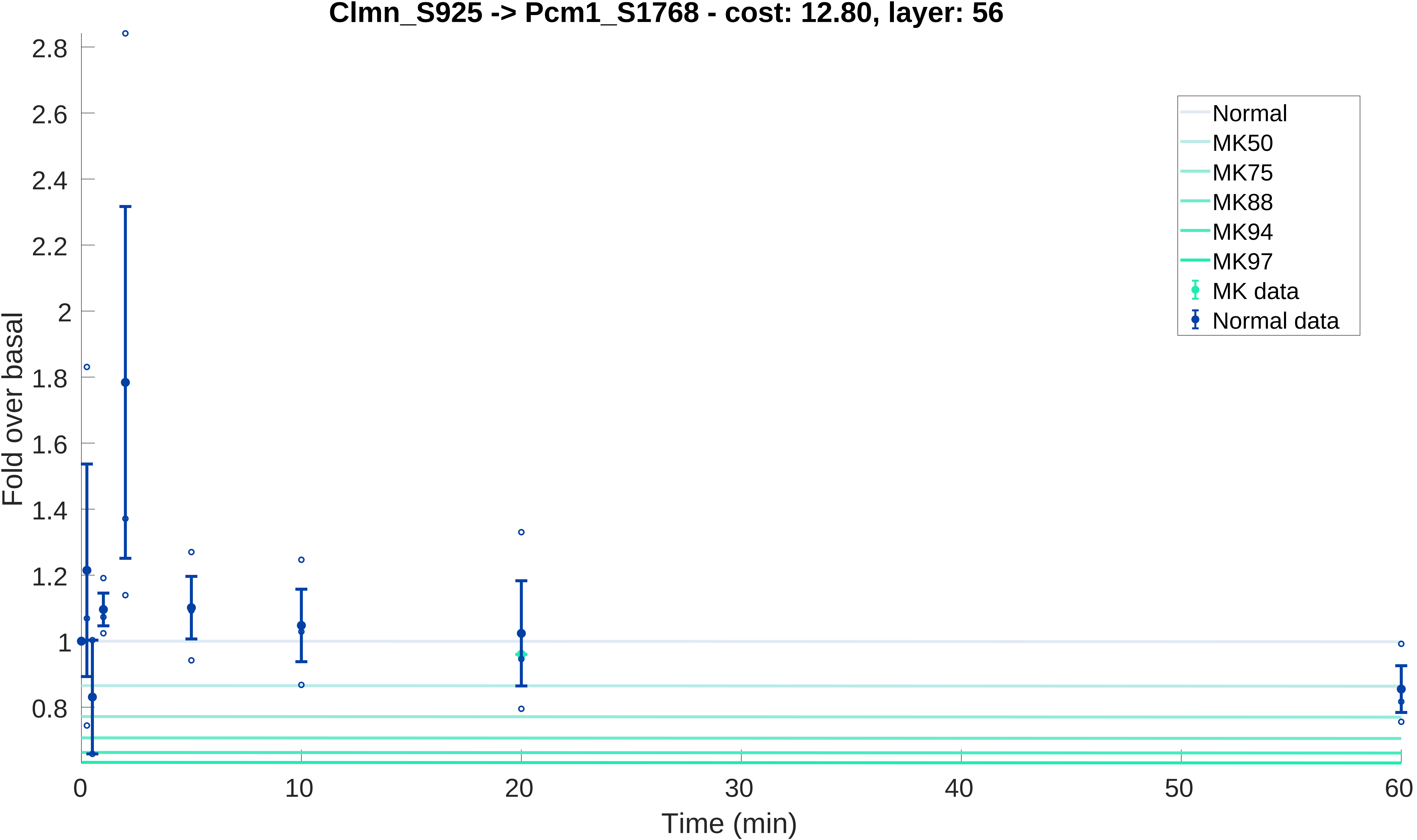

Clmn_S925 -> Pcm1_S1768 - cost: 12.80, layer: 56
Fold over basal
Time (min)
Normal
MK50
MK75
MK88
MK94
MK97
MK data
Normal data
0
10
20
30
40
50
60
0.8
1
1.2
1.4
1.6
1.8
2
2.2
2.4
2.6
2.8

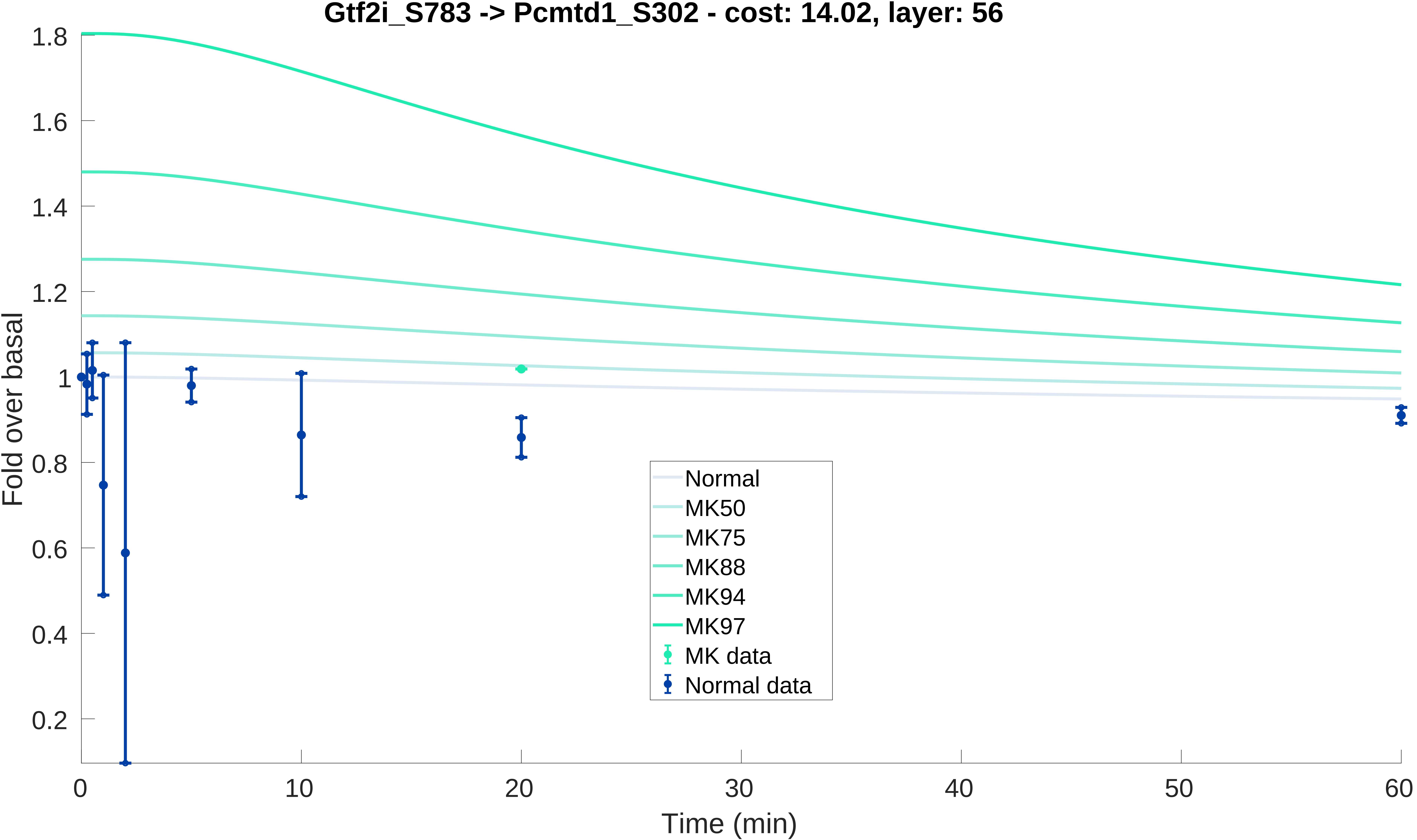

Gtf2i_S783 -> Pcmtd1_S302 - cost: 14.02, layer: 56
Fold over basal
Time (min)
1.8
1.6
1.4
1.2
1
0.8
0.6
0.4
0.2
0
10
20
30
40
50
60
Normal
MK50
MK75
MK88
MK94
MK97
MK data
Normal data

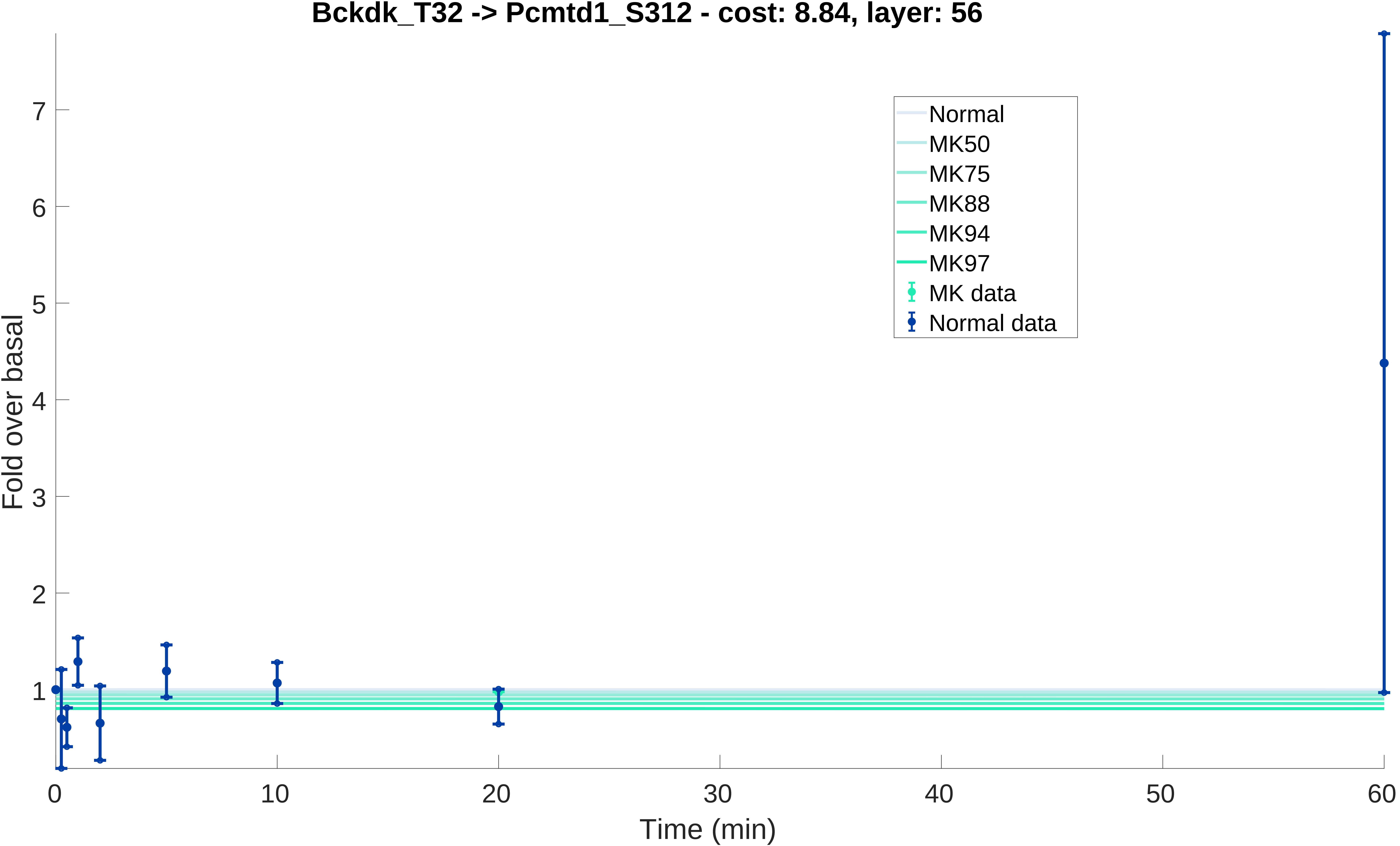

Bckdk_T32 -> Pcmtd1_S312 - cost: 8.84, layer: 56
Fold over basal
Time (min)
0
10
20
30
40
50
60
1
2
3
4
5
6
7
Normal
MK50
MK75
MK88
MK94
MK97
MK data
Normal data

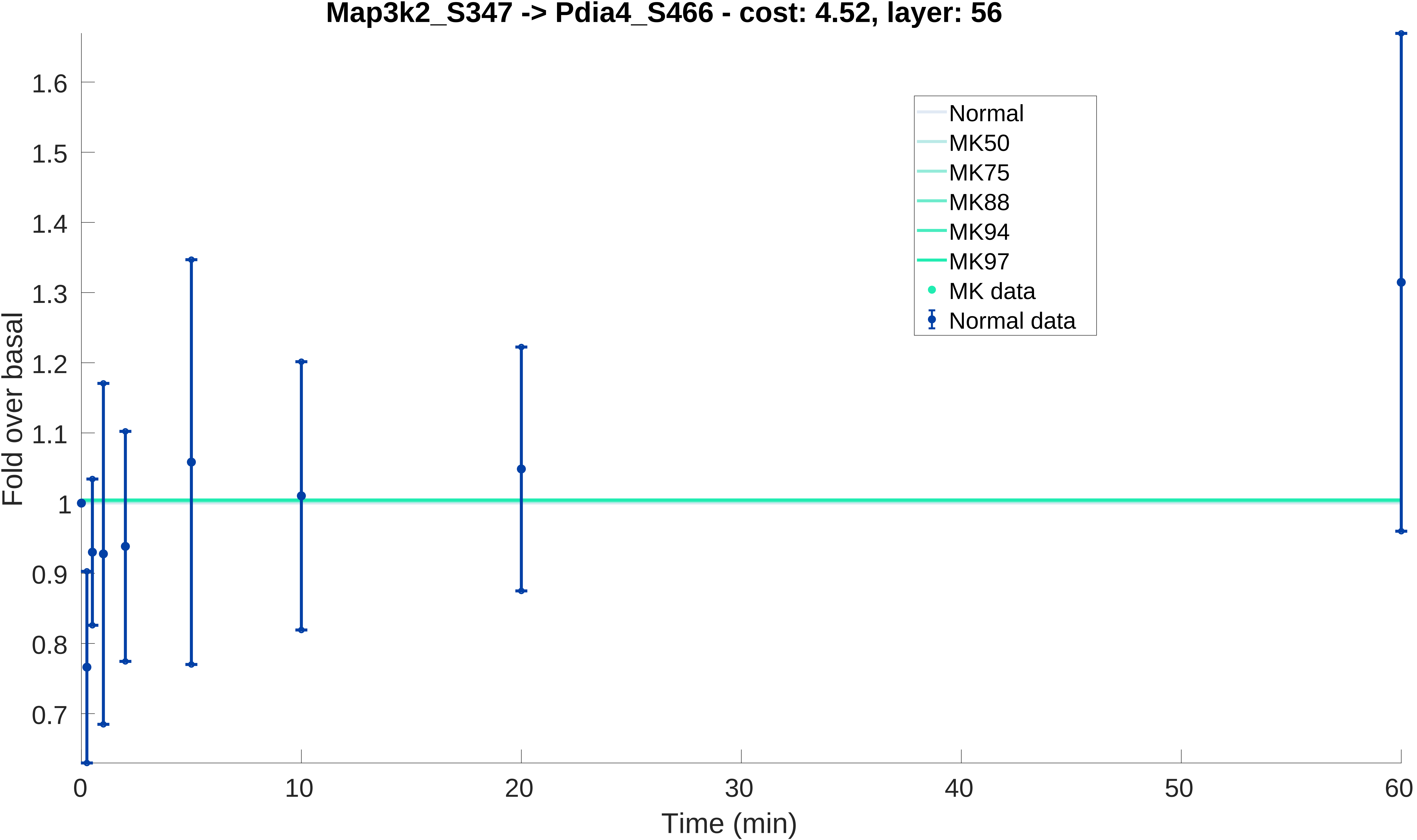

Map3k2_S347 -> Pdia4_S466 - cost: 4.52, layer: 56
Fold over basal
Time (min)
Normal
MK50
MK75
MK88
MK94
MK97
MK data
Normal data
1.6
1.5
1.4
1.3
1.2
1.1
1
0.9
0.8
0.7
0
10
20
30
40
50
60

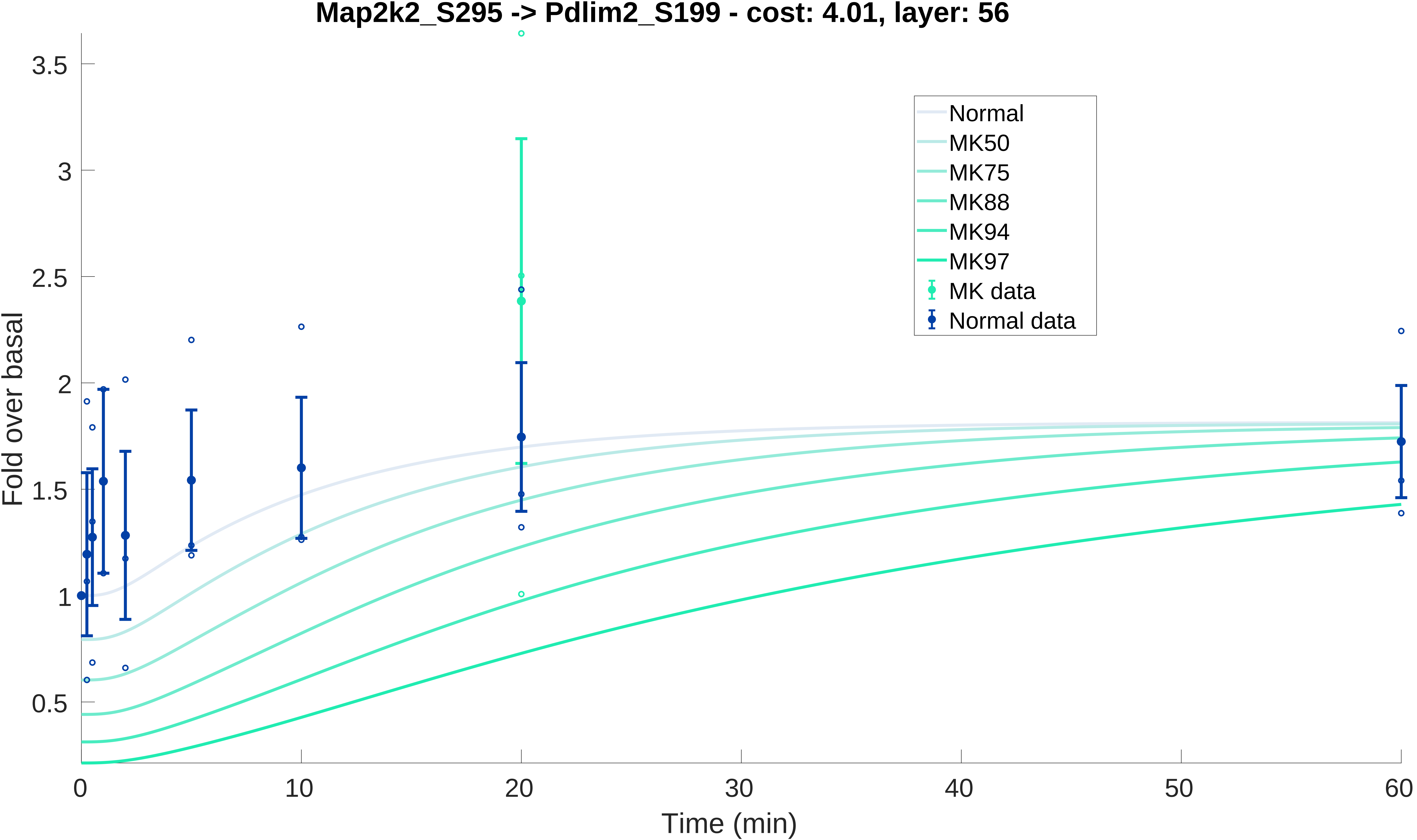

Map2k2_S295 -> Pdlim2_S199 - cost: 4.01, layer: 56
Fold over basal
3.5
3
2.5
2
1.5
1
0.5
0
10
20
30
40
50
60
Time (min)
Normal
MK50
MK75
MK88
MK94
MK97
MK data
Normal data

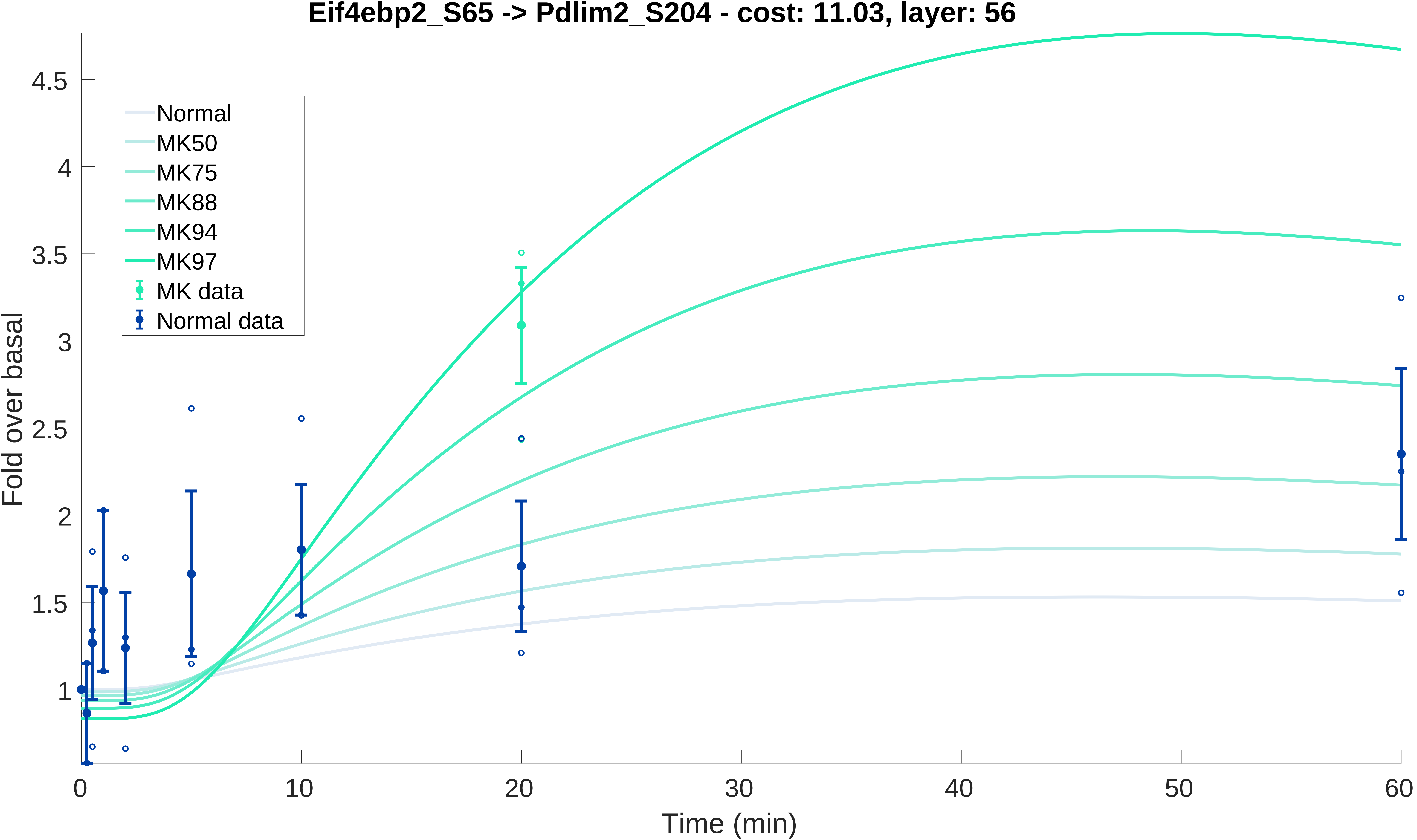
Eif4ebp2_S65 -> Pdlim2_S204 - cost: 11.03, layer: 56
Normal
MK50
MK75
MK88
MK94
MK97
MK data
Normal data
Fold over basal
Time (min)
4.5
4
3.5
3
2.5
2
1.5
1
0
10
20
30
40
50
60

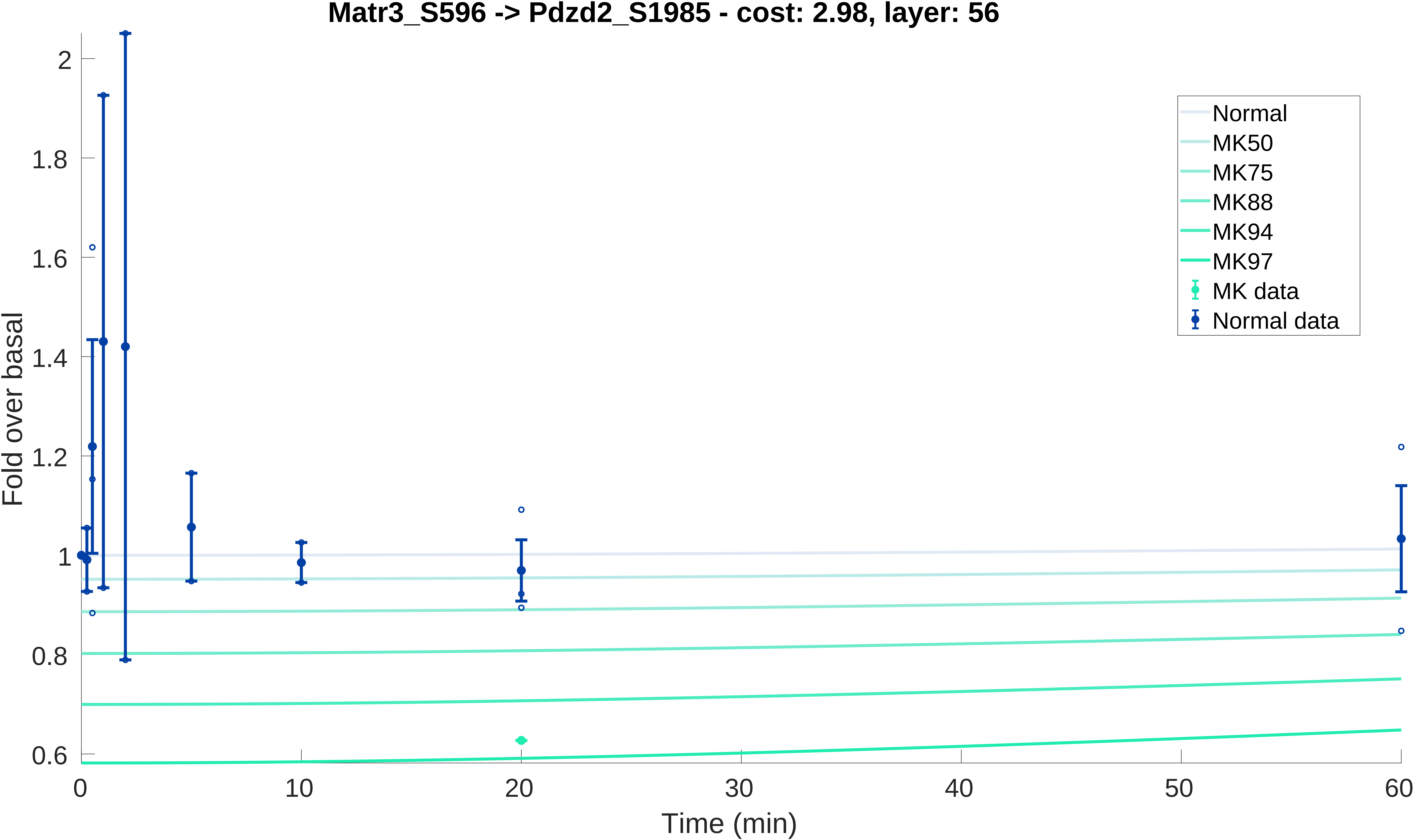
Matr3_S596 -> Pdzd2_S1985 - cost: 2.98, layer: 56
Fold over basal
Time (min)
0.6
0.8
1
1.2
1.4
1.6
1.8
2
0
10
20
30
40
50
60
Normal
MK50
MK75
MK88
MK94
MK97
MK data
Normal data

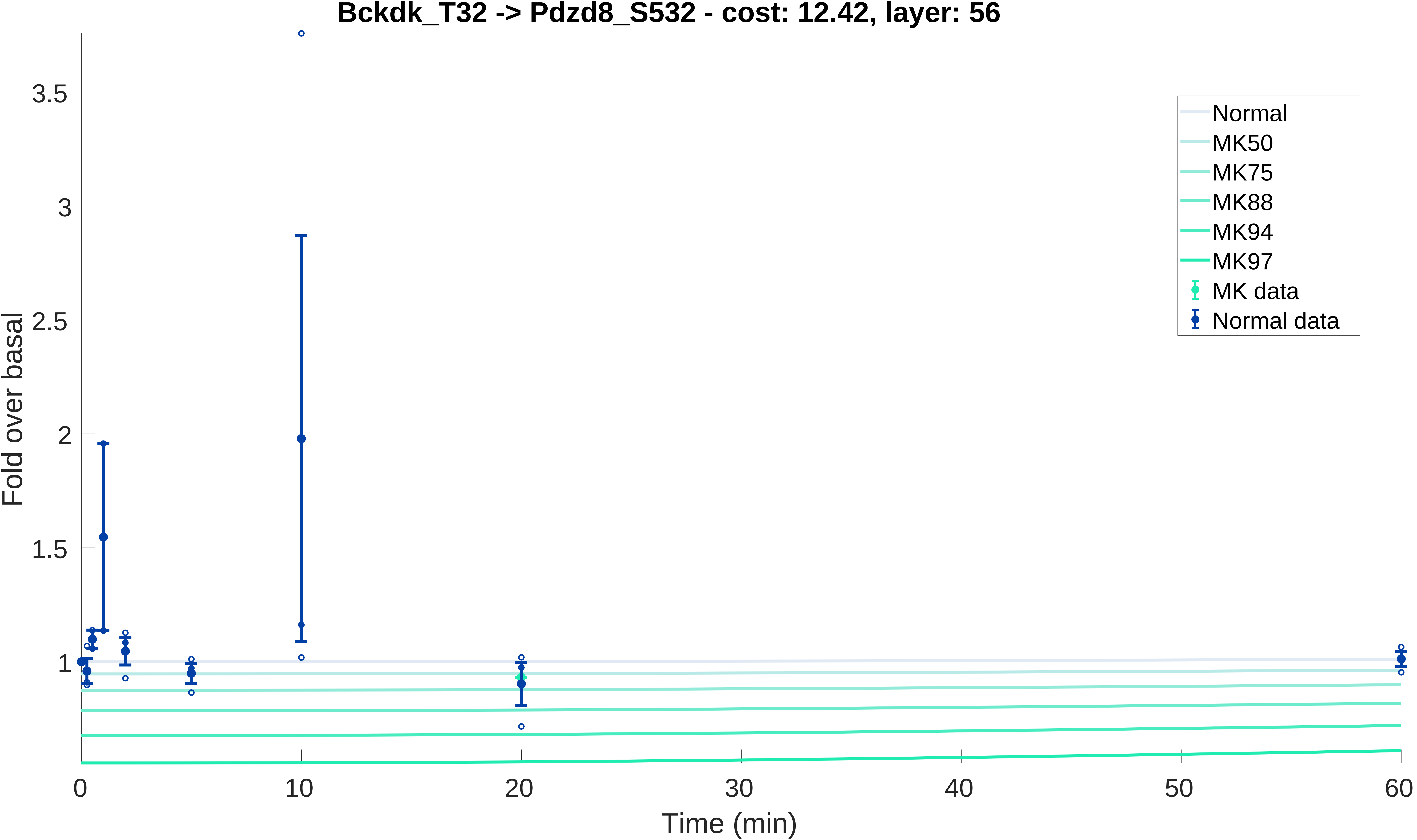
Bckdk_T32 -> Pdzd8_S532 - cost: 12.42, layer: 56
Fold over basal
Time (min)
3.5
3
2.5
2
1.5
1
0
10
20
30
40
50
60
Normal
MK50
MK75
MK88
MK94
MK97
MK data
Normal data

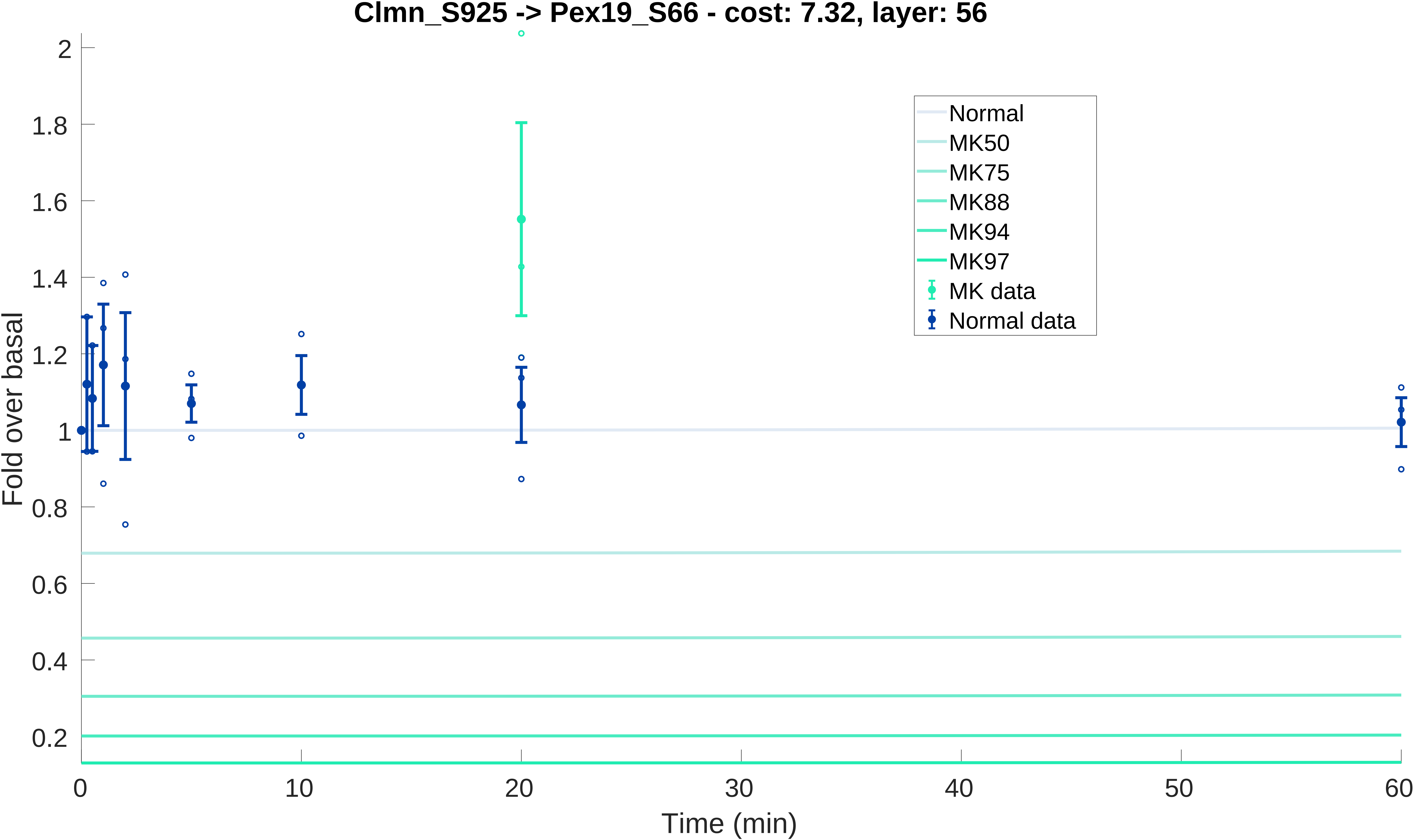

Clmn_S925 -> Pex19_S66 - cost: 7.32, layer: 56
Fold over basal
Time (min)
Normal
MK50
MK75
MK88
MK94
MK97
MK data
Normal data
2
1.8
1.6
1.4
1.2
1
0.8
0.6
0.4
0.2
0
10
20
30
40
50
60

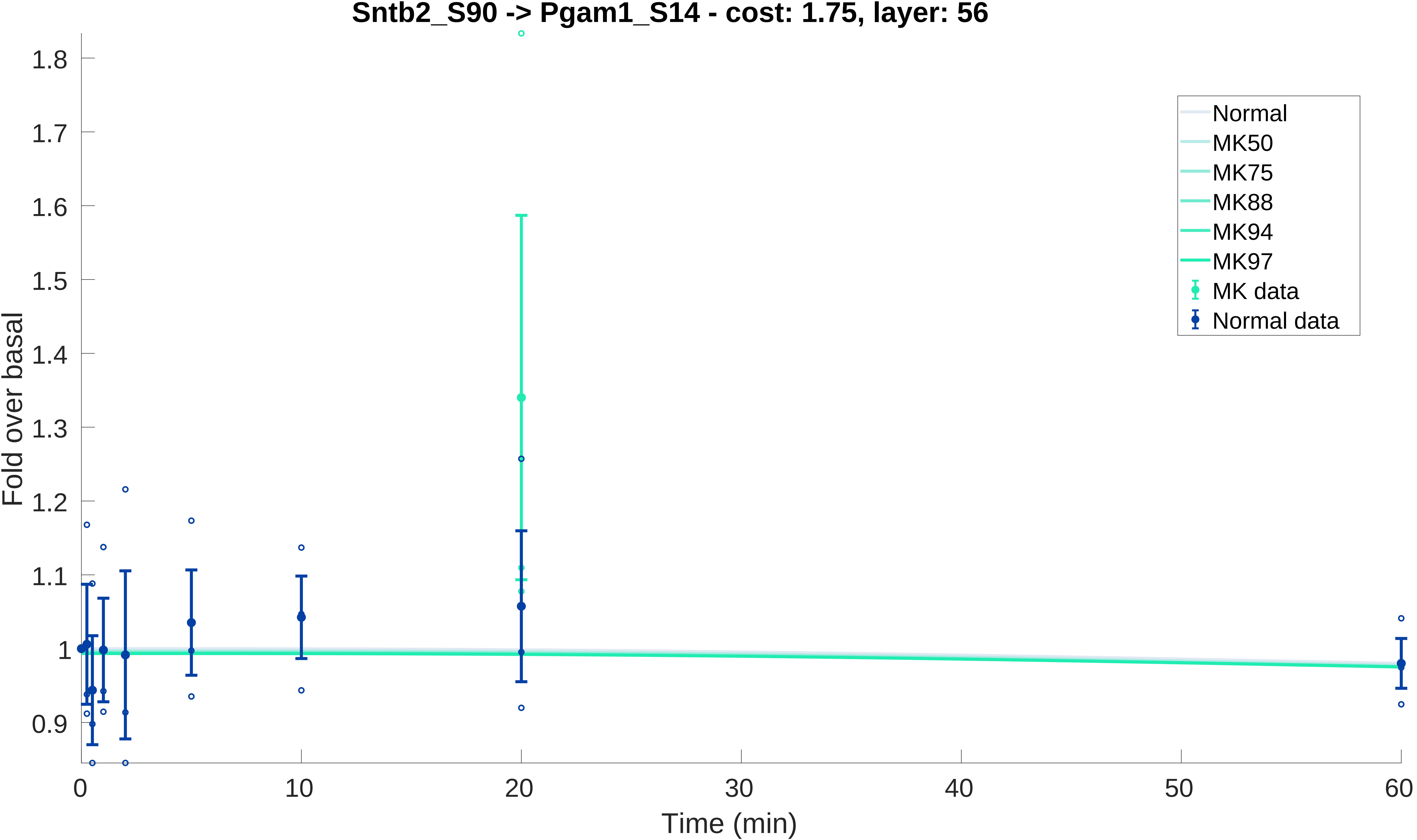

Sntb2_S90 -> Pgam1_S14 - cost: 1.75, layer: 56
Fold over basal
Time (min)
Normal
MK50
MK75
MK88
MK94
MK97
MK data
Normal data
0.9
1
1.1
1.2
1.3
1.4
1.5
1.6
1.7
1.8
0
10
20
30
40
50
60

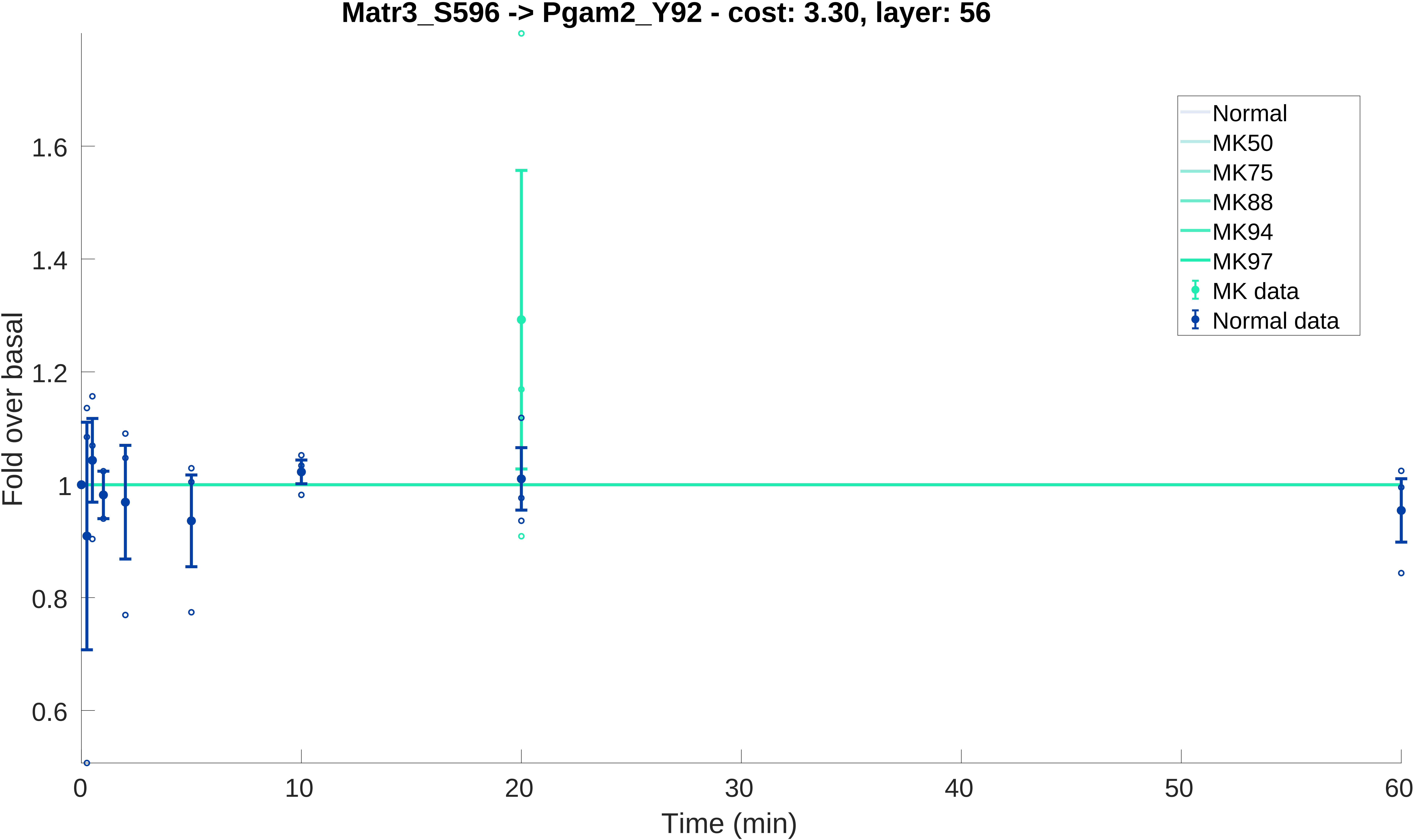

Matr3_S596 -> Pgam2_Y92 - cost: 3.30, layer: 56
Fold over basal
1.6
1.4
1.2
1
0.8
0.6
0
10
20
30
40
50
60
Time (min)
Normal
MK50
MK75
MK88
MK94
MK97
MK data
Normal data

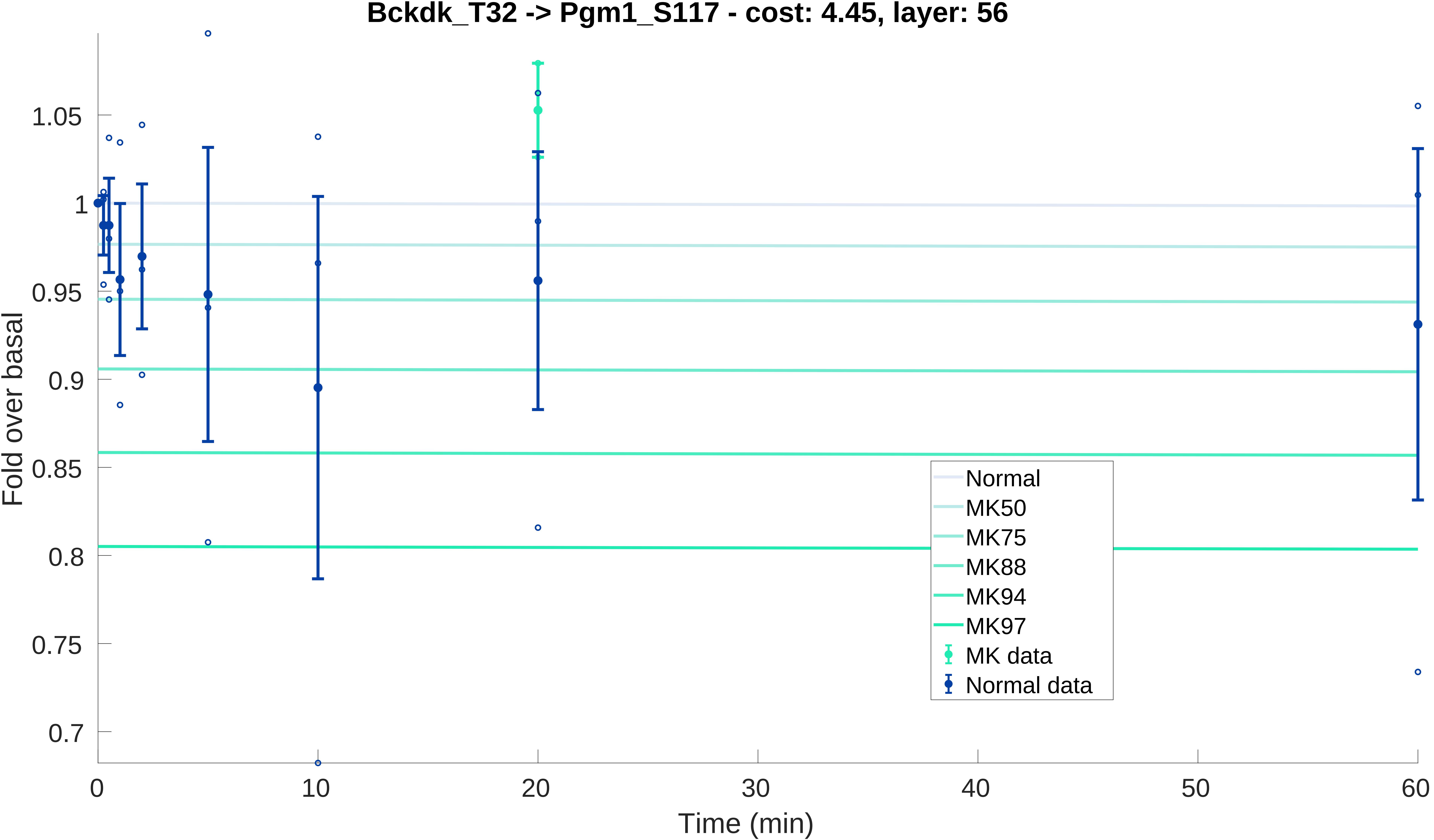

Bckdk_T32 -> Pgm1_S117 - cost: 4.45, layer: 56
Fold over basal
1.05
1
0.95
0.9
0.85
0.8
0.75
0.7
0
10
20
30
40
50
60
Time (min)
Normal
MK50
MK75
MK88
MK94
MK97
MK data
Normal data

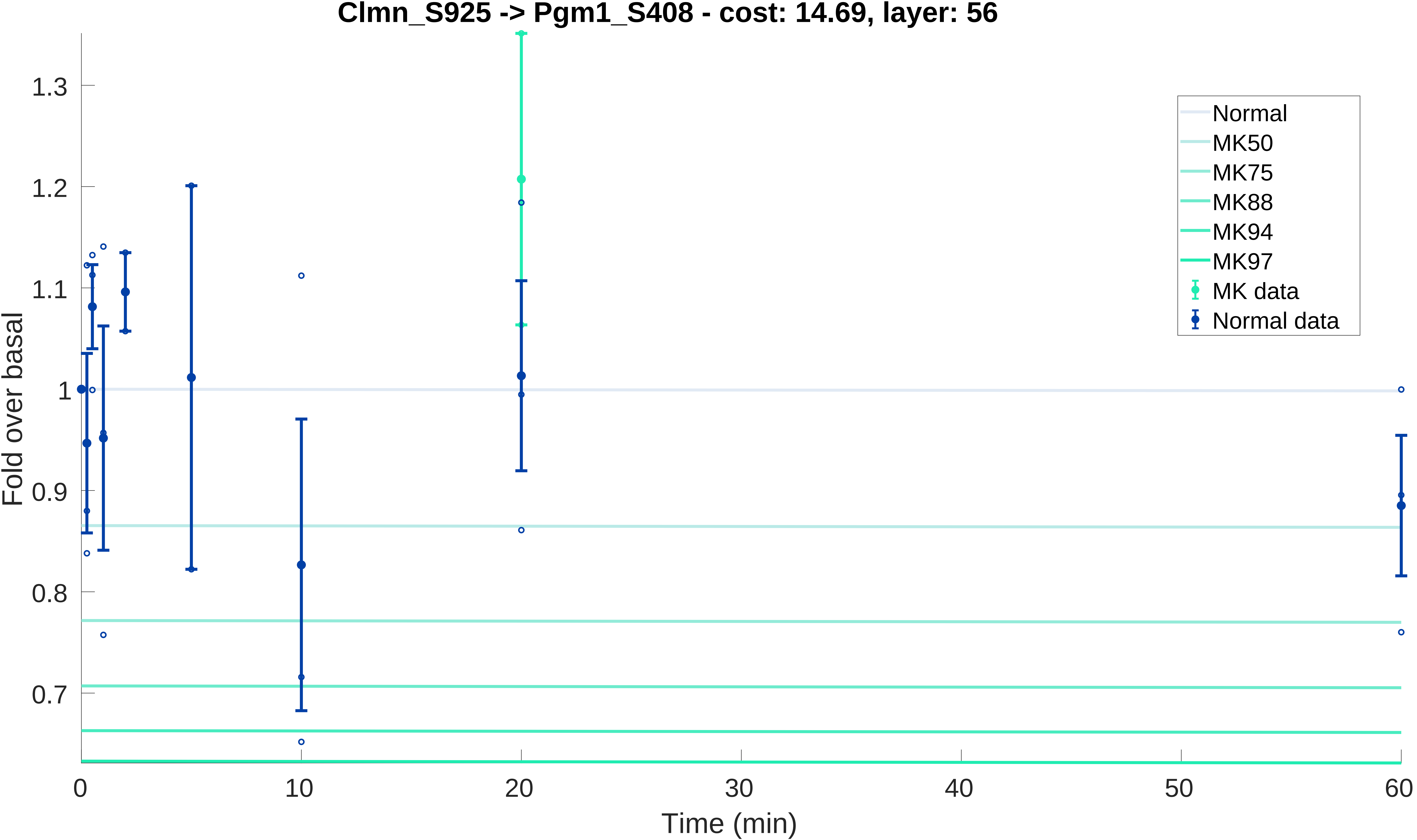

Clmn_S925 -> Pgm1_S408 - cost: 14.69, layer: 56
Fold over basal
1.3
1.2
1.1
1
0.9
0.8
0.7
0
10
20
30
40
50
60
Time (min)
Normal
MK50
MK75
MK88
MK94
MK97
MK data
Normal data

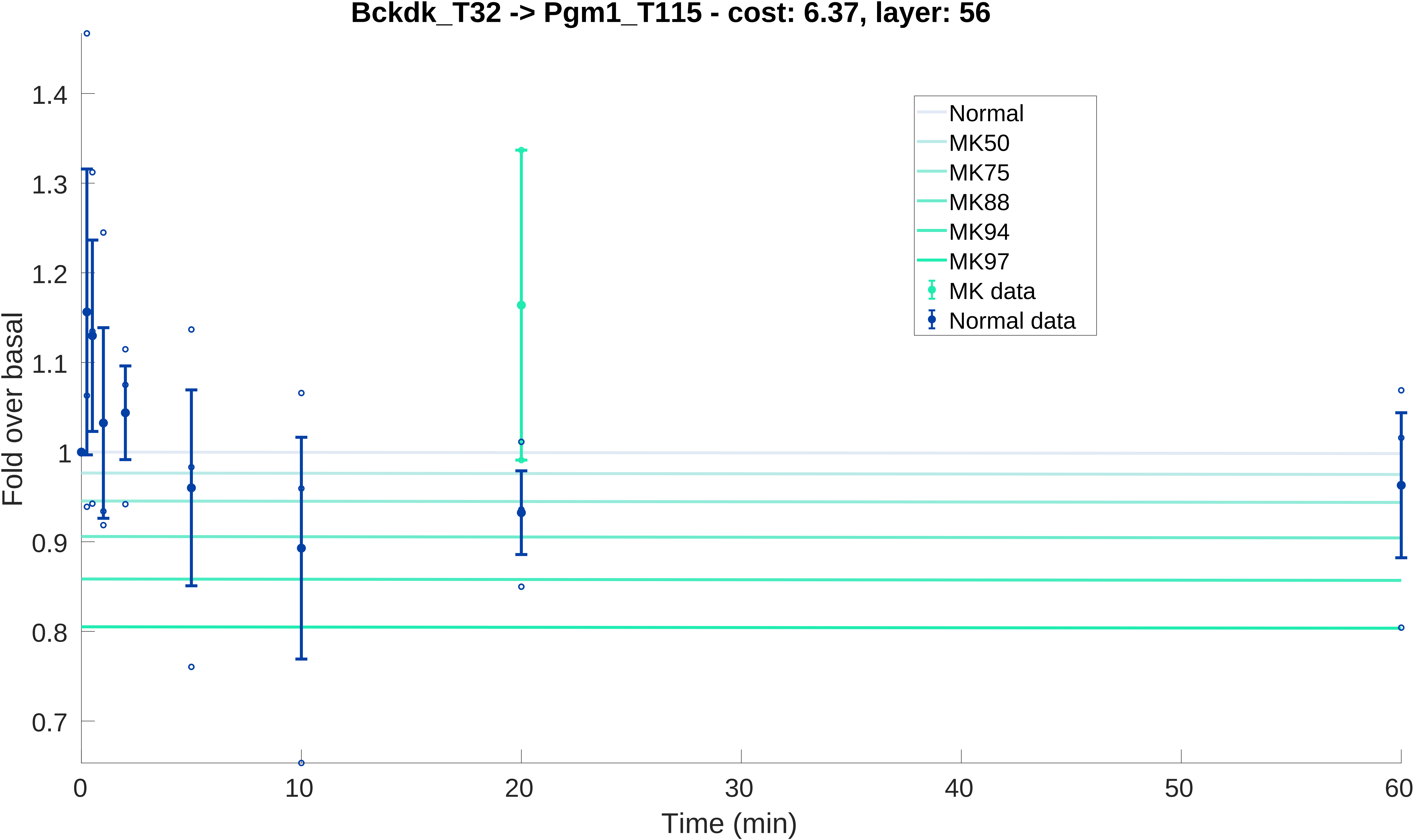

Bckdk_T32 -> Pgm1_T115 - cost: 6.37, layer: 56
Fold over basal
1.4
1.3
1.2
1.1
1
0.9
0.8
0.7
0
10
20
30
40
50
60
Time (min)
Normal
MK50
MK75
MK88
MK94
MK97
MK data
Normal data

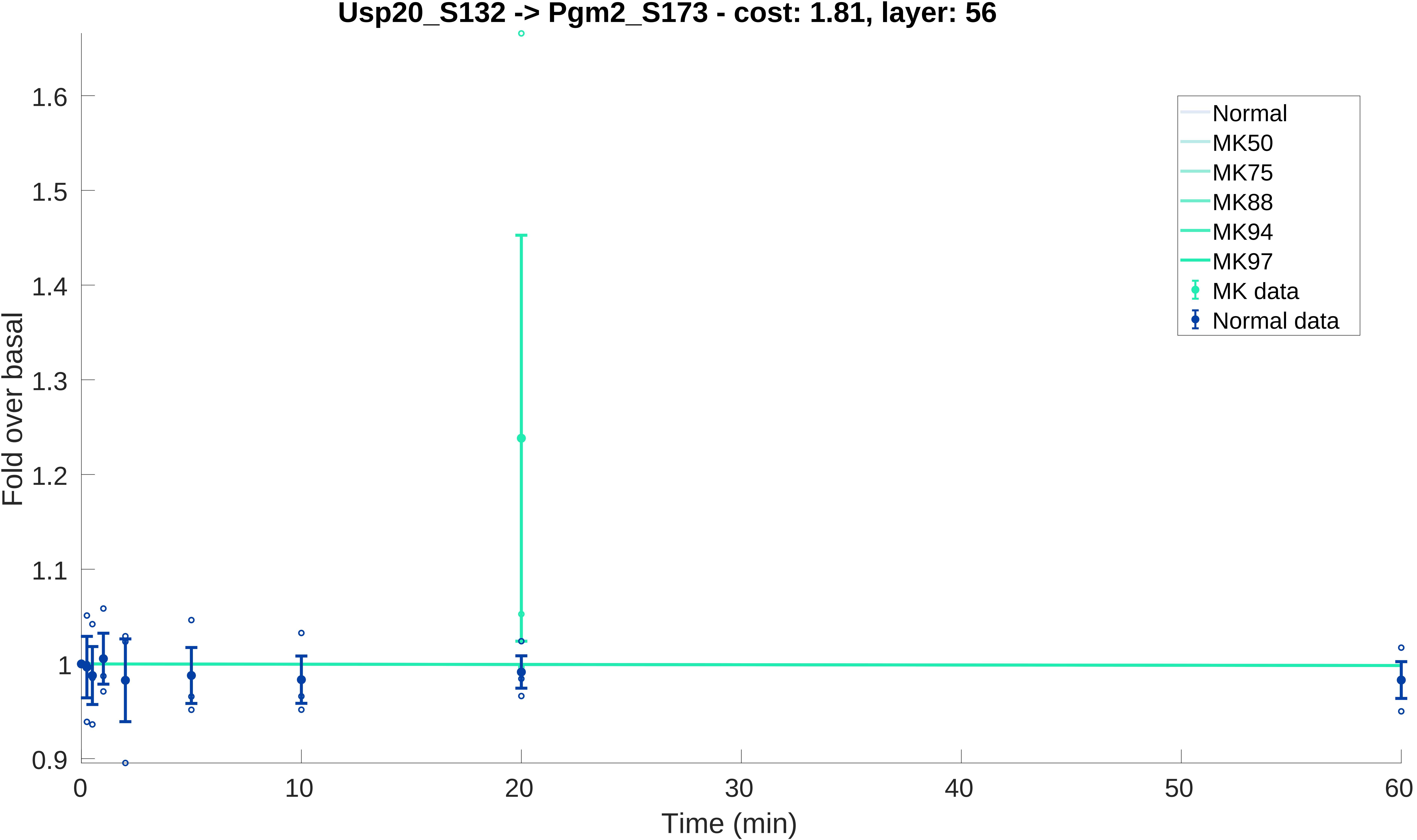

Usp20_S132 -> Pgm2_S173 - cost: 1.81, layer: 56
Fold over basal
Time (min)
0.9
1
1.1
1.2
1.3
1.4
1.5
1.6
0
10
20
30
40
50
60
Normal
MK50
MK75
MK88
MK94
MK97
MK data
Normal data

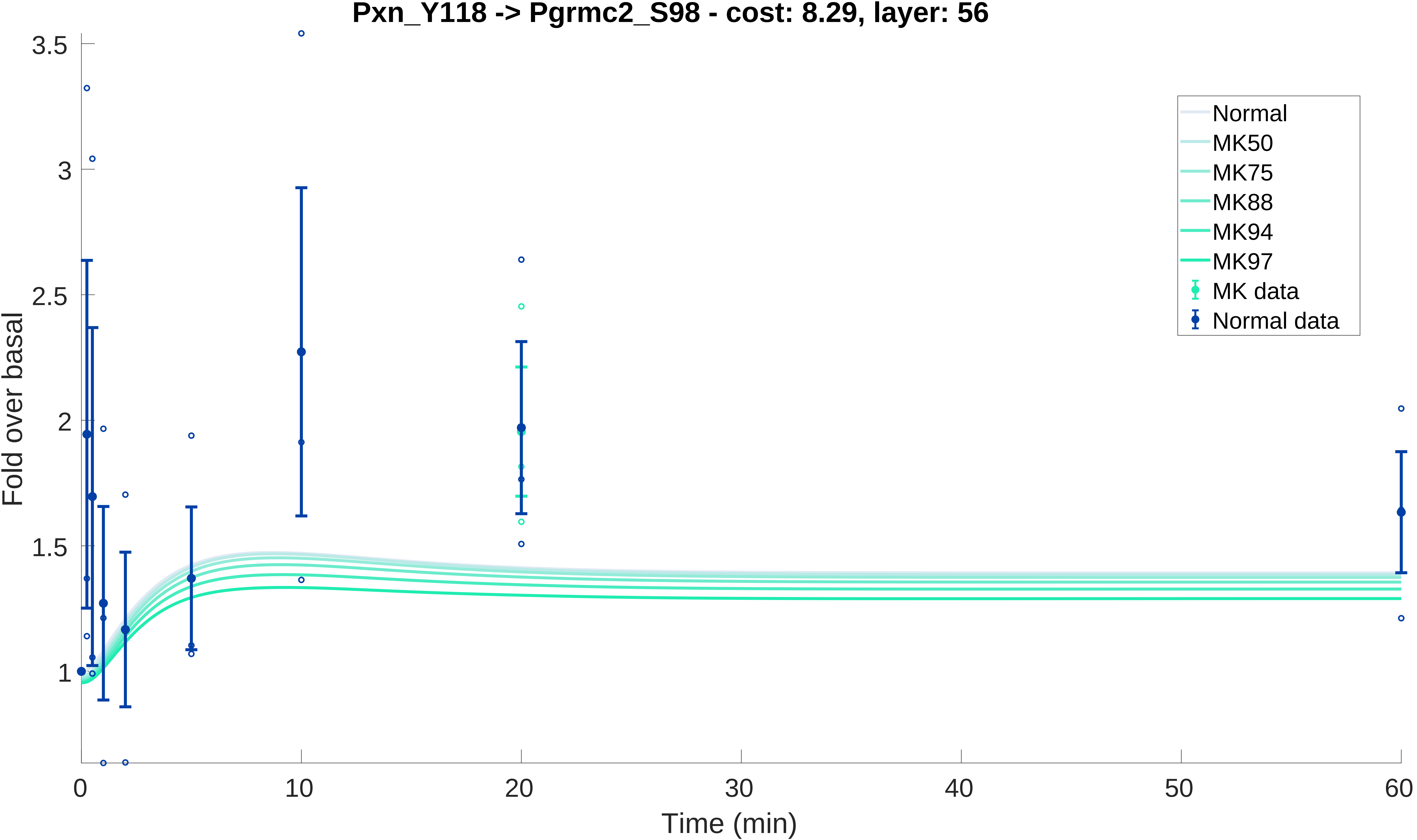

Pxn_Y118 -> Pgrmc2_S98 - cost: 8.29, layer: 56
Fold over basal
Time (min)
3.5
3
2.5
2
1.5
1
0
10
20
30
40
50
60
Normal
MK50
MK75
MK88
MK94
MK97
MK data
Normal data

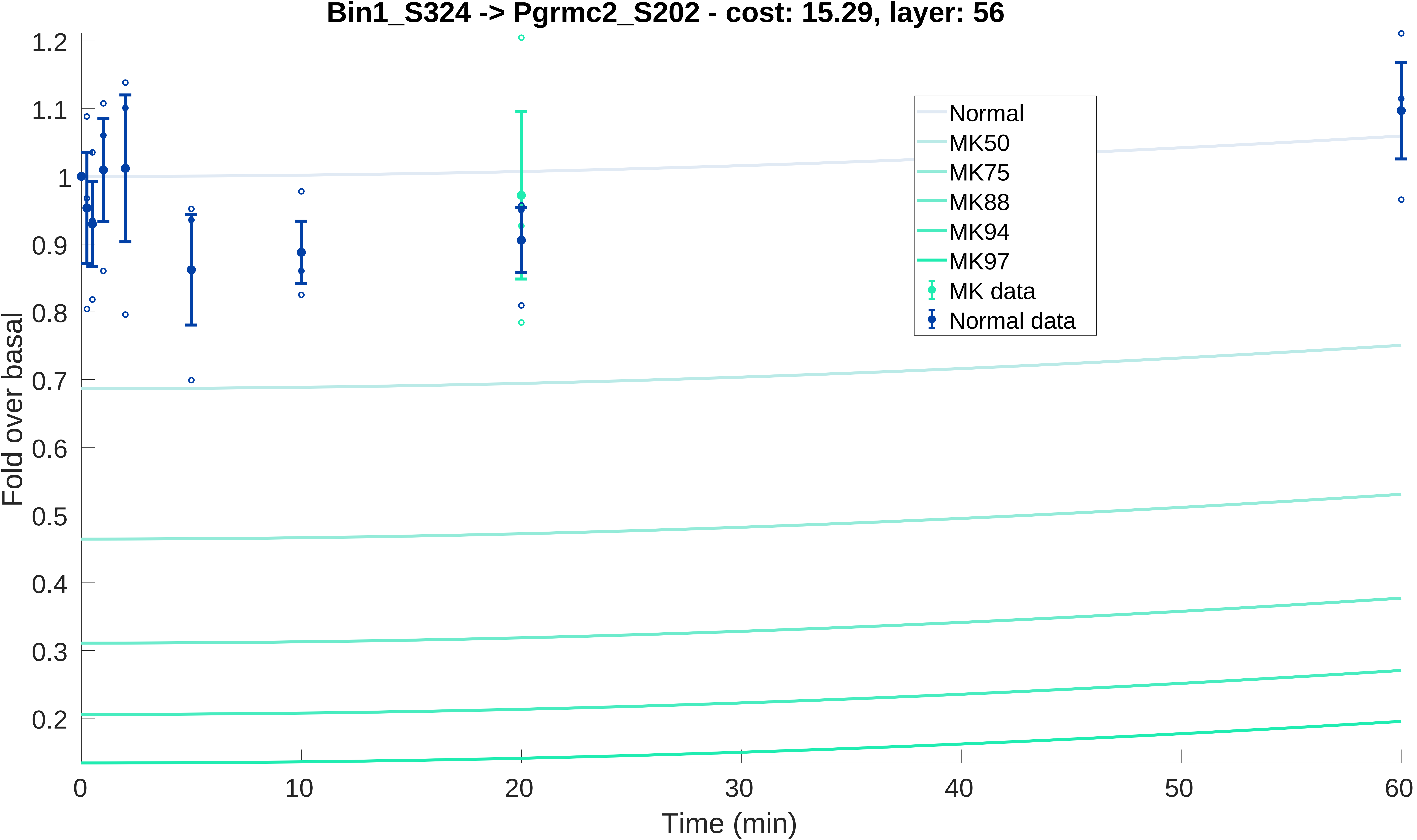

Bin1_S324 -> Pgrmc2_S202 - cost: 15.29, layer: 56
Fold over basal
Time (min)
1.2
1.1
1
0.9
0.8
0.7
0.6
0.5
0.4
0.3
0.2
0
10
20
30
40
50
60
Normal
MK50
MK75
MK88
MK94
MK97
MK data
Normal data

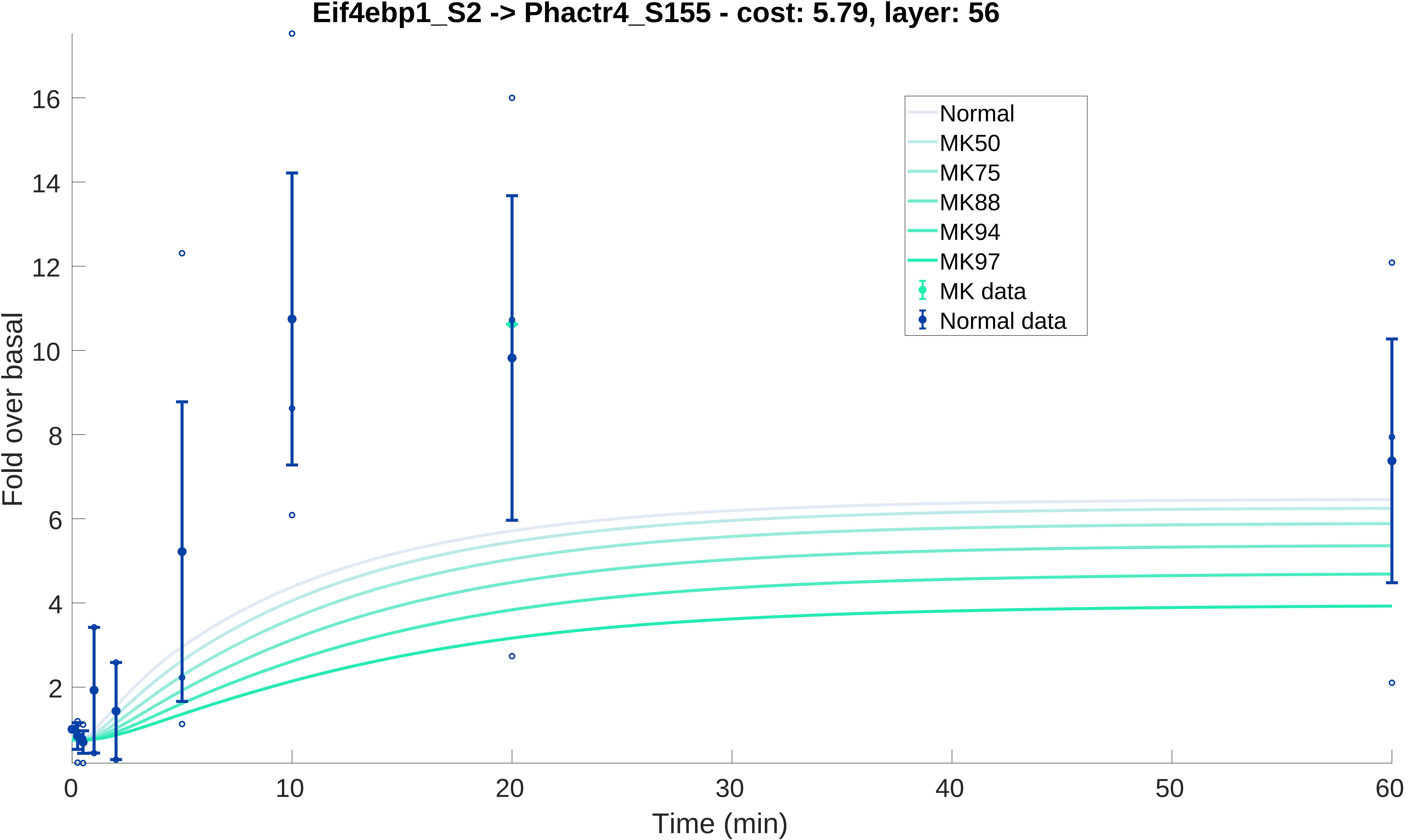

Eif4ebp1_S2 -> Phactr4_S155 - cost: 5.79, layer: 56
Fold over basal
Time (min)
0
10
20
30
40
50
60
2
4
6
8
10
12
14
16
Normal
MK50
MK75
MK88
MK94
MK97
MK data
Normal data

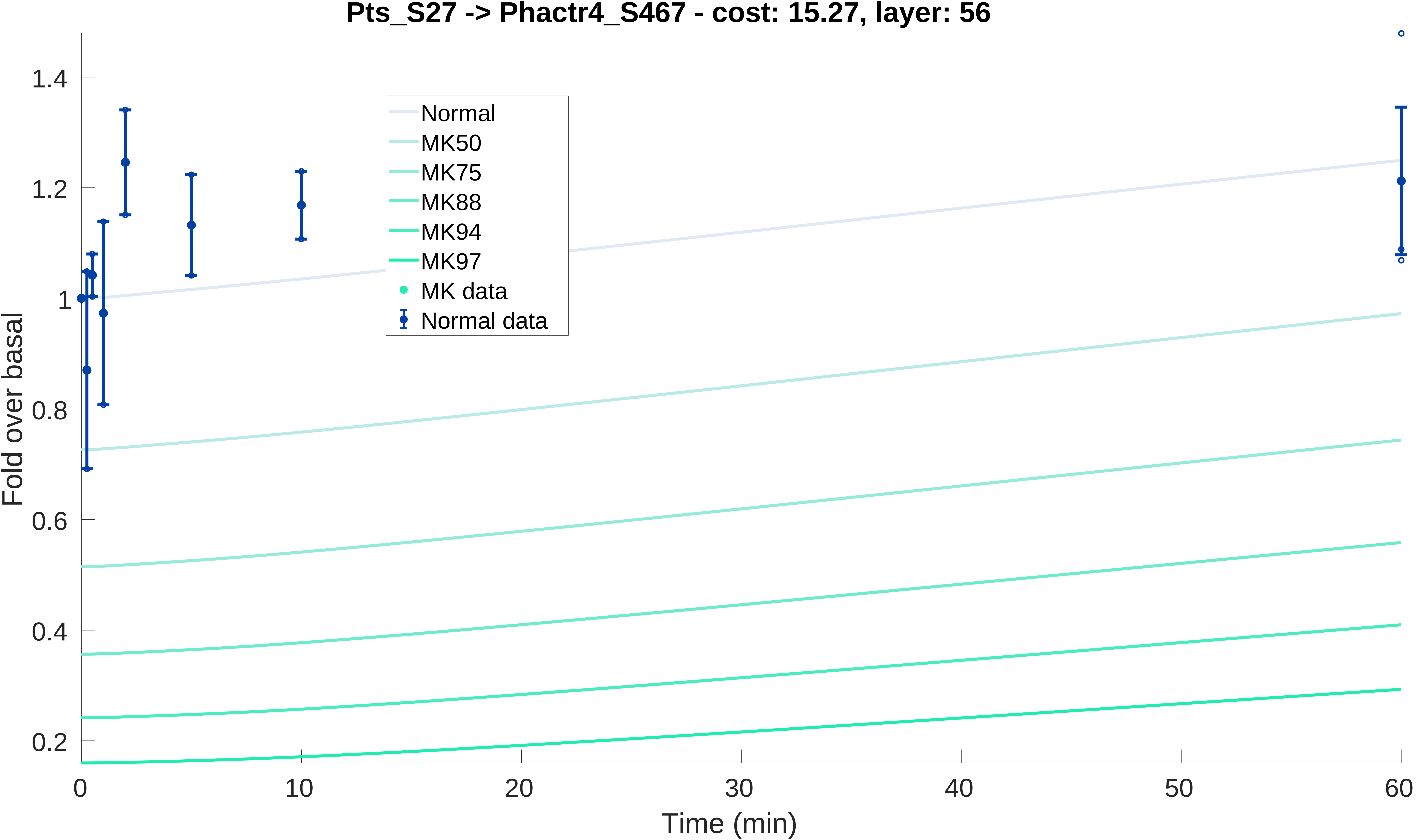

Pts_S27 -> Phactr4_S467 - cost: 15.27, layer: 56
Normal
MK50
MK75
MK88
MK94
MK97
MK data
Normal data
Fold over basal
Time (min)
0.2
0.4
0.6
0.8
1
1.2
1.4
0
10
20
30
40
50
60

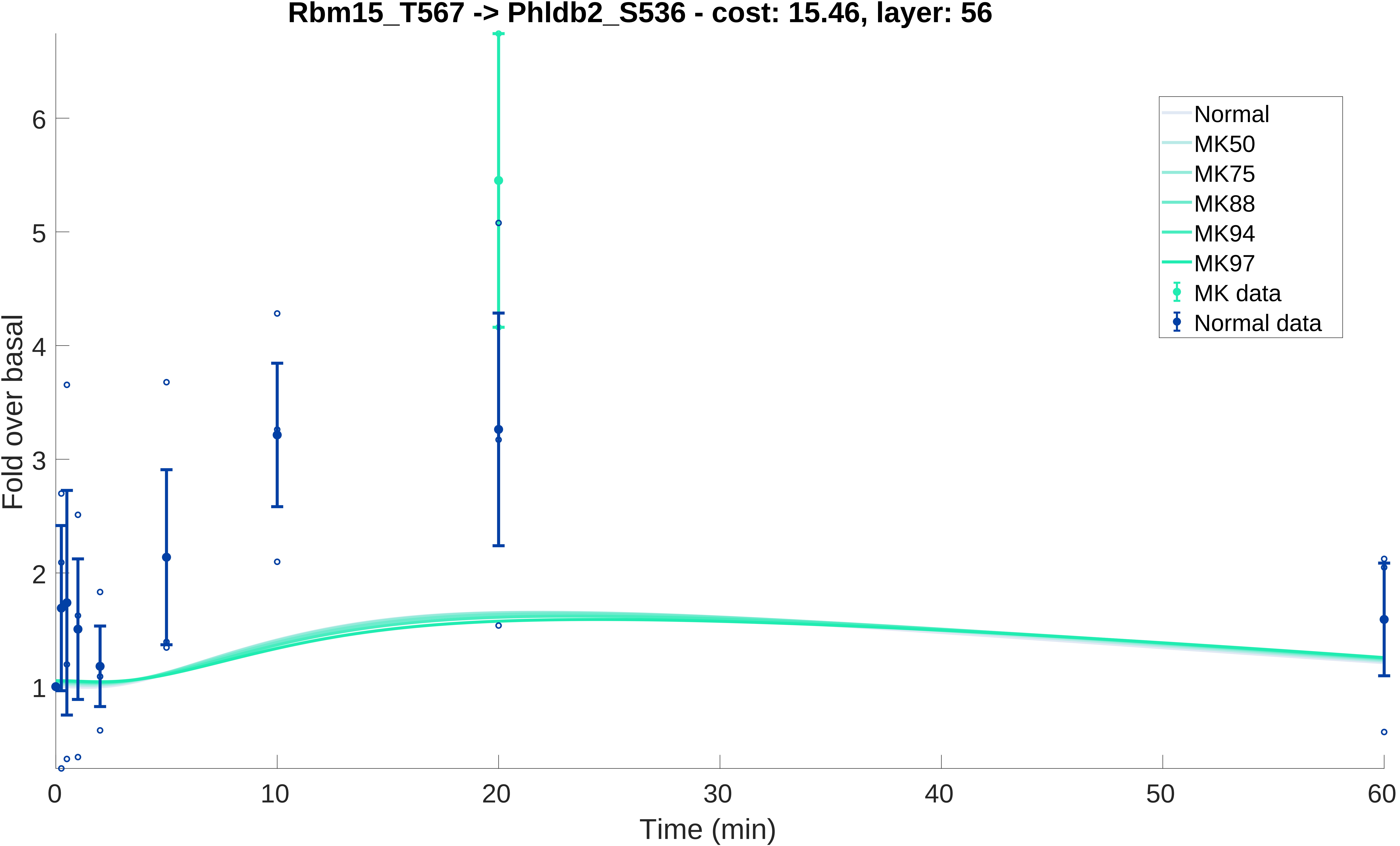

Rbm15_T567 -> Phldb2_S536 - cost: 15.46, layer: 56
Normal
MK50
MK75
MK88
MK94
MK97
MK data
Normal data
Fold over basal
Time (min)
1
2
3
4
5
6
0
10
20
30
40
50
60

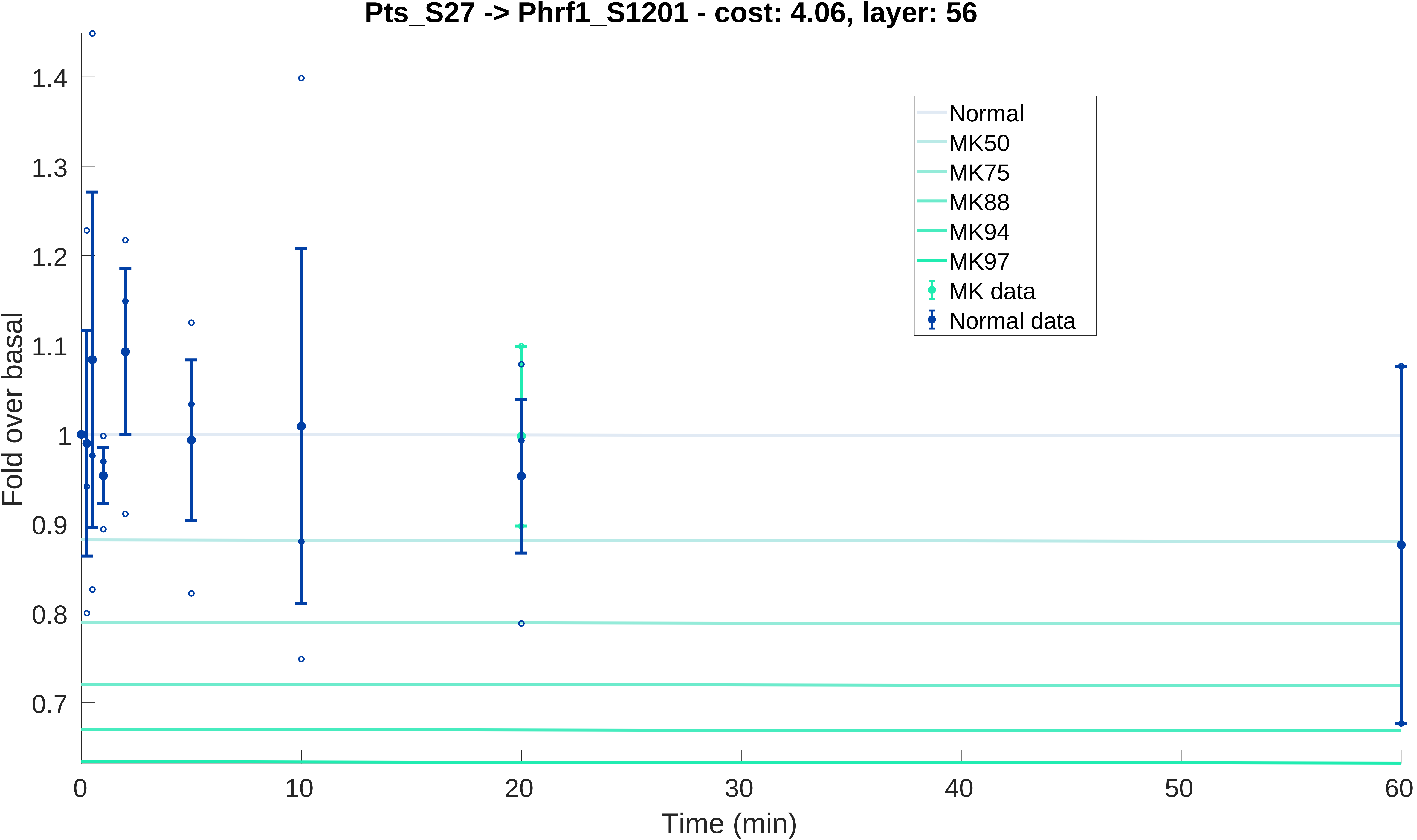
Pts_S27 -> Phrf1_S1201 - cost: 4.06, layer: 56
Fold over basal
Time (min)
Normal
MK50
MK75
MK88
MK94
MK97
MK data
Normal data
1.4
1.3
1.2
1.1
1
0.9
0.8
0.7
0
10
20
30
40
50
60

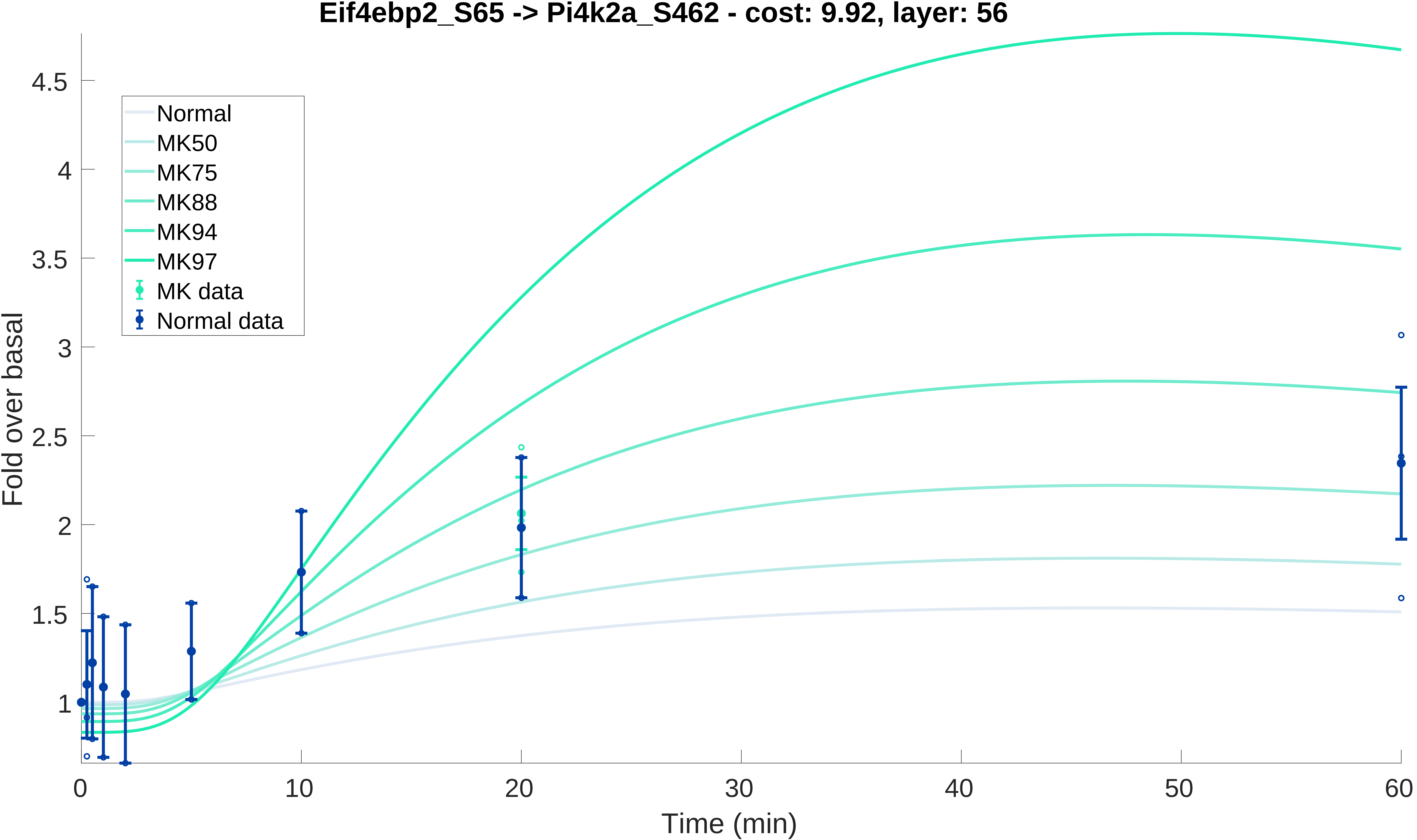
Eif4ebp2_S65 -> Pi4k2a_S462 - cost: 9.92, layer: 56
Fold over basal
Time (min)
4.5
4
3.5
3
2.5
2
1.5
1
0
10
20
30
40
50
60
Normal
MK50
MK75
MK88
MK94
MK97
MK data
Normal data

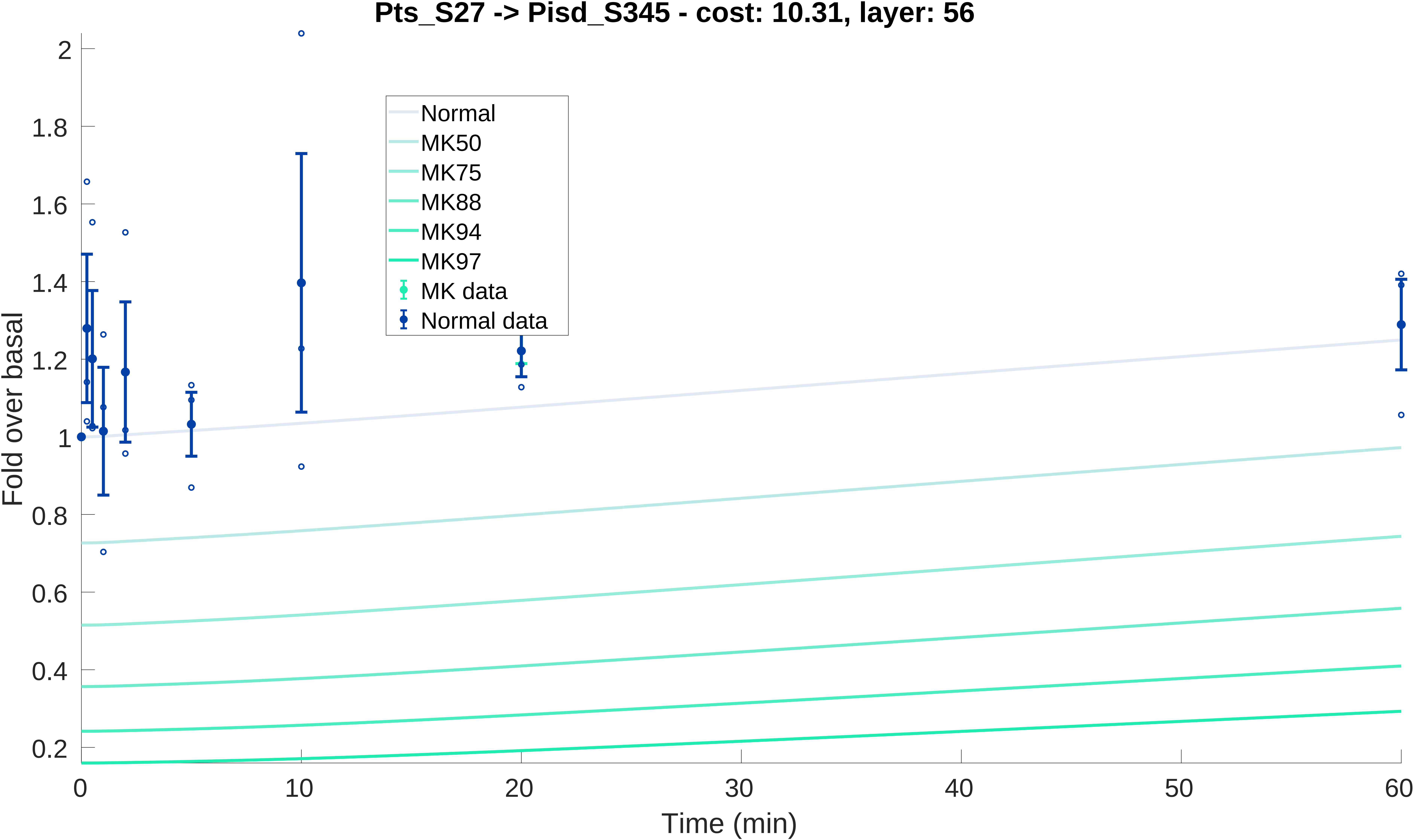

Pts_S27 -> Pisd_S345 - cost: 10.31, layer: 56
Fold over basal
Time (min)
Normal
MK50
MK75
MK88
MK94
MK97
MK data
Normal data
2
1.8
1.6
1.4
1.2
1
0.8
0.6
0.4
0.2
0
10
20
30
40
50
60

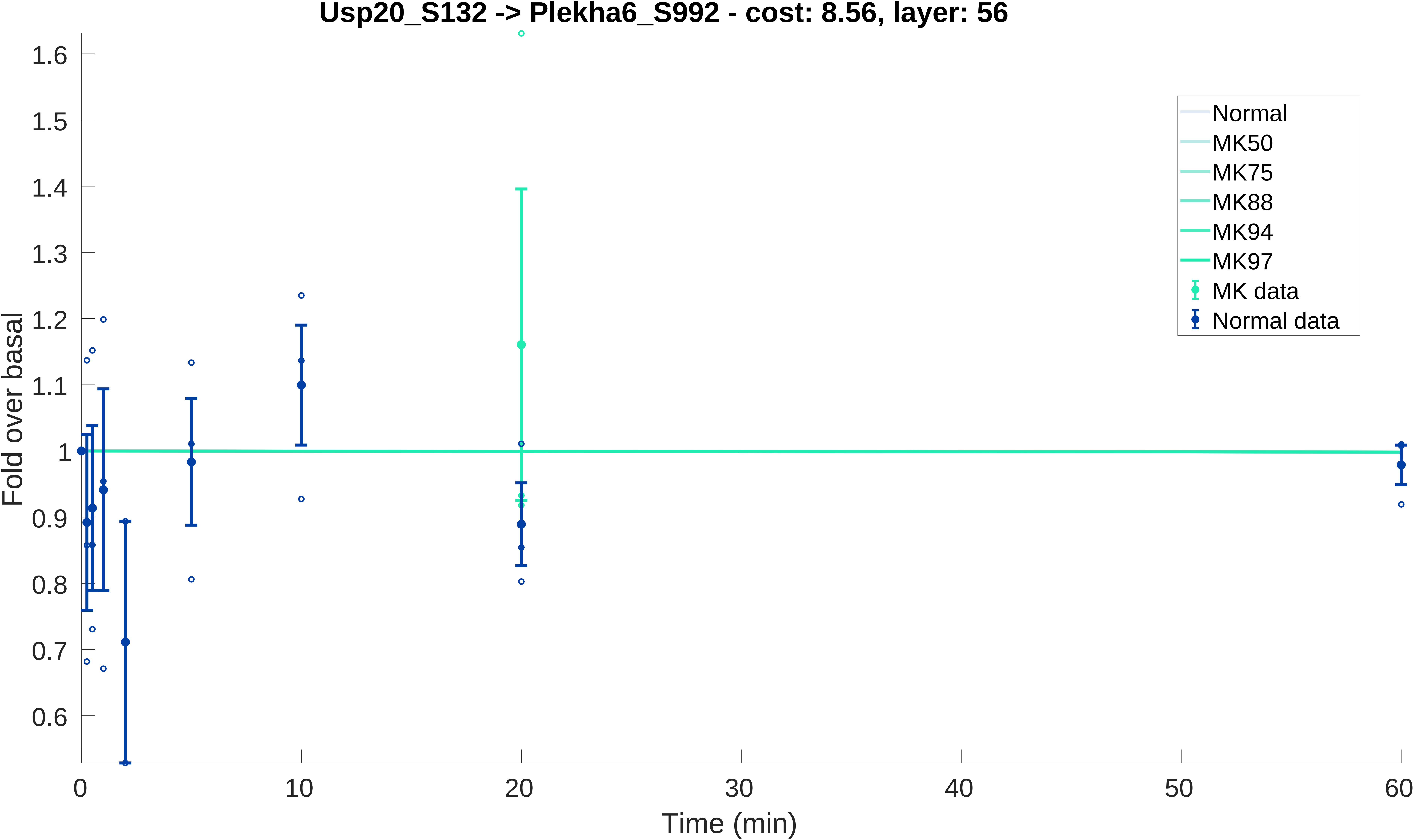

Usp20_S132 -> Plekha6_S992 - cost: 8.56, layer: 56
Fold over basal
Time (min)
1.6
1.5
1.4
1.3
1.2
1.1
1
0.9
0.8
0.7
0.6
0
10
20
30
40
50
60
Normal
MK50
MK75
MK88
MK94
MK97
MK data
Normal data

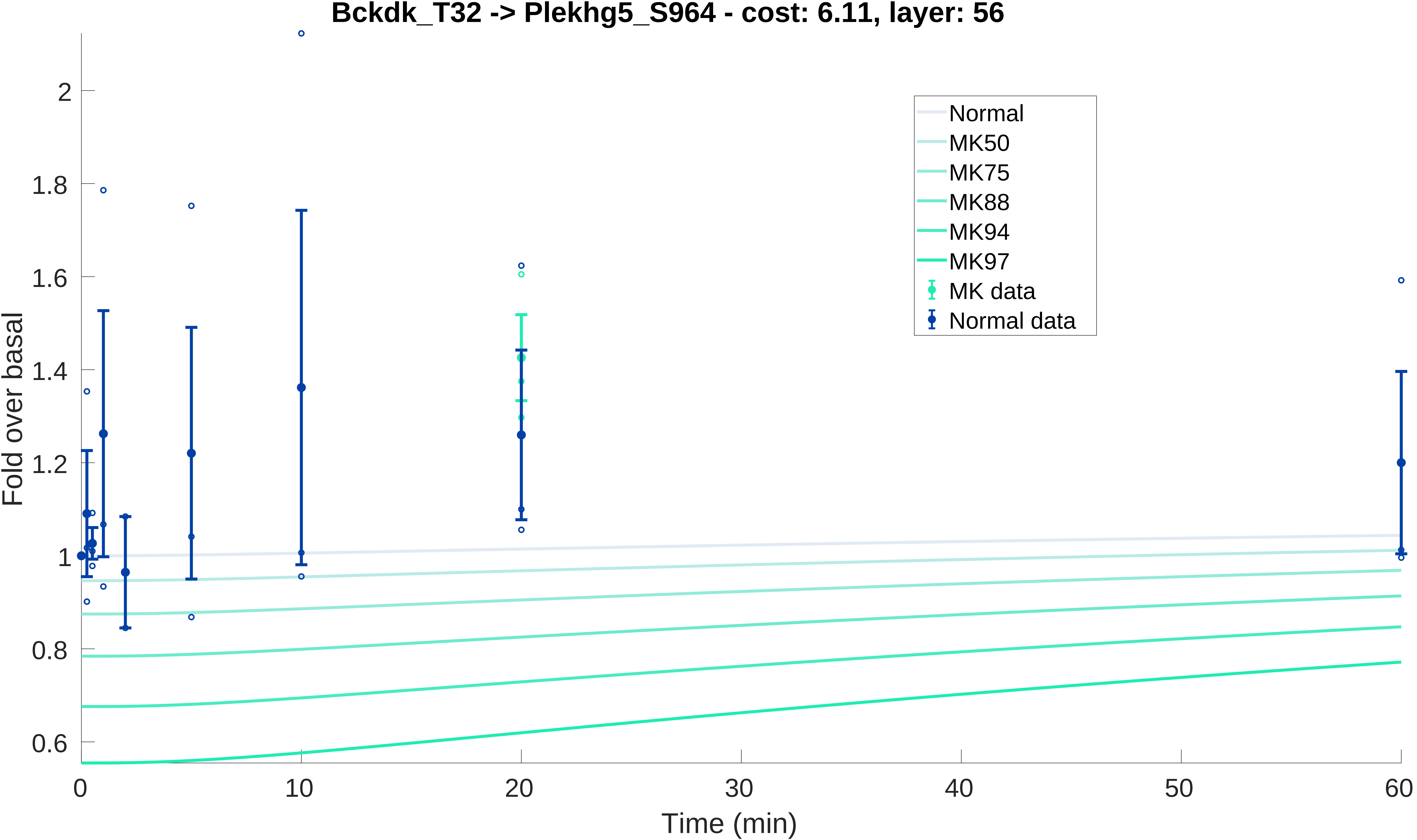

Bckdk_T32 -> Plekhg5_S964 - cost: 6.11, layer: 56
Fold over basal
Time (min)
0.6
0.8
1
1.2
1.4
1.6
1.8
2
0
10
20
30
40
50
60
Normal
MK50
MK75
MK88
MK94
MK97
MK data
Normal data

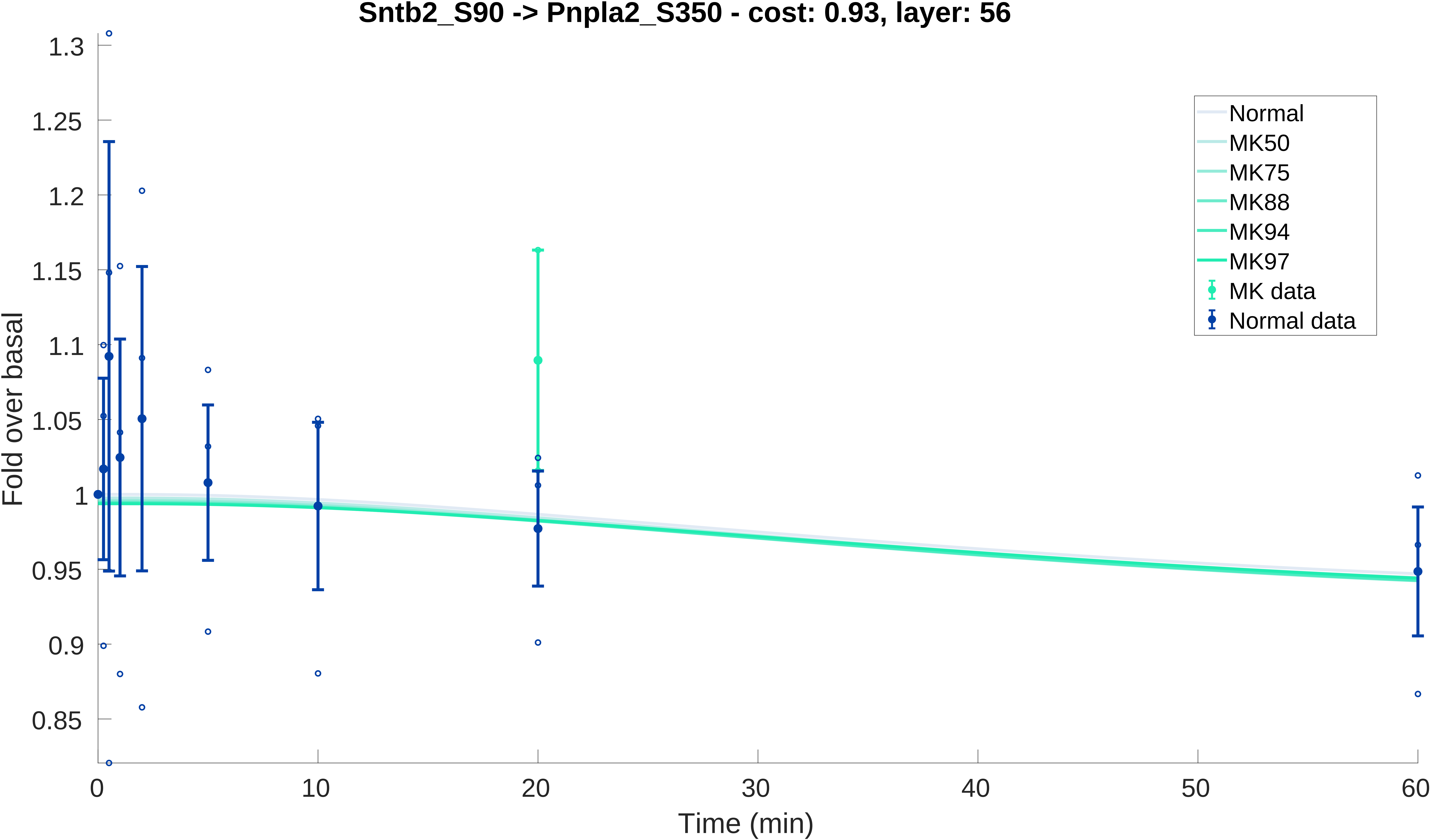

Sntb2_S90 -> Pnpla2_S350 - cost: 0.93, layer: 56
Fold over basal
Time (min)
0
10
20
30
40
50
60
0.85
0.9
0.95
1
1.05
1.1
1.15
1.2
1.25
1.3
Normal
MK50
MK75
MK88
MK94
MK97
MK data
Normal data

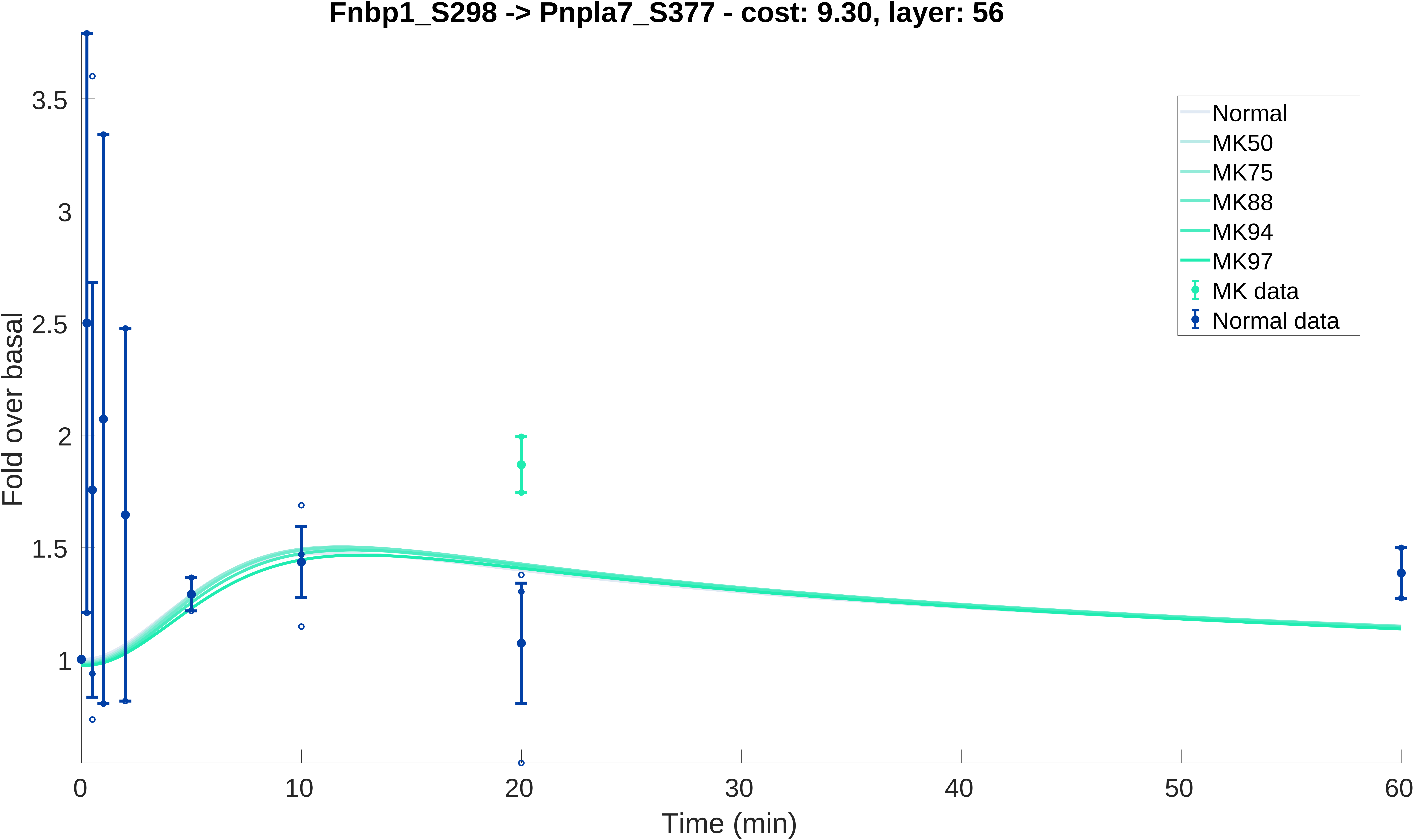

Fnbp1_S298 -> Pnpla7_S377 - cost: 9.30, layer: 56
Fold over basal
Time (min)
3.5
3
2.5
2
1.5
1
0
10
20
30
40
50
60
Normal
MK50
MK75
MK88
MK94
MK97
MK data
Normal data

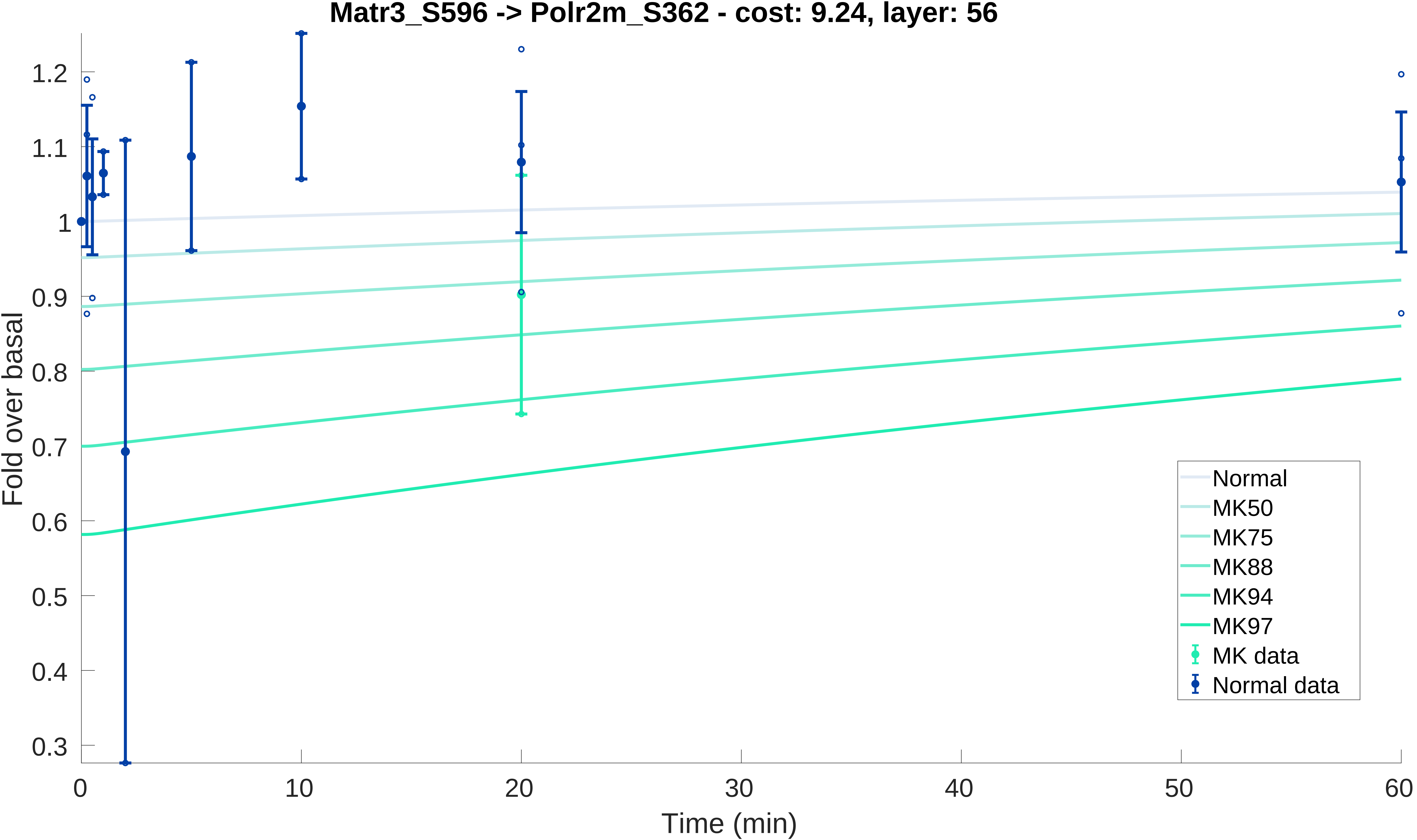

Matr3_S596 -> Polr2m_S362 - cost: 9.24, layer: 56
Fold over basal
Time (min)
0
10
20
30
40
50
60
0.3
0.4
0.5
0.6
0.7
0.8
0.9
1
1.1
1.2
Normal
MK50
MK75
MK88
MK94
MK97
MK data
Normal data

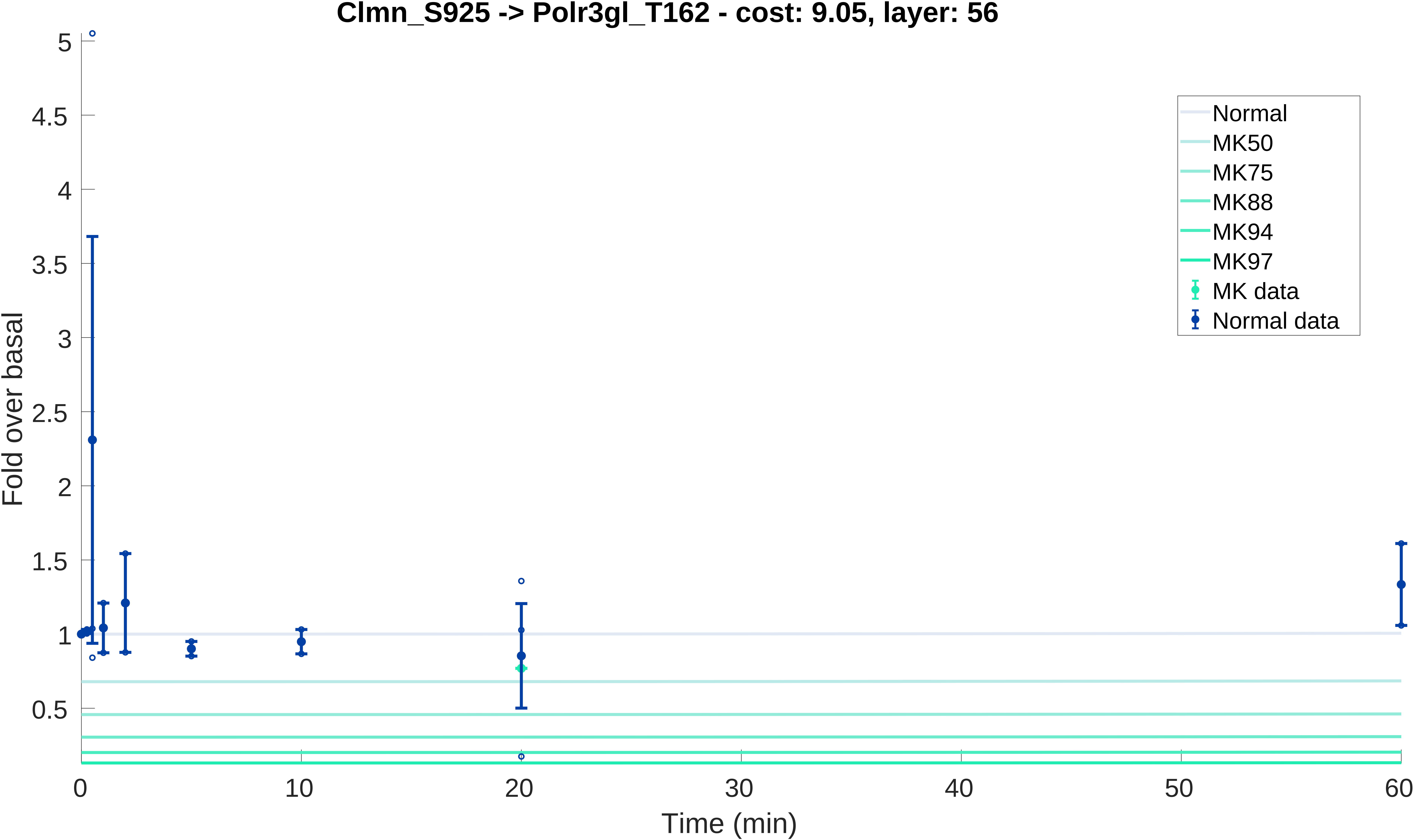

Clmn_S925 -> Polr3gl_T162 - cost: 9.05, layer: 56
Fold over basal
Time (min)
0.5
1
1.5
2
2.5
3
3.5
4
4.5
5
0
10
20
30
40
50
60
Normal
MK50
MK75
MK88
MK94
MK97
MK data
Normal data

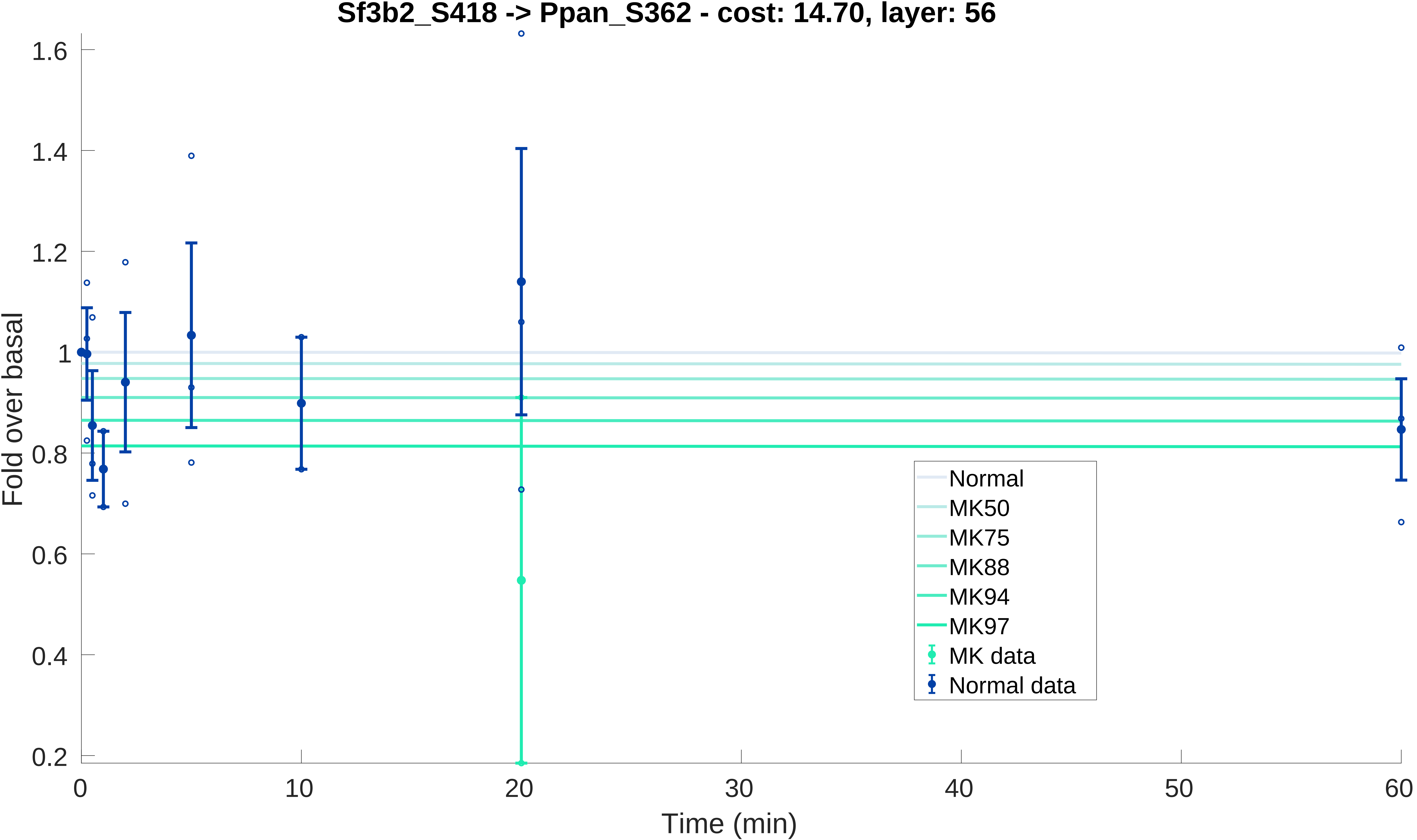

Sf3b2_S418 -> Ppan_S362 - cost: 14.70, layer: 56
Fold over basal
1.6
1.4
1.2
1
0.8
0.6
0.4
0.2
0
10
20
30
40
50
60
Time (min)
Normal
MK50
MK75
MK88
MK94
MK97
MK data
Normal data

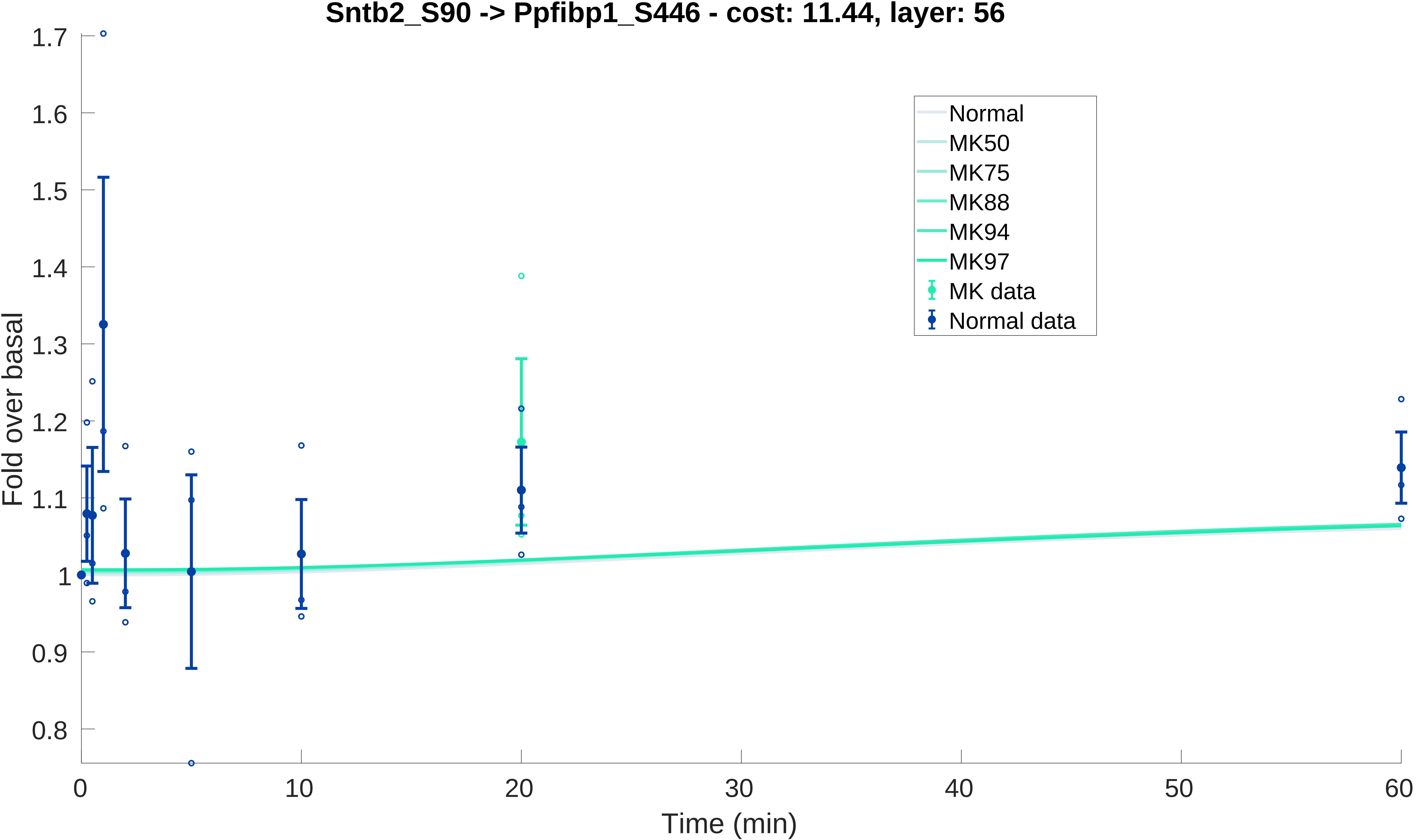

Sntb2_S90 -> Ppfibp1_S446 - cost: 11.44, layer: 56
Fold over basal
1.7
1.6
1.5
1.4
1.3
1.2
1.1
1
0.9
0.8
0
10
20
30
40
50
60
Time (min)
Normal
MK50
MK75
MK88
MK94
MK97
MK data
Normal data

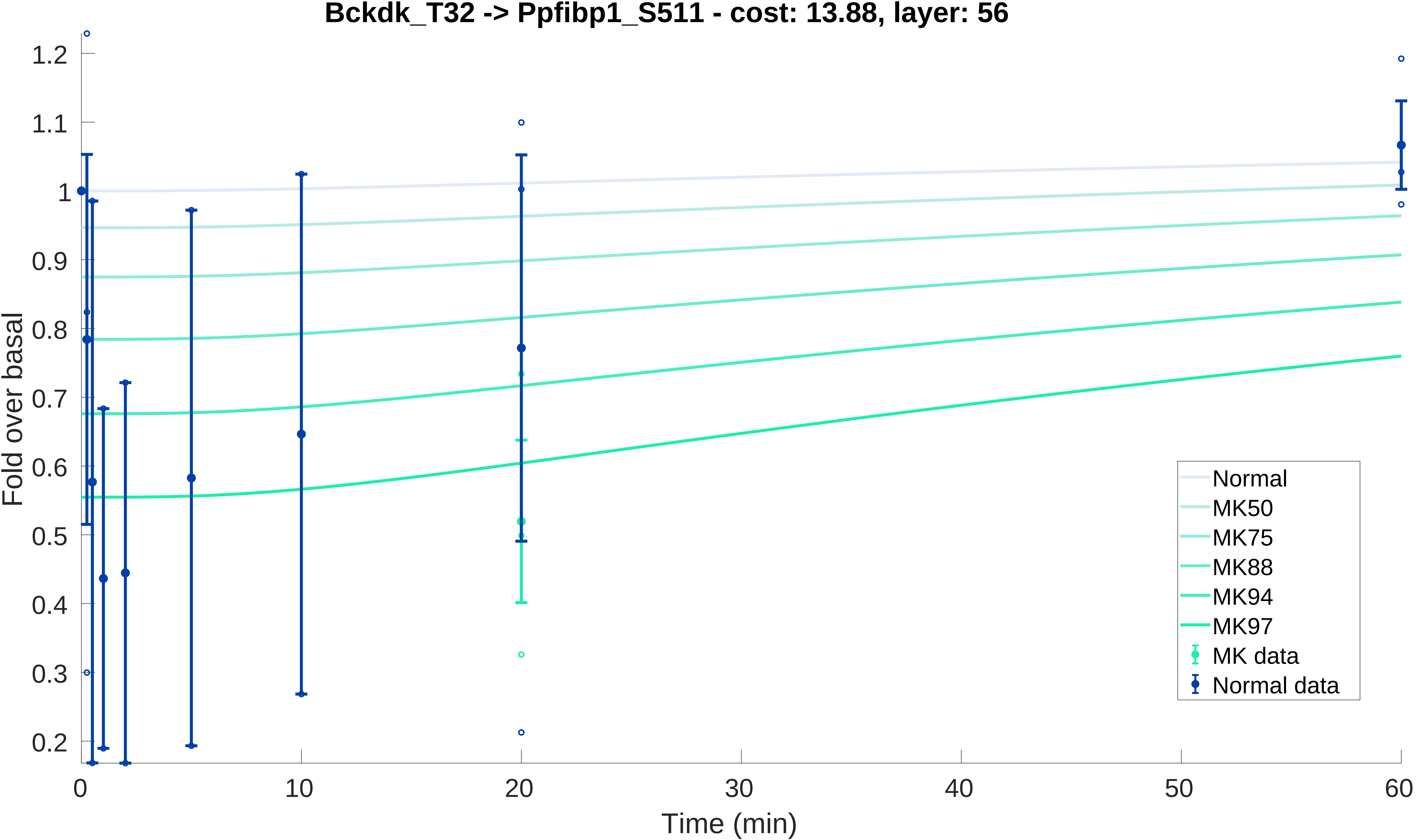

Bckdk_T32 -> Ppfibp1_S511 - cost: 13.88, layer: 56
Fold over basal
Time (min)
0
10
20
30
40
50
60
0.2
0.3
0.4
0.5
0.6
0.7
0.8
0.9
1
1.1
1.2
Normal
MK50
MK75
MK88
MK94
MK97
MK data
Normal data

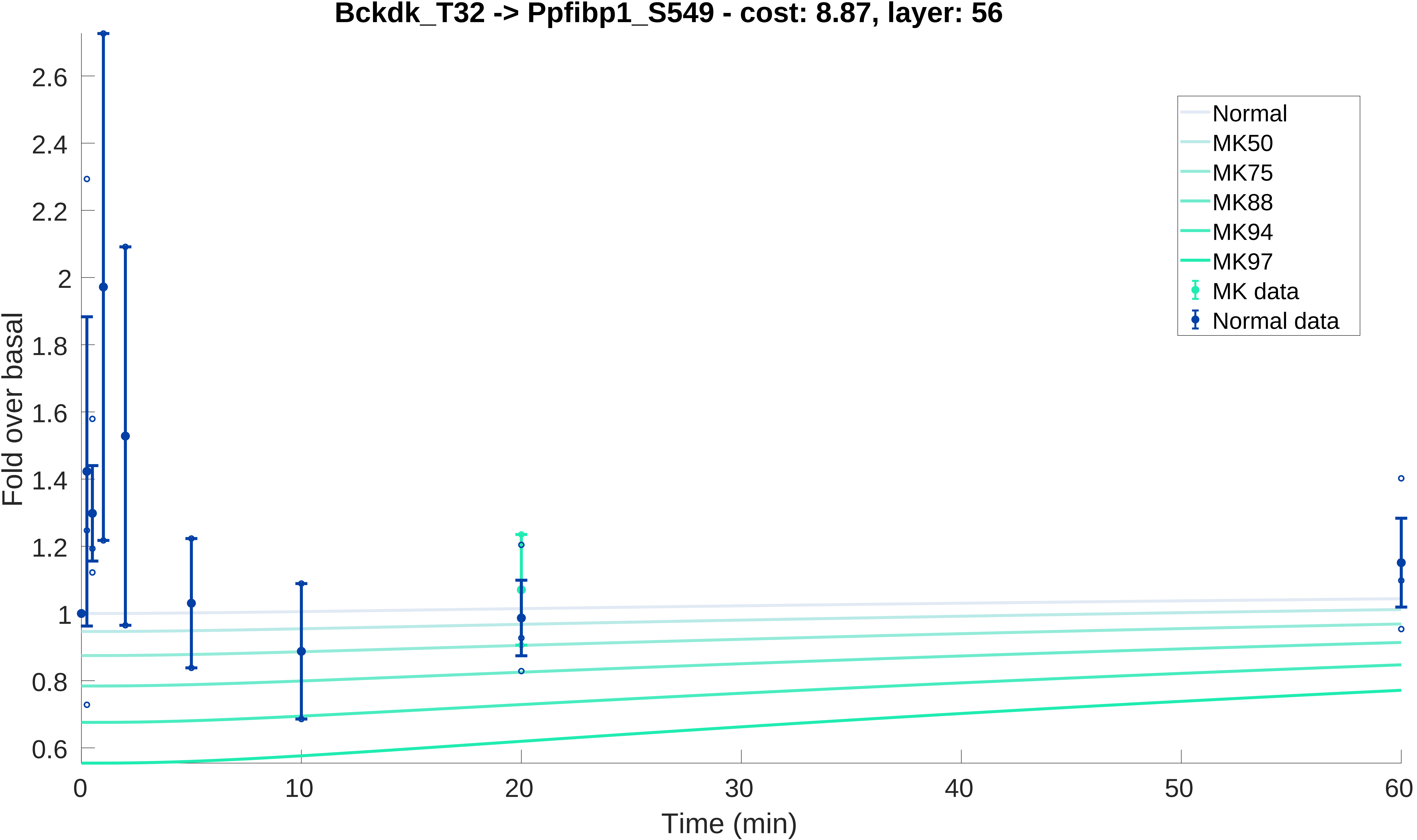

Bckdk_T32 -> Ppfibp1_S549 - cost: 8.87, layer: 56
Fold over basal
Time (min)
0.6
0.8
1
1.2
1.4
1.6
1.8
2
2.2
2.4
2.6
0
10
20
30
40
50
60
Normal
MK50
MK75
MK88
MK94
MK97
MK data
Normal data

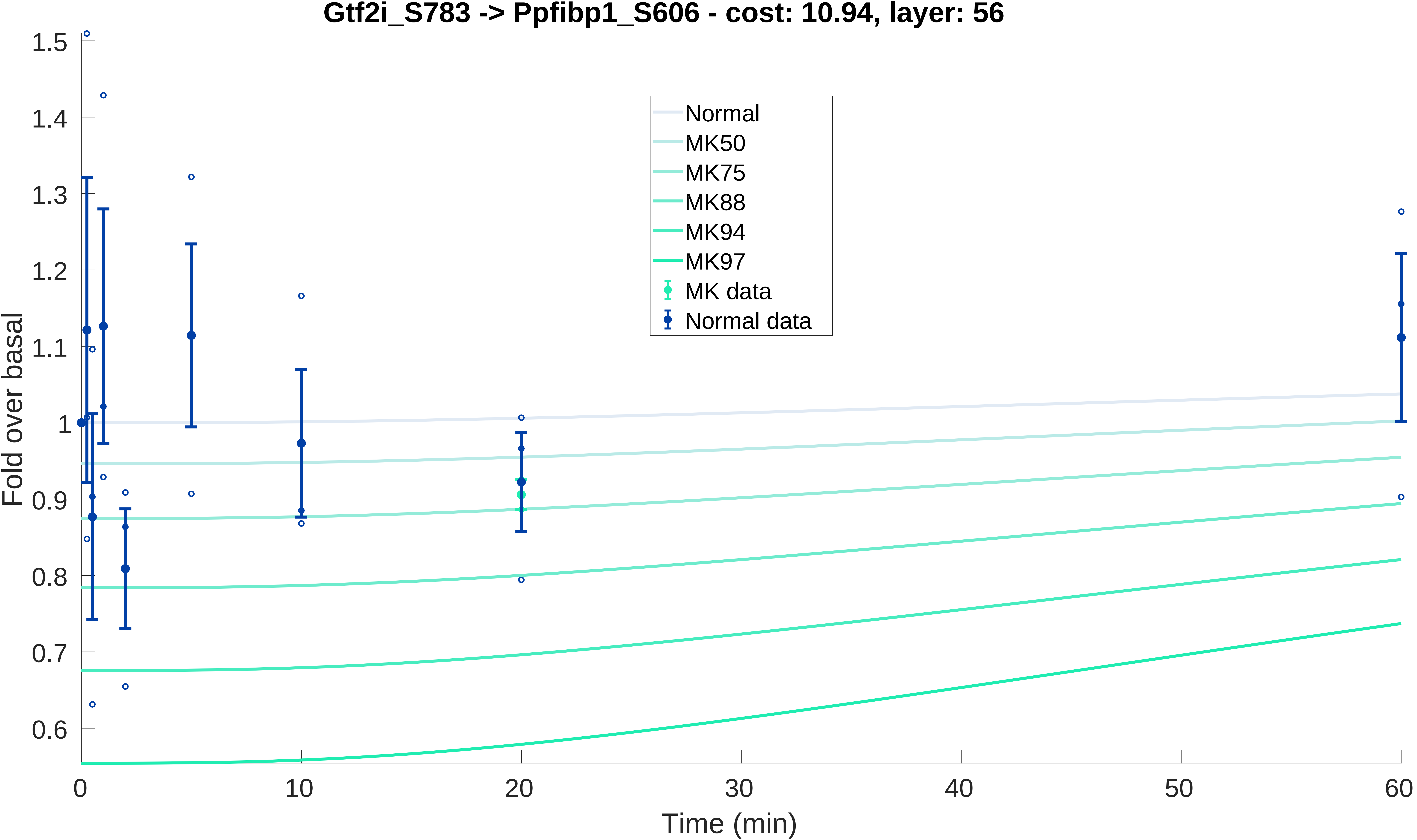

Gtf2i_S783 -> Ppfibp1_S606 - cost: 10.94, layer: 56
Fold over basal
Time (min)
Normal
MK50
MK75
MK88
MK94
MK97
MK data
Normal data
1.5
1.4
1.3
1.2
1.1
1
0.9
0.8
0.7
0.6
0
10
20
30
40
50
60

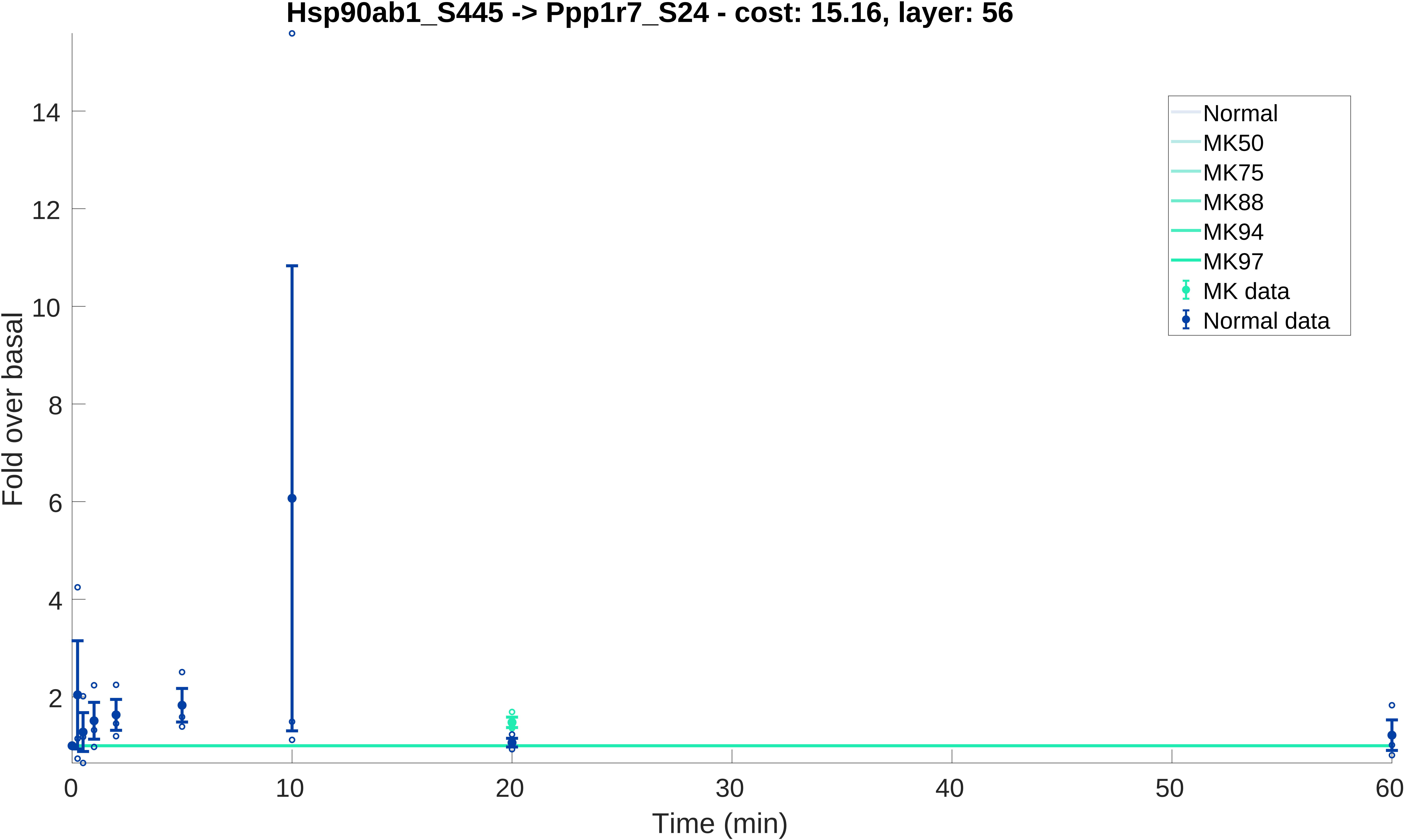

Hsp90ab1_S445 -> Ppp1r7_S24 - cost: 15.16, layer: 56
Fold over basal
14
12
10
8
6
4
2
0
10
20
30
40
50
60
Time (min)
Normal
MK50
MK75
MK88
MK94
MK97
MK data
Normal data

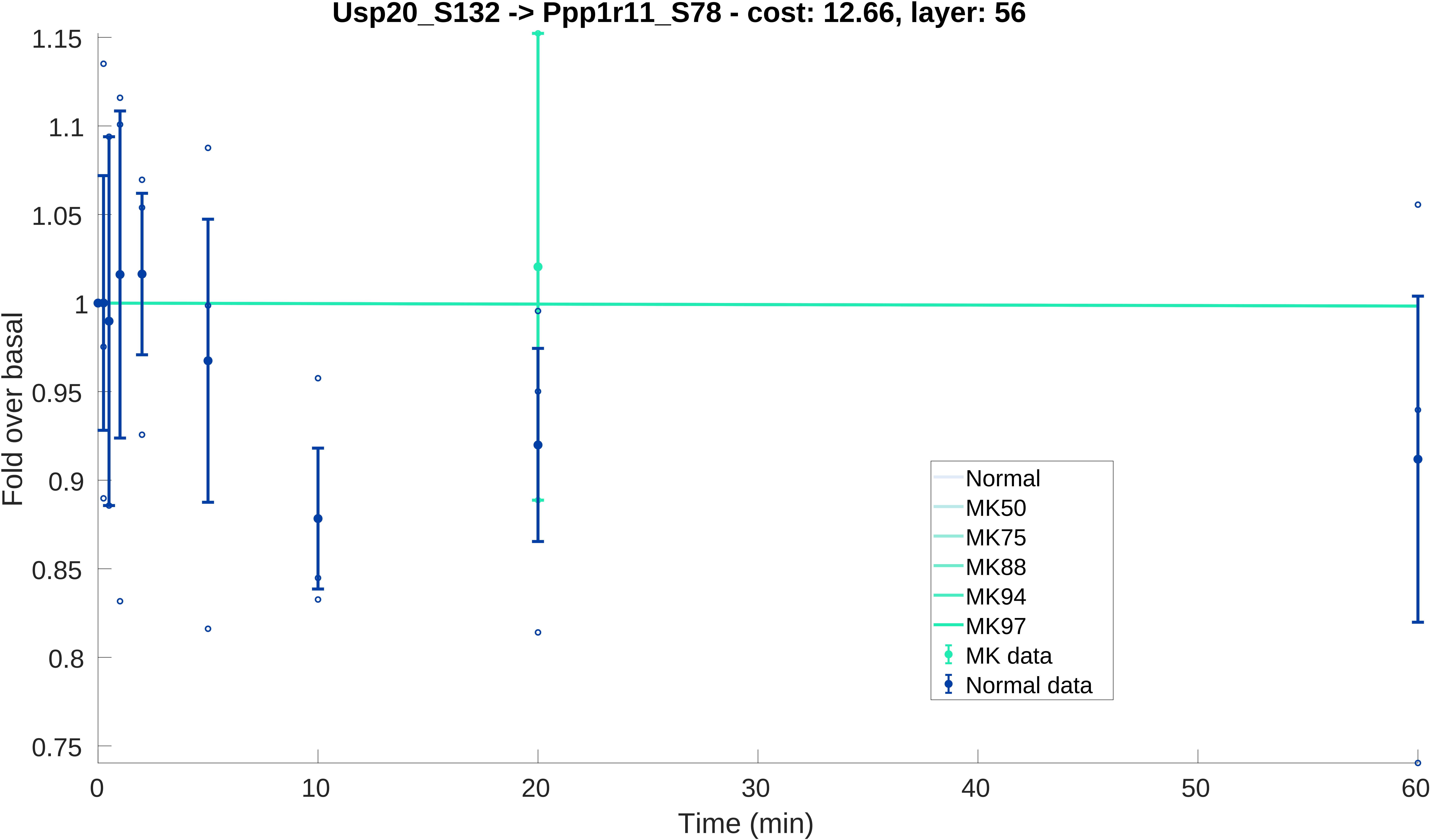
Usp20_S132 -> Ppp1r11_S78 - cost: 12.66, layer: 56
Fold over basal
Time (min)
1.15
1.1
1.05
1
0.95
0.9
0.85
0.8
0.75
0
10
20
30
40
50
60
Normal
MK50
MK75
MK88
MK94
MK97
MK data
Normal data

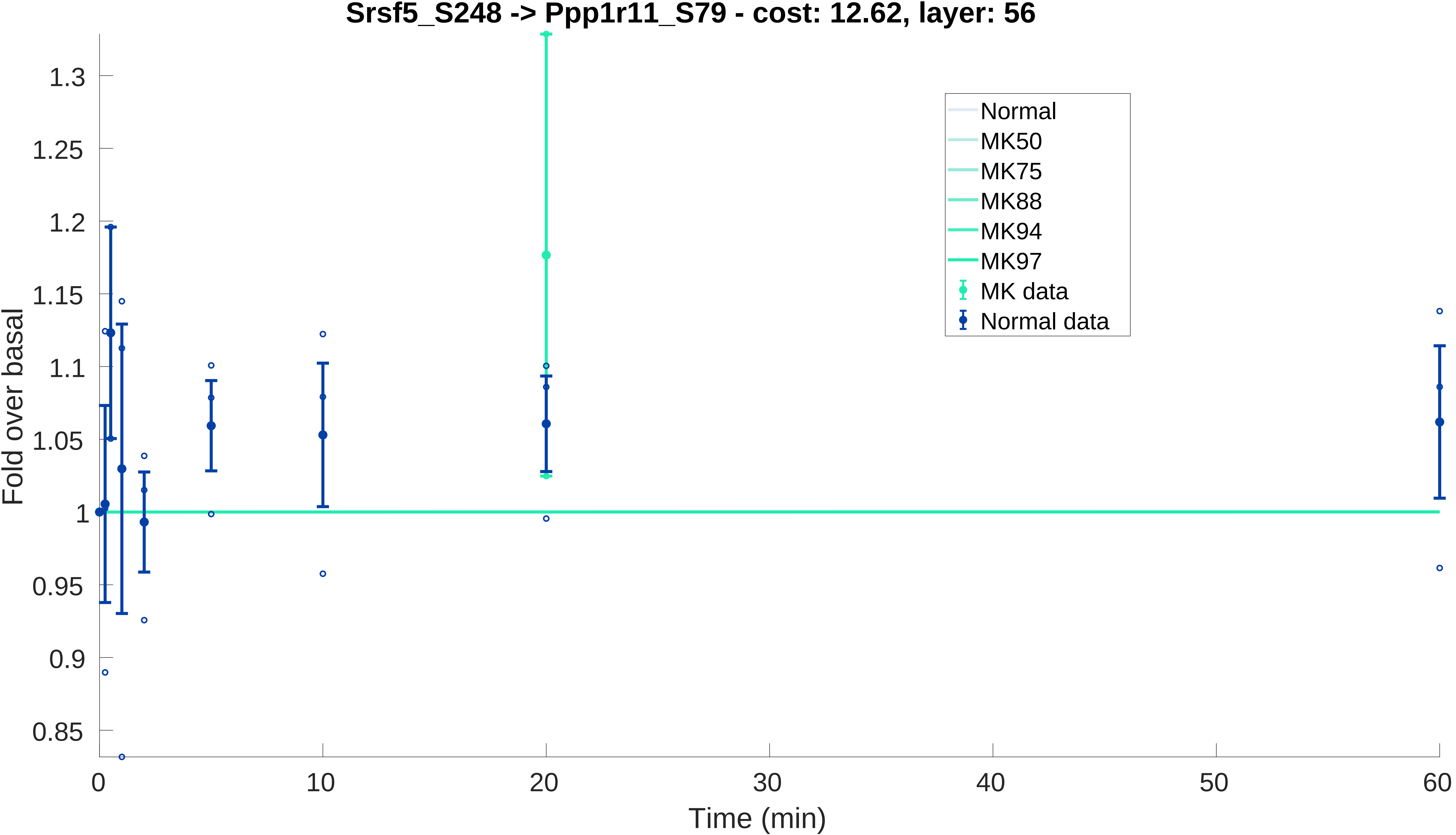
Srsf5_S248 -> Ppp1r11_S79 - cost: 12.62, layer: 56
Fold over basal
Time (min)
Normal
MK50
MK75
MK88
MK94
MK97
MK data
Normal data
1.3
1.25
1.2
1.15
1.1
1.05
1
0.95
0.9
0.85
0
10
20
30
40
50
60

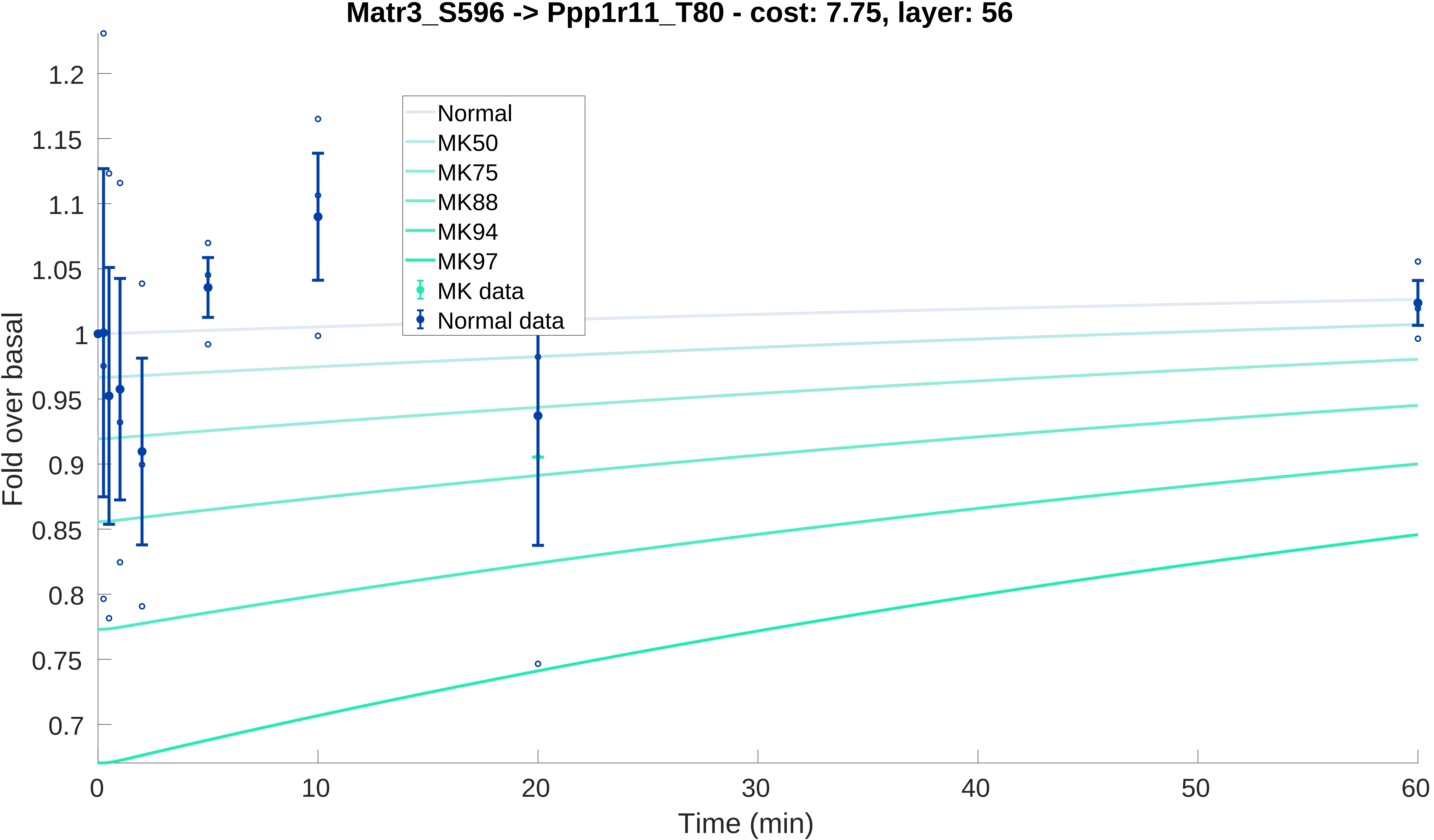

Matr3_S596 -> Ppp1r11_T80 - cost: 7.75, layer: 56
Fold over basal
Time (min)
Normal
MK50
MK75
MK88
MK94
MK97
MK data
Normal data
1.2
1.15
1.1
1.05
1
0.95
0.9
0.85
0.8
0.75
0.7
0
10
20
30
40
50
60

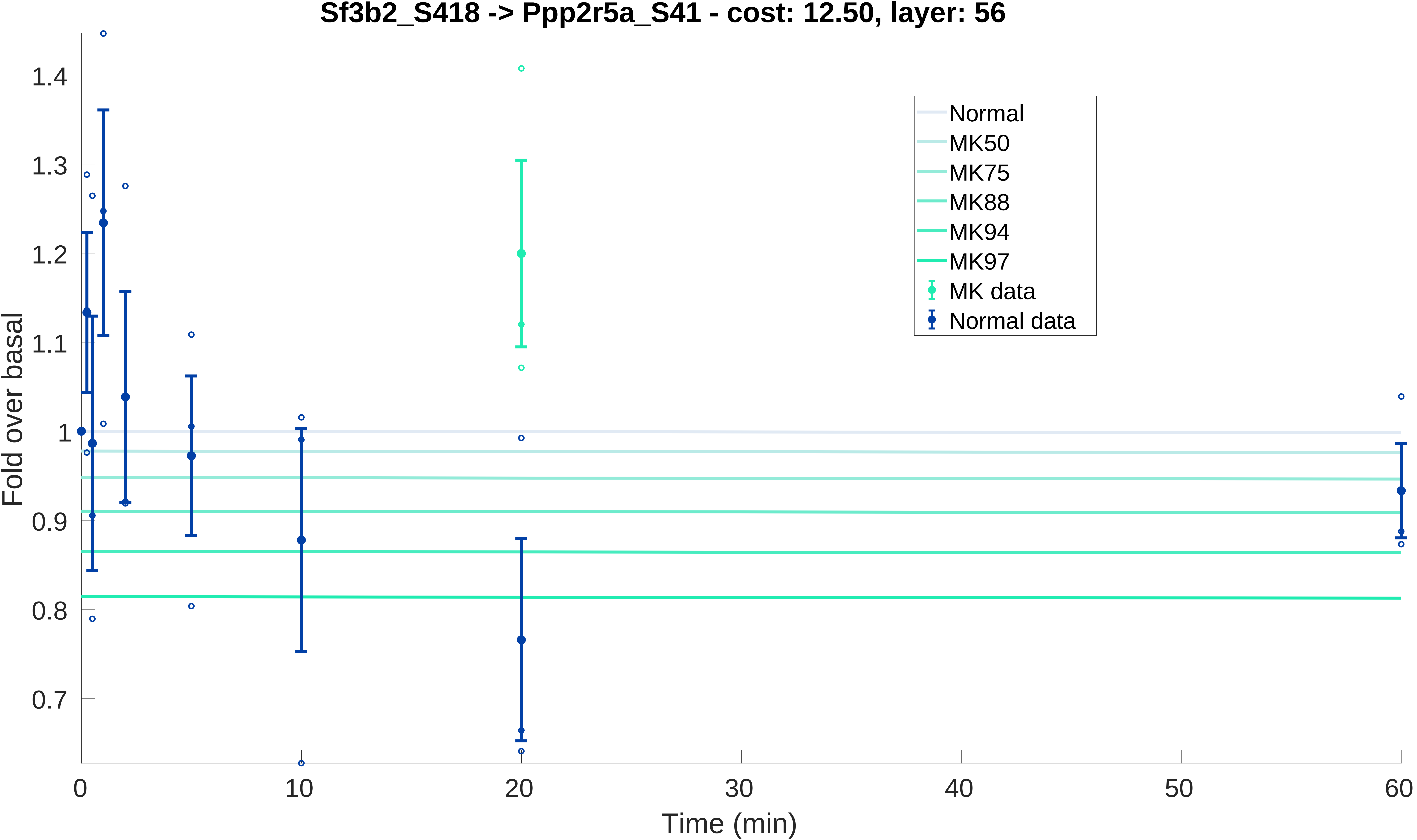

Sf3b2_S418 -> Ppp2r5a_S41 - cost: 12.50, layer: 56
Fold over basal
1.4
1.3
1.2
1.1
1
0.9
0.8
0.7
0
10
20
30
40
50
60
Time (min)
Normal
MK50
MK75
MK88
MK94
MK97
MK data
Normal data

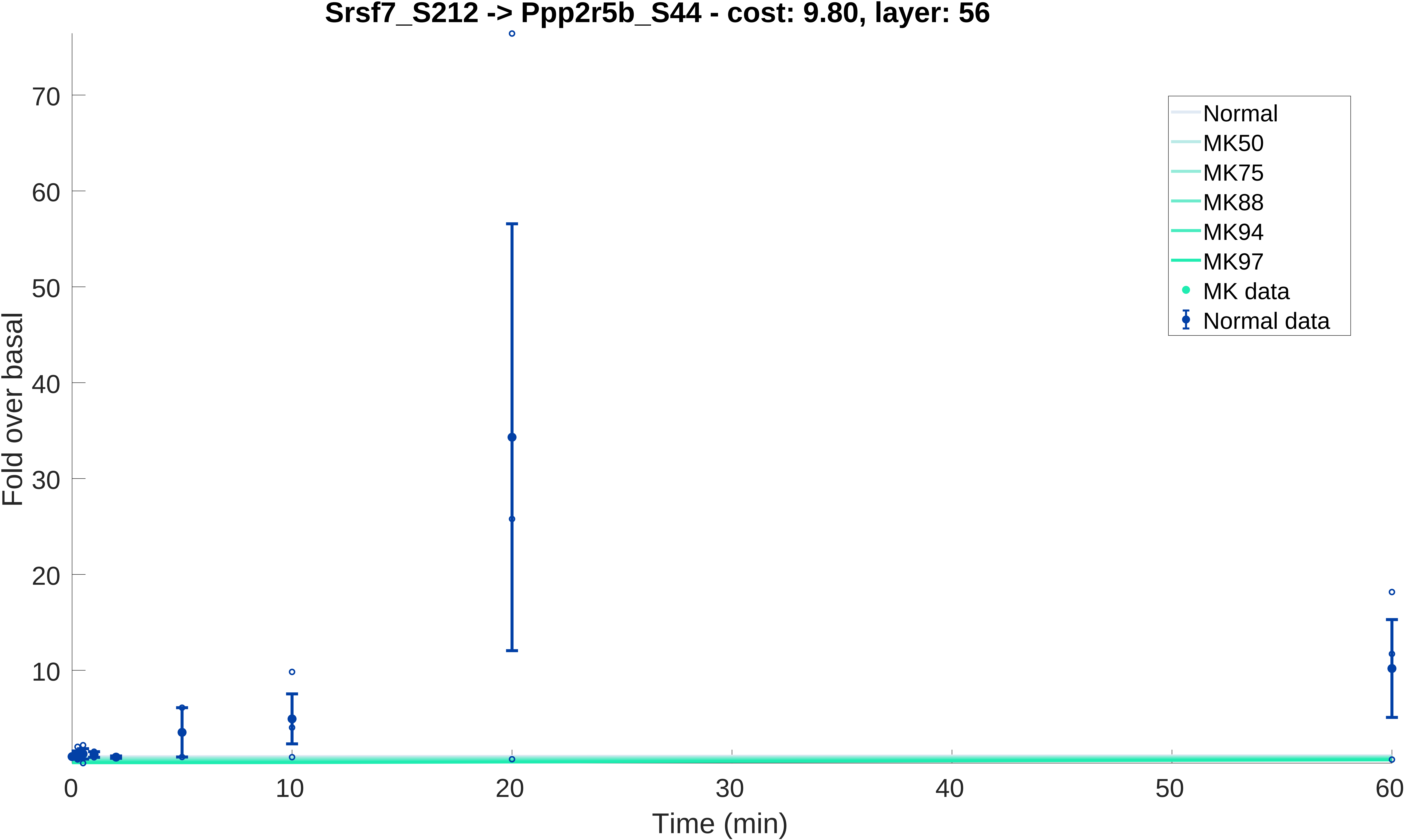
Srsf7_S212 -> Ppp2r5b_S44 - cost: 9.80, layer: 56
Fold over basal
Time (min)
0
10
20
30
40
50
60
70
Normal
MK50
MK75
MK88
MK94
MK97
MK data
Normal data

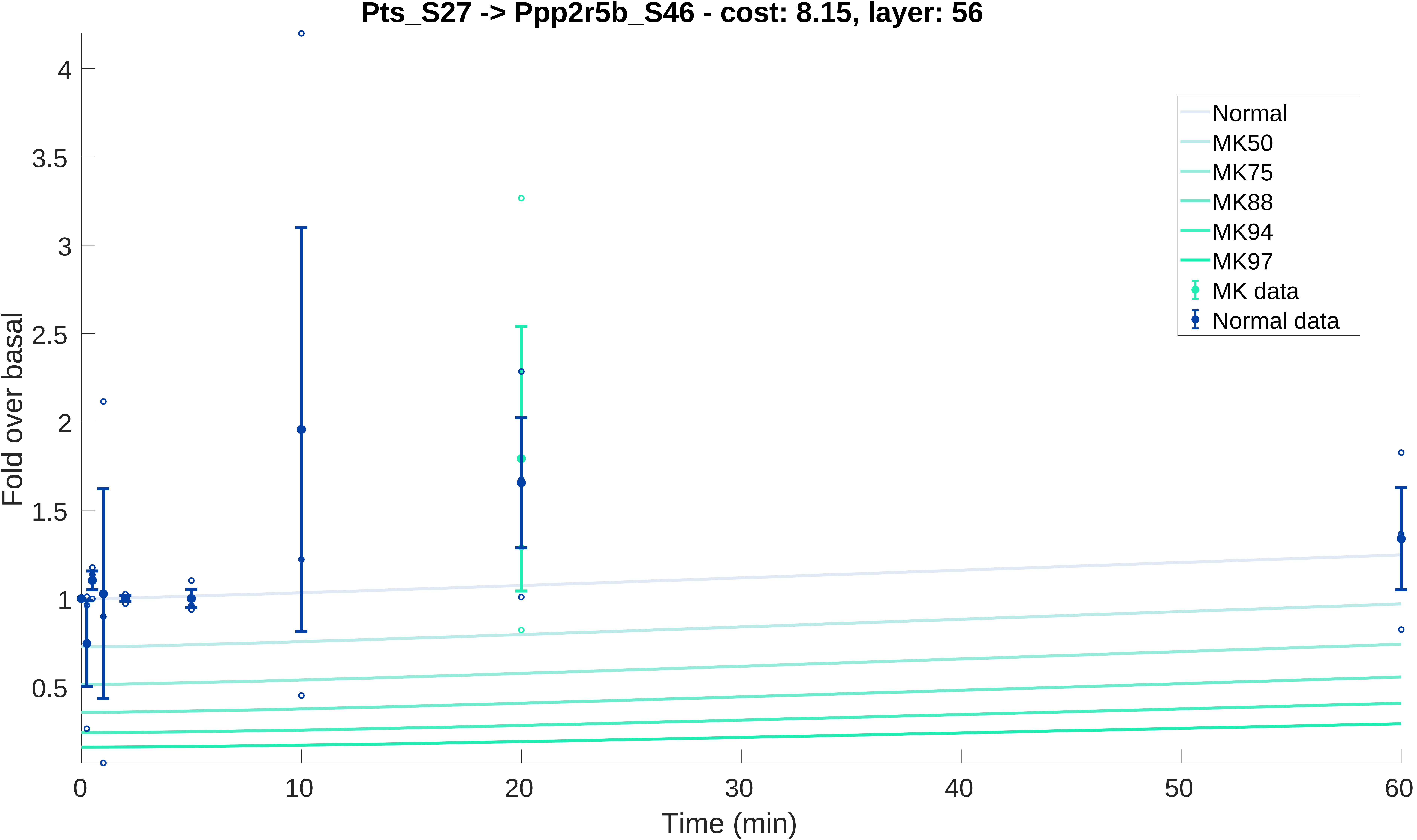

Pts_S27 -> Ppp2r5b_S46 - cost: 8.15, layer: 56
Fold over basal
Time (min)
0
10
20
30
40
50
60
0.5
1
1.5
2
2.5
3
3.5
4
Normal
MK50
MK75
MK88
MK94
MK97
MK data
Normal data

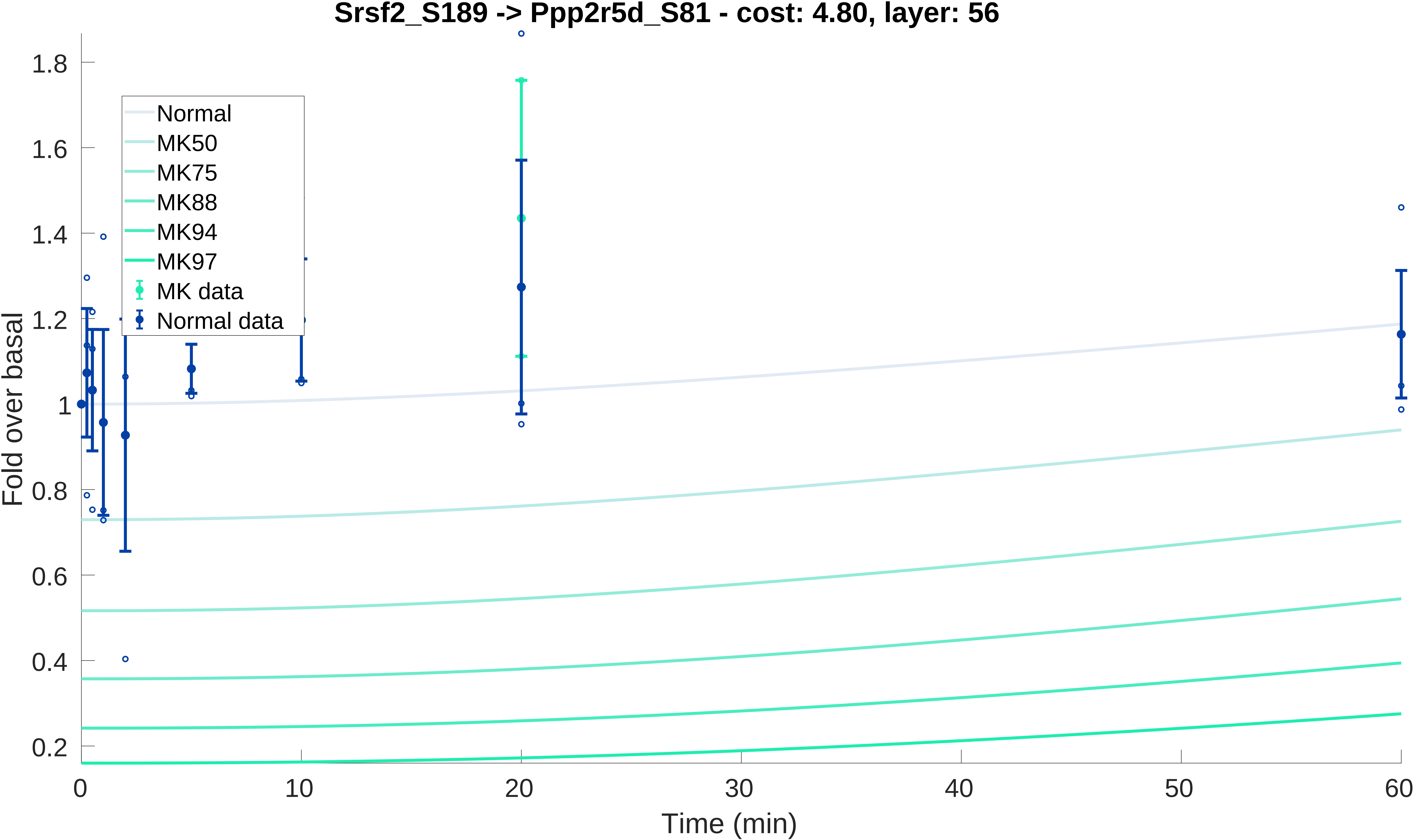

Srsf2_S189 -> Ppp2r5d_S81 - cost: 4.80, layer: 56
Normal
MK50
MK75
MK88
MK94
MK97
MK data
Normal data
Fold over basal
1.8
1.6
1.4
1.2
1
0.8
0.6
0.4
0.2
0
10
20
30
40
50
60
Time (min)

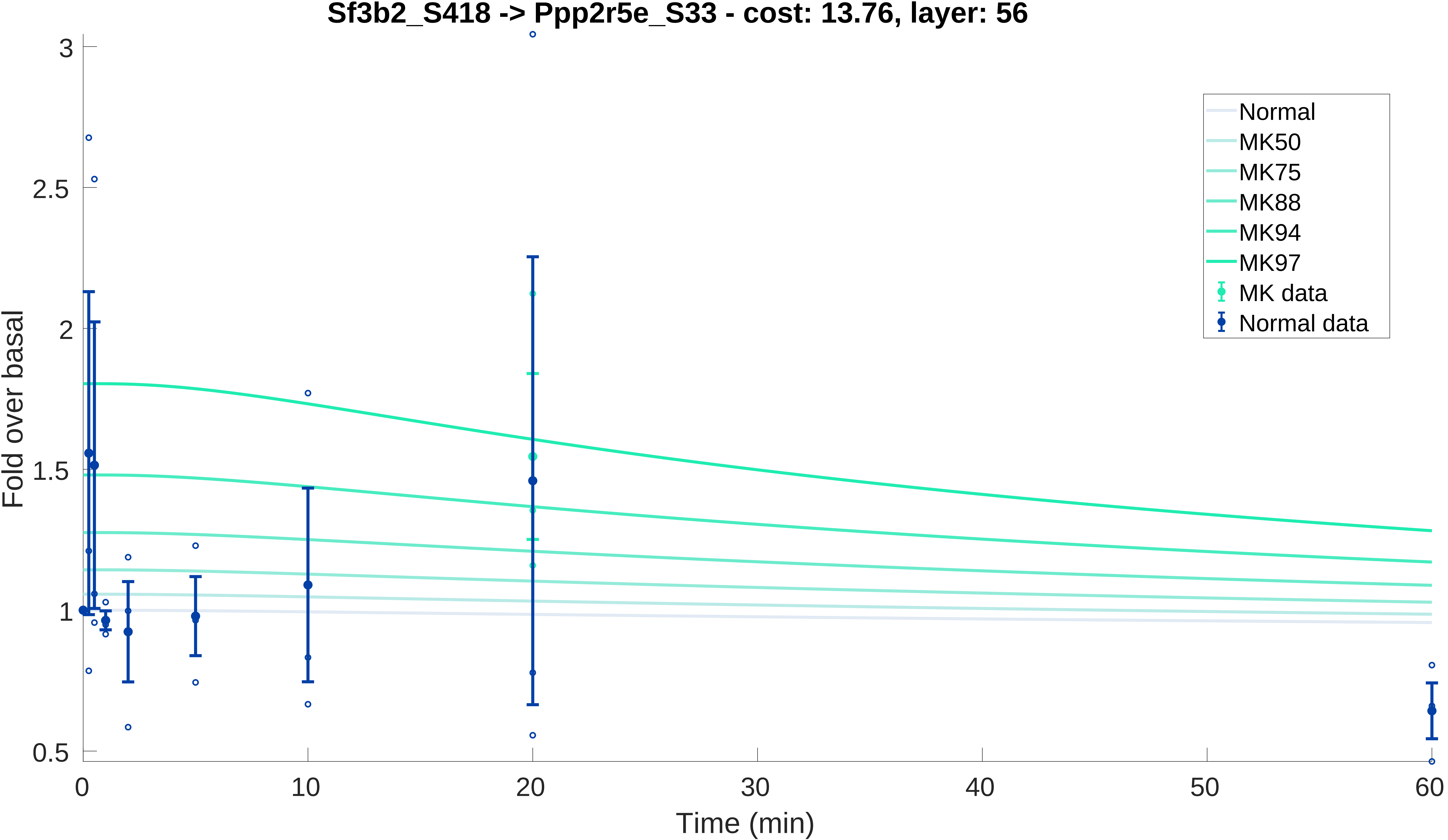
Sf3b2_S418 -> Ppp2r5e_S33 - cost: 13.76, layer: 56
Fold over basal
Time (min)
0.5
1
1.5
2
2.5
3
0
10
20
30
40
50
60
Normal
MK50
MK75
MK88
MK94
MK97
MK data
Normal data

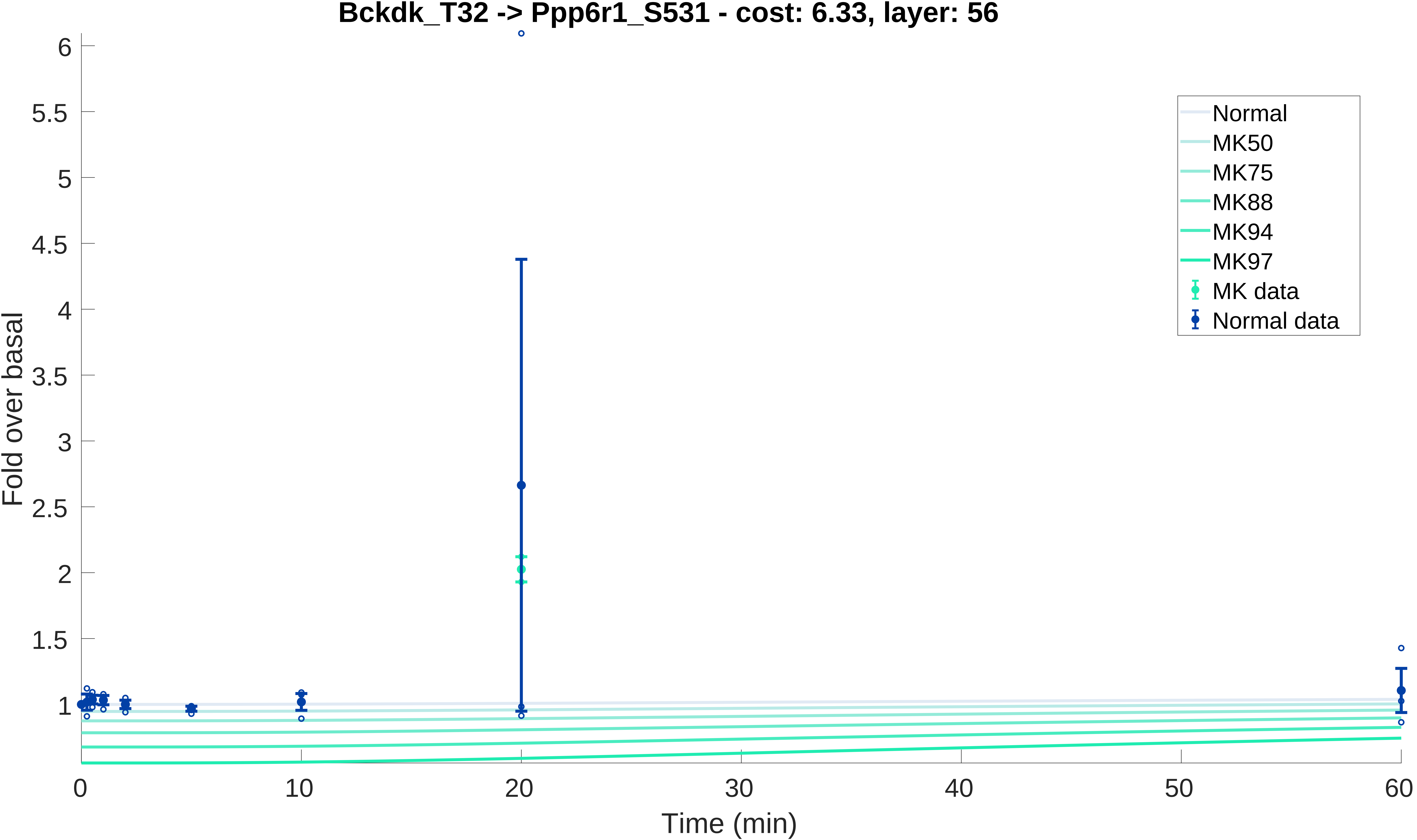
Bckdk_T32 -> Ppp6r1_S531 - cost: 6.33, layer: 56
Fold over basal
Time (min)
Normal
MK50
MK75
MK88
MK94
MK97
MK data
Normal data
6
5.5
5
4.5
4
3.5
3
2.5
2
1.5
1
0
10
20
30
40
50
60

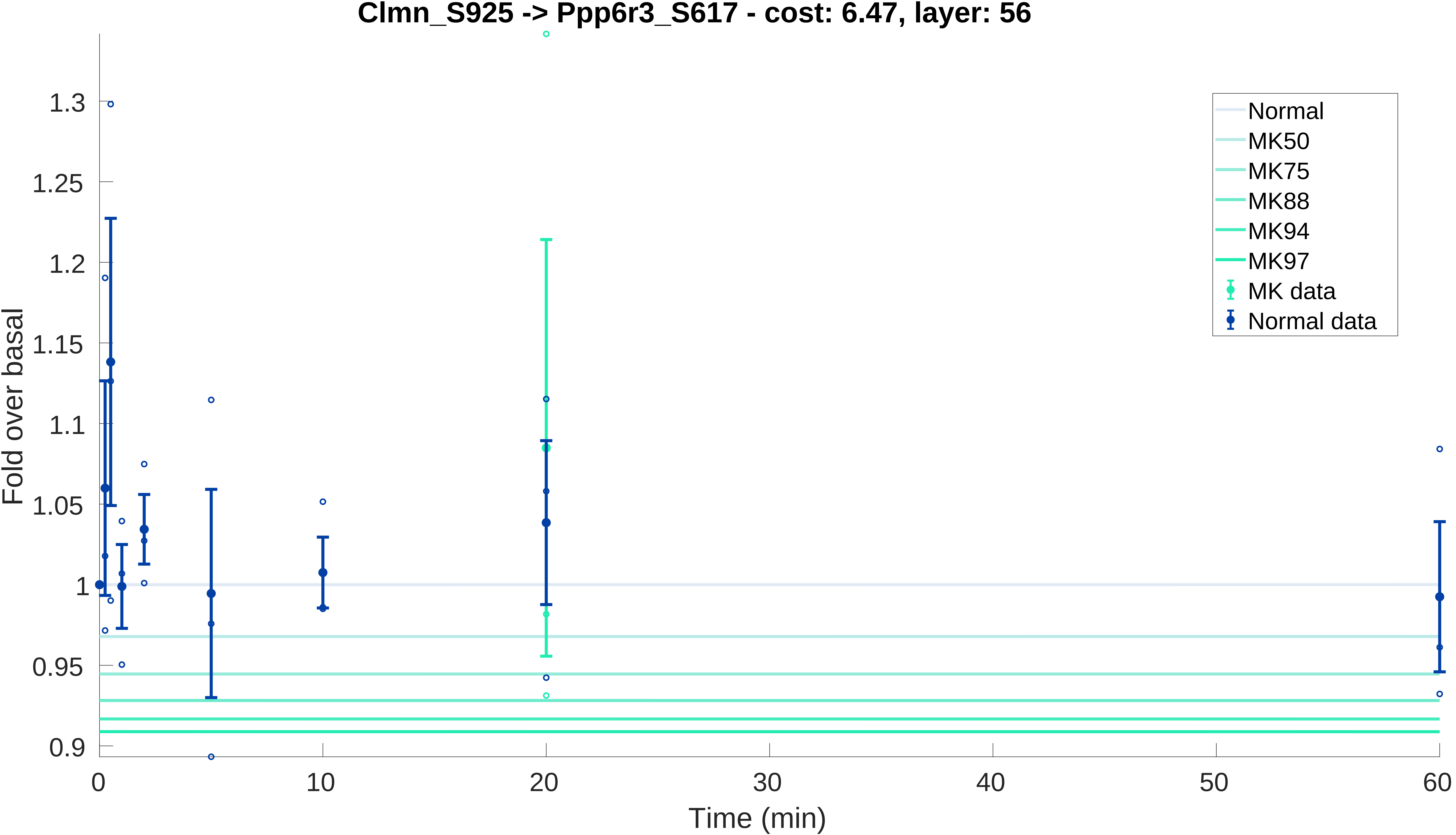

Clmn_S925 -> Ppp6r3_S617 - cost: 6.47, layer: 56
Fold over basal
Time (min)
1.3
1.25
1.2
1.15
1.1
1.05
1
0.95
0.9
0
10
20
30
40
50
60
Normal
MK50
MK75
MK88
MK94
MK97
MK data
Normal data

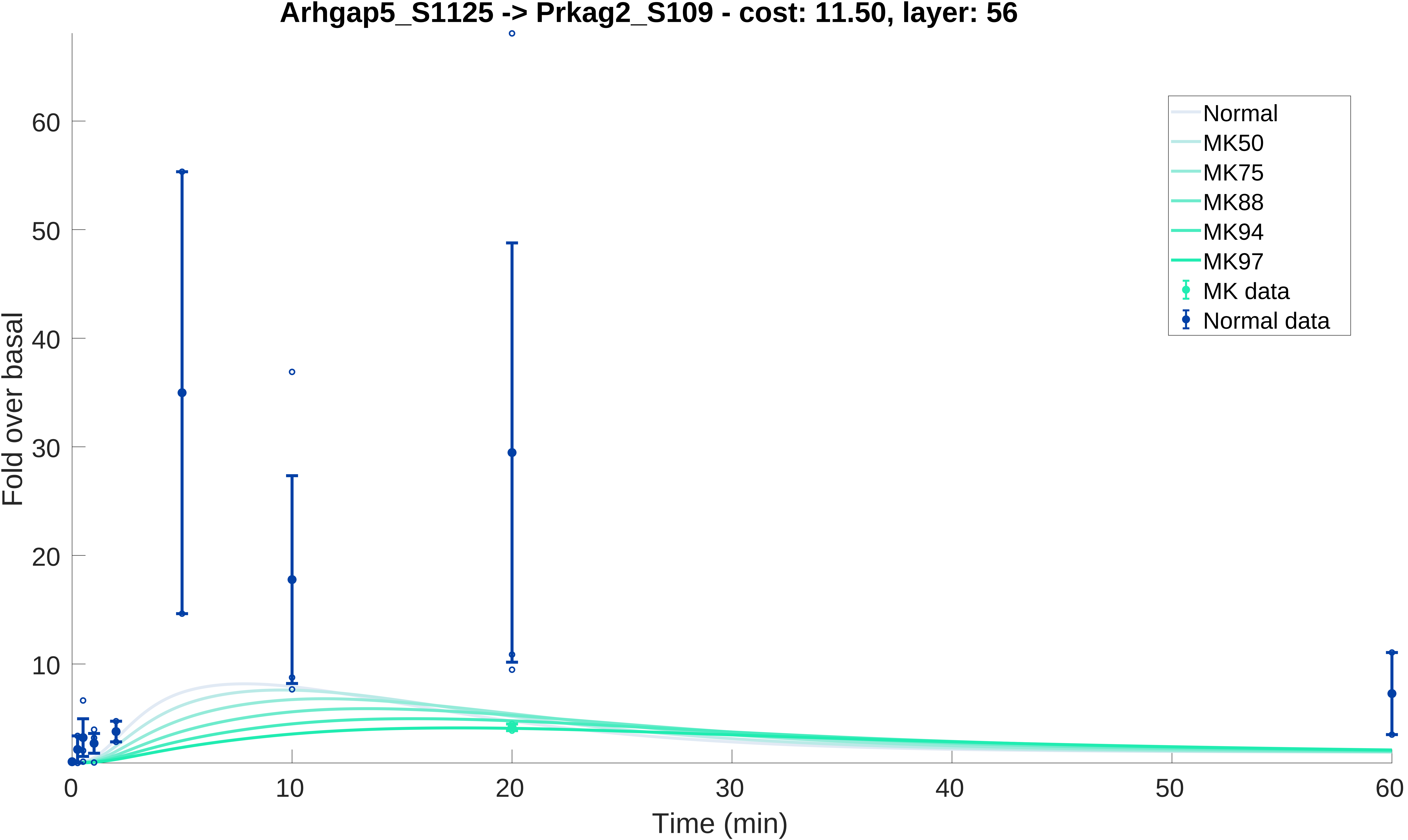

Arhgap5_S1125 -> Prkag2_S109 - cost: 11.50, layer: 56
Fold over basal
Time (min)
0
10
20
30
40
50
60
Normal
MK50
MK75
MK88
MK94
MK97
MK data
Normal data

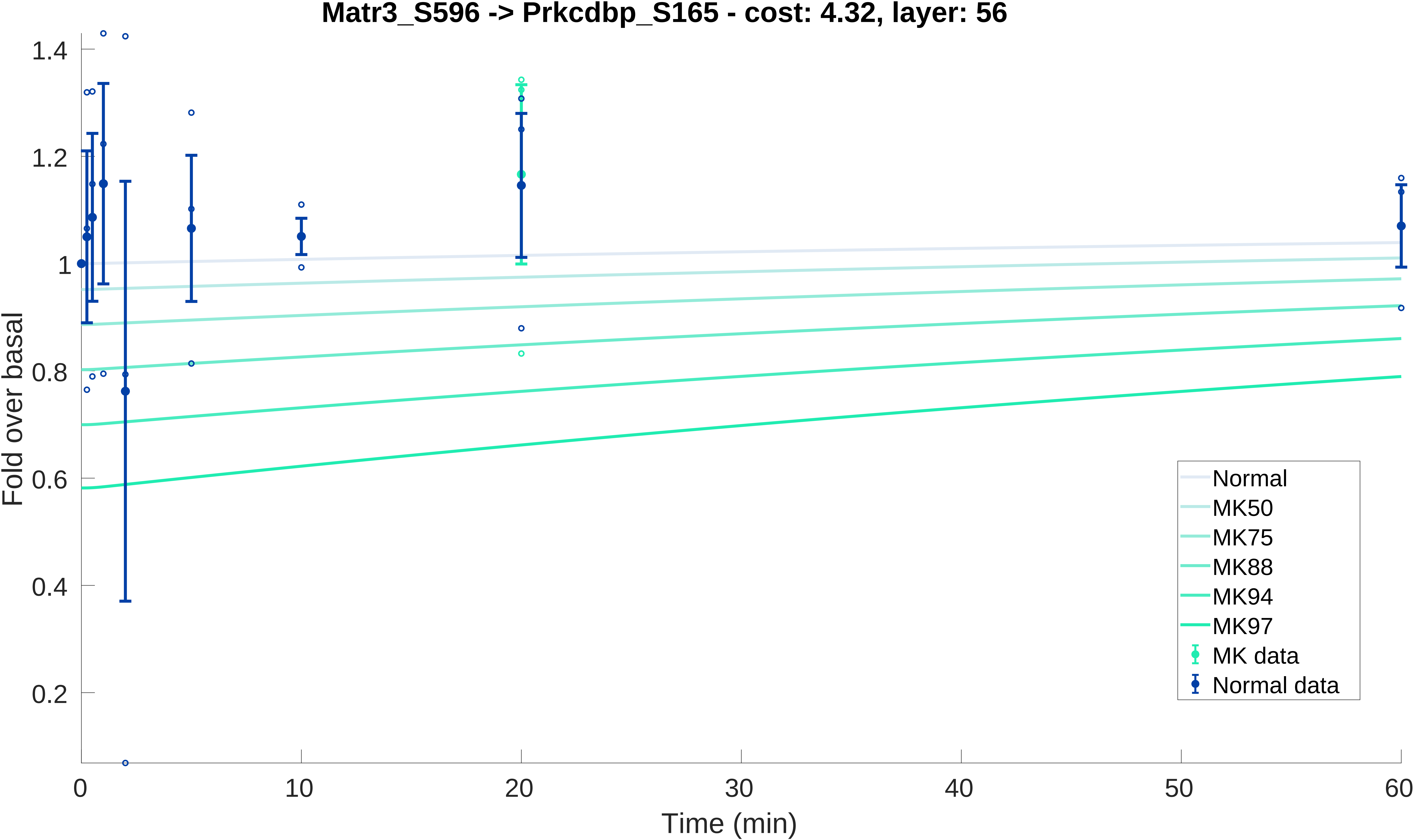

Matr3_S596 -> Prkcdbp_S165 - cost: 4.32, layer: 56
Fold over basal
Time (min)
1.4
1.2
1
0.8
0.6
0.4
0.2
0
10
20
30
40
50
60
Normal
MK50
MK75
MK88
MK94
MK97
MK data
Normal data

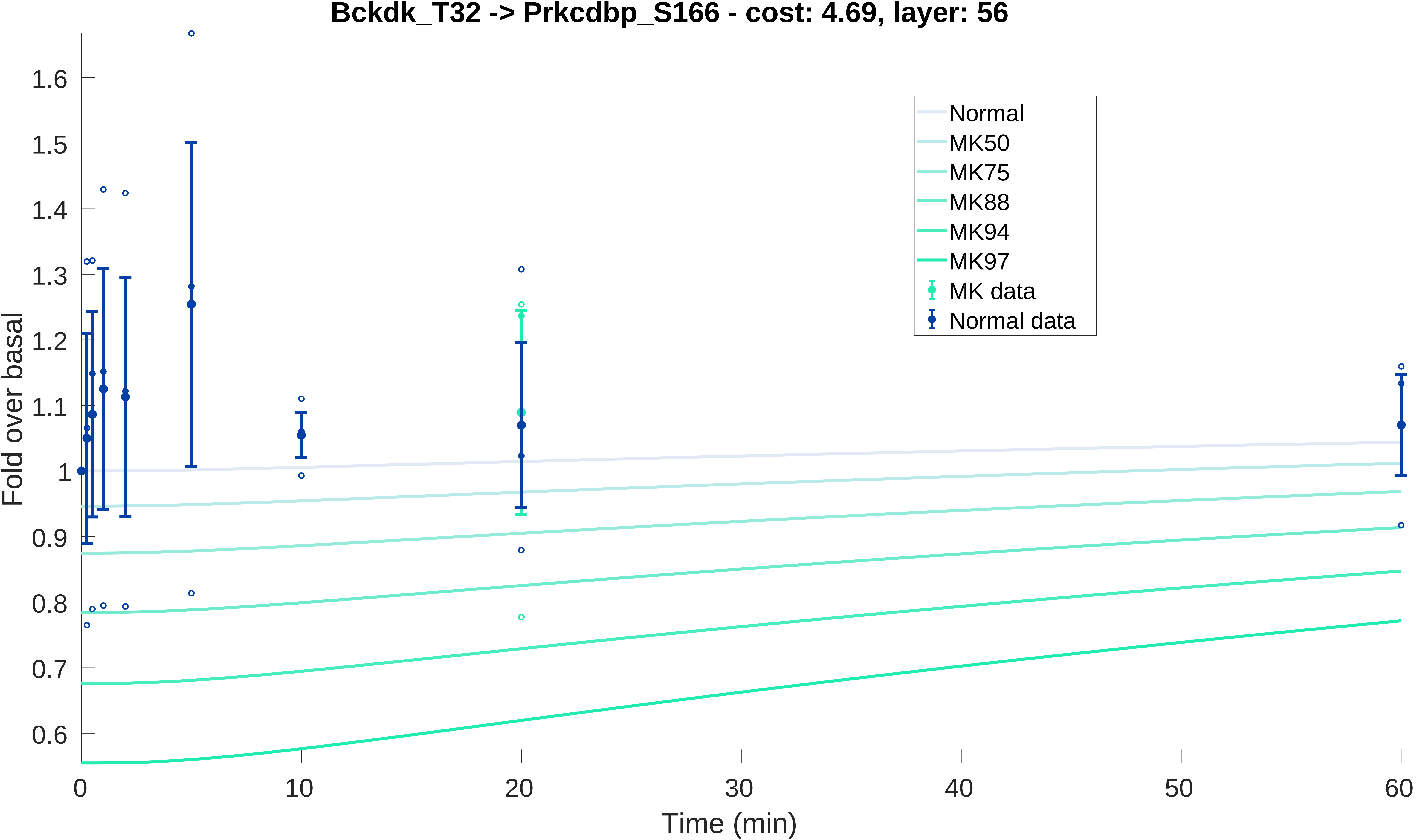
Bckdk_T32 -> Prkcdbp_S166 - cost: 4.69, layer: 56
Fold over basal
Time (min)
0
10
20
30
40
50
60
0.6
0.7
0.8
0.9
1
1.1
1.2
1.3
1.4
1.5
1.6
Normal
MK50
MK75
MK88
MK94
MK97
MK data
Normal data

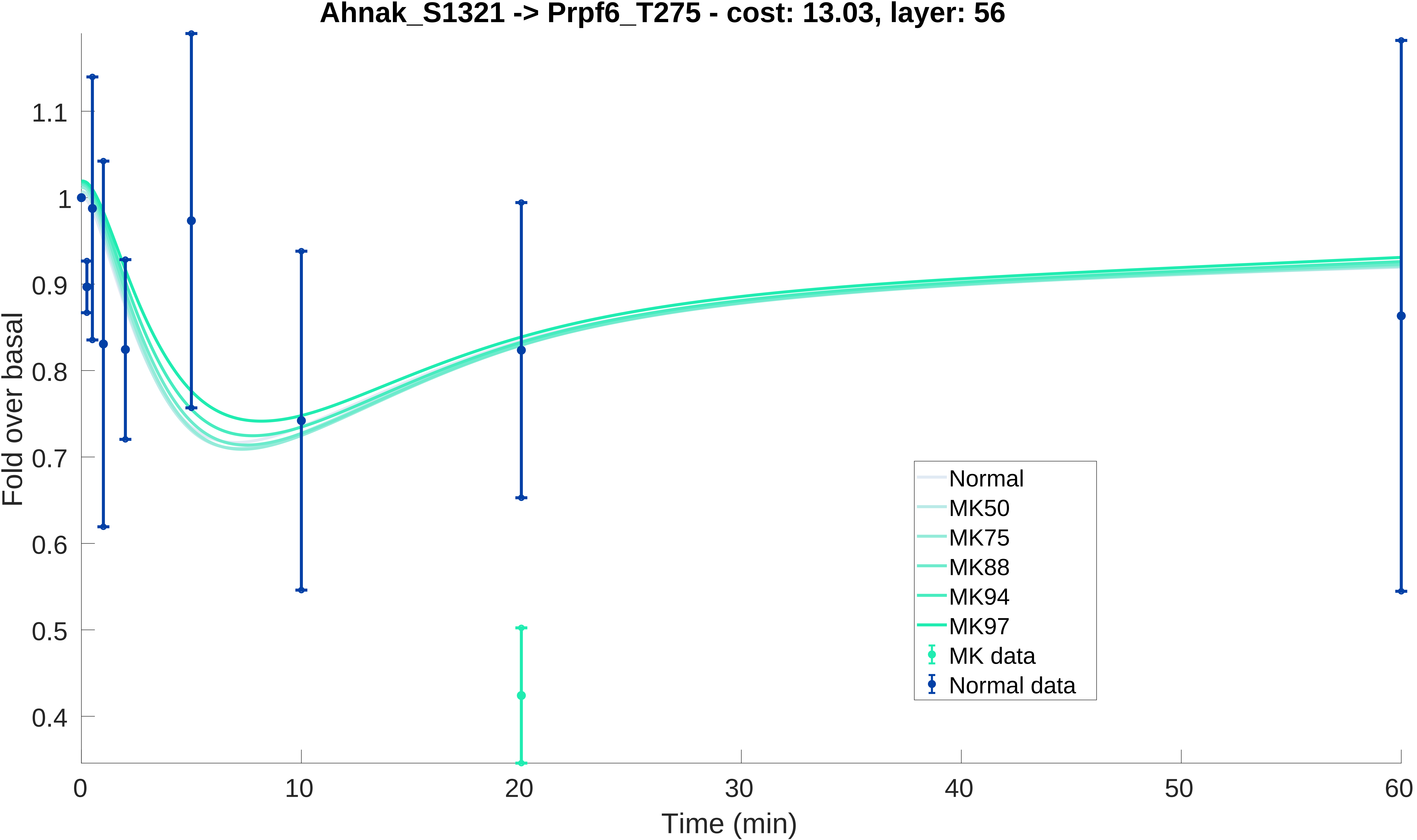

Ahnak_S1321 -> Prpf6_T275 - cost: 13.03, layer: 56
Fold over basal
1.1
1
0.9
0.8
0.7
0.6
0.5
0.4
0
10
20
30
40
50
60
Time (min)
Normal
MK50
MK75
MK88
MK94
MK97
MK data
Normal data

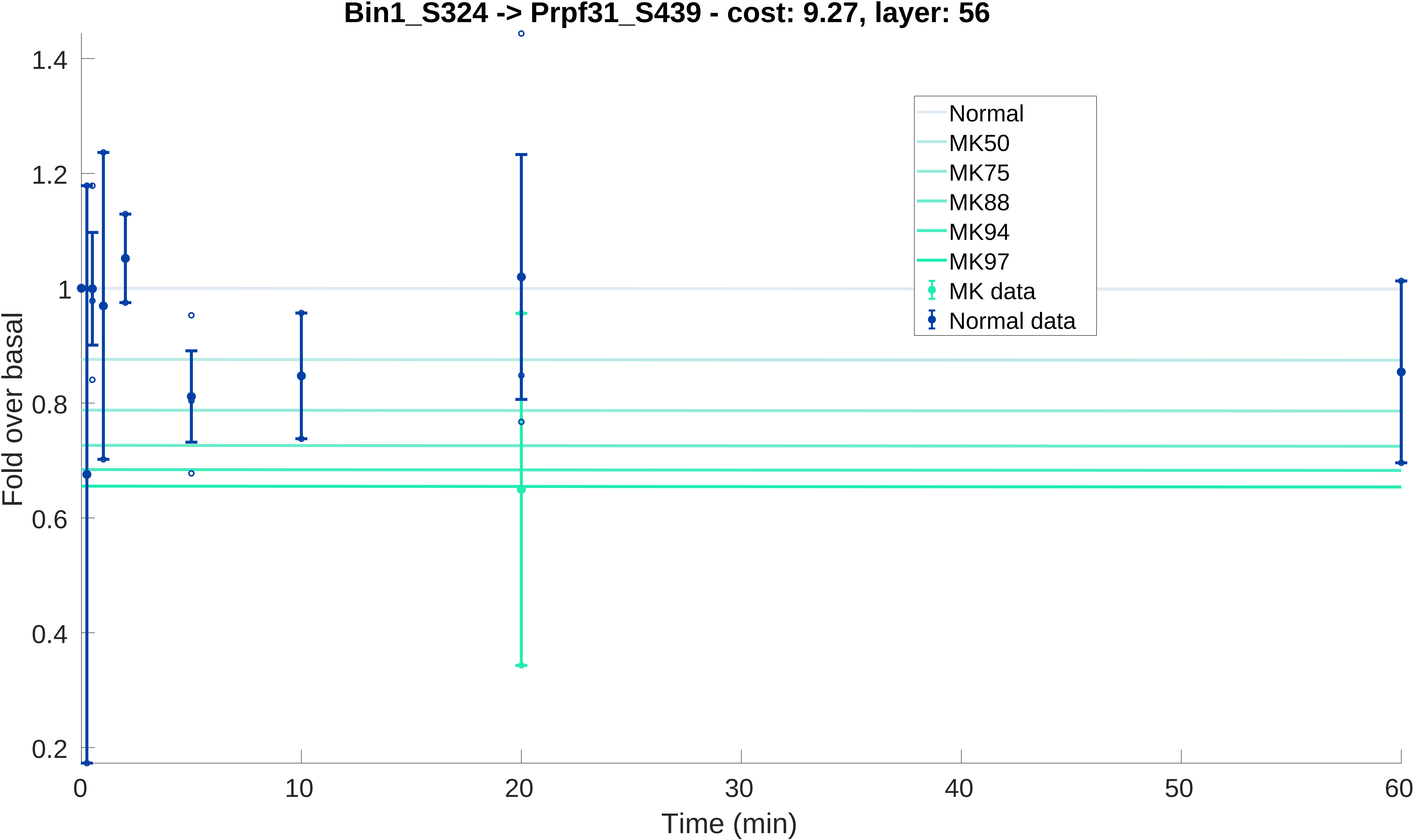

Bin1_S324 -> Prpf31_S439 - cost: 9.27, layer: 56
Normal
MK50
MK75
MK88
MK94
MK97
MK data
Normal data
Fold over basal
Time (min)
0
10
20
30
40
50
60
0.2
0.4
0.6
0.8
1
1.2
1.4

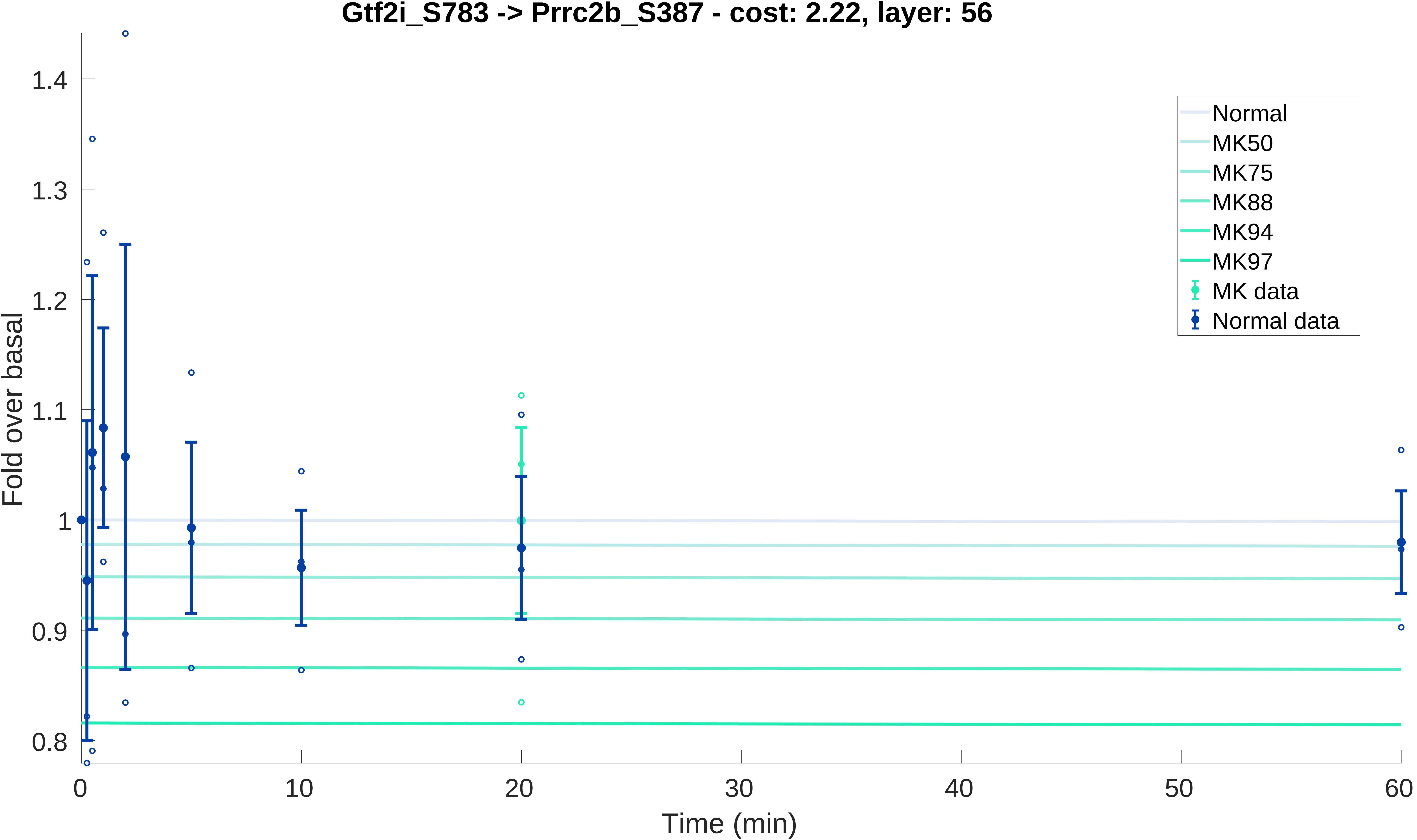
Gtf2i_S783 -> Prrc2b_S387 - cost: 2.22, layer: 56
Fold over basal
1.4
1.3
1.2
1.1
1
0.9
0.8
0
10
20
30
40
50
60
Time (min)
Normal
MK50
MK75
MK88
MK94
MK97
MK data
Normal data

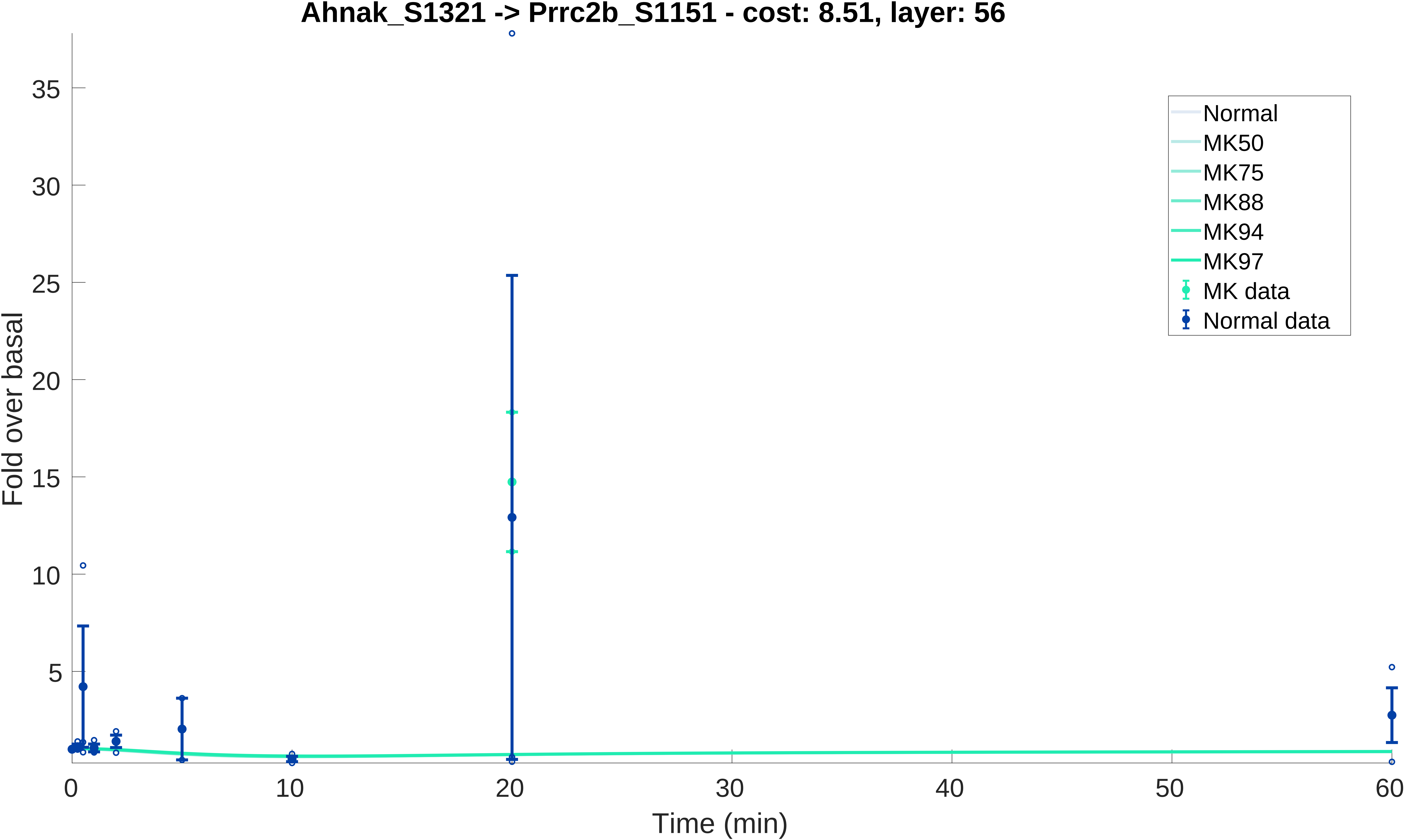

Ahnak_S1321 -> Prrc2b_S1151 - cost: 8.51, layer: 56
Fold over basal
Time (min)
0
10
20
30
40
50
60
5
10
15
20
25
30
35
Normal
MK50
MK75
MK88
MK94
MK97
MK data
Normal data

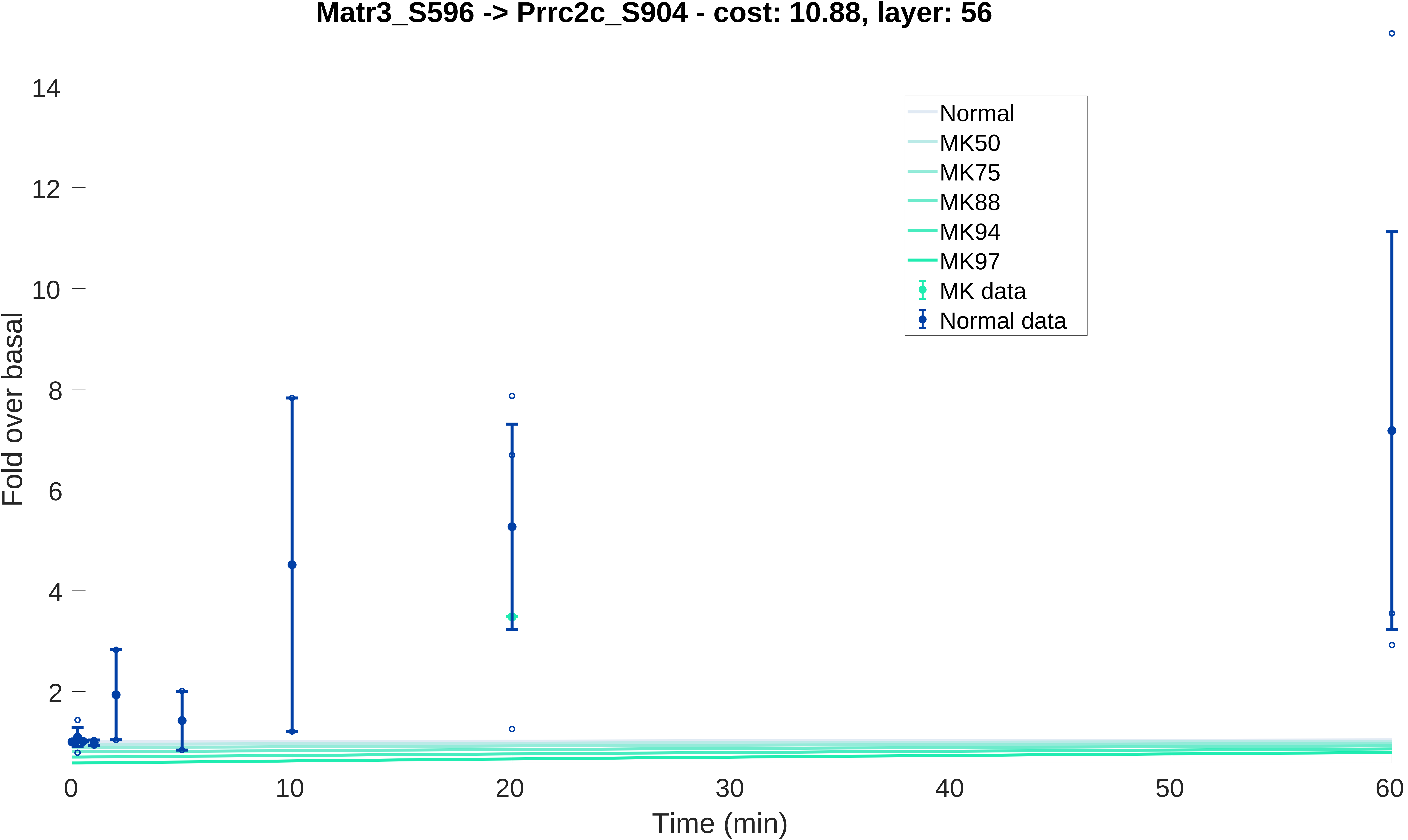

Matr3_S596 -> Prrc2c_S904 - cost: 10.88, layer: 56
Fold over basal
Time (min)
0
10
20
30
40
50
60
2
4
6
8
10
12
14
Normal
MK50
MK75
MK88
MK94
MK97
MK data
Normal data

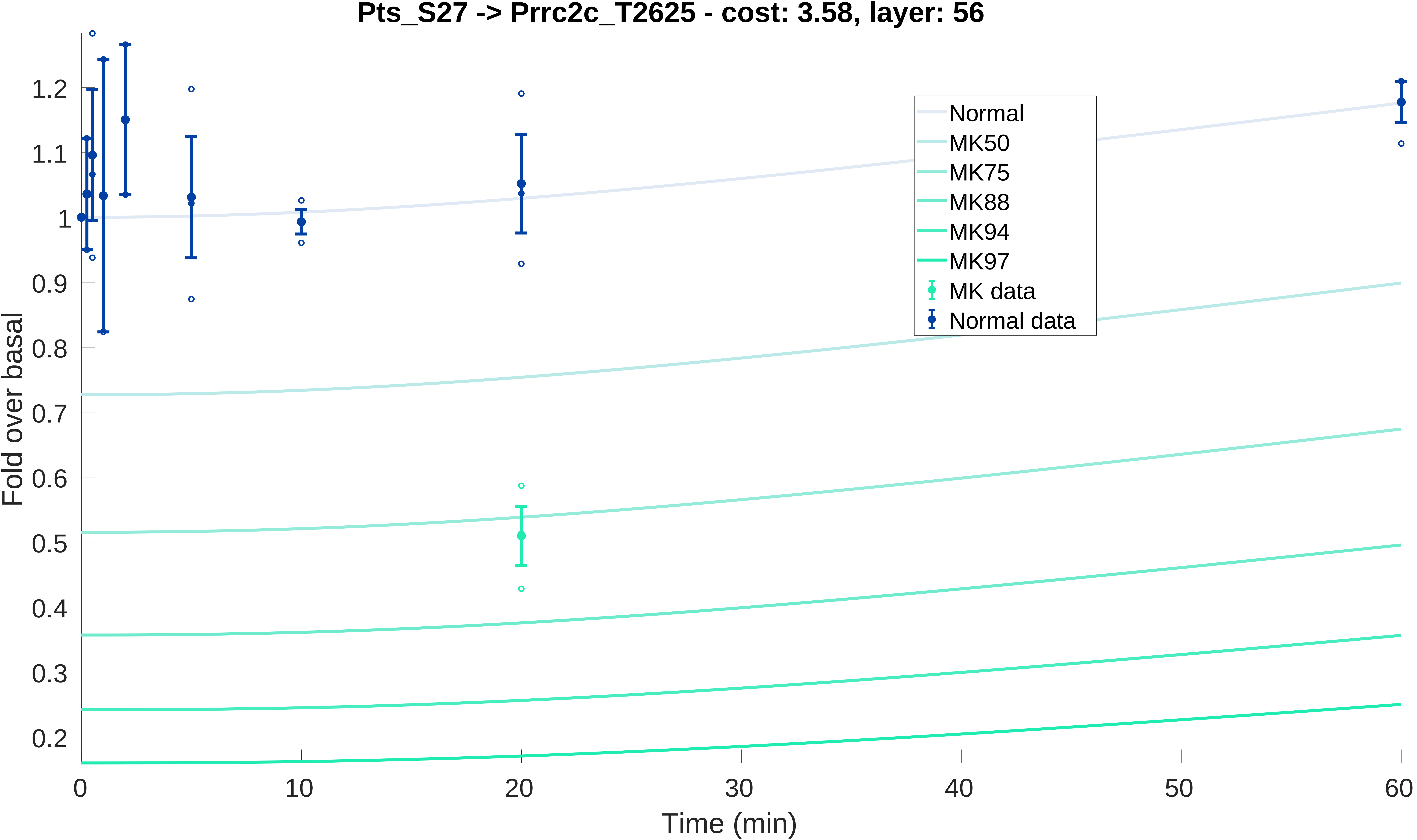
Pts_S27 -> Prrc2c_T2625 - cost: 3.58, layer: 56
Fold over basal
Time (min)
0
10
20
30
40
50
60
0.2
0.3
0.4
0.5
0.6
0.7
0.8
0.9
1
1.1
1.2
Normal
MK50
MK75
MK88
MK94
MK97
MK data
Normal data

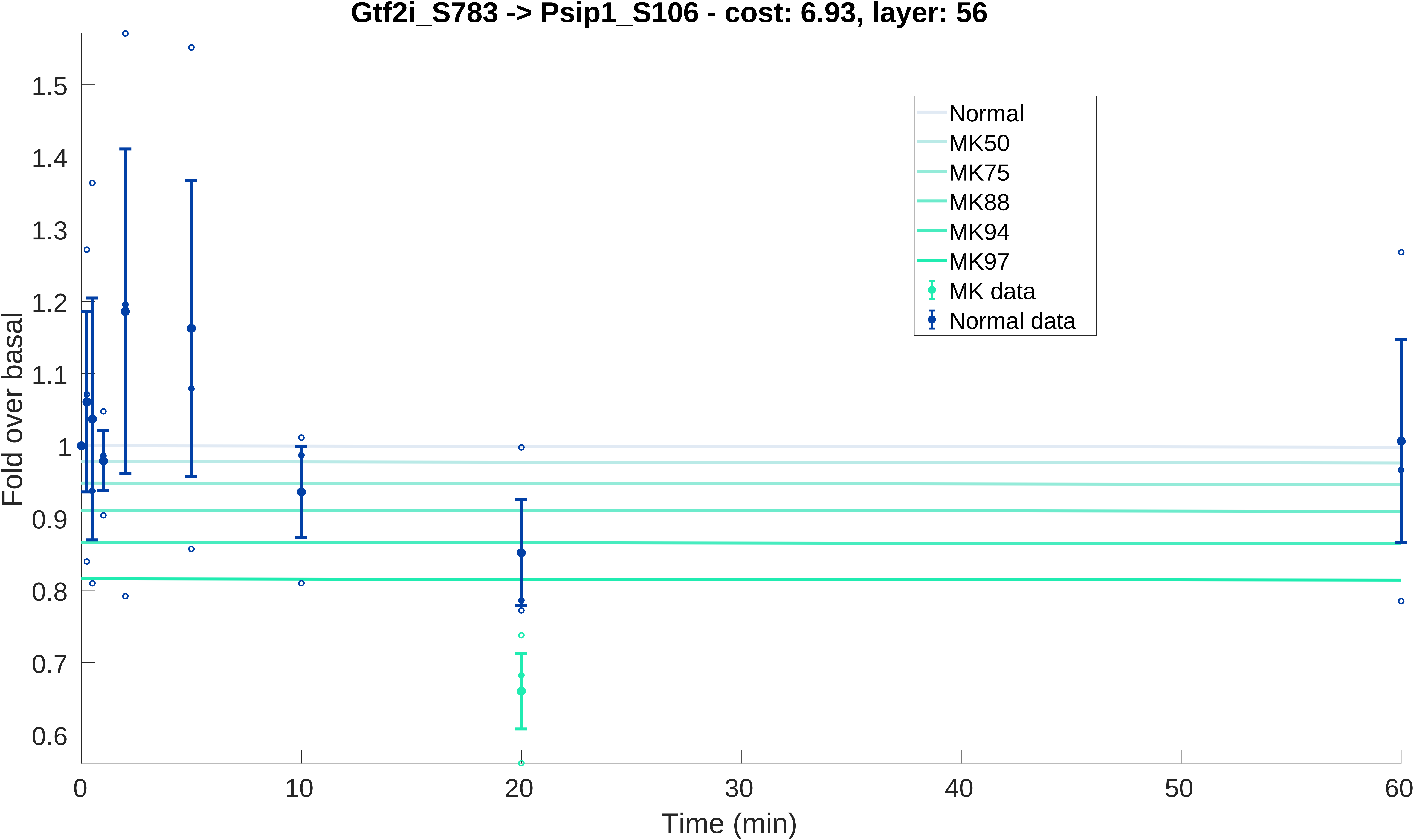

Gtf2i_S783 -> Psip1_S106 - cost: 6.93, layer: 56
Normal
MK50
MK75
MK88
MK94
MK97
MK data
Normal data
Fold over basal
Time (min)
1.5
1.4
1.3
1.2
1.1
1
0.9
0.8
0.7
0.6
0
10
20
30
40
50
60

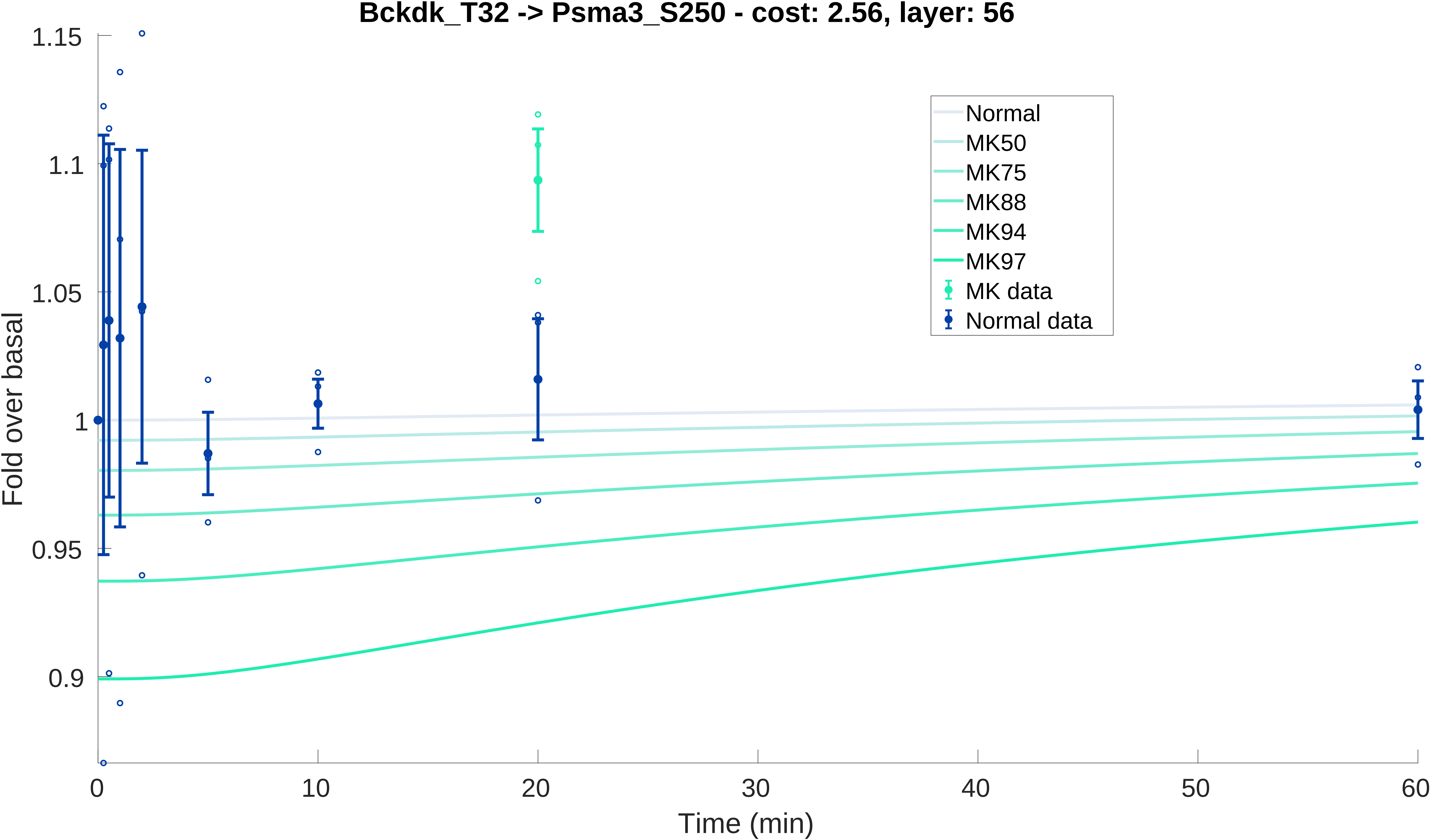

Bckdk_T32 -> Psma3_S250 - cost: 2.56, layer: 56
Fold over basal
Time (min)
1.15
1.1
1.05
1
0.95
0.9
0
10
20
30
40
50
60
Normal
MK50
MK75
MK88
MK94
MK97
MK data
Normal data

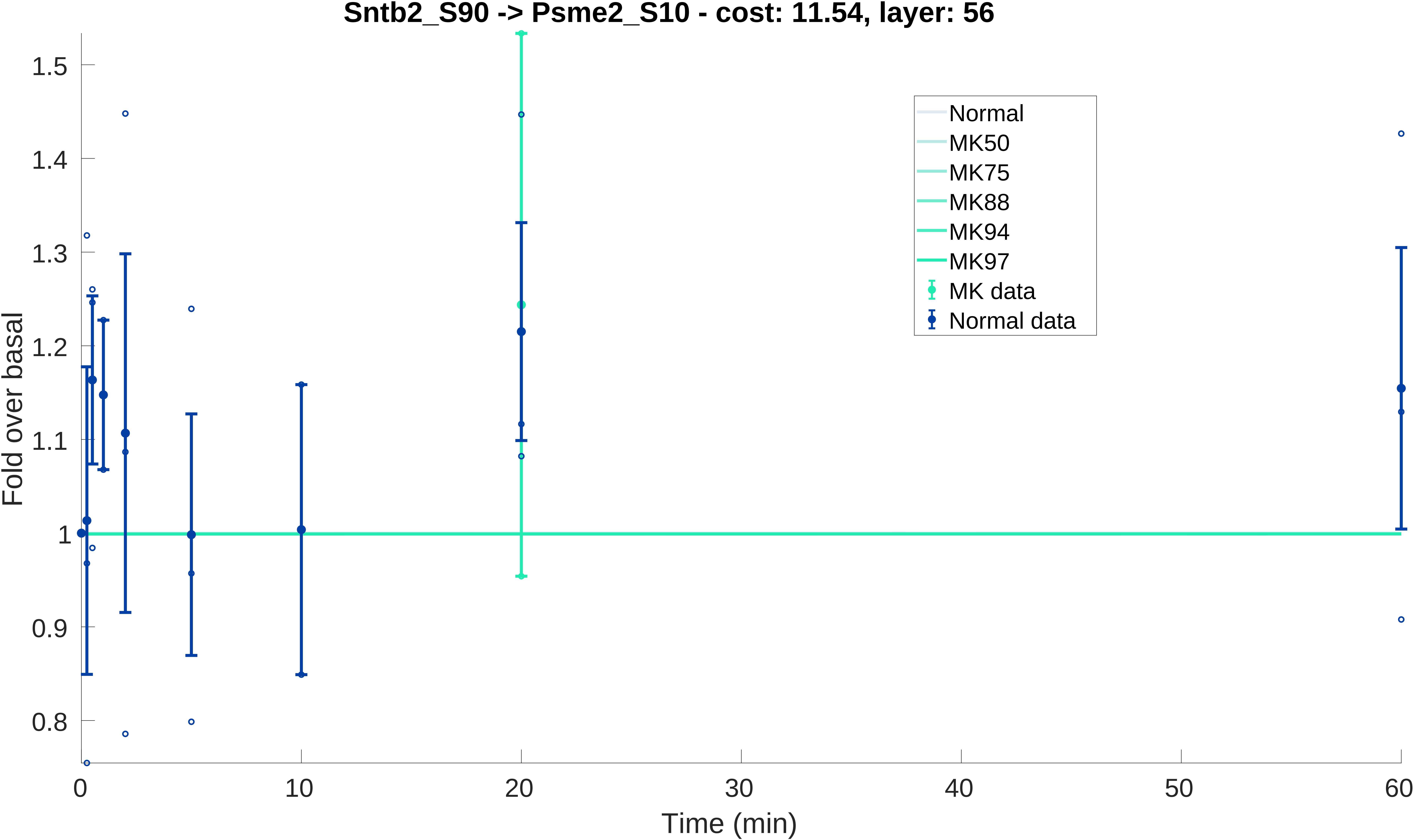

Sntb2_S90 -> Psme2_S10 - cost: 11.54, layer: 56
Normal
MK50
MK75
MK88
MK94
MK97
MK data
Normal data
Fold over basal
Time (min)
0.8
0.9
1
1.1
1.2
1.3
1.4
1.5
0
10
20
30
40
50
60

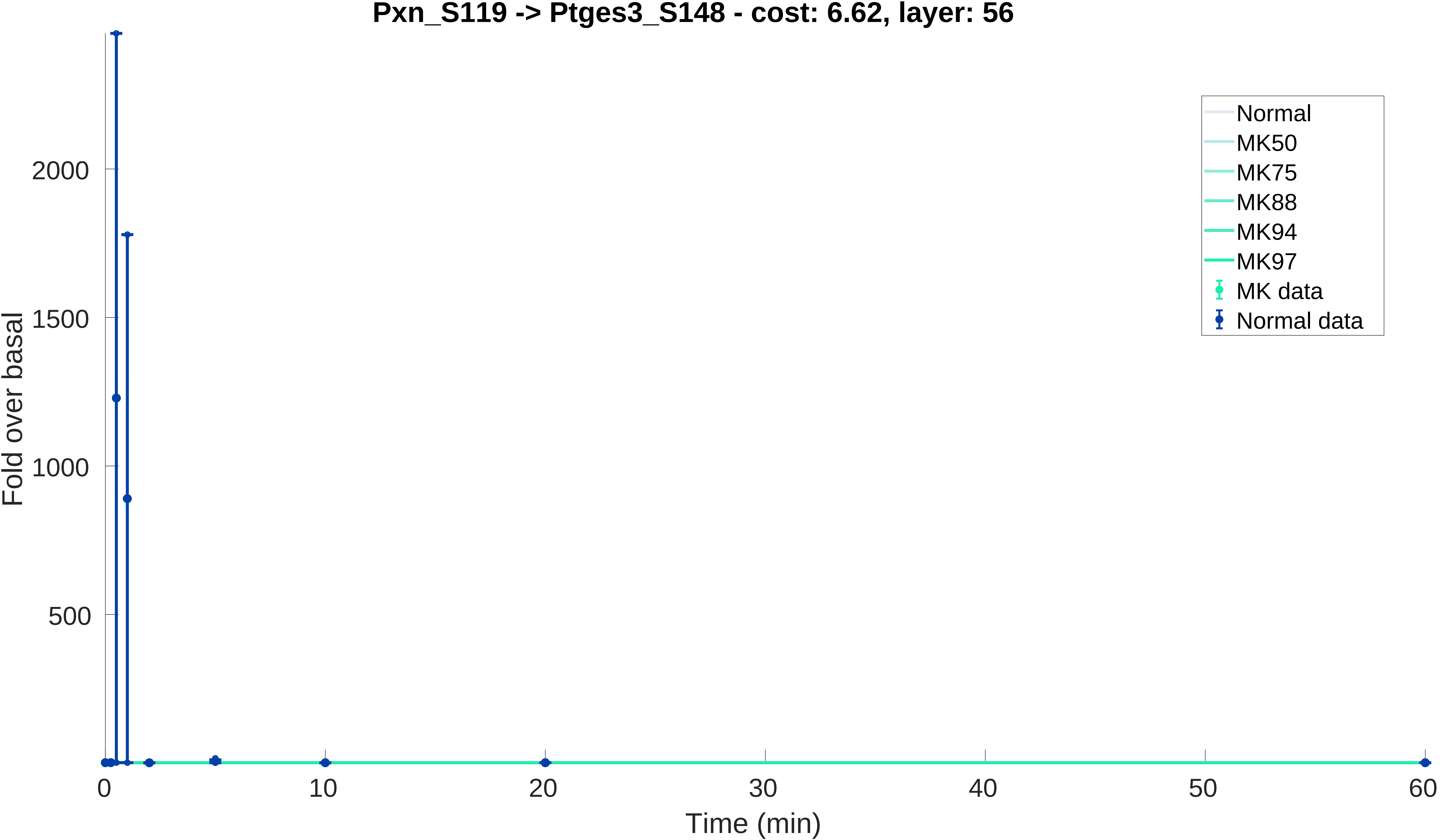

Pxn_S119 -> Ptges3_S148 - cost: 6.62, layer: 56
Fold over basal
2000
1500
1000
500
0
10
20
30
40
50
60
Time (min)
Normal
MK50
MK75
MK88
MK94
MK97
MK data
Normal data

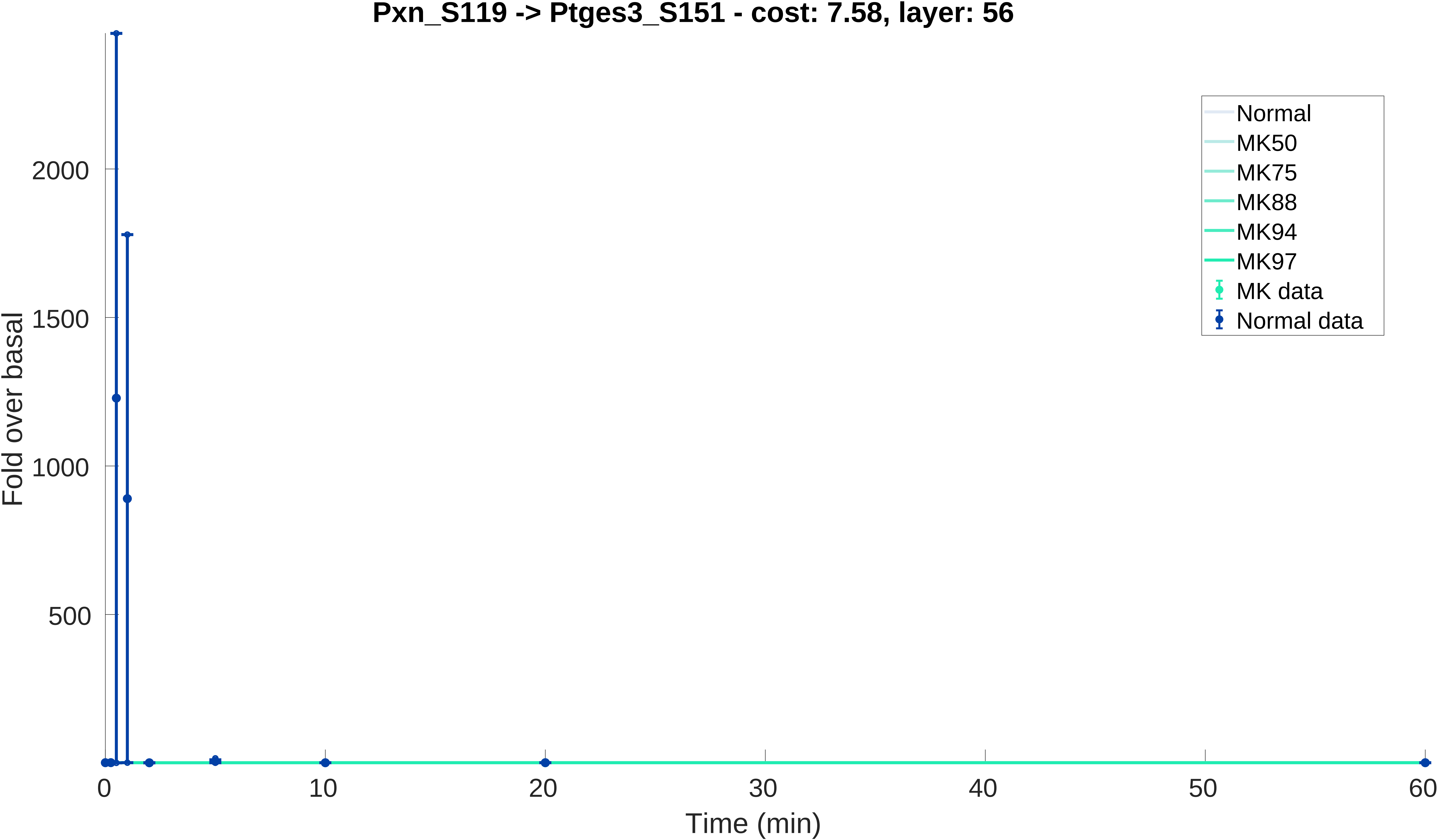
Pxn_S119 -> Ptges3_S151 - cost: 7.58, layer: 56
Fold over basal
2000
1500
1000
500
0
10
20
30
40
50
60
Time (min)
Normal
MK50
MK75
MK88
MK94
MK97
MK data
Normal data

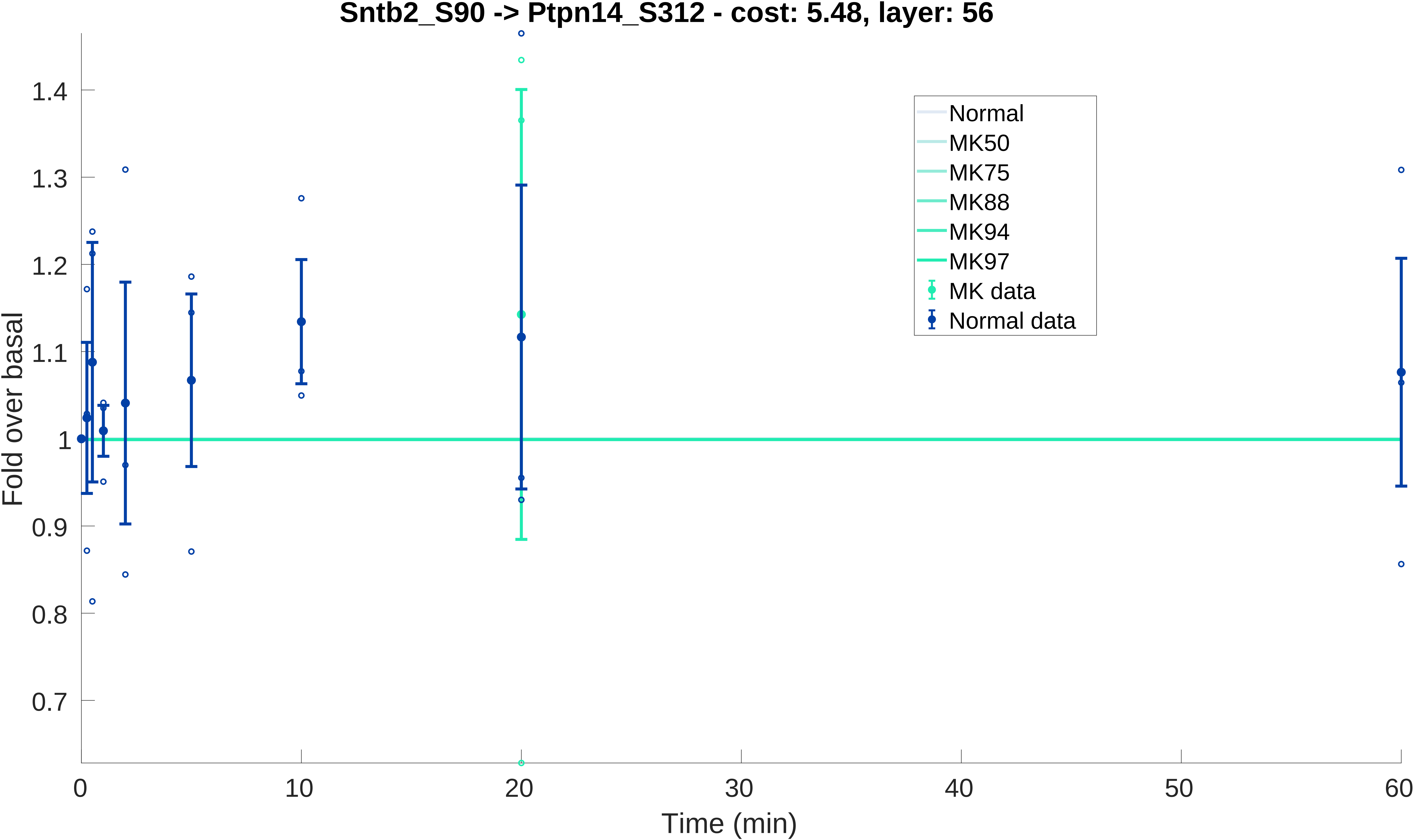

Sntb2_S90 -> Ptpn14_S312 - cost: 5.48, layer: 56
Fold over basal
Time (min)
1.4
1.3
1.2
1.1
1
0.9
0.8
0.7
0
10
20
30
40
50
60
Normal
MK50
MK75
MK88
MK94
MK97
MK data
Normal data

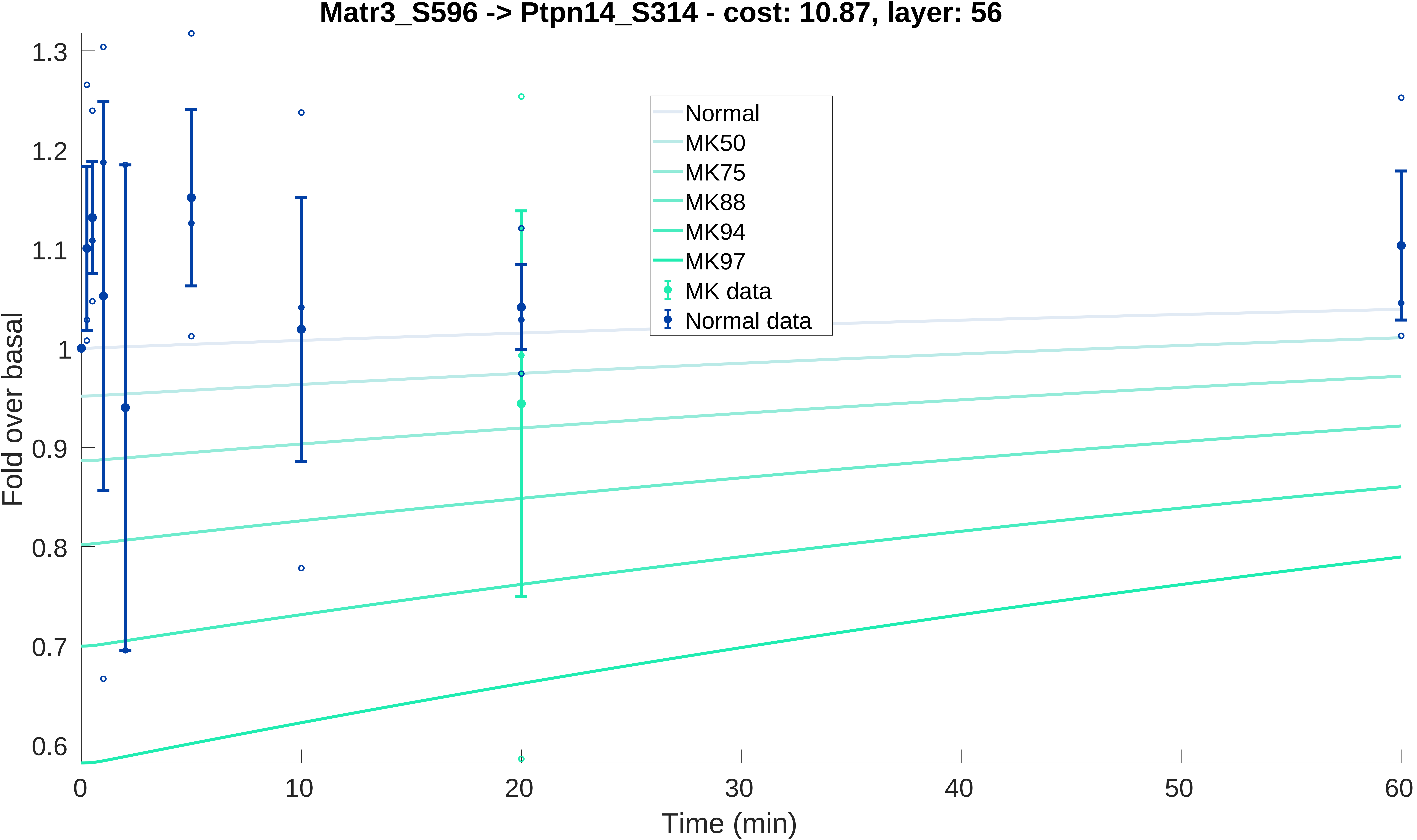
Matr3_S596 -> Ptpn14_S314 - cost: 10.87, layer: 56
Fold over basal
Time (min)
1.3
1.2
1.1
1
0.9
0.8
0.7
0.6
0
10
20
30
40
50
60
Normal
MK50
MK75
MK88
MK94
MK97
MK data
Normal data

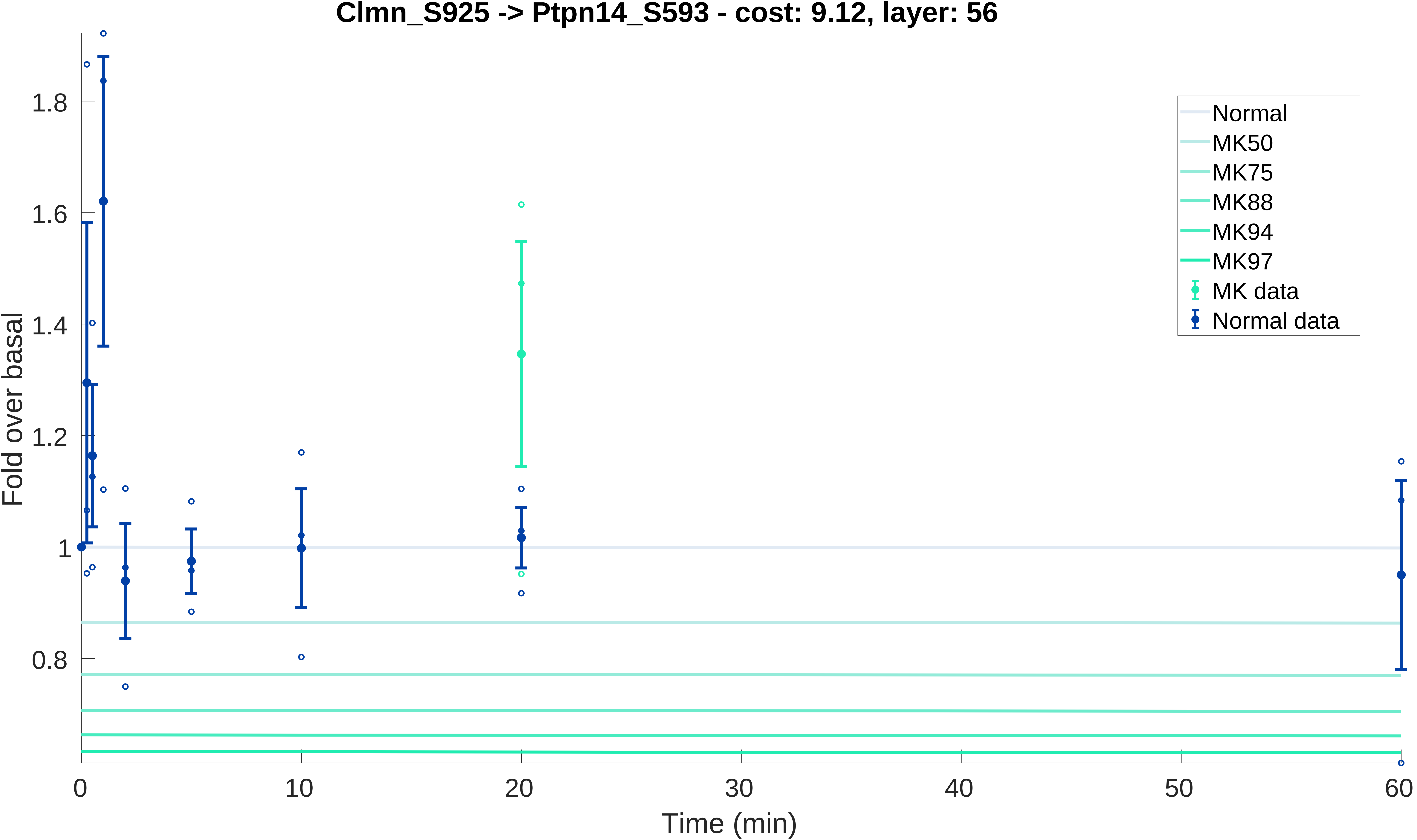
Clmn_S925 -> Ptpn14_S593 - cost: 9.12, layer: 56
Fold over basal
Time (min)
0.8
1
1.2
1.4
1.6
1.8
0
10
20
30
40
50
60
Normal
MK50
MK75
MK88
MK94
MK97
MK data
Normal data

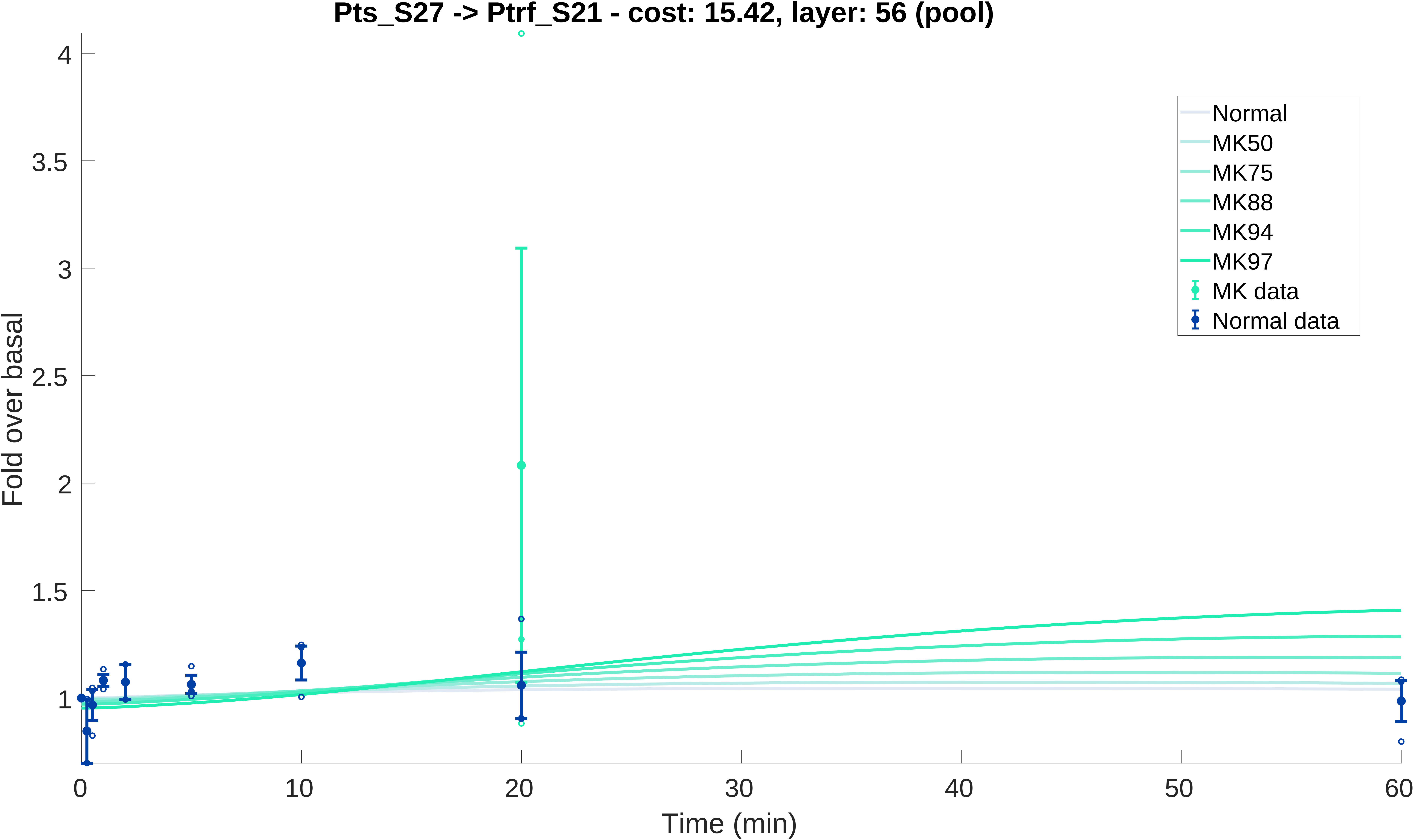

Pts_S27 -> Ptrf_S21 - cost: 15.42, layer: 56 (pool)
Fold over basal
Time (min)
Normal
MK50
MK75
MK88
MK94
MK97
MK data
Normal data
4
3.5
3
2.5
2
1.5
1
0
10
20
30
40
50
60

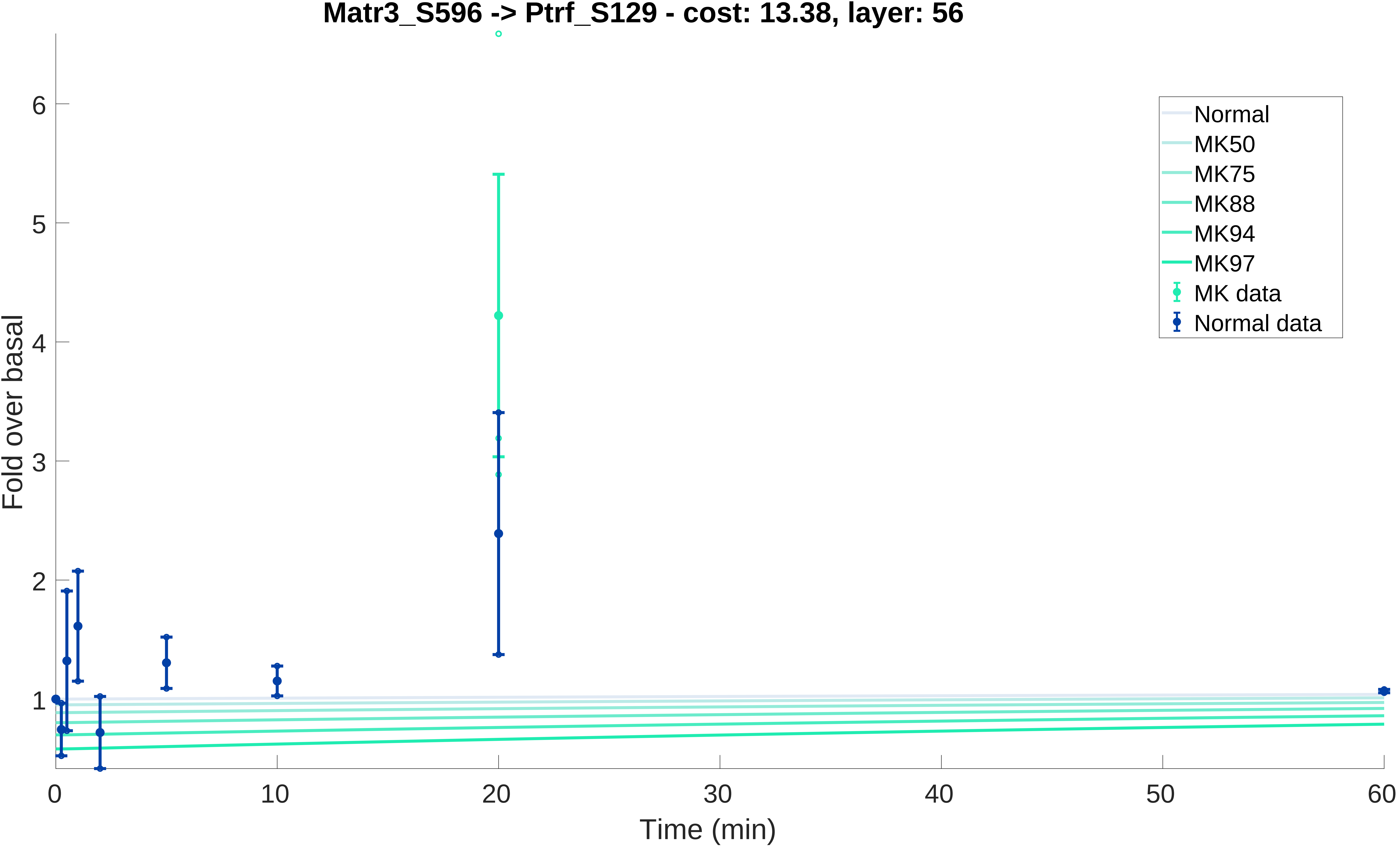

Matr3_S596 -> Ptrf_S129 - cost: 13.38, layer: 56
Fold over basal
Time (min)
0
10
20
30
40
50
60
1
2
3
4
5
6
Normal
MK50
MK75
MK88
MK94
MK97
MK data
Normal data

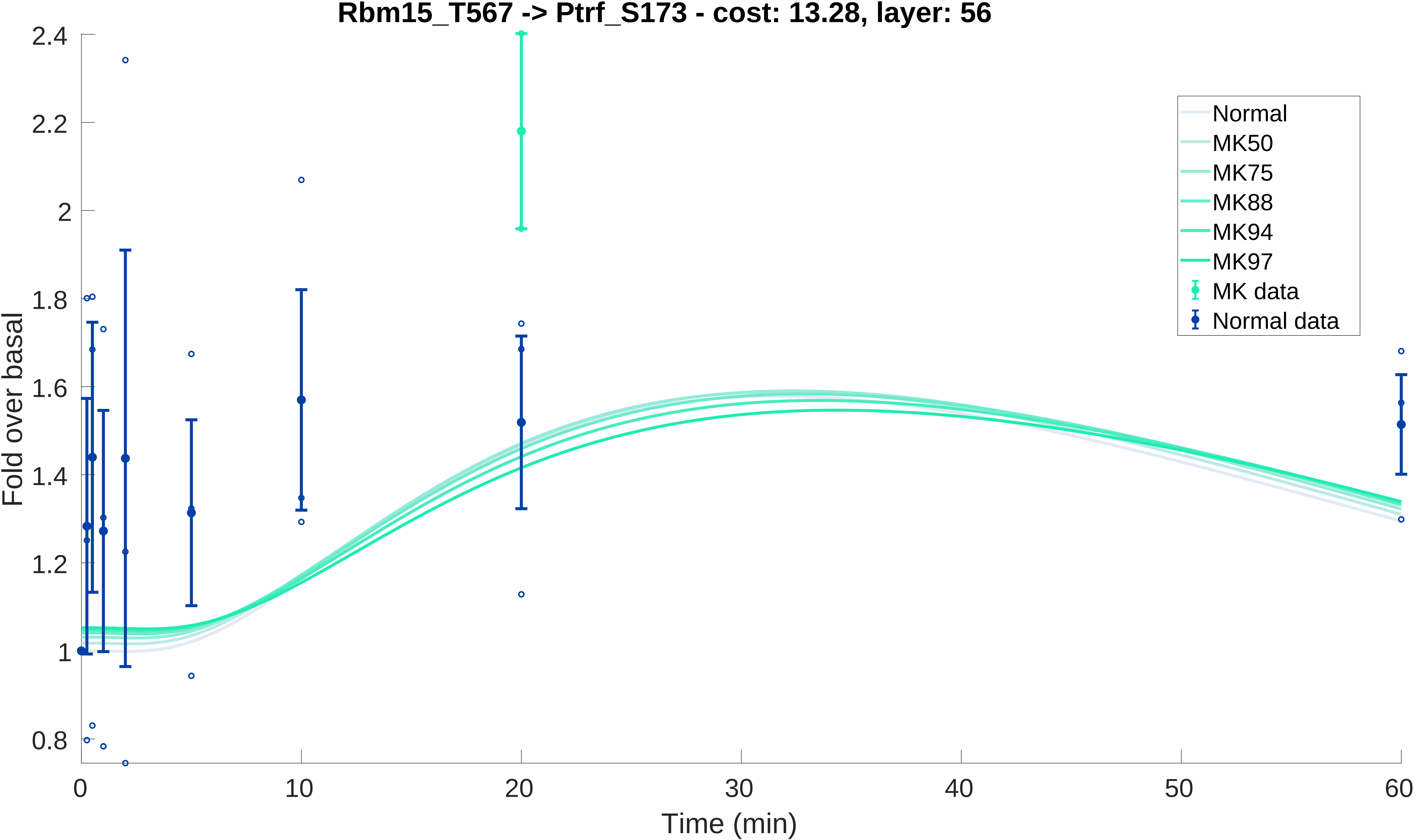

Rbm15_T567 -> Ptrf_S173 - cost: 13.28, layer: 56
Fold over basal
Time (min)
Normal
MK50
MK75
MK88
MK94
MK97
MK data
Normal data
2.4
2.2
2
1.8
1.6
1.4
1.2
1
0.8
0
10
20
30
40
50
60

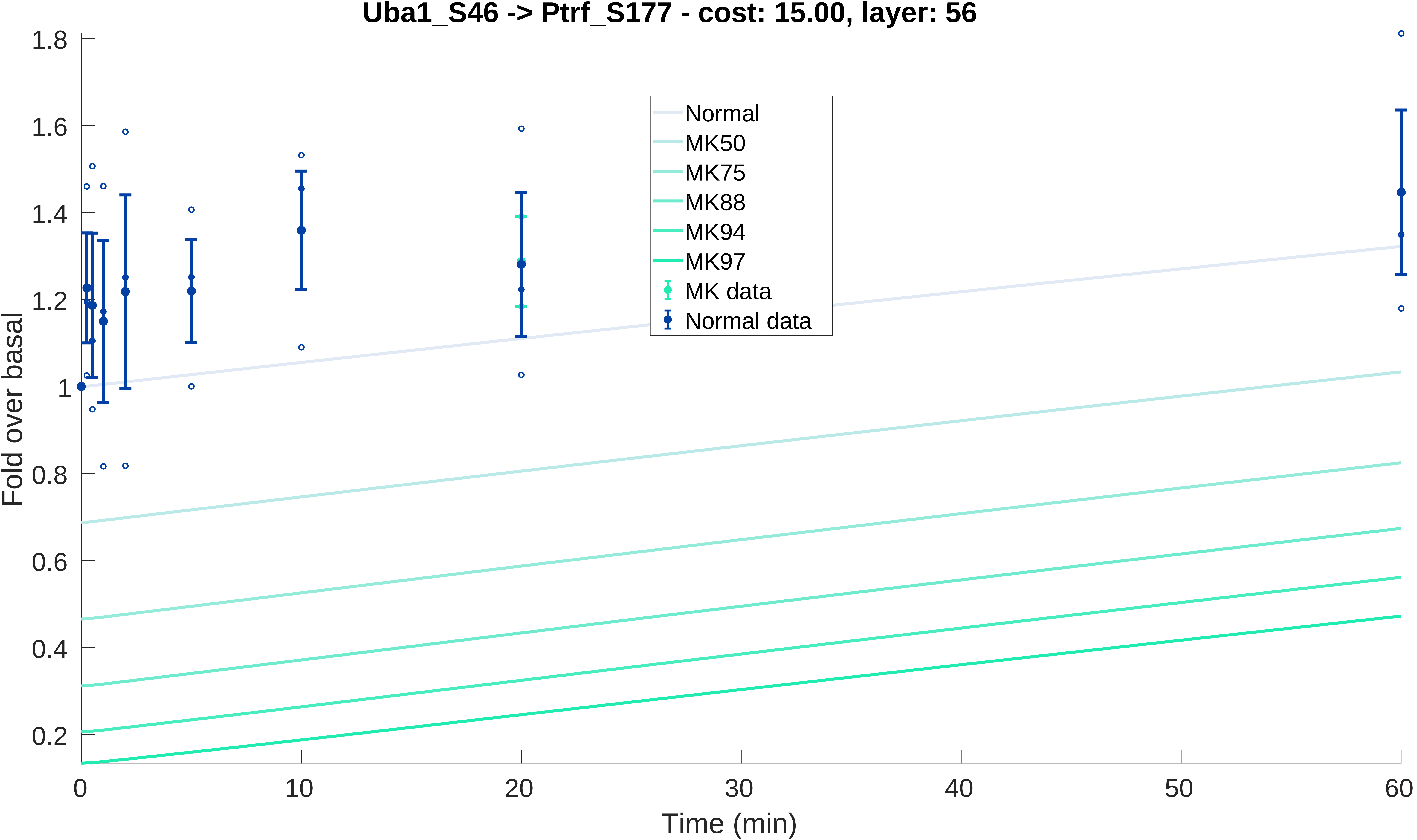

Uba1_S46 -> Ptrf_S177 - cost: 15.00, layer: 56
Fold over basal
Time (min)
Normal
MK50
MK75
MK88
MK94
MK97
MK data
Normal data
0
10
20
30
40
50
60
0.2
0.4
0.6
0.8
1
1.2
1.4
1.6
1.8

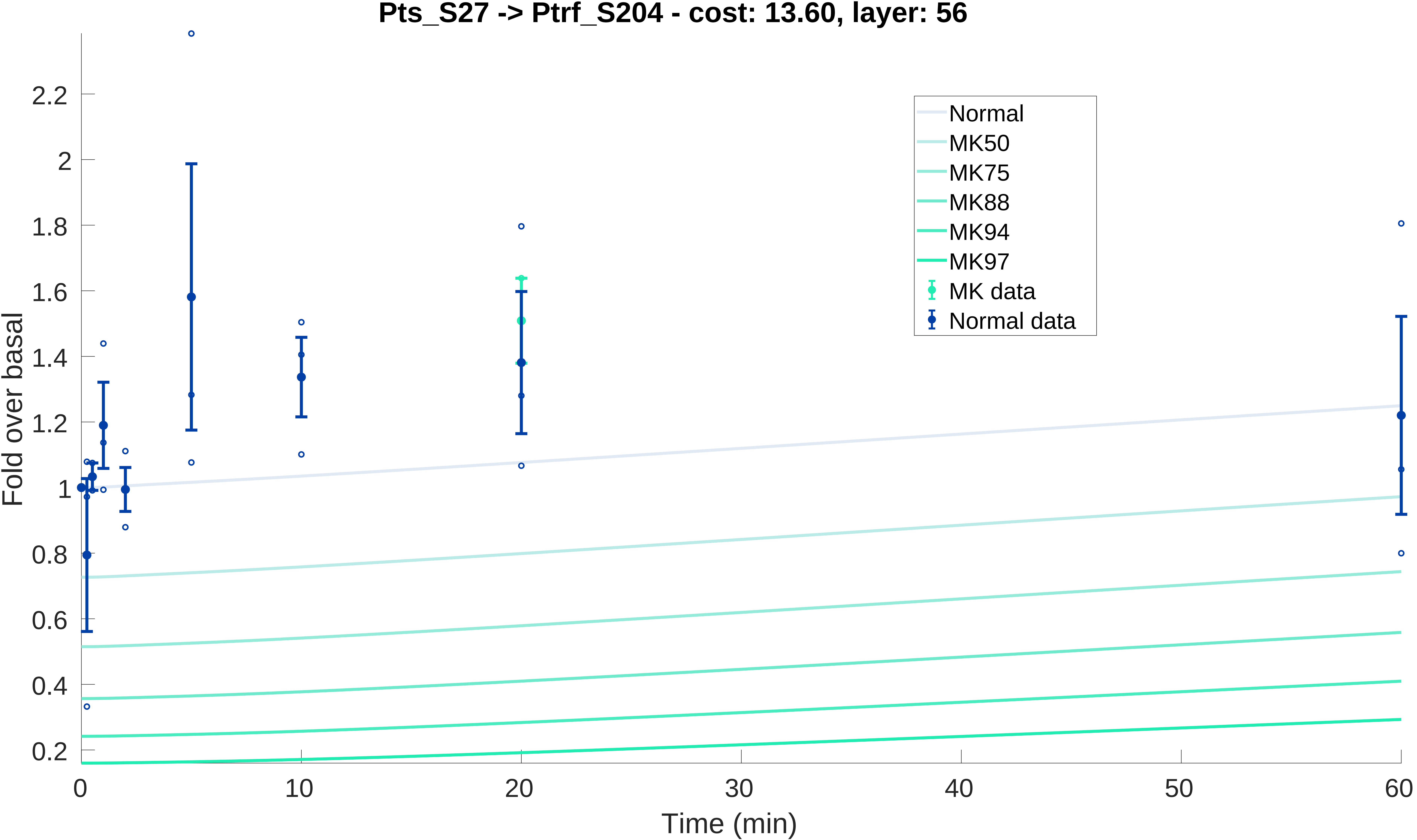
Pts_S27 -> Ptrf_S204 - cost: 13.60, layer: 56
Fold over basal
Time (min)
Normal
MK50
MK75
MK88
MK94
MK97
MK data
Normal data
0.2
0.4
0.6
0.8
1
1.2
1.4
1.6
1.8
2
2.2
0
10
20
30
40
50
60

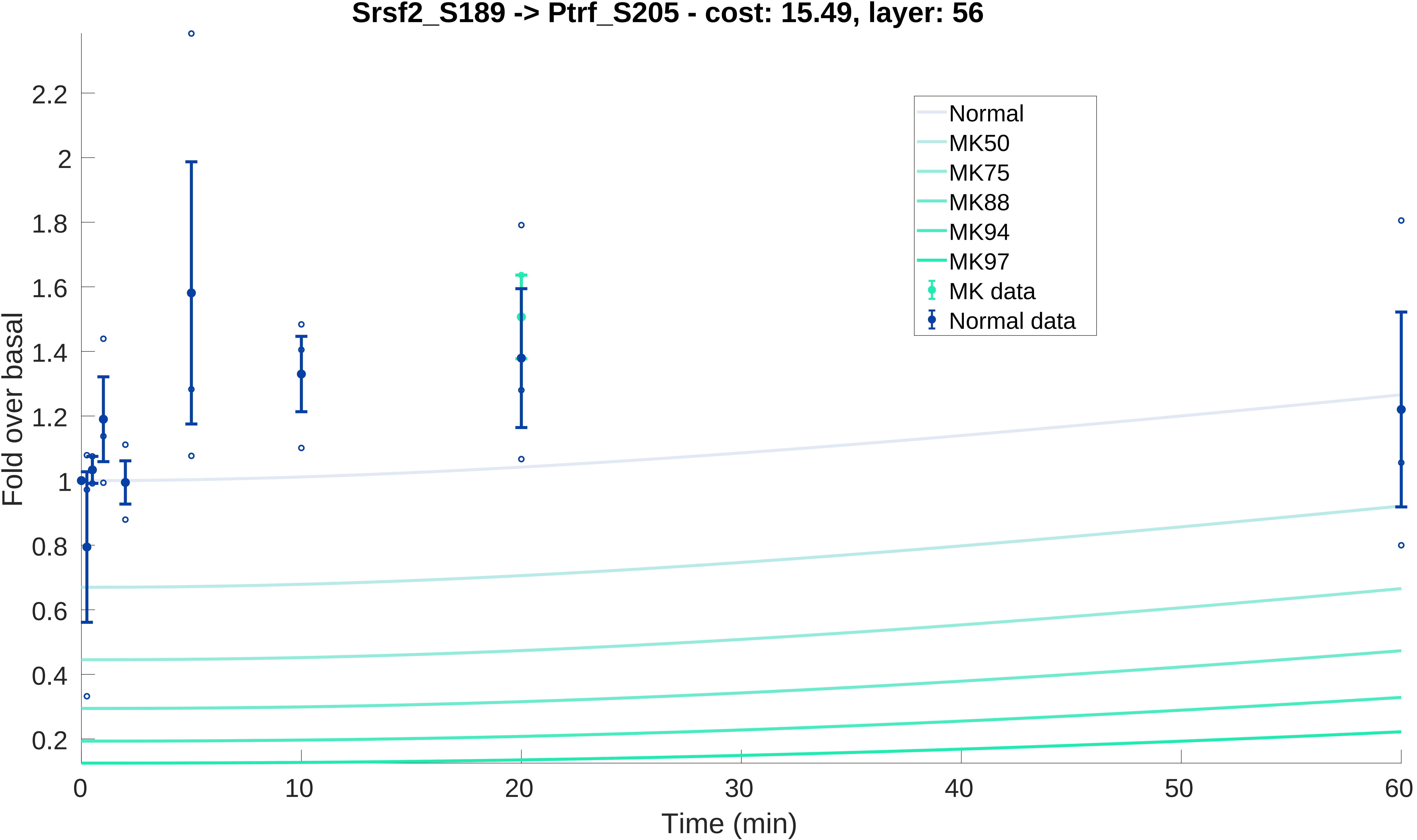

Srsf2_S189 -> Ptrf_S205 - cost: 15.49, layer: 56
Fold over basal
Time (min)
0
10
20
30
40
50
60
0.2
0.4
0.6
0.8
1
1.2
1.4
1.6
1.8
2
2.2
Normal
MK50
MK75
MK88
MK94
MK97
MK data
Normal data

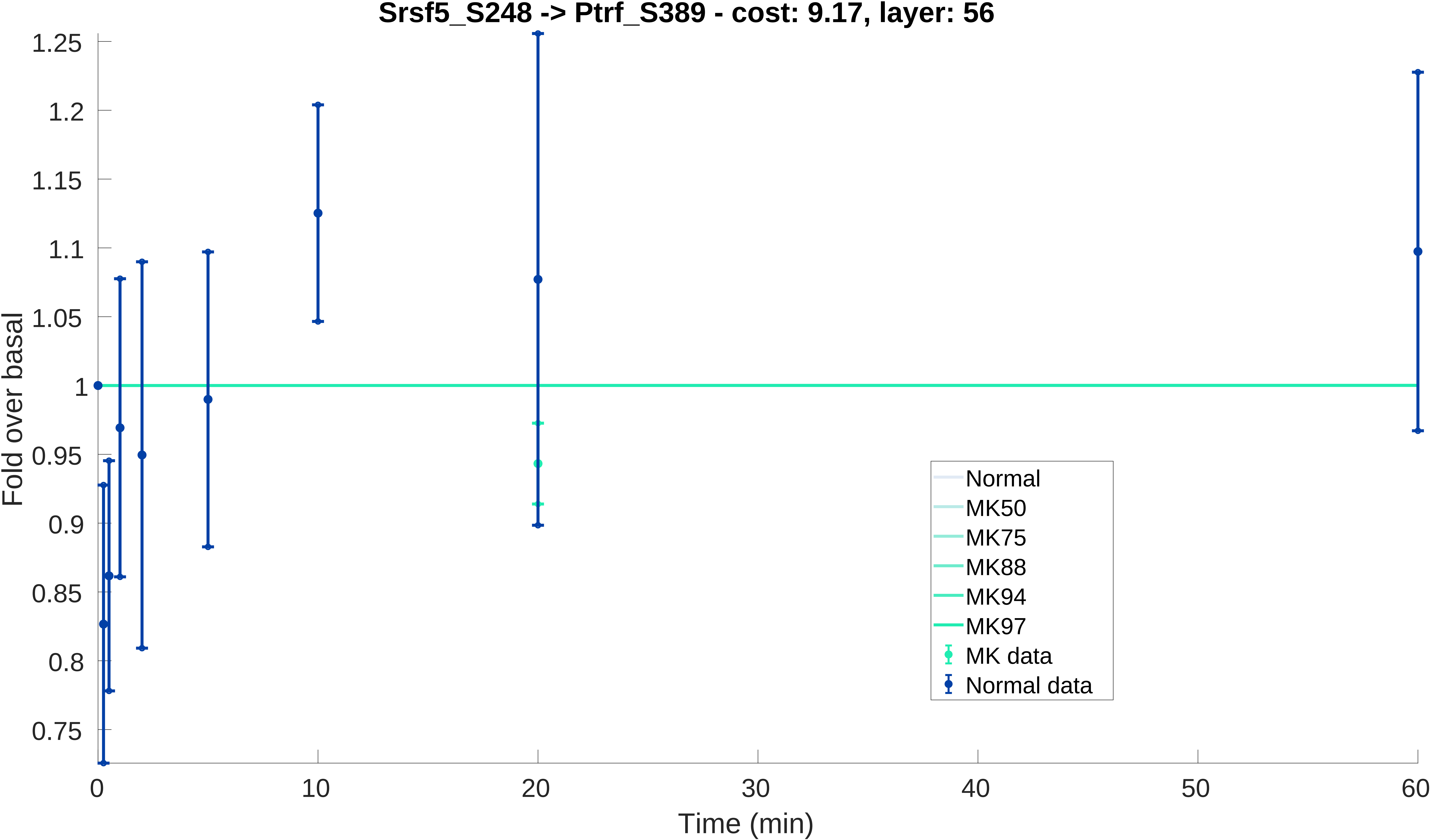
Srsf5_S248 -> Ptrf_S389 - cost: 9.17, layer: 56
Fold over basal
Time (min)
1.25
1.2
1.15
1.1
1.05
1
0.95
0.9
0.85
0.8
0.75
0
10
20
30
40
50
60
Normal
MK50
MK75
MK88
MK94
MK97
MK data
Normal data

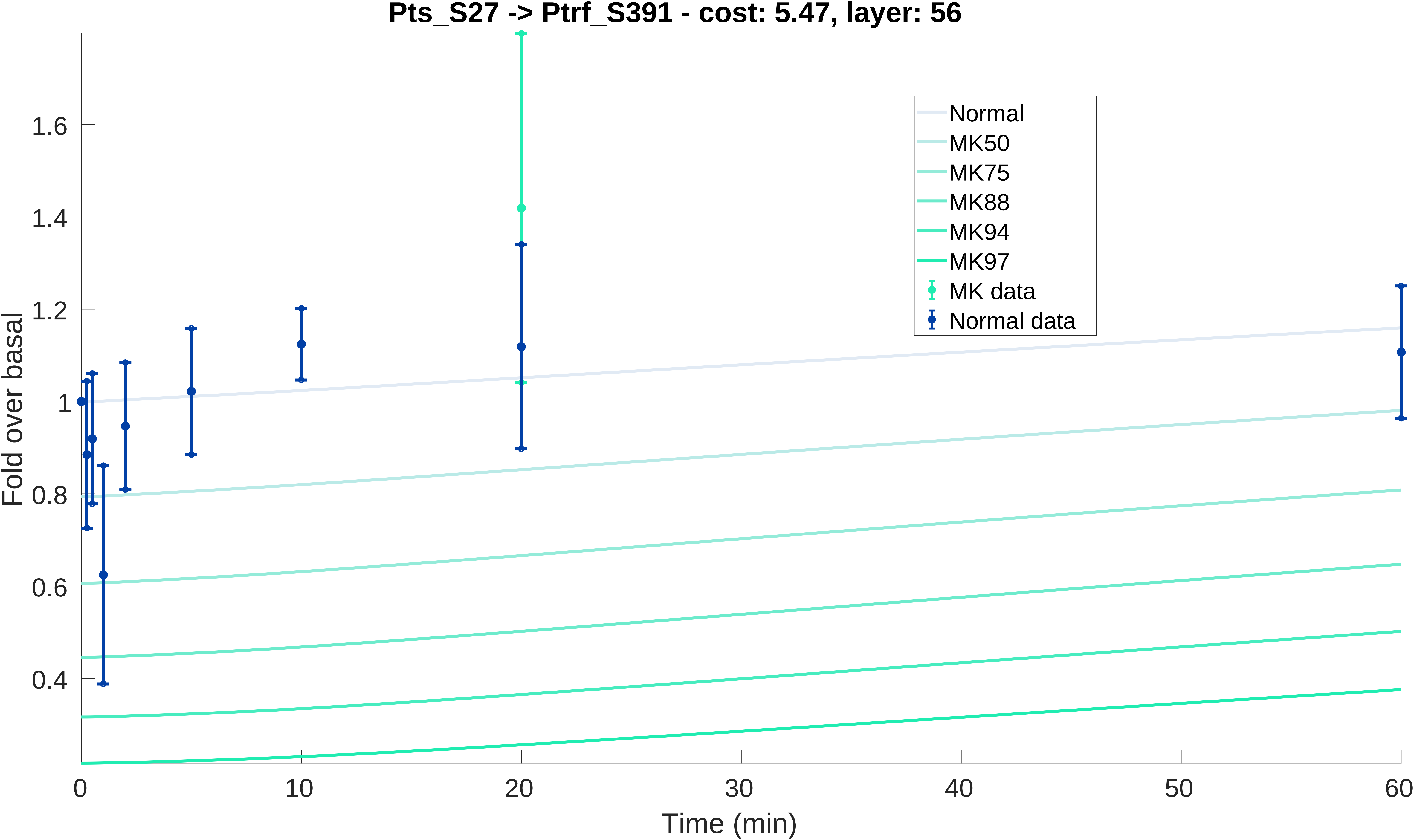
Pts_S27 -> Ptrf_S391 - cost: 5.47, layer: 56
Fold over basal
Time (min)
Normal
MK50
MK75
MK88
MK94
MK97
MK data
Normal data
0
10
20
30
40
50
60
0.4
0.6
0.8
1
1.2
1.4
1.6

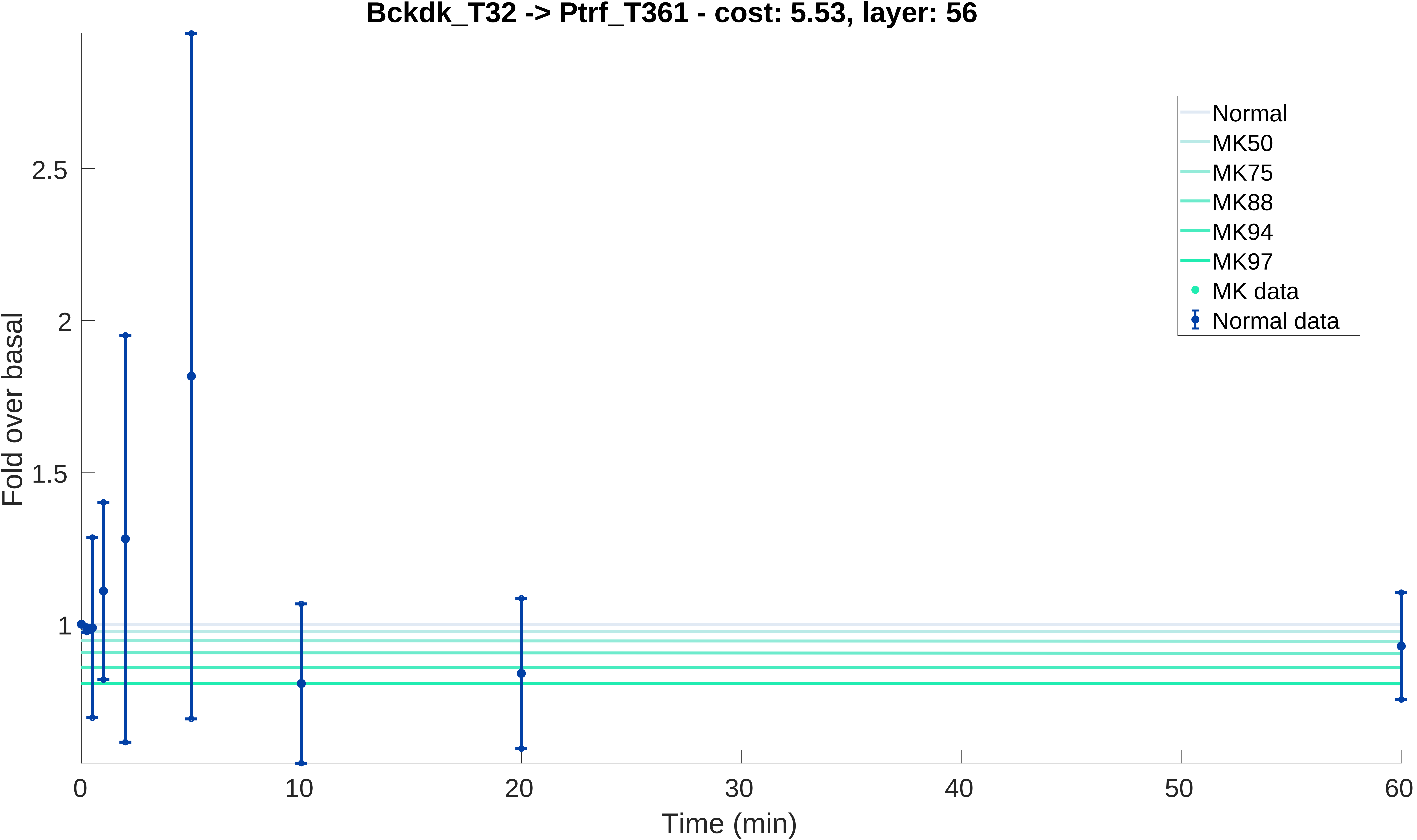

Bckdk_T32 -> Ptrf_T361 - cost: 5.53, layer: 56
Fold over basal
2.5
2
1.5
1
0
10
20
30
40
50
60
Time (min)
Normal
MK50
MK75
MK88
MK94
MK97
MK data
Normal data

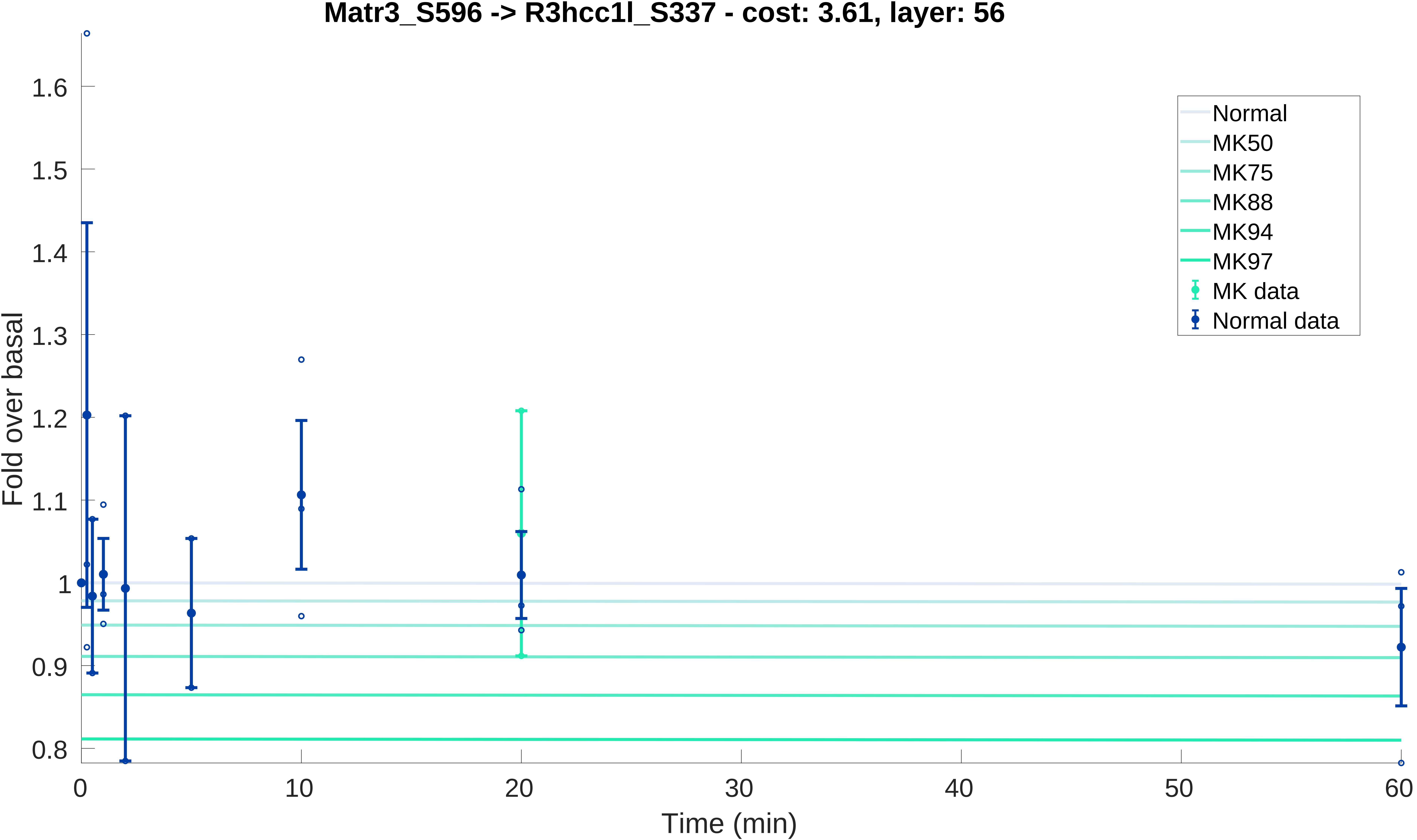

Matr3_S596 -> R3hcc1l_S337 - cost: 3.61, layer: 56
Fold over basal
1.6
1.5
1.4
1.3
1.2
1.1
1
0.9
0.8
0
10
20
30
40
50
60
Time (min)
Normal
MK50
MK75
MK88
MK94
MK97
MK data
Normal data

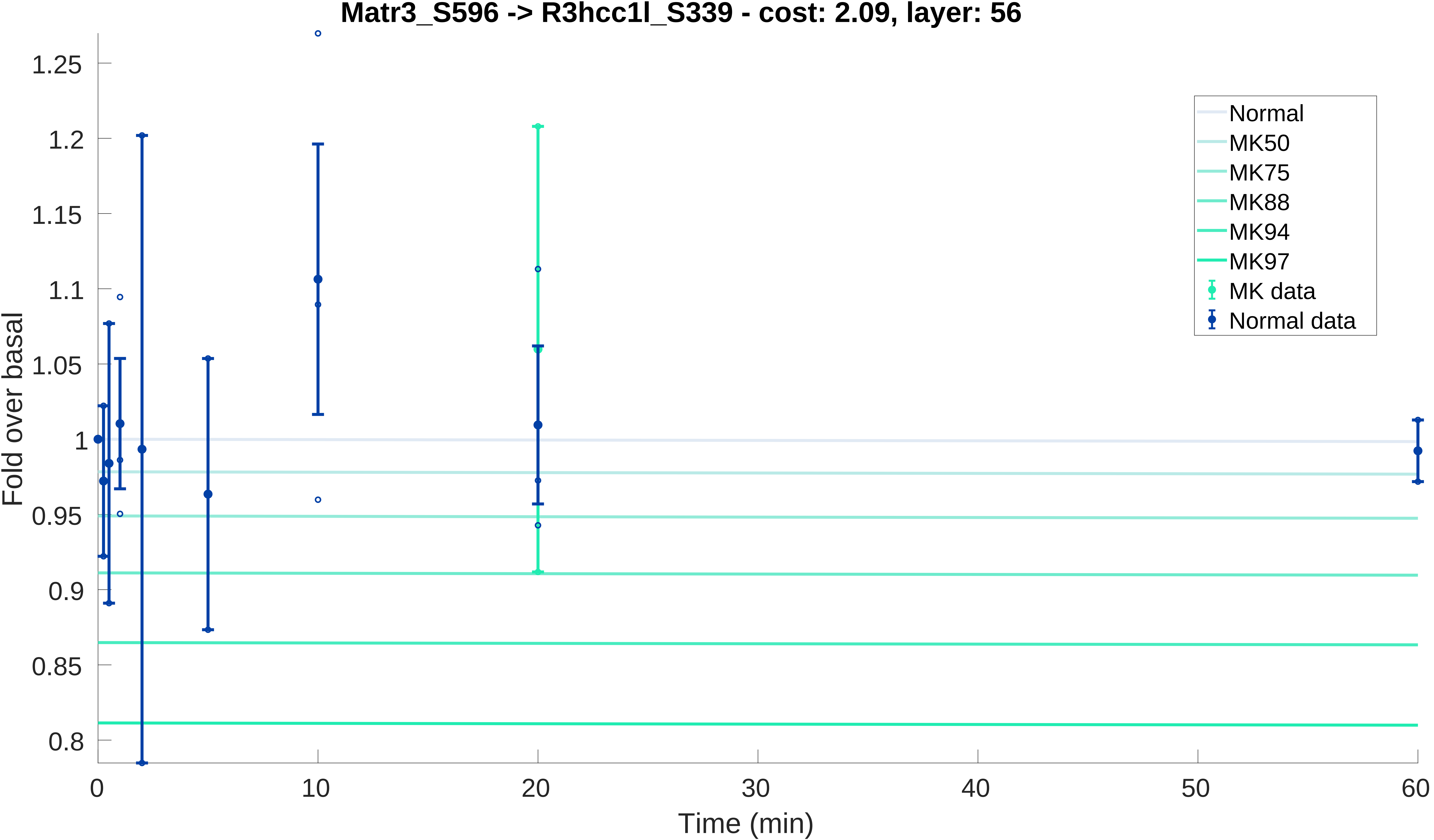

Matr3_S596 -> R3hcc1l_S339 - cost: 2.09, layer: 56
Fold over basal
Time (min)
Normal
MK50
MK75
MK88
MK94
MK97
MK data
Normal data
1.25
1.2
1.15
1.1
1.05
1
0.95
0.9
0.85
0.8
0
10
20
30
40
50
60

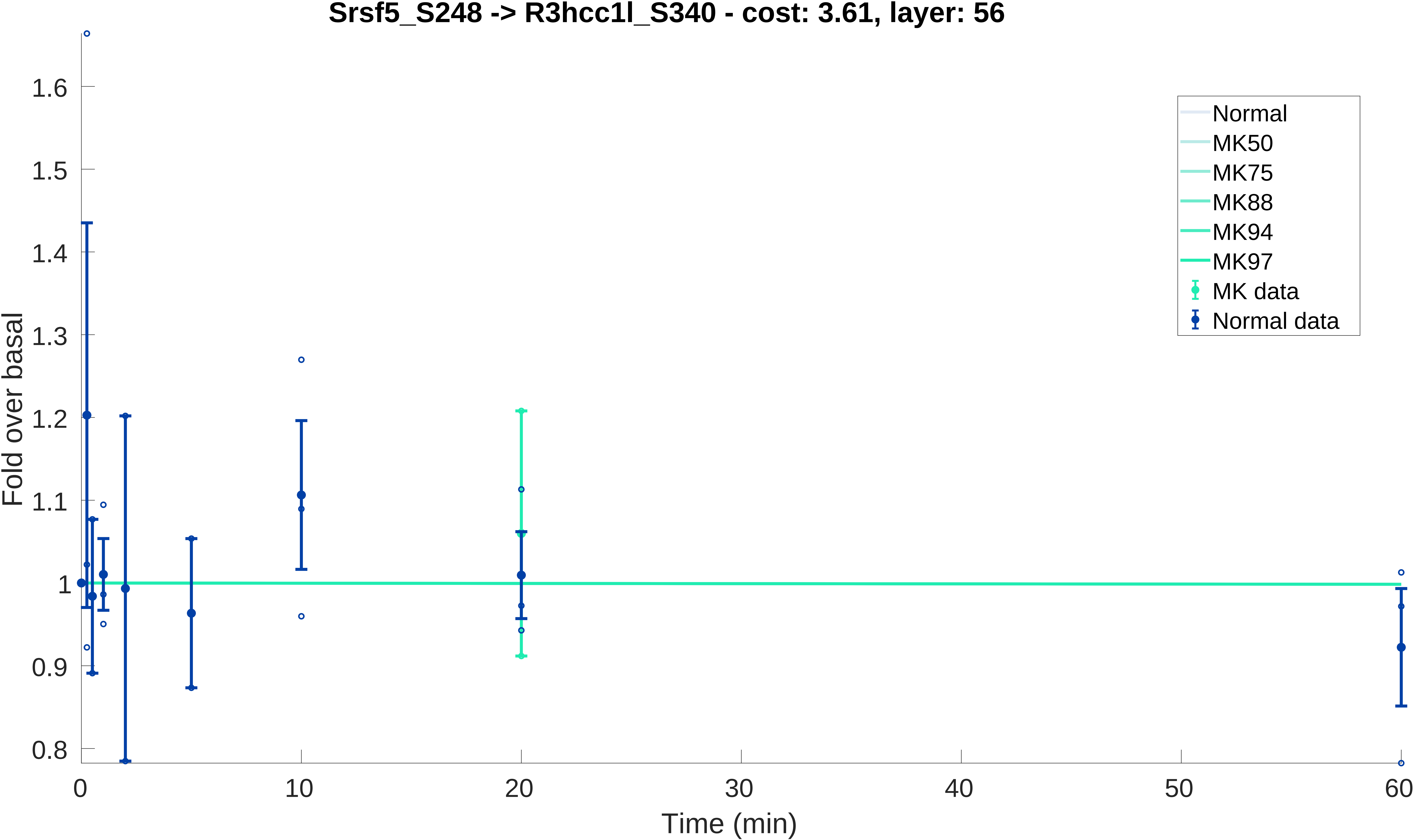
Srsf5_S248 -> R3hcc1l_S340 - cost: 3.61, layer: 56
Fold over basal
1.6
1.5
1.4
1.3
1.2
1.1
1
0.9
0.8
0
10
20
30
40
50
60
Time (min)
Normal
MK50
MK75
MK88
MK94
MK97
MK data
Normal data

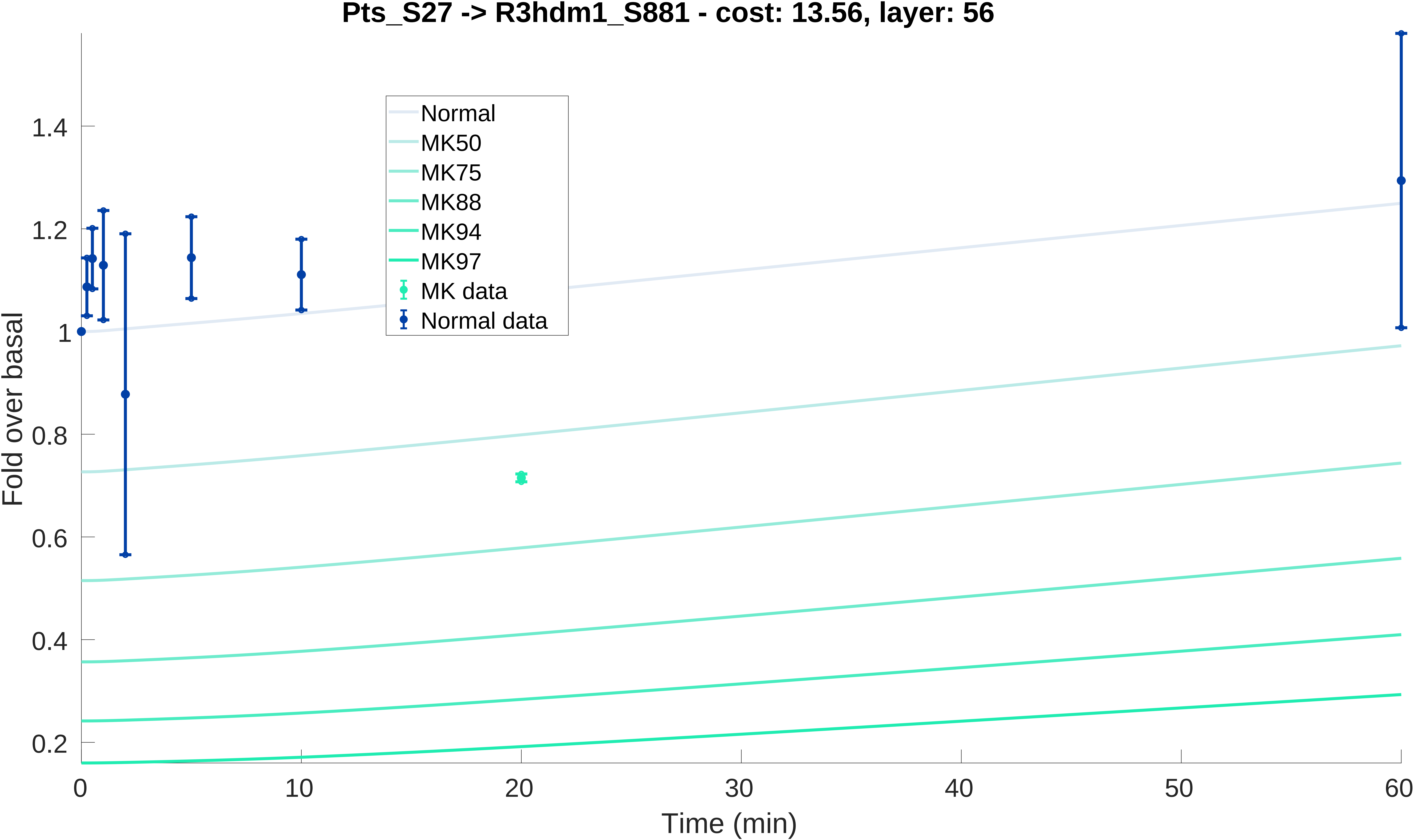
Pts_S27 -> R3hdm1_S881 - cost: 13.56, layer: 56
Normal
MK50
MK75
MK88
MK94
MK97
MK data
Normal data
Fold over basal
0.2
0.4
0.6
0.8
1
1.2
1.4
0
10
20
30
40
50
60
Time (min)

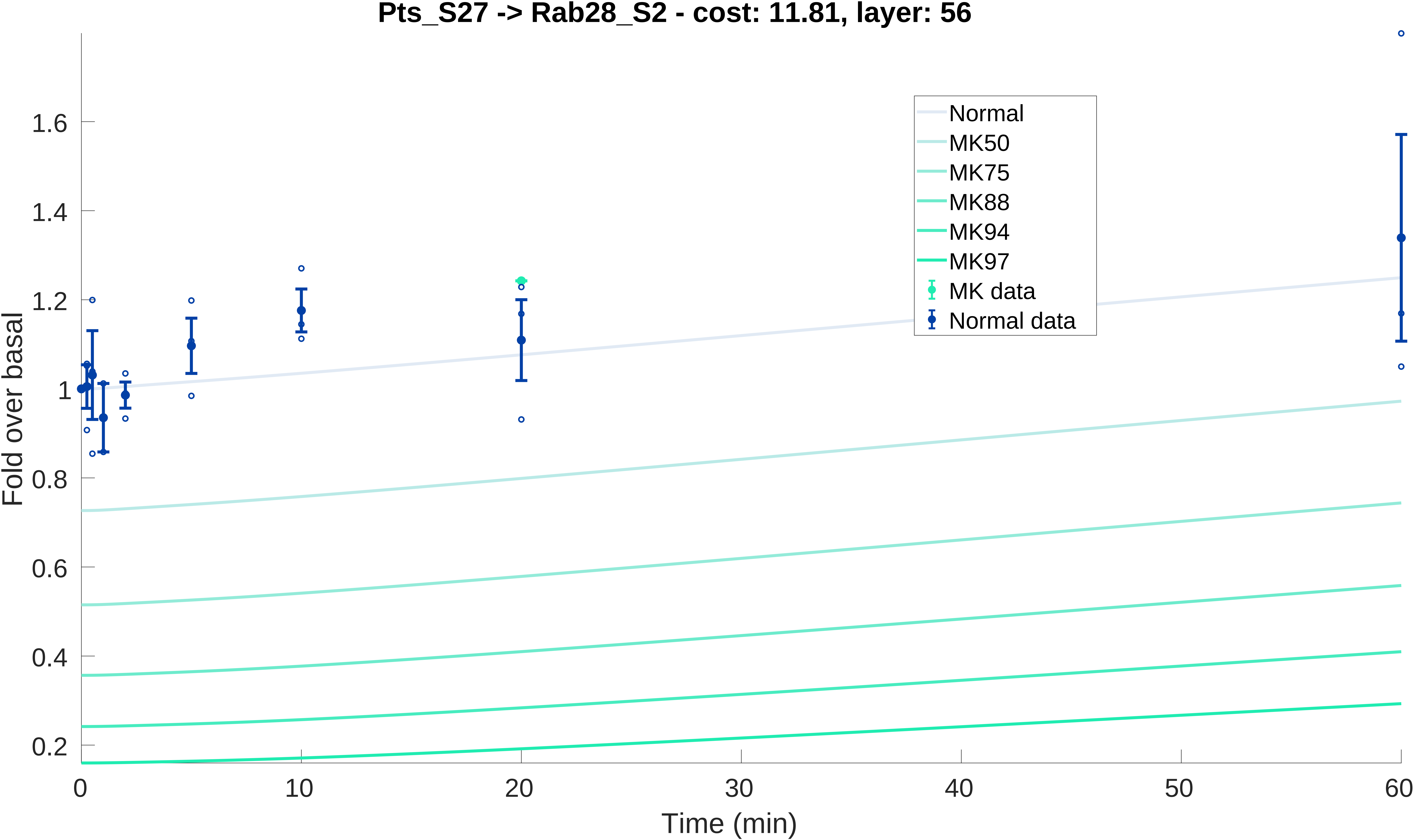

Pts_S27 -> Rab28_S2 - cost: 11.81, layer: 56
Fold over basal
Time (min)
0.2
0.4
0.6
0.8
1
1.2
1.4
1.6
0
10
20
30
40
50
60
Normal
MK50
MK75
MK88
MK94
MK97
MK data
Normal data

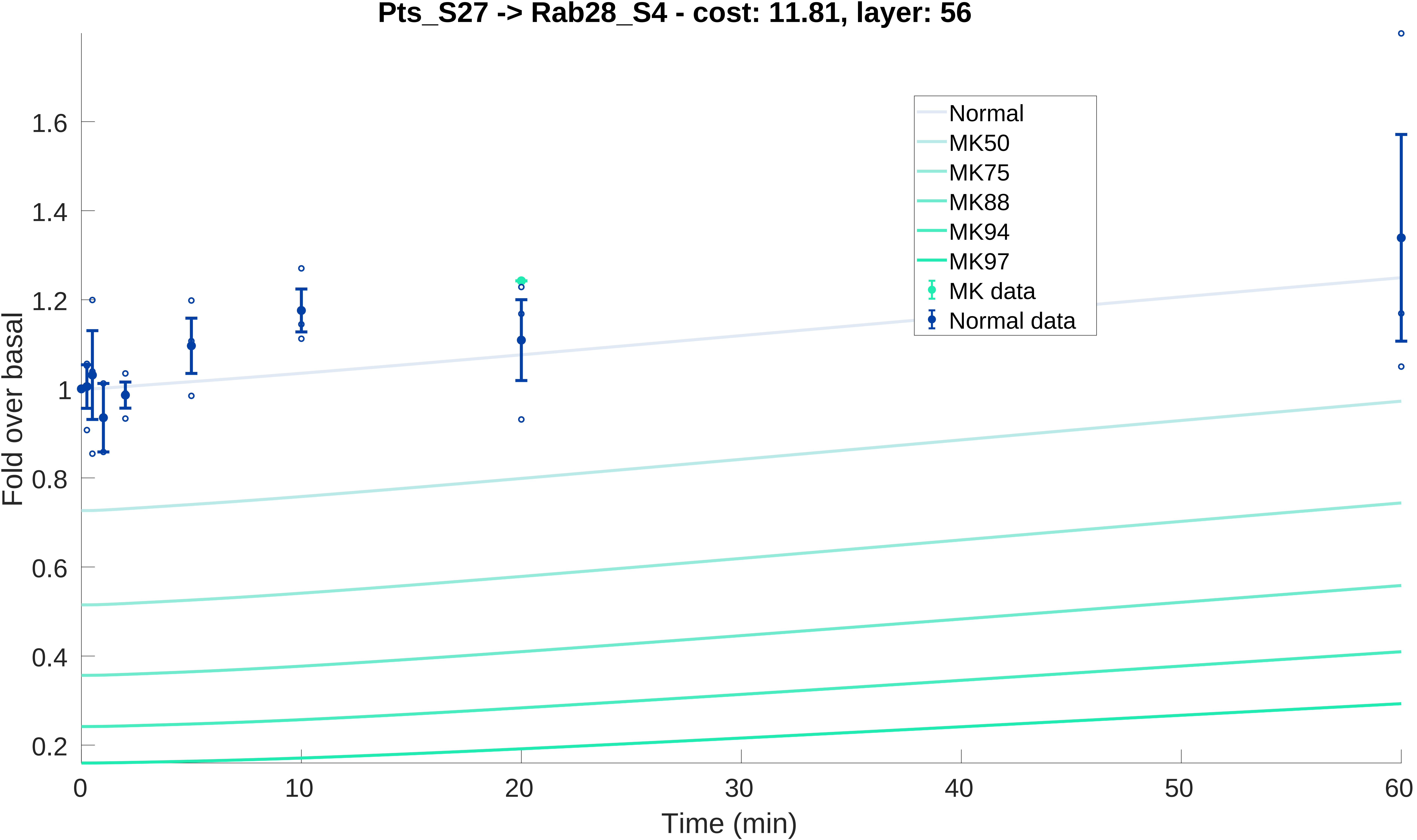

Pts_S27 -> Rab28_S4 - cost: 11.81, layer: 56
Fold over basal
Time (min)
0.2
0.4
0.6
0.8
1
1.2
1.4
1.6
0
10
20
30
40
50
60
Normal
MK50
MK75
MK88
MK94
MK97
MK data
Normal data

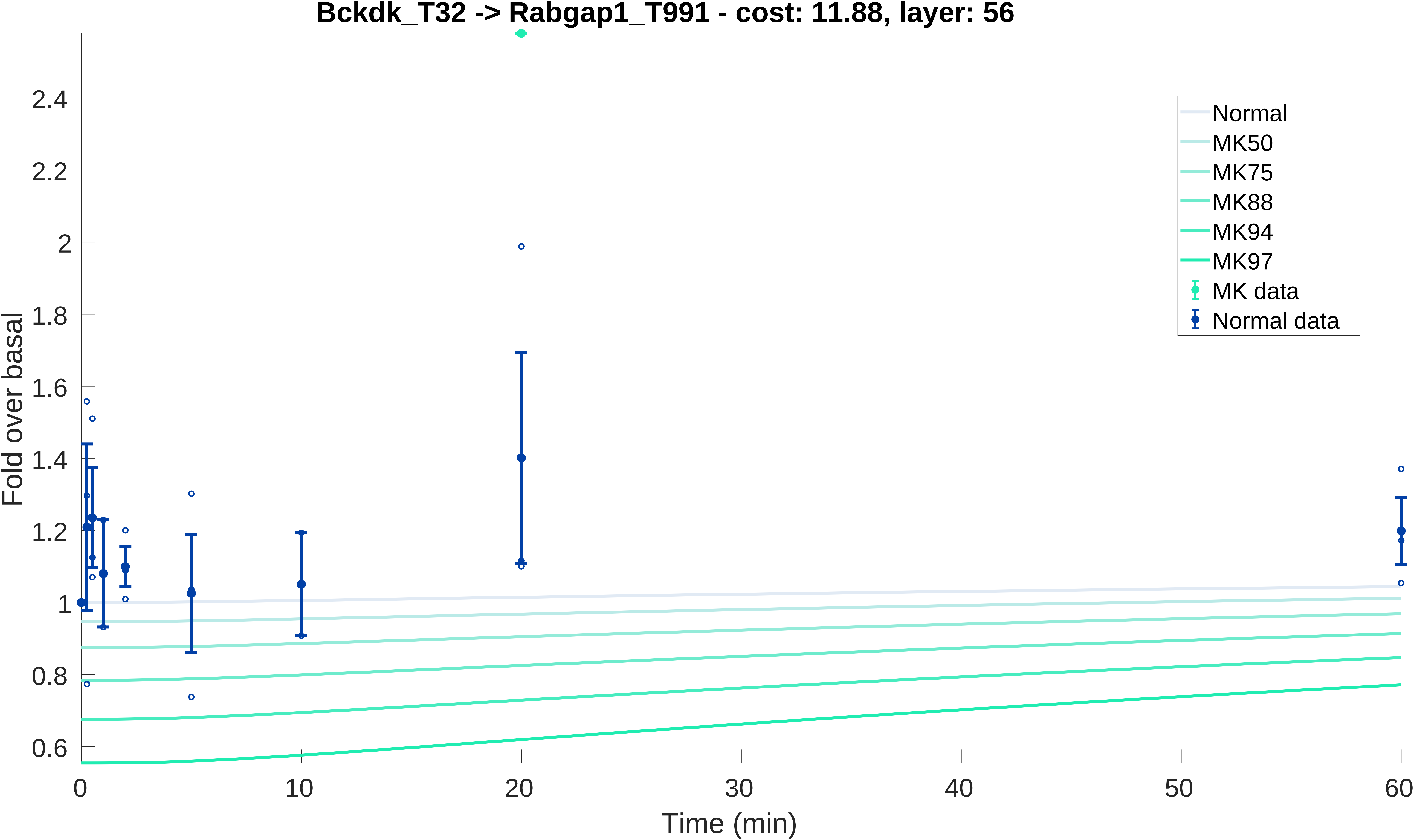
Bckdk_T32 -> Rabgap1_T991 - cost: 11.88, layer: 56
Fold over basal
Time (min)
0.6
0.8
1
1.2
1.4
1.6
1.8
2
2.2
2.4
0
10
20
30
40
50
60
Normal
MK50
MK75
MK88
MK94
MK97
MK data
Normal data

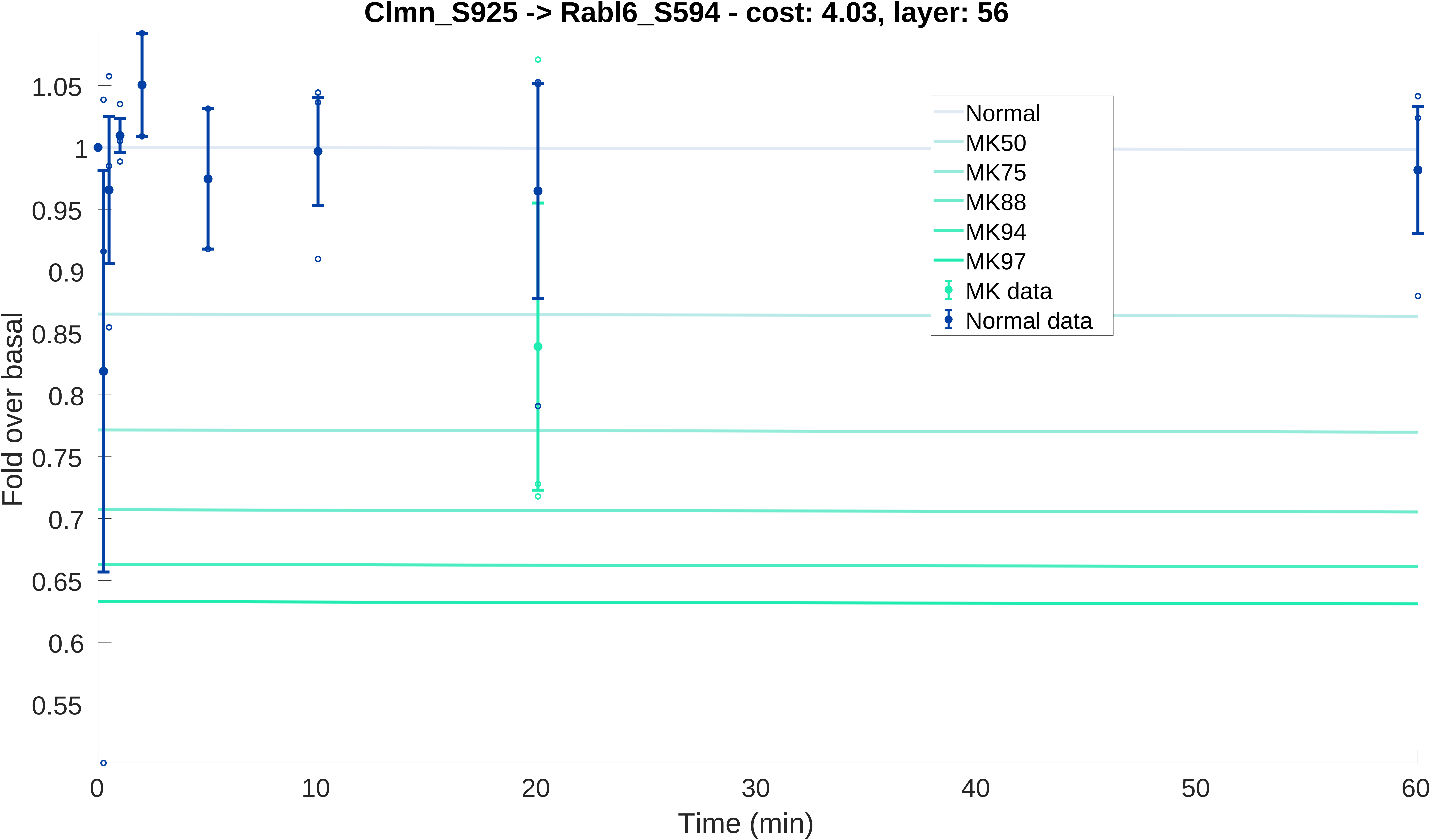
Clmn_S925 -> Rabl6_S594 - cost: 4.03, layer: 56
Fold over basal
Time (min)
1.05
1
0.95
0.9
0.85
0.8
0.75
0.7
0.65
0.6
0.55
0
10
20
30
40
50
60
Normal
MK50
MK75
MK88
MK94
MK97
MK data
Normal data

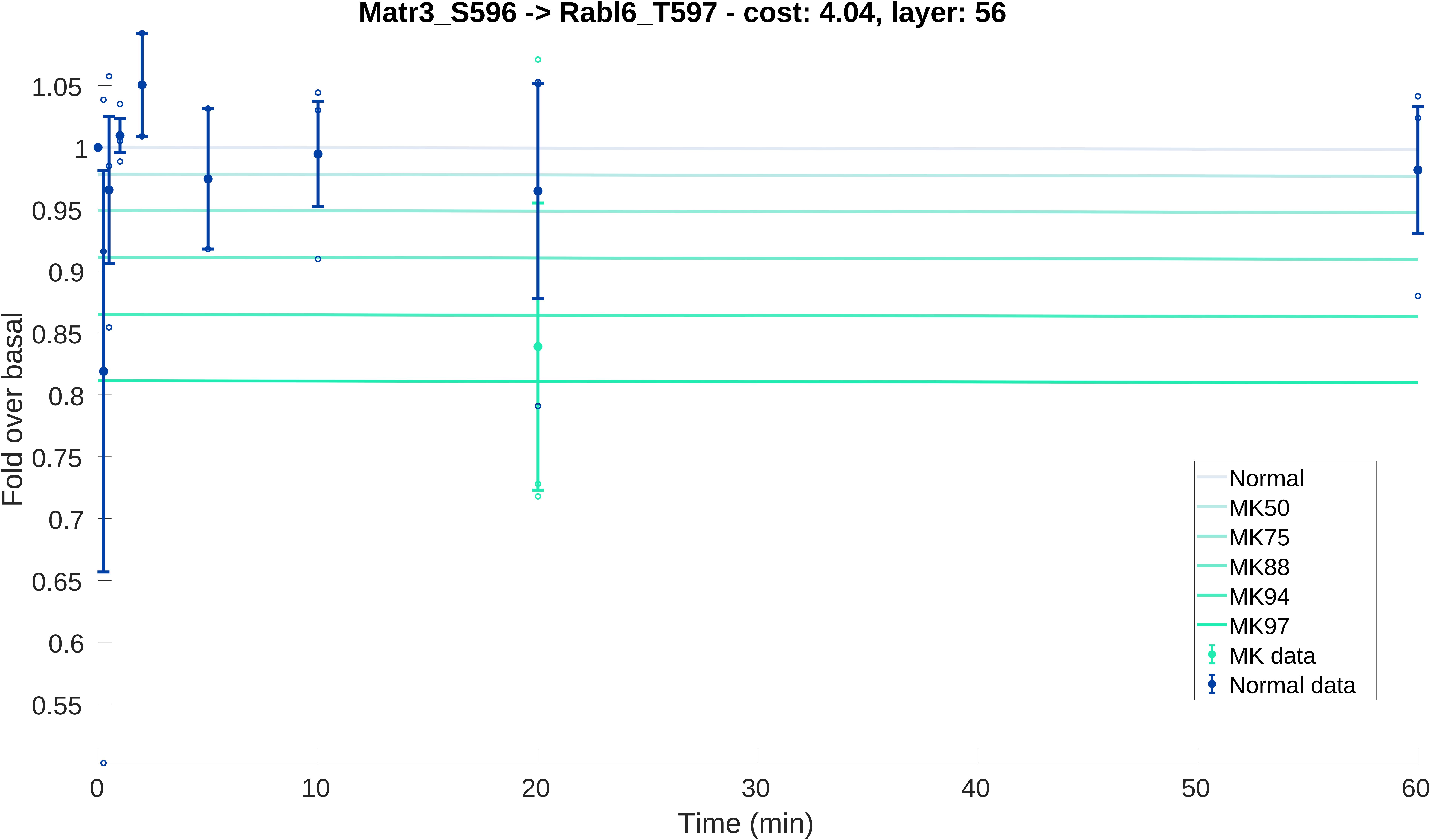

Matr3_S596 -> Rabl6_T597 - cost: 4.04, layer: 56
Fold over basal
1.05
1
0.95
0.9
0.85
0.8
0.75
0.7
0.65
0.6
0.55
0
10
20
30
40
50
60
Time (min)
Normal
MK50
MK75
MK88
MK94
MK97
MK data
Normal data

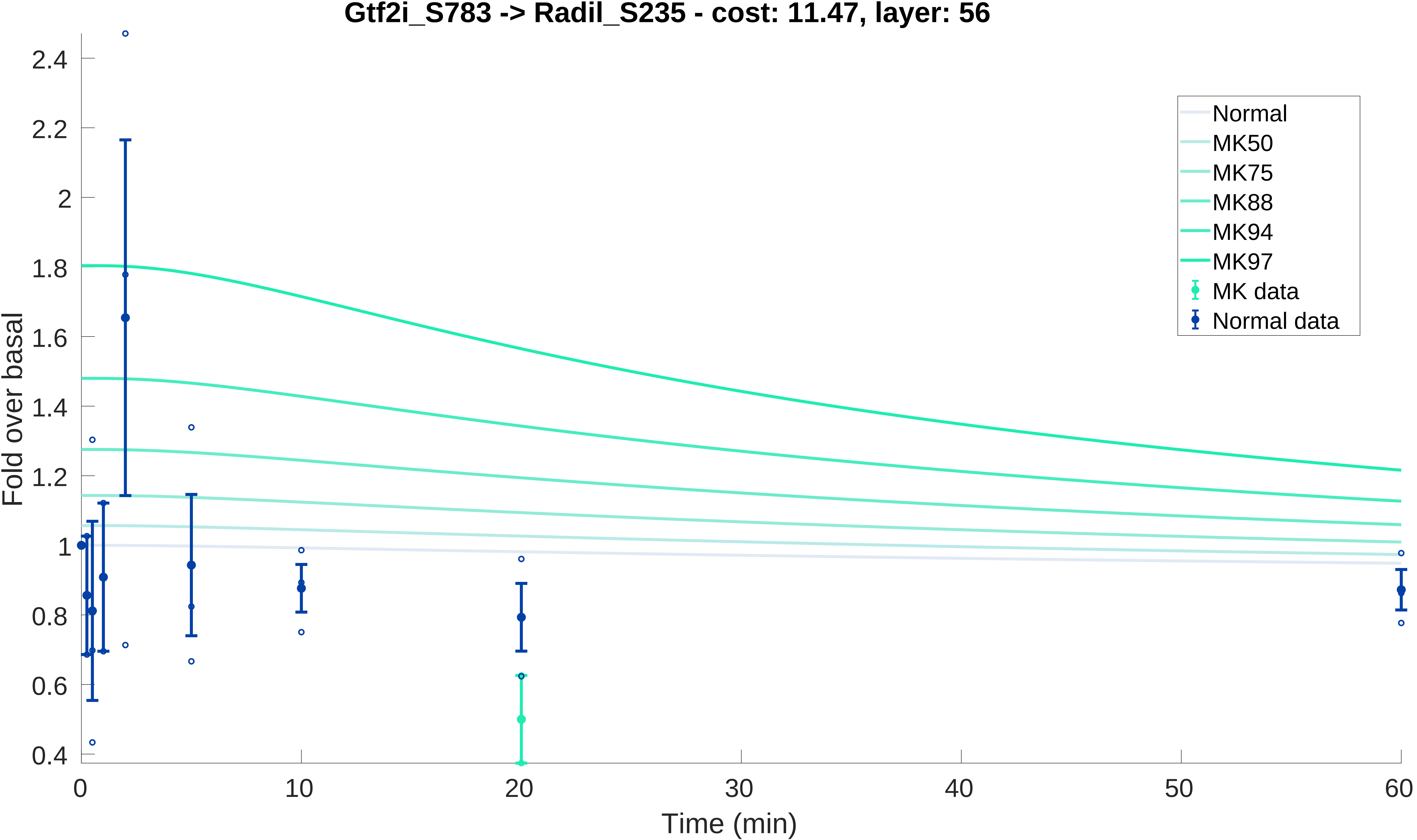

Gtf2i_S783 -> Radil_S235 - cost: 11.47, layer: 56
Fold over basal
2.4
2.2
2
1.8
1.6
1.4
1.2
1
0.8
0.6
0.4
0
10
20
30
40
50
60
Time (min)
Normal
MK50
MK75
MK88
MK94
MK97
MK data
Normal data

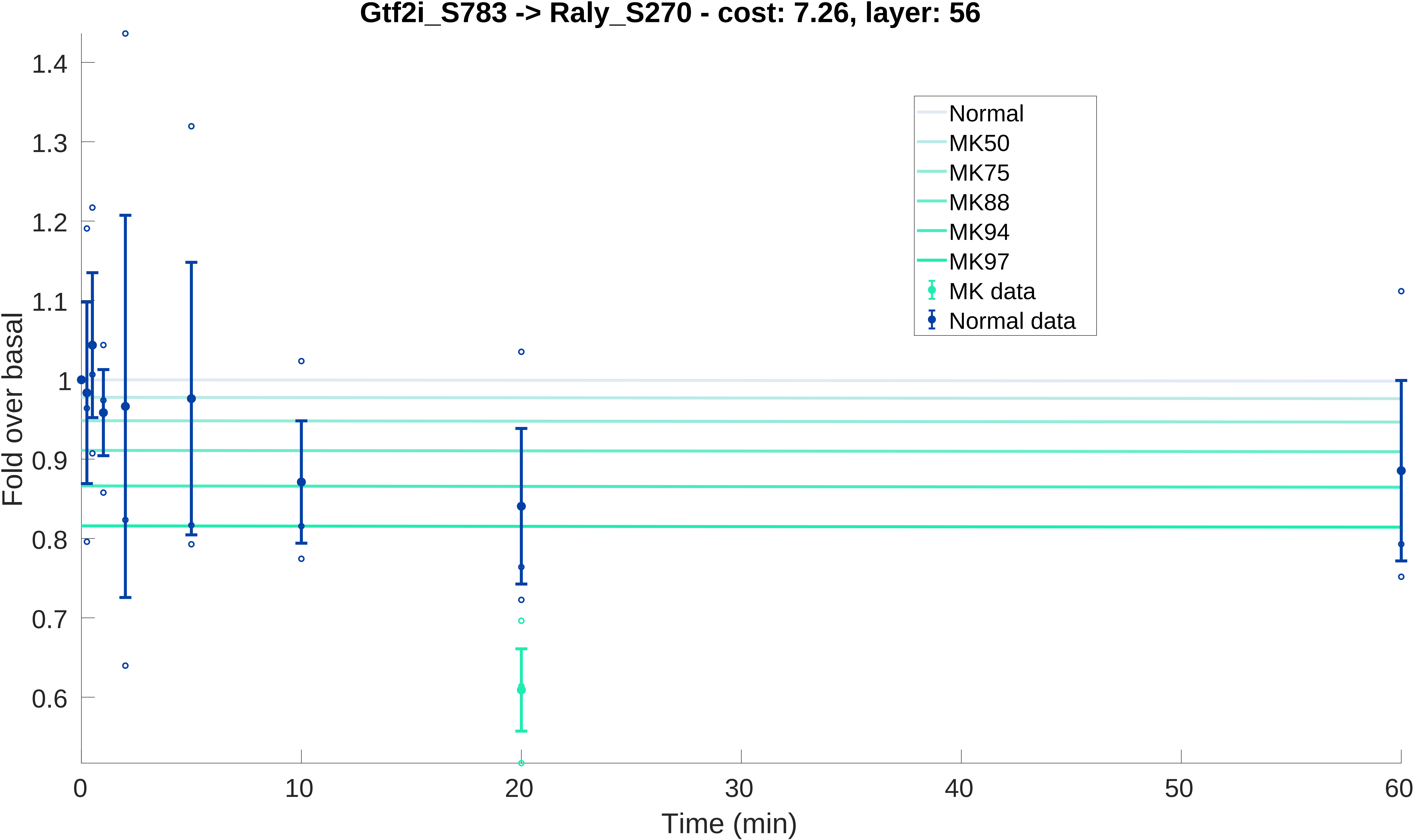

Gtf2i_S783 -> Raly_S270 - cost: 7.26, layer: 56
Fold over basal
Time (min)
Normal
MK50
MK75
MK88
MK94
MK97
MK data
Normal data
1.4
1.3
1.2
1.1
1
0.9
0.8
0.7
0.6
0
10
20
30
40
50
60

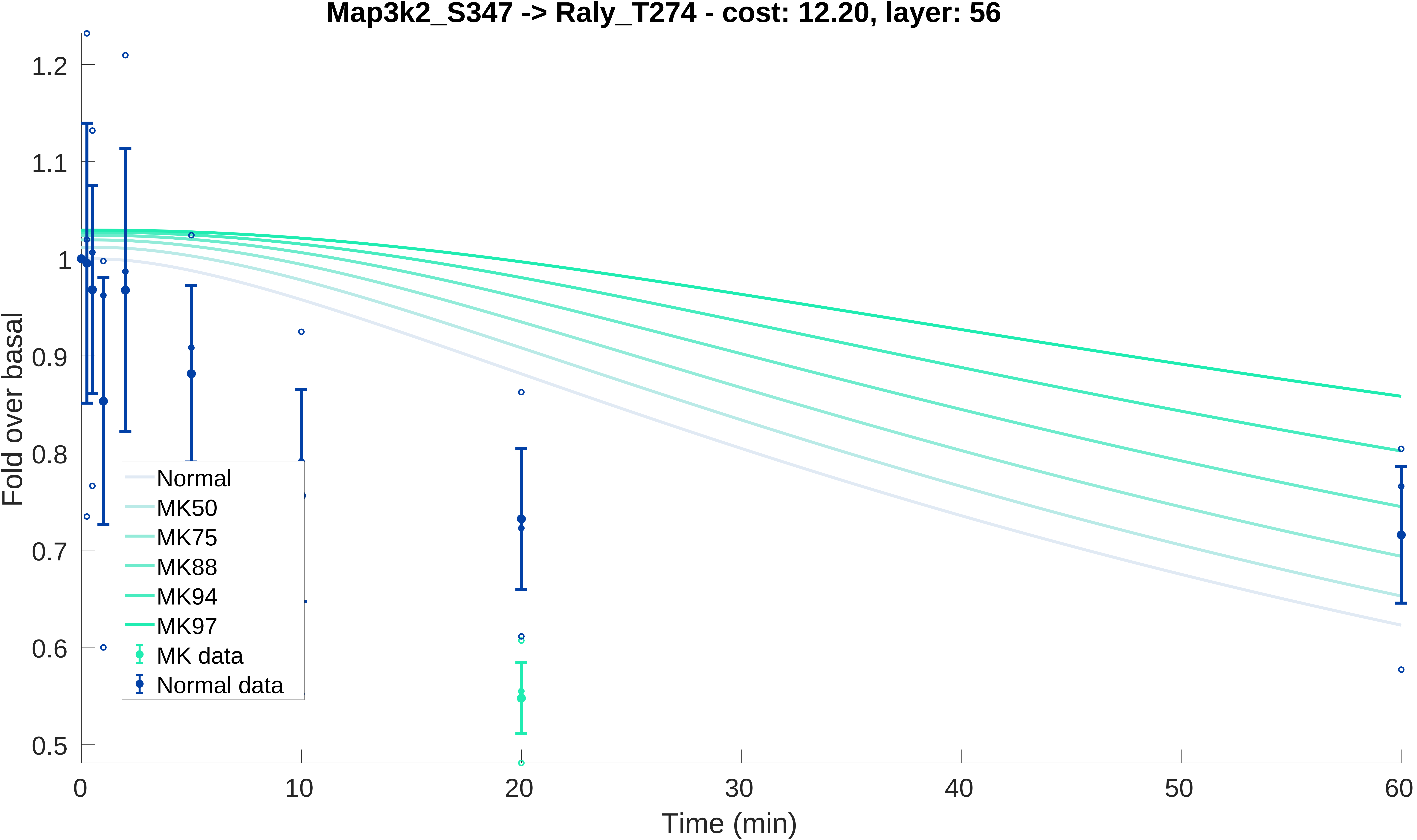

Map3k2_S347 -> Raly_T274 - cost: 12.20, layer: 56
Fold over basal
Time (min)
1.2
1.1
1
0.9
0.8
0.7
0.6
0.5
0
10
20
30
40
50
60
Normal
MK50
MK75
MK88
MK94
MK97
MK data
Normal data

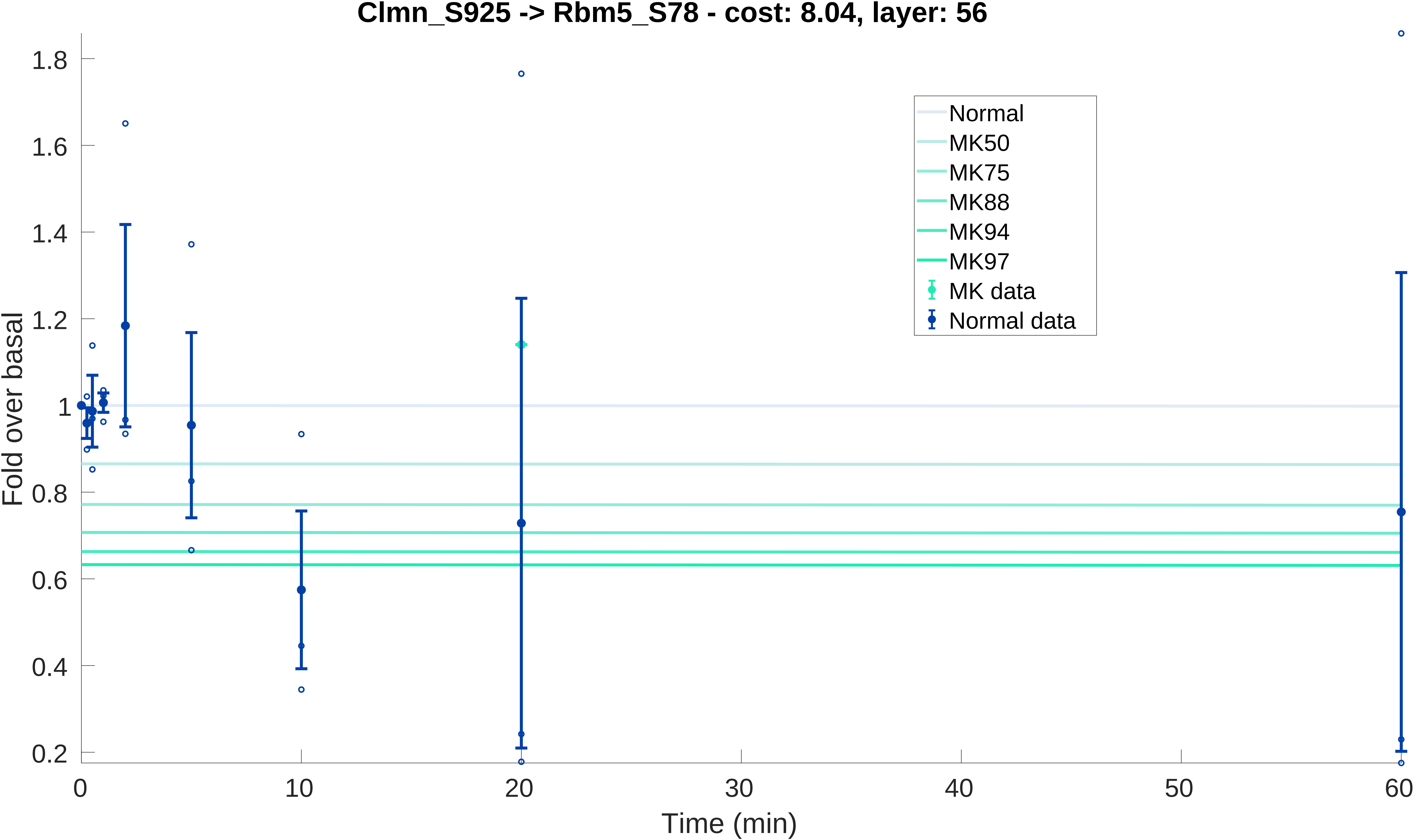
Clmn_S925 -> Rbm5_S78 - cost: 8.04, layer: 56
Fold over basal
Time (min)
Normal
MK50
MK75
MK88
MK94
MK97
MK data
Normal data
0.2
0.4
0.6
0.8
1
1.2
1.4
1.6
1.8
0
10
20
30
40
50
60

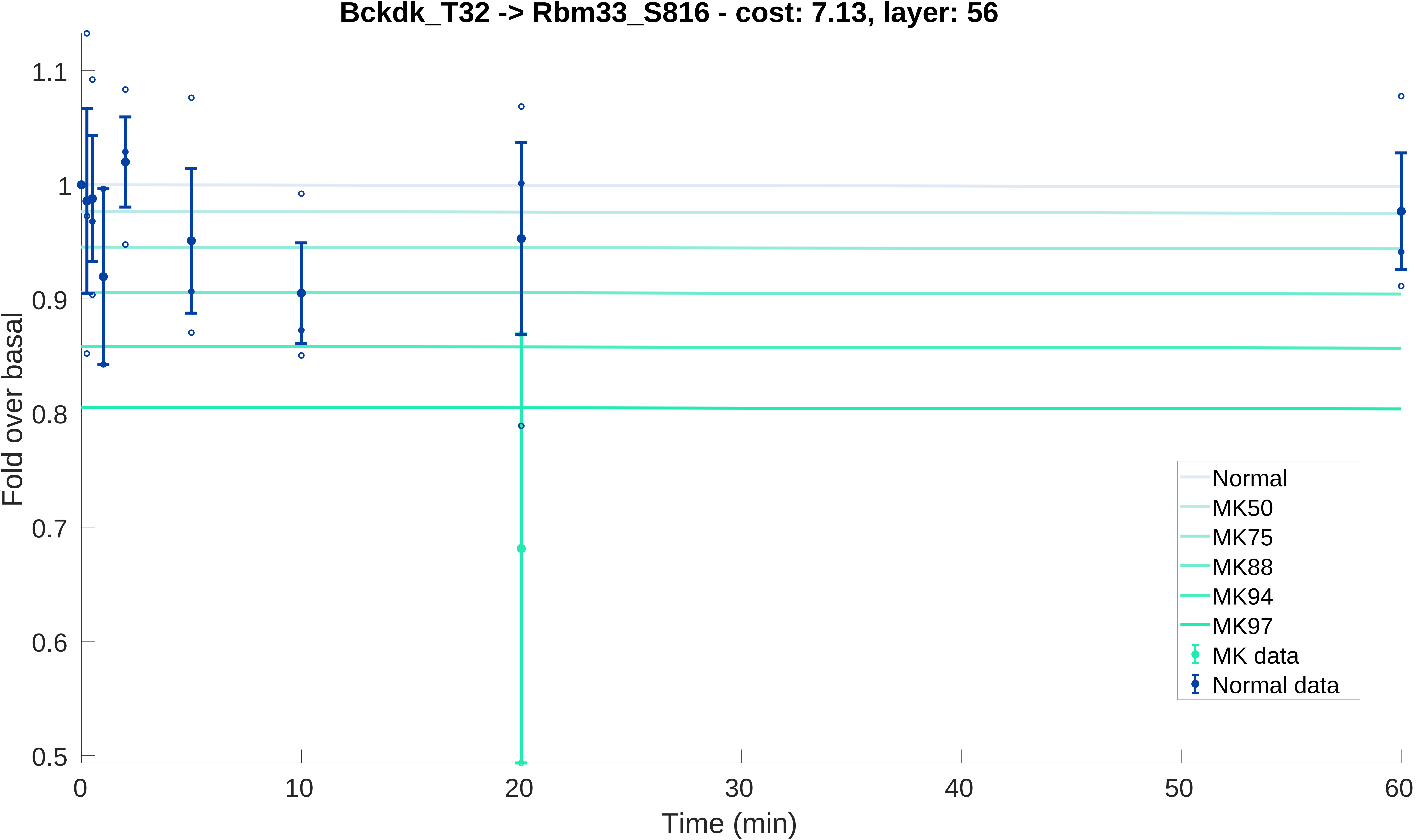

Bckdk_T32 -> Rbm33_S816 - cost: 7.13, layer: 56
Fold over basal
Time (min)
1.1
1
0.9
0.8
0.7
0.6
0.5
0
10
20
30
40
50
60
Normal
MK50
MK75
MK88
MK94
MK97
MK data
Normal data

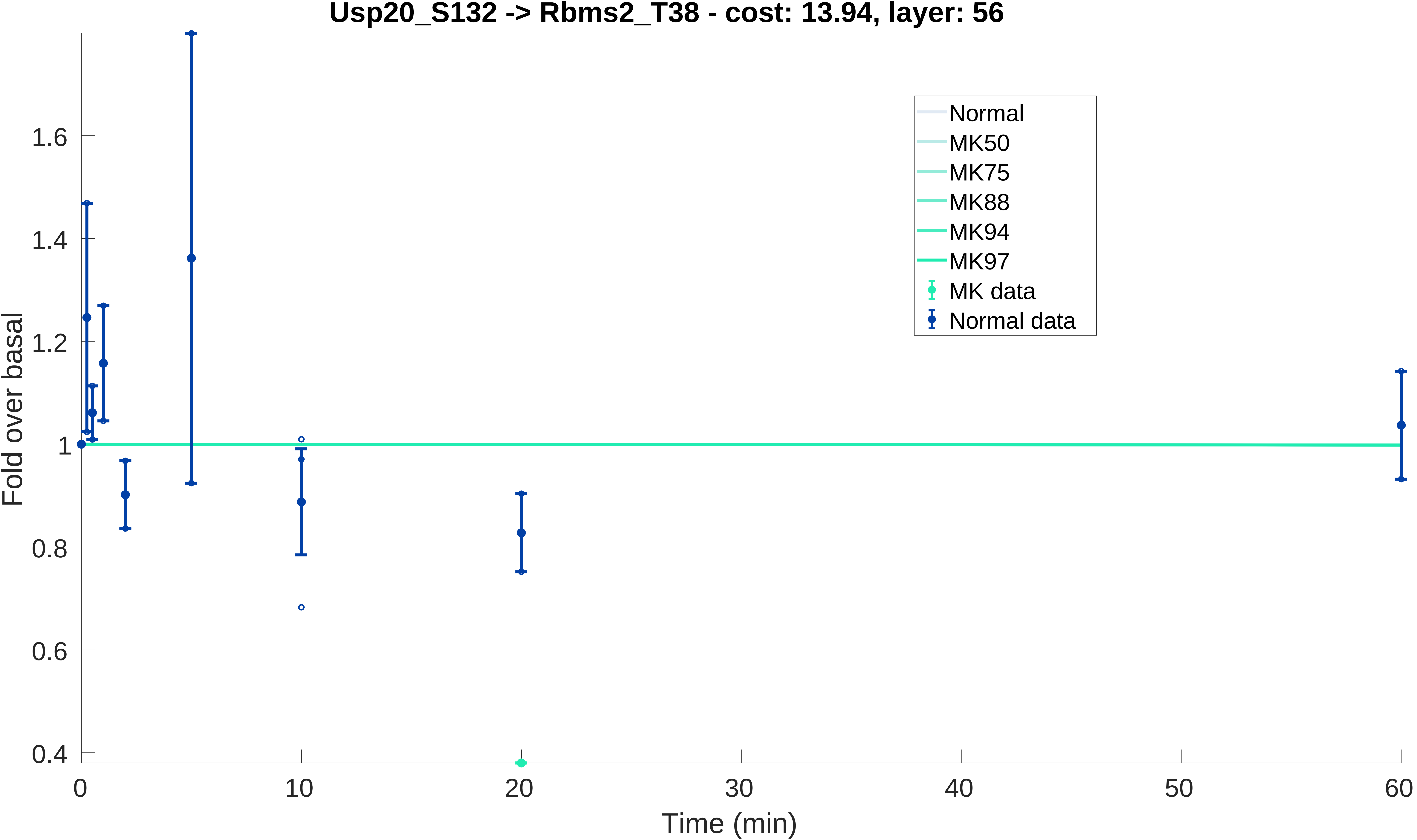

Usp20_S132 -> Rbms2_T38 - cost: 13.94, layer: 56
Fold over basal
Time (min)
0.4
0.6
0.8
1
1.2
1.4
1.6
0
10
20
30
40
50
60
Normal
MK50
MK75
MK88
MK94
MK97
MK data
Normal data

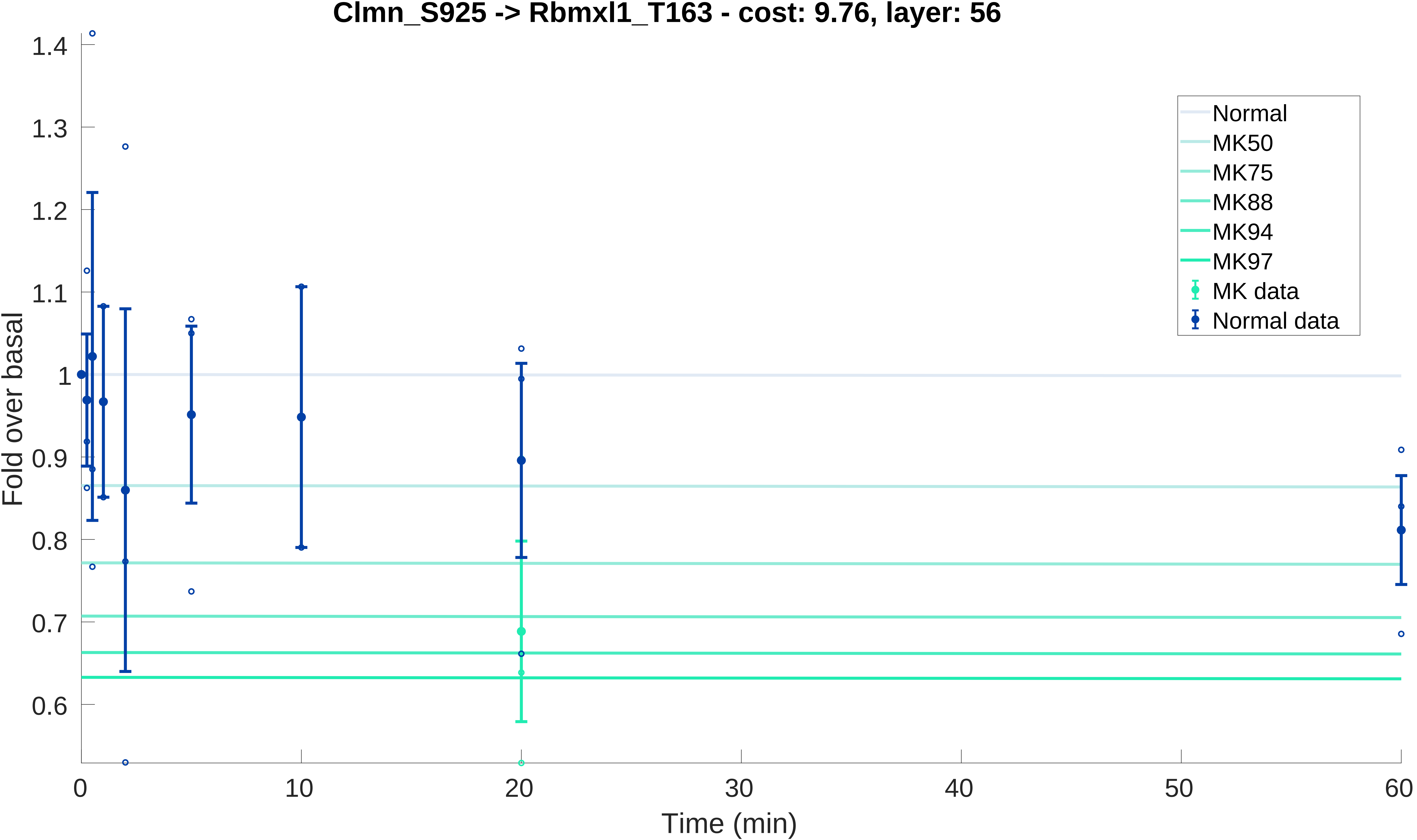

Clmn_S925 -> Rbmxl1_T163 - cost: 9.76, layer: 56
Fold over basal
Time (min)
1.4
1.3
1.2
1.1
1
0.9
0.8
0.7
0.6
0
10
20
30
40
50
60
Normal
MK50
MK75
MK88
MK94
MK97
MK data
Normal data

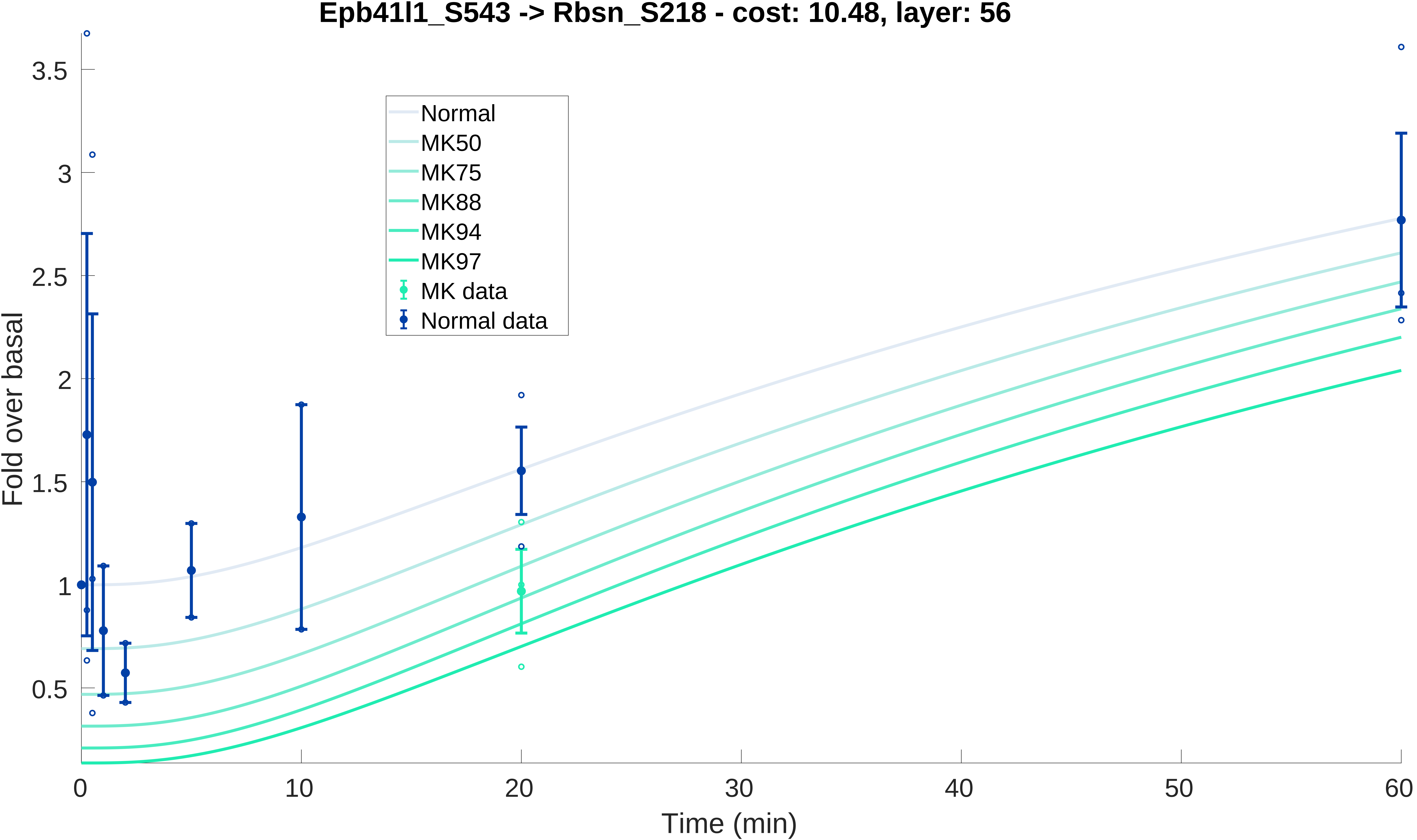

Epb41l1_S543 -> Rbsn_S218 - cost: 10.48, layer: 56
Normal
MK50
MK75
MK88
MK94
MK97
MK data
Normal data
Fold over basal
0.5
1
1.5
2
2.5
3
3.5
0
10
20
30
40
50
60
Time (min)

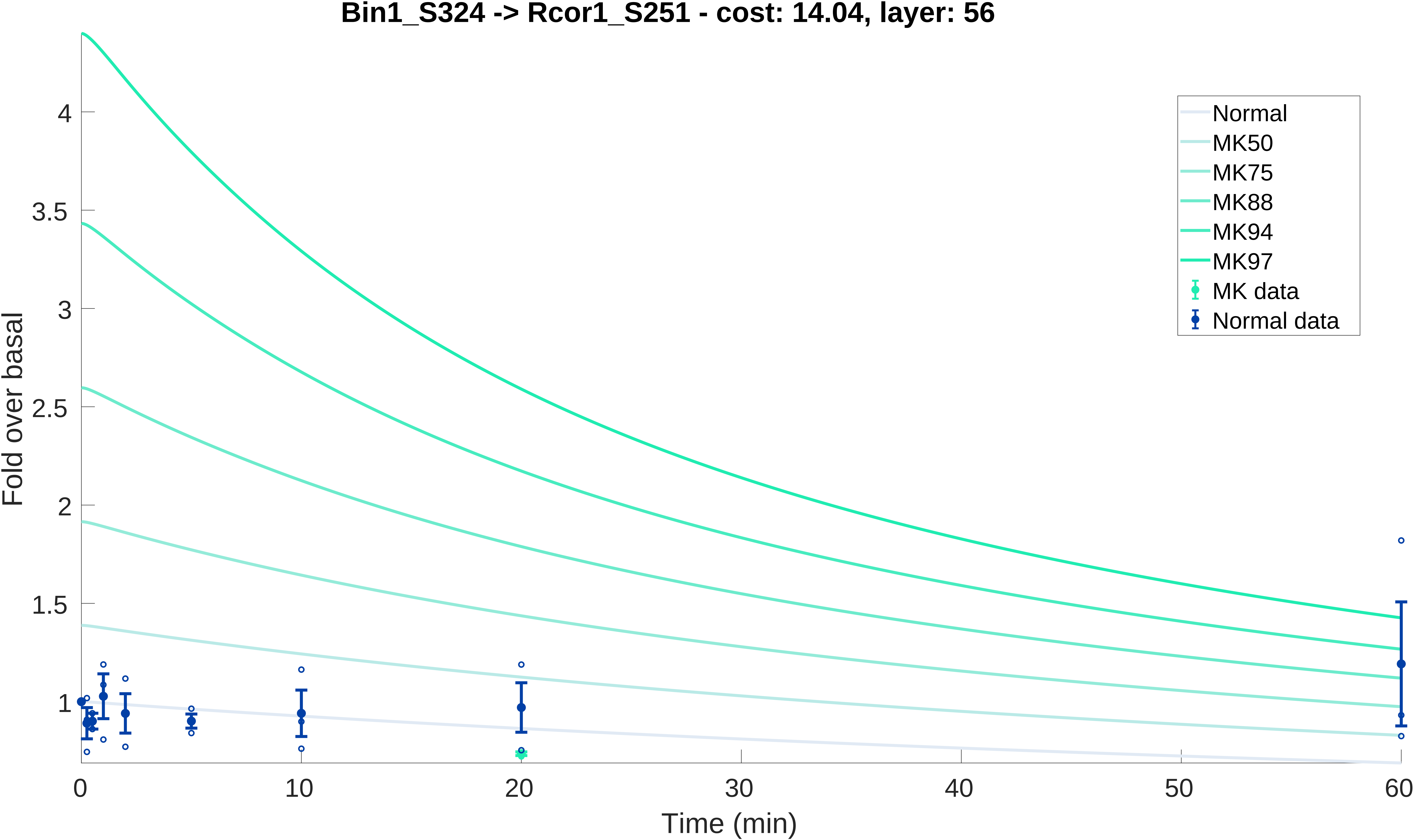

Bin1_S324 -> Rcor1_S251 - cost: 14.04, layer: 56
Fold over basal
Time (min)
0
10
20
30
40
50
60
1
1.5
2
2.5
3
3.5
4
Normal
MK50
MK75
MK88
MK94
MK97
MK data
Normal data

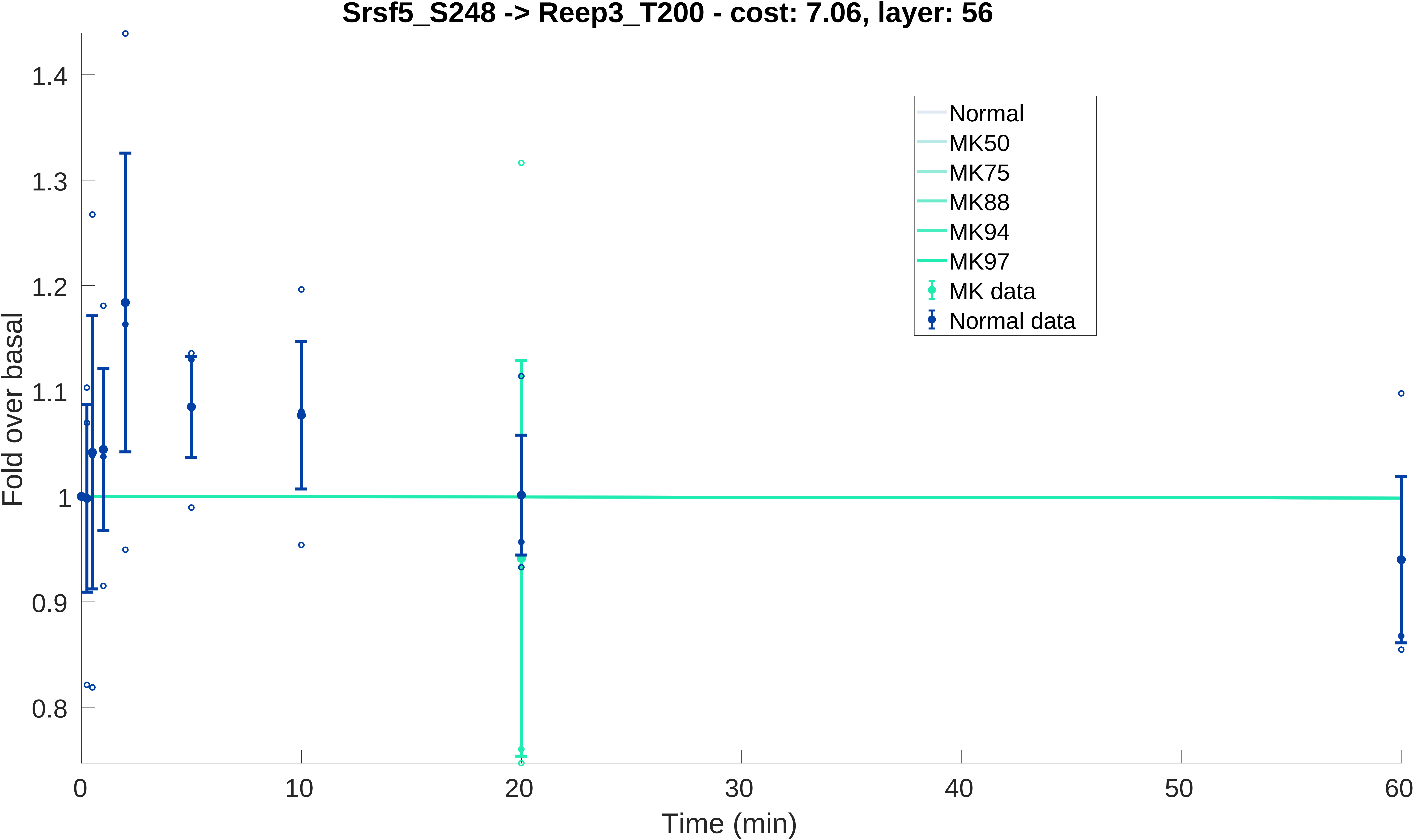
Srsf5_S248 -> Reep3_T200 - cost: 7.06, layer: 56
Normal
MK50
MK75
MK88
MK94
MK97
MK data
Normal data
Fold over basal
Time (min)
0
10
20
30
40
50
60
0.8
0.9
1
1.1
1.2
1.3
1.4

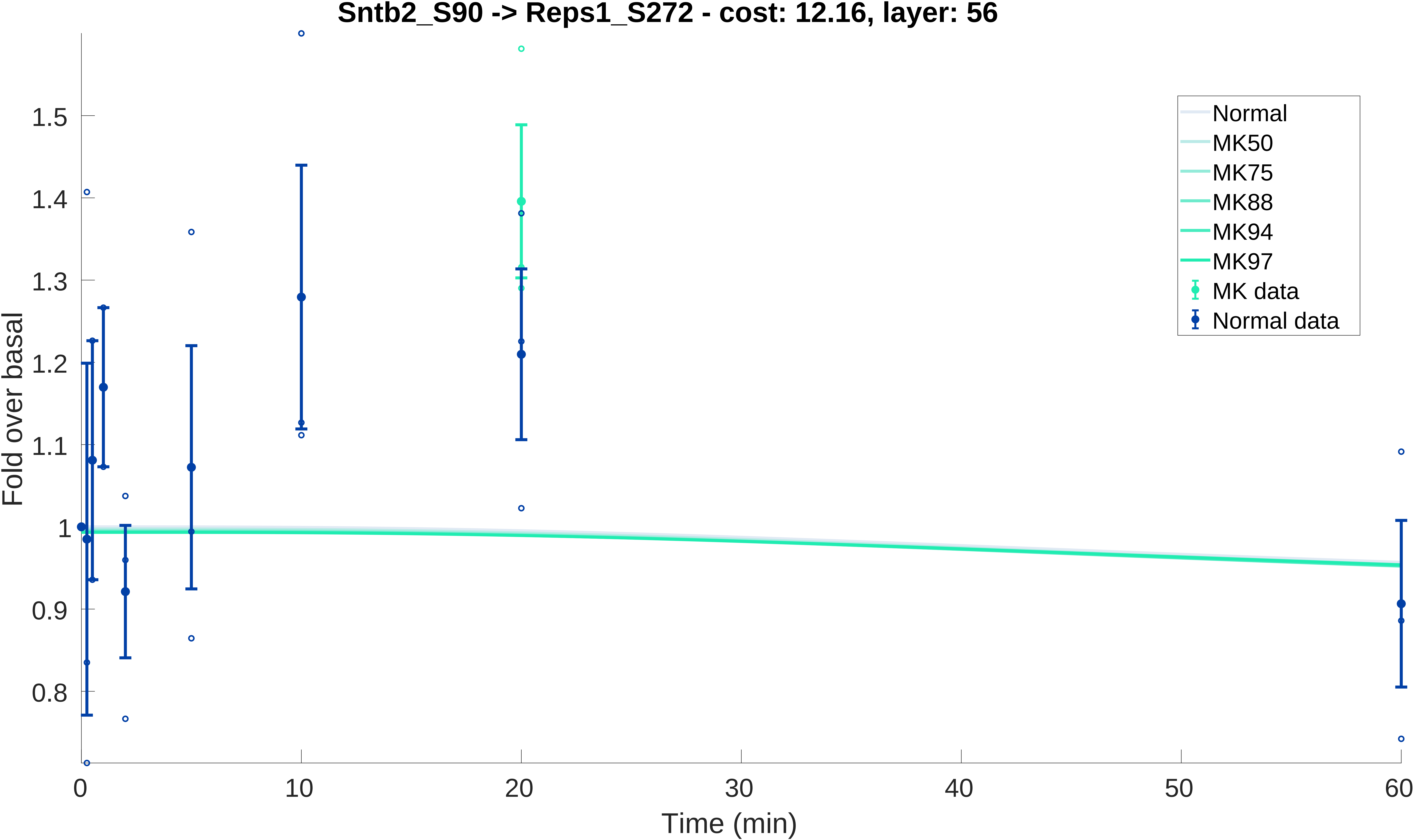

Sntb2_S90 -> Reps1_S272 - cost: 12.16, layer: 56
Fold over basal
Time (min)
1.5
1.4
1.3
1.2
1.1
1
0.9
0.8
0
10
20
30
40
50
60
Normal
MK50
MK75
MK88
MK94
MK97
MK data
Normal data

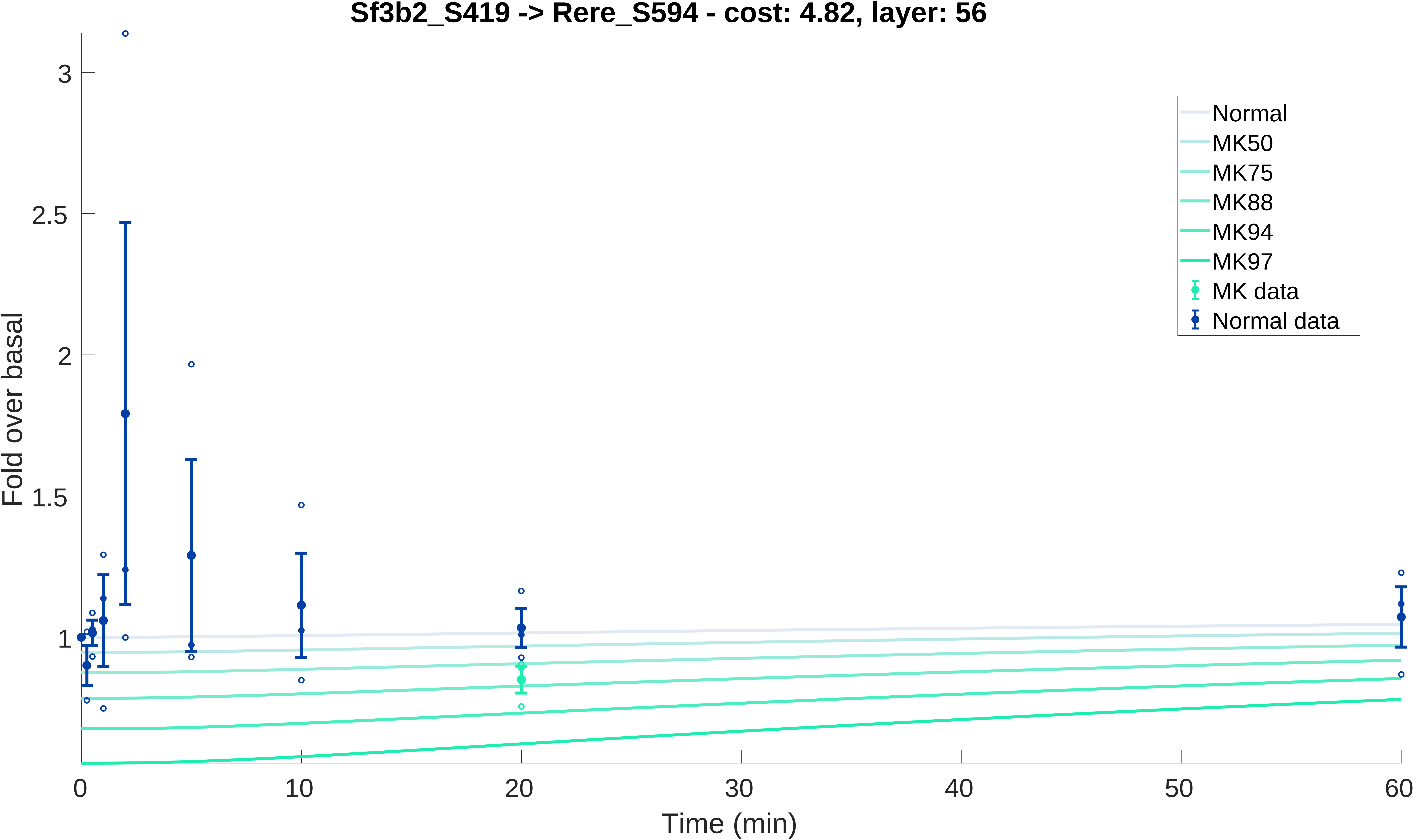

Sf3b2_S419 -> Rere_S594 - cost: 4.82, layer: 56
Fold over basal
Time (min)
0
10
20
30
40
50
60
1
1.5
2
2.5
3
Normal
MK50
MK75
MK88
MK94
MK97
MK data
Normal data

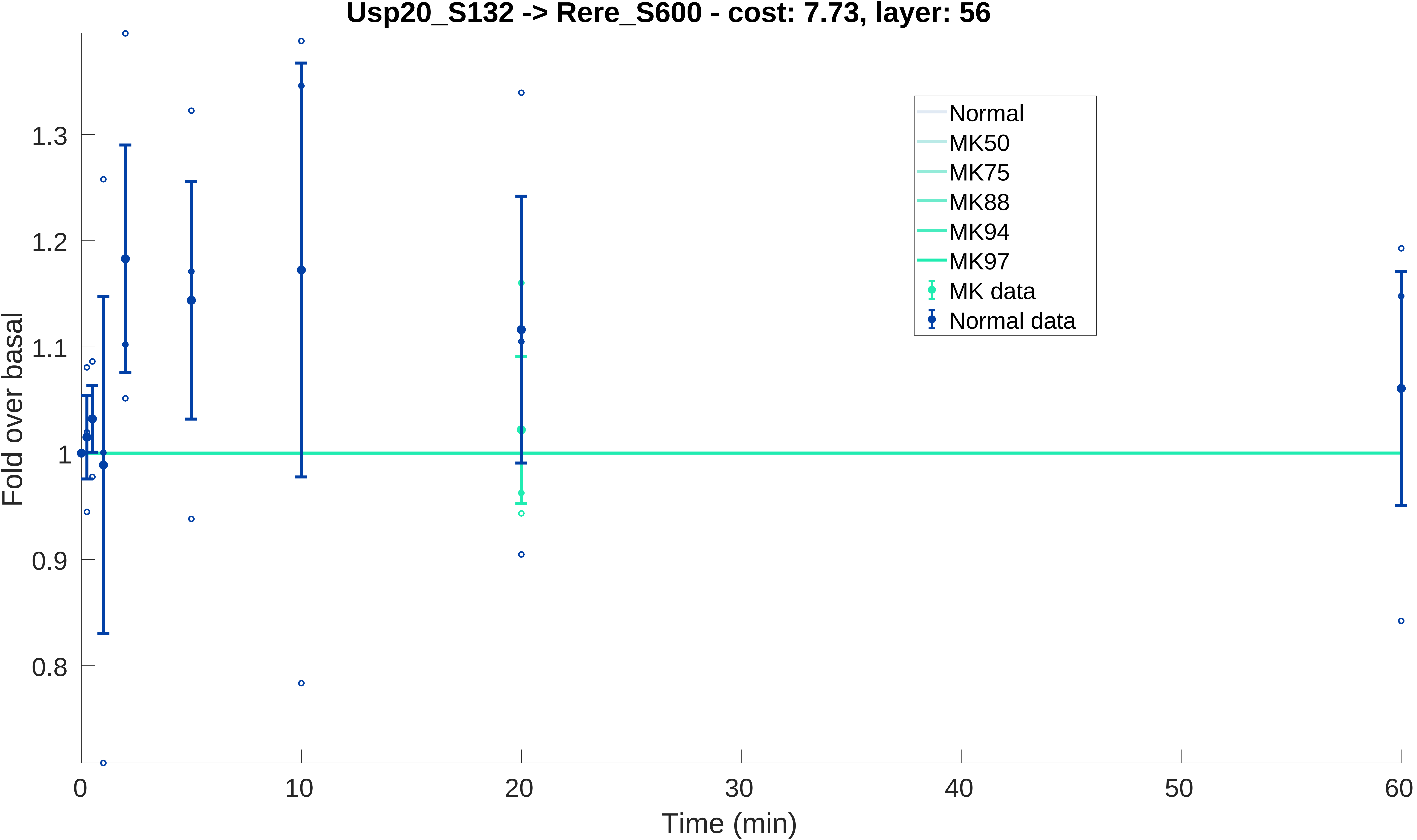
Usp20_S132 -> Rere_S600 - cost: 7.73, layer: 56
Fold over basal
Time (min)
0.8
0.9
1
1.1
1.2
1.3
0
10
20
30
40
50
60
Normal
MK50
MK75
MK88
MK94
MK97
MK data
Normal data

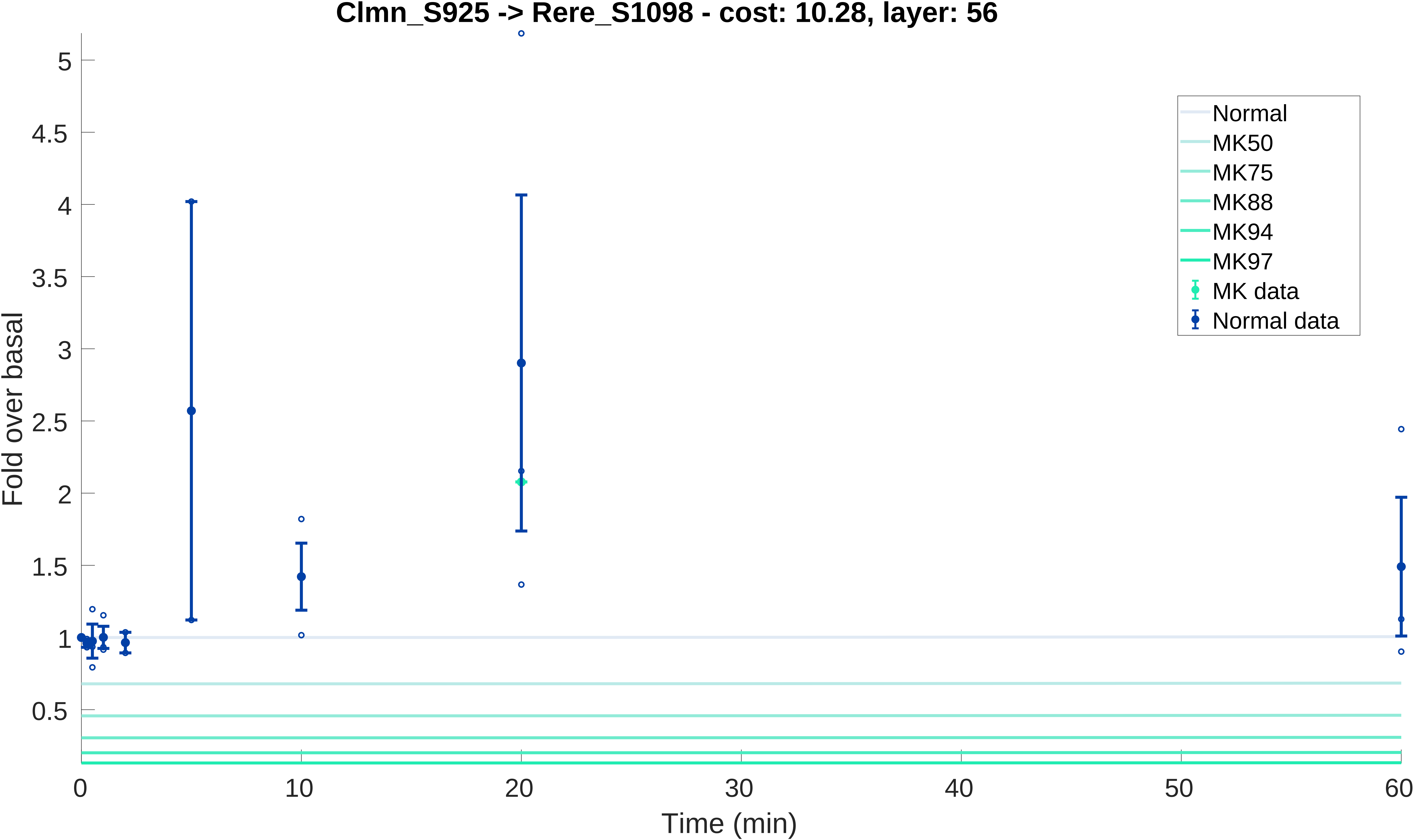
Clmn_S925 -> Rere_S1098 - cost: 10.28, layer: 56
Fold over basal
Time (min)
0
10
20
30
40
50
60
0.5
1
1.5
2
2.5
3
3.5
4
4.5
5
Normal
MK50
MK75
MK88
MK94
MK97
MK data
Normal data

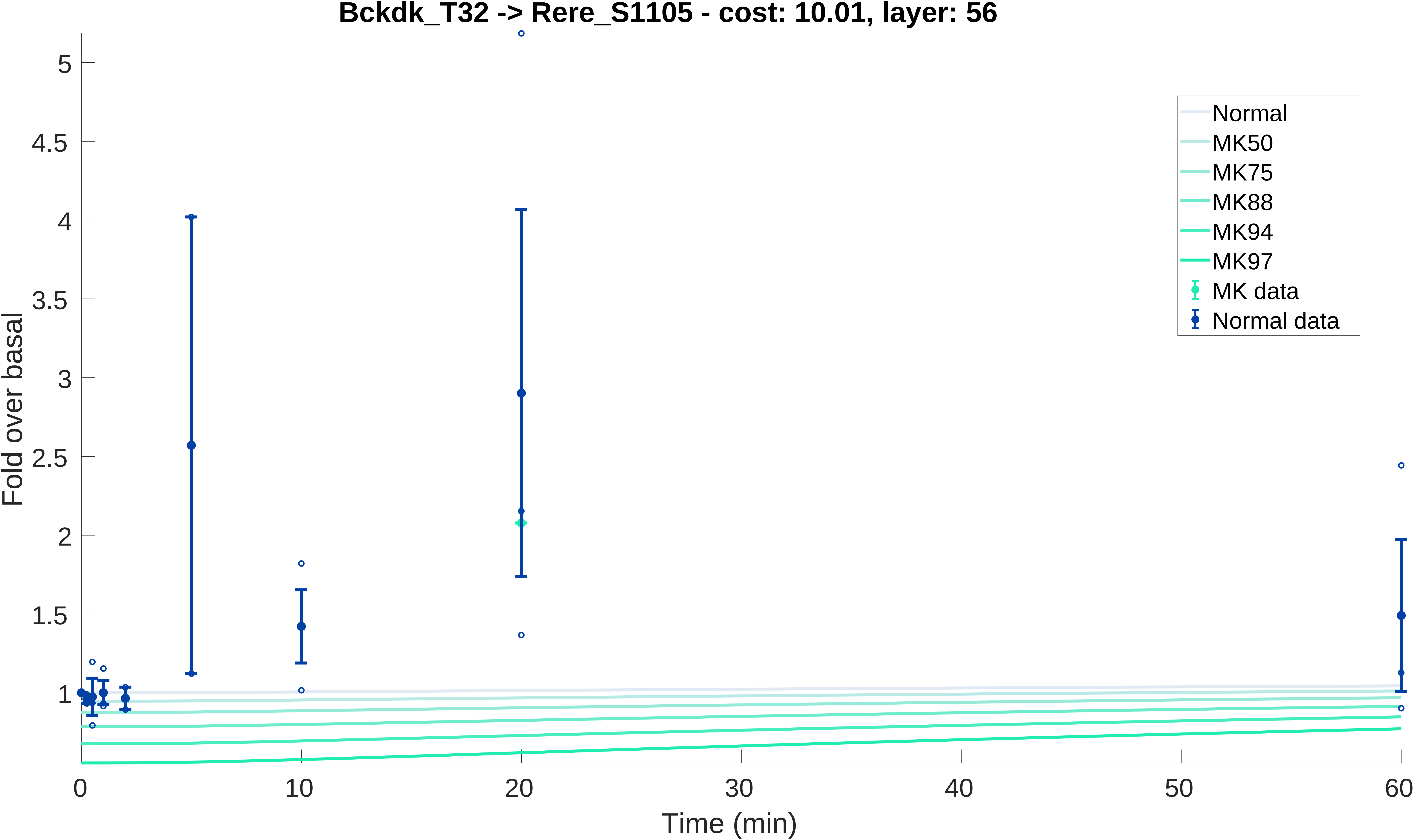

Bckdk_T32 -> Rere_S1105 - cost: 10.01, layer: 56
Fold over basal
5
4.5
4
3.5
3
2.5
2
1.5
1
0
10
20
30
40
50
60
Time (min)
Normal
MK50
MK75
MK88
MK94
MK97
MK data
Normal data

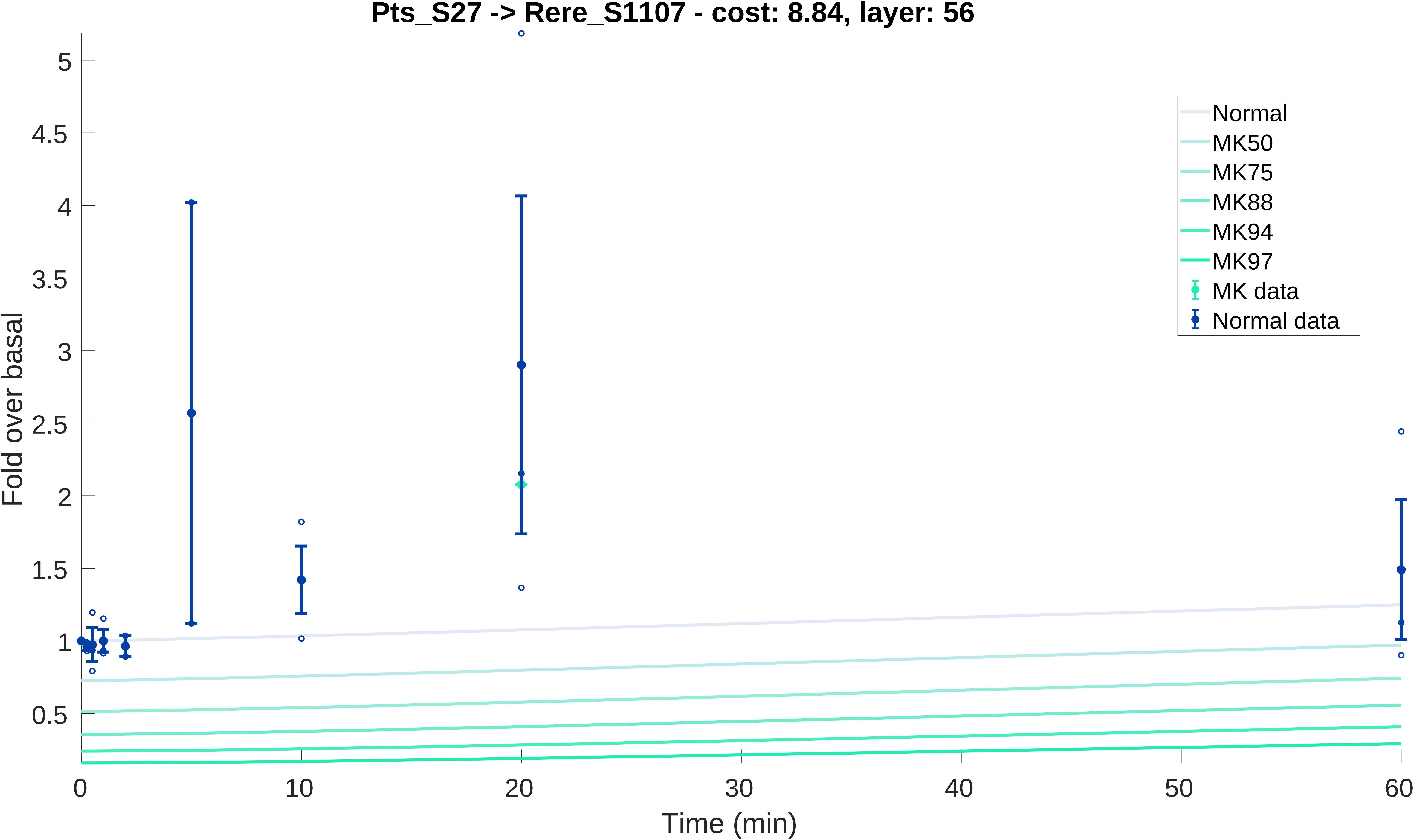

Pts_S27 -> Rere_S1107 - cost: 8.84, layer: 56
Fold over basal
Time (min)
0.5
1
1.5
2
2.5
3
3.5
4
4.5
5
0
10
20
30
40
50
60
Normal
MK50
MK75
MK88
MK94
MK97
MK data
Normal data

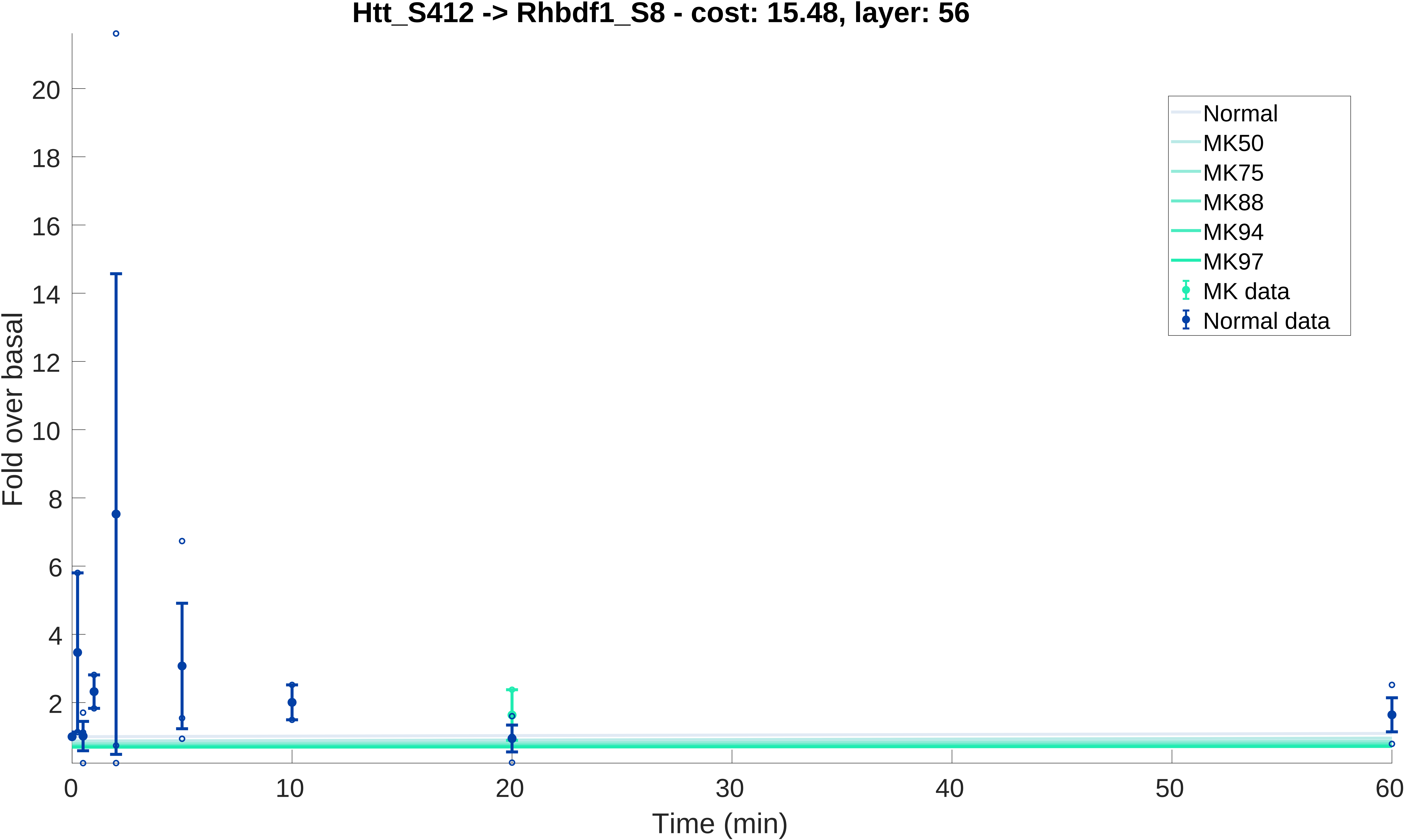

Htt_S412 -> Rhbdf1_S8 - cost: 15.48, layer: 56
Fold over basal
Time (min)
0
10
20
30
40
50
60
2
4
6
8
10
12
14
16
18
20
Normal
MK50
MK75
MK88
MK94
MK97
MK data
Normal data

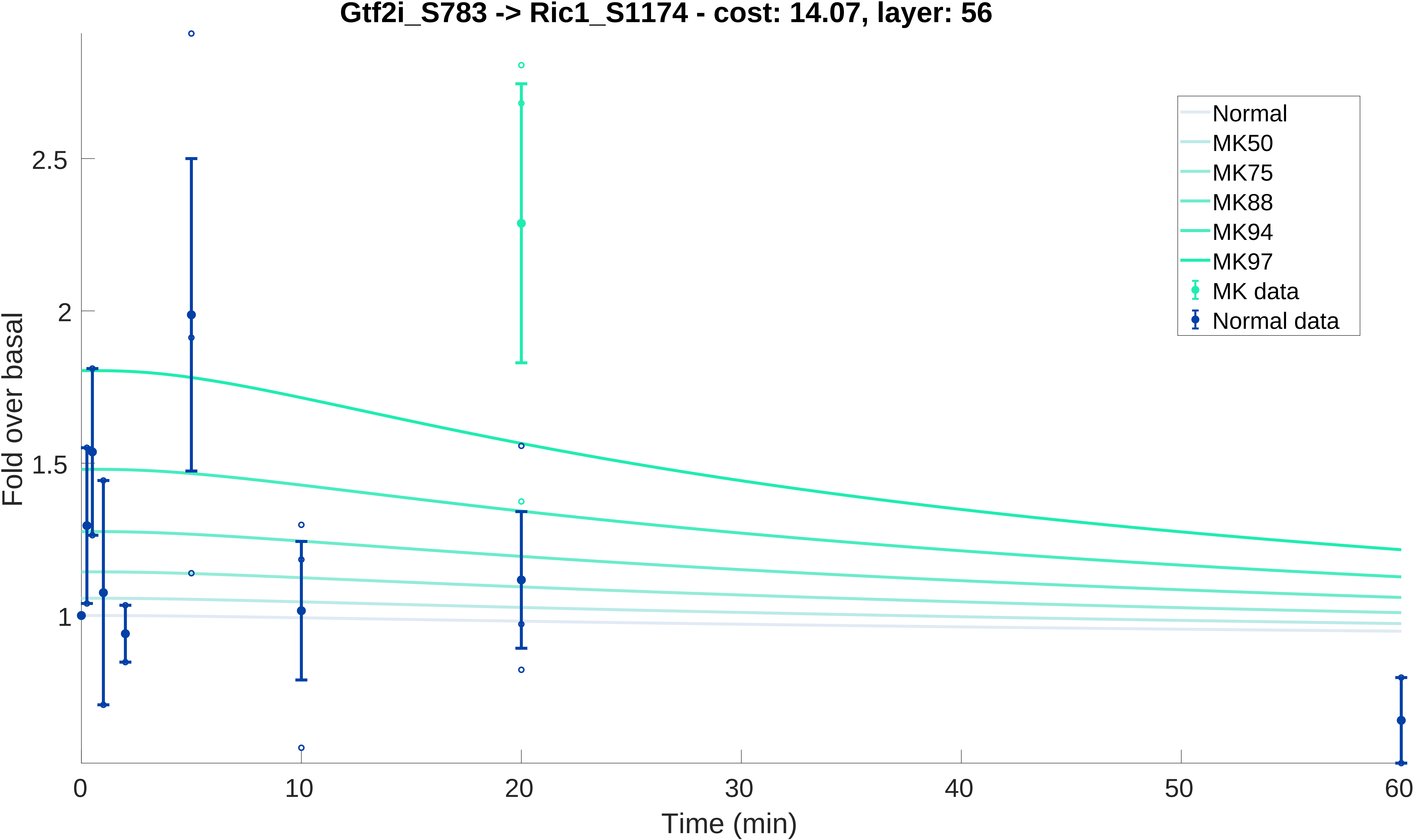

Gtf2i_S783 -> Ric1_S1174 - cost: 14.07, layer: 56
Fold over basal
Time (min)
2.5
2
1.5
1
0
10
20
30
40
50
60
Normal
MK50
MK75
MK88
MK94
MK97
MK data
Normal data

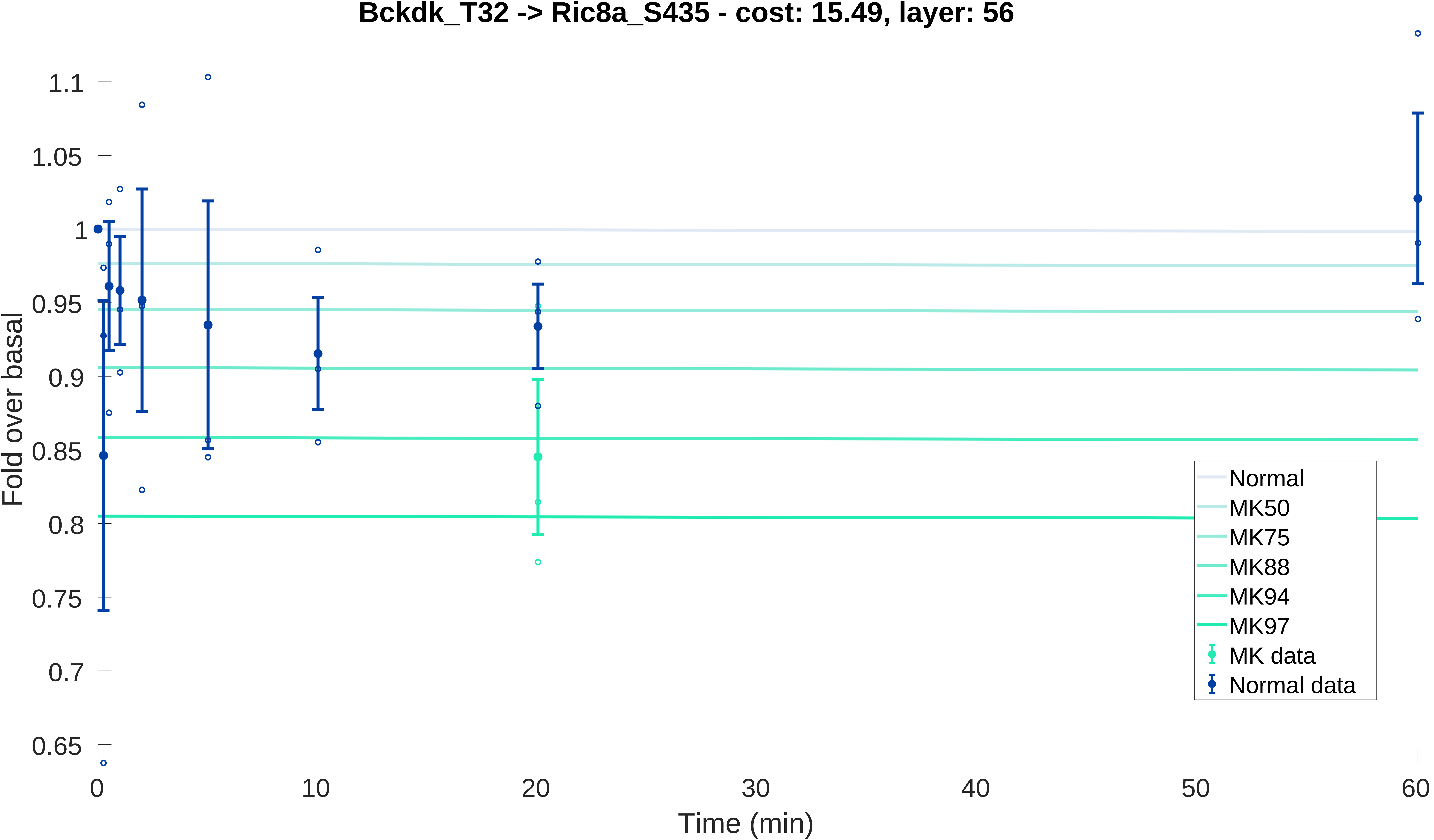

Bckdk_T32 -> Ric8a_S435 - cost: 15.49, layer: 56
Fold over basal
Time (min)
1.1
1.05
1
0.95
0.9
0.85
0.8
0.75
0.7
0.65
0
10
20
30
40
50
60
Normal
MK50
MK75
MK88
MK94
MK97
MK data
Normal data

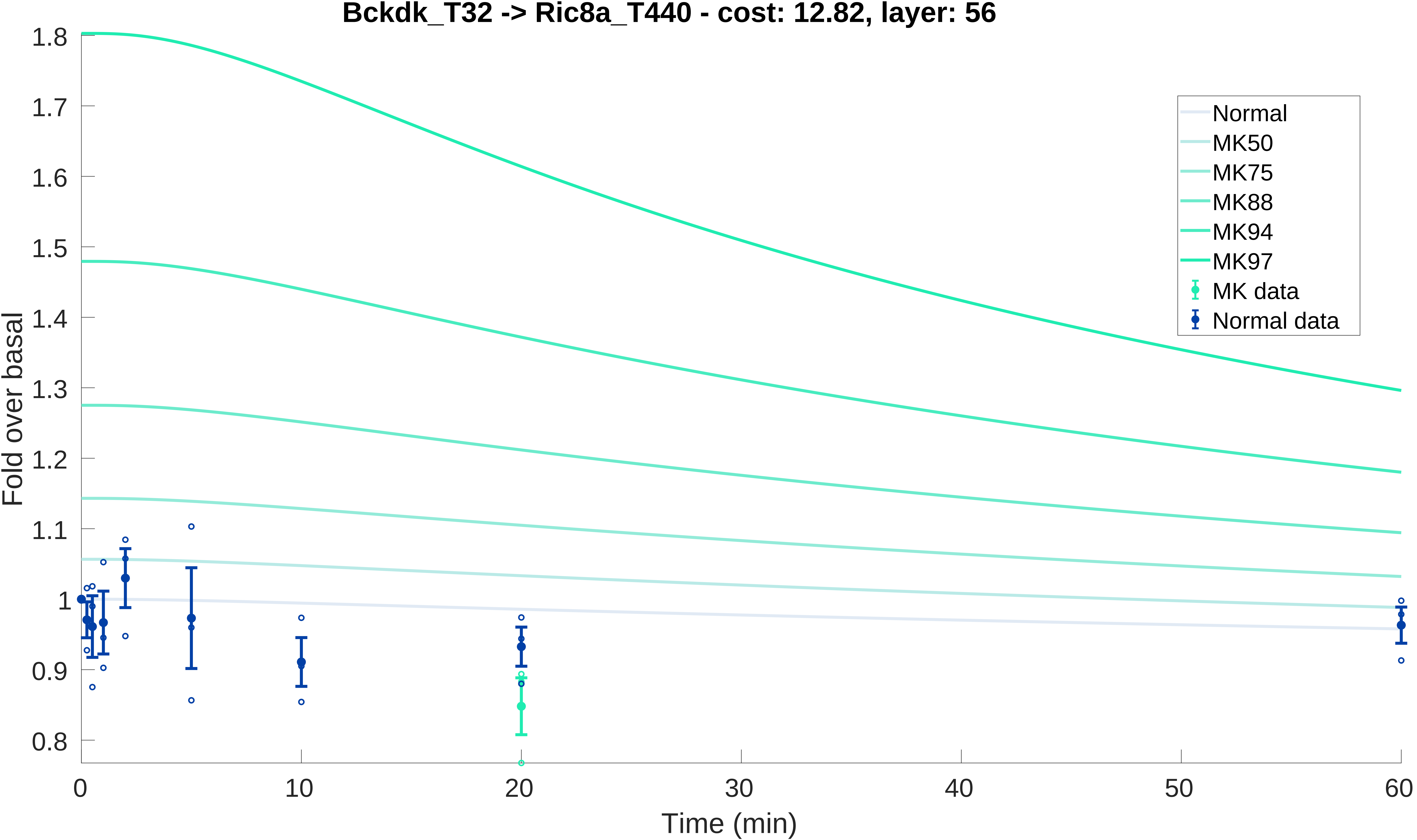

Bckdk_T32 -> Ric8a_T440 - cost: 12.82, layer: 56
Fold over basal
1.8
1.7
1.6
1.5
1.4
1.3
1.2
1.1
1
0.9
0.8
0
10
20
30
40
50
60
Time (min)
Normal
MK50
MK75
MK88
MK94
MK97
MK data
Normal data

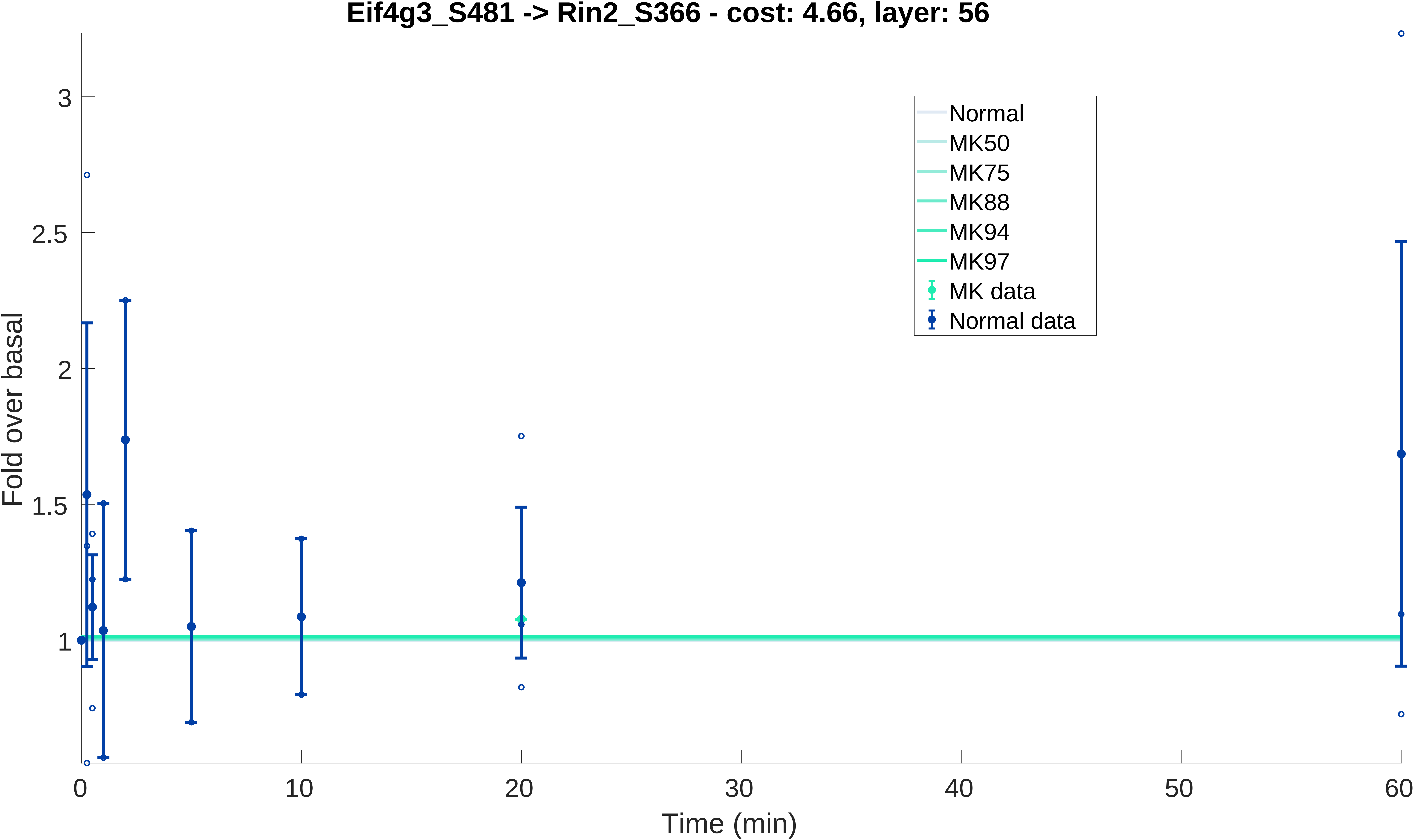
Eif4g3_S481 -> Rin2_S366 - cost: 4.66, layer: 56
Fold over basal
Time (min)
0
10
20
30
40
50
60
1
1.5
2
2.5
3
Normal
MK50
MK75
MK88
MK94
MK97
MK data
Normal data

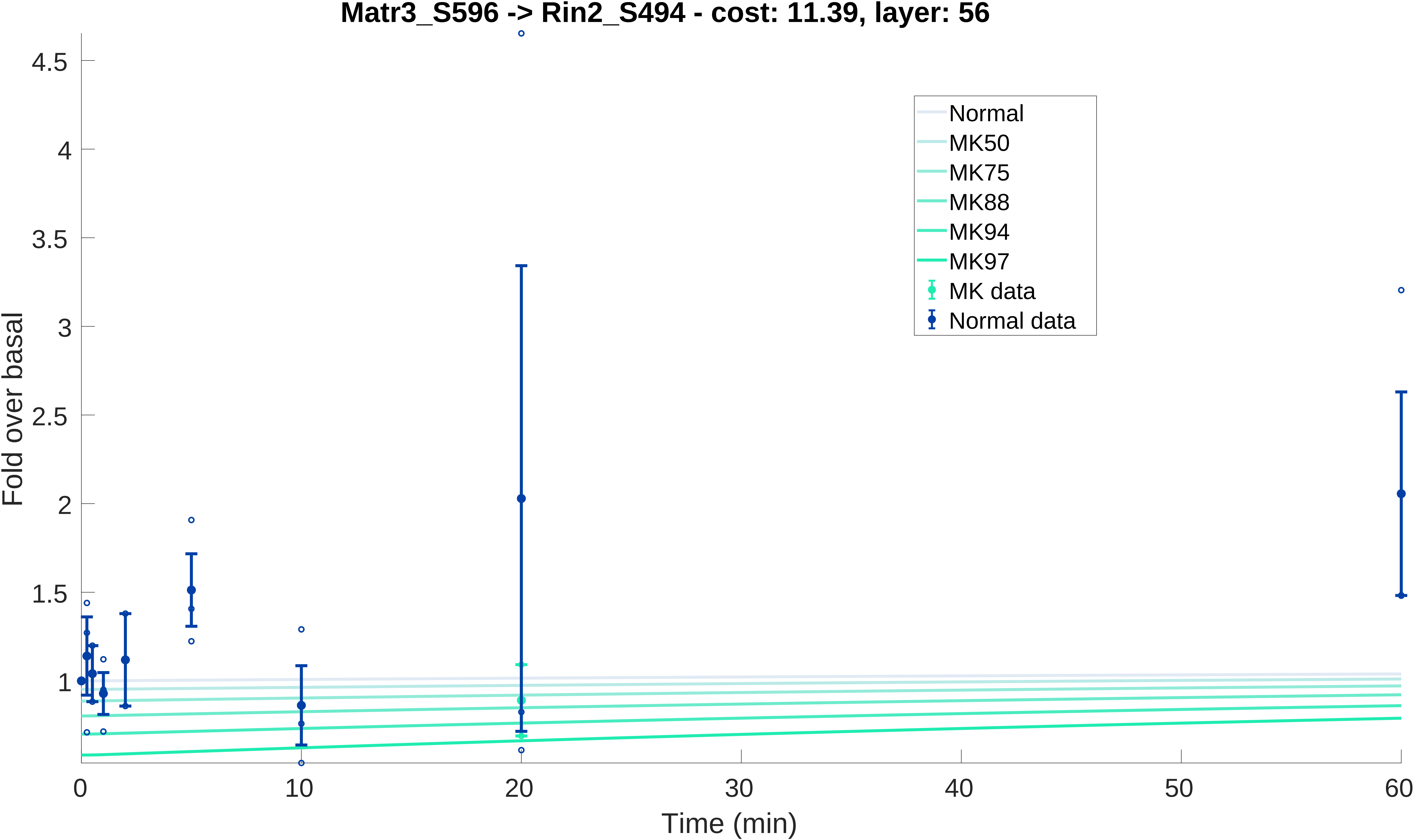

Matr3_S596 -> Rin2_S494 - cost: 11.39, layer: 56
Fold over basal
Time (min)
0
10
20
30
40
50
60
1
1.5
2
2.5
3
3.5
4
4.5
Normal
MK50
MK75
MK88
MK94
MK97
MK data
Normal data

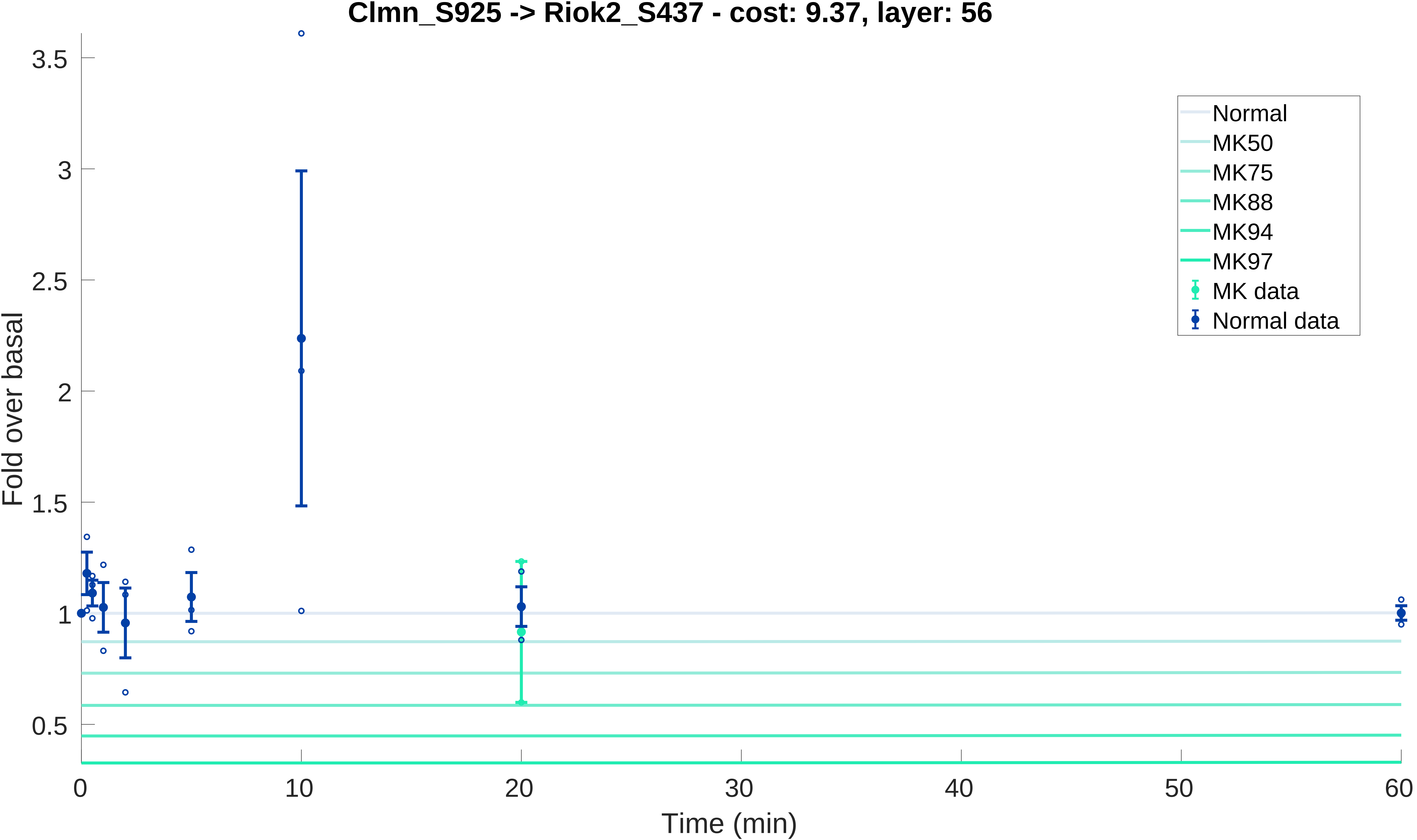
Clmn_S925 -> Riok2_S437 - cost: 9.37, layer: 56
Fold over basal
Time (min)
0.5
1
1.5
2
2.5
3
3.5
0
10
20
30
40
50
60
Normal
MK50
MK75
MK88
MK94
MK97
MK data
Normal data

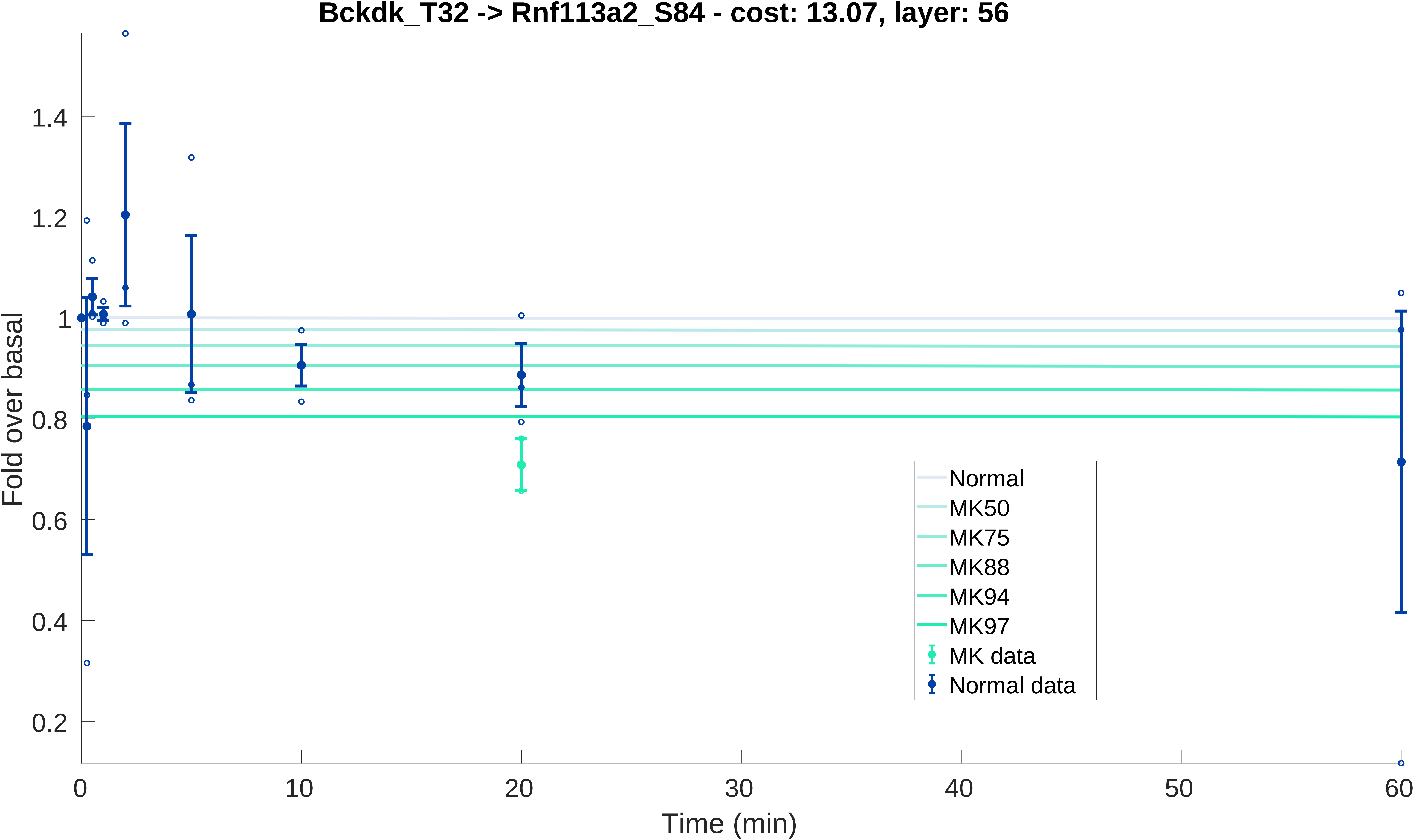

Bckdk_T32 -> Rnf113a2_S84 - cost: 13.07, layer: 56
Fold over basal
Time (min)
0.2
0.4
0.6
0.8
1
1.2
1.4
0
10
20
30
40
50
60
Normal
MK50
MK75
MK88
MK94
MK97
MK data
Normal data

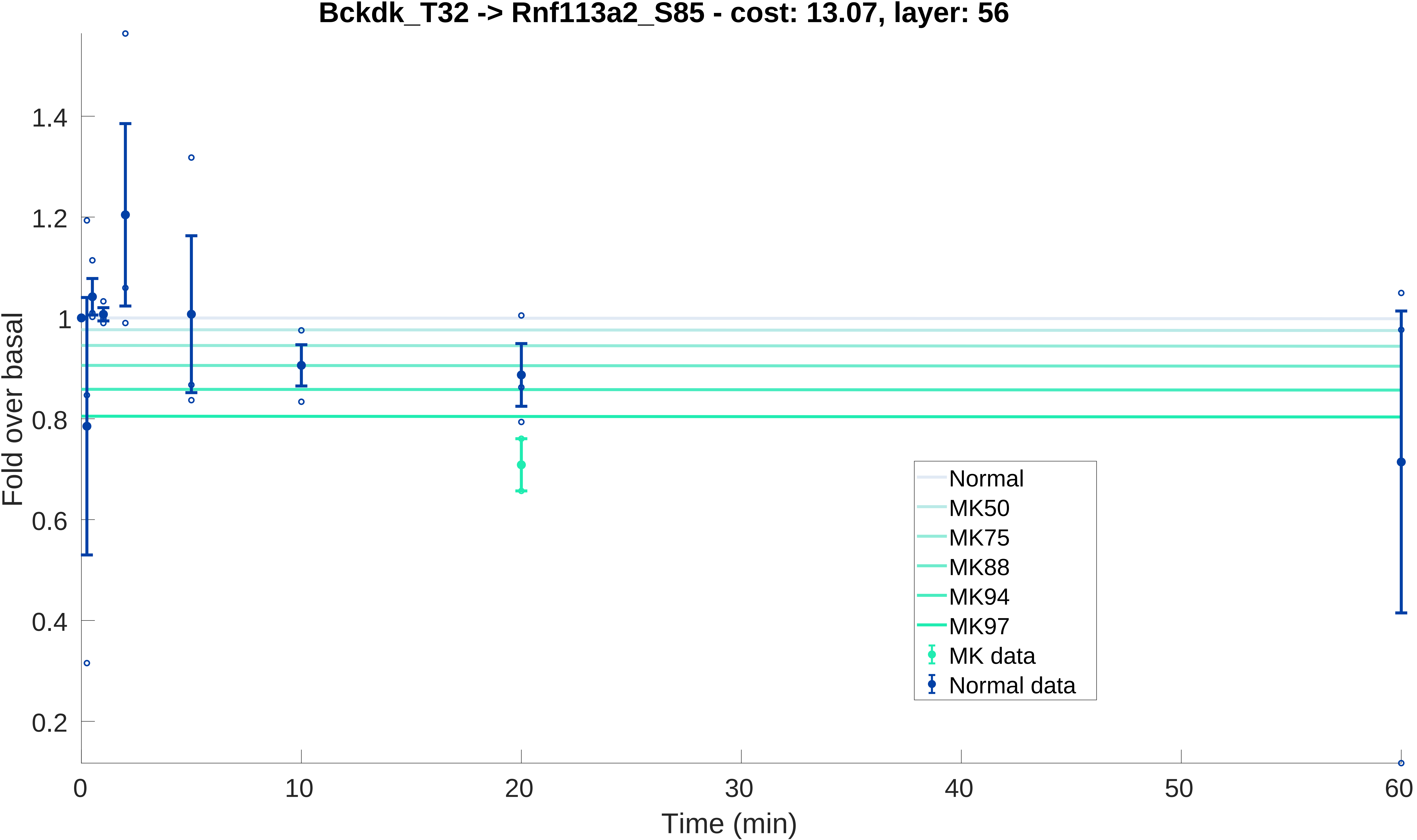

Bckdk_T32 -> Rnf113a2_S85 - cost: 13.07, layer: 56
Fold over basal
Time (min)
0
10
20
30
40
50
60
0.2
0.4
0.6
0.8
1
1.2
1.4
Normal
MK50
MK75
MK88
MK94
MK97
MK data
Normal data

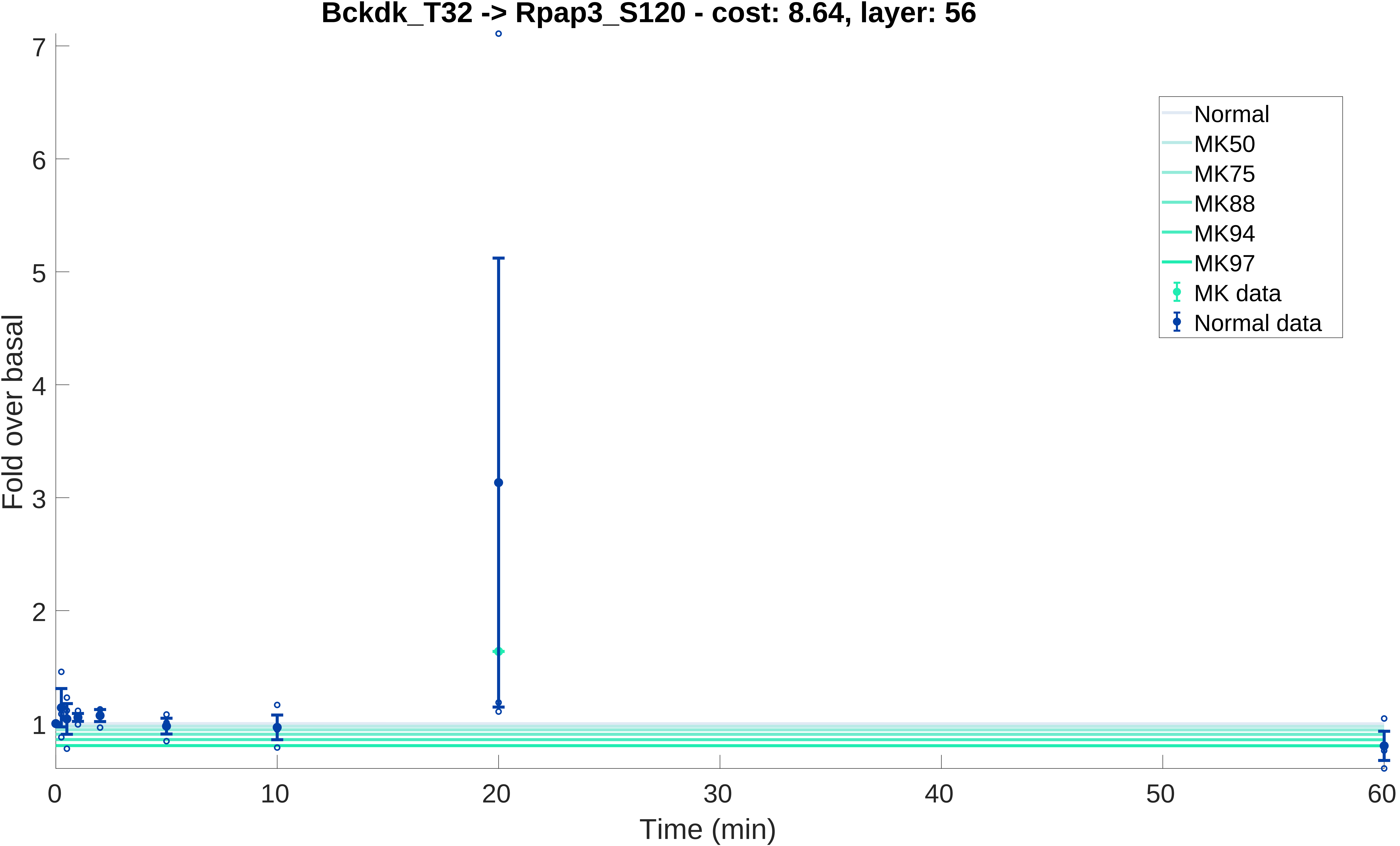

Bckdk_T32 -> Rpap3_S120 - cost: 8.64, layer: 56
Fold over basal
Time (min)
1
2
3
4
5
6
7
0
10
20
30
40
50
60
Normal
MK50
MK75
MK88
MK94
MK97
MK data
Normal data

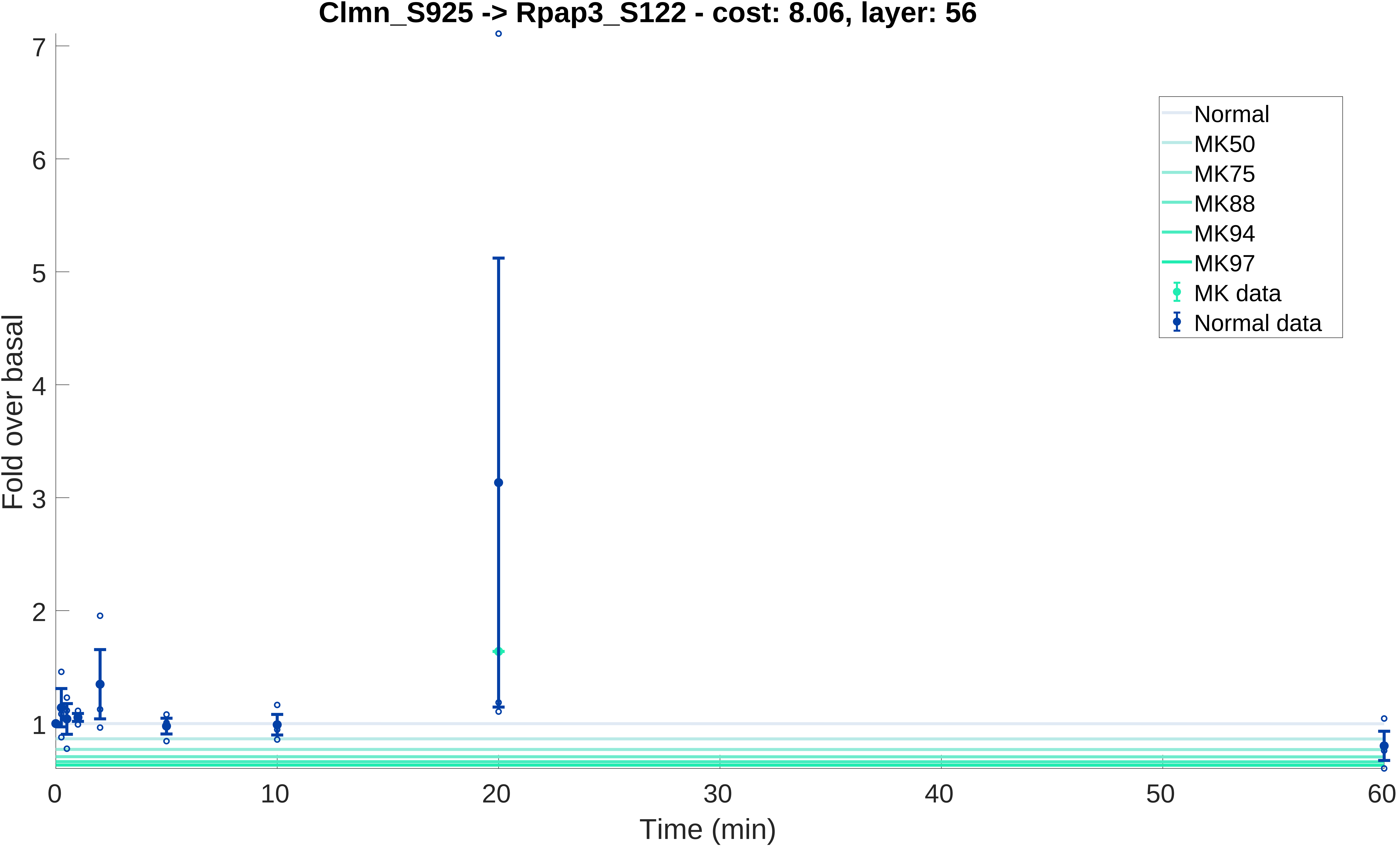

Clmn_S925 -> Rpap3_S122 - cost: 8.06, layer: 56
Fold over basal
Time (min)
Normal
MK50
MK75
MK88
MK94
MK97
MK data
Normal data

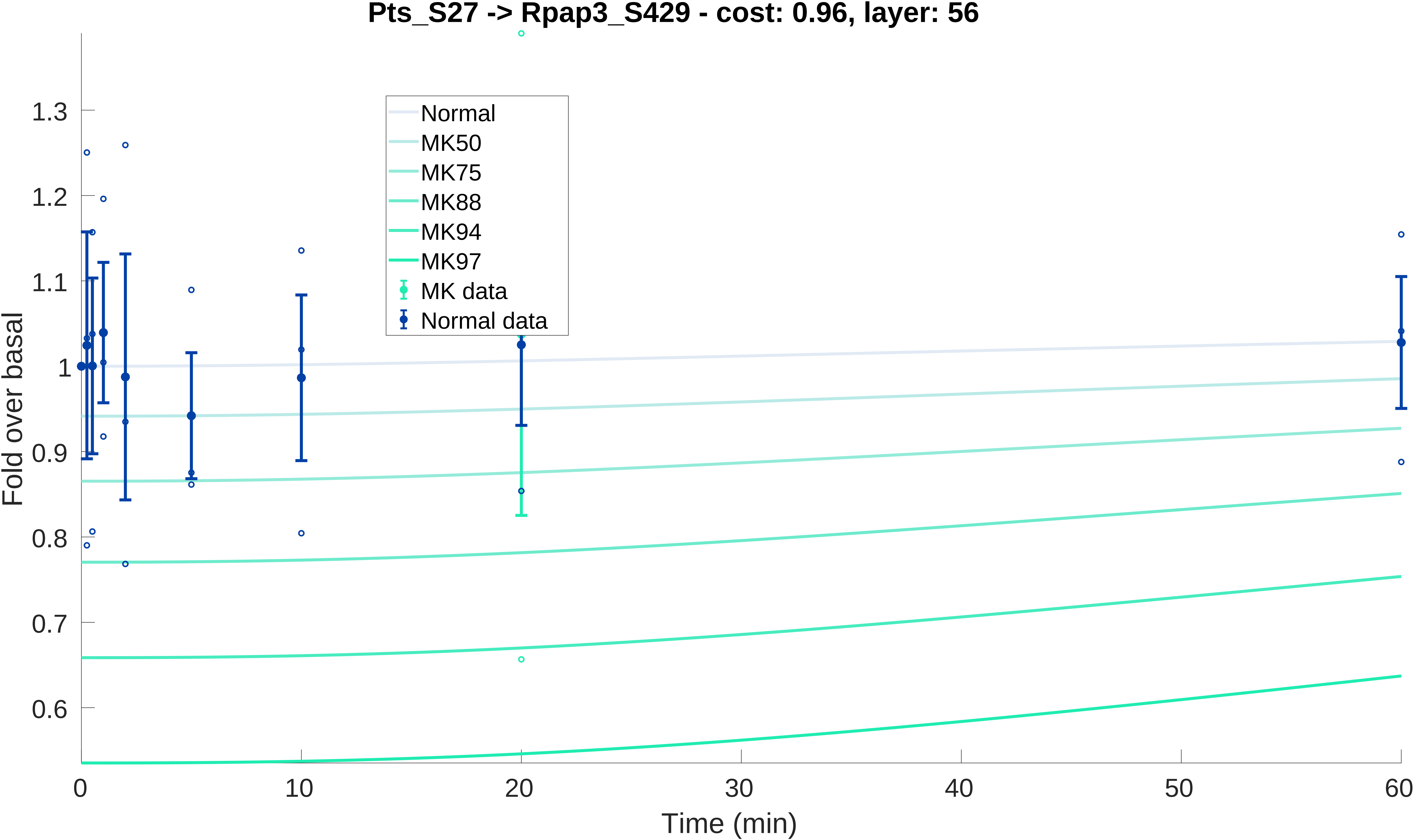

Pts_S27 -> Rpap3_S429 - cost: 0.96, layer: 56
Normal
MK50
MK75
MK88
MK94
MK97
MK data
Normal data
Fold over basal
Time (min)
1.3
1.2
1.1
1
0.9
0.8
0.7
0.6
0
10
20
30
40
50
60

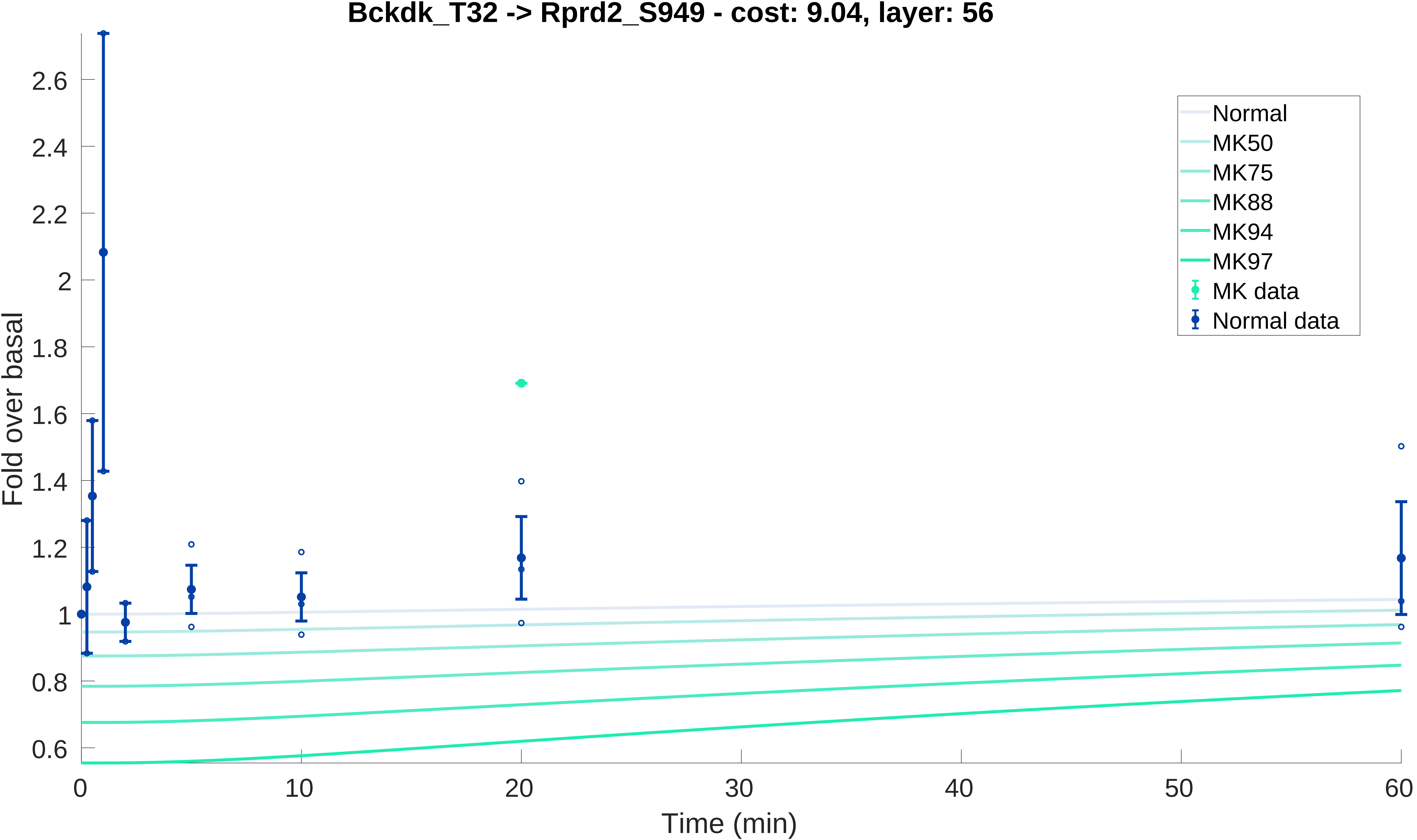
Bckdk_T32 -> Rprd2_S949 - cost: 9.04, layer: 56
Fold over basal
Time (min)
0.6
0.8
1
1.2
1.4
1.6
1.8
2
2.2
2.4
2.6
0
10
20
30
40
50
60
Normal
MK50
MK75
MK88
MK94
MK97
MK data
Normal data

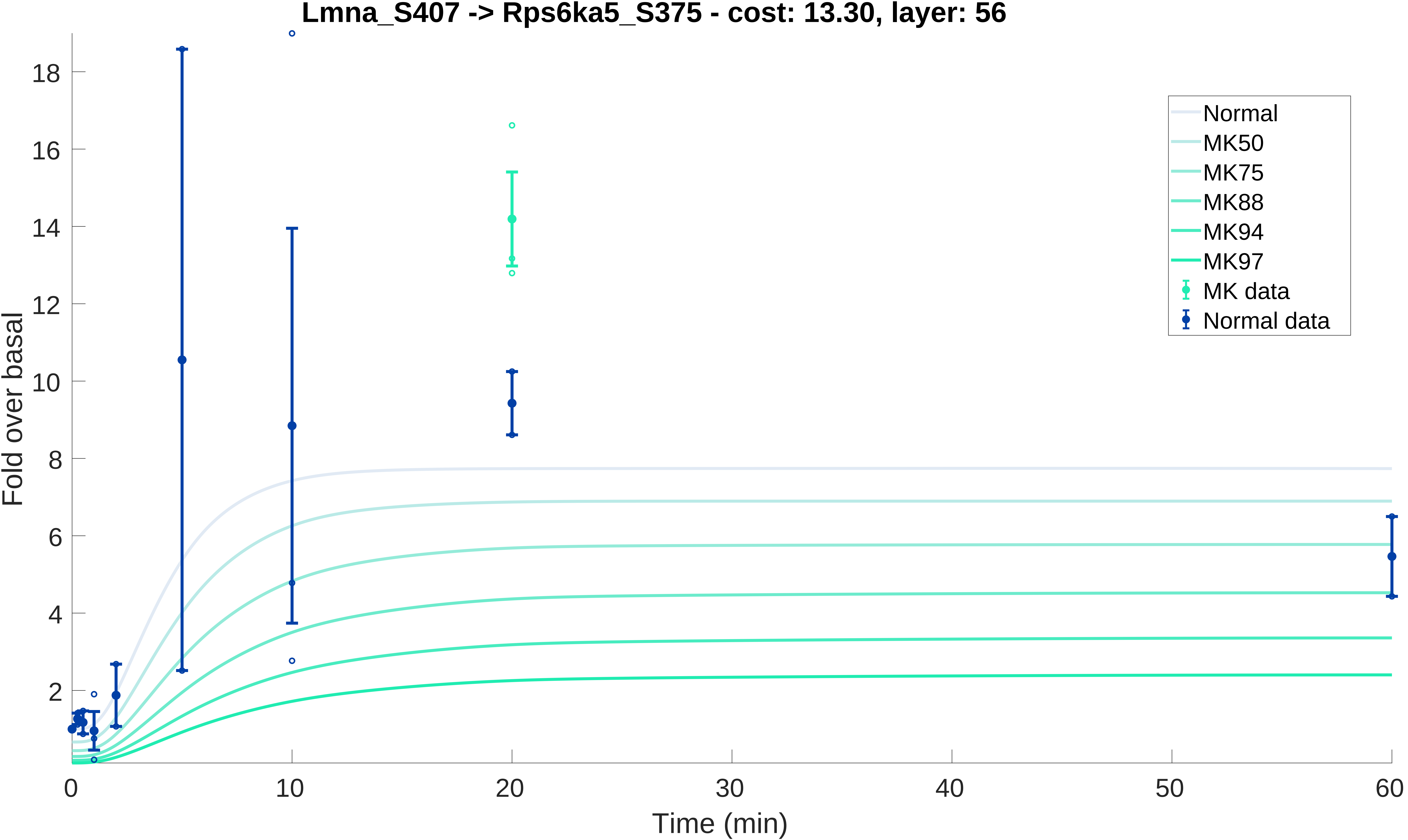

Lmna_S407 -> Rps6ka5_S375 - cost: 13.30, layer: 56
Fold over basal
Time (min)
Normal
MK50
MK75
MK88
MK94
MK97
MK data
Normal data

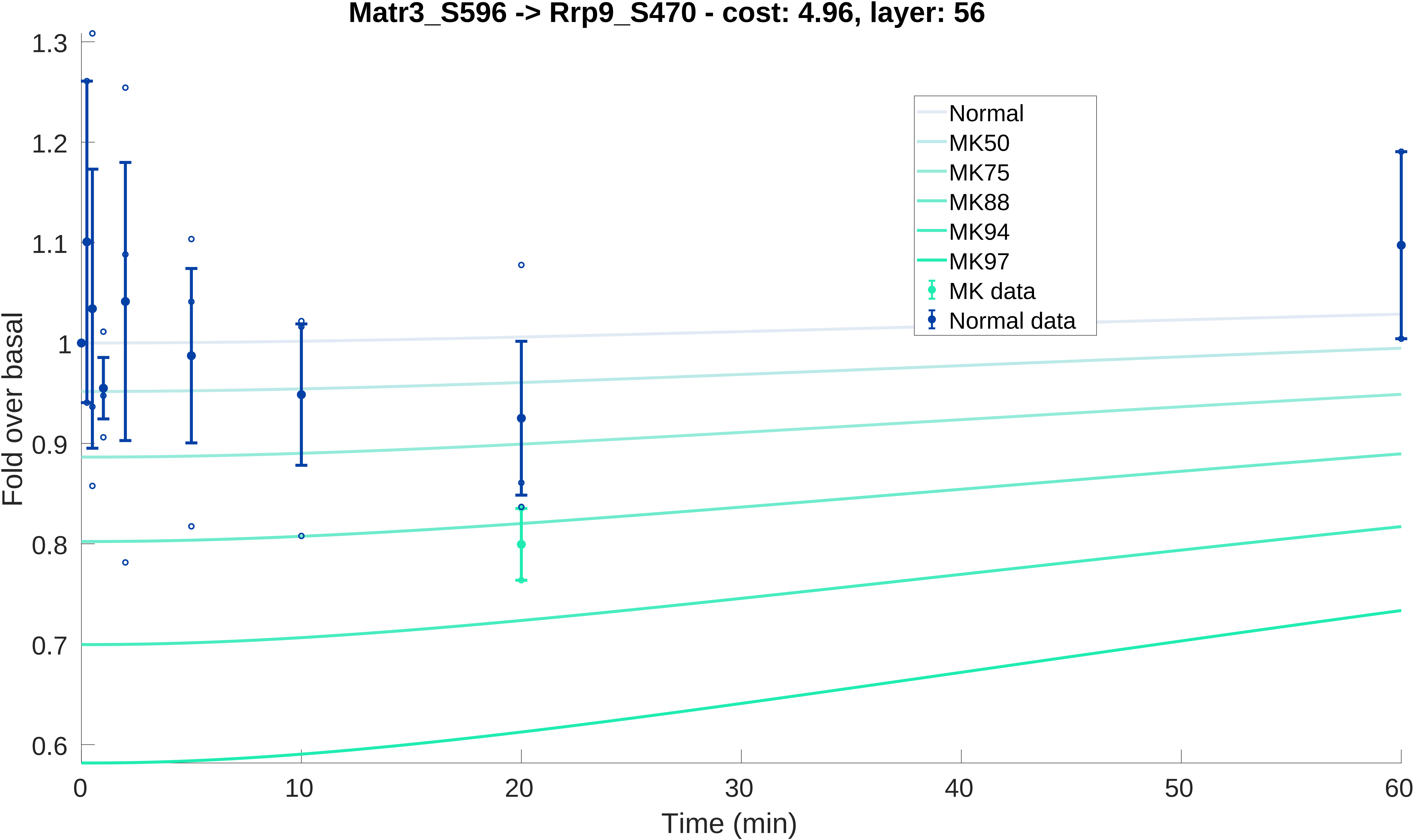

Matr3_S596 -> Rrp9_S470 - cost: 4.96, layer: 56
Fold over basal
Time (min)
Normal
MK50
MK75
MK88
MK94
MK97
MK data
Normal data
0
10
20
30
40
50
60
0.6
0.7
0.8
0.9
1
1.1
1.2
1.3

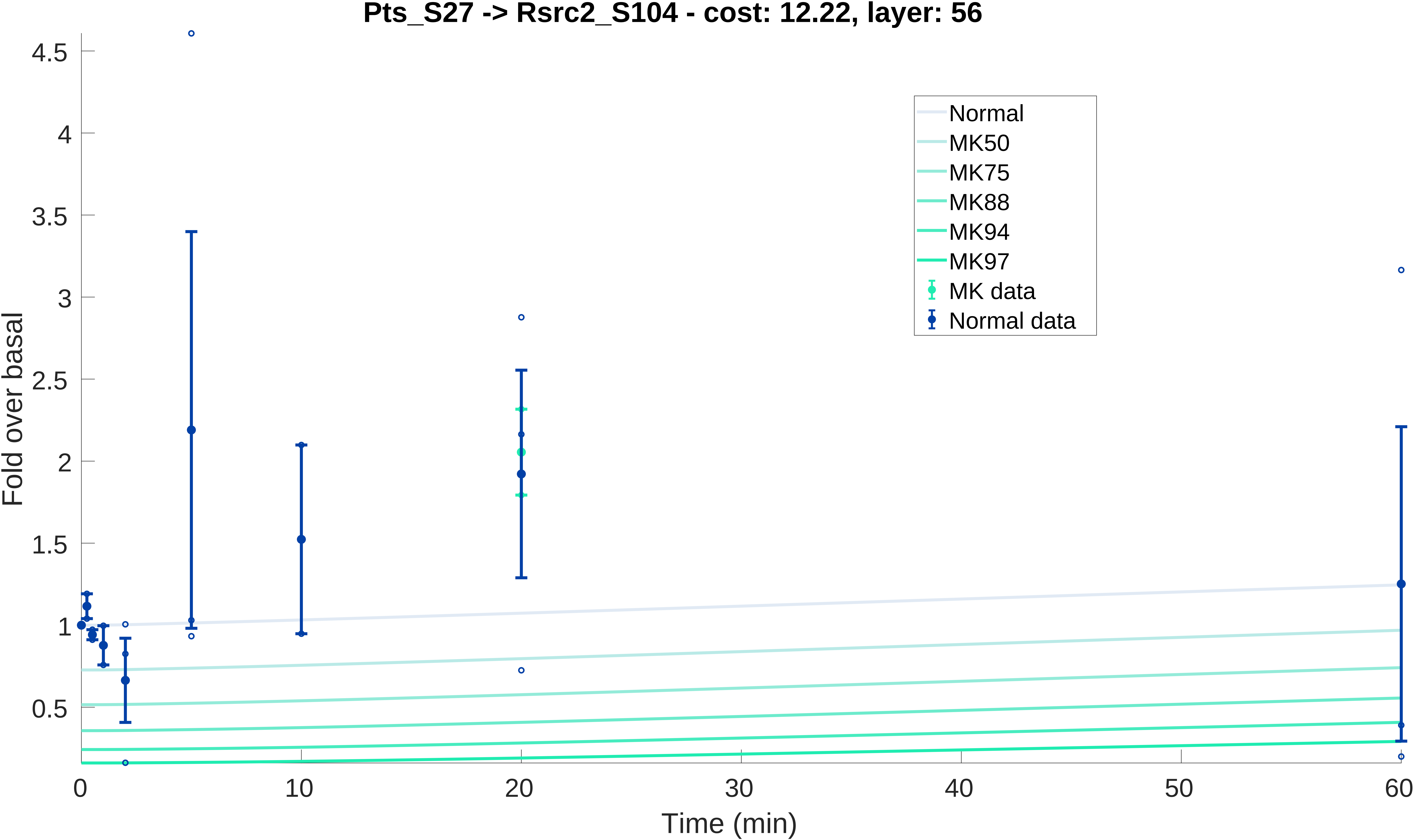

Pts_S27 -> Rsrc2_S104 - cost: 12.22, layer: 56
Fold over basal
Time (min)
0
10
20
30
40
50
60
0.5
1
1.5
2
2.5
3
3.5
4
4.5
Normal
MK50
MK75
MK88
MK94
MK97
MK data
Normal data

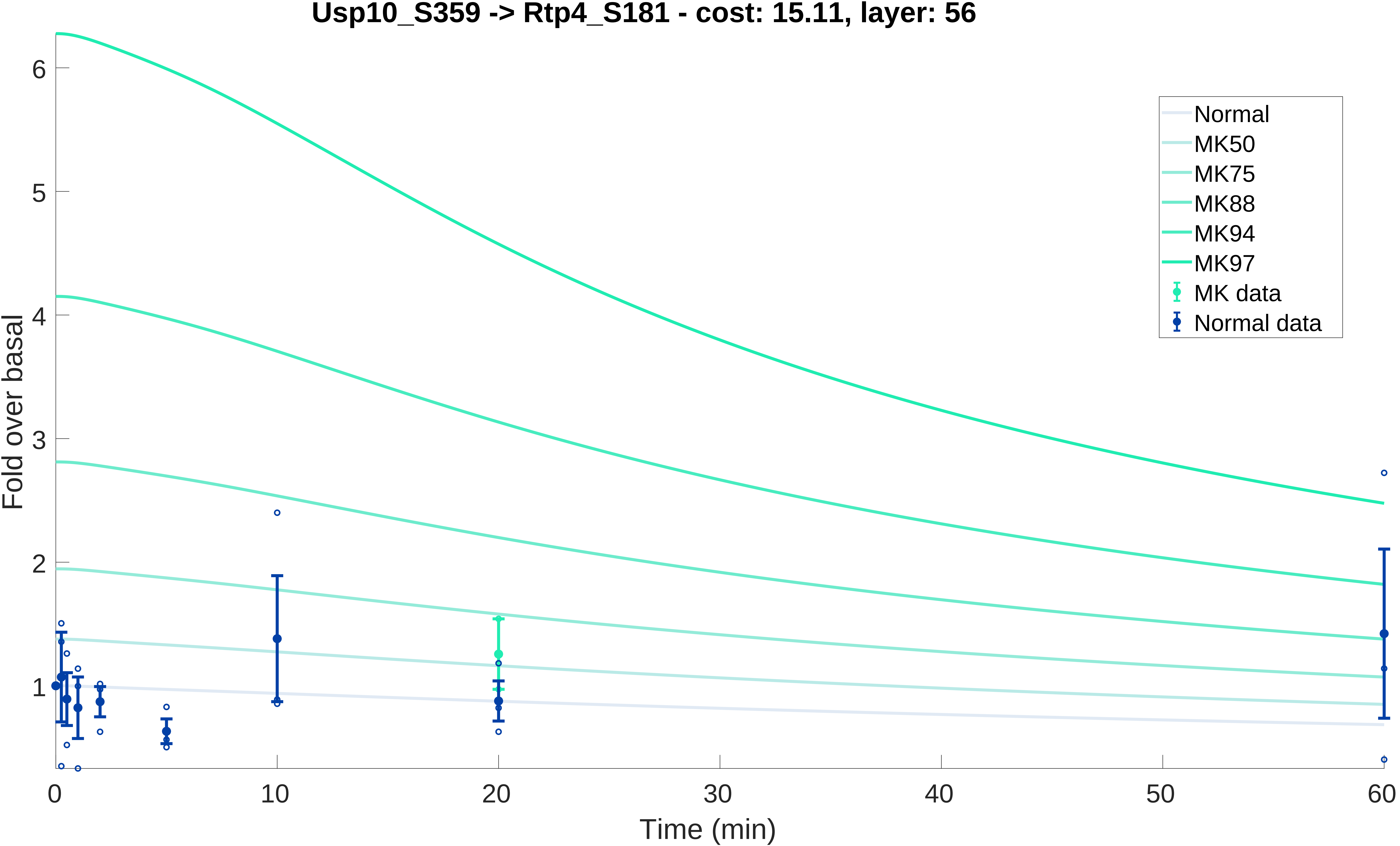

Usp10_S359 -> Rtp4_S181 - cost: 15.11, layer: 56
Fold over basal
Time (min)
0
10
20
30
40
50
60
1
2
3
4
5
6
Normal
MK50
MK75
MK88
MK94
MK97
MK data
Normal data

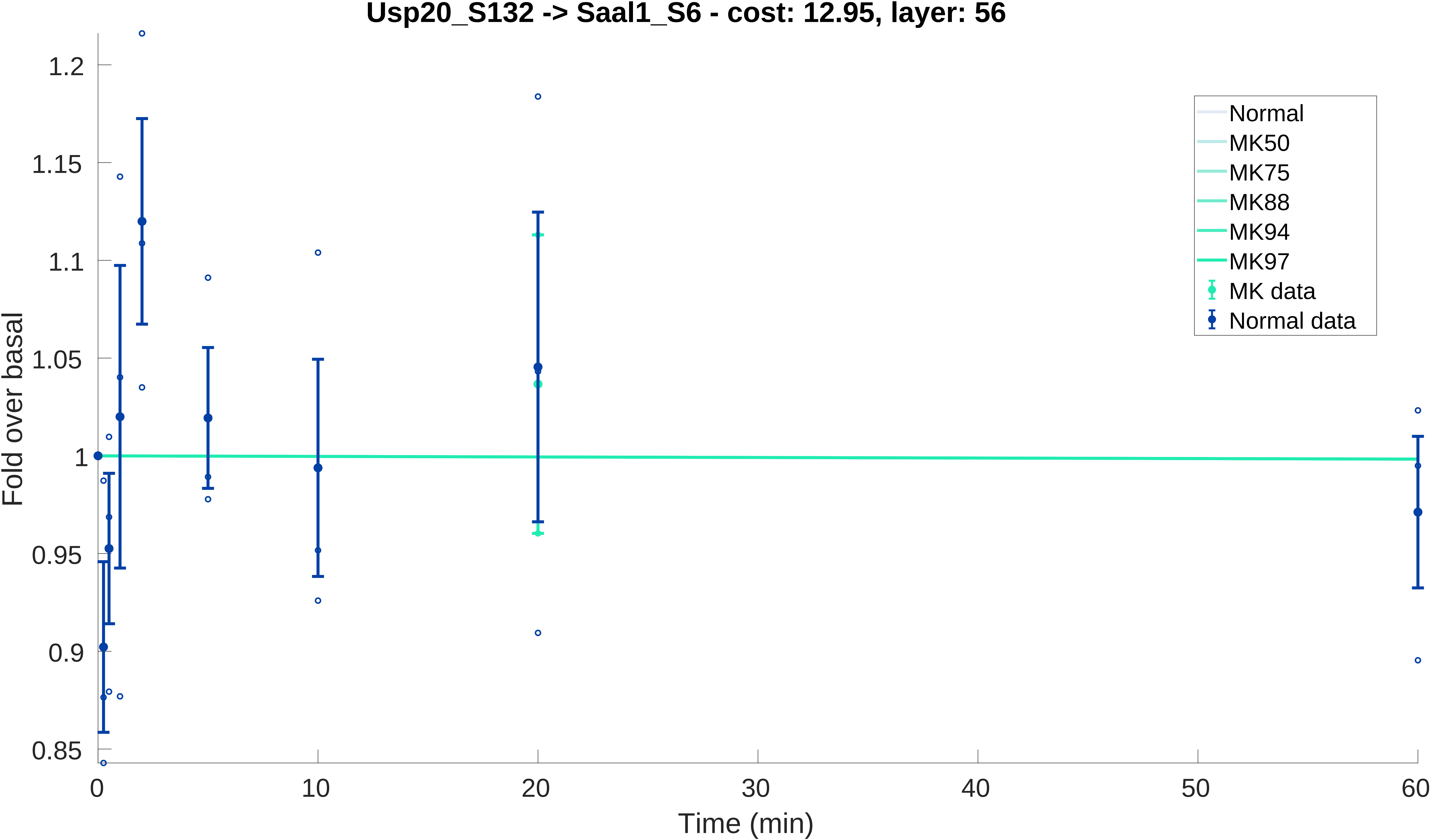
Usp20_S132 -> Saal1_S6 - cost: 12.95, layer: 56
Fold over basal
1.2
1.15
1.1
1.05
1
0.95
0.9
0.85
Time (min)
0
10
20
30
40
50
60
Normal
MK50
MK75
MK88
MK94
MK97
MK data
Normal data

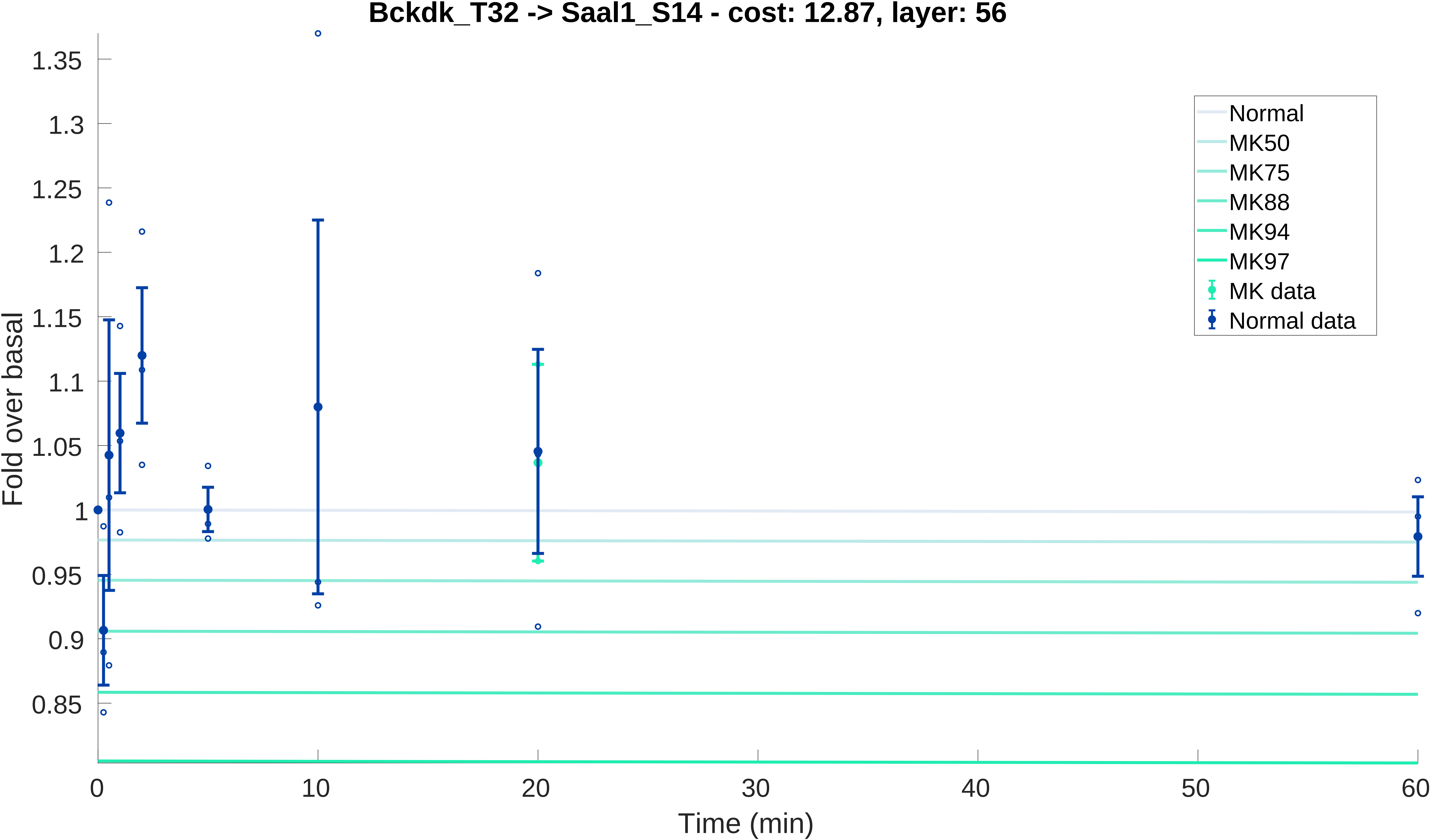
Bckdk_T32 -> Saal1_S14 - cost: 12.87, layer: 56
Fold over basal
1.35
1.3
1.25
1.2
1.15
1.1
1.05
1
0.95
0.9
0.85
0
10
20
30
40
50
60
Time (min)
Normal
MK50
MK75
MK88
MK94
MK97
MK data
Normal data

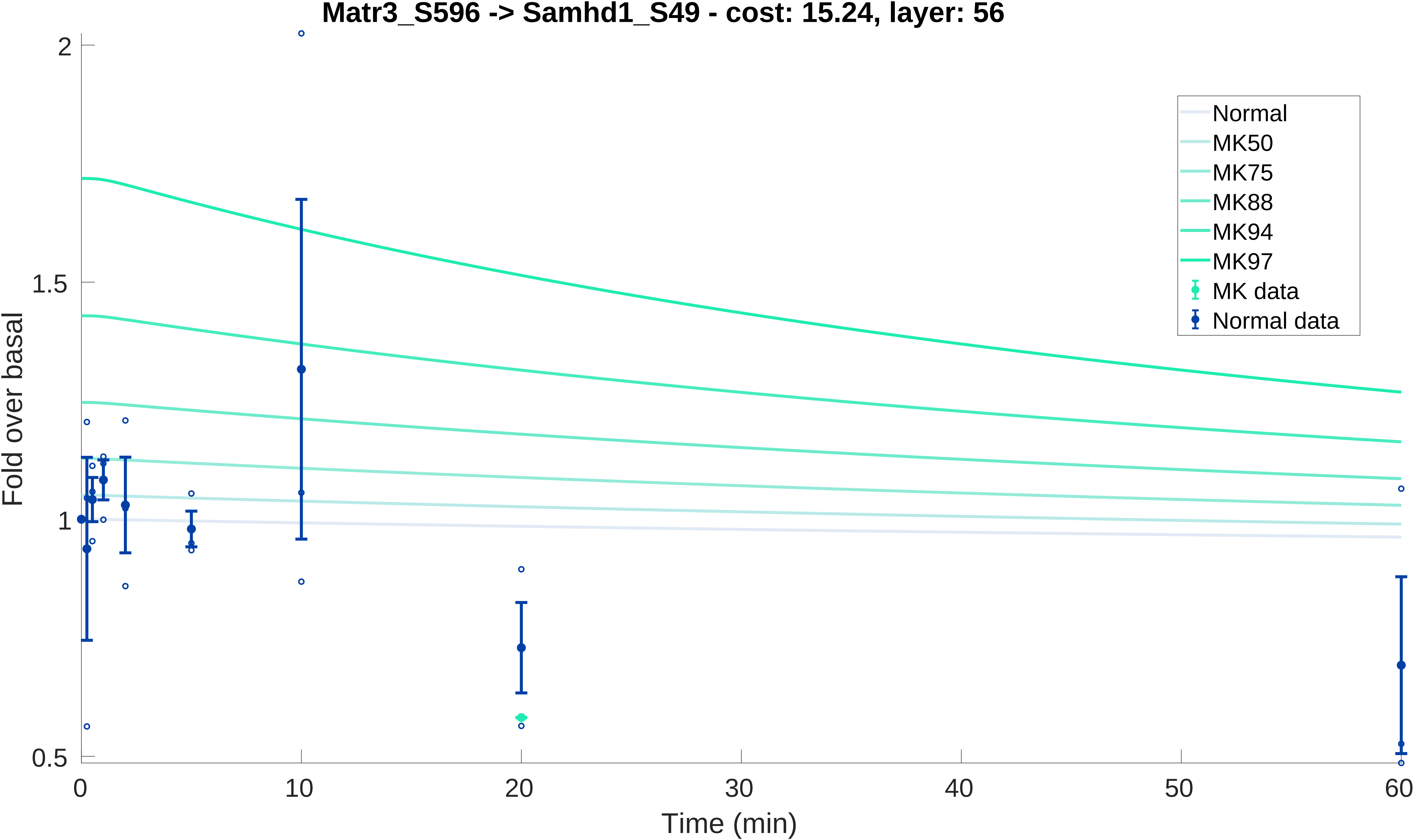

Matr3_S596 -> Samhd1_S49 - cost: 15.24, layer: 56
Fold over basal
Time (min)
Normal
MK50
MK75
MK88
MK94
MK97
MK data
Normal data
0.5
1
1.5
2
0
10
20
30
40
50
60

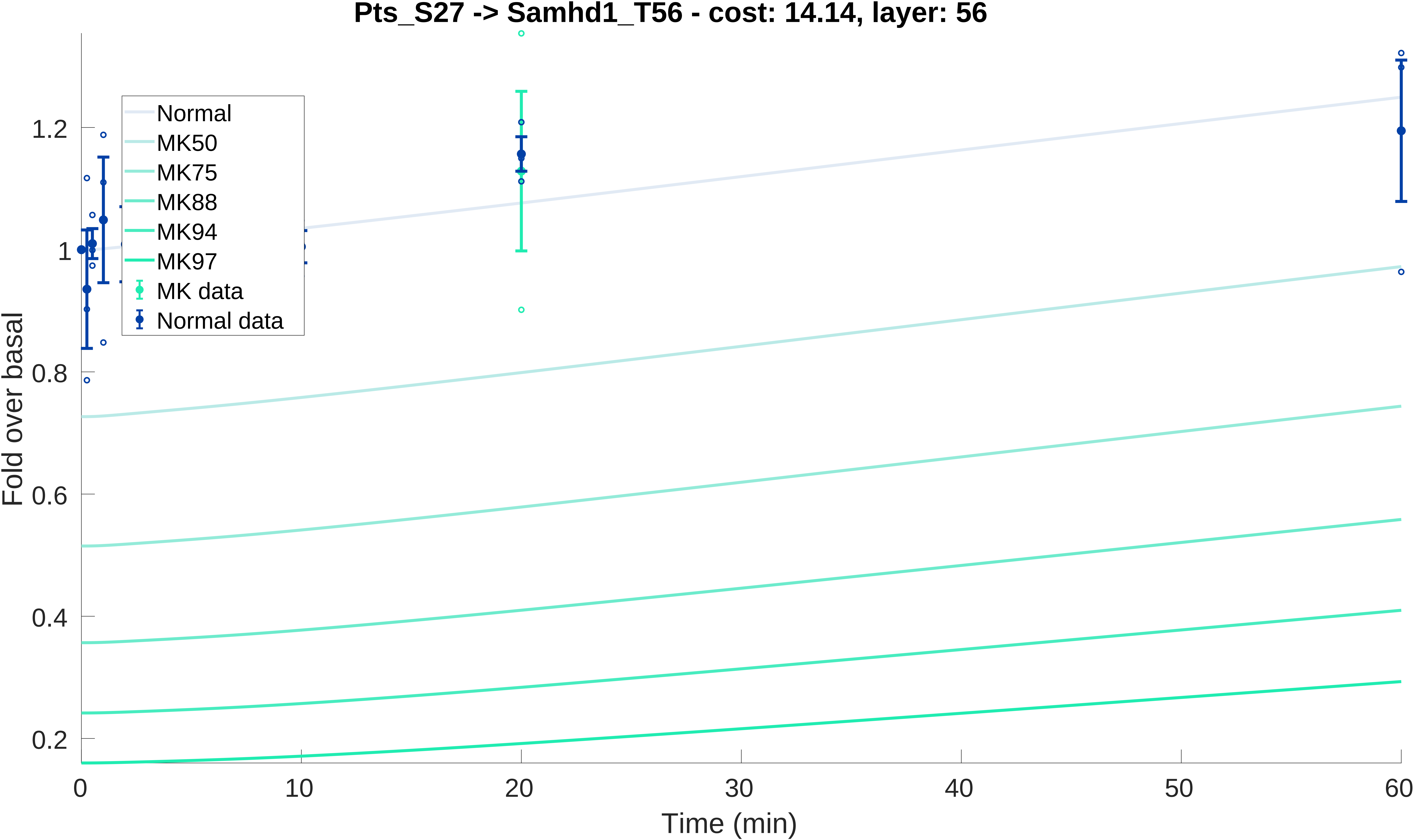

Pts_S27 -> Samhd1_T56 - cost: 14.14, layer: 56
Normal
MK50
MK75
MK88
MK94
MK97
MK data
Normal data
Fold over basal
Time (min)
0
10
20
30
40
50
60
0.2
0.4
0.6
0.8
1
1.2

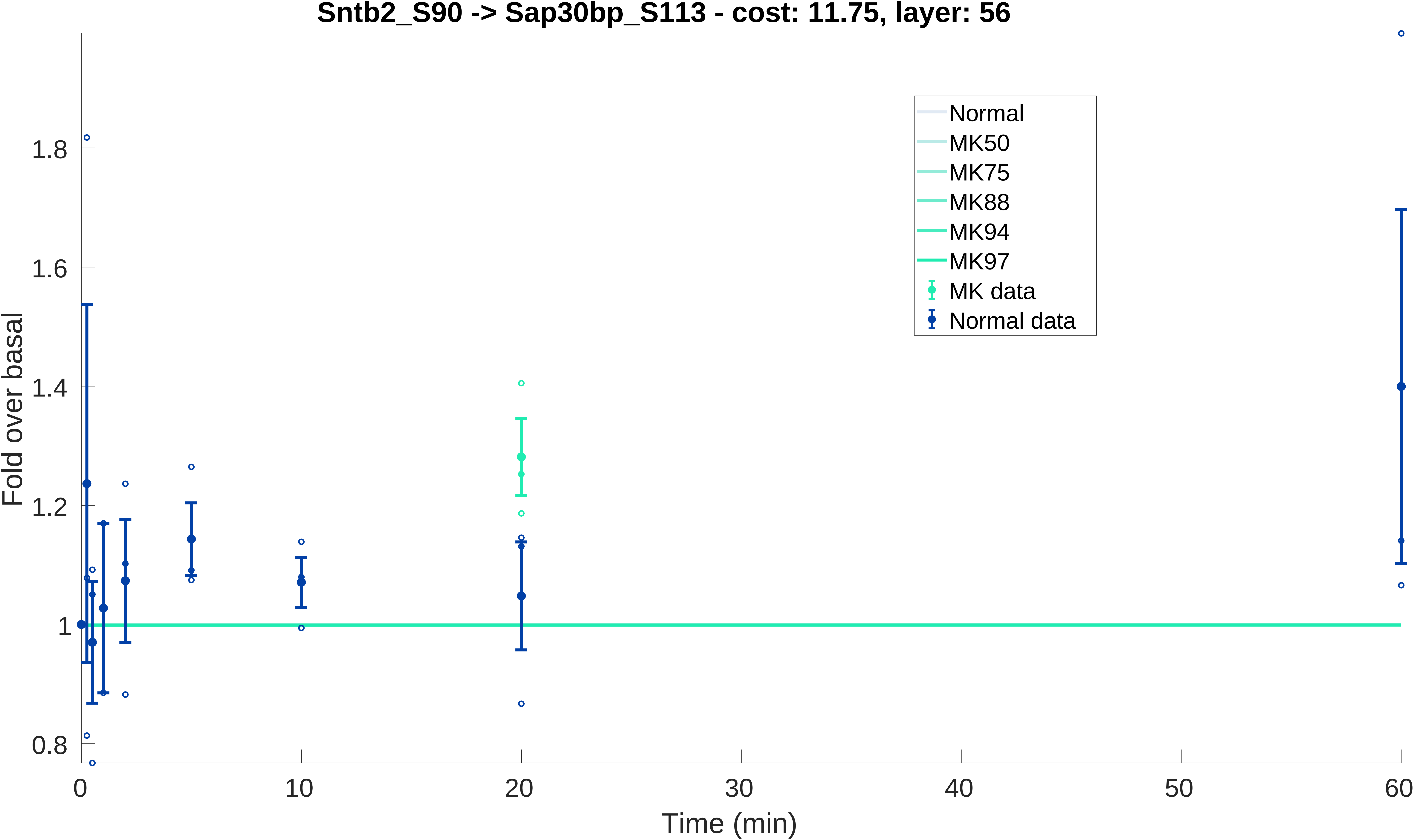

Sntb2_S90 -> Sap30bp_S113 - cost: 11.75, layer: 56
Fold over basal
Time (min)
0.8
1
1.2
1.4
1.6
1.8
0
10
20
30
40
50
60
Normal
MK50
MK75
MK88
MK94
MK97
MK data
Normal data

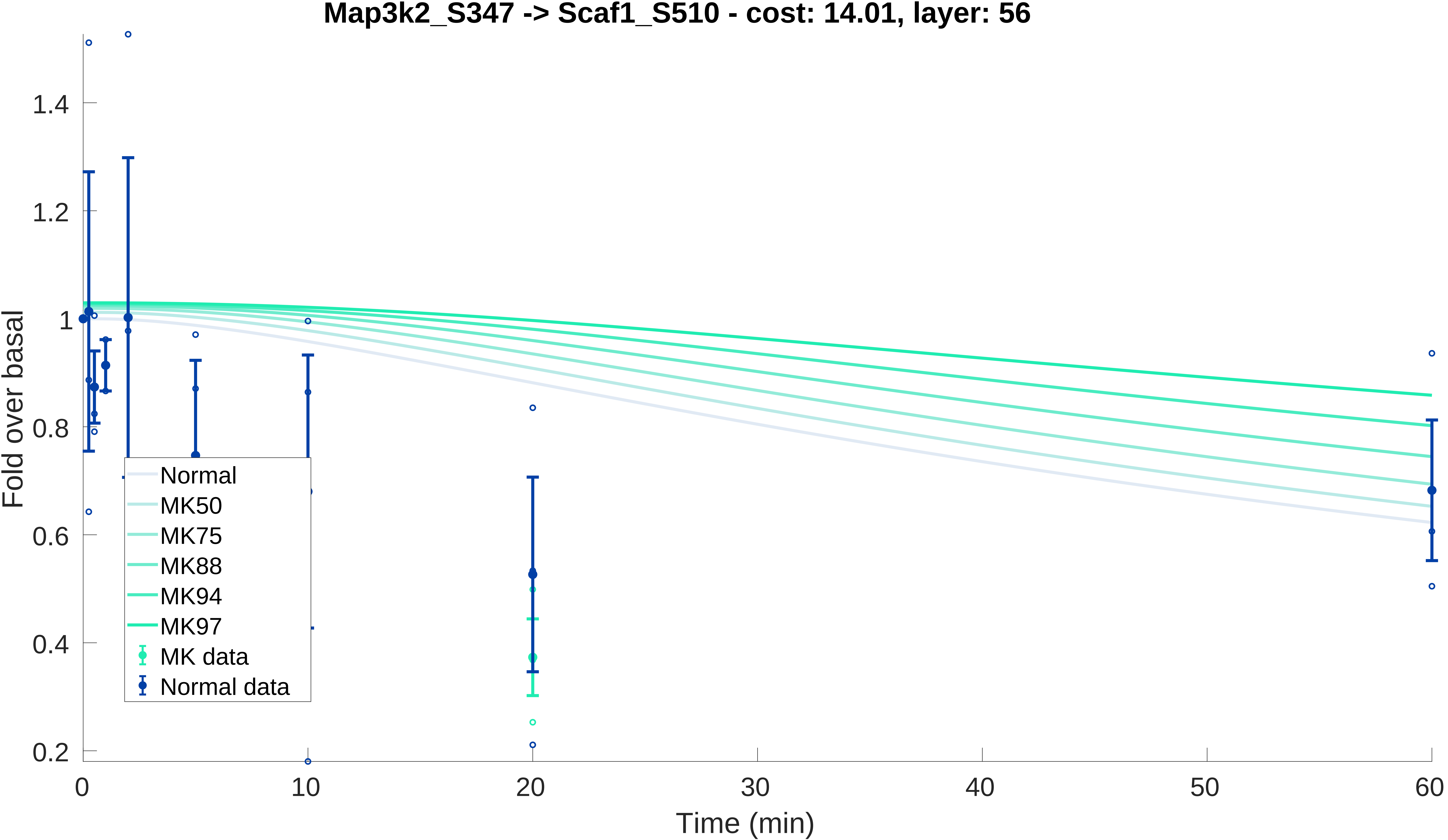

Map3k2_S347 -> Scaf1_S510 - cost: 14.01, layer: 56
Fold over basal
Time (min)
0.2
0.4
0.6
0.8
1
1.2
1.4
0
10
20
30
40
50
60
Normal
MK50
MK75
MK88
MK94
MK97
MK data
Normal data

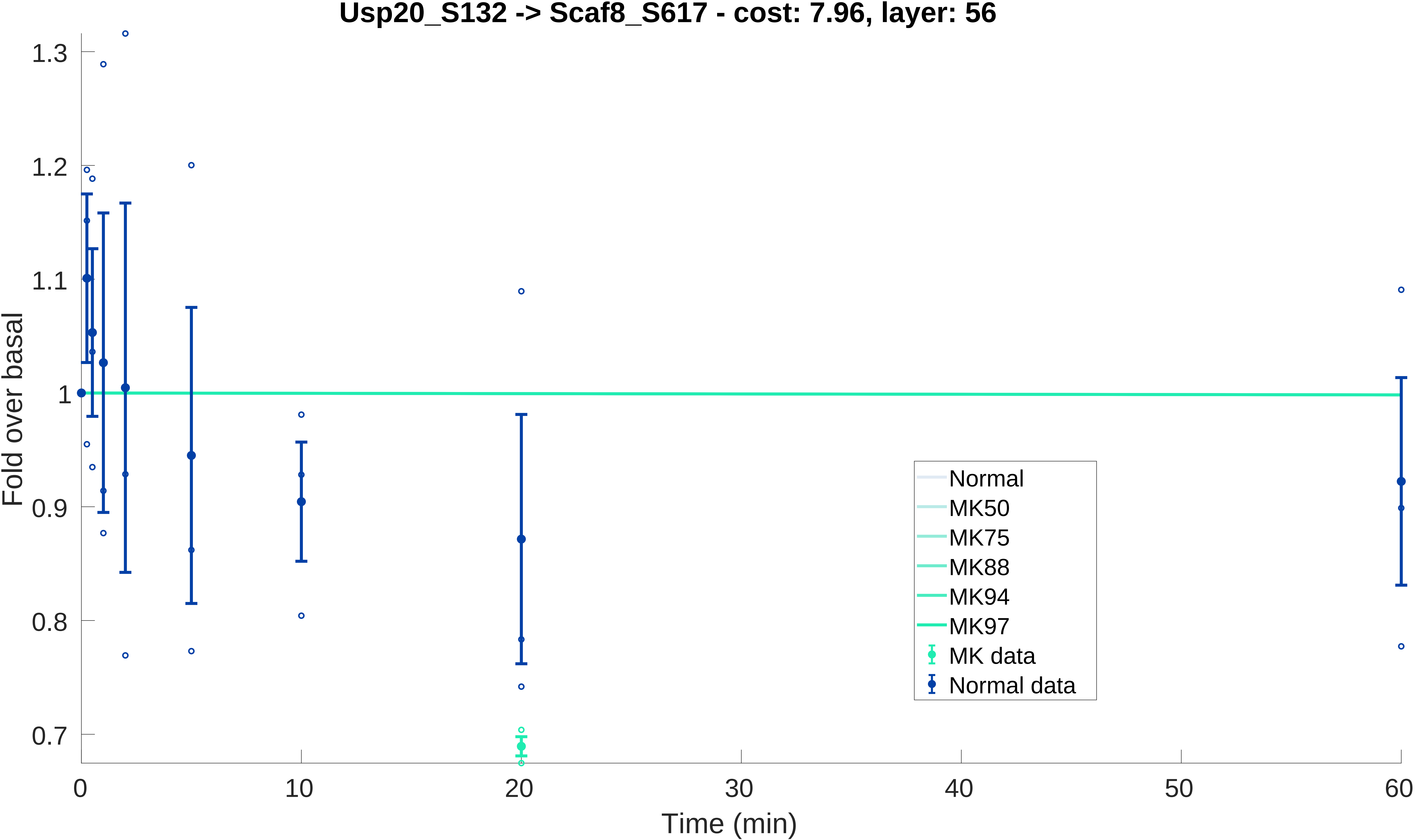
Usp20_S132 -> Scaf8_S617 - cost: 7.96, layer: 56
Fold over basal
Time (min)
1.3
1.2
1.1
1
0.9
0.8
0.7
0
10
20
30
40
50
60
Normal
MK50
MK75
MK88
MK94
MK97
MK data
Normal data

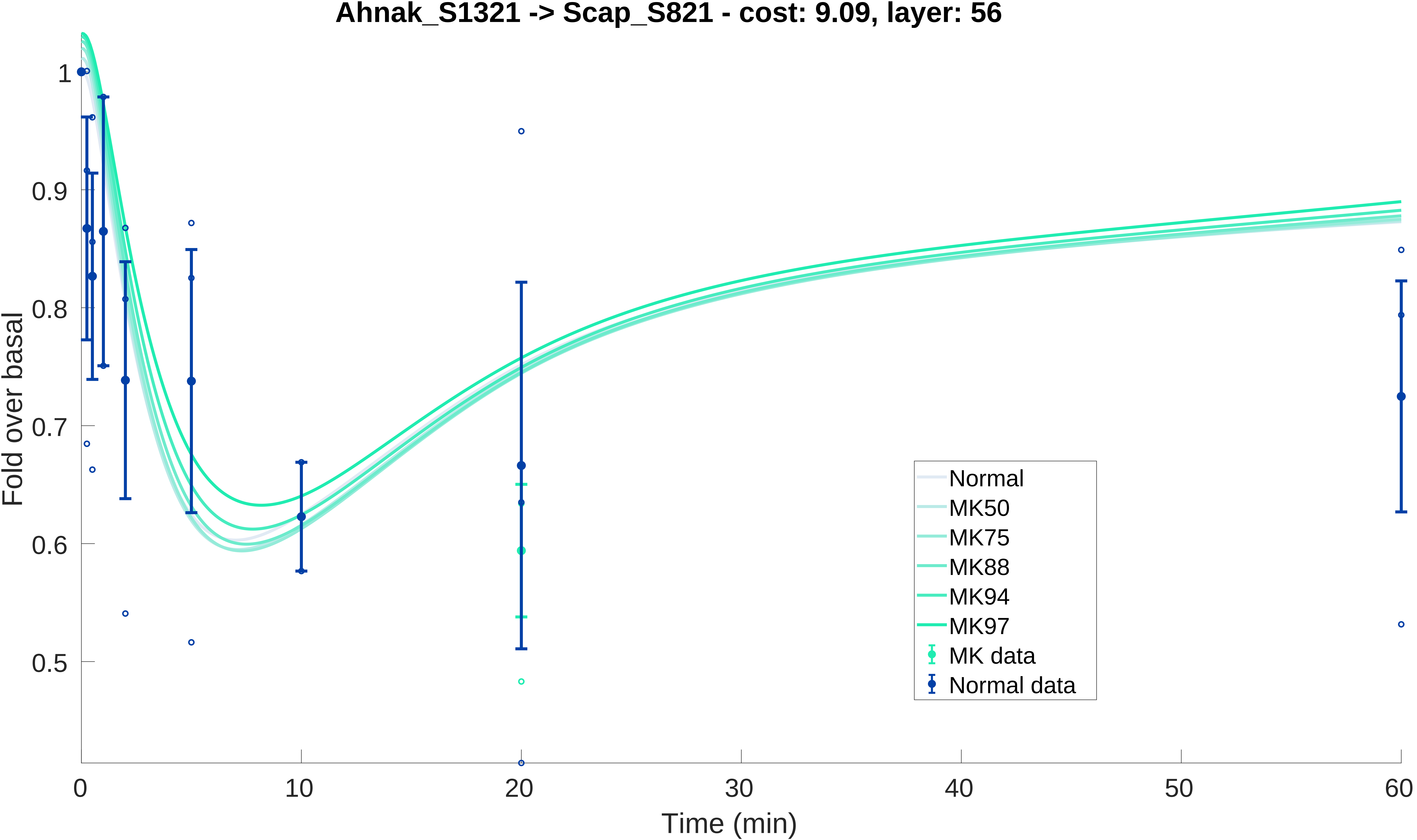
Ahnak_S1321 -> Scap_S821 - cost: 9.09, layer: 56
Fold over basal
Time (min)
1
0.9
0.8
0.7
0.6
0.5
0
10
20
30
40
50
60
Normal
MK50
MK75
MK88
MK94
MK97
MK data
Normal data

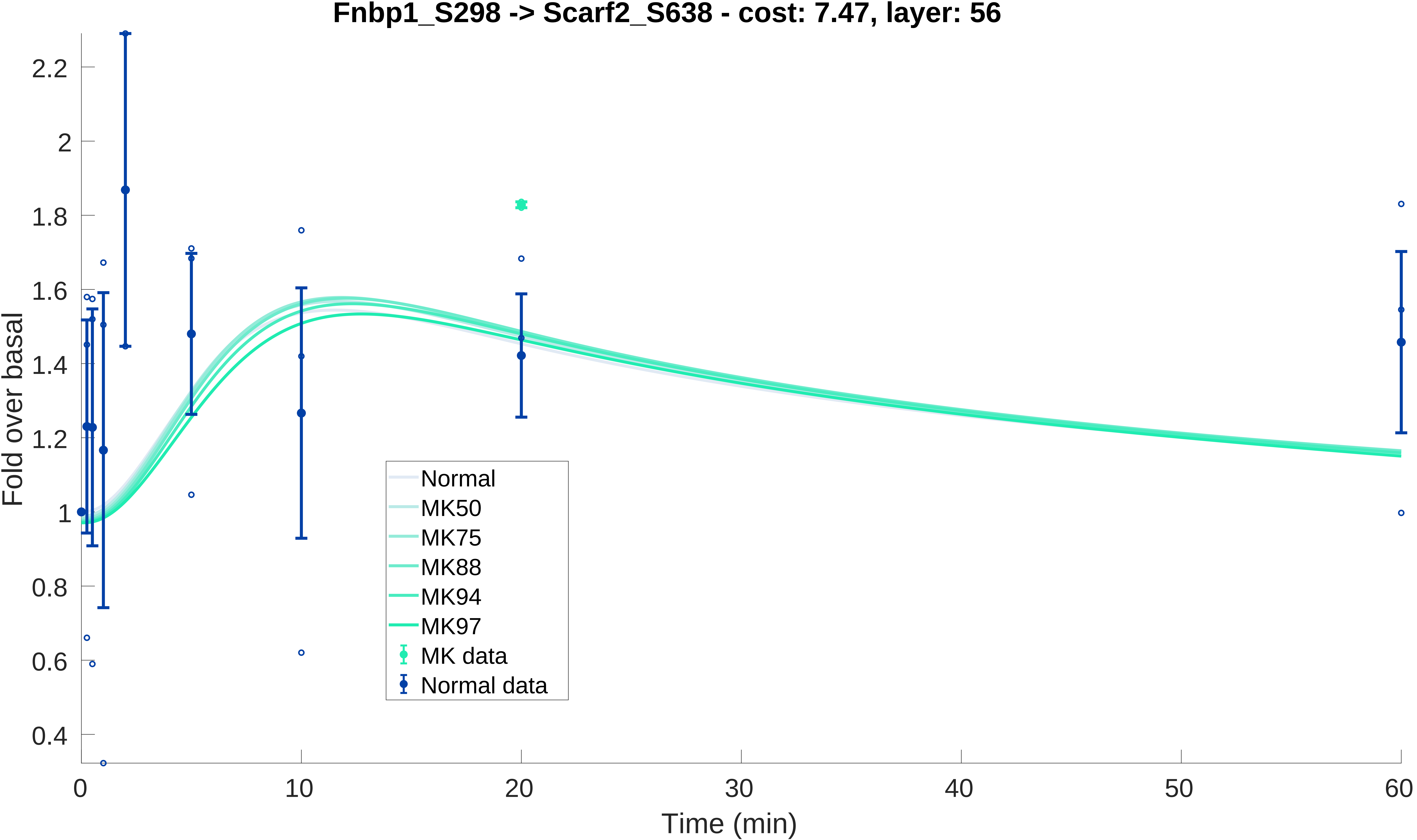

Fnbp1_S298 -> Scarf2_S638 - cost: 7.47, layer: 56
Fold over basal
Time (min)
0.4
0.6
0.8
1
1.2
1.4
1.6
1.8
2
2.2
0
10
20
30
40
50
60
Normal
MK50
MK75
MK88
MK94
MK97
MK data
Normal data

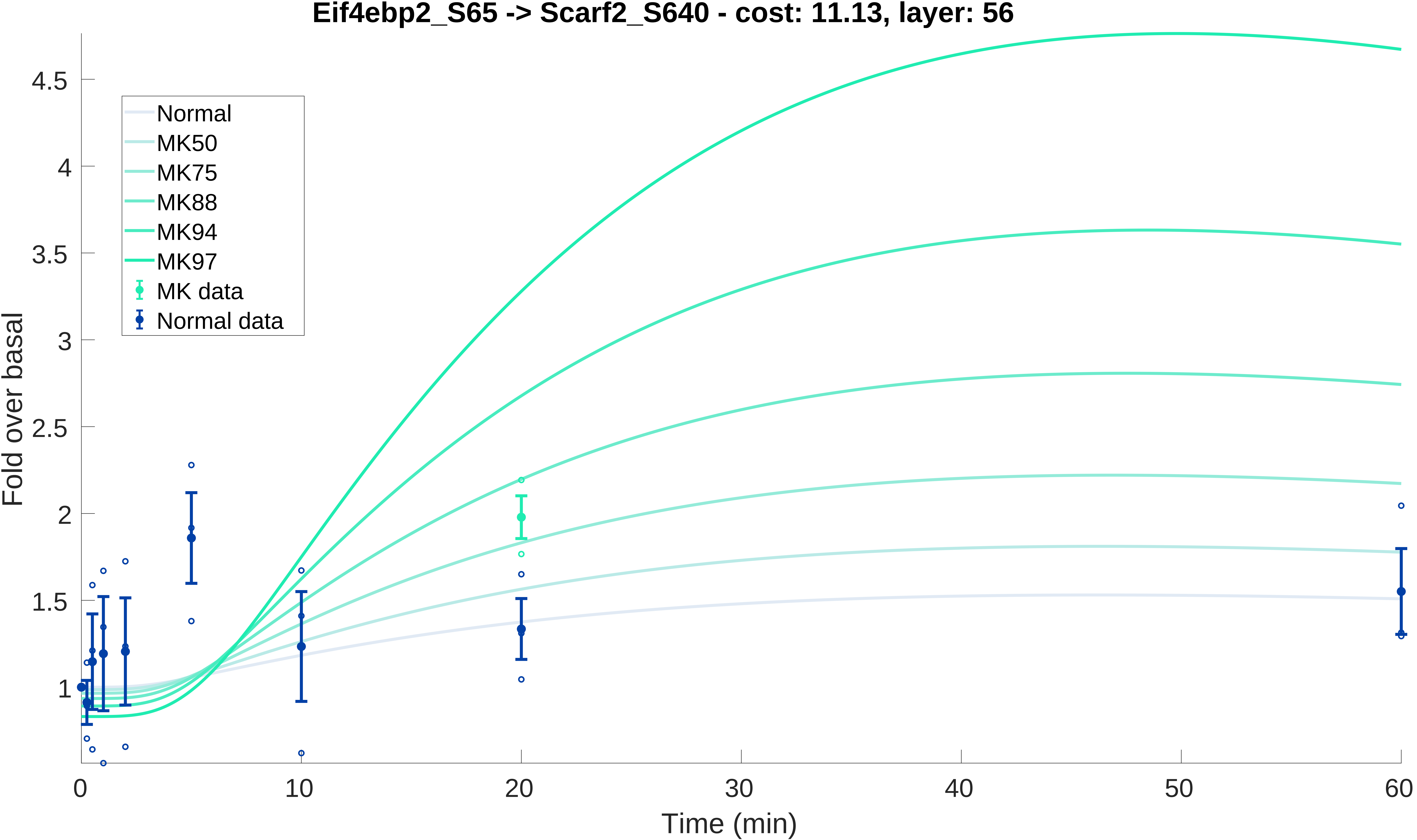

Eif4ebp2_S65 -> Scarf2_S640 - cost: 11.13, layer: 56
Normal
MK50
MK75
MK88
MK94
MK97
MK data
Normal data
Fold over basal
Time (min)
4.5
4
3.5
3
2.5
2
1.5
1
0
10
20
30
40
50
60

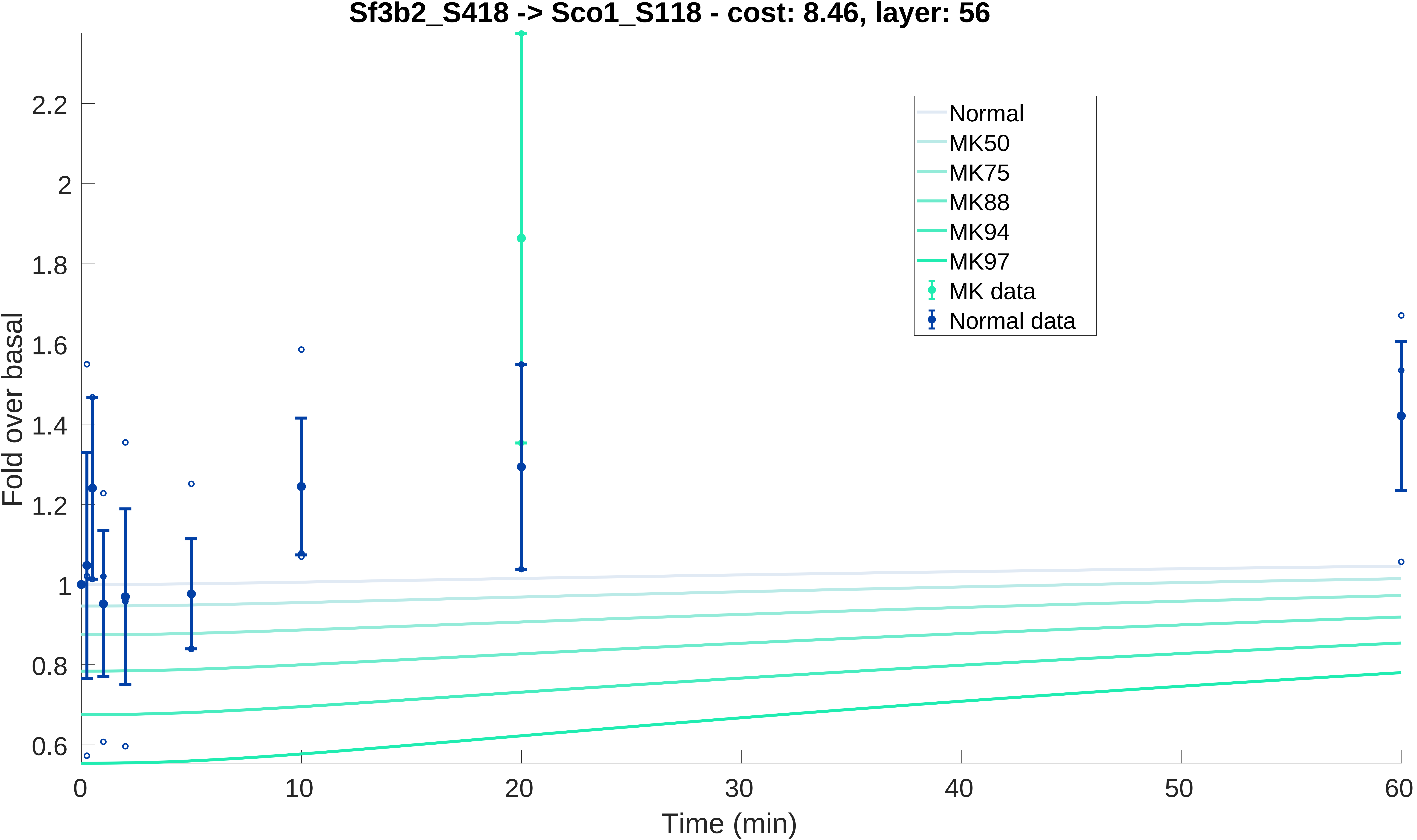

Sf3b2_S418 -> Sco1_S118 - cost: 8.46, layer: 56
Fold over basal
Time (min)
0.6
0.8
1
1.2
1.4
1.6
1.8
2
2.2
0
10
20
30
40
50
60
Normal
MK50
MK75
MK88
MK94
MK97
MK data
Normal data

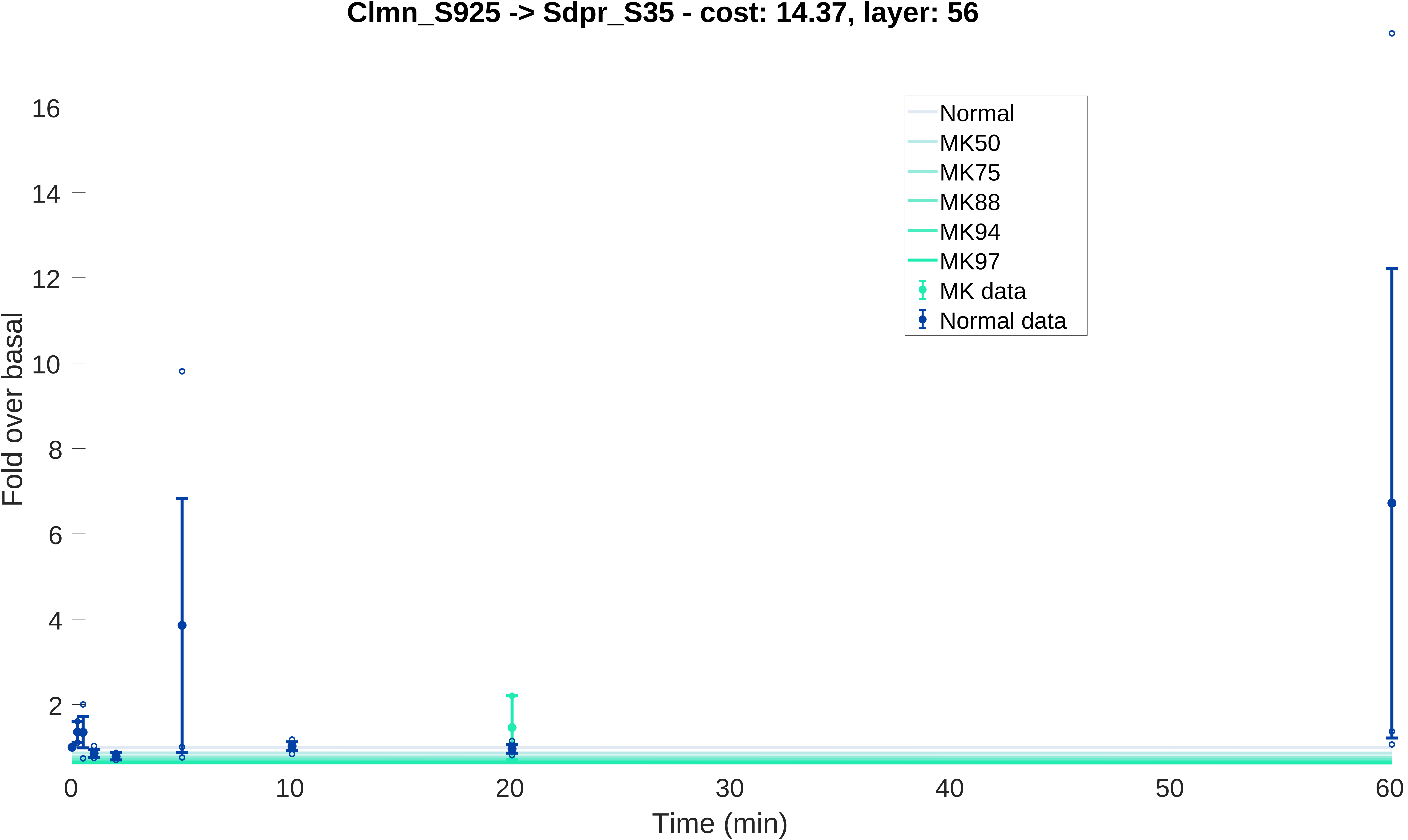

Clmn_S925 -> Sdpr_S35 - cost: 14.37, layer: 56
Fold over basal
Time (min)
0
10
20
30
40
50
60
2
4
6
8
10
12
14
16
Normal
MK50
MK75
MK88
MK94
MK97
MK data
Normal data

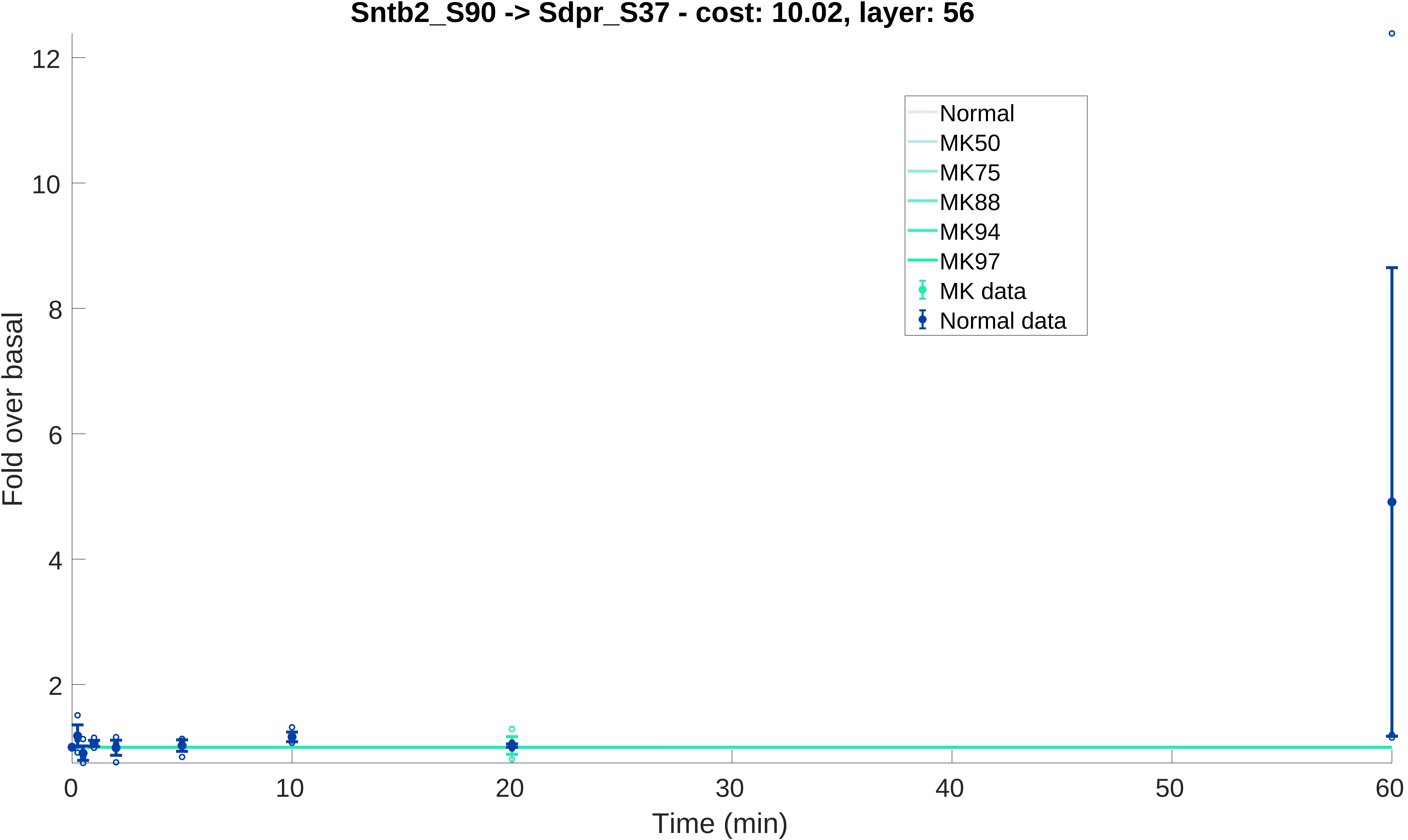

Sntb2_S90 -> Sdpr_S37 - cost: 10.02, layer: 56
Fold over basal
Time (min)
0
2
4
6
8
10
12
0
10
20
30
40
50
60
Normal
MK50
MK75
MK88
MK94
MK97
MK data
Normal data

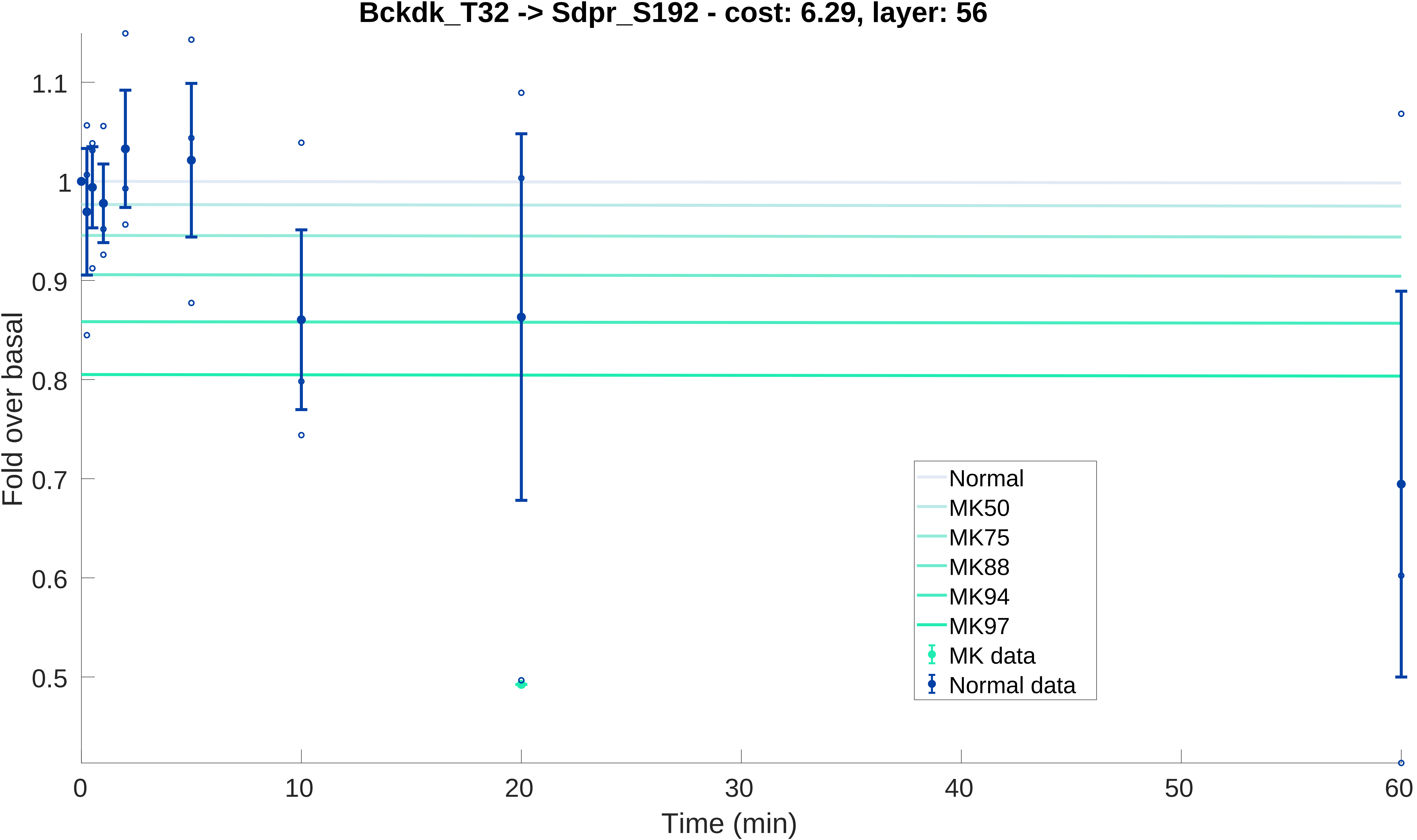

Bckdk_T32 -> Sdpr_S192 - cost: 6.29, layer: 56
Fold over basal
Time (min)
0
10
20
30
40
50
60
0.5
0.6
0.7
0.8
0.9
1
1.1
Normal
MK50
MK75
MK88
MK94
MK97
MK data
Normal data

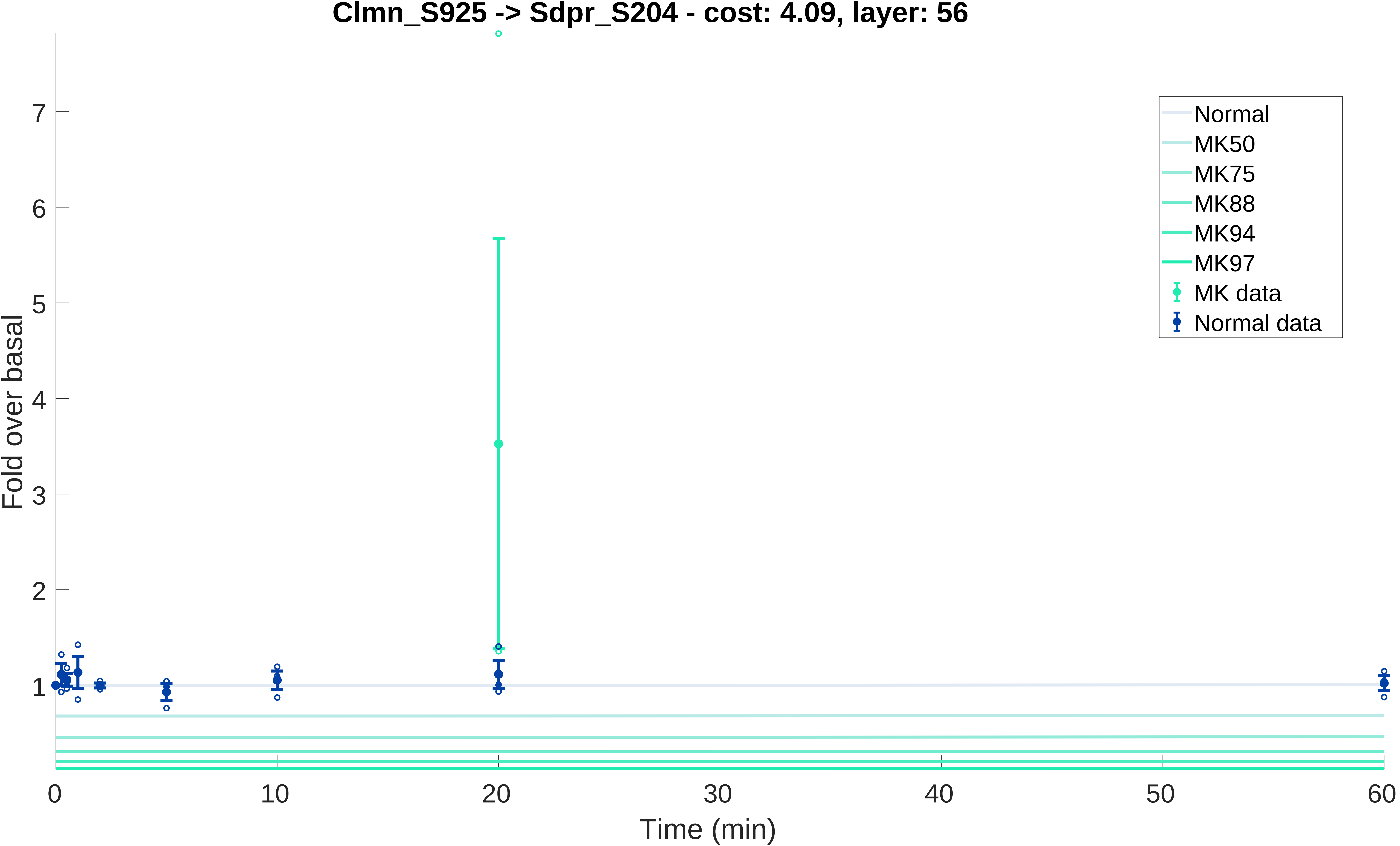

Clmn_S925 -> Sdpr_S204 - cost: 4.09, layer: 56
Fold over basal
Time (min)
Normal
MK50
MK75
MK88
MK94
MK97
MK data
Normal data
1
2
3
4
5
6
7
0
10
20
30
40
50
60

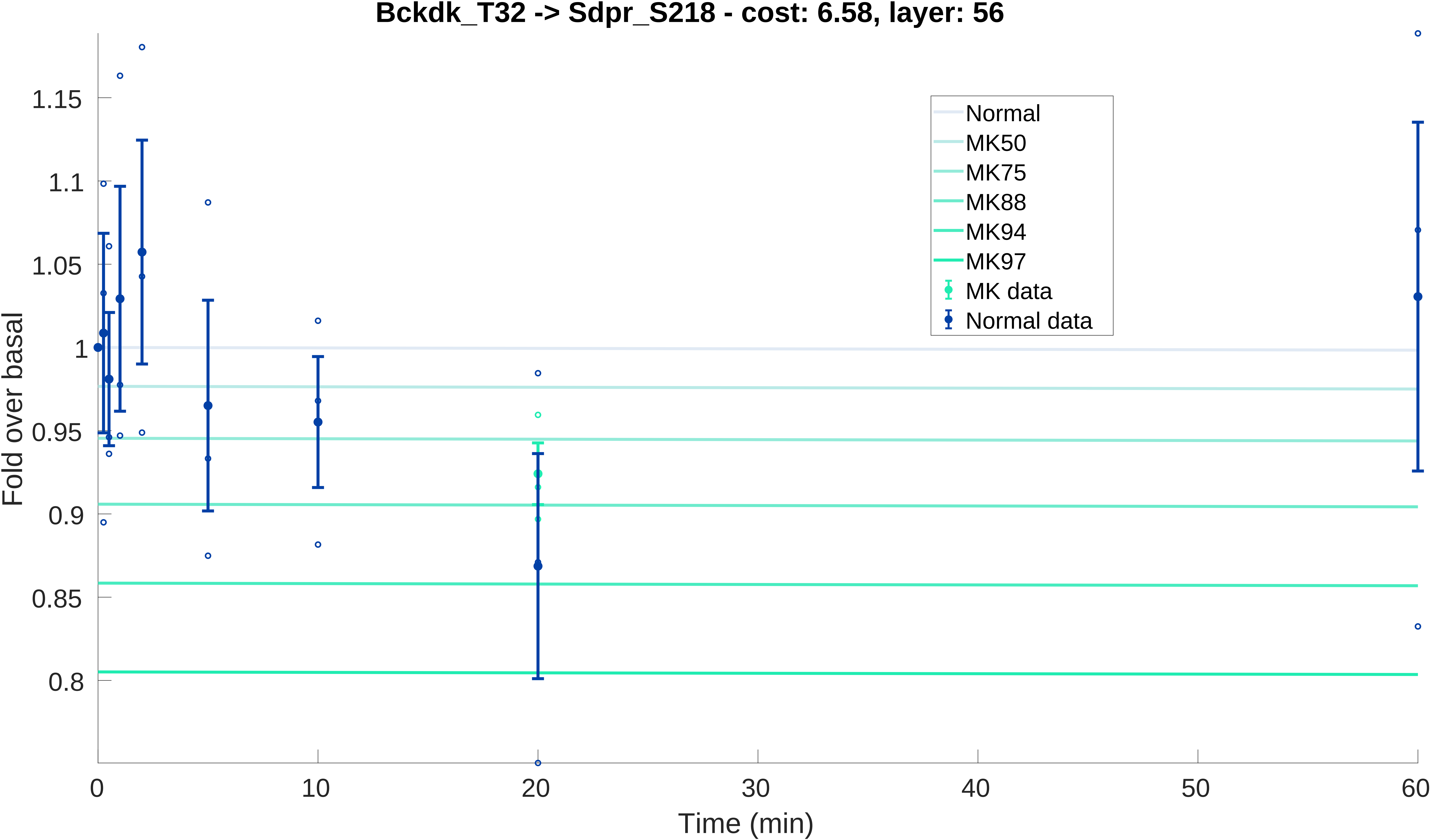

Bckdk_T32 -> Sdpr_S218 - cost: 6.58, layer: 56
Normal
MK50
MK75
MK88
MK94
MK97
MK data
Normal data
Fold over basal
Time (min)
1.15
1.1
1.05
1
0.95
0.9
0.85
0.8
0
10
20
30
40
50
60

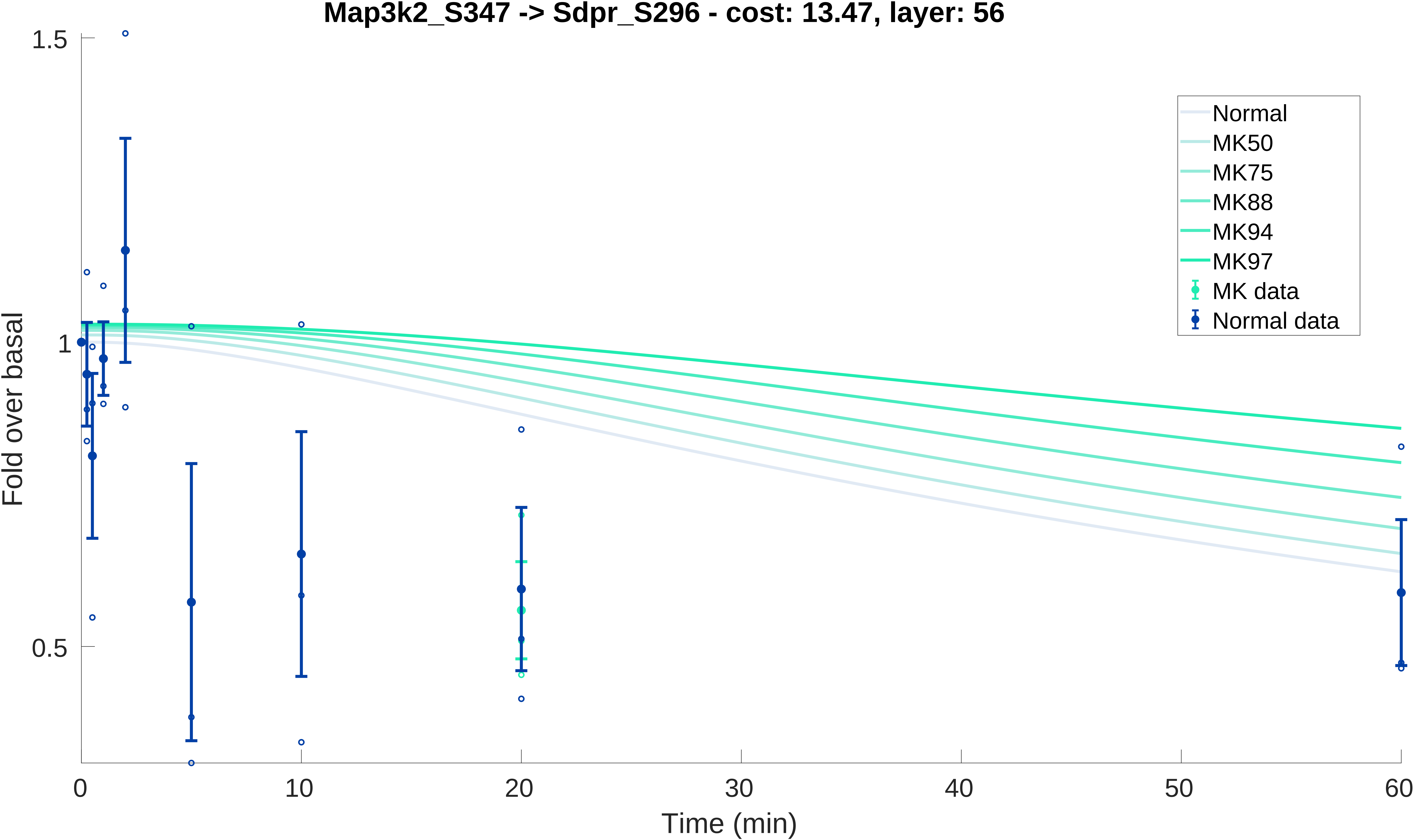

Map3k2_S347 -> Sdpr_S296 - cost: 13.47, layer: 56
Fold over basal
1.5
1
0.5
Time (min)
0
10
20
30
40
50
60
Normal
MK50
MK75
MK88
MK94
MK97
MK data
Normal data

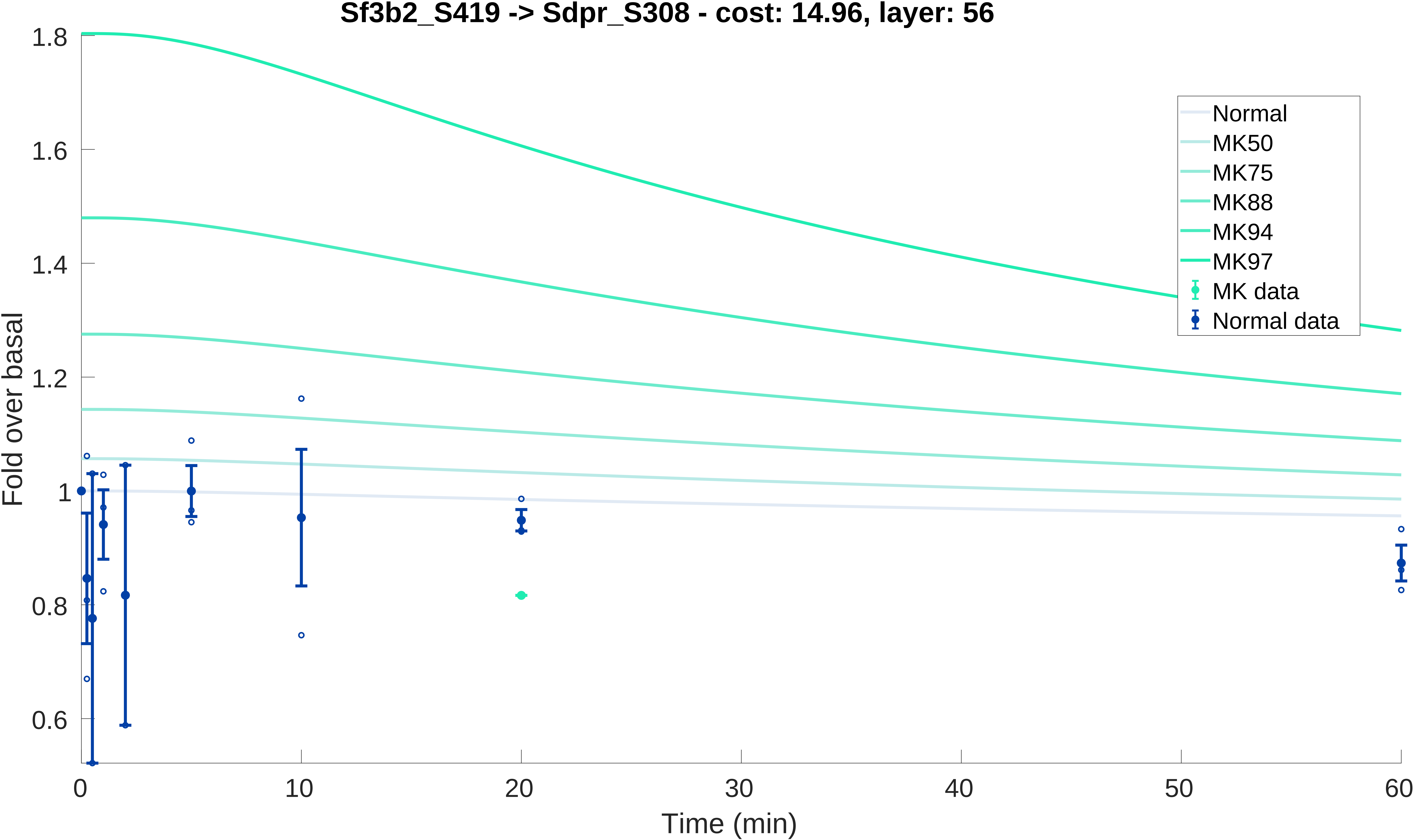

Sf3b2_S419 -> Sdpr_S308 - cost: 14.96, layer: 56
Fold over basal
1.8
1.6
1.4
1.2
1
0.8
0.6
0
10
20
30
40
50
60
Time (min)
Normal
MK50
MK75
MK88
MK94
MK97
MK data
Normal data

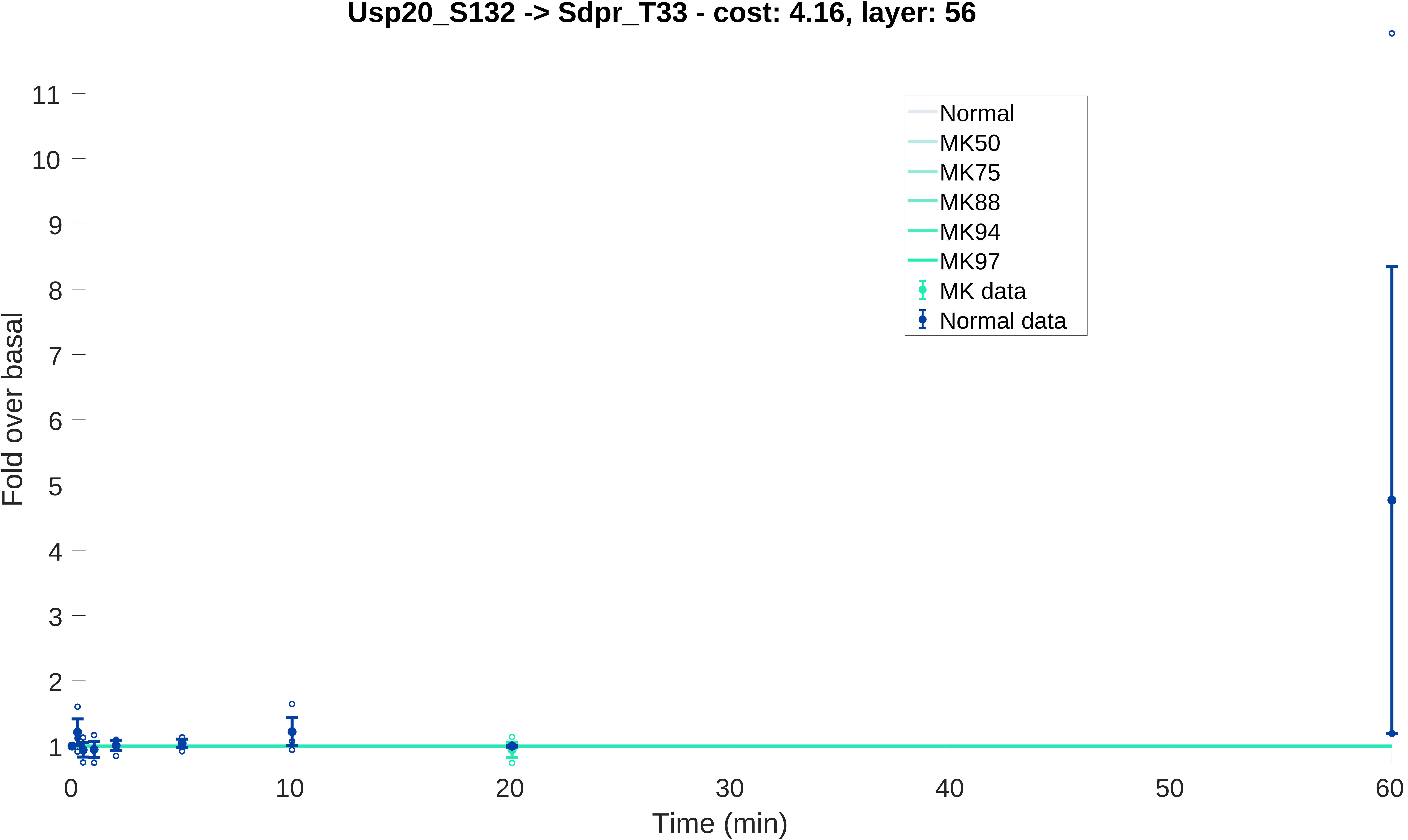

Usp20_S132 -> Sdpr_T33 - cost: 4.16, layer: 56
Fold over basal
Time (min)
0
10
20
30
40
50
60
1
2
3
4
5
6
7
8
9
10
11
Normal
MK50
MK75
MK88
MK94
MK97
MK data
Normal data

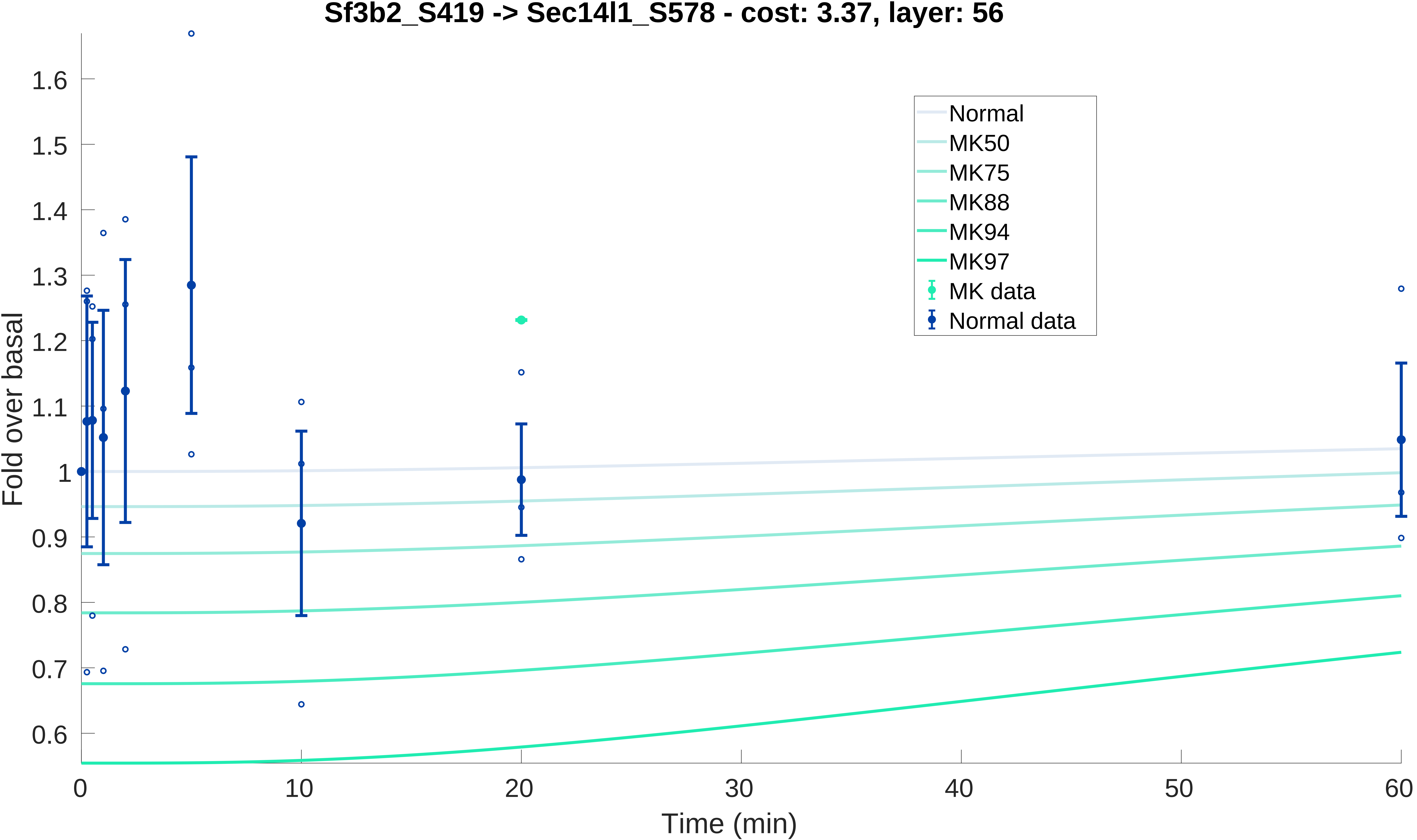

Sf3b2_S419 -> Sec14l1_S578 - cost: 3.37, layer: 56
Fold over basal
Time (min)
1.6
1.5
1.4
1.3
1.2
1.1
1
0.9
0.8
0.7
0.6
0
10
20
30
40
50
60
Normal
MK50
MK75
MK88
MK94
MK97
MK data
Normal data

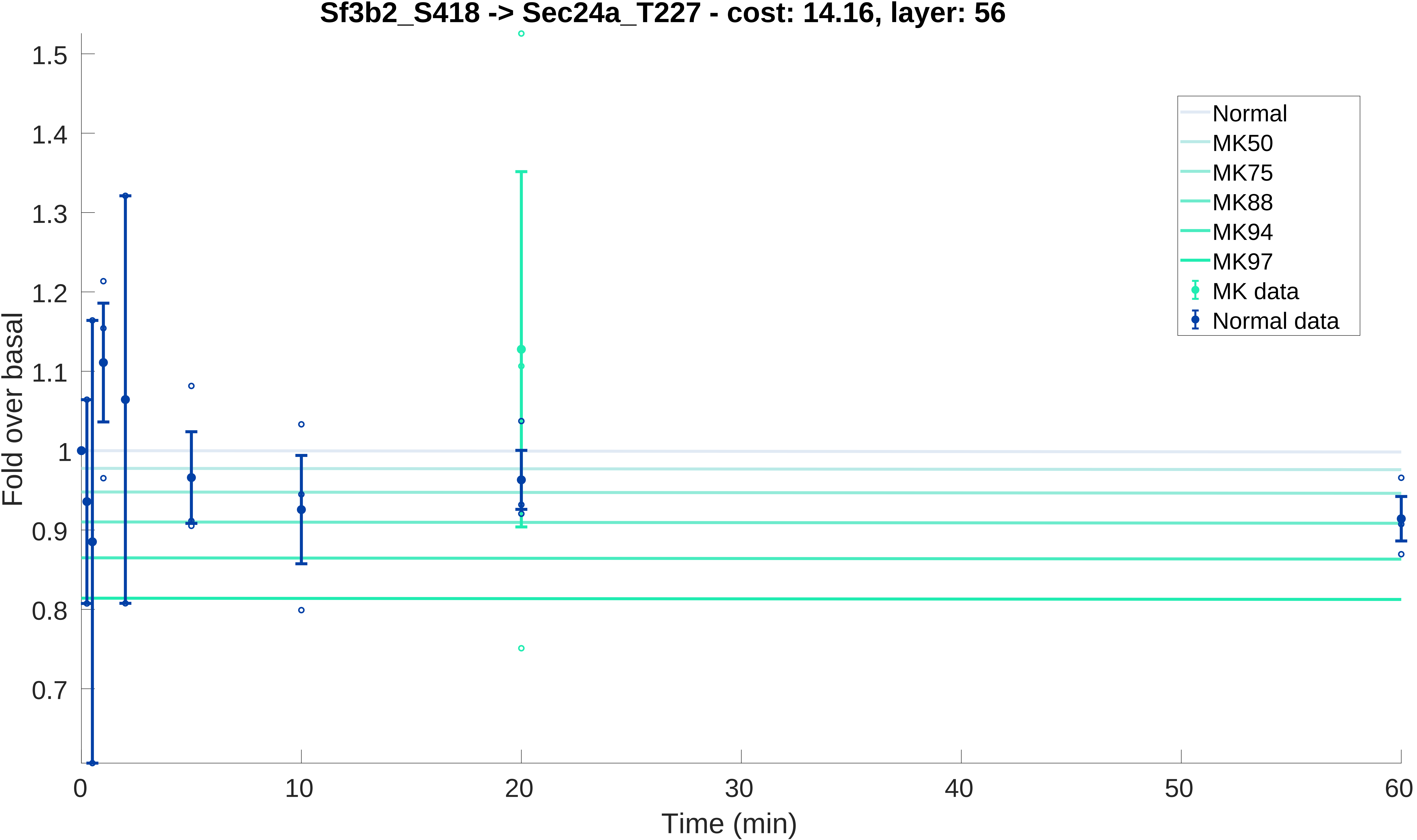

Sf3b2_S418 -> Sec24a_T227 - cost: 14.16, layer: 56
Fold over basal
1.5
1.4
1.3
1.2
1.1
1
0.9
0.8
0.7
Time (min)
0
10
20
30
40
50
60
Normal
MK50
MK75
MK88
MK94
MK97
MK data
Normal data

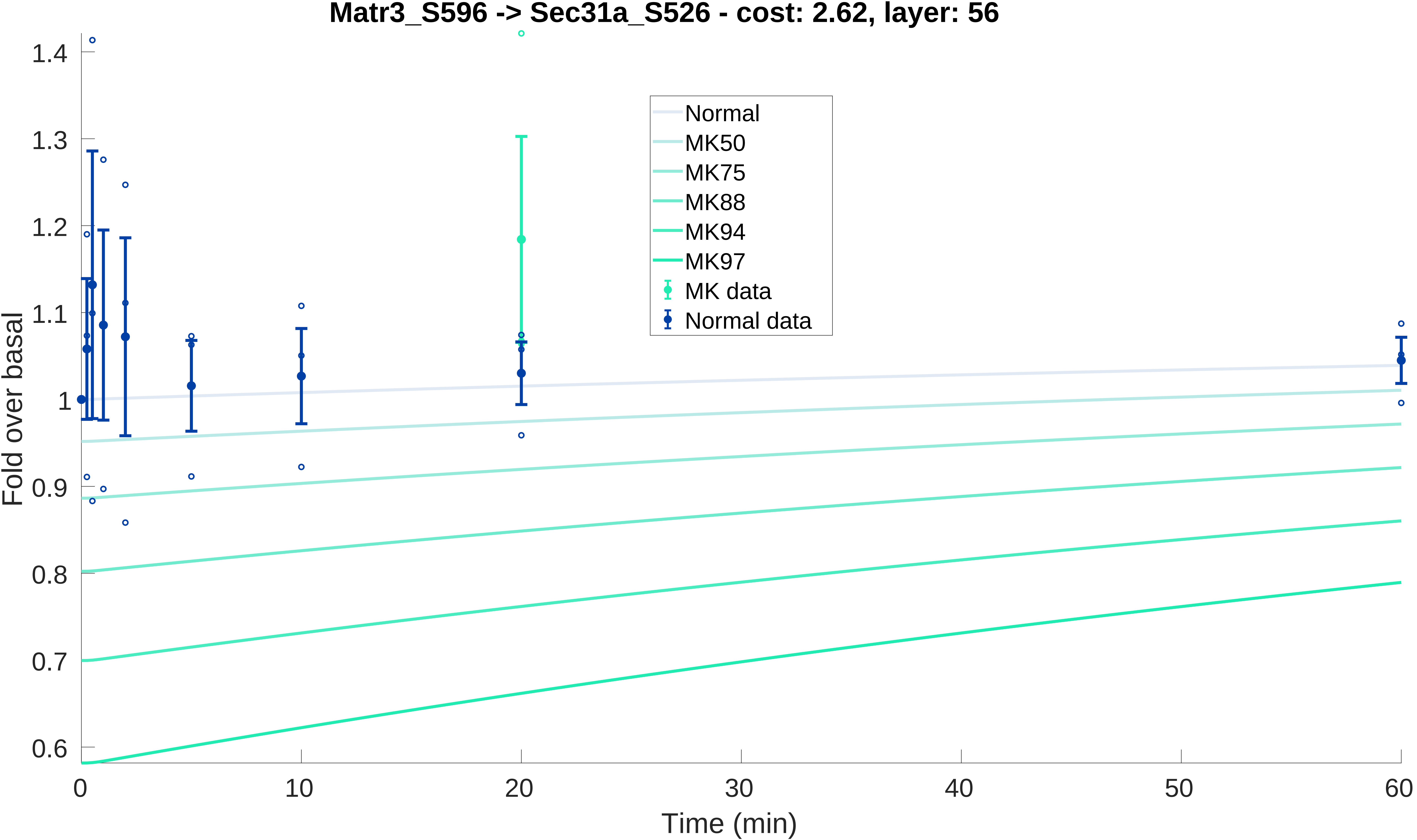

Matr3_S596 -> Sec31a_S526 - cost: 2.62, layer: 56
Fold over basal
1.4
1.3
1.2
1.1
1
0.9
0.8
0.7
0.6
0
10
20
30
40
50
60
Time (min)
Normal
MK50
MK75
MK88
MK94
MK97
MK data
Normal data

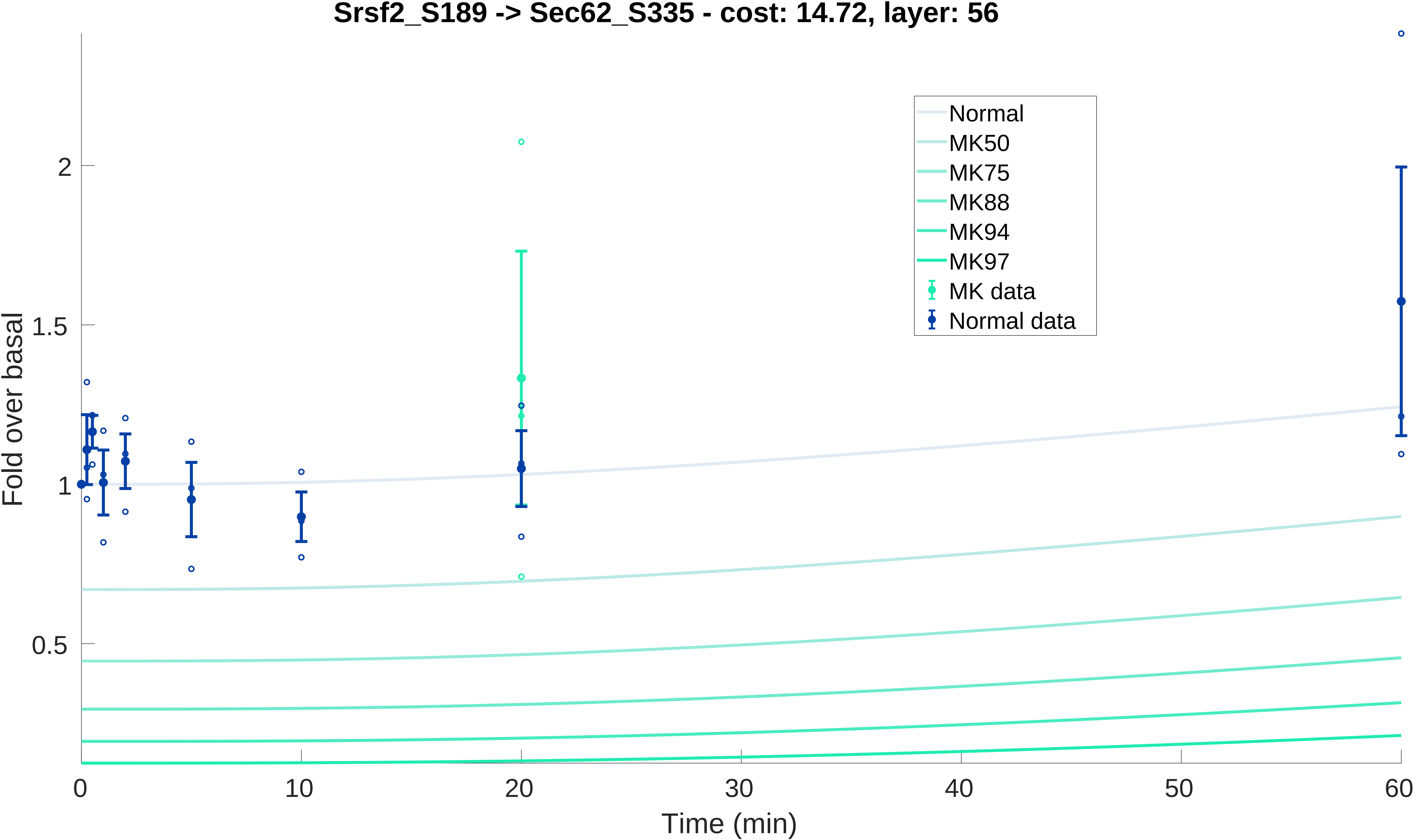

Srsf2_S189 -> Sec62_S335 - cost: 14.72, layer: 56
Fold over basal
Time (min)
0.5
1
1.5
2
0
10
20
30
40
50
60
Normal
MK50
MK75
MK88
MK94
MK97
MK data
Normal data

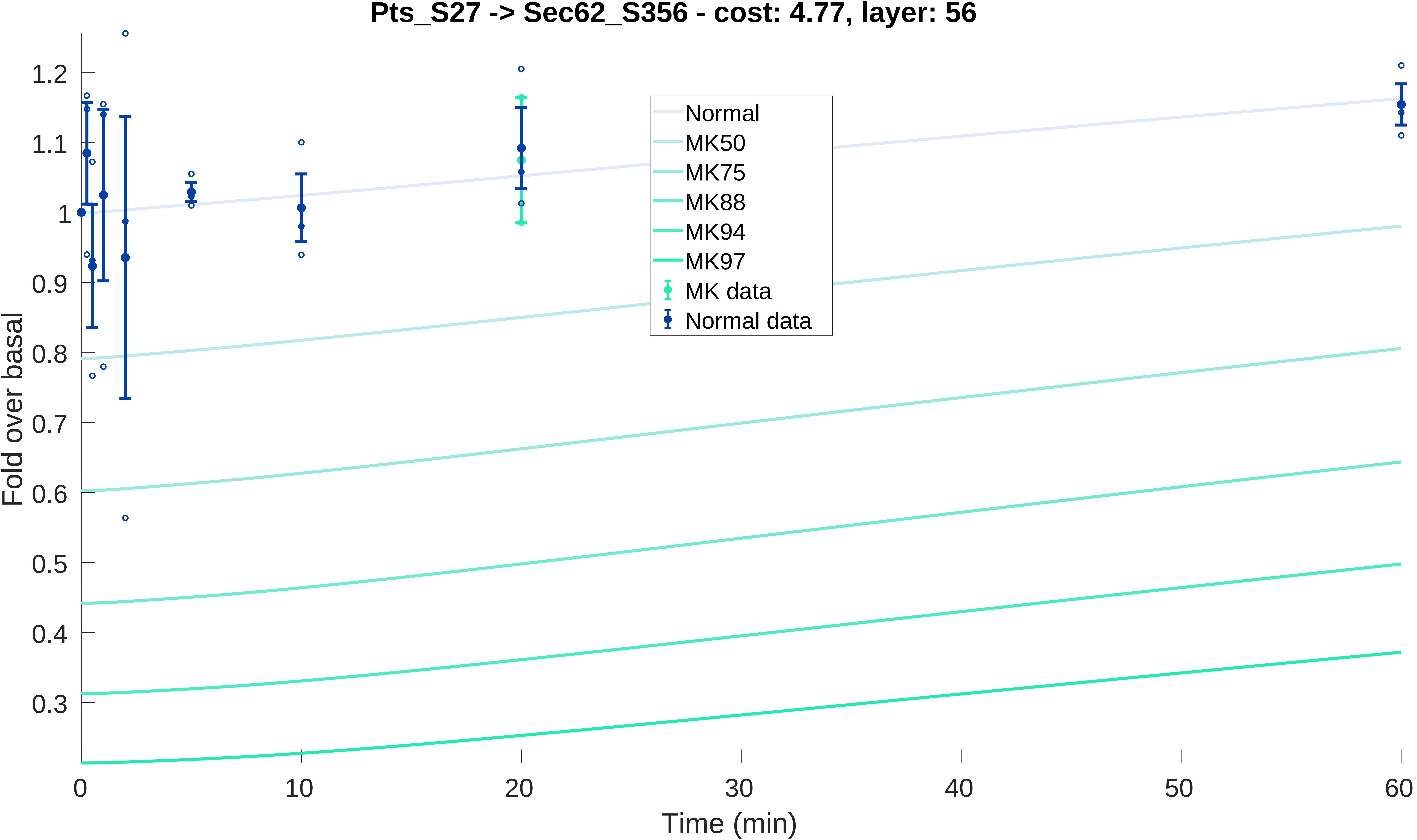
Pts_S27 -> Sec62_S356 - cost: 4.77, layer: 56
Fold over basal
Time (min)
1.2
1.1
1
0.9
0.8
0.7
0.6
0.5
0.4
0.3
0
10
20
30
40
50
60
Normal
MK50
MK75
MK88
MK94
MK97
MK data
Normal data

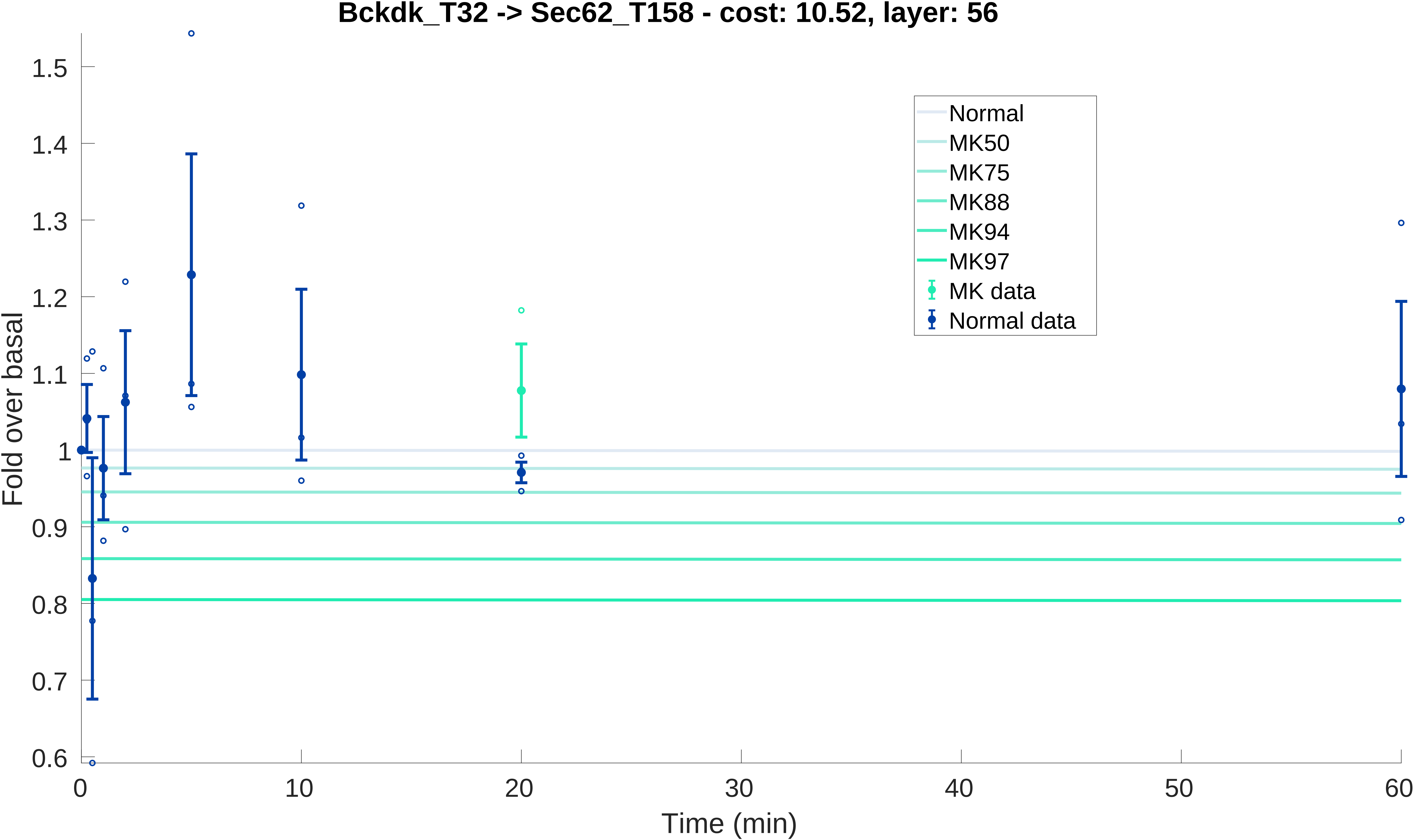

Bckdk_T32 -> Sec62_T158 - cost: 10.52, layer: 56
Fold over basal
Time (min)
1.5
1.4
1.3
1.2
1.1
1
0.9
0.8
0.7
0.6
0
10
20
30
40
50
60
Normal
MK50
MK75
MK88
MK94
MK97
MK data
Normal data

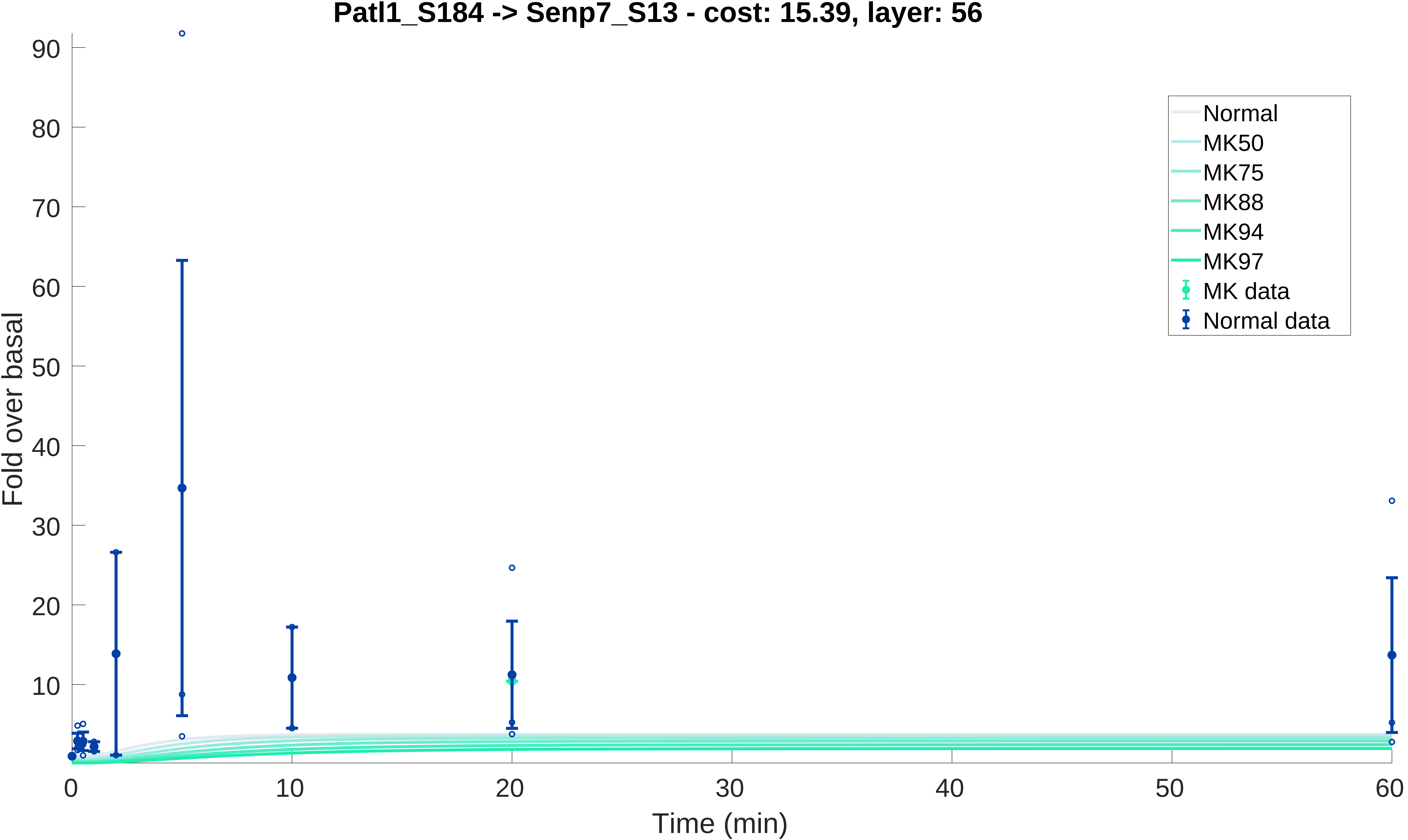

Patl1_S184 -> Senp7_S13 - cost: 15.39, layer: 56
Fold over basal
Time (min)
0
10
20
30
40
50
60
70
80
90
Normal
MK50
MK75
MK88
MK94
MK97
MK data
Normal data

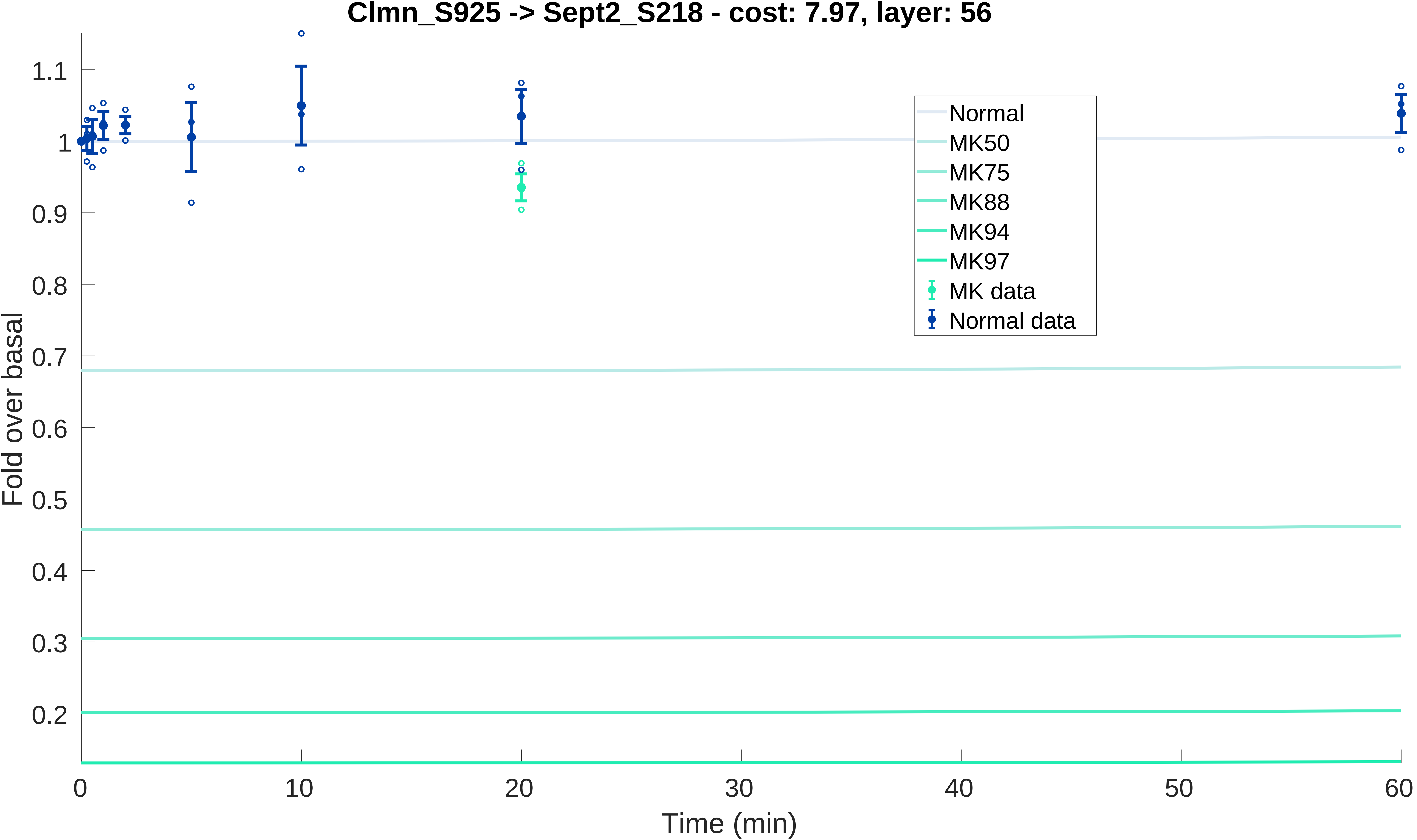

Clmn_S925 -> Sept2_S218 - cost: 7.97, layer: 56
Fold over basal
Time (min)
Normal
MK50
MK75
MK88
MK94
MK97
MK data
Normal data
1.1
1
0.9
0.8
0.7
0.6
0.5
0.4
0.3
0.2
0
10
20
30
40
50
60

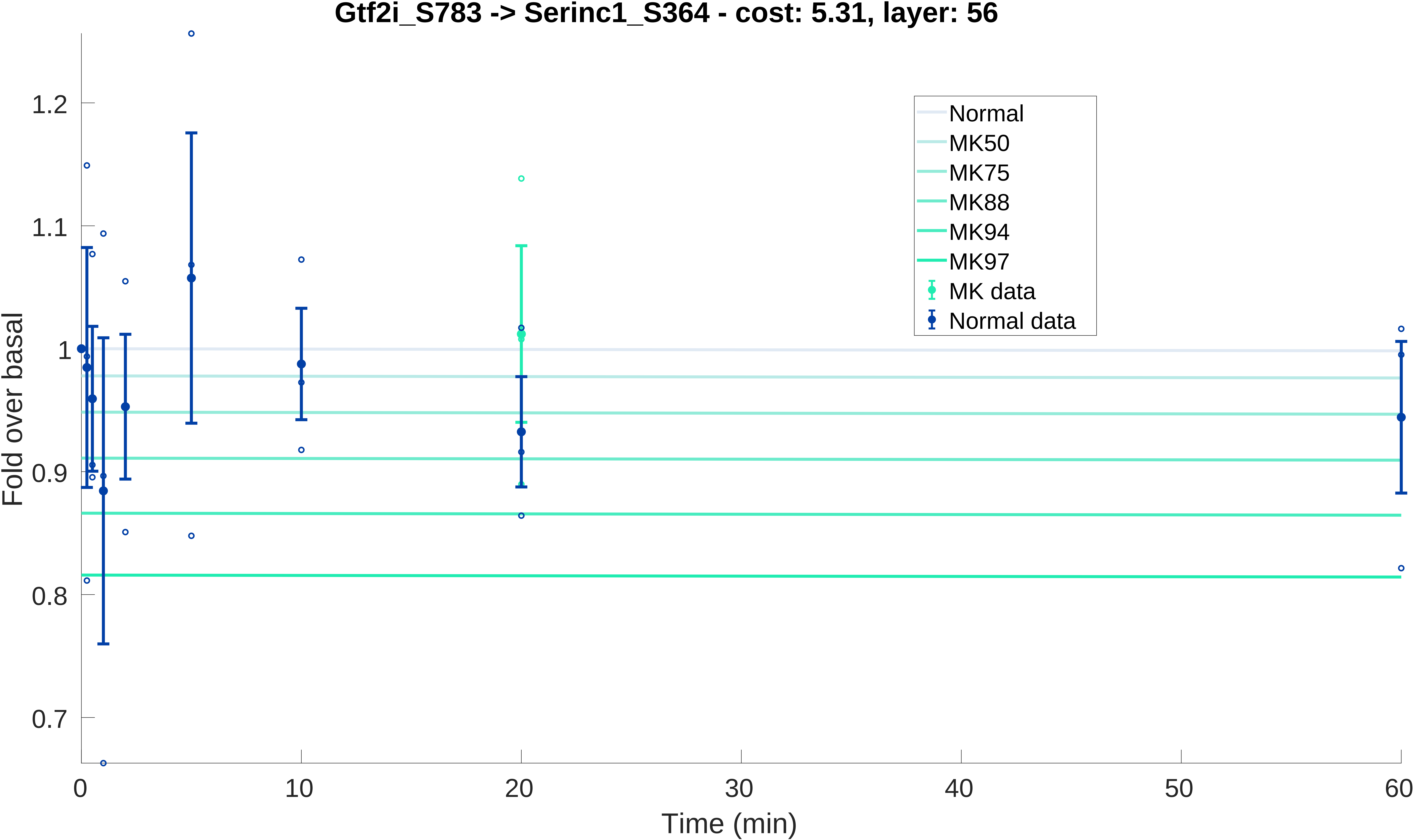

Gtf2i_S783 -> Serinc1_S364 - cost: 5.31, layer: 56
Fold over basal
Time (min)
0.7
0.8
0.9
1
1.1
1.2
0
10
20
30
40
50
60
Normal
MK50
MK75
MK88
MK94
MK97
MK data
Normal data

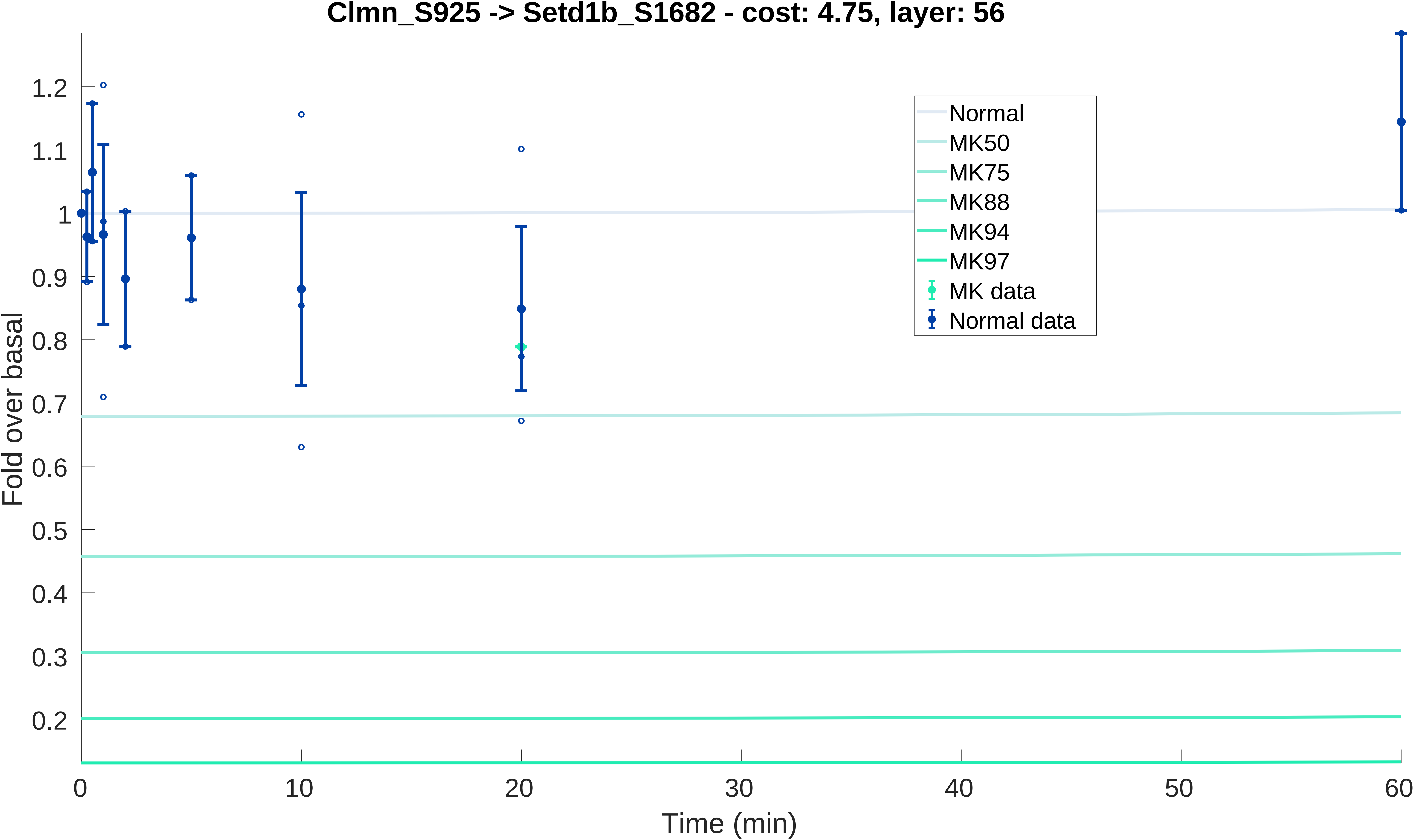

Clmn_S925 -> Setd1b_S1682 - cost: 4.75, layer: 56
Fold over basal
Time (min)
Normal
MK50
MK75
MK88
MK94
MK97
MK data
Normal data
0.2
0.3
0.4
0.5
0.6
0.7
0.8
0.9
1
1.1
1.2
0
10
20
30
40
50
60

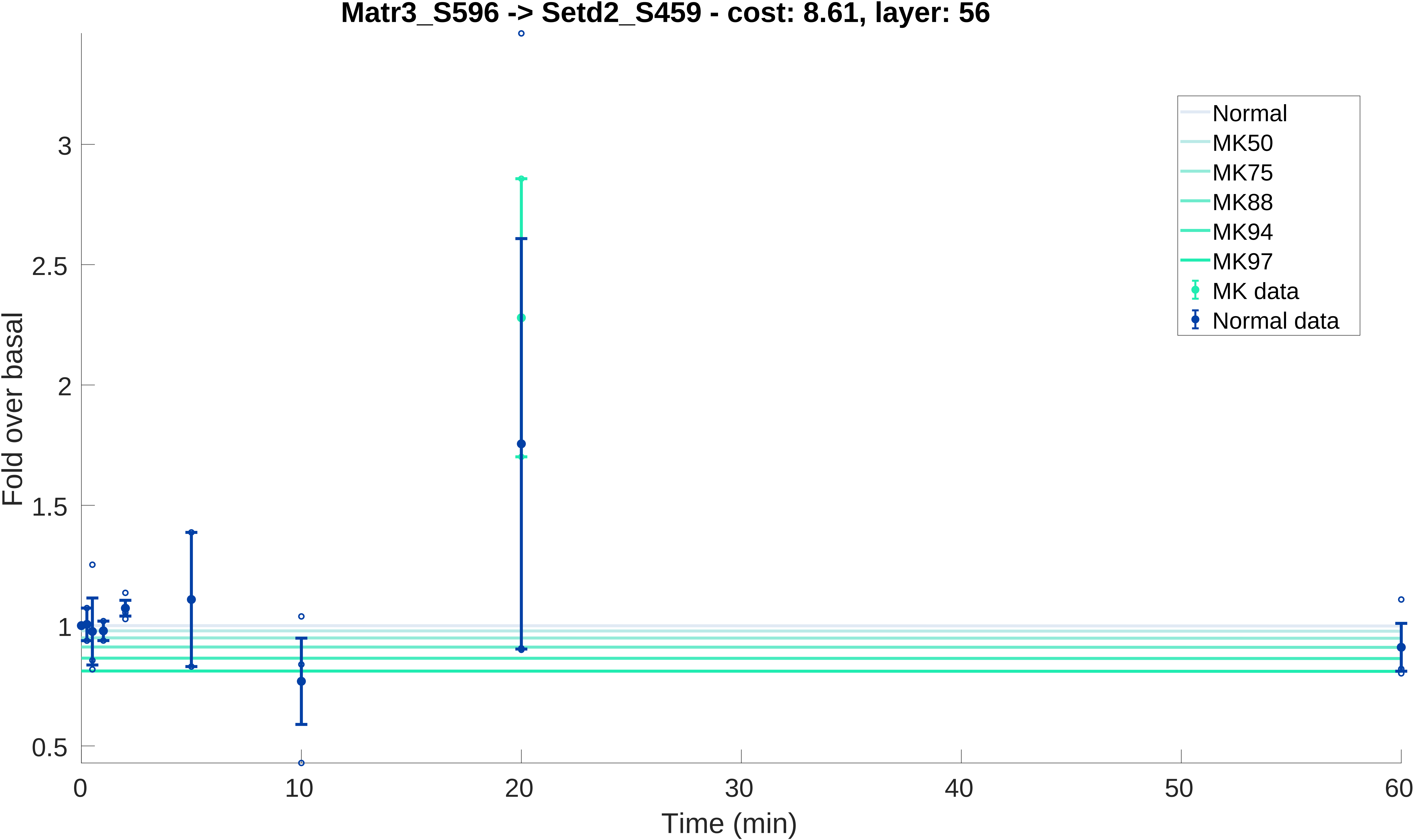

Matr3_S596 -> Setd2_S459 - cost: 8.61, layer: 56
Fold over basal
Time (min)
0.5
1
1.5
2
2.5
3
0
10
20
30
40
50
60
Normal
MK50
MK75
MK88
MK94
MK97
MK data
Normal data

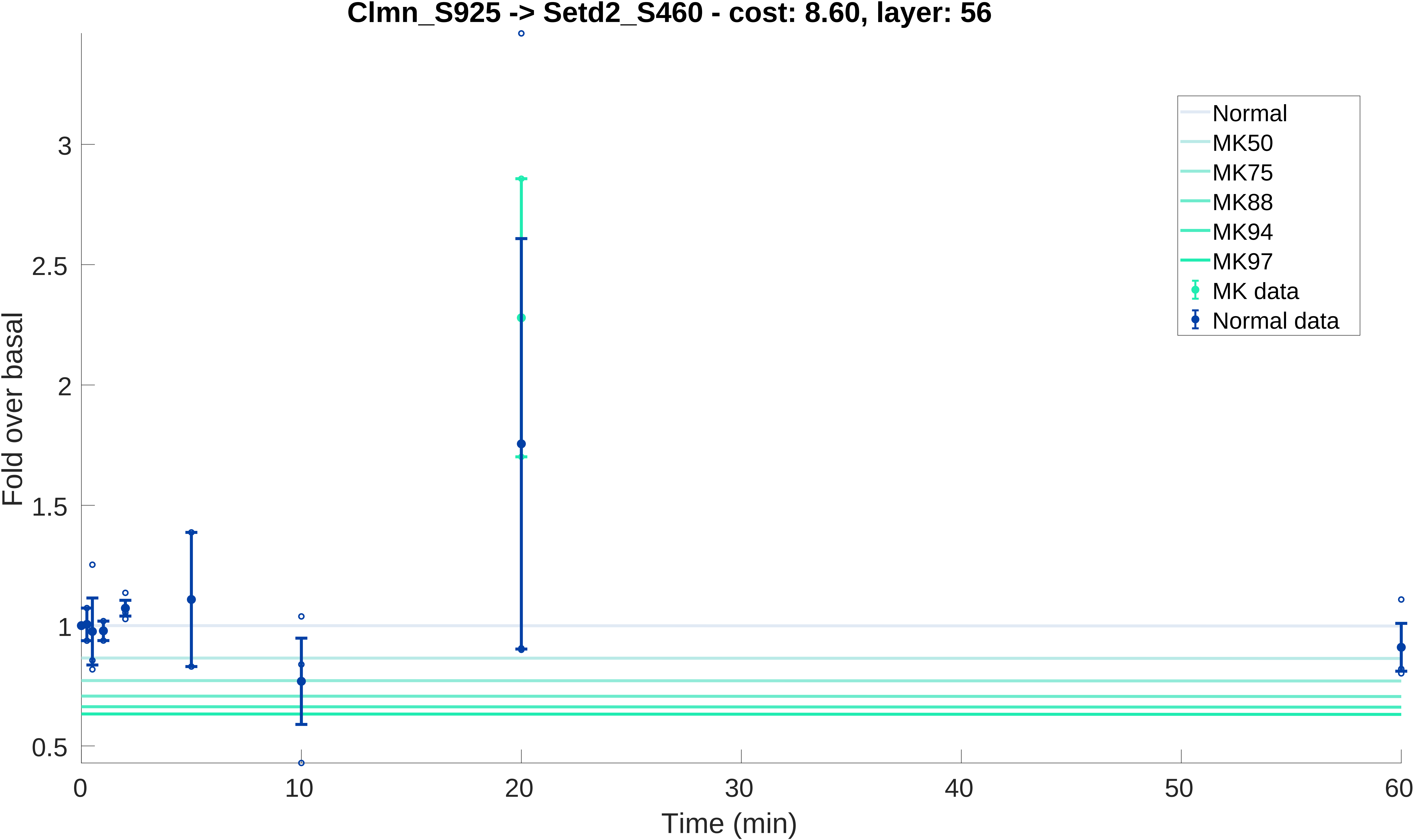
Clmn_S925 -> Setd2_S460 - cost: 8.60, layer: 56
Fold over basal
Time (min)
0.5
1
1.5
2
2.5
3
0
10
20
30
40
50
60
Normal
MK50
MK75
MK88
MK94
MK97
MK data
Normal data

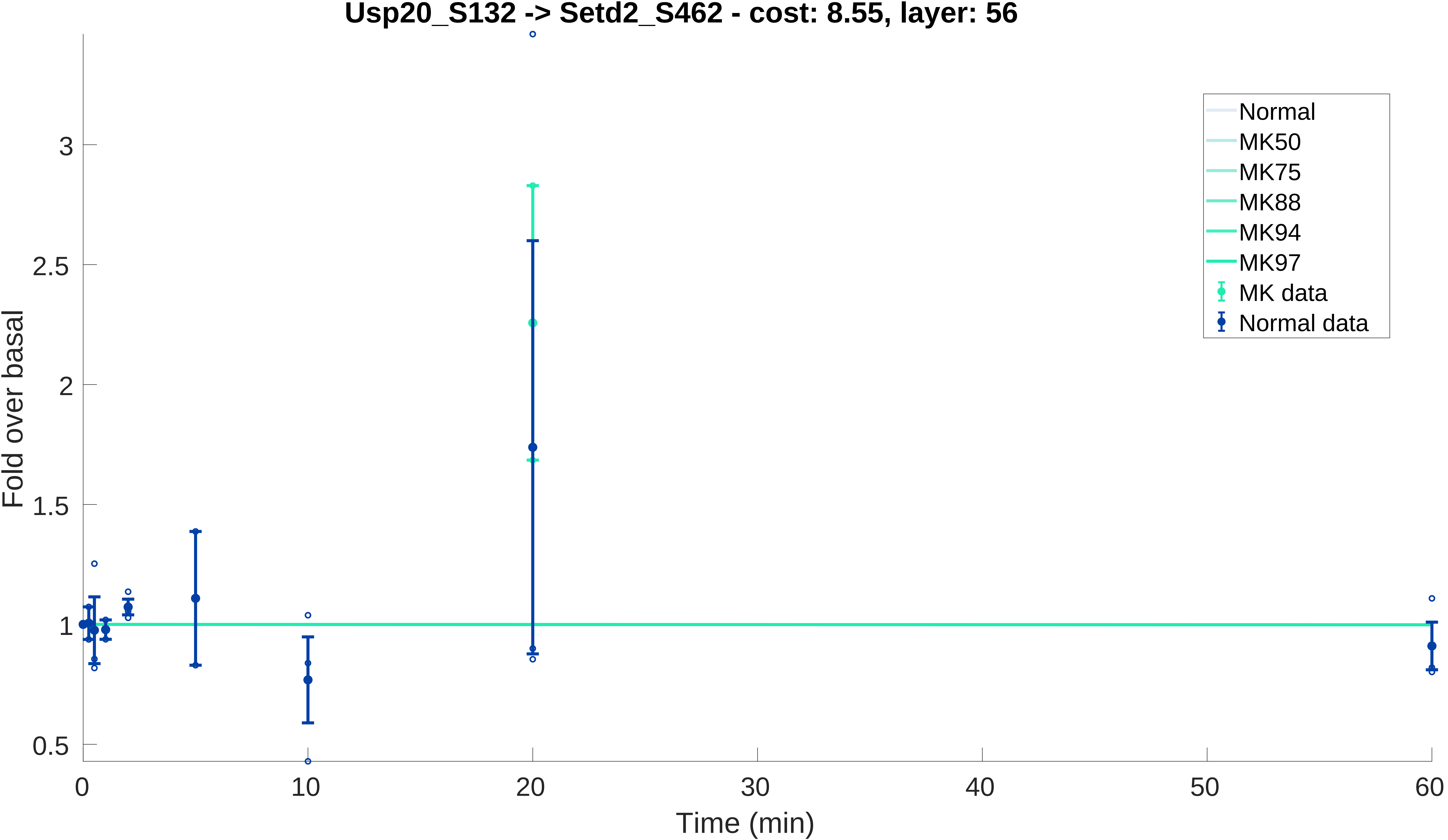

Usp20_S132 -> Setd2_S462 - cost: 8.55, layer: 56
Fold over basal
Time (min)
0.5
1
1.5
2
2.5
3
0
10
20
30
40
50
60
Normal
MK50
MK75
MK88
MK94
MK97
MK data
Normal data

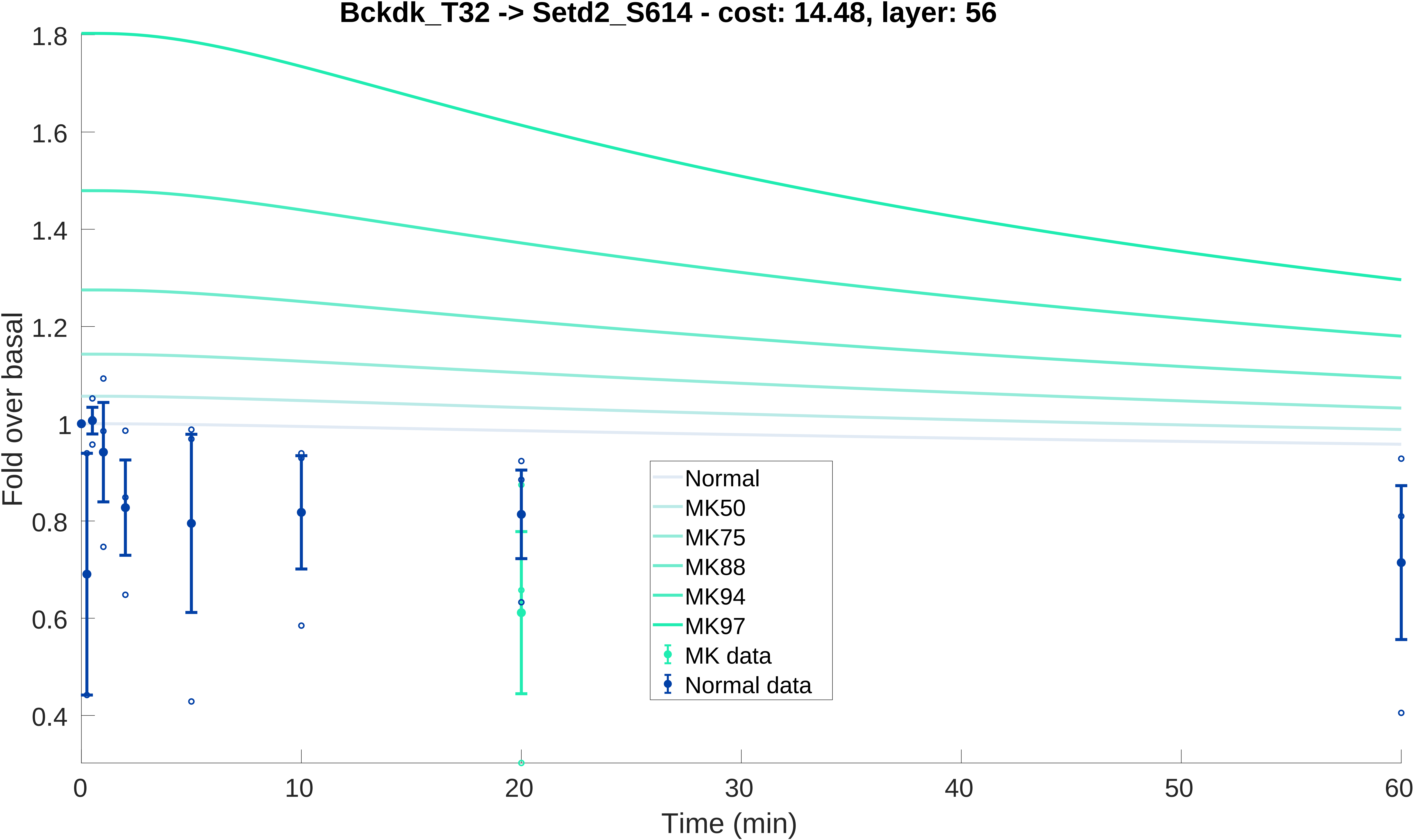

Bckdk_T32 -> Setd2_S614 - cost: 14.48, layer: 56
Fold over basal
1.8
1.6
1.4
1.2
1
0.8
0.6
0.4
0
10
20
30
40
50
60
Time (min)
Normal
MK50
MK75
MK88
MK94
MK97
MK data
Normal data

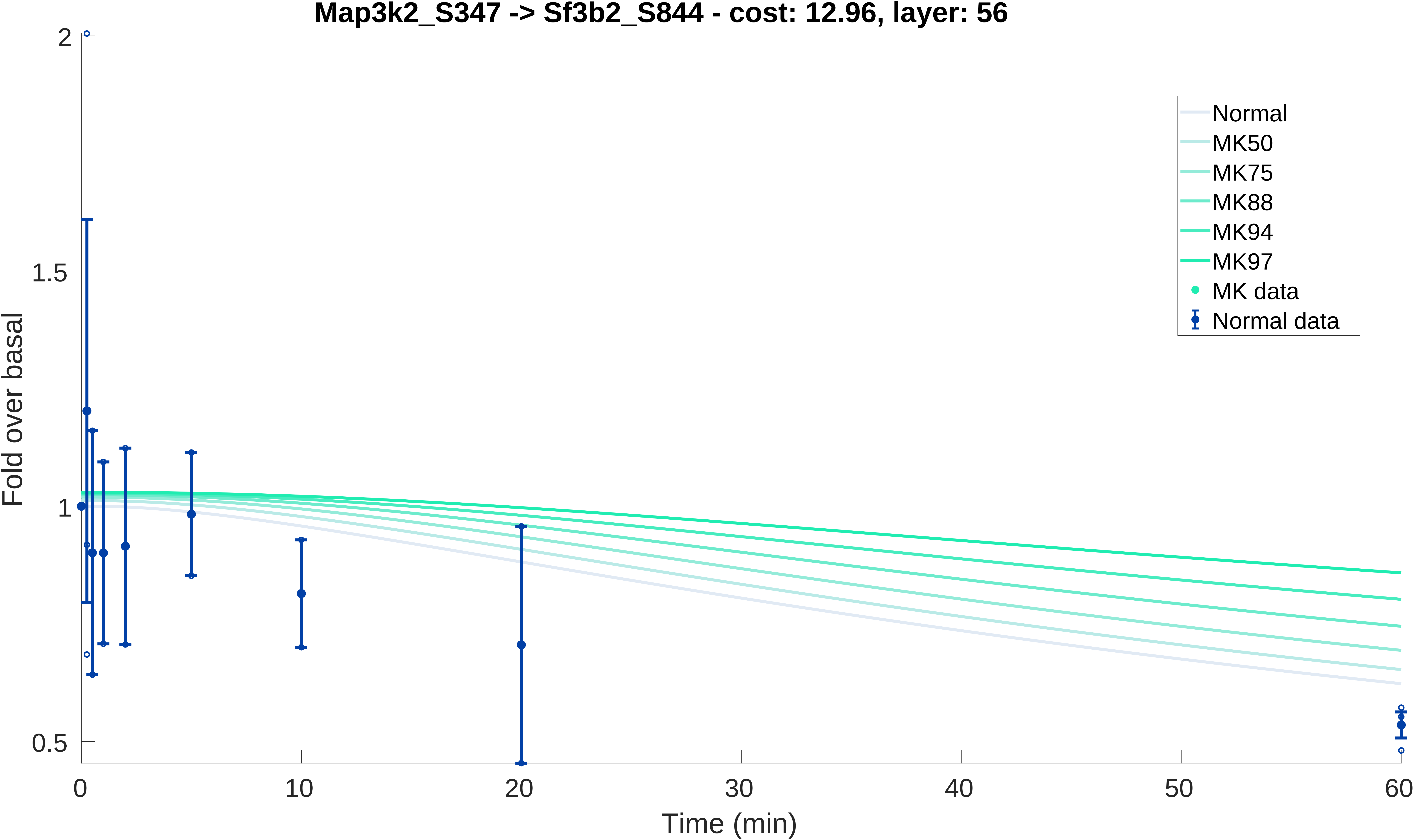

Map3k2_S347 -> Sf3b2_S844 - cost: 12.96, layer: 56
Fold over basal
2
1.5
1
0.5
0
10
20
30
40
50
60
Time (min)
Normal
MK50
MK75
MK88
MK94
MK97
MK data
Normal data

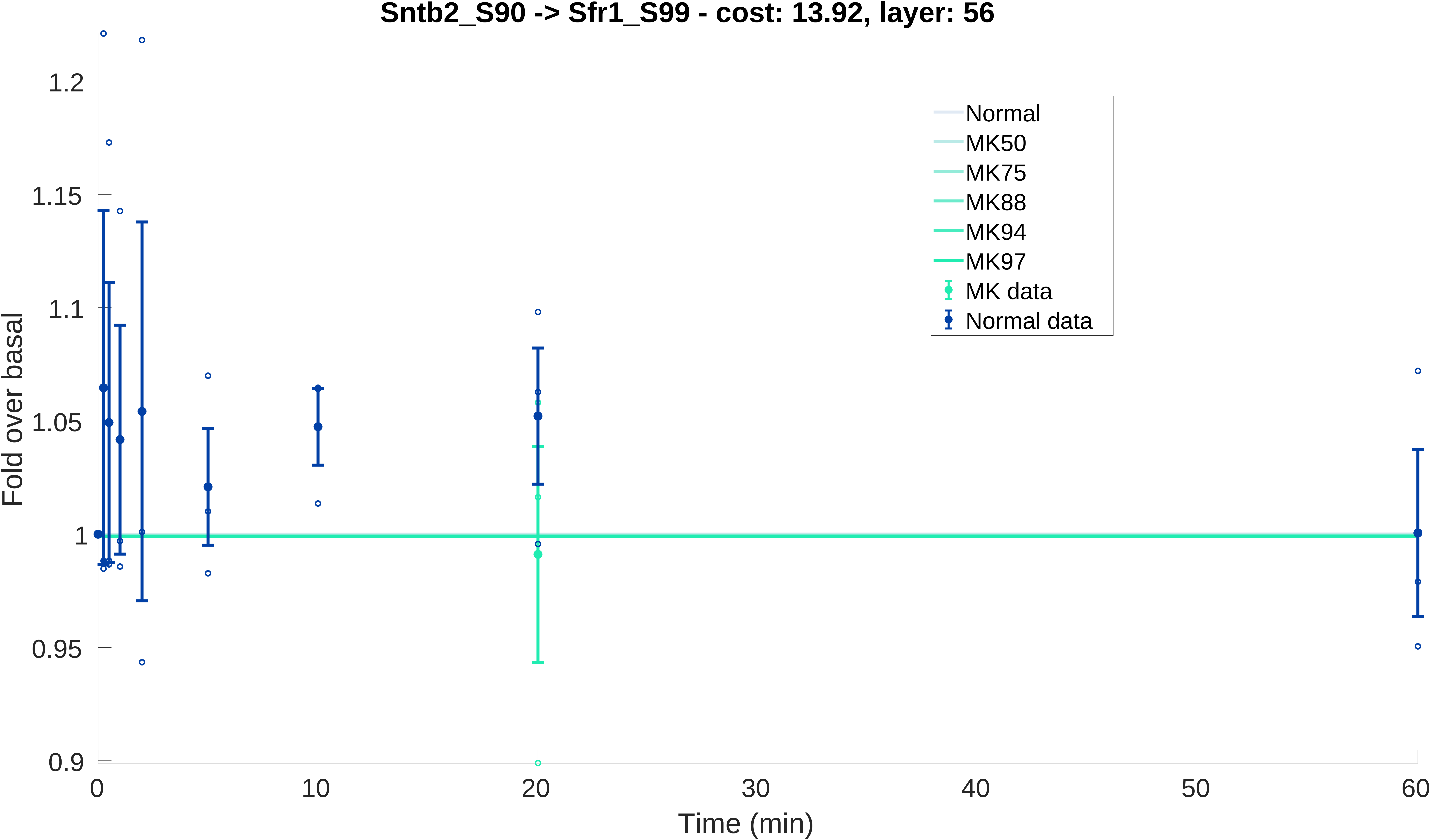

Sntb2_S90 -> Sfr1_S99 - cost: 13.92, layer: 56
Fold over basal
Time (min)
Normal
MK50
MK75
MK88
MK94
MK97
MK data
Normal data
1.2
1.15
1.1
1.05
1
0.95
0.9
0
10
20
30
40
50
60

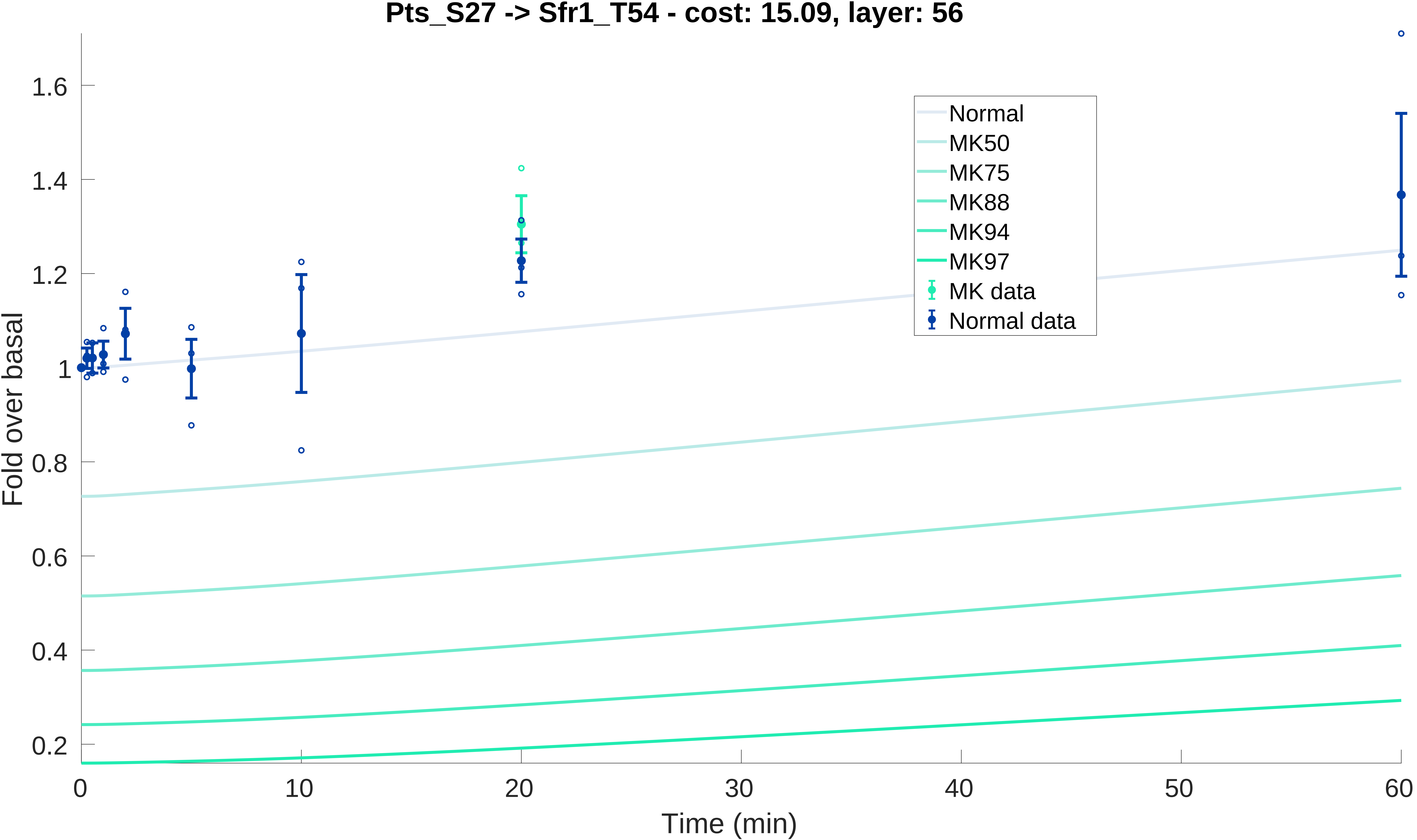

Pts_S27 -> Sfr1_T54 - cost: 15.09, layer: 56
Normal
MK50
MK75
MK88
MK94
MK97
MK data
Normal data
Fold over basal
Time (min)
0.2
0.4
0.6
0.8
1
1.2
1.4
1.6
0
10
20
30
40
50
60

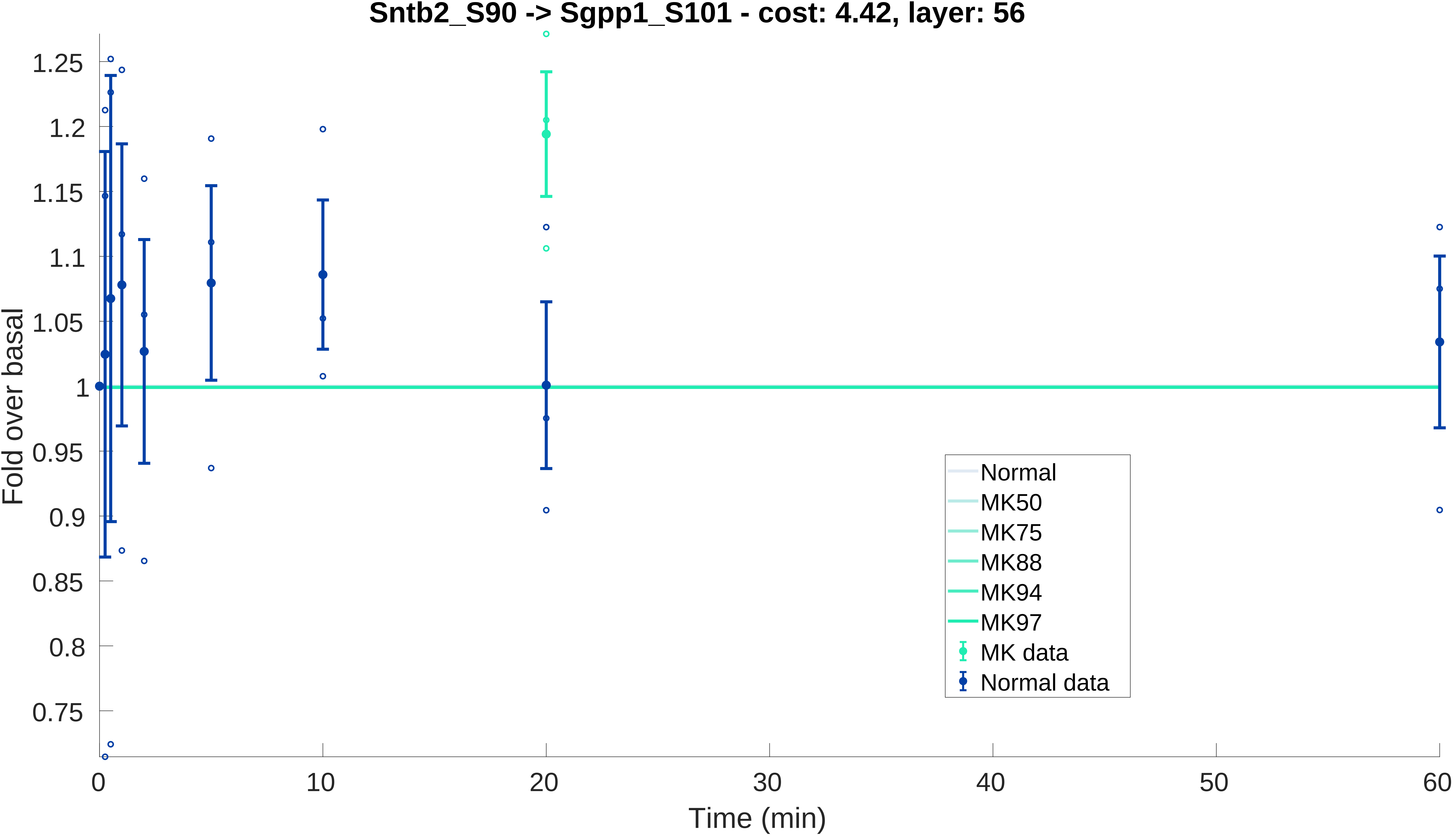

Sntb2_S90 -> Sgpp1_S101 - cost: 4.42, layer: 56
Fold over basal
Time (min)
1.25
1.2
1.15
1.1
1.05
1
0.95
0.9
0.85
0.8
0.75
0
10
20
30
40
50
60
Normal
MK50
MK75
MK88
MK94
MK97
MK data
Normal data

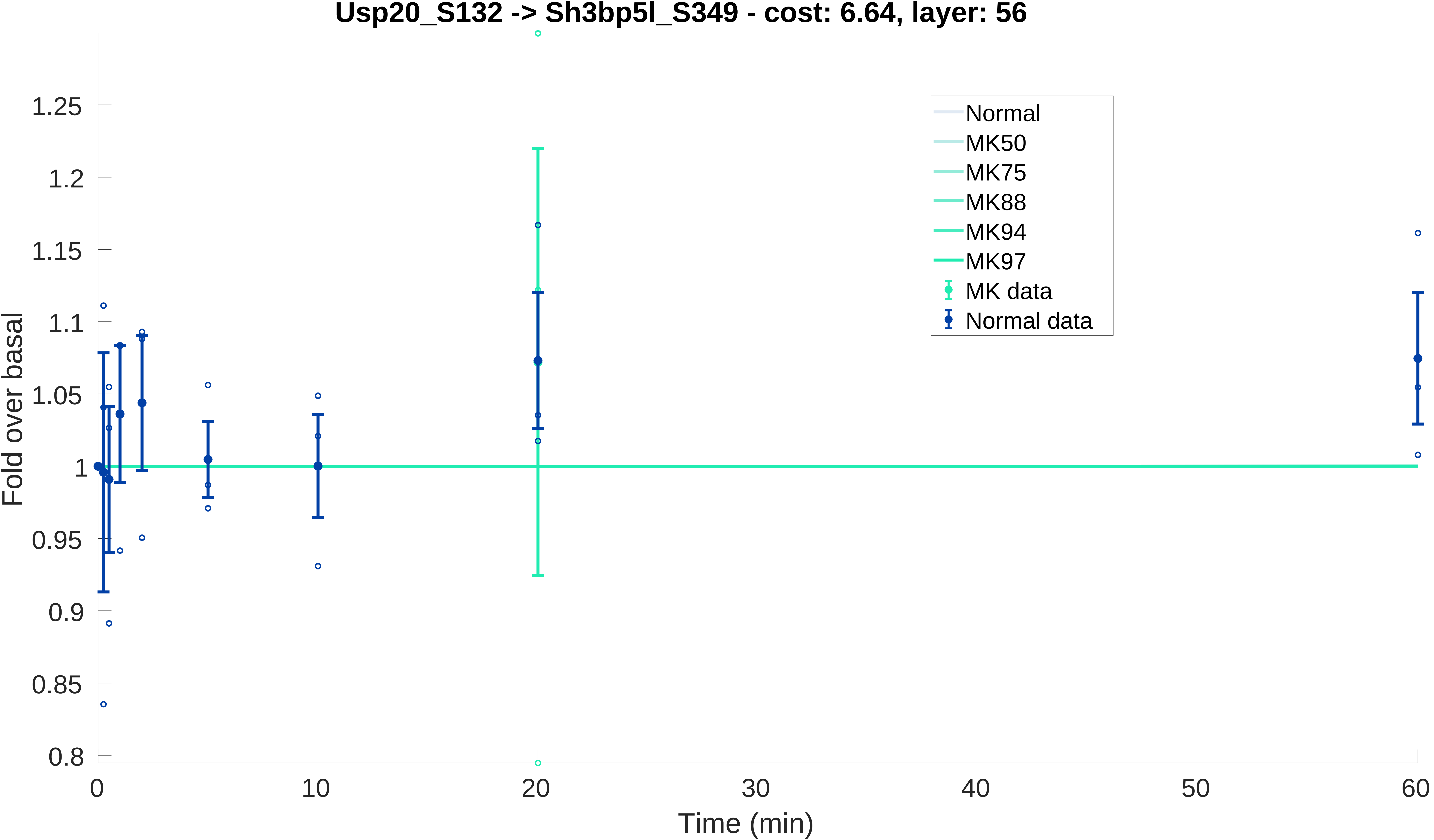
Usp20_S132 -> Sh3bp5l_S349 - cost: 6.64, layer: 56
Fold over basal
1.25
1.2
1.15
1.1
1.05
1
0.95
0.9
0.85
0.8
0
10
20
30
40
50
60
Time (min)
Normal
MK50
MK75
MK88
MK94
MK97
MK data
Normal data

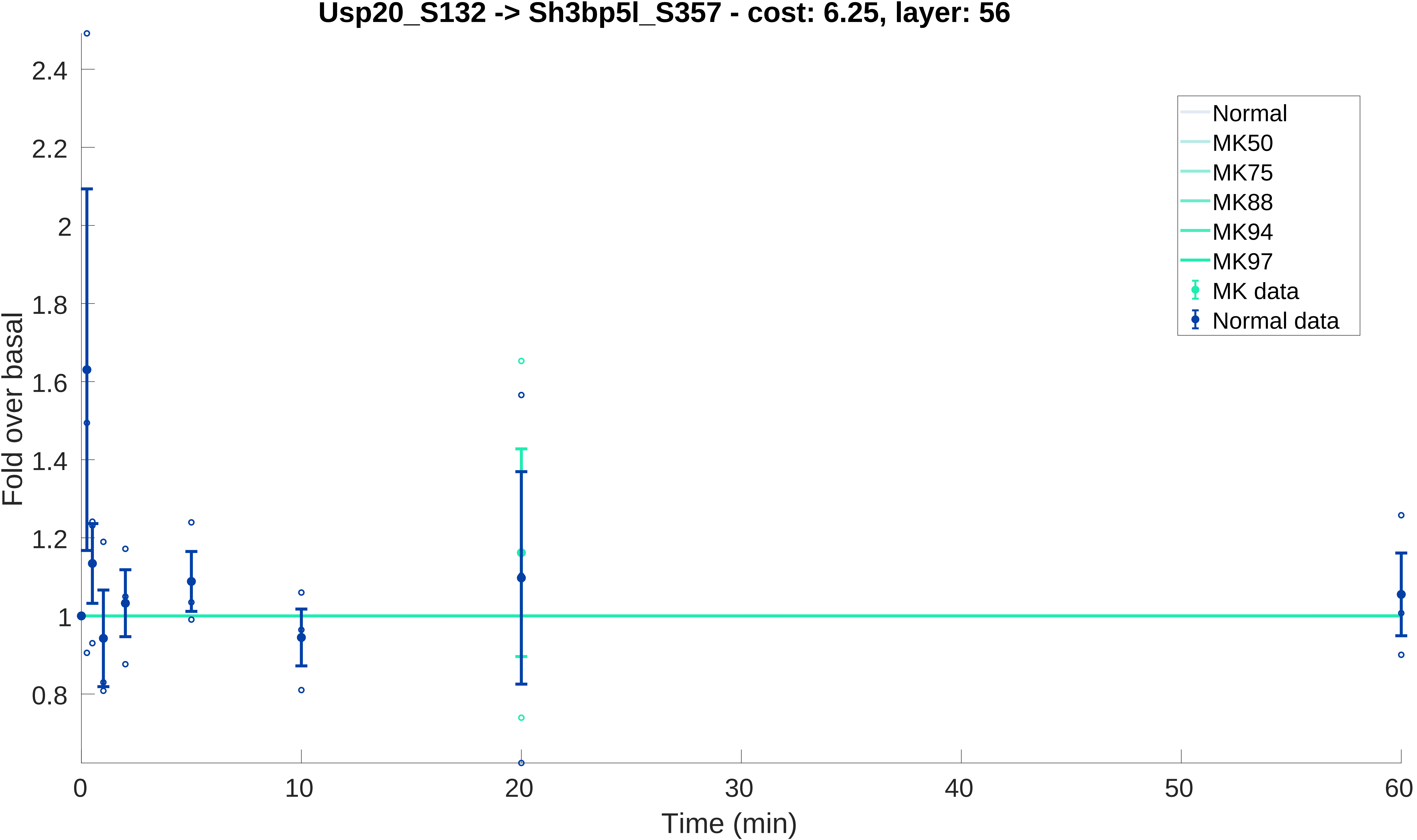

Usp20_S132 -> Sh3bp5l_S357 - cost: 6.25, layer: 56
Fold over basal
Time (min)
2.4
2.2
2
1.8
1.6
1.4
1.2
1
0.8
0
10
20
30
40
50
60
Normal
MK50
MK75
MK88
MK94
MK97
MK data
Normal data

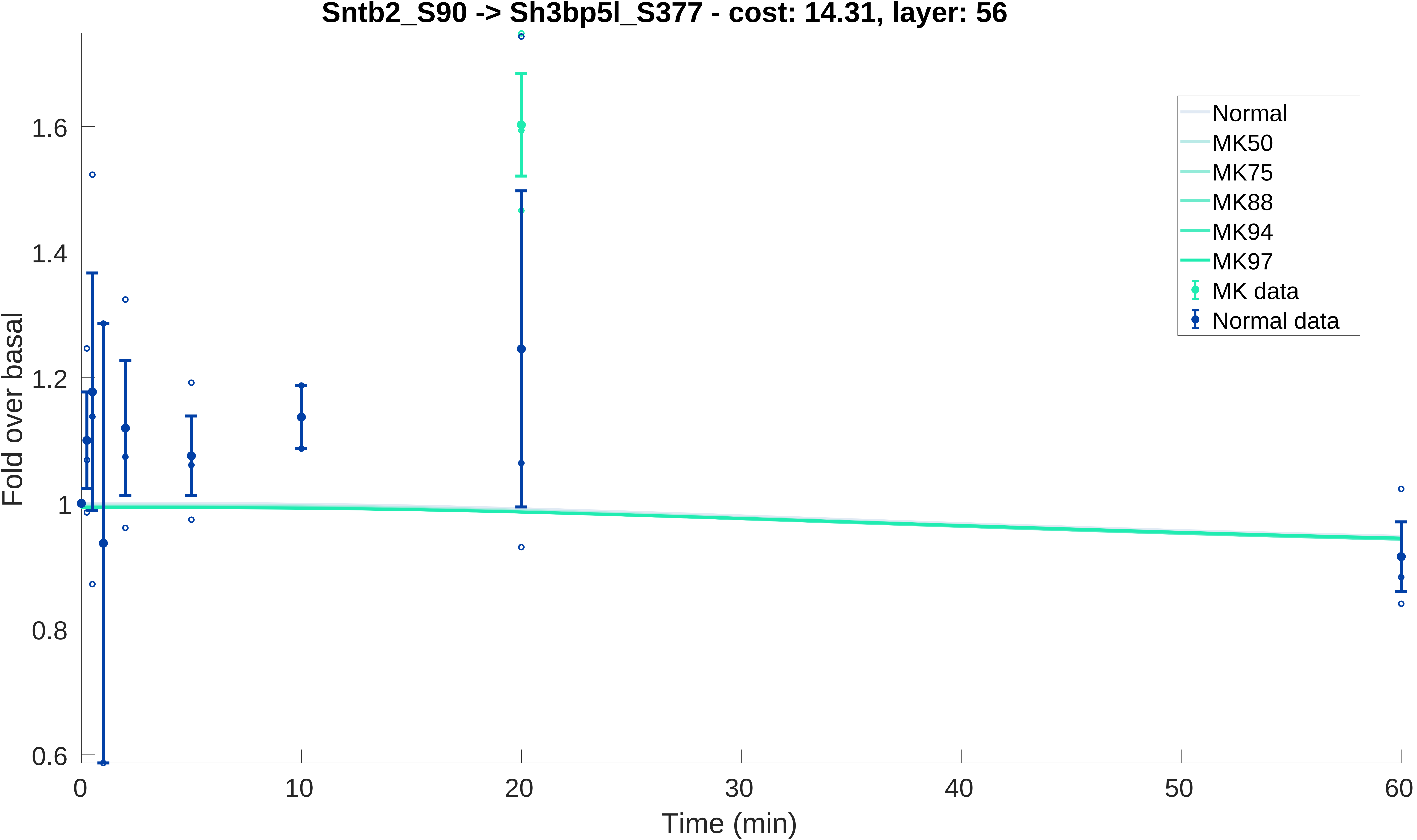

Sntb2_S90 -> Sh3bp5l_S377 - cost: 14.31, layer: 56
Fold over basal
Time (min)
Normal
MK50
MK75
MK88
MK94
MK97
MK data
Normal data
0.6
0.8
1
1.2
1.4
1.6
0
10
20
30
40
50
60

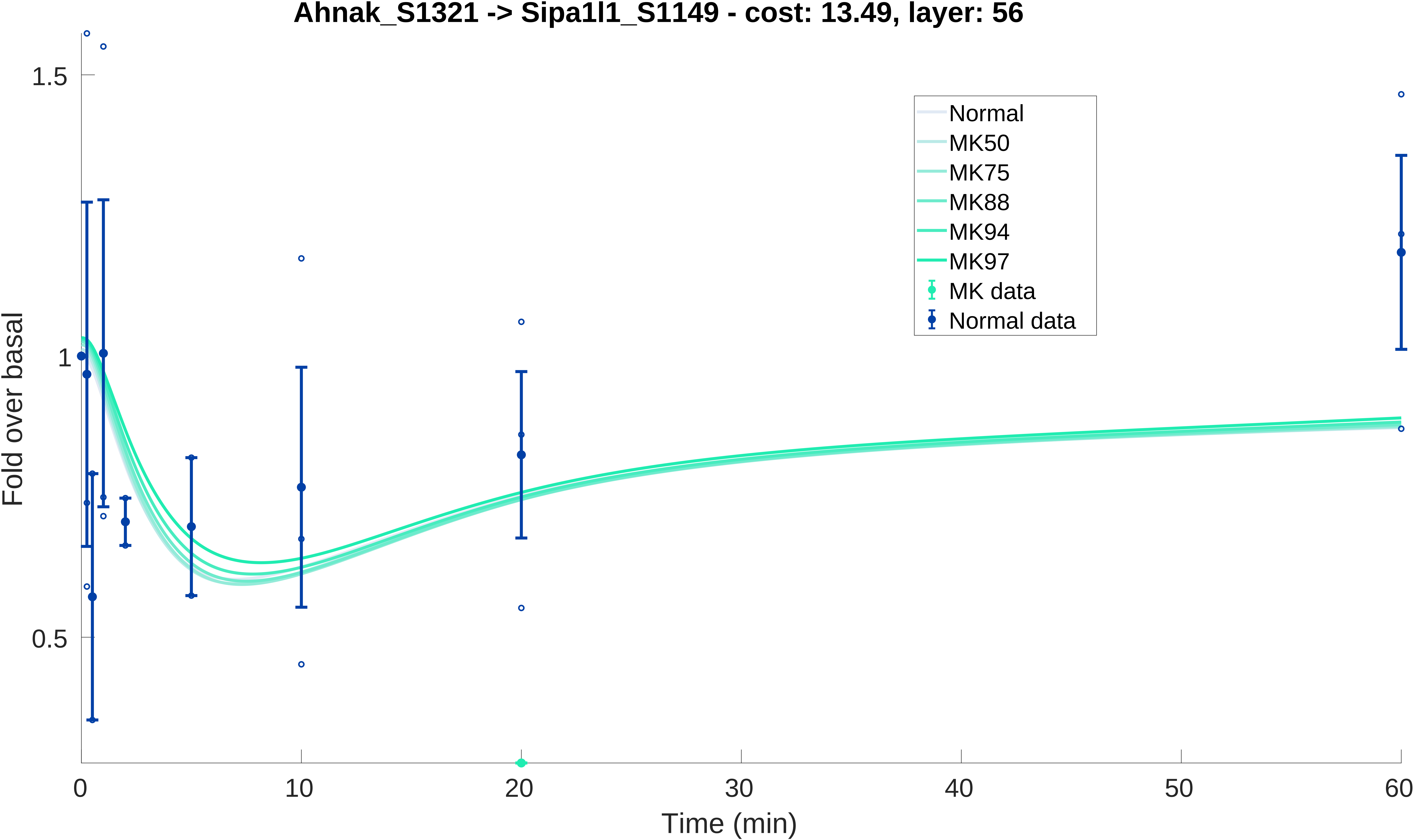

Ahnak_S1321 -> Sipa1l1_S1149 - cost: 13.49, layer: 56
Fold over basal
Time (min)
1.5
1
0.5
0
10
20
30
40
50
60
Normal
MK50
MK75
MK88
MK94
MK97
MK data
Normal data

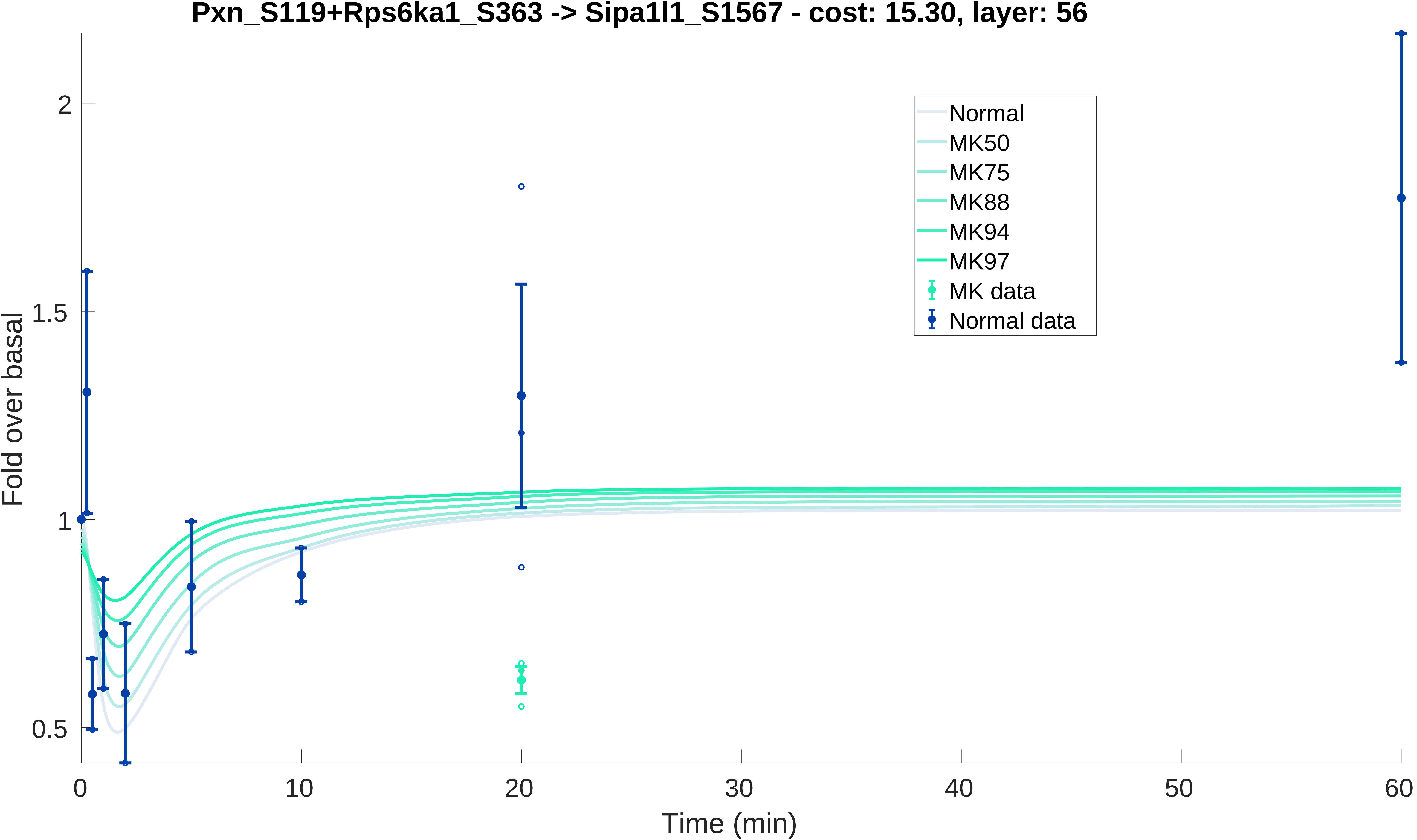

Pxn_S119+Rps6ka1_S363 -> Sipa1l1_S1567 - cost: 15.30, layer: 56
Fold over basal
Time (min)
0.5
1
1.5
2
0
10
20
30
40
50
60
Normal
MK50
MK75
MK88
MK94
MK97
MK data
Normal data

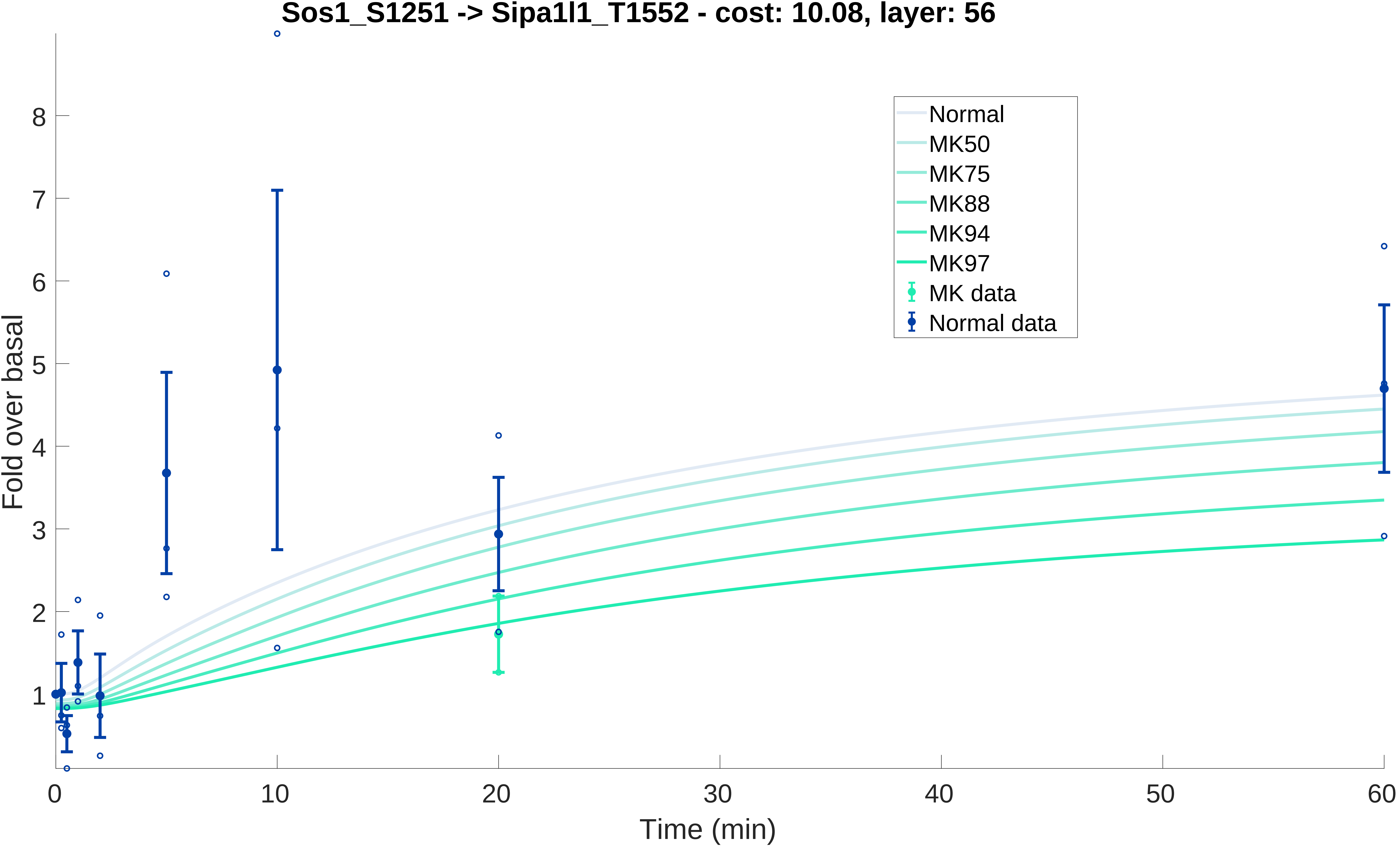

Sos1_S1251 -> Sipa1l1_T1552 - cost: 10.08, layer: 56
Normal
MK50
MK75
MK88
MK94
MK97
MK data
Normal data
Fold over basal
Time (min)
1
2
3
4
5
6
7
8
0
10
20
30
40
50
60

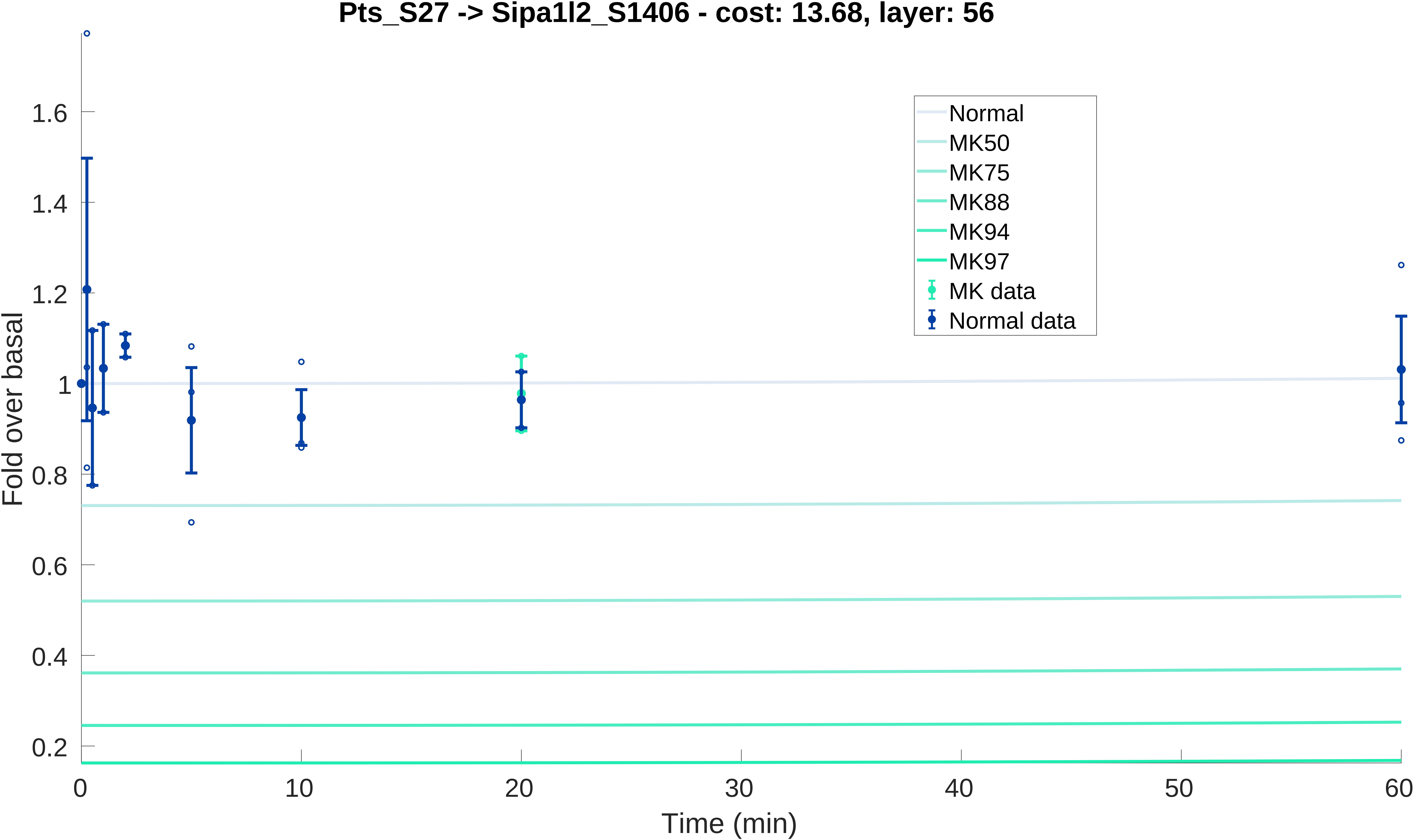

Pts_S27 -> Sipa1l2_S1406 - cost: 13.68, layer: 56
Normal
MK50
MK75
MK88
MK94
MK97
MK data
Normal data
Fold over basal
Time (min)
0
10
20
30
40
50
60
0.2
0.4
0.6
0.8
1
1.2
1.4
1.6

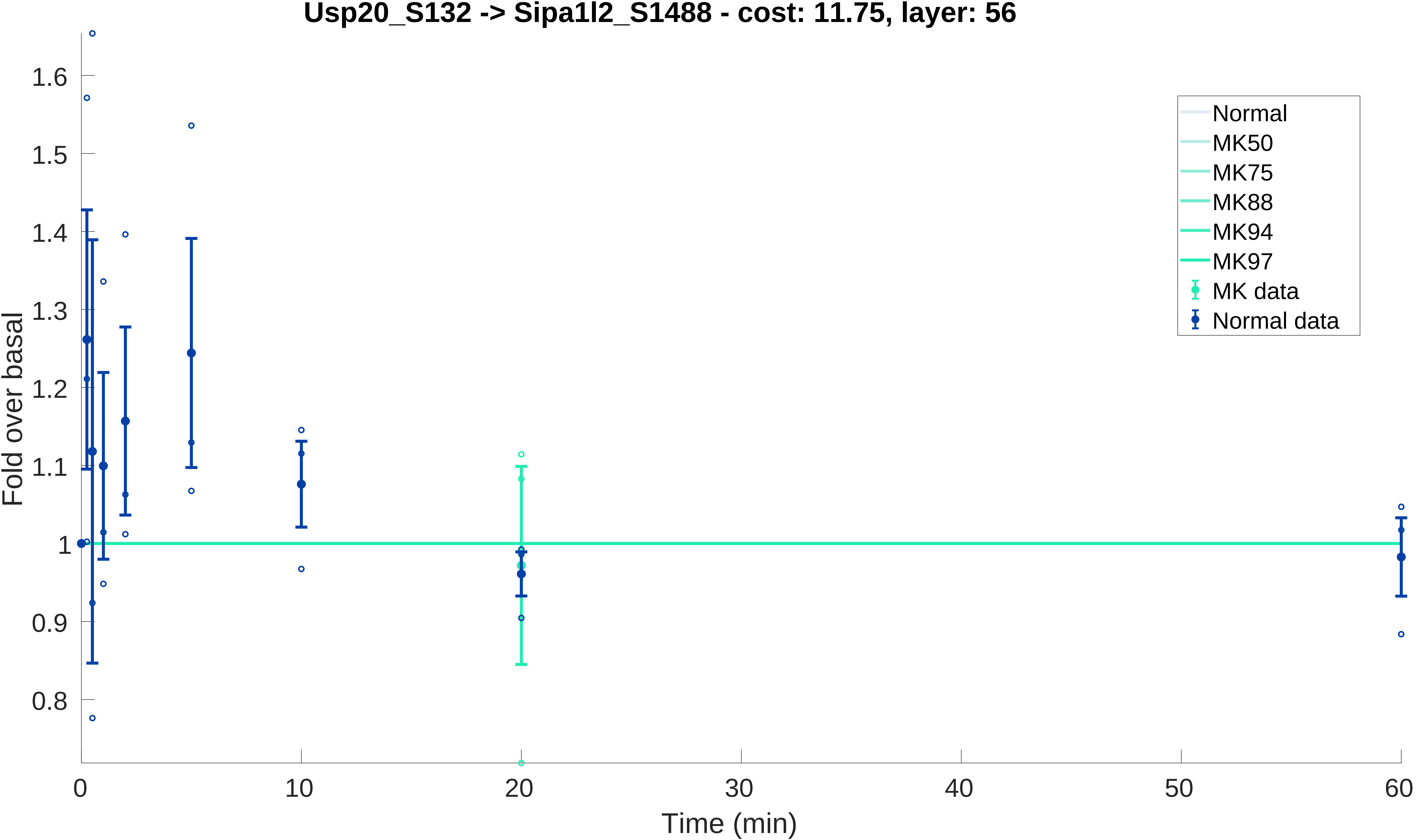

Usp20_S132 -> Sipa1l2_S1488 - cost: 11.75, layer: 56
Fold over basal
1.6
1.5
1.4
1.3
1.2
1.1
1
0.9
0.8
Time (min)
0
10
20
30
40
50
60
Normal
MK50
MK75
MK88
MK94
MK97
MK data
Normal data

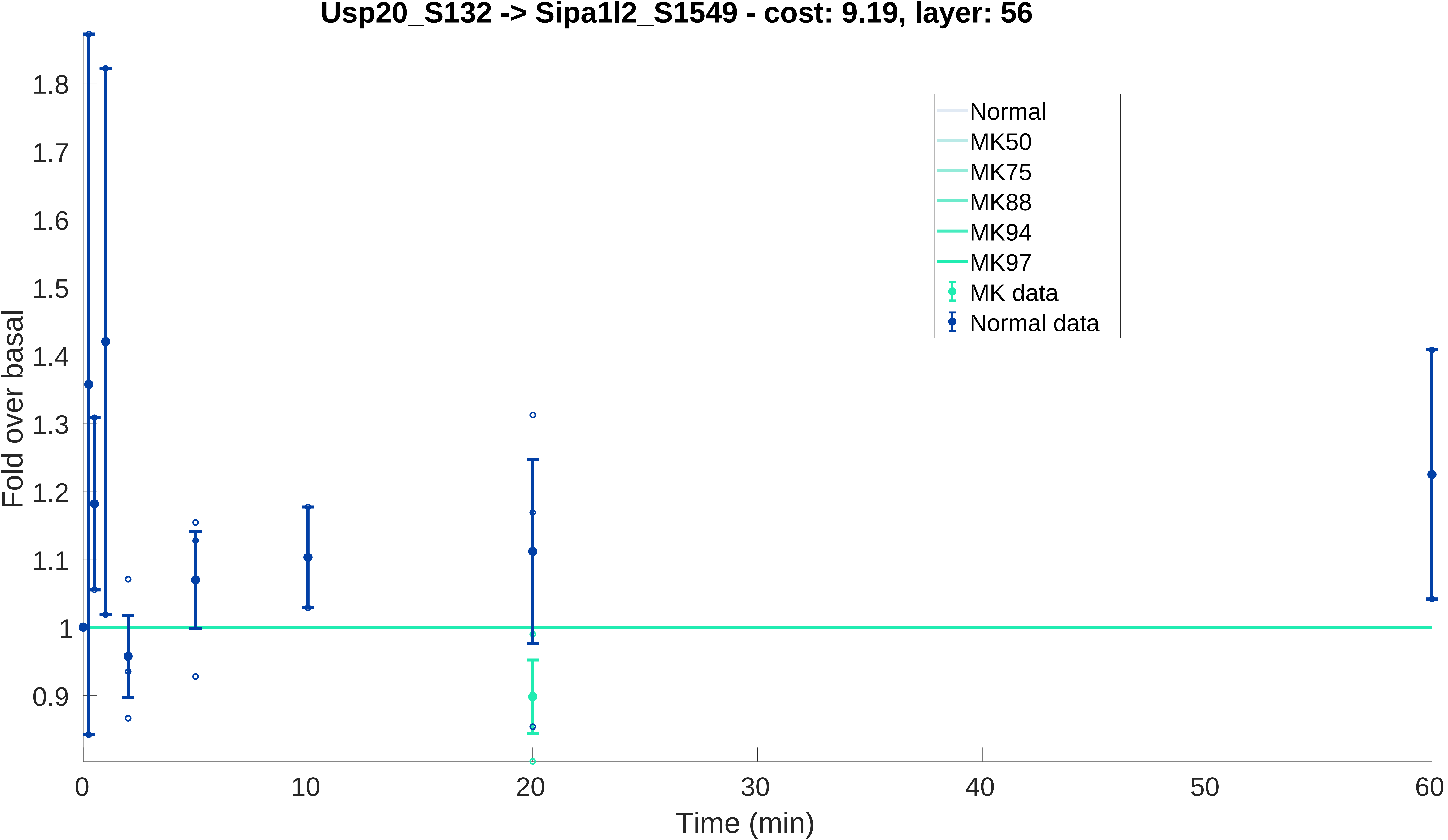
Usp20_S132 -> Sipa1l2_S1549 - cost: 9.19, layer: 56
Fold over basal
1.8
1.7
1.6
1.5
1.4
1.3
1.2
1.1
1
0.9
0
10
20
30
40
50
60
Time (min)
Normal
MK50
MK75
MK88
MK94
MK97
MK data
Normal data

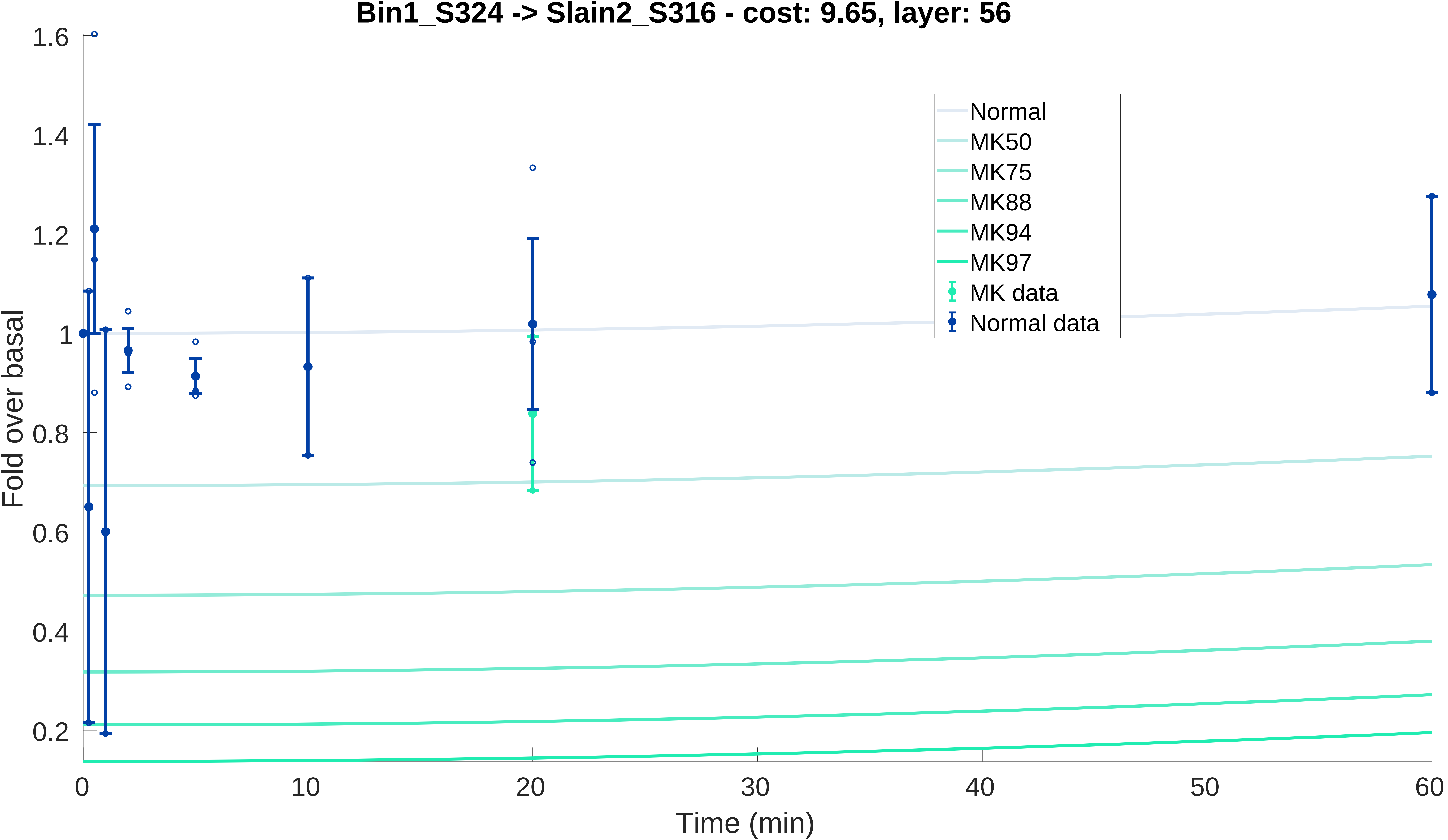
Bin1_S324 -> Slain2_S316 - cost: 9.65, layer: 56
Fold over basal
Time (min)
0
10
20
30
40
50
60
0.2
0.4
0.6
0.8
1
1.2
1.4
1.6
Normal
MK50
MK75
MK88
MK94
MK97
MK data
Normal data

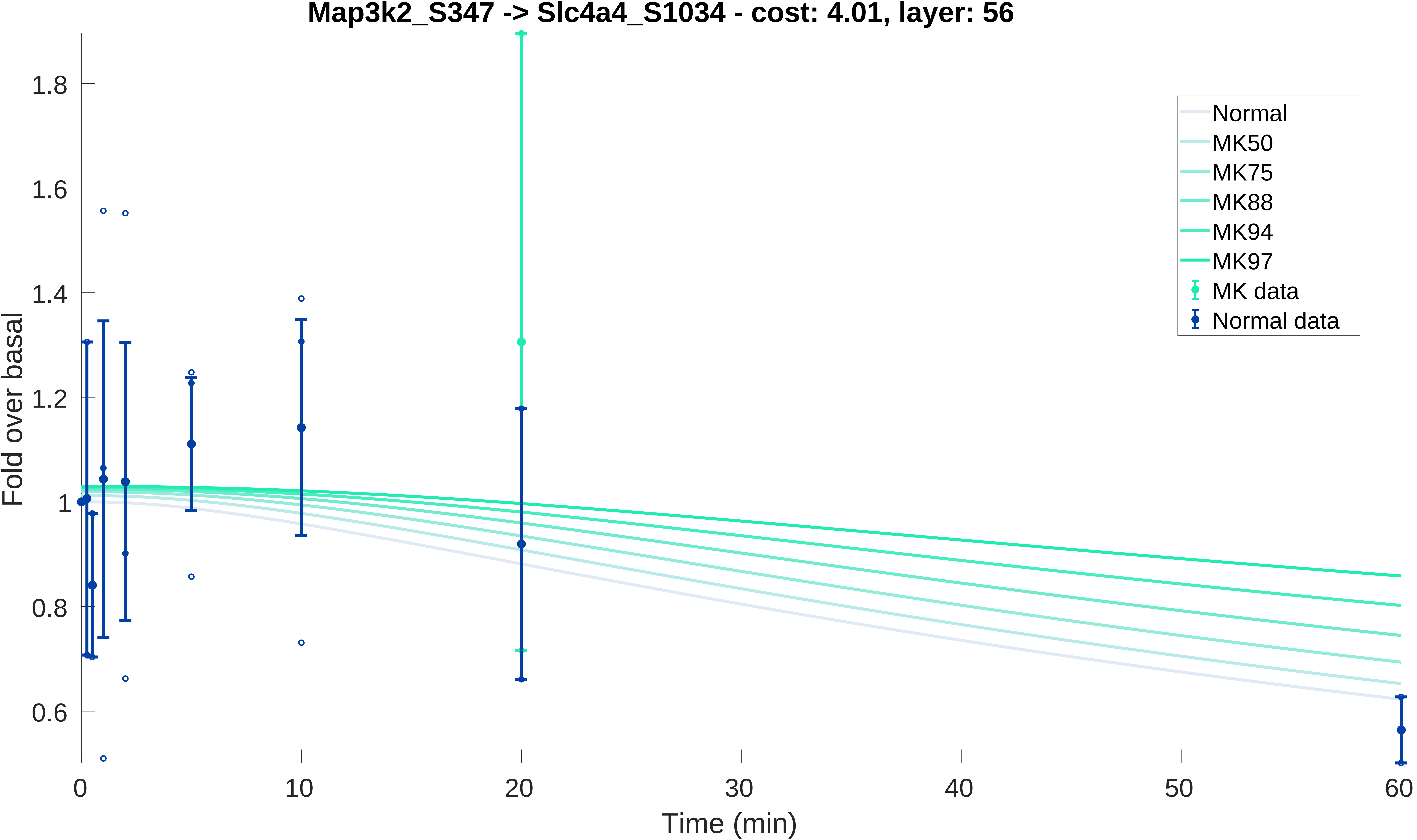

Map3k2_S347 -> Slc4a4_S1034 - cost: 4.01, layer: 56
Fold over basal
Time (min)
0
10
20
30
40
50
60
0.6
0.8
1
1.2
1.4
1.6
1.8
Normal
MK50
MK75
MK88
MK94
MK97
MK data
Normal data

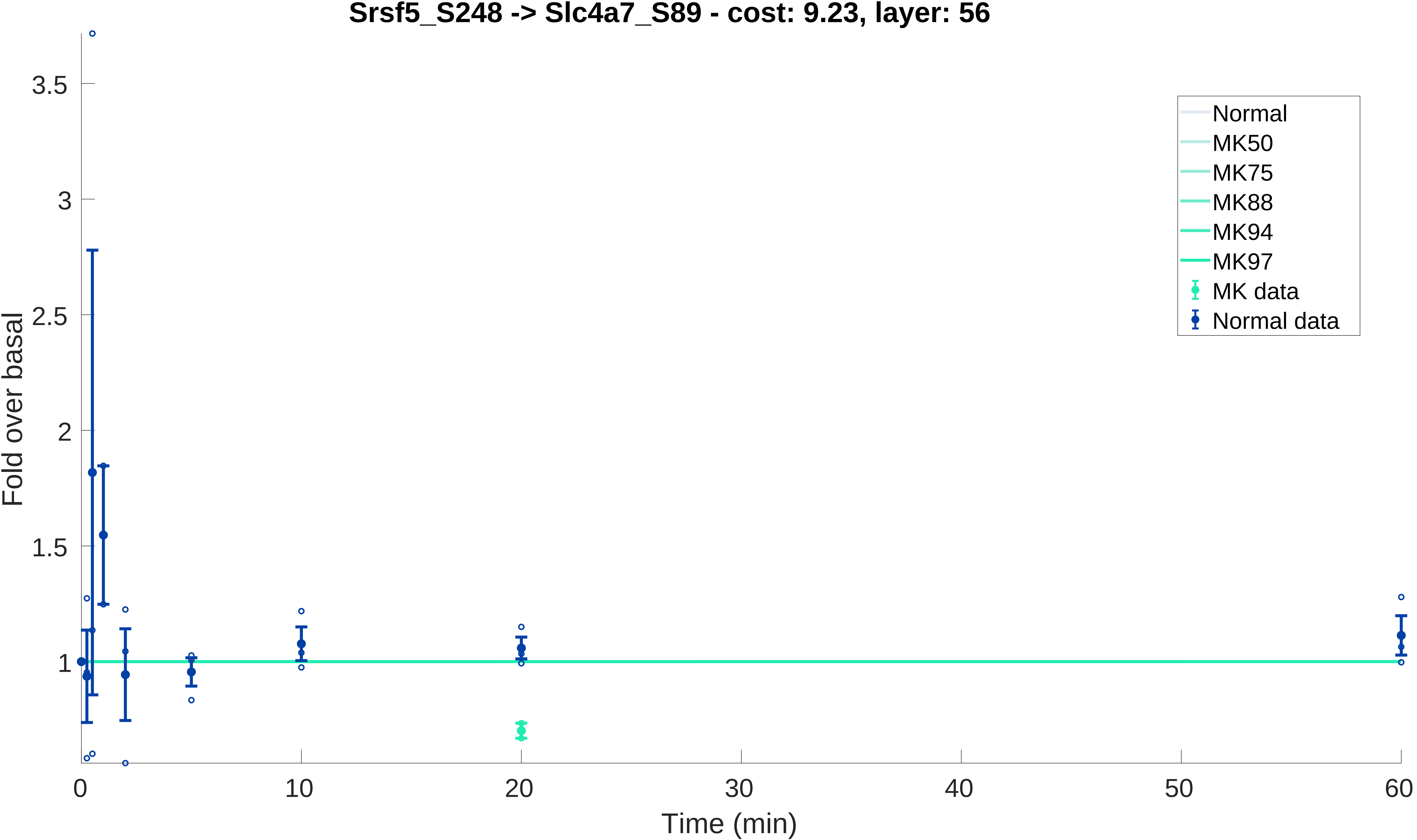
Srsf5_S248 -> Slc4a7_S89 - cost: 9.23, layer: 56
Fold over basal
3.5
3
2.5
2
1.5
1
Time (min)
0
10
20
30
40
50
60
Normal
MK50
MK75
MK88
MK94
MK97
MK data
Normal data

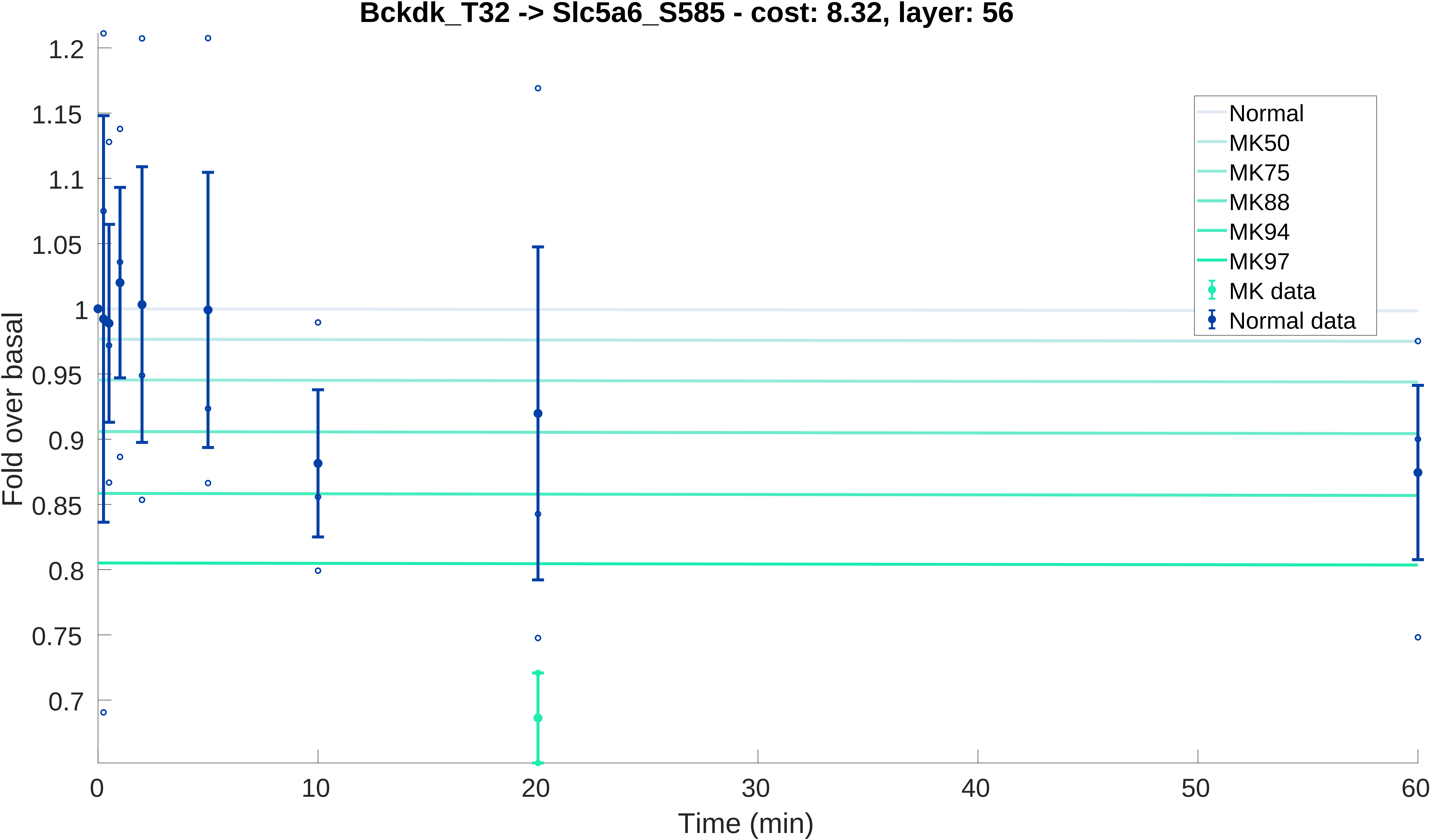

Bckdk_T32 -> Slc5a6_S585 - cost: 8.32, layer: 56
Fold over basal
Time (min)
1.2
1.15
1.1
1.05
1
0.95
0.9
0.85
0.8
0.75
0.7
0
10
20
30
40
50
60
Normal
MK50
MK75
MK88
MK94
MK97
MK data
Normal data

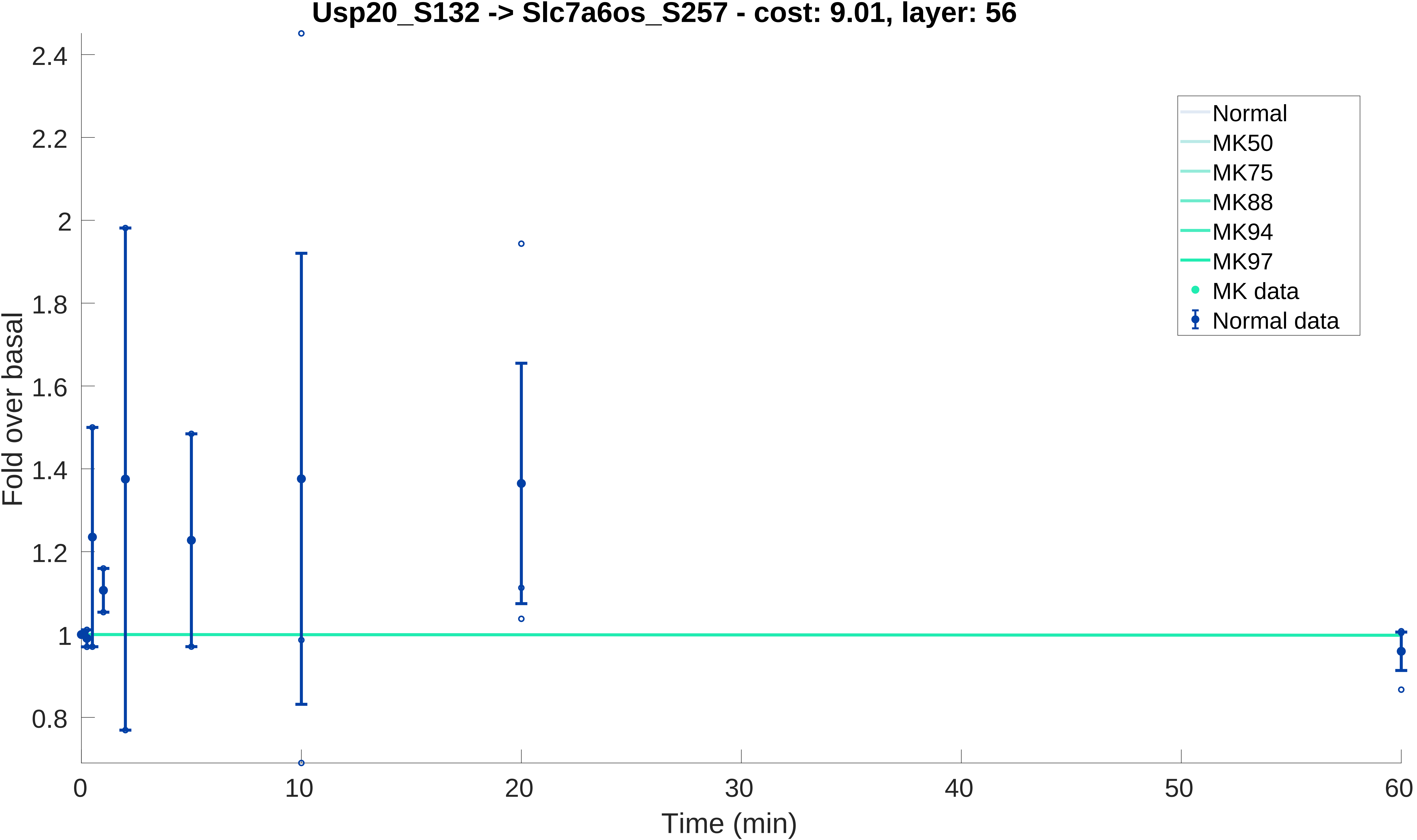

Usp20_S132 -> Slc7a6os_S257 - cost: 9.01, layer: 56
Fold over basal
Time (min)
Normal
MK50
MK75
MK88
MK94
MK97
MK data
Normal data
0.8
1
1.2
1.4
1.6
1.8
2
2.2
2.4
0
10
20
30
40
50
60

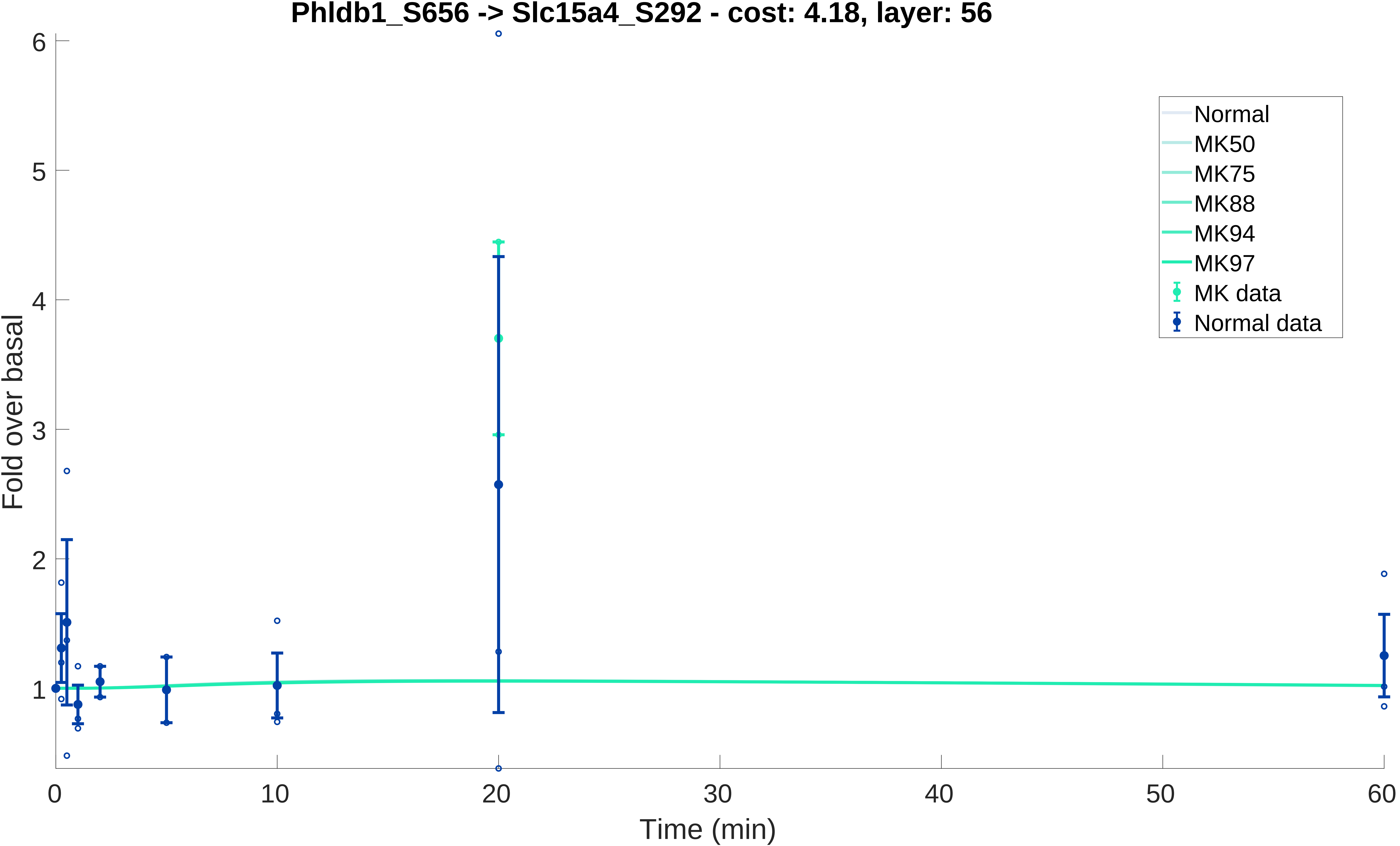
Phldb1_S656 -> Slc15a4_S292 - cost: 4.18, layer: 56
Fold over basal
Time (min)
0
10
20
30
40
50
60
1
2
3
4
5
6
Normal
MK50
MK75
MK88
MK94
MK97
MK data
Normal data

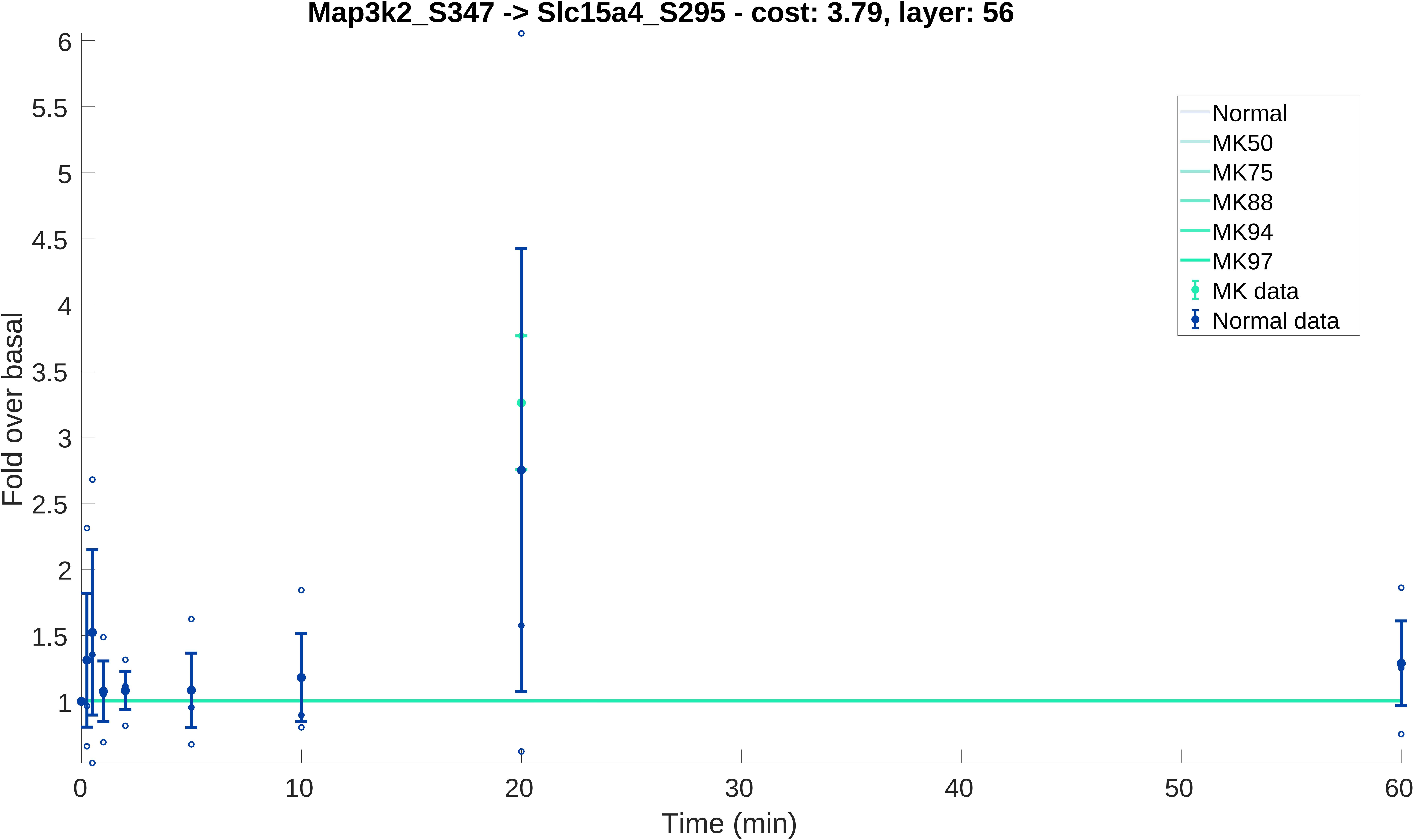

Map3k2_S347 -> Slc15a4_S295 - cost: 3.79, layer: 56
Fold over basal
Time (min)
Normal
MK50
MK75
MK88
MK94
MK97
MK data
Normal data
0
10
20
30
40
50
60
1
1.5
2
2.5
3
3.5
4
4.5
5
5.5
6

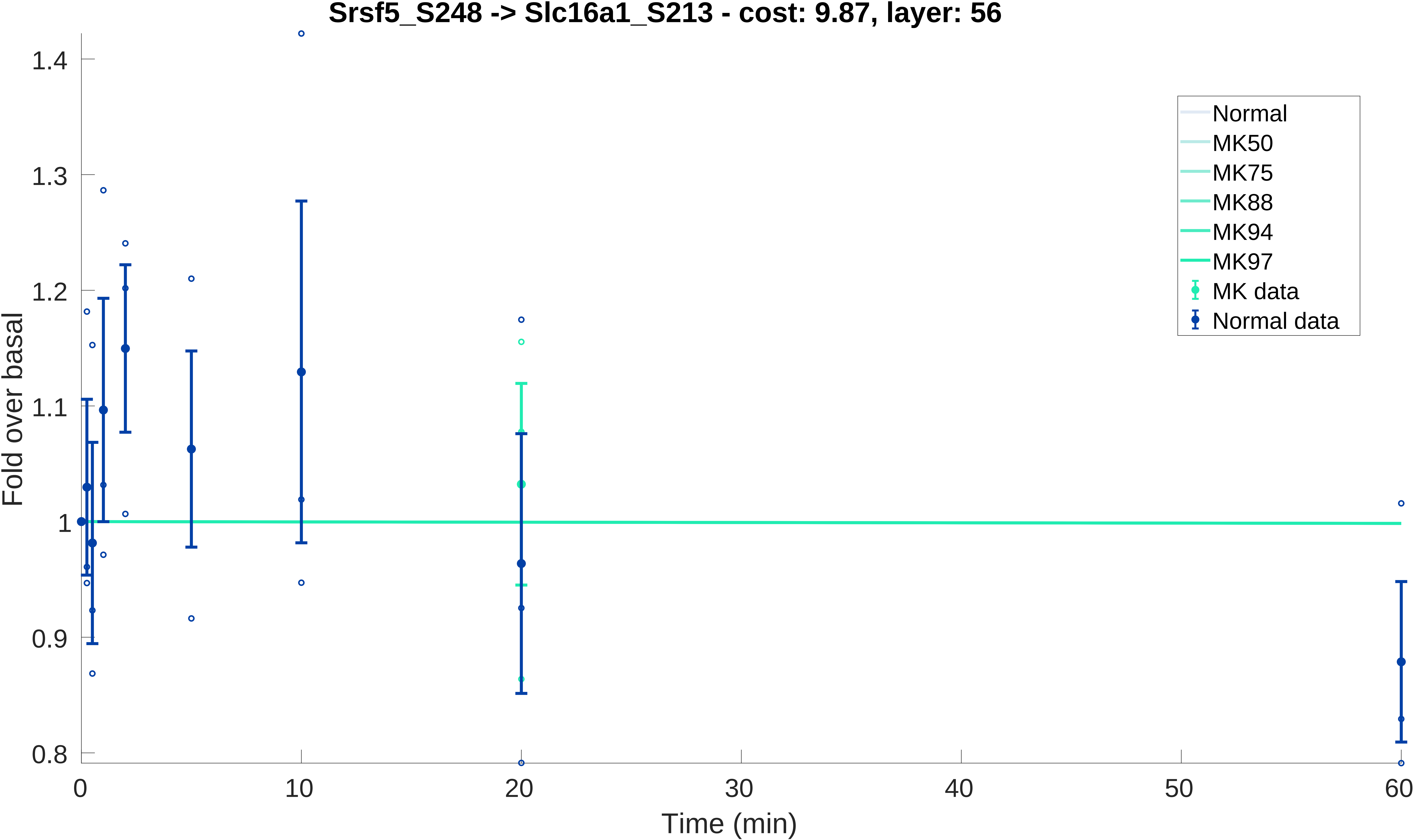

Srsf5_S248 -> Slc16a1_S213 - cost: 9.87, layer: 56
Fold over basal
Time (min)
1.4
1.3
1.2
1.1
1
0.9
0.8
0
10
20
30
40
50
60
Normal
MK50
MK75
MK88
MK94
MK97
MK data
Normal data

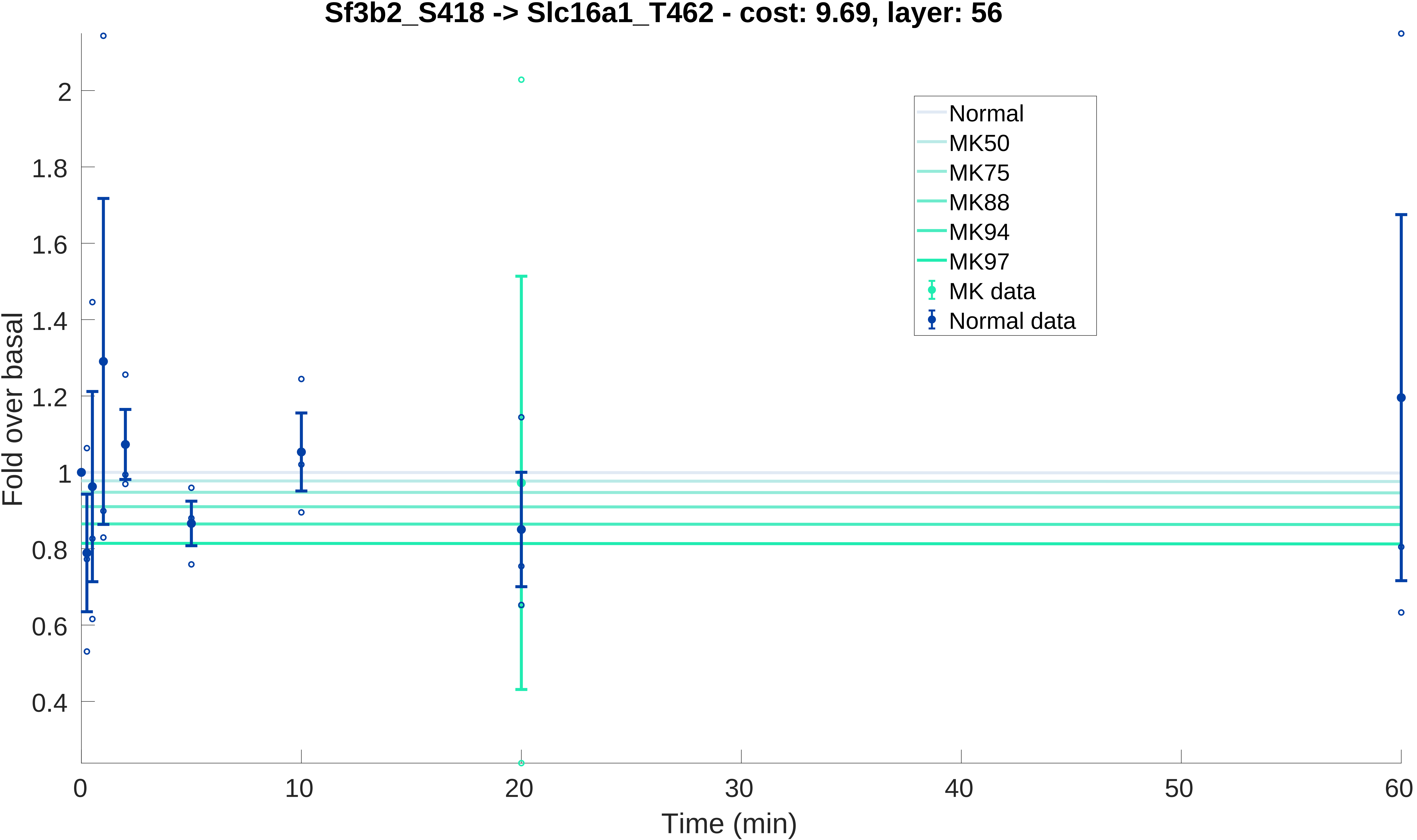

Sf3b2_S418 -> Slc16a1_T462 - cost: 9.69, layer: 56
Fold over basal
Time (min)
0.4
0.6
0.8
1
1.2
1.4
1.6
1.8
2
0
10
20
30
40
50
60
Normal
MK50
MK75
MK88
MK94
MK97
MK data
Normal data

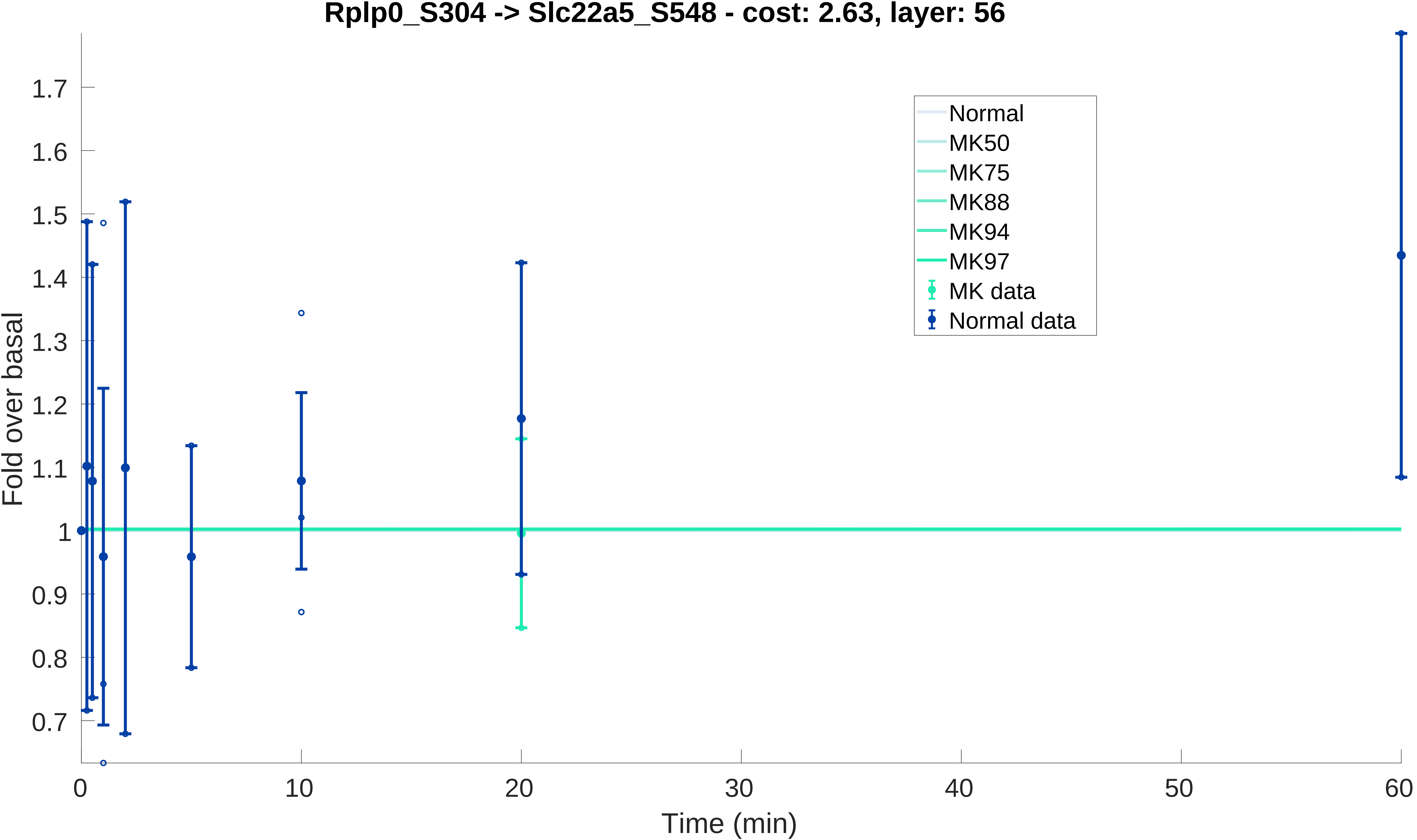

Rplp0_S304 -> Slc22a5_S548 - cost: 2.63, layer: 56
Fold over basal
1.7
1.6
1.5
1.4
1.3
1.2
1.1
1
0.9
0.8
0.7
0
10
20
30
40
50
60
Time (min)
Normal
MK50
MK75
MK88
MK94
MK97
MK data
Normal data

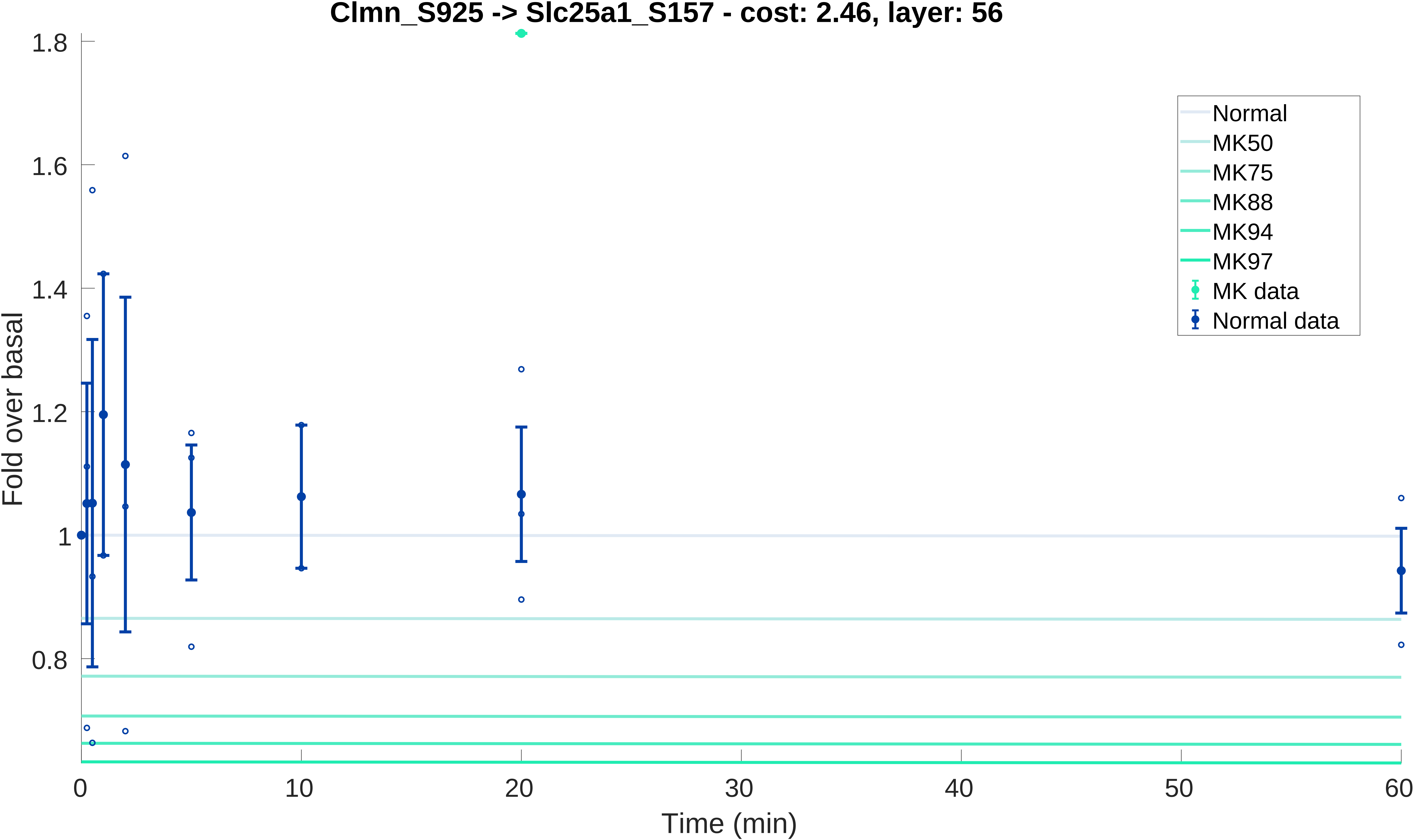
Clmn_S925 -> Slc25a1_S157 - cost: 2.46, layer: 56
Fold over basal
Time (min)
Normal
MK50
MK75
MK88
MK94
MK97
MK data
Normal data
1.8
1.6
1.4
1.2
1
0.8
0
10
20
30
40
50
60

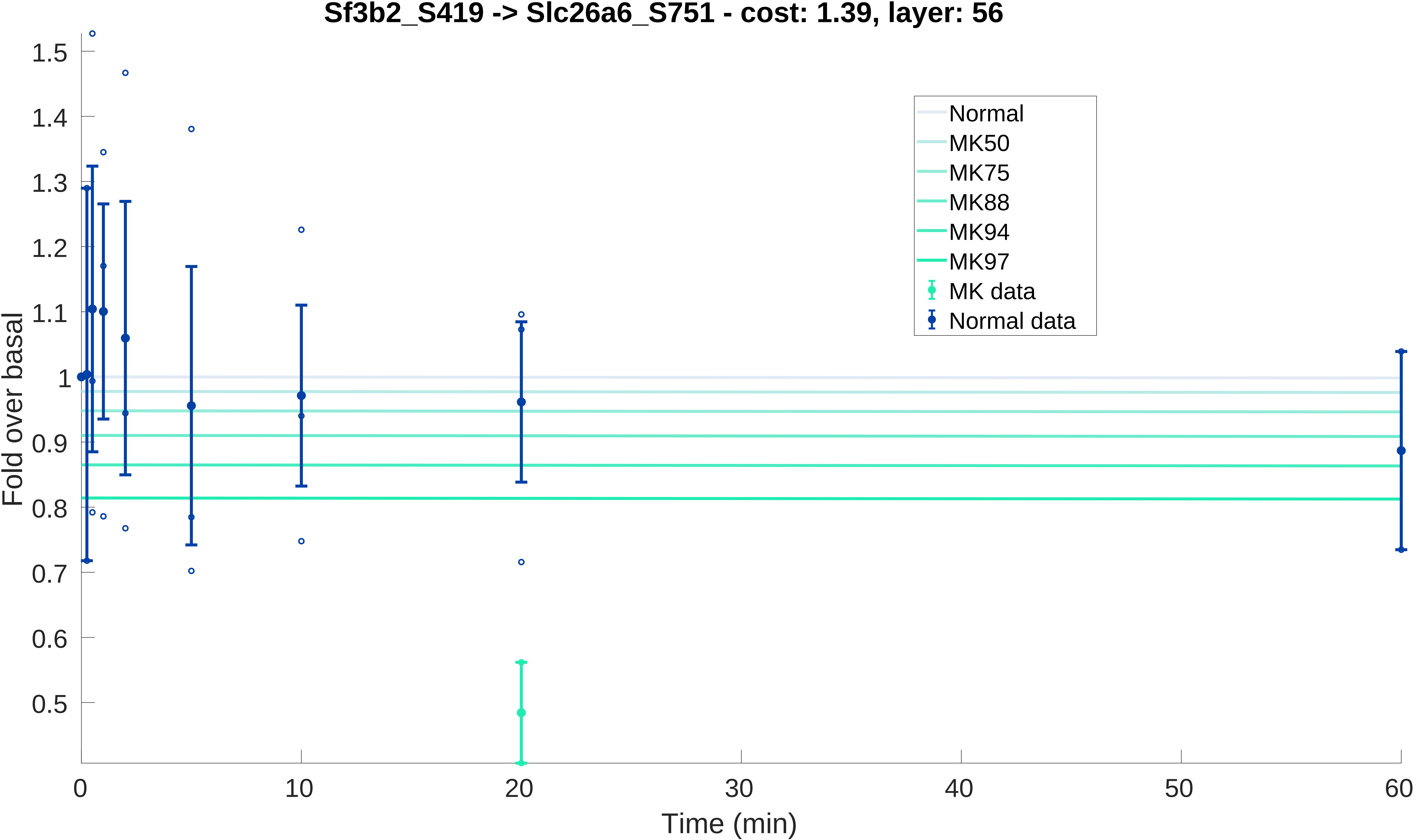
Sf3b2_S419 -> Slc26a6_S751 - cost: 1.39, layer: 56
Fold over basal
Time (min)
1.5
1.4
1.3
1.2
1.1
1
0.9
0.8
0.7
0.6
0.5
0
10
20
30
40
50
60
Normal
MK50
MK75
MK88
MK94
MK97
MK data
Normal data

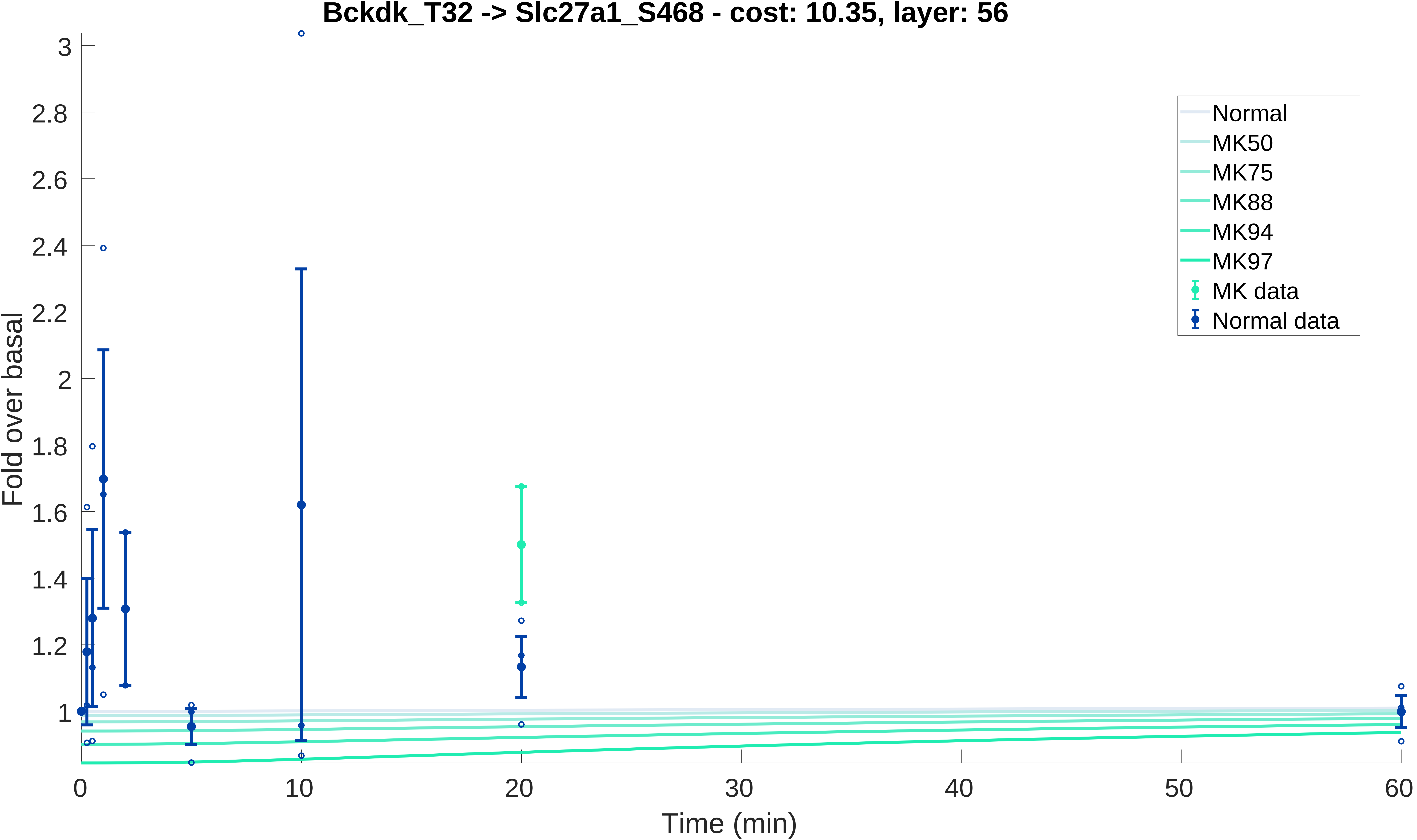

Bckdk_T32 -> Slc27a1_S468 - cost: 10.35, layer: 56
Fold over basal
Time (min)
Normal
MK50
MK75
MK88
MK94
MK97
MK data
Normal data
3
2.8
2.6
2.4
2.2
2
1.8
1.6
1.4
1.2
1
0
10
20
30
40
50
60

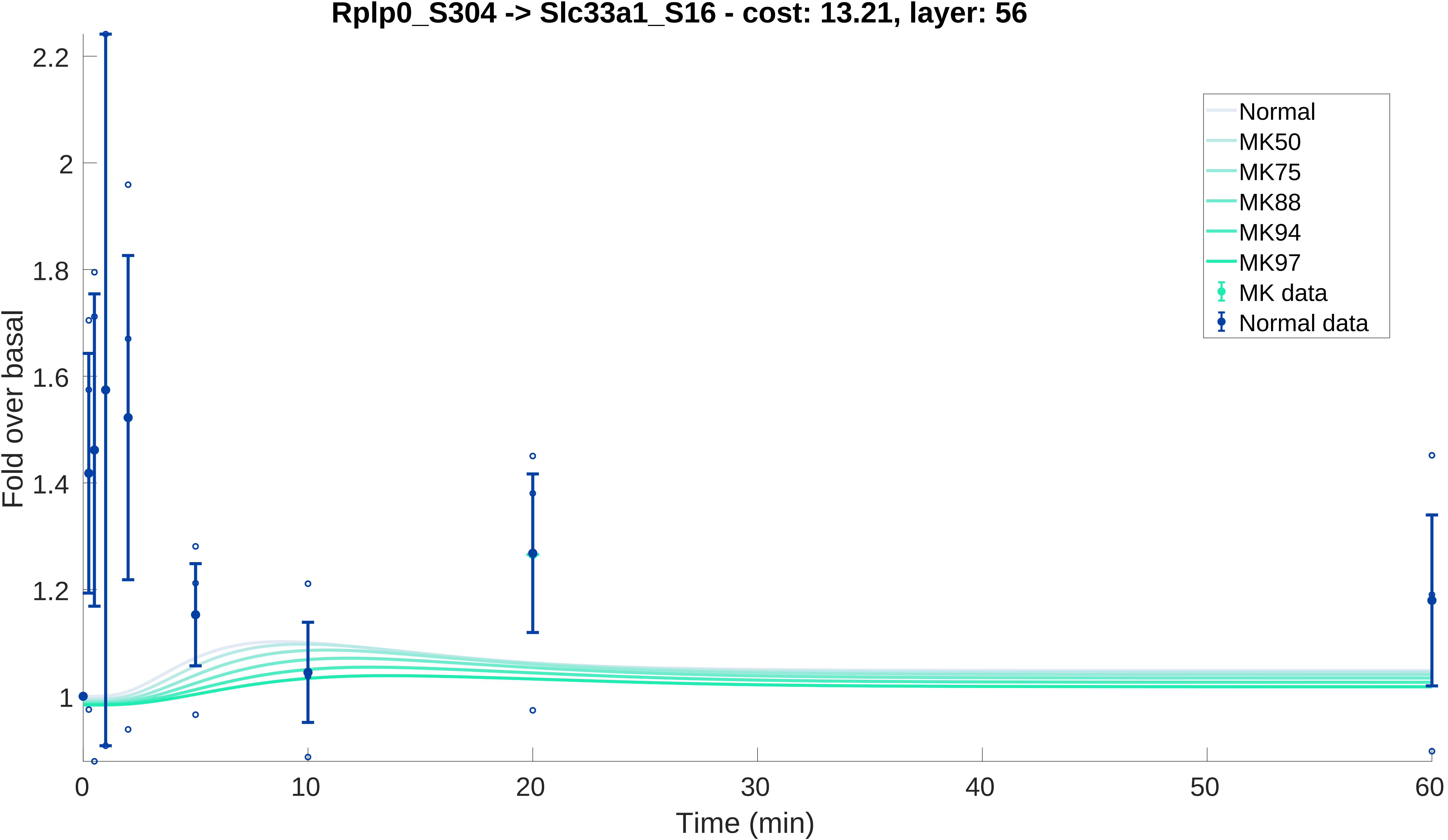

Rplp0_S304 -> Slc33a1_S16 - cost: 13.21, layer: 56
Fold over basal
Time (min)
2.2
2
1.8
1.6
1.4
1.2
1
0
10
20
30
40
50
60
Normal
MK50
MK75
MK88
MK94
MK97
MK data
Normal data

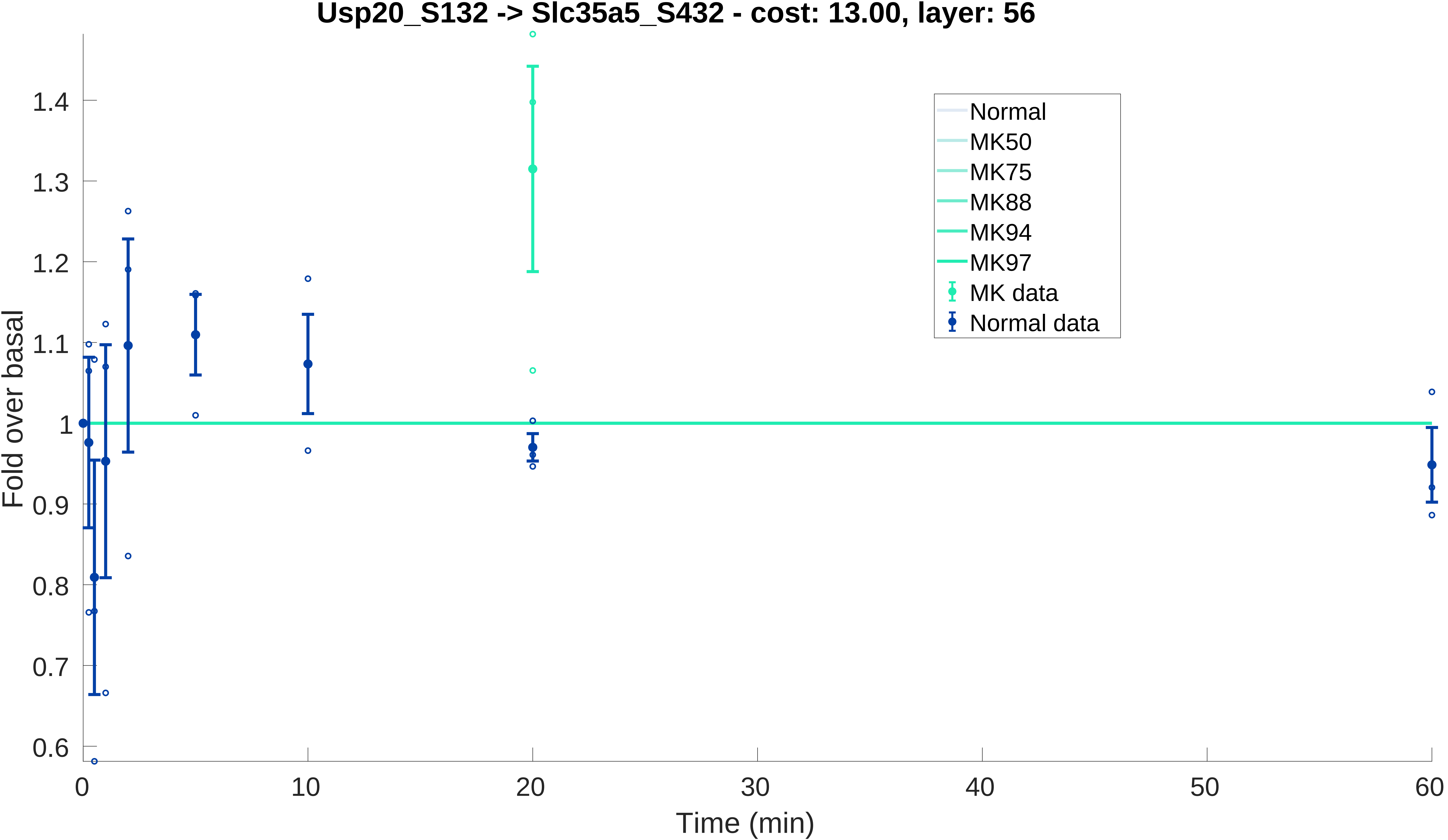

Usp20_S132 -> Slc35a5_S432 - cost: 13.00, layer: 56
Fold over basal
Time (min)
0.6
0.7
0.8
0.9
1
1.1
1.2
1.3
1.4
0
10
20
30
40
50
60
Normal
MK50
MK75
MK88
MK94
MK97
MK data
Normal data

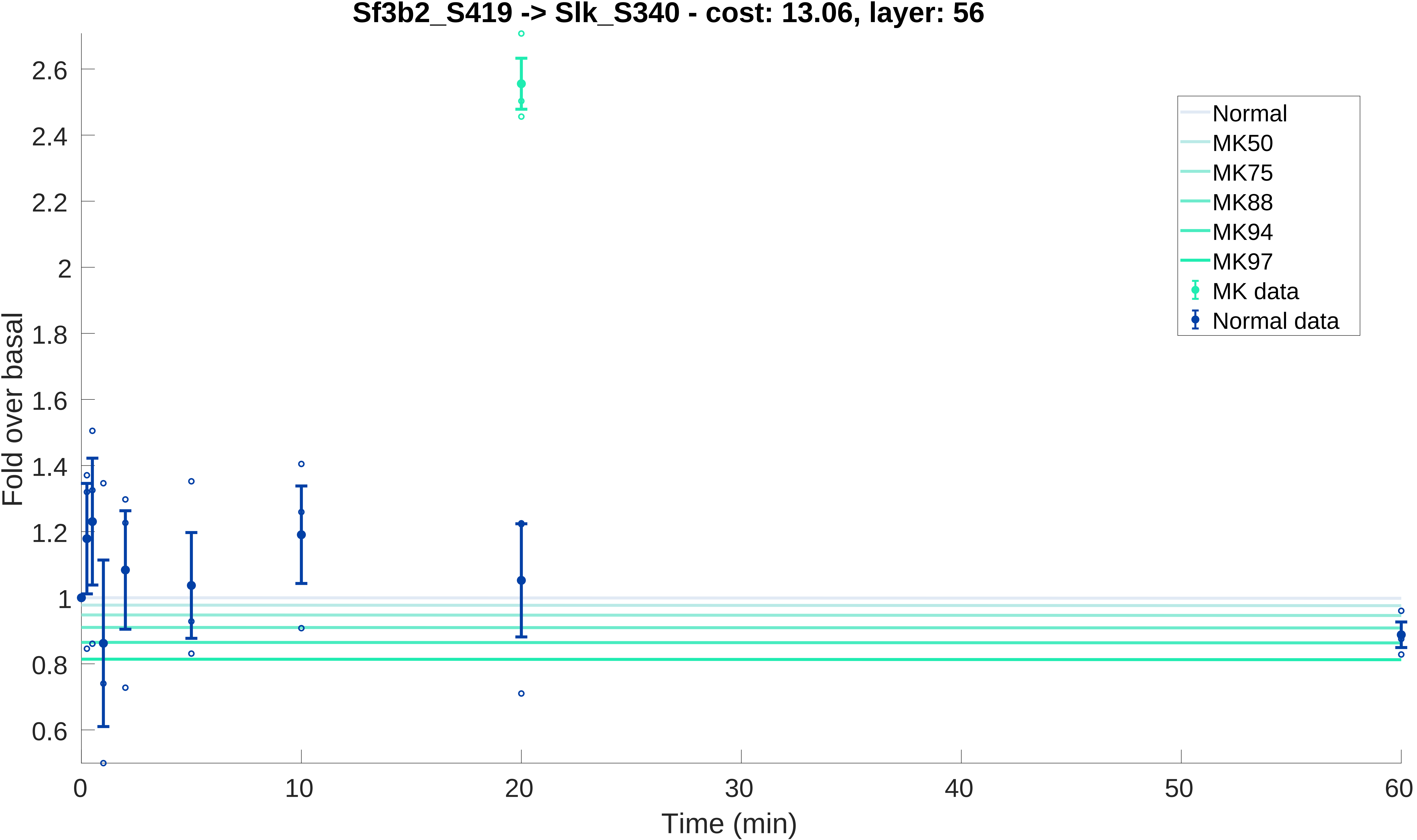

Sf3b2_S419 -> Slk_S340 - cost: 13.06, layer: 56
Fold over basal
Time (min)
0
10
20
30
40
50
60
0.6
0.8
1
1.2
1.4
1.6
1.8
2
2.2
2.4
2.6
Normal
MK50
MK75
MK88
MK94
MK97
MK data
Normal data

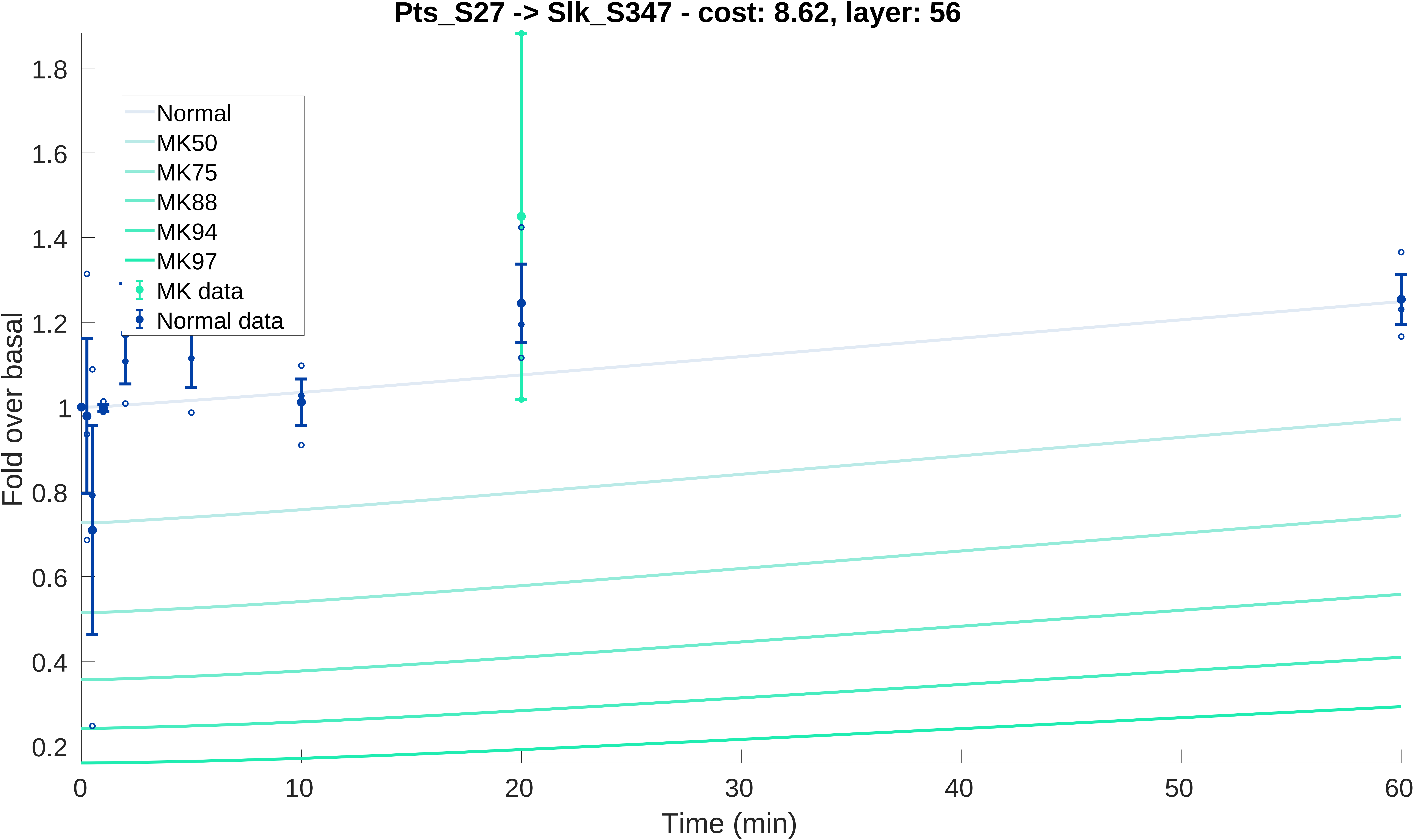

Pts_S27 -> Slk_S347 - cost: 8.62, layer: 56
Normal
MK50
MK75
MK88
MK94
MK97
MK data
Normal data
Fold over basal
Time (min)
1.8
1.6
1.4
1.2
1
0.8
0.6
0.4
0.2
0
10
20
30
40
50
60

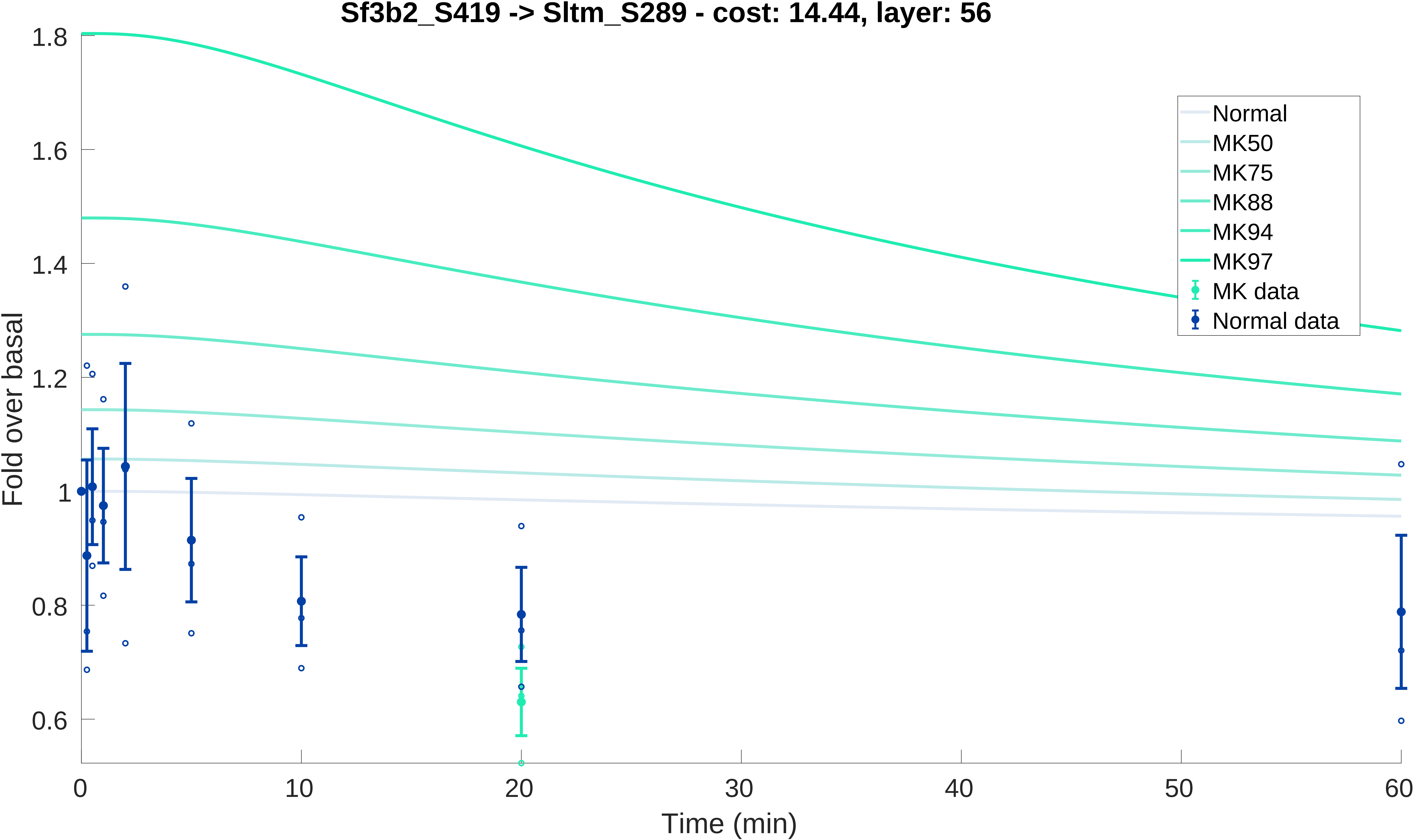
Sf3b2_S419 -> Sltm_S289 - cost: 14.44, layer: 56
Fold over basal
1.8
1.6
1.4
1.2
1
0.8
0.6
0
10
20
30
40
50
60
Time (min)
Normal
MK50
MK75
MK88
MK94
MK97
MK data
Normal data

Bin1_S324 -> Sltm_S550 - cost: 10.99, layer: 56

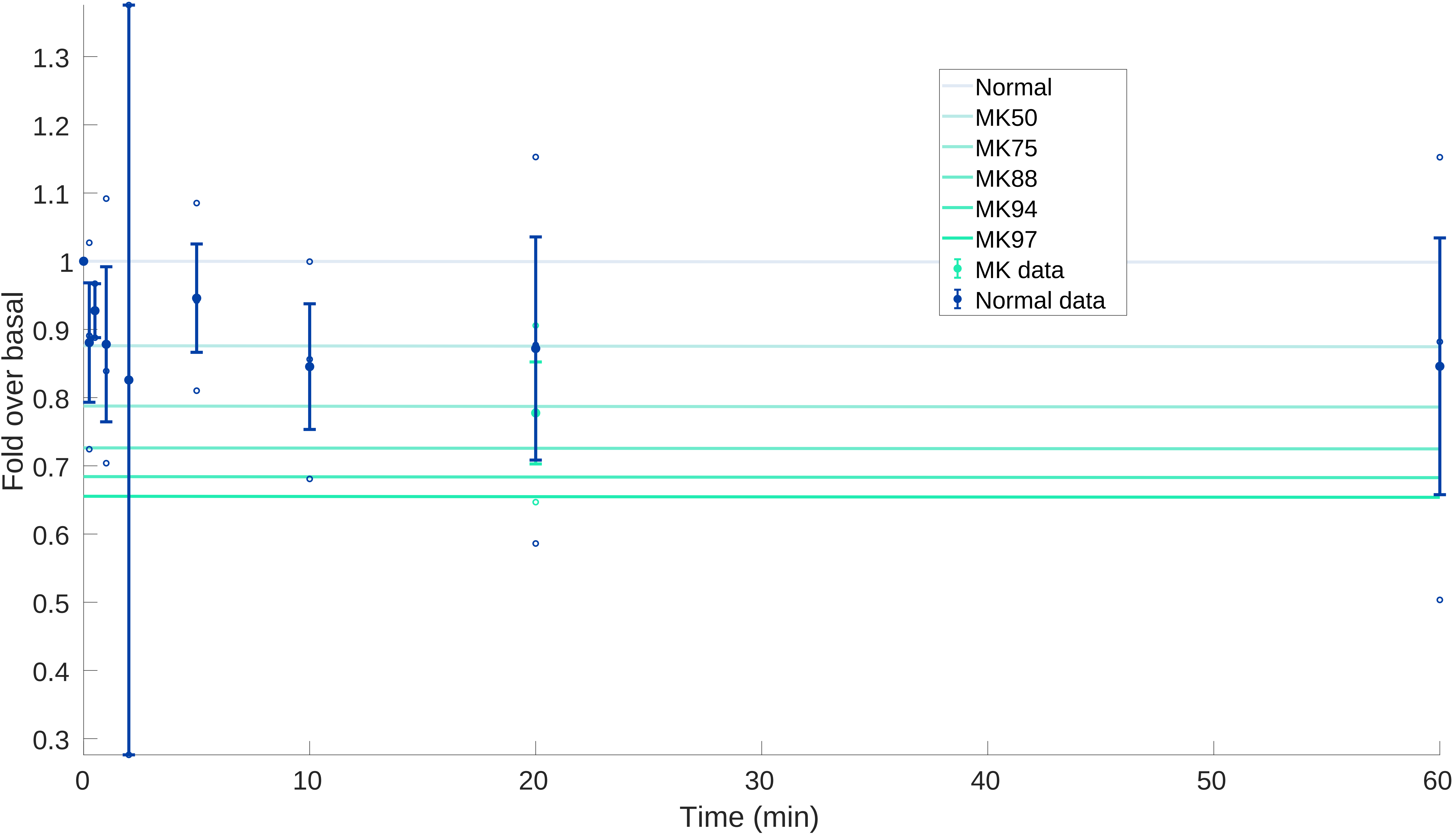

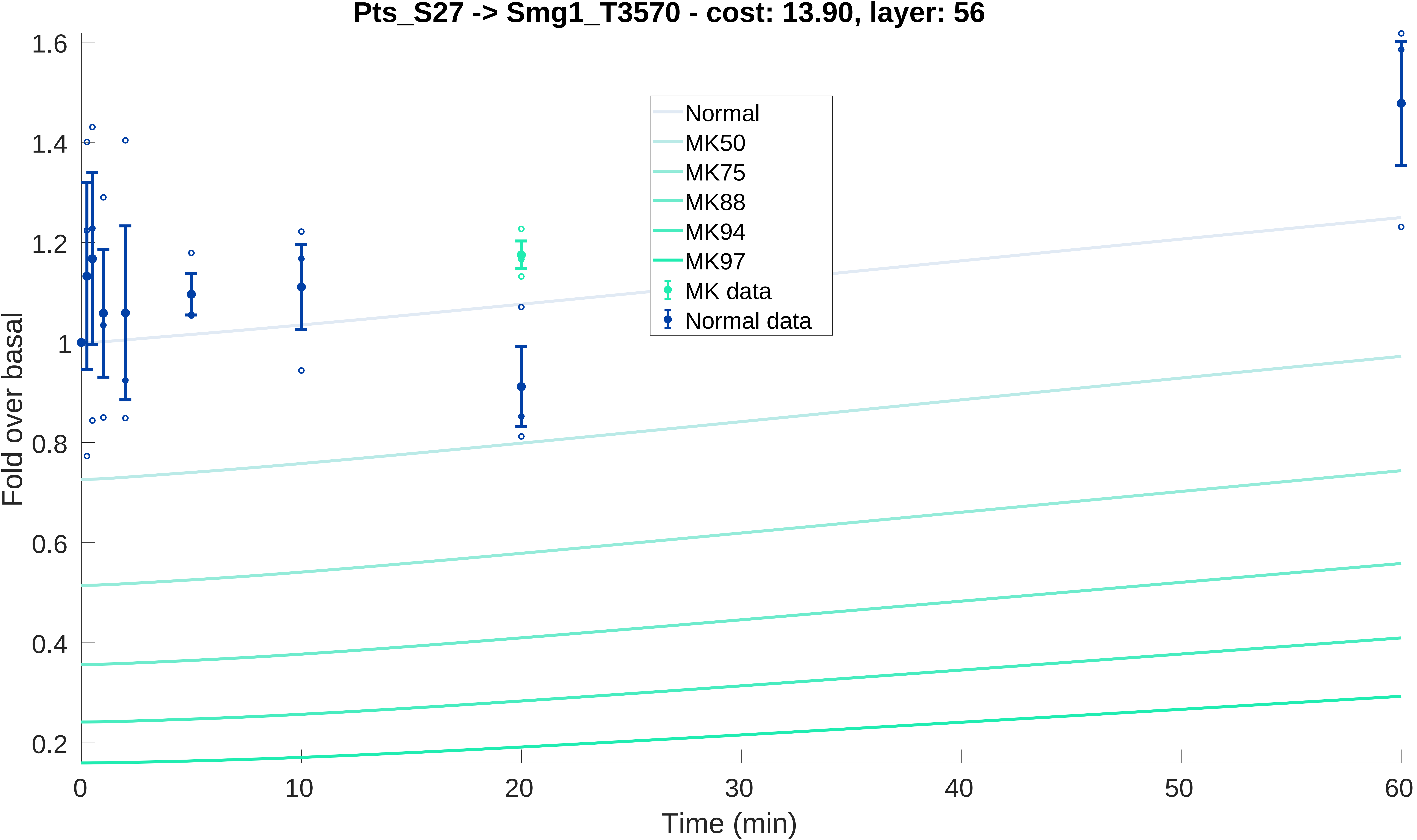

Pts_S27 -> Smg1_T3570 - cost: 13.90, layer: 56
Fold over basal
Time (min)
1.6
1.4
1.2
1
0.8
0.6
0.4
0.2
0
10
20
30
40
50
60
Normal
MK50
MK75
MK88
MK94
MK97
MK data
Normal data

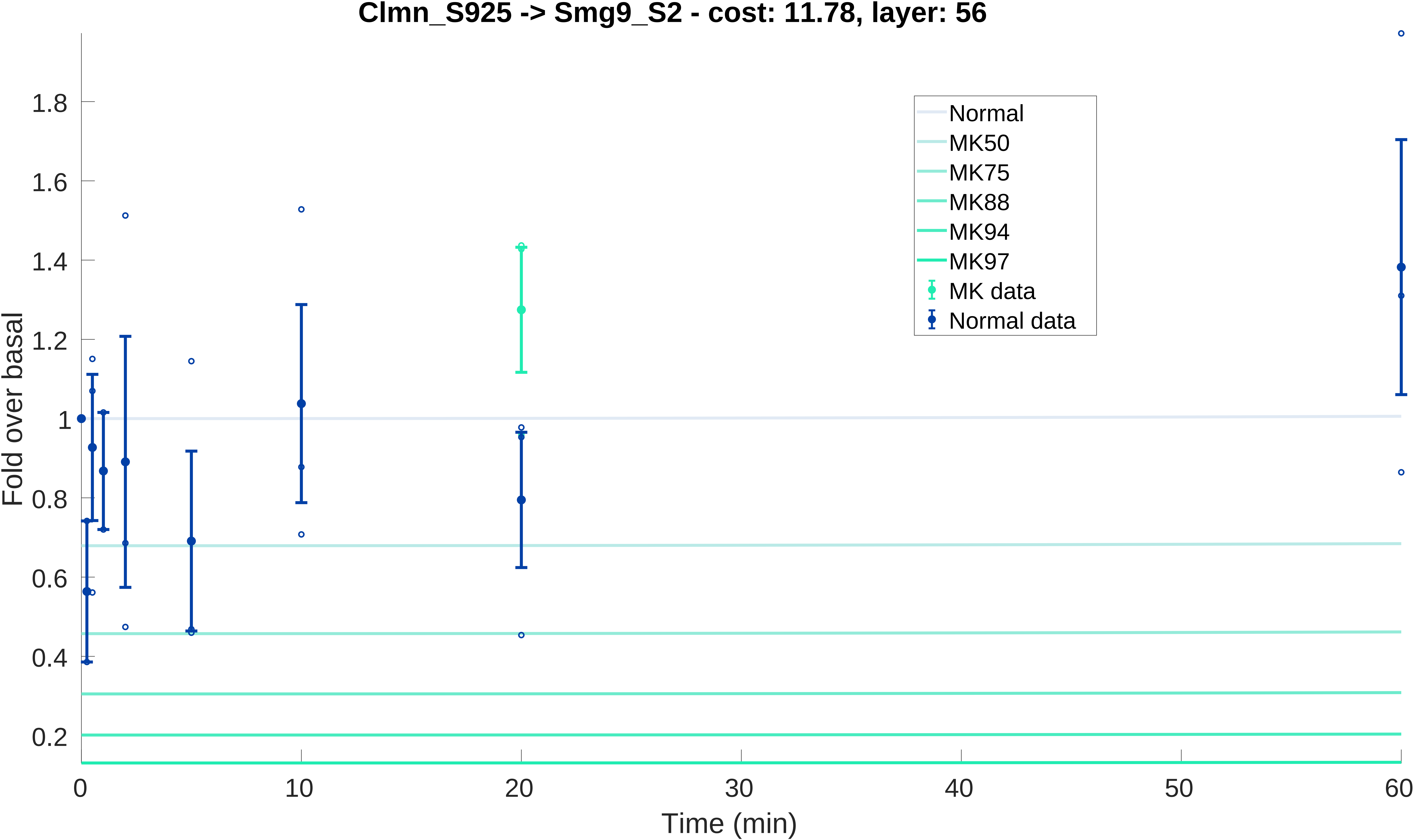
Clmn_S925 -> Smg9_S2 - cost: 11.78, layer: 56
Normal
MK50
MK75
MK88
MK94
MK97
MK data
Normal data
Fold over basal
Time (min)
1.8
1.6
1.4
1.2
1
0.8
0.6
0.4
0.2
0
10
20
30
40
50
60

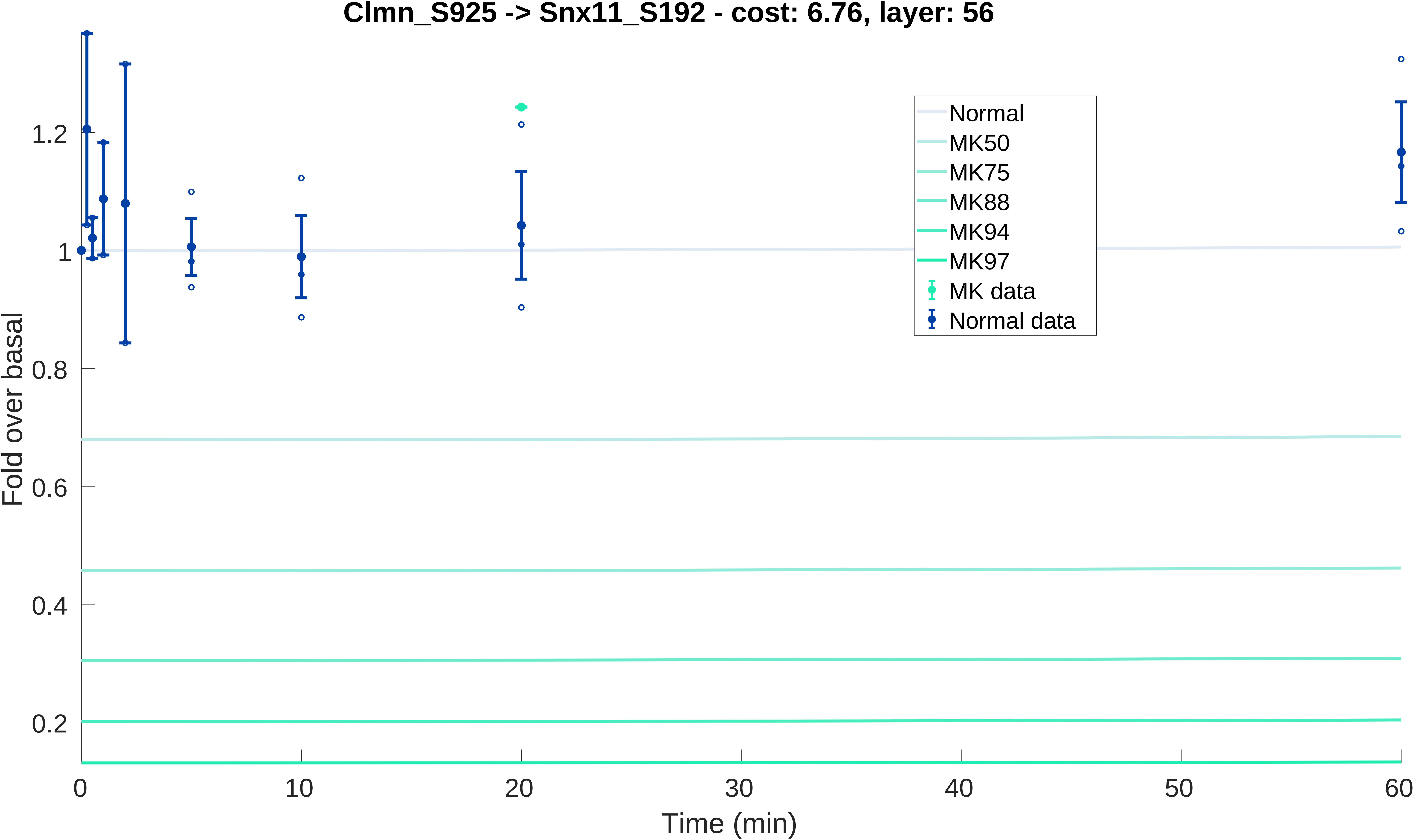

Clmn_S925 -> Snx11_S192 - cost: 6.76, layer: 56
Fold over basal
Time (min)
Normal
MK50
MK75
MK88
MK94
MK97
MK data
Normal data
0
10
20
30
40
50
60
0.2
0.4
0.6
0.8
1
1.2

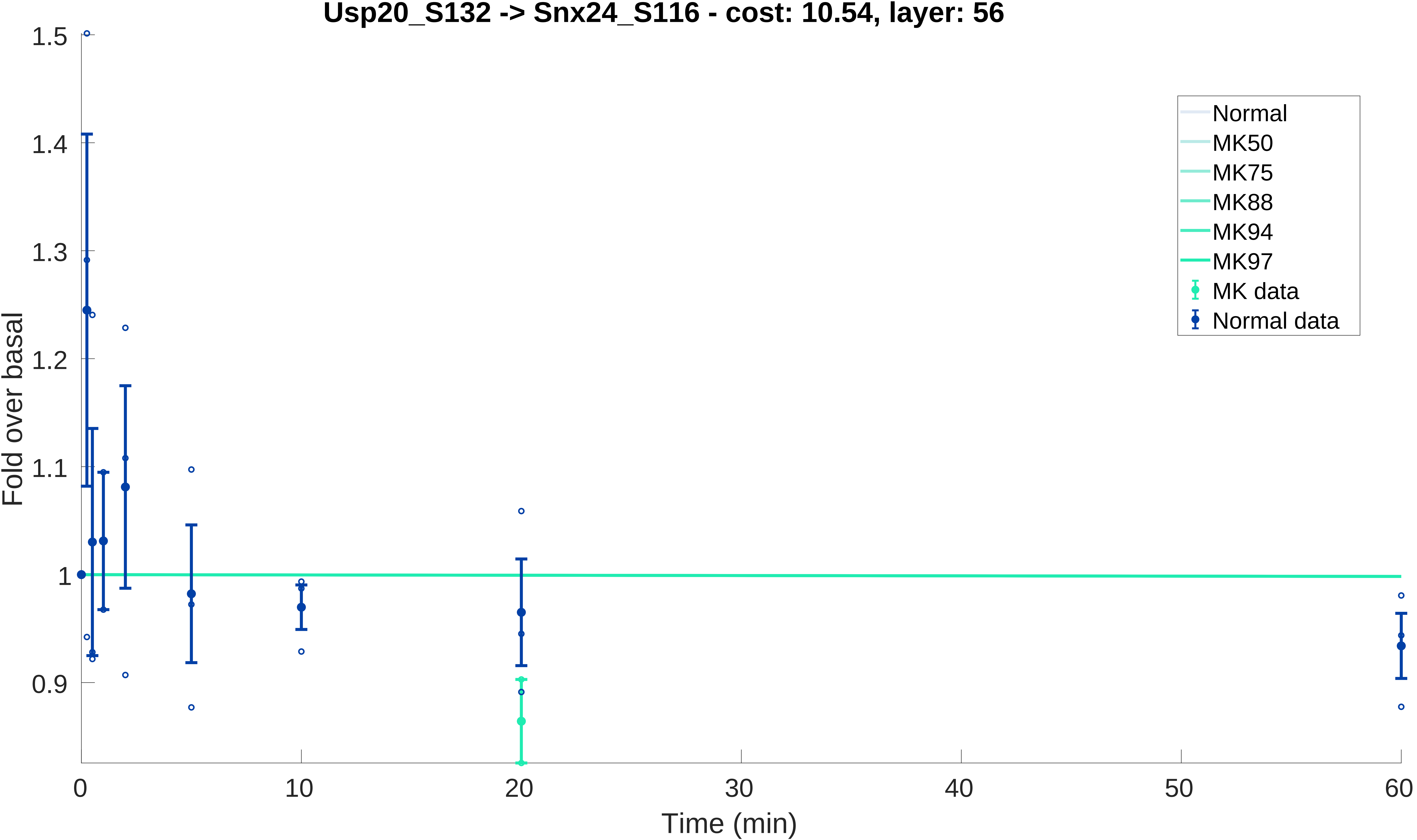
Usp20_S132 -> Snx24_S116 - cost: 10.54, layer: 56
Fold over basal
Time (min)
1.5
1.4
1.3
1.2
1.1
1
0.9
0
10
20
30
40
50
60
Normal
MK50
MK75
MK88
MK94
MK97
MK data
Normal data

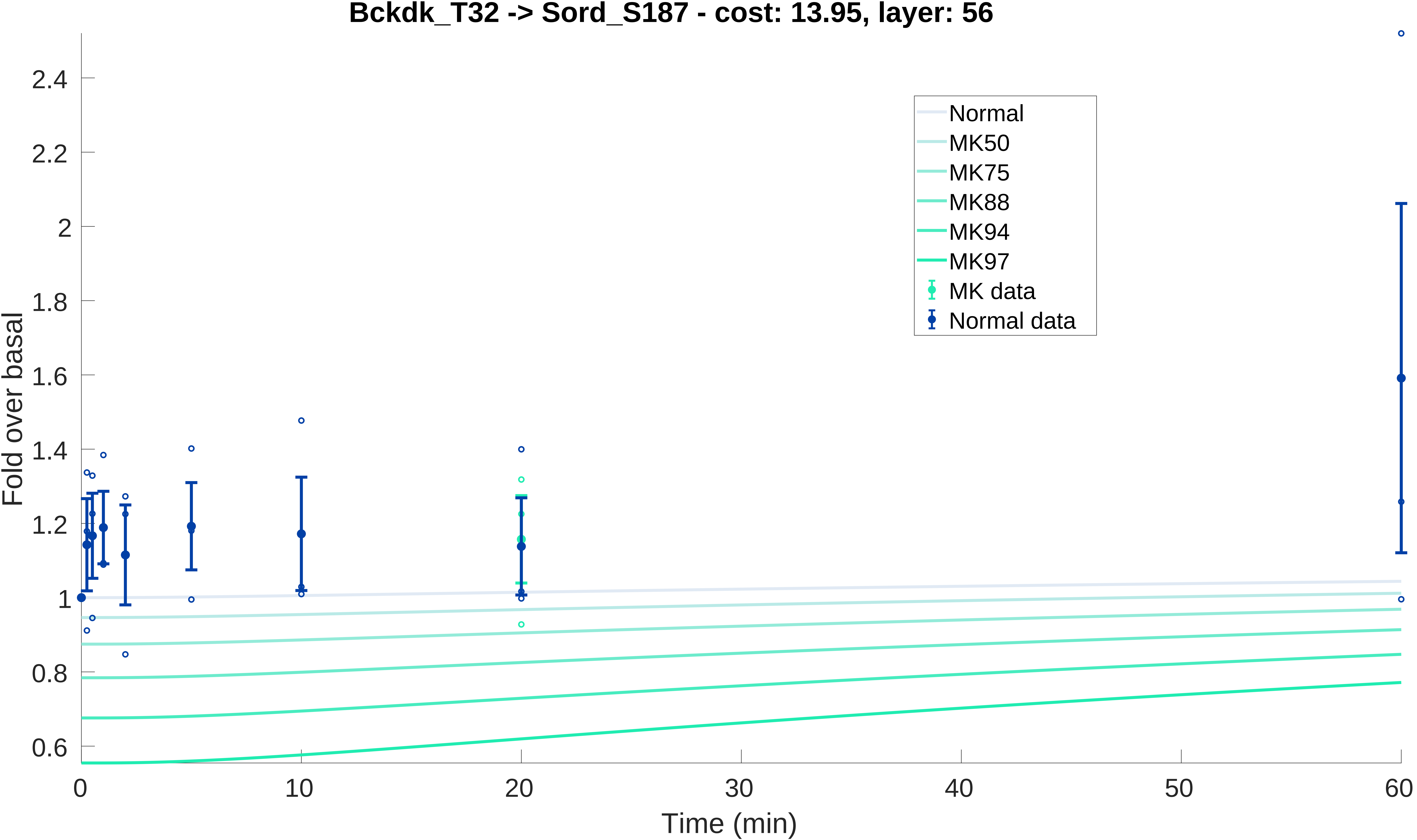

Bckdk_T32 -> Sord_S187 - cost: 13.95, layer: 56
Fold over basal
Time (min)
0.6
0.8
1
1.2
1.4
1.6
1.8
2
2.2
2.4
0
10
20
30
40
50
60
Normal
MK50
MK75
MK88
MK94
MK97
MK data
Normal data

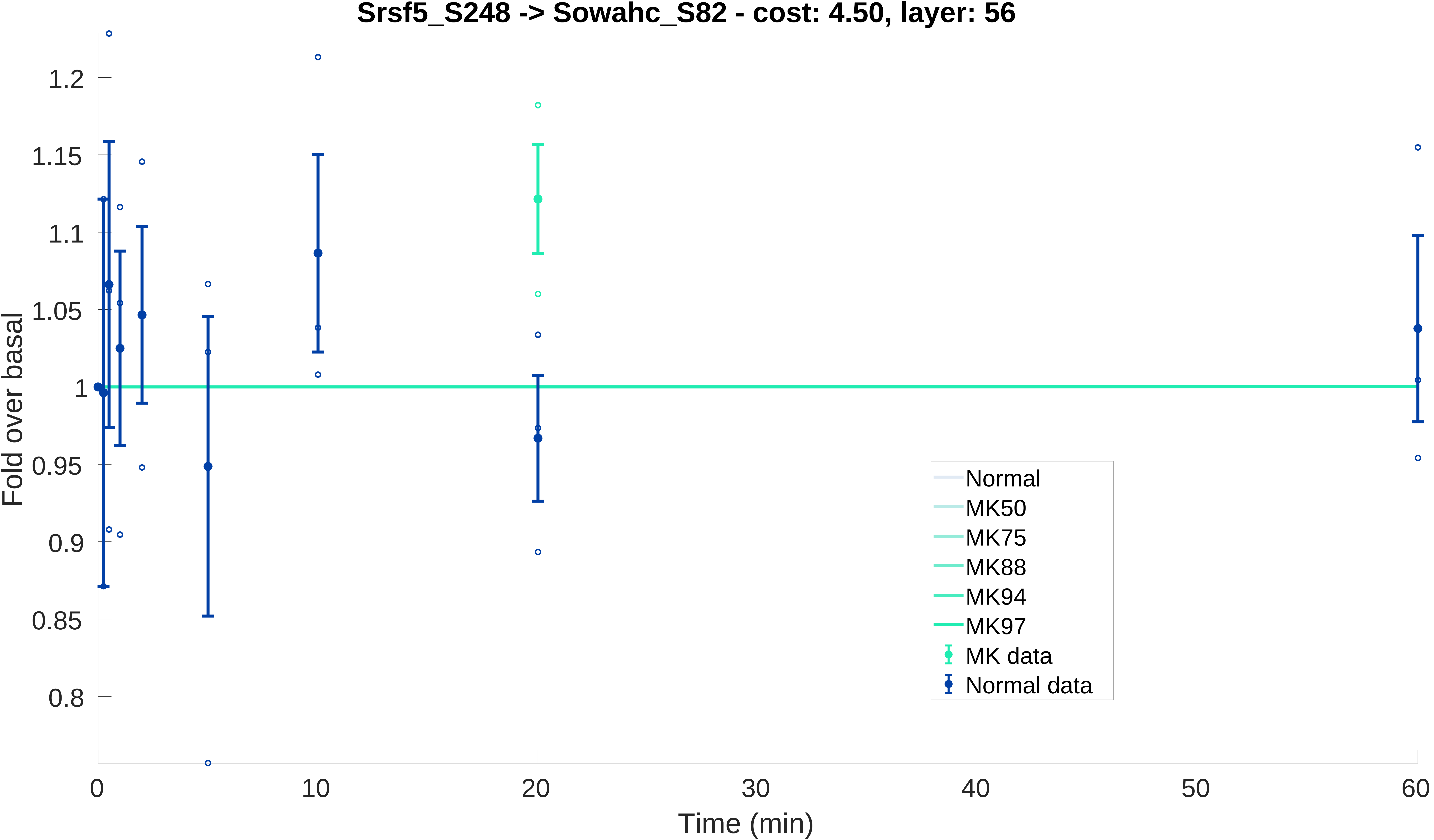

Srsf5_S248 -> Sowahc_S82 - cost: 4.50, layer: 56
Fold over basal
1.2
1.15
1.1
1.05
1
0.95
0.9
0.85
0.8
0
10
20
30
40
50
60
Time (min)
Normal
MK50
MK75
MK88
MK94
MK97
MK data
Normal data

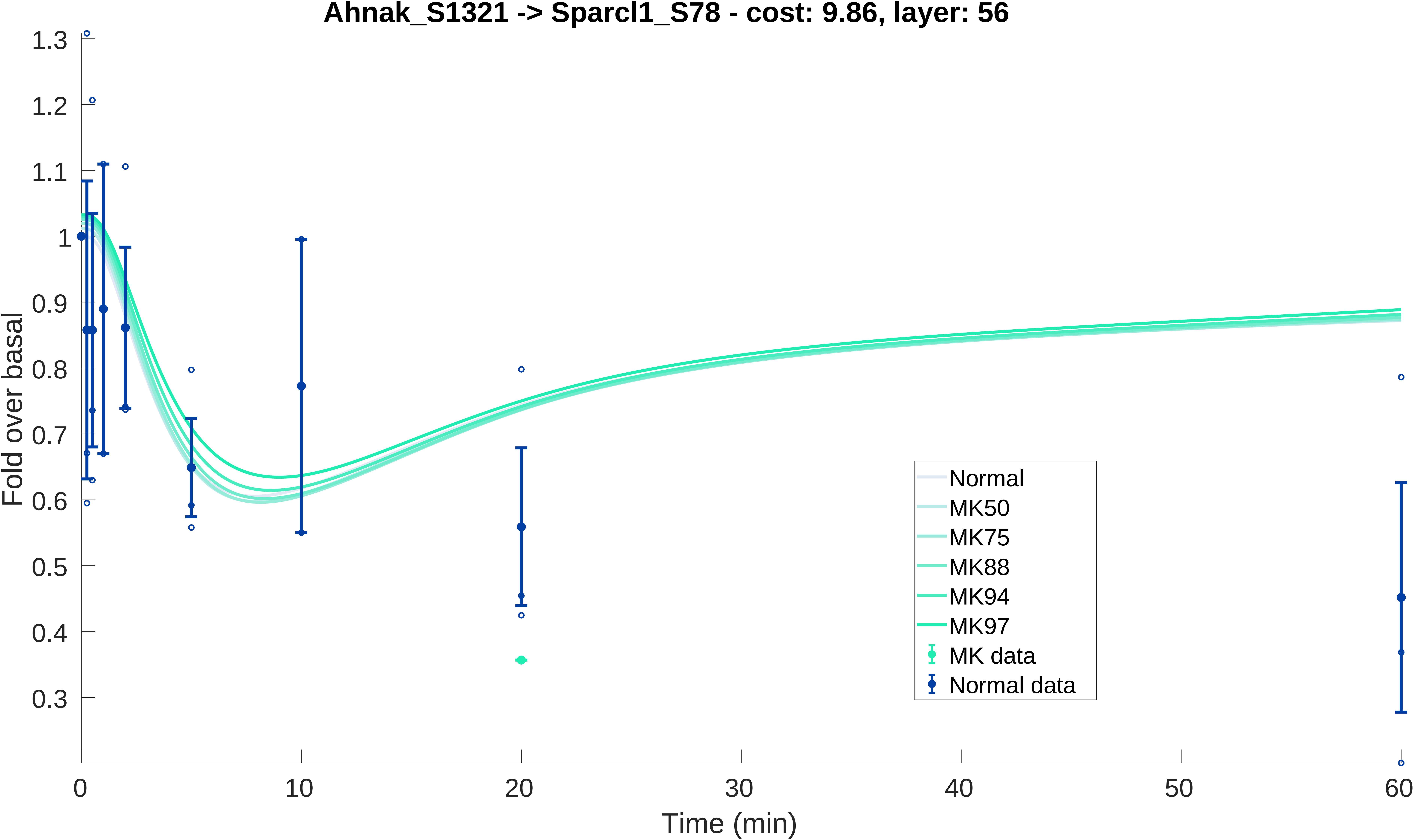
Ahnak_S1321 -> Sparcl1_S78 - cost: 9.86, layer: 56
Fold over basal
Time (min)
0
10
20
30
40
50
60
0.3
0.4
0.5
0.6
0.7
0.8
0.9
1
1.1
1.2
1.3
Normal
MK50
MK75
MK88
MK94
MK97
MK data
Normal data

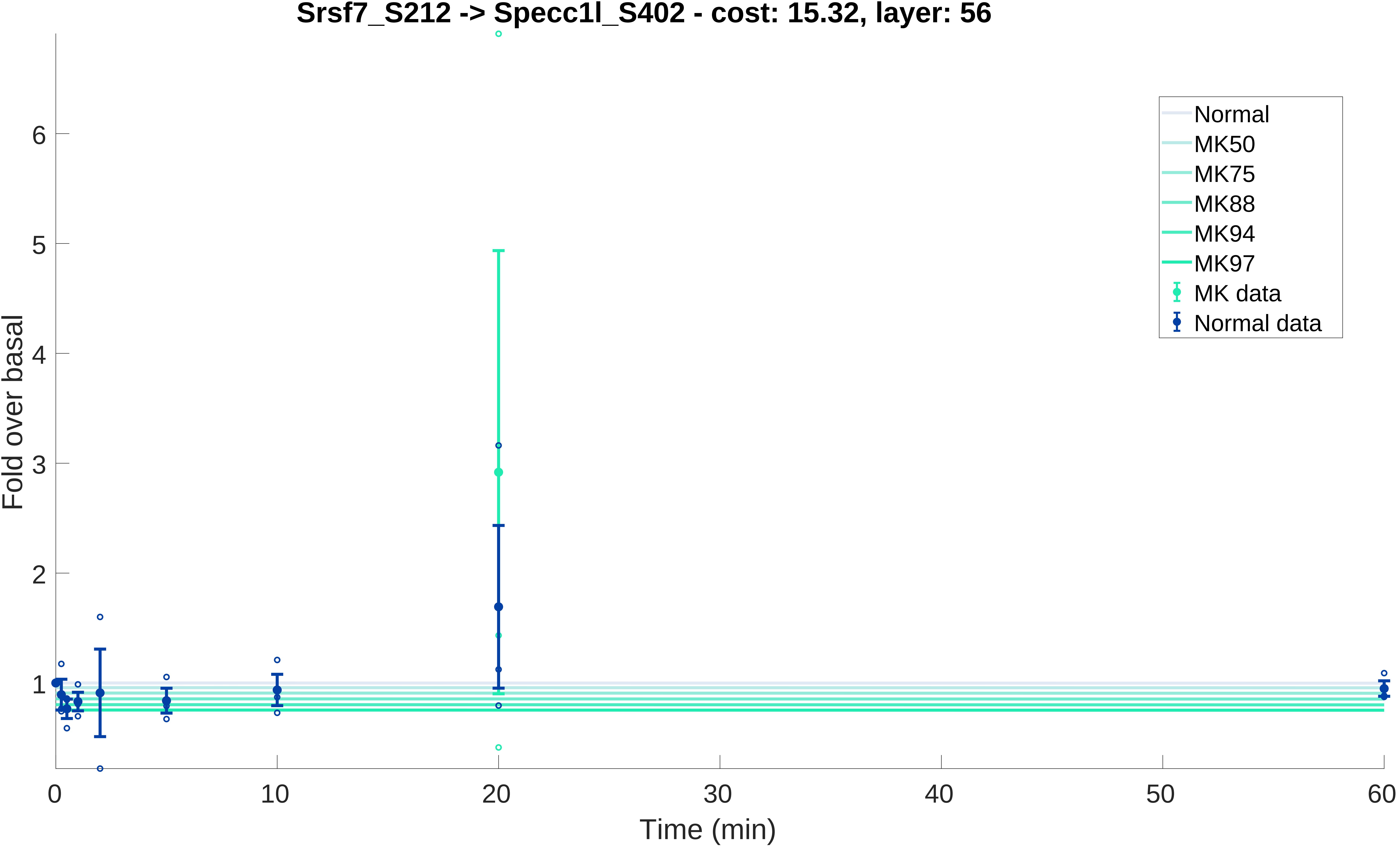
Srsf7_S212 -> Specc1l_S402 - cost: 15.32, layer: 56
Fold over basal
Time (min)
0
10
20
30
40
50
60
1
2
3
4
5
6
Normal
MK50
MK75
MK88
MK94
MK97
MK data
Normal data

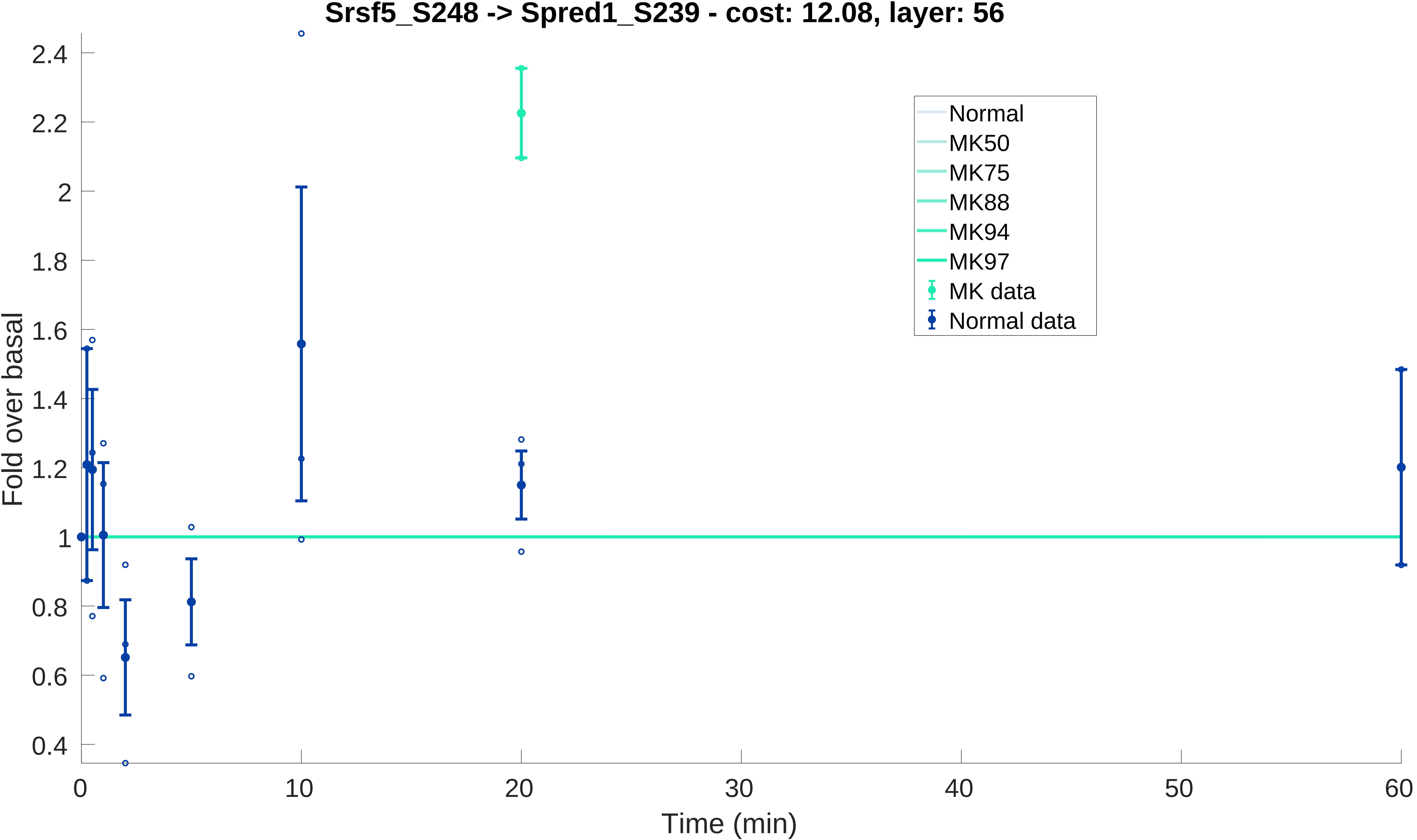

Srsf5_S248 -> Spred1_S239 - cost: 12.08, layer: 56
Normal
MK50
MK75
MK88
MK94
MK97
MK data
Normal data
Fold over basal
Time (min)
2.4
2.2
2
1.8
1.6
1.4
1.2
1
0.8
0.6
0.4
0
10
20
30
40
50
60

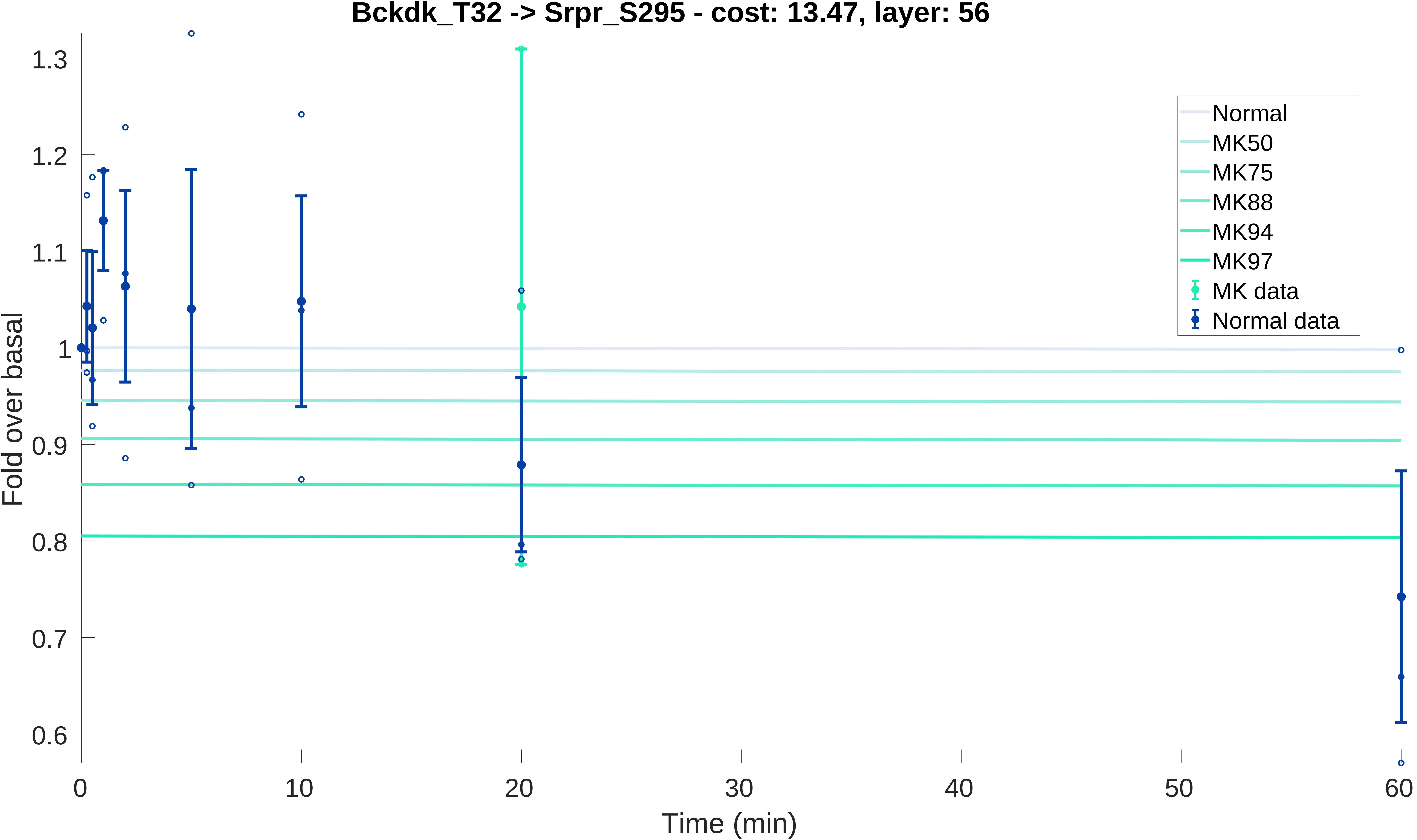

Bckdk_T32 -> Srpr_S295 - cost: 13.47, layer: 56
Fold over basal
Time (min)
0.6
0.7
0.8
0.9
1
1.1
1.2
1.3
0
10
20
30
40
50
60
Normal
MK50
MK75
MK88
MK94
MK97
MK data
Normal data

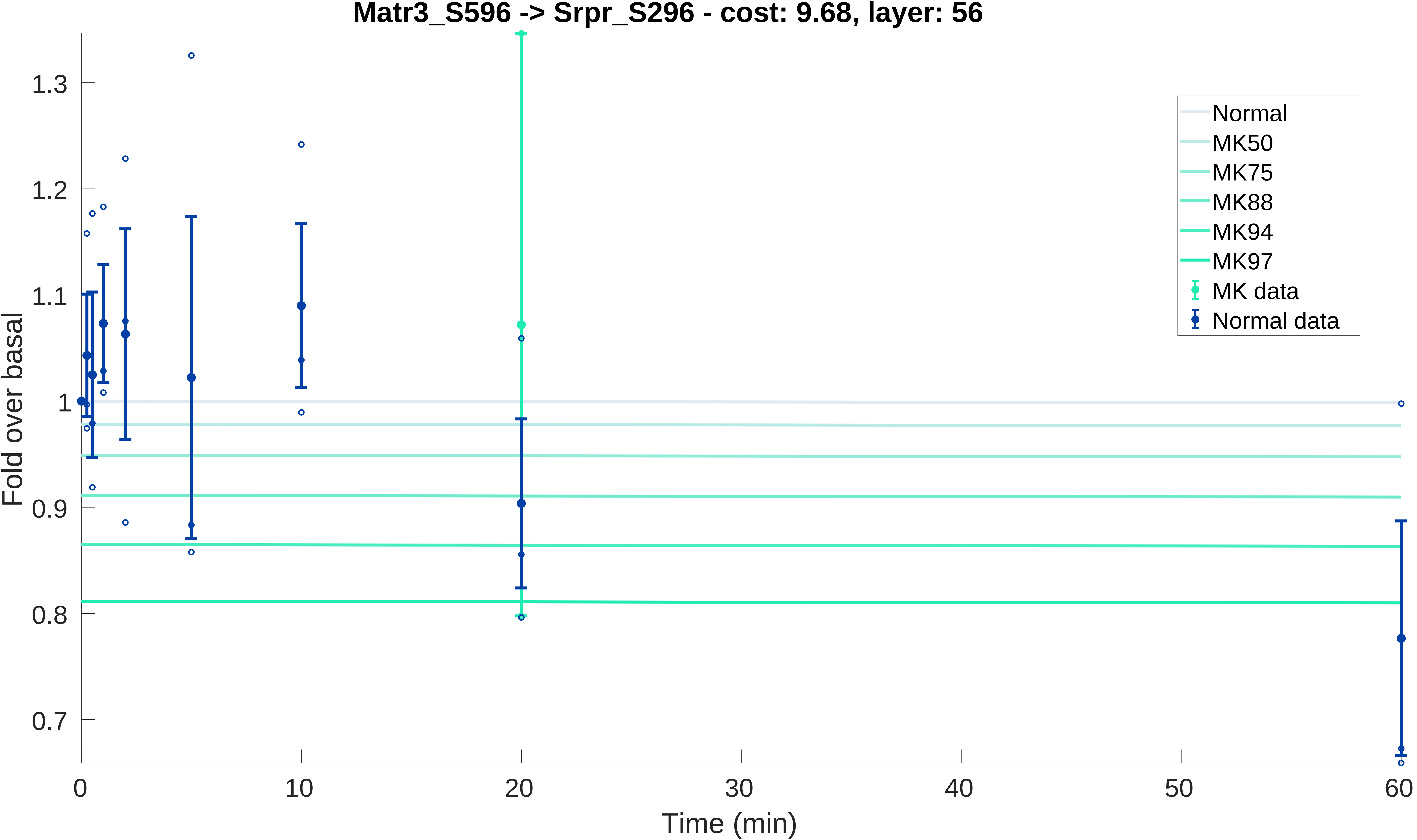
Matr3_S596 -> Srpr_S296 - cost: 9.68, layer: 56
Fold over basal
1.3
1.2
1.1
1
0.9
0.8
0.7
0
10
20
30
40
50
60
Time (min)
Normal
MK50
MK75
MK88
MK94
MK97
MK data
Normal data

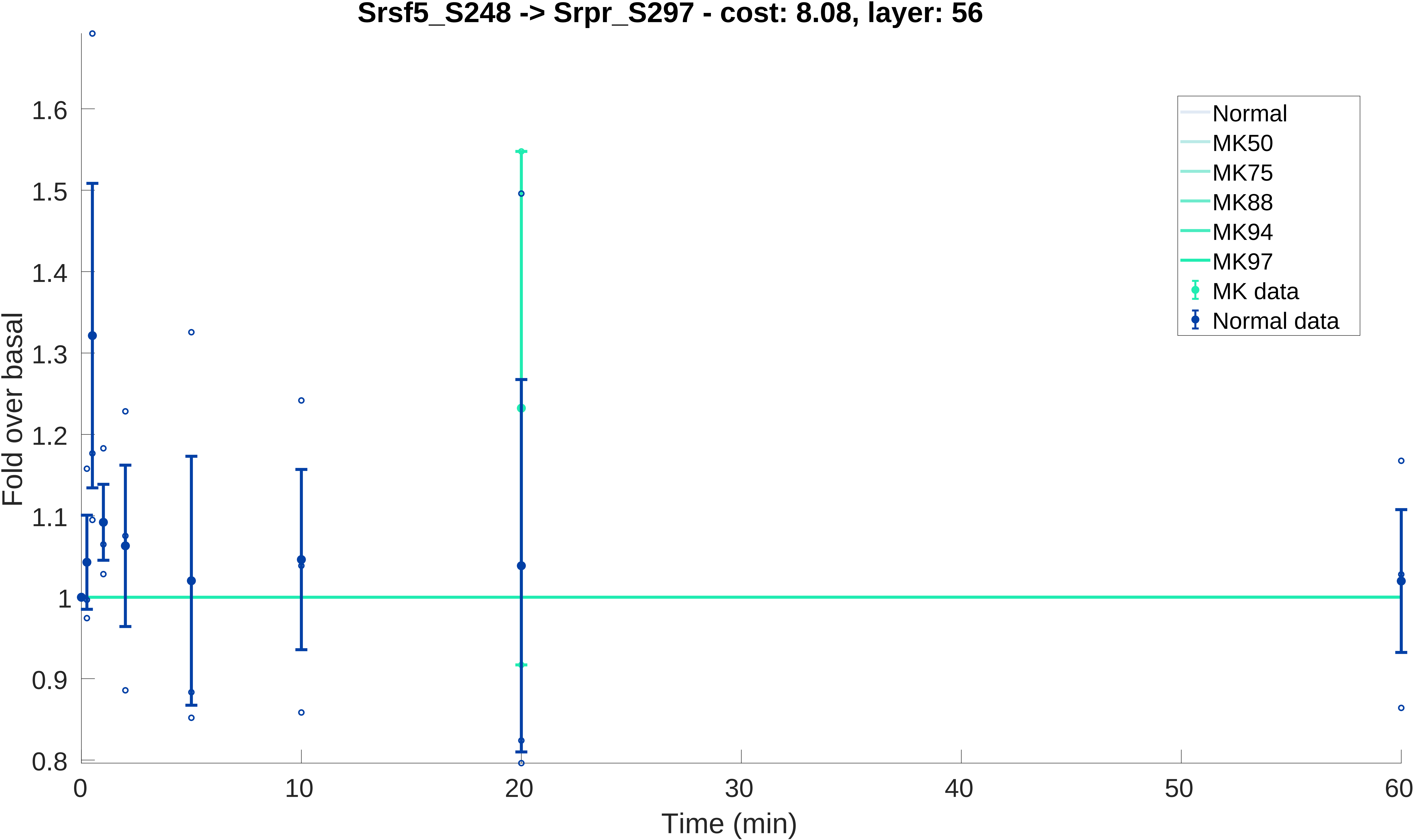
Srsf5_S248 -> Srpr_S297 - cost: 8.08, layer: 56
Fold over basal
Time (min)
0.8
0.9
1
1.1
1.2
1.3
1.4
1.5
1.6
0
10
20
30
40
50
60
Normal
MK50
MK75
MK88
MK94
MK97
MK data
Normal data

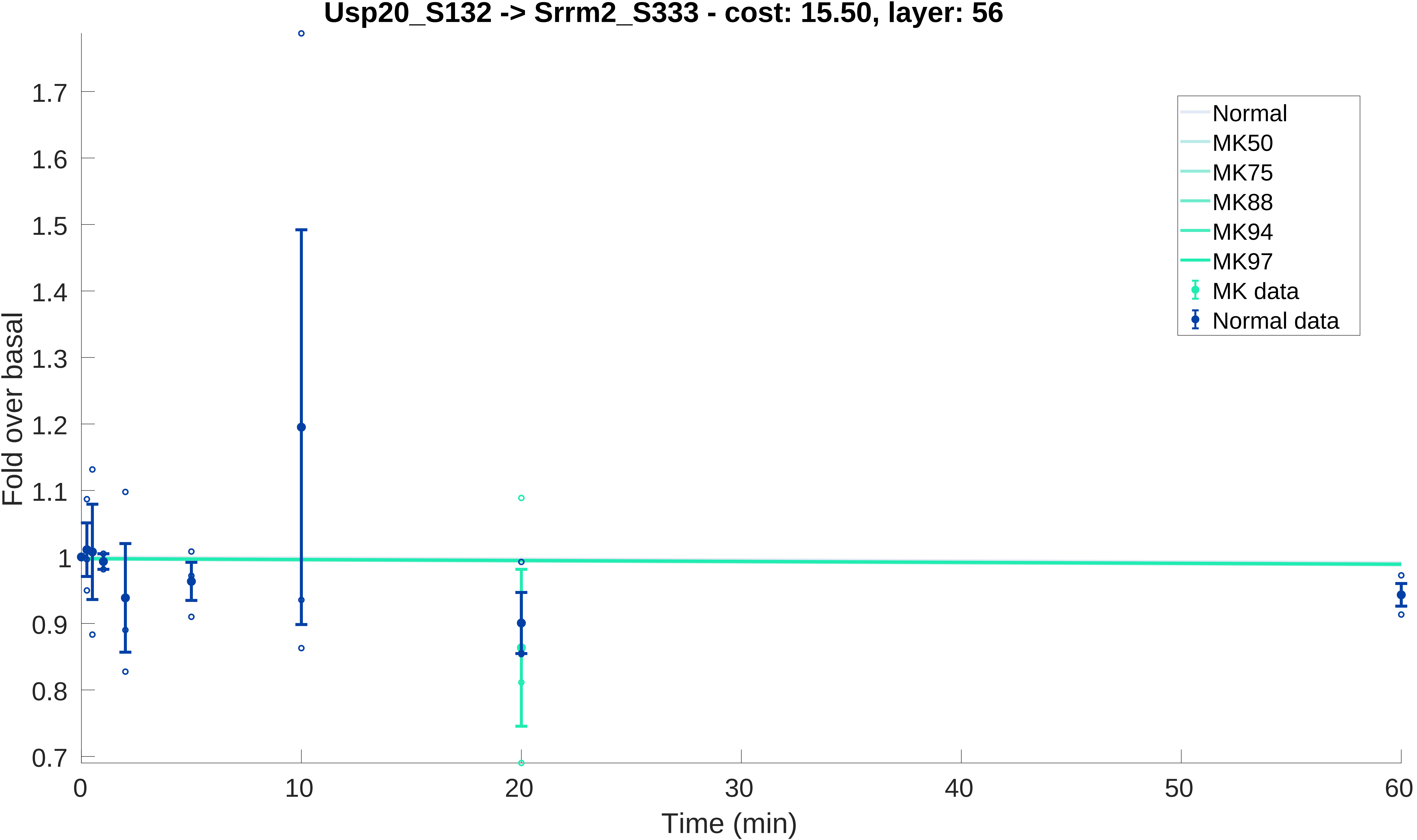

Usp20_S132 -> Srrm2_S333 - cost: 15.50, layer: 56
Fold over basal
Time (min)
0.7
0.8
0.9
1
1.1
1.2
1.3
1.4
1.5
1.6
1.7
0
10
20
30
40
50
60
Normal
MK50
MK75
MK88
MK94
MK97
MK data
Normal data

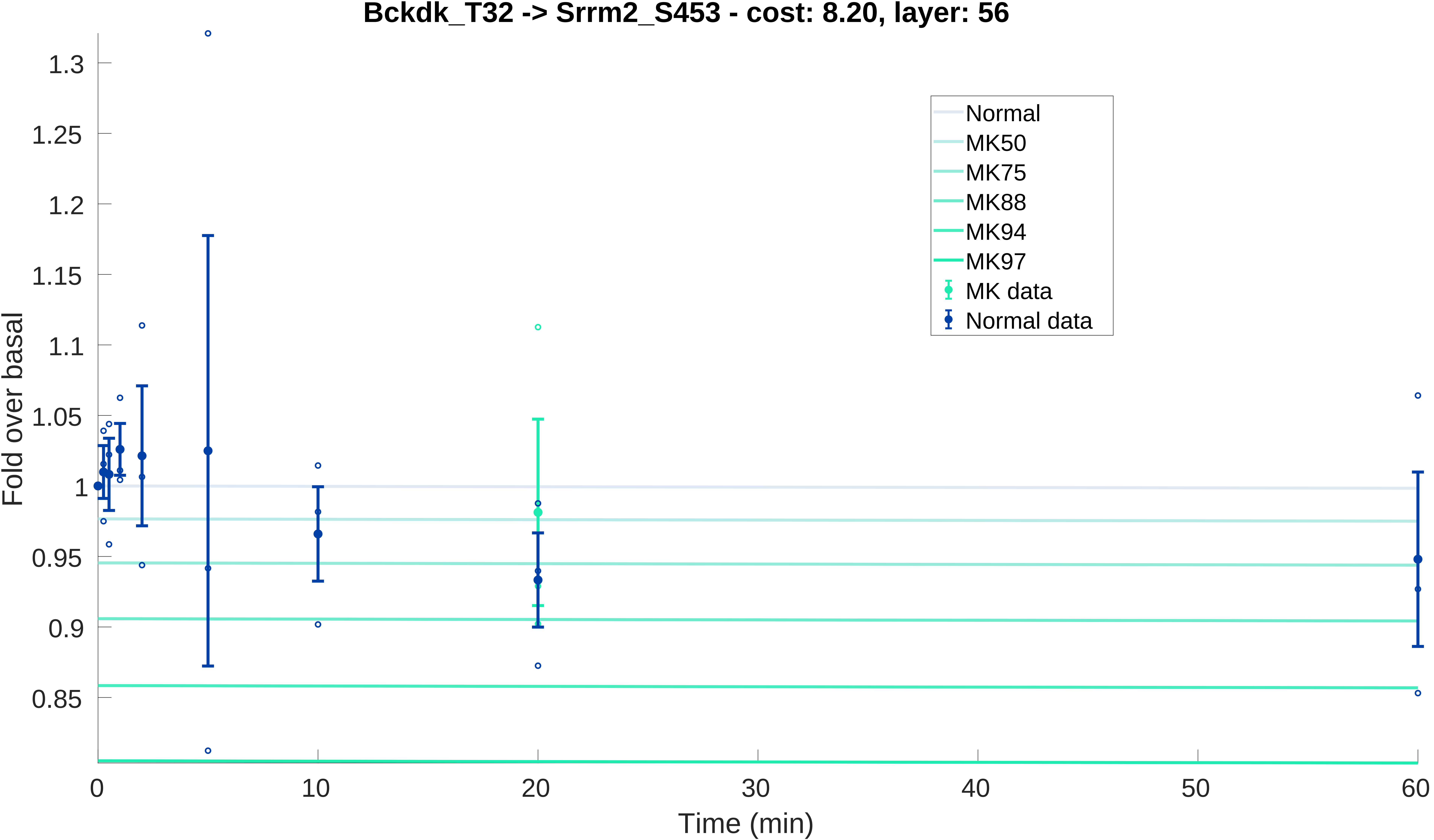

Bckdk_T32 -> Srrm2_S453 - cost: 8.20, layer: 56
Fold over basal
Time (min)
1.3
1.25
1.2
1.15
1.1
1.05
1
0.95
0.9
0.85
0
10
20
30
40
50
60
Normal
MK50
MK75
MK88
MK94
MK97
MK data
Normal data

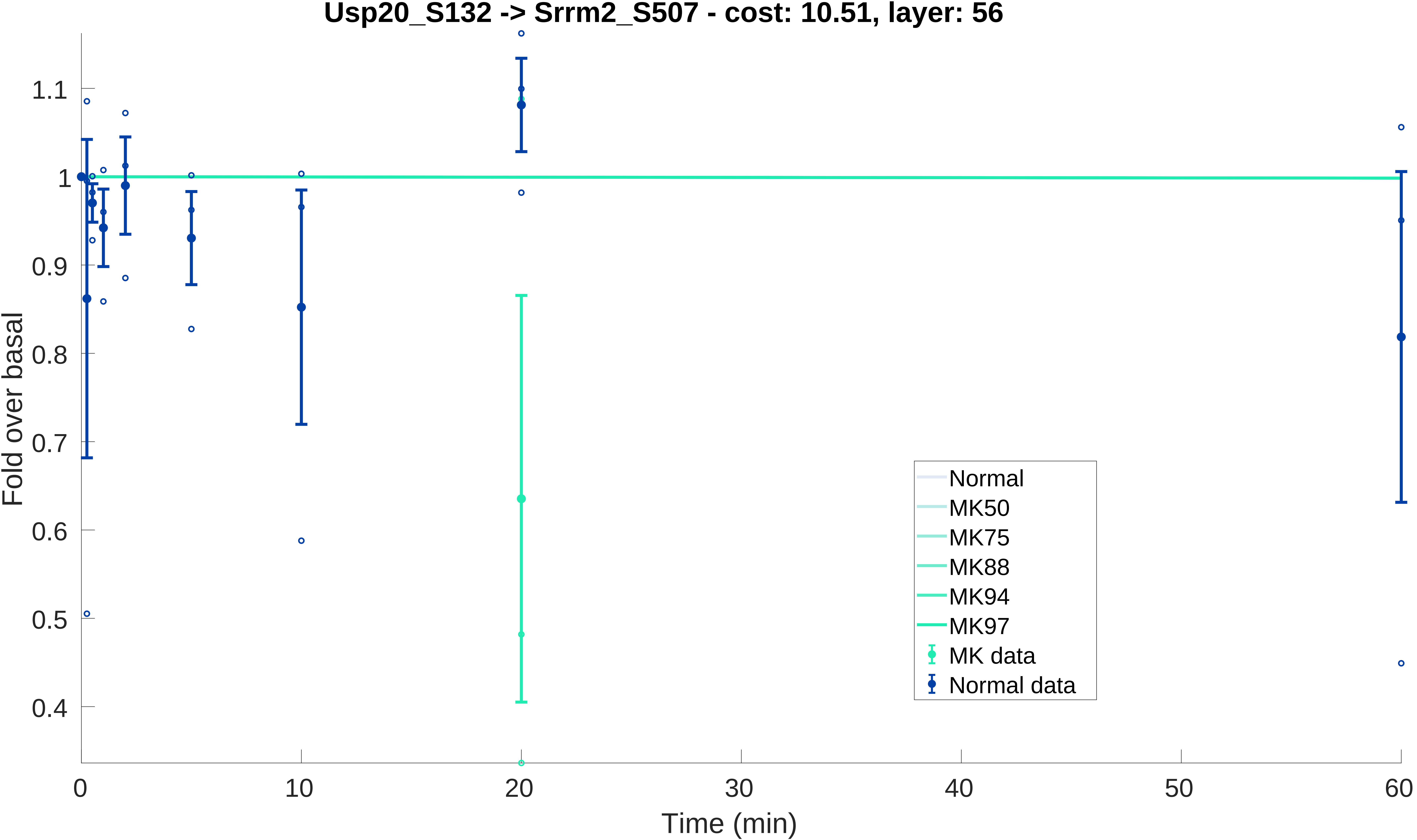

Usp20_S132 -> Srrm2_S507 - cost: 10.51, layer: 56
Fold over basal
1.1
1
0.9
0.8
0.7
0.6
0.5
0.4
0
10
20
30
40
50
60
Time (min)
Normal
MK50
MK75
MK88
MK94
MK97
MK data
Normal data

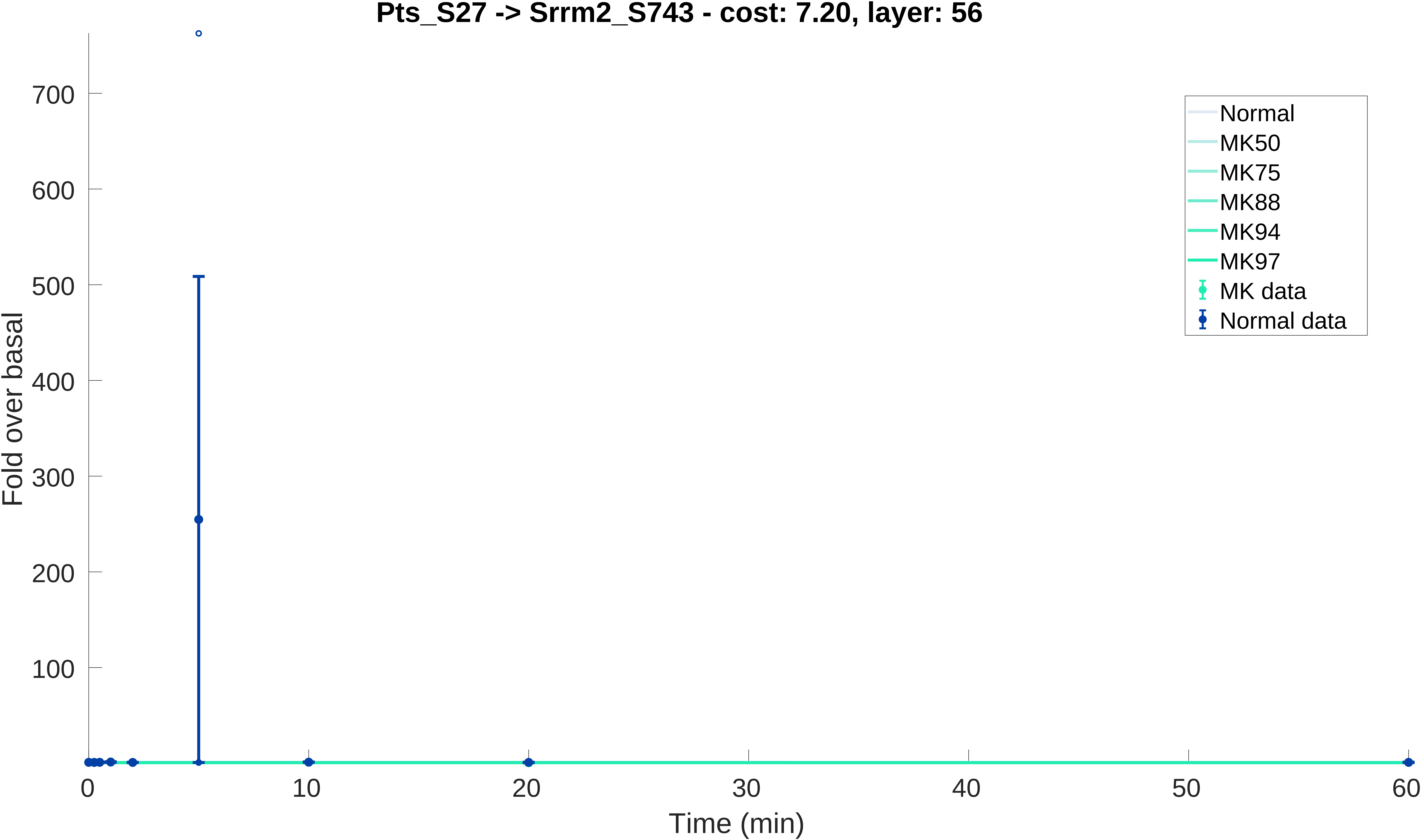

Pts_S27 -> Srrm2_S743 - cost: 7.20, layer: 56
Fold over basal
700
600
500
400
300
200
100
0
10
20
30
40
50
60
Time (min)
Normal
MK50
MK75
MK88
MK94
MK97
MK data
Normal data

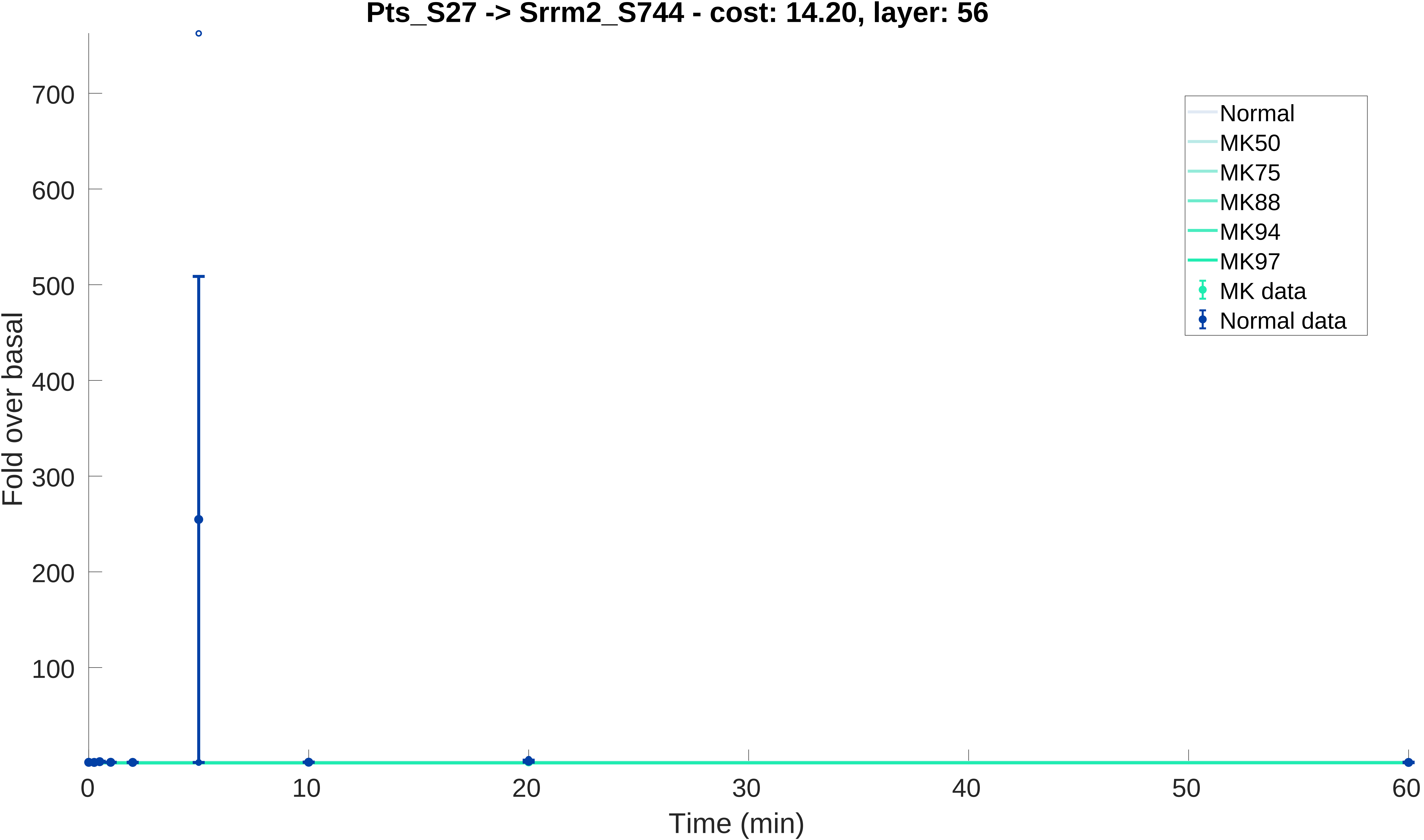

Pts_S27 -> Srrm2_S744 - cost: 14.20, layer: 56
Fold over basal
Time (min)
Normal
MK50
MK75
MK88
MK94
MK97
MK data
Normal data

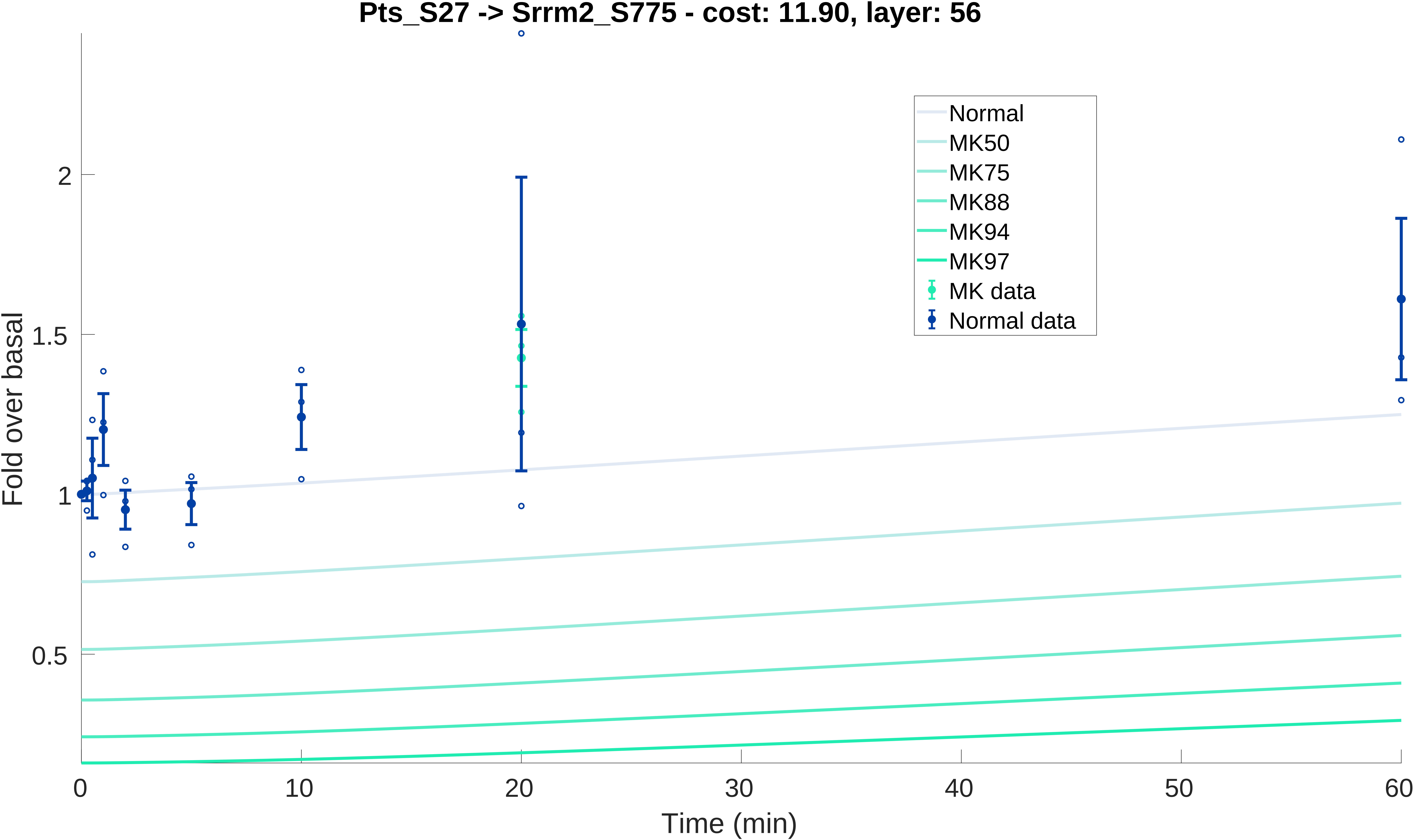

Pts_S27 -> Srrm2_S775 - cost: 11.90, layer: 56
Normal
MK50
MK75
MK88
MK94
MK97
MK data
Normal data
Fold over basal
0.5
1
1.5
2
0
10
20
30
40
50
60
Time (min)

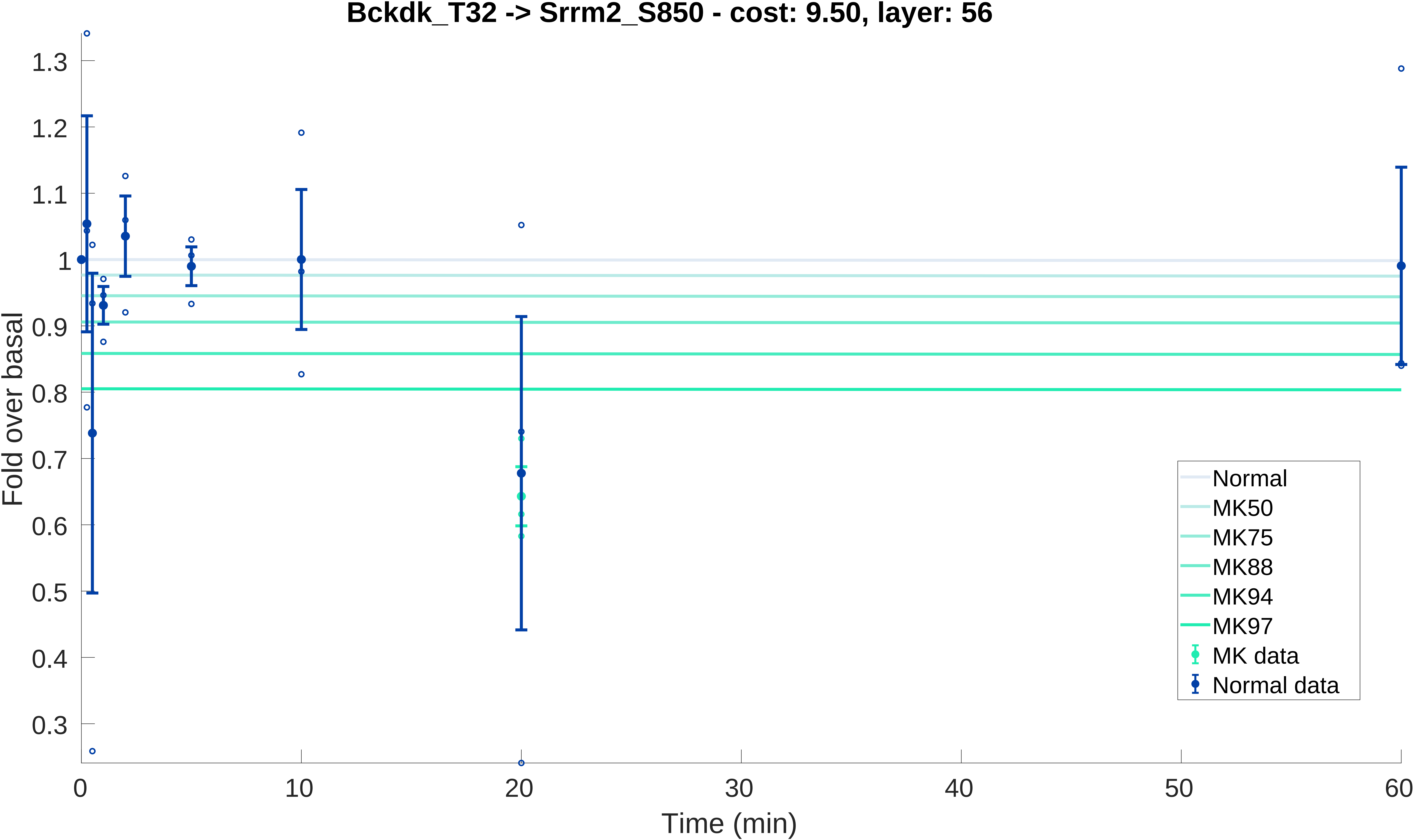
Bckdk_T32 -> Srrm2_S850 - cost: 9.50, layer: 56
Fold over basal
1.3
1.2
1.1
1
0.9
0.8
0.7
0.6
0.5
0.4
0.3
0
10
20
30
40
50
60
Time (min)
Normal
MK50
MK75
MK88
MK94
MK97
MK data
Normal data

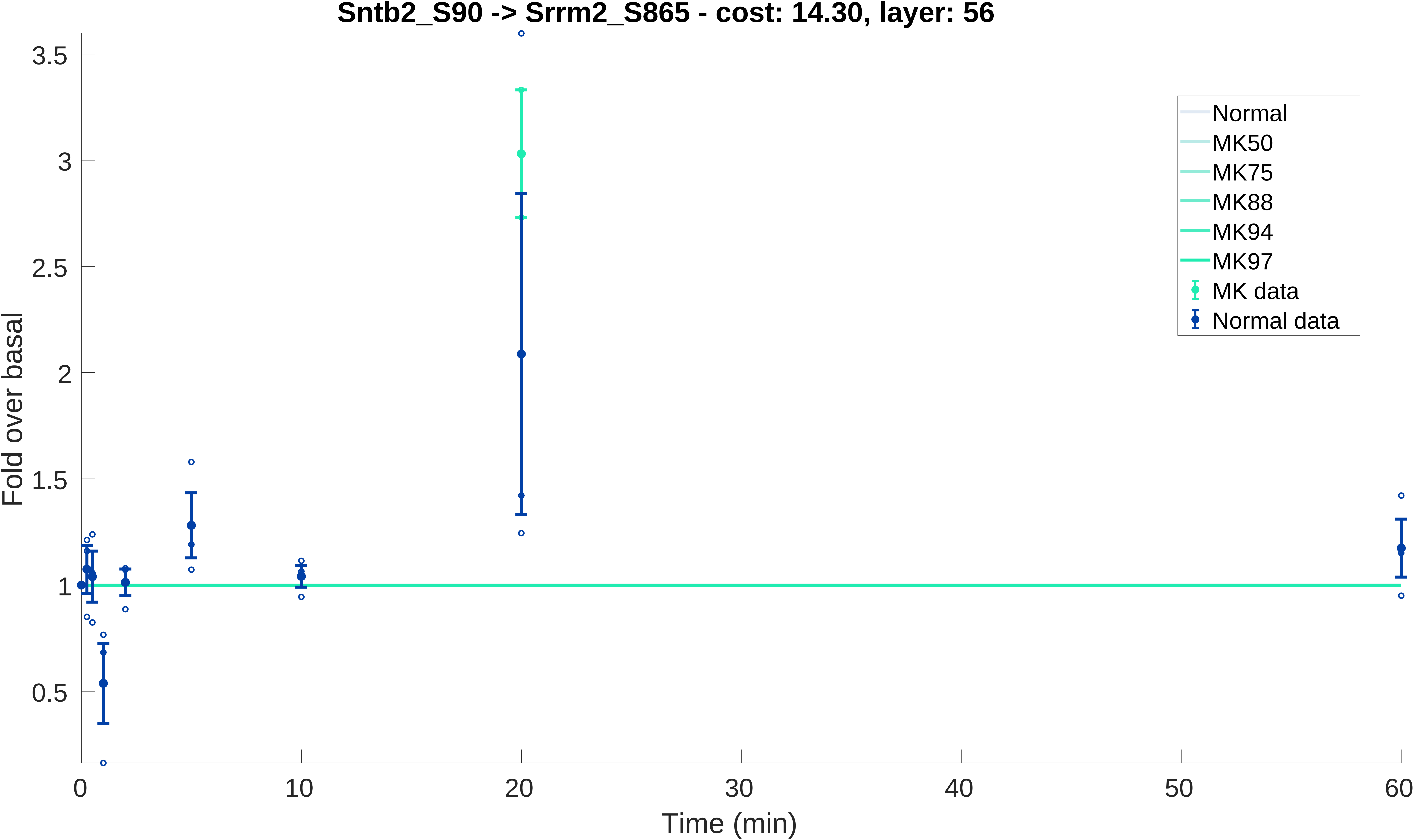
Sntb2_S90 -> Srrm2_S865 - cost: 14.30, layer: 56
Fold over basal
Time (min)
3.5
3
2.5
2
1.5
1
0.5
0
10
20
30
40
50
60
Normal
MK50
MK75
MK88
MK94
MK97
MK data
Normal data

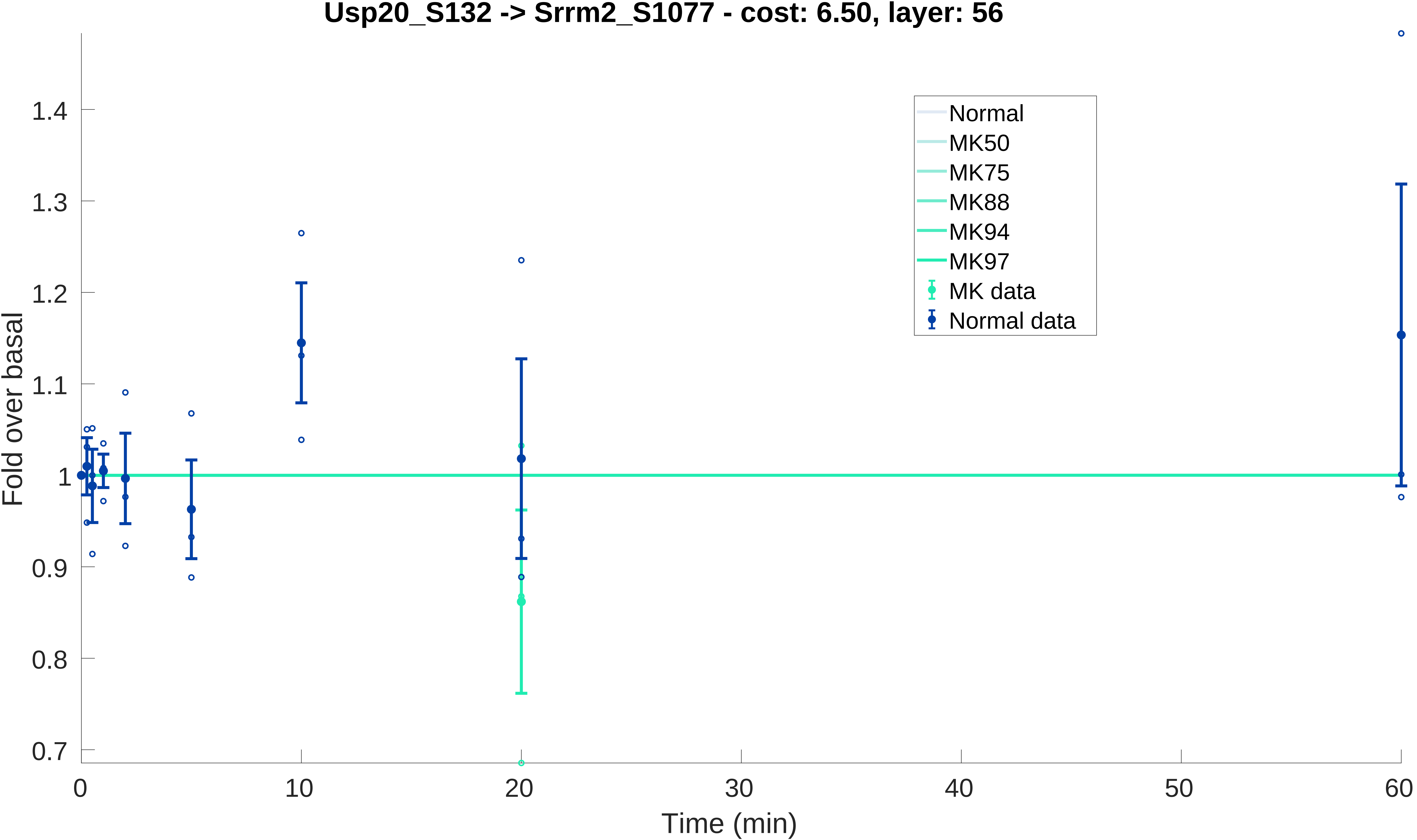

Usp20_S132 -> Srrm2_S1077 - cost: 6.50, layer: 56
Normal
MK50
MK75
MK88
MK94
MK97
MK data
Normal data
Fold over basal
Time (min)
0
10
20
30
40
50
60
0.7
0.8
0.9
1
1.1
1.2
1.3
1.4

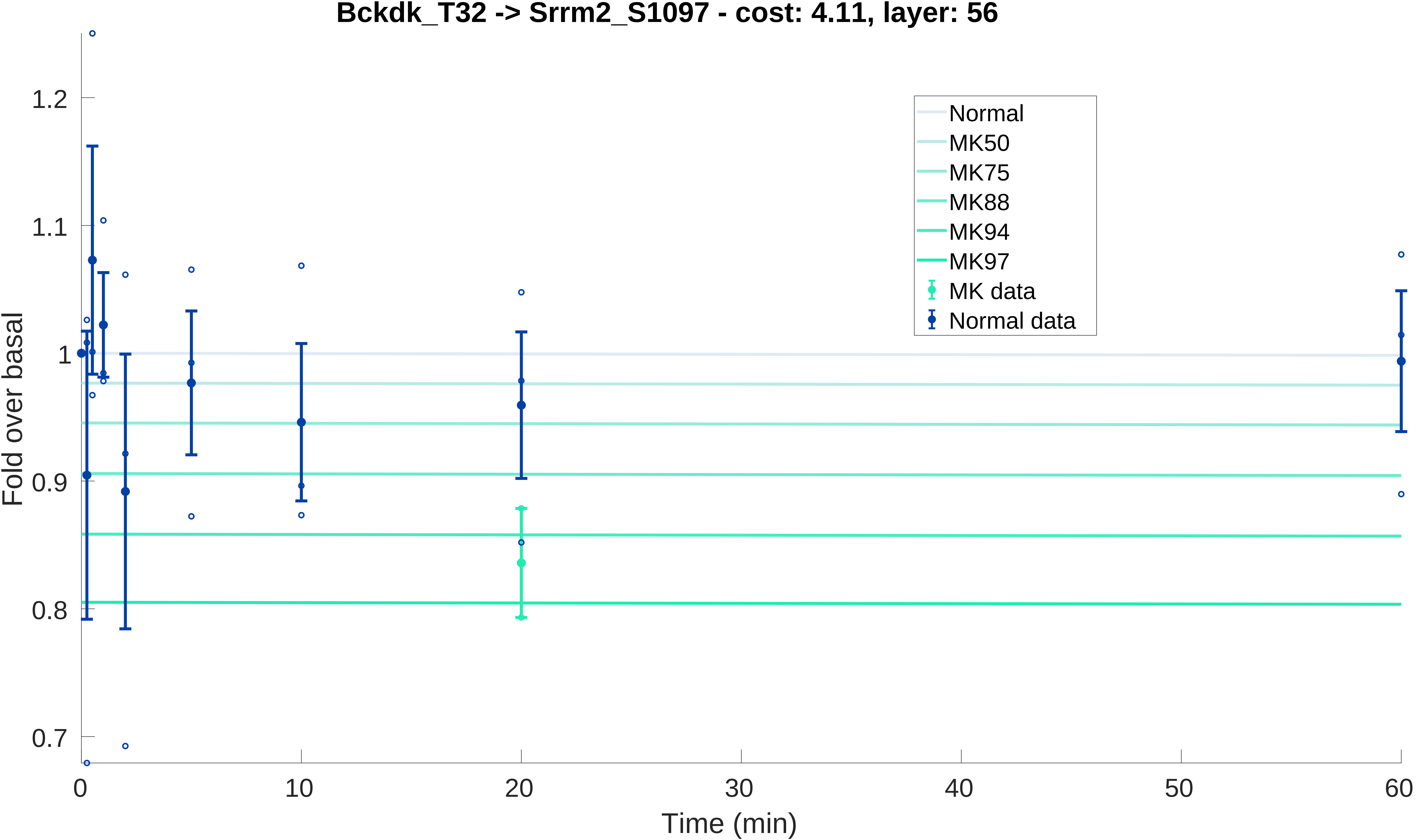
Bckdk_T32 -> Srrm2_S1097 - cost: 4.11, layer: 56
Fold over basal
1.2
1.1
1
0.9
0.8
0.7
0
10
20
30
40
50
60
Time (min)
Normal
MK50
MK75
MK88
MK94
MK97
MK data
Normal data

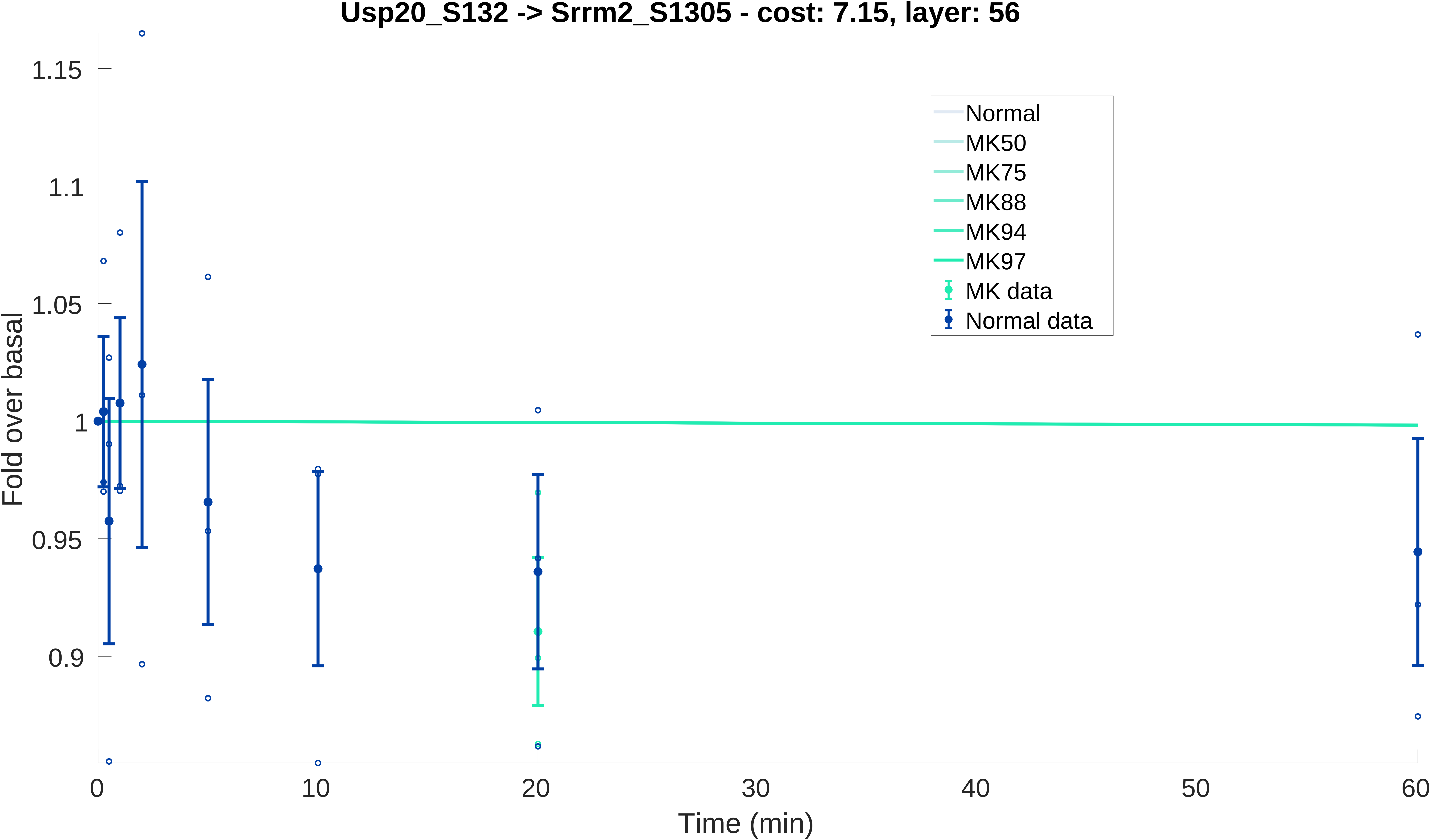
Usp20_S132 -> Srrm2_S1305 - cost: 7.15, layer: 56
Fold over basal
Time (min)
1.15
1.1
1.05
1
0.95
0.9
0
10
20
30
40
50
60
Normal
MK50
MK75
MK88
MK94
MK97
MK data
Normal data

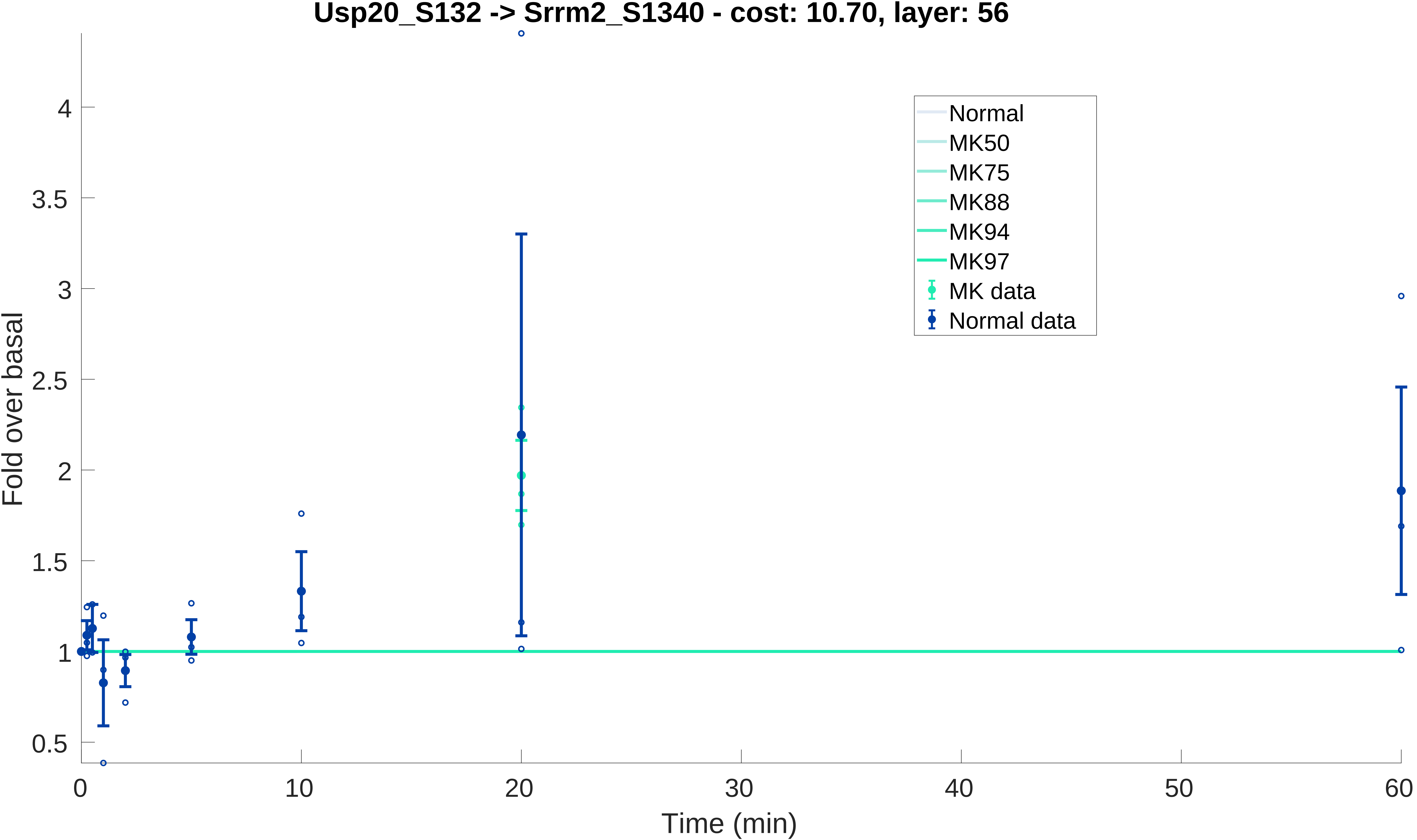

Usp20_S132 -> Srrm2_S1340 - cost: 10.70, layer: 56
Fold over basal
Time (min)
0.5
1
1.5
2
2.5
3
3.5
4
0
10
20
30
40
50
60
Normal
MK50
MK75
MK88
MK94
MK97
MK data
Normal data

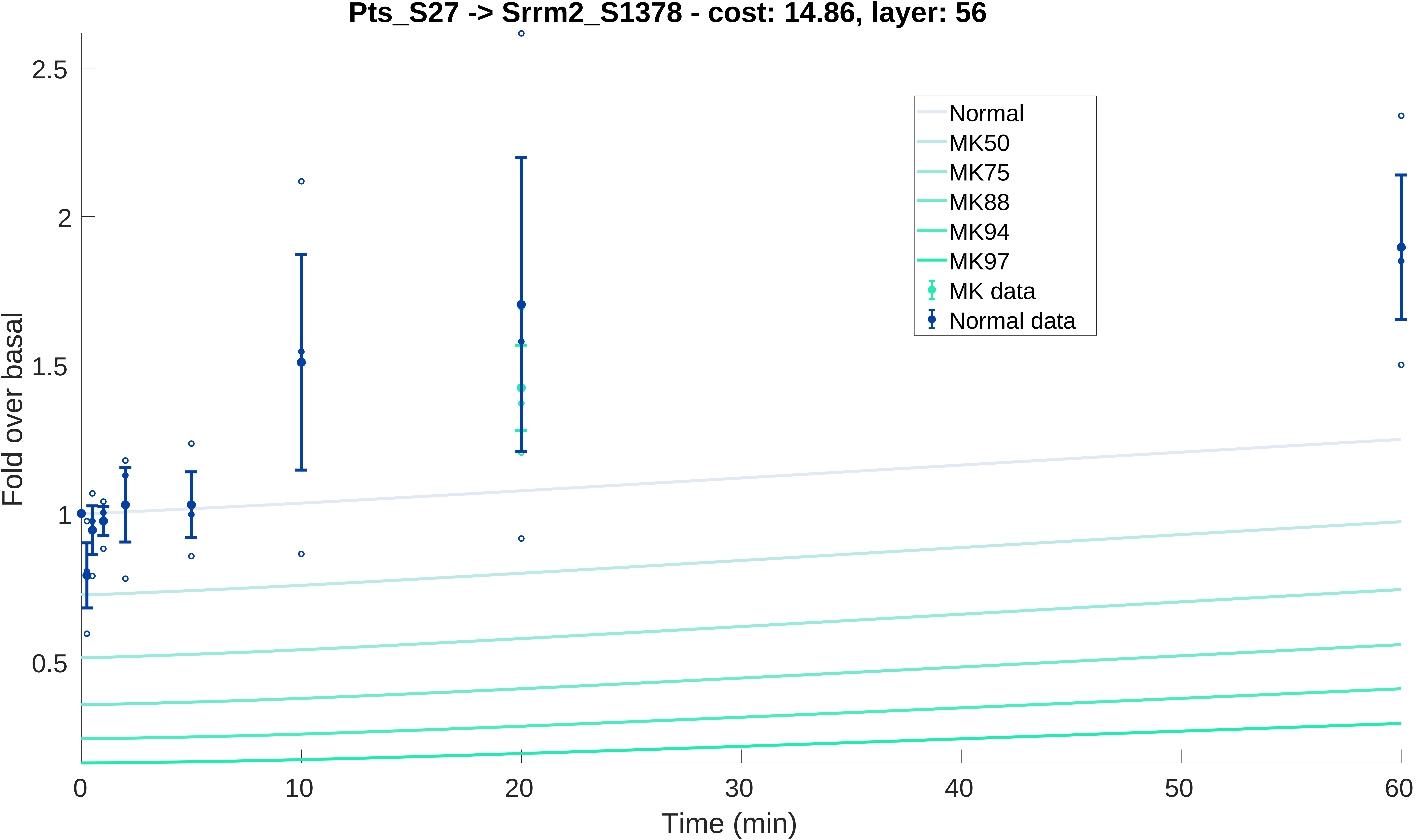
Pts_S27 -> Srrm2_S1378 - cost: 14.86, layer: 56
Fold over basal
Time (min)
2.5
2
1.5
1
0.5
0
10
20
30
40
50
60
Normal
MK50
MK75
MK88
MK94
MK97
MK data
Normal data

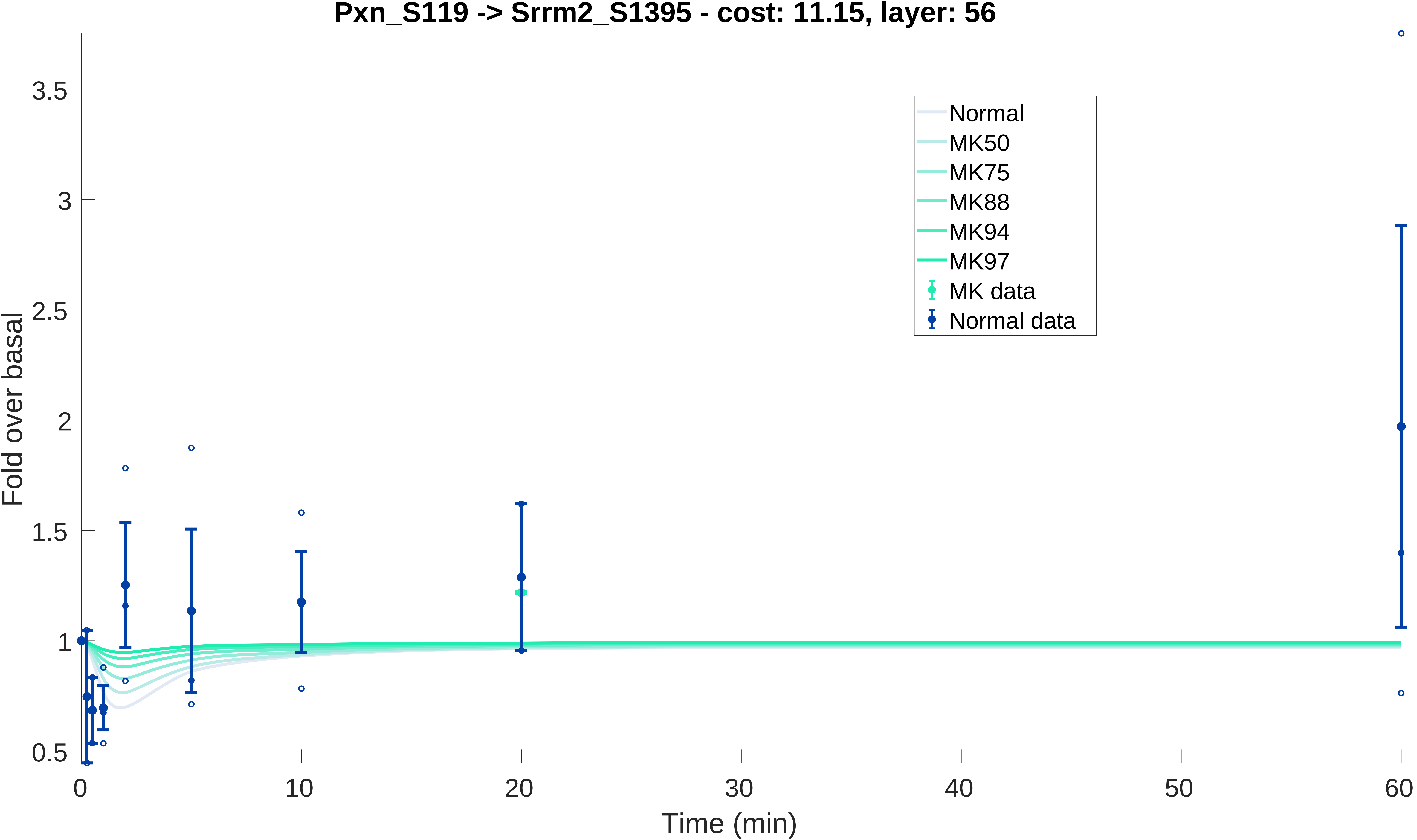

Pxn_S119 -> Srrm2_S1395 - cost: 11.15, layer: 56
Fold over basal
Time (min)
0.5
1
1.5
2
2.5
3
3.5
0
10
20
30
40
50
60
Normal
MK50
MK75
MK88
MK94
MK97
MK data
Normal data

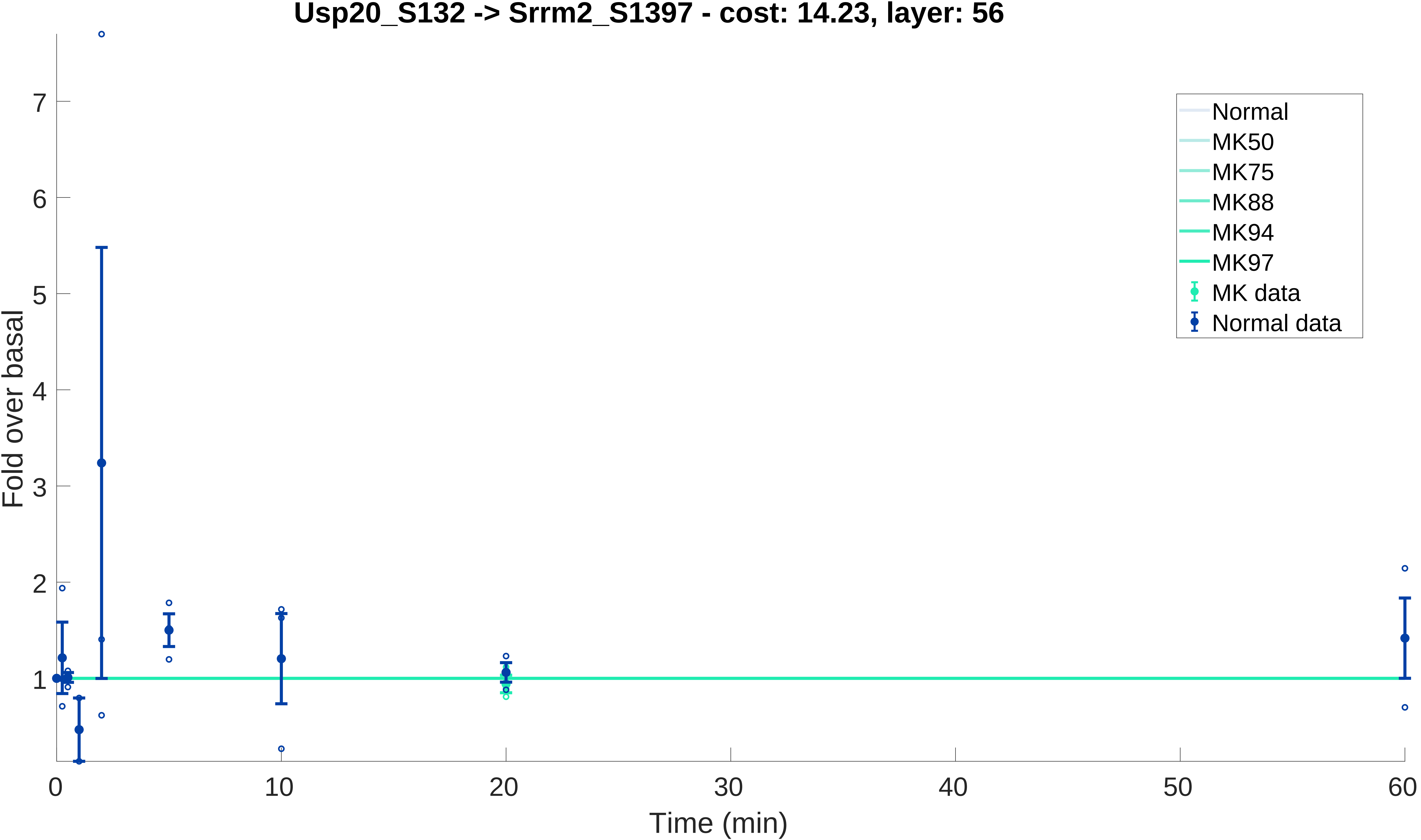

Usp20_S132 -> Srrm2_S1397 - cost: 14.23, layer: 56
Fold over basal
Time (min)
1
2
3
4
5
6
7
0
10
20
30
40
50
60
Normal
MK50
MK75
MK88
MK94
MK97
MK data
Normal data

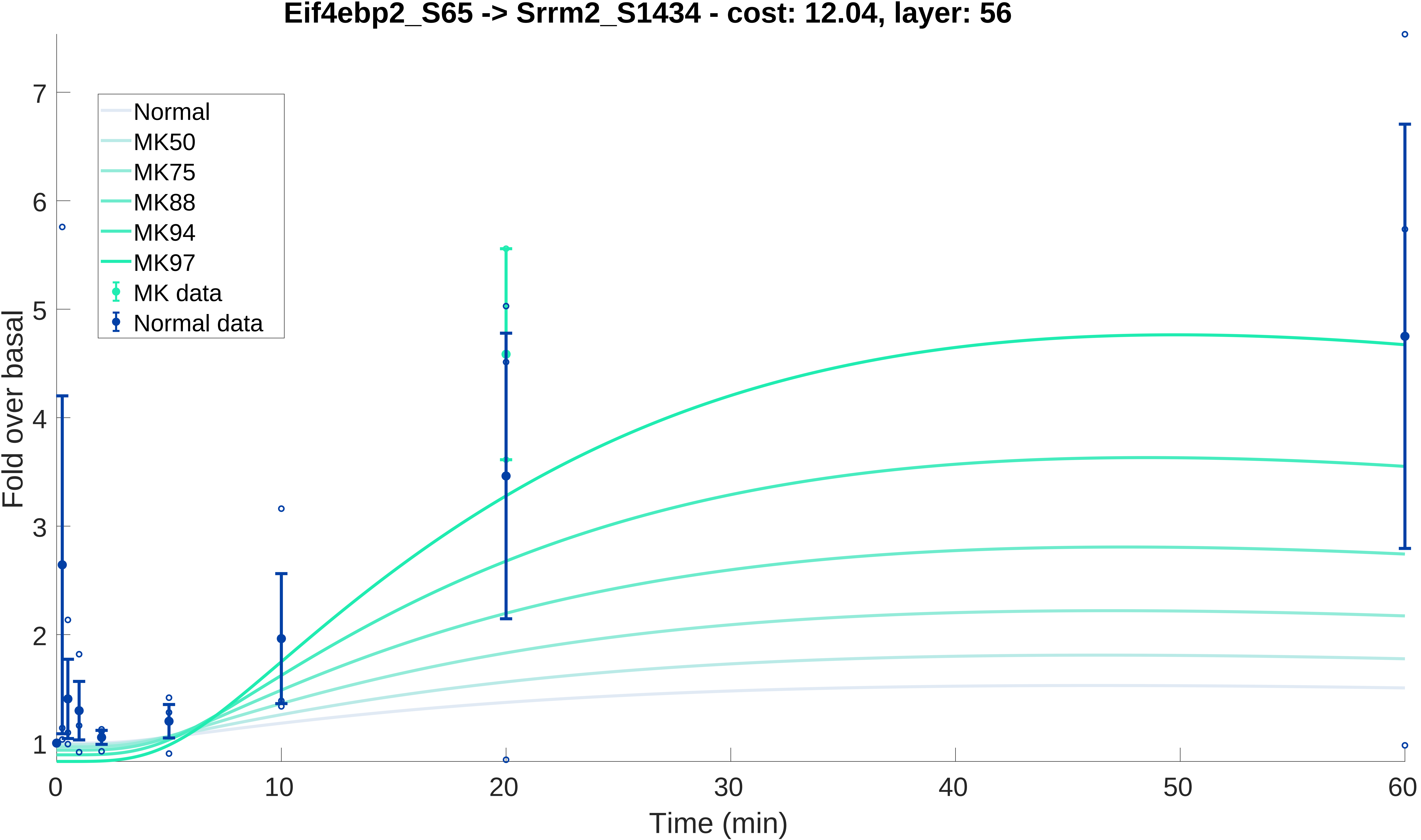
Eif4ebp2_S65 -> Srrm2_S1434 - cost: 12.04, layer: 56
Normal
MK50
MK75
MK88
MK94
MK97
MK data
Normal data
Fold over basal
Time (min)
1
2
3
4
5
6
7
0
10
20
30
40
50
60

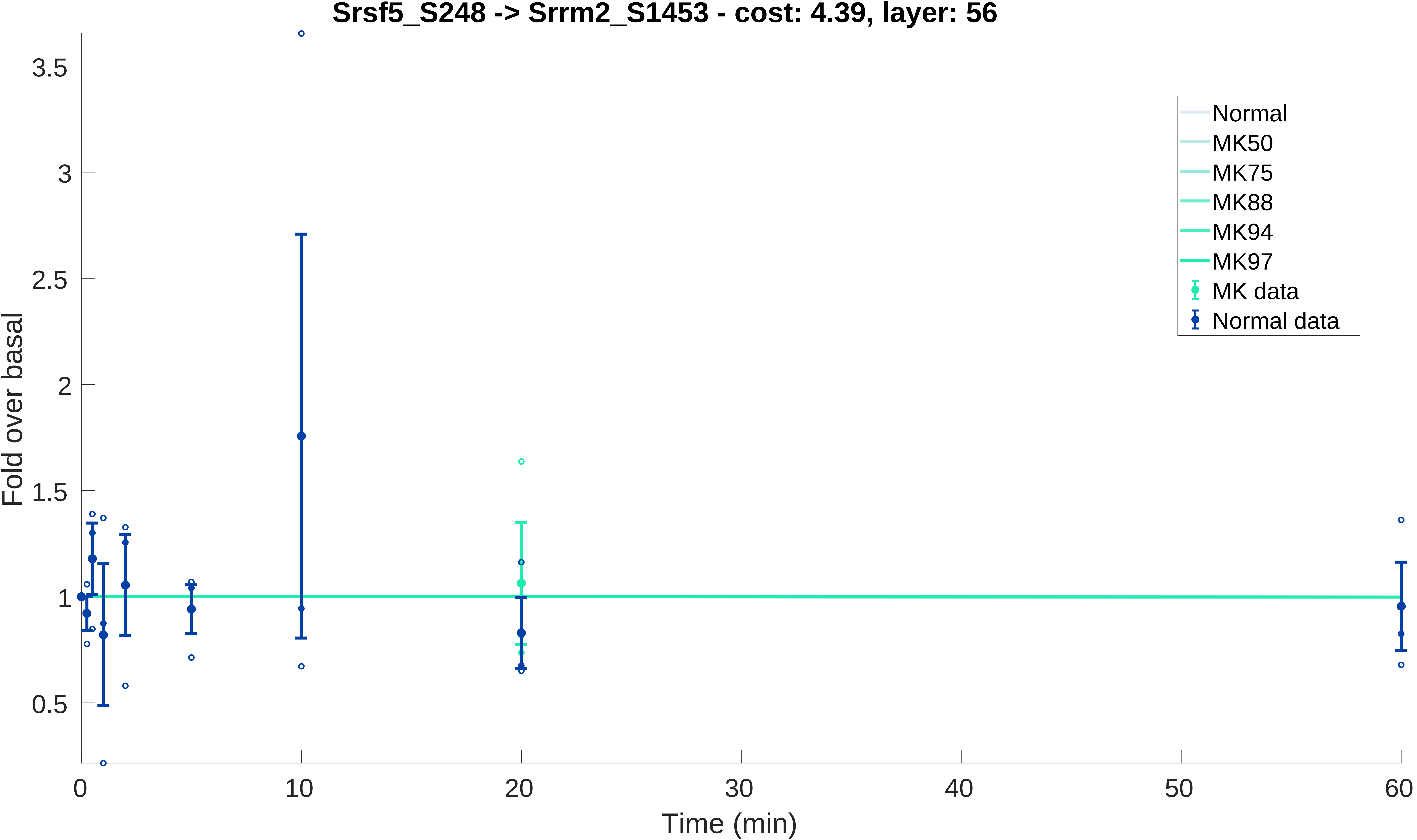

Srsf5_S248 -> Srrm2_S1453 - cost: 4.39, layer: 56
Fold over basal
Time (min)
0
10
20
30
40
50
60
0.5
1
1.5
2
2.5
3
3.5
Normal
MK50
MK75
MK88
MK94
MK97
MK data
Normal data

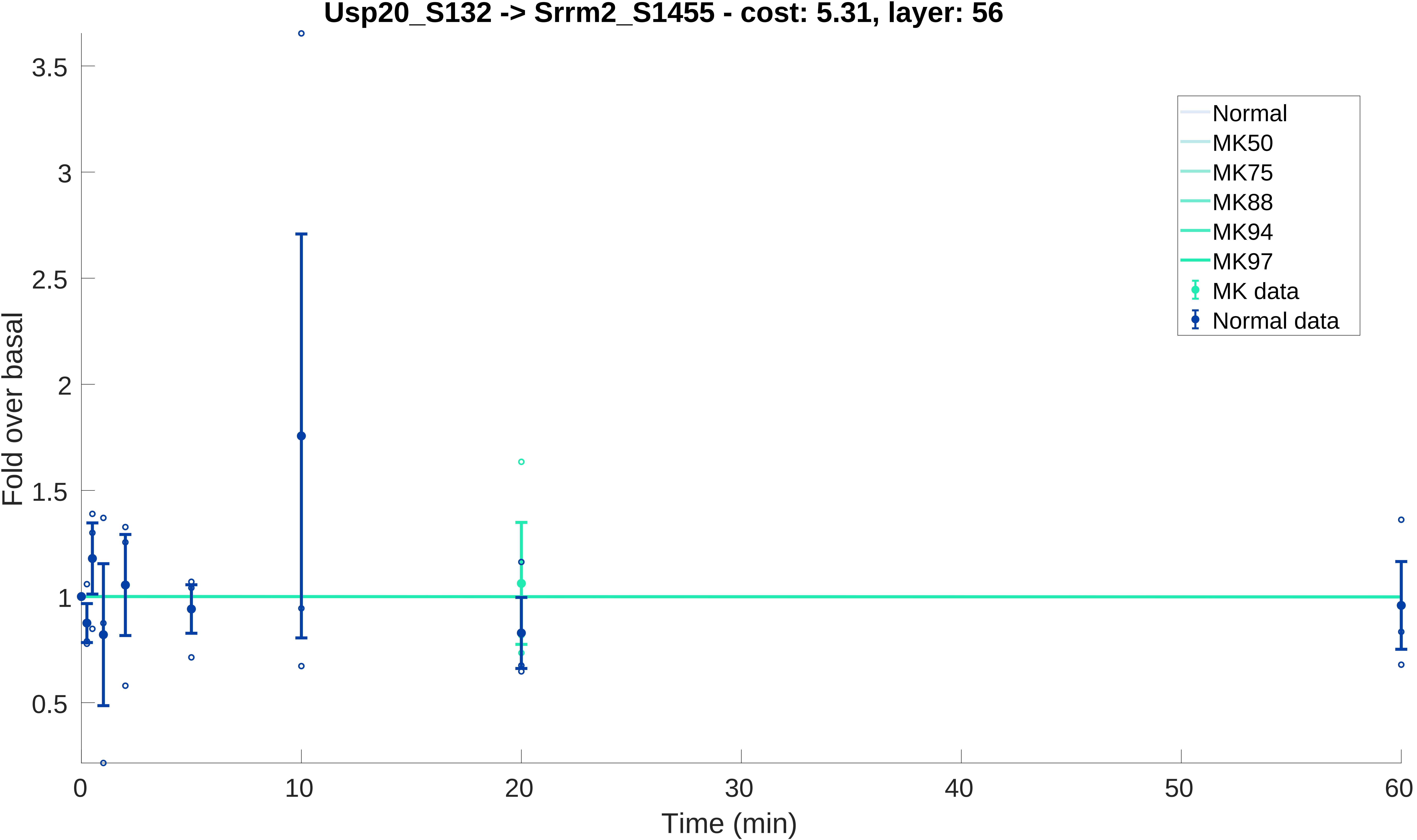

Usp20_S132 -> Srrm2_S1455 - cost: 5.31, layer: 56
Fold over basal
Time (min)
0.5
1
1.5
2
2.5
3
3.5
0
10
20
30
40
50
60
Normal
MK50
MK75
MK88
MK94
MK97
MK data
Normal data

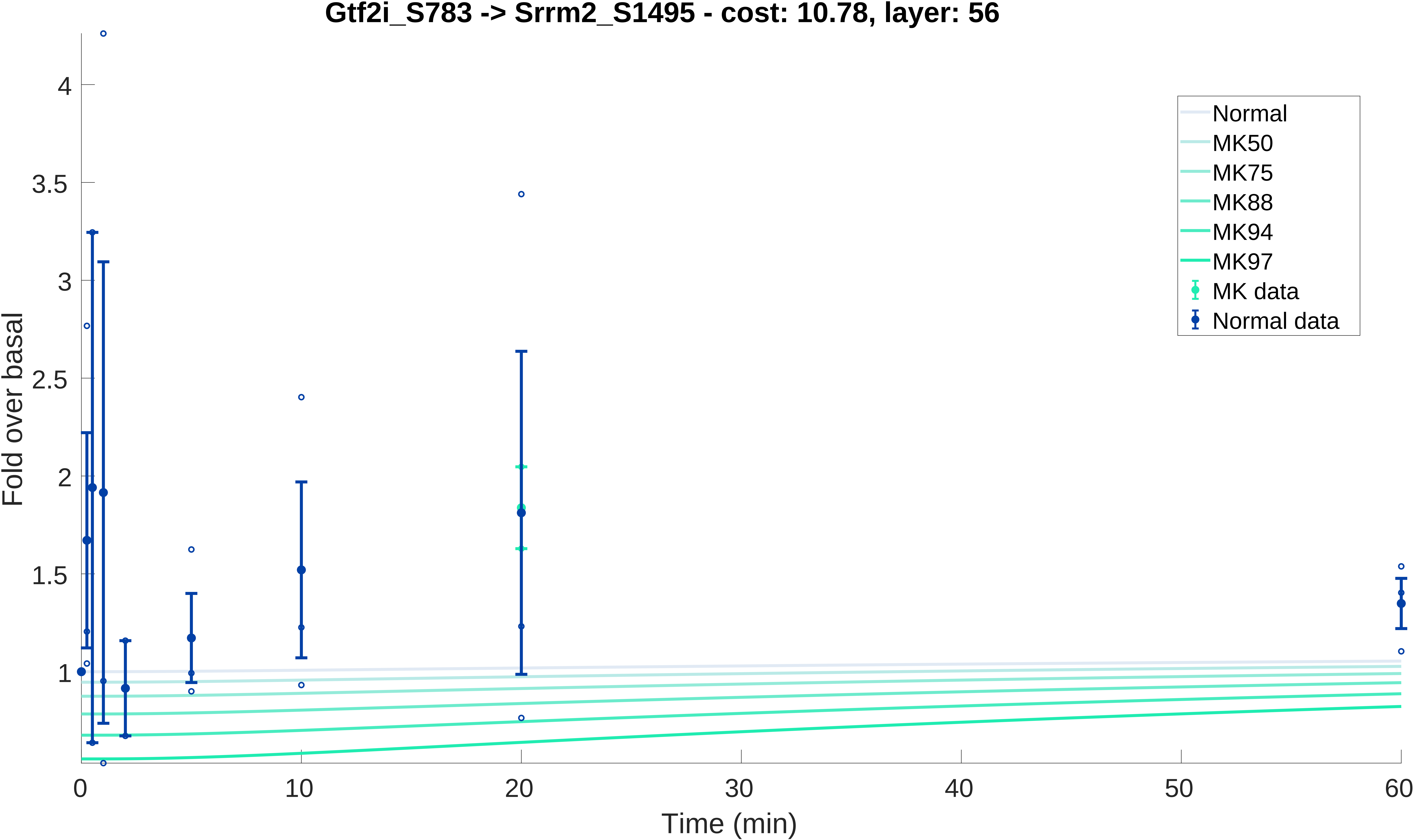

Gtf2i_S783 -> Srrm2_S1495 - cost: 10.78, layer: 56
Fold over basal
Time (min)
Normal
MK50
MK75
MK88
MK94
MK97
MK data
Normal data

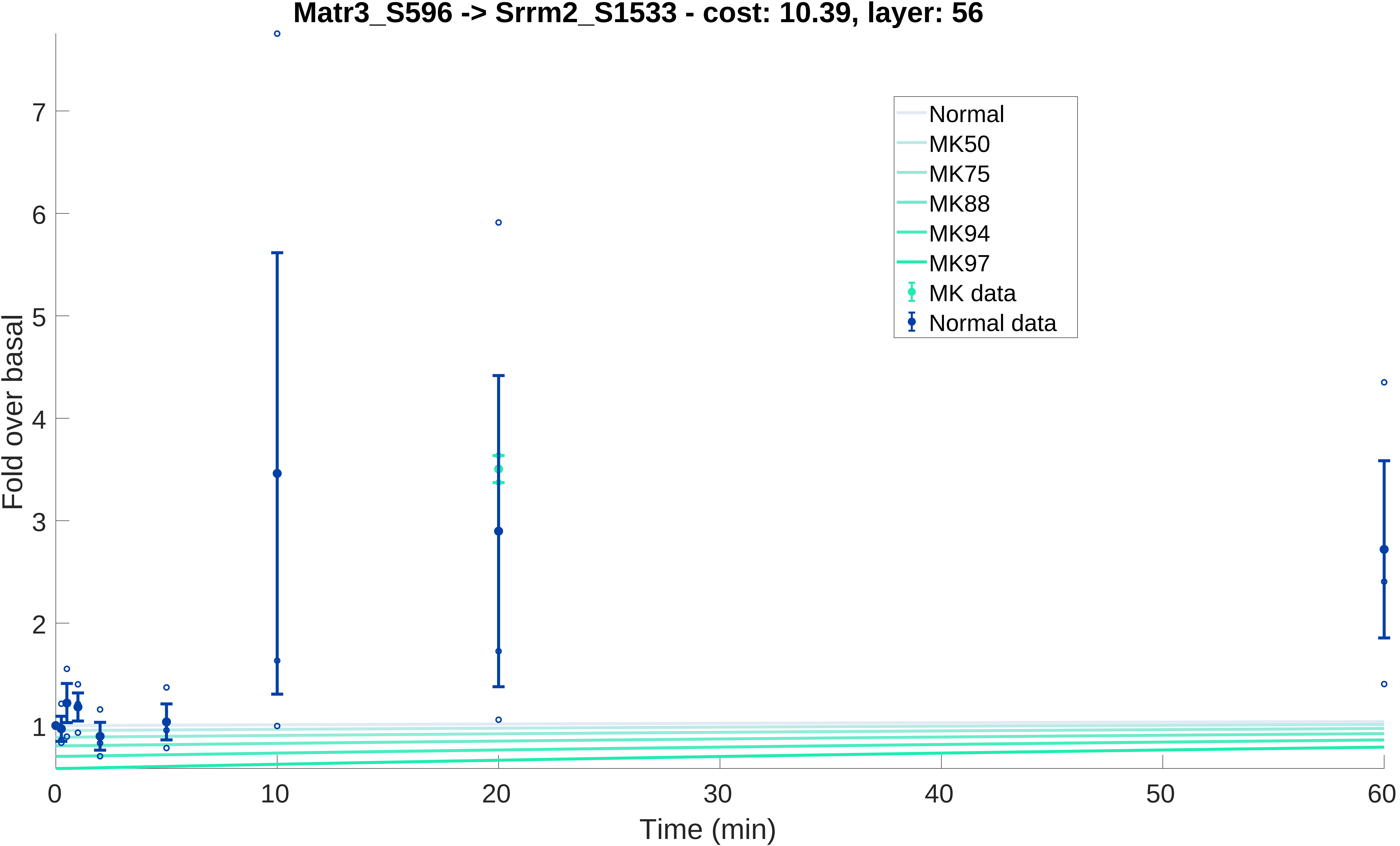

Matr3_S596 -> Srrm2_S1533 - cost: 10.39, layer: 56
Fold over basal
Time (min)
0
10
20
30
40
50
60
1
2
3
4
5
6
7
Normal
MK50
MK75
MK88
MK94
MK97
MK data
Normal data

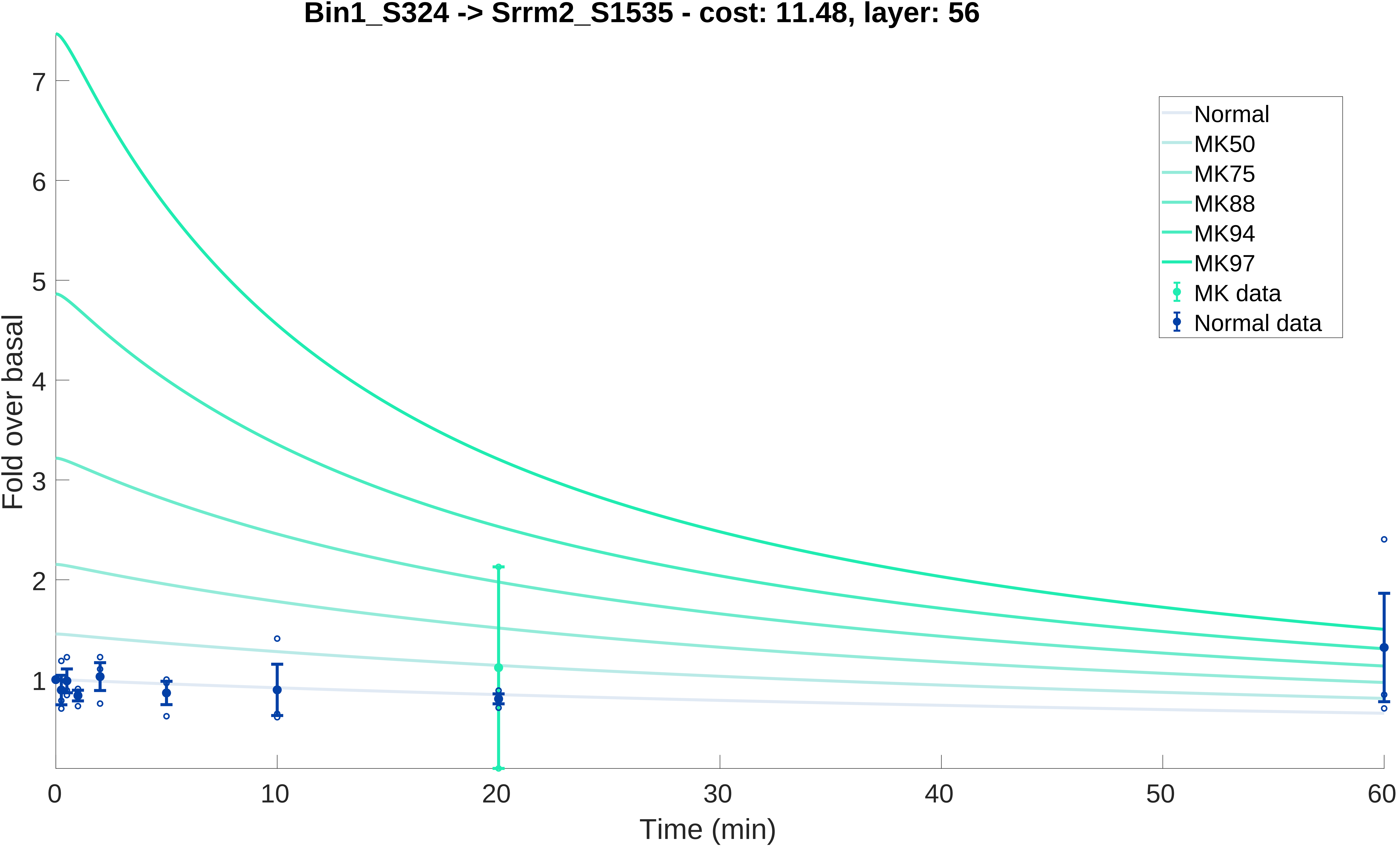

Bin1_S324 -> Srrm2_S1535 - cost: 11.48, layer: 56
Fold over basal
Time (min)
0
10
20
30
40
50
60
1
2
3
4
5
6
7
Normal
MK50
MK75
MK88
MK94
MK97
MK data
Normal data

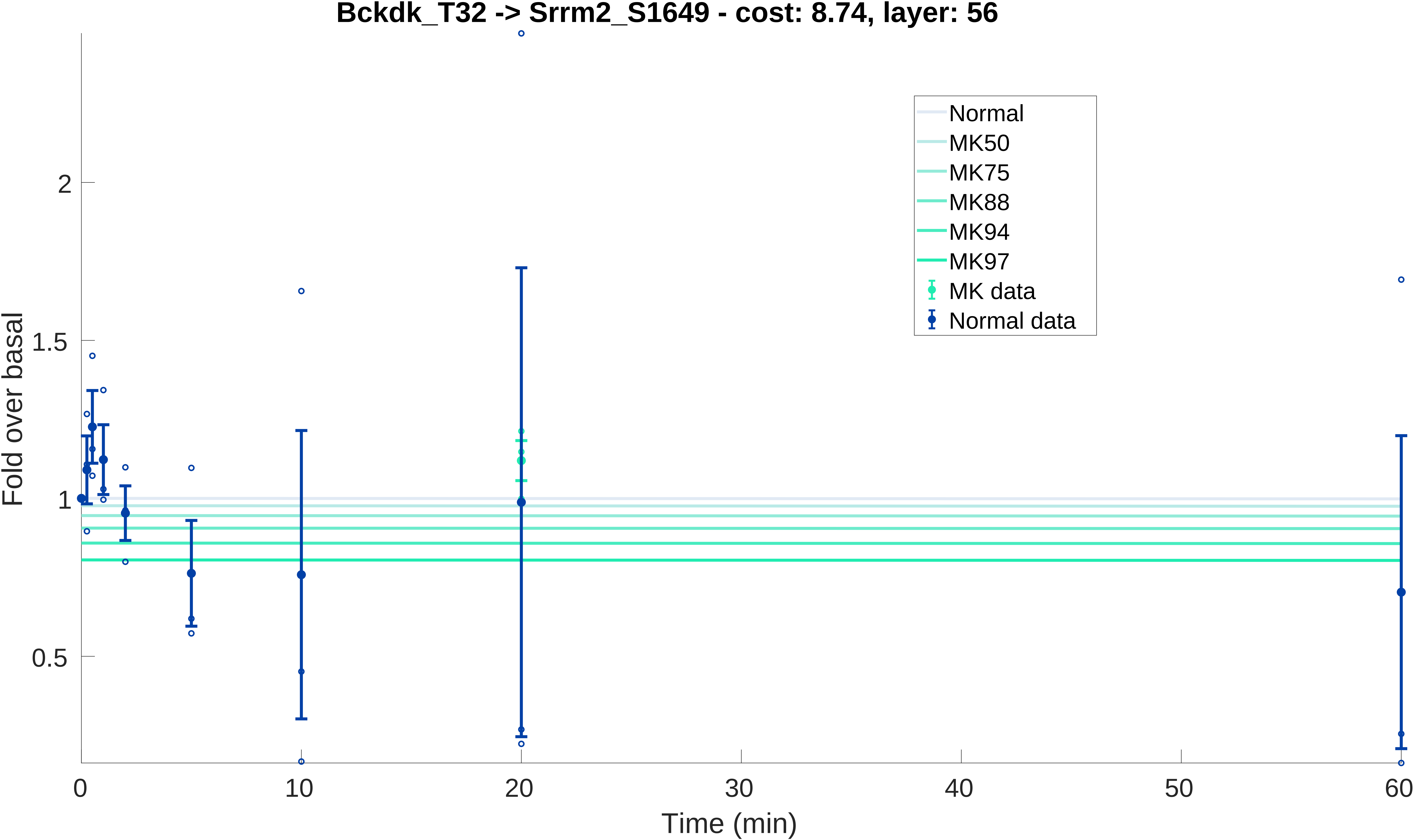

Bckdk_T32 -> Srrm2_S1649 - cost: 8.74, layer: 56
Fold over basal
Time (min)
0
10
20
30
40
50
60
0.5
1
1.5
2
Normal
MK50
MK75
MK88
MK94
MK97
MK data
Normal data

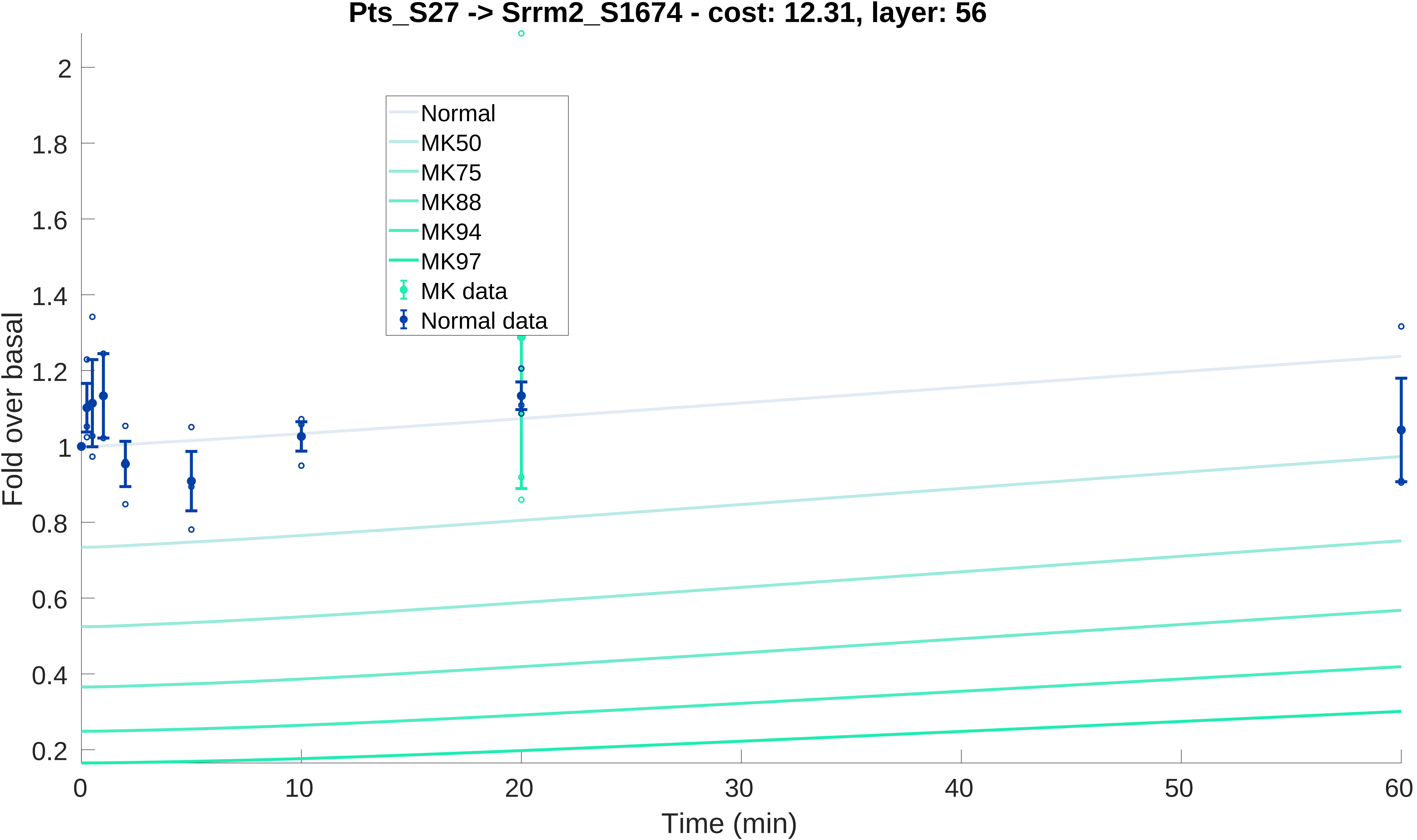

Pts_S27 -> Srrm2_S1674 - cost: 12.31, layer: 56
Fold over basal
Time (min)
0
10
20
30
40
50
60
0.2
0.4
0.6
0.8
1
1.2
1.4
1.6
1.8
2
Normal
MK50
MK75
MK88
MK94
MK97
MK data
Normal data

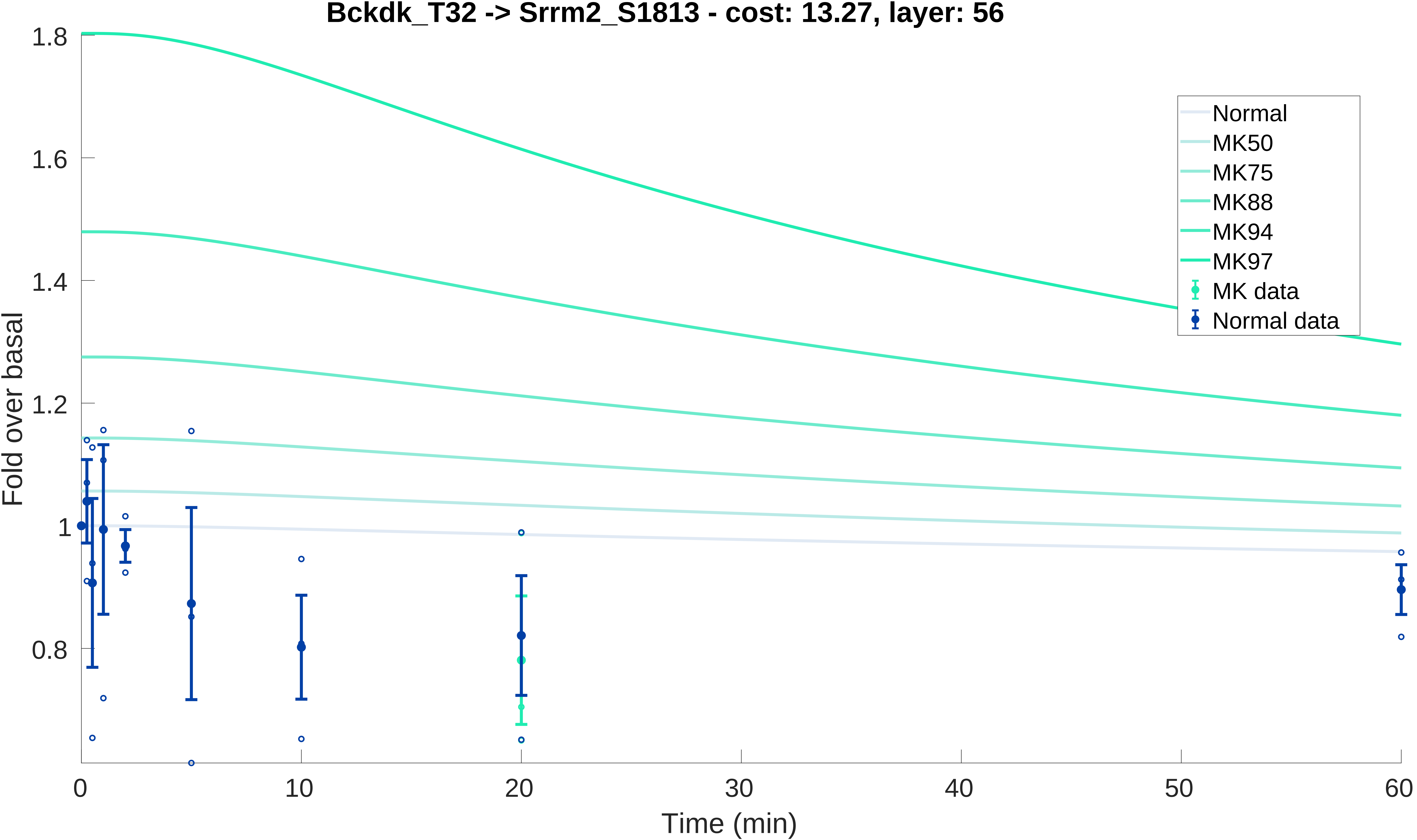

Bckdk_T32 -> Srrm2_S1813 - cost: 13.27, layer: 56
Fold over basal
Time (min)
1.8
1.6
1.4
1.2
1
0.8
0
10
20
30
40
50
60
Normal
MK50
MK75
MK88
MK94
MK97
MK data
Normal data

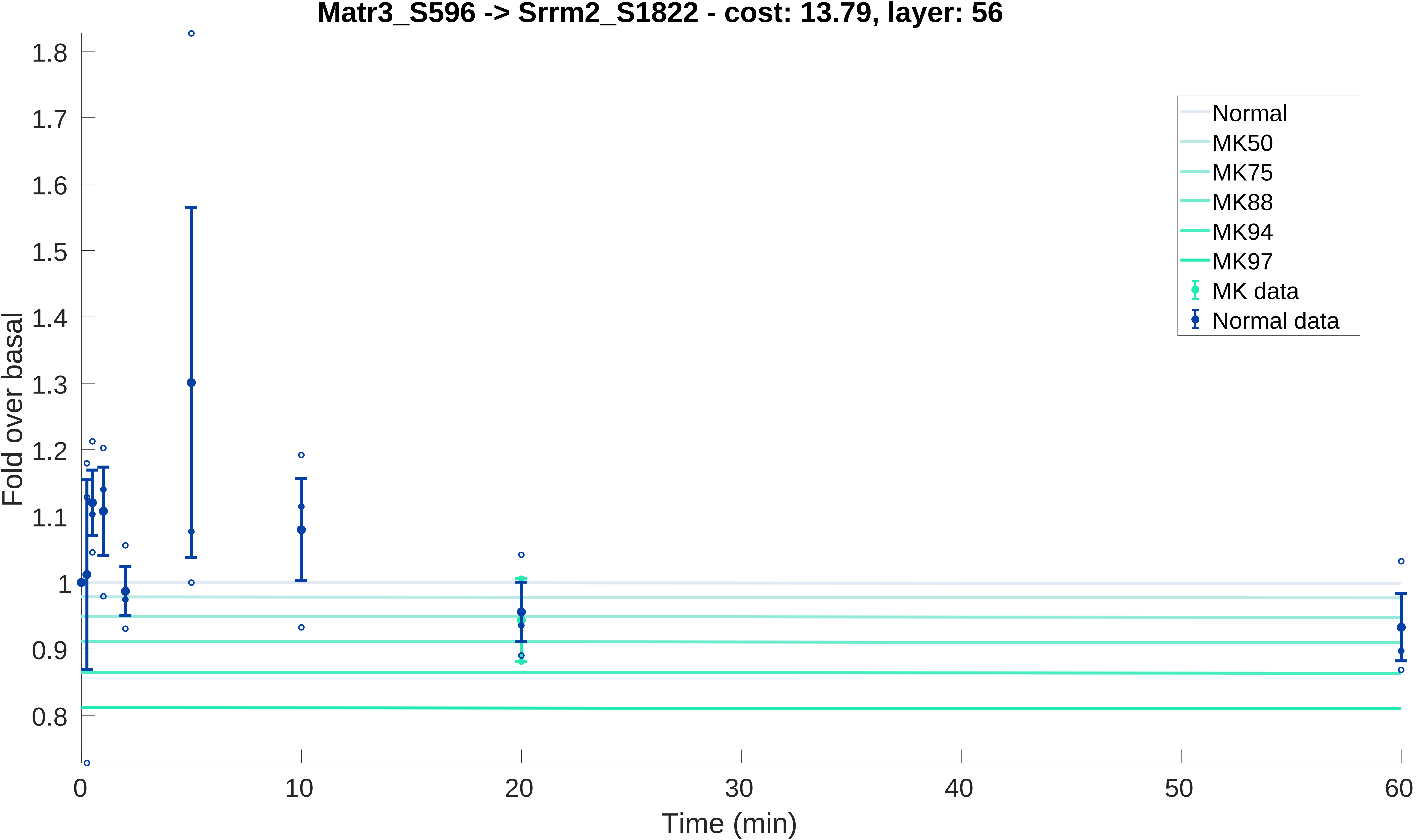

Matr3_S596 -> Srrm2_S1822 - cost: 13.79, layer: 56
Fold over basal
1.8
1.7
1.6
1.5
1.4
1.3
1.2
1.1
1
0.9
0.8
Time (min)
0
10
20
30
40
50
60
Normal
MK50
MK75
MK88
MK94
MK97
MK data
Normal data

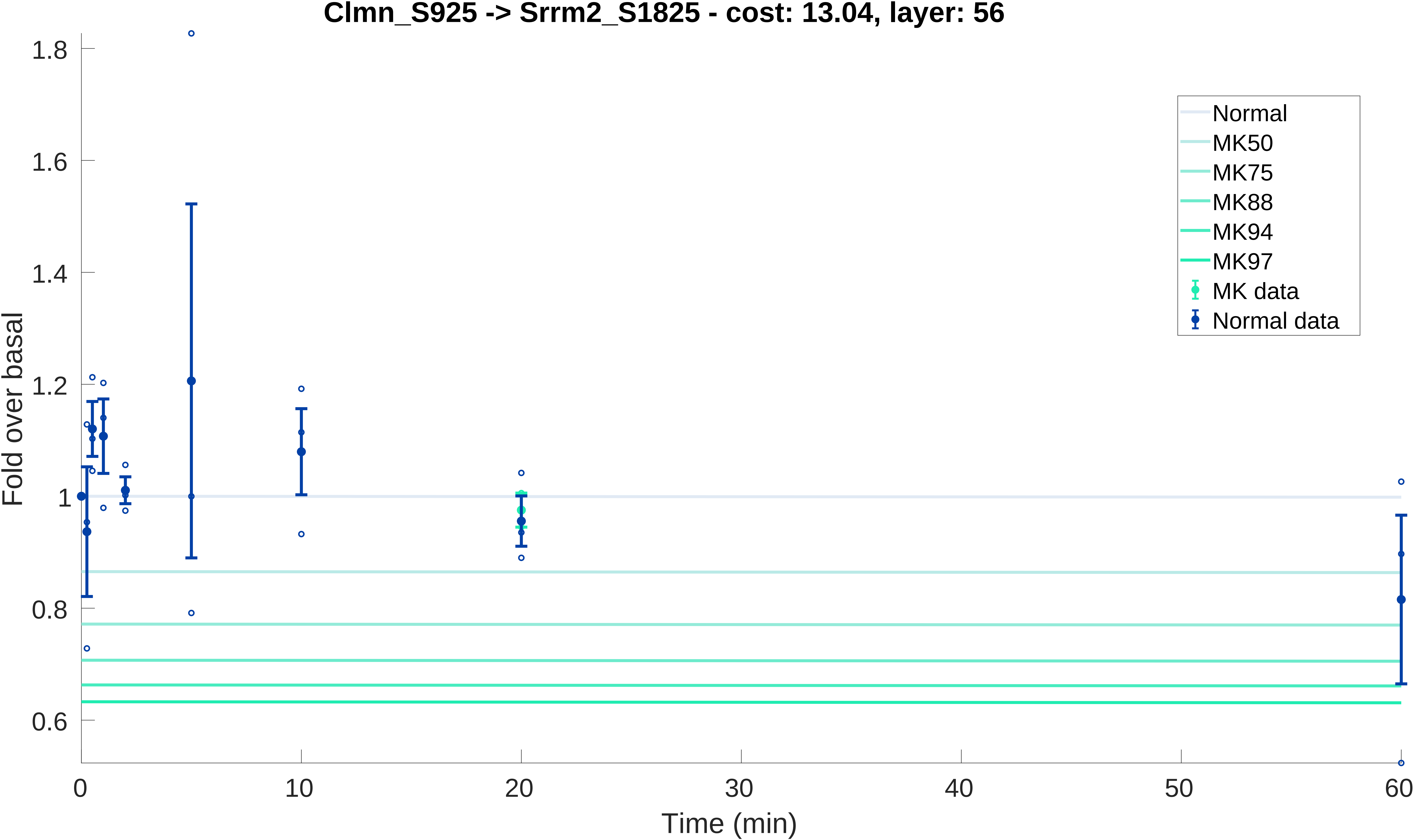

Clmn_S925 -> Srrm2_S1825 - cost: 13.04, layer: 56
1.8
1.6
1.4
1.2
1
0.8
0.6
Fold over basal
0
10
20
30
40
50
60
Time (min)
Normal
MK50
MK75
MK88
MK94
MK97
MK data
Normal data

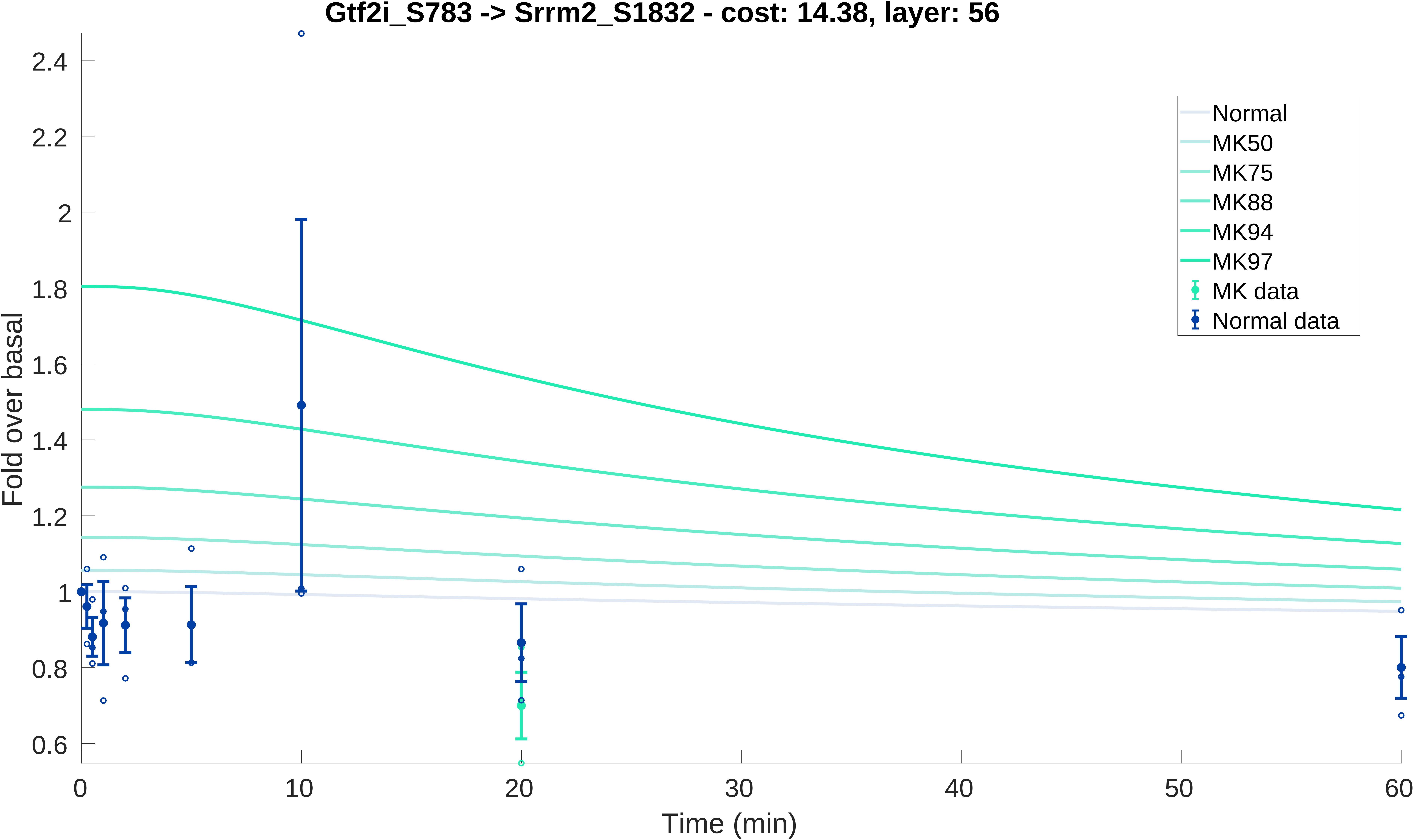

Gtf2i_S783 -> Srrm2_S1832 - cost: 14.38, layer: 56
Fold over basal
Time (min)
2.4
2.2
2
1.8
1.6
1.4
1.2
1
0.8
0.6
0
10
20
30
40
50
60
Normal
MK50
MK75
MK88
MK94
MK97
MK data
Normal data

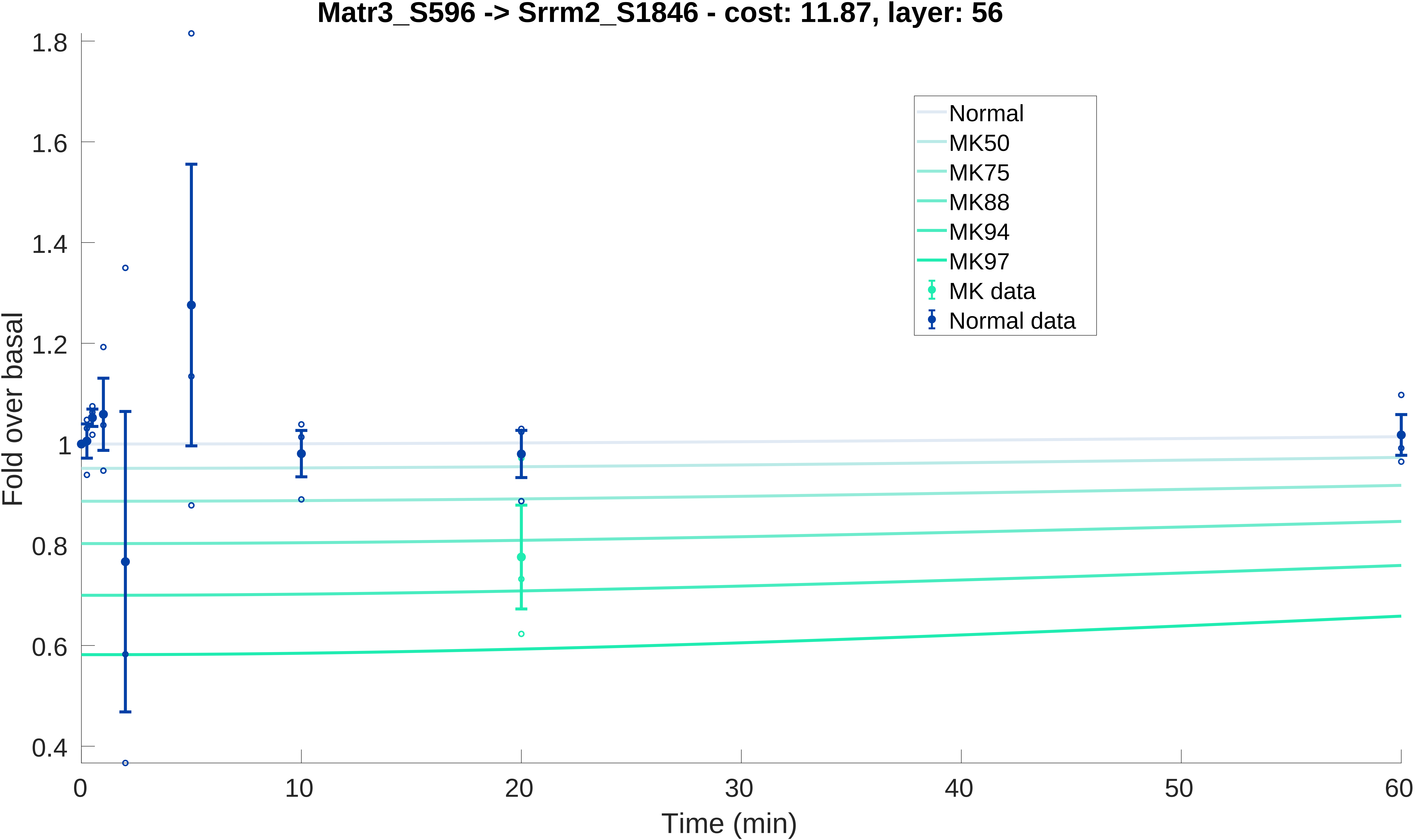

Matr3_S596 -> Srrm2_S1846 - cost: 11.87, layer: 56
Fold over basal
Time (min)
1.8
1.6
1.4
1.2
1
0.8
0.6
0.4
0
10
20
30
40
50
60
Normal
MK50
MK75
MK88
MK94
MK97
MK data
Normal data

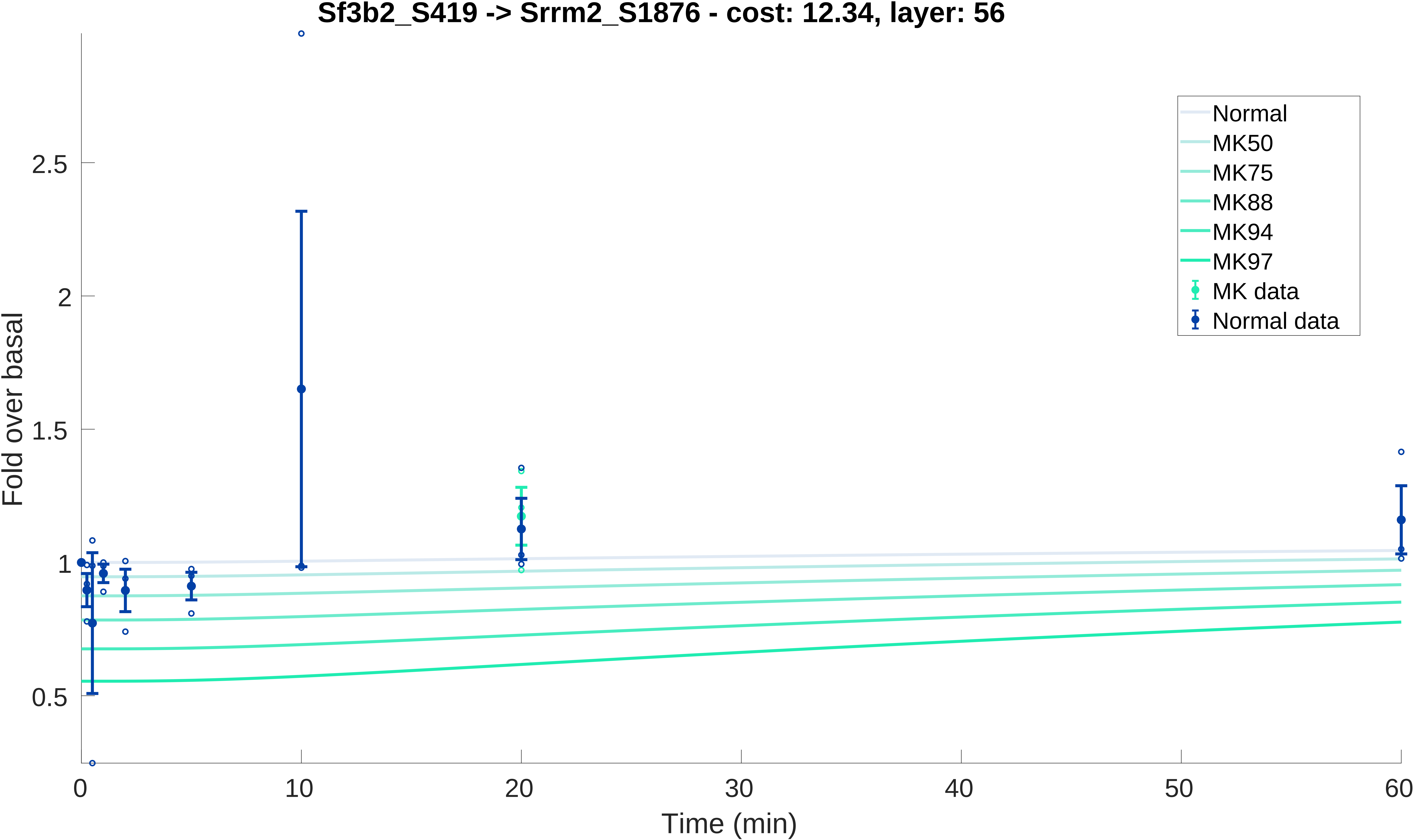
Sf3b2_S419 -> Srrm2_S1876 - cost: 12.34, layer: 56
Fold over basal
Time (min)
0.5
1
1.5
2
2.5
0
10
20
30
40
50
60
Normal
MK50
MK75
MK88
MK94
MK97
MK data
Normal data

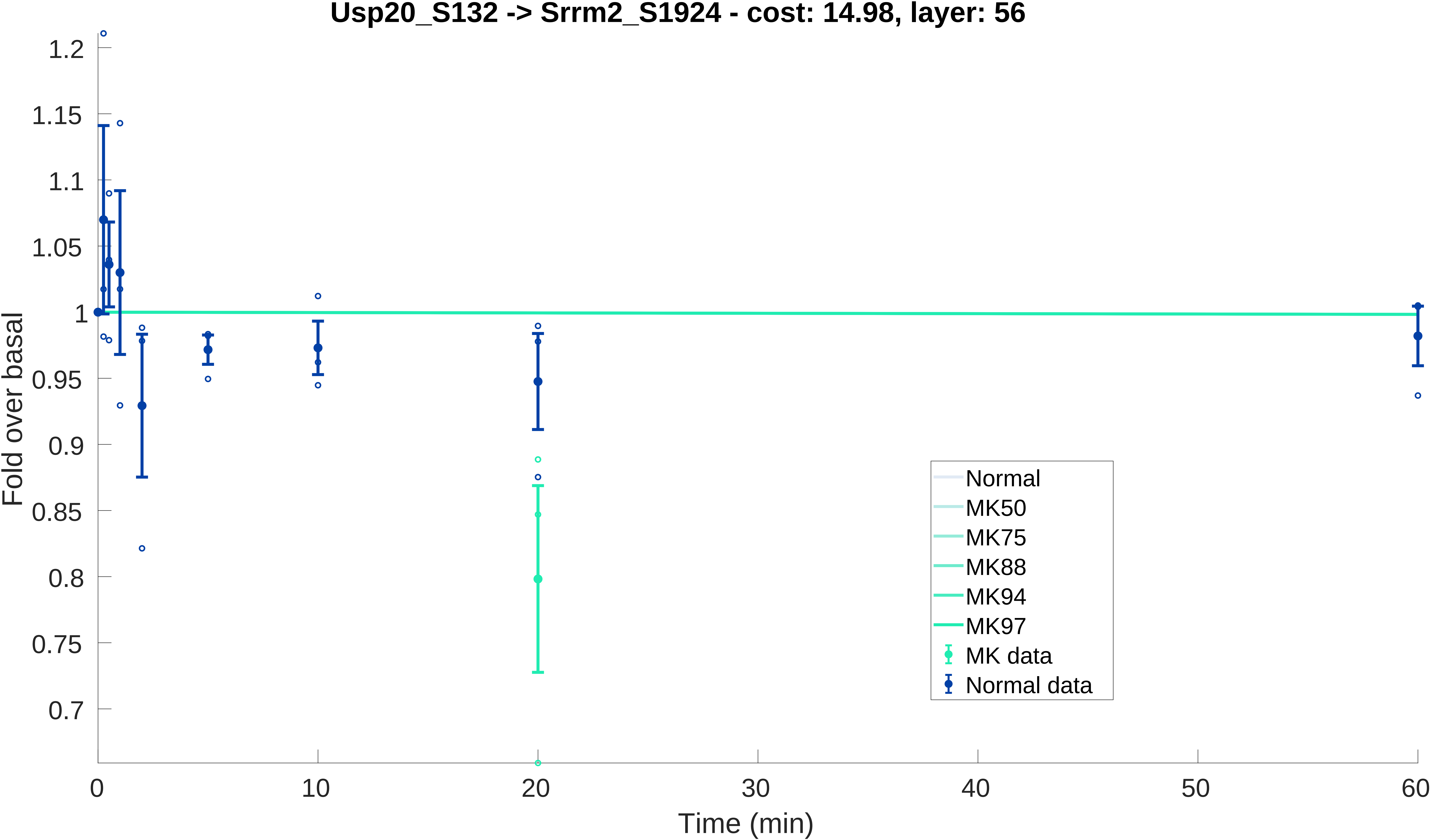

Usp20_S132 -> Srrm2_S1924 - cost: 14.98, layer: 56
1.2
1.15
1.1
1.05
1
0.95
0.9
0.85
0.8
0.75
0.7
Fold over basal
0
10
20
30
40
50
60
Time (min)
Normal
MK50
MK75
MK88
MK94
MK97
MK data
Normal data

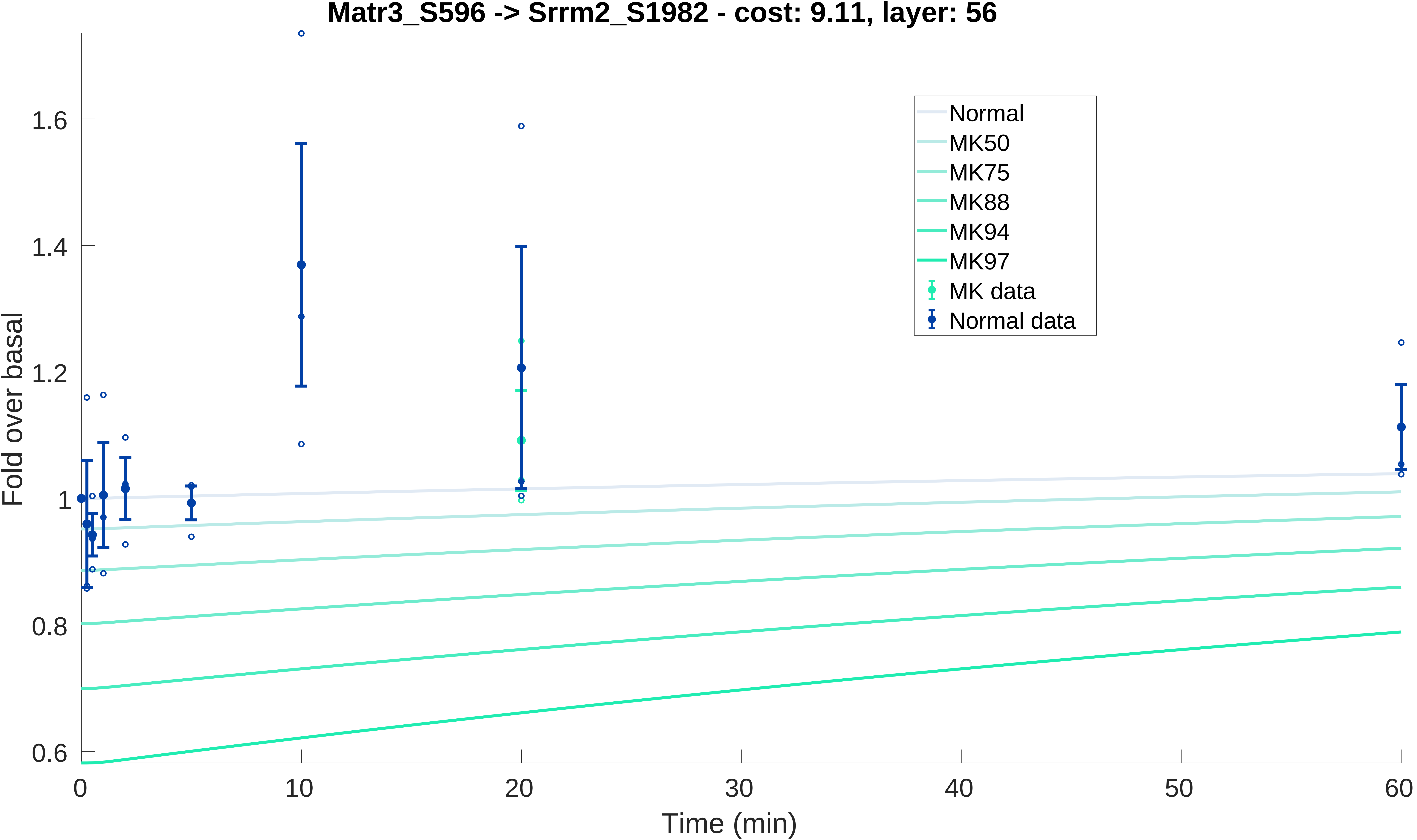

Matr3_S596 -> Srrm2_S1982 - cost: 9.11, layer: 56
Fold over basal
Time (min)
0.6
0.8
1
1.2
1.4
1.6
0
10
20
30
40
50
60
Normal
MK50
MK75
MK88
MK94
MK97
MK data
Normal data

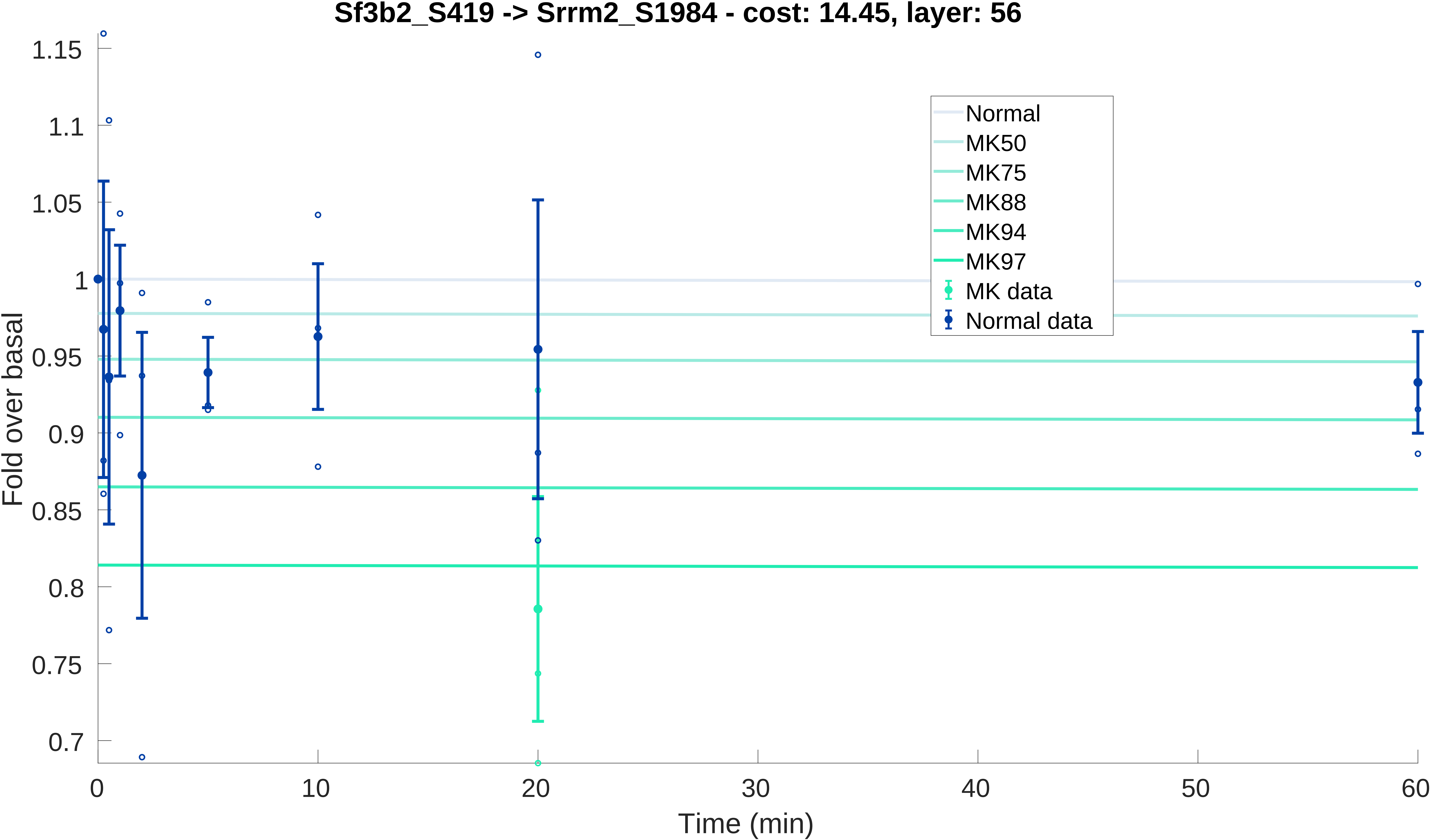

Sf3b2_S419 -> Srrm2_S1984 - cost: 14.45, layer: 56
Fold over basal
1.15
1.1
1.05
1
0.95
0.9
0.85
0.8
0.75
0.7
Time (min)
0
10
20
30
40
50
60
Normal
MK50
MK75
MK88
MK94
MK97
MK data
Normal data

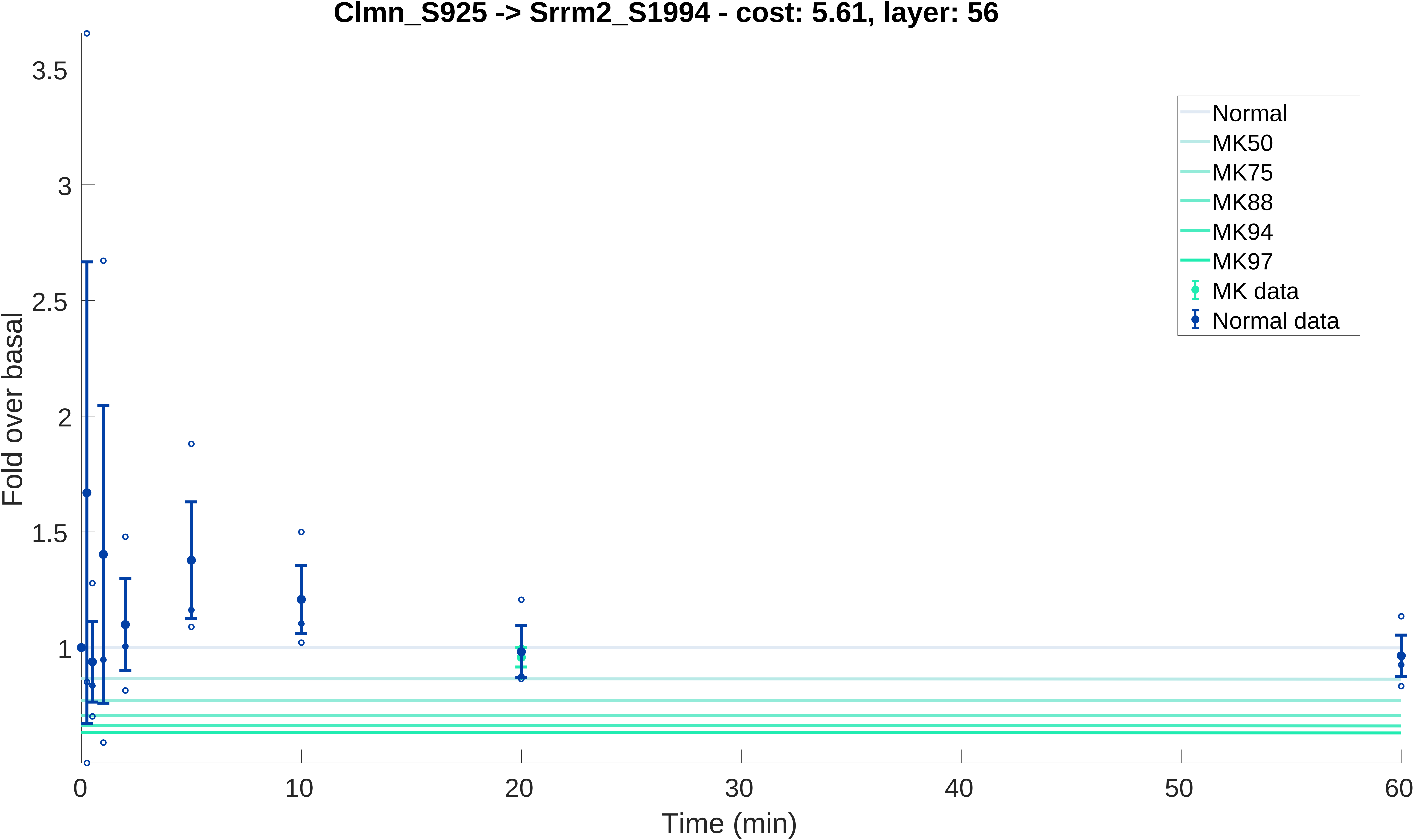

Clmn_S925 -> Srrm2_S1994 - cost: 5.61, layer: 56
Fold over basal
Time (min)
Normal
MK50
MK75
MK88
MK94
MK97
MK data
Normal data
3.5
3
2.5
2
1.5
1
0
10
20
30
40
50
60

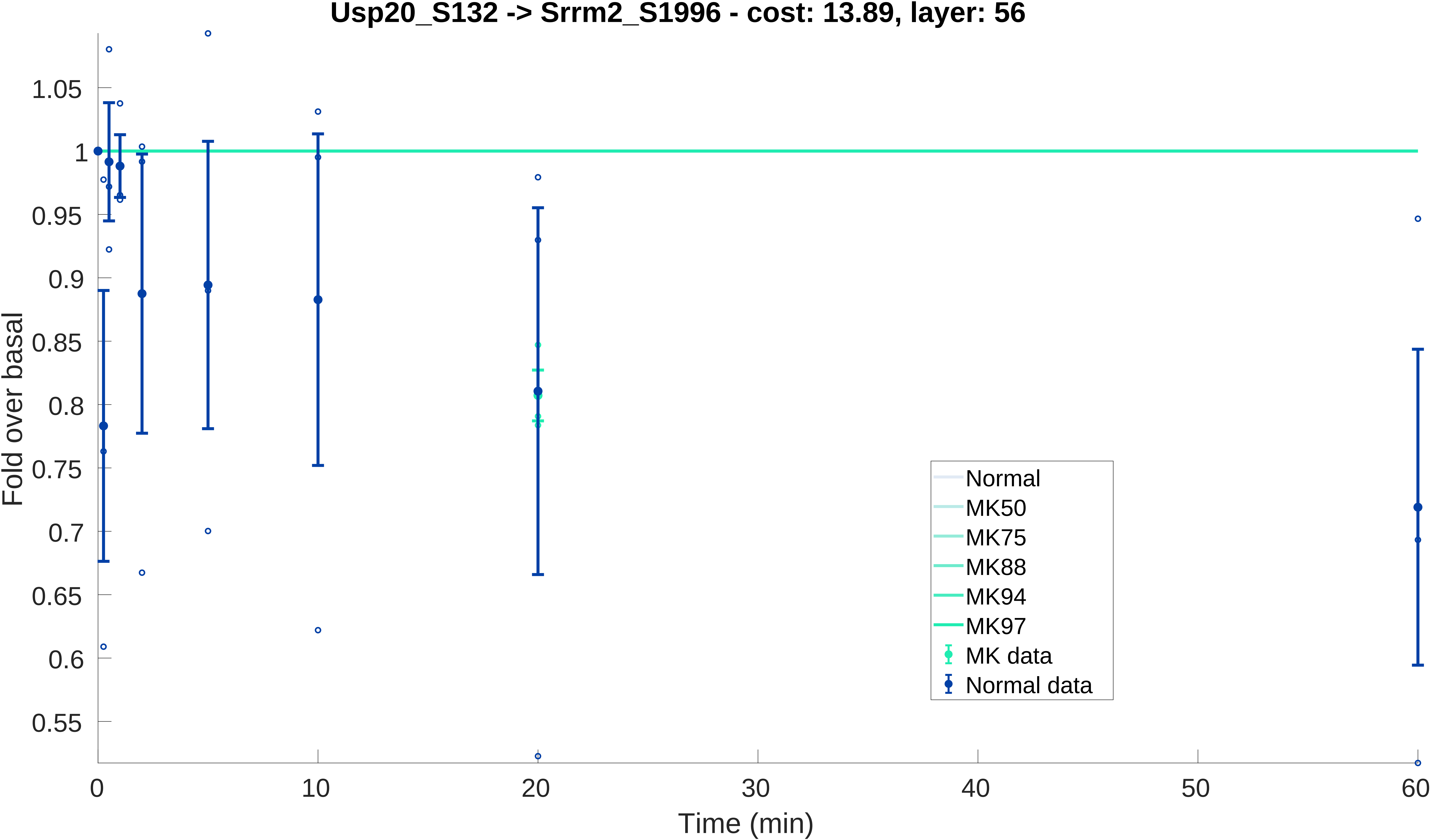
Usp20_S132 -> Srrm2_S1996 - cost: 13.89, layer: 56
Fold over basal
Time (min)
1.05
1
0.95
0.9
0.85
0.8
0.75
0.7
0.65
0.6
0.55
0
10
20
30
40
50
60
Normal
MK50
MK75
MK88
MK94
MK97
MK data
Normal data

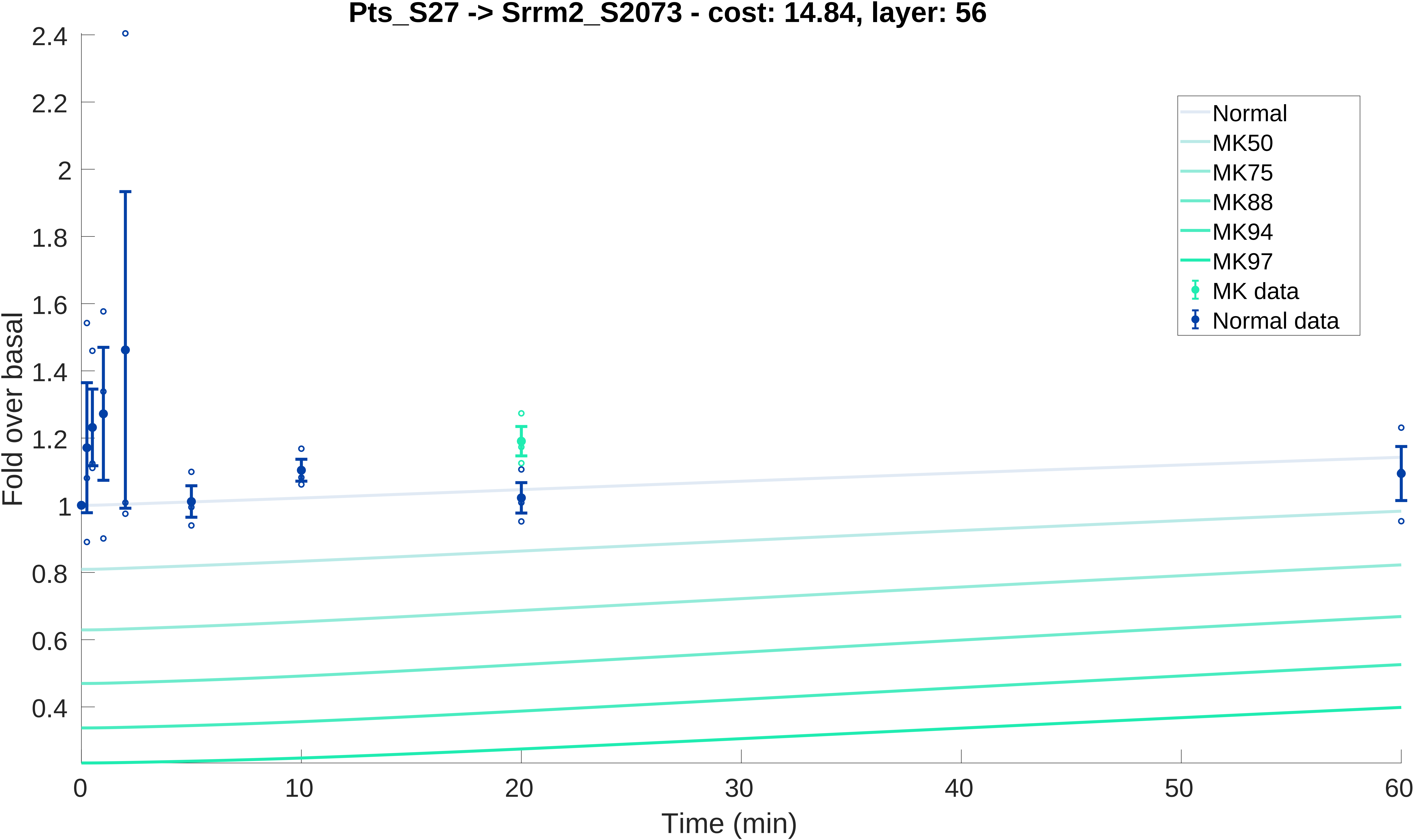
Pts_S27 -> Srrm2_S2073 - cost: 14.84, layer: 56
Fold over basal
Time (min)
2.4
2.2
2
1.8
1.6
1.4
1.2
1
0.8
0.6
0.4
0
10
20
30
40
50
60
Normal
MK50
MK75
MK88
MK94
MK97
MK data
Normal data

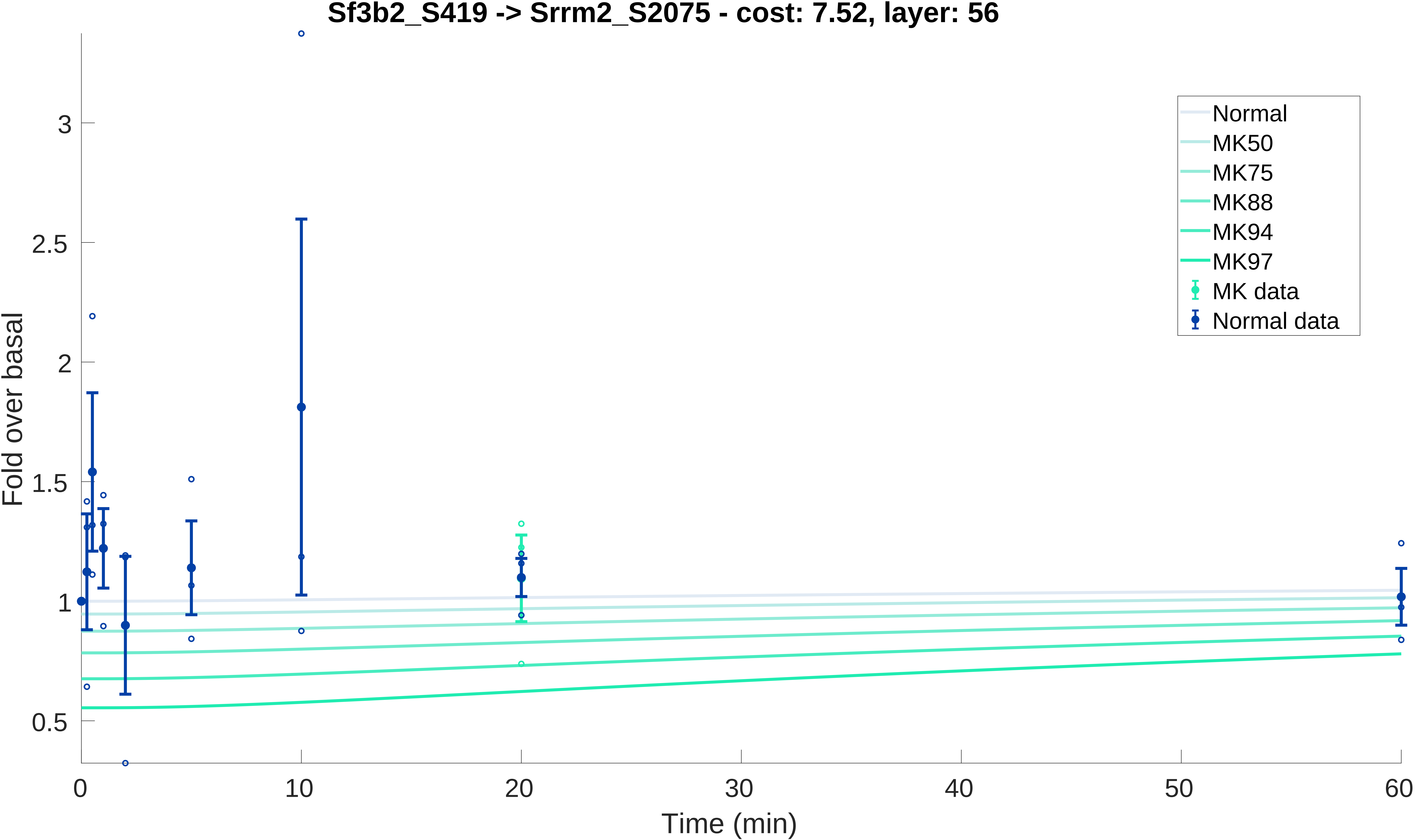
Sf3b2_S419 -> Srrm2_S2075 - cost: 7.52, layer: 56
Fold over basal
Time (min)
0.5
1
1.5
2
2.5
3
0
10
20
30
40
50
60
Normal
MK50
MK75
MK88
MK94
MK97
MK data
Normal data

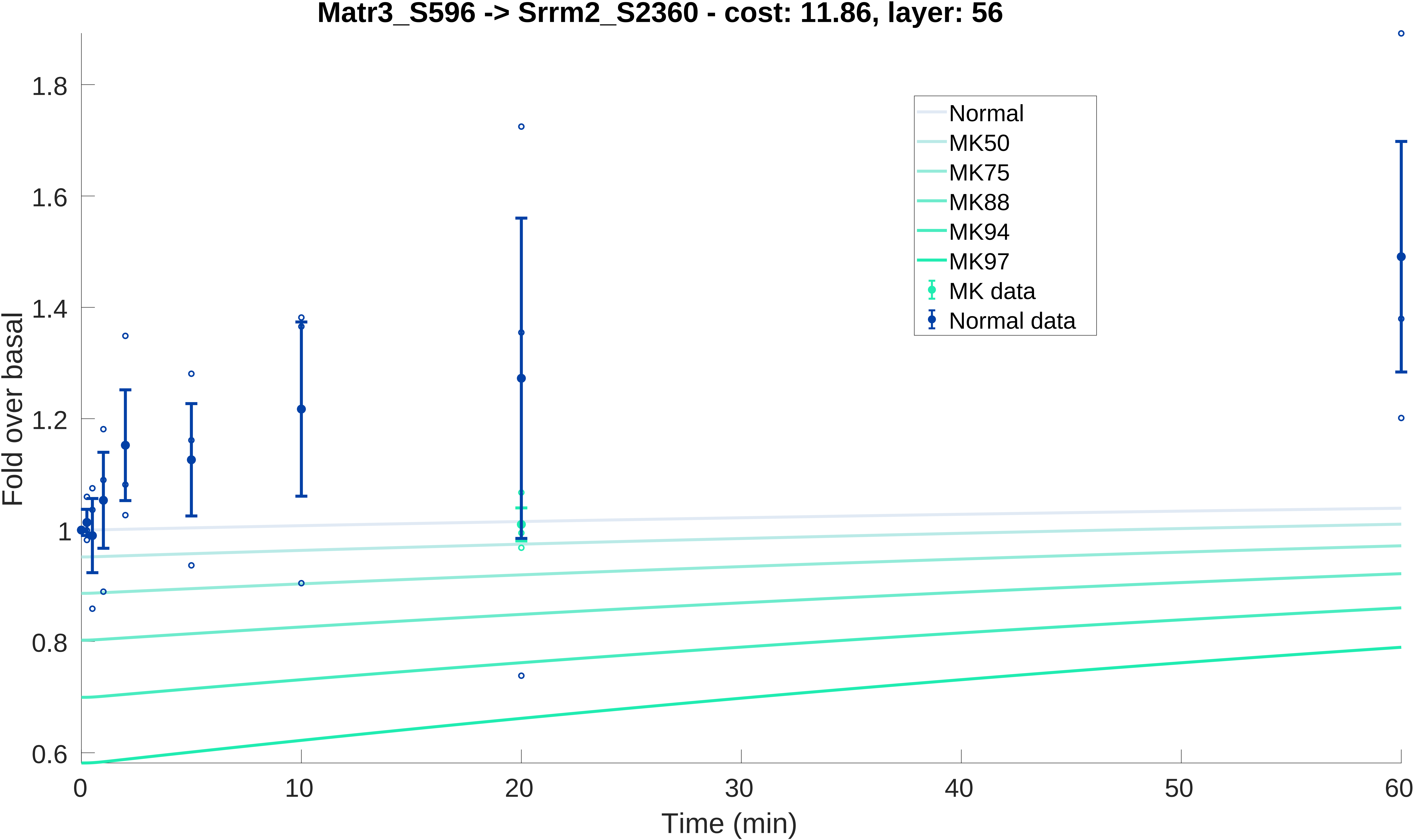
Matr3_S596 -> Srrm2_S2360 - cost: 11.86, layer: 56
Fold over basal
1.8
1.6
1.4
1.2
1
0.8
0.6
0
10
20
30
40
50
60
Time (min)
Normal
MK50
MK75
MK88
MK94
MK97
MK data
Normal data

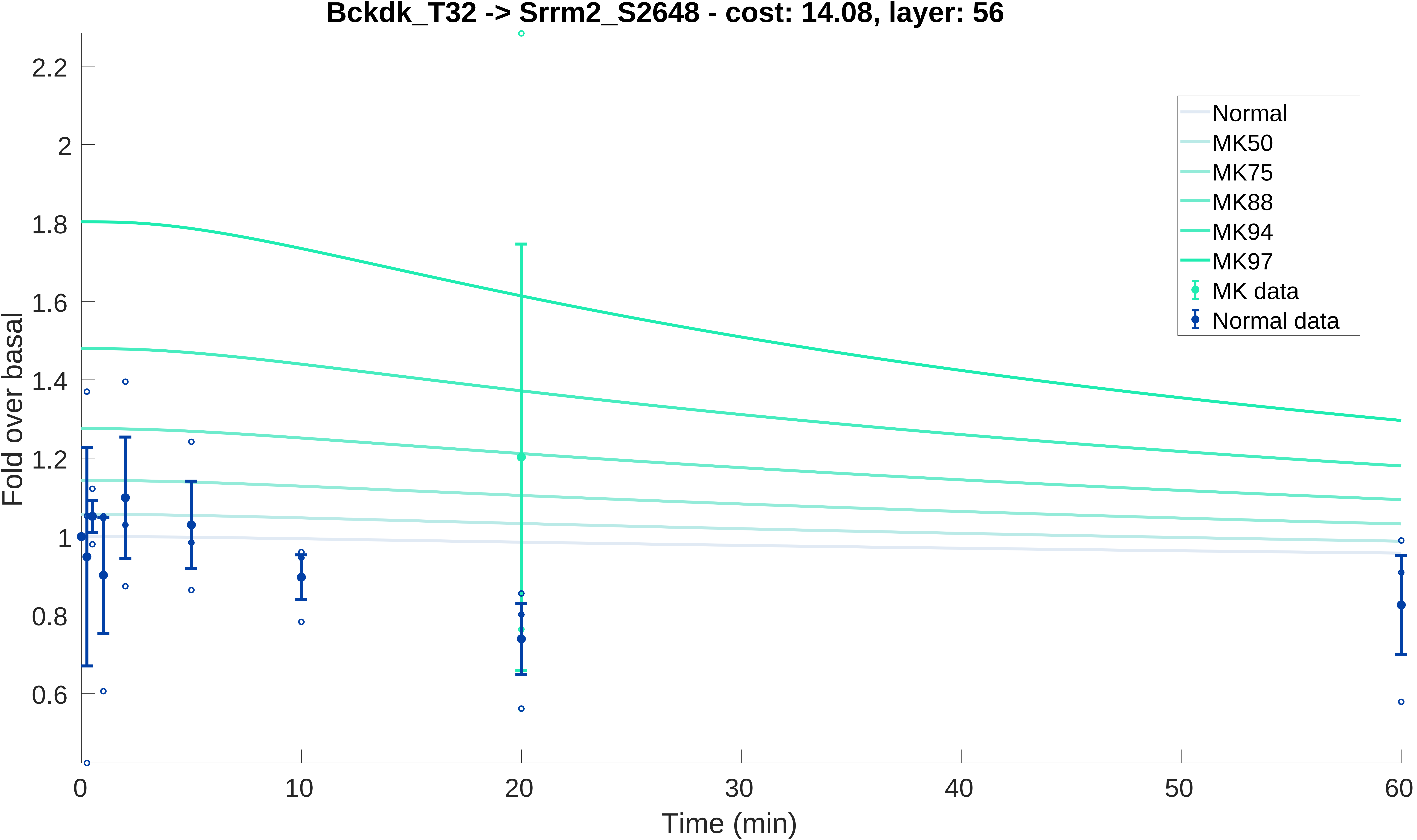

Bckdk_T32 -> Srrm2_S2648 - cost: 14.08, layer: 56
Fold over basal
Time (min)
Normal
MK50
MK75
MK88
MK94
MK97
MK data
Normal data
2.2
2
1.8
1.6
1.4
1.2
1
0.8
0.6
0
10
20
30
40
50
60

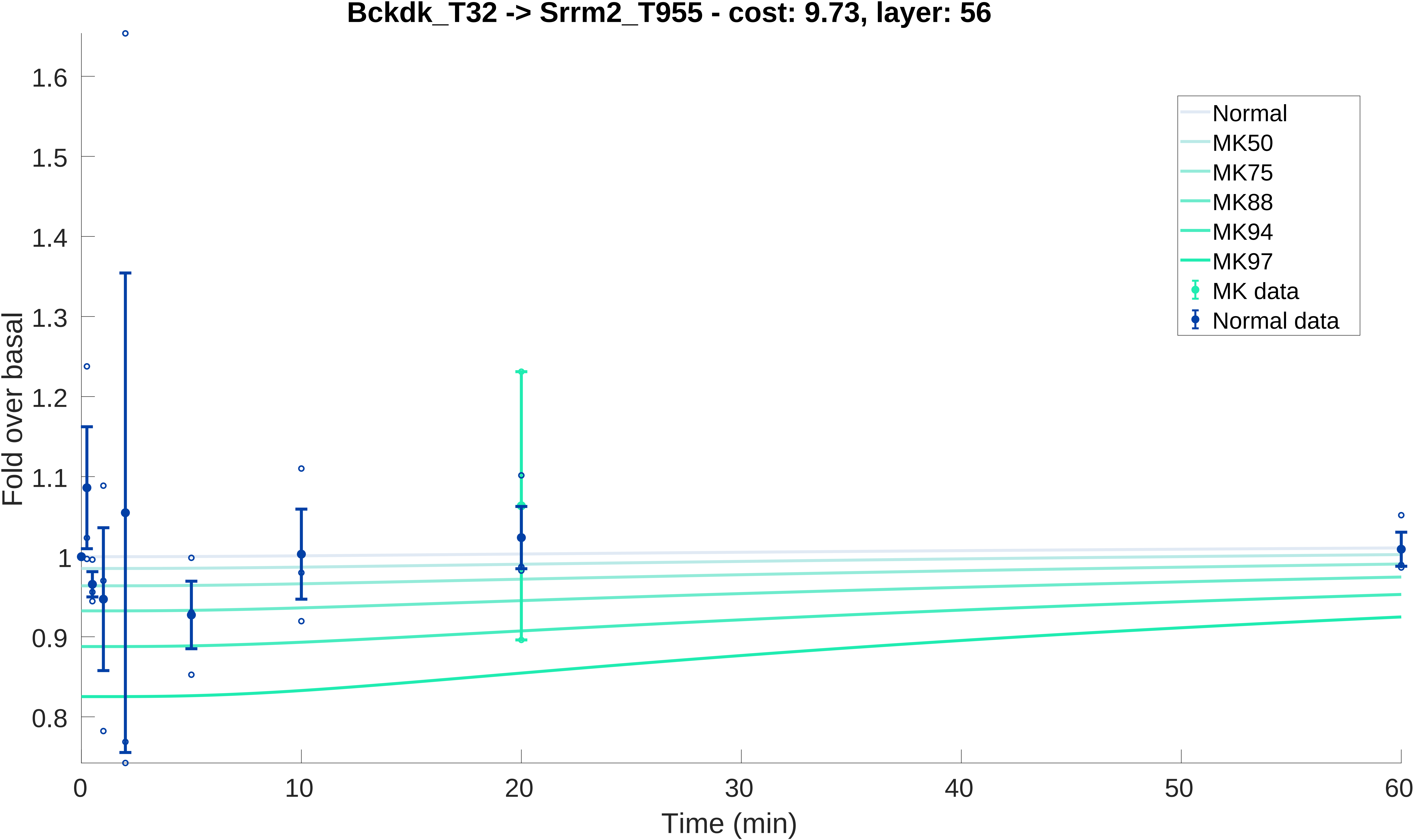
Bckdk_T32 -> Srrm2_T955 - cost: 9.73, layer: 56
Fold over basal
Time (min)
Normal
MK50
MK75
MK88
MK94
MK97
MK data
Normal data
0.8
0.9
1
1.1
1.2
1.3
1.4
1.5
1.6
0
10
20
30
40
50
60

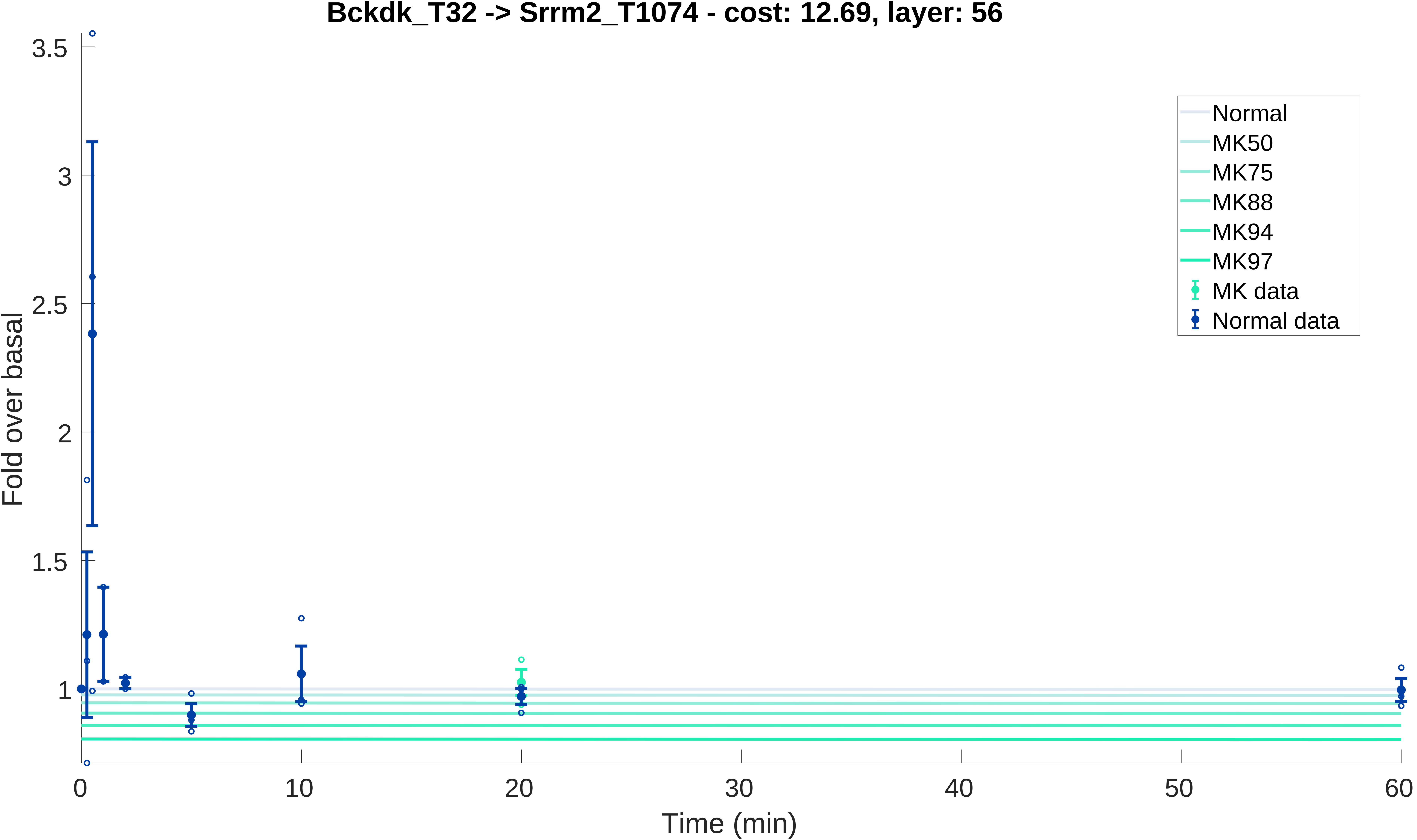

Bckdk_T32 -> Srrm2_T1074 - cost: 12.69, layer: 56
Fold over basal
3.5
3
2.5
2
1.5
1
0
10
20
30
40
50
60
Time (min)
Normal
MK50
MK75
MK88
MK94
MK97
MK data
Normal data

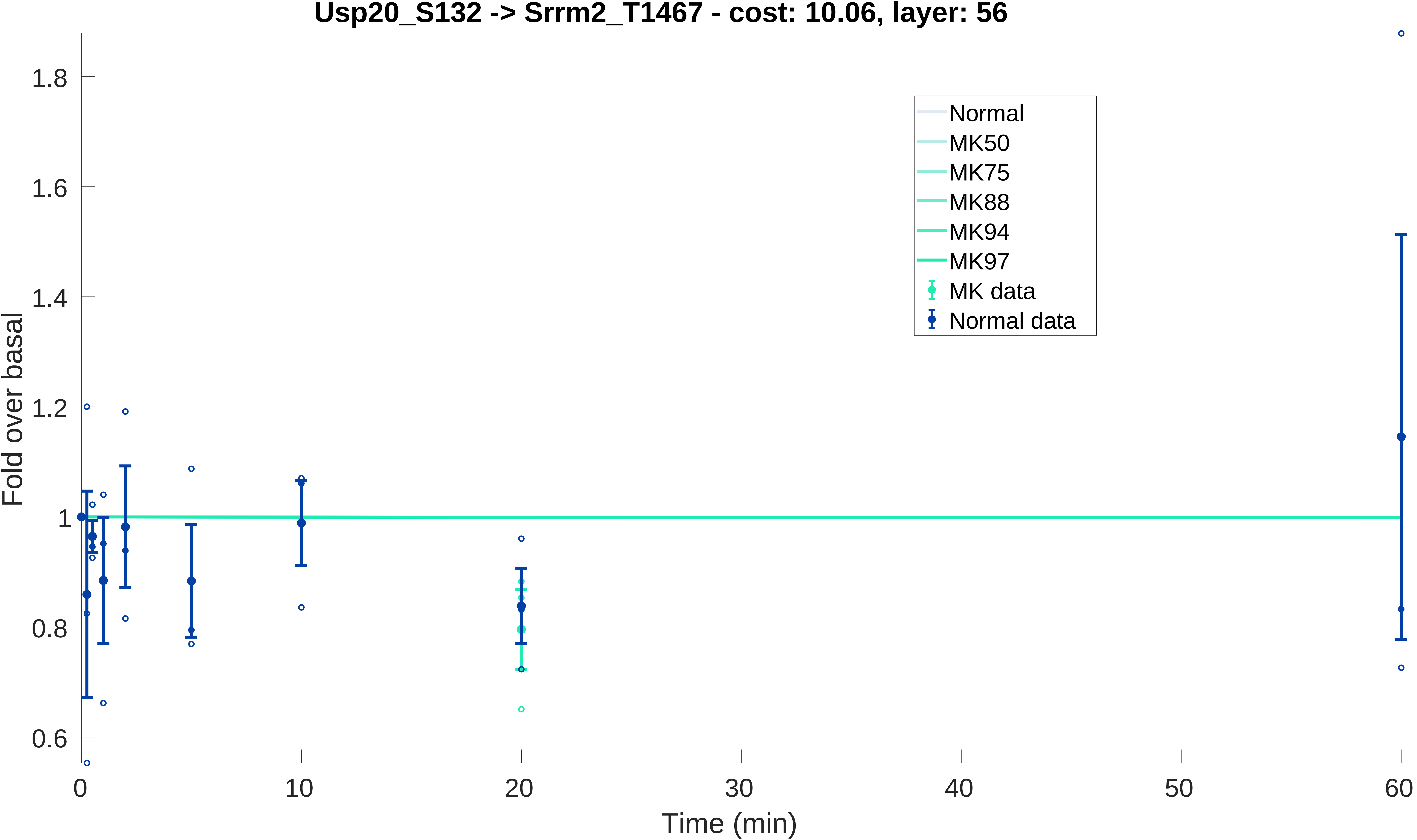

Usp20_S132 -> Srrm2_T1467 - cost: 10.06, layer: 56
Fold over basal
1.8
1.6
1.4
1.2
1
0.8
0.6
0
10
20
30
40
50
60
Time (min)
Normal
MK50
MK75
MK88
MK94
MK97
MK data
Normal data

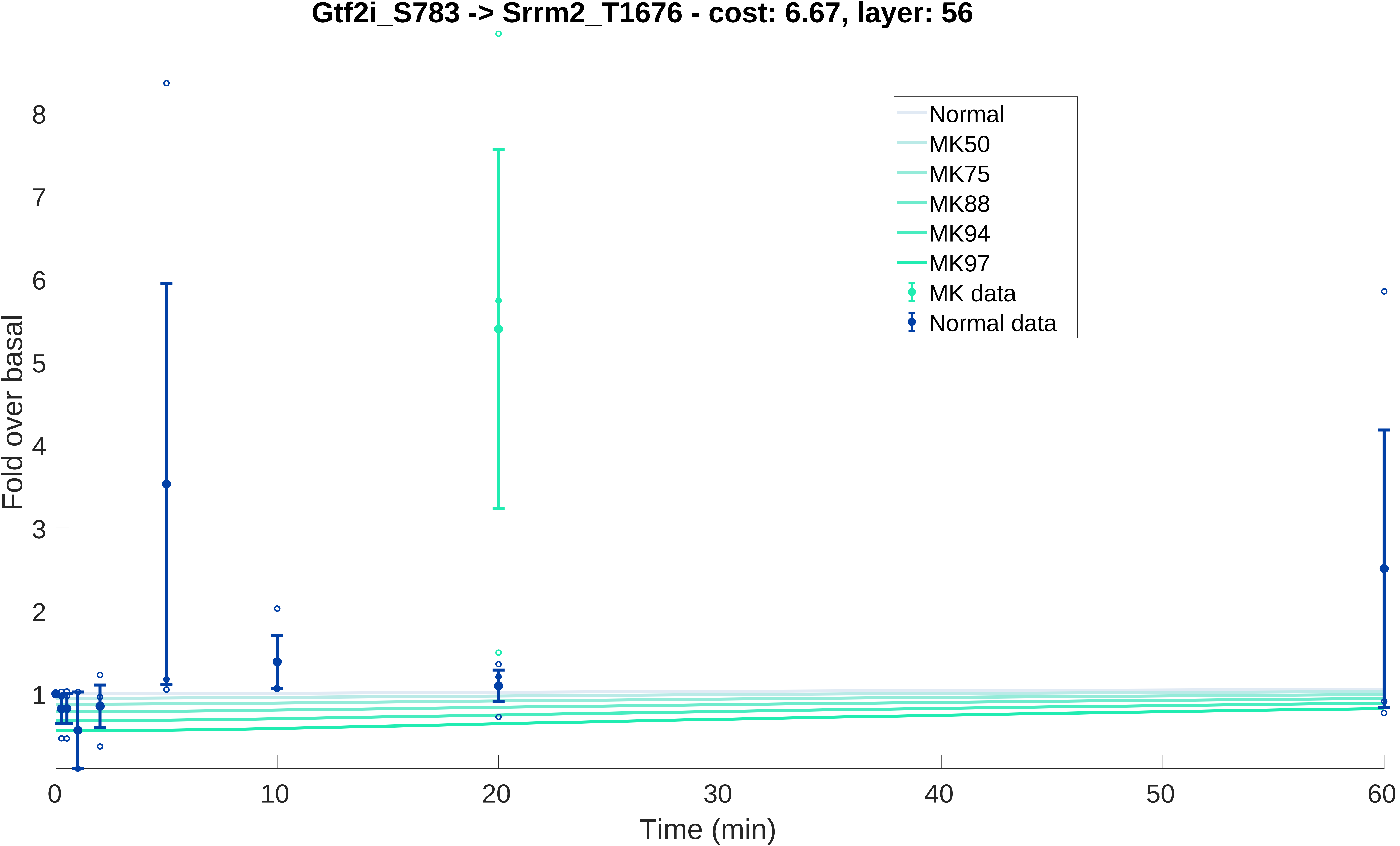

Gtf2i_S783 -> Srrm2_T1676 - cost: 6.67, layer: 56
Fold over basal
Time (min)
0
10
20
30
40
50
60
1
2
3
4
5
6
7
8
Normal
MK50
MK75
MK88
MK94
MK97
MK data
Normal data

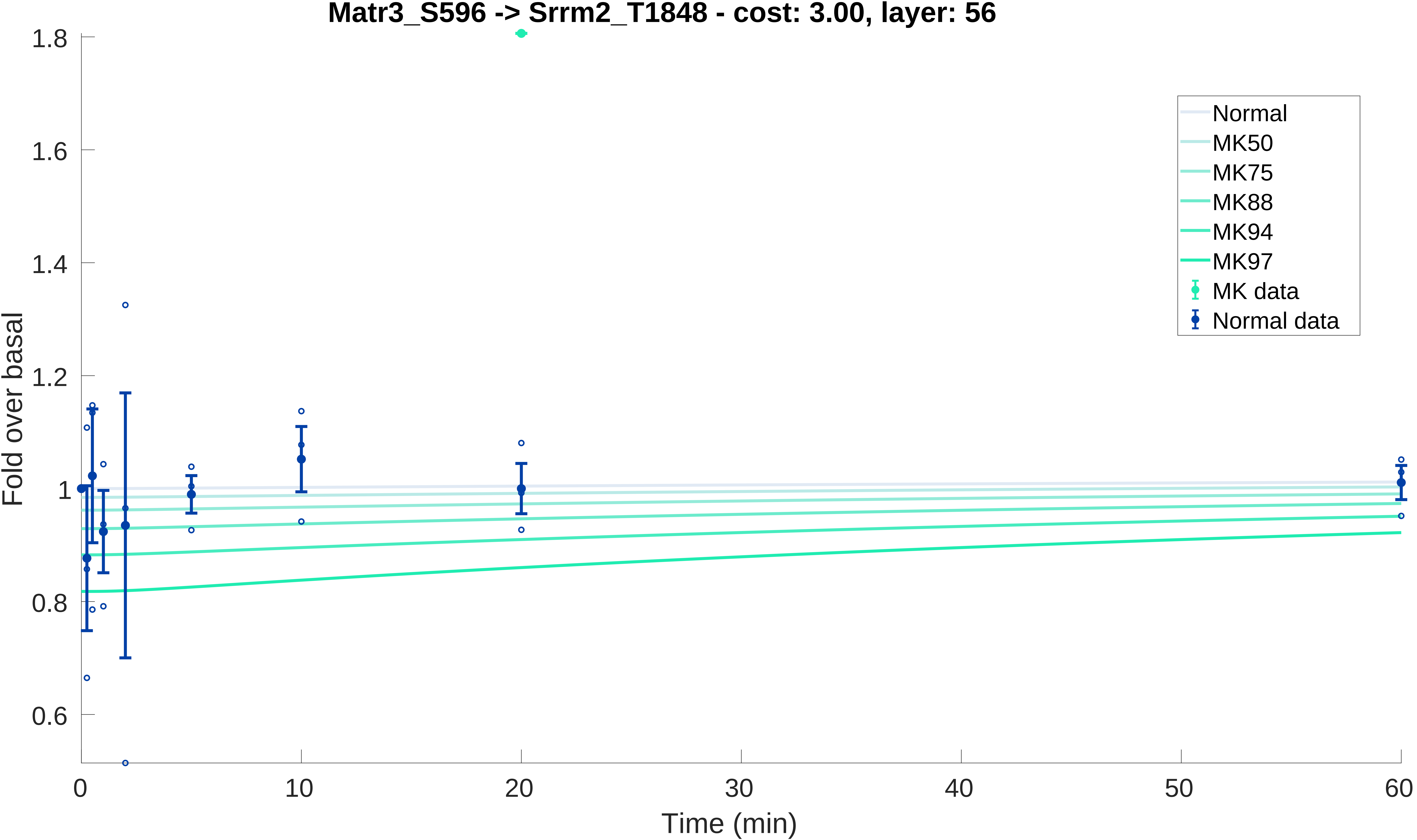
Matr3_S596 -> Srrm2_T1848 - cost: 3.00, layer: 56
Fold over basal
Time (min)
1.8
1.6
1.4
1.2
1
0.8
0.6
0
10
20
30
40
50
60
Normal
MK50
MK75
MK88
MK94
MK97
MK data
Normal data

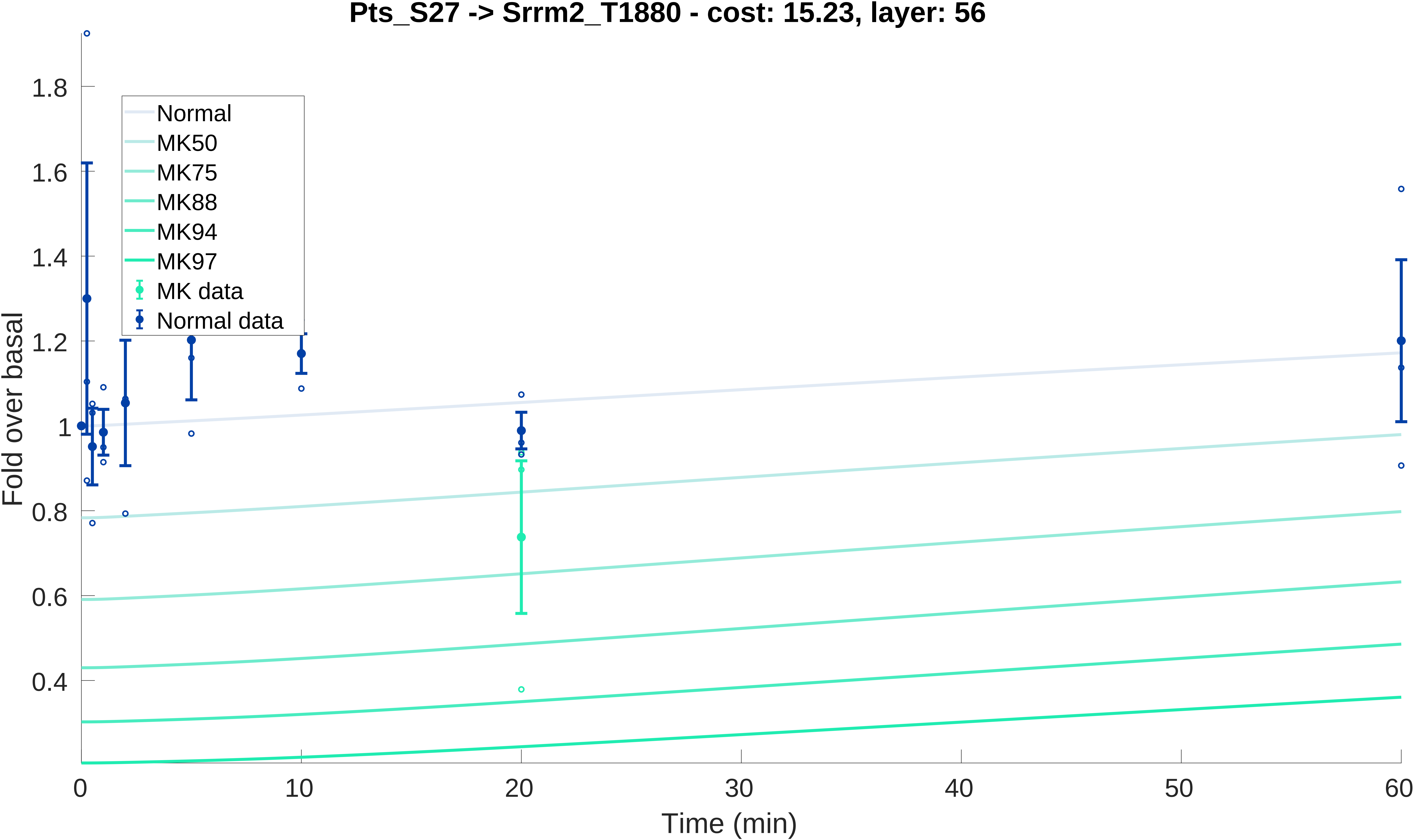

Pts_S27 -> Srrm2_T1880 - cost: 15.23, layer: 56
Normal
MK50
MK75
MK88
MK94
MK97
MK data
Normal data
Fold over basal
Time (min)
0.4
0.6
0.8
1
1.2
1.4
1.6
1.8
0
10
20
30
40
50
60

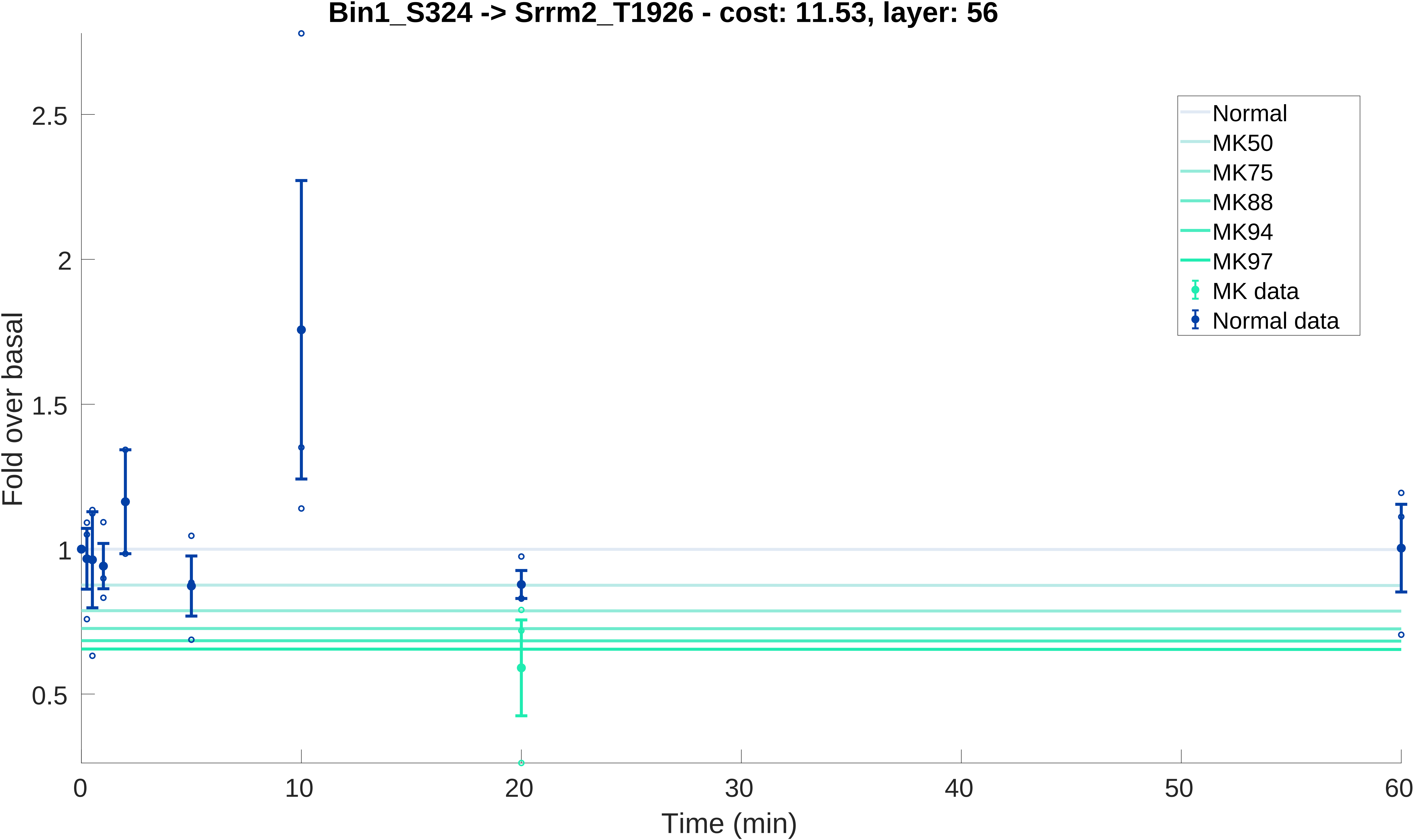

Bin1_S324 -> Srrm2_T1926 - cost: 11.53, layer: 56
Fold over basal
0.5
1
1.5
2
2.5
0
10
20
30
40
50
60
Time (min)
Normal
MK50
MK75
MK88
MK94
MK97
MK data
Normal data

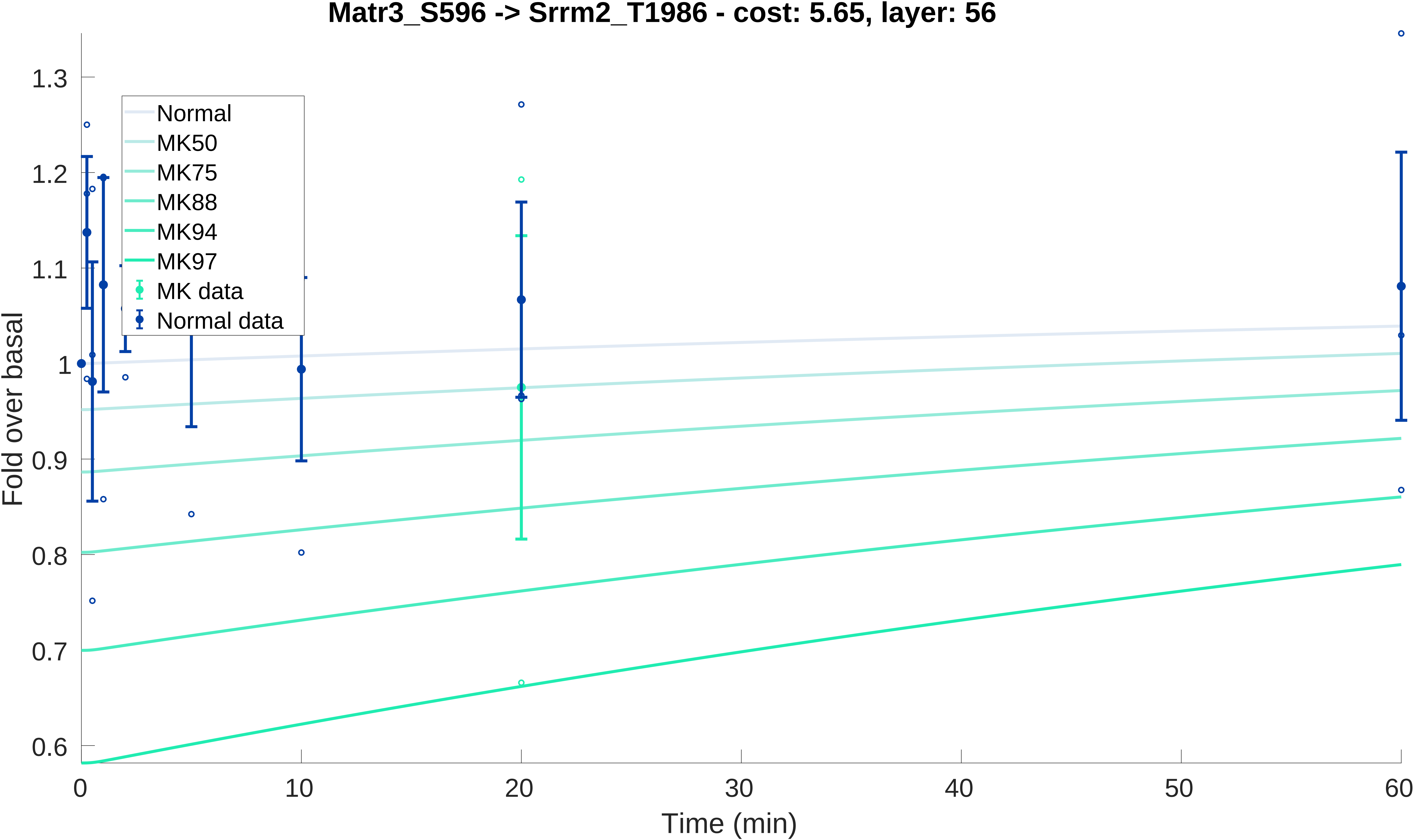

Matr3_S596 -> Srrm2_T1986 - cost: 5.65, layer: 56
Normal
MK50
MK75
MK88
MK94
MK97
MK data
Normal data
Fold over basal
1.3
1.2
1.1
1
0.9
0.8
0.7
0.6
0
10
20
30
40
50
60
Time (min)

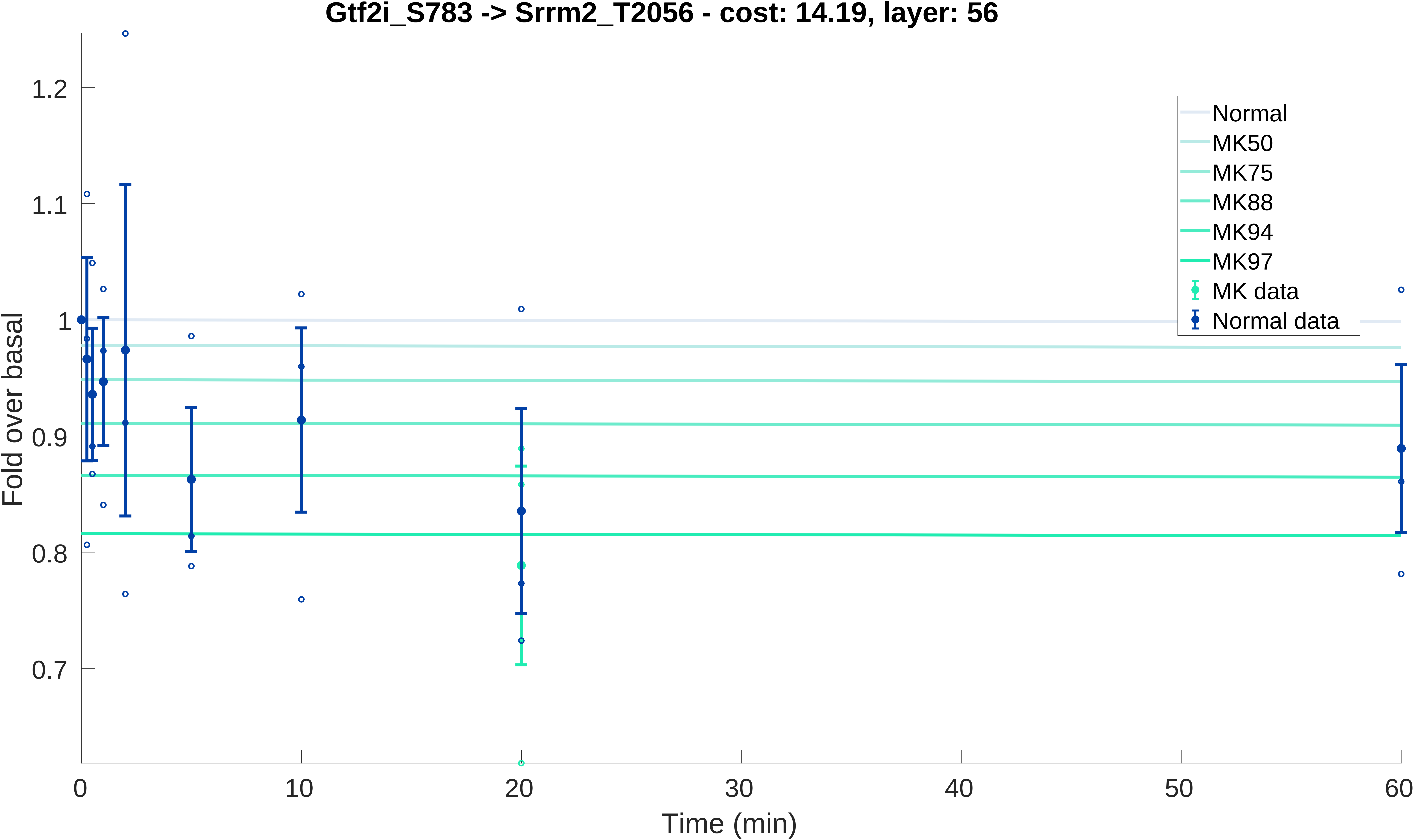

Gtf2i_S783 -> Srrm2_T2056 - cost: 14.19, layer: 56
Fold over basal
Time (min)
Normal
MK50
MK75
MK88
MK94
MK97
MK data
Normal data
1.2
1.1
1
0.9
0.8
0.7
0
10
20
30
40
50
60

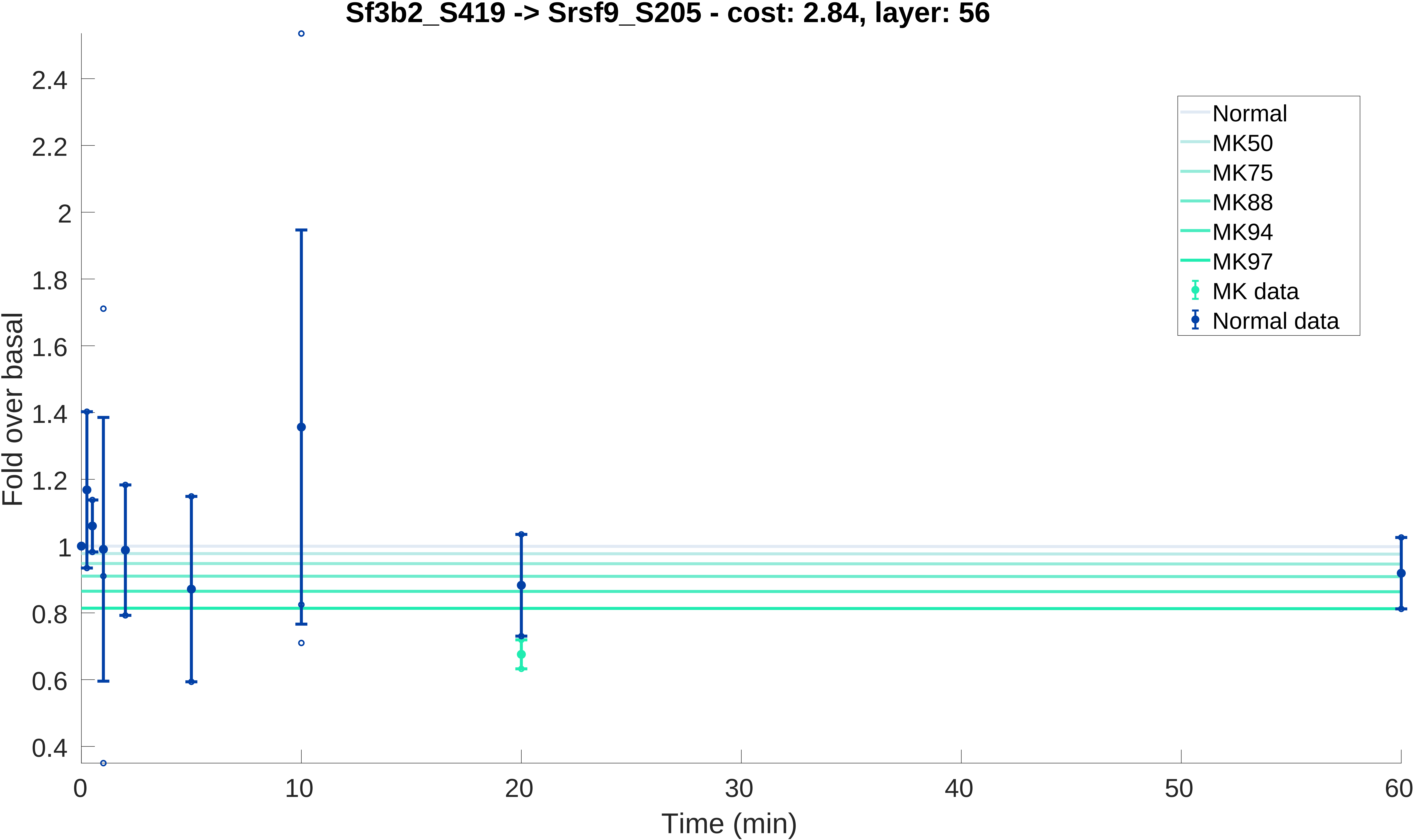

Sf3b2_S419 -> Srsf9_S205 - cost: 2.84, layer: 56
Fold over basal
Time (min)
2.4
2.2
2
1.8
1.6
1.4
1.2
1
0.8
0.6
0.4
0
10
20
30
40
50
60
Normal
MK50
MK75
MK88
MK94
MK97
MK data
Normal data

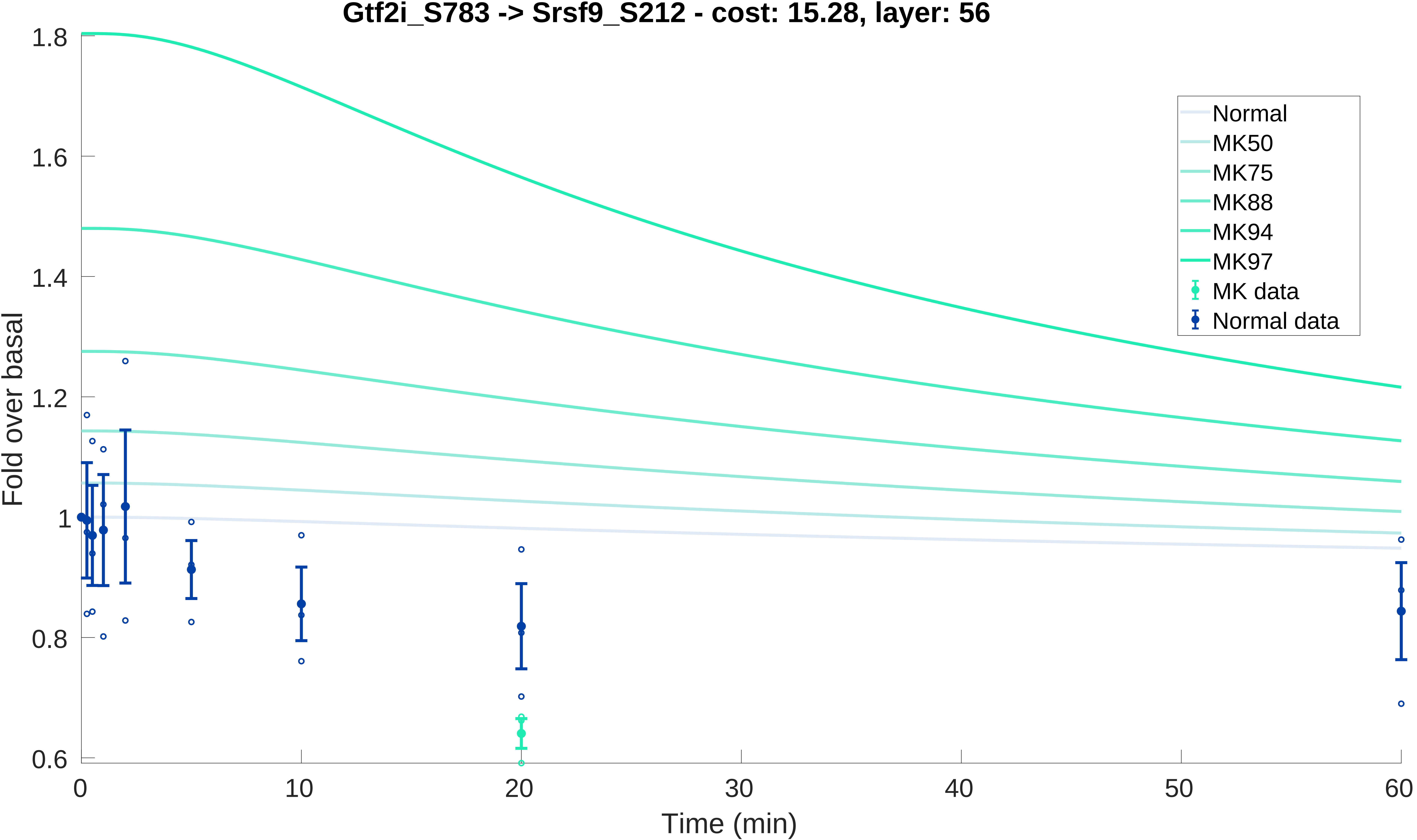
Gtf2i_S783 -> Srsf9_S212 - cost: 15.28, layer: 56
Fold over basal
Time (min)
1.8
1.6
1.4
1.2
1
0.8
0.6
0
10
20
30
40
50
60
Normal
MK50
MK75
MK88
MK94
MK97
MK data
Normal data

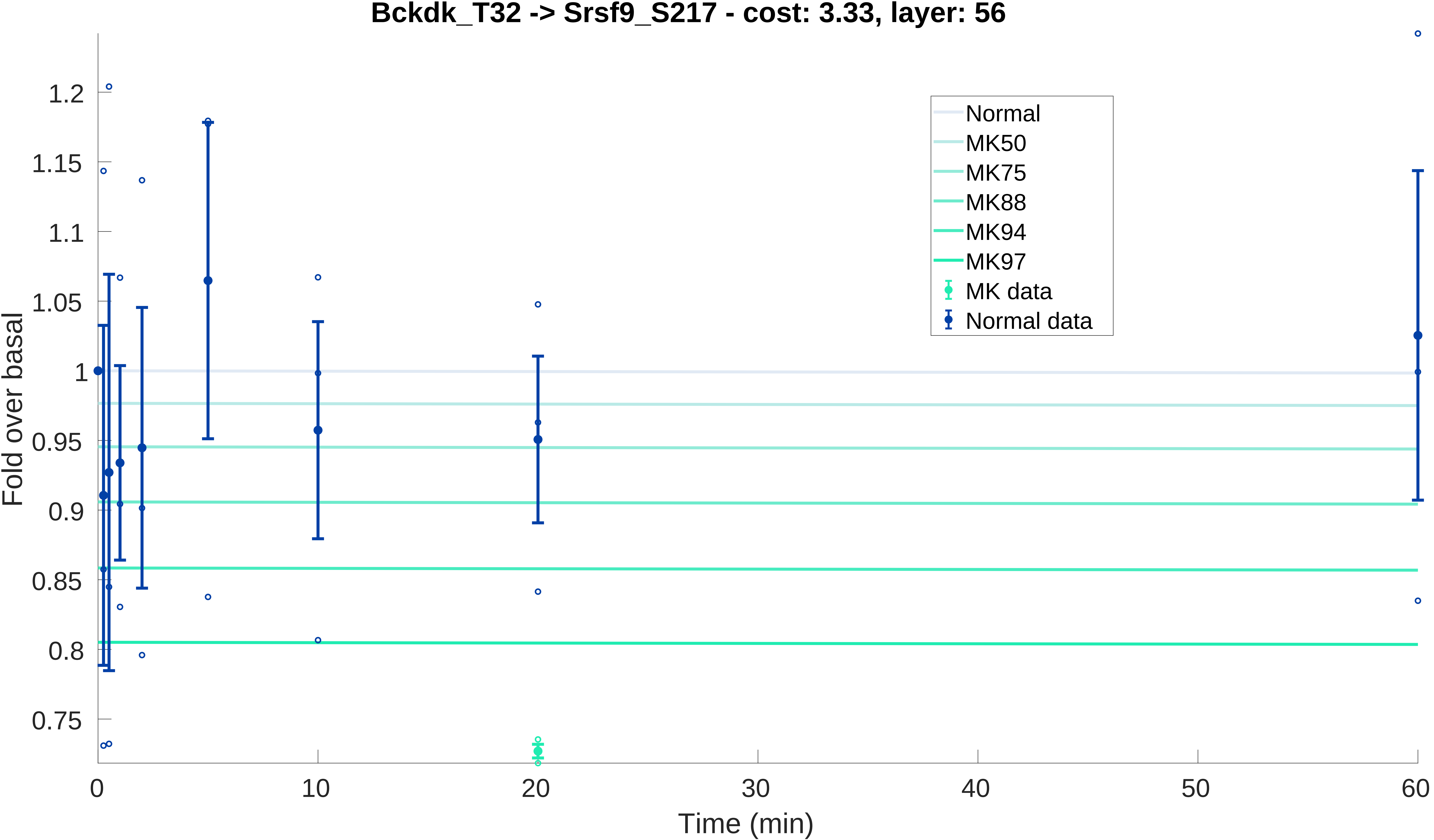
Bckdk_T32 -> Srsf9_S217 - cost: 3.33, layer: 56
Normal
MK50
MK75
MK88
MK94
MK97
MK data
Normal data
Fold over basal
Time (min)
1.2
1.15
1.1
1.05
1
0.95
0.9
0.85
0.8
0.75
0
10
20
30
40
50
60

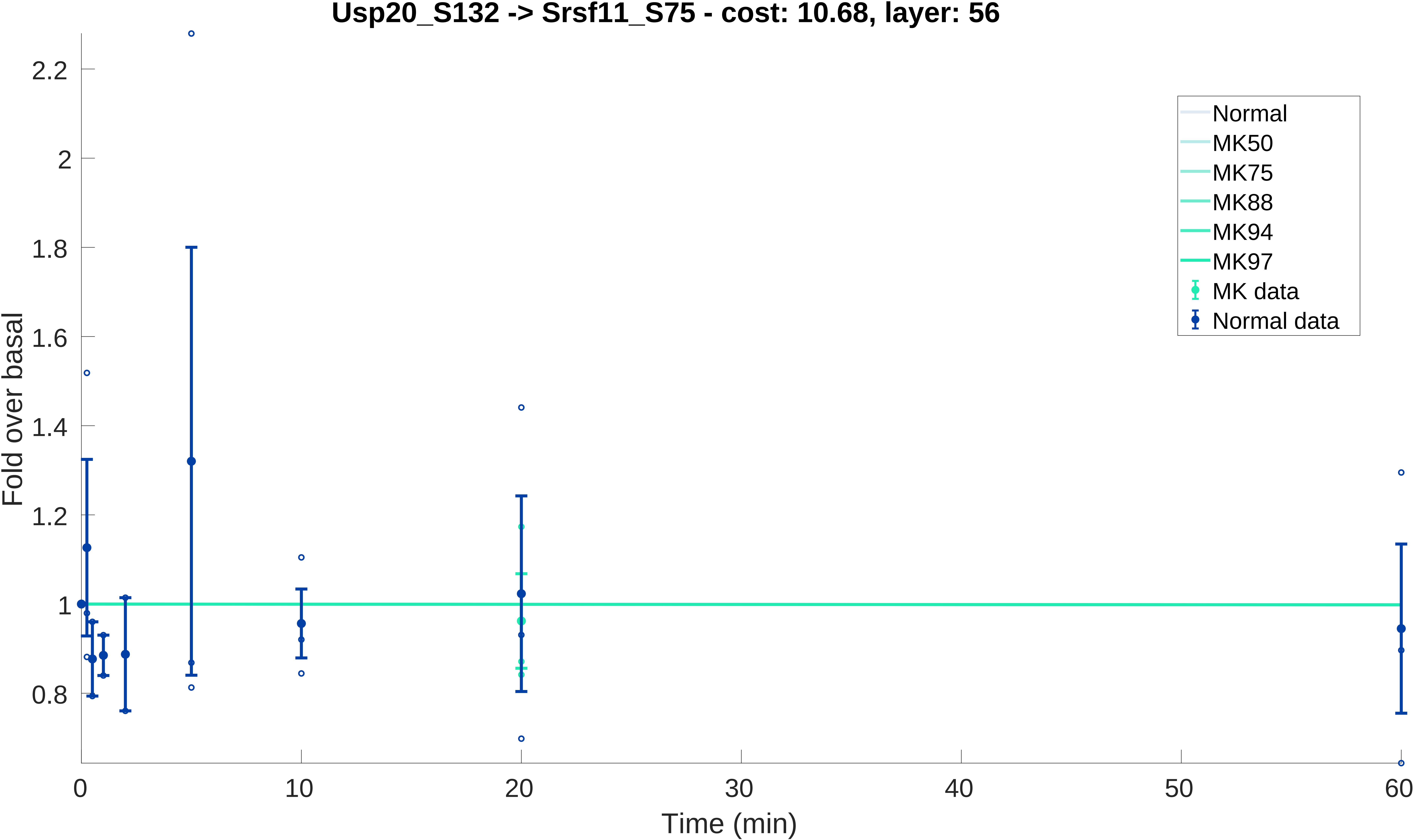

Usp20_S132 -> Srsf11_S75 - cost: 10.68, layer: 56
Fold over basal
Time (min)
0.8
1
1.2
1.4
1.6
1.8
2
2.2
0
10
20
30
40
50
60
Normal
MK50
MK75
MK88
MK94
MK97
MK data
Normal data

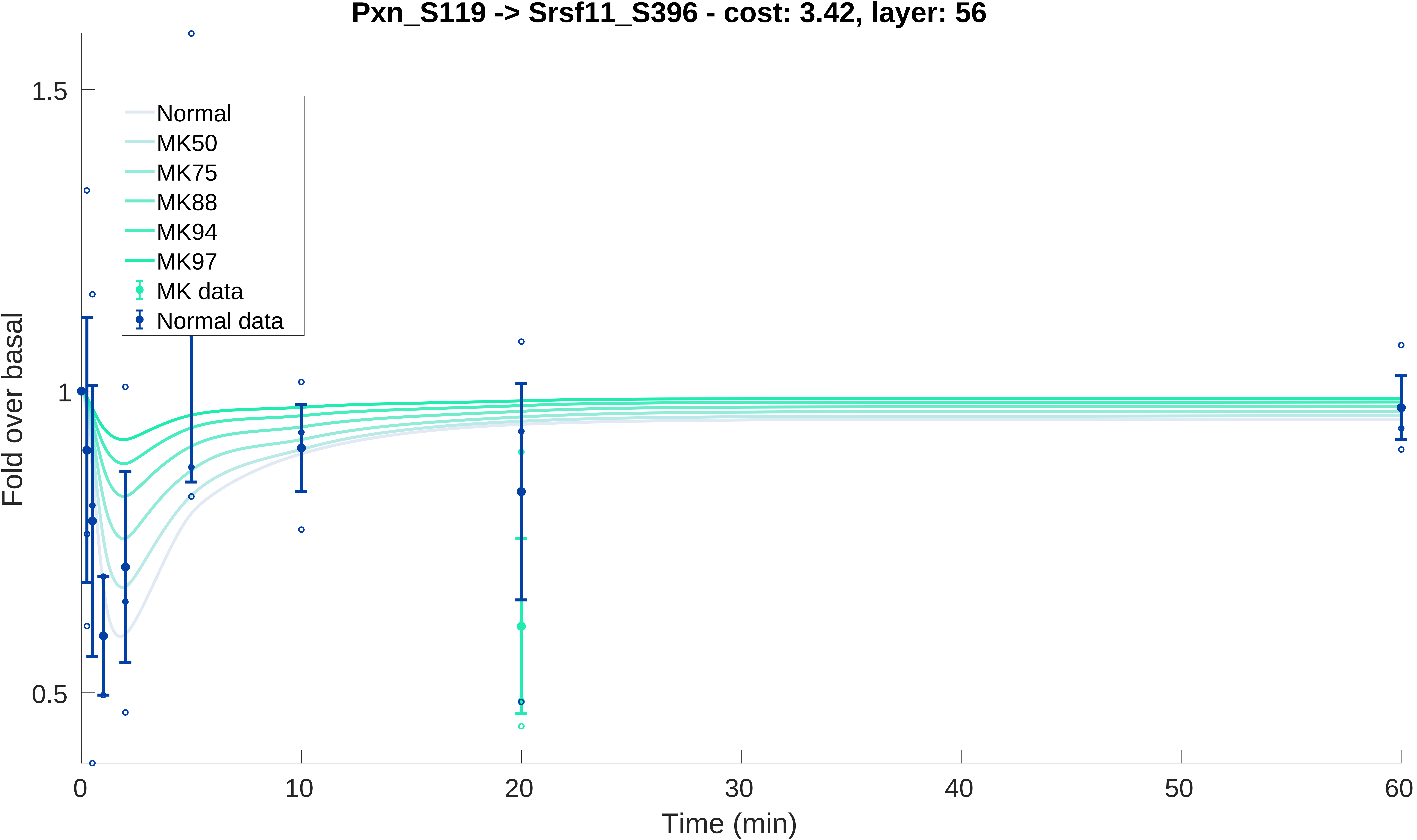

Pxn_S119 -> Srsf11_S396 - cost: 3.42, layer: 56
Fold over basal
Time (min)
1.5
1
0.5
0
10
20
30
40
50
60
Normal
MK50
MK75
MK88
MK94
MK97
MK data
Normal data

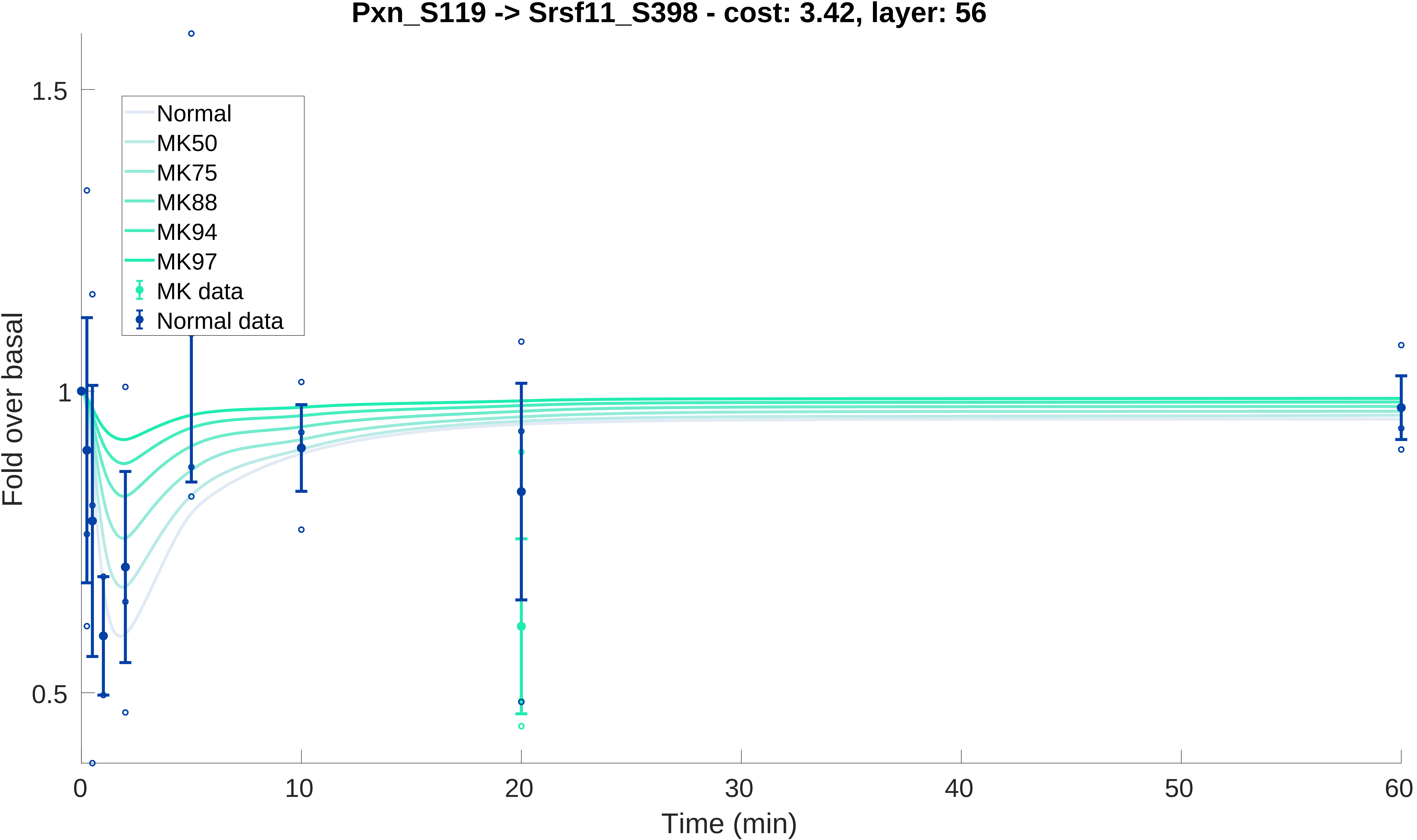

Pxn_S119 -> Srsf11_S398 - cost: 3.42, layer: 56
Fold over basal
1.5
1
0.5
Time (min)
0
10
20
30
40
50
60
Normal
MK50
MK75
MK88
MK94
MK97
MK data
Normal data

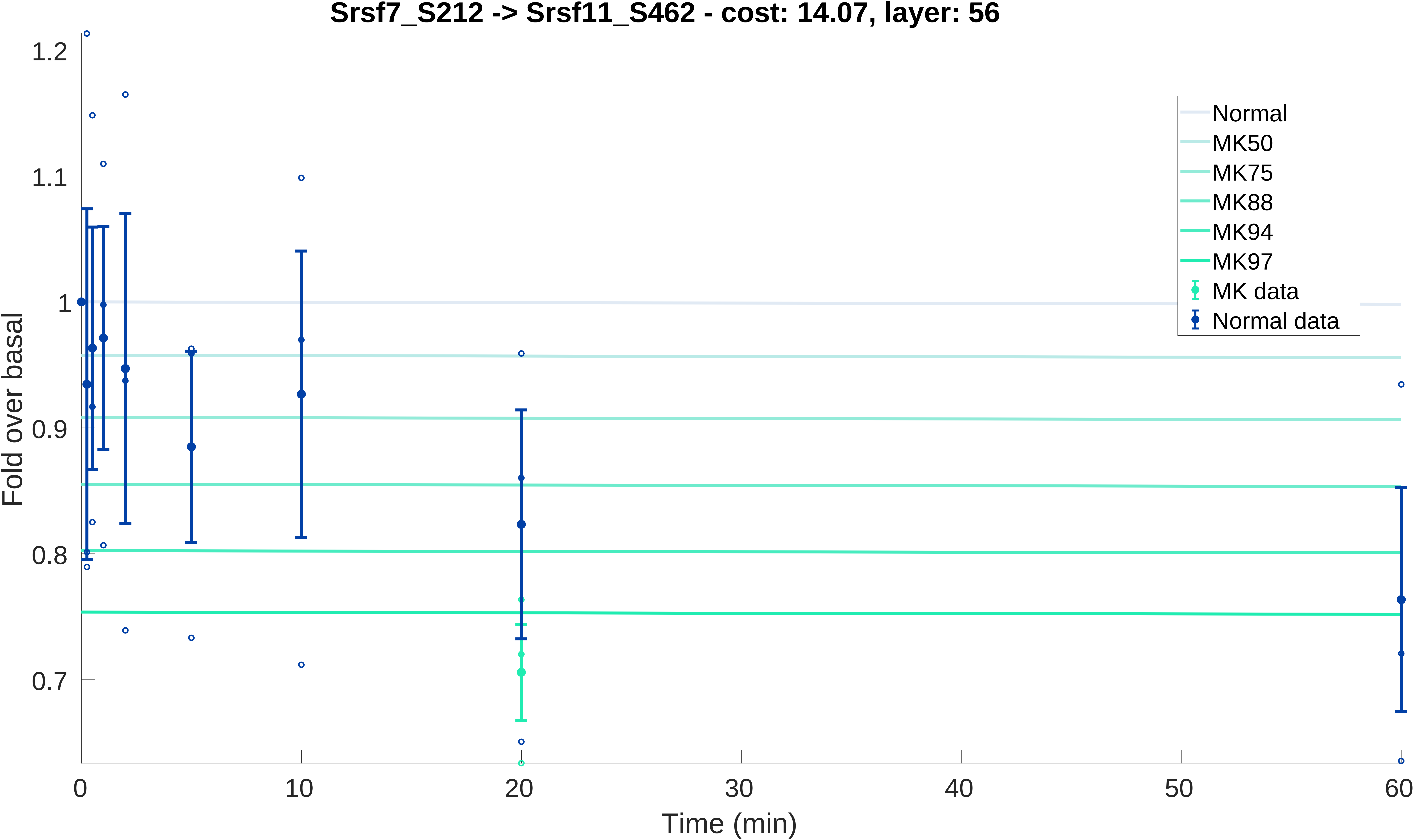

Srsf7_S212 -> Srsf11_S462 - cost: 14.07, layer: 56
Fold over basal
Time (min)
1.2
1.1
1
0.9
0.8
0.7
0
10
20
30
40
50
60
Normal
MK50
MK75
MK88
MK94
MK97
MK data
Normal data

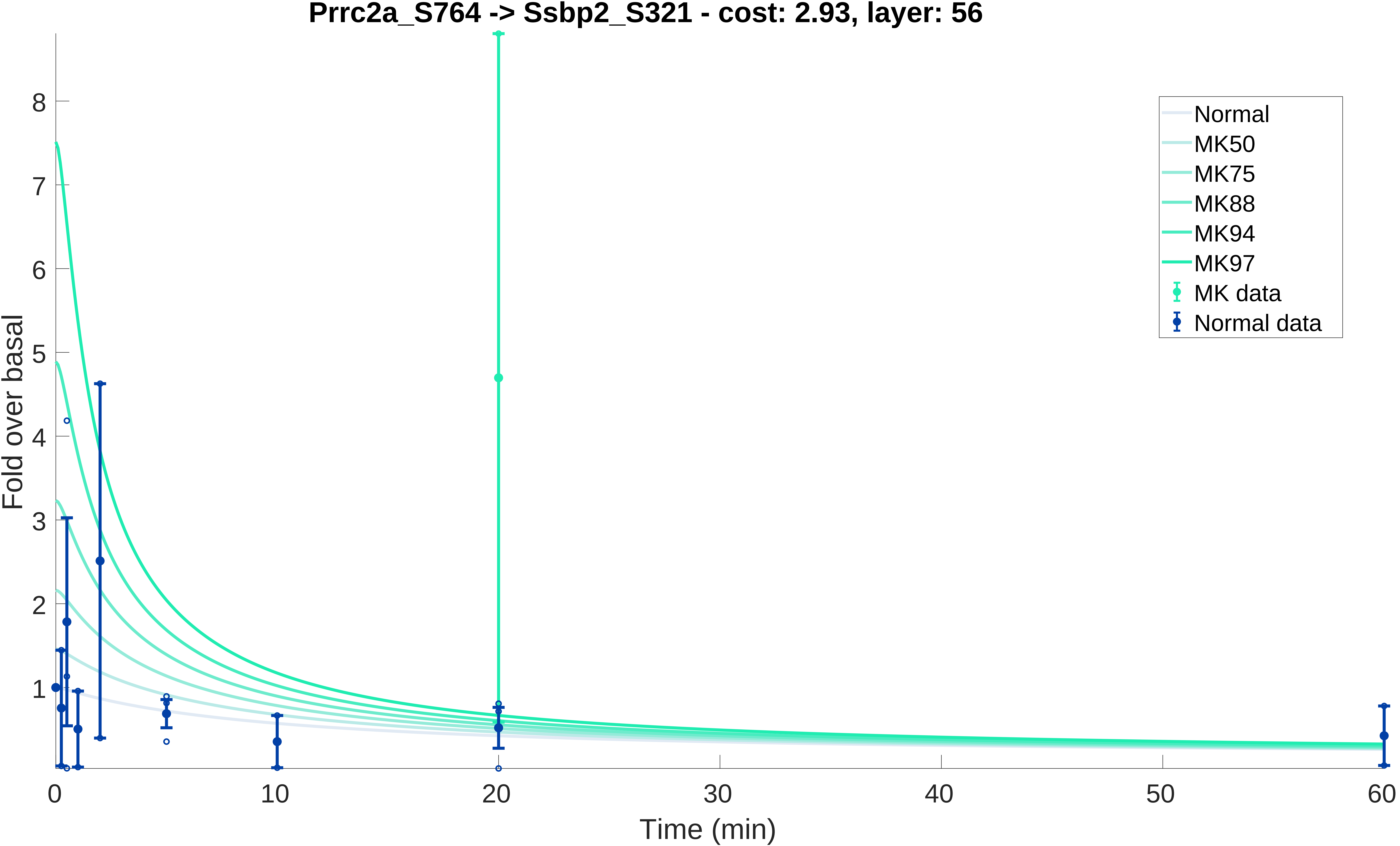

Prrc2a_S764 -> Ssbp2_S321 - cost: 2.93, layer: 56
Fold over basal
Time (min)
0
10
20
30
40
50
60
1
2
3
4
5
6
7
8
Normal
MK50
MK75
MK88
MK94
MK97
MK data
Normal data

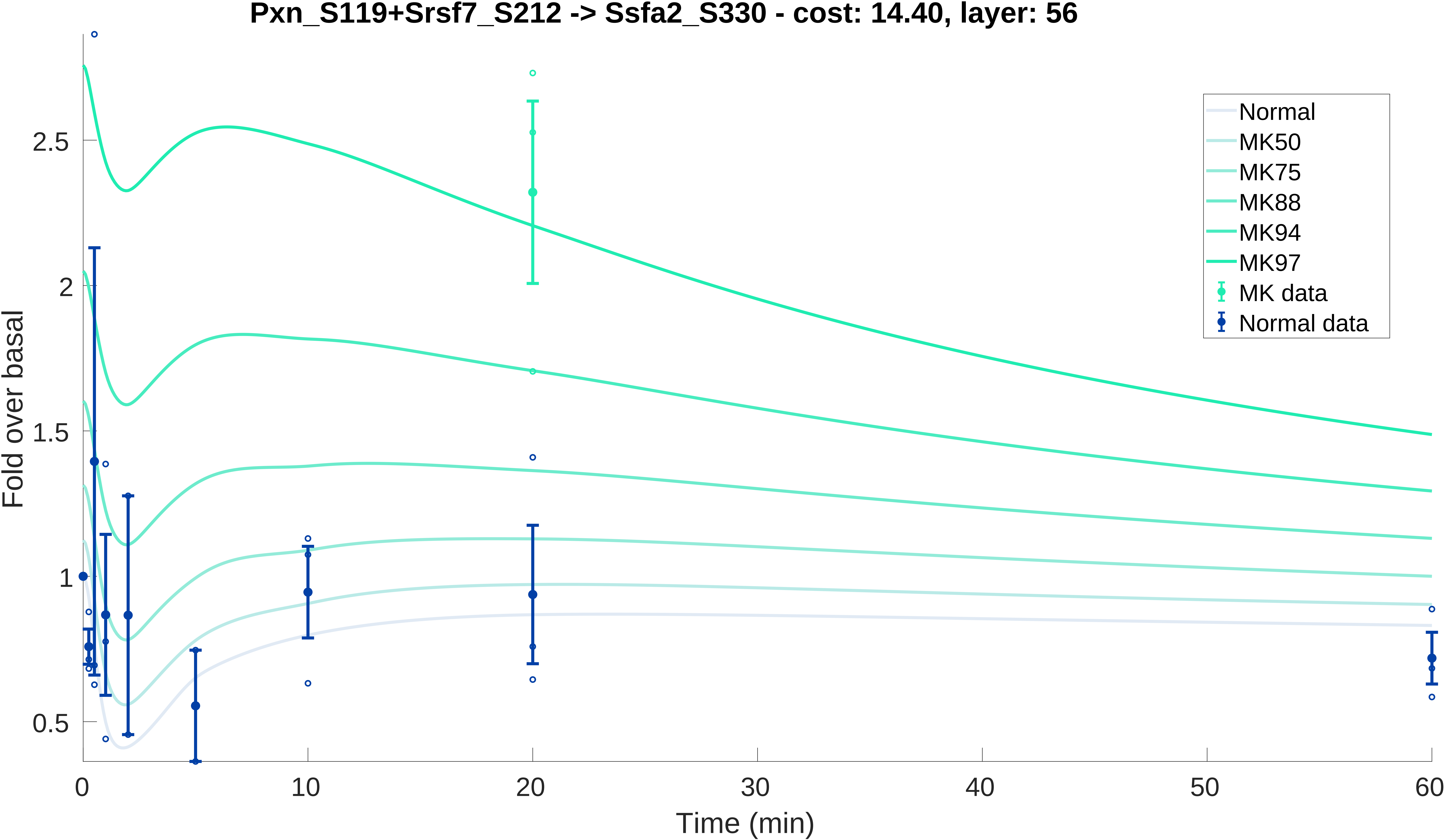

Pxn_S119+Srsf7_S212 -> Ssfa2_S330 - cost: 14.40, layer: 56
Fold over basal
0.5
1
1.5
2
2.5
0
10
20
30
40
50
60
Time (min)
Normal
MK50
MK75
MK88
MK94
MK97
MK data
Normal data

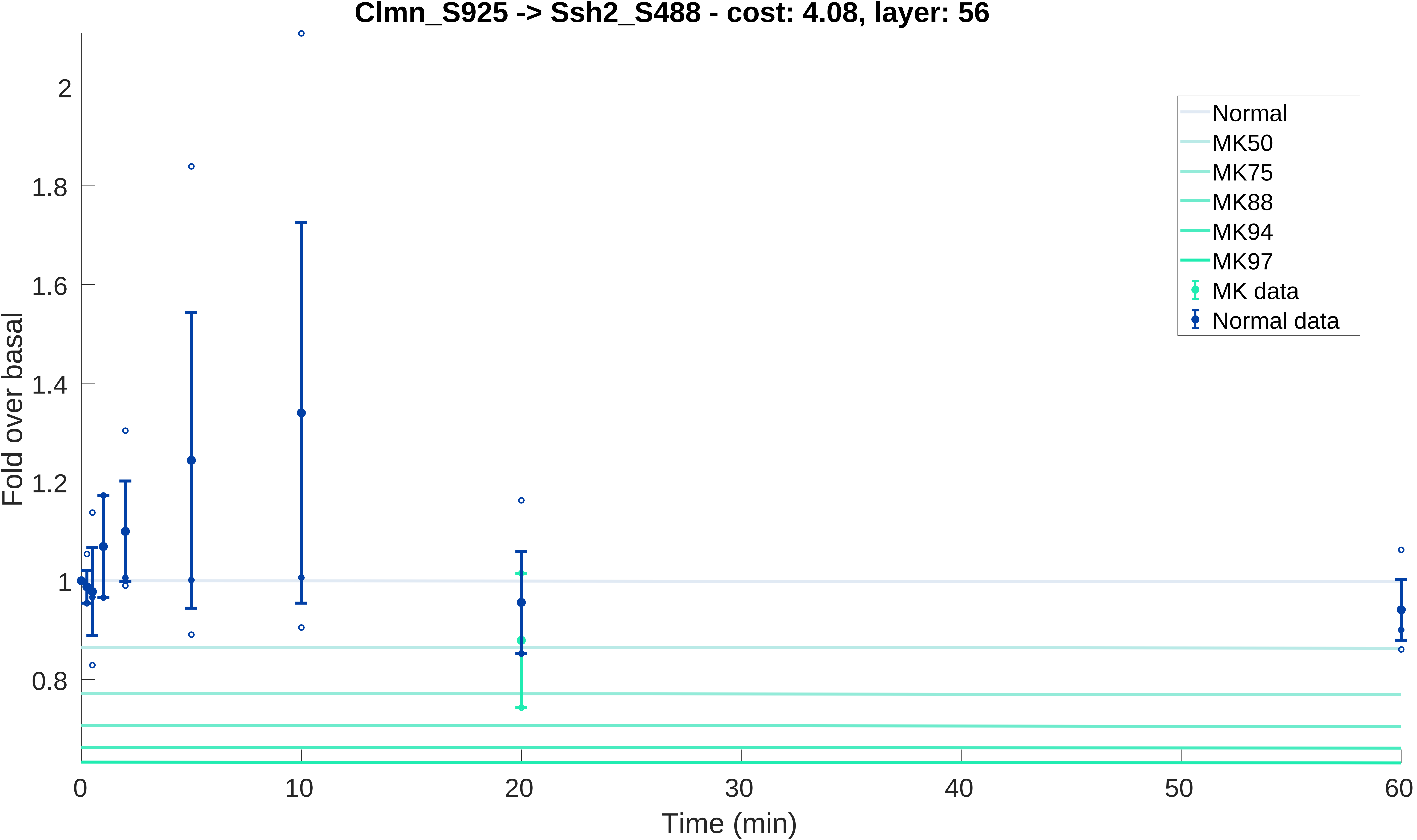

Clmn_S925 -> Ssh2_S488 - cost: 4.08, layer: 56
Fold over basal
2
1.8
1.6
1.4
1.2
1
0.8
0
10
20
30
40
50
60
Time (min)
Normal
MK50
MK75
MK88
MK94
MK97
MK data
Normal data

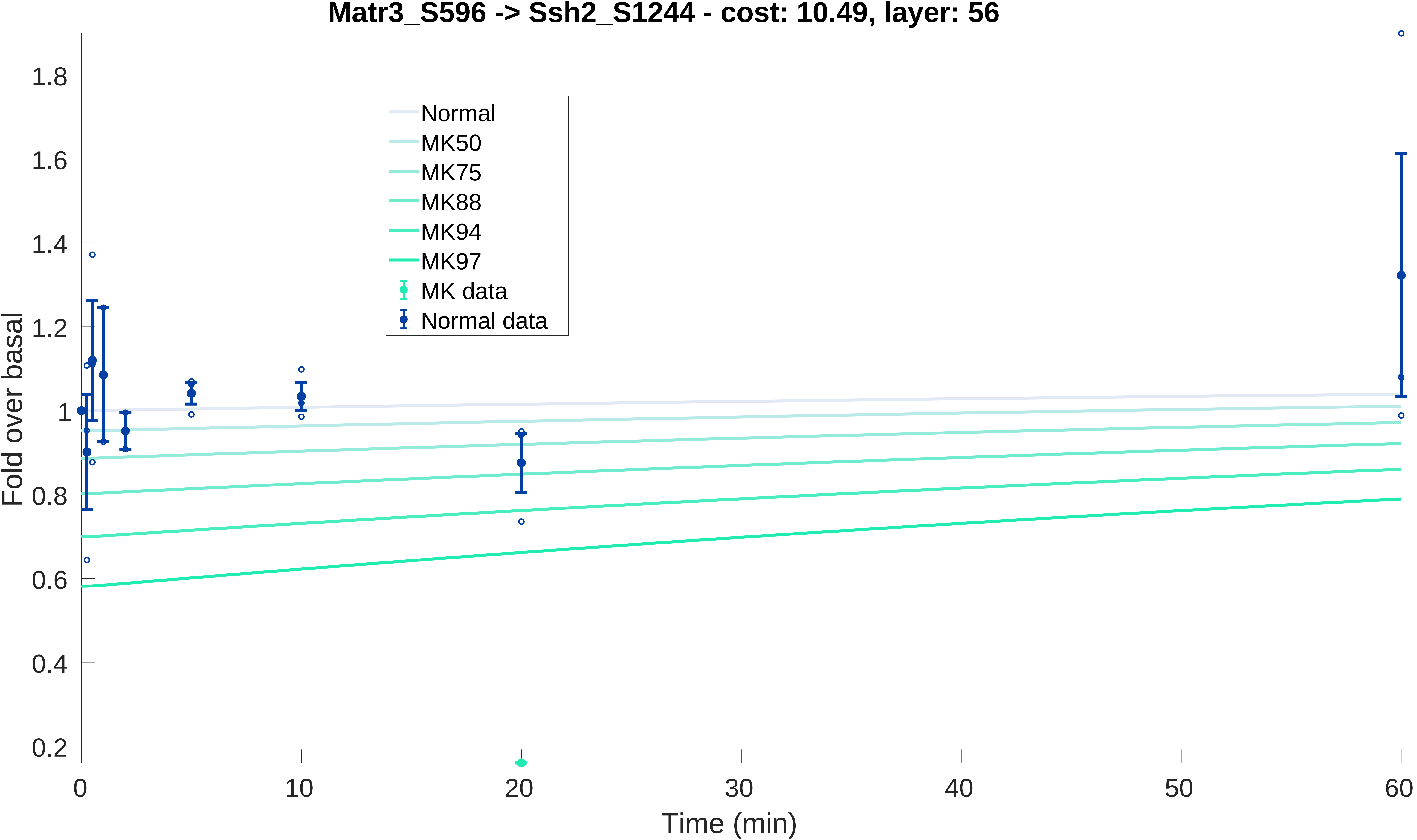

Matr3_S596 -> Ssh2_S1244 - cost: 10.49, layer: 56
Normal
MK50
MK75
MK88
MK94
MK97
MK data
Normal data
Fold over basal
1.8
1.6
1.4
1.2
1
0.8
0.6
0.4
0.2
0
10
20
30
40
50
60
Time (min)

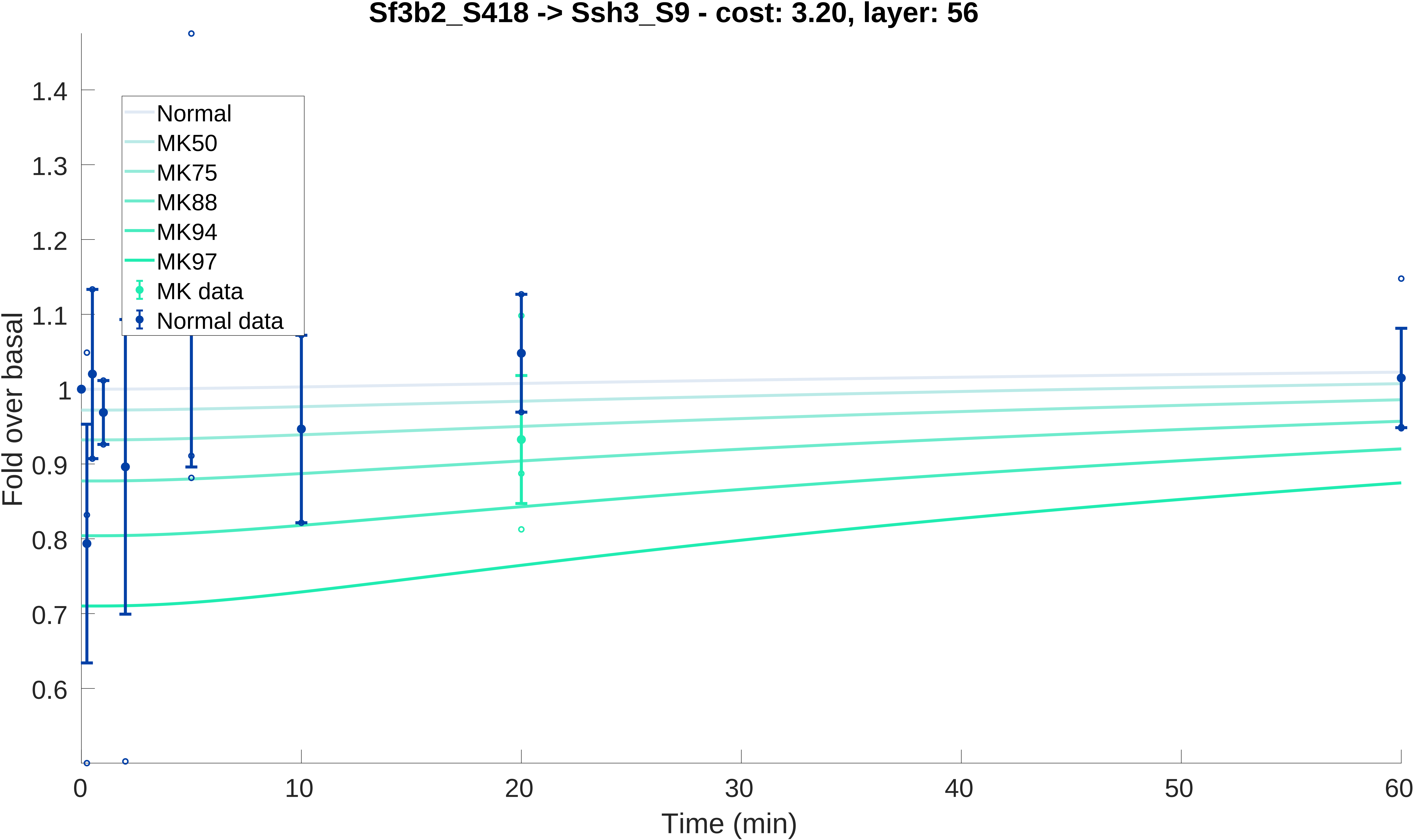
Sf3b2_S418 -> Ssh3_S9 - cost: 3.20, layer: 56
Normal
MK50
MK75
MK88
MK94
MK97
MK data
Normal data
Fold over basal
Time (min)
1.4
1.3
1.2
1.1
1
0.9
0.8
0.7
0.6
0
10
20
30
40
50
60

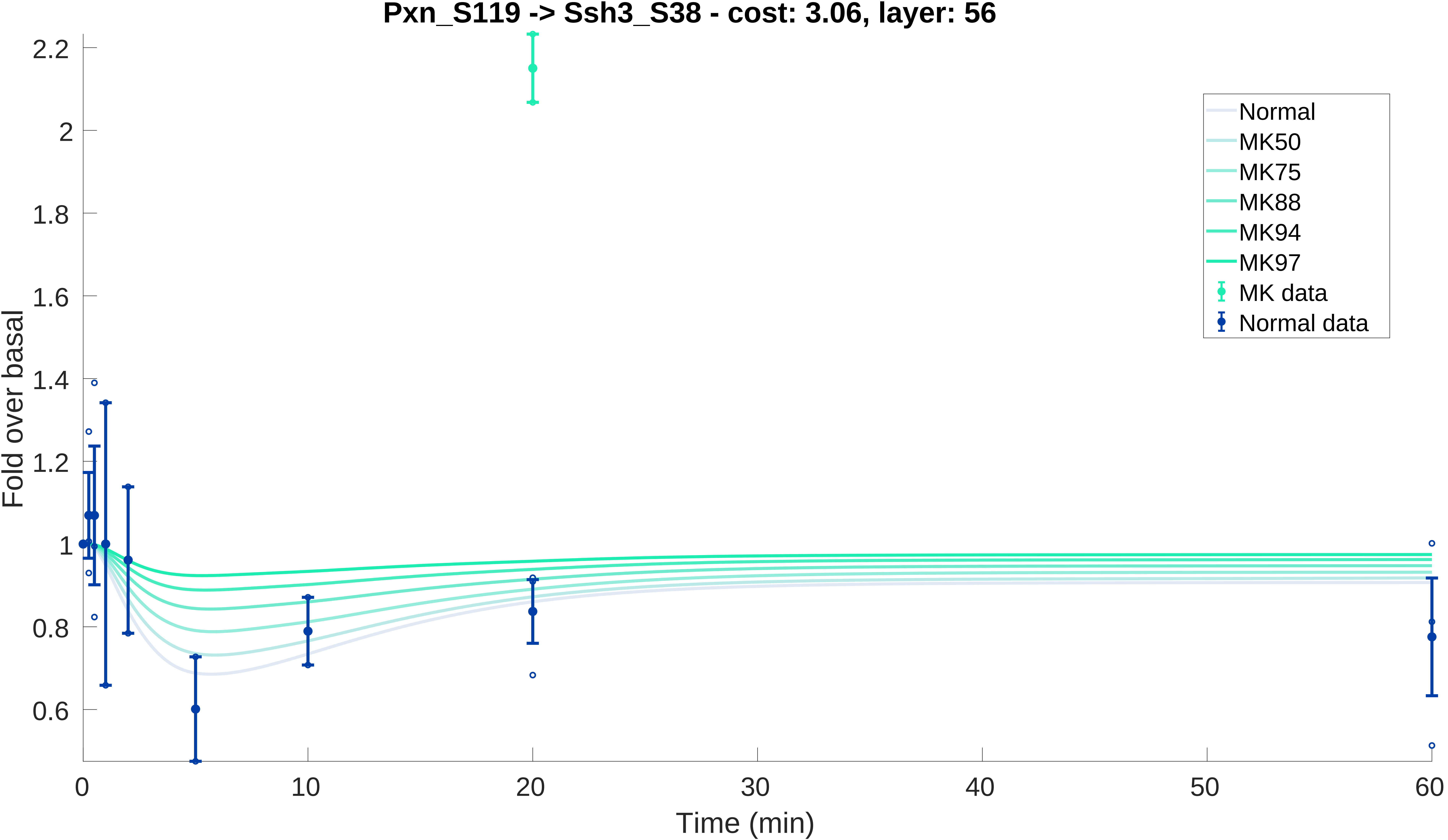

Pxn_S119 -> Ssh3_S38 - cost: 3.06, layer: 56
Fold over basal
Time (min)
Normal
MK50
MK75
MK88
MK94
MK97
MK data
Normal data
0.6
0.8
1
1.2
1.4
1.6
1.8
2
2.2
0
10
20
30
40
50
60

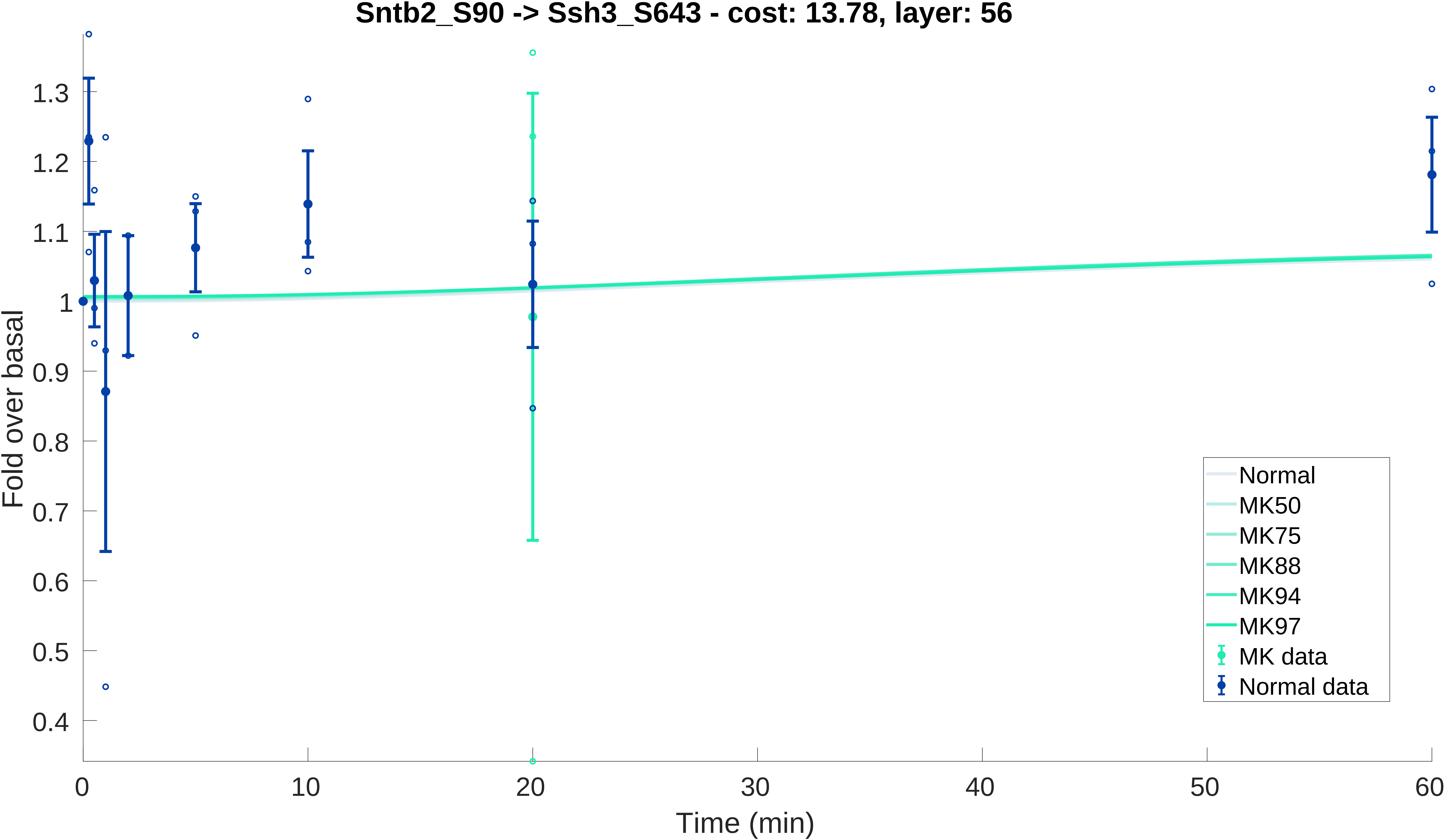

Sntb2_S90 -> Ssh3_S643 - cost: 13.78, layer: 56
Fold over basal
Time (min)
0
10
20
30
40
50
60
0.4
0.5
0.6
0.7
0.8
0.9
1
1.1
1.2
1.3
Normal
MK50
MK75
MK88
MK94
MK97
MK data
Normal data

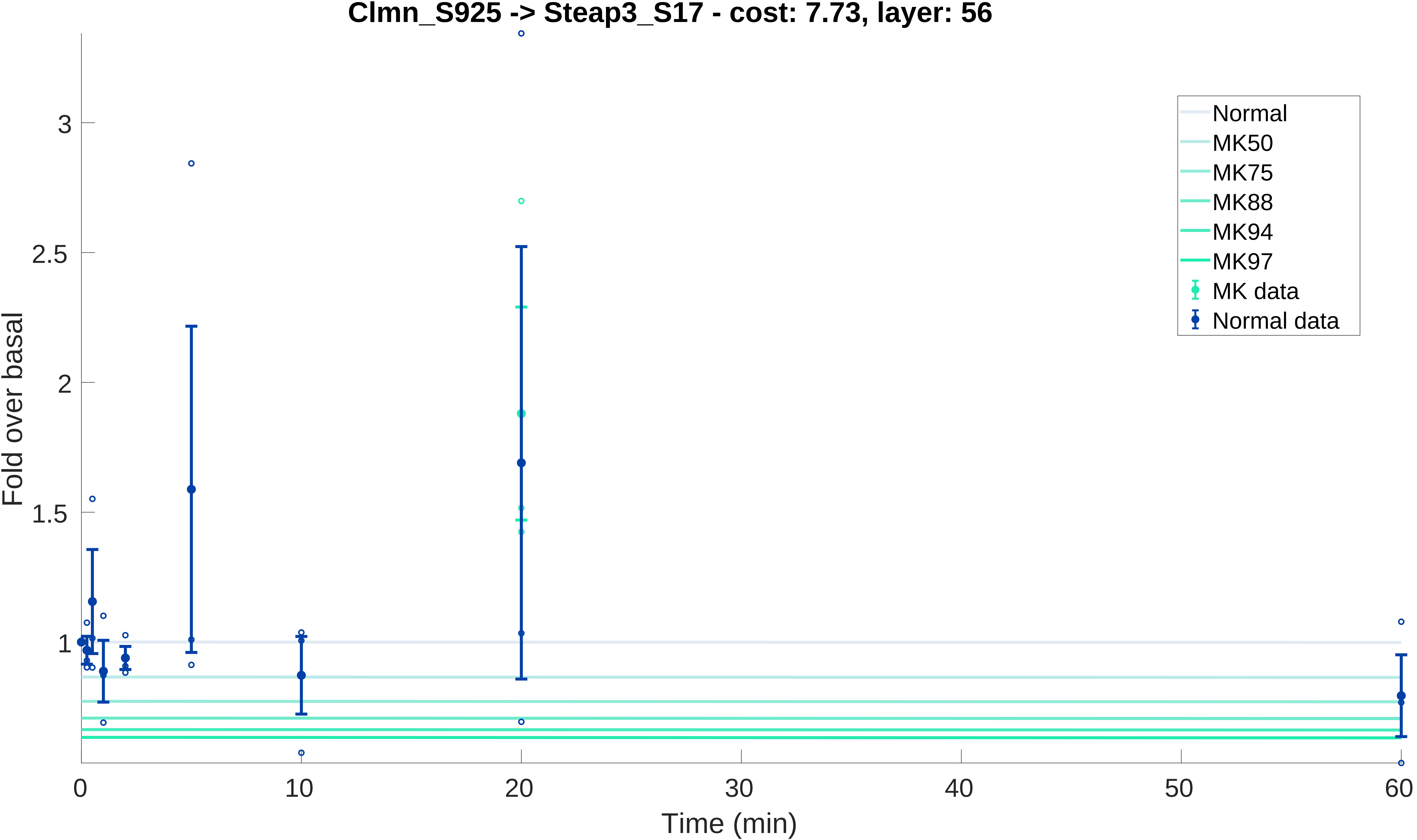
Clmn_S925 -> Steap3_S17 - cost: 7.73, layer: 56
Fold over basal
Time (min)
0
10
20
30
40
50
60
1
1.5
2
2.5
3
Normal
MK50
MK75
MK88
MK94
MK97
MK data
Normal data

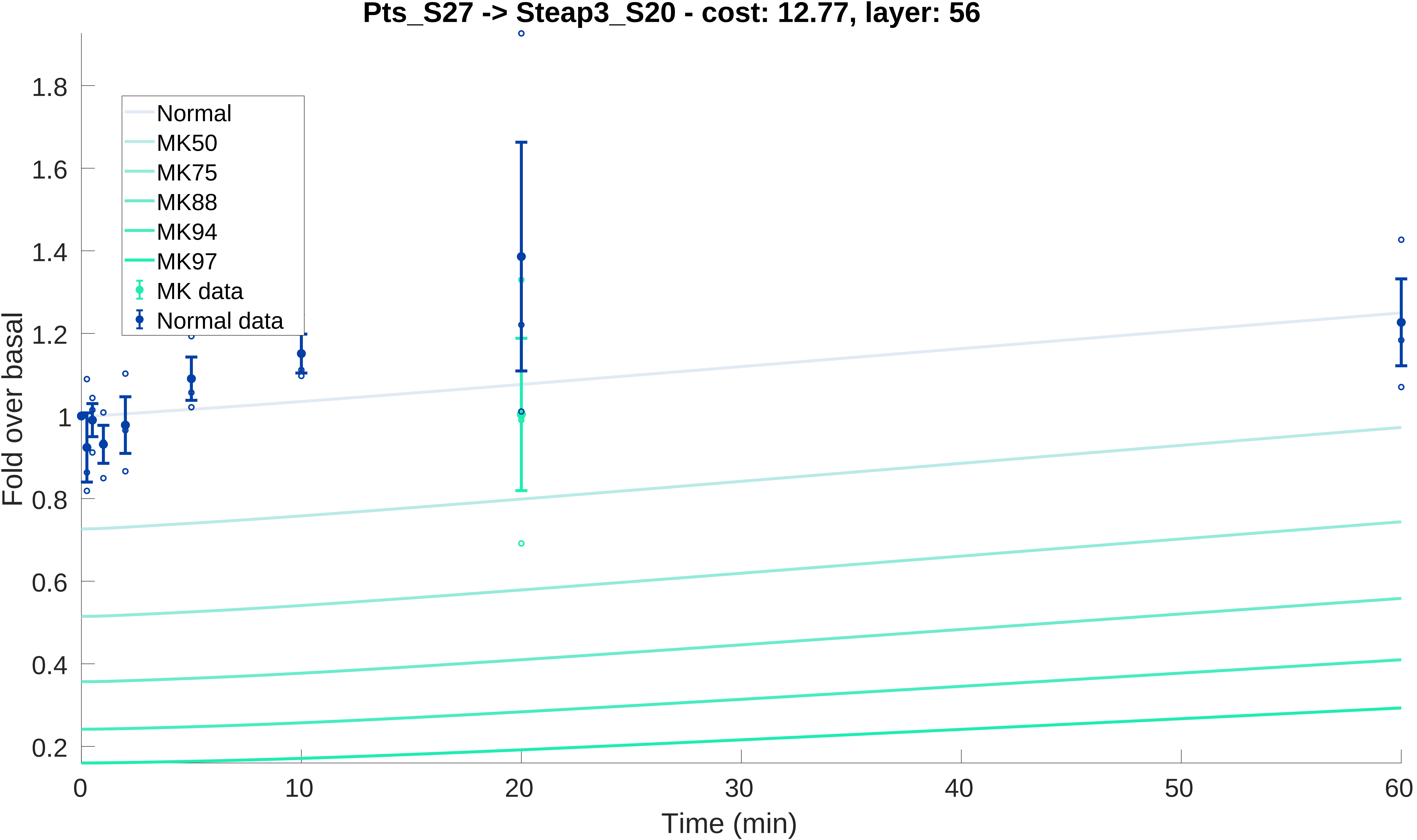
Pts_S27 -> Steap3_S20 - cost: 12.77, layer: 56
Normal
MK50
MK75
MK88
MK94
MK97
MK data
Normal data
Fold over basal
Time (min)
0.2
0.4
0.6
0.8
1
1.2
1.4
1.6
1.8
0
10
20
30
40
50
60

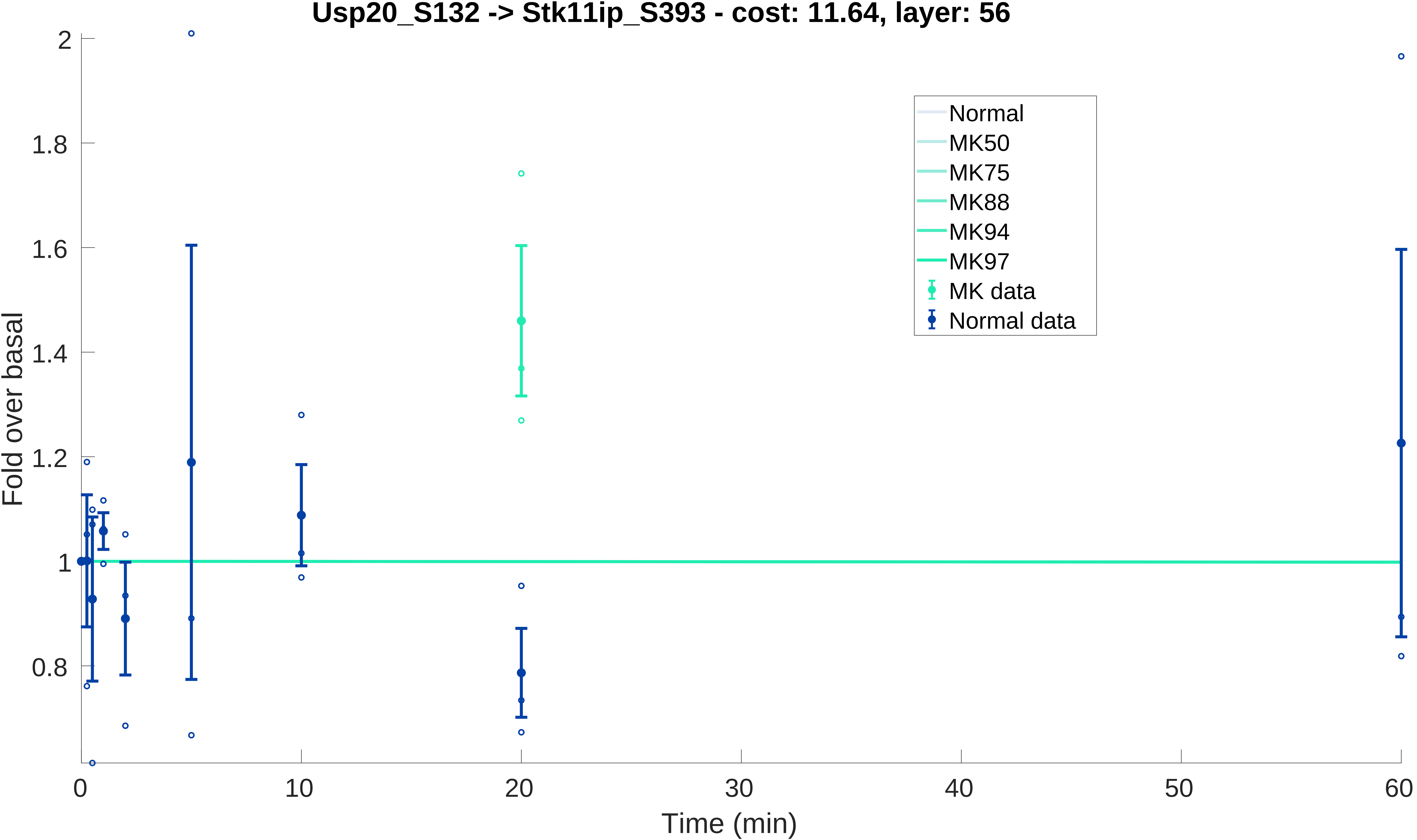

Usp20_S132 -> Stk11ip_S393 - cost: 11.64, layer: 56
Fold over basal
Time (min)
2
1.8
1.6
1.4
1.2
1
0.8
0
10
20
30
40
50
60
Normal
MK50
MK75
MK88
MK94
MK97
MK data
Normal data

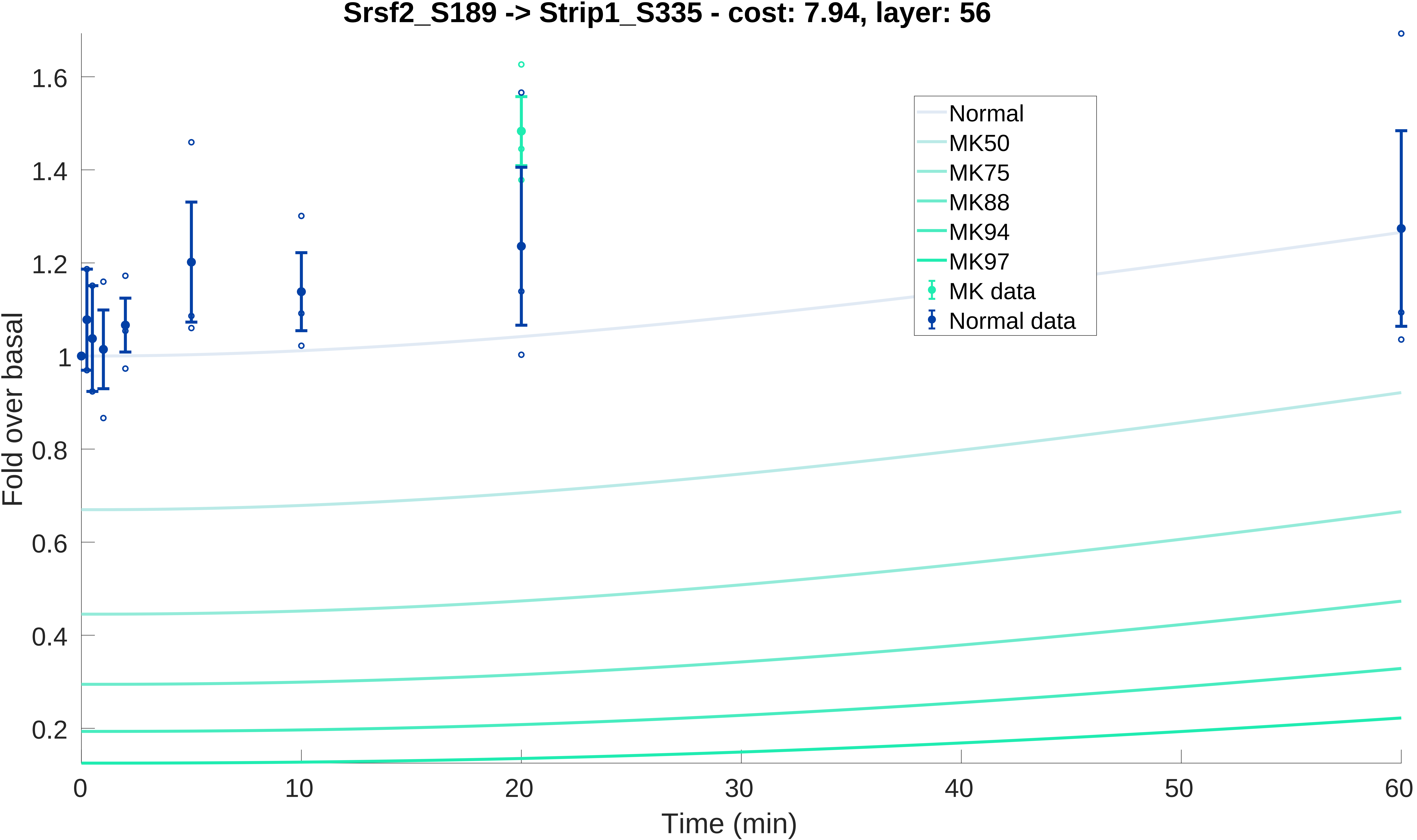

Srsf2_S189 -> Strip1_S335 - cost: 7.94, layer: 56
Fold over basal
Time (min)
Normal
MK50
MK75
MK88
MK94
MK97
MK data
Normal data
0.2
0.4
0.6
0.8
1
1.2
1.4
1.6
0
10
20
30
40
50
60

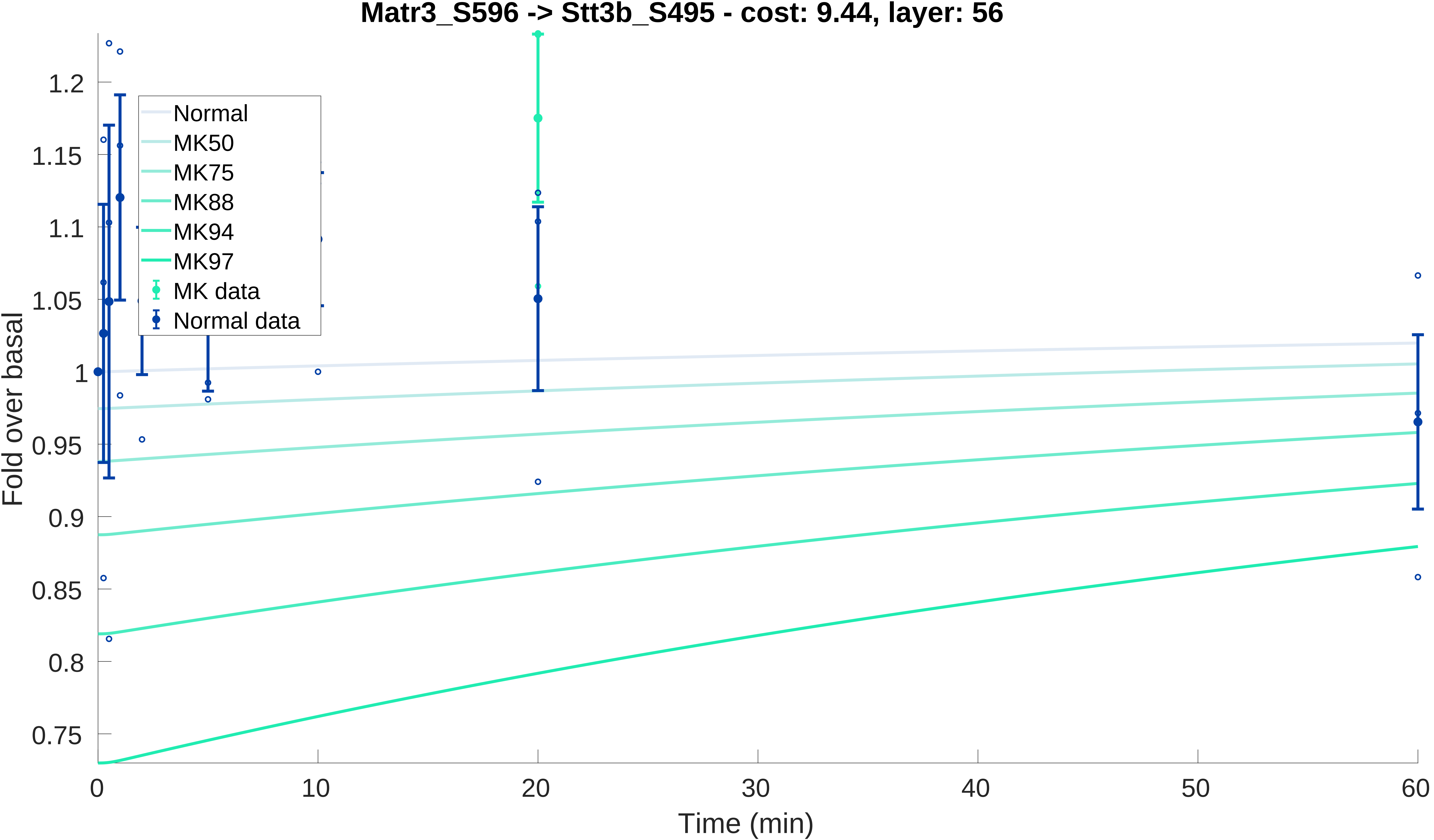

Matr3_S596 -> Stt3b_S495 - cost: 9.44, layer: 56
Normal
MK50
MK75
MK88
MK94
MK97
MK data
Normal data
Fold over basal
Time (min)
1.2
1.15
1.1
1.05
1
0.95
0.9
0.85
0.8
0.75
0
10
20
30
40
50
60

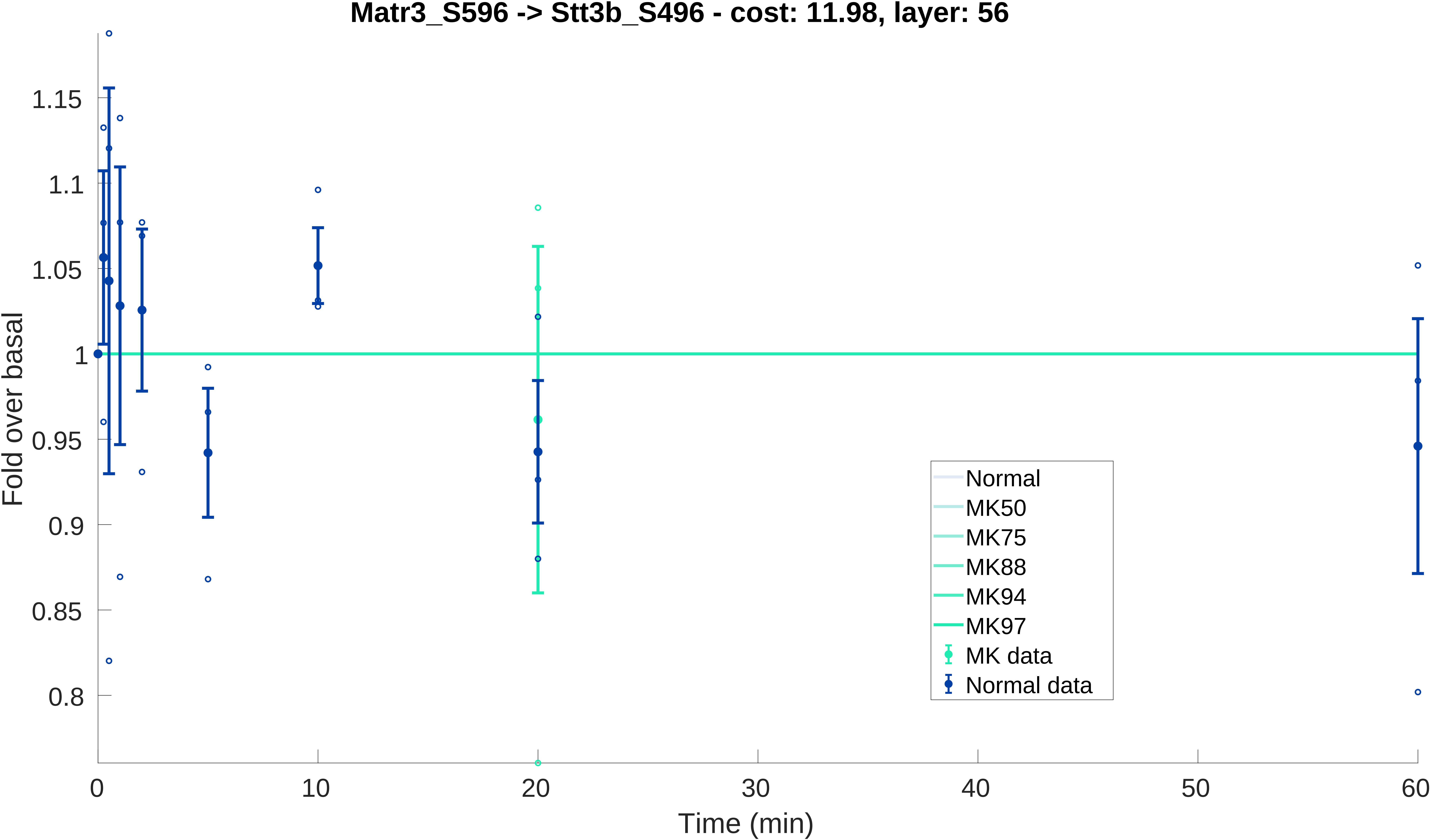

Matr3_S596 -> Stt3b_S496 - cost: 11.98, layer: 56
Fold over basal
Time (min)
1.15
1.1
1.05
1
0.95
0.9
0.85
0.8
0
10
20
30
40
50
60
Normal
MK50
MK75
MK88
MK94
MK97
MK data
Normal data

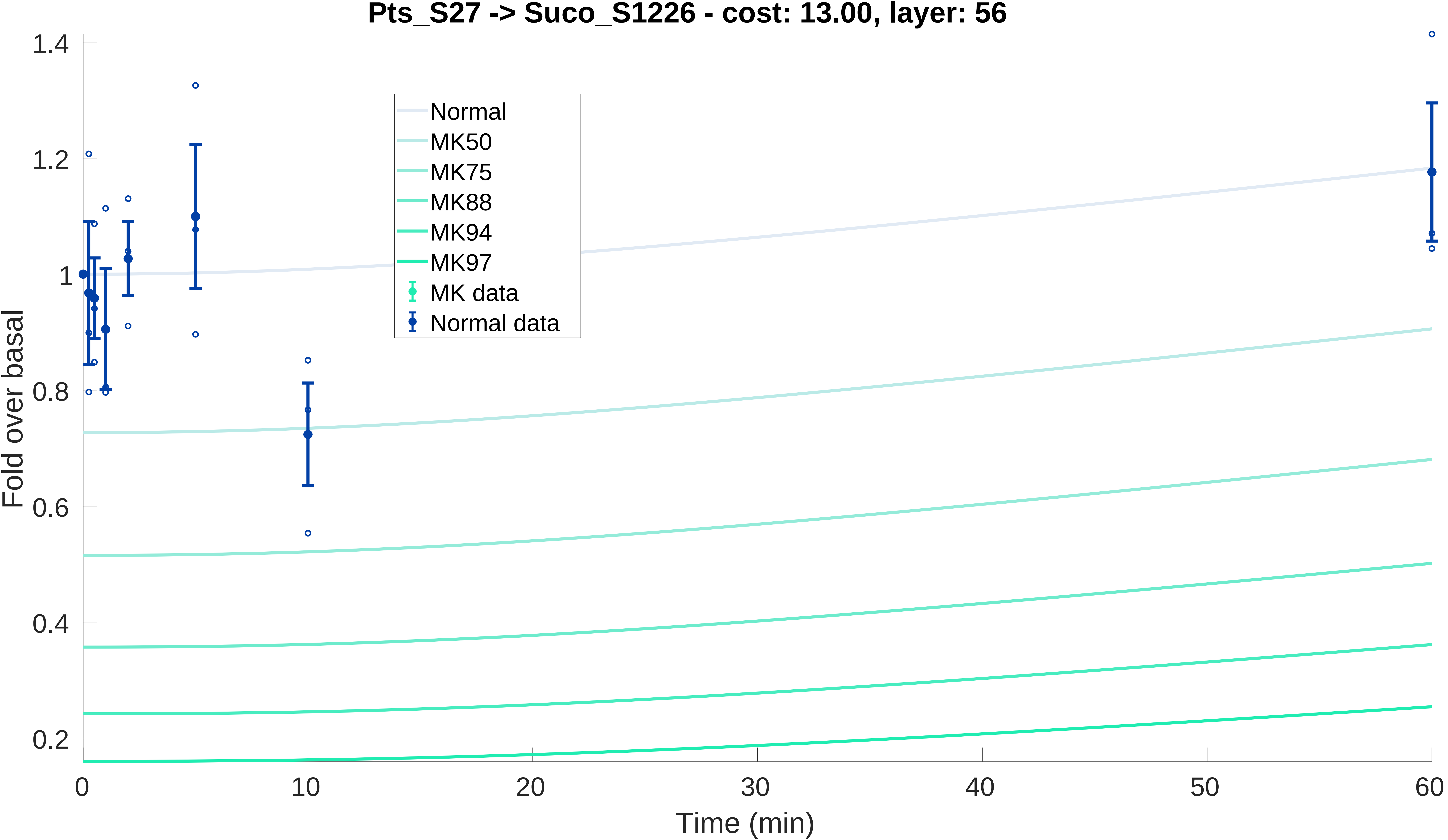

Pts_S27 -> Suco_S1226 - cost: 13.00, layer: 56
Fold over basal
Time (min)
0
10
20
30
40
50
60
0.2
0.4
0.6
0.8
1
1.2
1.4
Normal
MK50
MK75
MK88
MK94
MK97
MK data
Normal data

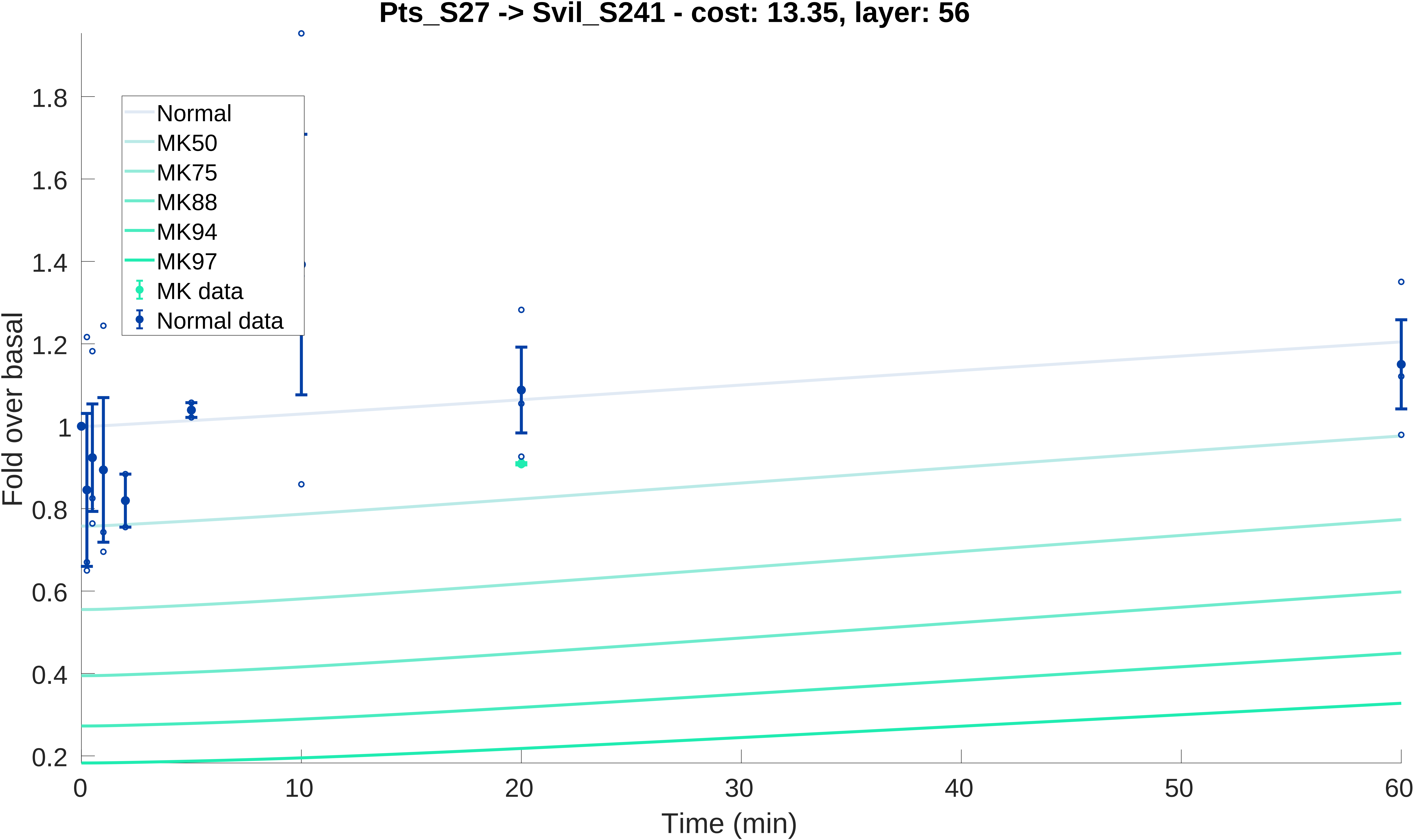
Pts_S27 -> Svil_S241 - cost: 13.35, layer: 56
Normal
MK50
MK75
MK88
MK94
MK97
MK data
Normal data
Fold over basal
Time (min)
0.2
0.4
0.6
0.8
1
1.2
1.4
1.6
1.8
0
10
20
30
40
50
60

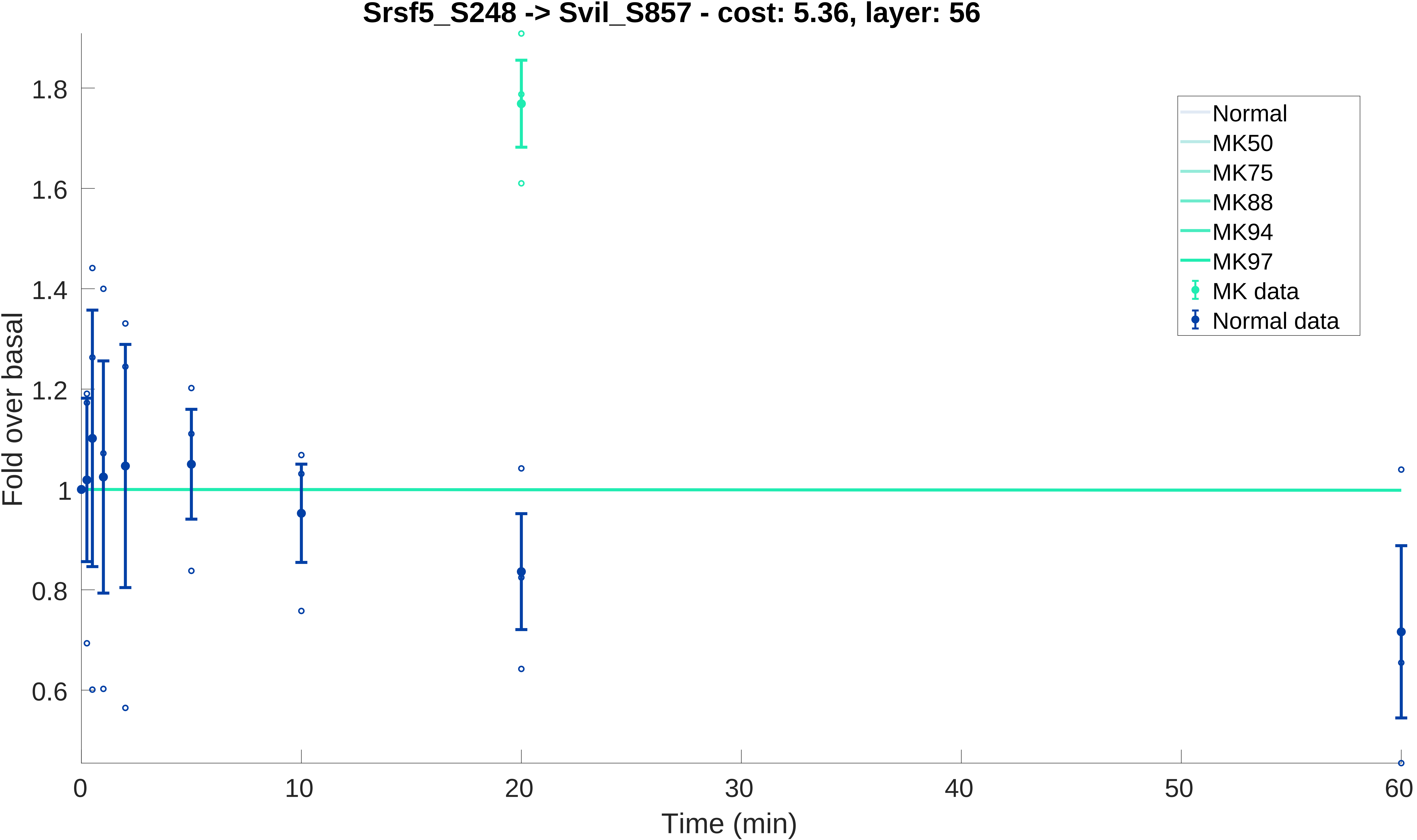
Srsf5_S248 -> Svil_S857 - cost: 5.36, layer: 56
Fold over basal
Time (min)
0.6
0.8
1
1.2
1.4
1.6
1.8
0
10
20
30
40
50
60
Normal
MK50
MK75
MK88
MK94
MK97
MK data
Normal data

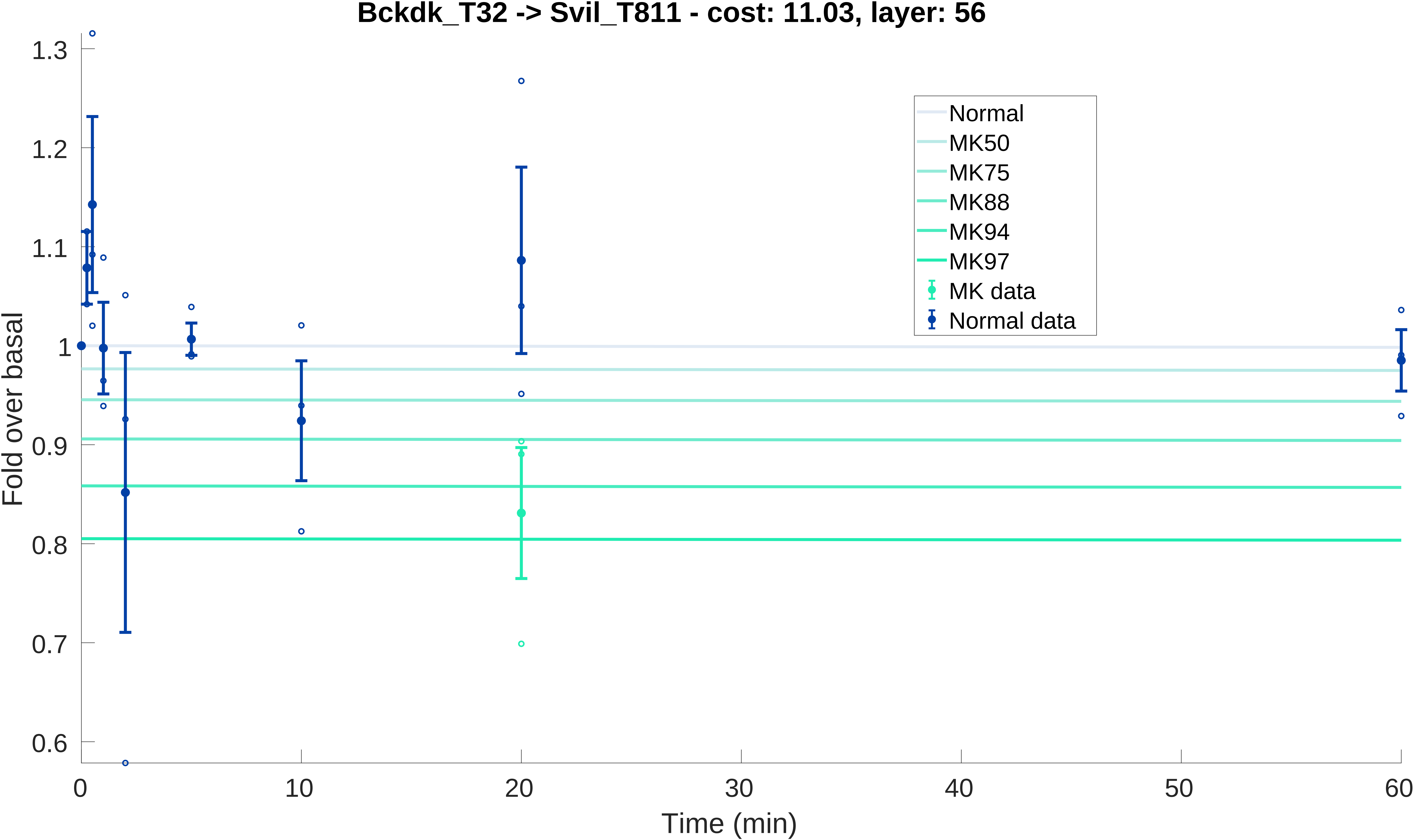
Bckdk_T32 -> Svil_T811 - cost: 11.03, layer: 56
Fold over basal
Time (min)
1.3
1.2
1.1
1
0.9
0.8
0.7
0.6
0
10
20
30
40
50
60
Normal
MK50
MK75
MK88
MK94
MK97
MK data
Normal data

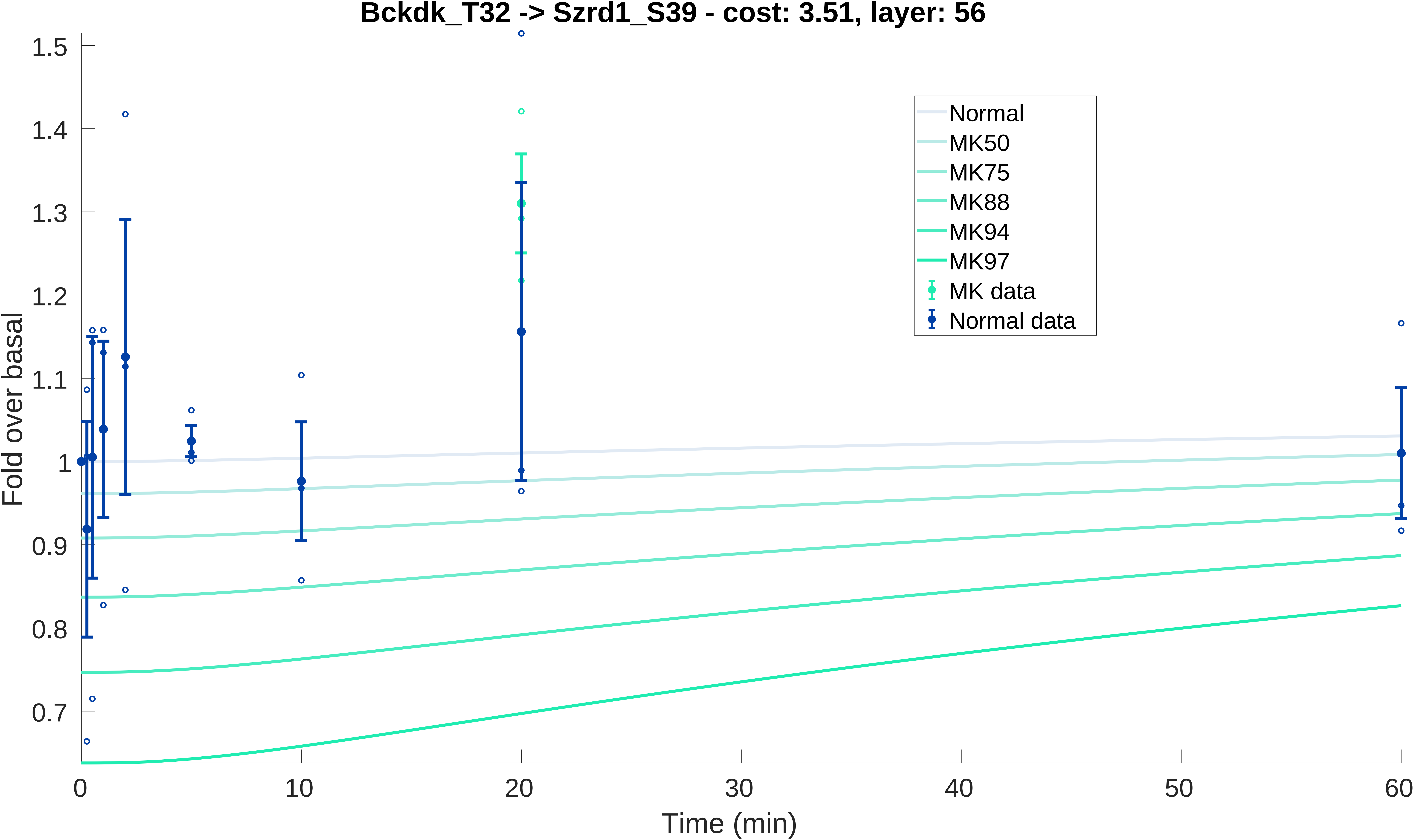

Bckdk_T32 -> Szrd1_S39 - cost: 3.51, layer: 56
Fold over basal
1.5
1.4
1.3
1.2
1.1
1
0.9
0.8
0.7
Time (min)
0
10
20
30
40
50
60
Normal
MK50
MK75
MK88
MK94
MK97
MK data
Normal data

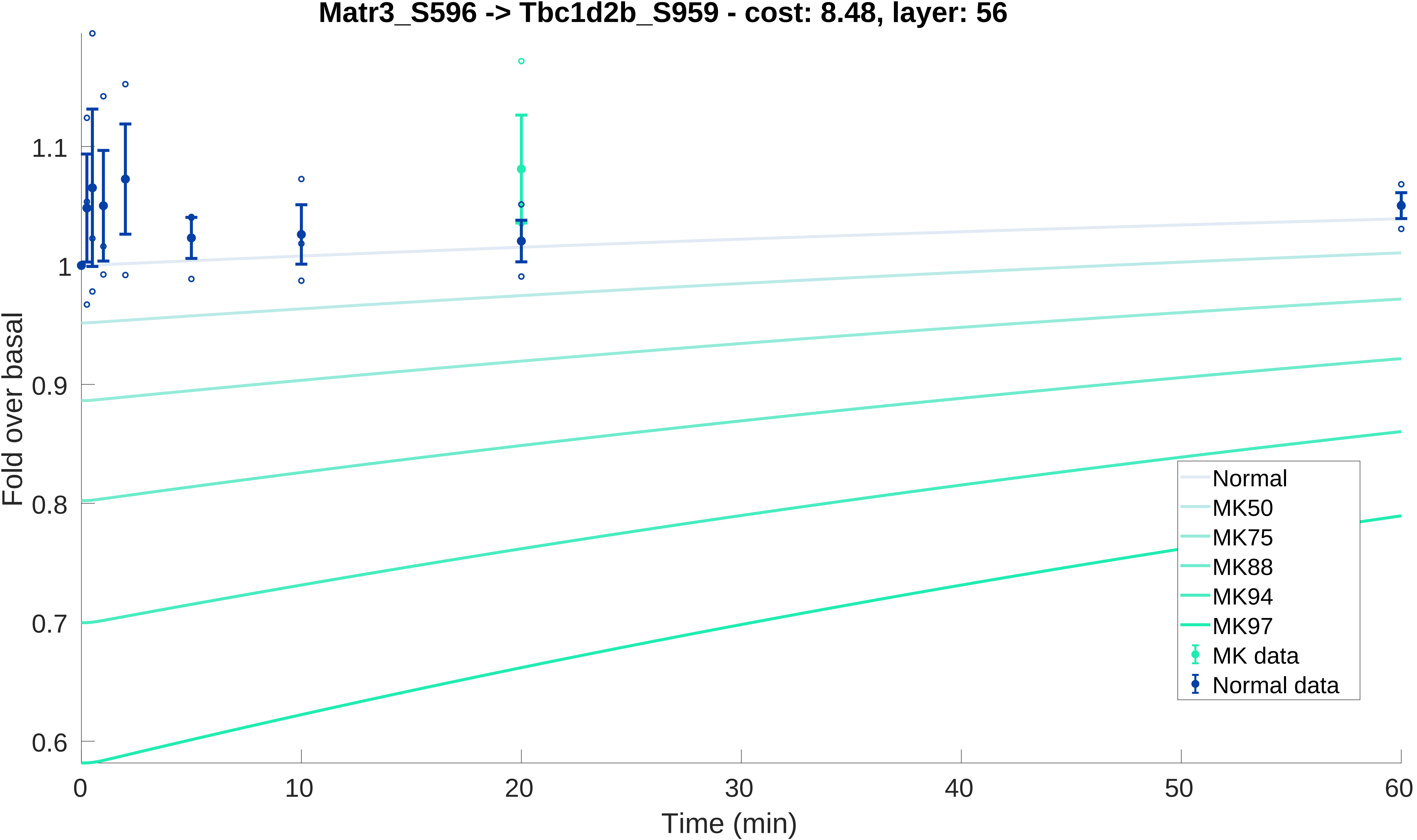

Matr3_S596 -> Tbc1d2b_S959 - cost: 8.48, layer: 56
Fold over basal
1.1
1
0.9
0.8
0.7
0.6
0
10
20
30
40
50
60
Time (min)
Normal
MK50
MK75
MK88
MK94
MK97
MK data
Normal data

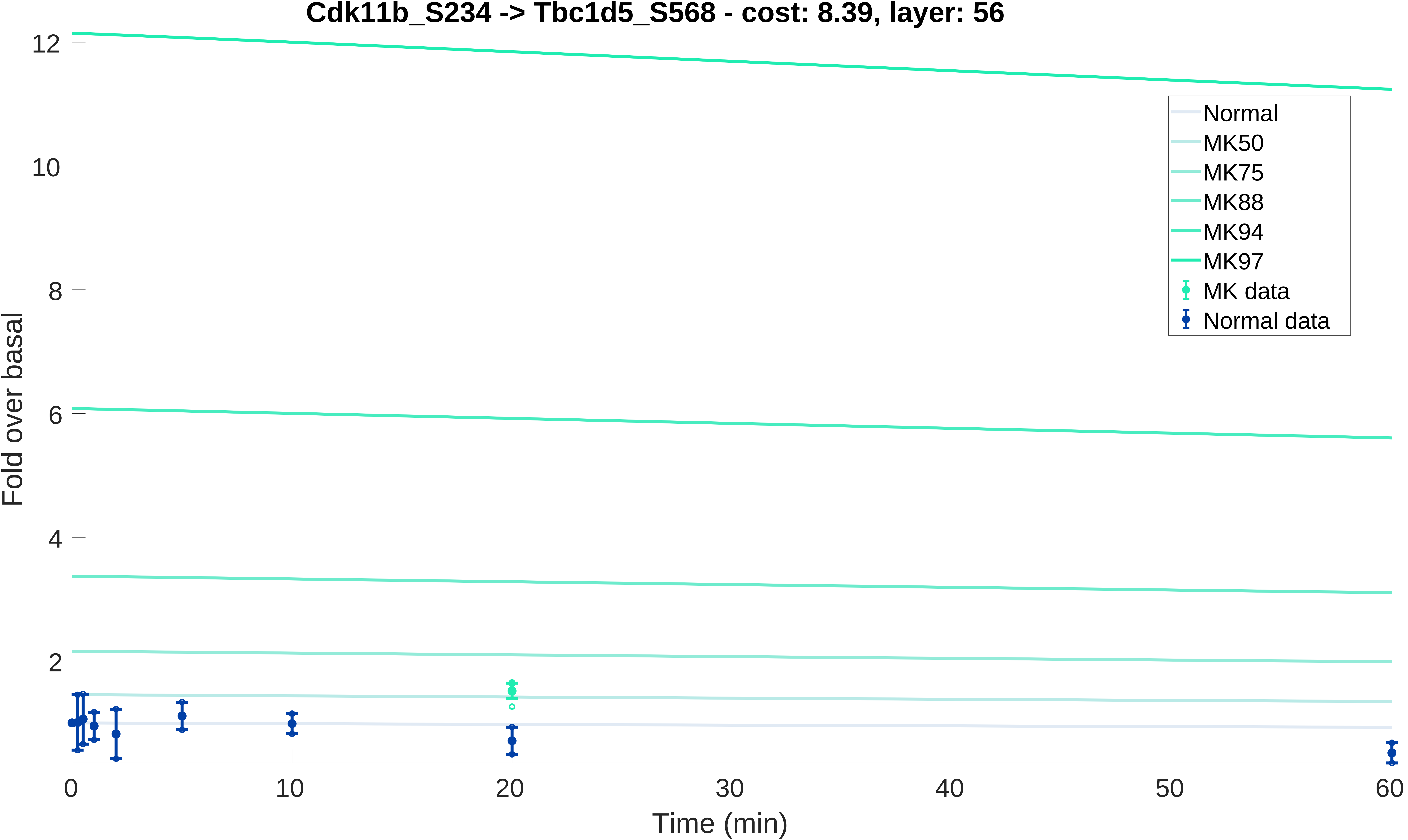

Cdk11b_S234 -> Tbc1d5_S568 - cost: 8.39, layer: 56
Fold over basal
Time (min)
Normal
MK50
MK75
MK88
MK94
MK97
MK data
Normal data
0
10
20
30
40
50
60
2
4
6
8
10
12

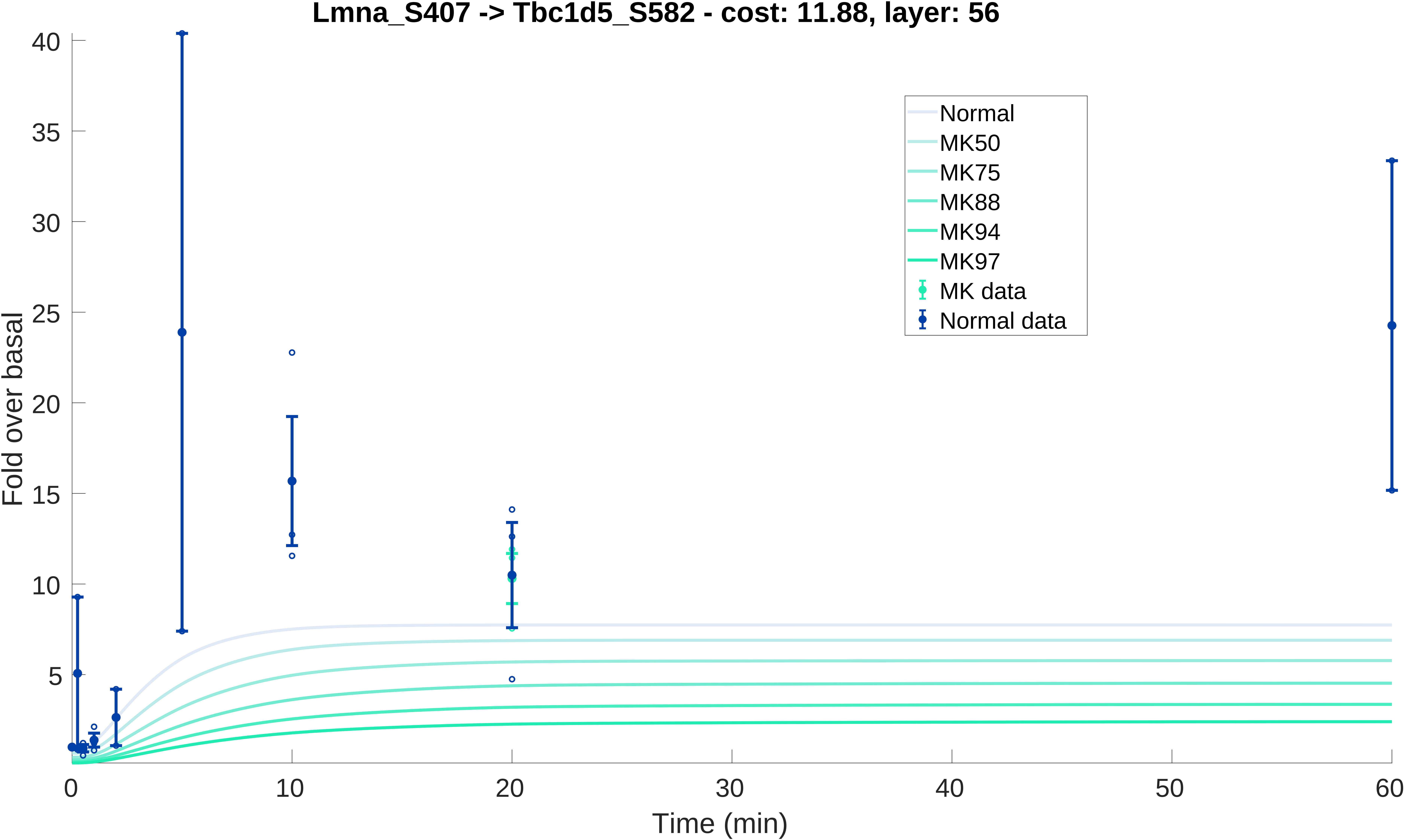

Lmna_S407 -> Tbc1d5_S582 - cost: 11.88, layer: 56
Fold over basal
Time (min)
0
10
20
30
40
50
60
5
10
15
20
25
30
35
40
Normal
MK50
MK75
MK88
MK94
MK97
MK data
Normal data

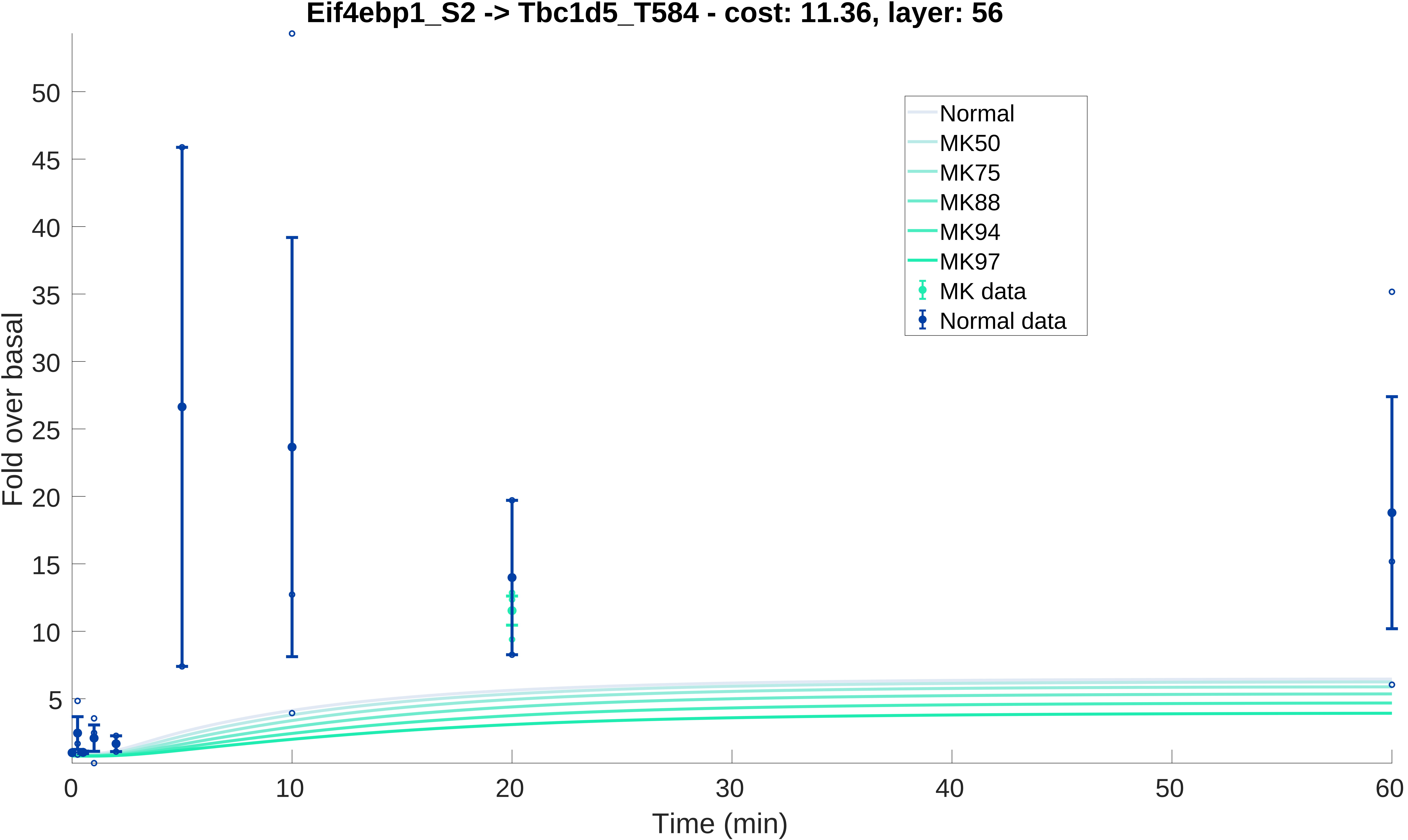

Eif4ebp1_S2 -> Tbc1d5_T584 - cost: 11.36, layer: 56
Fold over basal
Time (min)
0
10
20
30
40
50
60
5
10
15
20
25
30
35
40
45
50
Normal
MK50
MK75
MK88
MK94
MK97
MK data
Normal data

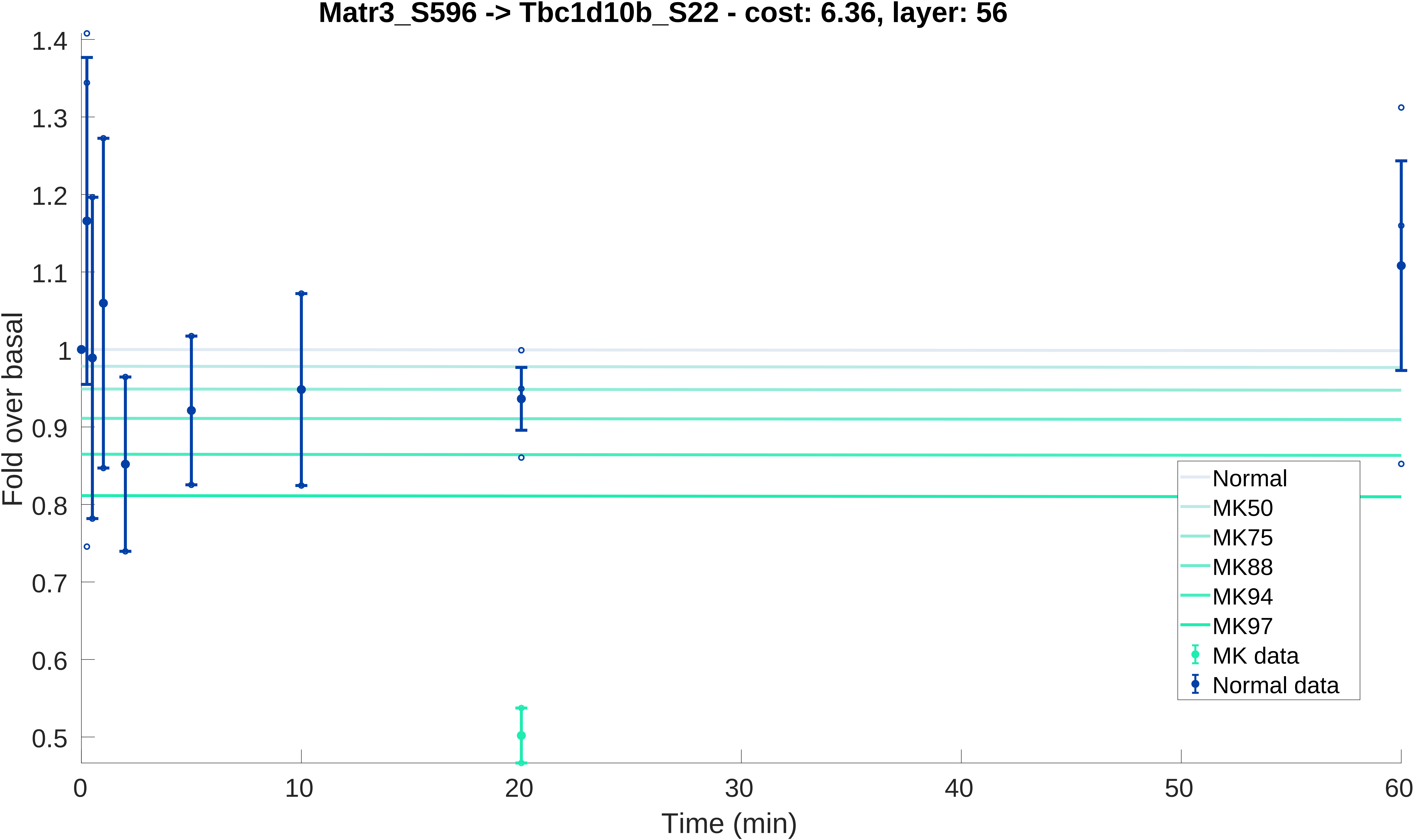

Matr3_S596 -> Tbc1d10b_S22 - cost: 6.36, layer: 56
Fold over basal
Time (min)
1.4
1.3
1.2
1.1
1
0.9
0.8
0.7
0.6
0.5
0
10
20
30
40
50
60
Normal
MK50
MK75
MK88
MK94
MK97
MK data
Normal data

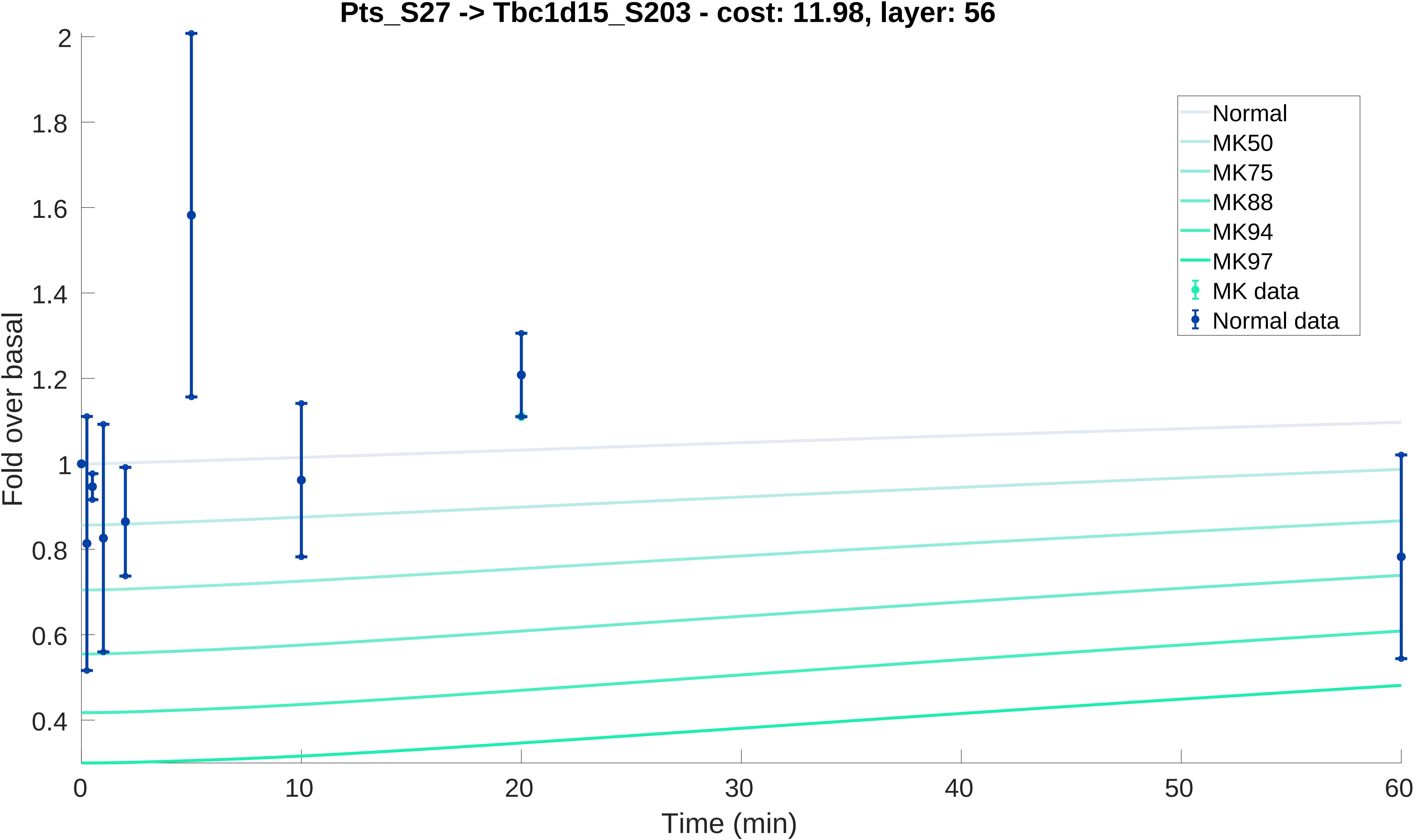

Pts_S27 -> Tbc1d15_S203 - cost: 11.98, layer: 56
Fold over basal
Time (min)
0
10
20
30
40
50
60
0.4
0.6
0.8
1
1.2
1.4
1.6
1.8
2
Normal
MK50
MK75
MK88
MK94
MK97
MK data
Normal data

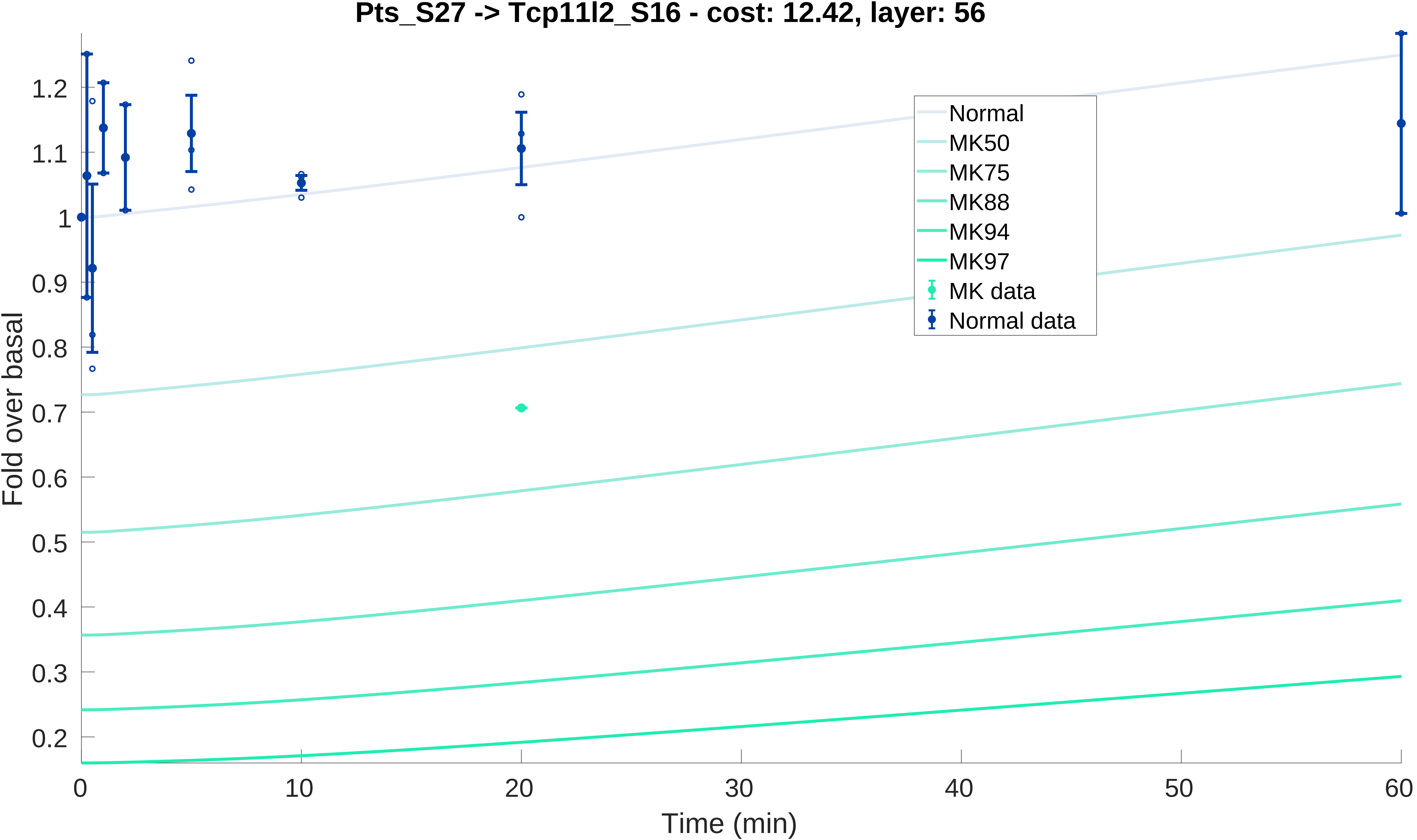

Pts_S27 -> Tcp11l2_S16 - cost: 12.42, layer: 56
Fold over basal
Time (min)
0
10
20
30
40
50
60
0.2
0.3
0.4
0.5
0.6
0.7
0.8
0.9
1
1.1
1.2
Normal
MK50
MK75
MK88
MK94
MK97
MK data
Normal data

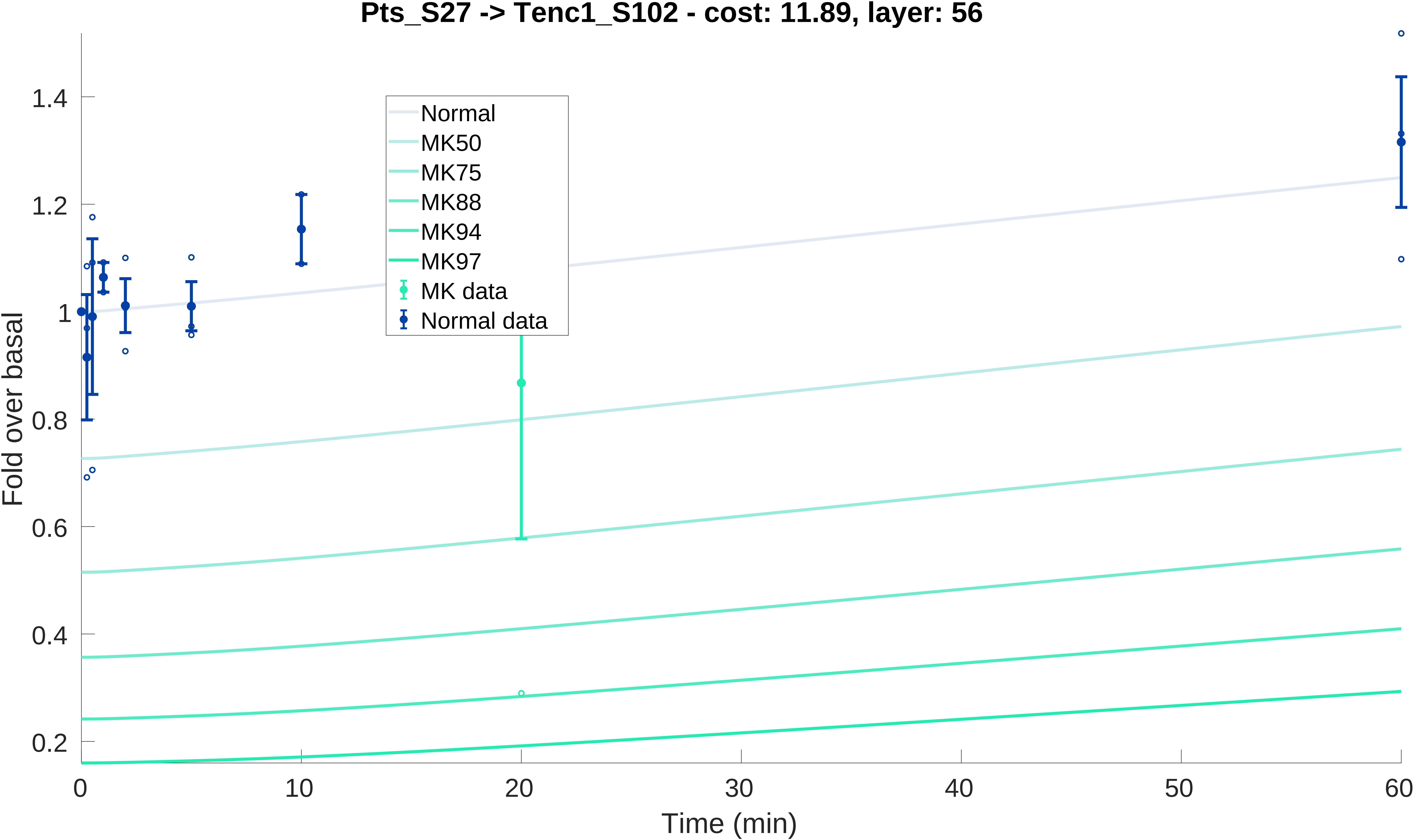

Pts_S27 -> Tenc1_S102 - cost: 11.89, layer: 56
Normal
MK50
MK75
MK88
MK94
MK97
MK data
Normal data
Fold over basal
1.4
1.2
1
0.8
0.6
0.4
0.2
0
10
20
30
40
50
60
Time (min)

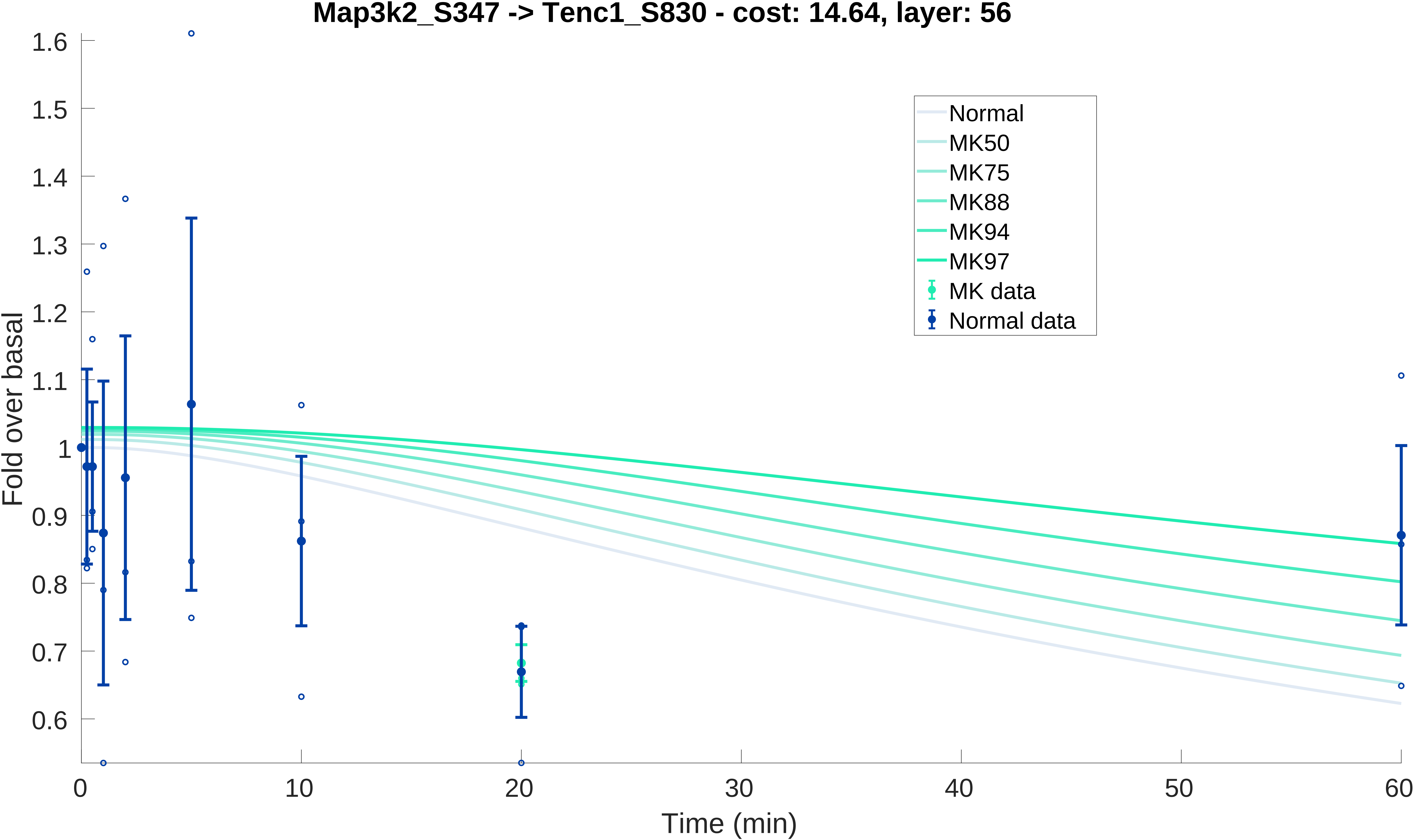
Map3k2_S347 -> Tenc1_S830 - cost: 14.64, layer: 56
Fold over basal
1.6
1.5
1.4
1.3
1.2
1.1
1
0.9
0.8
0.7
0.6
0
10
20
30
40
50
60
Time (min)
Normal
MK50
MK75
MK88
MK94
MK97
MK data
Normal data

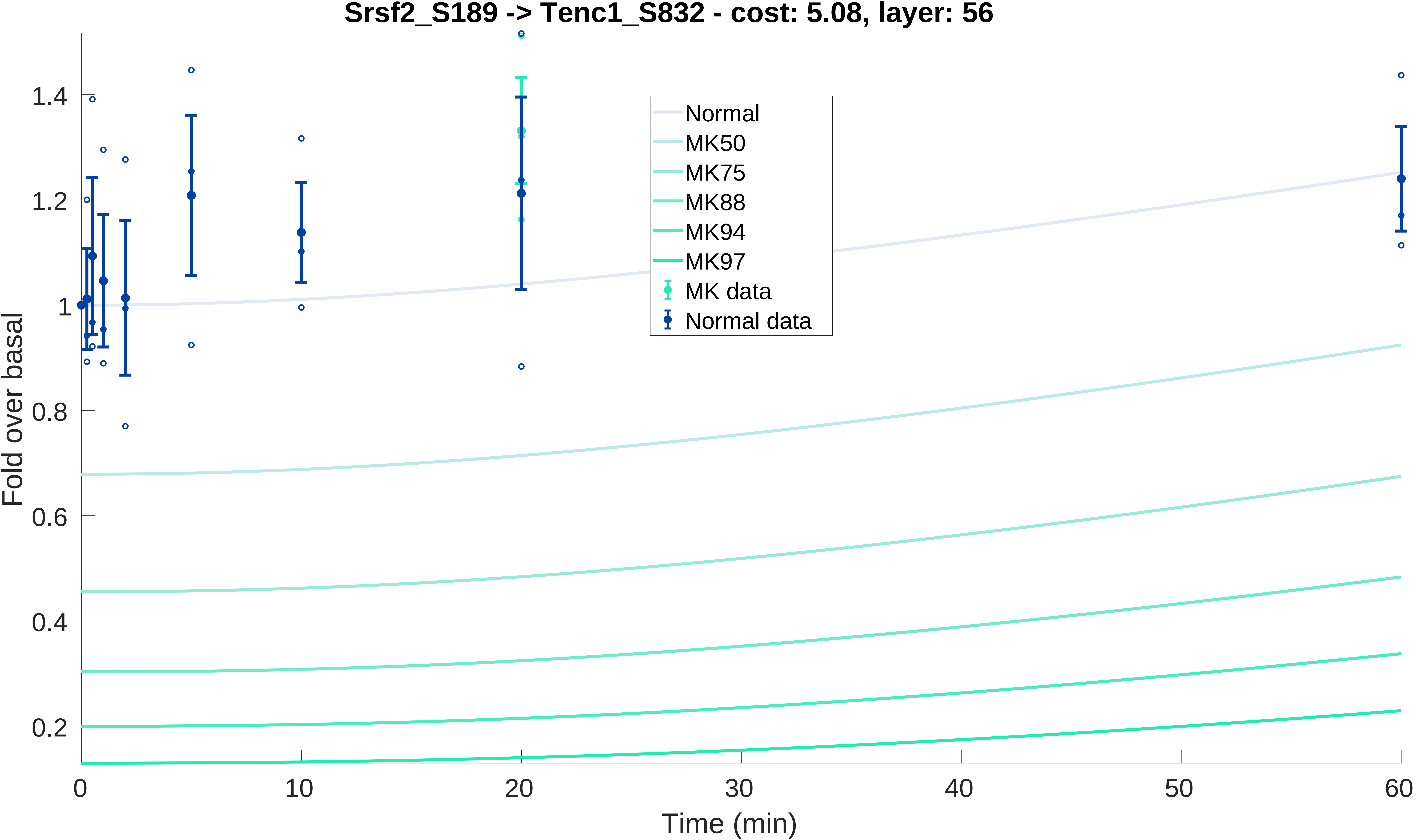
Srsf2_S189 -> Tenc1_S832 - cost: 5.08, layer: 56
Normal
MK50
MK75
MK88
MK94
MK97
MK data
Normal data
Fold over basal
Time (min)
0
10
20
30
40
50
60
0.2
0.4
0.6
0.8
1
1.2
1.4

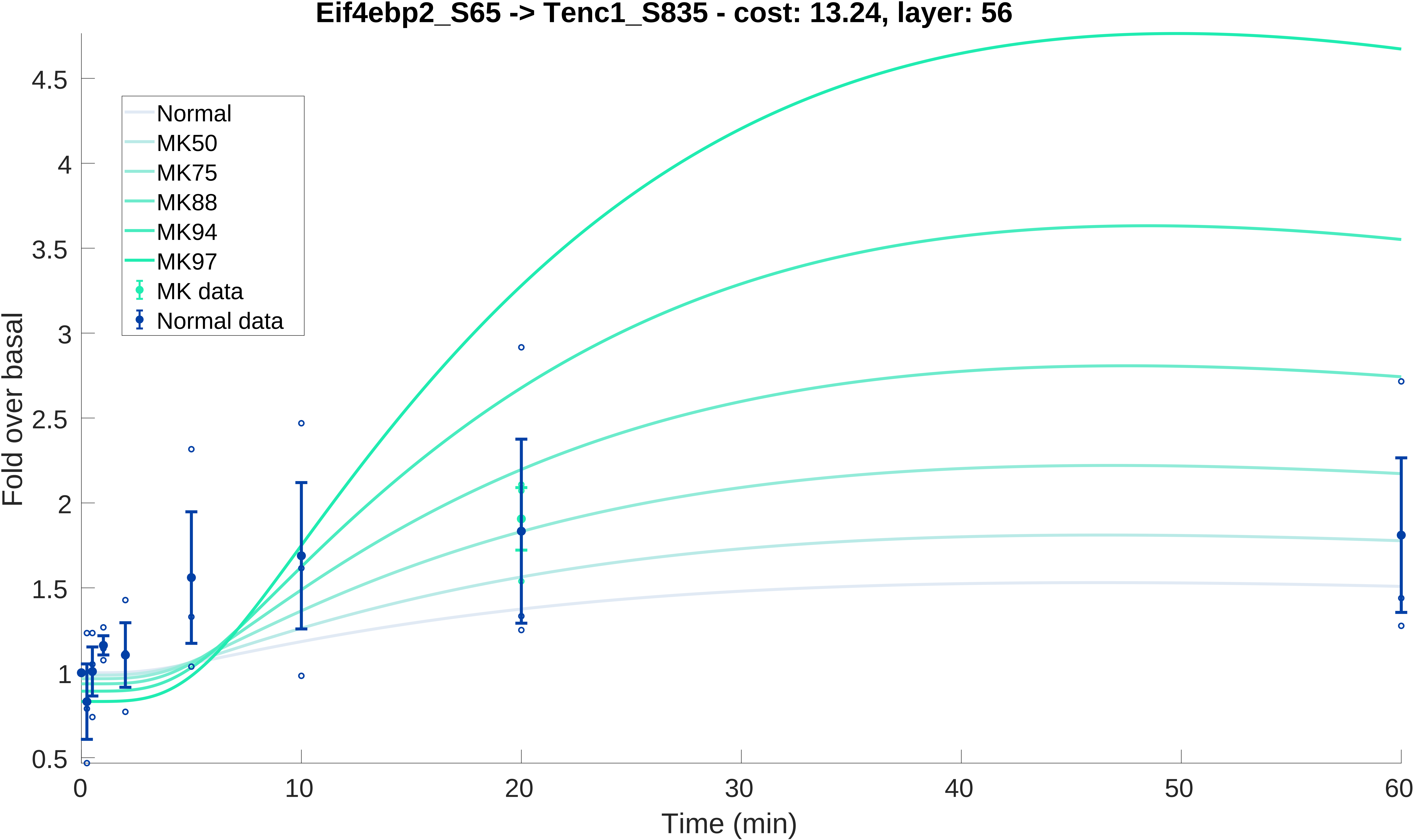

Eif4ebp2_S65 -> Tenc1_S835 - cost: 13.24, layer: 56
Normal
MK50
MK75
MK88
MK94
MK97
MK data
Normal data
Fold over basal
Time (min)
0.5
1
1.5
2
2.5
3
3.5
4
4.5
0
10
20
30
40
50
60

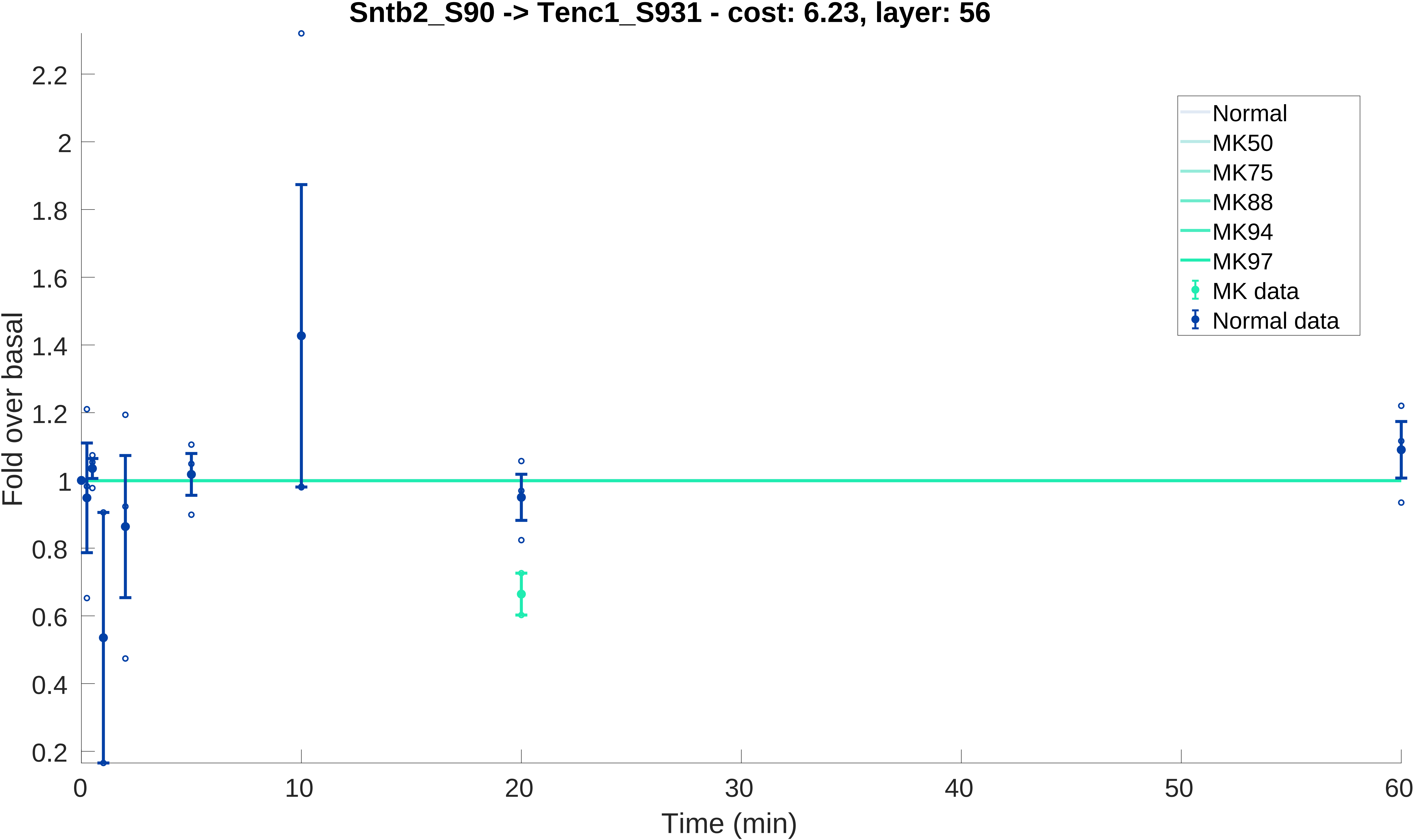
Sntb2_S90 -> Tenc1_S931 - cost: 6.23, layer: 56
Fold over basal
Time (min)
2.2
2
1.8
1.6
1.4
1.2
1
0.8
0.6
0.4
0.2
0
10
20
30
40
50
60
Normal
MK50
MK75
MK88
MK94
MK97
MK data
Normal data

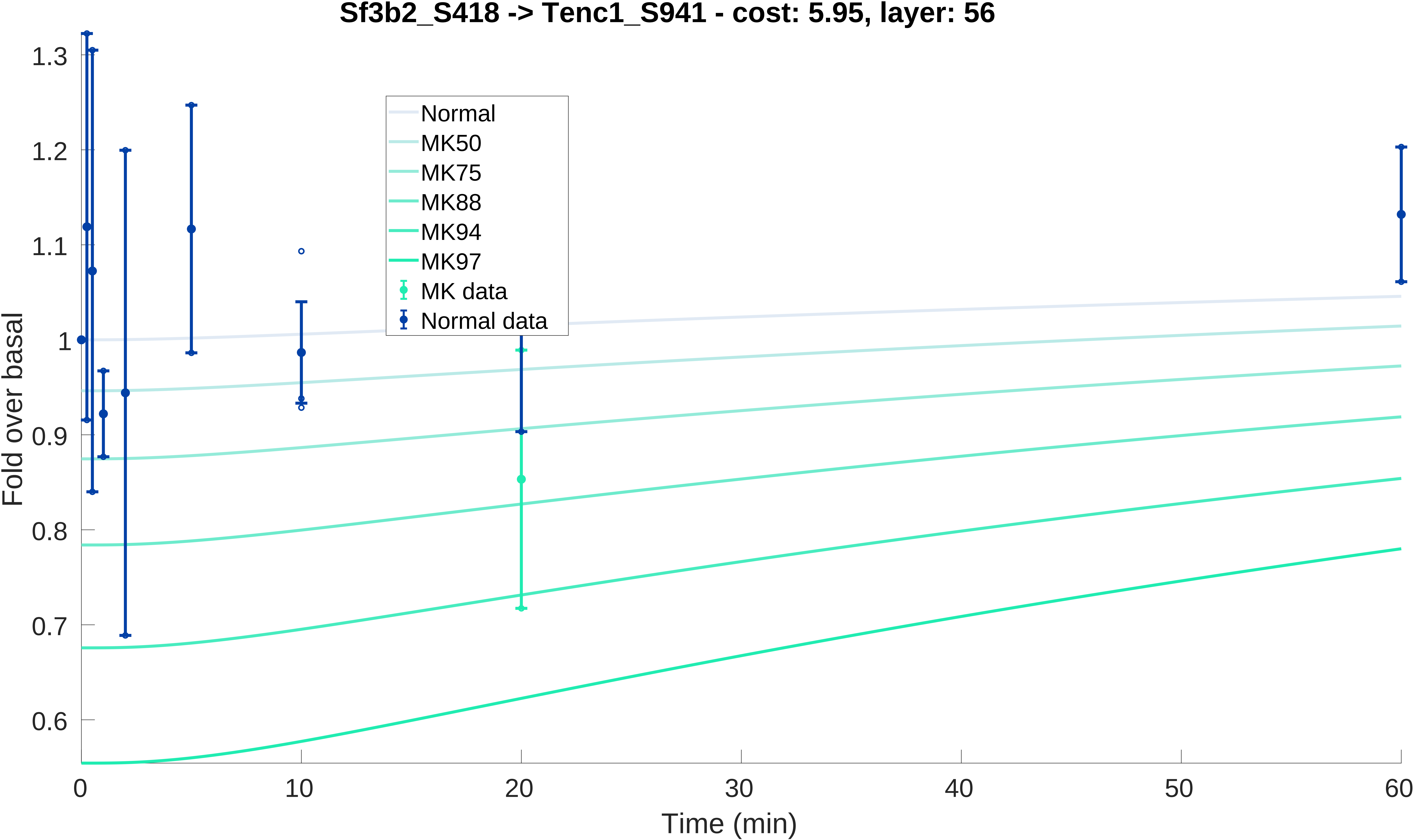

Sf3b2_S418 -> Tenc1_S941 - cost: 5.95, layer: 56
Normal
MK50
MK75
MK88
MK94
MK97
MK data
Normal data
Fold over basal
Time (min)
1.3
1.2
1.1
1
0.9
0.8
0.7
0.6
0
10
20
30
40
50
60

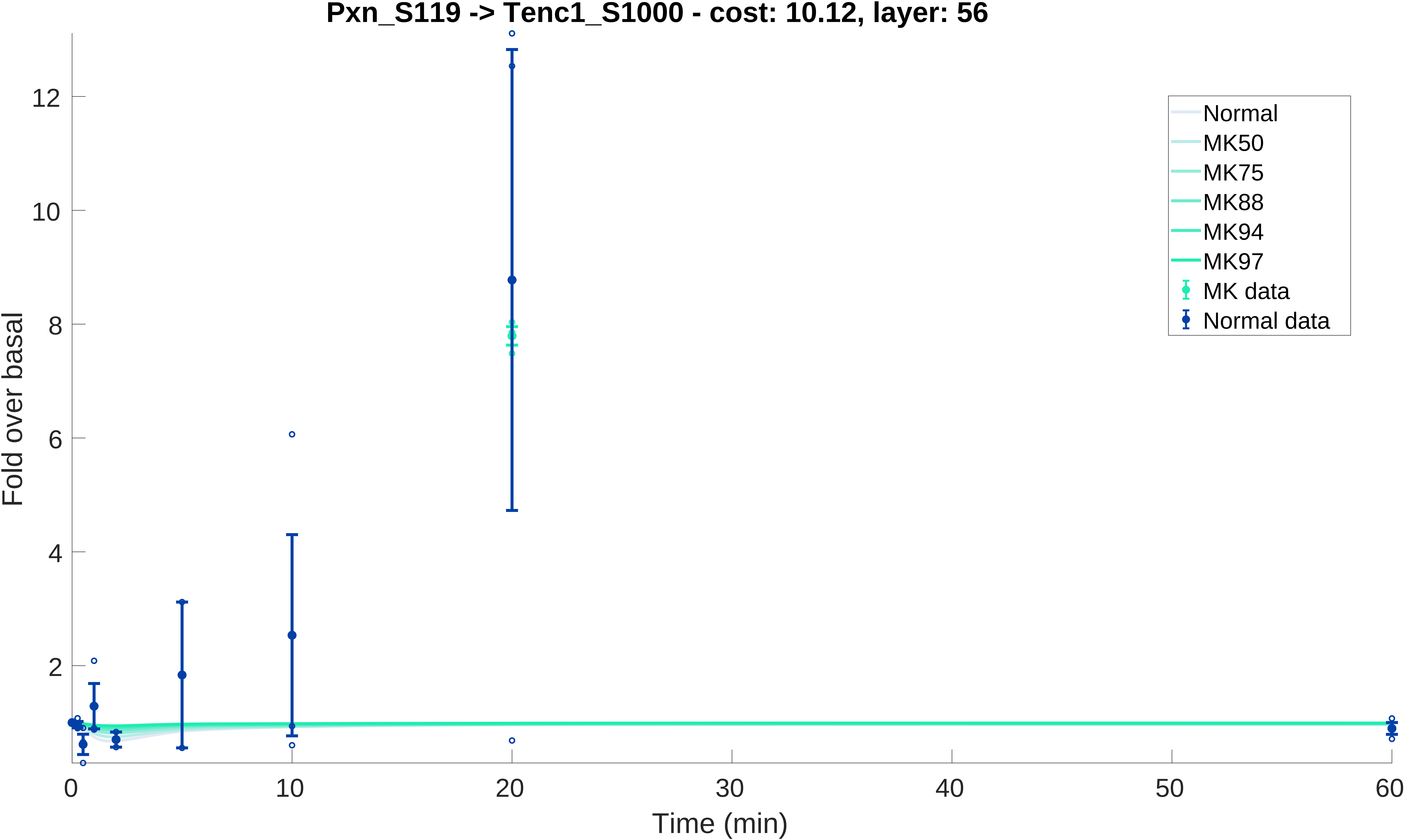

Pxn_S119 -> Tenc1_S1000 - cost: 10.12, layer: 56
Fold over basal
Time (min)
0
10
20
30
40
50
60
2
4
6
8
10
12
Normal
MK50
MK75
MK88
MK94
MK97
MK data
Normal data

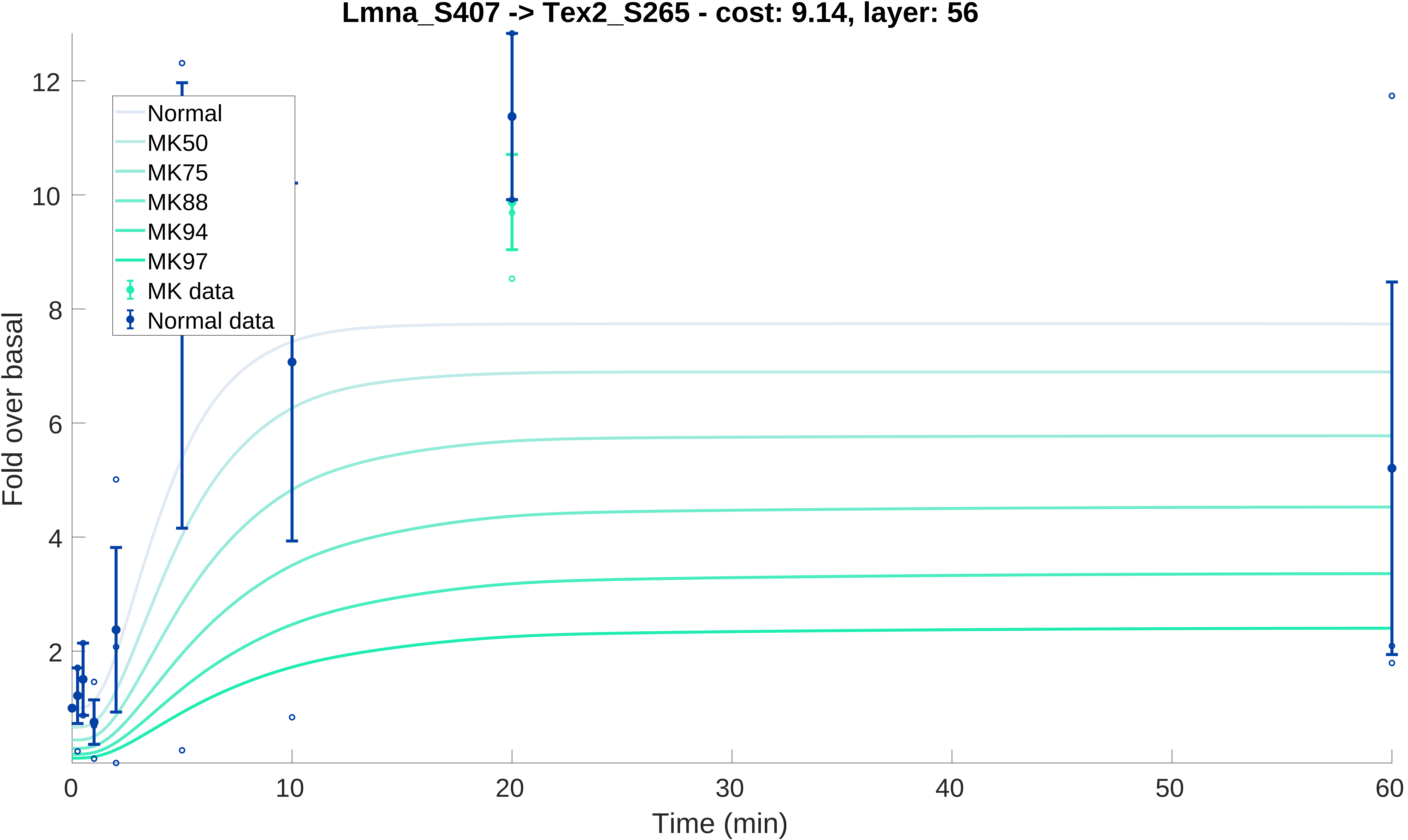

Lmna_S407 -> Tex2_S265 - cost: 9.14, layer: 56
Normal
MK50
MK75
MK88
MK94
MK97
MK data
Normal data
Fold over basal
Time (min)
0
10
20
30
40
50
60
2
4
6
8
10
12

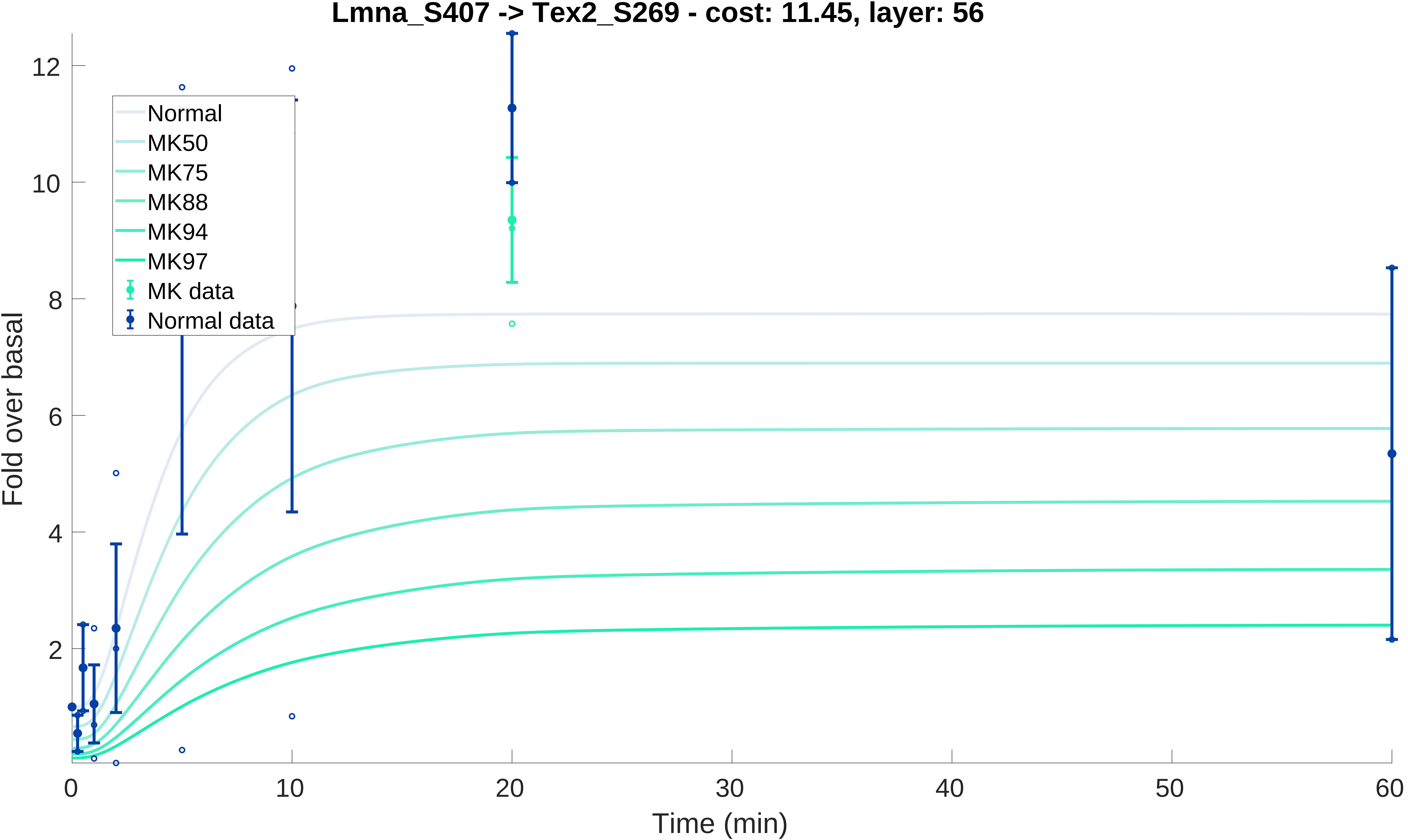
Lmna_S407 -> Tex2_S269 - cost: 11.45, layer: 56
Fold over basal
Time (min)
Normal
MK50
MK75
MK88
MK94
MK97
MK data
Normal data
0
10
20
30
40
50
60
2
4
6
8
10
12

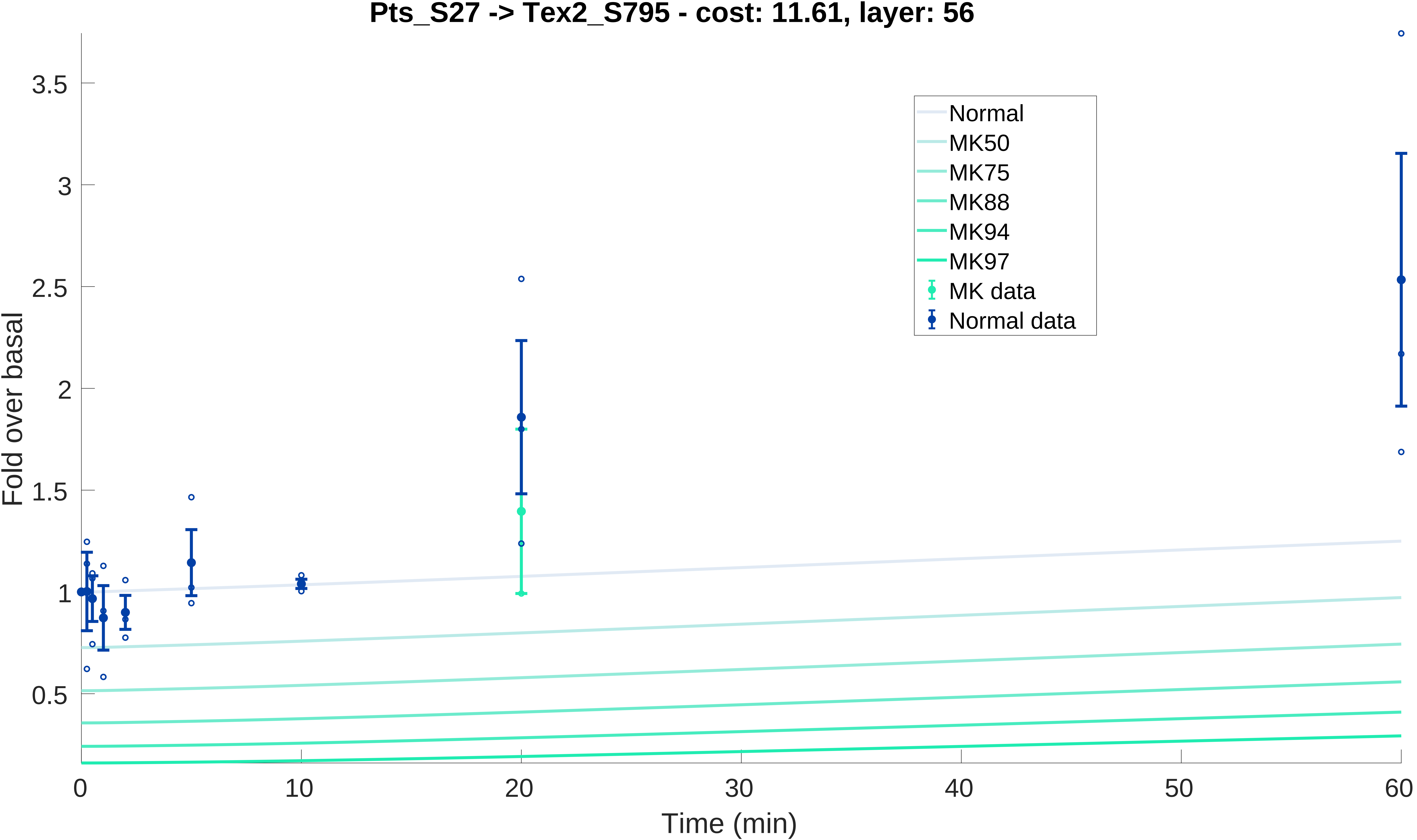

Pts_S27 -> Tex2_S795 - cost: 11.61, layer: 56
Fold over basal
Time (min)
3.5
3
2.5
2
1.5
1
0.5
0
10
20
30
40
50
60
Normal
MK50
MK75
MK88
MK94
MK97
MK data
Normal data

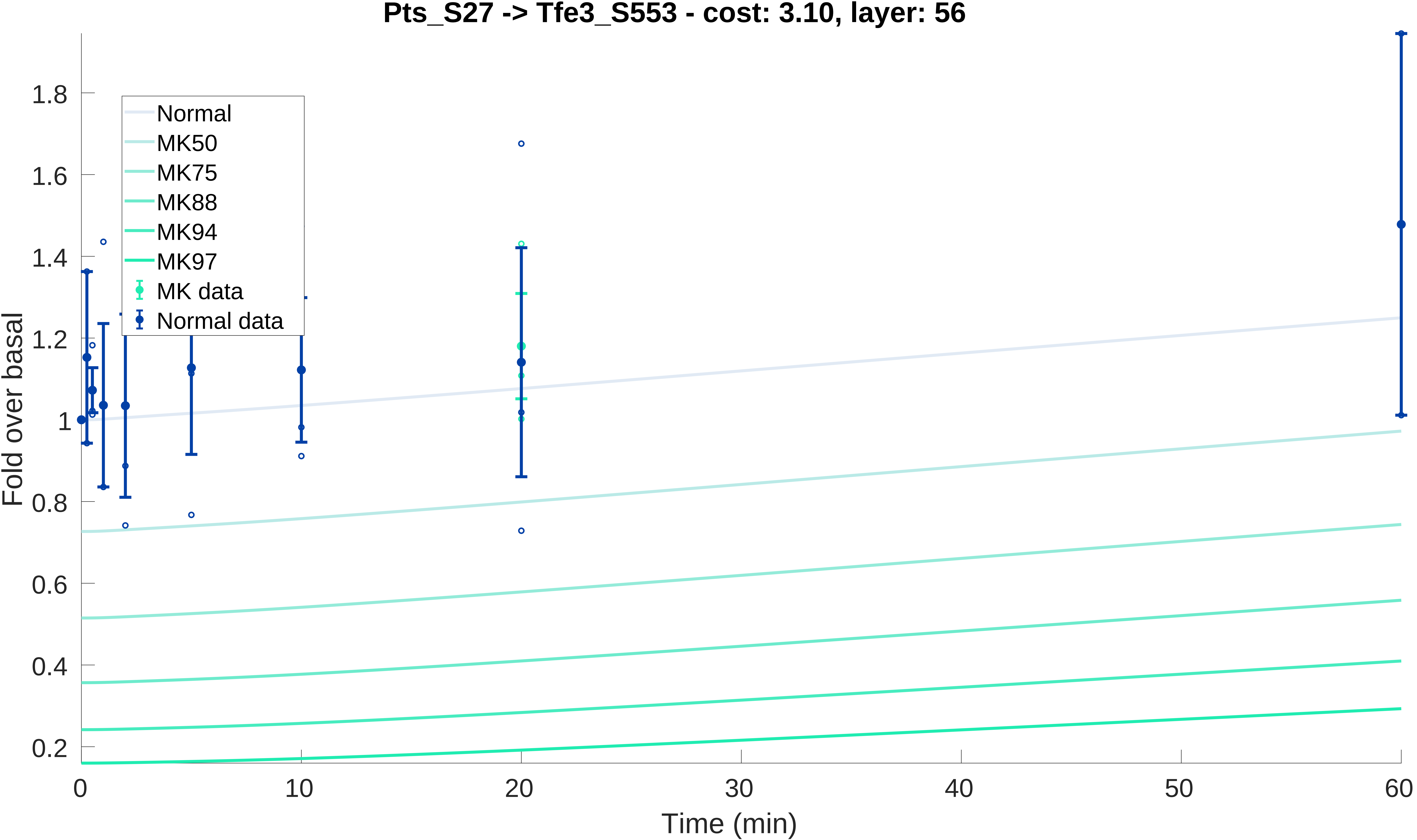

Pts_S27 -> Tfe3_S553 - cost: 3.10, layer: 56
Fold over basal
1.8
1.6
1.4
1.2
1
0.8
0.6
0.4
0.2
0
10
20
30
40
50
60
Time (min)
Normal
MK50
MK75
MK88
MK94
MK97
MK data
Normal data

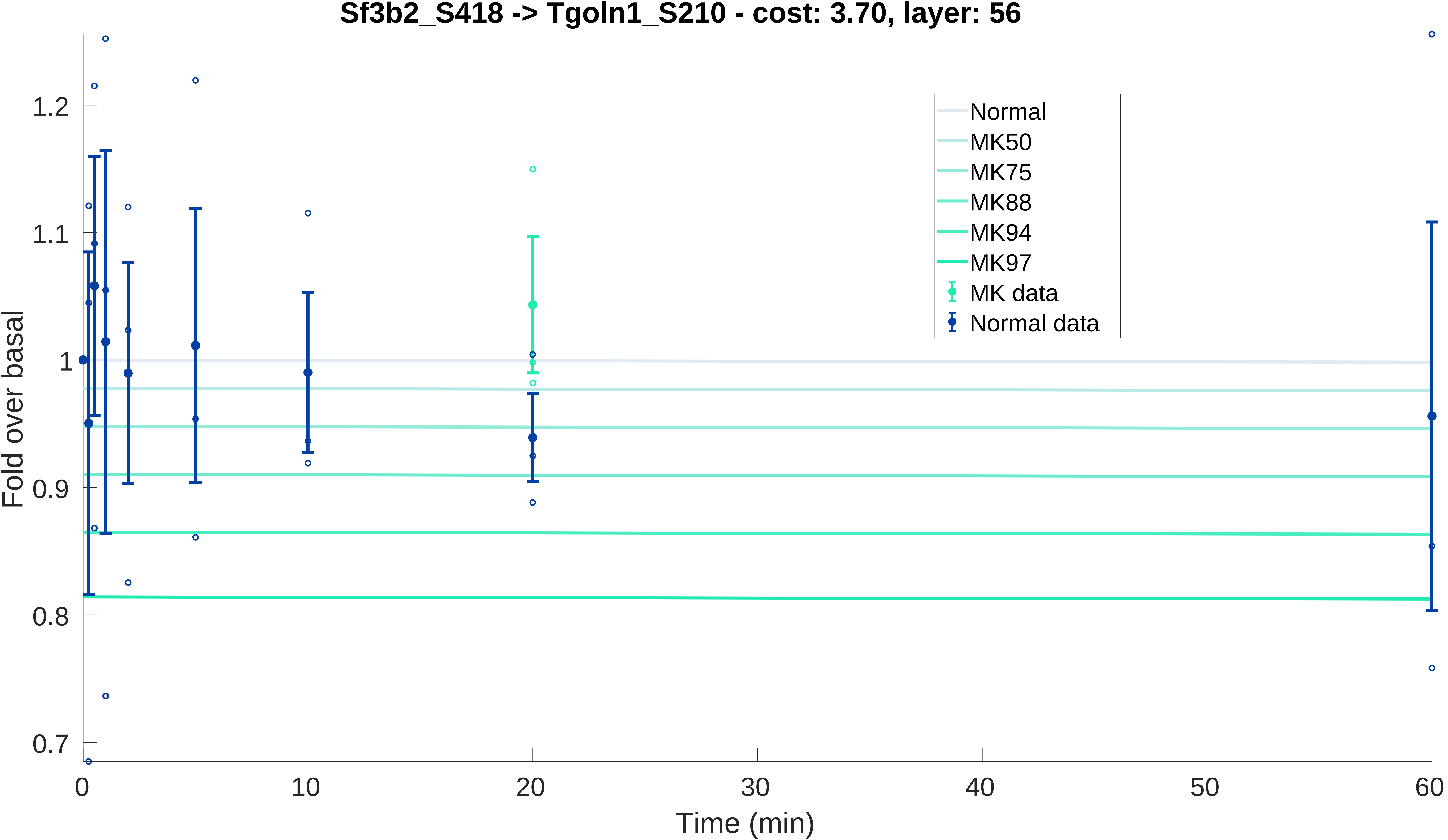

Sf3b2_S418 -> Tgoln1_S210 - cost: 3.70, layer: 56
Normal
MK50
MK75
MK88
MK94
MK97
MK data
Normal data
Fold over basal
1.2
1.1
1
0.9
0.8
0.7
0
10
20
30
40
50
60
Time (min)

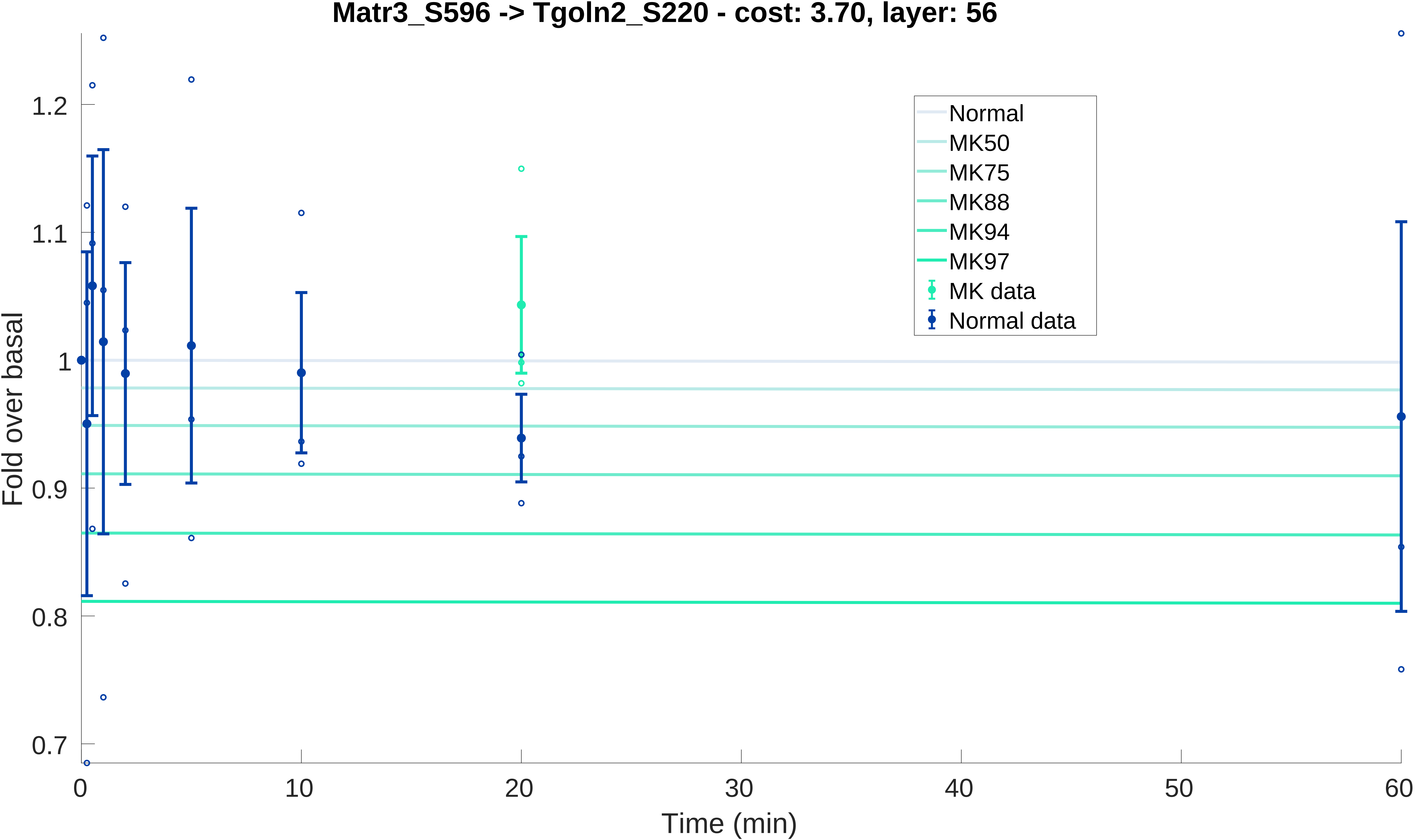
Matr3_S596 -> Tgoln2_S220 - cost: 3.70, layer: 56
Fold over basal
Time (min)
1.2
1.1
1
0.9
0.8
0.7
0
10
20
30
40
50
60
Normal
MK50
MK75
MK88
MK94
MK97
MK data
Normal data

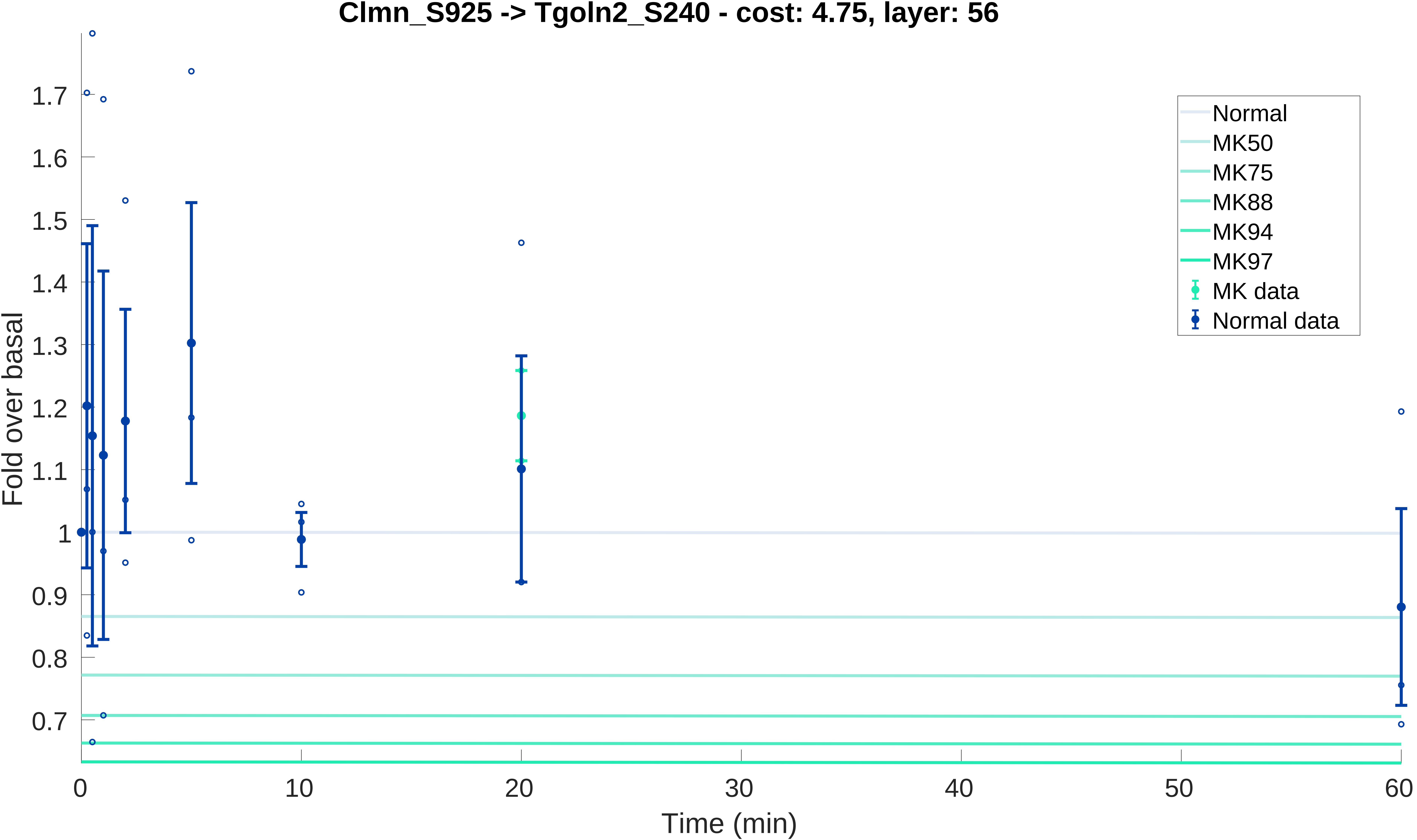

Clmn_S925 -> Tgoln2_S240 - cost: 4.75, layer: 56
Fold over basal
Time (min)
Normal
MK50
MK75
MK88
MK94
MK97
MK data
Normal data
1.7
1.6
1.5
1.4
1.3
1.2
1.1
1
0.9
0.8
0.7
0
10
20
30
40
50
60

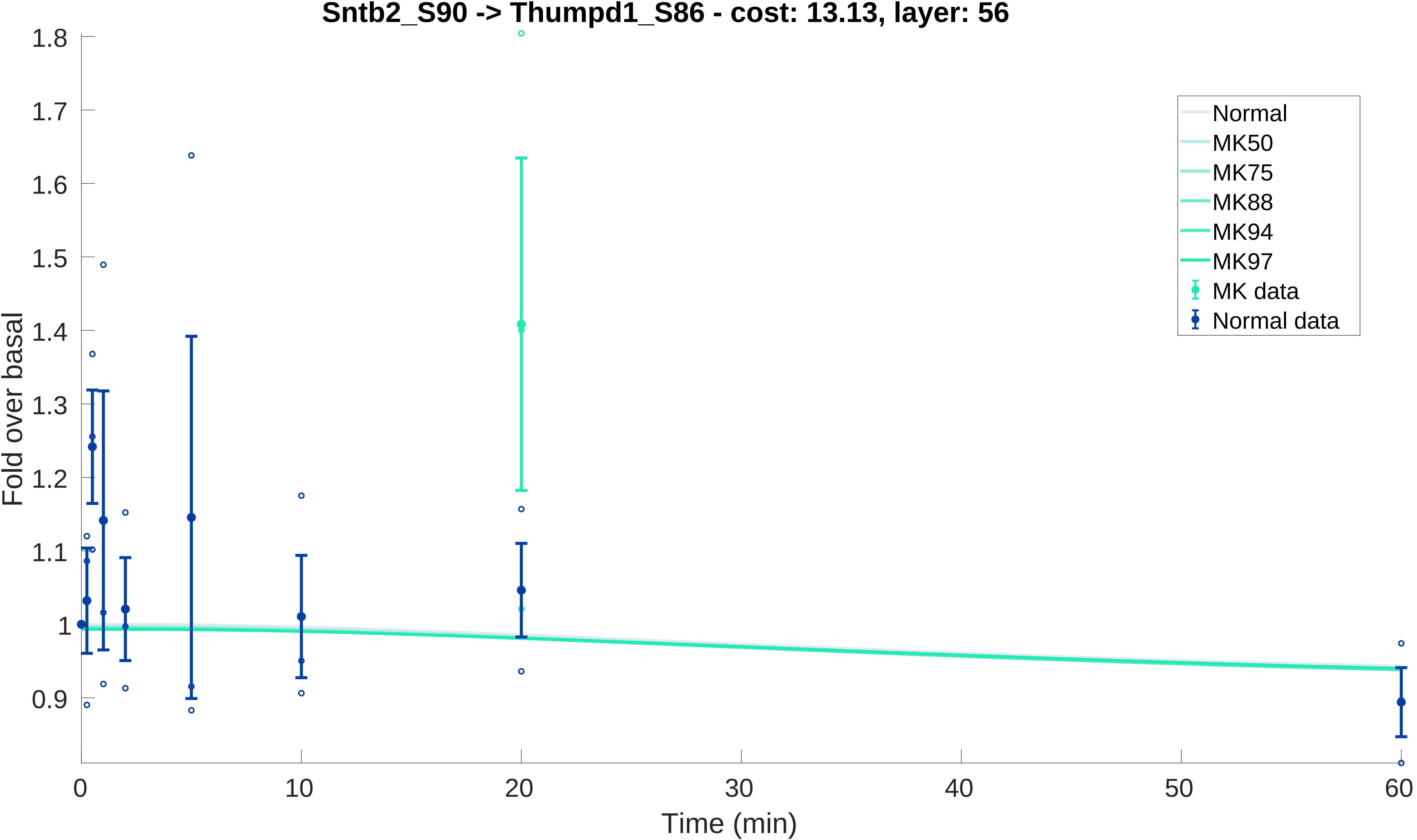
Sntb2_S90 -> Thumpd1_S86 - cost: 13.13, layer: 56
Fold over basal
1.8
1.7
1.6
1.5
1.4
1.3
1.2
1.1
1
0.9
0
10
20
30
40
50
60
Time (min)
Normal
MK50
MK75
MK88
MK94
MK97
MK data
Normal data

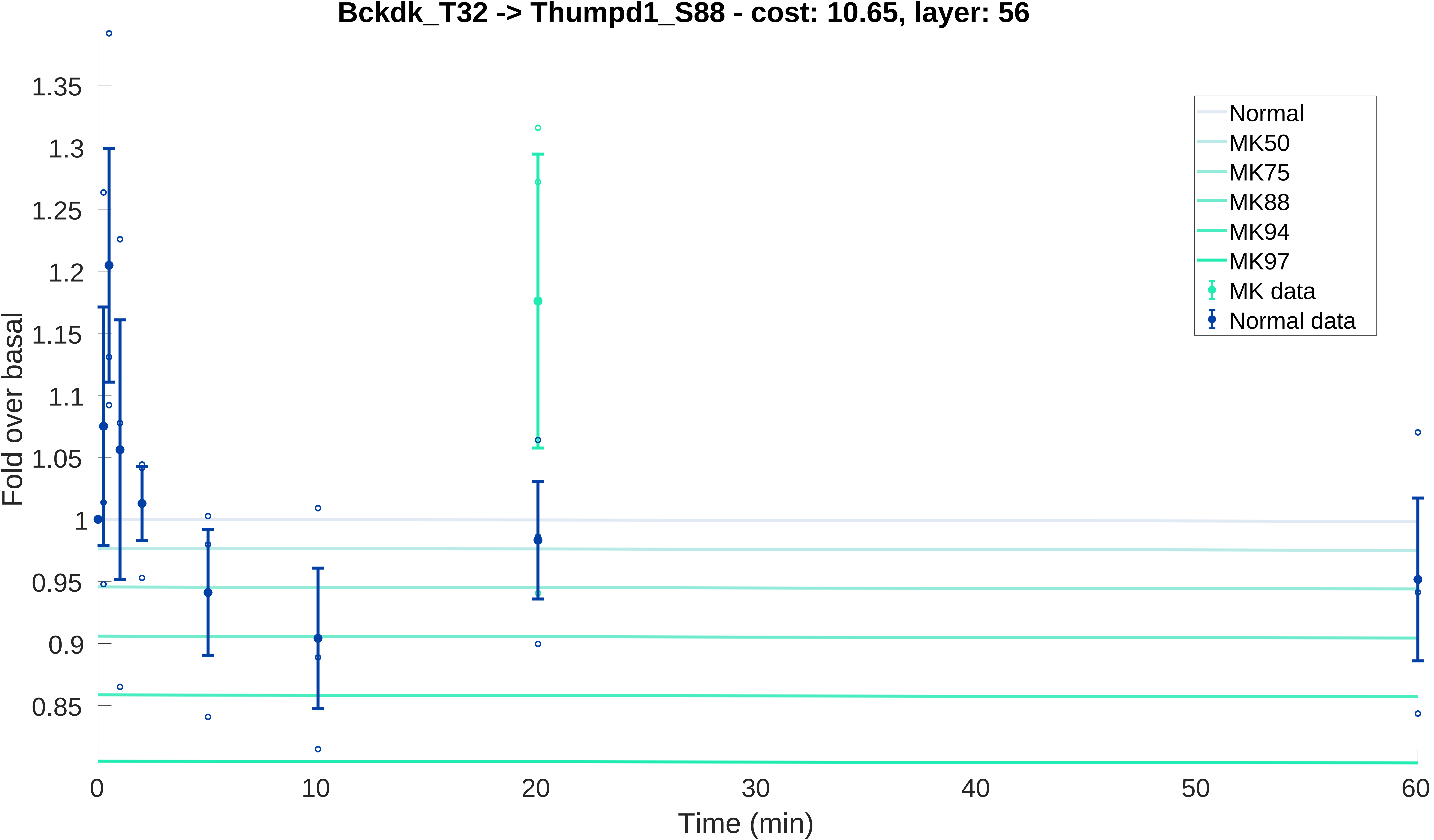
Bckdk_T32 -> Thumpd1_S88 - cost: 10.65, layer: 56
Fold over basal
Time (min)
1.35
1.3
1.25
1.2
1.15
1.1
1.05
1
0.95
0.9
0.85
0
10
20
30
40
50
60
Normal
MK50
MK75
MK88
MK94
MK97
MK data
Normal data

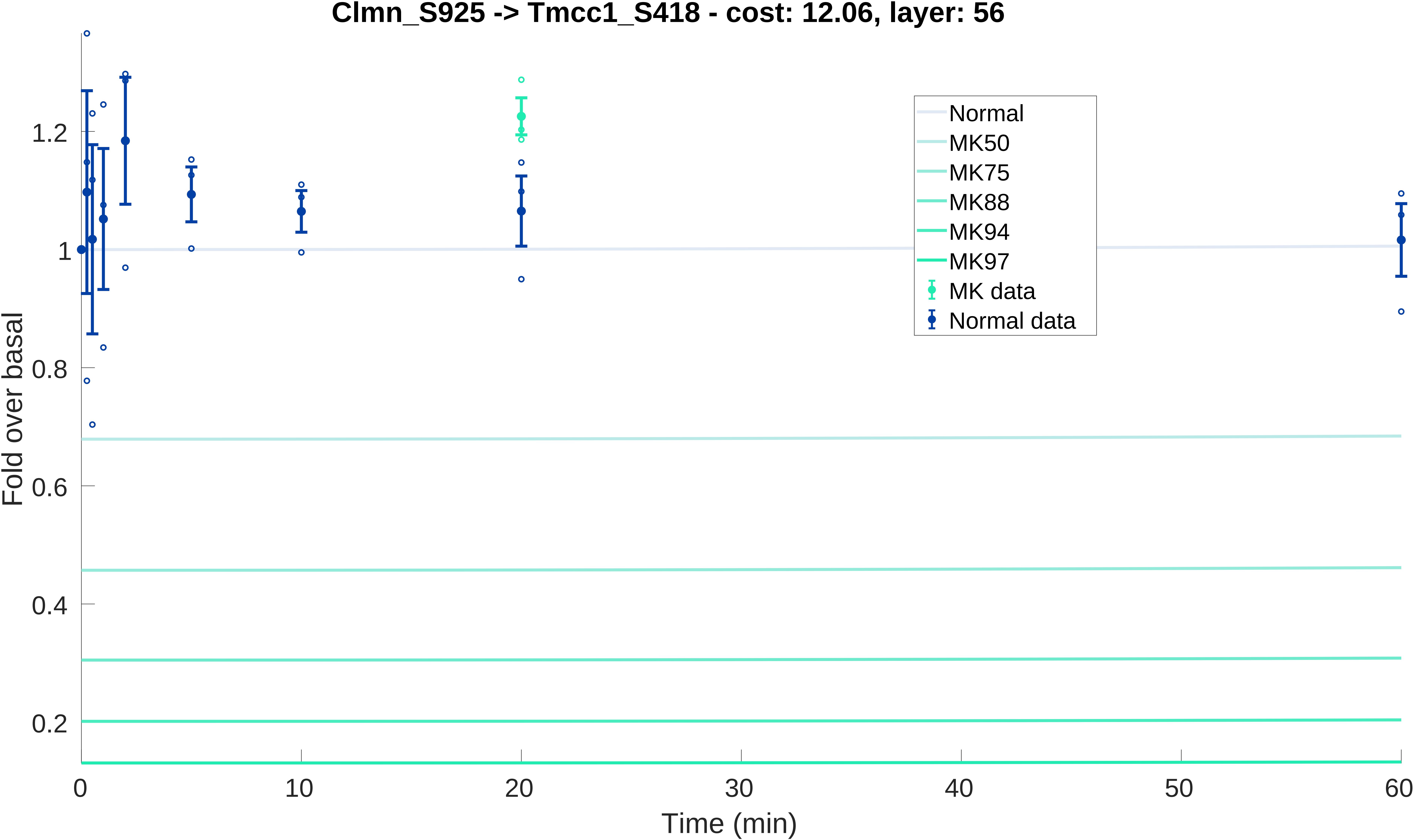

Clmn_S925 -> Tmcc1_S418 - cost: 12.06, layer: 56
Fold over basal
Time (min)
Normal
MK50
MK75
MK88
MK94
MK97
MK data
Normal data
0
10
20
30
40
50
60
0.2
0.4
0.6
0.8
1
1.2

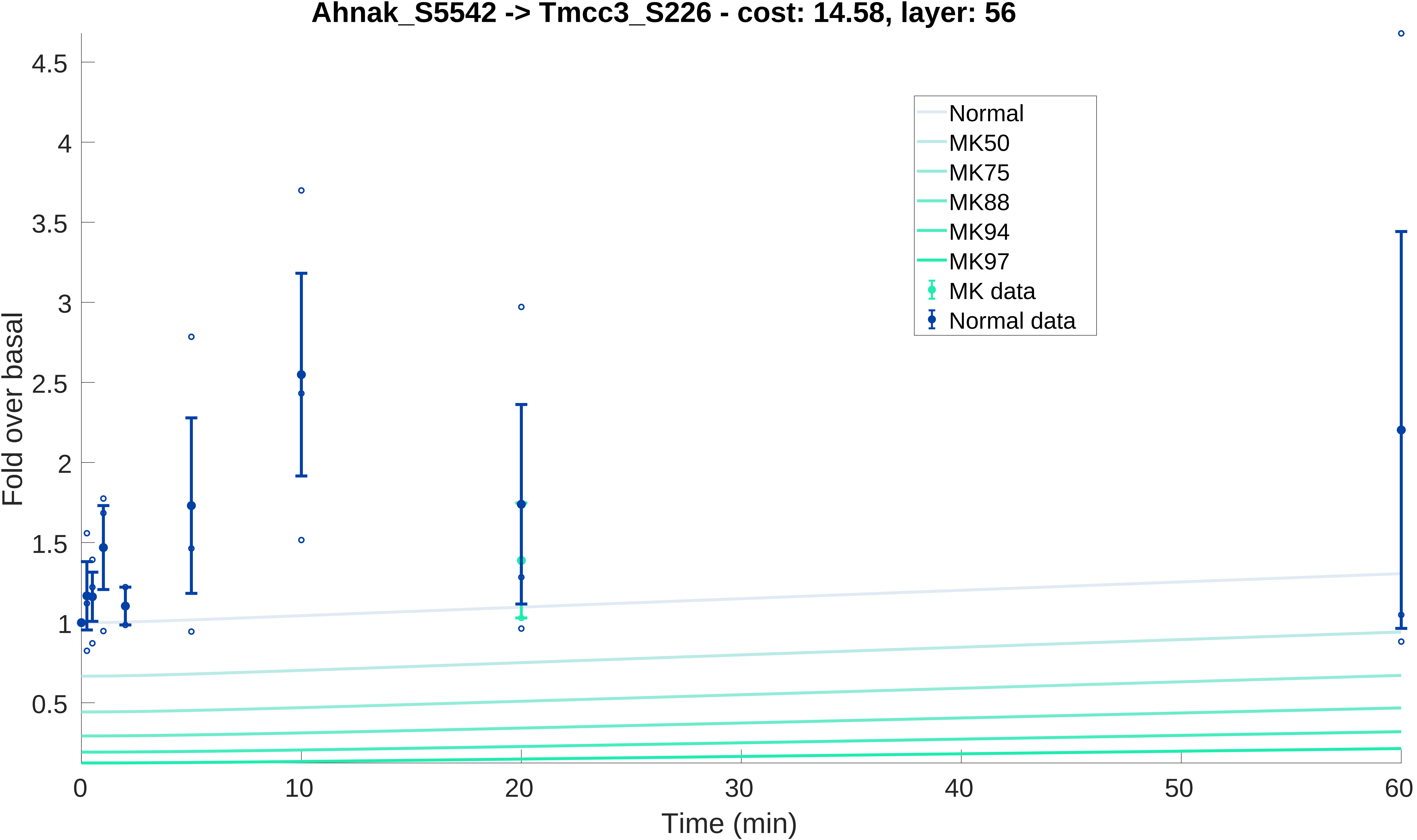

Ahnak_S5542 -> Tmcc3_S226 - cost: 14.58, layer: 56
Fold over basal
Time (min)
0
10
20
30
40
50
60
0.5
1
1.5
2
2.5
3
3.5
4
4.5
Normal
MK50
MK75
MK88
MK94
MK97
MK data
Normal data

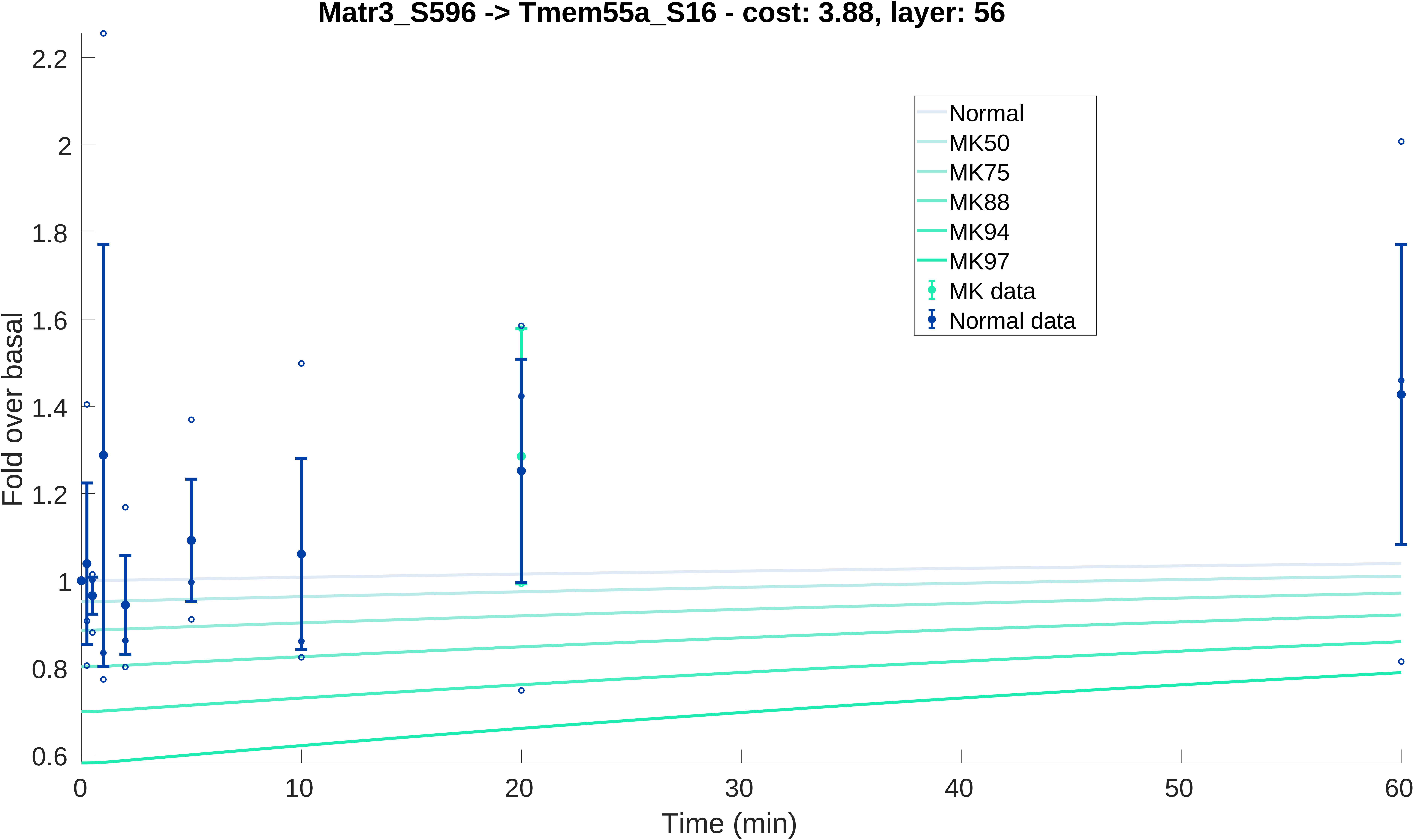

Matr3_S596 -> Tmem55a_S16 - cost: 3.88, layer: 56
Fold over basal
Time (min)
Normal
MK50
MK75
MK88
MK94
MK97
MK data
Normal data
2.2
2
1.8
1.6
1.4
1.2
1
0.8
0.6
0
10
20
30
40
50
60

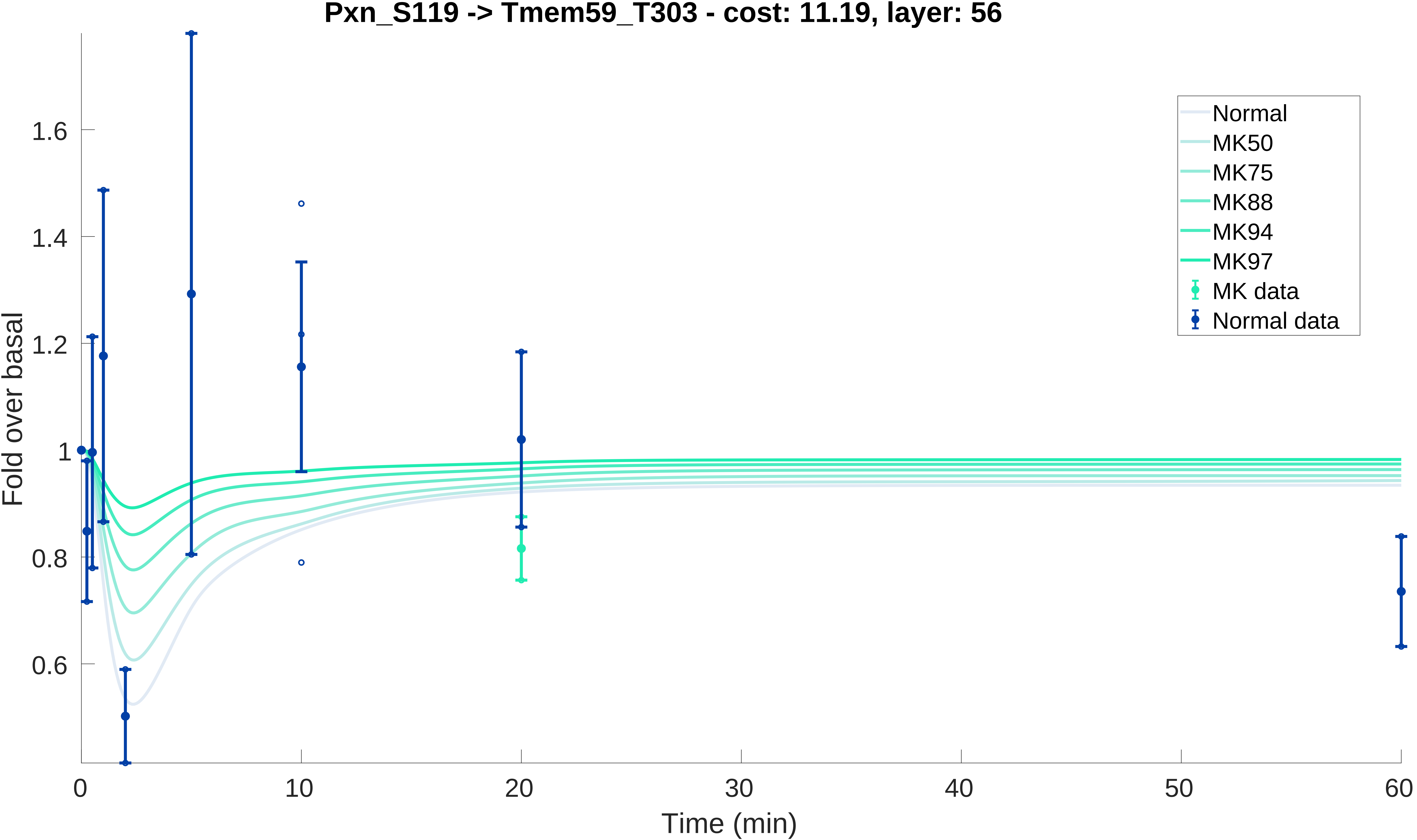

Pxn_S119 -> Tmem59_T303 - cost: 11.19, layer: 56
Fold over basal
Time (min)
0.6
0.8
1
1.2
1.4
1.6
0
10
20
30
40
50
60
Normal
MK50
MK75
MK88
MK94
MK97
MK data
Normal data

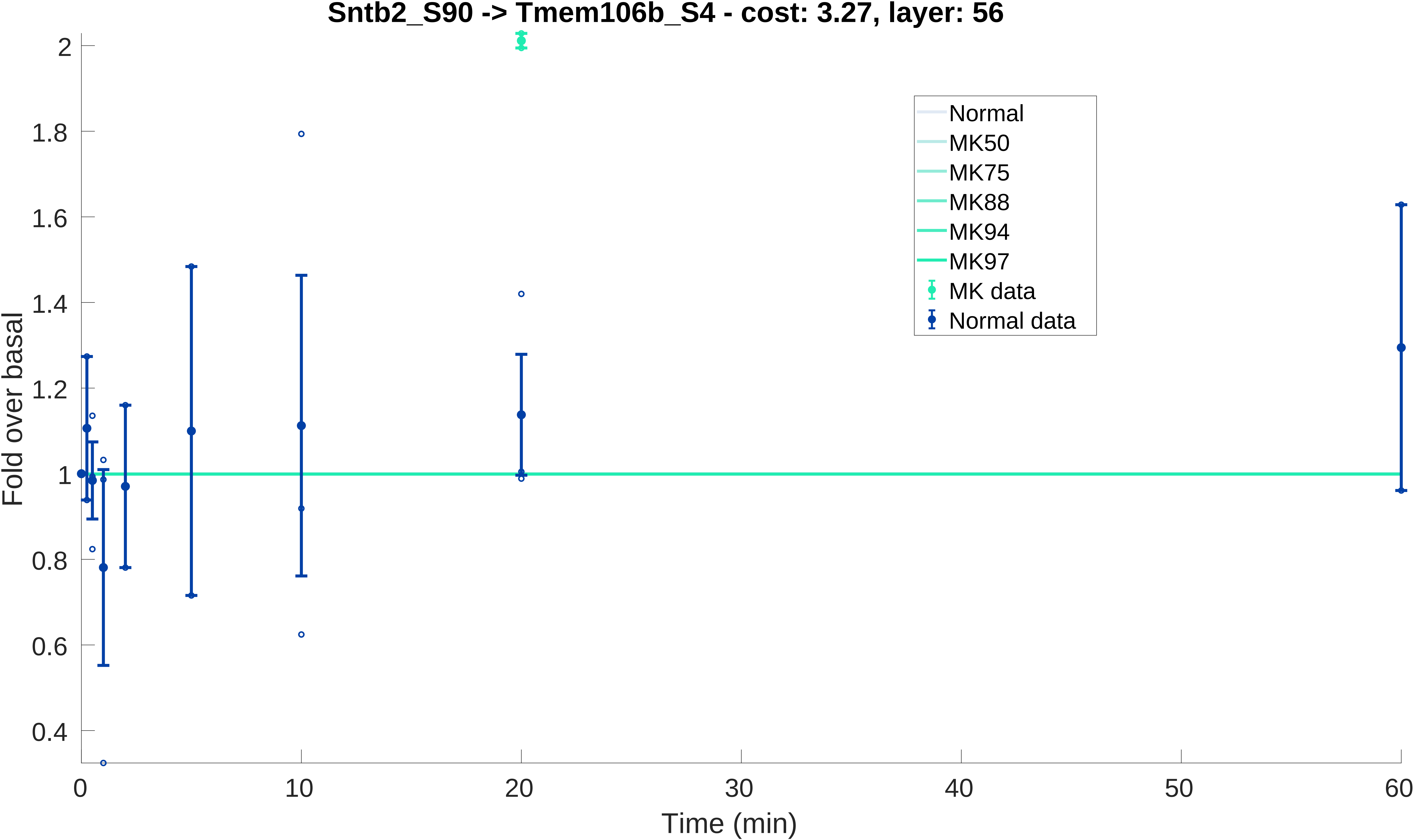
Sntb2_S90 -> Tmem106b_S4 - cost: 3.27, layer: 56
Fold over basal
Time (min)
Normal
MK50
MK75
MK88
MK94
MK97
MK data
Normal data
2
1.8
1.6
1.4
1.2
1
0.8
0.6
0.4
0
10
20
30
40
50
60

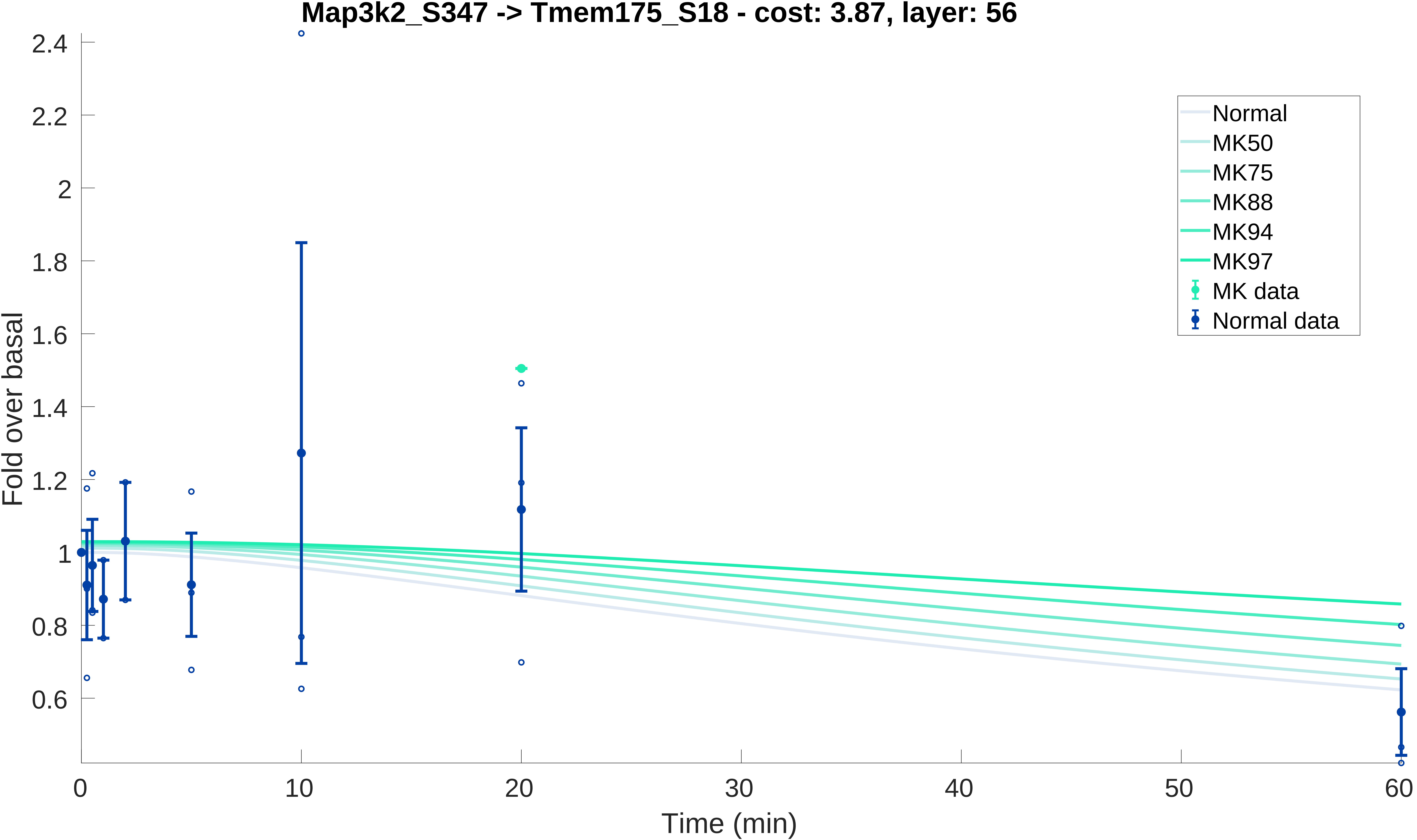
Map3k2_S347 -> Tmem175_S18 - cost: 3.87, layer: 56
Fold over basal
2.4
2.2
2
1.8
1.6
1.4
1.2
1
0.8
0.6
0
10
20
30
40
50
60
Time (min)
Normal
MK50
MK75
MK88
MK94
MK97
MK data
Normal data

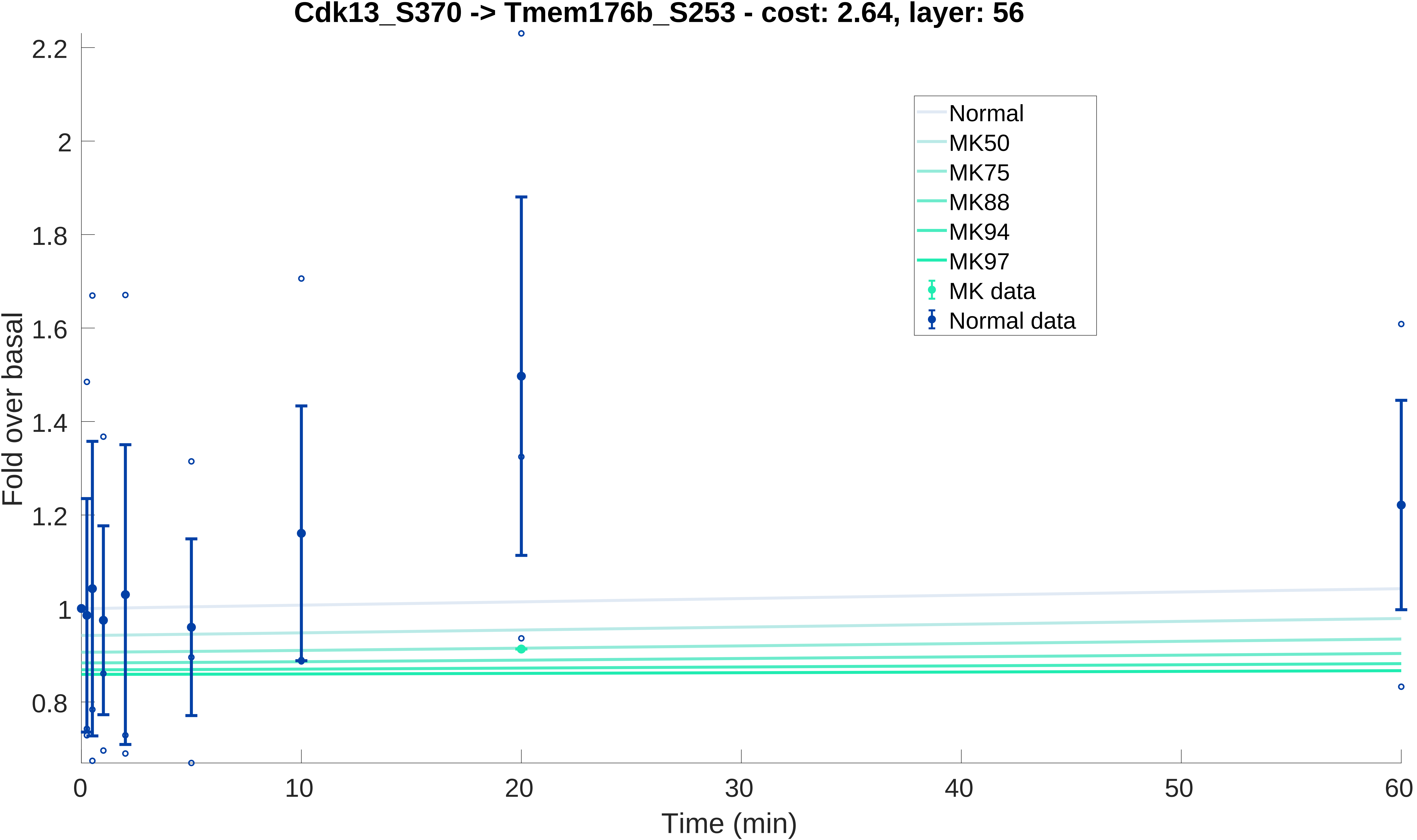

Cdk13_S370 -> Tmem176b_S253 - cost: 2.64, layer: 56
Fold over basal
Time (min)
Normal
MK50
MK75
MK88
MK94
MK97
MK data
Normal data
2.2
2
1.8
1.6
1.4
1.2
1
0.8
0
10
20
30
40
50
60

Ahnak_S1321 -> Tmem184b_S384 - cost: 7.26, layer: 56

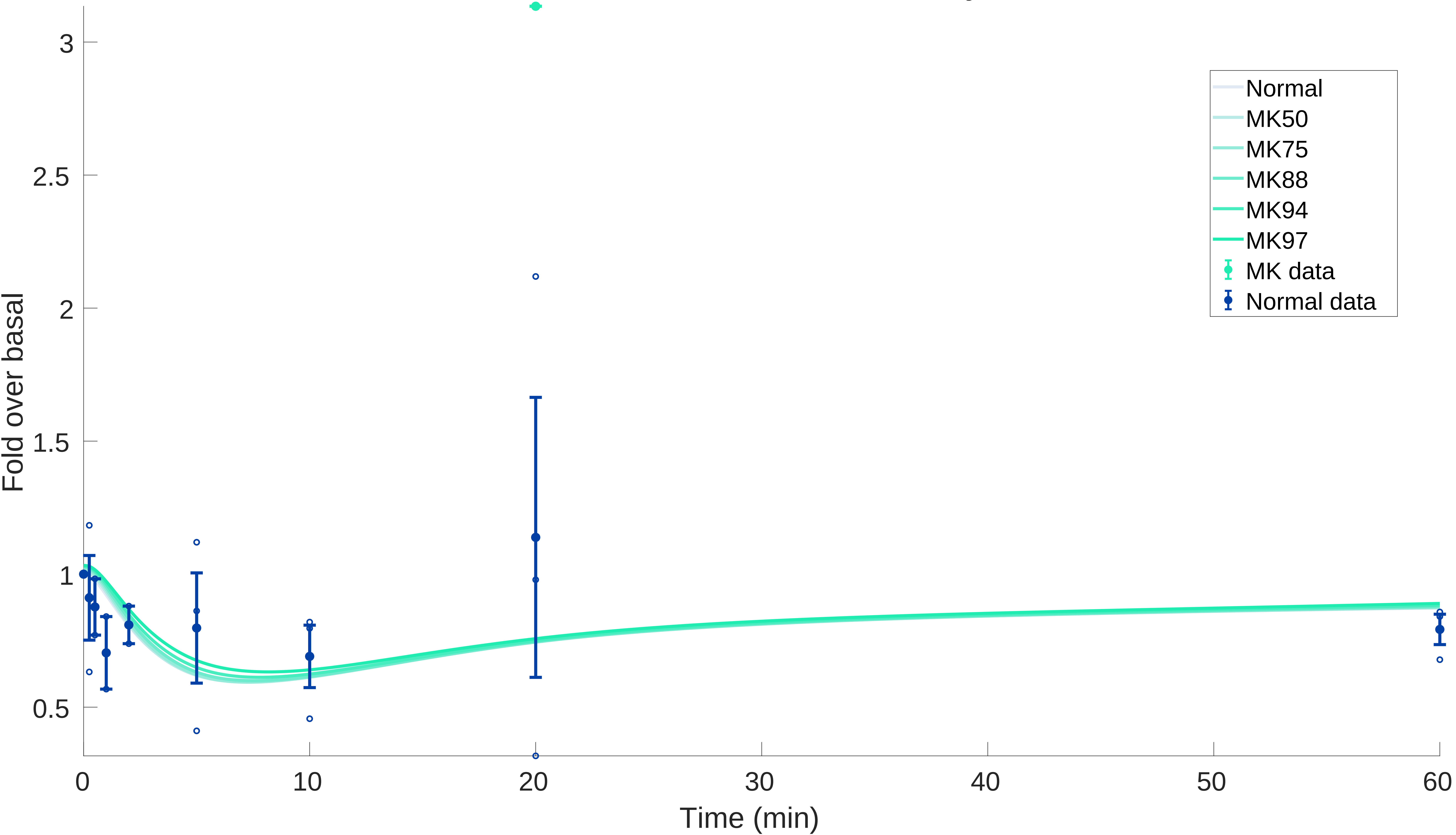

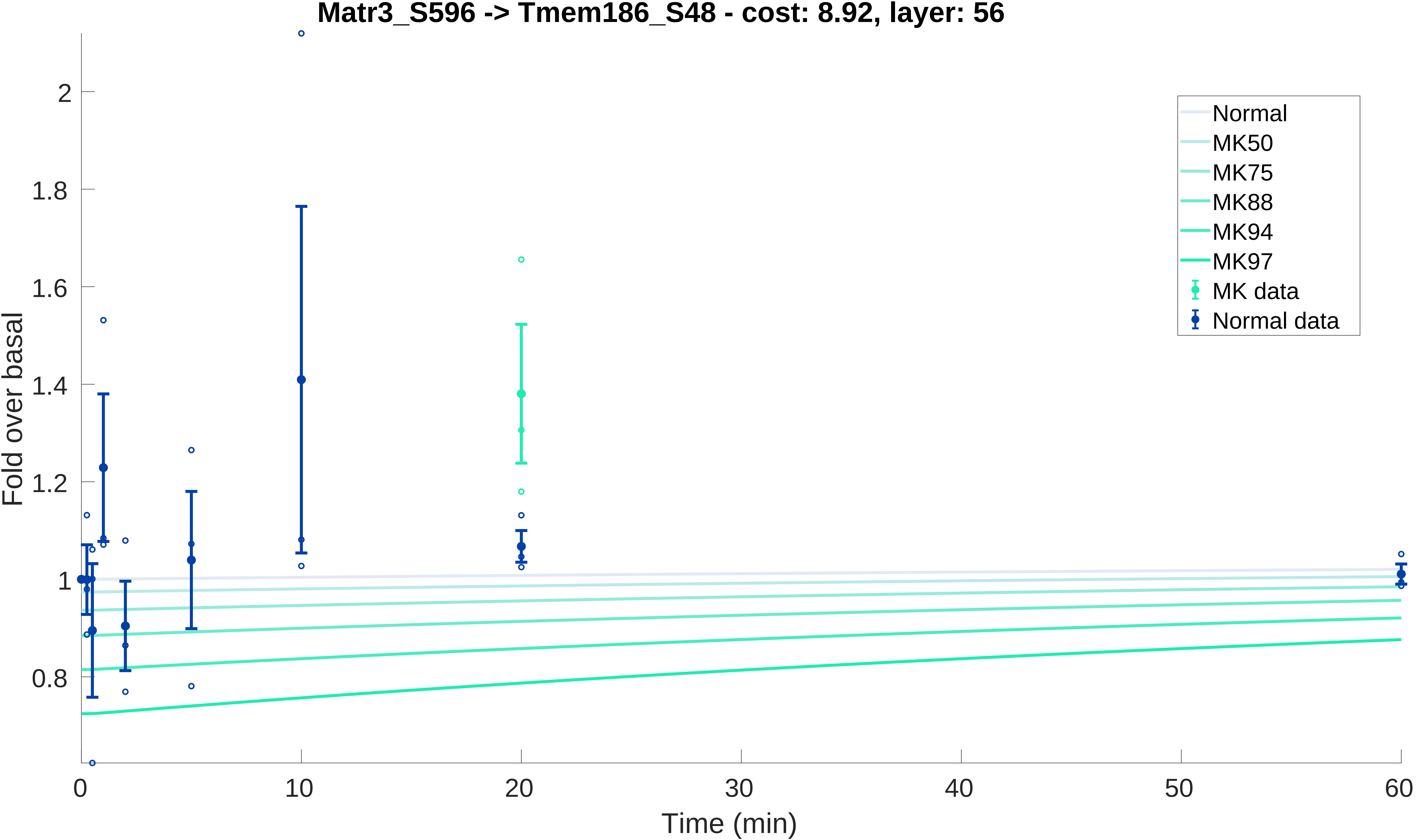
Matr3_S596 -> Tmem186_S48 - cost: 8.92, layer: 56
Fold over basal
Time (min)
0.8
1
1.2
1.4
1.6
1.8
2
0
10
20
30
40
50
60
Normal
MK50
MK75
MK88
MK94
MK97
MK data
Normal data

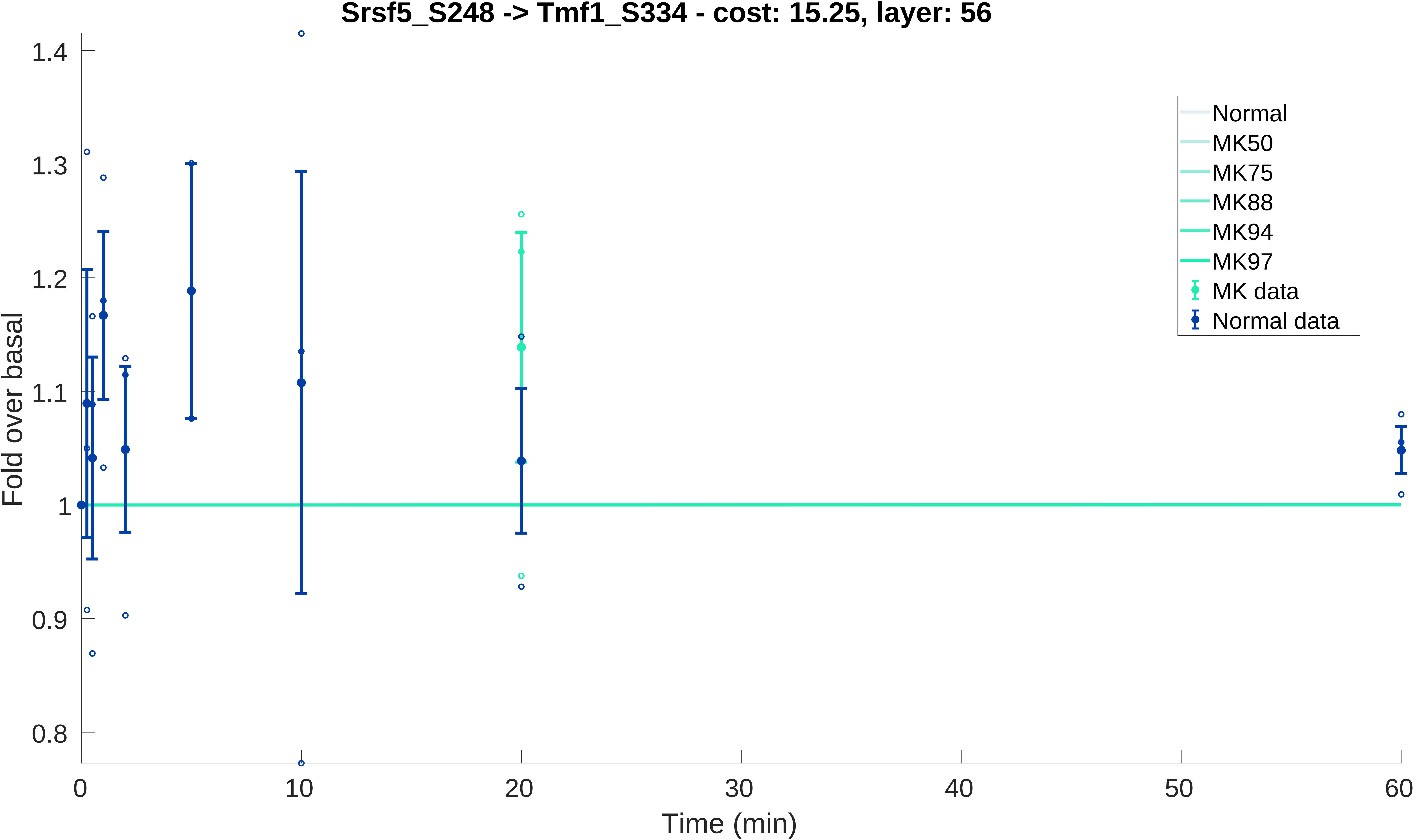

Srsf5_S248 -> Tmf1_S334 - cost: 15.25, layer: 56
Fold over basal
Time (min)
Normal
MK50
MK75
MK88
MK94
MK97
MK data
Normal data
1.4
1.3
1.2
1.1
1
0.9
0.8
0
10
20
30
40
50
60

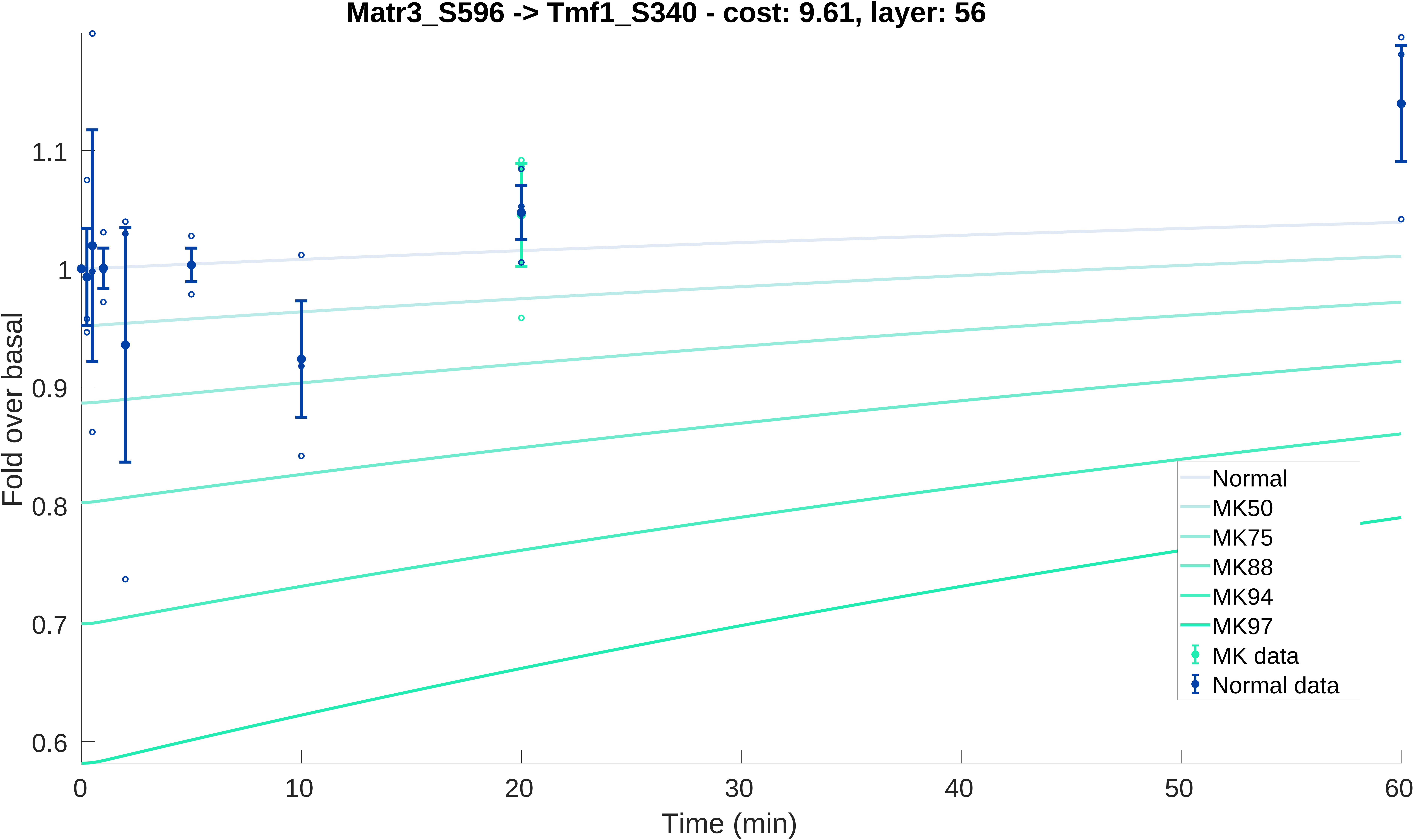

Matr3_S596 -> Tmf1_S340 - cost: 9.61, layer: 56
Fold over basal
1.1
1
0.9
0.8
0.7
0.6
0
10
20
30
40
50
60
Time (min)
Normal
MK50
MK75
MK88
MK94
MK97
MK data
Normal data

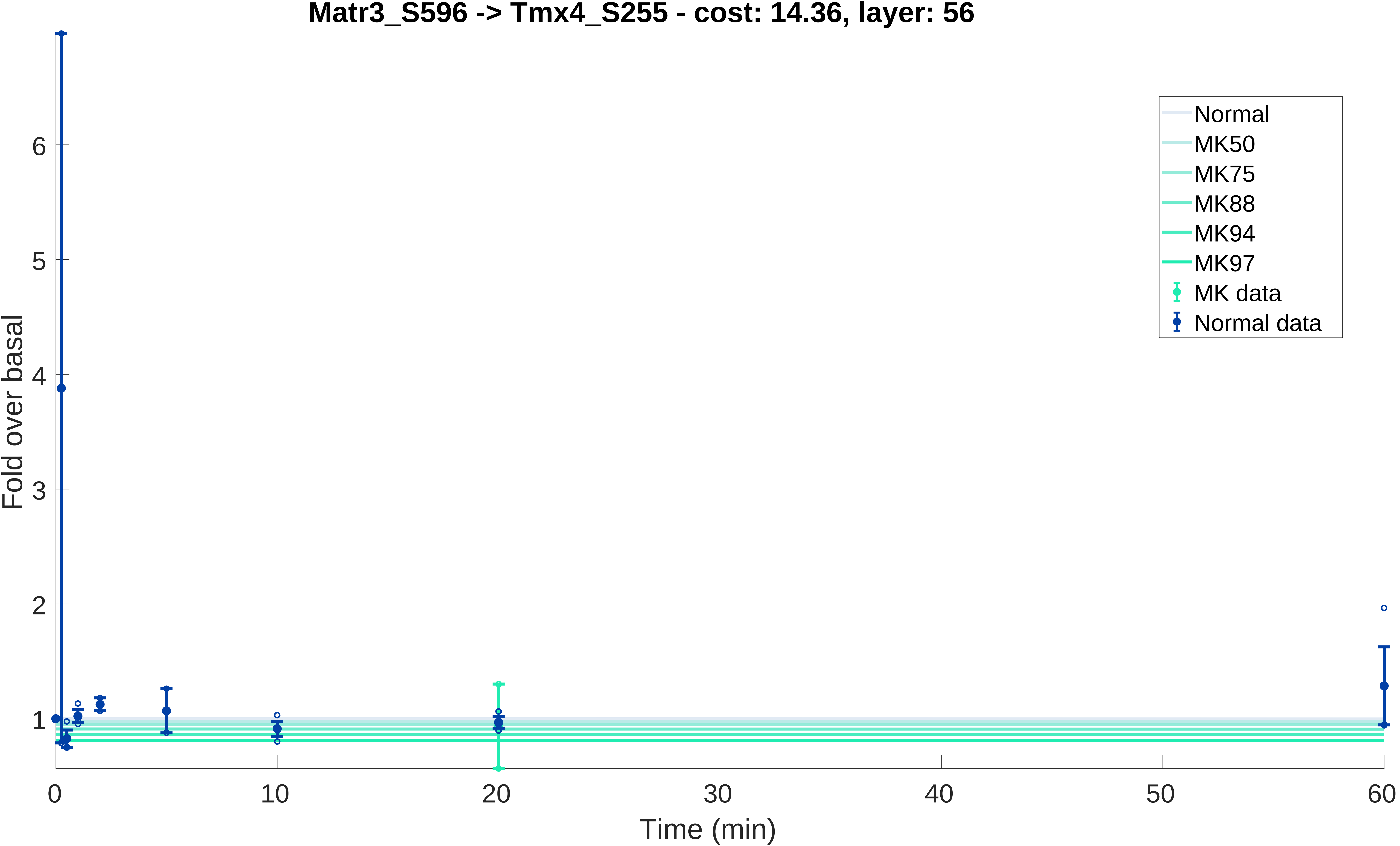

Matr3_S596 -> Tmx4_S255 - cost: 14.36, layer: 56
Fold over basal
Time (min)
0
10
20
30
40
50
60
1
2
3
4
5
6
Normal
MK50
MK75
MK88
MK94
MK97
MK data
Normal data

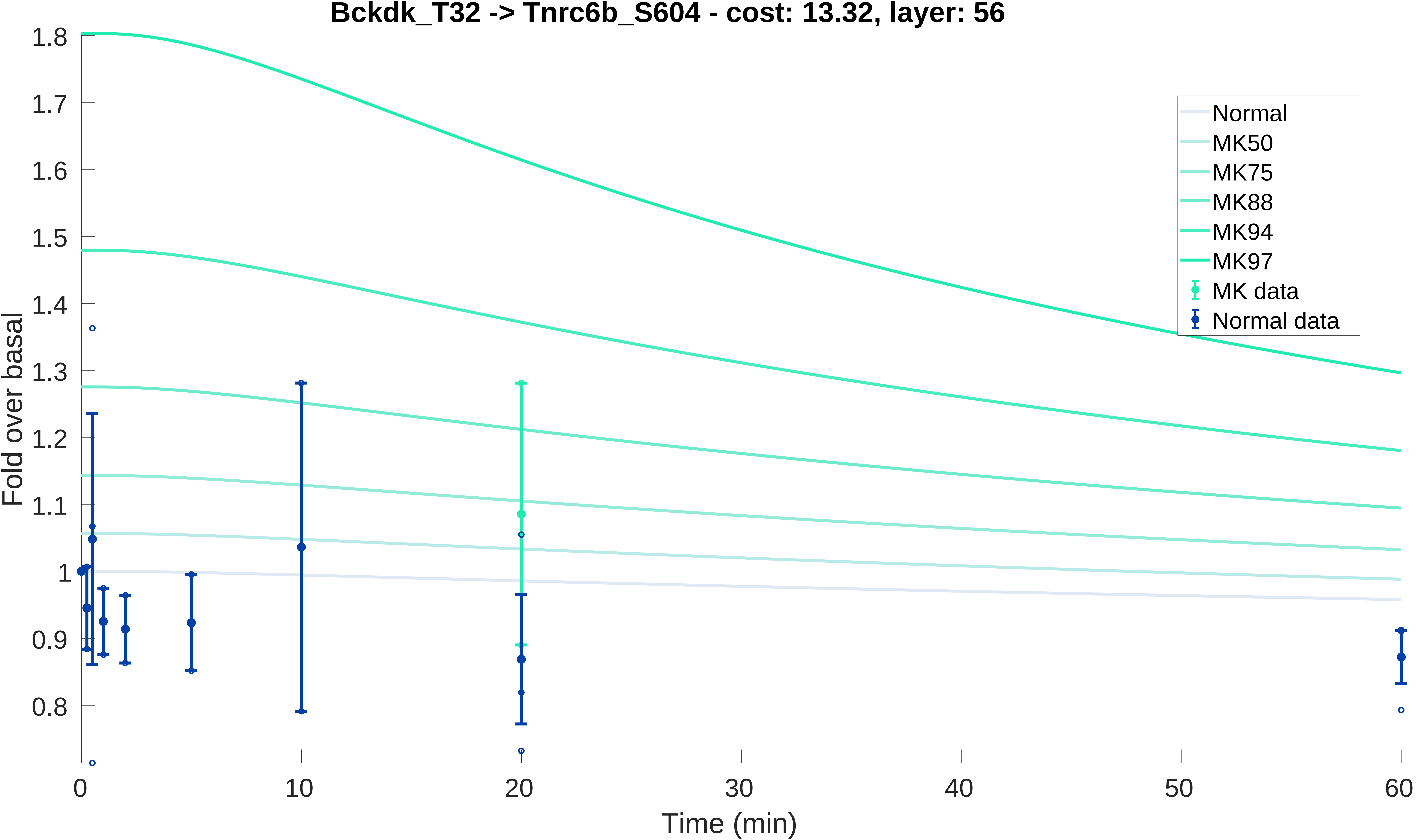

Bckdk_T32 -> Tnrc6b_S604 - cost: 13.32, layer: 56
Fold over basal
Time (min)
Normal
MK50
MK75
MK88
MK94
MK97
MK data
Normal data
1.8
1.7
1.6
1.5
1.4
1.3
1.2
1.1
1
0.9
0.8
0
10
20
30
40
50
60

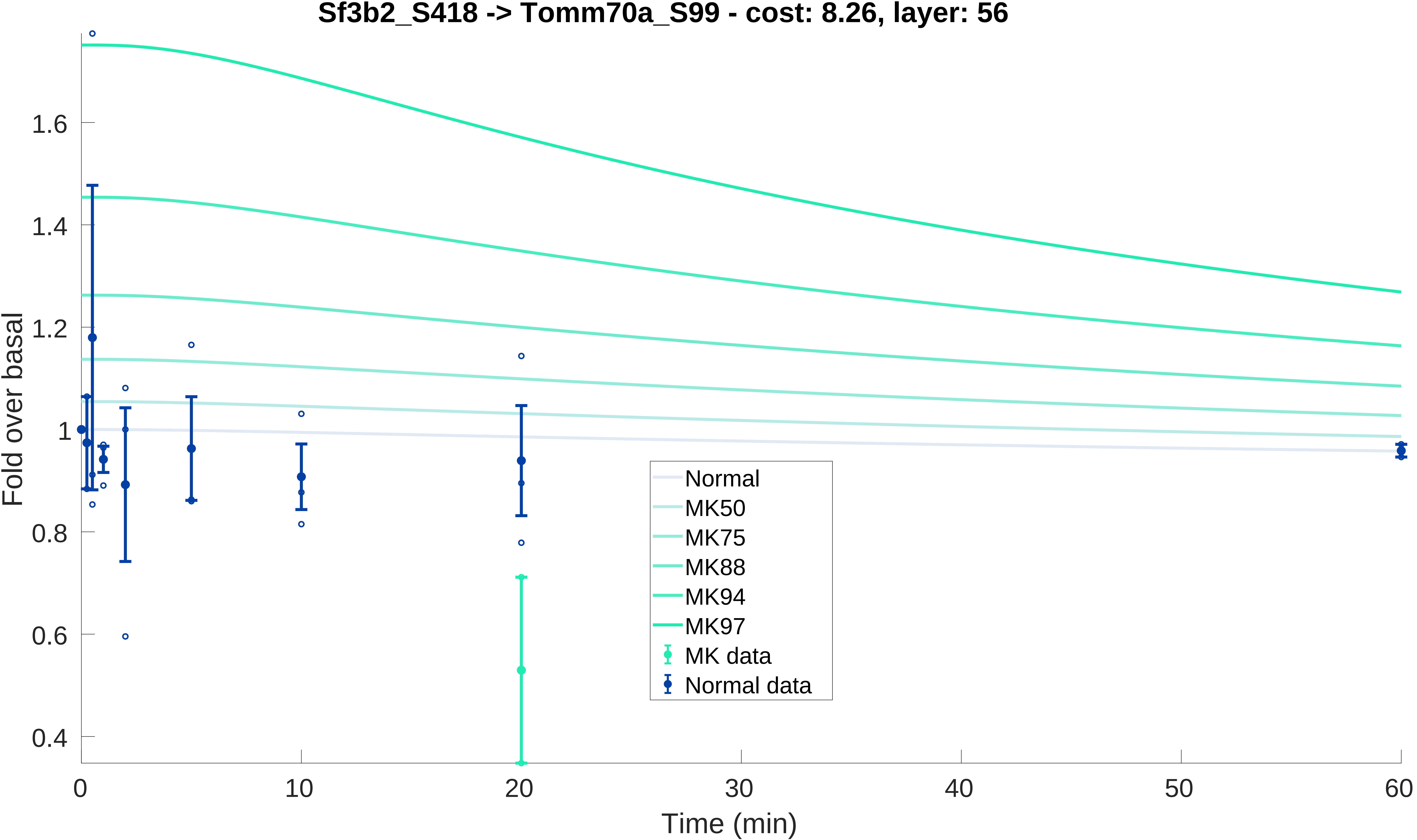

Sf3b2_S418 -> Tomm70a_S99 - cost: 8.26, layer: 56
Fold over basal
1.6
1.4
1.2
1
0.8
0.6
0.4
0
10
20
30
40
50
60
Time (min)
Normal
MK50
MK75
MK88
MK94
MK97
MK data
Normal data

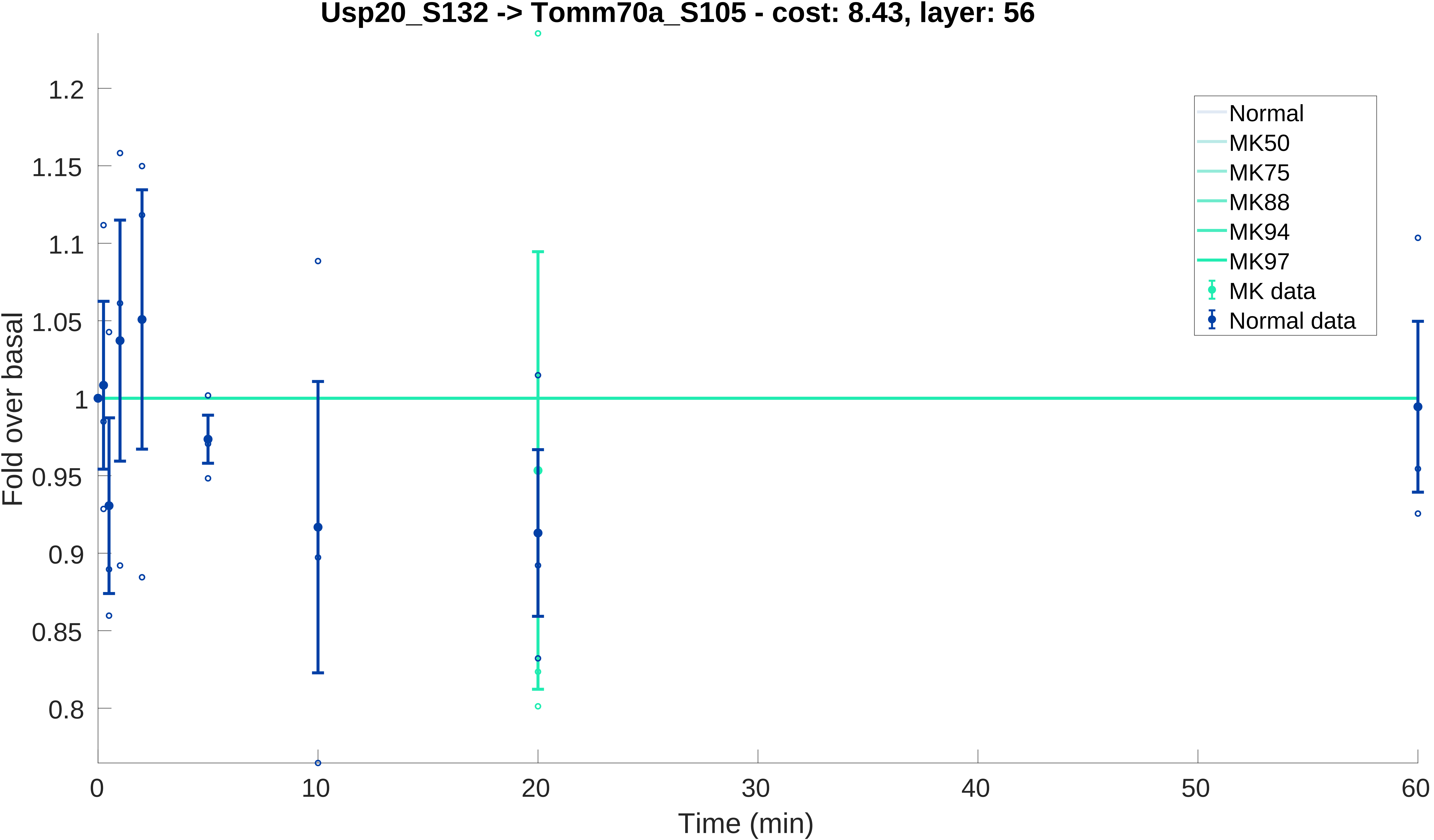
Usp20_S132 -> Tomm70a_S105 - cost: 8.43, layer: 56
Fold over basal
Time (min)
1.2
1.15
1.1
1.05
1
0.95
0.9
0.85
0.8
0
10
20
30
40
50
60
Normal
MK50
MK75
MK88
MK94
MK97
MK data
Normal data

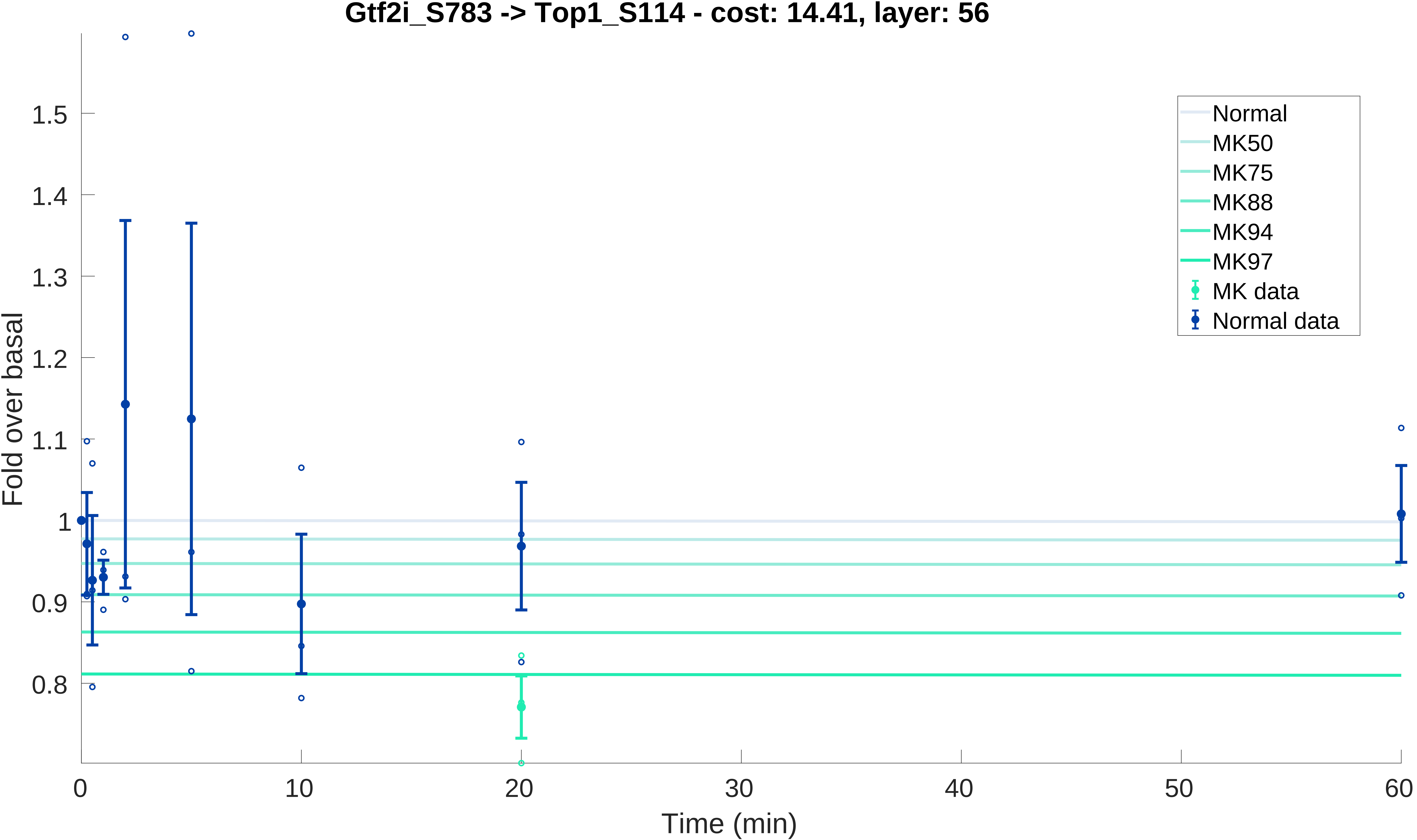

Gtf2i_S783 -> Top1_S114 - cost: 14.41, layer: 56
Fold over basal
1.5
1.4
1.3
1.2
1.1
1
0.9
0.8
Time (min)
0
10
20
30
40
50
60
Normal
MK50
MK75
MK88
MK94
MK97
MK data
Normal data

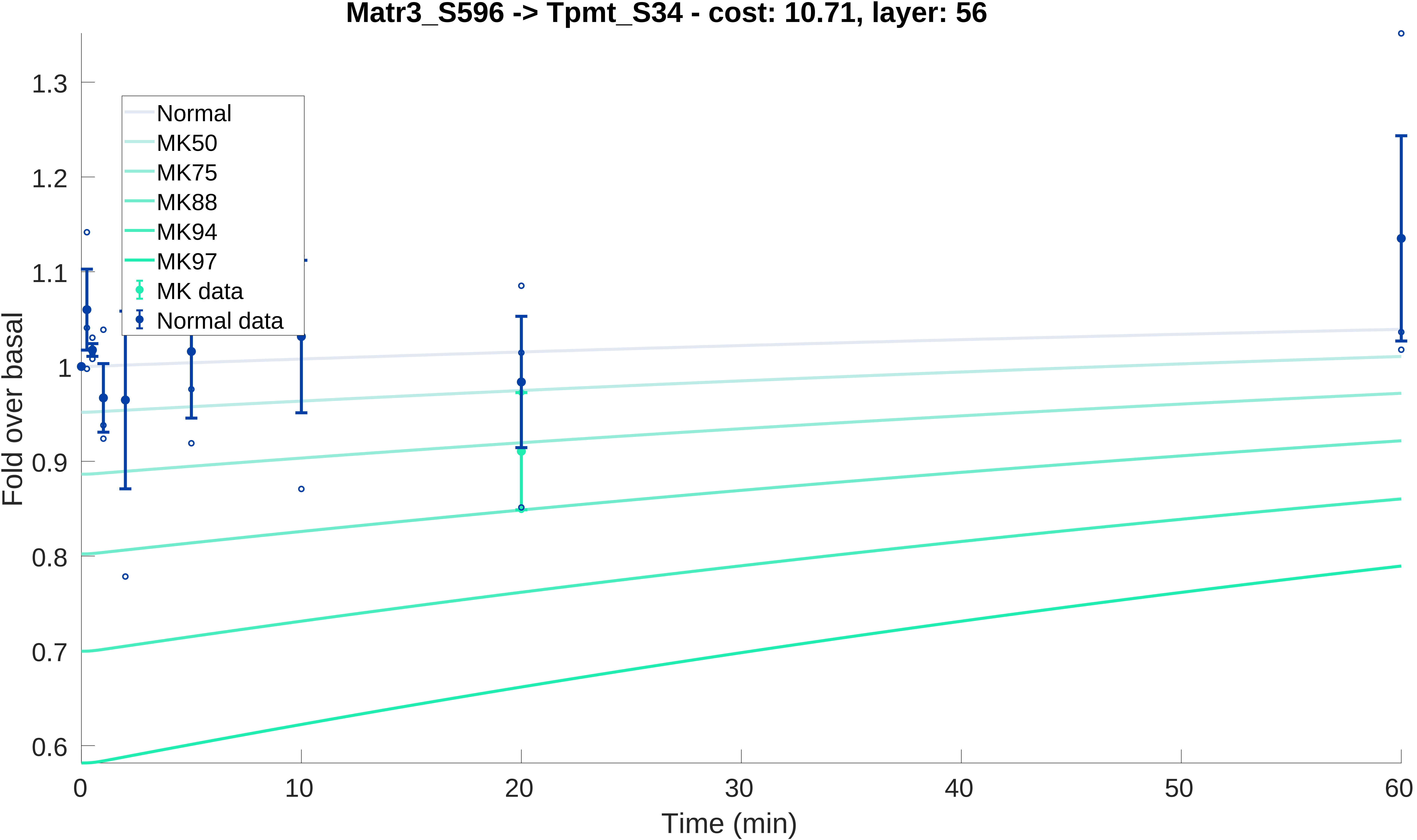
Matr3_S596 -> Tpmt_S34 - cost: 10.71, layer: 56
Normal
MK50
MK75
MK88
MK94
MK97
MK data
Normal data
Fold over basal
Time (min)
0.6
0.7
0.8
0.9
1
1.1
1.2
1.3
0
10
20
30
40
50
60

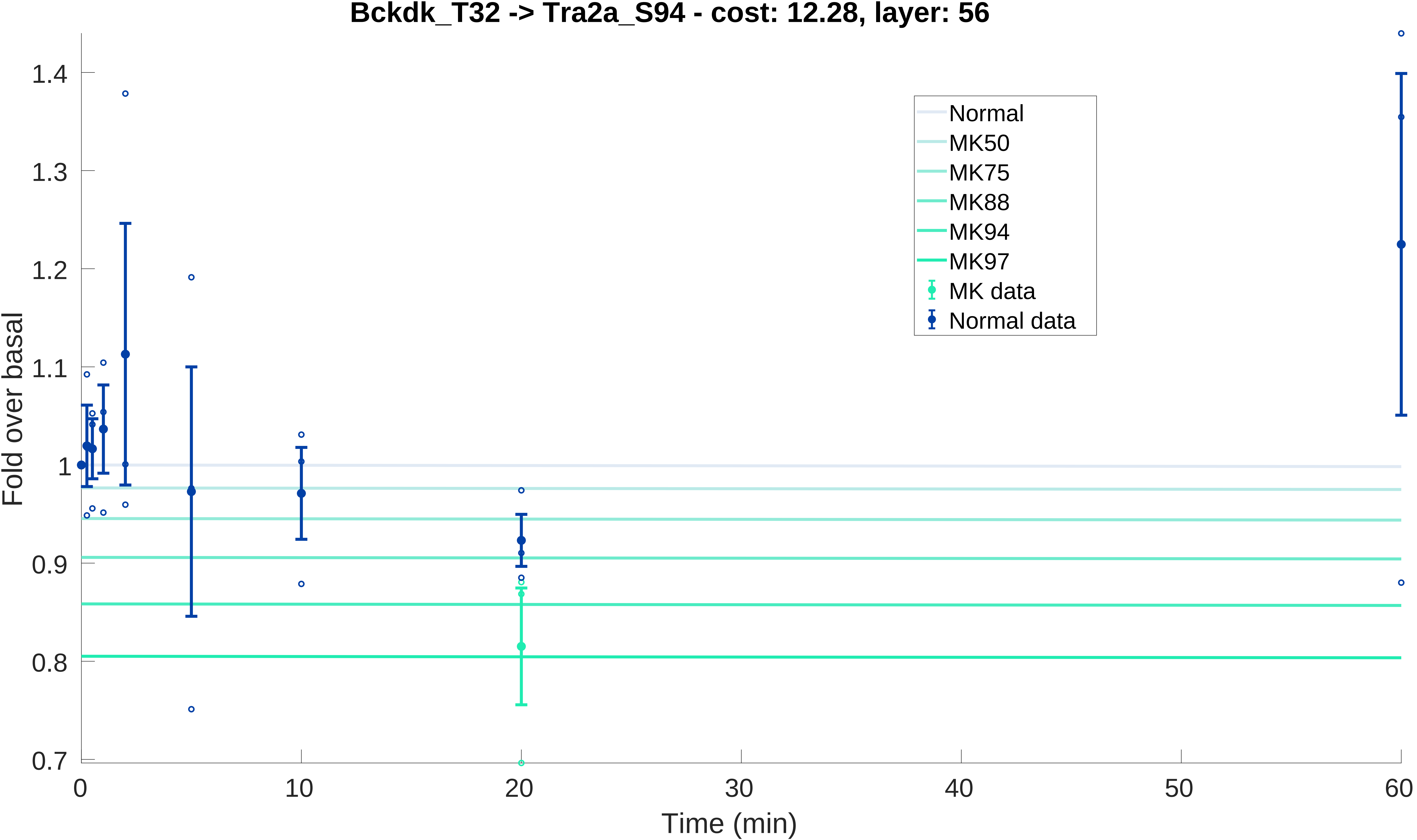

Bckdk_T32 -> Tra2a_S94 - cost: 12.28, layer: 56
Fold over basal
Time (min)
1.4
1.3
1.2
1.1
1
0.9
0.8
0.7
0
10
20
30
40
50
60
Normal
MK50
MK75
MK88
MK94
MK97
MK data
Normal data

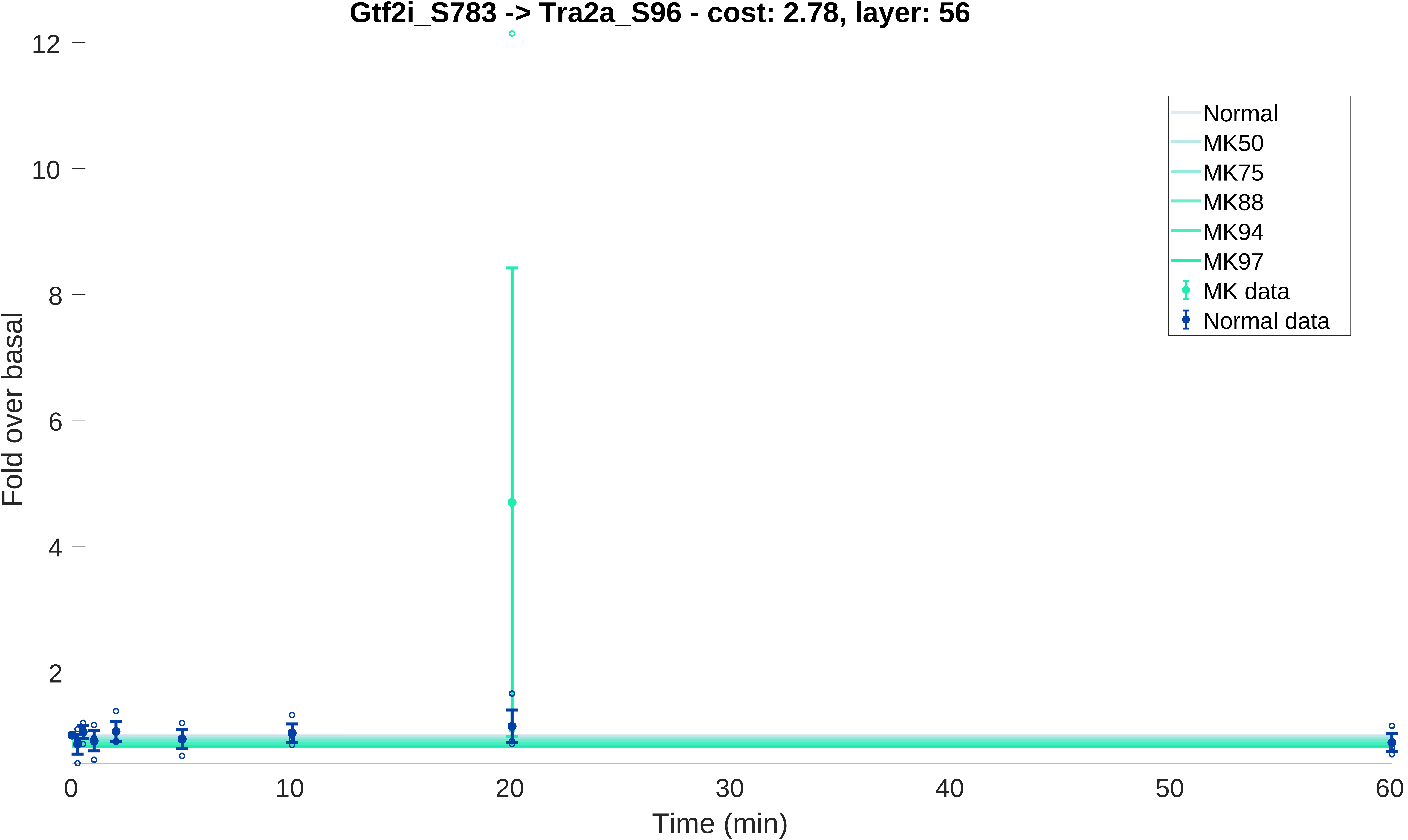

Gtf2i_S783 -> Tra2a_S96 - cost: 2.78, layer: 56
Fold over basal
Time (min)
Normal
MK50
MK75
MK88
MK94
MK97
MK data
Normal data
0
2
4
6
8
10
12
10
20
30
40
50
60

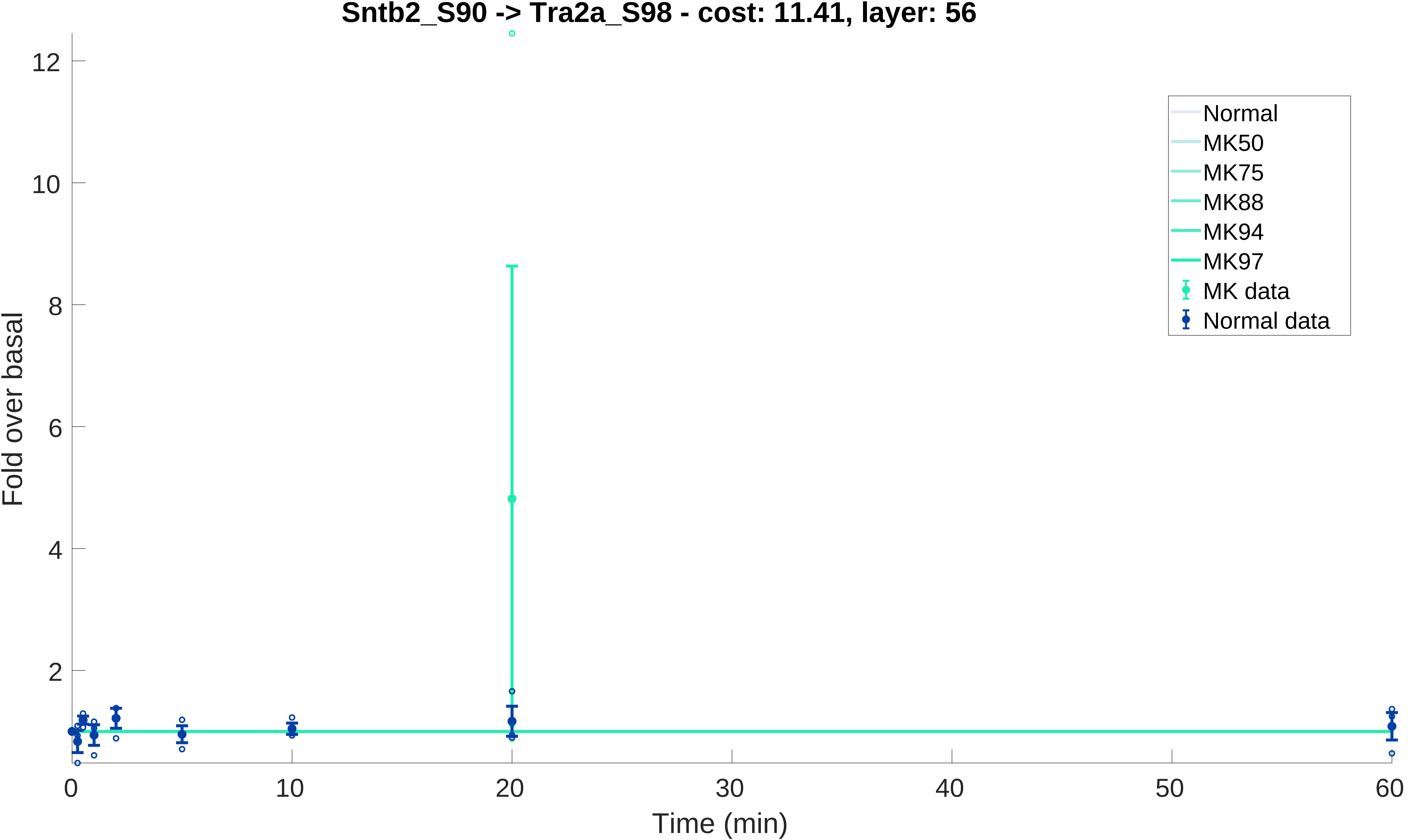

Sntb2_S90 -> Tra2a_S98 - cost: 11.41, layer: 56
Fold over basal
Time (min)
0
10
20
30
40
50
60
2
4
6
8
10
12
Normal
MK50
MK75
MK88
MK94
MK97
MK data
Normal data

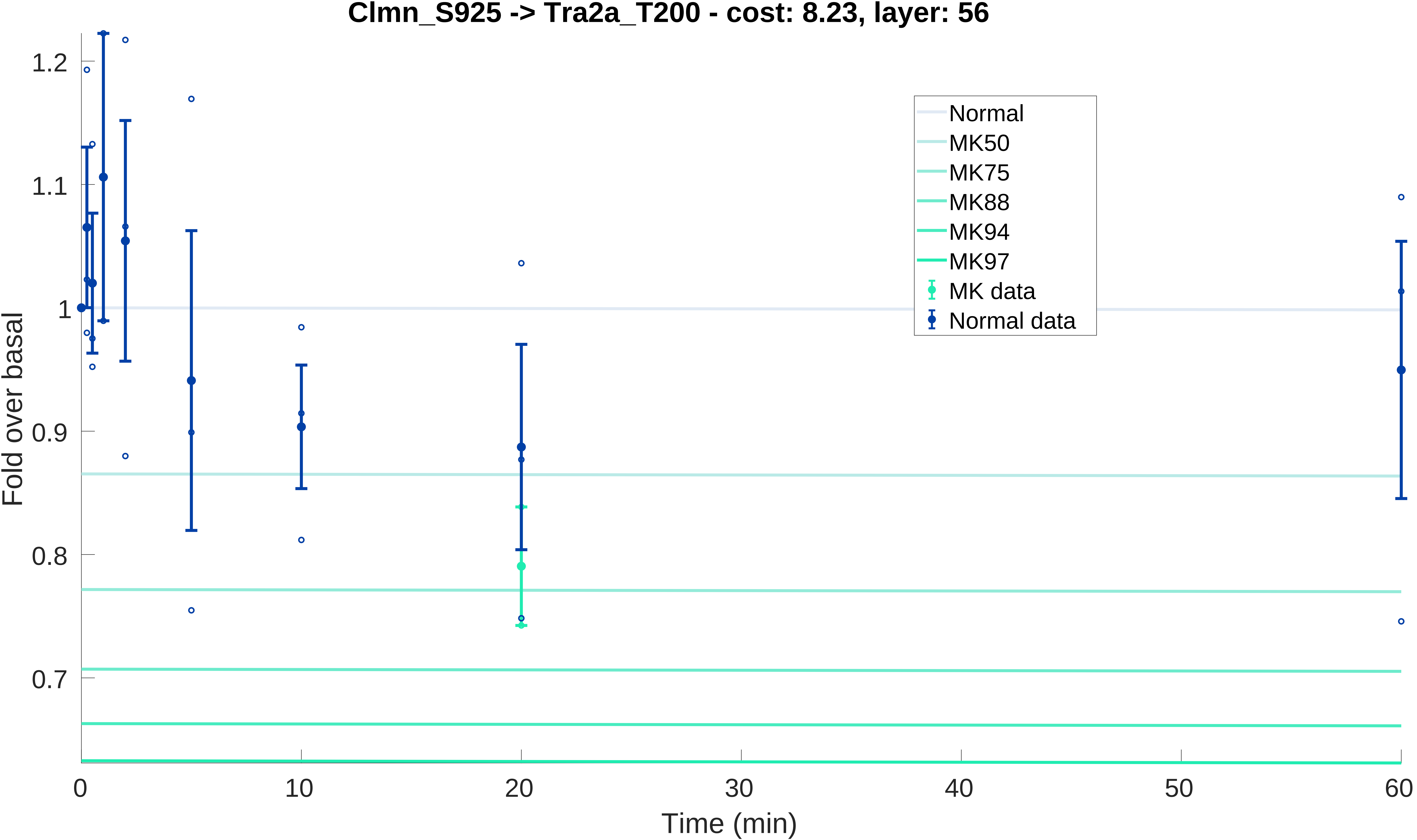

Clmn_S925 -> Tra2a_T200 - cost: 8.23, layer: 56
Normal
MK50
MK75
MK88
MK94
MK97
MK data
Normal data
Fold over basal
Time (min)
1.2
1.1
1
0.9
0.8
0.7
0
10
20
30
40
50
60

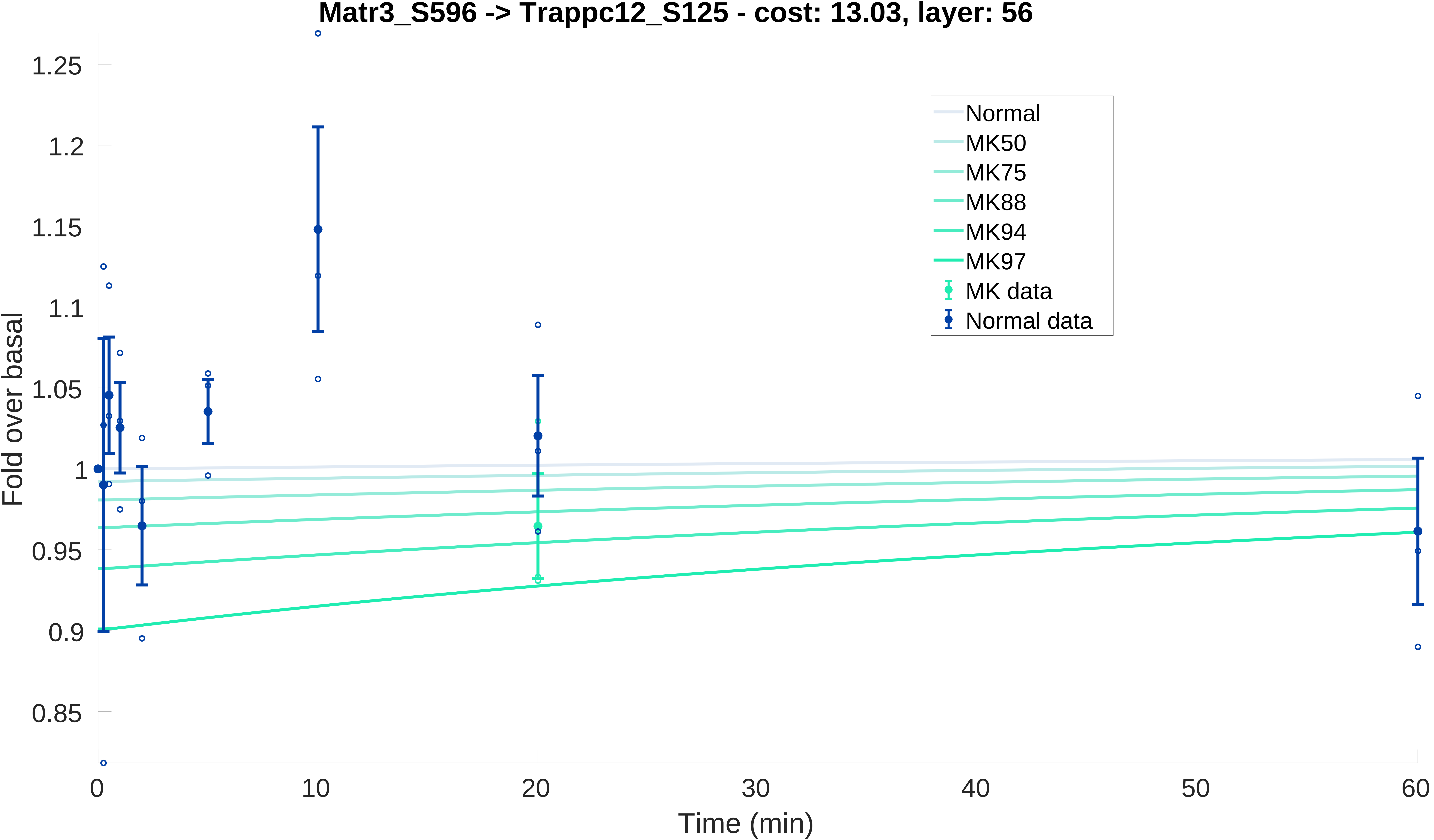

Matr3_S596 -> Trappc12_S125 - cost: 13.03, layer: 56
Fold over basal
Time (min)
1.25
1.2
1.15
1.1
1.05
1
0.95
0.9
0.85
0
10
20
30
40
50
60
Normal
MK50
MK75
MK88
MK94
MK97
MK data
Normal data

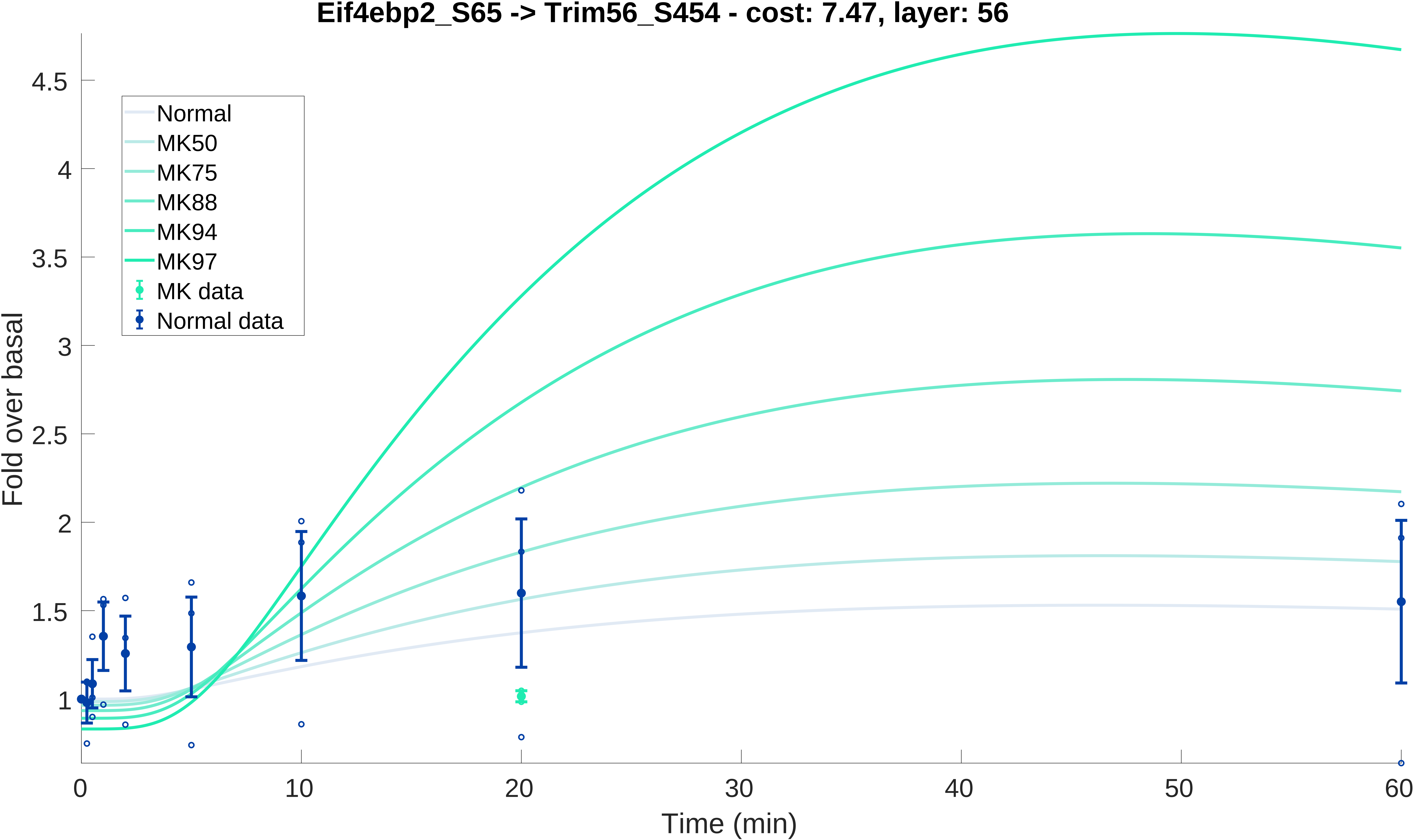

Eif4ebp2_S65 -> Trim56_S454 - cost: 7.47, layer: 56
Normal
MK50
MK75
MK88
MK94
MK97
MK data
Normal data
Fold over basal
Time (min)
4.5
4
3.5
3
2.5
2
1.5
1
0
10
20
30
40
50
60

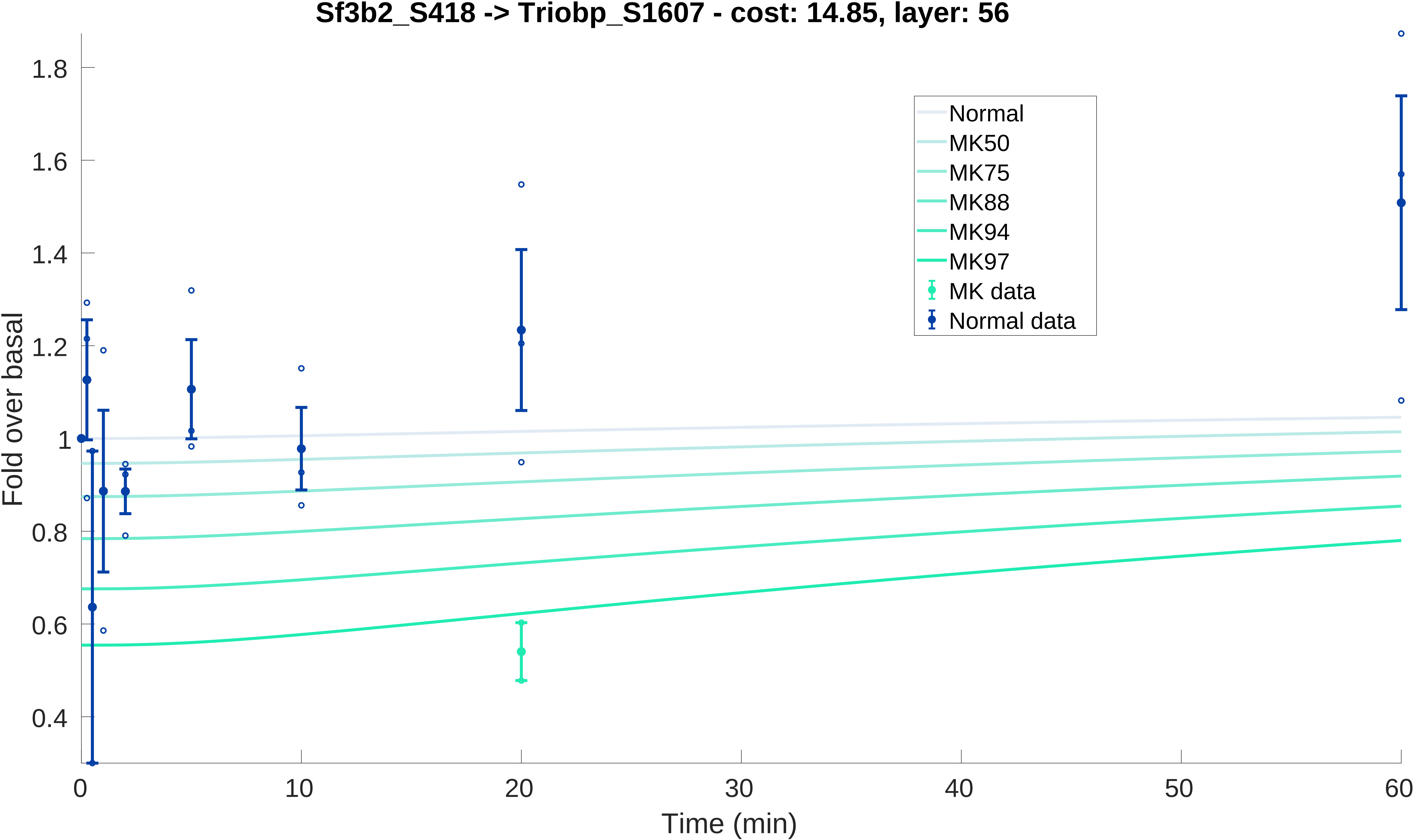

Sf3b2_S418 -> Triobp_S1607 - cost: 14.85, layer: 56
Fold over basal
Time (min)
0.4
0.6
0.8
1
1.2
1.4
1.6
1.8
0
10
20
30
40
50
60
Normal
MK50
MK75
MK88
MK94
MK97
MK data
Normal data

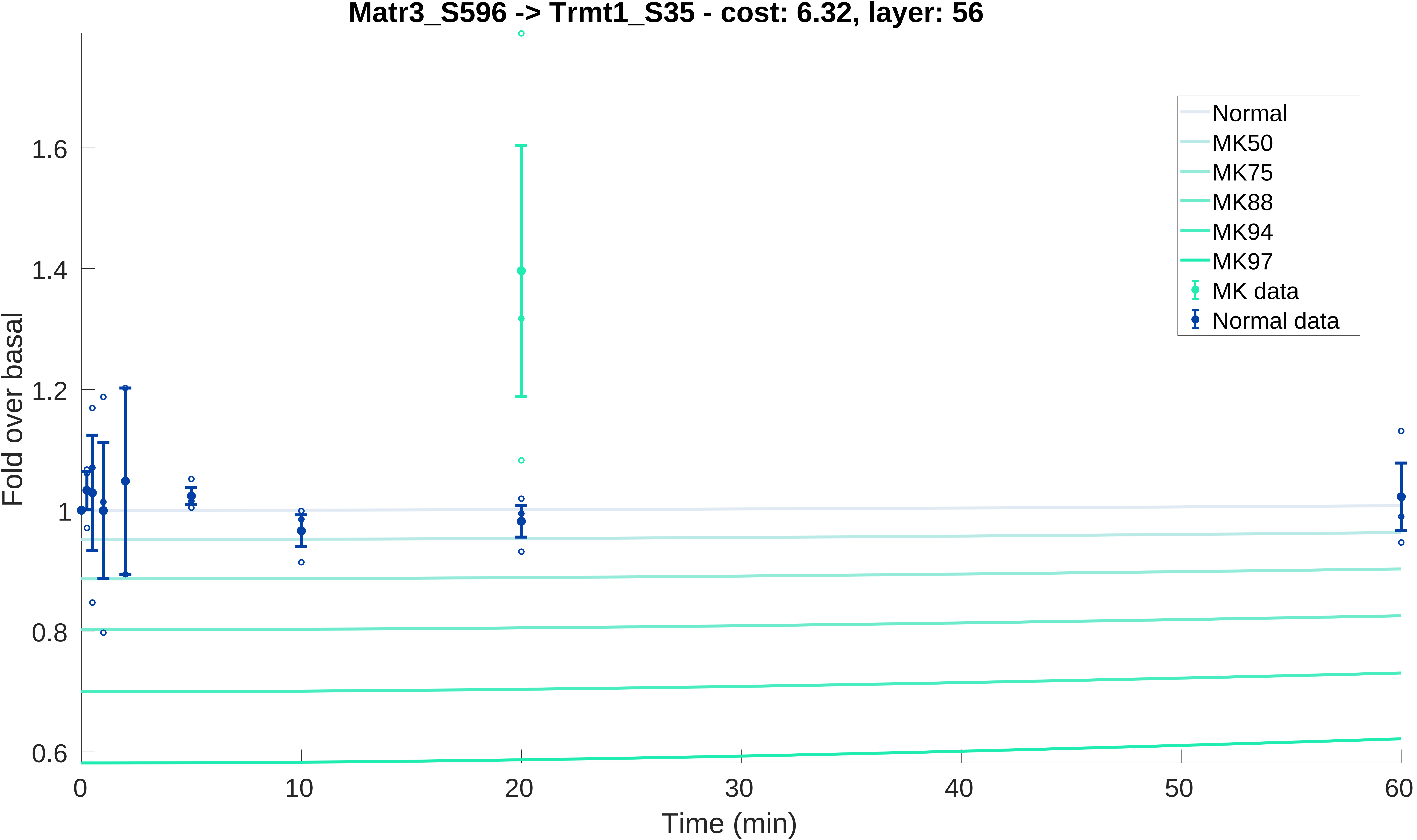

Matr3_S596 -> Trmt1_S35 - cost: 6.32, layer: 56
Fold over basal
Time (min)
1.6
1.4
1.2
1
0.8
0.6
0
10
20
30
40
50
60
Normal
MK50
MK75
MK88
MK94
MK97
MK data
Normal data

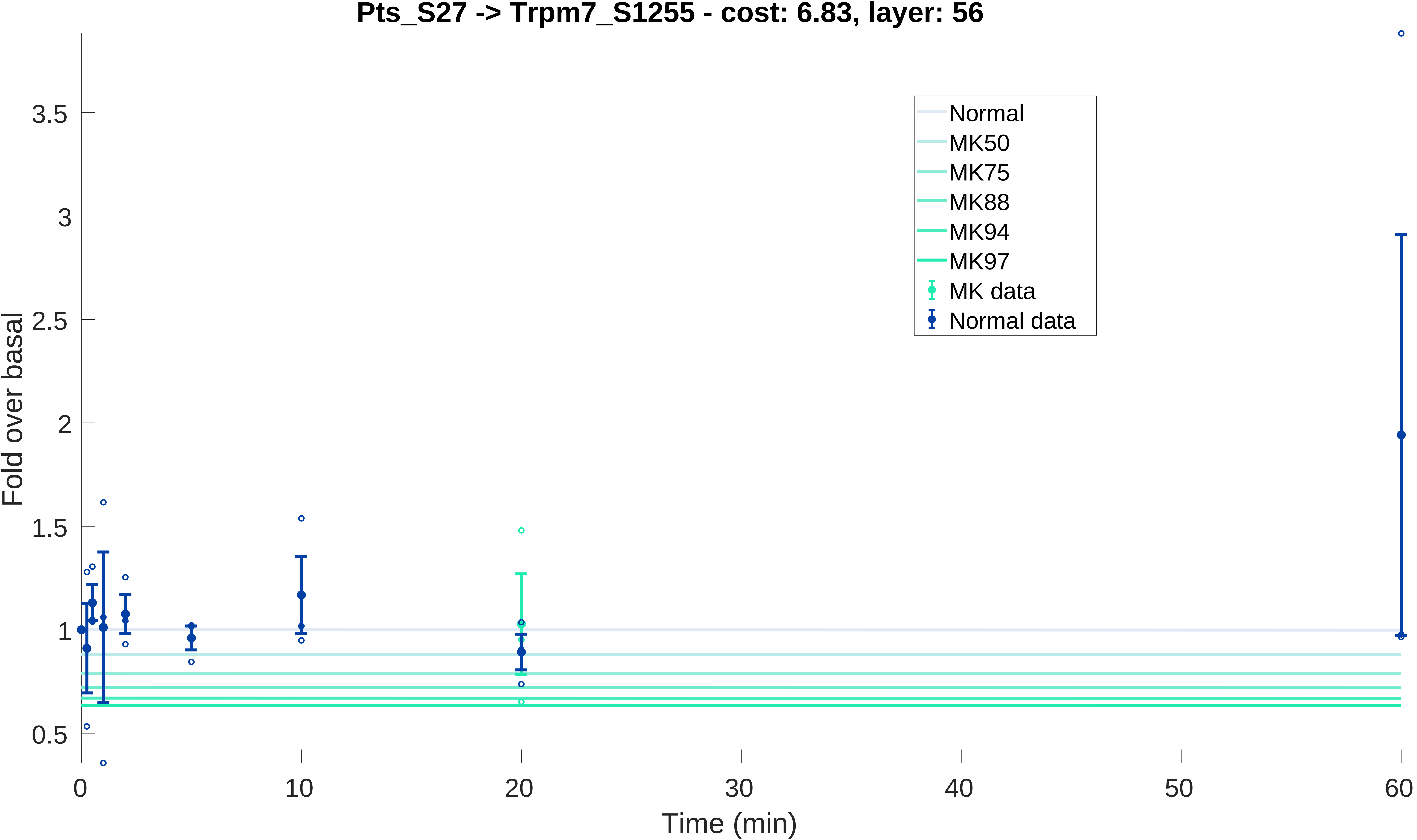

Pts_S27 -> Trpm7_S1255 - cost: 6.83, layer: 56
Fold over basal
3.5
3
2.5
2
1.5
1
0.5
0
10
20
30
40
50
60
Time (min)
Normal
MK50
MK75
MK88
MK94
MK97
MK data
Normal data

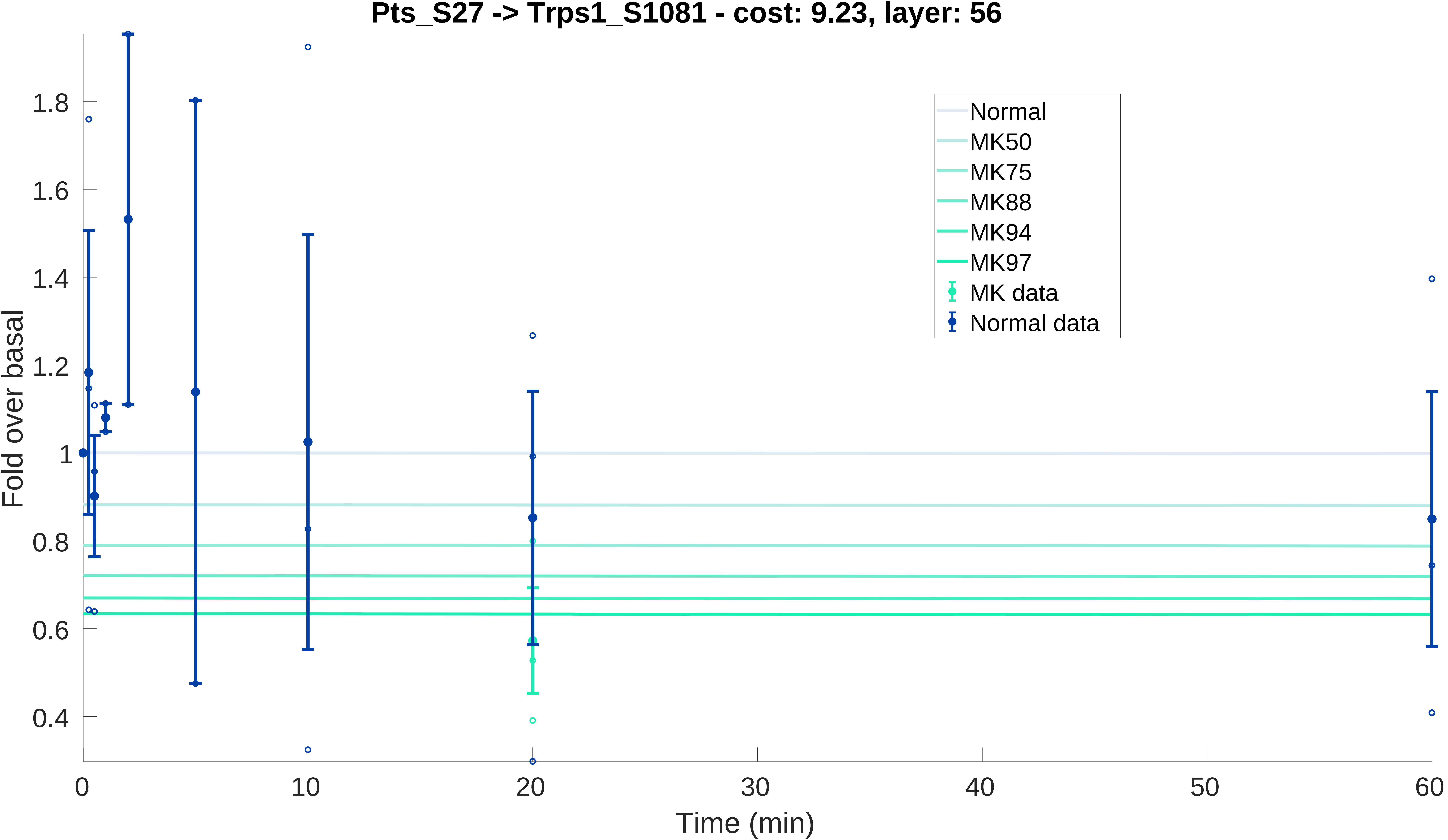
Pts_S27 -> Trps1_S1081 - cost: 9.23, layer: 56
Fold over basal
Time (min)
Normal
MK50
MK75
MK88
MK94
MK97
MK data
Normal data
1.8
1.6
1.4
1.2
1
0.8
0.6
0.4
0
10
20
30
40
50
60

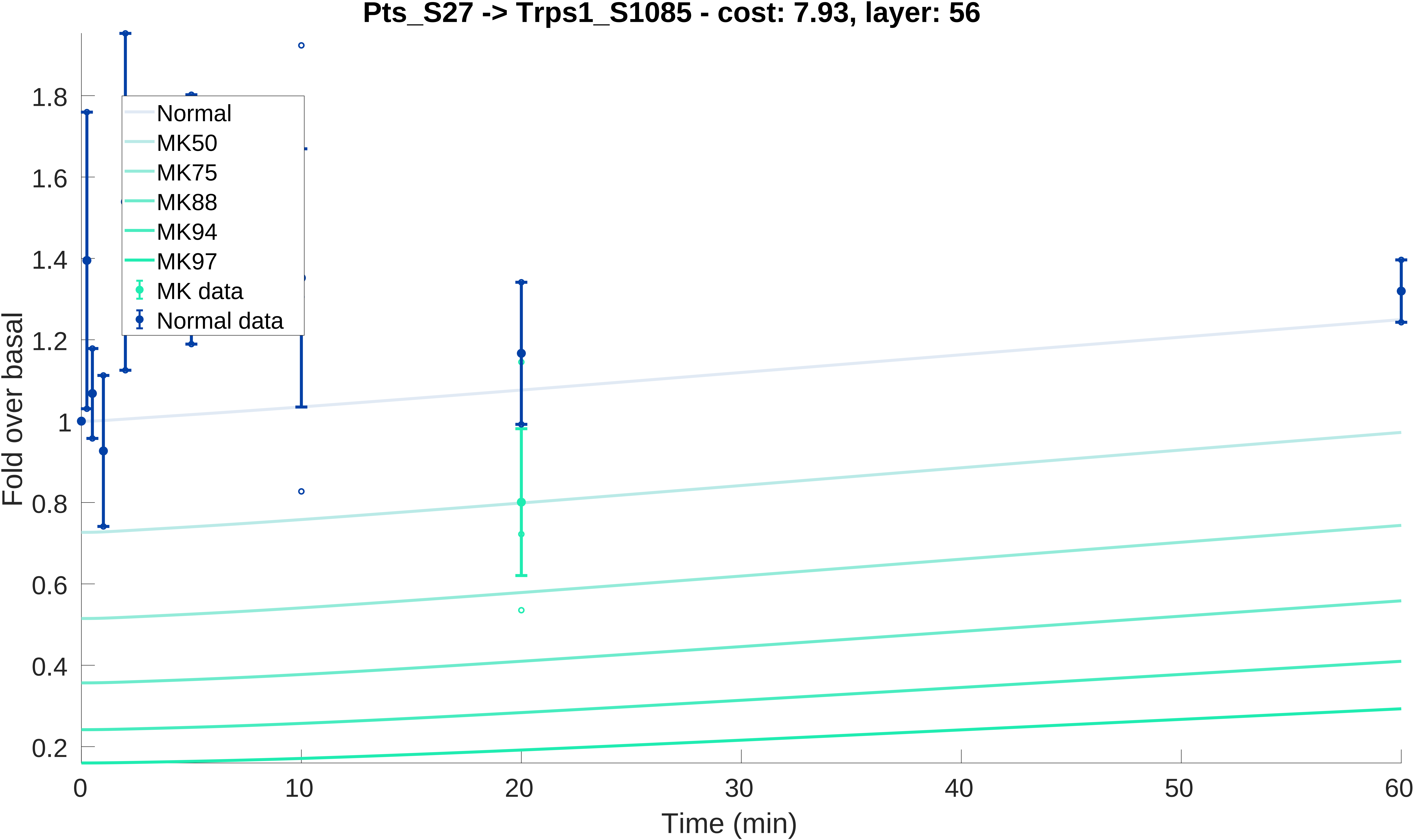

Pts_S27 -> Trps1_S1085 - cost: 7.93, layer: 56
Normal
MK50
MK75
MK88
MK94
MK97
MK data
Normal data
Fold over basal
Time (min)
0.2
0.4
0.6
0.8
1
1.2
1.4
1.6
1.8
0
10
20
30
40
50
60

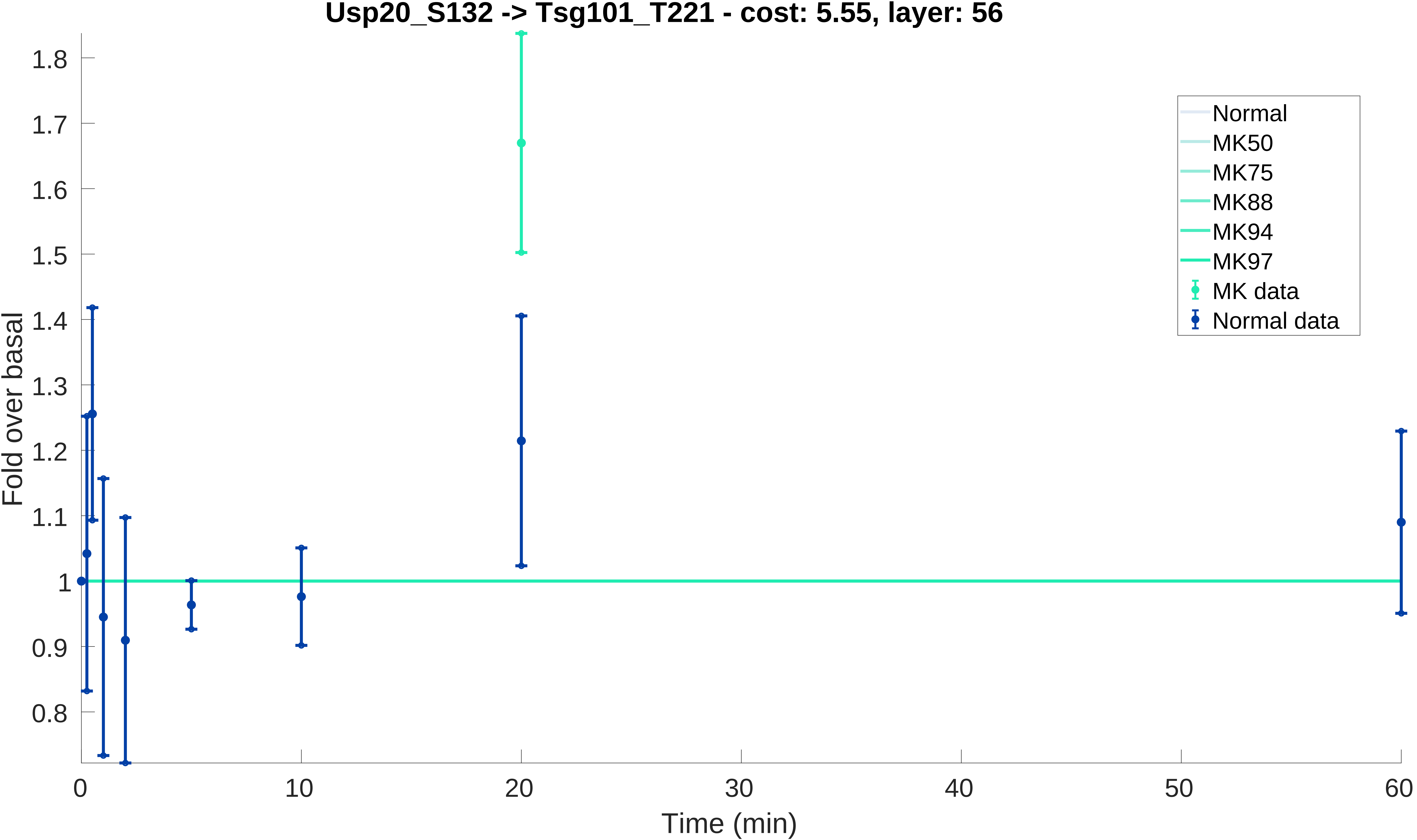

Usp20_S132 -> Tsg101_T221 - cost: 5.55, layer: 56
Fold over basal
Time (min)
1.8
1.7
1.6
1.5
1.4
1.3
1.2
1.1
1
0.9
0.8
0
10
20
30
40
50
60
Normal
MK50
MK75
MK88
MK94
MK97
MK data
Normal data

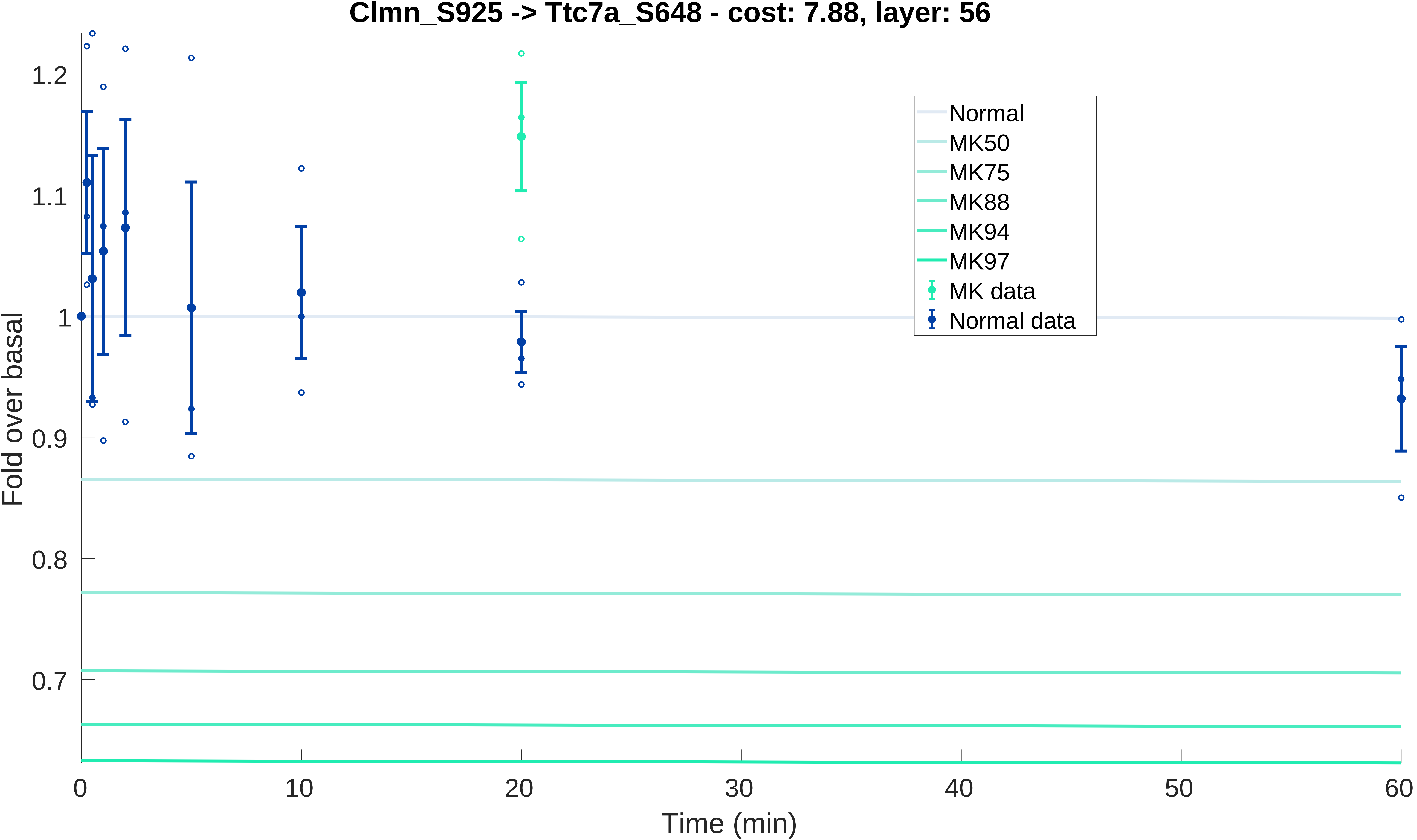

Clmn_S925 -> Ttc7a_S648 - cost: 7.88, layer: 56
Fold over basal
Time (min)
1.2
1.1
1
0.9
0.8
0.7
0
10
20
30
40
50
60
Normal
MK50
MK75
MK88
MK94
MK97
MK data
Normal data

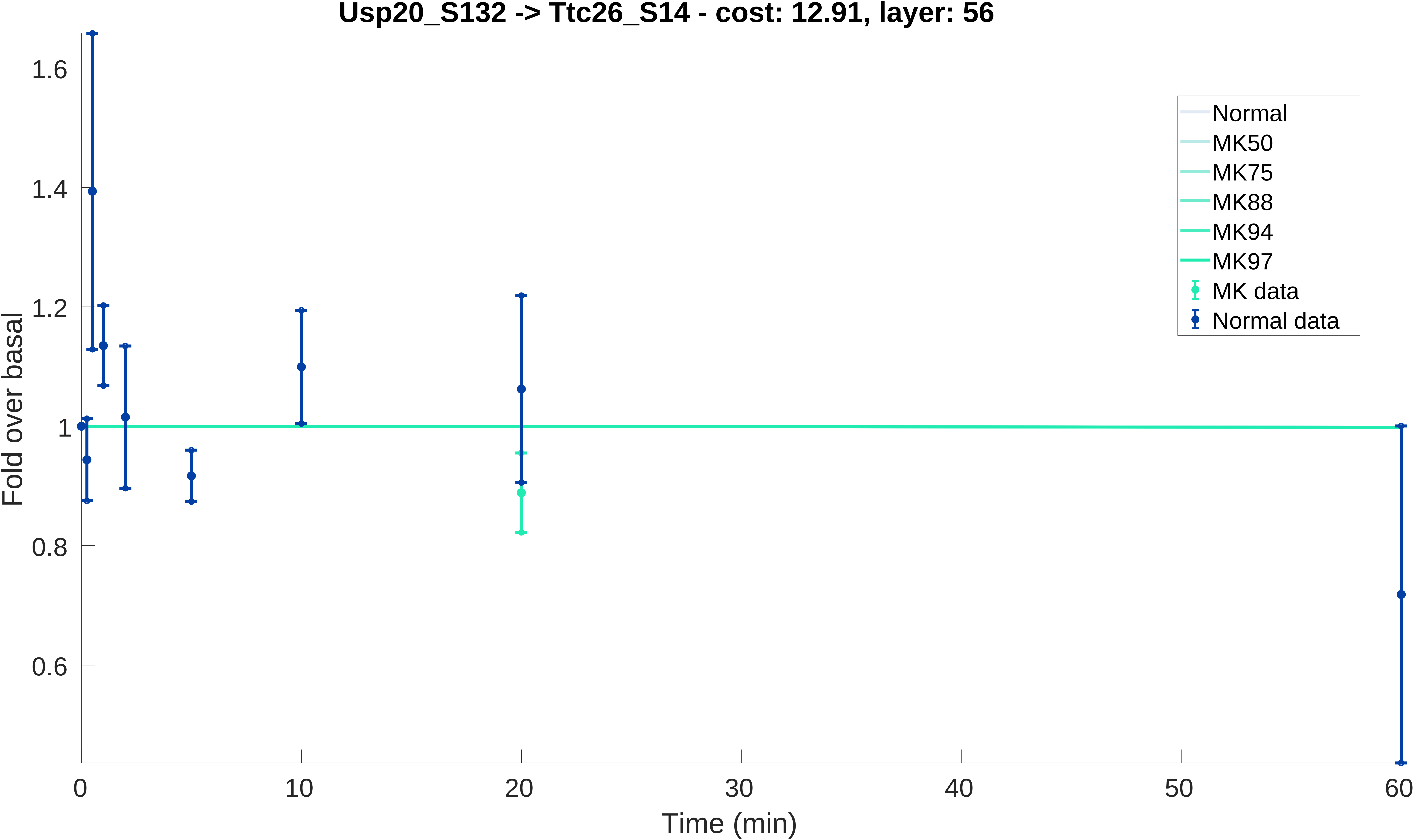
Usp20_S132 -> Ttc26_S14 - cost: 12.91, layer: 56
Fold over basal
Time (min)
1.6
1.4
1.2
1
0.8
0.6
0
10
20
30
40
50
60
Normal
MK50
MK75
MK88
MK94
MK97
MK data
Normal data

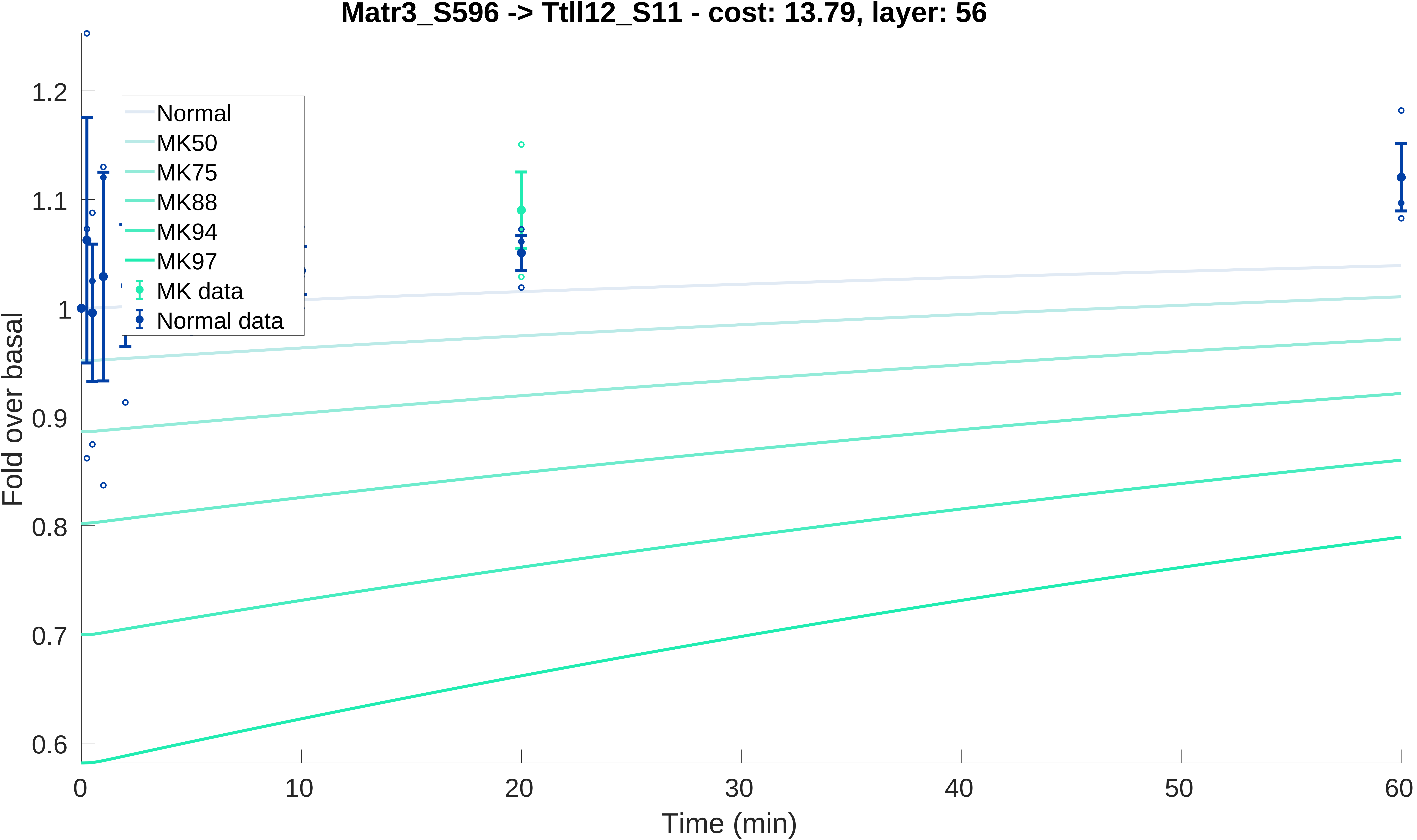
Matr3_S596 -> Ttll12_S11 - cost: 13.79, layer: 56
Fold over basal
Time (min)
Normal
MK50
MK75
MK88
MK94
MK97
MK data
Normal data
1.2
1.1
1
0.9
0.8
0.7
0.6
0
10
20
30
40
50
60

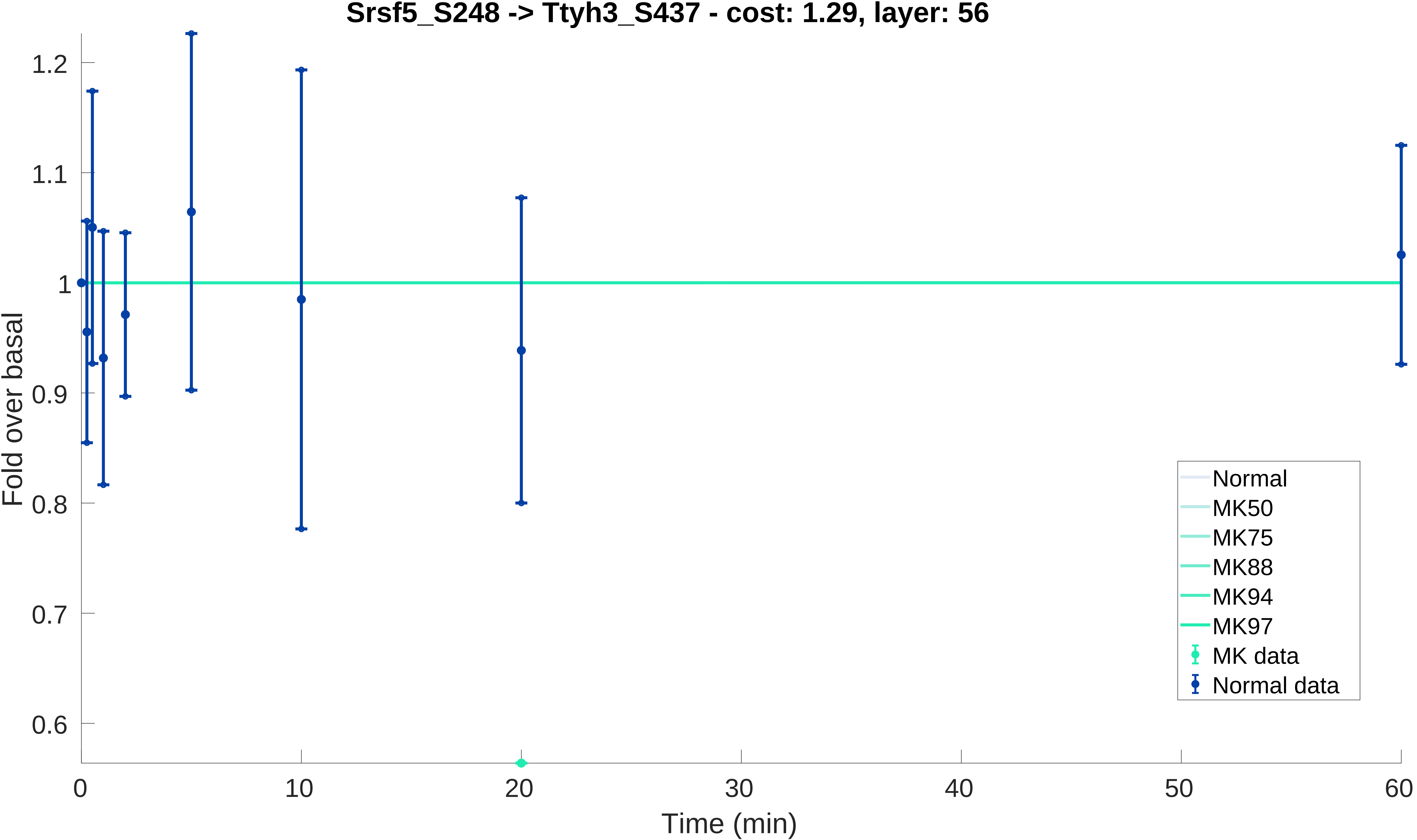

Srsf5_S248 -> Ttyh3_S437 - cost: 1.29, layer: 56
Fold over basal
Time (min)
0.6
0.7
0.8
0.9
1
1.1
1.2
0
10
20
30
40
50
60
Normal
MK50
MK75
MK88
MK94
MK97
MK data
Normal data

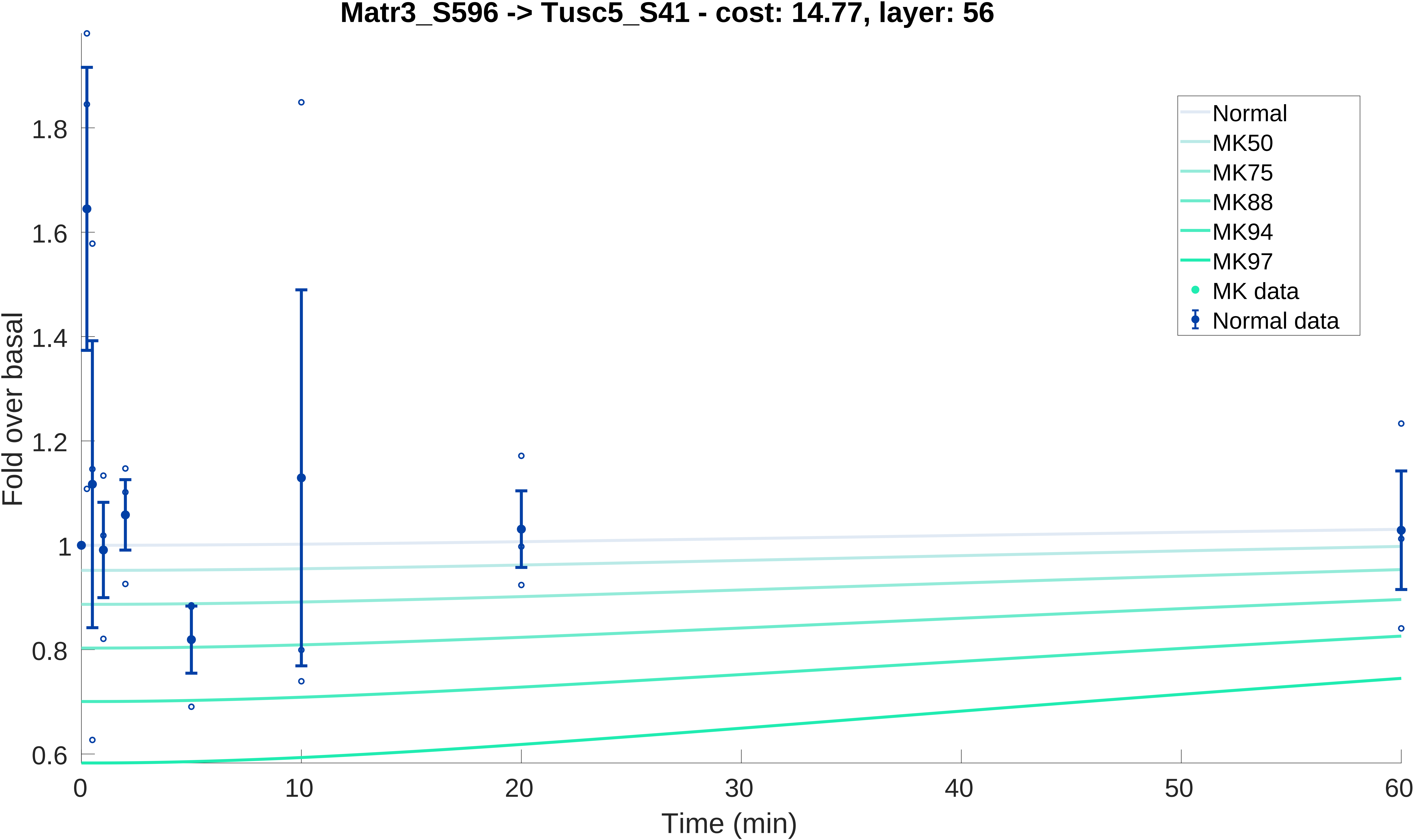
Matr3_S596 -> Tusc5_S41 - cost: 14.77, layer: 56
Fold over basal
Time (min)
0.6
0.8
1
1.2
1.4
1.6
1.8
0
10
20
30
40
50
60
Normal
MK50
MK75
MK88
MK94
MK97
MK data
Normal data

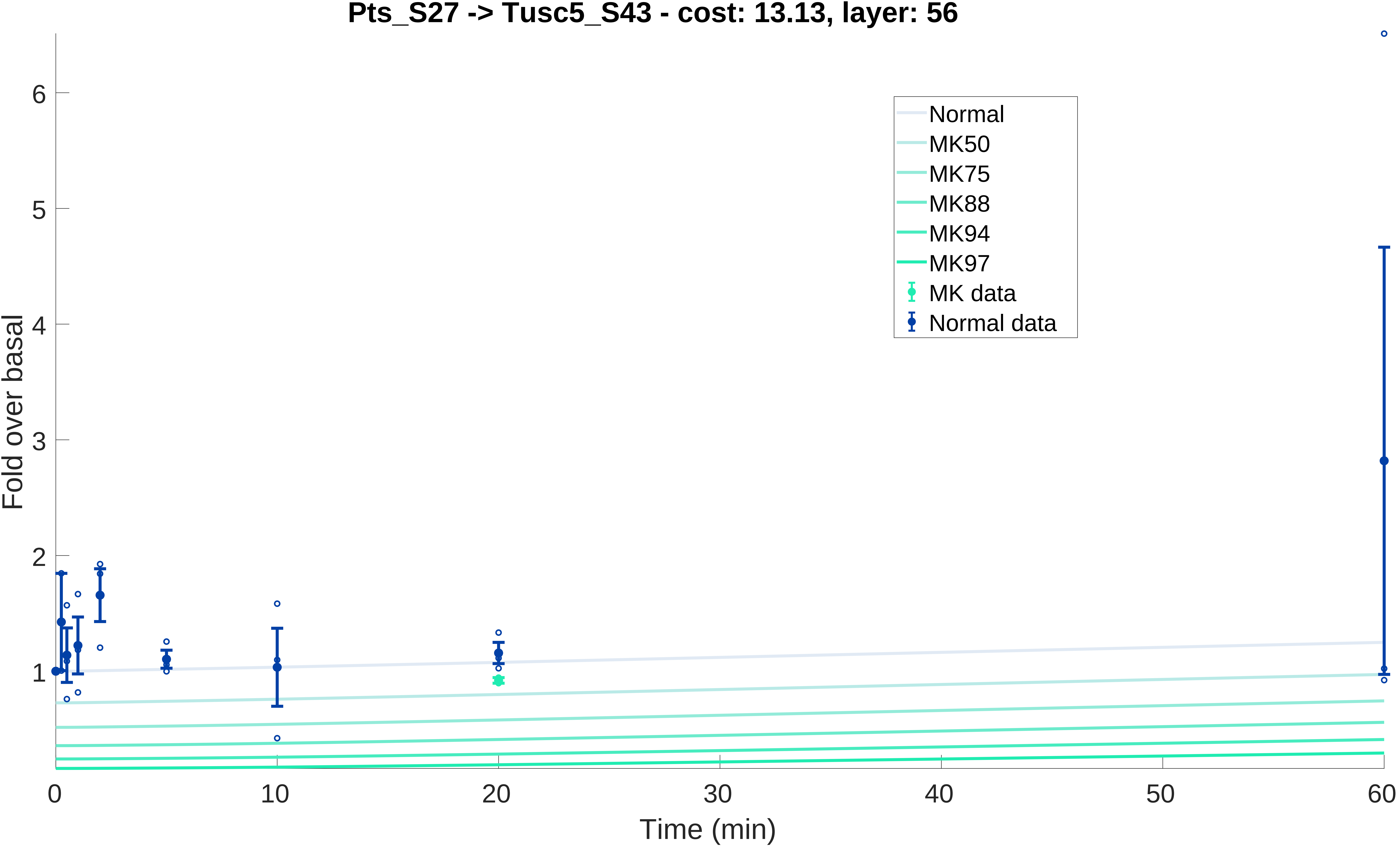

Pts_S27 -> Tusc5_S43 - cost: 13.13, layer: 56
Fold over basal
Time (min)
Normal
MK50
MK75
MK88
MK94
MK97
MK data
Normal data
0
10
20
30
40
50
60
1
2
3
4
5
6

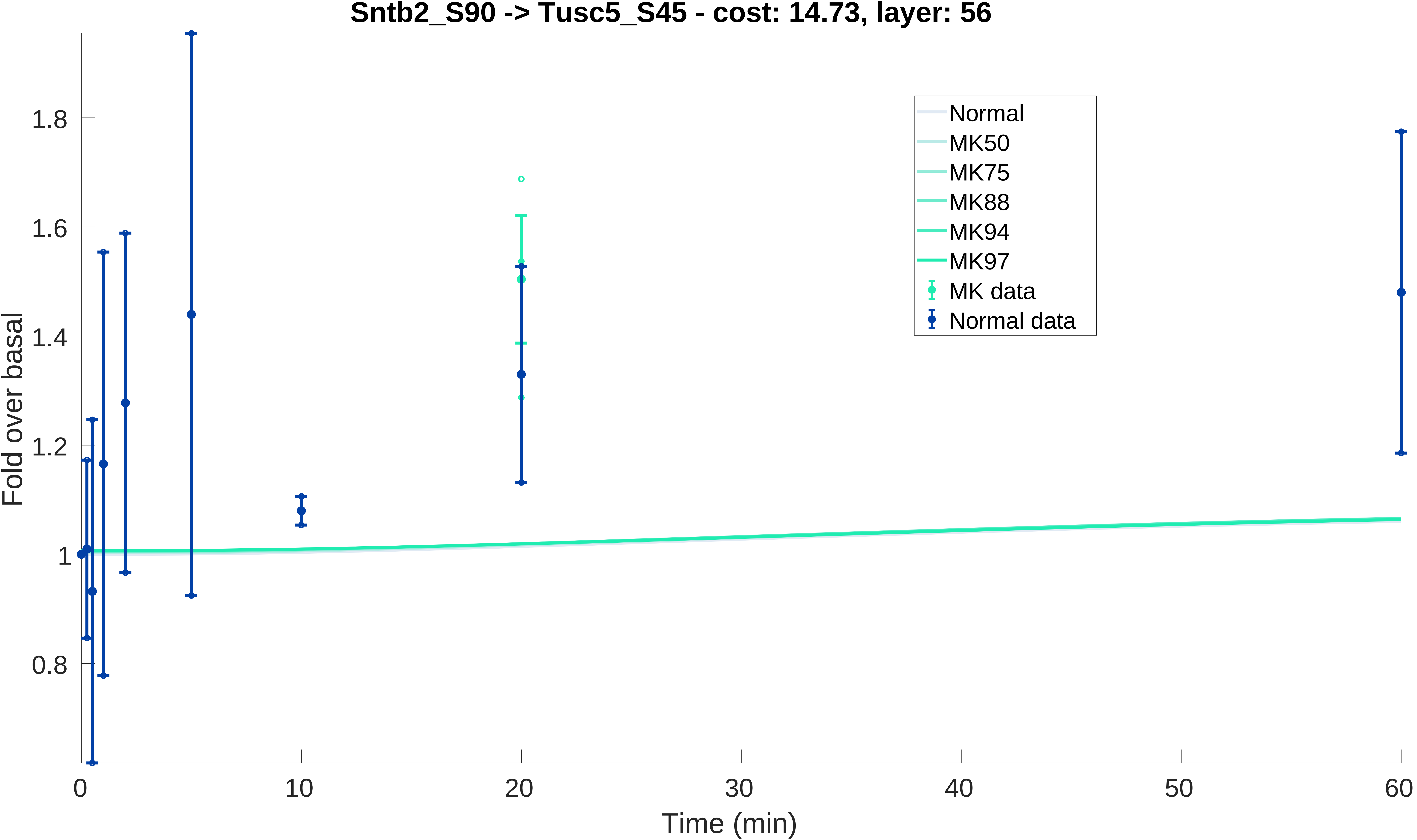

Sntb2_S90 -> Tusc5_S45 - cost: 14.73, layer: 56
Fold over basal
Time (min)
0.8
1
1.2
1.4
1.6
1.8
0
10
20
30
40
50
60
Normal
MK50
MK75
MK88
MK94
MK97
MK data
Normal data

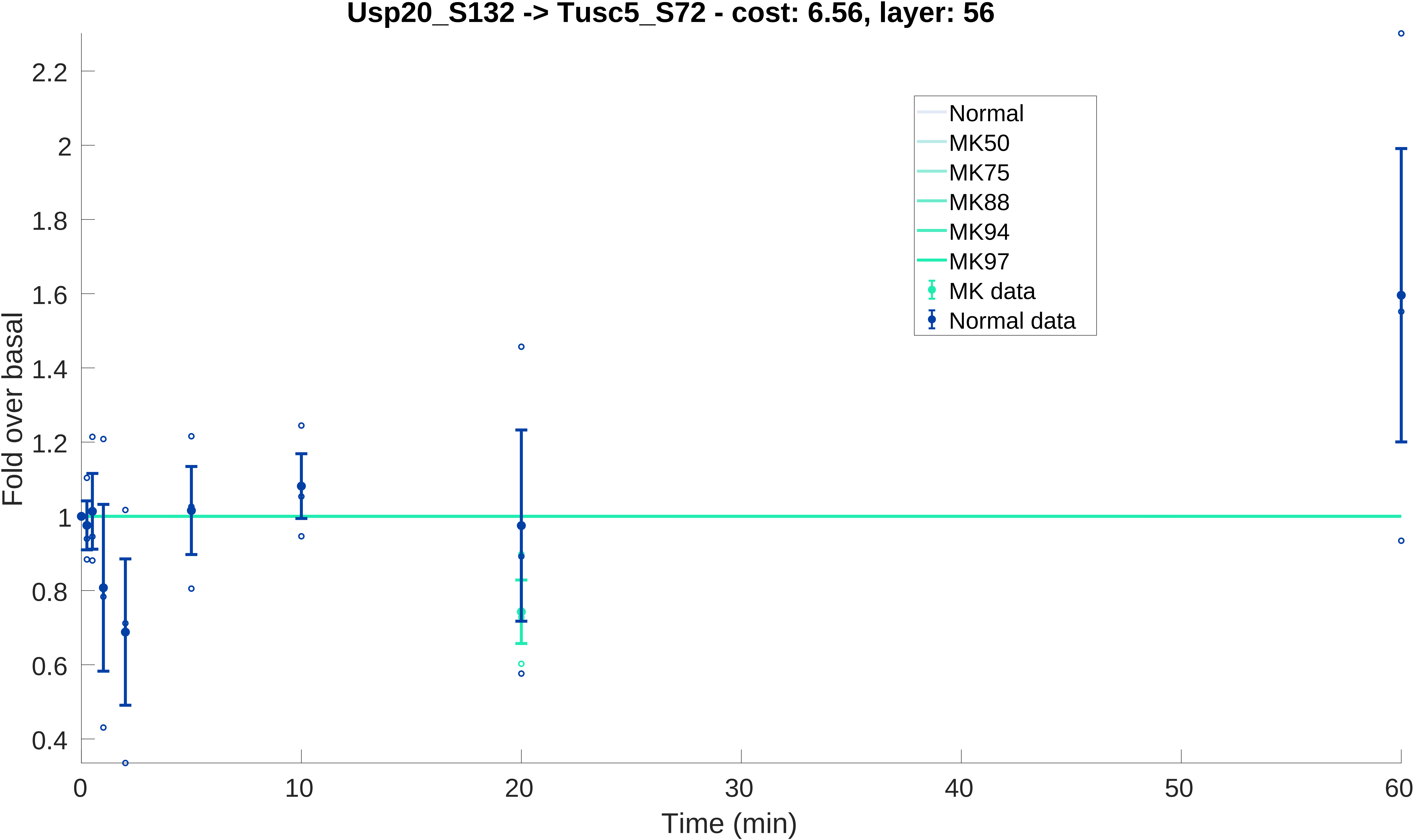

Usp20_S132 -> Tusc5_S72 - cost: 6.56, layer: 56
Normal
MK50
MK75
MK88
MK94
MK97
MK data
Normal data
Fold over basal
Time (min)
0.4
0.6
0.8
1
1.2
1.4
1.6
1.8
2
2.2
0
10
20
30
40
50
60

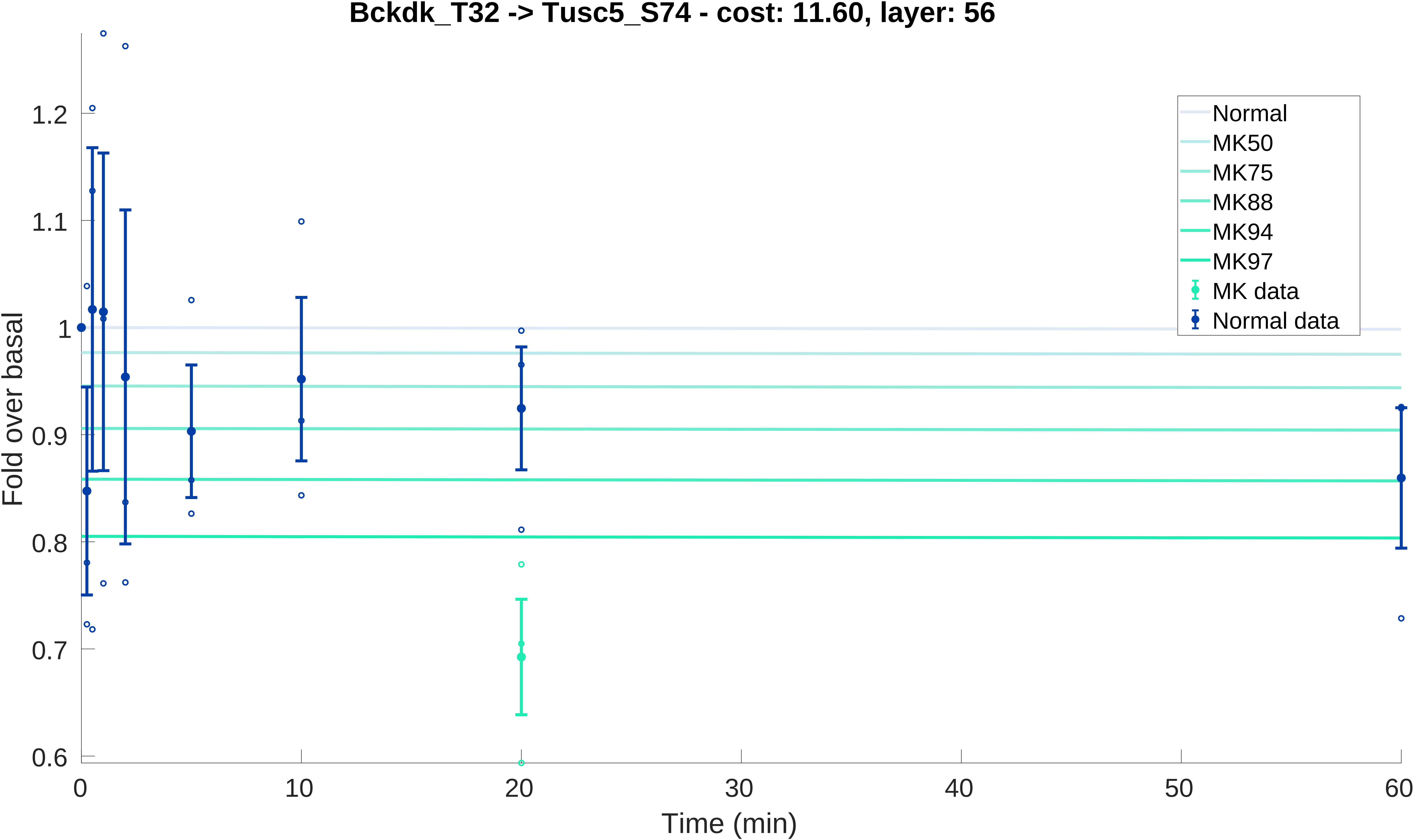

Bckdk_T32 -> Tusc5_S74 - cost: 11.60, layer: 56
Fold over basal
Time (min)
1.2
1.1
1
0.9
0.8
0.7
0.6
0
10
20
30
40
50
60
Normal
MK50
MK75
MK88
MK94
MK97
MK data
Normal data

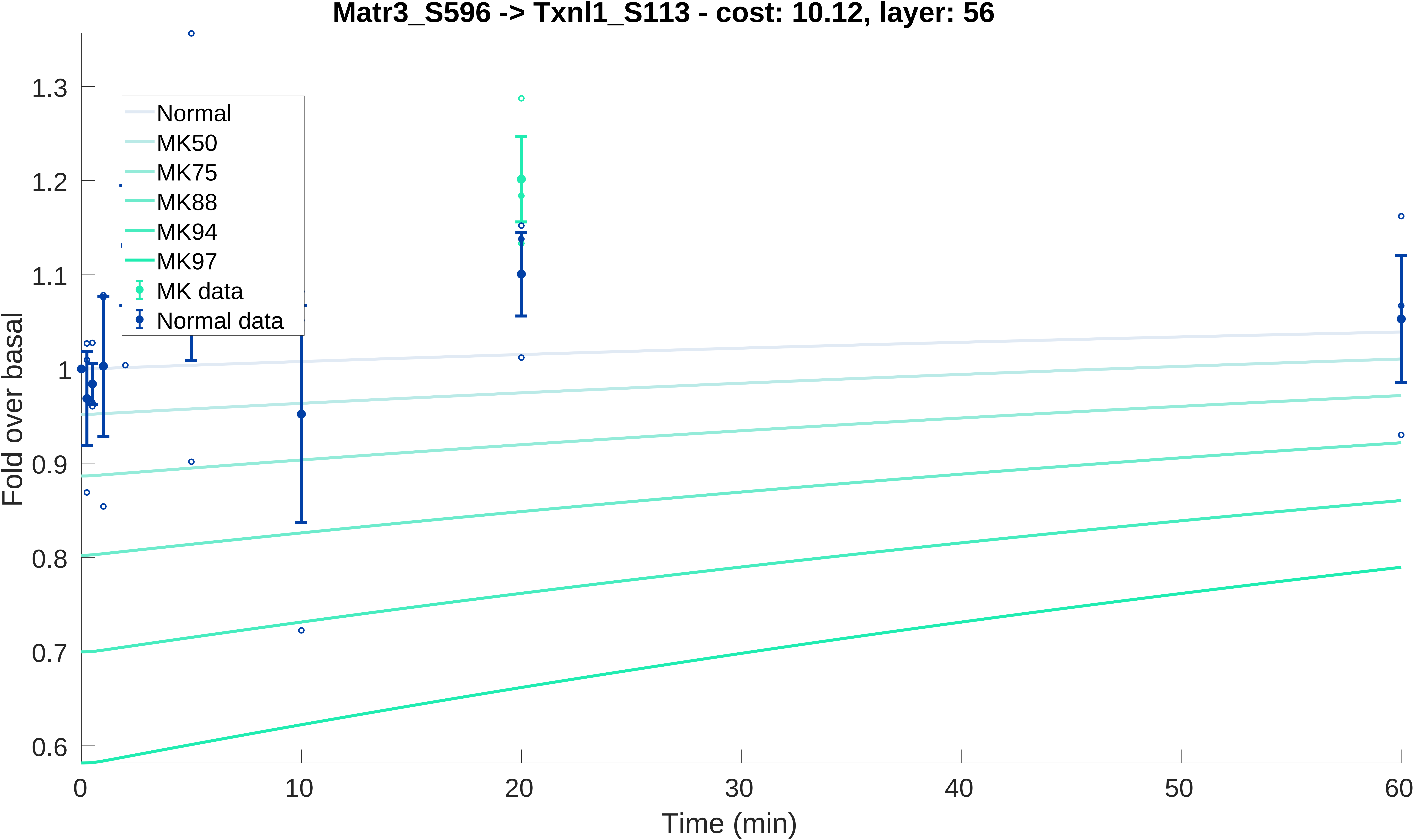

Matr3_S596 -> Txnl1_S113 - cost: 10.12, layer: 56
Fold over basal
Time (min)
Normal
MK50
MK75
MK88
MK94
MK97
MK data
Normal data
1.3
1.2
1.1
1
0.9
0.8
0.7
0.6
0
10
20
30
40
50
60

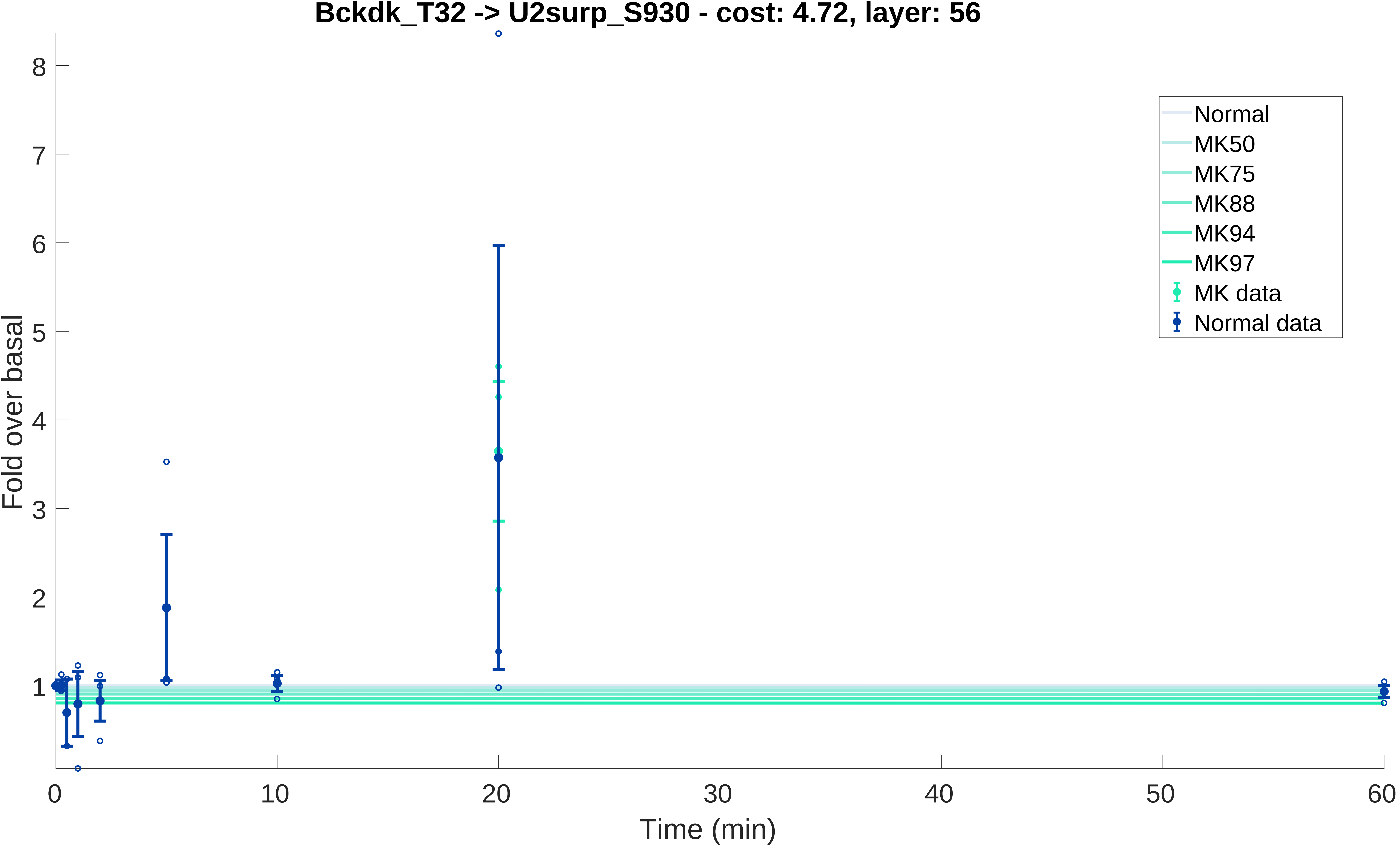

Bckdk_T32 -> U2surp_S930 - cost: 4.72, layer: 56
Fold over basal
Time (min)
0
10
20
30
40
50
60
1
2
3
4
5
6
7
8
Normal
MK50
MK75
MK88
MK94
MK97
MK data
Normal data

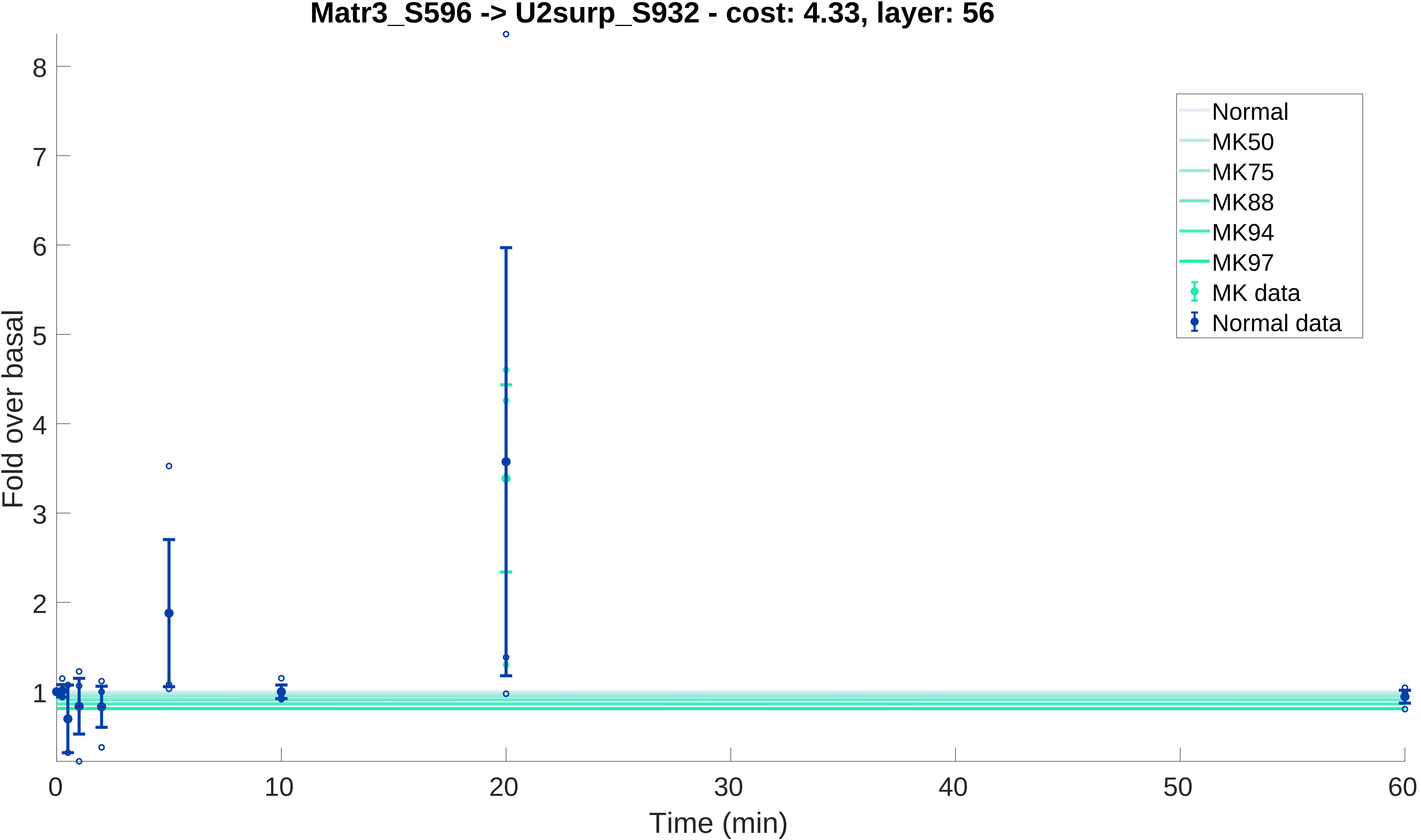

Matr3_S596 -> U2surp_S932 - cost: 4.33, layer: 56
Fold over basal
Time (min)
Normal
MK50
MK75
MK88
MK94
MK97
MK data
Normal data
0
10
20
30
40
50
60
1
2
3
4
5
6
7
8

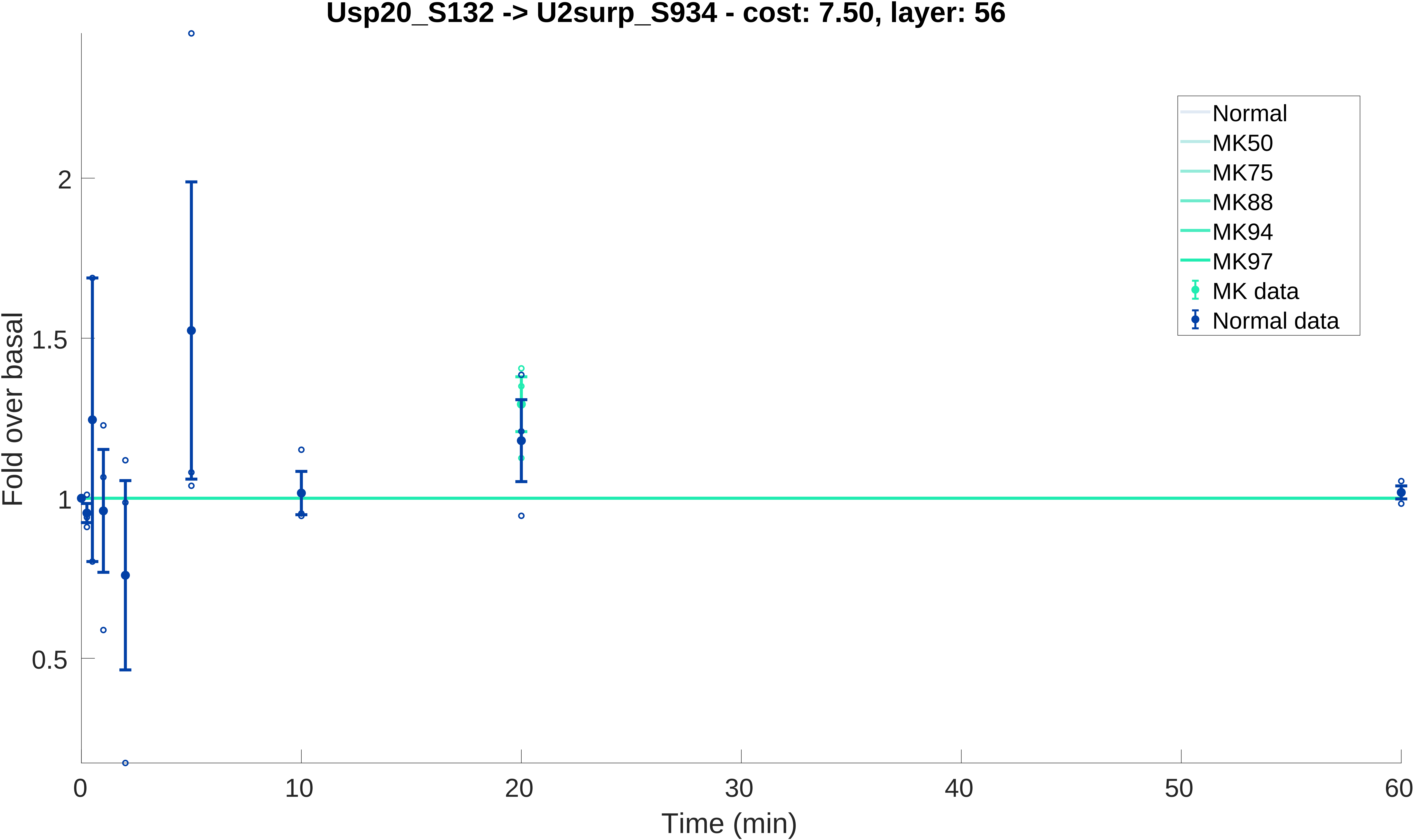

Usp20_S132 -> U2surp_S934 - cost: 7.50, layer: 56
Fold over basal
Time (min)
0
10
20
30
40
50
60
0.5
1
1.5
2
Normal
MK50
MK75
MK88
MK94
MK97
MK data
Normal data

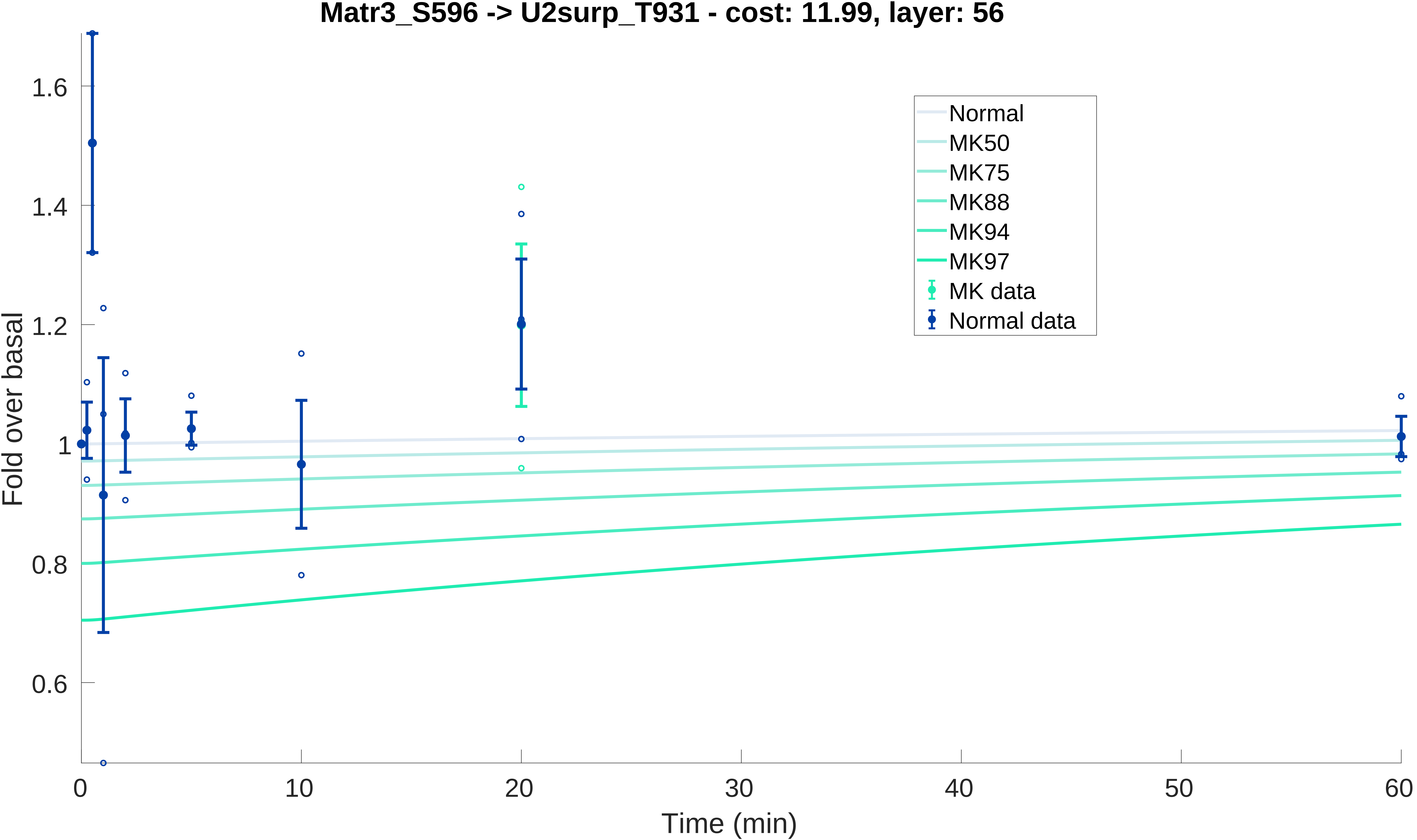
Matr3_S596 -> U2surp_T931 - cost: 11.99, layer: 56
Fold over basal
1.6
1.4
1.2
1
0.8
0.6
0
10
20
30
40
50
60
Time (min)
Normal
MK50
MK75
MK88
MK94
MK97
MK data
Normal data

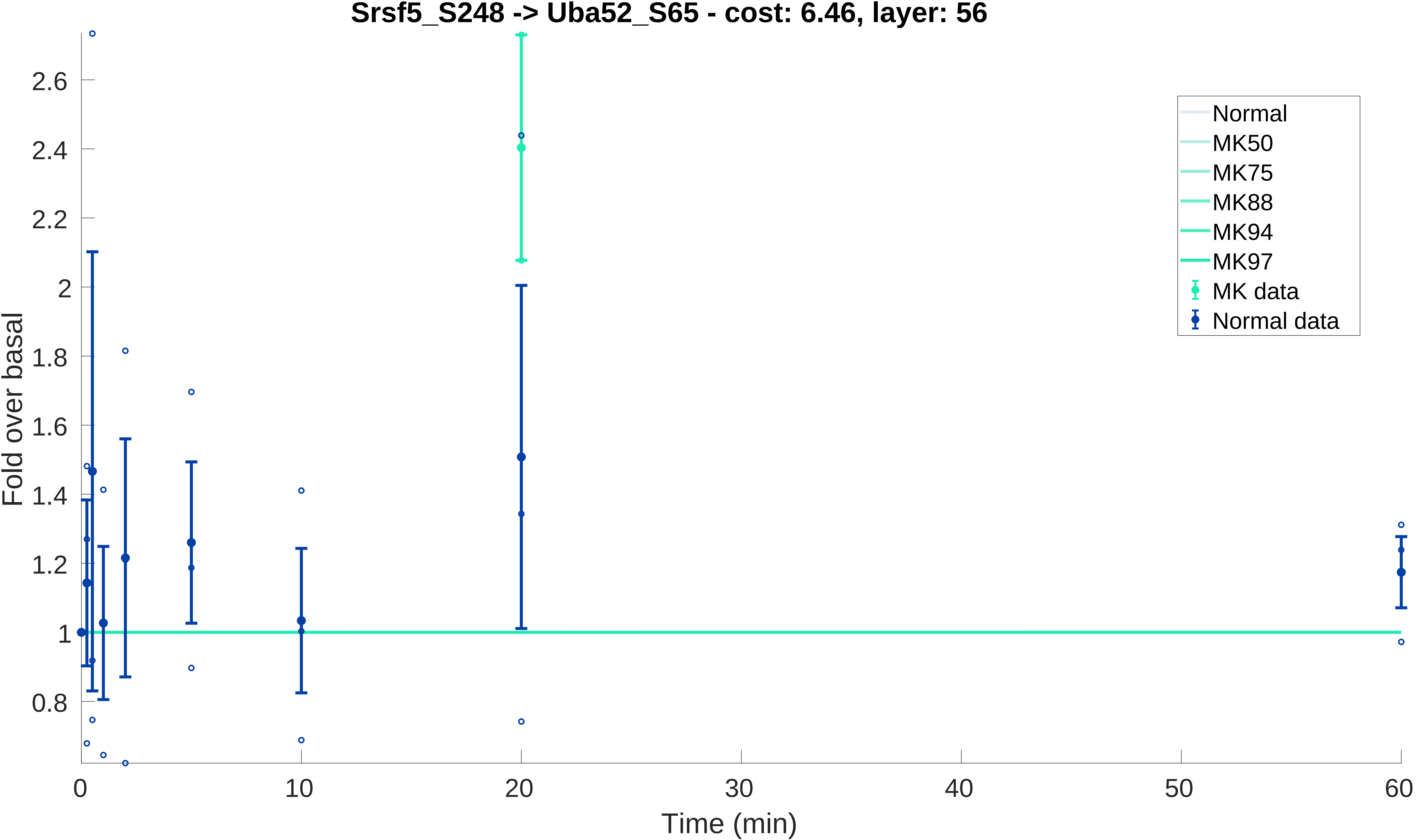

Srsf5_S248 -> Uba52_S65 - cost: 6.46, layer: 56
Fold over basal
Time (min)
2.6
2.4
2.2
2
1.8
1.6
1.4
1.2
1
0.8
0
10
20
30
40
50
60
Normal
MK50
MK75
MK88
MK94
MK97
MK data
Normal data

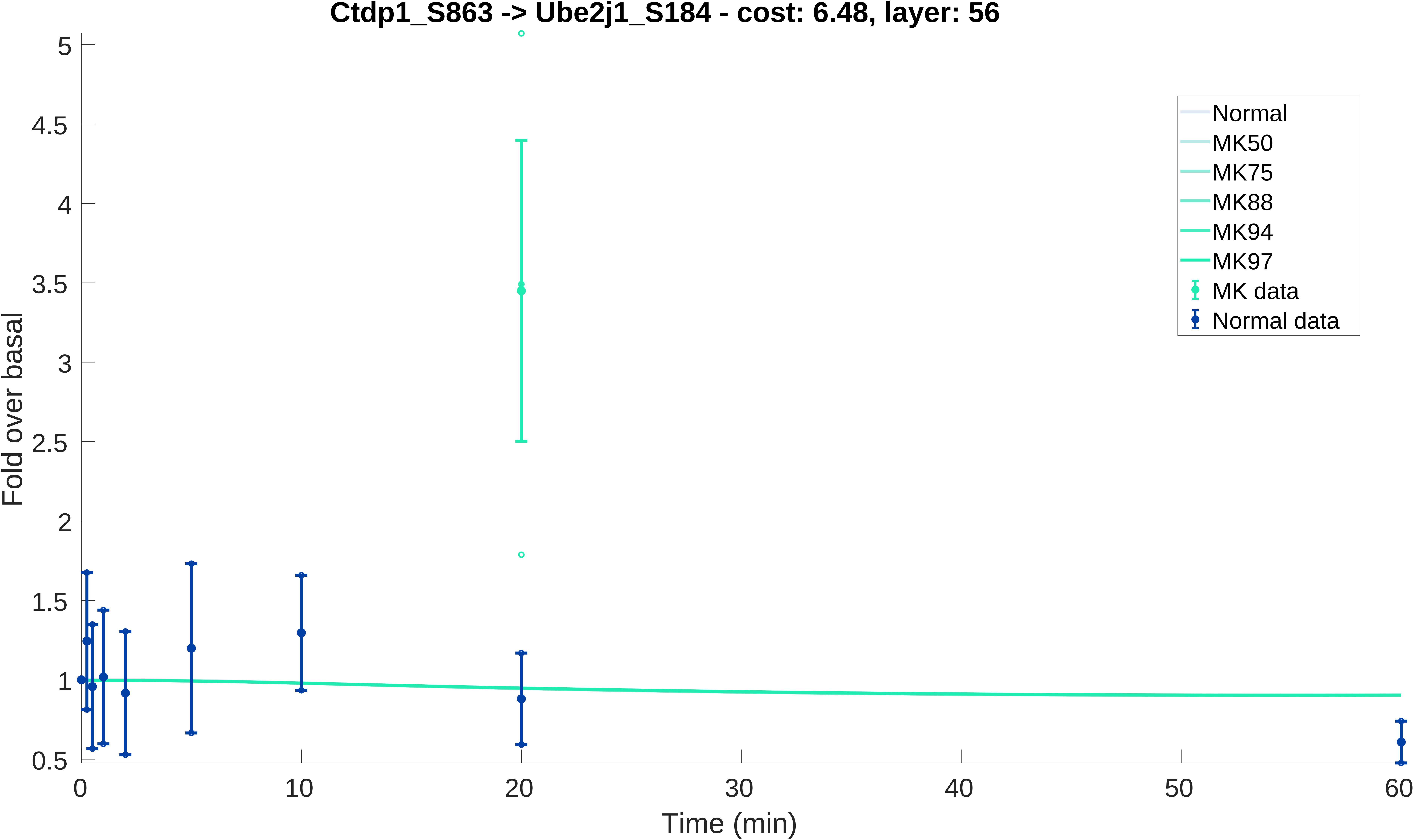

Ctdp1_S863 -> Ube2j1_S184 - cost: 6.48, layer: 56
Fold over basal
Time (min)
0
10
20
30
40
50
60
0.5
1
1.5
2
2.5
3
3.5
4
4.5
5
Normal
MK50
MK75
MK88
MK94
MK97
MK data
Normal data

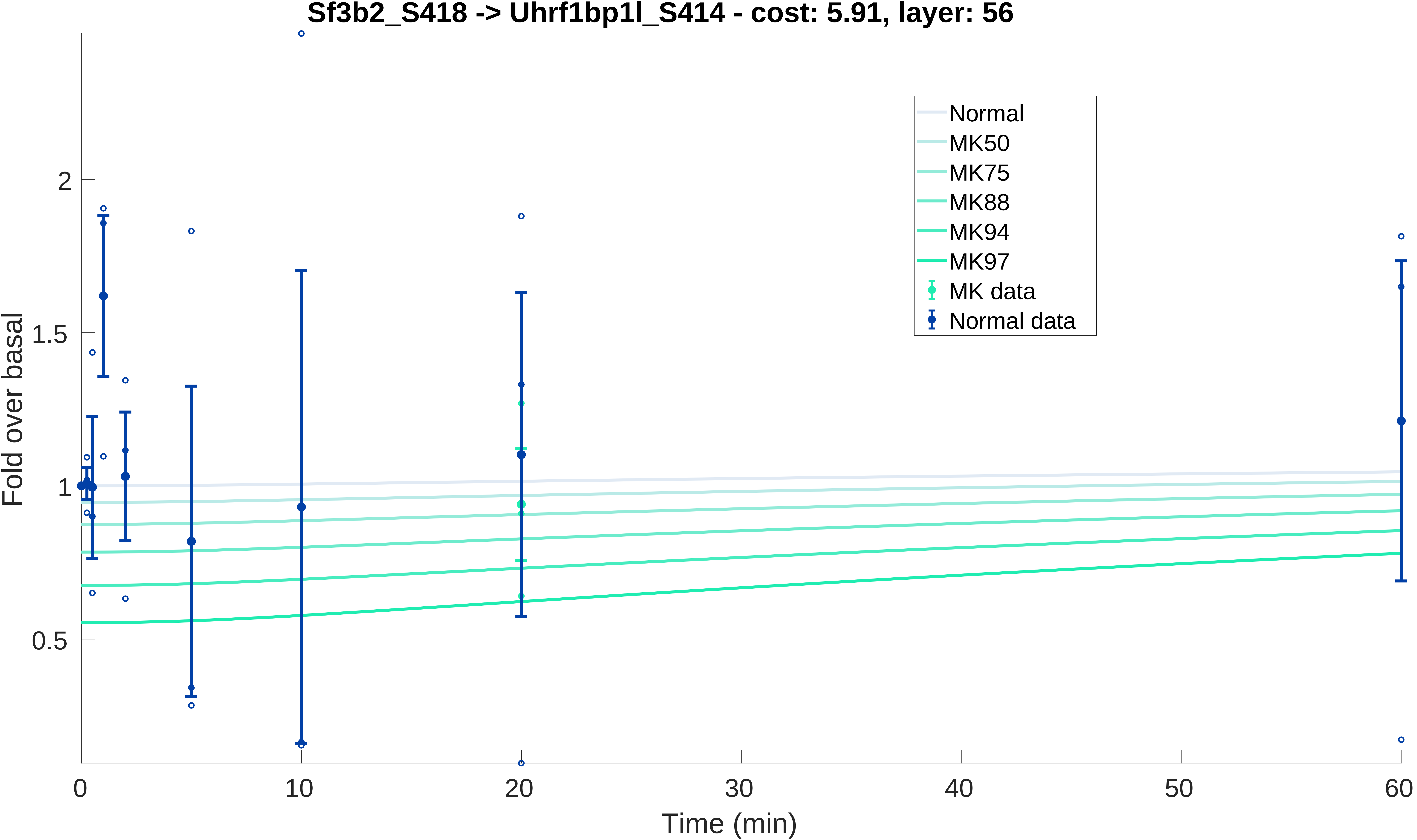

Sf3b2_S418 -> Uhrf1bp1l_S414 - cost: 5.91, layer: 56
Fold over basal
Time (min)
0.5
1
1.5
2
0
10
20
30
40
50
60
Normal
MK50
MK75
MK88
MK94
MK97
MK data
Normal data

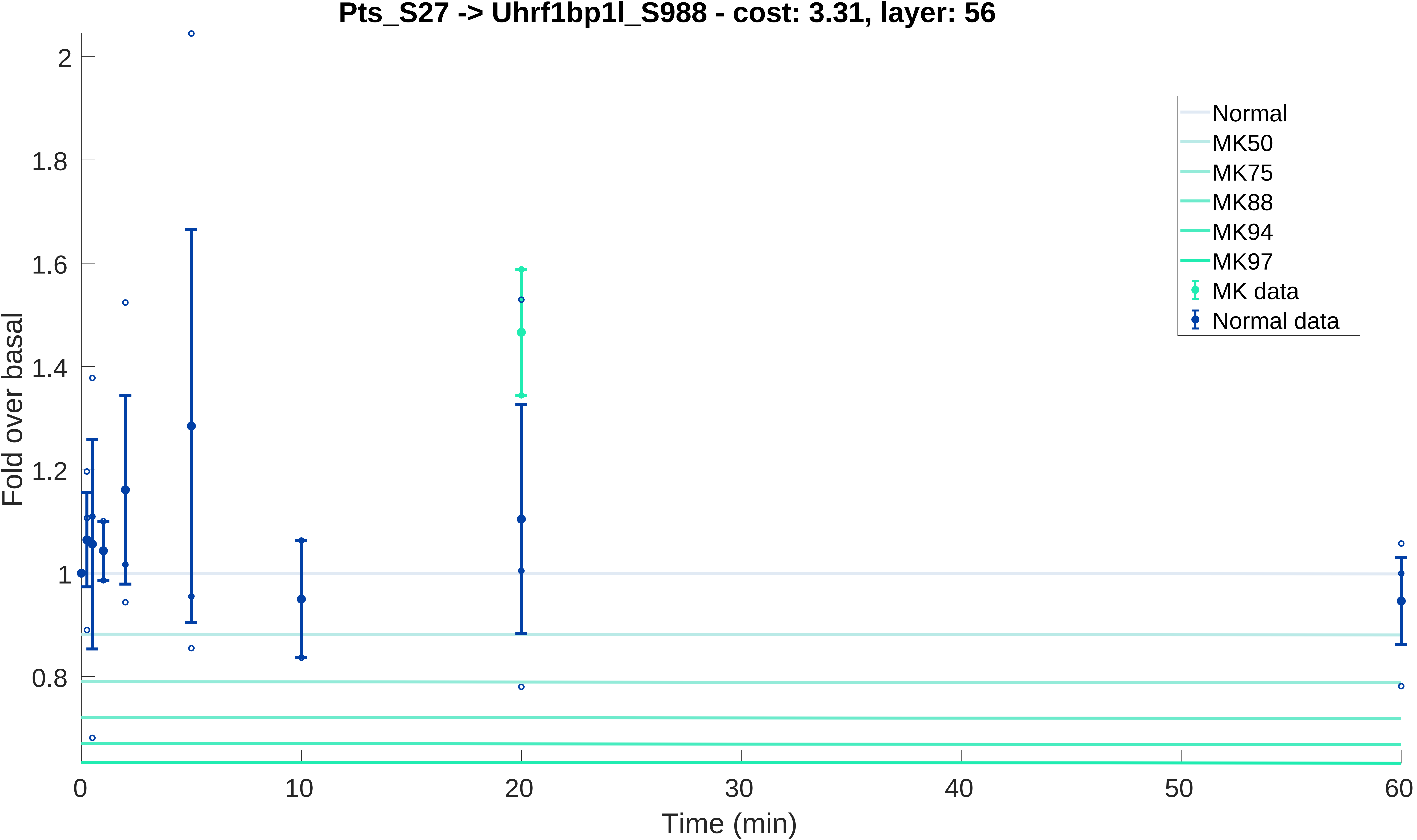
Pts_S27 -> Uhrf1bp1l_S988 - cost: 3.31, layer: 56
Fold over basal
2
1.8
1.6
1.4
1.2
1
0.8
0
10
20
30
40
50
60
Time (min)
Normal
MK50
MK75
MK88
MK94
MK97
MK data
Normal data

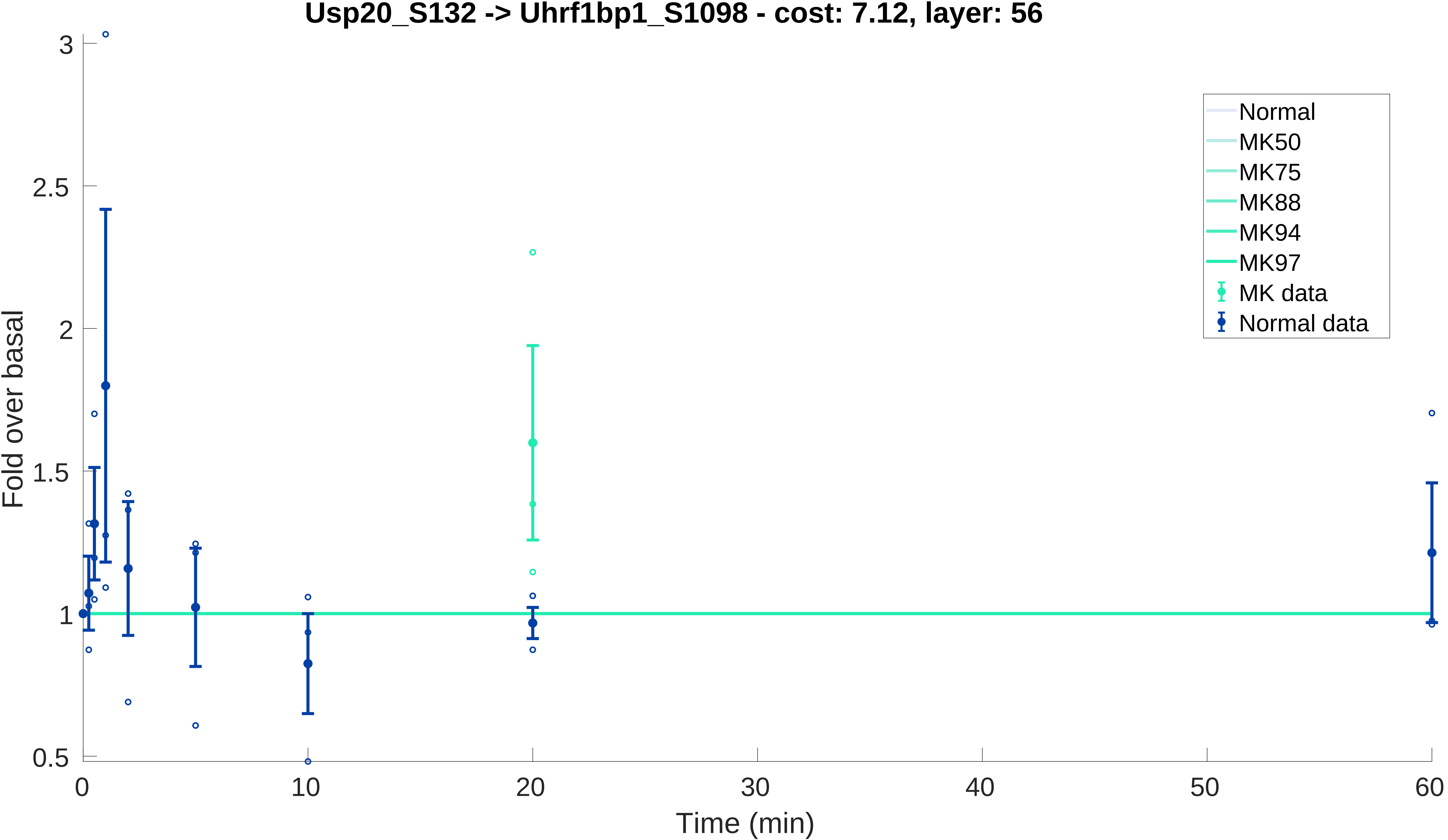

Usp20_S132 -> Uhrf1bp1_S1098 - cost: 7.12, layer: 56
Fold over basal
Time (min)
0.5
1
1.5
2
2.5
3
0
10
20
30
40
50
60
Normal
MK50
MK75
MK88
MK94
MK97
MK data
Normal data

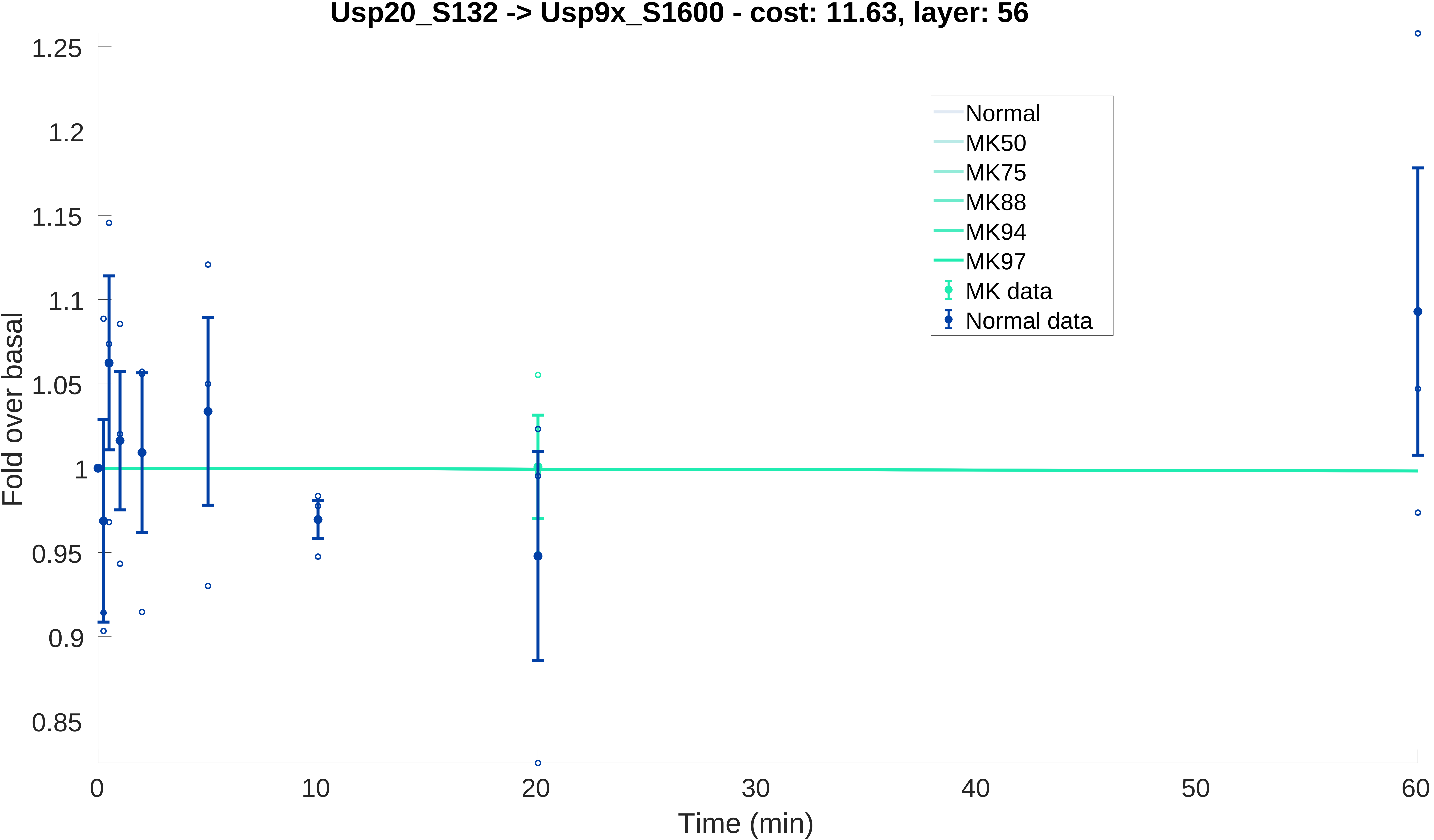

Usp20_S132 -> Usp9x_S1600 - cost: 11.63, layer: 56
Fold over basal
Time (min)
1.25
1.2
1.15
1.1
1.05
1
0.95
0.9
0.85
0
10
20
30
40
50
60
Normal
MK50
MK75
MK88
MK94
MK97
MK data
Normal data

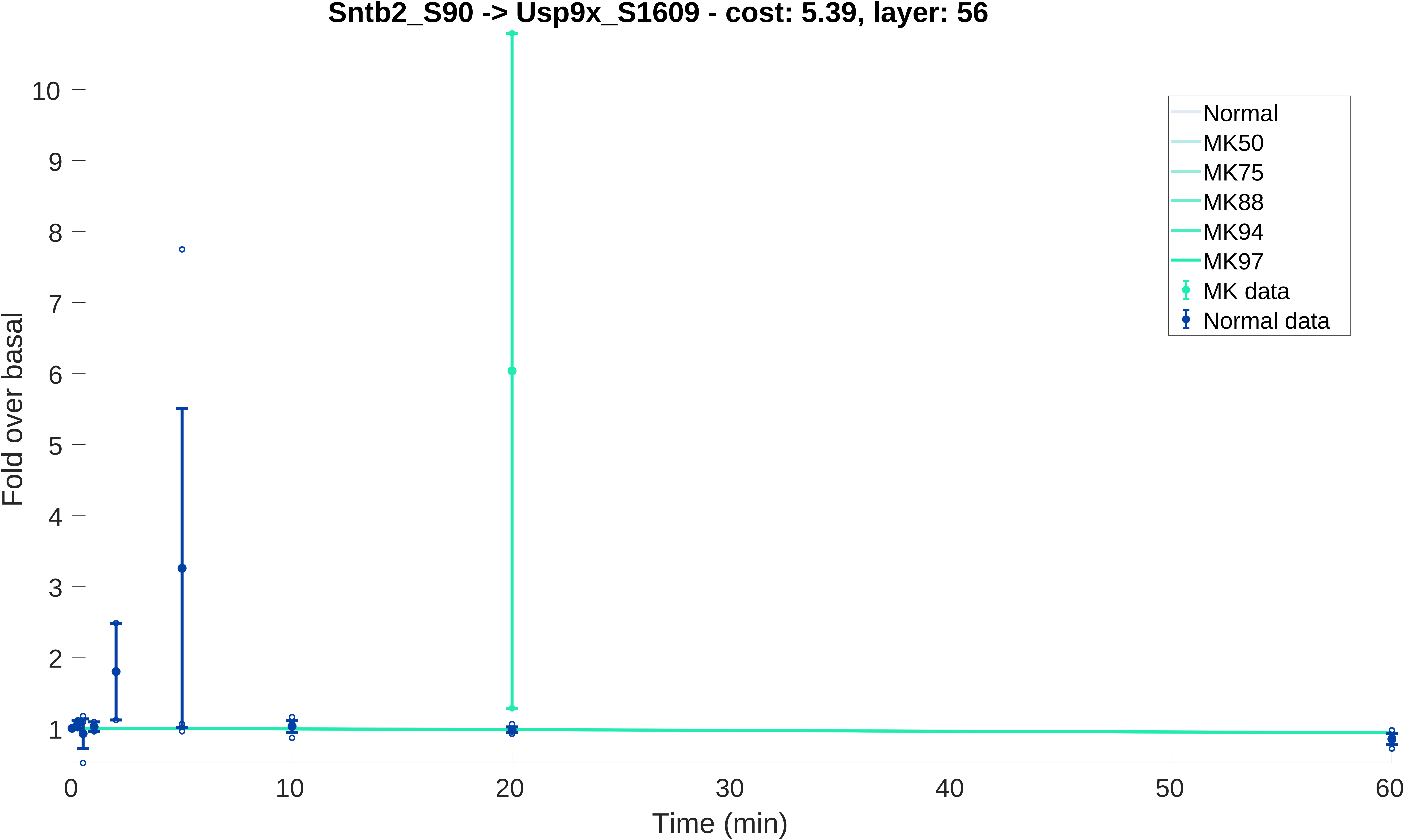
Sntb2_S90 -> Usp9x_S1609 - cost: 5.39, layer: 56
Fold over basal
Time (min)
0
10
20
30
40
50
60
1
2
3
4
5
6
7
8
9
10
Normal
MK50
MK75
MK88
MK94
MK97
MK data
Normal data

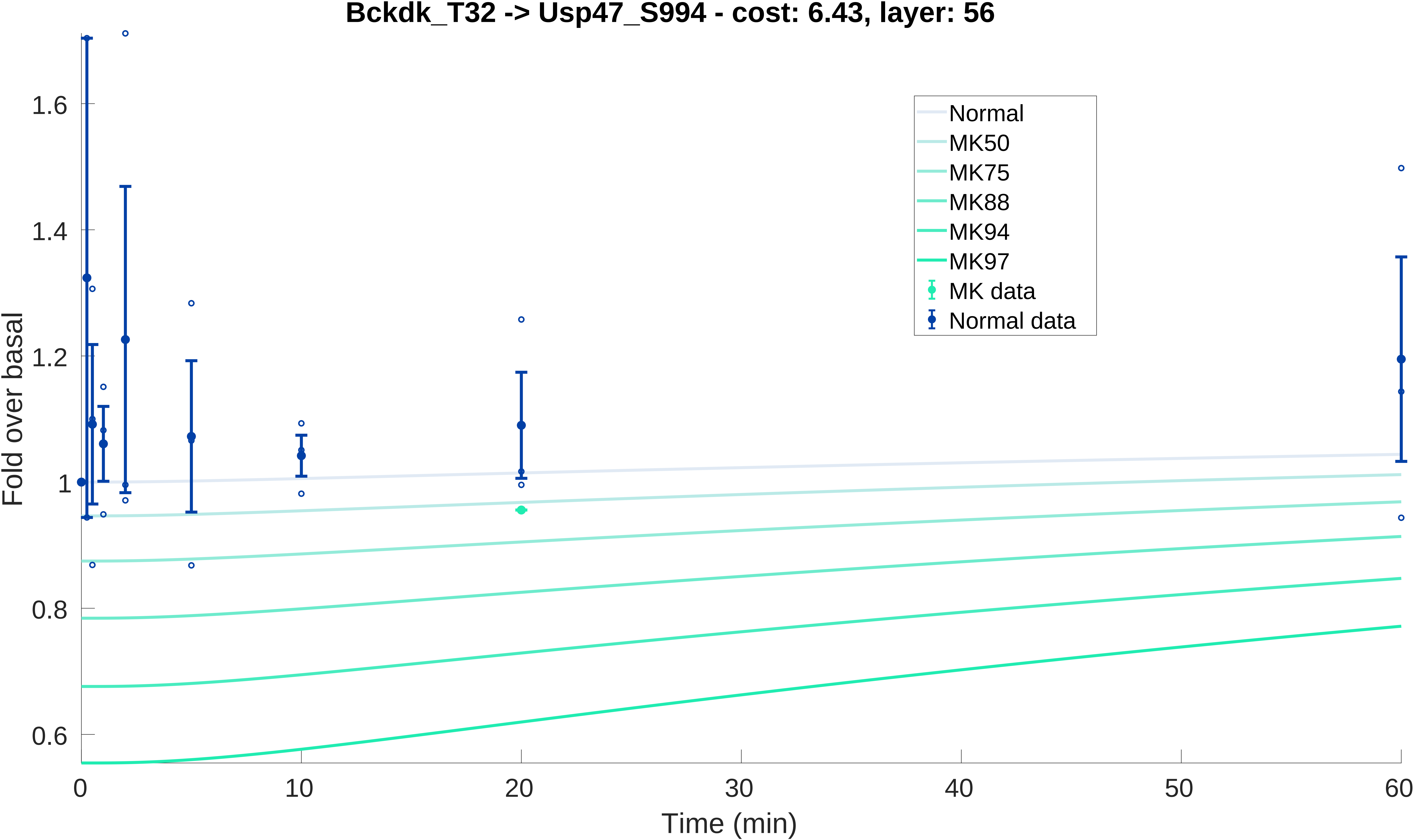

Bckdk_T32 -> Usp47_S994 - cost: 6.43, layer: 56
Fold over basal
Time (min)
0.6
0.8
1
1.2
1.4
1.6
0
10
20
30
40
50
60
Normal
MK50
MK75
MK88
MK94
MK97
MK data
Normal data

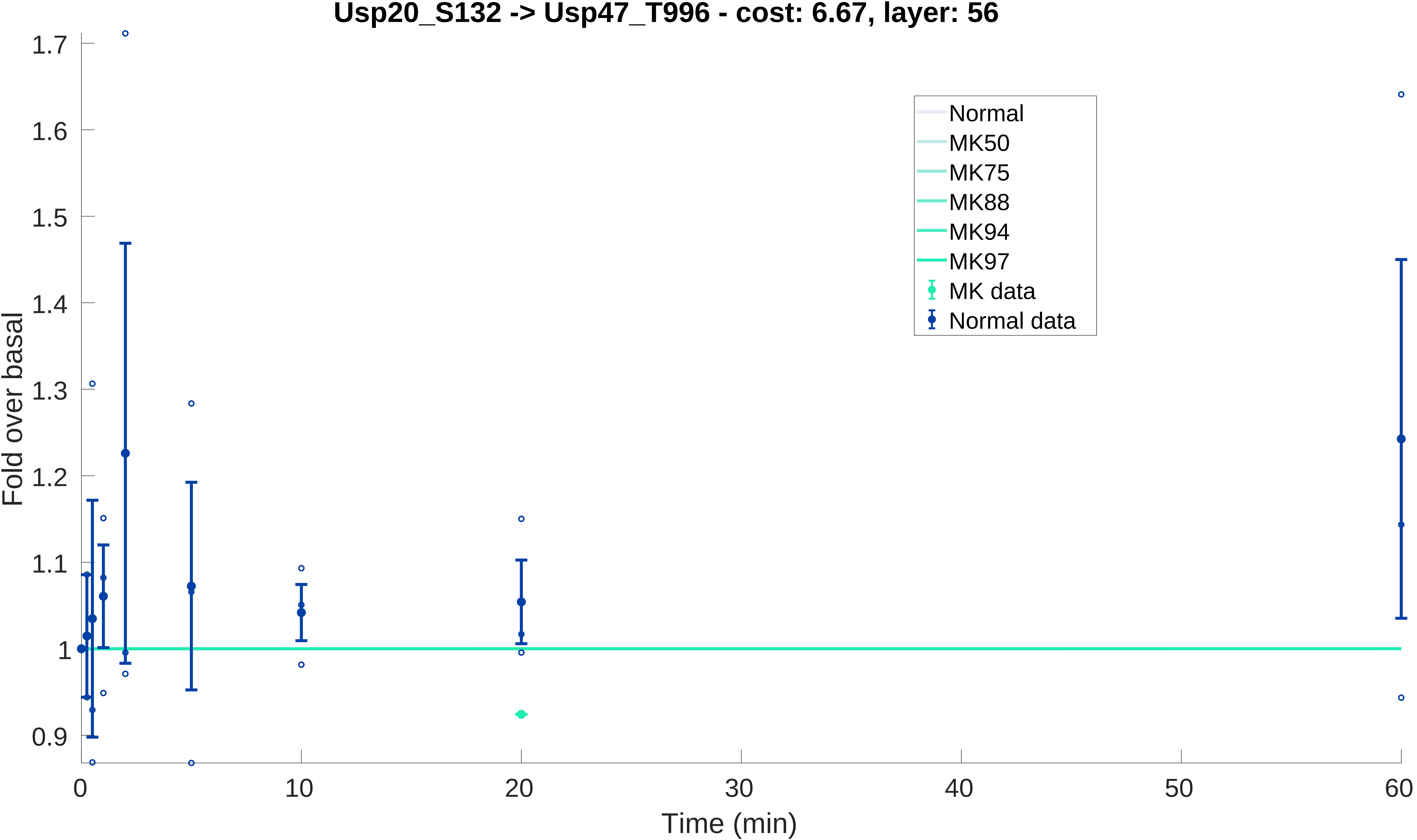

Usp20_S132 -> Usp47_T996 - cost: 6.67, layer: 56
Fold over basal
Time (min)
Normal
MK50
MK75
MK88
MK94
MK97
MK data
Normal data
1.7
1.6
1.5
1.4
1.3
1.2
1.1
1
0.9
0
10
20
30
40
50
60

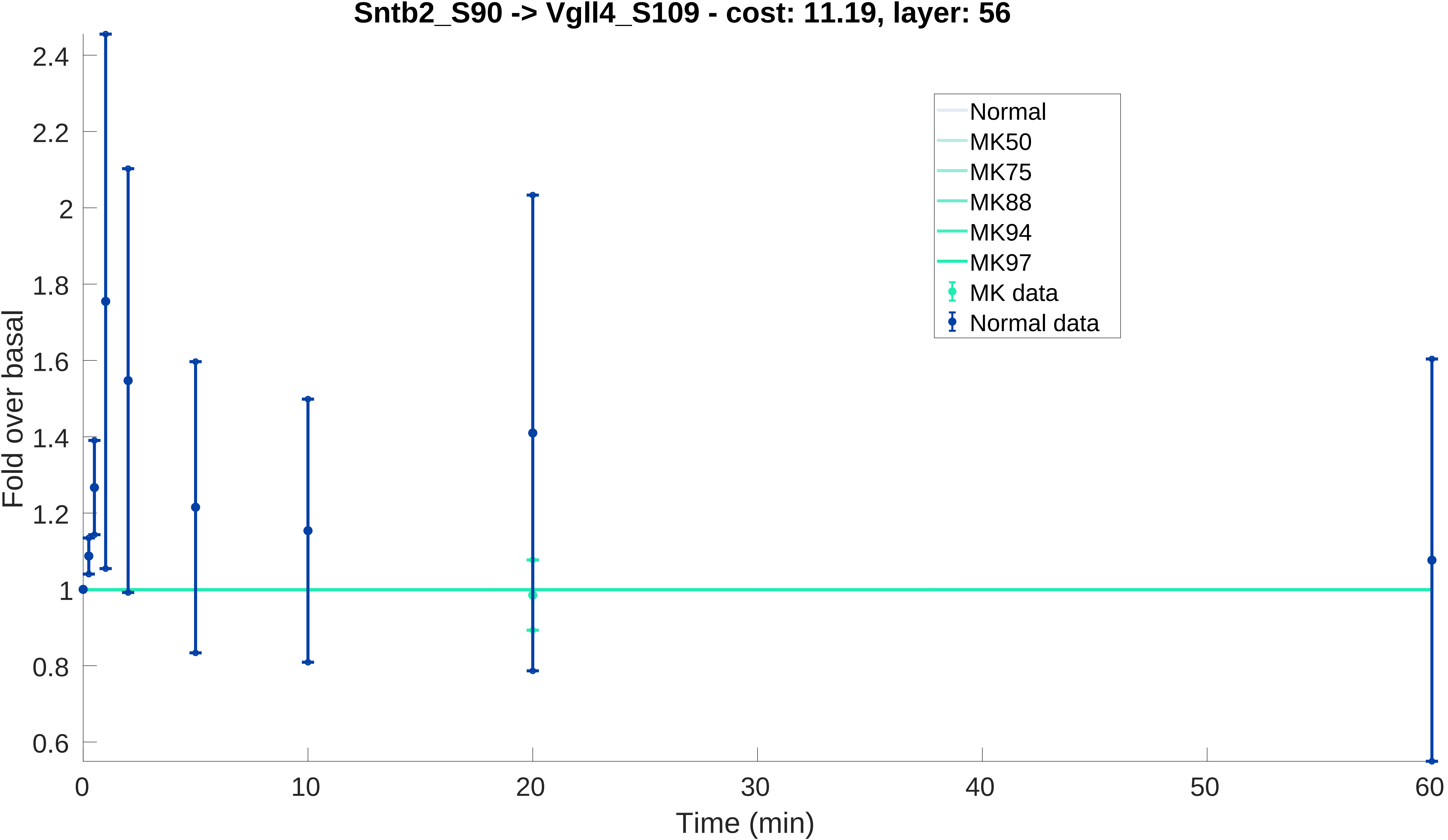

Sntb2_S90 -> Vgll4_S109 - cost: 11.19, layer: 56
Fold over basal
Time (min)
0.6
0.8
1
1.2
1.4
1.6
1.8
2
2.2
2.4
0
10
20
30
40
50
60
Normal
MK50
MK75
MK88
MK94
MK97
MK data
Normal data

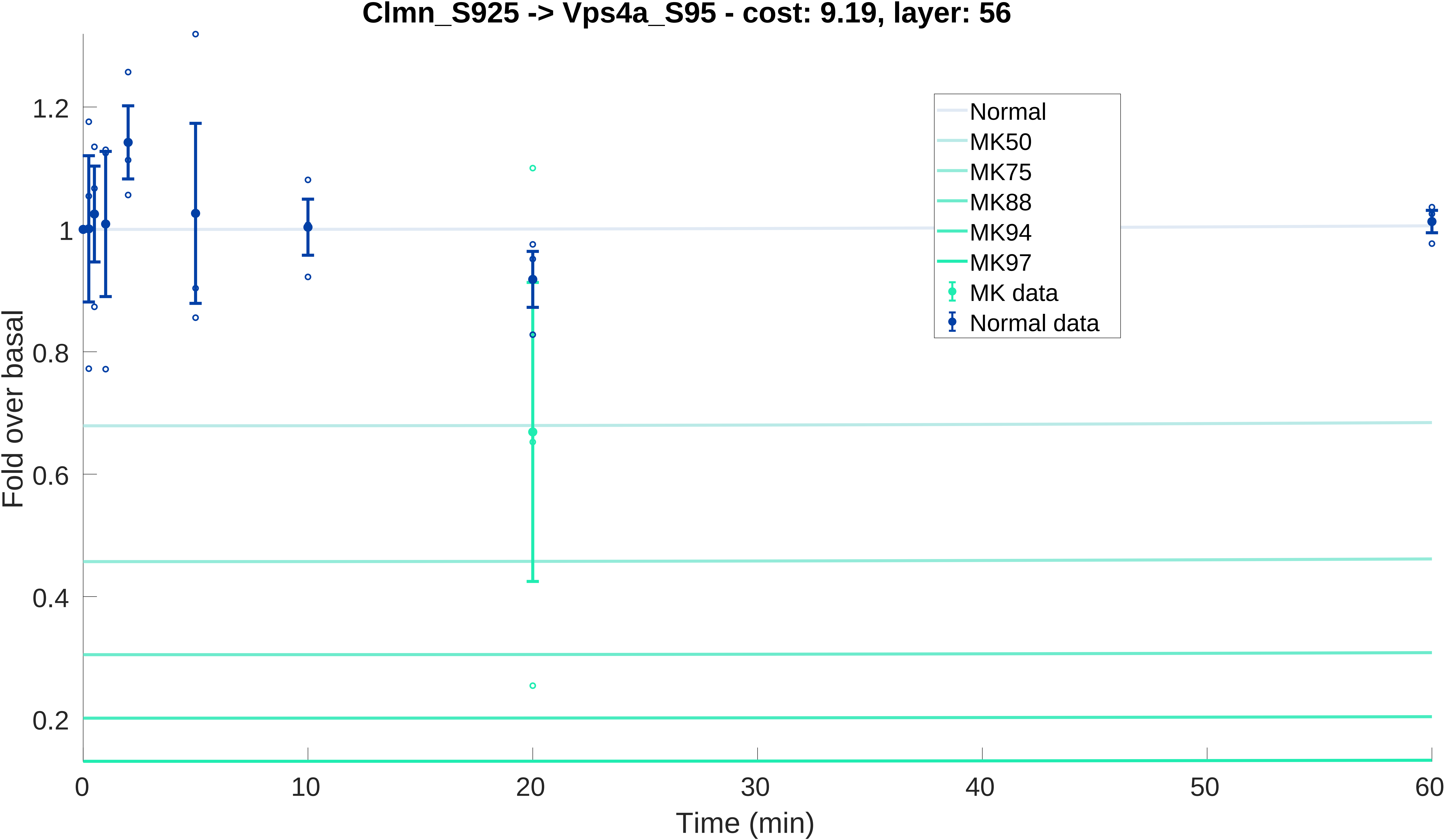

Clmn_S925 -> Vps4a_S95 - cost: 9.19, layer: 56
Fold over basal
Time (min)
0.2
0.4
0.6
0.8
1
1.2
0
10
20
30
40
50
60
Normal
MK50
MK75
MK88
MK94
MK97
MK data
Normal data

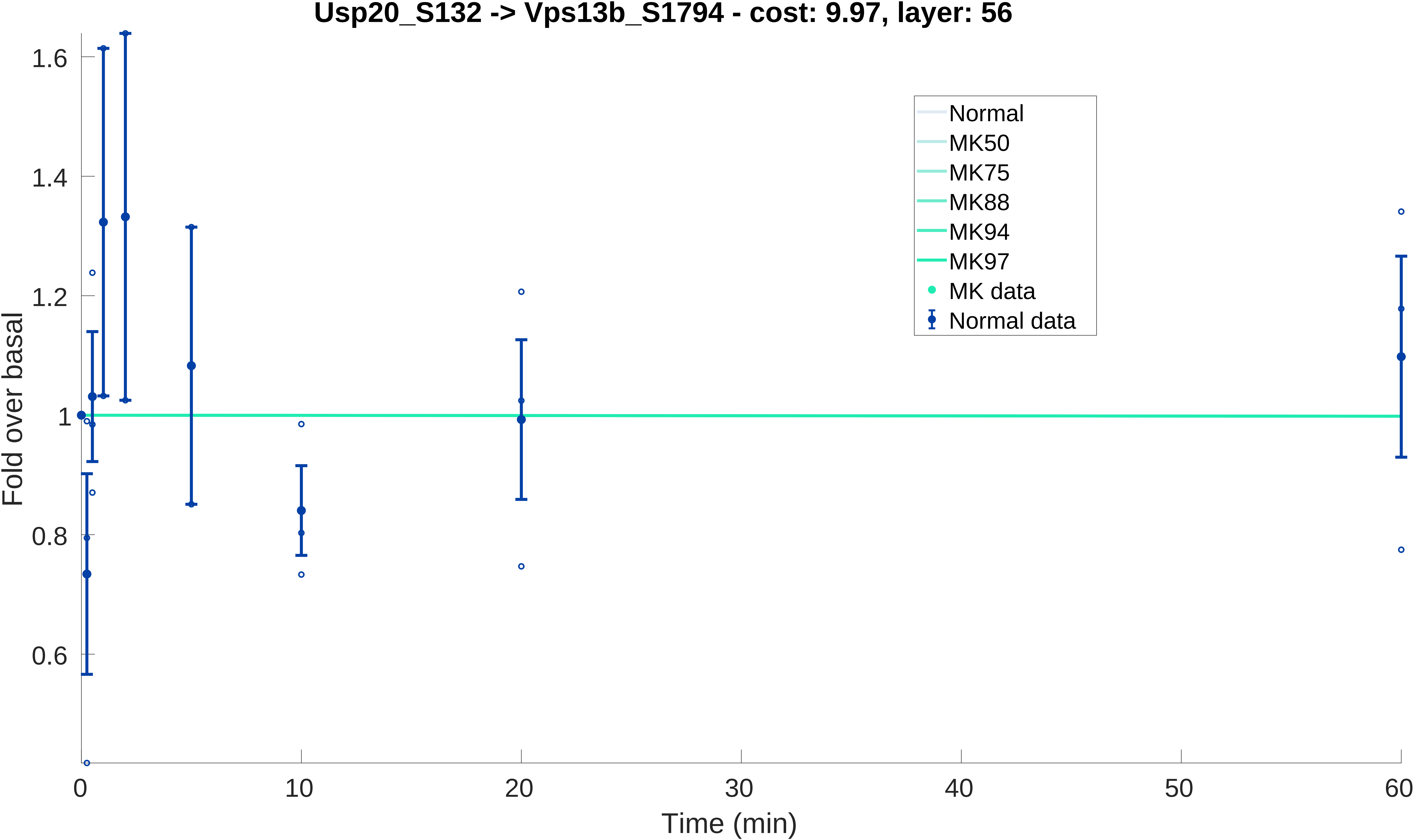

Usp20_S132 -> Vps13b_S1794 - cost: 9.97, layer: 56
Fold over basal
1.6
1.4
1.2
1
0.8
0.6
0
10
20
30
40
50
60
Time (min)
Normal
MK50
MK75
MK88
MK94
MK97
MK data
Normal data

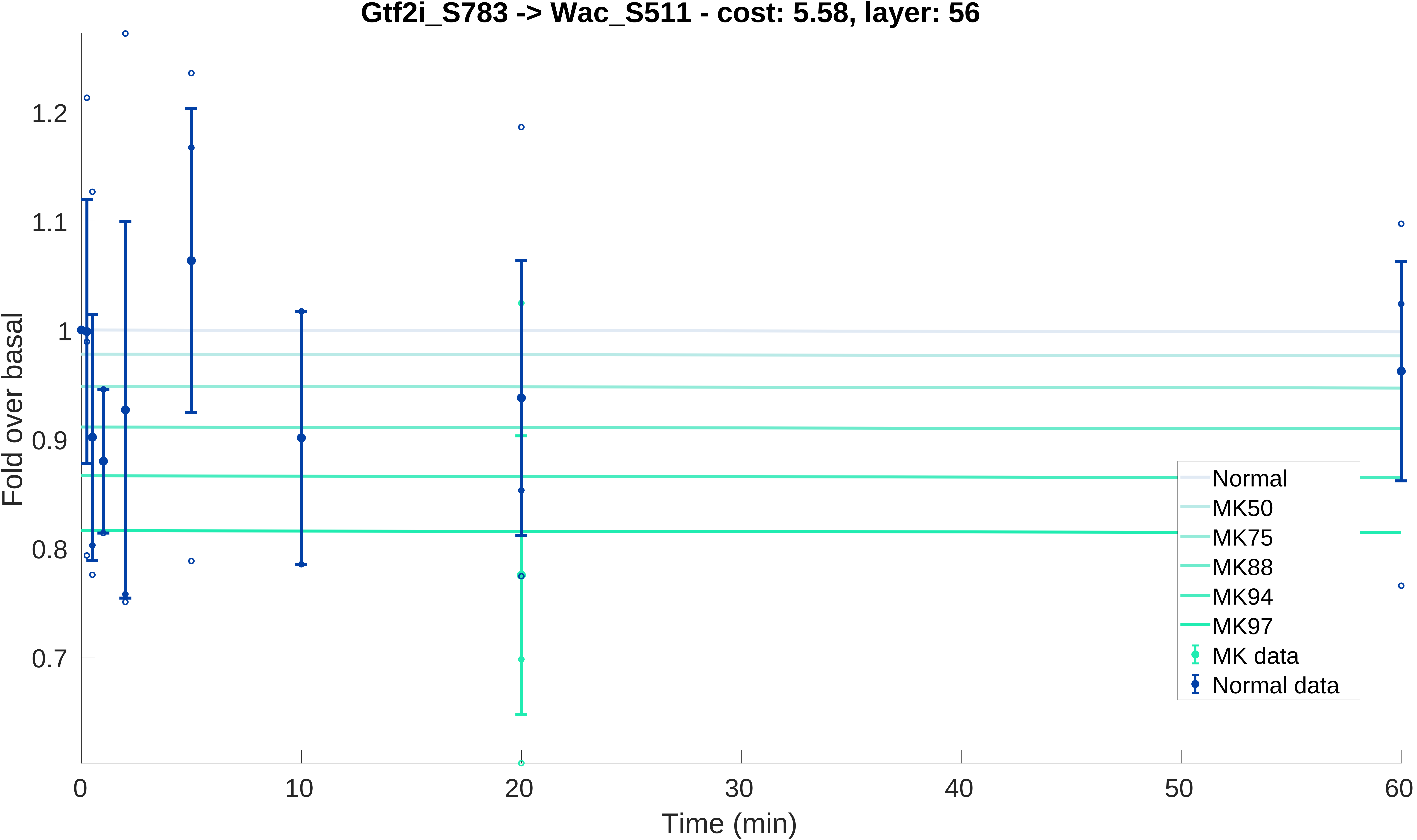
Gtf2i_S783 -> Wac_S511 - cost: 5.58, layer: 56
Fold over basal
Time (min)
1.2
1.1
1
0.9
0.8
0.7
0
10
20
30
40
50
60
Normal
MK50
MK75
MK88
MK94
MK97
MK data
Normal data

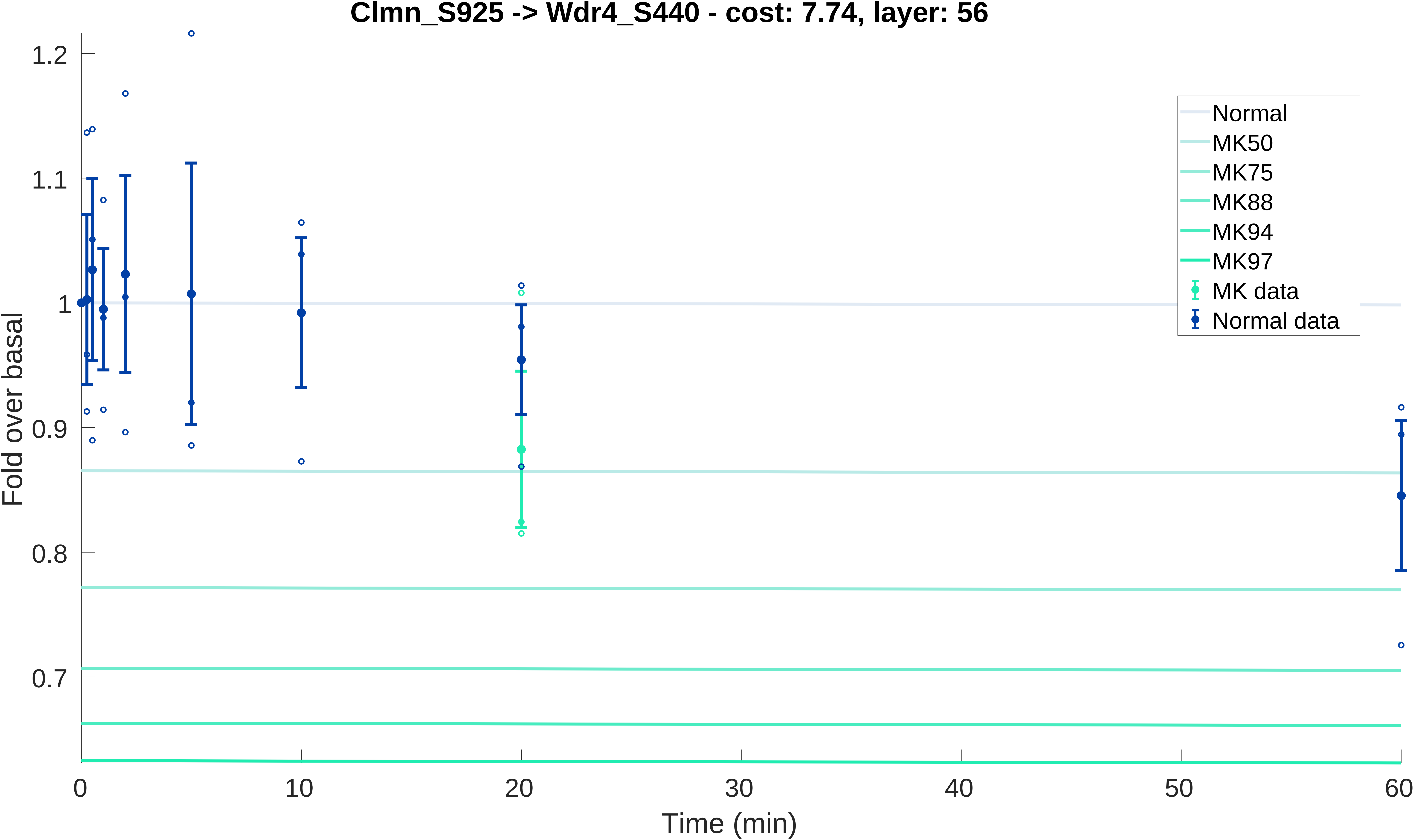

Clmn_S925 -> Wdr4_S440 - cost: 7.74, layer: 56
Fold over basal
Time (min)
1.2
1.1
1
0.9
0.8
0.7
0
10
20
30
40
50
60
Normal
MK50
MK75
MK88
MK94
MK97
MK data
Normal data

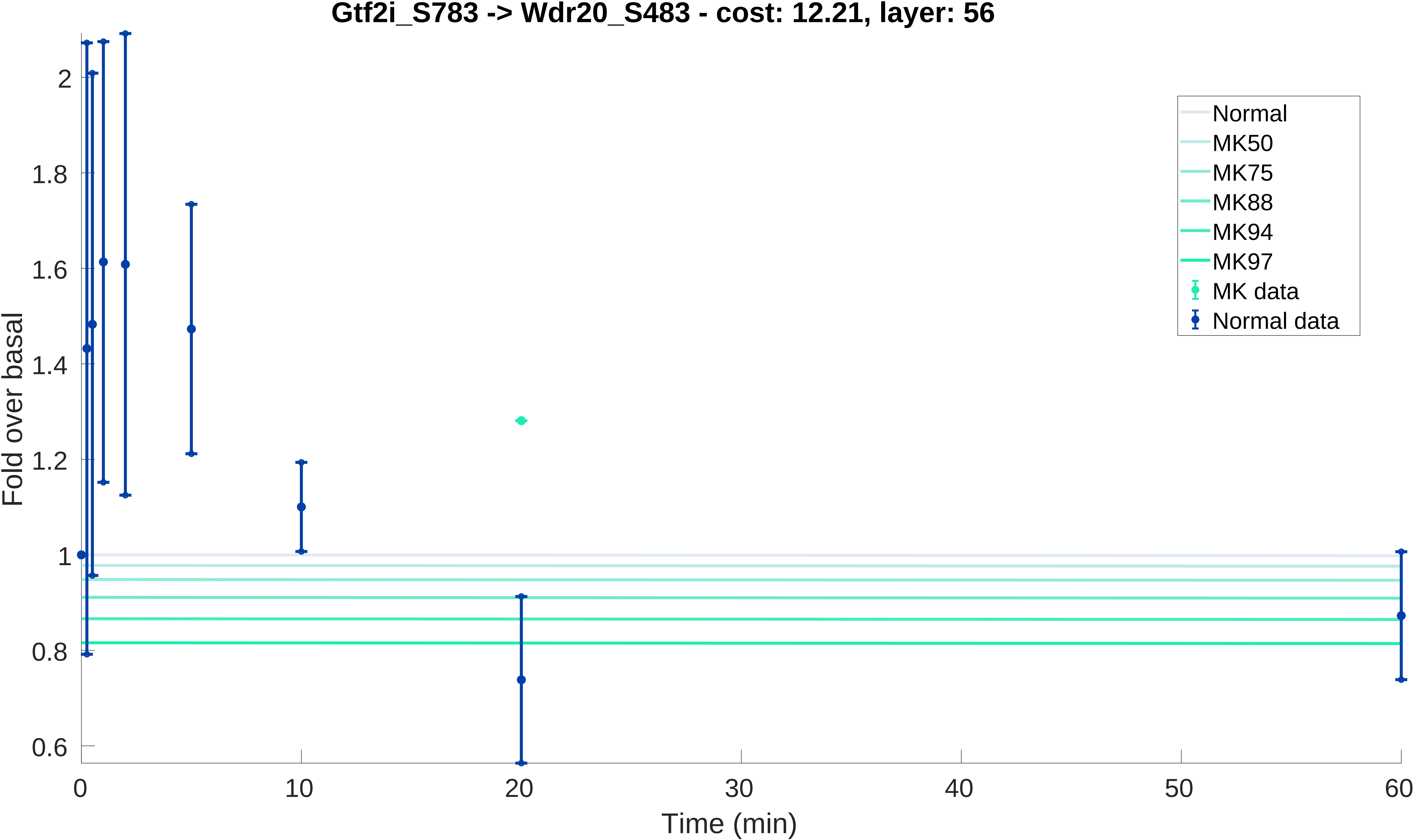

Gtf2i_S783 -> Wdr20_S483 - cost: 12.21, layer: 56
Fold over basal
Time (min)
0.6
0.8
1
1.2
1.4
1.6
1.8
2
0
10
20
30
40
50
60
Normal
MK50
MK75
MK88
MK94
MK97
MK data
Normal data

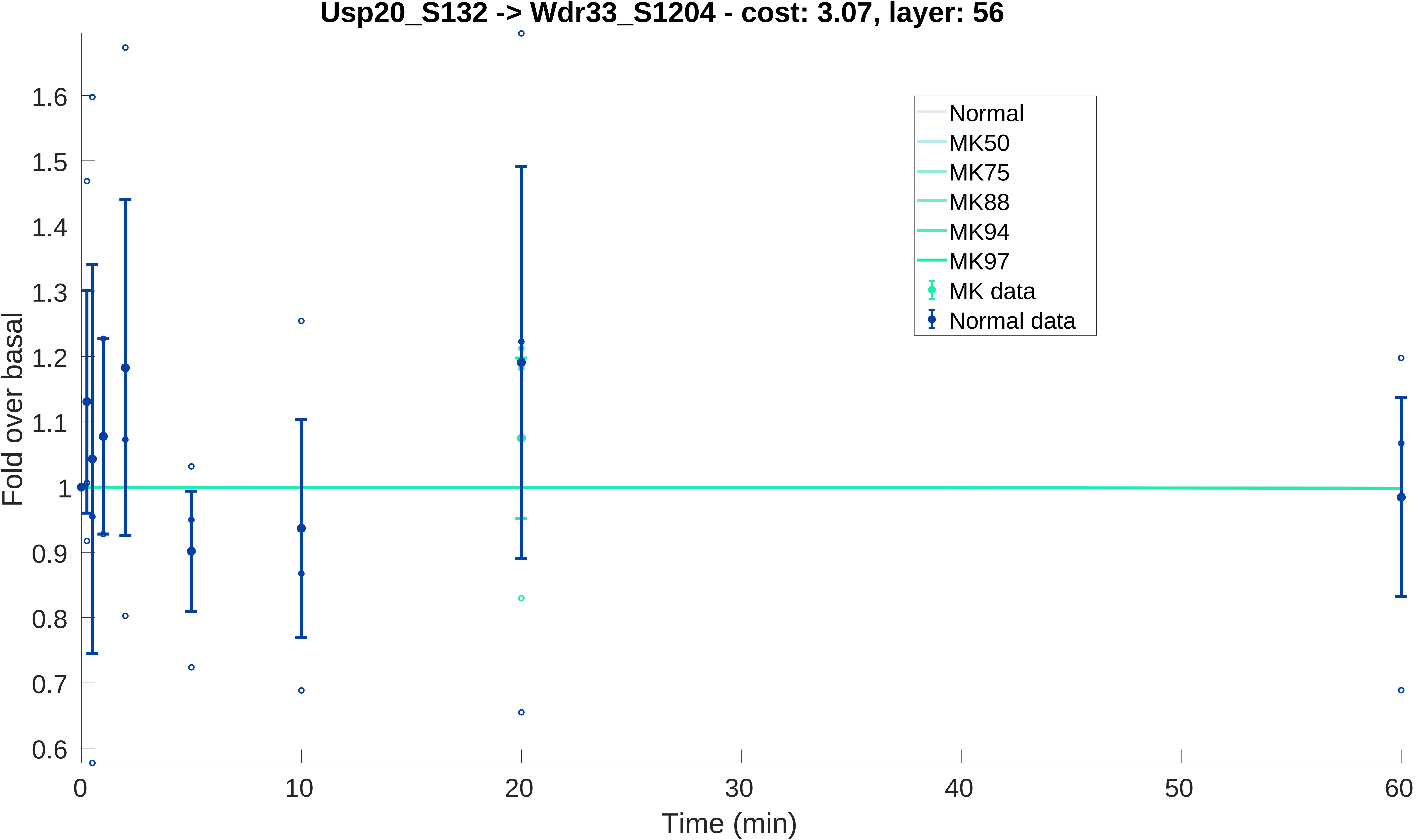

Usp20_S132 -> Wdr33_S1204 - cost: 3.07, layer: 56
Fold over basal
1.6
1.5
1.4
1.3
1.2
1.1
1
0.9
0.8
0.7
0.6
0
10
20
30
40
50
60
Time (min)
Normal
MK50
MK75
MK88
MK94
MK97
MK data
Normal data

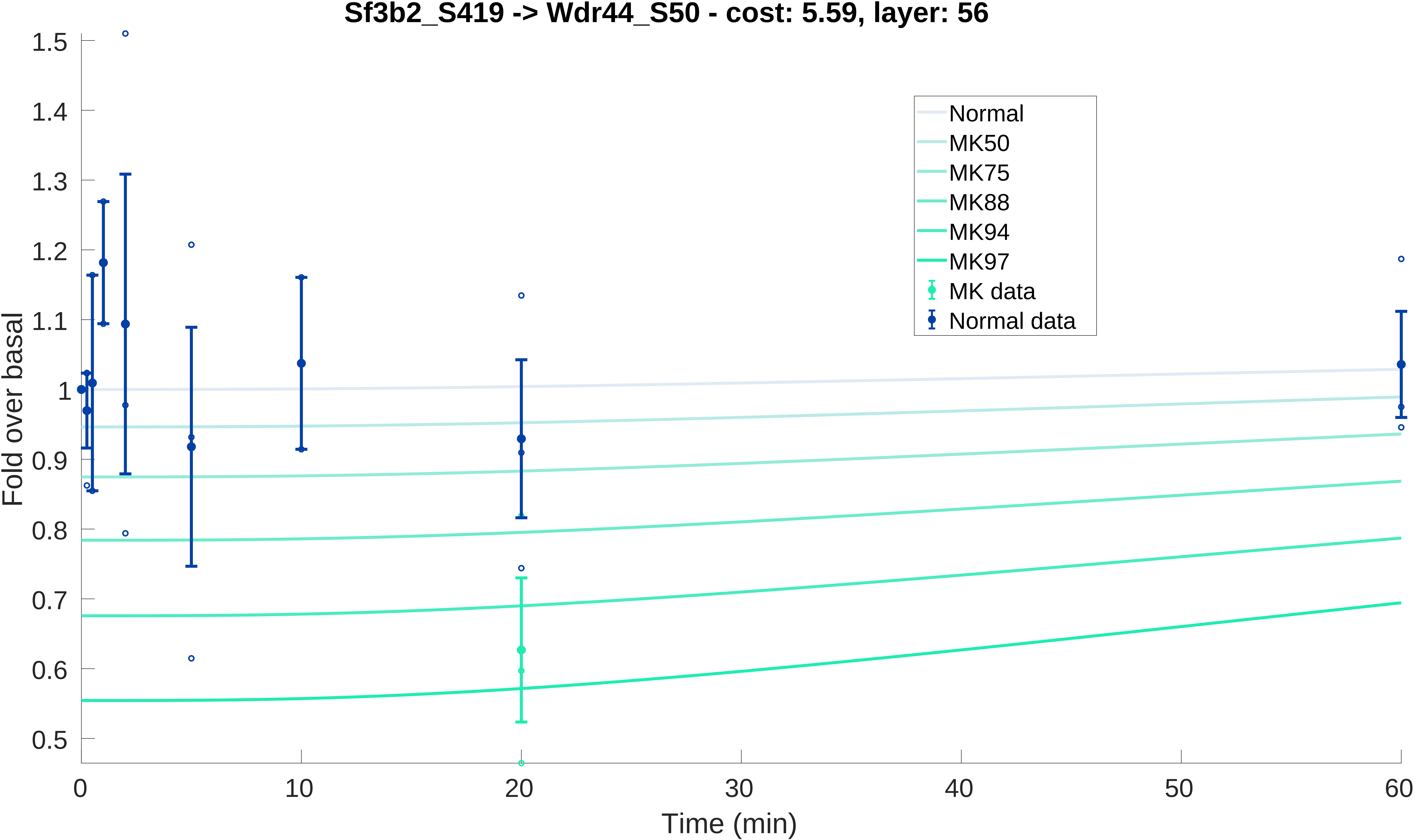

Sf3b2_S419 -> Wdr44_S50 - cost: 5.59, layer: 56
Fold over basal
1.5
1.4
1.3
1.2
1.1
1
0.9
0.8
0.7
0.6
0.5
0
10
20
30
40
50
60
Time (min)
Normal
MK50
MK75
MK88
MK94
MK97
MK data
Normal data

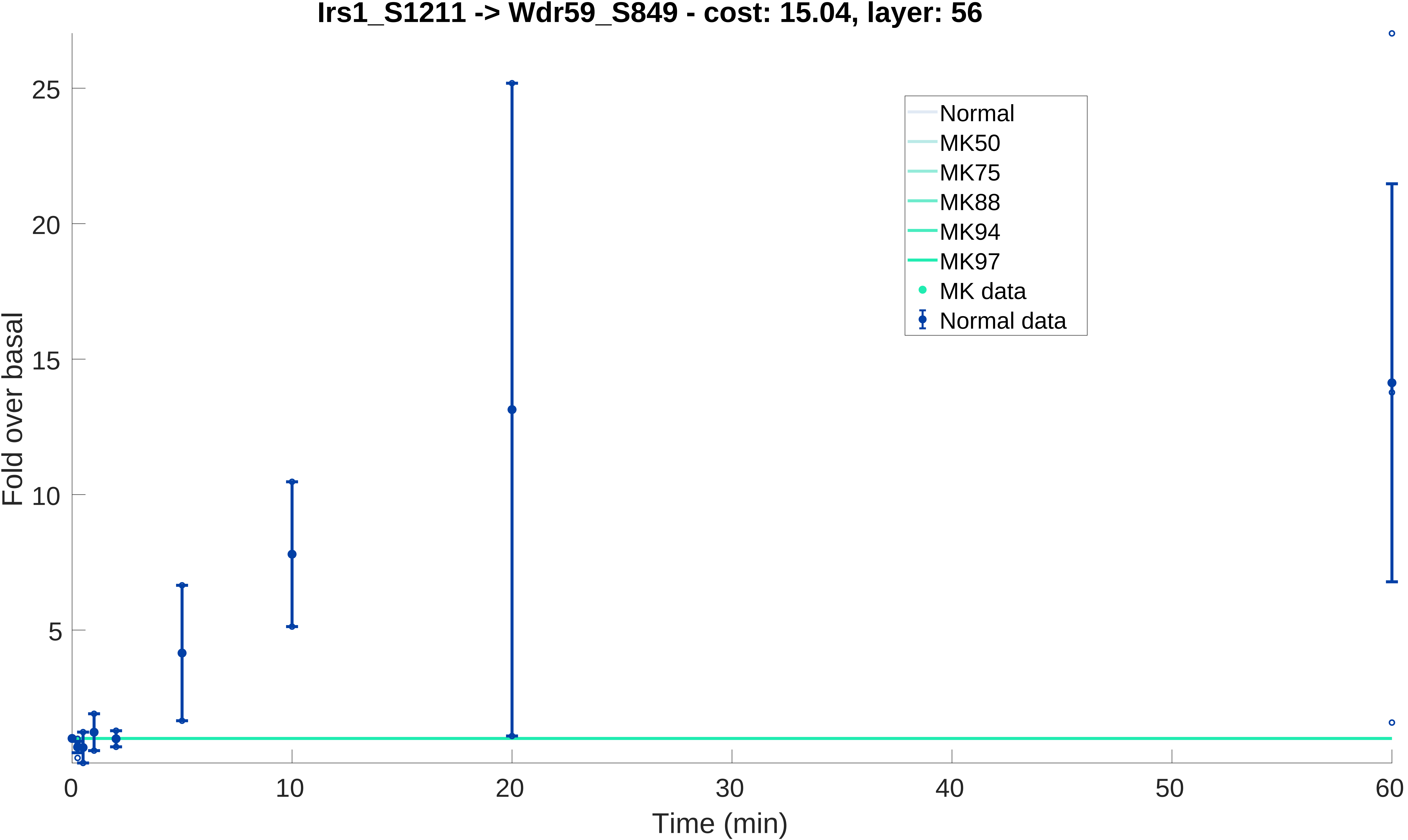

Irs1_S1211 -> Wdr59_S849 - cost: 15.04, layer: 56
Fold over basal
Time (min)
0
5
10
15
20
25
30
40
50
60
Normal
MK50
MK75
MK88
MK94
MK97
MK data
Normal data

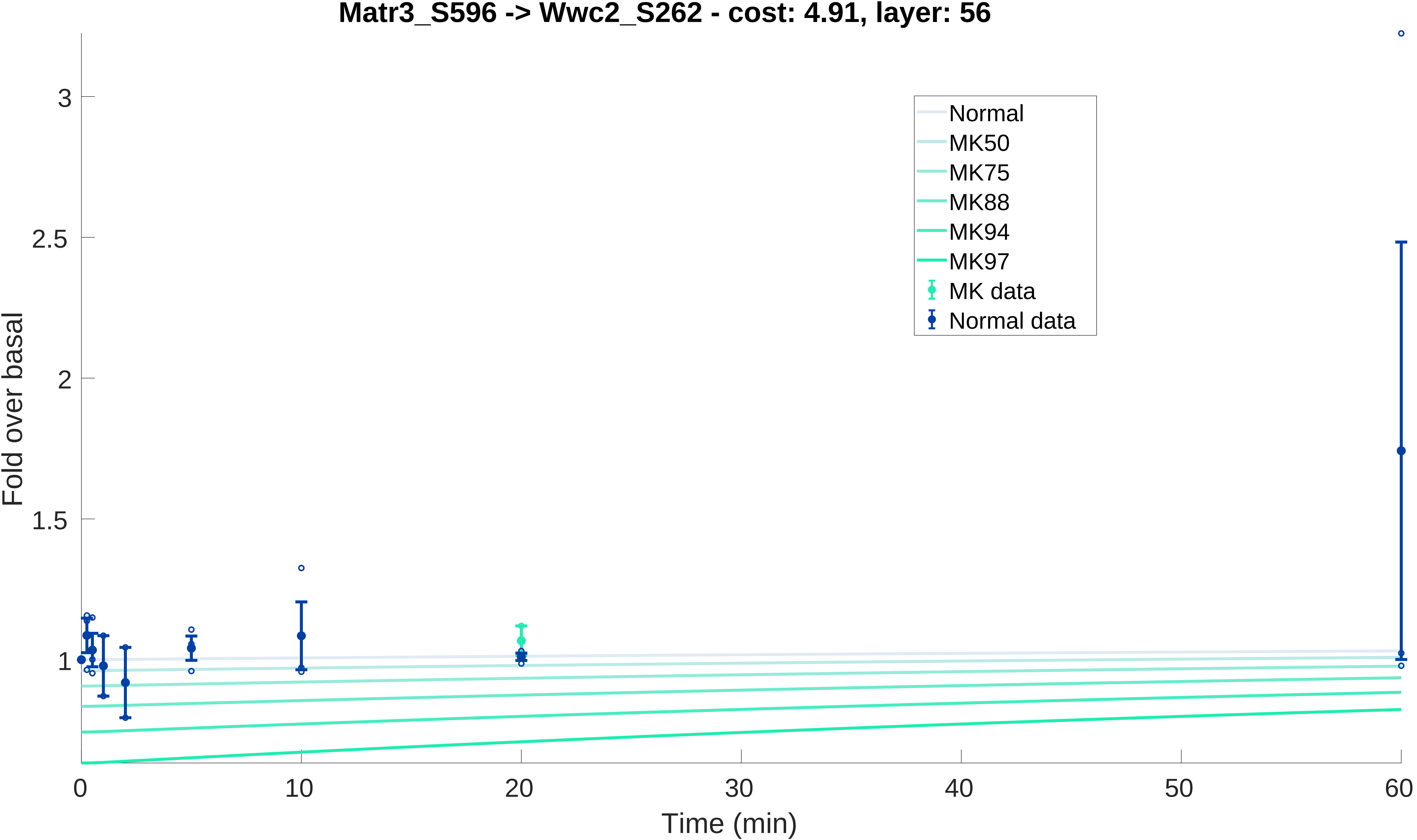
Matr3_S596 -> Wwc2_S262 - cost: 4.91, layer: 56
Fold over basal
Time (min)
0
10
20
30
40
50
60
1
1.5
2
2.5
3
Normal
MK50
MK75
MK88
MK94
MK97
MK data
Normal data

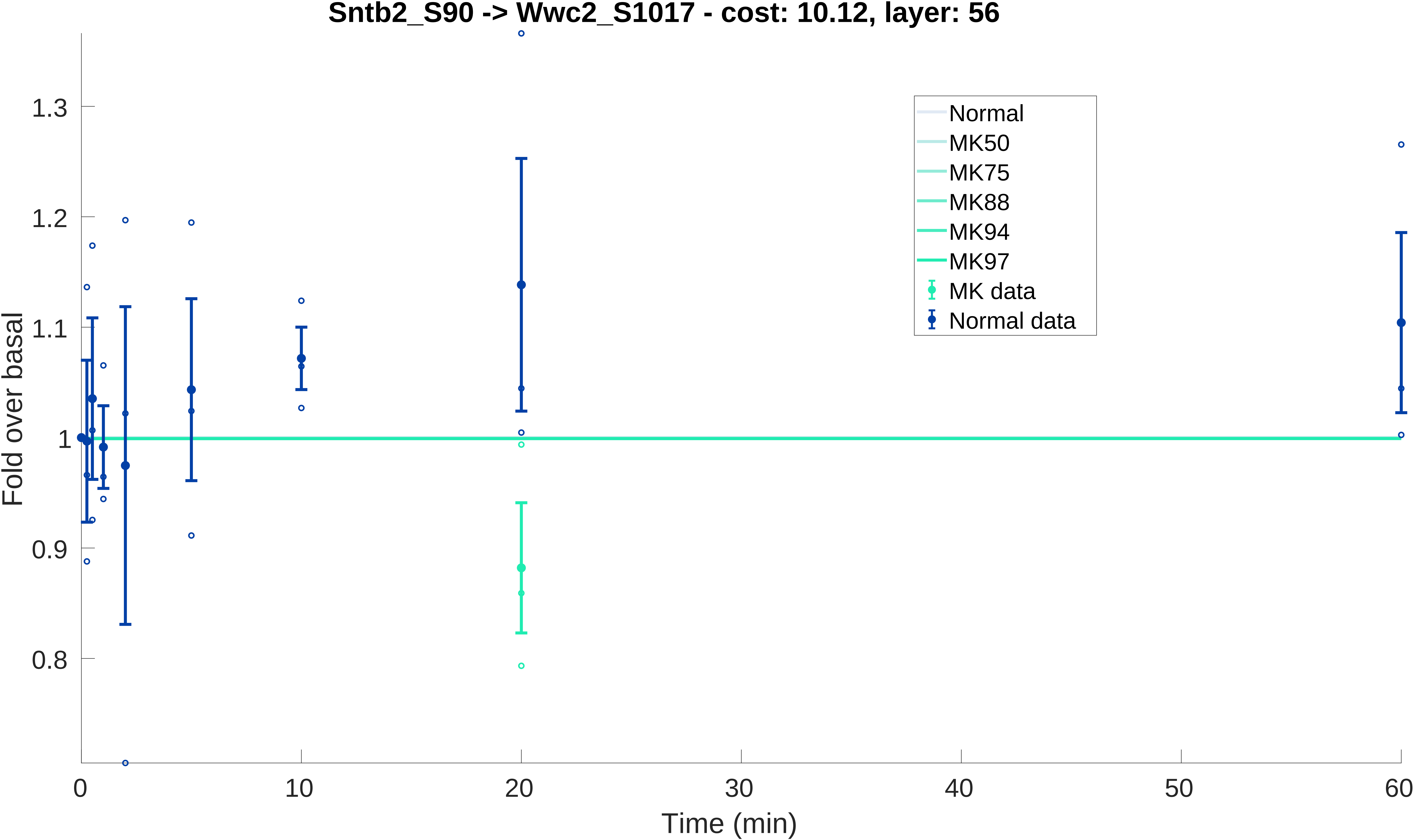

Sntb2_S90 -> Wwc2_S1017 - cost: 10.12, layer: 56
Fold over basal
Time (min)
0.8
0.9
1
1.1
1.2
1.3
0
10
20
30
40
50
60
Normal
MK50
MK75
MK88
MK94
MK97
MK data
Normal data

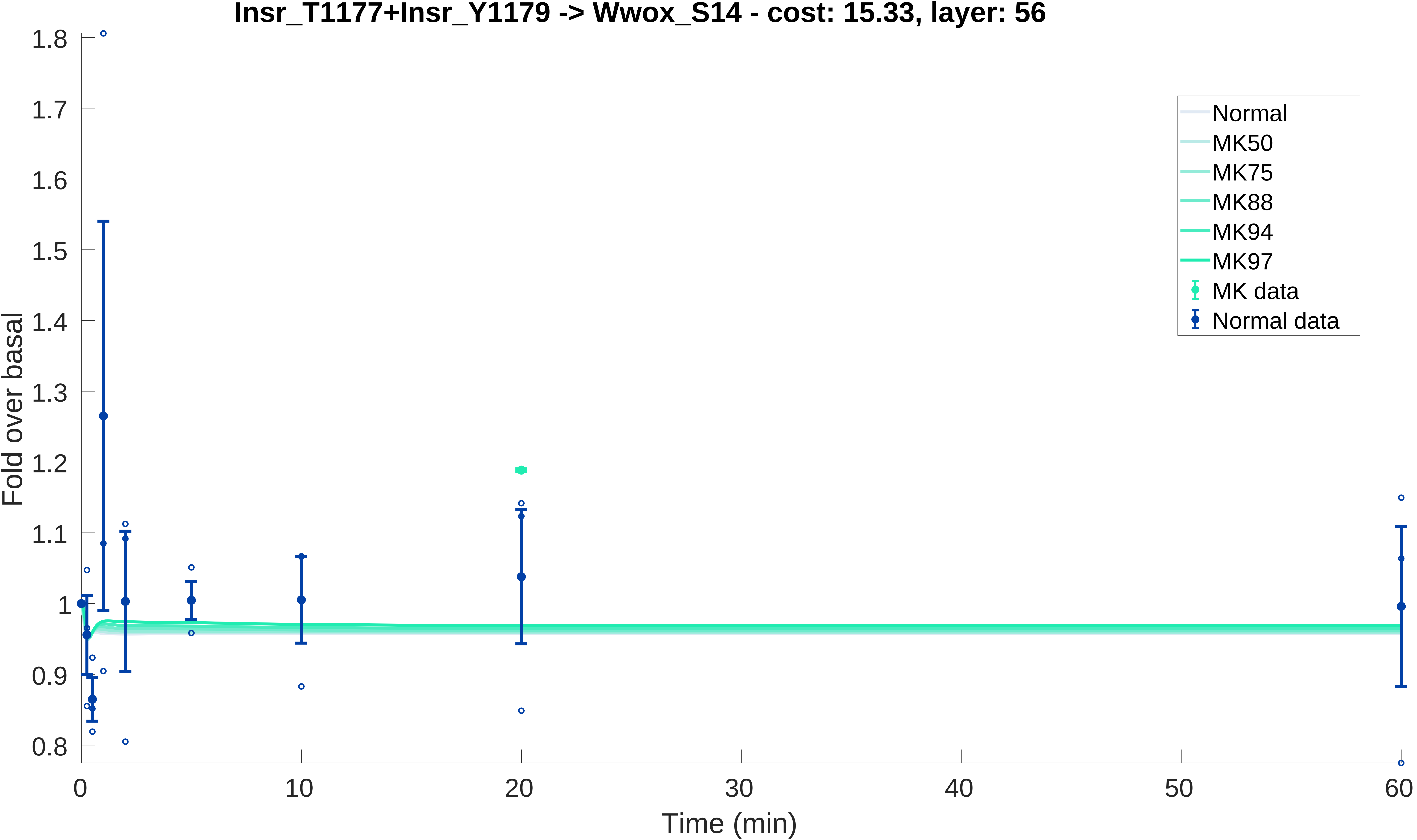
Insr_T1177+Insr_Y1179 -> Wwox_S14 - cost: 15.33, layer: 56
Fold over basal
Time (min)
Normal
MK50
MK75
MK88
MK94
MK97
MK data
Normal data
1.8
1.7
1.6
1.5
1.4
1.3
1.2
1.1
1
0.9
0.8
0
10
20
30
40
50
60

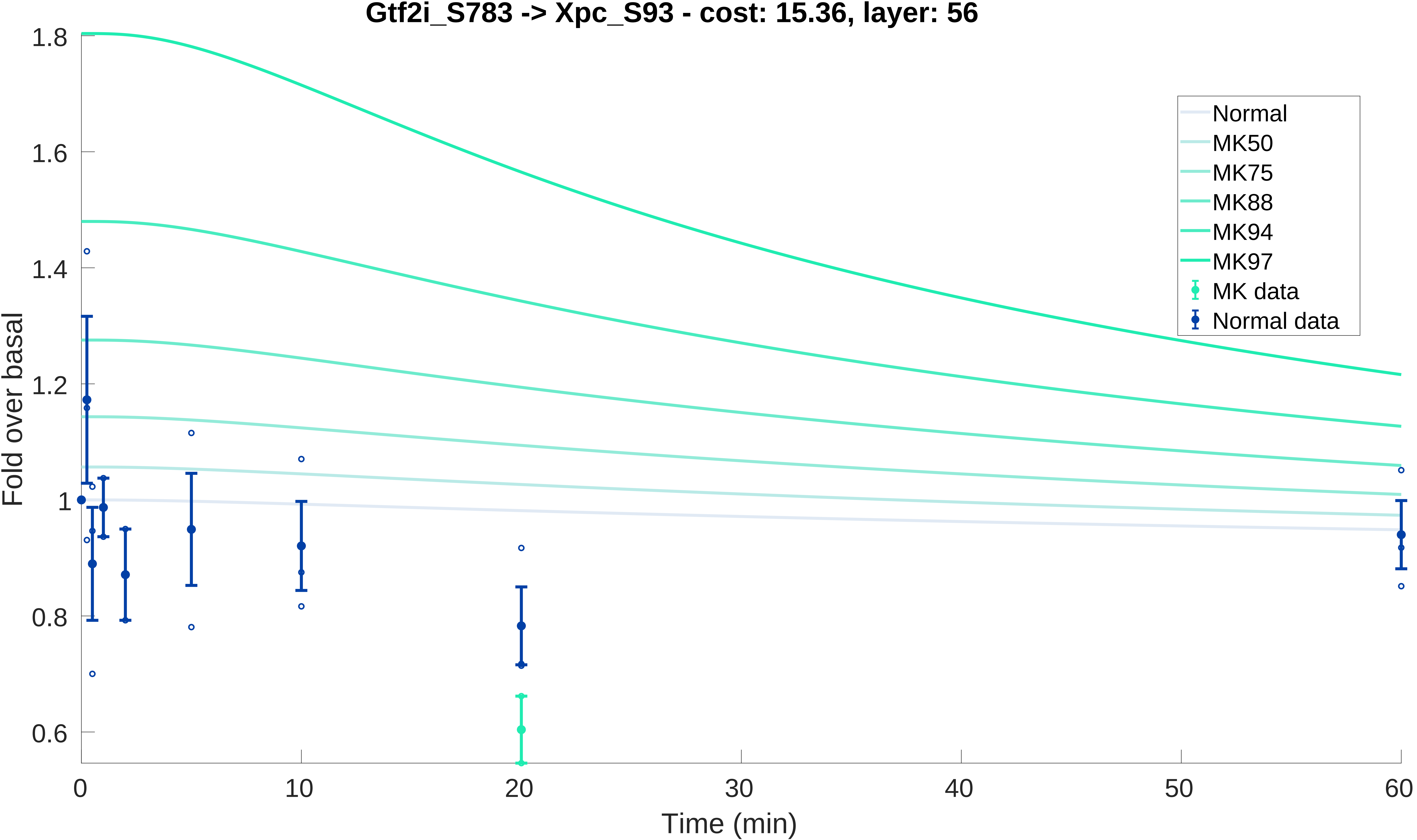

Gtf2i_S783 -> Xpc_S93 - cost: 15.36, layer: 56
Fold over basal
1.8
1.6
1.4
1.2
1
0.8
0.6
0
10
20
30
40
50
60
Time (min)
Normal
MK50
MK75
MK88
MK94
MK97
MK data
Normal data

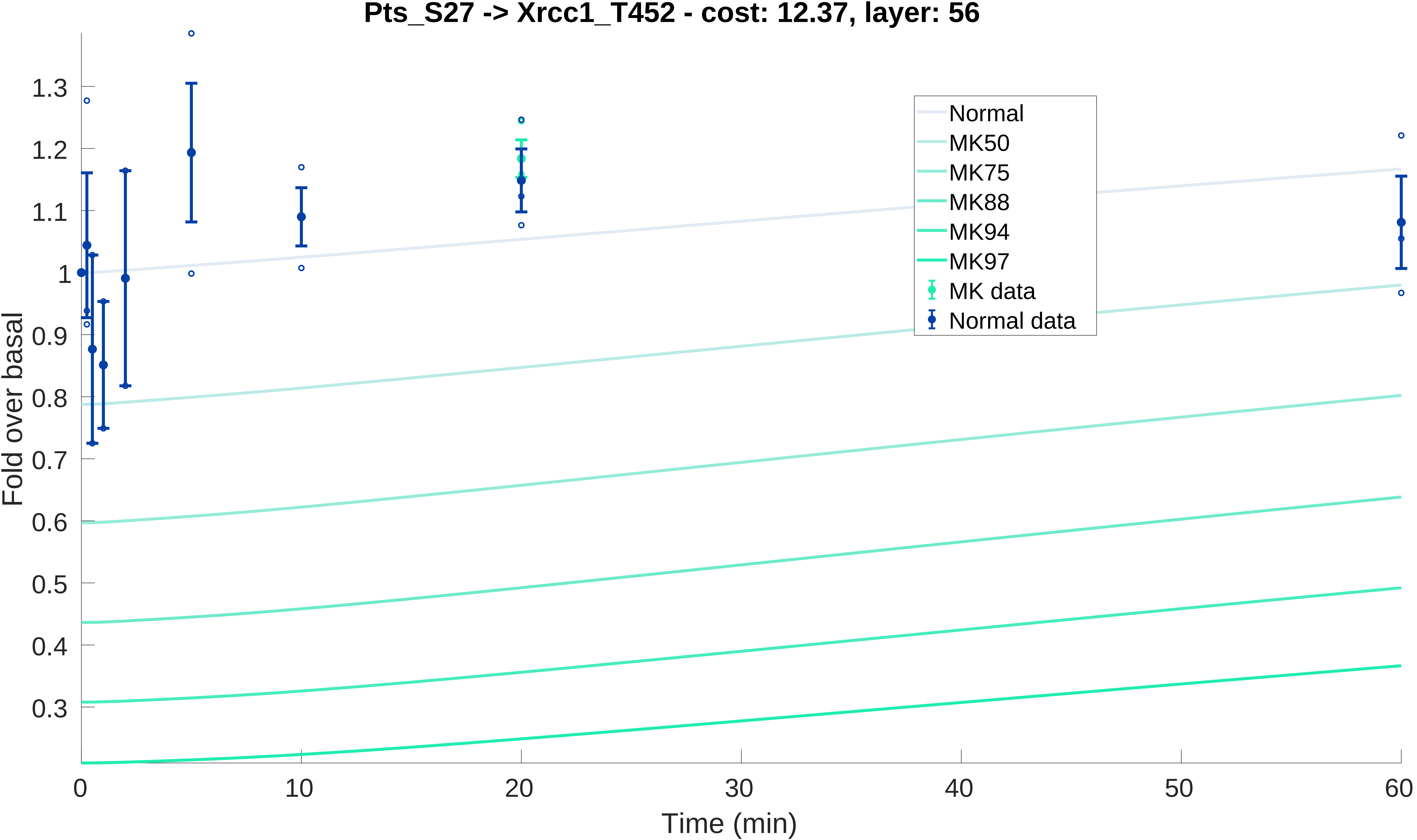

Pts_S27 -> Xrcc1_T452 - cost: 12.37, layer: 56
Fold over basal
Time (min)
1.3
1.2
1.1
1
0.9
0.8
0.7
0.6
0.5
0.4
0.3
0
10
20
30
40
50
60
Normal
MK50
MK75
MK88
MK94
MK97
MK data
Normal data

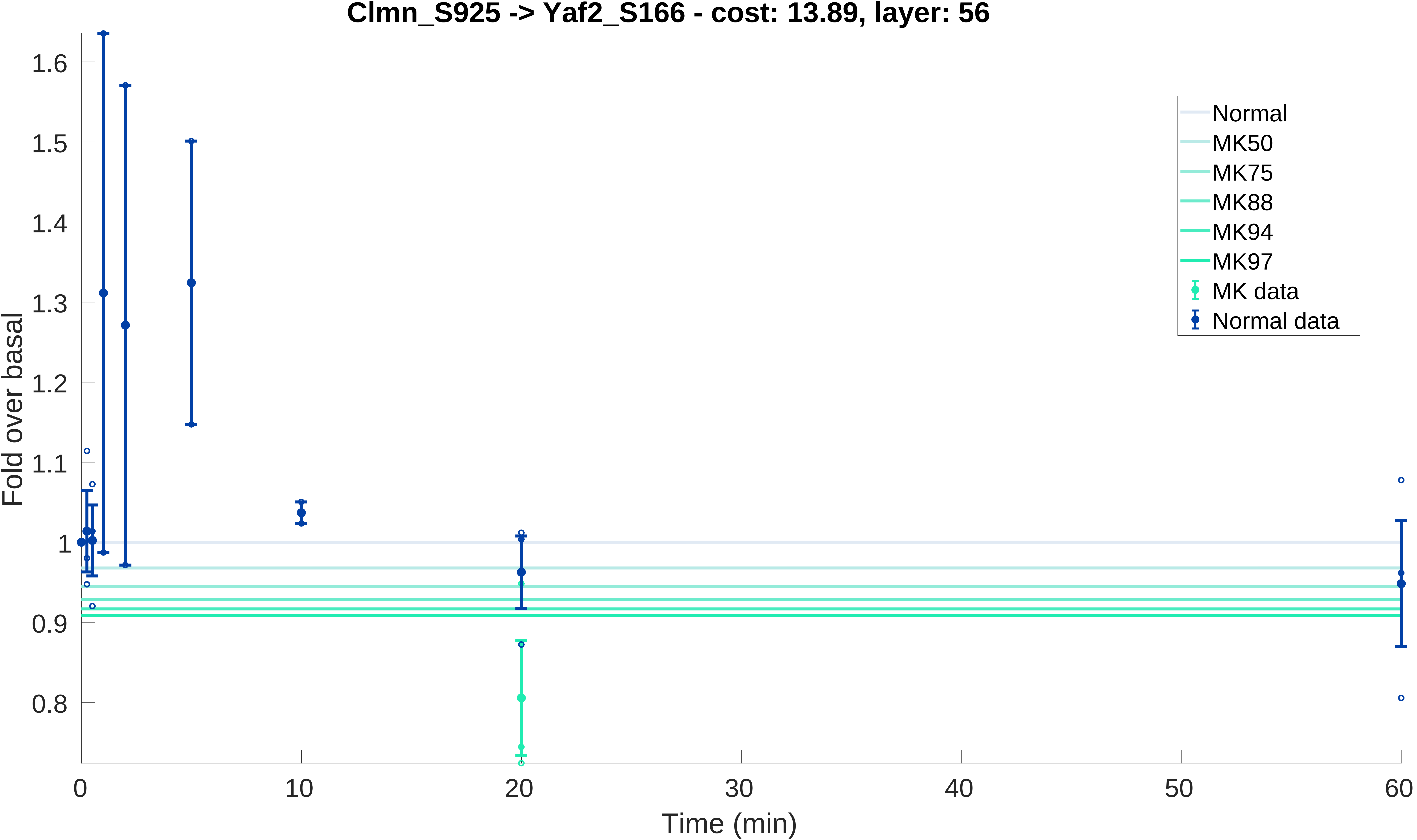

Clmn_S925 -> Yaf2_S166 - cost: 13.89, layer: 56
Fold over basal
Time (min)
Normal
MK50
MK75
MK88
MK94
MK97
MK data
Normal data
0
10
20
30
40
50
60
0.8
0.9
1
1.1
1.2
1.3
1.4
1.5
1.6

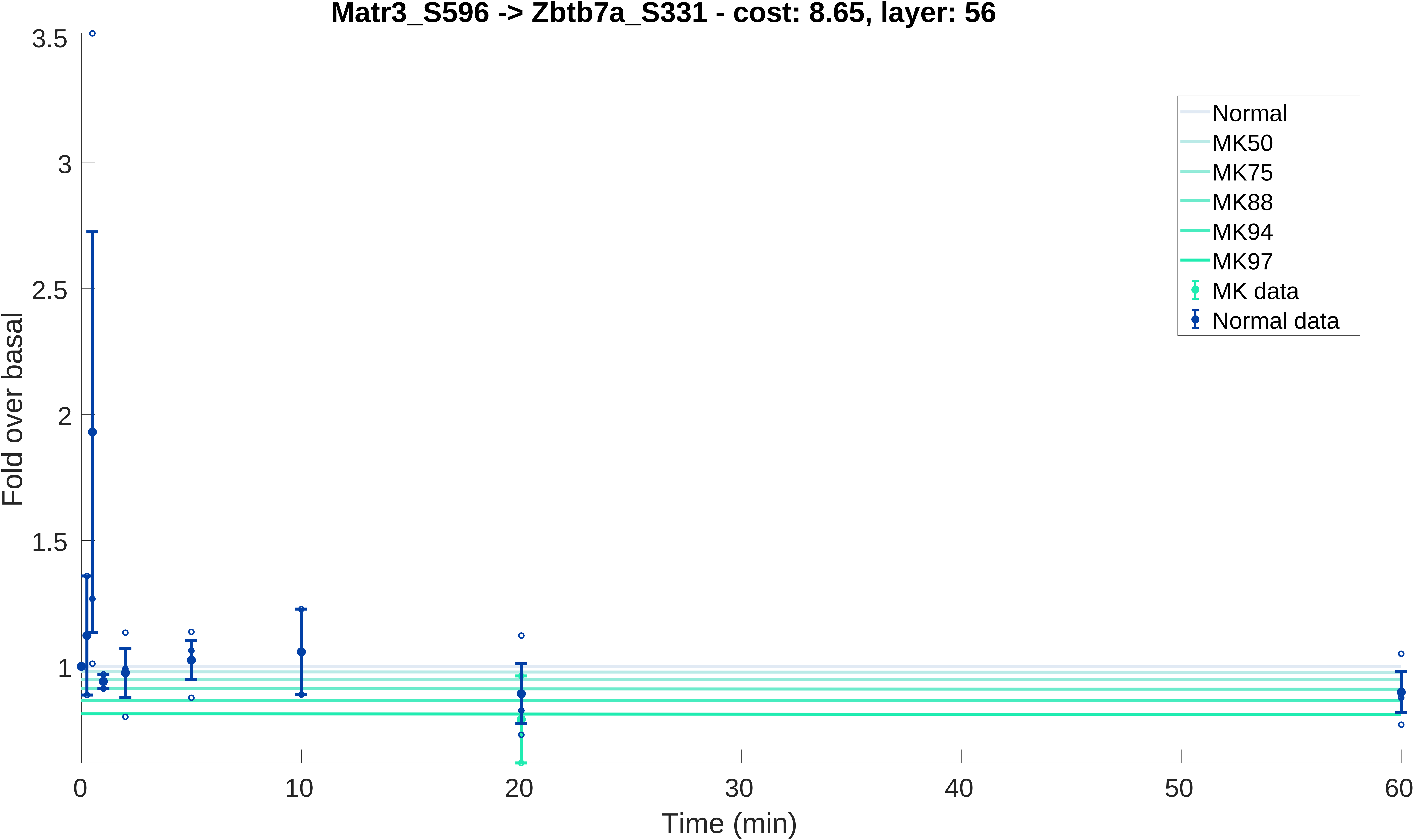
Matr3_S596 -> Zbtb7a_S331 - cost: 8.65, layer: 56
Fold over basal
Time (min)
3.5
3
2.5
2
1.5
1
0
10
20
30
40
50
60
Normal
MK50
MK75
MK88
MK94
MK97
MK data
Normal data

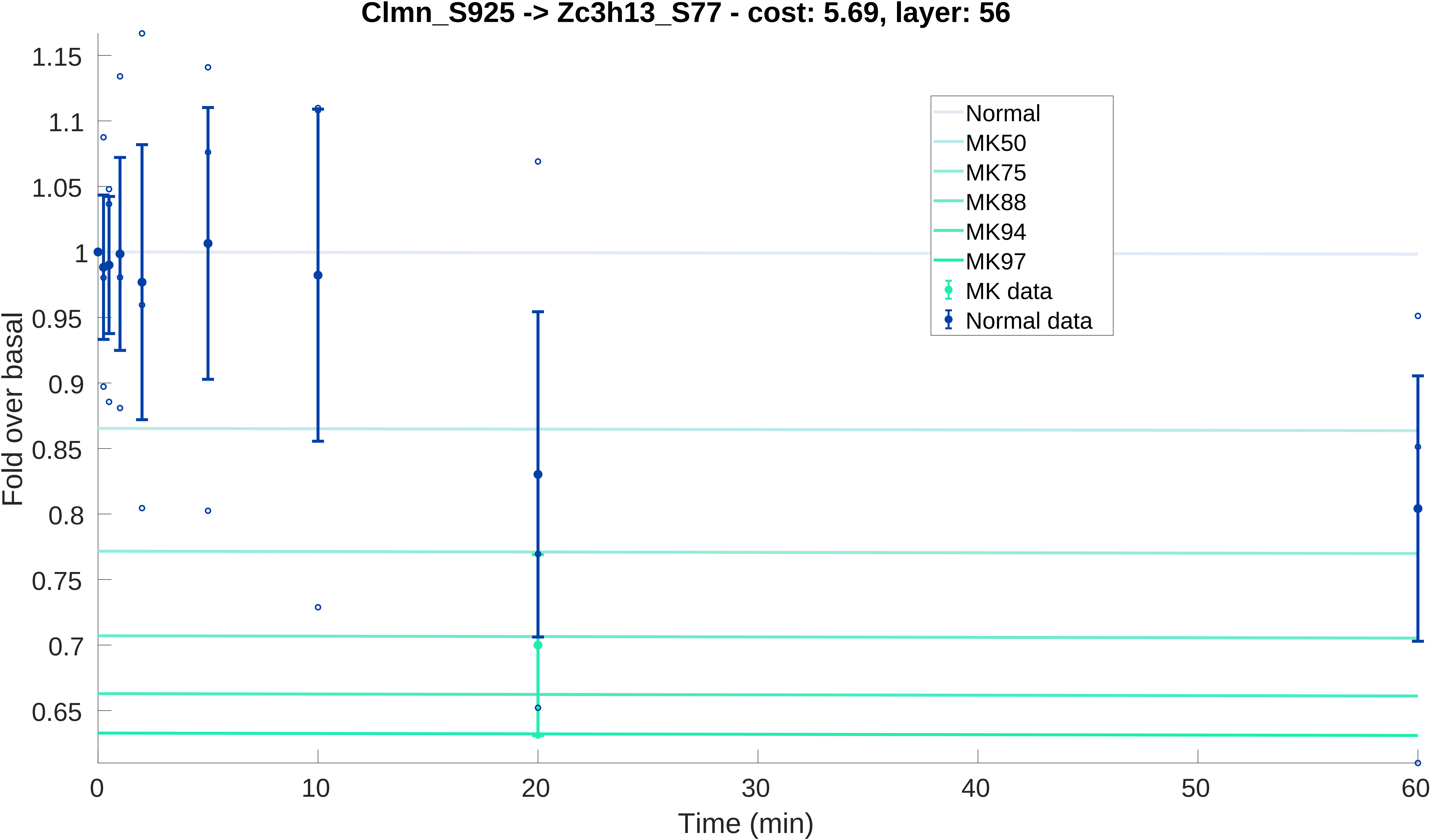
Clmn_S925 -> Zc3h13_S77 - cost: 5.69, layer: 56
Fold over basal
1.15
1.1
1.05
1
0.95
0.9
0.85
0.8
0.75
0.7
0.65
0
10
20
30
40
50
60
Time (min)
Normal
MK50
MK75
MK88
MK94
MK97
MK data
Normal data

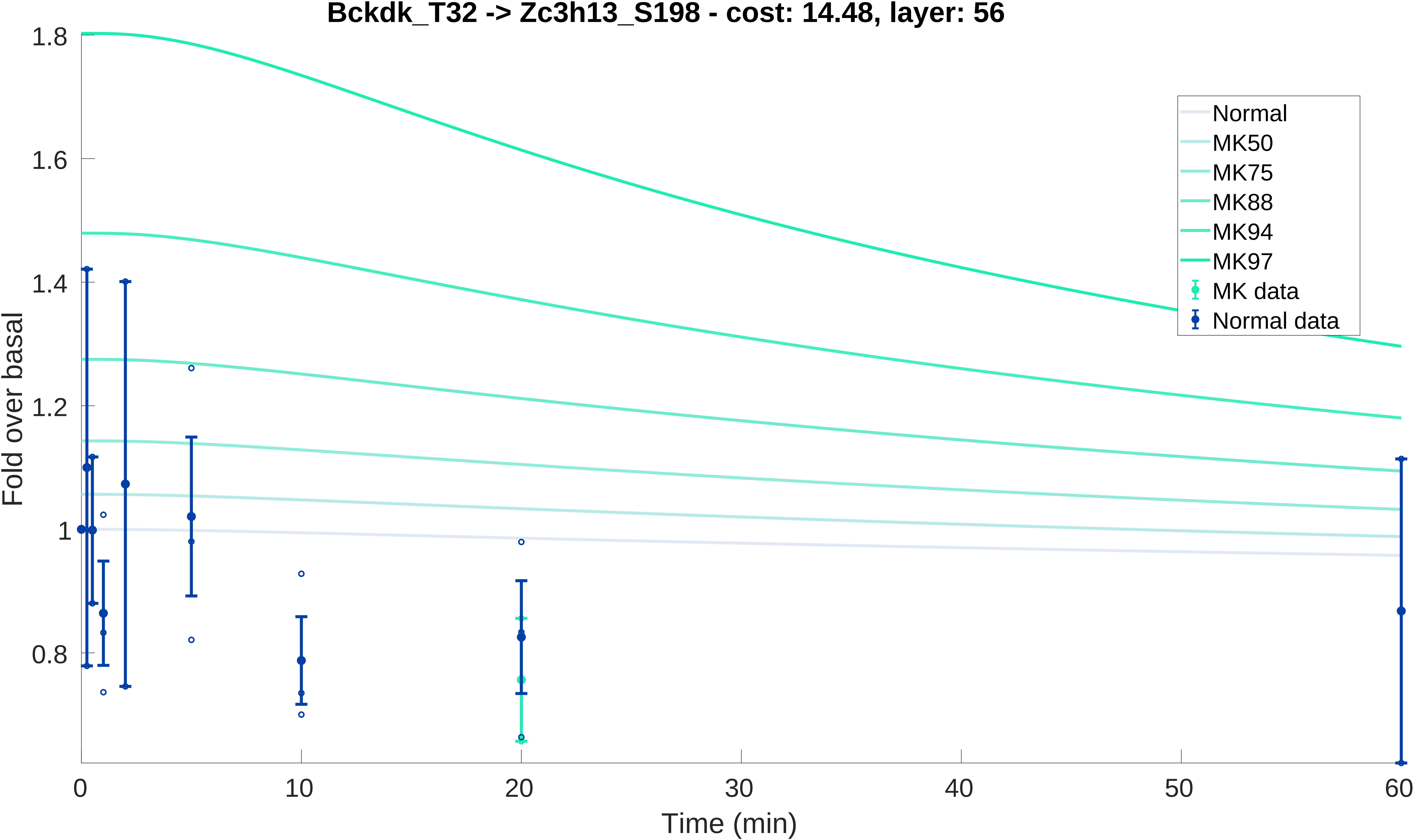

Bckdk_T32 -> Zc3h13_S198 - cost: 14.48, layer: 56
Fold over basal
Time (min)
1.8
1.6
1.4
1.2
1
0.8
0
10
20
30
40
50
60
Normal
MK50
MK75
MK88
MK94
MK97
MK data
Normal data

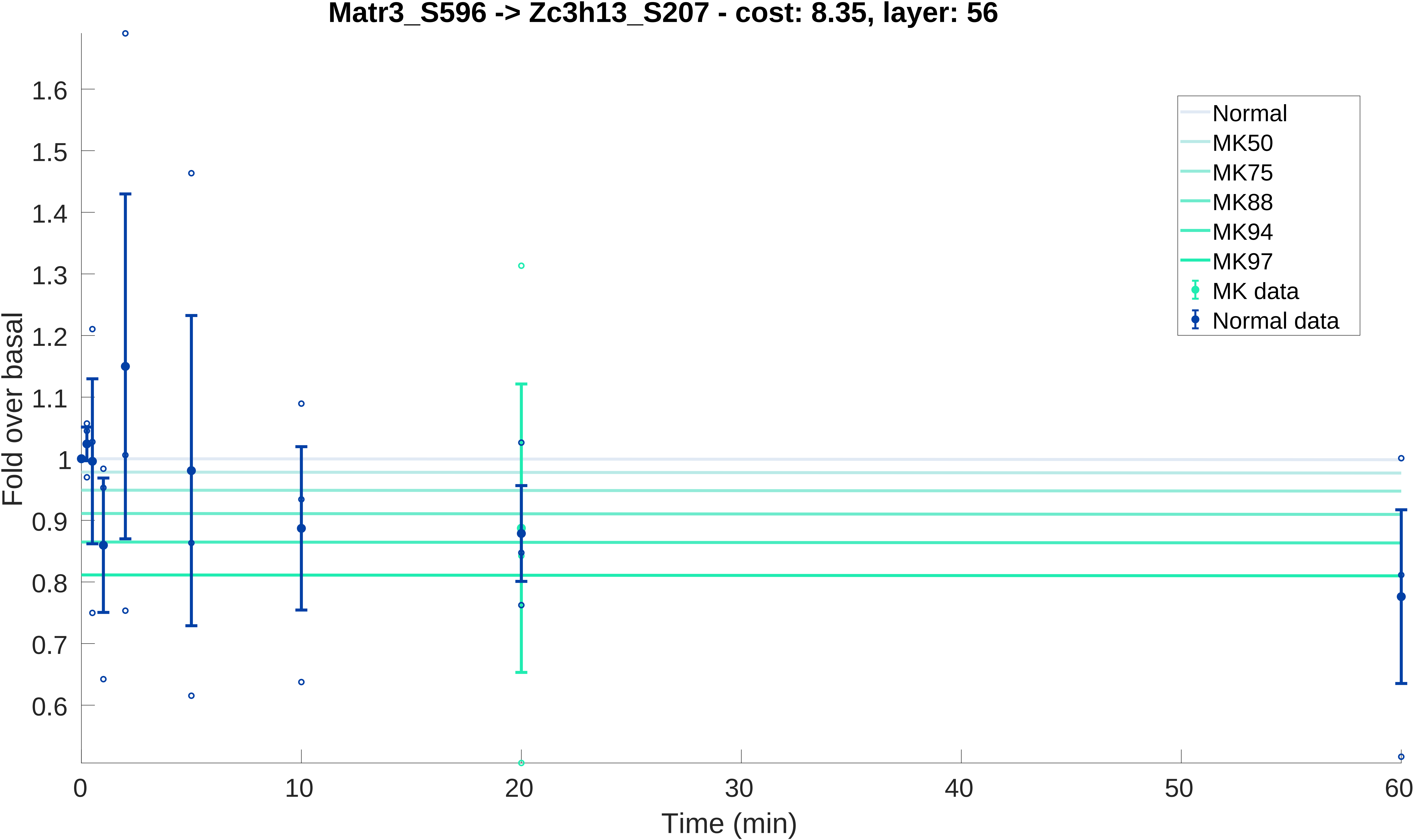

Matr3_S596 -> Zc3h13_S207 - cost: 8.35, layer: 56
Fold over basal
Time (min)
1.6
1.5
1.4
1.3
1.2
1.1
1
0.9
0.8
0.7
0.6
0
10
20
30
40
50
60
Normal
MK50
MK75
MK88
MK94
MK97
MK data
Normal data

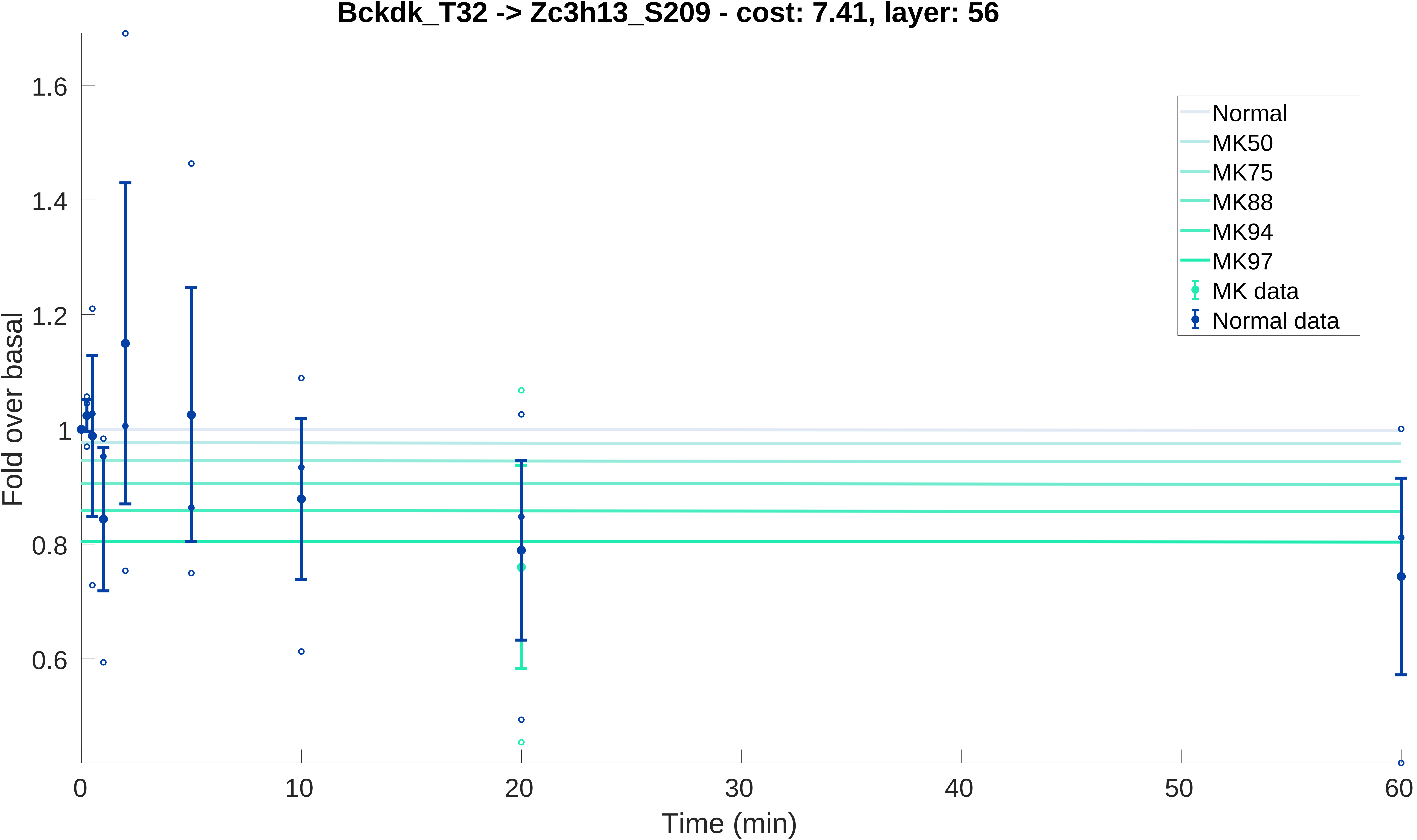

Bckdk_T32 -> Zc3h13_S209 - cost: 7.41, layer: 56
Fold over basal
Time (min)
1.6
1.4
1.2
1
0.8
0.6
0
10
20
30
40
50
60
Normal
MK50
MK75
MK88
MK94
MK97
MK data
Normal data

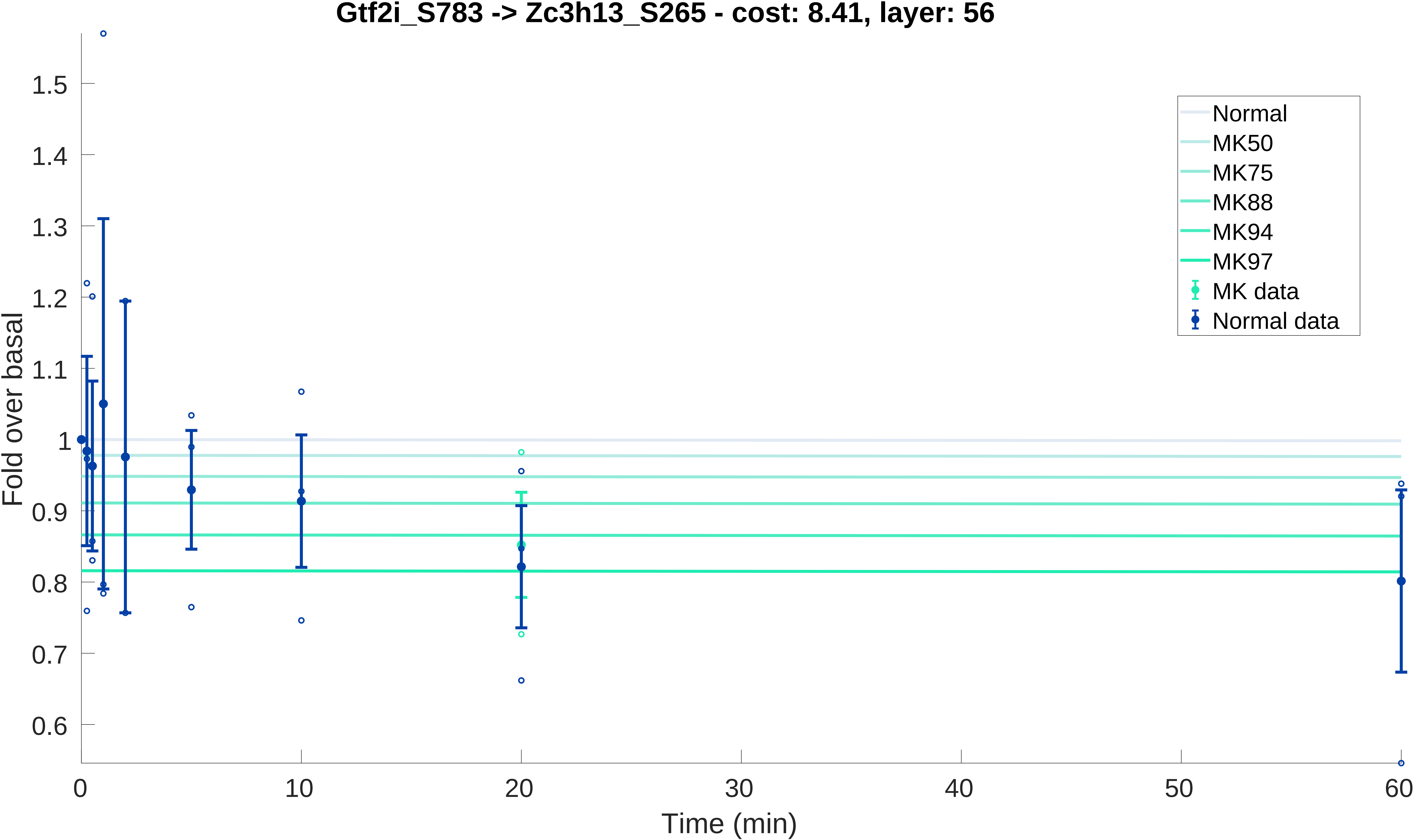

Gtf2i_S783 -> Zc3h13_S265 - cost: 8.41, layer: 56
Fold over basal
Time (min)
1.5
1.4
1.3
1.2
1.1
1
0.9
0.8
0.7
0.6
0
10
20
30
40
50
60
Normal
MK50
MK75
MK88
MK94
MK97
MK data
Normal data

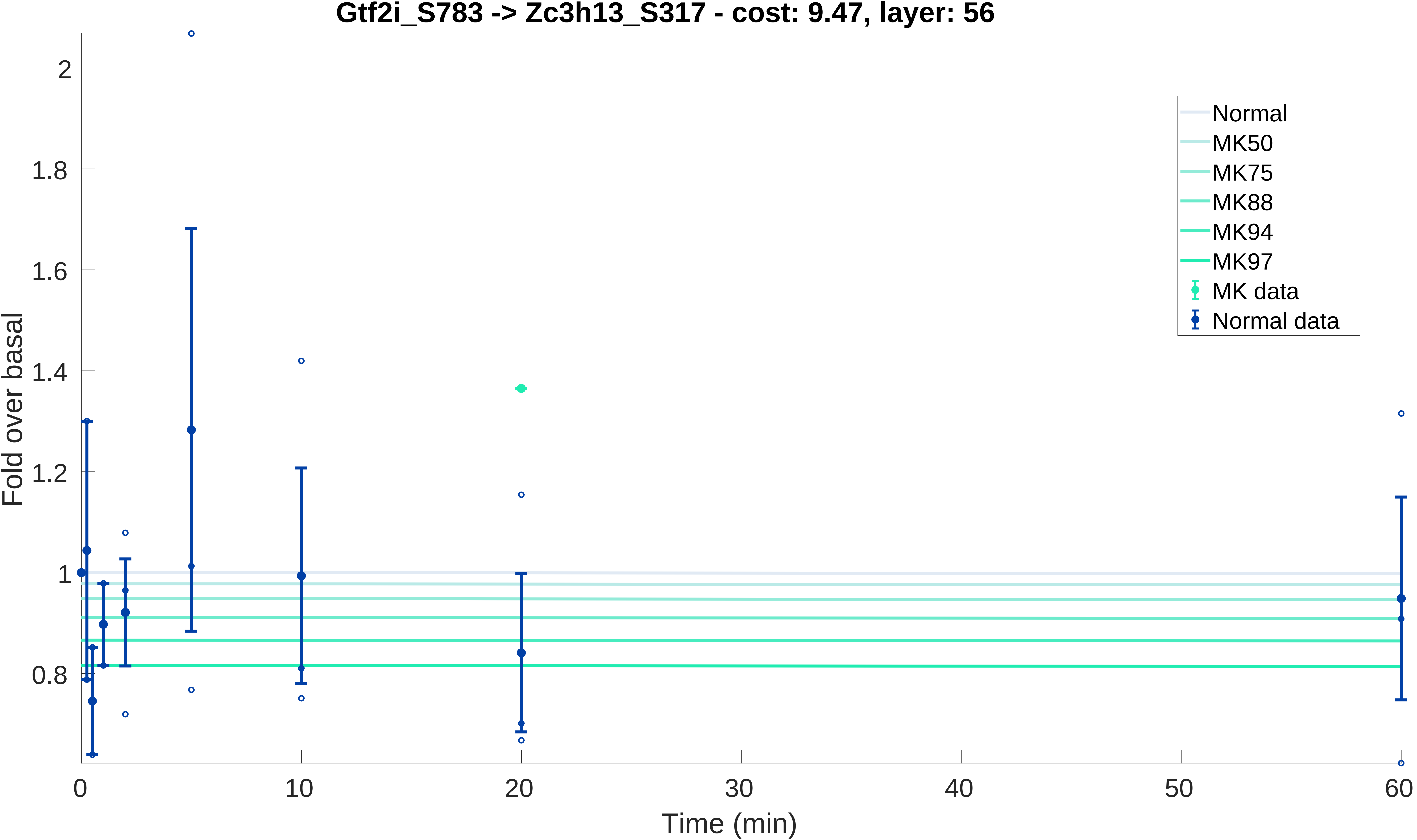
Gtf2i_S783 -> Zc3h13_S317 - cost: 9.47, layer: 56
Fold over basal
Time (min)
Normal
MK50
MK75
MK88
MK94
MK97
MK data
Normal data
0
10
20
30
40
50
60
0.8
1
1.2
1.4
1.6
1.8
2

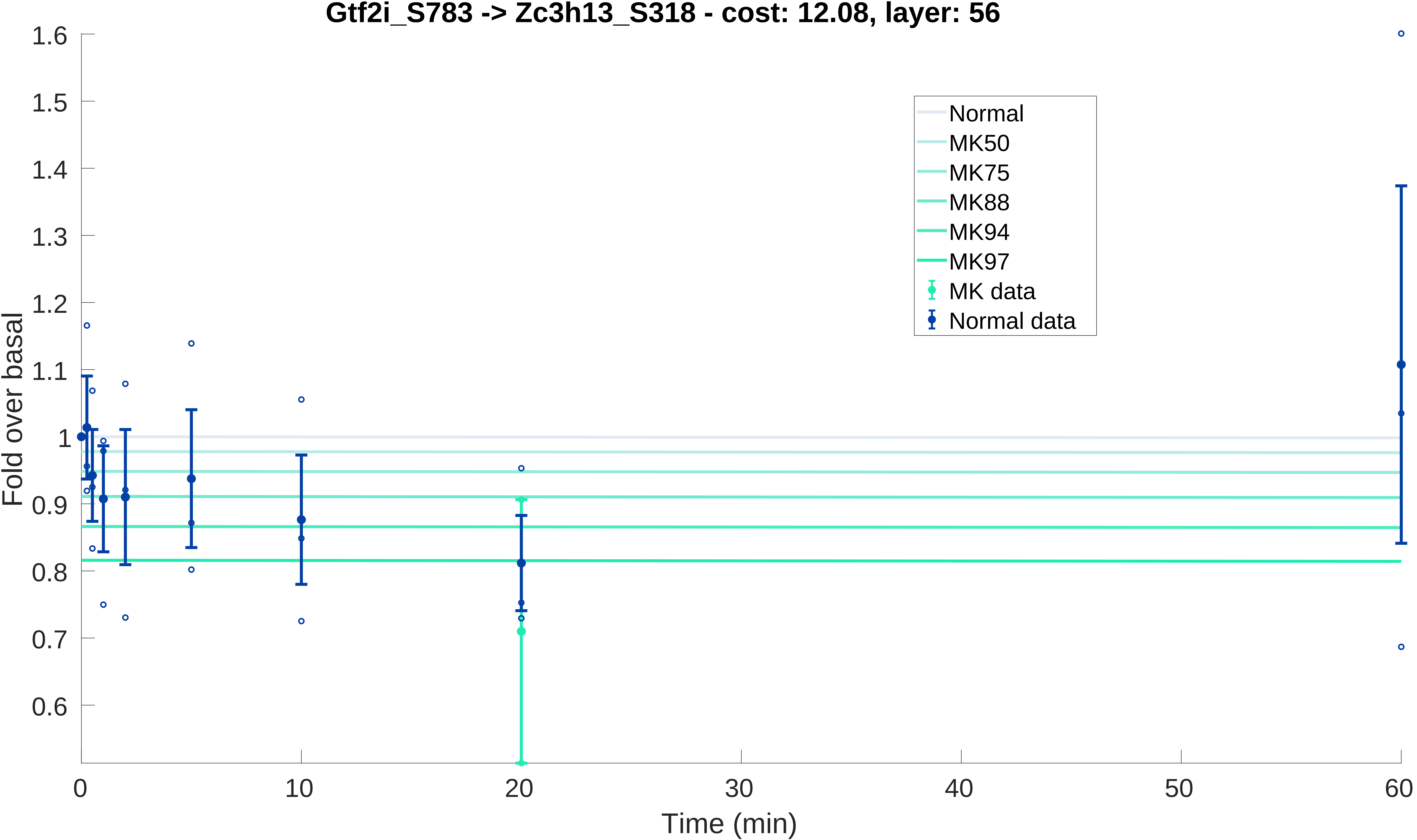

Gtf2i_S783 -> Zc3h13_S318 - cost: 12.08, layer: 56
Fold over basal
Time (min)
1.6
1.5
1.4
1.3
1.2
1.1
1
0.9
0.8
0.7
0.6
0
10
20
30
40
50
60
Normal
MK50
MK75
MK88
MK94
MK97
MK data
Normal data

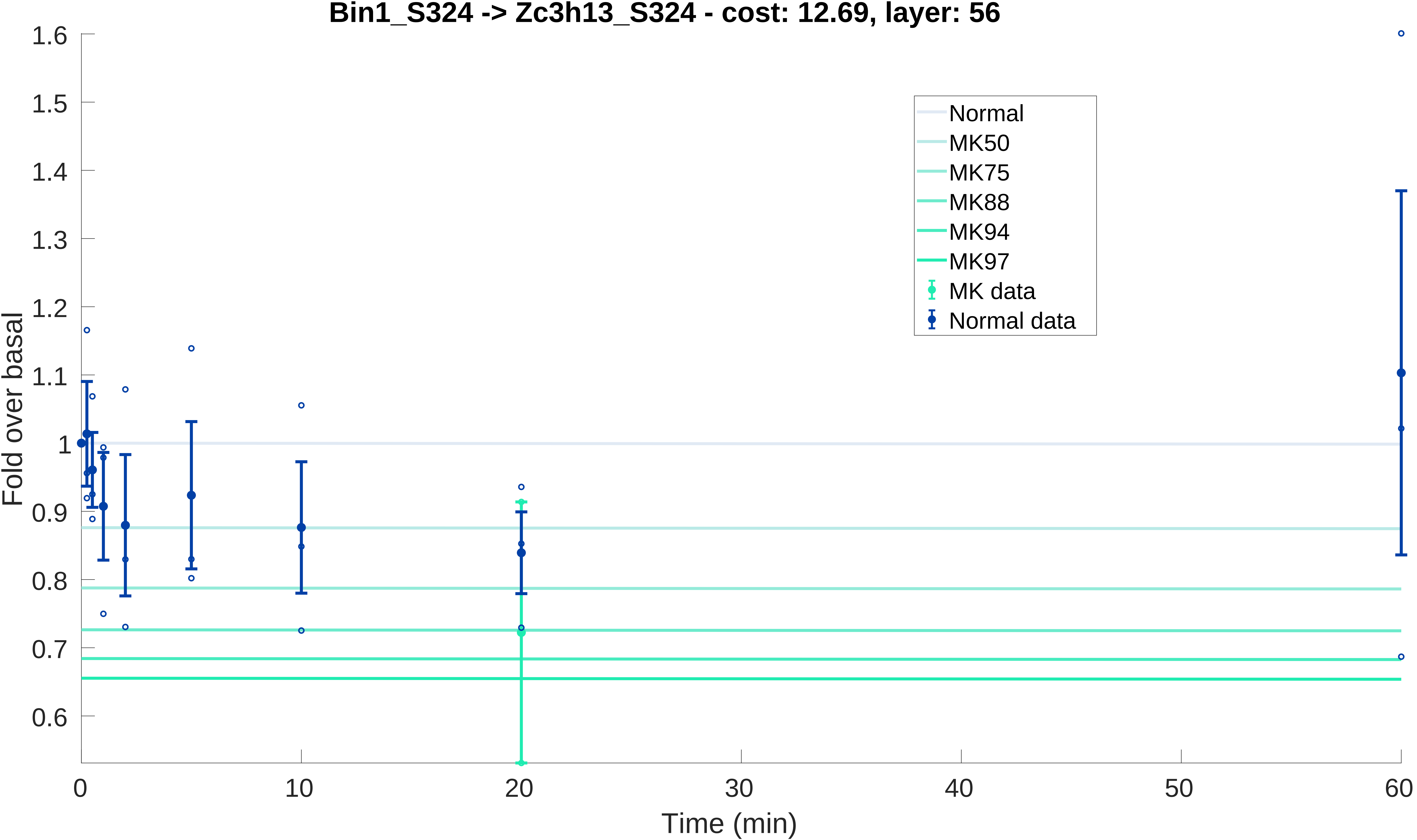

Bin1_S324 -> Zc3h13_S324 - cost: 12.69, layer: 56
Fold over basal
Time (min)
0.6
0.7
0.8
0.9
1
1.1
1.2
1.3
1.4
1.5
1.6
0
10
20
30
40
50
60
Normal
MK50
MK75
MK88
MK94
MK97
MK data
Normal data

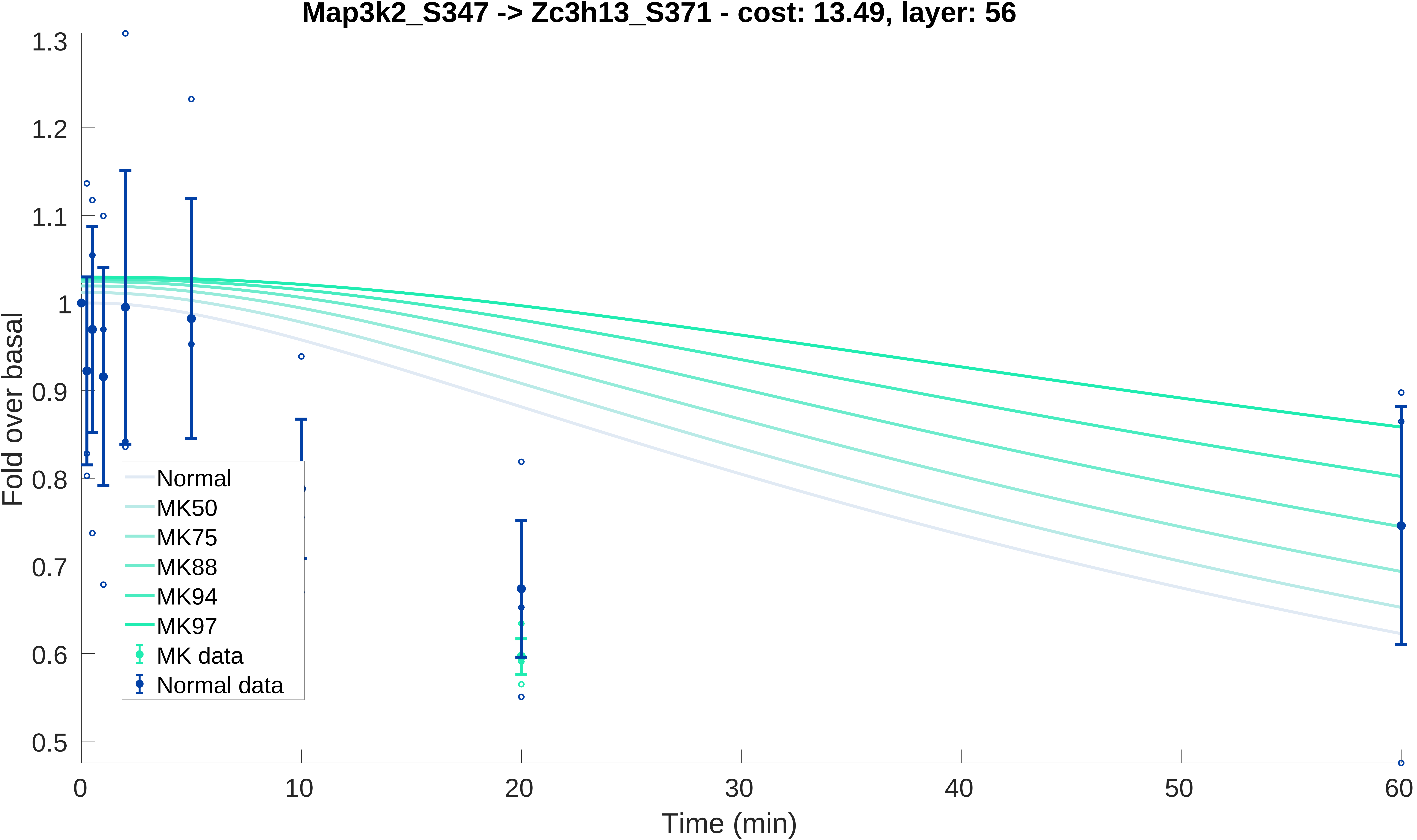

Map3k2_S347 -> Zc3h13_S371 - cost: 13.49, layer: 56
Fold over basal
Time (min)
1.3
1.2
1.1
1
0.9
0.8
0.7
0.6
0.5
0
10
20
30
40
50
60
Normal
MK50
MK75
MK88
MK94
MK97
MK data
Normal data

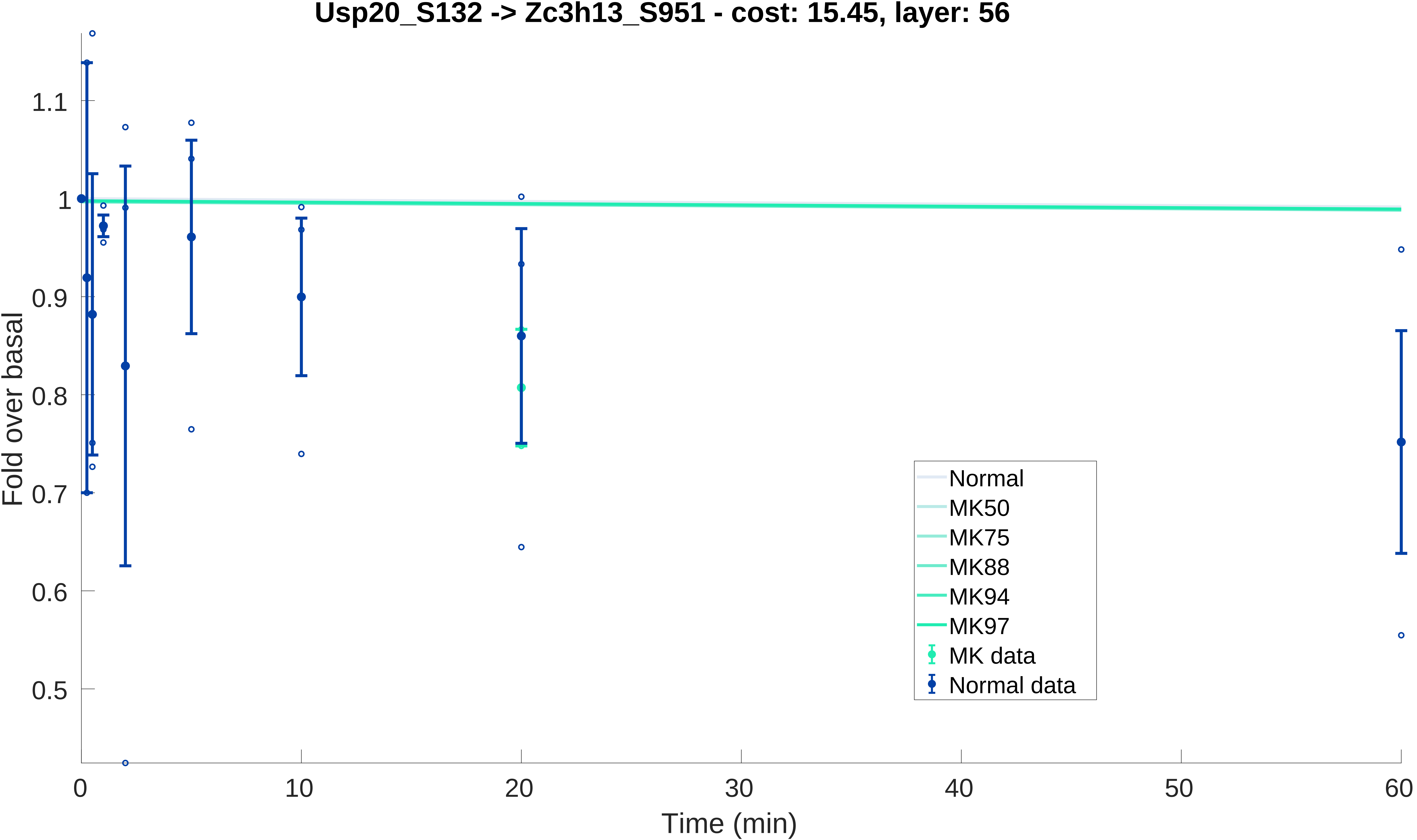

Usp20_S132 -> Zc3h13_S951 - cost: 15.45, layer: 56
Fold over basal
Time (min)
1.1
1
0.9
0.8
0.7
0.6
0.5
0
10
20
30
40
50
60
Normal
MK50
MK75
MK88
MK94
MK97
MK data
Normal data

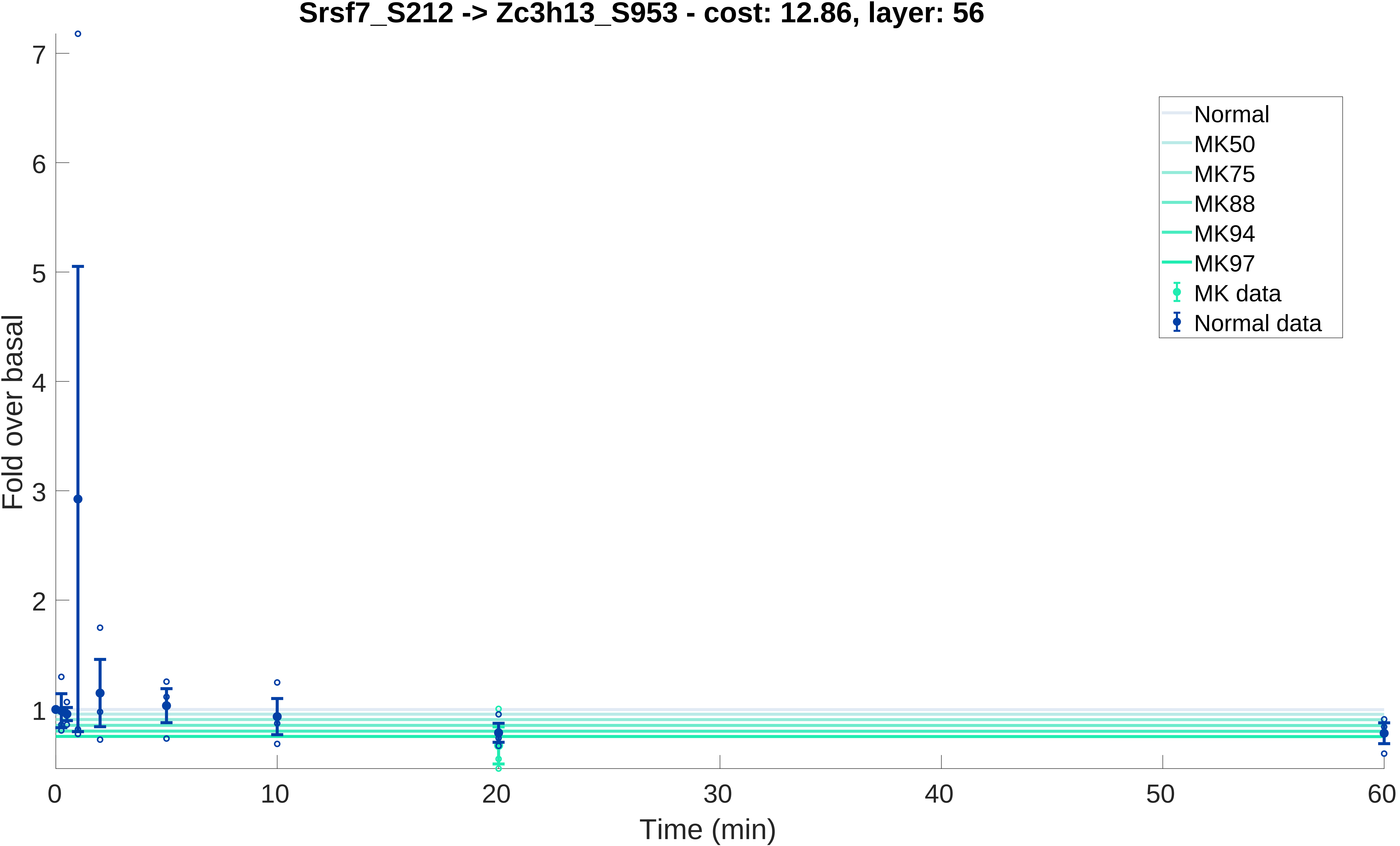
Srsf7_S212 -> Zc3h13_S953 - cost: 12.86, layer: 56
Fold over basal
Time (min)
0
10
20
30
40
50
60
1
2
3
4
5
6
7
Normal
MK50
MK75
MK88
MK94
MK97
MK data
Normal data

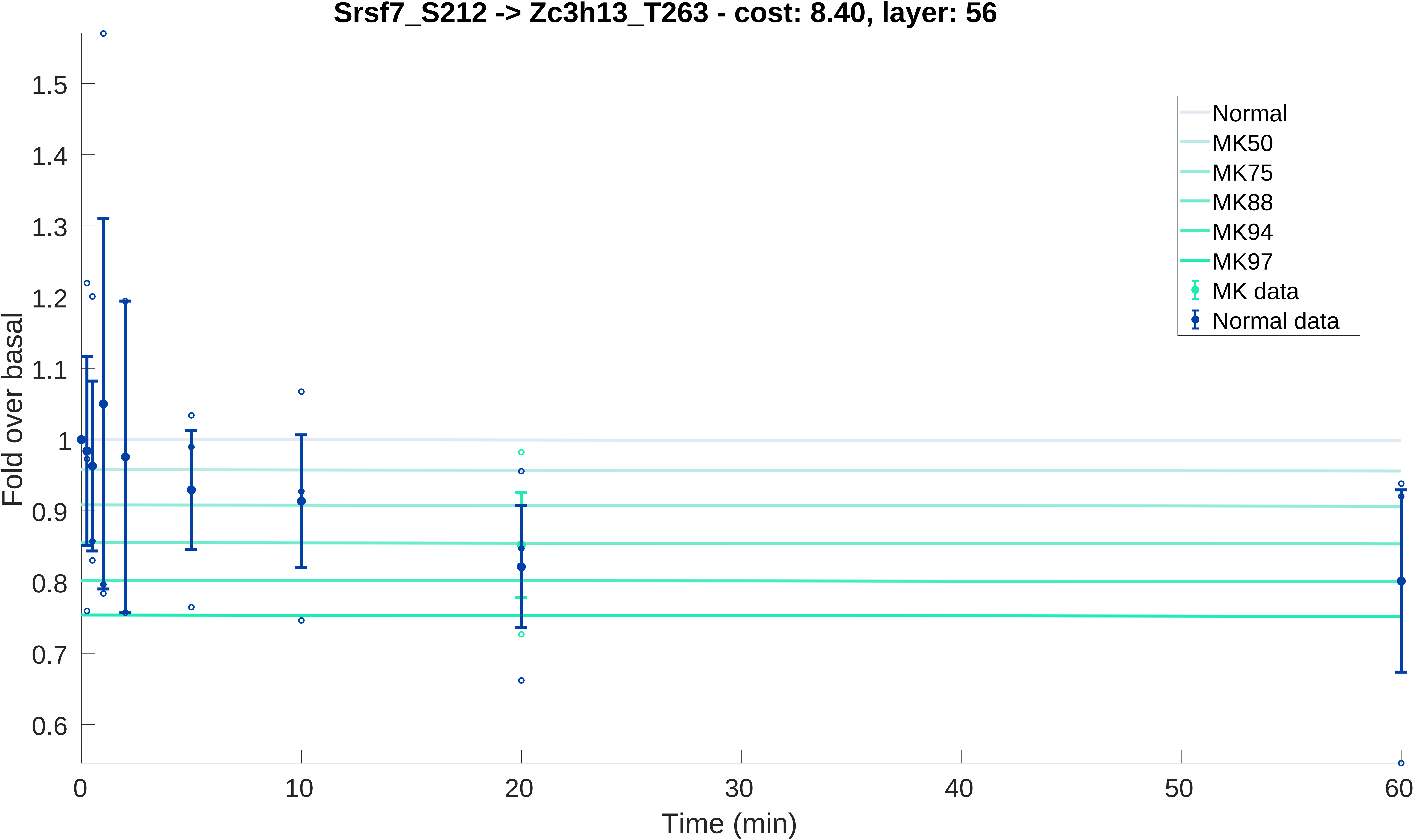
Srsf7_S212 -> Zc3h13_T263 - cost: 8.40, layer: 56
Fold over basal
Time (min)
Normal
MK50
MK75
MK88
MK94
MK97
MK data
Normal data
1.5
1.4
1.3
1.2
1.1
1
0.9
0.8
0.7
0.6
0
10
20
30
40
50
60

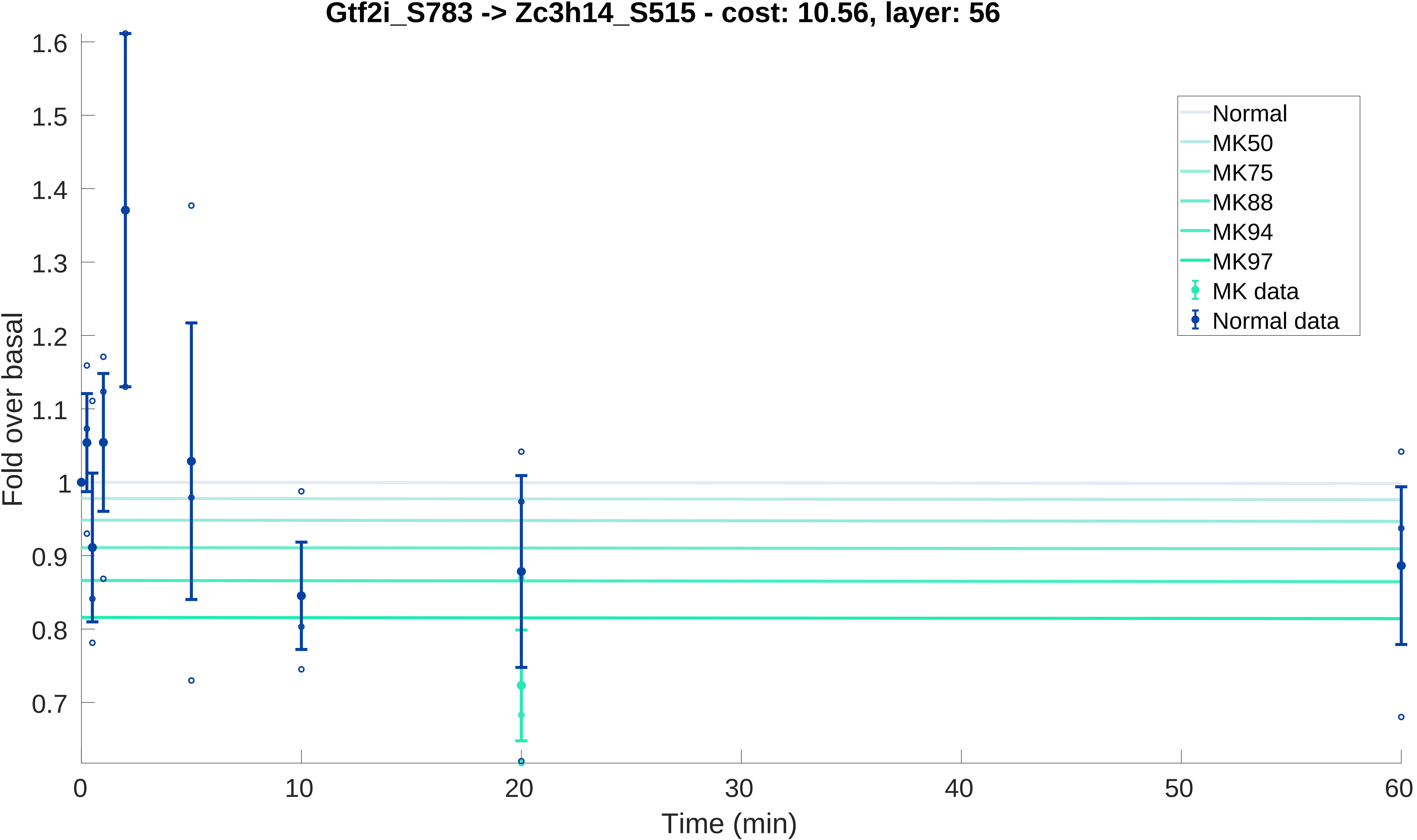

Gtf2i_S783 -> Zc3h14_S515 - cost: 10.56, layer: 56
Fold over basal
Time (min)
1.6
1.5
1.4
1.3
1.2
1.1
1
0.9
0.8
0.7
0
10
20
30
40
50
60
Normal
MK50
MK75
MK88
MK94
MK97
MK data
Normal data

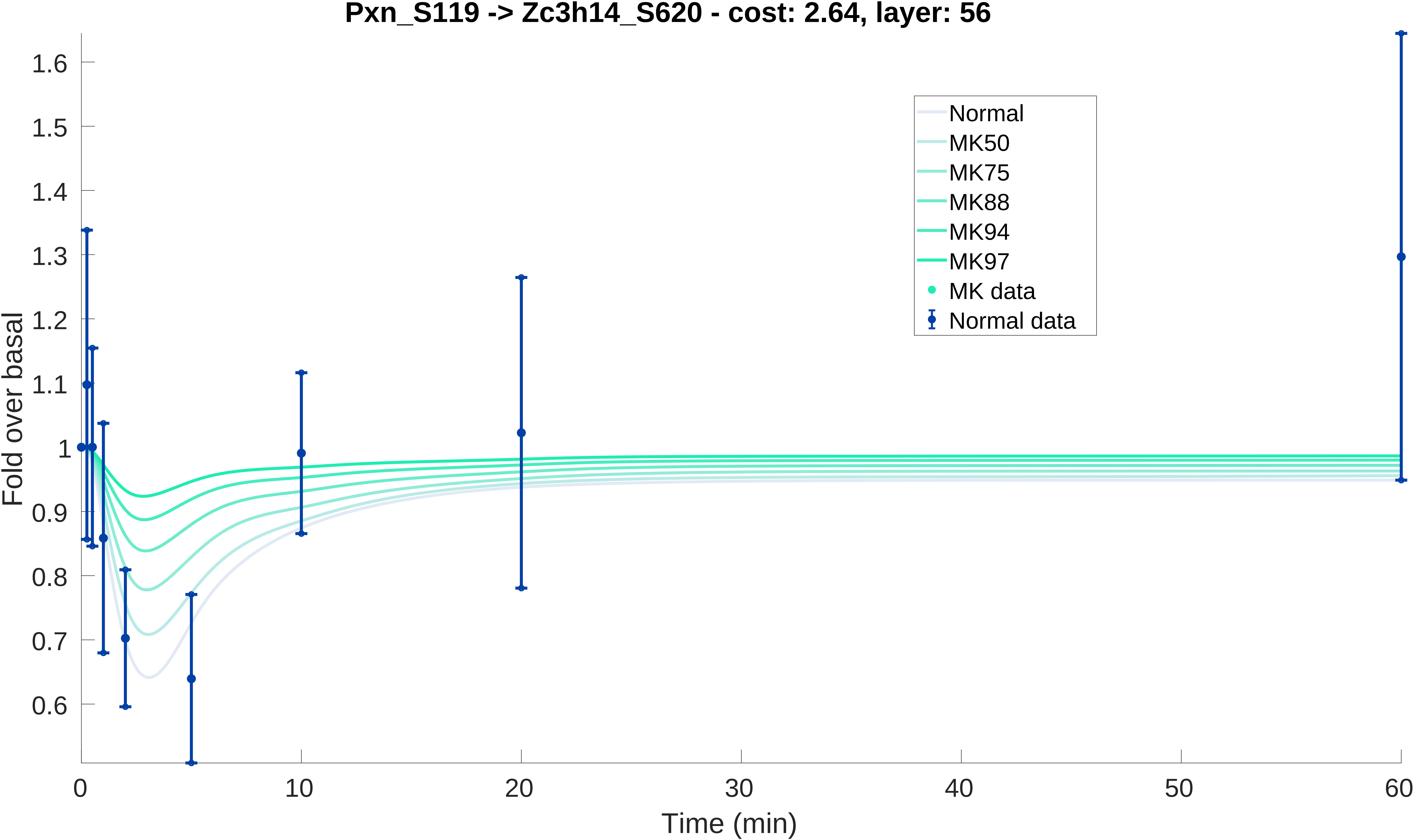

Pxn_S119 -> Zc3h14_S620 - cost: 2.64, layer: 56
Fold over basal
Time (min)
1.6
1.5
1.4
1.3
1.2
1.1
1
0.9
0.8
0.7
0.6
0
10
20
30
40
50
60
Normal
MK50
MK75
MK88
MK94
MK97
MK data
Normal data

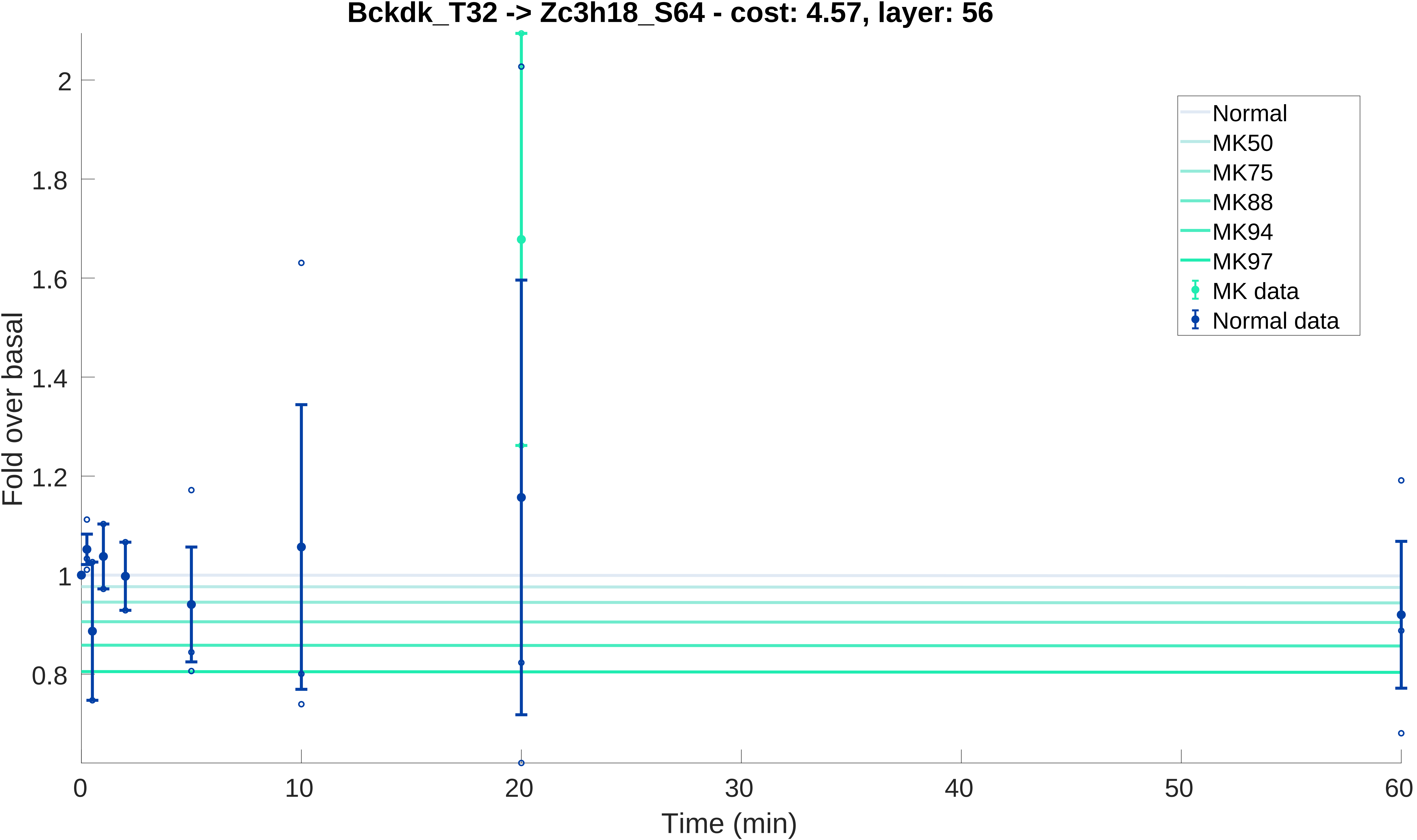

Bckdk_T32 -> Zc3h18_S64 - cost: 4.57, layer: 56
Fold over basal
Time (min)
0
10
20
30
40
50
60
0.8
1
1.2
1.4
1.6
1.8
2
Normal
MK50
MK75
MK88
MK94
MK97
MK data
Normal data

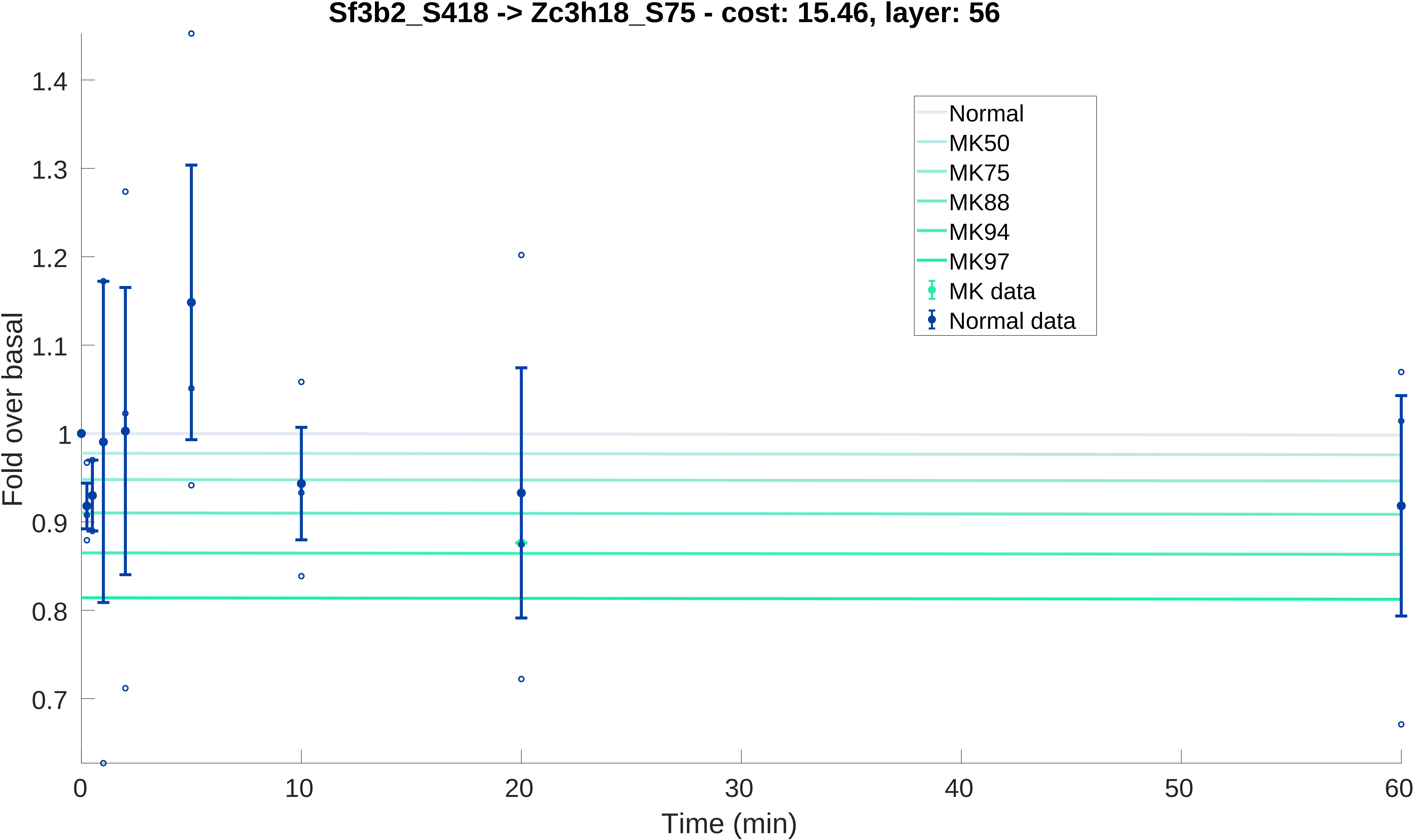

Sf3b2_S418 -> Zc3h18_S75 - cost: 15.46, layer: 56
Fold over basal
Time (min)
1.4
1.3
1.2
1.1
1
0.9
0.8
0.7
0
10
20
30
40
50
60
Normal
MK50
MK75
MK88
MK94
MK97
MK data
Normal data

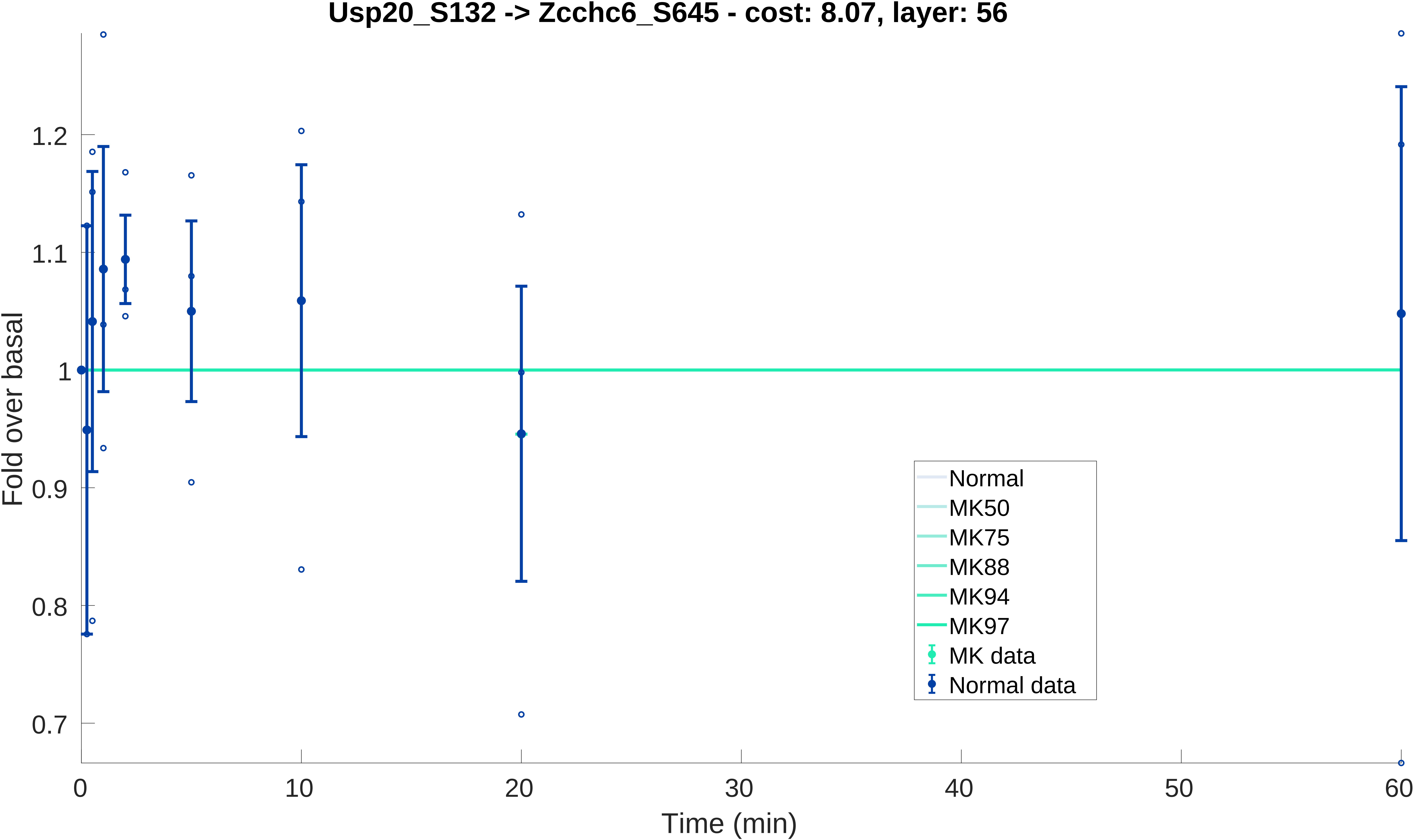

Usp20_S132 -> Zcchc6_S645 - cost: 8.07, layer: 56
Fold over basal
1.2
1.1
1
0.9
0.8
0.7
0
10
20
30
40
50
60
Time (min)
Normal
MK50
MK75
MK88
MK94
MK97
MK data
Normal data

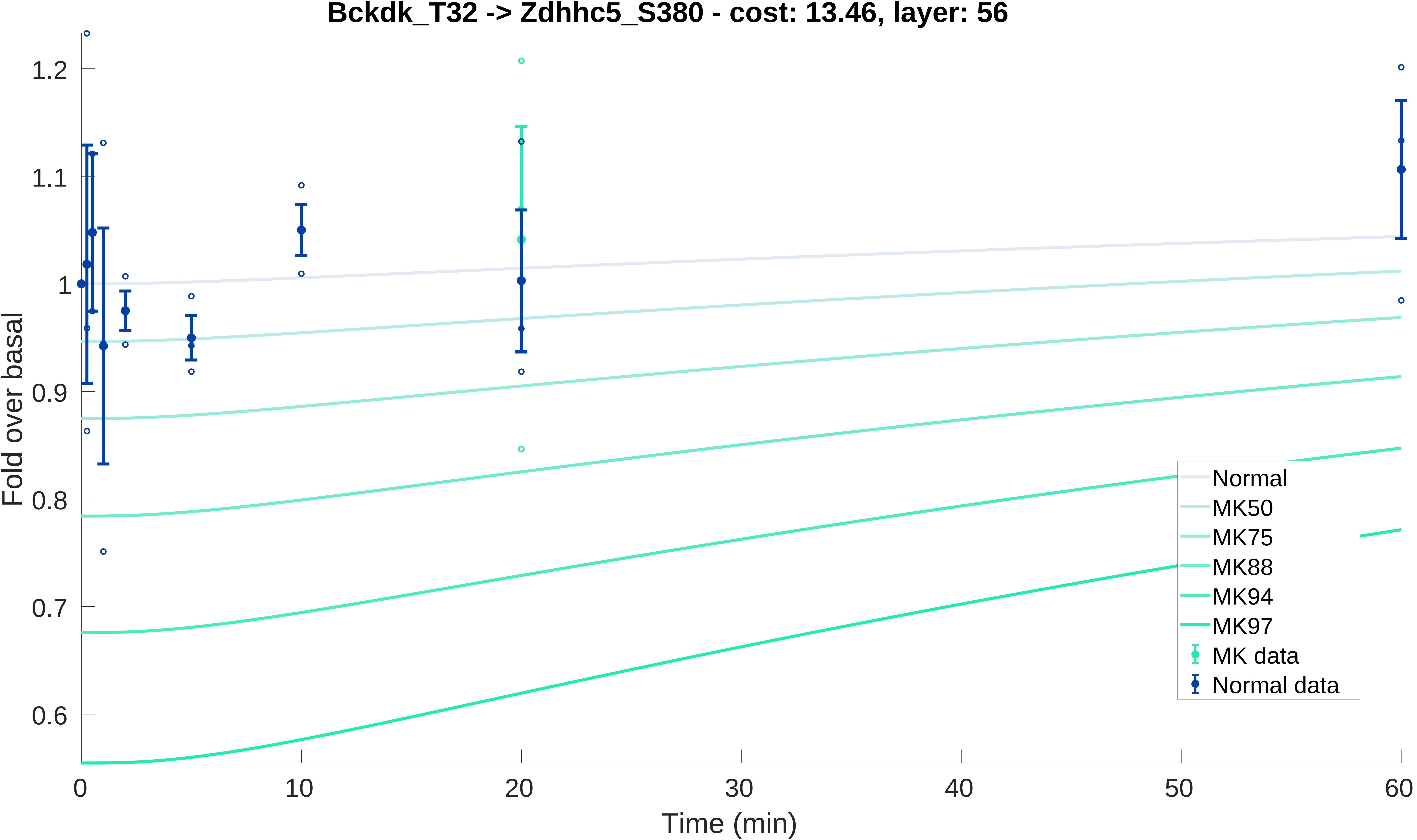

Bckdk_T32 -> Zdhhc5_S380 - cost: 13.46, layer: 56
Fold over basal
Time (min)
1.2
1.1
1
0.9
0.8
0.7
0.6
0
10
20
30
40
50
60
Normal
MK50
MK75
MK88
MK94
MK97
MK data
Normal data

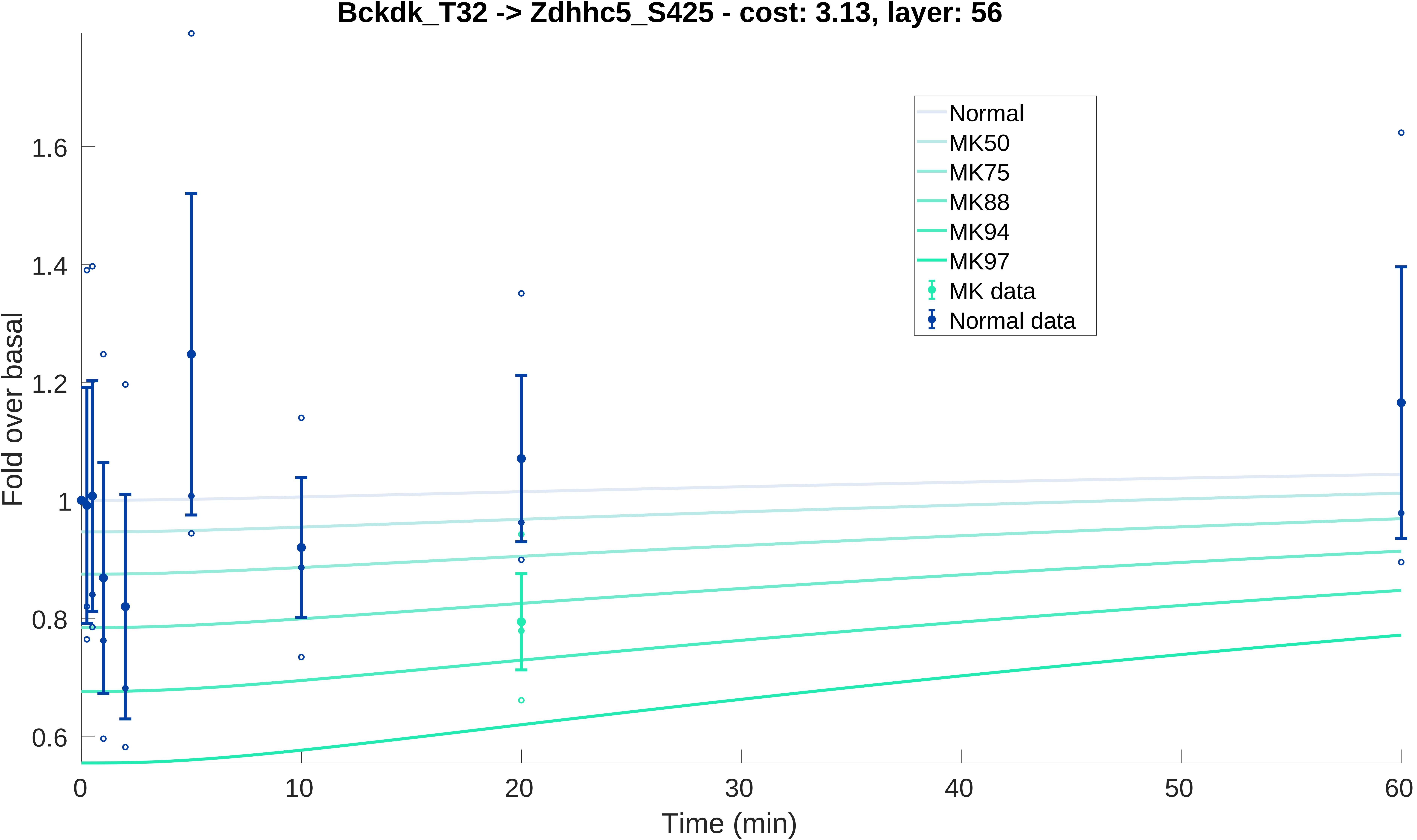

Bckdk_T32 -> Zdhhc5_S425 - cost: 3.13, layer: 56
Fold over basal
Time (min)
0.6
0.8
1
1.2
1.4
1.6
0
10
20
30
40
50
60
Normal
MK50
MK75
MK88
MK94
MK97
MK data
Normal data

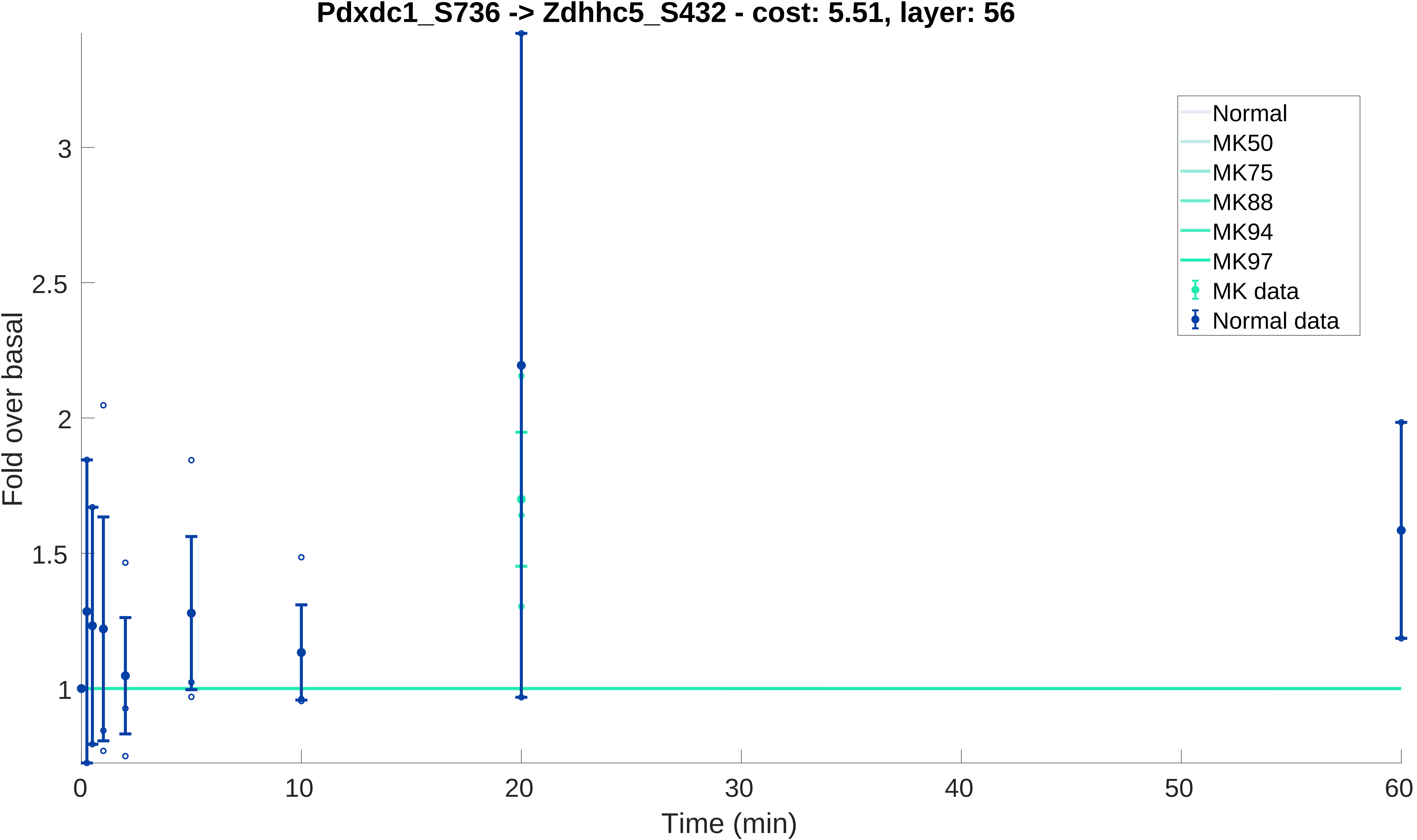

Pdxdc1_S736 -> Zdhhc5_S432 - cost: 5.51, layer: 56
Fold over basal
Time (min)
0
10
20
30
40
50
60
1
1.5
2
2.5
3
Normal
MK50
MK75
MK88
MK94
MK97
MK data
Normal data

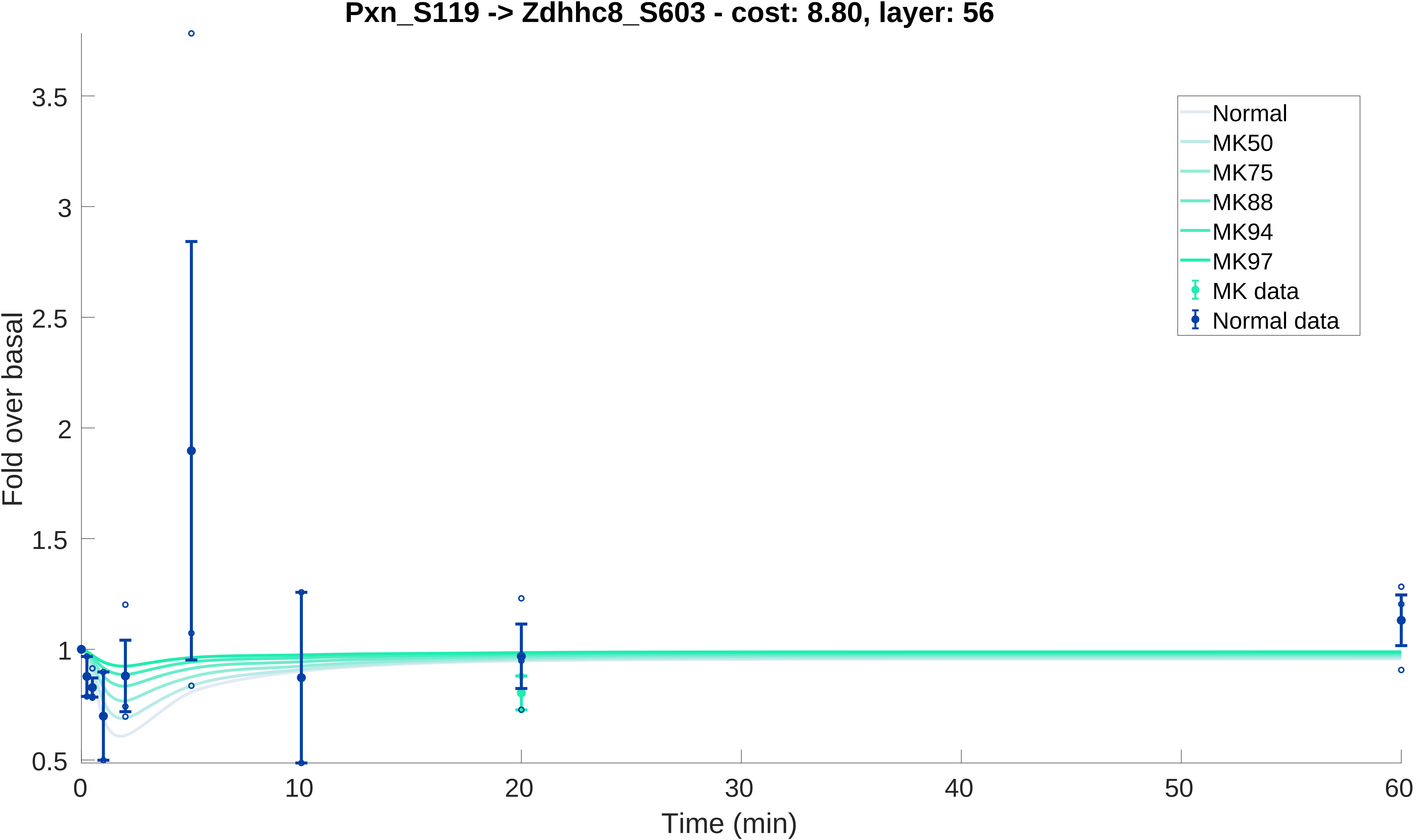
Pxn_S119 -> Zdhhc8_S603 - cost: 8.80, layer: 56
Fold over basal
Time (min)
0.5
1
1.5
2
2.5
3
3.5
0
10
20
30
40
50
60
Normal
MK50
MK75
MK88
MK94
MK97
MK data
Normal data

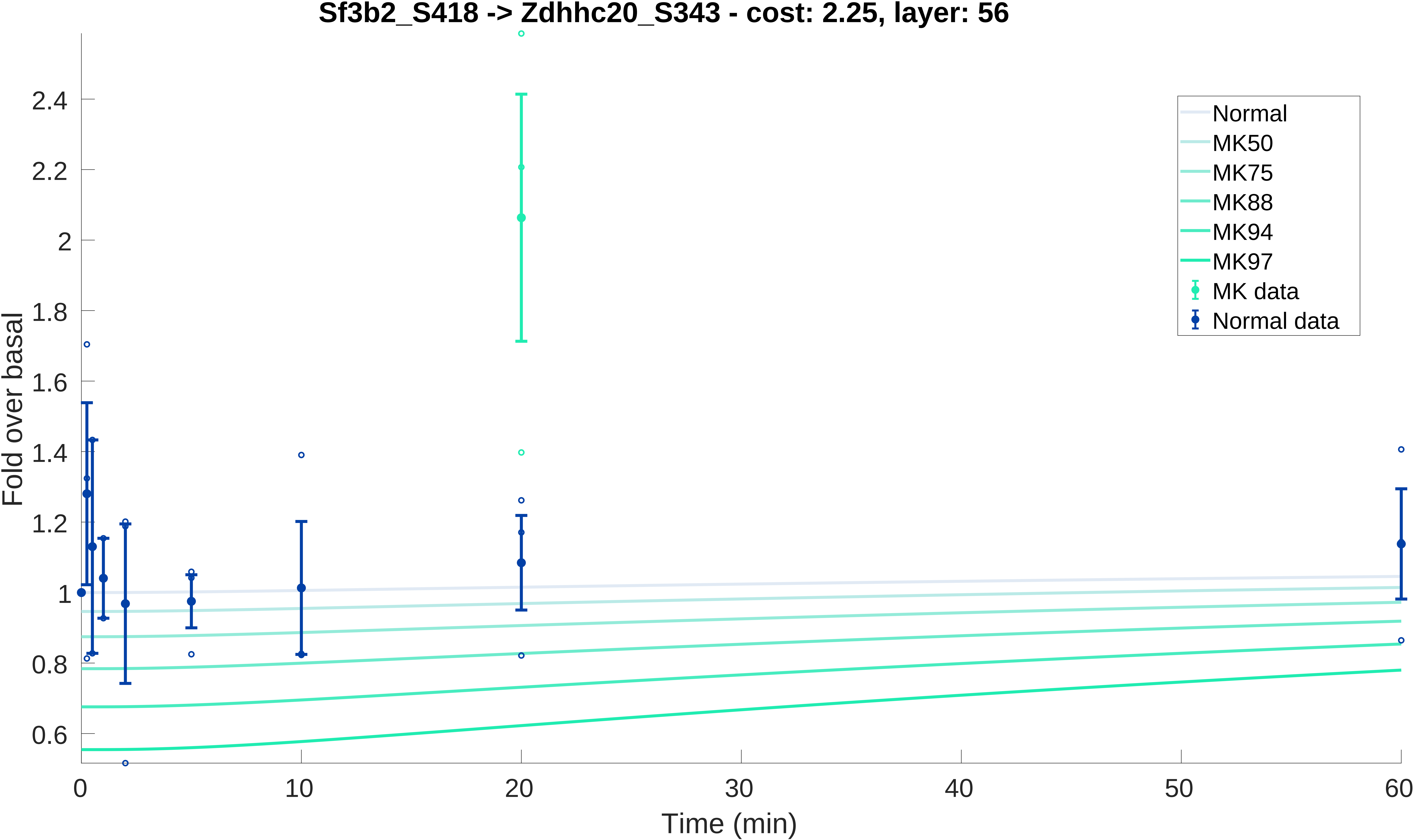
Sf3b2_S418 -> Zdhhc20_S343 - cost: 2.25, layer: 56
Fold over basal
2.4
2.2
2
1.8
1.6
1.4
1.2
1
0.8
0.6
0
10
20
30
40
50
60
Time (min)
Normal
MK50
MK75
MK88
MK94
MK97
MK data
Normal data

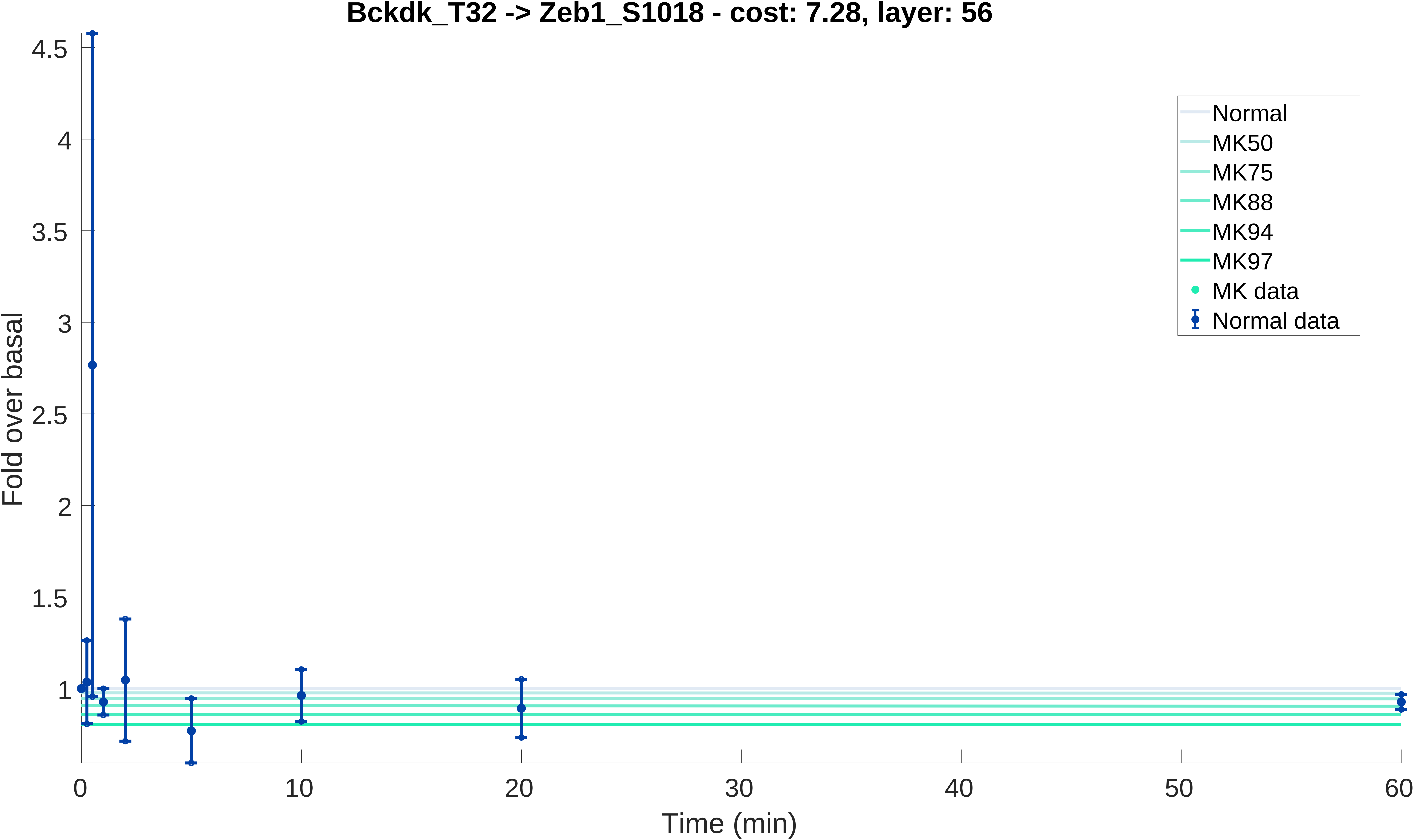

Bckdk_T32 -> Zeb1_S1018 - cost: 7.28, layer: 56
Fold over basal
4.5
4
3.5
3
2.5
2
1.5
1
0
10
20
30
40
50
60
Time (min)
Normal
MK50
MK75
MK88
MK94
MK97
MK data
Normal data

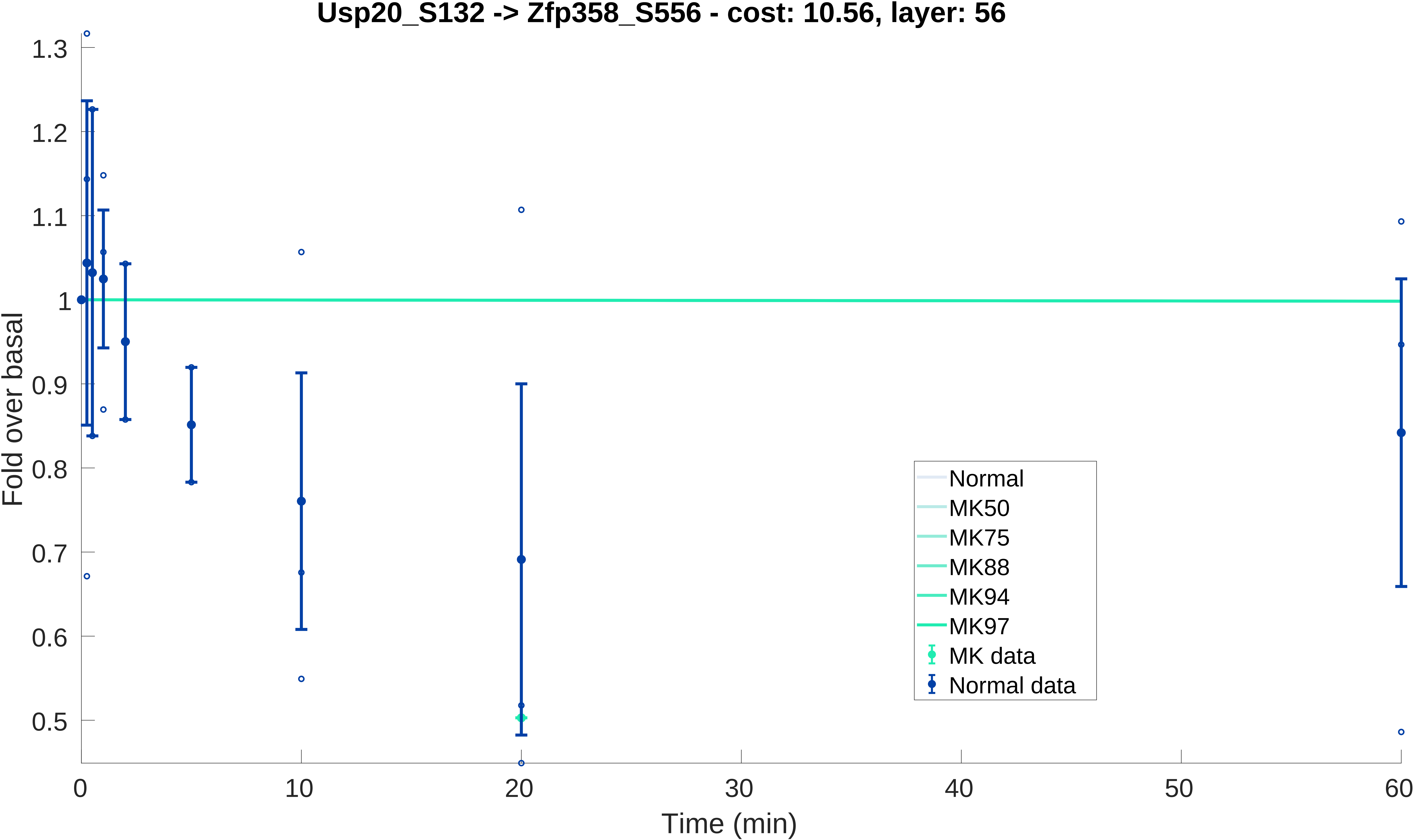
Usp20_S132 -> Zfp358_S556 - cost: 10.56, layer: 56
Fold over basal
Time (min)
1.3
1.2
1.1
1
0.9
0.8
0.7
0.6
0.5
0
10
20
30
40
50
60
Normal
MK50
MK75
MK88
MK94
MK97
MK data
Normal data

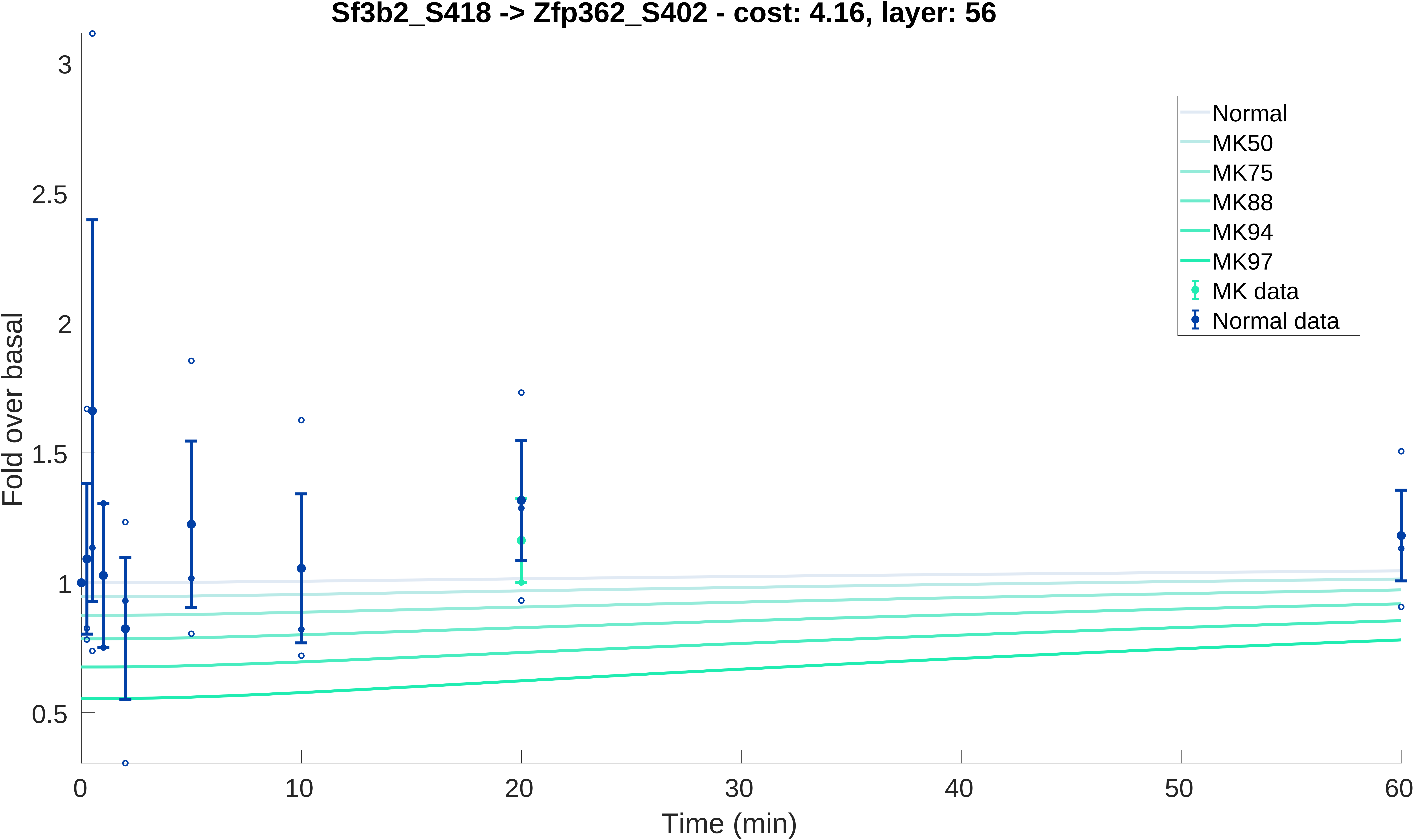

Sf3b2_S418 -> Zfp362_S402 - cost: 4.16, layer: 56
Fold over basal
Time (min)
0.5
1
1.5
2
2.5
3
0
10
20
30
40
50
60
Normal
MK50
MK75
MK88
MK94
MK97
MK data
Normal data

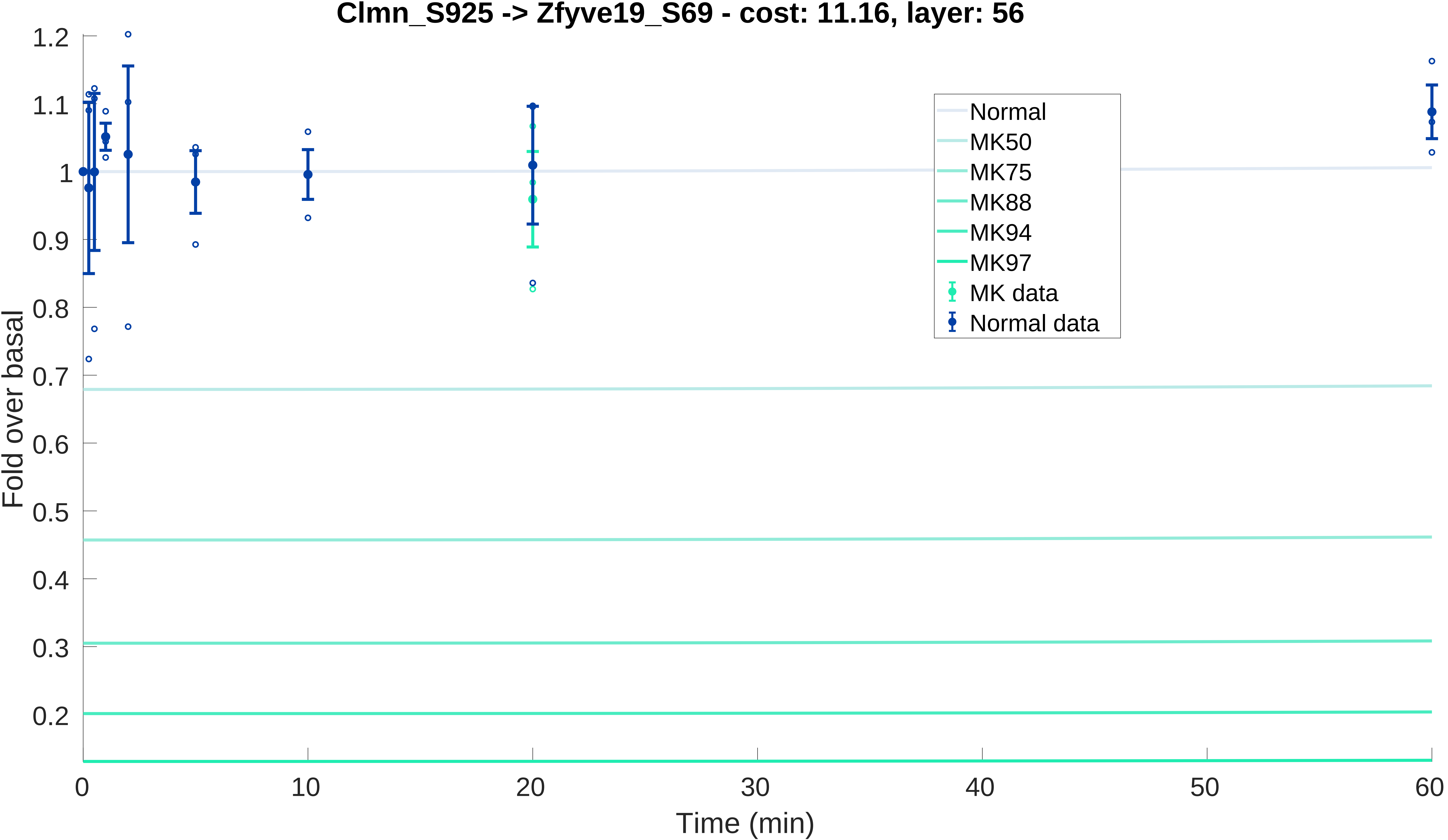

Clmn_S925 -> Zfyve19_S69 - cost: 11.16, layer: 56
Fold over basal
Time (min)
0
10
20
30
40
50
60
0.2
0.3
0.4
0.5
0.6
0.7
0.8
0.9
1
1.1
1.2
Normal
MK50
MK75
MK88
MK94
MK97
MK data
Normal data

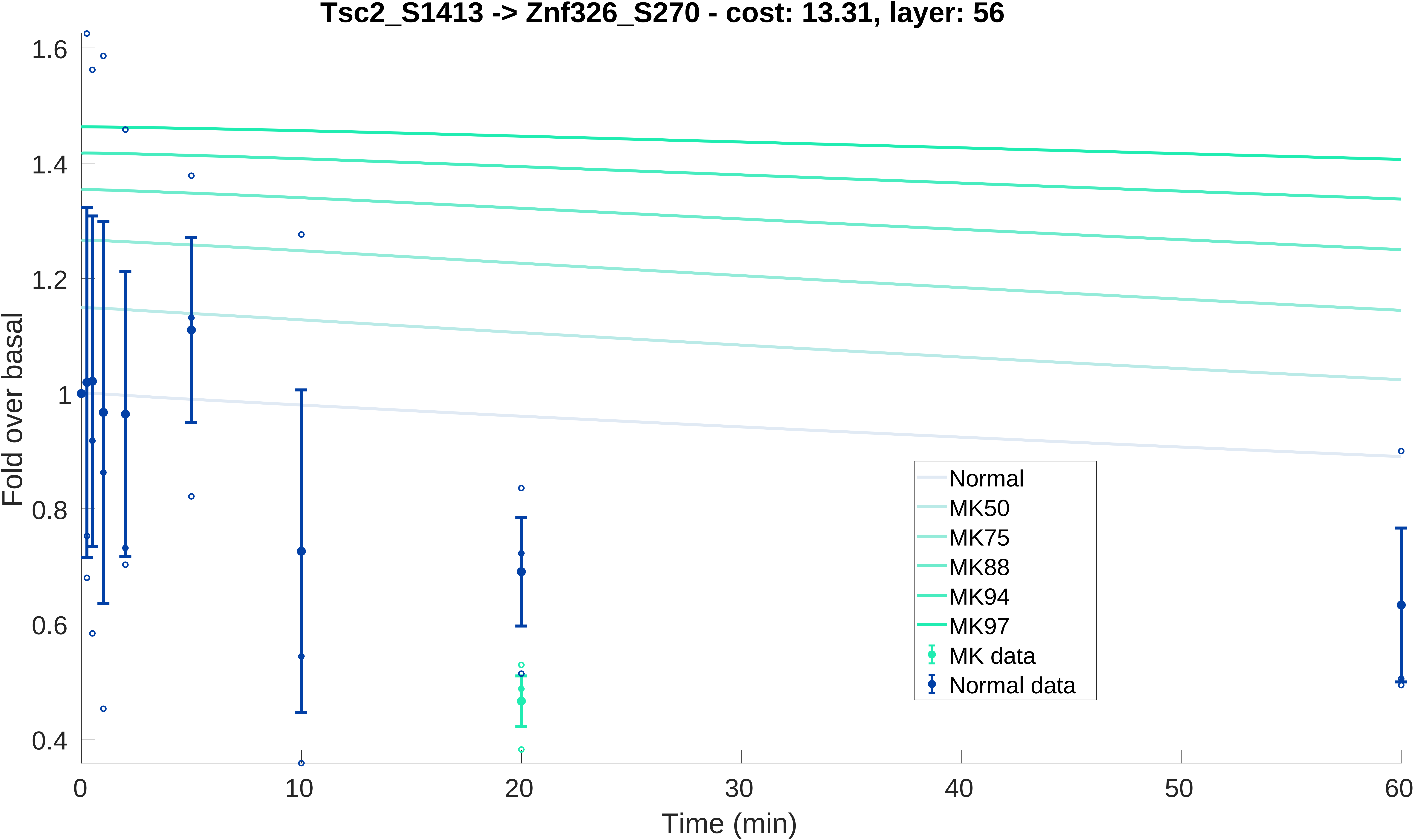

Tsc2_S1413 -> Znf326_S270 - cost: 13.31, layer: 56
Fold over basal
Time (min)
1.6
1.4
1.2
1
0.8
0.6
0.4
0
10
20
30
40
50
60
Normal
MK50
MK75
MK88
MK94
MK97
MK data
Normal data

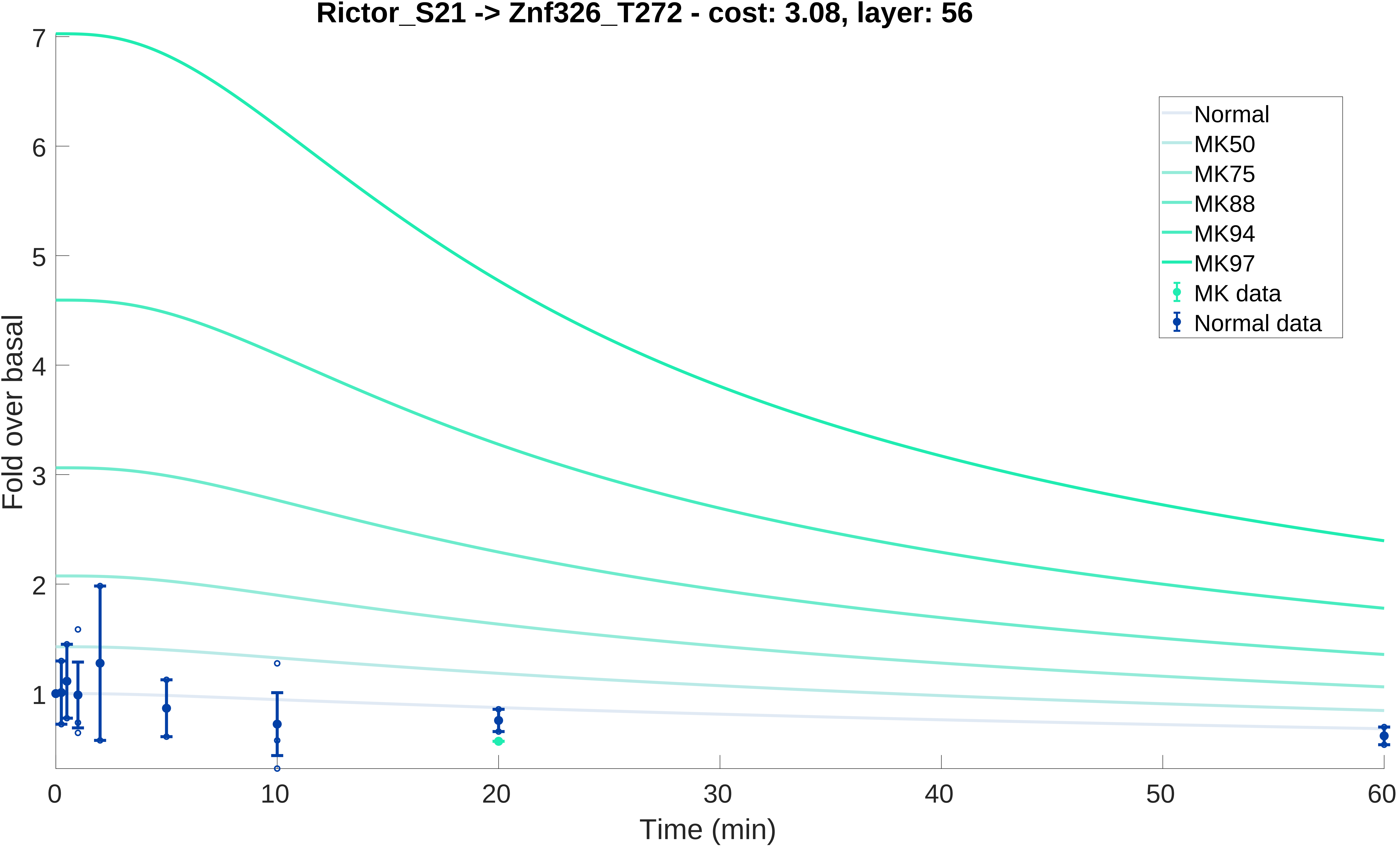

Rictor_S21 -> Znf326_T272 - cost: 3.08, layer: 56
Fold over basal
Time (min)
0
1
2
3
4
5
6
7
10
20
30
40
50
60
Normal
MK50
MK75
MK88
MK94
MK97
MK data
Normal data

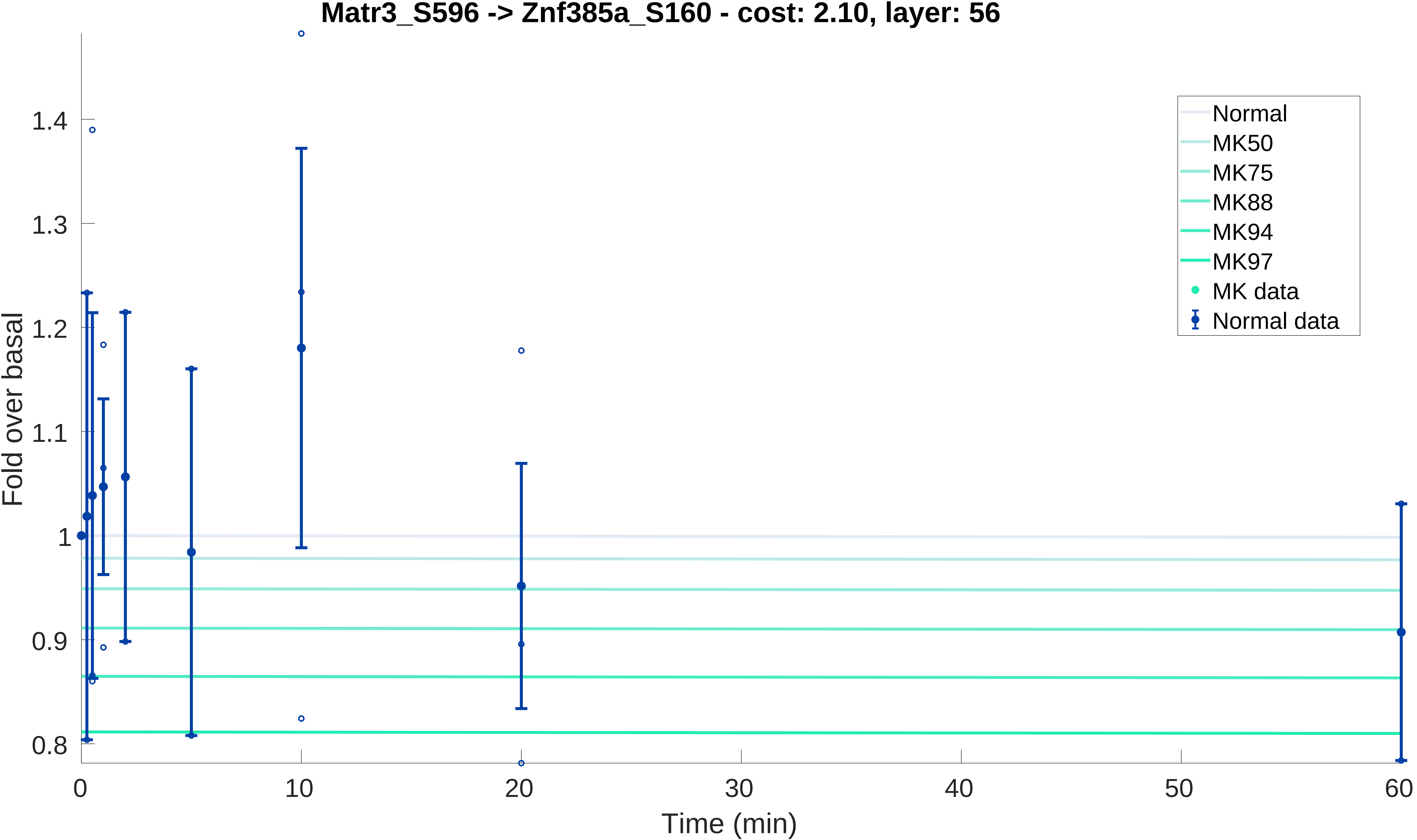

Matr3_S596 -> Znf385a_S160 - cost: 2.10, layer: 56
Fold over basal
Time (min)
0.8
0.9
1
1.1
1.2
1.3
1.4
0
10
20
30
40
50
60
Normal
MK50
MK75
MK88
MK94
MK97
MK data
Normal data

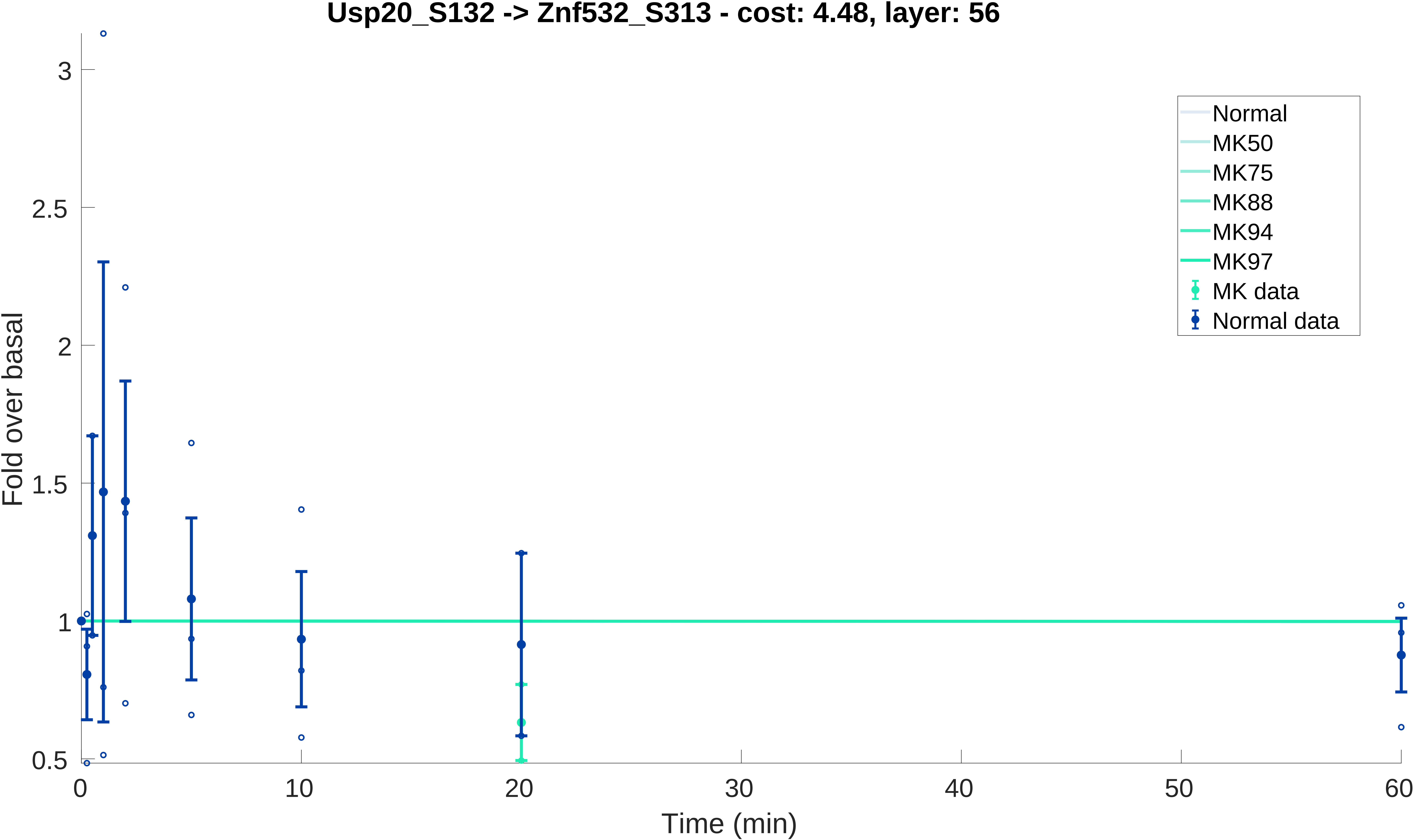

Usp20_S132 -> Znf532_S313 - cost: 4.48, layer: 56
Fold over basal
Time (min)
Normal
MK50
MK75
MK88
MK94
MK97
MK data
Normal data
0.5
1
1.5
2
2.5
3
0
10
20
30
40
50
60

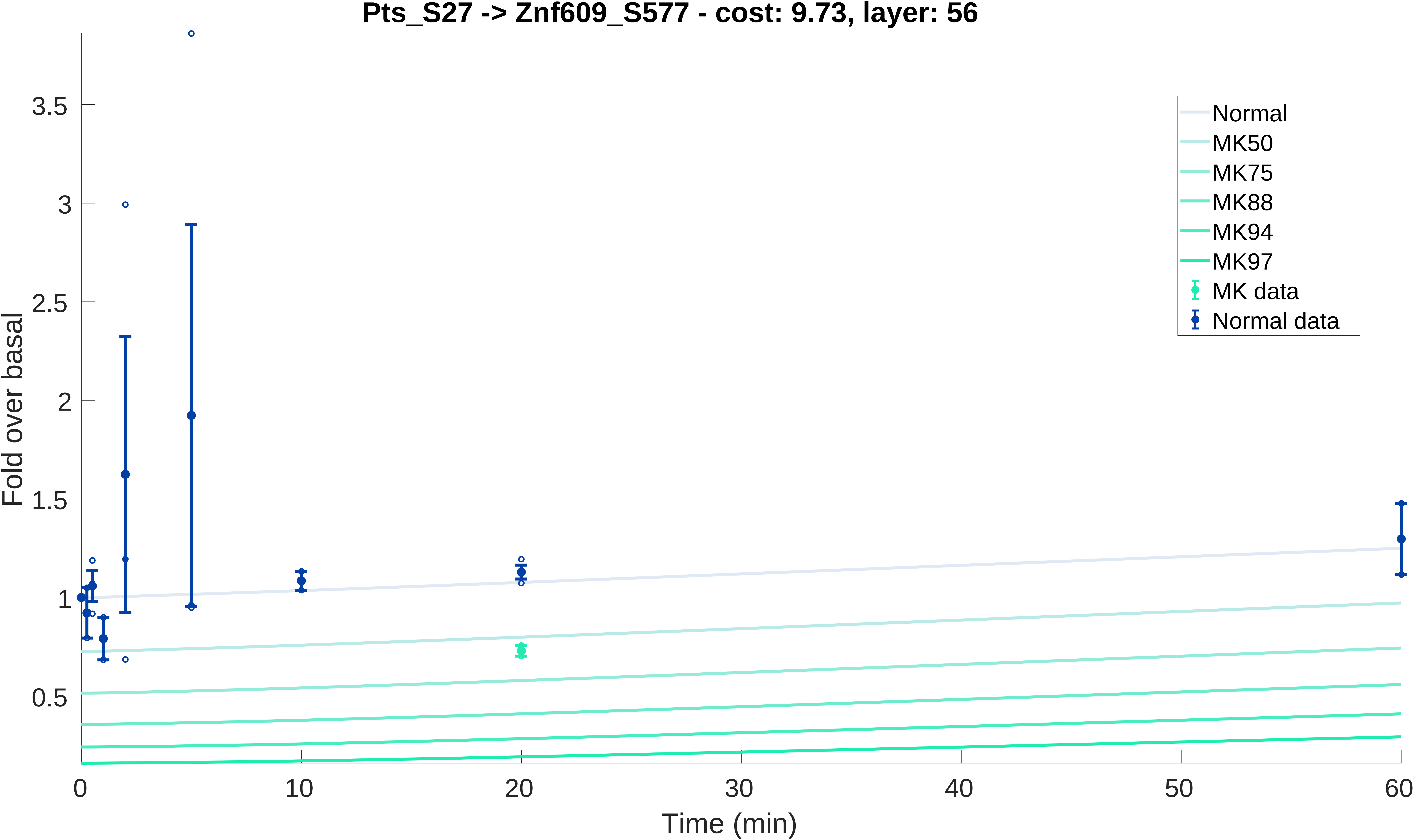

Pts_S27 -> Znf609_S577 - cost: 9.73, layer: 56
Fold over basal
Time (min)
3.5
3
2.5
2
1.5
1
0.5
0
10
20
30
40
50
60
Normal
MK50
MK75
MK88
MK94
MK97
MK data
Normal data

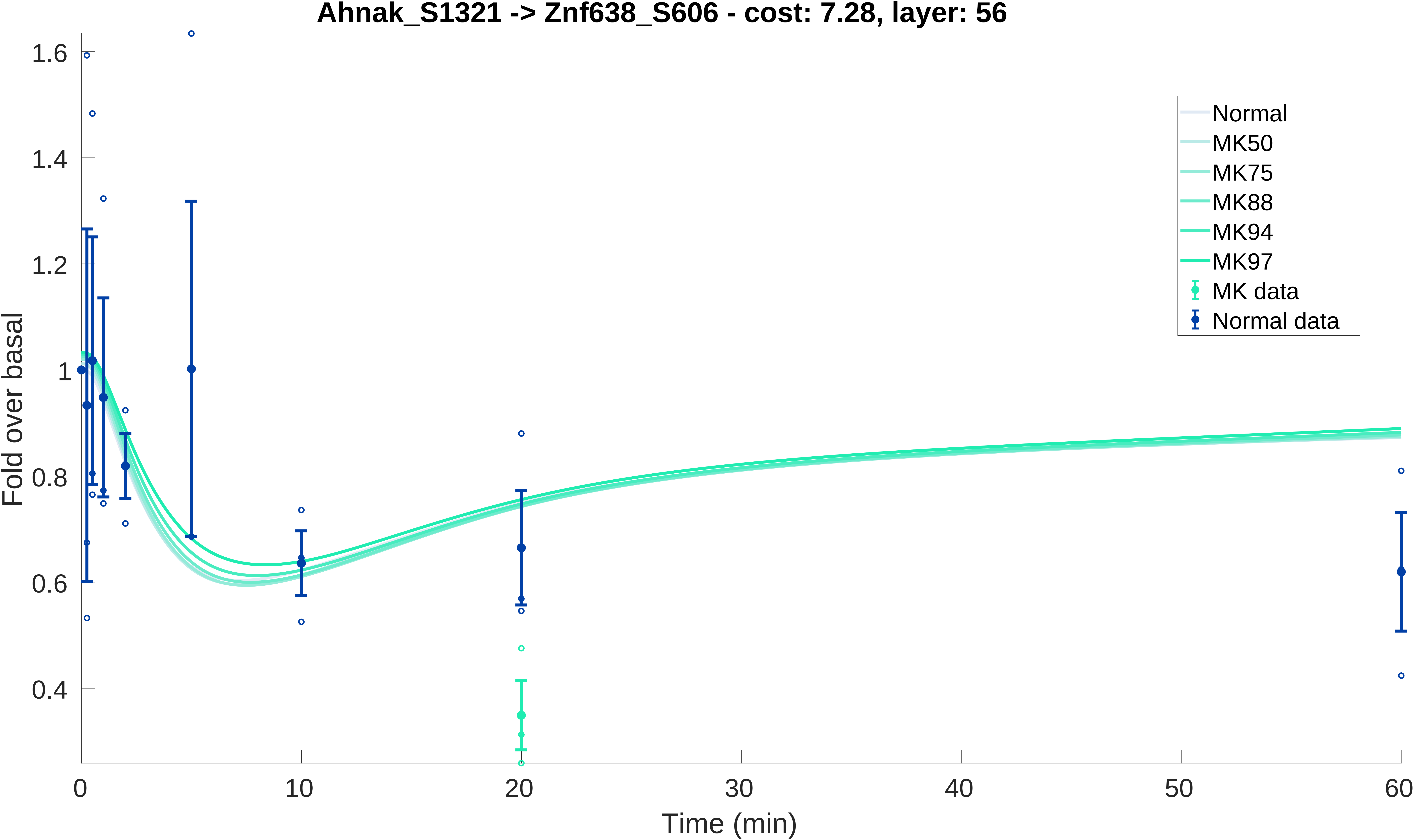

Ahnak_S1321 -> Znf638_S606 - cost: 7.28, layer: 56
Fold over basal
Time (min)
1.6
1.4
1.2
1
0.8
0.6
0.4
0
10
20
30
40
50
60
Normal
MK50
MK75
MK88
MK94
MK97
MK data
Normal data

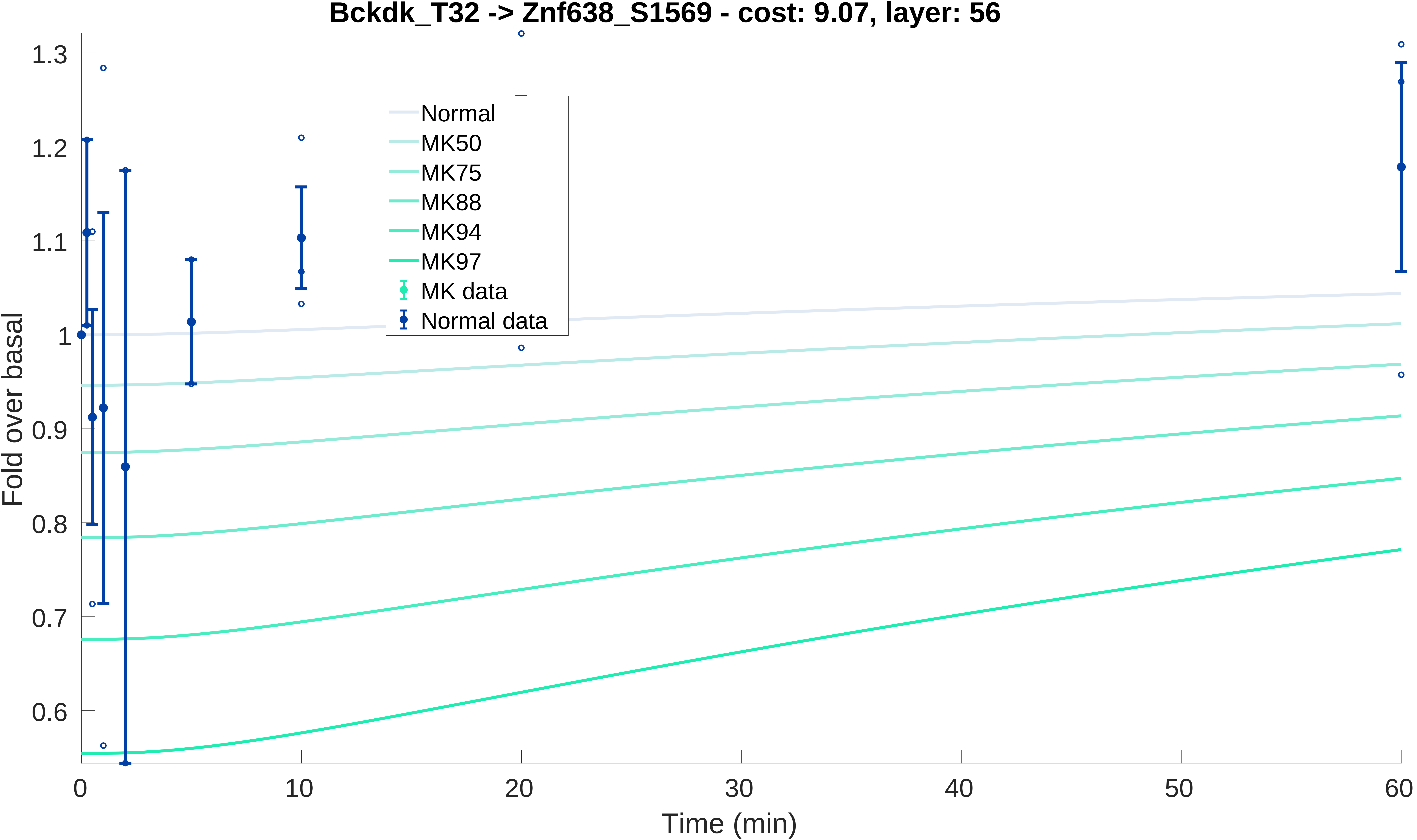

Bckdk_T32 -> Znf638_S1569 - cost: 9.07, layer: 56
Normal
MK50
MK75
MK88
MK94
MK97
MK data
Normal data
Fold over basal
Time (min)
1.3
1.2
1.1
1
0.9
0.8
0.7
0.6
0
10
20
30
40
50
60

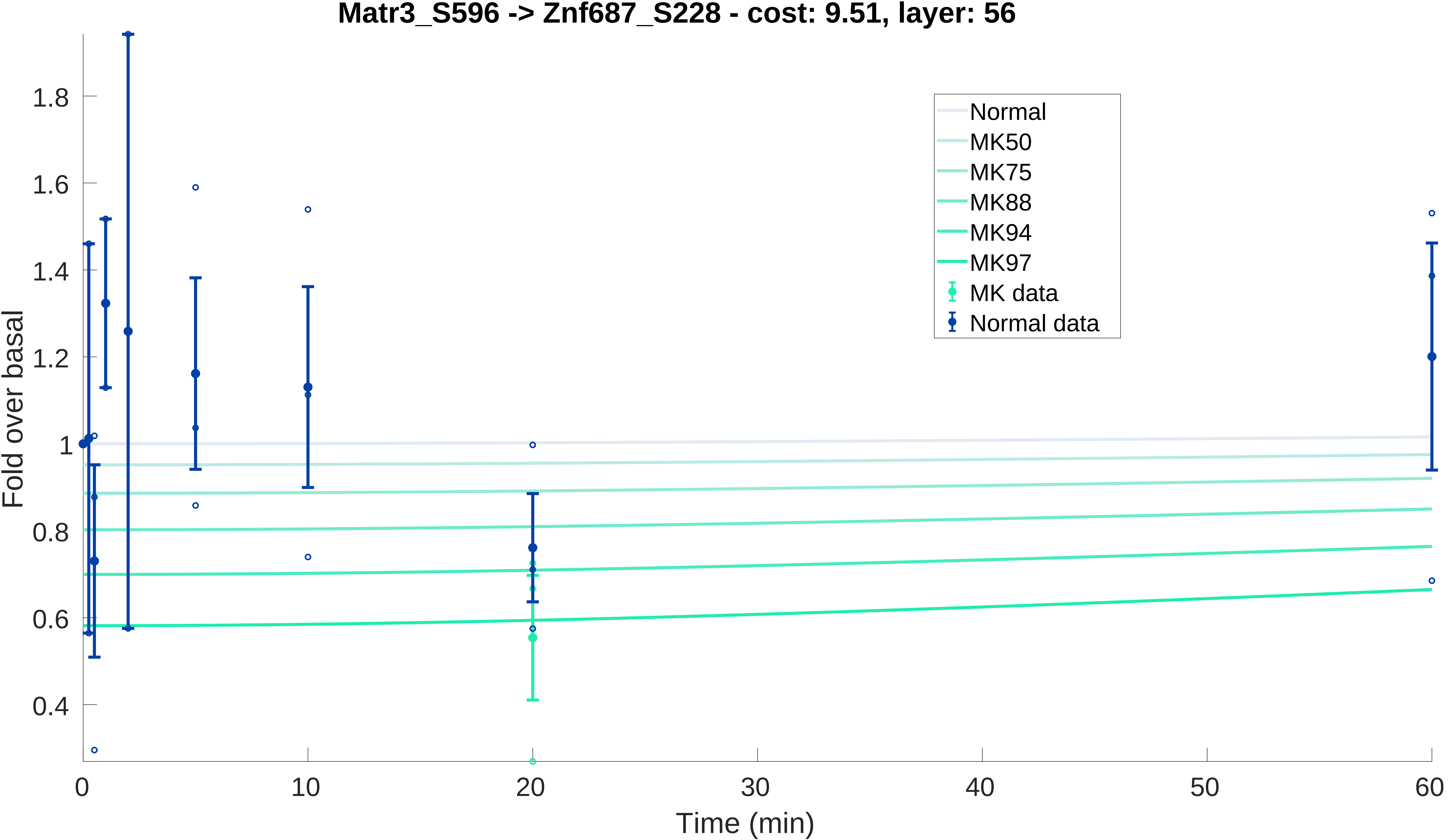
Matr3_S596 -> Znf687_S228 - cost: 9.51, layer: 56
Fold over basal
Time (min)
0
10
20
30
40
50
60
0.4
0.6
0.8
1
1.2
1.4
1.6
1.8
Normal
MK50
MK75
MK88
MK94
MK97
MK data
Normal data

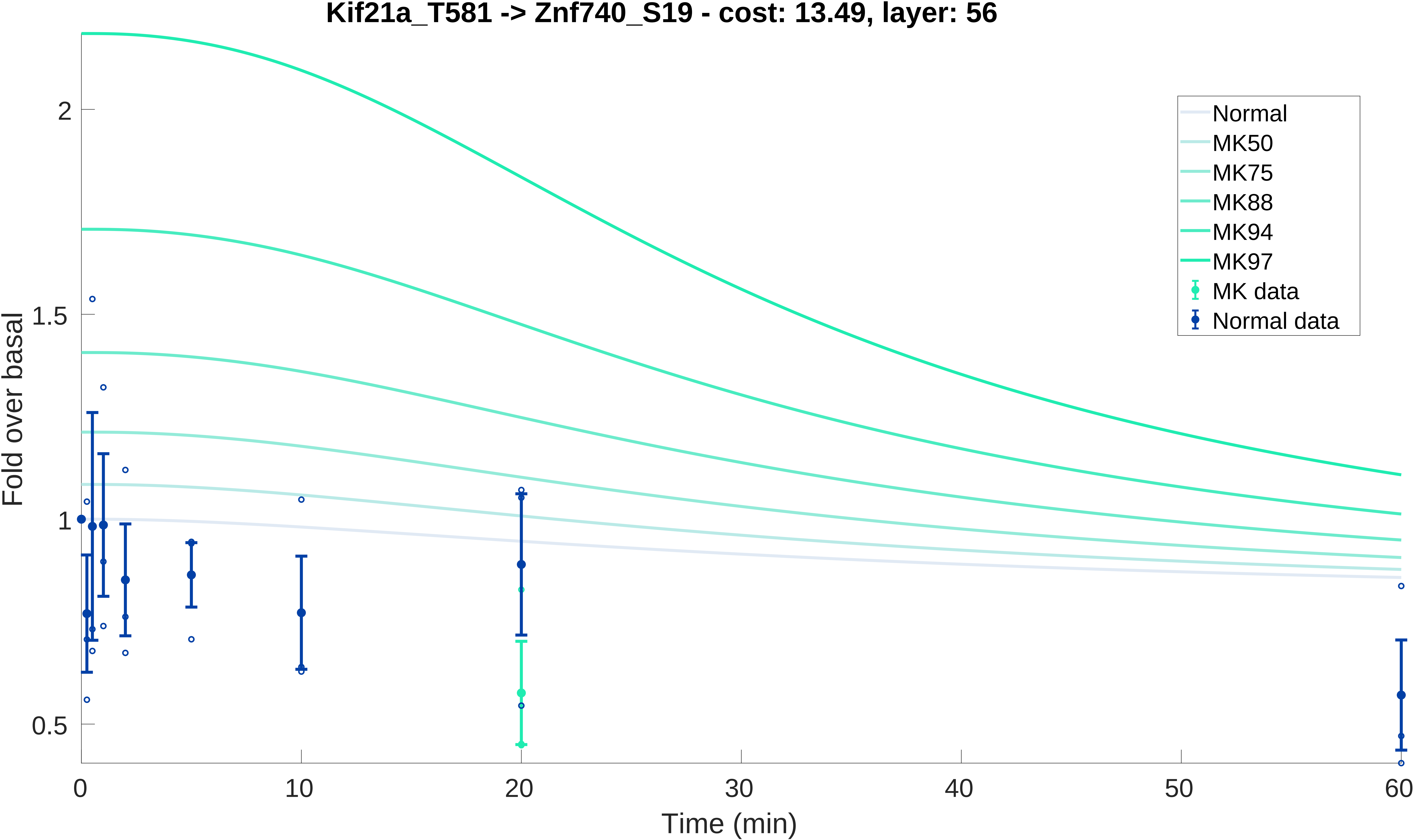

Kif21a_T581 -> Znf740_S19 - cost: 13.49, layer: 56
Fold over basal
Time (min)
Normal
MK50
MK75
MK88
MK94
MK97
MK data
Normal data
0.5
1
1.5
2
0
10
20
30
40
50
60

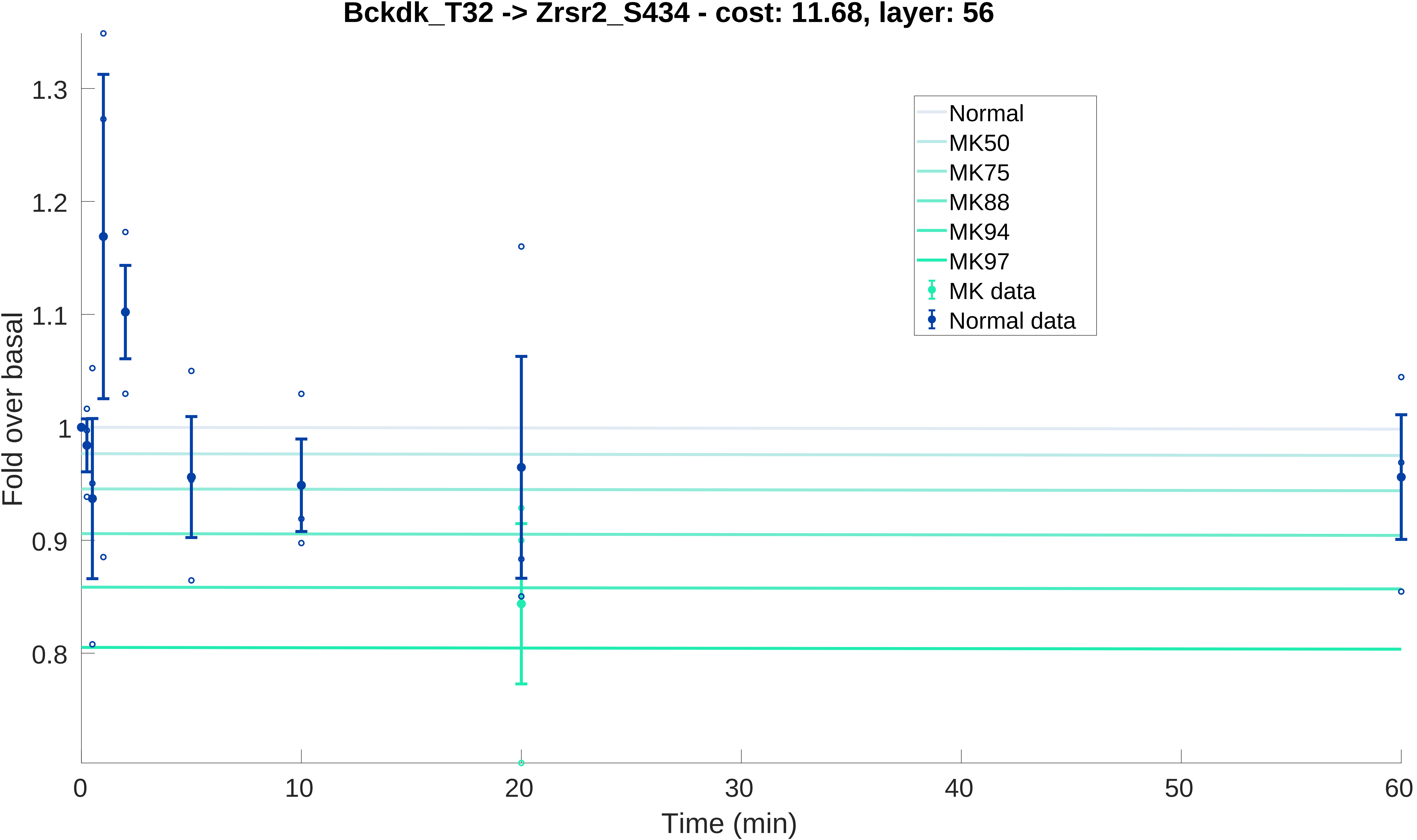
Bckdk_T32 -> Zrsr2_S434 - cost: 11.68, layer: 56
Normal
MK50
MK75
MK88
MK94
MK97
MK data
Normal data
Fold over basal
Time (min)
1.3
1.2
1.1
1
0.9
0.8
0
10
20
30
40
50
60

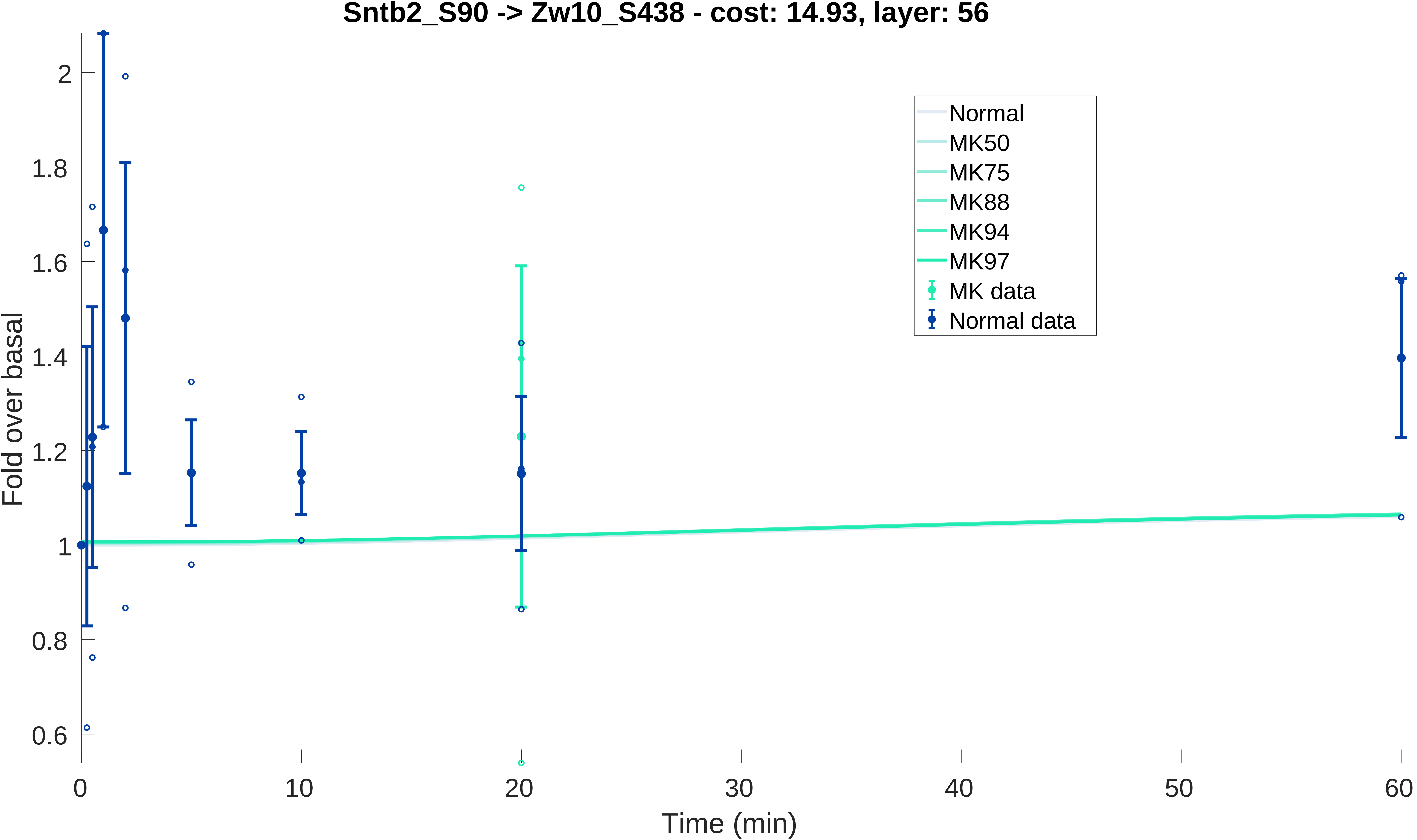

Sntb2_S90 -> Zw10_S438 - cost: 14.93, layer: 56
Fold over basal
Time (min)
0.6
0.8
1
1.2
1.4
1.6
1.8
2
0
10
20
30
40
50
60
Normal
MK50
MK75
MK88
MK94
MK97
MK data
Normal data

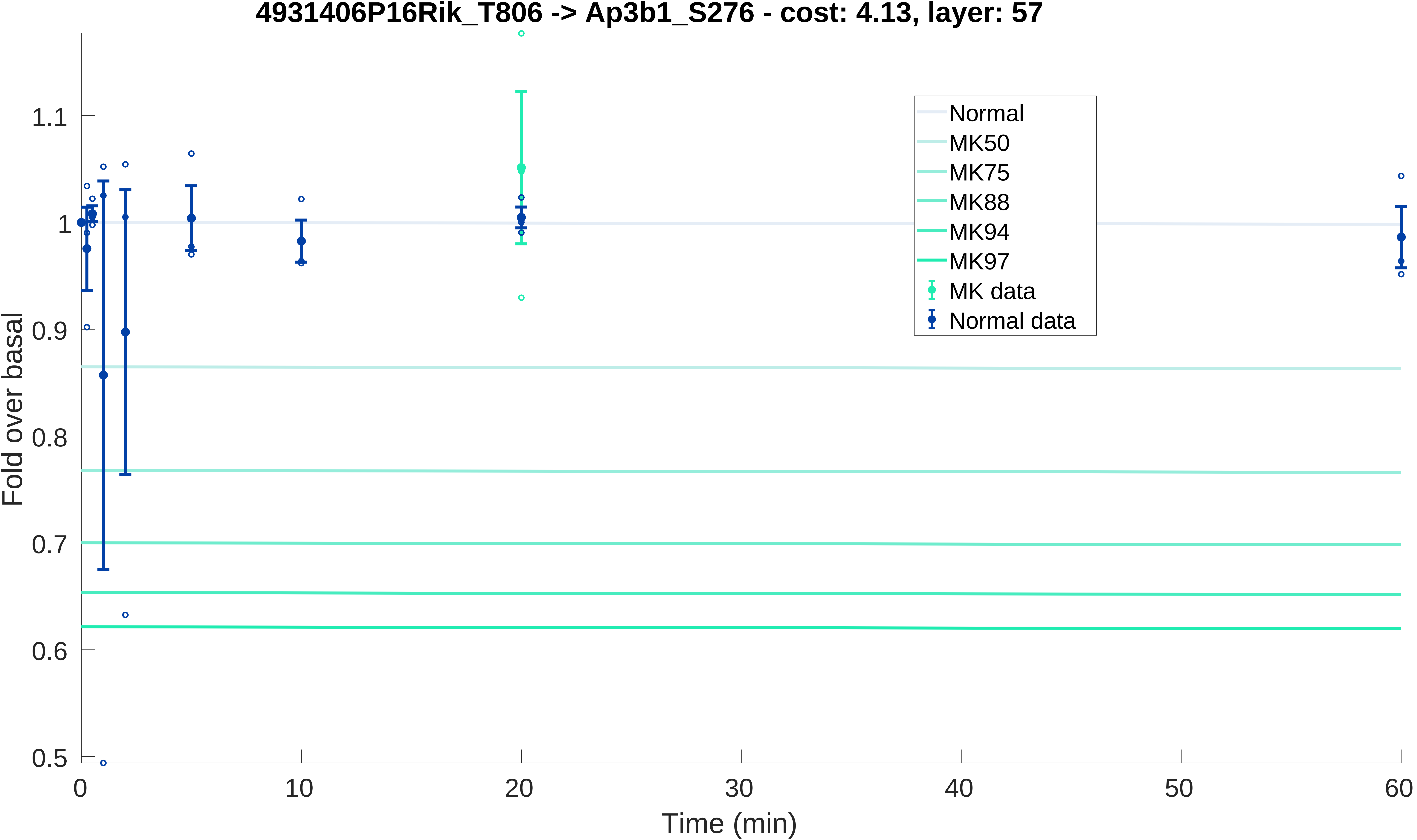
4931406P16Rik_T806 -> Ap3b1_S276 - cost: 4.13, layer: 57
Fold over basal
Time (min)
Normal
MK50
MK75
MK88
MK94
MK97
MK data
Normal data
1.1
1
0.9
0.8
0.7
0.6
0.5
0
10
20
30
40
50
60

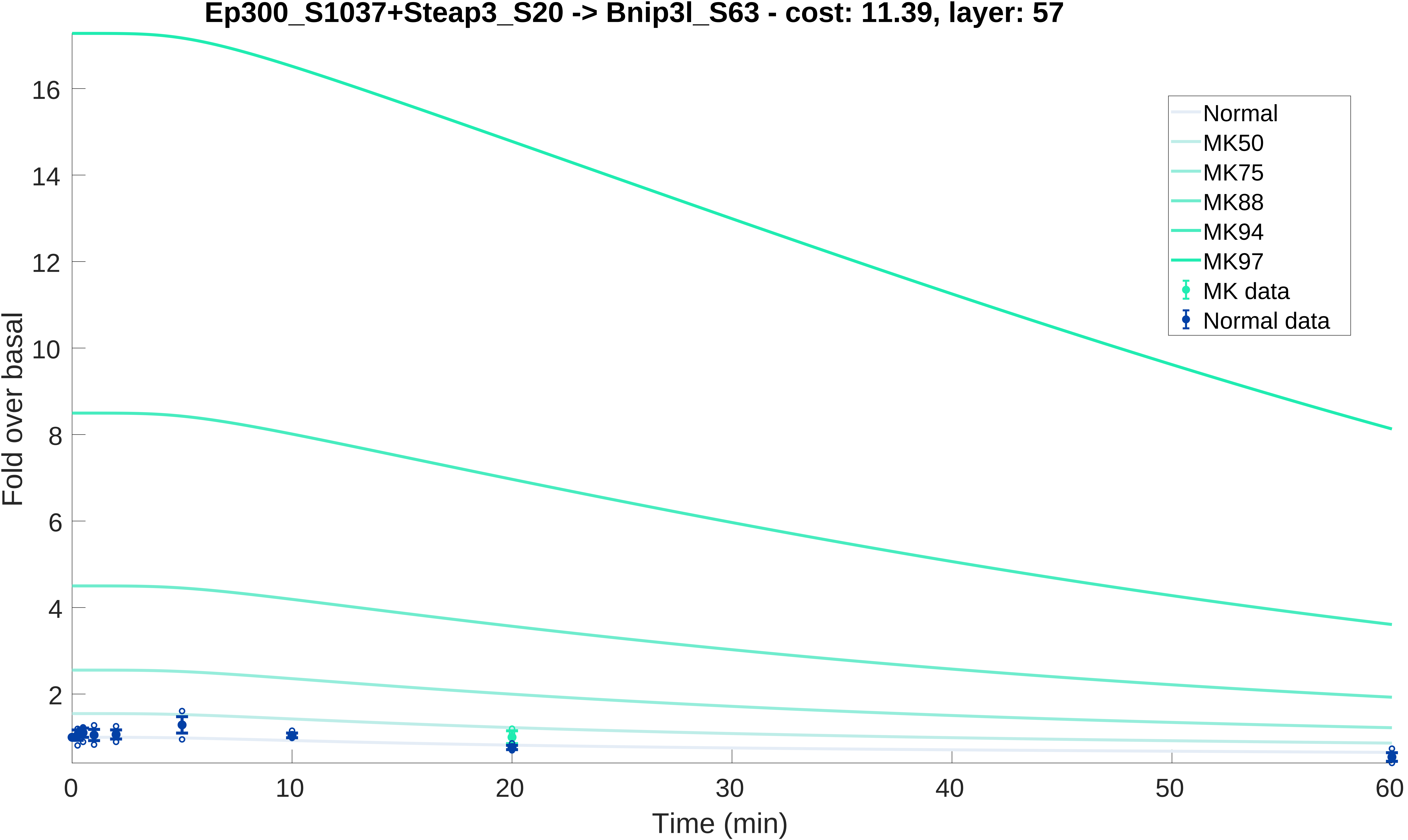

Ep300_S1037+Steap3_S20 -> Bnip3l_S63 - cost: 11.39, layer: 57
Fold over basal
Time (min)
Normal
MK50
MK75
MK88
MK94
MK97
MK data
Normal data

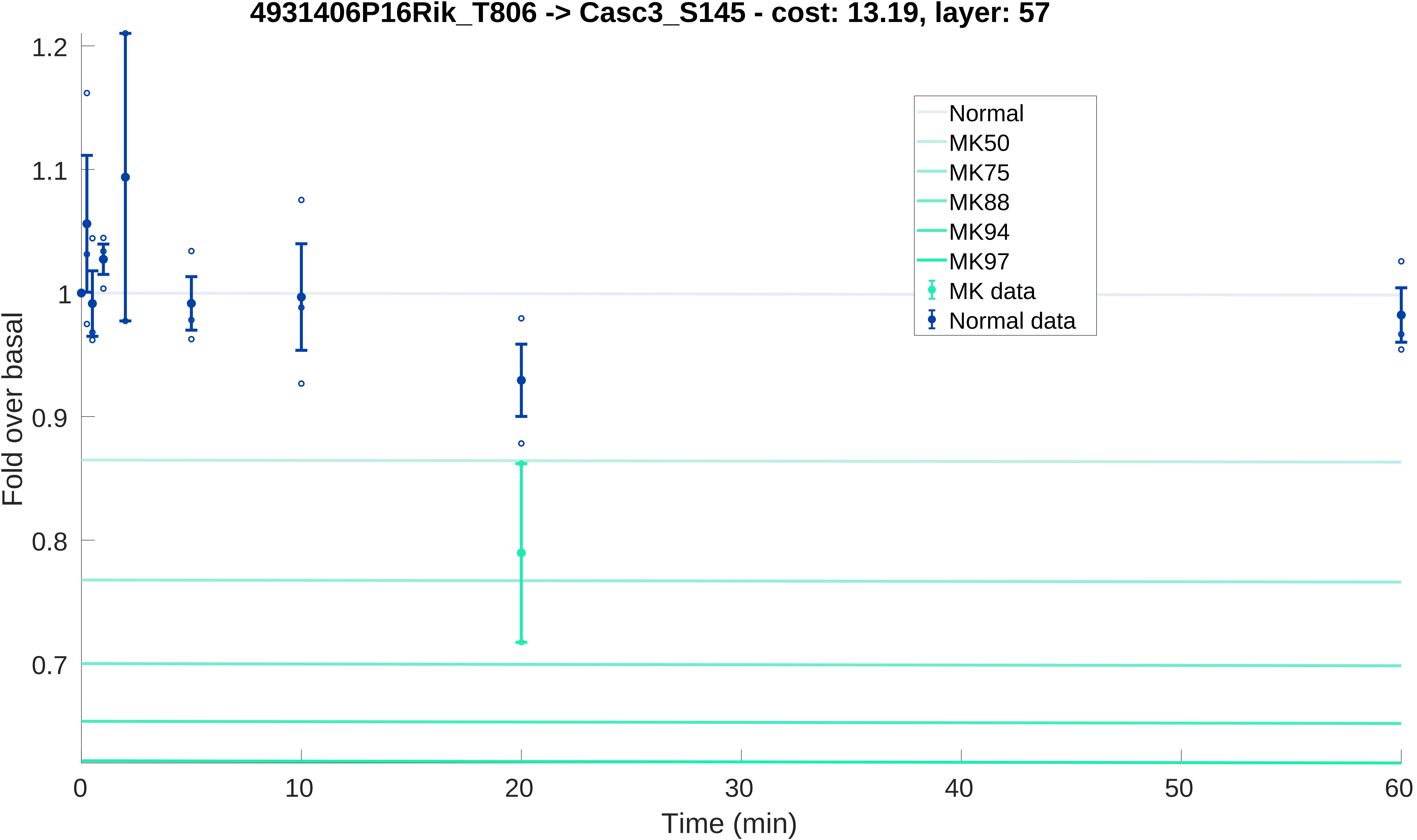
4931406P16Rik_T806 -> Casc3_S145 - cost: 13.19, layer: 57
Normal
MK50
MK75
MK88
MK94
MK97
MK data
Normal data
Fold over basal
Time (min)
1.2
1.1
1
0.9
0.8
0.7
0
10
20
30
40
50
60

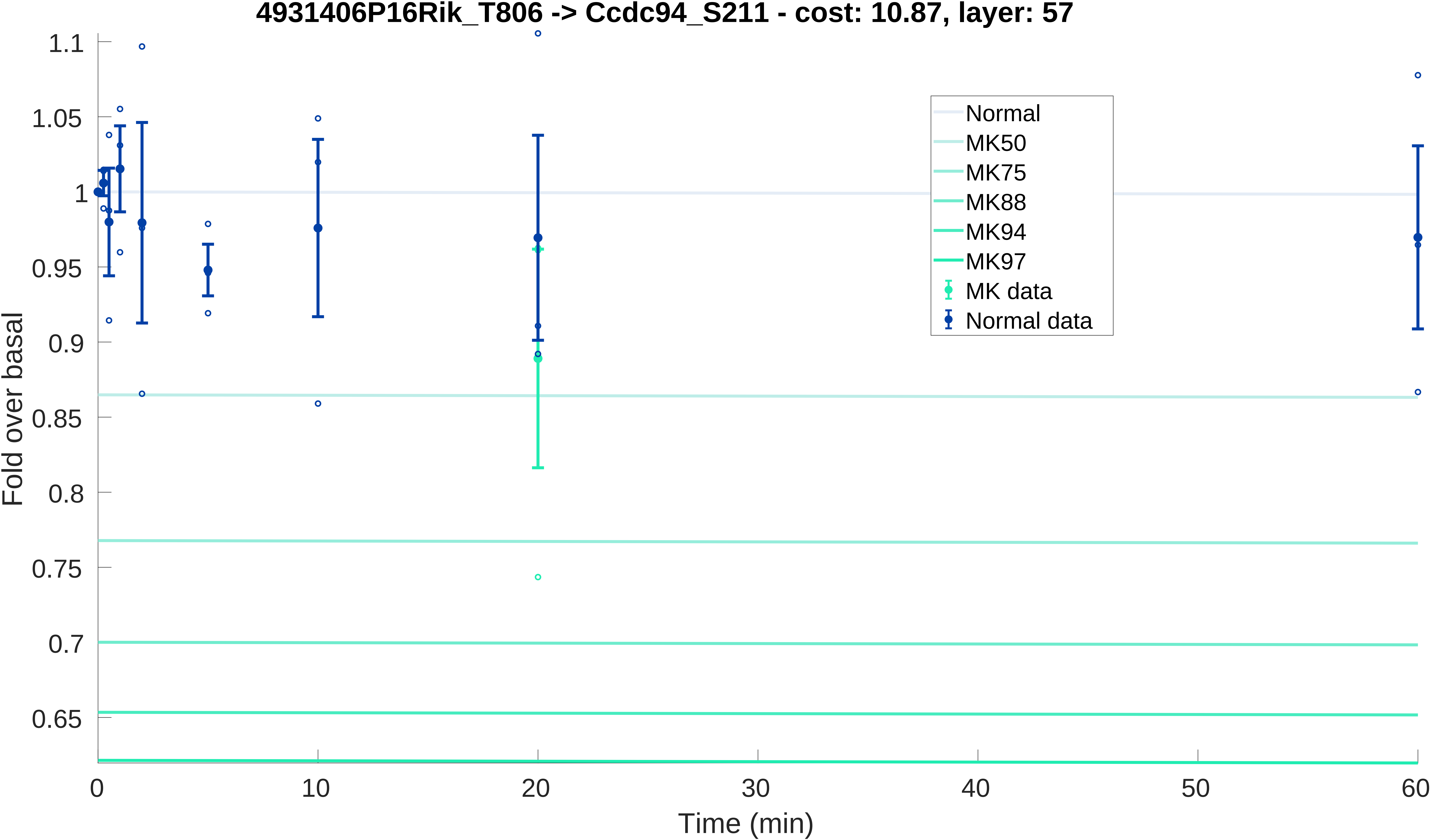

4931406P16Rik_T806 -> Ccdc94_S211 - cost: 10.87, layer: 57
Fold over basal
Time (min)
Normal
MK50
MK75
MK88
MK94
MK97
MK data
Normal data
1.1
1.05
1
0.95
0.9
0.85
0.8
0.75
0.7
0.65
0
10
20
30
40
50
60

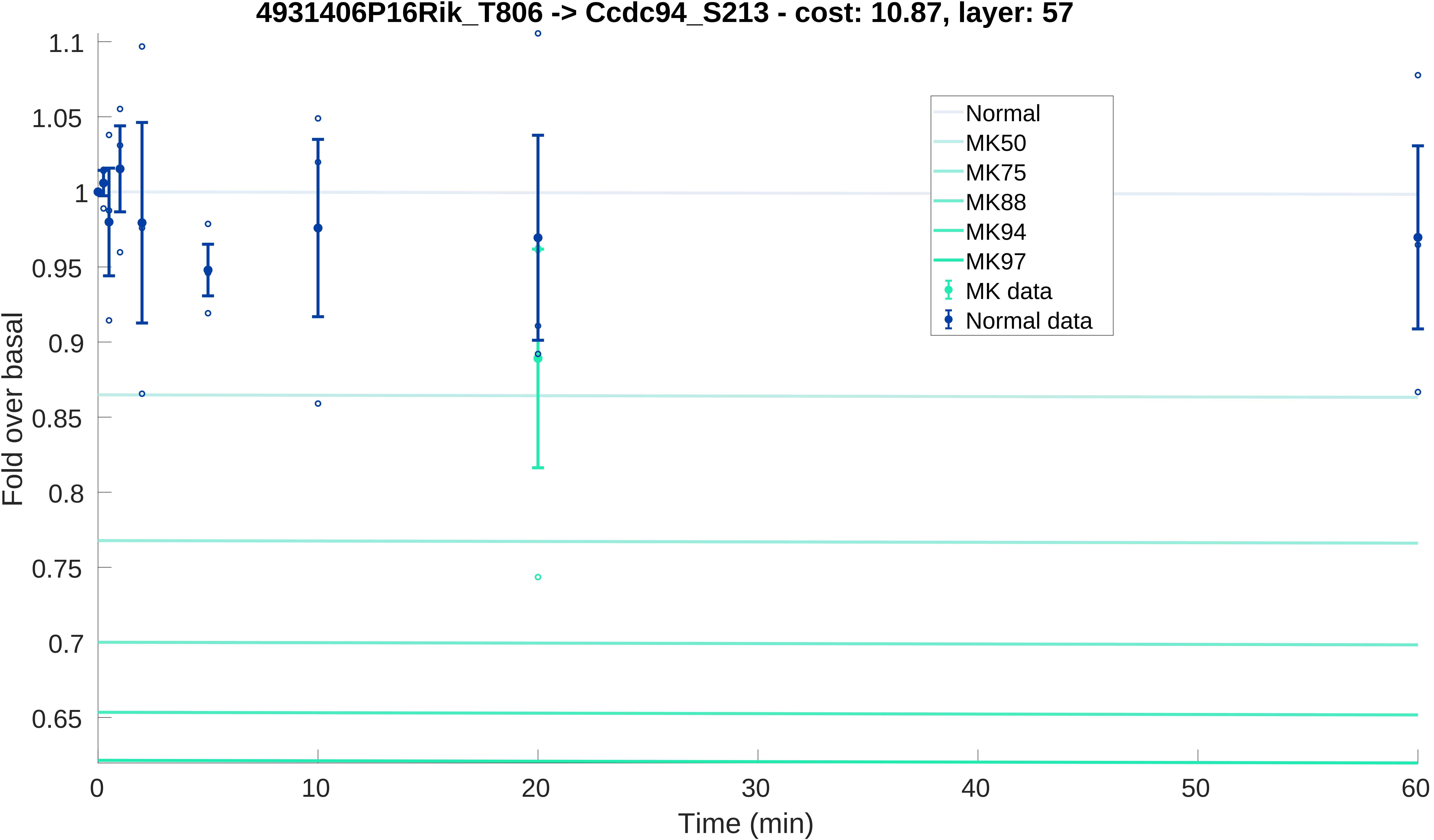

4931406P16Rik_T806 -> Ccdc94_S213 - cost: 10.87, layer: 57
Fold over basal
Time (min)
1.1
1.05
1
0.95
0.9
0.85
0.8
0.75
0.7
0.65
0
10
20
30
40
50
60
Normal
MK50
MK75
MK88
MK94
MK97
MK data
Normal data

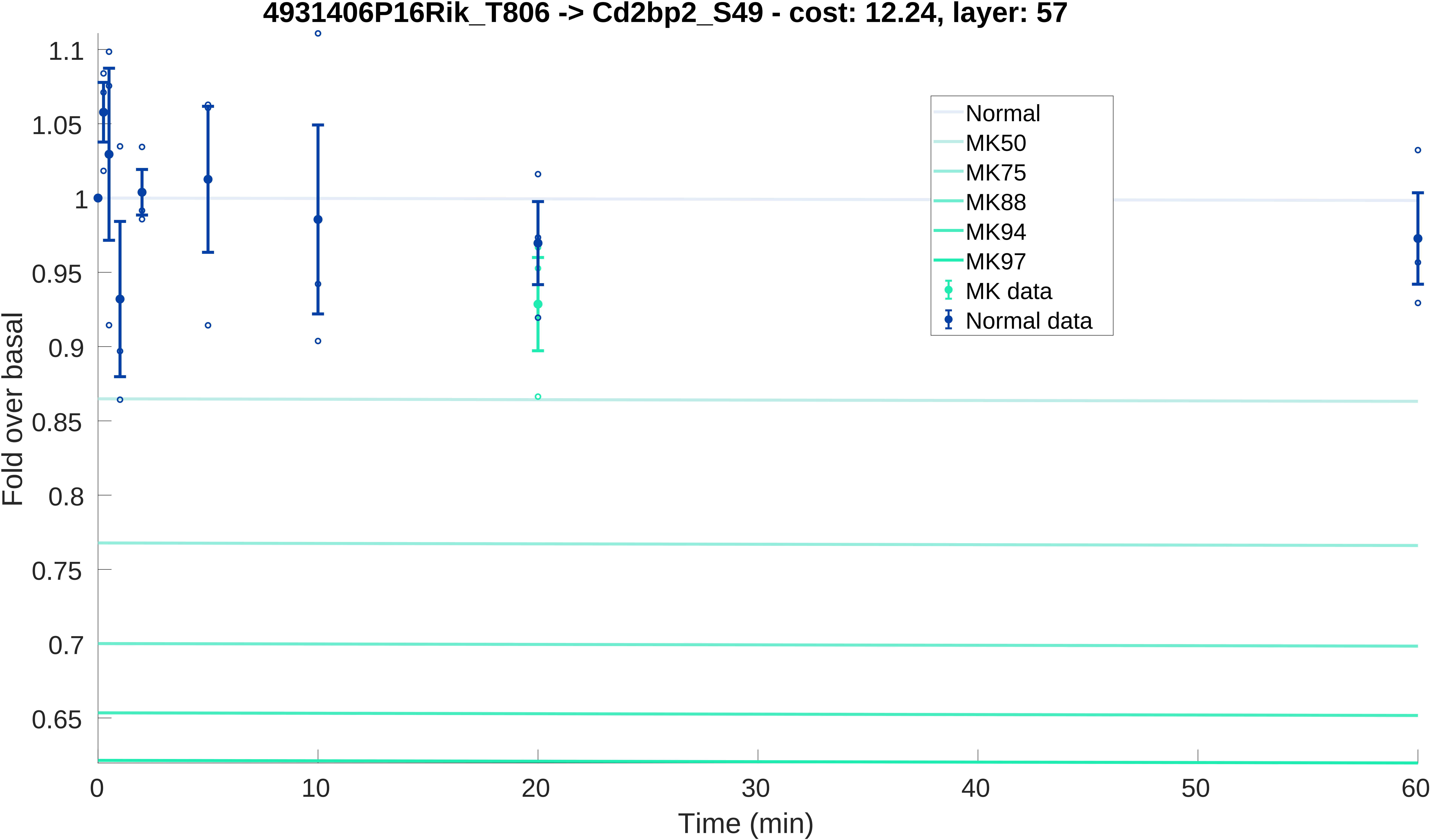

4931406P16Rik_T806 -> Cd2bp2_S49 - cost: 12.24, layer: 57
Fold over basal
Time (min)
1.1
1.05
1
0.95
0.9
0.85
0.8
0.75
0.7
0.65
0
10
20
30
40
50
60
Normal
MK50
MK75
MK88
MK94
MK97
MK data
Normal data

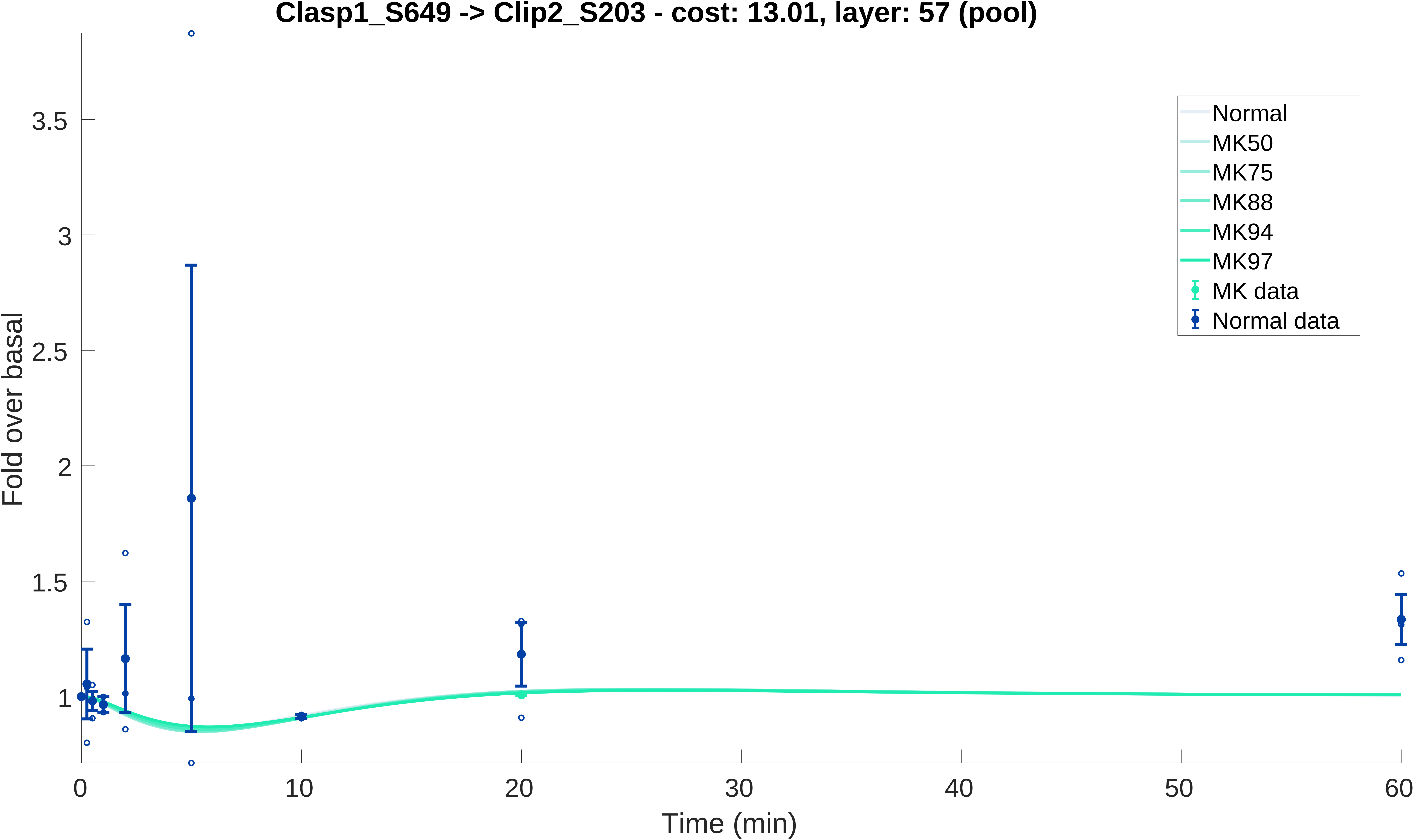
Clasp1_S649 -> Clip2_S203 - cost: 13.01, layer: 57 (pool)
Fold over basal
3.5
3
2.5
2
1.5
1
0
10
20
30
40
50
60
Time (min)
Normal
MK50
MK75
MK88
MK94
MK97
MK data
Normal data

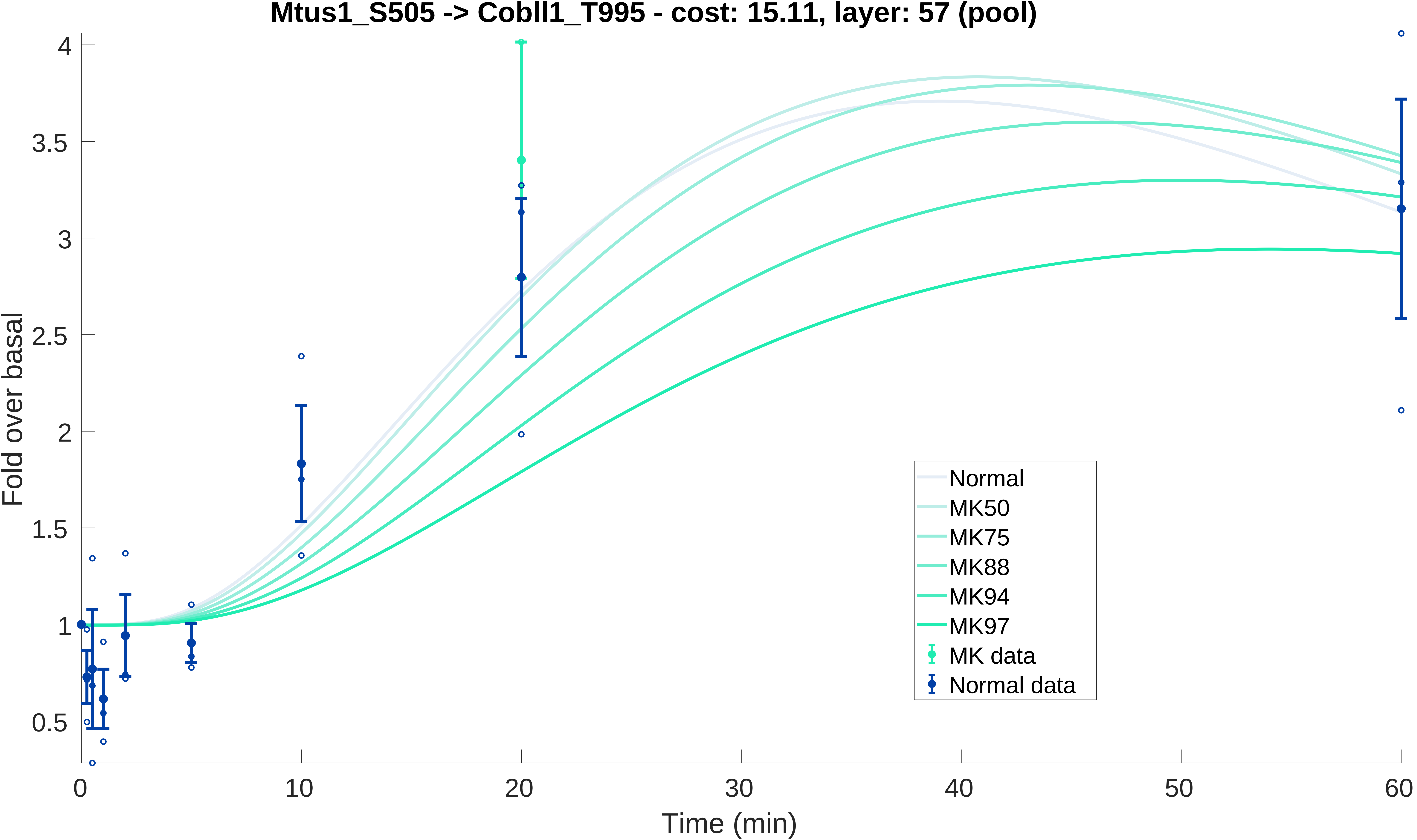
Mtus1_S505 -> Cobll1_T995 - cost: 15.11, layer: 57 (pool)
Fold over basal
Time (min)
0.5
1
1.5
2
2.5
3
3.5
4
0
10
20
30
40
50
60
Normal
MK50
MK75
MK88
MK94
MK97
MK data
Normal data

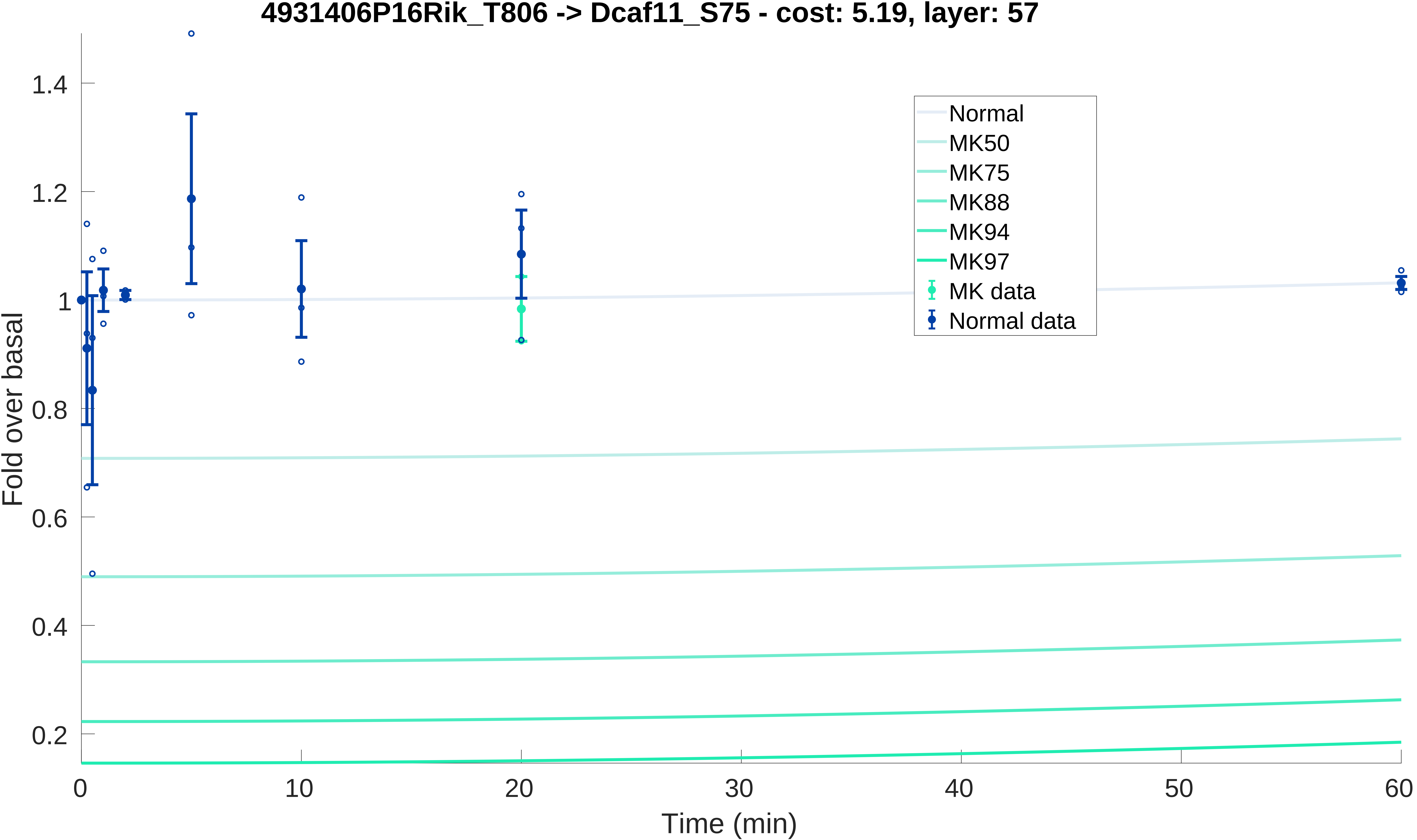

4931406P16Rik_T806 -> Dcaf11_S75 - cost: 5.19, layer: 57
Fold over basal
Time (min)
0
10
20
30
40
50
60
0.2
0.4
0.6
0.8
1
1.2
1.4
Normal
MK50
MK75
MK88
MK94
MK97
MK data
Normal data

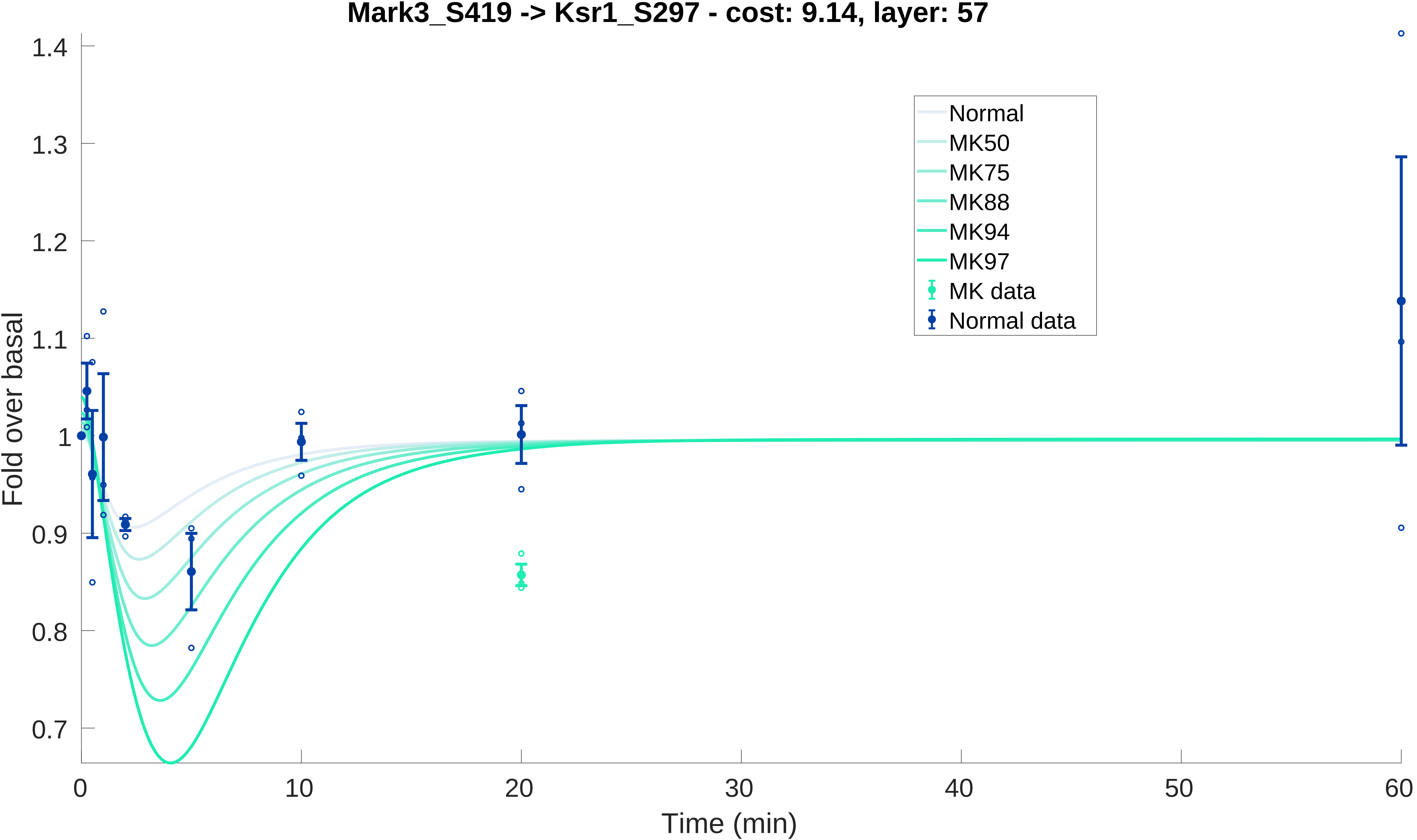

Mark3_S419 -> Ksr1_S297 - cost: 9.14, layer: 57
Fold over basal
Time (min)
1.4
1.3
1.2
1.1
1
0.9
0.8
0.7
0
10
20
30
40
50
60
Normal
MK50
MK75
MK88
MK94
MK97
MK data
Normal data

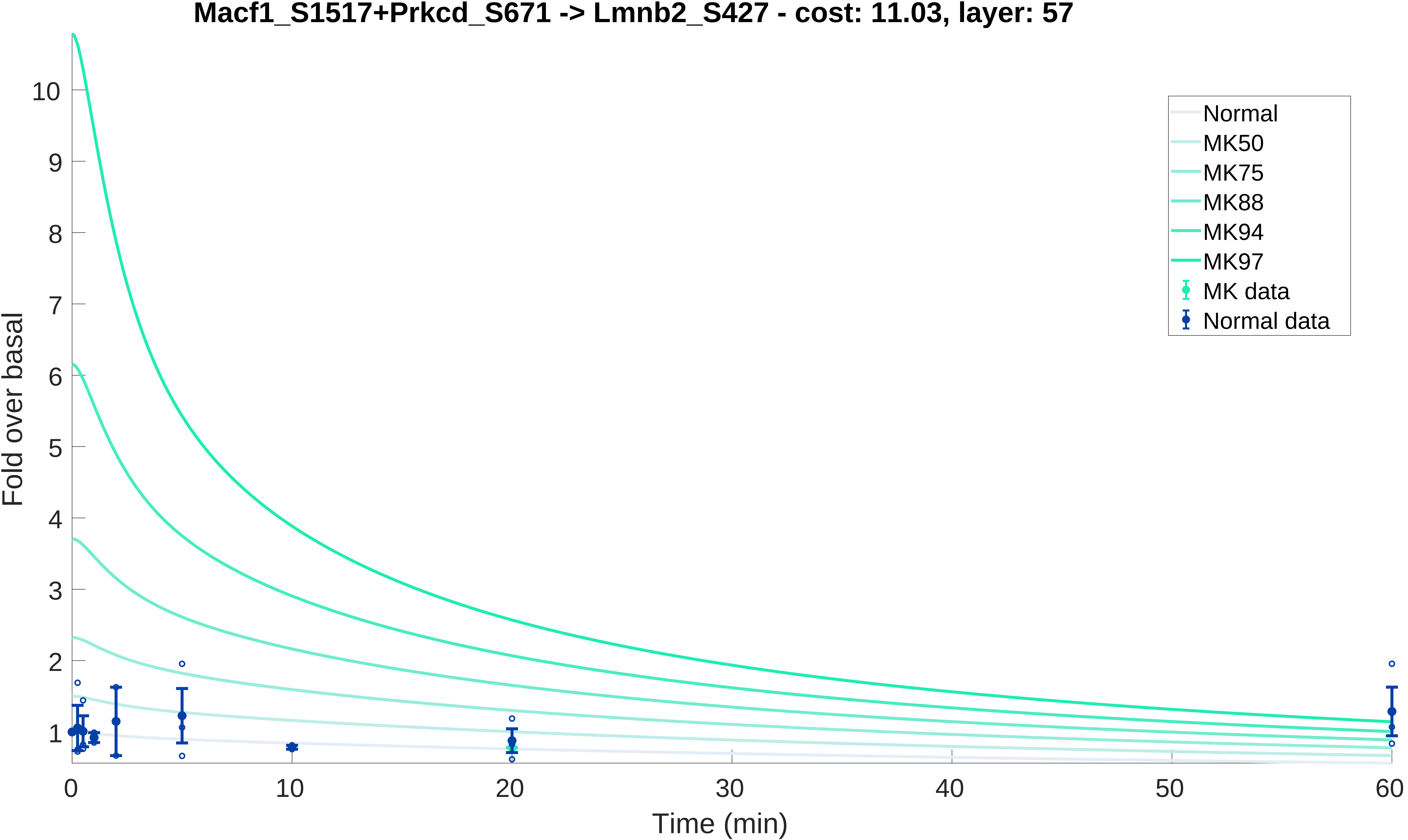

Macf1_S1517+Prkcd_S671 -> Lmnb2_S427 - cost: 11.03, layer: 57
Fold over basal
Time (min)
Normal
MK50
MK75
MK88
MK94
MK97
MK data
Normal data
0
10
20
30
40
50
60
1
2
3
4
5
6
7
8
9
10

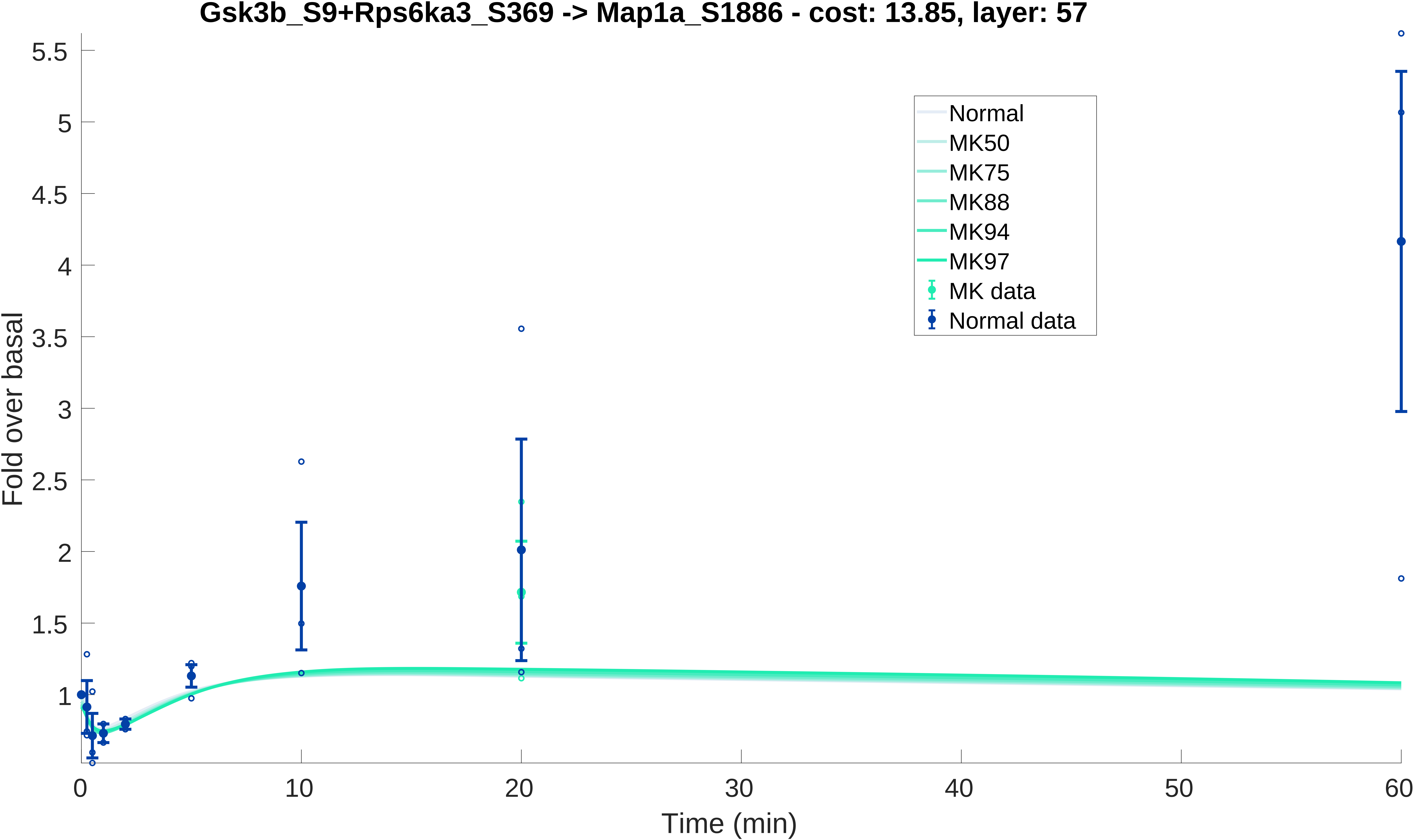

Gsk3b_S9+Rps6ka3_S369 -> Map1a_S1886 - cost: 13.85, layer: 57
Fold over basal
Time (min)
5.5
5
4.5
4
3.5
3
2.5
2
1.5
1
0
10
20
30
40
50
60
Normal
MK50
MK75
MK88
MK94
MK97
MK data
Normal data

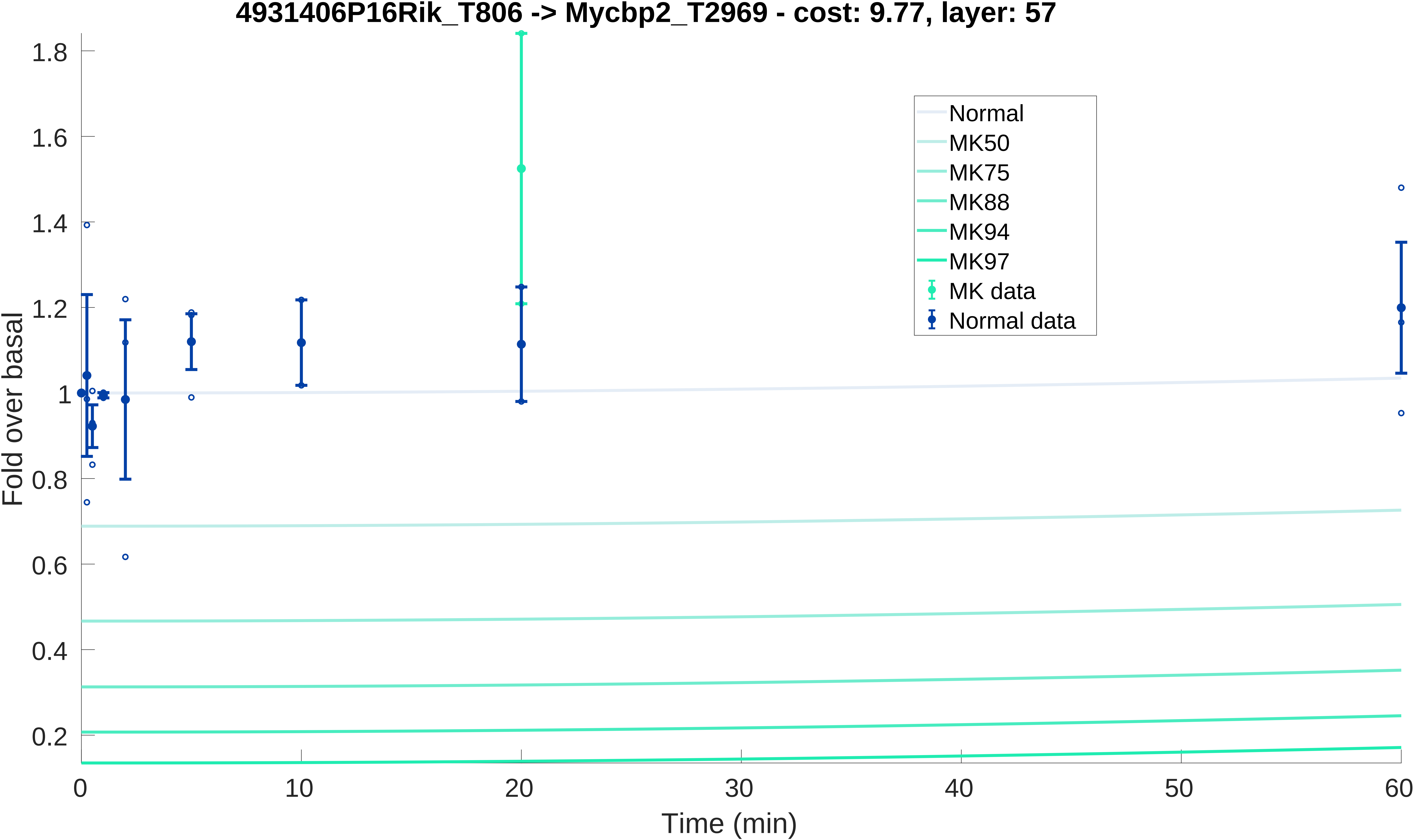
4931406P16Rik_T806 -> Mycbp2_T2969 - cost: 9.77, layer: 57
Fold over basal
Time (min)
1.8
1.6
1.4
1.2
1
0.8
0.6
0.4
0.2
0
10
20
30
40
50
60
Normal
MK50
MK75
MK88
MK94
MK97
MK data
Normal data

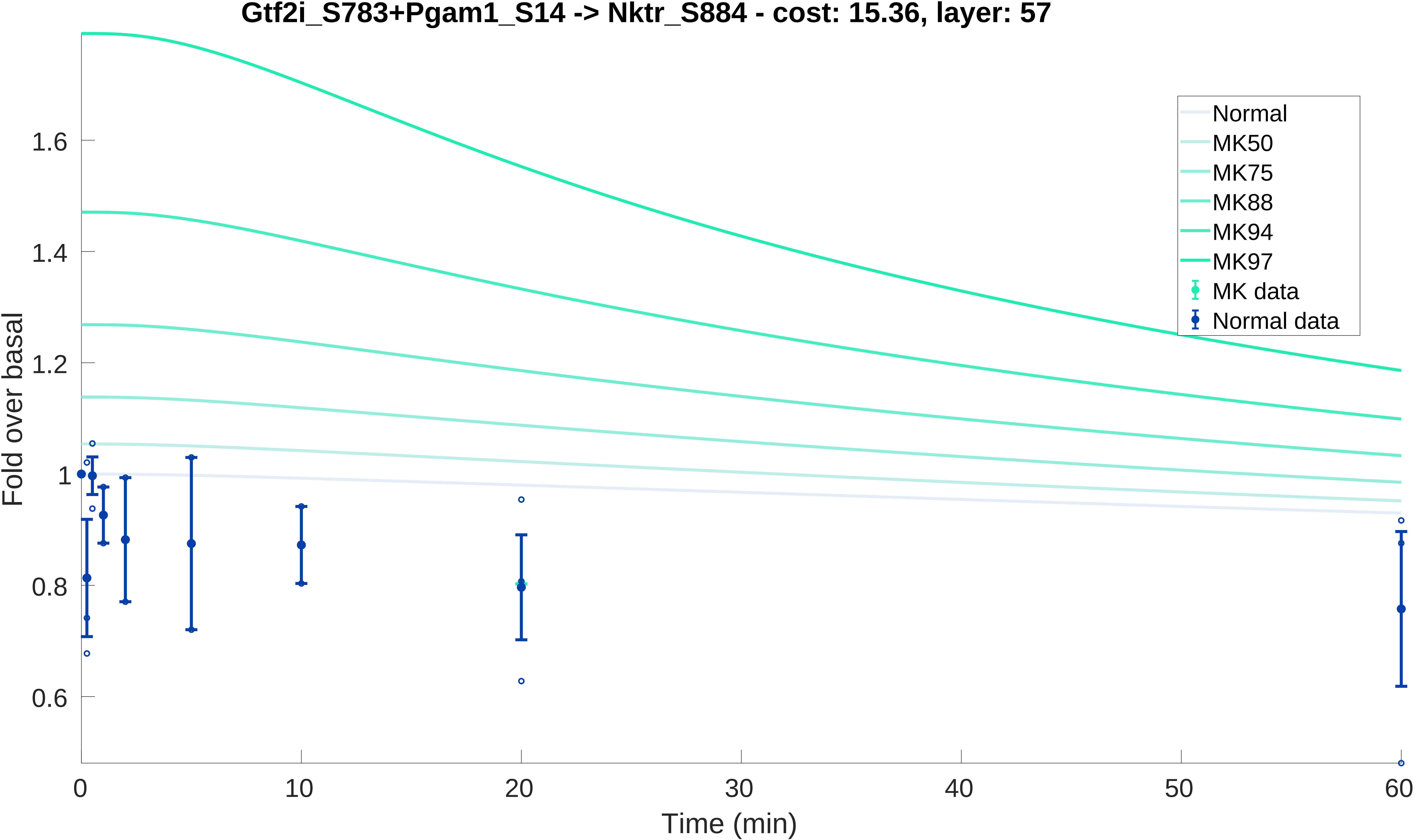

Gtf2i_S783+Pgam1_S14 -> Nktr_S884 - cost: 15.36, layer: 57
Fold over basal
1.6
1.4
1.2
1
0.8
0.6
0
10
20
30
40
50
60
Time (min)
Normal
MK50
MK75
MK88
MK94
MK97
MK data
Normal data

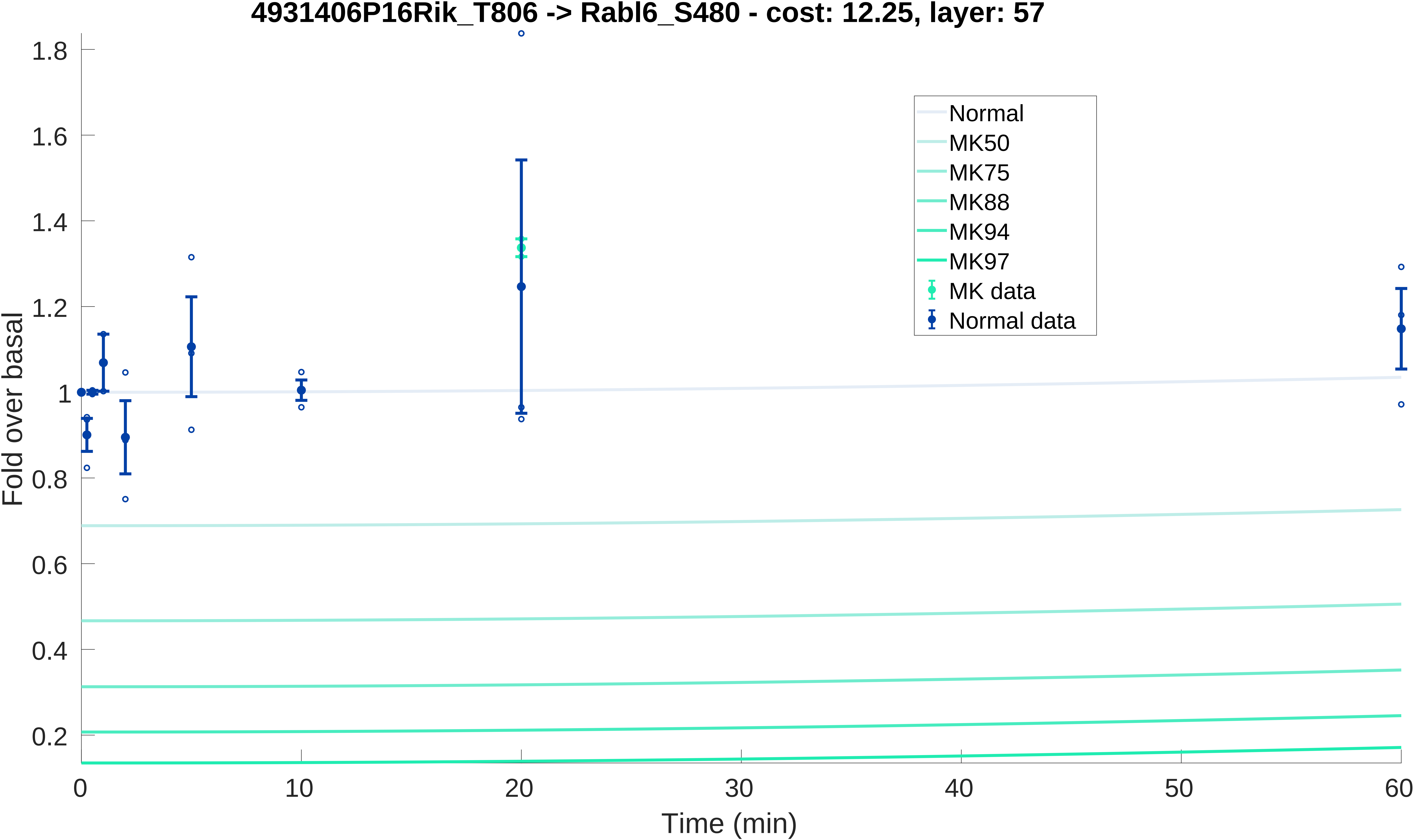

4931406P16Rik_T806 -> Rabl6_S480 - cost: 12.25, layer: 57
Fold over basal
1.8
1.6
1.4
1.2
1
0.8
0.6
0.4
0.2
0
10
20
30
40
50
60
Time (min)
Normal
MK50
MK75
MK88
MK94
MK97
MK data
Normal data

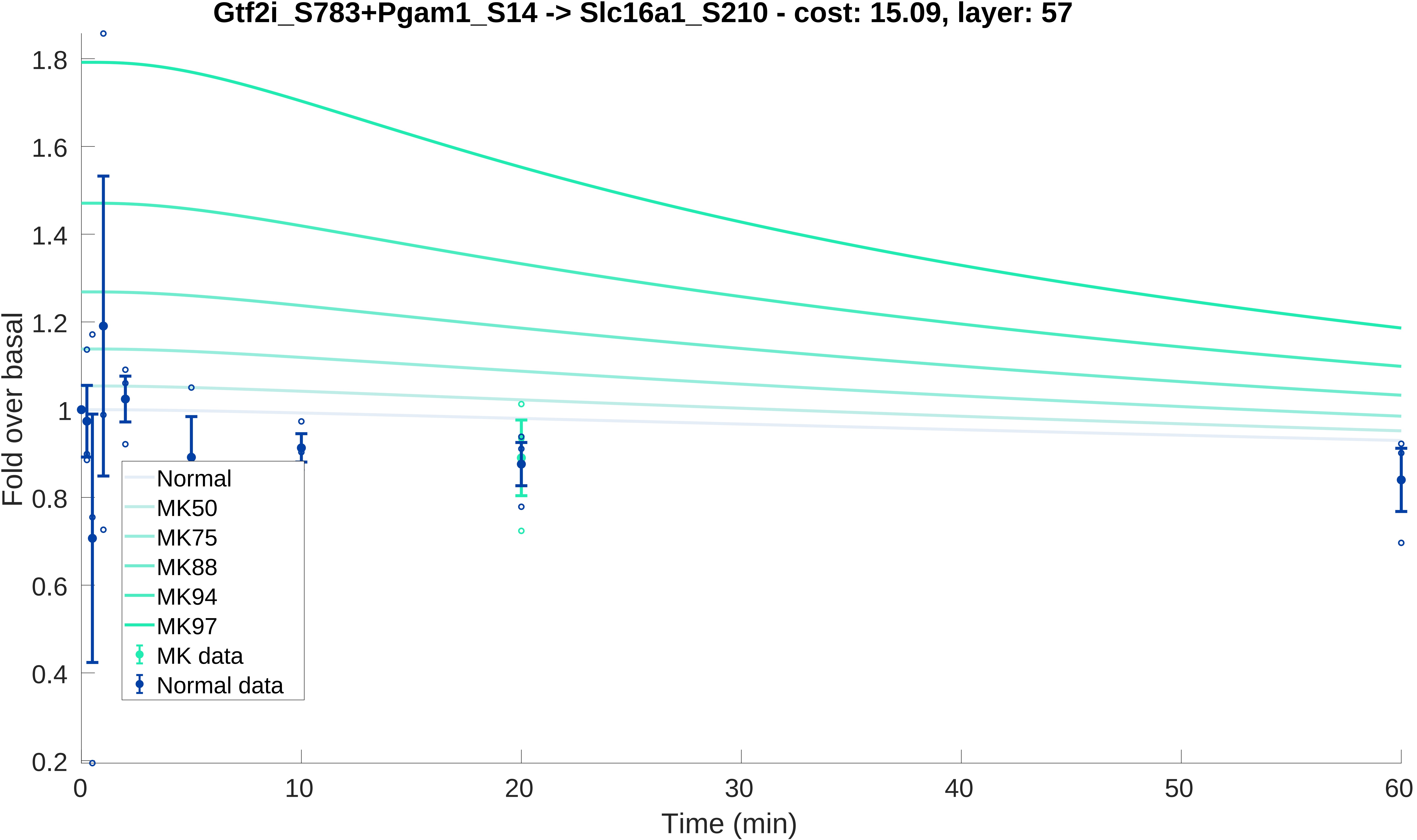

Gtf2i_S783+Pgam1_S14 -> Slc16a1_S210 - cost: 15.09, layer: 57
Fold over basal
Time (min)
1.8
1.6
1.4
1.2
1
0.8
0.6
0.4
0.2
0
10
20
30
40
50
60
Normal
MK50
MK75
MK88
MK94
MK97
MK data
Normal data

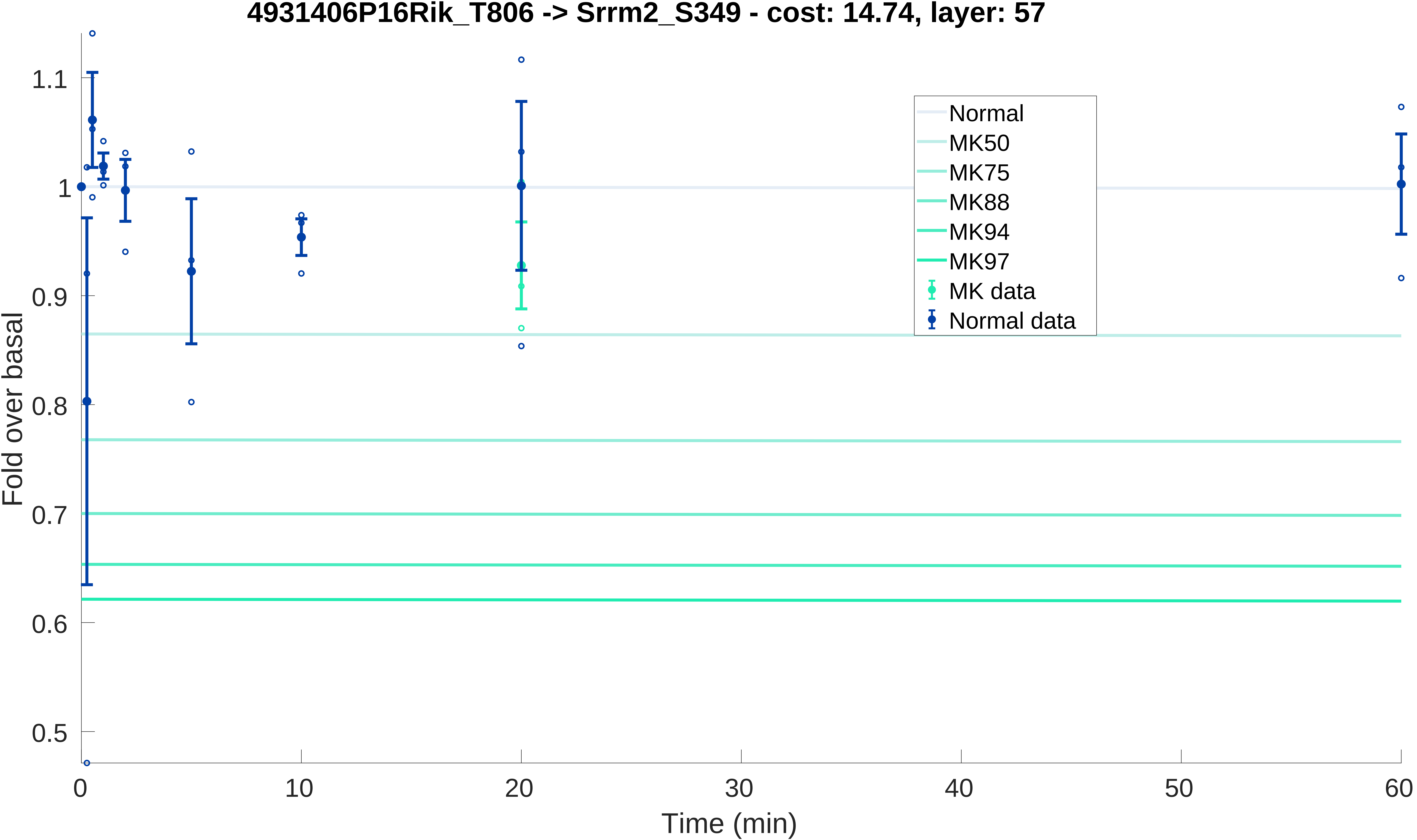

4931406P16Rik_T806 -> Srrm2_S349 - cost: 14.74, layer: 57
Fold over basal
Time (min)
1.1
1
0.9
0.8
0.7
0.6
0.5
0
10
20
30
40
50
60
Normal
MK50
MK75
MK88
MK94
MK97
MK data
Normal data

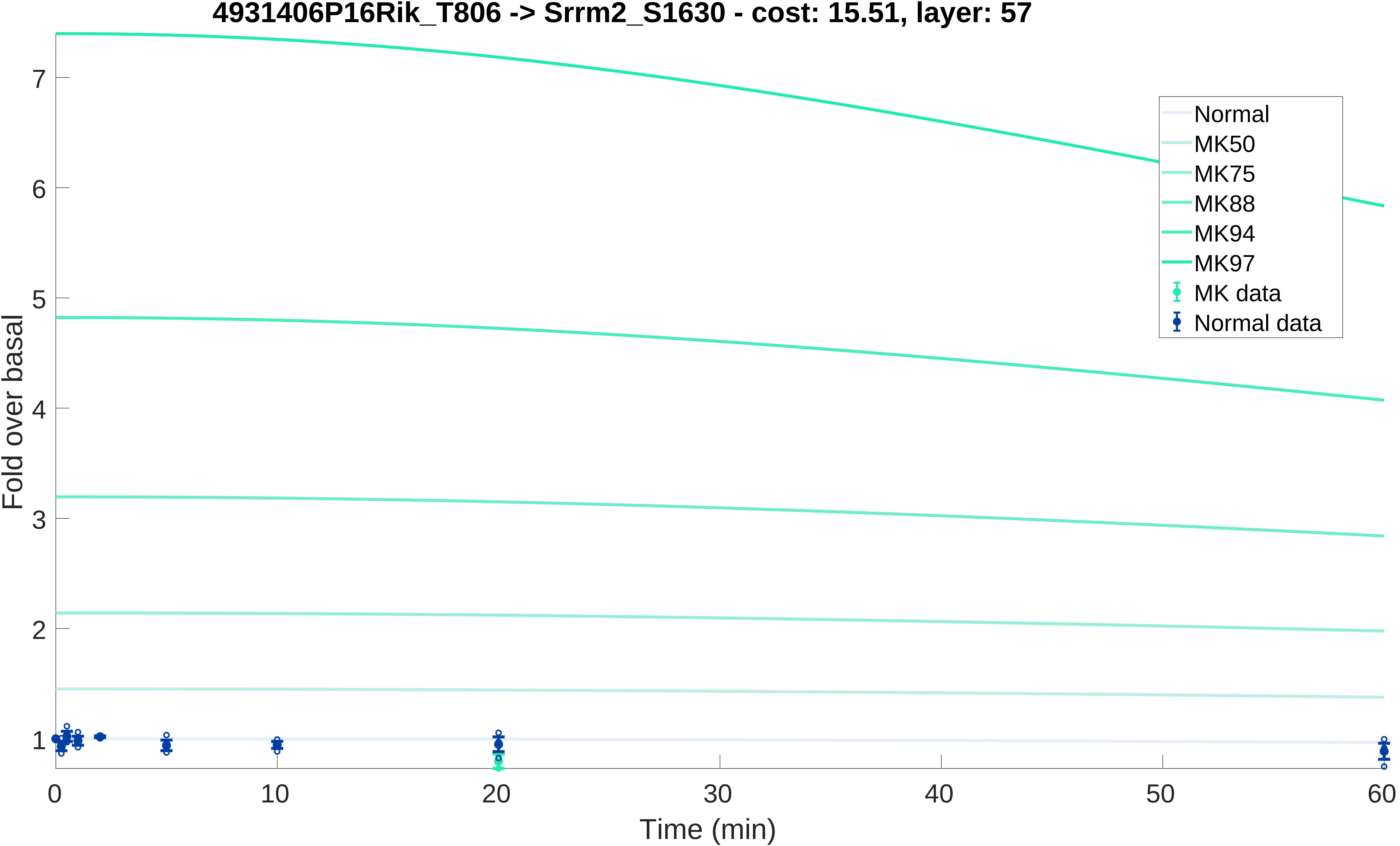
4931406P16Rik_T806 -> Srrm2_S1630 - cost: 15.51, layer: 57
Fold over basal
Time (min)
1
2
3
4
5
6
7
0
10
20
30
40
50
60
Normal
MK50
MK75
MK88
MK94
MK97
MK data
Normal data

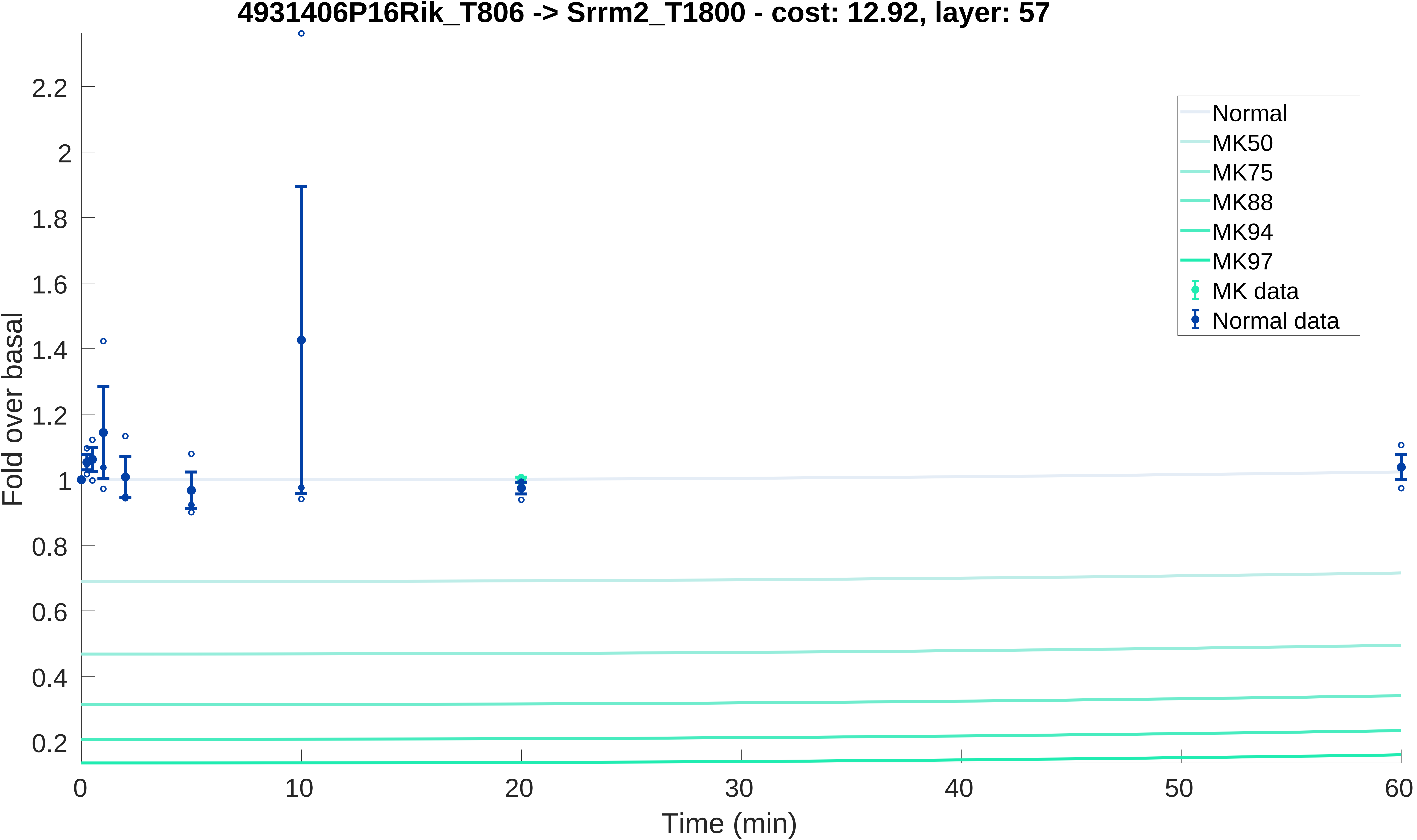

4931406P16Rik_T806 -> Srrm2_T1800 - cost: 12.92, layer: 57
Fold over basal
Time (min)
0
10
20
30
40
50
60
0.2
0.4
0.6
0.8
1
1.2
1.4
1.6
1.8
2
2.2
Normal
MK50
MK75
MK88
MK94
MK97
MK data
Normal data

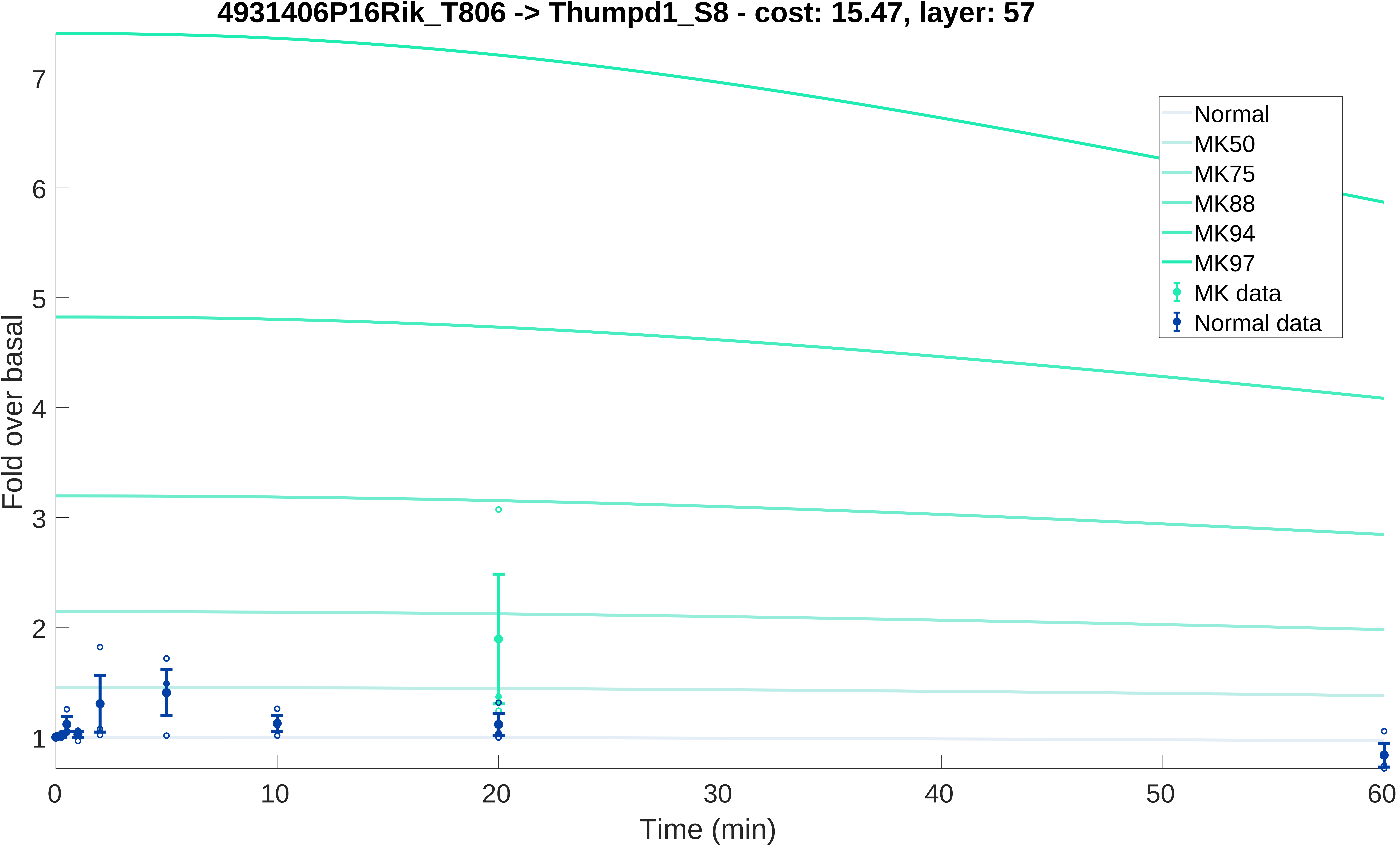

4931406P16Rik_T806 -> Thumpd1_S8 - cost: 15.47, layer: 57
Fold over basal
Time (min)
0
10
20
30
40
50
60
1
2
3
4
5
6
7
Normal
MK50
MK75
MK88
MK94
MK97
MK data
Normal data

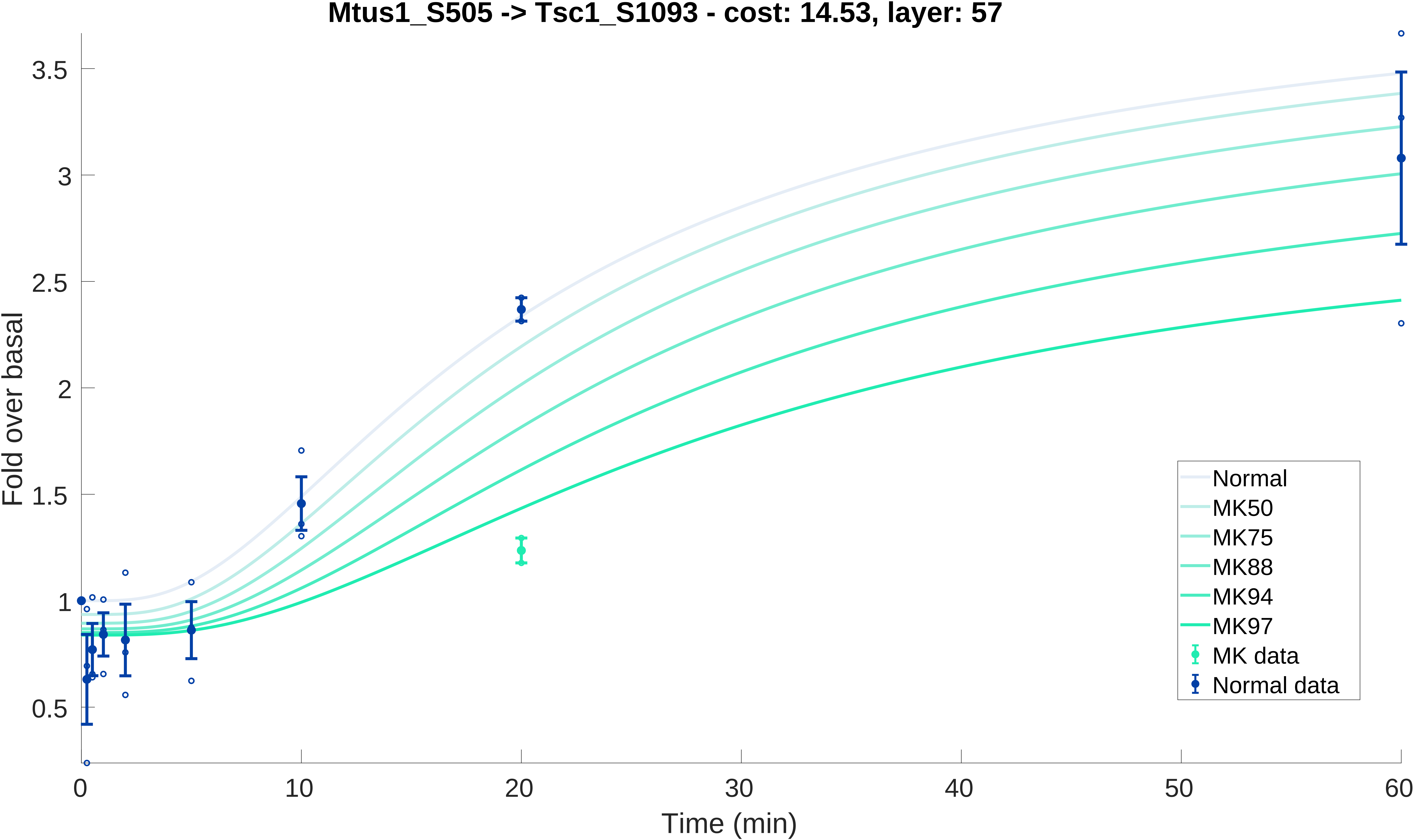

Mtus1_S505 -> Tsc1_S1093 - cost: 14.53, layer: 57
Fold over basal
Time (min)
0.5
1
1.5
2
2.5
3
3.5
0
10
20
30
40
50
60
Normal
MK50
MK75
MK88
MK94
MK97
MK data
Normal data

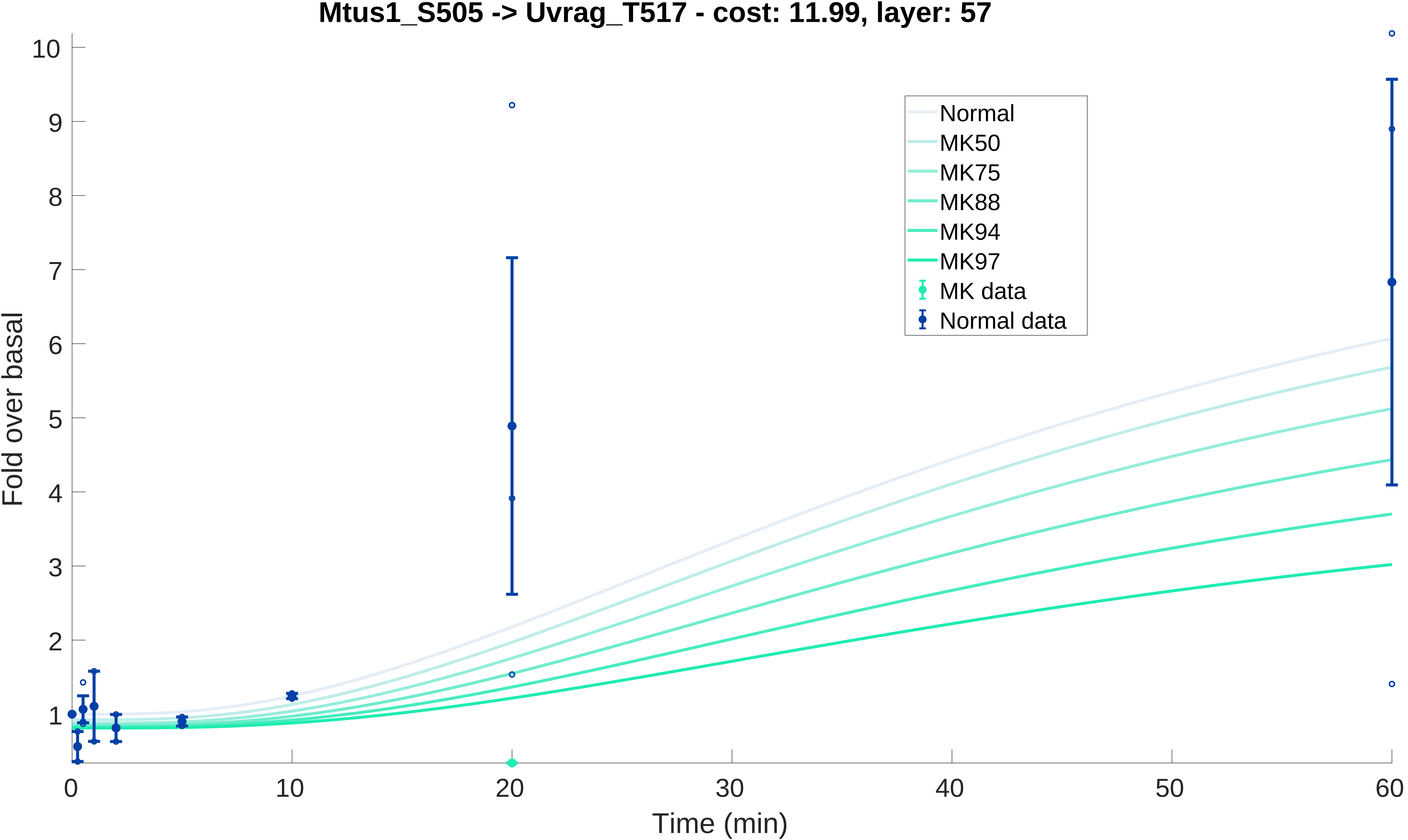

Mtus1_S505 -> Uvrag_T517 - cost: 11.99, layer: 57
Fold over basal
Time (min)
Normal
MK50
MK75
MK88
MK94
MK97
MK data
Normal data
0
10
20
30
40
50
60
1
2
3
4
5
6
7
8
9
10

3.4 Insulin stimulation, normal and with the PI3K inhibitor

The following pages give all simulations of insulin stimulation normally and with the PI3K inhibitor LY294002.

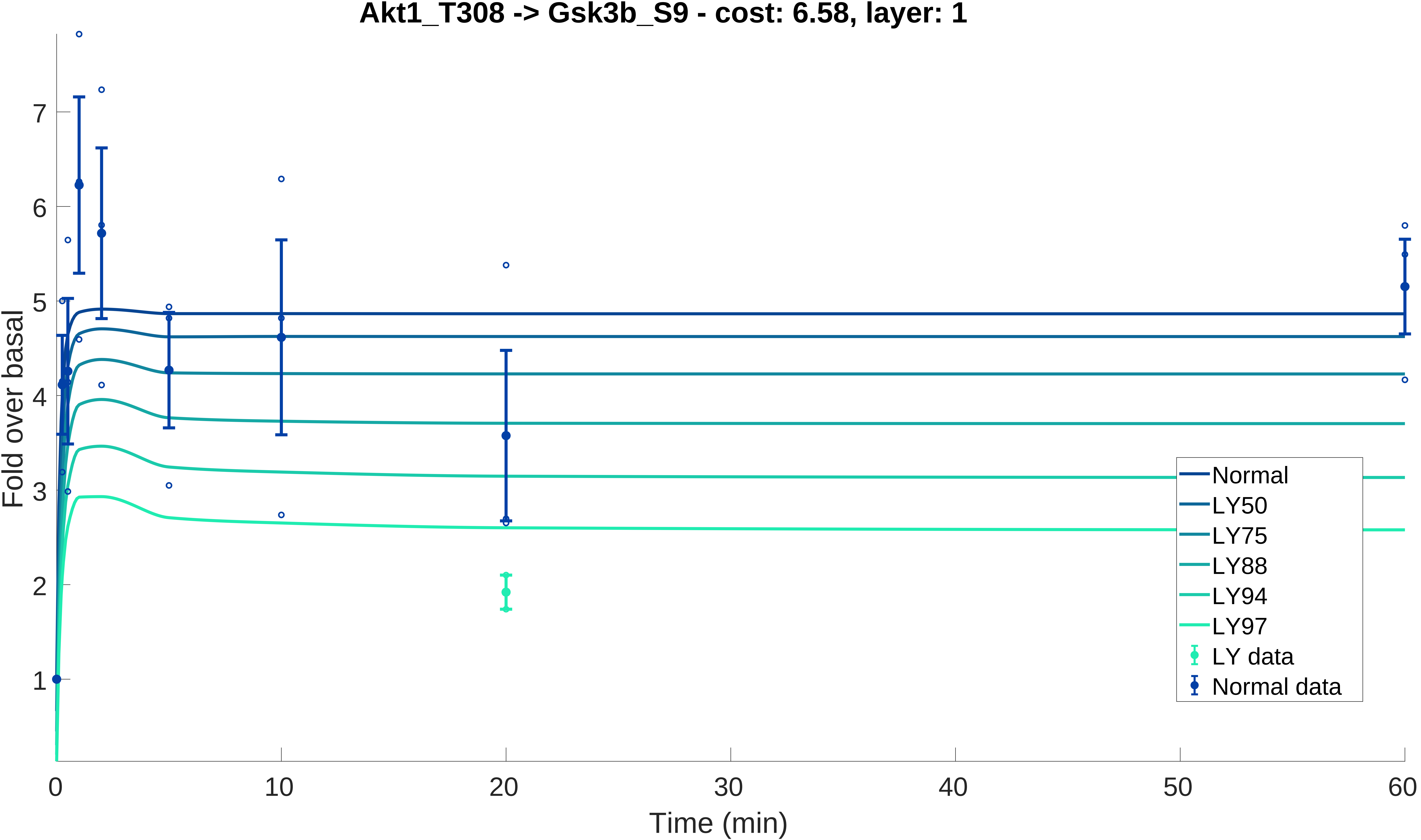
Akt1_T308 -> Gsk3b_S9 - cost: 6.58, layer: 1
Fold over basal
Time (min)
0
10
20
30
40
50
60
1
2
3
4
5
6
7
Normal
LY50
LY75
LY88
LY94
LY97
LY data
Normal data

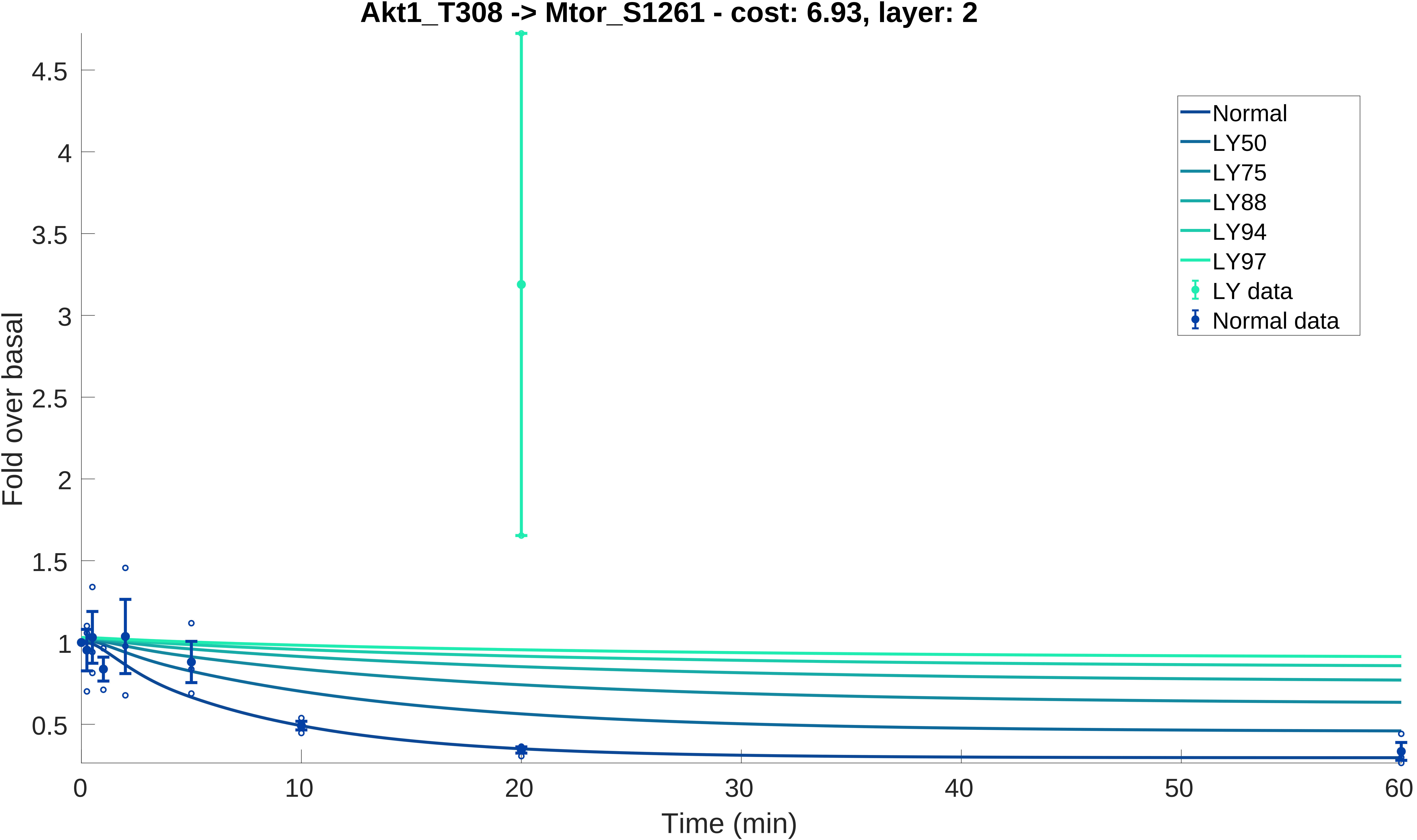

Akt1_T308 -> Mtor_S1261 - cost: 6.93, layer: 2
Fold over basal
4.5
4
3.5
3
2.5
2
1.5
1
0.5
0
10
20
30
40
50
60
Time (min)
Normal
LY50
LY75
LY88
LY94
LY97
LY data
Normal data

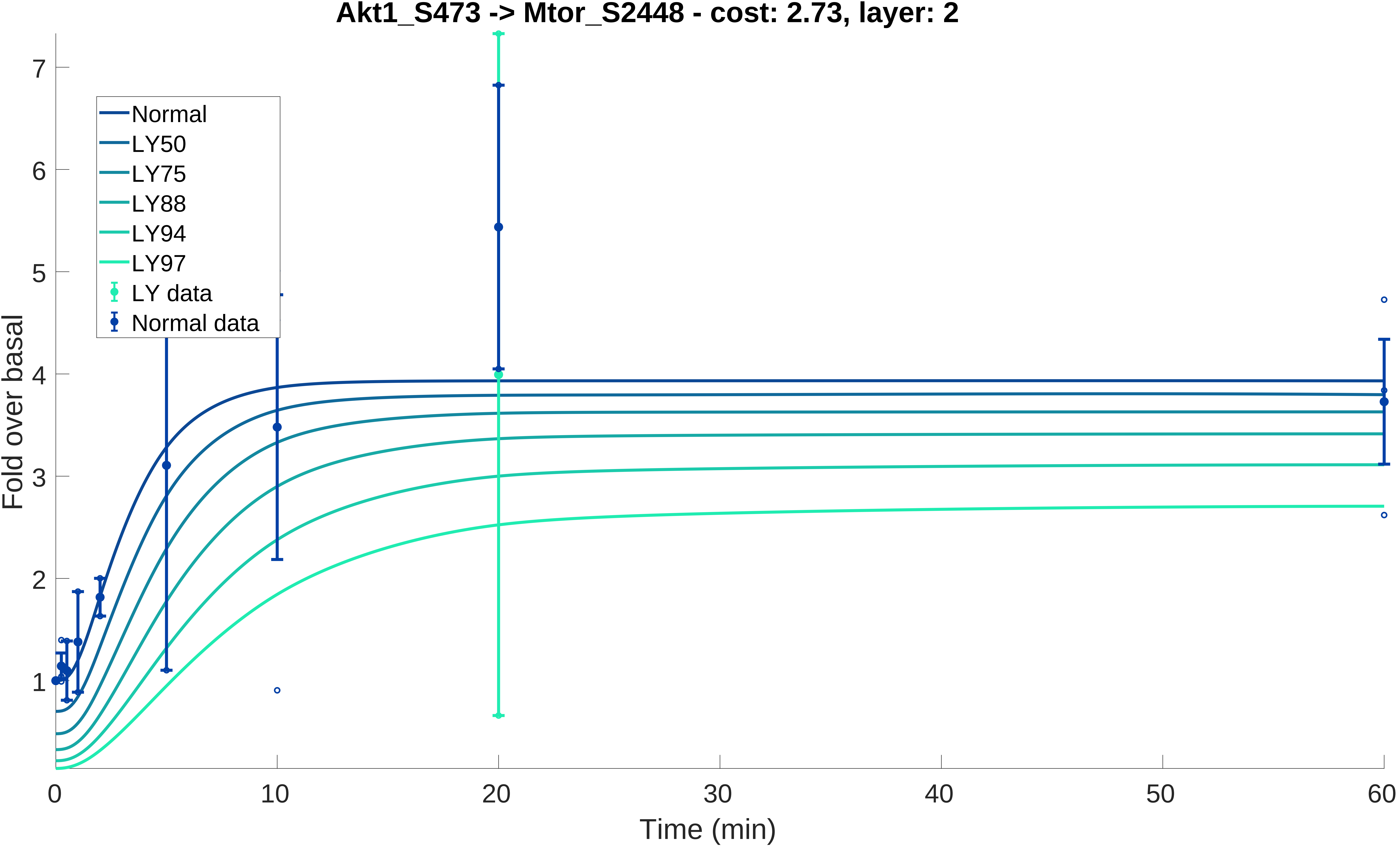

Akt1_S473 -> Mtor_S2448 - cost: 2.73, layer: 2
Normal
LY50
LY75
LY88
LY94
LY97
LY data
Normal data
Fold over basal
Time (min)
0
10
20
30
40
50
60
1
2
3
4
5
6
7

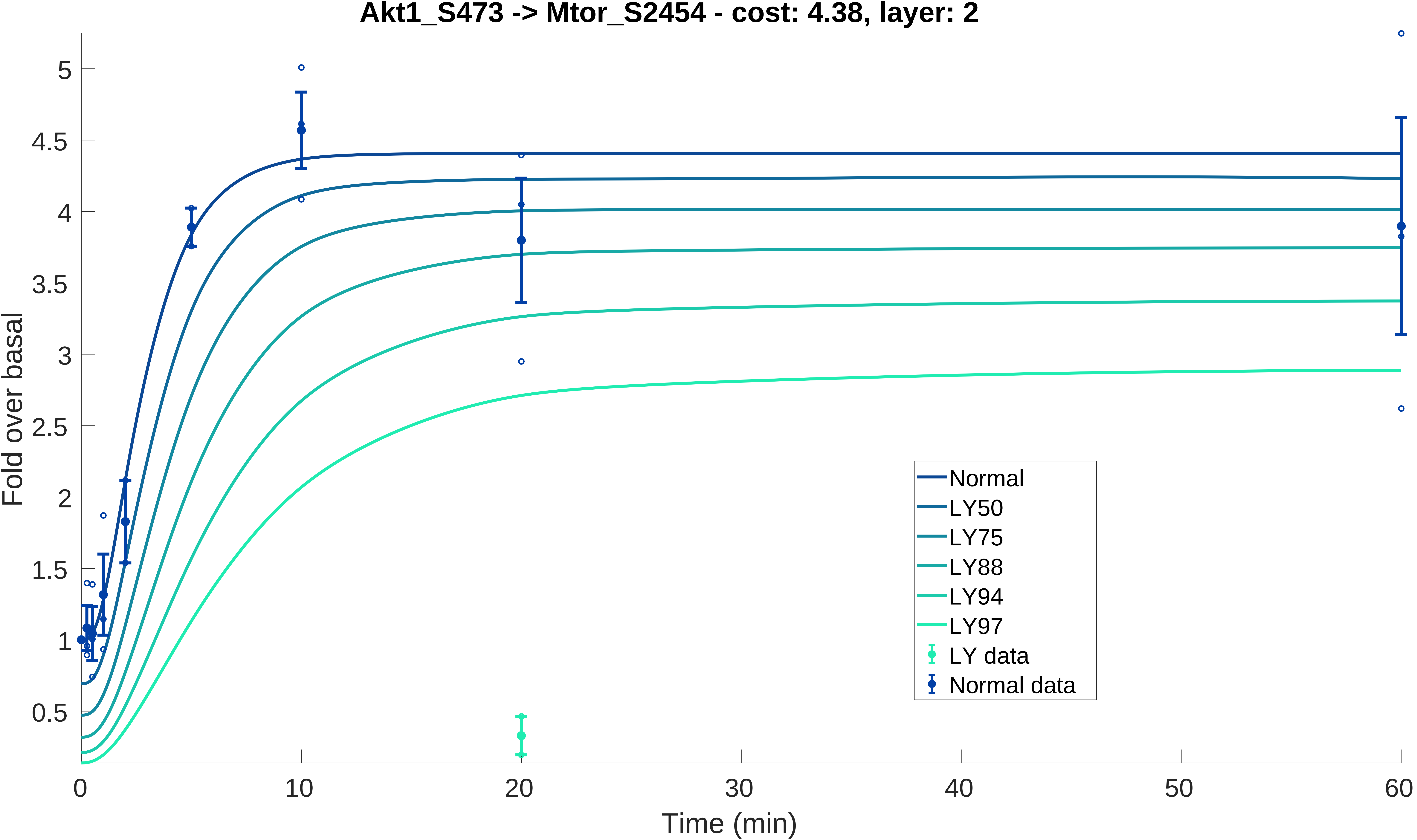

Akt1_S473 -> Mtor_S2454 - cost: 4.38, layer: 2
Fold over basal
Time (min)
0
10
20
30
40
50
60
0.5
1
1.5
2
2.5
3
3.5
4
4.5
5
Normal
LY50
LY75
LY88
LY94
LY97
LY data
Normal data

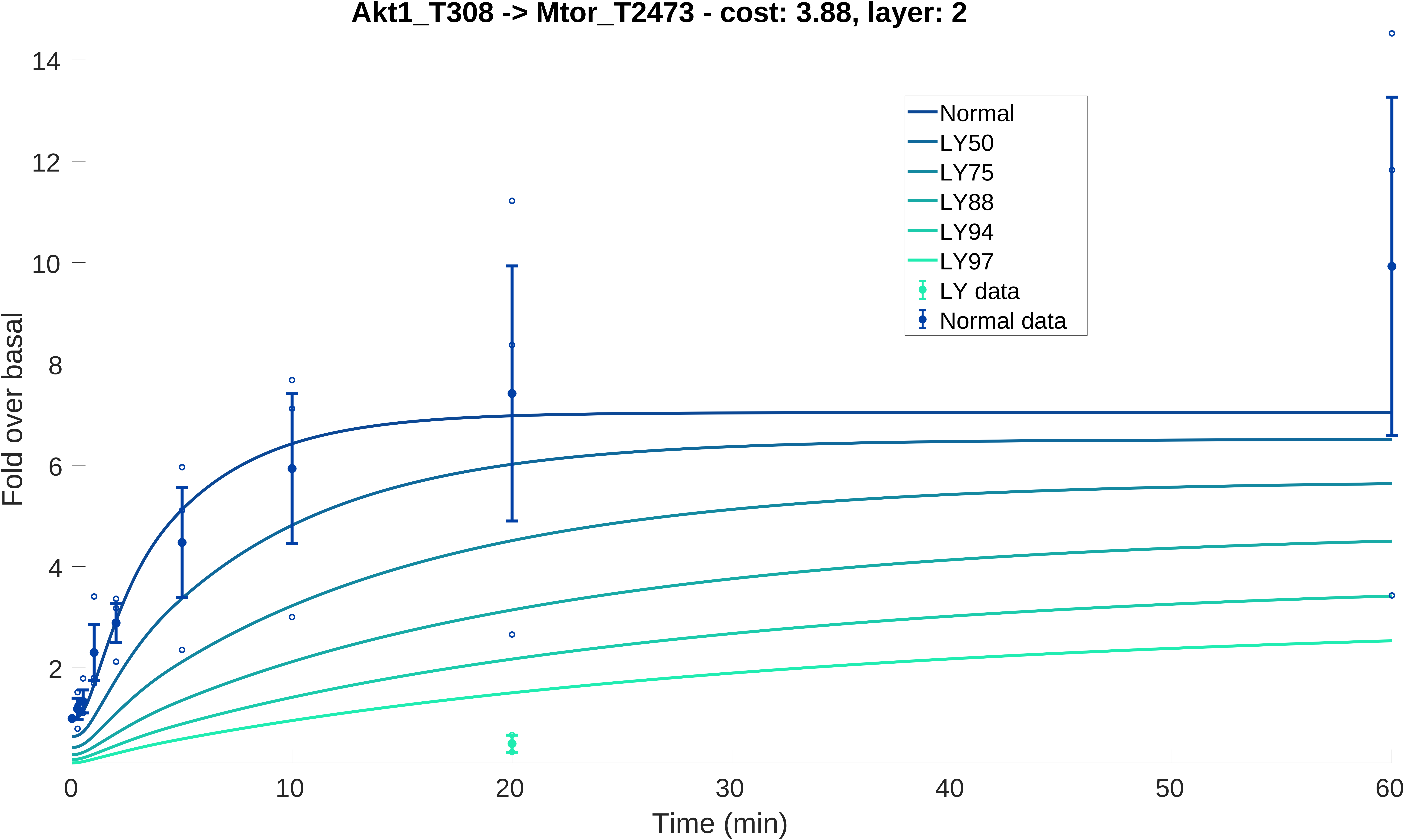

Akt1_T308 -> Mtor_T2473 - cost: 3.88, layer: 2
Fold over basal
Time (min)
0
10
20
30
40
50
60
2
4
6
8
10
12
14
Normal
LY50
LY75
LY88
LY94
LY97
LY data
Normal data

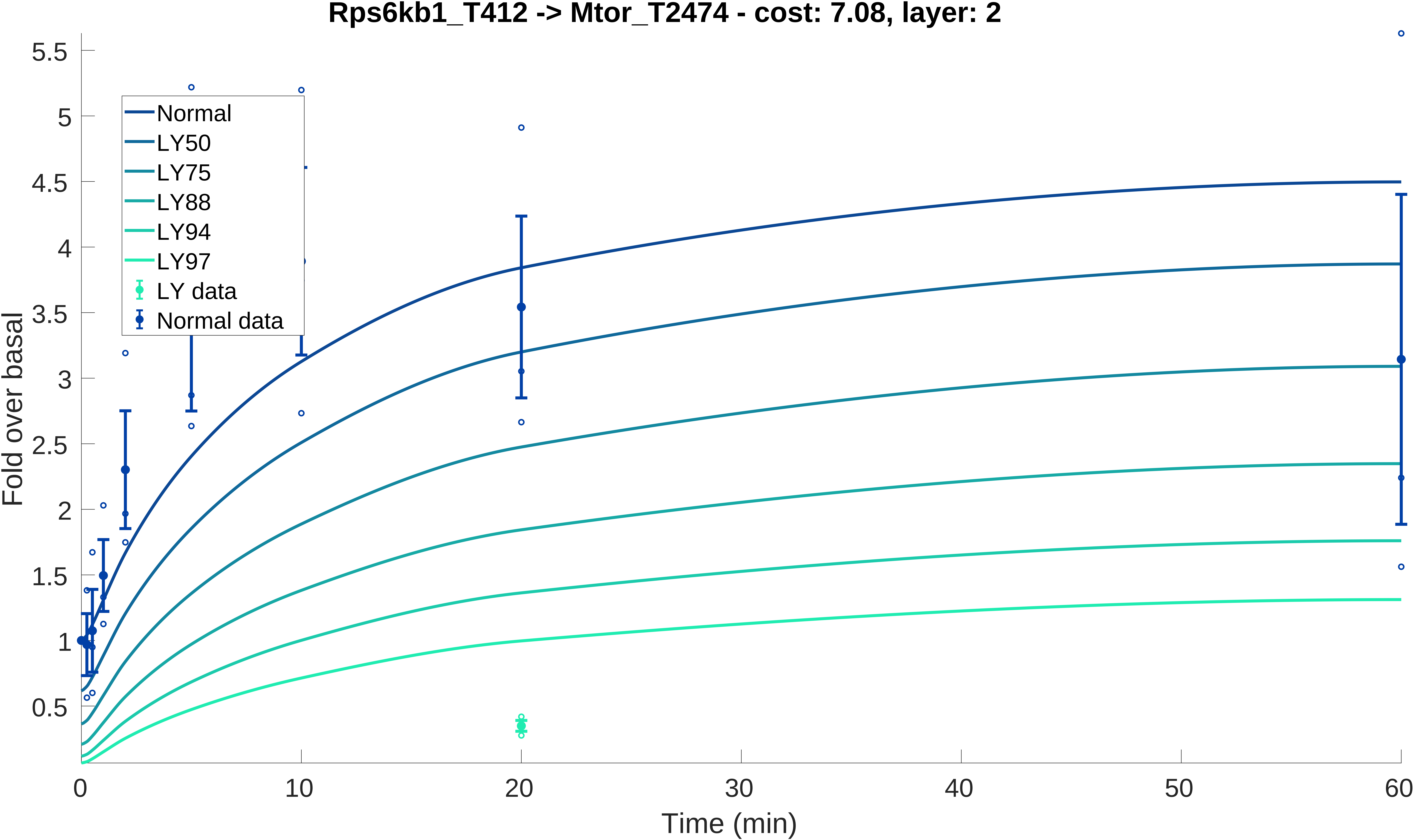

Rps6kb1_T412 -> Mtor_T2474 - cost: 7.08, layer: 2
Normal
LY50
LY75
LY88
LY94
LY97
LY data
Normal data
Fold over basal
Time (min)
0
10
20
30
40
50
60
0.5
1
1.5
2
2.5
3
3.5
4
4.5
5
5.5

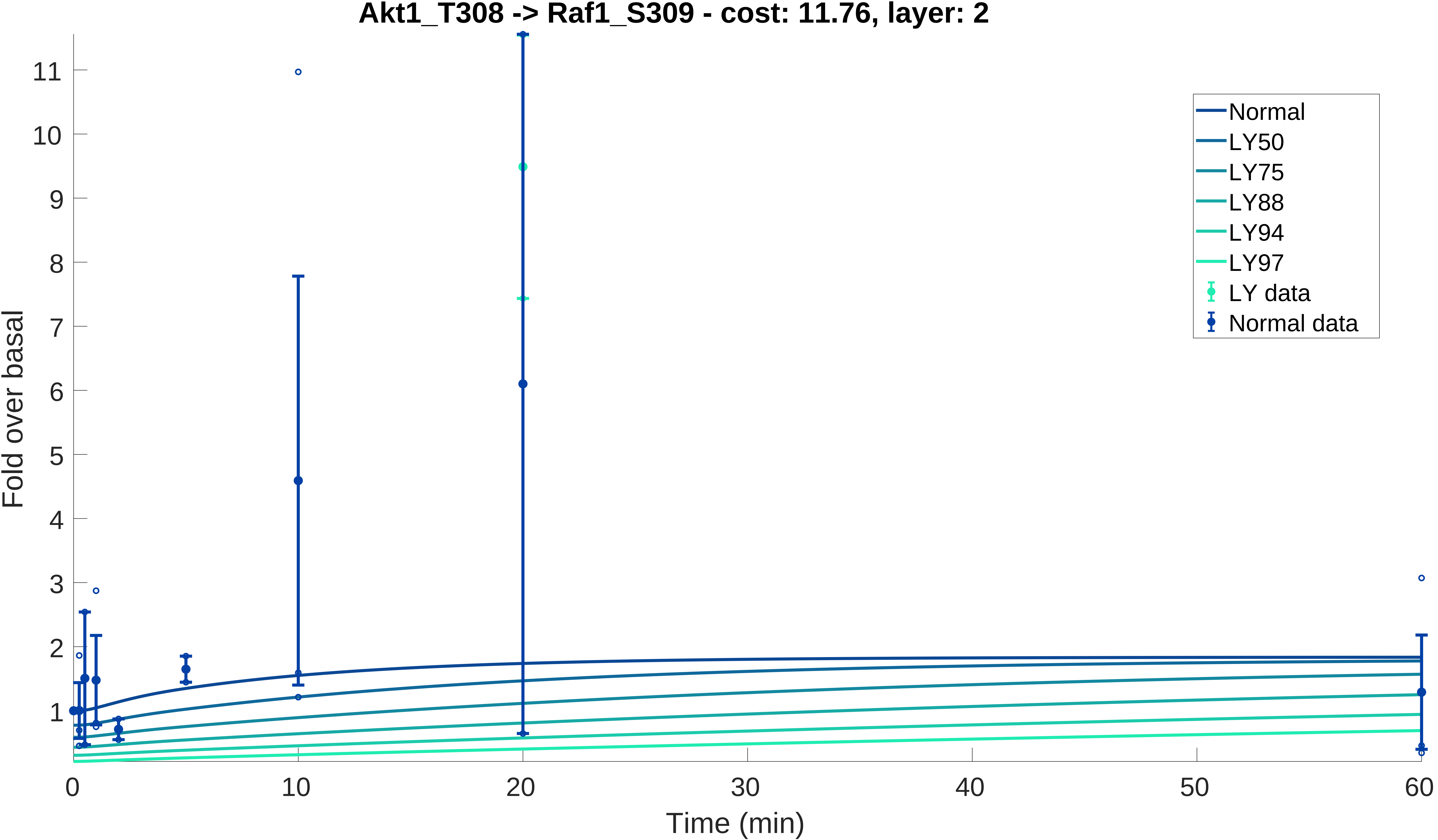
Akt1_T308 -> Raf1_S309 - cost: 11.76, layer: 2
Fold over basal
Time (min)
0
10
20
30
40
50
60
1
2
3
4
5
6
7
8
9
10
11
Normal
LY50
LY75
LY88
LY94
LY97
LY data
Normal data

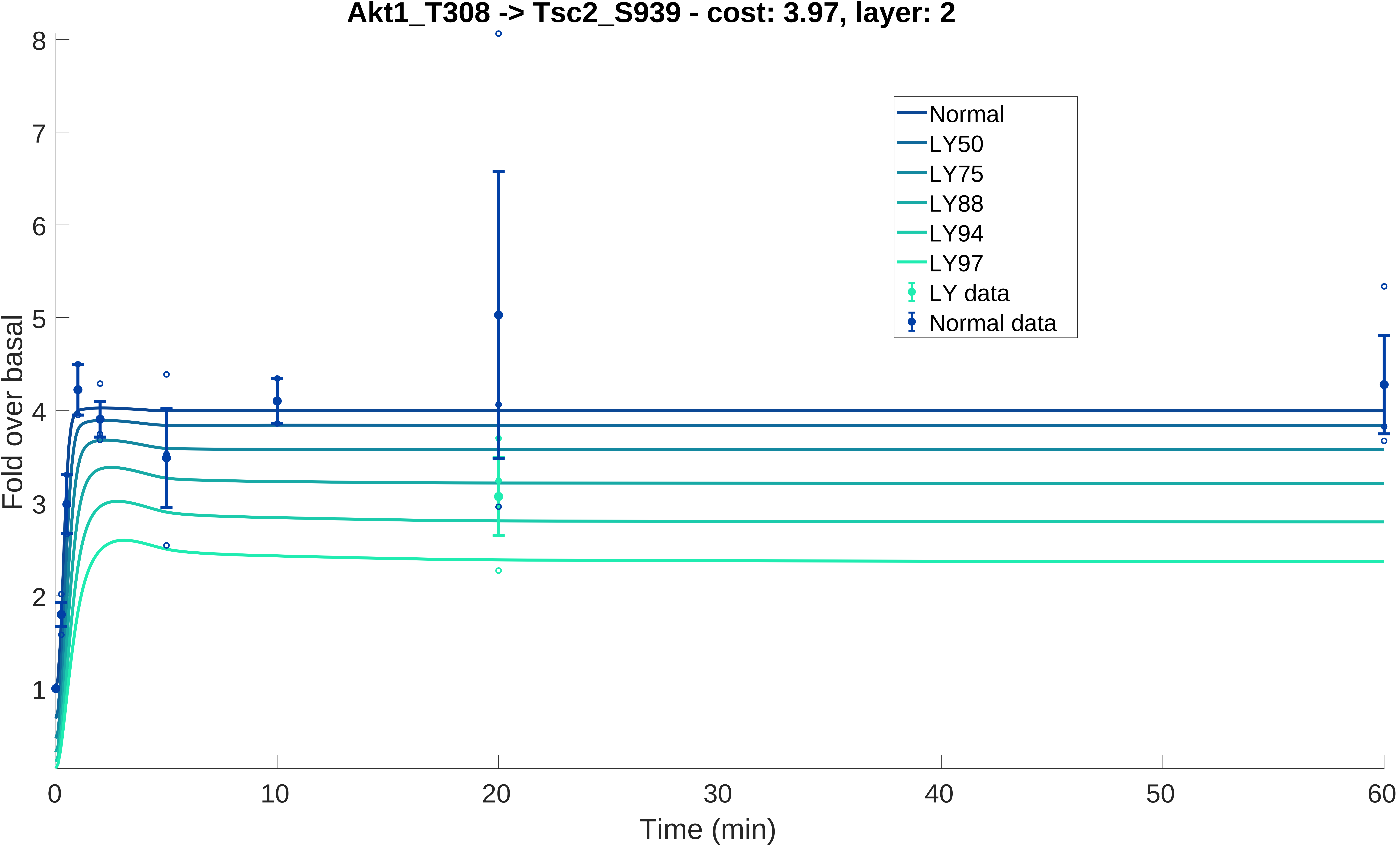

Akt1_T308 -> Tsc2_S939 - cost: 3.97, layer: 2
Fold over basal
Time (min)
Normal
LY50
LY75
LY88
LY94
LY97
LY data
Normal data
0
10
20
30
40
50
60
1
2
3
4
5
6
7
8

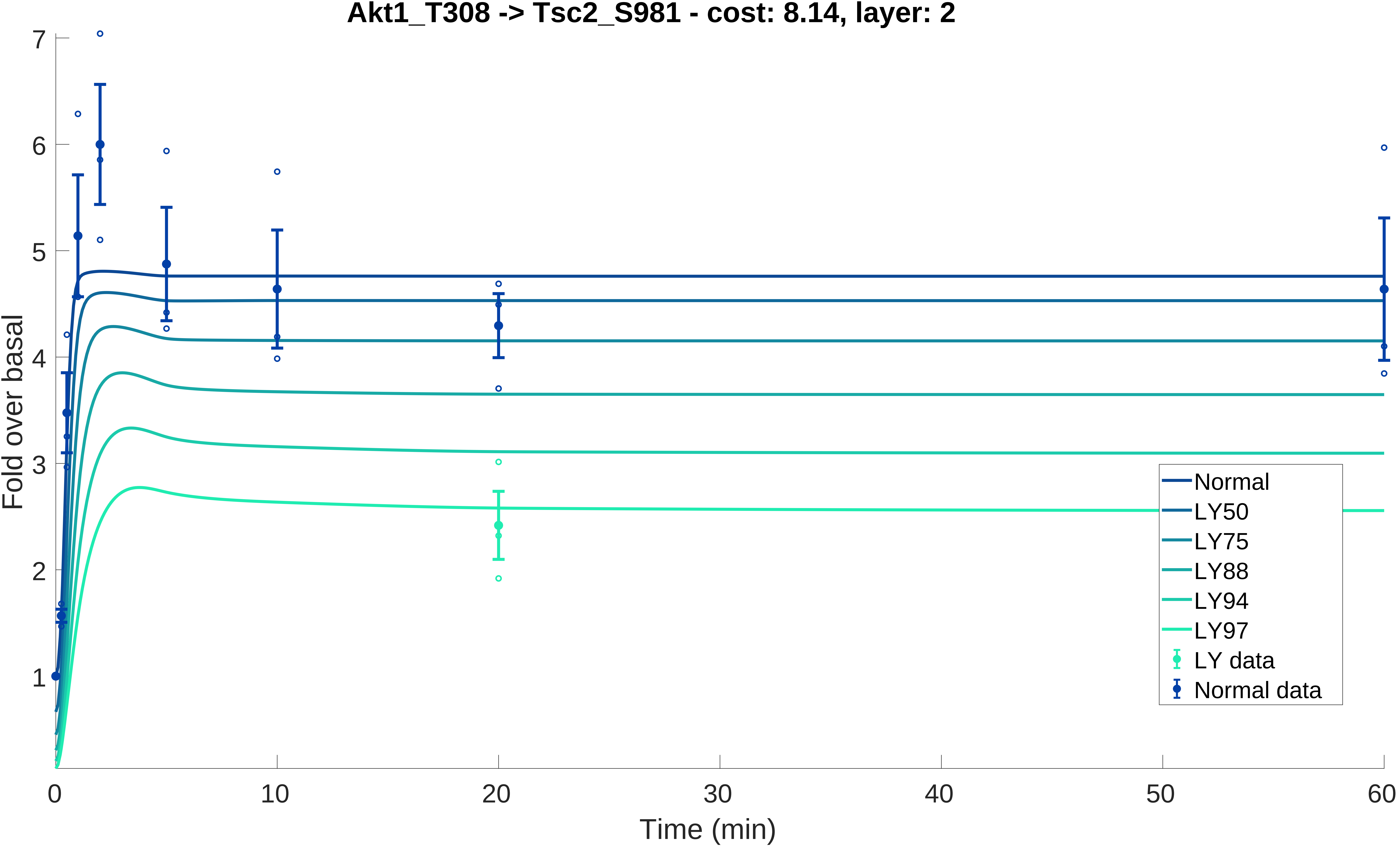

Akt1_T308 -> Tsc2_S981 - cost: 8.14, layer: 2
Fold over basal
Time (min)
0
10
20
30
40
50
60
1
2
3
4
5
6
7
Normal
LY50
LY75
LY88
LY94
LY97
LY data
Normal data

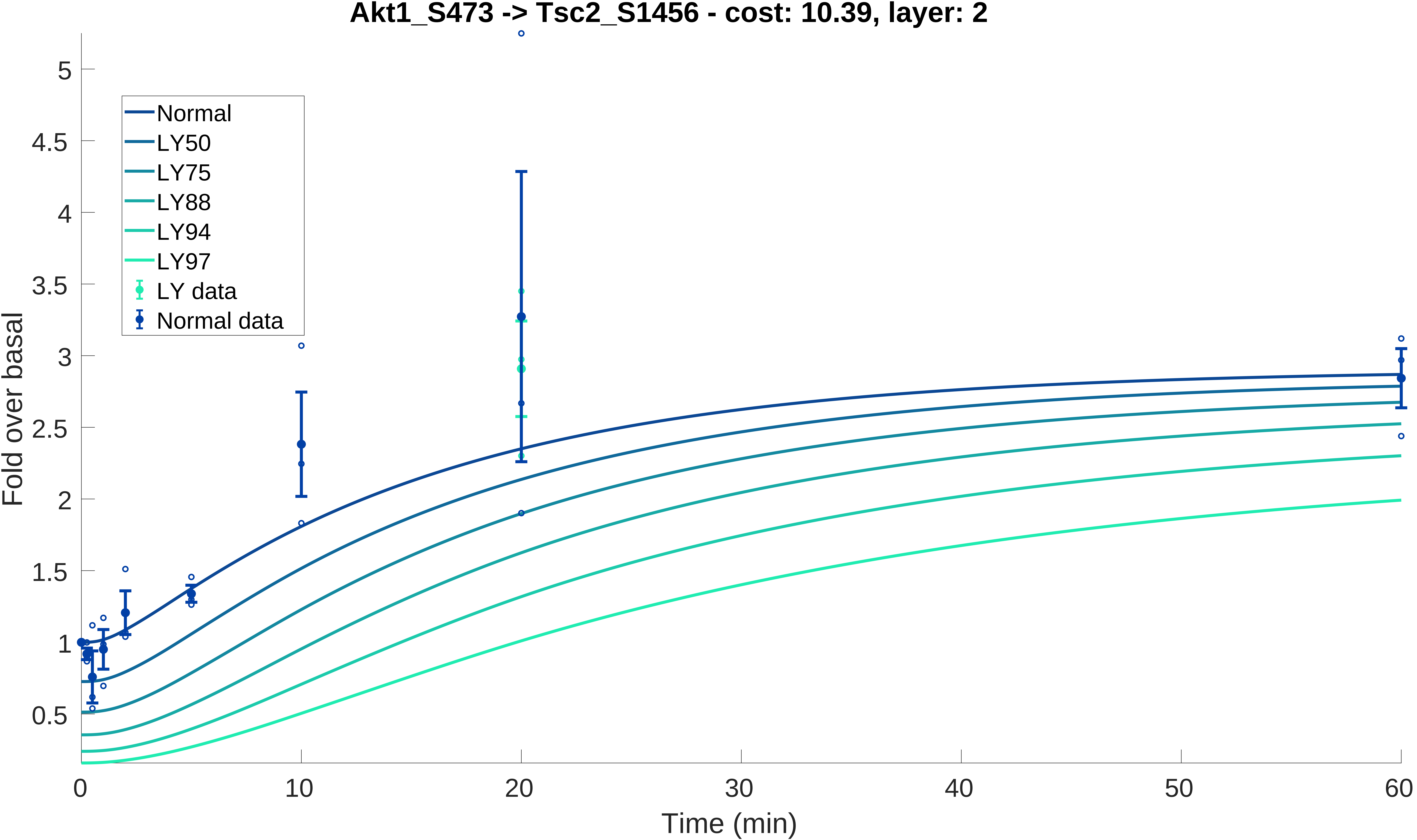

Akt1_S473 -> Tsc2_S1456 - cost: 10.39, layer: 2
Normal
LY50
LY75
LY88
LY94
LY97
LY data
Normal data
Fold over basal
Time (min)
0.5
1
1.5
2
2.5
3
3.5
4
4.5
5
0
10
20
30
40
50
60

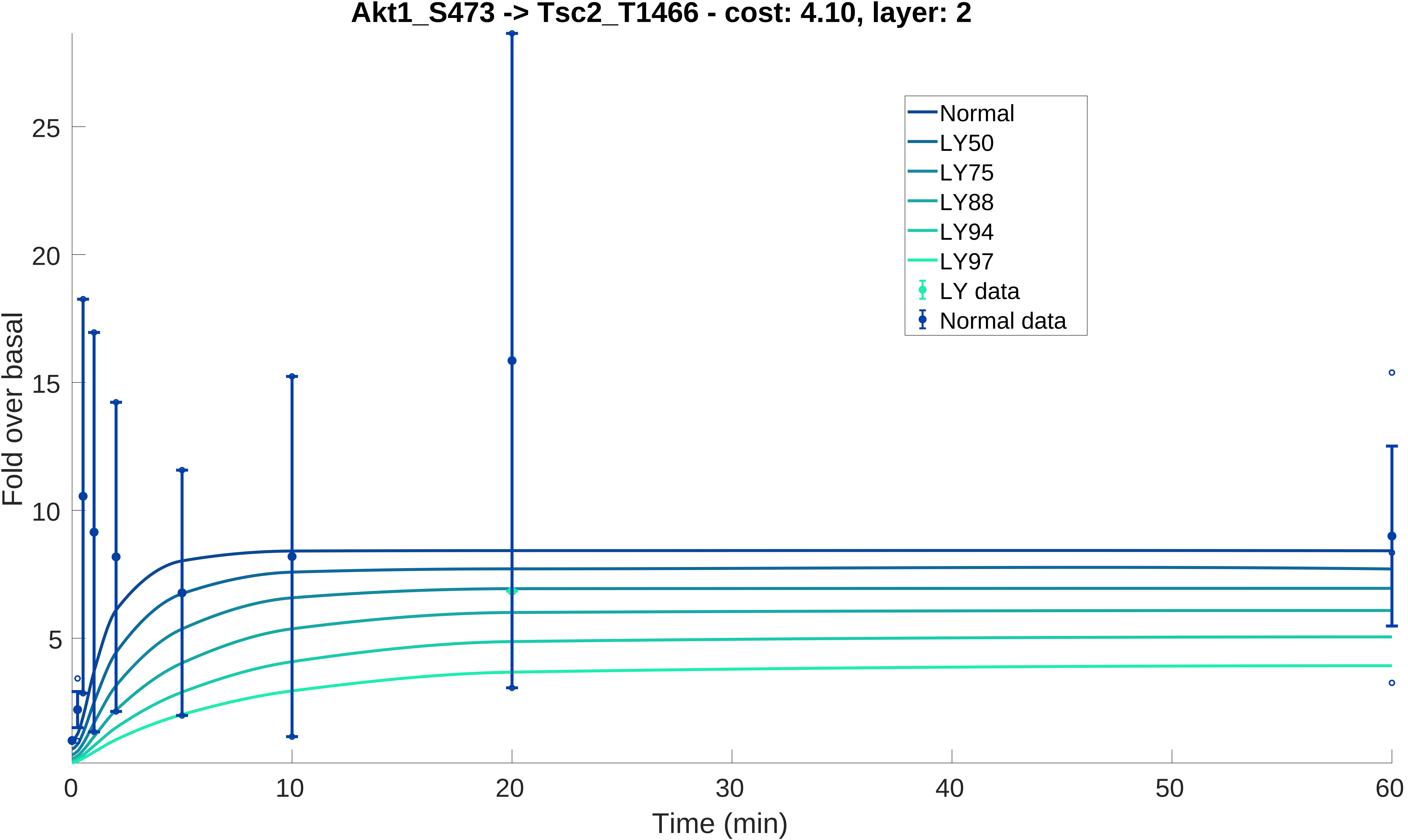
Akt1_S473 -> Tsc2_T1466 - cost: 4.10, layer: 2
Fold over basal
Time (min)
Normal
LY50
LY75
LY88
LY94
LY97
LY data
Normal data
0
10
20
30
40
50
60
5
10
15
20
25

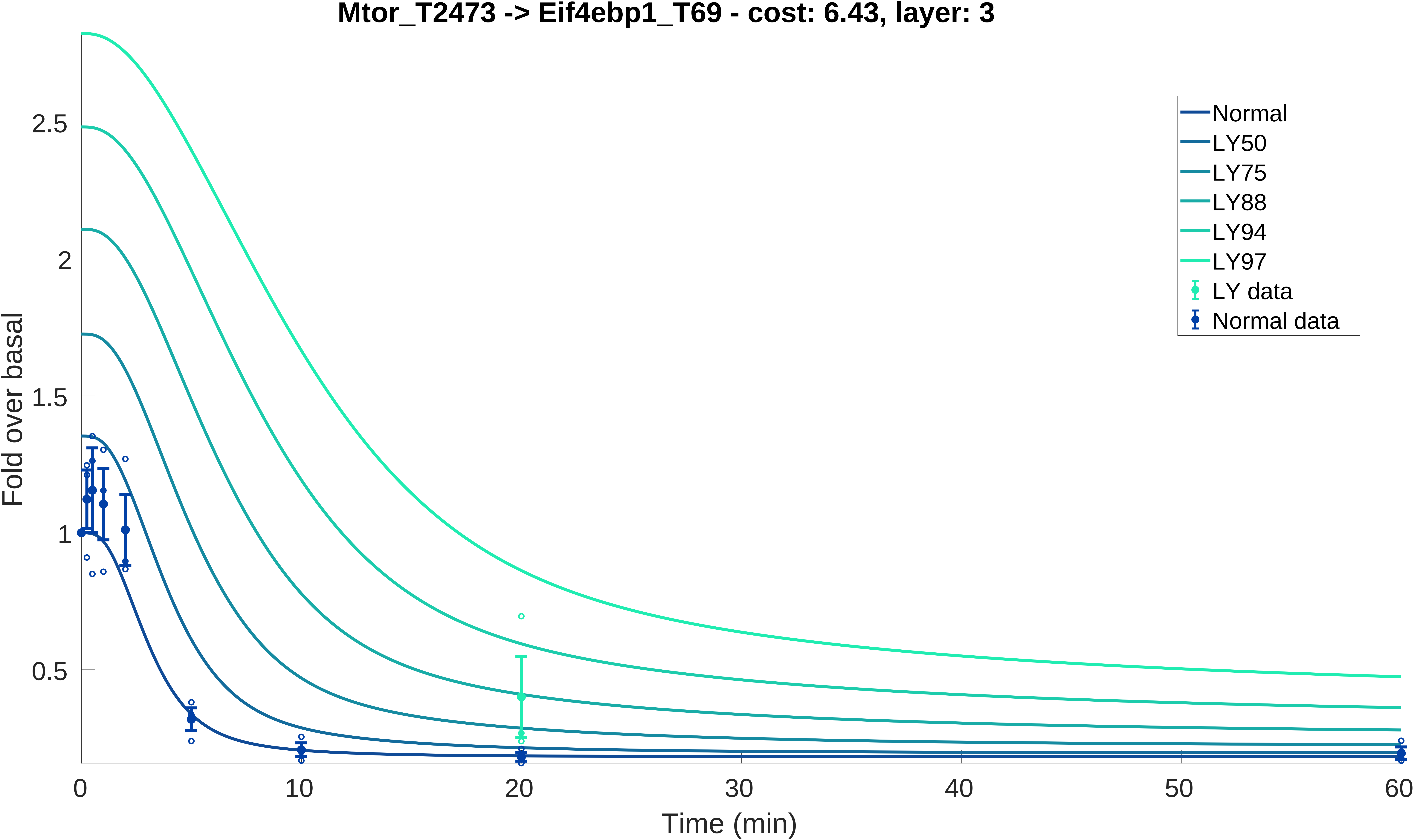

Mtor_T2473 -> Eif4ebp1_T69 - cost: 6.43, layer: 3
Normal
LY50
LY75
LY88
LY94
LY97
LY data
Normal data
Fold over basal
Time (min)
0
10
20
30
40
50
60
0.5
1
1.5
2
2.5

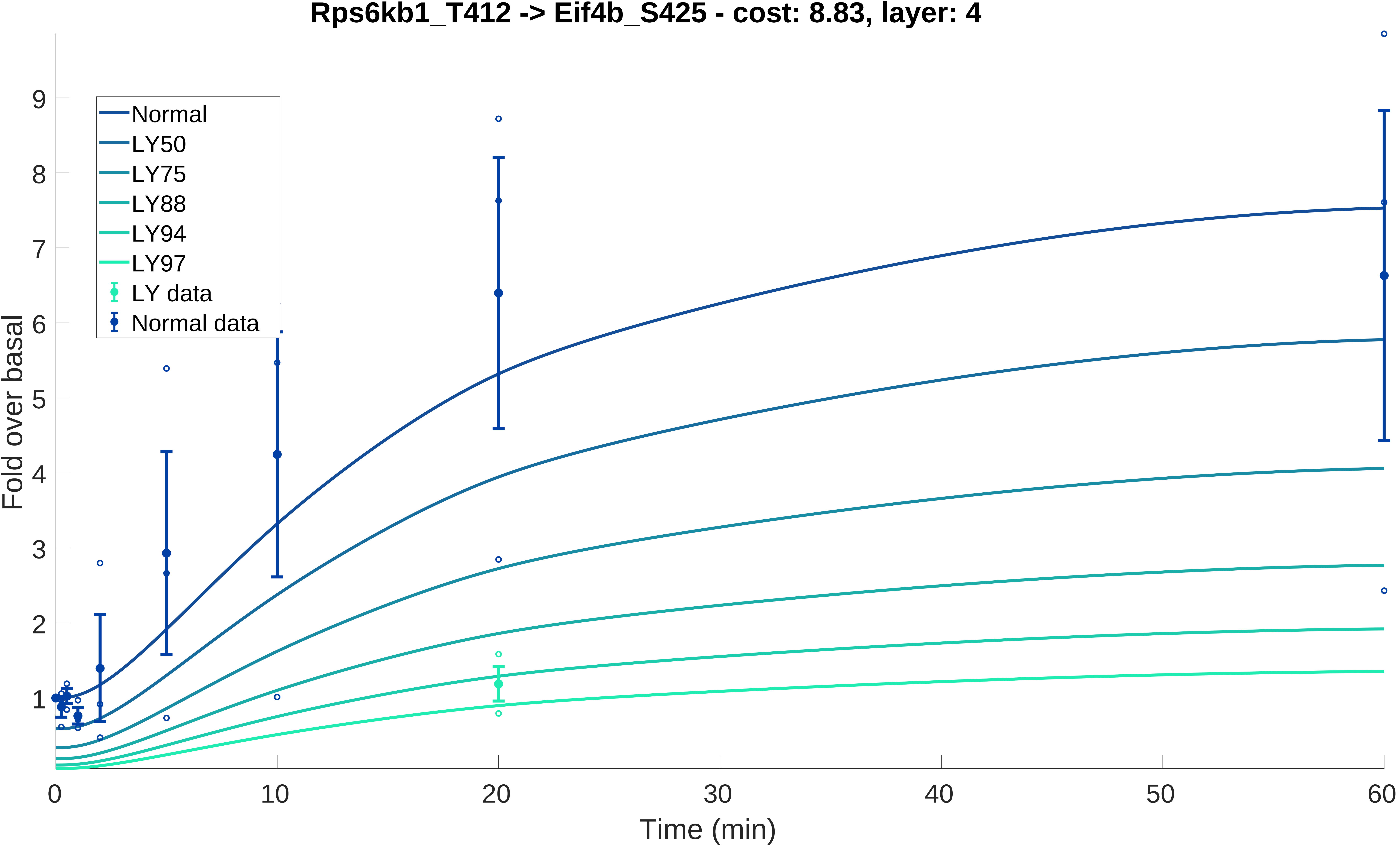

Rps6kb1_T412 -> Eif4b_S425 - cost: 8.83, layer: 4
Normal
LY50
LY75
LY88
LY94
LY97
LY data
Normal data
Fold over basal
Time (min)
0
10
20
30
40
50
60
1
2
3
4
5
6
7
8
9

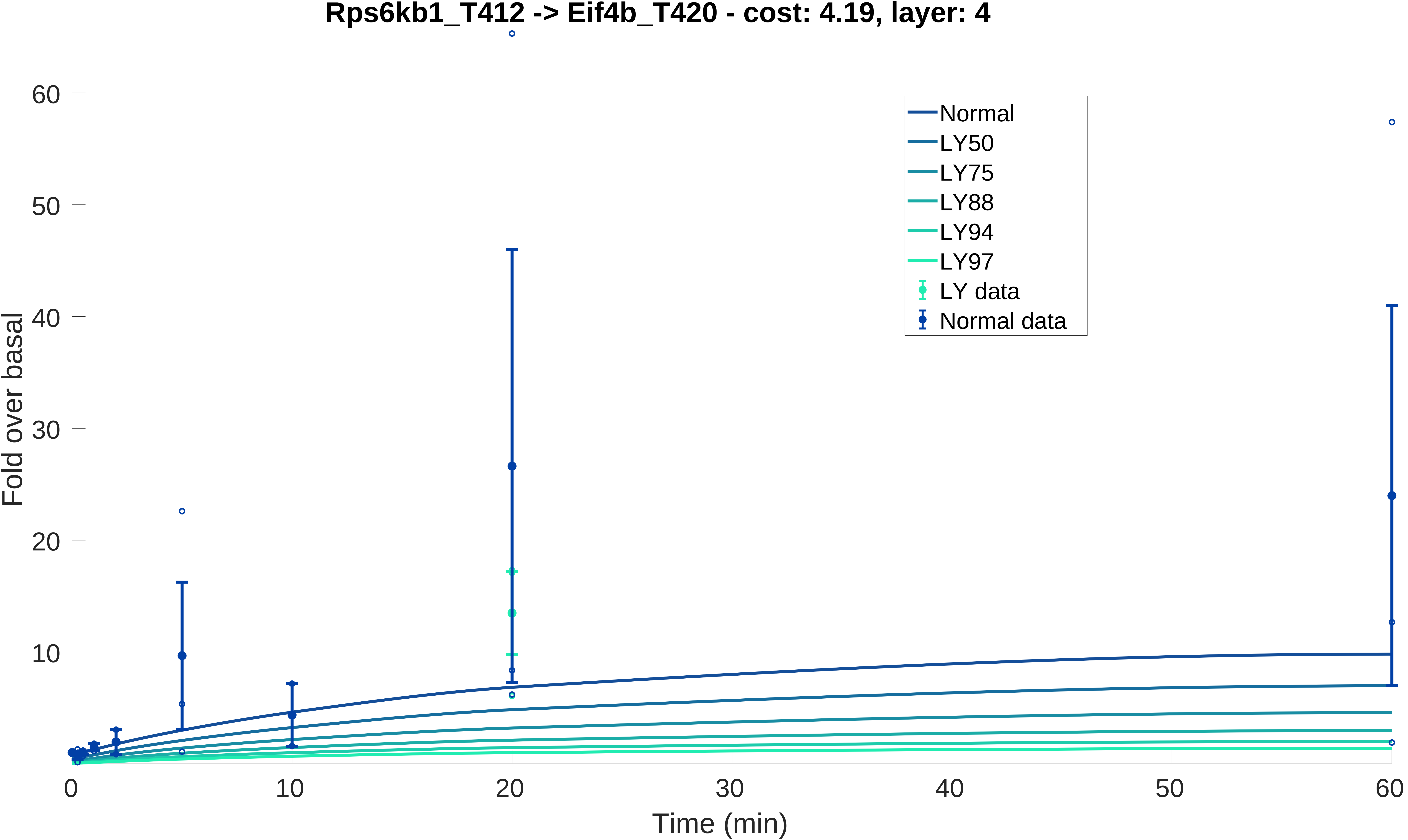

Rps6kb1_T412 -> Eif4b_T420 - cost: 4.19, layer: 4
Fold over basal
Time (min)
0
10
20
30
40
50
60
Normal
LY50
LY75
LY88
LY94
LY97
LY data
Normal data

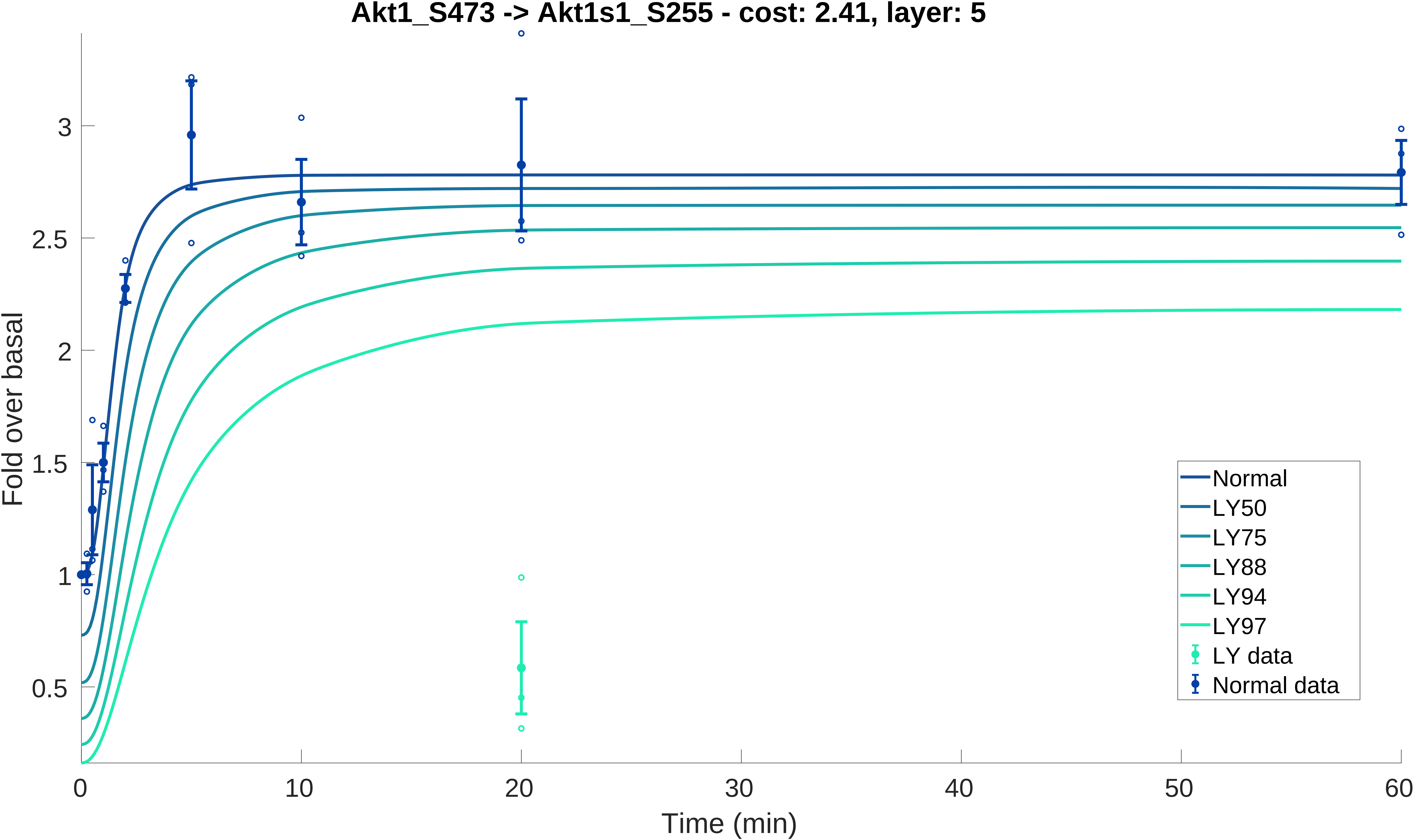

Akt1_S473 -> Akt1s1_S255 - cost: 2.41, layer: 5
Fold over basal
Time (min)
0
10
20
30
40
50
60
0.5
1
1.5
2
2.5
3
Normal
LY50
LY75
LY88
LY94
LY97
LY data
Normal data

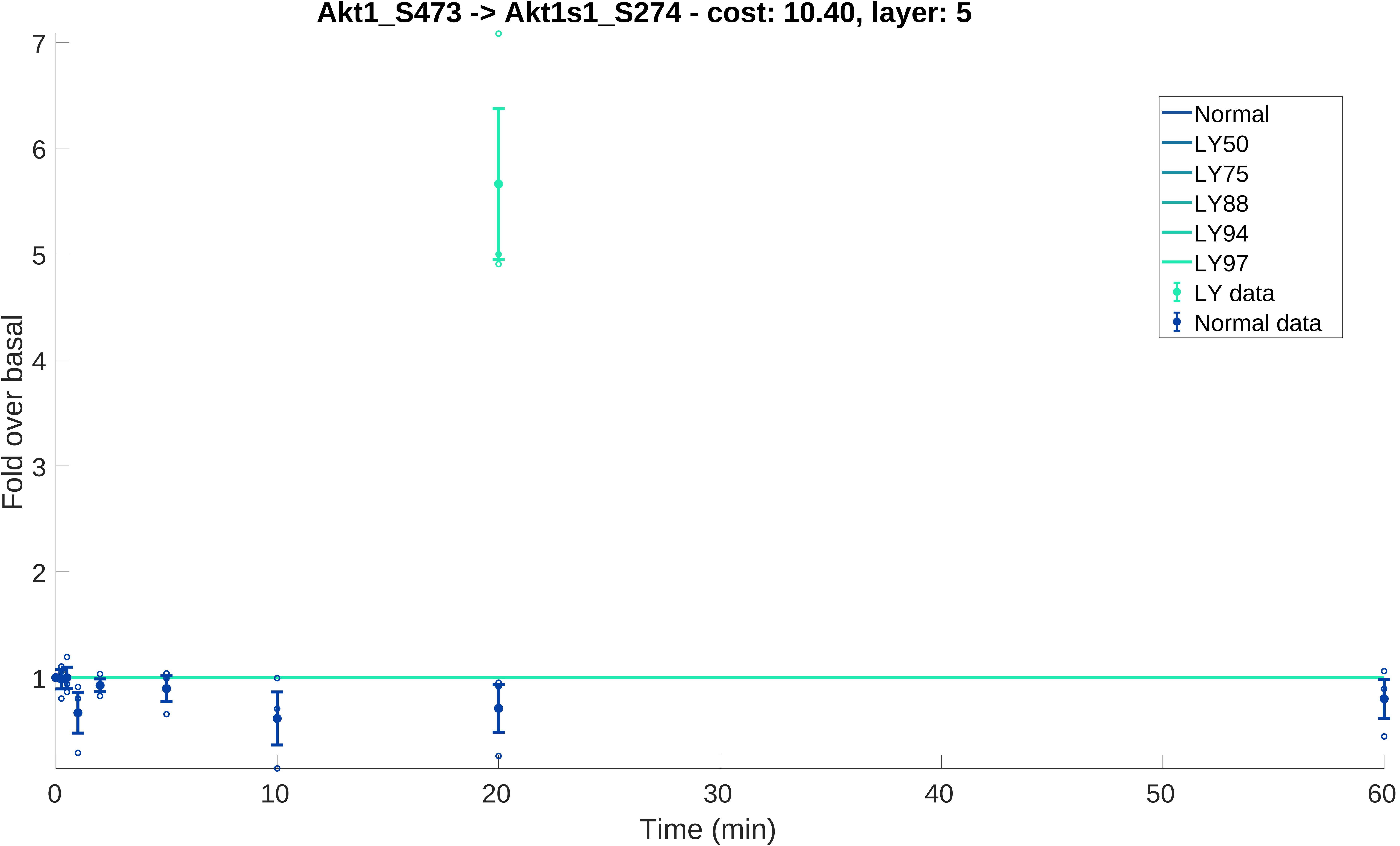

Akt1_S473 -> Akt1s1_S274 - cost: 10.40, layer: 5
Fold over basal
Time (min)
0
10
20
30
40
50
60
1
2
3
4
5
6
7
Normal
LY50
LY75
LY88
LY94
LY97
LY data
Normal data

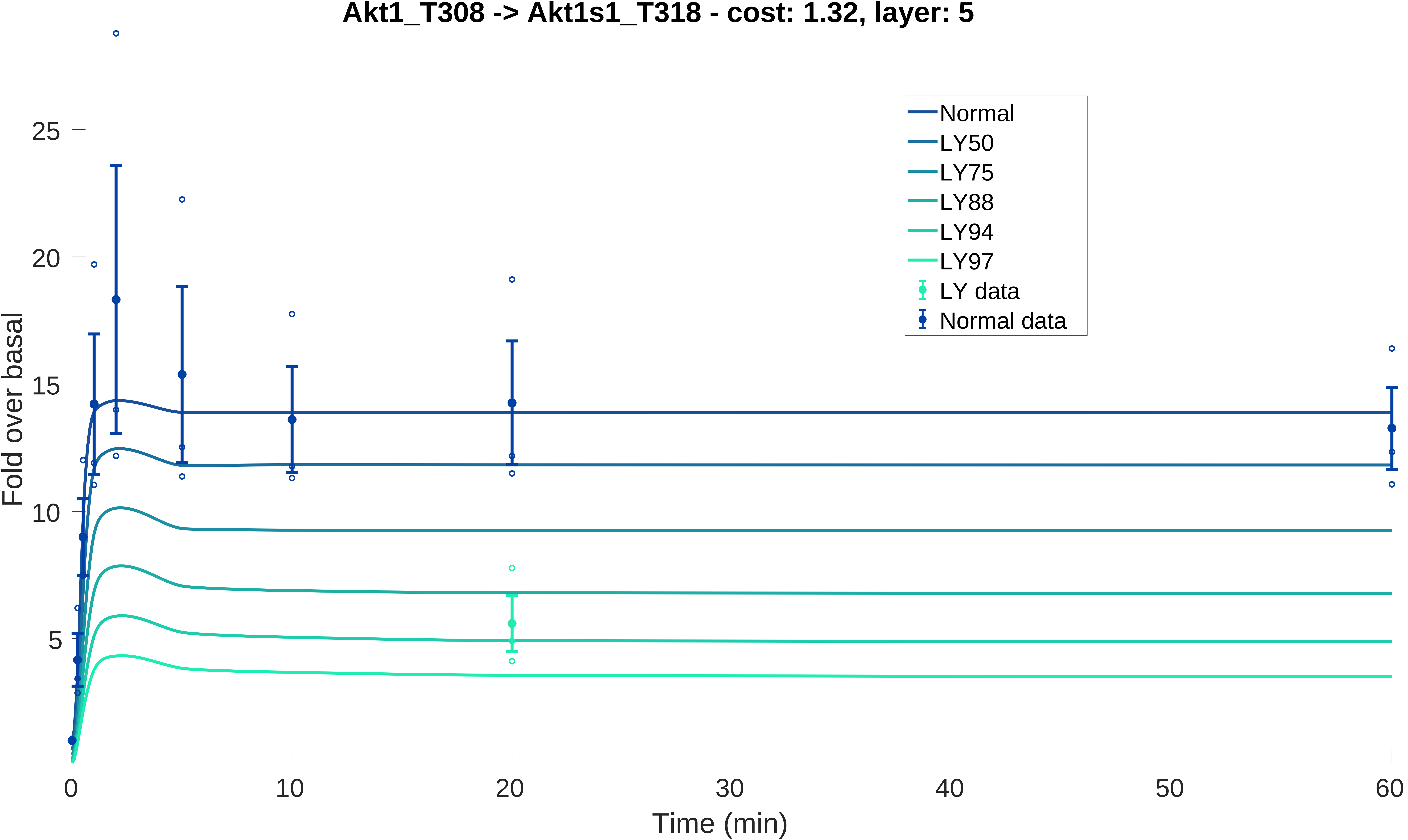

Akt1_T308 -> Akt1s1_T318 - cost: 1.32, layer: 5
Fold over basal
Time (min)
0
10
20
30
40
50
60
5
10
15
20
25
Normal
LY50
LY75
LY88
LY94
LY97
LY data
Normal data

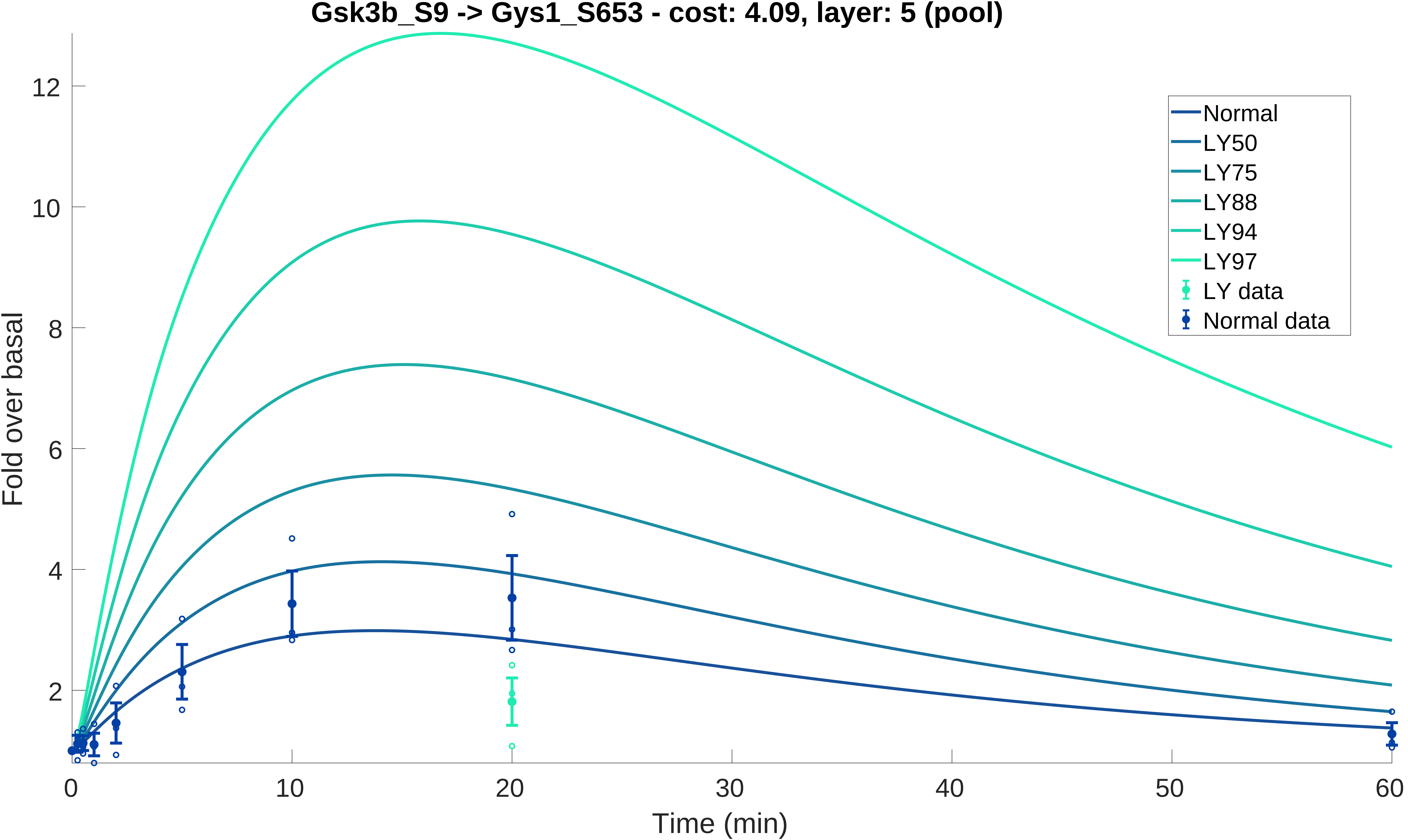

Gsk3b_S9 -> Gys1_S653 - cost: 4.09, layer: 5 (pool)
Fold over basal
Time (min)
0
10
20
30
40
50
60
2
4
6
8
10
12
Normal
LY50
LY75
LY88
LY94
LY97
LY data
Normal data

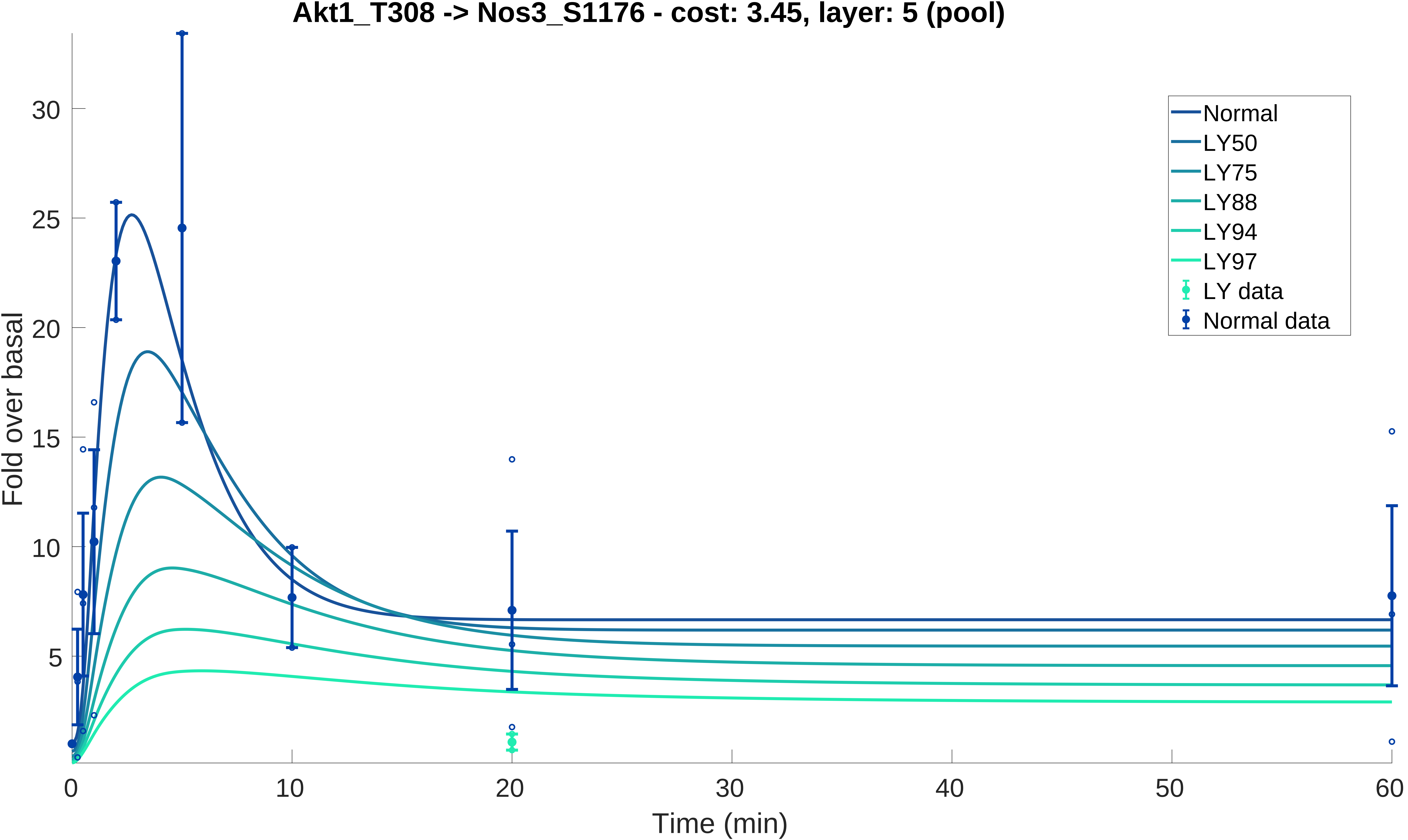

Akt1_T308 -> Nos3_S1176 - cost: 3.45, layer: 5 (pool)
Fold over basal
Time (min)
0
10
20
30
40
50
60
5
10
15
20
25
30
Normal
LY50
LY75
LY88
LY94
LY97
LY data
Normal data

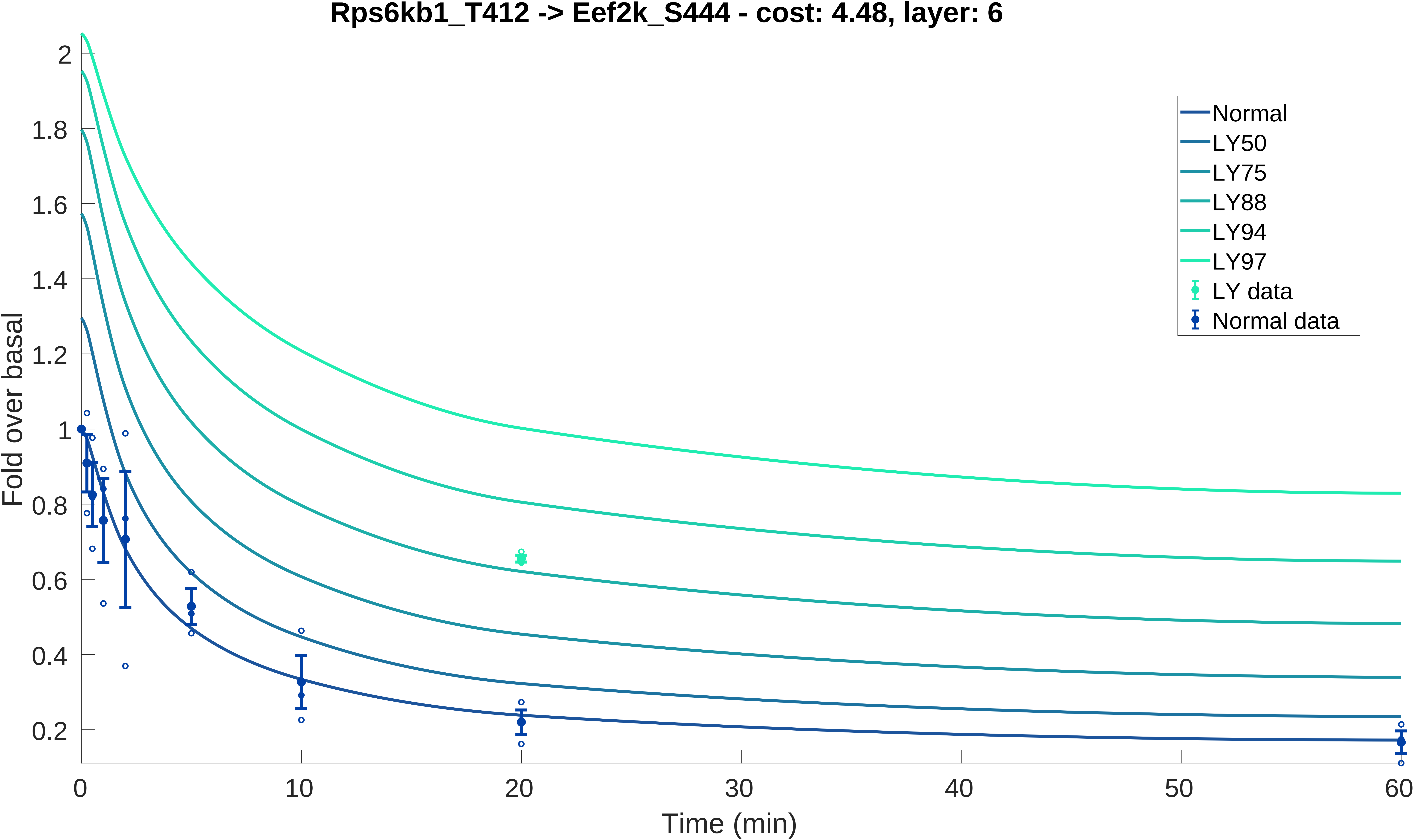

Rps6kb1_T412 -> Eef2k_S444 - cost: 4.48, layer: 6
Fold over basal
Time (min)
0
10
20
30
40
50
60
0.2
0.4
0.6
0.8
1
1.2
1.4
1.6
1.8
2
Normal
LY50
LY75
LY88
LY94
LY97
LY data
Normal data

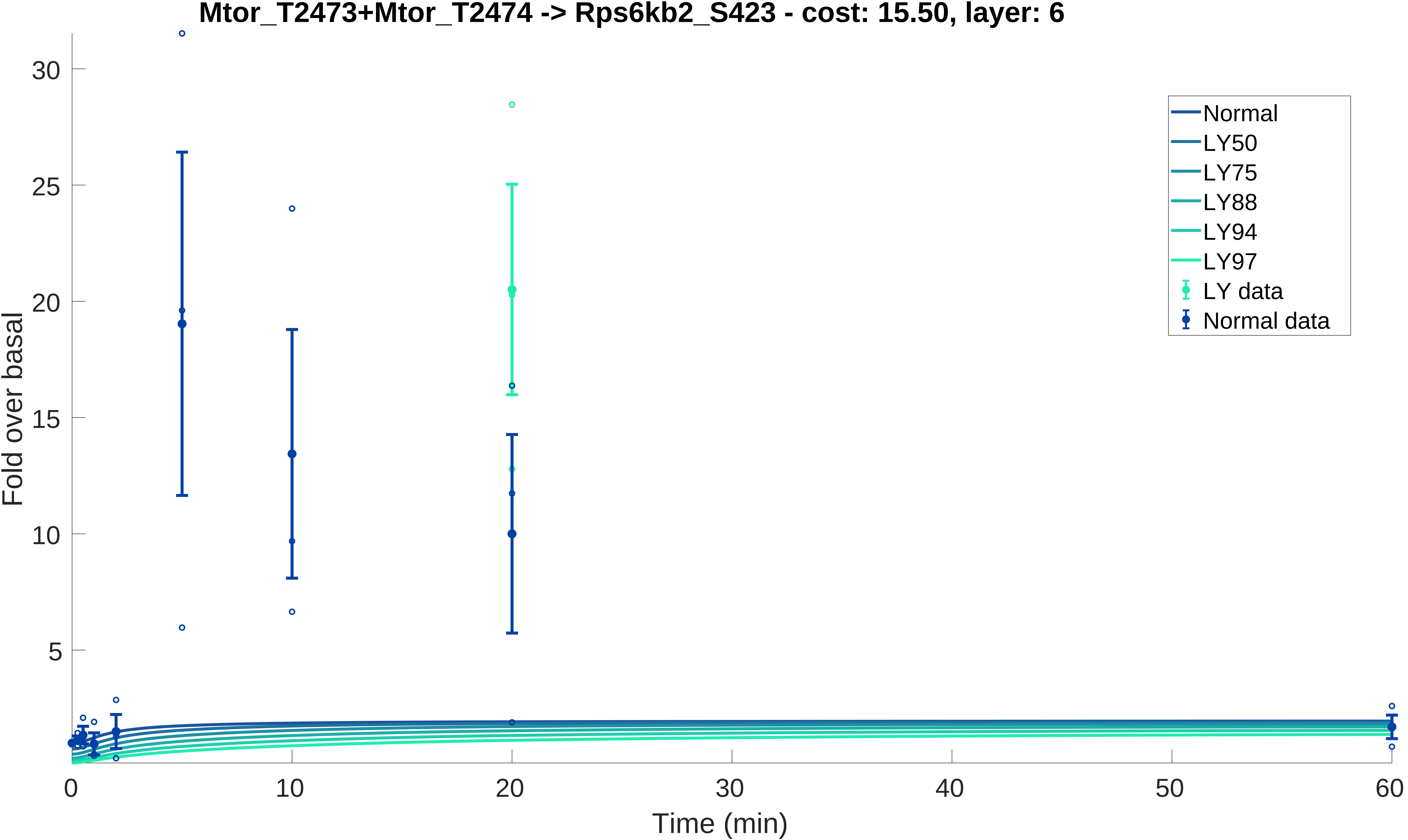
Mtor_T2473+Mtor_T2474 -> Rps6kb2_S423 - cost: 15.50, layer: 6
Fold over basal
Time (min)
0
10
20
30
40
50
60
5
10
15
20
25
30
Normal
LY50
LY75
LY88
LY94
LY97
LY data
Normal data

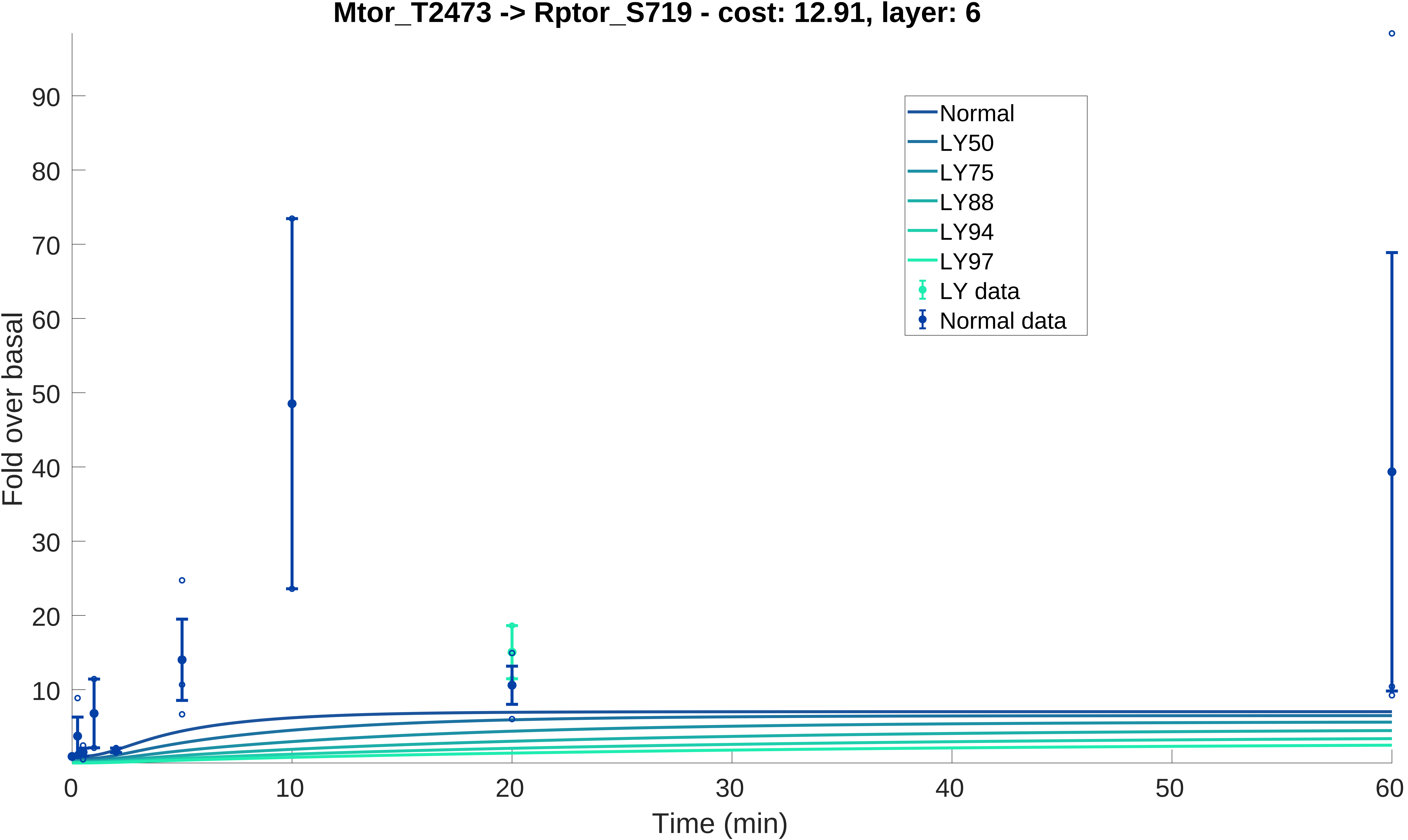

Mtor_T2473 -> Rptor_S719 - cost: 12.91, layer: 6
Fold over basal
Time (min)
0
10
20
30
40
50
60
70
80
90
Normal
LY50
LY75
LY88
LY94
LY97
LY data
Normal data

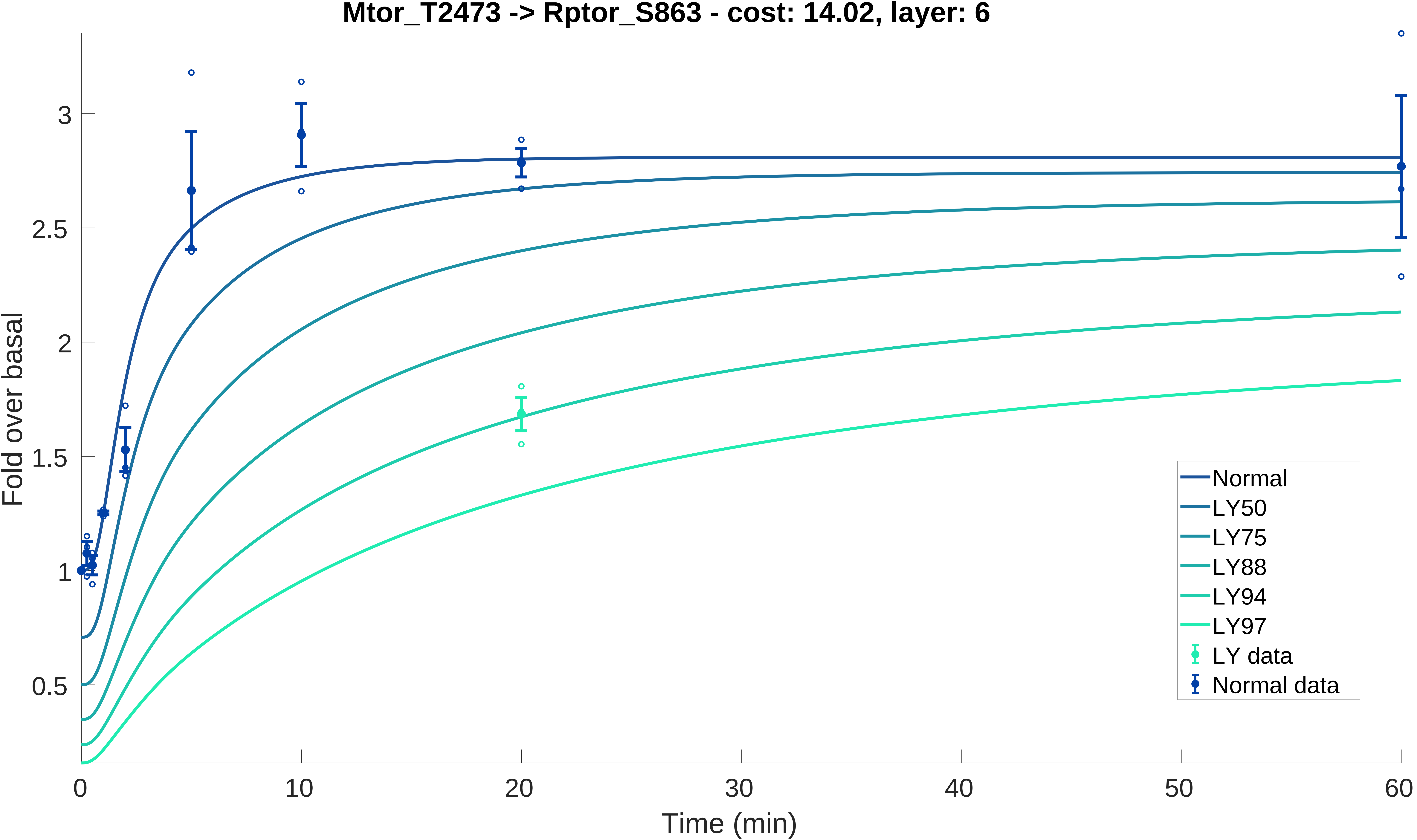

Mtor_T2473 -> Rptor_S863 - cost: 14.02, layer: 6
Fold over basal
Time (min)
0
10
20
30
40
50
60
0.5
1
1.5
2
2.5
3
Normal
LY50
LY75
LY88
LY94
LY97
LY data
Normal data

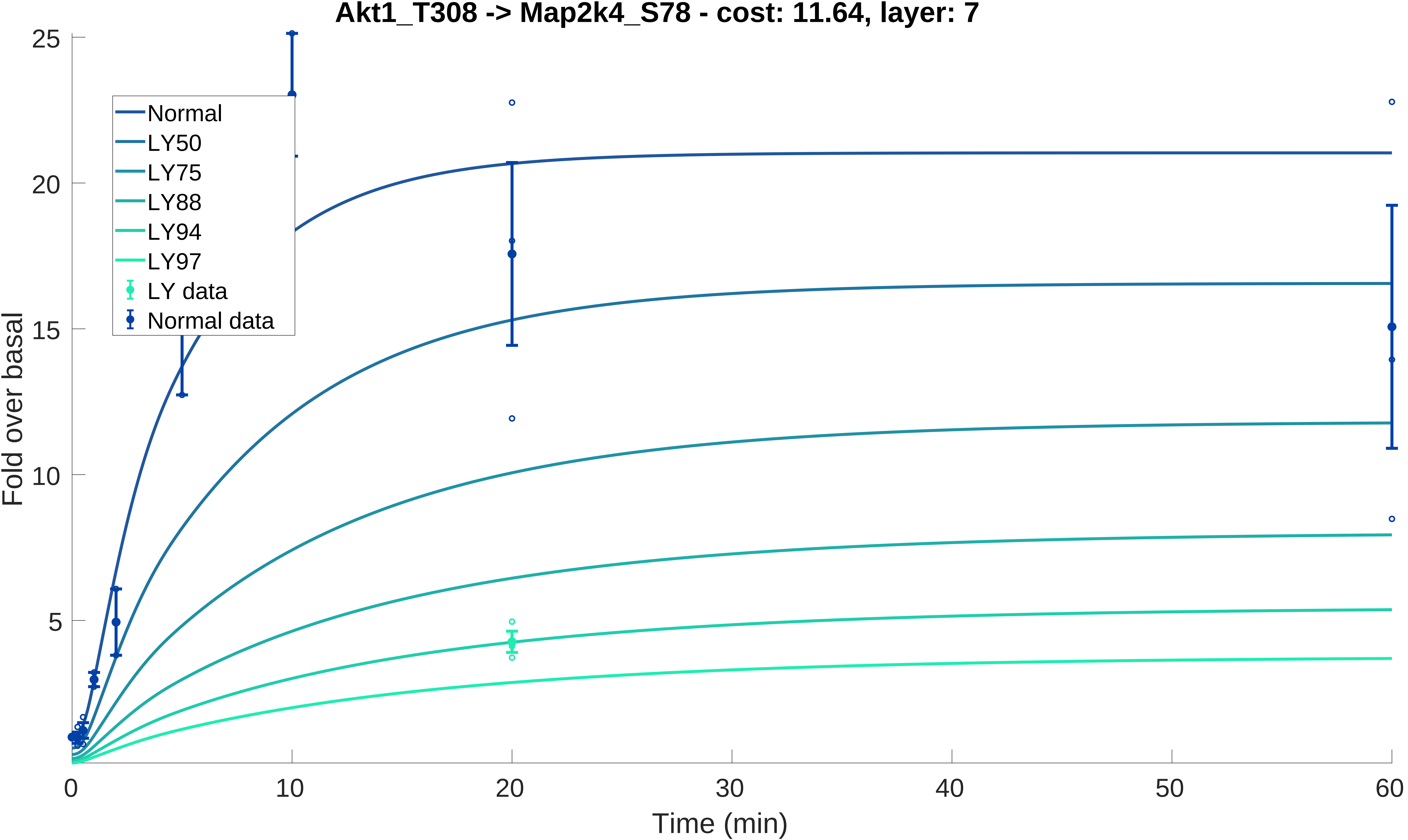

Akt1_T308 -> Map2k4_S78 - cost: 11.64, layer: 7
Fold over basal
Time (min)
0
5
10
15
20
25
30
40
50
60
Normal
LY50
LY75
LY88
LY94
LY97
LY data
Normal data

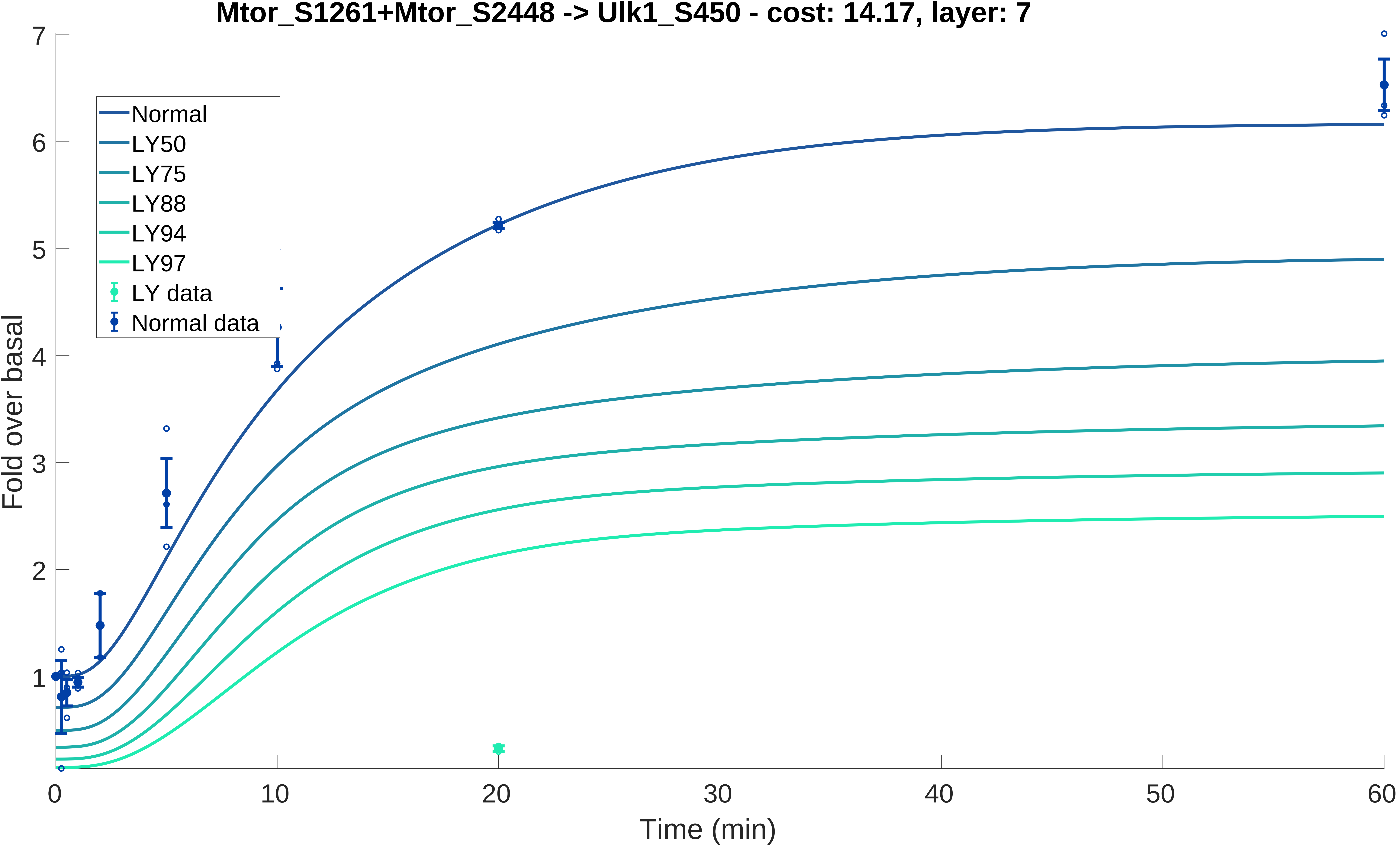

Mtor_S1261+Mtor_S2448 -> Ulk1_S450 - cost: 14.17, layer: 7
Fold over basal
Time (min)
Normal
LY50
LY75
LY88
LY94
LY97
LY data
Normal data
0
10
20
30
40
50
60
1
2
3
4
5
6
7

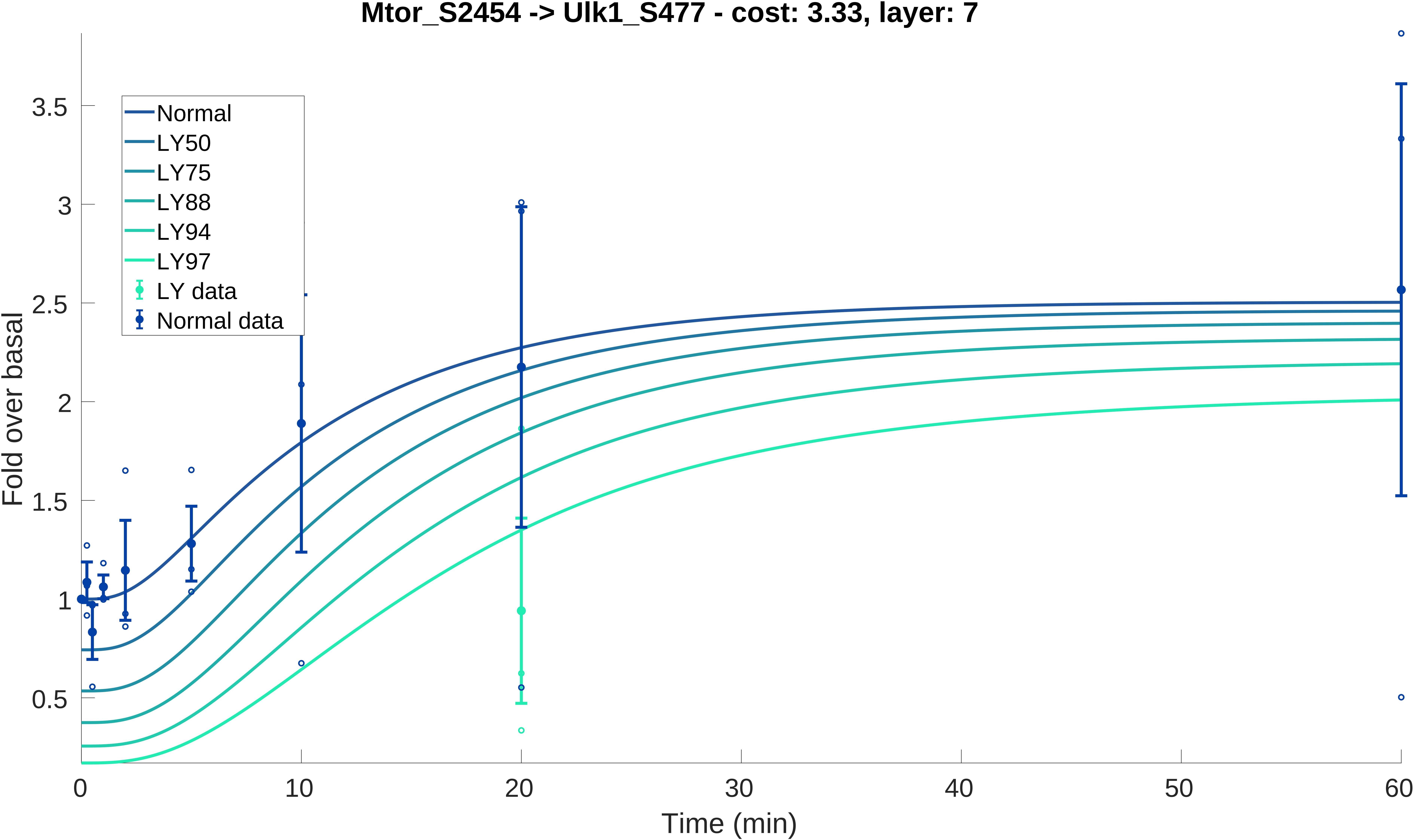
Mtor_S2454 -> Ulk1_S477 - cost: 3.33, layer: 7
Normal
LY50
LY75
LY88
LY94
LY97
LY data
Normal data
Fold over basal
3.5
3
2.5
2
1.5
1
0.5
0
10
20
30
40
50
60
Time (min)

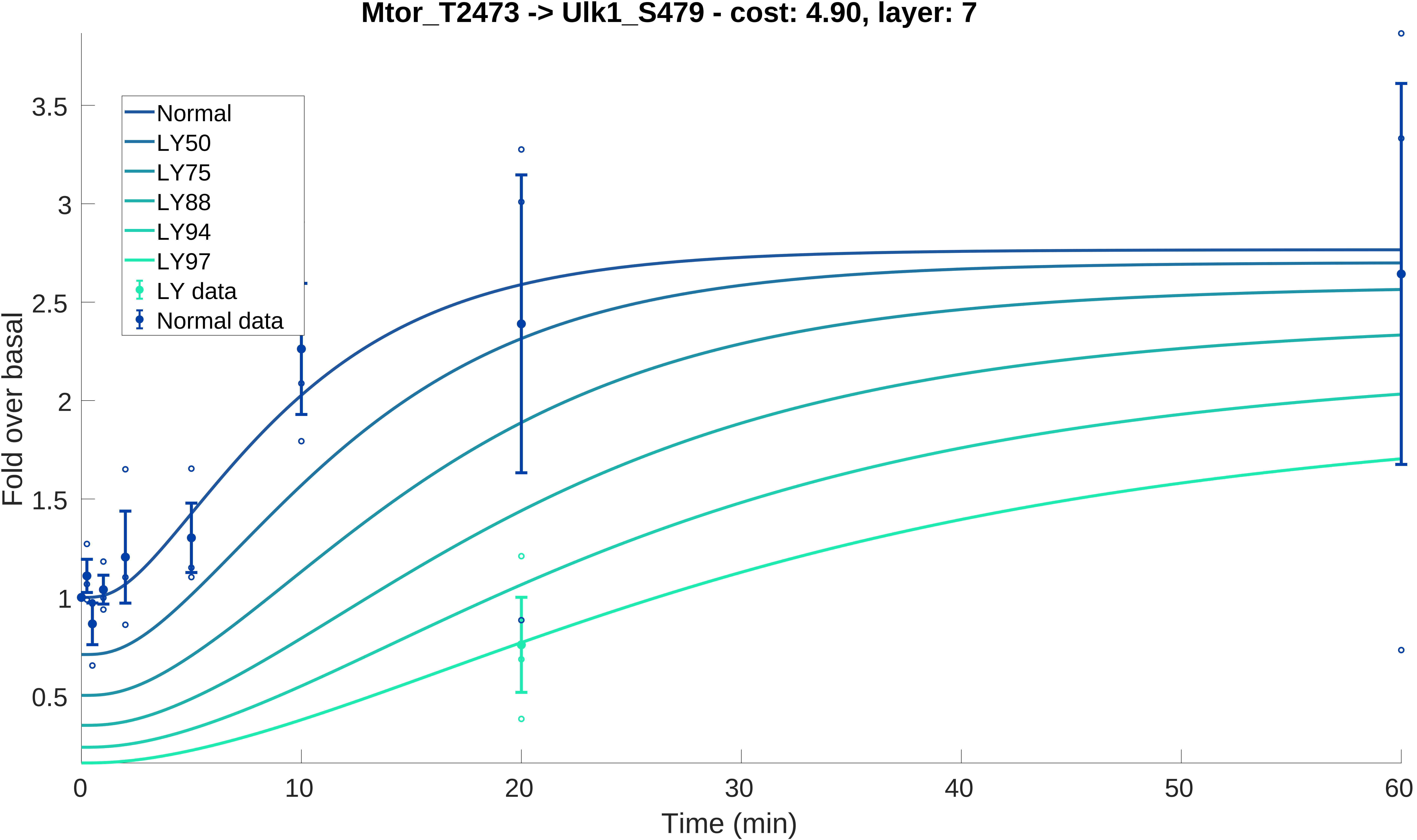

Mtor_T2473 -> Ulk1_S479 - cost: 4.90, layer: 7
Normal
LY50
LY75
LY88
LY94
LY97
LY data
Normal data
Fold over basal
3.5
3
2.5
2
1.5
1
0.5
0
10
20
30
40
50
60
Time (min)

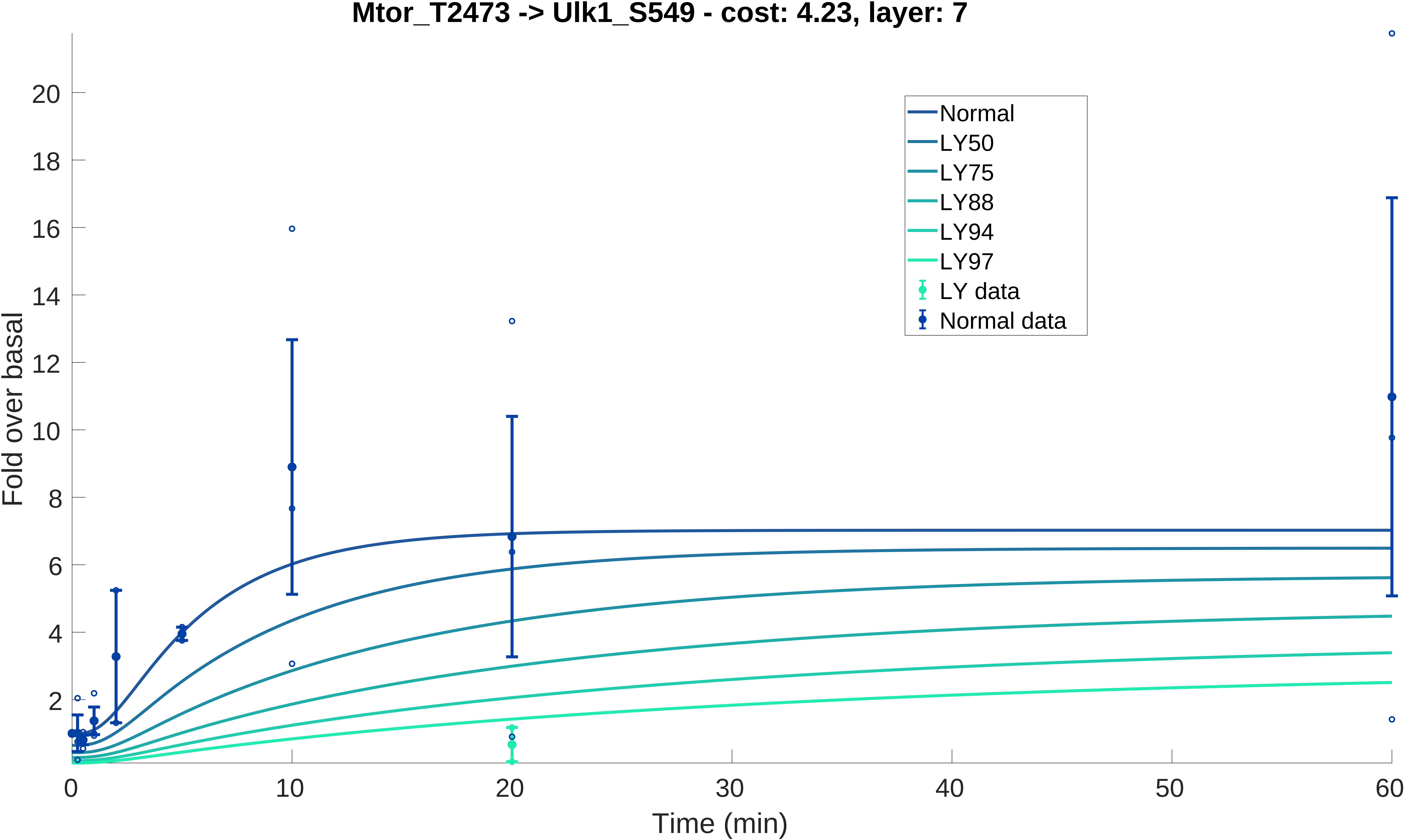

Mtor_T2473 -> Ulk1_S549 - cost: 4.23, layer: 7
Fold over basal
Time (min)
0
10
20
30
40
50
60
2
4
6
8
10
12
14
16
18
20
Normal
LY50
LY75
LY88
LY94
LY97
LY data
Normal data

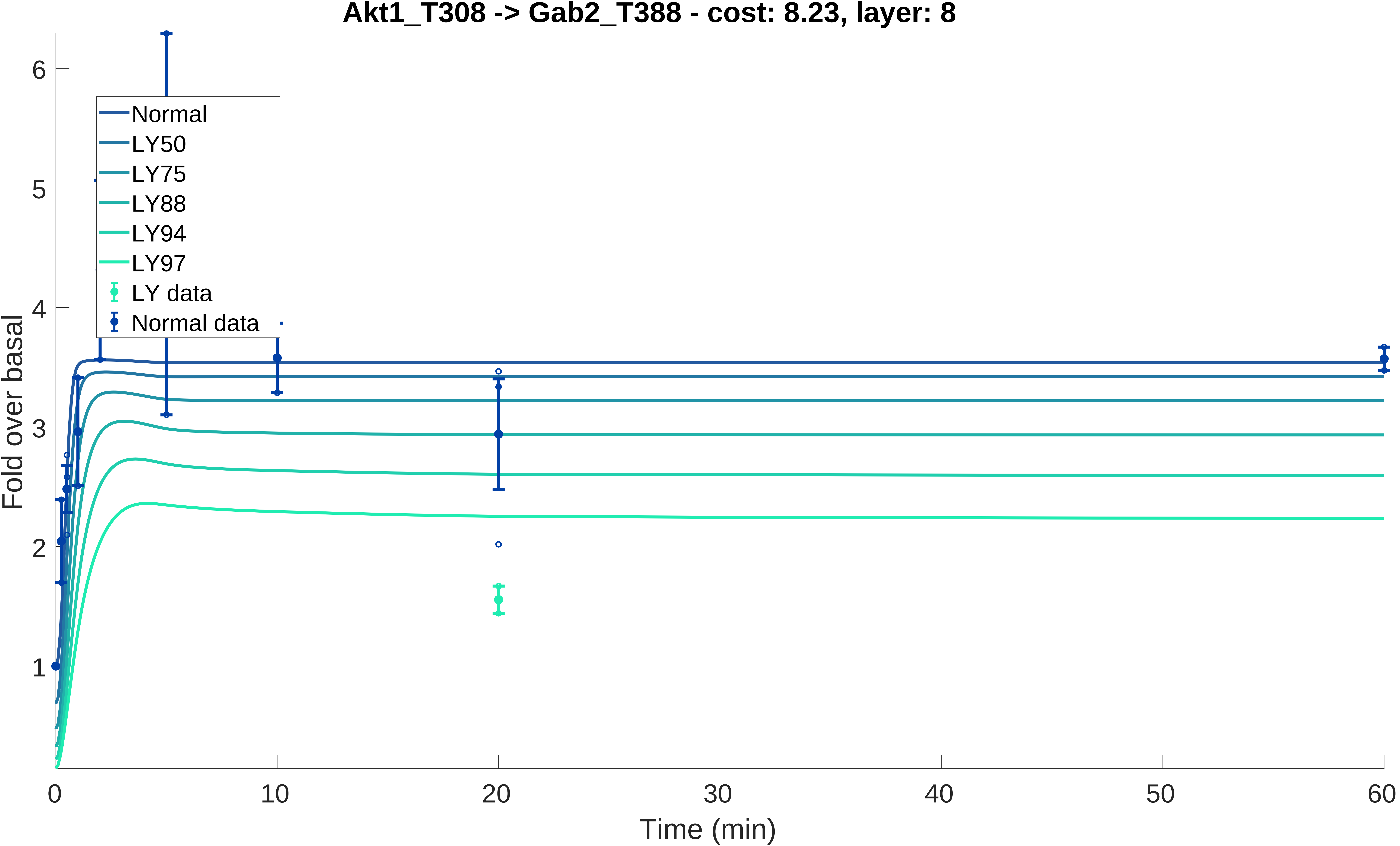

Akt1_T308 -> Gab2_T388 - cost: 8.23, layer: 8
Normal
LY50
LY75
LY88
LY94
LY97
LY data
Normal data
Fold over basal
1
2
3
4
5
6
0
10
20
30
40
50
60
Time (min)

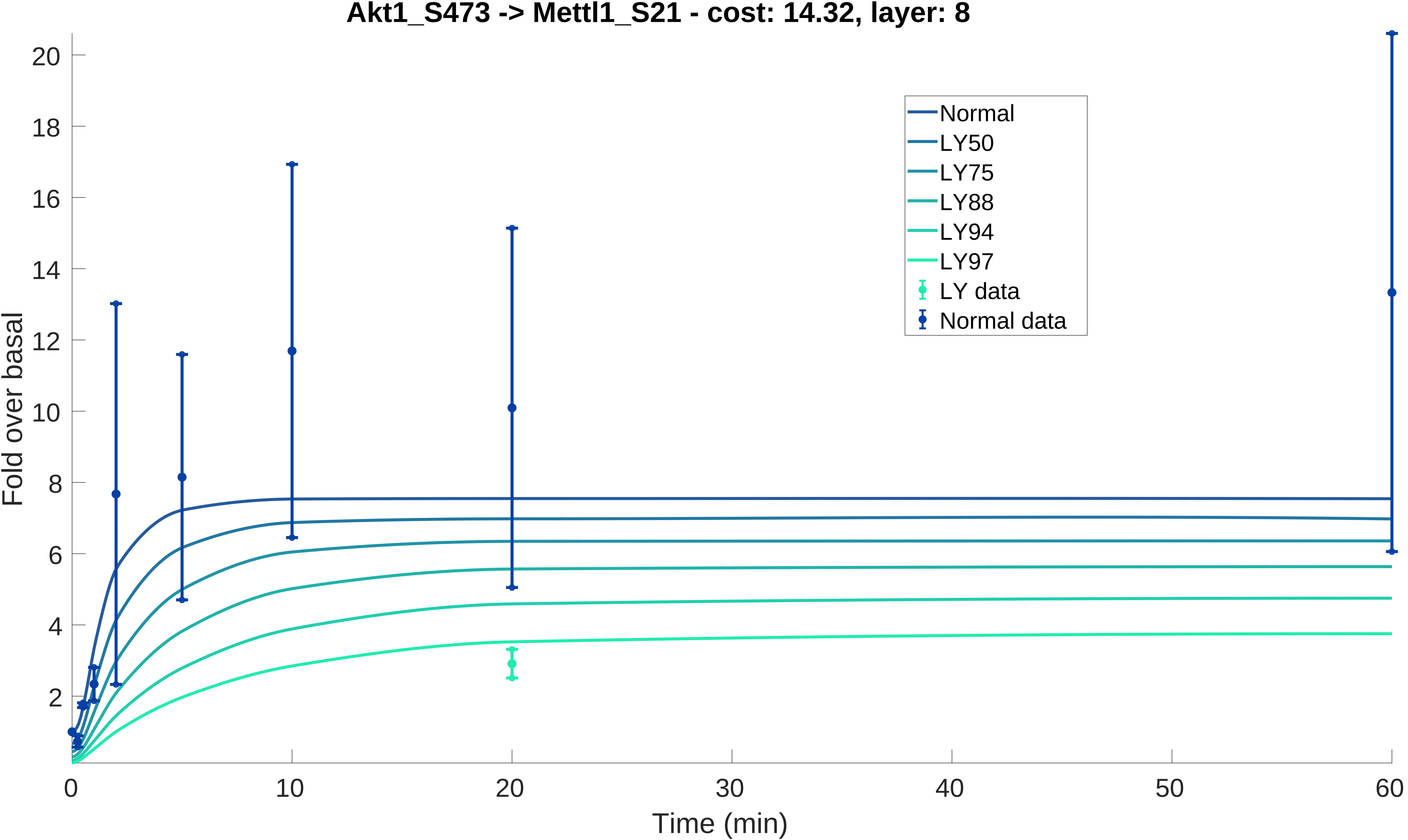

Akt1_S473 -> Mettl1_S21 - cost: 14.32, layer: 8
Fold over basal
Time (min)
Normal
LY50
LY75
LY88
LY94
LY97
LY data
Normal data
0
10
20
30
40
50
60
2
4
6
8
10
12
14
16
18
20

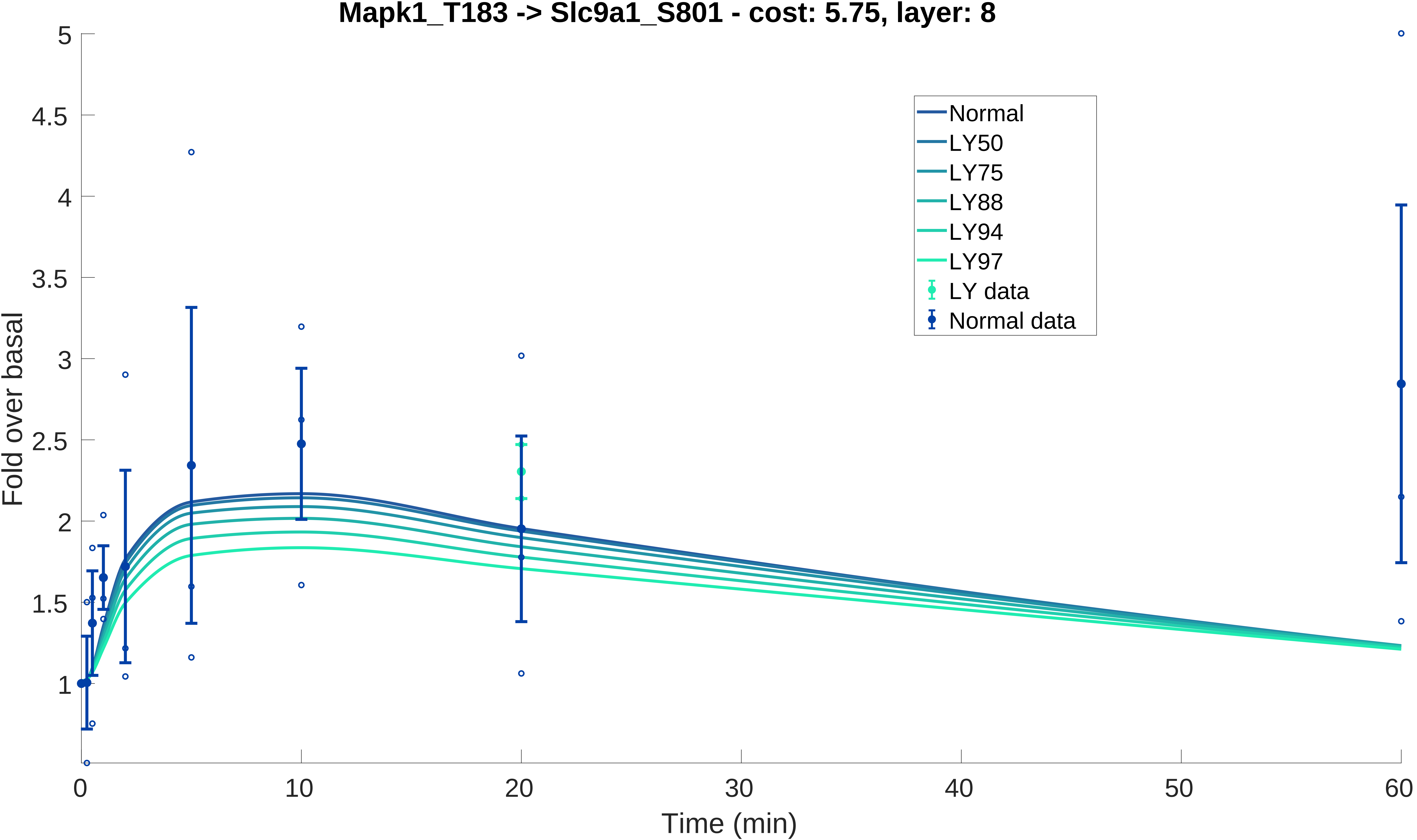

Mapk1_T183 -> Slc9a1_S801 - cost: 5.75, layer: 8
Fold over basal
Time (min)
0
10
20
30
40
50
60
1
1.5
2
2.5
3
3.5
4
4.5
5
Normal
LY50
LY75
LY88
LY94
LY97
LY data
Normal data

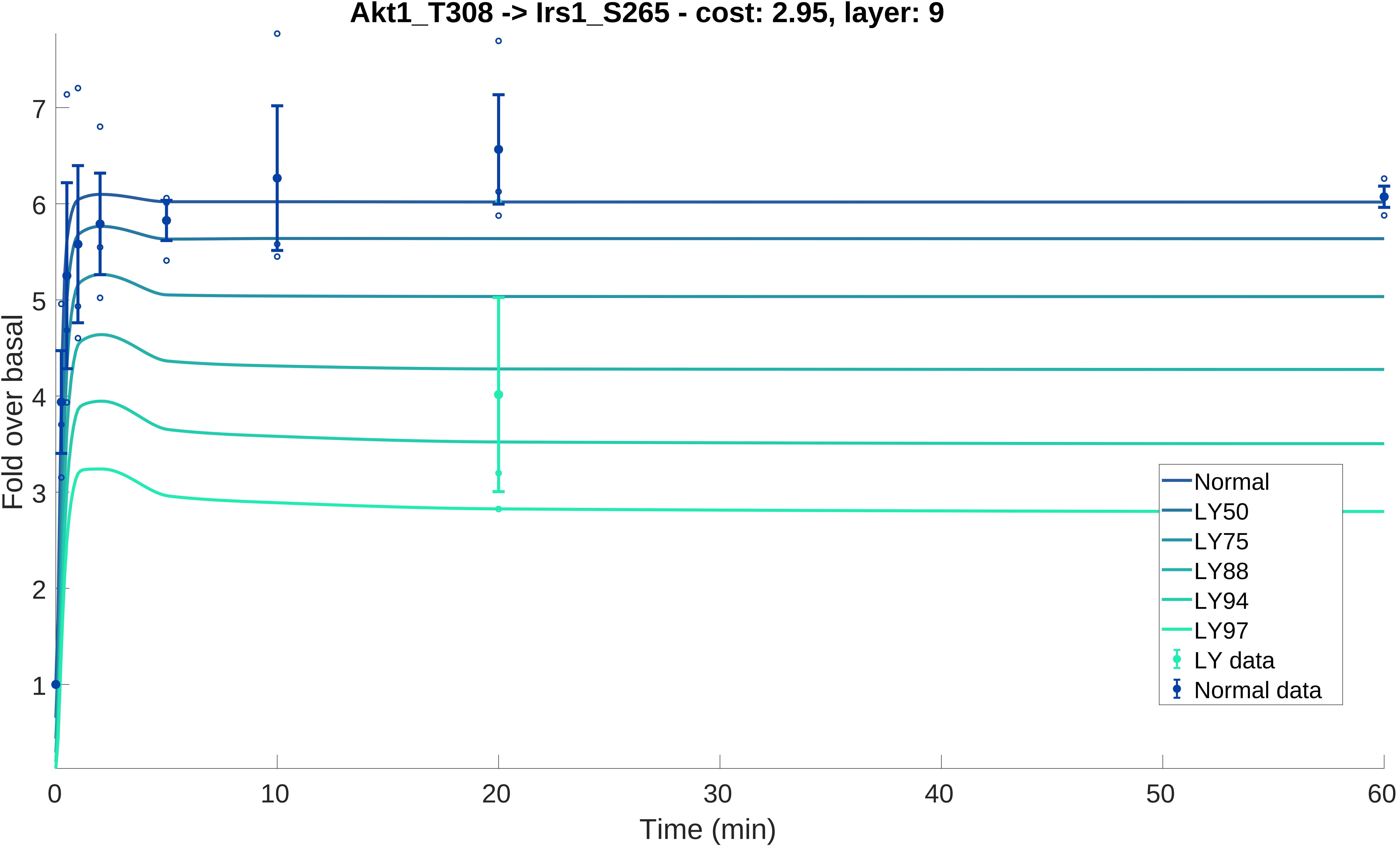

Akt1_T308 -> Irs1_S265 - cost: 2.95, layer: 9
Fold over basal
Time (min)
0
10
20
30
40
50
60
1
2
3
4
5
6
7
Normal
LY50
LY75
LY88
LY94
LY97
LY data
Normal data

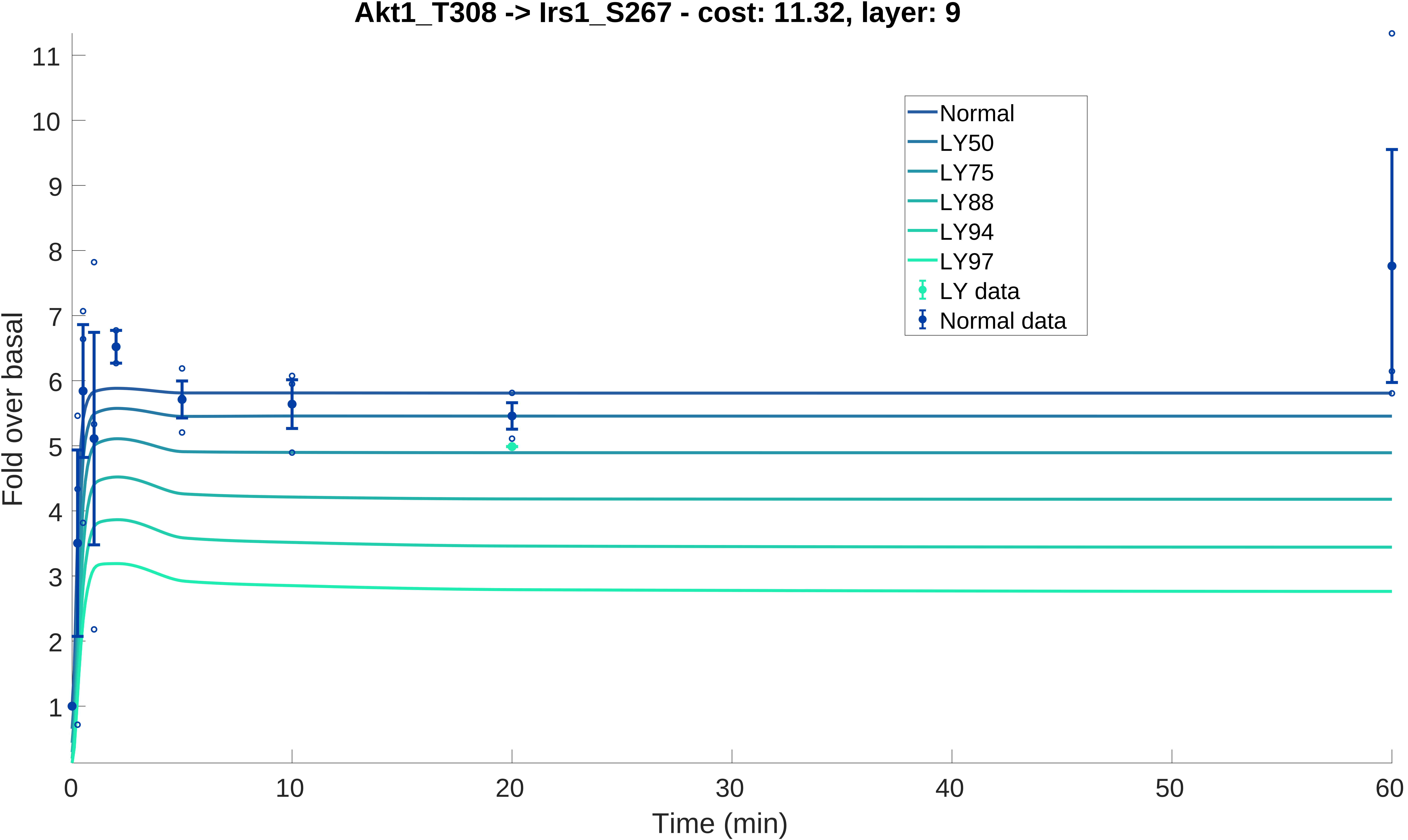
Akt1_T308 -> Irs1_S267 - cost: 11.32, layer: 9
Fold over basal
Time (min)
Normal
LY50
LY75
LY88
LY94
LY97
LY data
Normal data
0
10
20
30
40
50
60
1
2
3
4
5
6
7
8
9
10
11

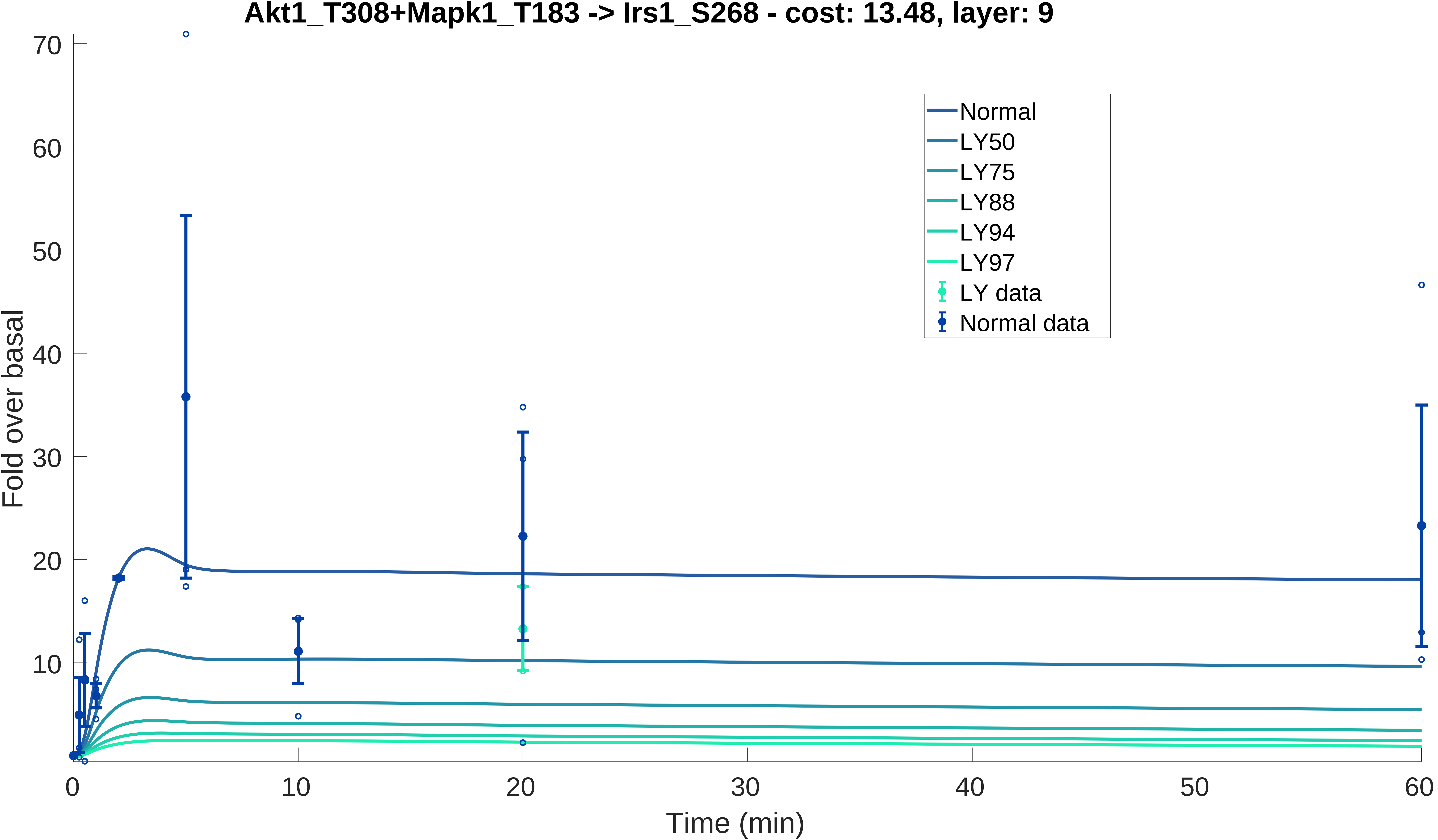

Akt1_T308+Mapk1_T183 -> Irs1_S268 - cost: 13.48, layer: 9
Fold over basal
Time (min)
0
10
20
30
40
50
60
70
Normal
LY50
LY75
LY88
LY94
LY97
LY data
Normal data

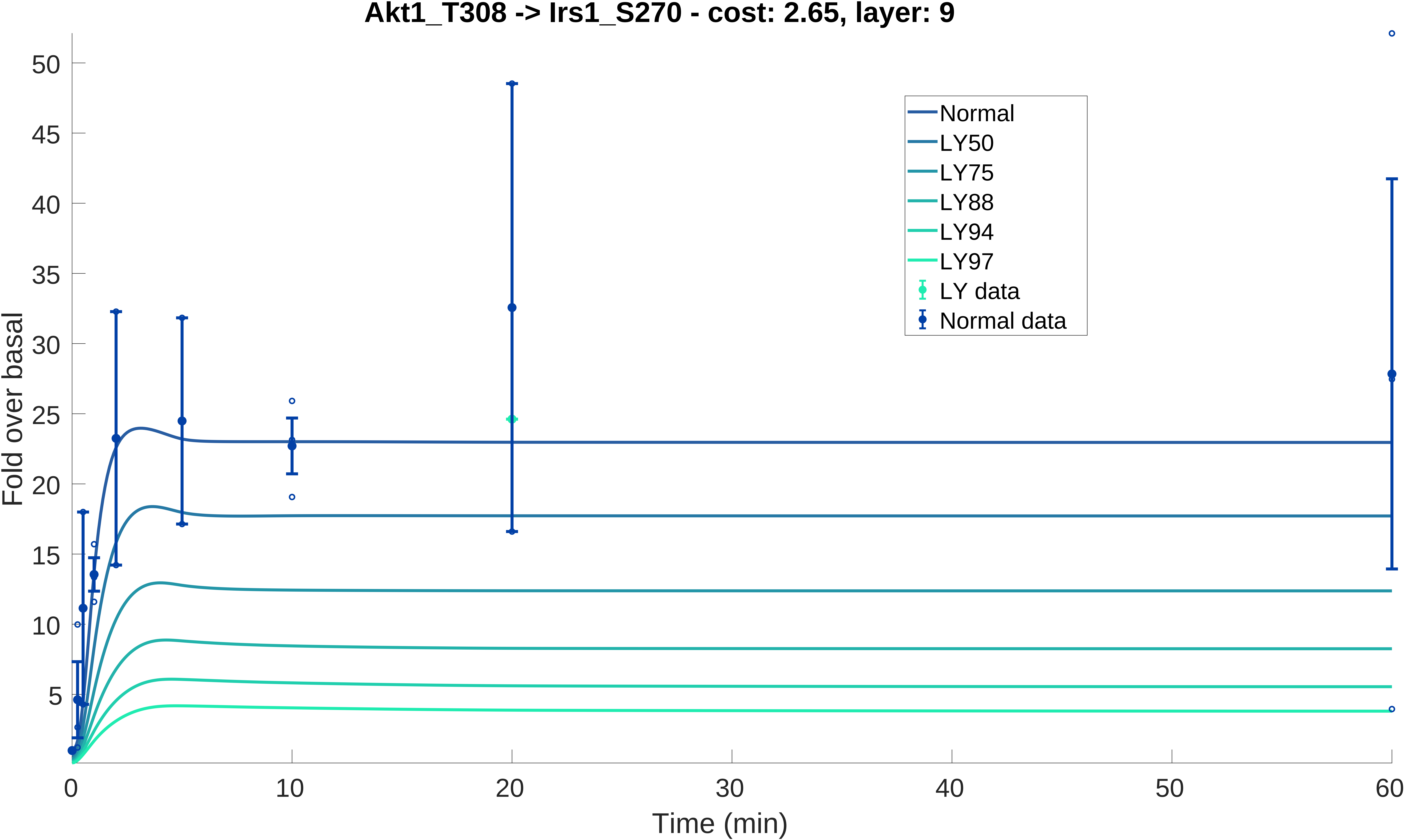
Akt1_T308 -> Irs1_S270 - cost: 2.65, layer: 9
Fold over basal
Time (min)
0
10
20
30
40
50
60
5
10
15
20
25
30
35
40
45
50
Normal
LY50
LY75
LY88
LY94
LY97
LY data
Normal data

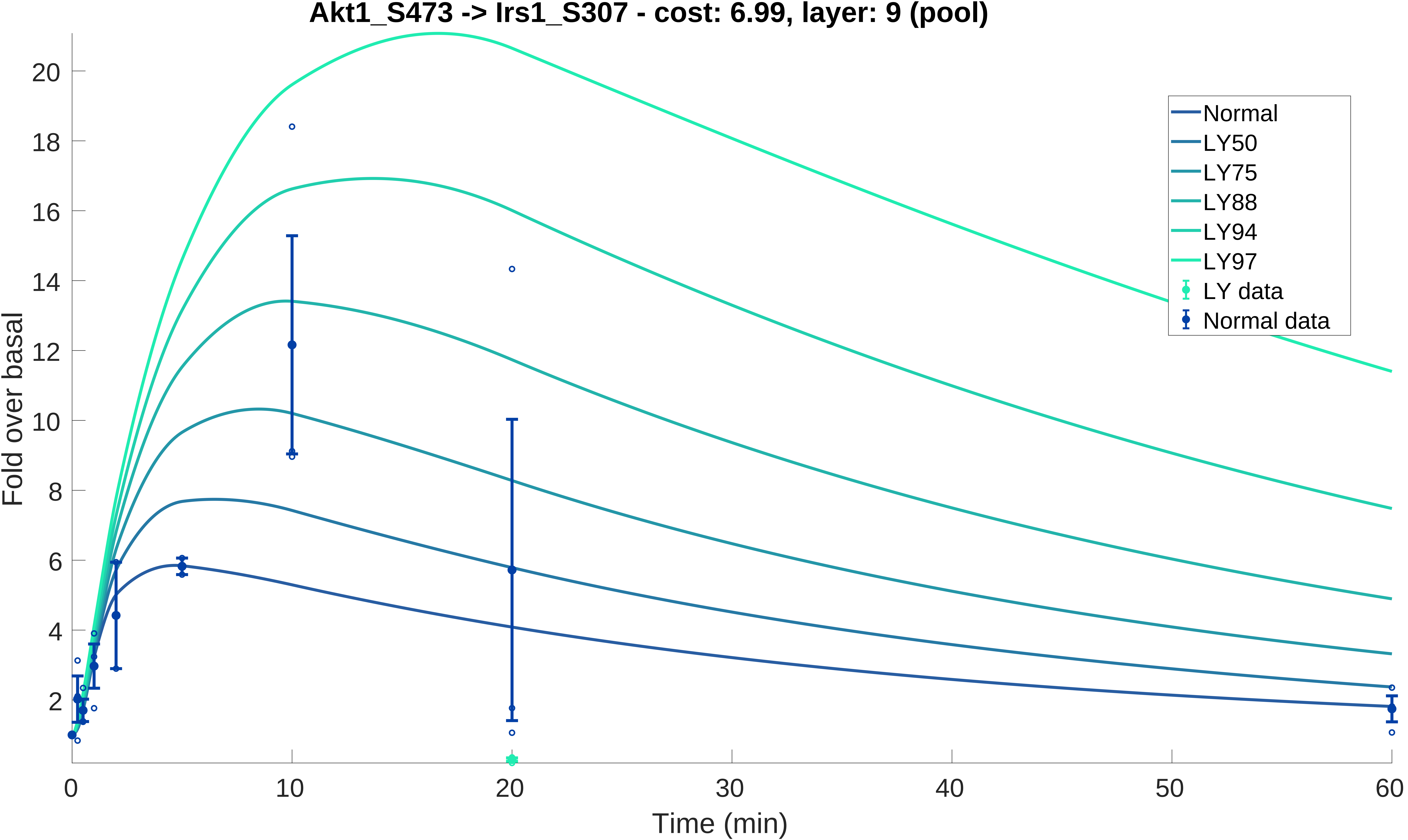

Akt1_S473 -> Irs1_S307 - cost: 6.99, layer: 9 (pool)
Fold over basal
Time (min)
0
10
20
30
40
50
60
2
4
6
8
10
12
14
16
18
20
Normal
LY50
LY75
LY88
LY94
LY97
LY data
Normal data

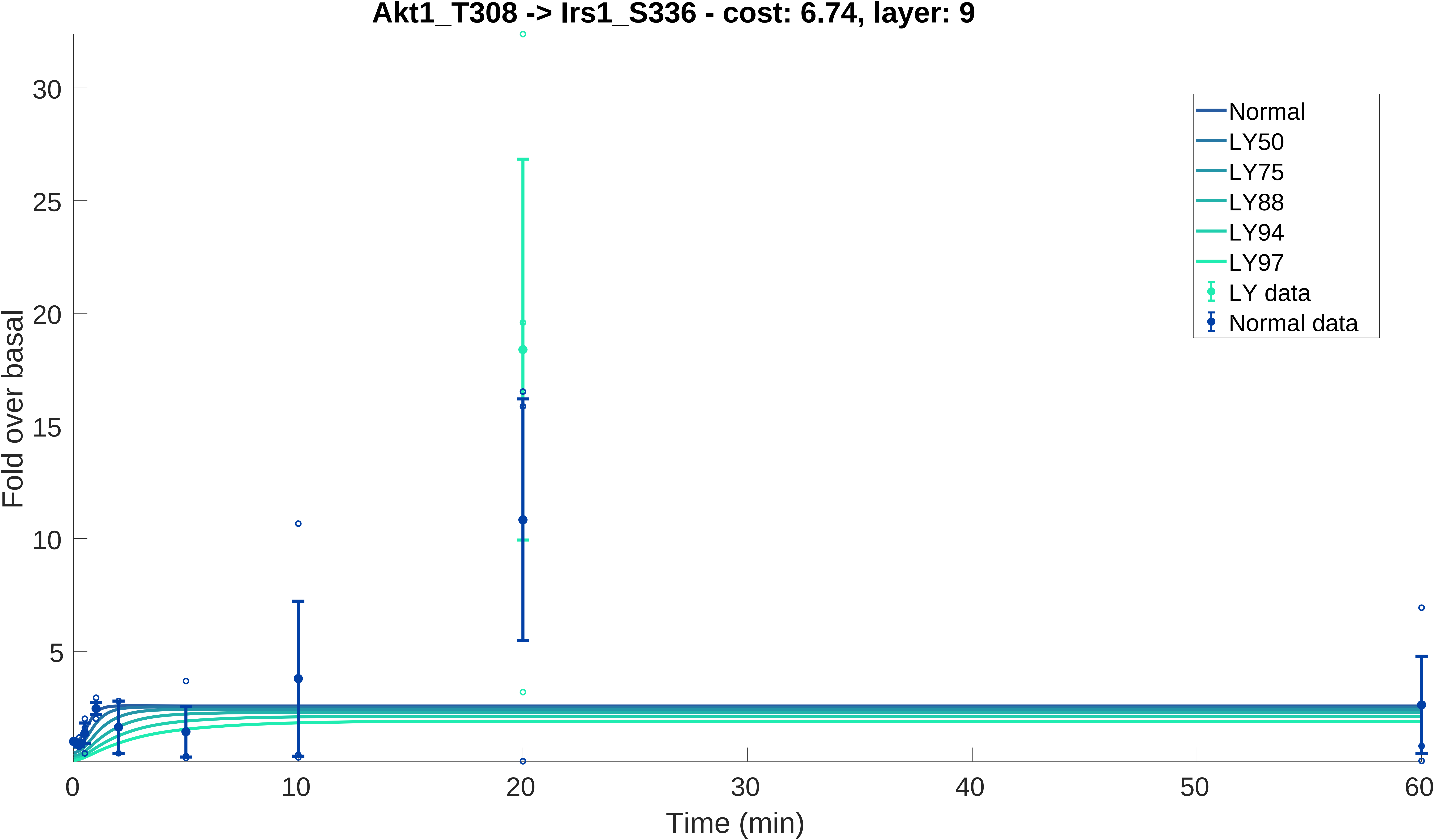
Akt1_T308 -> Irs1_S336 - cost: 6.74, layer: 9
Fold over basal
Time (min)
0
10
20
30
40
50
60
5
10
15
20
25
30
Normal
LY50
LY75
LY88
LY94
LY97
LY data
Normal data

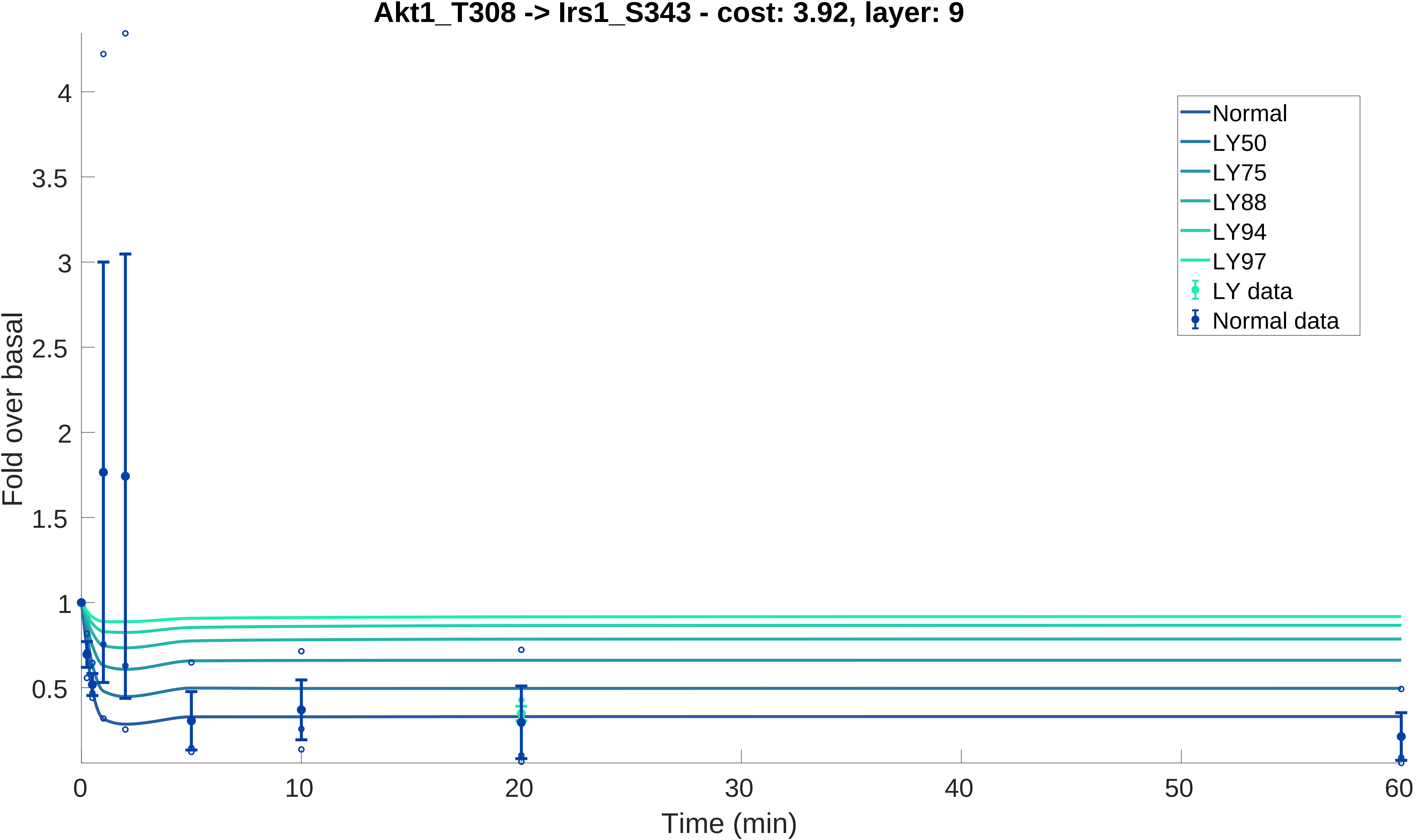

Akt1_T308 -> Irs1_S343 - cost: 3.92, layer: 9
Fold over basal
Time (min)
0
10
20
30
40
50
60
0.5
1
1.5
2
2.5
3
3.5
4
Normal
LY50
LY75
LY88
LY94
LY97
LY data
Normal data

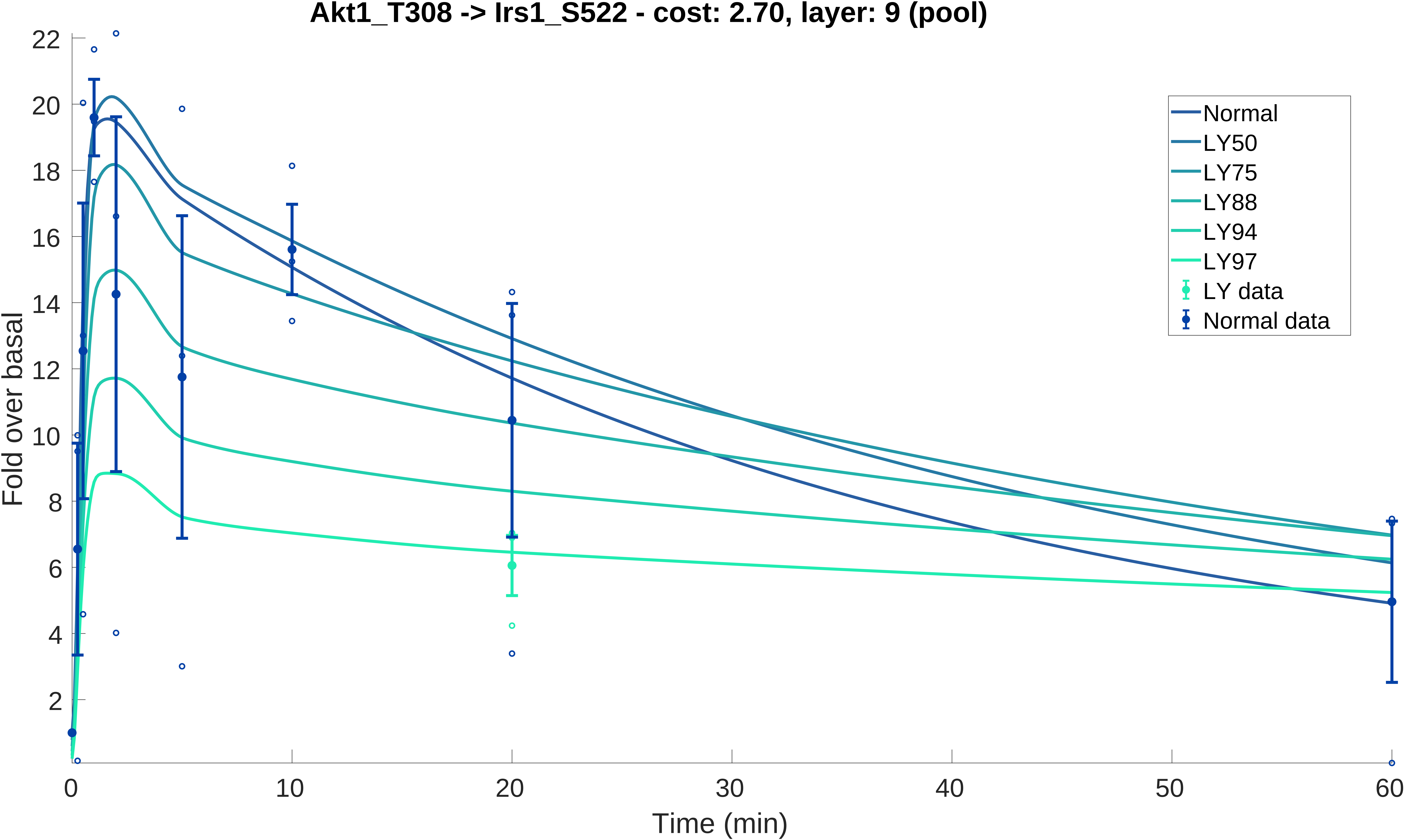

Akt1_T308 -> Irs1_S522 - cost: 2.70, layer: 9 (pool)
Fold over basal
Time (min)
0
10
20
30
40
50
60
2
4
6
8
10
12
14
16
18
20
22
Normal
LY50
LY75
LY88
LY94
LY97
LY data
Normal data

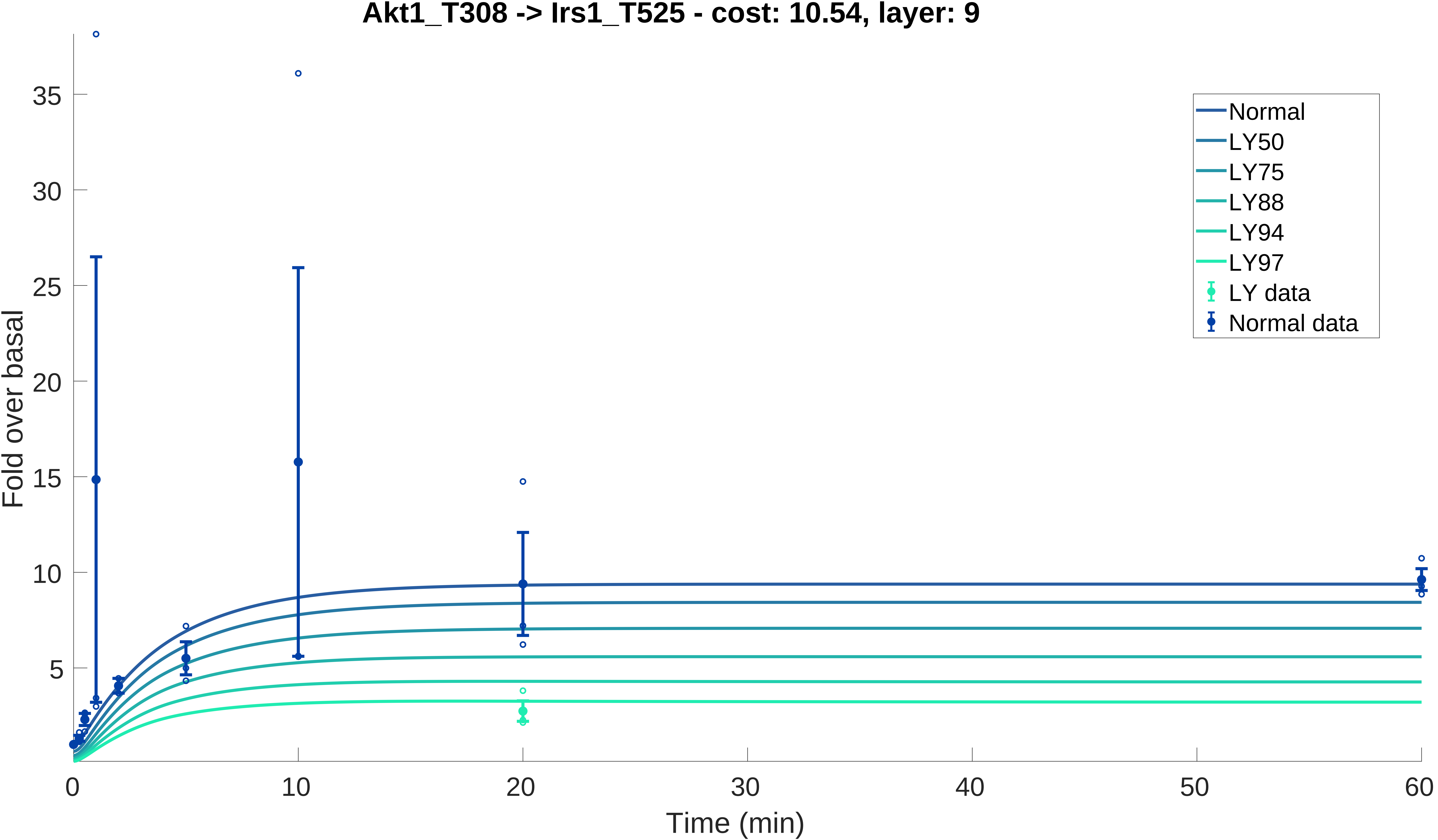
Akt1_T308 -> Irs1_T525 - cost: 10.54, layer: 9
Fold over basal
Time (min)
0
10
20
30
40
50
60
5
10
15
20
25
30
35
Normal
LY50
LY75
LY88
LY94
LY97
LY data
Normal data

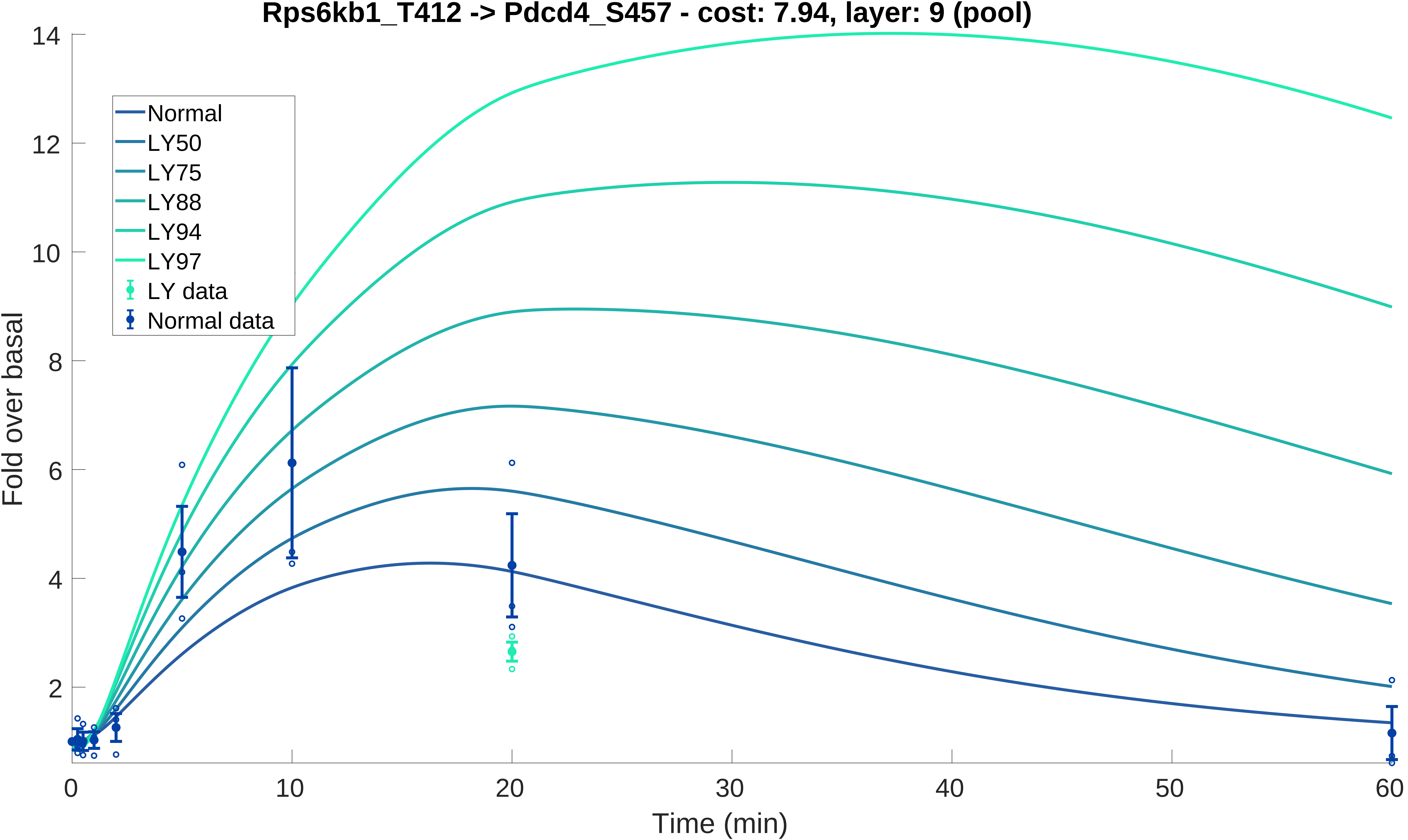

Rps6kb1_T412 -> Pdcd4_S457 - cost: 7.94, layer: 9 (pool)
Normal
LY50
LY75
LY88
LY94
LY97
LY data
Normal data
Fold over basal
Time (min)
0
10
20
30
40
50
60
2
4
6
8
10
12
14

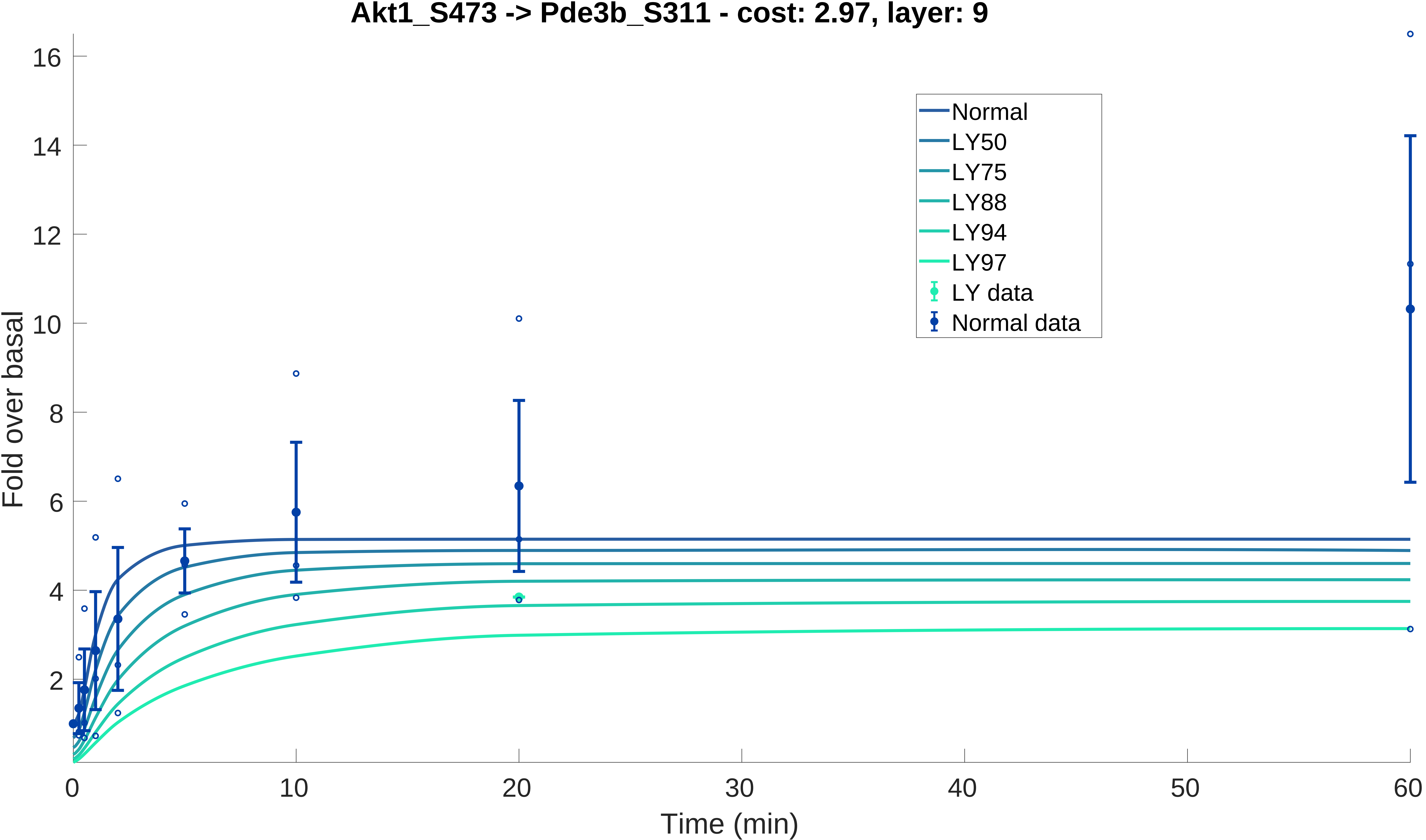
Akt1_S473 -> Pde3b_S311 - cost: 2.97, layer: 9
Fold over basal
Time (min)
Normal
LY50
LY75
LY88
LY94
LY97
LY data
Normal data
0
10
20
30
40
50
60
2
4
6
8
10
12
14
16

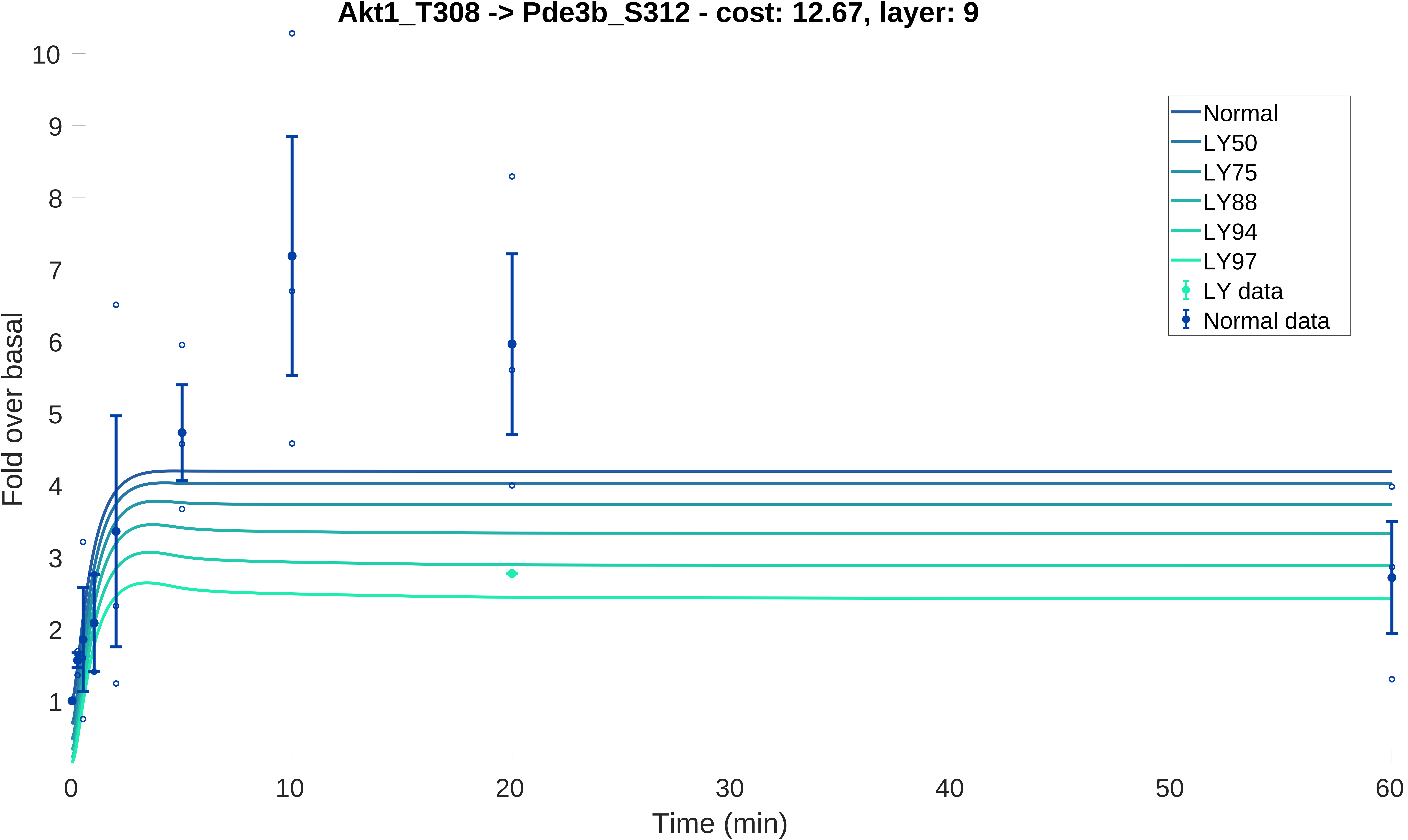
Akt1_T308 -> Pde3b_S312 - cost: 12.67, layer: 9
Fold over basal
Time (min)
Normal
LY50
LY75
LY88
LY94
LY97
LY data
Normal data
10
9
8
7
6
5
4
3
2
1
0
10
20
30
40
50
60

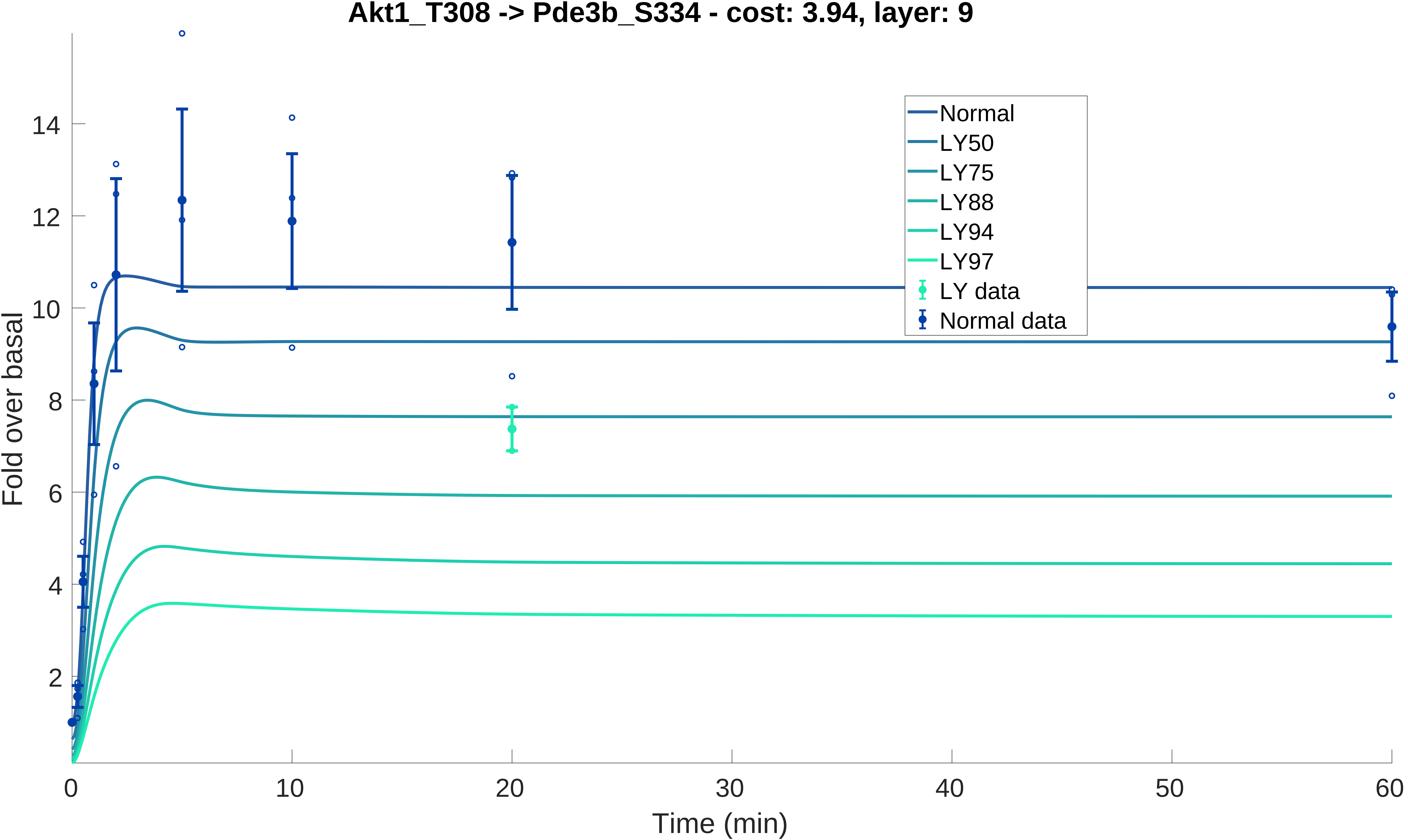
Akt1_T308 -> Pde3b_S334 - cost: 3.94, layer: 9
Normal
LY50
LY75
LY88
LY94
LY97
LY data
Normal data
Fold over basal
Time (min)
2
4
6
8
10
12
14
0
10
20
30
40
50
60

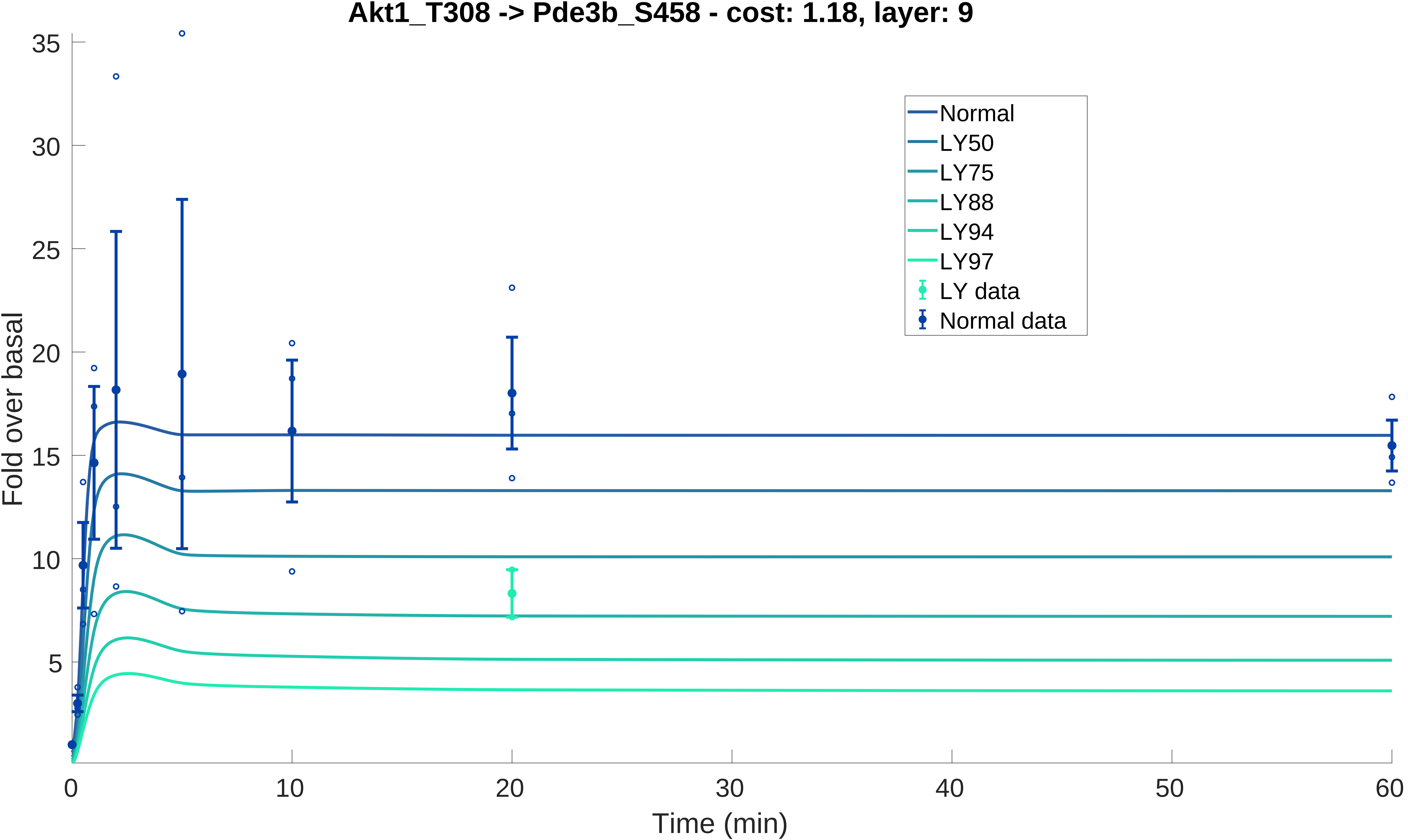

Akt1_T308 -> Pde3b_S458 - cost: 1.18, layer: 9
Fold over basal
Time (min)
0
10
20
30
40
50
60
5
10
15
20
25
30
35
Normal
LY50
LY75
LY88
LY94
LY97
LY data
Normal data

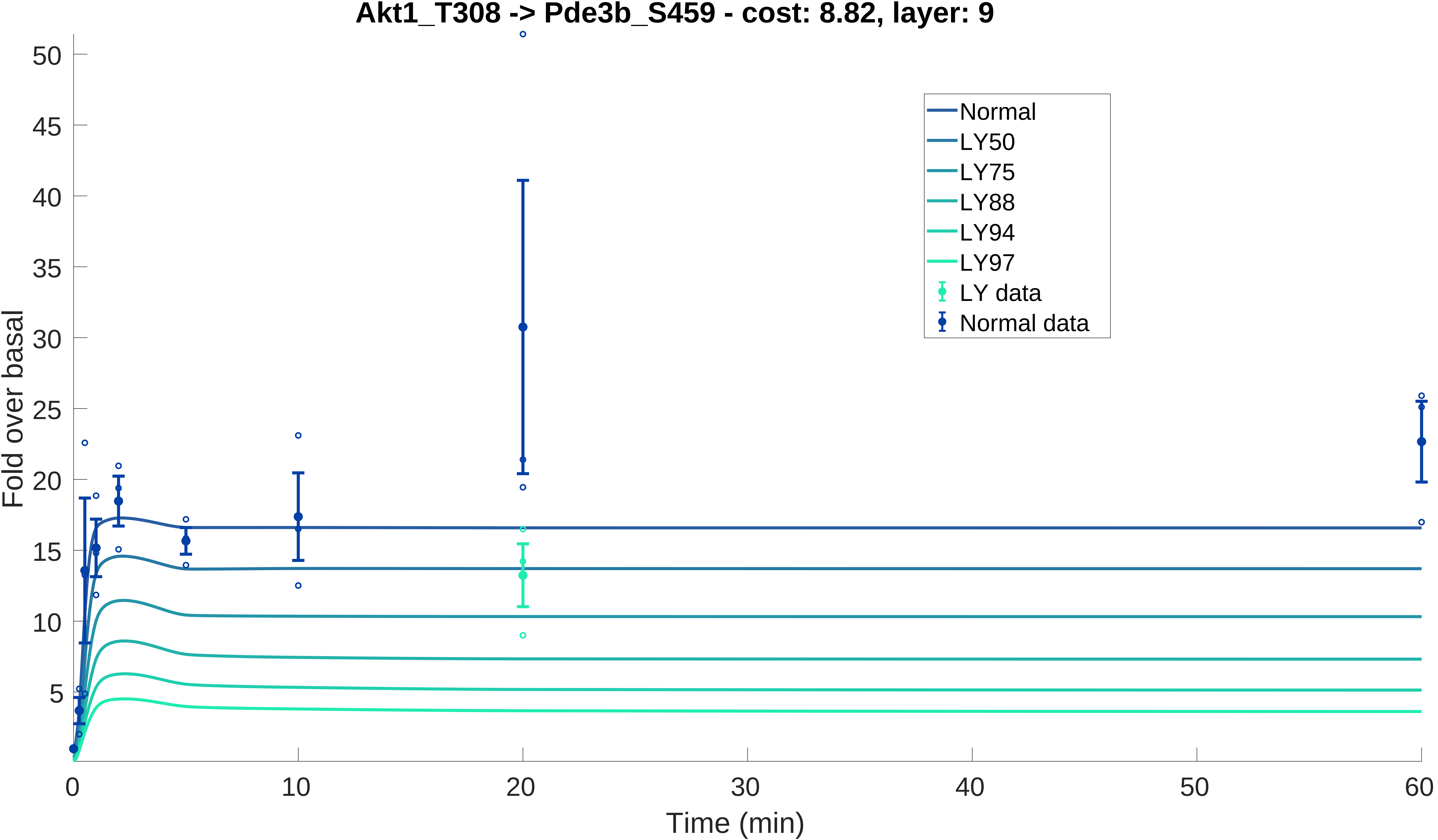

Akt1_T308 -> Pde3b_S459 - cost: 8.82, layer: 9
Fold over basal
Time (min)
0
10
20
30
40
50
60
5
10
15
20
25
30
35
40
45
50
Normal
LY50
LY75
LY88
LY94
LY97
LY data
Normal data

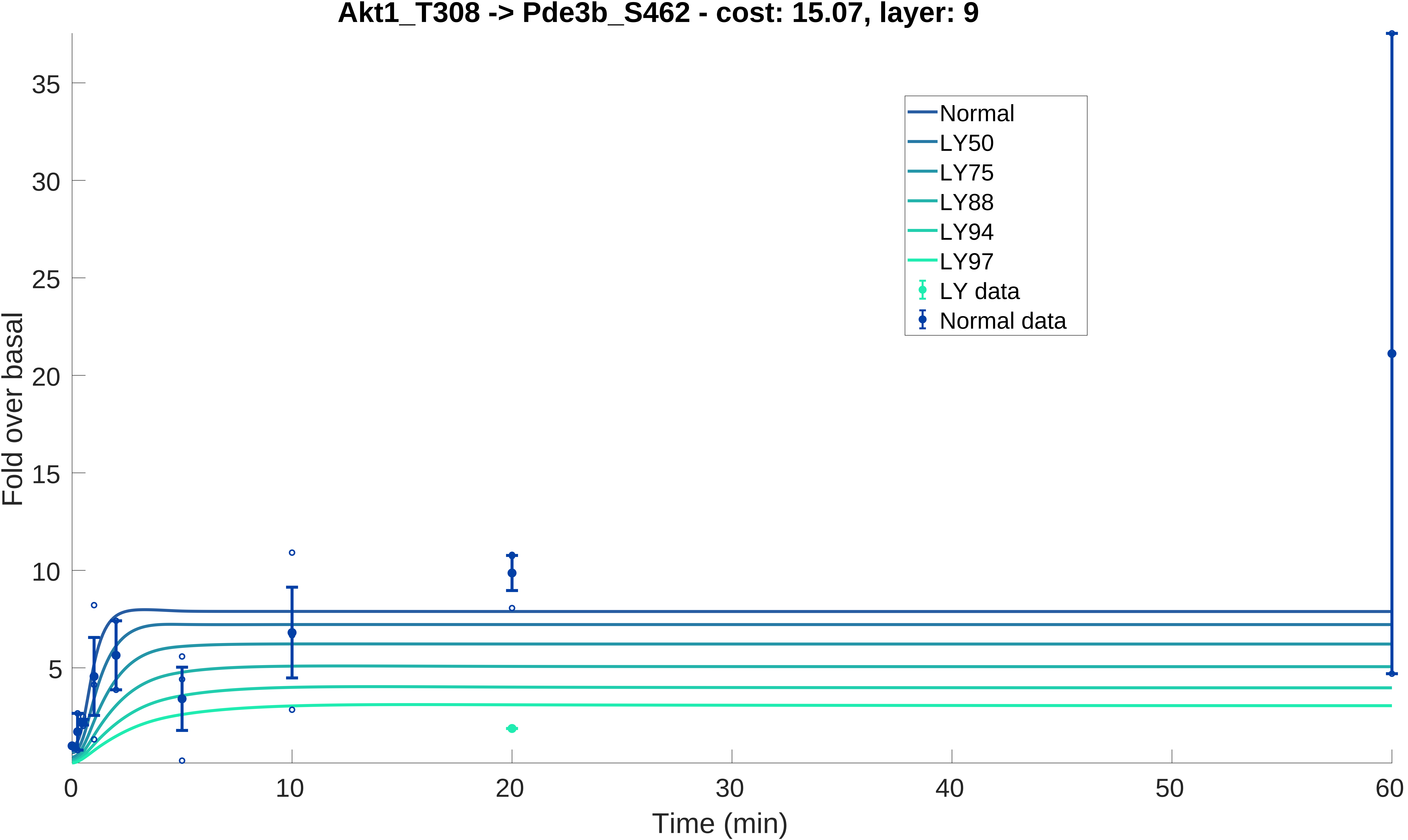
Akt1_T308 -> Pde3b_S462 - cost: 15.07, layer: 9
Fold over basal
Time (min)
0
5
10
15
20
25
30
35
40
50
60
Normal
LY50
LY75
LY88
LY94
LY97
LY data
Normal data

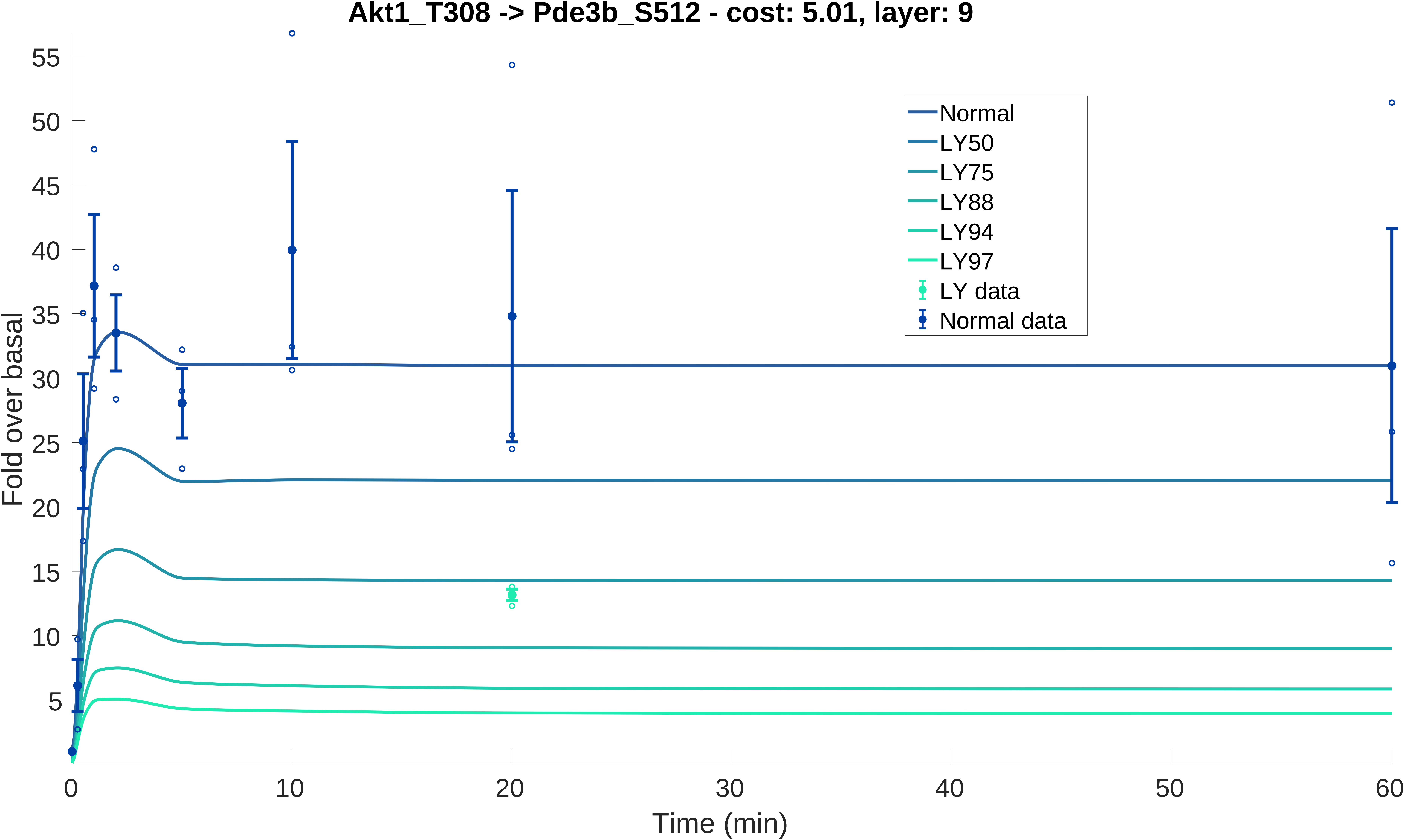
Akt1_T308 -> Pde3b_S512 - cost: 5.01, layer: 9
Fold over basal
Time (min)
Normal
LY50
LY75
LY88
LY94
LY97
LY data
Normal data
55
50
45
40
35
30
25
20
15
10
5
0
10
20
30
40
50
60

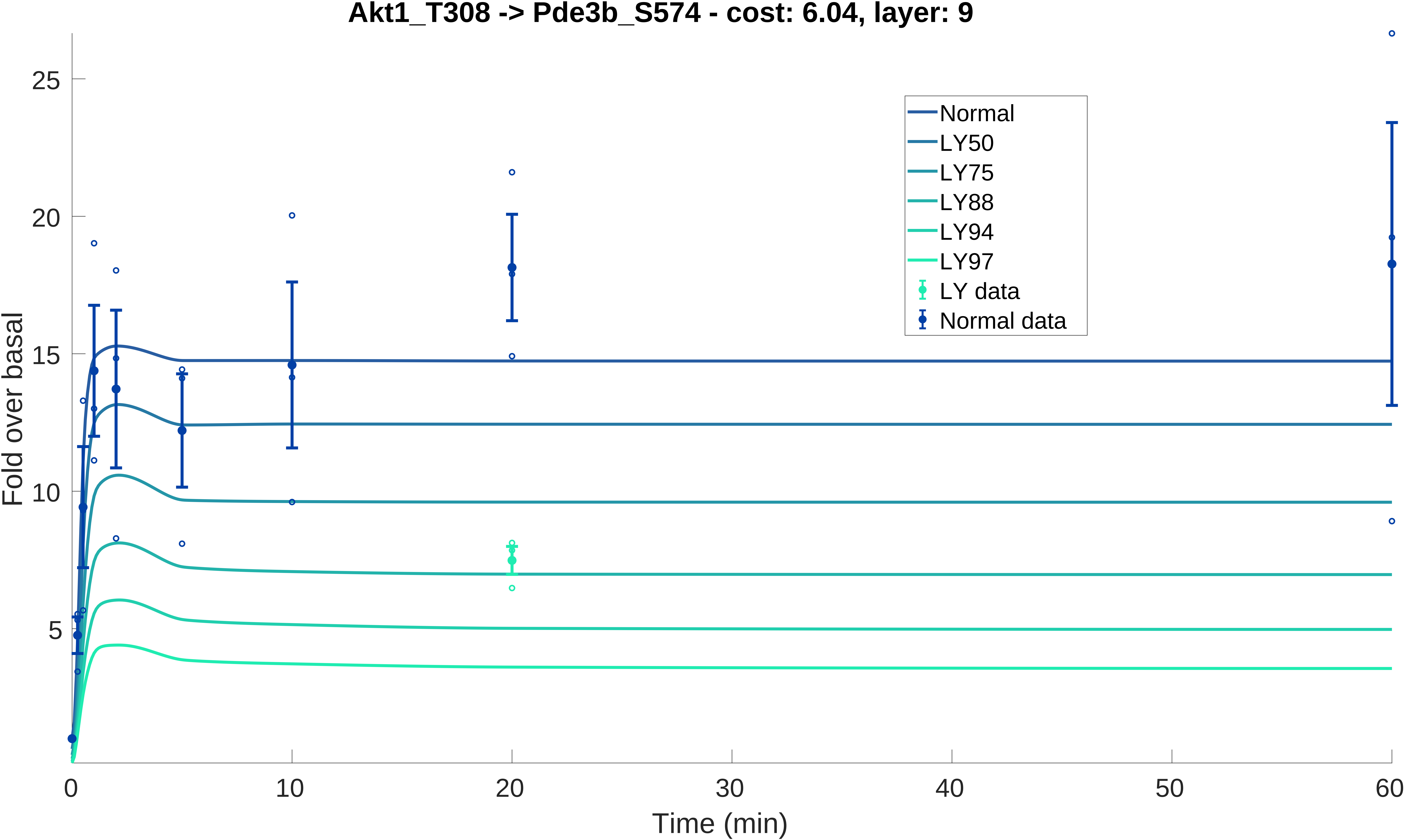
Akt1_T308 -> Pde3b_S574 - cost: 6.04, layer: 9
Fold over basal
Time (min)
Normal
LY50
LY75
LY88
LY94
LY97
LY data
Normal data
0
10
20
30
40
50
60
5
10
15
20
25

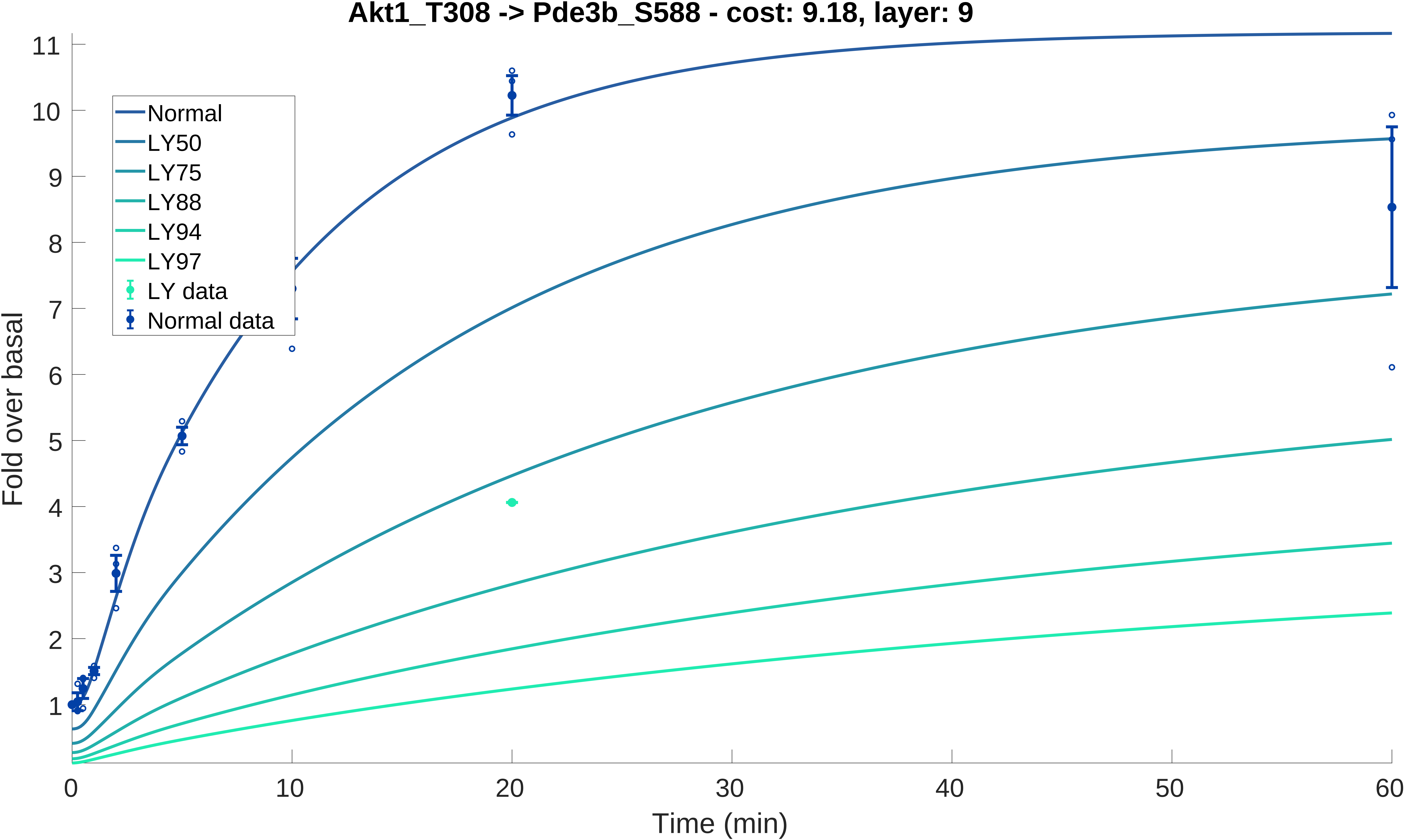

Akt1_T308 -> Pde3b_S588 - cost: 9.18, layer: 9
Normal
LY50
LY75
LY88
LY94
LY97
LY data
Normal data
Fold over basal
Time (min)
0
10
20
30
40
50
60
1
2
3
4
5
6
7
8
9
10
11

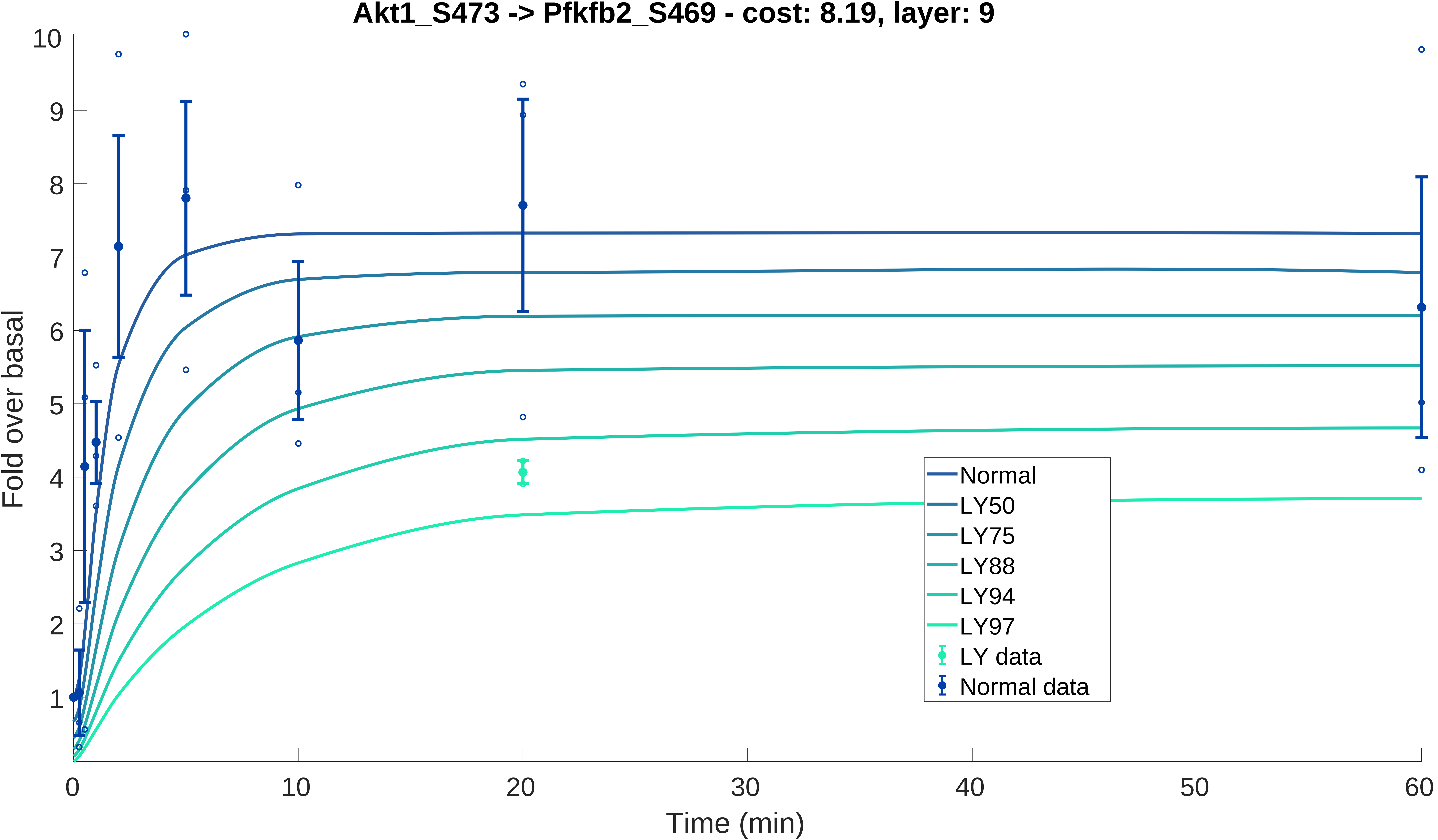

Akt1_S473 -> Pfkfb2_S469 - cost: 8.19, layer: 9
Fold over basal
Time (min)
0
10
20
30
40
50
60
1
2
3
4
5
6
7
8
9
10
Normal
LY50
LY75
LY88
LY94
LY97
LY data
Normal data

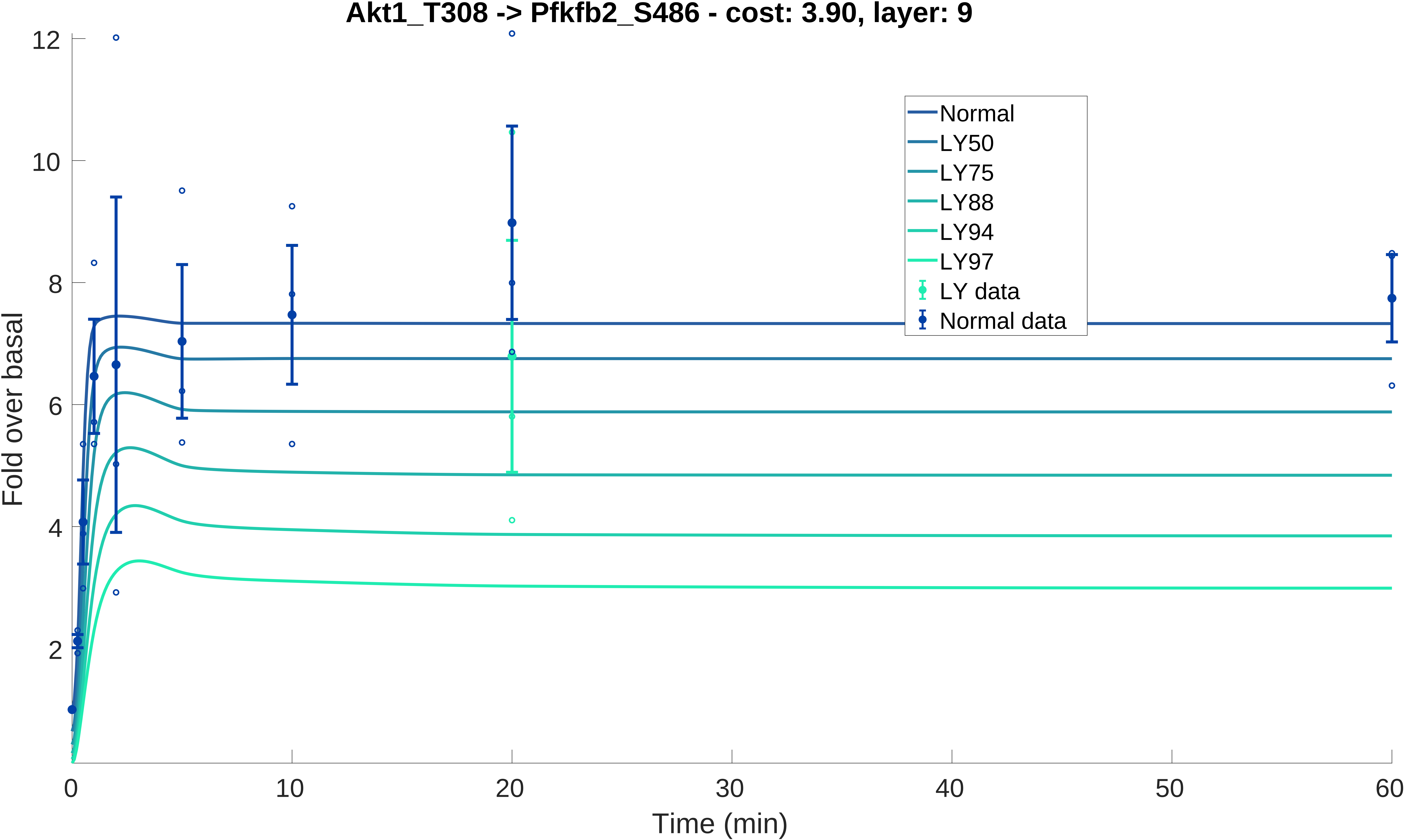
Akt1_T308 -> Pfkfb2_S486 - cost: 3.90, layer: 9
Fold over basal
Time (min)
0
10
20
30
40
50
60
2
4
6
8
10
12
Normal
LY50
LY75
LY88
LY94
LY97
LY data
Normal data

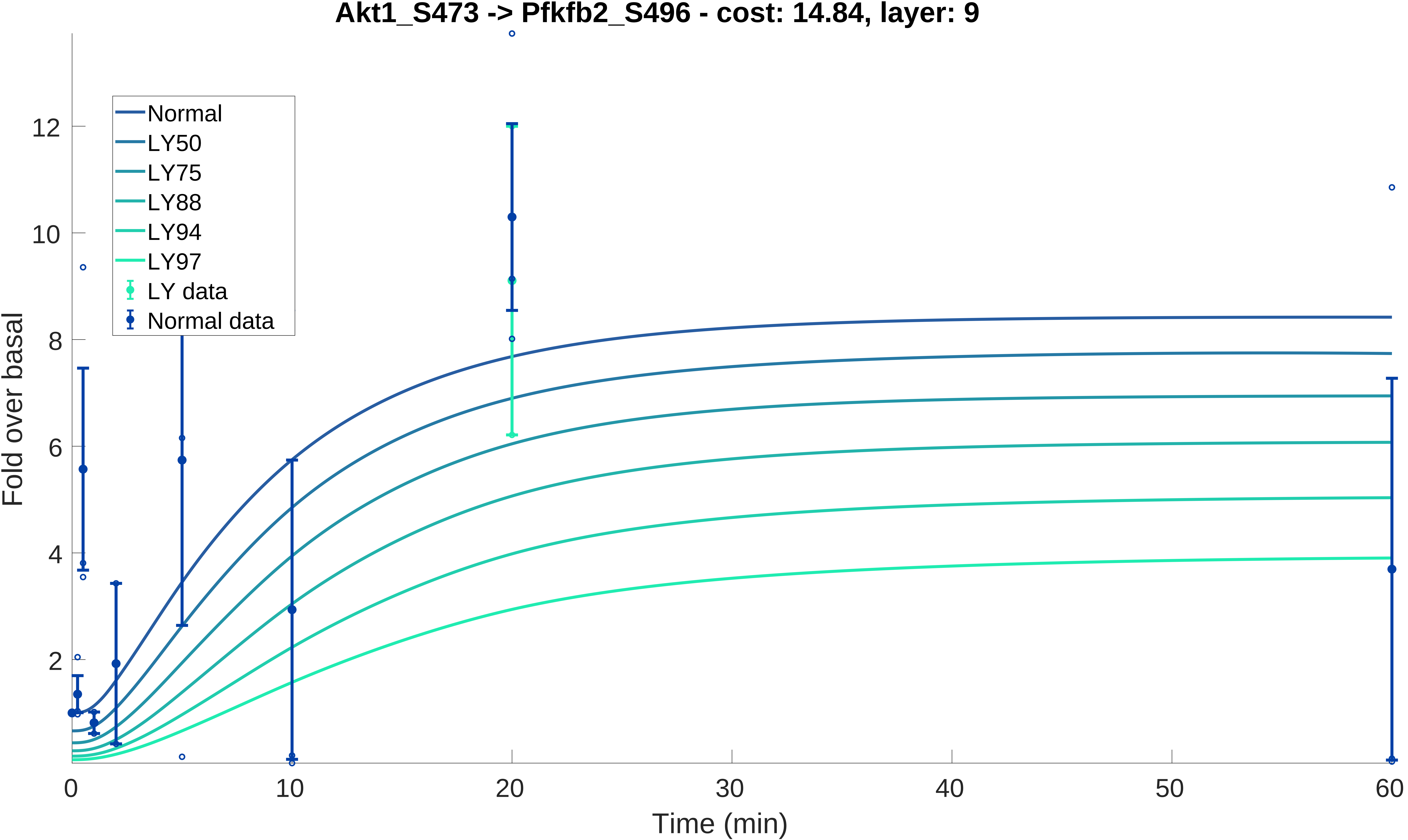

Akt1_S473 -> Pfkfb2_S496 - cost: 14.84, layer: 9
Normal
LY50
LY75
LY88
LY94
LY97
LY data
Normal data
Fold over basal
Time (min)
0
2
4
6
8
10
12
0
10
20
30
40
50
60

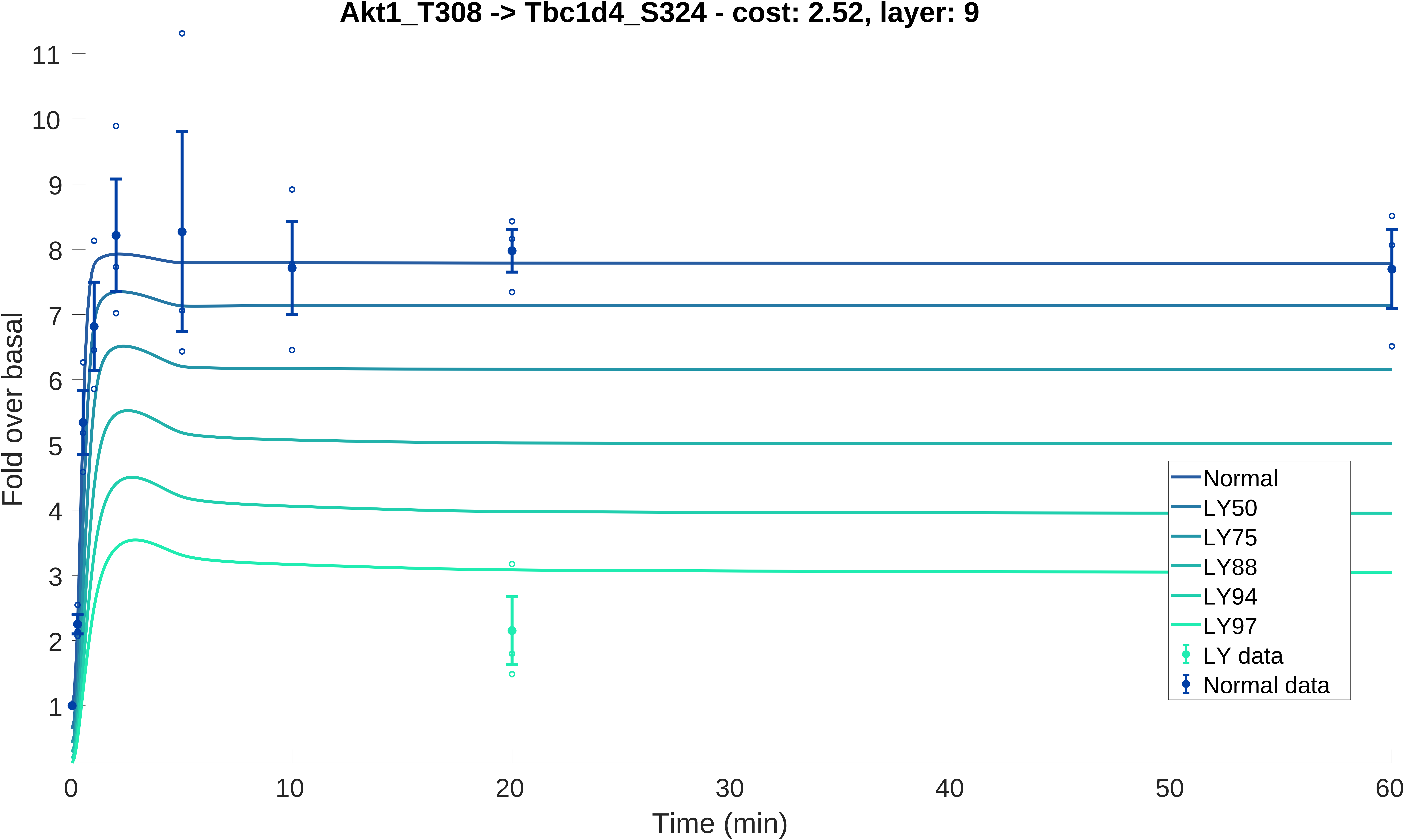

Akt1_T308 -> Tbc1d4_S324 - cost: 2.52, layer: 9
Fold over basal
Time (min)
1
2
3
4
5
6
7
8
9
10
11
0
10
20
30
40
50
60
Normal
LY50
LY75
LY88
LY94
LY97
LY data
Normal data

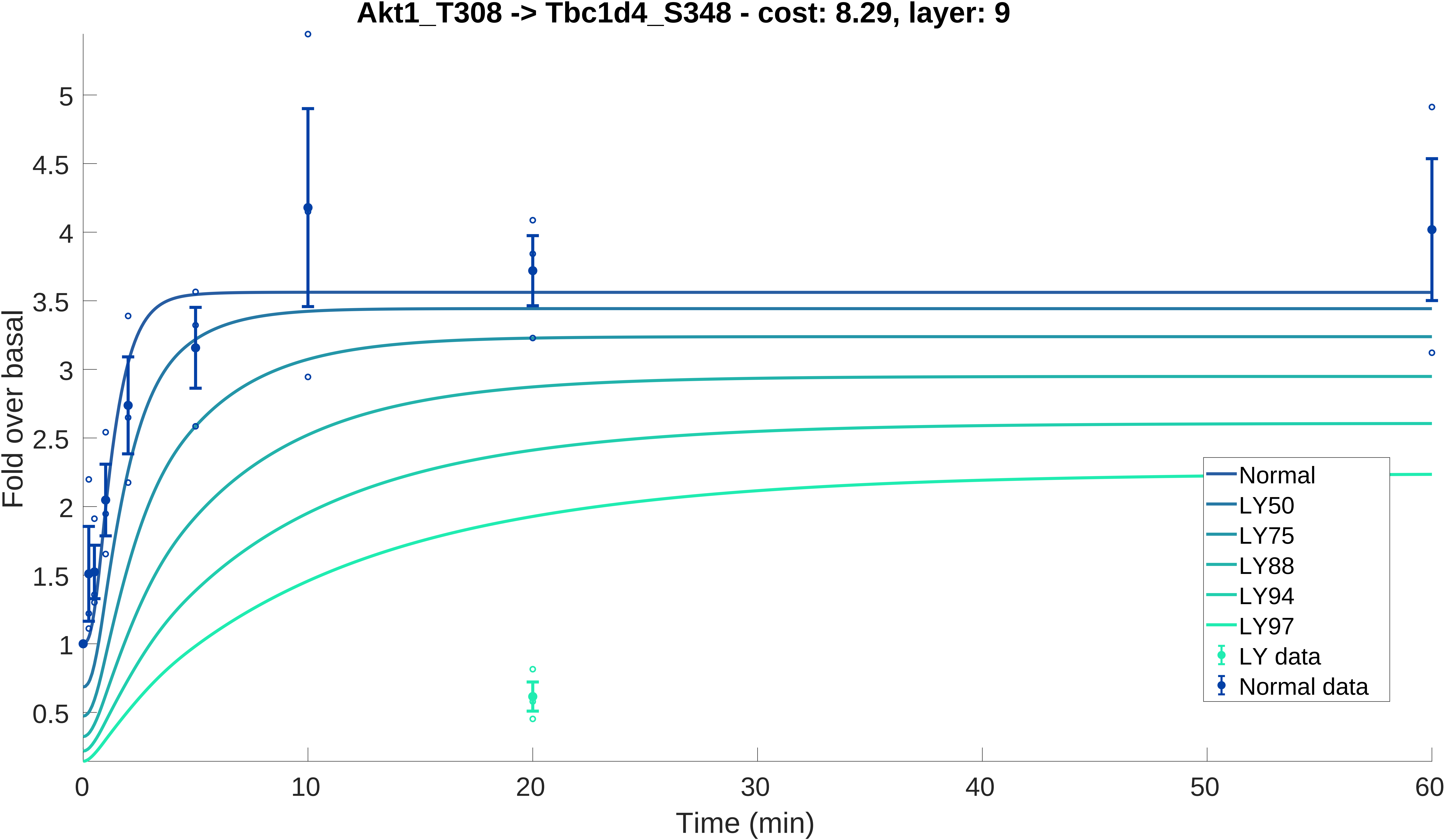

Akt1_T308 -> Tbc1d4_S348 - cost: 8.29, layer: 9
Fold over basal
Time (min)
0.5
1
1.5
2
2.5
3
3.5
4
4.5
5
0
10
20
30
40
50
60
Normal
LY50
LY75
LY88
LY94
LY97
LY data
Normal data

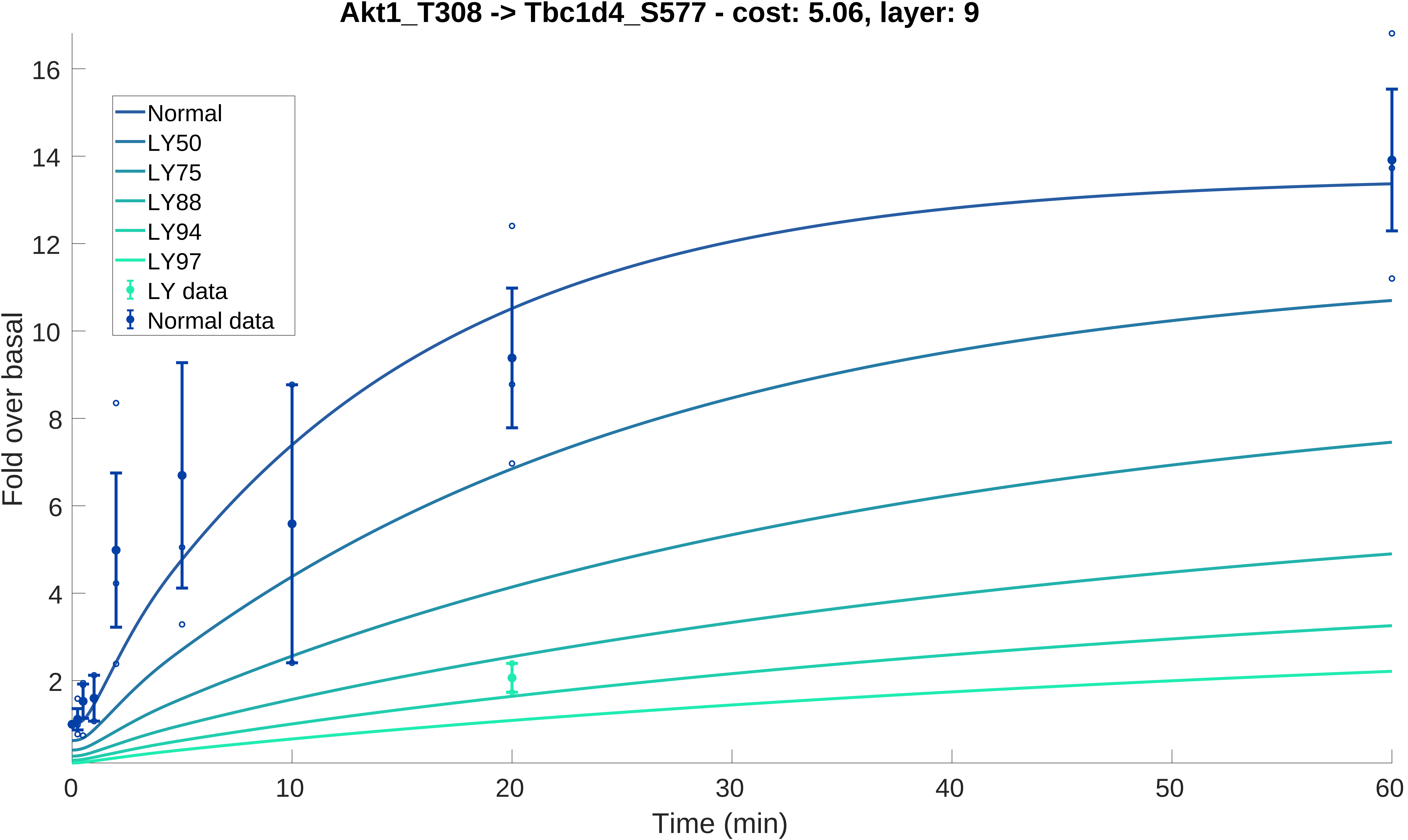

Akt1_T308 -> Tbc1d4_S577 - cost: 5.06, layer: 9
Normal
LY50
LY75
LY88
LY94
LY97
LY data
Normal data
Fold over basal
Time (min)
0
10
20
30
40
50
60
2
4
6
8
10
12
14
16

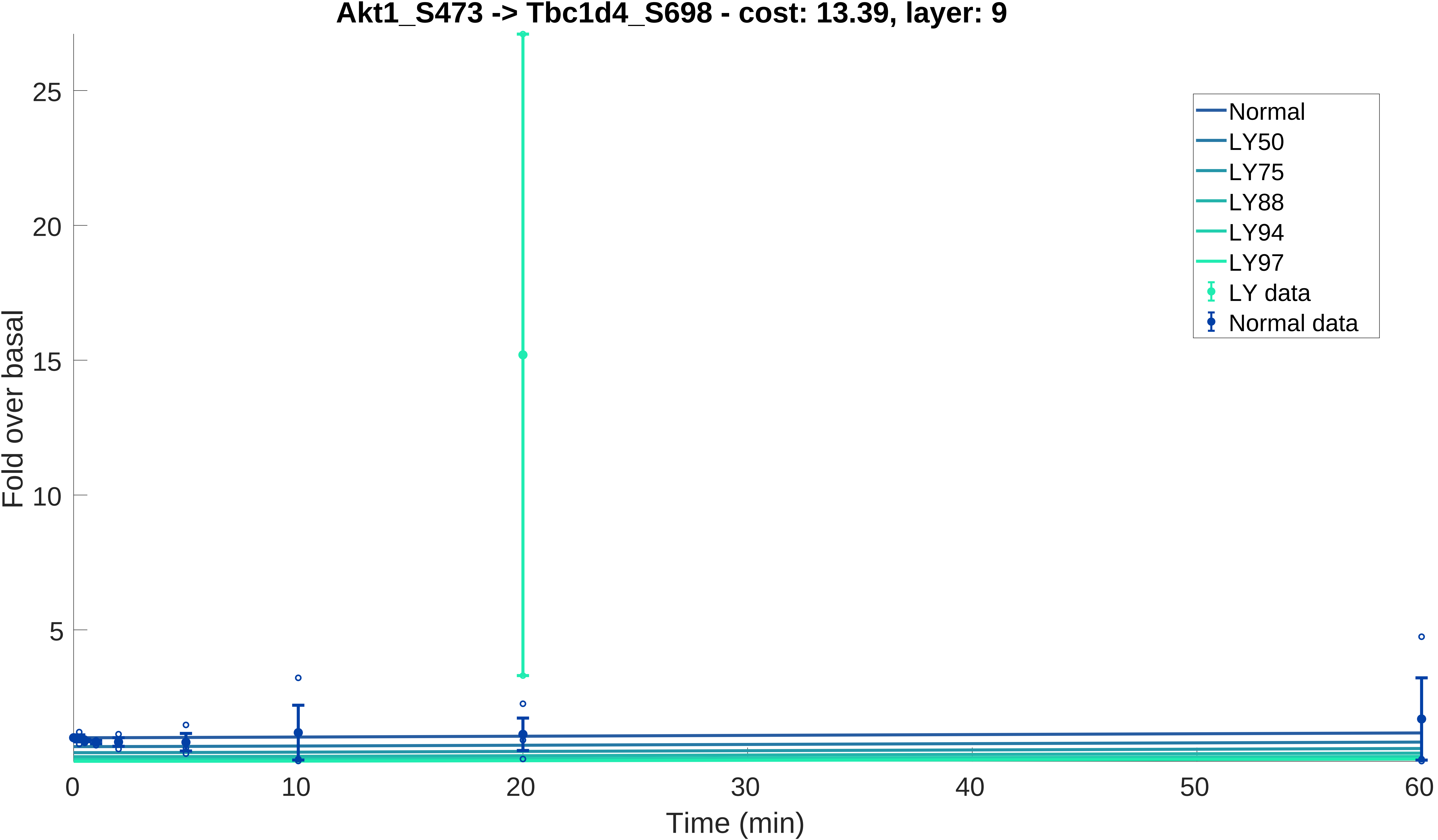
Akt1_S473 -> Tbc1d4_S698 - cost: 13.39, layer: 9
Fold over basal
Time (min)
0
10
20
30
40
50
60
5
10
15
20
25
Normal
LY50
LY75
LY88
LY94
LY97
LY data
Normal data

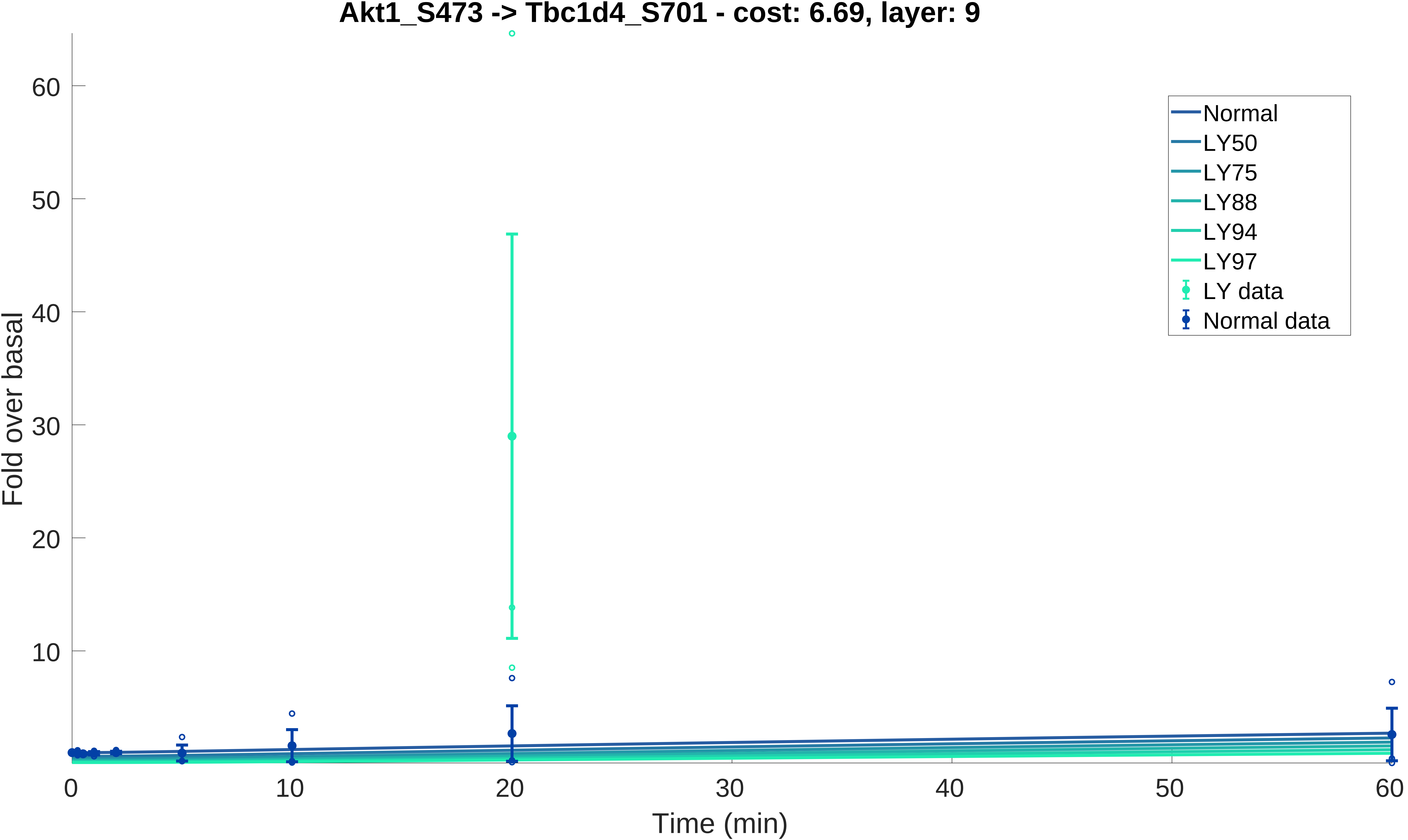

Akt1_S473 -> Tbc1d4_S701 - cost: 6.69, layer: 9
Fold over basal
Time (min)
0
10
20
30
40
50
60
Normal
LY50
LY75
LY88
LY94
LY97
LY data
Normal data

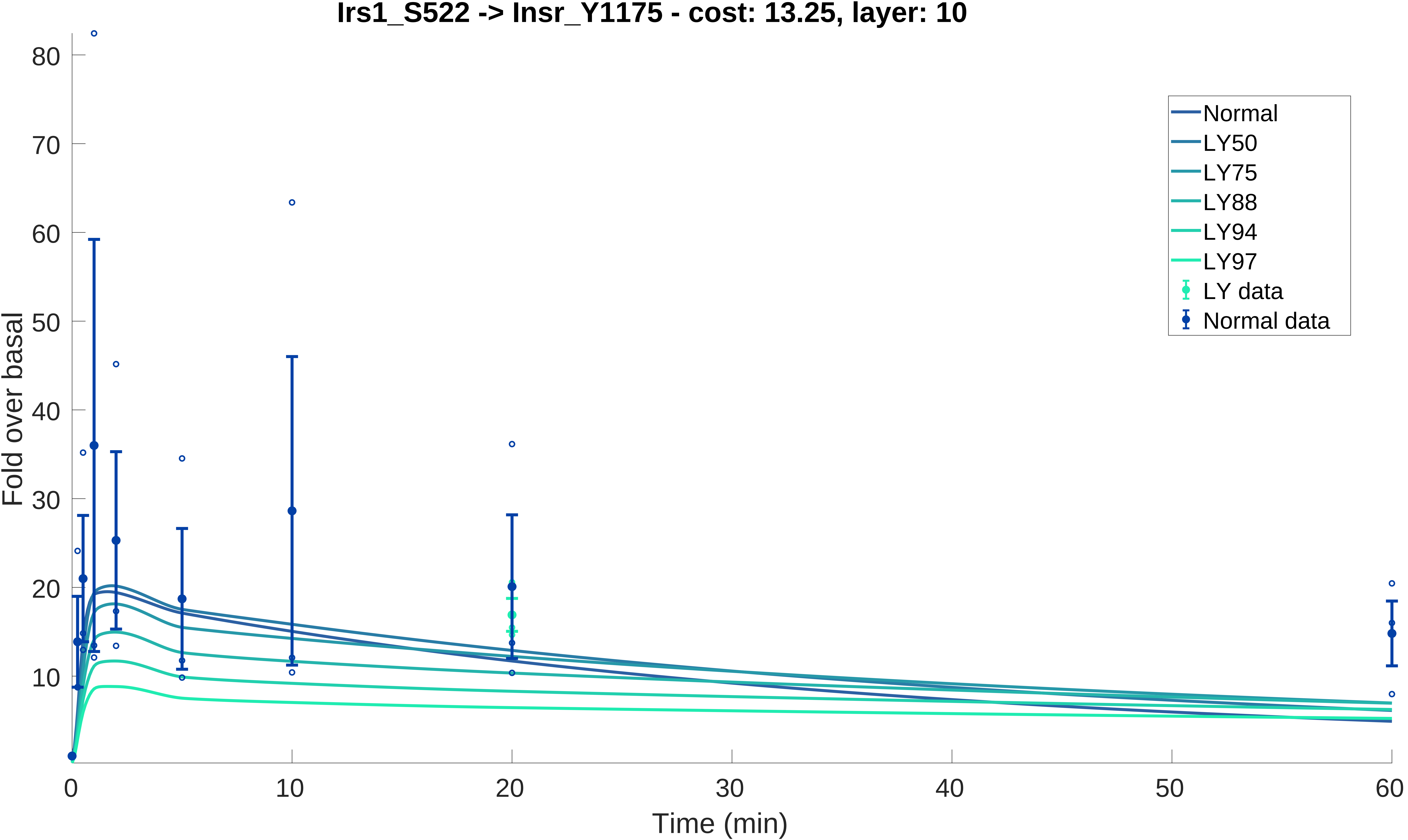

Irs1_S522 -> Insr_Y1175 - cost: 13.25, layer: 10
Fold over basal
Time (min)
0
10
20
30
40
50
60
70
80
Normal
LY50
LY75
LY88
LY94
LY97
LY data
Normal data

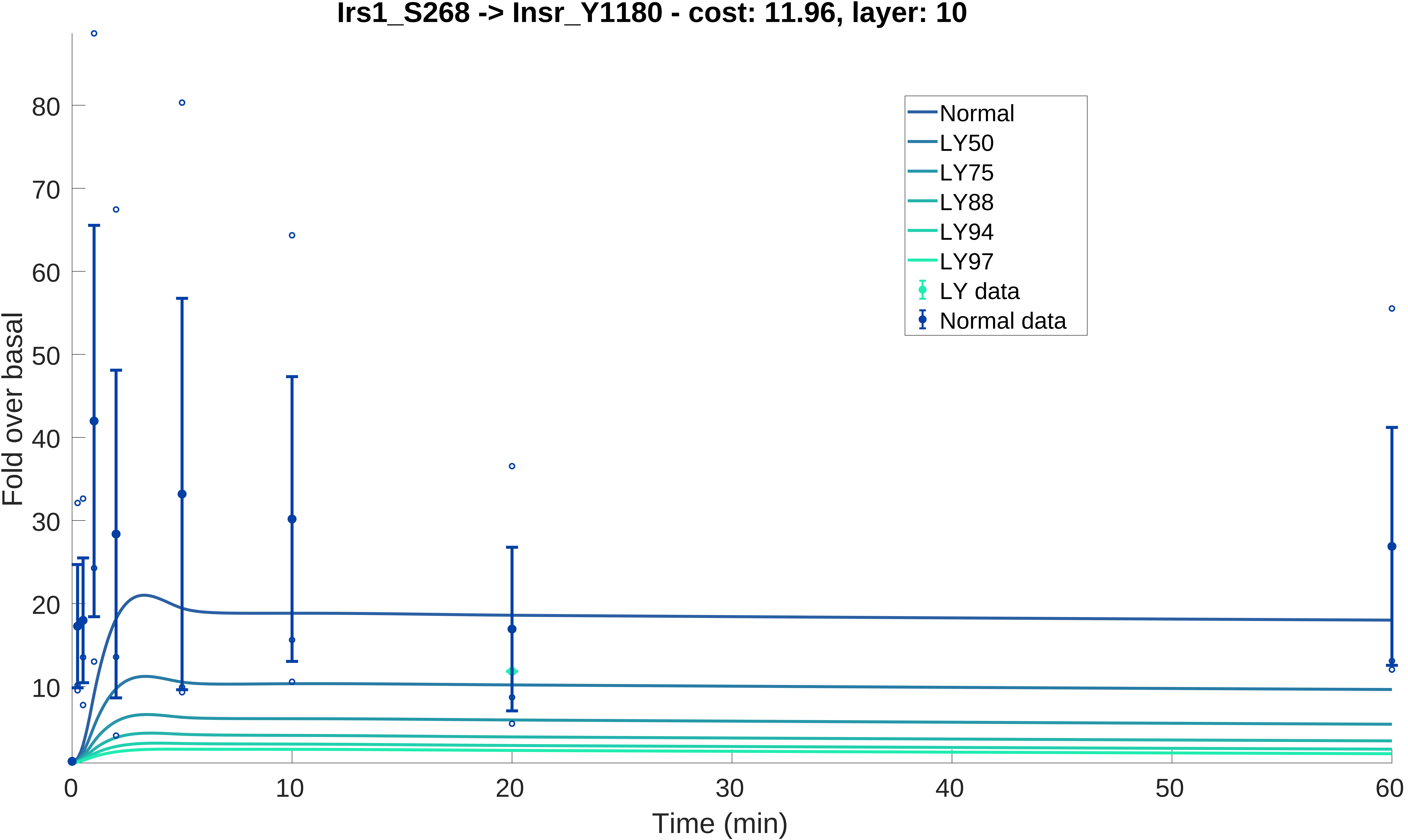

Irs1_S268 -> Insr_Y1180 - cost: 11.96, layer: 10
Fold over basal
80
70
60
50
40
30
20
10
0
10
20
30
40
50
60
Time (min)
Normal
LY50
LY75
LY88
LY94
LY97
LY data
Normal data

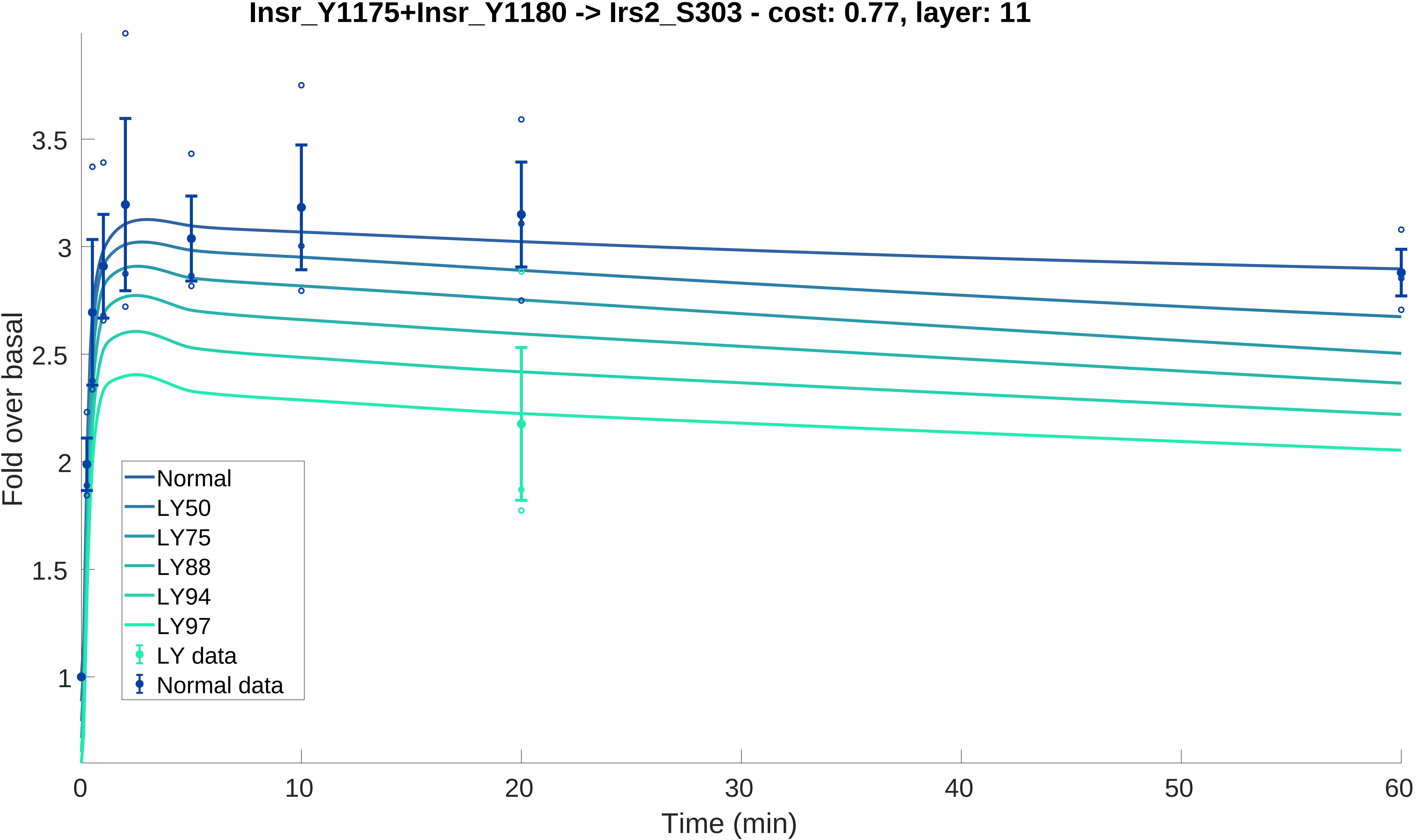

Insr_Y1175+Insr_Y1180 -> Irs2_S303 - cost: 0.77, layer: 11
Fold over basal
3.5
3
2.5
2
1.5
1
0
10
20
30
40
50
60
Time (min)
Normal
LY50
LY75
LY88
LY94
LY97
LY data
Normal data

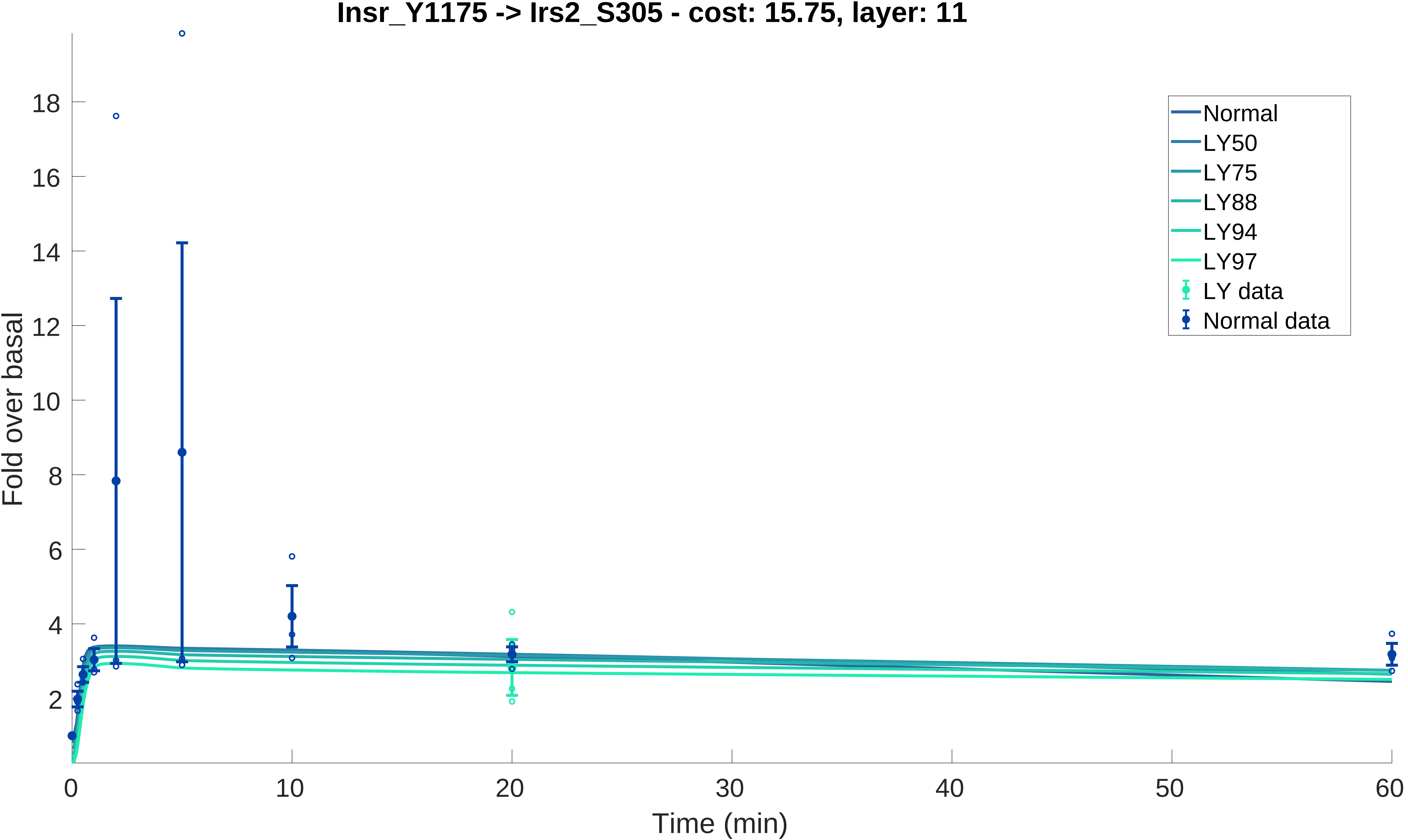
Insr_Y1175 -> Irs2_S305 - cost: 15.75, layer: 11
Fold over basal
Time (min)
0
10
20
30
40
50
60
2
4
6
8
10
12
14
16
18
Normal
LY50
LY75
LY88
LY94
LY97
LY data
Normal data

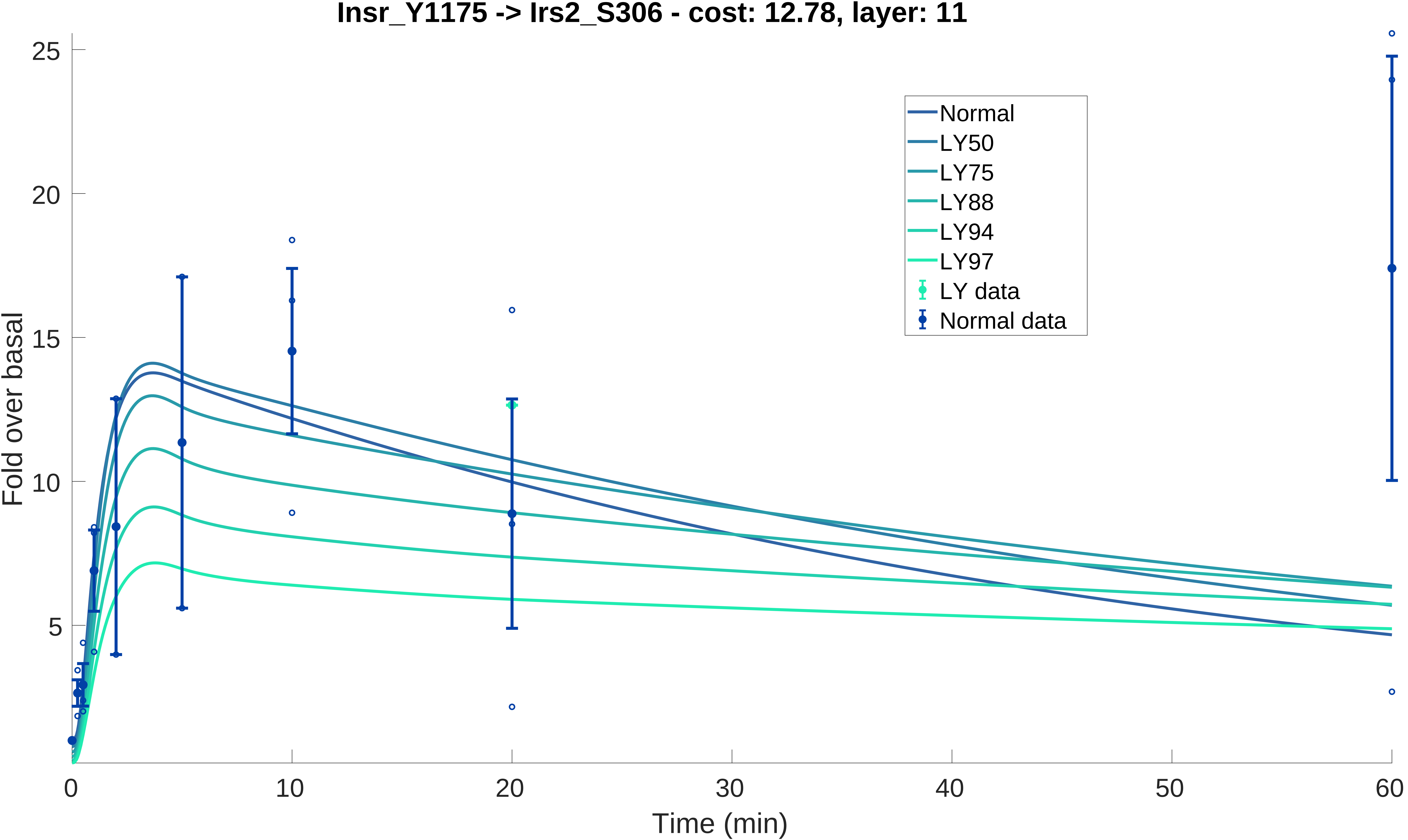
Insr_Y1175 -> Irs2_S306 - cost: 12.78, layer: 11
Fold over basal
Time (min)
0
10
20
30
40
50
60
5
10
15
20
25
Normal
LY50
LY75
LY88
LY94
LY97
LY data
Normal data

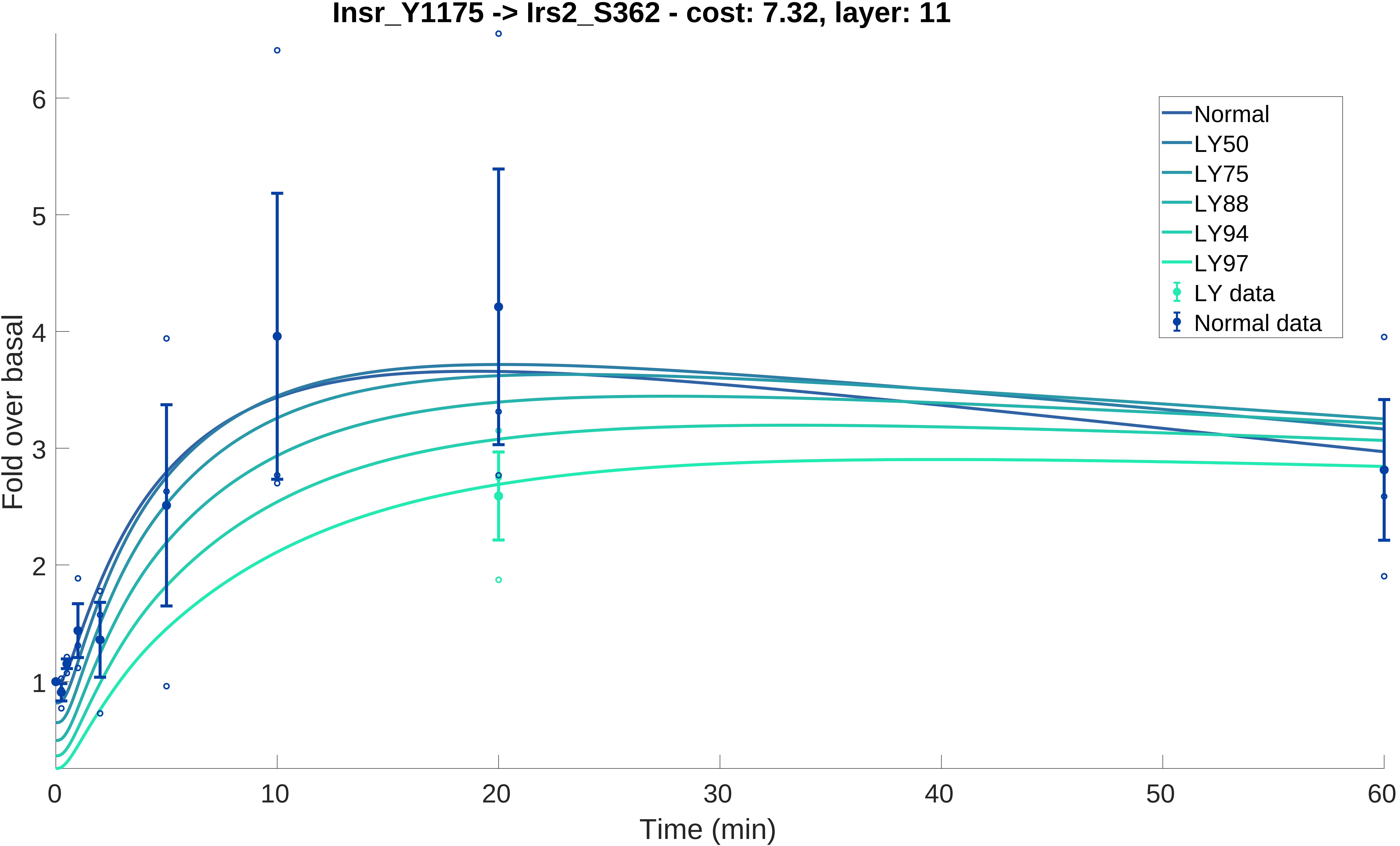
Insr_Y1175 -> Irs2_S362 - cost: 7.32, layer: 11
Fold over basal
Time (min)
0
10
20
30
40
50
60
1
2
3
4
5
6
Normal
LY50
LY75
LY88
LY94
LY97
LY data
Normal data

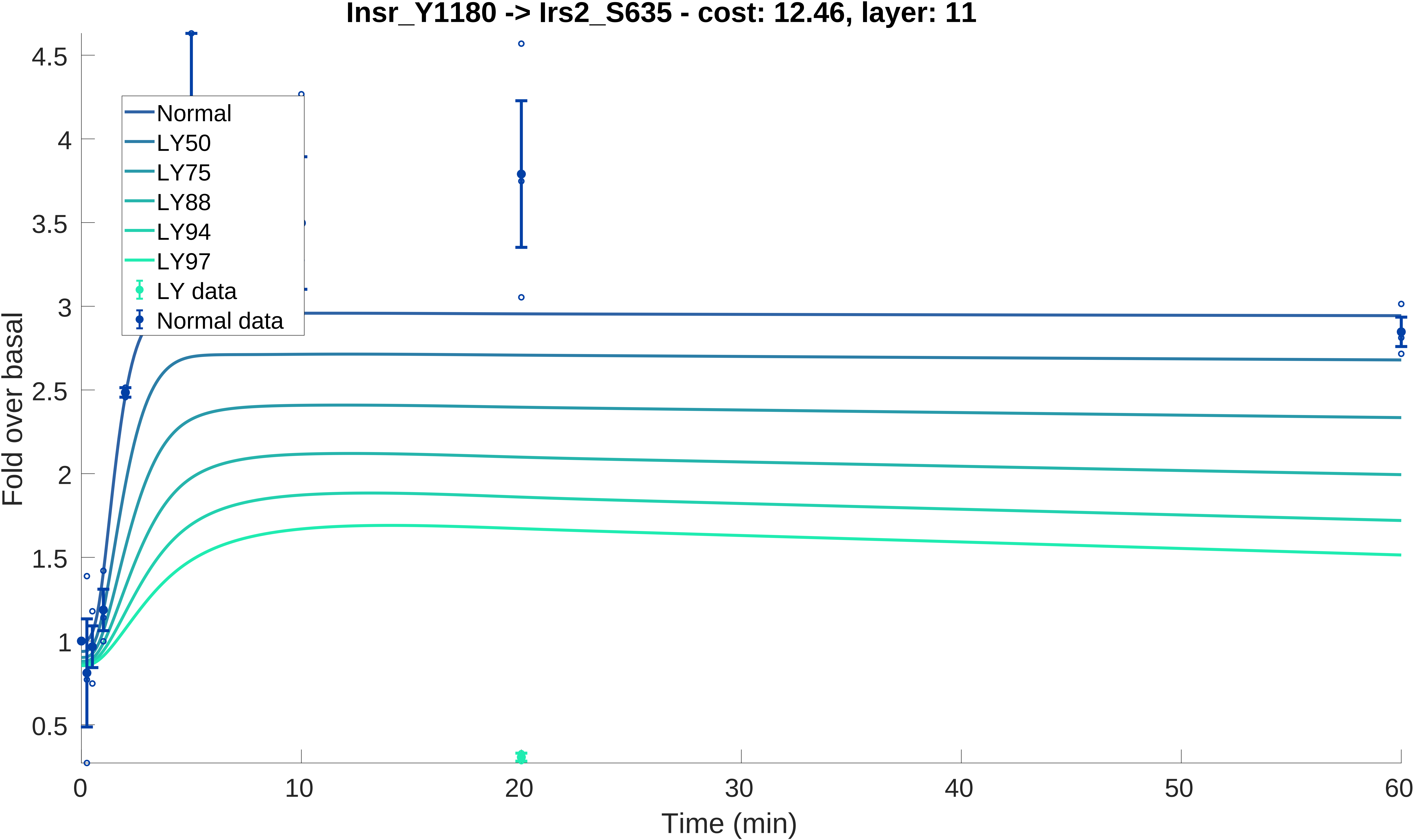

Insr_Y1180 -> Irs2_S635 - cost: 12.46, layer: 11
Normal
LY50
LY75
LY88
LY94
LY97
LY data
Normal data
Fold over basal
Time (min)
4.5
4
3.5
3
2.5
2
1.5
1
0.5
0
10
20
30
40
50
60

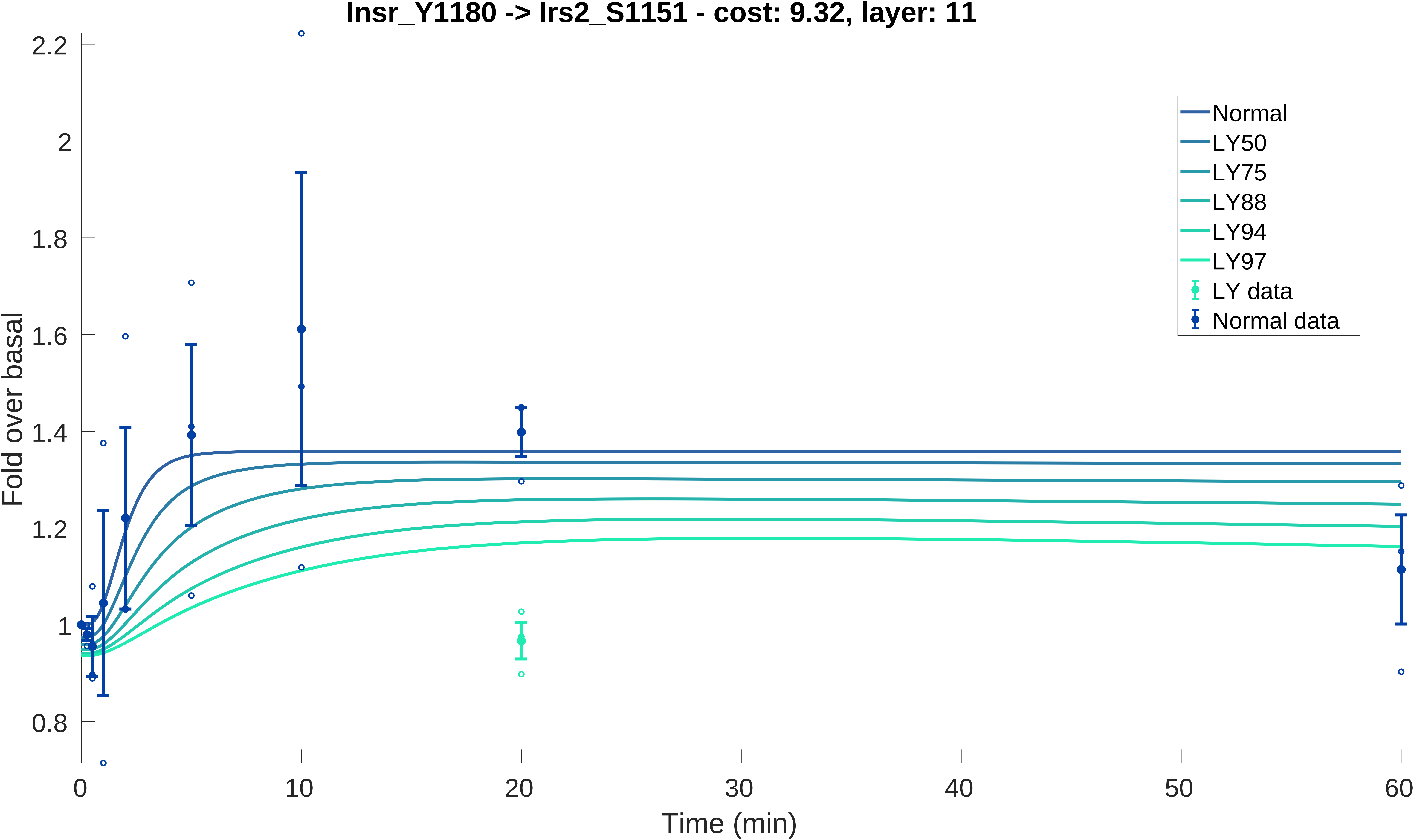

Insr_Y1180 -> Irs2_S1151 - cost: 9.32, layer: 11
Fold over basal
Time (min)
0.8
1
1.2
1.4
1.6
1.8
2
2.2
0
10
20
30
40
50
60
Normal
LY50
LY75
LY88
LY94
LY97
LY data
Normal data

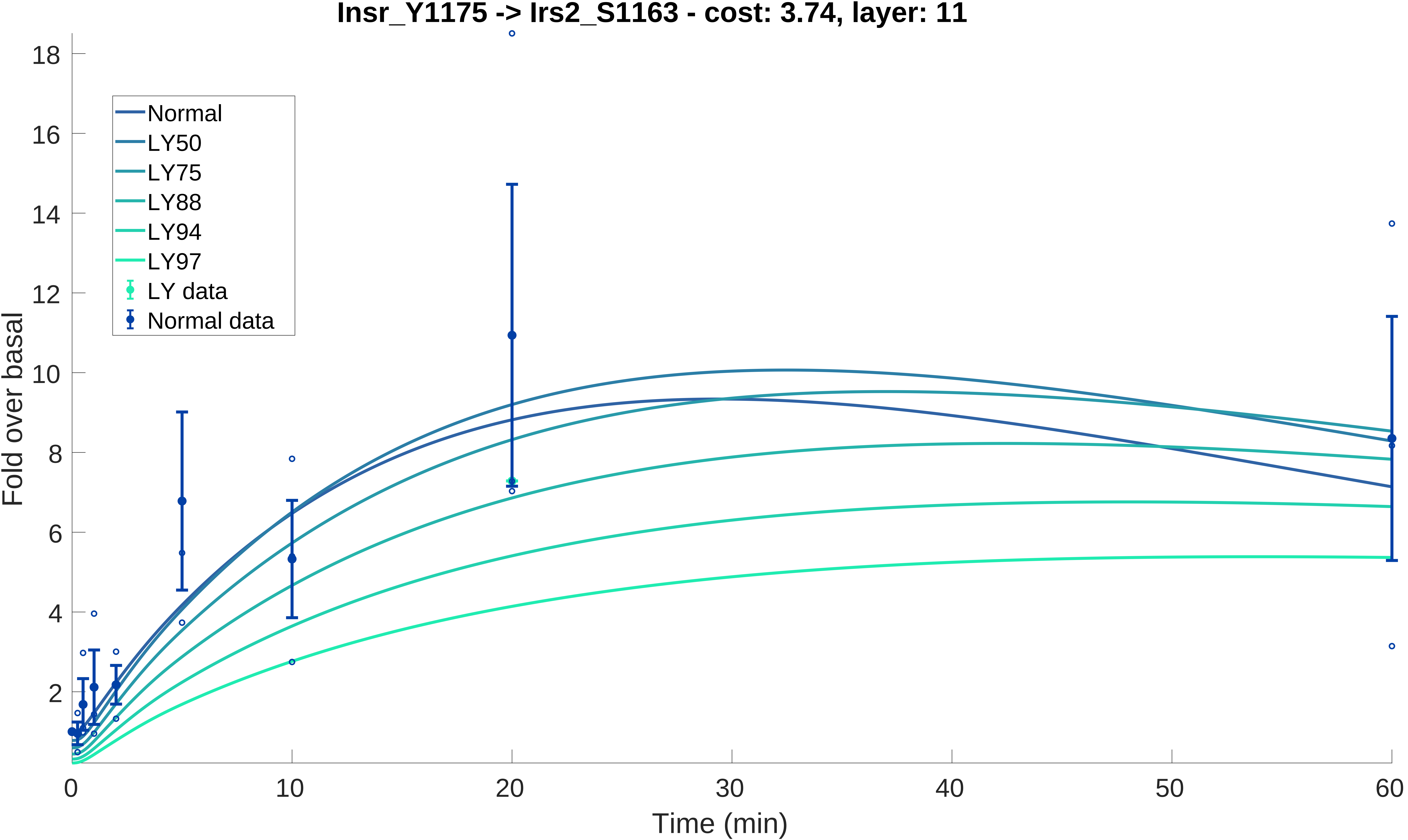

Insr_Y1175 -> Irs2_S1163 - cost: 3.74, layer: 11
Fold over basal
Time (min)
Normal
LY50
LY75
LY88
LY94
LY97
LY data
Normal data
0
10
20
30
40
50
60
2
4
6
8
10
12
14
16
18

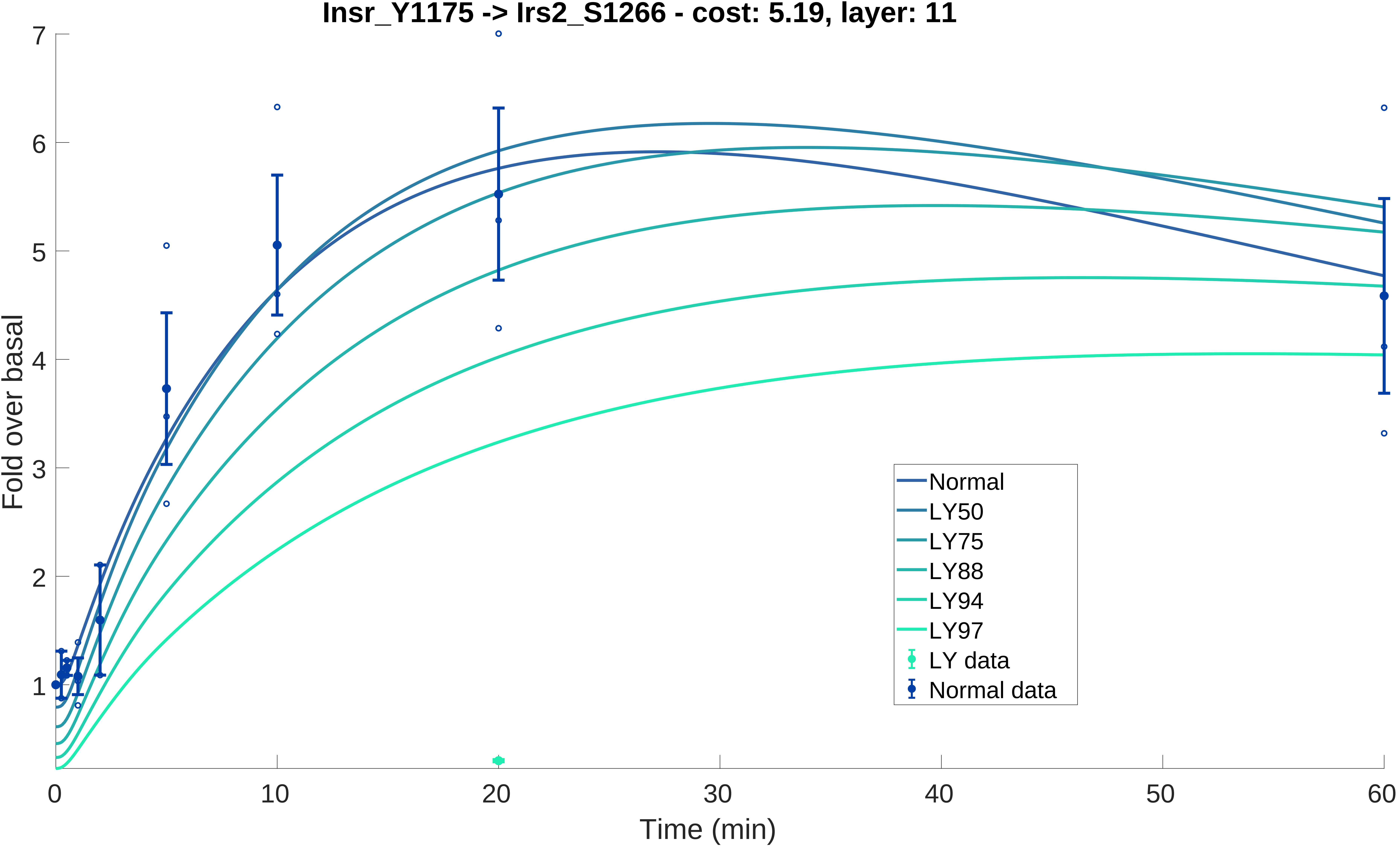

Insr_Y1175 -> Irs2_S1266 - cost: 5.19, layer: 11
Fold over basal
Time (min)
0
10
20
30
40
50
60
1
2
3
4
5
6
7
Normal
LY50
LY75
LY88
LY94
LY97
LY data
Normal data

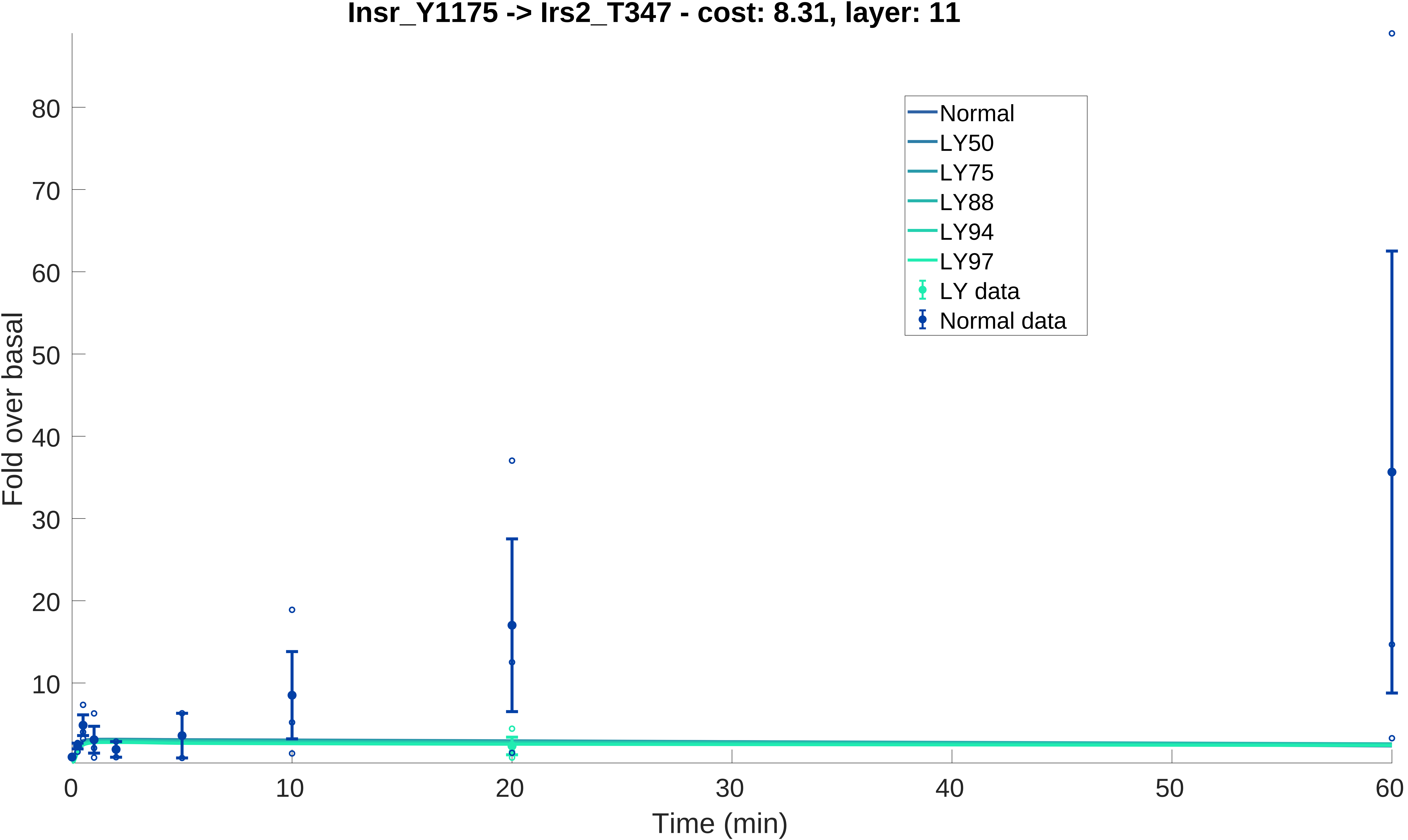

Insr_Y1175 -> Irs2_T347 - cost: 8.31, layer: 11
Normal
LY50
LY75
LY88
LY94
LY97
LY data
Normal data
Fold over basal
Time (min)
0
10
20
30
40
50
60
70
80

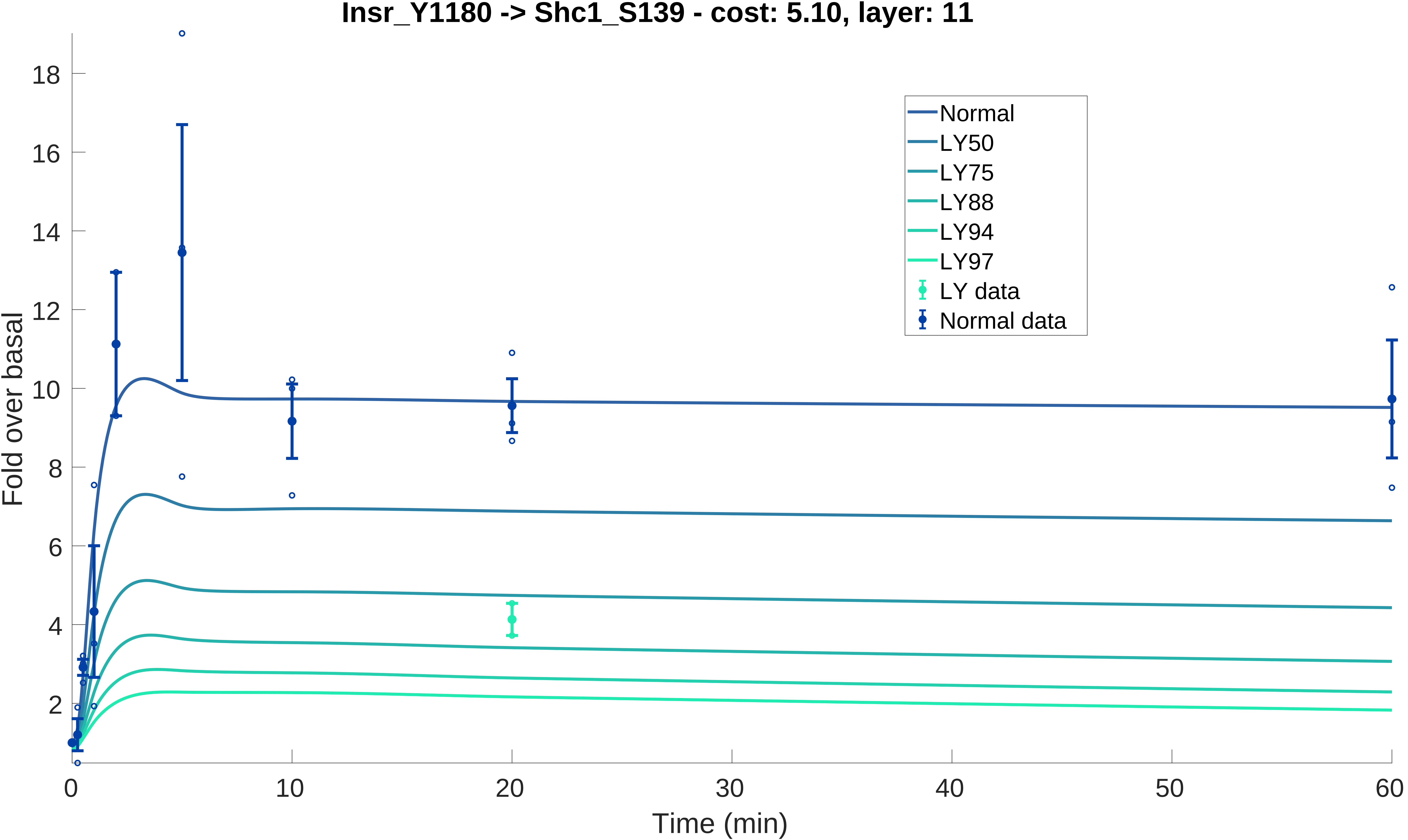

Insr_Y1180 -> Shc1_S139 - cost: 5.10, layer: 11
Normal
LY50
LY75
LY88
LY94
LY97
LY data
Normal data
Fold over basal
Time (min)
0
10
20
30
40
50
60
2
4
6
8
10
12
14
16
18

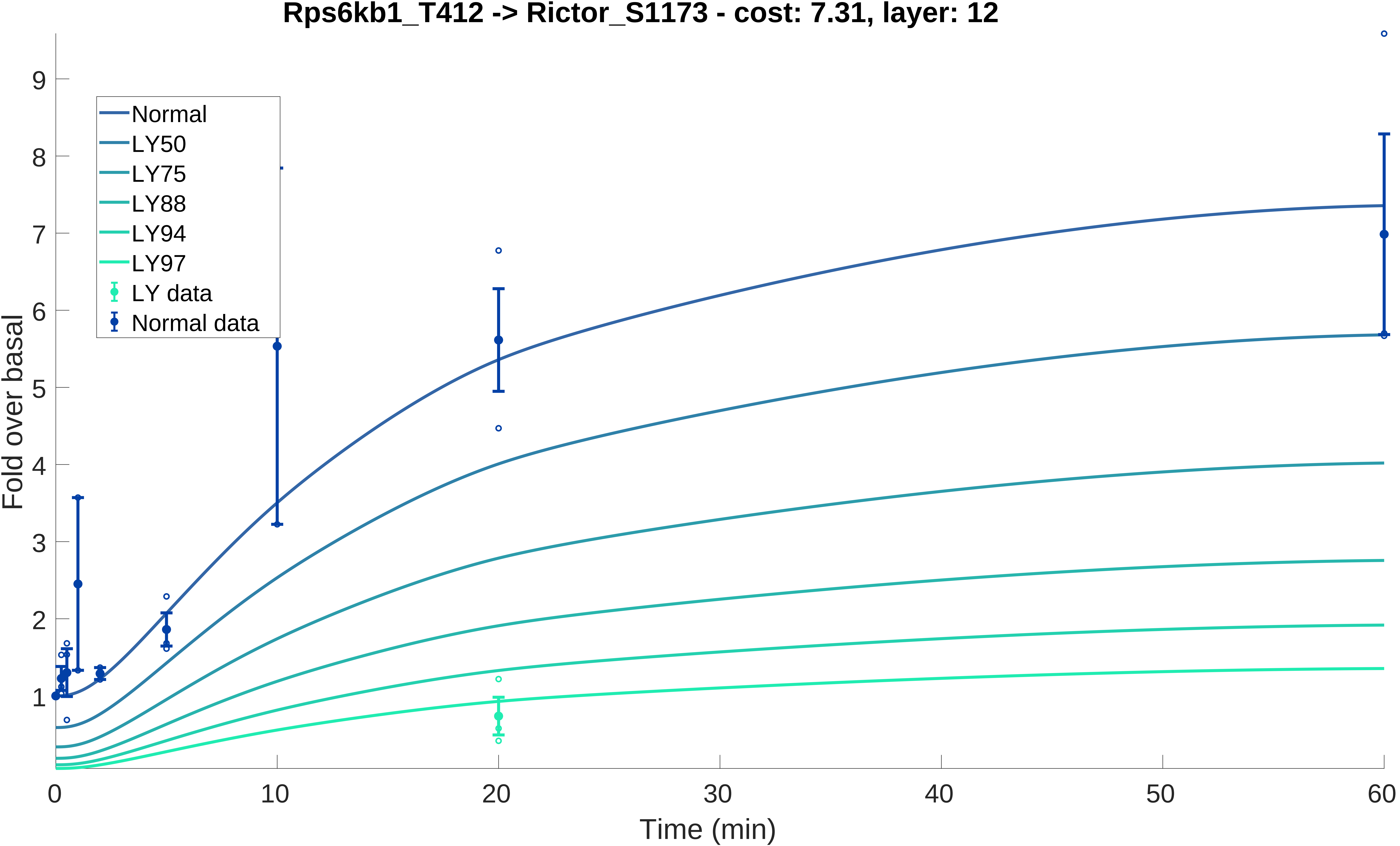
Rps6kb1_T412 -> Rictor_S1173 - cost: 7.31, layer: 12
Normal
LY50
LY75
LY88
LY94
LY97
LY data
Normal data
Fold over basal
Time (min)
0
10
20
30
40
50
60
1
2
3
4
5
6
7
8
9

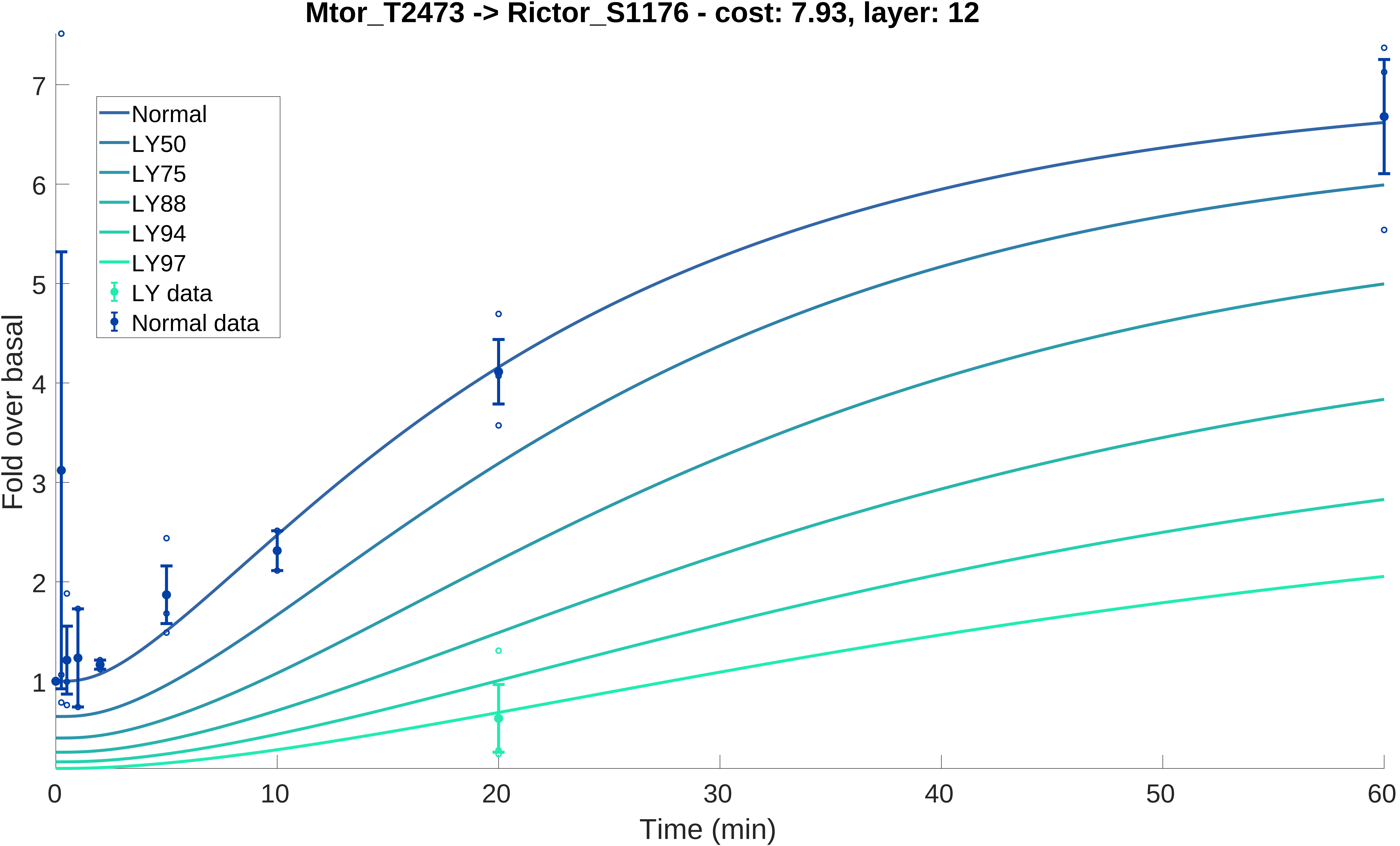

Mtor_T2473 -> Rictor_S1176 - cost: 7.93, layer: 12
Normal
LY50
LY75
LY88
LY94
LY97
LY data
Normal data
Fold over basal
Time (min)
1
2
3
4
5
6
7
0
10
20
30
40
50
60

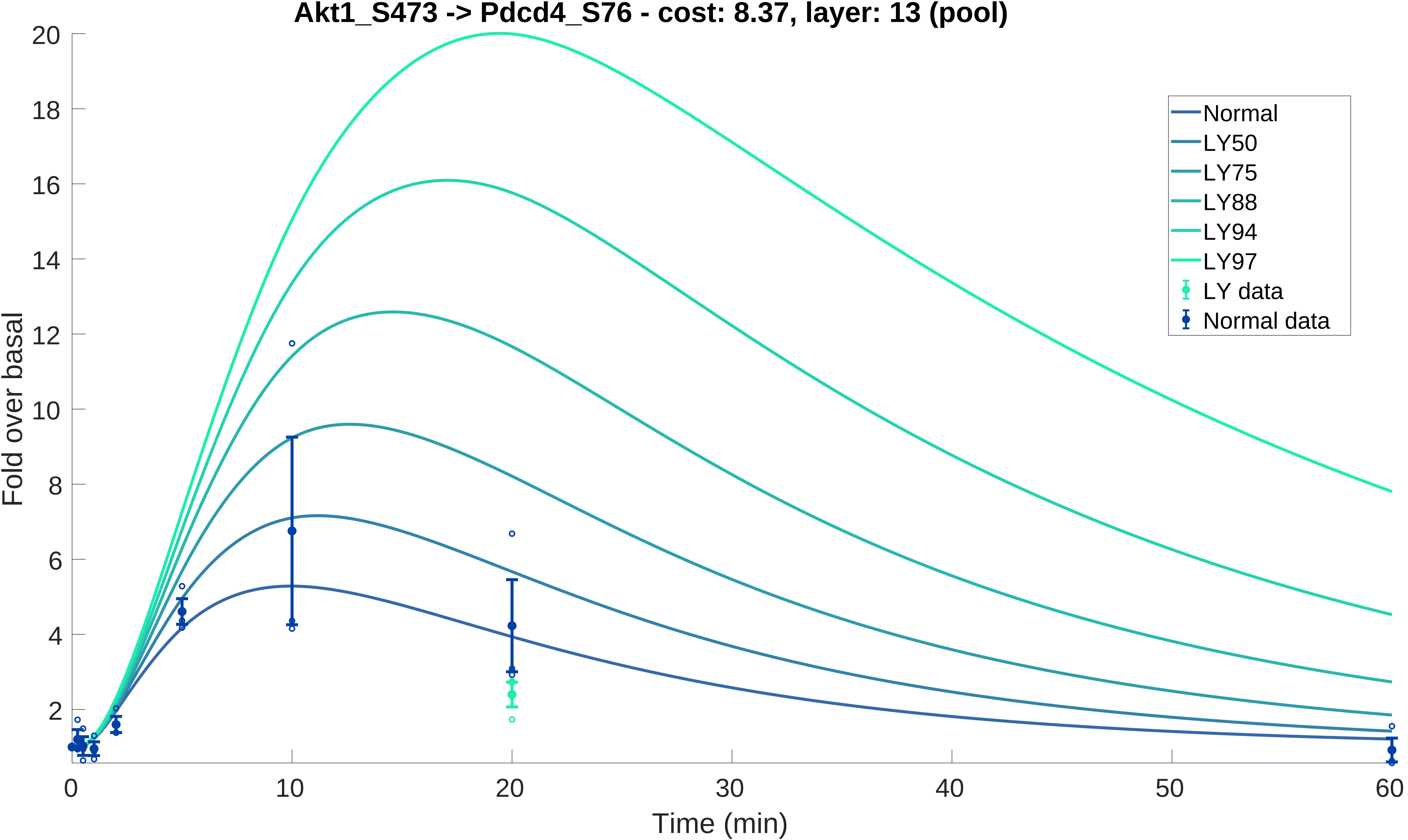

Akt1_S473 -> Pdcd4_S76 - cost: 8.37, layer: 13 (pool)
Fold over basal
Time (min)
Normal
LY50
LY75
LY88
LY94
LY97
LY data
Normal data
0
10
20
30
40
50
60
2
4
6
8
10
12
14
16
18
20

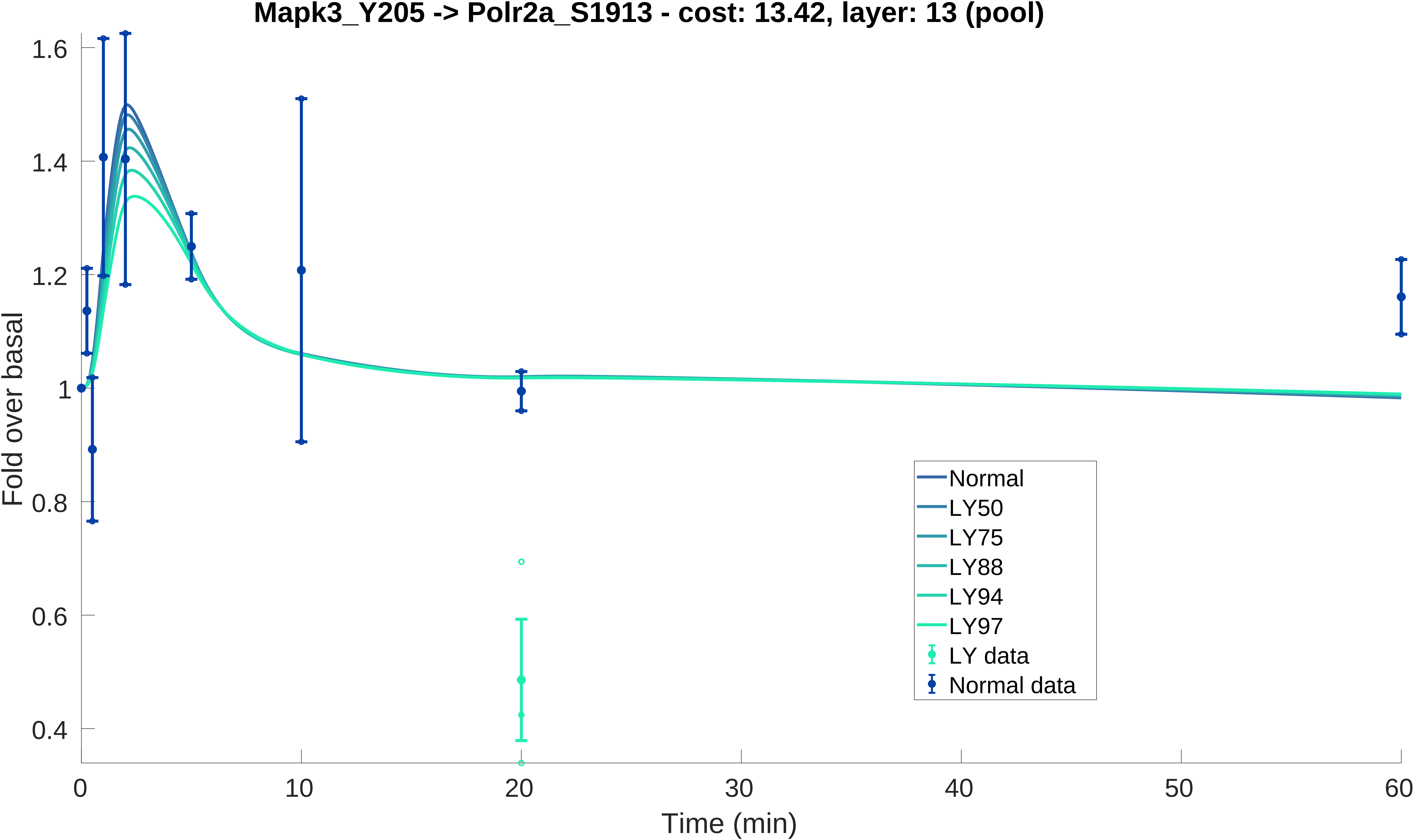

Mapk3_Y205 -> Polr2a_S1913 - cost: 13.42, layer: 13 (pool)
Fold over basal
Time (min)
1.6
1.4
1.2
1
0.8
0.6
0.4
0
10
20
30
40
50
60
Normal
LY50
LY75
LY88
LY94
LY97
LY data
Normal data

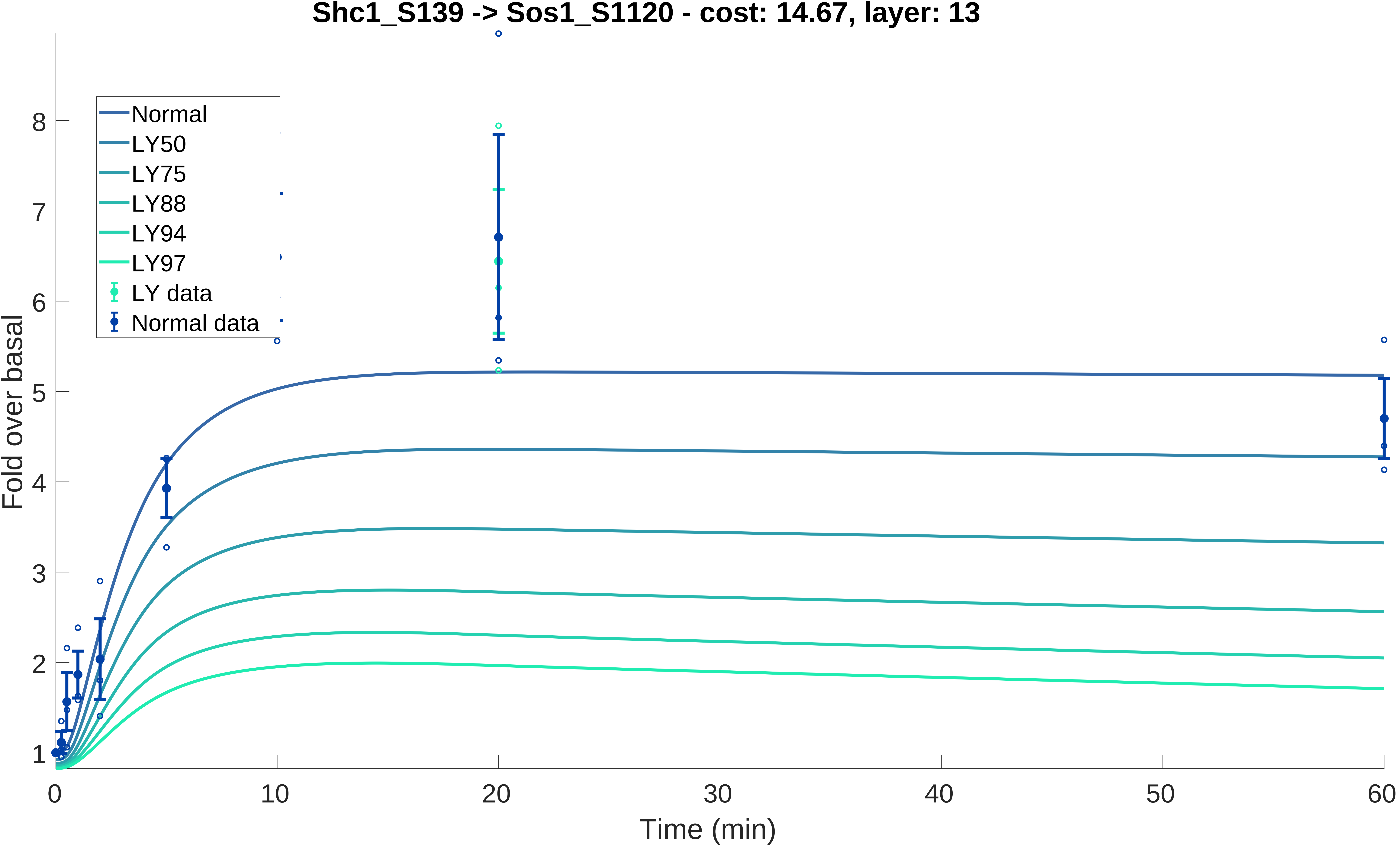
Shc1_S139 -> Sos1_S1120 - cost: 14.67, layer: 13
Normal
LY50
LY75
LY88
LY94
LY97
LY data
Normal data
Fold over basal
Time (min)
1
2
3
4
5
6
7
8
0
10
20
30
40
50
60

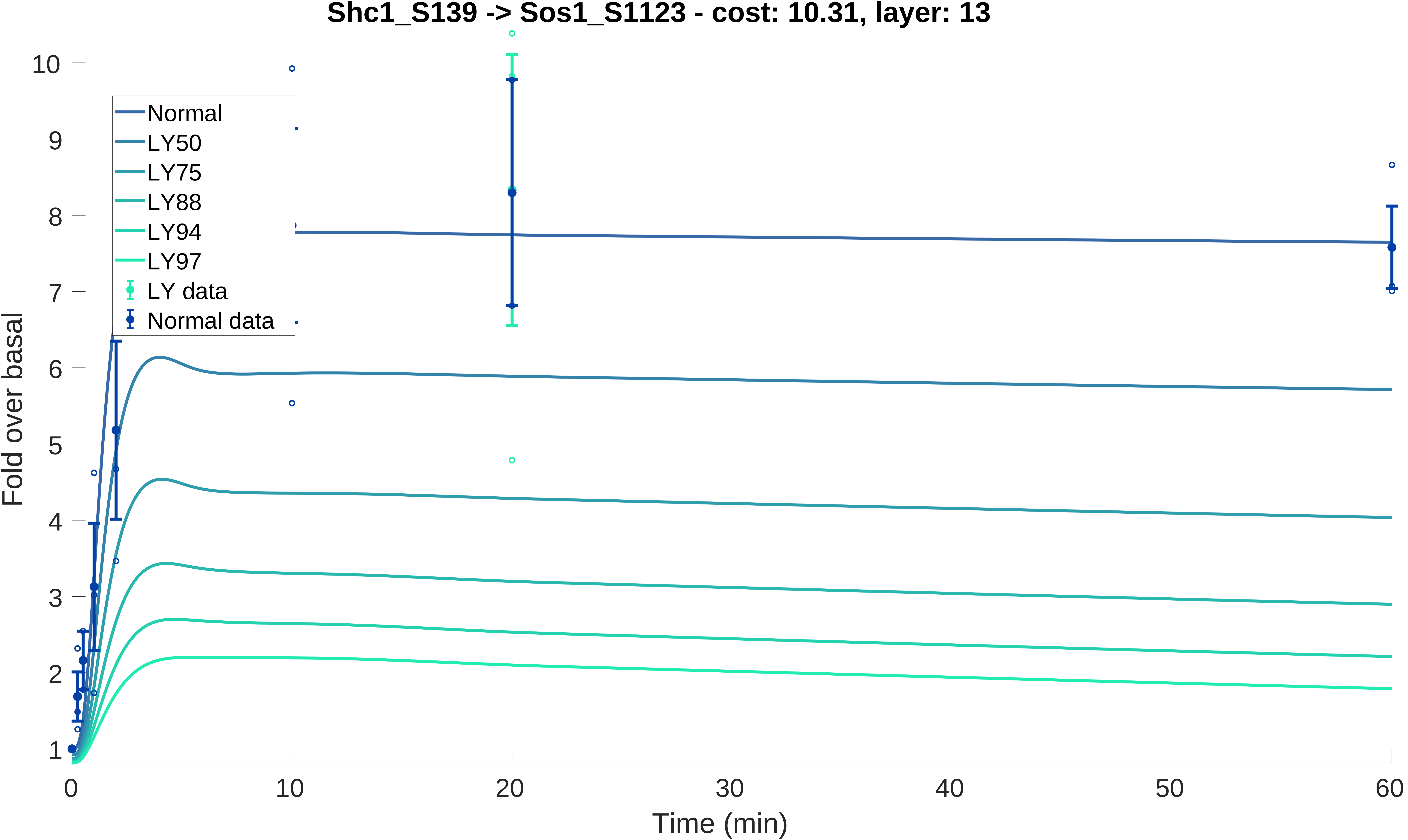

Shc1_S139 -> Sos1_S1123 - cost: 10.31, layer: 13
Normal
LY50
LY75
LY88
LY94
LY97
LY data
Normal data
Fold over basal
Time (min)
1
2
3
4
5
6
7
8
9
10
0
10
20
30
40
50
60

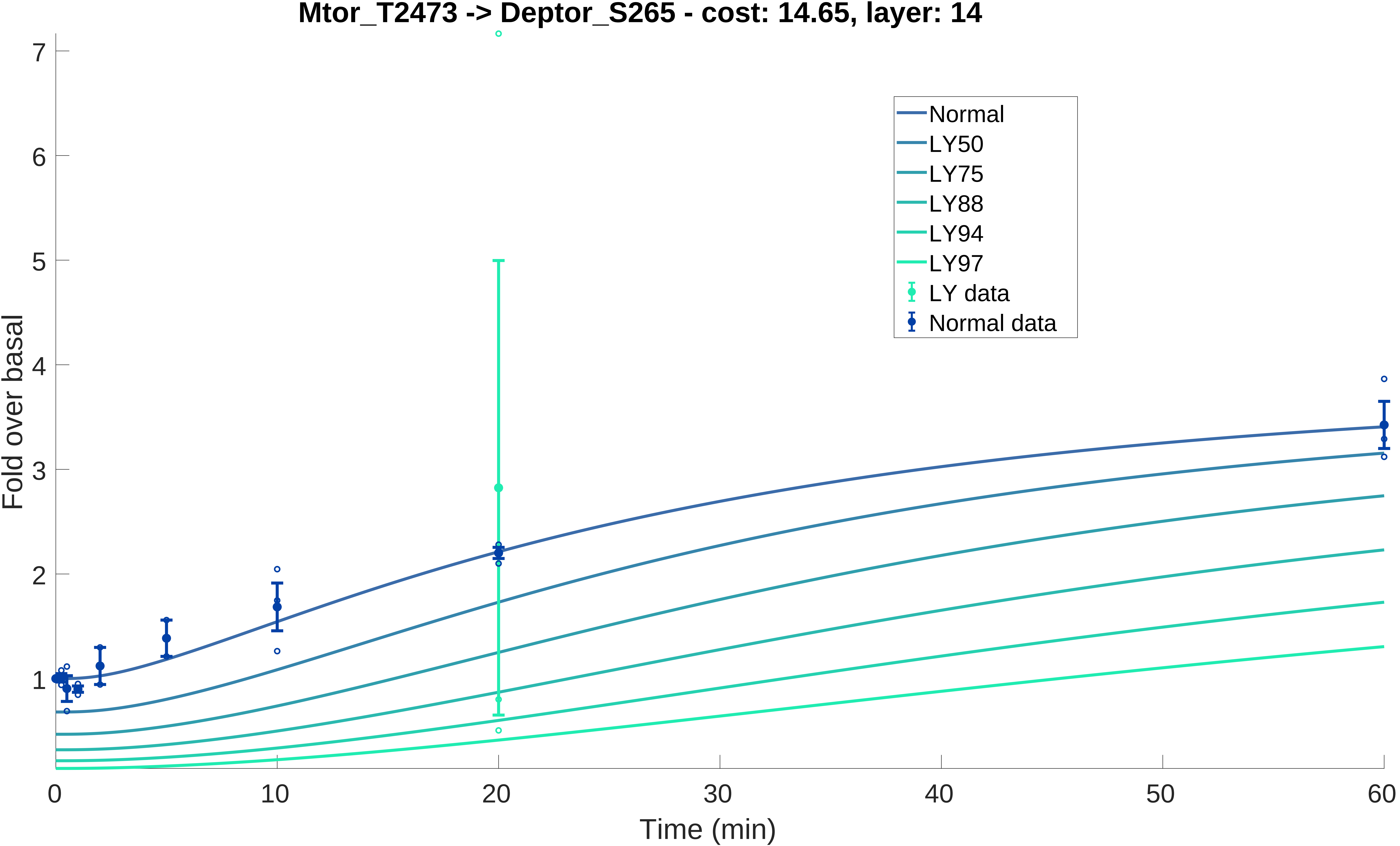

Mtor_T2473 -> Deptor_S265 - cost: 14.65, layer: 14
Fold over basal
Time (min)
Normal
LY50
LY75
LY88
LY94
LY97
LY data
Normal data
0
10
20
30
40
50
60
1
2
3
4
5
6
7

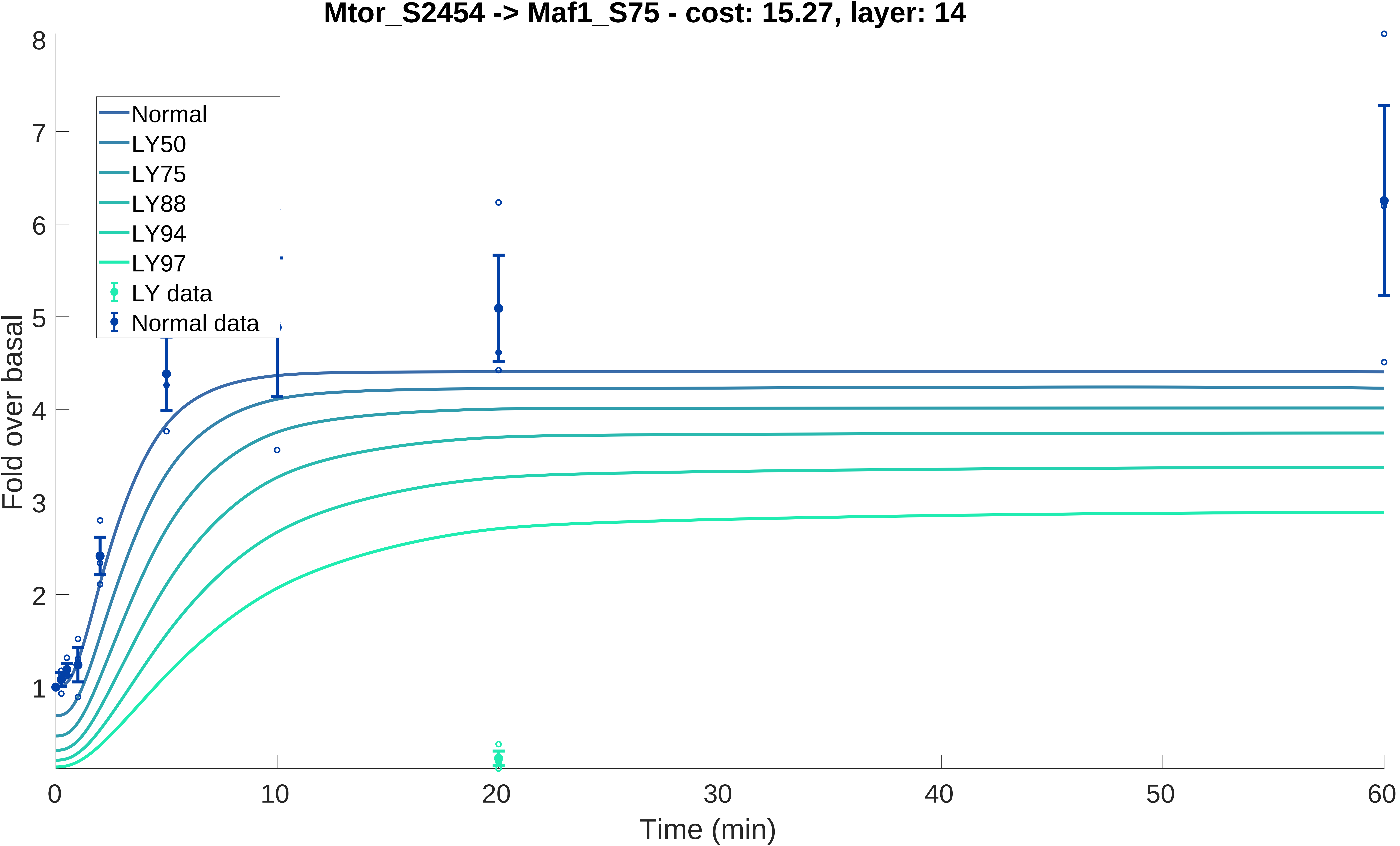

Mtor_S2454 -> Maf1_S75 - cost: 15.27, layer: 14
Normal
LY50
LY75
LY88
LY94
LY97
LY data
Normal data
Fold over basal
Time (min)
0
10
20
30
40
50
60
1
2
3
4
5
6
7
8

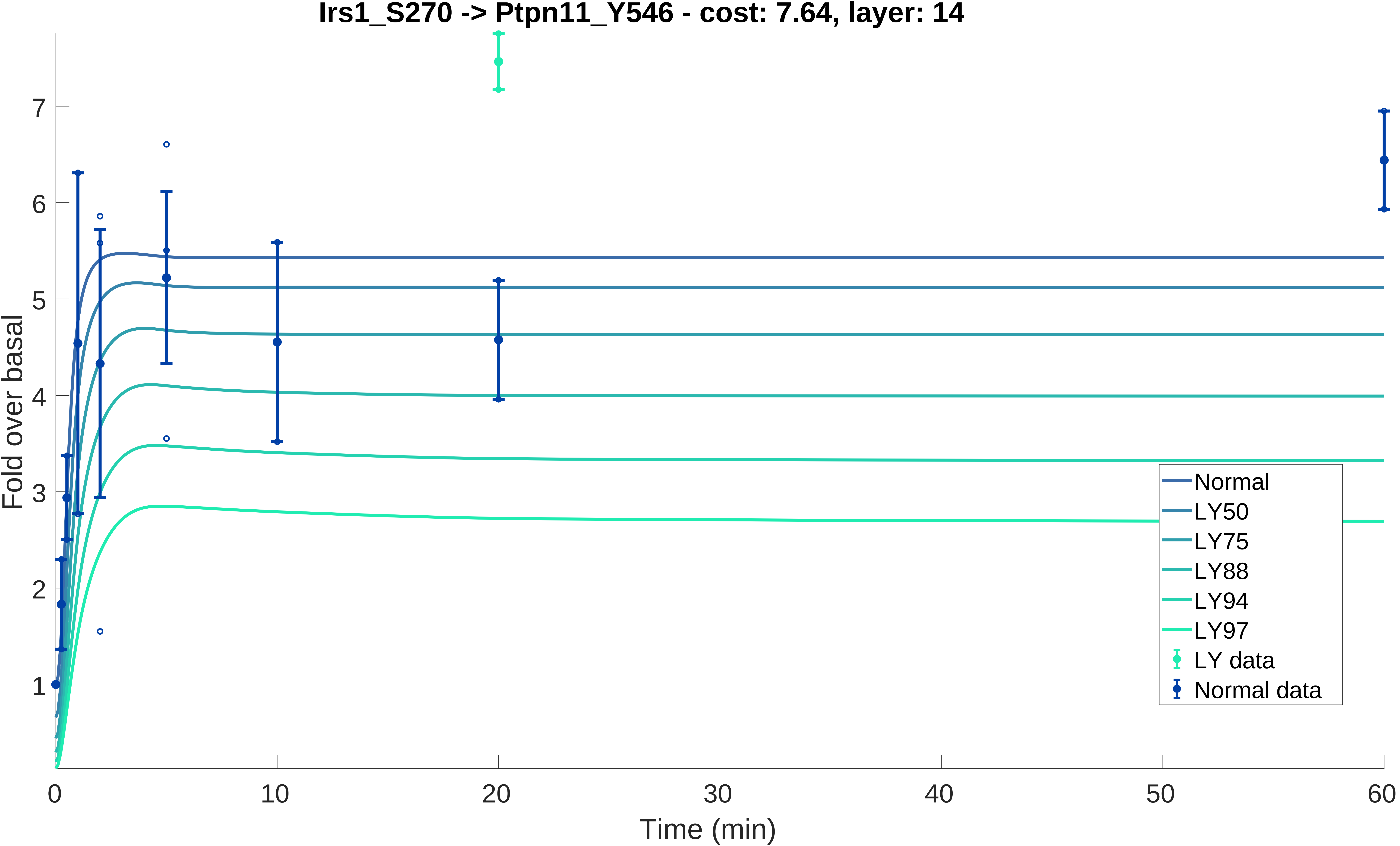

Irs1_S270 -> Ptpn11_Y546 - cost: 7.64, layer: 14
Fold over basal
Time (min)
0
10
20
30
40
50
60
1
2
3
4
5
6
7
Normal
LY50
LY75
LY88
LY94
LY97
LY data
Normal data

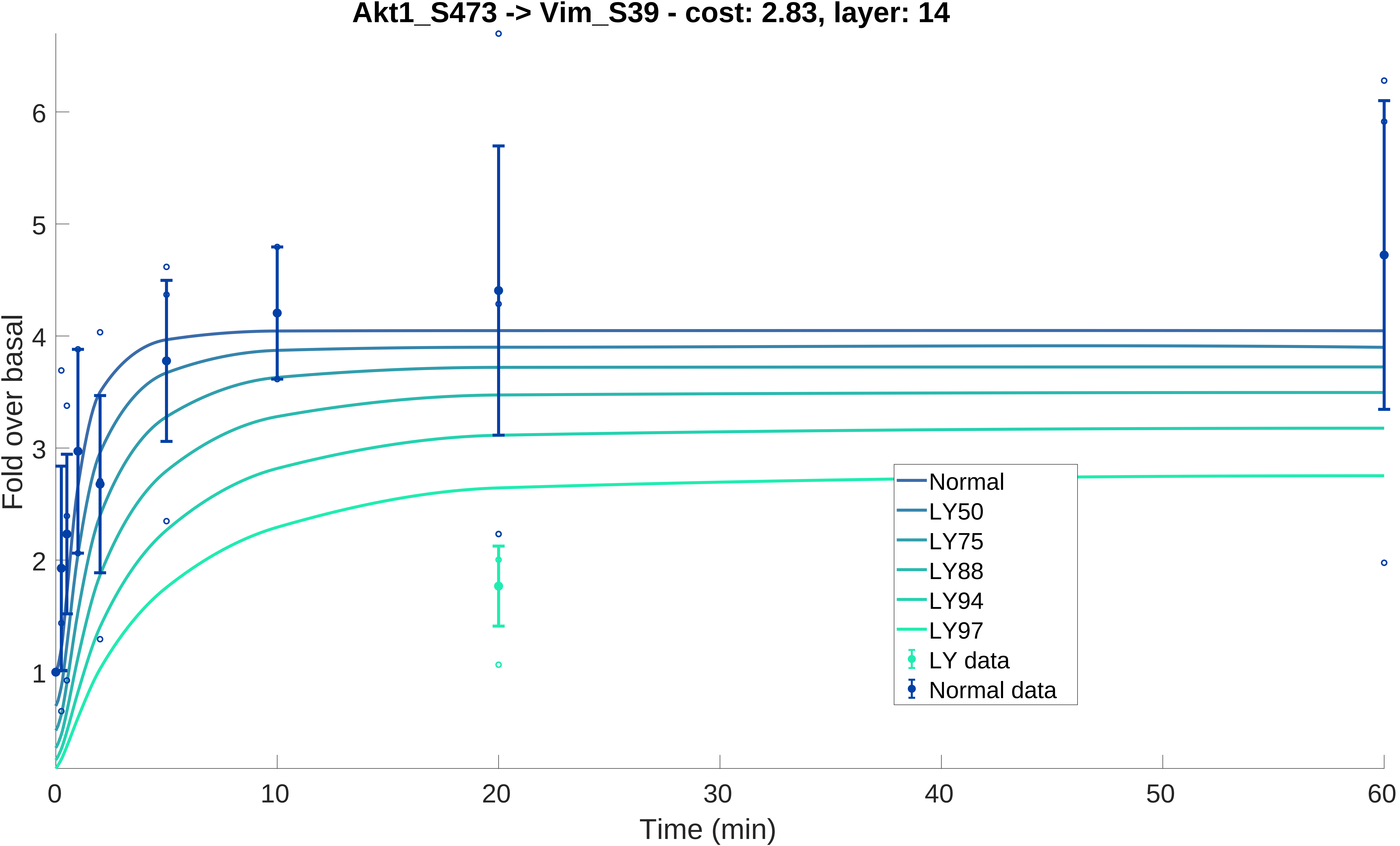

Akt1_S473 -> Vim_S39 - cost: 2.83, layer: 14
Fold over basal
Time (min)
0
10
20
30
40
50
60
1
2
3
4
5
6
Normal
LY50
LY75
LY88
LY94
LY97
LY data
Normal data

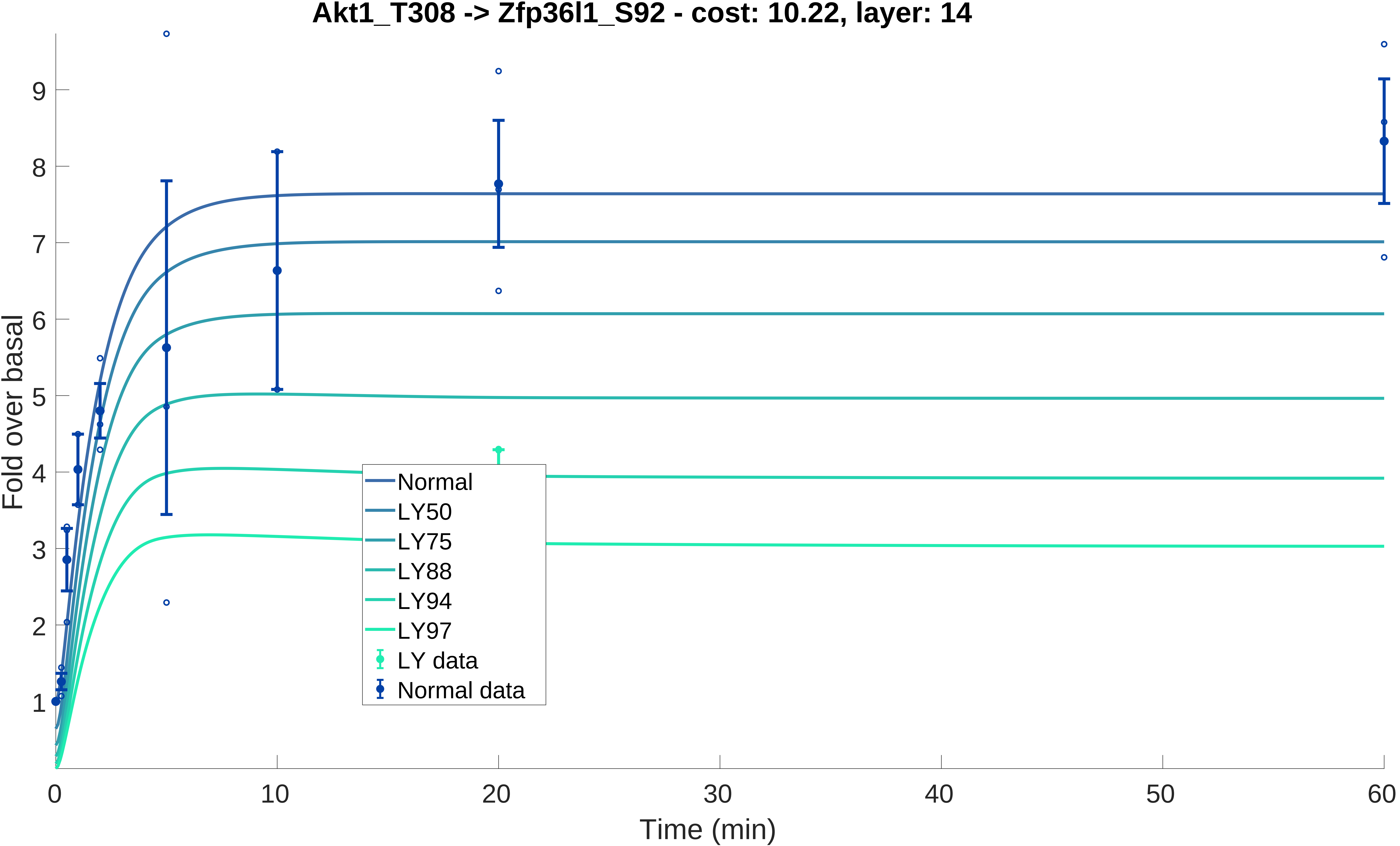

Akt1_T308 -> Zfp36l1_S92 - cost: 10.22, layer: 14
Fold over basal
Time (min)
1
2
3
4
5
6
7
8
9
0
10
20
30
40
50
60
Normal
LY50
LY75
LY88
LY94
LY97
LY data
Normal data

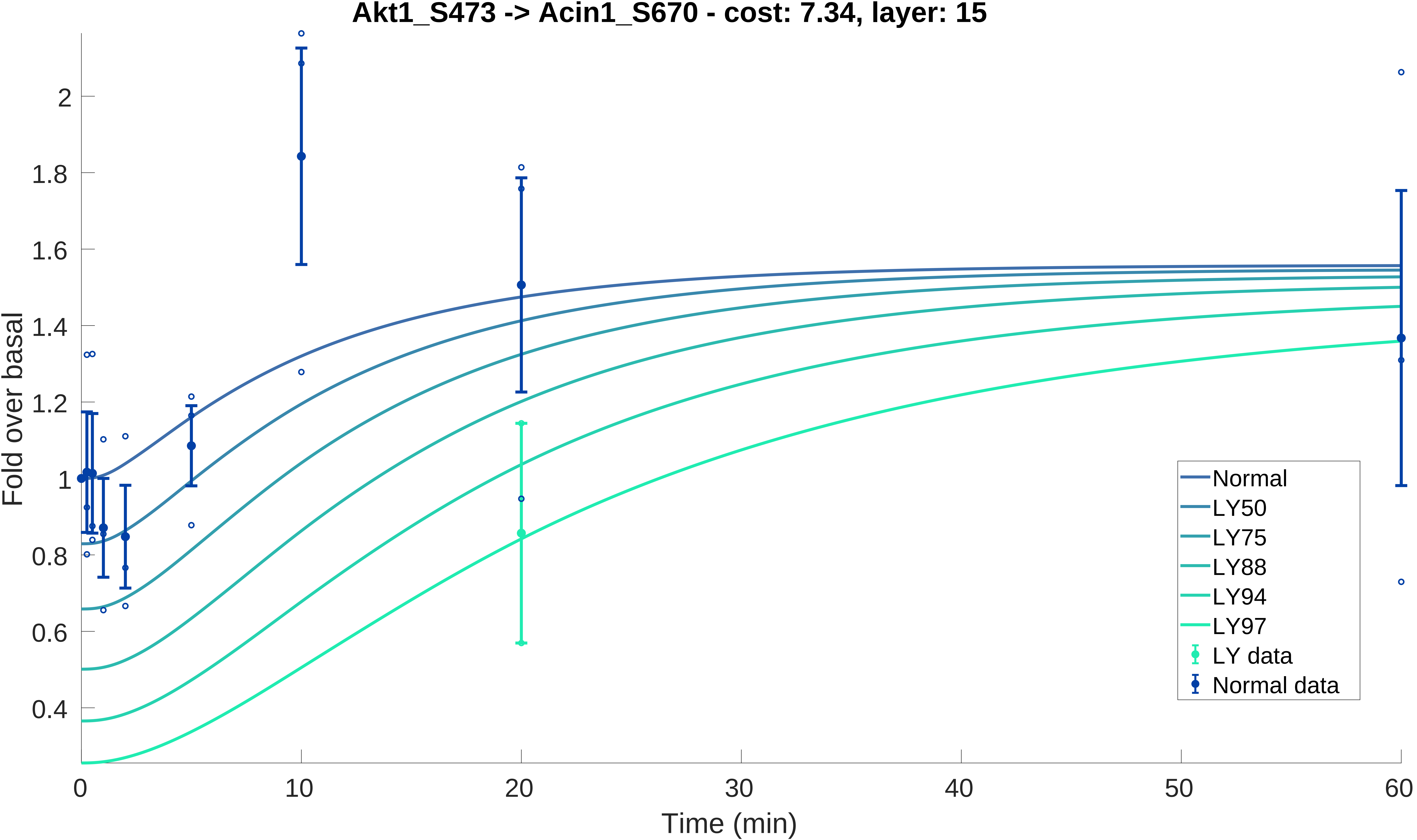
Akt1_S473 -> Acin1_S670 - cost: 7.34, layer: 15
Fold over basal
Time (min)
0
10
20
30
40
50
60
0.4
0.6
0.8
1
1.2
1.4
1.6
1.8
2
Normal
LY50
LY75
LY88
LY94
LY97
LY data
Normal data

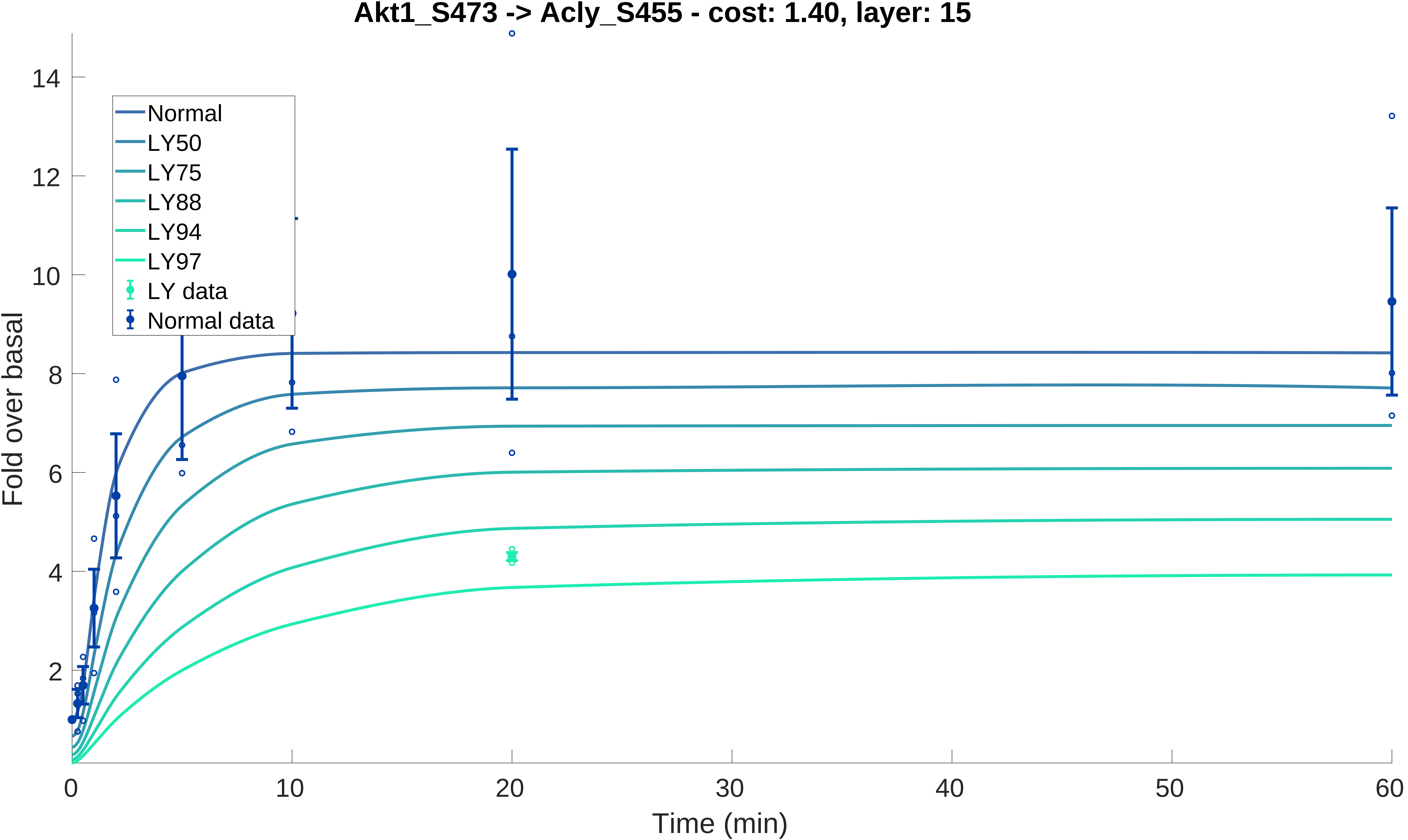
Akt1_S473 -> Acly_S455 - cost: 1.40, layer: 15
Fold over basal
Time (min)
Normal
LY50
LY75
LY88
LY94
LY97
LY data
Normal data
0
10
20
30
40
50
60
2
4
6
8
10
12
14

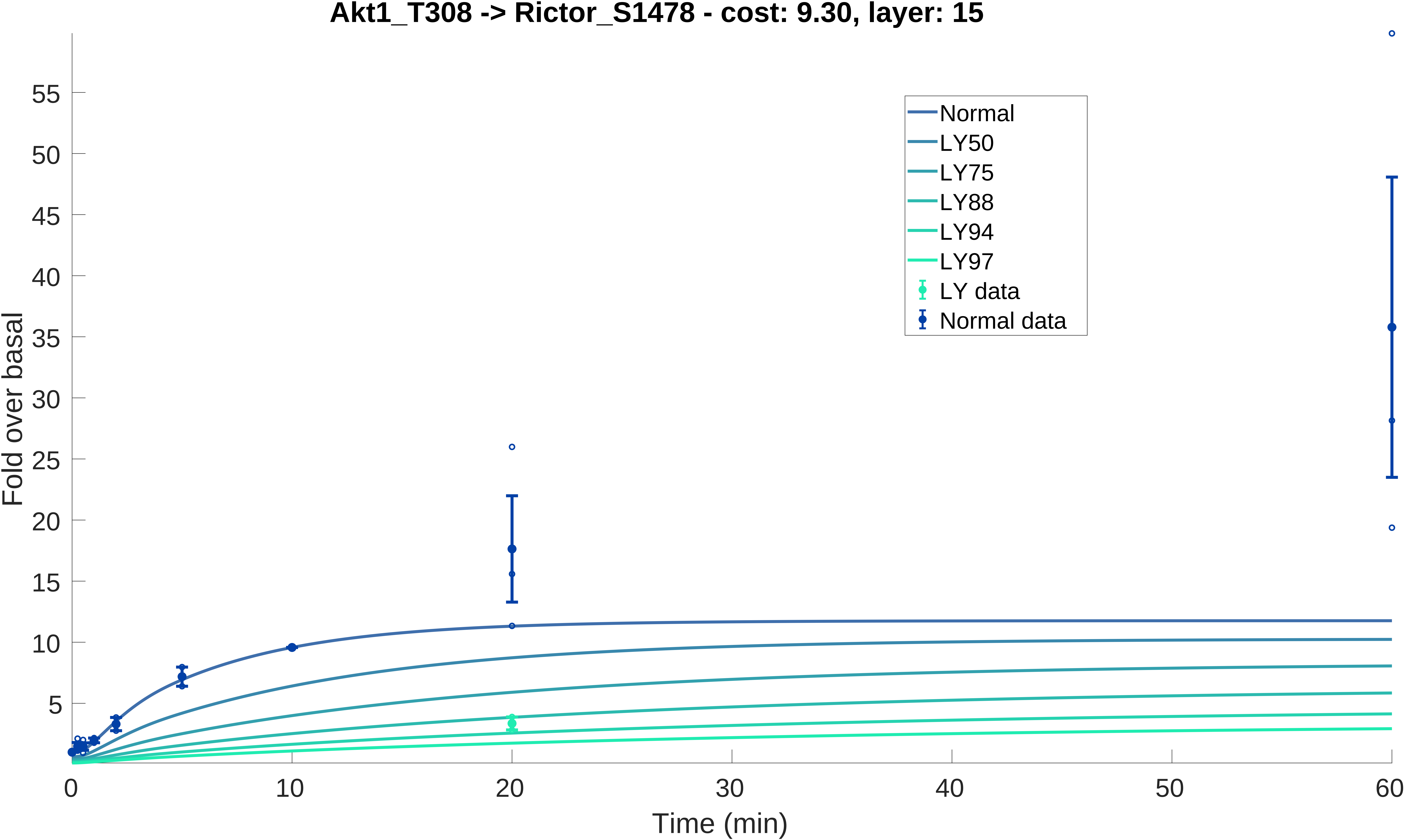

Akt1_T308 -> Rictor_S1478 - cost: 9.30, layer: 15
Fold over basal
Time (min)
Normal
LY50
LY75
LY88
LY94
LY97
LY data
Normal data
0
10
20
30
40
50
60
5
10
15
20
25
30
35
40
45
50
55

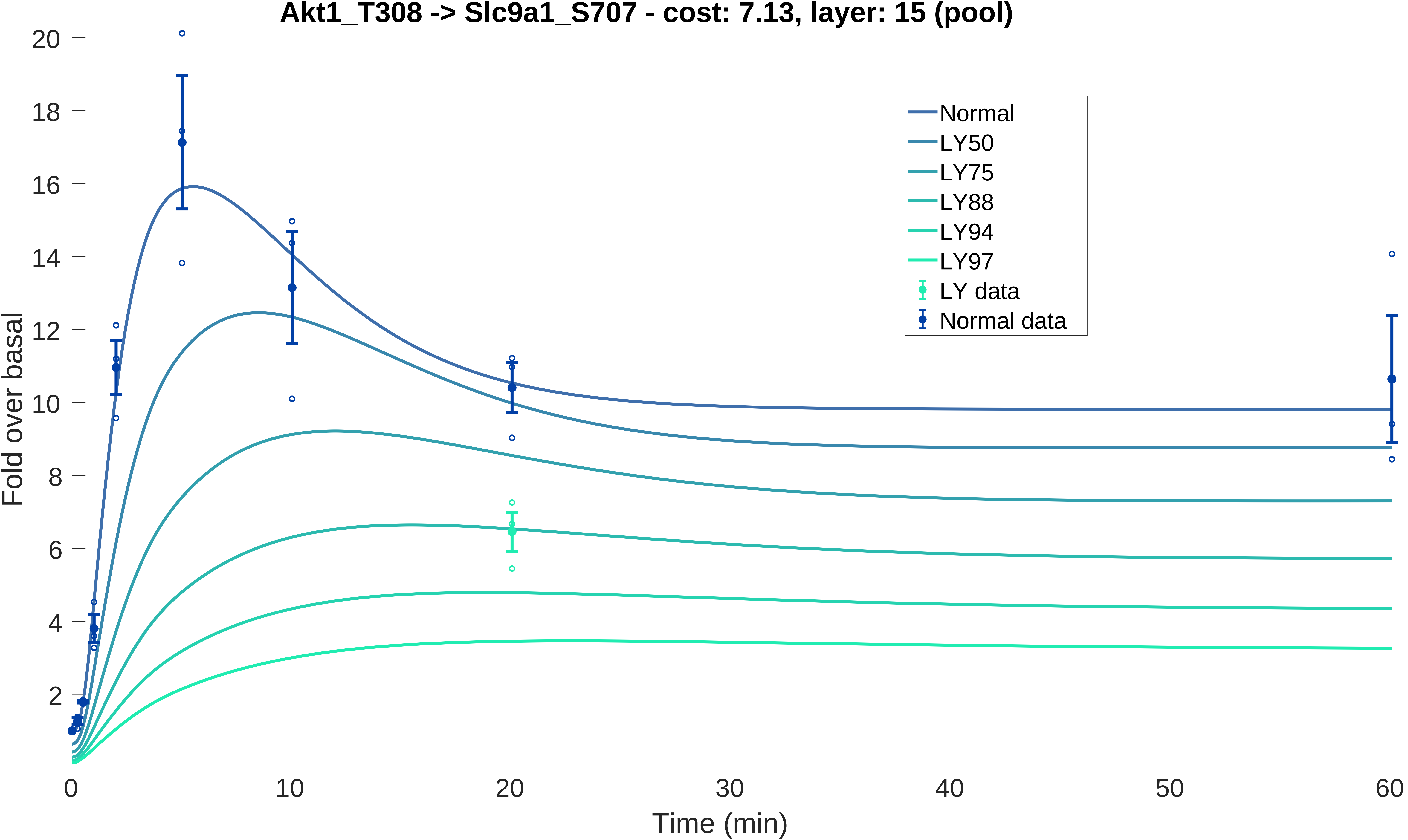

Akt1_T308 -> Slc9a1_S707 - cost: 7.13, layer: 15 (pool)
Normal
LY50
LY75
LY88
LY94
LY97
LY data
Normal data
Fold over basal
Time (min)
0
10
20
30
40
50
60
2
4
6
8
10
12
14
16
18
20

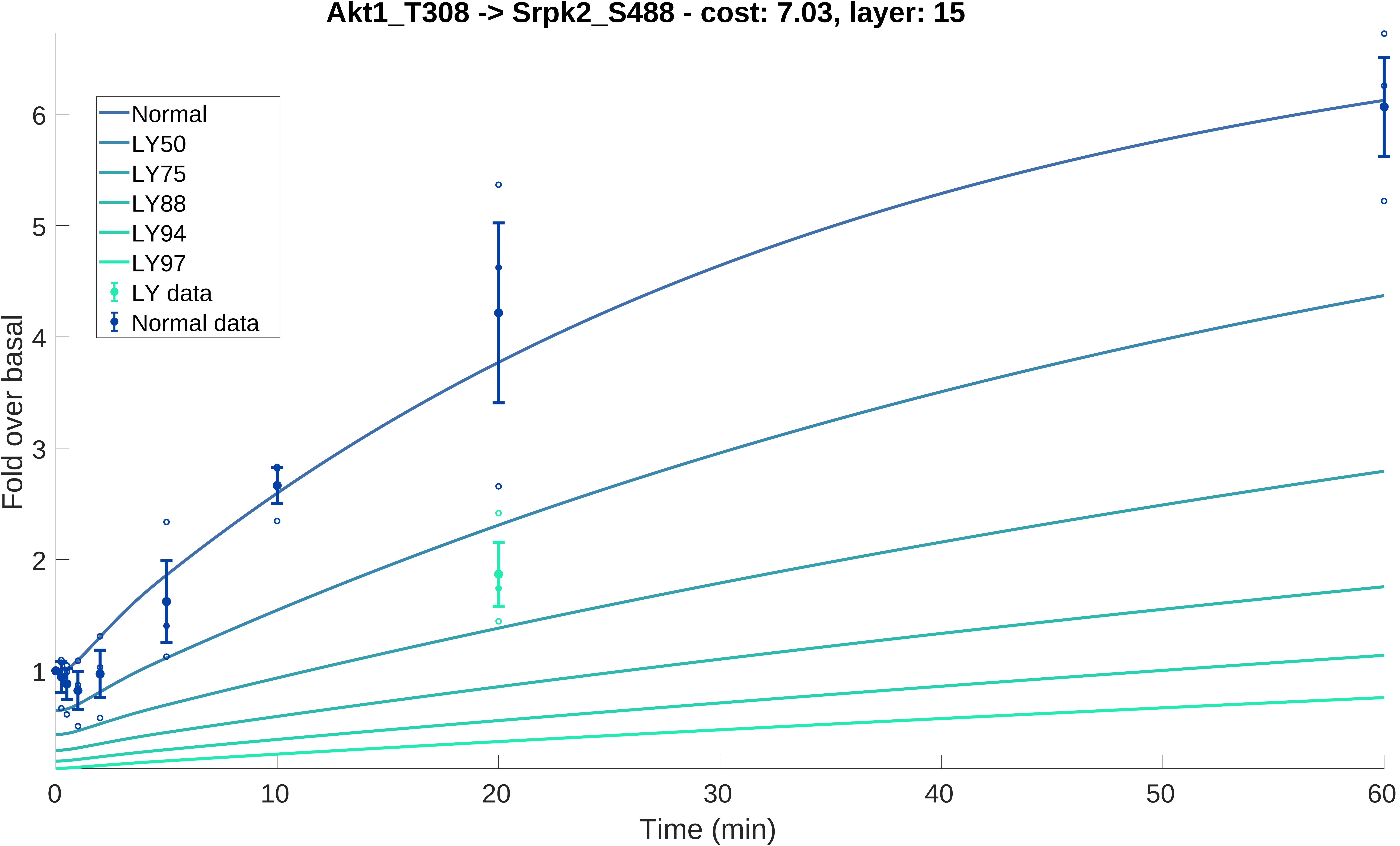
Akt1_T308 -> Srpk2_S488 - cost: 7.03, layer: 15
Normal
LY50
LY75
LY88
LY94
LY97
LY data
Normal data
Fold over basal
Time (min)
1
2
3
4
5
6
0
10
20
30
40
50
60

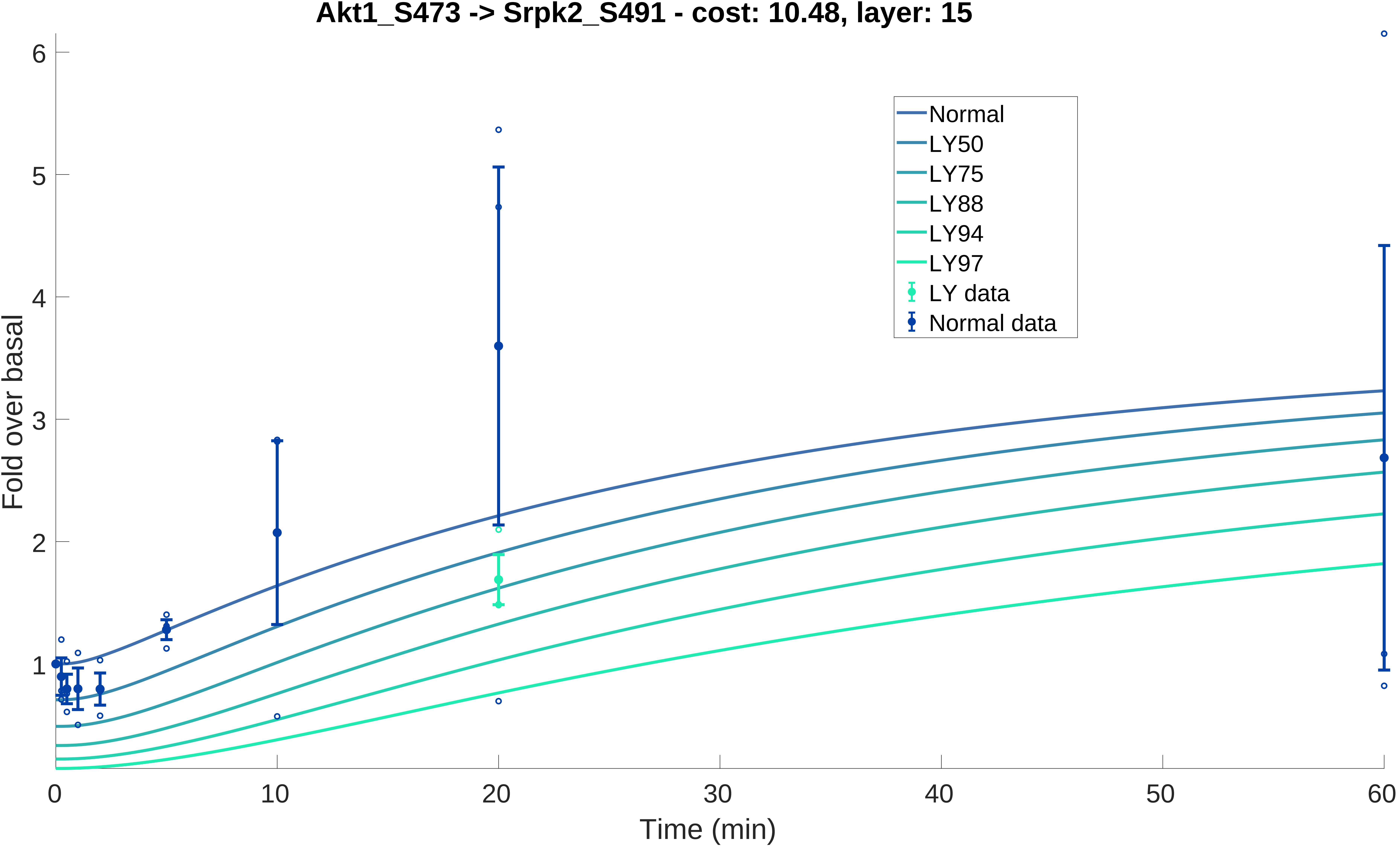

Akt1_S473 -> Srpk2_S491 - cost: 10.48, layer: 15
Fold over basal
Time (min)
0
1
2
3
4
5
6
10
20
30
40
50
60
Normal
LY50
LY75
LY88
LY94
LY97
LY data
Normal data

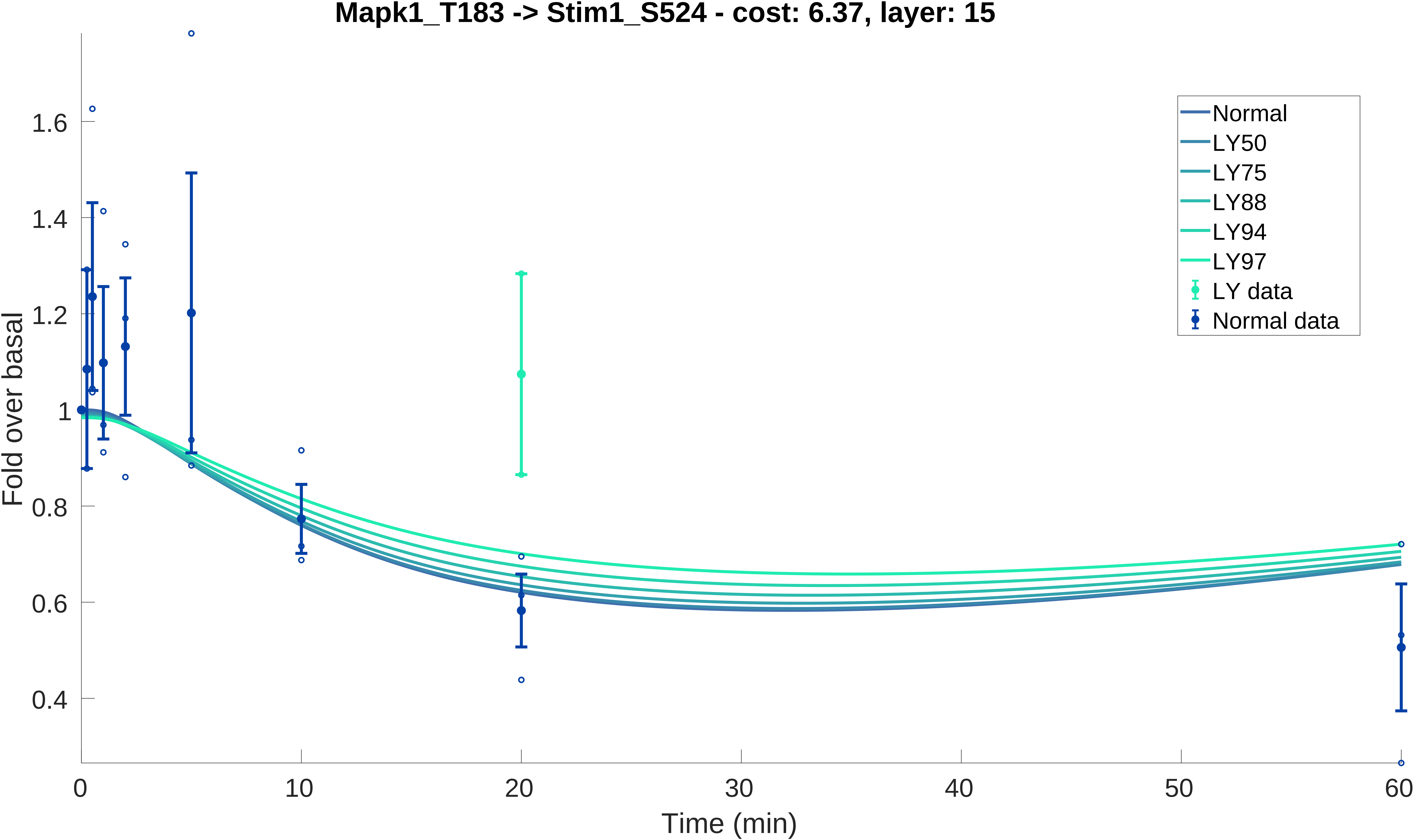
Mapk1_T183 -> Stim1_S524 - cost: 6.37, layer: 15
Fold over basal
Time (min)
0
10
20
30
40
50
60
0.4
0.6
0.8
1
1.2
1.4
1.6
Normal
LY50
LY75
LY88
LY94
LY97
LY data
Normal data

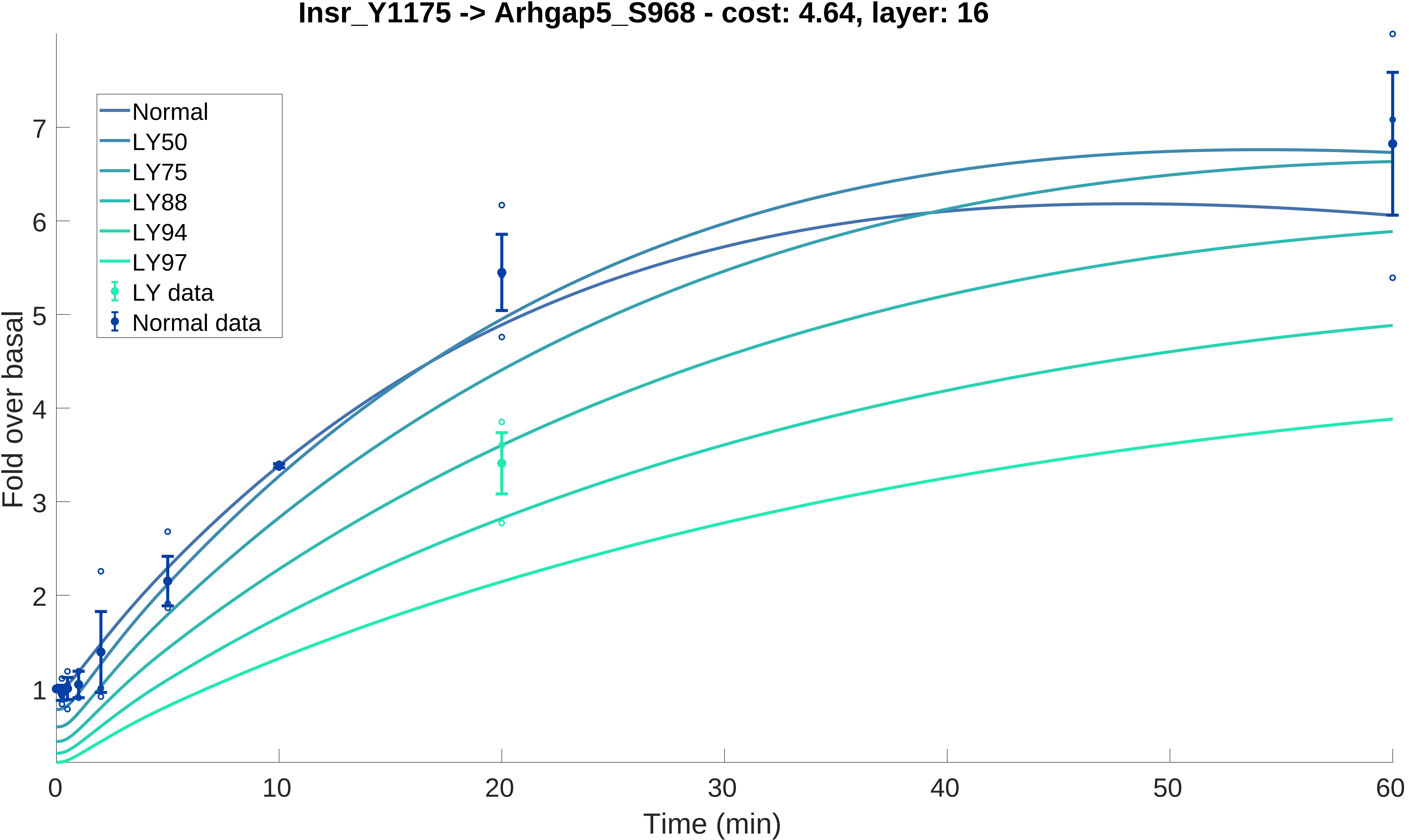

Insr_Y1175 -> Arhgap5_S968 - cost: 4.64, layer: 16
Normal
LY50
LY75
LY88
LY94
LY97
LY data
Normal data
Fold over basal
Time (min)
1
2
3
4
5
6
7
0
10
20
30
40
50
60

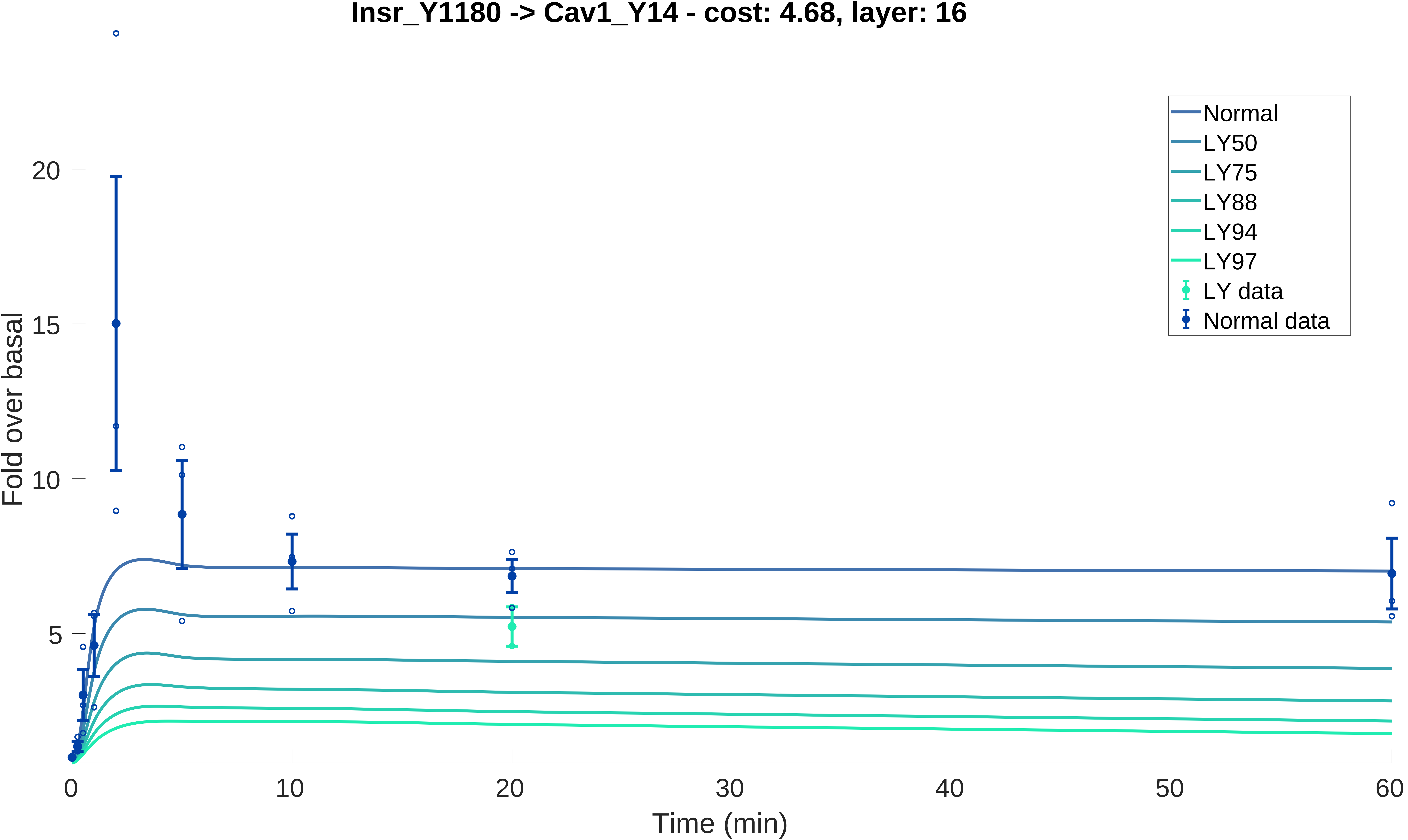

Insr_Y1180 -> Cav1_Y14 - cost: 4.68, layer: 16
Fold over basal
Time (min)
0
10
20
30
40
50
60
5
10
15
20
Normal
LY50
LY75
LY88
LY94
LY97
LY data
Normal data

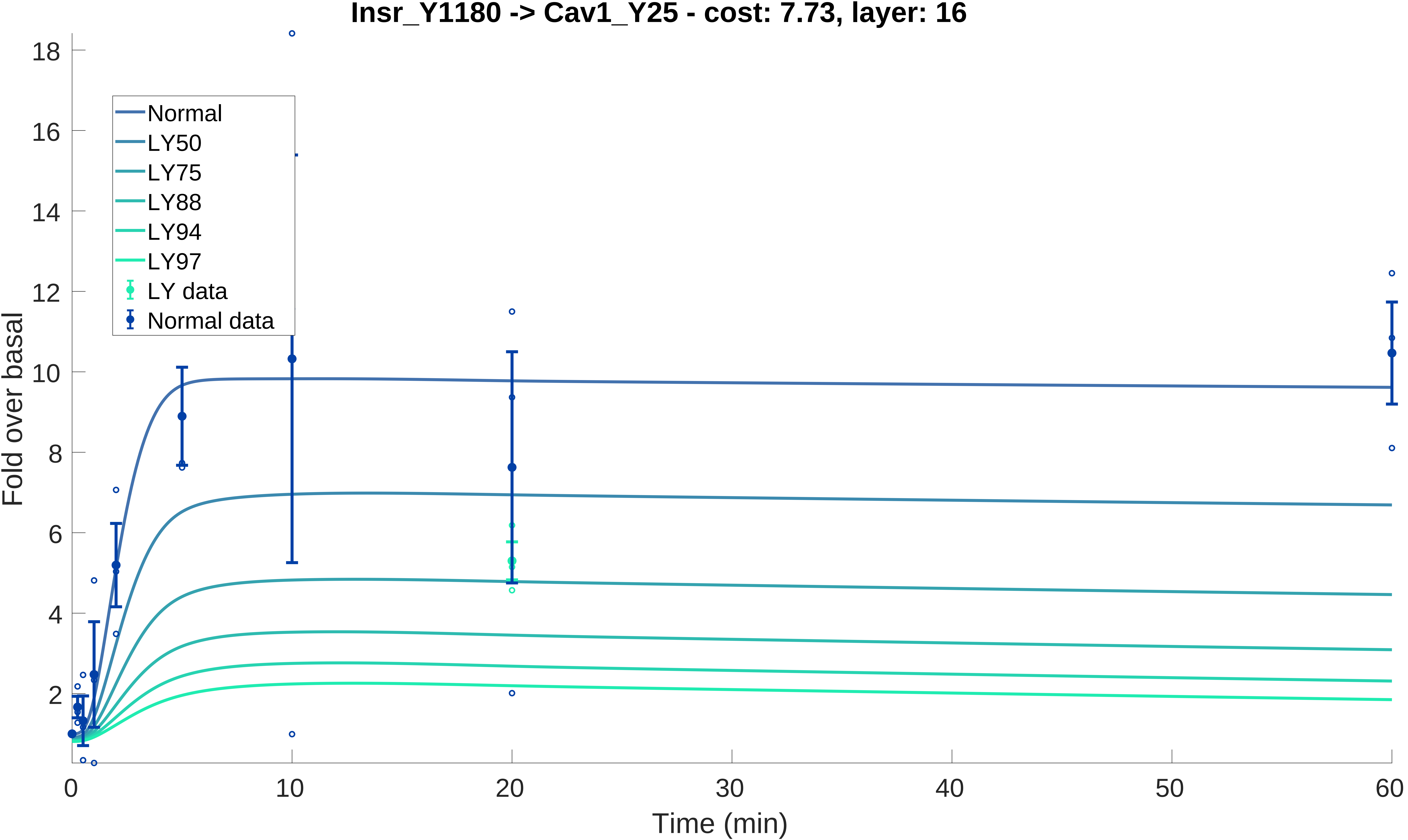
Insr_Y1180 -> Cav1_Y25 - cost: 7.73, layer: 16
Fold over basal
Time (min)
Normal
LY50
LY75
LY88
LY94
LY97
LY data
Normal data
18
16
14
12
10
8
6
4
2
0
10
20
30
40
50
60

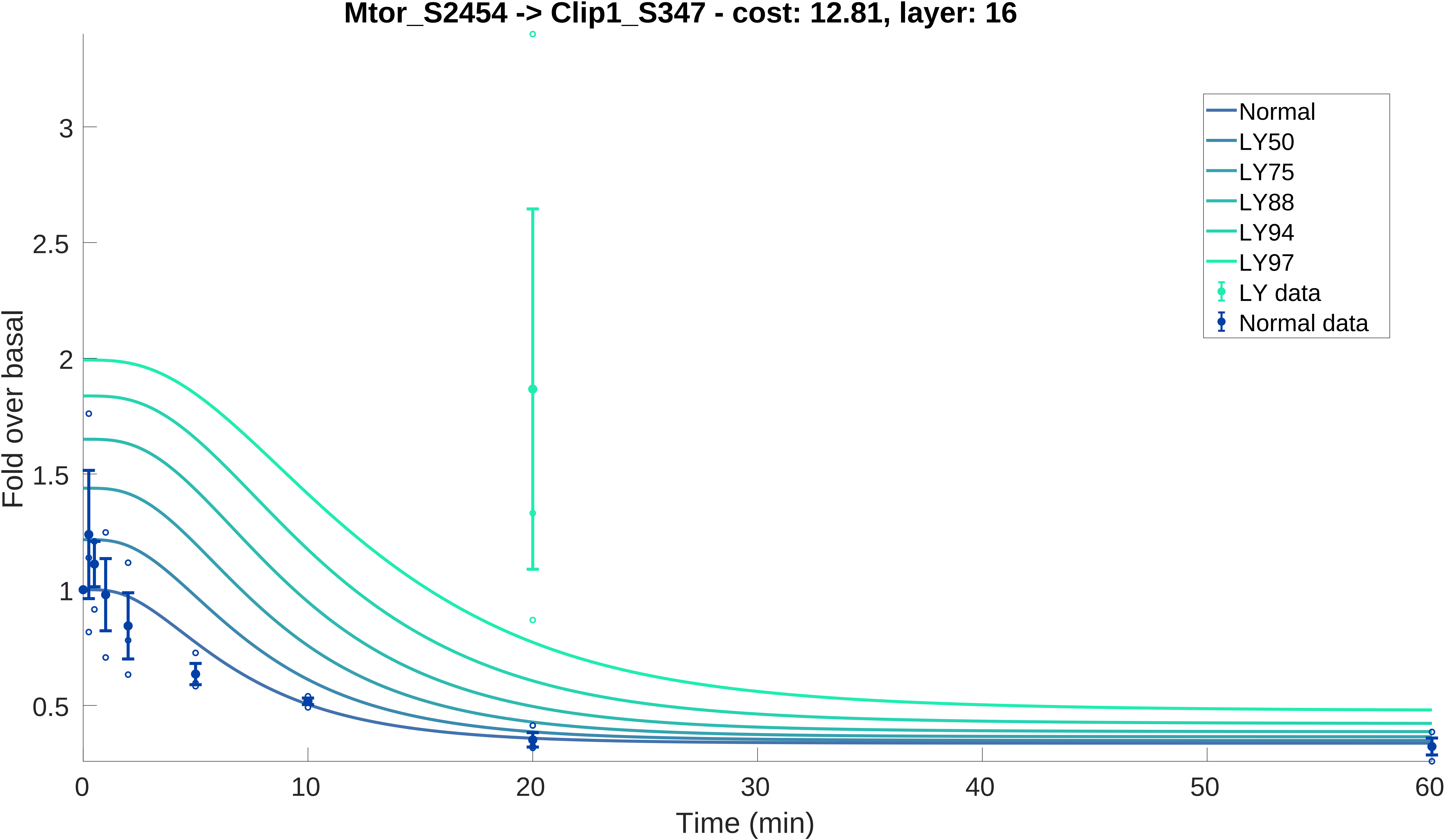

Mtor_S2454 -> Clip1_S347 - cost: 12.81, layer: 16
Fold over basal
Time (min)
0.5
1
1.5
2
2.5
3
0
10
20
30
40
50
60
Normal
LY50
LY75
LY88
LY94
LY97
LY data
Normal data

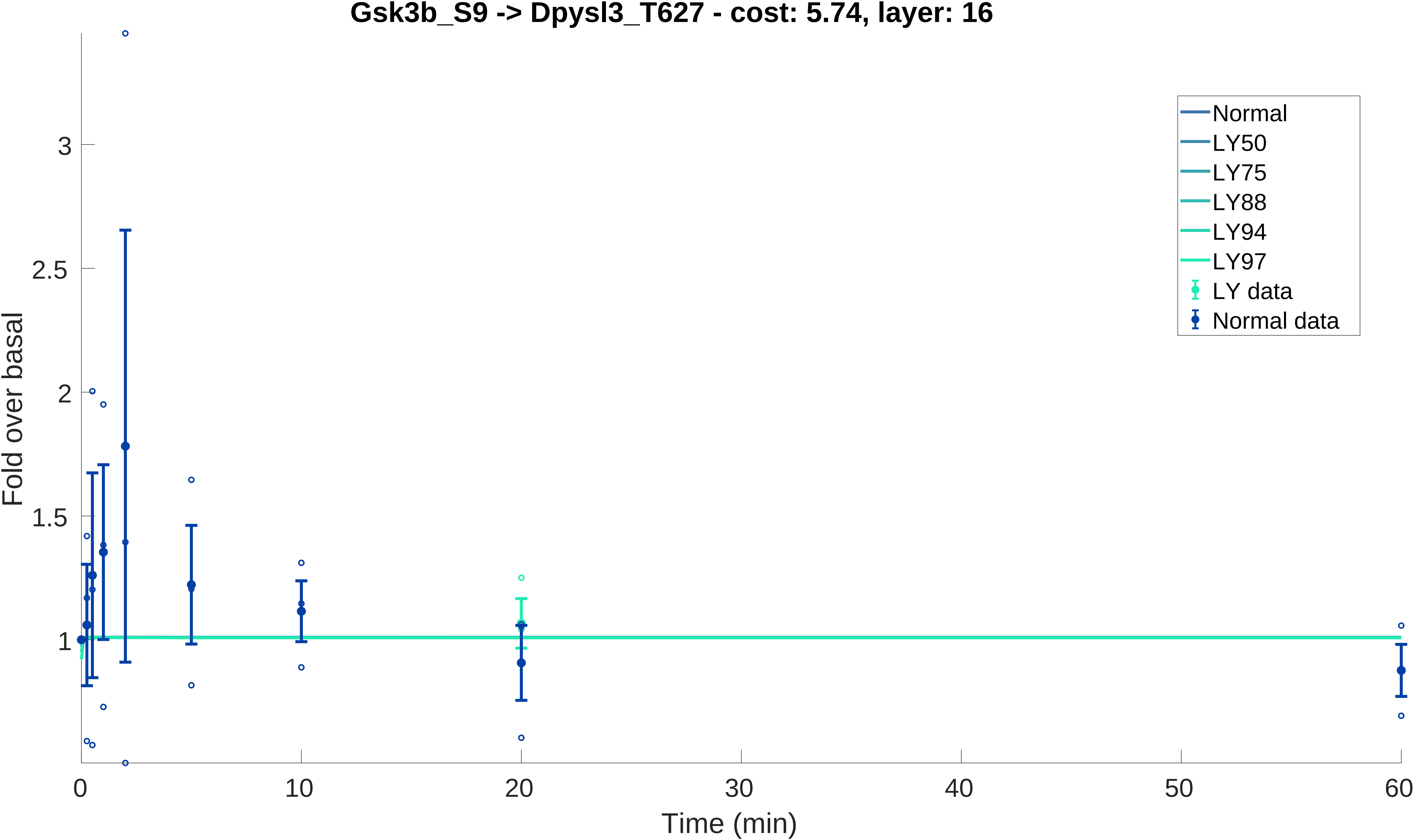

Gsk3b_S9 -> Dpysl3_T627 - cost: 5.74, layer: 16
Fold over basal
Time (min)
3
2.5
2
1.5
1
0
10
20
30
40
50
60
Normal
LY50
LY75
LY88
LY94
LY97
LY data
Normal data

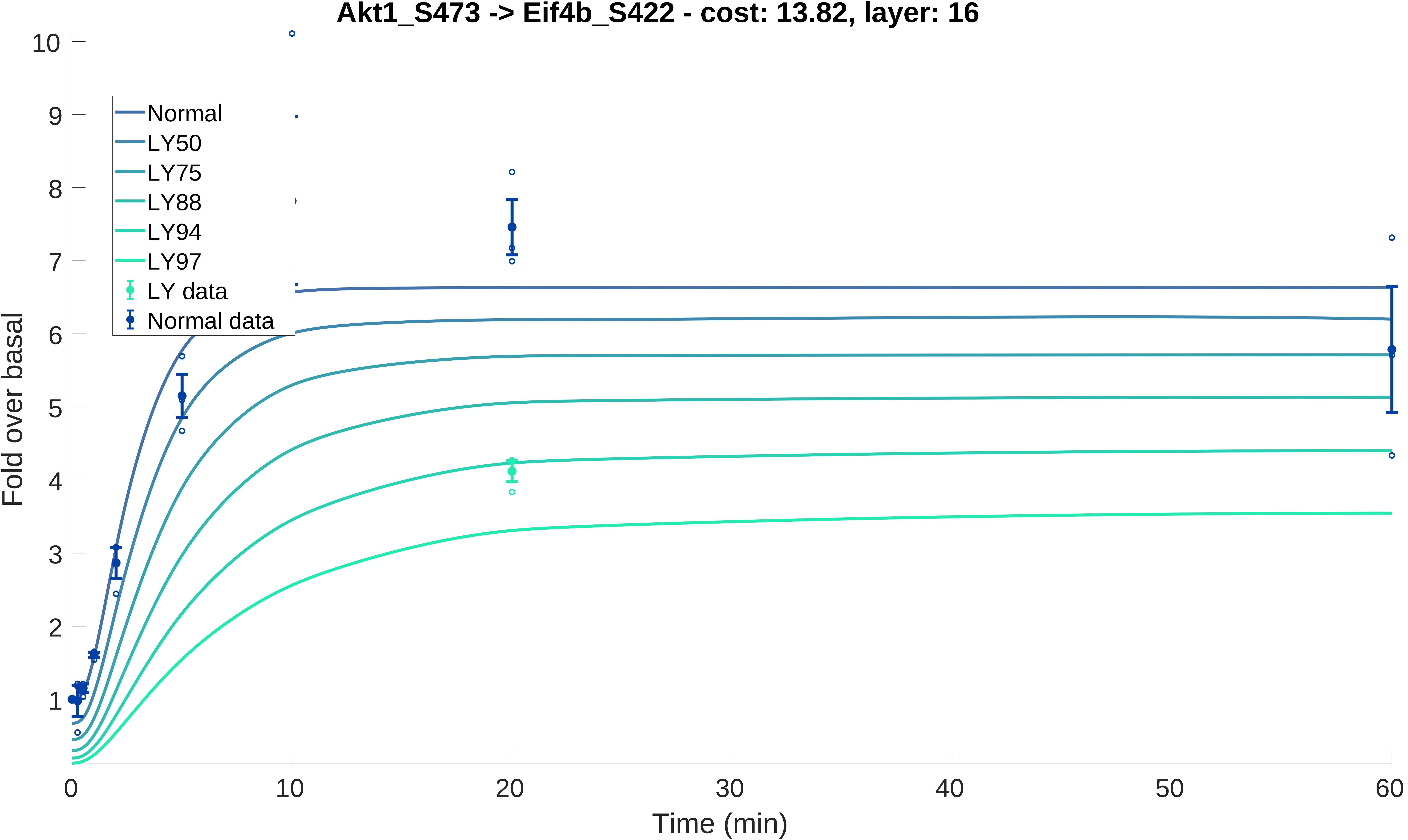

Akt1_S473 -> Eif4b_S422 - cost: 13.82, layer: 16
Normal
LY50
LY75
LY88
LY94
LY97
LY data
Normal data
Fold over basal
Time (min)
0
10
20
30
40
50
60
1
2
3
4
5
6
7
8
9
10

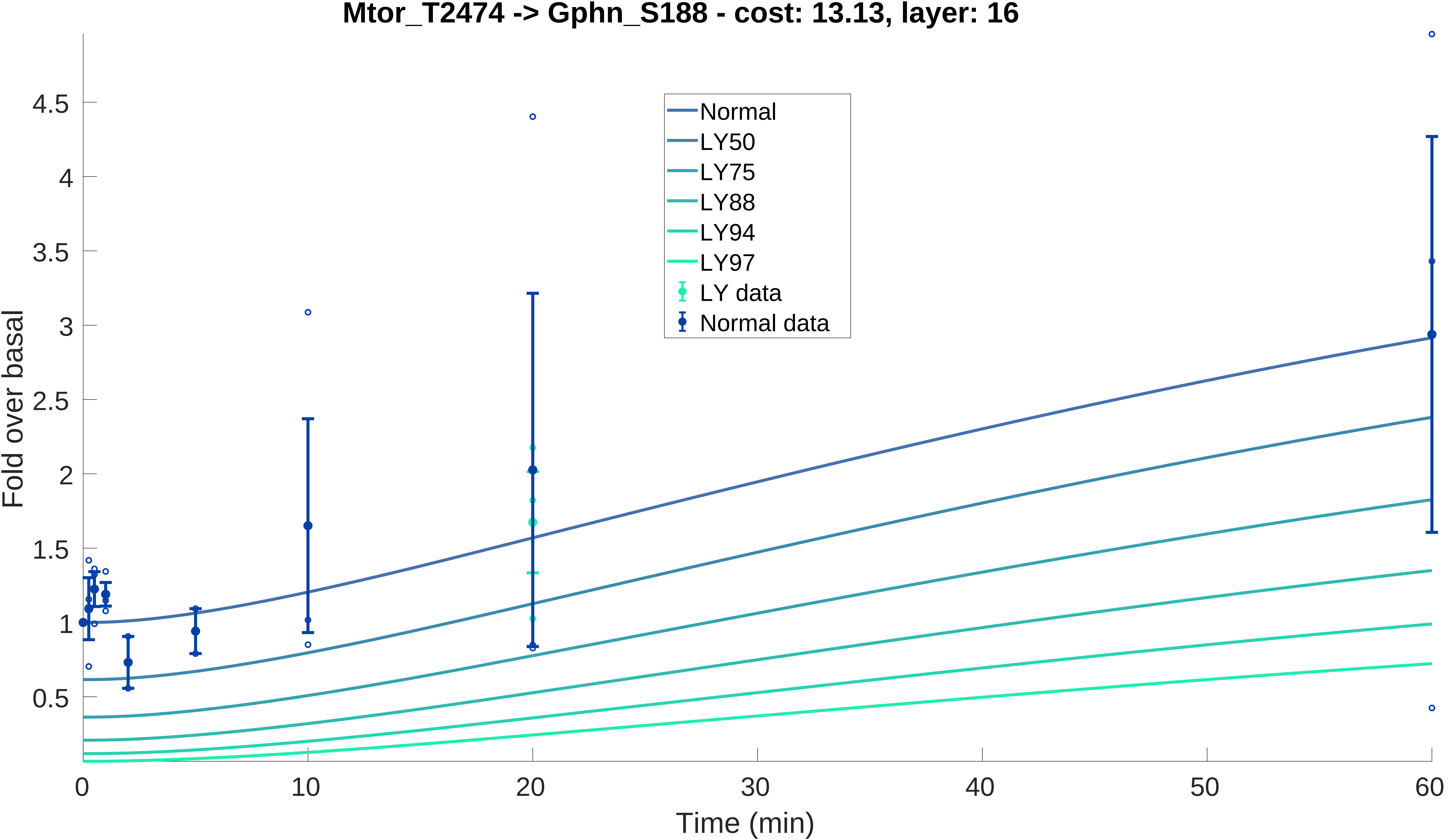

Mtor_T2474 -> Gphn_S188 - cost: 13.13, layer: 16
Fold over basal
Time (min)
0
10
20
30
40
50
60
0.5
1
1.5
2
2.5
3
3.5
4
4.5
Normal
LY50
LY75
LY88
LY94
LY97
LY data
Normal data

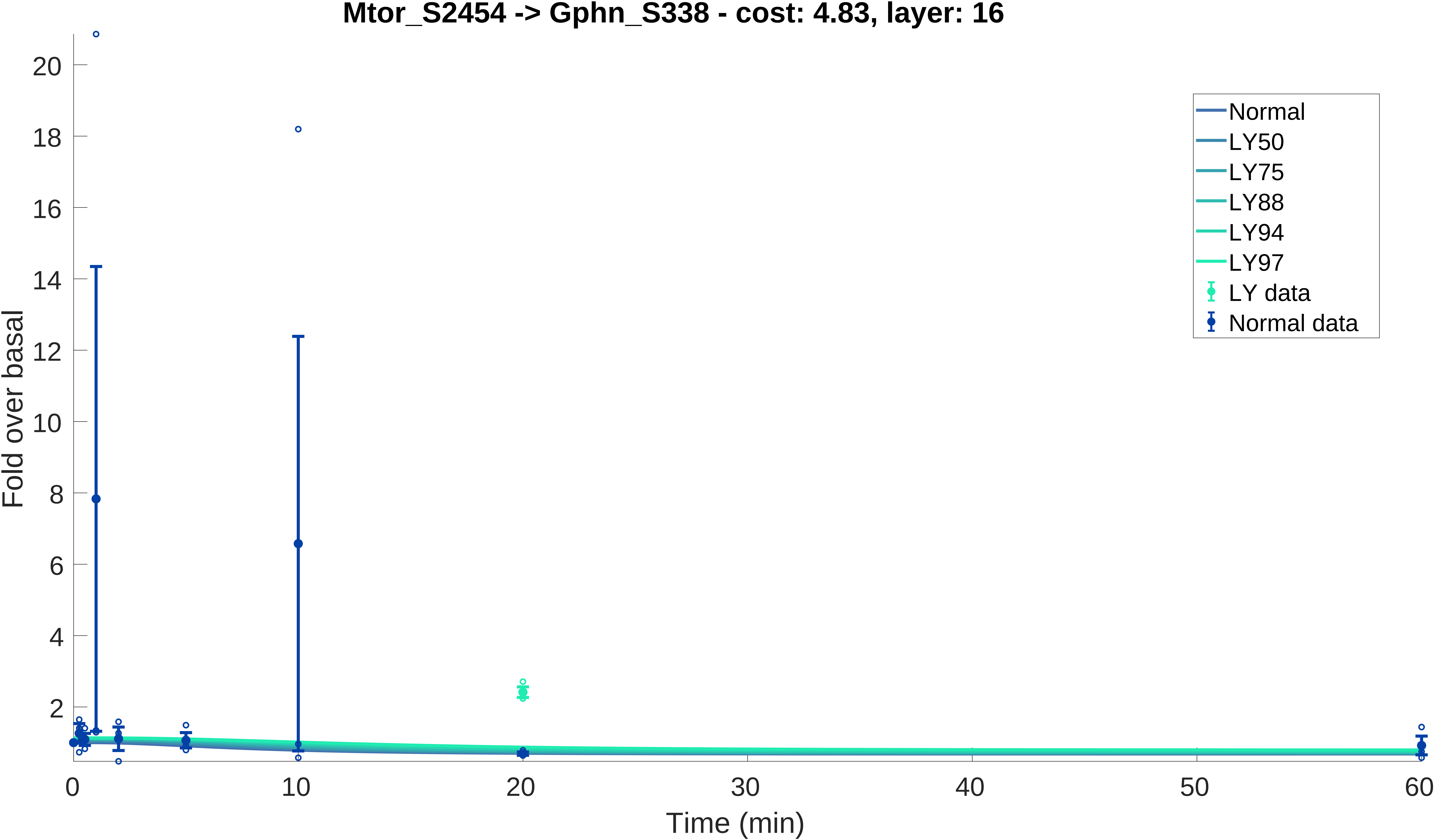

Mtor_S2454 -> Gphn_S338 - cost: 4.83, layer: 16
Fold over basal
Time (min)
0
10
20
30
40
50
60
2
4
6
8
10
12
14
16
18
20
Normal
LY50
LY75
LY88
LY94
LY97
LY data
Normal data

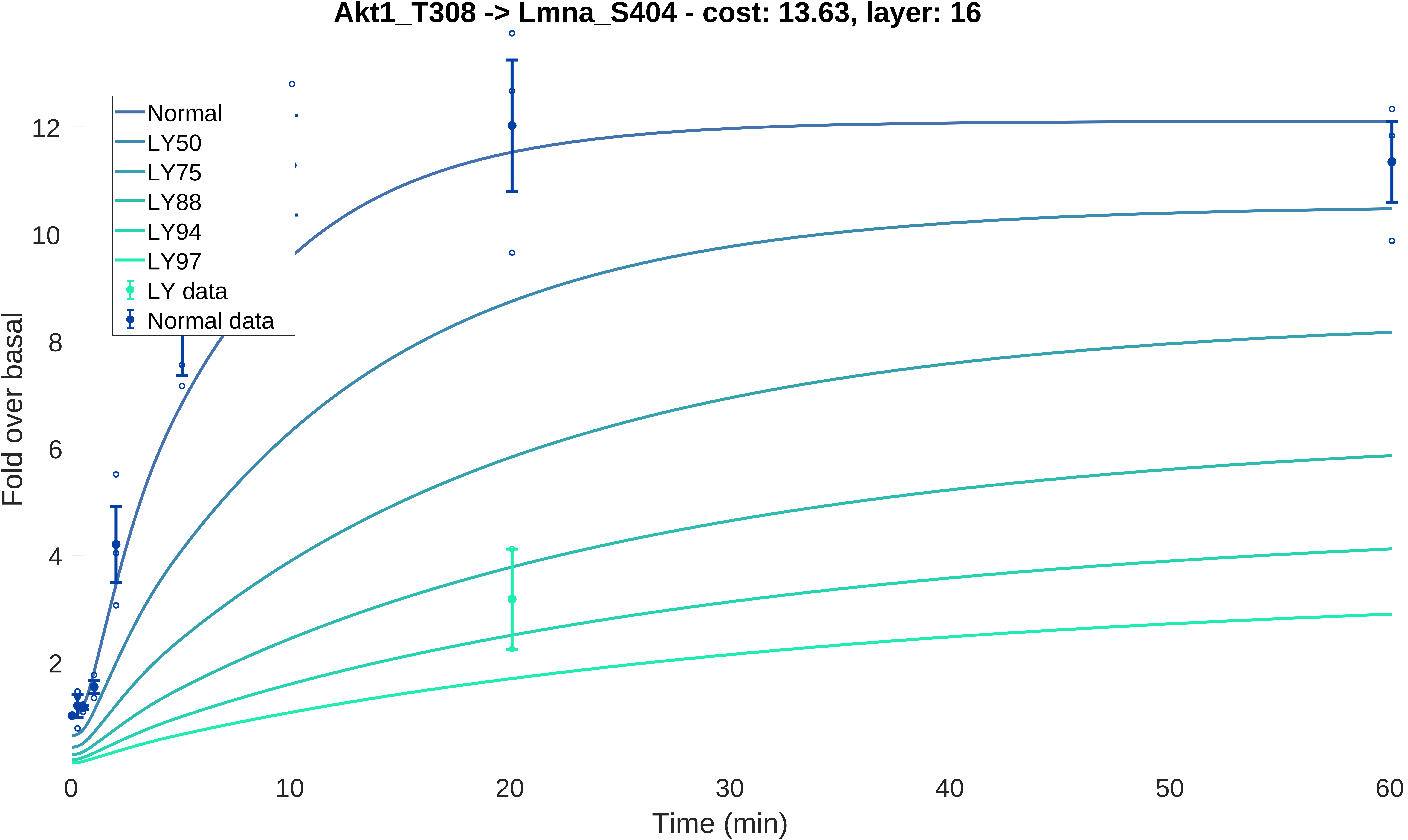

Akt1_T308 -> Lmna_S404 - cost: 13.63, layer: 16
Normal
LY50
LY75
LY88
LY94
LY97
LY data
Normal data
Fold over basal
Time (min)
0
10
20
30
40
50
60
2
4
6
8
10
12

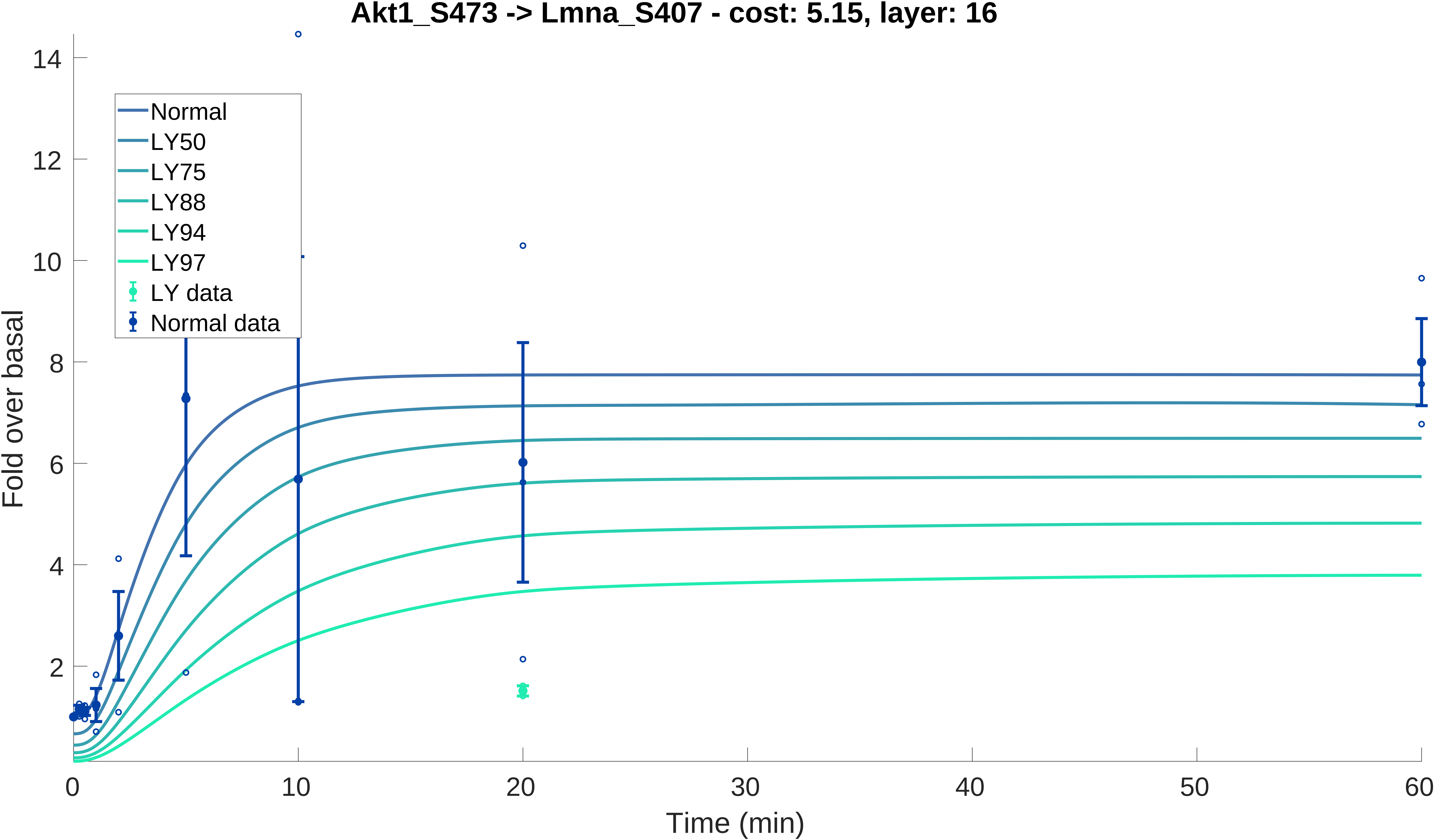

Akt1_S473 -> Lmna_S407 - cost: 5.15, layer: 16
Fold over basal
Time (min)
0
10
20
30
40
50
60
2
4
6
8
10
12
14
Normal
LY50
LY75
LY88
LY94
LY97
LY data
Normal data

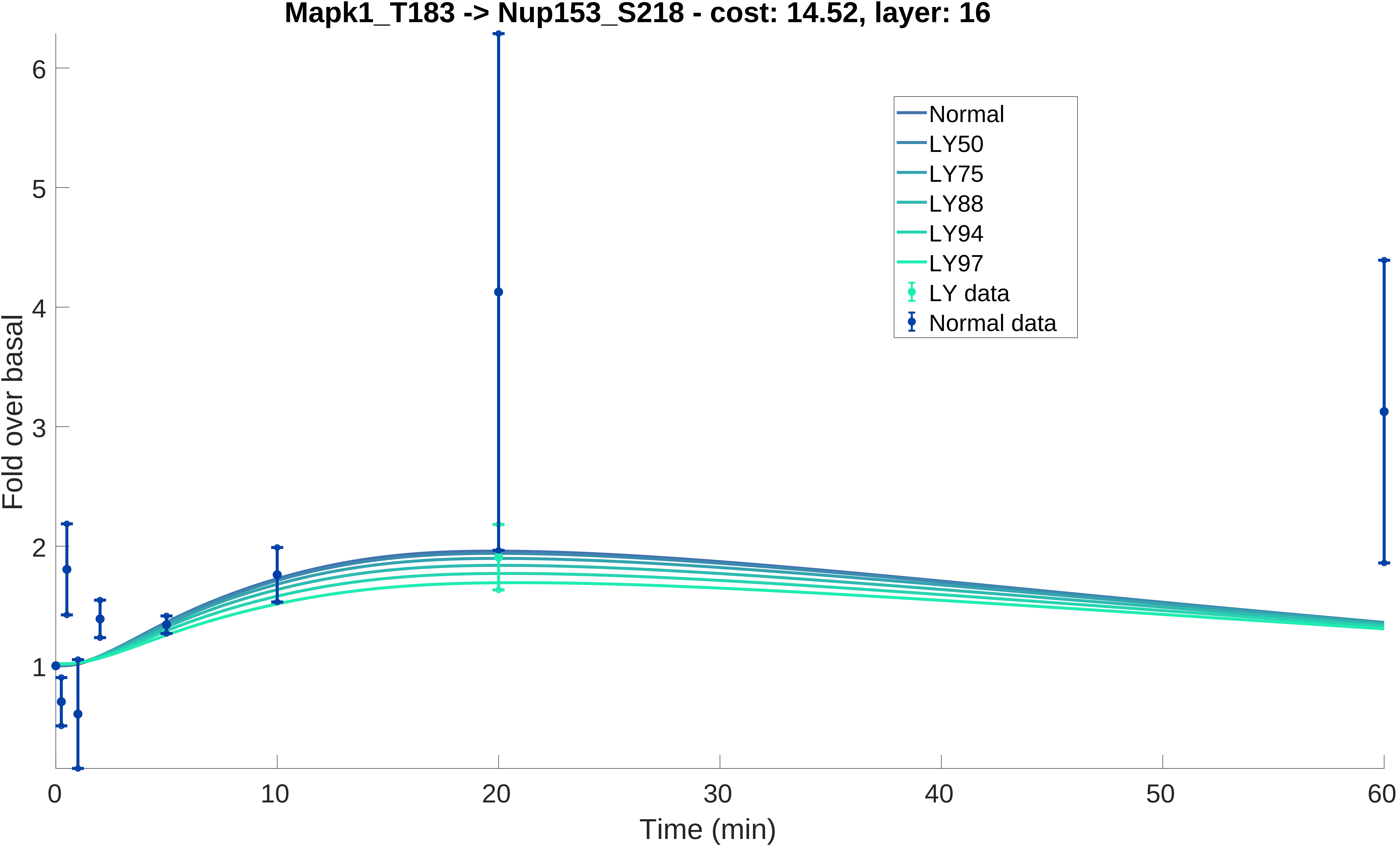

Mapk1_T183 -> Nup153_S218 - cost: 14.52, layer: 16
Fold over basal
Time (min)
0
10
20
30
40
50
60
1
2
3
4
5
6
Normal
LY50
LY75
LY88
LY94
LY97
LY data
Normal data

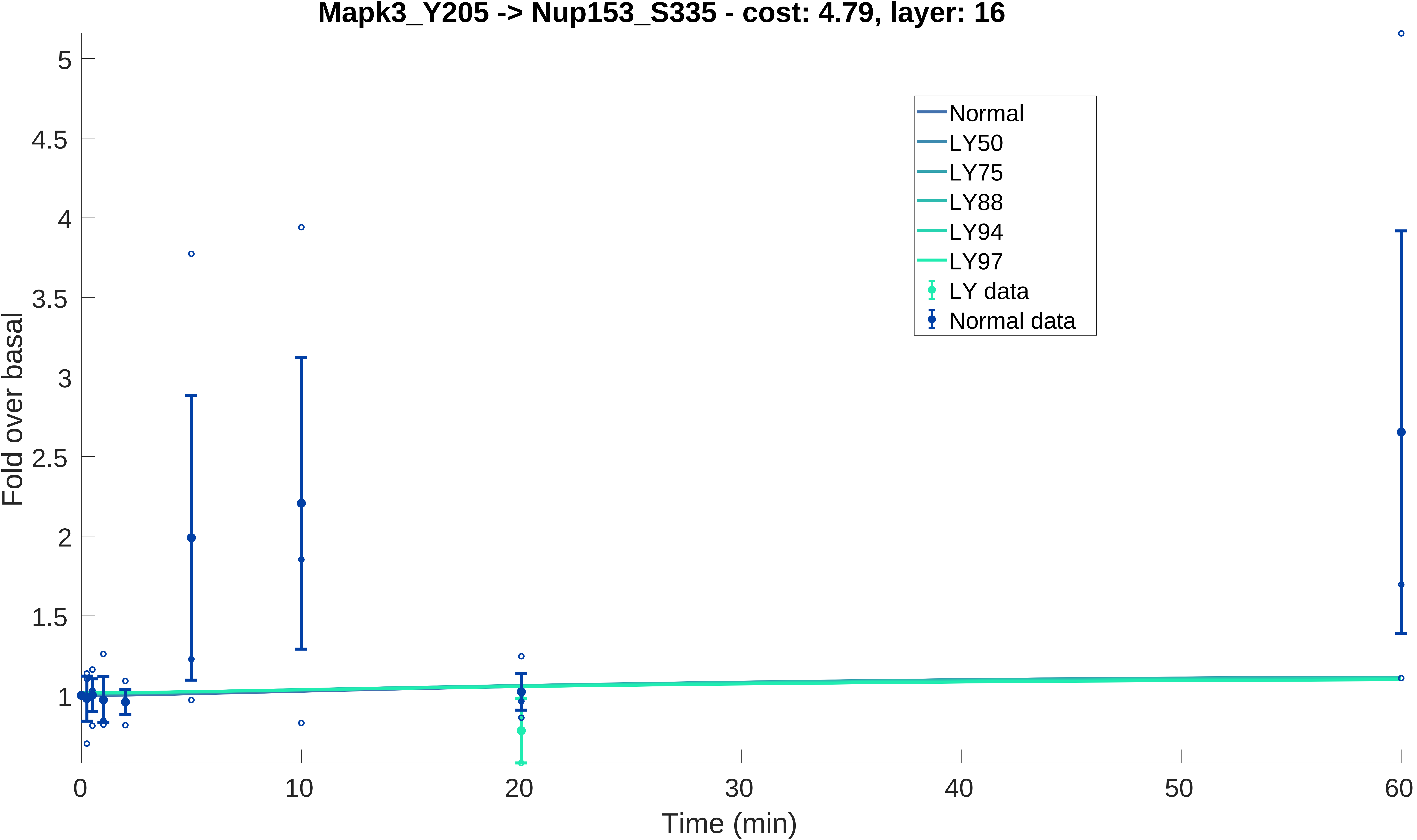

Mapk3_Y205 -> Nup153_S335 - cost: 4.79, layer: 16
Fold over basal
5
4.5
4
3.5
3
2.5
2
1.5
1
0
10
20
30
40
50
60
Time (min)
Normal
LY50
LY75
LY88
LY94
LY97
LY data
Normal data

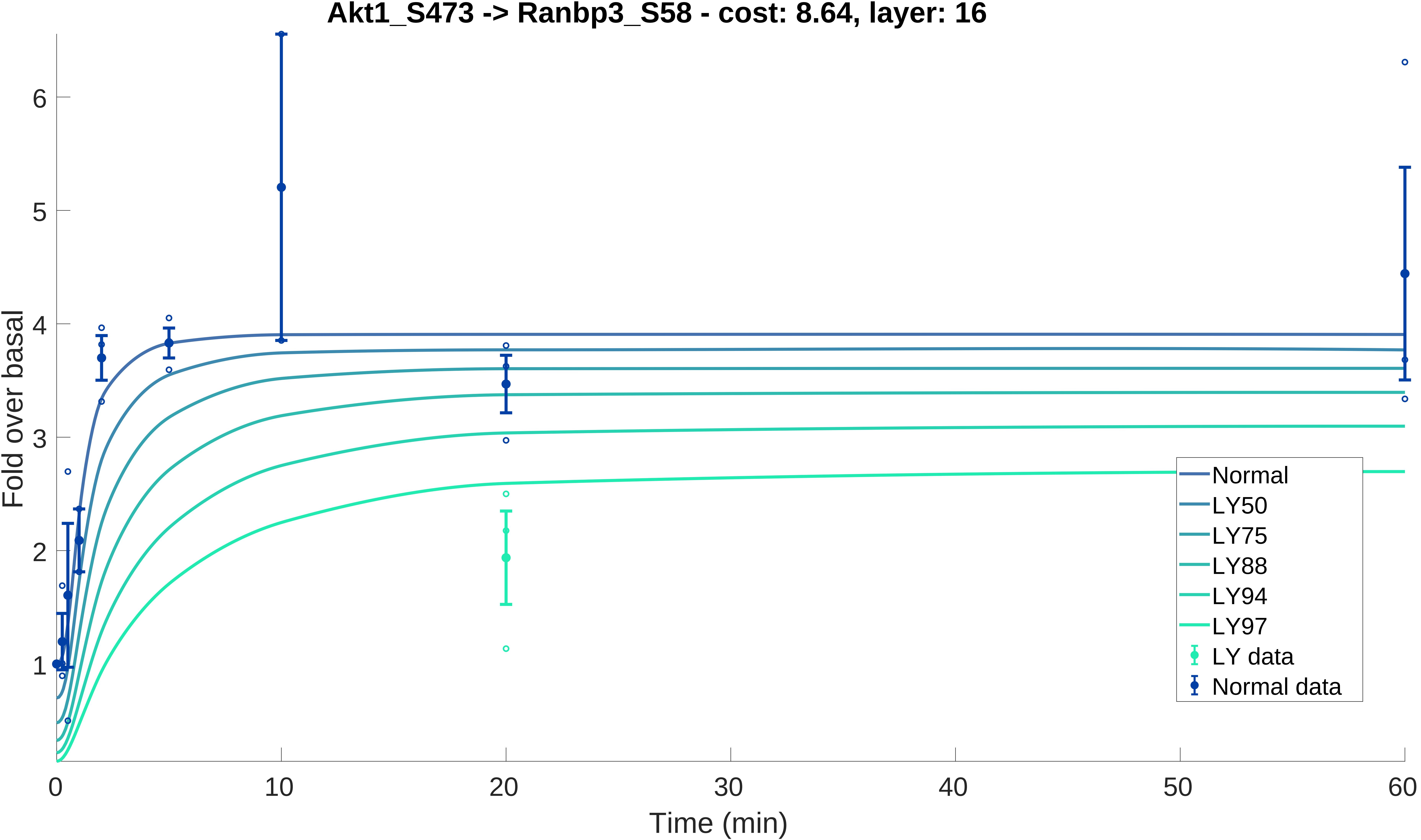

Akt1_S473 -> Ranbp3_S58 - cost: 8.64, layer: 16
Fold over basal
Time (min)
0
10
20
30
40
50
60
1
2
3
4
5
6
Normal
LY50
LY75
LY88
LY94
LY97
LY data
Normal data

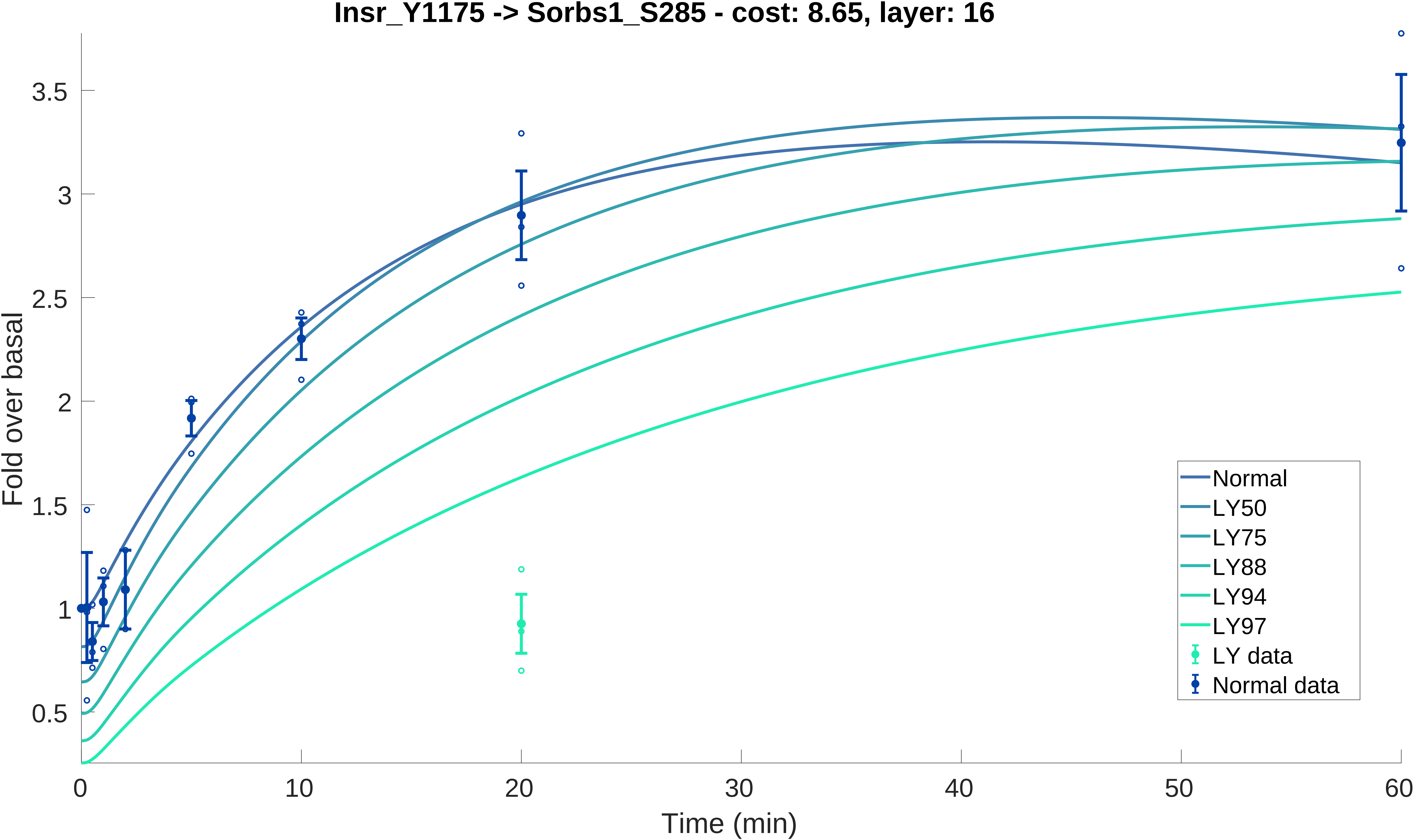

Insr_Y1175 -> Sorbs1_S285 - cost: 8.65, layer: 16
Fold over basal
Time (min)
0.5
1
1.5
2
2.5
3
3.5
0
10
20
30
40
50
60
Normal
LY50
LY75
LY88
LY94
LY97
LY data
Normal data

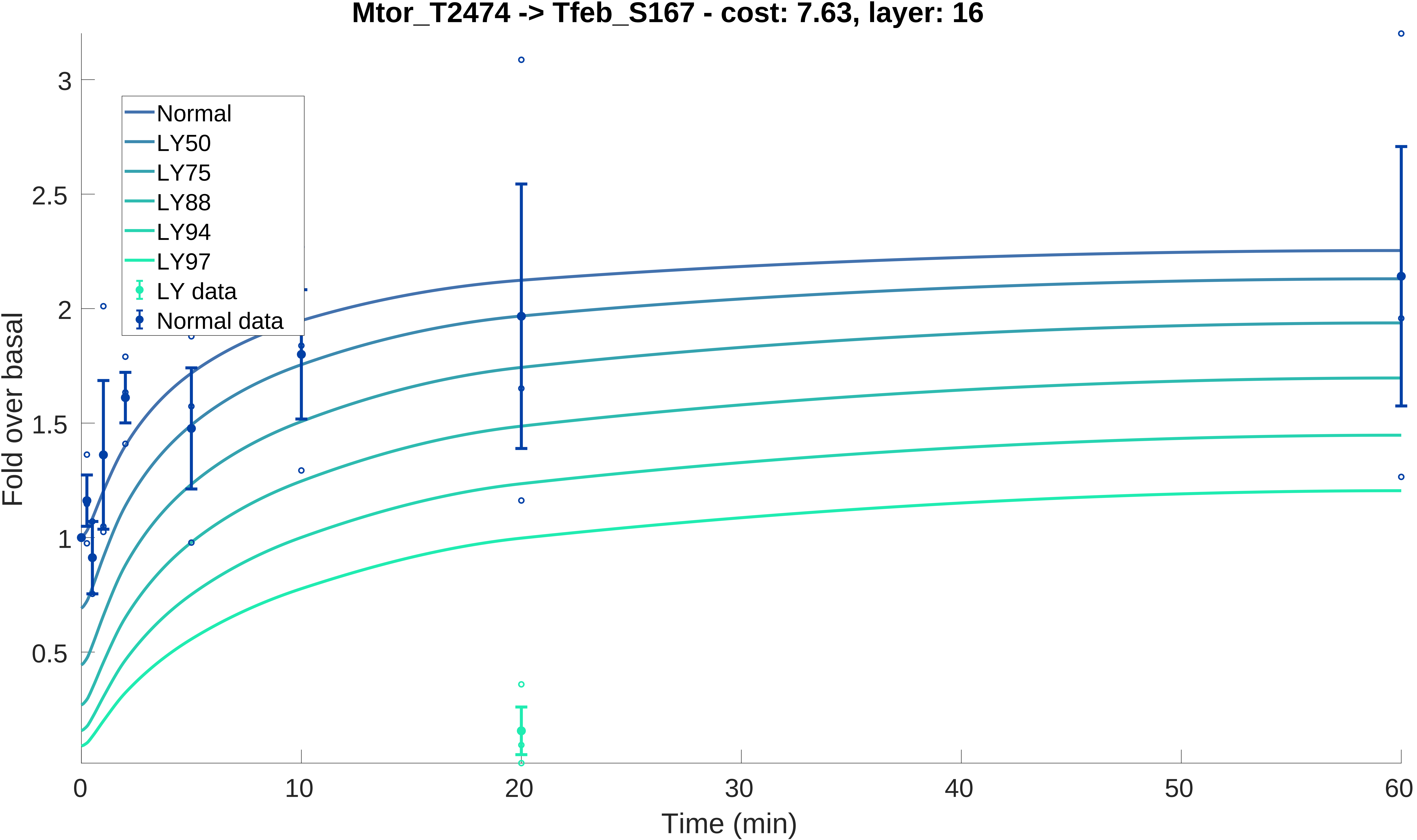

Mtor_T2474 -> Tfeb_S167 - cost: 7.63, layer: 16
Normal
LY50
LY75
LY88
LY94
LY97
LY data
Normal data
Fold over basal
Time (min)
0
10
20
30
40
50
60
0.5
1
1.5
2
2.5
3

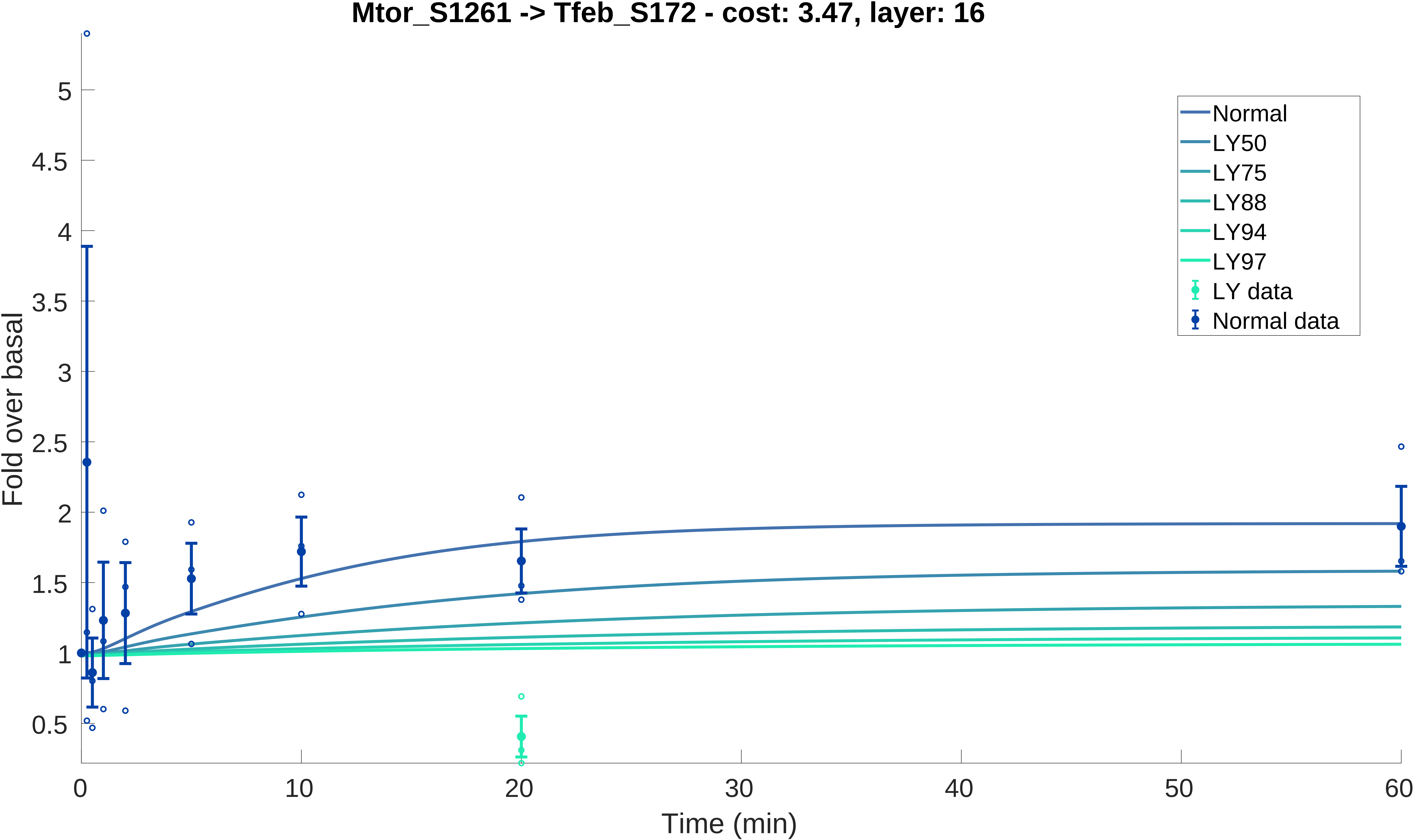

Mtor_S1261 -> Tfeb_S172 - cost: 3.47, layer: 16
Fold over basal
Time (min)
0.5
1
1.5
2
2.5
3
3.5
4
4.5
5
0
10
20
30
40
50
60
Normal
LY50
LY75
LY88
LY94
LY97
LY data
Normal data

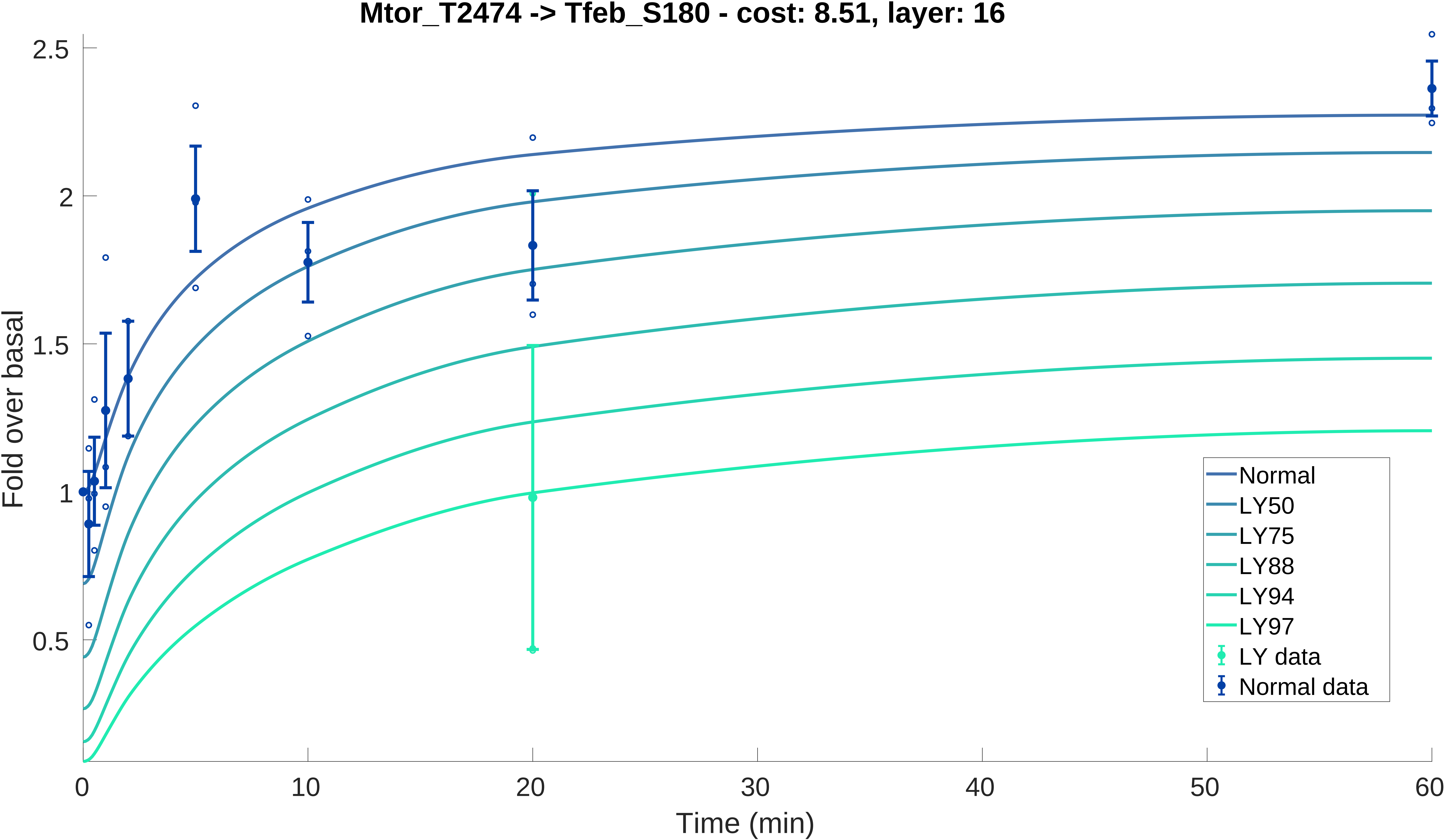
Mtor_T2474 -> Tfeb_S180 - cost: 8.51, layer: 16
Fold over basal
2.5
2
1.5
1
0.5
0
10
20
30
40
50
60
Time (min)
Normal
LY50
LY75
LY88
LY94
LY97
LY data
Normal data

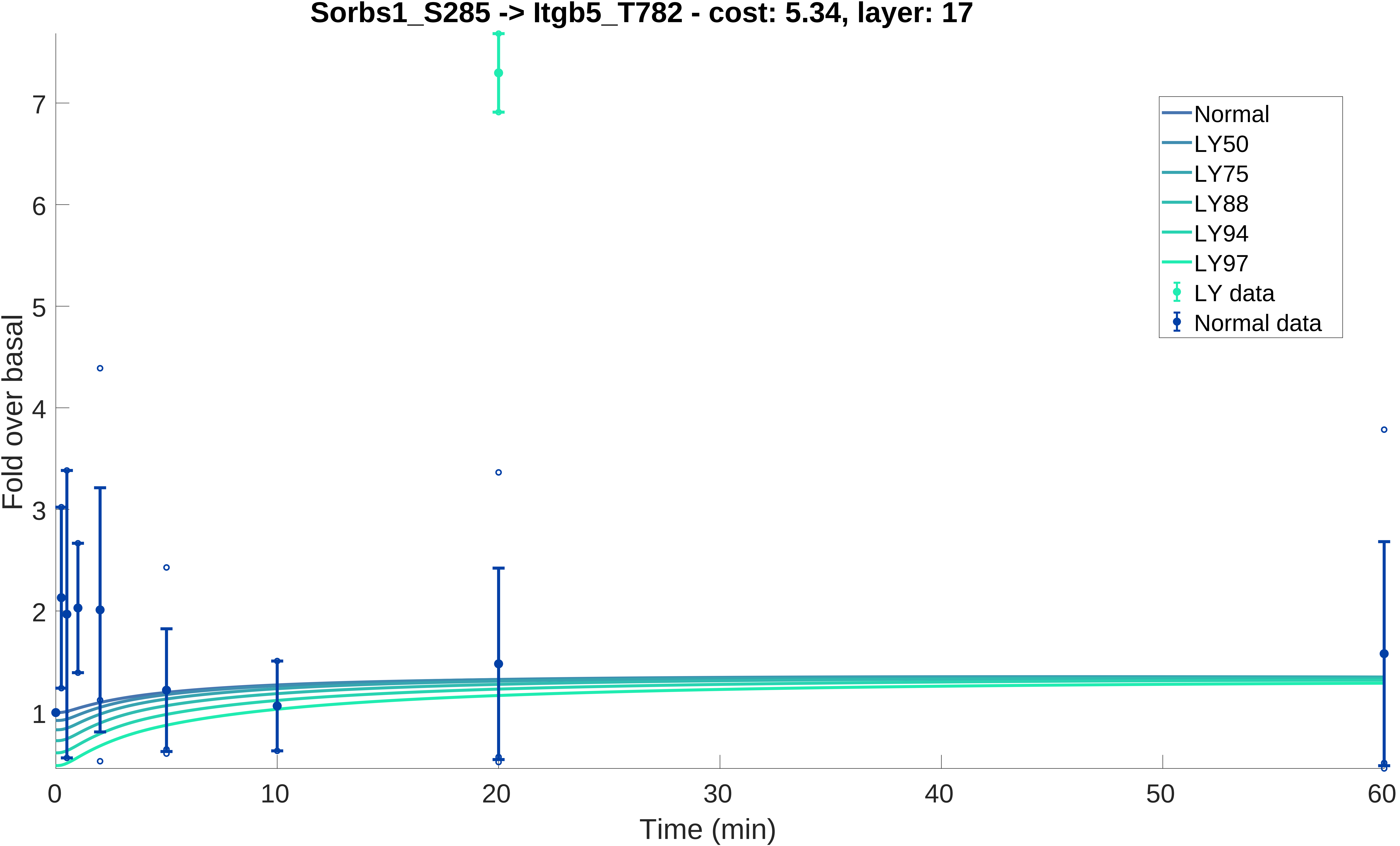

Sorbs1_S285 -> Itgb5_T782 - cost: 5.34, layer: 17
Fold over basal
Time (min)
0
10
20
30
40
50
60
1
2
3
4
5
6
7
Normal
LY50
LY75
LY88
LY94
LY97
LY data
Normal data

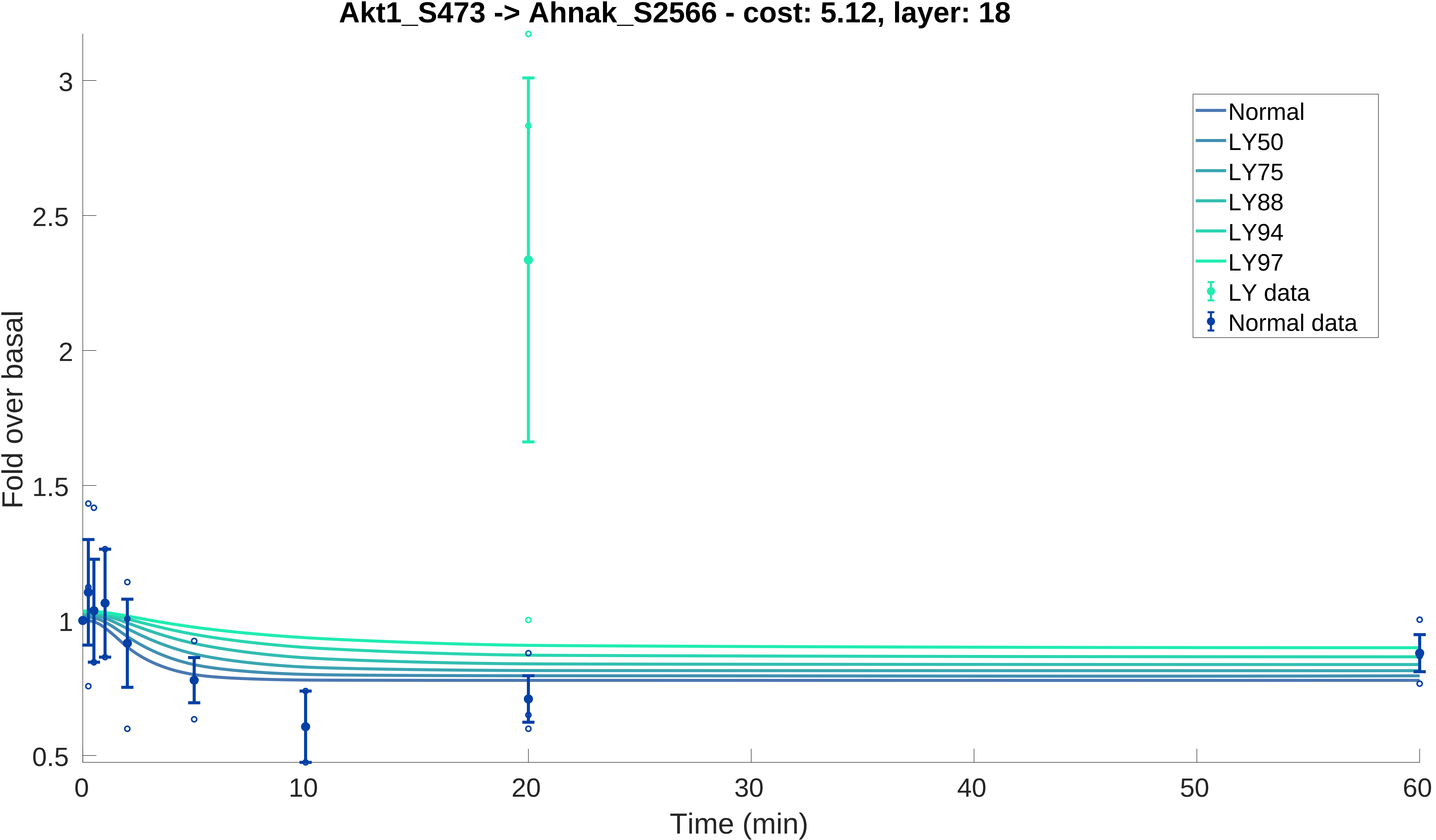

Akt1_S473 -> Ahnak_S2566 - cost: 5.12, layer: 18
Fold over basal
3
2.5
2
1.5
1
0.5
0
10
20
30
40
50
60
Time (min)
Normal
LY50
LY75
LY88
LY94
LY97
LY data
Normal data

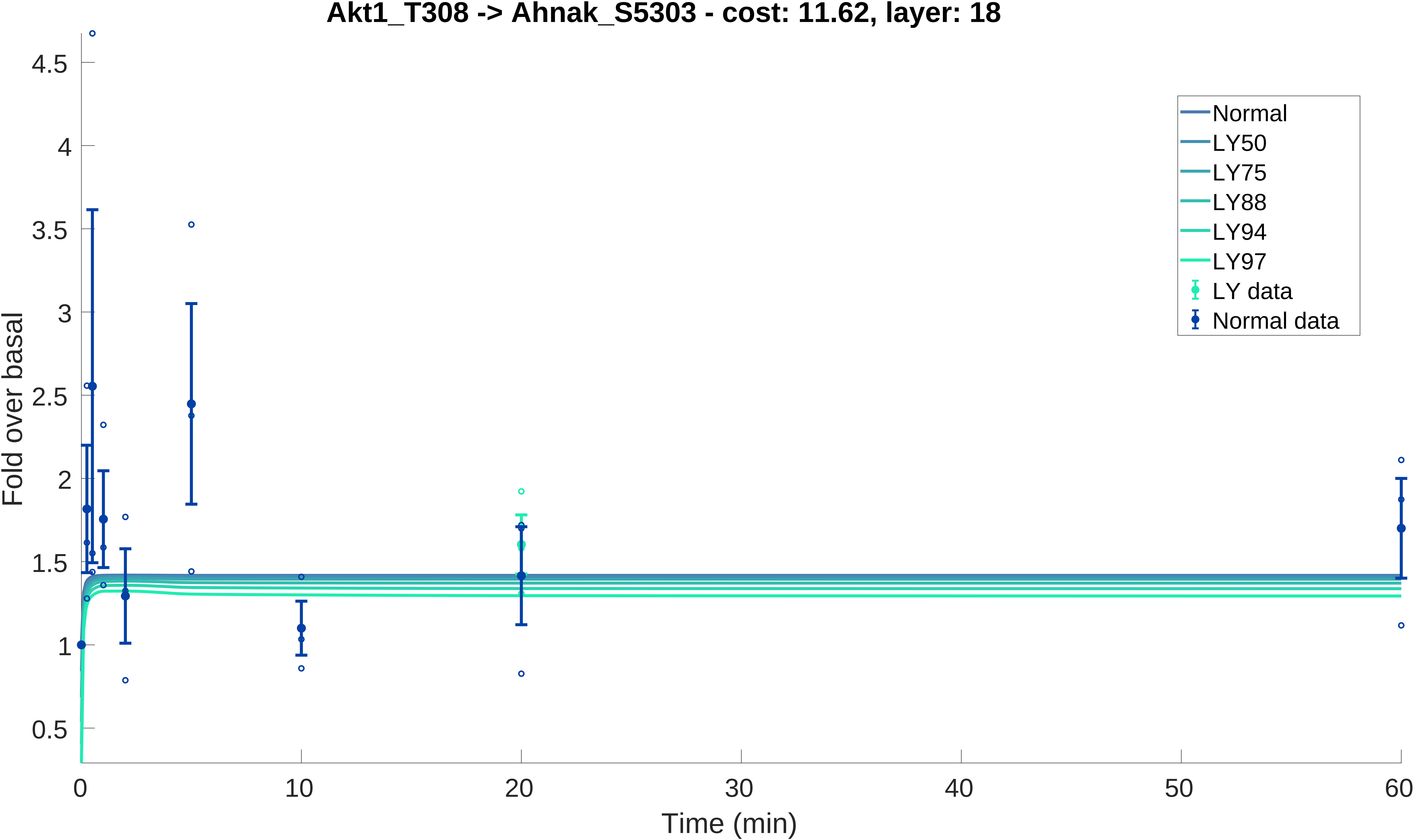

Akt1_T308 -> Ahnak_S5303 - cost: 11.62, layer: 18
Fold over basal
Time (min)
4.5
4
3.5
3
2.5
2
1.5
1
0.5
0
10
20
30
40
50
60
Normal
LY50
LY75
LY88
LY94
LY97
LY data
Normal data

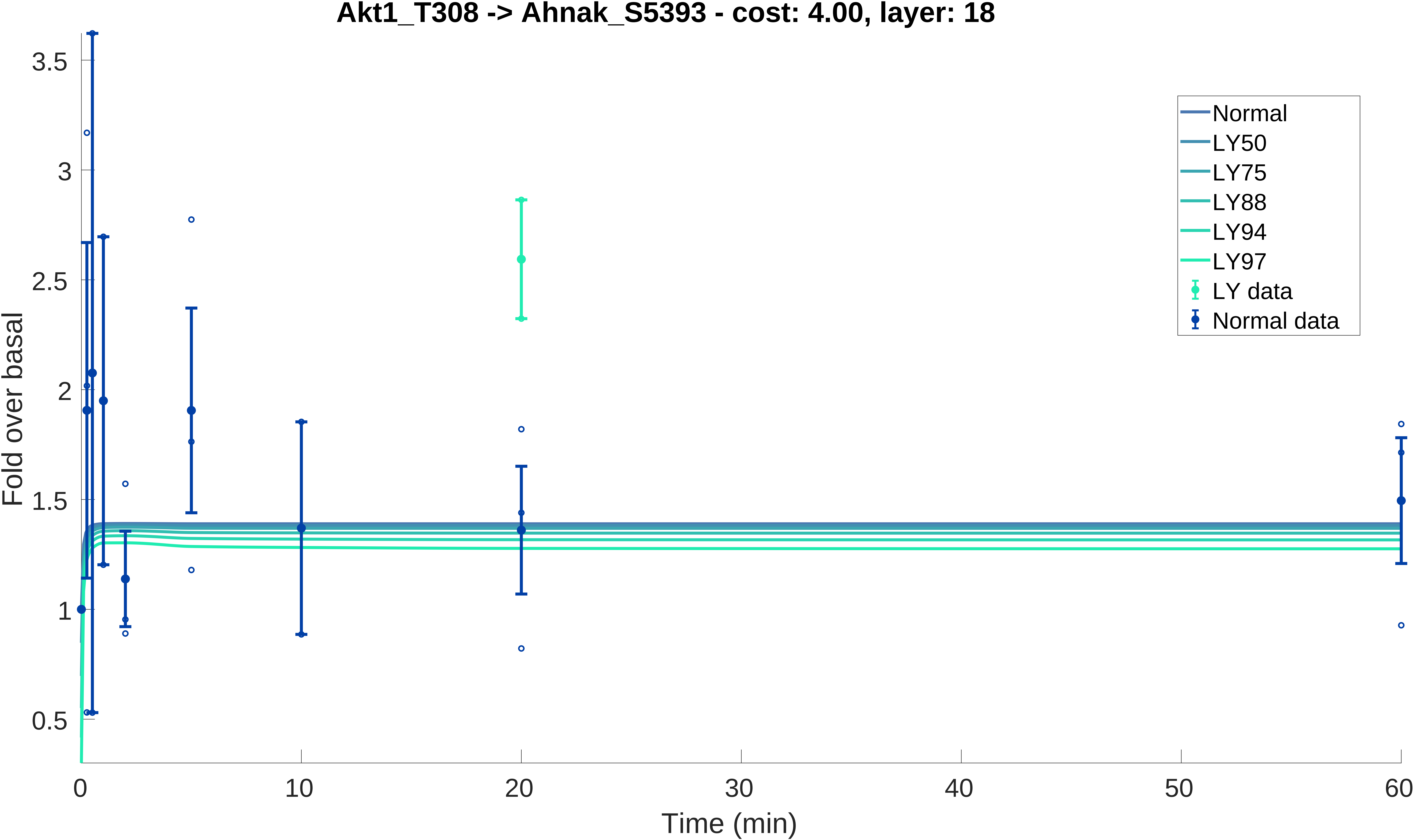

Akt1_T308 -> Ahnak_S5393 - cost: 4.00, layer: 18
Fold over basal
Time (min)
0.5
1
1.5
2
2.5
3
3.5
0
10
20
30
40
50
60
Normal
LY50
LY75
LY88
LY94
LY97
LY data
Normal data

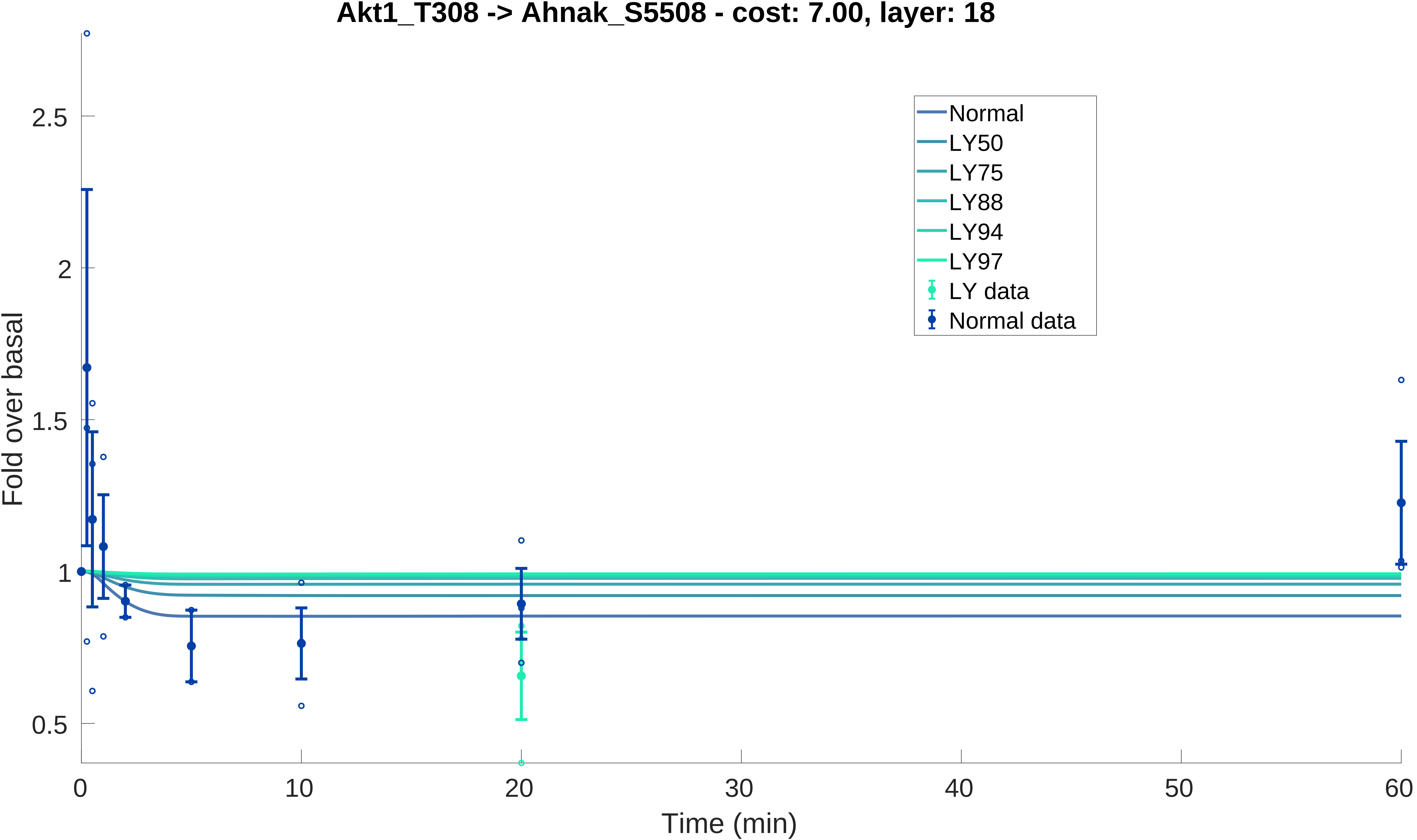
Akt1_T308 -> Ahnak_S5508 - cost: 7.00, layer: 18
Fold over basal
Time (min)
2.5
2
1.5
1
0.5
0
10
20
30
40
50
60
Normal
LY50
LY75
LY88
LY94
LY97
LY data
Normal data

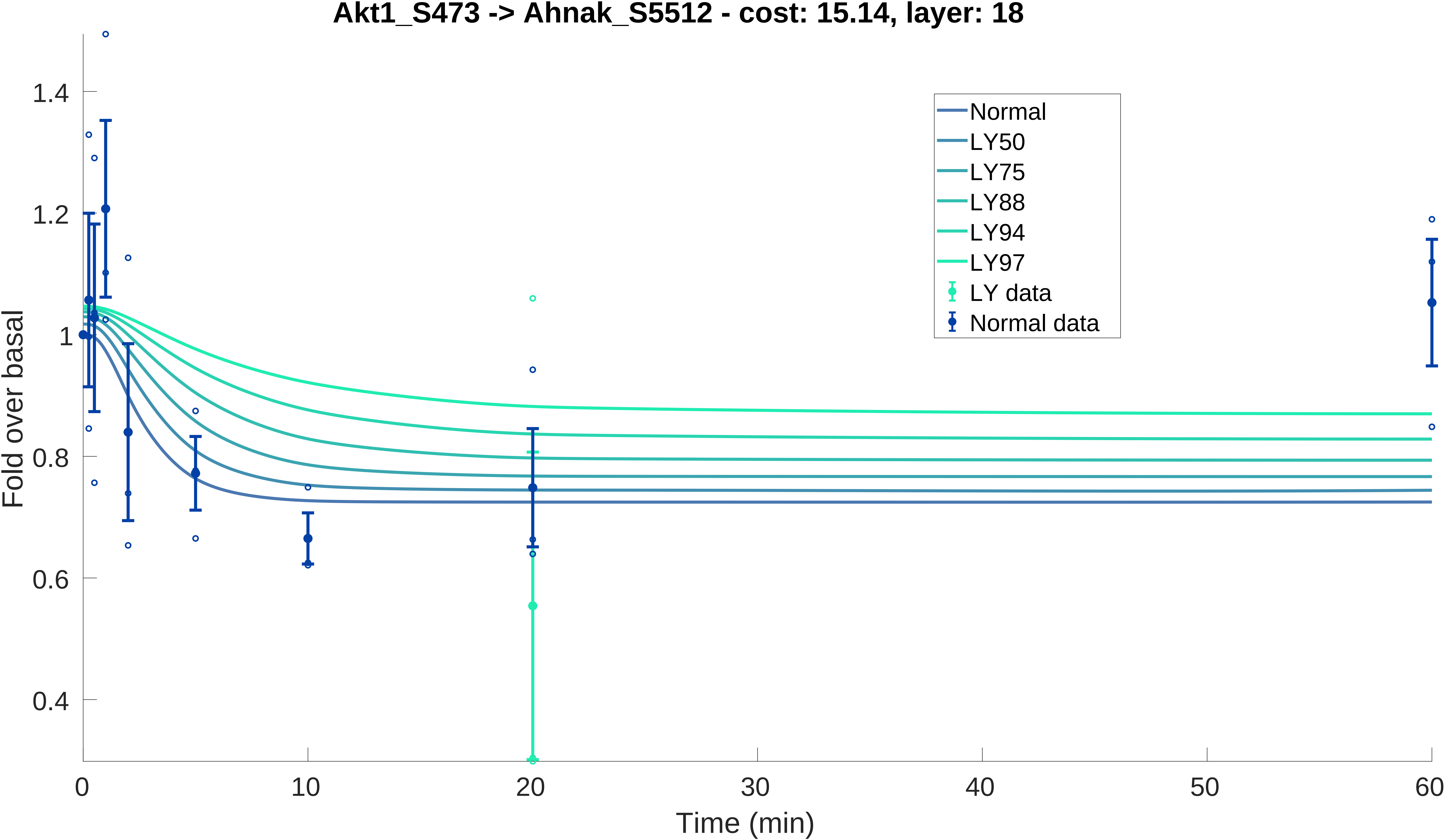
Akt1_S473 -> Ahnak_S5512 - cost: 15.14, layer: 18
Fold over basal
1.4
1.2
1
0.8
0.6
0.4
0
10
20
30
40
50
60
Time (min)
Normal
LY50
LY75
LY88
LY94
LY97
LY data
Normal data

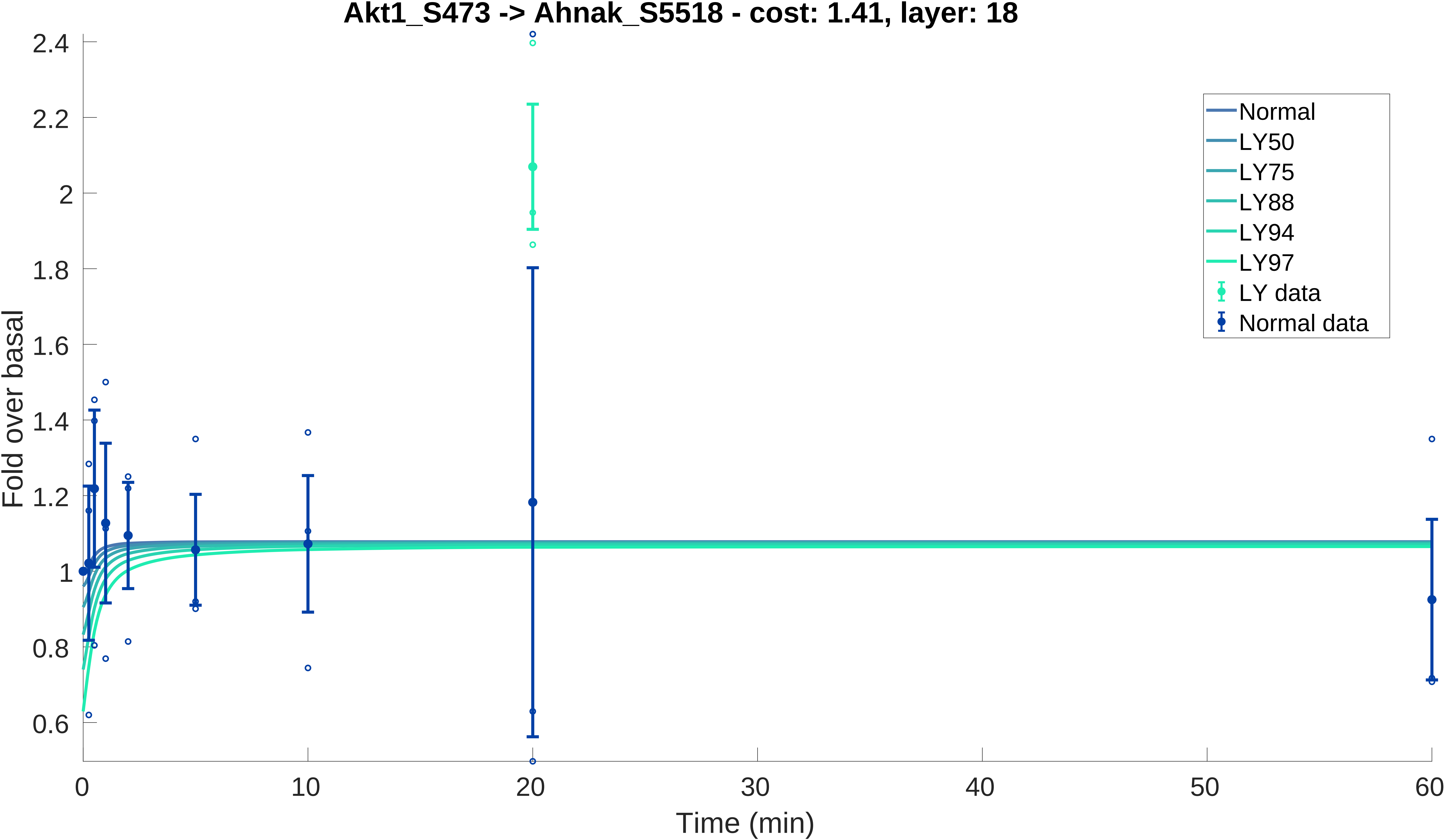
Akt1_S473 -> Ahnak_S5518 - cost: 1.41, layer: 18
Fold over basal
Time (min)
0
10
20
30
40
50
60
0.6
0.8
1
1.2
1.4
1.6
1.8
2
2.2
2.4
Normal
LY50
LY75
LY88
LY94
LY97
LY data
Normal data

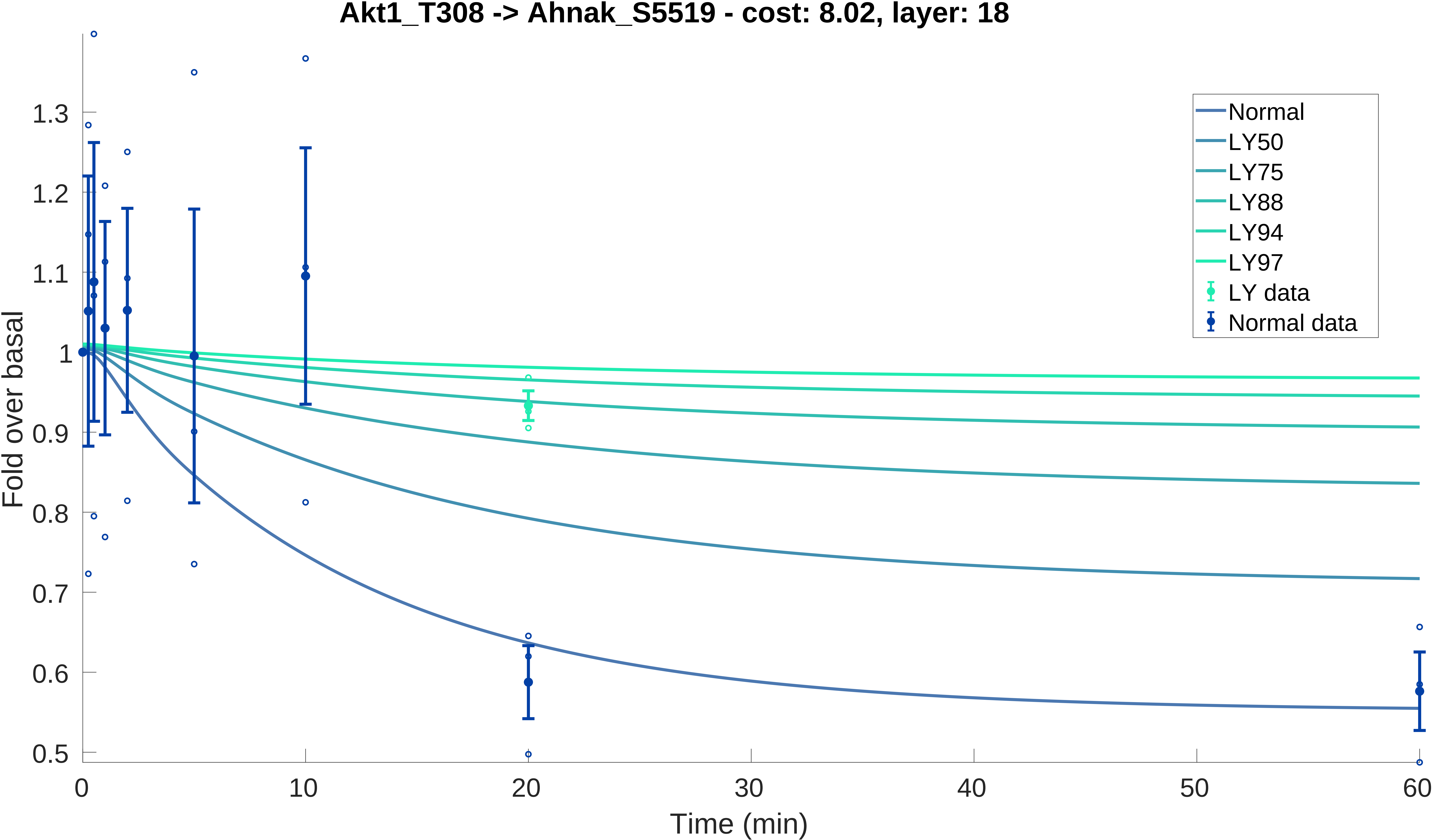
Akt1_T308 -> Ahnak_S5519 - cost: 8.02, layer: 18
Fold over basal
Time (min)
Normal
LY50
LY75
LY88
LY94
LY97
LY data
Normal data
0.5
0.6
0.7
0.8
0.9
1
1.1
1.2
1.3
0
10
20
30
40
50
60

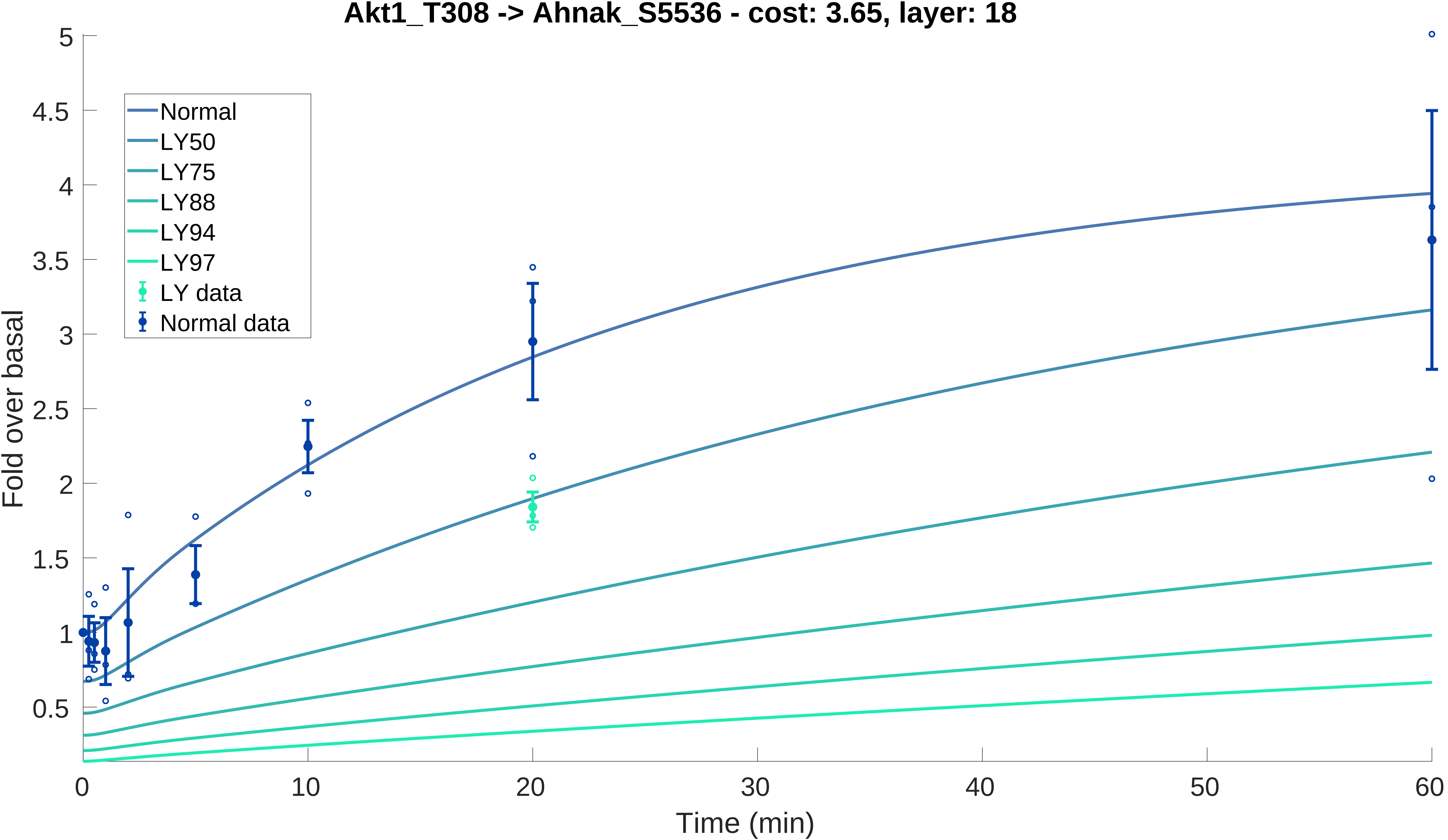
Akt1_T308 -> Ahnak_S5536 - cost: 3.65, layer: 18
Normal
LY50
LY75
LY88
LY94
LY97
LY data
Normal data
Fold over basal
Time (min)
0
10
20
30
40
50
60
0.5
1
1.5
2
2.5
3
3.5
4
4.5
5

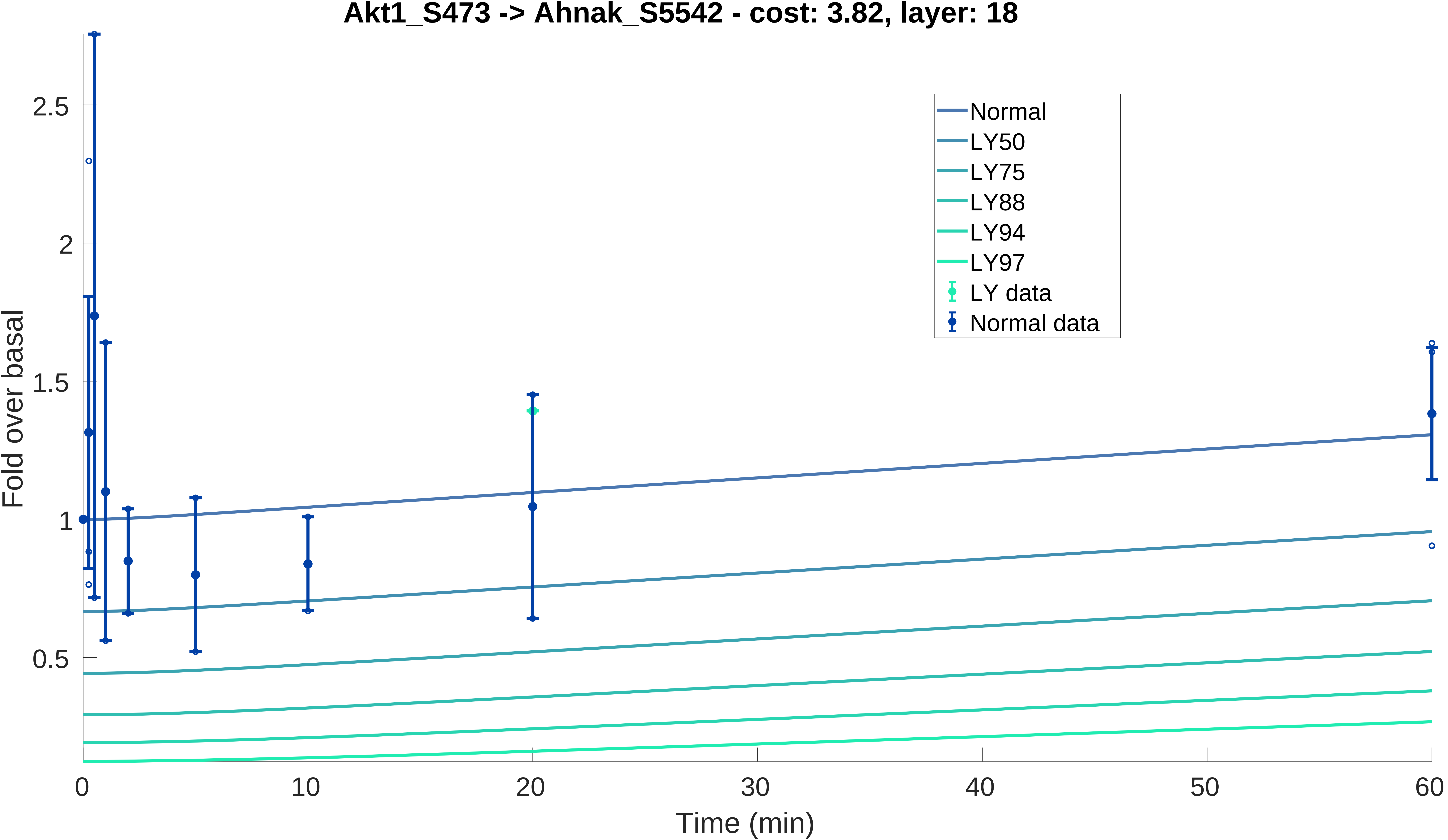
Akt1_S473 -> Ahnak_S5542 - cost: 3.82, layer: 18
Fold over basal
Time (min)
2.5
2
1.5
1
0.5
0
10
20
30
40
50
60
Normal
LY50
LY75
LY88
LY94
LY97
LY data
Normal data

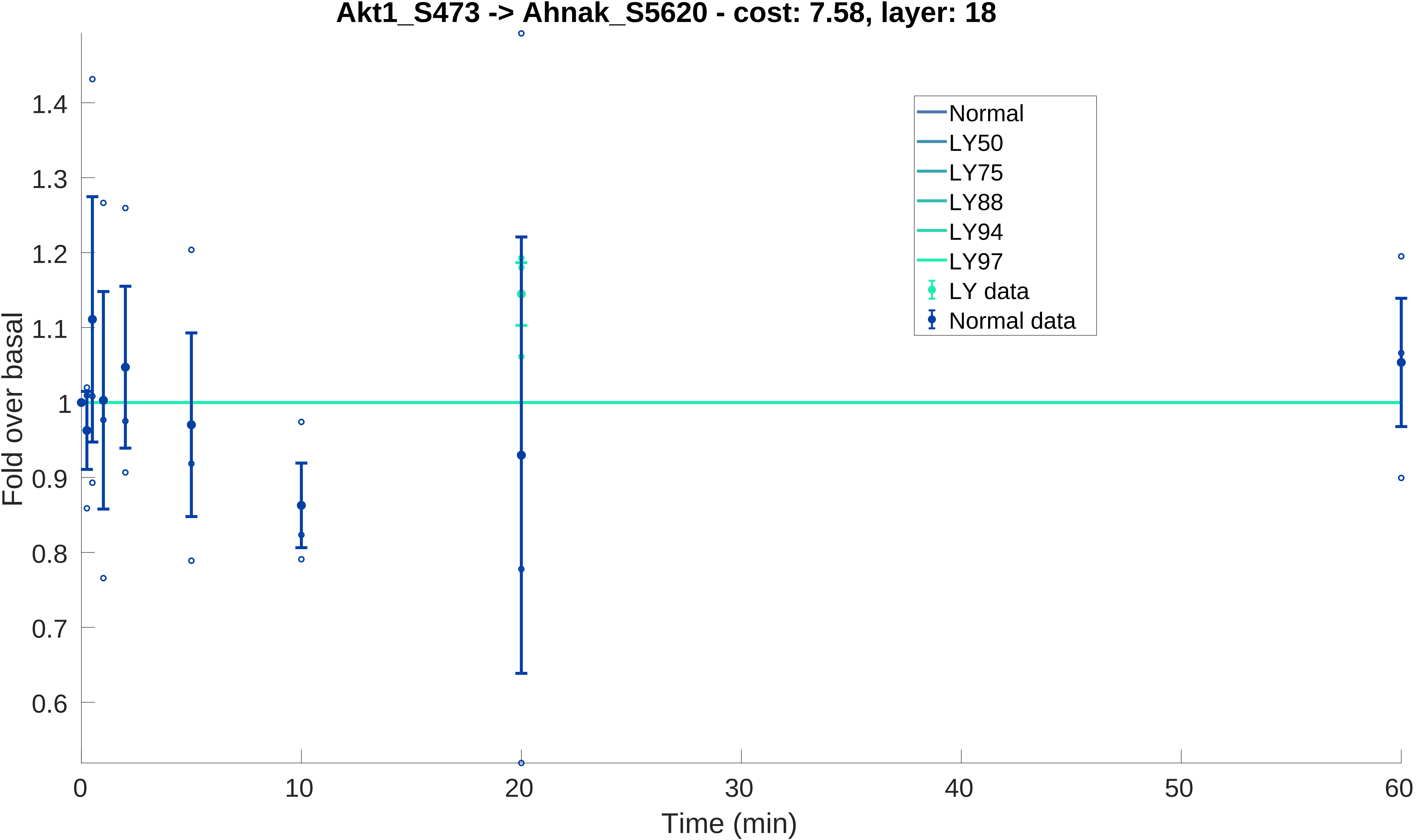

Akt1_S473 -> Ahnak_S5620 - cost: 7.58, layer: 18
Fold over basal
Time (min)
Normal
LY50
LY75
LY88
LY94
LY97
LY data
Normal data
1.4
1.3
1.2
1.1
1
0.9
0.8
0.7
0.6
0
10
20
30
40
50
60

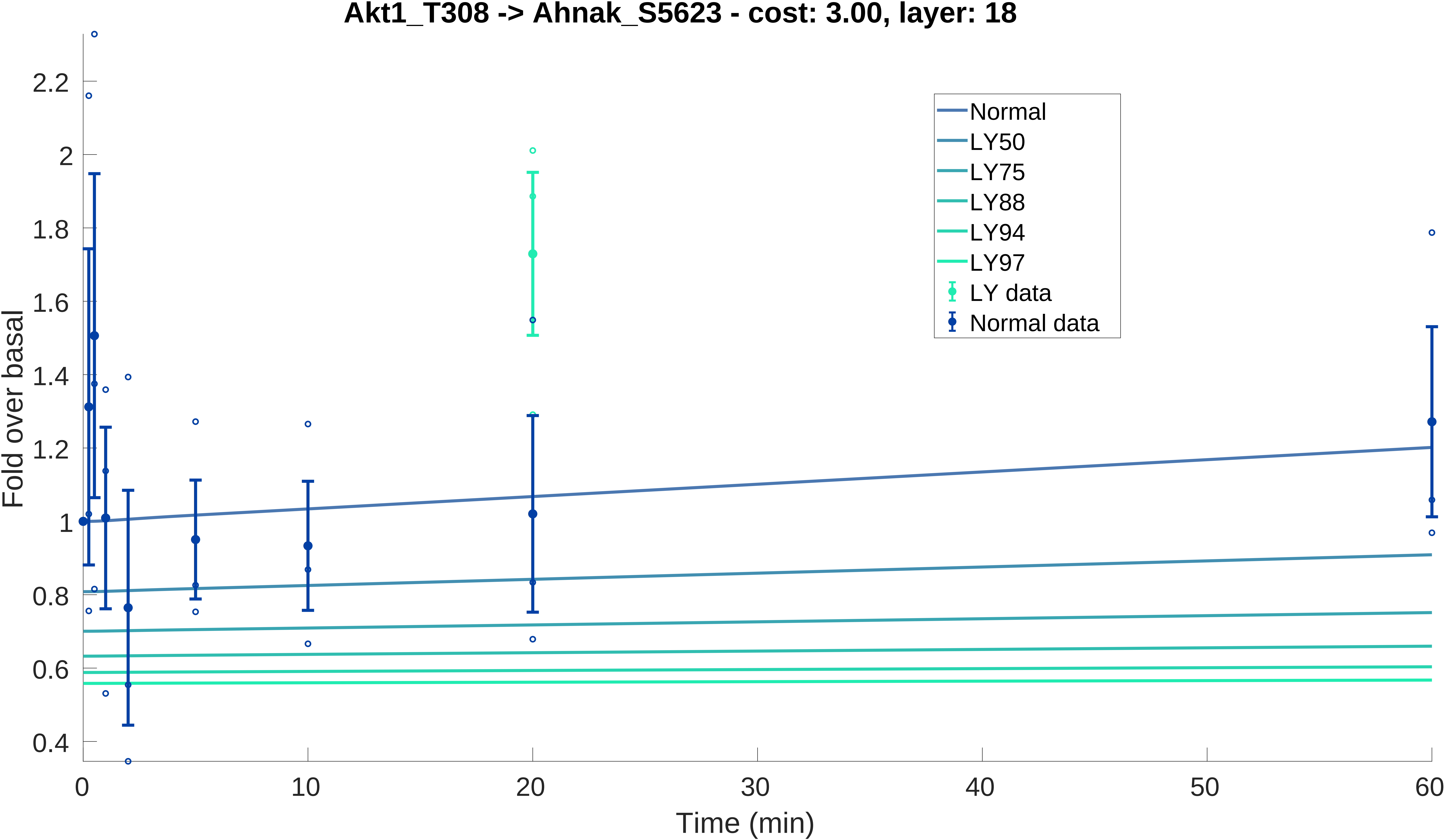

Akt1_T308 -> Ahnak_S5623 - cost: 3.00, layer: 18
Fold over basal
Time (min)
Normal
LY50
LY75
LY88
LY94
LY97
LY data
Normal data
0.4
0.6
0.8
1
1.2
1.4
1.6
1.8
2
2.2
0
10
20
30
40
50
60

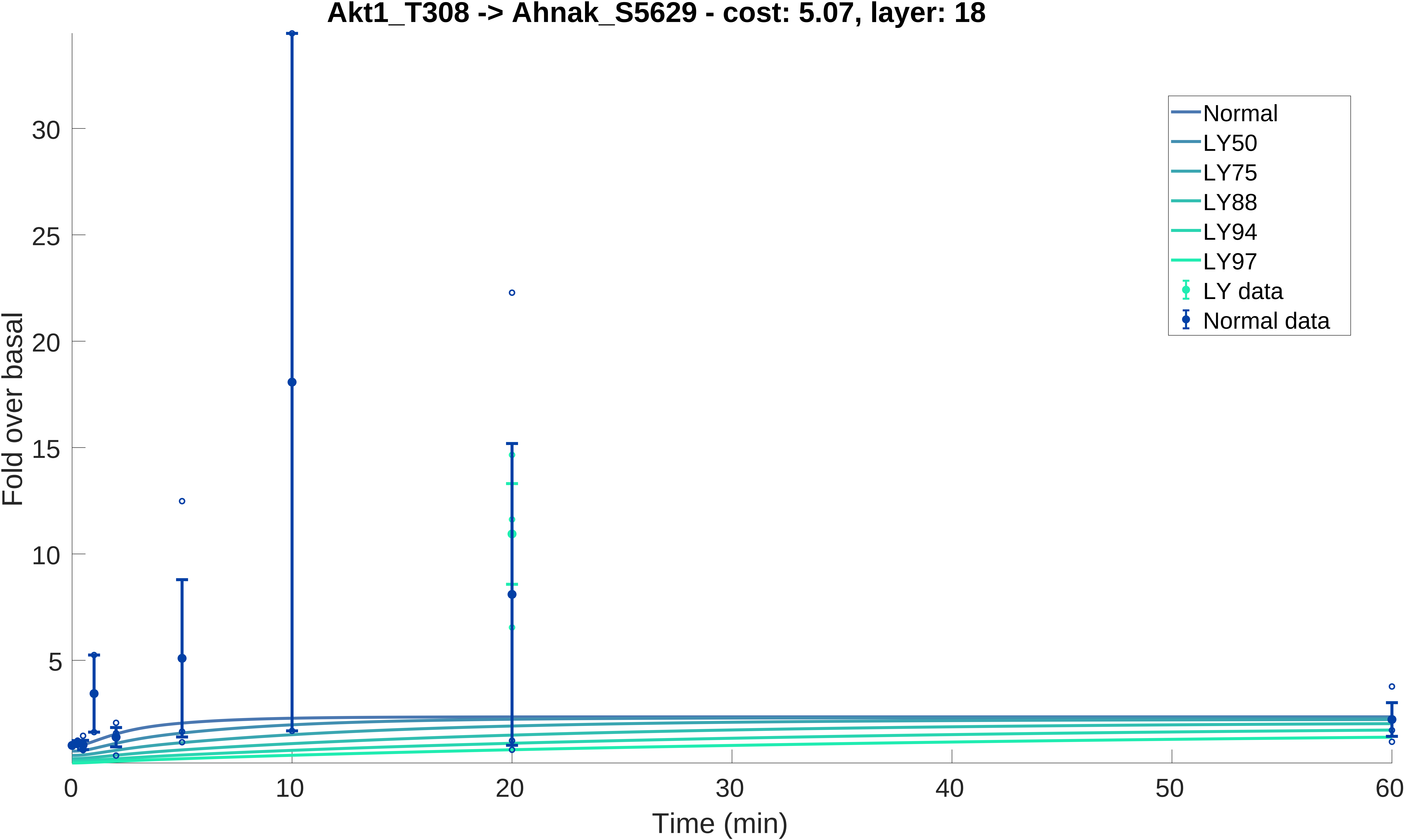
Akt1_T308 -> Ahnak_S5629 - cost: 5.07, layer: 18
Fold over basal
Time (min)
0
10
20
30
40
50
60
5
10
15
20
25
30
Normal
LY50
LY75
LY88
LY94
LY97
LY data
Normal data

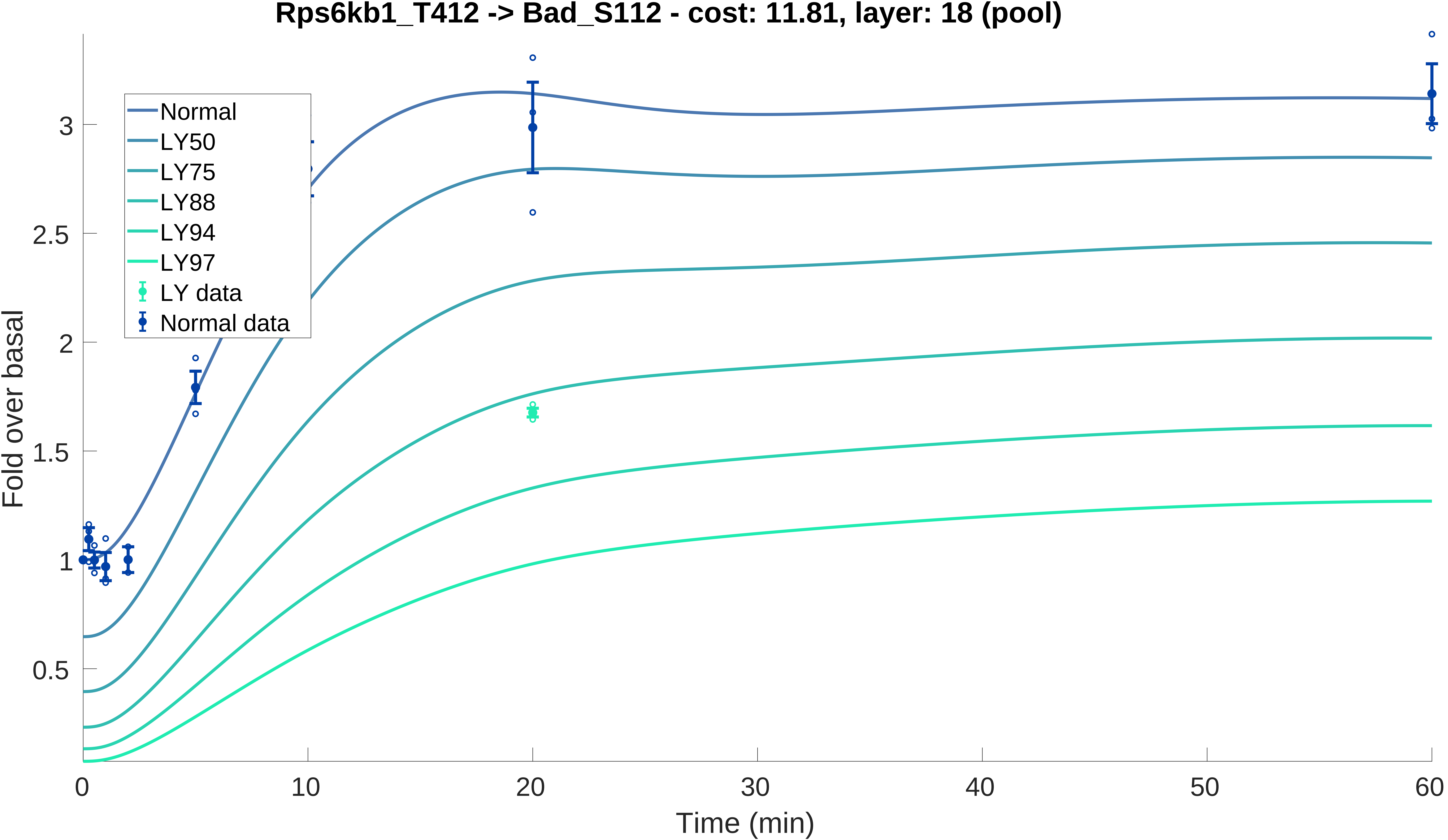
Rps6kb1_T412 -> Bad_S112 - cost: 11.81, layer: 18 (pool)
Fold over basal
Time (min)
Normal
LY50
LY75
LY88
LY94
LY97
LY data
Normal data
0
10
20
30
40
50
60
0.5
1
1.5
2
2.5
3

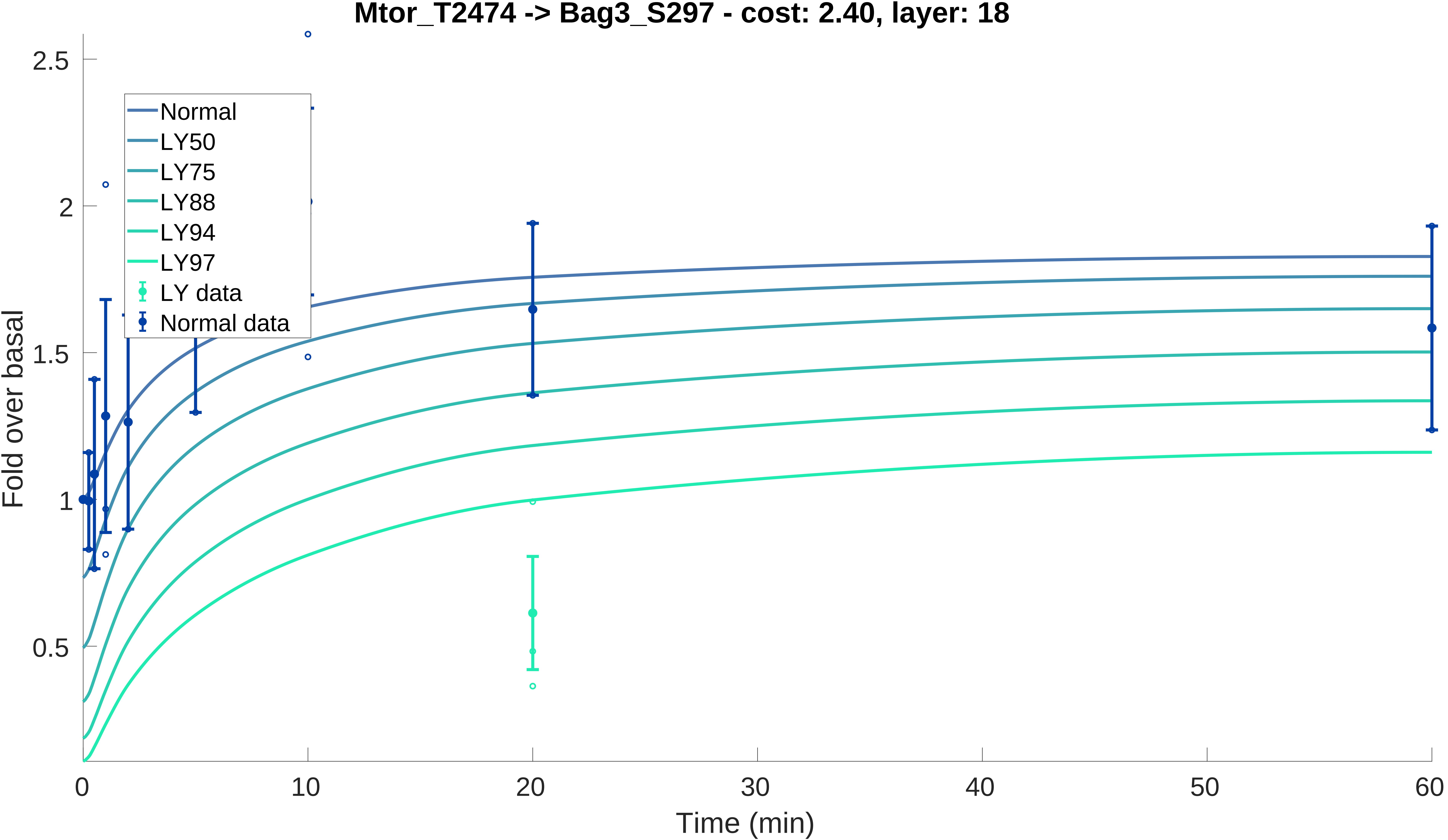
Mtor_T2474 -> Bag3_S297 - cost: 2.40, layer: 18
Normal
LY50
LY75
LY88
LY94
LY97
LY data
Normal data
Fold over basal
2.5
2
1.5
1
0.5
0
10
20
30
40
50
60
Time (min)

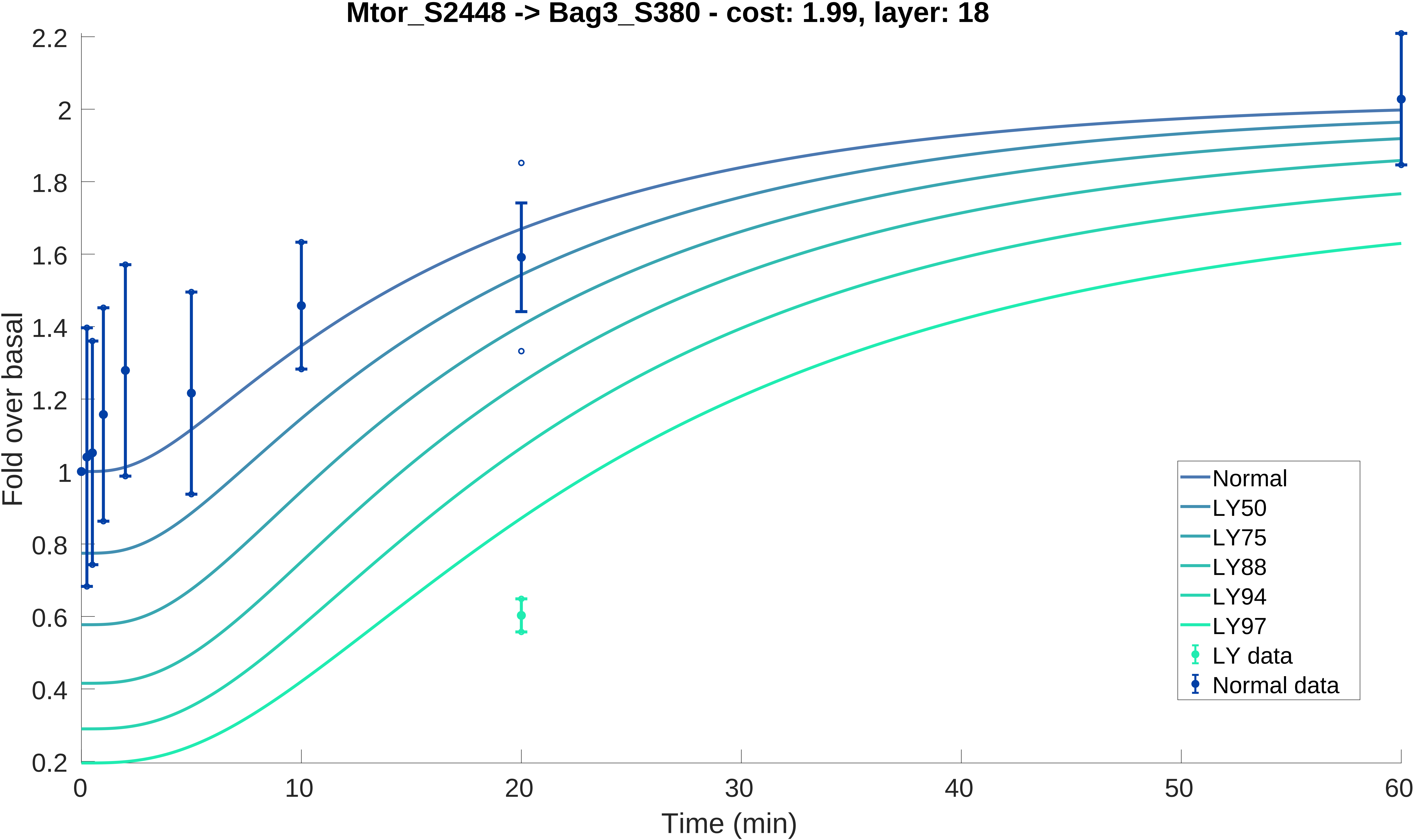
Mtor_S2448 -> Bag3_S380 - cost: 1.99, layer: 18
Fold over basal
Time (min)
2.2
2
1.8
1.6
1.4
1.2
1
0.8
0.6
0.4
0.2
0
10
20
30
40
50
60
Normal
LY50
LY75
LY88
LY94
LY97
LY data
Normal data

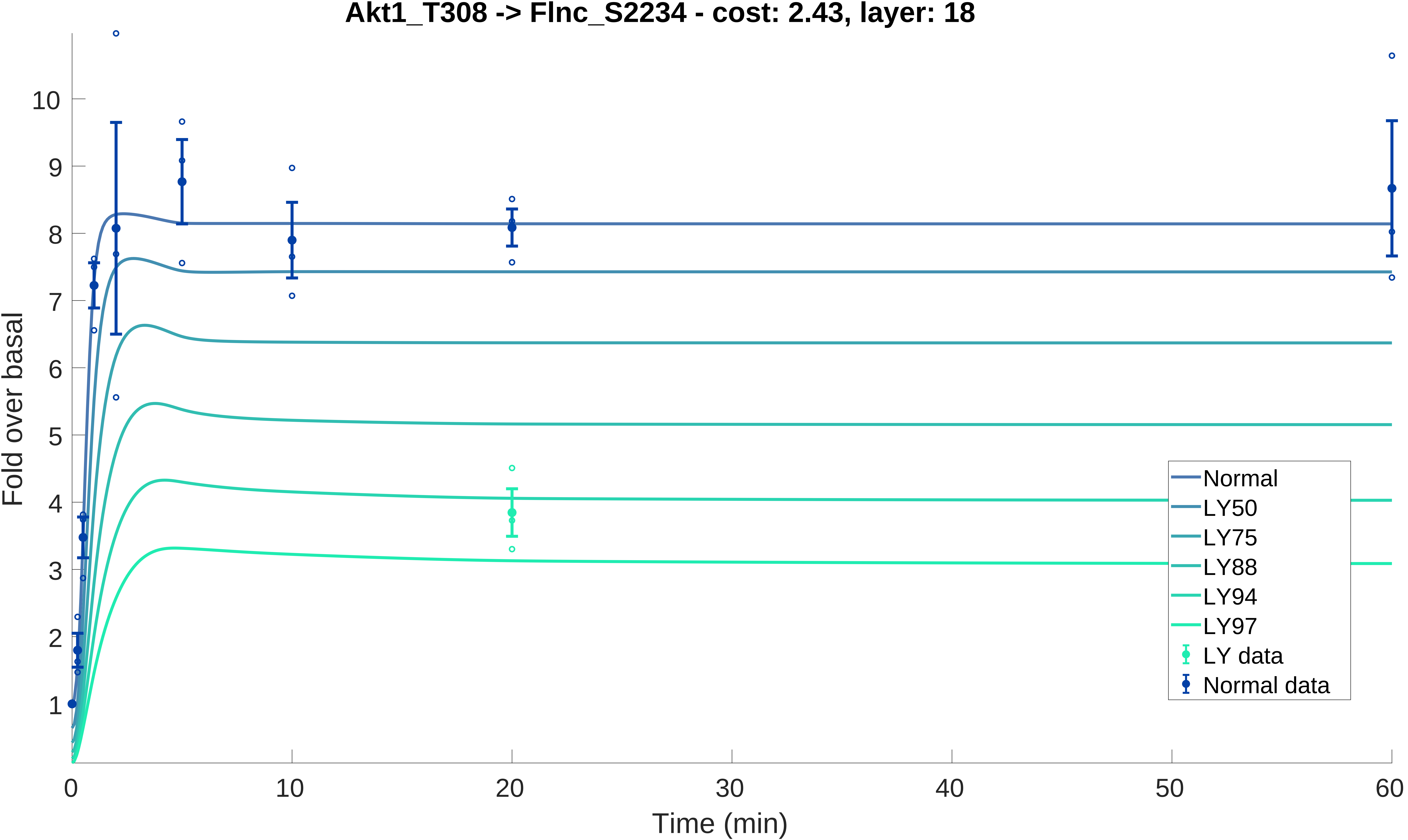
Akt1_T308 -> Flnc_S2234 - cost: 2.43, layer: 18
Fold over basal
Time (min)
0
10
20
30
40
50
60
1
2
3
4
5
6
7
8
9
10
Normal
LY50
LY75
LY88
LY94
LY97
LY data
Normal data

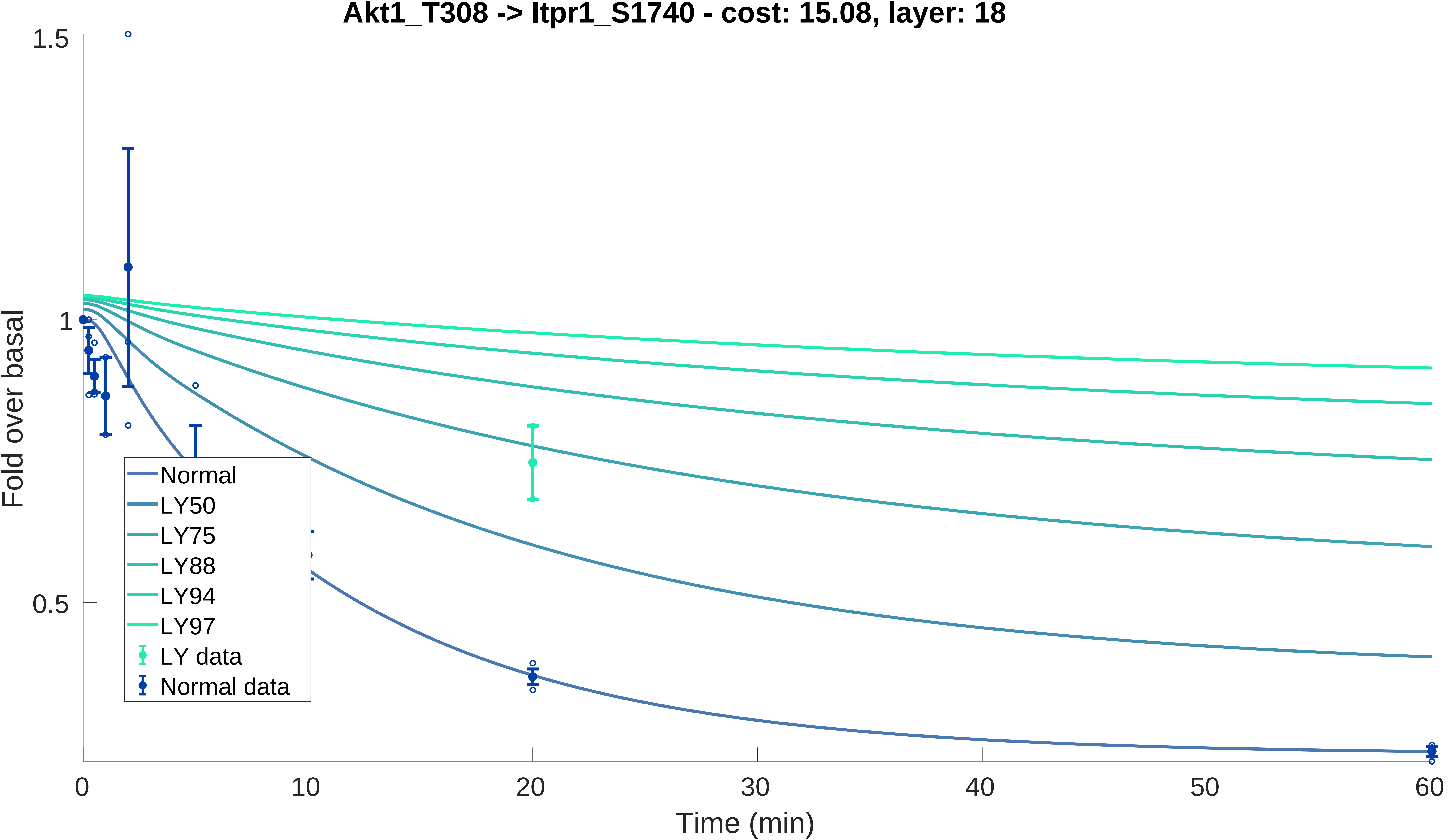

Akt1_T308 -> Itpr1_S1740 - cost: 15.08, layer: 18
1.5
1
0.5
Fold over basal
0
10
20
30
40
50
60
Time (min)
Normal
LY50
LY75
LY88
LY94
LY97
LY data
Normal data

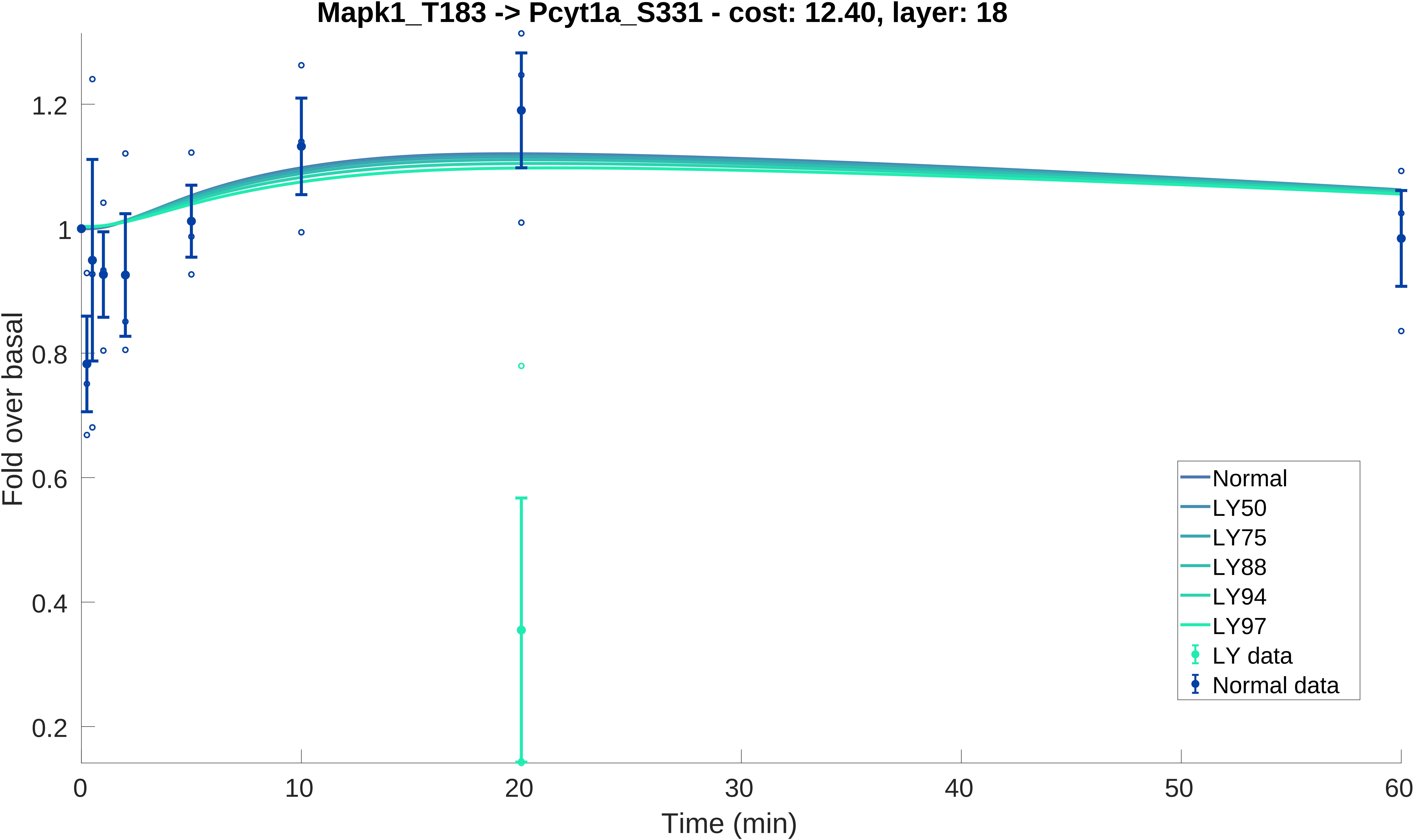

Mapk1_T183 -> Pcyt1a_S331 - cost: 12.40, layer: 18
Fold over basal
Time (min)
0.2
0.4
0.6
0.8
1
1.2
0
10
20
30
40
50
60
Normal
LY50
LY75
LY88
LY94
LY97
LY data
Normal data

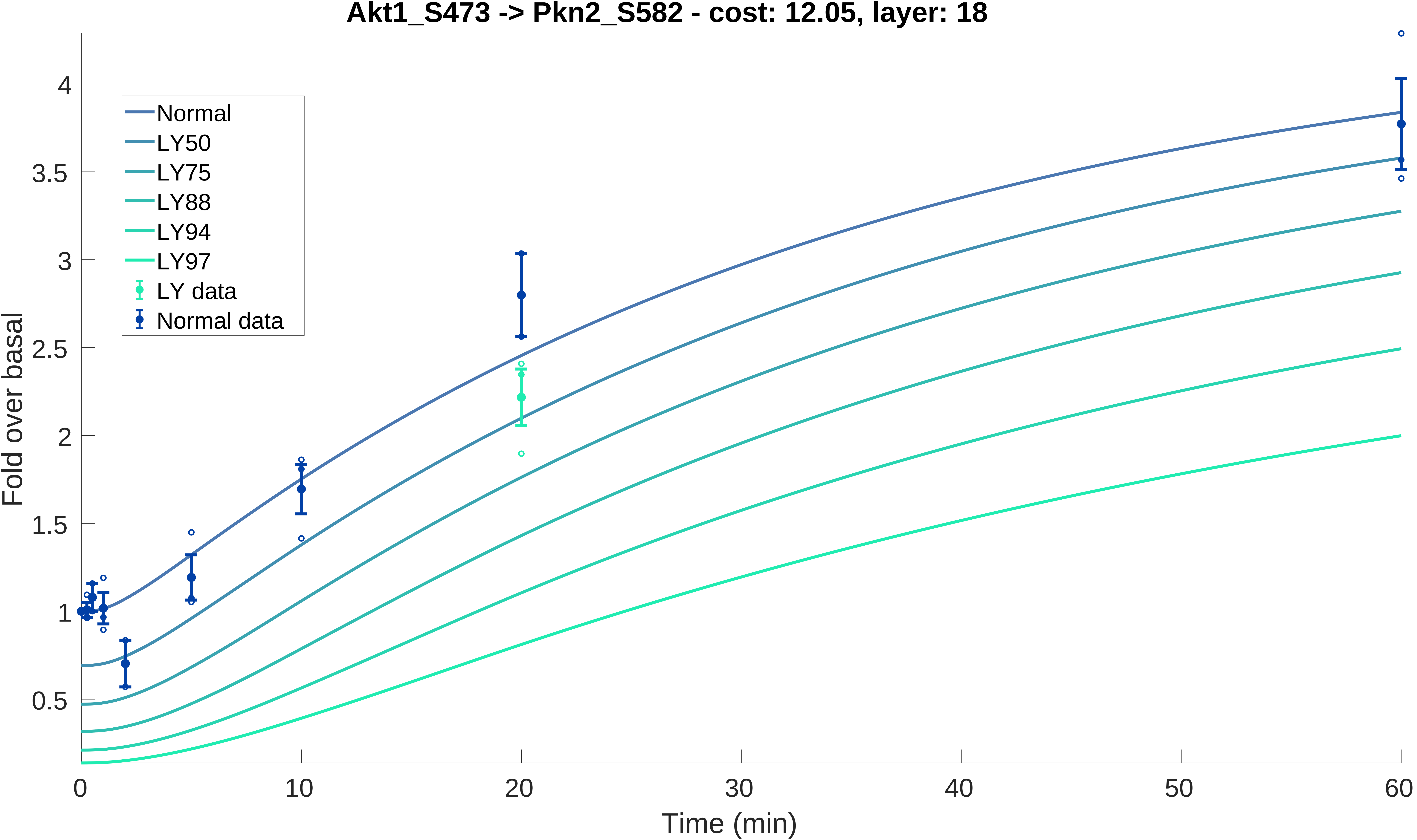

Akt1_S473 -> Pkn2_S582 - cost: 12.05, layer: 18
Normal
LY50
LY75
LY88
LY94
LY97
LY data
Normal data
Fold over basal
Time (min)
0
10
20
30
40
50
60
0.5
1
1.5
2
2.5
3
3.5
4

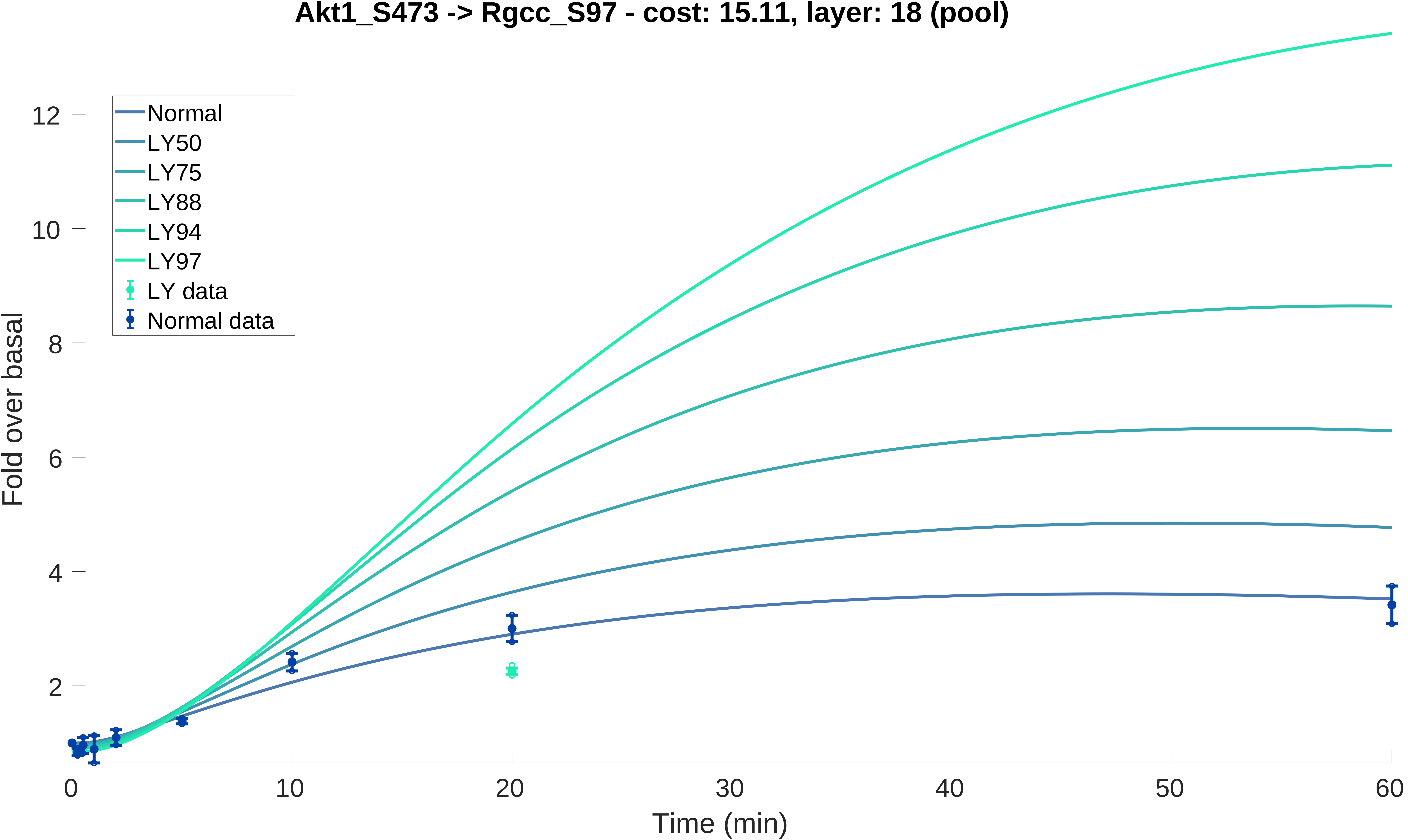
Akt1_S473 -> Rgcc_S97 - cost: 15.11, layer: 18 (pool)
Normal
LY50
LY75
LY88
LY94
LY97
LY data
Normal data
Fold over basal
Time (min)
0
10
20
30
40
50
60
2
4
6
8
10
12

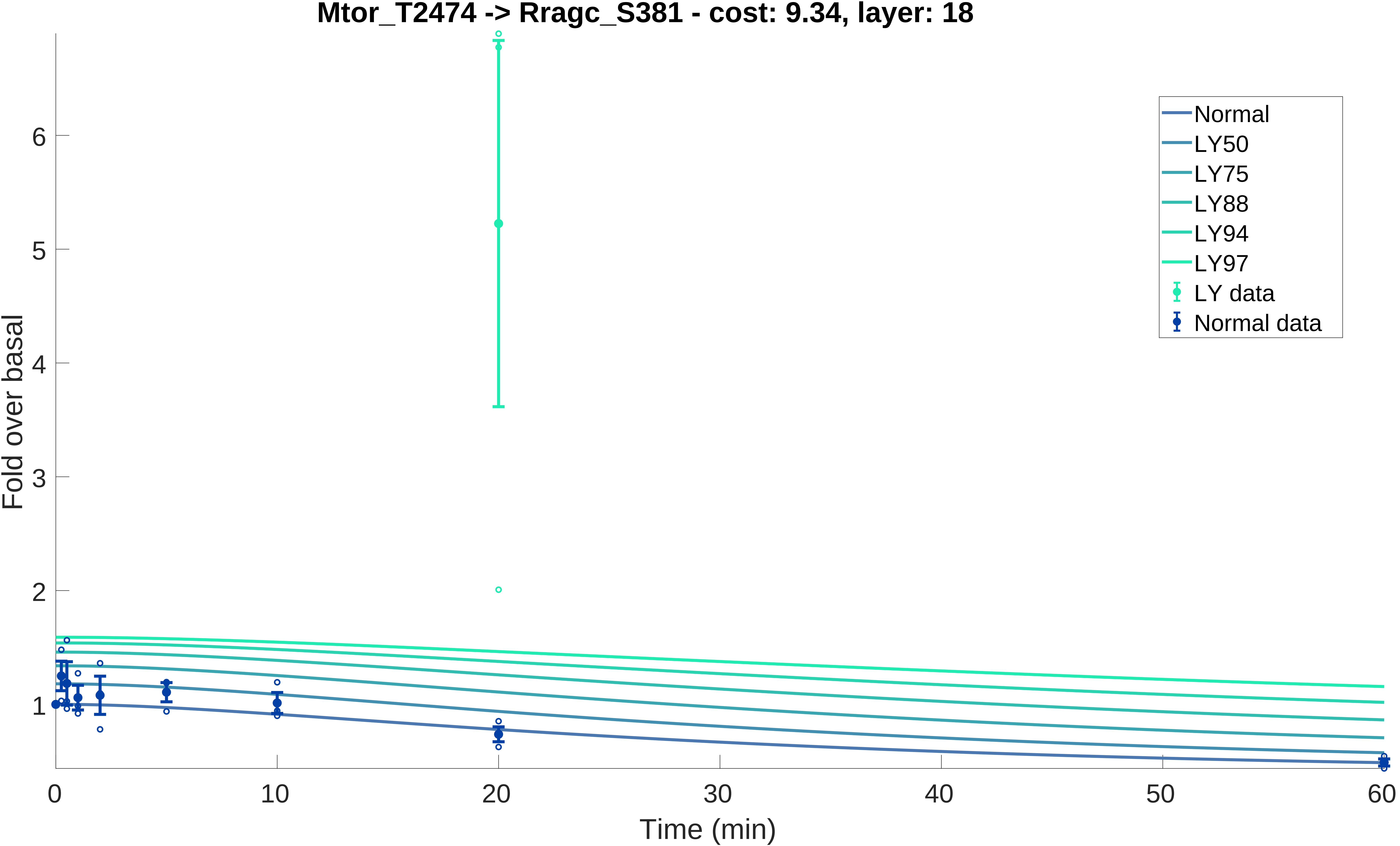

Mtor_T2474 -> Rragc_S381 - cost: 9.34, layer: 18
Fold over basal
Time (min)
0
10
20
30
40
50
60
1
2
3
4
5
6
Normal
LY50
LY75
LY88
LY94
LY97
LY data
Normal data

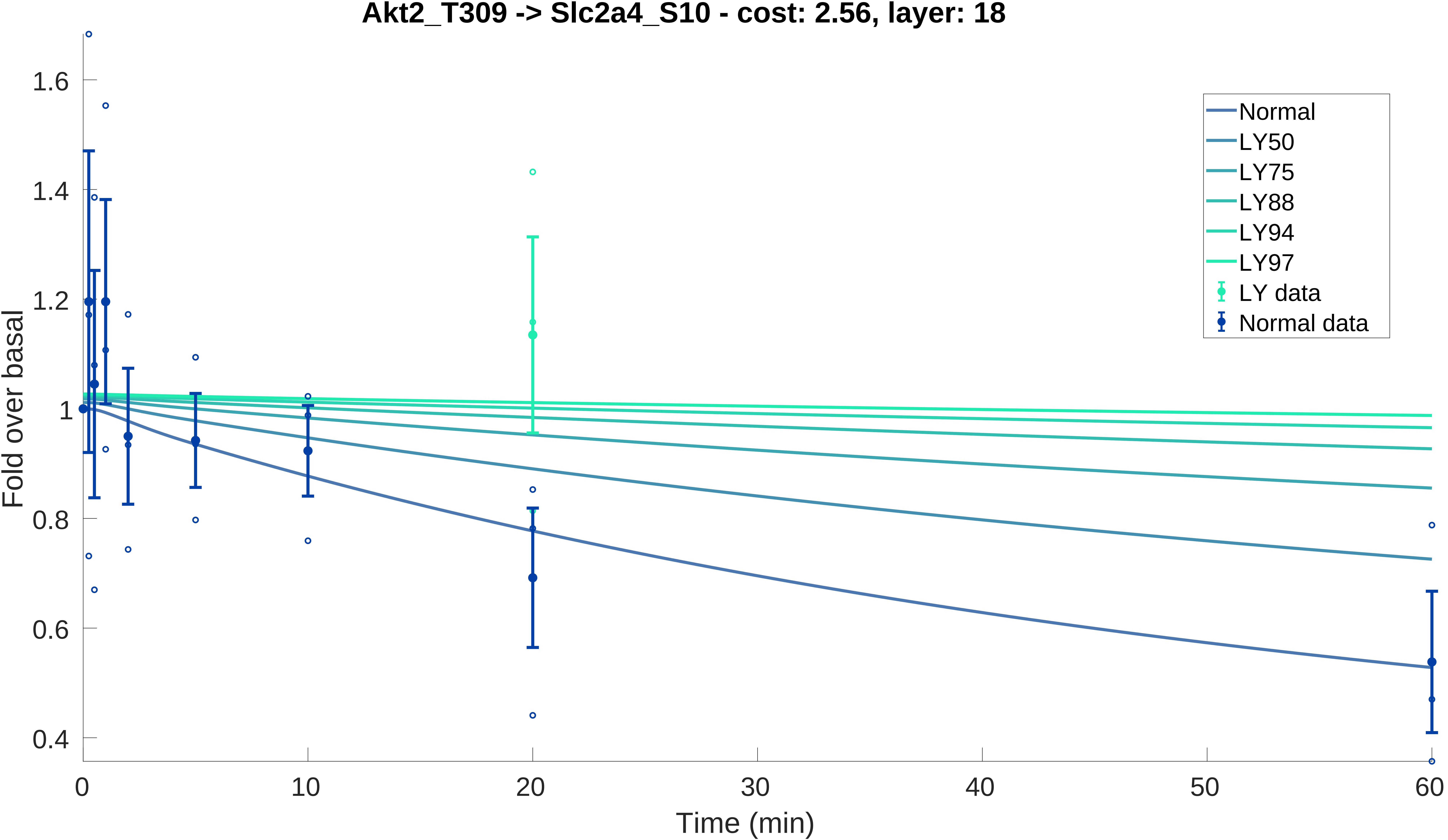

Akt2_T309 -> Slc2a4_S10 - cost: 2.56, layer: 18
Fold over basal
1.6
1.4
1.2
1
0.8
0.6
0.4
0
10
20
30
40
50
60
Time (min)
Normal
LY50
LY75
LY88
LY94
LY97
LY data
Normal data

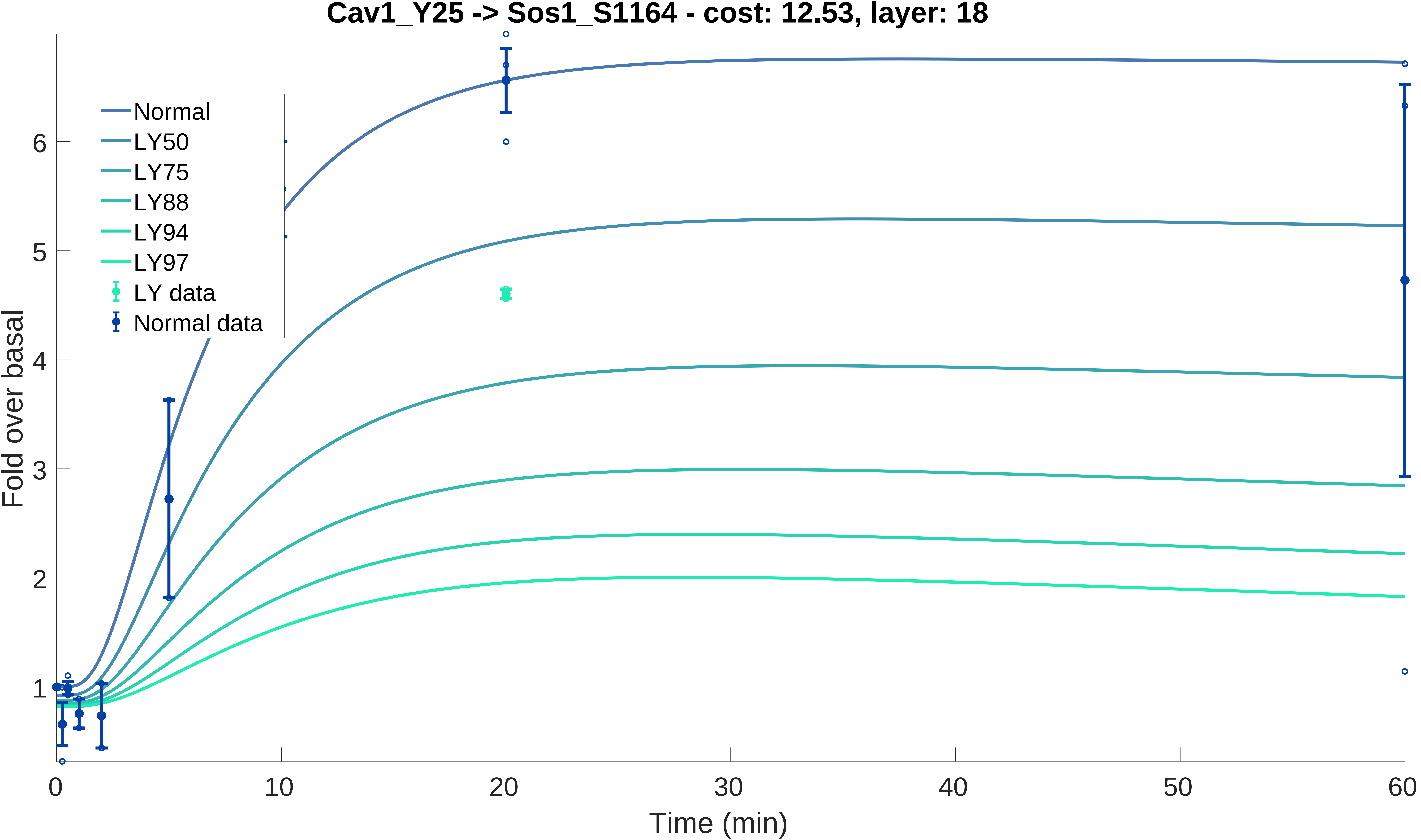
Cav1_Y25 -> Sos1_S1164 - cost: 12.53, layer: 18
Normal
LY50
LY75
LY88
LY94
LY97
LY data
Normal data
Fold over basal
Time (min)
0
10
20
30
40
50
60
1
2
3
4
5
6

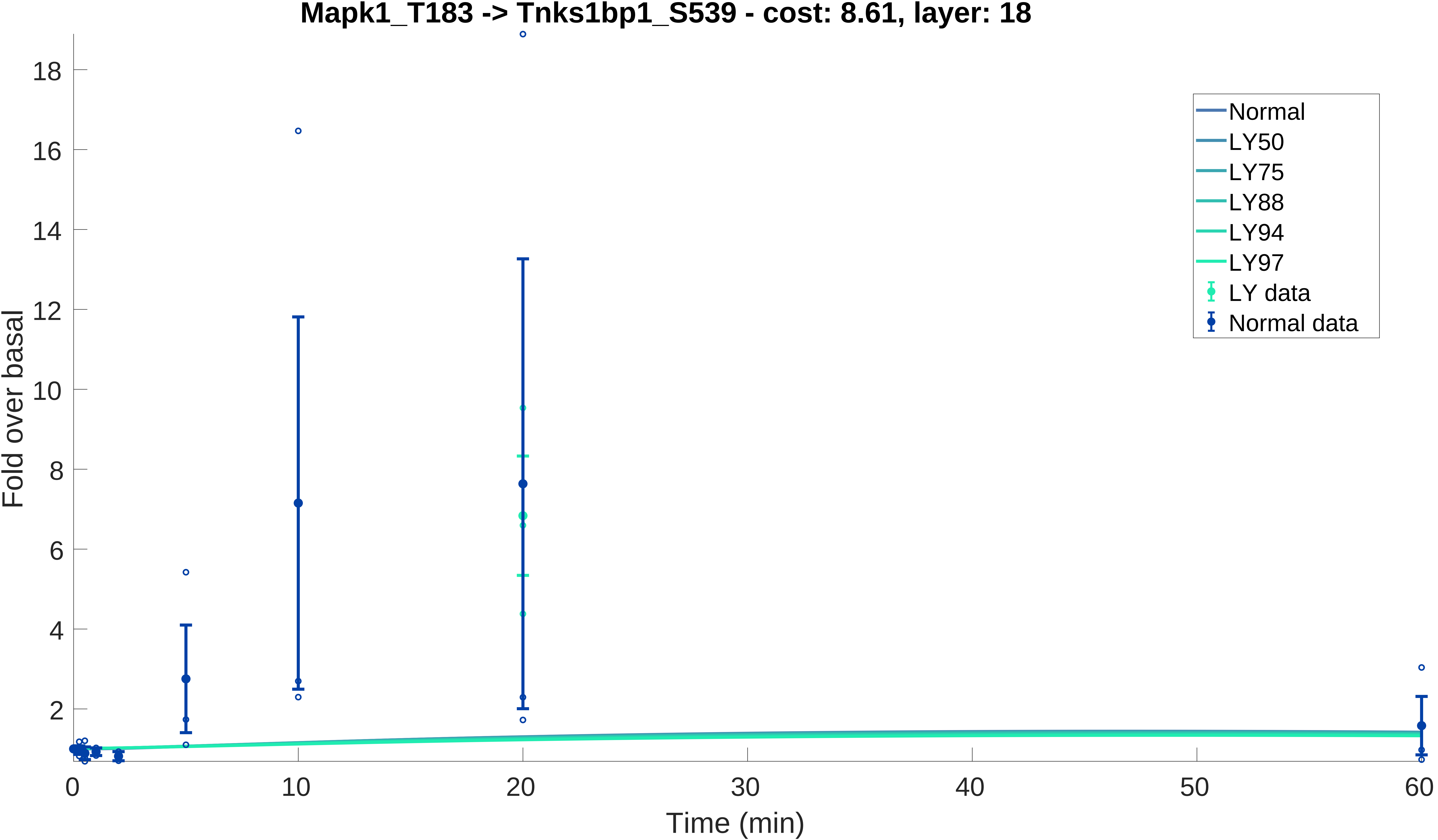

Mapk1_T183 -> Tnks1bp1_S539 - cost: 8.61, layer: 18
Fold over basal
Time (min)
Normal
LY50
LY75
LY88
LY94
LY97
LY data
Normal data
0
10
20
30
40
50
60
2
4
6
8
10
12
14
16
18

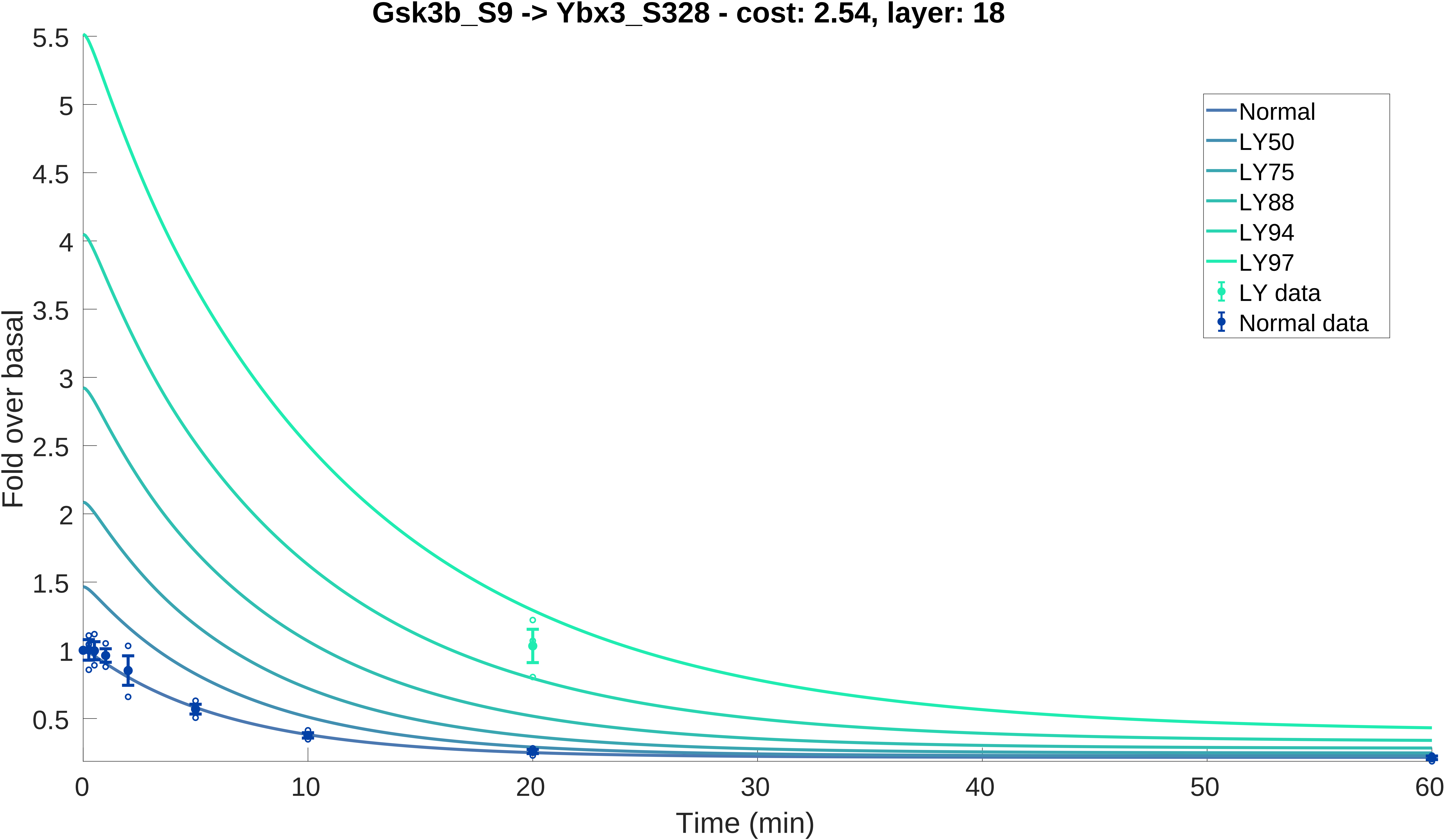
Gsk3b_S9 -> Ybx3_S328 - cost: 2.54, layer: 18
Fold over basal
5.5
5
4.5
4
3.5
3
2.5
2
1.5
1
0.5
0
10
20
30
40
50
60
Time (min)
Normal
LY50
LY75
LY88
LY94
LY97
LY data
Normal data

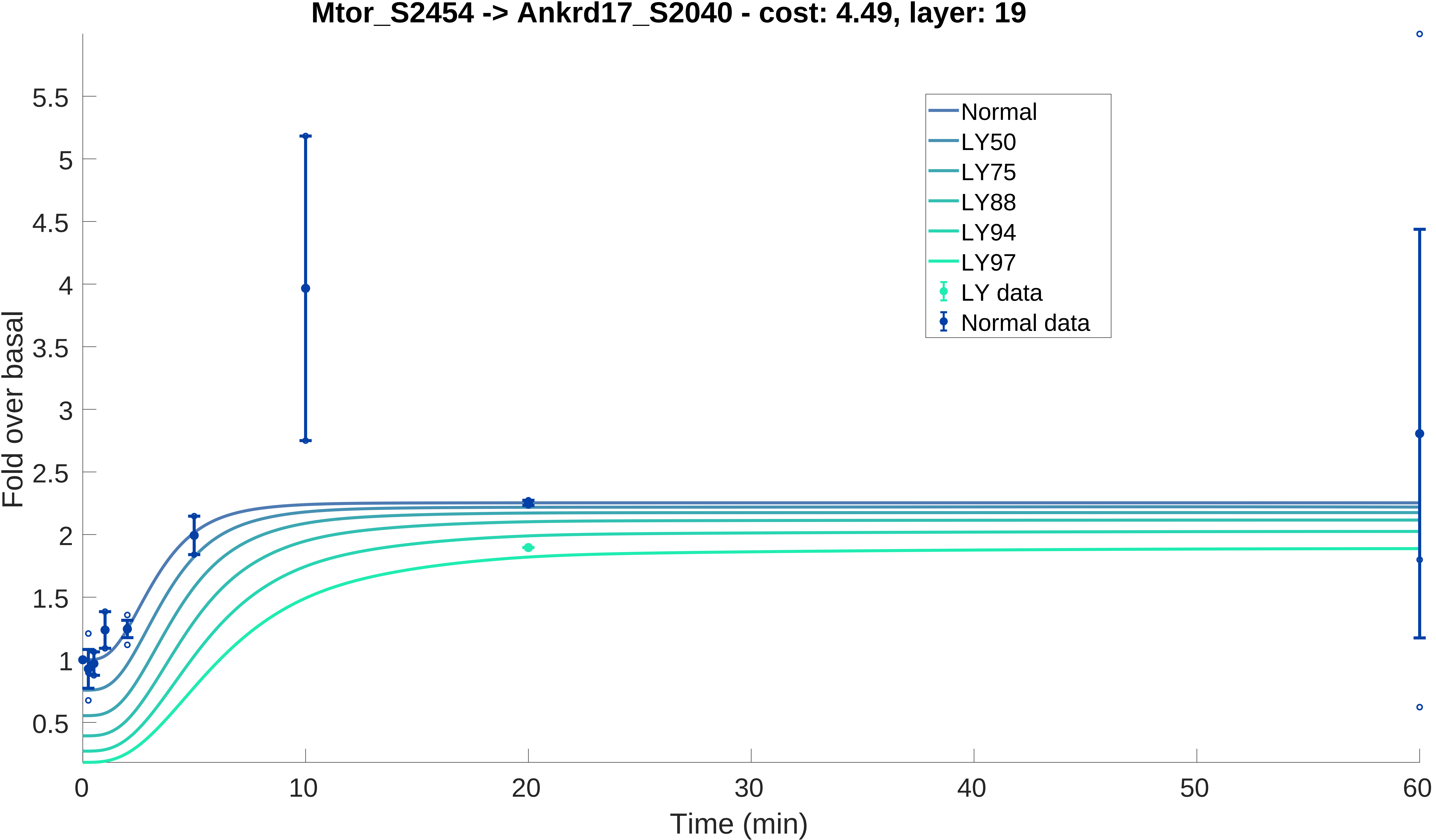

Mtor_S2454 -> Ankrd17_S2040 - cost: 4.49, layer: 19
Fold over basal
Time (min)
0.5
1
1.5
2
2.5
3
3.5
4
4.5
5
5.5
0
10
20
30
40
50
60
Normal
LY50
LY75
LY88
LY94
LY97
LY data
Normal data

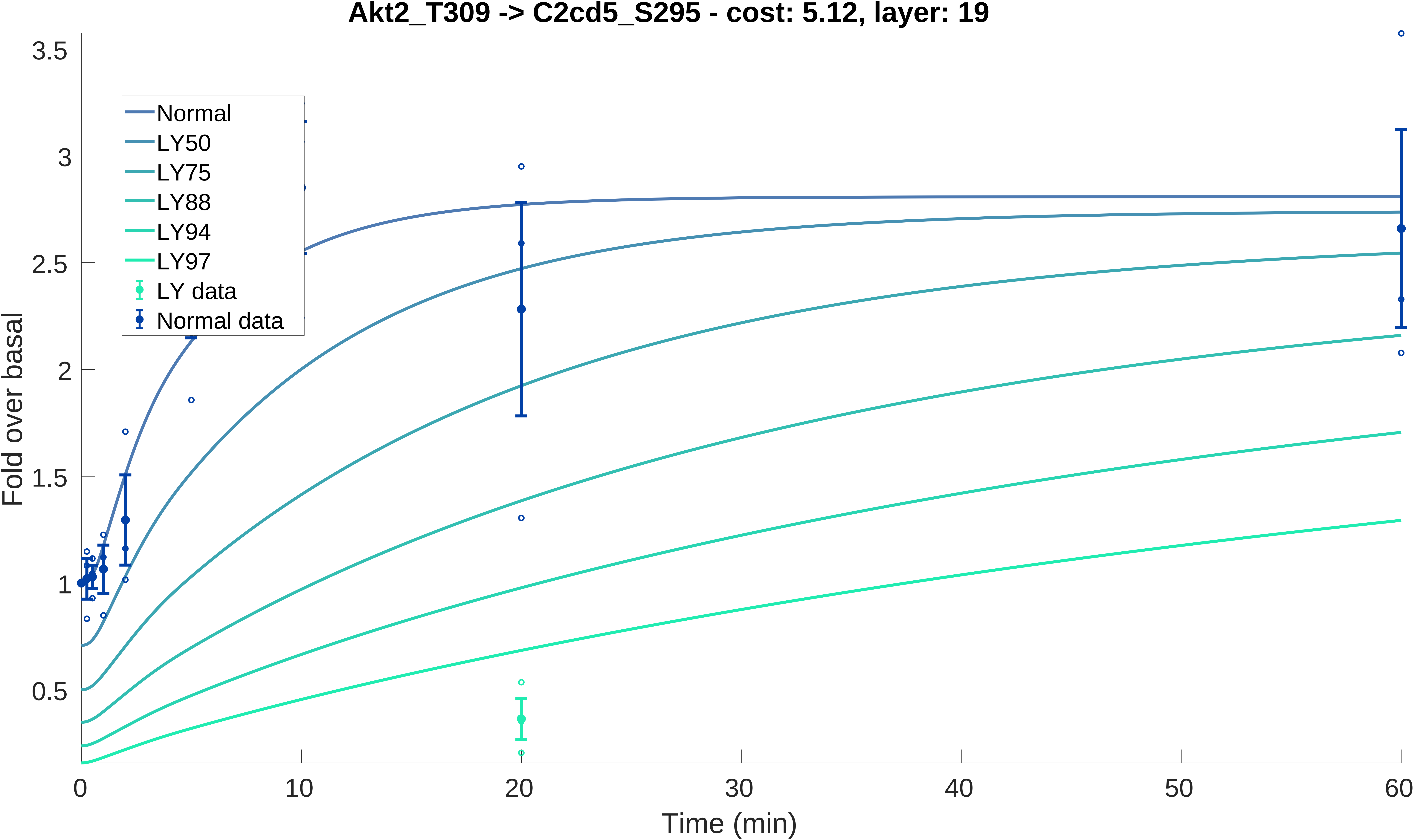

Akt2_T309 -> C2cd5_S295 - cost: 5.12, layer: 19
Normal
LY50
LY75
LY88
LY94
LY97
LY data
Normal data
Fold over basal
Time (min)
3.5
3
2.5
2
1.5
1
0.5
0
10
20
30
40
50
60

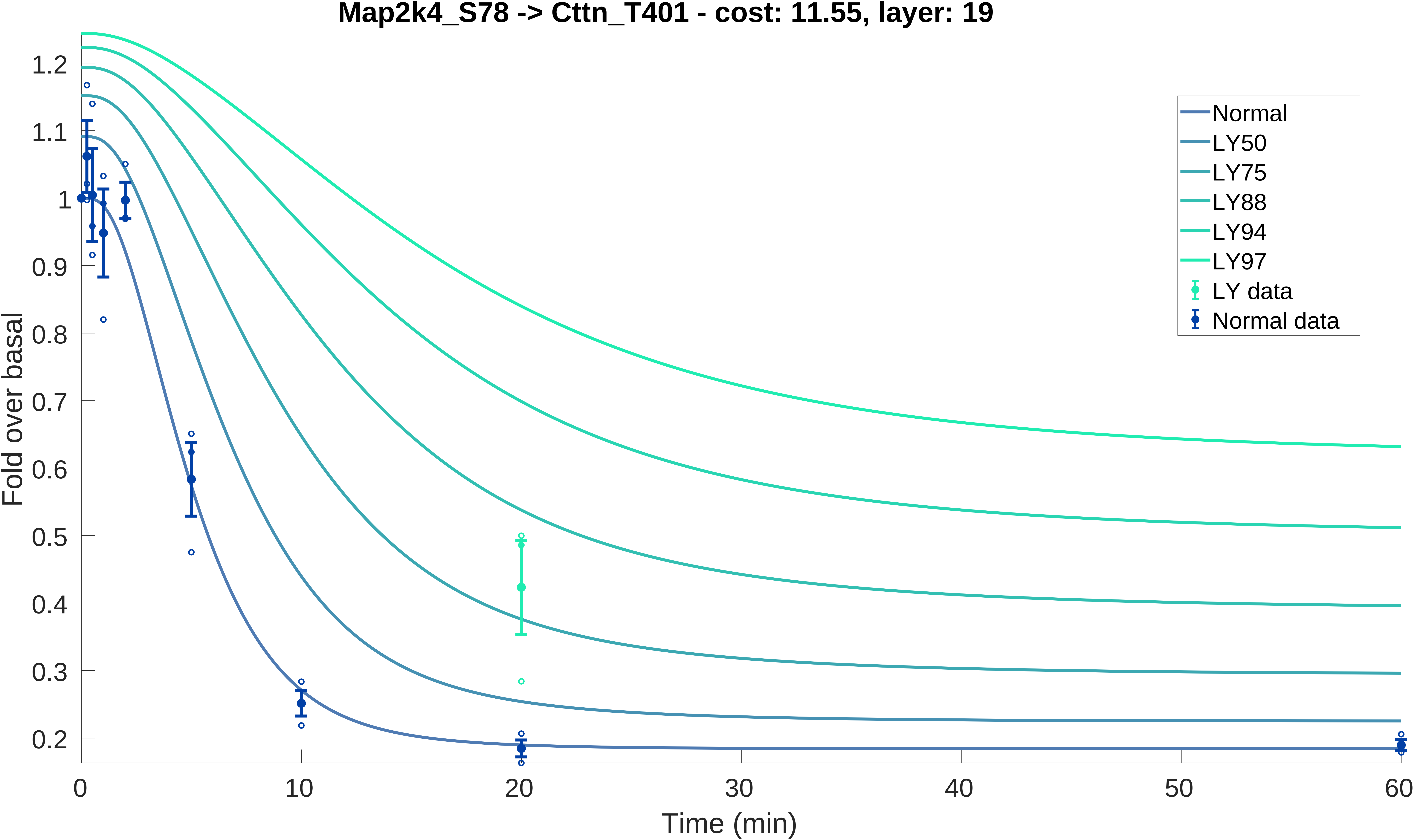

Map2k4_S78 -> Cttn_T401 - cost: 11.55, layer: 19
Fold over basal
Time (min)
0
10
20
30
40
50
60
0.2
0.3
0.4
0.5
0.6
0.7
0.8
0.9
1
1.1
1.2
Normal
LY50
LY75
LY88
LY94
LY97
LY data
Normal data

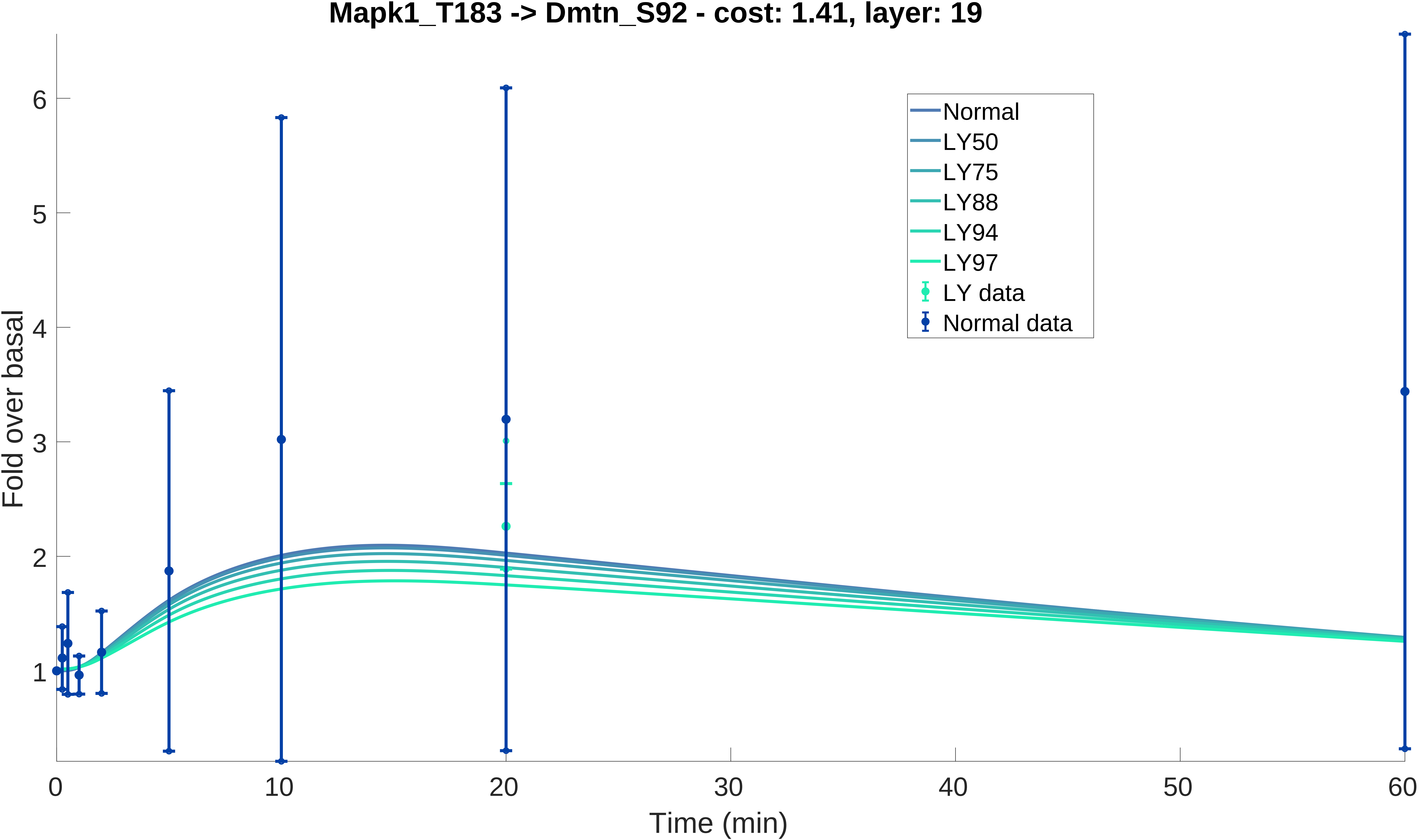

Mapk1_T183 -> Dmtn_S92 - cost: 1.41, layer: 19
Fold over basal
Time (min)
0
10
20
30
40
50
60
1
2
3
4
5
6
Normal
LY50
LY75
LY88
LY94
LY97
LY data
Normal data

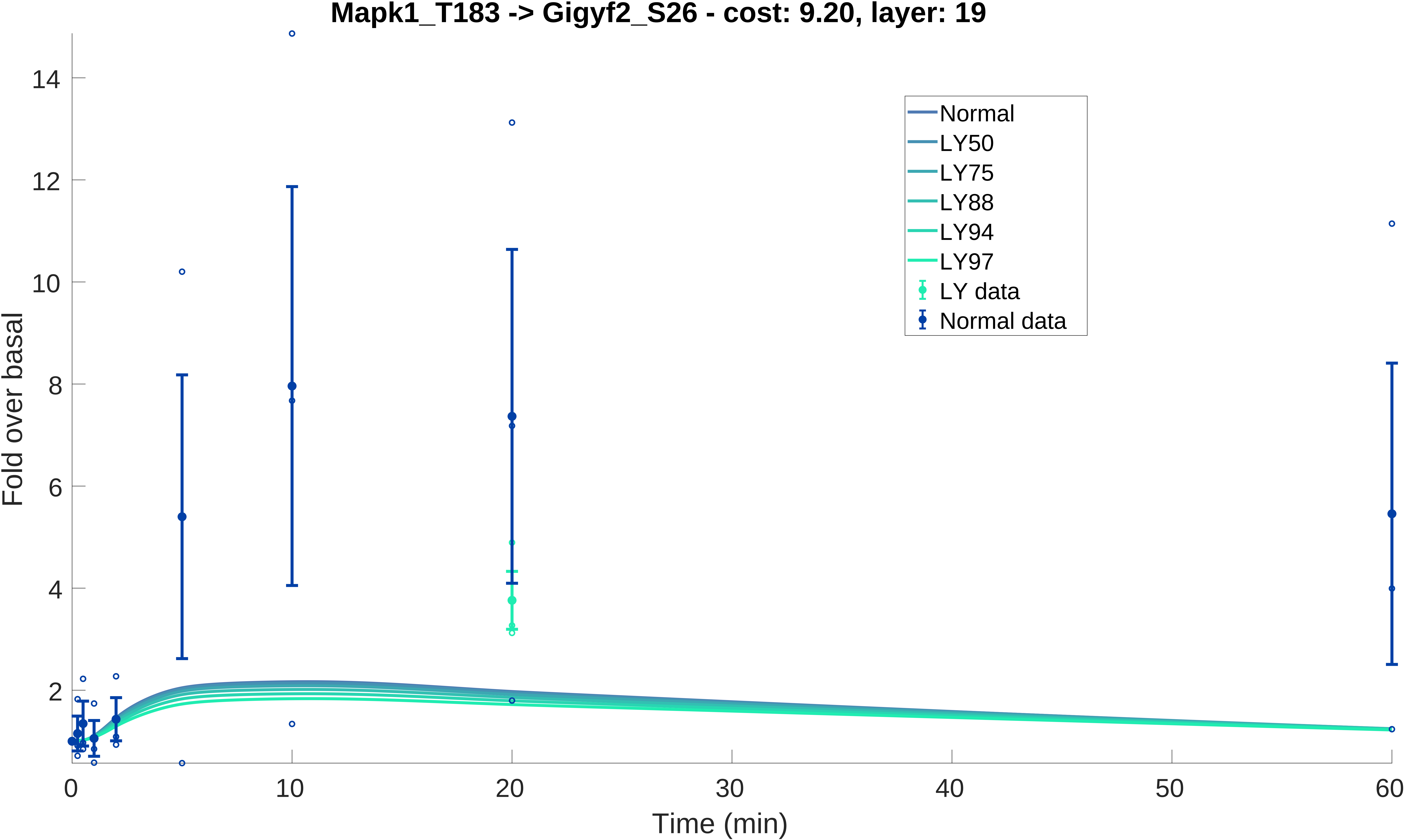
Mapk1_T183 -> Gigyf2_S26 - cost: 9.20, layer: 19
Fold over basal
Time (min)
0
10
20
30
40
50
60
2
4
6
8
10
12
14
Normal
LY50
LY75
LY88
LY94
LY97
LY data
Normal data

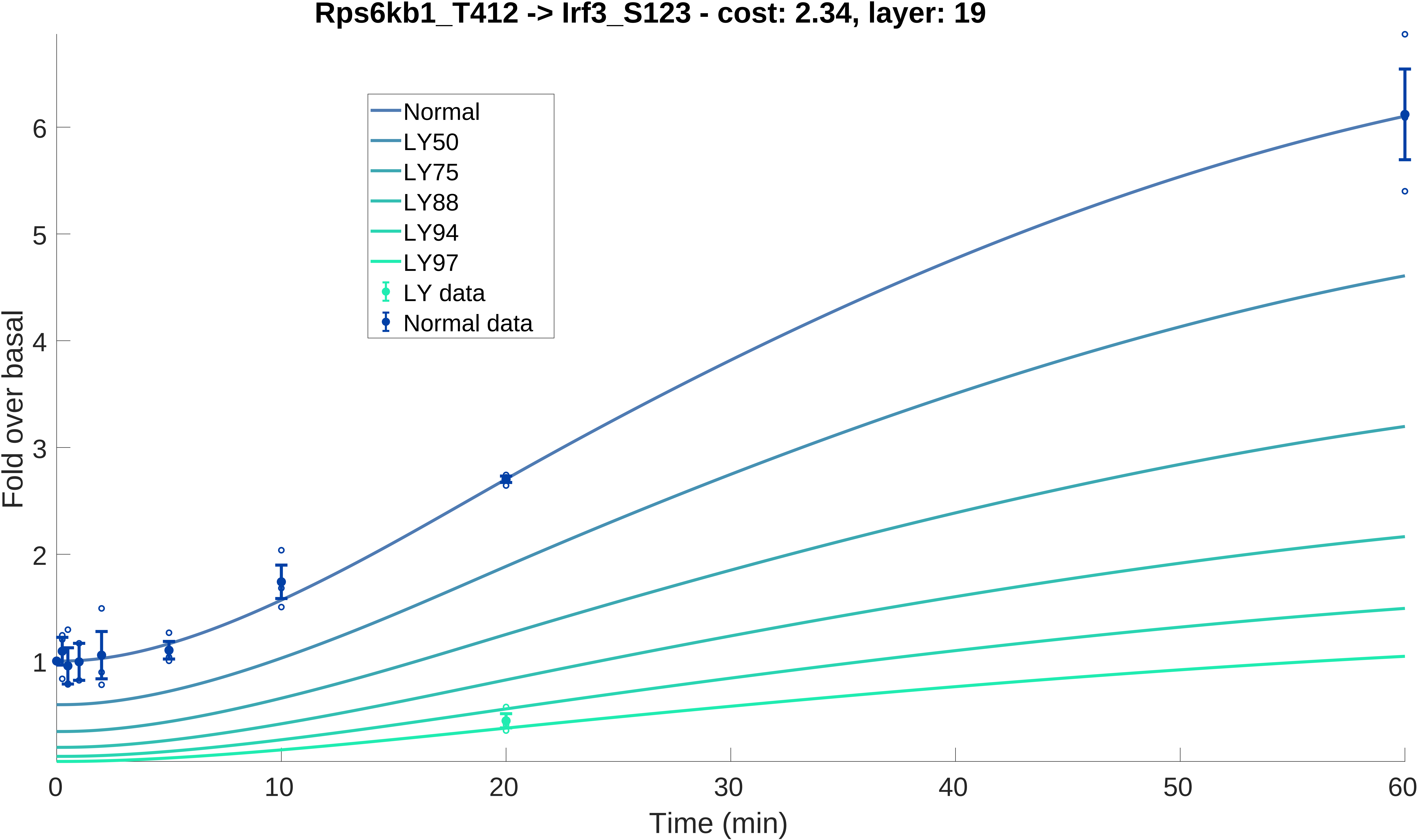

Rps6kb1_T412 -> Irf3_S123 - cost: 2.34, layer: 19
Normal
LY50
LY75
LY88
LY94
LY97
LY data
Normal data
Fold over basal
1
2
3
4
5
6
0
10
20
30
40
50
60
Time (min)

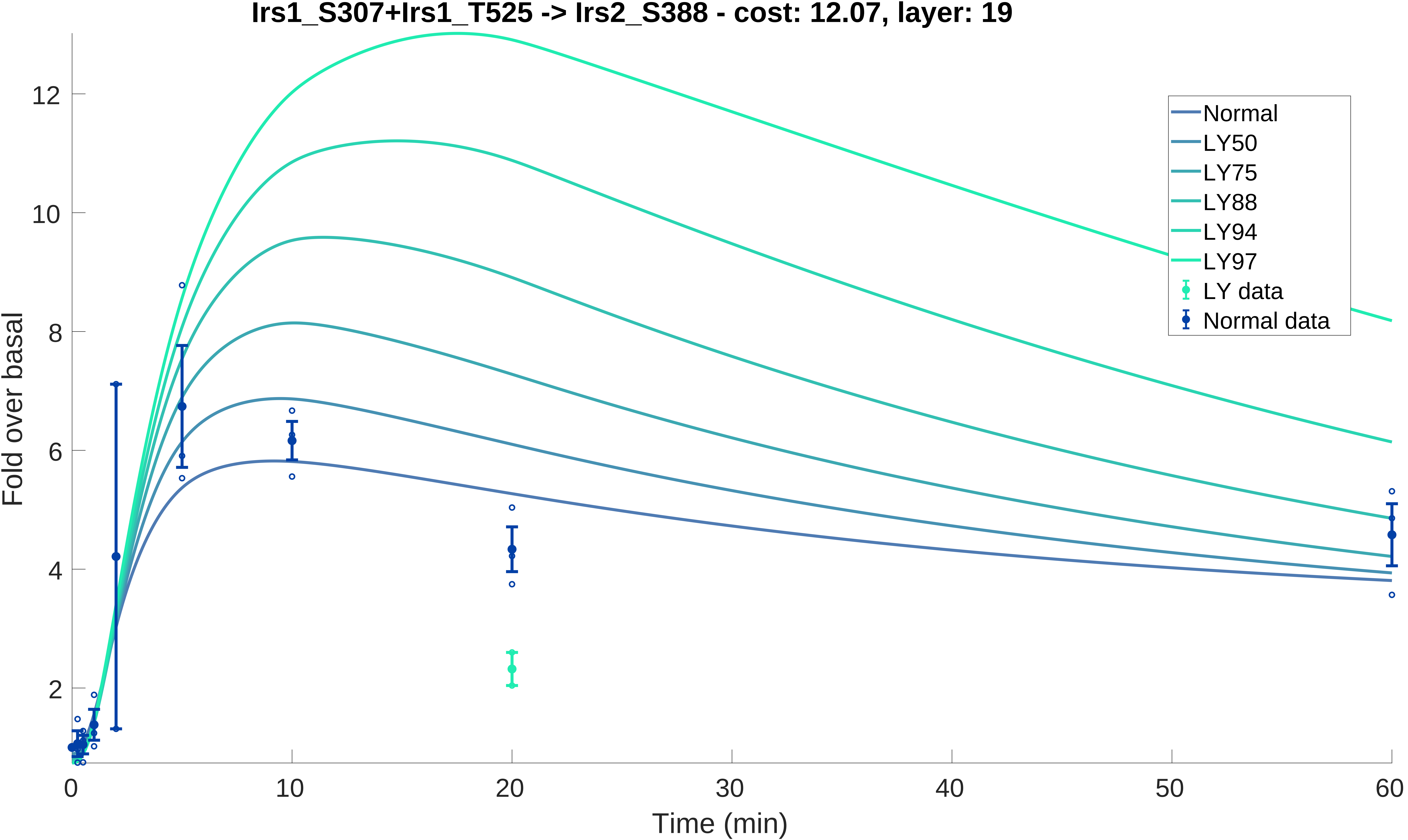
Irs1_S307+Irs1_T525 -> Irs2_S388 - cost: 12.07, layer: 19
Fold over basal
Time (min)
Normal
LY50
LY75
LY88
LY94
LY97
LY data
Normal data
0
10
20
30
40
50
60
2
4
6
8
10
12

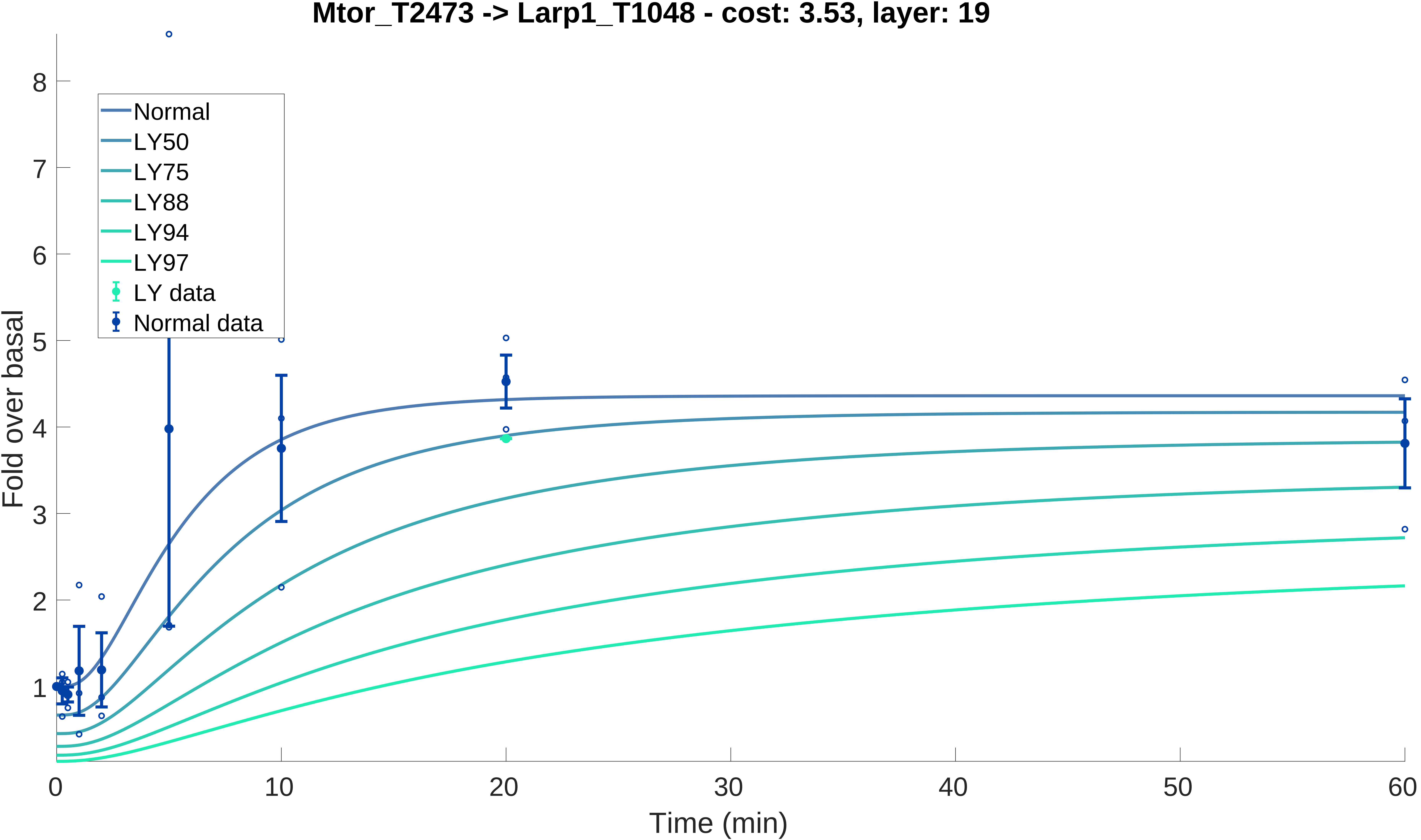

Mtor_T2473 -> Larp1_T1048 - cost: 3.53, layer: 19
Normal
LY50
LY75
LY88
LY94
LY97
LY data
Normal data
Fold over basal
Time (min)
1
2
3
4
5
6
7
8
0
10
20
30
40
50
60

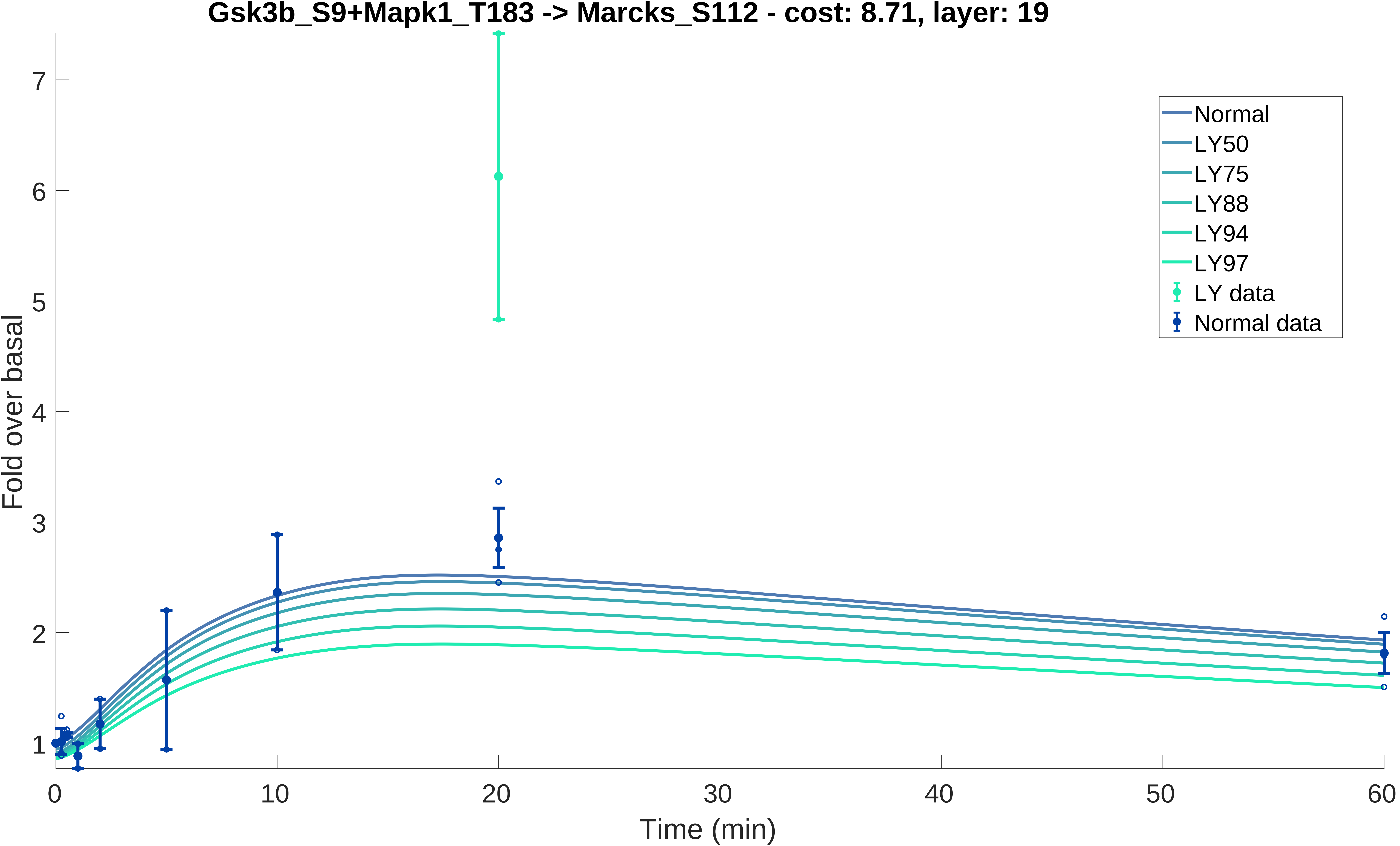

Gsk3b_S9+Mapk1_T183 -> Marcks_S112 - cost: 8.71, layer: 19
Fold over basal
Time (min)
Normal
LY50
LY75
LY88
LY94
LY97
LY data
Normal data
1
2
3
4
5
6
7
0
10
20
30
40
50
60

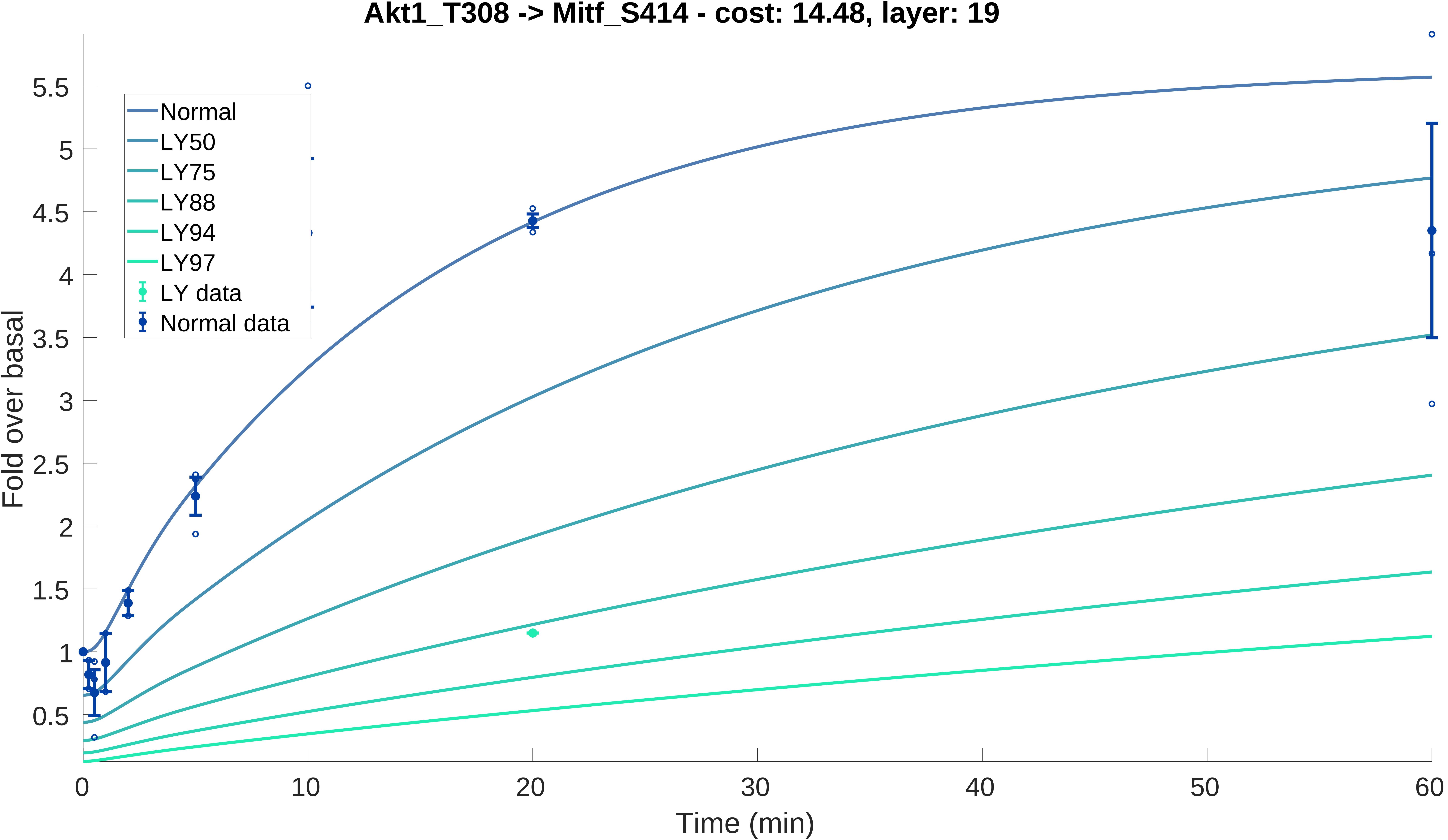
Akt1_T308 -> Mitf_S414 - cost: 14.48, layer: 19
Normal
LY50
LY75
LY88
LY94
LY97
LY data
Normal data
Fold over basal
Time (min)
0
10
20
30
40
50
60
0.5
1
1.5
2
2.5
3
3.5
4
4.5
5
5.5

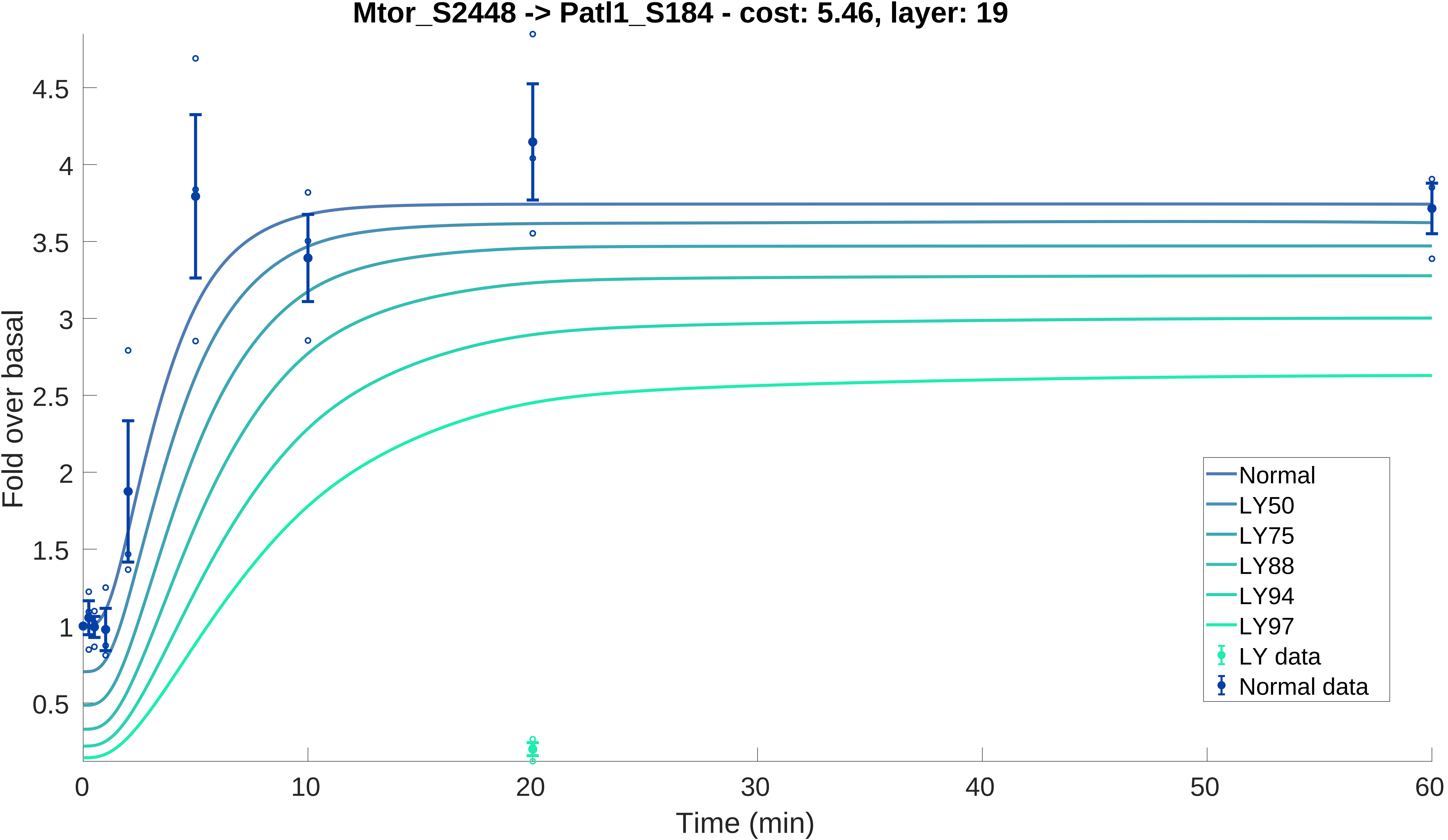

Mtor_S2448 -> Patl1_S184 - cost: 5.46, layer: 19
Fold over basal
Time (min)
0.5
1
1.5
2
2.5
3
3.5
4
4.5
0
10
20
30
40
50
60
Normal
LY50
LY75
LY88
LY94
LY97
LY data
Normal data

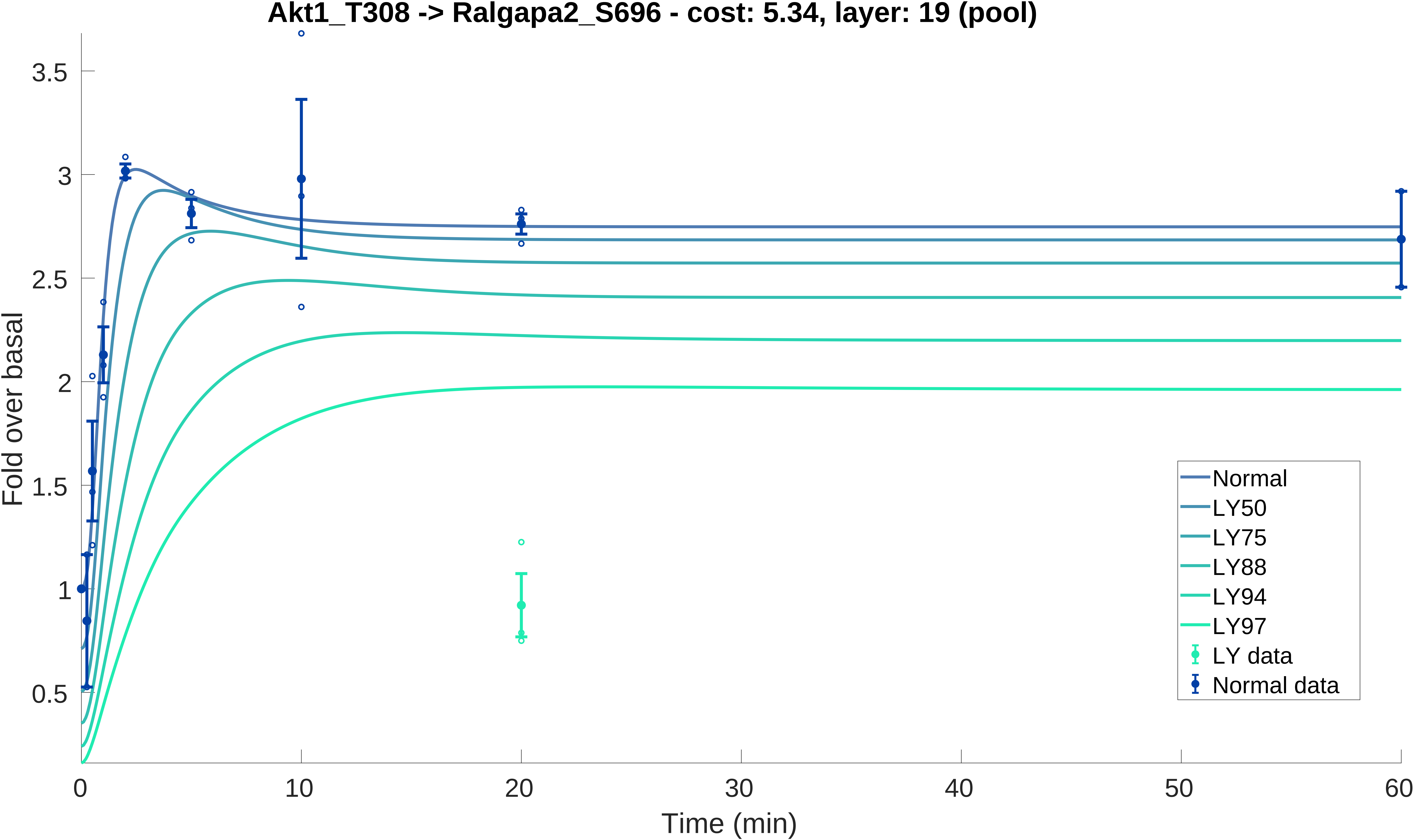
Akt1_T308 -> Ralgapa2_S696 - cost: 5.34, layer: 19 (pool)
Fold over basal
Time (min)
0.5
1
1.5
2
2.5
3
3.5
0
10
20
30
40
50
60
Normal
LY50
LY75
LY88
LY94
LY97
LY data
Normal data

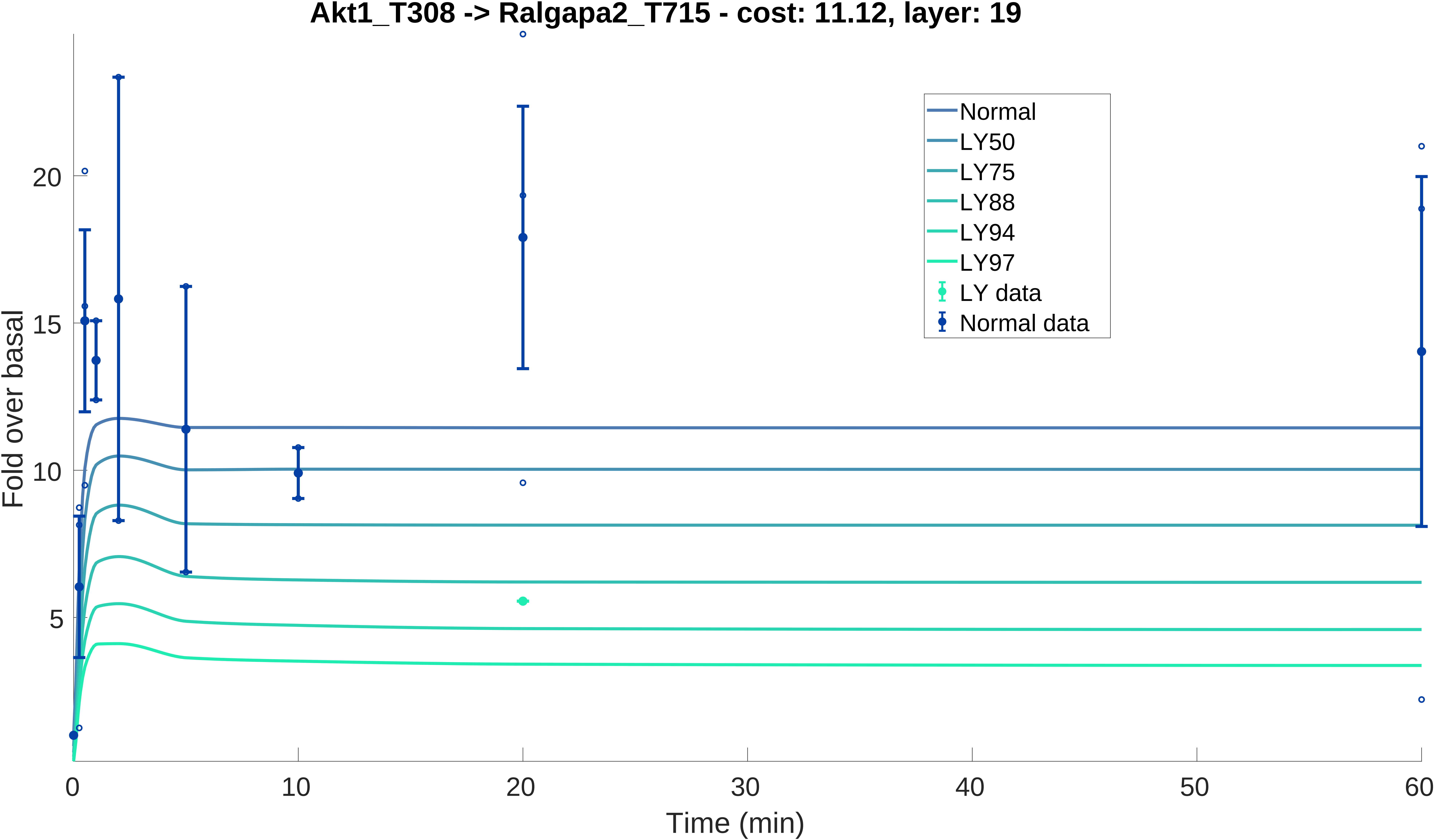
Akt1_T308 -> Ralgapa2_T715 - cost: 11.12, layer: 19
Fold over basal
Time (min)
0
10
20
30
40
50
60
5
10
15
20
Normal
LY50
LY75
LY88
LY94
LY97
LY data
Normal data

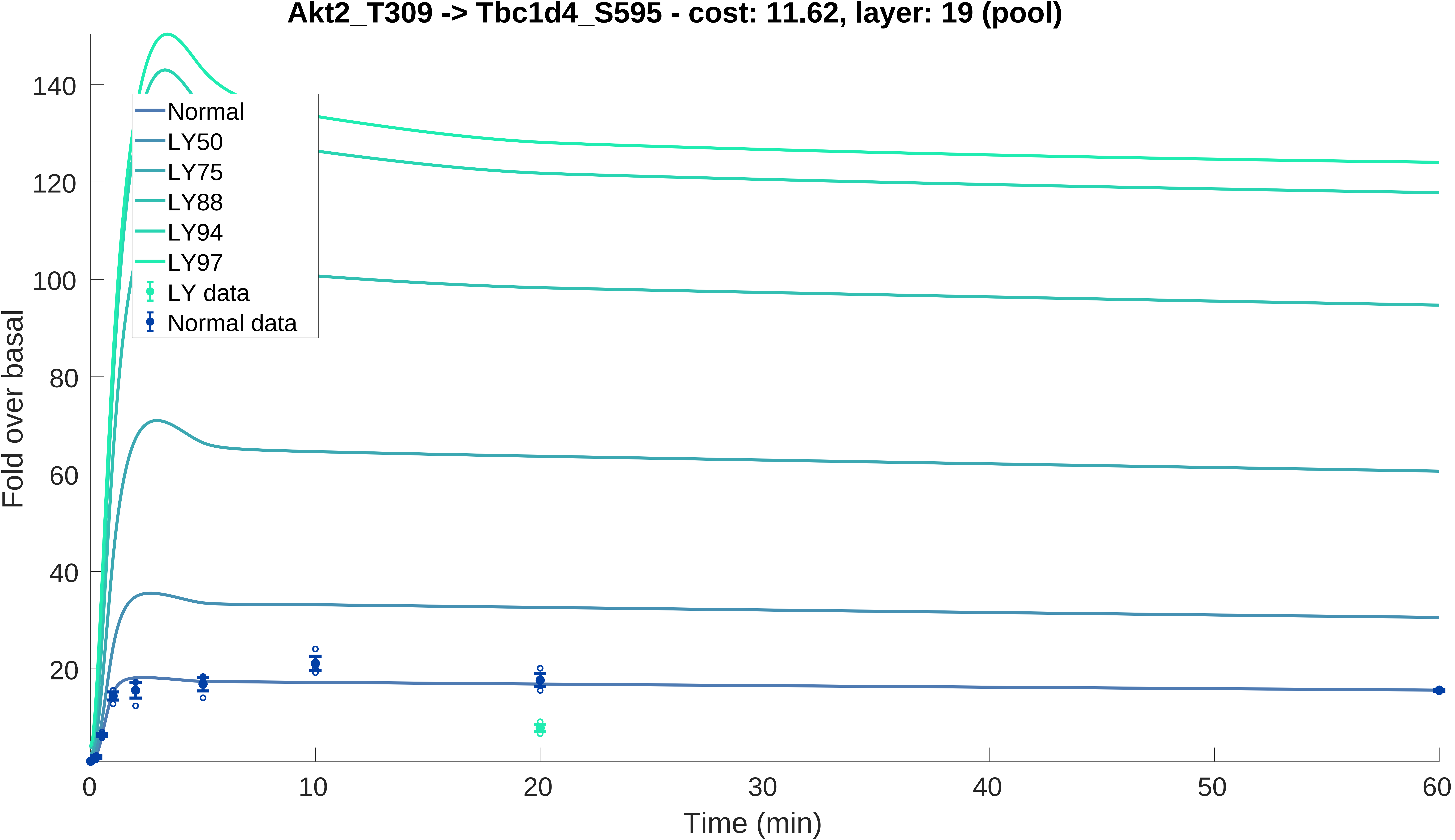

Akt2_T309 -> Tbc1d4_S595 - cost: 11.62, layer: 19 (pool)
Normal
LY50
LY75
LY88
LY94
LY97
LY data
Normal data
Fold over basal
Time (min)
0
10
20
30
40
50
60
20
40
60
80
100
120
140

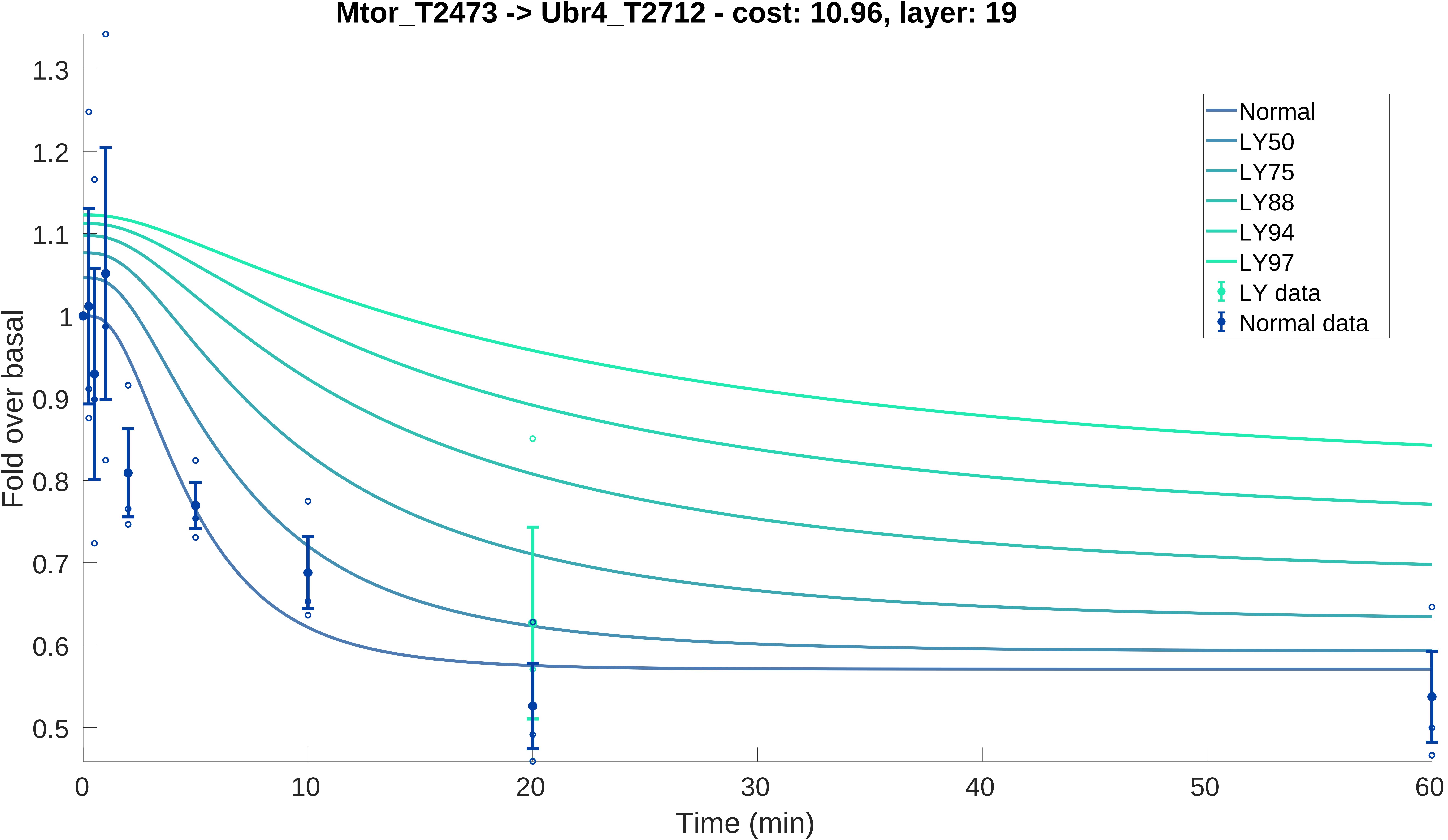
Mtor_T2473 -> Ubr4_T2712 - cost: 10.96, layer: 19
Fold over basal
1.3
1.2
1.1
1
0.9
0.8
0.7
0.6
0.5
0
10
20
30
40
50
60
Time (min)
Normal
LY50
LY75
LY88
LY94
LY97
LY data
Normal data

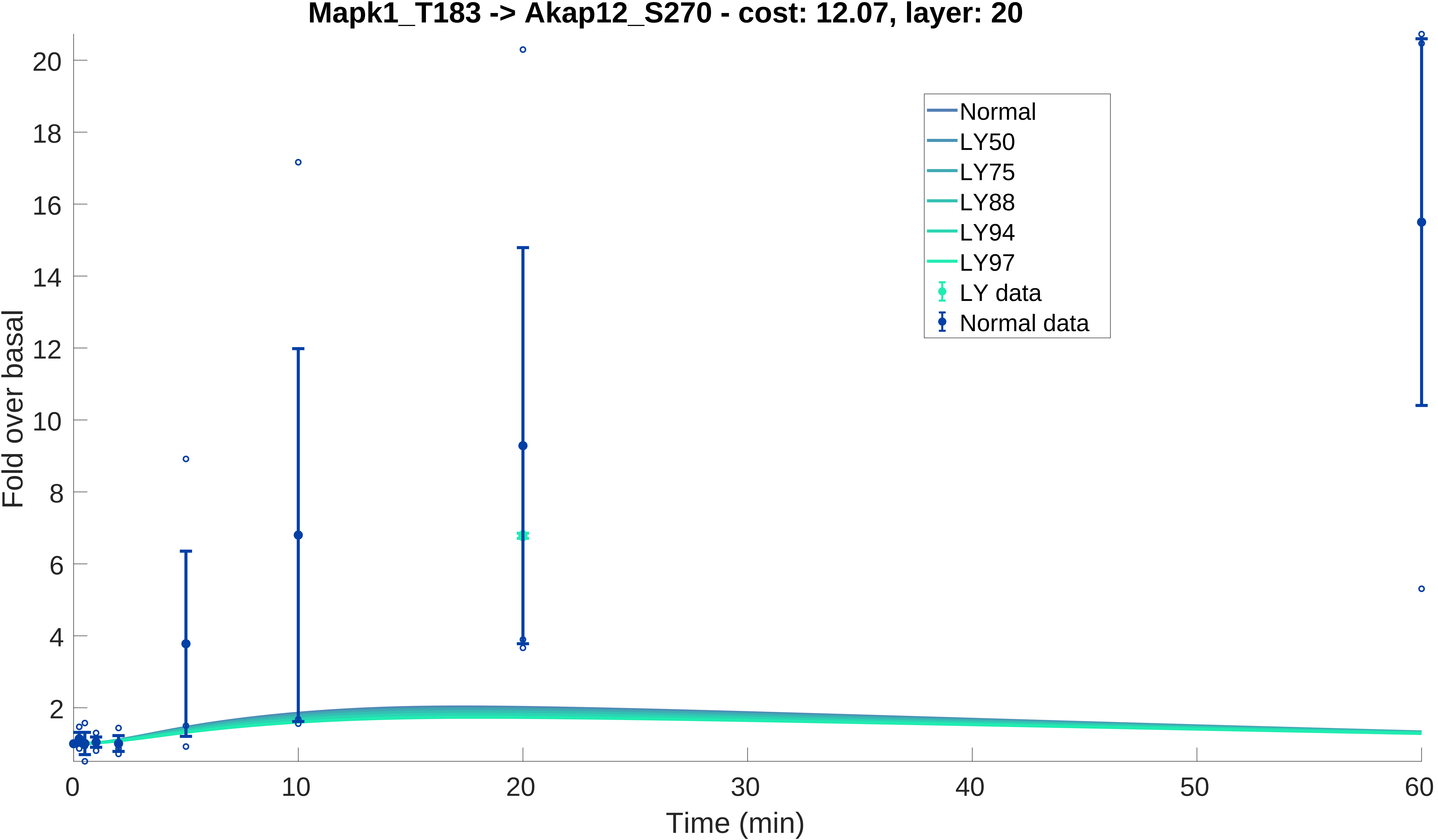

Mapk1_T183 -> Akap12_S270 - cost: 12.07, layer: 20
Fold over basal
Time (min)
Normal
LY50
LY75
LY88
LY94
LY97
LY data
Normal data
0
10
20
30
40
50
60
2
4
6
8
10
12
14
16
18
20

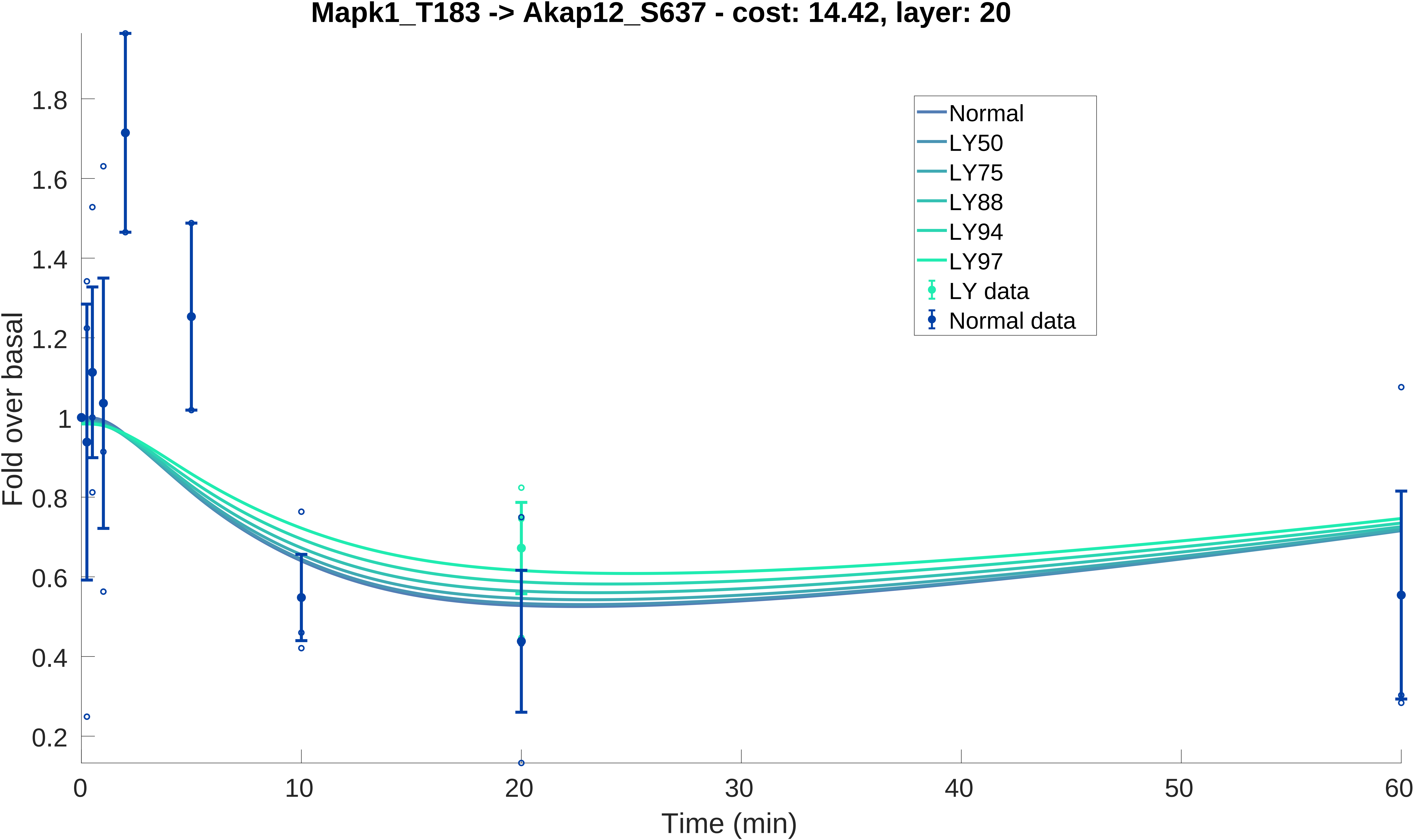

Mapk1_T183 -> Akap12_S637 - cost: 14.42, layer: 20
Fold over basal
Time (min)
0.2
0.4
0.6
0.8
1
1.2
1.4
1.6
1.8
0
10
20
30
40
50
60
Normal
LY50
LY75
LY88
LY94
LY97
LY data
Normal data

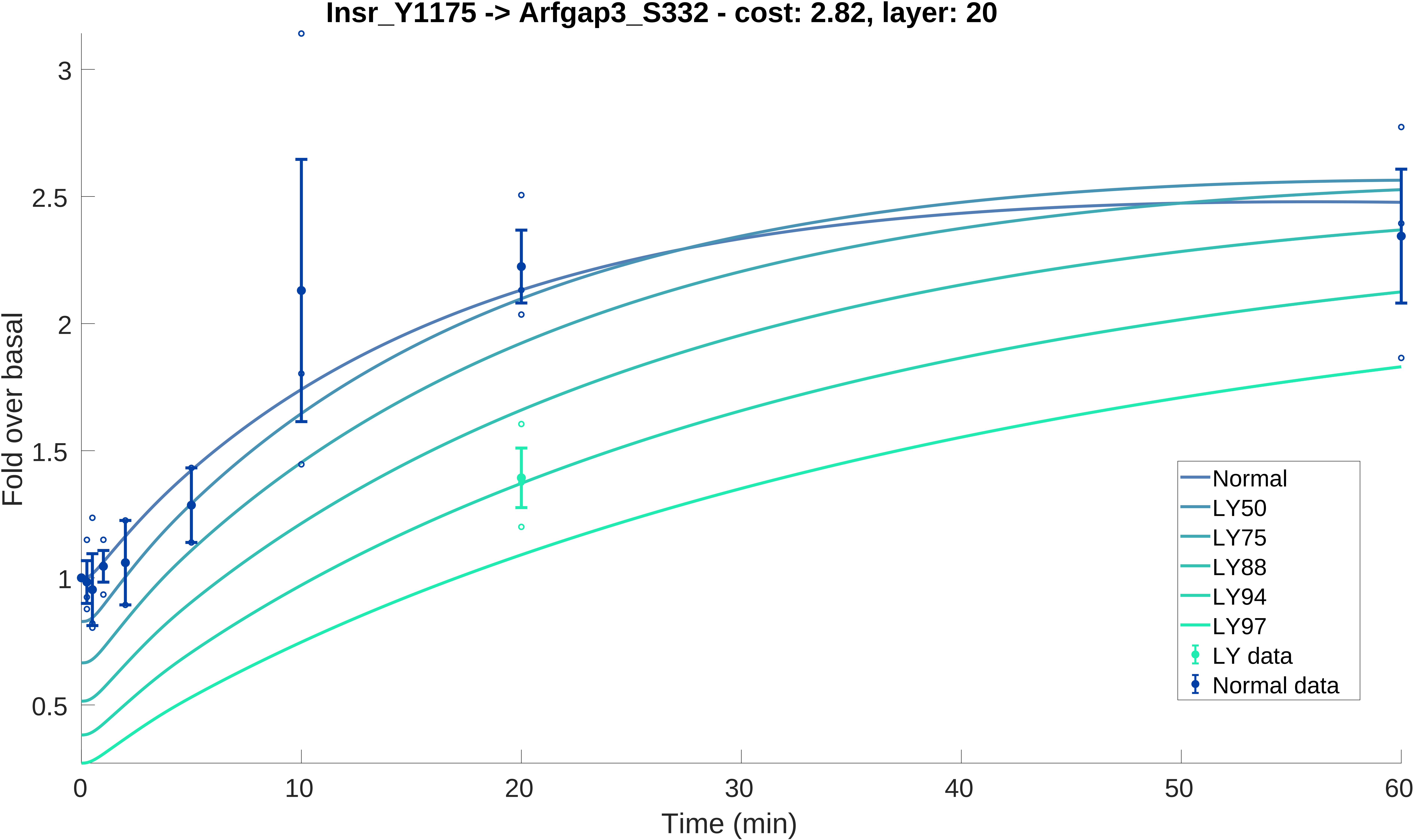
Insr_Y1175 -> Arfgap3_S332 - cost: 2.82, layer: 20
Fold over basal
3
2.5
2
1.5
1
0.5
0
10
20
30
40
50
60
Time (min)
Normal
LY50
LY75
LY88
LY94
LY97
LY data
Normal data

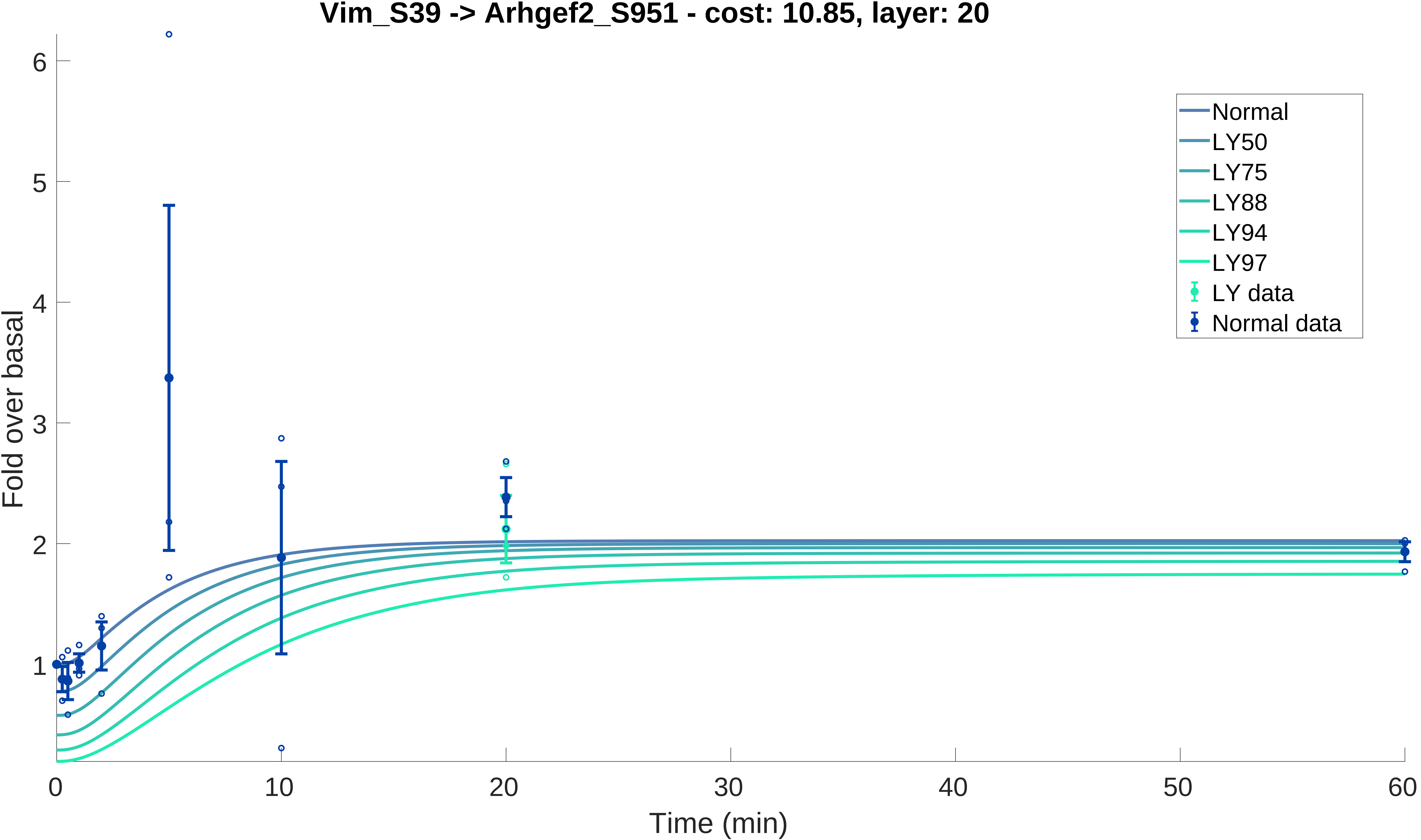
Vim_S39 -> Arhgef2_S951 - cost: 10.85, layer: 20
Fold over basal
Time (min)
0
10
20
30
40
50
60
1
2
3
4
5
6
Normal
LY50
LY75
LY88
LY94
LY97
LY data
Normal data

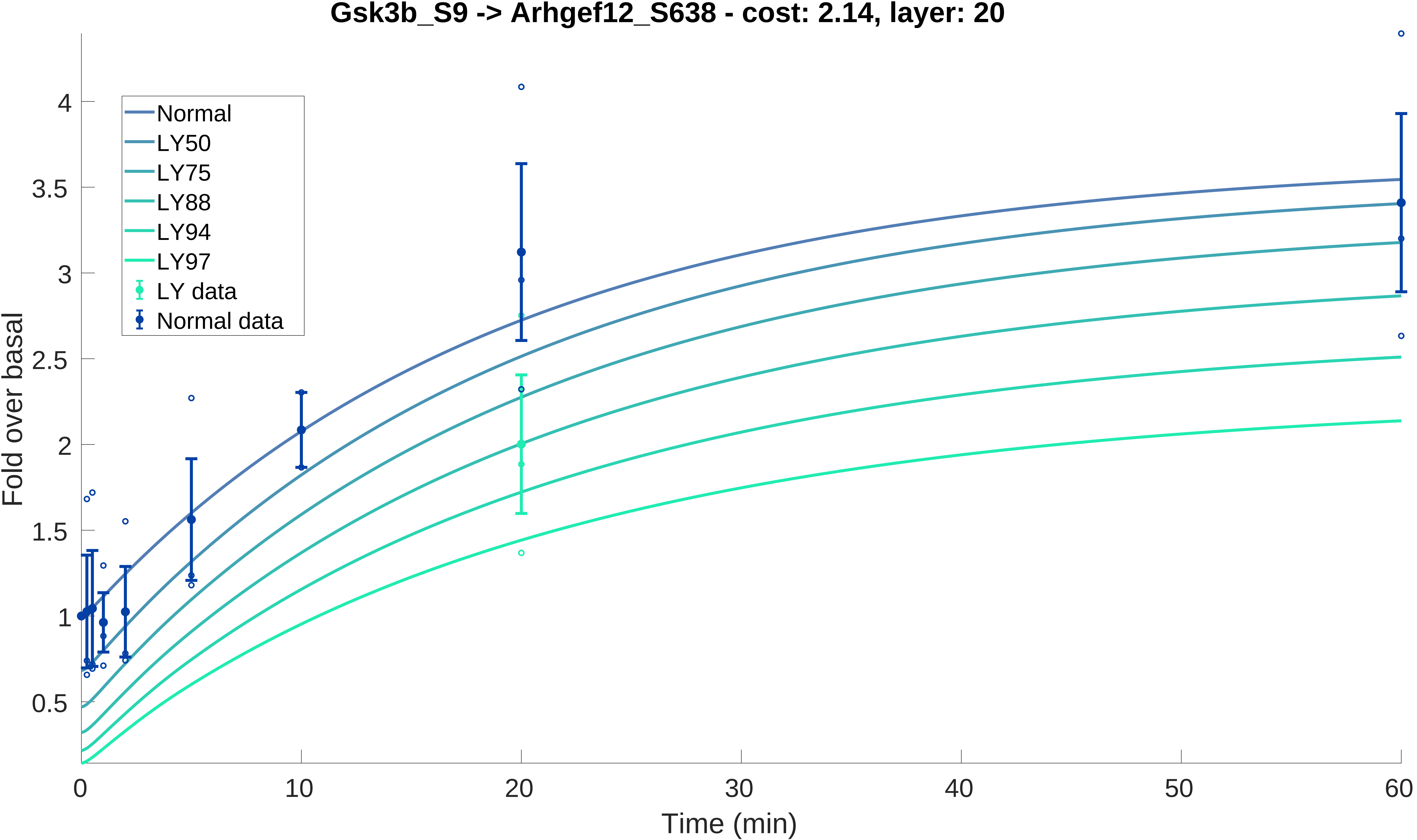

Gsk3b_S9 -> Arhgef12_S638 - cost: 2.14, layer: 20
Normal
LY50
LY75
LY88
LY94
LY97
LY data
Normal data
Fold over basal
Time (min)
0
10
20
30
40
50
60
0.5
1
1.5
2
2.5
3
3.5
4

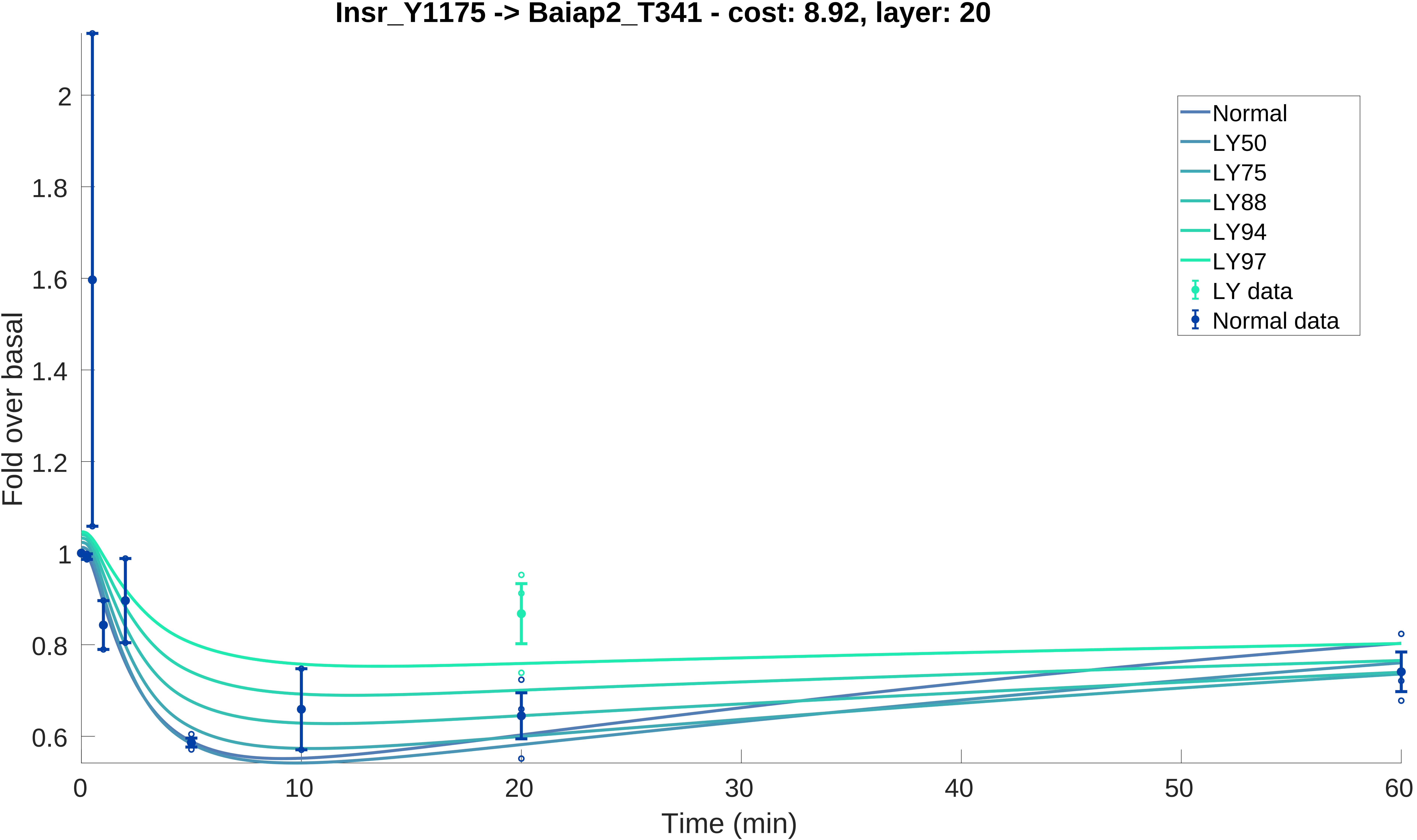

Insr_Y1175 -> Baiap2_T341 - cost: 8.92, layer: 20
Fold over basal
Time (min)
Normal
LY50
LY75
LY88
LY94
LY97
LY data
Normal data

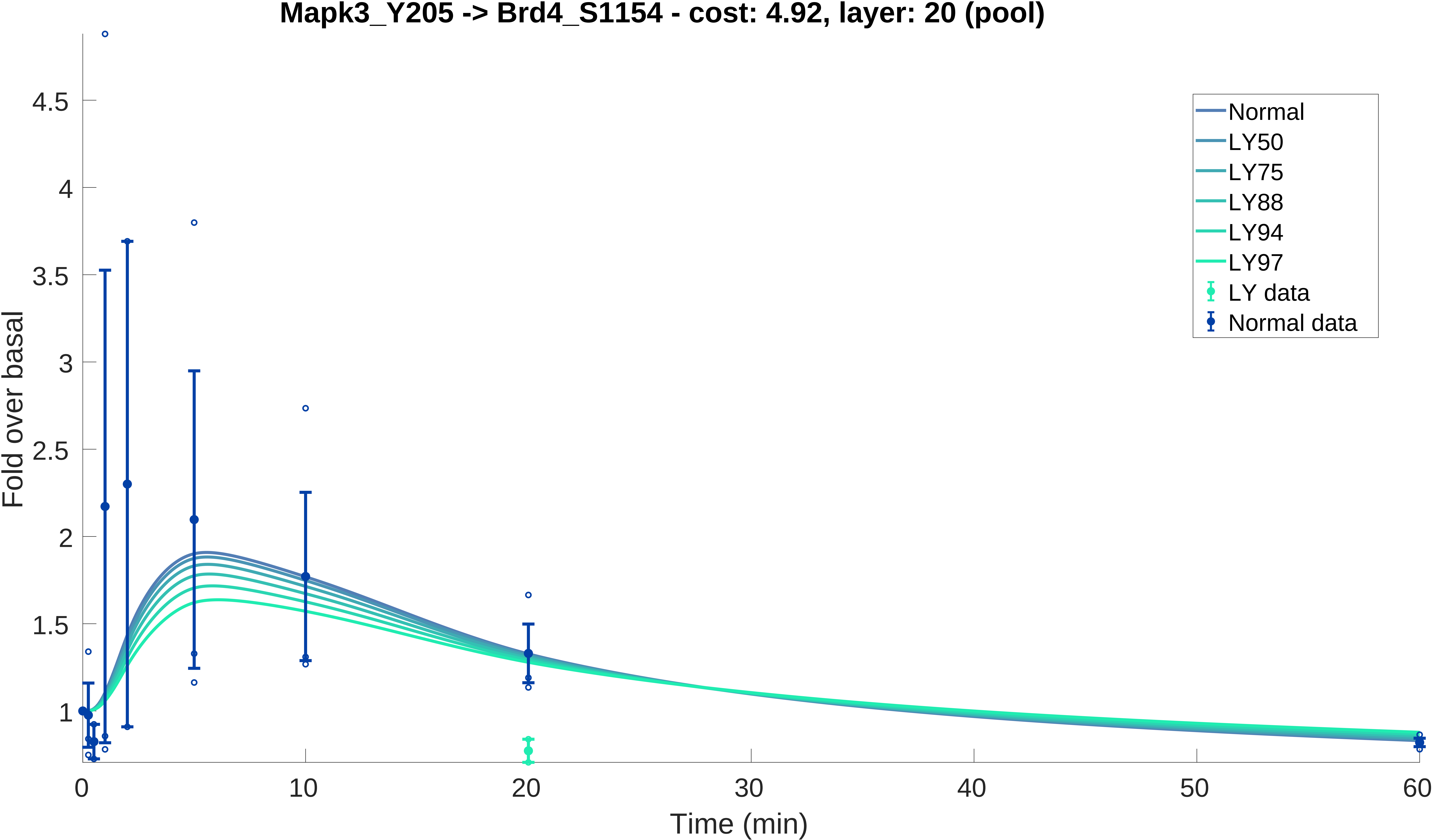

Mapk3_Y205 -> Brd4_S1154 - cost: 4.92, layer: 20 (pool)
Fold over basal
Time (min)
Normal
LY50
LY75
LY88
LY94
LY97
LY data
Normal data
4.5
4
3.5
3
2.5
2
1.5
1
0
10
20
30
40
50
60

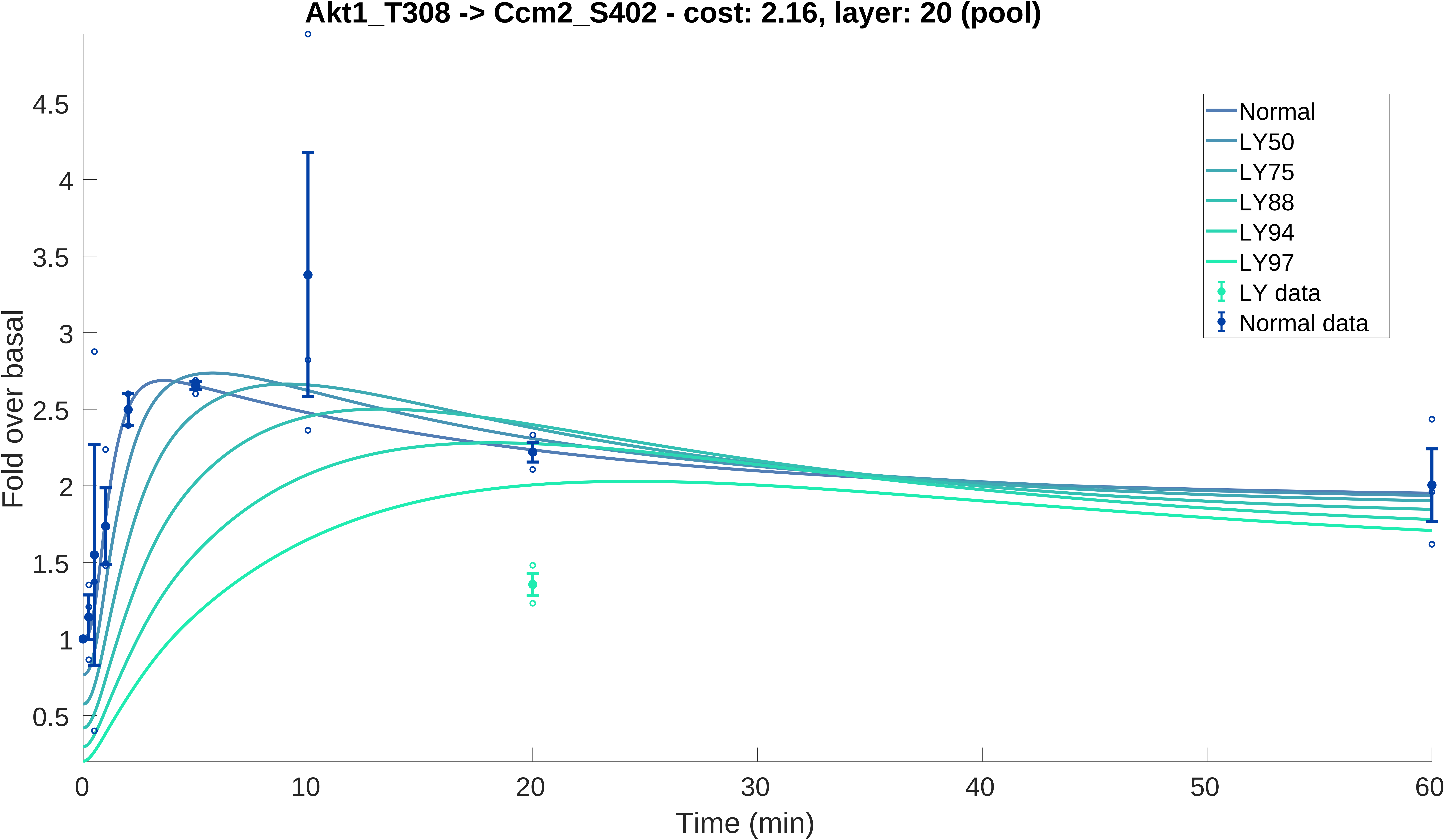

Akt1_T308 -> Ccm2_S402 - cost: 2.16, layer: 20 (pool)
Fold over basal
Time (min)
0.5
1
1.5
2
2.5
3
3.5
4
4.5
0
10
20
30
40
50
60
Normal
LY50
LY75
LY88
LY94
LY97
LY data
Normal data

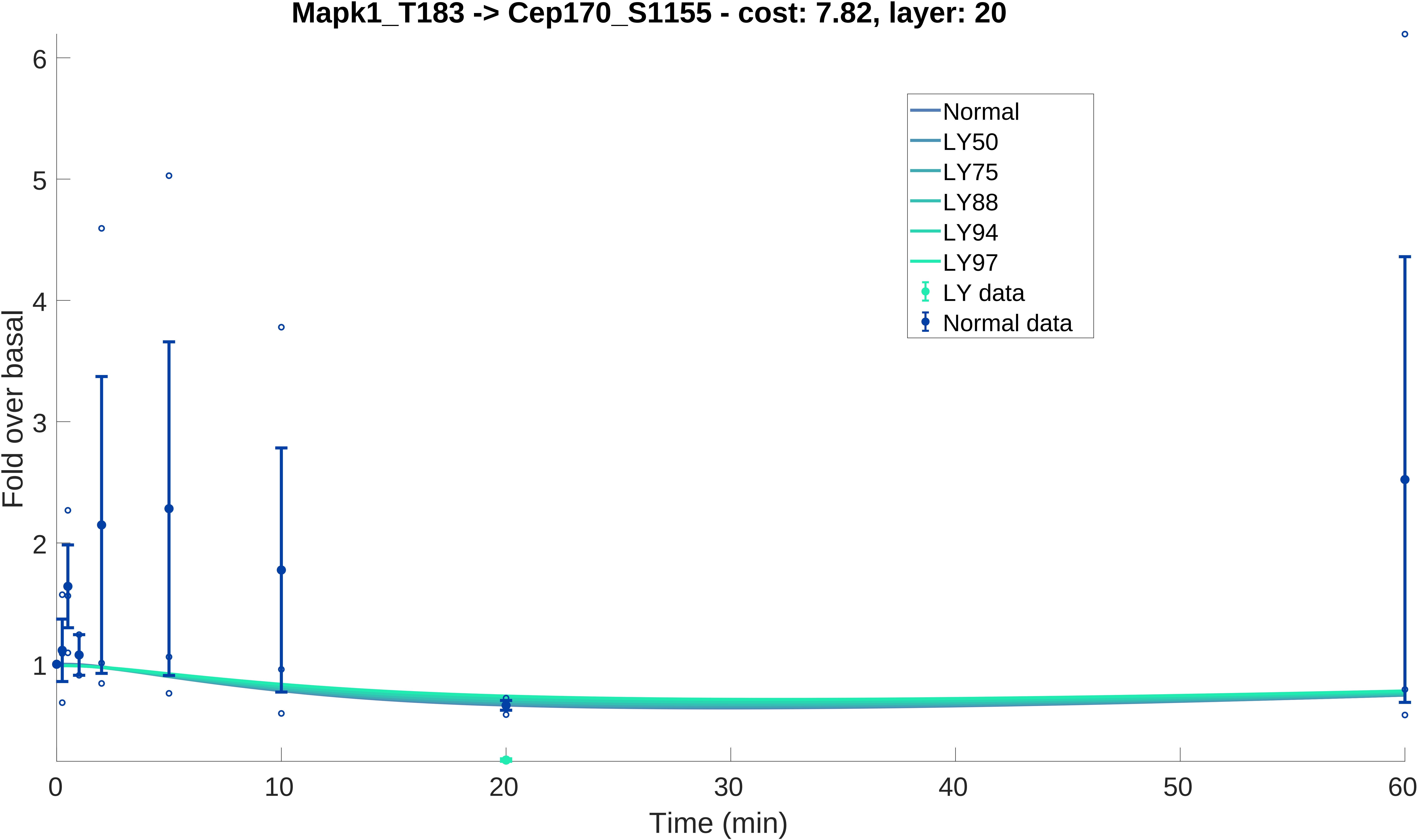
Mapk1_T183 -> Cep170_S1155 - cost: 7.82, layer: 20
Fold over basal
Time (min)
0
10
20
30
40
50
60
1
2
3
4
5
6
Normal
LY50
LY75
LY88
LY94
LY97
LY data
Normal data

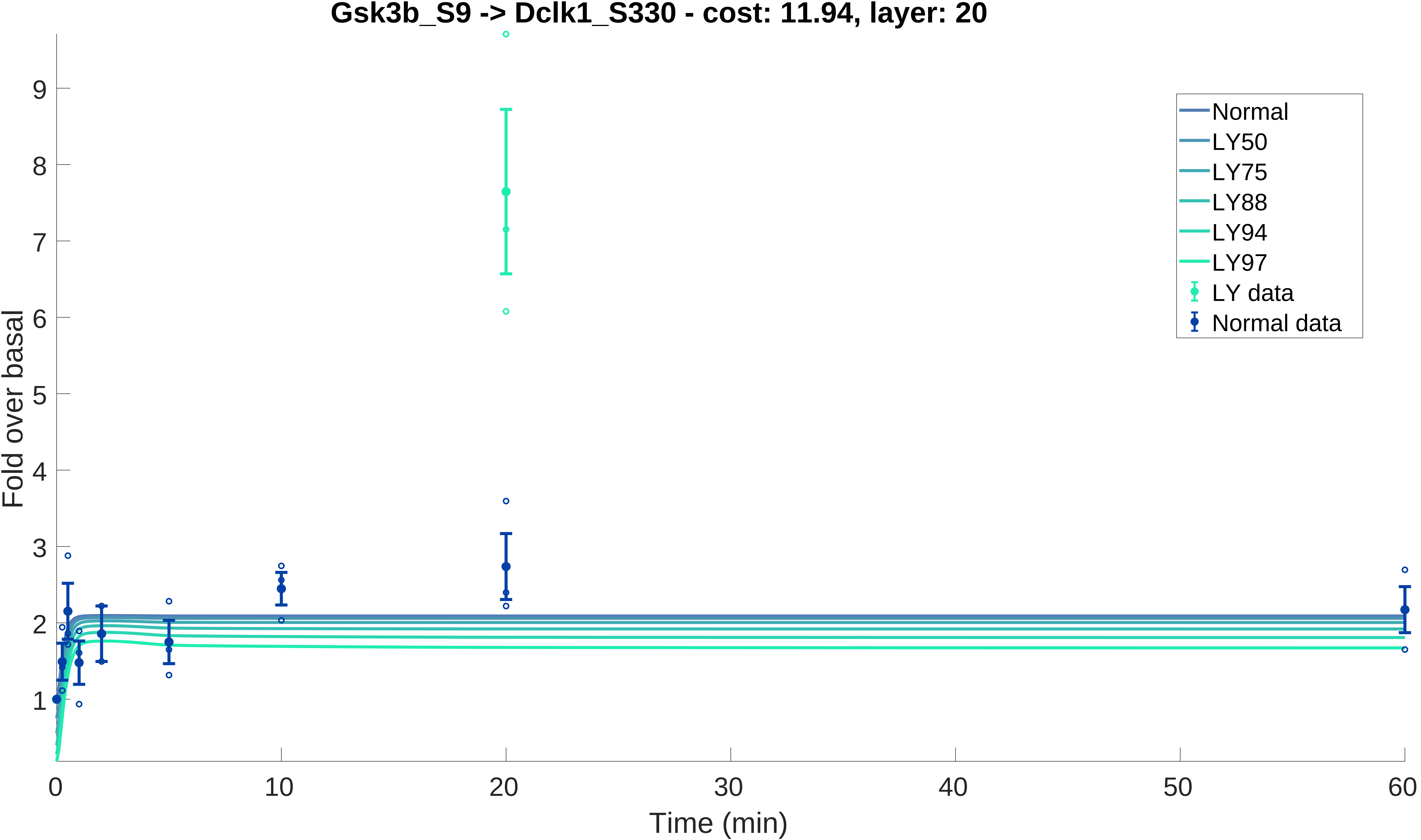
Gsk3b_S9 -> Dclk1_S330 - cost: 11.94, layer: 20
Fold over basal
Time (min)
Normal
LY50
LY75
LY88
LY94
LY97
LY data
Normal data

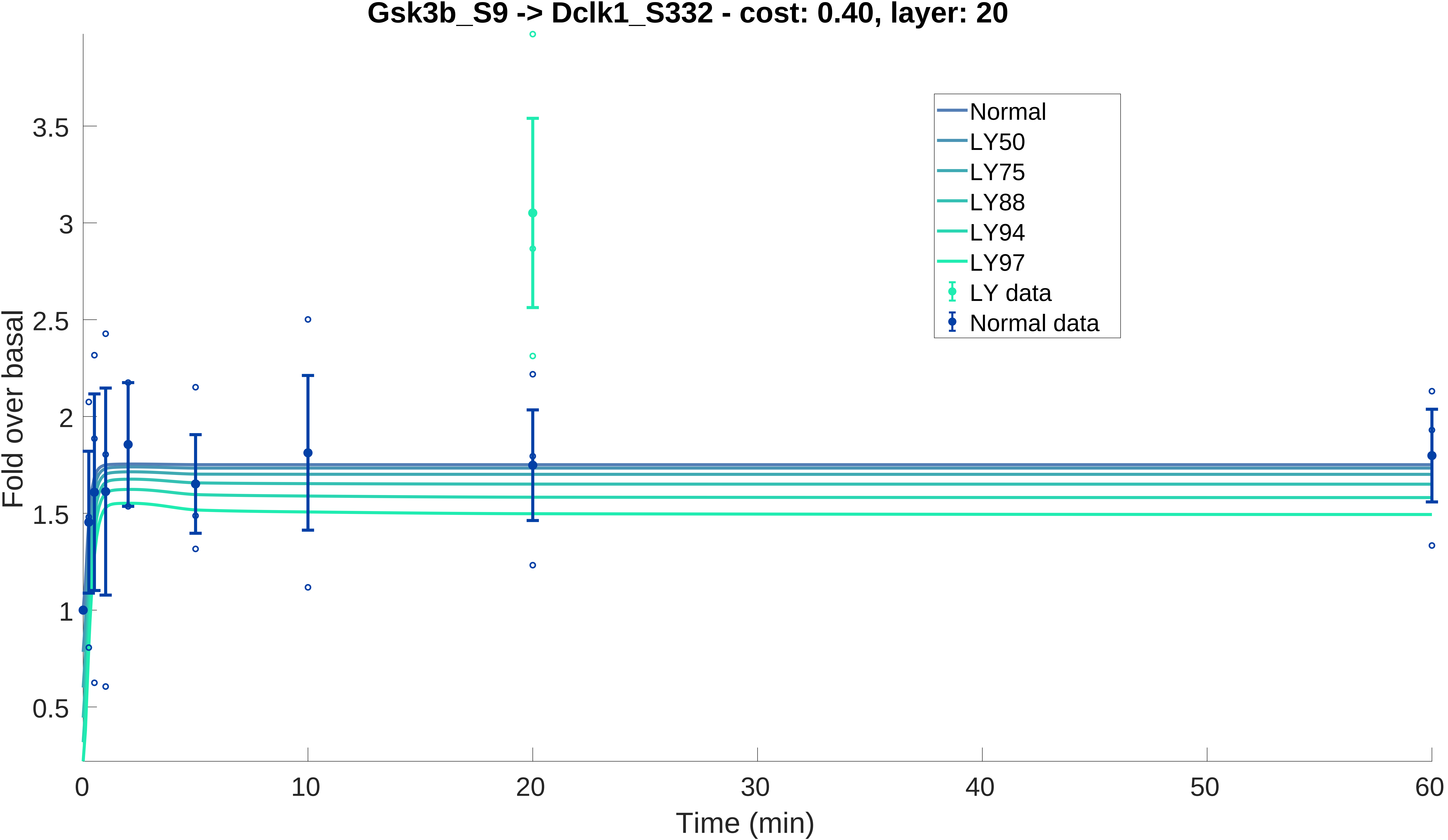

Gsk3b_S9 -> Dclk1_S332 - cost: 0.40, layer: 20
Fold over basal
Time (min)
0.5
1
1.5
2
2.5
3
3.5
0
10
20
30
40
50
60
Normal
LY50
LY75
LY88
LY94
LY97
LY data
Normal data

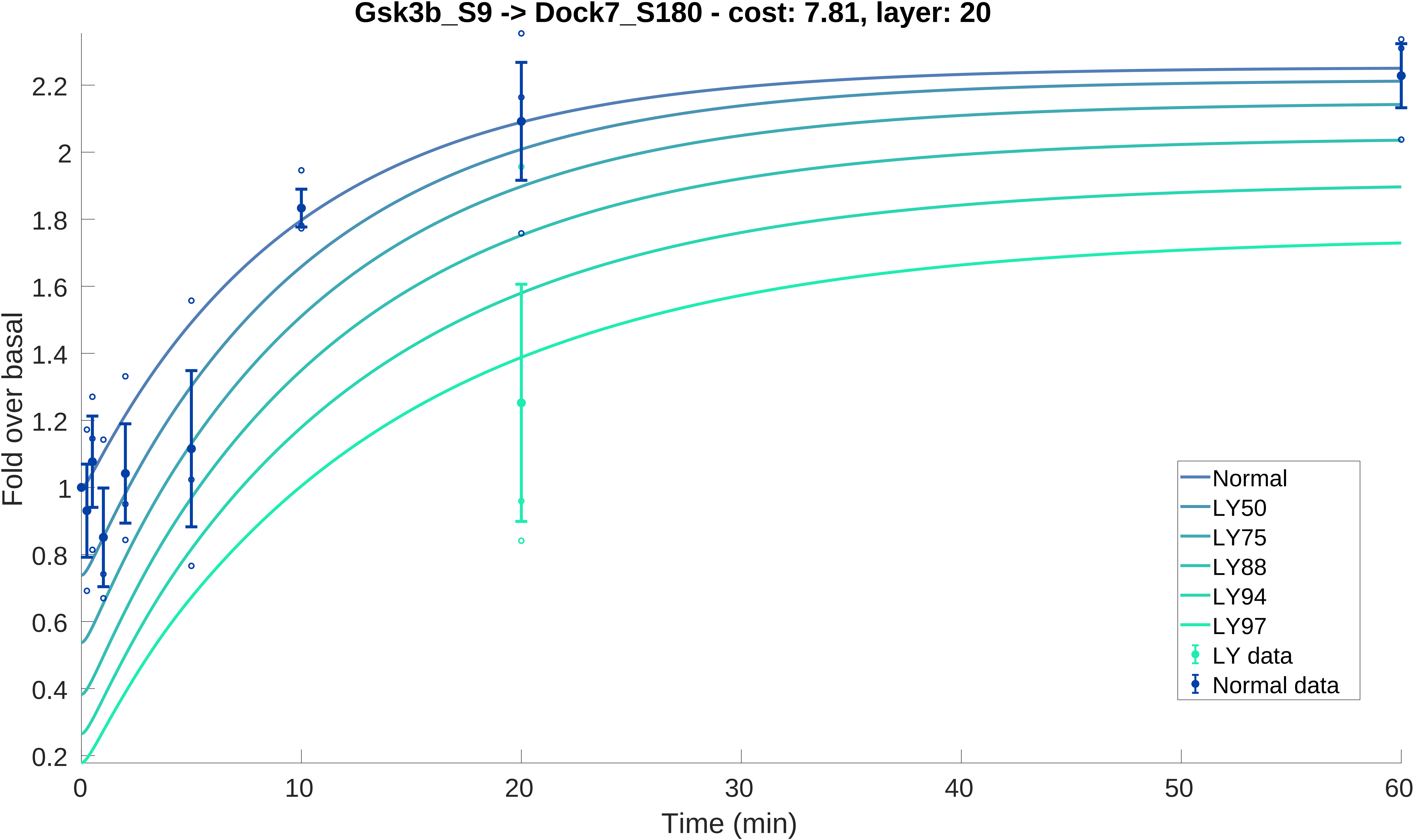
Gsk3b_S9 -> Dock7_S180 - cost: 7.81, layer: 20
Fold over basal
Time (min)
0.2
0.4
0.6
0.8
1
1.2
1.4
1.6
1.8
2
2.2
0
10
20
30
40
50
60
Normal
LY50
LY75
LY88
LY94
LY97
LY data
Normal data

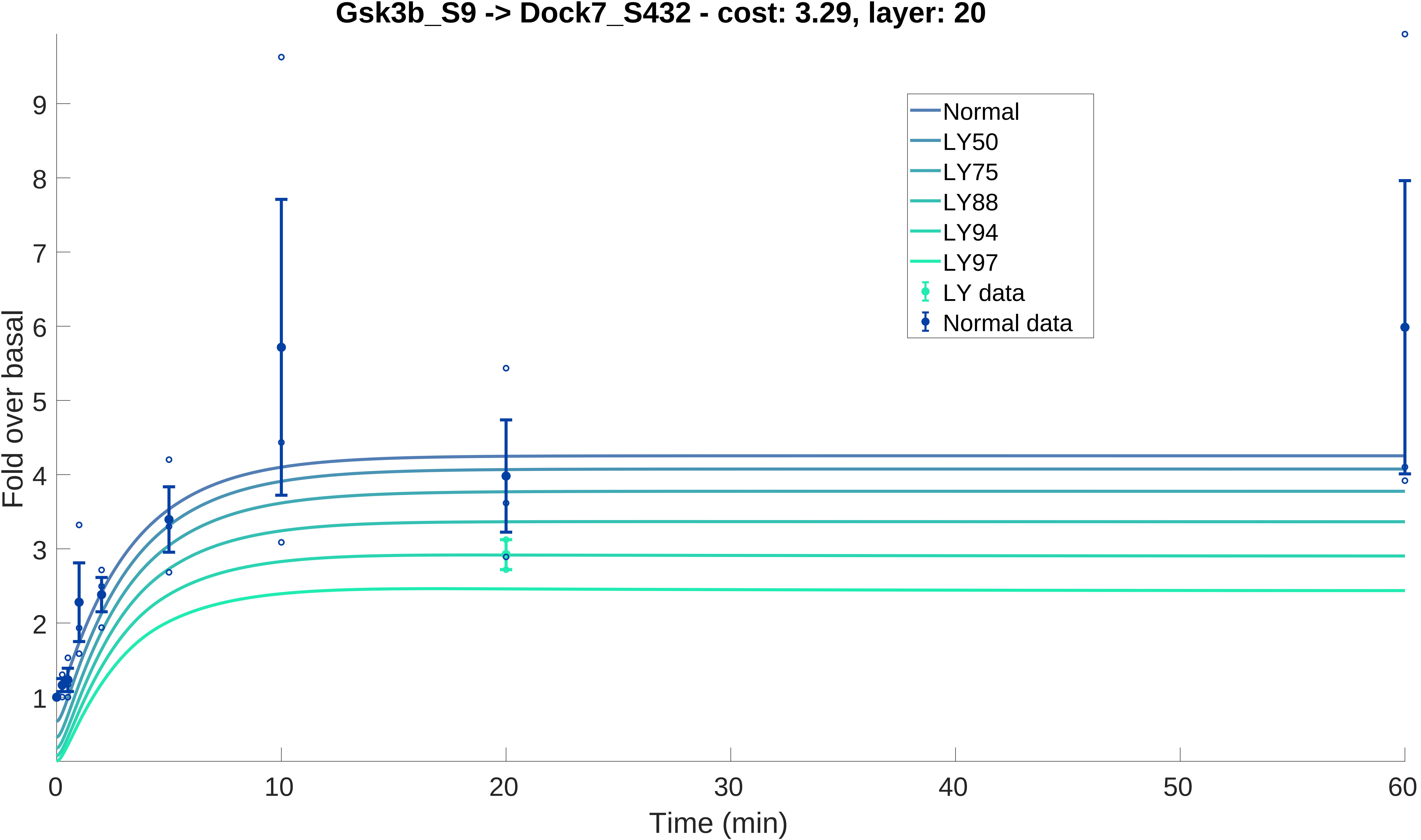
Gsk3b_S9 -> Dock7_S432 - cost: 3.29, layer: 20
Normal
LY50
LY75
LY88
LY94
LY97
LY data
Normal data
Fold over basal
Time (min)
0
10
20
30
40
50
60
1
2
3
4
5
6
7
8
9

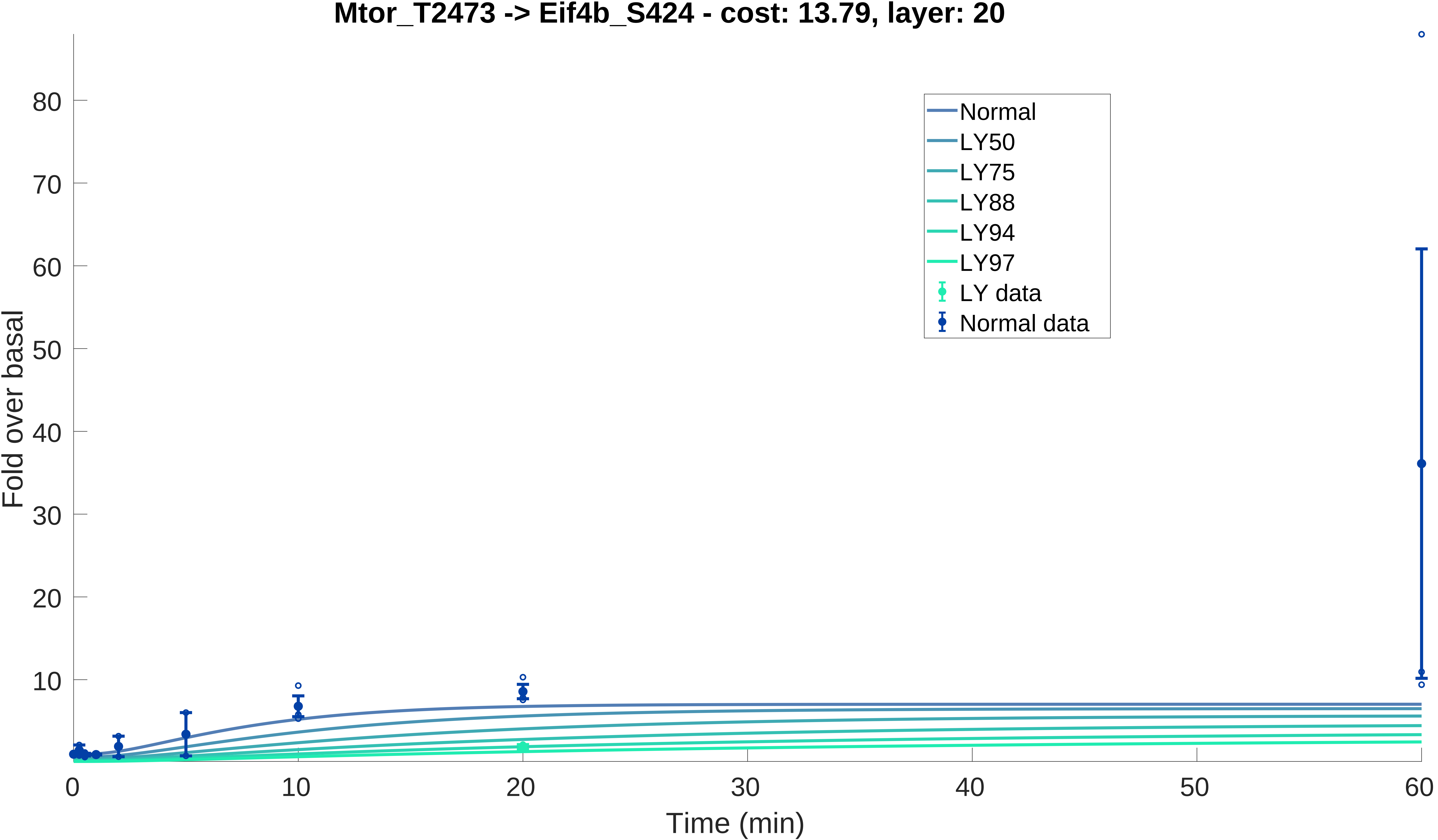
Mtor_T2473 -> Eif4b_S424 - cost: 13.79, layer: 20
Fold over basal
80
70
60
50
40
30
20
10
0
10
20
30
40
50
60
Time (min)
Normal
LY50
LY75
LY88
LY94
LY97
LY data
Normal data

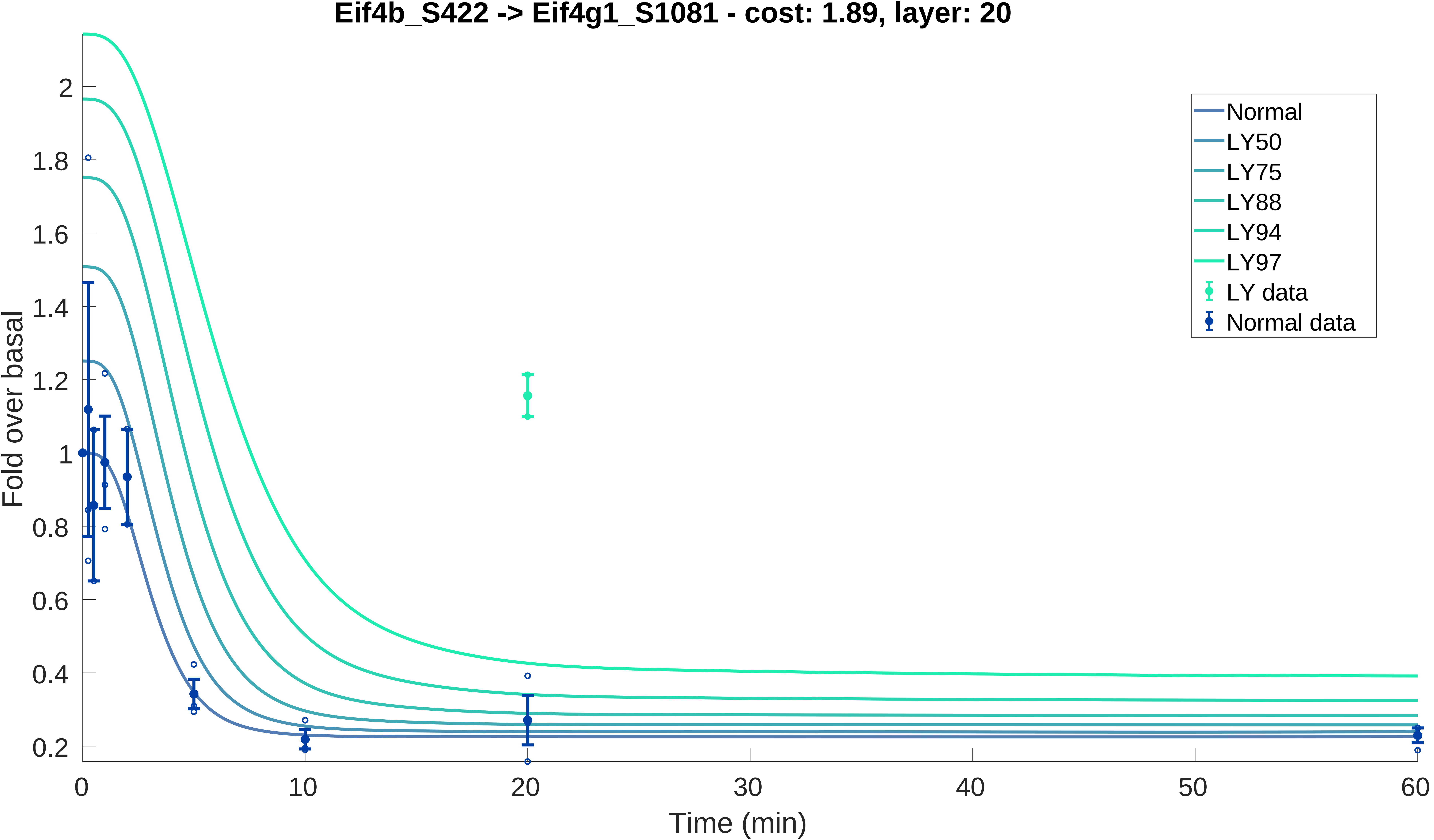

Eif4b_S422 -> Eif4g1_S1081 - cost: 1.89, layer: 20
Fold over basal
Time (min)
0
10
20
30
40
50
60
0.2
0.4
0.6
0.8
1
1.2
1.4
1.6
1.8
2
Normal
LY50
LY75
LY88
LY94
LY97
LY data
Normal data

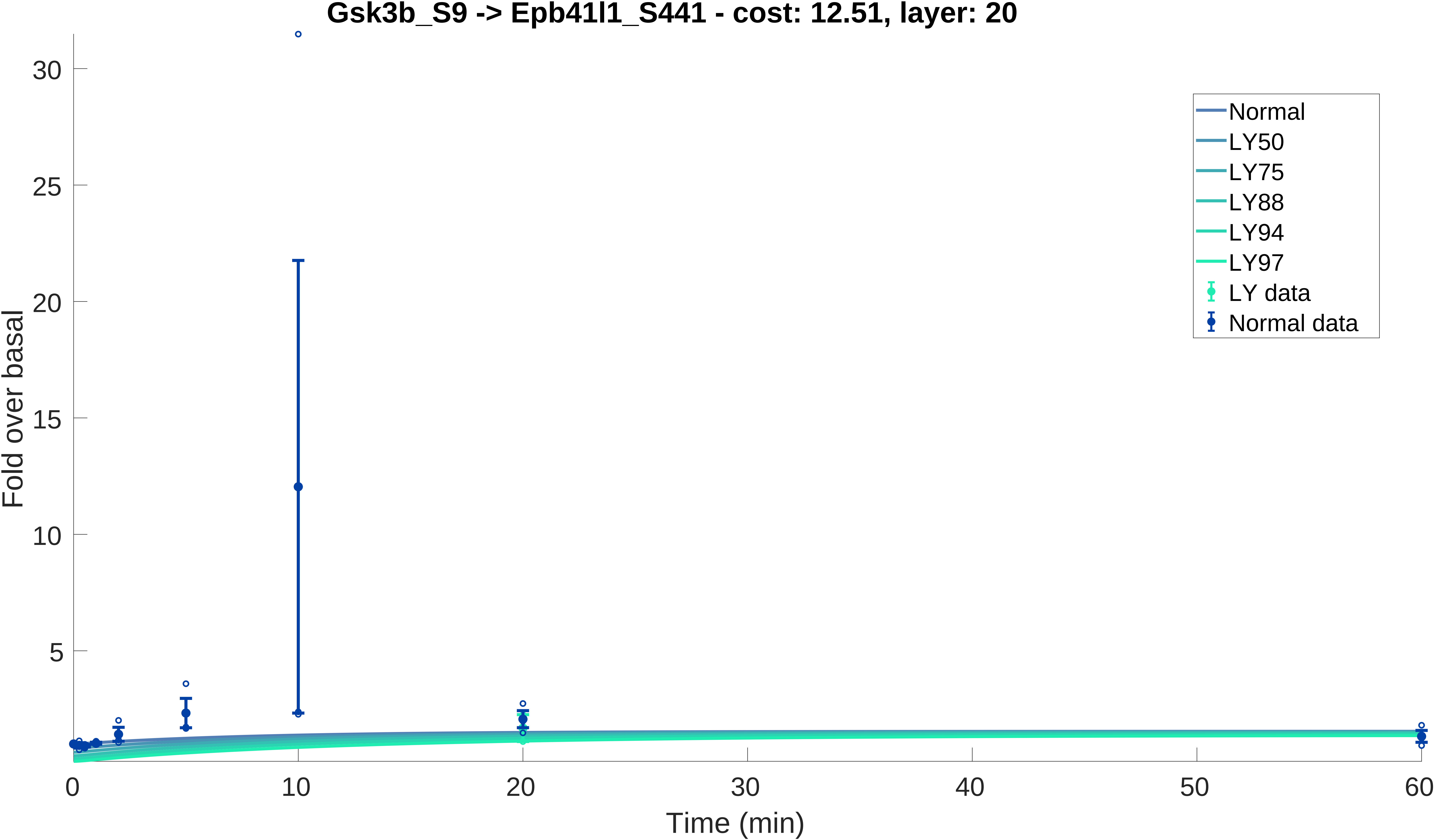

Gsk3b_S9 -> Epb41l1_S441 - cost: 12.51, layer: 20
Fold over basal
Time (min)
0
10
20
30
40
50
60
5
10
15
20
25
30
Normal
LY50
LY75
LY88
LY94
LY97
LY data
Normal data

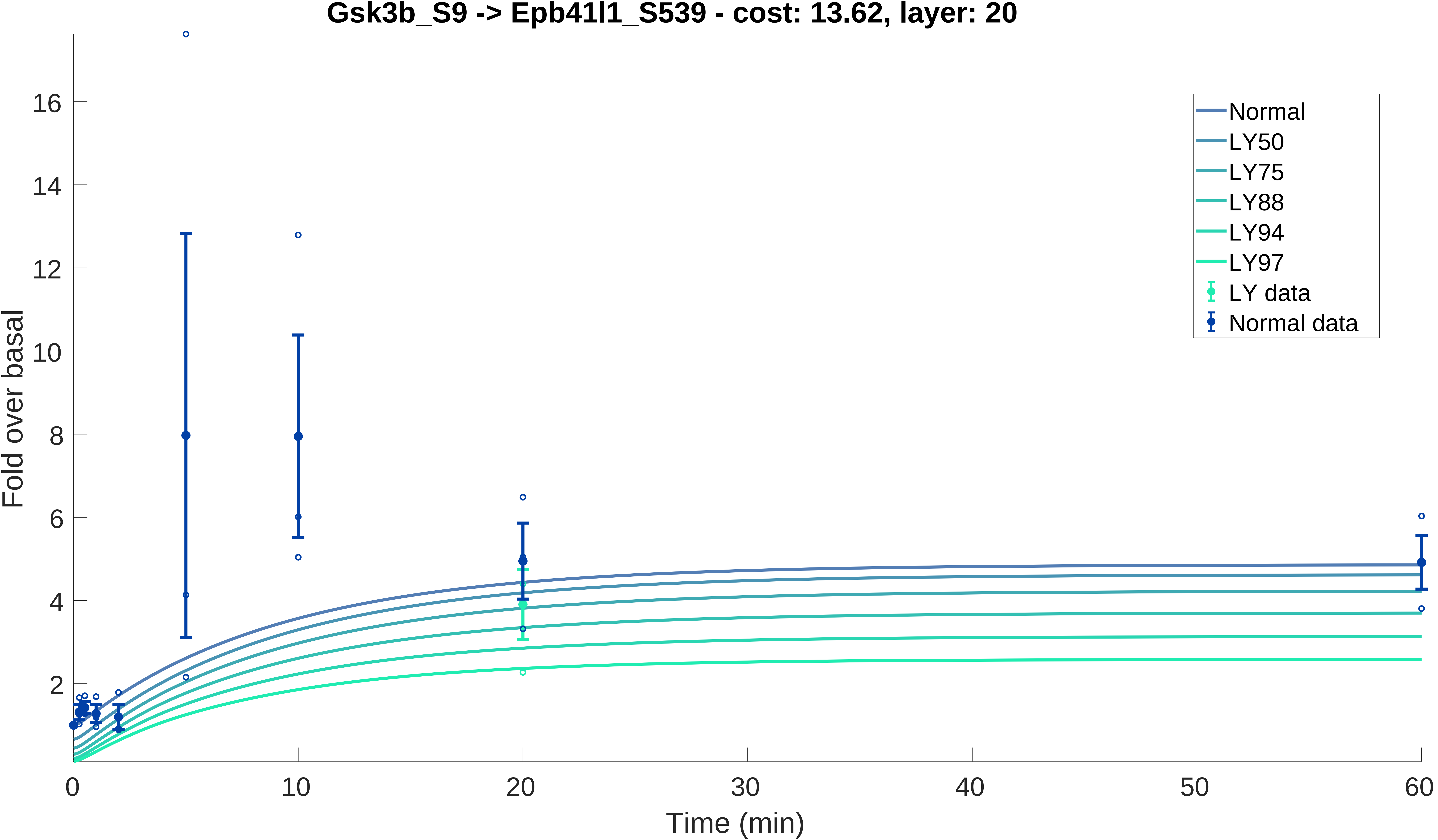

Gsk3b_S9 -> Epb41l1_S539 - cost: 13.62, layer: 20
Fold over basal
Time (min)
Normal
LY50
LY75
LY88
LY94
LY97
LY data
Normal data
0
10
20
30
40
50
60
2
4
6
8
10
12
14
16

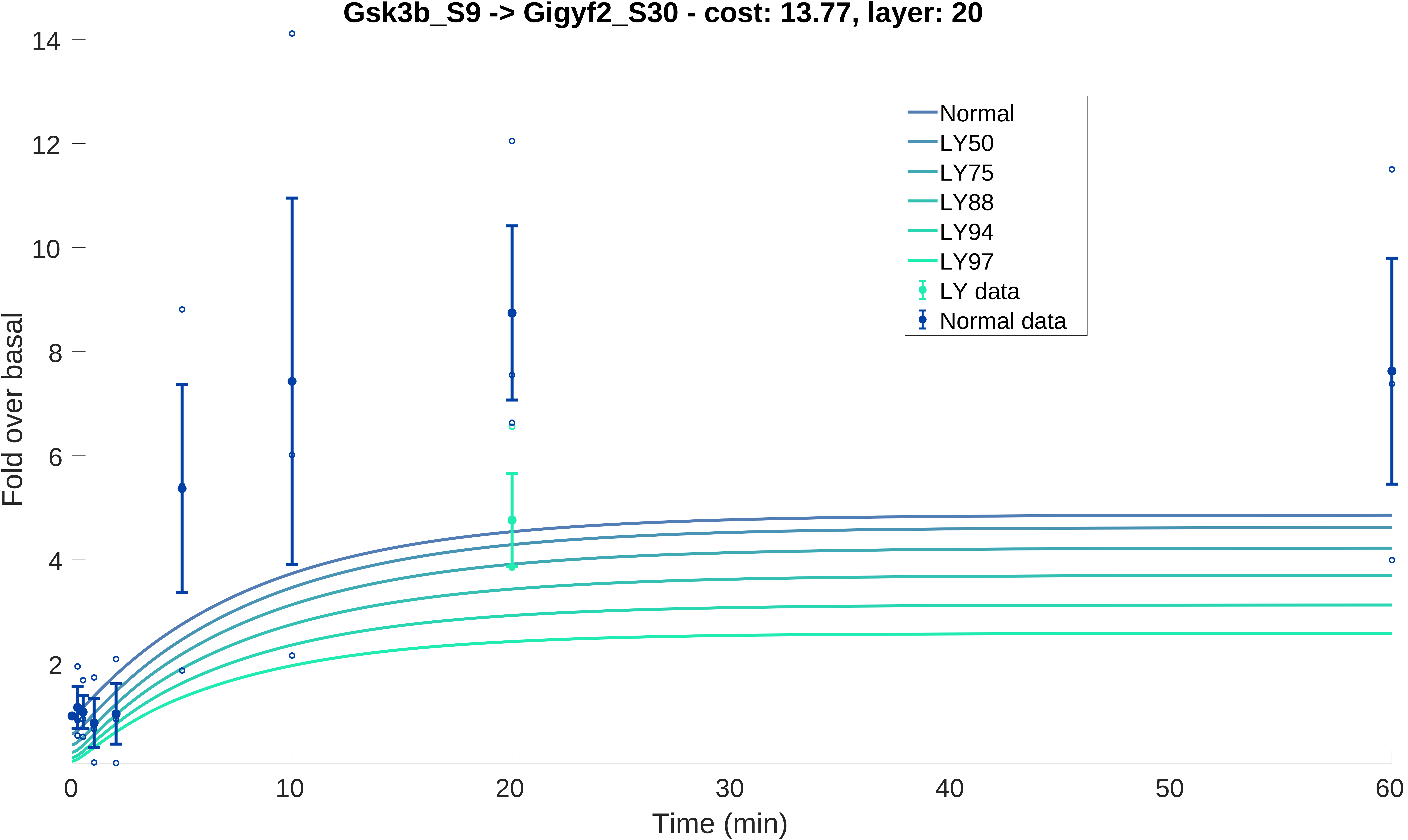
Gsk3b_S9 -> Gigyf2_S30 - cost: 13.77, layer: 20
Fold over basal
Time (min)
0
10
20
30
40
50
60
2
4
6
8
10
12
14
Normal
LY50
LY75
LY88
LY94
LY97
LY data
Normal data

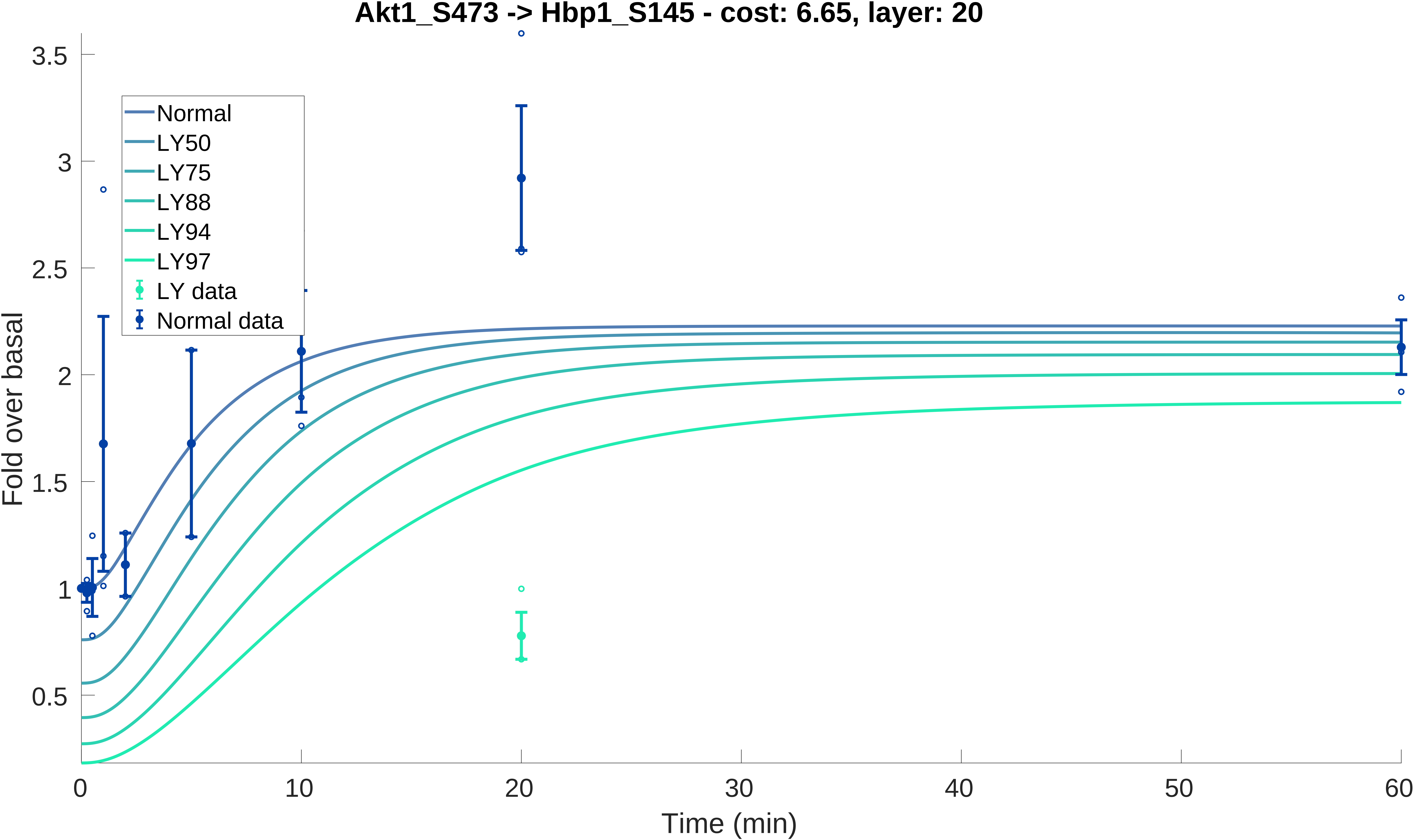

Akt1_S473 -> Hbp1_S145 - cost: 6.65, layer: 20
Fold over basal
Time (min)
Normal
LY50
LY75
LY88
LY94
LY97
LY data
Normal data
0.5
1
1.5
2
2.5
3
3.5
0
10
20
30
40
50
60

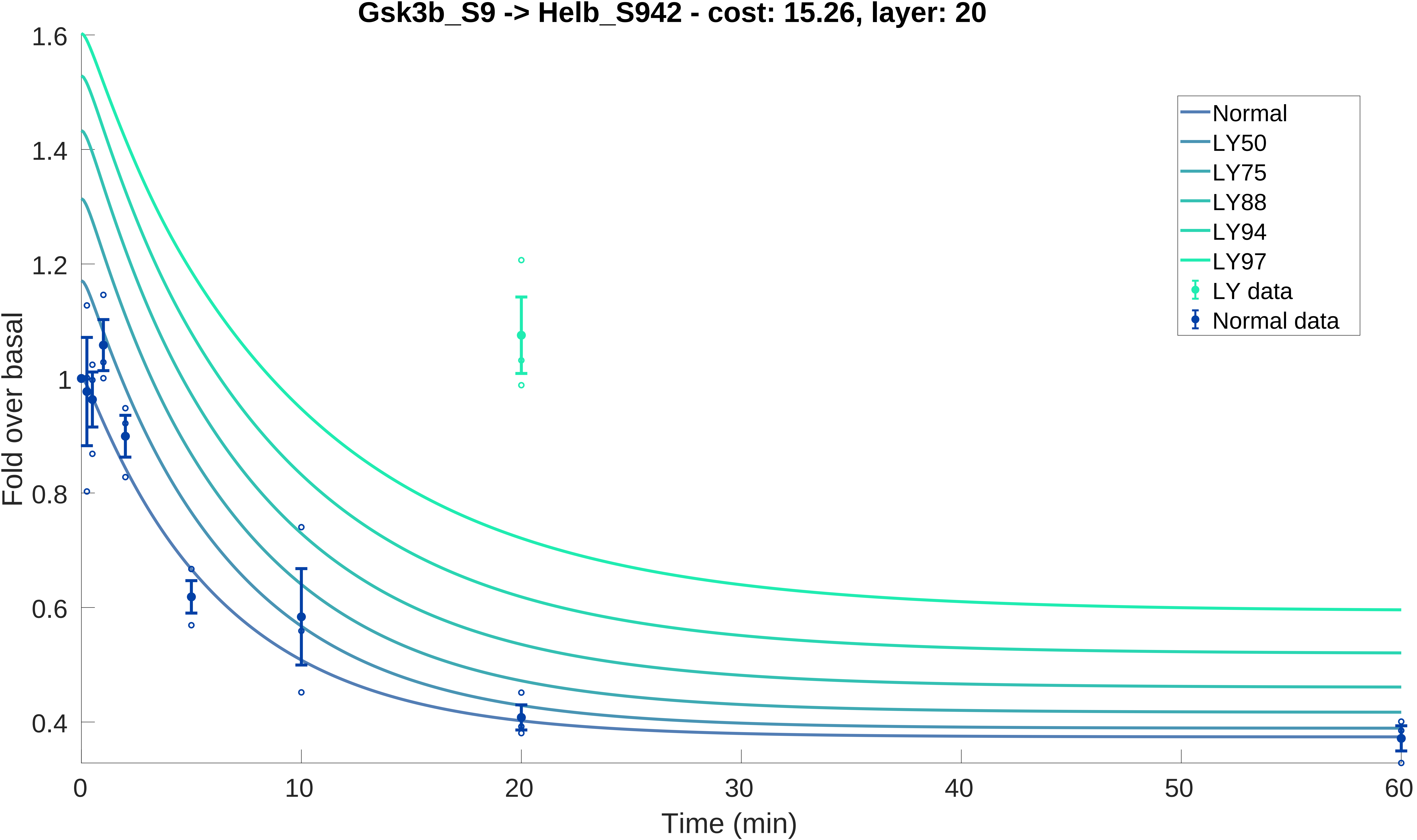

Gsk3b_S9 -> Helb_S942 - cost: 15.26, layer: 20
Fold over basal
1.6
1.4
1.2
1
0.8
0.6
0.4
0
10
20
30
40
50
60
Time (min)
Normal
LY50
LY75
LY88
LY94
LY97
LY data
Normal data

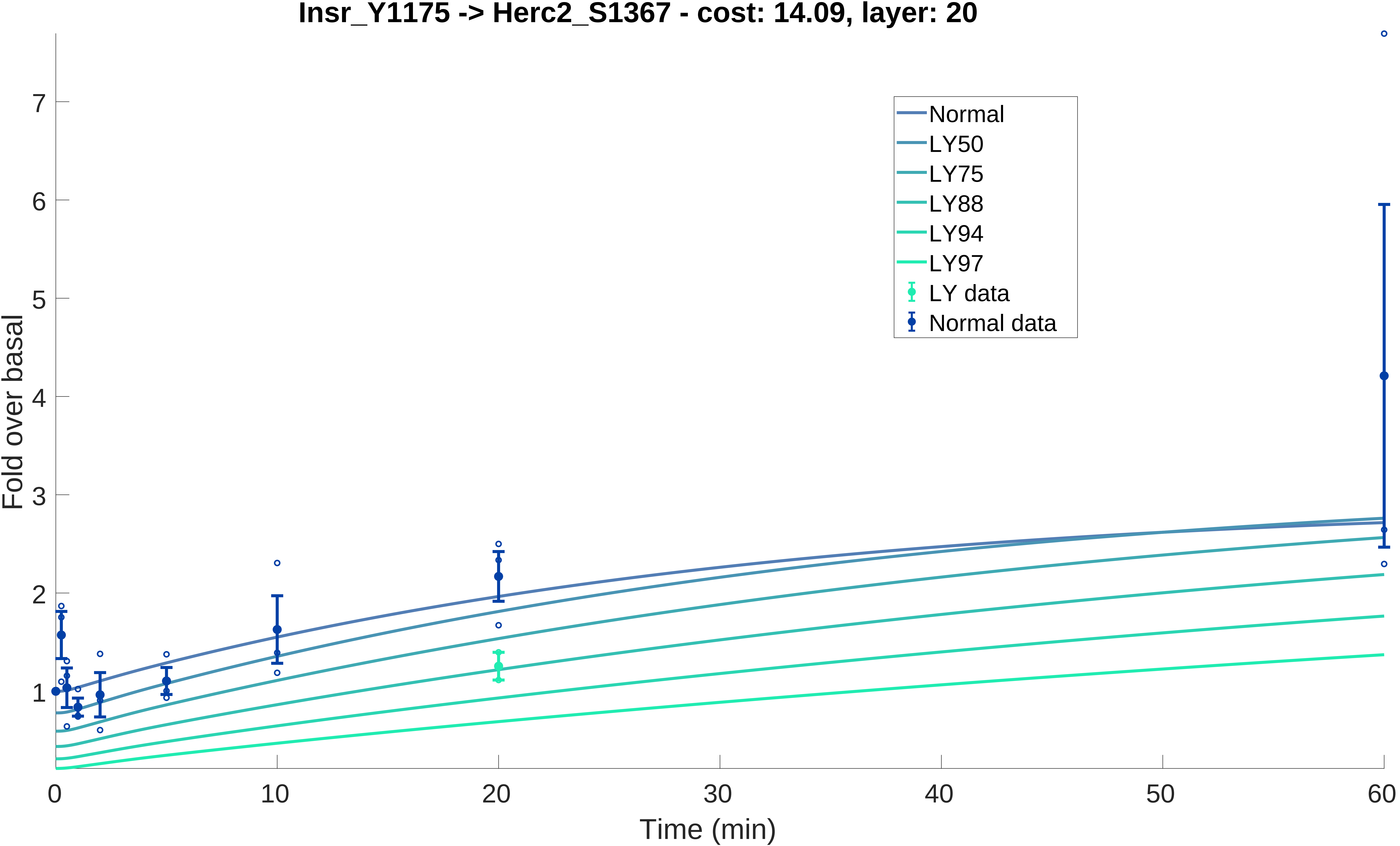
Insr_Y1175 -> Herc2_S1367 - cost: 14.09, layer: 20
Fold over basal
Time (min)
0
10
20
30
40
50
60
1
2
3
4
5
6
7
Normal
LY50
LY75
LY88
LY94
LY97
LY data
Normal data

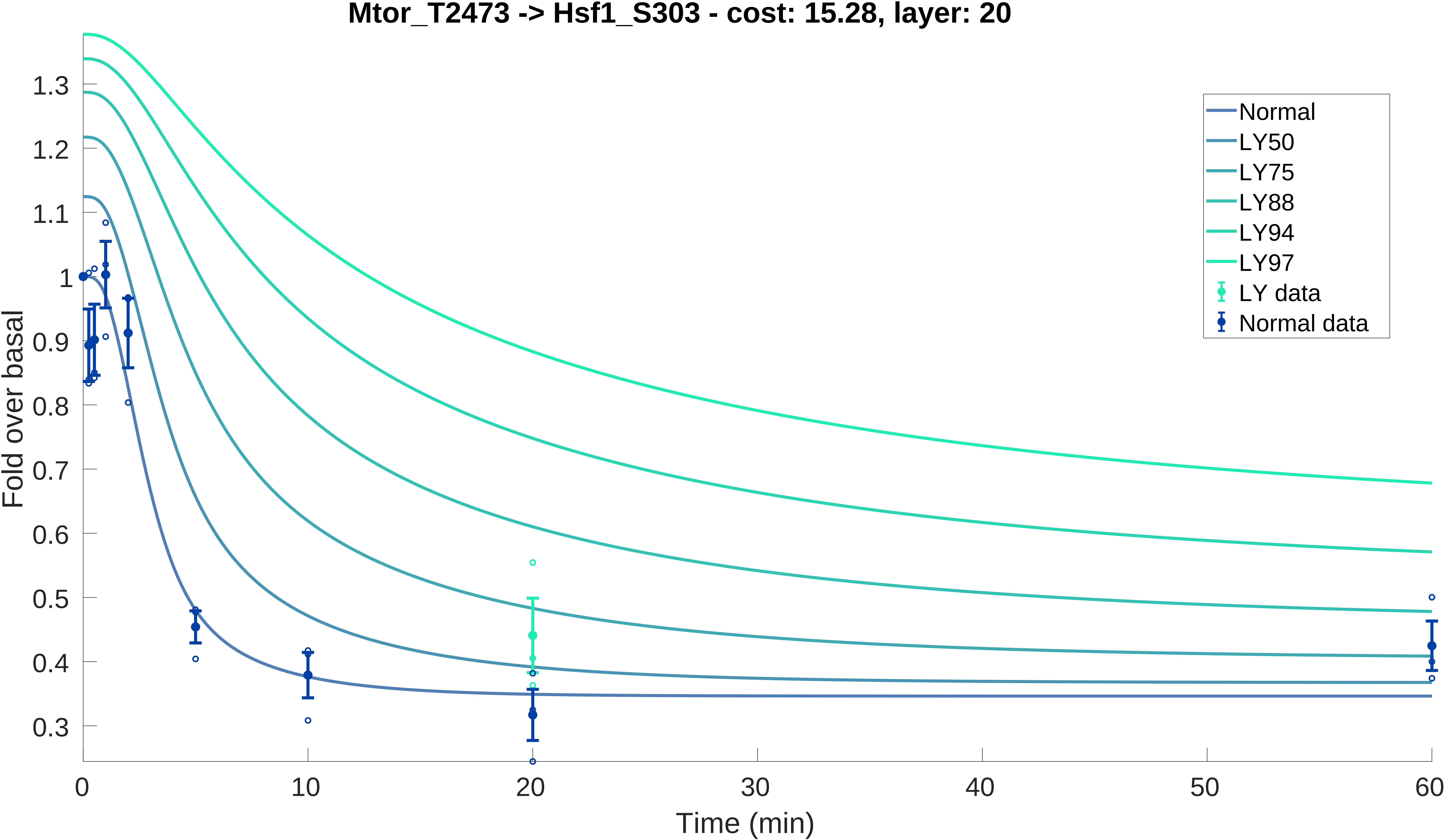

Mtor_T2473 -> Hsf1_S303 - cost: 15.28, layer: 20
Fold over basal
Time (min)
1.3
1.2
1.1
1
0.9
0.8
0.7
0.6
0.5
0.4
0.3
0
10
20
30
40
50
60
Normal
LY50
LY75
LY88
LY94
LY97
LY data
Normal data

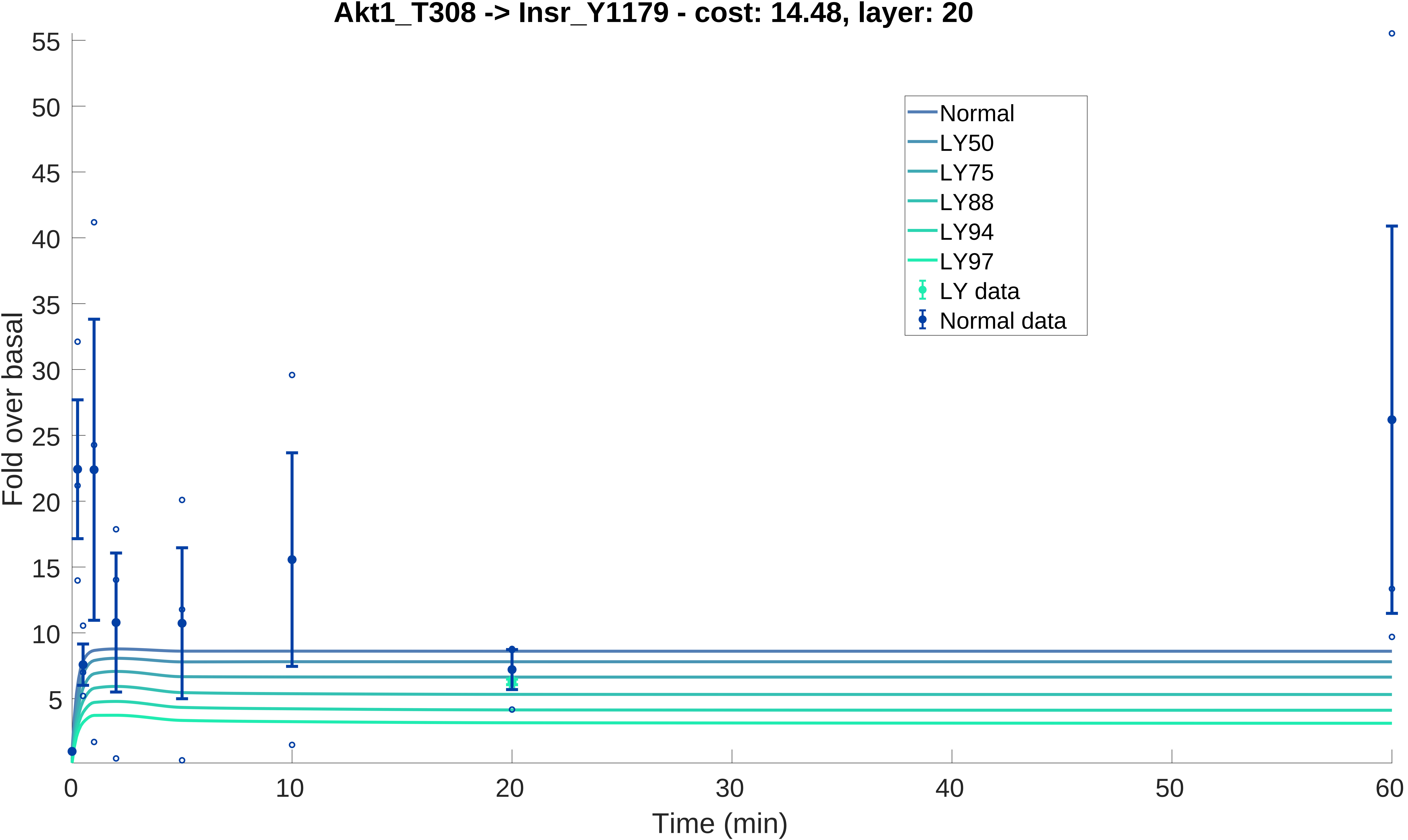
Akt1_T308 -> Insr_Y1179 - cost: 14.48, layer: 20
Fold over basal
Time (min)
Normal
LY50
LY75
LY88
LY94
LY97
LY data
Normal data
0
10
20
30
40
50
60
5
10
15
20
25
30
35
40
45
50
55

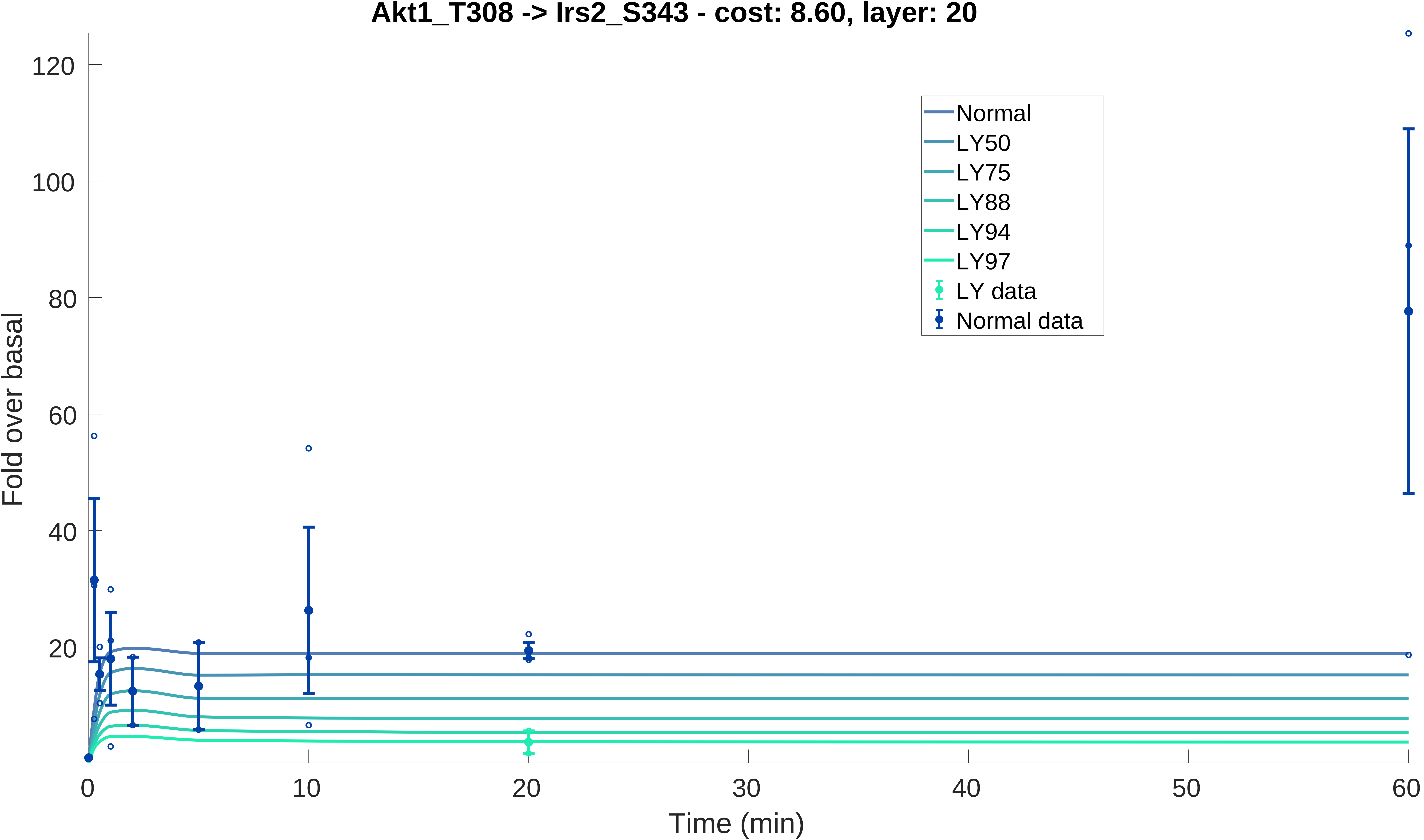

Akt1_T308 -> Irs2_S343 - cost: 8.60, layer: 20
Fold over basal
Time (min)
0
10
20
30
40
50
60
20
40
60
80
100
120
Normal
LY50
LY75
LY88
LY94
LY97
LY data
Normal data

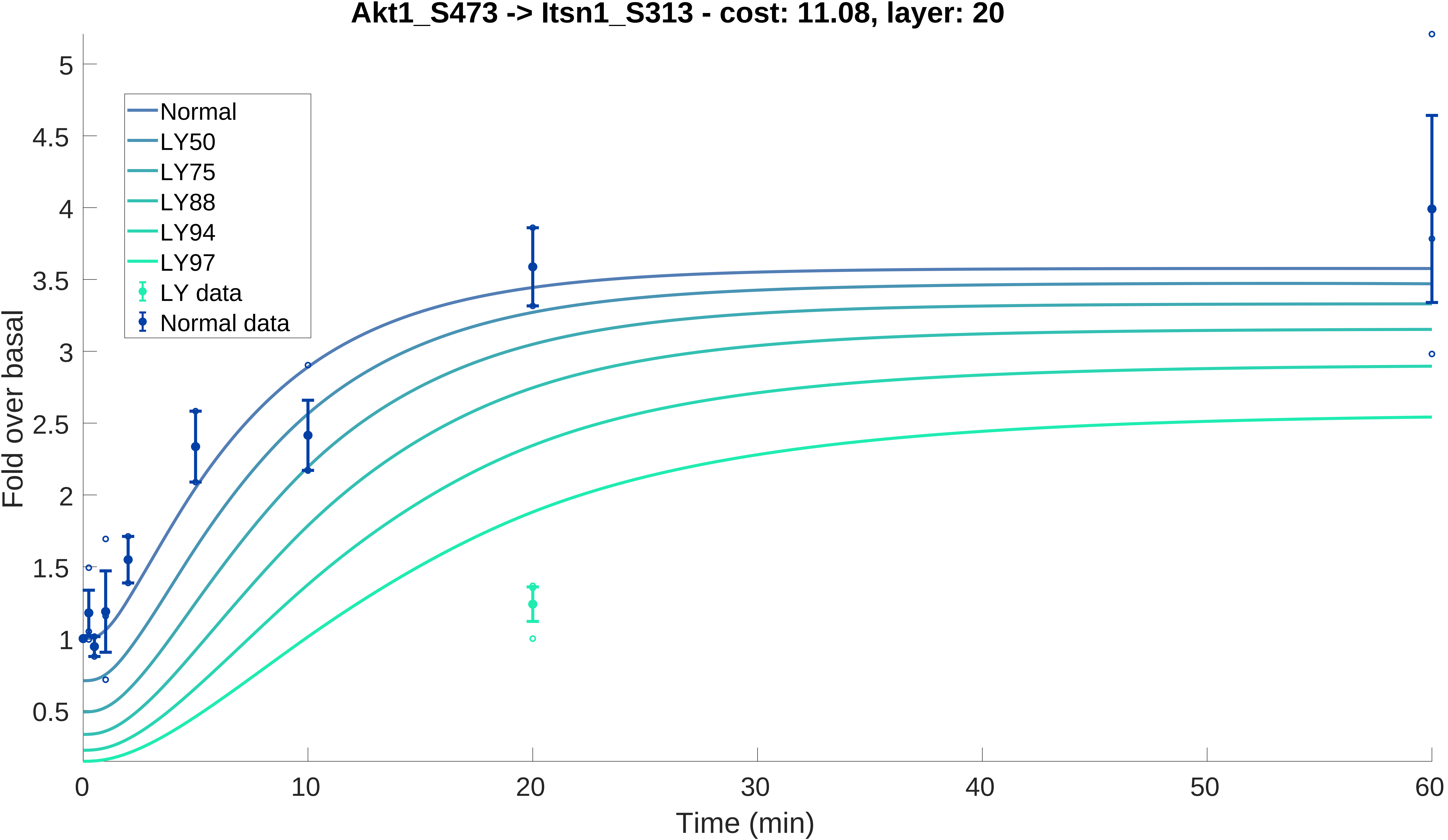

Akt1_S473 -> Itsn1_S313 - cost: 11.08, layer: 20
Normal
LY50
LY75
LY88
LY94
LY97
LY data
Normal data
Fold over basal
Time (min)
0
10
20
30
40
50
60
0.5
1
1.5
2
2.5
3
3.5
4
4.5
5

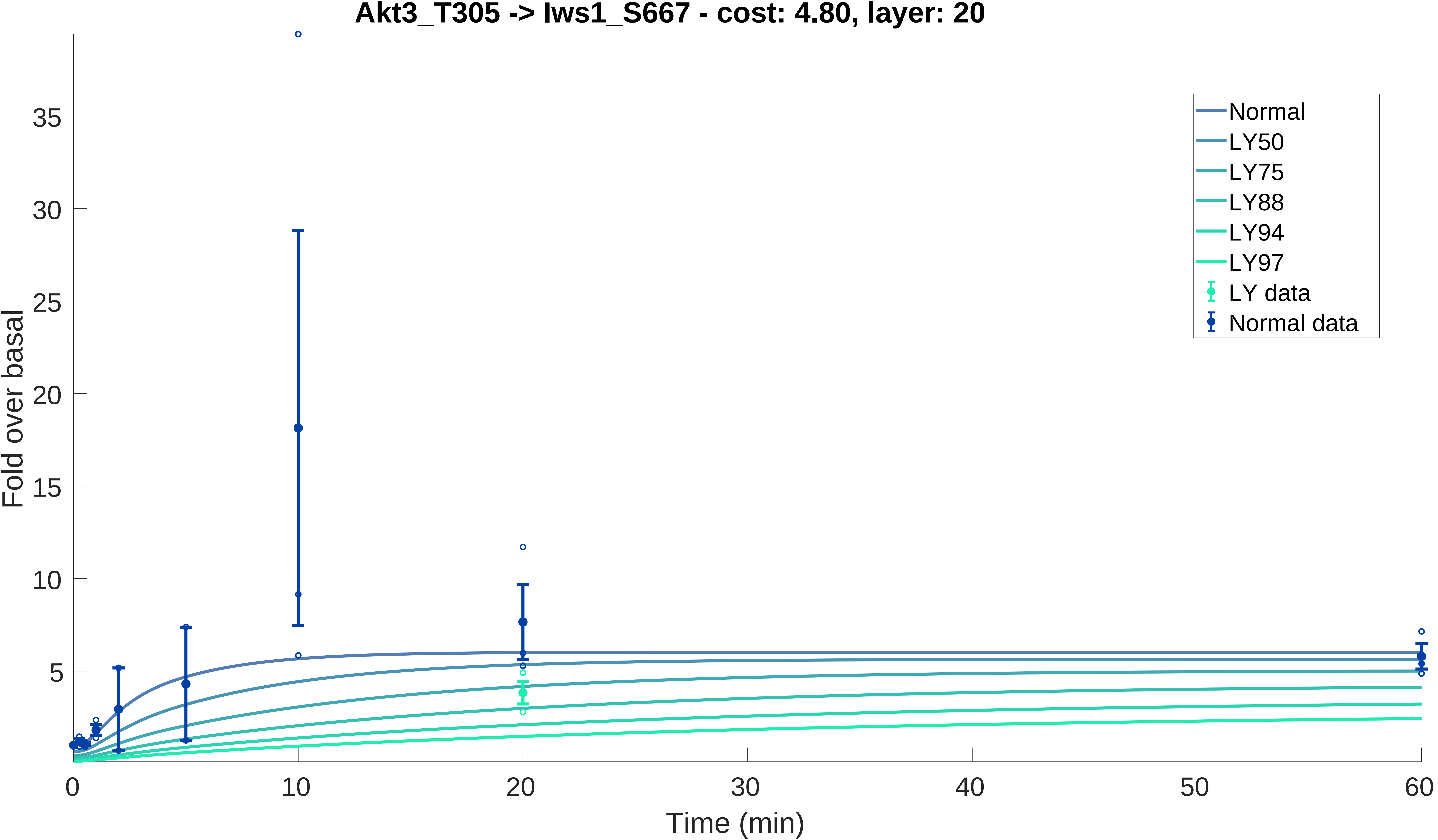

Akt3_T305 -> Iws1_S667 - cost: 4.80, layer: 20
Normal
LY50
LY75
LY88
LY94
LY97
LY data
Normal data
Fold over basal
Time (min)
0
10
20
30
40
50
60
5
10
15
20
25
30
35

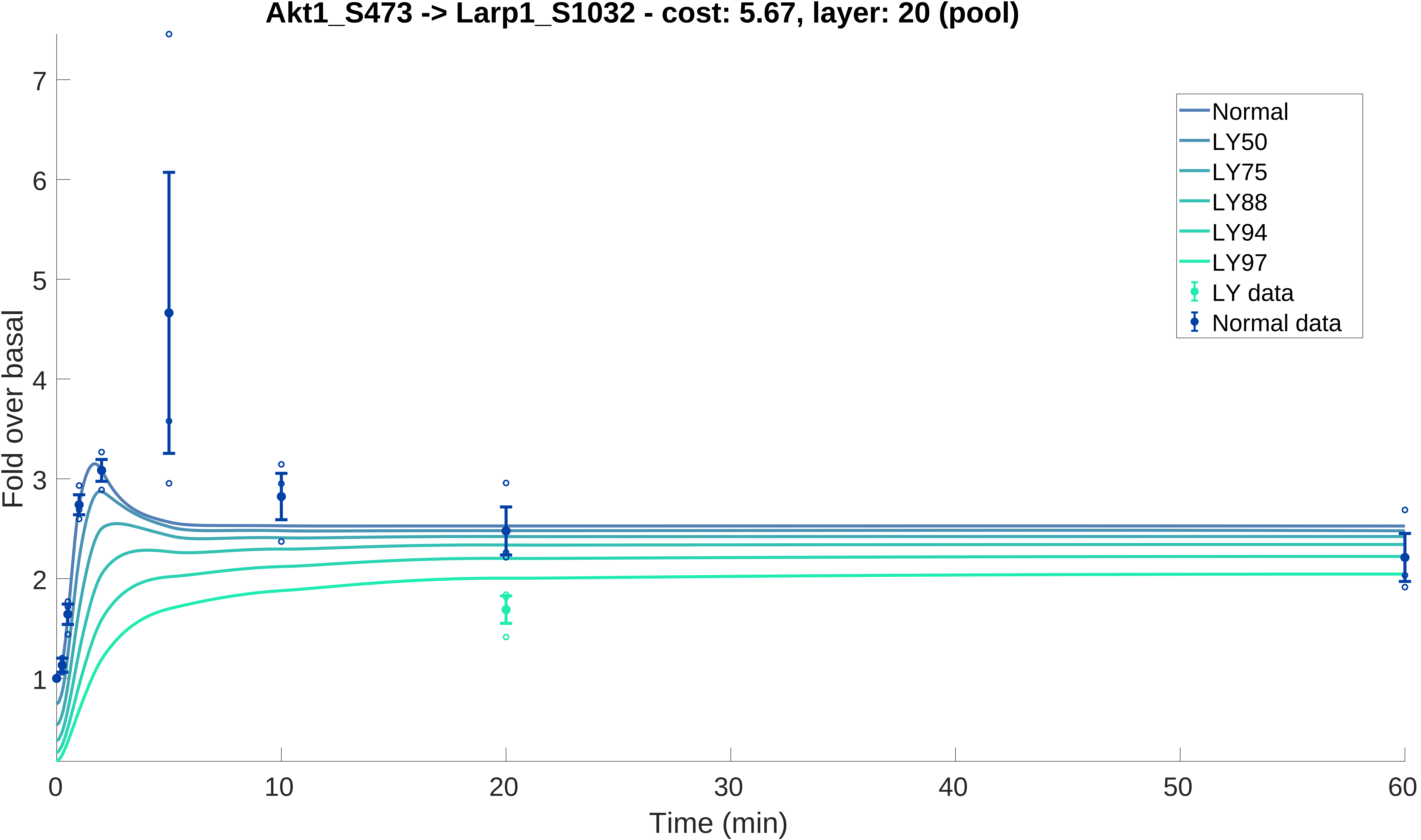
Akt1_S473 -> Larp1_S1032 - cost: 5.67, layer: 20 (pool)
Fold over basal
Time (min)
0
10
20
30
40
50
60
1
2
3
4
5
6
7
Normal
LY50
LY75
LY88
LY94
LY97
LY data
Normal data

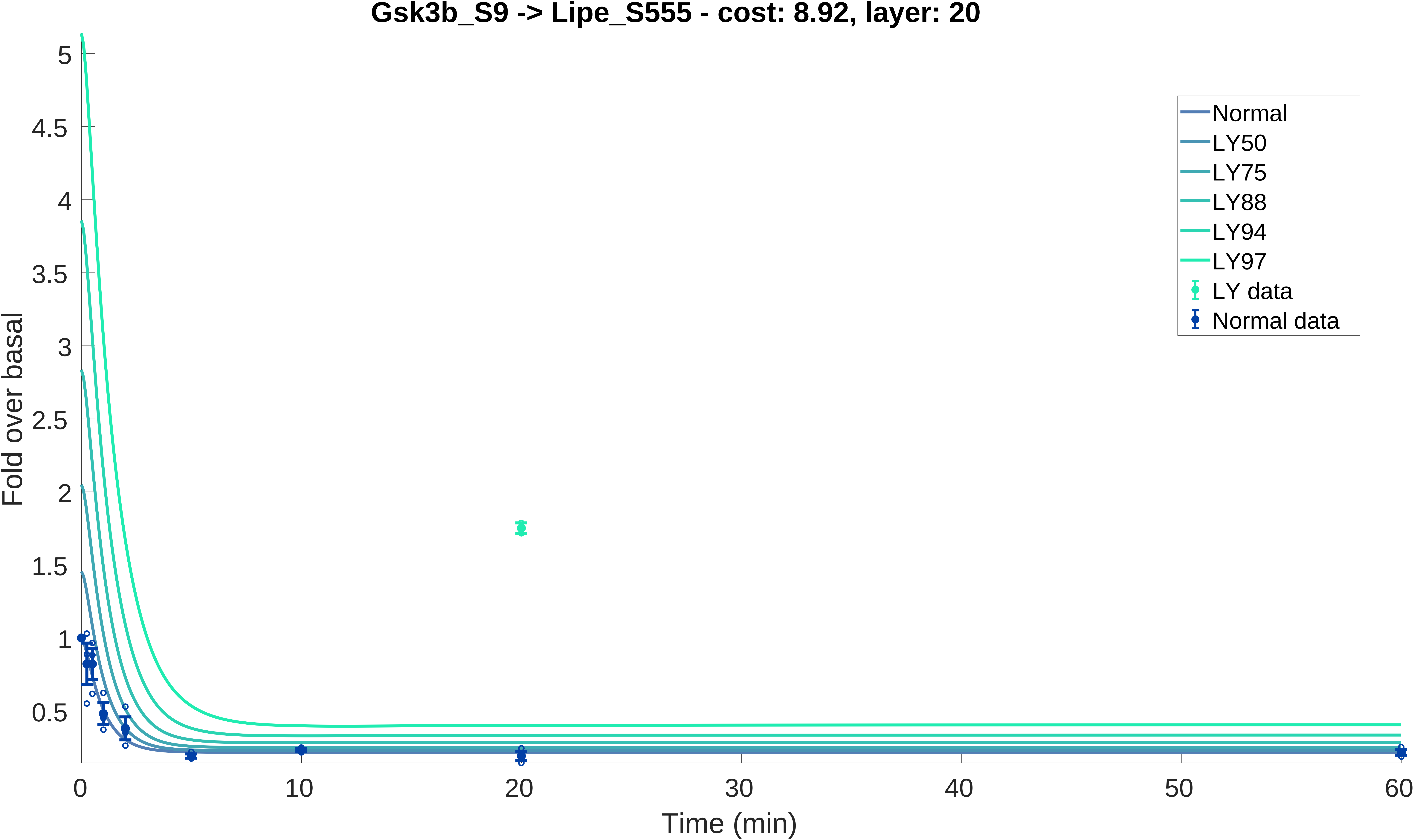
Gsk3b_S9 -> Lipe_S555 - cost: 8.92, layer: 20
Fold over basal
Time (min)
0
10
20
30
40
50
60
0.5
1
1.5
2
2.5
3
3.5
4
4.5
5
Normal
LY50
LY75
LY88
LY94
LY97
LY data
Normal data

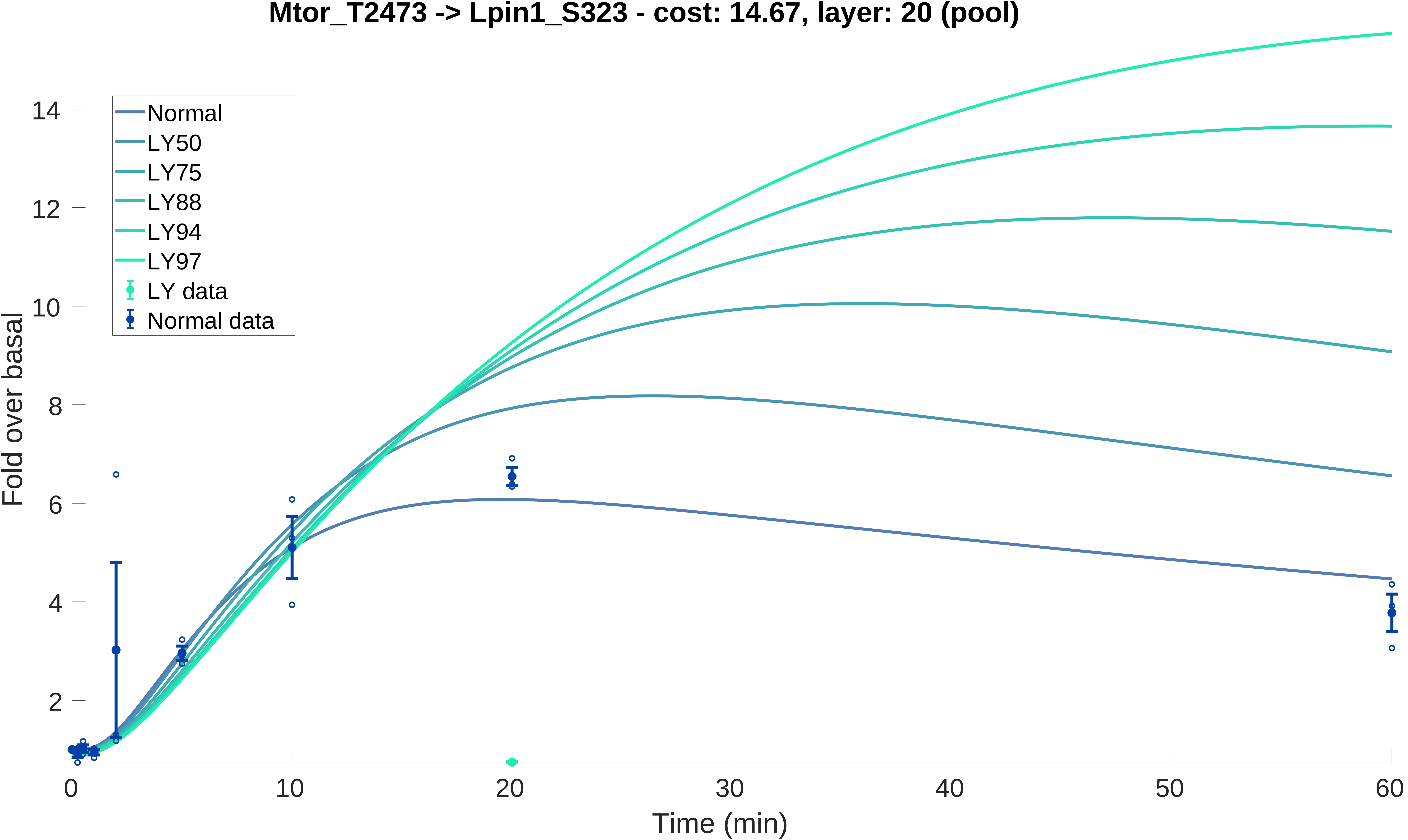

Mtor_T2473 -> Lpin1_S323 - cost: 14.67, layer: 20 (pool)
Normal
LY50
LY75
LY88
LY94
LY97
LY data
Normal data
Fold over basal
Time (min)
0
10
20
30
40
50
60
2
4
6
8
10
12
14

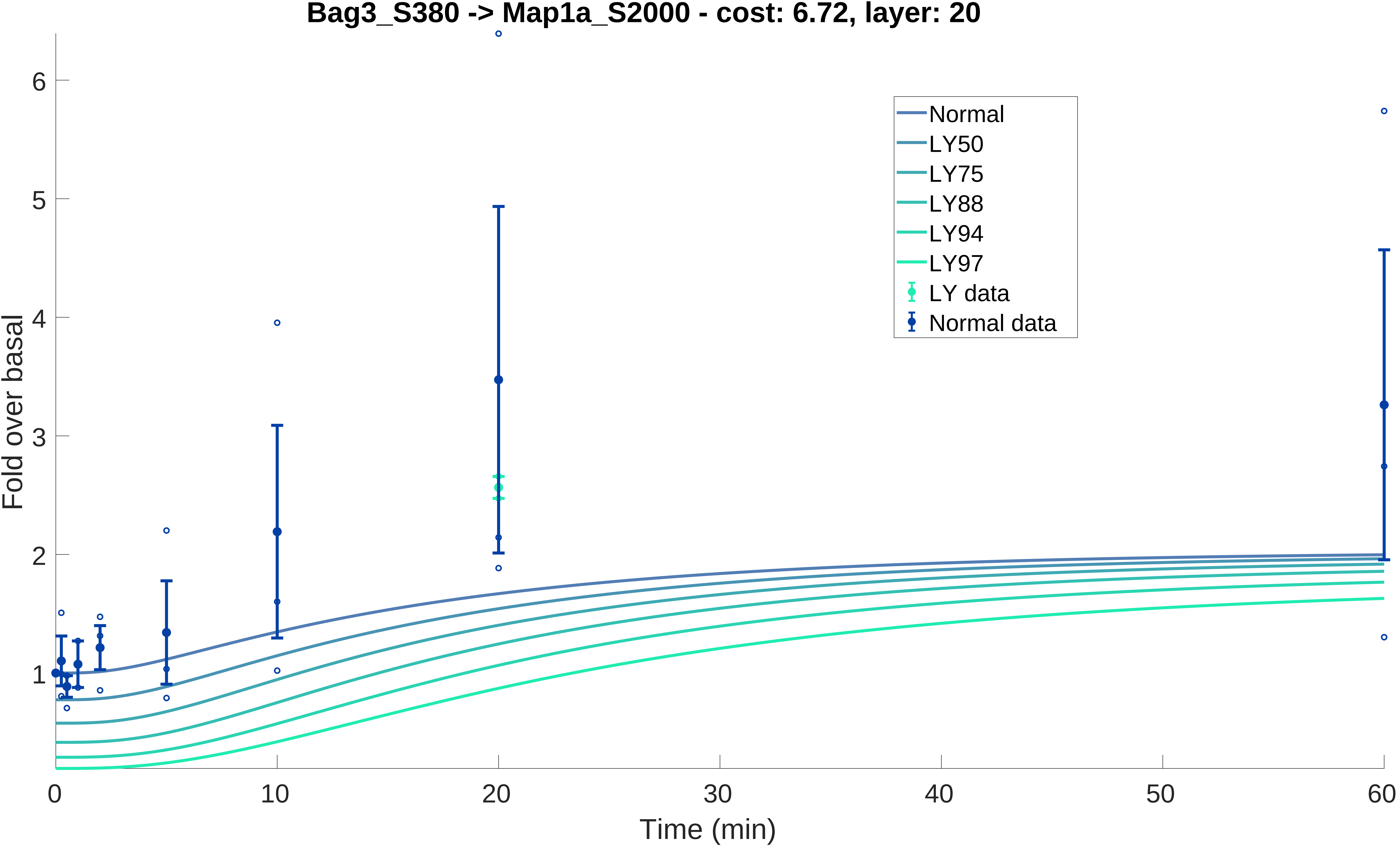

Bag3_S380 -> Map1a_S2000 - cost: 6.72, layer: 20
Normal
LY50
LY75
LY88
LY94
LY97
LY data
Normal data
Fold over basal
Time (min)
1
2
3
4
5
6
0
10
20
30
40
50
60

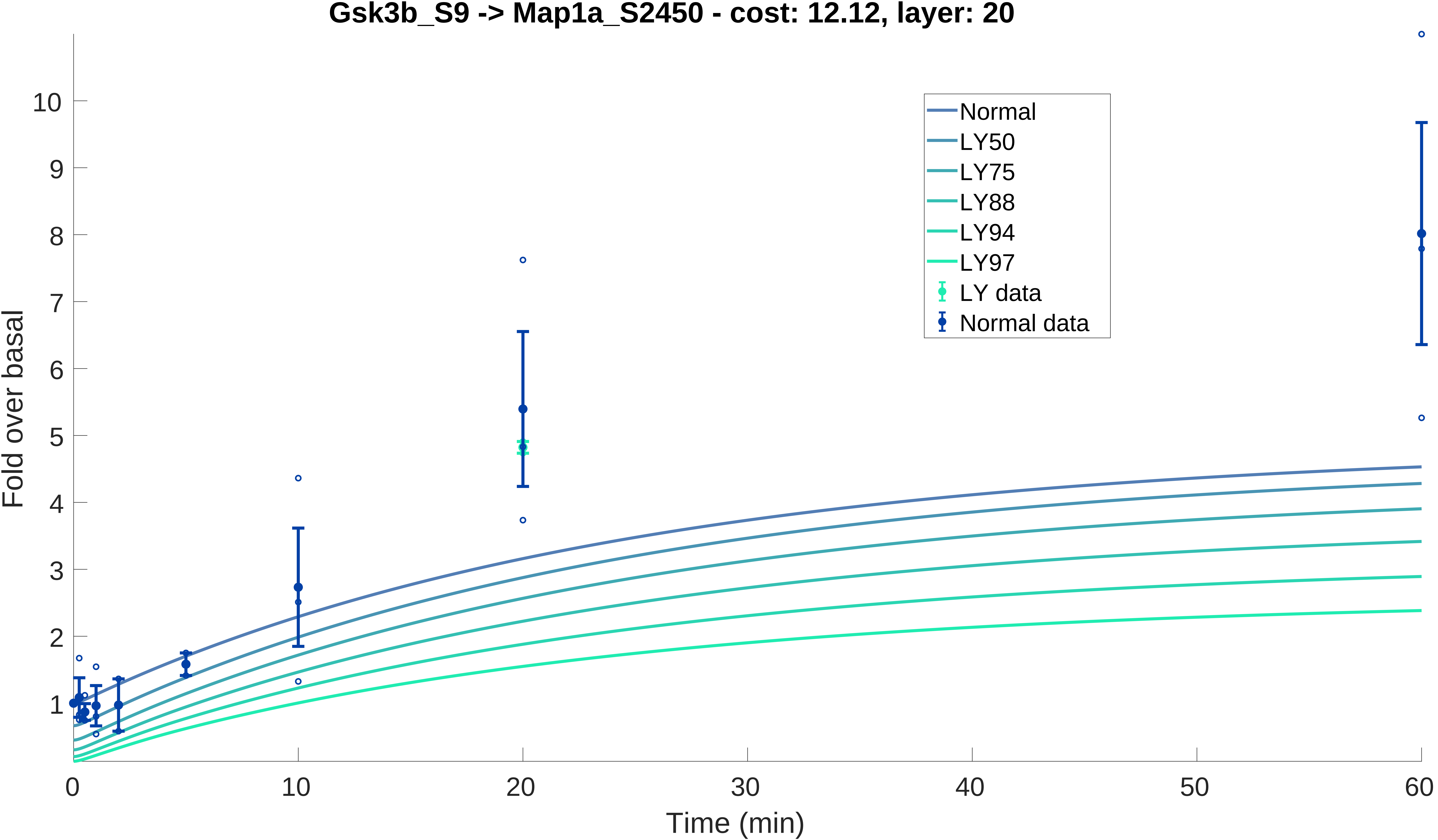

Gsk3b_S9 -> Map1a_S2450 - cost: 12.12, layer: 20
Fold over basal
Time (min)
Normal
LY50
LY75
LY88
LY94
LY97
LY data
Normal data
0
10
20
30
40
50
60
1
2
3
4
5
6
7
8
9
10

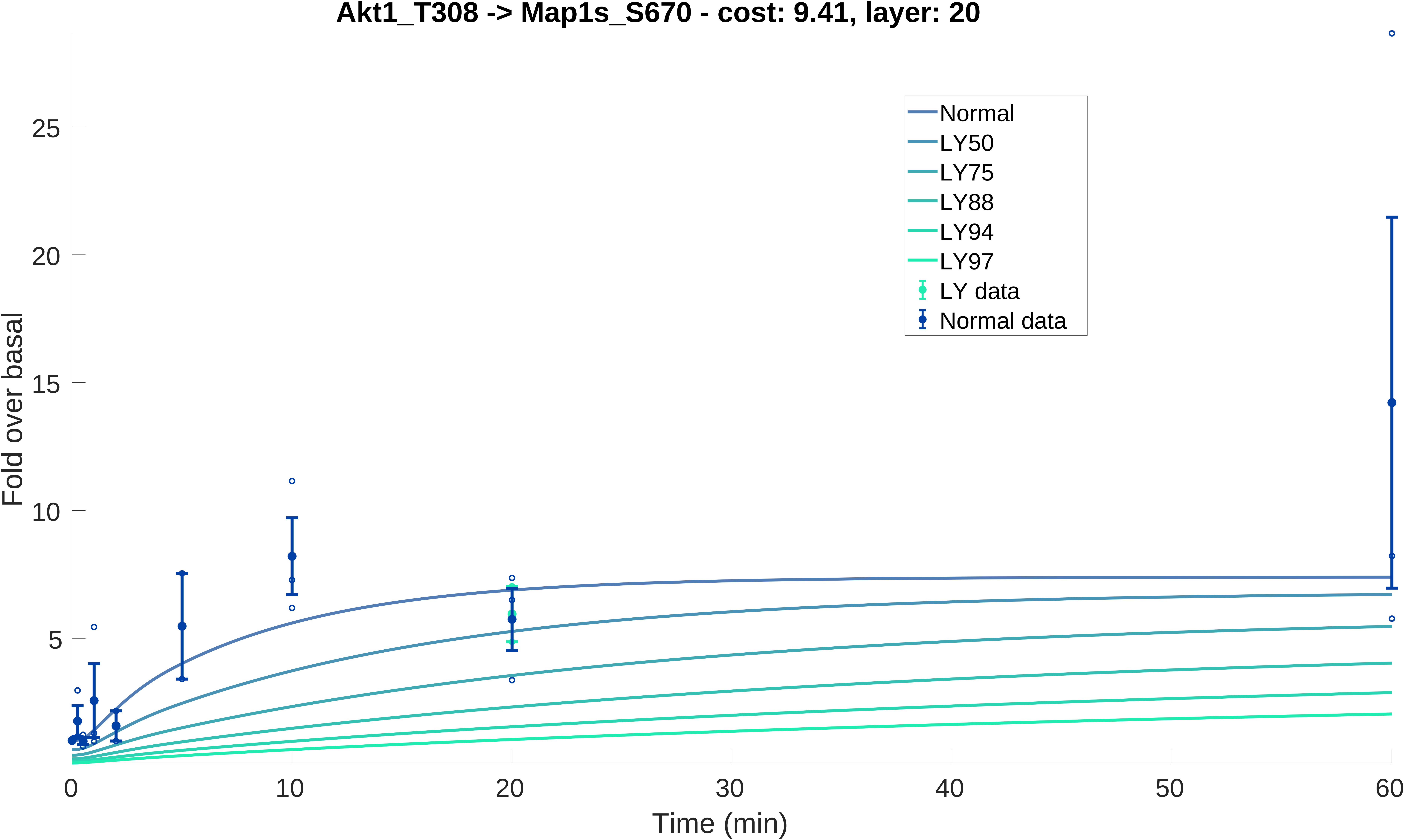
Akt1_T308 -> Map1s_S670 - cost: 9.41, layer: 20
Fold over basal
Time (min)
0
10
20
30
40
50
60
5
10
15
20
25
Normal
LY50
LY75
LY88
LY94
LY97
LY data
Normal data

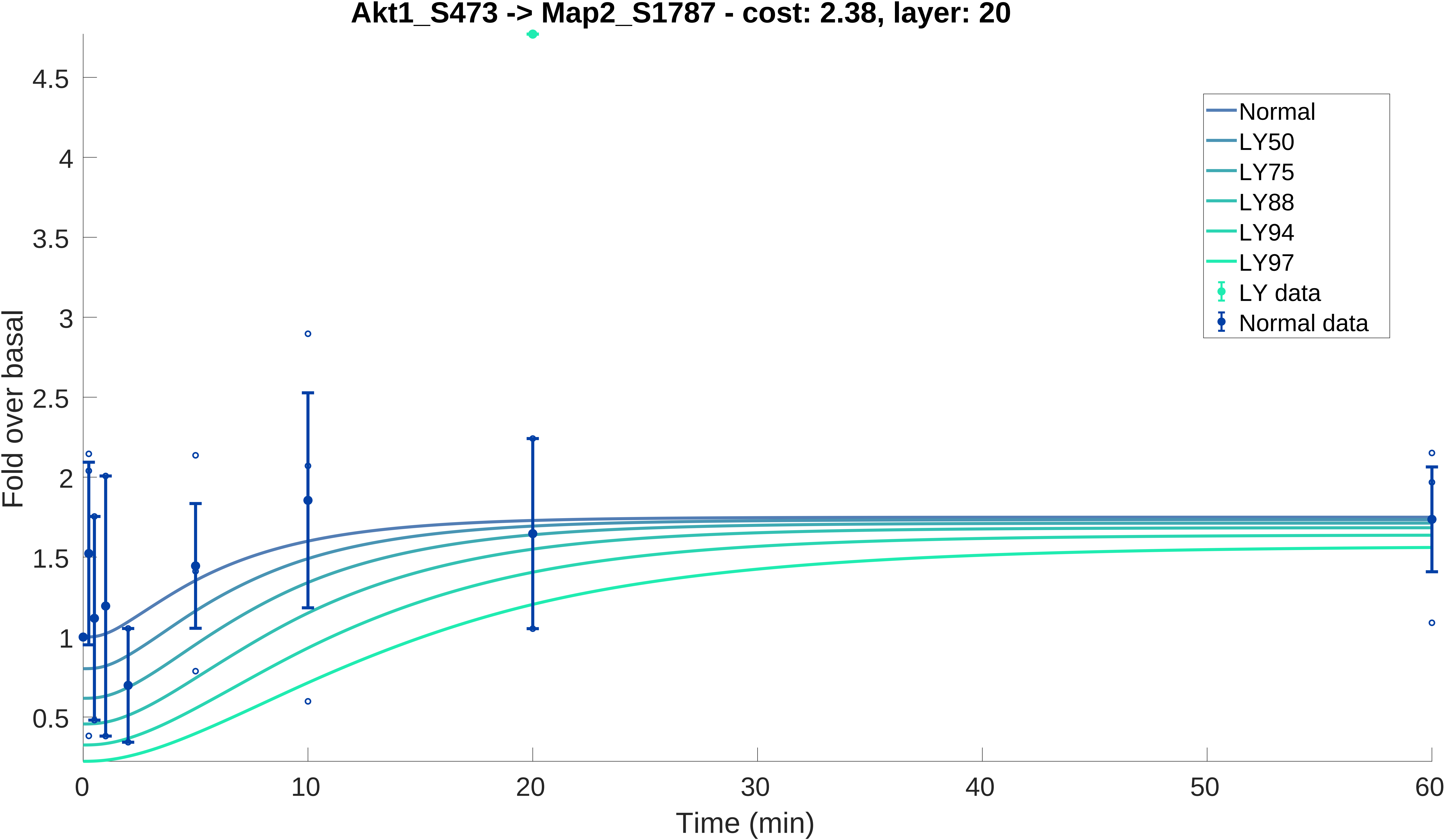
Akt1_S473 -> Map2_S1787 - cost: 2.38, layer: 20
Fold over basal
Time (min)
Normal
LY50
LY75
LY88
LY94
LY97
LY data
Normal data
0.5
1
1.5
2
2.5
3
3.5
4
4.5
0
10
20
30
40
50
60

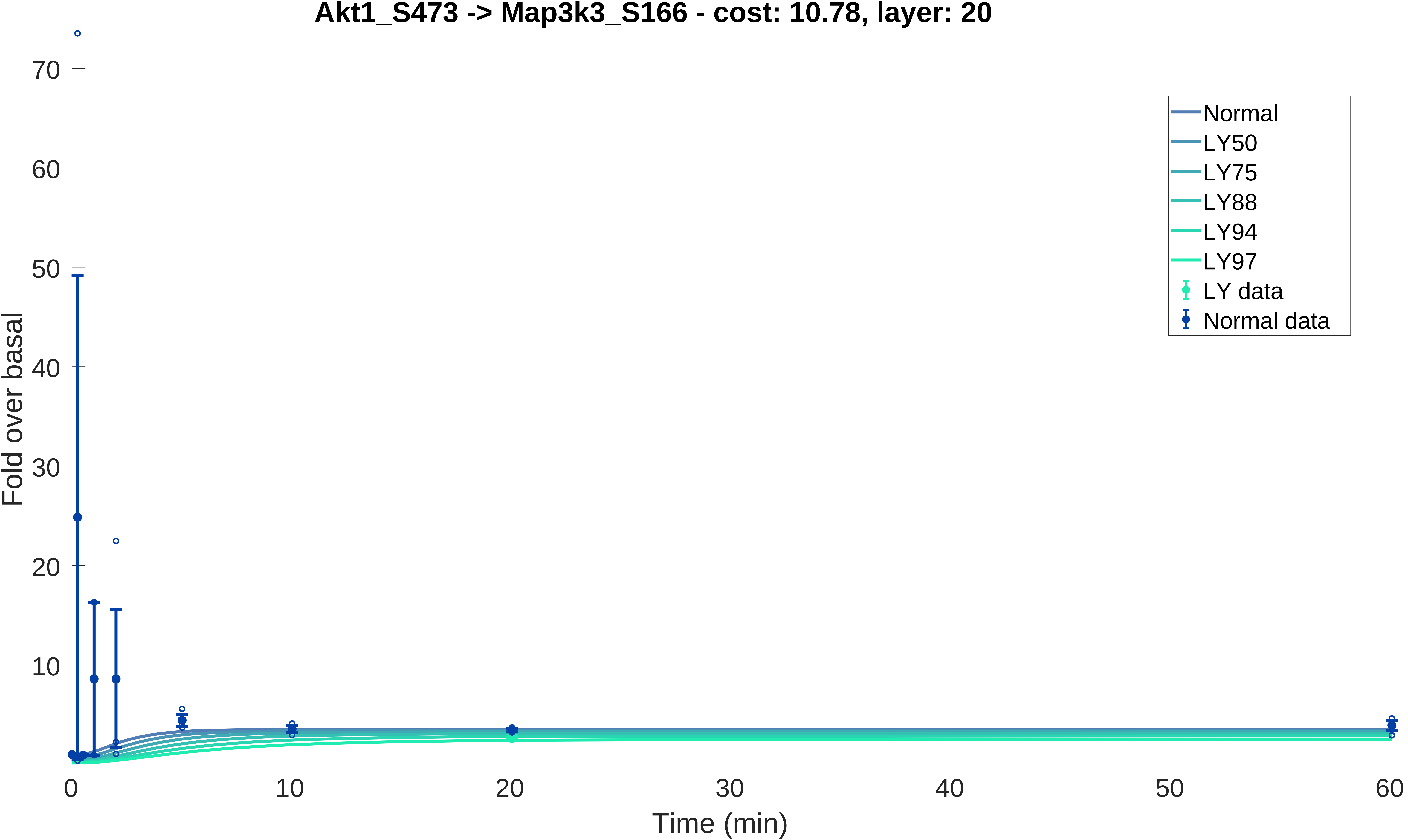

Akt1_S473 -> Map3k3_S166 - cost: 10.78, layer: 20
Fold over basal
70
60
50
40
30
20
10
0
10
20
30
40
50
60
Time (min)
Normal
LY50
LY75
LY88
LY94
LY97
LY data
Normal data

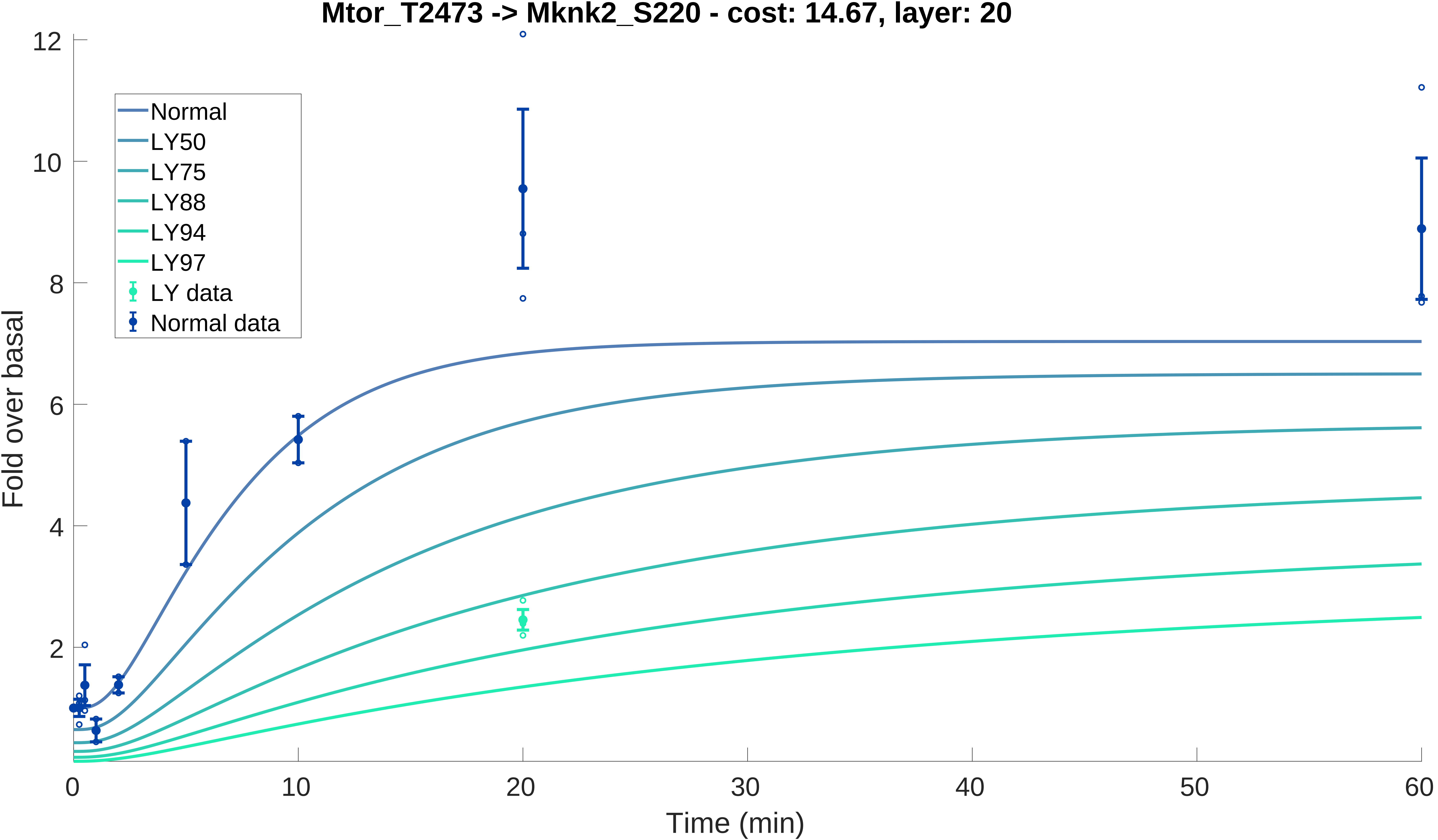
Mtor_T2473 -> Mknk2_S220 - cost: 14.67, layer: 20
Normal
LY50
LY75
LY88
LY94
LY97
LY data
Normal data
Fold over basal
Time (min)
0
10
20
30
40
50
60
2
4
6
8
10
12

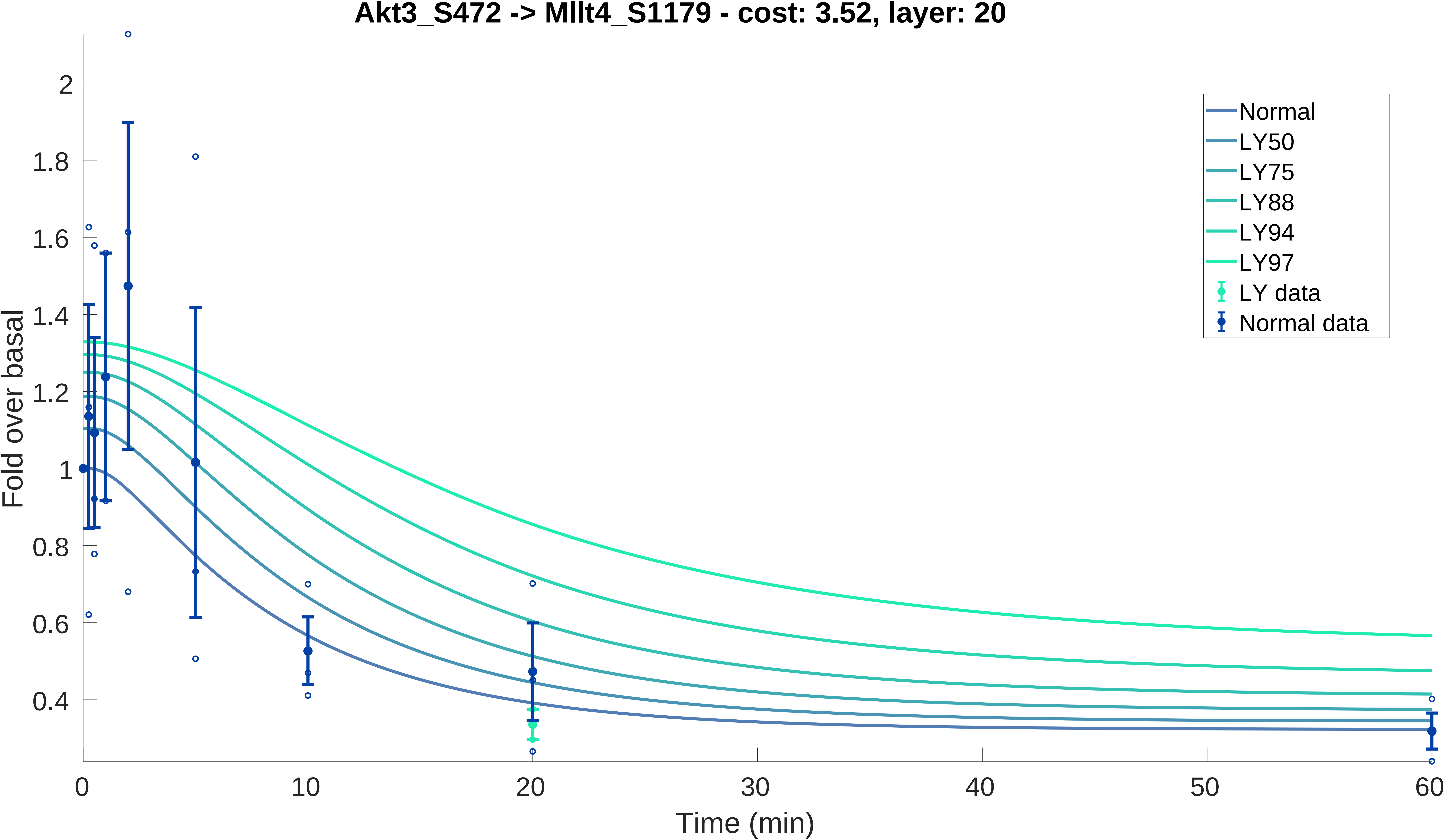

Akt3_S472 -> Mllt4_S1179 - cost: 3.52, layer: 20
Fold over basal
Time (min)
0
10
20
30
40
50
60
0.4
0.6
0.8
1
1.2
1.4
1.6
1.8
2
Normal
LY50
LY75
LY88
LY94
LY97
LY data
Normal data

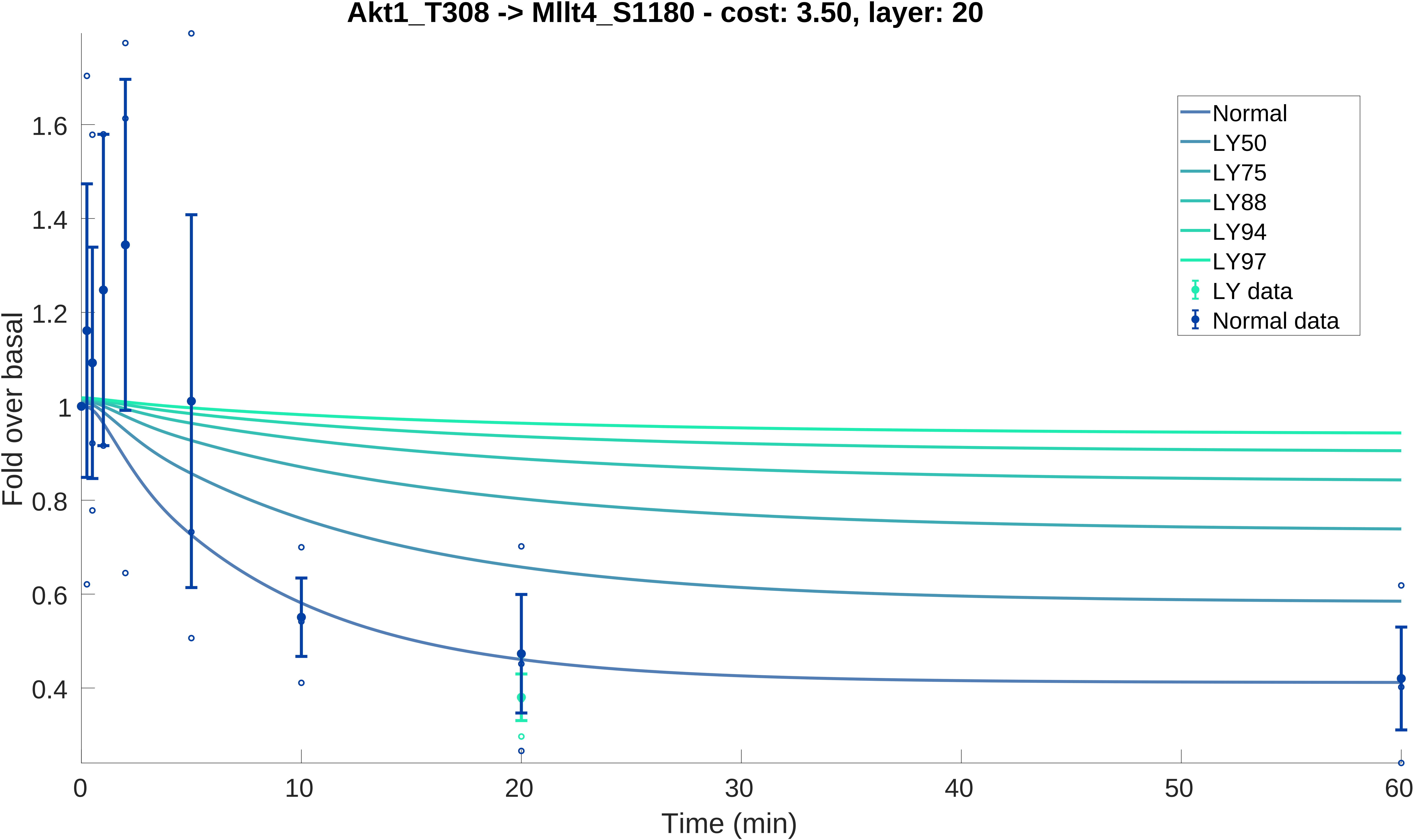

Akt1_T308 -> Mllt4_S1180 - cost: 3.50, layer: 20
Fold over basal
Time (min)
Normal
LY50
LY75
LY88
LY94
LY97
LY data
Normal data
0.4
0.6
0.8
1
1.2
1.4
1.6
0
10
20
30
40
50
60

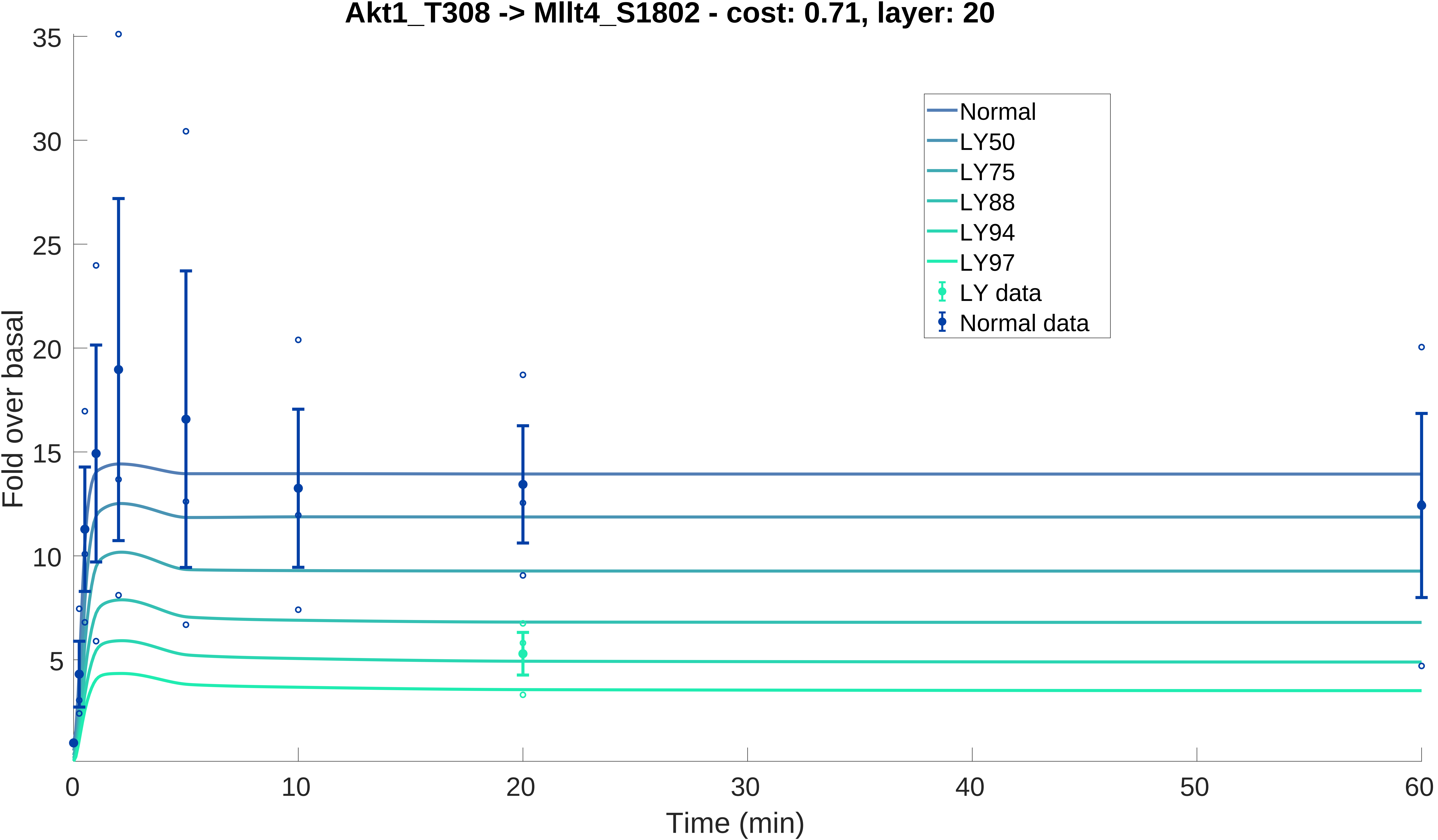

Akt1_T308 -> Mllt4_S1802 - cost: 0.71, layer: 20
Fold over basal
Time (min)
0
10
20
30
40
50
60
5
10
15
20
25
30
35
Normal
LY50
LY75
LY88
LY94
LY97
LY data
Normal data

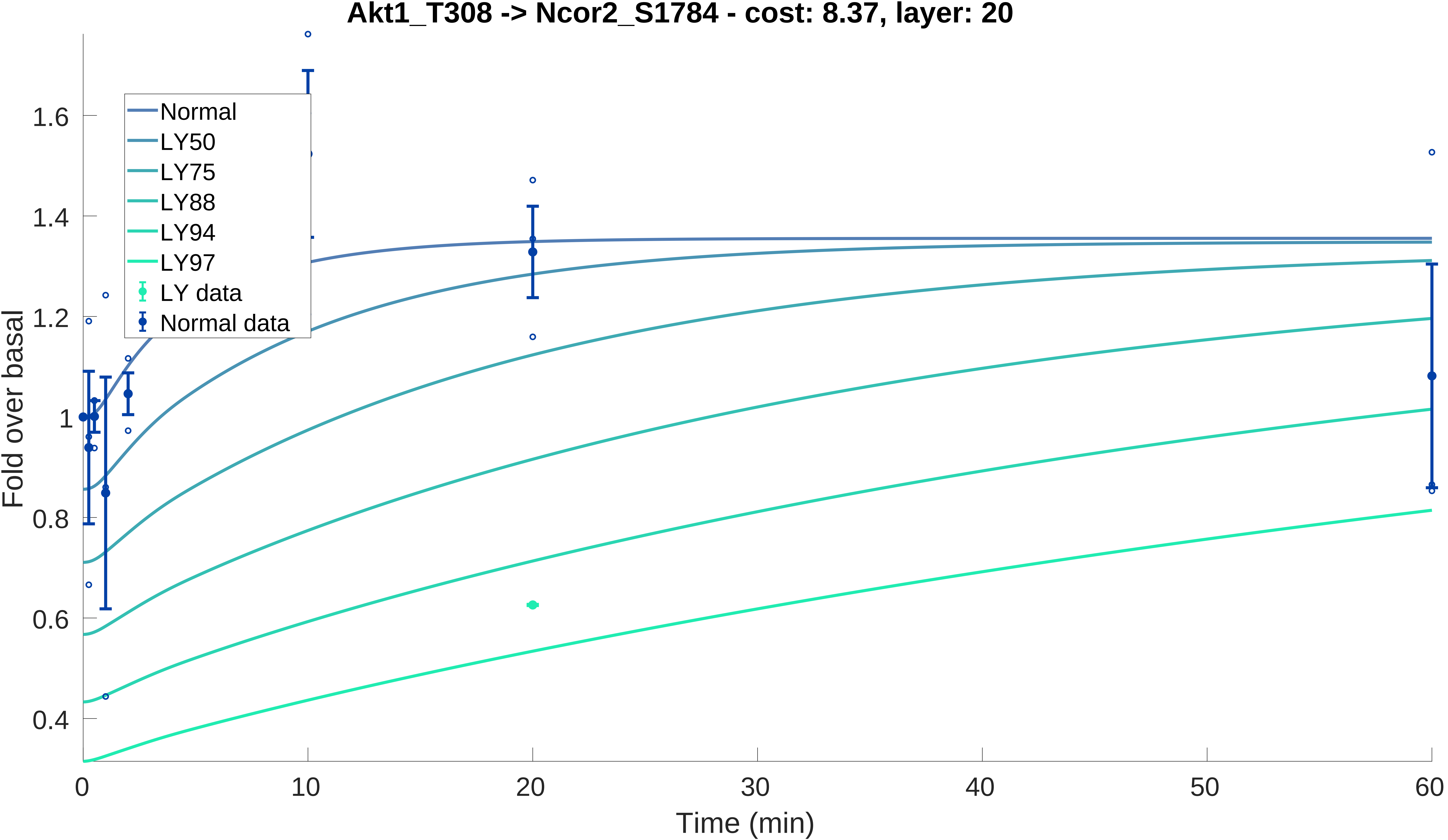

Akt1_T308 -> Ncor2_S1784 - cost: 8.37, layer: 20
Normal
LY50
LY75
LY88
LY94
LY97
LY data
Normal data
Fold over basal
1.6
1.4
1.2
1
0.8
0.6
0.4
0
10
20
30
40
50
60
Time (min)

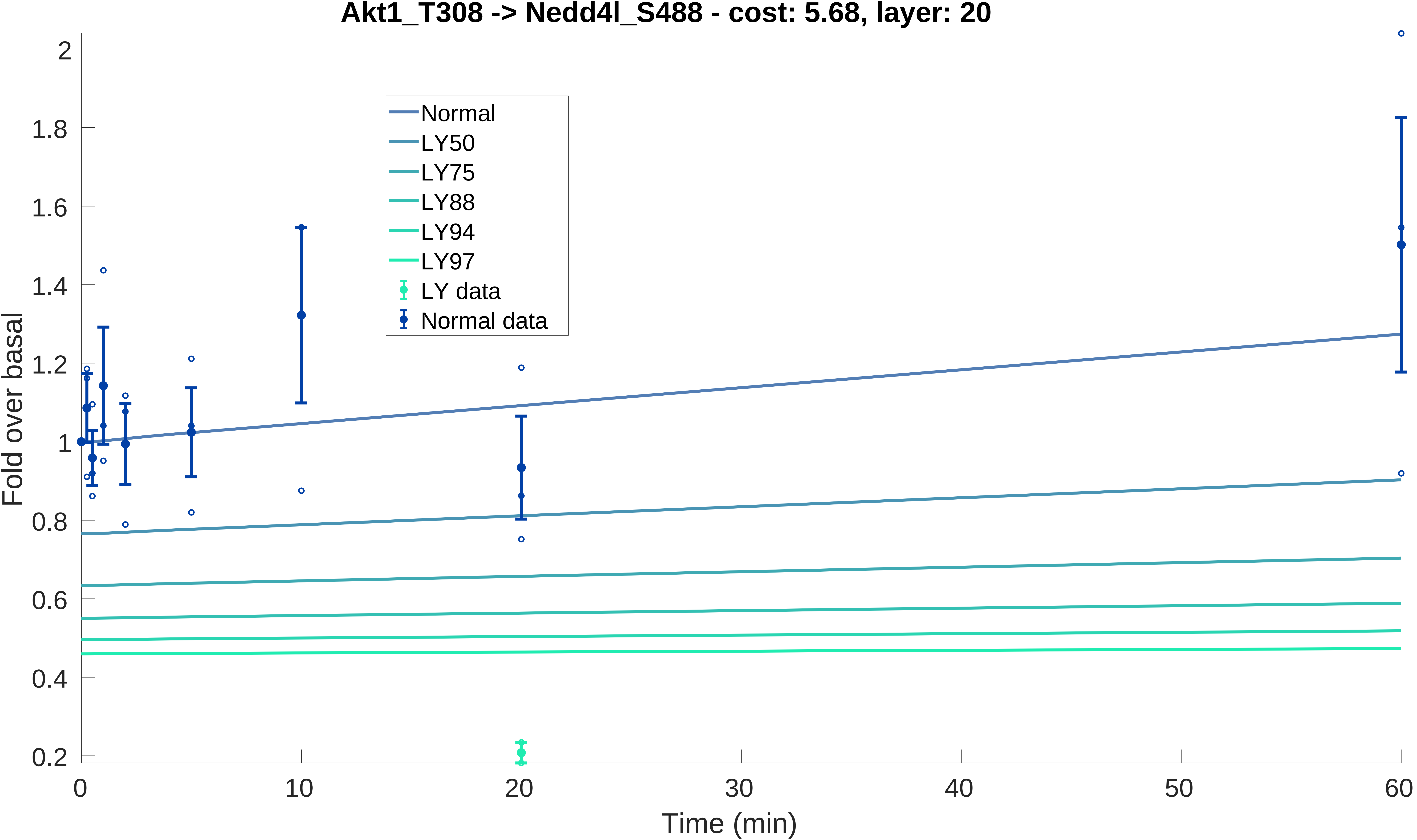

Akt1_T308 -> Nedd4l_S488 - cost: 5.68, layer: 20
Normal
LY50
LY75
LY88
LY94
LY97
LY data
Normal data
Fold over basal
Time (min)
0.2
0.4
0.6
0.8
1
1.2
1.4
1.6
1.8
2
0
10
20
30
40
50
60

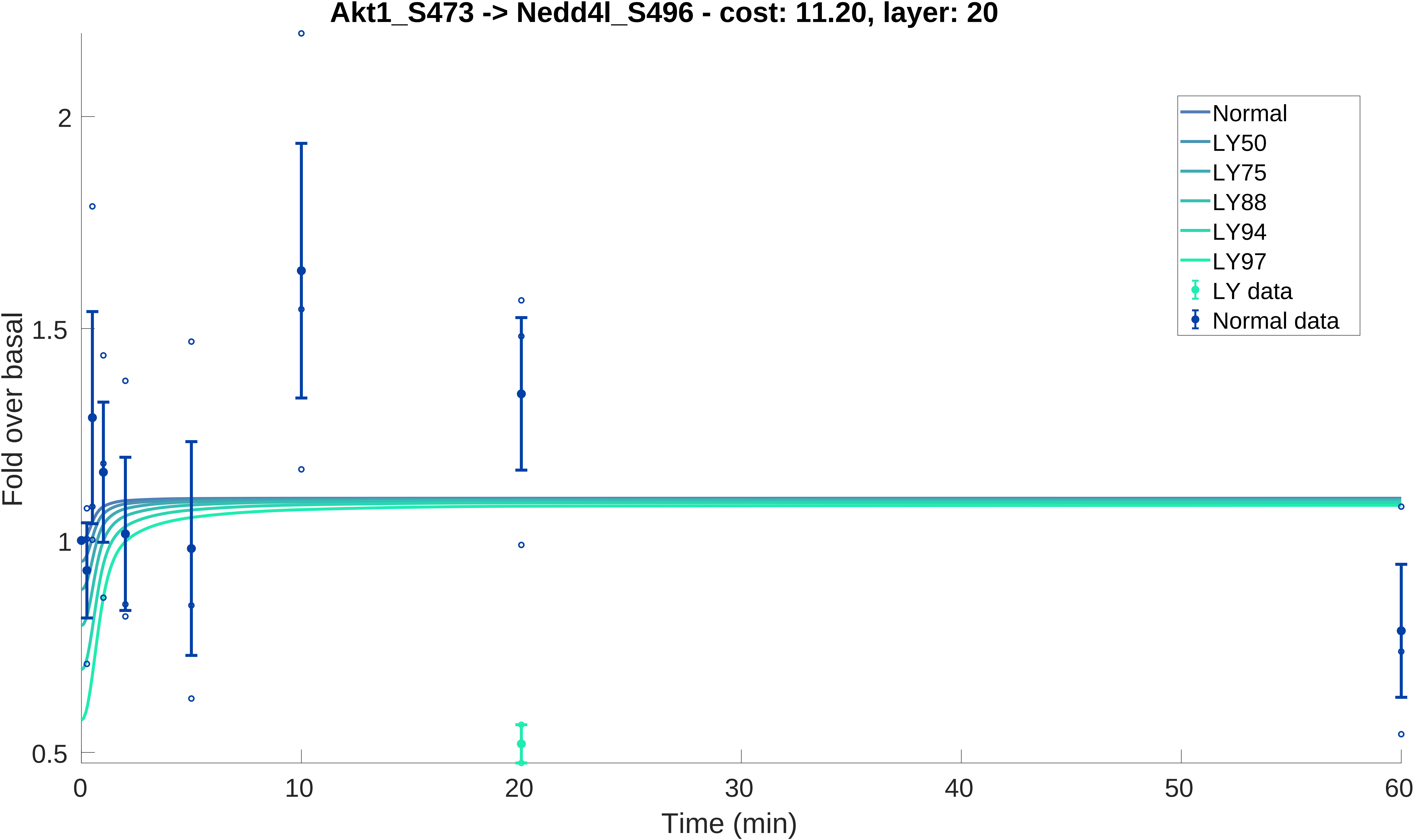
Akt1_S473 -> Nedd4l_S496 - cost: 11.20, layer: 20
Fold over basal
Time (min)
Normal
LY50
LY75
LY88
LY94
LY97
LY data
Normal data
0.5
1
1.5
2
0
10
20
30
40
50
60

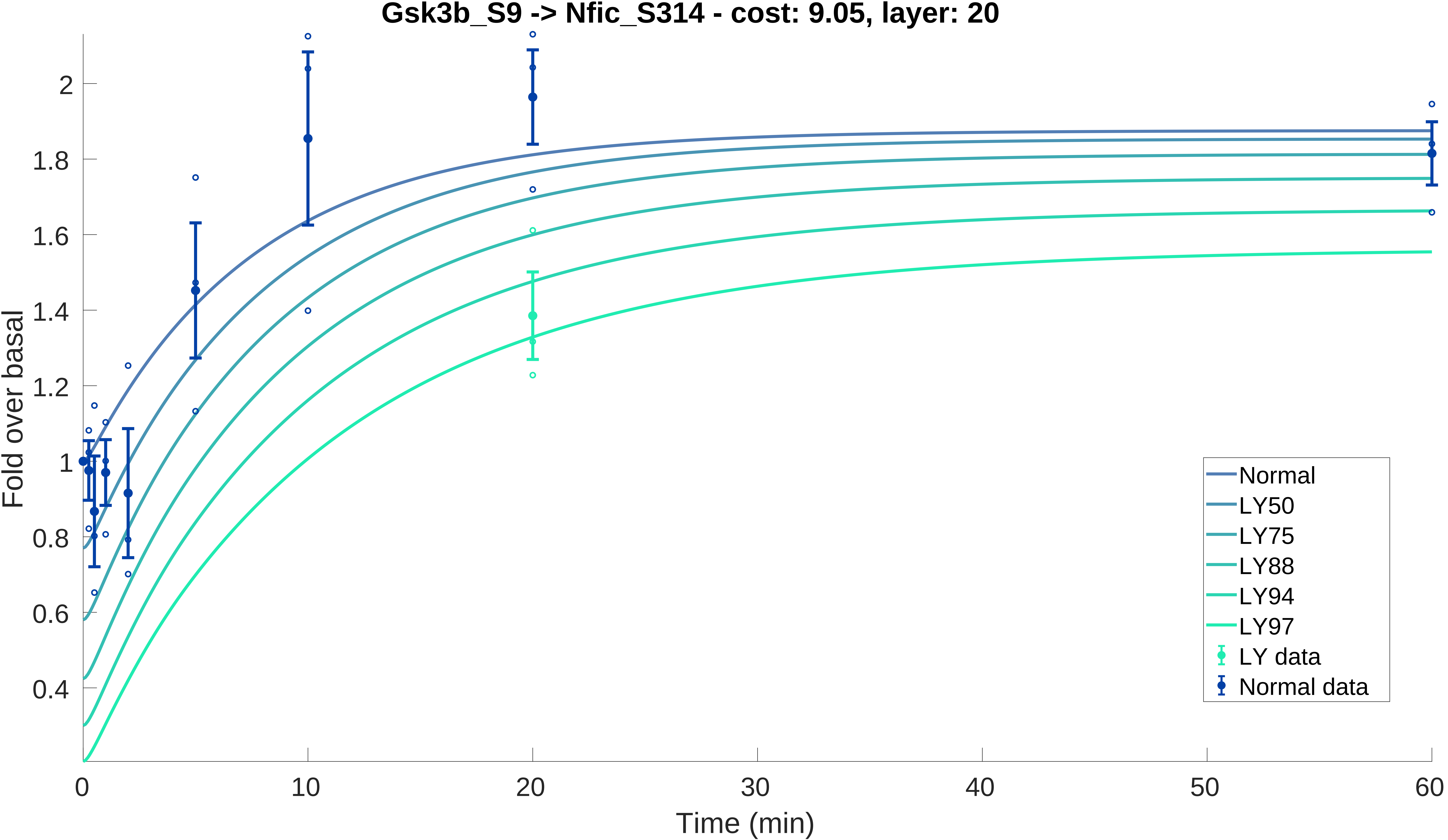

Gsk3b_S9 -> Nfic_S314 - cost: 9.05, layer: 20
Fold over basal
Time (min)
0
10
20
30
40
50
60
0.4
0.6
0.8
1
1.2
1.4
1.6
1.8
2
Normal
LY50
LY75
LY88
LY94
LY97
LY data
Normal data

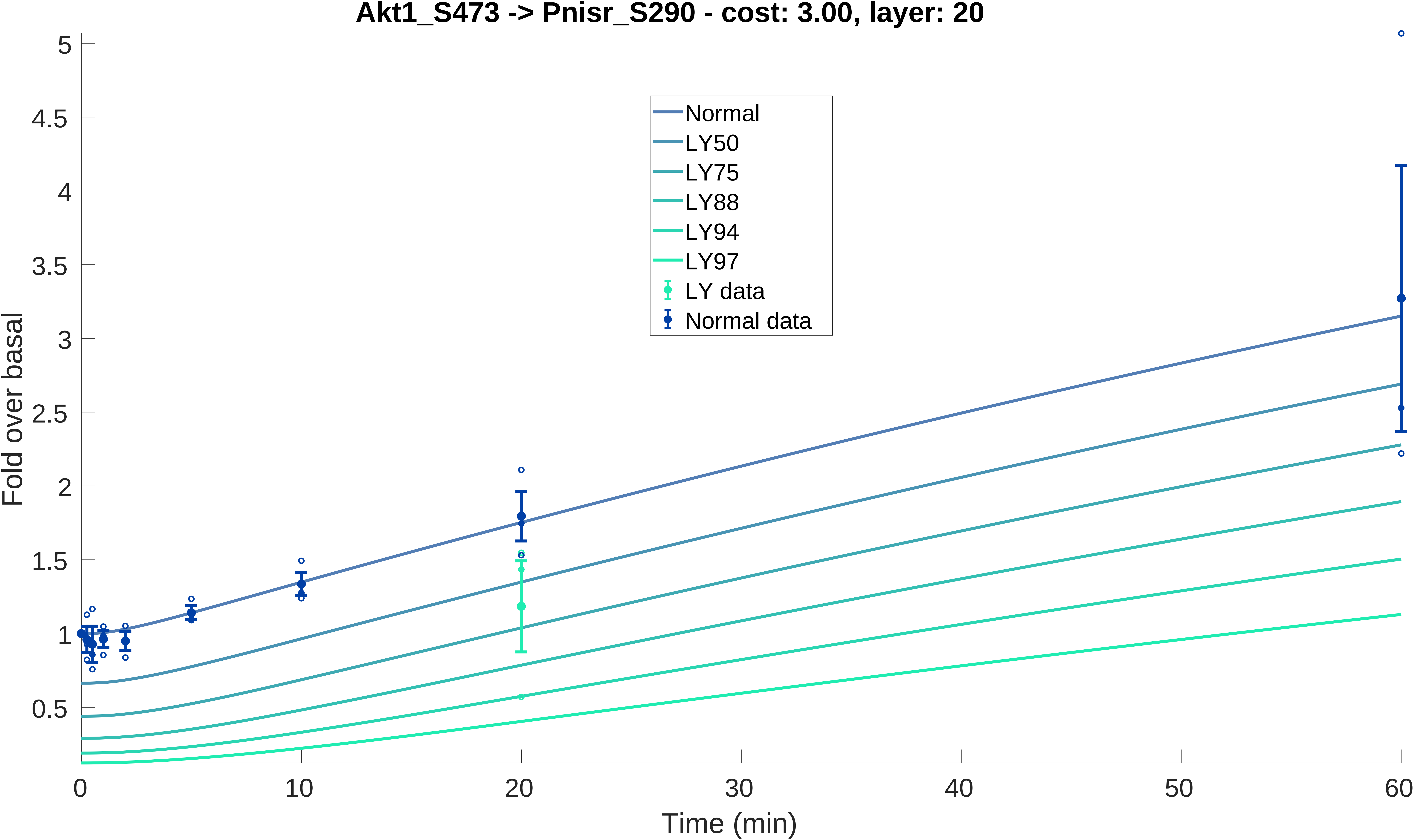

Akt1_S473 -> Pnisr_S290 - cost: 3.00, layer: 20
Fold over basal
Time (min)
Normal
LY50
LY75
LY88
LY94
LY97
LY data
Normal data
0.5
1
1.5
2
2.5
3
3.5
4
4.5
5
0
10
20
30
40
50
60

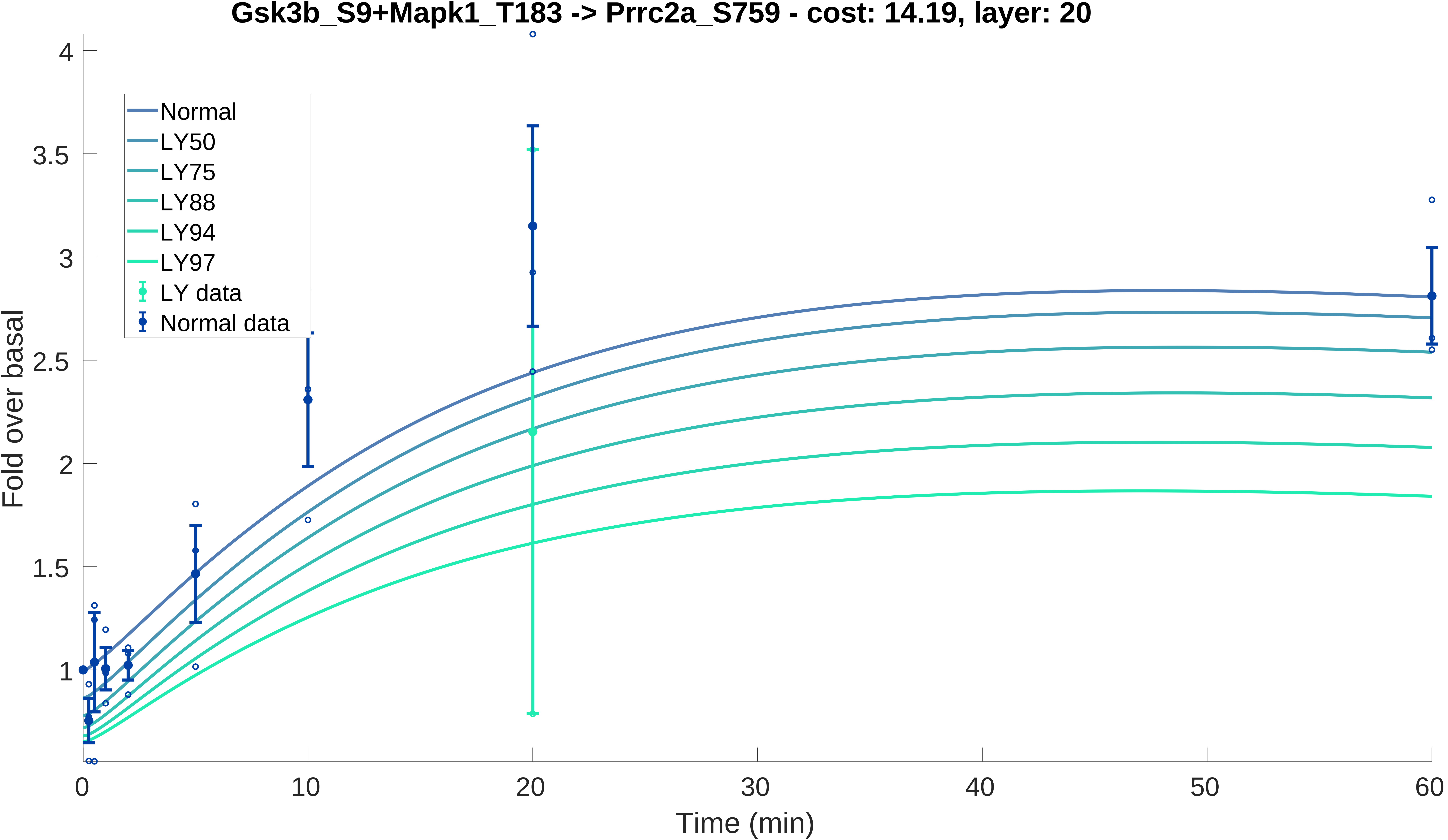
Gsk3b_S9+Mapk1_T183 -> Prrc2a_S759 - cost: 14.19, layer: 20
Normal
LY50
LY75
LY88
LY94
LY97
LY data
Normal data
Fold over basal
Time (min)
0
10
20
30
40
50
60
1
1.5
2
2.5
3
3.5
4

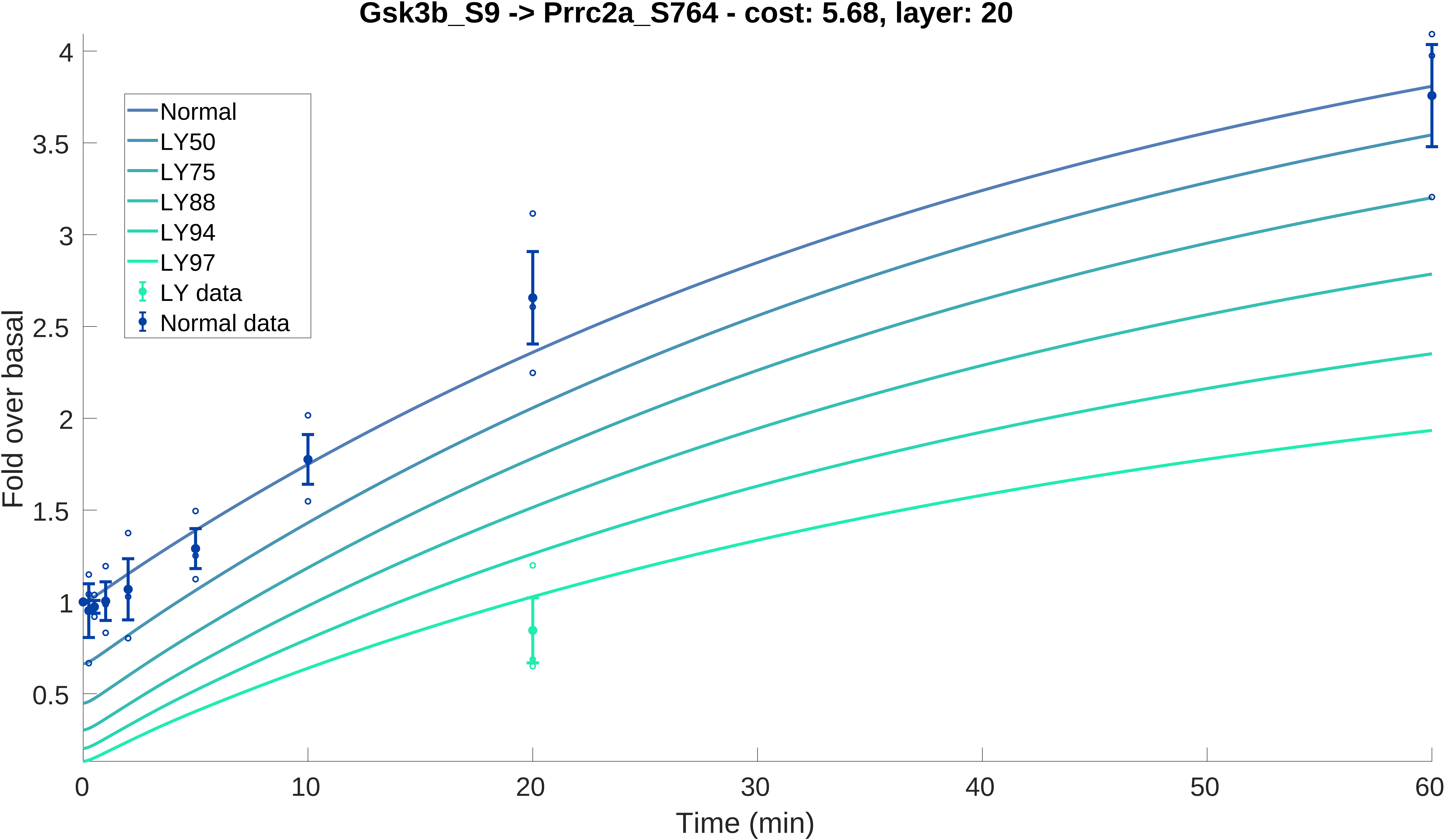

Gsk3b_S9 -> Prrc2a_S764 - cost: 5.68, layer: 20
Normal
LY50
LY75
LY88
LY94
LY97
LY data
Normal data
Fold over basal
Time (min)
0.5
1
1.5
2
2.5
3
3.5
4
0
10
20
30
40
50
60

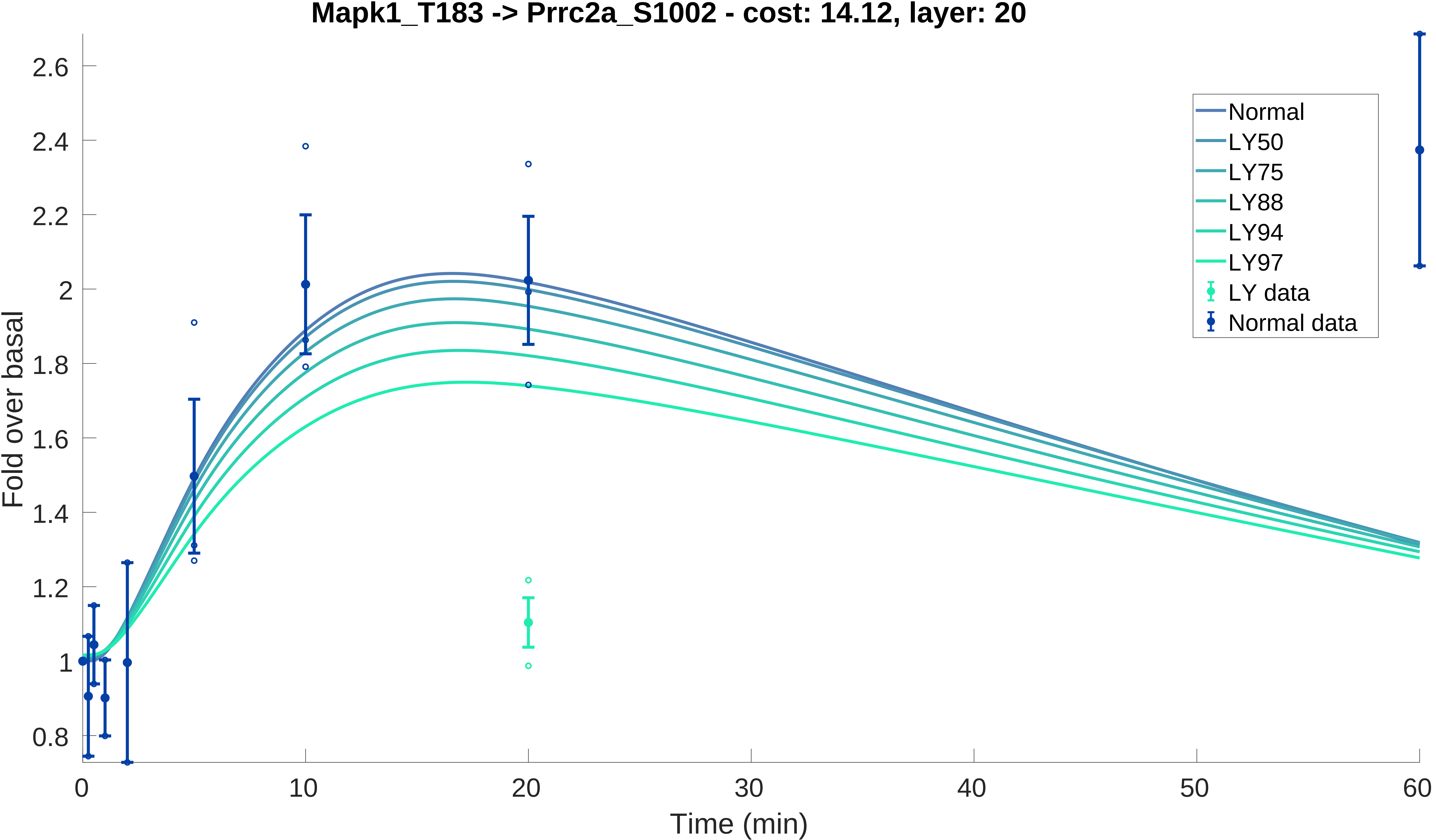

Mapk1_T183 -> Prrc2a_S1002 - cost: 14.12, layer: 20
Fold over basal
Time (min)
0.8
1
1.2
1.4
1.6
1.8
2
2.2
2.4
2.6
0
10
20
30
40
50
60
Normal
LY50
LY75
LY88
LY94
LY97
LY data
Normal data

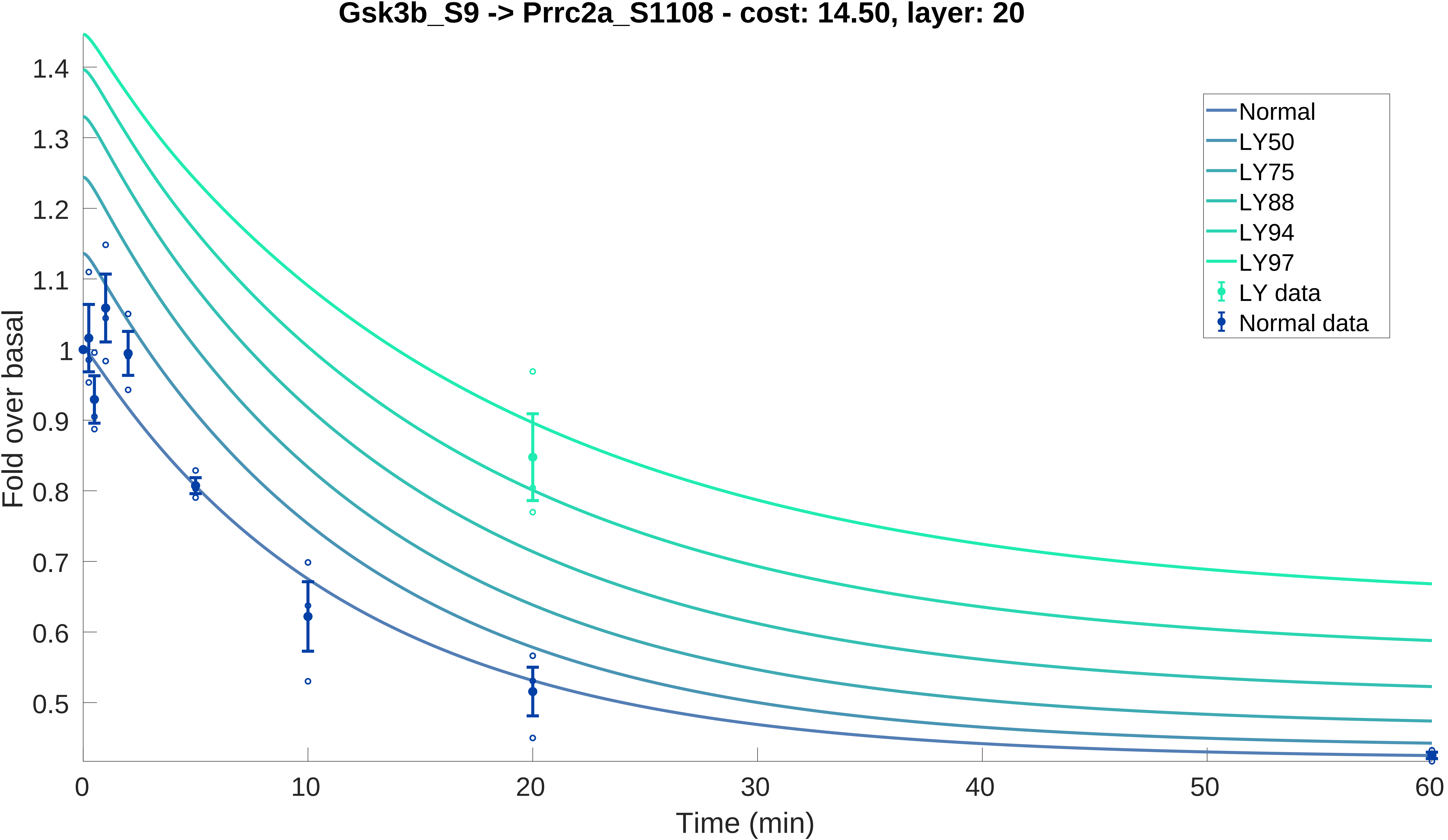

Gsk3b_S9 -> Prrc2a_S1108 - cost: 14.50, layer: 20
Fold over basal
Time (min)
Normal
LY50
LY75
LY88
LY94
LY97
LY data
Normal data
1.4
1.3
1.2
1.1
1
0.9
0.8
0.7
0.6
0.5
0
10
20
30
40
50
60

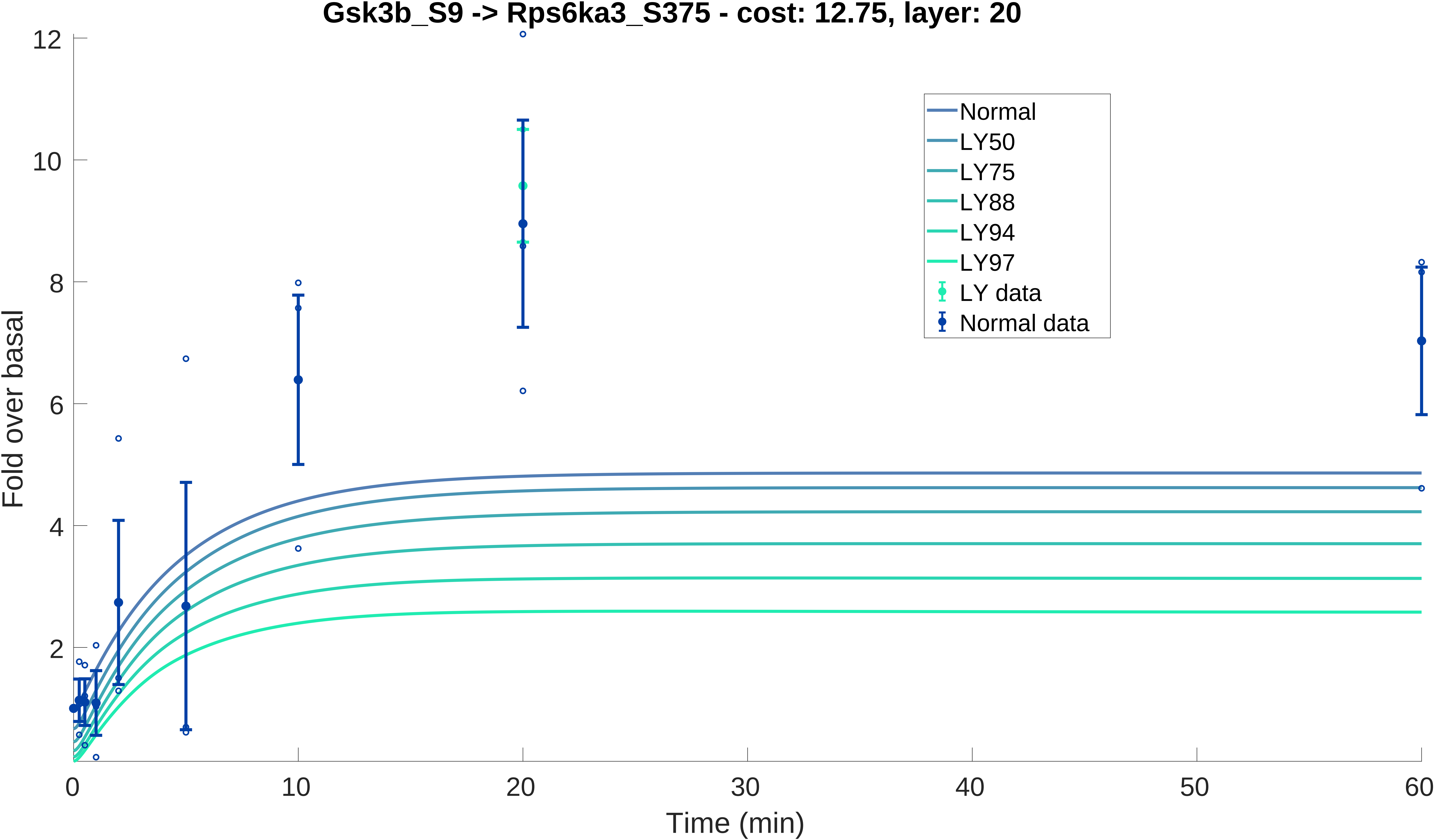

Gsk3b_S9 -> Rps6ka3_S375 - cost: 12.75, layer: 20
Fold over basal
Time (min)
0
10
20
30
40
50
60
2
4
6
8
10
12
Normal
LY50
LY75
LY88
LY94
LY97
LY data
Normal data

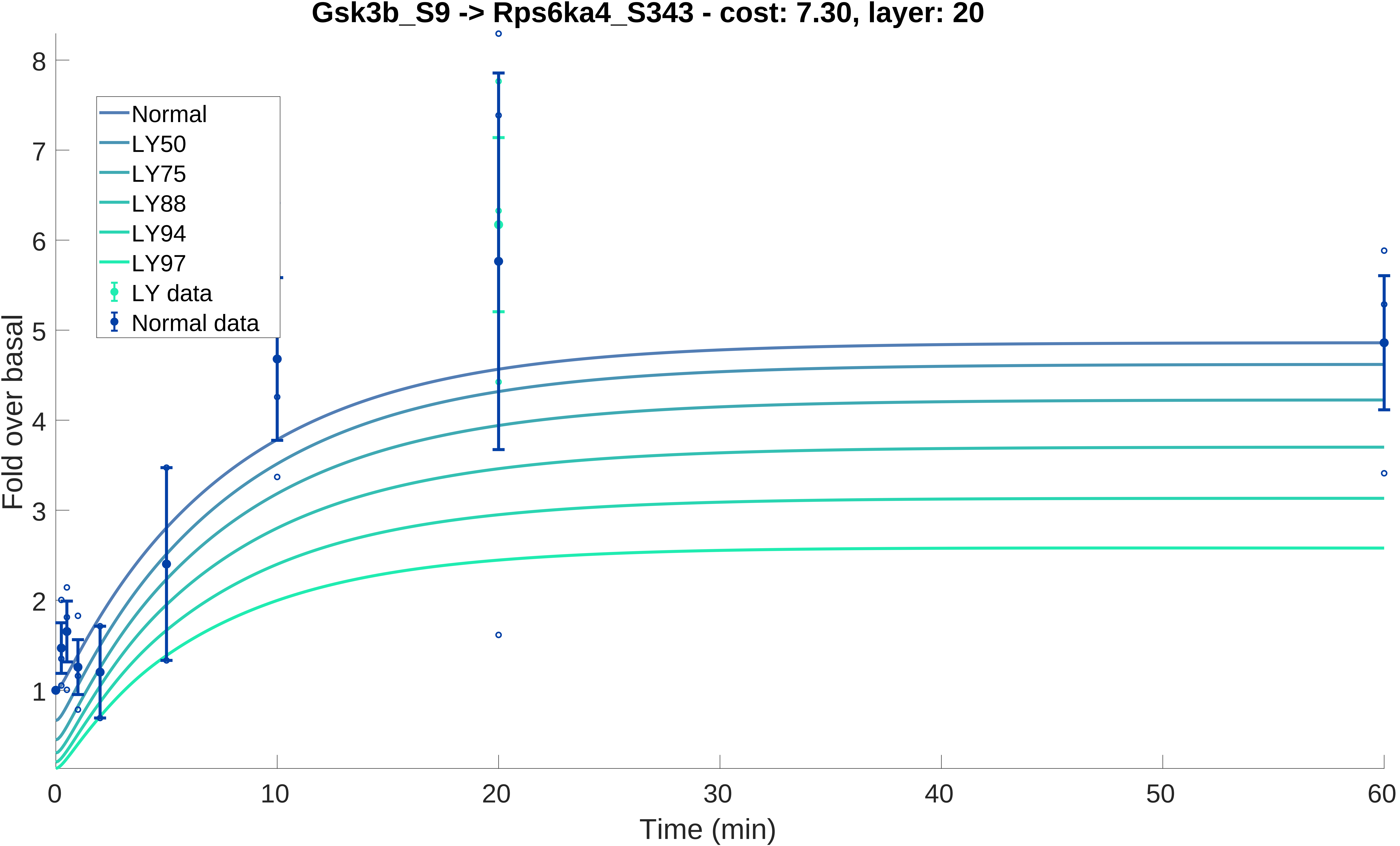

Gsk3b_S9 -> Rps6ka4_S343 - cost: 7.30, layer: 20
Fold over basal
Time (min)
Normal
LY50
LY75
LY88
LY94
LY97
LY data
Normal data
1
2
3
4
5
6
7
8
0
10
20
30
40
50
60

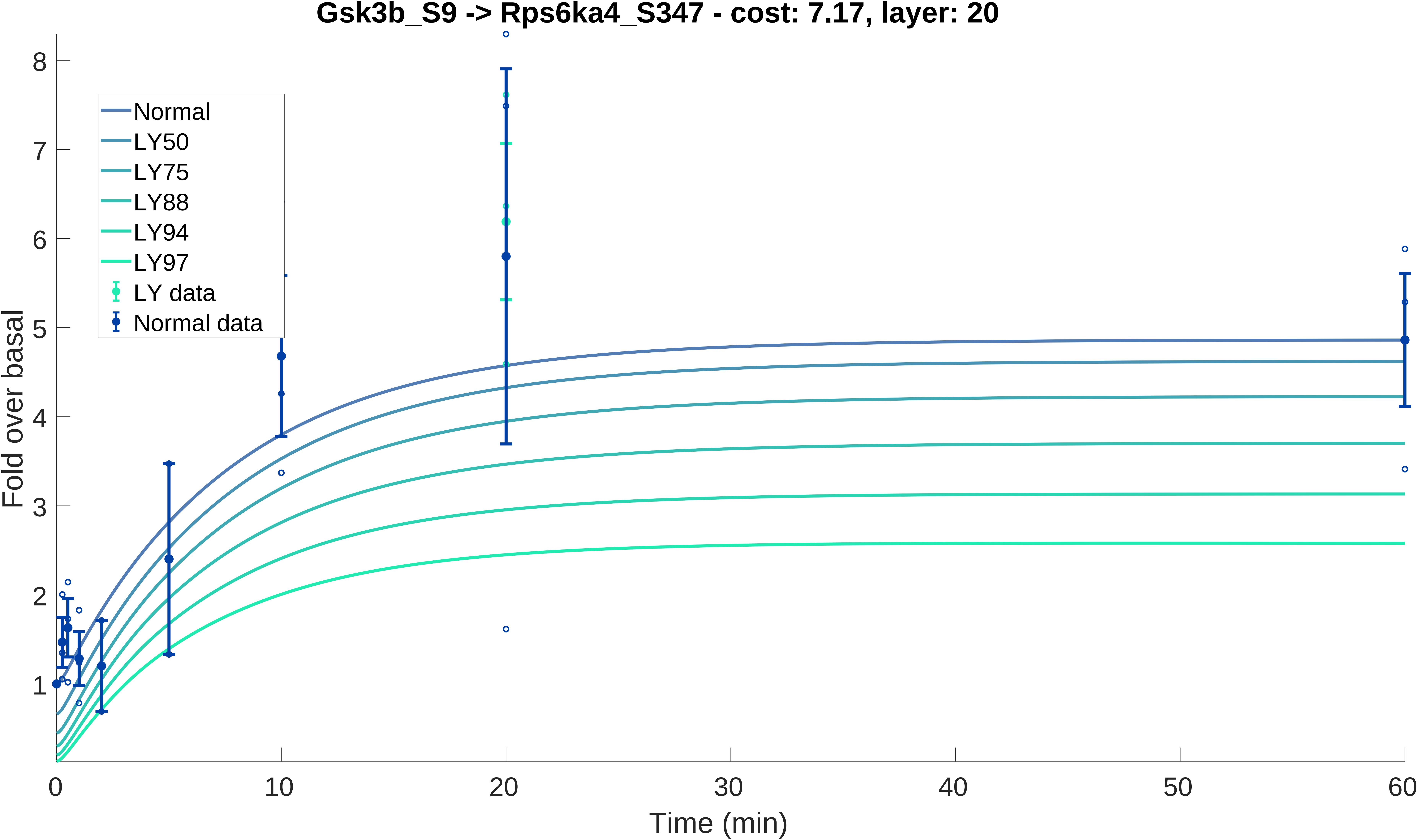

Gsk3b_S9 -> Rps6ka4_S347 - cost: 7.17, layer: 20
Normal
LY50
LY75
LY88
LY94
LY97
LY data
Normal data
Fold over basal
Time (min)
1
2
3
4
5
6
7
8
0
10
20
30
40
50
60

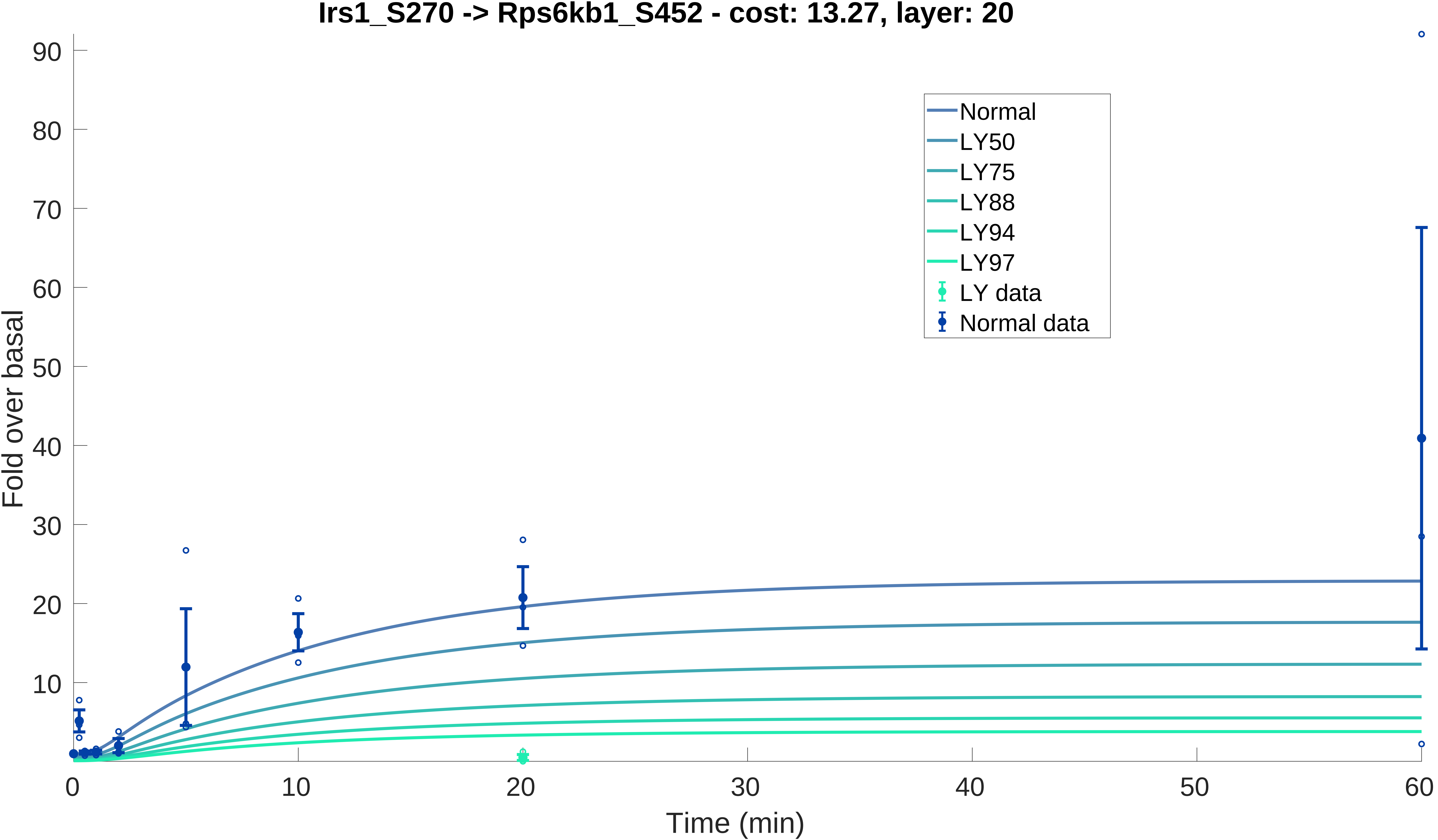

Irs1_S270 -> Rps6kb1_S452 - cost: 13.27, layer: 20
Fold over basal
Time (min)
0
10
20
30
40
50
60
70
80
90
Normal
LY50
LY75
LY88
LY94
LY97
LY data
Normal data

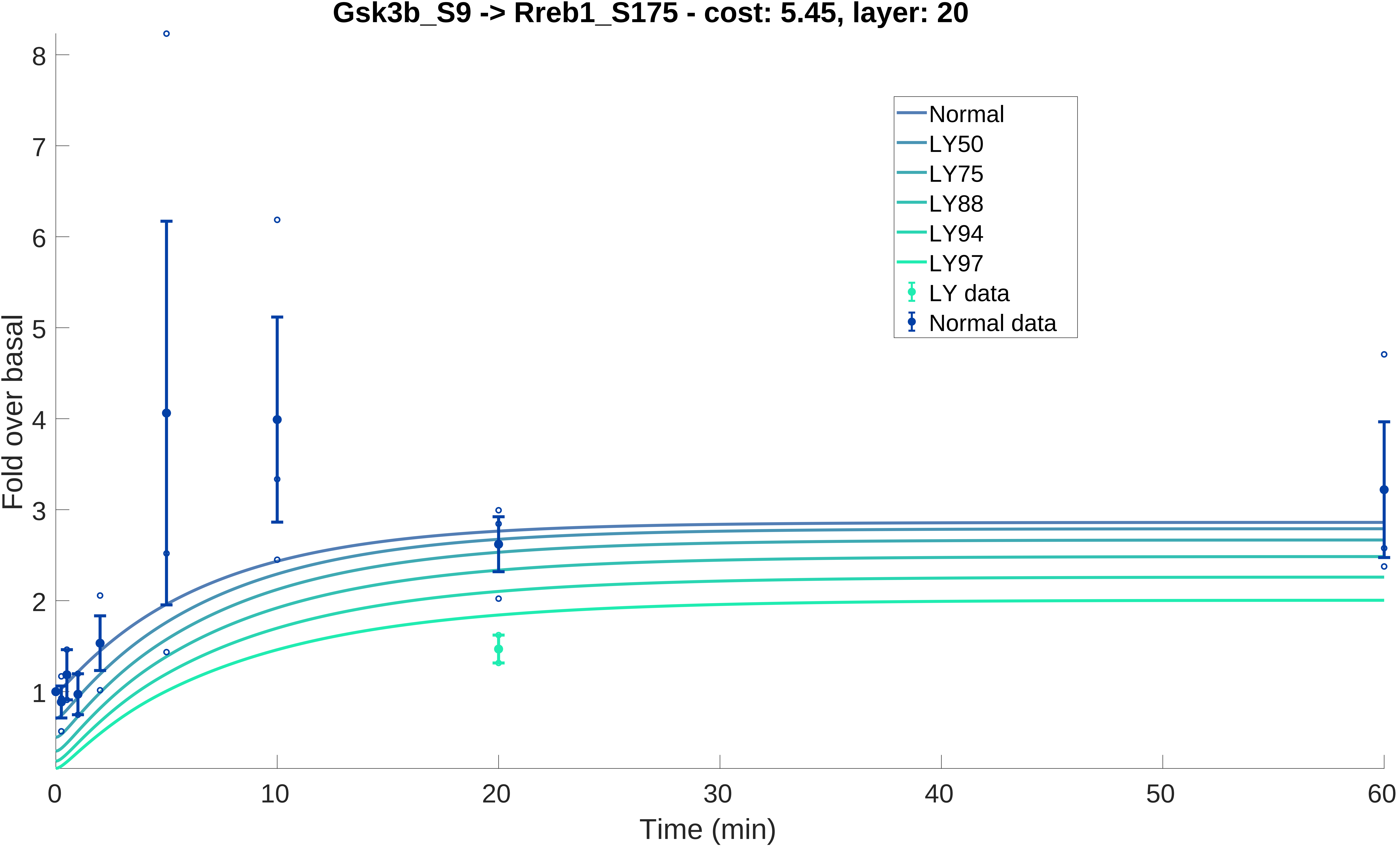

Gsk3b_S9 -> Rreb1_S175 - cost: 5.45, layer: 20
Fold over basal
Time (min)
0
10
20
30
40
50
60
1
2
3
4
5
6
7
8
Normal
LY50
LY75
LY88
LY94
LY97
LY data
Normal data

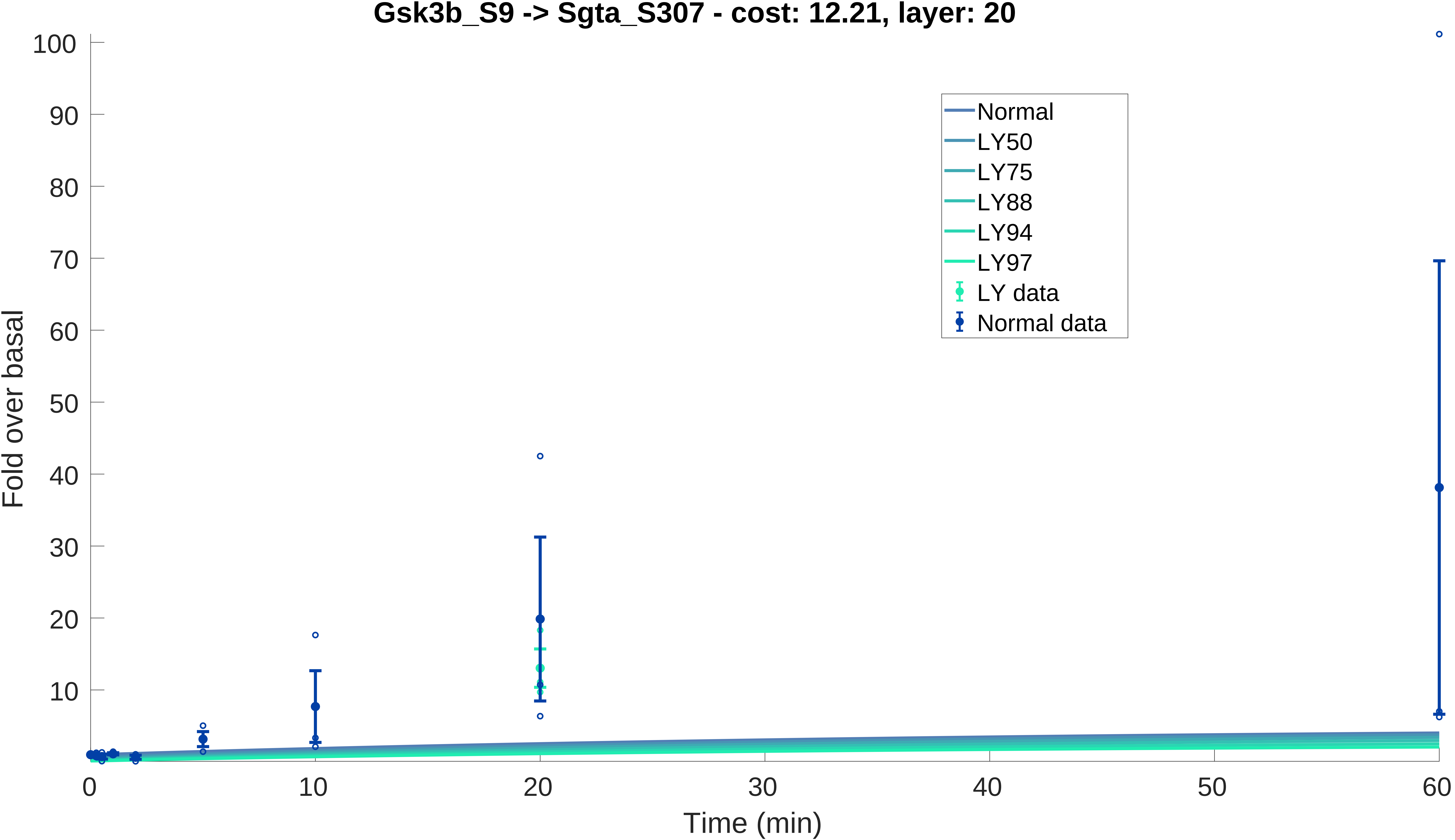

Gsk3b_S9 -> Sgta_S307 - cost: 12.21, layer: 20
Fold over basal
Time (min)
0
10
20
30
40
50
60
70
80
90
100
Normal
LY50
LY75
LY88
LY94
LY97
LY data
Normal data

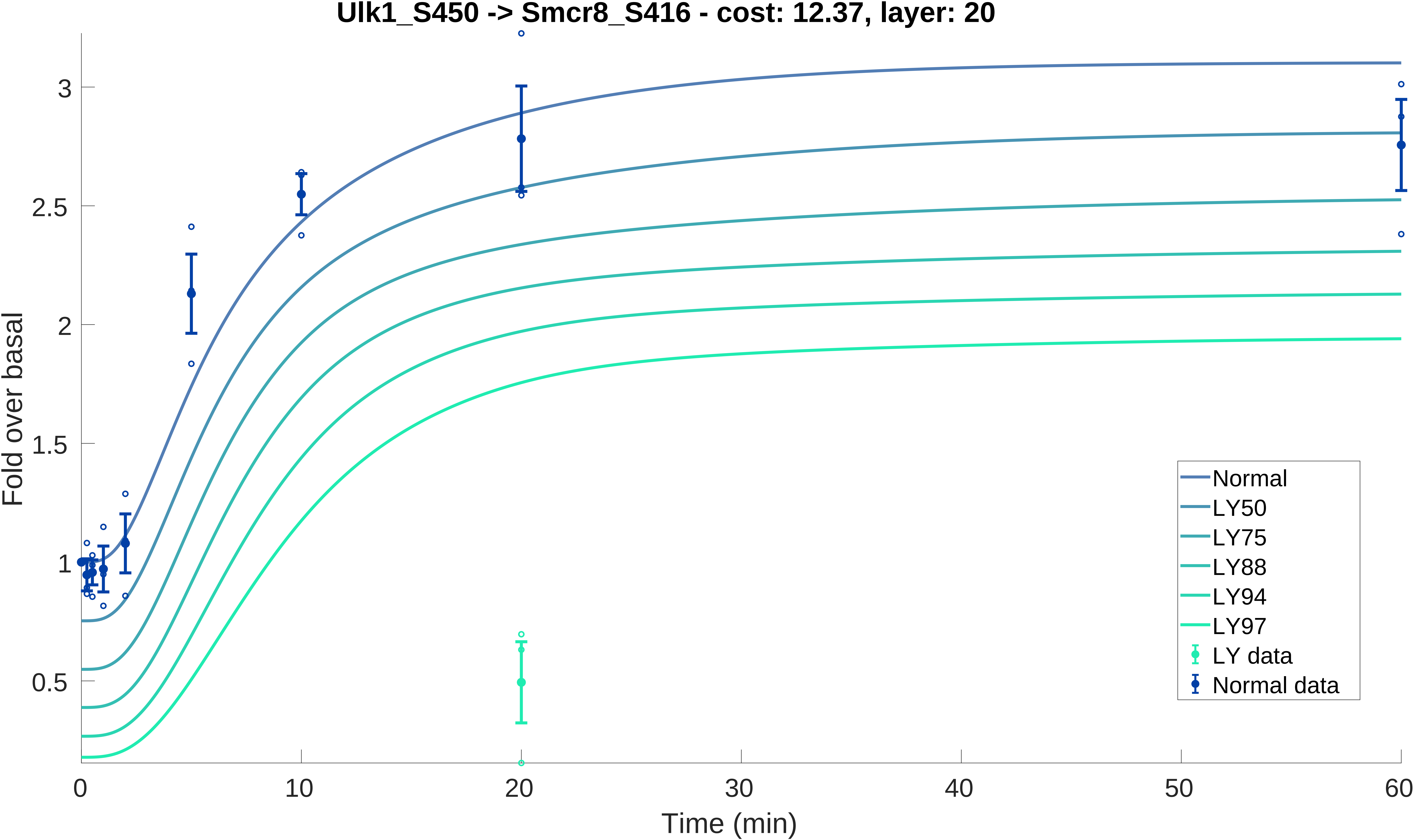
Ulk1_S450 -> Smcr8_S416 - cost: 12.37, layer: 20
Fold over basal
Time (min)
0.5
1
1.5
2
2.5
3
0
10
20
30
40
50
60
Normal
LY50
LY75
LY88
LY94
LY97
LY data
Normal data

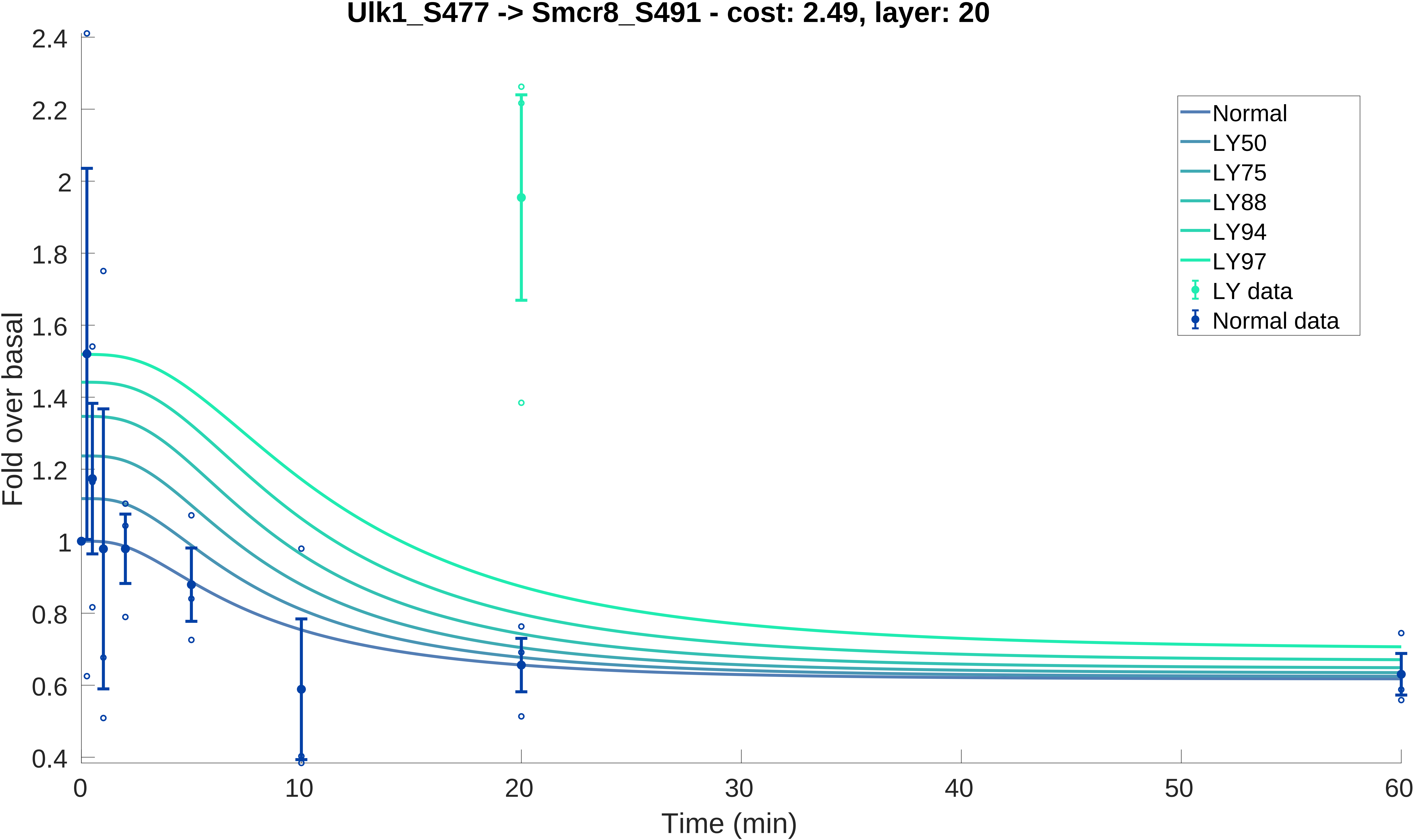

Ulk1_S477 -> Smcr8_S491 - cost: 2.49, layer: 20
Fold over basal
Time (min)
2.4
2.2
2
1.8
1.6
1.4
1.2
1
0.8
0.6
0.4
0
10
20
30
40
50
60
Normal
LY50
LY75
LY88
LY94
LY97
LY data
Normal data

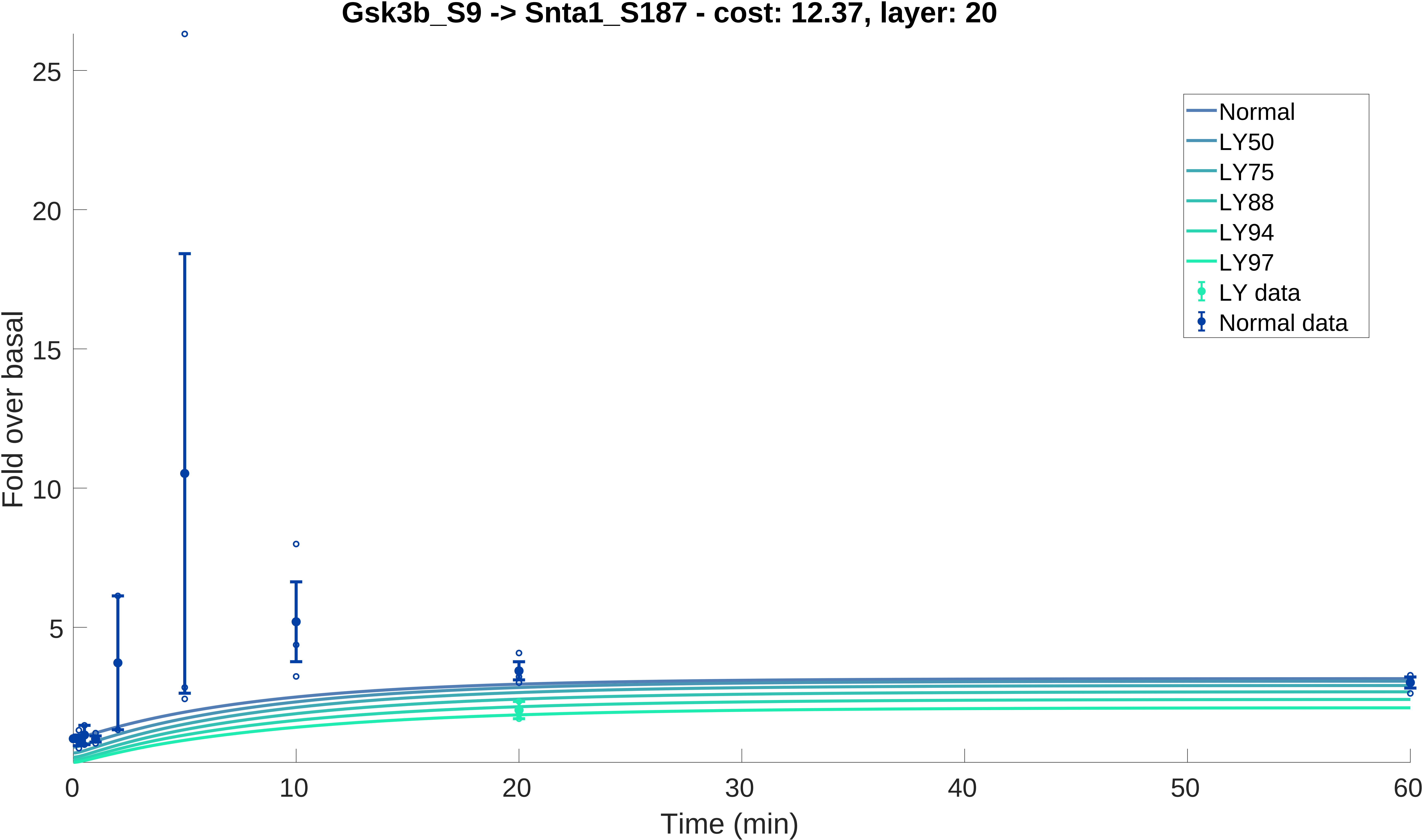

Gsk3b_S9 -> Snta1_S187 - cost: 12.37, layer: 20
Fold over basal
Time (min)
0
5
10
15
20
25
0
10
20
30
40
50
60
Normal
LY50
LY75
LY88
LY94
LY97
LY data
Normal data

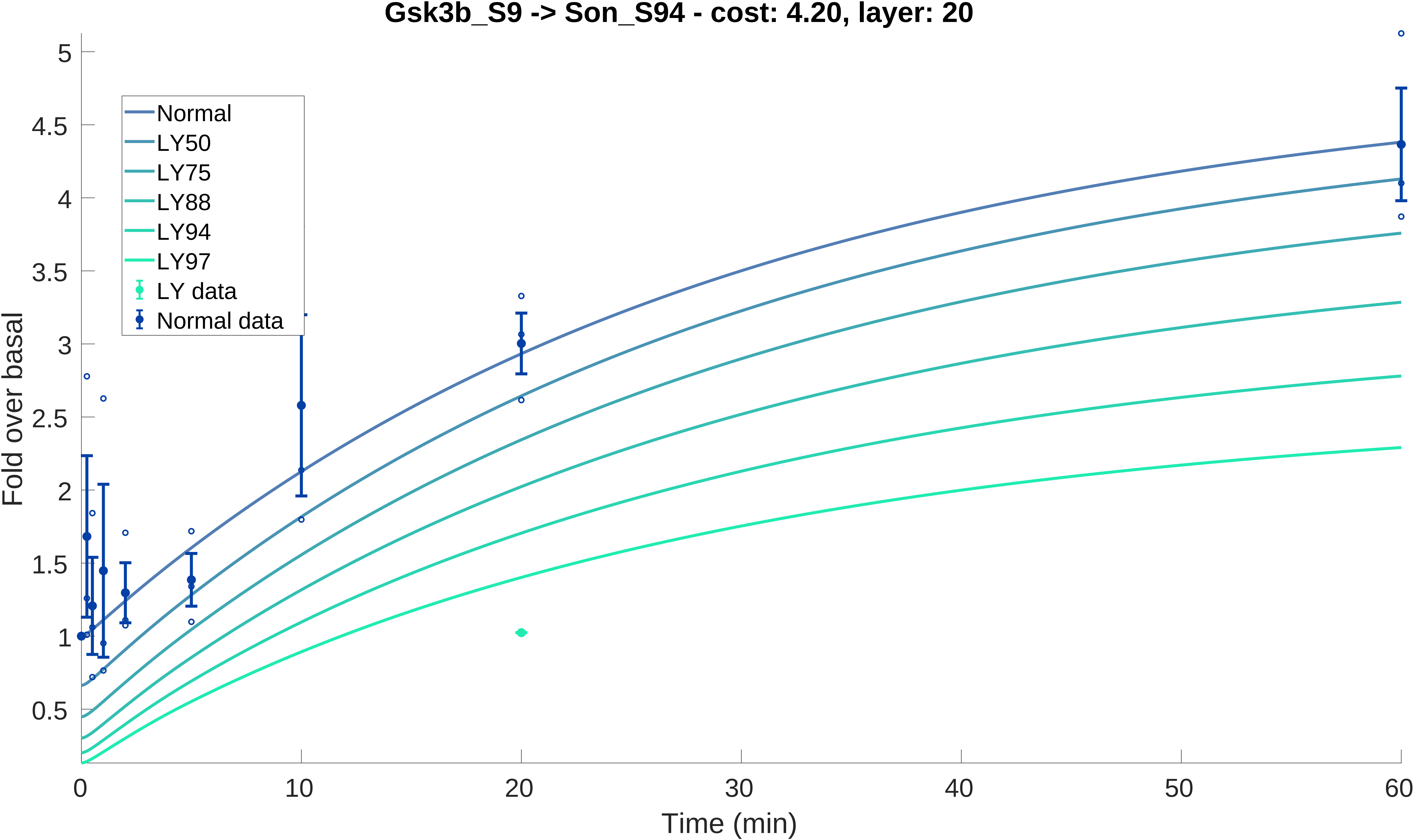

Gsk3b_S9 -> Son_S94 - cost: 4.20, layer: 20
Fold over basal
Time (min)
Normal
LY50
LY75
LY88
LY94
LY97
LY data
Normal data
0
10
20
30
40
50
60
0.5
1
1.5
2
2.5
3
3.5
4
4.5
5

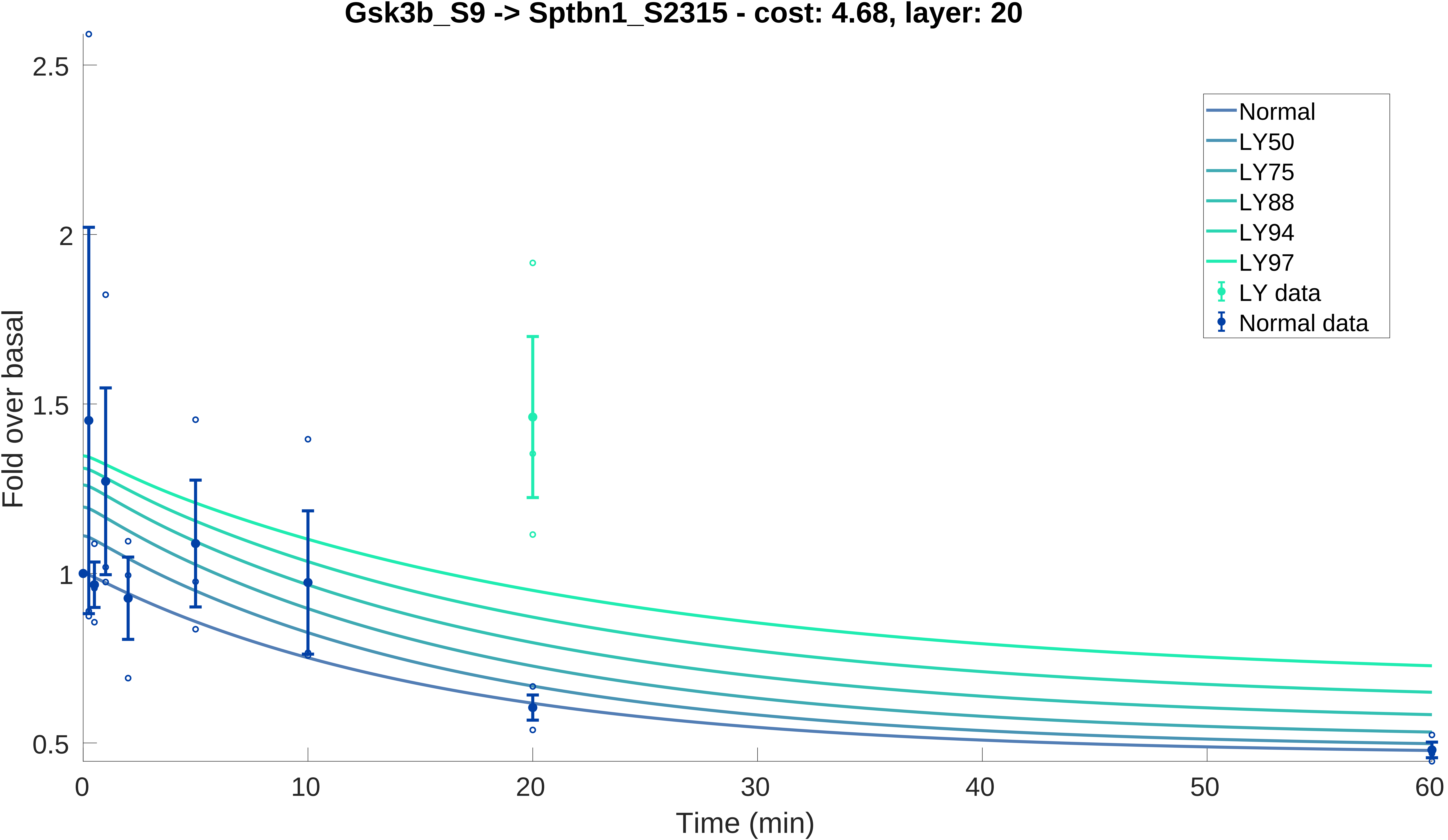

Gsk3b_S9 -> Sptbn1_S2315 - cost: 4.68, layer: 20
Fold over basal
Time (min)
0
10
20
30
40
50
60
0.5
1
1.5
2
2.5
Normal
LY50
LY75
LY88
LY94
LY97
LY data
Normal data

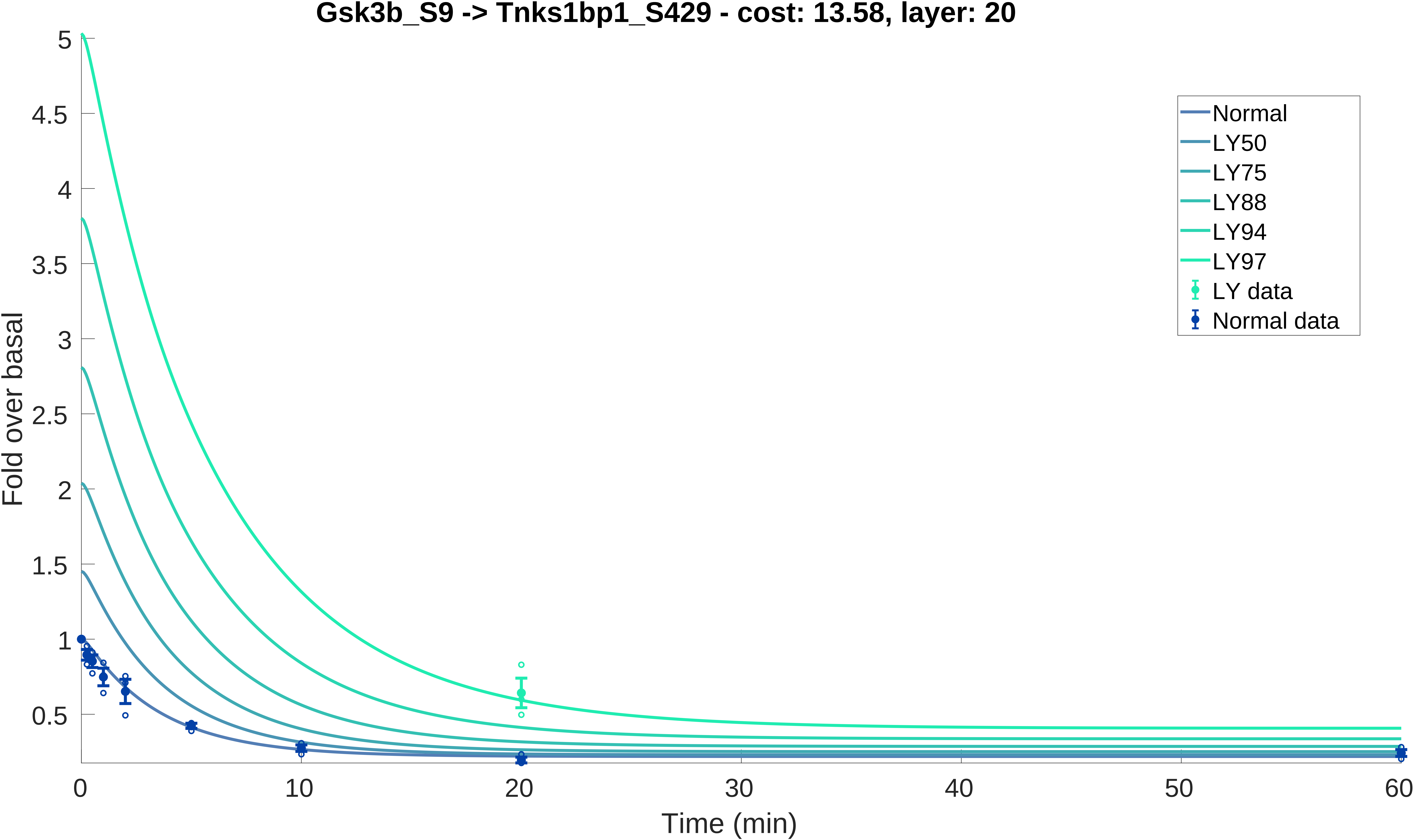

Gsk3b_S9 -> Tnks1bp1_S429 - cost: 13.58, layer: 20
Fold over basal
Time (min)
0
10
20
30
40
50
60
0.5
1
1.5
2
2.5
3
3.5
4
4.5
5
Normal
LY50
LY75
LY88
LY94
LY97
LY data
Normal data

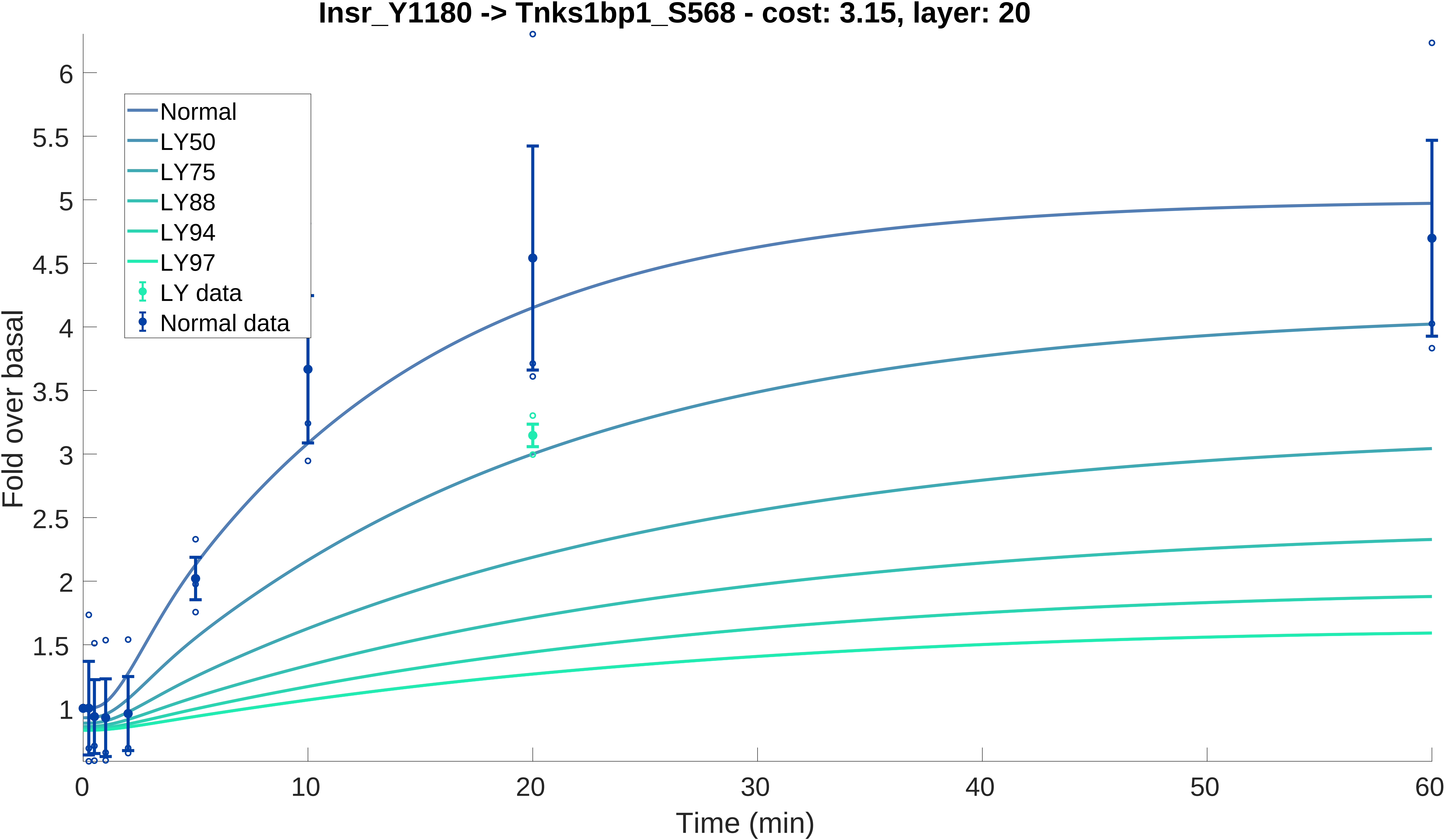

Insr_Y1180 -> Tnks1bp1_S568 - cost: 3.15, layer: 20
Fold over basal
Time (min)
Normal
LY50
LY75
LY88
LY94
LY97
LY data
Normal data
0
10
20
30
40
50
60
1
1.5
2
2.5
3
3.5
4
4.5
5
5.5
6

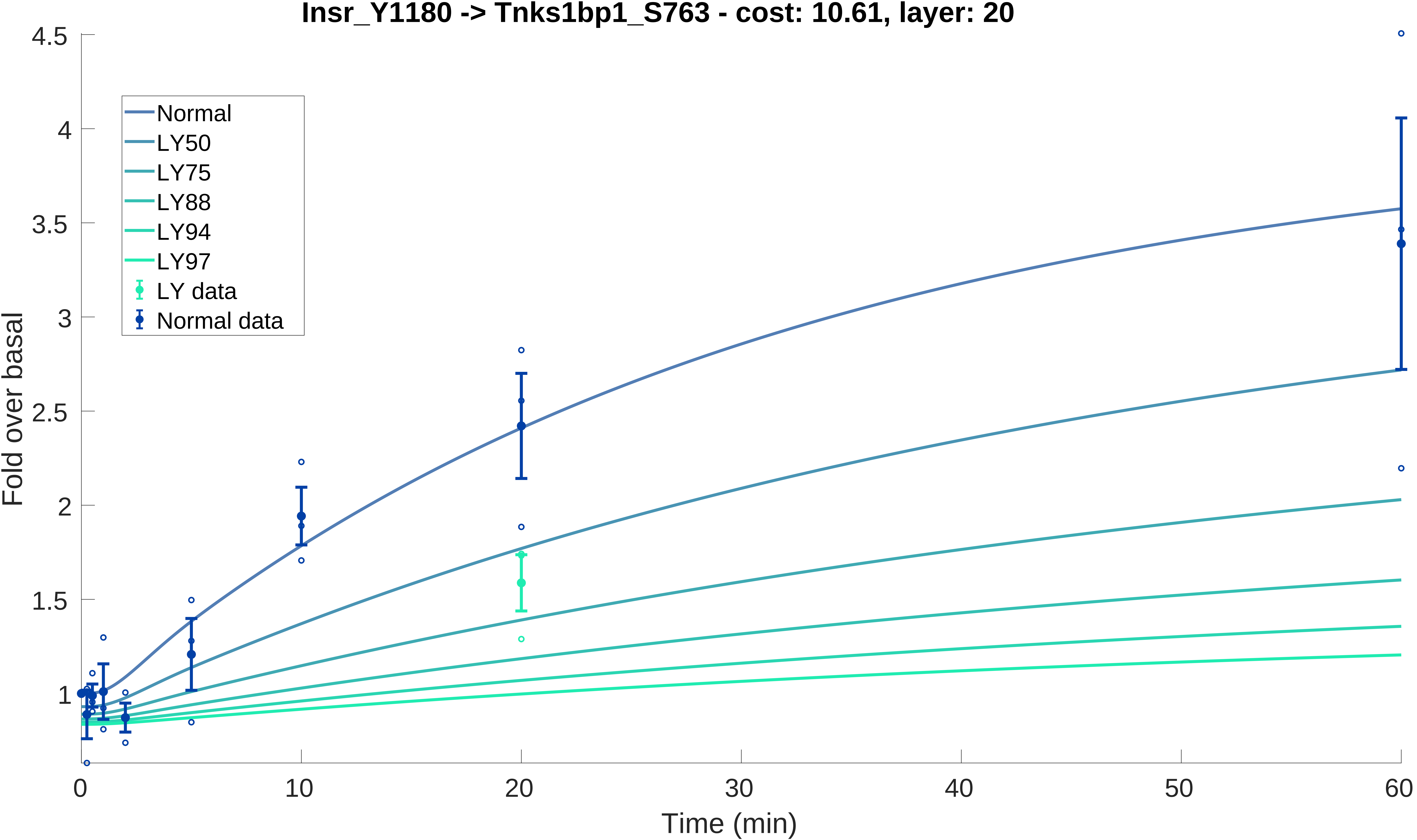
Insr_Y1180 -> Tnks1bp1_S763 - cost: 10.61, layer: 20
Fold over basal
Time (min)
0
10
20
30
40
50
60
1
1.5
2
2.5
3
3.5
4
4.5
Normal
LY50
LY75
LY88
LY94
LY97
LY data
Normal data

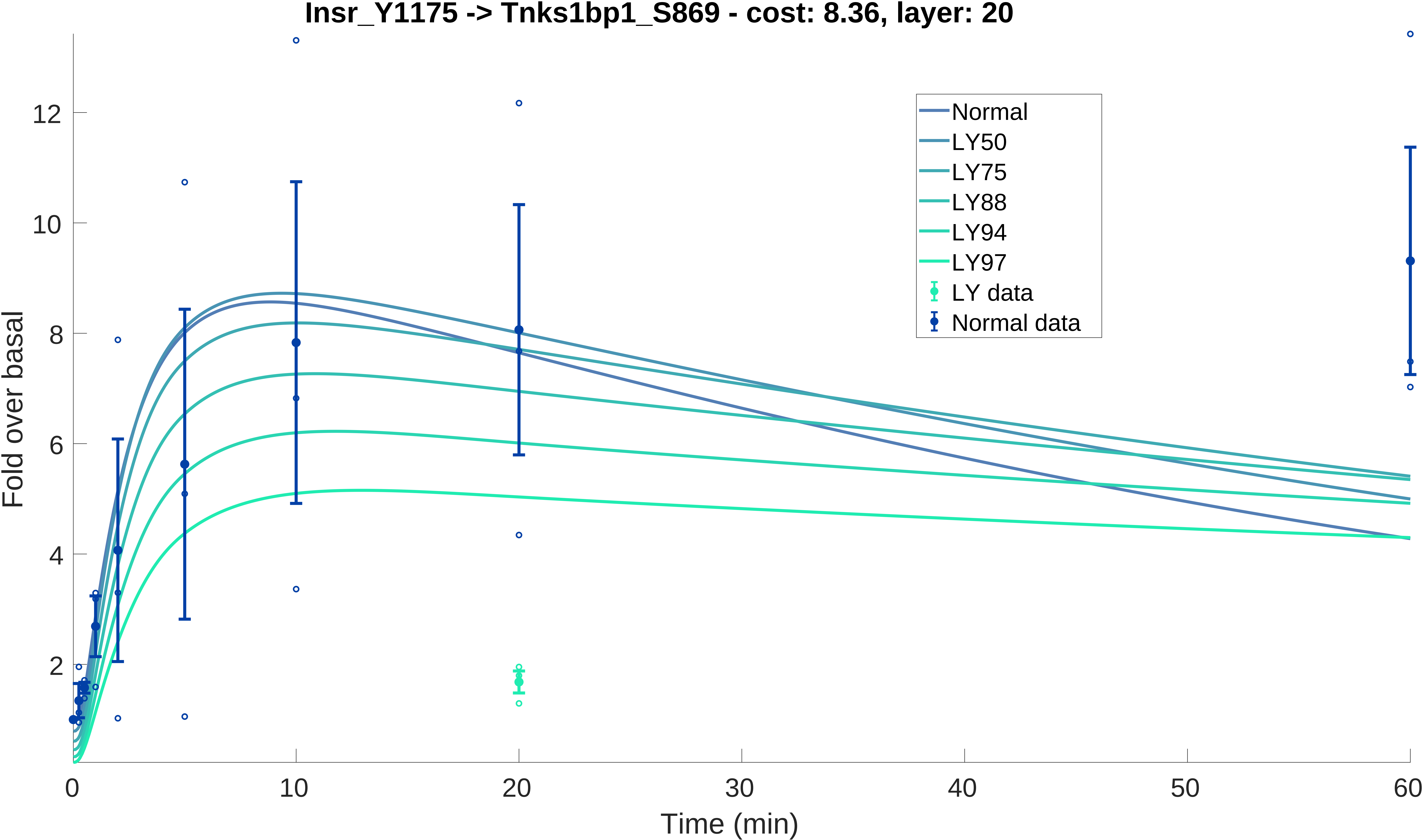
Insr_Y1175 -> Tnks1bp1_S869 - cost: 8.36, layer: 20
Normal
LY50
LY75
LY88
LY94
LY97
LY data
Normal data
Fold over basal
Time (min)
0
10
20
30
40
50
60
2
4
6
8
10
12

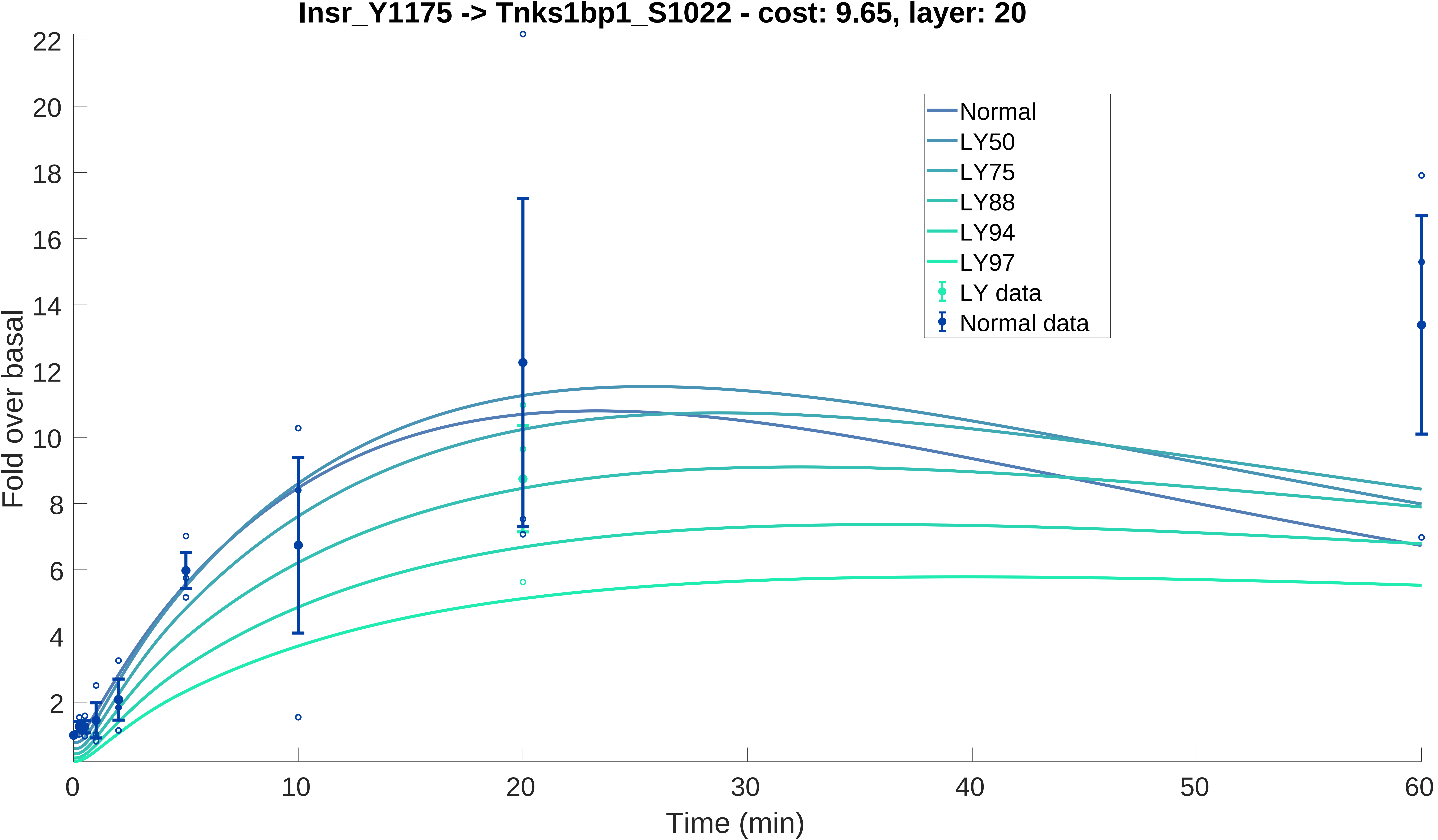

Insr_Y1175 -> Tnks1bp1_S1022 - cost: 9.65, layer: 20
Fold over basal
Time (min)
0
10
20
30
40
50
60
2
4
6
8
10
12
14
16
18
20
22
Normal
LY50
LY75
LY88
LY94
LY97
LY data
Normal data

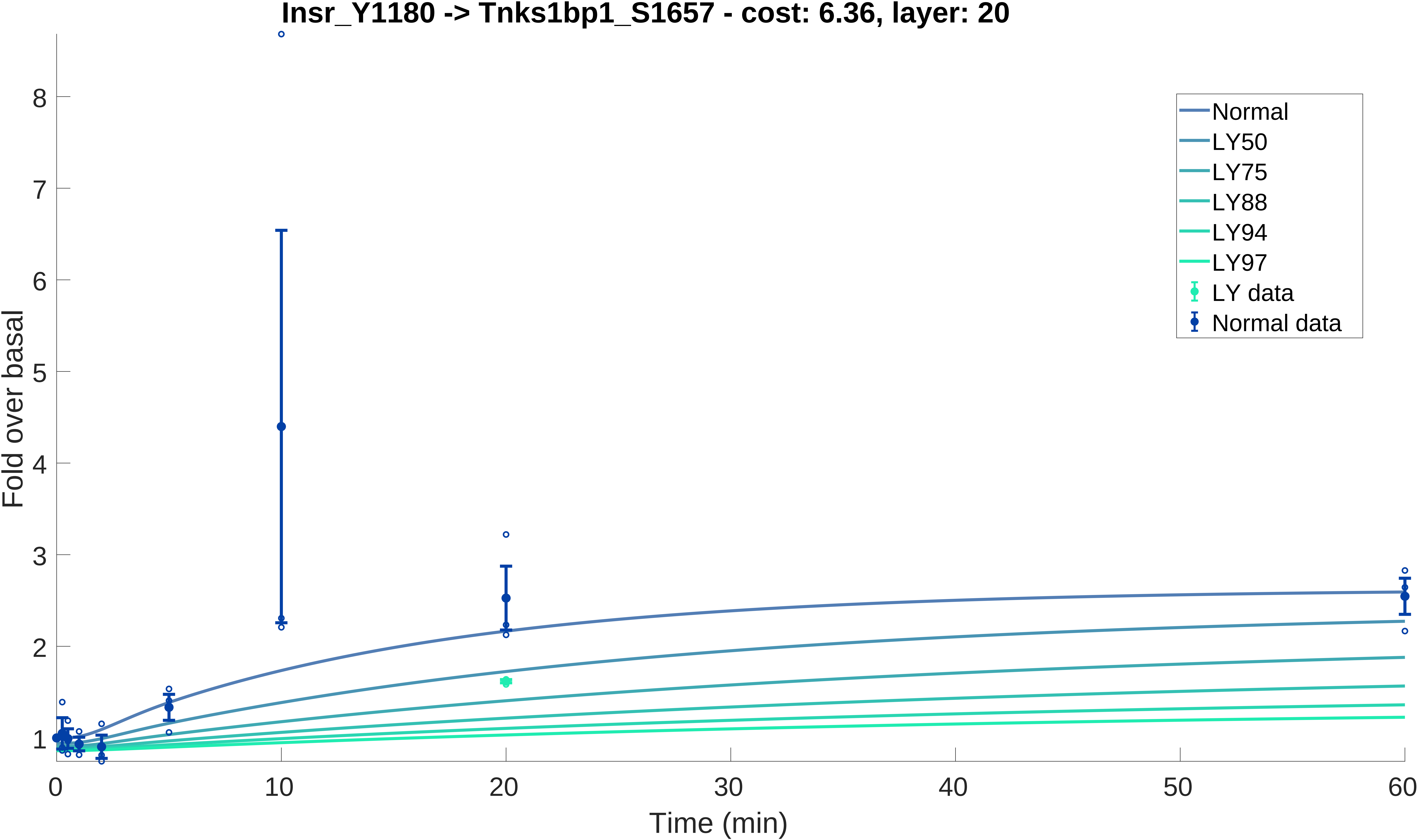

Insr_Y1180 -> Tnks1bp1_S1657 - cost: 6.36, layer: 20
Fold over basal
Time (min)
8
7
6
5
4
3
2
1
0
10
20
30
40
50
60
Normal
LY50
LY75
LY88
LY94
LY97
LY data
Normal data

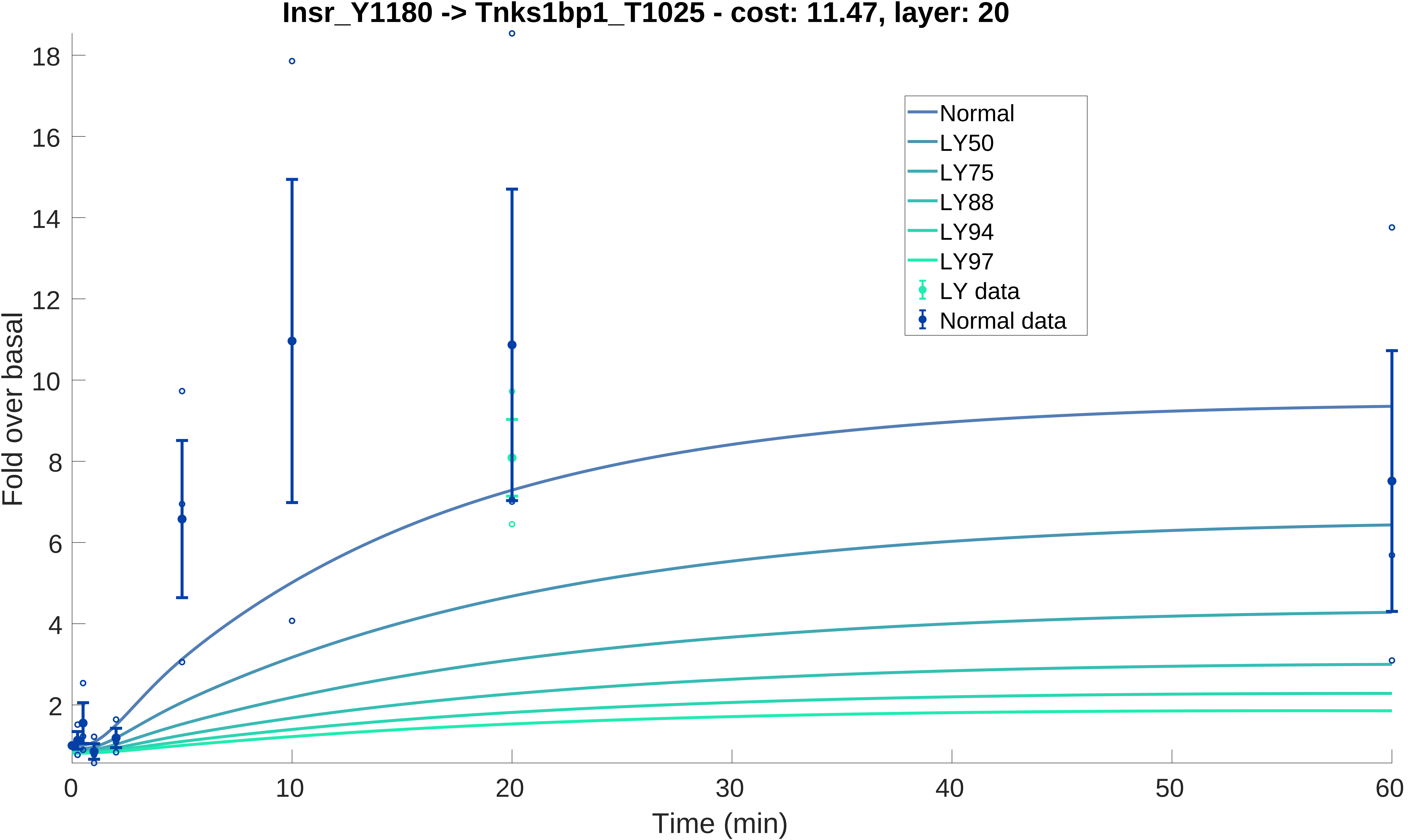

Insr_Y1180 -> Tnks1bp1_T1025 - cost: 11.47, layer: 20
Fold over basal
Time (min)
0
10
20
30
40
50
60
2
4
6
8
10
12
14
16
18
Normal
LY50
LY75
LY88
LY94
LY97
LY data
Normal data

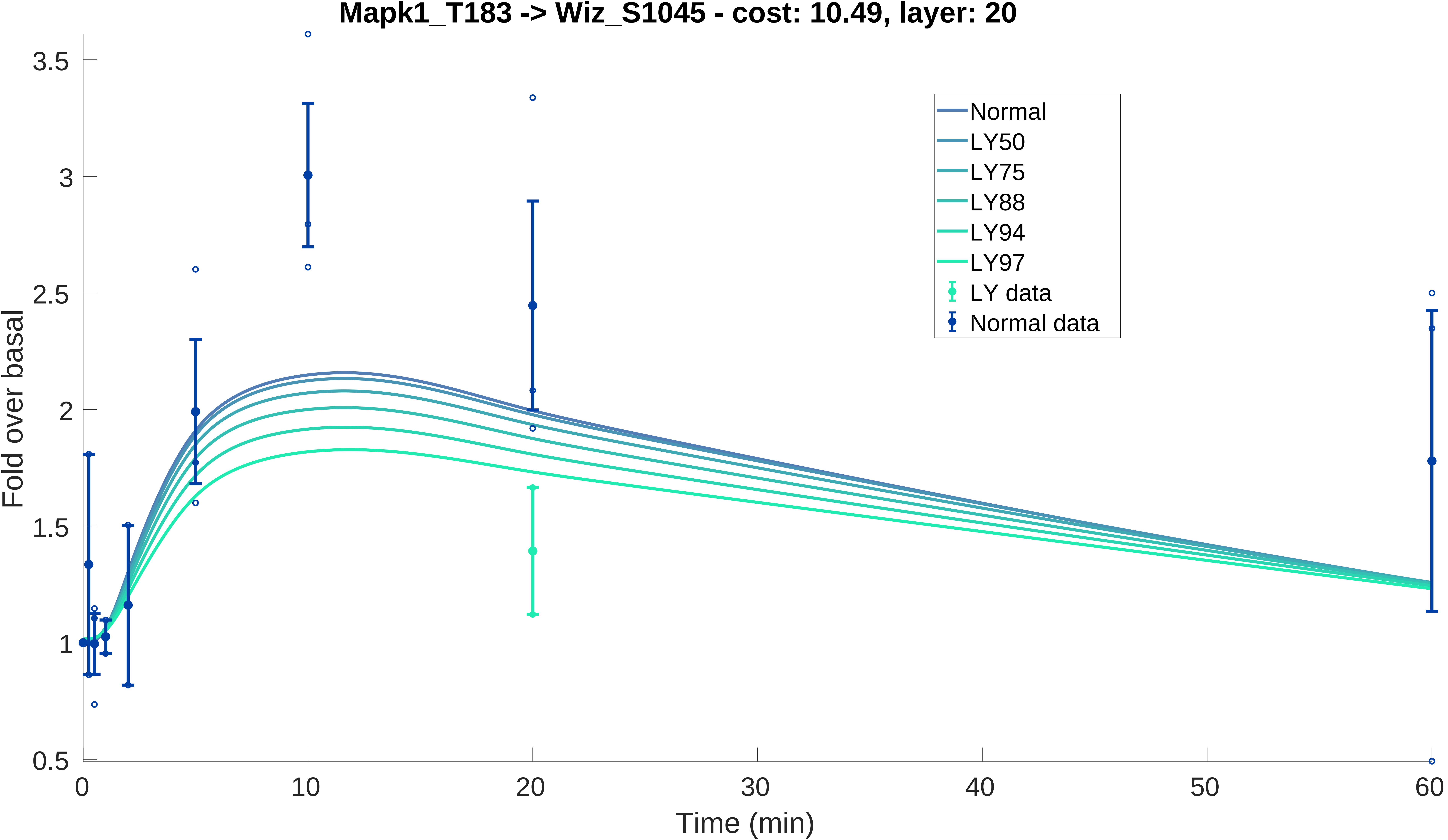

Mapk1_T183 -> Wiz_S1045 - cost: 10.49, layer: 20
Normal
LY50
LY75
LY88
LY94
LY97
LY data
Normal data
Fold over basal
Time (min)
0.5
1
1.5
2
2.5
3
3.5
0
10
20
30
40
50
60

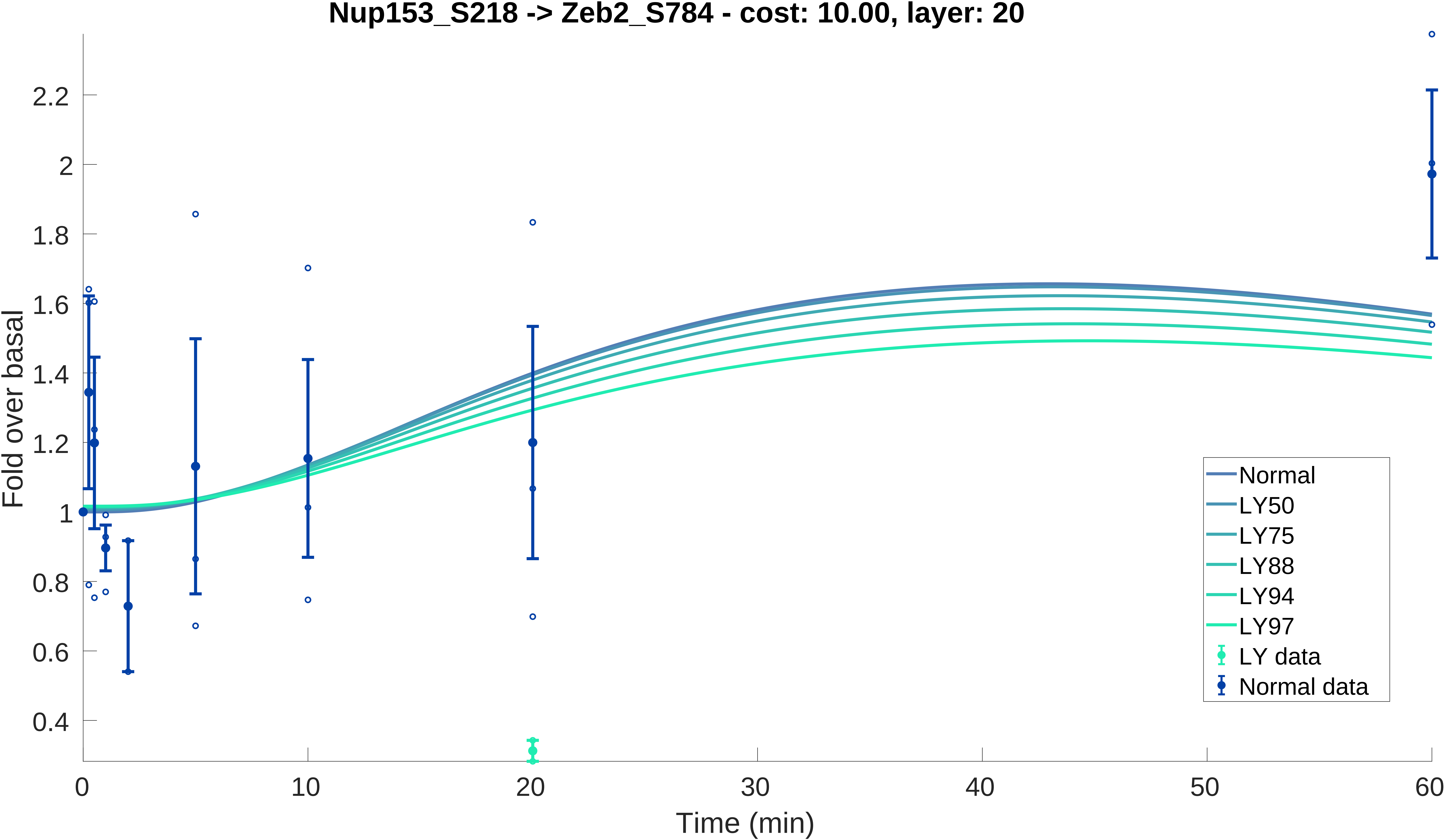
Nup153_S218 -> Zeb2_S784 - cost: 10.00, layer: 20
Fold over basal
Time (min)
0.4
0.6
0.8
1
1.2
1.4
1.6
1.8
2
2.2
0
10
20
30
40
50
60
Normal
LY50
LY75
LY88
LY94
LY97
LY data
Normal data

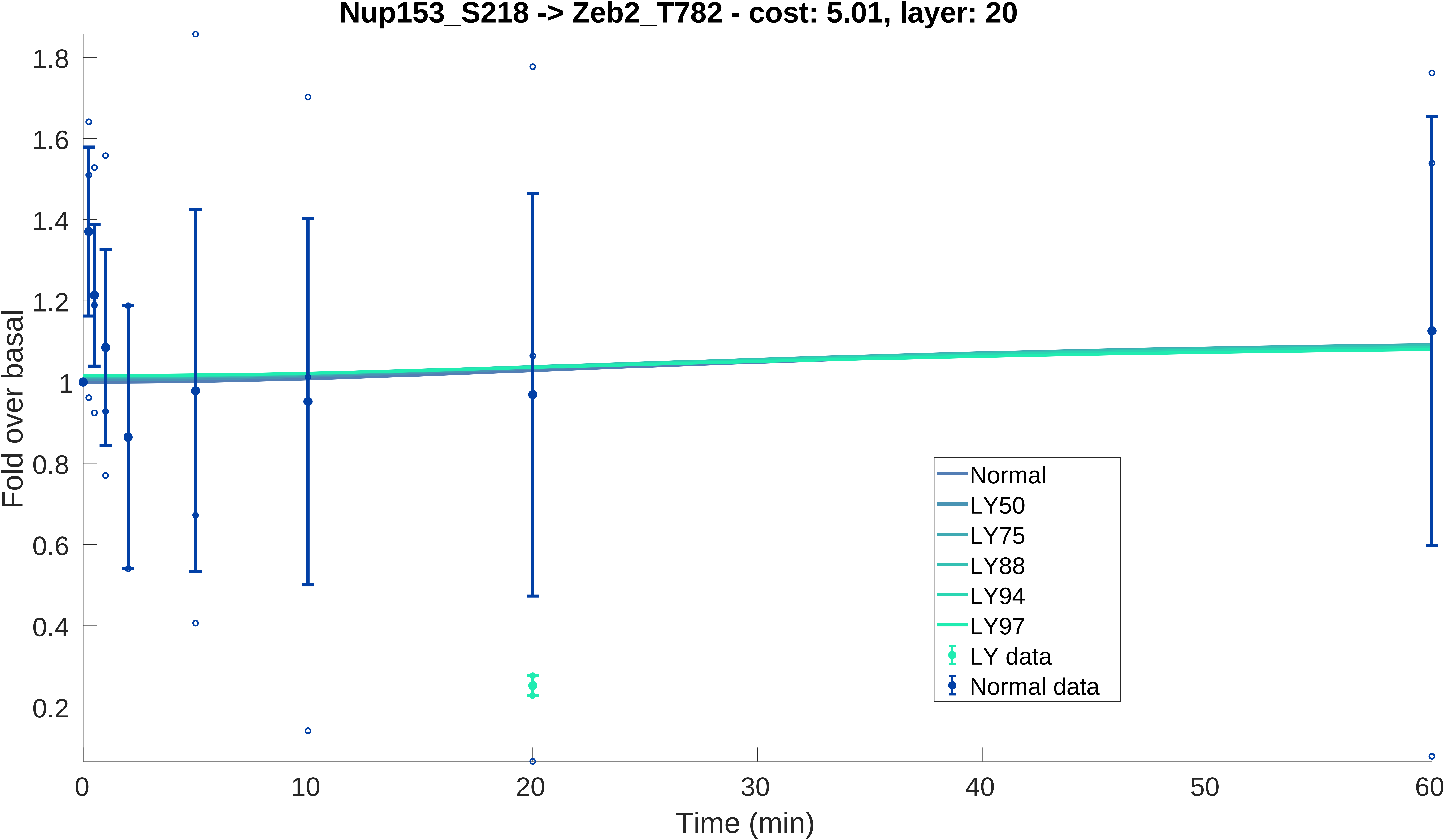

Nup153_S218 -> Zeb2_T782 - cost: 5.01, layer: 20
Fold over basal
1.8
1.6
1.4
1.2
1
0.8
0.6
0.4
0.2
0
10
20
30
40
50
60
Time (min)
Normal
LY50
LY75
LY88
LY94
LY97
LY data
Normal data

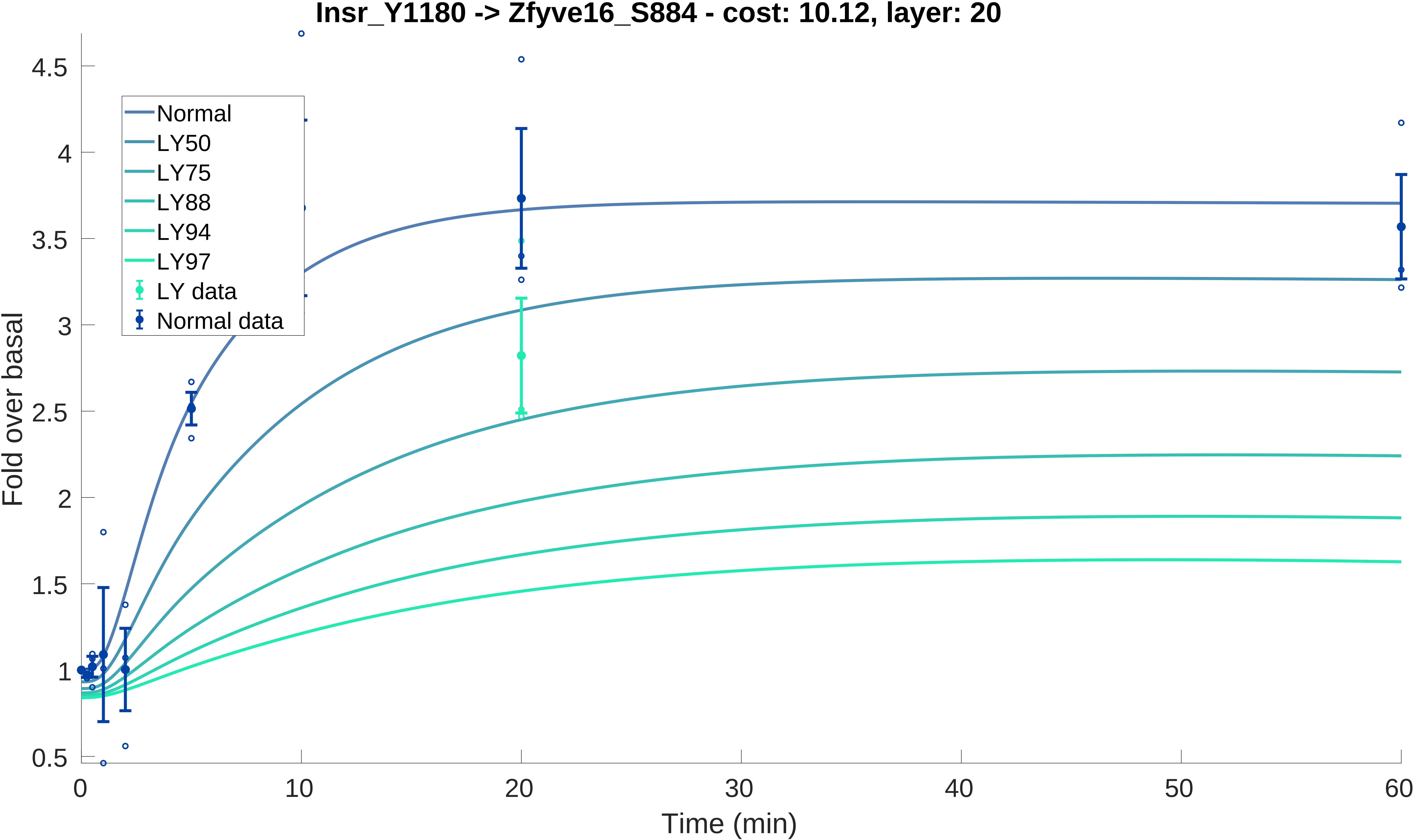

Insr_Y1180 -> Zfyve16_S884 - cost: 10.12, layer: 20
Normal
LY50
LY75
LY88
LY94
LY97
LY data
Normal data
Fold over basal
Time (min)
0.5
1
1.5
2
2.5
3
3.5
4
4.5
0
10
20
30
40
50
60

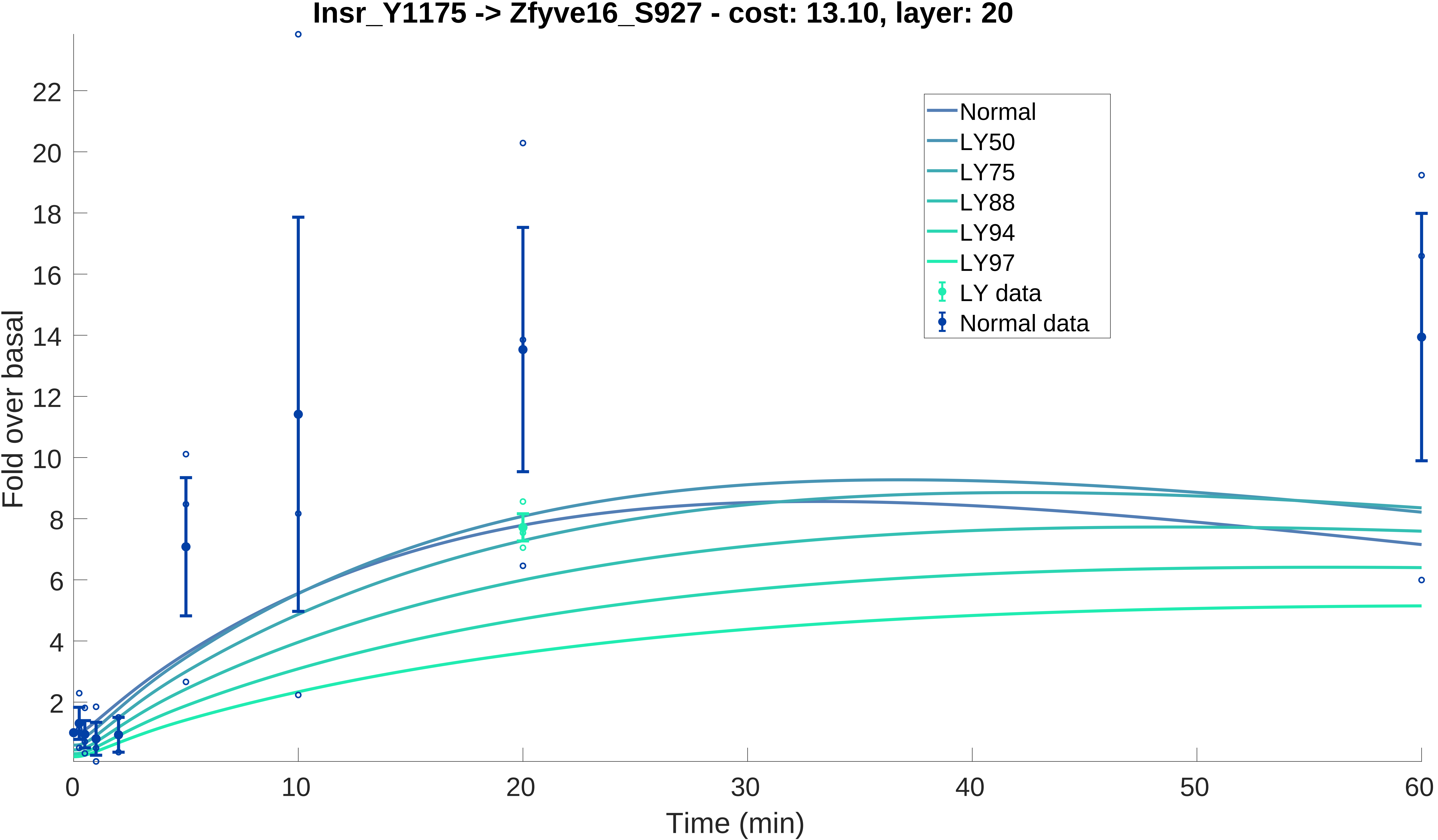

Insr_Y1175 -> Zfyve16_S927 - cost: 13.10, layer: 20
Fold over basal
Time (min)
0
10
20
30
40
50
60
2
4
6
8
10
12
14
16
18
20
22
Normal
LY50
LY75
LY88
LY94
LY97
LY data
Normal data

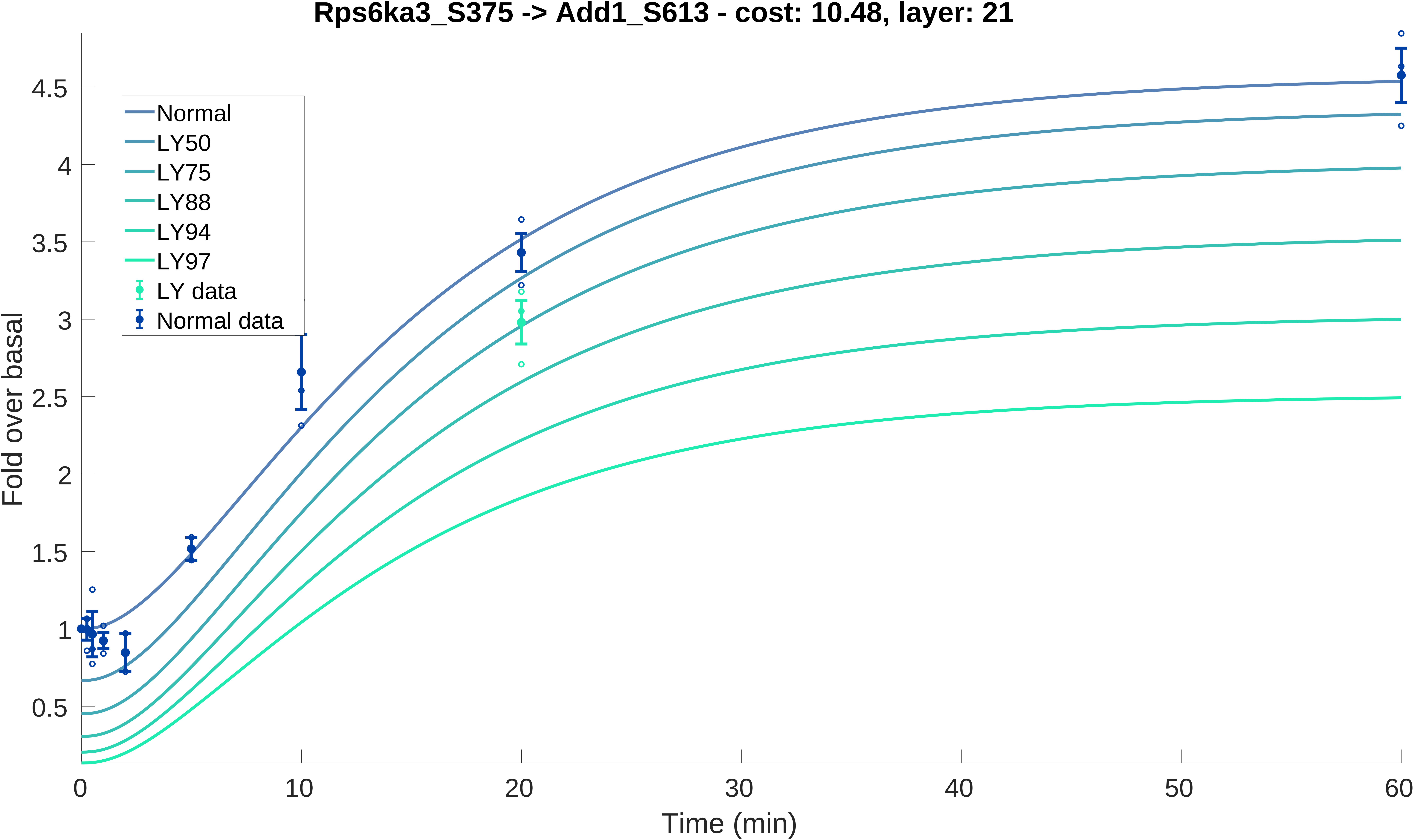

Rps6ka3_S375 -> Add1_S613 - cost: 10.48, layer: 21
Normal
LY50
LY75
LY88
LY94
LY97
LY data
Normal data
Fold over basal
Time (min)
0.5
1
1.5
2
2.5
3
3.5
4
4.5
0
10
20
30
40
50
60

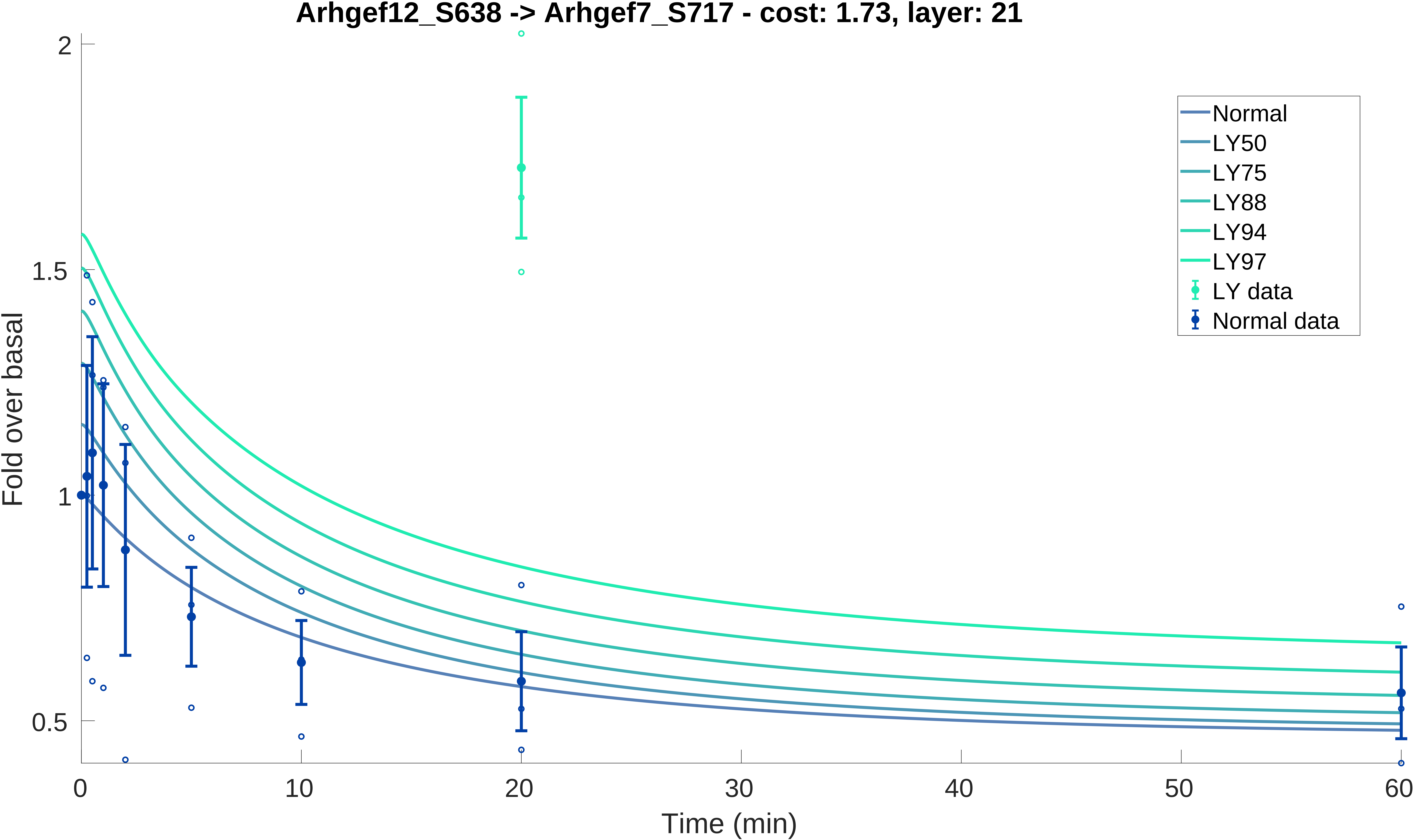

Arhgef12_S638 -> Arhgef7_S717 - cost: 1.73, layer: 21
Fold over basal
Time (min)
Normal
LY50
LY75
LY88
LY94
LY97
LY data
Normal data
0.5
1
1.5
2
0
10
20
30
40
50
60

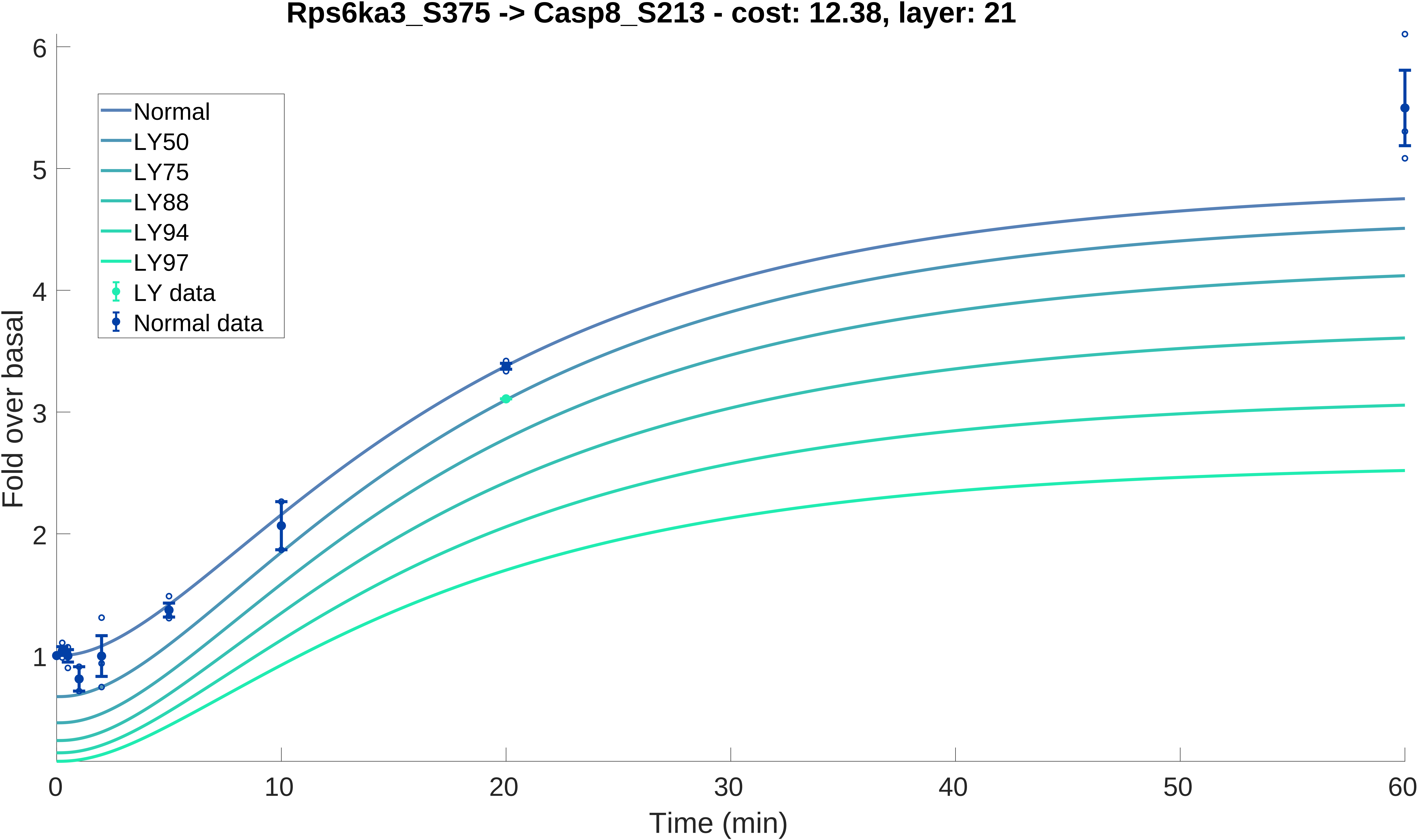

Rps6ka3_S375 -> Casp8_S213 - cost: 12.38, layer: 21
Normal
LY50
LY75
LY88
LY94
LY97
LY data
Normal data
Fold over basal
Time (min)
0
10
20
30
40
50
60
1
2
3
4
5
6

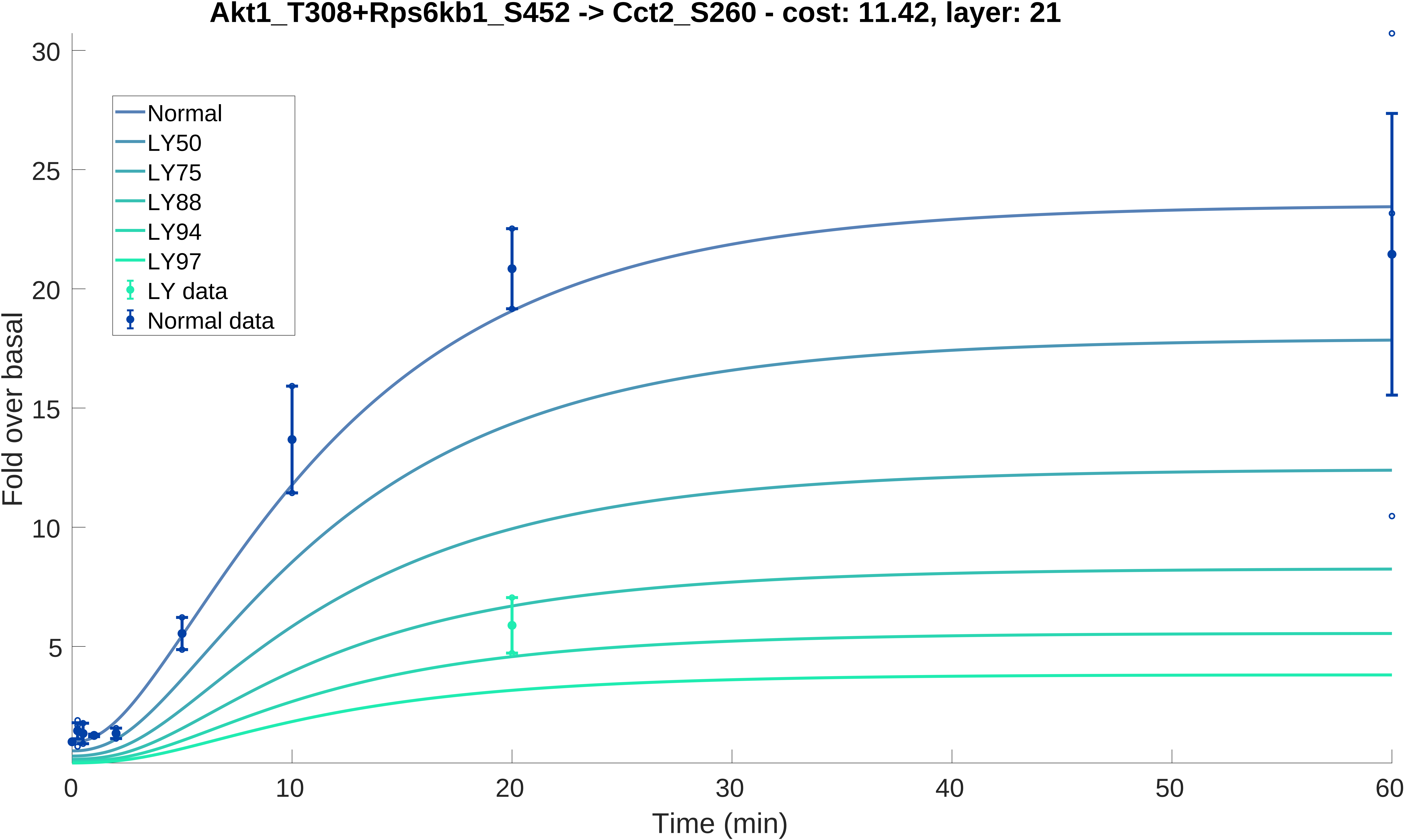

Akt1_T308+Rps6kb1_S452 -> Cct2_S260 - cost: 11.42, layer: 21
Normal
LY50
LY75
LY88
LY94
LY97
LY data
Normal data
Fold over basal
Time (min)
0
10
20
30
40
50
60
5
15
25

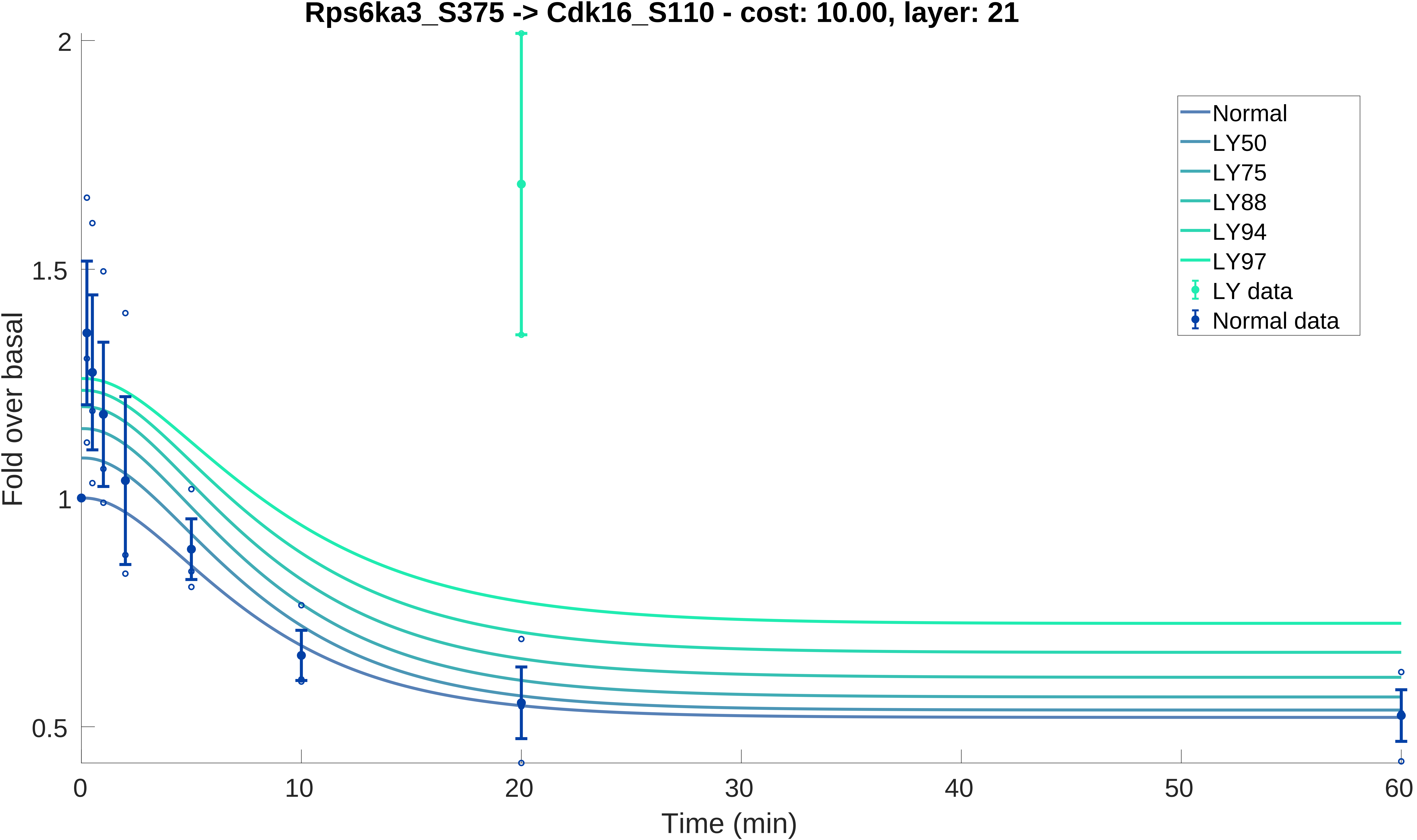
Rps6ka3_S375 -> Cdk16_S110 - cost: 10.00, layer: 21
Fold over basal
2
1.5
1
0.5
0
10
20
30
40
50
60
Time (min)
Normal
LY50
LY75
LY88
LY94
LY97
LY data
Normal data

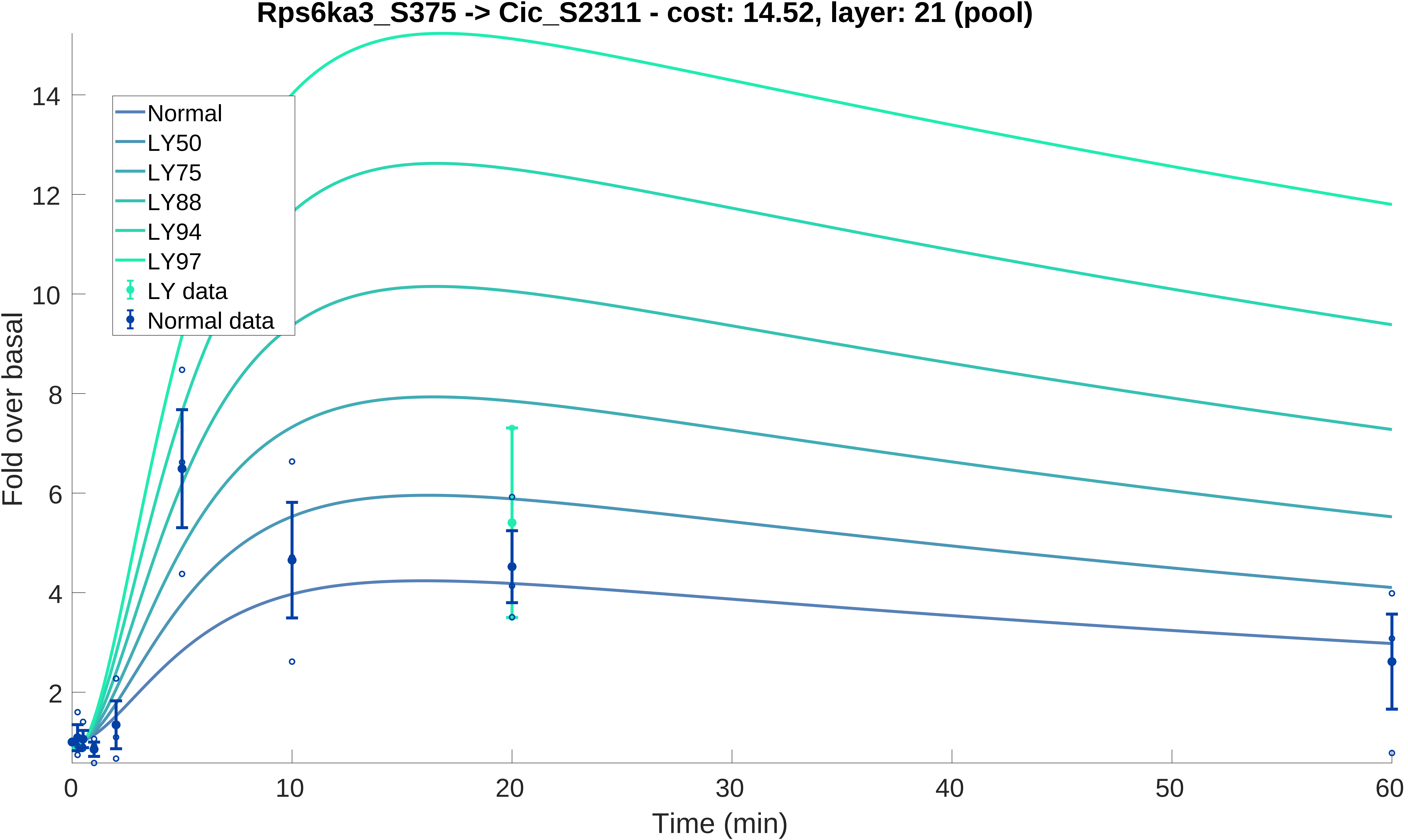

Rps6ka3_S375 -> Cic_S2311 - cost: 14.52, layer: 21 (pool)
Normal
LY50
LY75
LY88
LY94
LY97
LY data
Normal data
Fold over basal
Time (min)
0
10
20
30
40
50
60
2
4
6
8
10
12
14

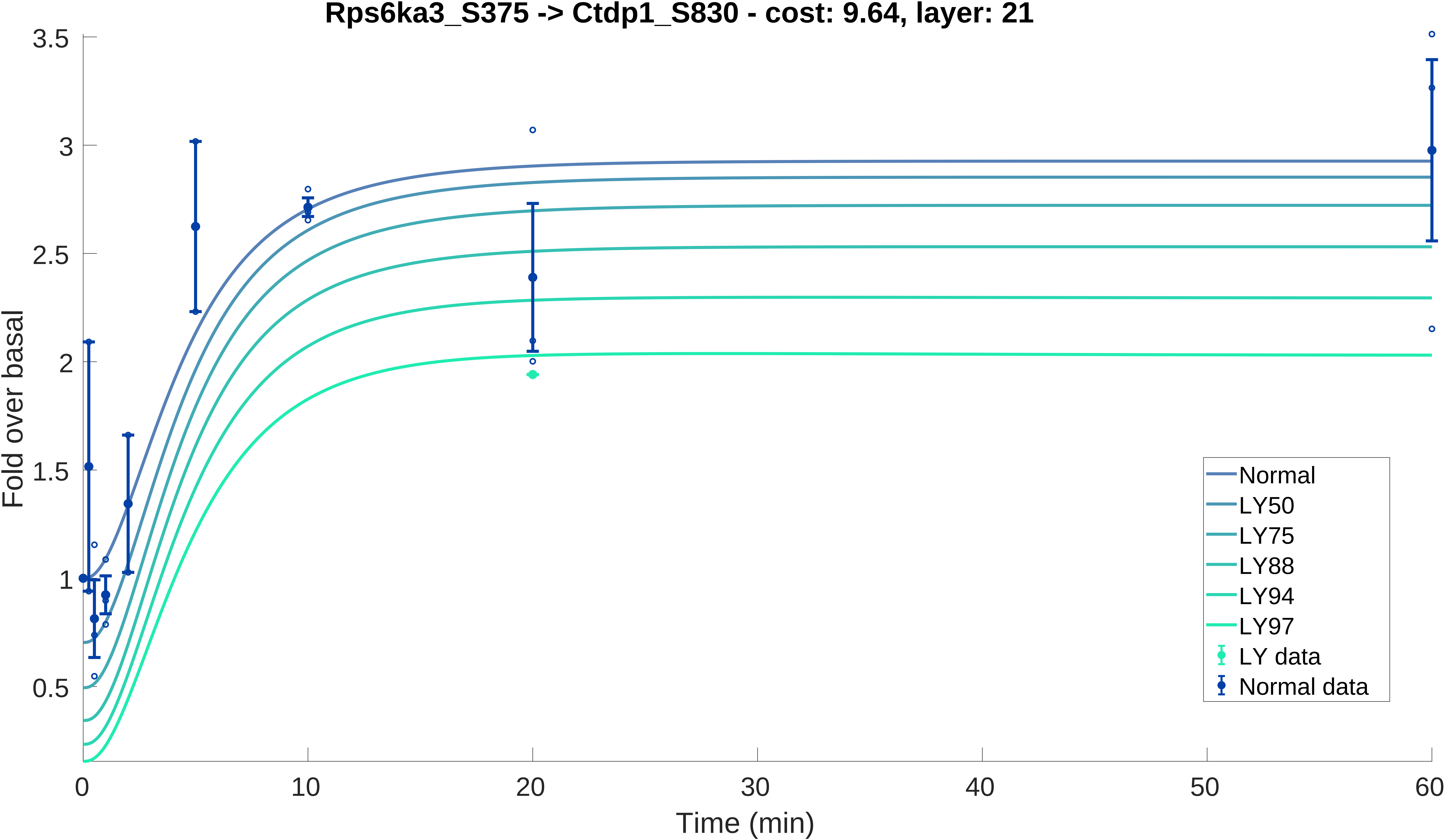

Rps6ka3_S375 -> Ctdp1_S830 - cost: 9.64, layer: 21
Fold over basal
Time (min)
0
10
20
30
40
50
60
0.5
1
1.5
2
2.5
3
3.5
Normal
LY50
LY75
LY88
LY94
LY97
LY data
Normal data

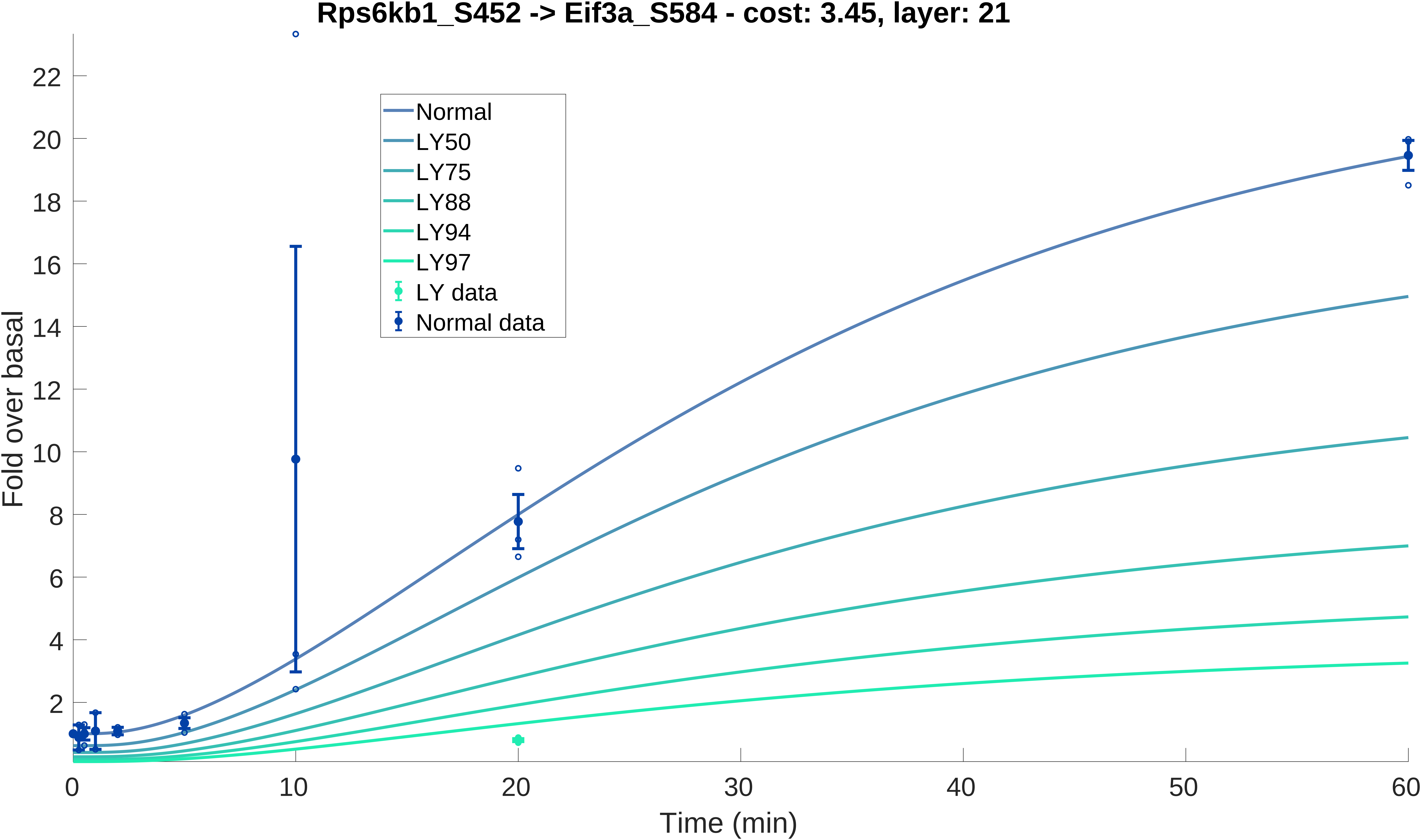

Rps6kb1_S452 -> Eif3a_S584 - cost: 3.45, layer: 21
Normal
LY50
LY75
LY88
LY94
LY97
LY data
Normal data
Fold over basal
Time (min)
0
10
20
30
40
50
60
2
4
6
8
10
12
14
16
18
20
22

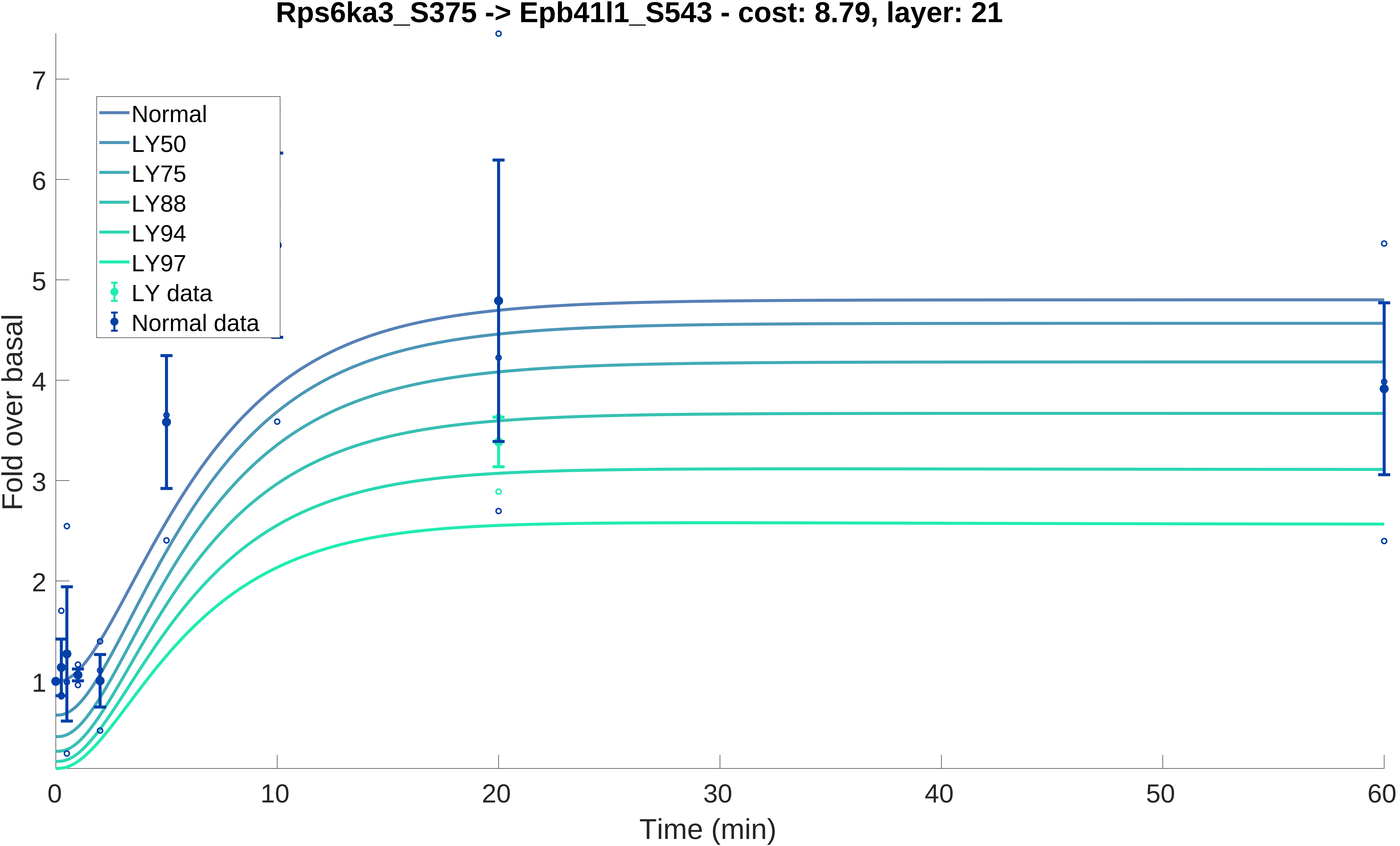

Rps6ka3_S375 -> Epb41l1_S543 - cost: 8.79, layer: 21
Normal
LY50
LY75
LY88
LY94
LY97
LY data
Normal data
Fold over basal
Time (min)
0
10
20
30
40
50
60
1
2
3
4
5
6
7

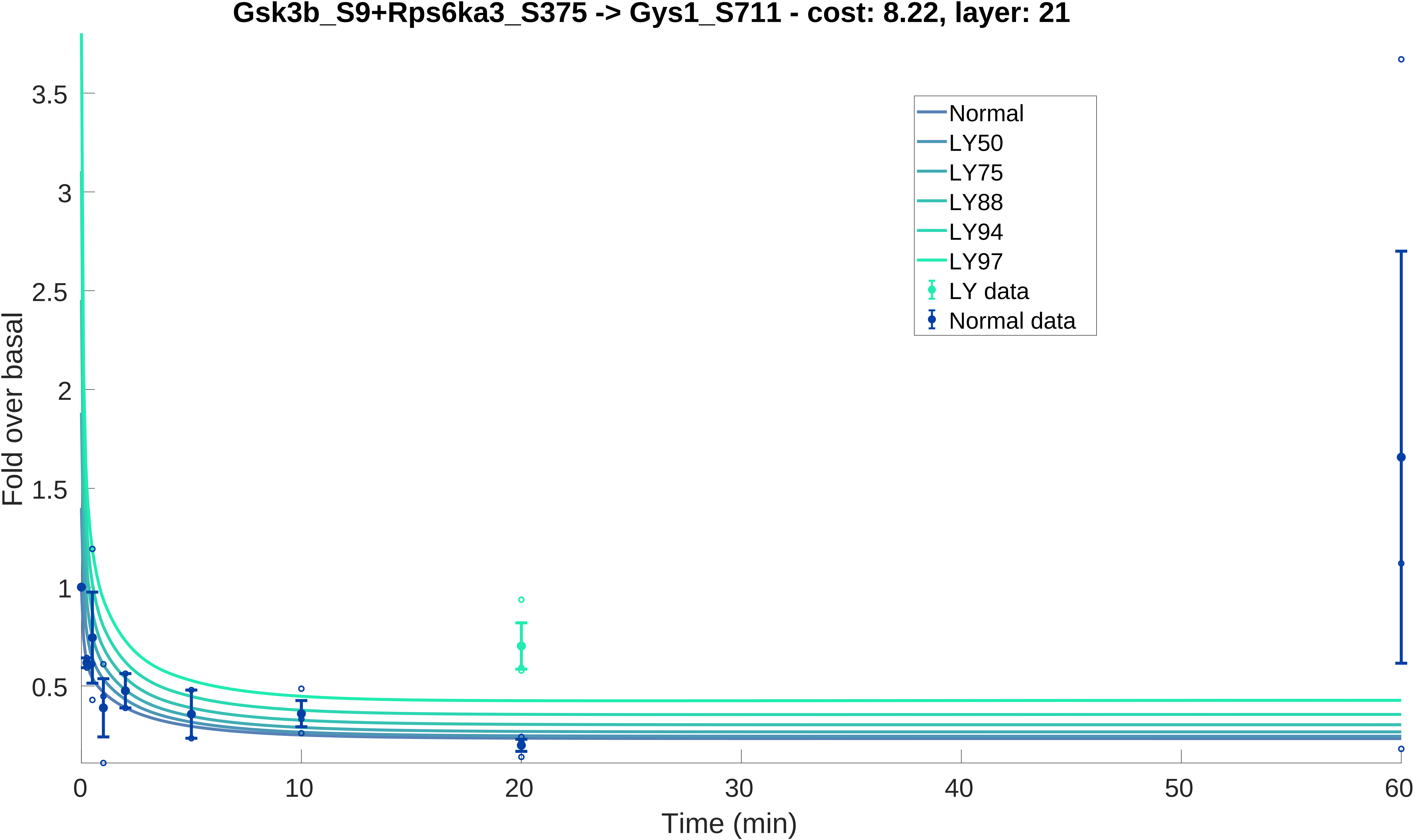

Gsk3b_S9+Rps6ka3_S375 -> Gys1_S711 - cost: 8.22, layer: 21
Fold over basal
Time (min)
Normal
LY50
LY75
LY88
LY94
LY97
LY data
Normal data
0.5
1
1.5
2
2.5
3
3.5
0
10
20
30
40
50
60

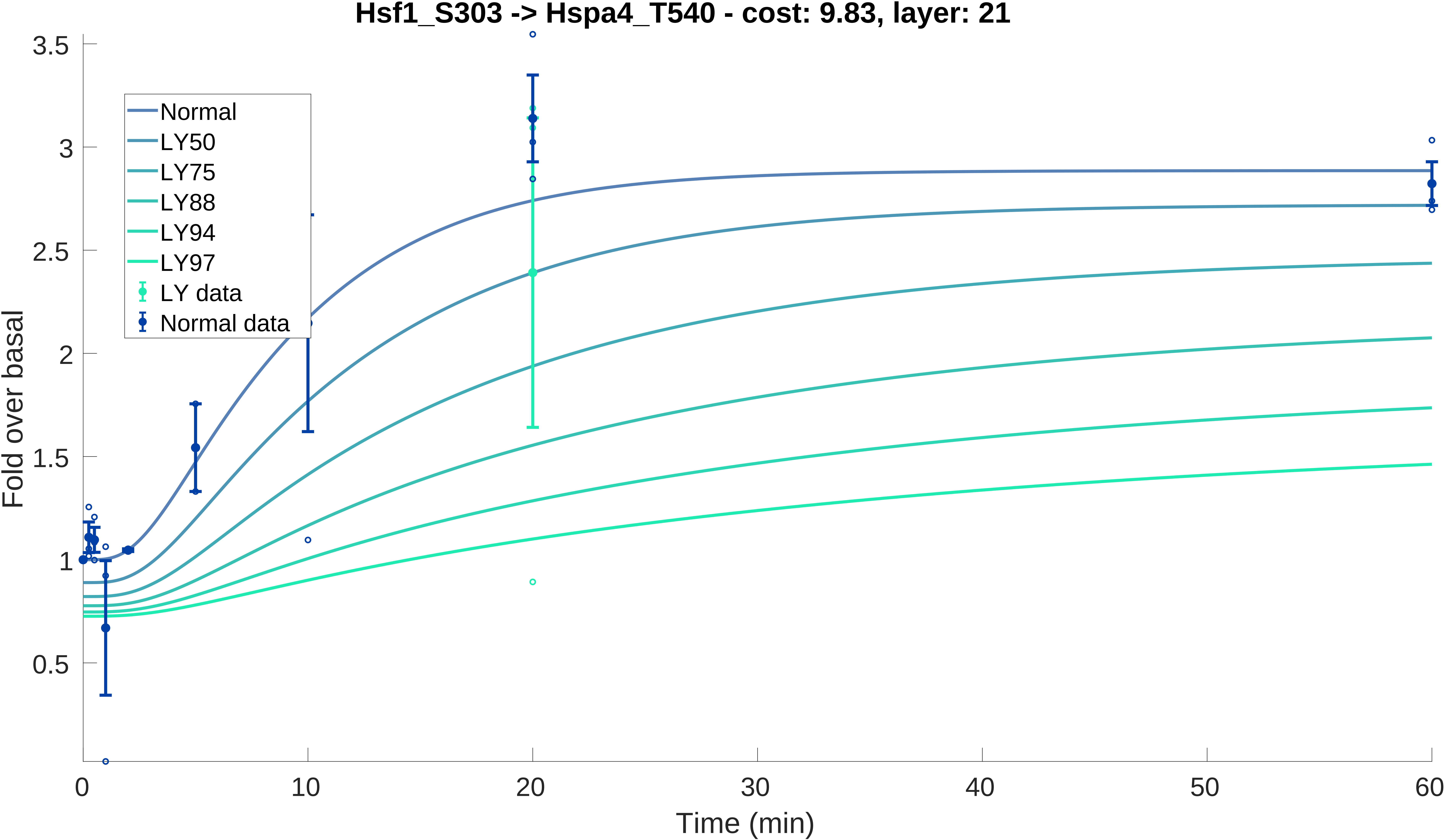
Hsf1_S303 -> Hspa4_T540 - cost: 9.83, layer: 21
Normal
LY50
LY75
LY88
LY94
LY97
LY data
Normal data
Fold over basal
Time (min)
0.5
1
1.5
2
2.5
3
3.5
0
10
20
30
40
50
60

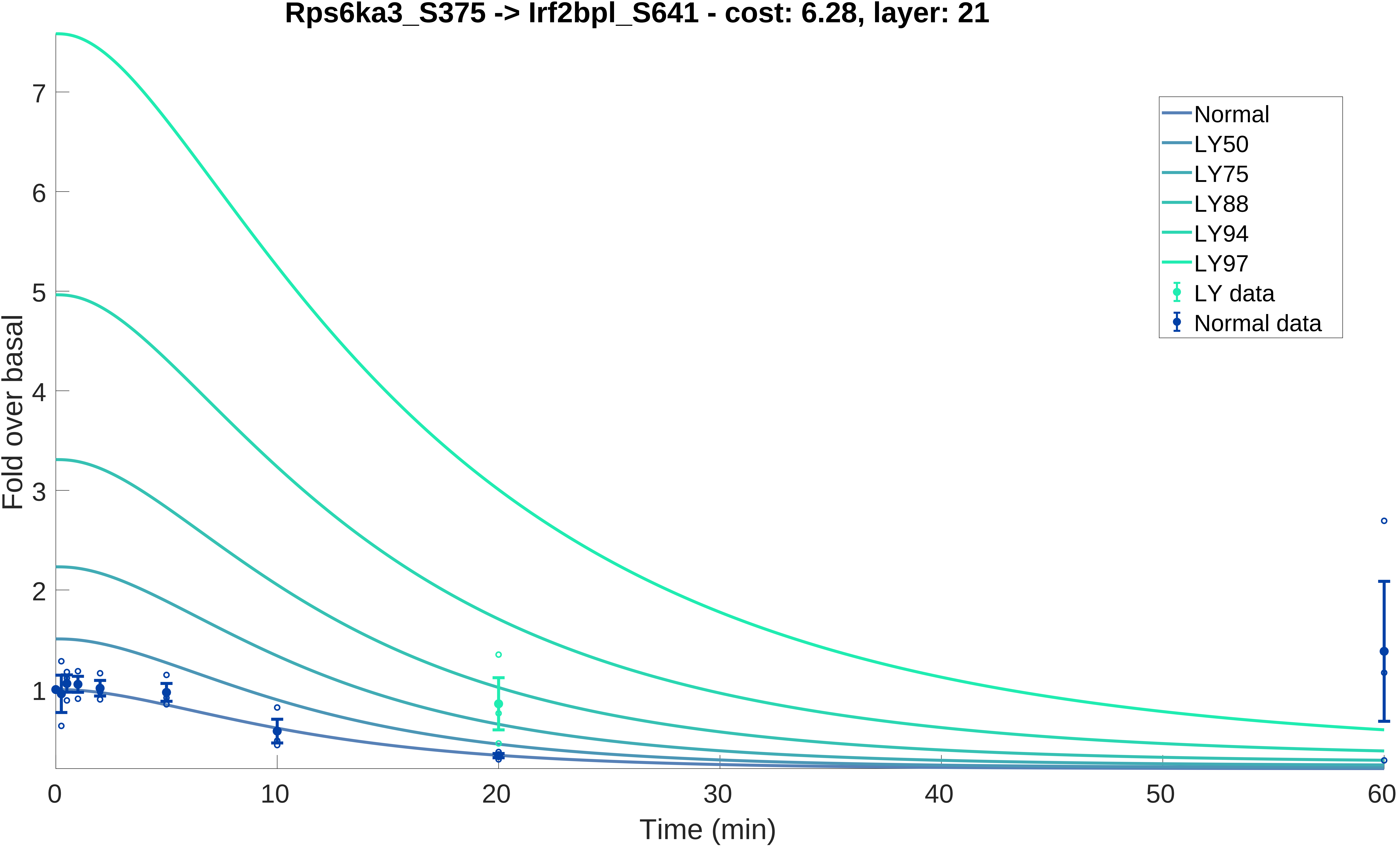

Rps6ka3_S375 -> Irf2bpl_S641 - cost: 6.28, layer: 21
Fold over basal
Time (min)
0
10
20
30
40
50
60
1
2
3
4
5
6
7
Normal
LY50
LY75
LY88
LY94
LY97
LY data
Normal data

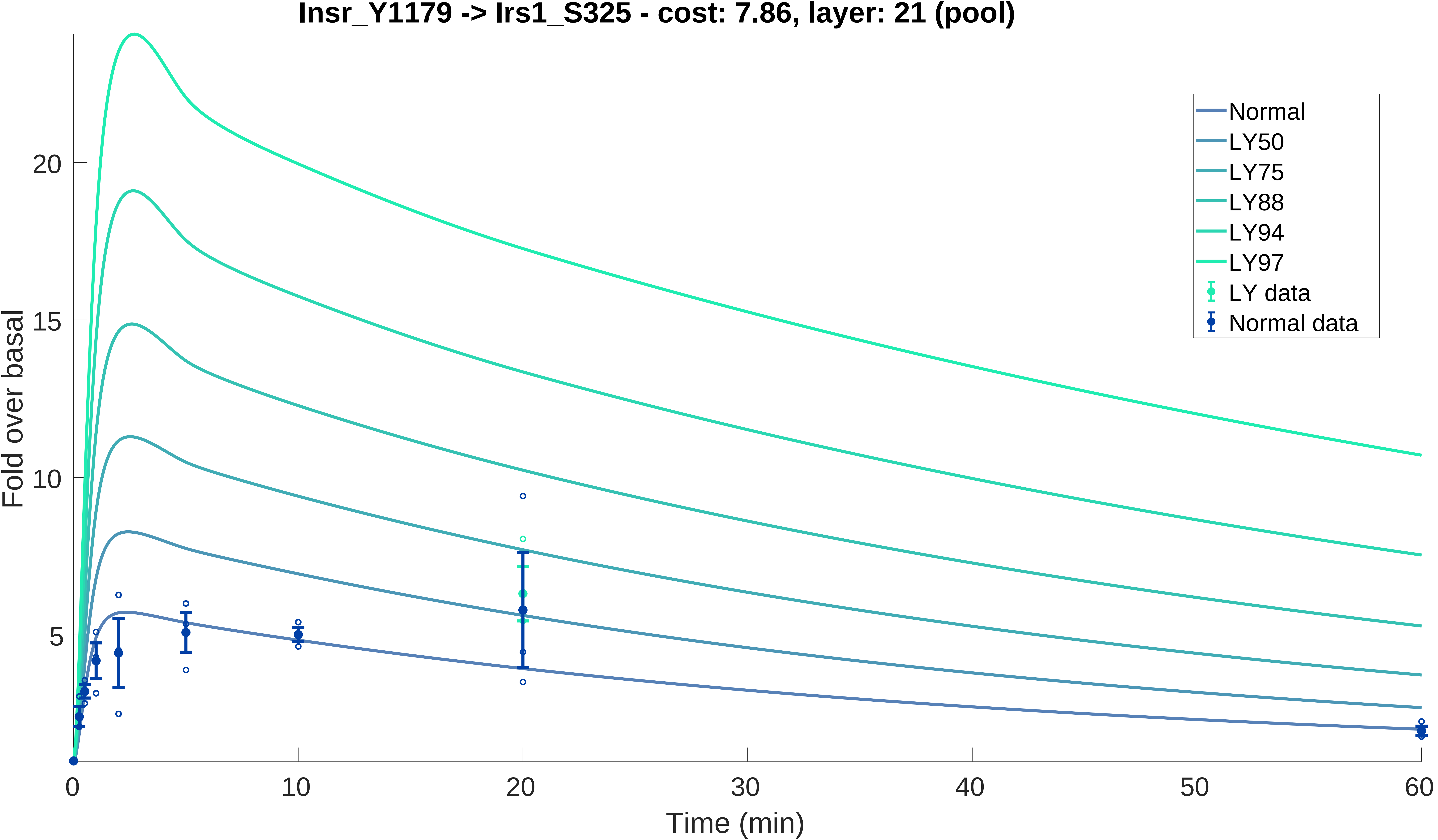
Insr_Y1179 -> Irs1_S325 - cost: 7.86, layer: 21 (pool)
Fold over basal
Time (min)
0
10
20
30
40
50
60
5
10
15
20
Normal
LY50
LY75
LY88
LY94
LY97
LY data
Normal data

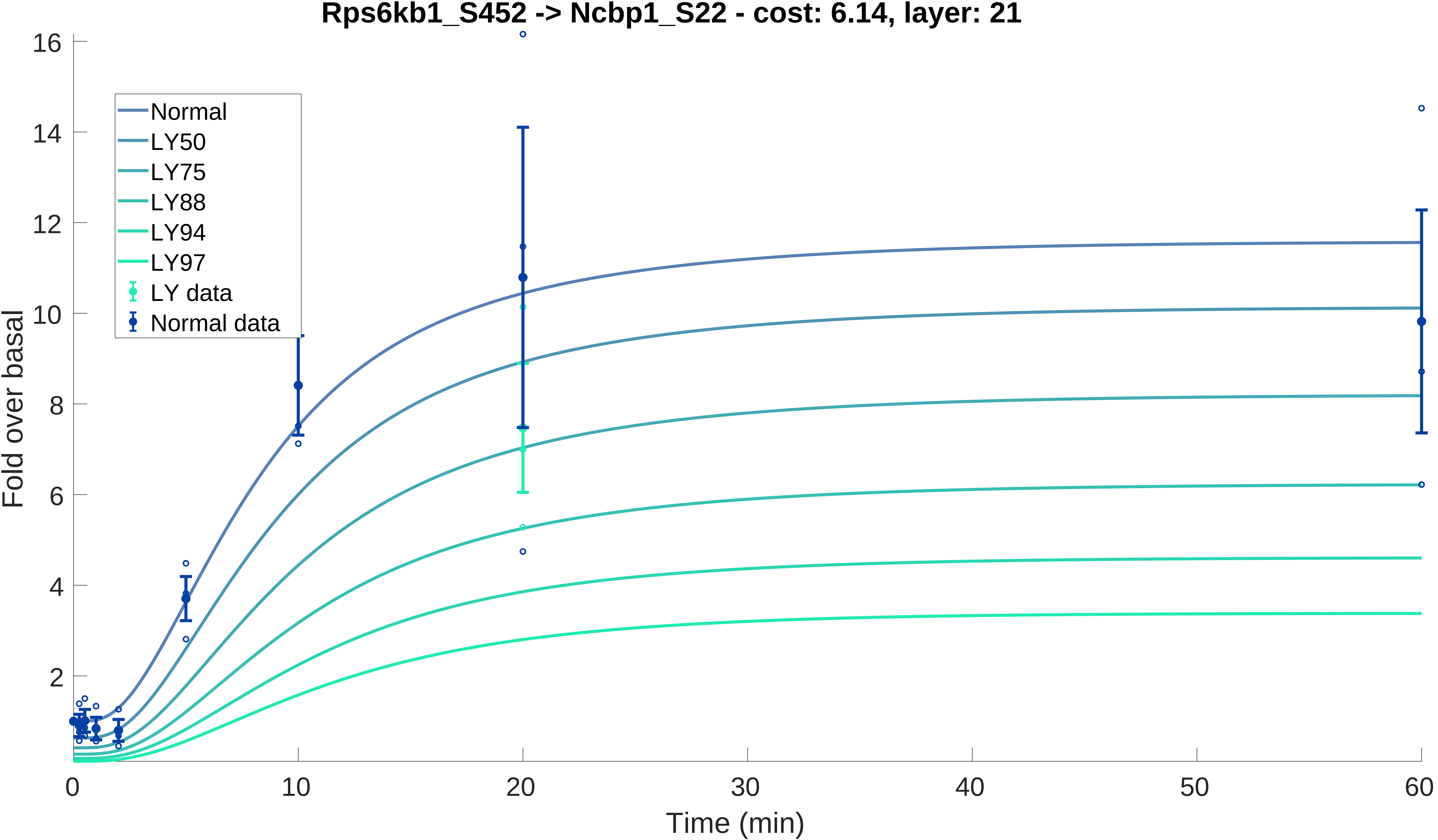

Rps6kb1_S452 -> Ncbp1_S22 - cost: 6.14, layer: 21
Normal
LY50
LY75
LY88
LY94
LY97
LY data
Normal data
Fold over basal
Time (min)
0
10
20
30
40
50
60
2
4
6
8
10
12
14
16

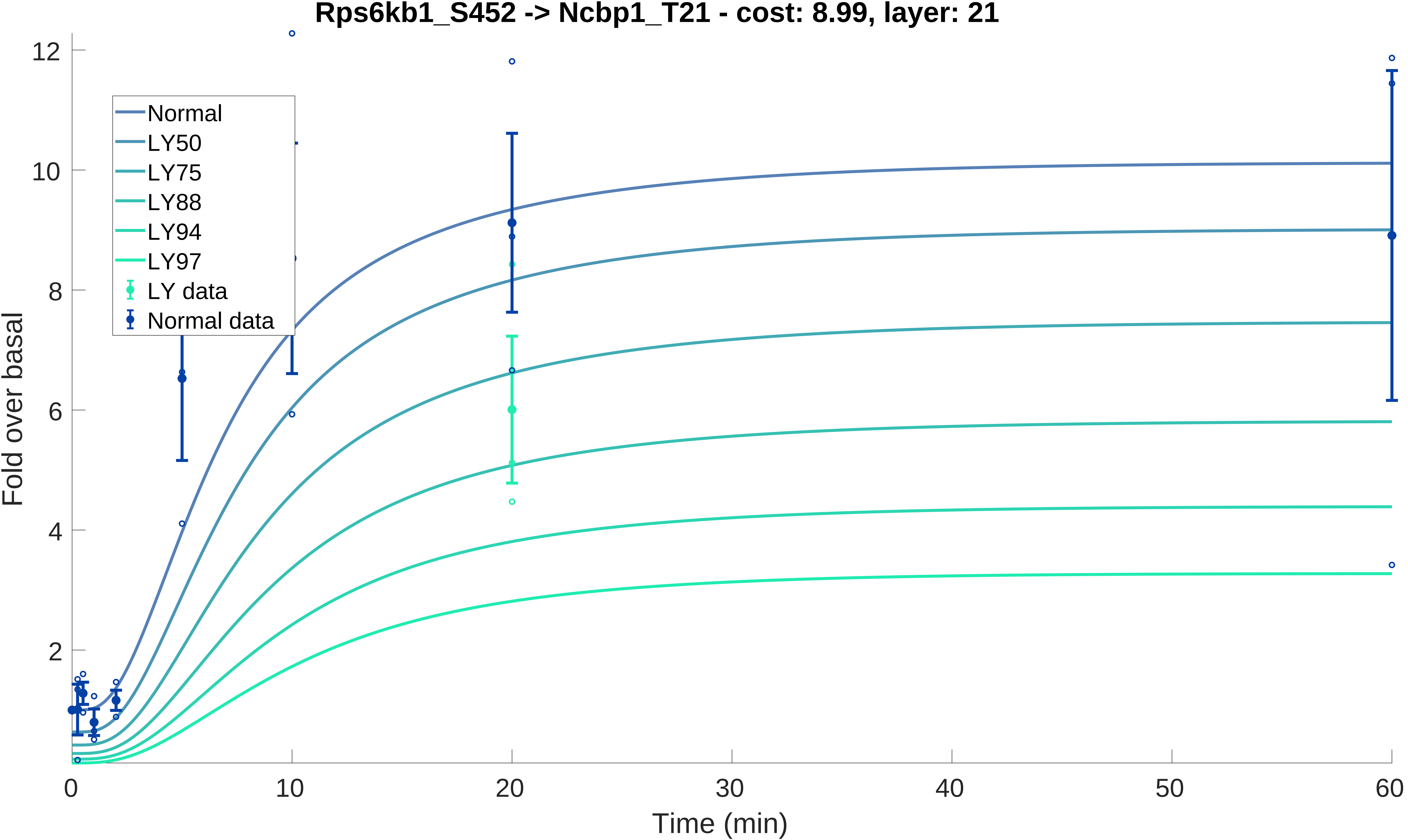

Rps6kb1_S452 -> Ncbp1_T21 - cost: 8.99, layer: 21
Fold over basal
Time (min)
Normal
LY50
LY75
LY88
LY94
LY97
LY data
Normal data
0
10
20
30
40
50
60
2
4
6
8
10
12

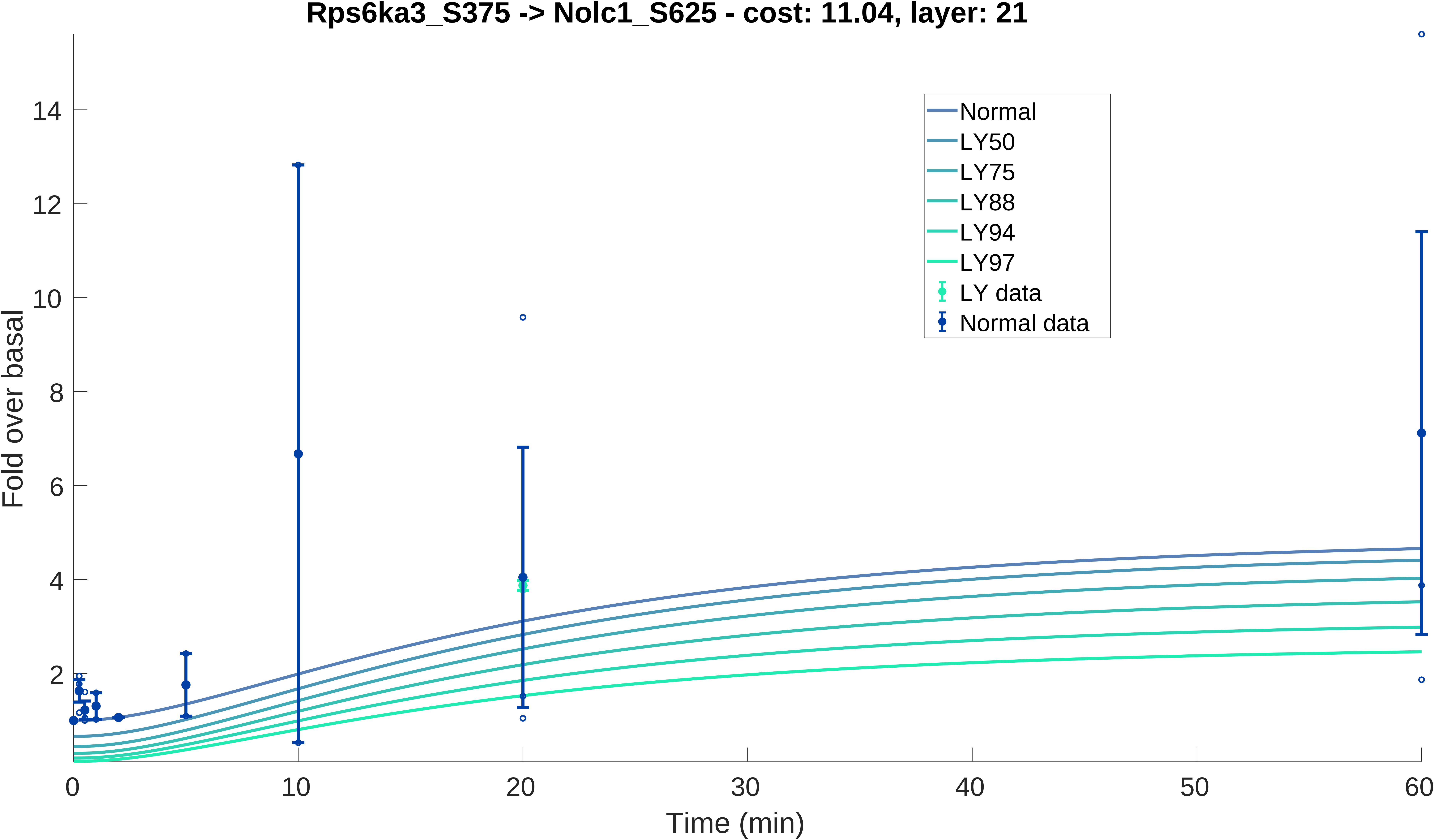
Rps6ka3_S375 -> Nolc1_S625 - cost: 11.04, layer: 21
Fold over basal
Time (min)
0
10
20
30
40
50
60
2
4
6
8
10
12
14
Normal
LY50
LY75
LY88
LY94
LY97
LY data
Normal data

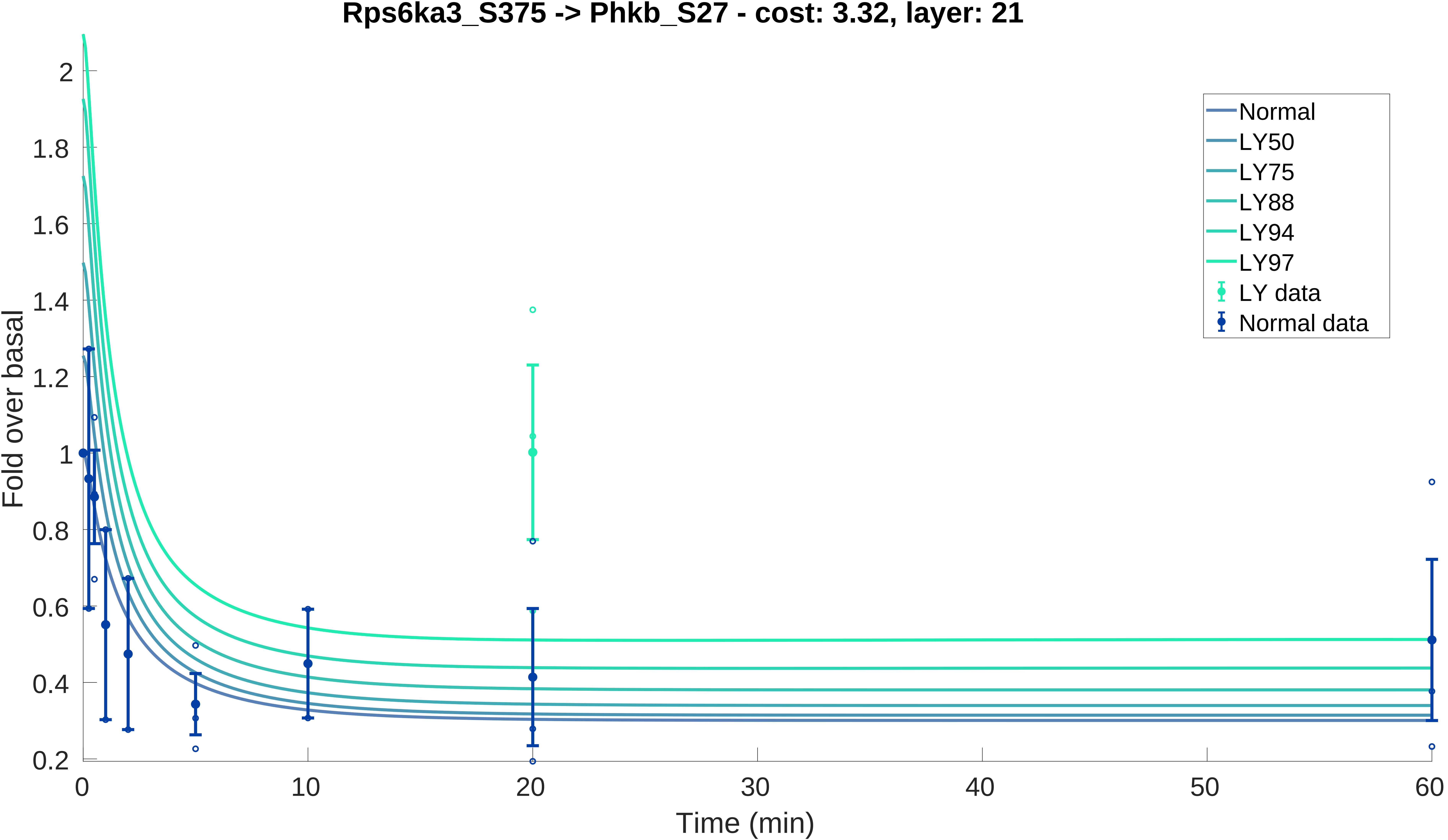

Rps6ka3_S375 -> Phkb_S27 - cost: 3.32, layer: 21
Fold over basal
Time (min)
Normal
LY50
LY75
LY88
LY94
LY97
LY data
Normal data
0.2
0.4
0.6
0.8
1
1.2
1.4
1.6
1.8
2
0
10
20
30
40
50
60

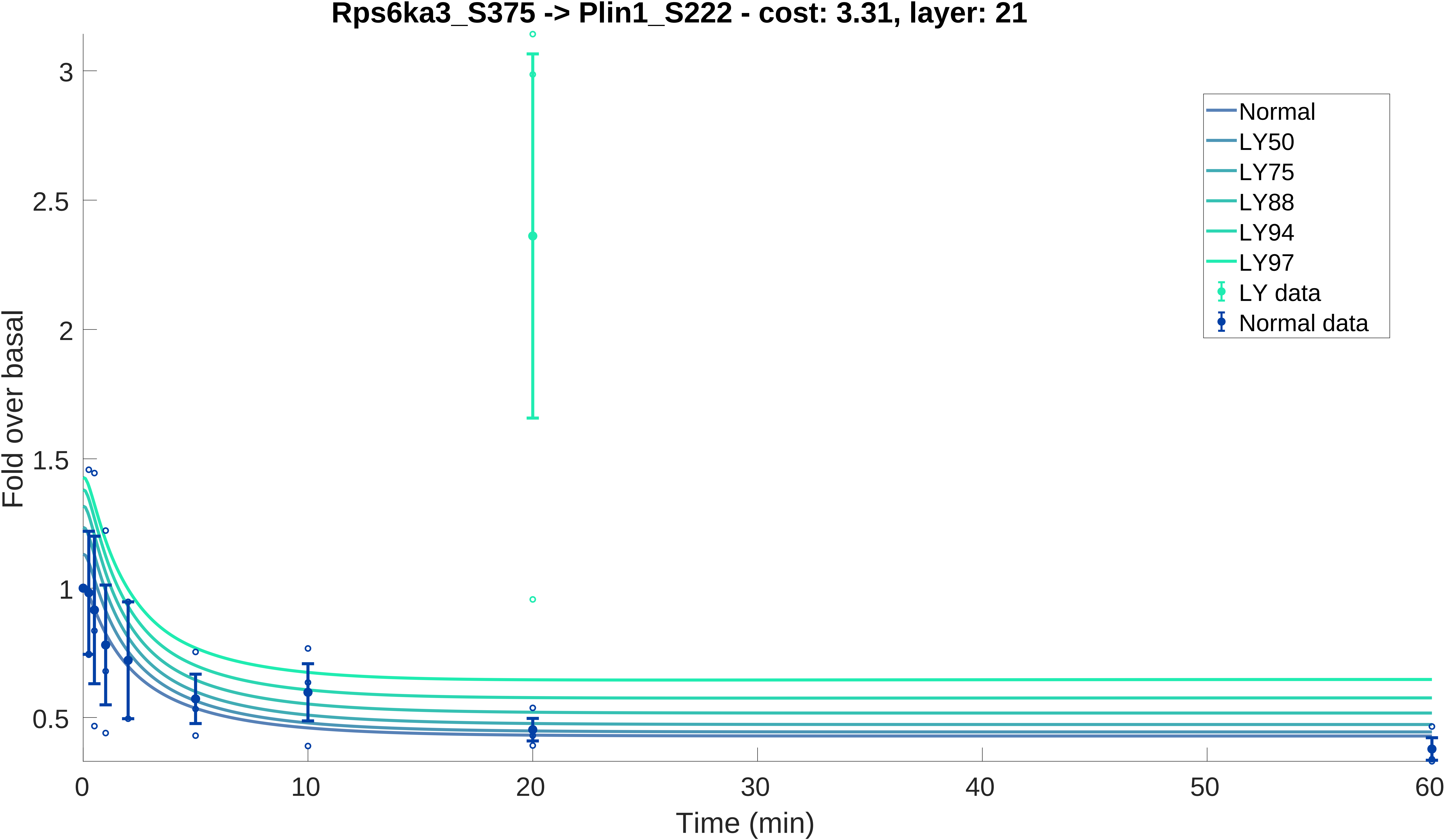

Rps6ka3_S375 -> Plin1_S222 - cost: 3.31, layer: 21
Fold over basal
Time (min)
0
10
20
30
40
50
60
0.5
1
1.5
2
2.5
3
Normal
LY50
LY75
LY88
LY94
LY97
LY data
Normal data

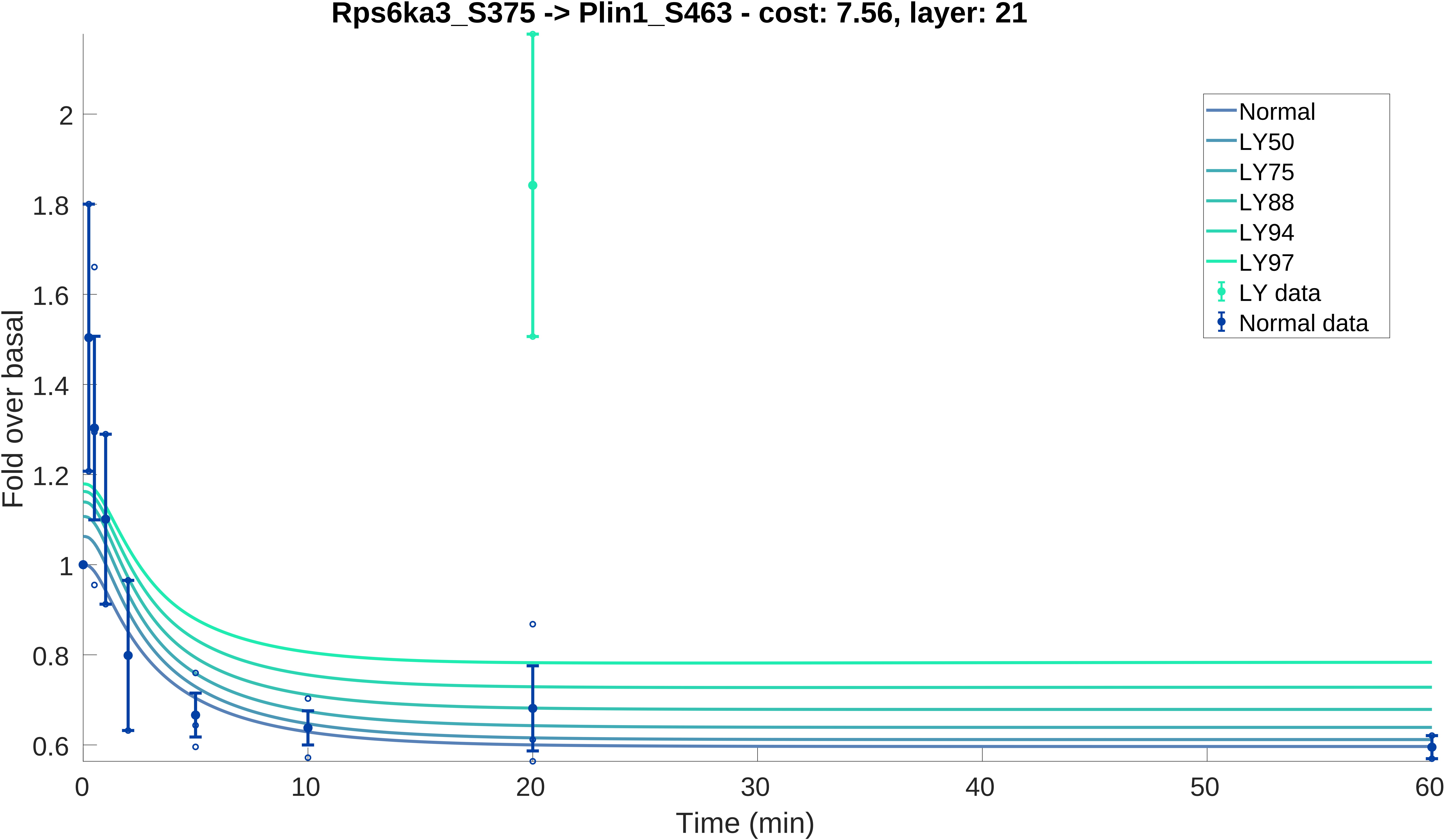

Rps6ka3_S375 -> Plin1_S463 - cost: 7.56, layer: 21
Fold over basal
Time (min)
0.6
0.8
1
1.2
1.4
1.6
1.8
2
0
10
20
30
40
50
60
Normal
LY50
LY75
LY88
LY94
LY97
LY data
Normal data

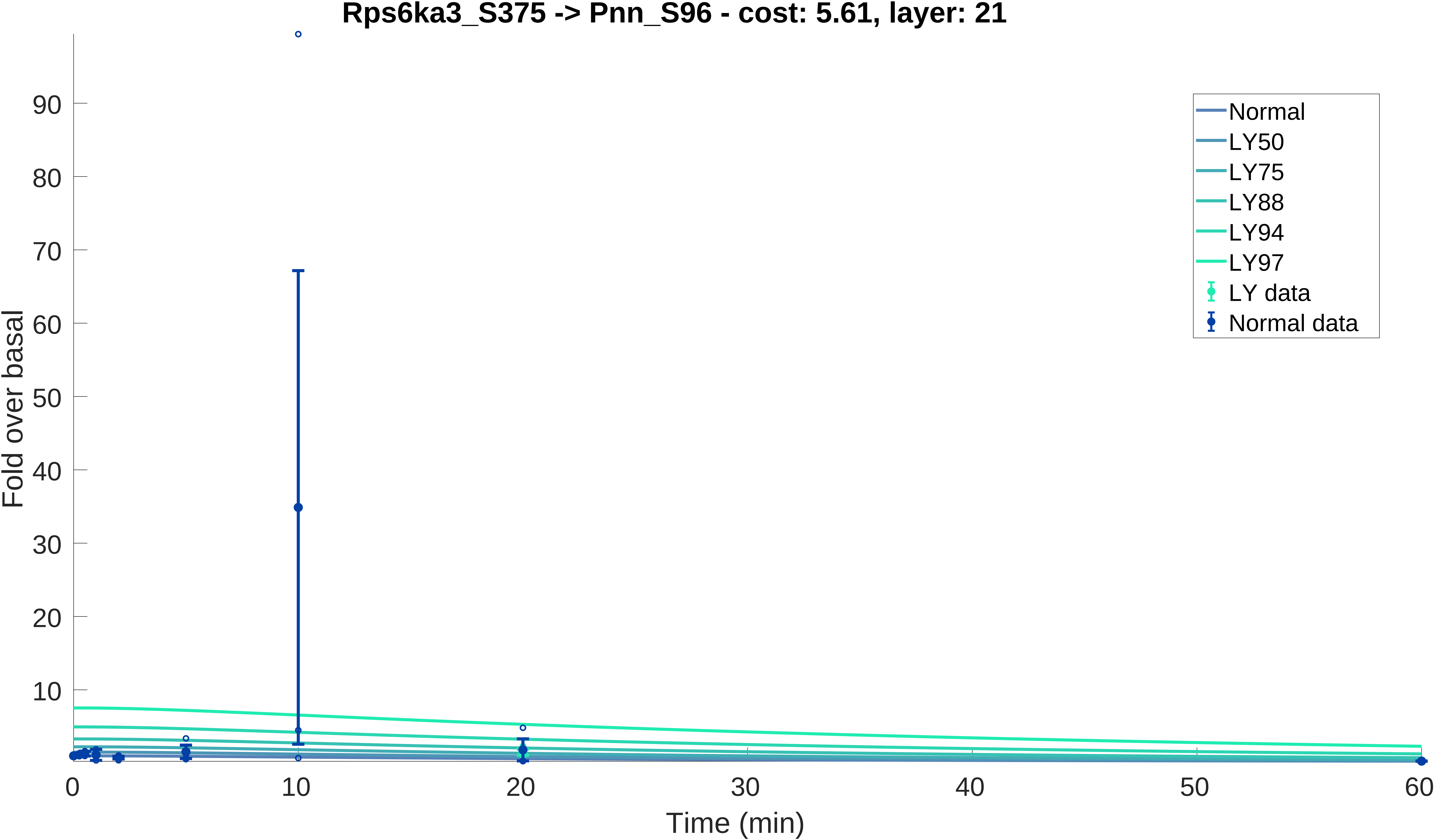
Rps6ka3_S375 -> Pnn_S96 - cost: 5.61, layer: 21
Fold over basal
Time (min)
0
10
20
30
40
50
60
70
80
90
Normal
LY50
LY75
LY88
LY94
LY97
LY data
Normal data

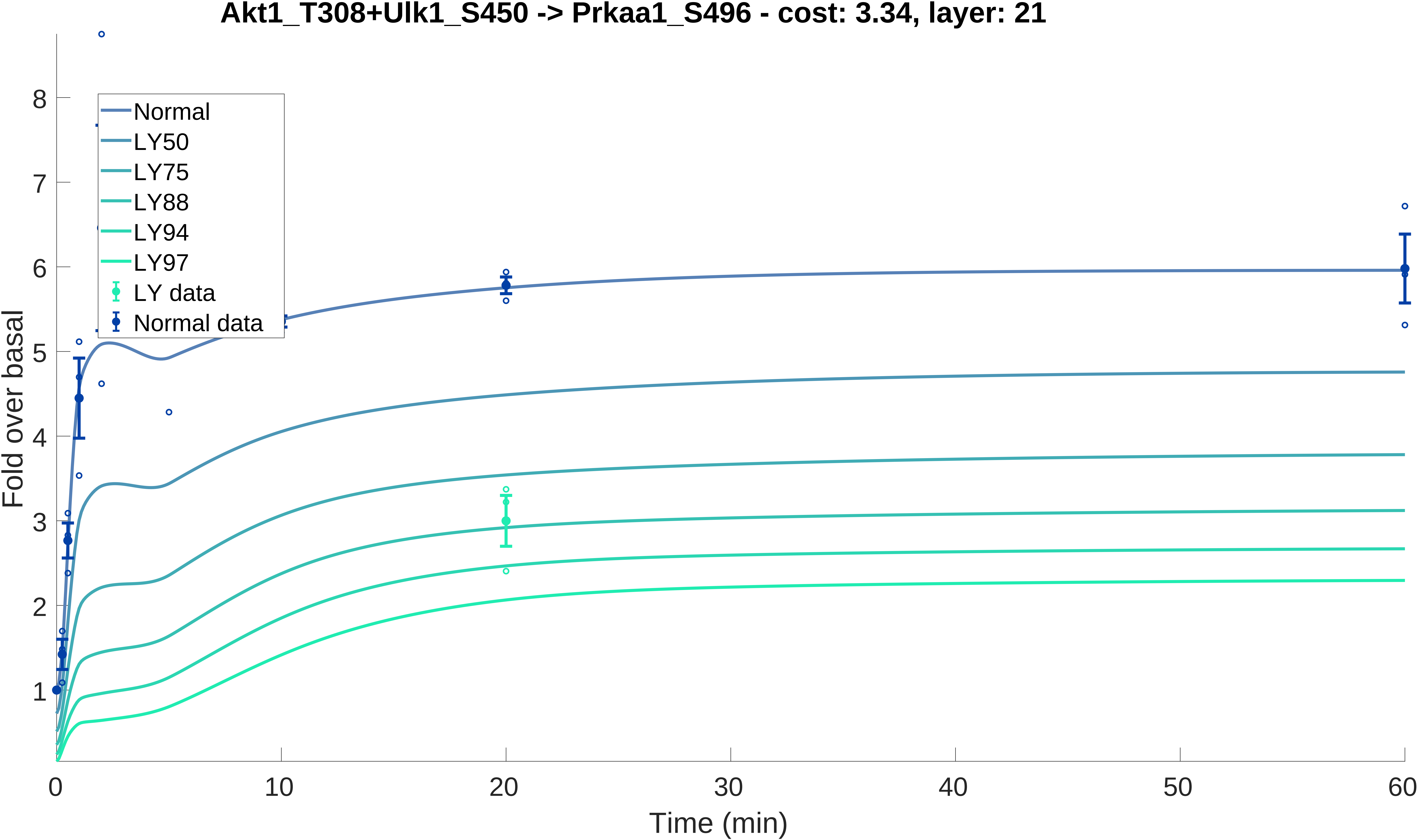

Akt1_T308+Ulk1_S450 -> Prkaa1_S496 - cost: 3.34, layer: 21
Normal
LY50
LY75
LY88
LY94
LY97
LY data
Normal data
Fold over basal
Time (min)
0
10
20
30
40
50
60
1
2
3
4
5
6
7
8

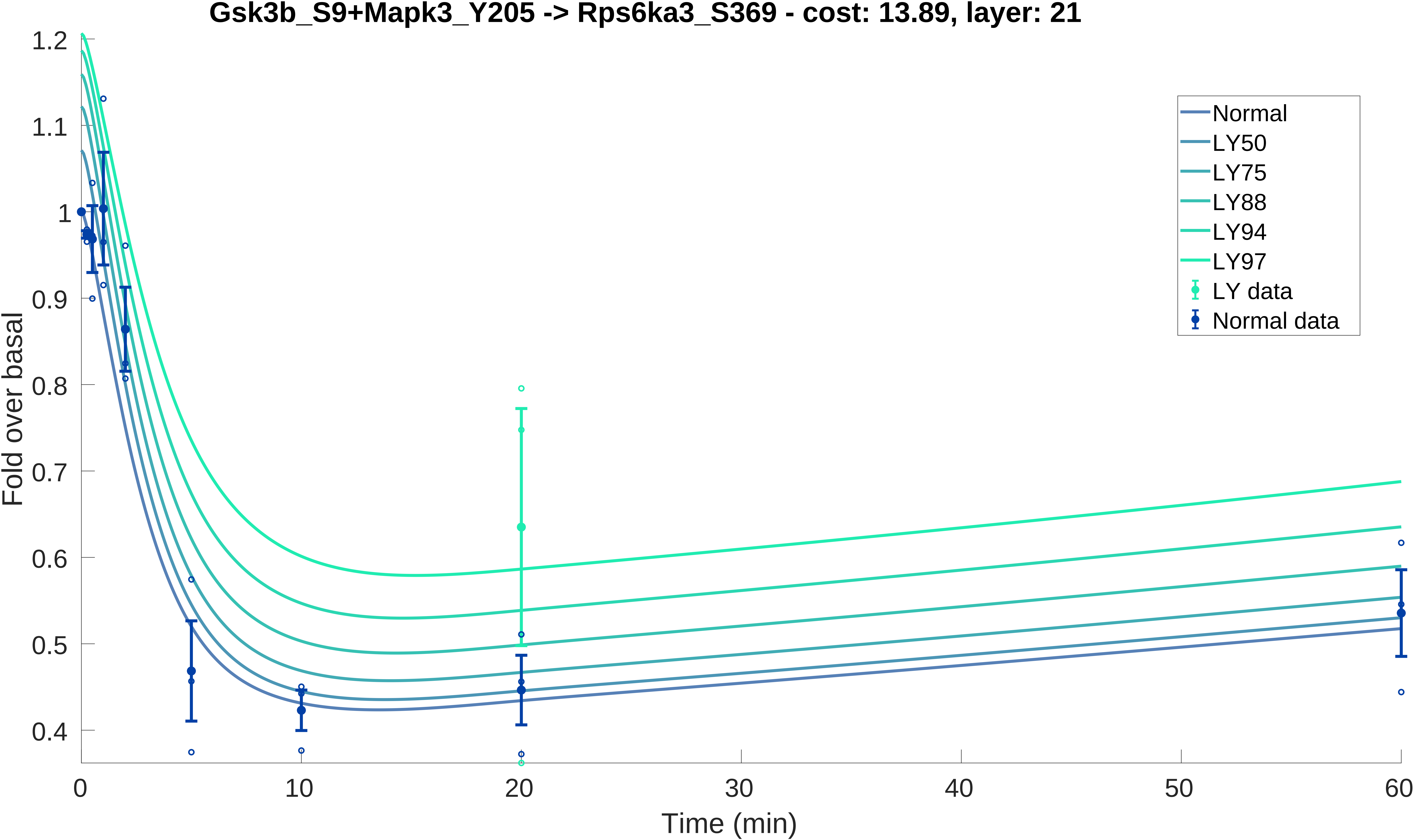

Gsk3b_S9+Mapk3_Y205 -> Rps6ka3_S369 - cost: 13.89, layer: 21
Fold over basal
Time (min)
1.2
1.1
1
0.9
0.8
0.7
0.6
0.5
0.4
0
10
20
30
40
50
60
Normal
LY50
LY75
LY88
LY94
LY97
LY data
Normal data

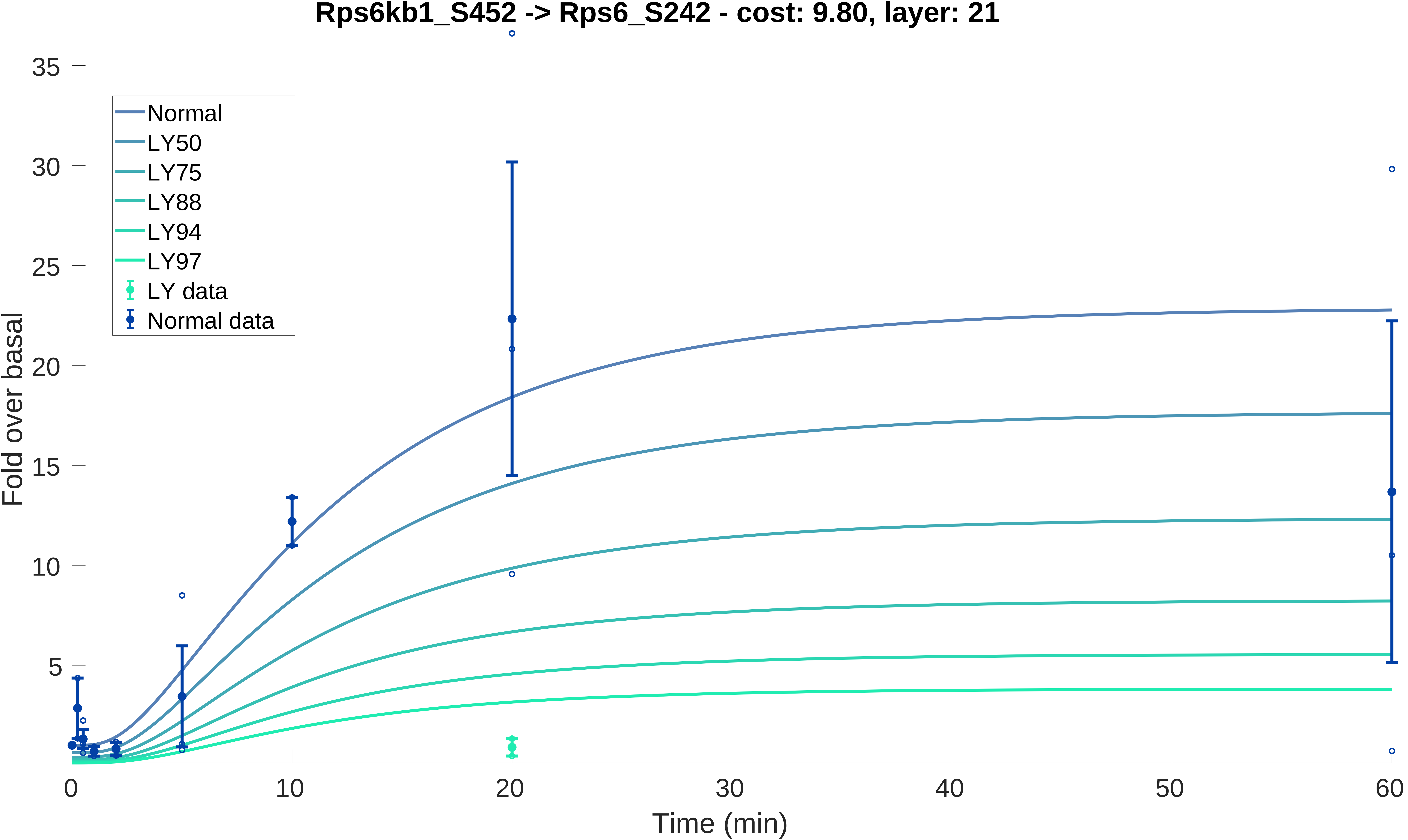

Rps6kb1_S452 -> Rps6_S242 - cost: 9.80, layer: 21
Fold over basal
Time (min)
35
30
25
20
15
10
5
0
10
20
30
40
50
60
Normal
LY50
LY75
LY88
LY94
LY97
LY data
Normal data

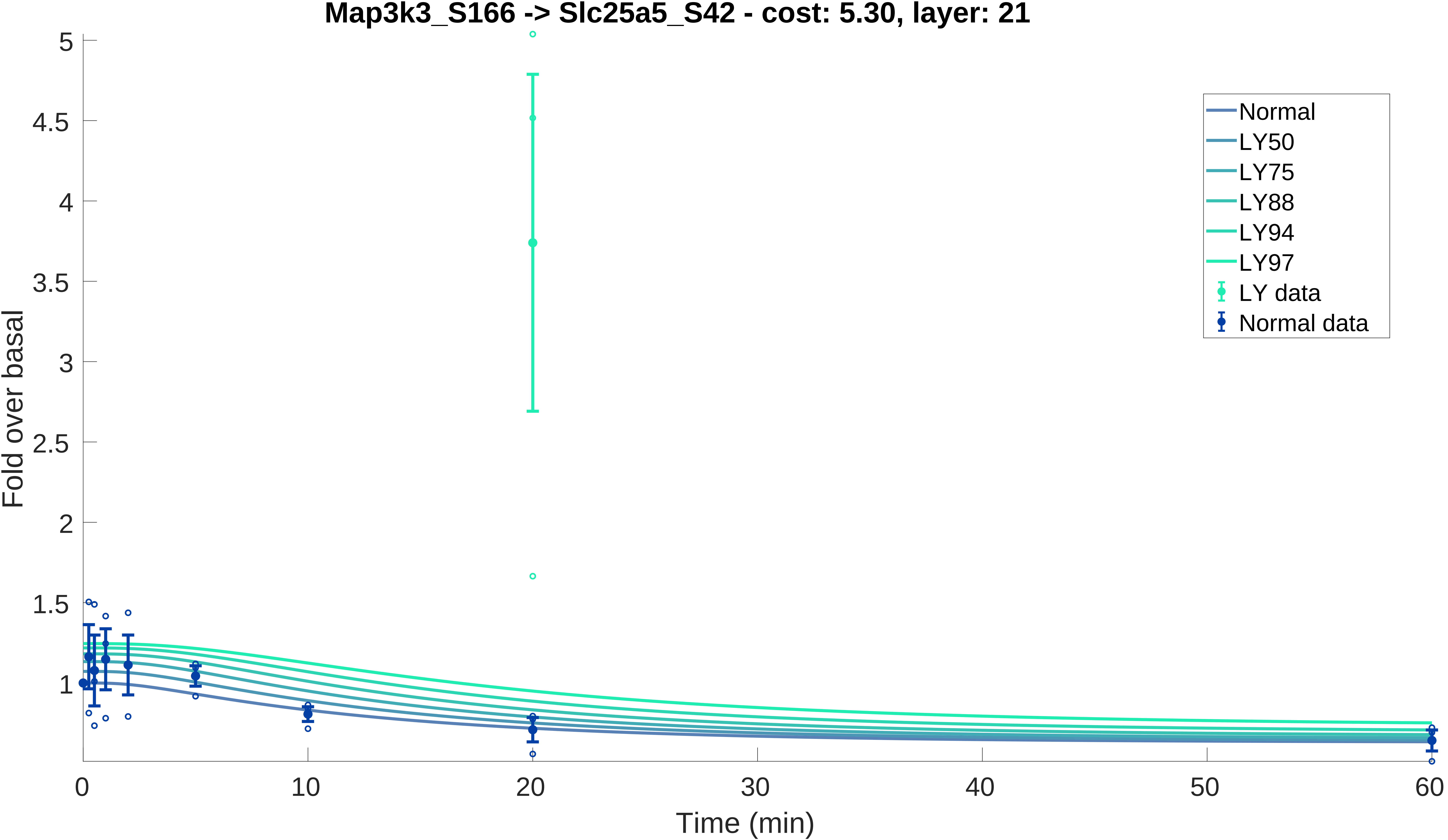

Map3k3_S166 -> Slc25a5_S42 - cost: 5.30, layer: 21
Fold over basal
Time (min)
0
10
20
30
40
50
60
1
1.5
2
2.5
3
3.5
4
4.5
5
Normal
LY50
LY75
LY88
LY94
LY97
LY data
Normal data

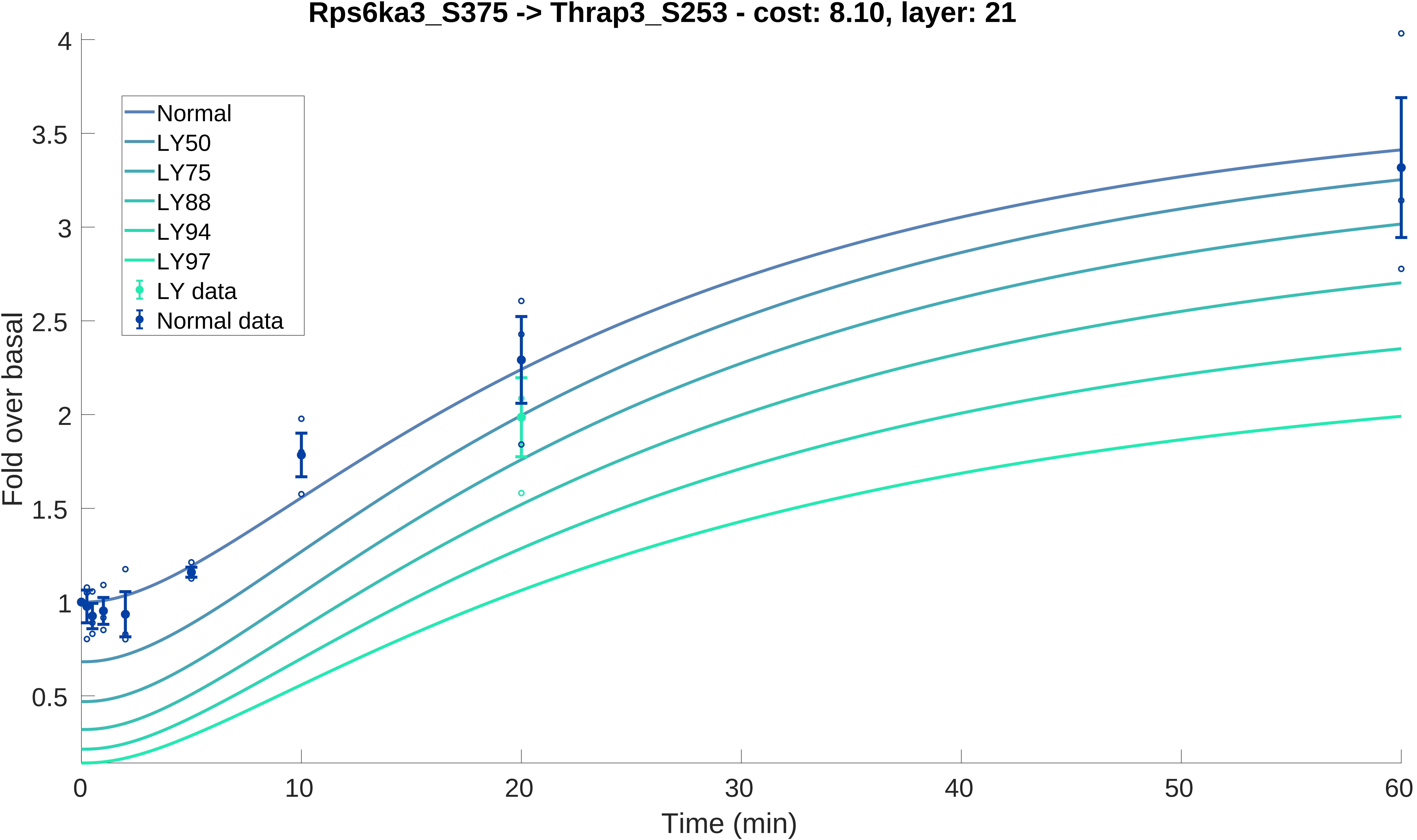

Rps6ka3_S375 -> Thrap3_S253 - cost: 8.10, layer: 21
Fold over basal
Time (min)
0.5
1
1.5
2
2.5
3
3.5
4
0
10
20
30
40
50
60
Normal
LY50
LY75
LY88
LY94
LY97
LY data
Normal data

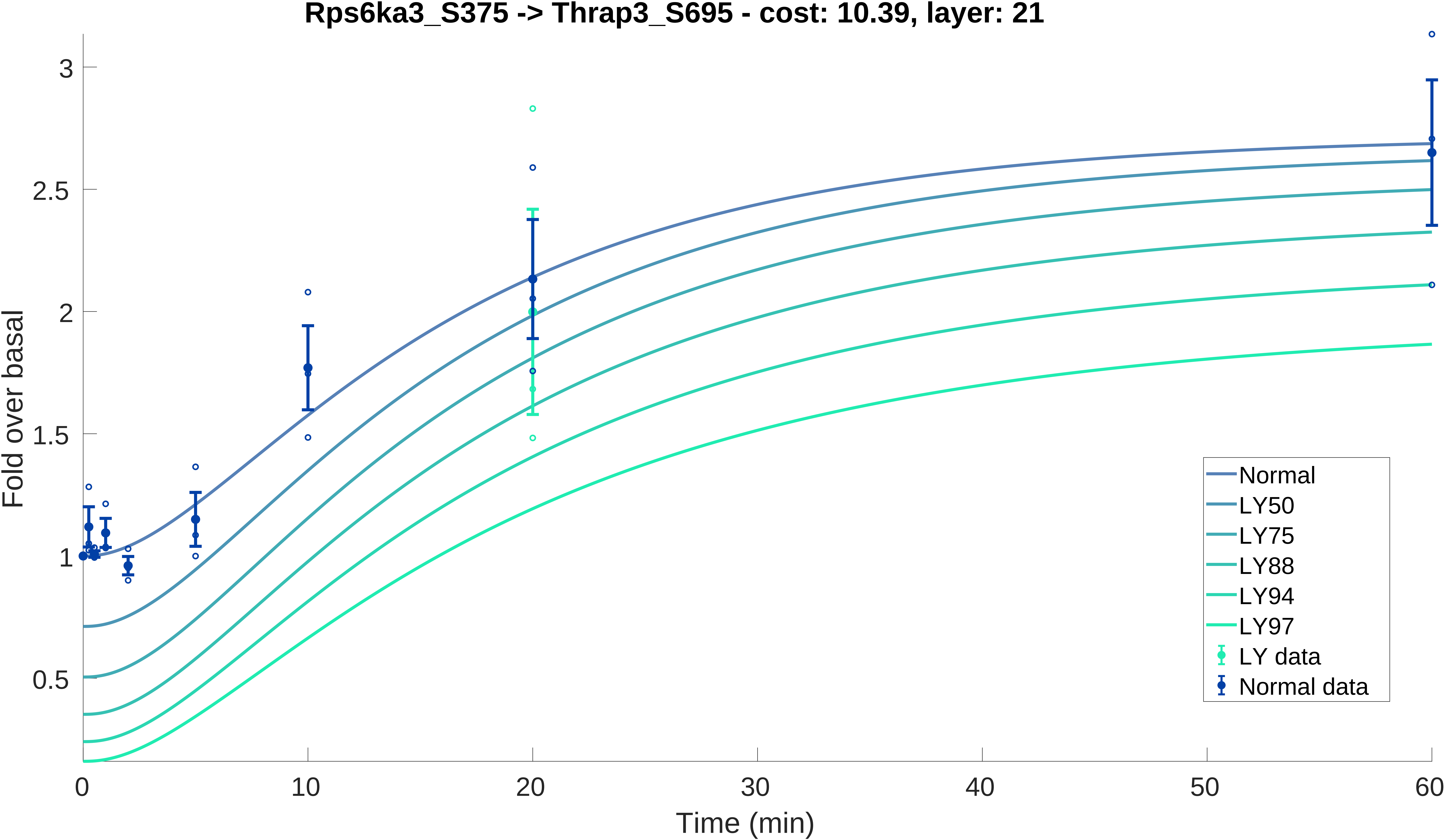

Rps6ka3_S375 -> Thrap3_S695 - cost: 10.39, layer: 21
Fold over basal
Time (min)
0.5
1
1.5
2
2.5
3
0
10
20
30
40
50
60
Normal
LY50
LY75
LY88
LY94
LY97
LY data
Normal data

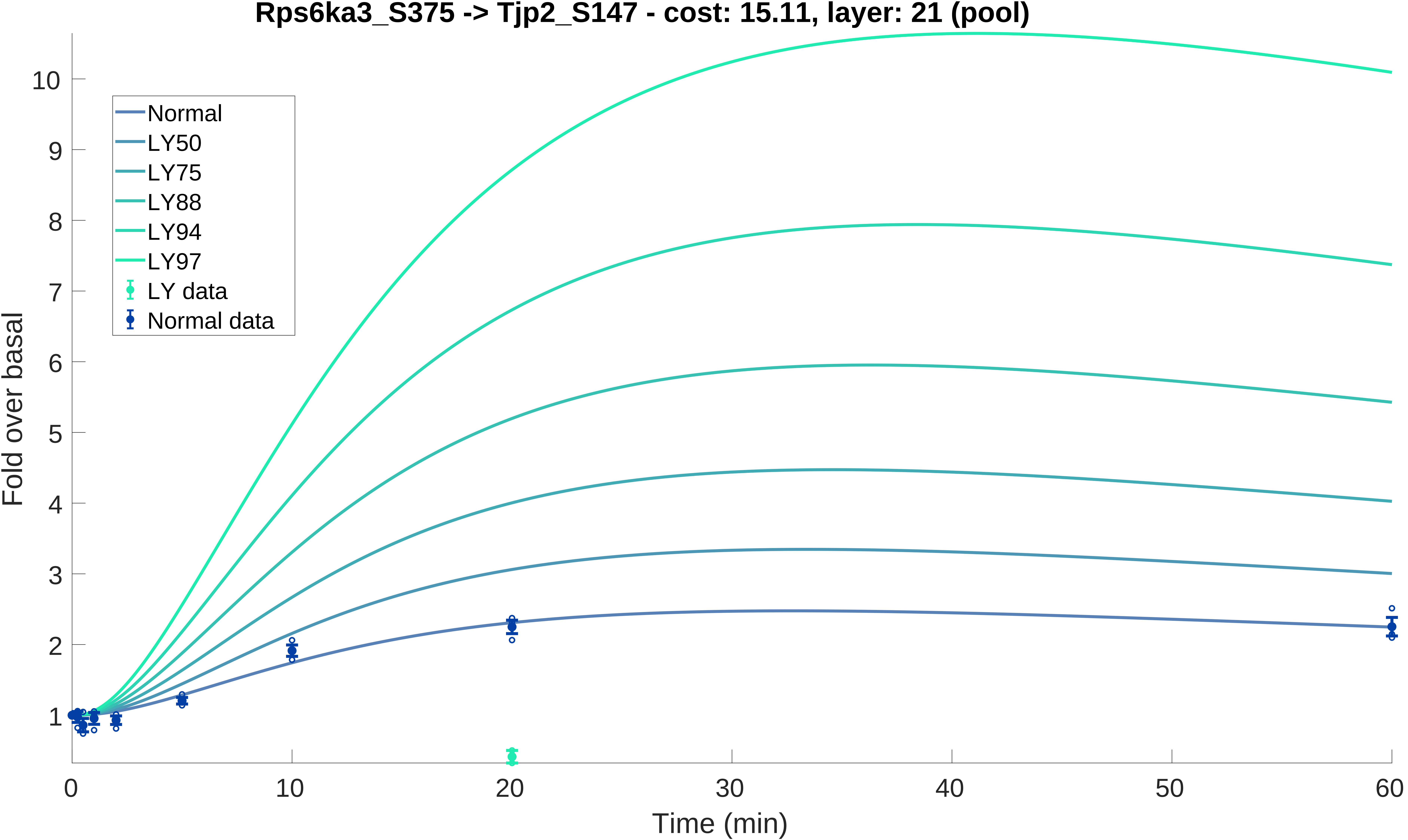

Rps6ka3_S375 -> Tjp2_S147 - cost: 15.11, layer: 21 (pool)
Normal
LY50
LY75
LY88
LY94
LY97
LY data
Normal data
Fold over basal
Time (min)
0
10
20
30
40
50
60
1
2
3
4
5
6
7
8
9
10

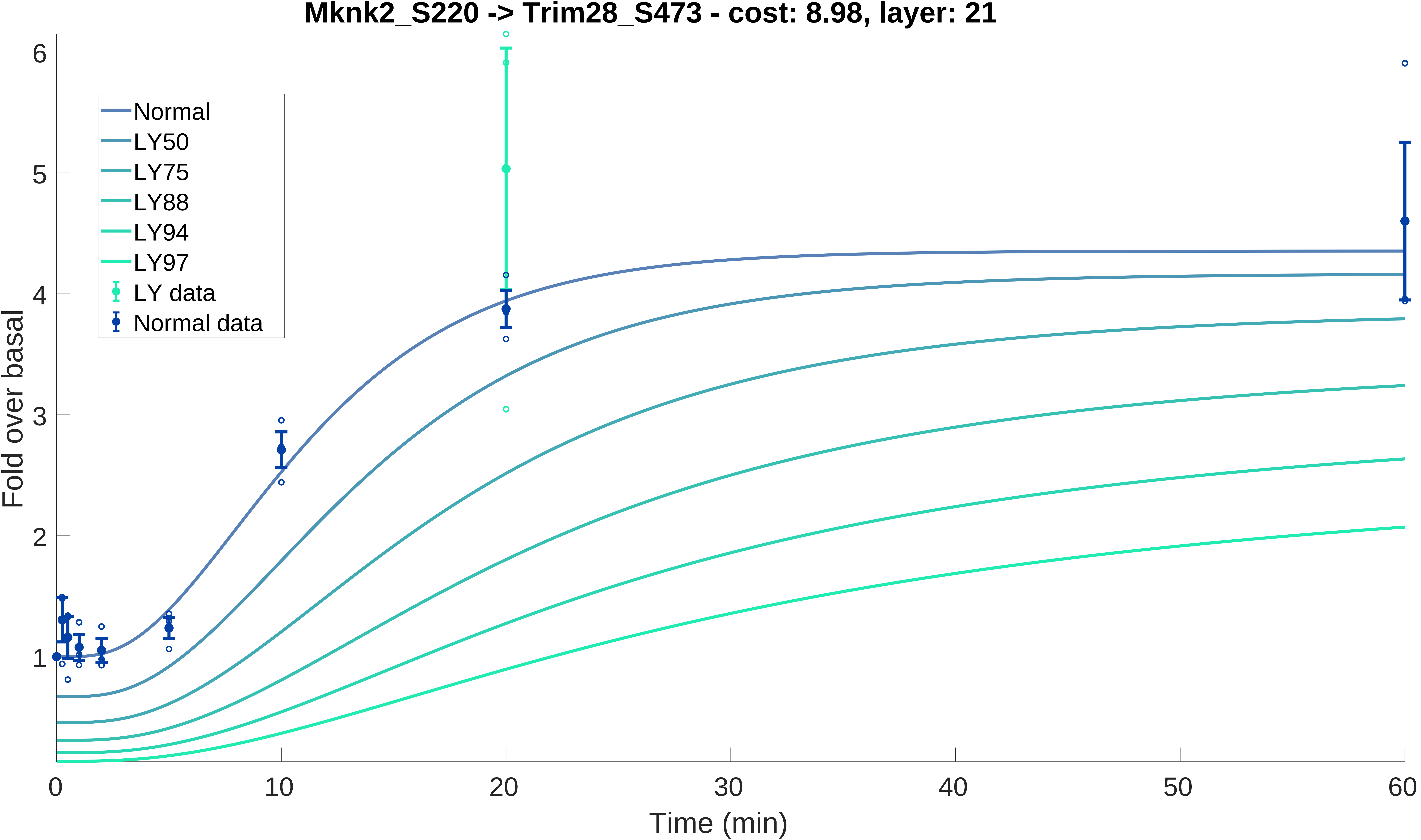

Mknk2_S220 -> Trim28_S473 - cost: 8.98, layer: 21
Normal
LY50
LY75
LY88
LY94
LY97
LY data
Normal data
Fold over basal
Time (min)
0
10
20
30
40
50
60
1
2
3
4
5
6

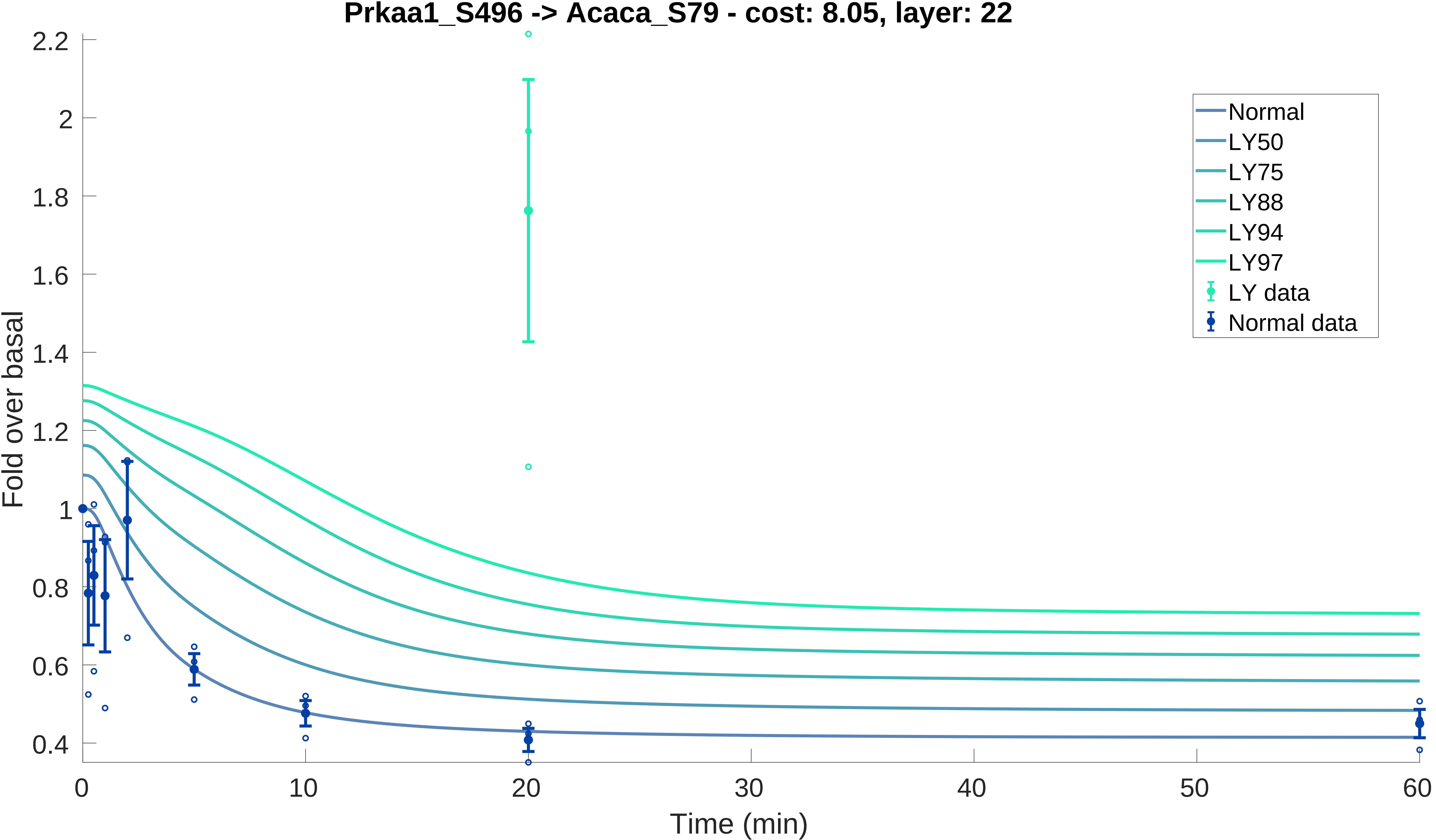

Prkaa1_S496 -> Acaca_S79 - cost: 8.05, layer: 22
Fold over basal
Time (min)
0.4
0.6
0.8
1
1.2
1.4
1.6
1.8
2
2.2
0
10
20
30
40
50
60
Normal
LY50
LY75
LY88
LY94
LY97
LY data
Normal data

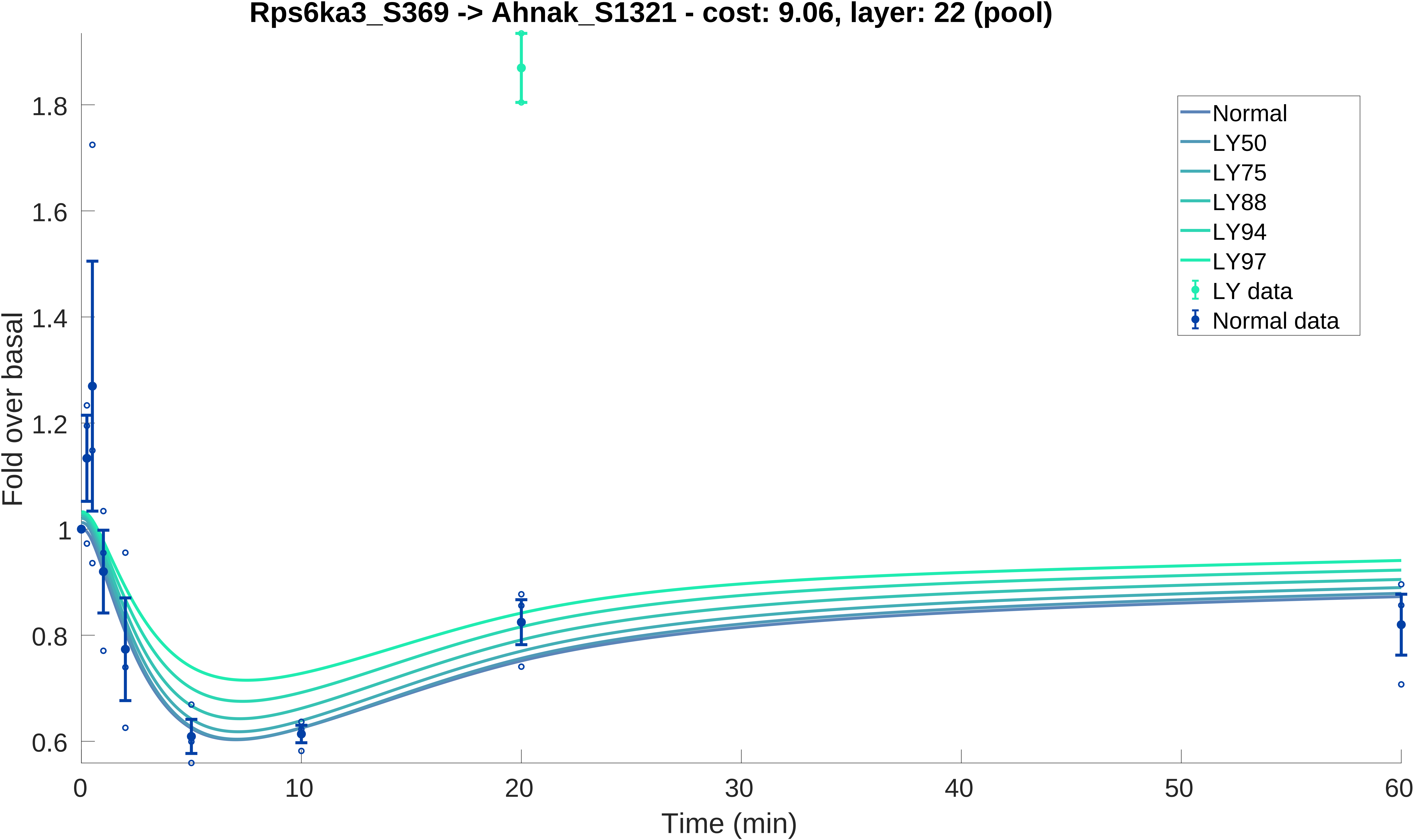

Rps6ka3_S369 -> Ahnak_S1321 - cost: 9.06, layer: 22 (pool)
Fold over basal
Time (min)
1.8
1.6
1.4
1.2
1
0.8
0.6
0
10
20
30
40
50
60
Normal
LY50
LY75
LY88
LY94
LY97
LY data
Normal data

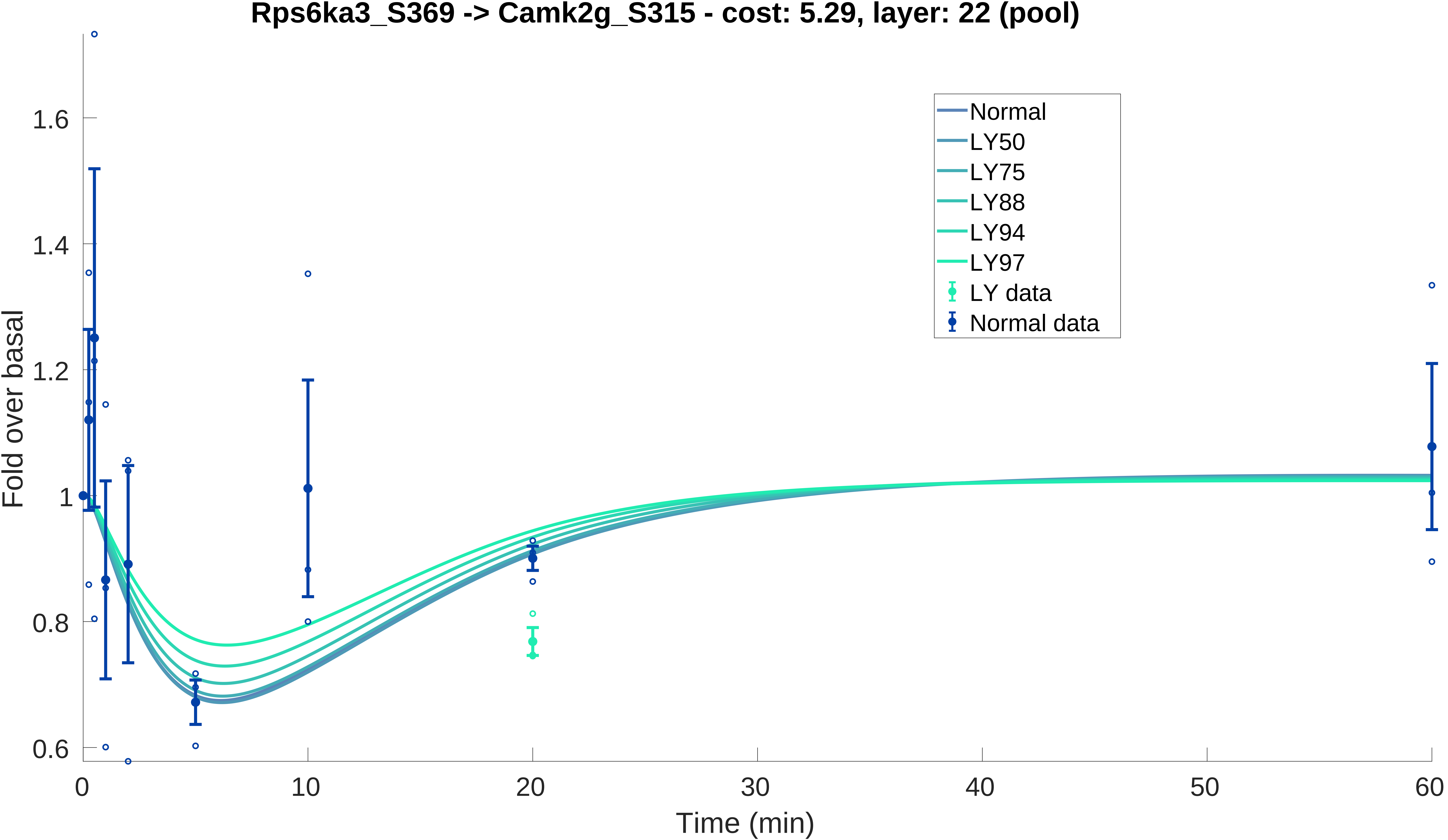

Rps6ka3_S369 -> Camk2g_S315 - cost: 5.29, layer: 22 (pool)
Normal
LY50
LY75
LY88
LY94
LY97
LY data
Normal data
Fold over basal
1.6
1.4
1.2
1
0.8
0.6
0
10
20
30
40
50
60
Time (min)

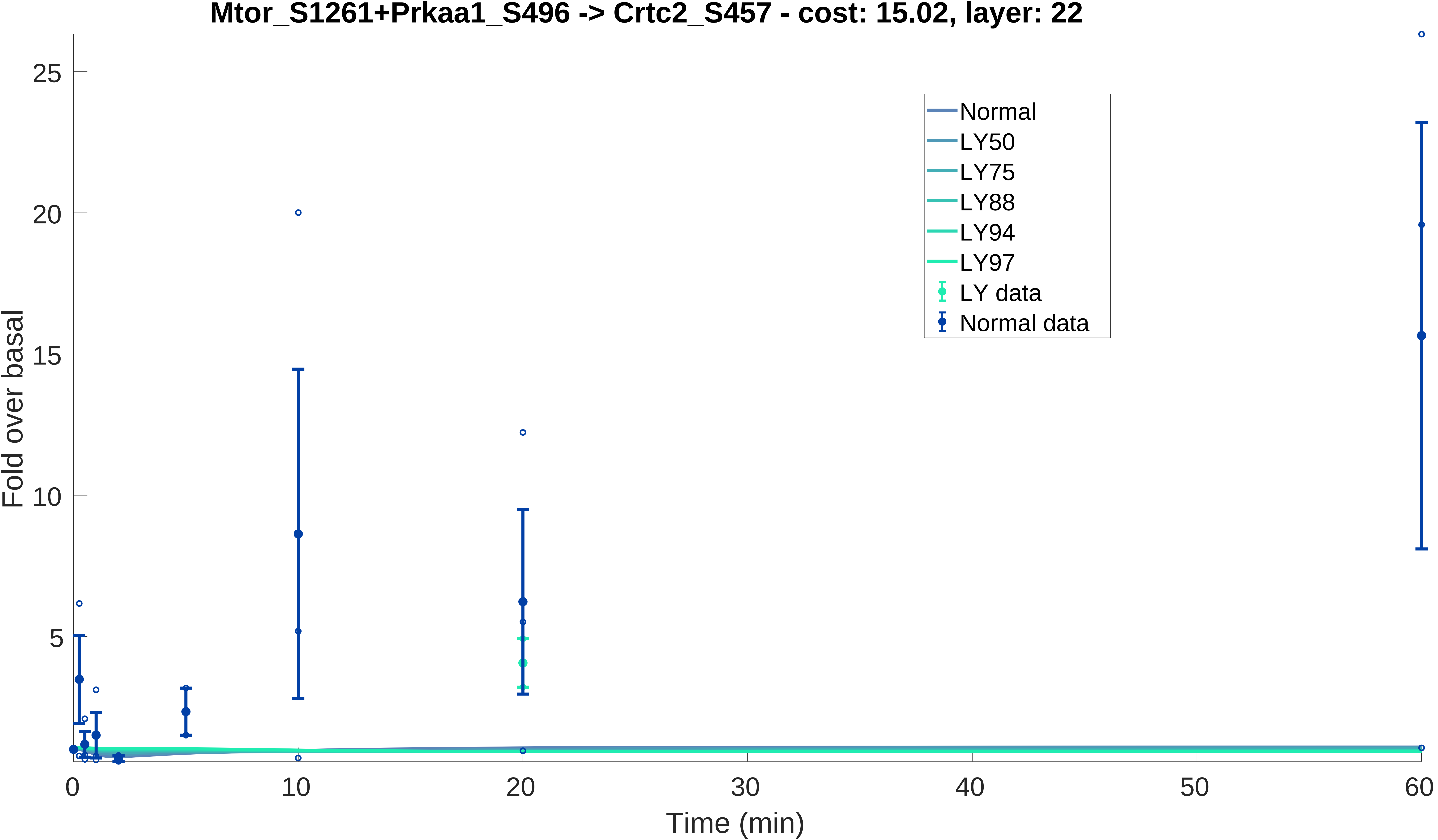

Mtor_S1261+Prkaa1_S496 -> Crtc2_S457 - cost: 15.02, layer: 22
Fold over basal
Time (min)
0
10
20
30
40
50
60
5
10
15
20
25
Normal
LY50
LY75
LY88
LY94
LY97
LY data
Normal data

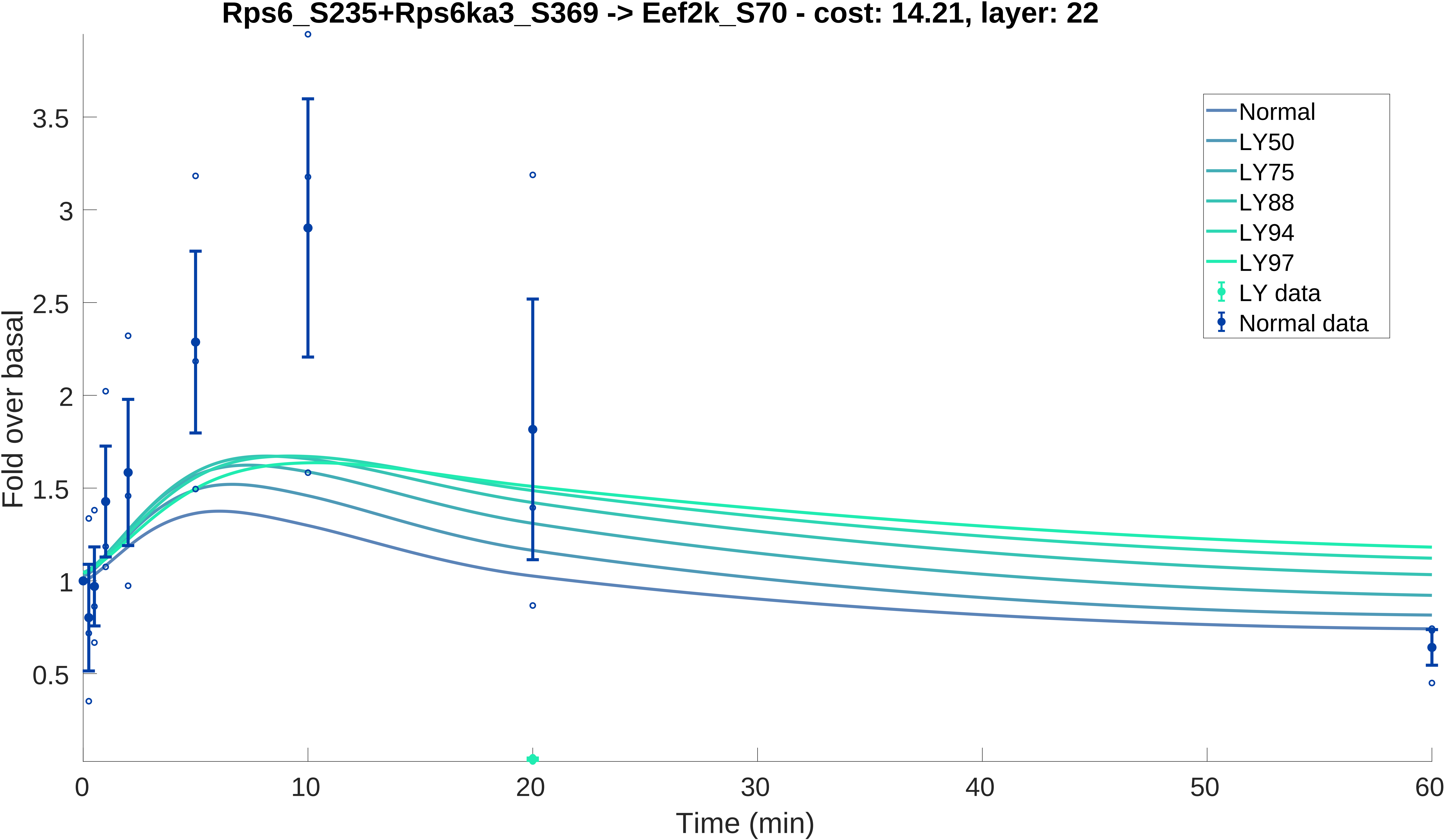
Rps6_S235+Rps6ka3_S369 -> Eef2k_S70 - cost: 14.21, layer: 22
Fold over basal
Time (min)
0
10
20
30
40
50
60
0.5
1
1.5
2
2.5
3
3.5
Normal
LY50
LY75
LY88
LY94
LY97
LY data
Normal data

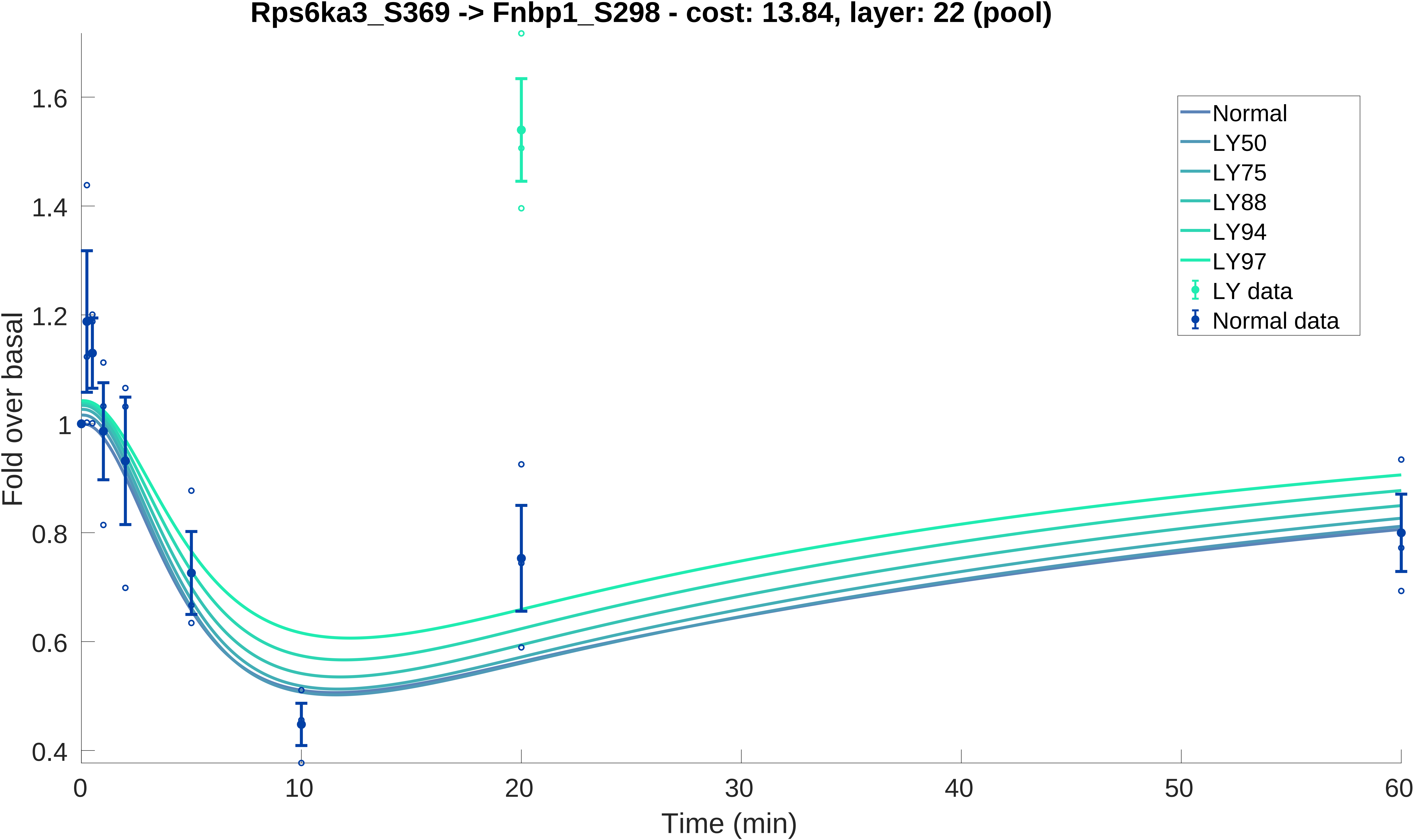

Rps6ka3_S369 -> Fnbp1_S298 - cost: 13.84, layer: 22 (pool)
Fold over basal
Time (min)
0
10
20
30
40
50
60
0.4
0.6
0.8
1
1.2
1.4
1.6
Normal
LY50
LY75
LY88
LY94
LY97
LY data
Normal data

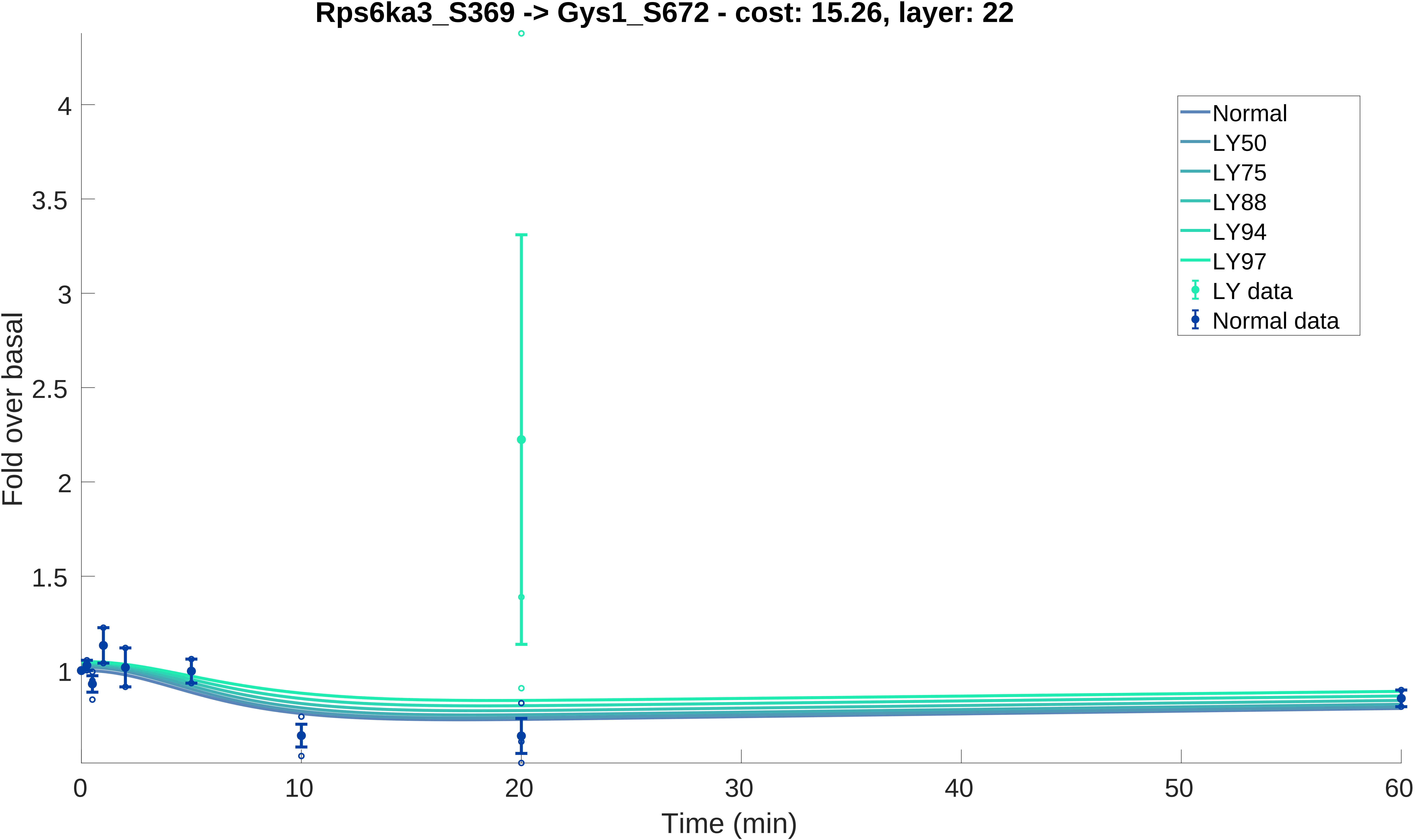
Rps6ka3_S369 -> Gys1_S672 - cost: 15.26, layer: 22
Fold over basal
Time (min)
0
10
20
30
40
50
60
1
1.5
2
2.5
3
3.5
4
Normal
LY50
LY75
LY88
LY94
LY97
LY data
Normal data

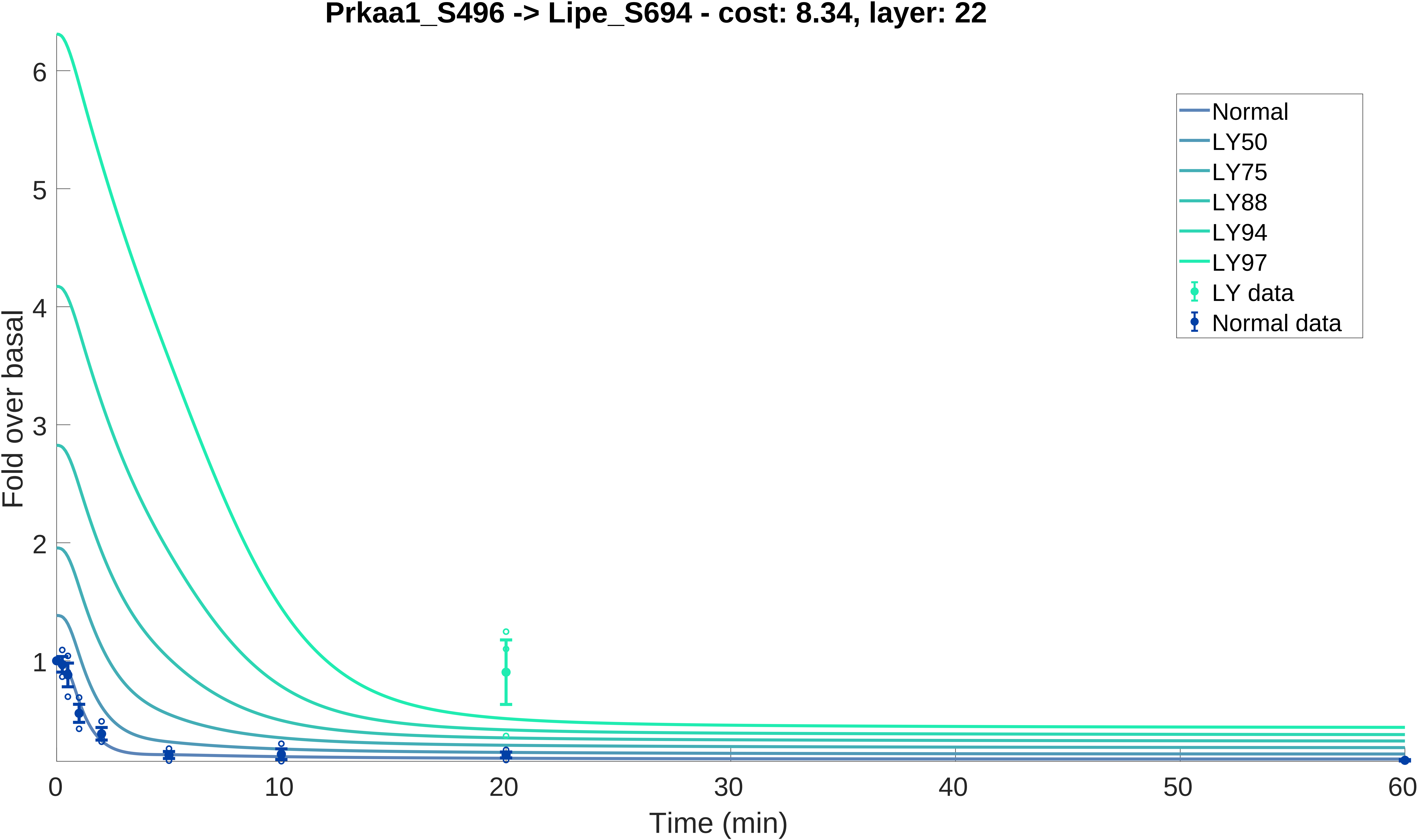

Prrkaa1_S496 -> Lipe_S694 - cost: 8.34, layer: 22
Fold over basal
Time (min)
0
10
20
30
40
50
60
1
2
3
4
5
6
Normal
LY50
LY75
LY88
LY94
LY97
LY data
Normal data

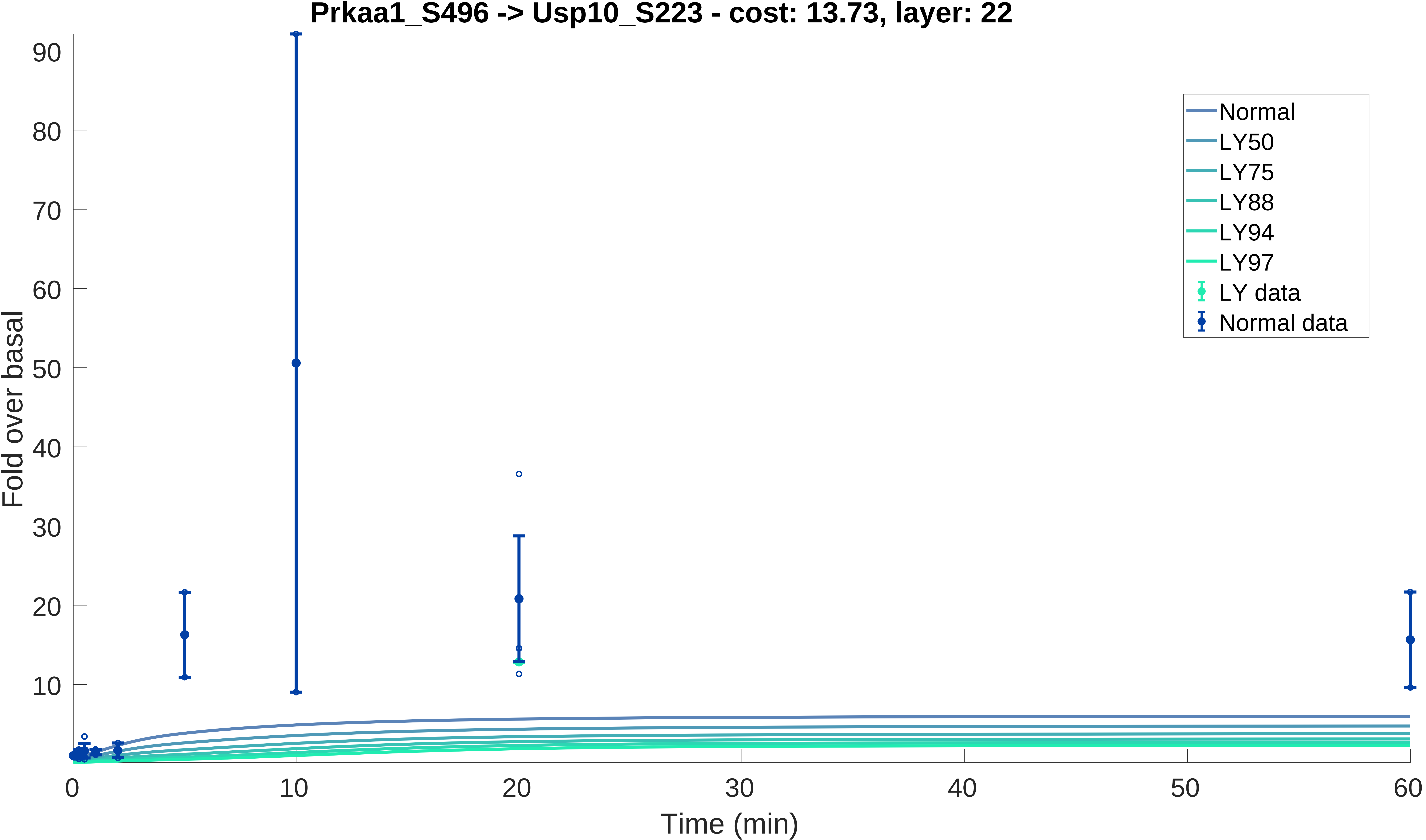

Prrkaa1_S496 -> Usp10_S223 - cost: 13.73, layer: 22
Fold over basal
Time (min)
Normal
LY50
LY75
LY88
LY94
LY97
LY data
Normal data

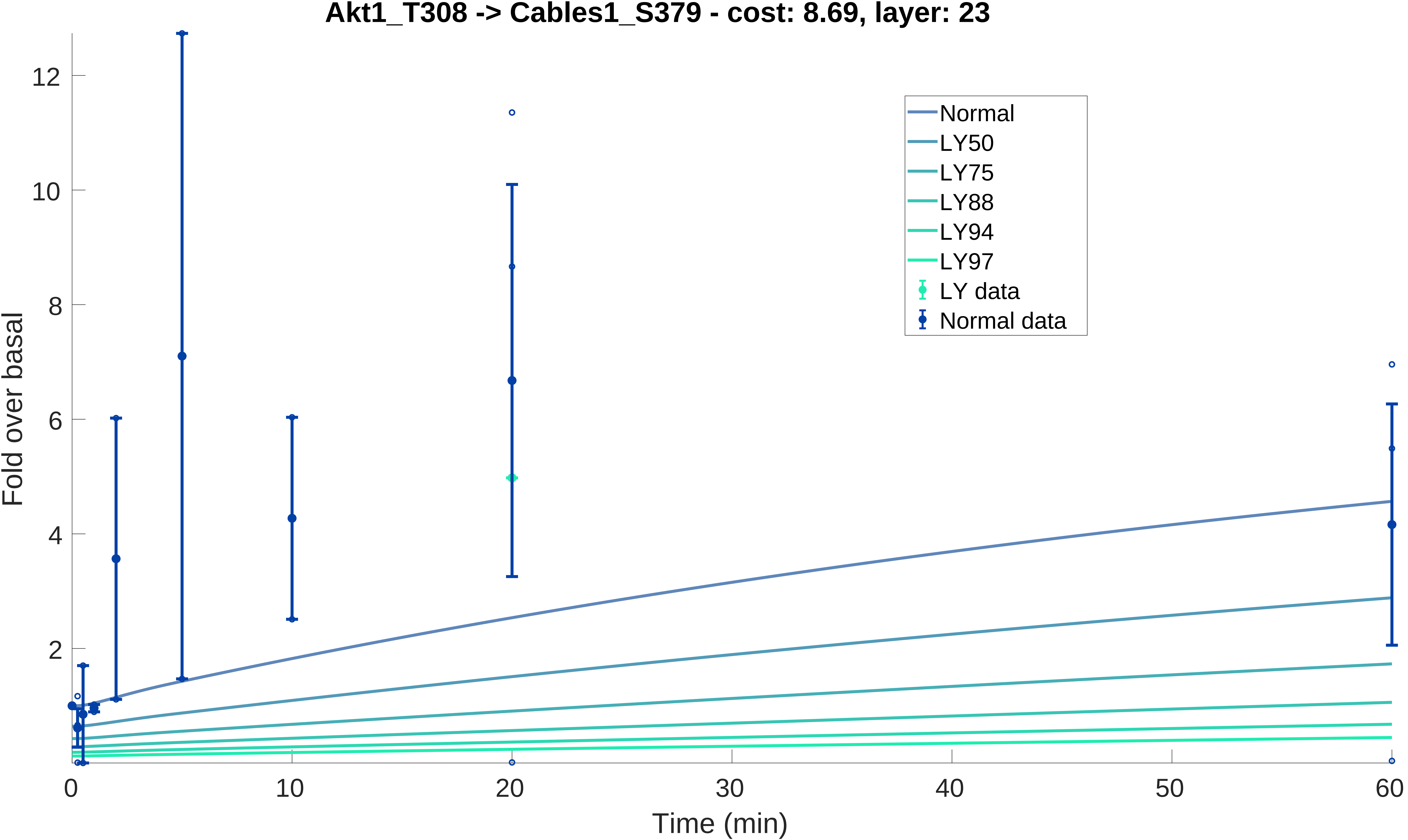

Akt1_T308 -> Cables1_S379 - cost: 8.69, layer: 23
Fold over basal
Time (min)
0
2
4
6
8
10
12
0
10
20
30
40
50
60
Normal
LY50
LY75
LY88
LY94
LY97
LY data
Normal data

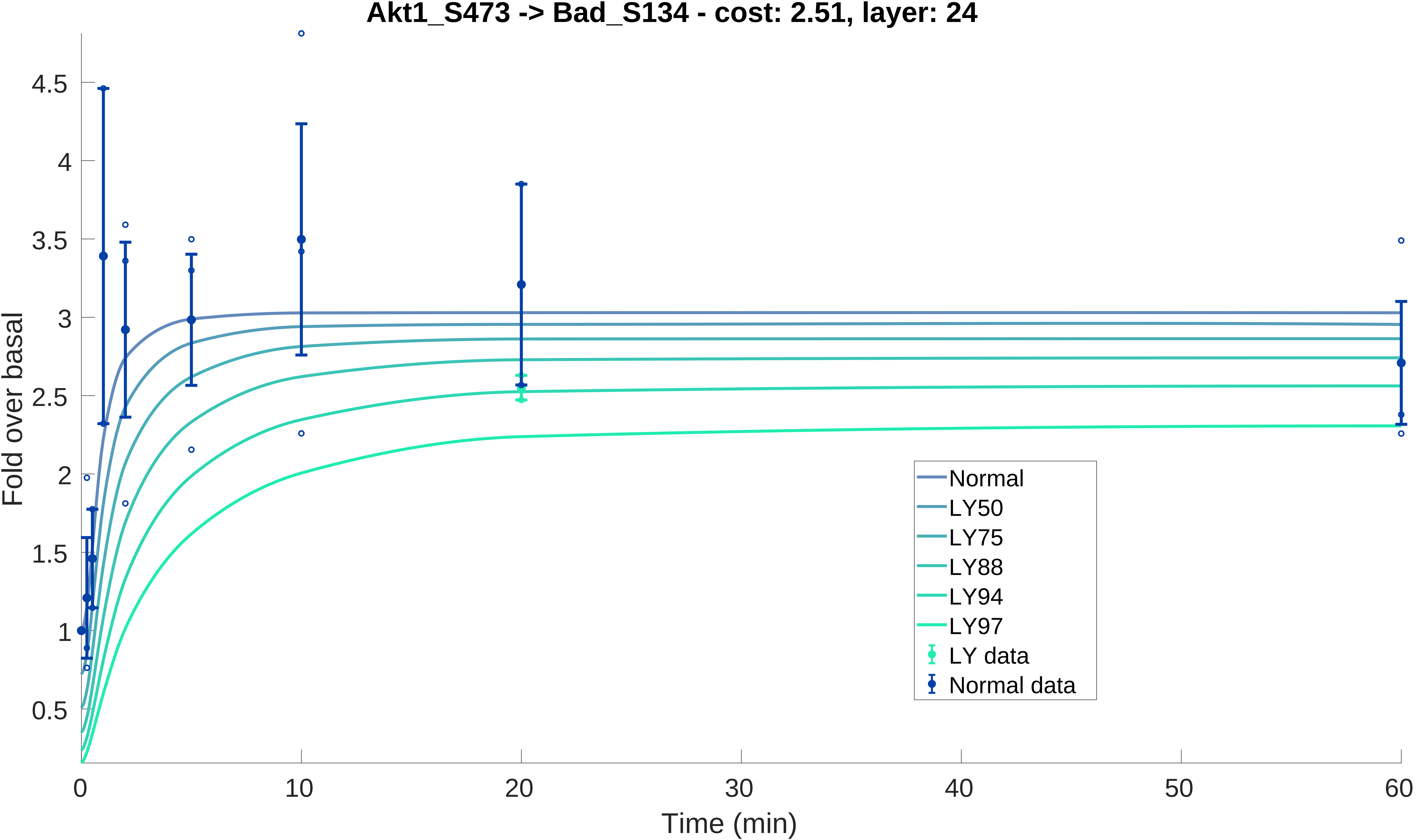

Akt1_S473 -> Bad_S134 - cost: 2.51, layer: 24
Fold over basal
Time (min)
0.5
1
1.5
2
2.5
3
3.5
4
4.5
0
10
20
30
40
50
60
Normal
LY50
LY75
LY88
LY94
LY97
LY data
Normal data

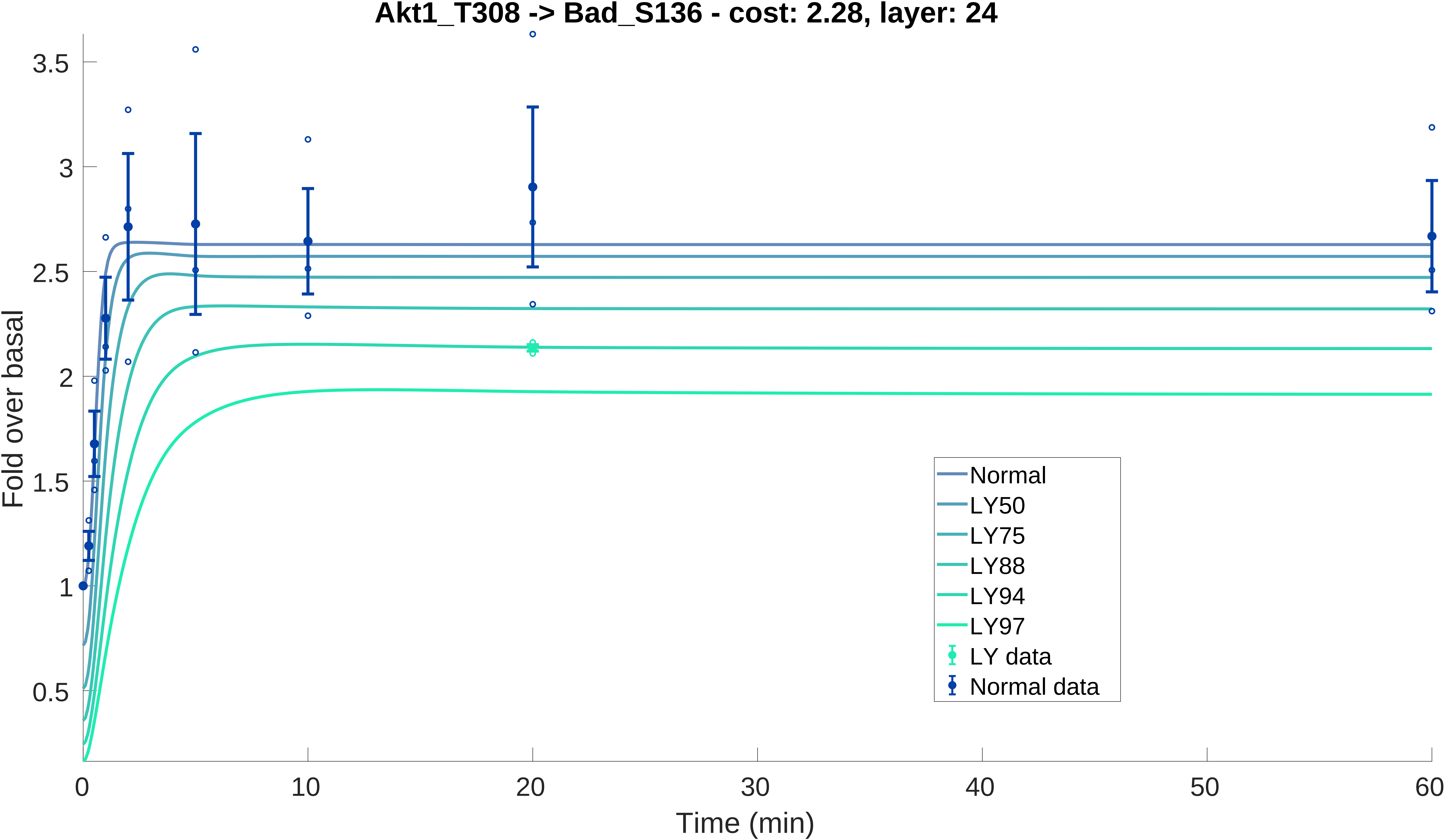

Akt1_T308 -> Bad_S136 - cost: 2.28, layer: 24
Fold over basal
Time (min)
0
10
20
30
40
50
60
0.5
1
1.5
2
2.5
3
3.5
Normal
LY50
LY75
LY88
LY94
LY97
LY data
Normal data

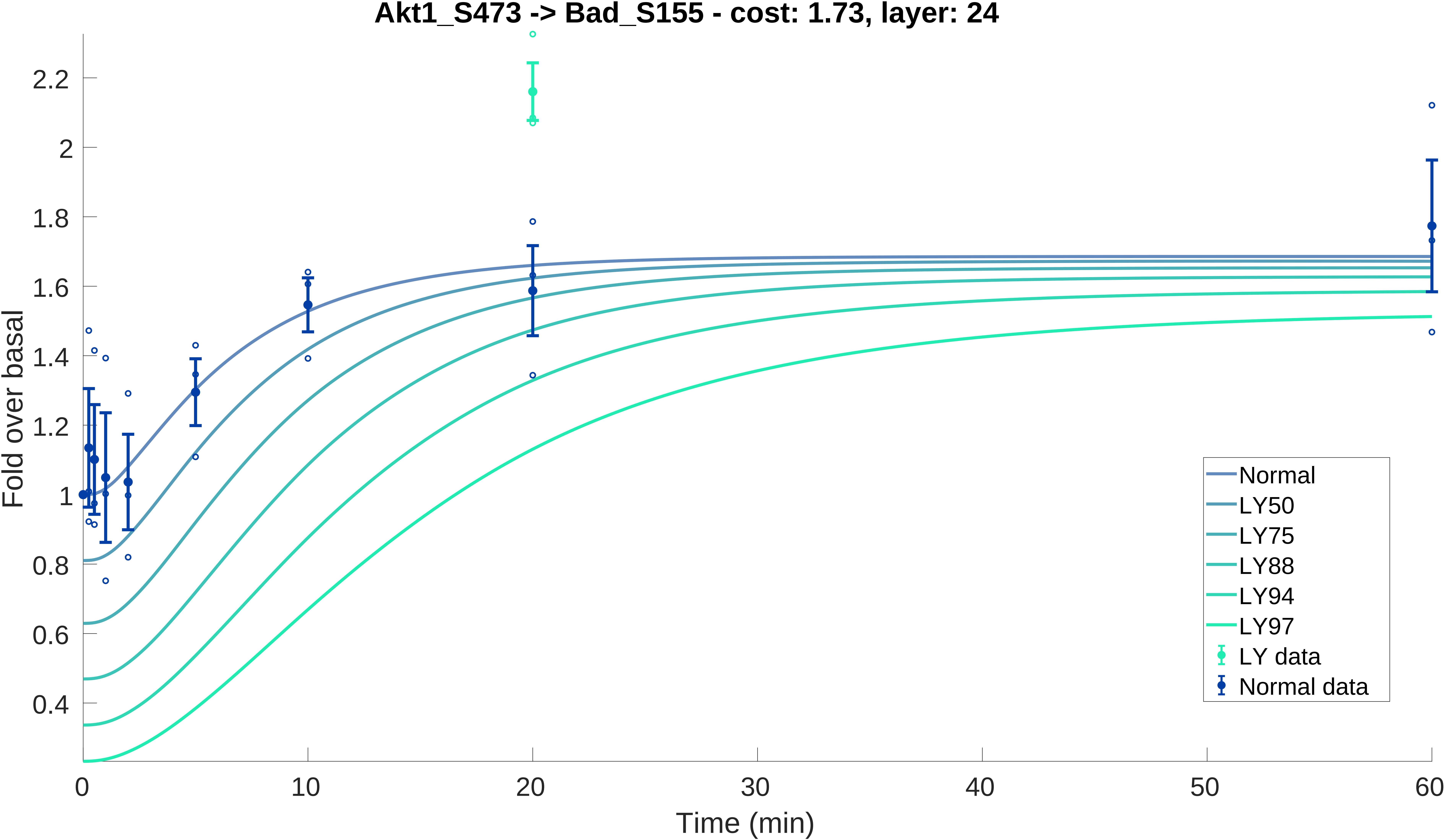

Akt1_S473 -> Bad_S155 - cost: 1.73, layer: 24
Fold over basal
2.2
2
1.8
1.6
1.4
1.2
1
0.8
0.6
0.4
0
10
20
30
40
50
60
Time (min)
Normal
LY50
LY75
LY88
LY94
LY97
LY data
Normal data

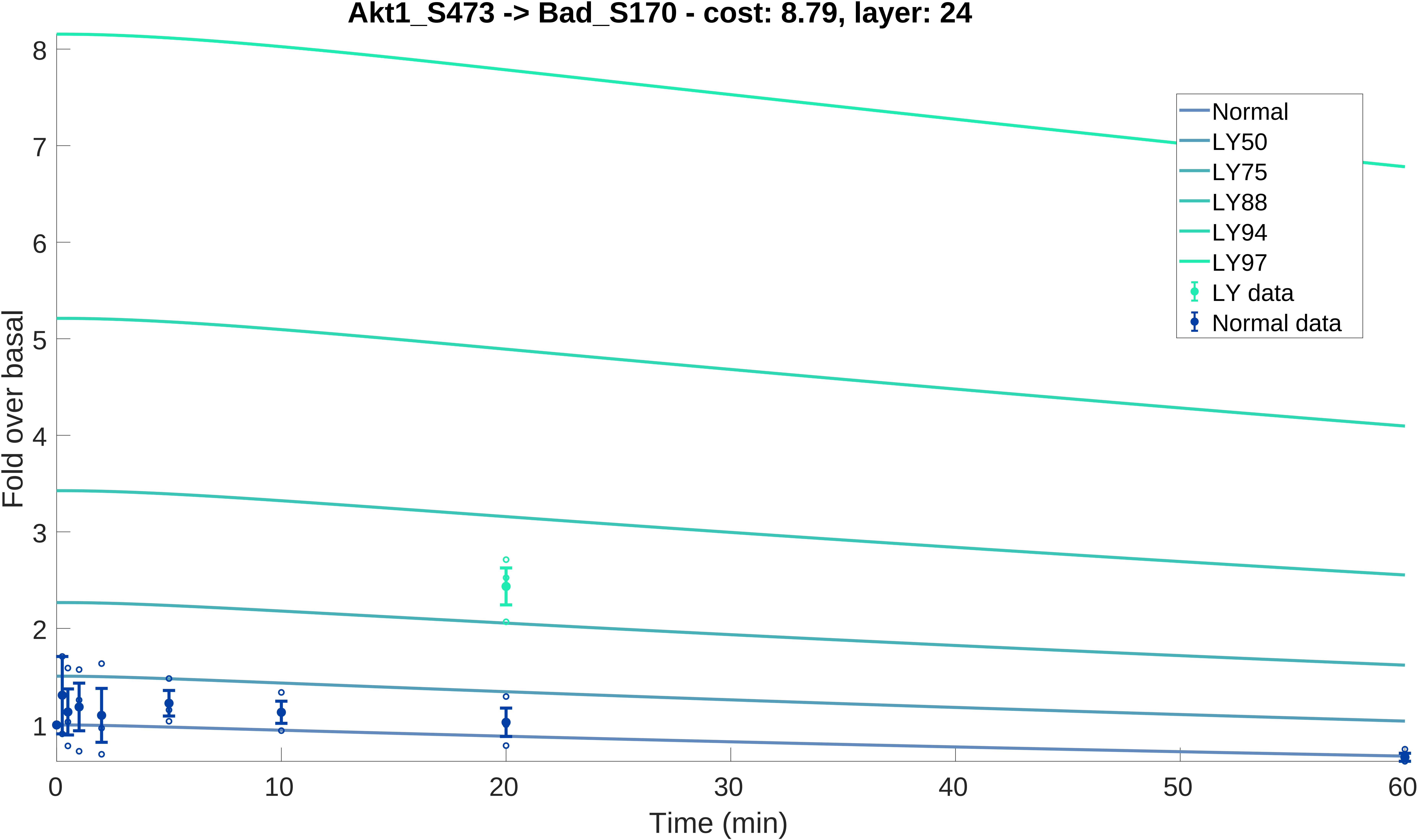

Akt1_S473 -> Bad_S170 - cost: 8.79, layer: 24
Fold over basal
Time (min)
1
2
3
4
5
6
7
8
0
10
20
30
40
50
60
Normal
LY50
LY75
LY88
LY94
LY97
LY data
Normal data

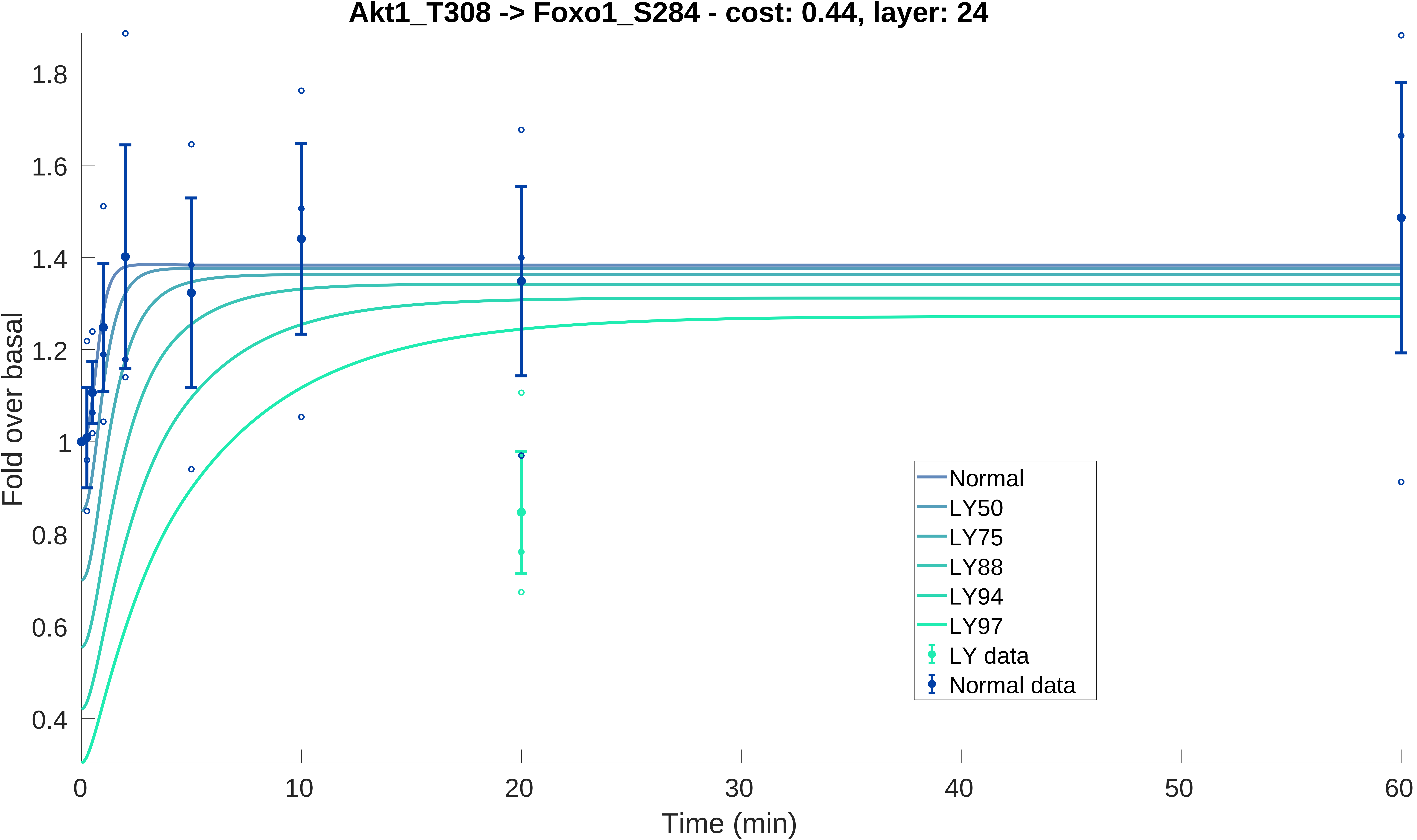
Akt1_T308 -> Foxo1_S284 - cost: 0.44, layer: 24
Fold over basal
Time (min)
0.4
0.6
0.8
1
1.2
1.4
1.6
1.8
0
10
20
30
40
50
60
Normal
LY50
LY75
LY88
LY94
LY97
LY data
Normal data

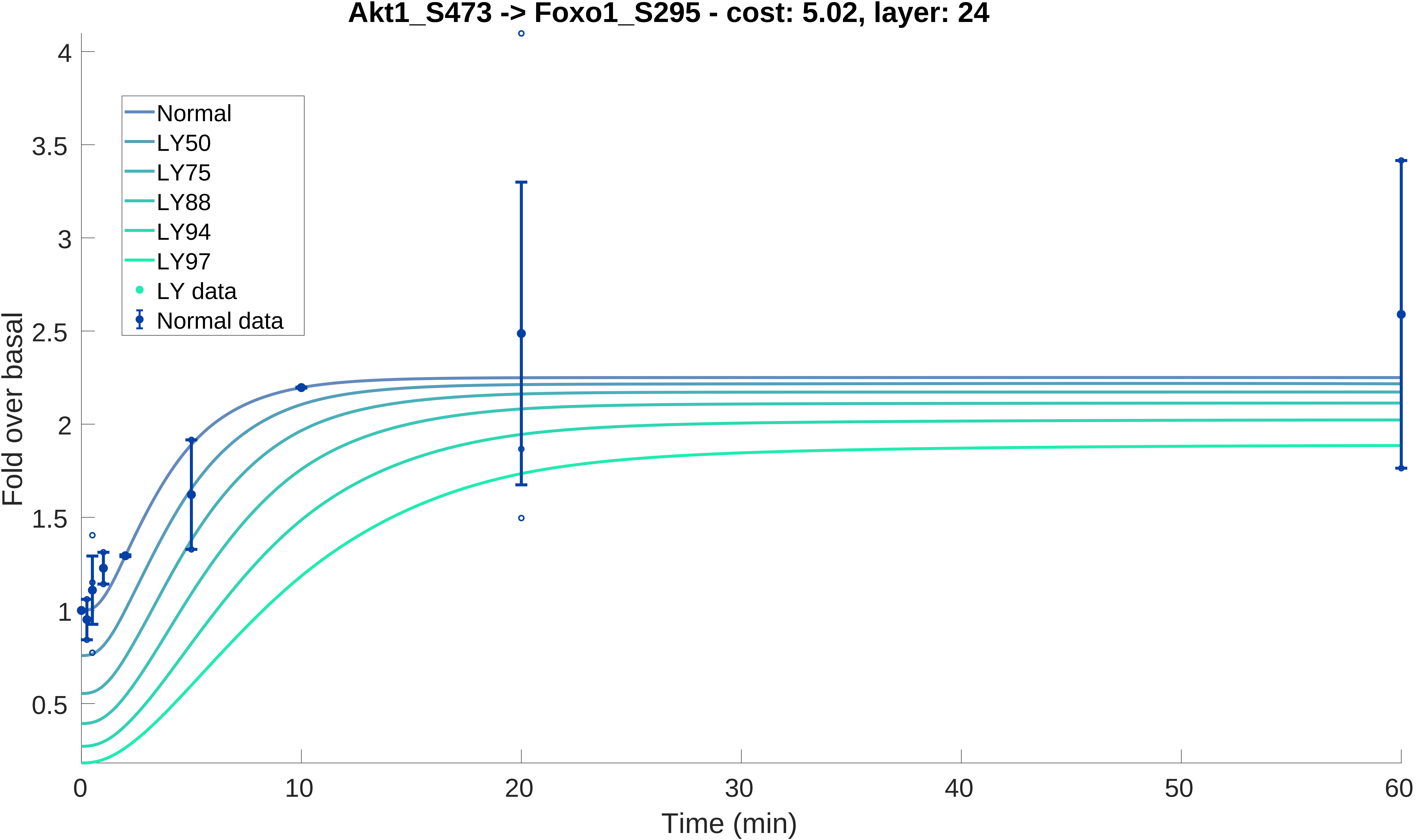

Akt1_S473 -> Foxo1_S295 - cost: 5.02, layer: 24
Normal
LY50
LY75
LY88
LY94
LY97
LY data
Normal data
Fold over basal
Time (min)
0.5
1
1.5
2
2.5
3
3.5
4
0
10
20
30
40
50
60

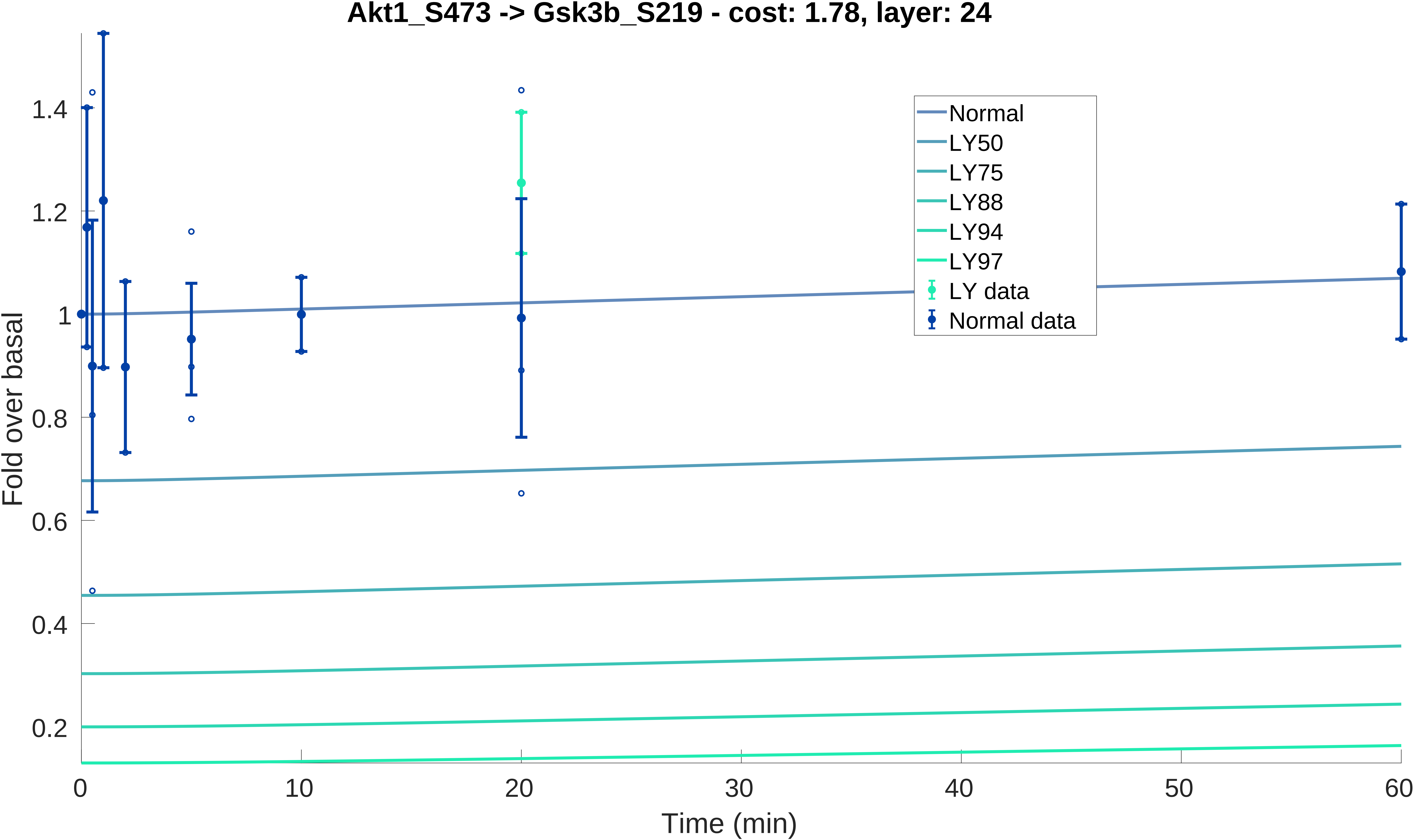

Akt1_S473 -> Gsk3b_S219 - cost: 1.78, layer: 24
Fold over basal
Time (min)
0.2
0.4
0.6
0.8
1
1.2
1.4
0
10
20
30
40
50
60
Normal
LY50
LY75
LY88
LY94
LY97
LY data
Normal data

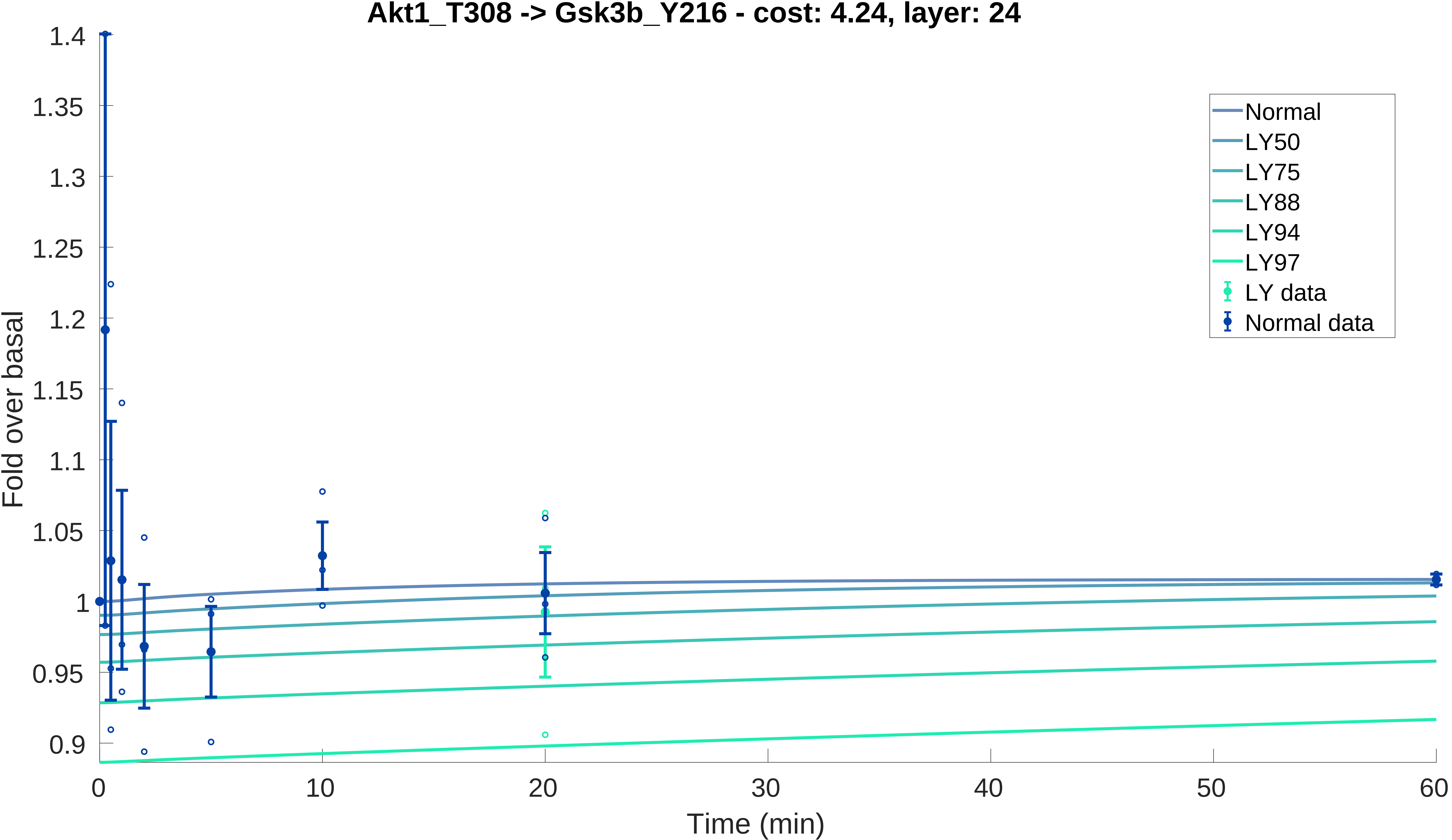

Akt1_T308 -> Gsk3b_Y216 - cost: 4.24, layer: 24
Fold over basal
1.4
1.35
1.3
1.25
1.2
1.15
1.1
1.05
1
0.95
0.9
0
10
20
30
40
50
60
Time (min)
Normal
LY50
LY75
LY88
LY94
LY97
LY data
Normal data

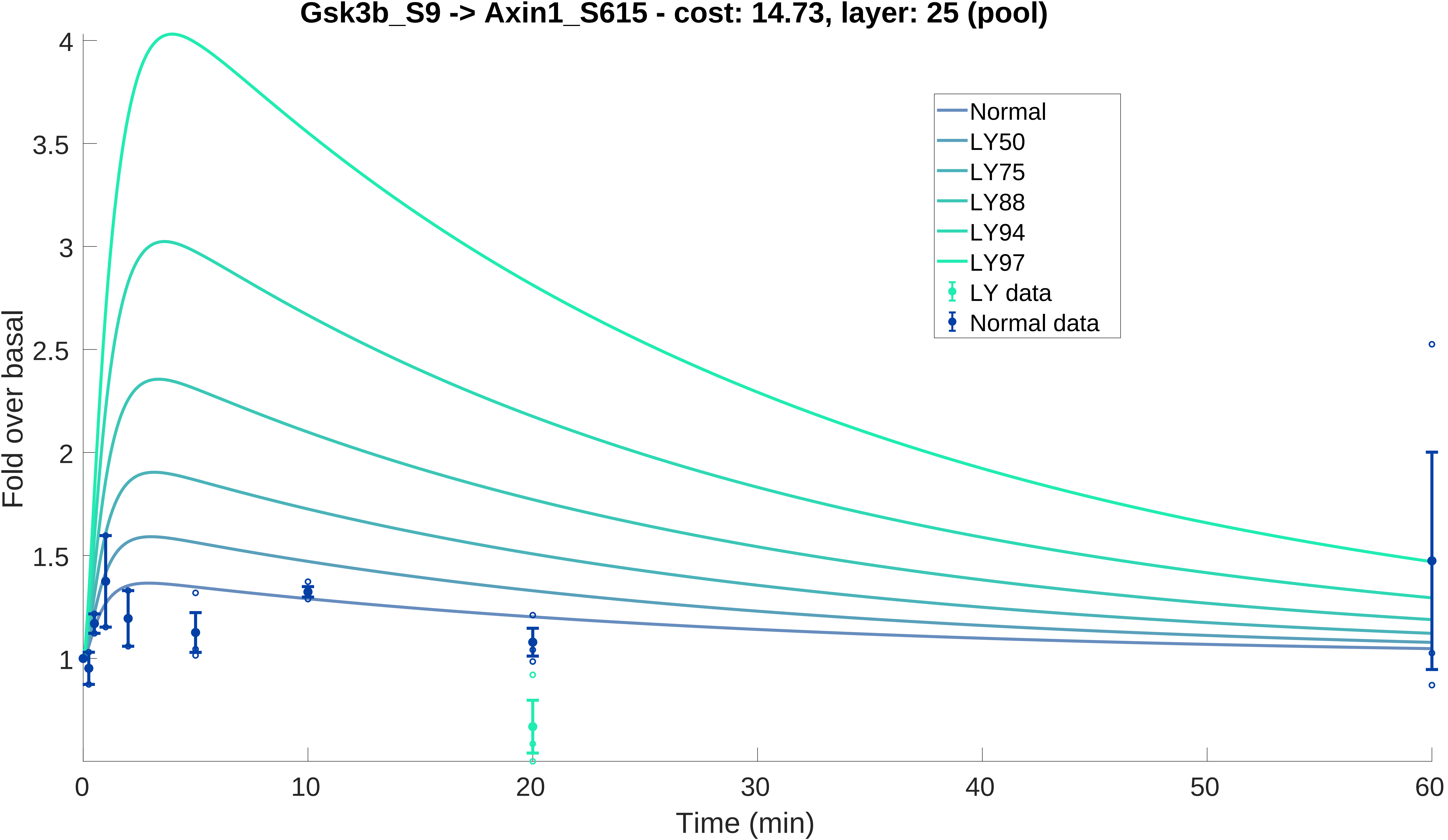

Gsk3b_S9 -> Axin1_S615 - cost: 14.73, layer: 25 (pool)
Fold over basal
Time (min)
4
3.5
3
2.5
2
1.5
1
0
10
20
30
40
50
60
Normal
LY50
LY75
LY88
LY94
LY97
LY data
Normal data

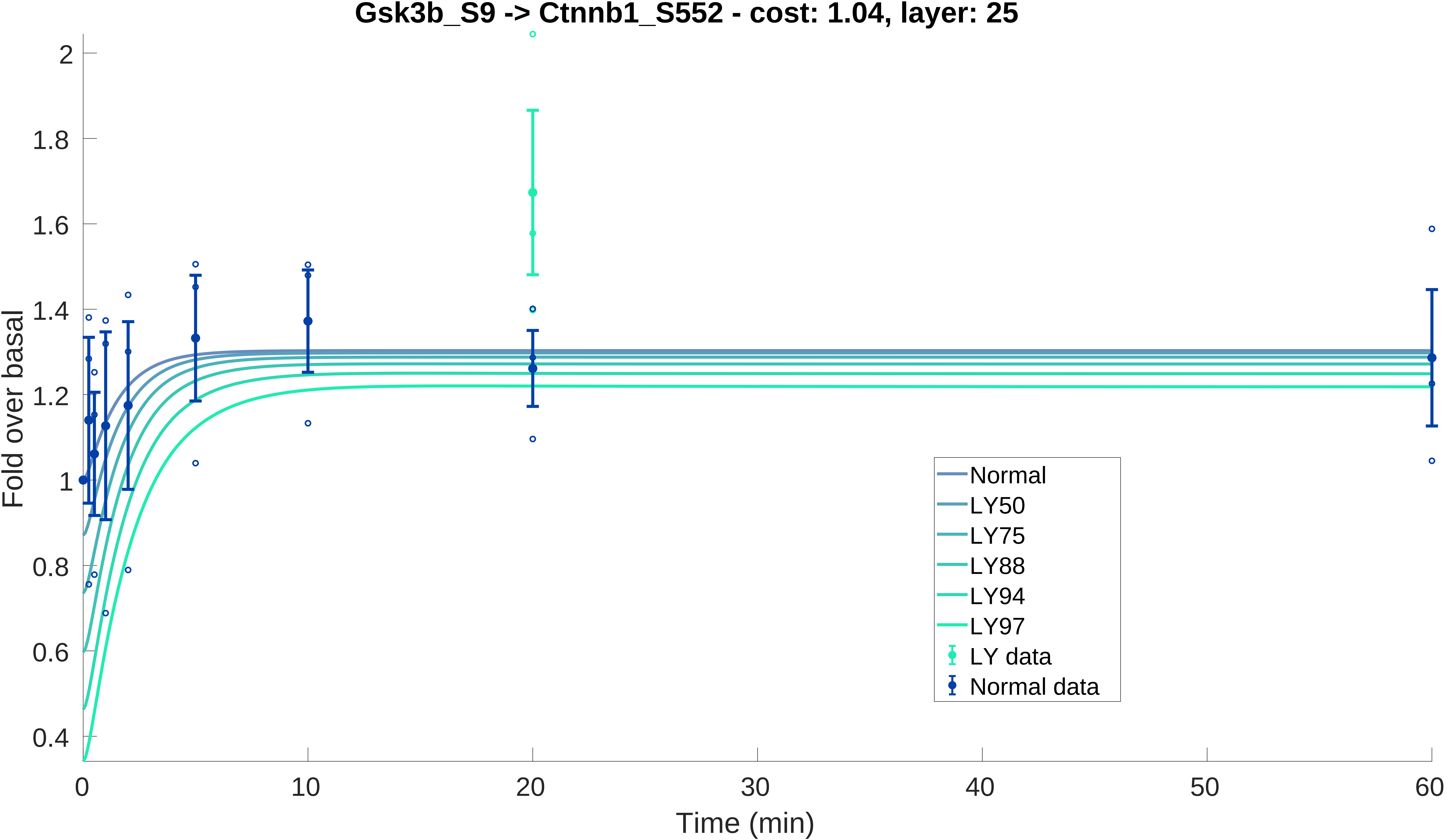

Gsk3b_S9 -> Ctnnb1_S552 - cost: 1.04, layer: 25
Fold over basal
Time (min)
2
1.8
1.6
1.4
1.2
1
0.8
0.6
0.4
0
10
20
30
40
50
60
Normal
LY50
LY75
LY88
LY94
LY97
LY data
Normal data

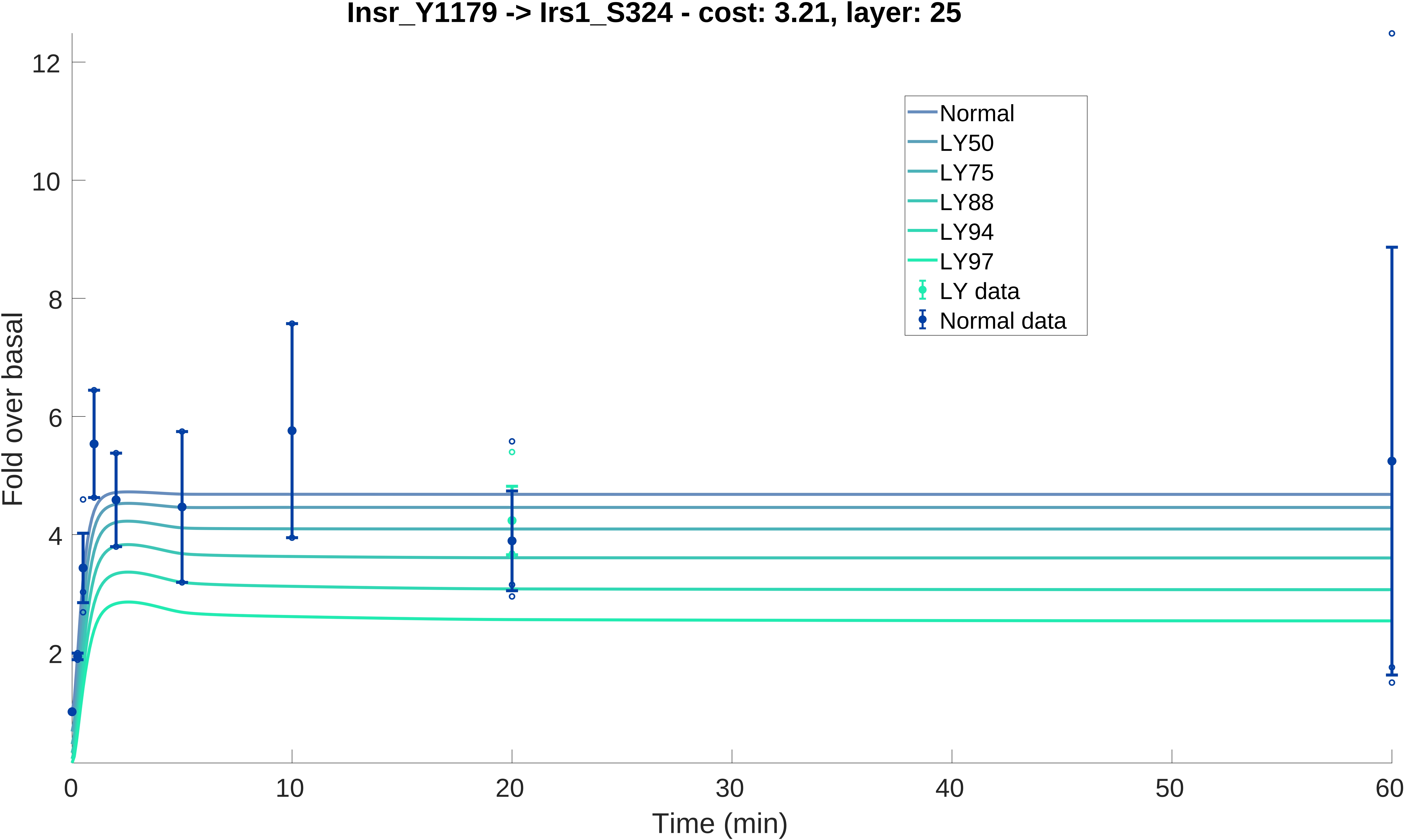

Insr_Y1179 -> Irs1_S324 - cost: 3.21, layer: 25
Normal
LY50
LY75
LY88
LY94
LY97
LY data
Normal data
Fold over basal
Time (min)
0
10
20
30
40
50
60
2
4
6
8
10
12

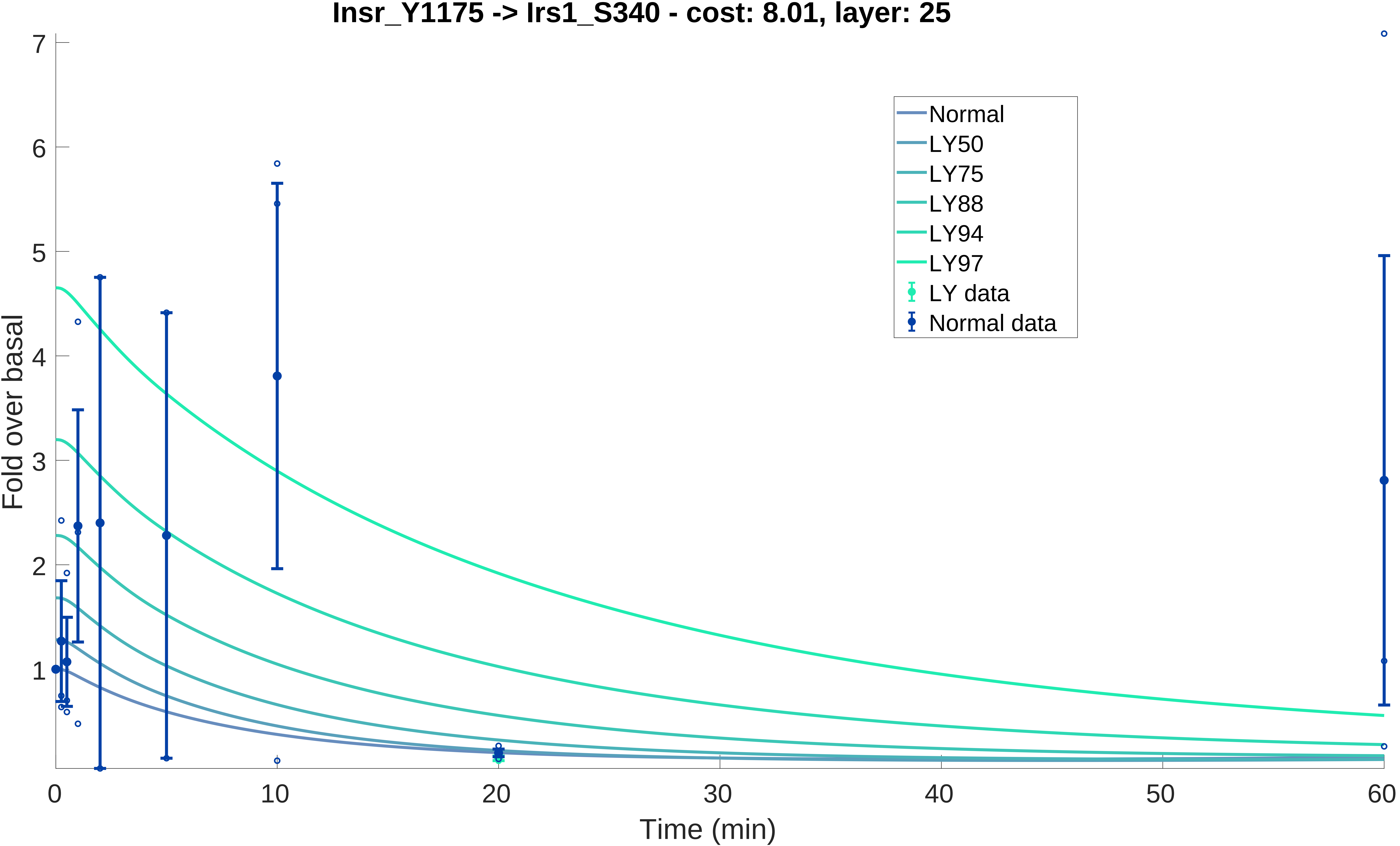

Insr_Y1175 -> Irs1_S340 - cost: 8.01, layer: 25
Fold over basal
Time (min)
0
10
20
30
40
50
60
1
2
3
4
5
6
7
Normal
LY50
LY75
LY88
LY94
LY97
LY data
Normal data

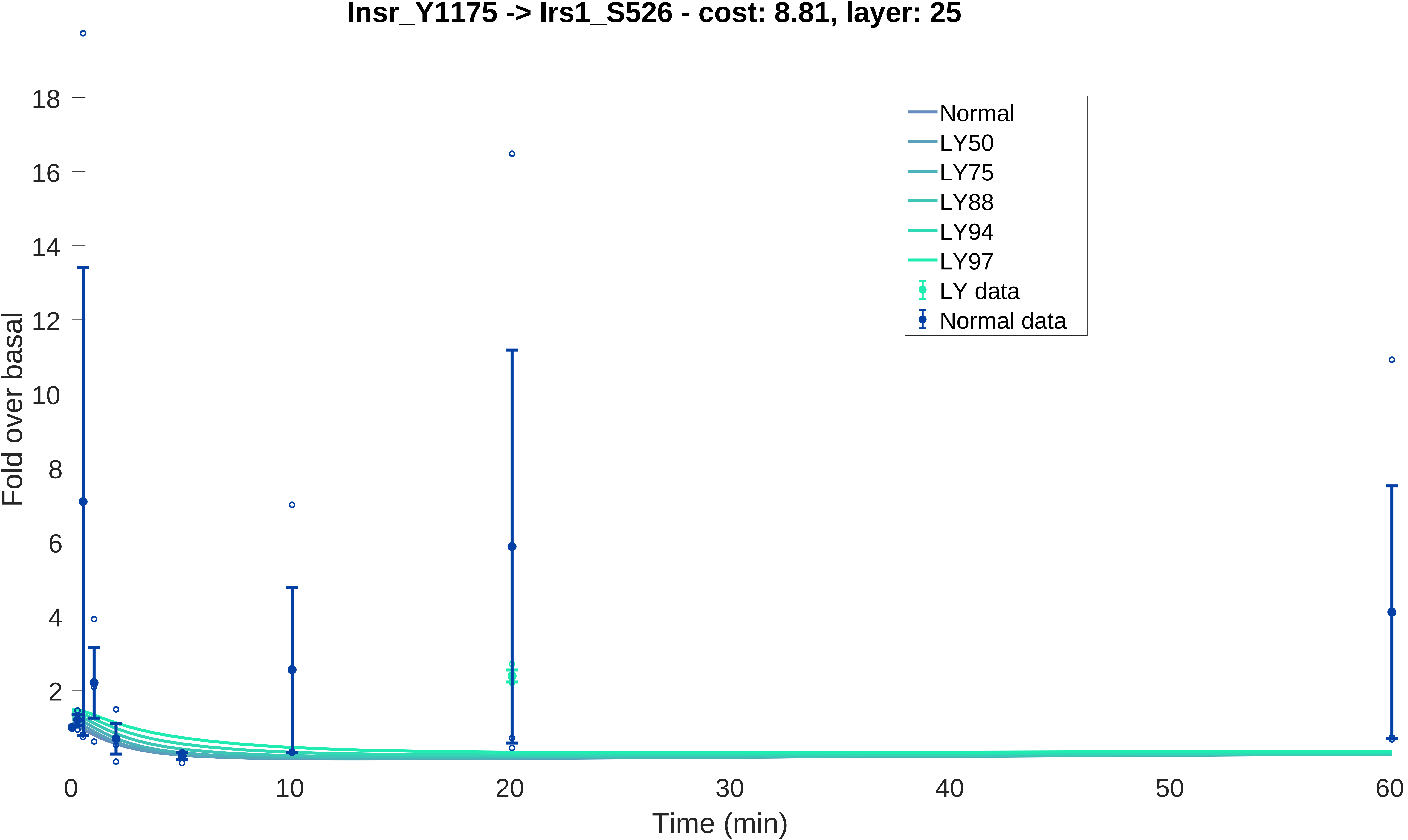

Insr_Y1175 -> Irs1_S526 - cost: 8.81, layer: 25
Fold over basal
Time (min)
Normal
LY50
LY75
LY88
LY94
LY97
LY data
Normal data
0
10
20
30
40
50
60
2
4
6
8
10
12
14
16
18

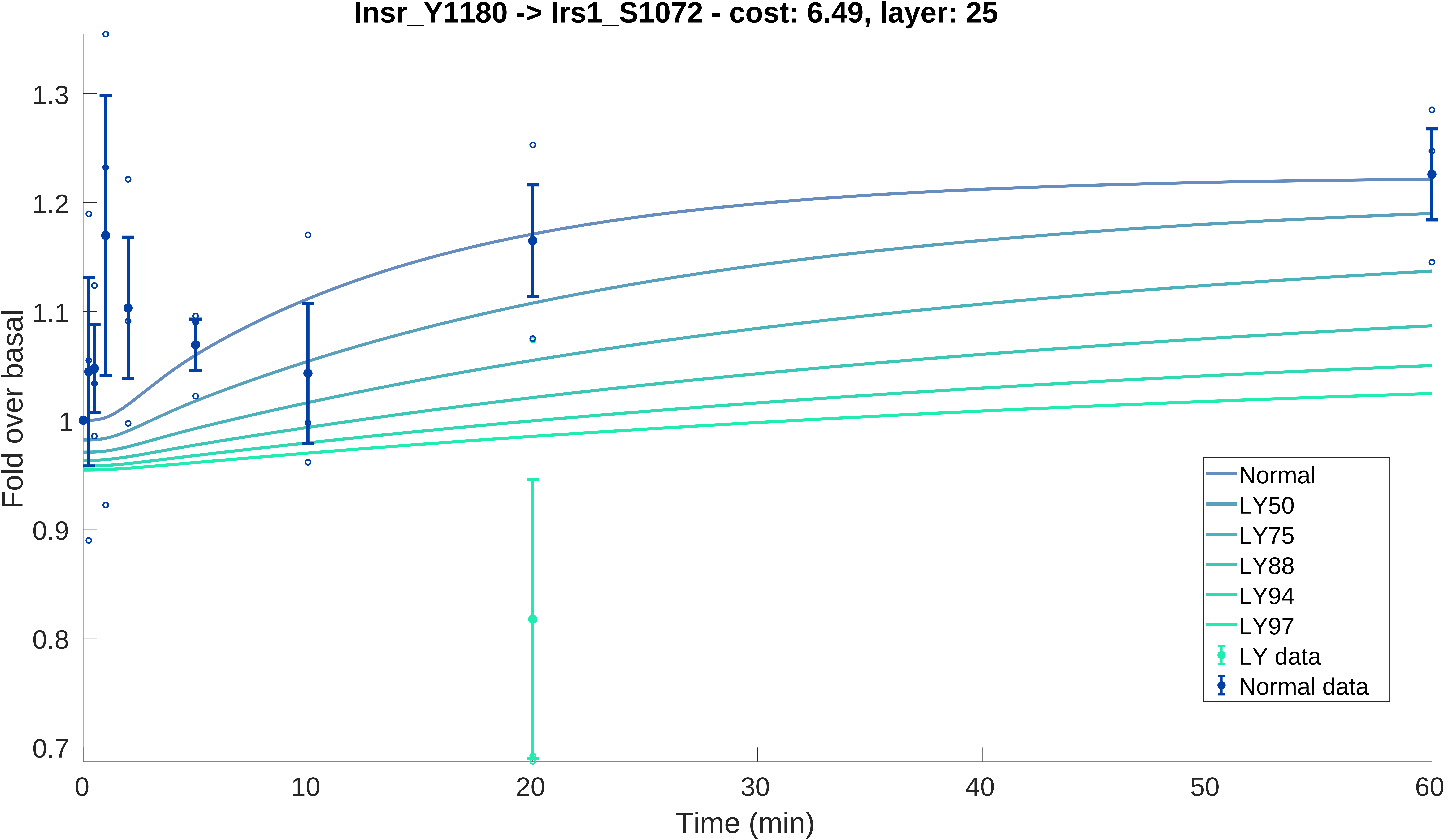
Insr_Y1180 -> Irs1_S1072 - cost: 6.49, layer: 25
Fold over basal
Time (min)
1.3
1.2
1.1
1
0.9
0.8
0.7
0
10
20
30
40
50
60
Normal
LY50
LY75
LY88
LY94
LY97
LY data
Normal data

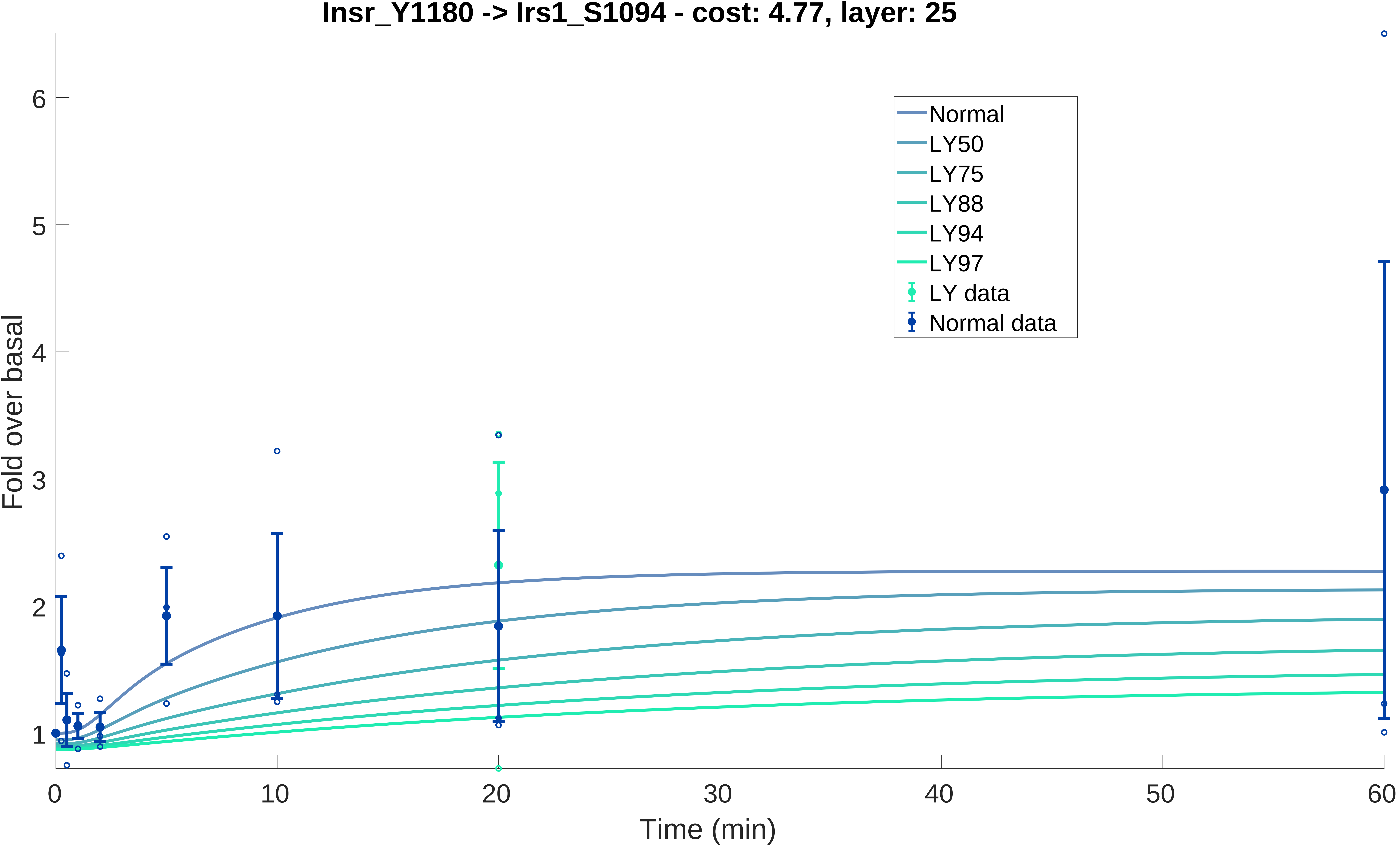

Insr_Y1180 -> Irs1_S1094 - cost: 4.77, layer: 25
Fold over basal
Time (min)
0
10
20
30
40
50
60
1
2
3
4
5
6
Normal
LY50
LY75
LY88
LY94
LY97
LY data
Normal data

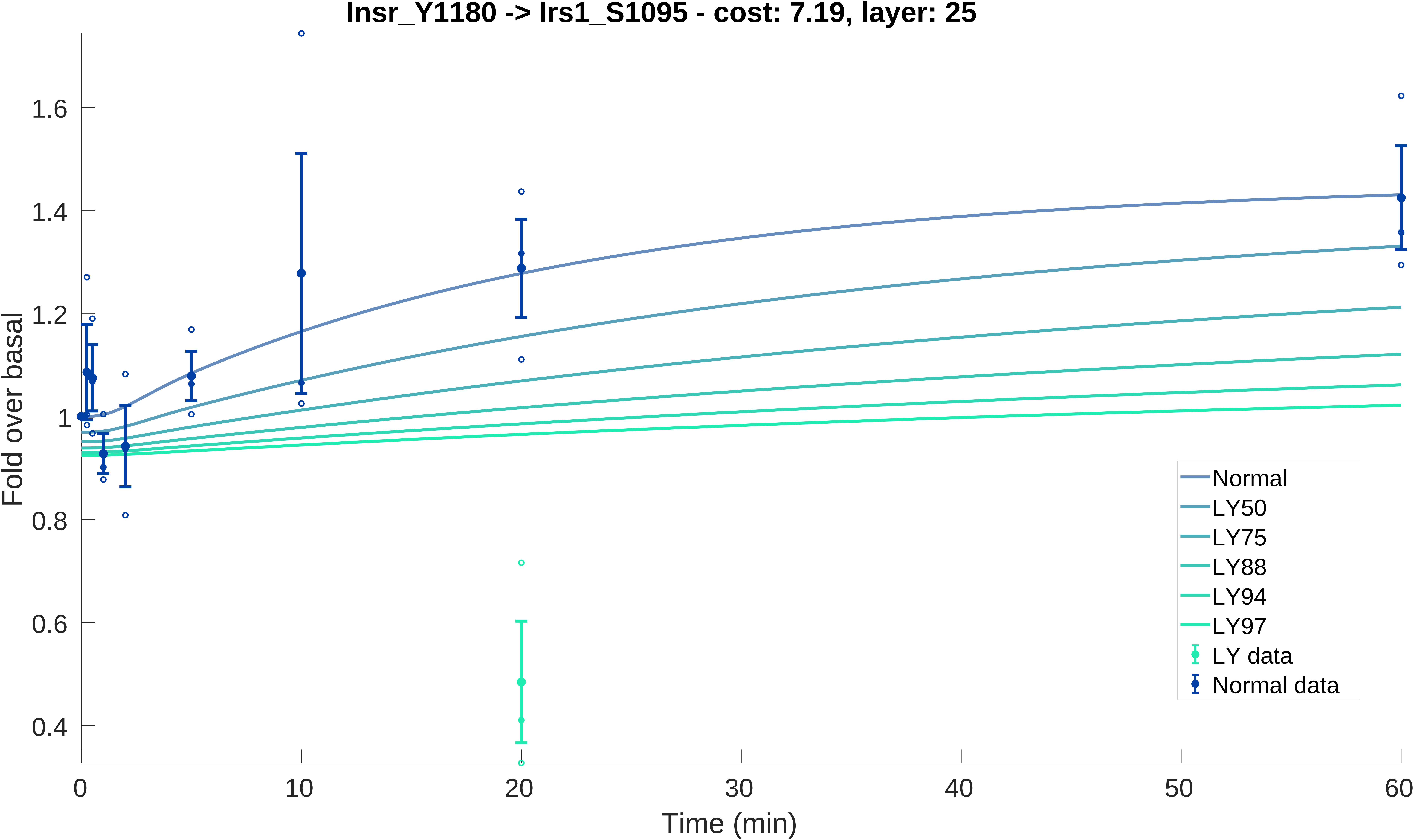
Insr_Y1180 -> Irs1_S1095 - cost: 7.19, layer: 25
Fold over basal
Time (min)
0.4
0.6
0.8
1
1.2
1.4
1.6
0
10
20
30
40
50
60
Normal
LY50
LY75
LY88
LY94
LY97
LY data
Normal data

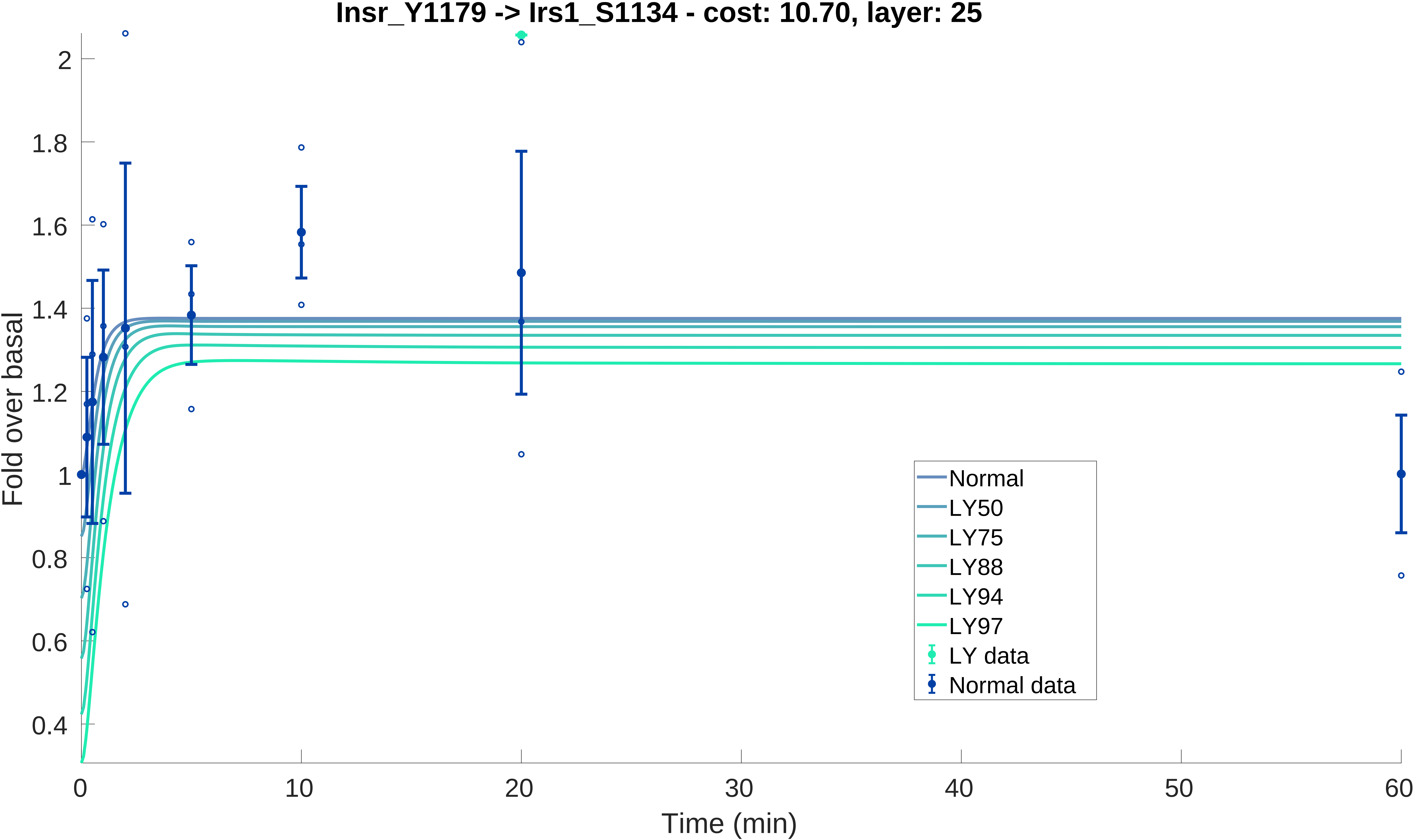

Insr_Y1179 -> Irs1_S1134 - cost: 10.70, layer: 25
Fold over basal
Time (min)
0
10
20
30
40
50
60
0.4
0.6
0.8
1
1.2
1.4
1.6
1.8
2
Normal
LY50
LY75
LY88
LY94
LY97
LY data
Normal data

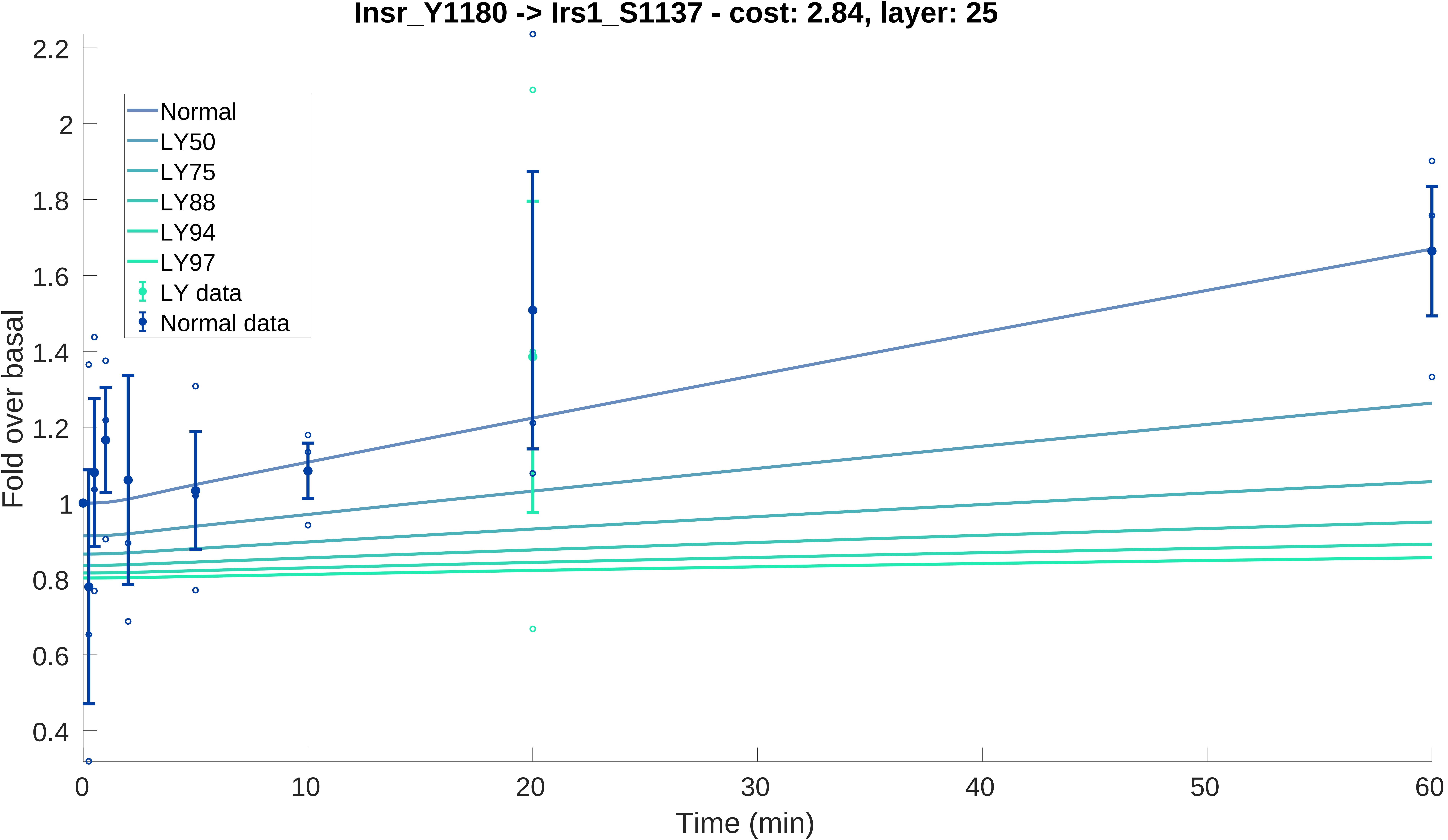
Insr_Y1180 -> Irs1_S1137 - cost: 2.84, layer: 25
Normal
LY50
LY75
LY88
LY94
LY97
LY data
Normal data
Fold over basal
Time (min)
2.2
2
1.8
1.6
1.4
1.2
1
0.8
0.6
0.4
0
10
20
30
40
50
60

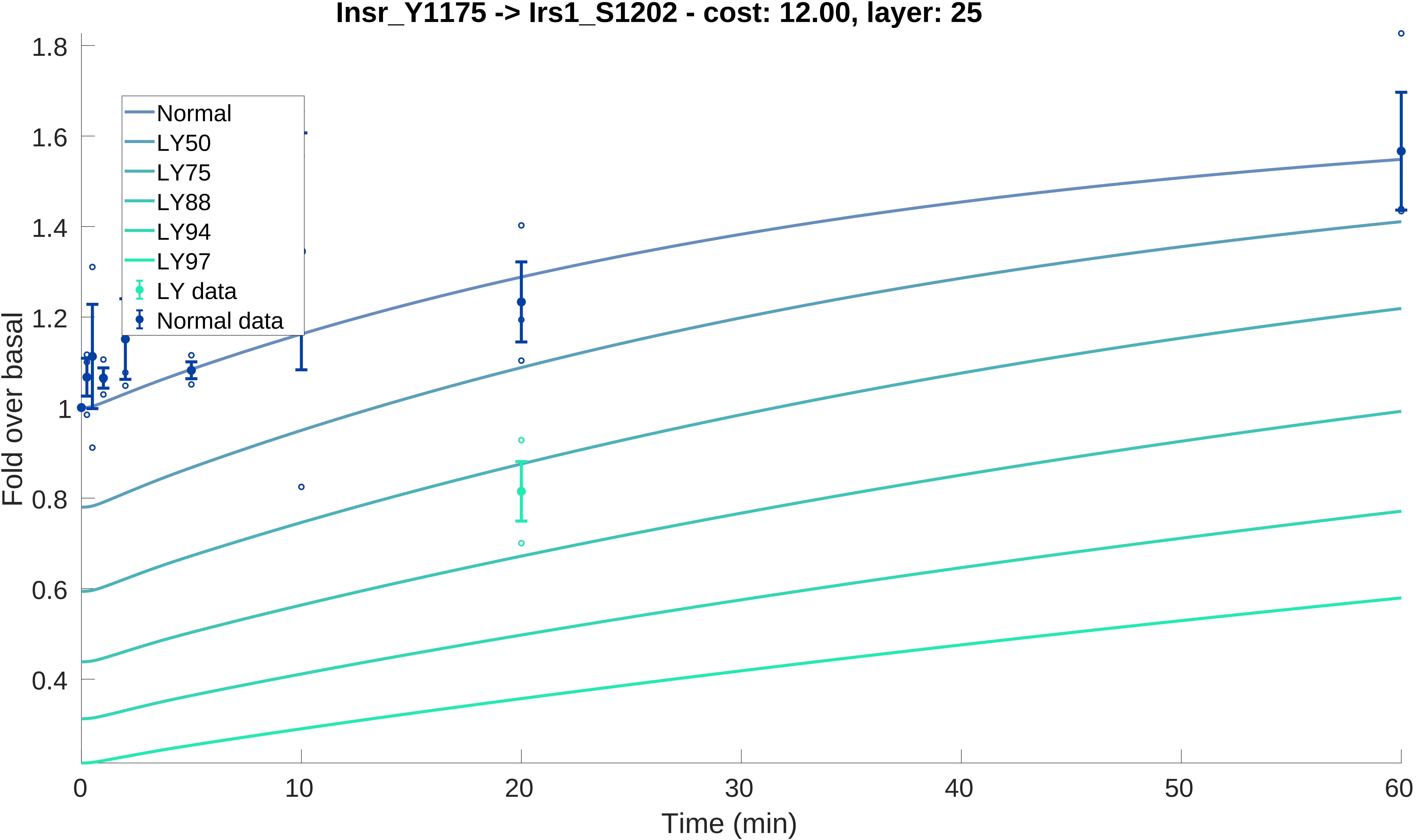

Insr_Y1175 -> Irs1_S1202 - cost: 12.00, layer: 25
Normal
LY50
LY75
LY88
LY94
LY97
LY data
Normal data
Fold over basal
Time (min)
0
10
20
30
40
50
60
0.4
0.6
0.8
1
1.2
1.4
1.6
1.8

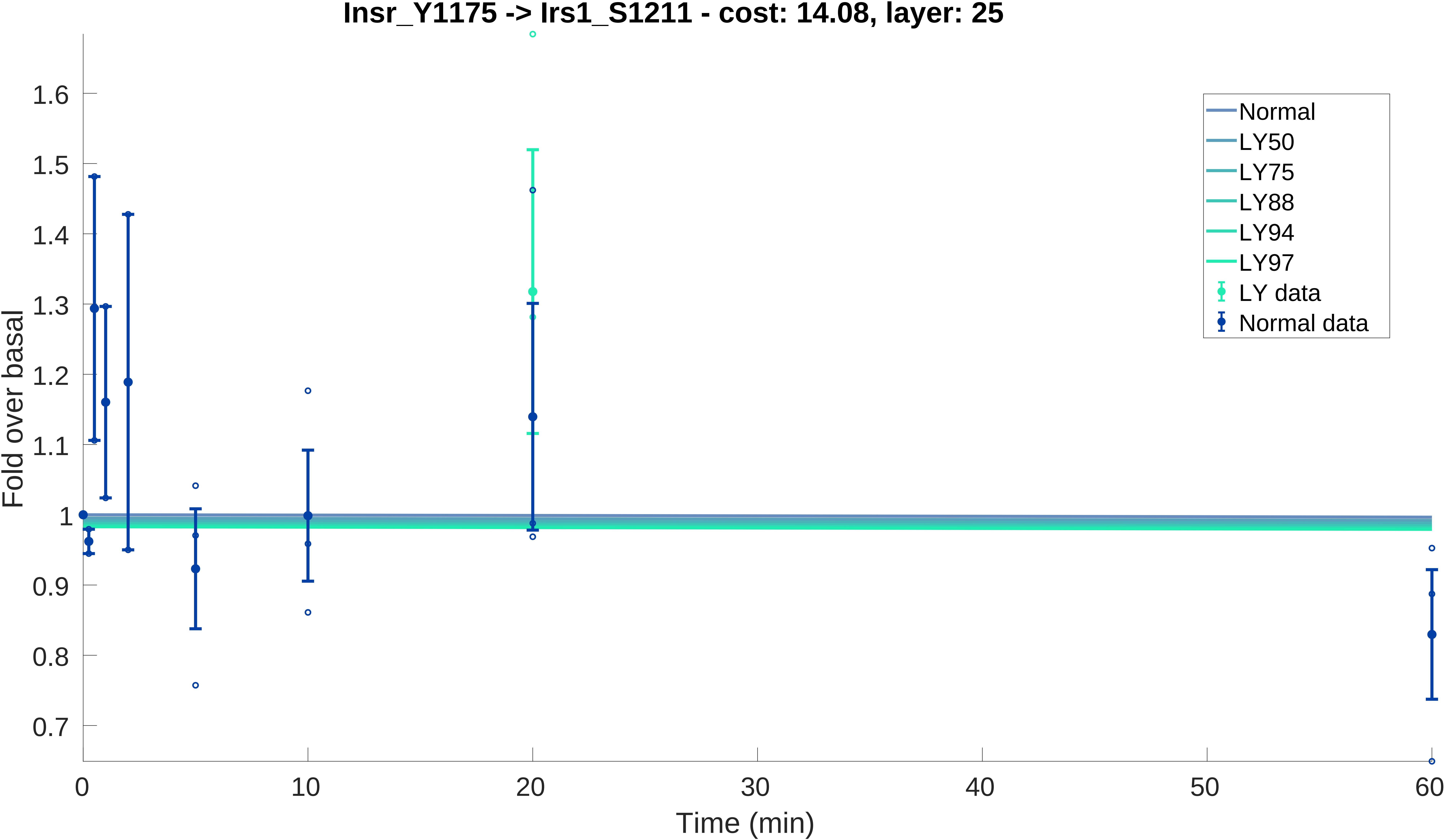
Insr_Y1175 -> Irs1_S1211 - cost: 14.08, layer: 25
Fold over basal
1.6
1.5
1.4
1.3
1.2
1.1
1
0.9
0.8
0.7
0
10
20
30
40
50
60
Time (min)
Normal
LY50
LY75
LY88
LY94
LY97
LY data
Normal data

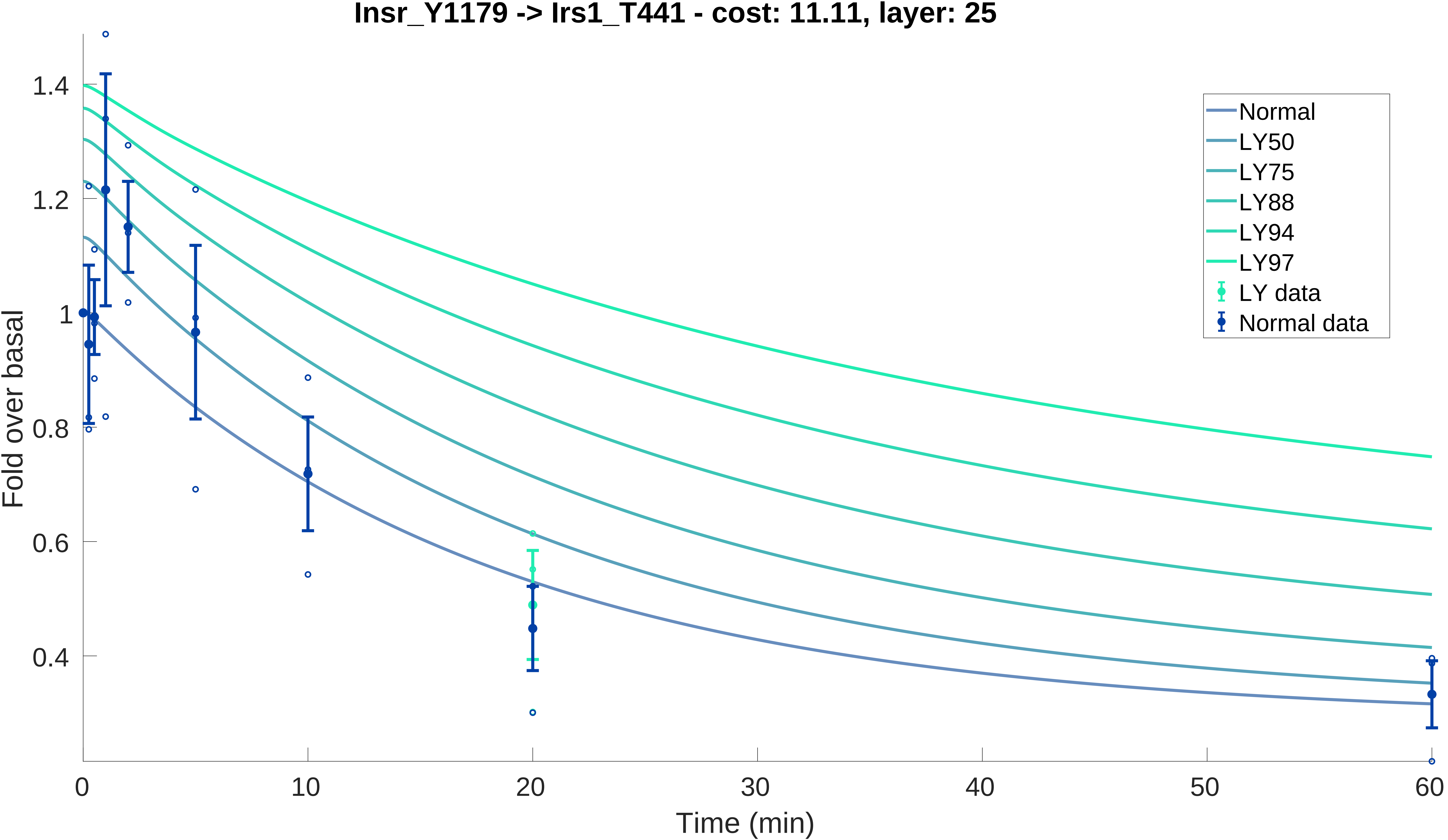

Insr_Y1179 -> Irs1_T441 - cost: 11.11, layer: 25
Fold over basal
1.4
1.2
1
0.8
0.6
0.4
0
10
20
30
40
50
60
Time (min)
Normal
LY50
LY75
LY88
LY94
LY97
LY data
Normal data

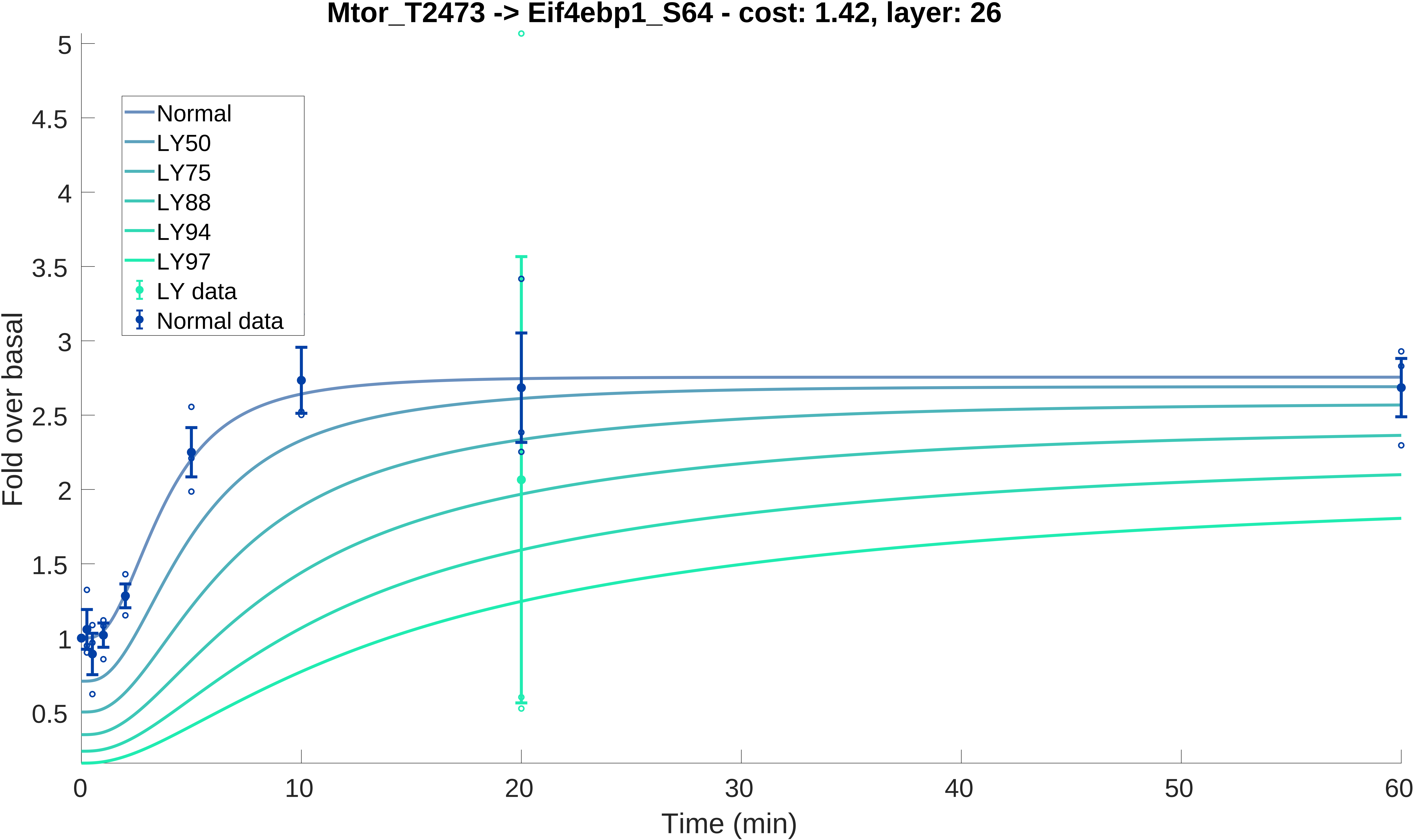

Mtor_T2473 -> Eif4ebp1_S64 - cost: 1.42, layer: 26
Normal
LY50
LY75
LY88
LY94
LY97
LY data
Normal data
Fold over basal
Time (min)
0.5
1
1.5
2
2.5
3
3.5
4
4.5
5
0
10
20
30
40
50
60

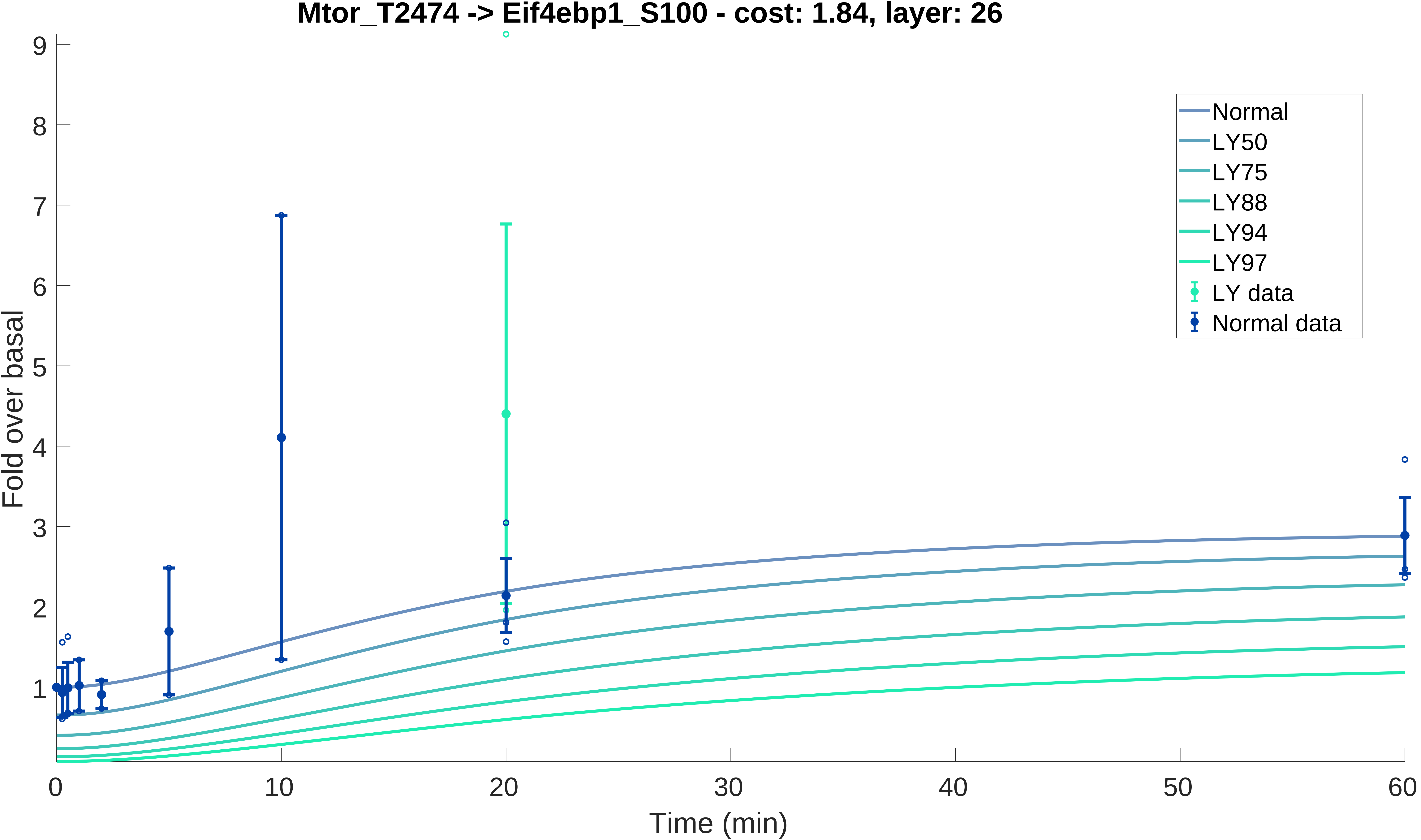

Mtor_T2474 -> Eif4ebp1_S100 - cost: 1.84, layer: 26
Fold over basal
Time (min)
Normal
LY50
LY75
LY88
LY94
LY97
LY data
Normal data

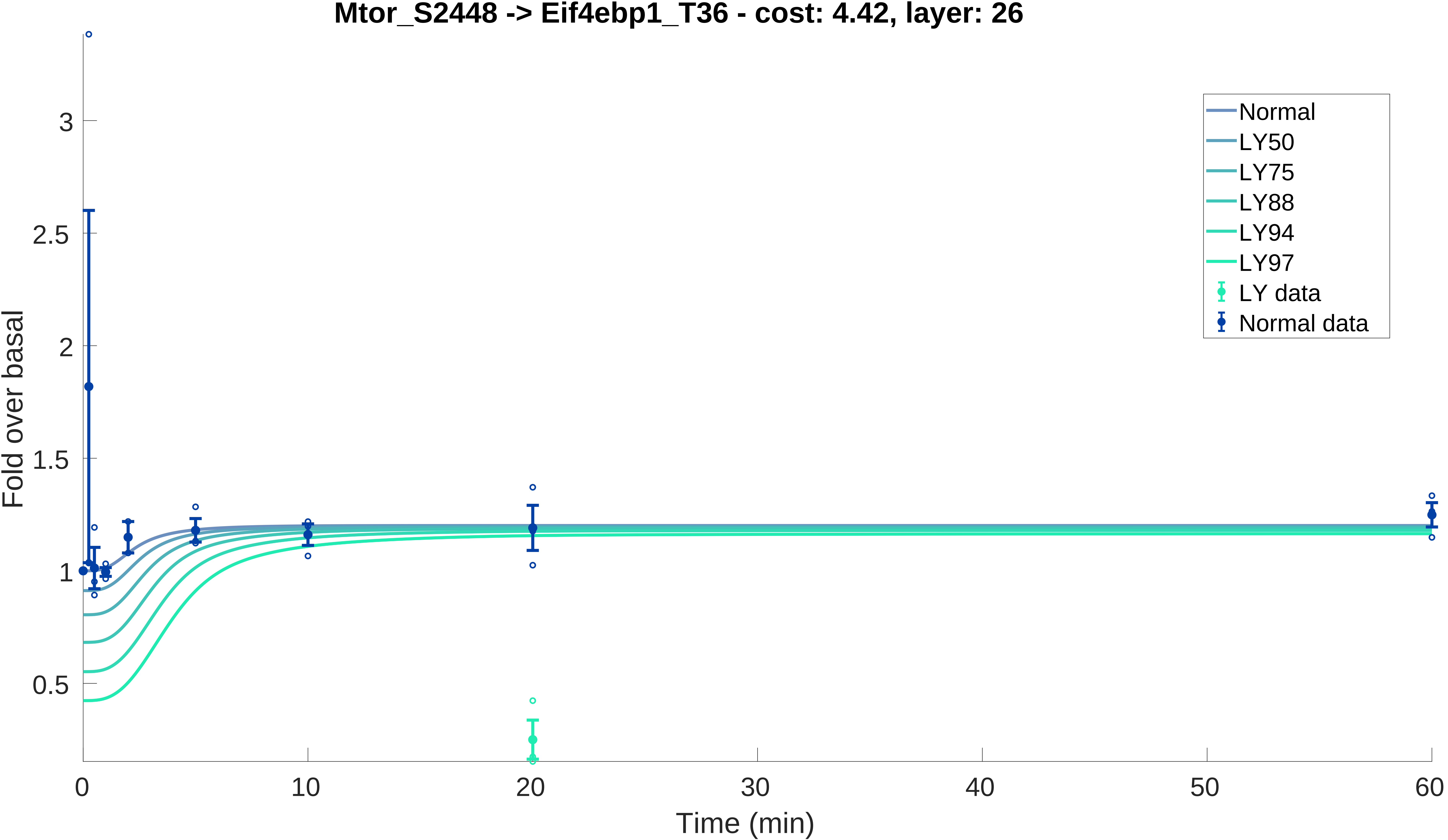
Mtor_S2448 -> Eif4ebp1_T36 - cost: 4.42, layer: 26
Fold over basal
Time (min)
0.5
1
1.5
2
2.5
3
0
10
20
30
40
50
60
Normal
LY50
LY75
LY88
LY94
LY97
LY data
Normal data

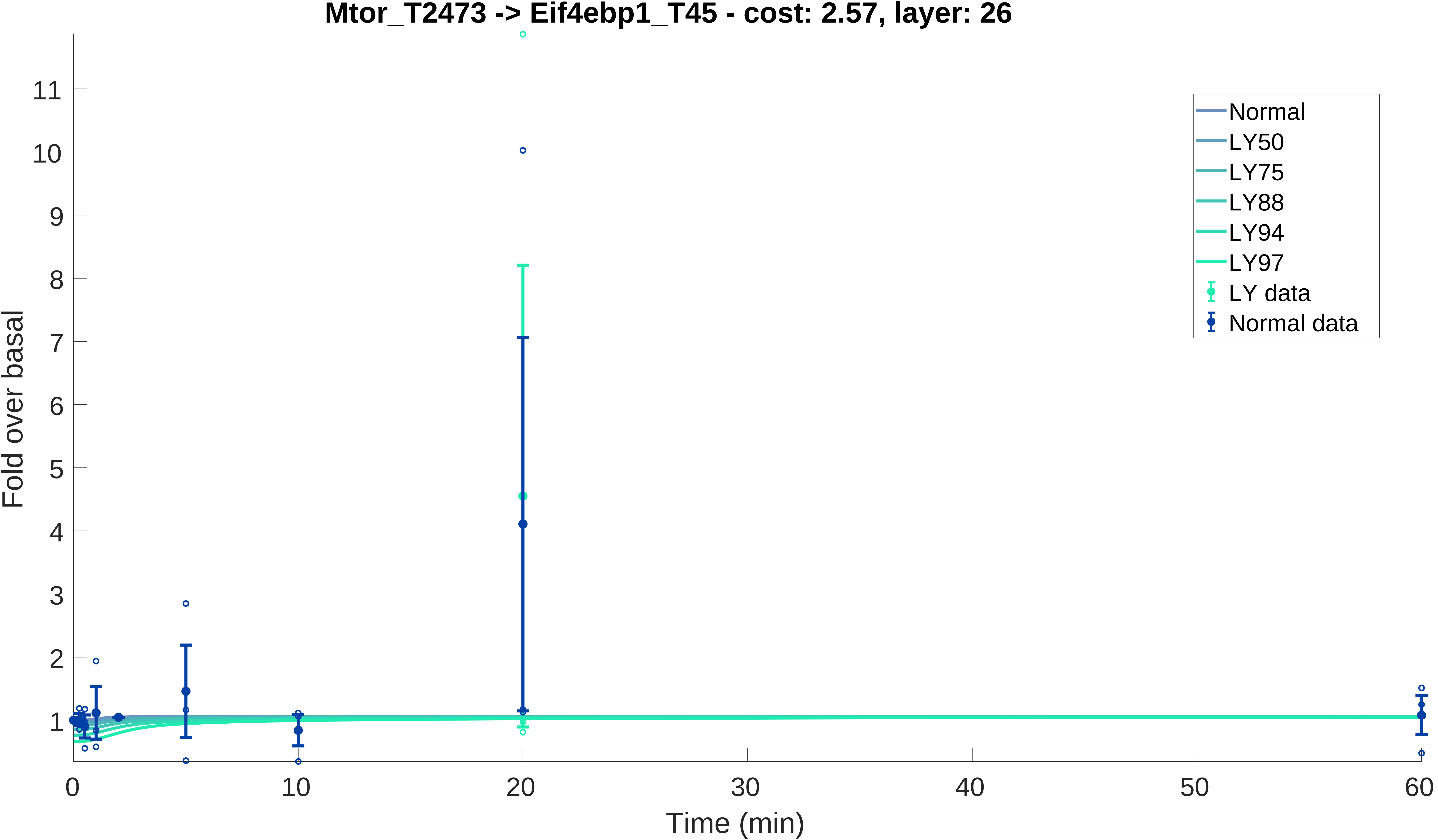

Mtor_T2473 -> Eif4ebp1_T45 - cost: 2.57, layer: 26
Fold over basal
Time (min)
Normal
LY50
LY75
LY88
LY94
LY97
LY data
Normal data
0
10
20
30
40
50
60
1
2
3
4
5
6
7
8
9
10
11

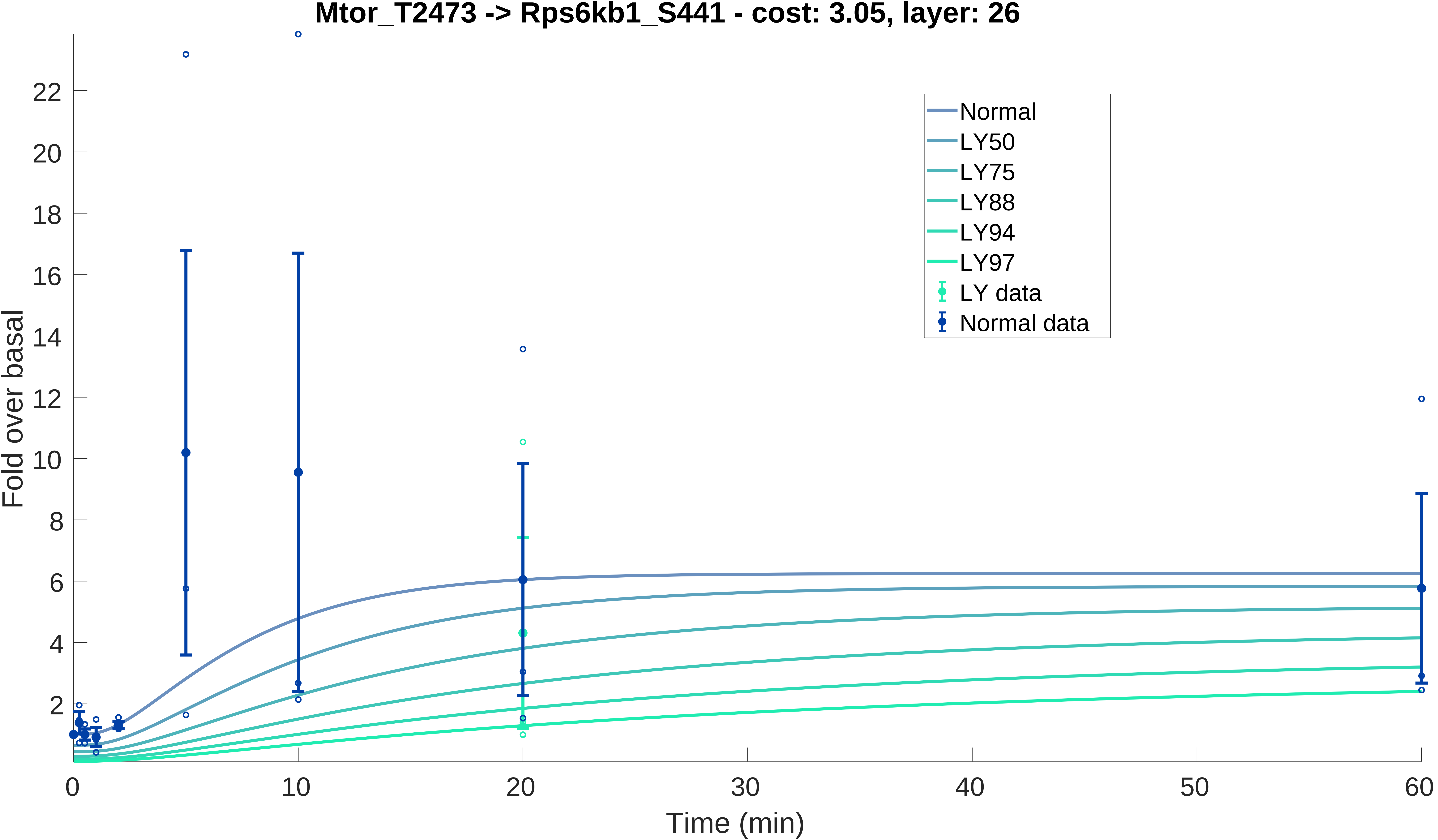

Mtor_T2473 -> Rps6kb1_S441 - cost: 3.05, layer: 26
Normal
LY50
LY75
LY88
LY94
LY97
LY data
Normal data
Fold over basal
Time (min)
0
10
20
30
40
50
60
2
4
6
8
10
12
14
16
18
20
22

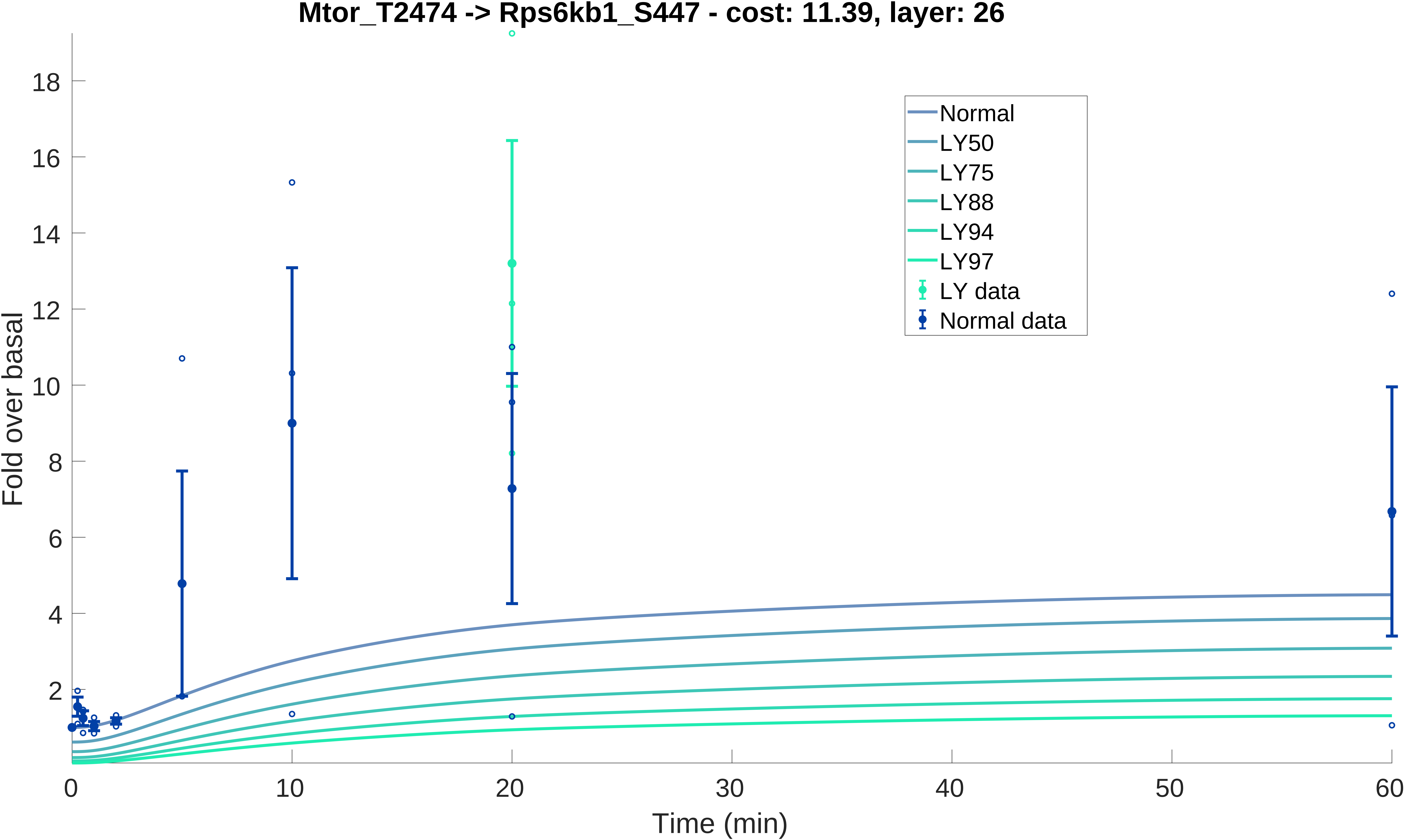

Mtor_T2474 -> Rps6kb1_S447 - cost: 11.39, layer: 26
Fold over basal
Time (min)
Normal
LY50
LY75
LY88
LY94
LY97
LY data
Normal data

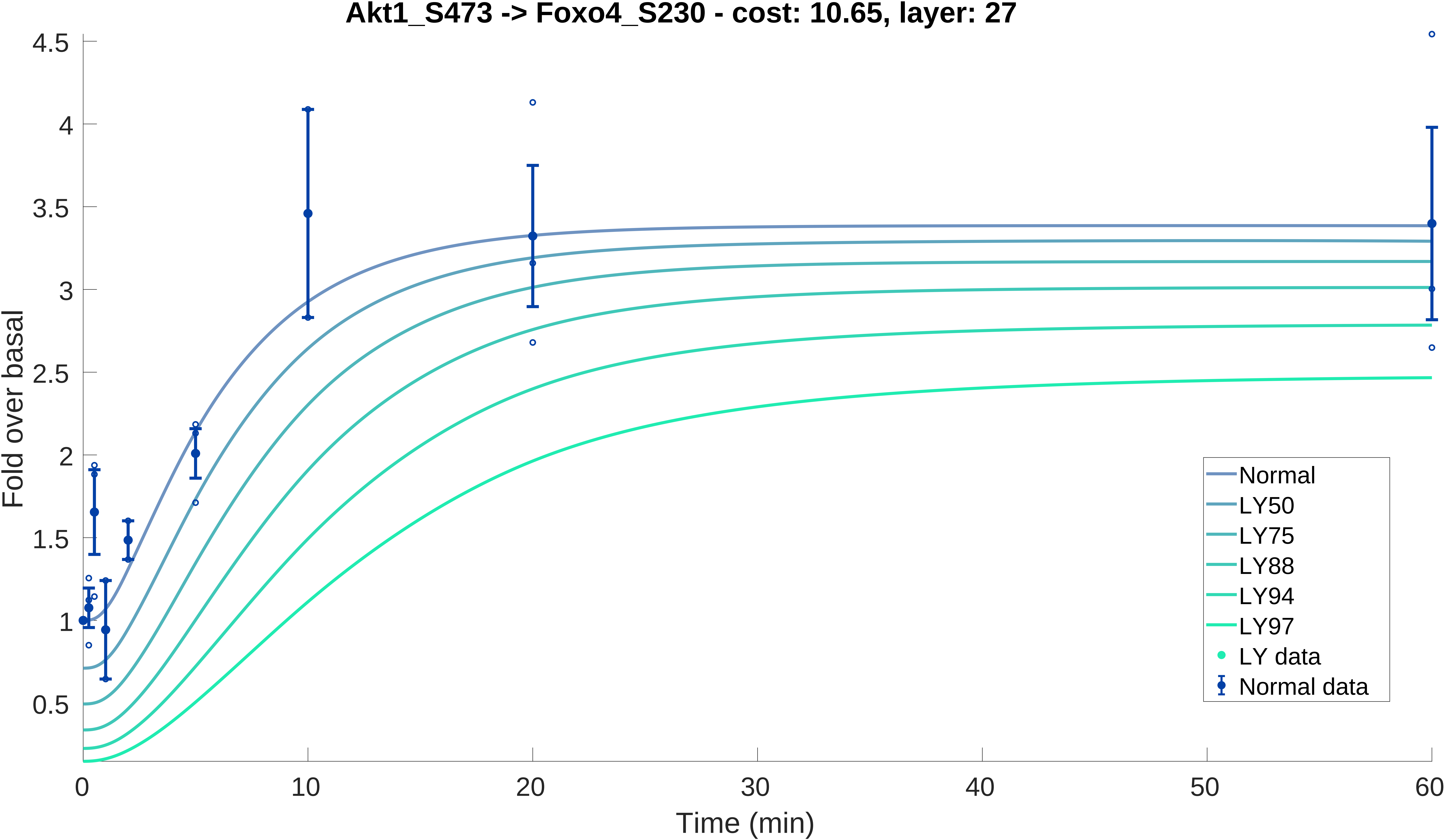
Akt1_S473 -> Foxo4_S230 - cost: 10.65, layer: 27
Fold over basal
4.5
4
3.5
3
2.5
2
1.5
1
0.5
0
10
20
30
40
50
60
Time (min)
Normal
LY50
LY75
LY88
LY94
LY97
LY data
Normal data

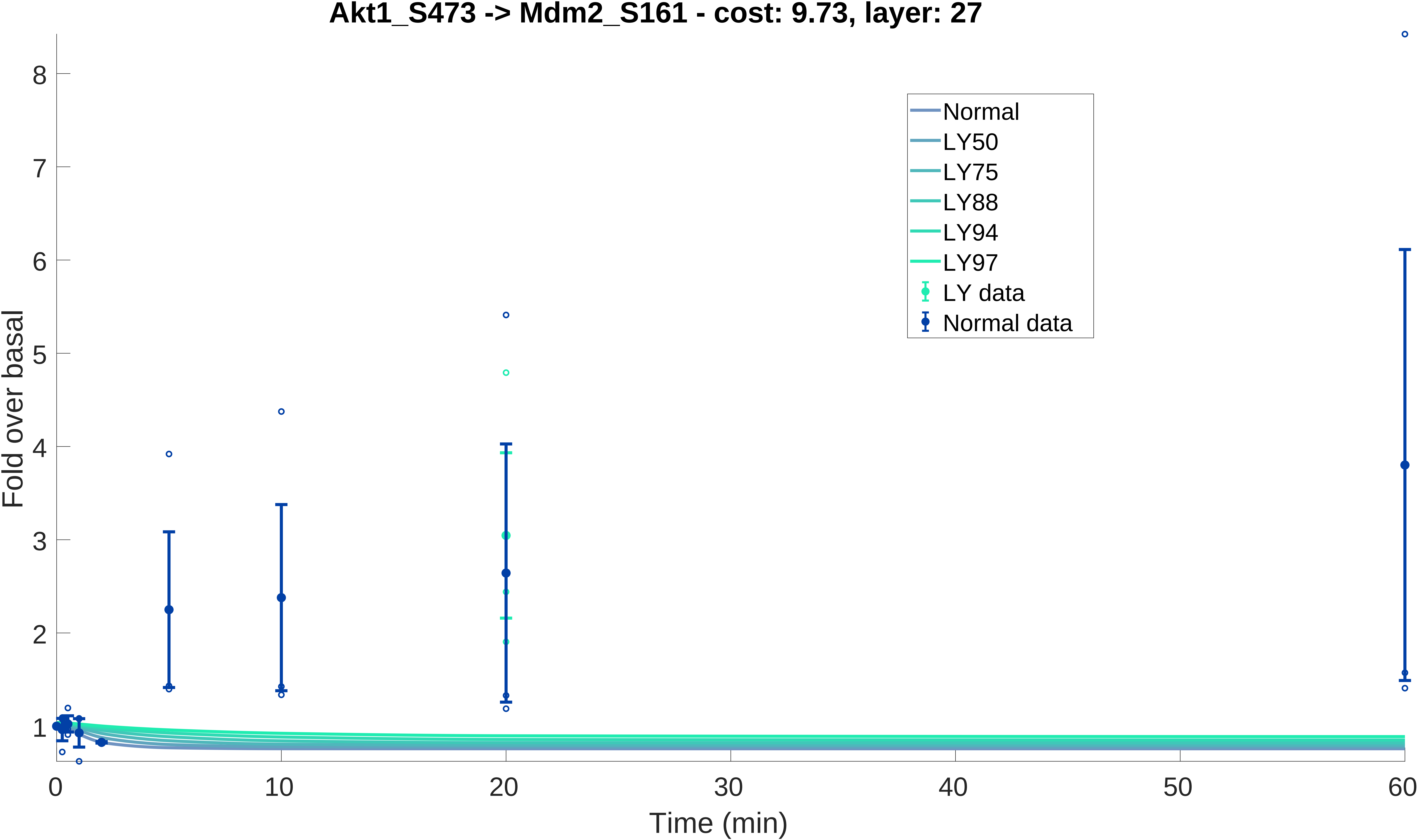

Akt1_S473 -> Mdm2_S161 - cost: 9.73, layer: 27
Fold over basal
Time (min)
Normal
LY50
LY75
LY88
LY94
LY97
LY data
Normal data
1
2
3
4
5
6
7
8
0
10
20
30
40
50
60

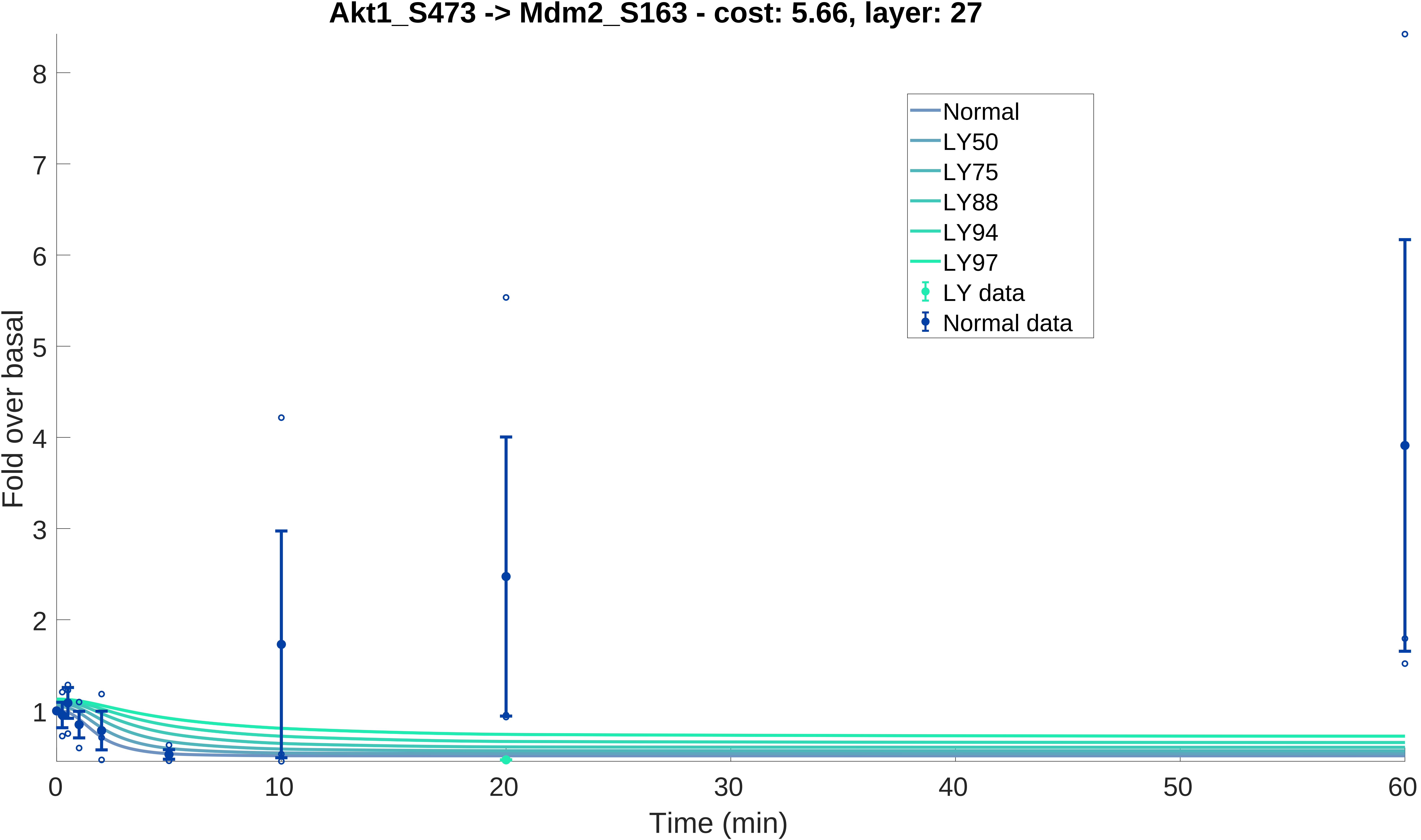

Akt1_S473 -> Mdm2_S163 - cost: 5.66, layer: 27
Fold over basal
Time (min)
Normal
LY50
LY75
LY88
LY94
LY97
LY data
Normal data
0
10
20
30
40
50
60
1
2
3
4
5
6
7
8

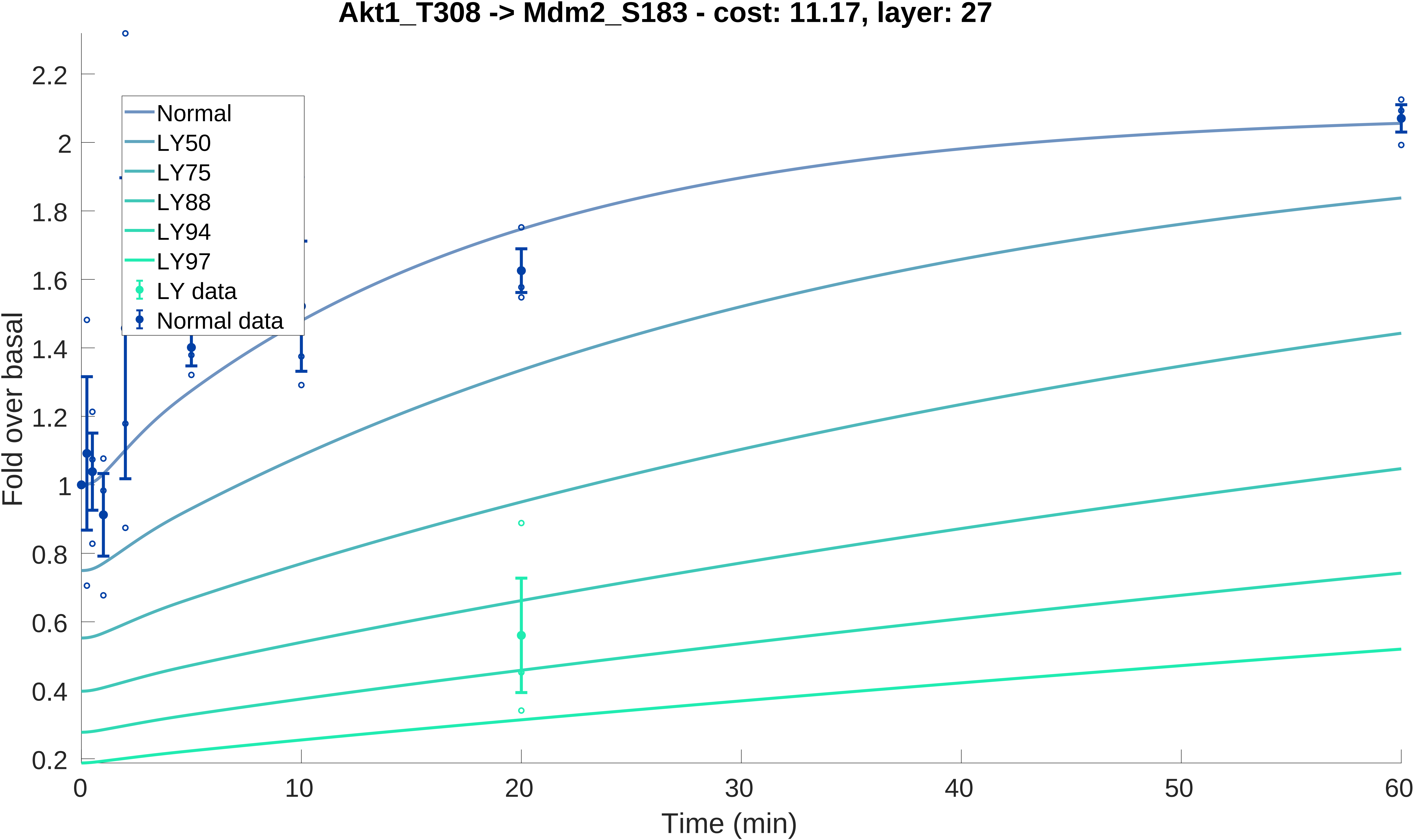

Akt1_T308 -> Mdm2_S183 - cost: 11.17, layer: 27
Normal
LY50
LY75
LY88
LY94
LY97
LY data
Normal data
Fold over basal
Time (min)
0
10
20
30
40
50
60
0.2
0.4
0.6
0.8
1
1.2
1.4
1.6
1.8
2
2.2

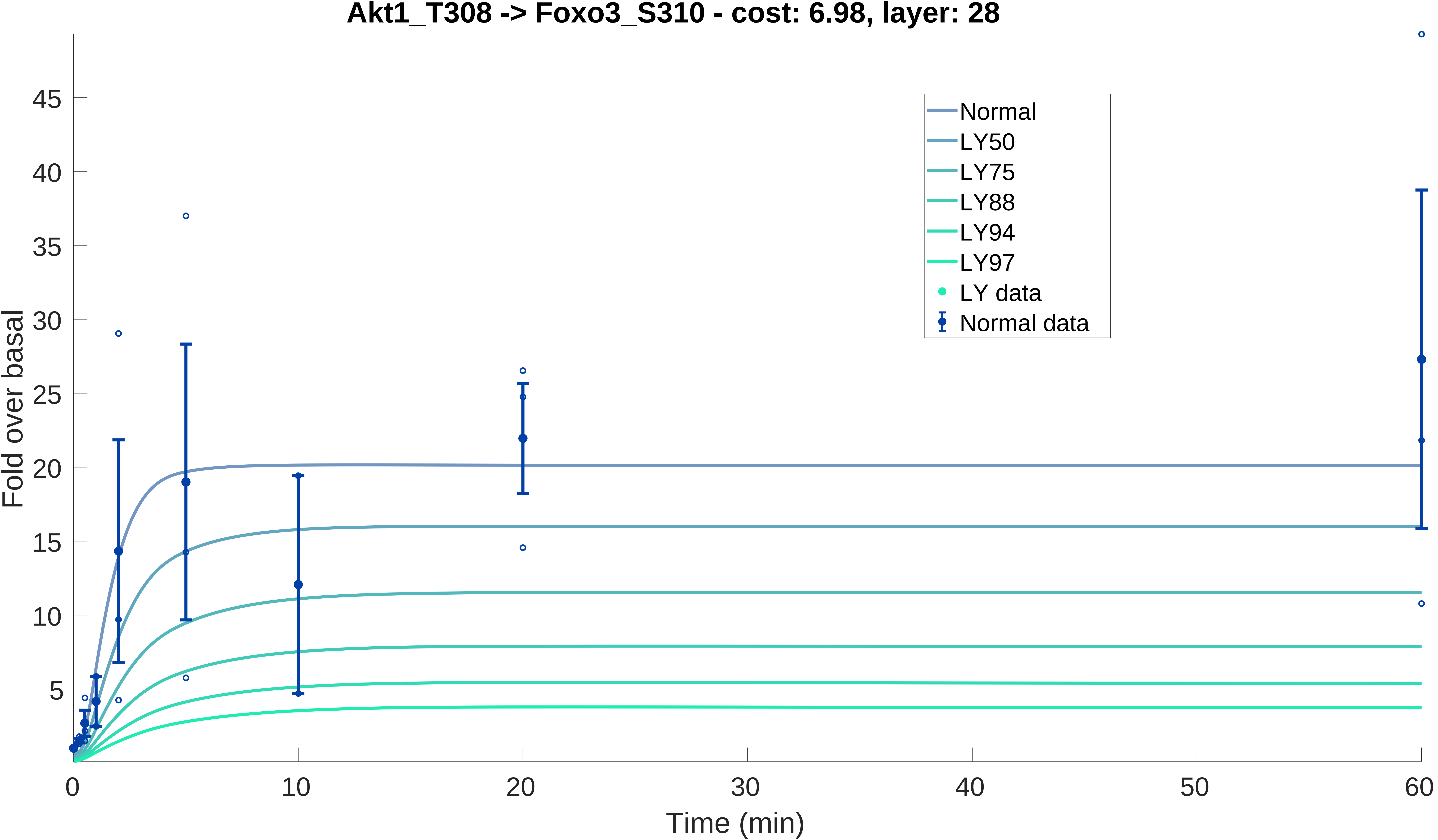
Akt1_T308 -> Foxo3_S310 - cost: 6.98, layer: 28
Fold over basal
Time (min)
0
10
20
30
40
50
60
5
10
15
20
25
30
35
40
45
Normal
LY50
LY75
LY88
LY94
LY97
LY data
Normal data

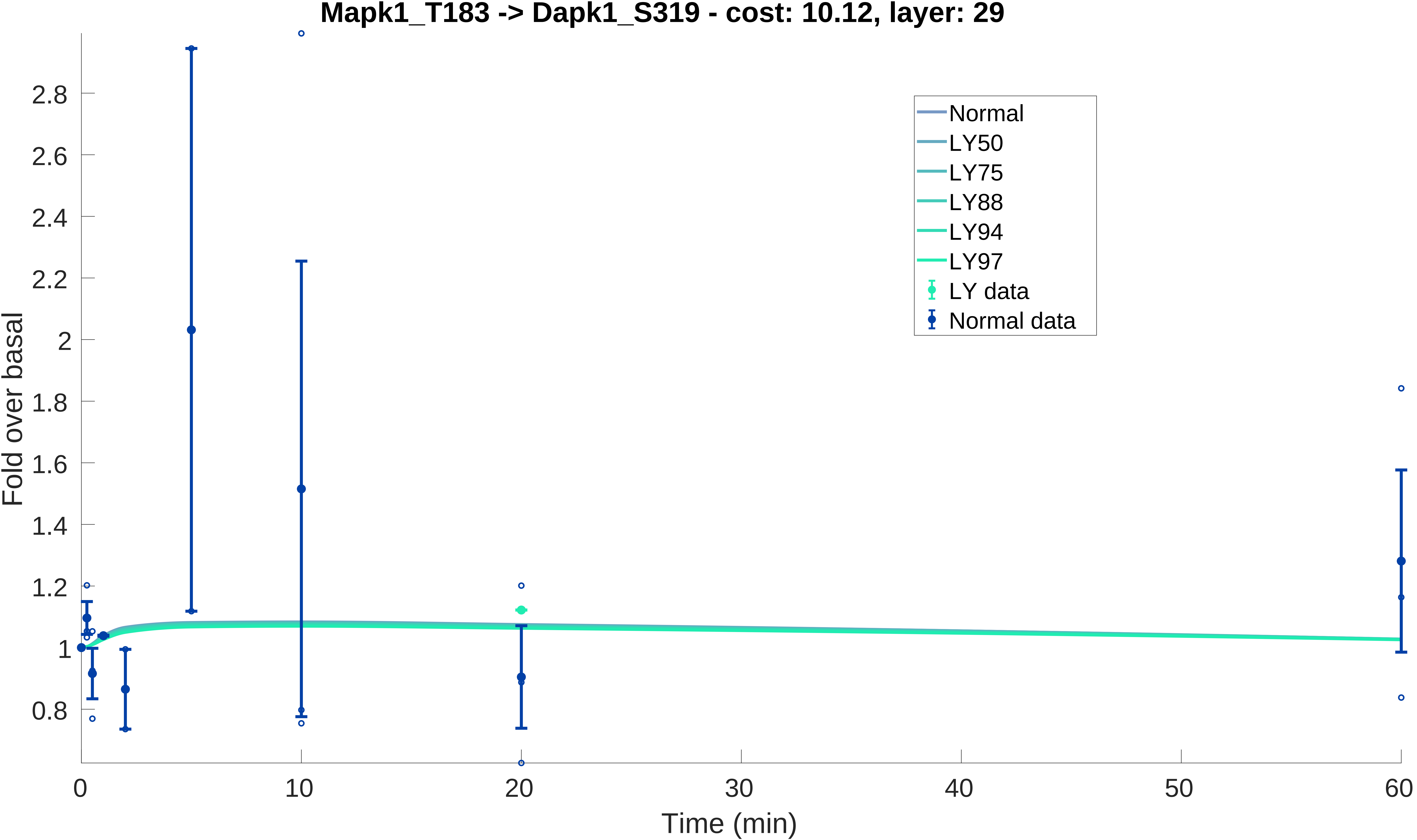

Mapk1_T183 -> Dapk1_S319 - cost: 10.12, layer: 29
Fold over basal
2.8
2.6
2.4
2.2
2
1.8
1.6
1.4
1.2
1
0.8
0
10
20
30
40
50
60
Time (min)
Normal
LY50
LY75
LY88
LY94
LY97
LY data
Normal data

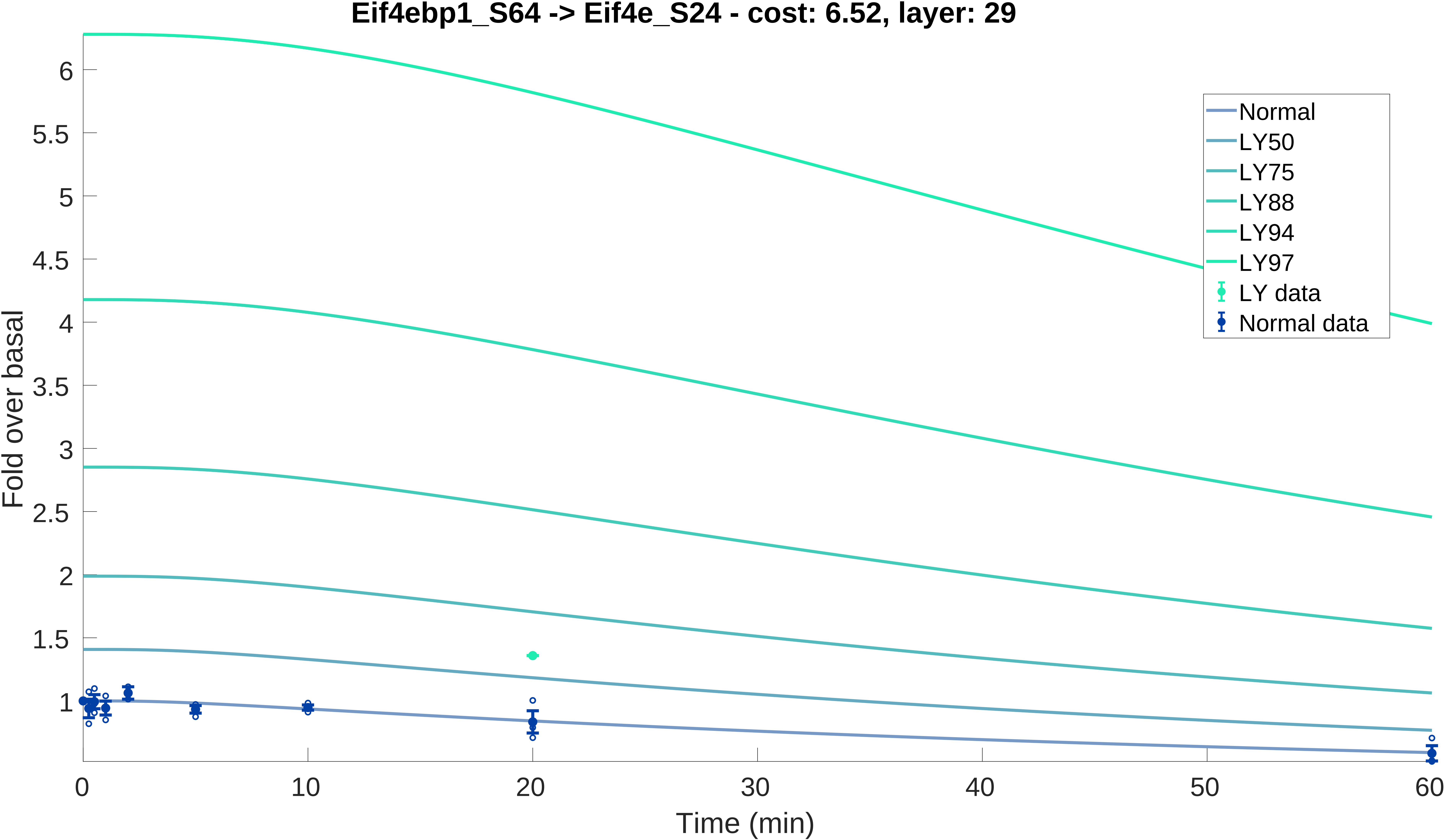

Eif4ebp1_S64 -> Eif4e_S24 - cost: 6.52, layer: 29
Fold over basal
Time (min)
0
10
20
30
40
50
60
1
1.5
2
2.5
3
3.5
4
4.5
5
5.5
6
Normal
LY50
LY75
LY88
LY94
LY97
LY data
Normal data

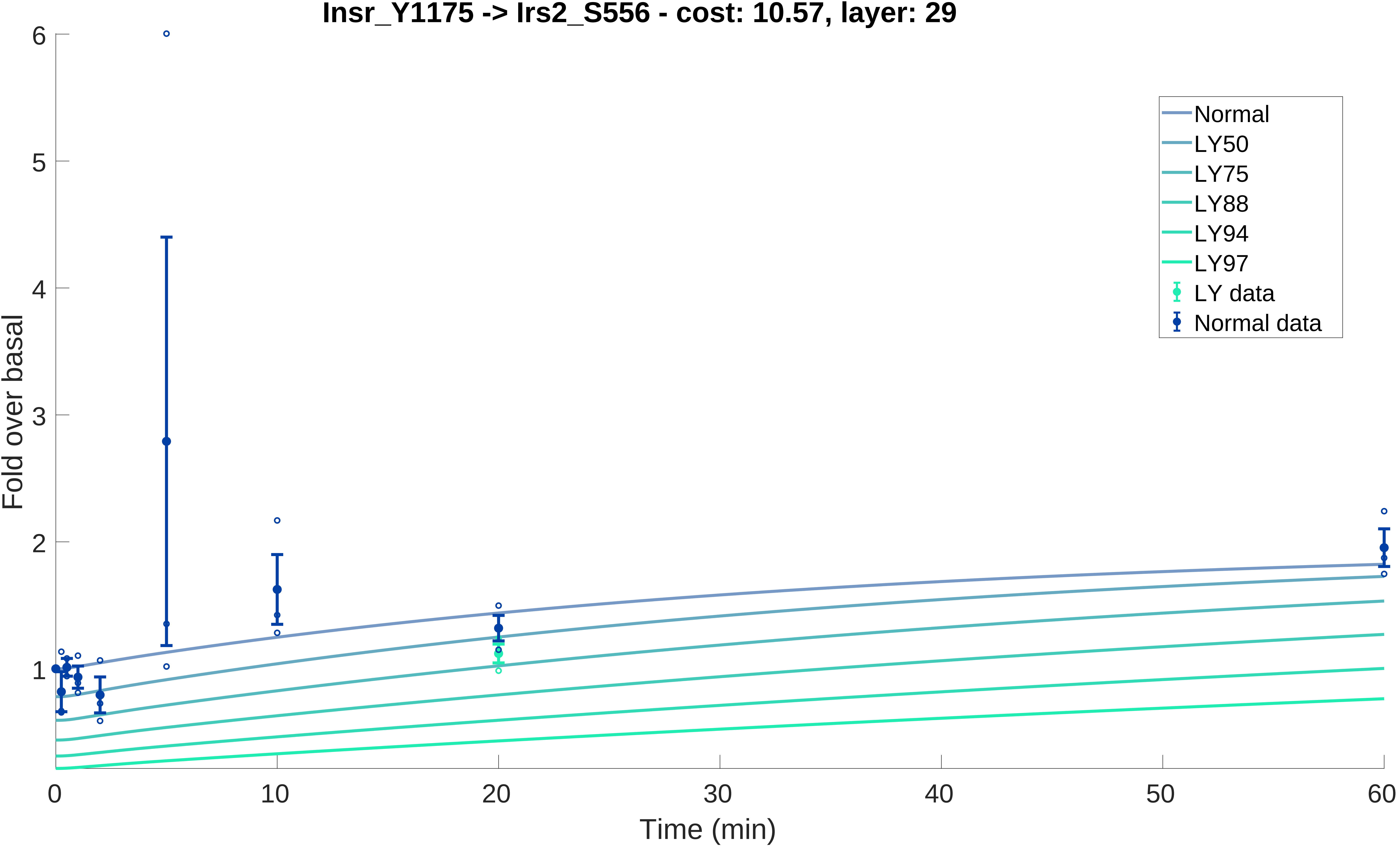

Insr_Y1175 -> Irs2_S556 - cost: 10.57, layer: 29
Fold over basal
Time (min)
0
10
20
30
40
50
60
1
2
3
4
5
6
Normal
LY50
LY75
LY88
LY94
LY97
LY data
Normal data

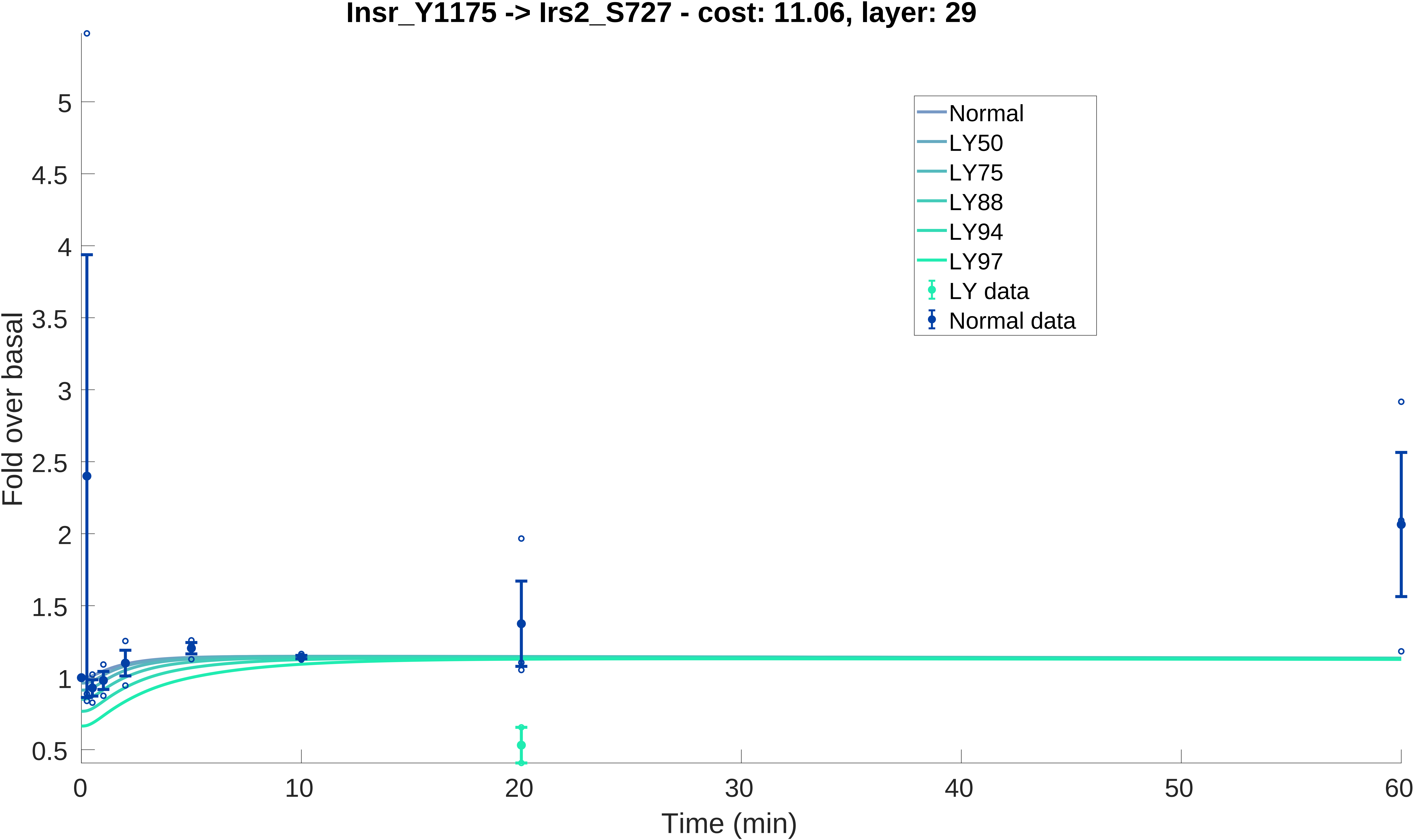

Insr_Y1175 -> Irs2_S727 - cost: 11.06, layer: 29
Fold over basal
Time (min)
Normal
LY50
LY75
LY88
LY94
LY97
LY data
Normal data
0.5
1
1.5
2
2.5
3
3.5
4
4.5
5
0
10
20
30
40
50
60

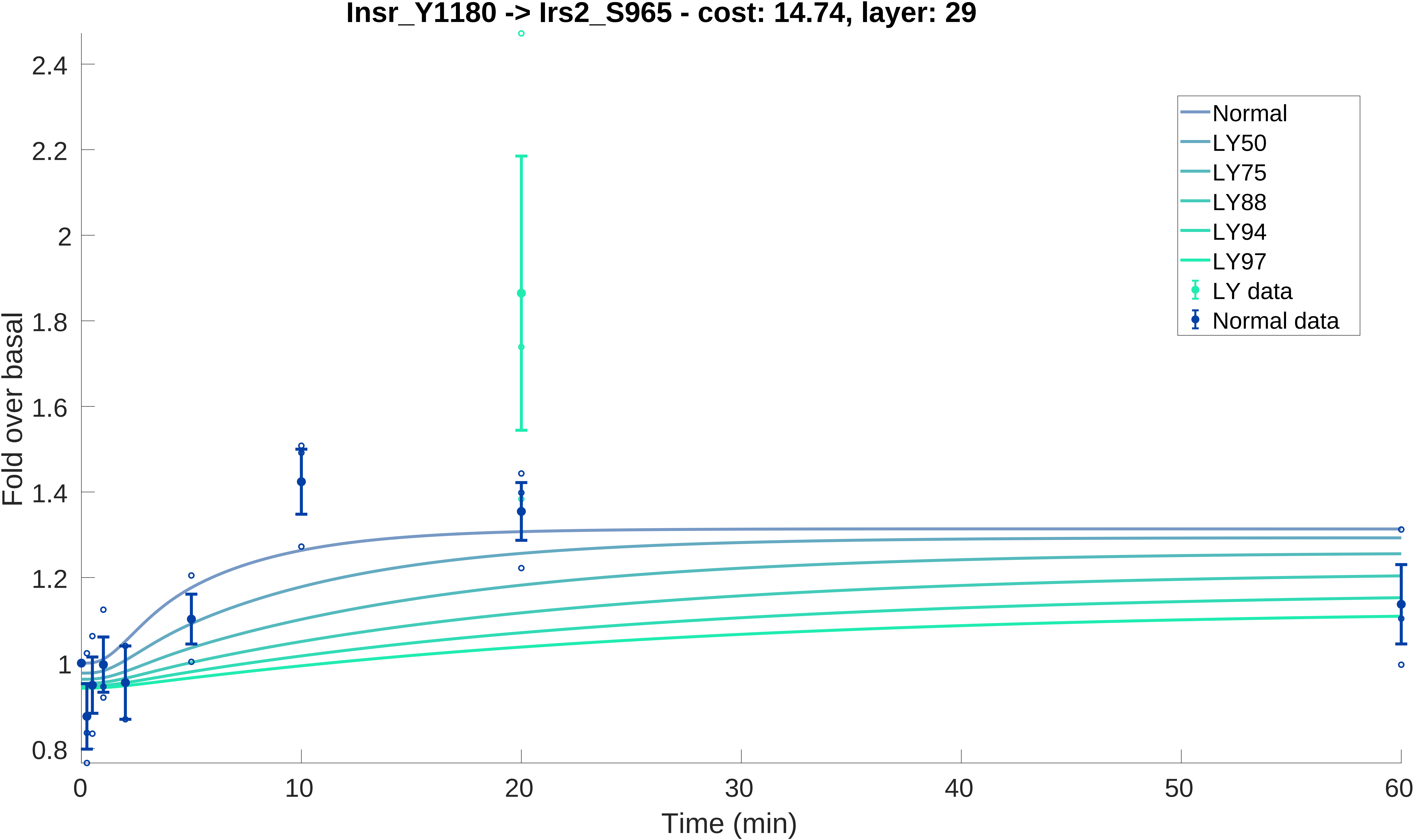
Insr_Y1180 -> Irs2_S965 - cost: 14.74, layer: 29
Fold over basal
Time (min)
0.8
1
1.2
1.4
1.6
1.8
2
2.2
2.4
0
10
20
30
40
50
60
Normal
LY50
LY75
LY88
LY94
LY97
LY data
Normal data

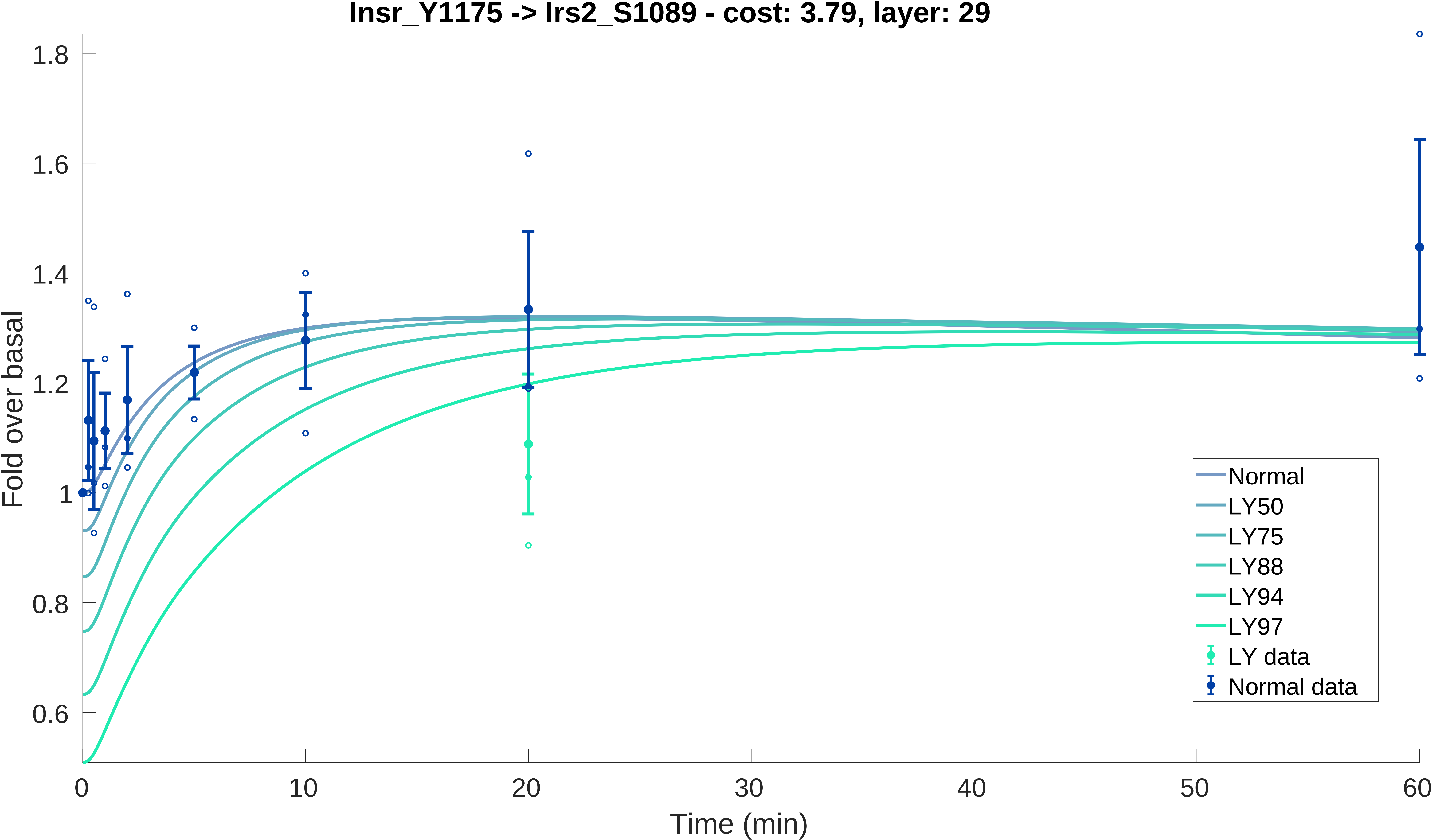
Insr_Y1175 -> Irs2_S1089 - cost: 3.79, layer: 29
Fold over basal
Time (min)
0
10
20
30
40
50
60
0.6
0.8
1
1.2
1.4
1.6
1.8
Normal
LY50
LY75
LY88
LY94
LY97
LY data
Normal data

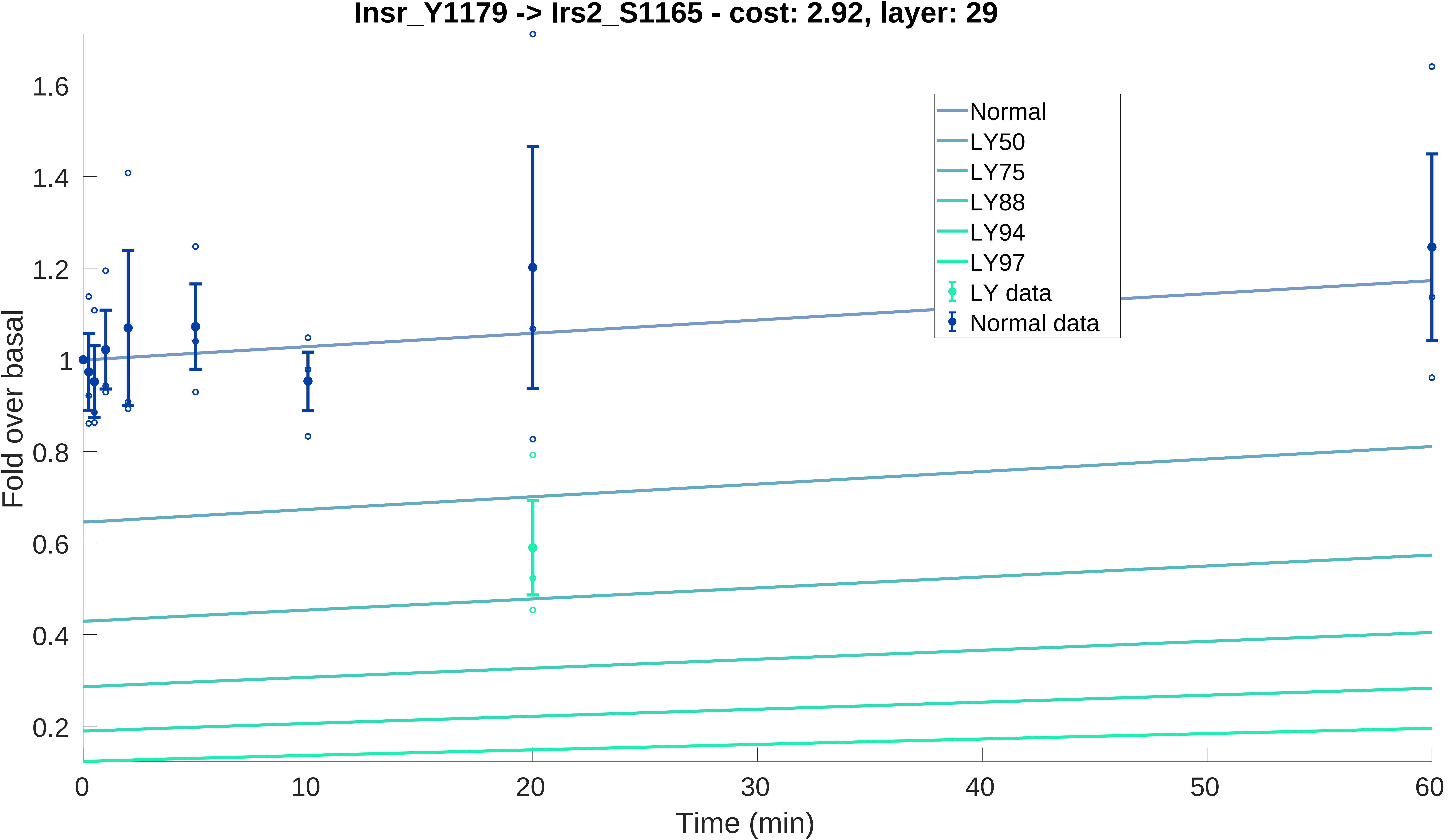
Insr_Y1179 -> Irs2_S1165 - cost: 2.92, layer: 29
Fold over basal
1.6
1.4
1.2
1
0.8
0.6
0.4
0.2
0
10
20
30
40
50
60
Time (min)
Normal
LY50
LY75
LY88
LY94
LY97
LY data
Normal data

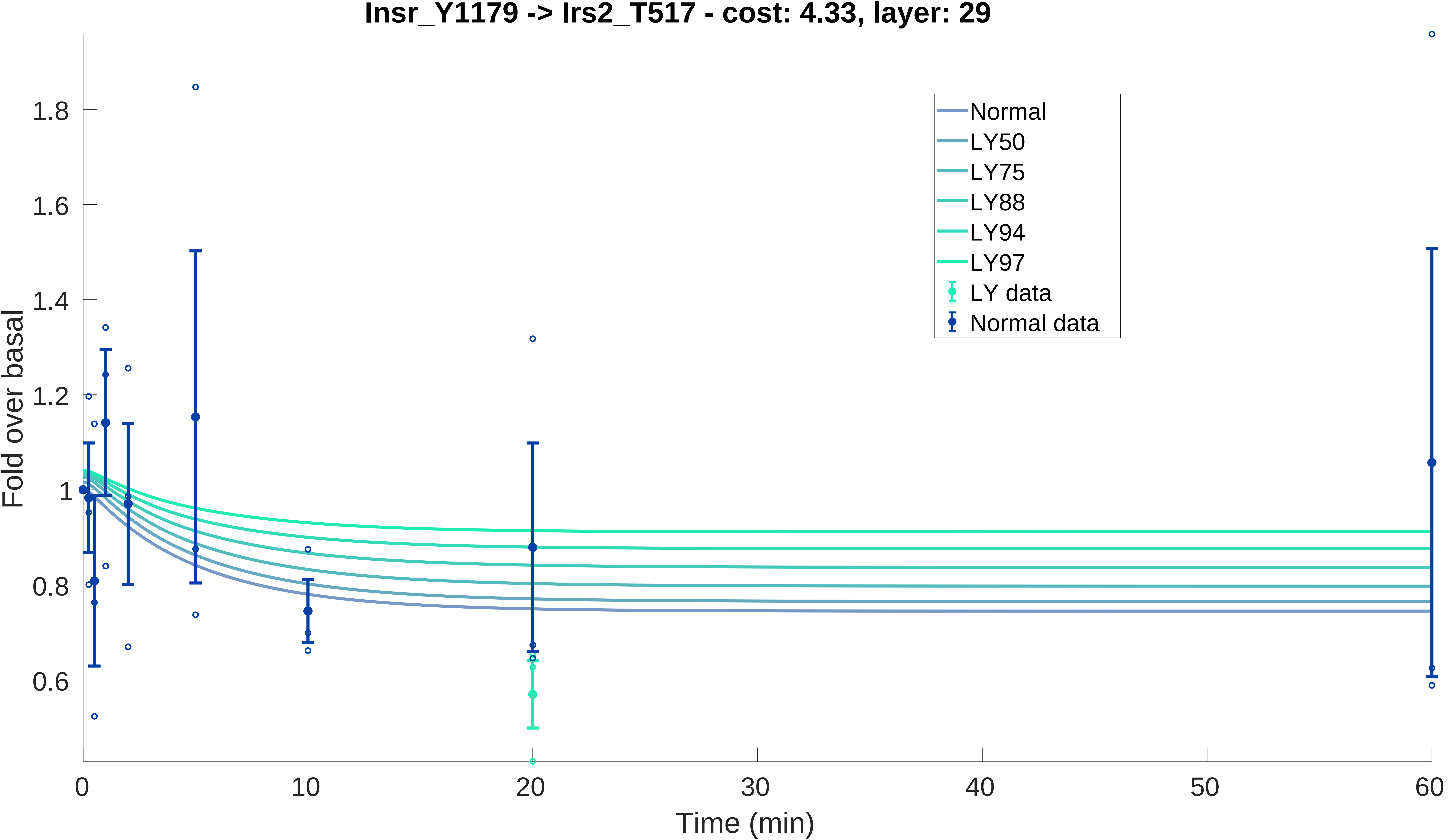
Insr_Y1179 -> Irs2_T517 - cost: 4.33, layer: 29
Fold over basal
1.8
1.6
1.4
1.2
1
0.8
0.6
0
10
20
30
40
50
60
Time (min)
Normal
LY50
LY75
LY88
LY94
LY97
LY data
Normal data

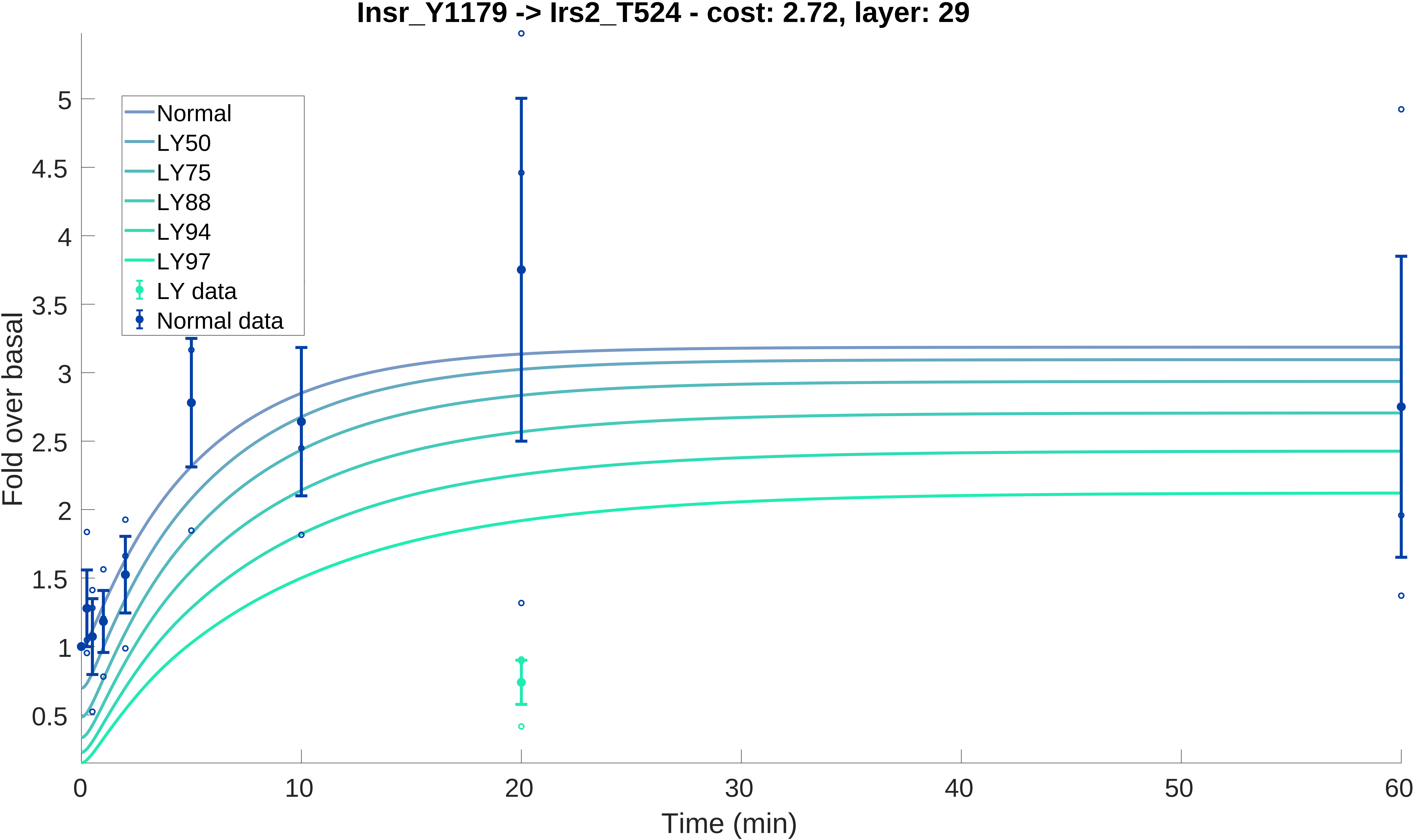

Insr_Y1179 -> Irs2_T524 - cost: 2.72, layer: 29
Normal
LY50
LY75
LY88
LY94
LY97
LY data
Normal data
Fold over basal
Time (min)
0.5
1
1.5
2
2.5
3
3.5
4
4.5
5
0
10
20
30
40
50
60

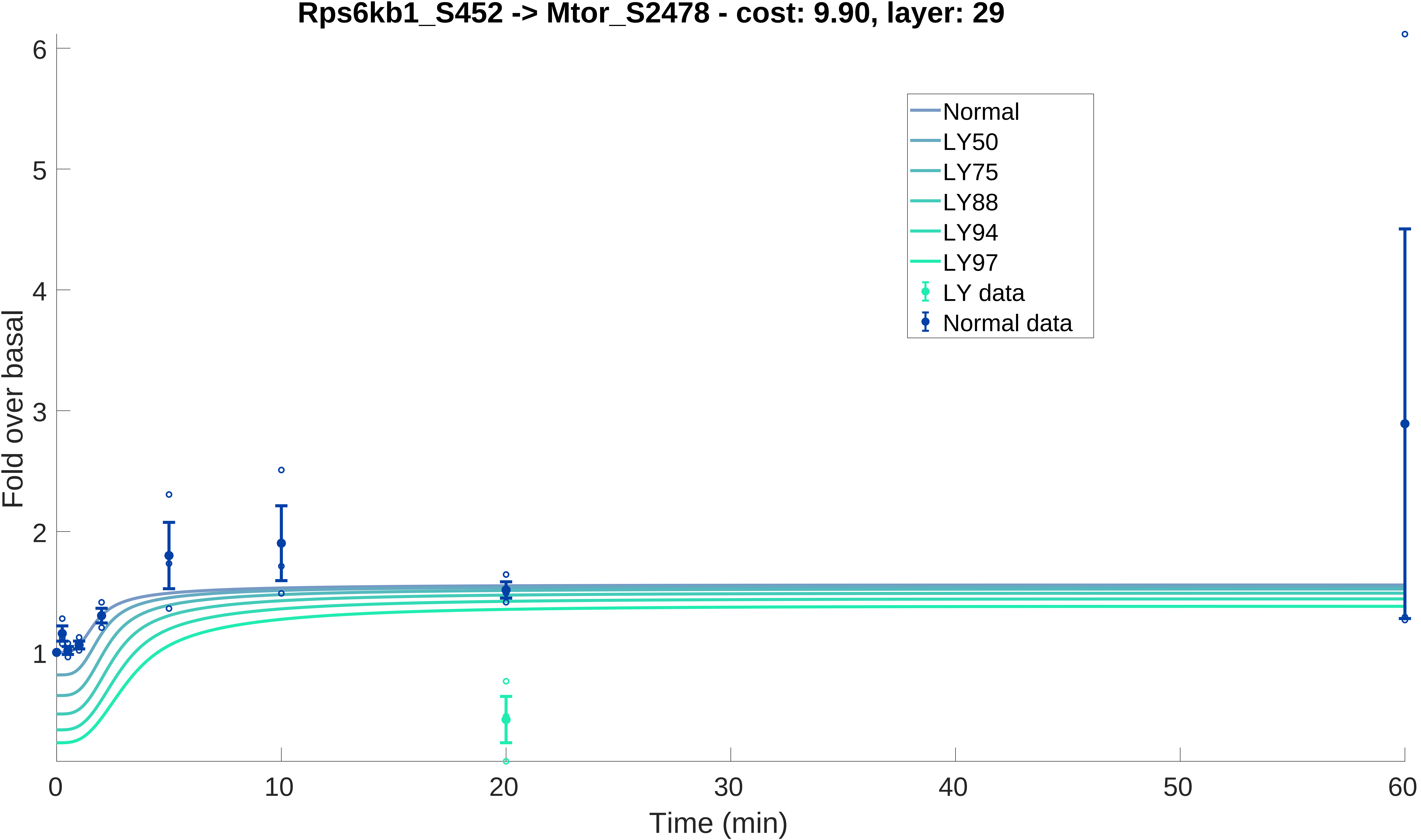

Rps6kb1_S452 -> Mtor_S2478 - cost: 9.90, layer: 29
Fold over basal
Time (min)
0
10
20
30
40
50
60
1
2
3
4
5
6
Normal
LY50
LY75
LY88
LY94
LY97
LY data
Normal data

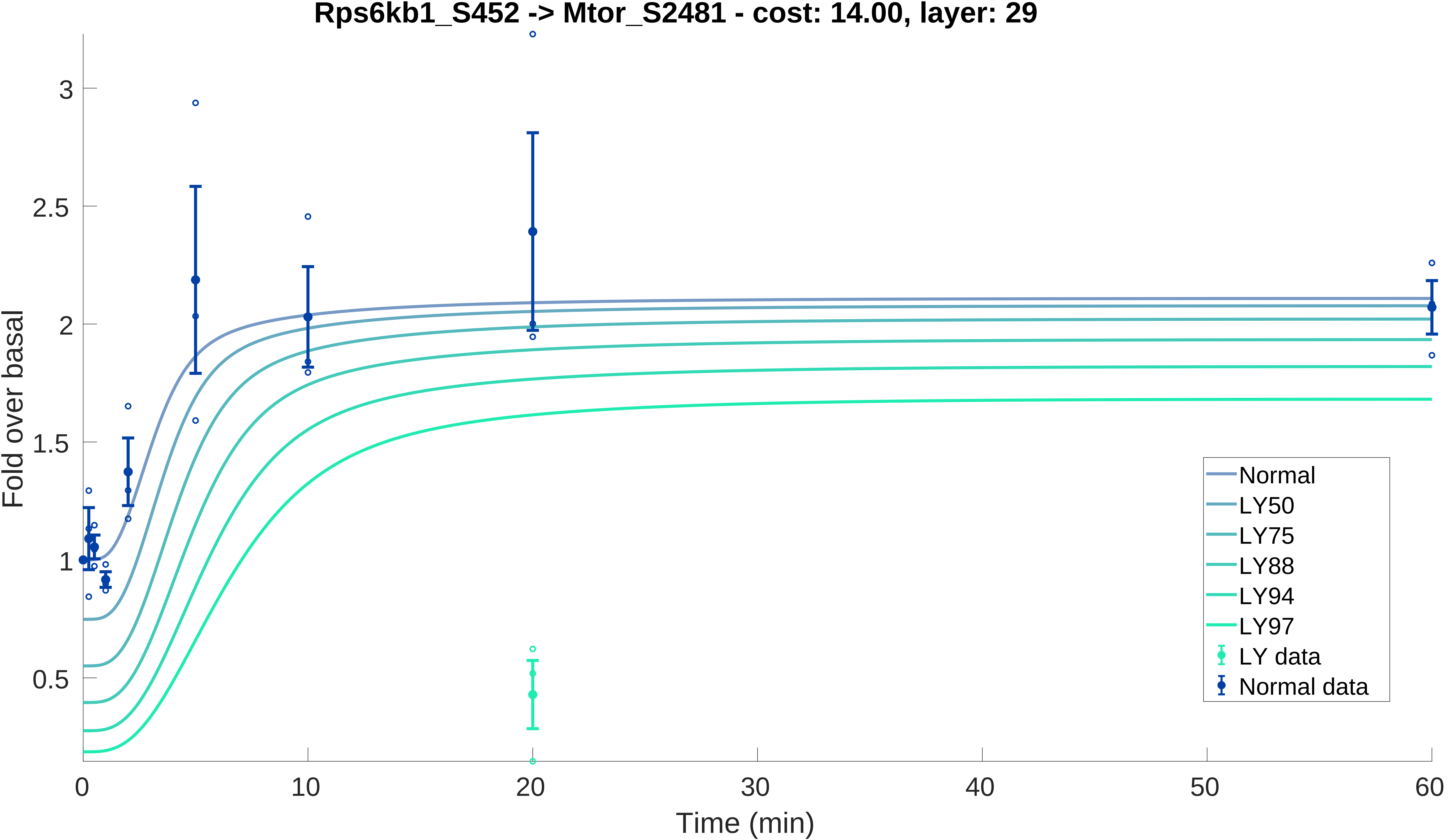

Rps6kb1_S452 -> Mtor_S2481 - cost: 14.00, layer: 29
Fold over basal
Time (min)
0
10
20
30
40
50
60
0.5
1
1.5
2
2.5
3
Normal
LY50
LY75
LY88
LY94
LY97
LY data
Normal data

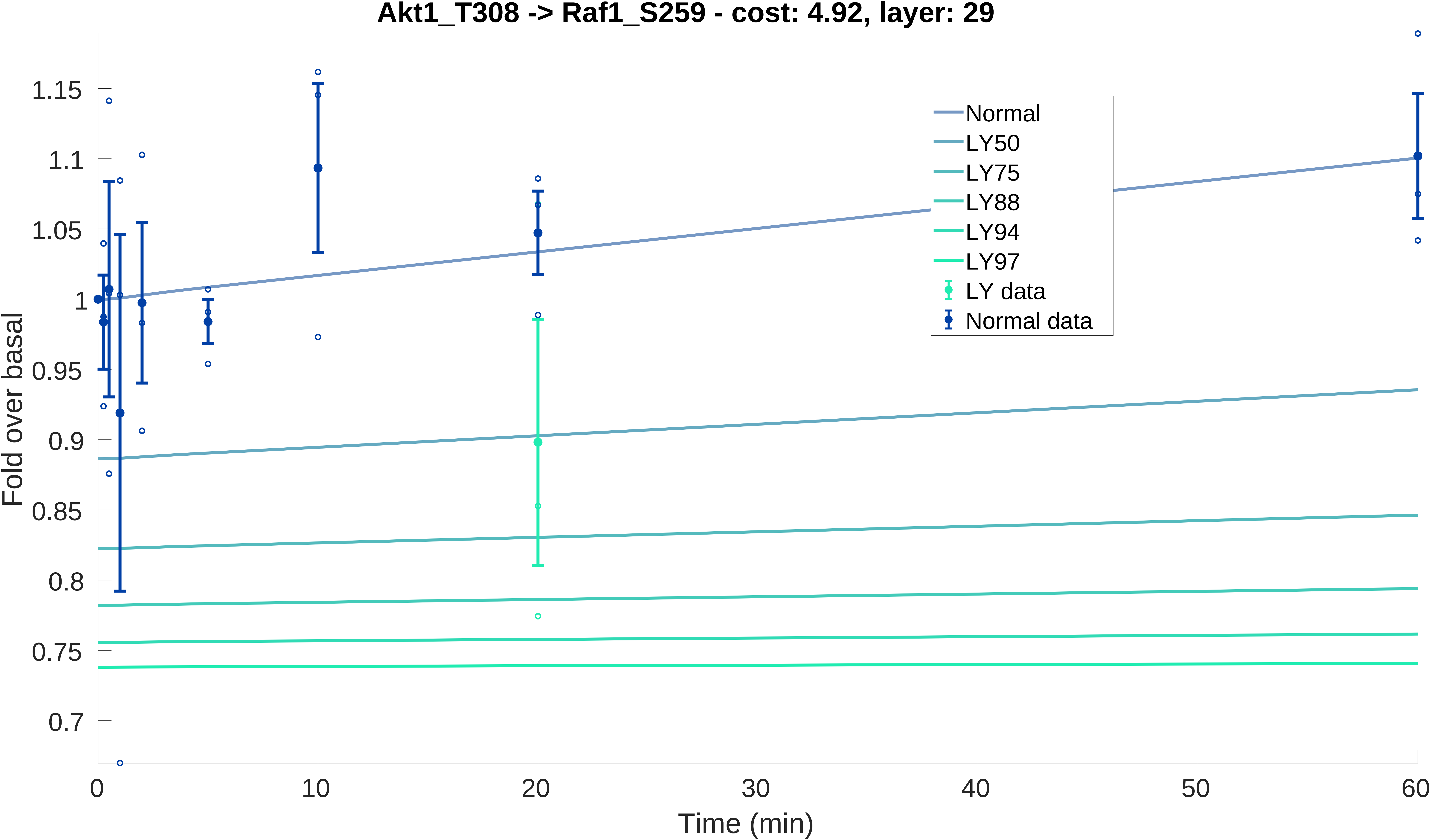
Akt1_T308 -> Raf1_S259 - cost: 4.92, layer: 29
Fold over basal
Time (min)
Normal
LY50
LY75
LY88
LY94
LY97
LY data
Normal data
1.15
1.1
1.05
1
0.95
0.9
0.85
0.8
0.75
0.7
0
10
20
30
40
50
60

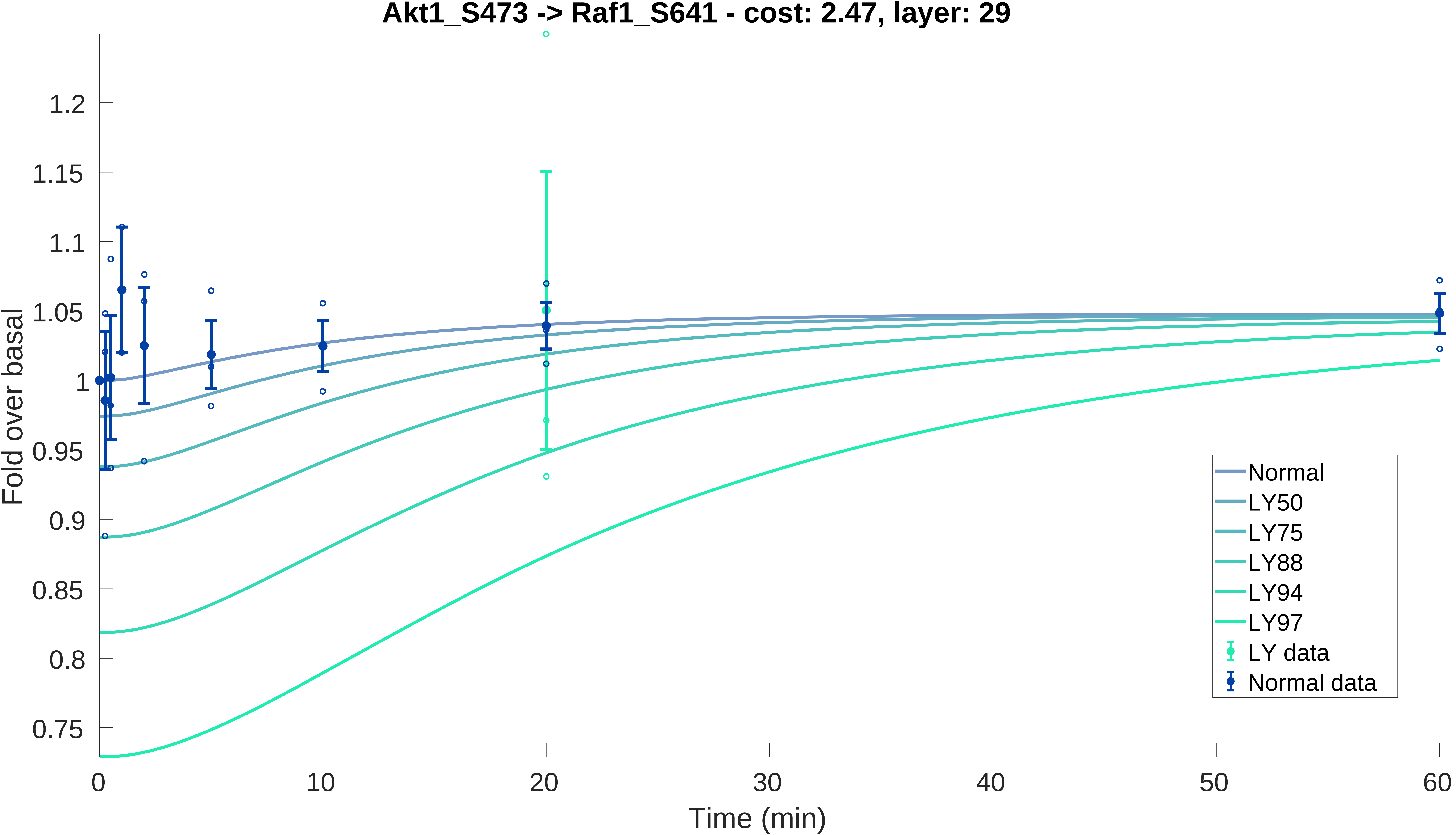

Akt1_S473 -> Raf1_S641 - cost: 2.47, layer: 29
Fold over basal
1.2
1.15
1.1
1.05
1
0.95
0.9
0.85
0.8
0.75
0
10
20
30
40
50
60
Time (min)
Normal
LY50
LY75
LY88
LY94
LY97
LY data
Normal data

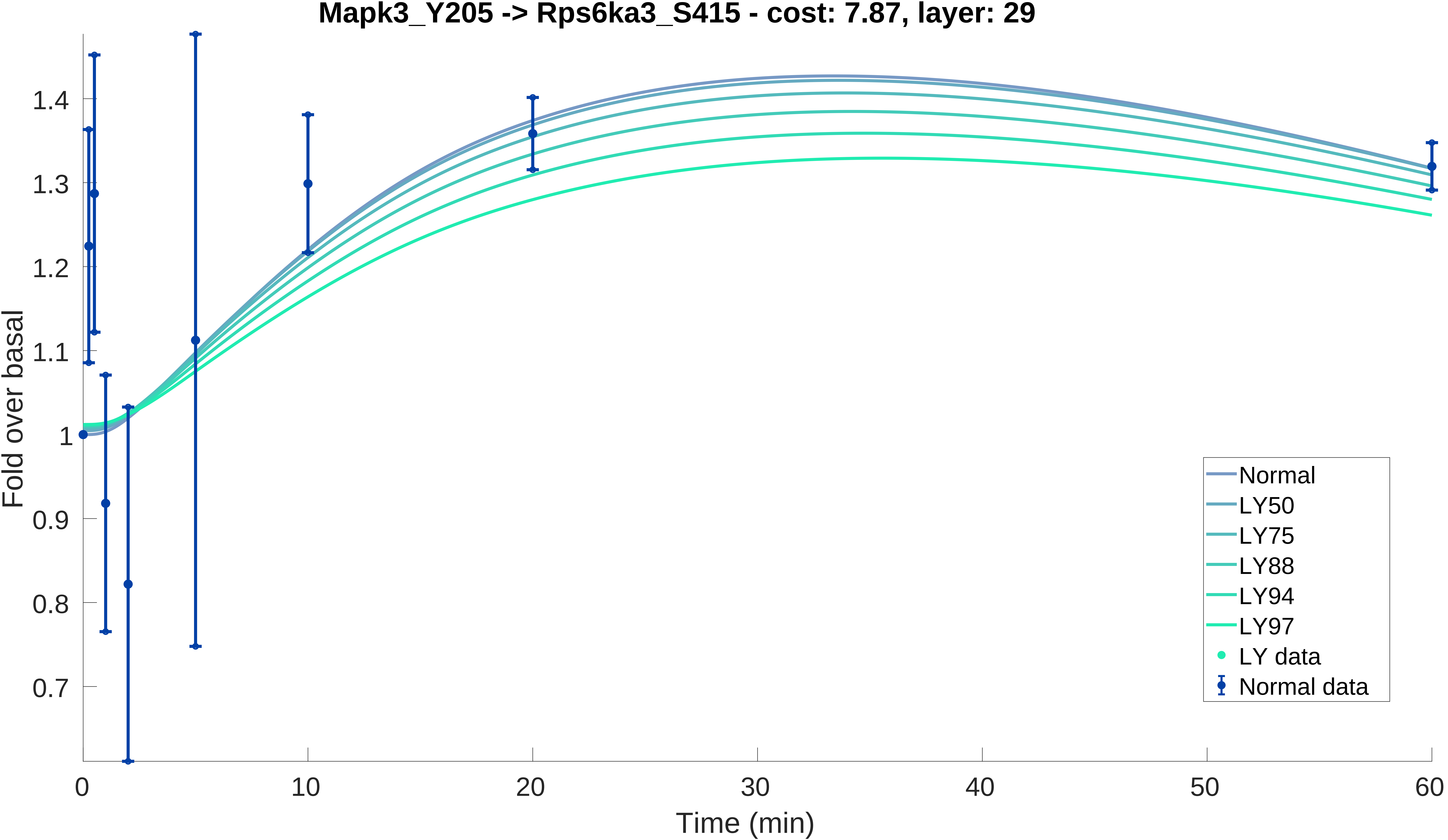
Mapk3_Y205 -> Rps6ka3_S415 - cost: 7.87, layer: 29
Fold over basal
Time (min)
0
10
20
30
40
50
60
0.7
0.8
0.9
1
1.1
1.2
1.3
1.4
Normal
LY50
LY75
LY88
LY94
LY97
LY data
Normal data

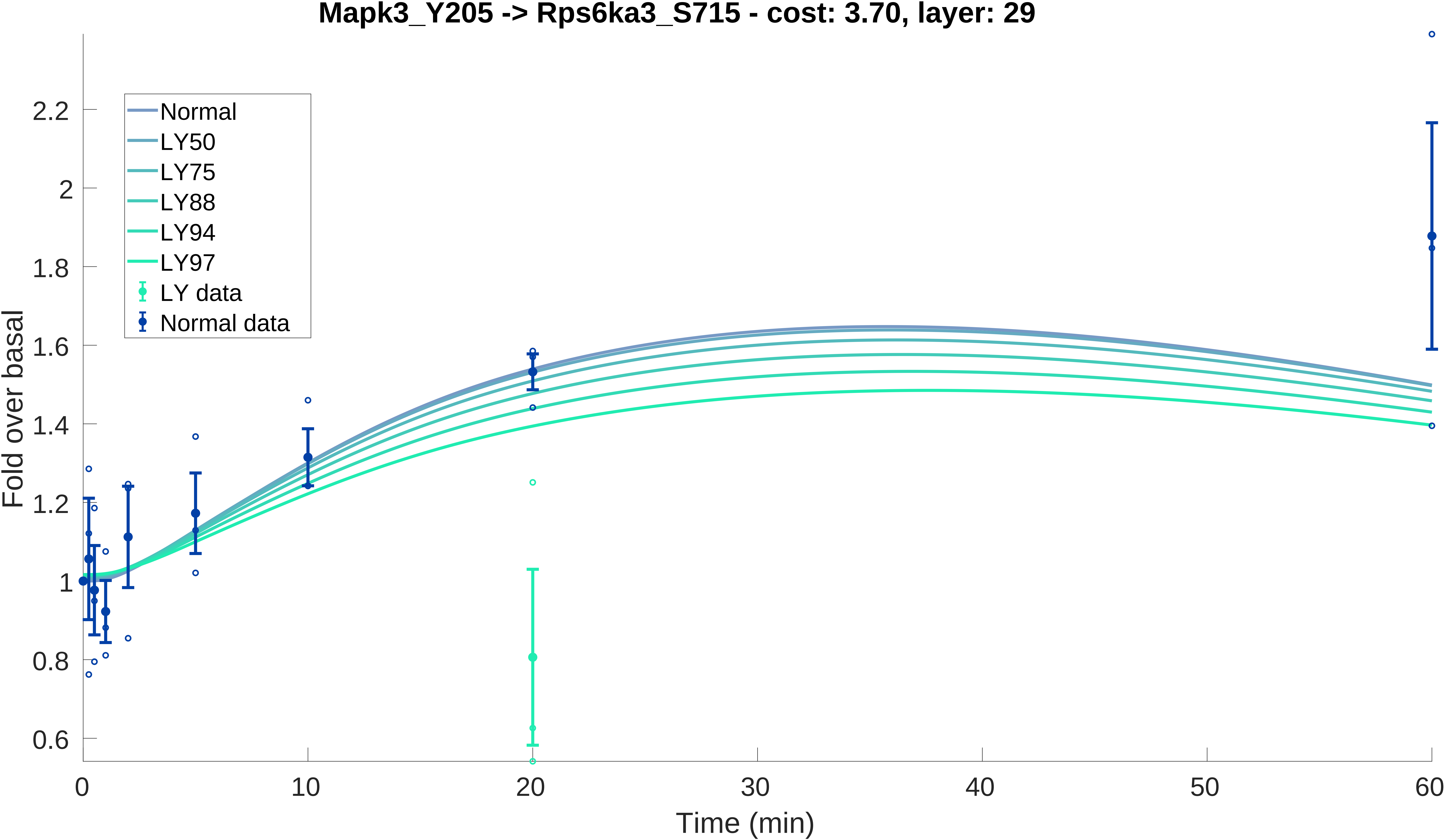

Mapk3_Y205 -> Rps6ka3_S715 - cost: 3.70, layer: 29
Normal
LY50
LY75
LY88
LY94
LY97
LY data
Normal data
Fold over basal
Time (min)
0.6
0.8
1
1.2
1.4
1.6
1.8
2
2.2
0
10
20
30
40
50
60

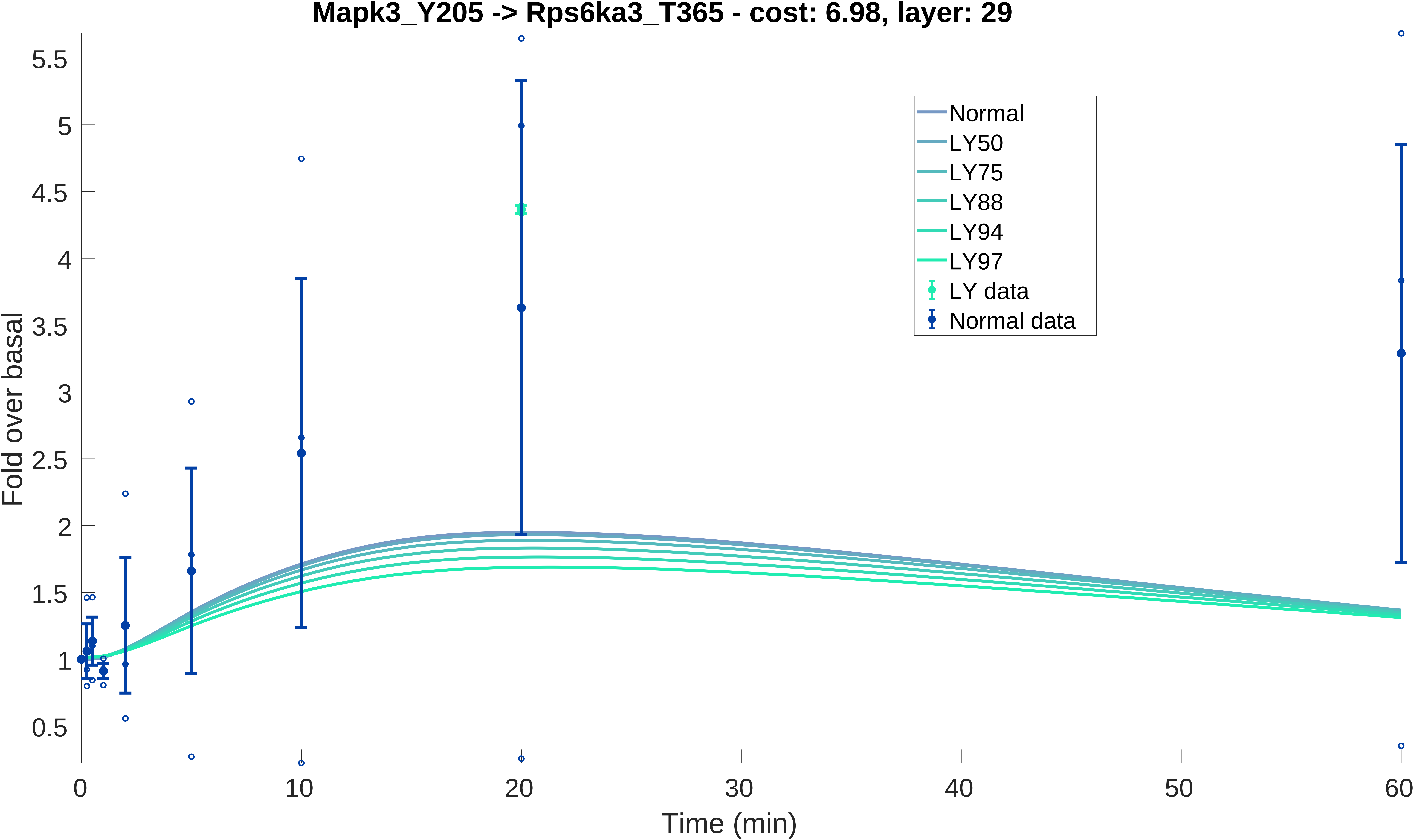

Mapk3_Y205 -> Rps6ka3_T365 - cost: 6.98, layer: 29
Fold over basal
Time (min)
0
10
20
30
40
50
60
0.5
1
1.5
2
2.5
3
3.5
4
4.5
5
5.5
Normal
LY50
LY75
LY88
LY94
LY97
LY data
Normal data

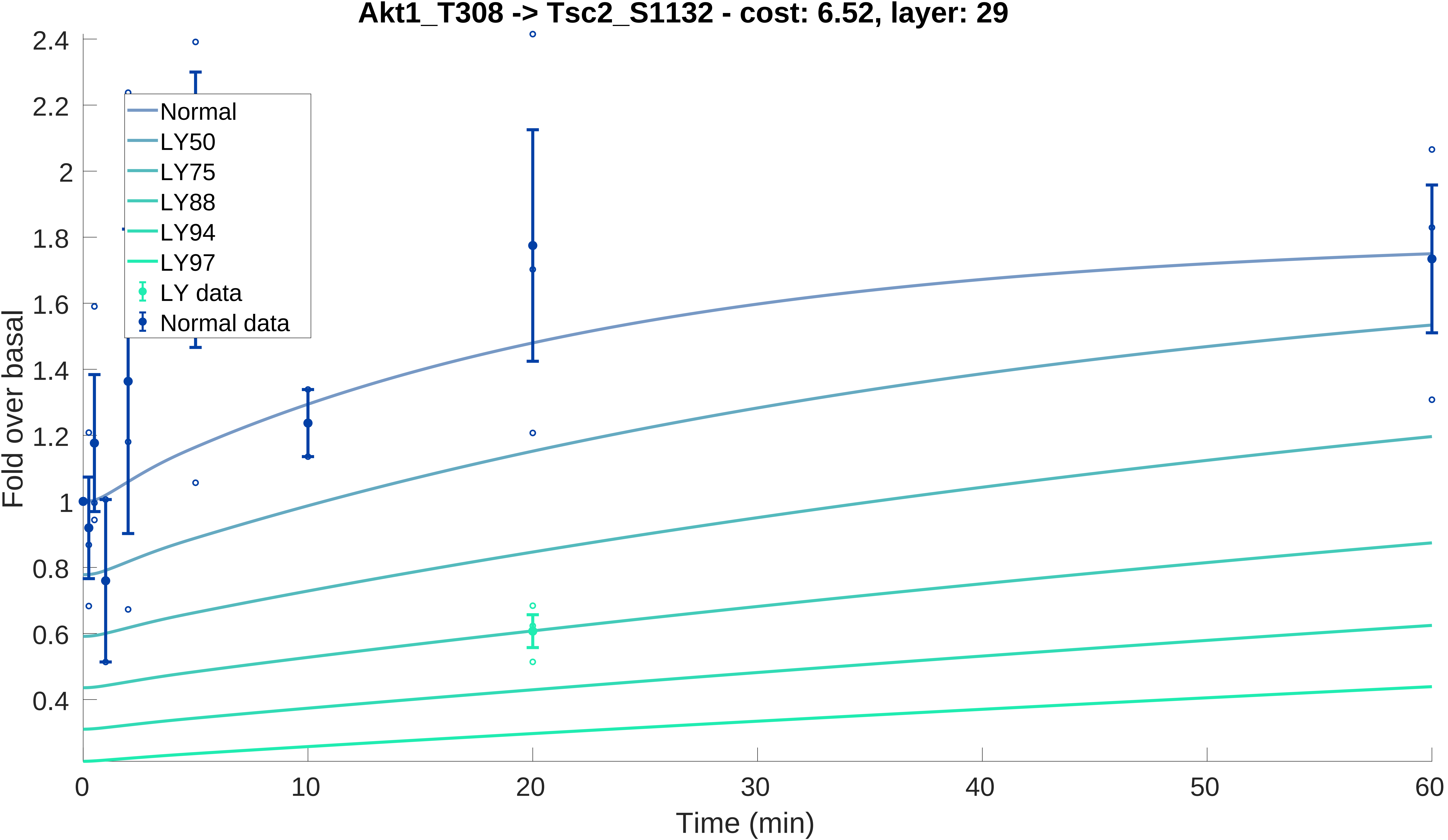

Akt1_T308 -> Tsc2_S1132 - cost: 6.52, layer: 29
Fold over basal
Time (min)
Normal
LY50
LY75
LY88
LY94
LY97
LY data
Normal data
0.4
0.6
0.8
1
1.2
1.4
1.6
1.8
2
2.2
2.4
0
10
20
30
40
50
60

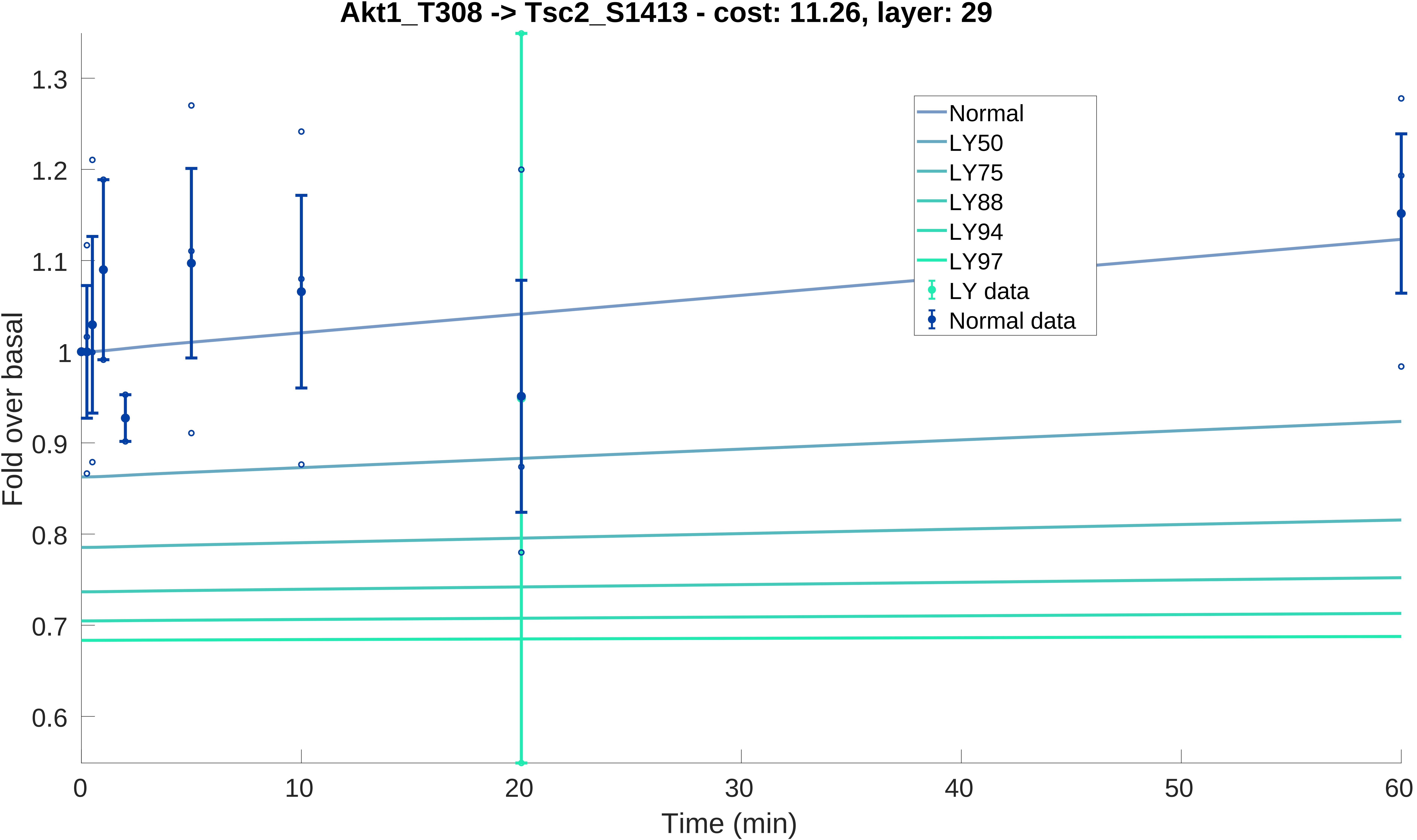
Akt1_T308 -> Tsc2_S1413 - cost: 11.26, layer: 29
Fold over basal
Time (min)
1.3
1.2
1.1
1
0.9
0.8
0.7
0.6
0
10
20
30
40
50
60
Normal
LY50
LY75
LY88
LY94
LY97
LY data
Normal data

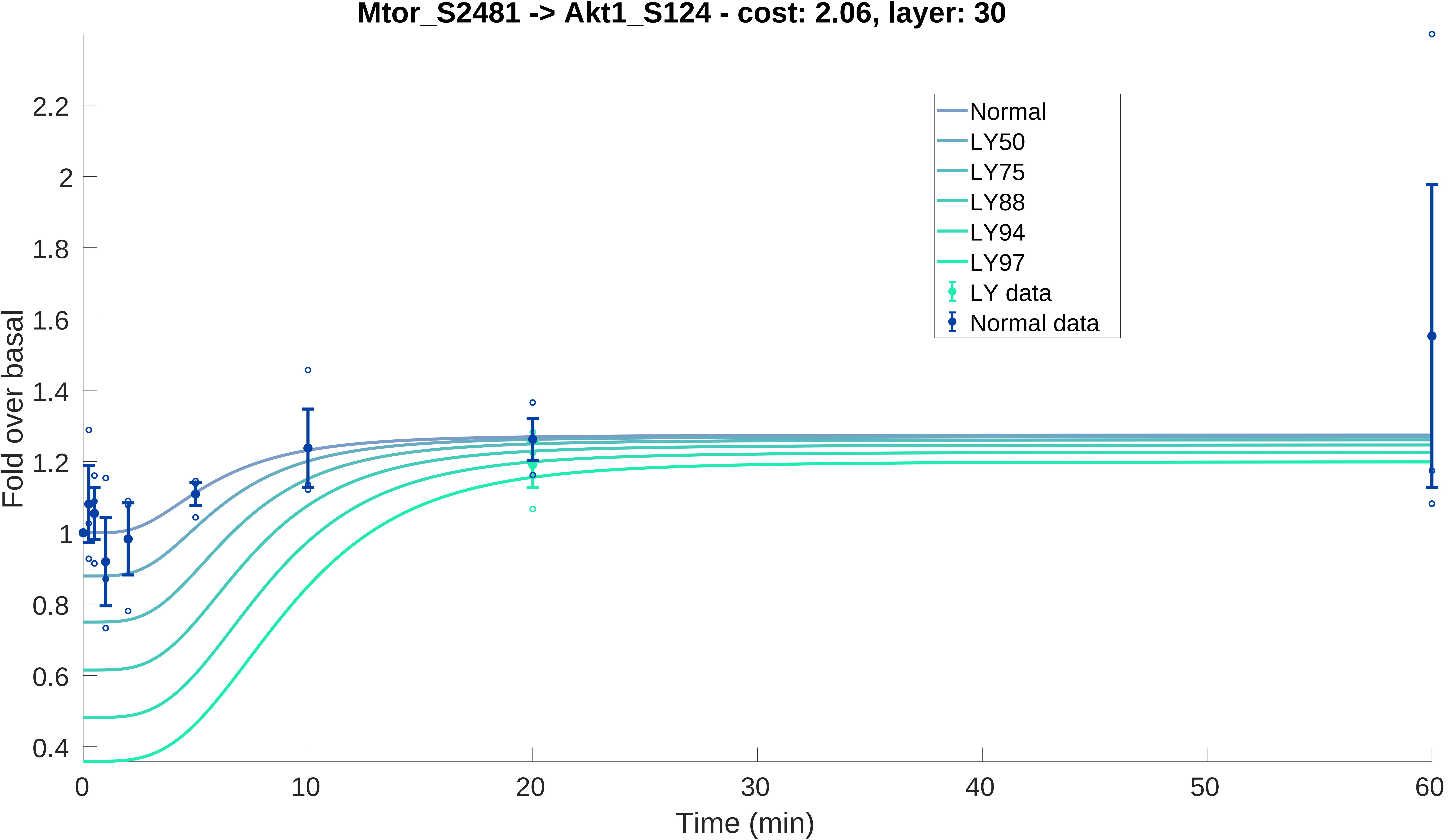

Mtor_S2481 -> Akt1_S124 - cost: 2.06, layer: 30
Fold over basal
Time (min)
0
10
20
30
40
50
60
0.4
0.6
0.8
1
1.2
1.4
1.6
1.8
2
2.2
Normal
LY50
LY75
LY88
LY94
LY97
LY data
Normal data

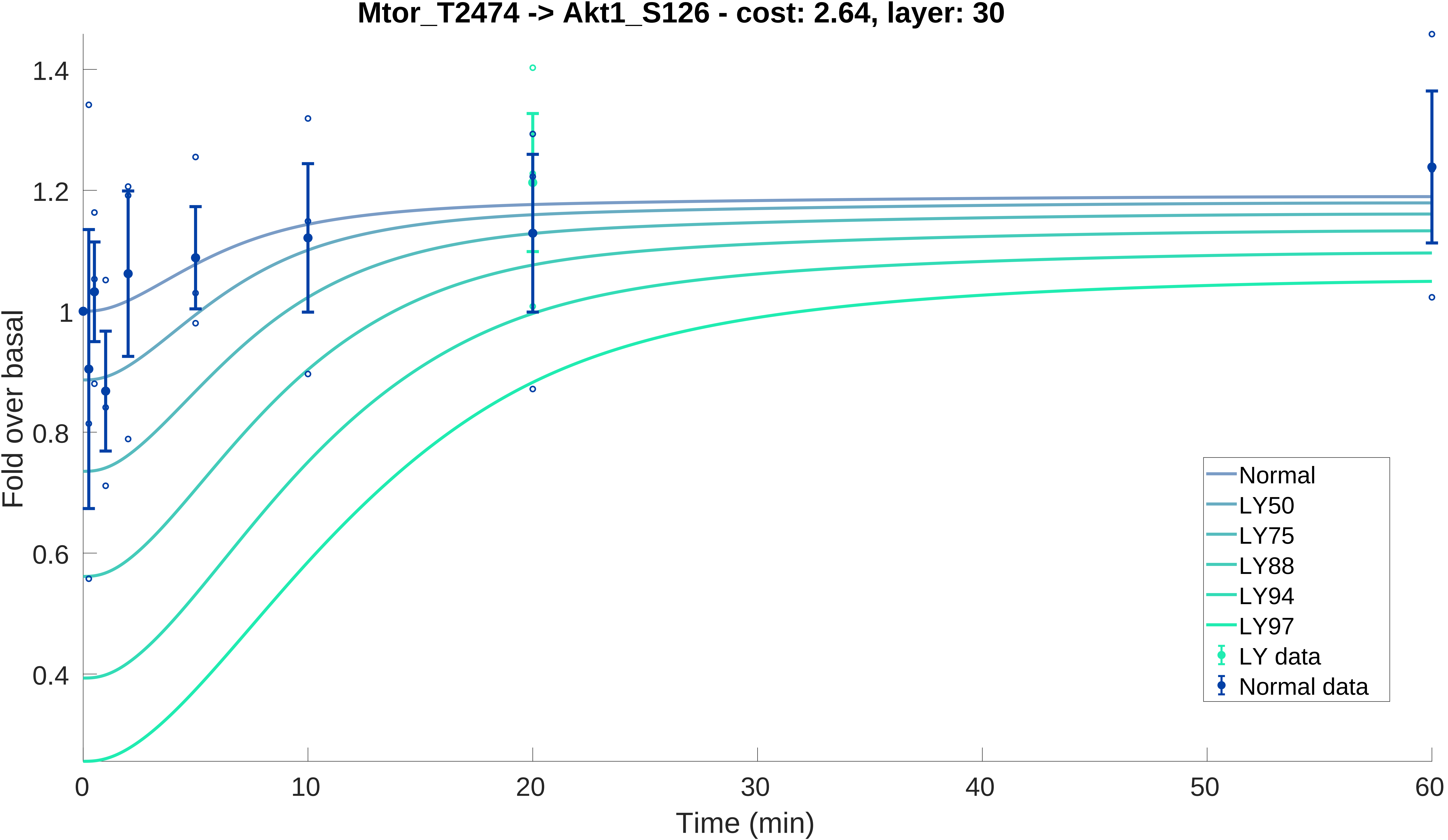
Mtor_T2474 -> Akt1_S126 - cost: 2.64, layer: 30
Fold over basal
1.4
1.2
1
0.8
0.6
0.4
0
10
20
30
40
50
60
Time (min)
Normal
LY50
LY75
LY88
LY94
LY97
LY data
Normal data

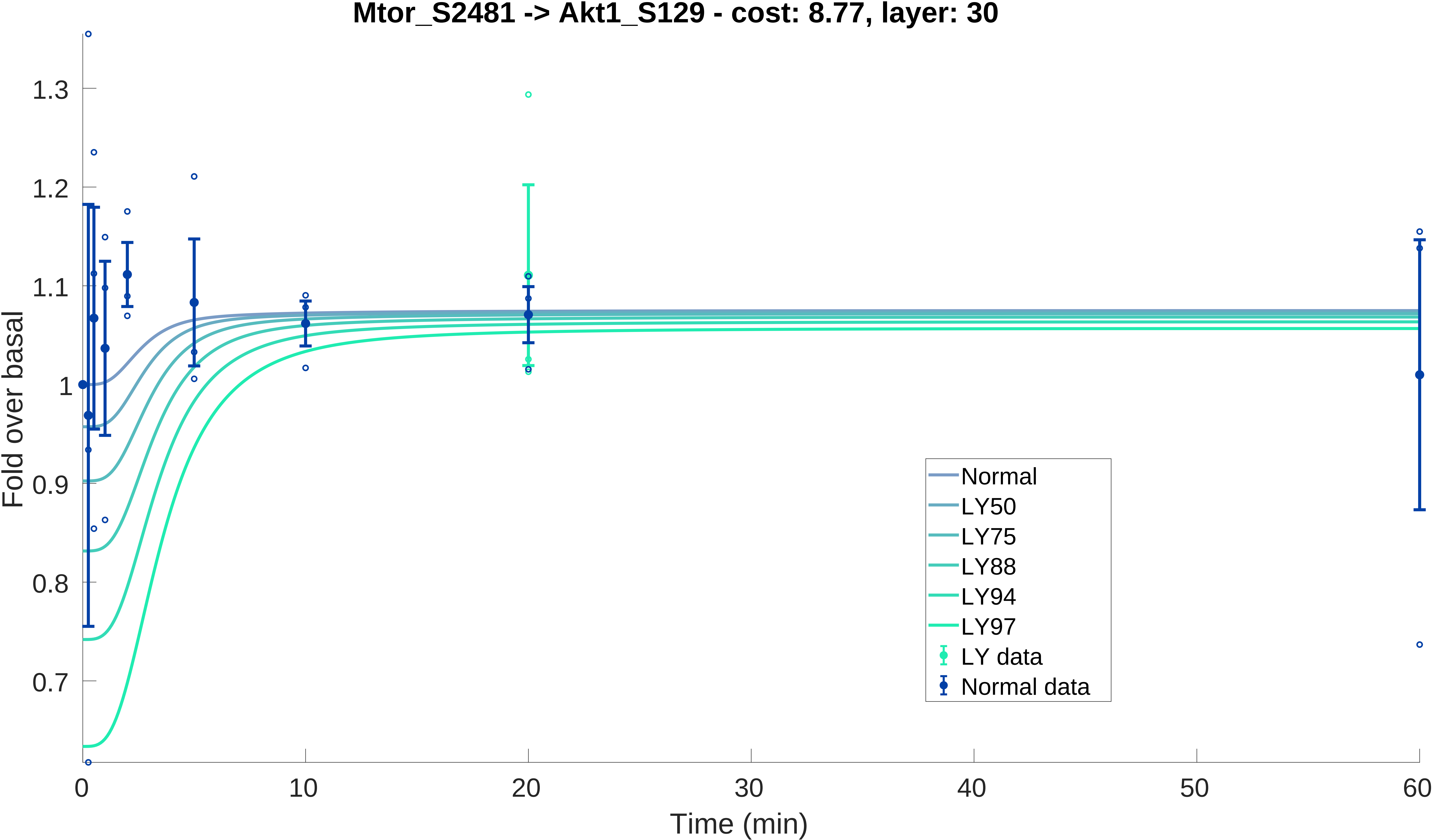

Mtor_S2481 -> Akt1_S129 - cost: 8.77, layer: 30
Fold over basal
1.3
1.2
1.1
1
0.9
0.8
0.7
0
10
20
30
40
50
60
Time (min)
Normal
LY50
LY75
LY88
LY94
LY97
LY data
Normal data

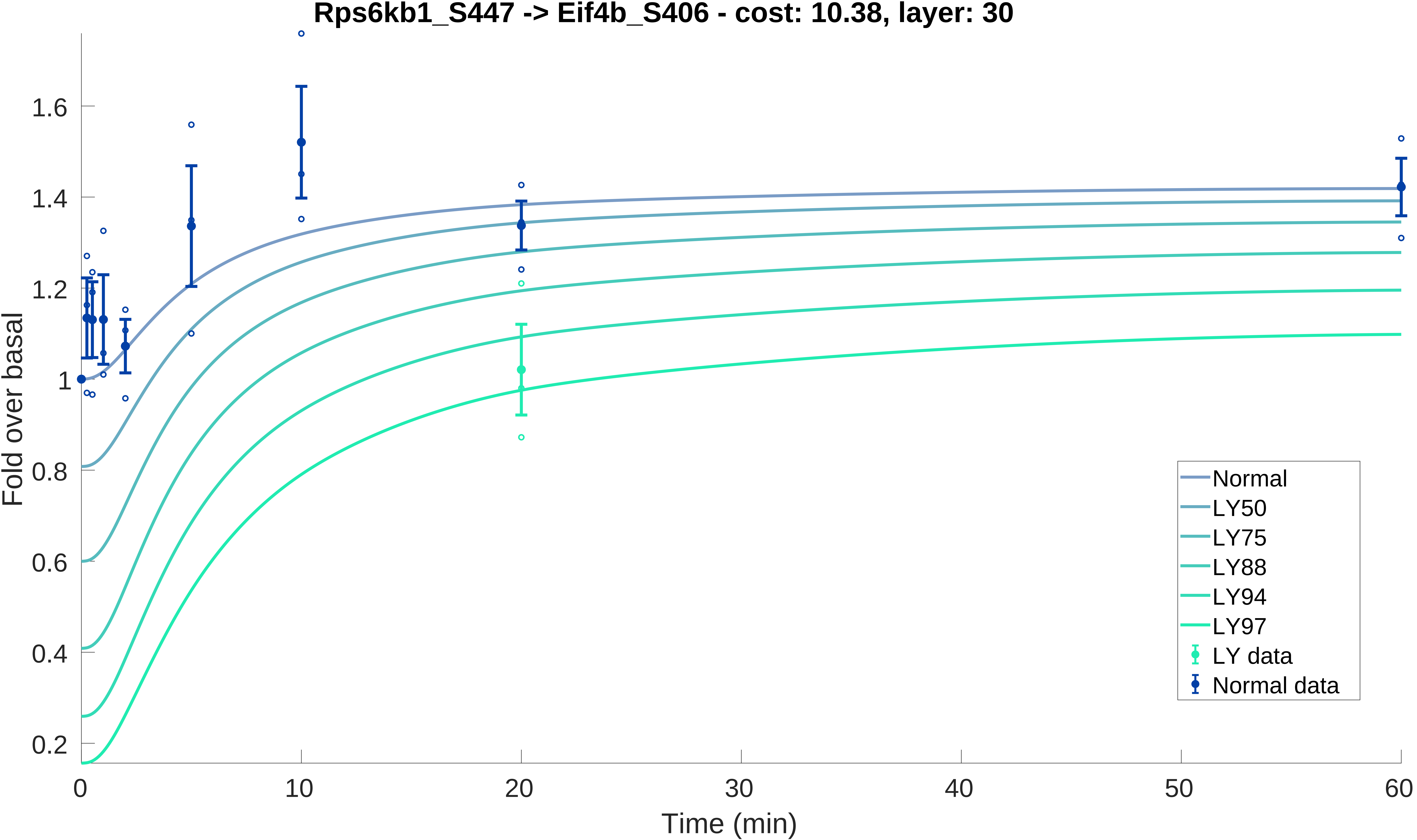

Rps6kb1_S447 -> Eif4b_S406 - cost: 10.38, layer: 30
Fold over basal
Time (min)
0
10
20
30
40
50
60
0.2
0.4
0.6
0.8
1
1.2
1.4
1.6
Normal
LY50
LY75
LY88
LY94
LY97
LY data
Normal data

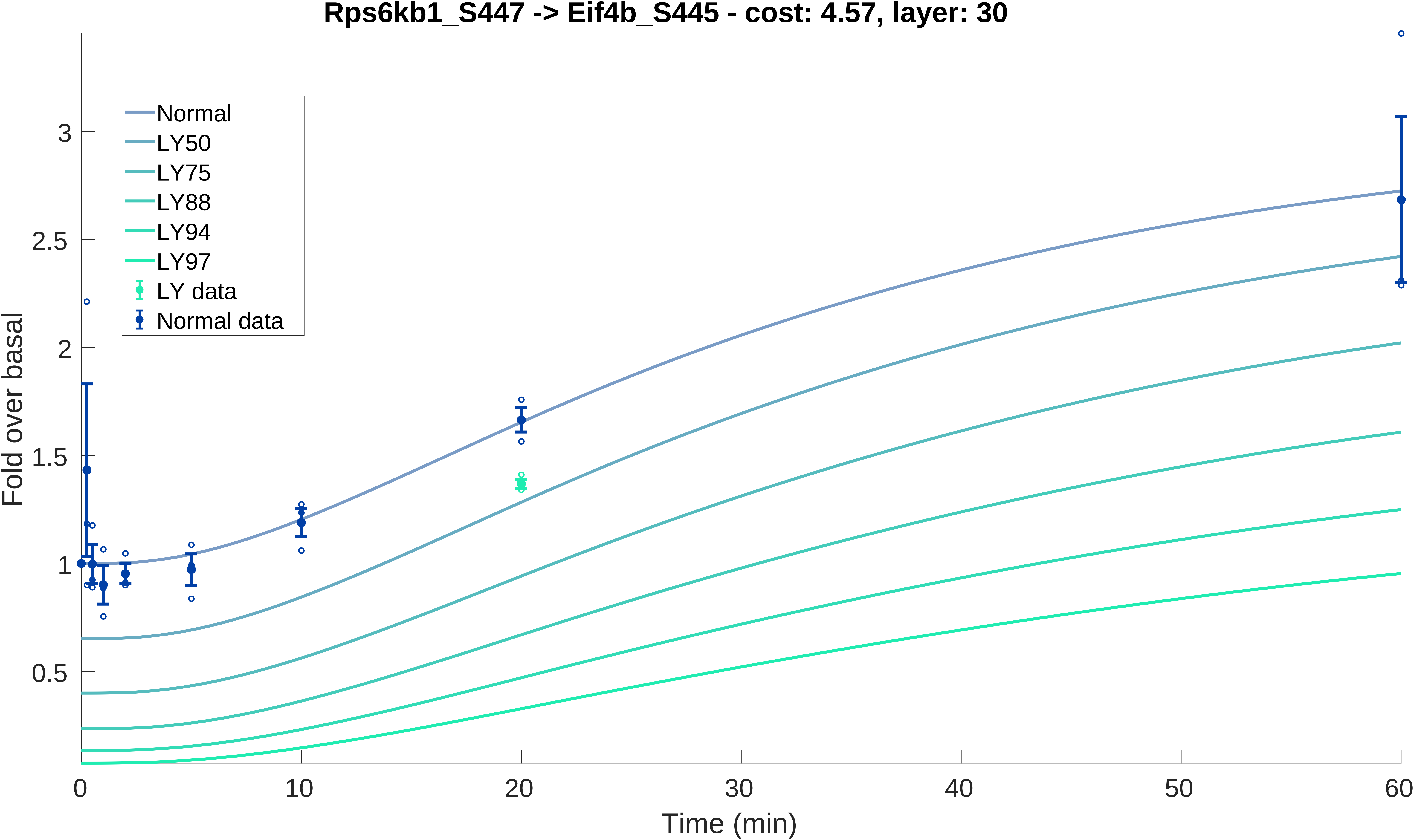

Rps6kb1_S447 -> Eif4b_S445 - cost: 4.57, layer: 30
Normal
LY50
LY75
LY88
LY94
LY97
LY data
Normal data
Fold over basal
Time (min)
0.5
1
1.5
2
2.5
3
0
10
20
30
40
50
60

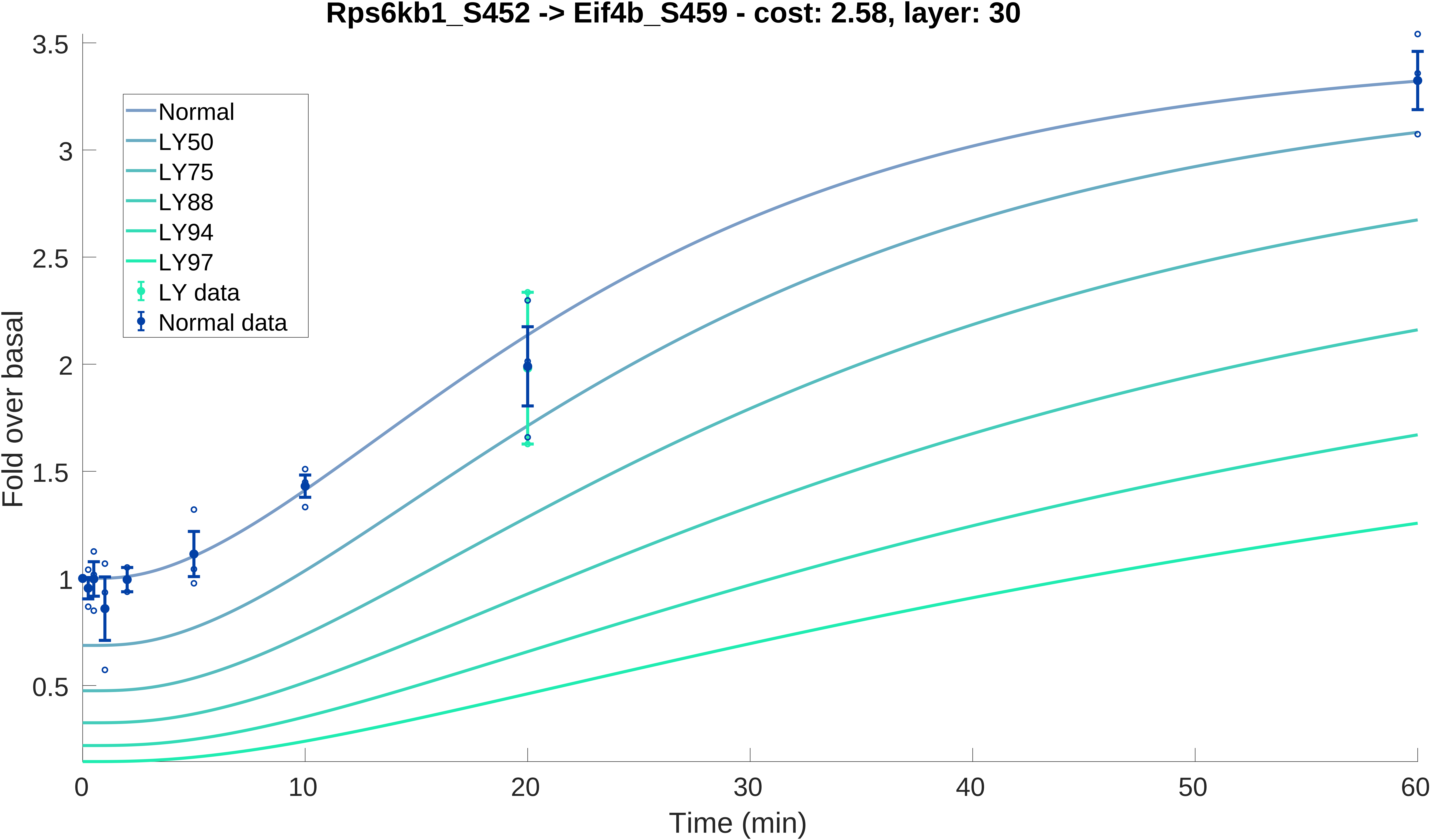

Rps6kb1_S452 -> Eif4b_S459 - cost: 2.58, layer: 30
Fold over basal
Time (min)
3.5
3
2.5
2
1.5
1
0.5
0
10
20
30
40
50
60
Normal
LY50
LY75
LY88
LY94
LY97
LY data
Normal data

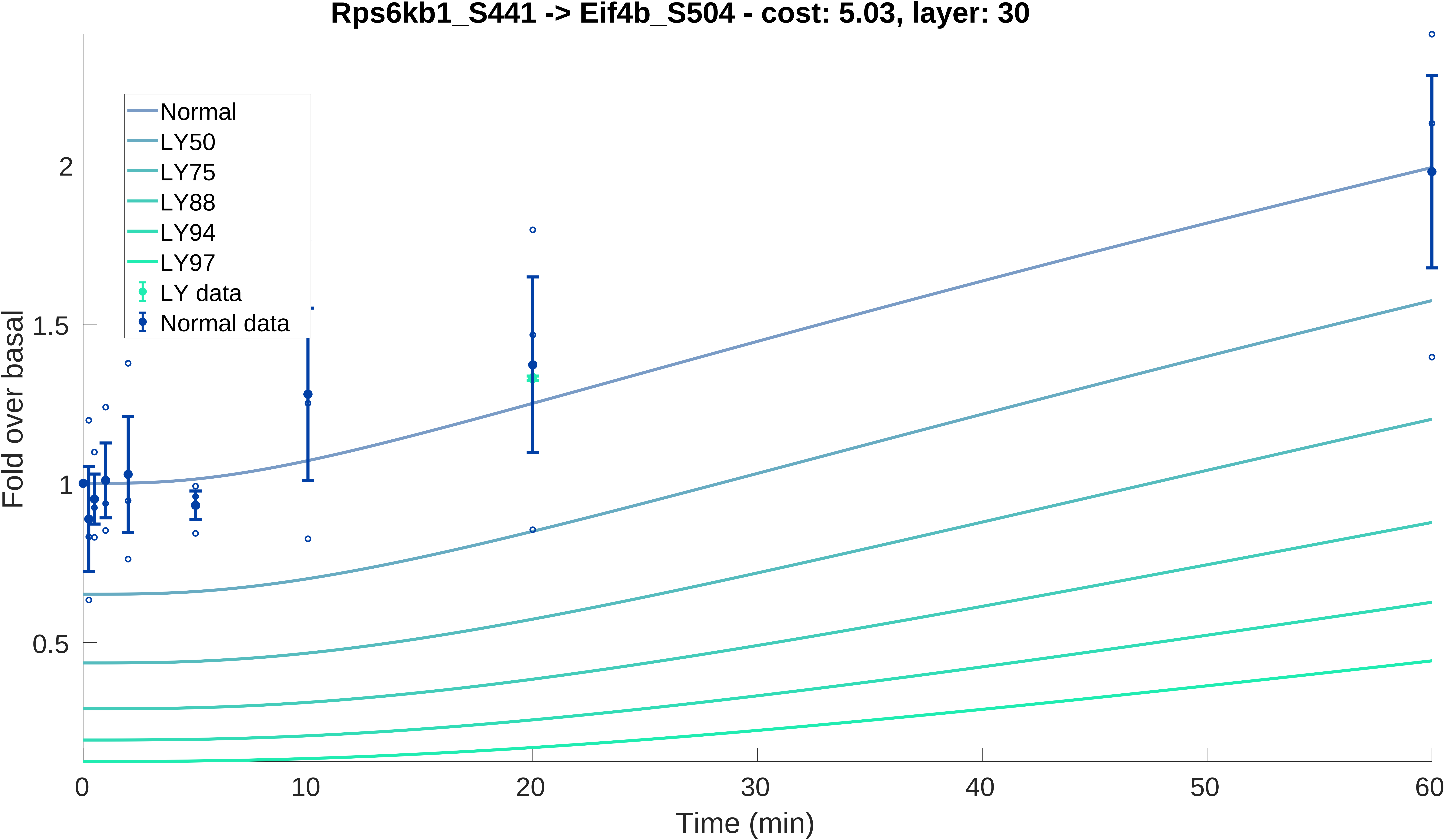

Rps6kb1_S441 -> Eif4b_S504 - cost: 5.03, layer: 30
Normal
LY50
LY75
LY88
LY94
LY97
LY data
Normal data
Fold over basal
0.5
1
1.5
2
0
10
20
30
40
50
60
Time (min)

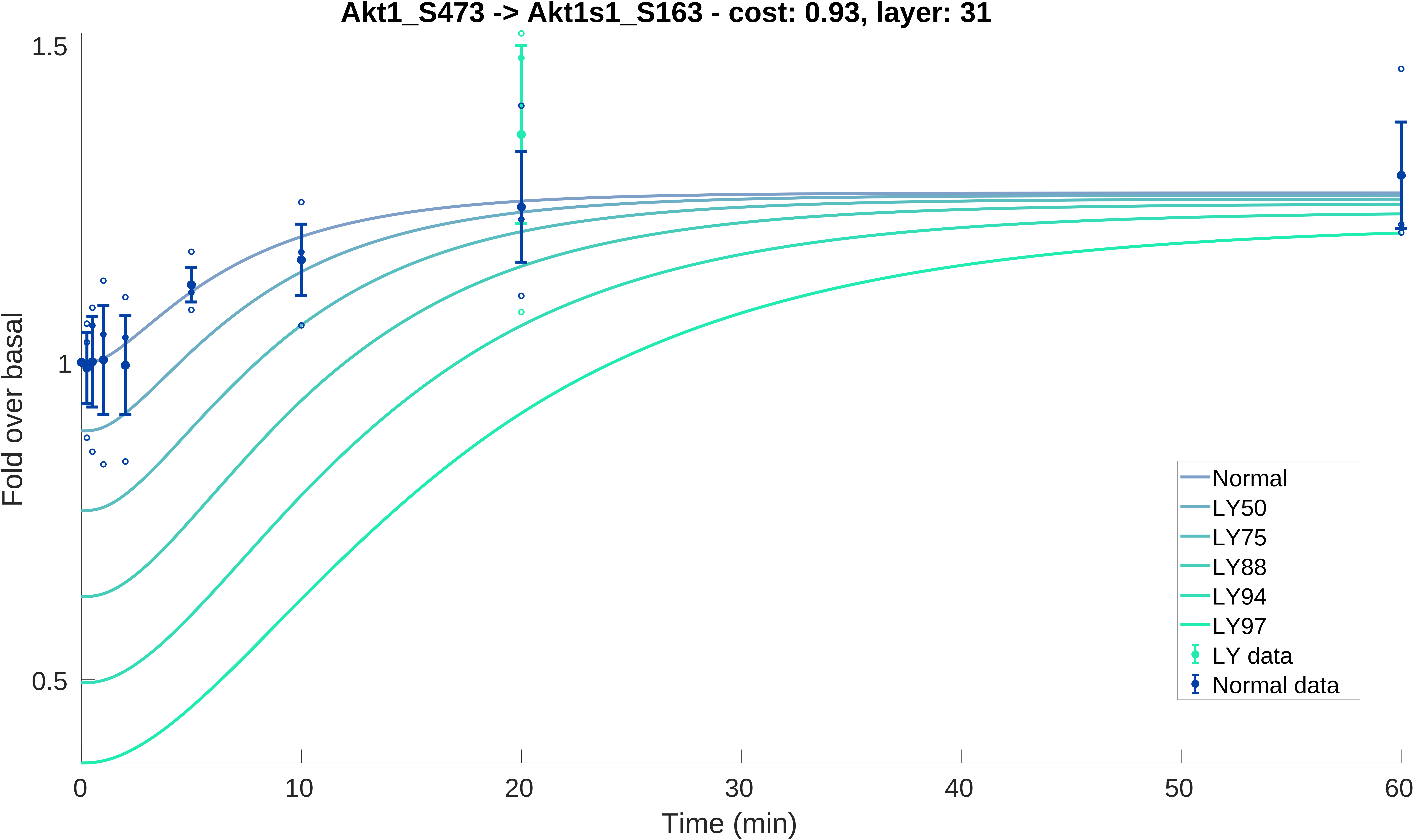
Akt1_S473 -> Akt1s1_S163 - cost: 0.93, layer: 31
Fold over basal
1.5
1
0.5
0
10
20
30
40
50
60
Time (min)
Normal
LY50
LY75
LY88
LY94
LY97
LY data
Normal data

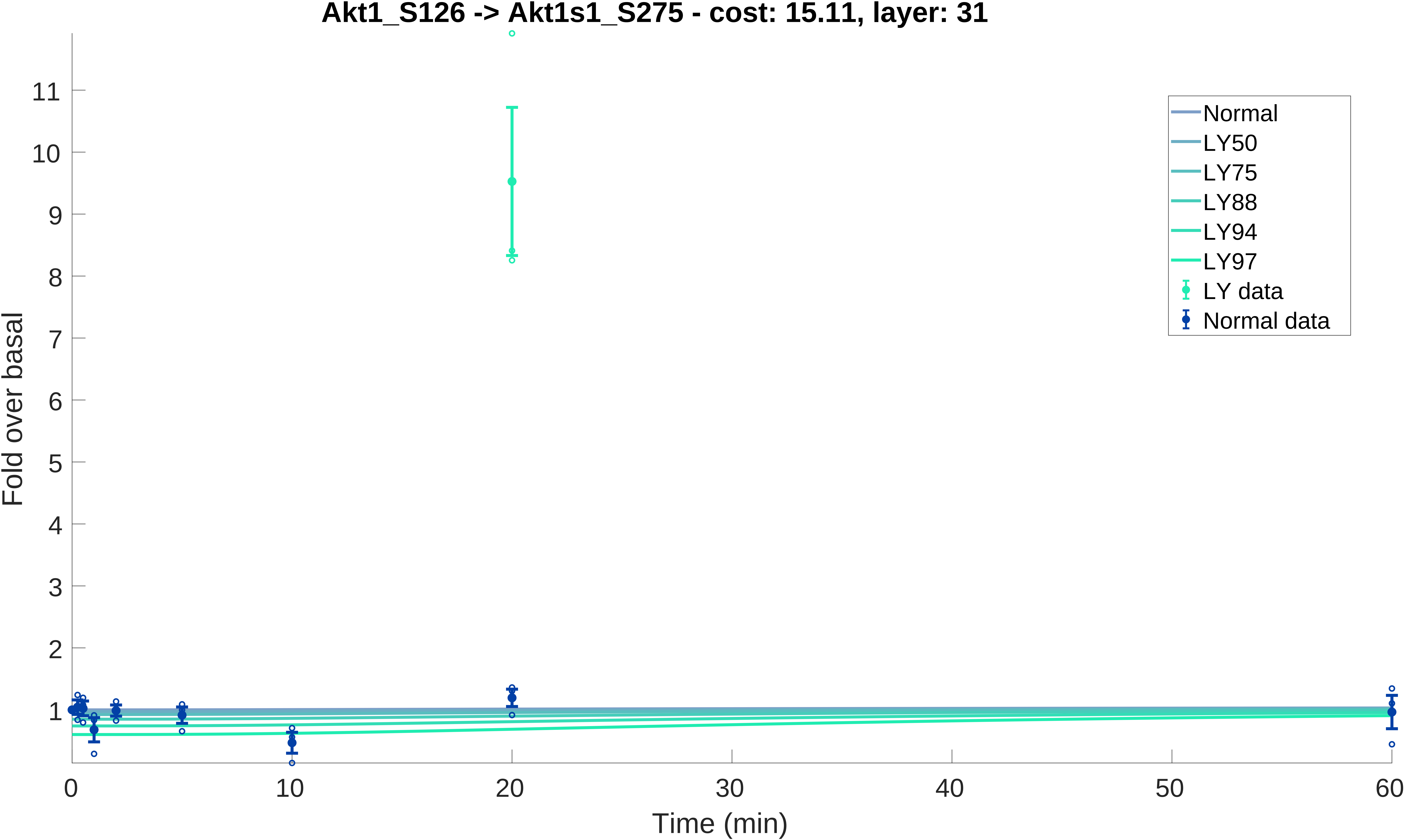
Akt1_S126 -> Akt1s1_S275 - cost: 15.11, layer: 31
Fold over basal
Time (min)
1
2
3
4
5
6
7
8
9
10
11
0
10
20
30
40
50
60
Normal
LY50
LY75
LY88
LY94
LY97
LY data
Normal data

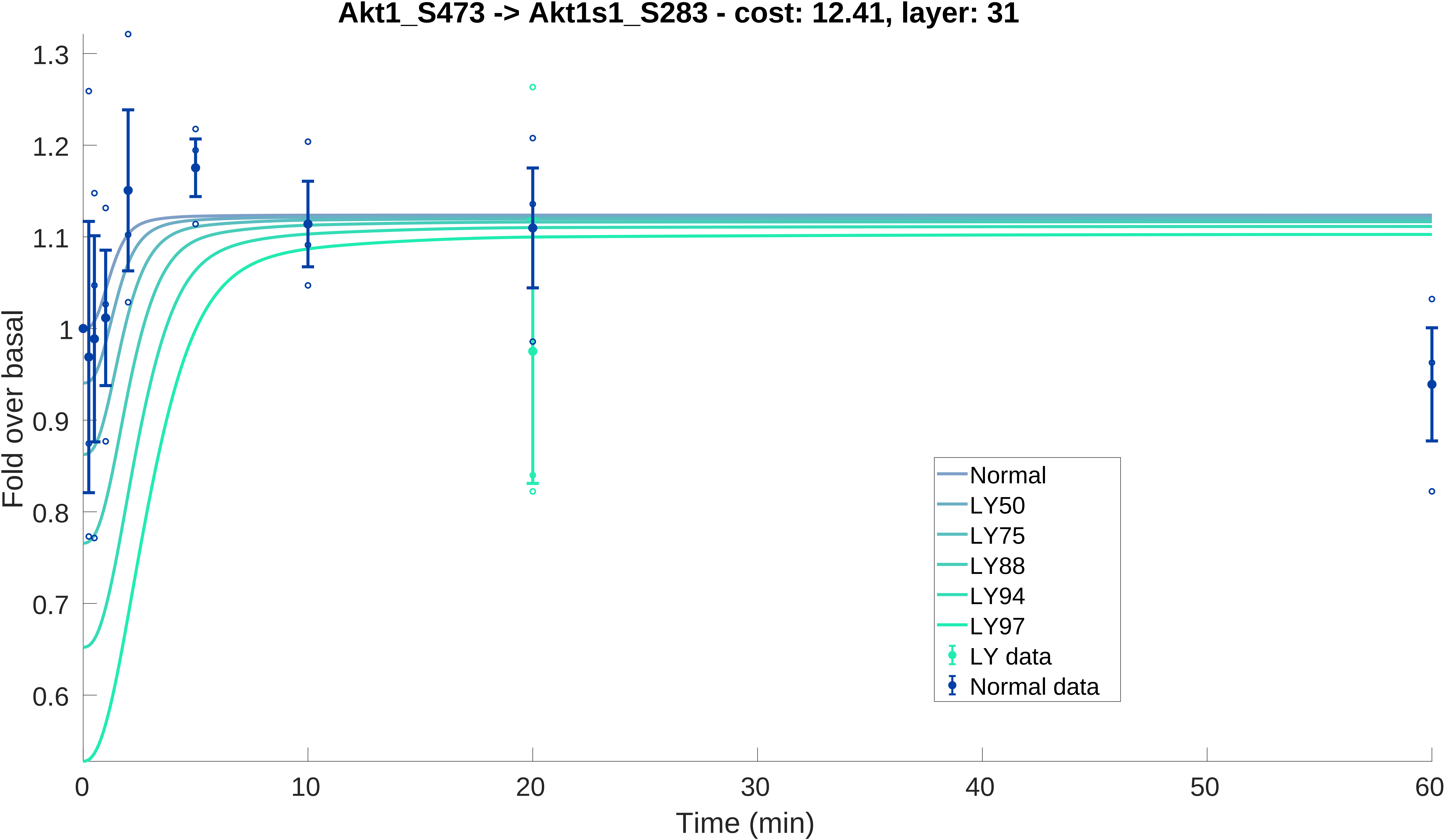

Akt1_S473 -> Akt1s1_S283 - cost: 12.41, layer: 31
Fold over basal
1.3
1.2
1.1
1
0.9
0.8
0.7
0.6
0
10
20
30
40
50
60
Time (min)
Normal
LY50
LY75
LY88
LY94
LY97
LY data
Normal data

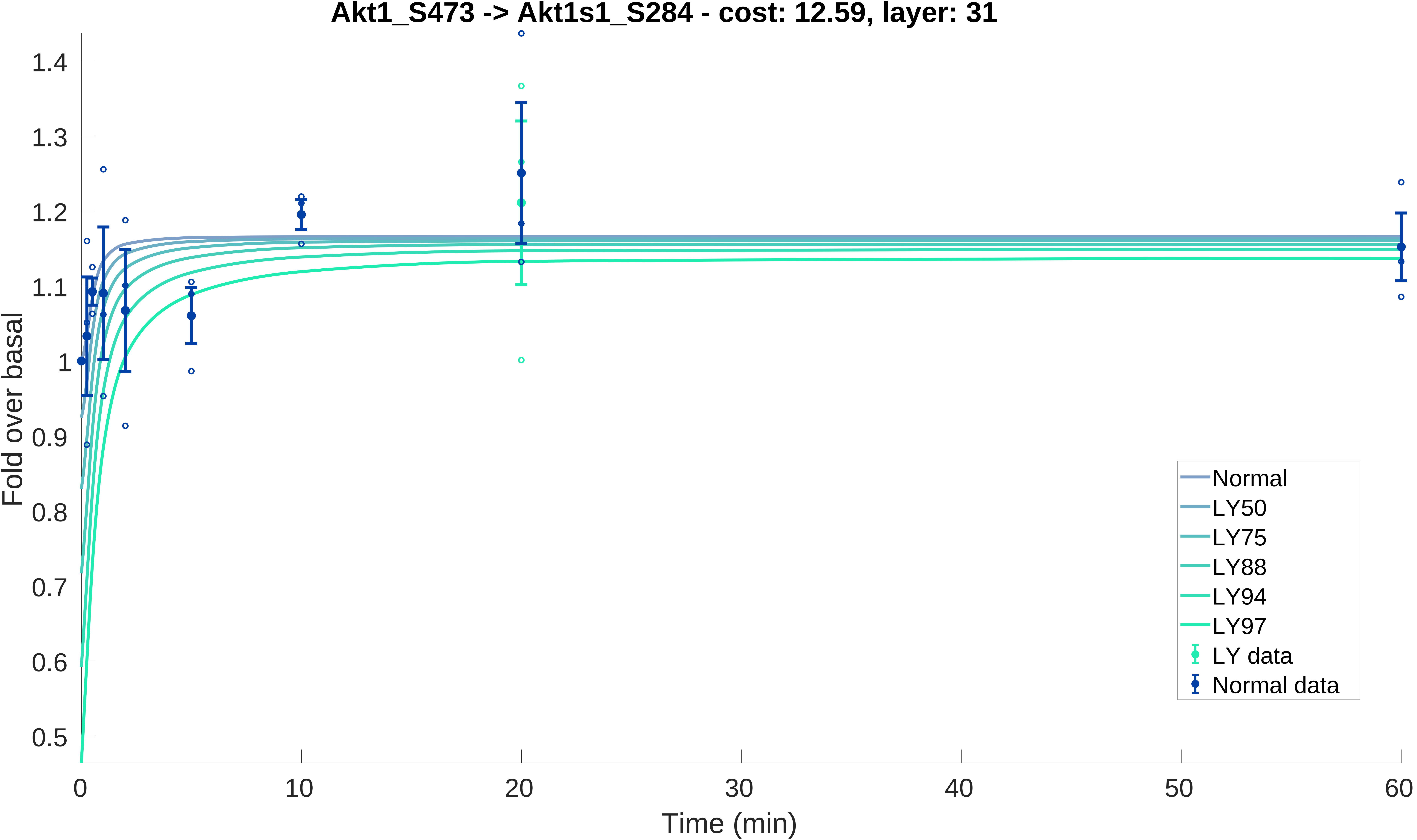

Akt1_S473 -> Akt1s1_S284 - cost: 12.59, layer: 31
Fold over basal
1.4
1.3
1.2
1.1
1
0.9
0.8
0.7
0.6
0.5
0
10
20
30
40
50
60
Time (min)
Normal
LY50
LY75
LY88
LY94
LY97
LY data
Normal data

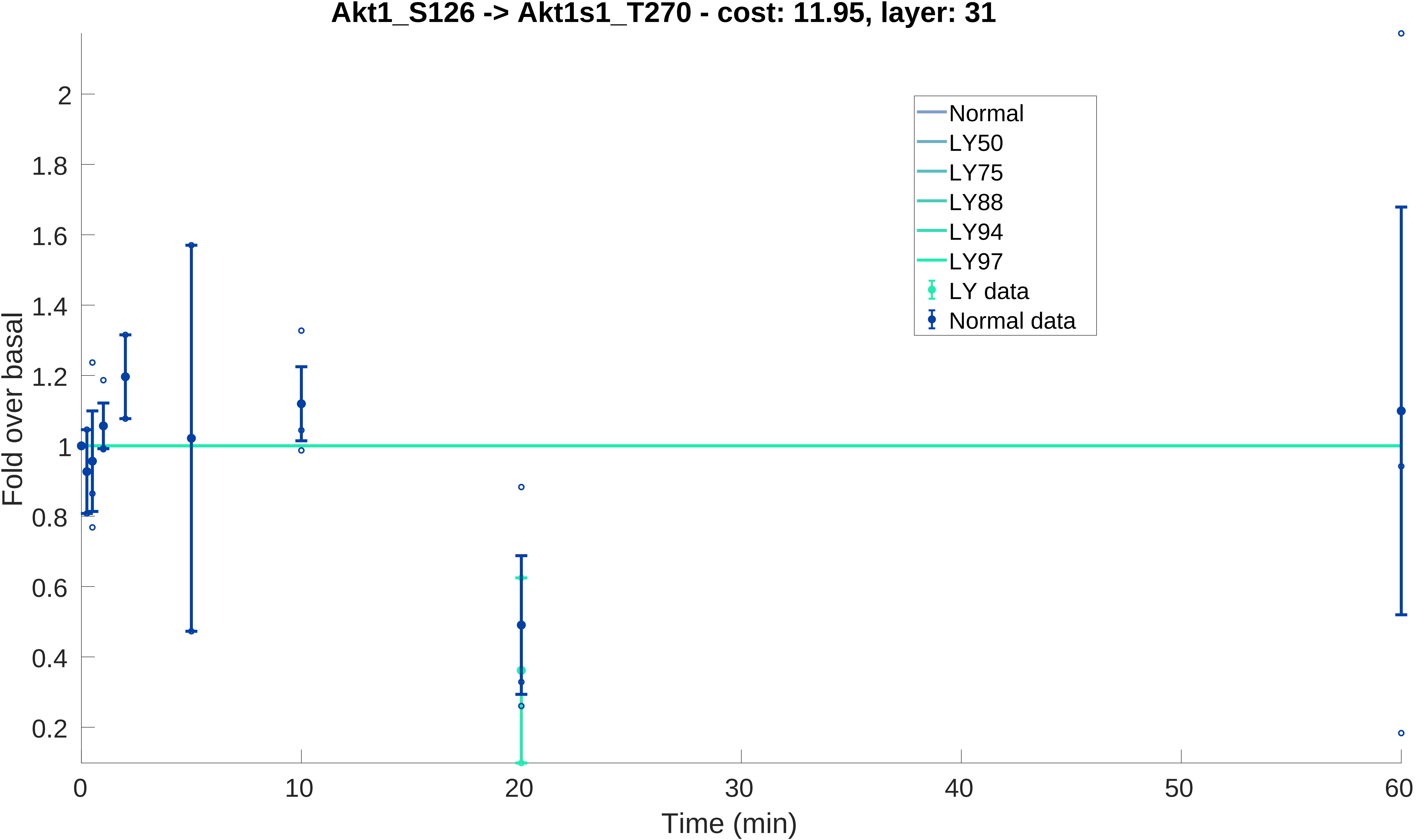

Akt1_S126 -> Akt1s1_T270 - cost: 11.95, layer: 31
Fold over basal
Time (min)
Normal
LY50
LY75
LY88
LY94
LY97
LY data
Normal data
2
1.8
1.6
1.4
1.2
1
0.8
0.6
0.4
0.2
0
10
20
30
40
50
60

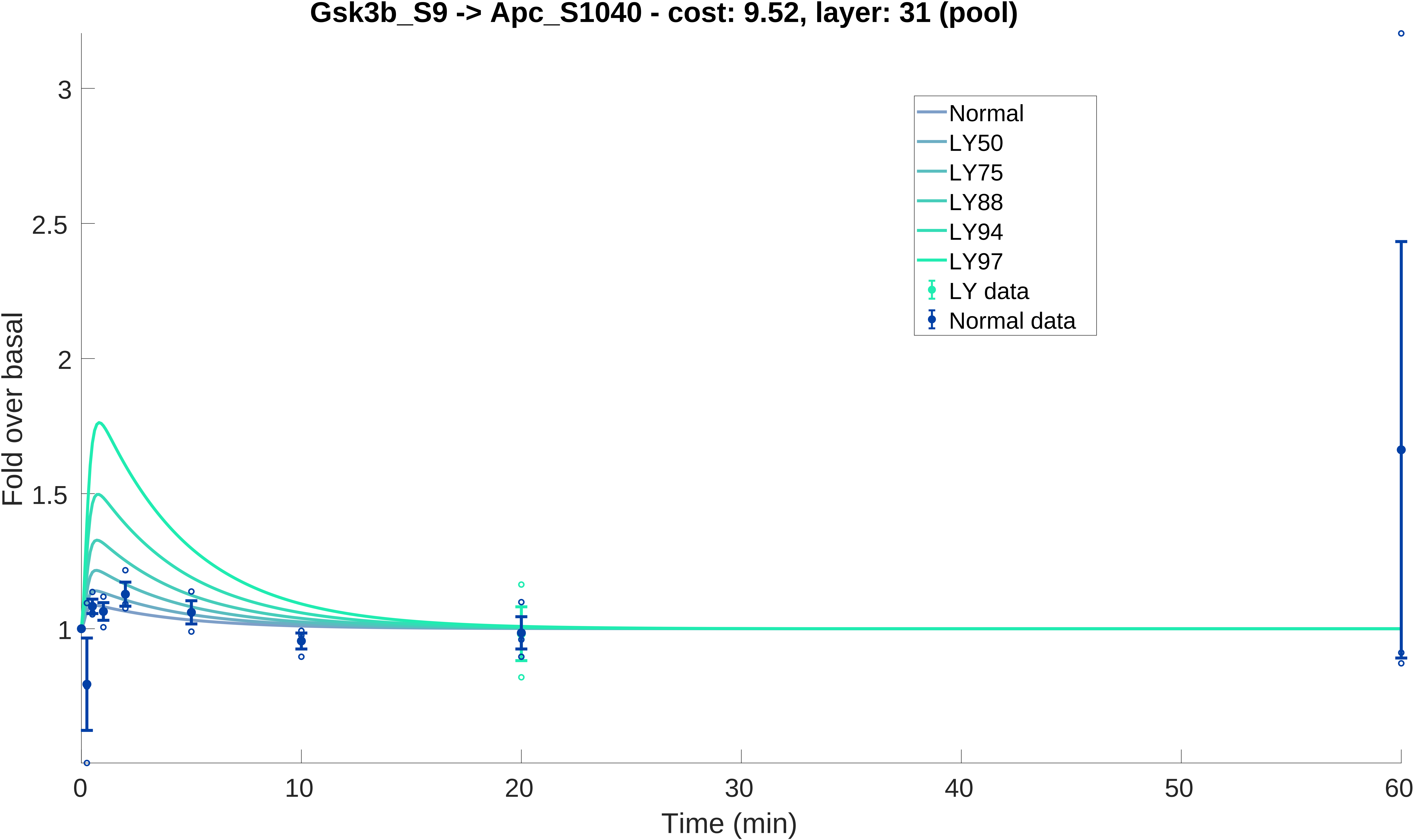

Gsk3b_S9 -> Apc_S1040 - cost: 9.52, layer: 31 (pool)
Normal
LY50
LY75
LY88
LY94
LY97
LY data
Normal data
Fold over basal
Time (min)
3
2.5
2
1.5
1
0
10
20
30
40
50
60

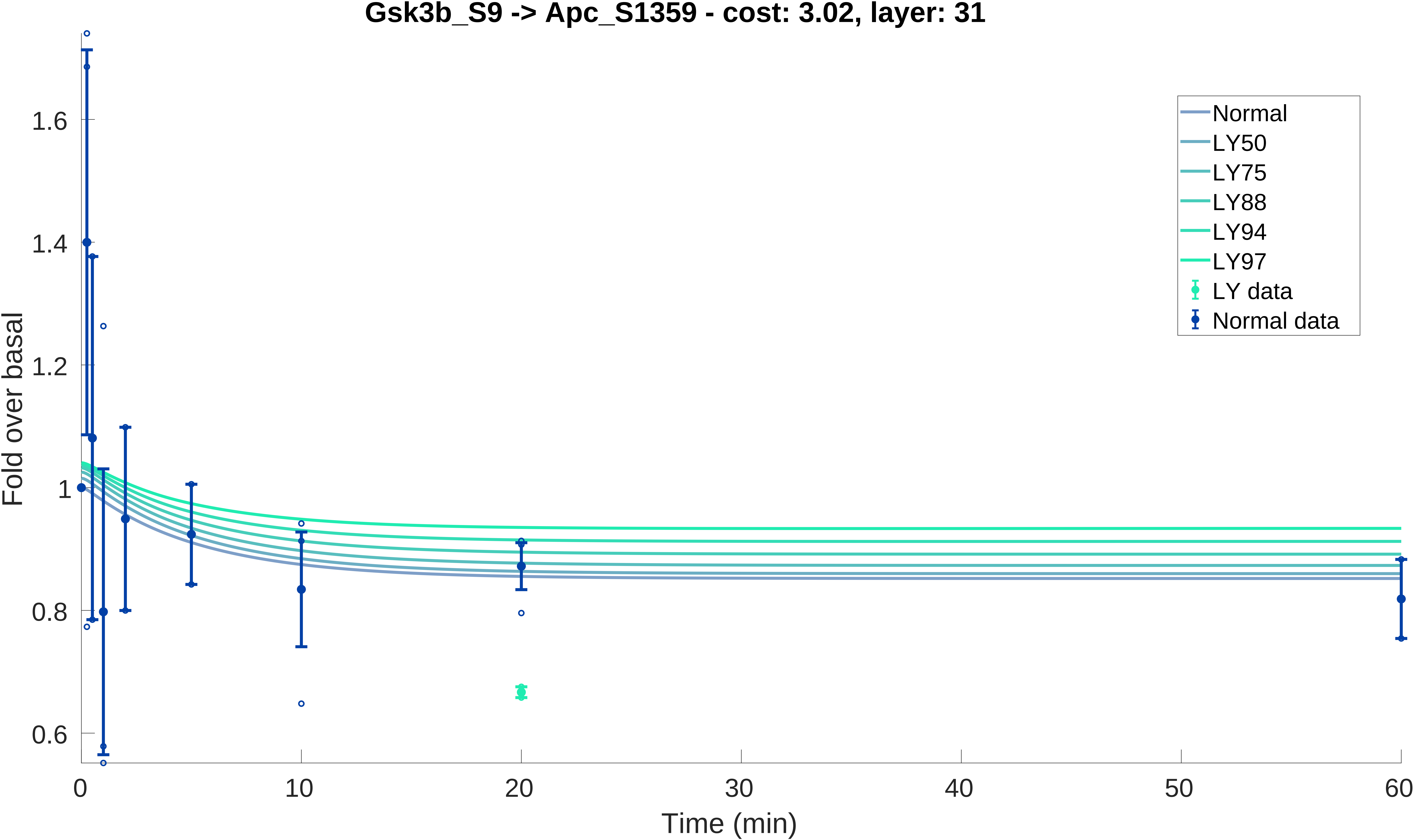

Gsk3b_S9 -> Apc_S1359 - cost: 3.02, layer: 31
Fold over basal
Time (min)
0.6
0.8
1
1.2
1.4
1.6
0
10
20
30
40
50
60
Normal
LY50
LY75
LY88
LY94
LY97
LY data
Normal data

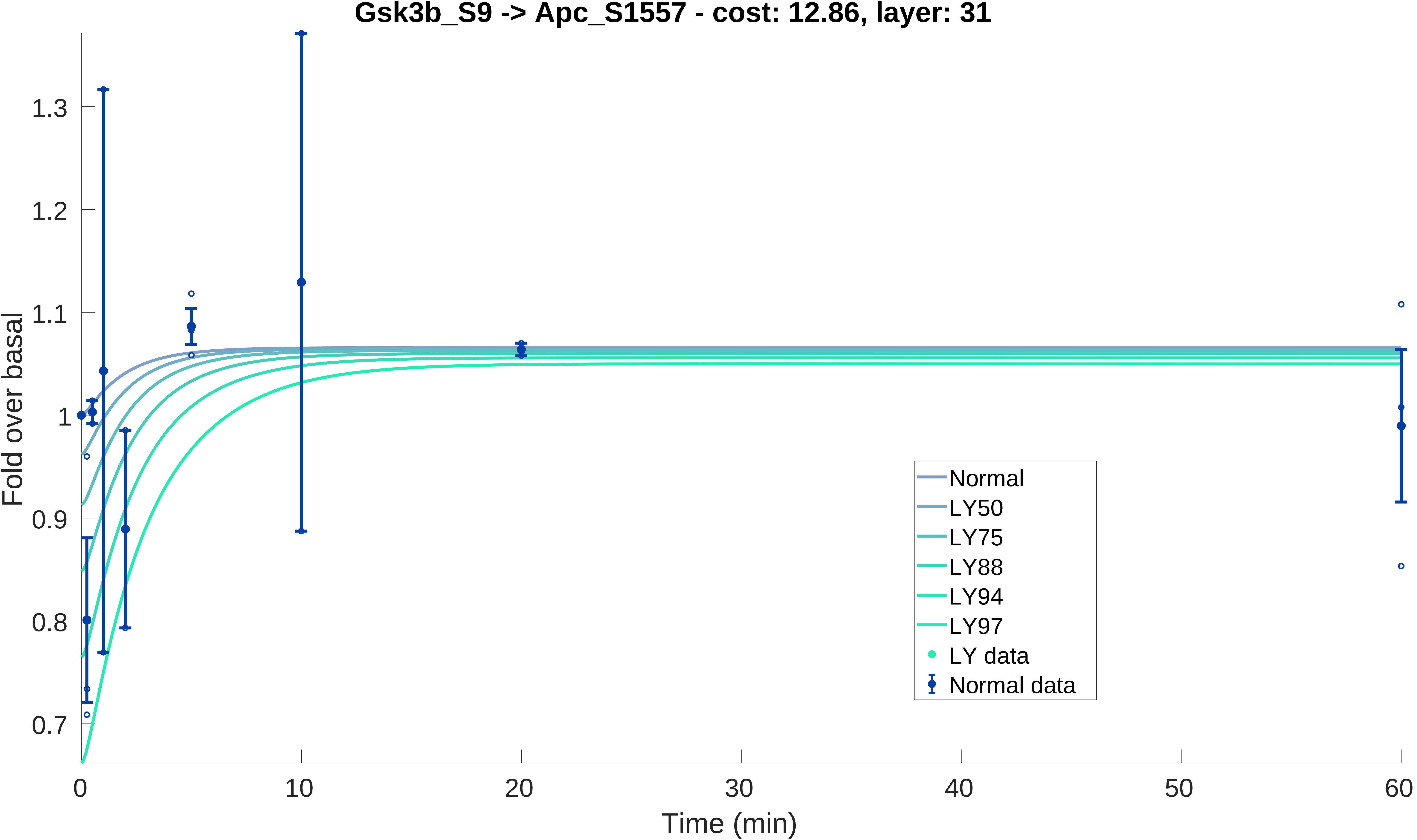
Gsk3b_S9 -> Apc_S1557 - cost: 12.86, layer: 31
Fold over basal
1.3
1.2
1.1
1
0.9
0.8
0.7
0
10
20
30
40
50
60
Time (min)
Normal
LY50
LY75
LY88
LY94
LY97
LY data
Normal data

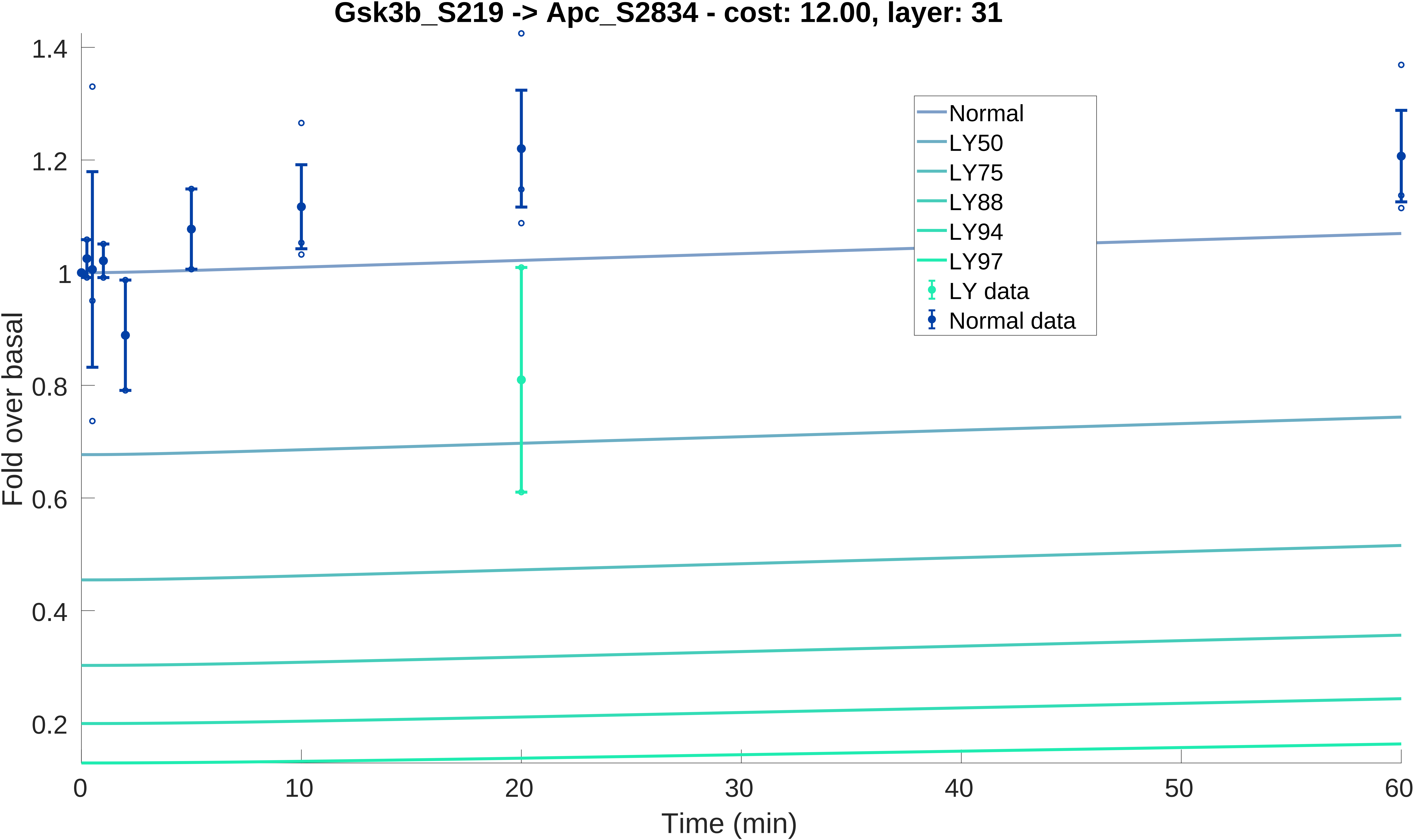
Gsk3b_S219 -> Apc_S2834 - cost: 12.00, layer: 31
Fold over basal
Time (min)
0
10
20
30
40
50
60
0.2
0.4
0.6
0.8
1
1.2
1.4
Normal
LY50
LY75
LY88
LY94
LY97
LY data
Normal data

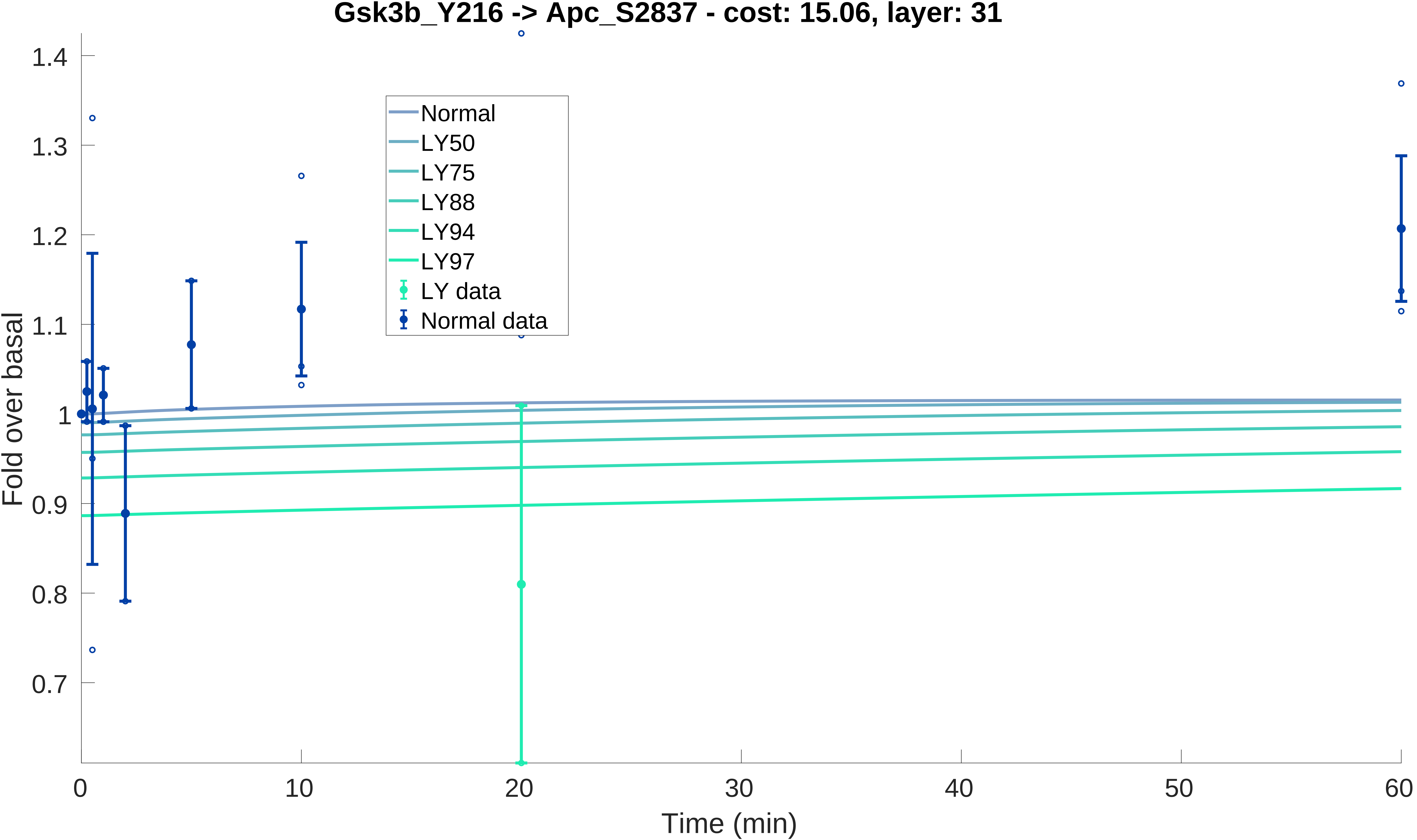

Gsk3b_Y216 -> Apc_S2837 - cost: 15.06, layer: 31
Normal
LY50
LY75
LY88
LY94
LY97
LY data
Normal data
Fold over basal
Time (min)
1.4
1.3
1.2
1.1
1
0.9
0.8
0.7
0
10
20
30
40
50
60

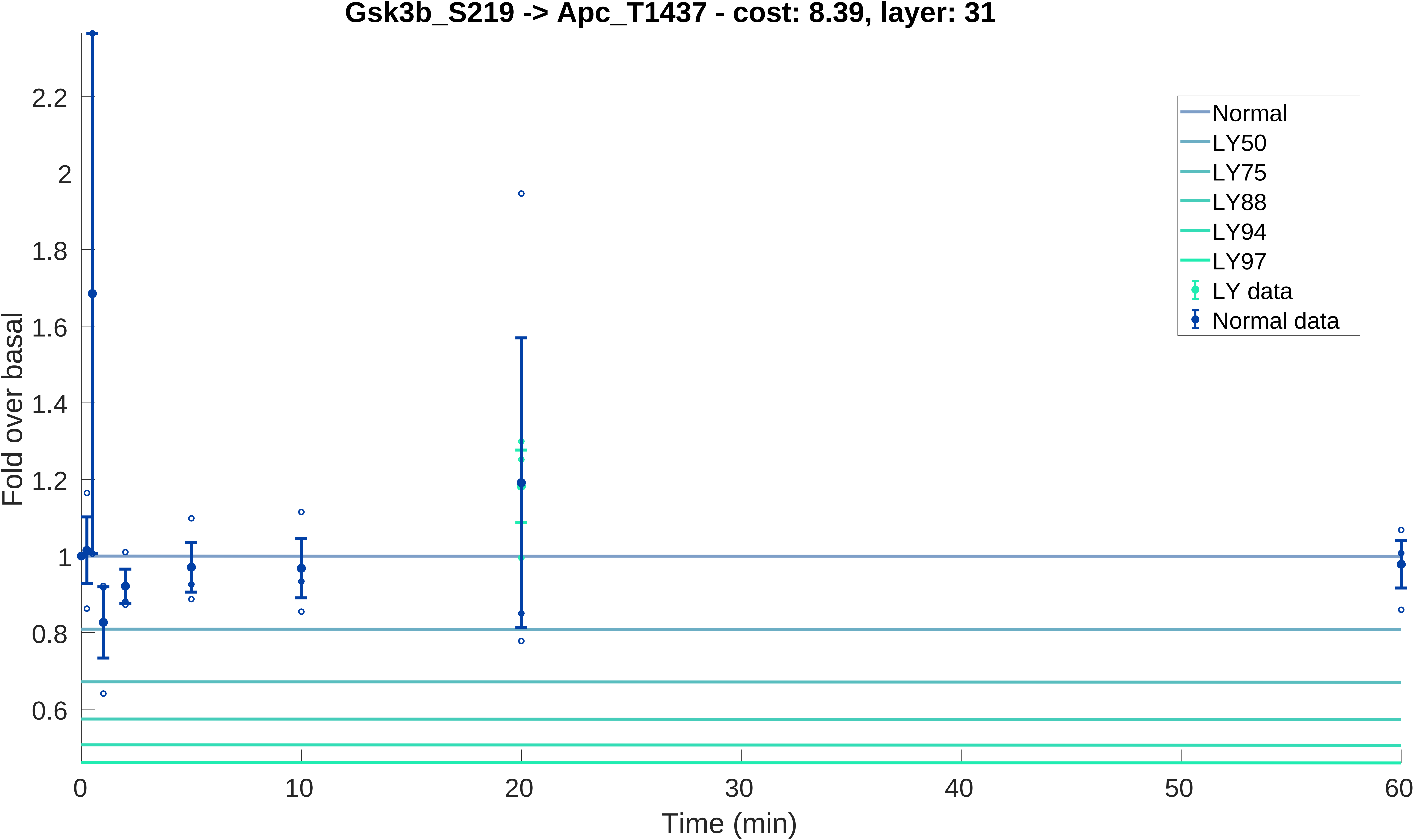

Gsk3b_S219 -> Apc_T1437 - cost: 8.39, layer: 31
Fold over basal
Time (min)
2.2
2
1.8
1.6
1.4
1.2
1
0.8
0.6
0
10
20
30
40
50
60
Normal
LY50
LY75
LY88
LY94
LY97
LY data
Normal data

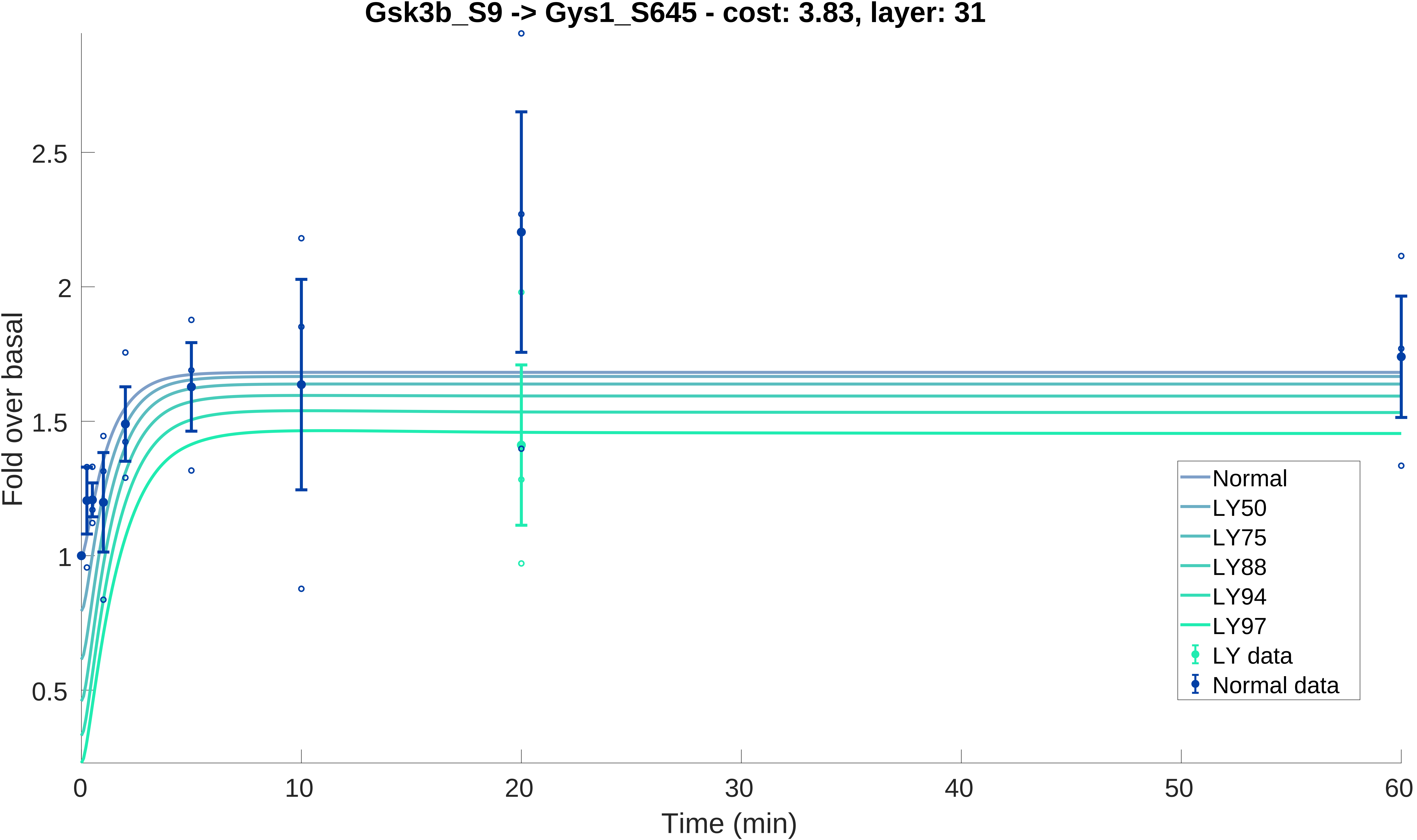

Gsk3b_S9 -> Gys1_S645 - cost: 3.83, layer: 31
Fold over basal
Time (min)
0.5
1
1.5
2
2.5
0
10
20
30
40
50
60
Normal
LY50
LY75
LY88
LY94
LY97
LY data
Normal data

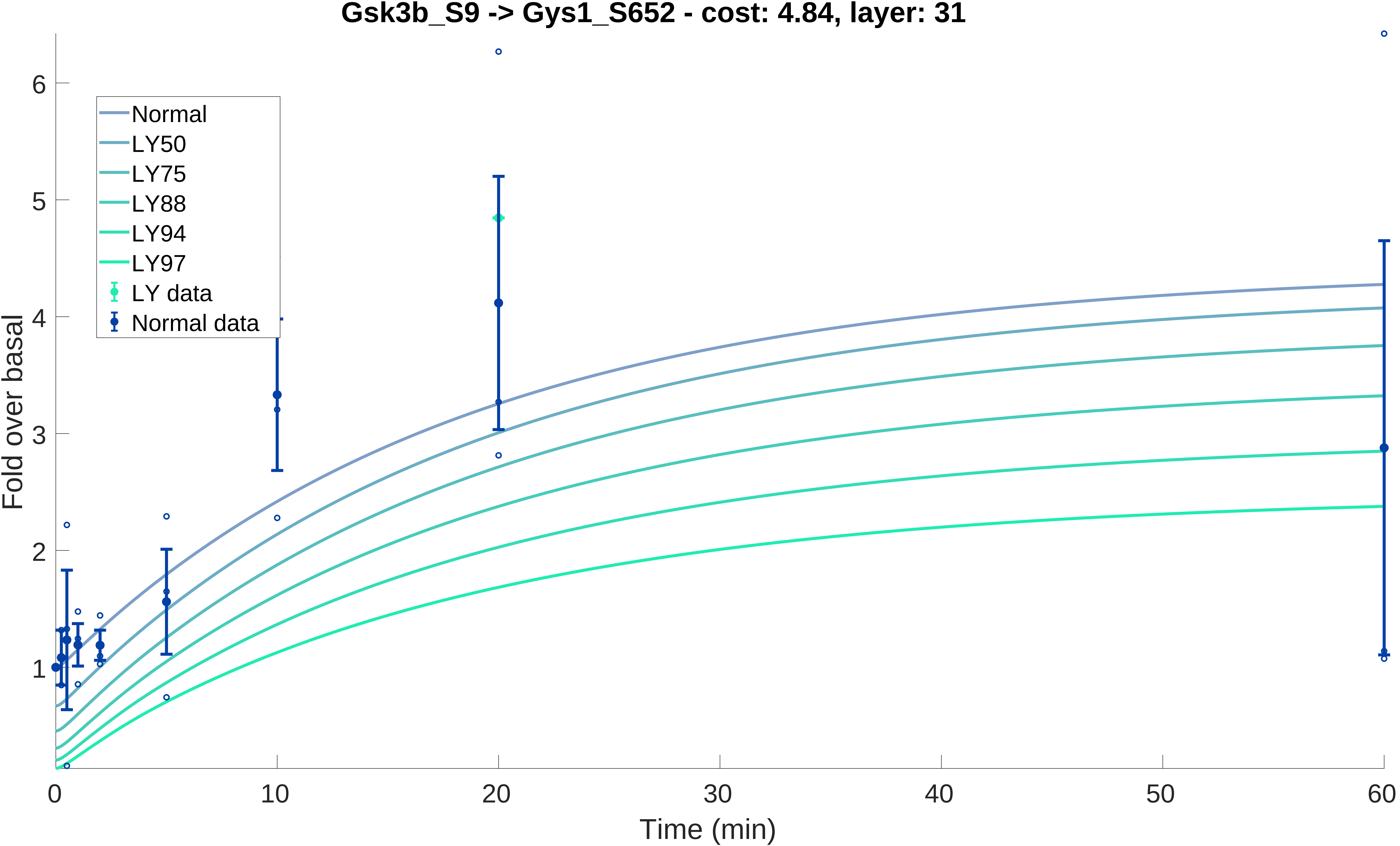

Gsk3b_S9 -> Gys1_S652 - cost: 4.84, layer: 31
Normal
LY50
LY75
LY88
LY94
LY97
LY data
Normal data
Fold over basal
Time (min)
0
10
20
30
40
50
60
1
2
3
4
5
6

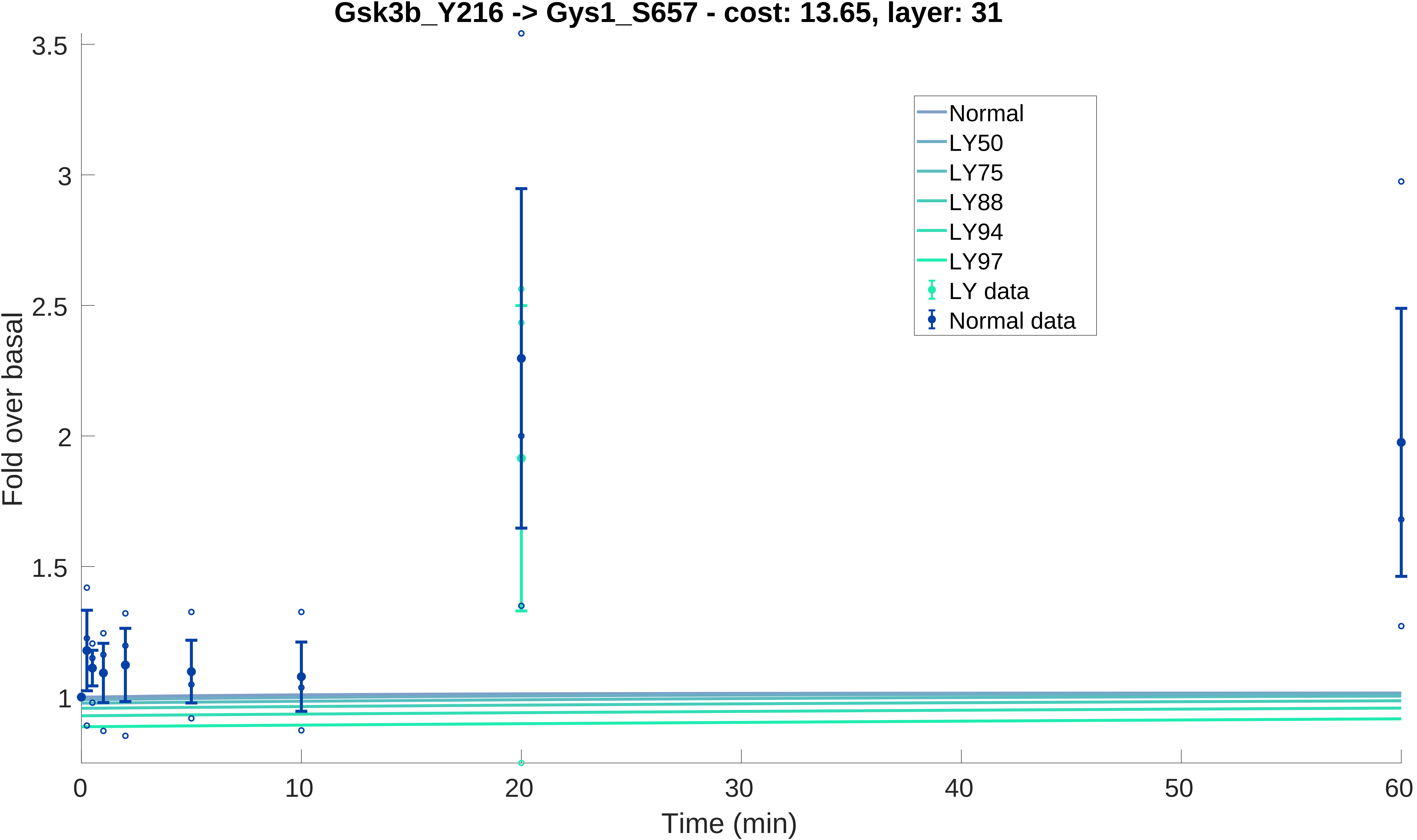

Gsk3b_Y216 -> Gys1_S657 - cost: 13.65, layer: 31
Fold over basal
Time (min)
3.5
3
2.5
2
1.5
1
0
10
20
30
40
50
60
Normal
LY50
LY75
LY88
LY94
LY97
LY data
Normal data

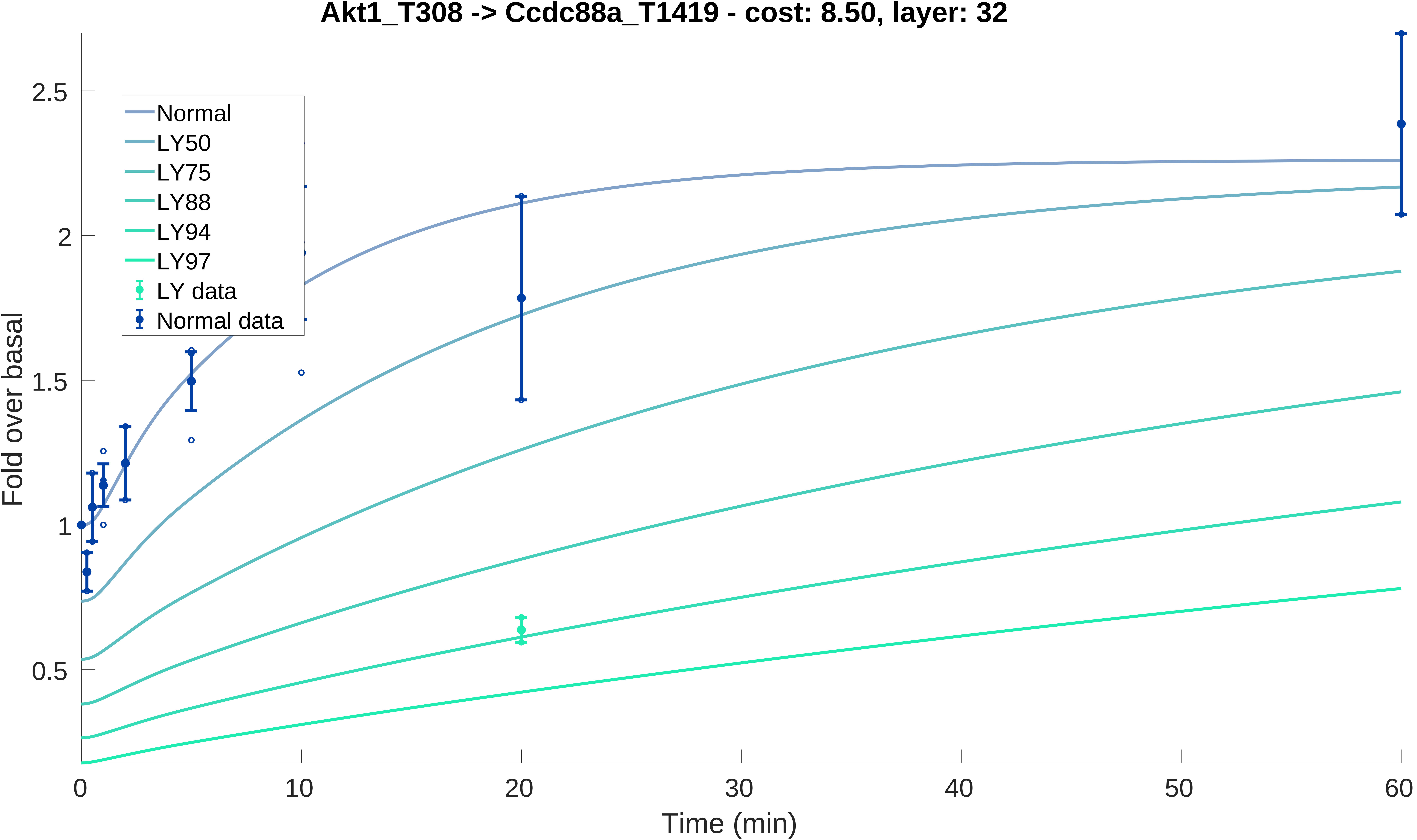

Akt1_T308 -> Ccdc88a_T1419 - cost: 8.50, layer: 32
Normal
LY50
LY75
LY88
LY94
LY97
LY data
Normal data
Fold over basal
Time (min)
0.5
1
1.5
2
2.5
0
10
20
30
40
50
60

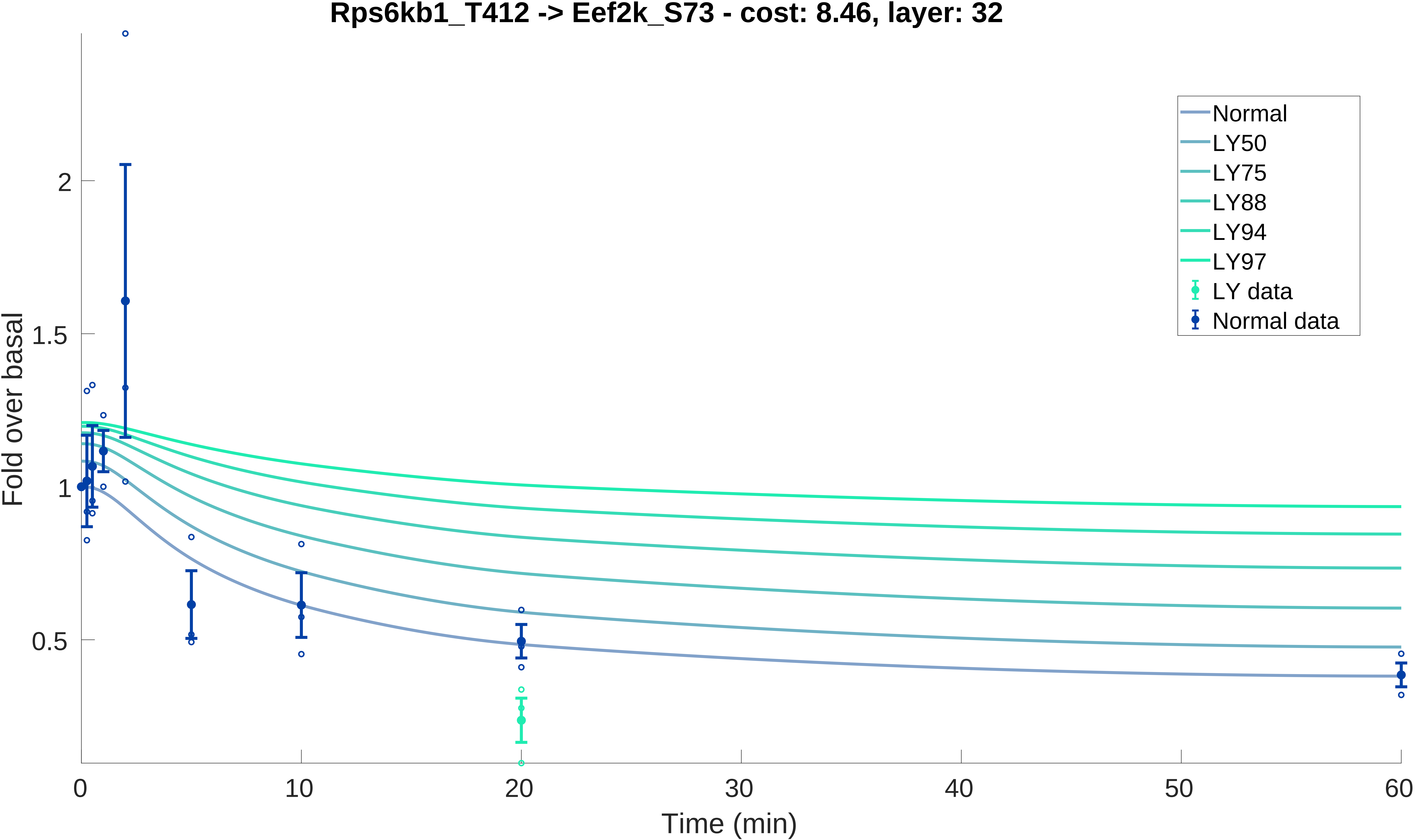

Rps6kb1_T412 -> Eef2k_S73 - cost: 8.46, layer: 32
Fold over basal
Time (min)
0.5
1
1.5
2
0
10
20
30
40
50
60
Normal
LY50
LY75
LY88
LY94
LY97
LY data
Normal data

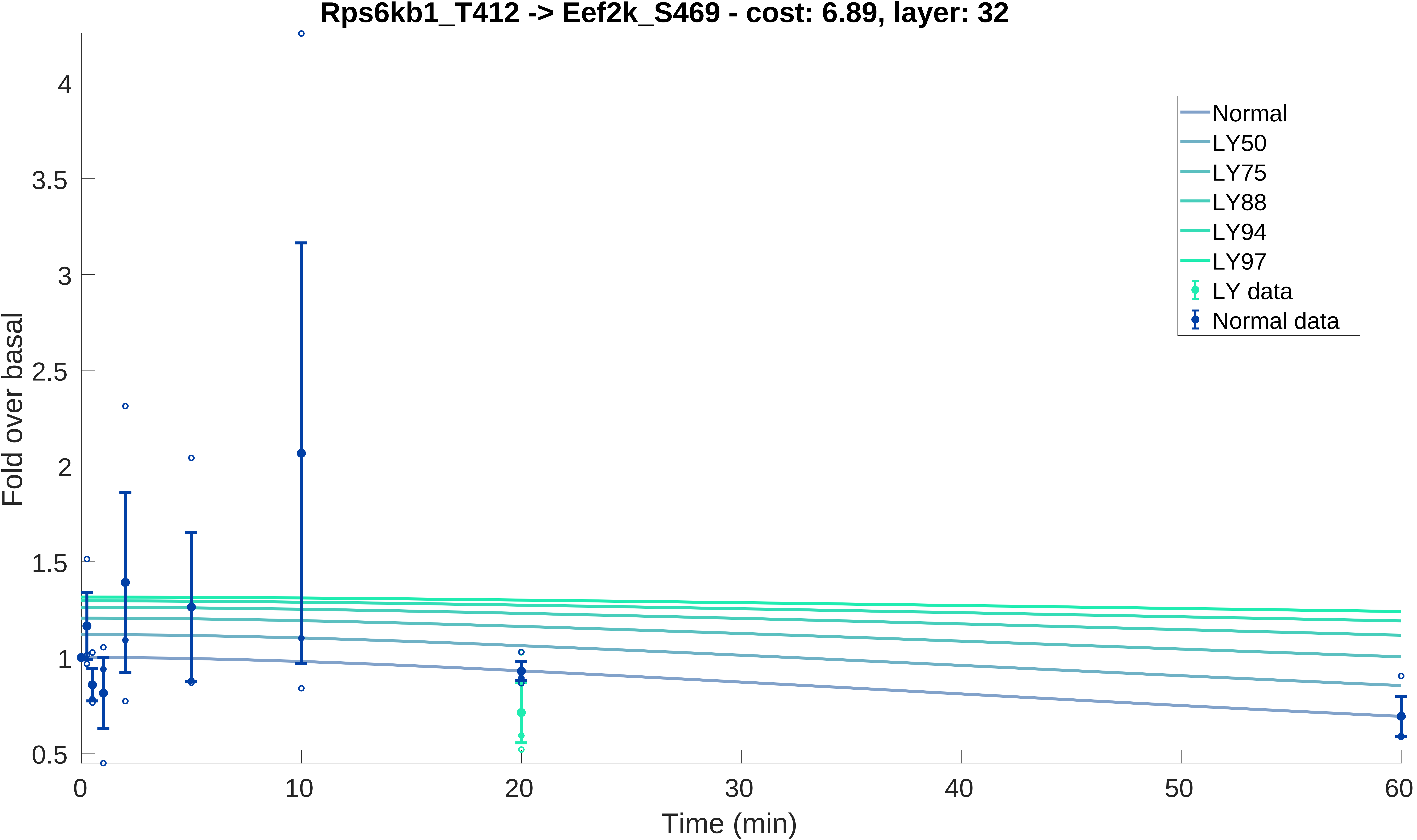

Rps6kb1_T412 -> Eef2k_S469 - cost: 6.89, layer: 32
Fold over basal
Time (min)
0
10
20
30
40
50
60
0.5
1
1.5
2
2.5
3
3.5
4
Normal
LY50
LY75
LY88
LY94
LY97
LY data
Normal data

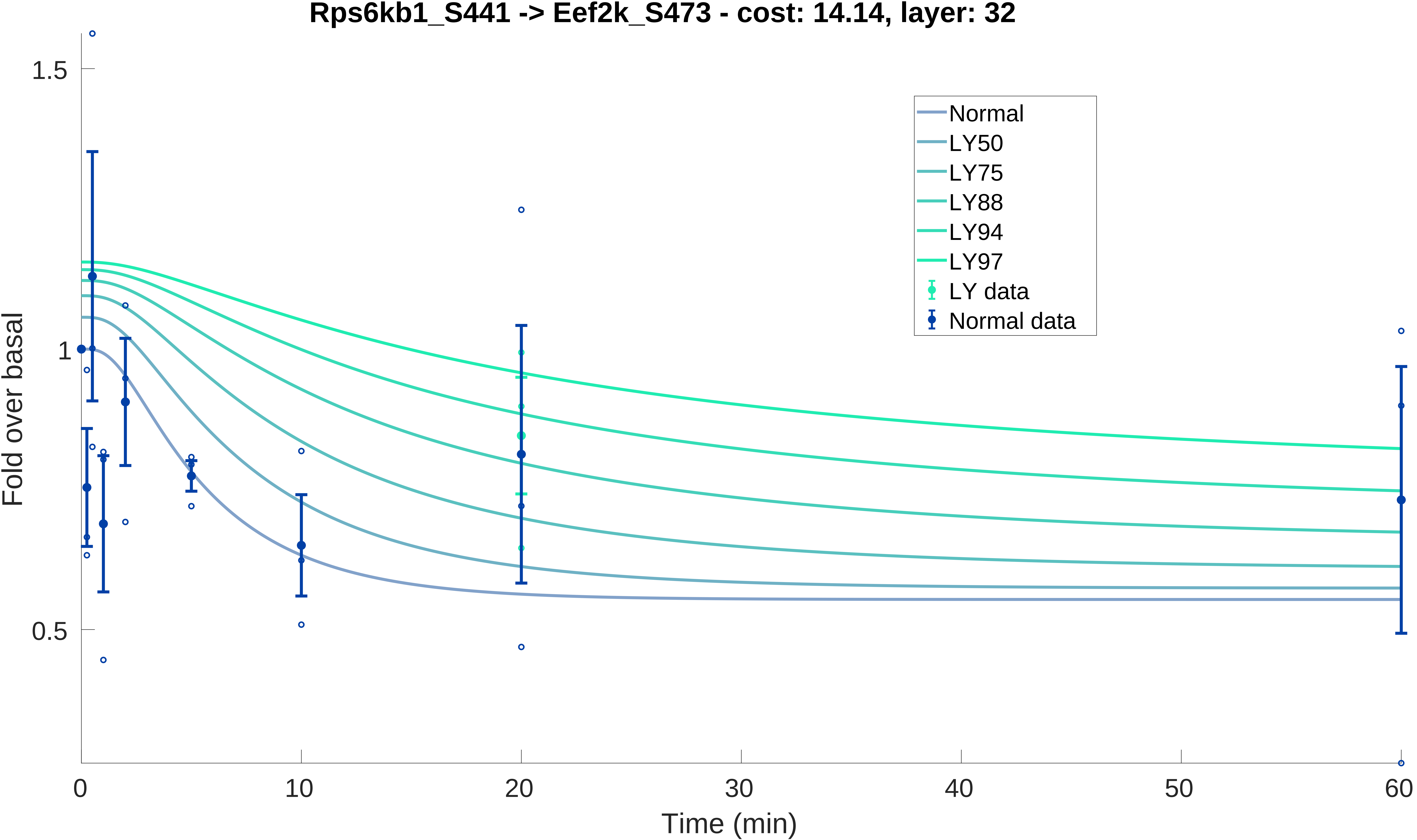

Rps6kb1_S441 -> Eef2k_S473 - cost: 14.14, layer: 32
Fold over basal
1.5
1
0.5
0
10
20
30
40
50
60
Time (min)
Normal
LY50
LY75
LY88
LY94
LY97
LY data
Normal data

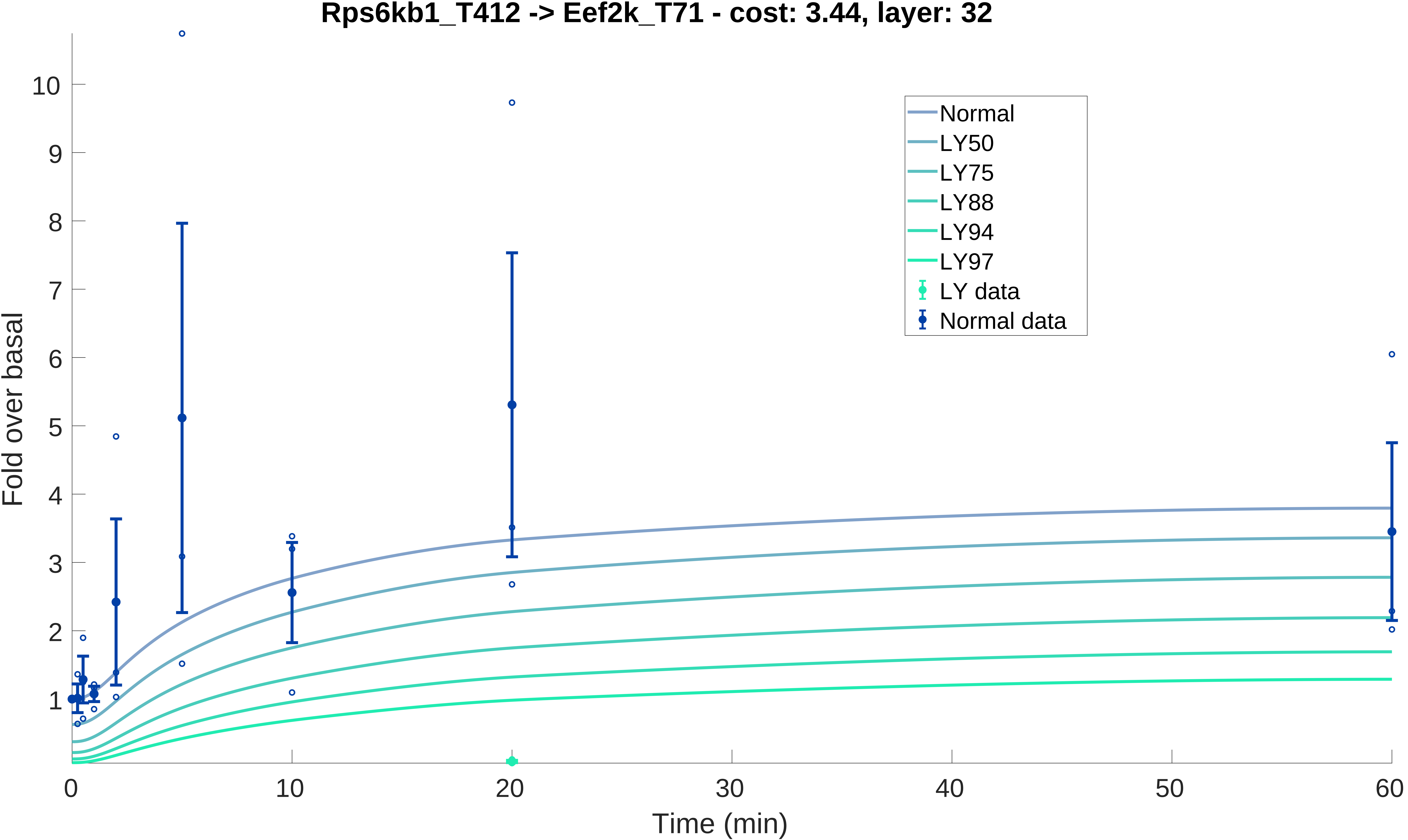
Rps6kb1_T412 -> Eef2k_T71 - cost: 3.44, layer: 32
Fold over basal
Time (min)
Normal
LY50
LY75
LY88
LY94
LY97
LY data
Normal data
0
10
20
30
40
50
60
1
2
3
4
5
6
7
8
9
10

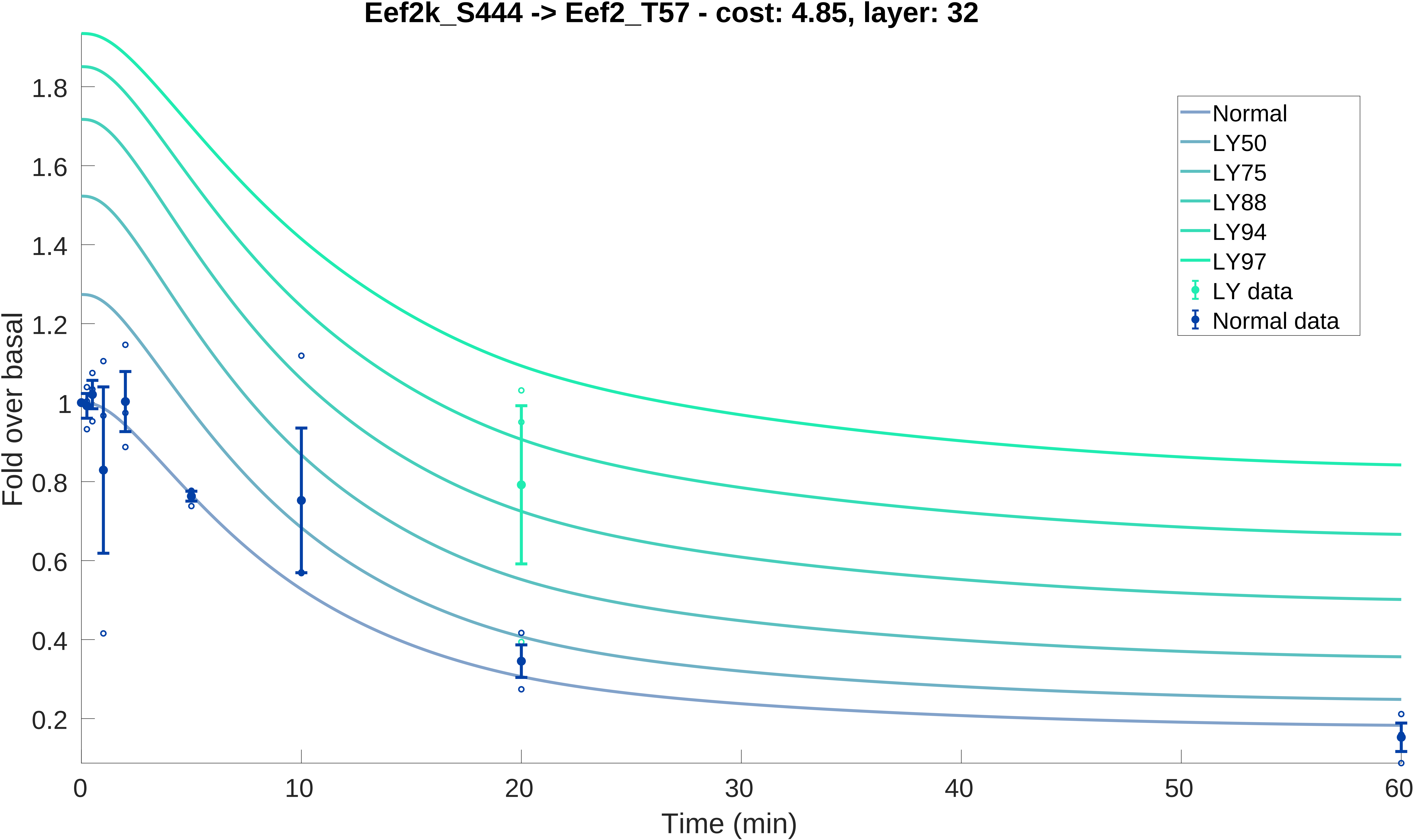

Eef2k_S444 -> Eef2_T57 - cost: 4.85, layer: 32
Fold over basal
Time (min)
0.2
0.4
0.6
0.8
1
1.2
1.4
1.6
1.8
0
10
20
30
40
50
60
Normal
LY50
LY75
LY88
LY94
LY97
LY data
Normal data

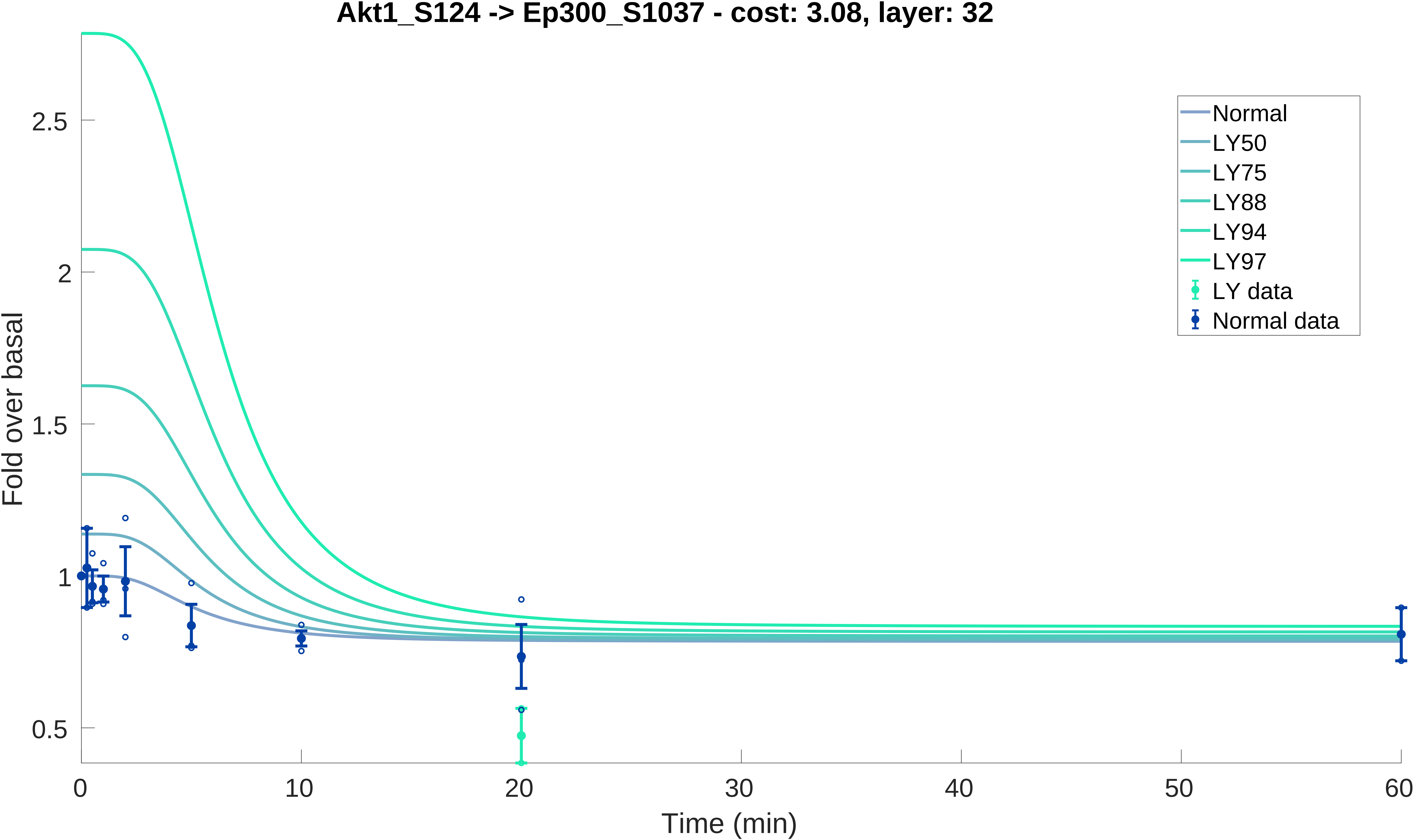

Akt1_S124 -> Ep300_S1037 - cost: 3.08, layer: 32
Fold over basal
2.5
2
1.5
1
0.5
0
10
20
30
40
50
60
Time (min)
Normal
LY50
LY75
LY88
LY94
LY97
LY data
Normal data

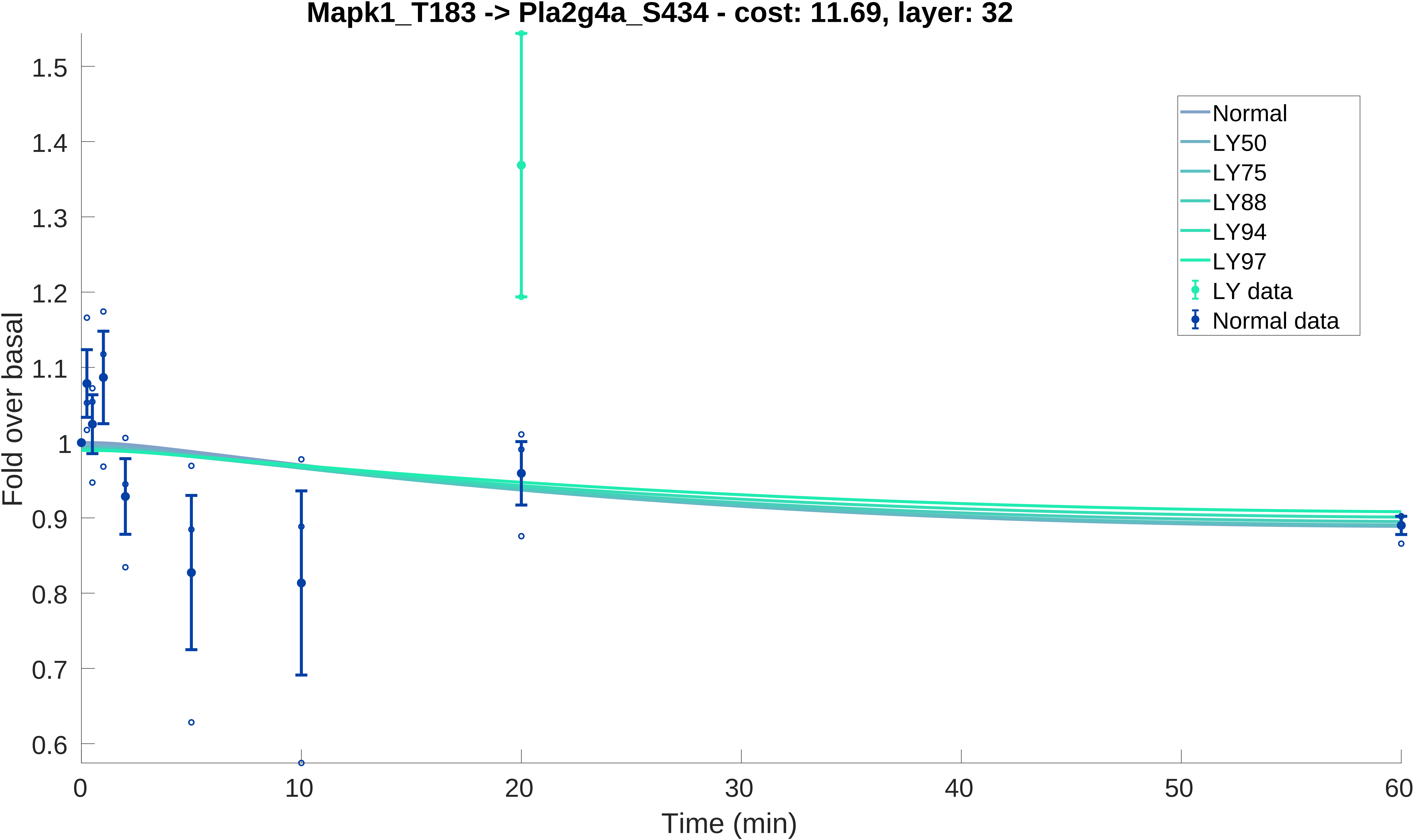

Mapk1_T183 -> Pla2g4a_S434 - cost: 11.69, layer: 32
Fold over basal
Time (min)
1.5
1.4
1.3
1.2
1.1
1
0.9
0.8
0.7
0.6
0
10
20
30
40
50
60
Normal
LY50
LY75
LY88
LY94
LY97
LY data
Normal data

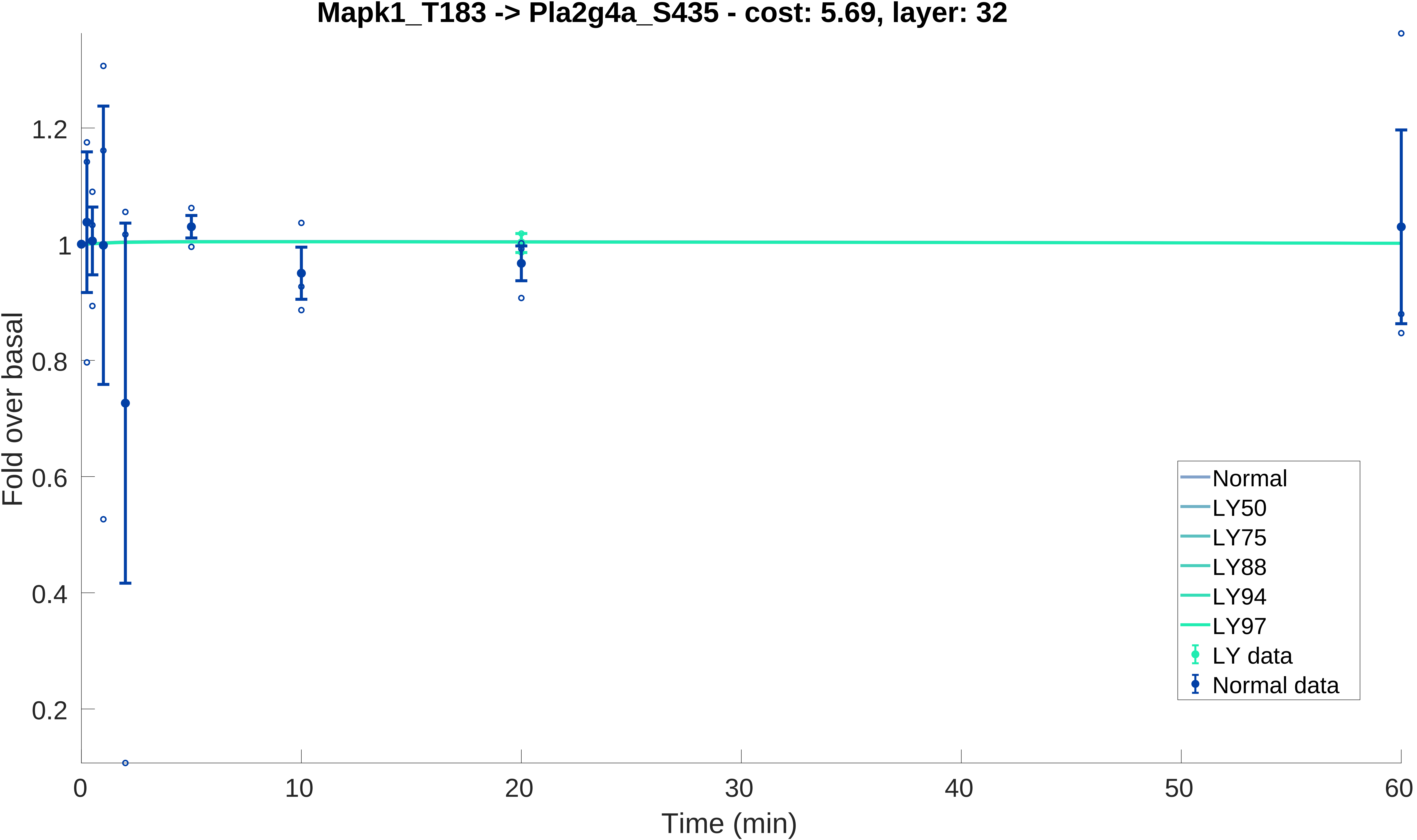

Mapk1_T183 -> Pla2g4a_S435 - cost: 5.69, layer: 32
Fold over basal
Time (min)
0.2
0.4
0.6
0.8
1
1.2
0
10
20
30
40
50
60
Normal
LY50
LY75
LY88
LY94
LY97
LY data
Normal data

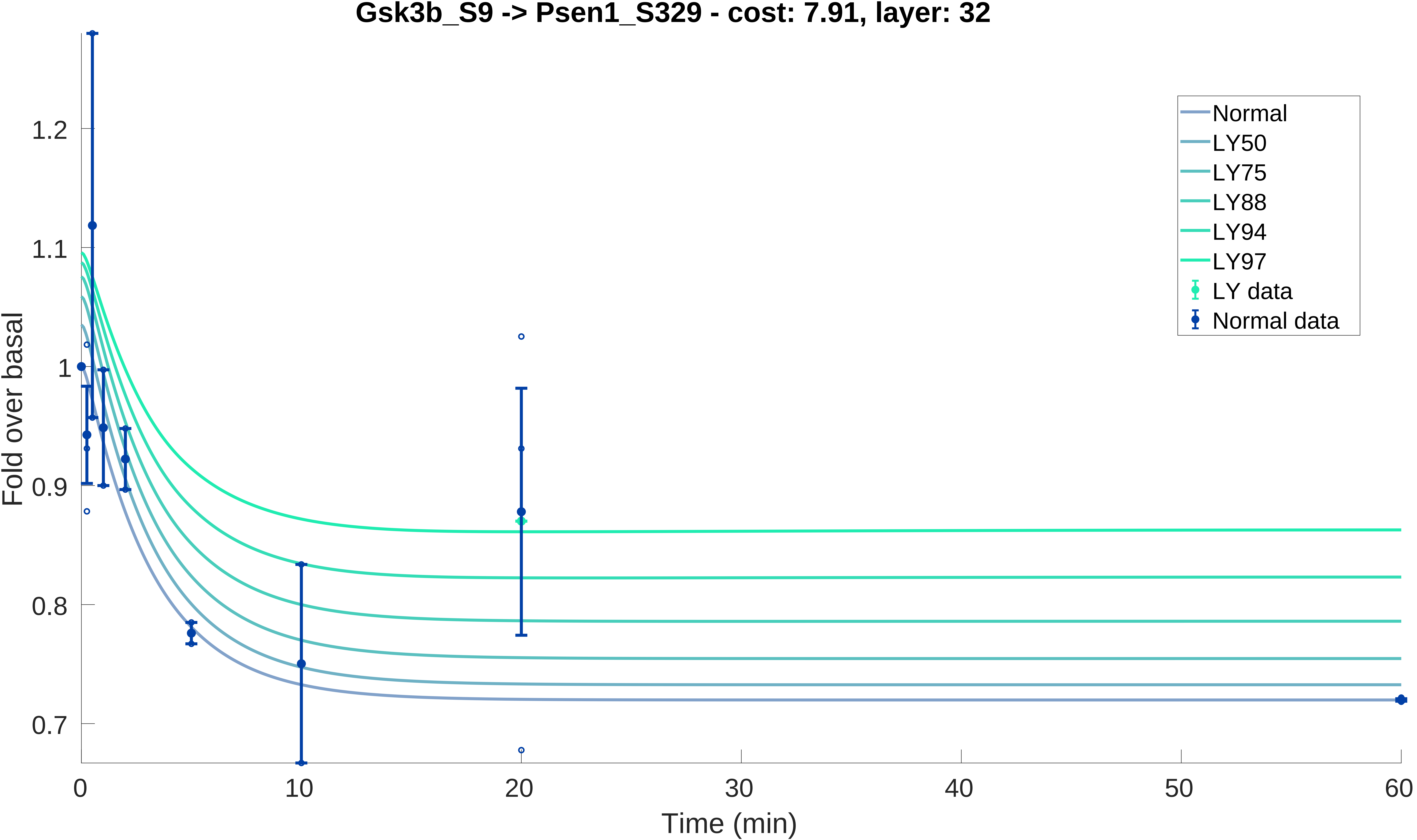

Gsk3b_S9 -> Psen1_S329 - cost: 7.91, layer: 32
Fold over basal
Time (min)
Normal
LY50
LY75
LY88
LY94
LY97
LY data
Normal data
1.2
1.1
1
0.9
0.8
0.7
0
10
20
30
40
50
60

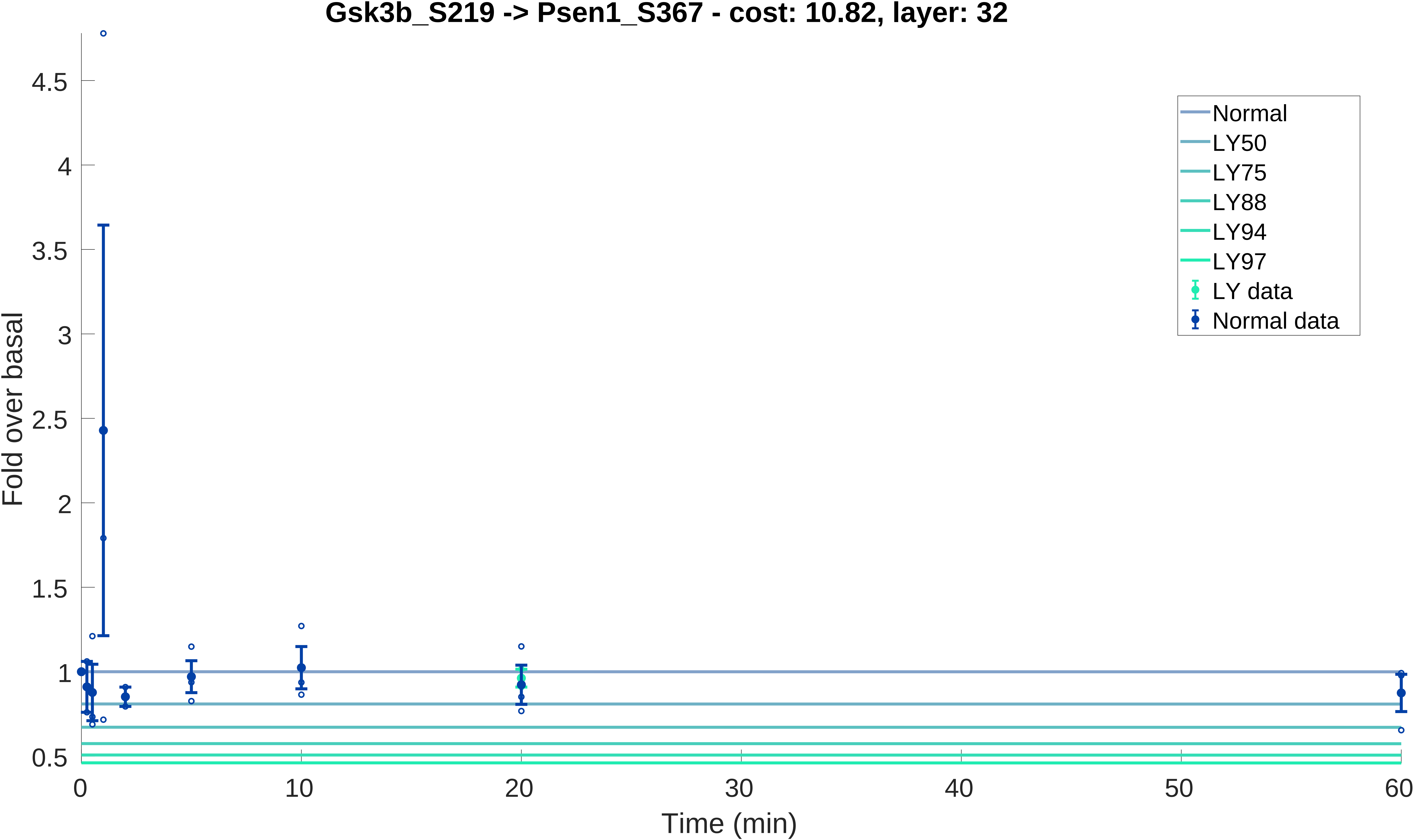
Gsk3b_S219 -> Psen1_S367 - cost: 10.82, layer: 32
Fold over basal
Time (min)
0.5
1
1.5
2
2.5
3
3.5
4
4.5
0
10
20
30
40
50
60
Normal
LY50
LY75
LY88
LY94
LY97
LY data
Normal data

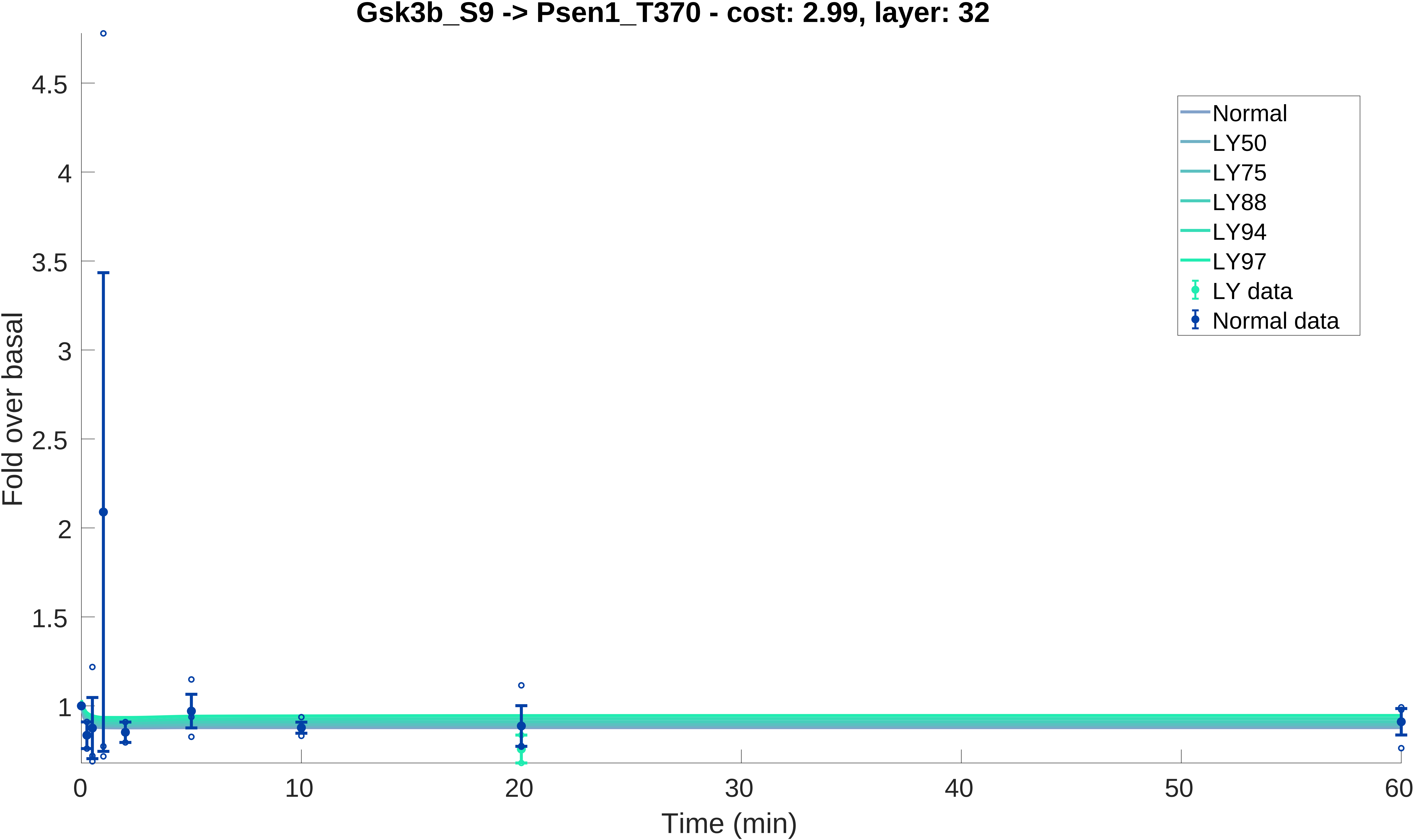

Gsk3b_S9 -> Psen1_T370 - cost: 2.99, layer: 32
Fold over basal
Time (min)
Normal
LY50
LY75
LY88
LY94
LY97
LY data
Normal data
4.5
4
3.5
3
2.5
2
1.5
1
0
10
20
30
40
50
60

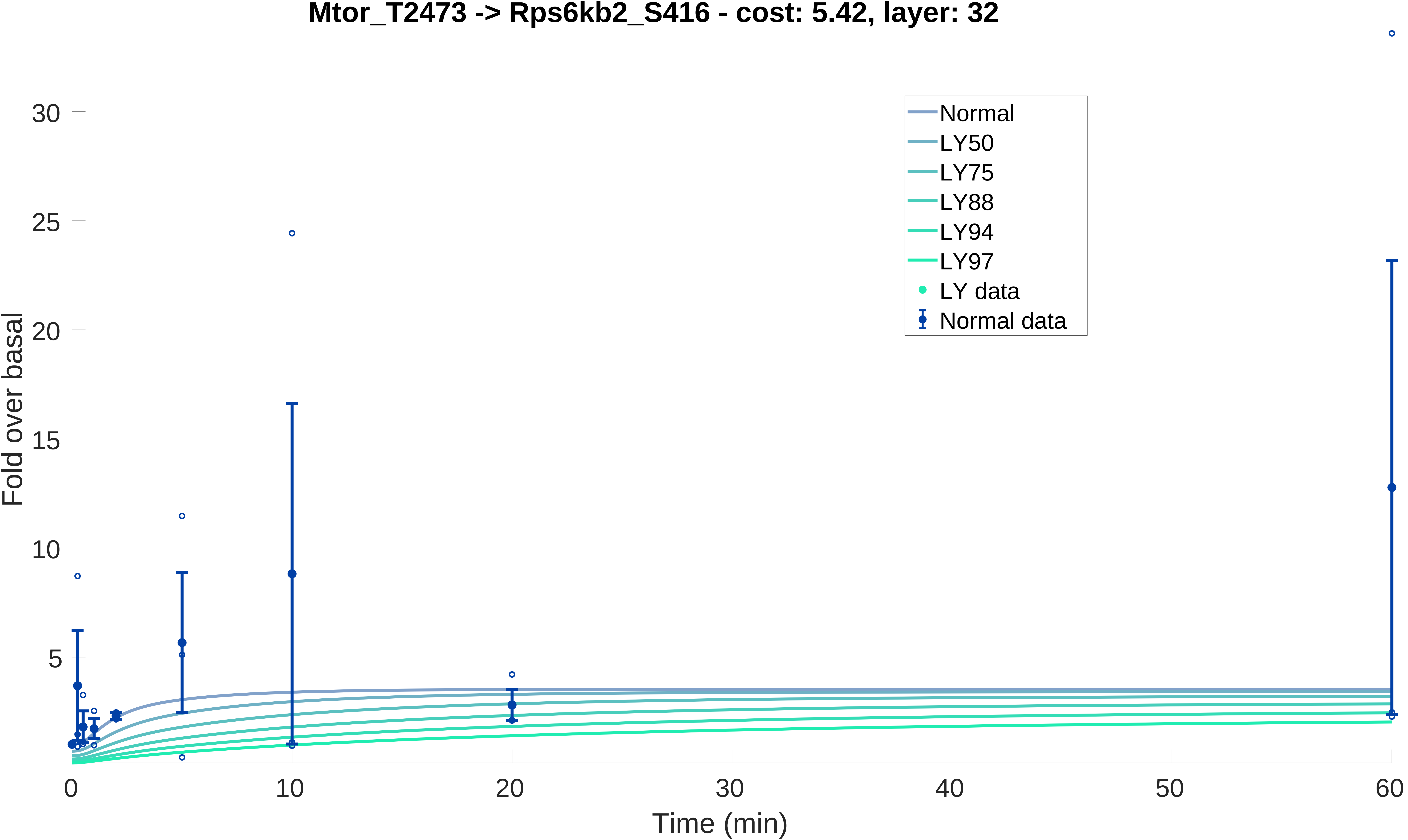

Mtor_T2473 -> Rps6kb2_S416 - cost: 5.42, layer: 32
Fold over basal
Time (min)
0
10
20
30
40
50
60
5
10
15
20
25
30
Normal
LY50
LY75
LY88
LY94
LY97
LY data
Normal data

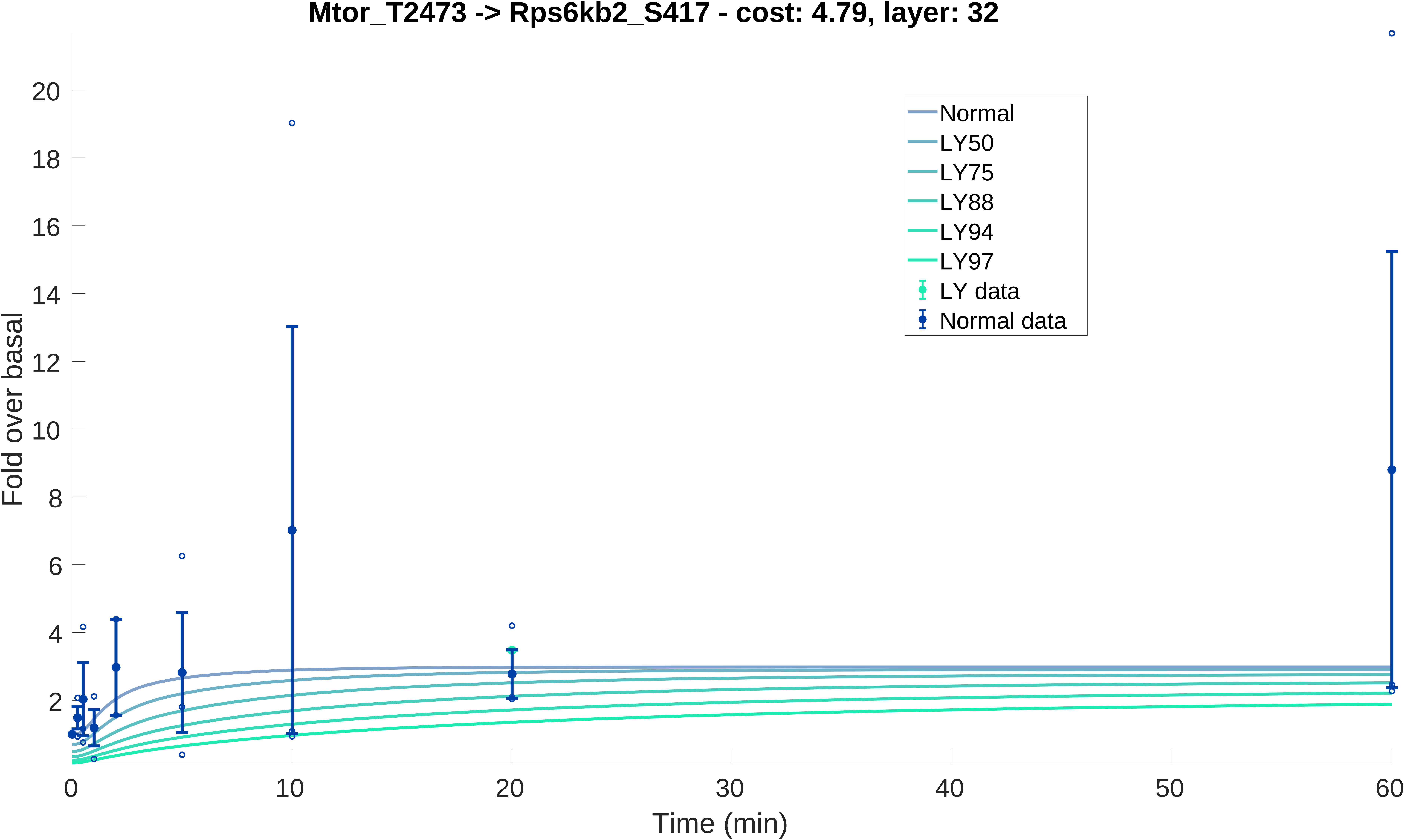
Mtor_T2473 -> Rps6kb2_S417 - cost: 4.79, layer: 32
Fold over basal
Time (min)
0
10
20
30
40
50
60
2
4
6
8
10
12
14
16
18
20
Normal
LY50
LY75
LY88
LY94
LY97
LY data
Normal data

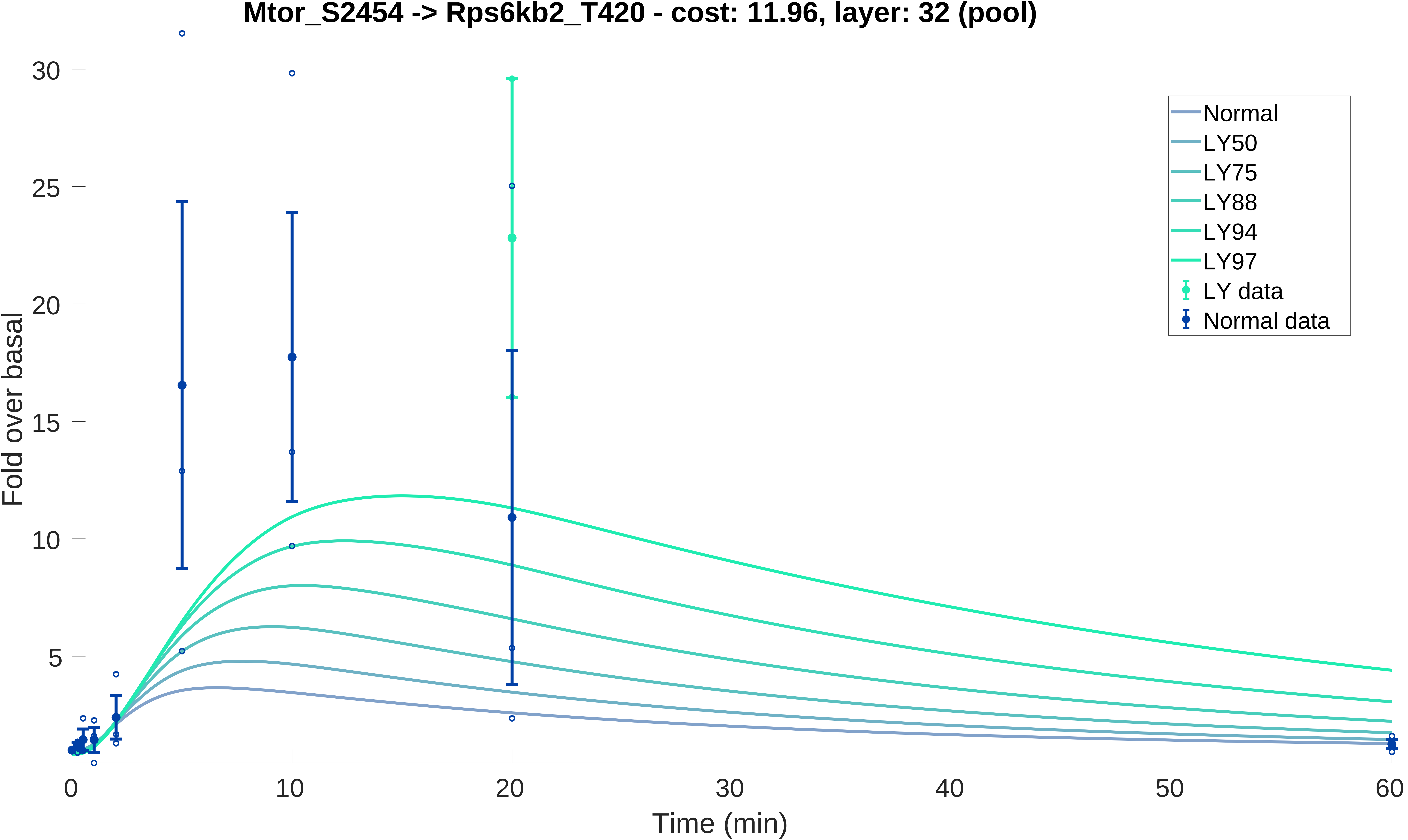
Mtor_S2454 -> Rps6kb2_T420 - cost: 11.96, layer: 32 (pool)
Fold over basal
Time (min)
0
10
20
30
40
50
60
5
10
15
20
25
30
Normal
LY50
LY75
LY88
LY94
LY97
LY data
Normal data

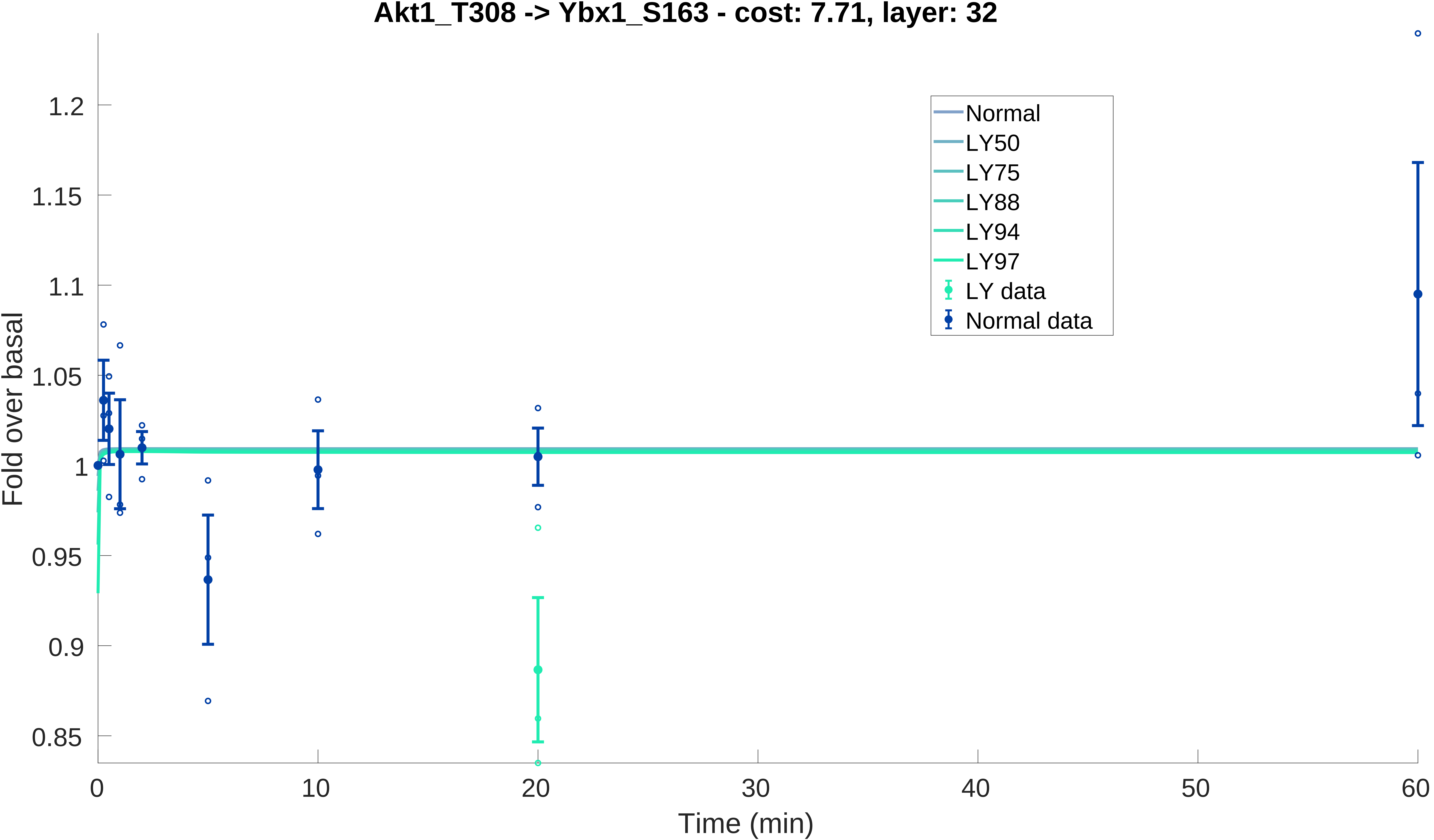

Akt1_T308 -> Ybx1_S163 - cost: 7.71, layer: 32
Fold over basal
Time (min)
1.2
1.15
1.1
1.05
1
0.95
0.9
0.85
0
10
20
30
40
50
60
Normal
LY50
LY75
LY88
LY94
LY97
LY data
Normal data

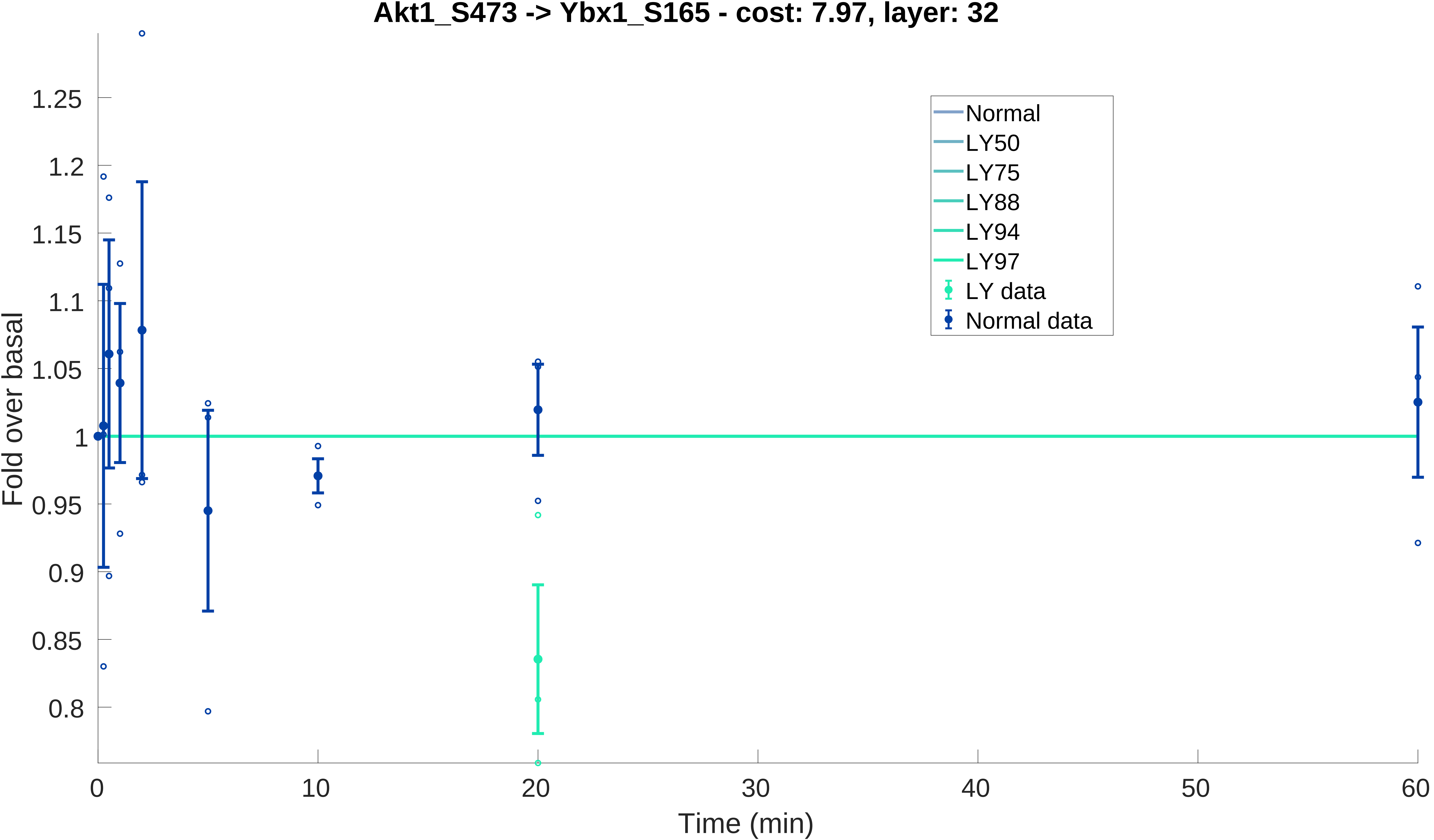

Akt1_S473 -> Ybx1_S165 - cost: 7.97, layer: 32
Fold over basal
1.25
1.2
1.15
1.1
1.05
1
0.95
0.9
0.85
0.8
0
10
20
30
40
50
60
Time (min)
Normal
LY50
LY75
LY88
LY94
LY97
LY data
Normal data

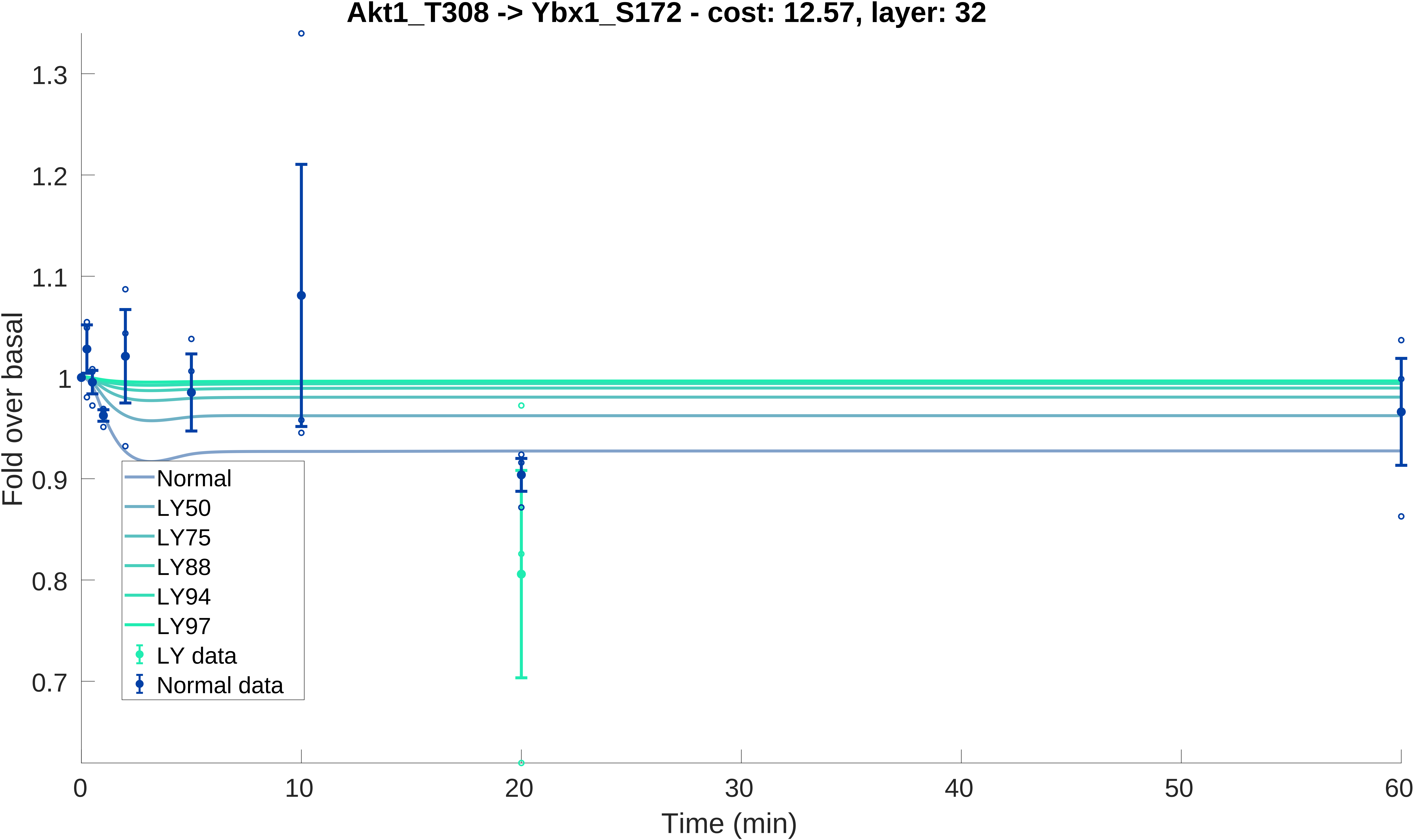

Akt1_T308 -> Ybx1_S172 - cost: 12.57, layer: 32
Fold over basal
1.3
1.2
1.1
1
0.9
0.8
0.7
Normal
LY50
LY75
LY88
LY94
LY97
LY data
Normal data
0
10
20
30
40
50
60
Time (min)

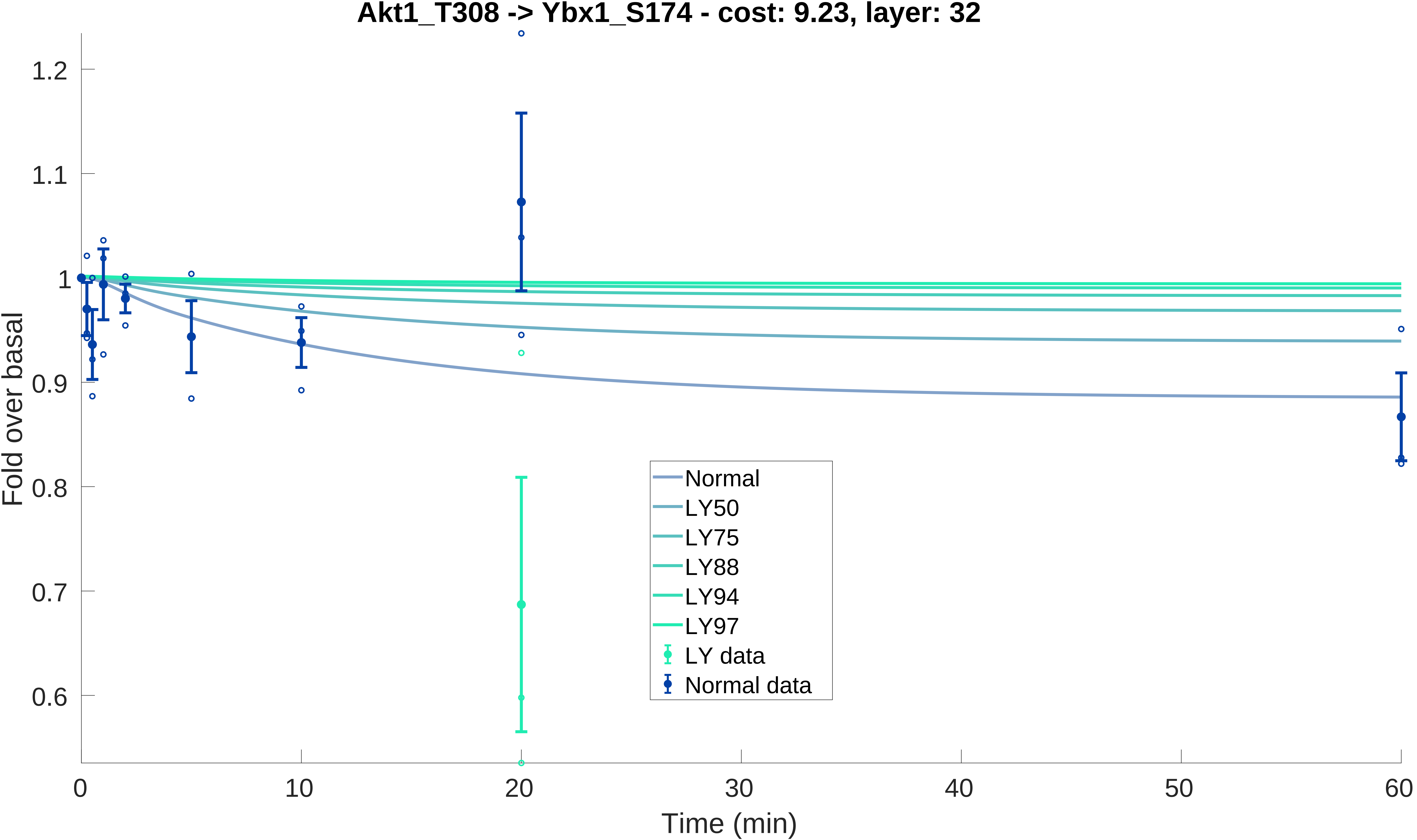

Akt1_T308 -> Ybx1_S174 - cost: 9.23, layer: 32
Fold over basal
1.2
1.1
1
0.9
0.8
0.7
0.6
0
10
20
30
40
50
60
Time (min)
Normal
LY50
LY75
LY88
LY94
LY97
LY data
Normal data

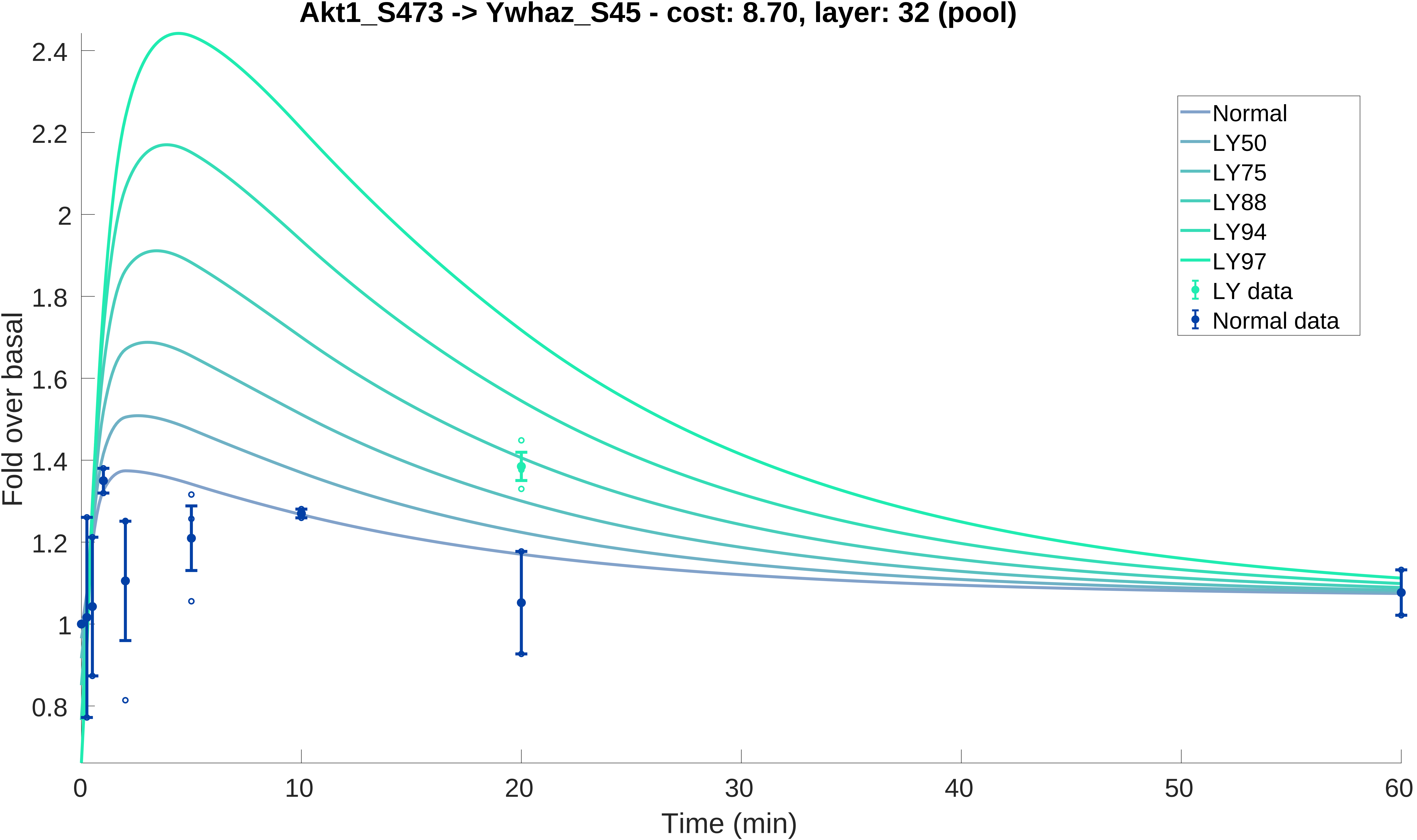

Akt1_S473 -> Ywhaz_S45 - cost: 8.70, layer: 32 (pool)
Fold over basal
Time (min)
2.4
2.2
2
1.8
1.6
1.4
1.2
1
0.8
0
10
20
30
40
50
60
Normal
LY50
LY75
LY88
LY94
LY97
LY data
Normal data

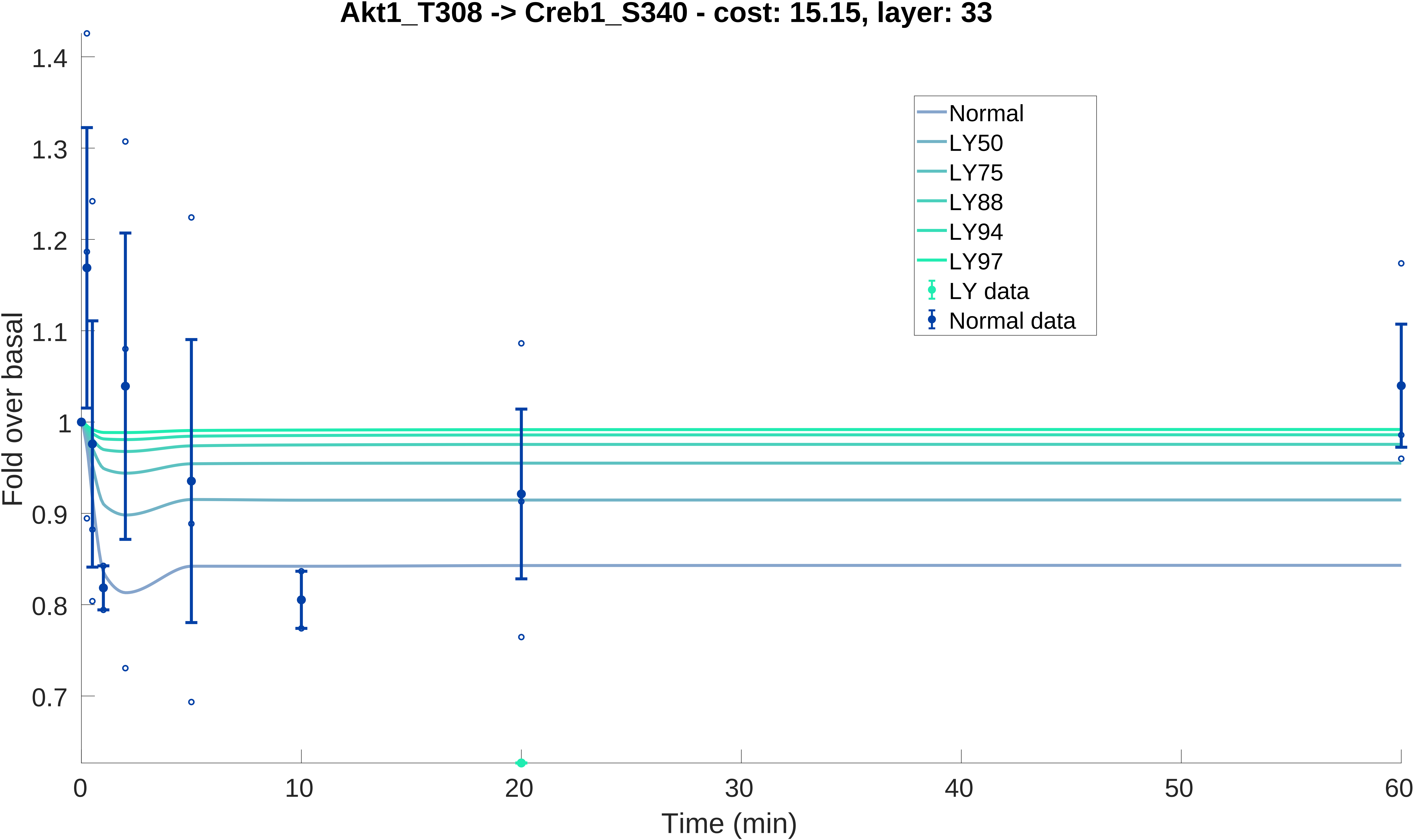

Akt1_T308 -> Creb1_S340 - cost: 15.15, layer: 33
Fold over basal
Time (min)
1.4
1.3
1.2
1.1
1
0.9
0.8
0.7
0
10
20
30
40
50
60
Normal
LY50
LY75
LY88
LY94
LY97
LY data
Normal data

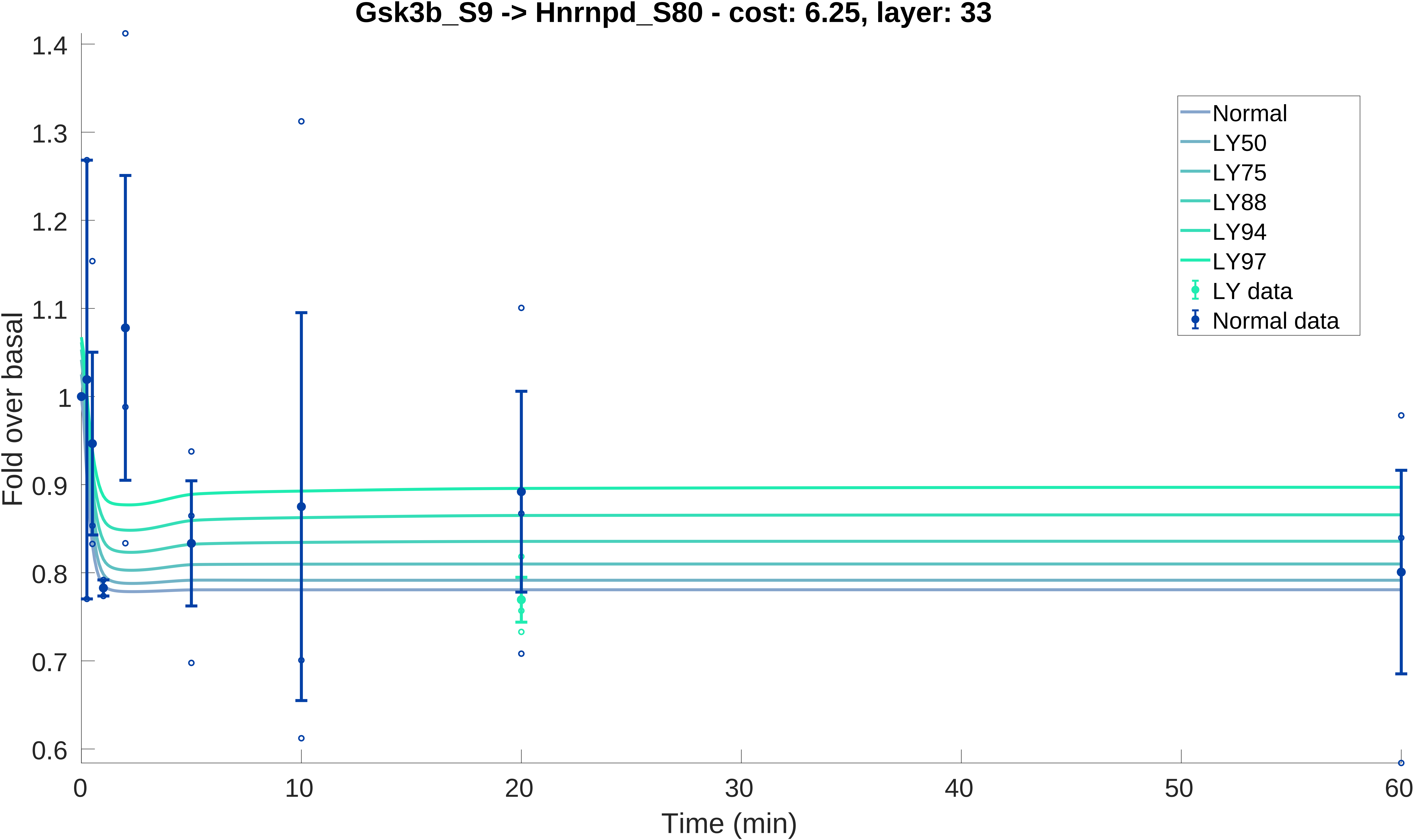

Gsk3b_S9 -> Hnrnpd_S80 - cost: 6.25, layer: 33
Fold over basal
Time (min)
1.4
1.3
1.2
1.1
1
0.9
0.8
0.7
0.6
0
10
20
30
40
50
60
Normal
LY50
LY75
LY88
LY94
LY97
LY data
Normal data

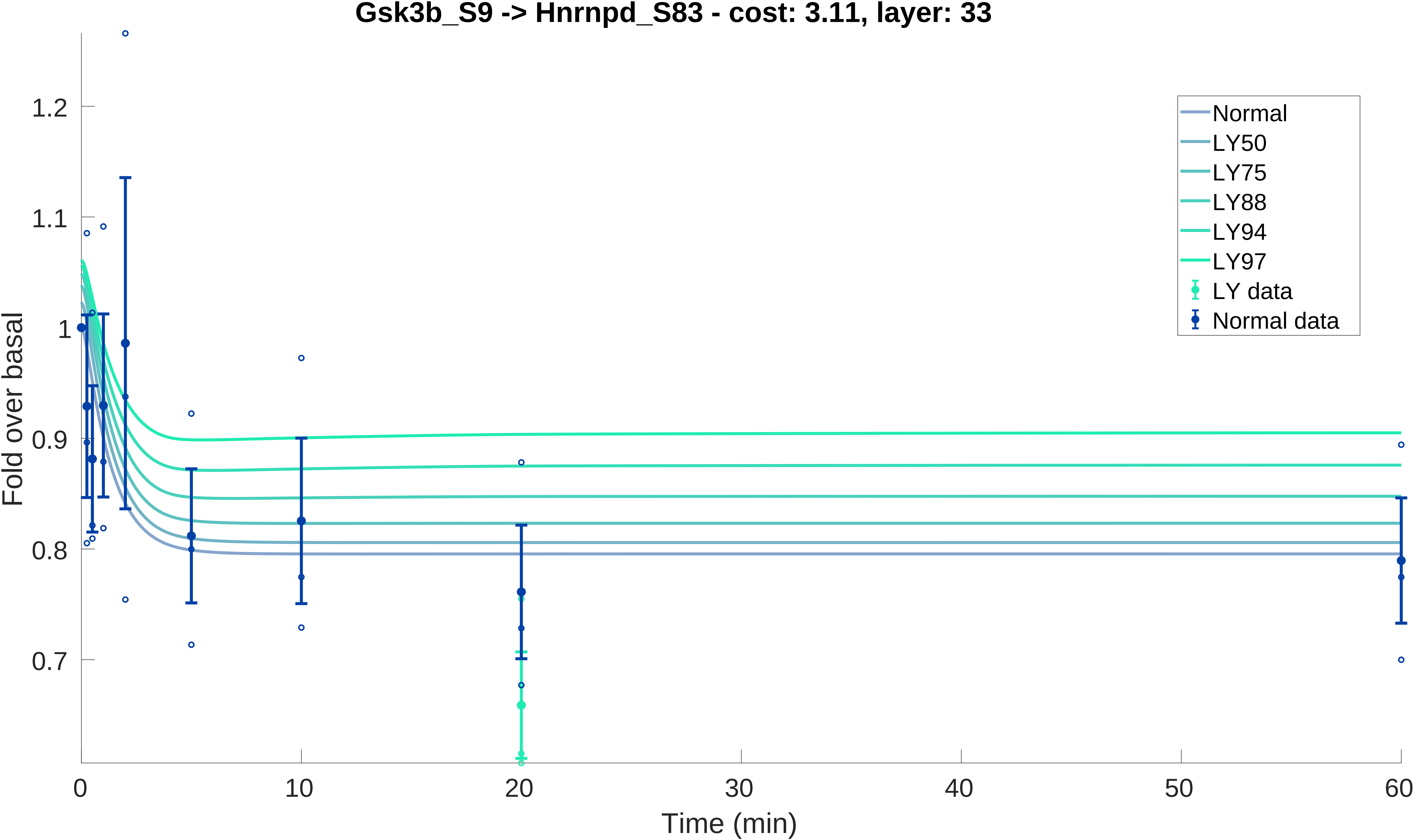

Gsk3b_S9 -> Hnrnpd_S83 - cost: 3.11, layer: 33
Fold over basal
1.2
1.1
1
0.9
0.8
0.7
0
10
20
30
40
50
60
Time (min)
Normal
LY50
LY75
LY88
LY94
LY97
LY data
Normal data

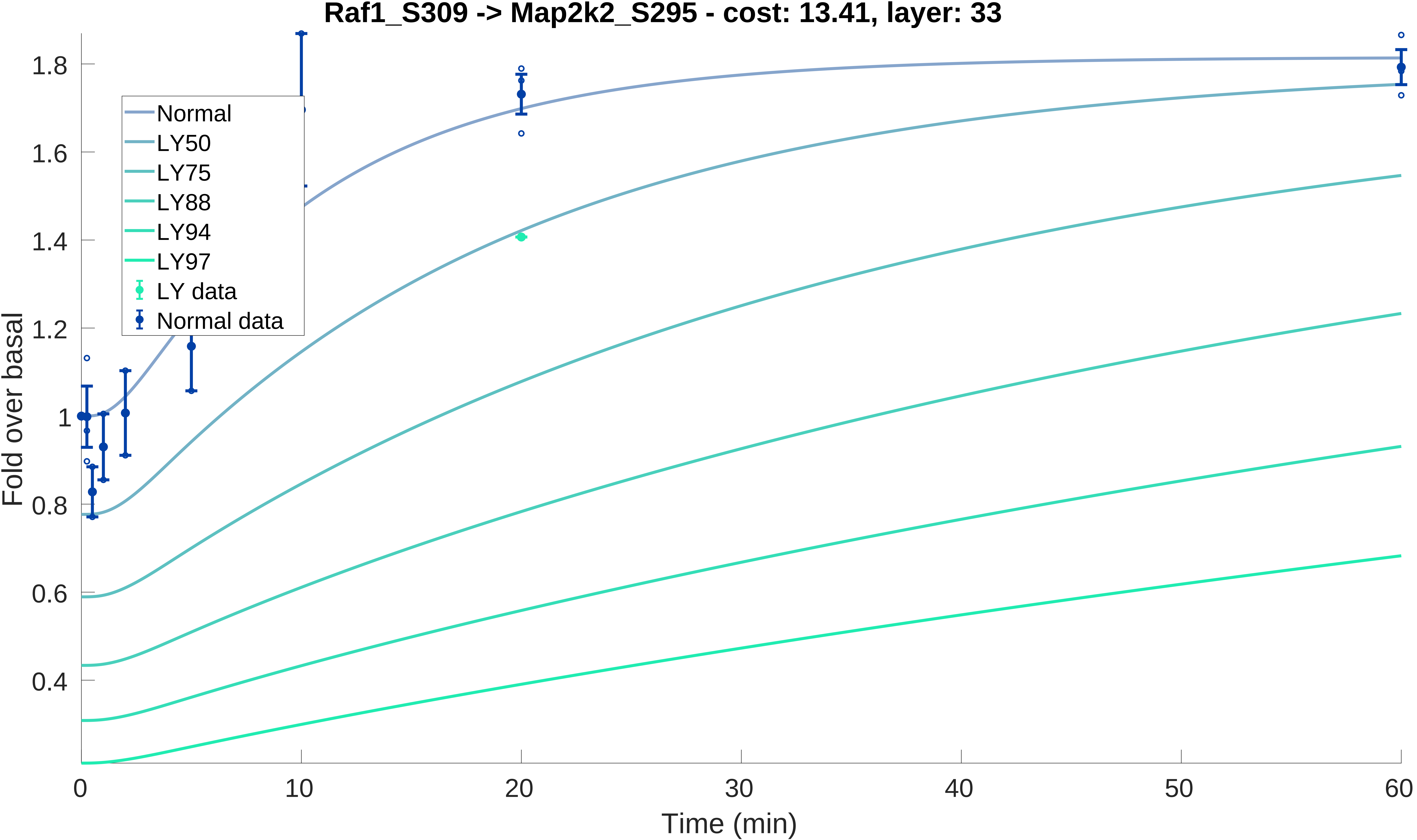
Raf1_S309 -> Map2k2_S295 - cost: 13.41, layer: 33
Normal
LY50
LY75
LY88
LY94
LY97
LY data
Normal data
Fold over basal
1.8
1.6
1.4
1.2
1
0.8
0.6
0.4
0
10
20
30
40
50
60
Time (min)

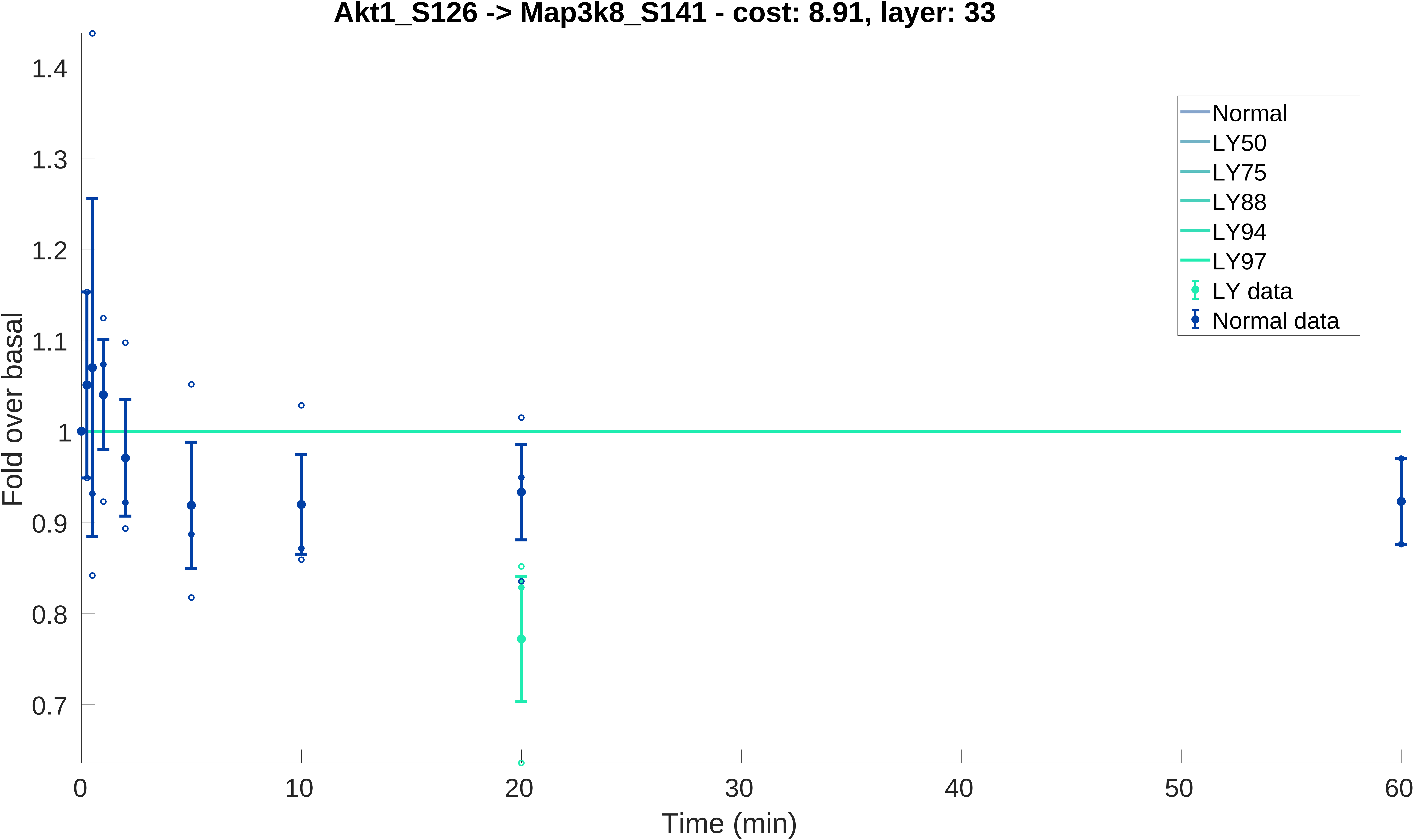

Akt1_S126 -> Map3k8_S141 - cost: 8.91, layer: 33
Fold over basal
Time (min)
0.7
0.8
0.9
1
1.1
1.2
1.3
1.4
0
10
20
30
40
50
60
Normal
LY50
LY75
LY88
LY94
LY97
LY data
Normal data

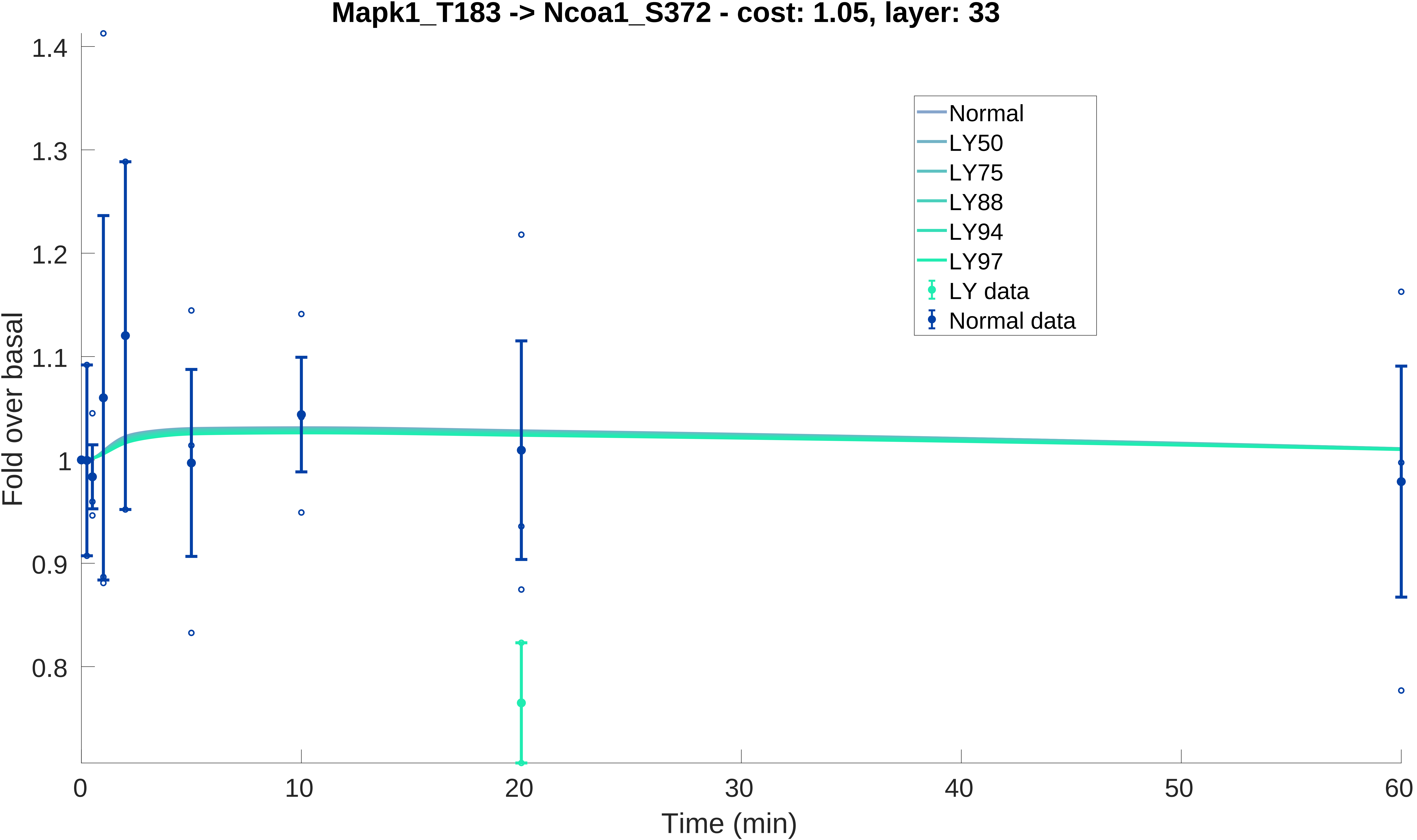
Mapk1_T183 -> Ncoa1_S372 - cost: 1.05, layer: 33
Fold over basal
Time (min)
Normal
LY50
LY75
LY88
LY94
LY97
LY data
Normal data
1.4
1.3
1.2
1.1
1
0.9
0.8
0
10
20
30
40
50
60

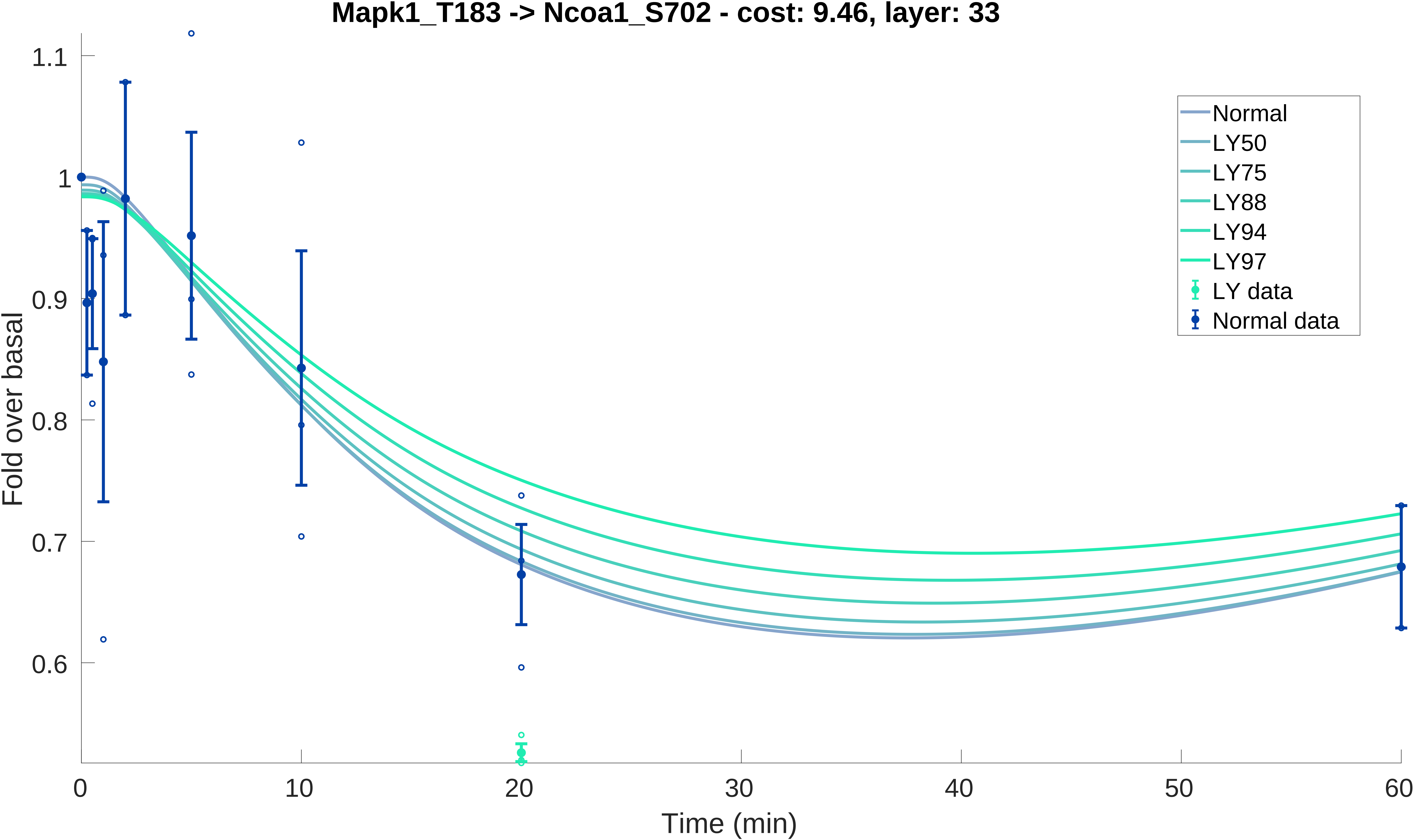

Mapk1_T183 -> Ncoa1_S702 - cost: 9.46, layer: 33
Fold over basal
Time (min)
1.1
1
0.9
0.8
0.7
0.6
0
10
20
30
40
50
60
Normal
LY50
LY75
LY88
LY94
LY97
LY data
Normal data

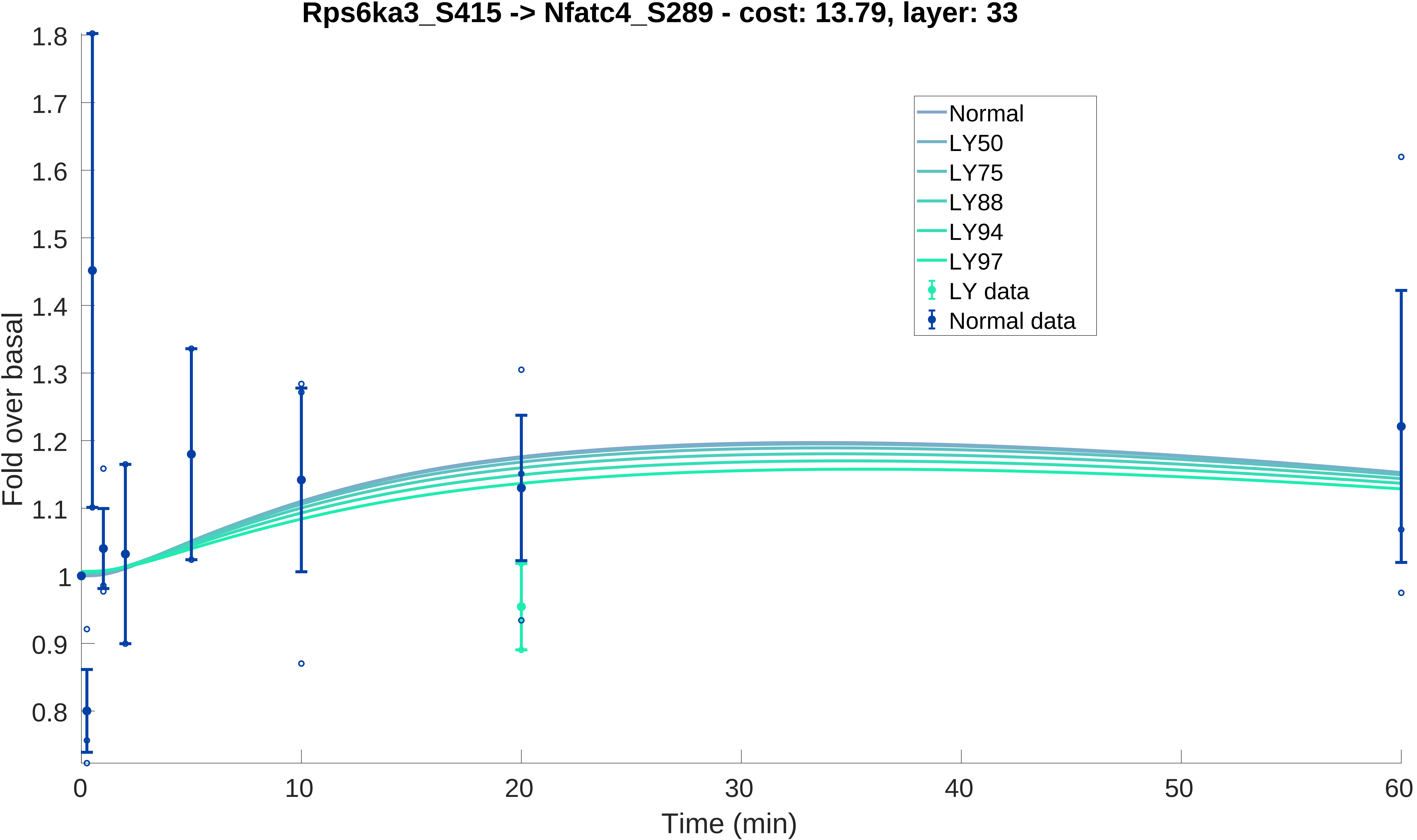

Rps6ka3_S415 -> Nfatc4_S289 - cost: 13.79, layer: 33
Fold over basal
Time (min)
Normal
LY50
LY75
LY88
LY94
LY97
LY data
Normal data
1.8
1.7
1.6
1.5
1.4
1.3
1.2
1.1
1
0.9
0.8
0
10
20
30
40
50
60

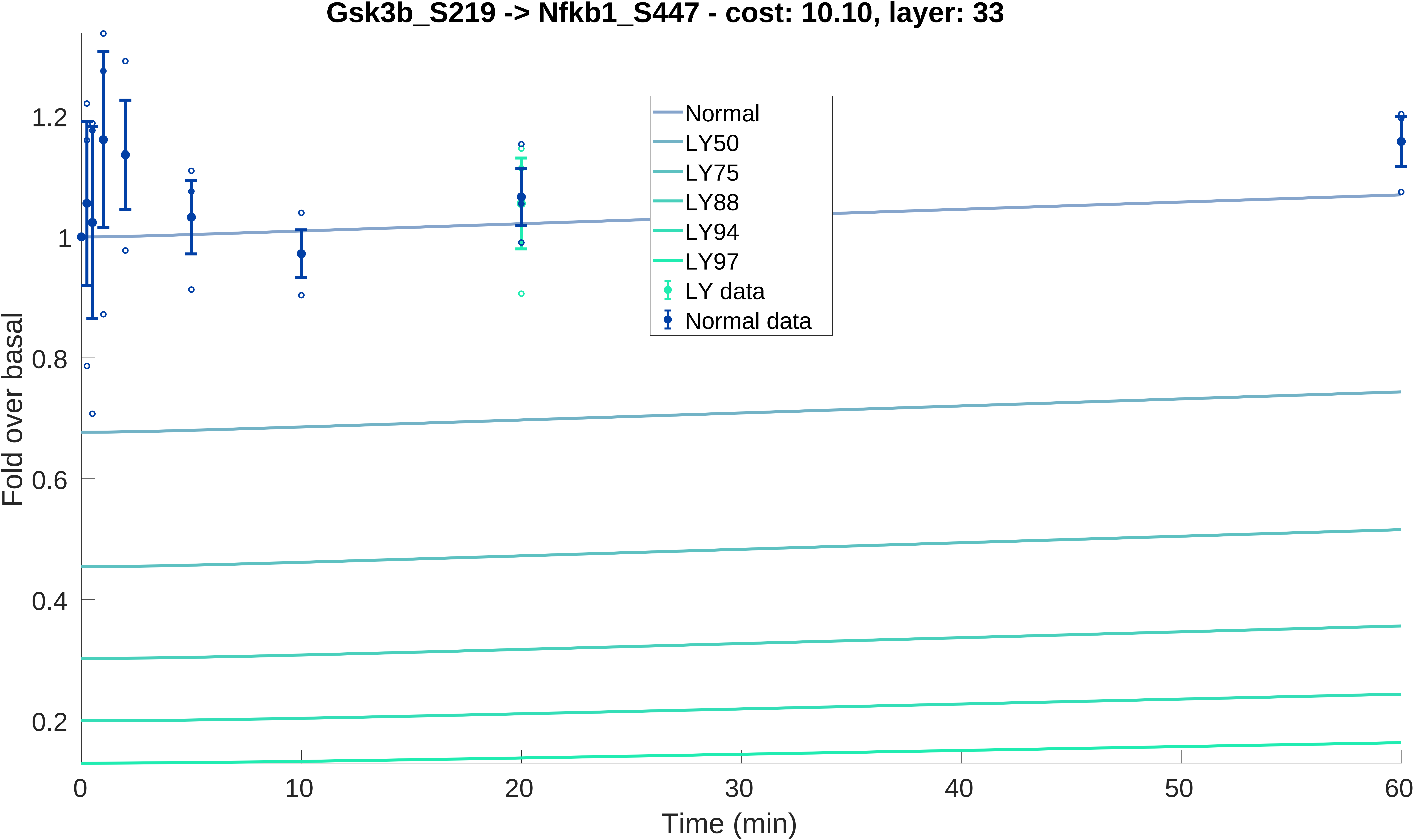

Gsk3b_S219 -> Nfkb1_S447 - cost: 10.10, layer: 33
Fold over basal
1.2
1
0.8
0.6
0.4
0.2
0
10
20
30
40
50
60
Time (min)
Normal
LY50
LY75
LY88
LY94
LY97
LY data
Normal data

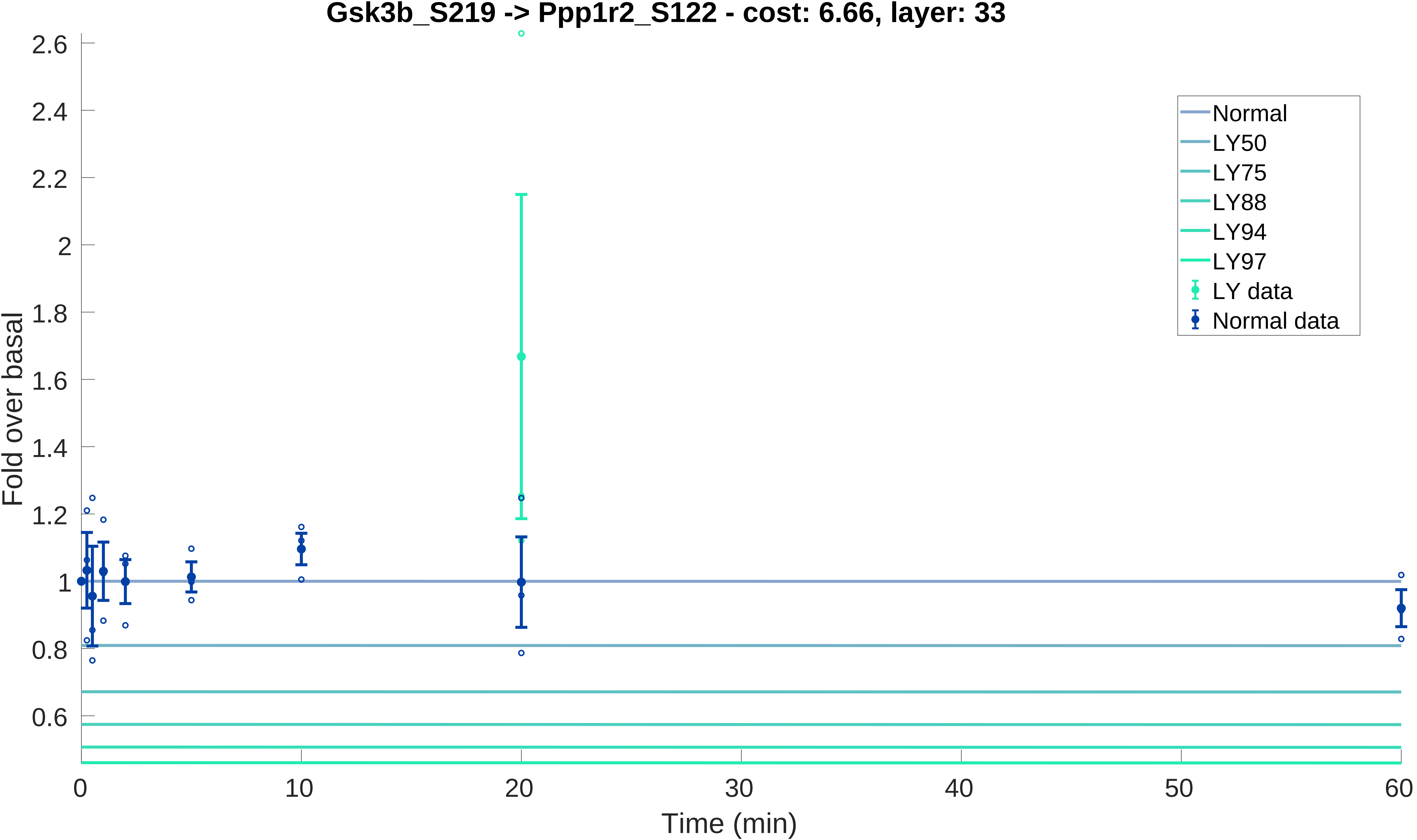
Gsk3b_S219 -> Ppp1r2_S122 - cost: 6.66, layer: 33
Fold over basal
Time (min)
2.6
2.4
2.2
2
1.8
1.6
1.4
1.2
1
0.8
0.6
0
10
20
30
40
50
60
Normal
LY50
LY75
LY88
LY94
LY97
LY data
Normal data

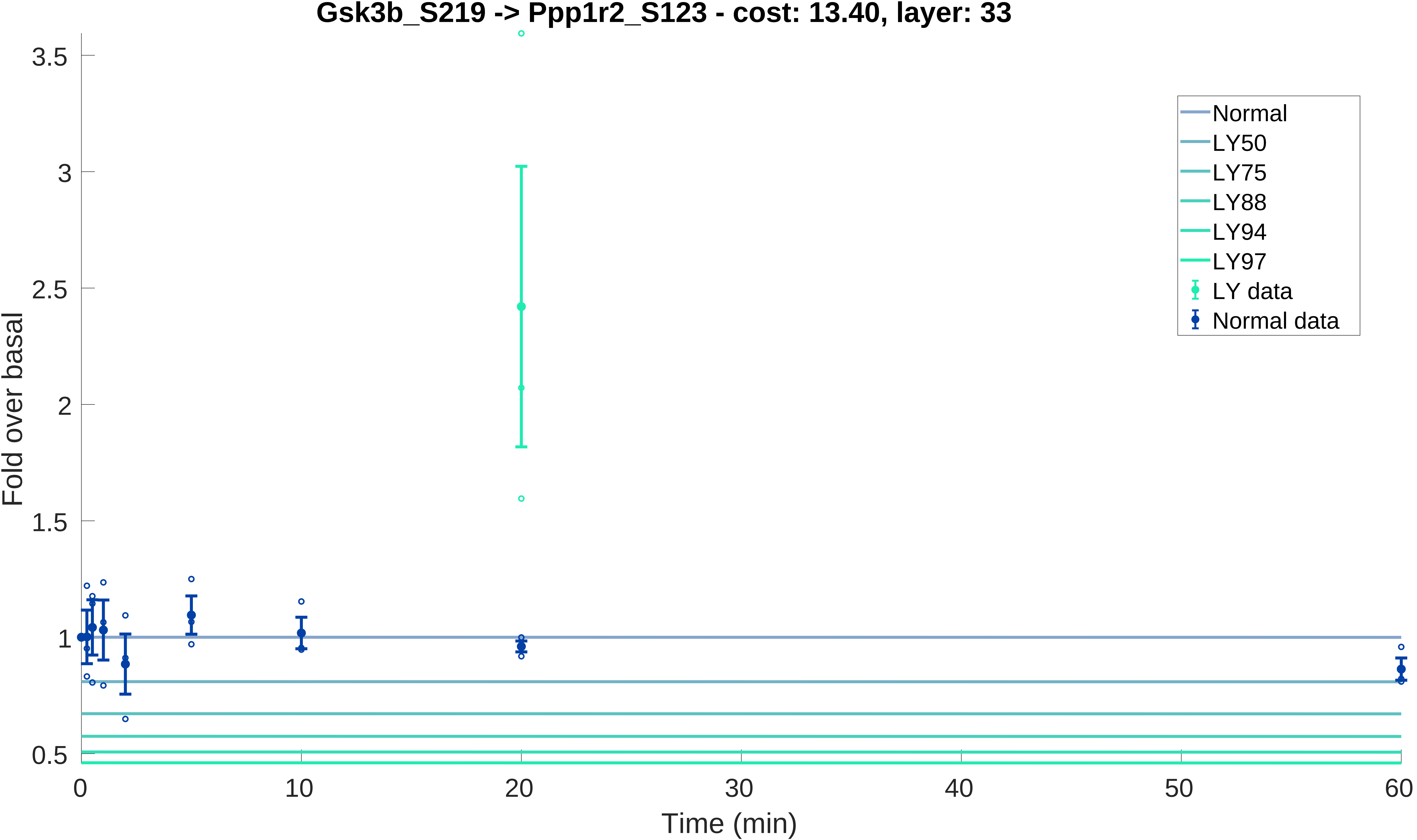

Gsk3b_S219 -> Ppp1r2_S123 - cost: 13.40, layer: 33
Fold over basal
3.5
3
2.5
2
1.5
1
0.5
0
10
20
30
40
50
60
Time (min)
Normal
LY50
LY75
LY88
LY94
LY97
LY data
Normal data

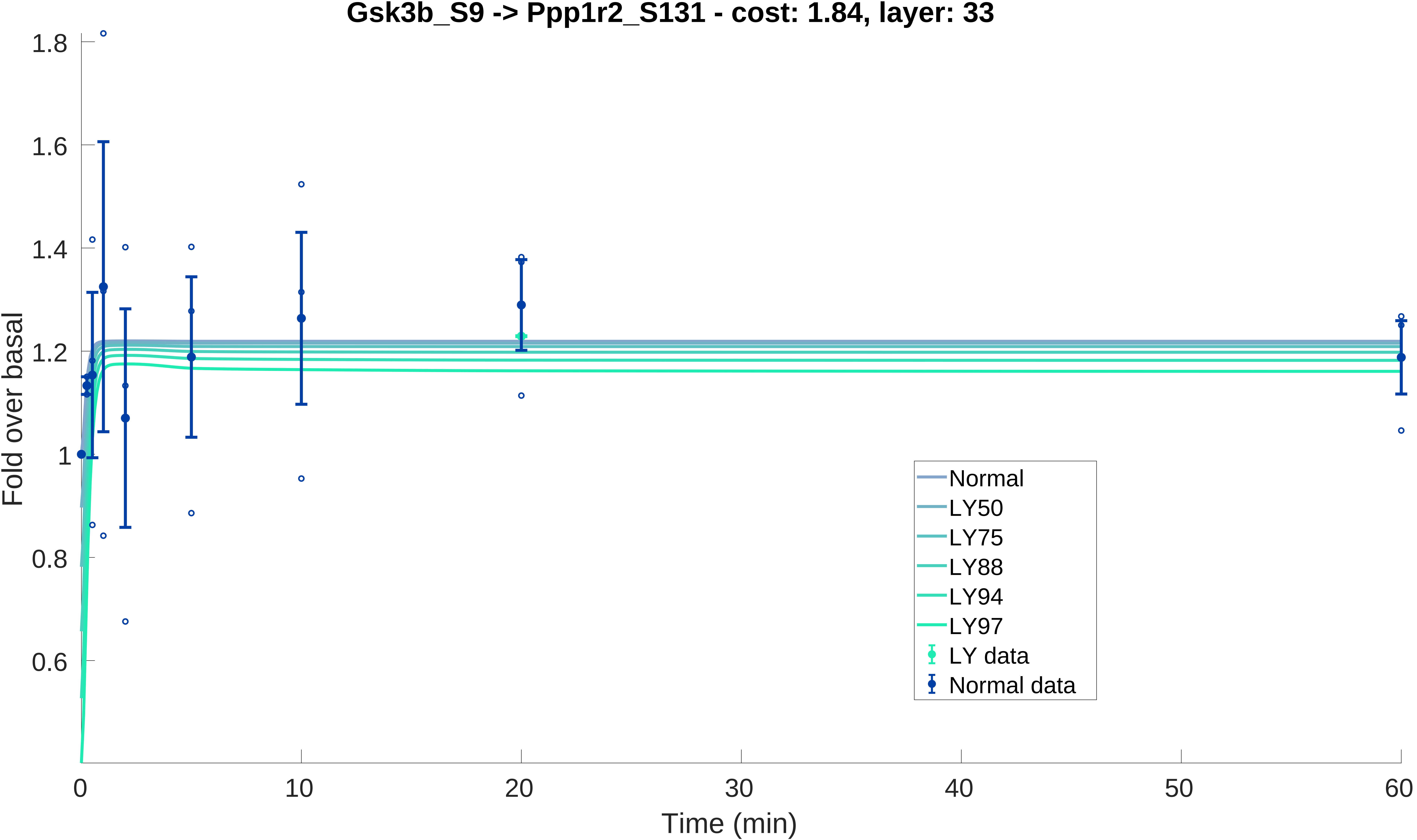
Gsk3b_S9 -> Ppp1r2_S131 - cost: 1.84, layer: 33
Fold over basal
Time (min)
1.8
1.6
1.4
1.2
1
0.8
0.6
0
10
20
30
40
50
60
Normal
LY50
LY75
LY88
LY94
LY97
LY data
Normal data

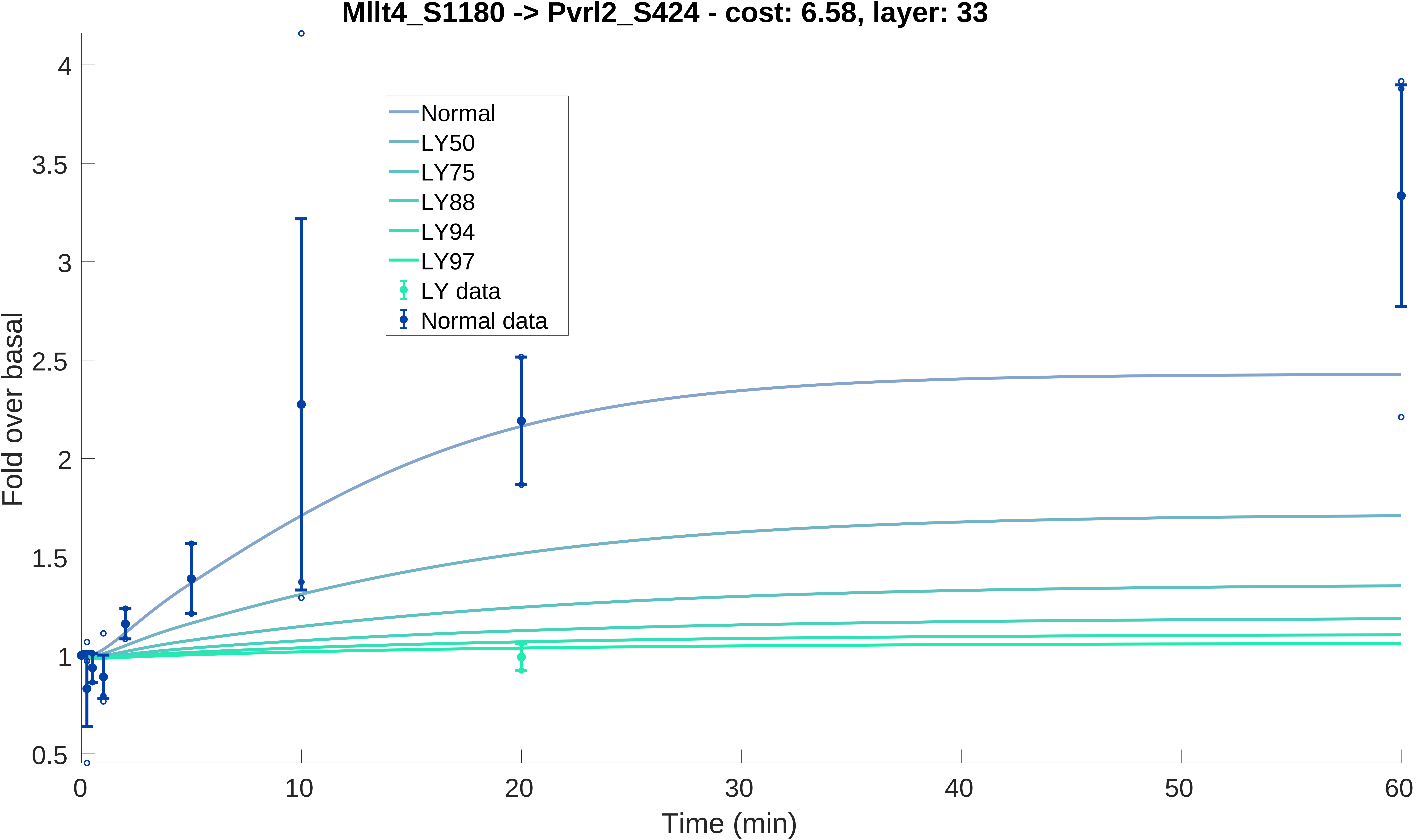

Mllt4_S1180 -> Pvrl2_S424 - cost: 6.58, layer: 33
Normal
LY50
LY75
LY88
LY94
LY97
LY data
Normal data
Fold over basal
Time (min)
0.5
1
1.5
2
2.5
3
3.5
4
0
10
20
30
40
50
60

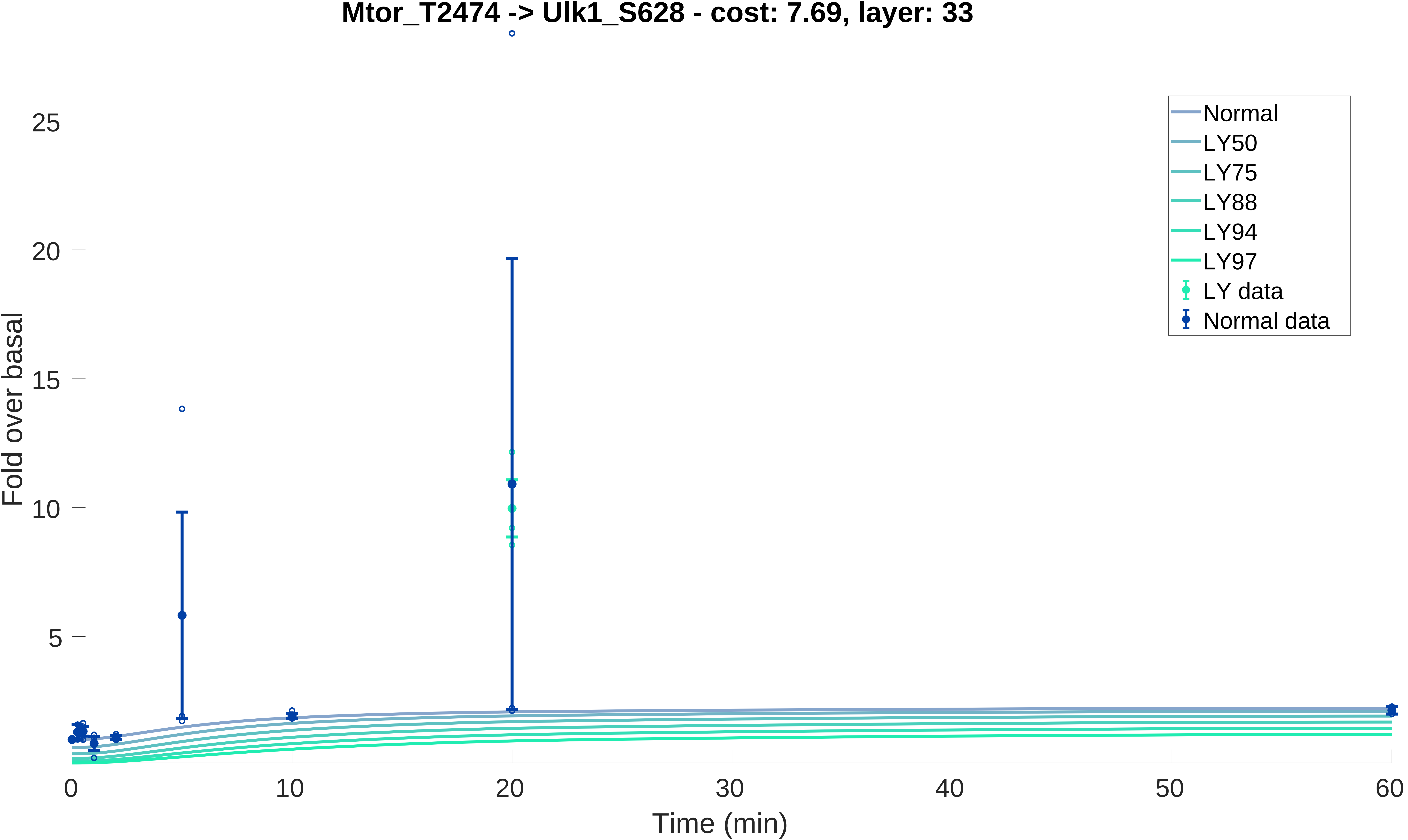

Mtor_T2474 -> Ulk1_S628 - cost: 7.69, layer: 33
Fold over basal
Time (min)
0
10
20
30
40
50
60
5
10
15
20
25
Normal
LY50
LY75
LY88
LY94
LY97
LY data
Normal data

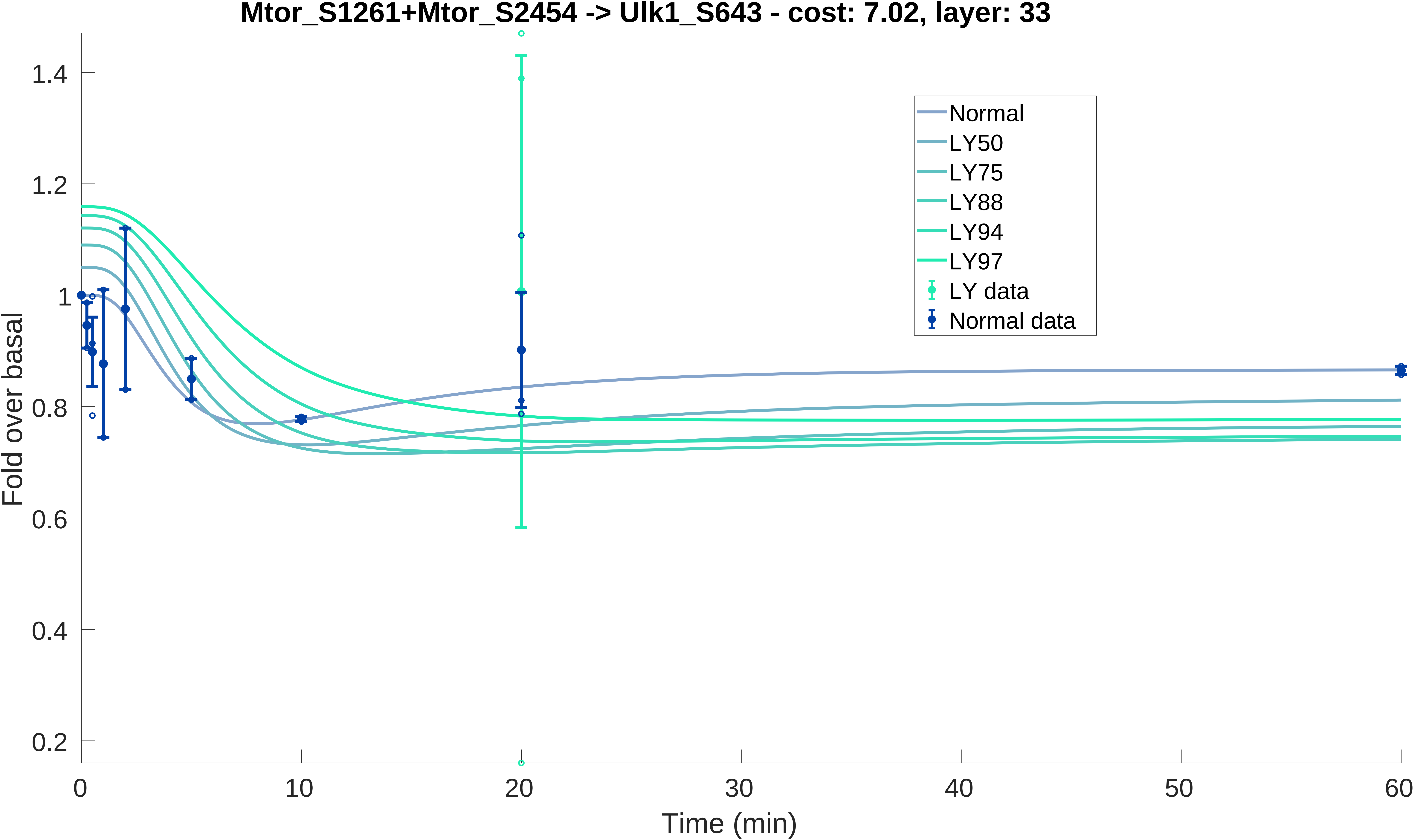
Mtor_S1261+Mtor_S2454 -> Ulk1_S643 - cost: 7.02, layer: 33
Fold over basal
Time (min)
0
10
20
30
40
50
60
0.2
0.4
0.6
0.8
1
1.2
1.4
Normal
LY50
LY75
LY88
LY94
LY97
LY data
Normal data

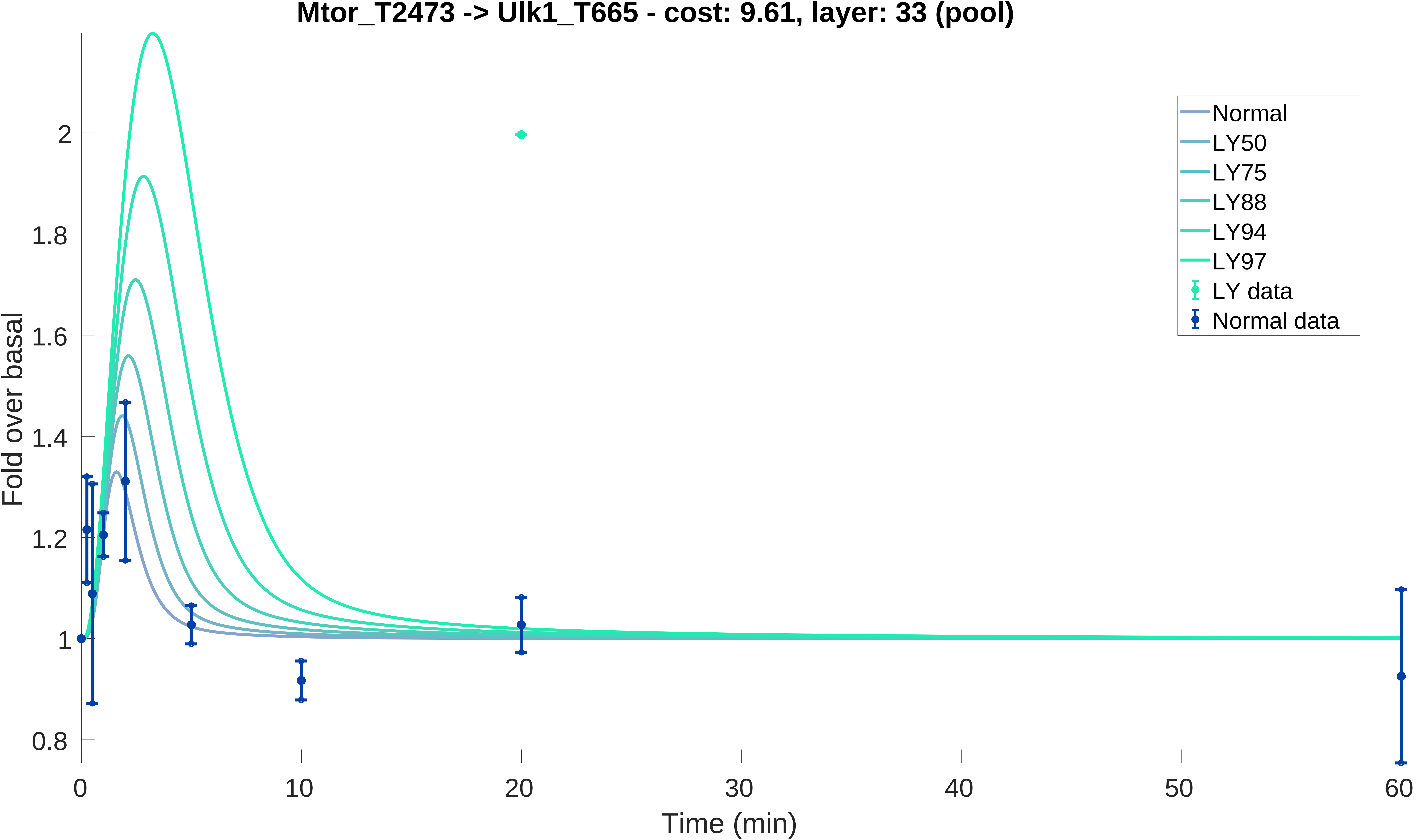

Mtor_T2473 -> Ulk1_T665 - cost: 9.61, layer: 33 (pool)
Fold over basal
Time (min)
0.8
1
1.2
1.4
1.6
1.8
2
0
10
20
30
40
50
60
Normal
LY50
LY75
LY88
LY94
LY97
LY data
Normal data

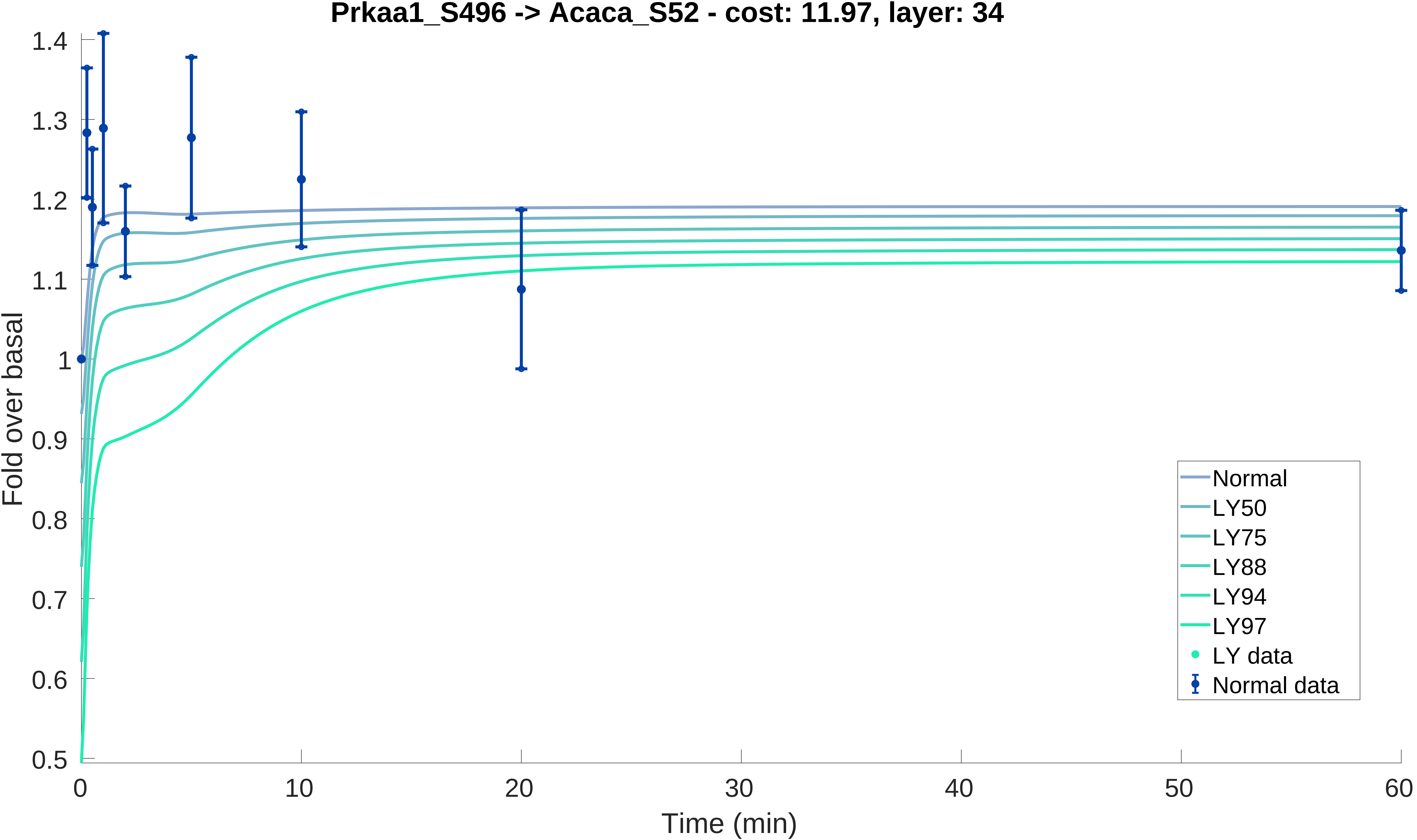

Prkaa1_S496 -> Acaca_S52 - cost: 11.97, layer: 34
Fold over basal
Time (min)
0.5
0.6
0.7
0.8
0.9
1
1.1
1.2
1.3
1.4
0
10
20
30
40
50
60
Normal
LY50
LY75
LY88
LY94
LY97
LY data
Normal data

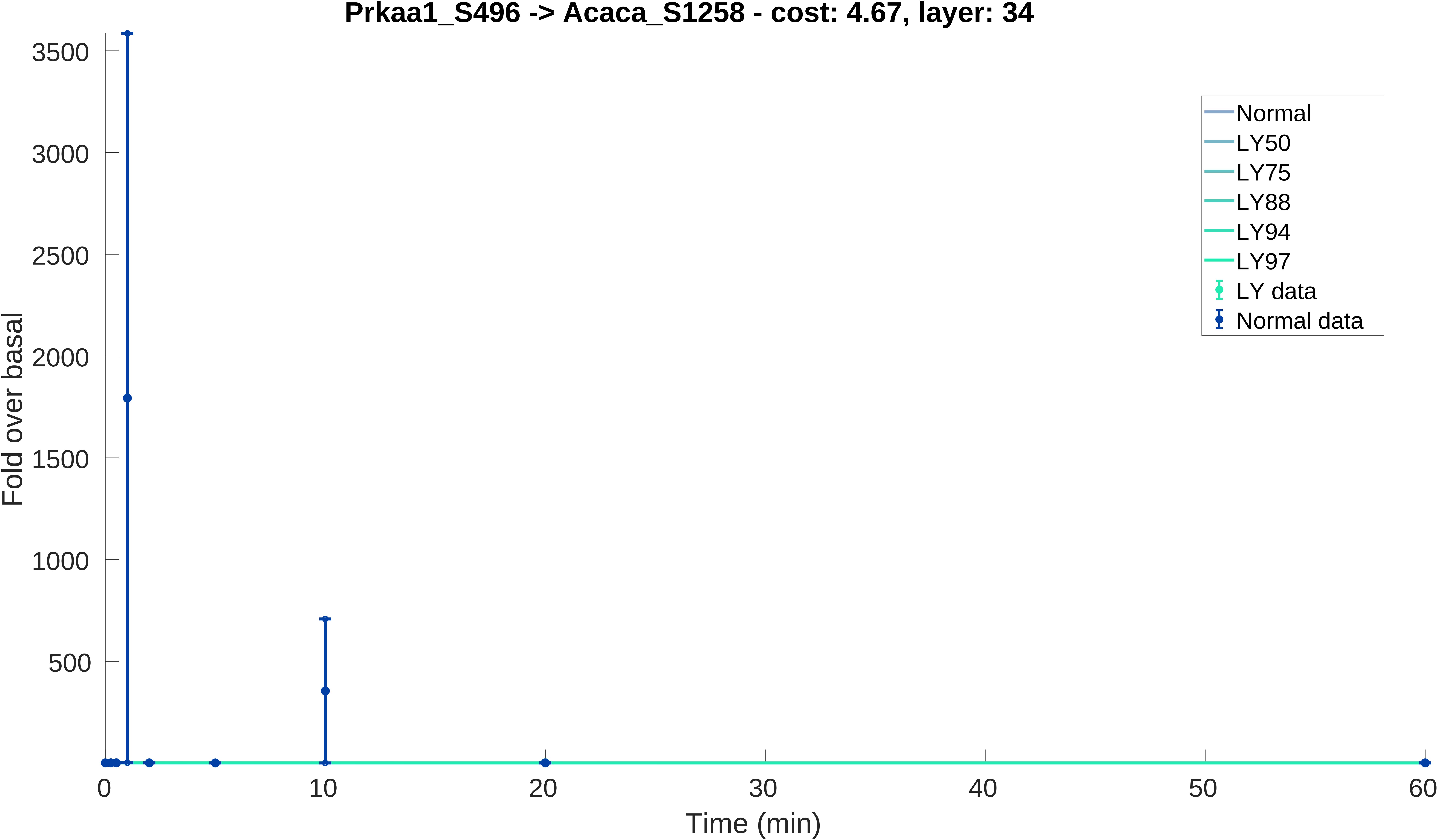
Prkaa1_S496 -> Acaca_S1258 - cost: 4.67, layer: 34
Fold over basal
3500
3000
2500
2000
1500
1000
500
0
10
20
30
40
50
60
Time (min)
Normal
LY50
LY75
LY88
LY94
LY97
LY data
Normal data

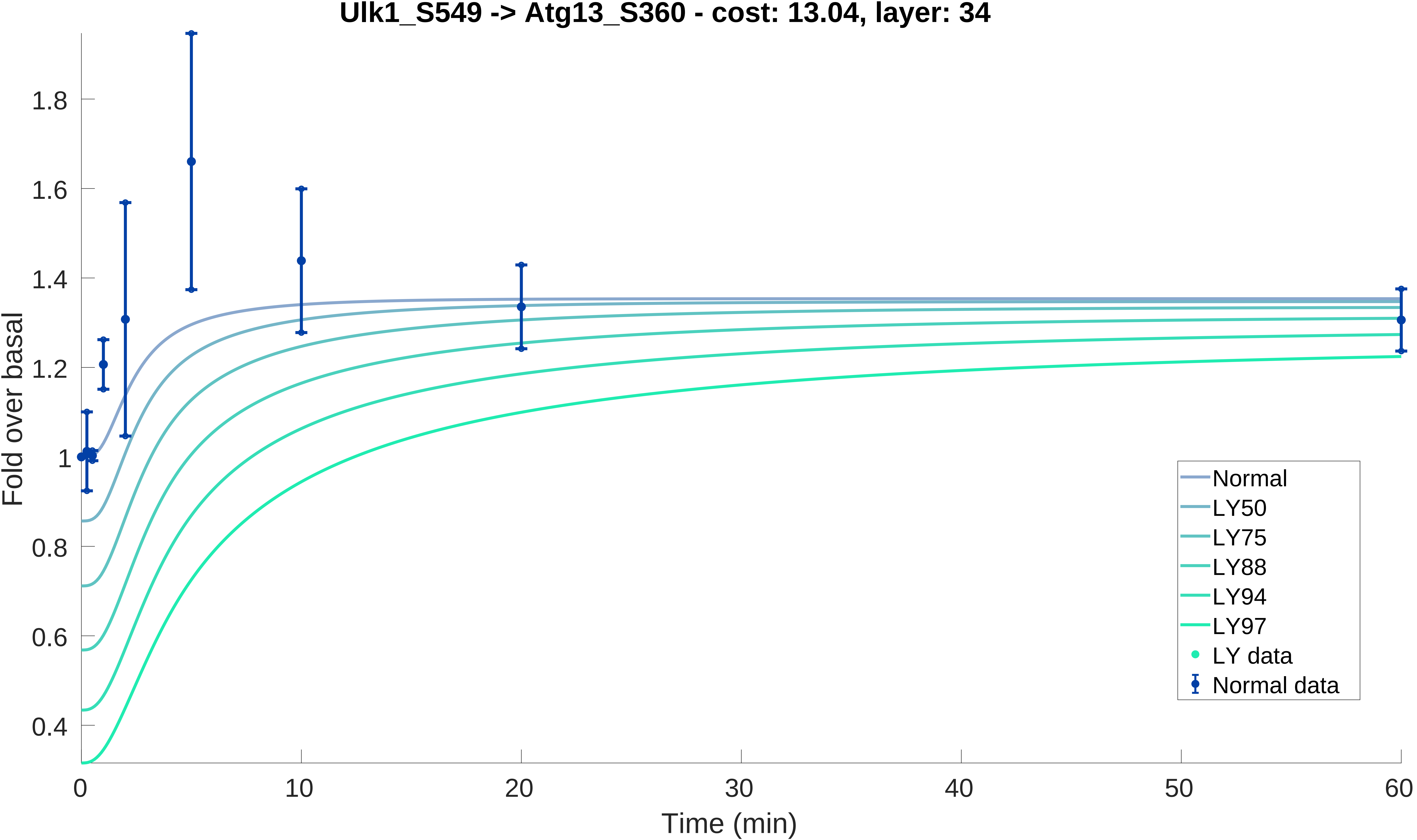

Ulk1_S549 -> Atg13_S360 - cost: 13.04, layer: 34
Fold over basal
1.8
1.6
1.4
1.2
1
0.8
0.6
0.4
0
10
20
30
40
50
60
Time (min)
Normal
LY50
LY75
LY88
LY94
LY97
LY data
Normal data

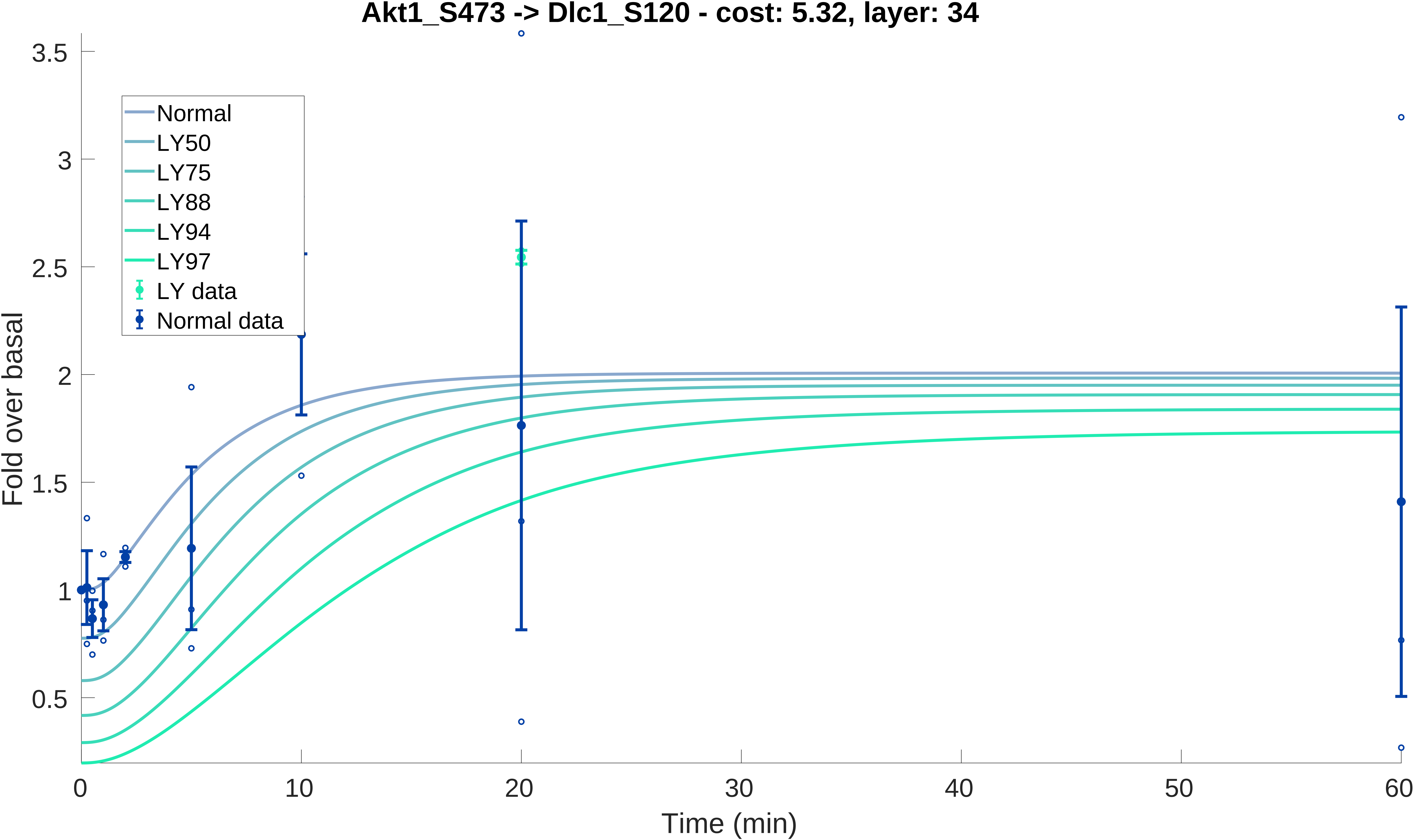

Akt1_S473 -> Dlc1_S120 - cost: 5.32, layer: 34
Normal
LY50
LY75
LY88
LY94
LY97
LY data
Normal data
Fold over basal
Time (min)
0
10
20
30
40
50
60
0.5
1
1.5
2
2.5
3
3.5

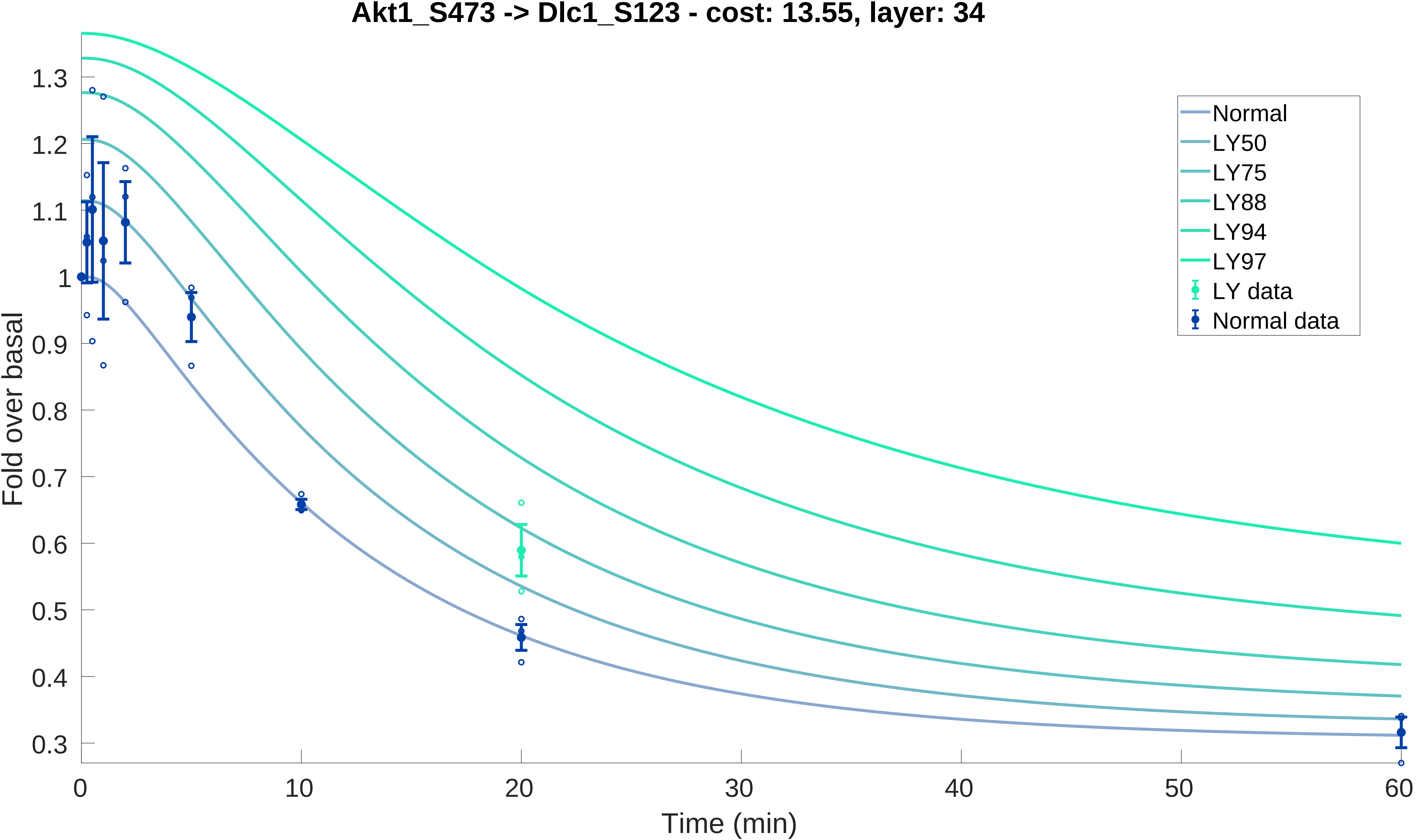
Akt1_S473 -> Dlc1_S123 - cost: 13.55, layer: 34
Fold over basal
Time (min)
1.3
1.2
1.1
1
0.9
0.8
0.7
0.6
0.5
0.4
0.3
0
10
20
30
40
50
60
Normal
LY50
LY75
LY88
LY94
LY97
LY data
Normal data

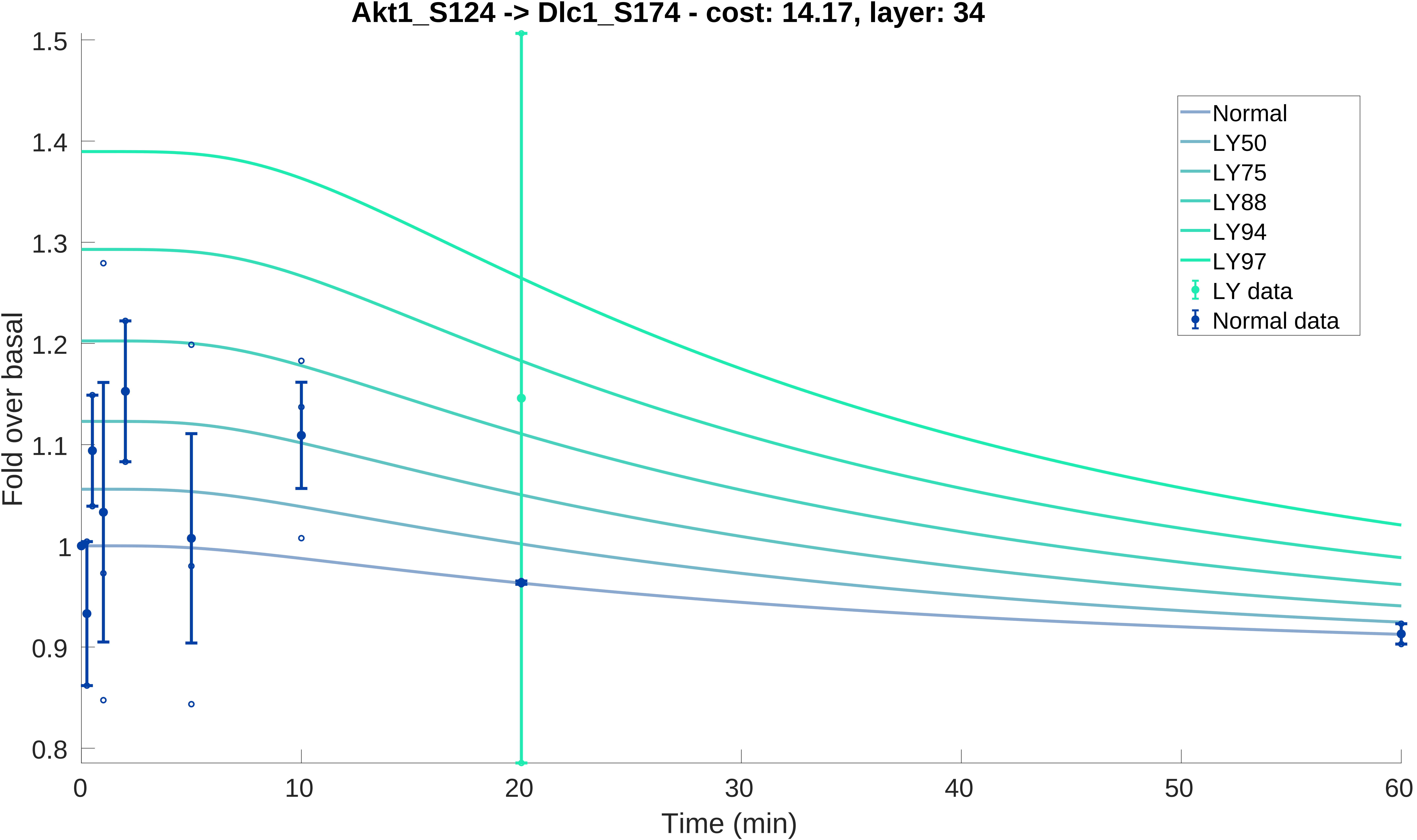

Akt1_S124 -> Dlc1_S174 - cost: 14.17, layer: 34
Fold over basal
Time (min)
0
10
20
30
40
50
60
0.8
0.9
1
1.1
1.2
1.3
1.4
1.5
Normal
LY50
LY75
LY88
LY94
LY97
LY data
Normal data

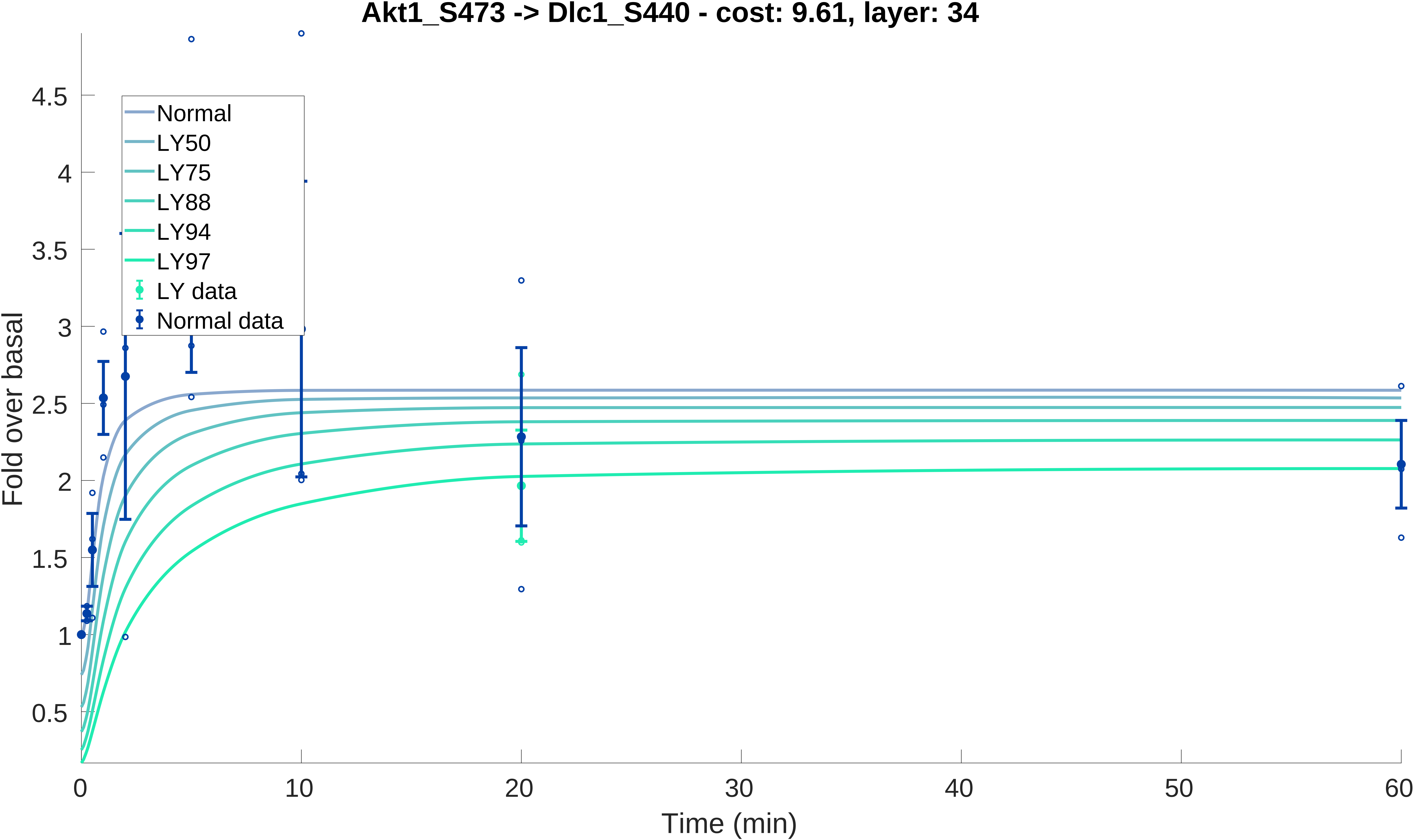

Akt1_S473 -> Dlc1_S440 - cost: 9.61, layer: 34
Normal
LY50
LY75
LY88
LY94
LY97
LY data
Normal data
Fold over basal
Time (min)
0
10
20
30
40
50
60
0.5
1
1.5
2
2.5
3
3.5
4
4.5

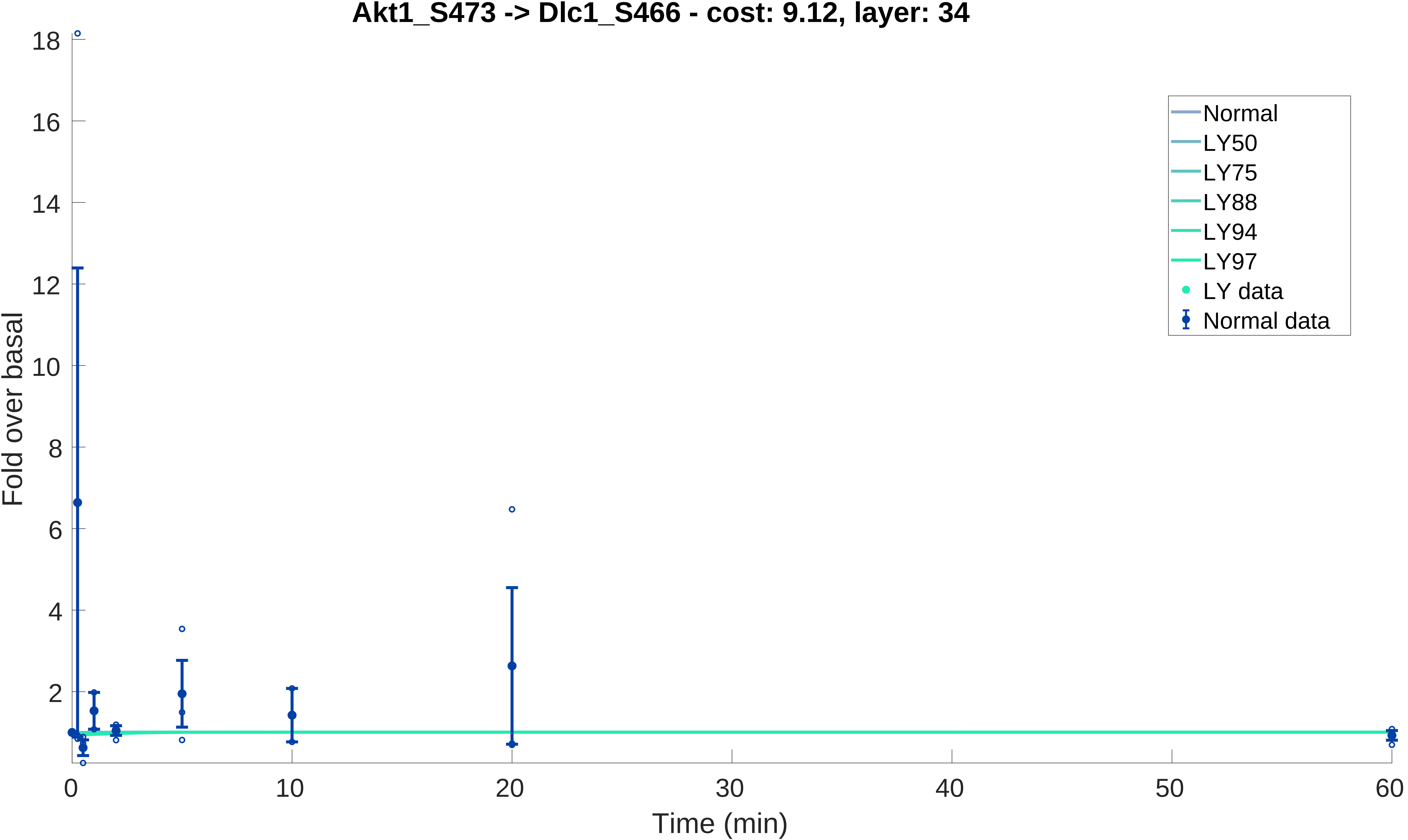

Akt1_S473 -> Dlc1_S466 - cost: 9.12, layer: 34
Fold over basal
Time (min)
0
10
20
30
40
50
60
2
4
6
8
10
12
14
16
18
Normal
LY50
LY75
LY88
LY94
LY97
LY data
Normal data

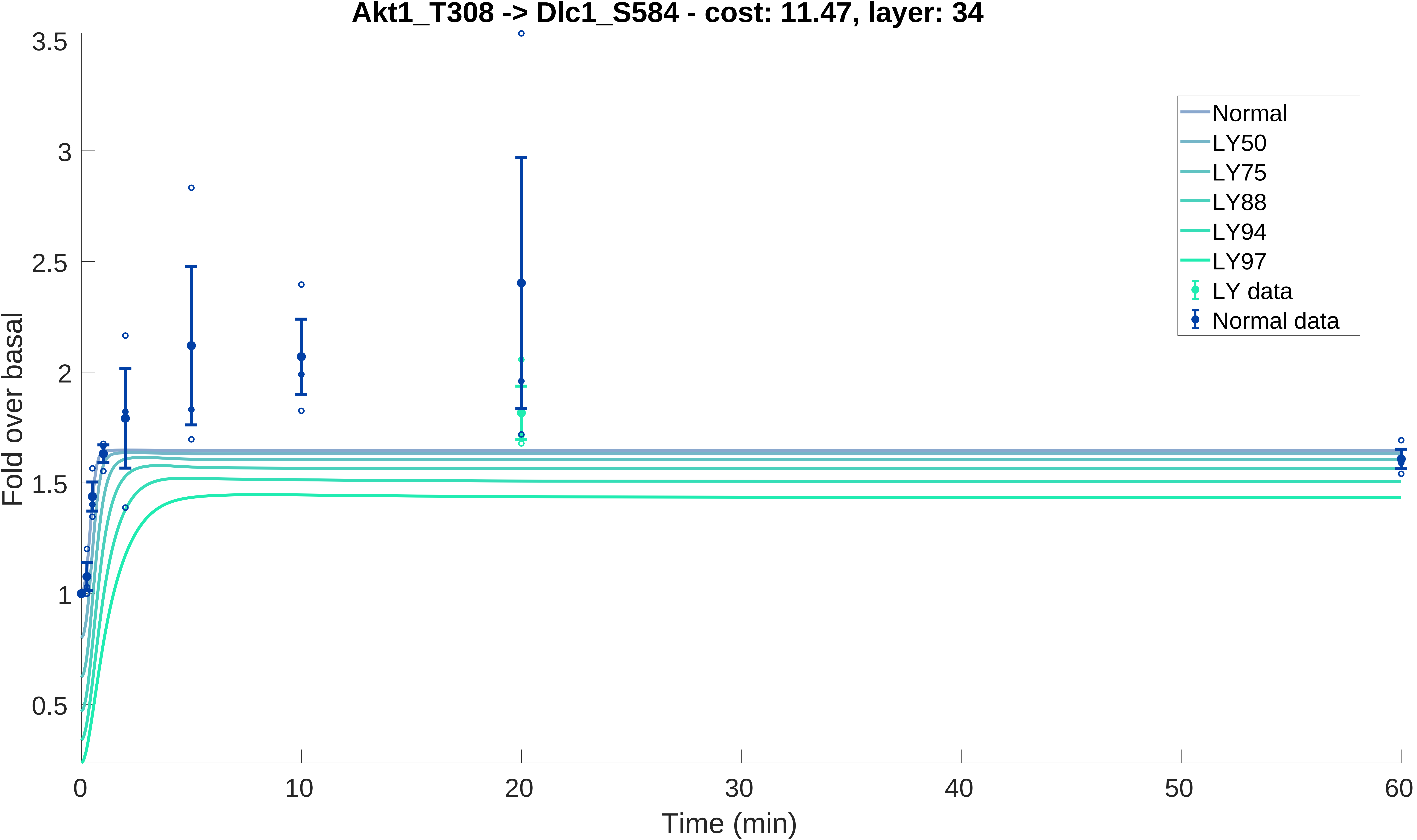

Akt1_T308 -> Dlc1_S584 - cost: 11.47, layer: 34
Fold over basal
Time (min)
Normal
LY50
LY75
LY88
LY94
LY97
LY data
Normal data
0
10
20
30
40
50
60
0.5
1
1.5
2
2.5
3
3.5

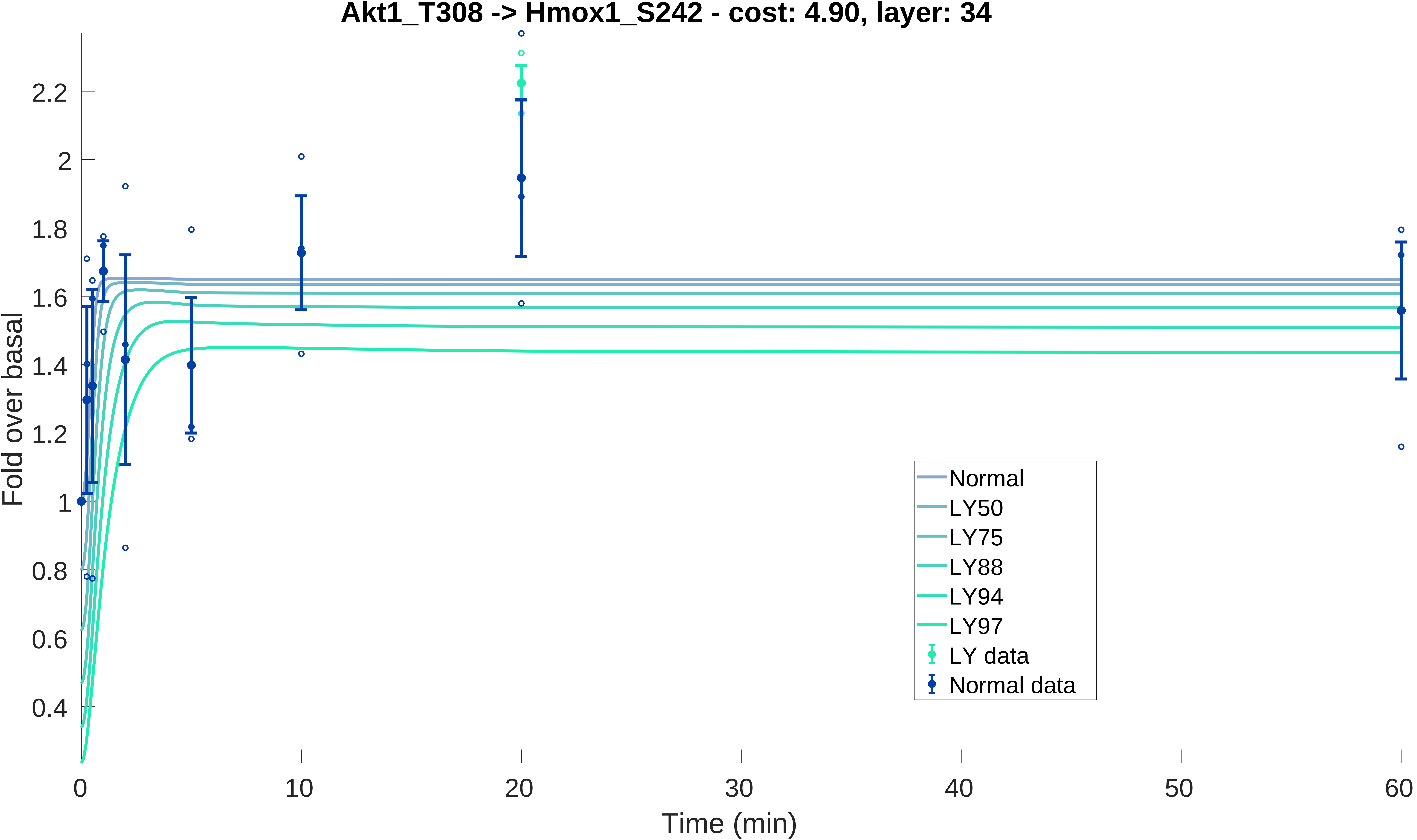

Akt1_T308 -> Hmox1_S242 - cost: 4.90, layer: 34
Fold over basal
Time (min)
2.2
2
1.8
1.6
1.4
1.2
1
0.8
0.6
0.4
0
10
20
30
40
50
60
Normal
LY50
LY75
LY88
LY94
LY97
LY data
Normal data

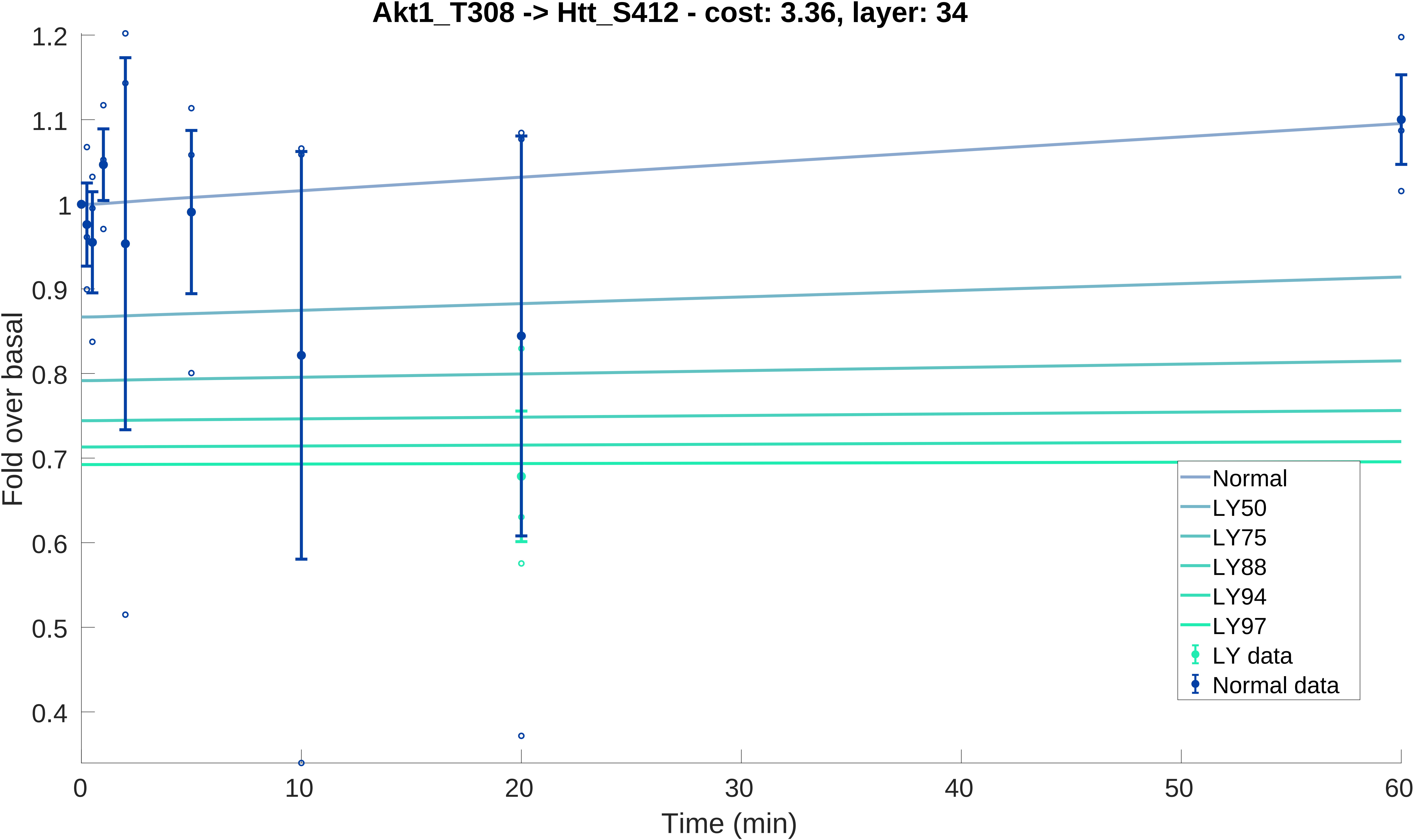
Akt1_T308 -> Htt_S412 - cost: 3.36, layer: 34
Fold over basal
Time (min)
1.2
1.1
1
0.9
0.8
0.7
0.6
0.5
0.4
0
10
20
30
40
50
60
Normal
LY50
LY75
LY88
LY94
LY97
LY data
Normal data

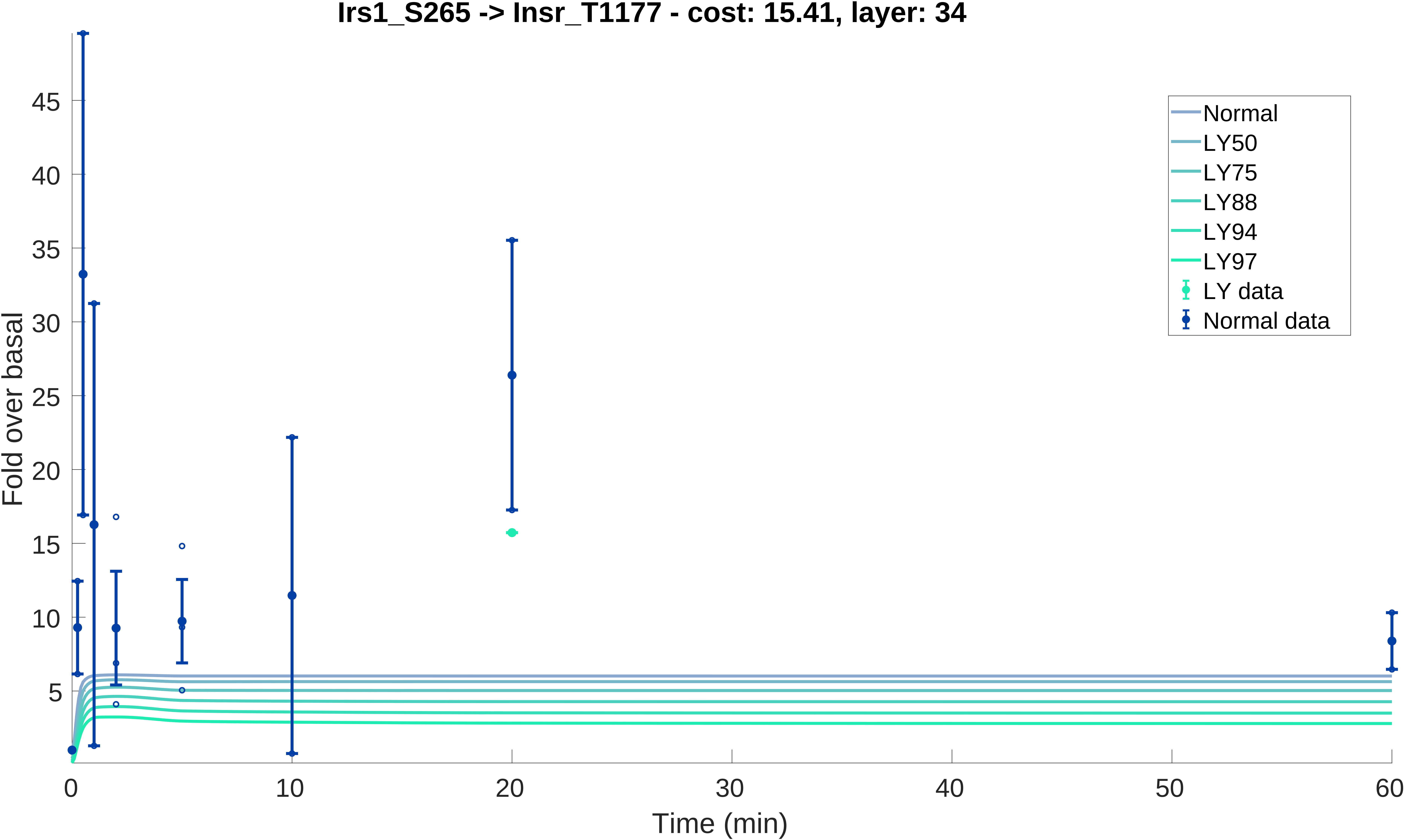

Irs1_S265 -> Insr_T1177 - cost: 15.41, layer: 34
Fold over basal
Time (min)
0
10
20
30
40
50
60
5
10
15
20
25
30
35
40
45
Normal
LY50
LY75
LY88
LY94
LY97
LY data
Normal data

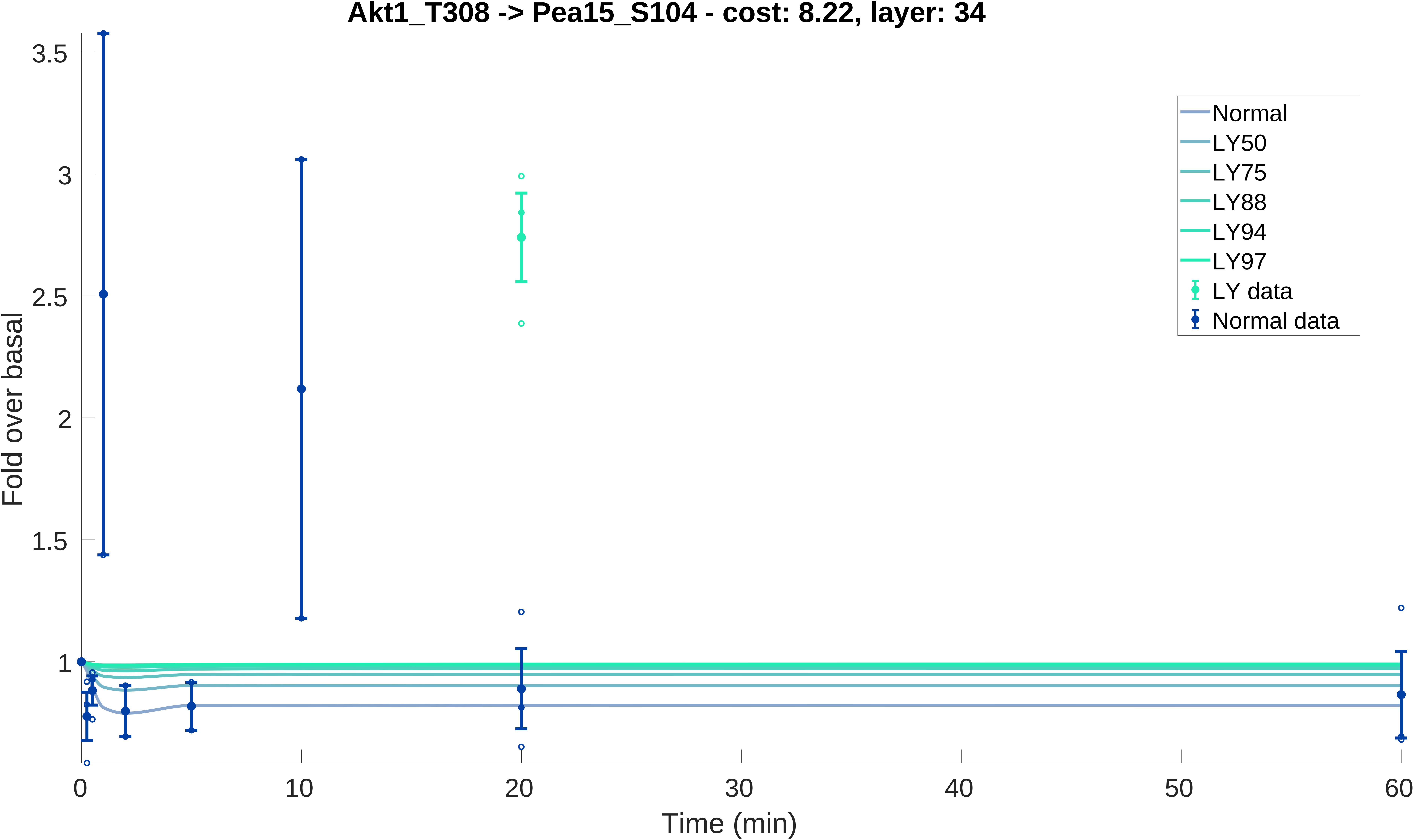
Akt1_T308 -> Pea15_S104 - cost: 8.22, layer: 34
Fold over basal
Time (min)
3.5
3
2.5
2
1.5
1
0
10
20
30
40
50
60
Normal
LY50
LY75
LY88
LY94
LY97
LY data
Normal data

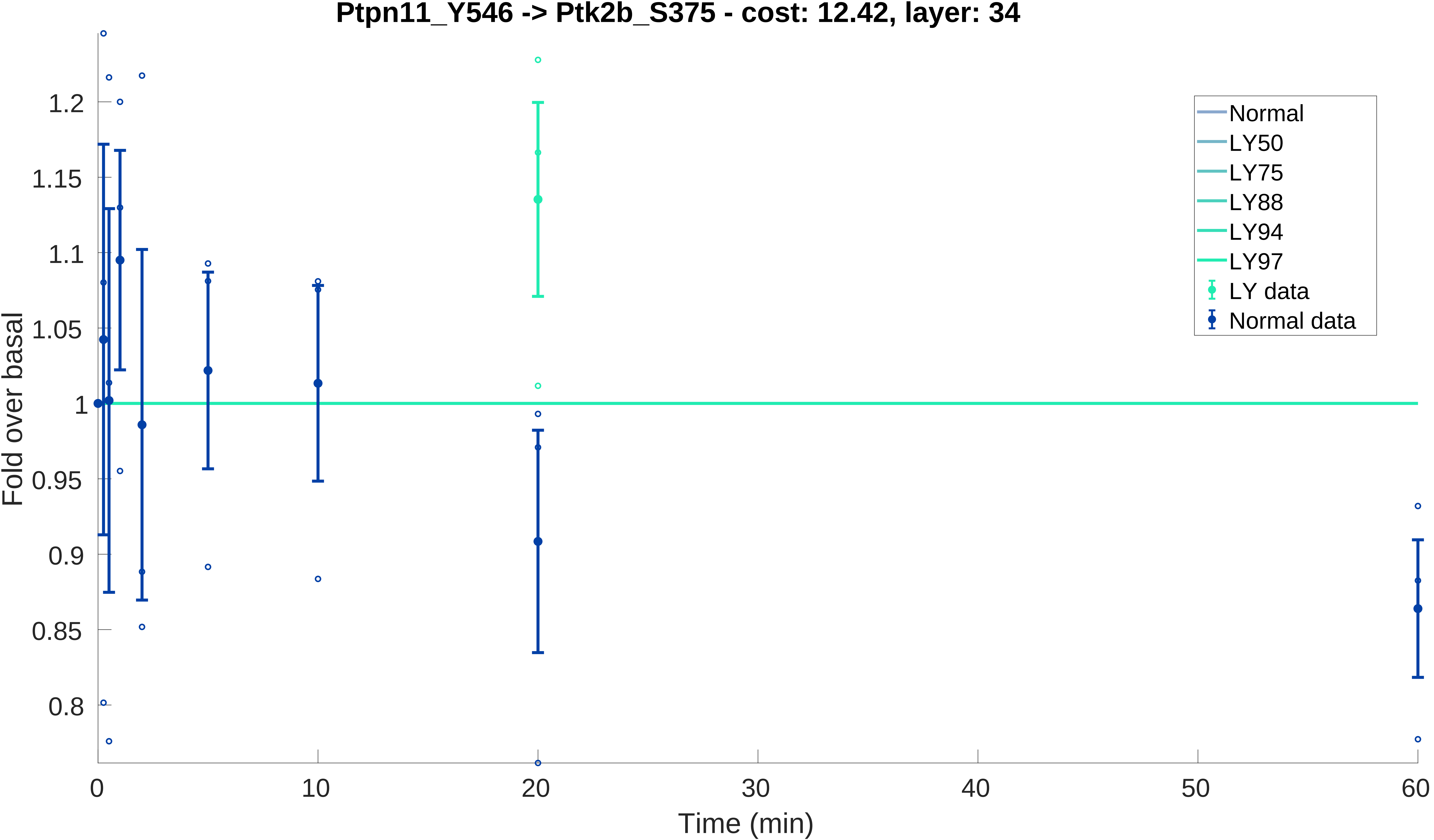
Ptpn11_Y546 -> Ptk2b_S375 - cost: 12.42, layer: 34
Fold over basal
Time (min)
Normal
LY50
LY75
LY88
LY94
LY97
LY data
Normal data
0
10
20
30
40
50
60
0.8
0.85
0.9
0.95
1
1.05
1.1
1.15
1.2

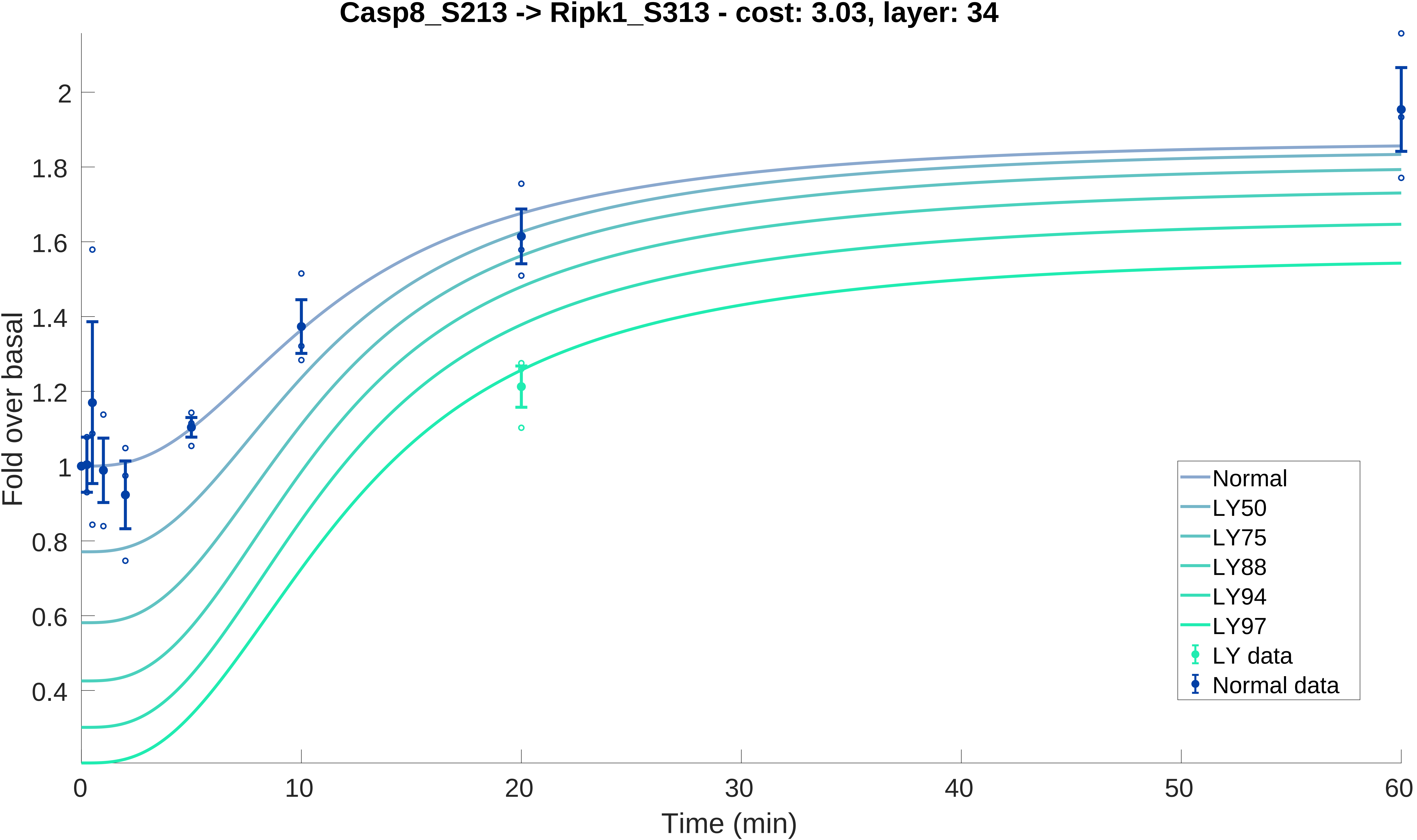

Casp8_S213 -> Ripk1_S313 - cost: 3.03, layer: 34
Fold over basal
Time (min)
0
10
20
30
40
50
60
0.4
0.6
0.8
1
1.2
1.4
1.6
1.8
2
Normal
LY50
LY75
LY88
LY94
LY97
LY data
Normal data

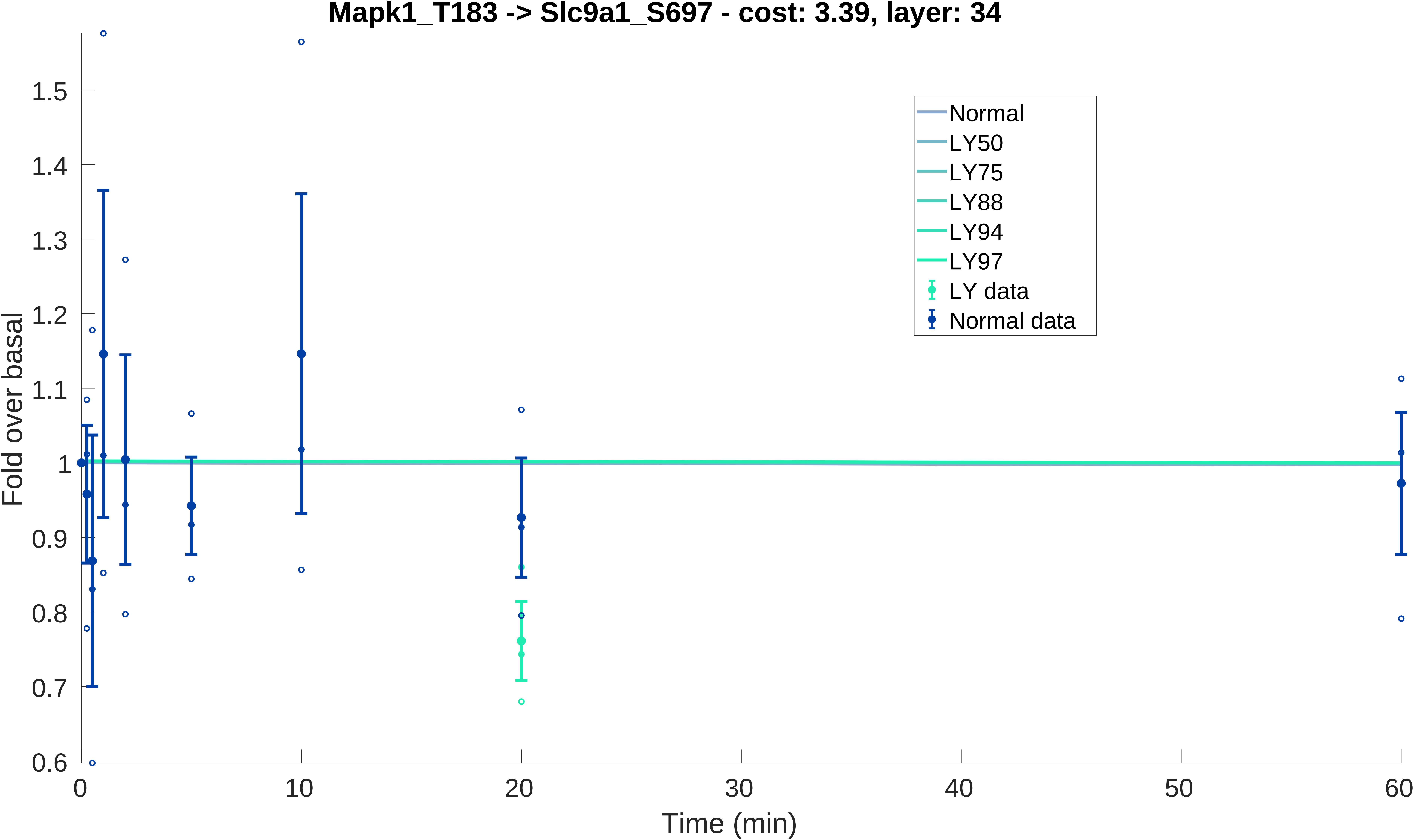

Mapk1_T183 -> Slc9a1_S697 - cost: 3.39, layer: 34
Fold over basal
1.5
1.4
1.3
1.2
1.1
1
0.9
0.8
0.7
0.6
0
10
20
30
40
50
60
Time (min)
Normal
LY50
LY75
LY88
LY94
LY97
LY data
Normal data

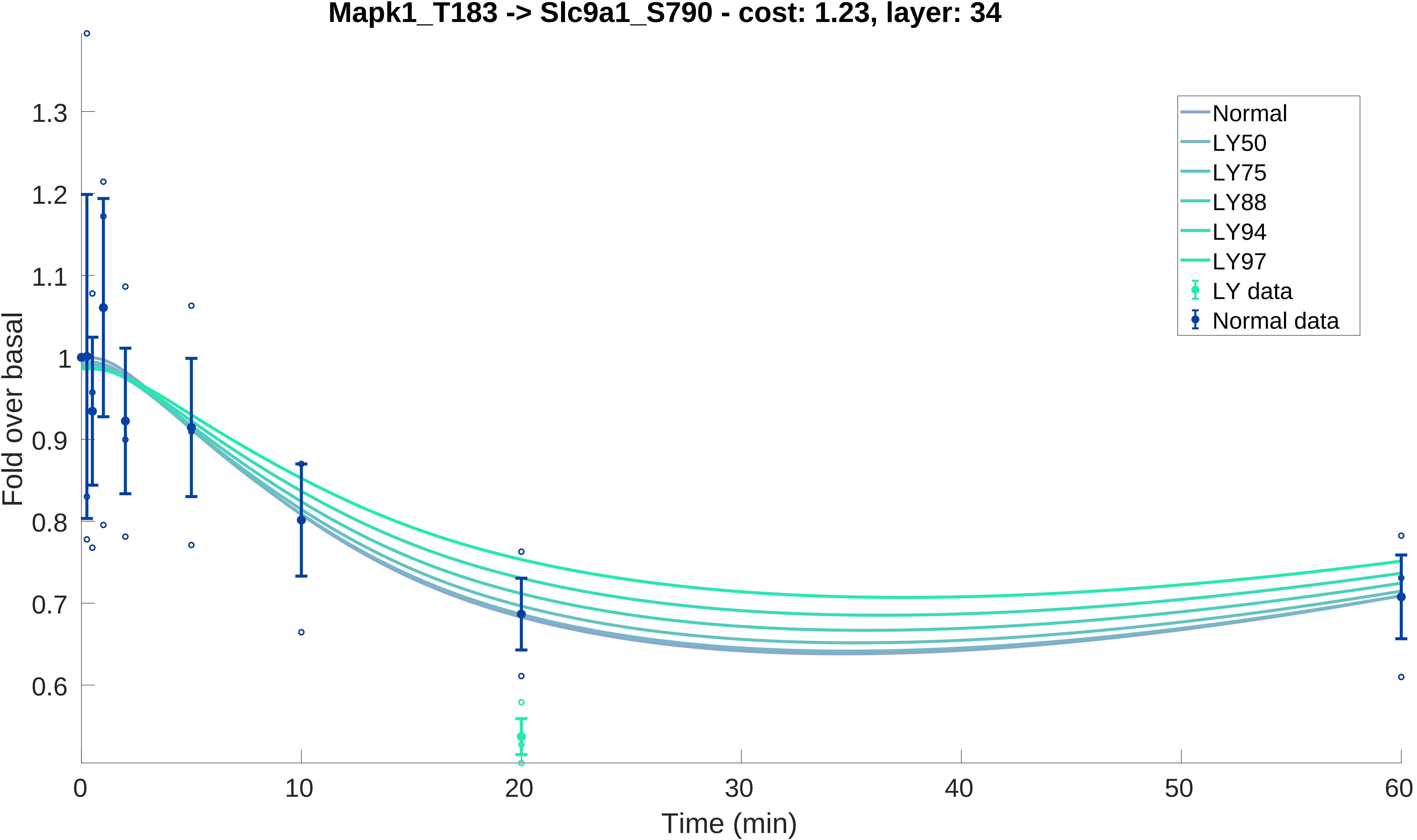
Mapk1_T183 -> Slc9a1_S790 - cost: 1.23, layer: 34
Fold over basal
1.3
1.2
1.1
1
0.9
0.8
0.7
0.6
0
10
20
30
40
50
60
Time (min)
Normal
LY50
LY75
LY88
LY94
LY97
LY data
Normal data

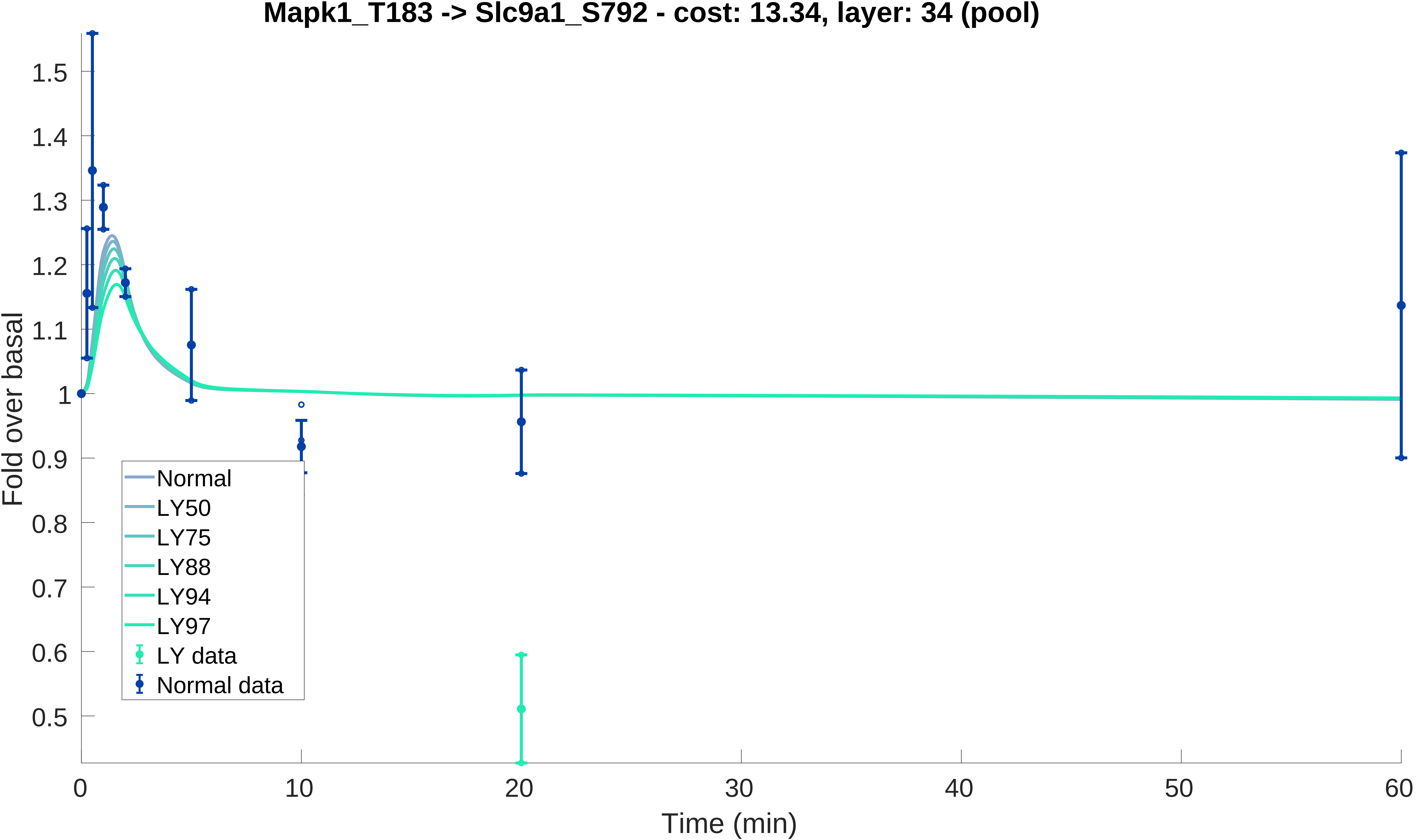

Mapk1_T183 -> Slc9a1_S792 - cost: 13.34, layer: 34 (pool)
Fold over basal
Time (min)
1.5
1.4
1.3
1.2
1.1
1
0.9
0.8
0.7
0.6
0.5
0
10
20
30
40
50
60
Normal
LY50
LY75
LY88
LY94
LY97
LY data
Normal data

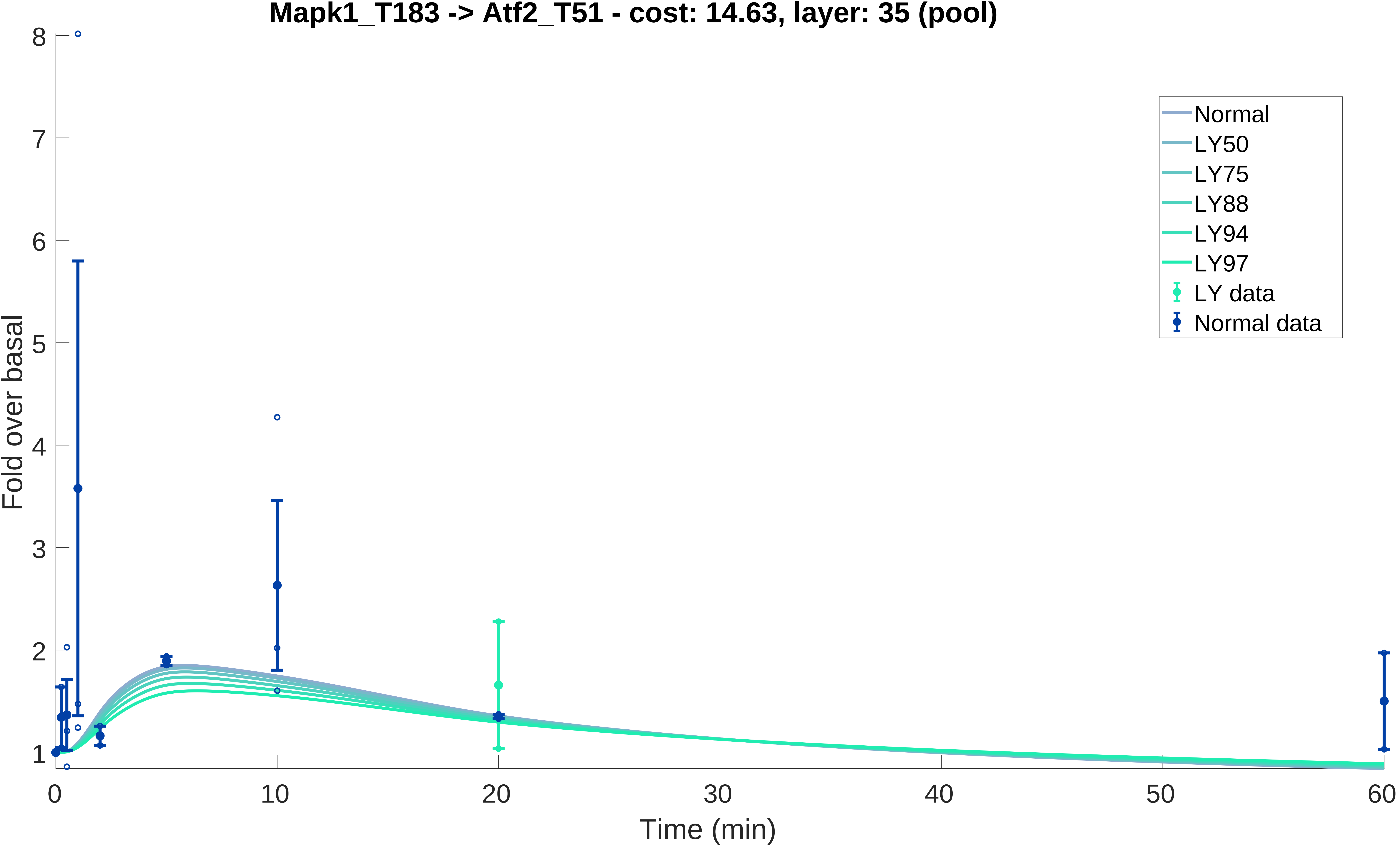

Mapk1_T183 -> Atf2_T51 - cost: 14.63, layer: 35 (pool)
Fold over basal
Time (min)
Normal
LY50
LY75
LY88
LY94
LY97
LY data
Normal data
1
2
3
4
5
6
7
8
0
10
20
30
40
50
60

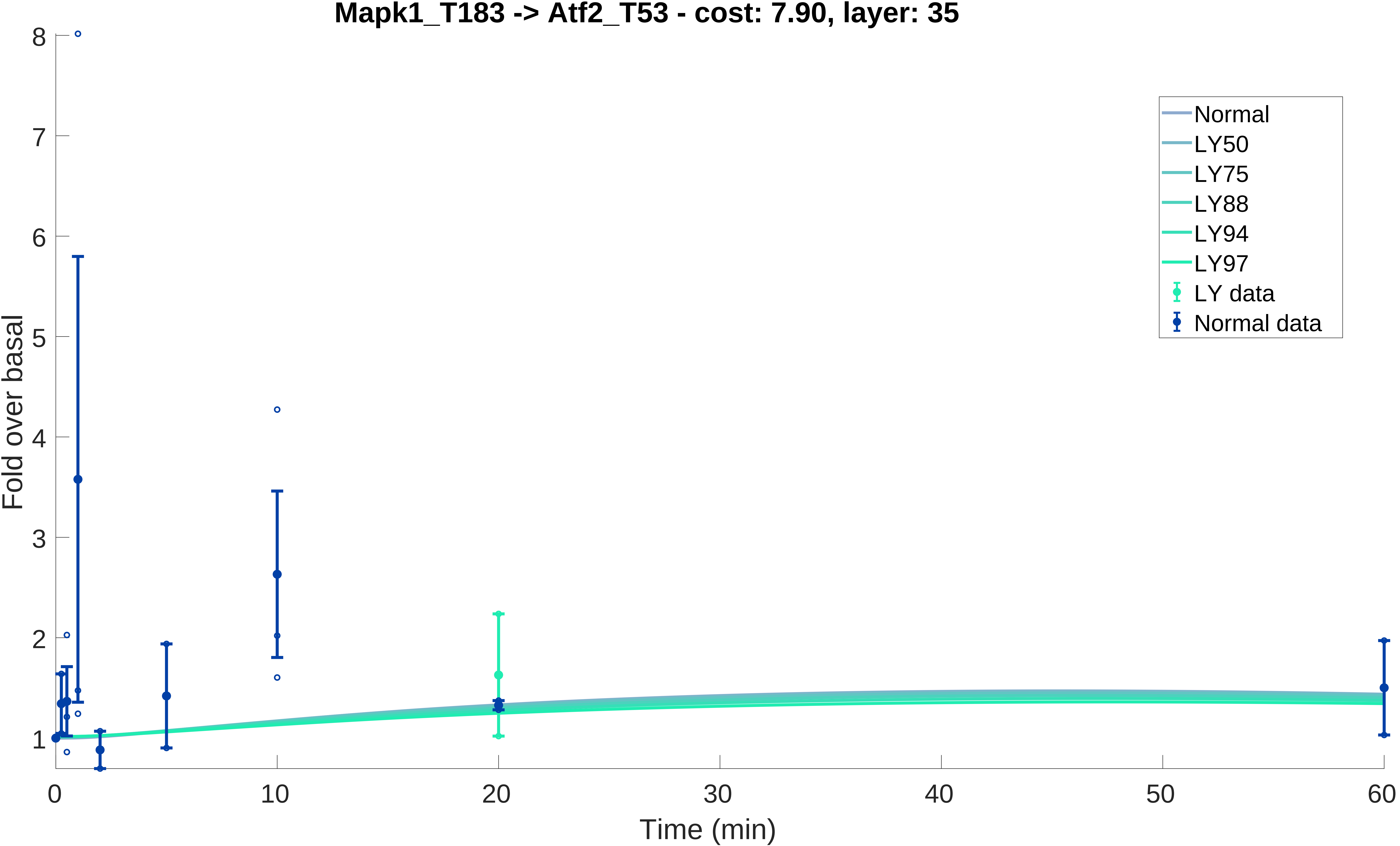
Mapk1_T183 -> Atf2_T53 - cost: 7.90, layer: 35
Fold over basal
Time (min)
0
10
20
30
40
50
60
1
2
3
4
5
6
7
8
Normal
LY50
LY75
LY88
LY94
LY97
LY data
Normal data

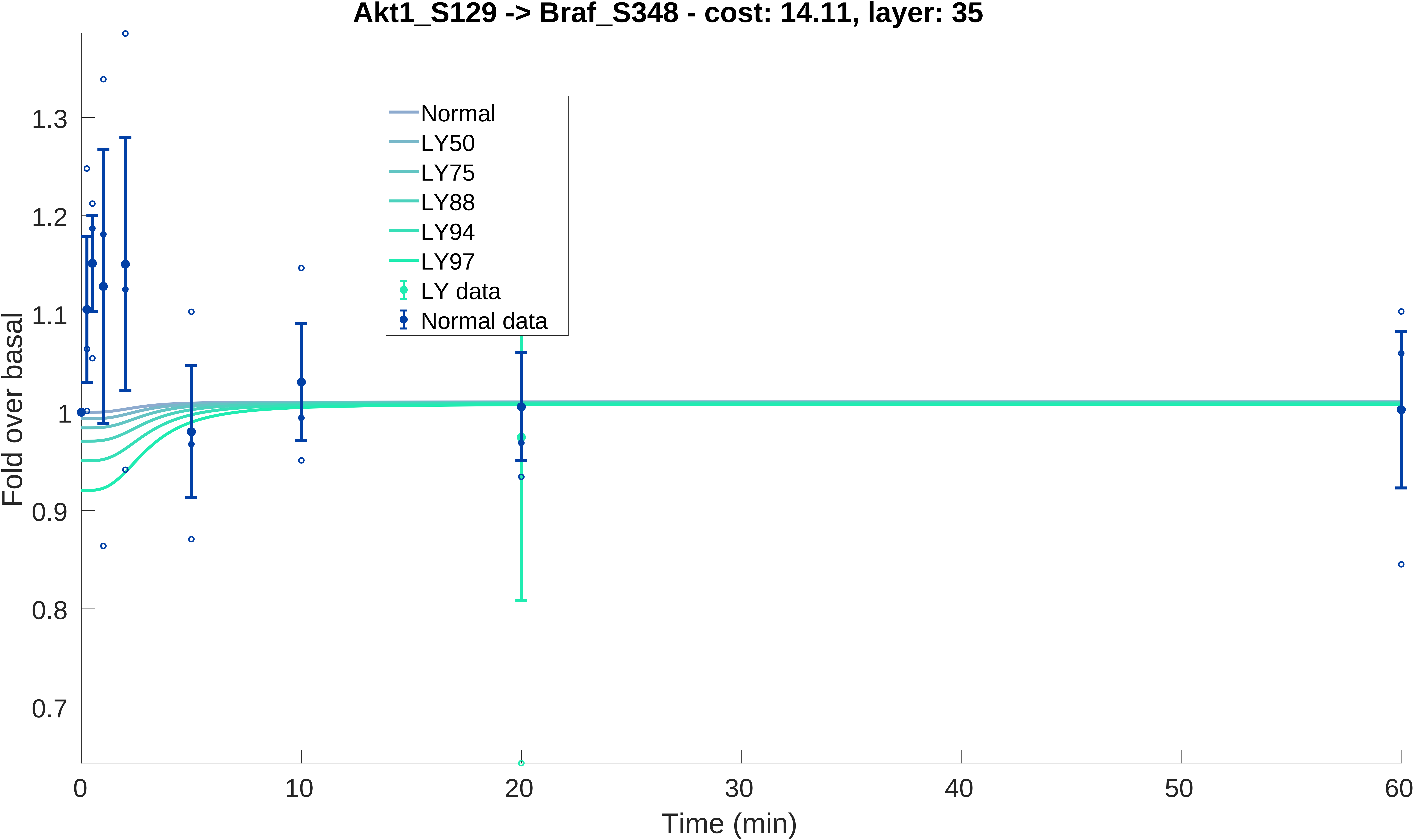
Akt1_S129 -> Braf_S348 - cost: 14.11, layer: 35
Normal
LY50
LY75
LY88
LY94
LY97
LY data
Normal data
Fold over basal
Time (min)
1.3
1.2
1.1
1
0.9
0.8
0.7
0
10
20
30
40
50
60

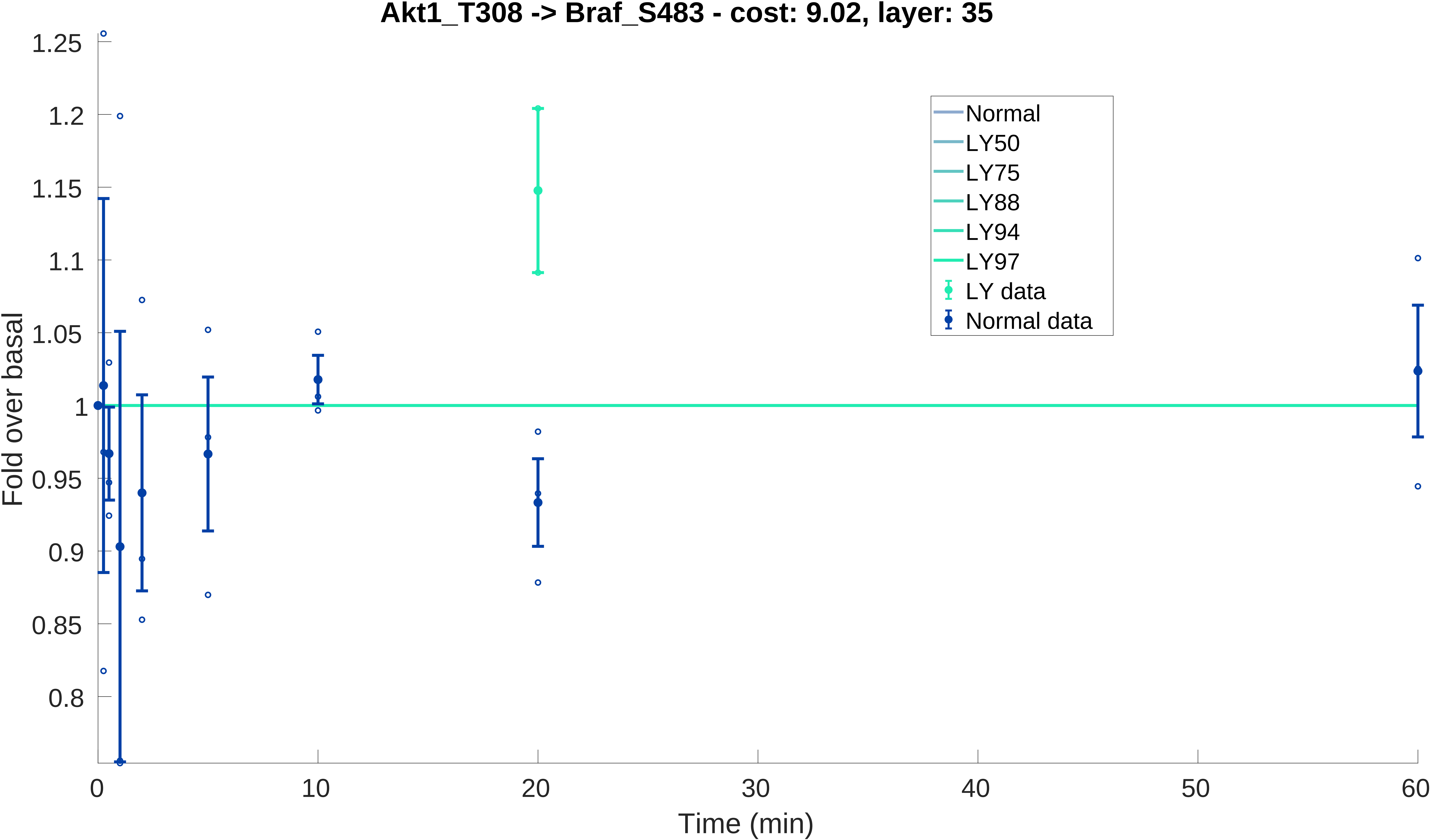

Akt1_T308 -> Braf_S483 - cost: 9.02, layer: 35
Fold over basal
Time (min)
1.25
1.2
1.15
1.1
1.05
1
0.95
0.9
0.85
0.8
0
10
20
30
40
50
60
Normal
LY50
LY75
LY88
LY94
LY97
LY data
Normal data

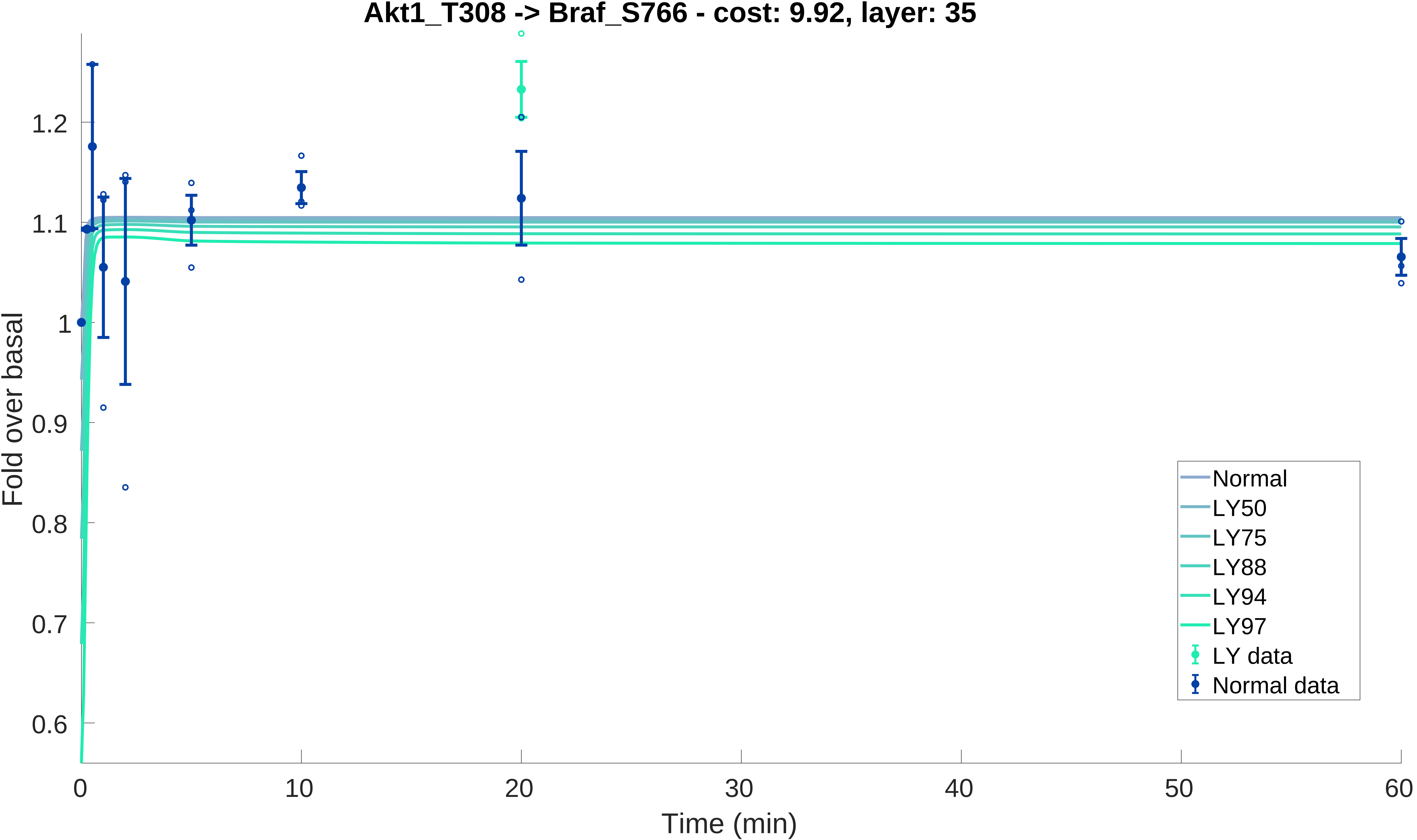
Akt1_T308 -> Braf_S766 - cost: 9.92, layer: 35
Fold over basal
Time (min)
1.2
1.1
1
0.9
0.8
0.7
0.6
0
10
20
30
40
50
60
Normal
LY50
LY75
LY88
LY94
LY97
LY data
Normal data

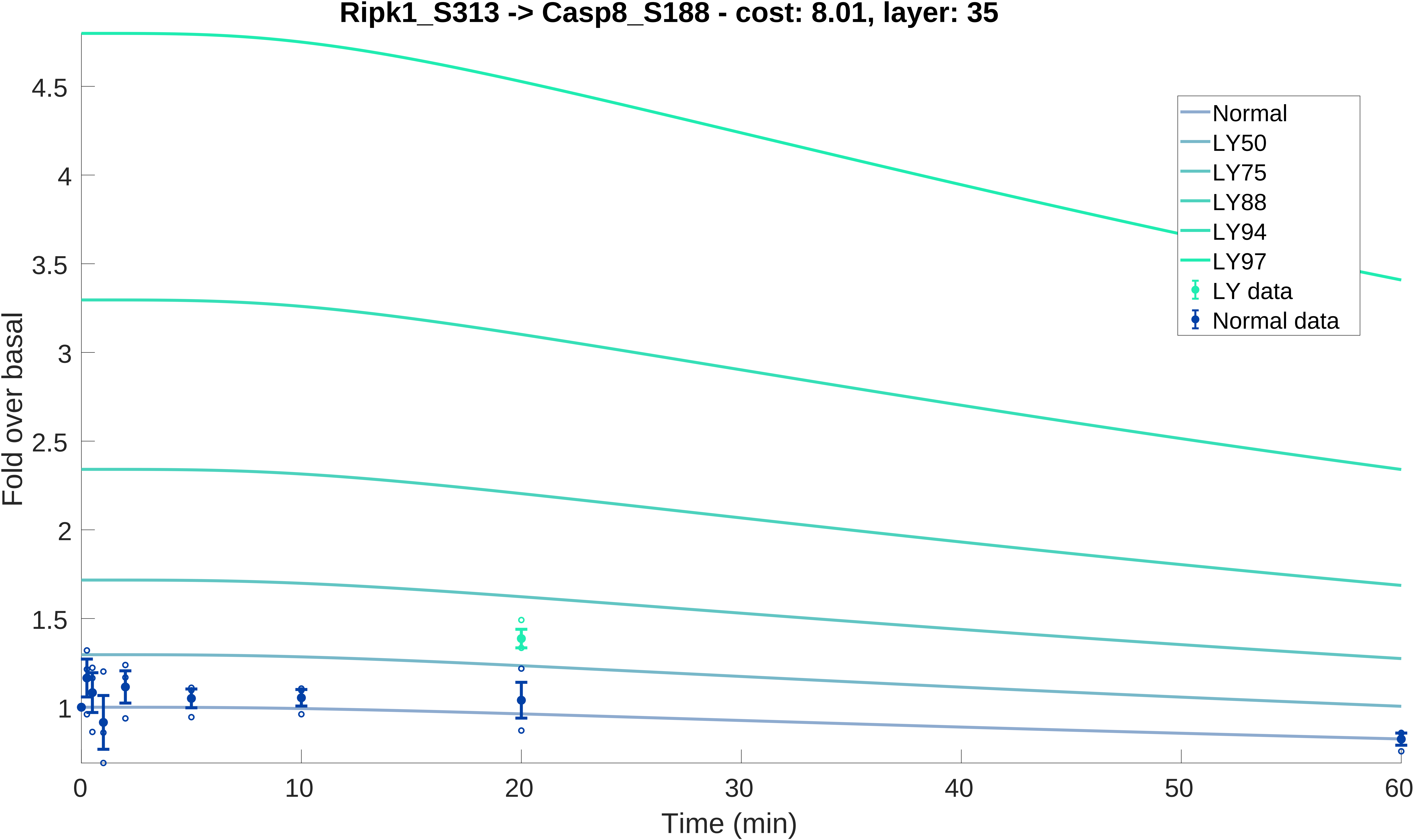

Ripk1_S313 -> Casp8_S188 - cost: 8.01, layer: 35
Fold over basal
4.5
4
3.5
3
2.5
2
1.5
1
0
10
20
30
40
50
60
Time (min)
Normal
LY50
LY75
LY88
LY94
LY97
LY data
Normal data

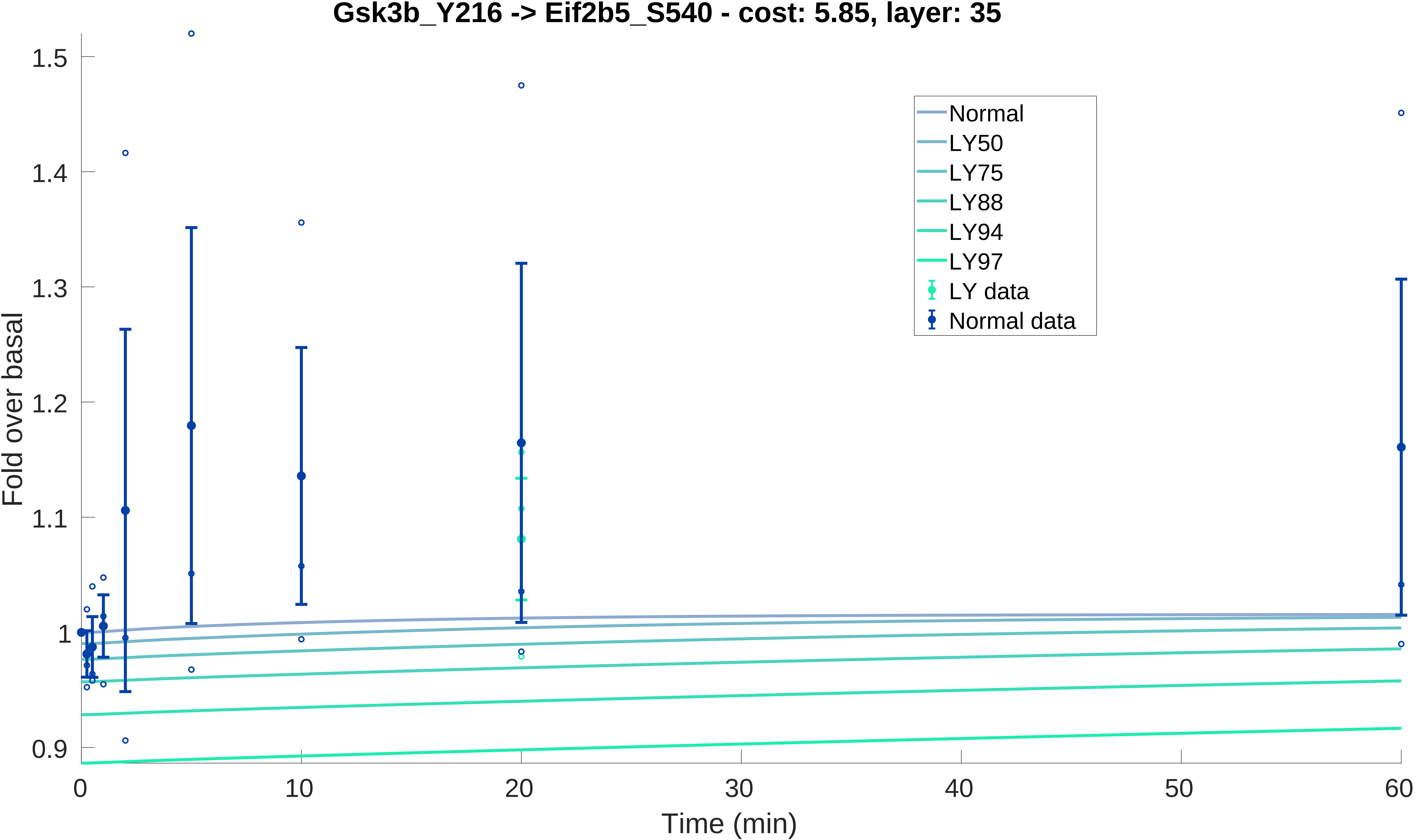

Gsk3b_Y216 -> Eif2b5_S540 - cost: 5.85, layer: 35
Fold over basal
Time (min)
1.5
1.4
1.3
1.2
1.1
1
0.9
0
10
20
30
40
50
60
Normal
LY50
LY75
LY88
LY94
LY97
LY data
Normal data

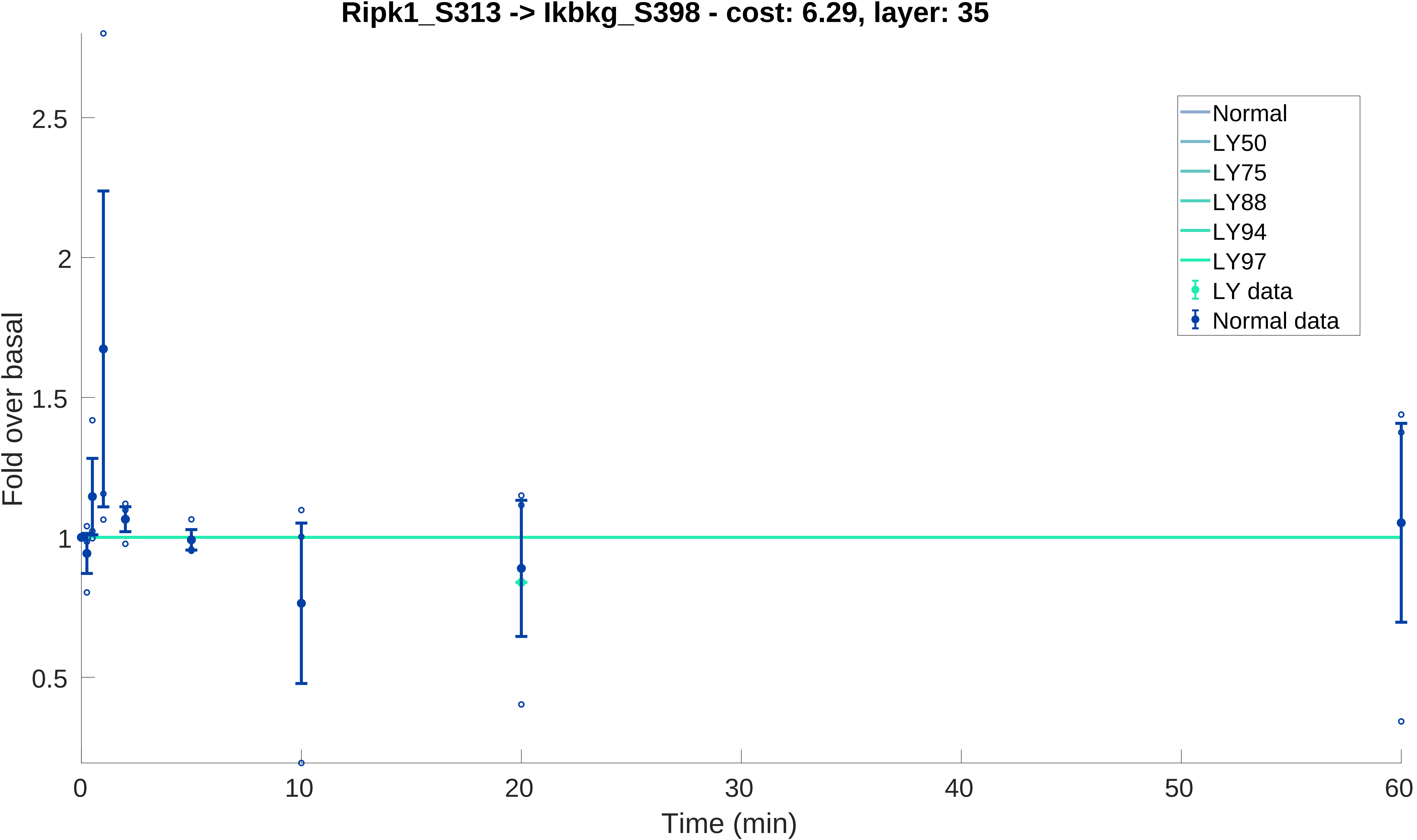

Ripk1_S313 -> Ikbkg_S398 - cost: 6.29, layer: 35
Fold over basal
Time (min)
2.5
2
1.5
1
0.5
0
10
20
30
40
50
60
Normal
LY50
LY75
LY88
LY94
LY97
LY data
Normal data

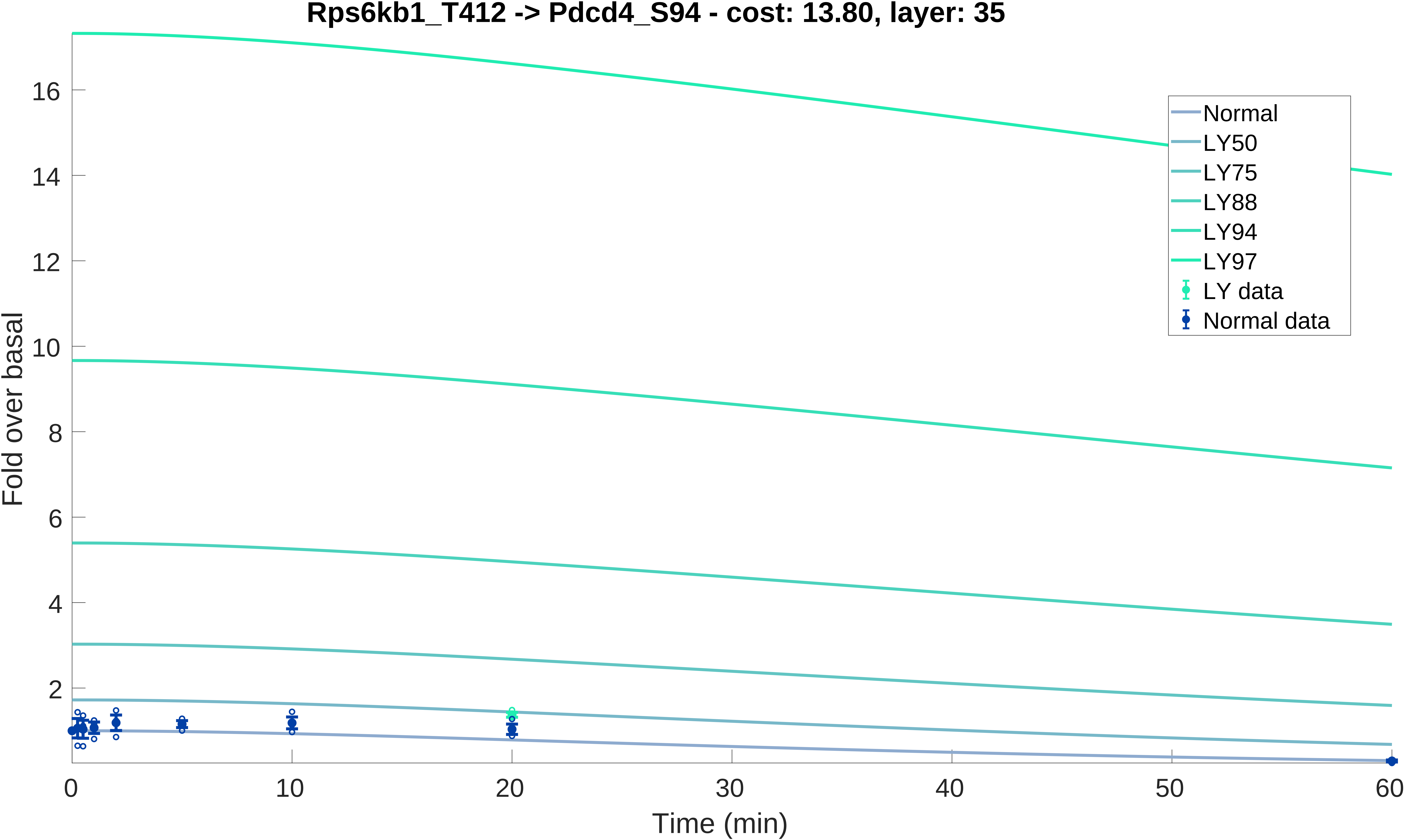

Rps6kb1_T412 -> Pdcd4_S94 - cost: 13.80, layer: 35
16
14
12
10
8
6
4
2
0
10
20
30
40
50
60
Time (min)
Fold over basal
Normal
LY50
LY75
LY88
LY94
LY97
LY data
Normal data

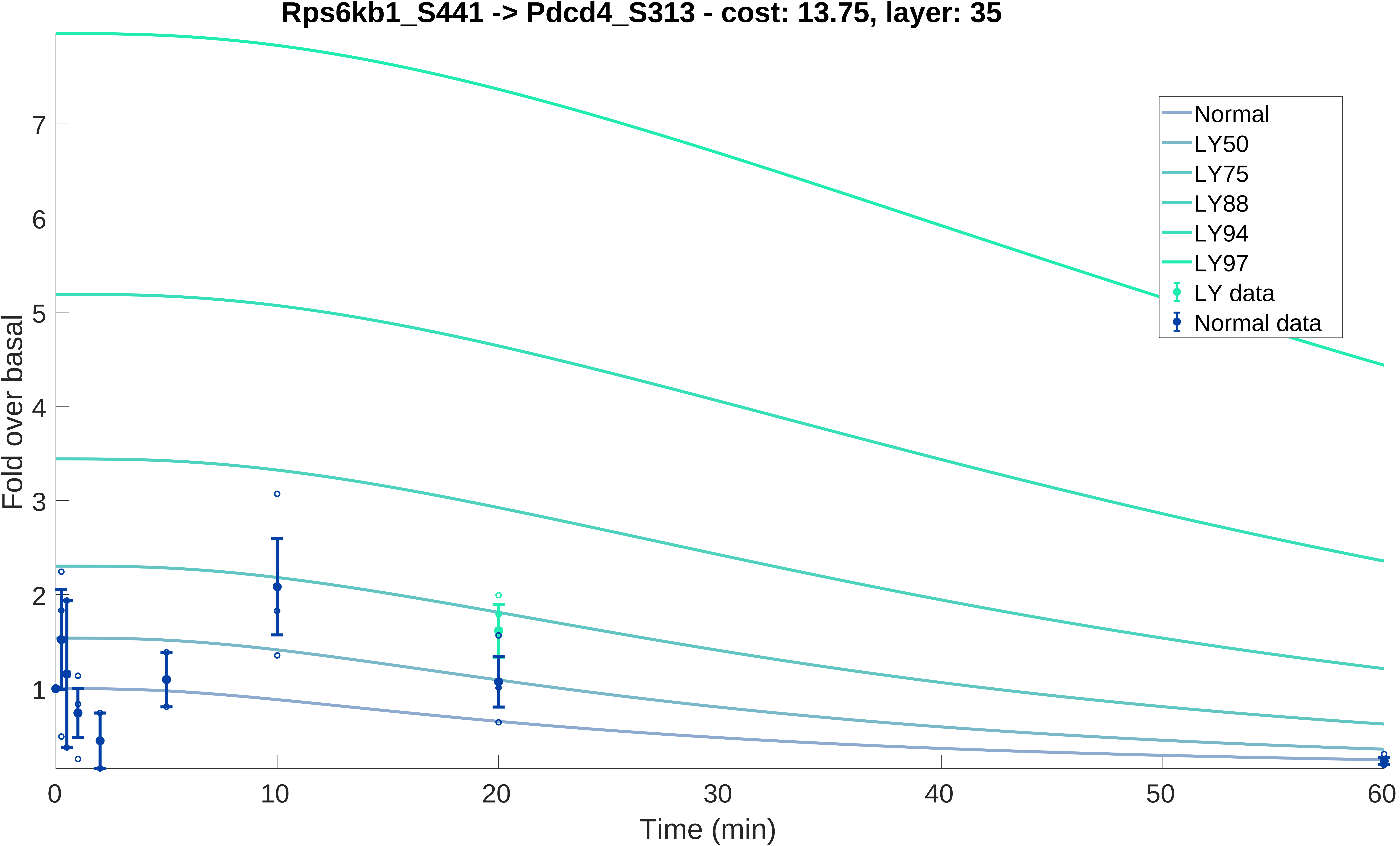
Rps6kb1_S441 -> Pdcd4_S313 - cost: 13.75, layer: 35
Fold over basal
7
6
5
4
3
2
1
0
10
20
30
40
50
60
Time (min)
Normal
LY50
LY75
LY88
LY94
LY97
LY data
Normal data

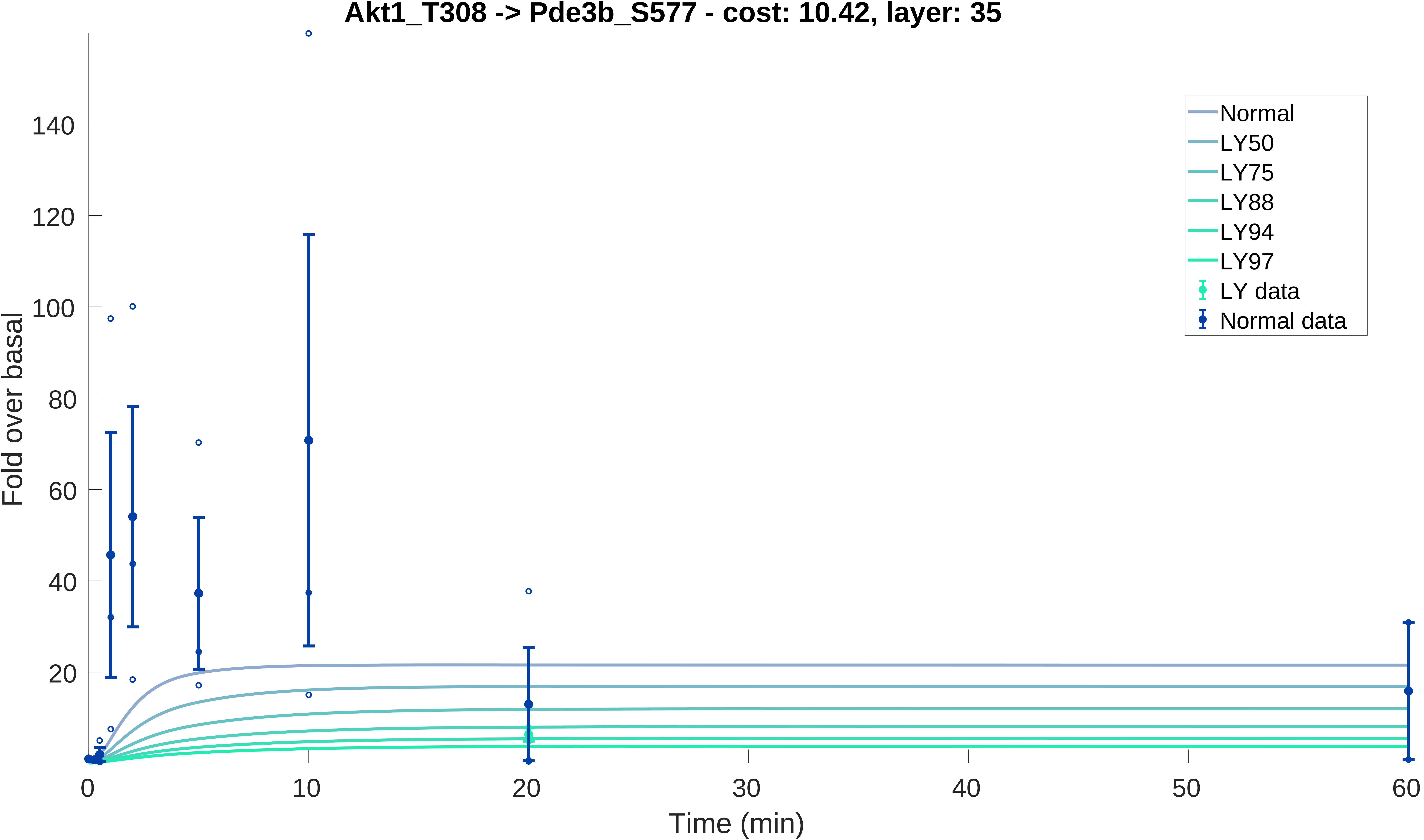

Akt1_T308 -> Pde3b_S577 - cost: 10.42, layer: 35
Fold over basal
Time (min)
0
10
20
30
40
50
60
20
40
60
80
100
120
140
Normal
LY50
LY75
LY88
LY94
LY97
LY data
Normal data

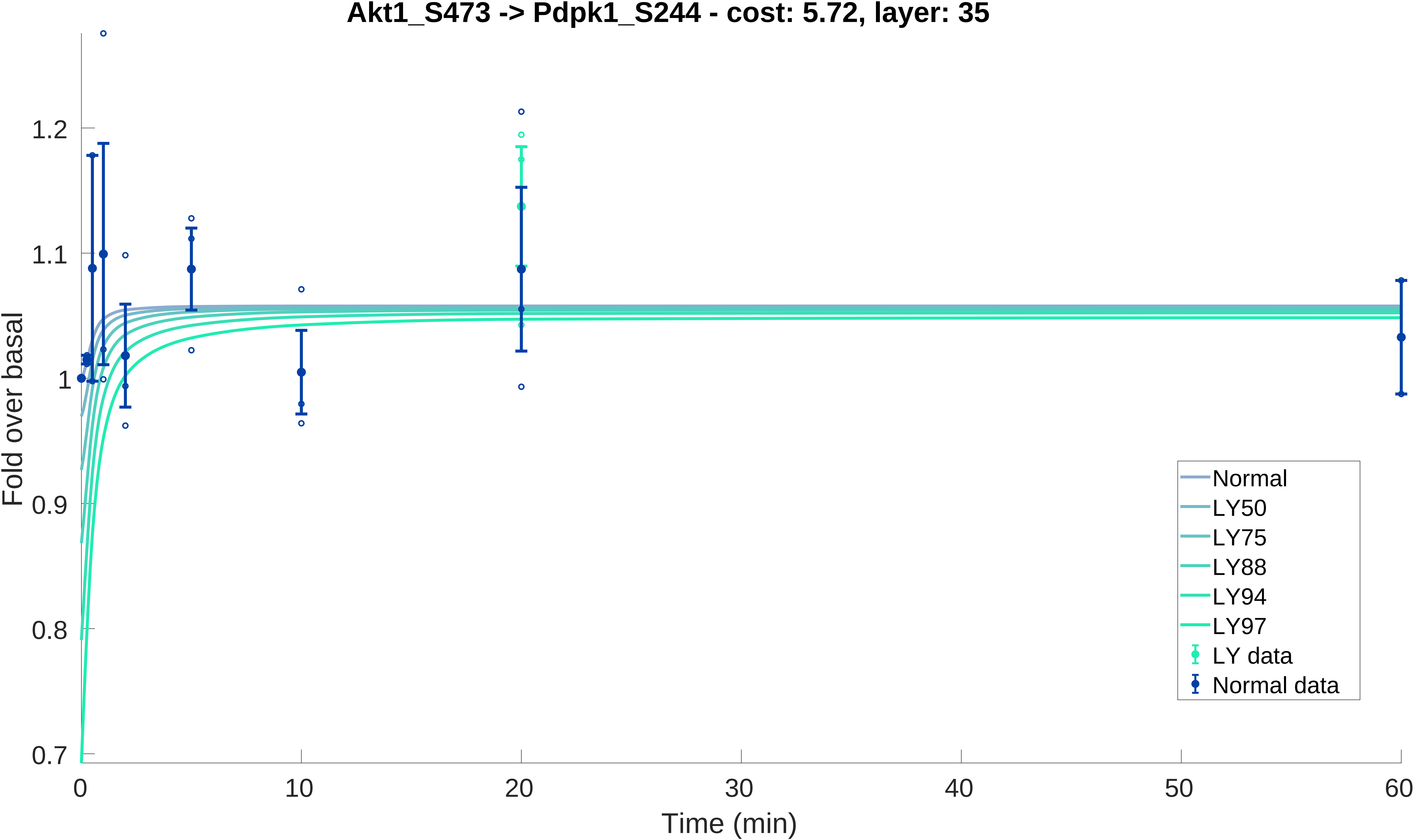
Akt1_S473 -> Pdpk1_S244 - cost: 5.72, layer: 35
Fold over basal
Time (min)
0.7
0.8
0.9
1
1.1
1.2
0
10
20
30
40
50
60
Normal
LY50
LY75
LY88
LY94
LY97
LY data
Normal data

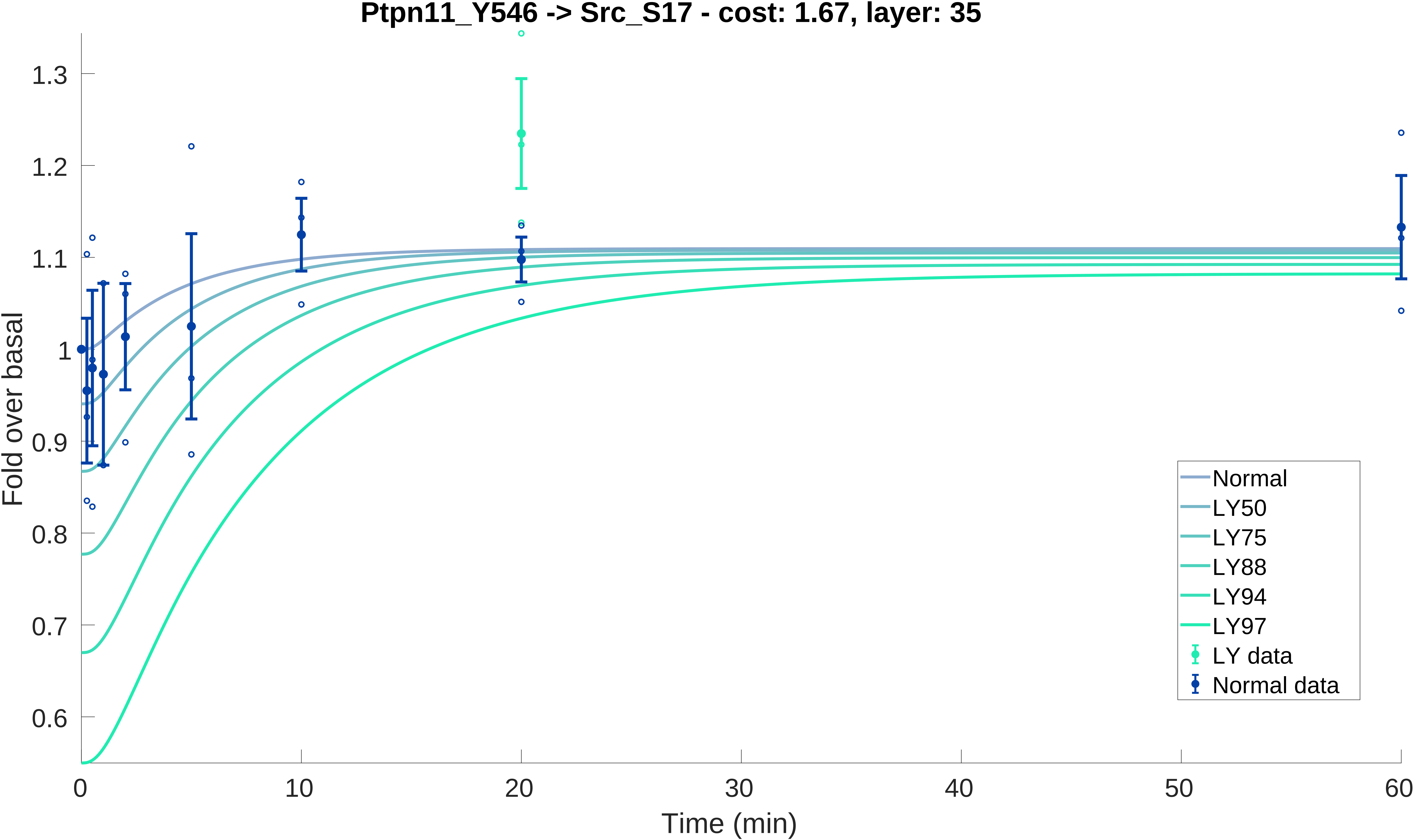

Ptpn11_Y546 -> Src_S17 - cost: 1.67, layer: 35
1.3
1.2
1.1
1
0.9
0.8
0.7
0.6
Fold over basal
0
10
20
30
40
50
60
Time (min)
Normal
LY50
LY75
LY88
LY94
LY97
LY data
Normal data

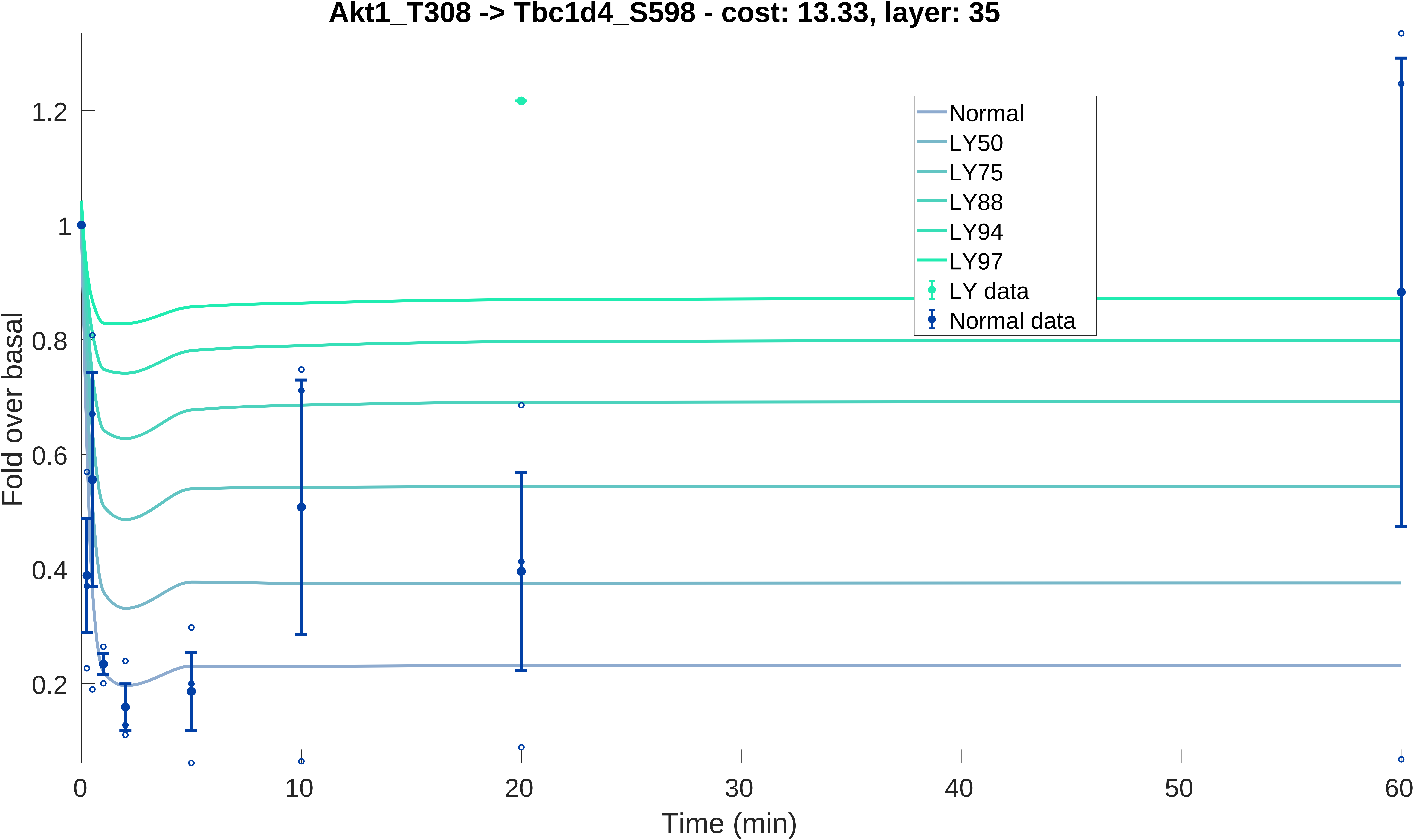

Akt1_T308 -> Tbc1d4_S598 - cost: 13.33, layer: 35
Fold over basal
Time (min)
0
10
20
30
40
50
60
0.2
0.4
0.6
0.8
1
1.2
Normal
LY50
LY75
LY88
LY94
LY97
LY data
Normal data

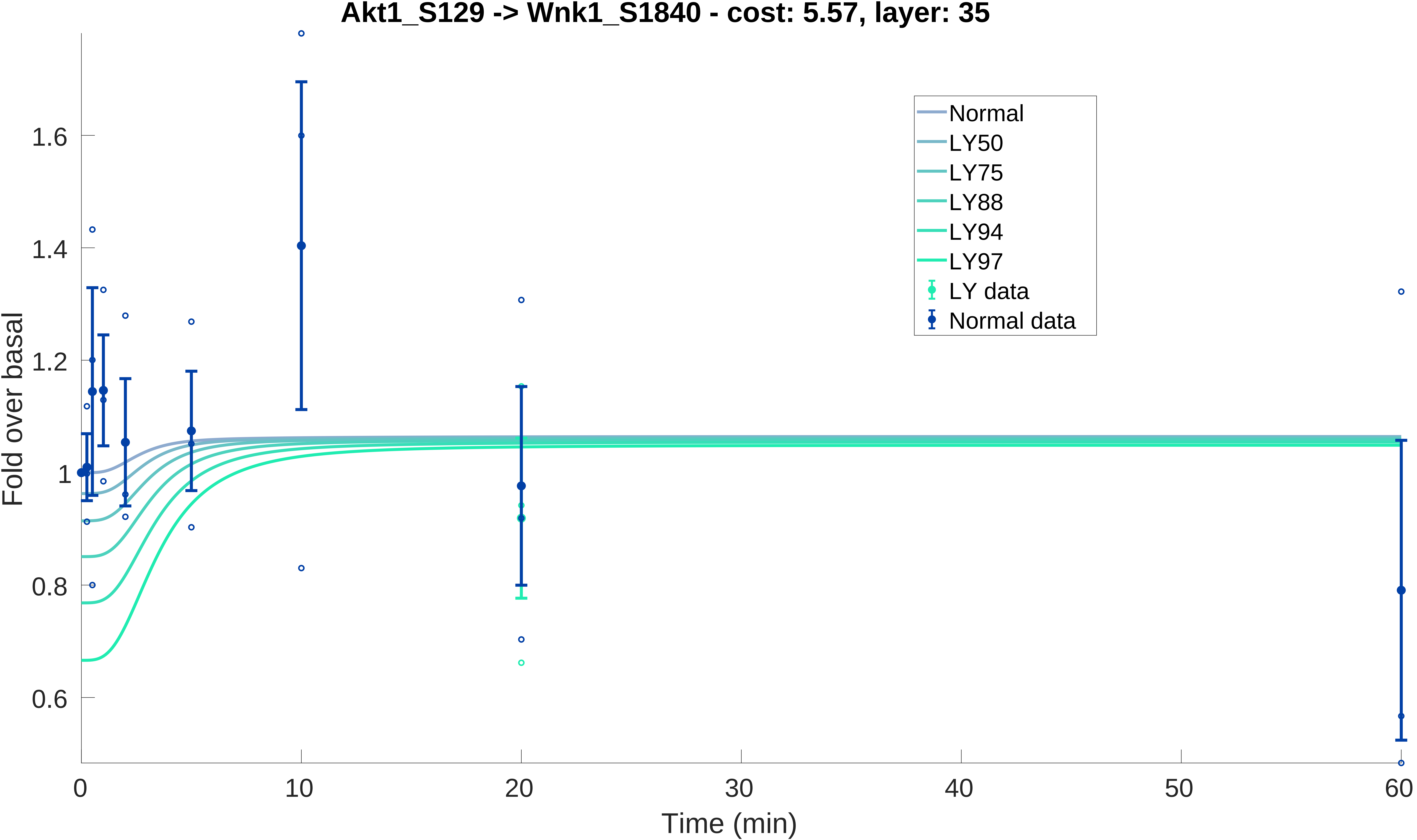

Akt1_S129 -> Wnk1_S1840 - cost: 5.57, layer: 35
Fold over basal
Time (min)
Normal
LY50
LY75
LY88
LY94
LY97
LY data
Normal data
0.6
0.8
1
1.2
1.4
1.6
0
10
20
30
40
50
60

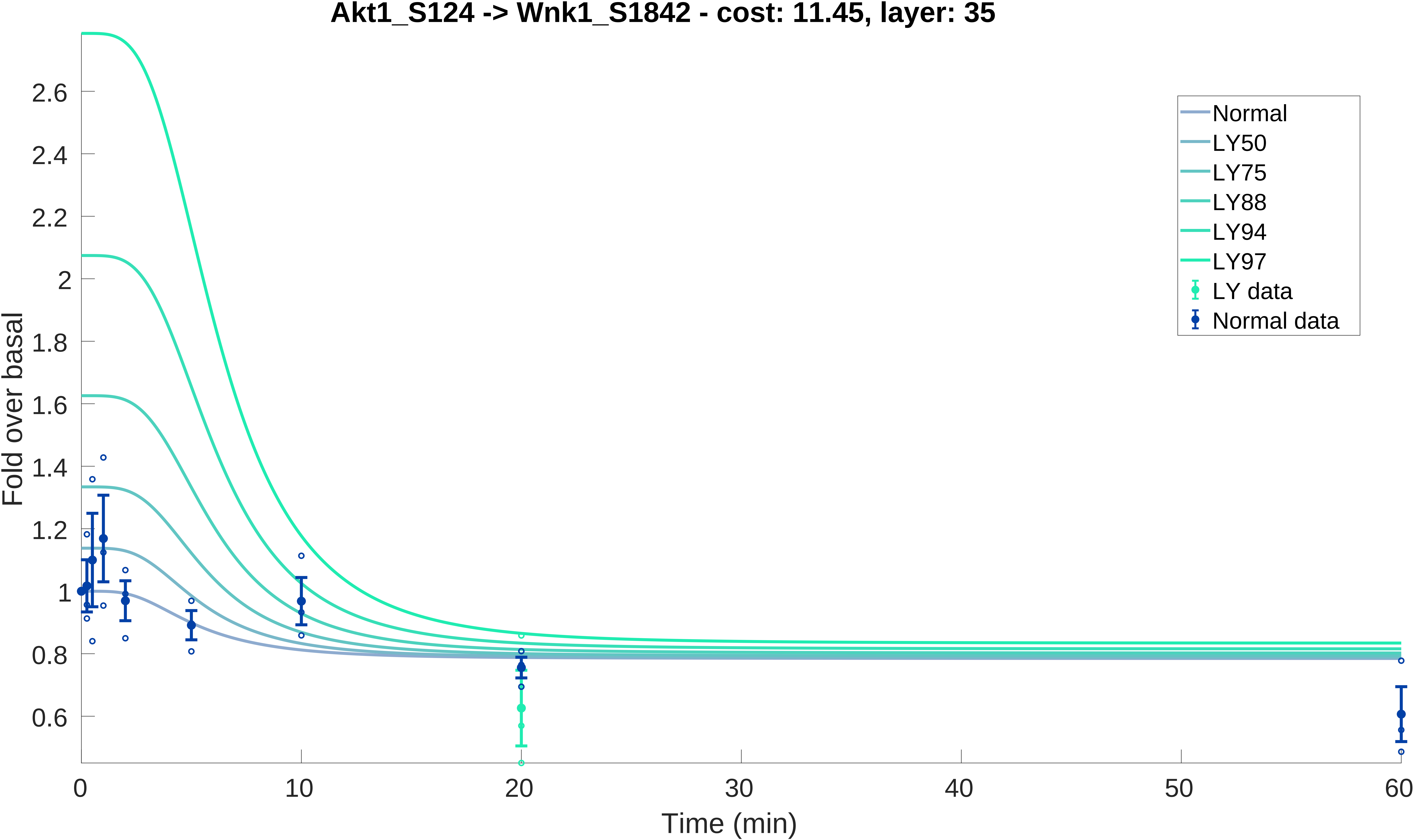

Akt1_S124 -> Wnk1_S1842 - cost: 11.45, layer: 35
Fold over basal
Time (min)
2.6
2.4
2.2
2
1.8
1.6
1.4
1.2
1
0.8
0.6
0
10
20
30
40
50
60
Normal
LY50
LY75
LY88
LY94
LY97
LY data
Normal data

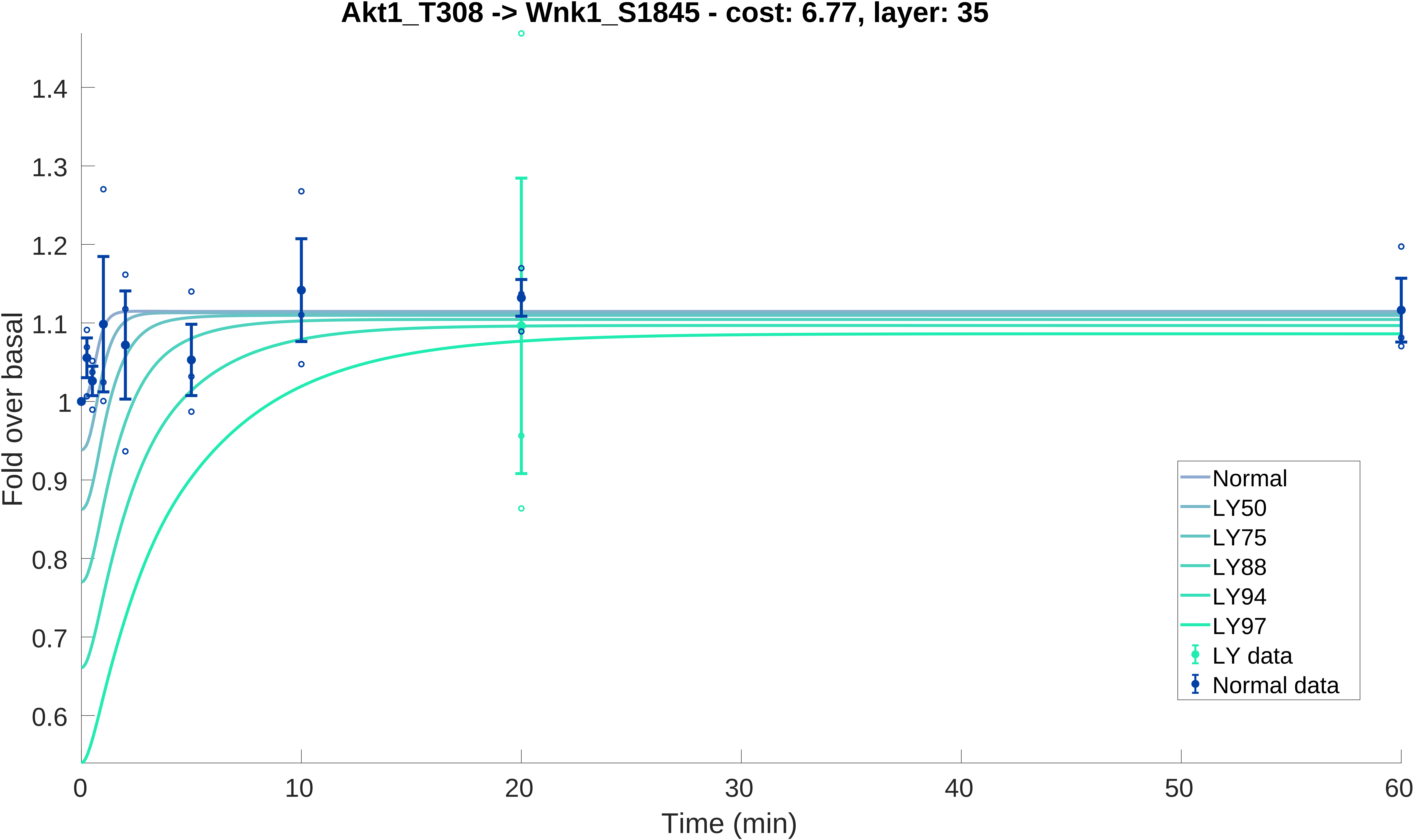

Akt1_T308 -> Wnk1_S1845 - cost: 6.77, layer: 35
Fold over basal
1.4
1.3
1.2
1.1
1
0.9
0.8
0.7
0.6
0
10
20
30
40
50
60
Time (min)
Normal
LY50
LY75
LY88
LY94
LY97
LY data
Normal data

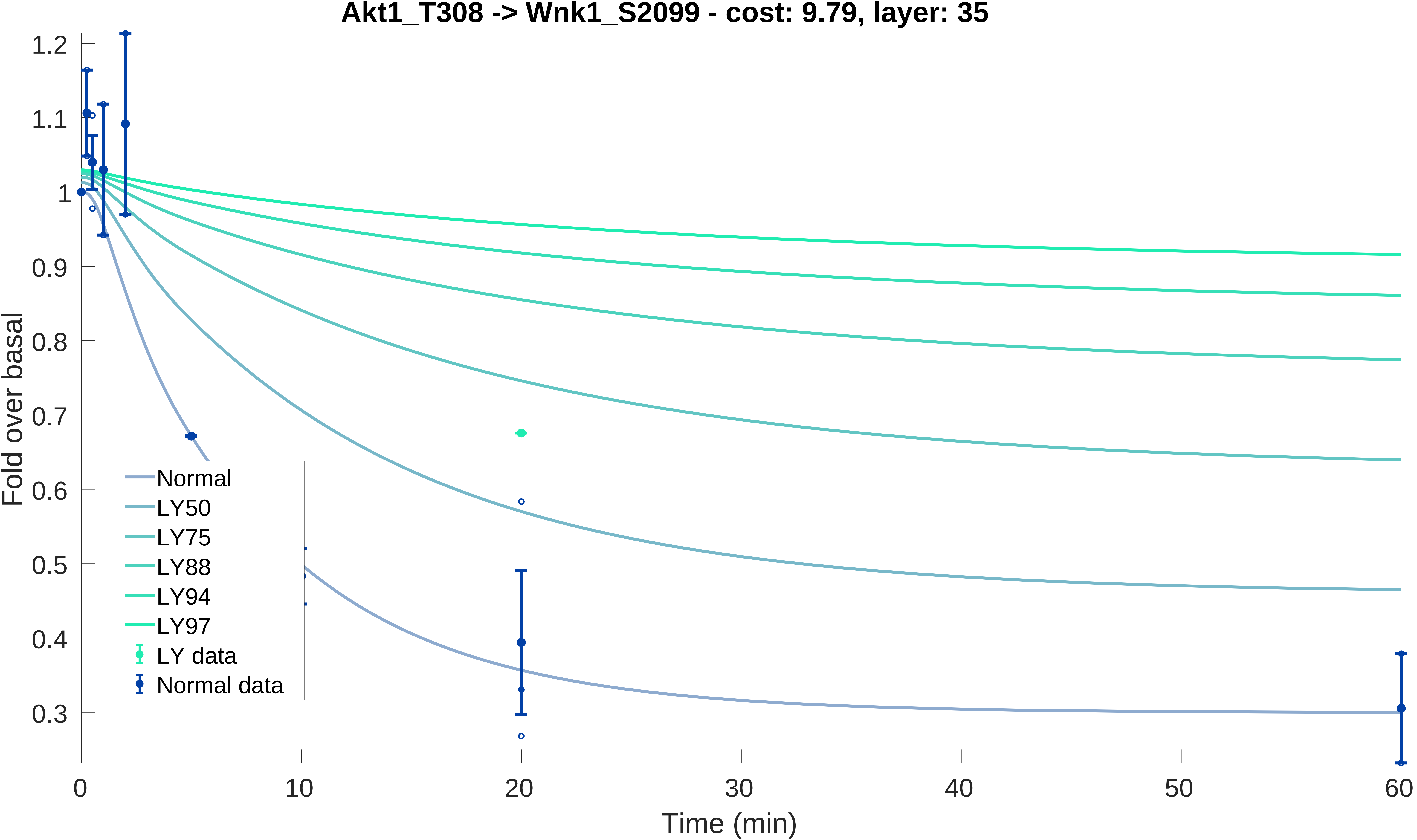
Akt1_T308 -> Wnk1_S2099 - cost: 9.79, layer: 35
Fold over basal
Time (min)
1.2
1.1
1
0.9
0.8
0.7
0.6
0.5
0.4
0.3
0
10
20
30
40
50
60
Normal
LY50
LY75
LY88
LY94
LY97
LY data
Normal data

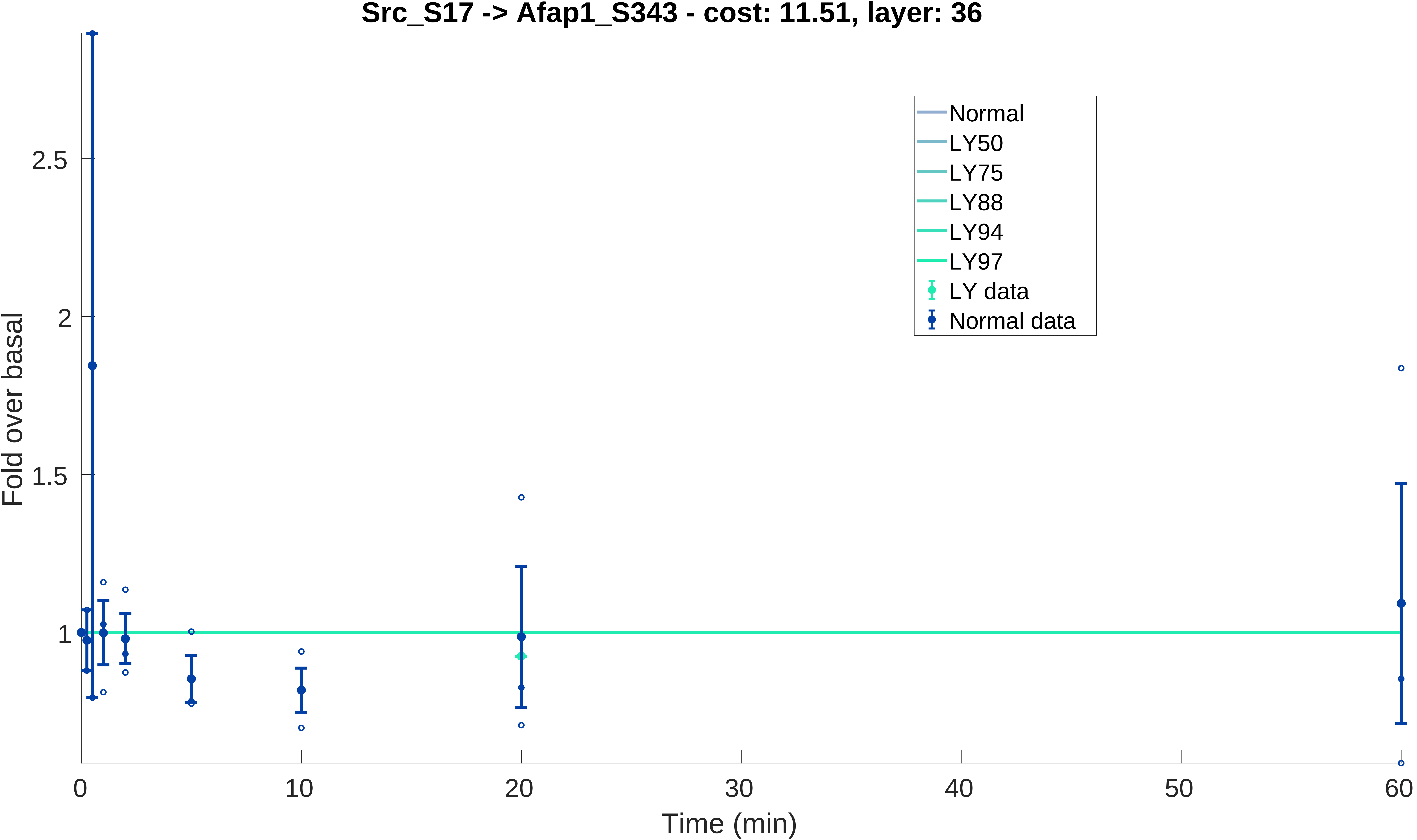

Src_S17 -> Afap1_S343 - cost: 11.51, layer: 36
Normal
LY50
LY75
LY88
LY94
LY97
LY data
Normal data
Fold over basal
2.5
2
1.5
1
0
10
20
30
40
50
60
Time (min)

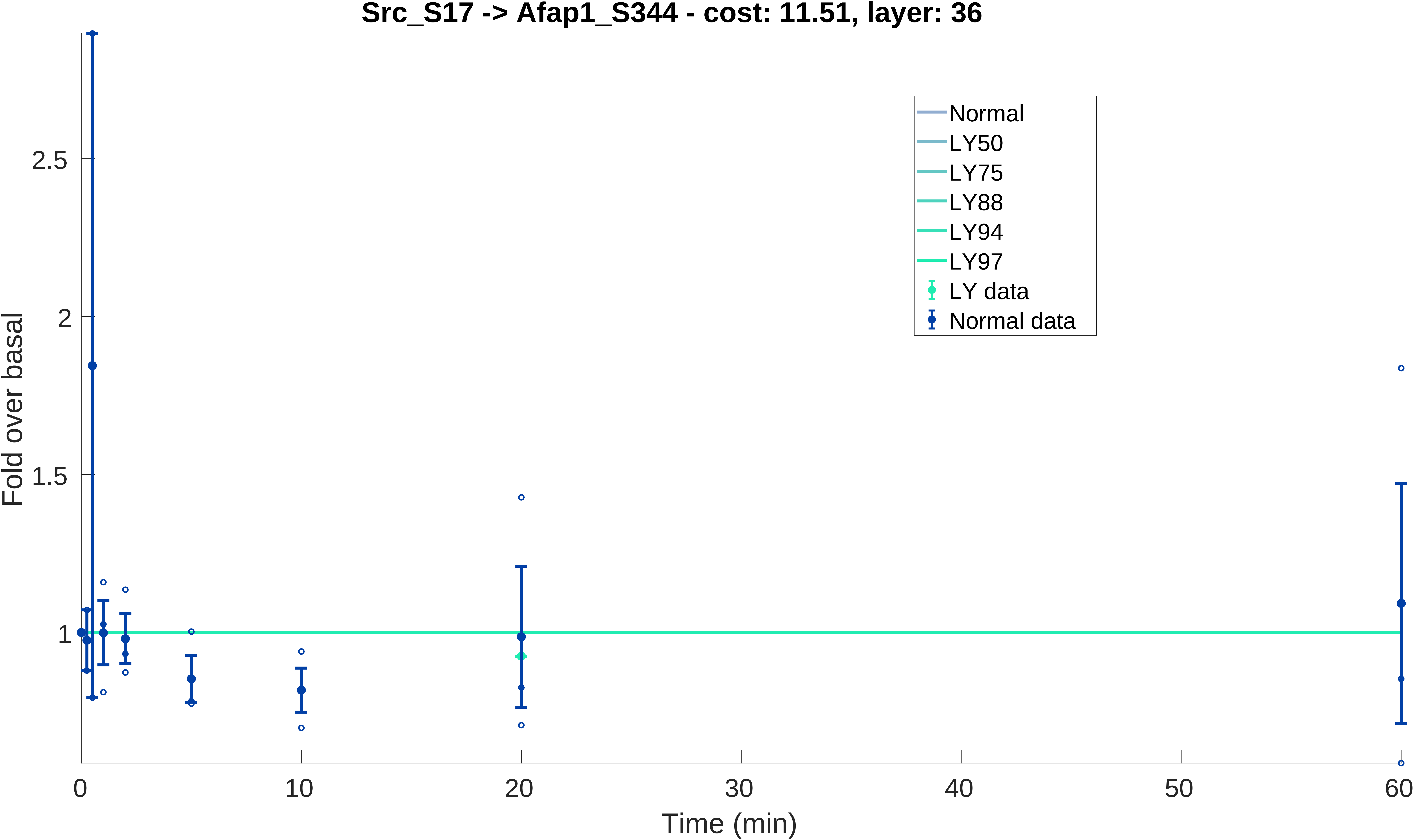

Src_S17 -> Afap1_S344 - cost: 11.51, layer: 36
Fold over basal
2.5
2
1.5
1
Time (min)
0
10
20
30
40
50
60
Normal
LY50
LY75
LY88
LY94
LY97
LY data
Normal data

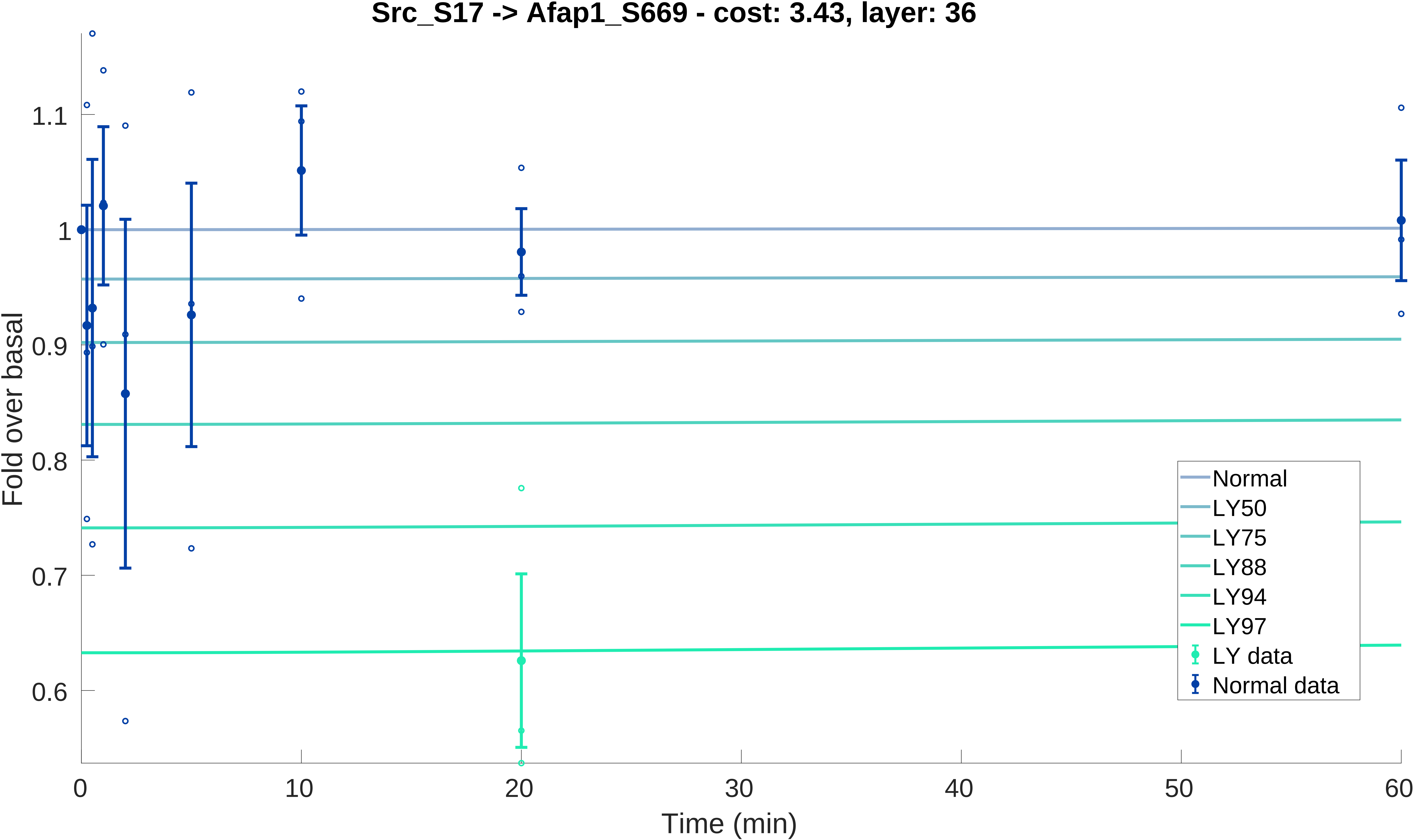

Src_S17 -> Afap1_S669 - cost: 3.43, layer: 36
Fold over basal
1.1
1
0.9
0.8
0.7
0.6
0
10
20
30
40
50
60
Time (min)
Normal
LY50
LY75
LY88
LY94
LY97
LY data
Normal data

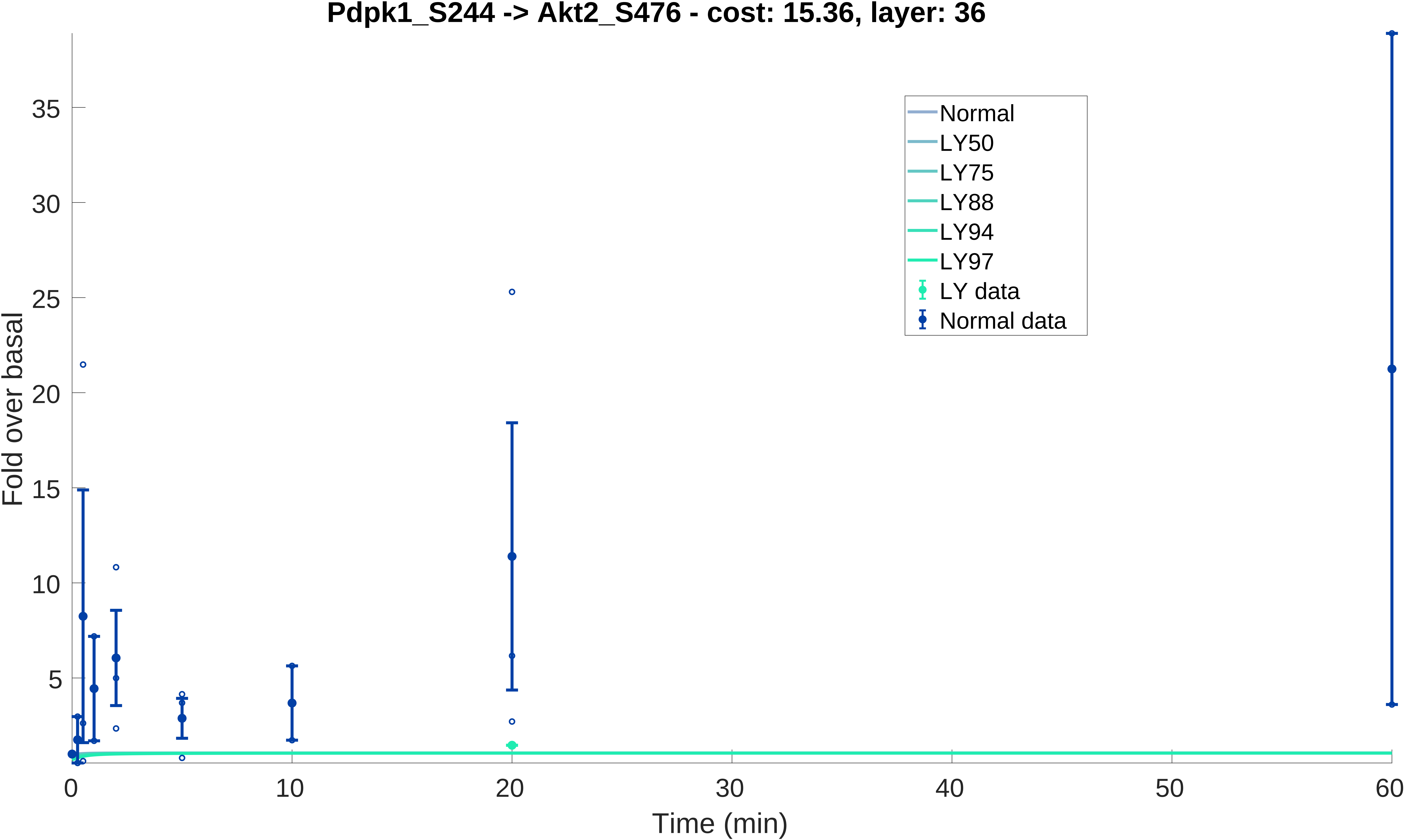
Pdpk1_S244 -> Akt2_S476 - cost: 15.36, layer: 36
Fold over basal
Time (min)
0
10
20
30
40
50
60
5
10
15
20
25
30
35
Normal
LY50
LY75
LY88
LY94
LY97
LY data
Normal data

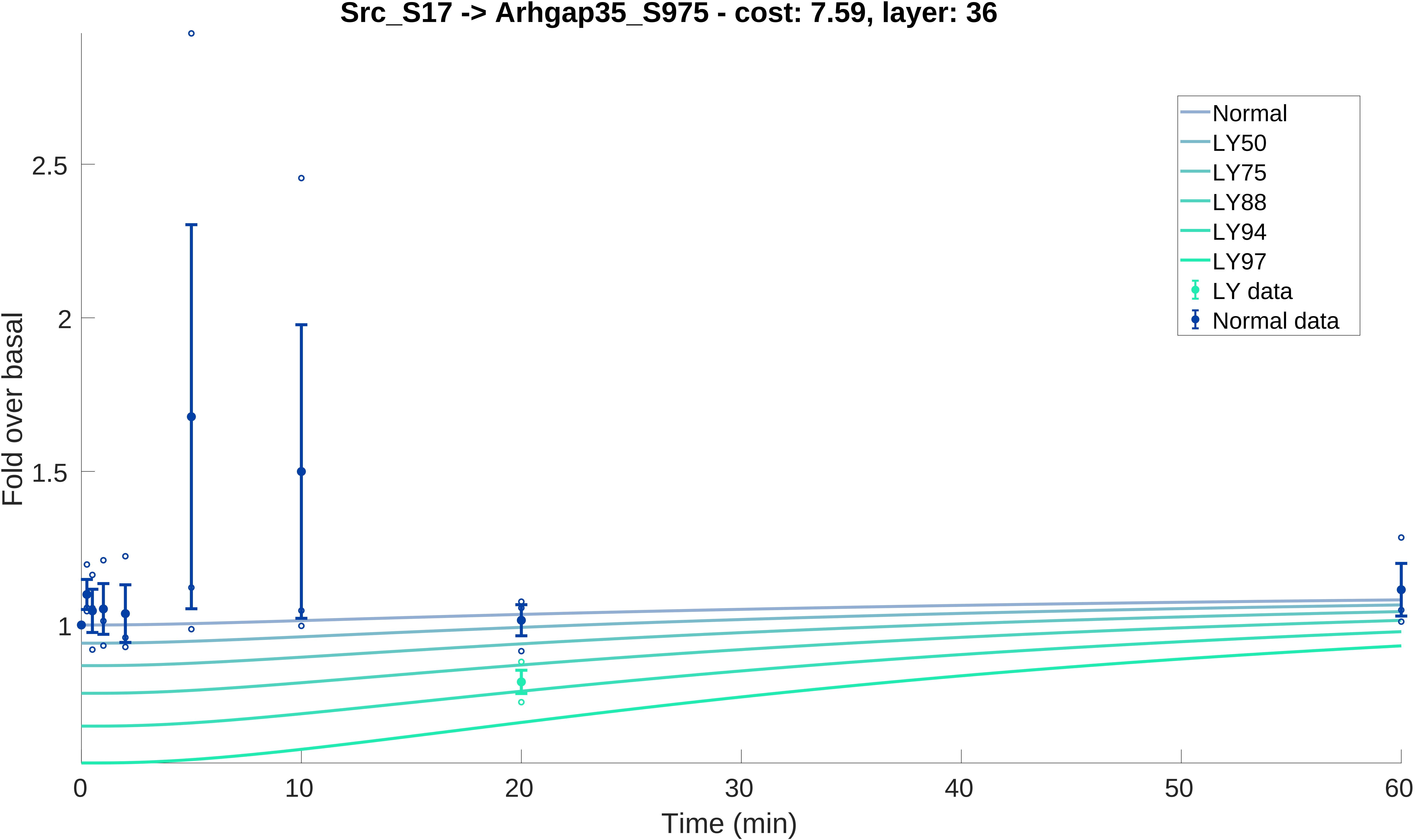

Src_S17 -> Arhgap35_S975 - cost: 7.59, layer: 36
Fold over basal
Time (min)
Normal
LY50
LY75
LY88
LY94
LY97
LY data
Normal data
2.5
2
1.5
1
0
10
20
30
40
50
60

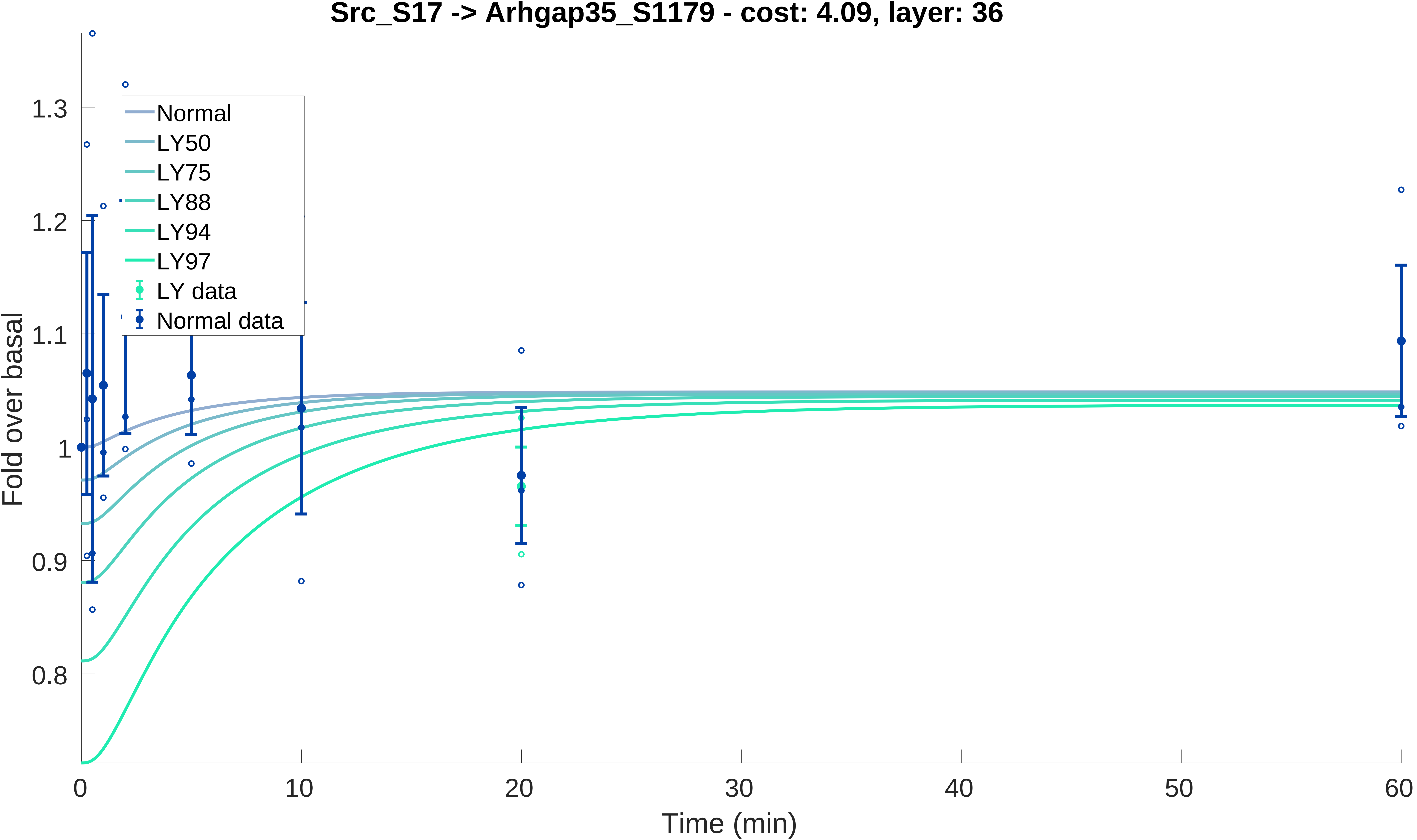
Src_S17 -> Arhgap35_S1179 - cost: 4.09, layer: 36
Fold over basal
1.3
1.2
1.1
1
0.9
0.8
0
10
20
30
40
50
60
Time (min)
Normal
LY50
LY75
LY88
LY94
LY97
LY data
Normal data

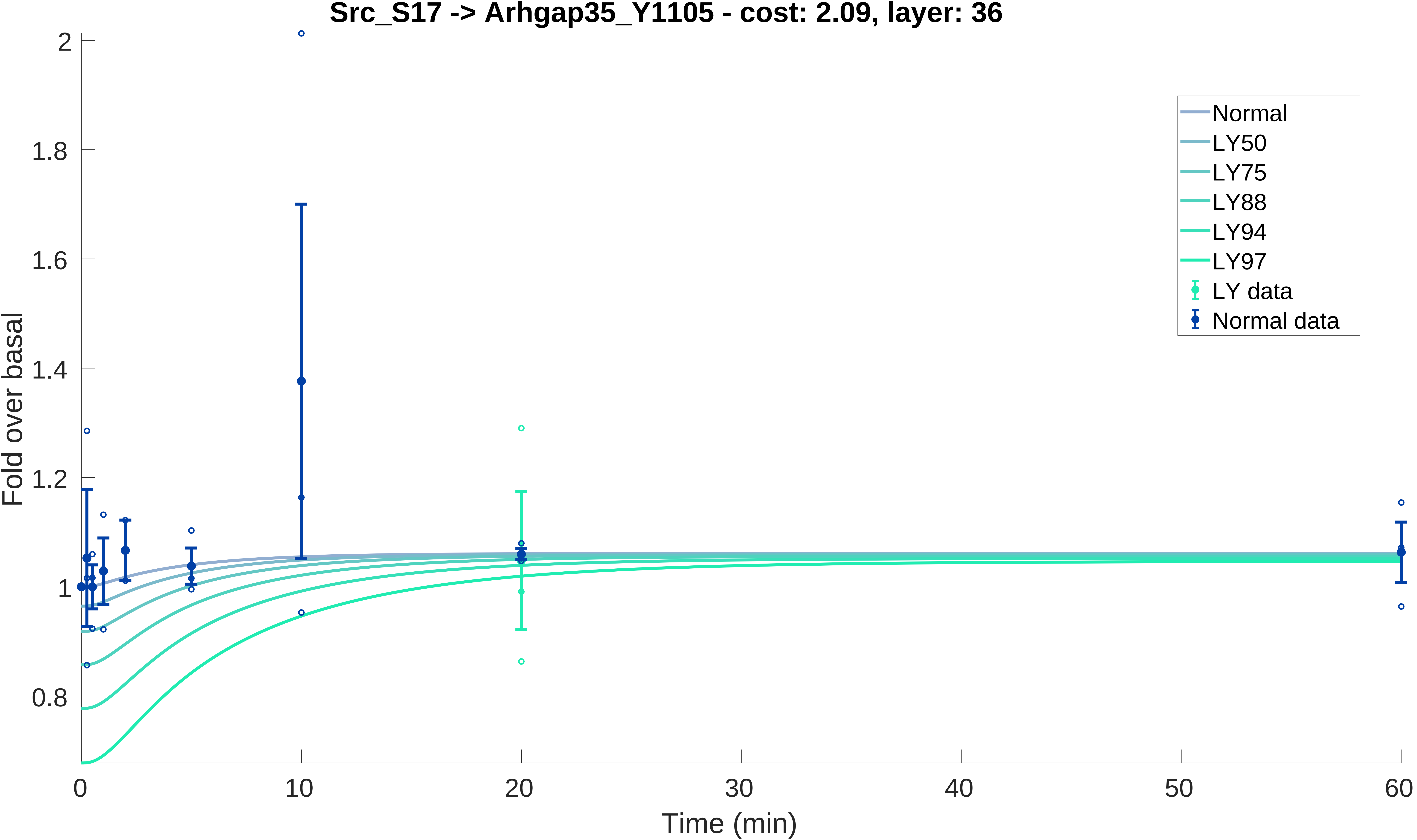
Src_S17 -> Arhgap35_Y1105 - cost: 2.09, layer: 36
Fold over basal
Time (min)
0.8
1
1.2
1.4
1.6
1.8
2
0
10
20
30
40
50
60
Normal
LY50
LY75
LY88
LY94
LY97
LY data
Normal data

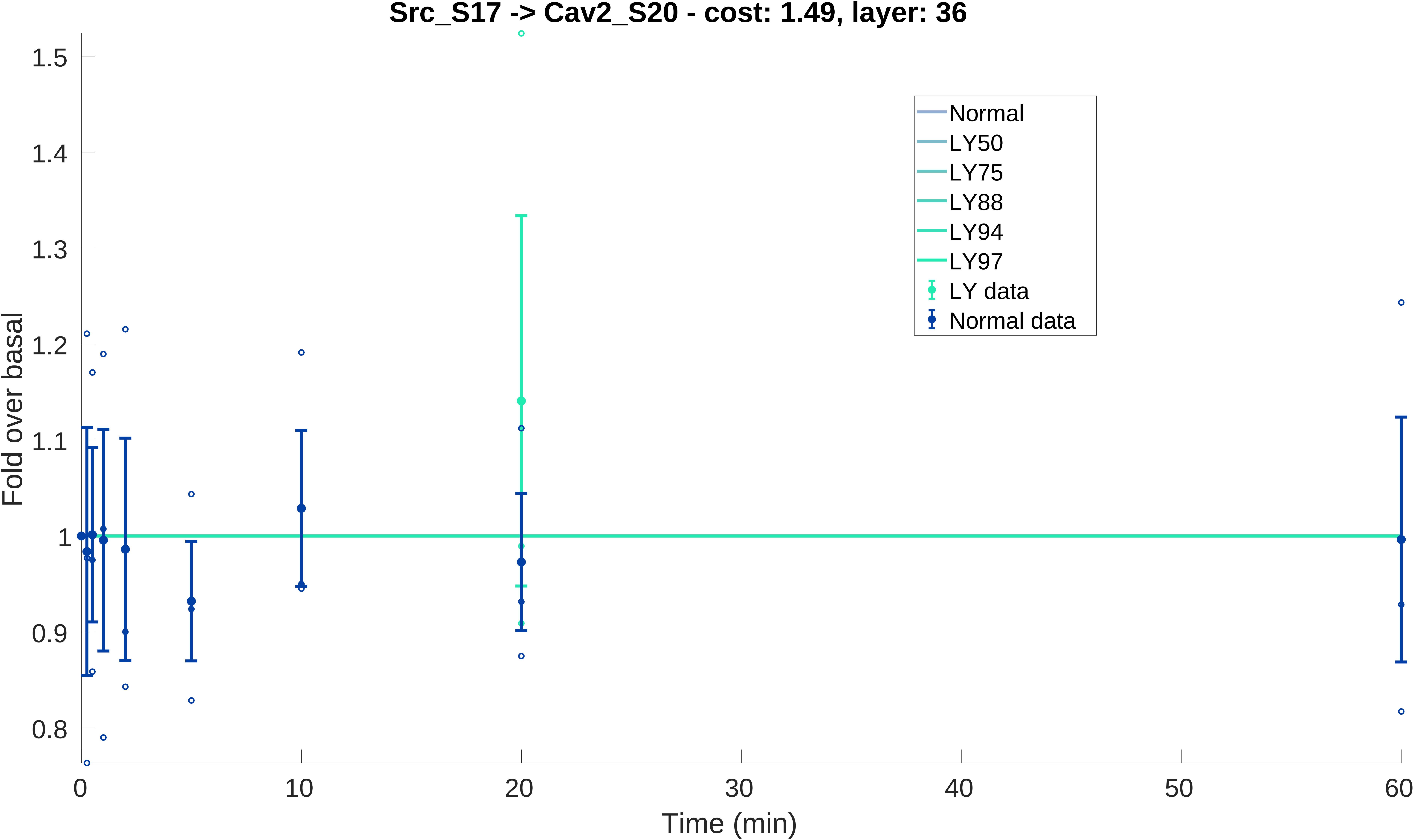

Src_S17 -> Cav2_S20 - cost: 1.49, layer: 36
Fold over basal
1.5
1.4
1.3
1.2
1.1
1
0.9
0.8
0
10
20
30
40
50
60
Time (min)
Normal
LY50
LY75
LY88
LY94
LY97
LY data
Normal data

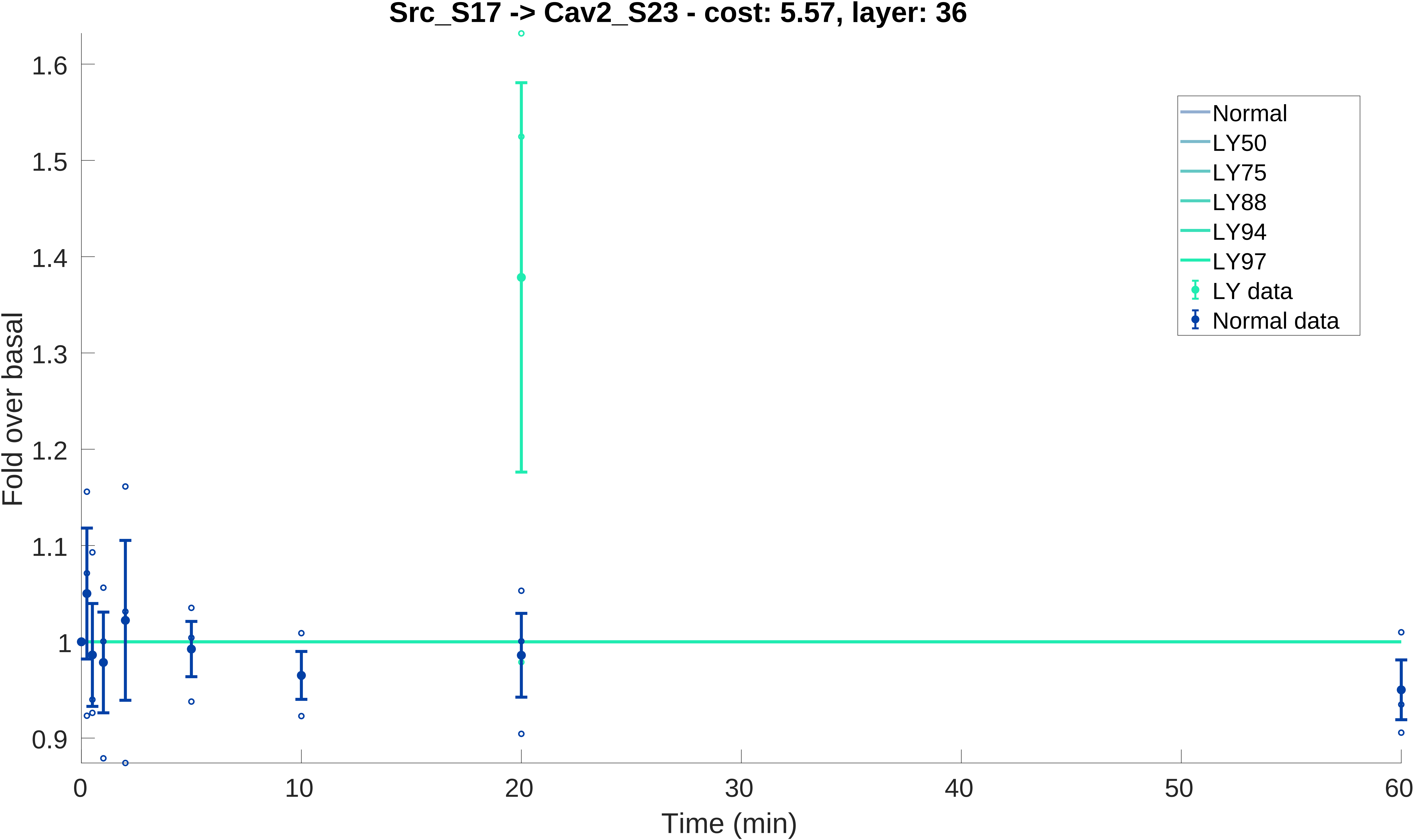
Src_S17 -> Cav2_S23 - cost: 5.57, layer: 36
Fold over basal
1.6
1.5
1.4
1.3
1.2
1.1
1
0.9
0
10
20
30
40
50
60
Time (min)
Normal
LY50
LY75
LY88
LY94
LY97
LY data
Normal data

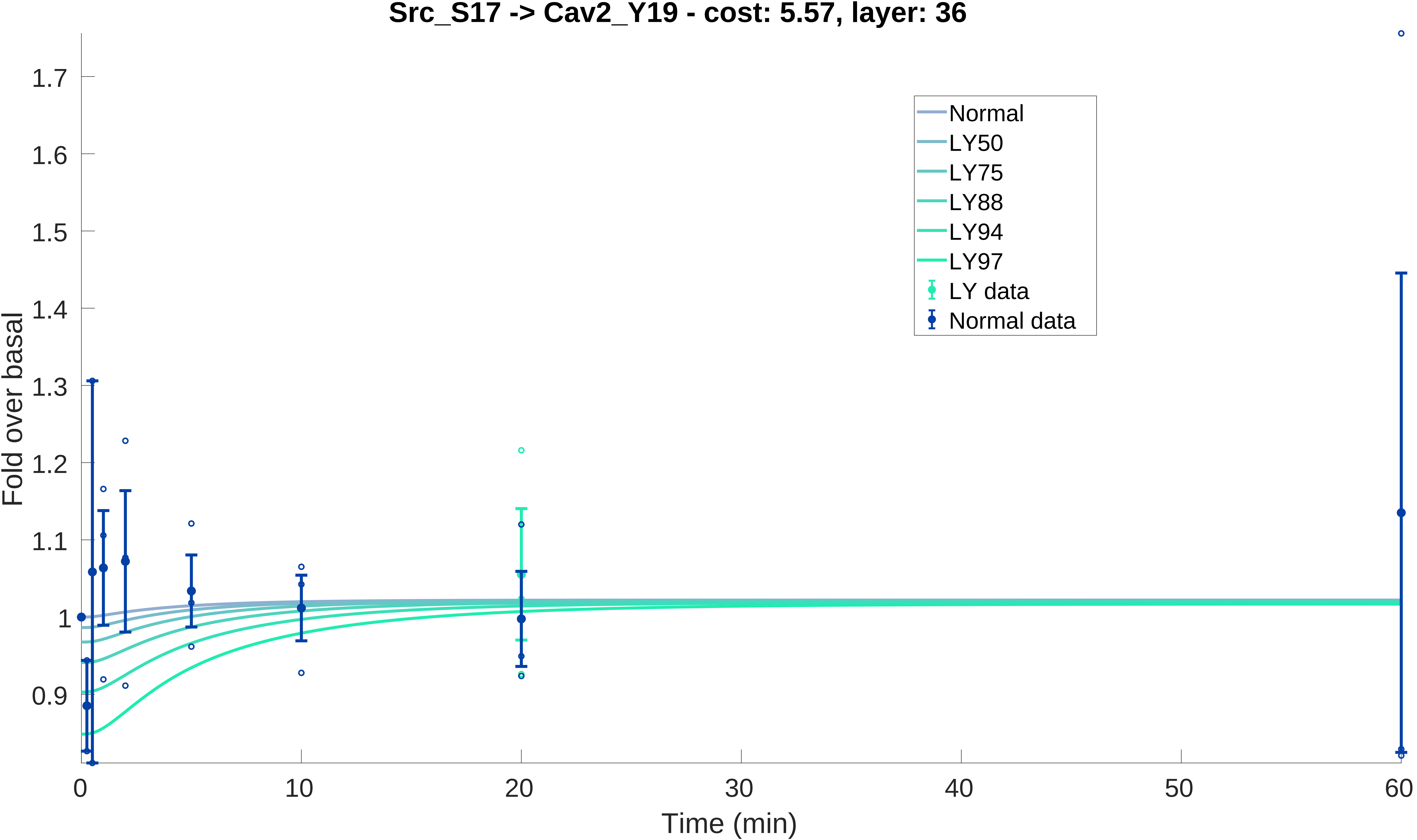
Src_S17 -> Cav2_Y19 - cost: 5.57, layer: 36
Fold over basal
Time (min)
0.9
1
1.1
1.2
1.3
1.4
1.5
1.6
1.7
0
10
20
30
40
50
60
Normal
LY50
LY75
LY88
LY94
LY97
LY data
Normal data

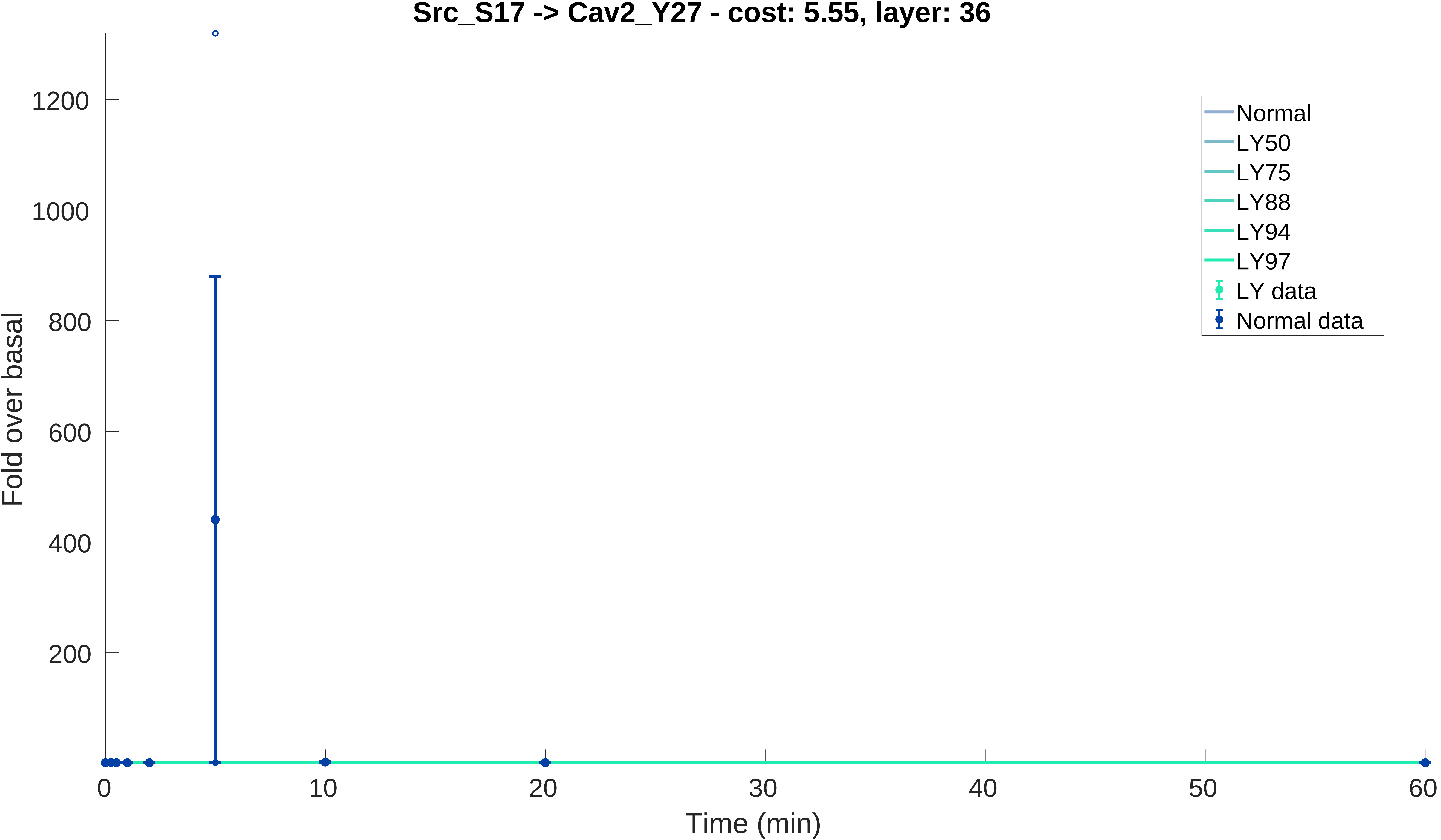

Src_S17 -> Cav2_Y27 - cost: 5.55, layer: 36
1200
1000
800
600
400
200
Fold over basal
0
10
20
30
40
50
60
Time (min)
Normal
LY50
LY75
LY88
LY94
LY97
LY data
Normal data

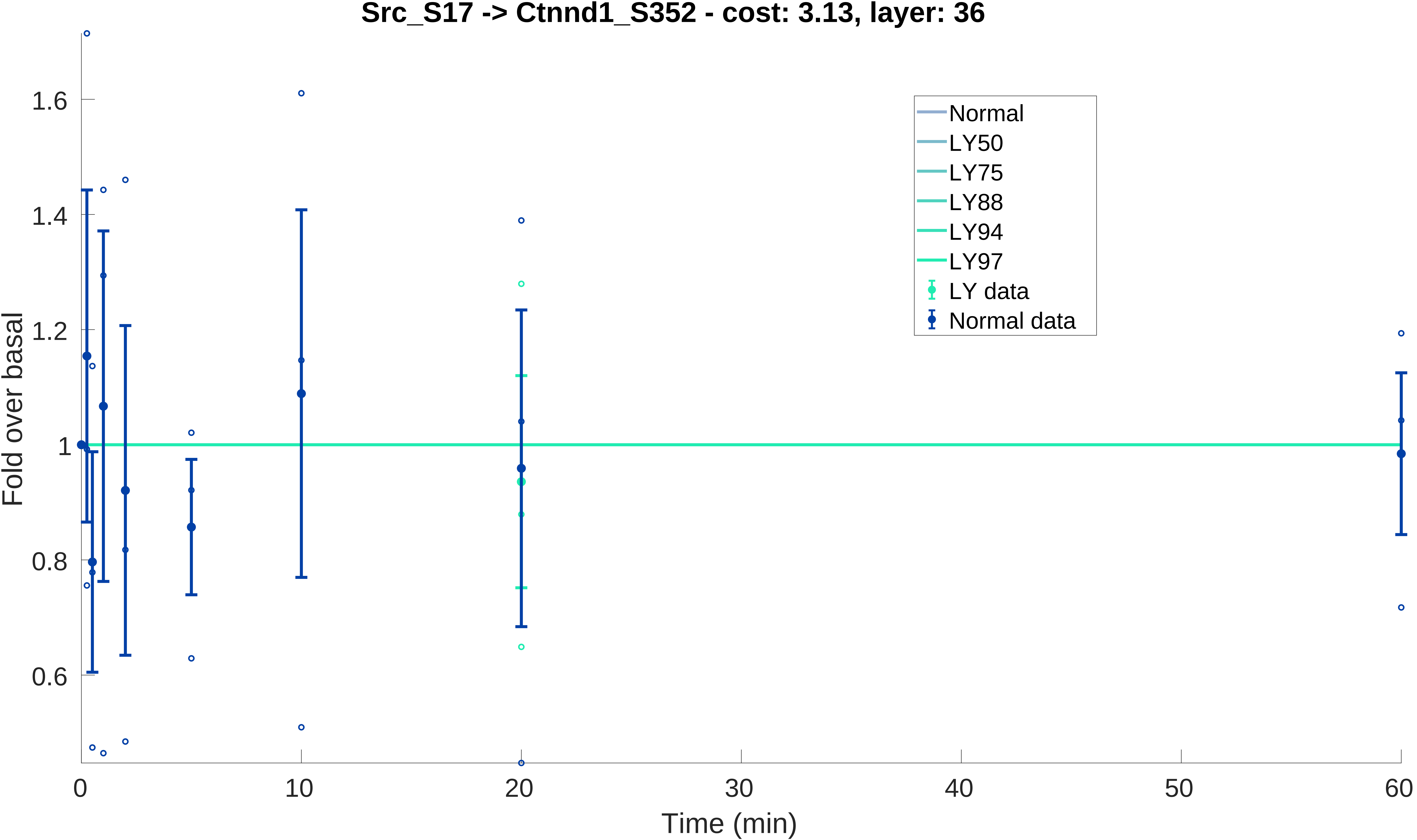

Src_S17 -> Ctnnd1_S352 - cost: 3.13, layer: 36
Fold over basal
Time (min)
1.6
1.4
1.2
1
0.8
0.6
0
10
20
30
40
50
60
Normal
LY50
LY75
LY88
LY94
LY97
LY data
Normal data

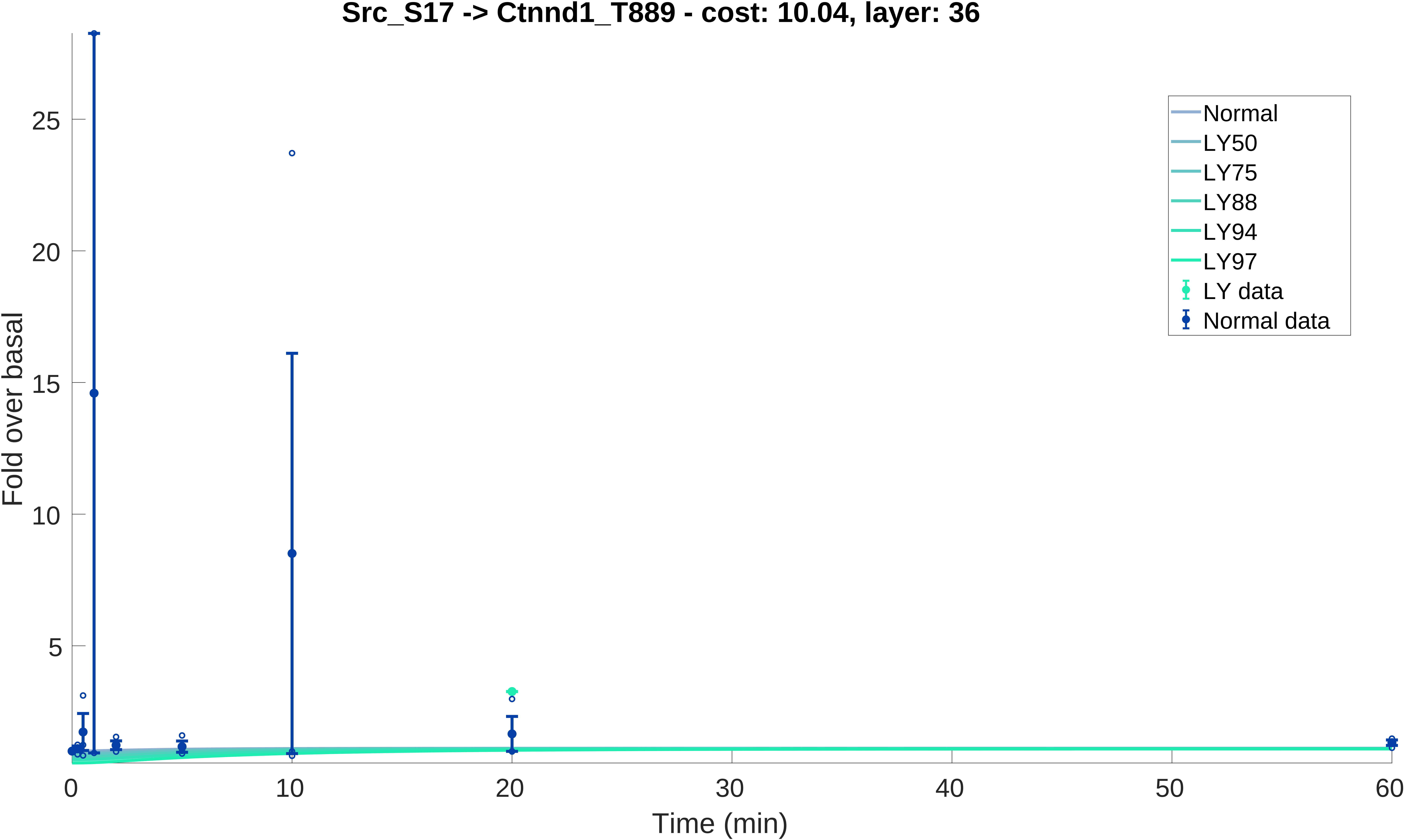

Src_S17 -> Ctnnd1_T889 - cost: 10.04, layer: 36
Fold over basal
Time (min)
0
10
20
30
40
50
60
5
10
15
20
25
Normal
LY50
LY75
LY88
LY94
LY97
LY data
Normal data

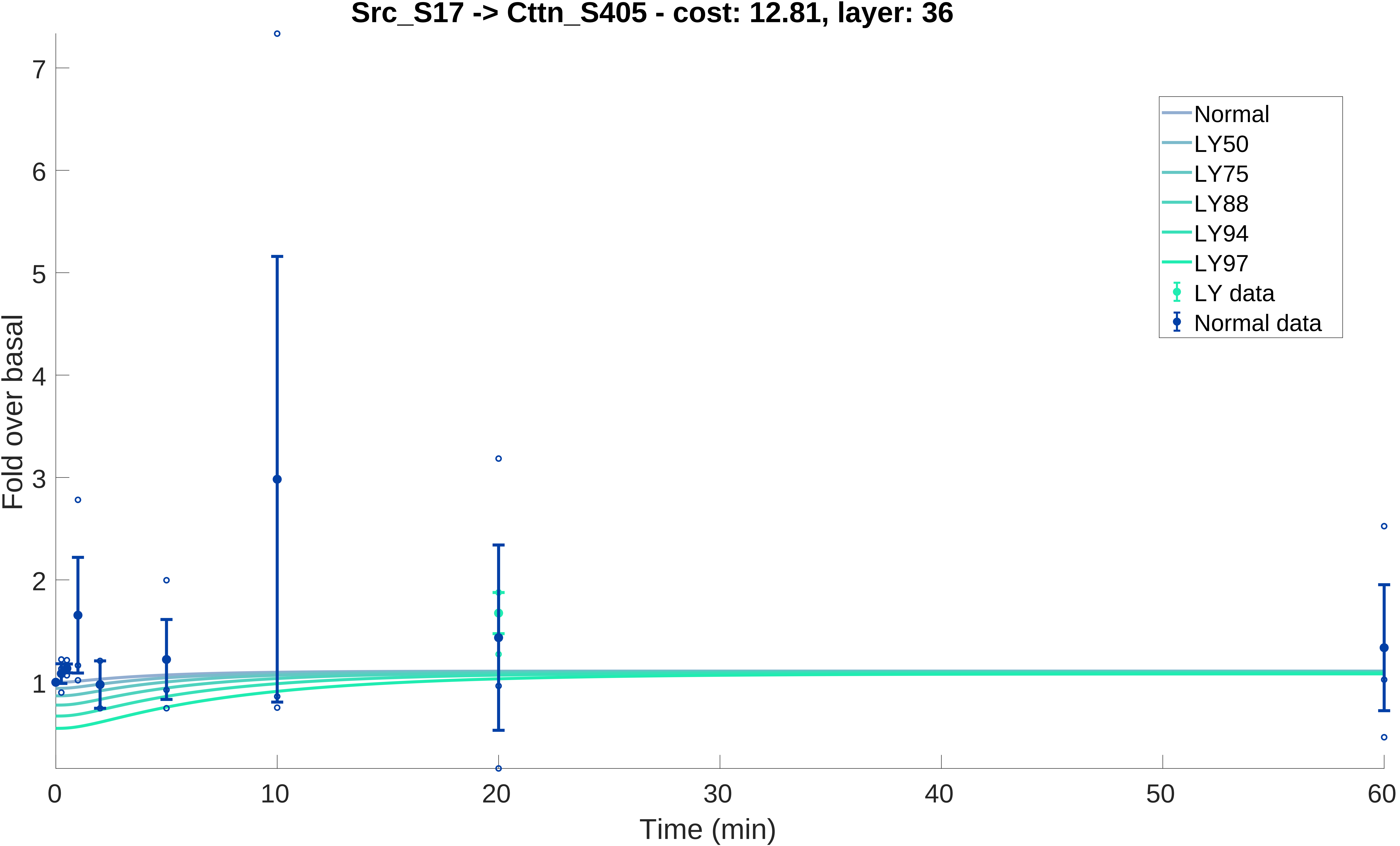

Src_S17 -> Cttn_S405 - cost: 12.81, layer: 36
Fold over basal
Time (min)
Normal
LY50
LY75
LY88
LY94
LY97
LY data
Normal data
0
10
20
30
40
50
60
1
2
3
4
5
6
7

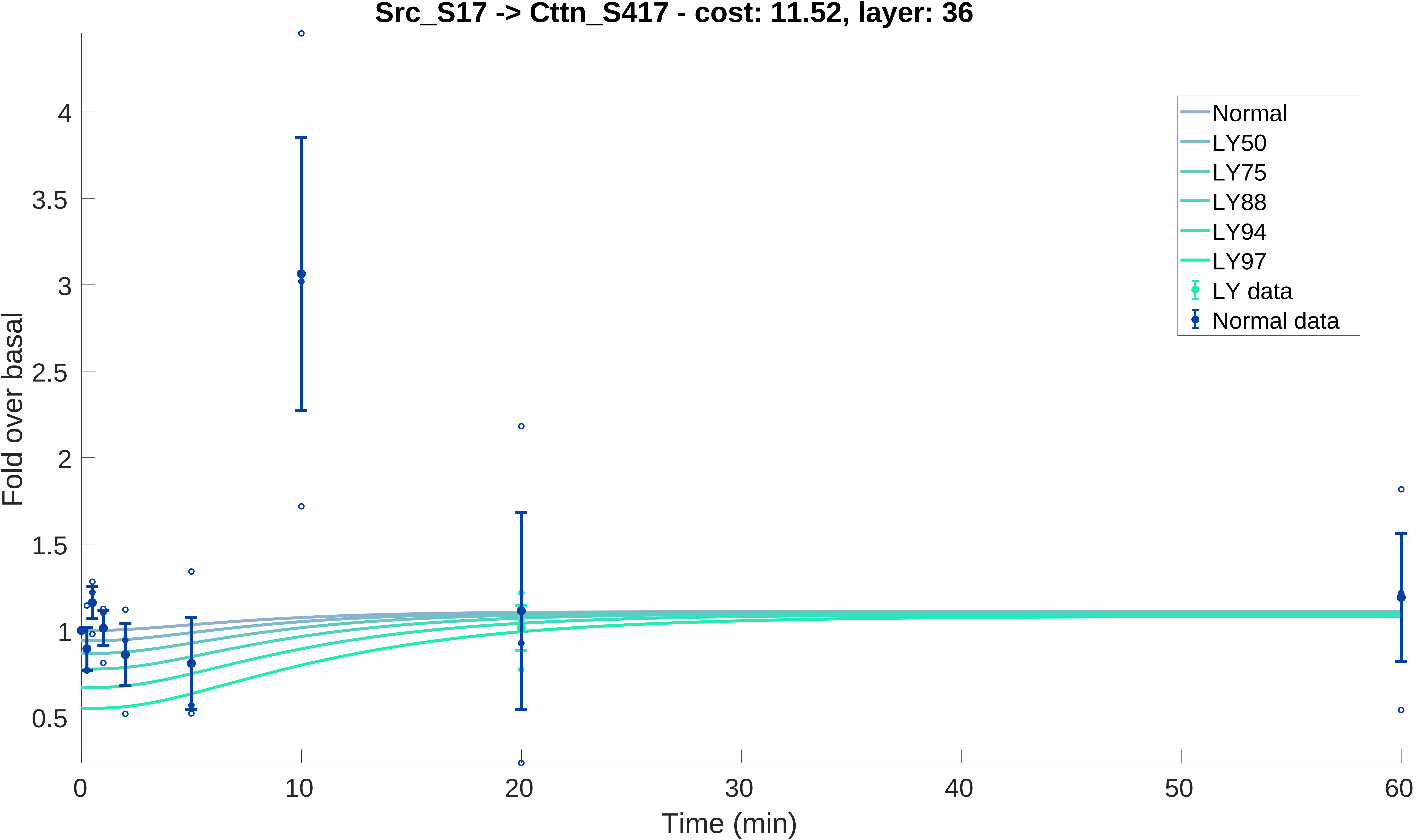
Src_S17 -> Cttn_S417 - cost: 11.52, layer: 36
Fold over basal
Time (min)
0
10
20
30
40
50
60
0.5
1
1.5
2
2.5
3
3.5
4
Normal
LY50
LY75
LY88
LY94
LY97
LY data
Normal data

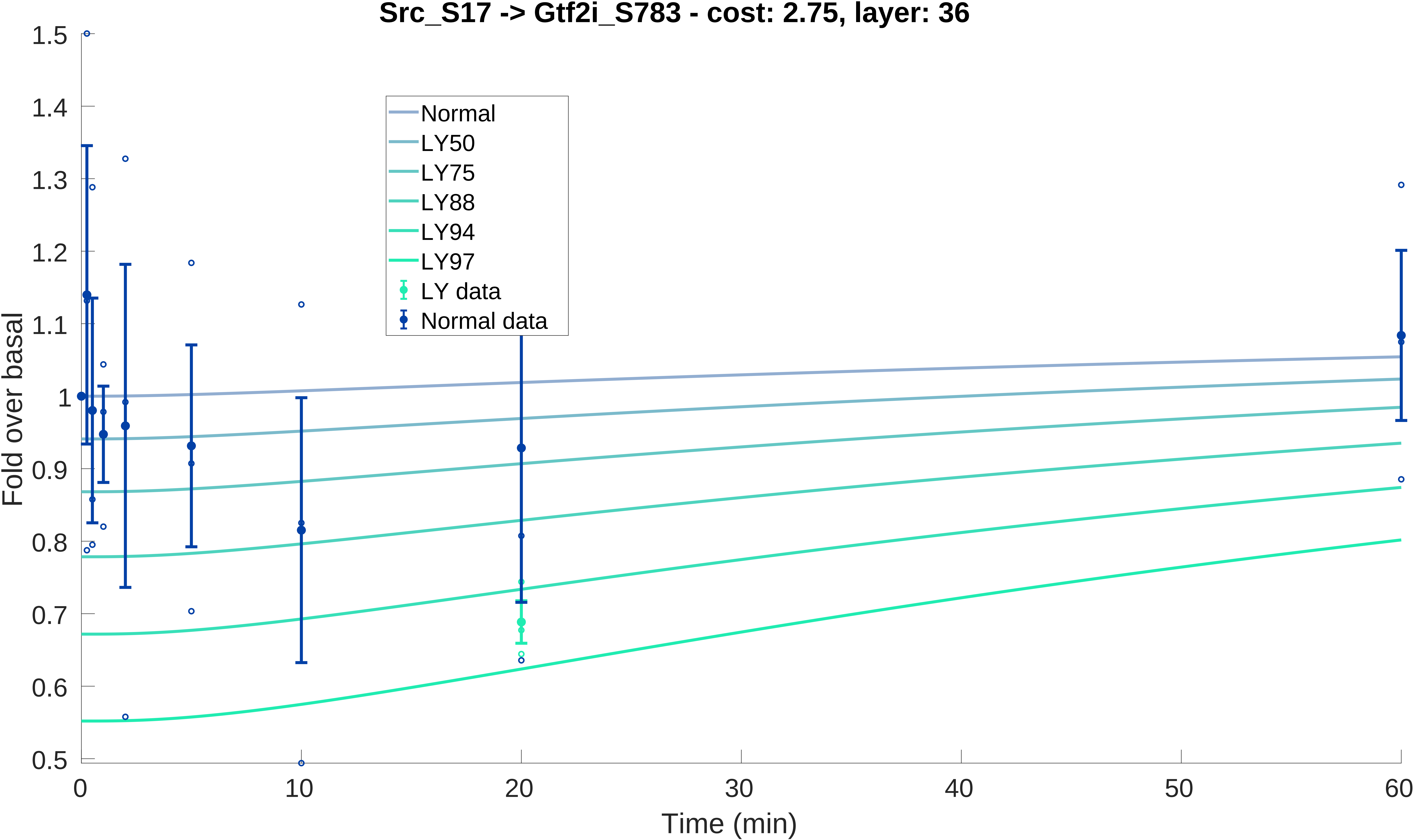

Src_S17 -> Gtf2i_S783 - cost: 2.75, layer: 36
Fold over basal
Time (min)
Normal
LY50
LY75
LY88
LY94
LY97
LY data
Normal data
1.5
1.4
1.3
1.2
1.1
1
0.9
0.8
0.7
0.6
0.5
0
10
20
30
40
50
60

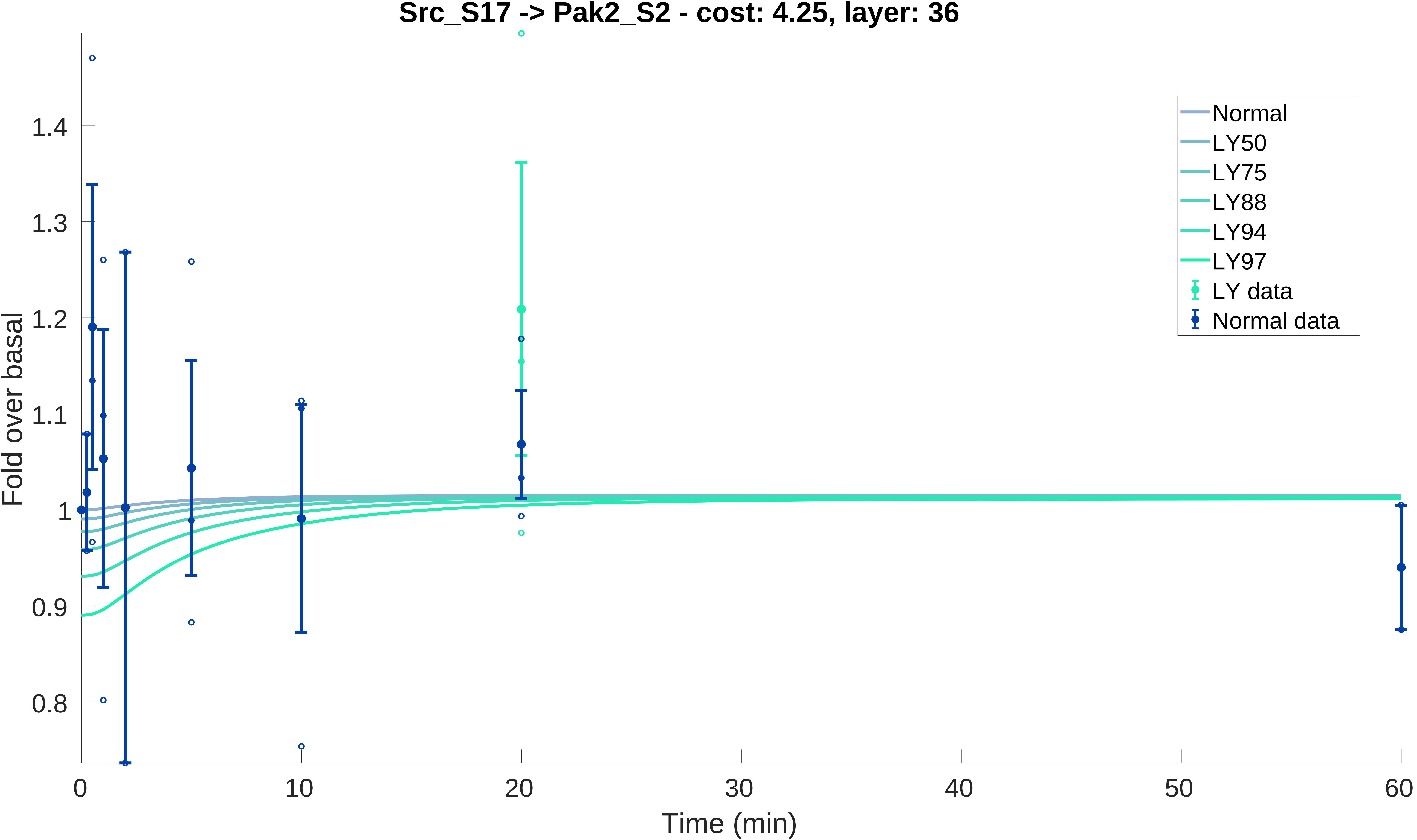

Src_S17 -> Pak2_S2 - cost: 4.25, layer: 36
Normal
LY50
LY75
LY88
LY94
LY97
LY data
Normal data
Fold over basal
1.4
1.3
1.2
1.1
1
0.9
0.8
0
10
20
30
40
50
60
Time (min)

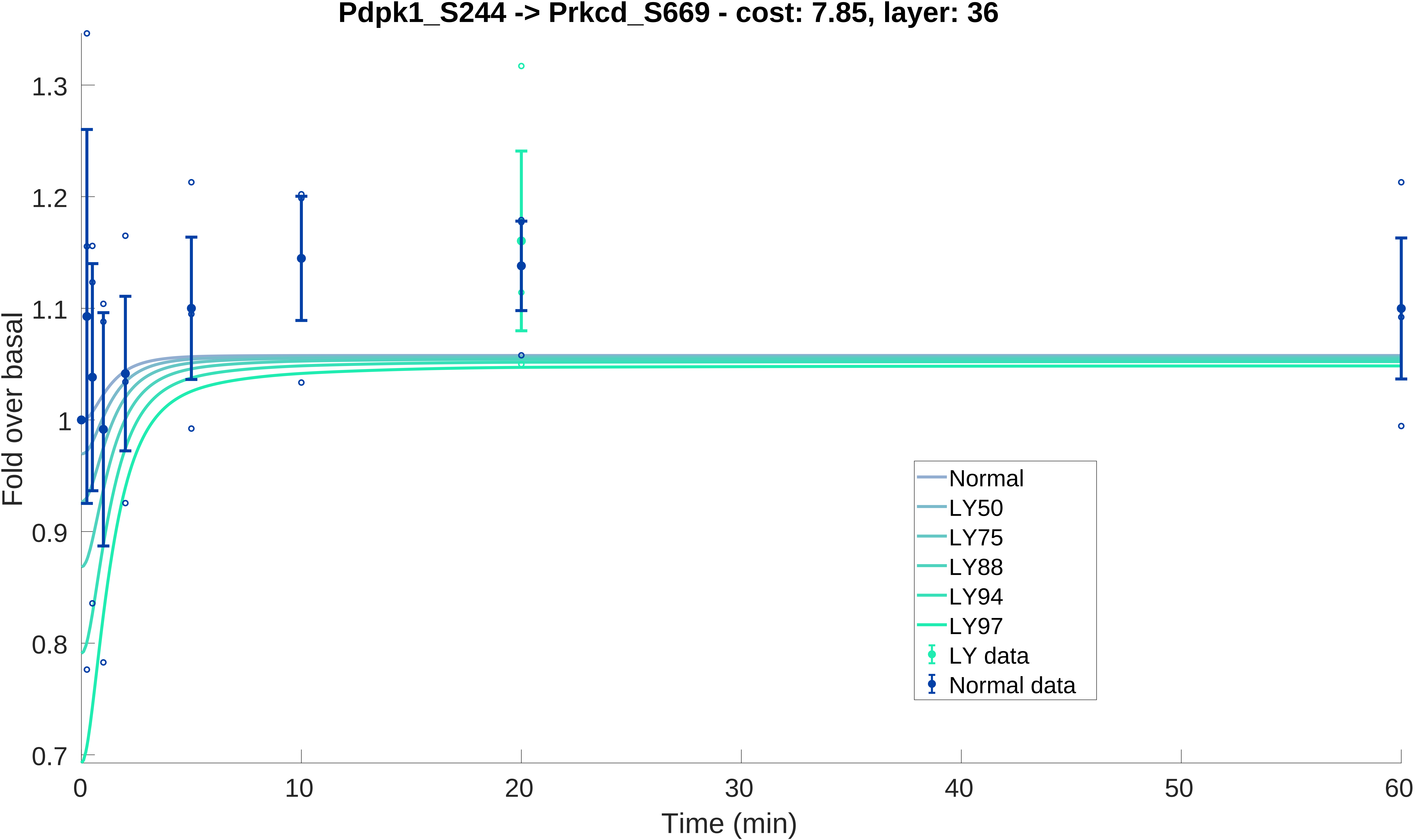

Pdpk1_S244 -> Prkcd_S669 - cost: 7.85, layer: 36
Fold over basal
Time (min)
1.3
1.2
1.1
1
0.9
0.8
0.7
0
10
20
30
40
50
60
Normal
LY50
LY75
LY88
LY94
LY97
LY data
Normal data

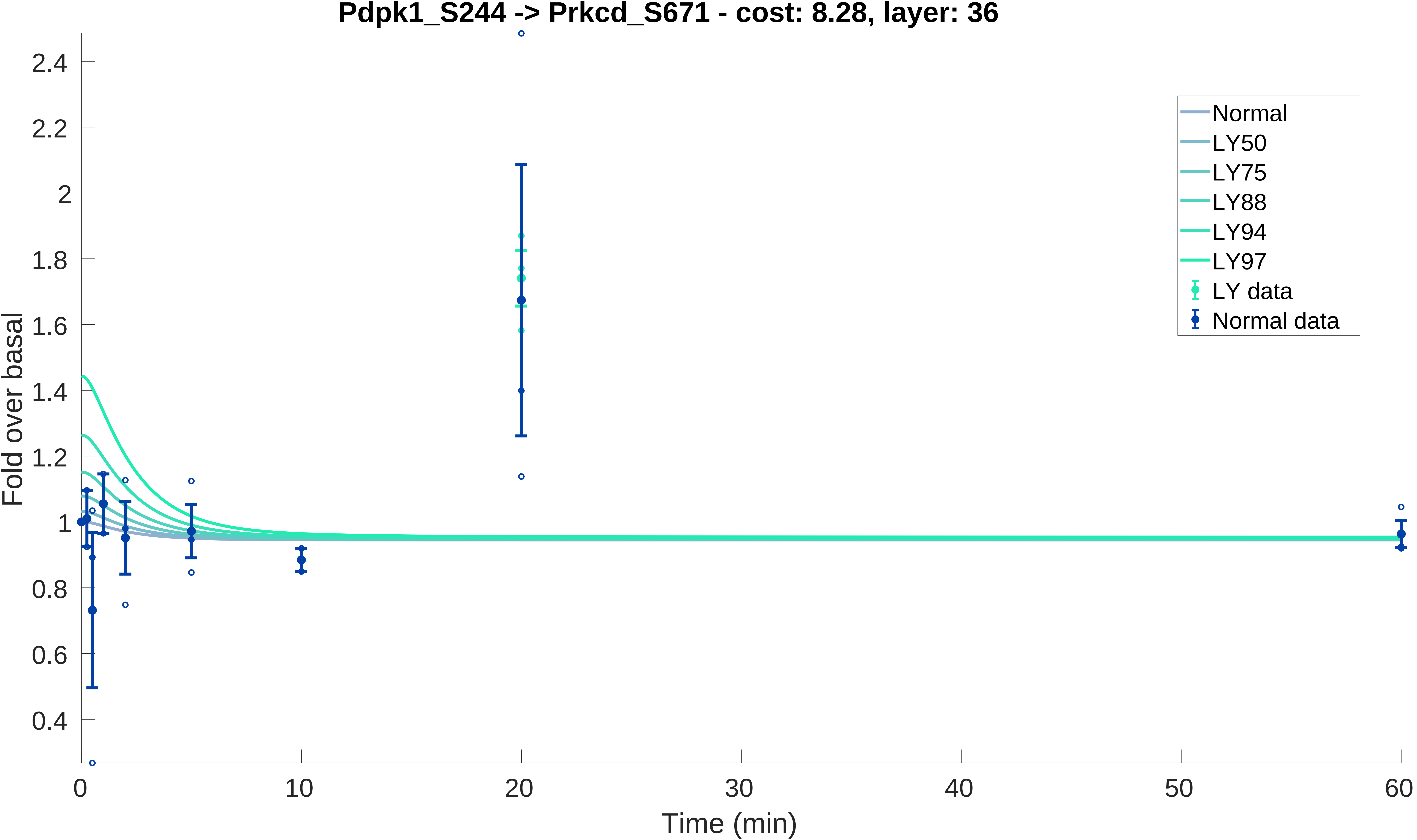

Pdpk1_S244 -> Prkcd_S671 - cost: 8.28, layer: 36
Fold over basal
Time (min)
Normal
LY50
LY75
LY88
LY94
LY97
LY data
Normal data
0.4
0.6
0.8
1
1.2
1.4
1.6
1.8
2
2.2
2.4
0
10
20
30
40
50
60

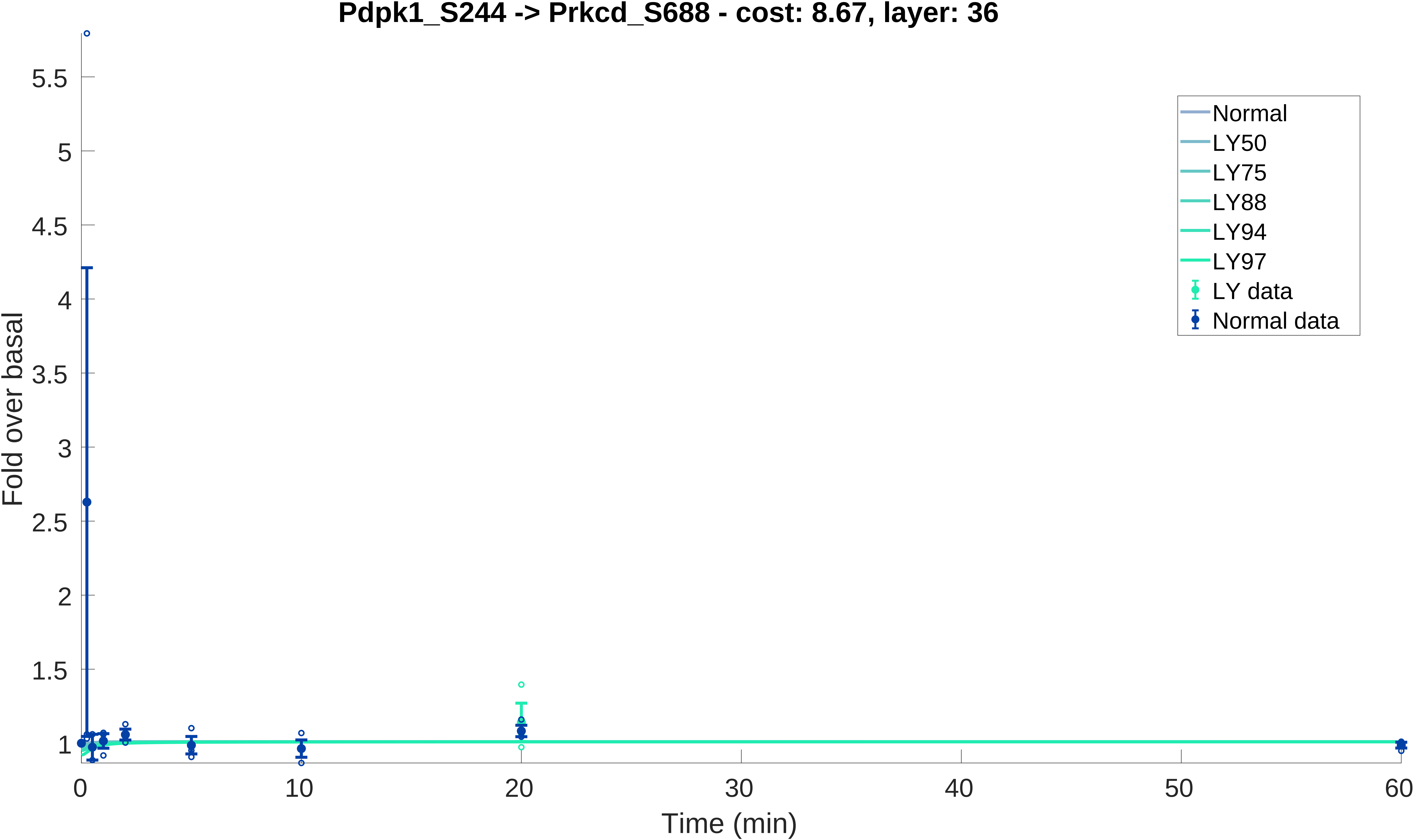

Pdpk1_S244 -> Prkcd_S688 - cost: 8.67, layer: 36
Fold over basal
Time (min)
0
10
20
30
40
50
60
1
1.5
2
2.5
3
3.5
4
4.5
5
5.5
Normal
LY50
LY75
LY88
LY94
LY97
LY data
Normal data

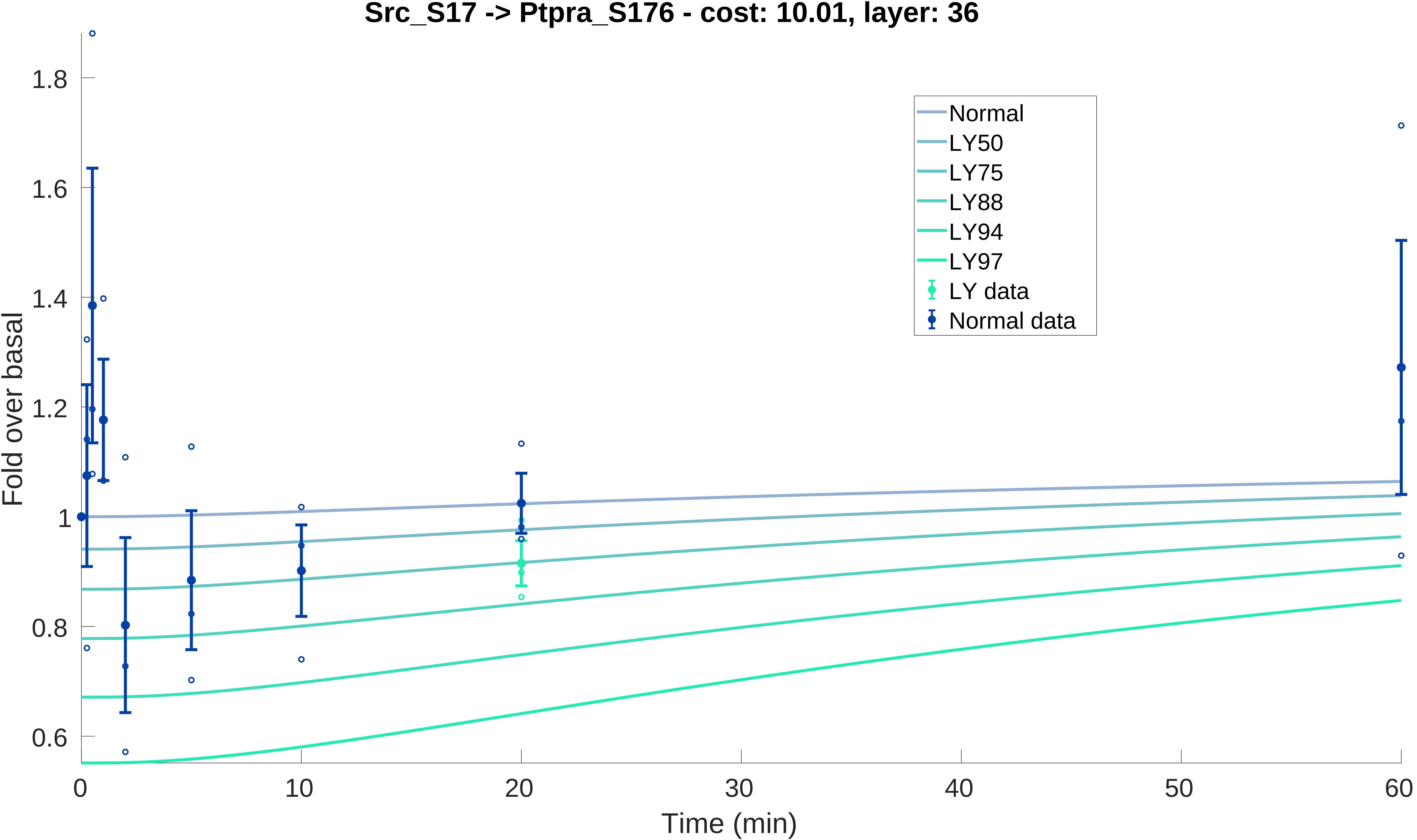

Src_S17 -> Ptpra_S176 - cost: 10.01, layer: 36
Fold over basal
Time (min)
0.6
0.8
1
1.2
1.4
1.6
1.8
0
10
20
30
40
50
60
Normal
LY50
LY75
LY88
LY94
LY97
LY data
Normal data

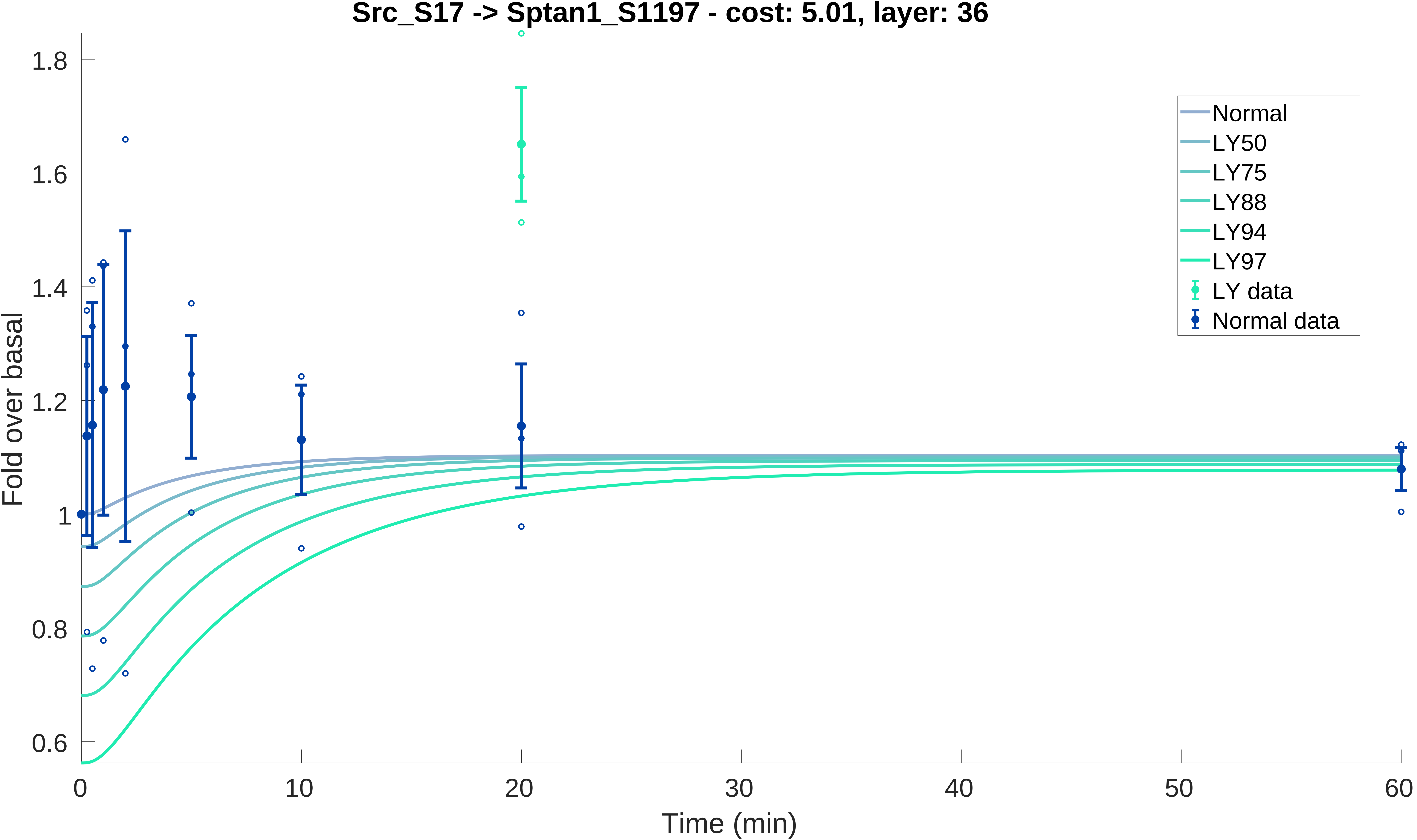
Src_S17 -> Sptan1_S1197 - cost: 5.01, layer: 36
Fold over basal
Time (min)
1.8
1.6
1.4
1.2
1
0.8
0.6
0
10
20
30
40
50
60
Normal
LY50
LY75
LY88
LY94
LY97
LY data
Normal data

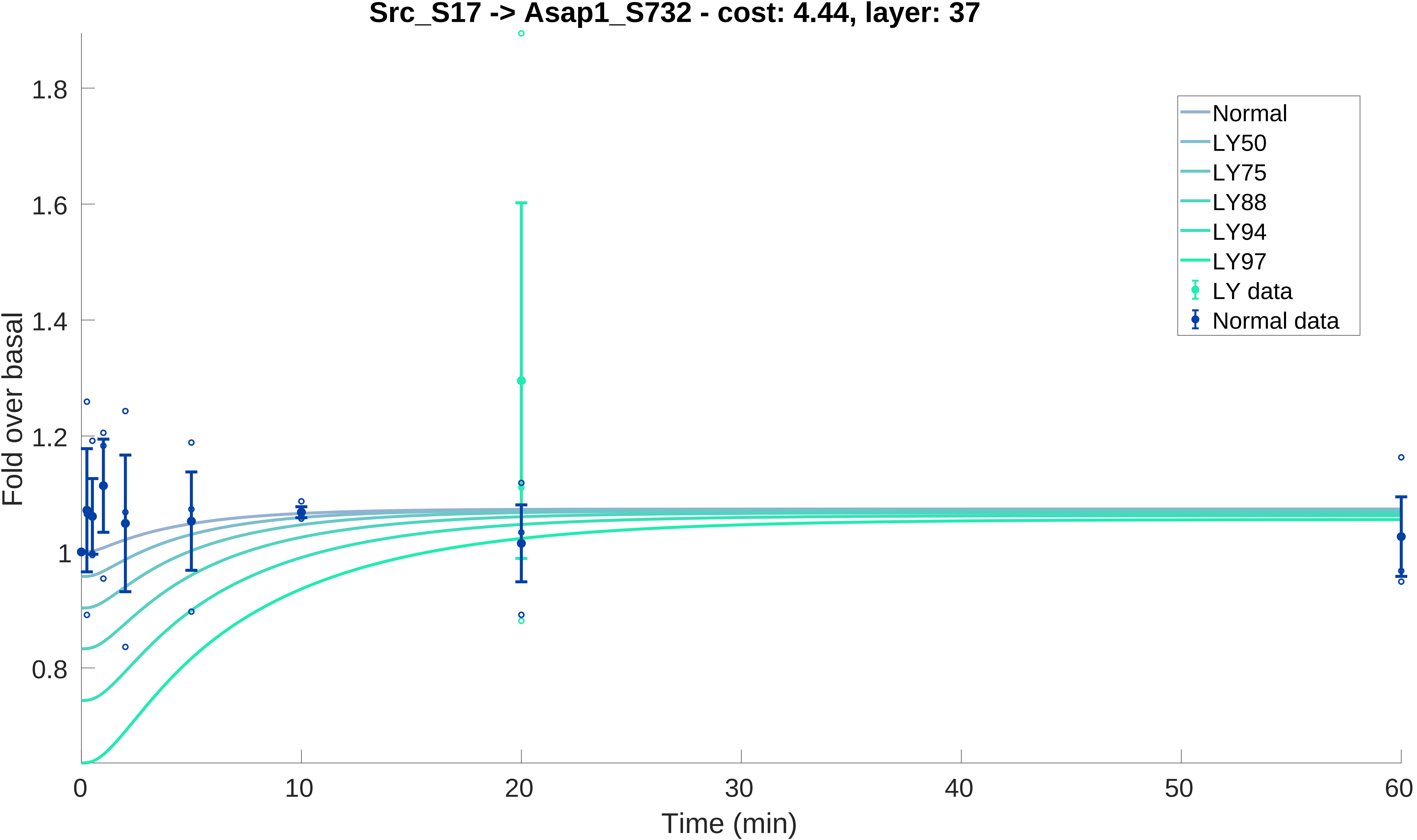

Src_S17 -> Asap1_S732 - cost: 4.44, layer: 37
Fold over basal
1.8
1.6
1.4
1.2
1
0.8
0
10
20
30
40
50
60
Time (min)
Normal
LY50
LY75
LY88
LY94
LY97
LY data
Normal data

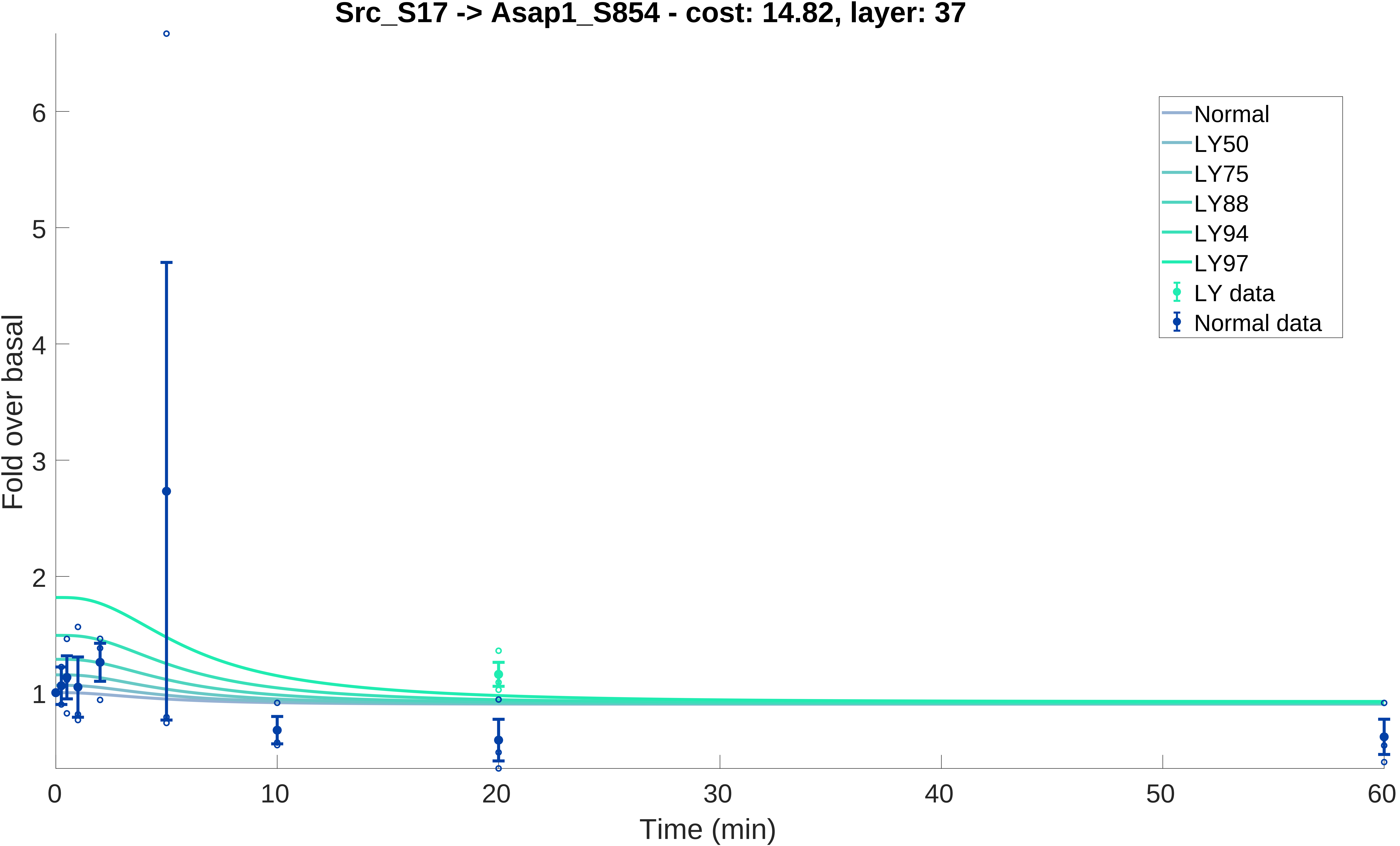

Src_S17 -> Asap1_S854 - cost: 14.82, layer: 37
Fold over basal
Time (min)
0
10
20
30
40
50
60
1
2
3
4
5
6
Normal
LY50
LY75
LY88
LY94
LY97
LY data
Normal data

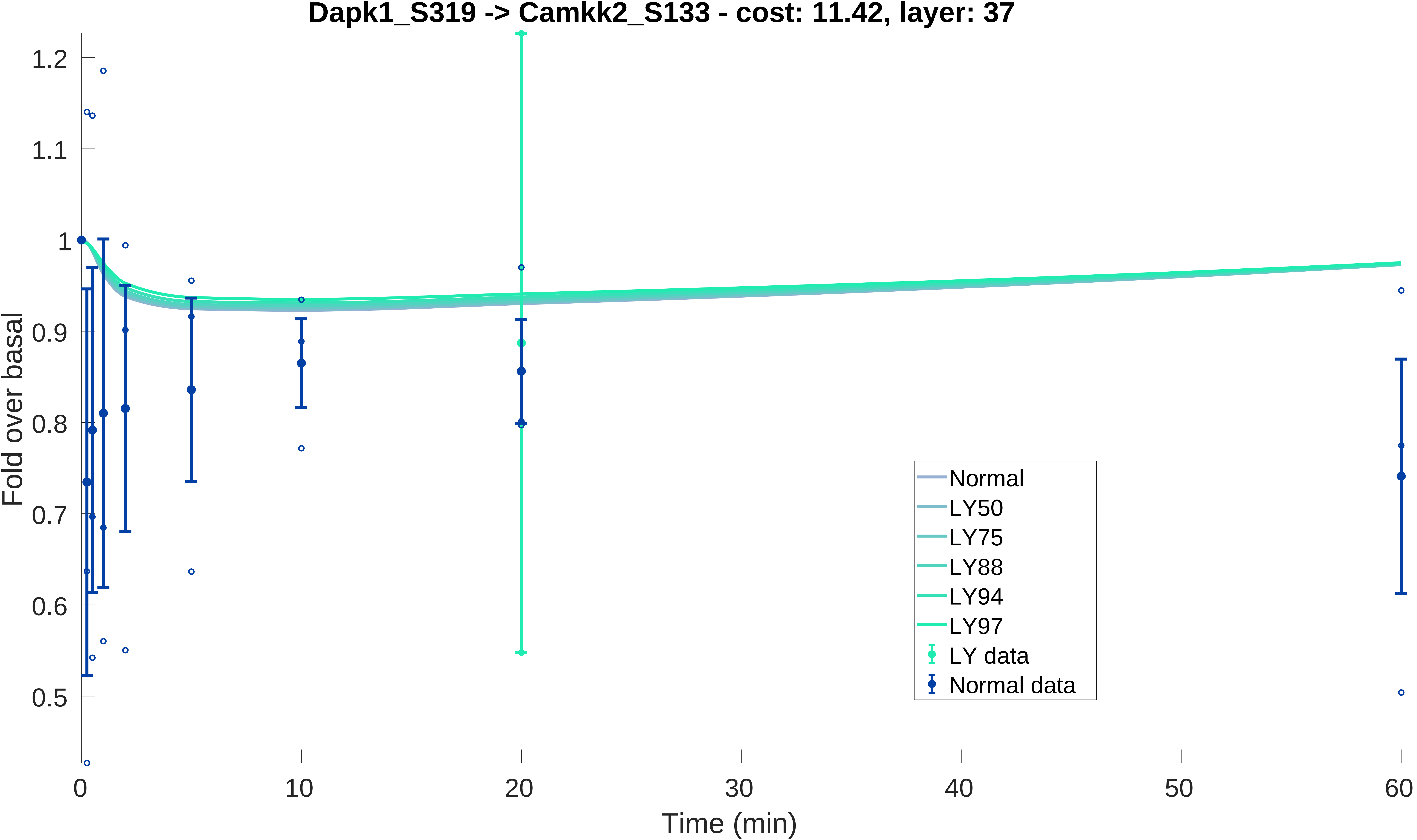

Dapk1_S319 -> Camkk2_S133 - cost: 11.42, layer: 37
Fold over basal
1.2
1.1
1
0.9
0.8
0.7
0.6
0.5
0
10
20
30
40
50
60
Time (min)
Normal
LY50
LY75
LY88
LY94
LY97
LY data
Normal data

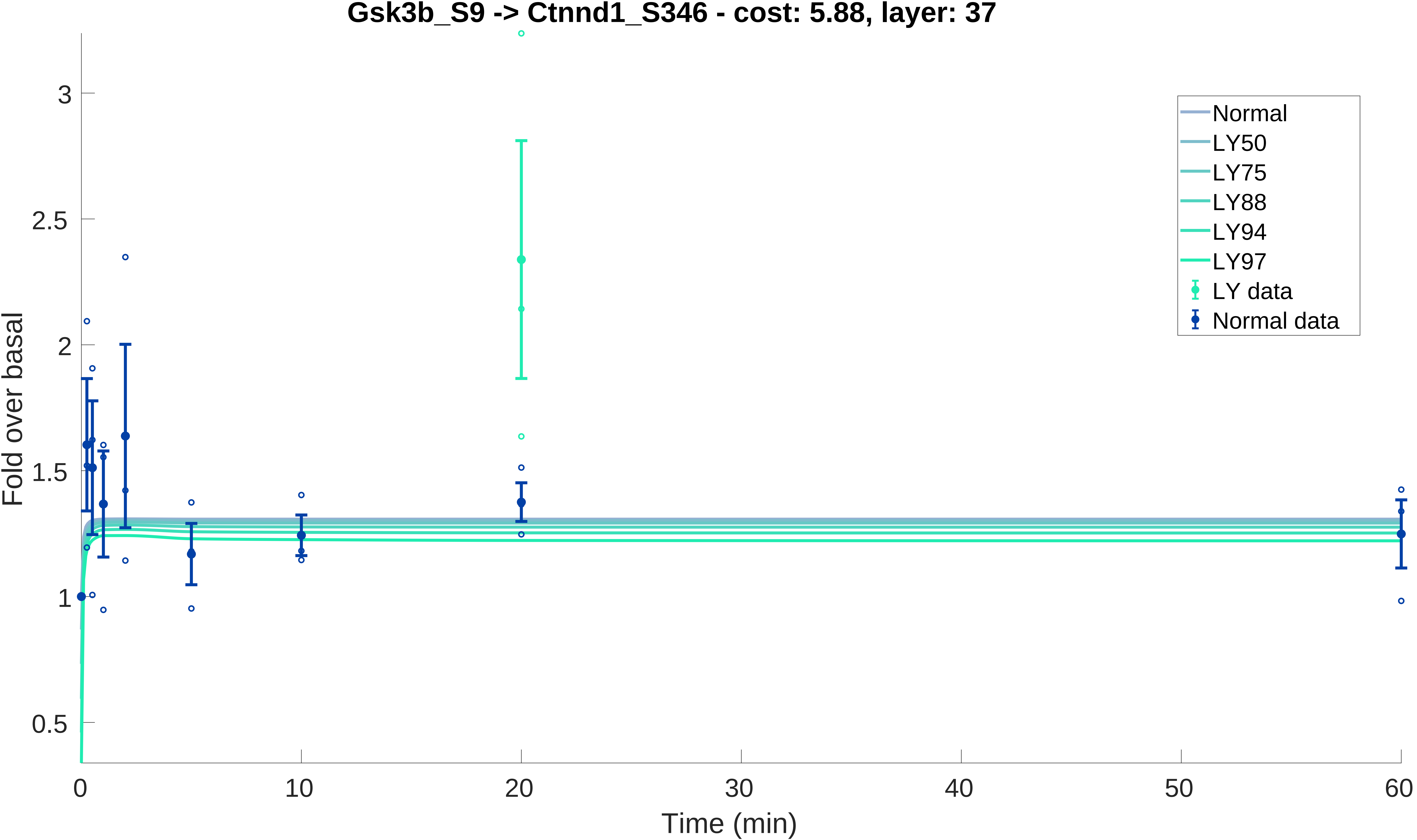

Gsk3b_S9 -> Ctnnd1_S346 - cost: 5.88, layer: 37
Fold over basal
Time (min)
0.5
1
1.5
2
2.5
3
0
10
20
30
40
50
60
Normal
LY50
LY75
LY88
LY94
LY97
LY data
Normal data

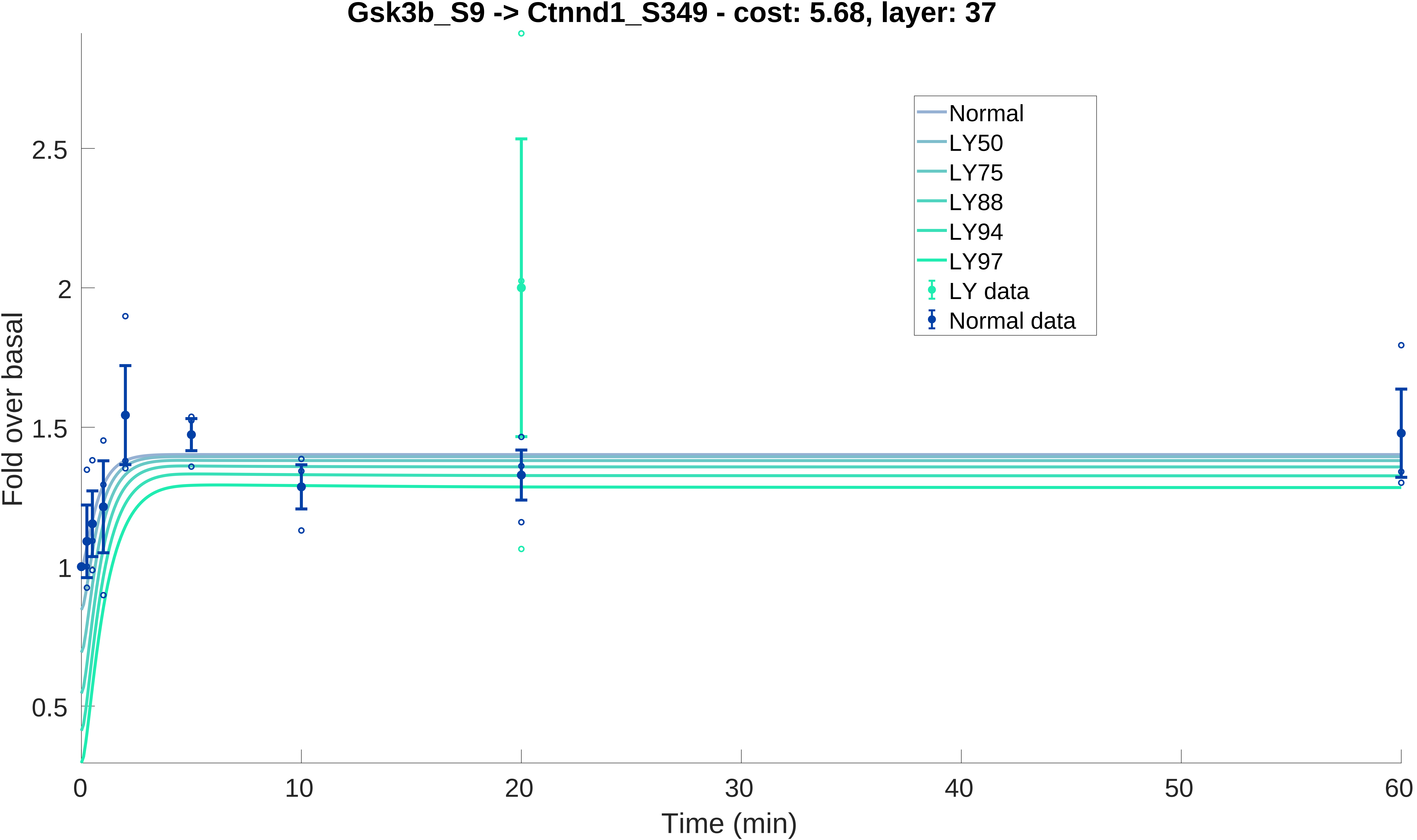

Gsk3b_S9 -> Ctnnd1_S349 - cost: 5.68, layer: 37
Fold over basal
0.5
1
1.5
2
2.5
0
10
20
30
40
50
60
Time (min)
Normal
LY50
LY75
LY88
LY94
LY97
LY data
Normal data

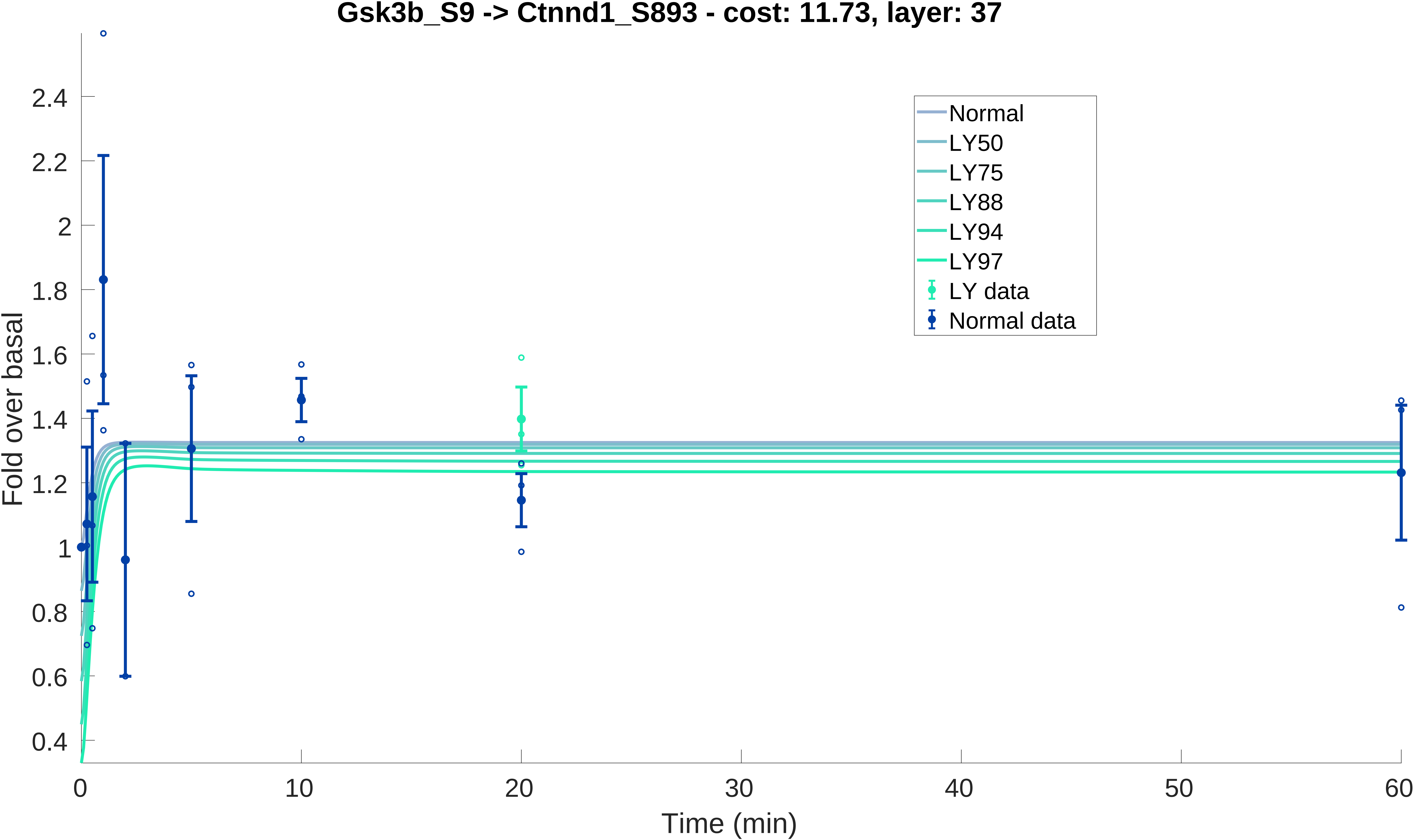

Gsk3b_S9 -> Ctnnd1_S893 - cost: 11.73, layer: 37
Fold over basal
Time (min)
Normal
LY50
LY75
LY88
LY94
LY97
LY data
Normal data
0.4
0.6
0.8
1
1.2
1.4
1.6
1.8
2
2.2
2.4
0
10
20
30
40
50
60

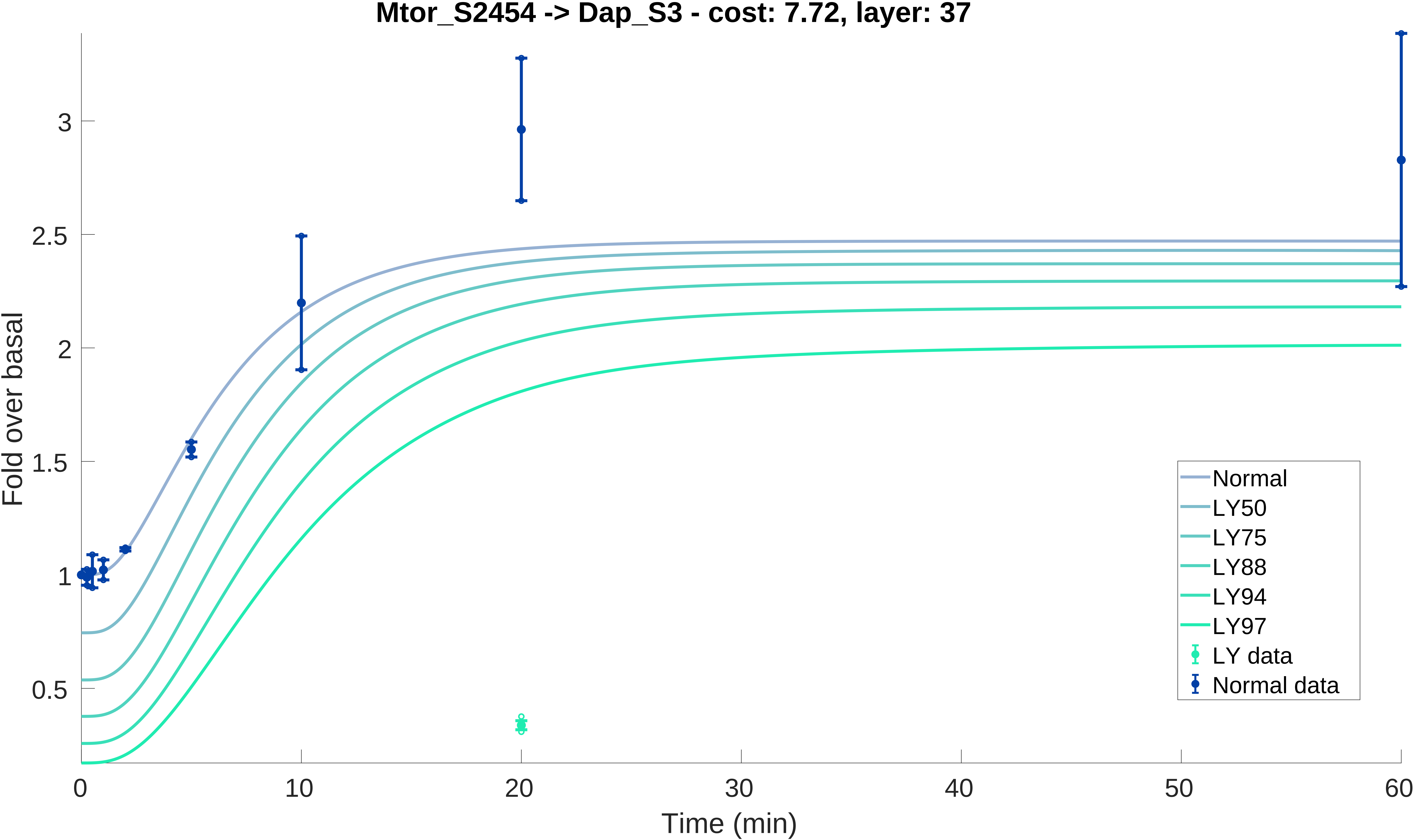

Mtor_S2454 -> Dap_S3 - cost: 7.72, layer: 37
Fold over basal
Time (min)
0.5
1
1.5
2
2.5
3
0
10
20
30
40
50
60
Normal
LY50
LY75
LY88
LY94
LY97
LY data
Normal data

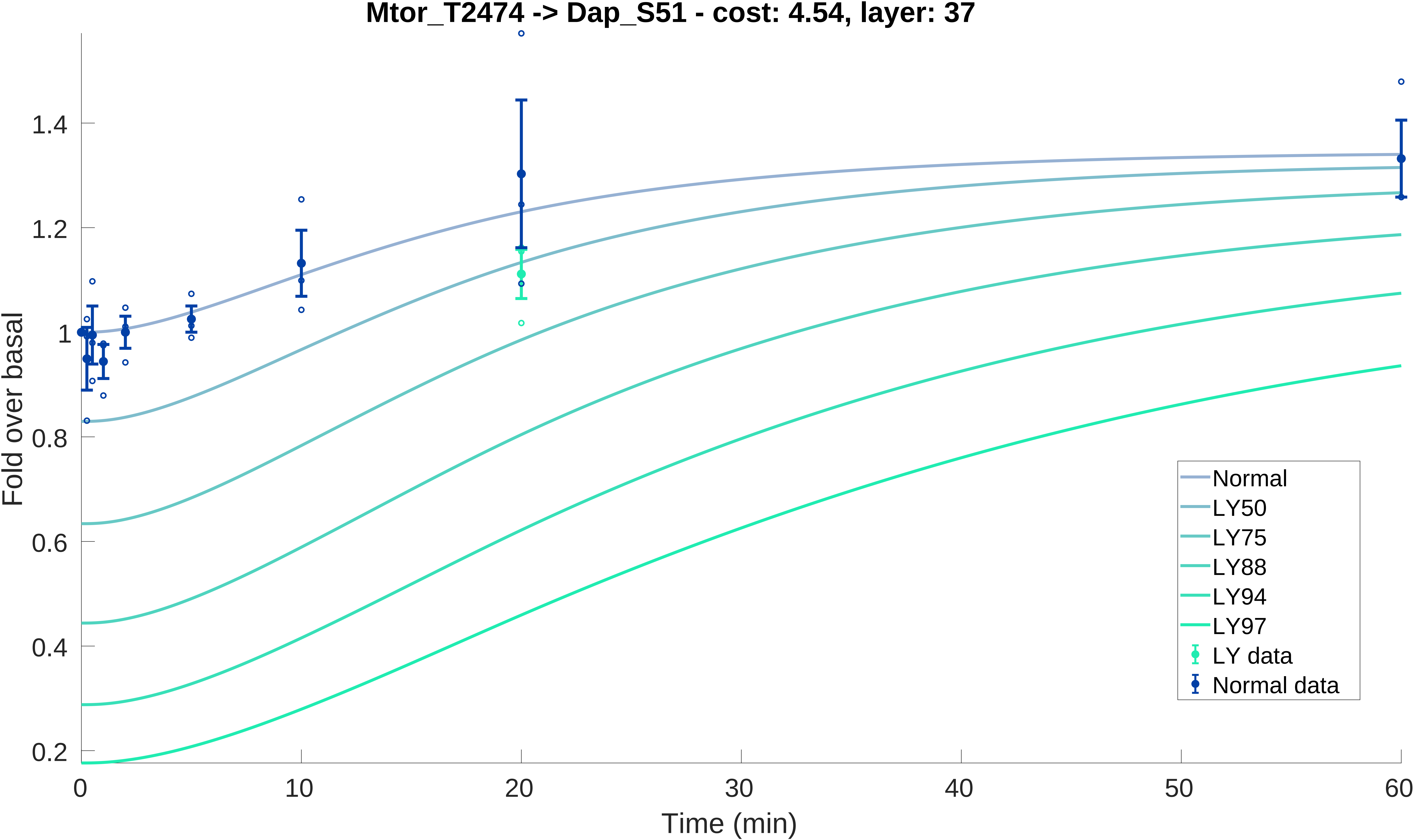
Mtor_T2474 -> Dap_S51 - cost: 4.54, layer: 37
Fold over basal
Time (min)
0.2
0.4
0.6
0.8
1
1.2
1.4
0
10
20
30
40
50
60
Normal
LY50
LY75
LY88
LY94
LY97
LY data
Normal data

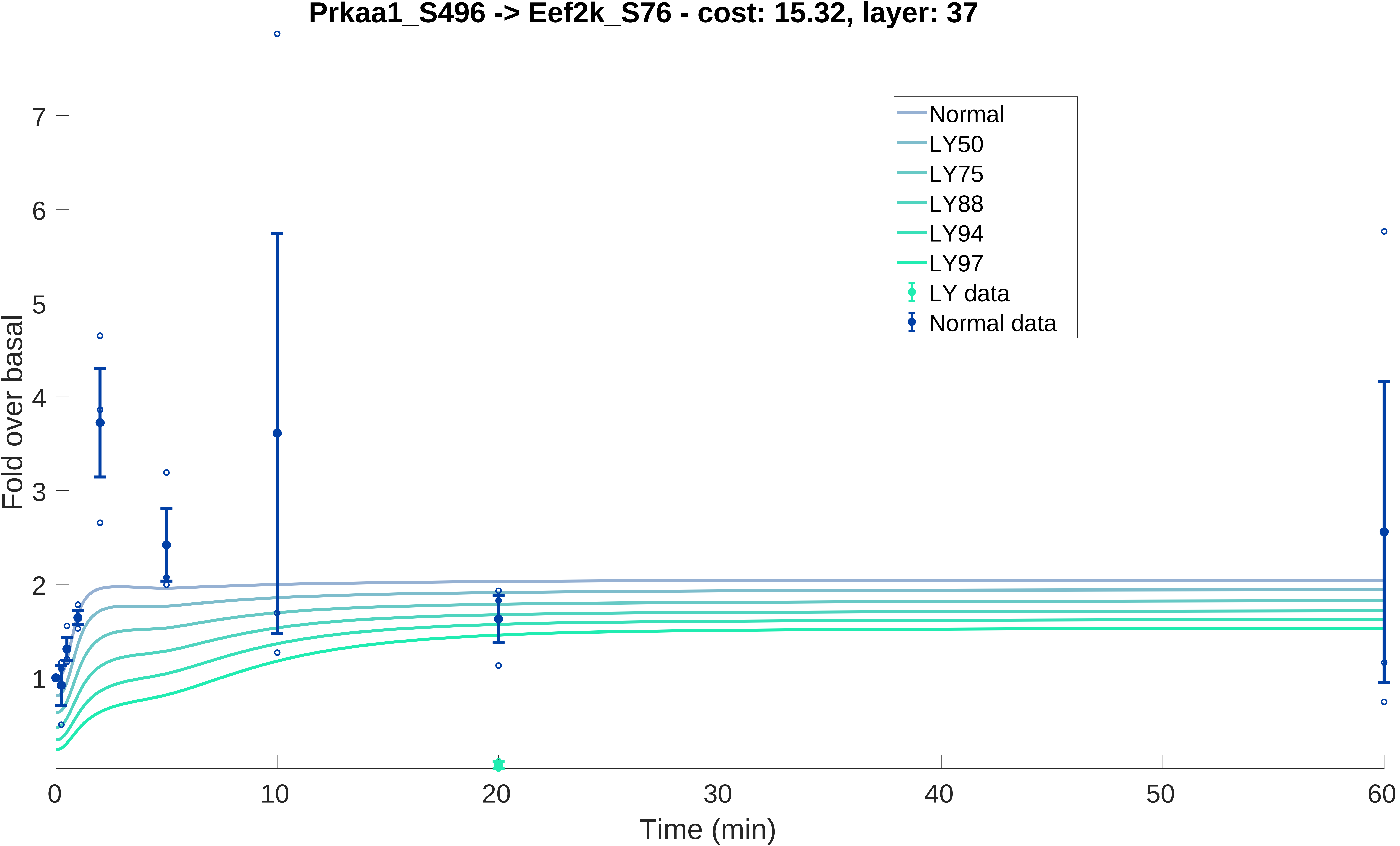

Prkaa1_S496 -> Eef2k_S76 - cost: 15.32, layer: 37
Normal
LY50
LY75
LY88
LY94
LY97
LY data
Normal data
Fold over basal
Time (min)
1
2
3
4
5
6
7
0
10
20
30
40
50
60

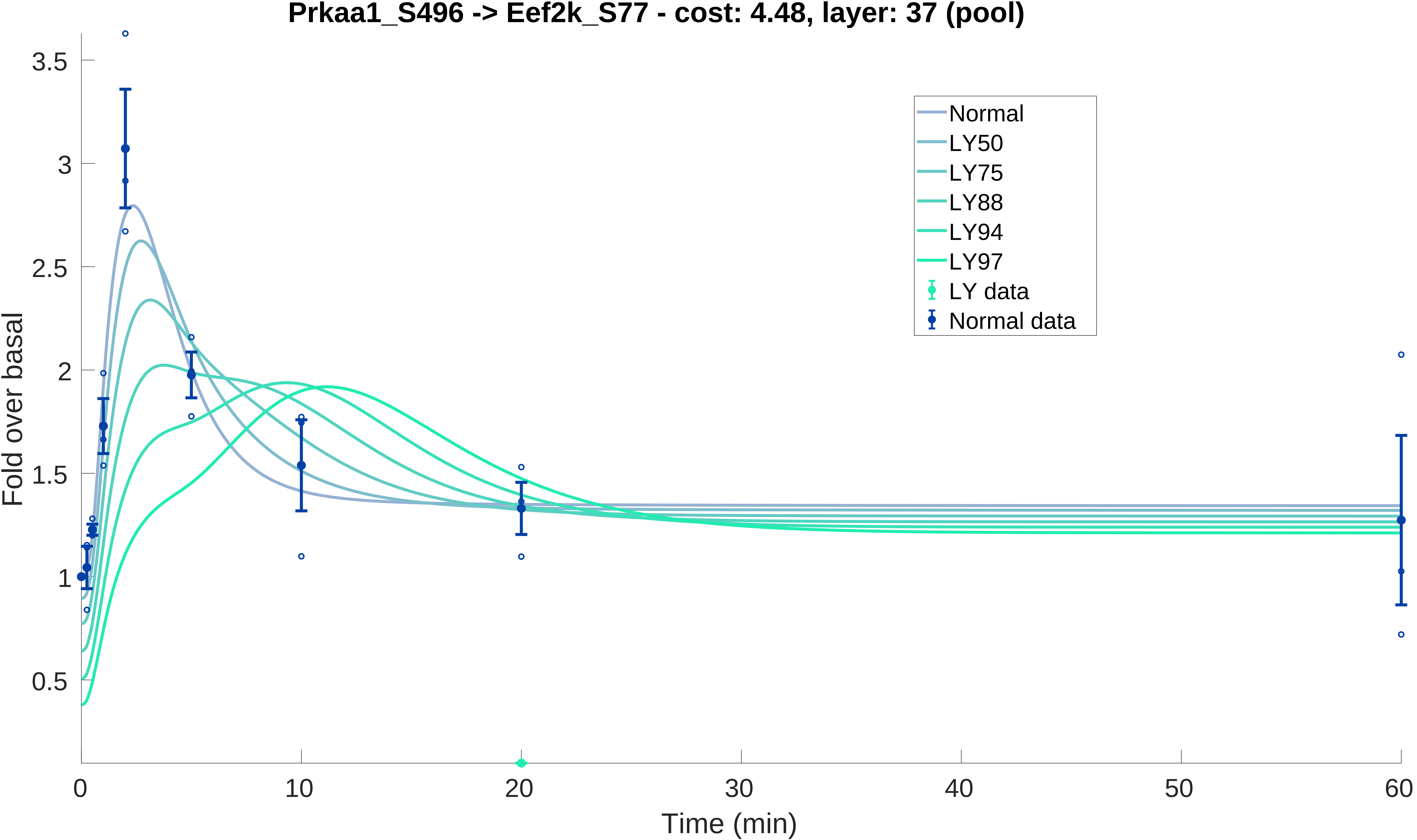

Prkaa1_S496 -> Eef2k_S77 - cost: 4.48, layer: 37 (pool)
Normal
LY50
LY75
LY88
LY94
LY97
LY data
Normal data
Fold over basal
Time (min)
0
10
20
30
40
50
60
0.5
1
1.5
2
2.5
3
3.5

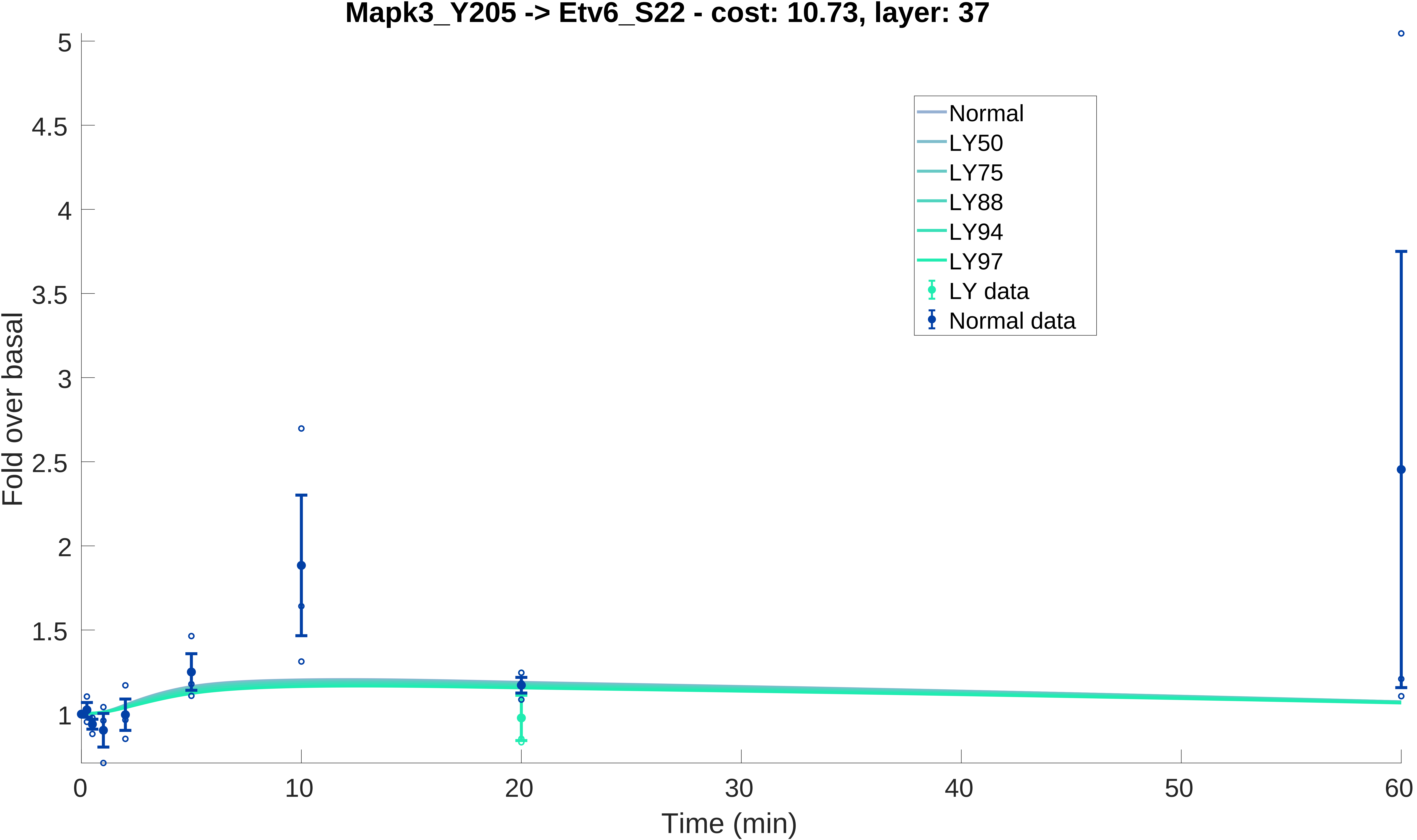

Mapk3_Y205 -> Etv6_S22 - cost: 10.73, layer: 37
Fold over basal
Time (min)
5
4.5
4
3.5
3
2.5
2
1.5
1
0
10
20
30
40
50
60
Normal
LY50
LY75
LY88
LY94
LY97
LY data
Normal data

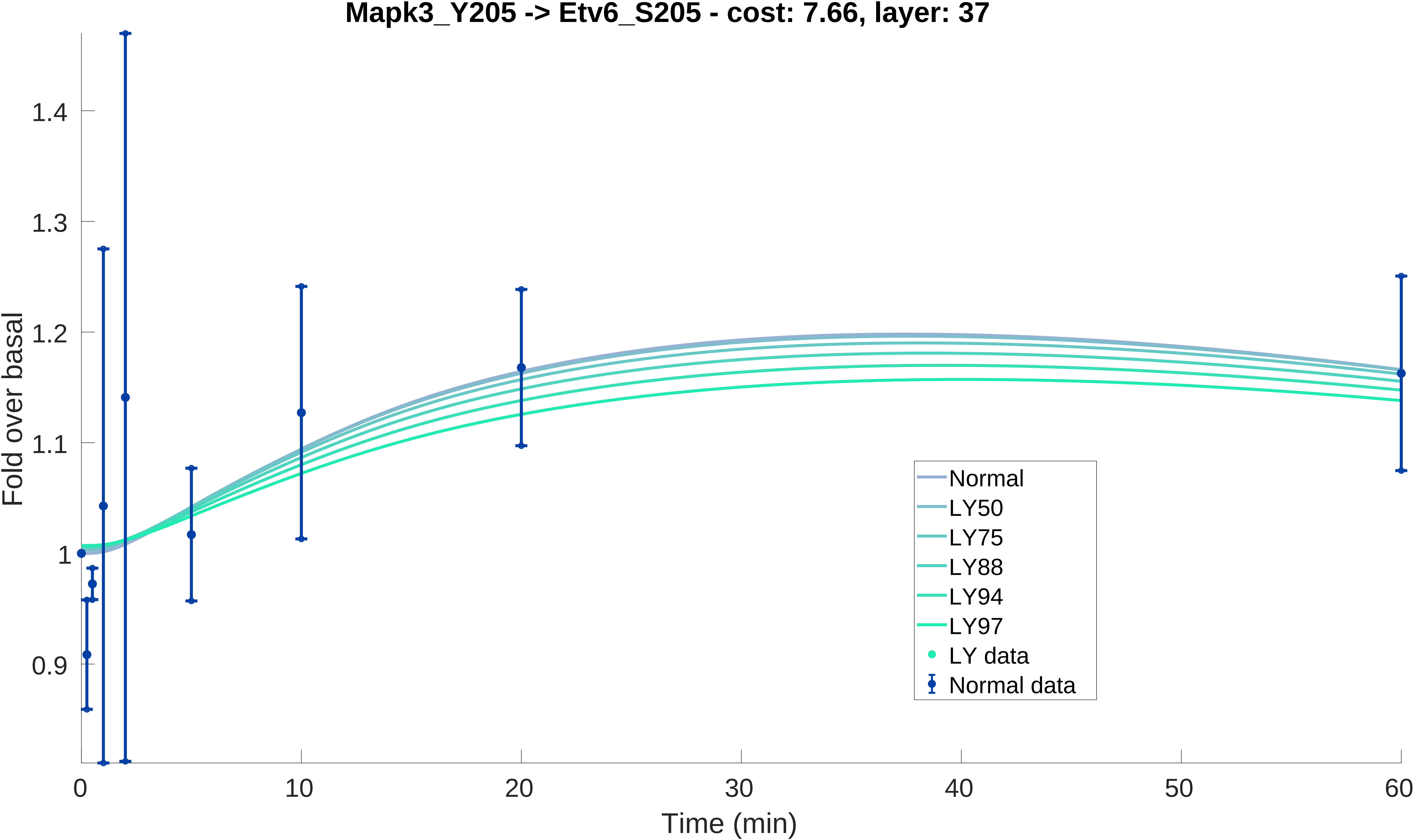

Mapk3_Y205 -> Etv6_S205 - cost: 7.66, layer: 37
Fold over basal
Time (min)
0.9
1
1.1
1.2
1.3
1.4
0
10
20
30
40
50
60
Normal
LY50
LY75
LY88
LY94
LY97
LY data
Normal data

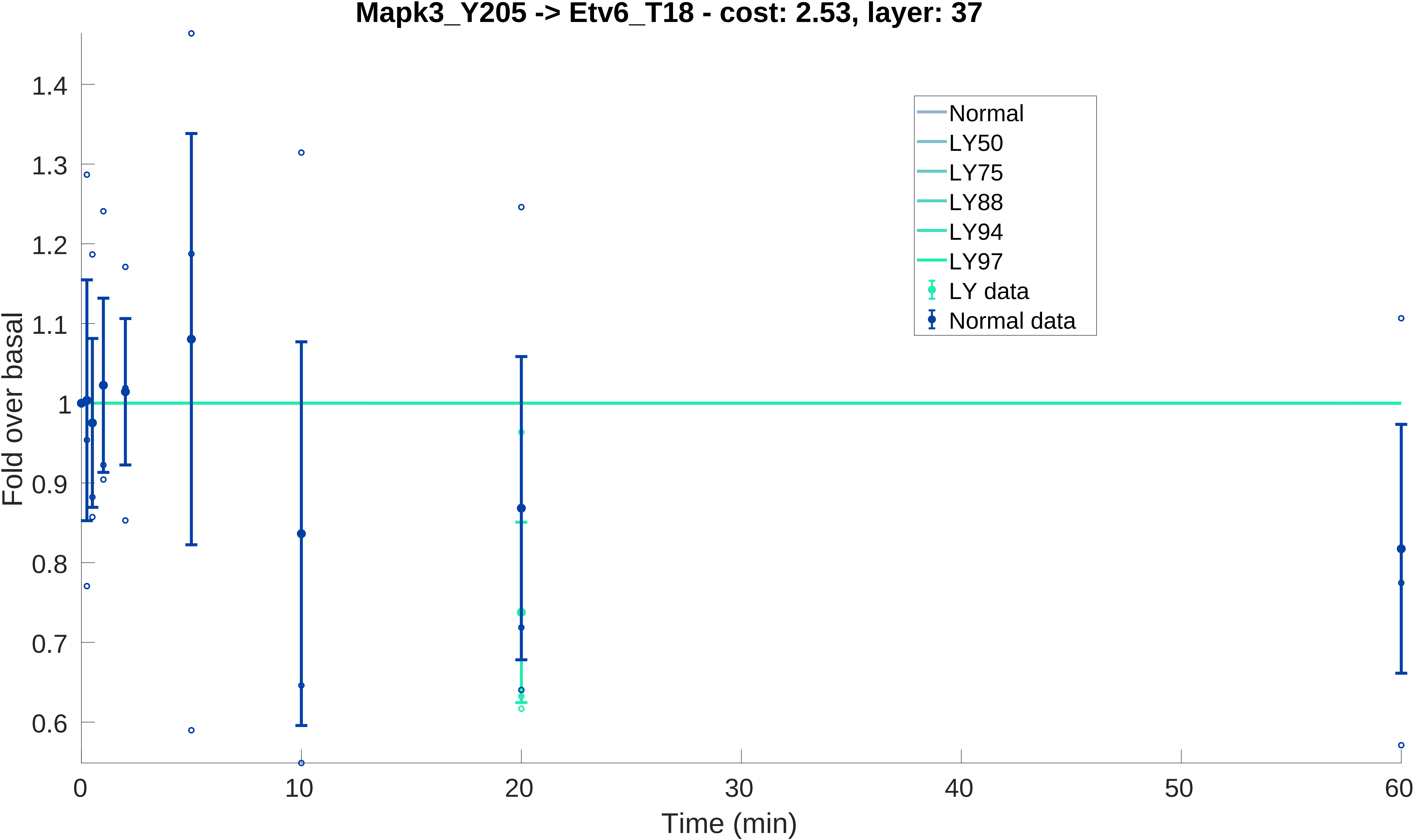

Mapk3_Y205 -> Etv6_T18 - cost: 2.53, layer: 37
Fold over basal
Time (min)
1.4
1.3
1.2
1.1
1
0.9
0.8
0.7
0.6
0
10
20
30
40
50
60
Normal
LY50
LY75
LY88
LY94
LY97
LY data
Normal data

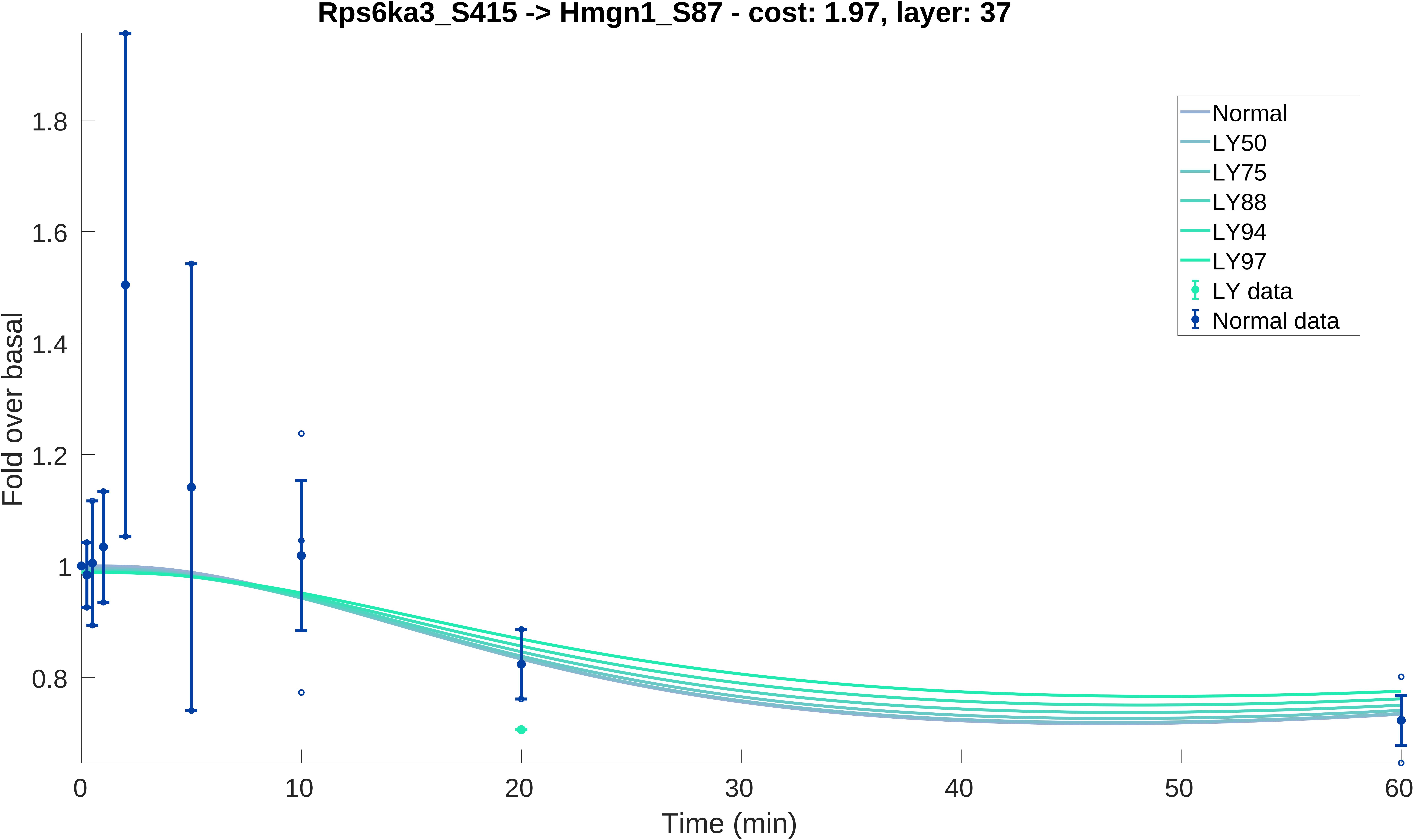

Rps6ka3_S415 -> Hmgn1_S87 - cost: 1.97, layer: 37
Fold over basal
Time (min)
Normal
LY50
LY75
LY88
LY94
LY97
LY data
Normal data
0.8
1
1.2
1.4
1.6
1.8
0
10
20
30
40
50
60

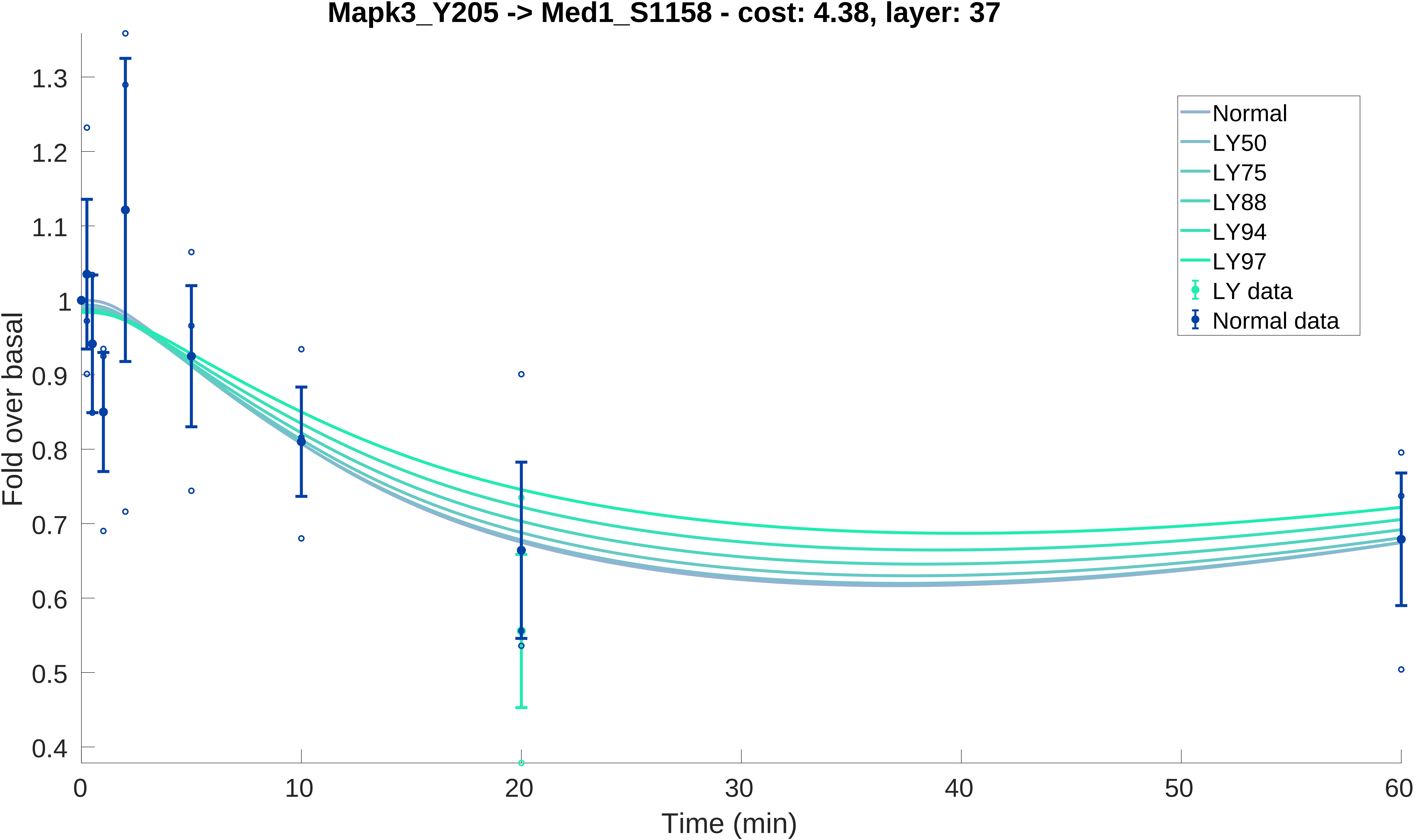
Mapk3_Y205 -> Med1_S1158 - cost: 4.38, layer: 37
Fold over basal
Time (min)
Normal
LY50
LY75
LY88
LY94
LY97
LY data
Normal data
1.3
1.2
1.1
1
0.9
0.8
0.7
0.6
0.5
0.4
0
10
20
30
40
50
60

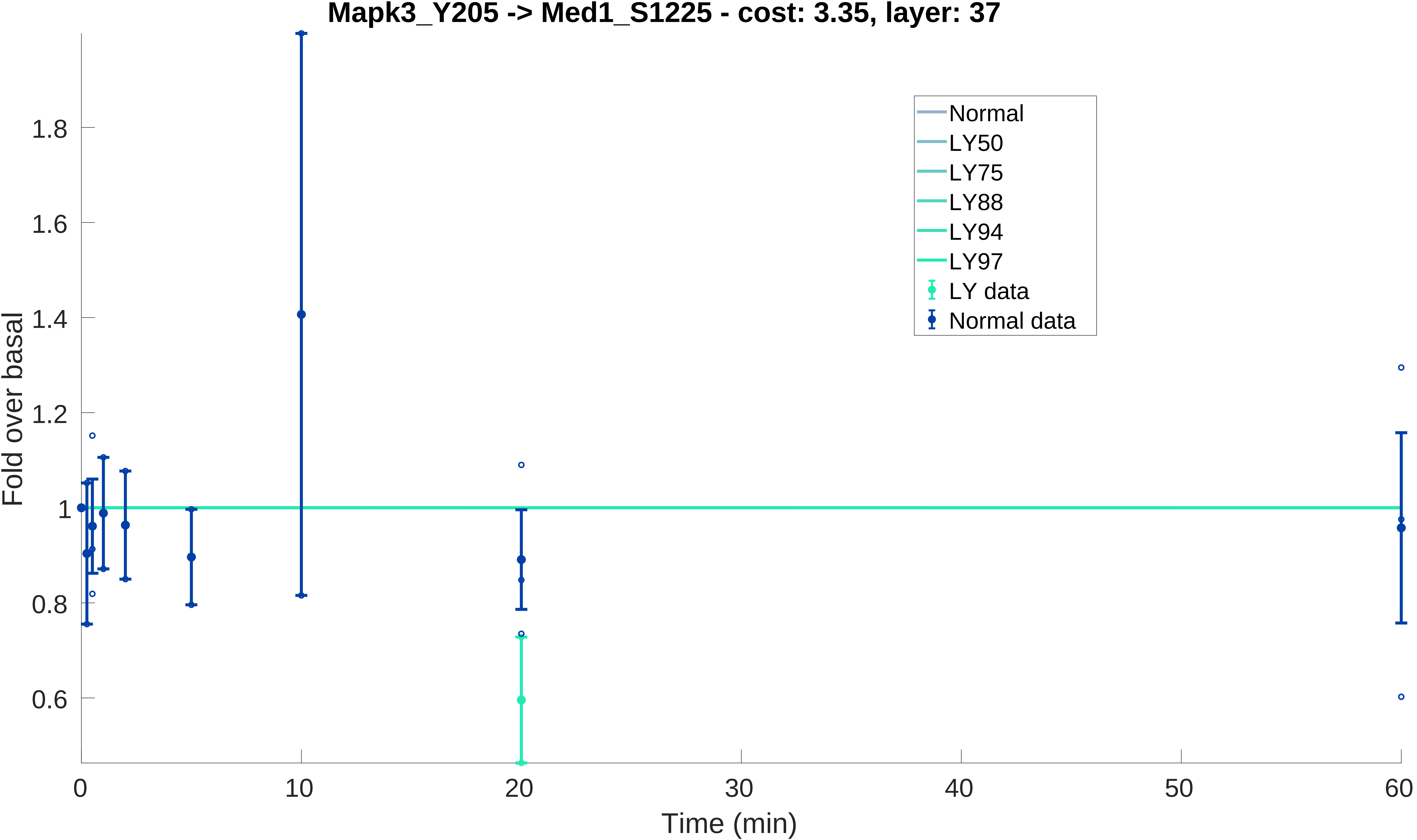

Mapk3_Y205 -> Med1_S1225 - cost: 3.35, layer: 37
Normal
LY50
LY75
LY88
LY94
LY97
LY data
Normal data
Fold over basal
Time (min)
0.6
0.8
1
1.2
1.4
1.6
1.8
0
10
20
30
40
50
60

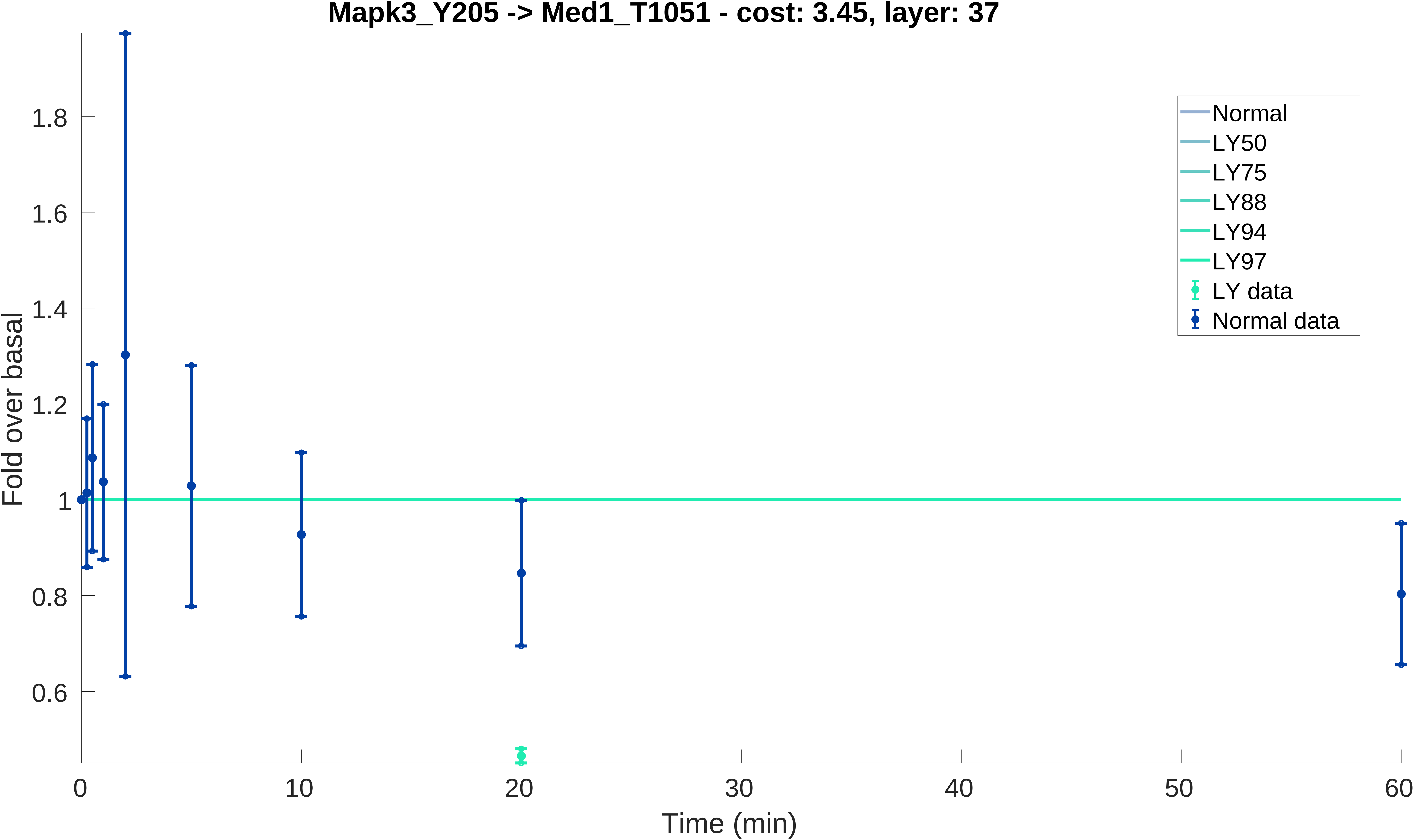

Mapk3_Y205 -> Med1_T1051 - cost: 3.45, layer: 37
Fold over basal
Time (min)
0.6
0.8
1
1.2
1.4
1.6
1.8
0
10
20
30
40
50
60
Normal
LY50
LY75
LY88
LY94
LY97
LY data
Normal data

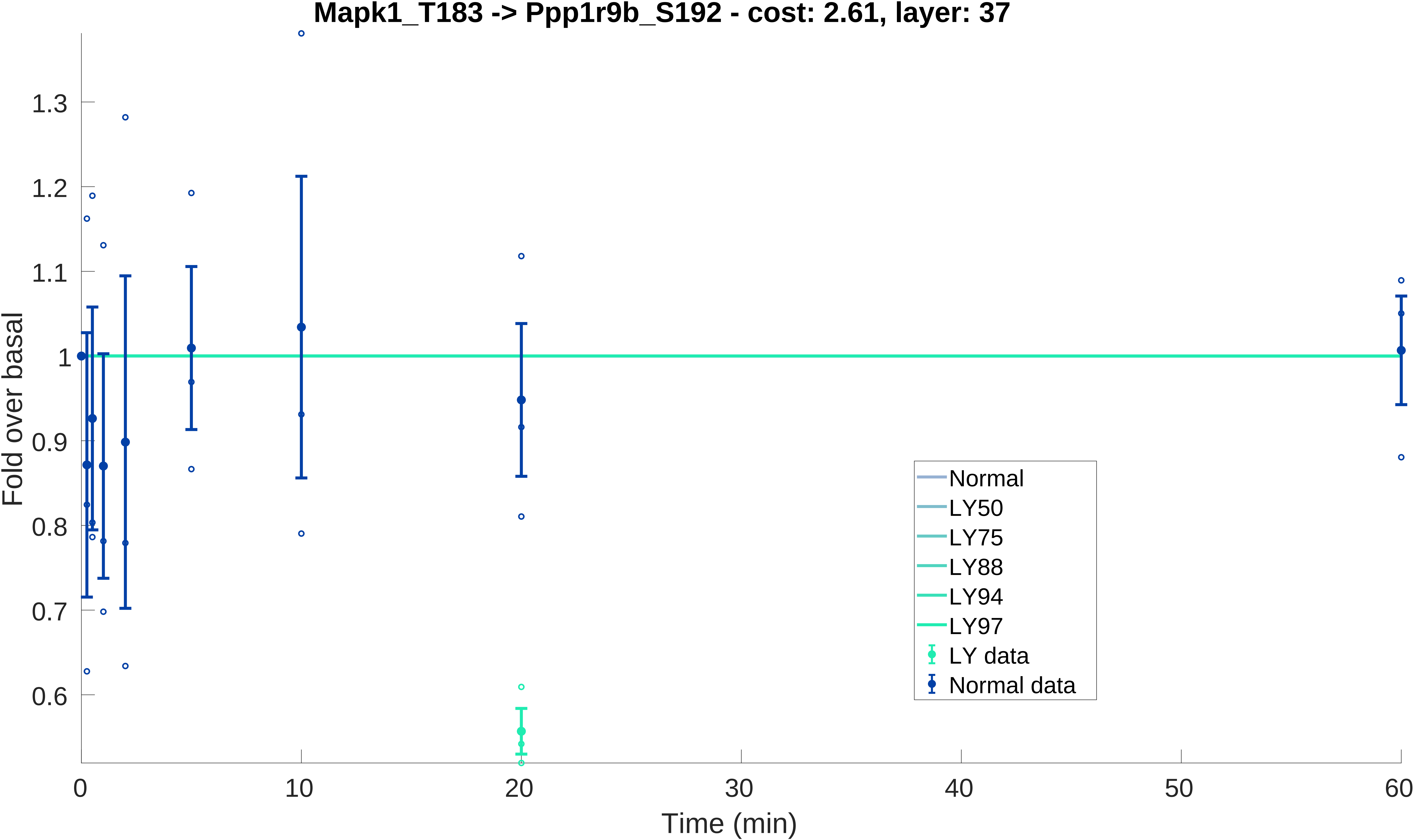
Mapk1_T183 -> Ppp1r9b_S192 - cost: 2.61, layer: 37
Fold over basal
Time (min)
1.3
1.2
1.1
1
0.9
0.8
0.7
0.6
0
10
20
30
40
50
60
Normal
LY50
LY75
LY88
LY94
LY97
LY data
Normal data

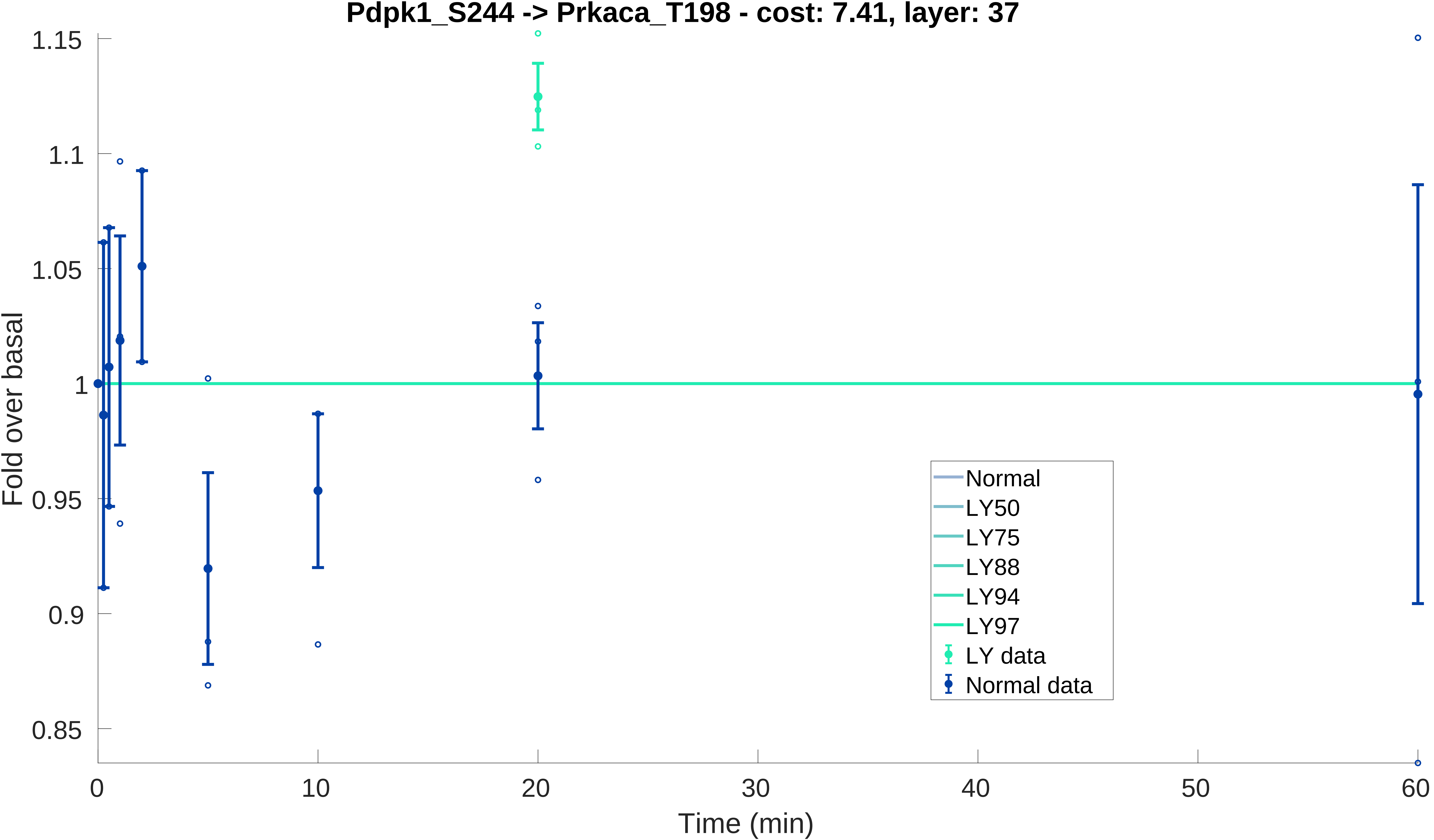

Pdpk1_S244 -> Prkaca_T198 - cost: 7.41, layer: 37
Fold over basal
1.15
1.1
1.05
1
0.95
0.9
0.85
0
10
20
30
40
50
60
Time (min)
Normal
LY50
LY75
LY88
LY94
LY97
LY data
Normal data

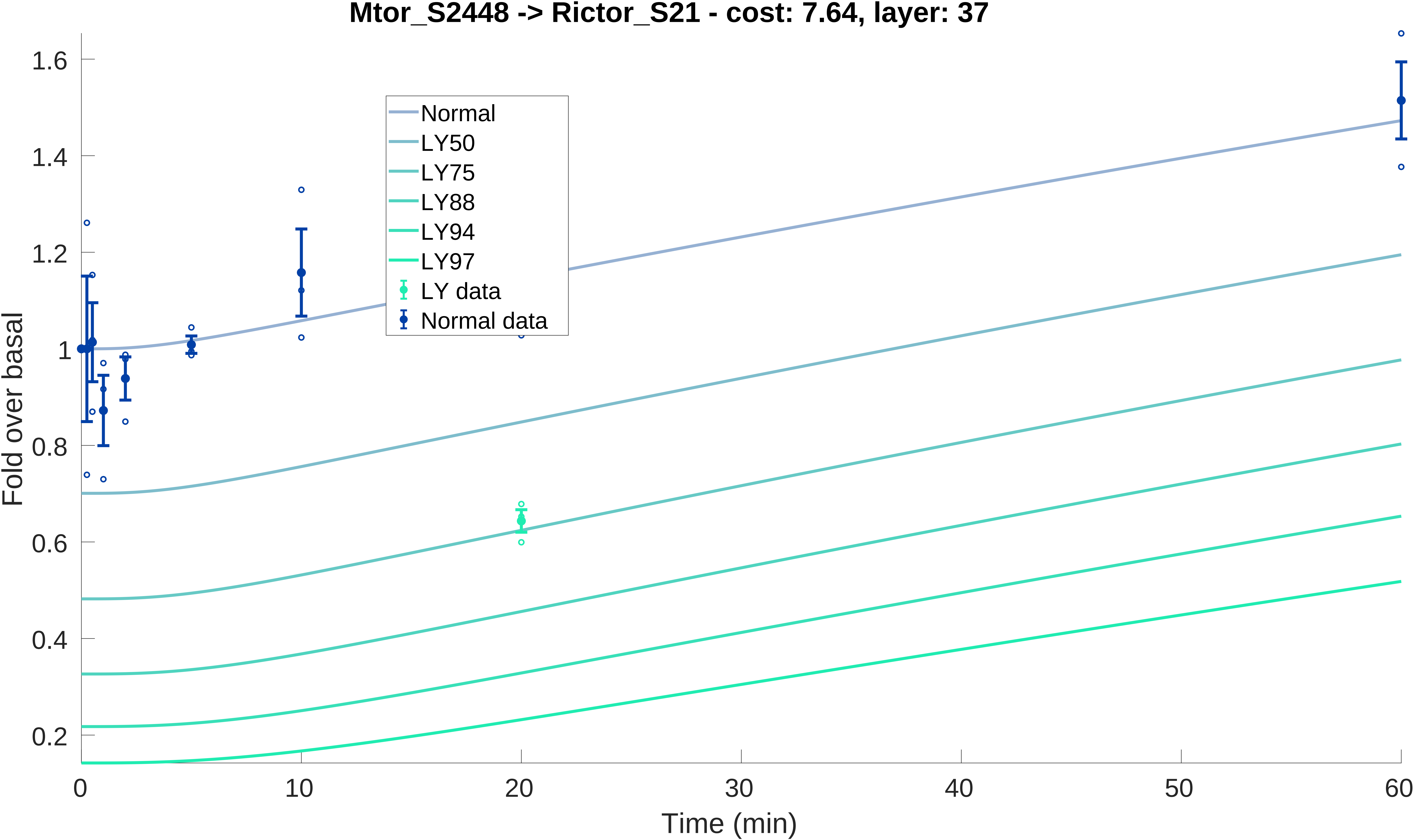
Mtor_S2448 -> Rictor_S21 - cost: 7.64, layer: 37
Normal
LY50
LY75
LY88
LY94
LY97
LY data
Normal data
Fold over basal
Time (min)
0.2
0.4
0.6
0.8
1
1.2
1.4
1.6
0
10
20
30
40
50
60

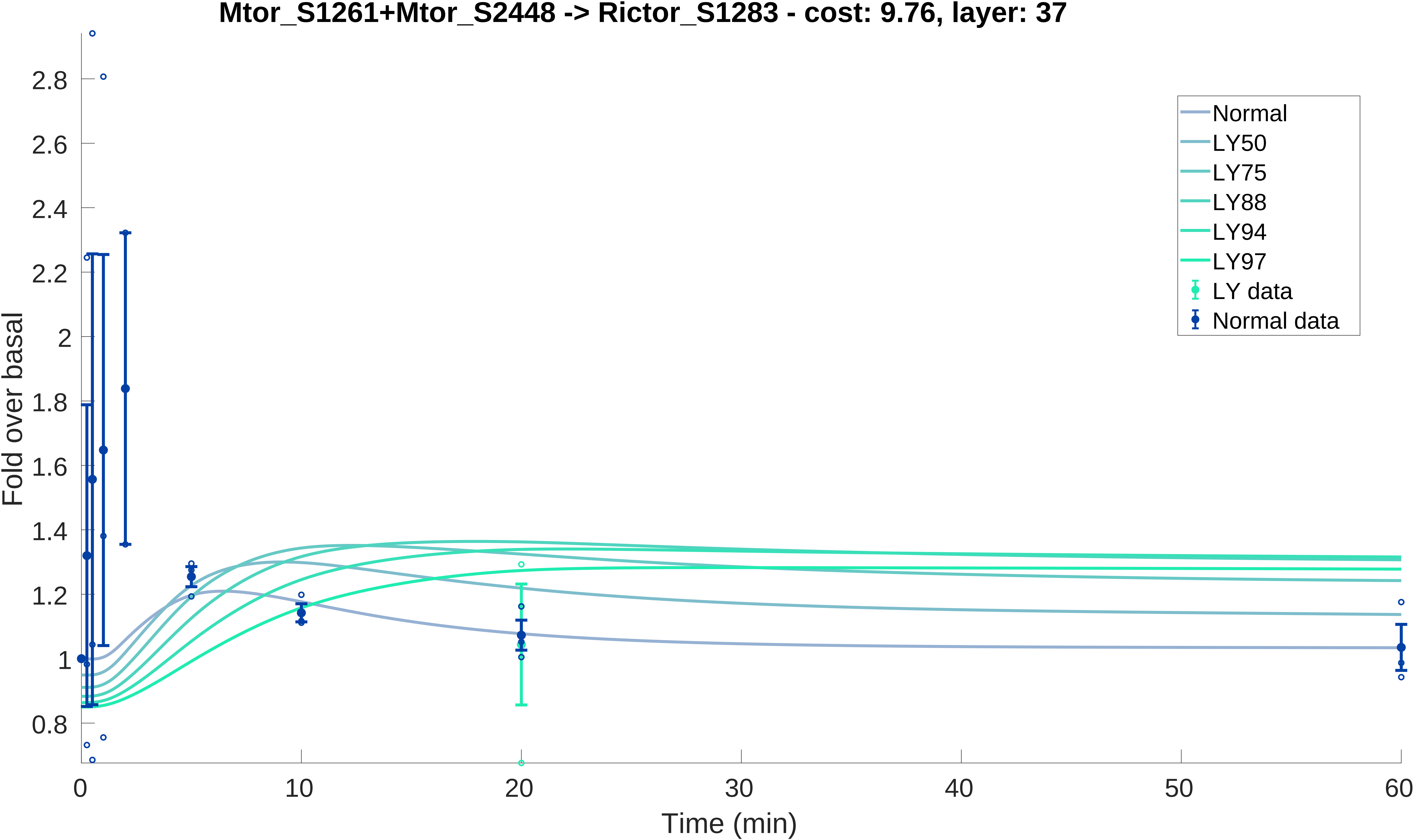

Mtor_S1261+Mtor_S2448 -> Rictor_S1283 - cost: 9.76, layer: 37
Fold over basal
Time (min)
0.8
1
1.2
1.4
1.6
1.8
2
2.2
2.4
2.6
2.8
0
10
20
30
40
50
60
Normal
LY50
LY75
LY88
LY94
LY97
LY data
Normal data

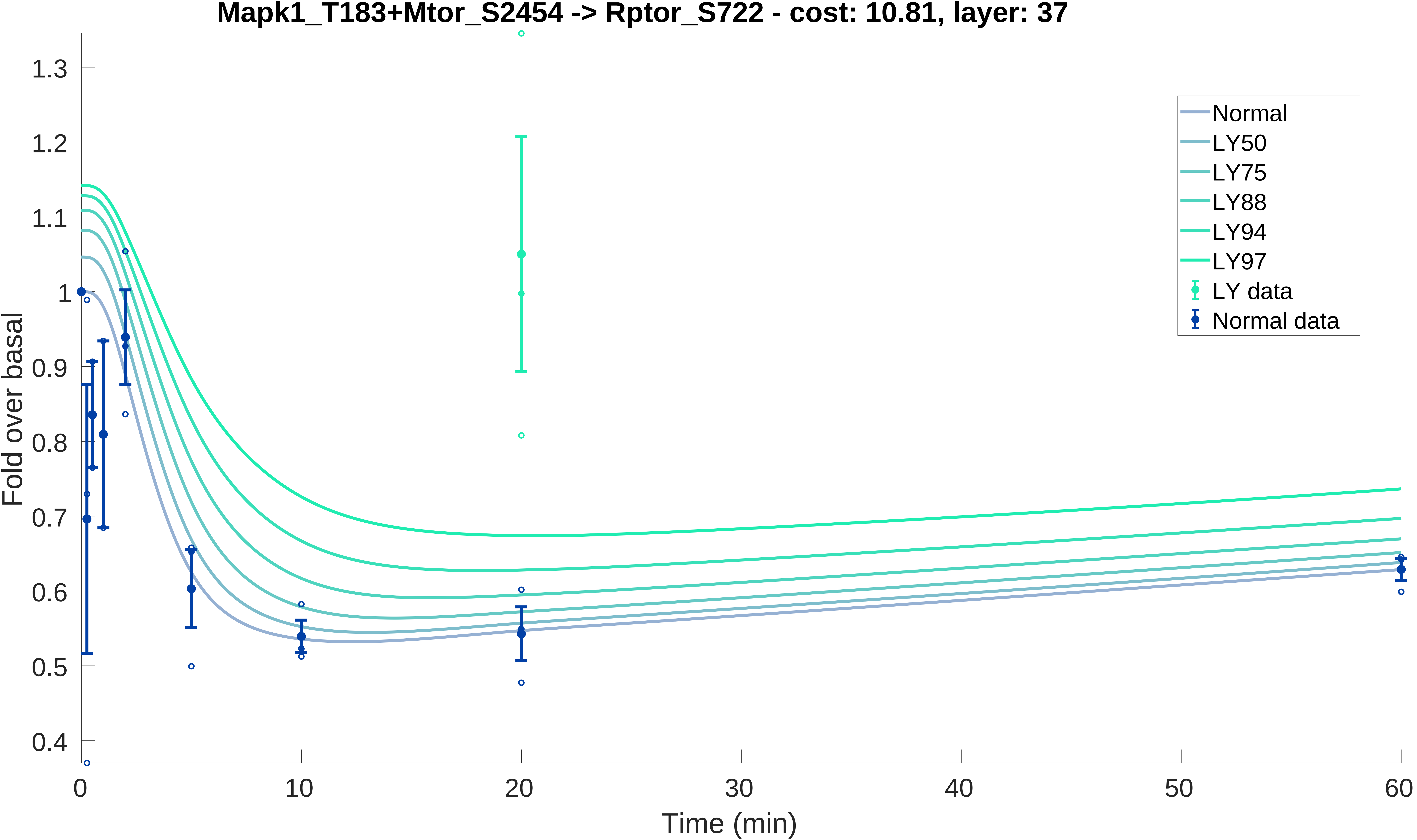

Mapk1_T183+Mtor_S2454 -> Rptor_S722 - cost: 10.81, layer: 37
Fold over basal
Time (min)
1.3
1.2
1.1
1
0.9
0.8
0.7
0.6
0.5
0.4
0
10
20
30
40
50
60
Normal
LY50
LY75
LY88
LY94
LY97
LY data
Normal data

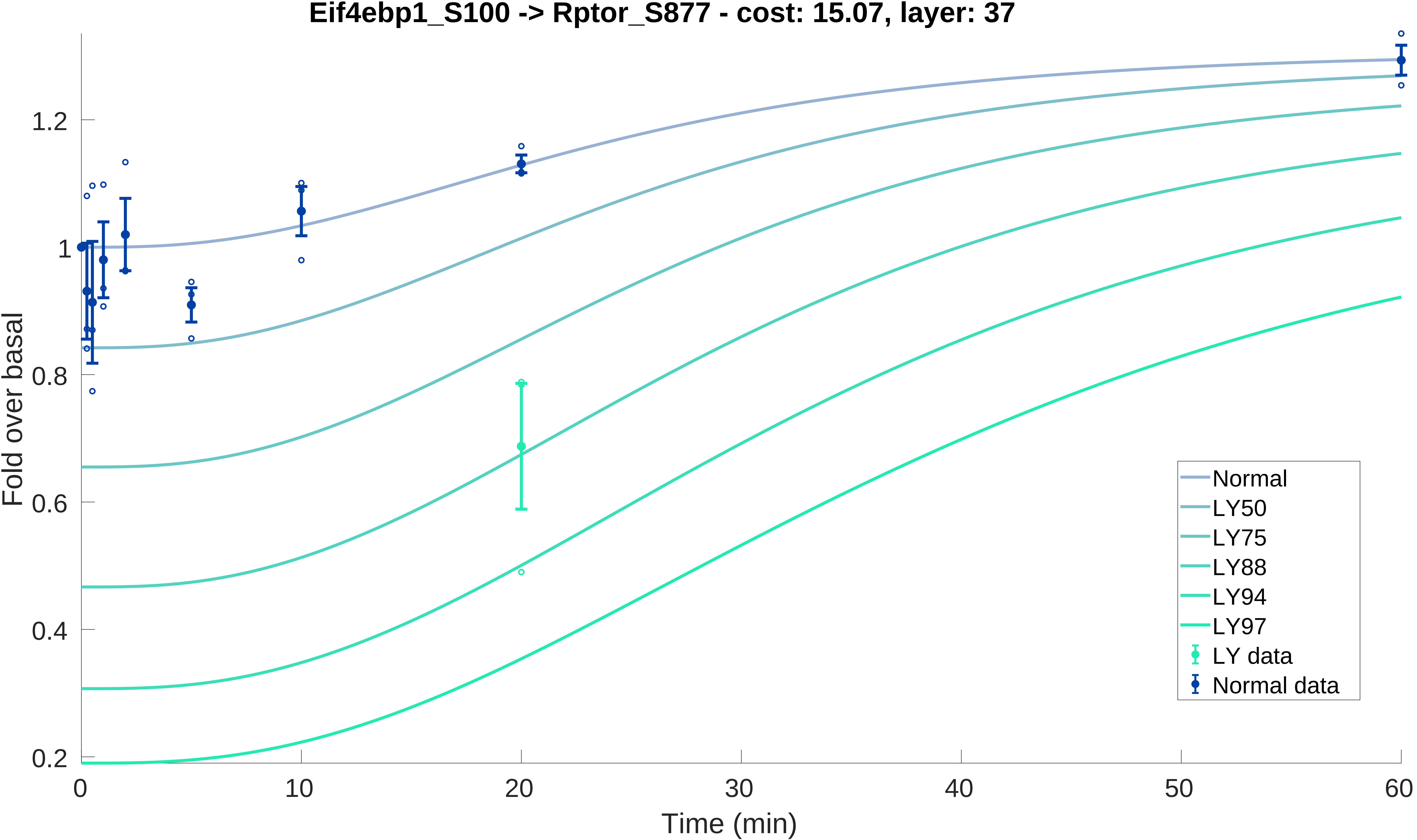
Eif4ebp1_S100 -> Rptor_S877 - cost: 15.07, layer: 37
Fold over basal
Time (min)
0.2
0.4
0.6
0.8
1
1.2
0
10
20
30
40
50
60
Normal
LY50
LY75
LY88
LY94
LY97
LY data
Normal data

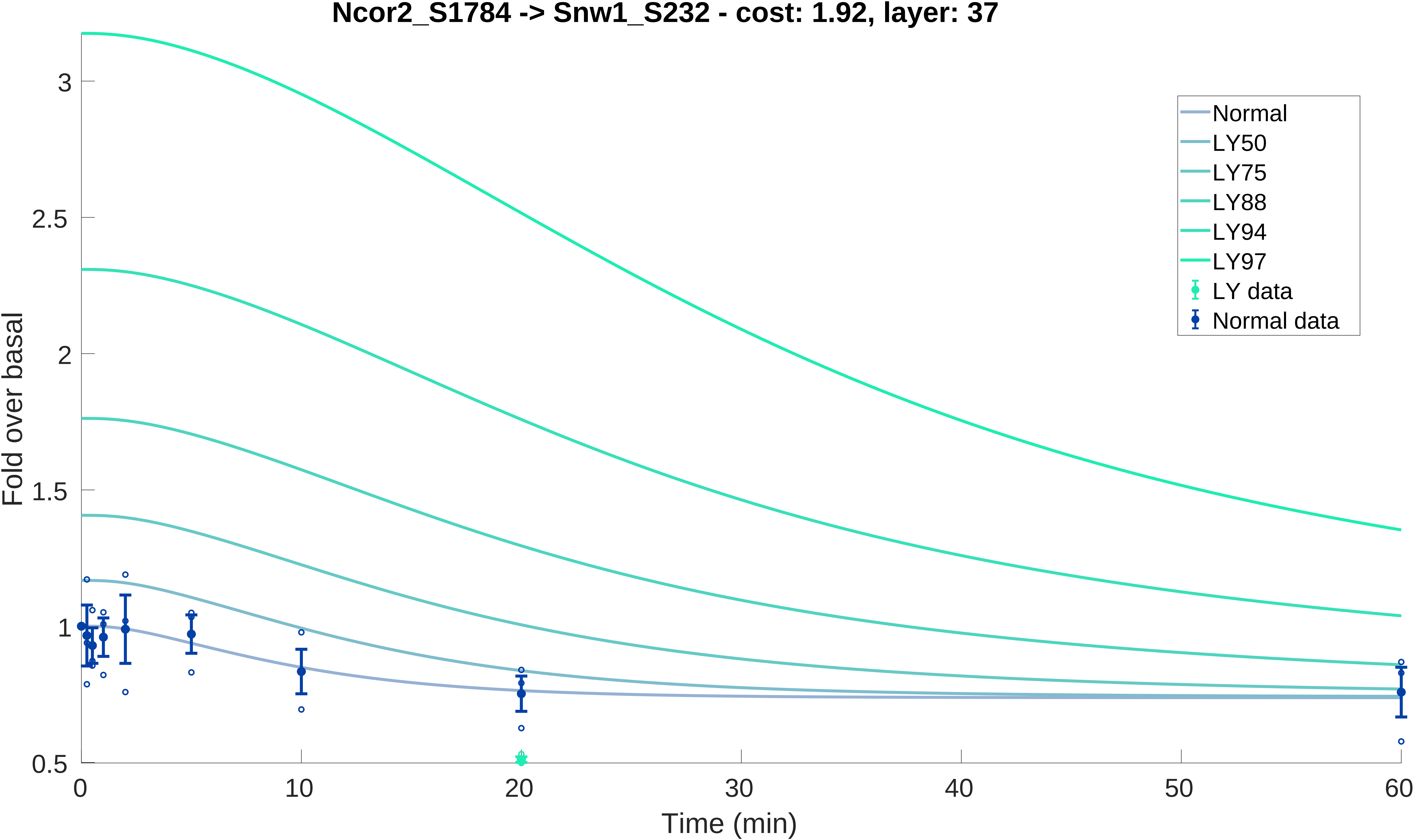

Ncor2_S1784 -> Snw1_S232 - cost: 1.92, layer: 37
Fold over basal
Time (min)
0.5
1
1.5
2
2.5
3
0
10
20
30
40
50
60
Normal
LY50
LY75
LY88
LY94
LY97
LY data
Normal data

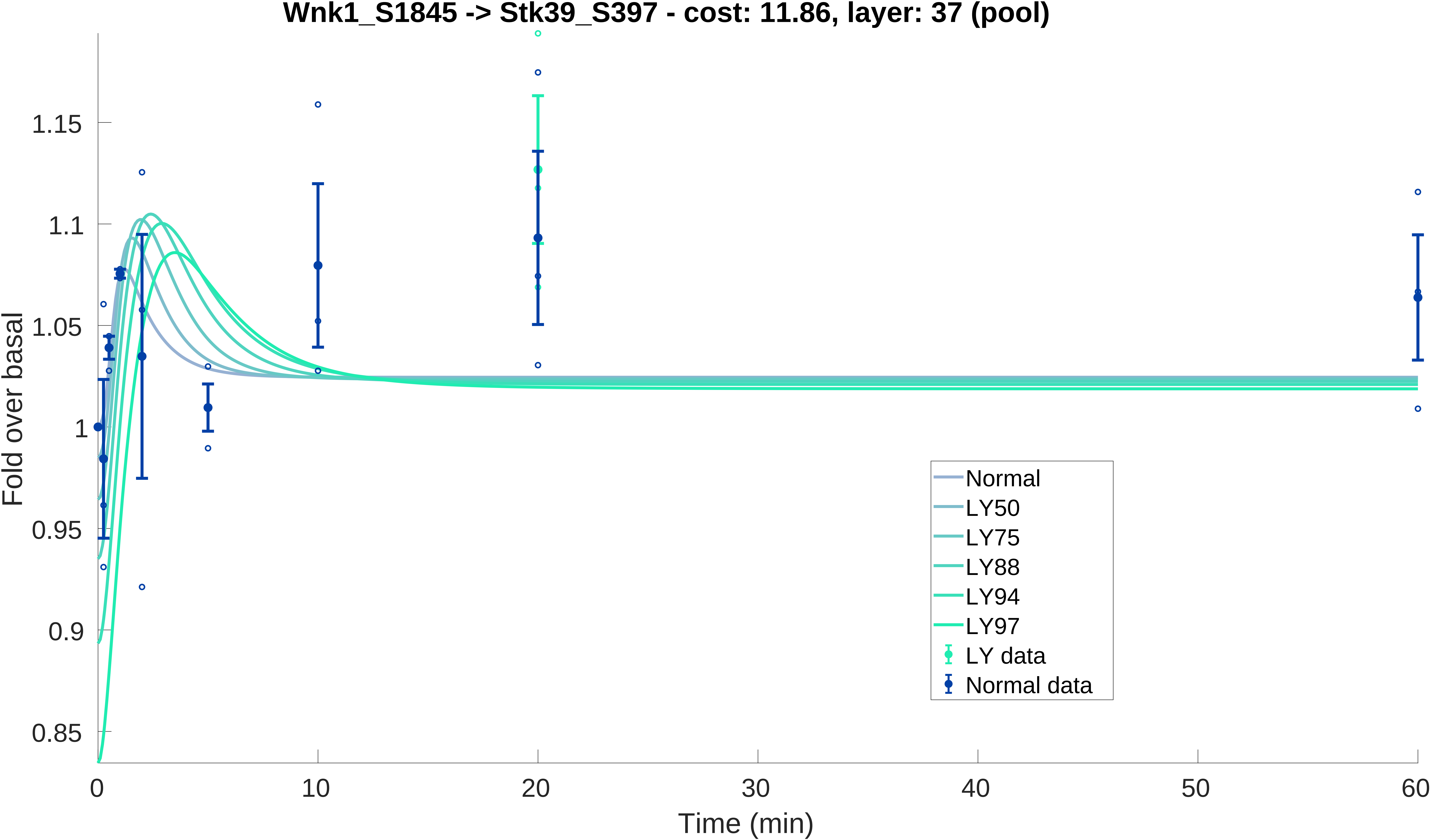
Wnk1_S1845 -> Stk39_S397 - cost: 11.86, layer: 37 (pool)
Fold over basal
1.15
1.1
1.05
1
0.95
0.9
0.85
0
10
20
30
40
50
60
Time (min)
Normal
LY50
LY75
LY88
LY94
LY97
LY data
Normal data

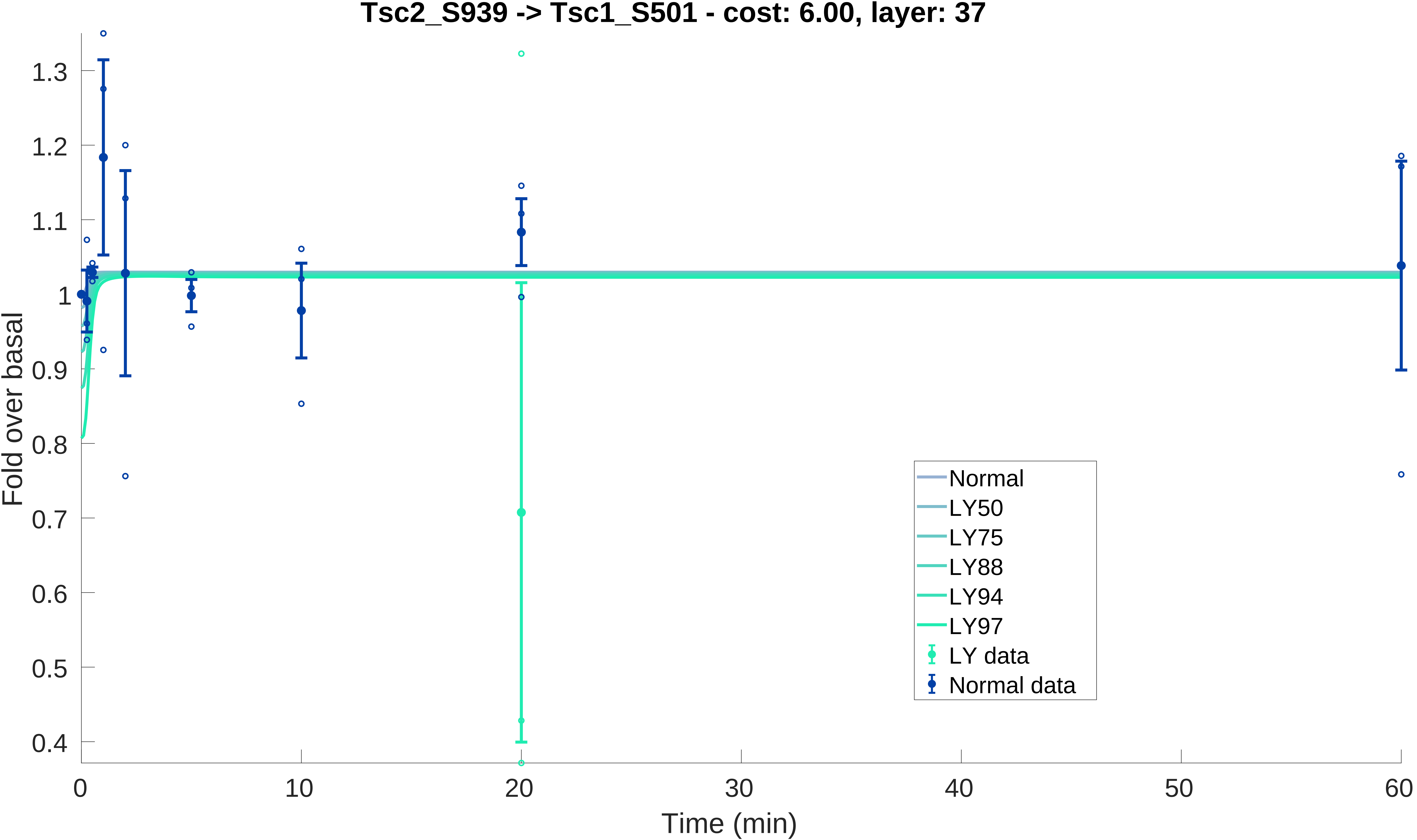

Tsc2_S939 -> Tsc1_S501 - cost: 6.00, layer: 37
Fold over basal
Time (min)
1.3
1.2
1.1
1
0.9
0.8
0.7
0.6
0.5
0.4
0
10
20
30
40
50
60
Normal
LY50
LY75
LY88
LY94
LY97
LY data
Normal data

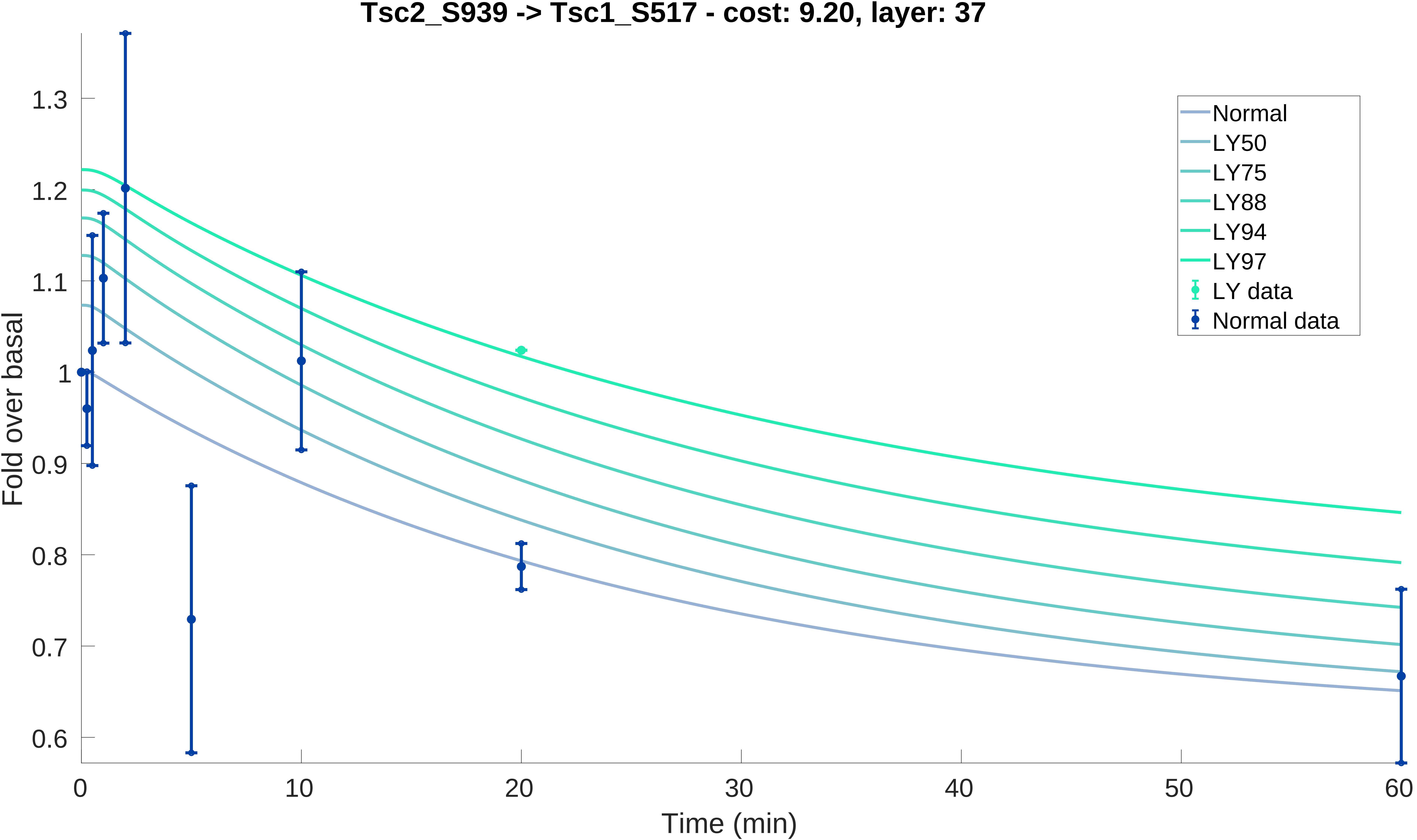

Tsc2_S939 -> Tsc1_S517 - cost: 9.20, layer: 37
Fold over basal
Time (min)
0.6
0.7
0.8
0.9
1
1.1
1.2
1.3
0
10
20
30
40
50
60
Normal
LY50
LY75
LY88
LY94
LY97
LY data
Normal data

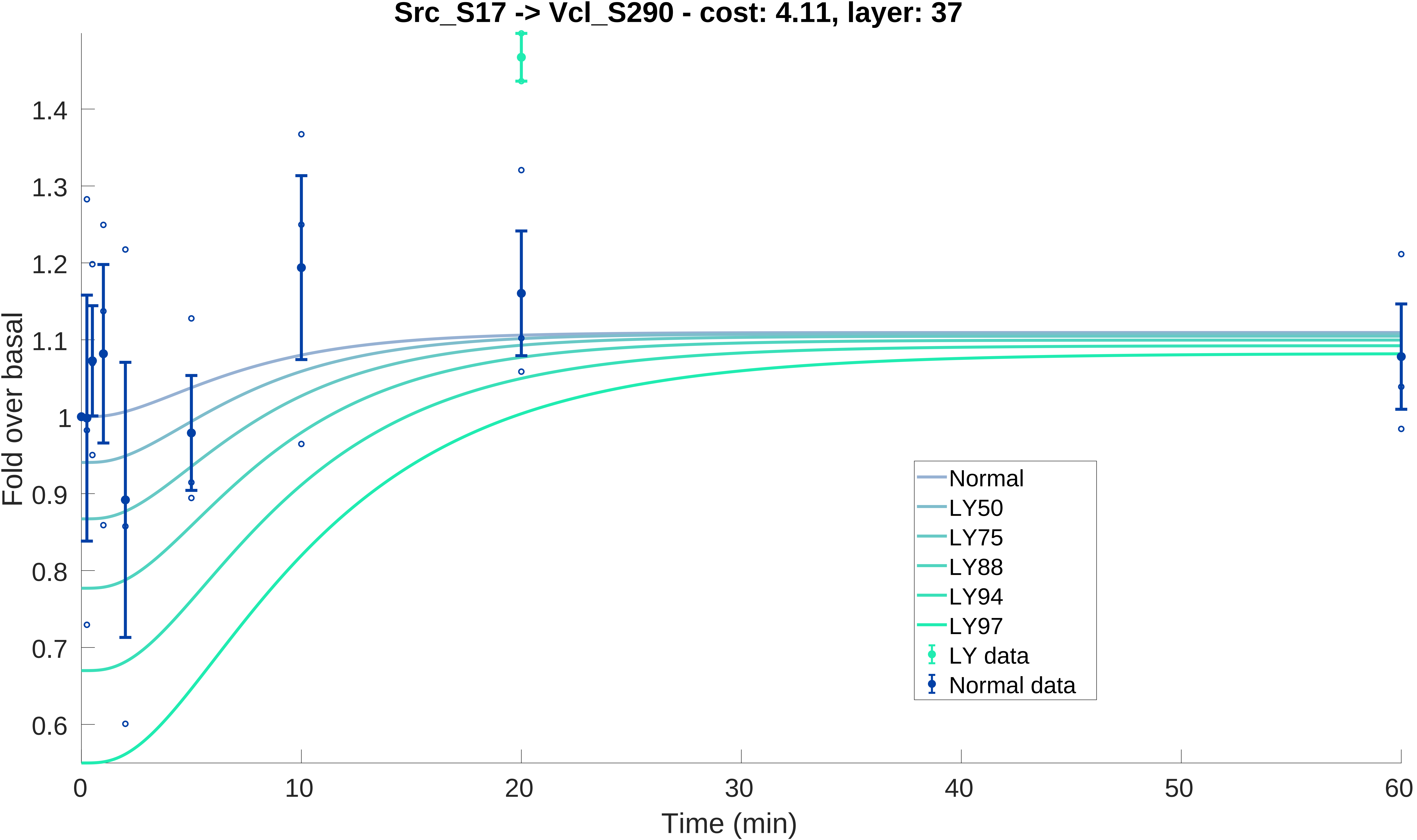

Src_S17 -> Vcl_S290 - cost: 4.11, layer: 37
Fold over basal
Time (min)
0.6
0.7
0.8
0.9
1
1.1
1.2
1.3
1.4
0
10
20
30
40
50
60
Normal
LY50
LY75
LY88
LY94
LY97
LY data
Normal data

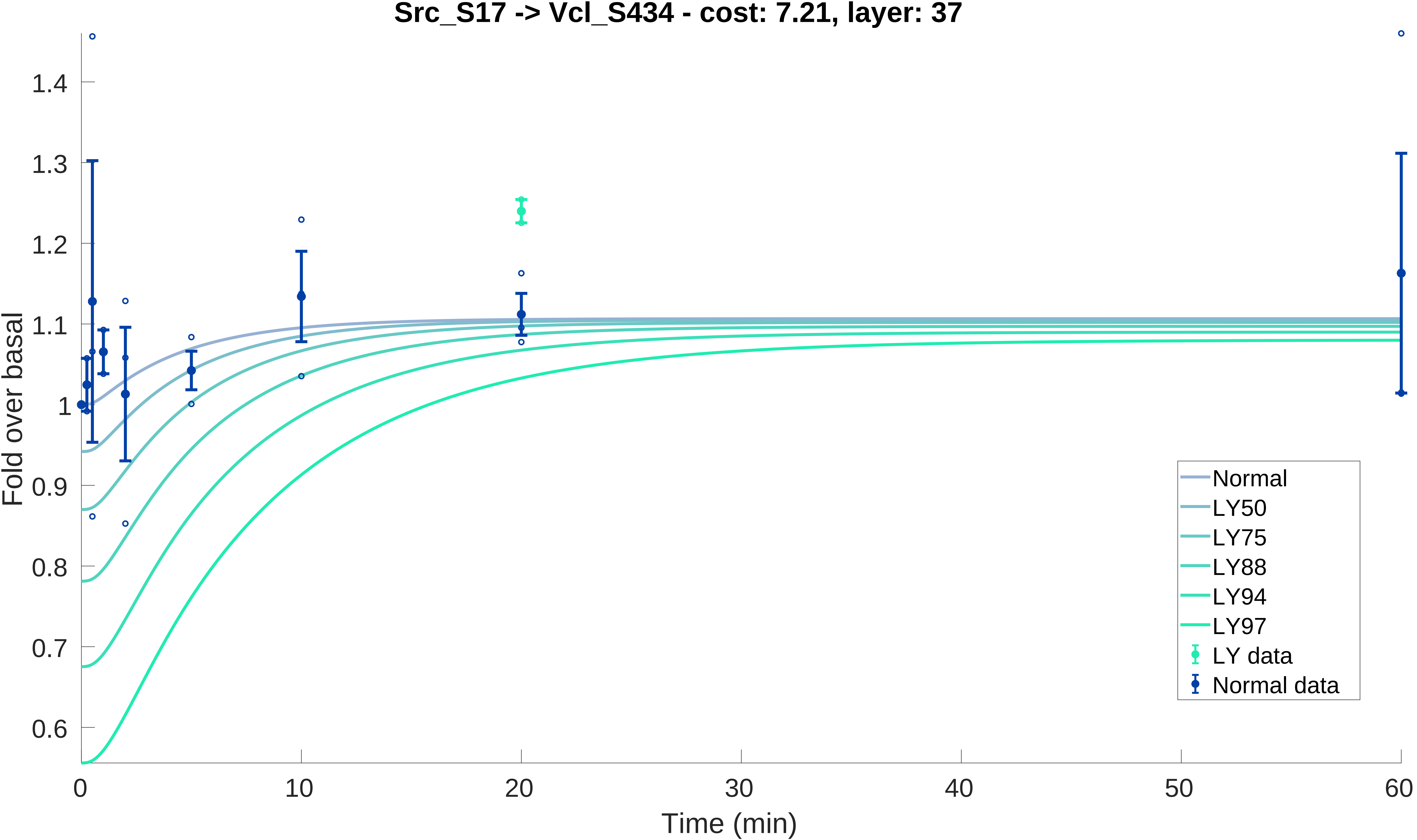

Src_S17 -> Vcl_S434 - cost: 7.21, layer: 37
Fold over basal
1.4
1.3
1.2
1.1
1
0.9
0.8
0.7
0.6
0
10
20
30
40
50
60
Time (min)
Normal
LY50
LY75
LY88
LY94
LY97
LY data
Normal data

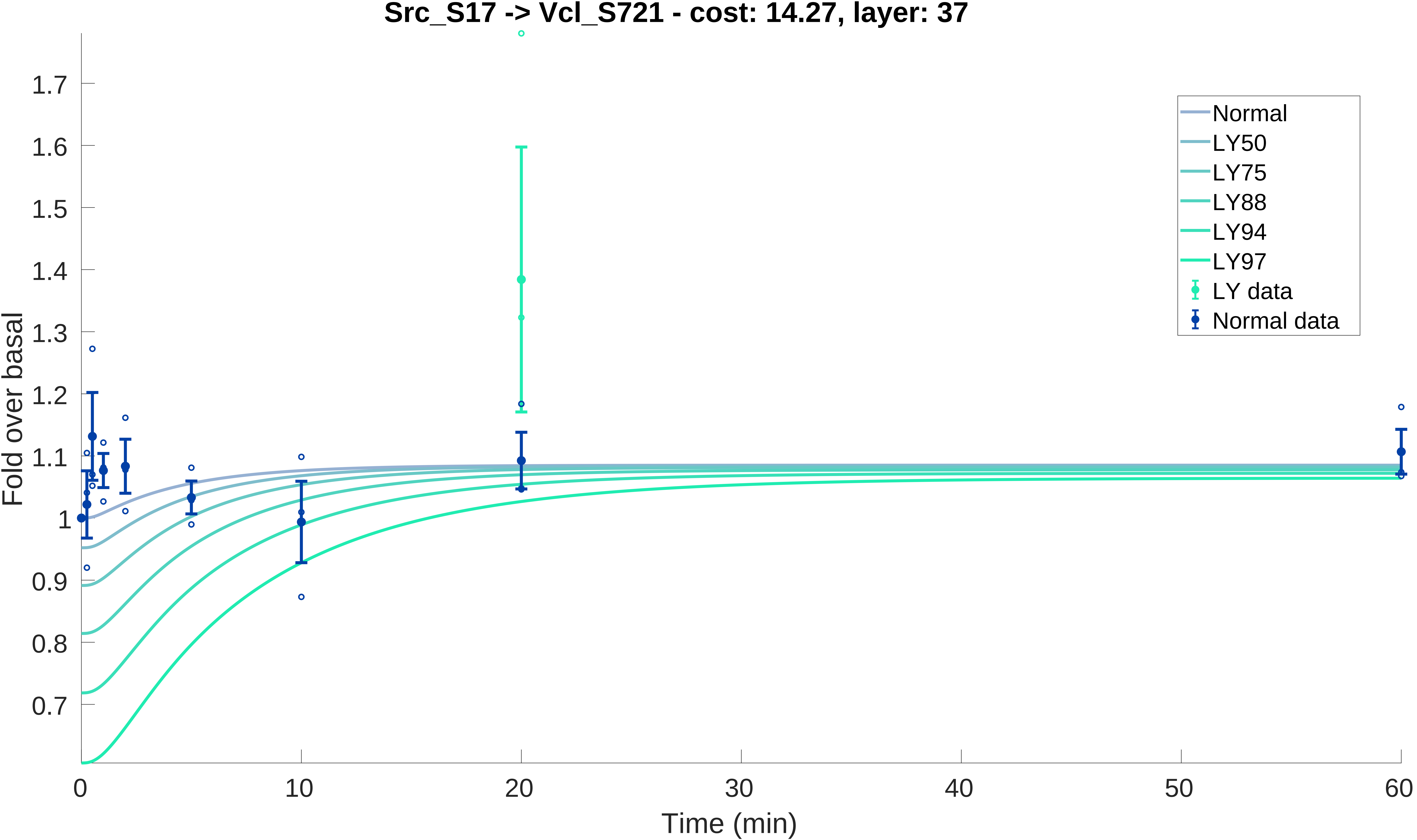

Src_S17 -> Vcl_S721 - cost: 14.27, layer: 37
Fold over basal
Time (min)
1.7
1.6
1.5
1.4
1.3
1.2
1.1
1
0.9
0.8
0.7
0
10
20
30
40
50
60
Normal
LY50
LY75
LY88
LY94
LY97
LY data
Normal data

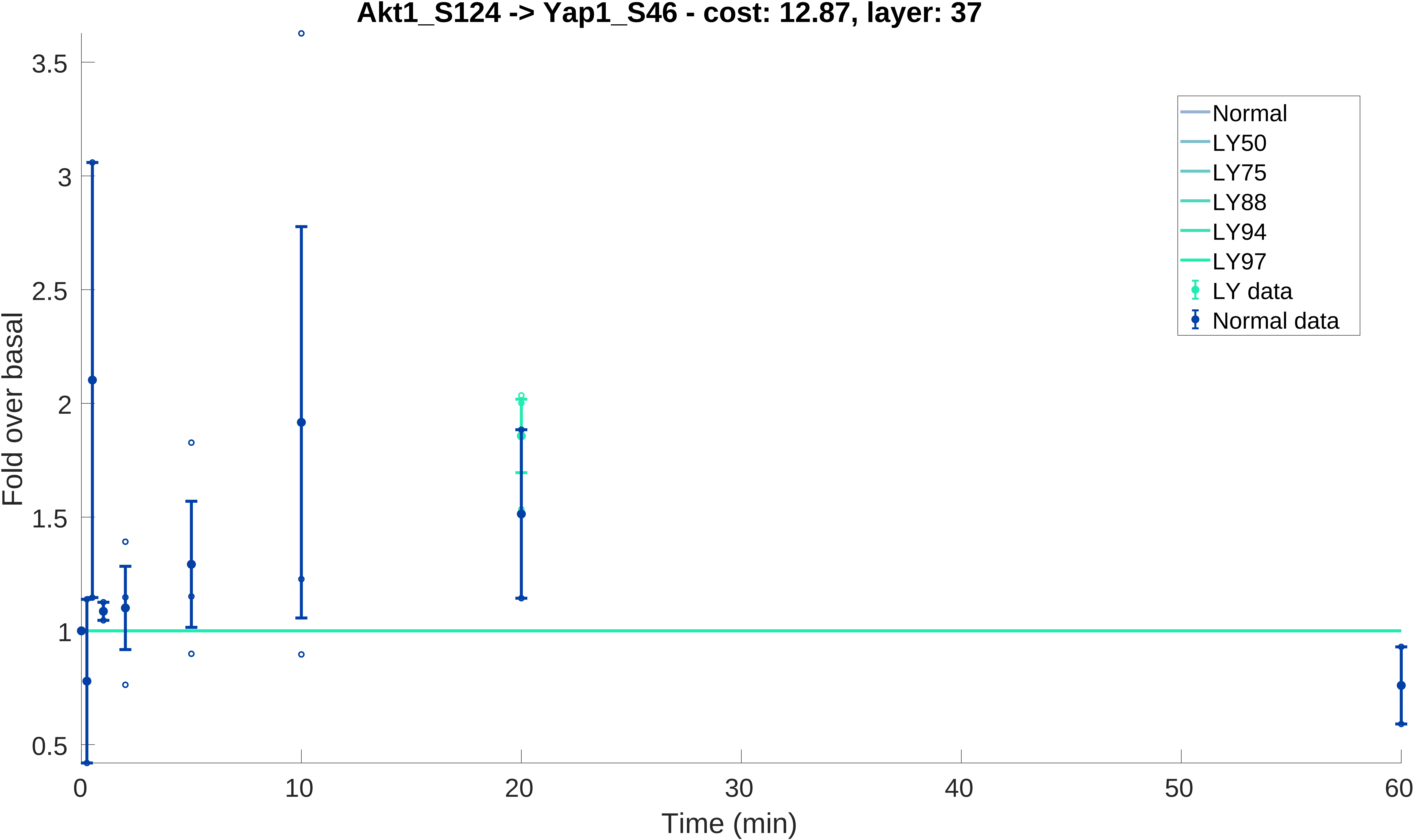

Akt1_S124 -> Yap1_S46 - cost: 12.87, layer: 37
Fold over basal
Time (min)
0
10
20
30
40
50
60
0.5
1
1.5
2
2.5
3
3.5
Normal
LY50
LY75
LY88
LY94
LY97
LY data
Normal data

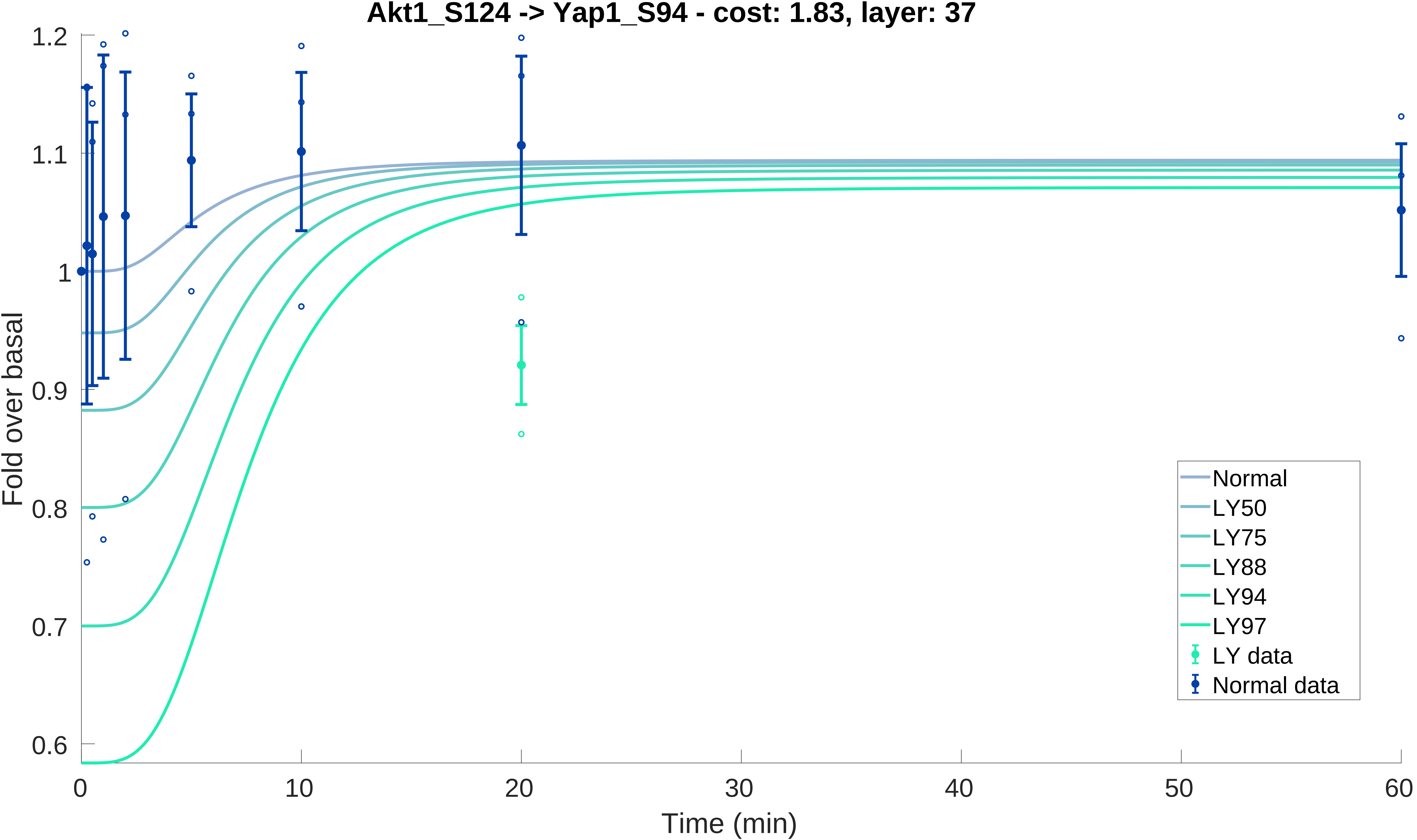
Akt1_S124 -> Yap1_S94 - cost: 1.83, layer: 37
Fold over basal
Time (min)
1.2
1.1
1
0.9
0.8
0.7
0.6
0
10
20
30
40
50
60
Normal
LY50
LY75
LY88
LY94
LY97
LY data
Normal data

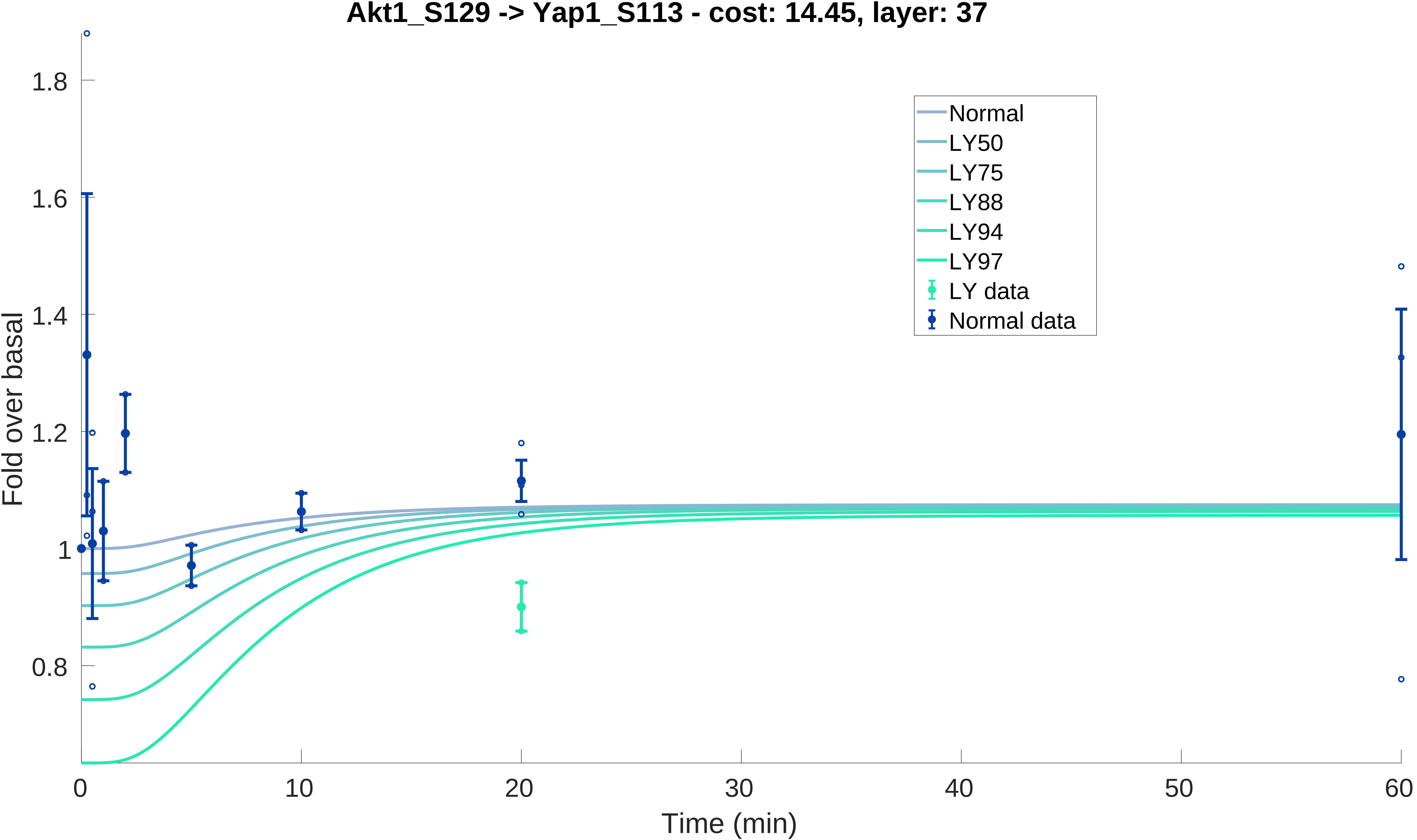

Akt1_S129 -> Yap1_S113 - cost: 14.45, layer: 37
Fold over basal
1.8
1.6
1.4
1.2
1
0.8
0
10
20
30
40
50
60
Time (min)
Normal
LY50
LY75
LY88
LY94
LY97
LY data
Normal data

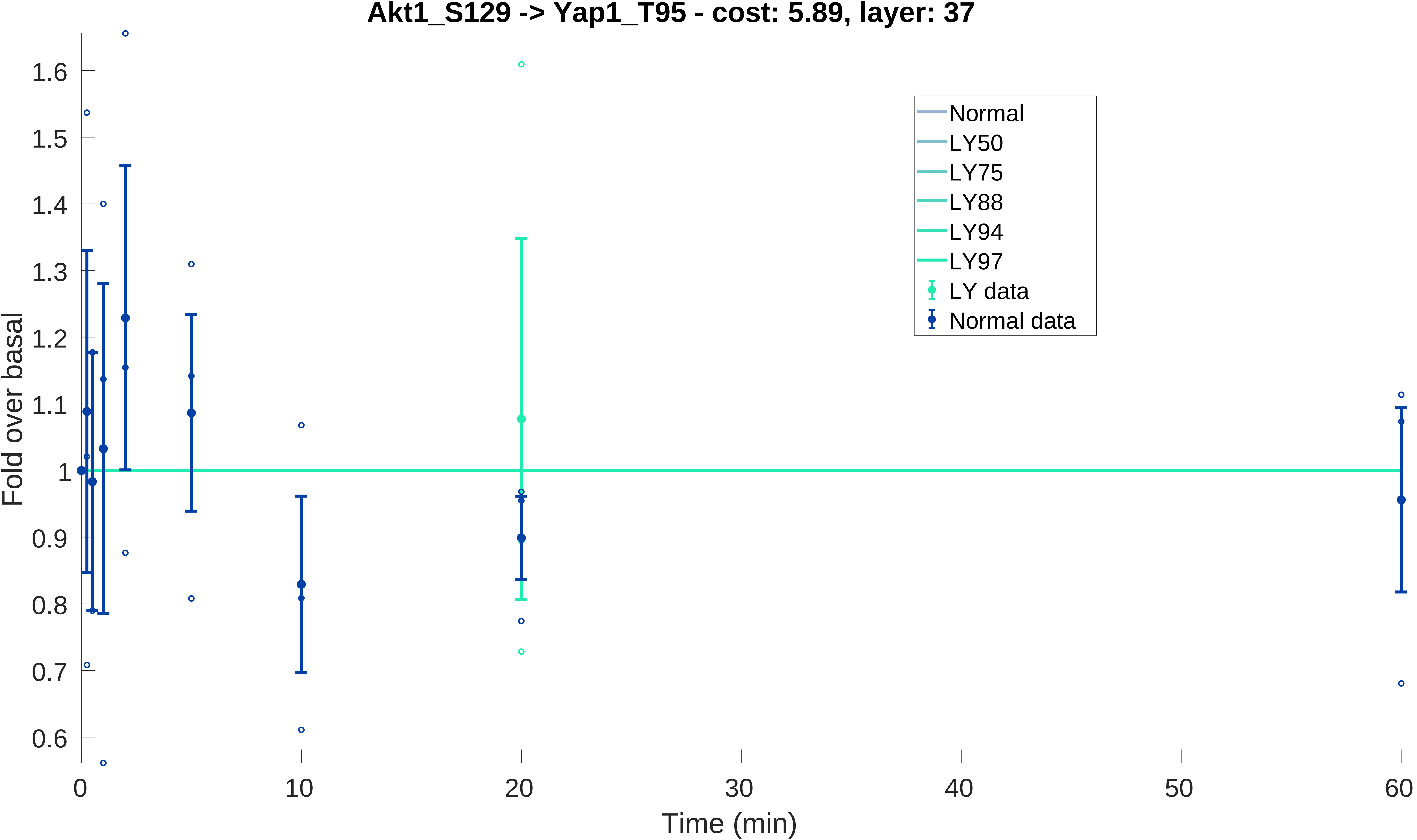

Akt1_S129 -> Yap1_T95 - cost: 5.89, layer: 37
Fold over basal
1.6
1.5
1.4
1.3
1.2
1.1
1
0.9
0.8
0.7
0.6
0
10
20
30
40
50
60
Time (min)
Normal
LY50
LY75
LY88
LY94
LY97
LY data
Normal data

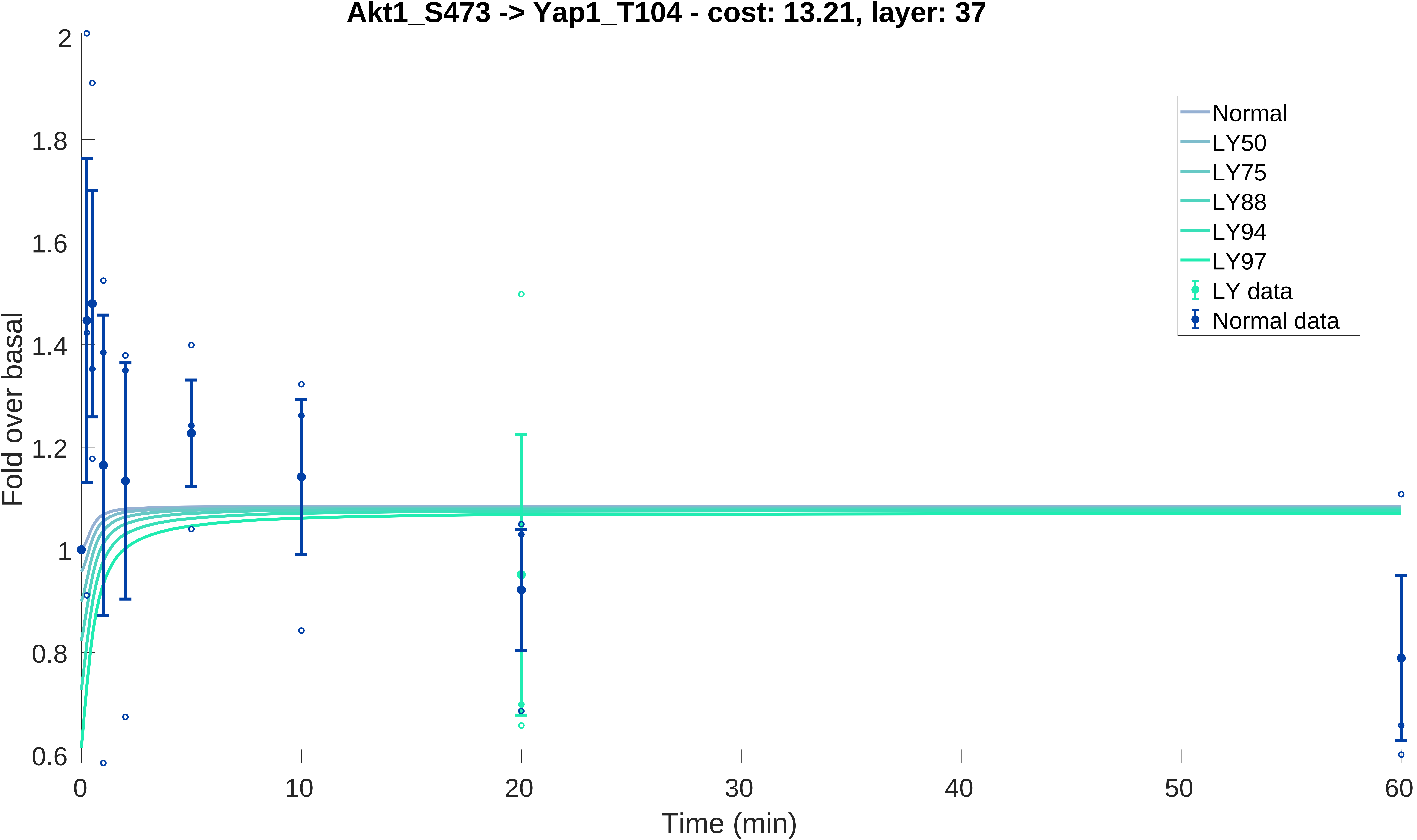

Akt1_S473 -> Yap1_T104 - cost: 13.21, layer: 37
Fold over basal
Time (min)
Normal
LY50
LY75
LY88
LY94
LY97
LY data
Normal data
2
1.8
1.6
1.4
1.2
1
0.8
0.6
0
10
20
30
40
50
60

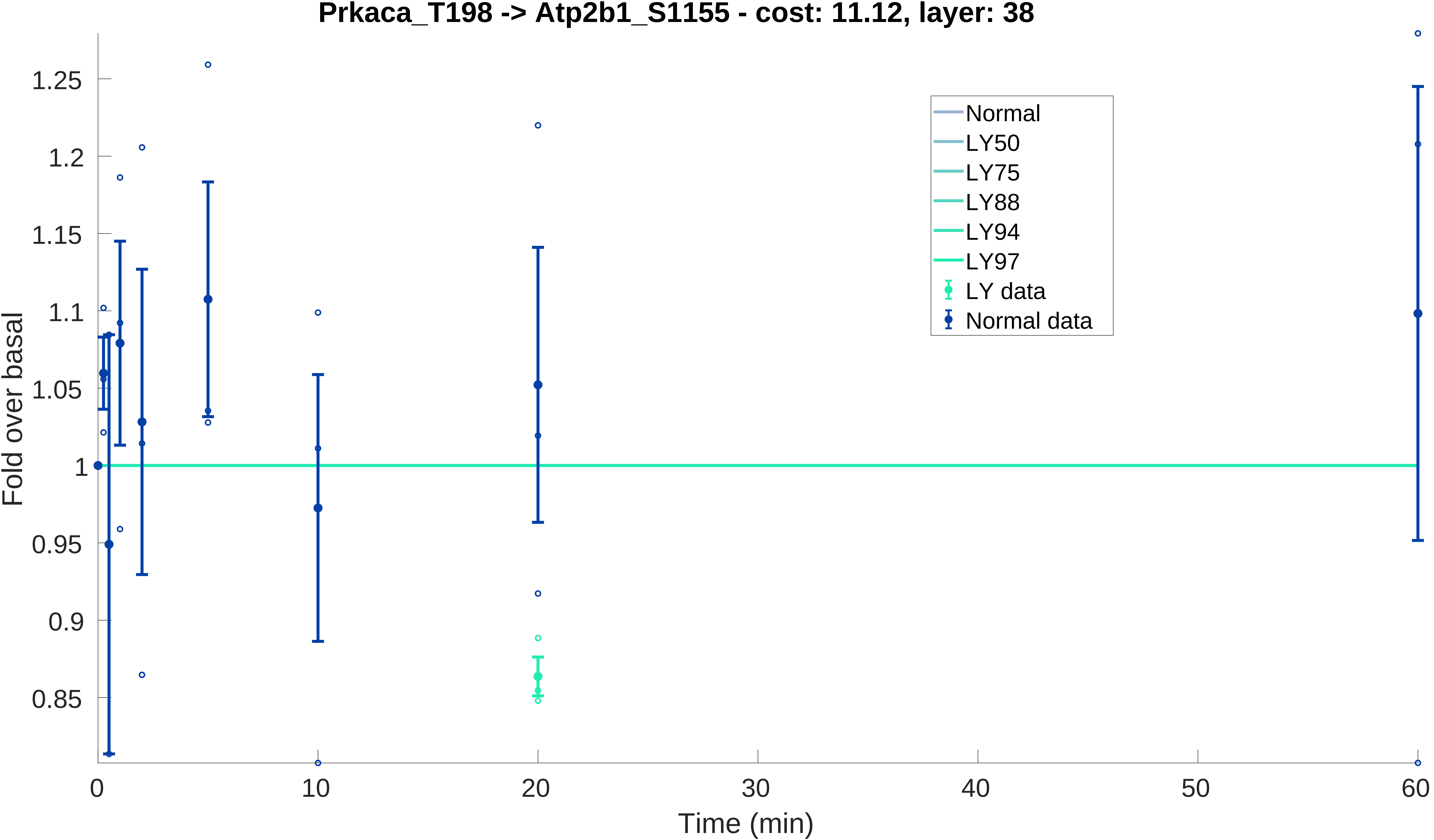

Prkaca_T198 -> Atp2b1_S1155 - cost: 11.12, layer: 38
Fold over basal
1.25
1.2
1.15
1.1
1.05
1
0.95
0.9
0.85
0
10
20
30
40
50
60
Time (min)
Normal
LY50
LY75
LY88
LY94
LY97
LY data
Normal data

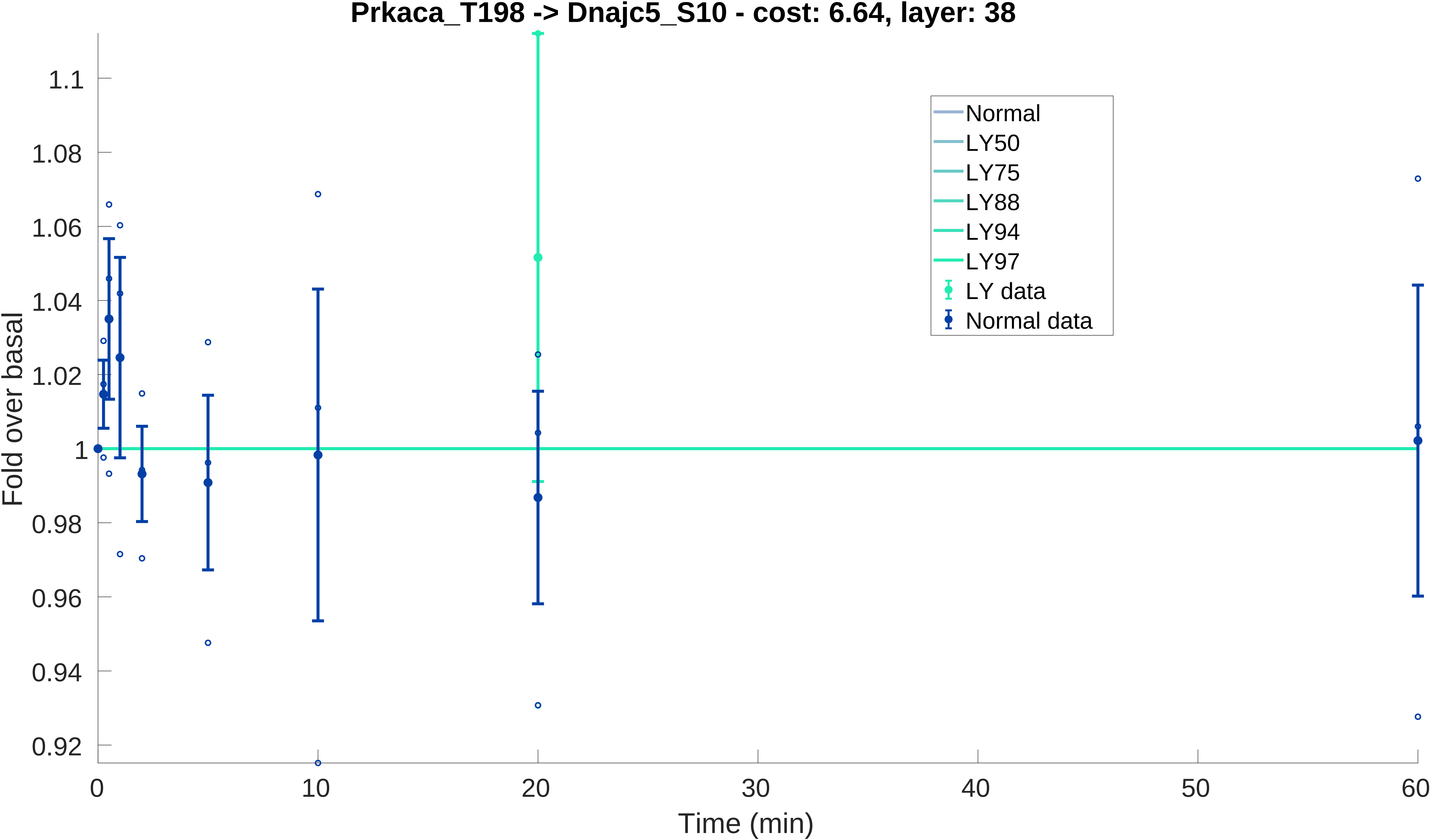

Prkaca_T198 -> Dnajc5_S10 - cost: 6.64, layer: 38
Fold over basal
Time (min)
1.1
1.08
1.06
1.04
1.02
1
0.98
0.96
0.94
0.92
0
10
20
30
40
50
60
Normal
LY50
LY75
LY88
LY94
LY97
LY data
Normal data

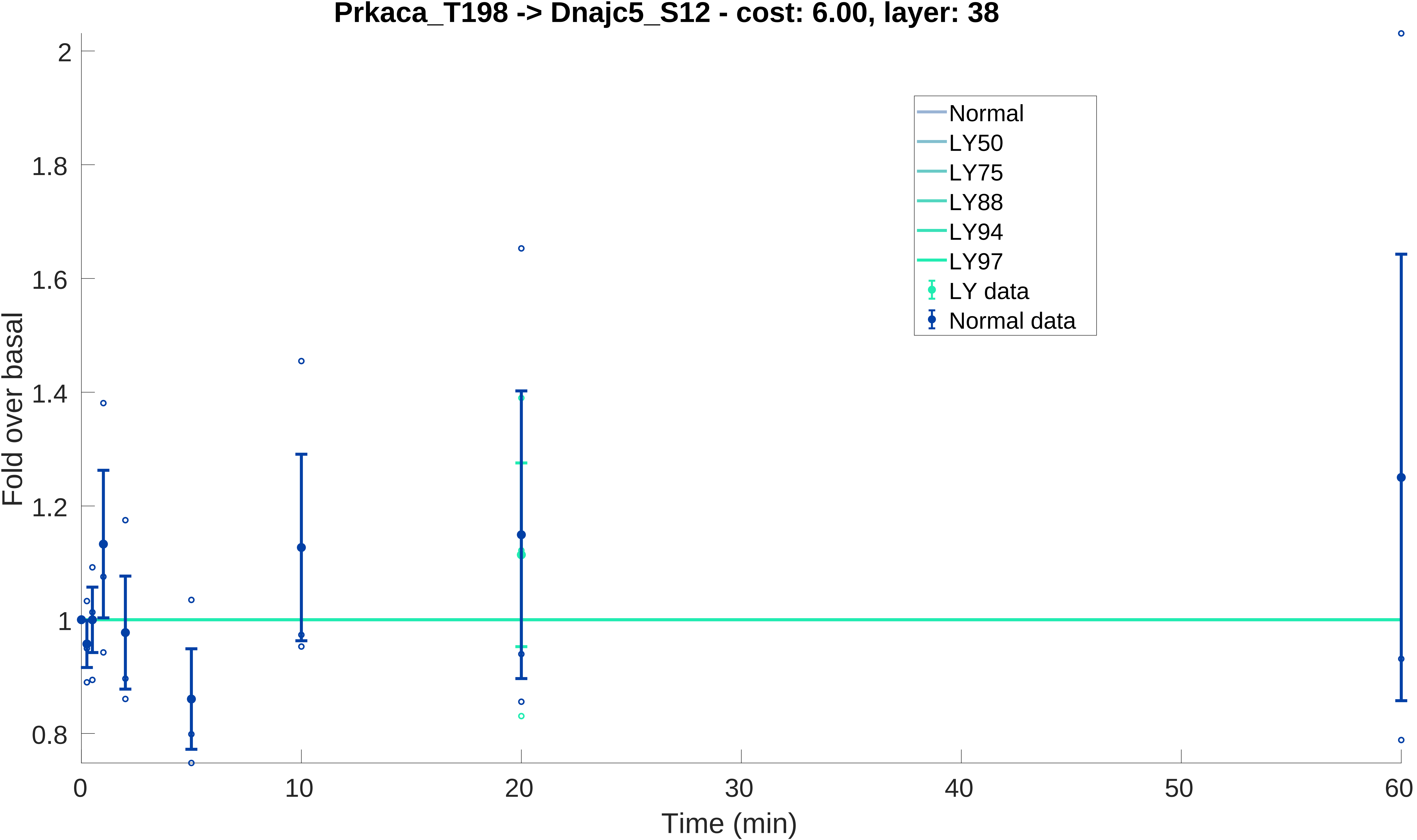

Prkaca_T198 -> Dnajc5_S12 - cost: 6.00, layer: 38
Fold over basal
Time (min)
0.8
1
1.2
1.4
1.6
1.8
2
0
10
20
30
40
50
60
Normal
LY50
LY75
LY88
LY94
LY97
LY data
Normal data

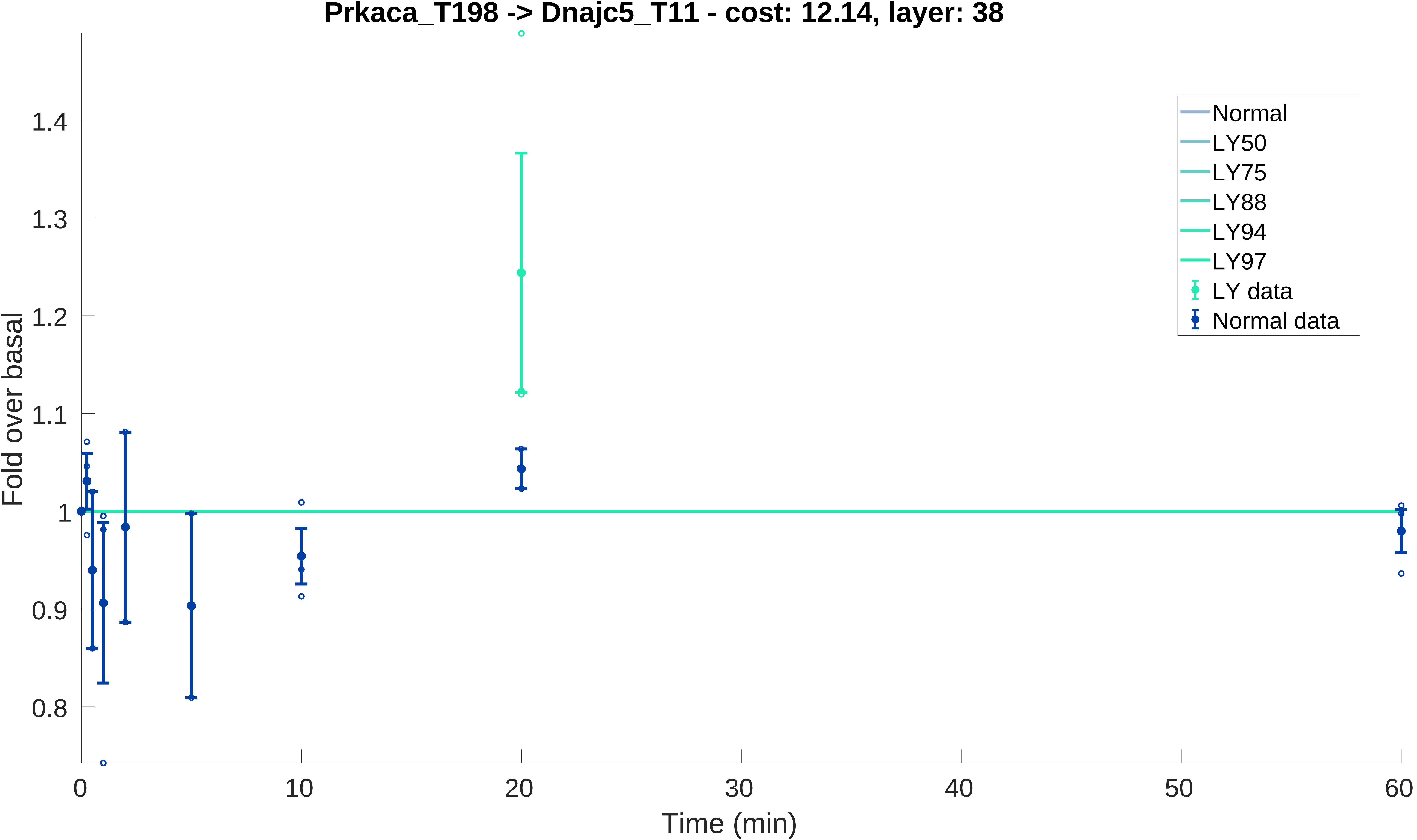

Prkaca_T198 -> Dnajc5_T11 - cost: 12.14, layer: 38
Fold over basal
Time (min)
0.8
0.9
1
1.1
1.2
1.3
1.4
0
10
20
30
40
50
60
Normal
LY50
LY75
LY88
LY94
LY97
LY data
Normal data

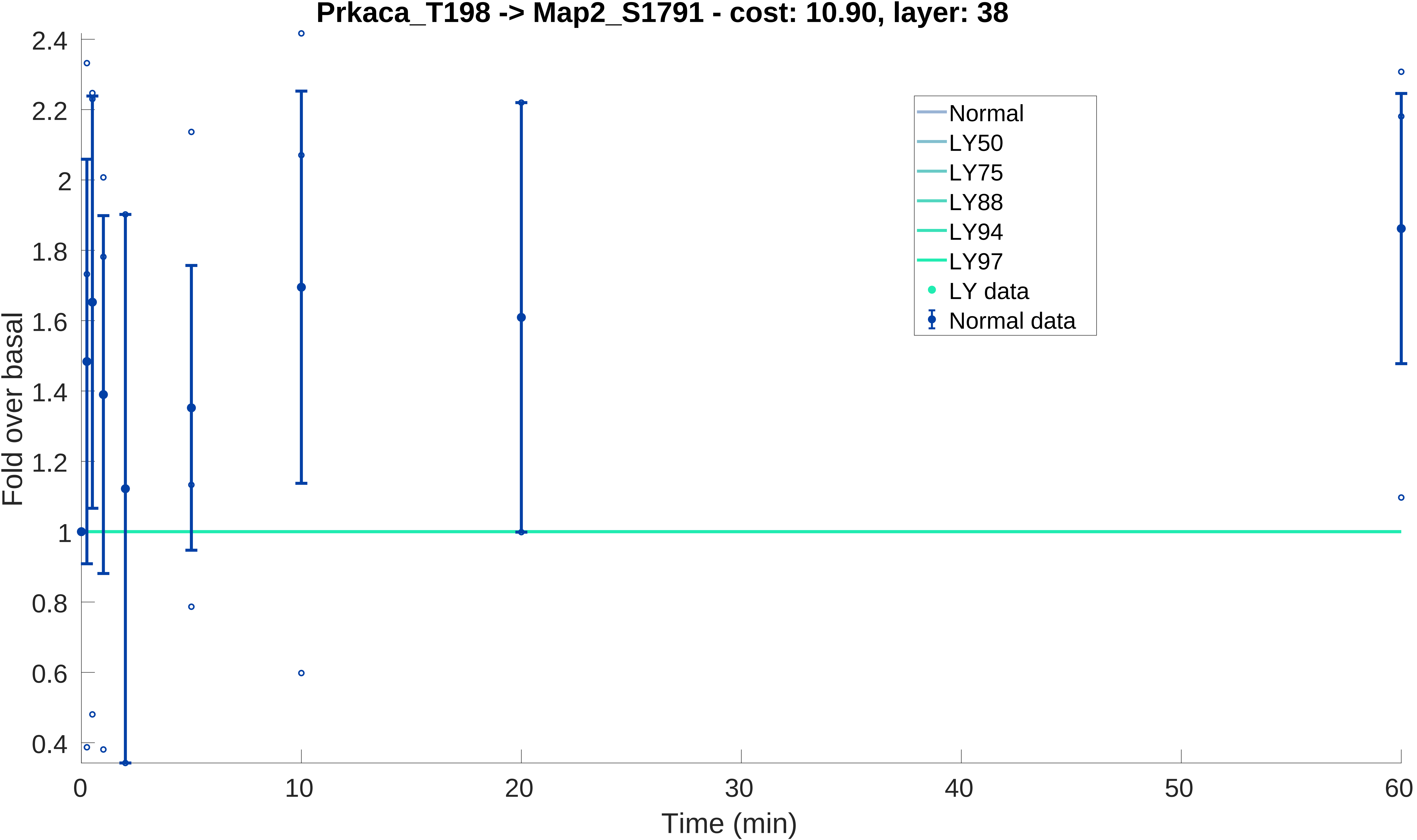

Prkaca_T198 -> Map2_S1791 - cost: 10.90, layer: 38
Fold over basal
Time (min)
Normal
LY50
LY75
LY88
LY94
LY97
LY data
Normal data
2.4
2.2
2
1.8
1.6
1.4
1.2
1
0.8
0.6
0.4
0
10
20
30
40
50
60

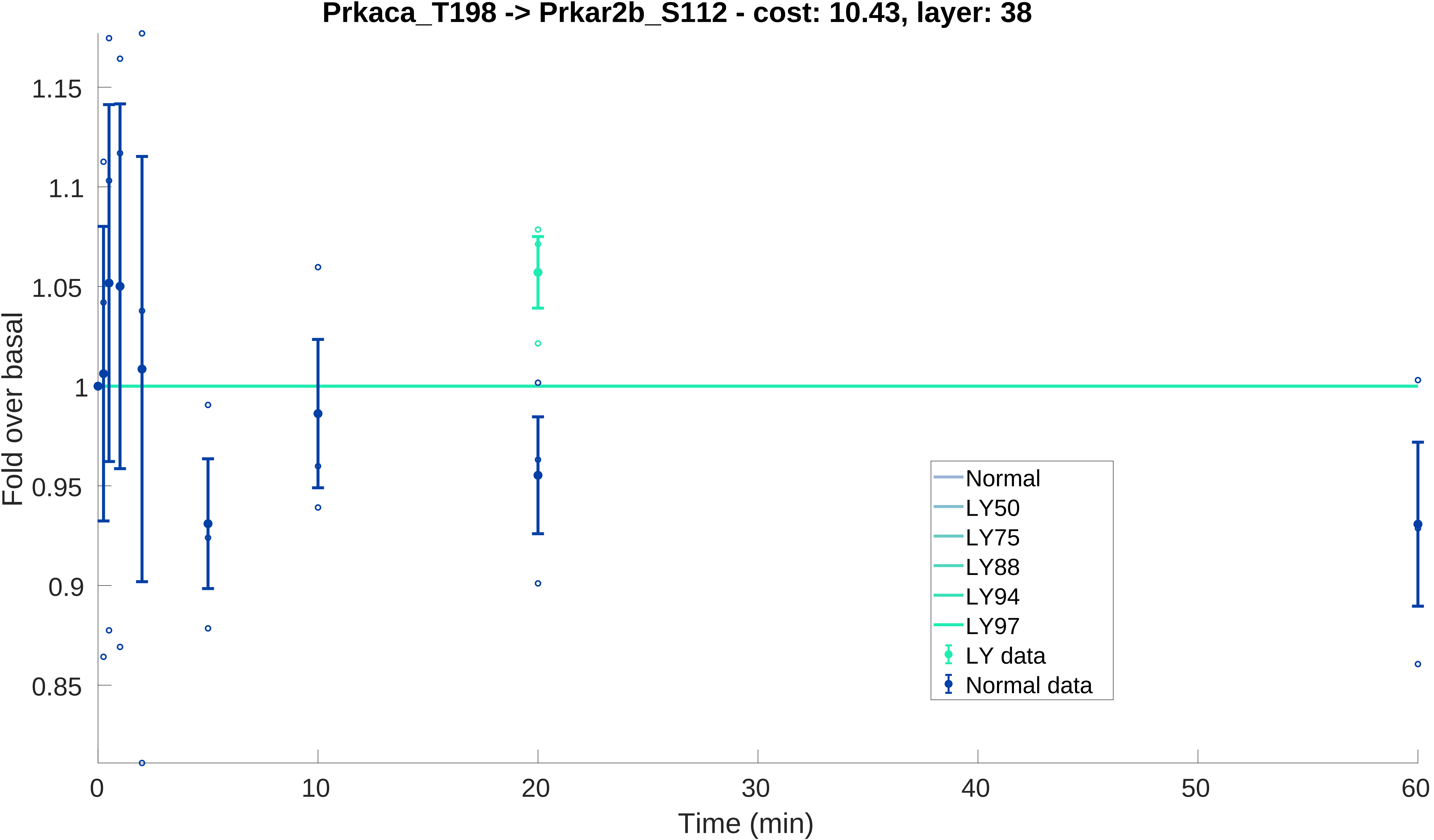

Prkaca_T198 -> Prkar2b_S112 - cost: 10.43, layer: 38
Fold over basal
1.15
1.1
1.05
1
0.95
0.9
0.85
0
10
20
30
40
50
60
Time (min)
Normal
LY50
LY75
LY88
LY94
LY97
LY data
Normal data

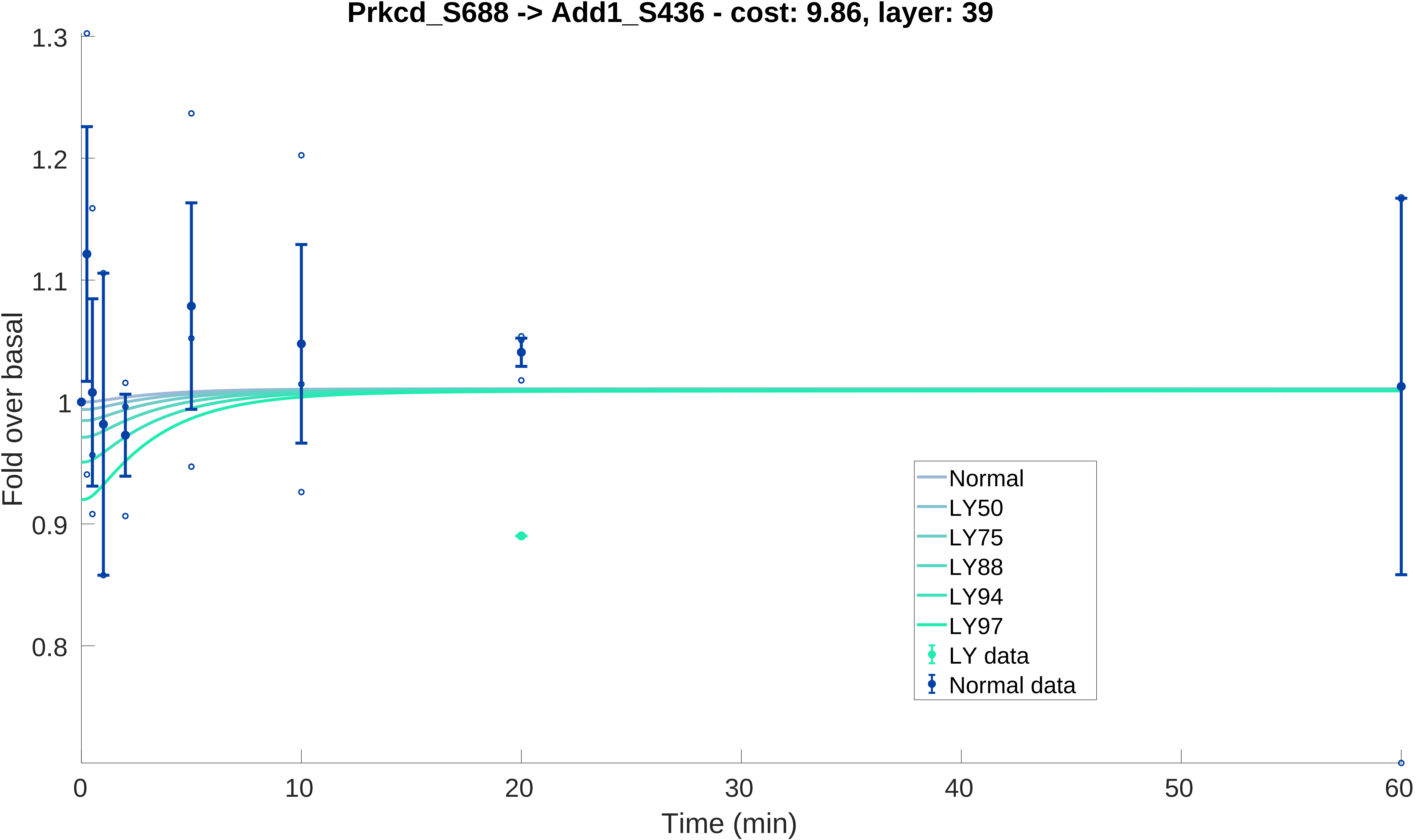

Prkcd_S688 -> Add1_S436 - cost: 9.86, layer: 39
Fold over basal
1.3
1.2
1.1
1
0.9
0.8
Time (min)
0
10
20
30
40
50
60
Normal
LY50
LY75
LY88
LY94
LY97
LY data
Normal data

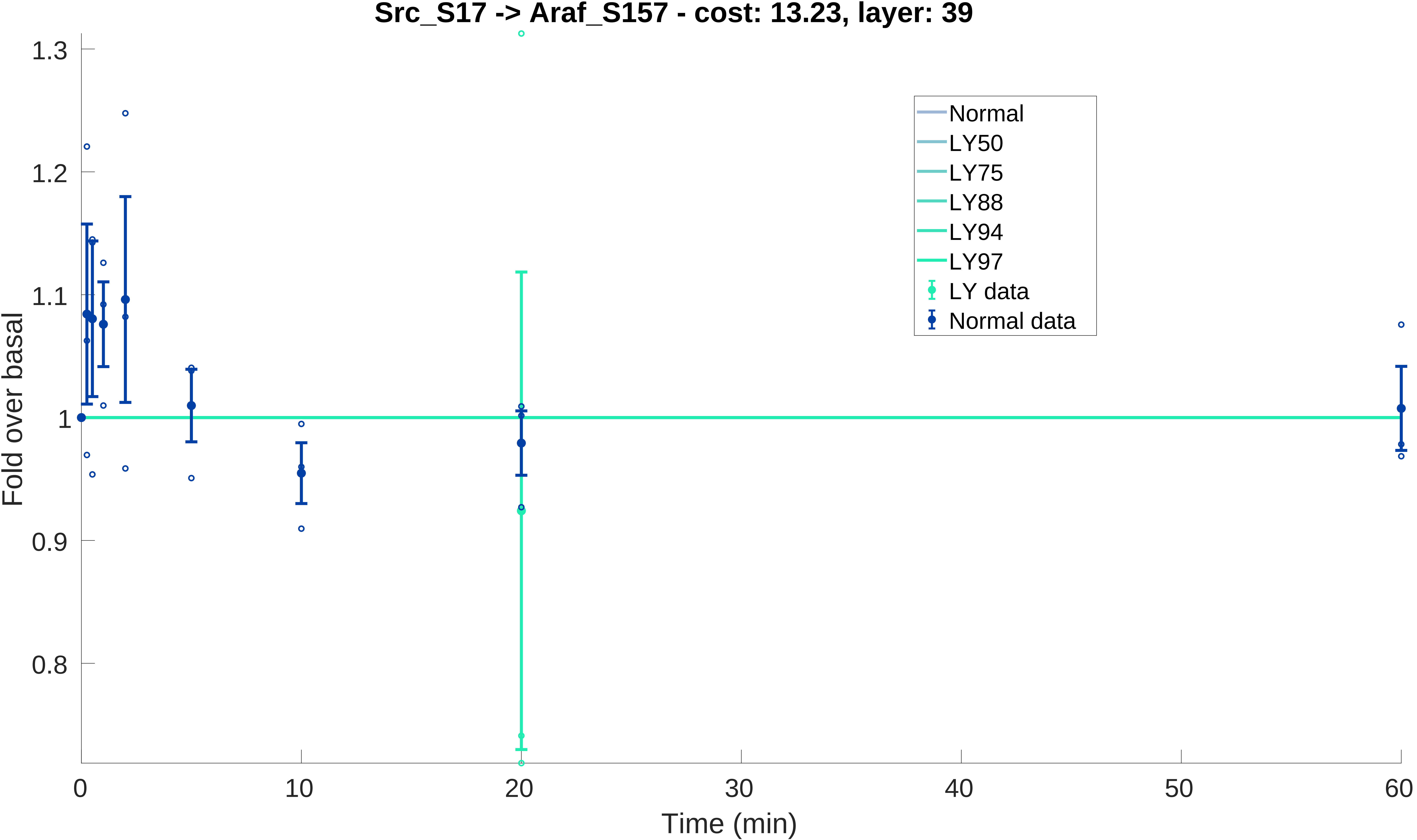

Src_S17 -> Araf_S157 - cost: 13.23, layer: 39
Fold over basal
1.3
1.2
1.1
1
0.9
0.8
0
10
20
30
40
50
60
Time (min)
Normal
LY50
LY75
LY88
LY94
LY97
LY data
Normal data

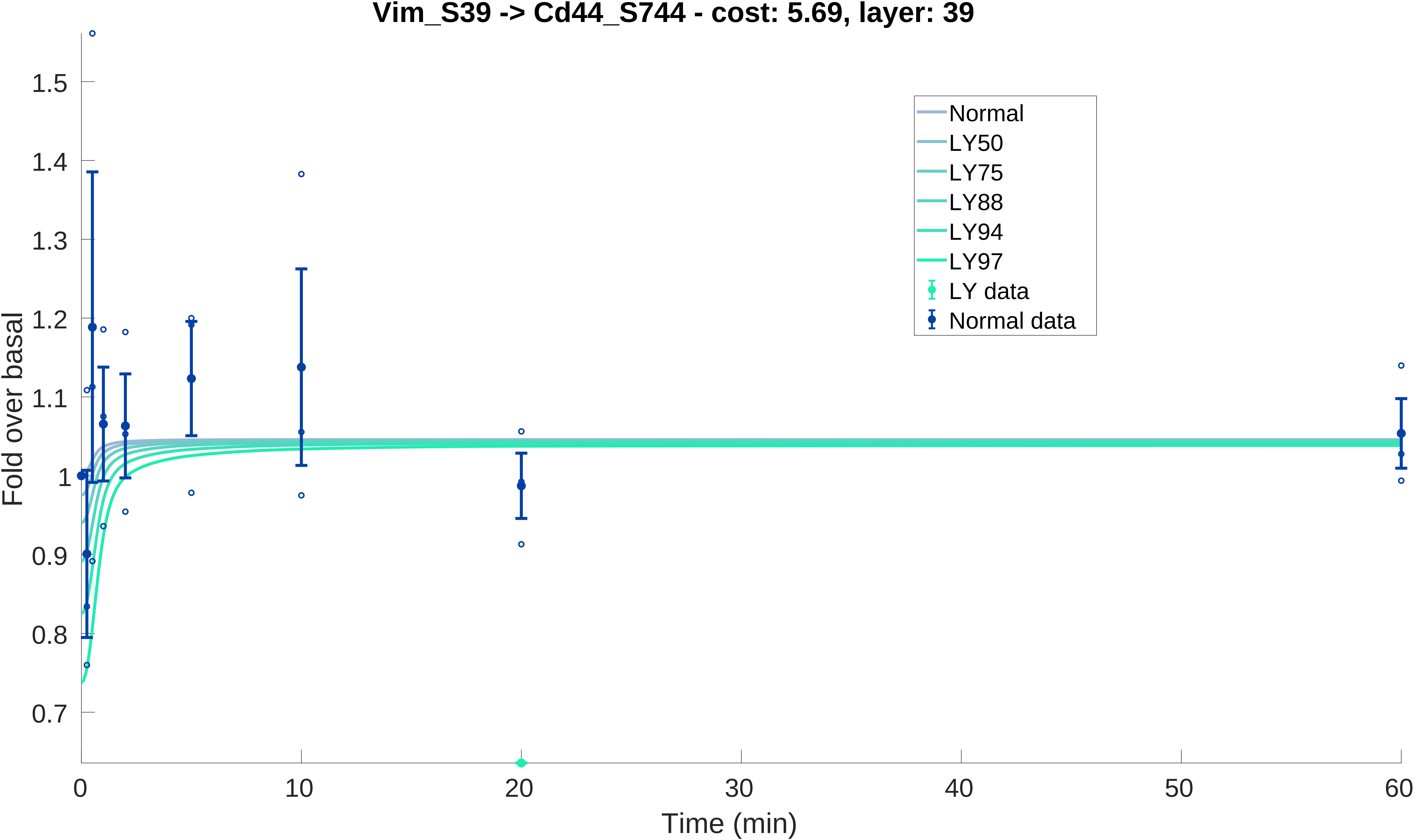

Vim_S39 -> Cd44_S744 - cost: 5.69, layer: 39
Fold over basal
Time (min)
Normal
LY50
LY75
LY88
LY94
LY97
LY data
Normal data
1.5
1.4
1.3
1.2
1.1
1
0.9
0.8
0.7
0
10
20
30
40
50
60

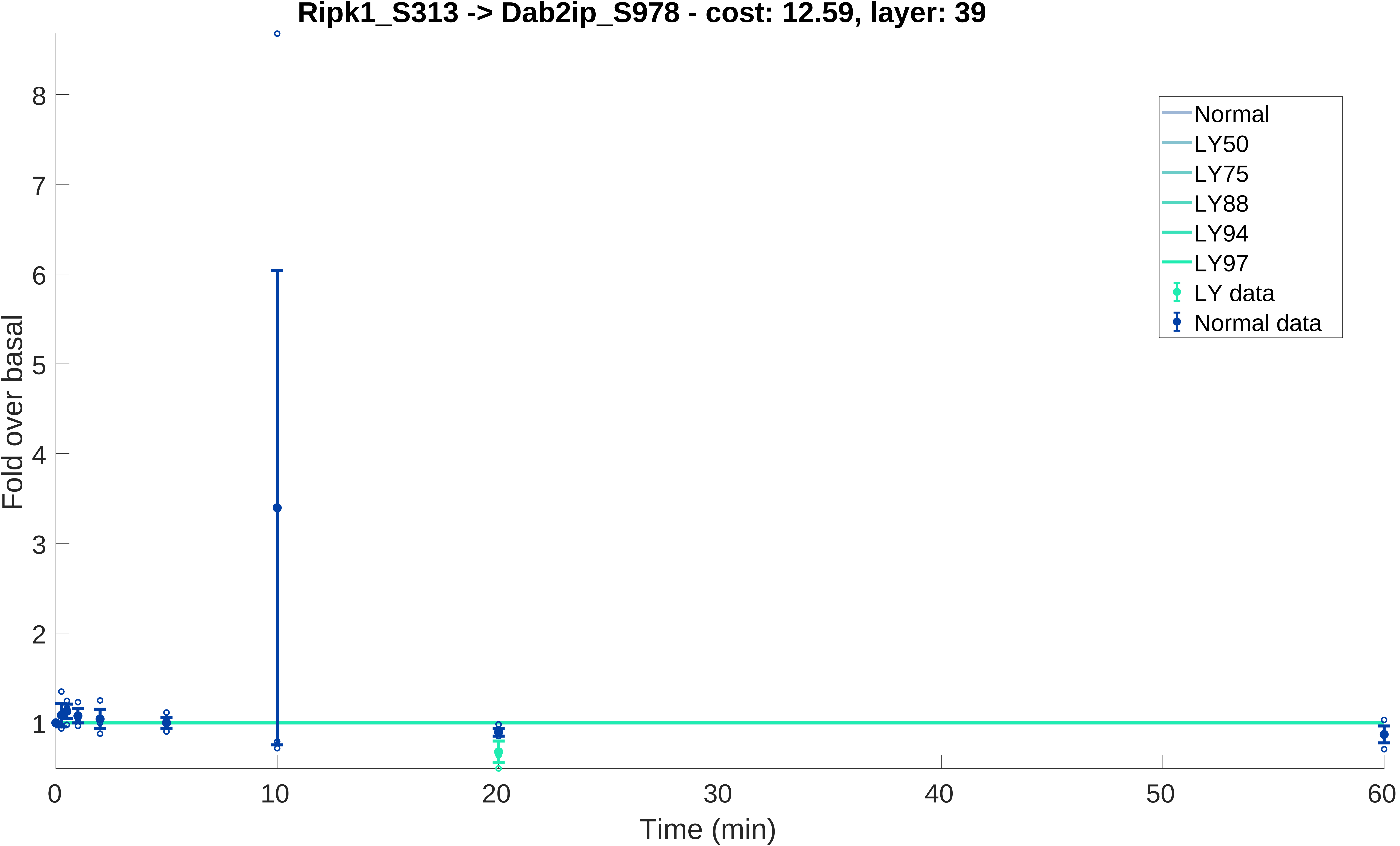

Ripk1_S313 -> Dab2ip_S978 - cost: 12.59, layer: 39
Fold over basal
Time (min)
Normal
LY50
LY75
LY88
LY94
LY97
LY data
Normal data
0
10
20
30
40
50
60
1
2
3
4
5
6
7
8

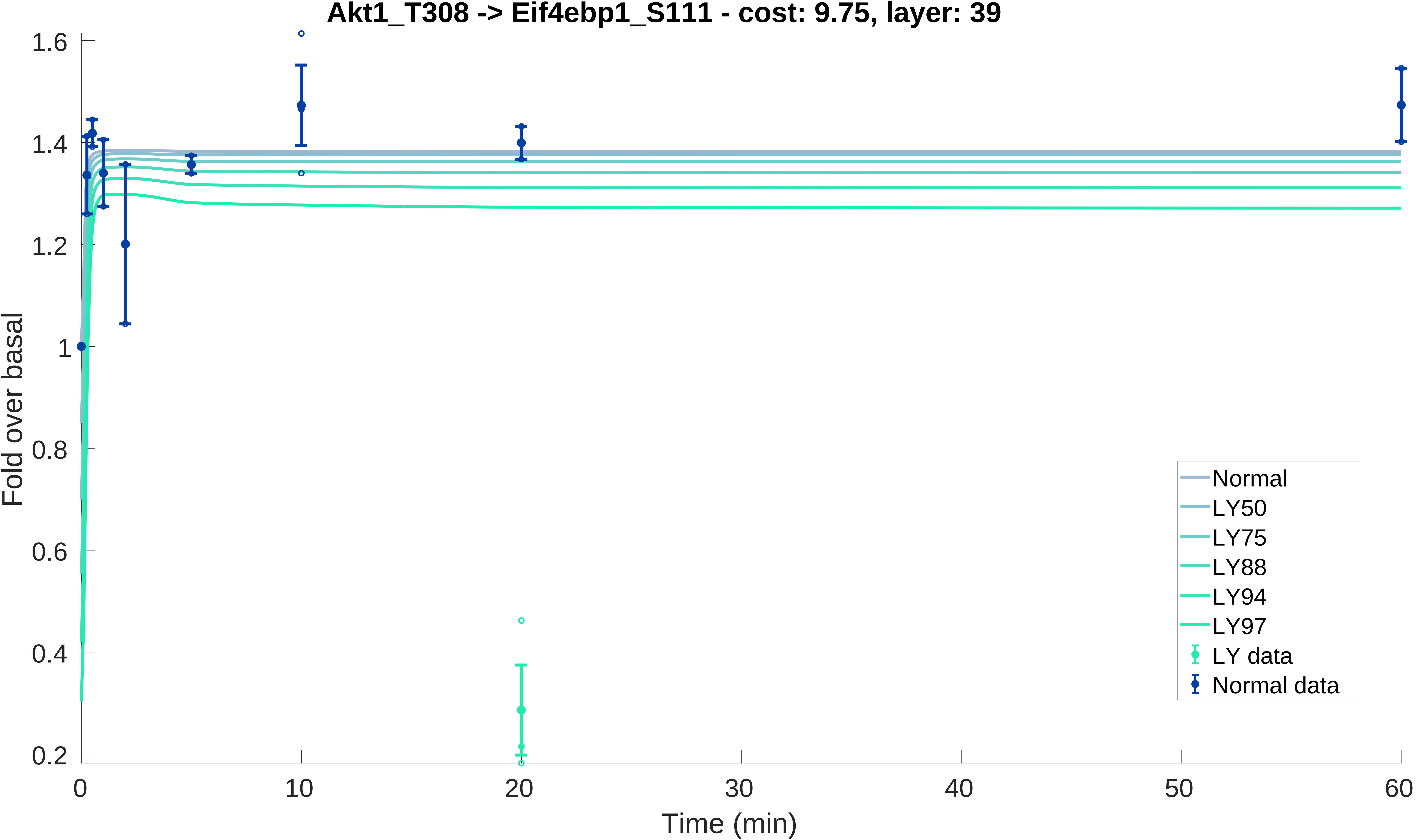

Akt1_T308 -> Eif4ebp1_S111 - cost: 9.75, layer: 39
Fold over basal
Time (min)
1.6
1.4
1.2
1
0.8
0.6
0.4
0.2
0
10
20
30
40
50
60
Normal
LY50
LY75
LY88
LY94
LY97
LY data
Normal data

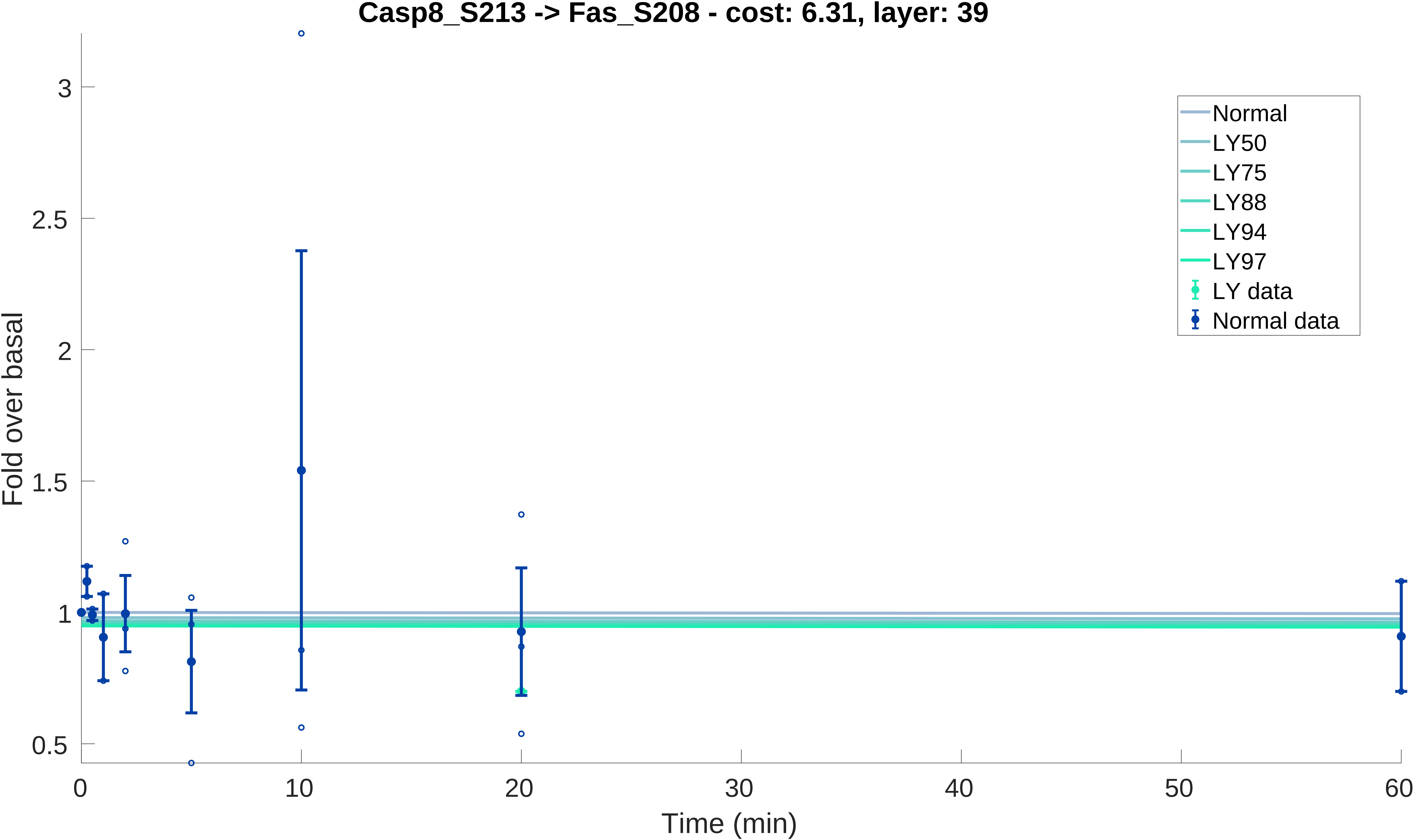

Casp8_S213 -> Fas_S208 - cost: 6.31, layer: 39
Fold over basal
Time (min)
0.5
1
1.5
2
2.5
3
0
10
20
30
40
50
60
Normal
LY50
LY75
LY88
LY94
LY97
LY data
Normal data

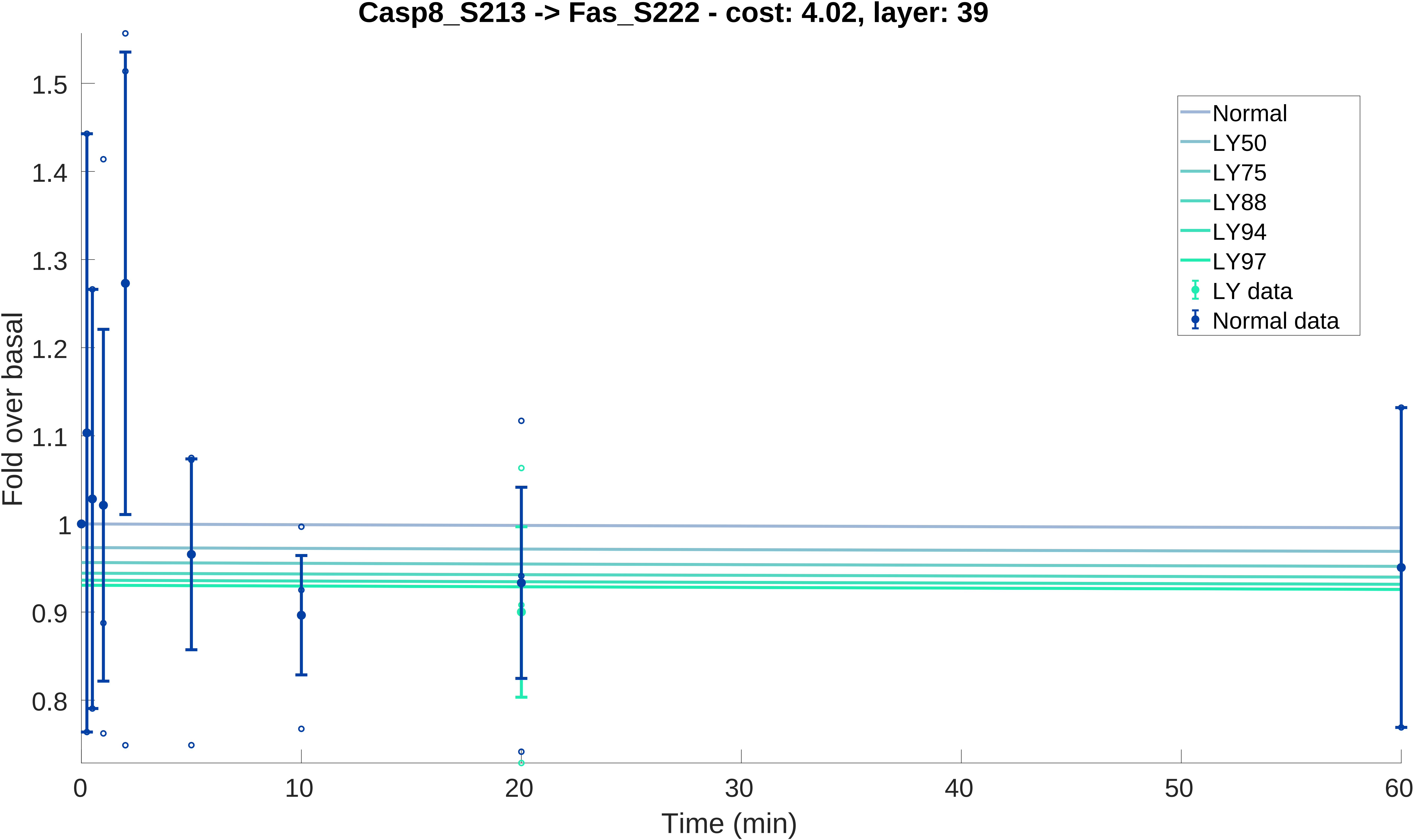

Casp8_S213 -> Fas_S222 - cost: 4.02, layer: 39
Fold over basal
Time (min)
0
10
20
30
40
50
60
0.8
0.9
1
1.1
1.2
1.3
1.4
1.5
Normal
LY50
LY75
LY88
LY94
LY97
LY data
Normal data

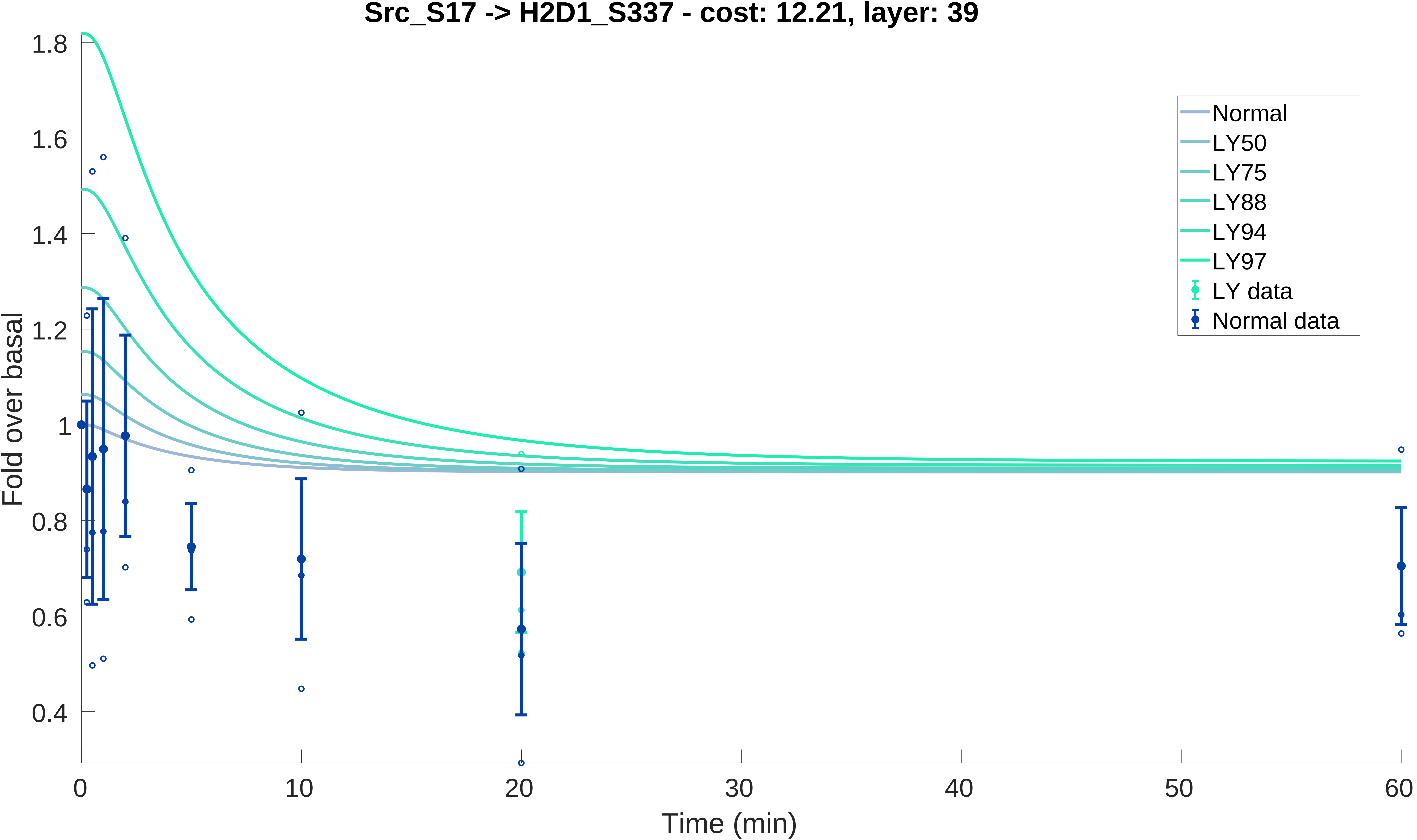

Src_S17 -> H2D1_S337 - cost: 12.21, layer: 39
Fold over basal
Time (min)
0
10
20
30
40
50
60
0.4
0.6
0.8
1
1.2
1.4
1.6
1.8
Normal
LY50
LY75
LY88
LY94
LY97
LY data
Normal data

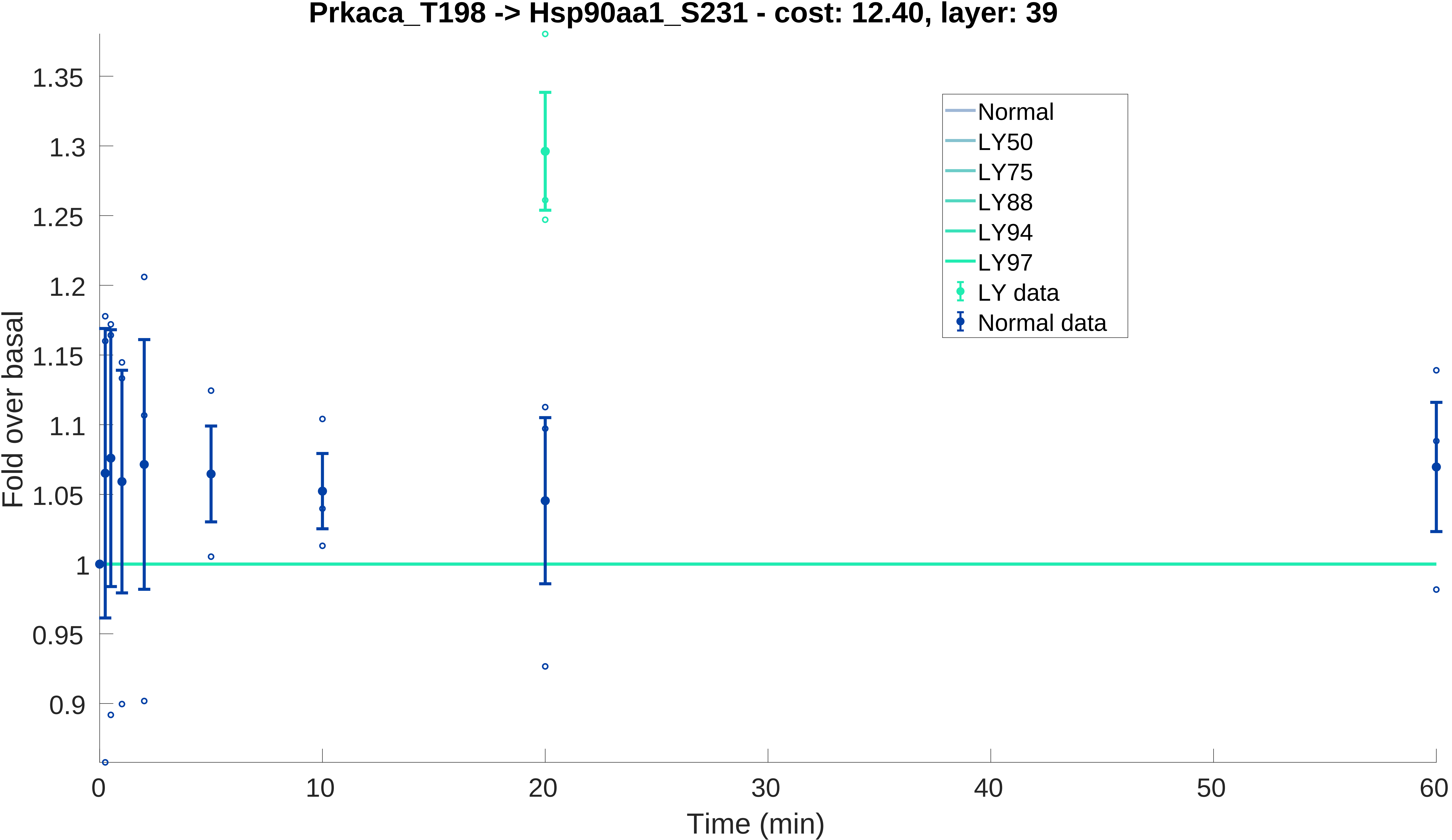

Prkaca_T198 -> Hsp90aa1_S231 - cost: 12.40, layer: 39
Fold over basal
Time (min)
1.35
1.3
1.25
1.2
1.15
1.1
1.05
1
0.95
0.9
0
10
20
30
40
50
60
Normal
LY50
LY75
LY88
LY94
LY97
LY data
Normal data

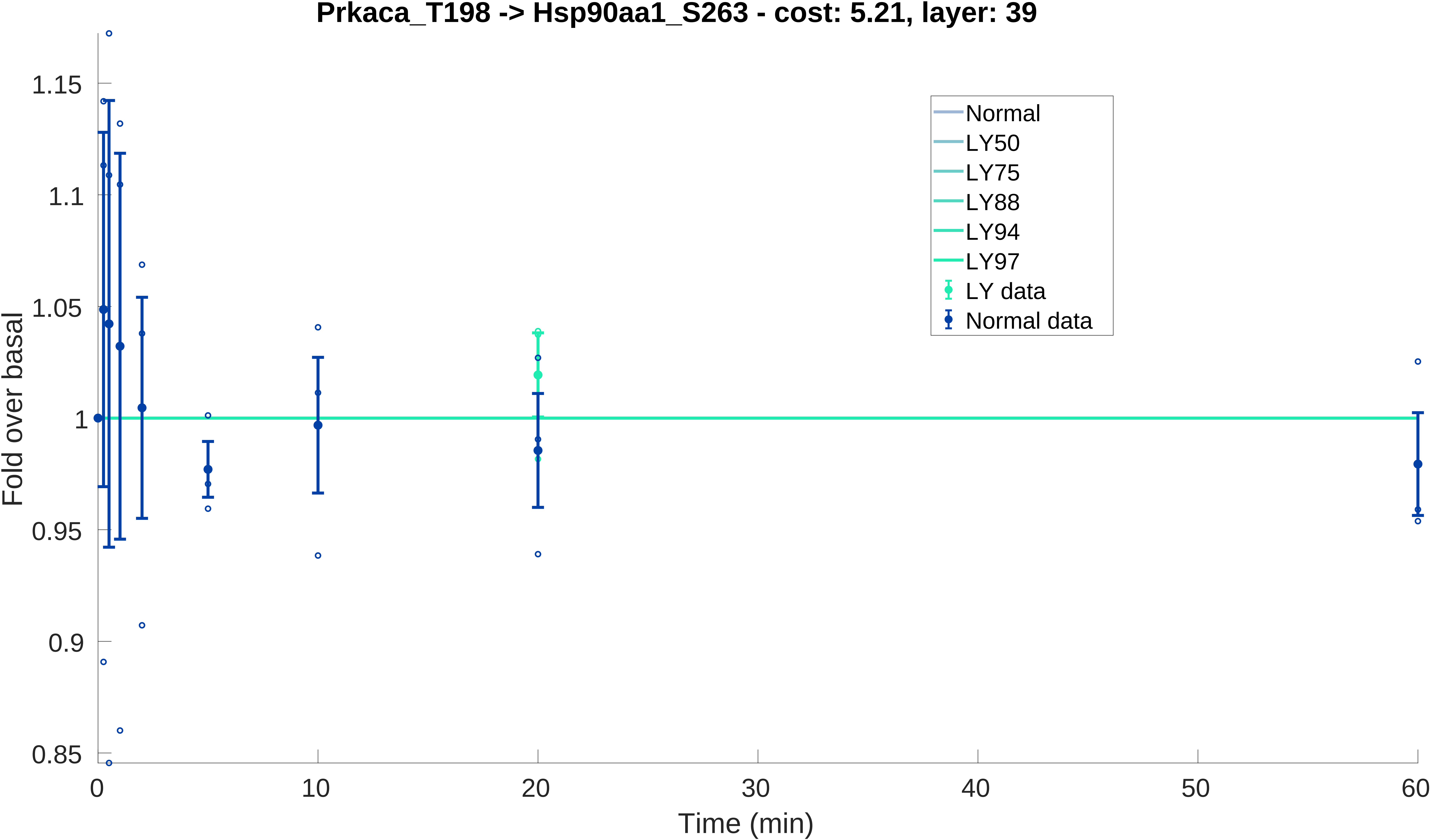

Prrkaca_T198 -> Hsp90aa1_S263 - cost: 5.21, layer: 39
Fold over basal
Time (min)
1.15
1.1
1.05
1
0.95
0.9
0.85
0
10
20
30
40
50
60
Normal
LY50
LY75
LY88
LY94
LY97
LY data
Normal data

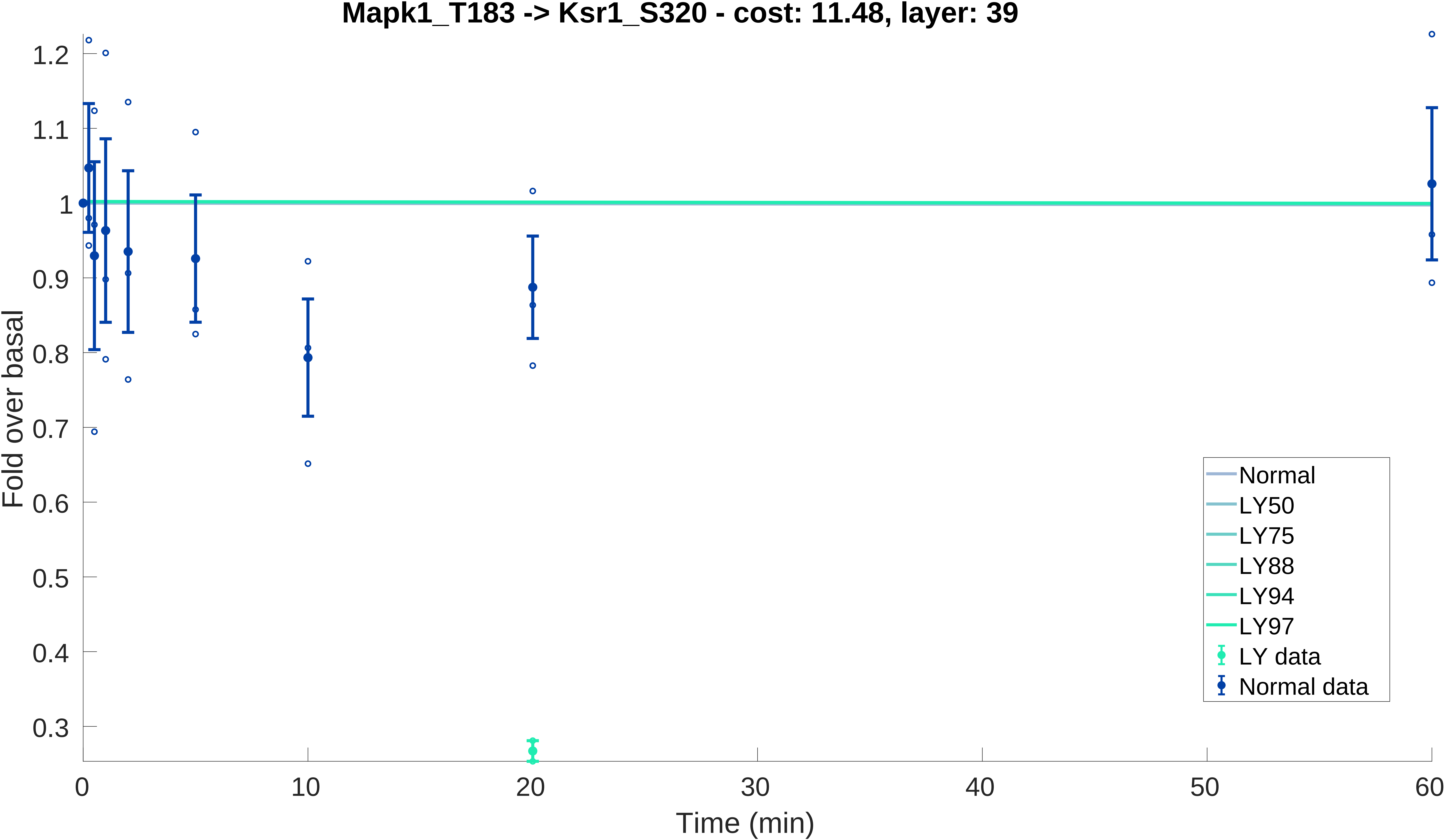

Mapk1_T183 -> Ksr1_S320 - cost: 11.48, layer: 39
Fold over basal
1.2
1.1
1
0.9
0.8
0.7
0.6
0.5
0.4
0.3
0
10
20
30
40
50
60
Time (min)
Normal
LY50
LY75
LY88
LY94
LY97
LY data
Normal data

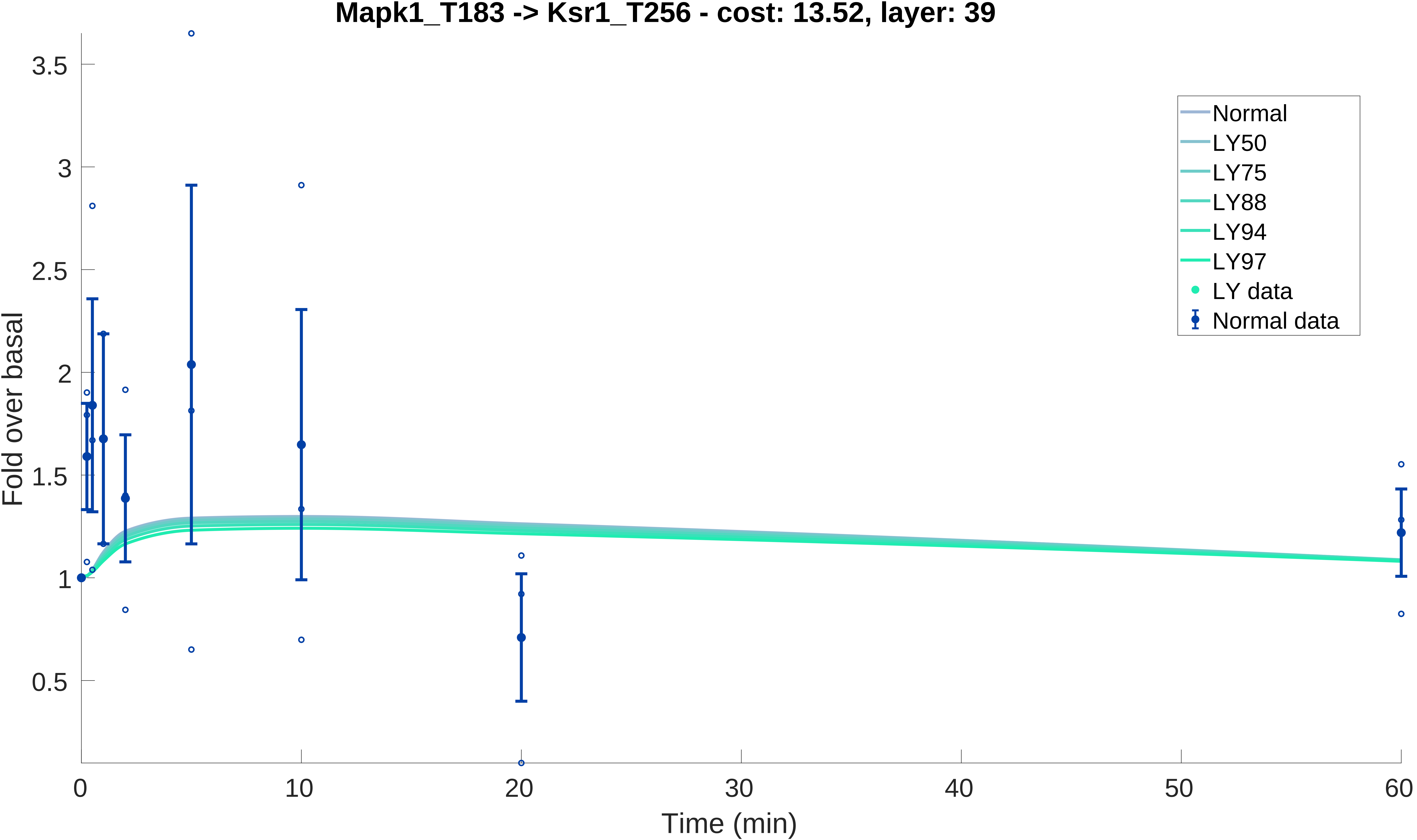

Mapk1_T183 -> Ksr1_T256 - cost: 13.52, layer: 39
Fold over basal
Time (min)
0.5
1
1.5
2
2.5
3
3.5
0
10
20
30
40
50
60
Normal
LY50
LY75
LY88
LY94
LY97
LY data
Normal data

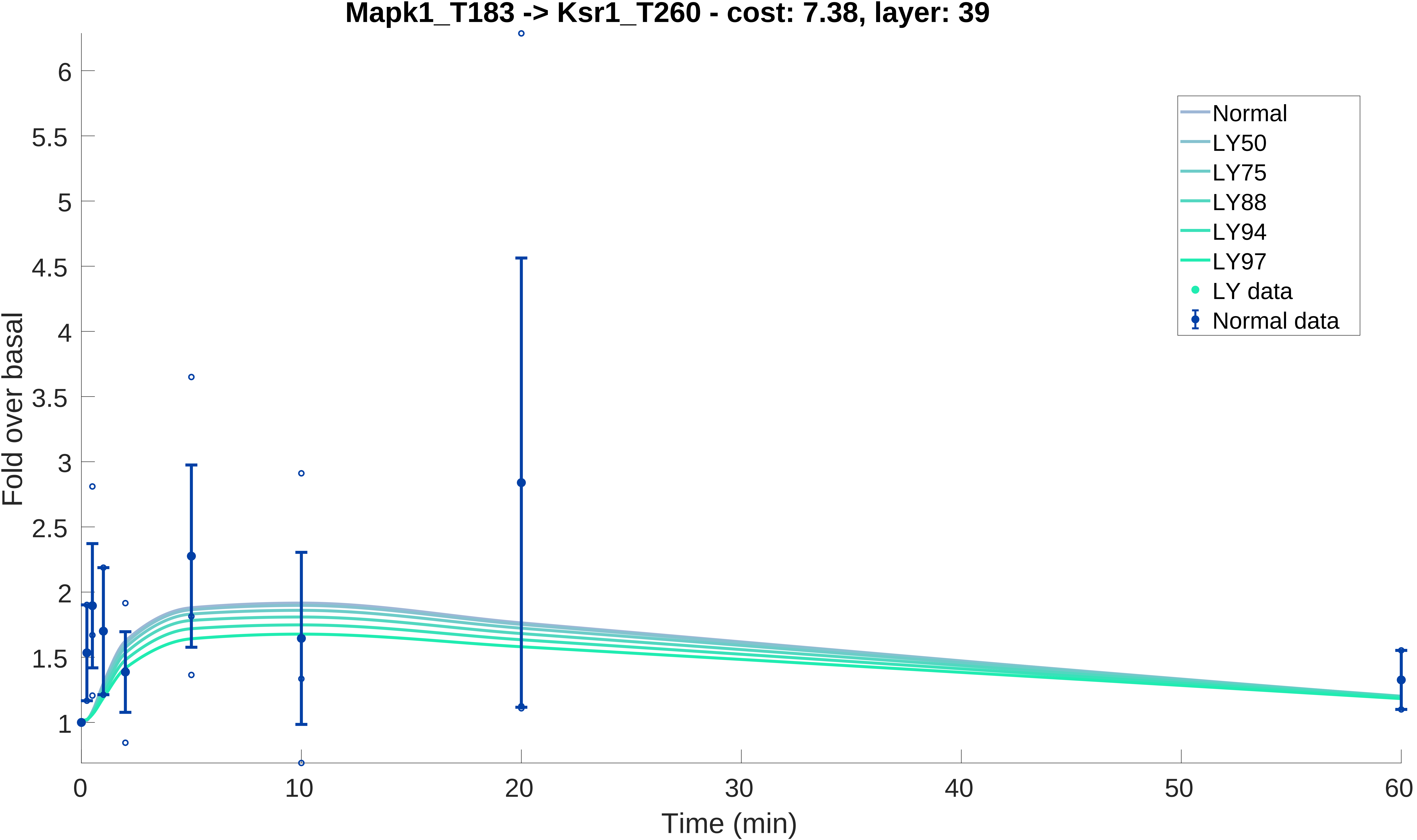

Mapk1_T183 -> Ksr1_T260 - cost: 7.38, layer: 39
Fold over basal
Time (min)
0
10
20
30
40
50
60
1
1.5
2
2.5
3
3.5
4
4.5
5
5.5
6
Normal
LY50
LY75
LY88
LY94
LY97
LY data
Normal data

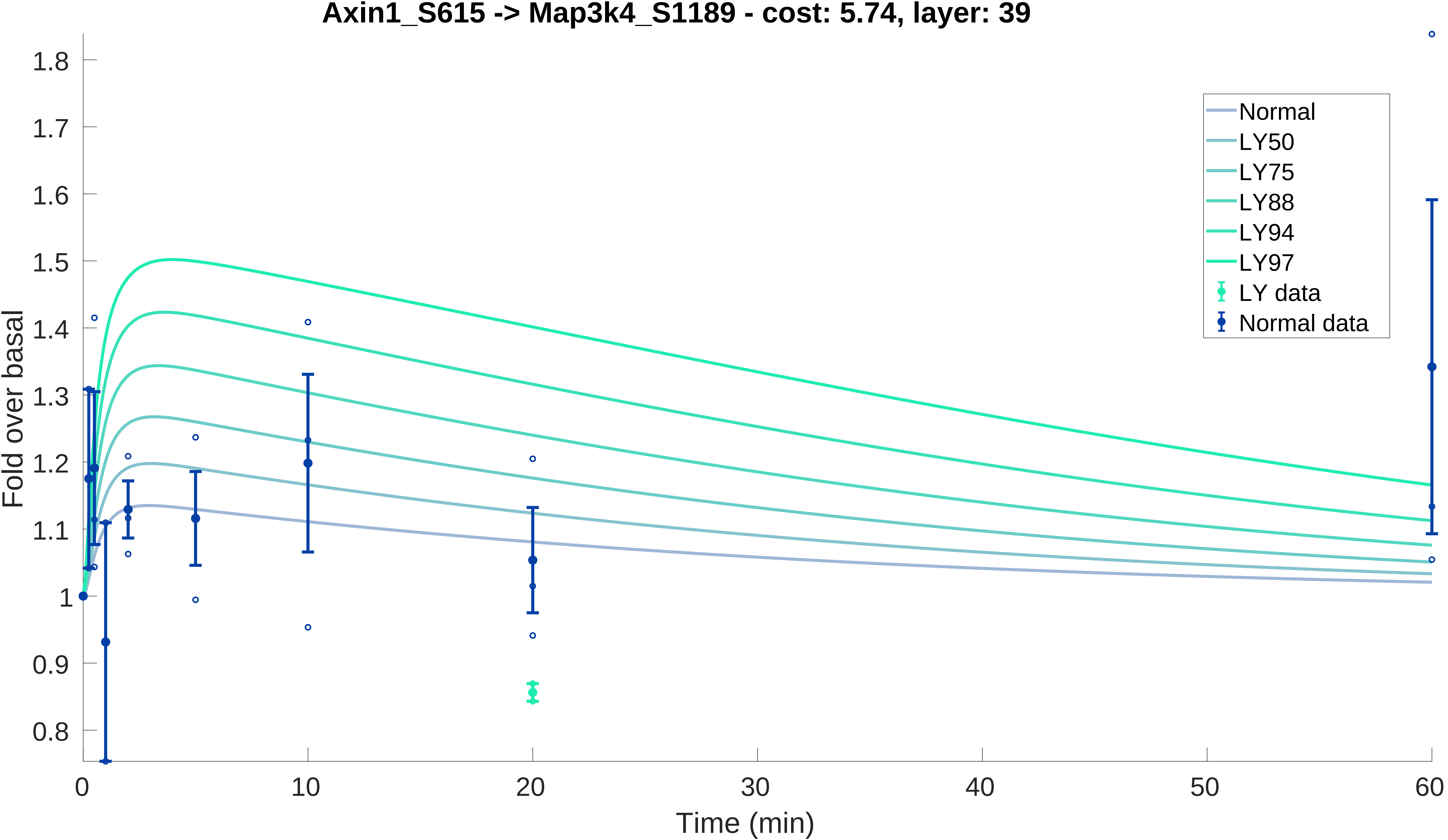

Axin1_S615 -> Map3k4_S1189 - cost: 5.74, layer: 39
Fold over basal
Time (min)
0
10
20
30
40
50
60
0.8
0.9
1
1.1
1.2
1.3
1.4
1.5
1.6
1.7
1.8
Normal
LY50
LY75
LY88
LY94
LY97
LY data
Normal data

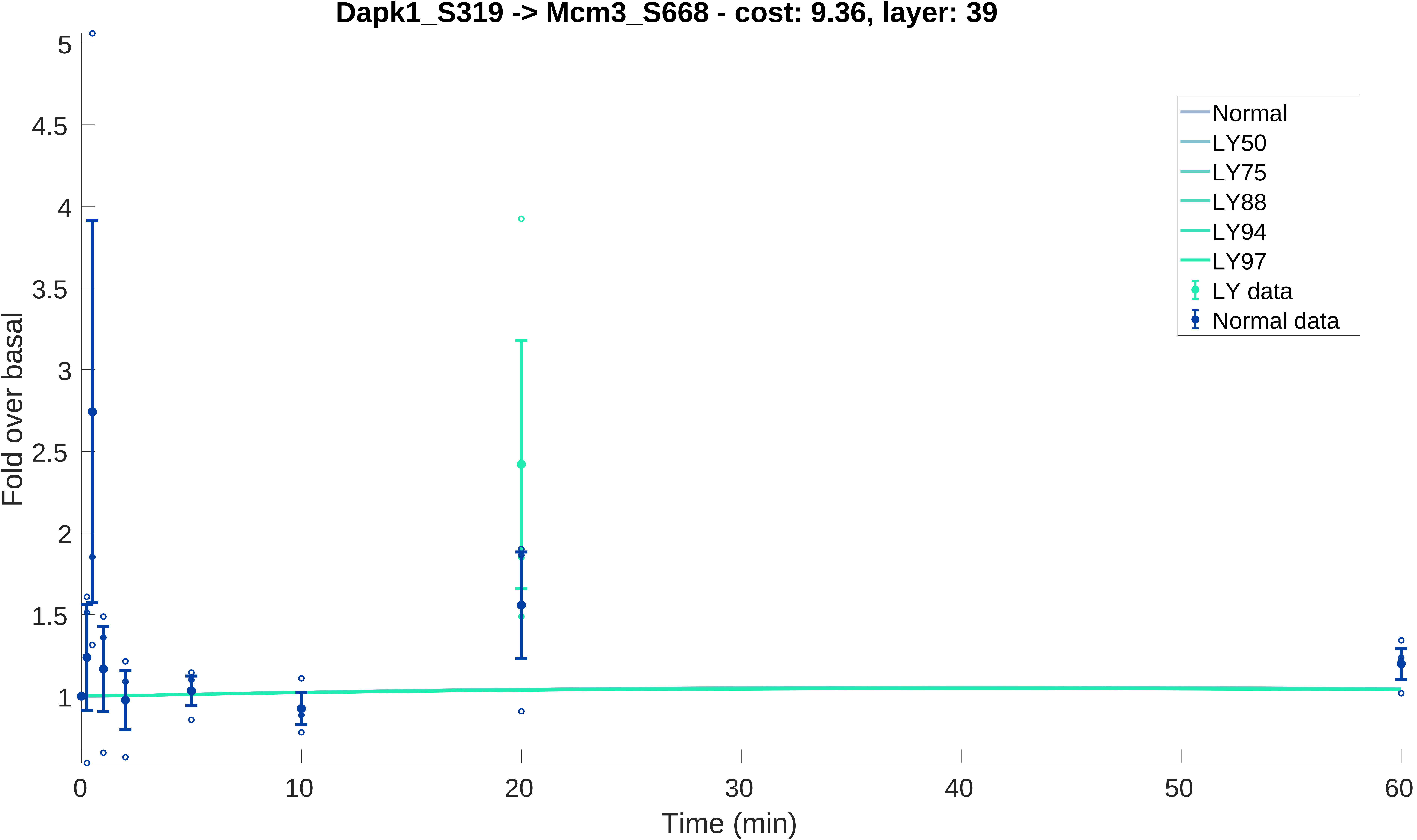

Dapk1_S319 -> Mcm3_S668 - cost: 9.36, layer: 39
Fold over basal
Time (min)
Normal
LY50
LY75
LY88
LY94
LY97
LY data
Normal data
0
10
20
30
40
50
60
1
1.5
2
2.5
3
3.5
4
4.5
5

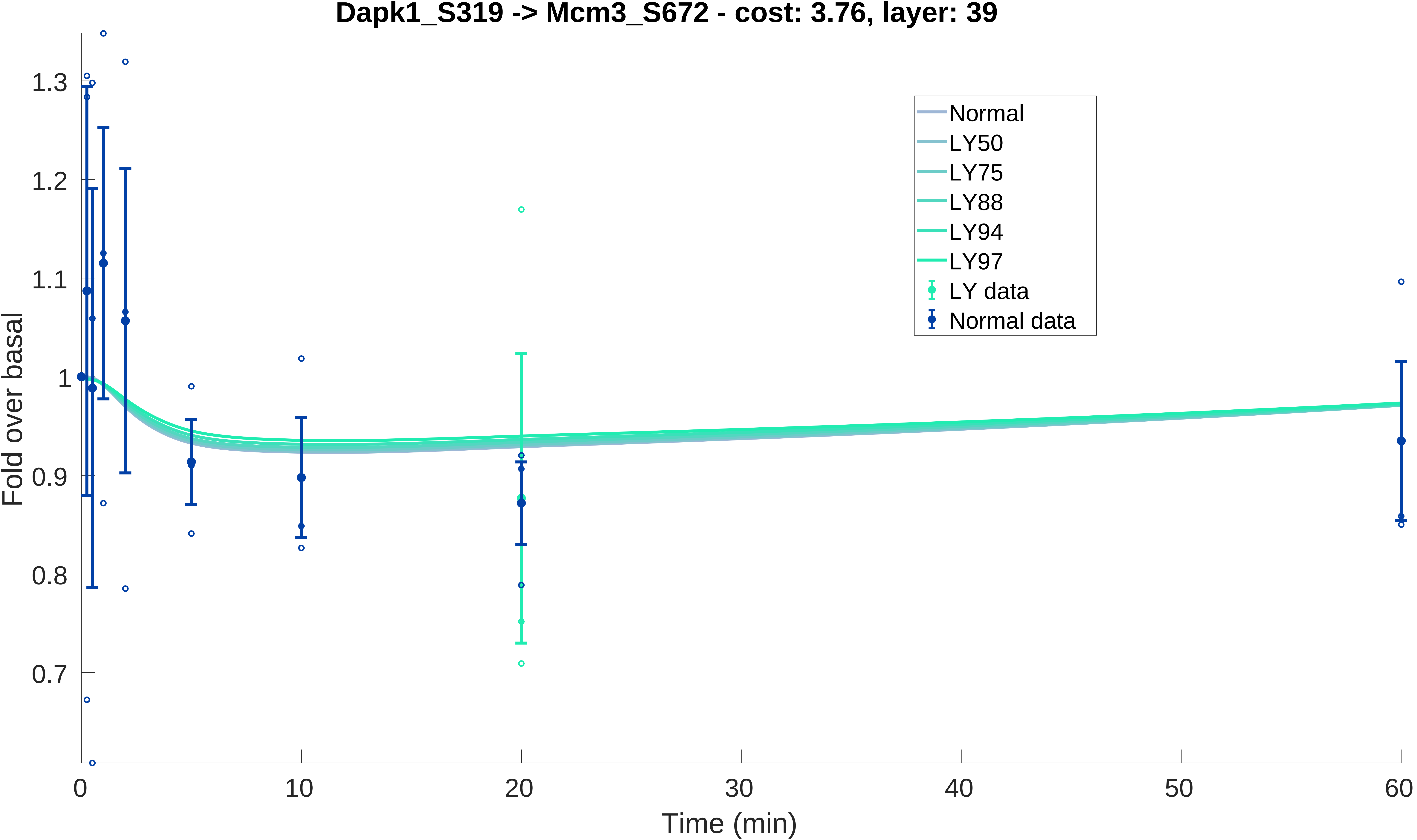

Dapk1_S319 -> Mcm3_S672 - cost: 3.76, layer: 39
Fold over basal
Time (min)
Normal
LY50
LY75
LY88
LY94
LY97
LY data
Normal data
0
10
20
30
40
50
60
0.7
0.8
0.9
1
1.1
1.2
1.3

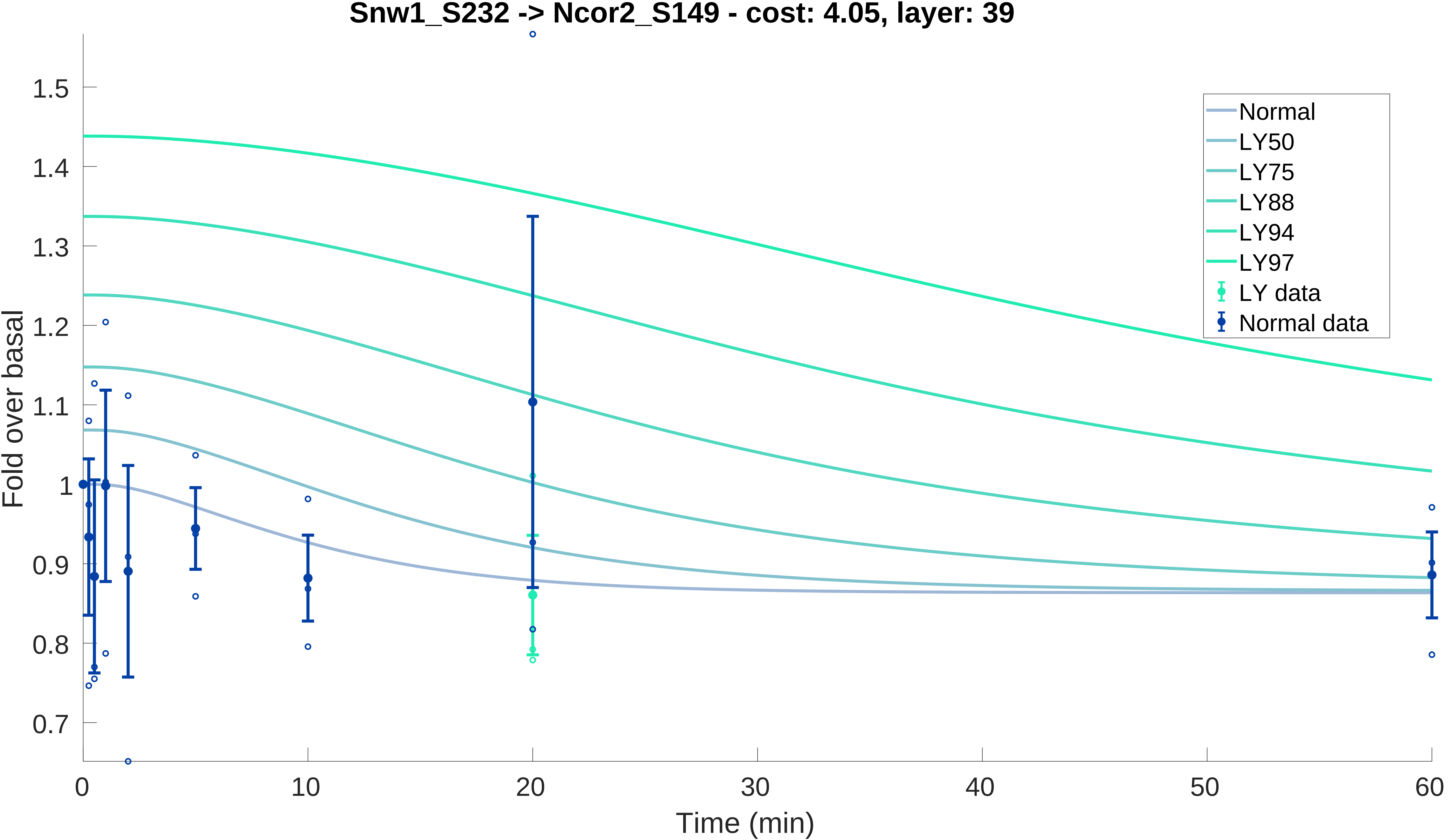

Snw1_S232 -> Ncor2_S149 - cost: 4.05, layer: 39
Fold over basal
Time (min)
Normal
LY50
LY75
LY88
LY94
LY97
LY data
Normal data
0.7
0.8
0.9
1
1.1
1.2
1.3
1.4
1.5
0
10
20
30
40
50
60

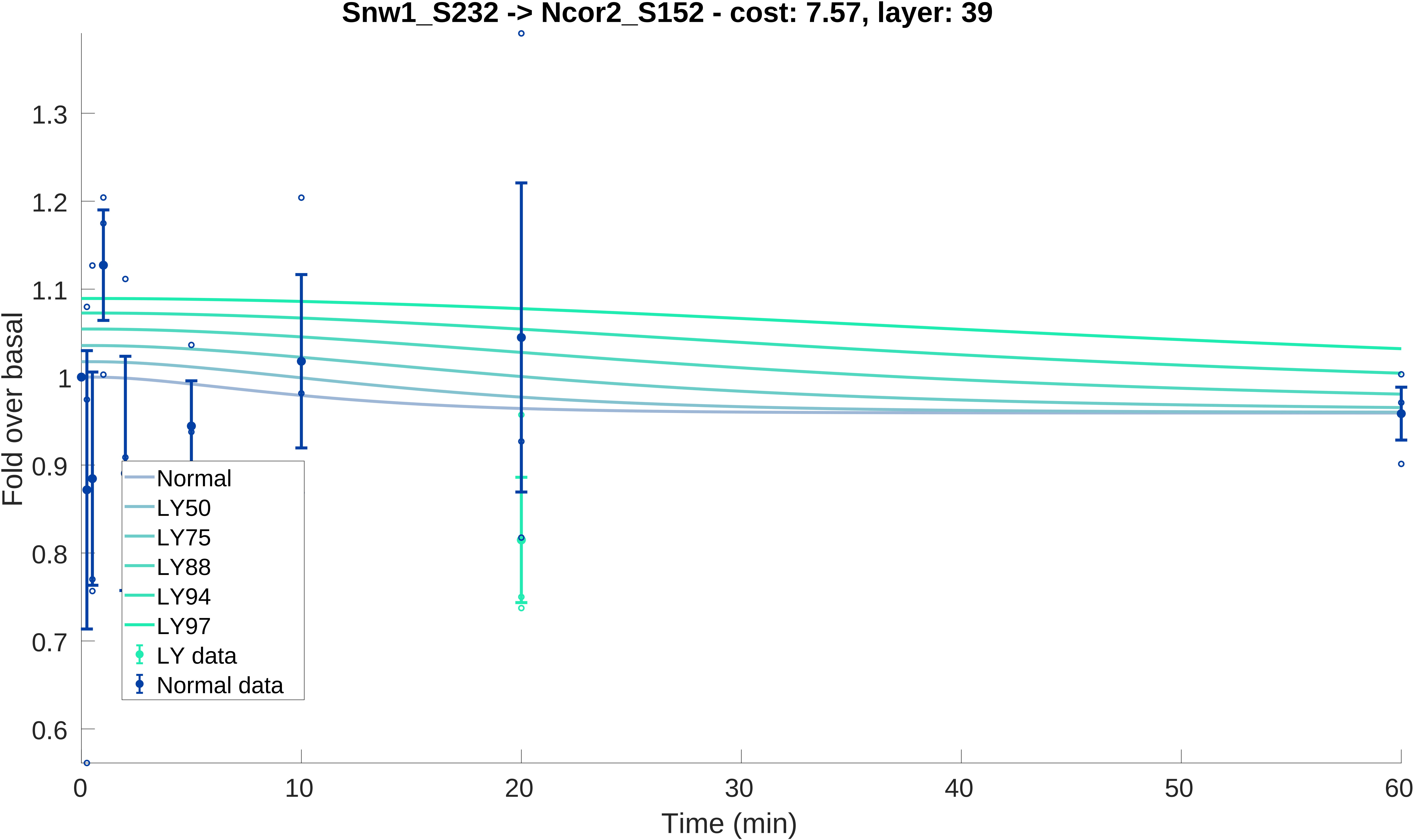

Snw1_S232 -> Ncor2_S152 - cost: 7.57, layer: 39
Fold over basal
Time (min)
0.6
0.7
0.8
0.9
1
1.1
1.2
1.3
0
10
20
30
40
50
60
Normal
LY50
LY75
LY88
LY94
LY97
LY data
Normal data

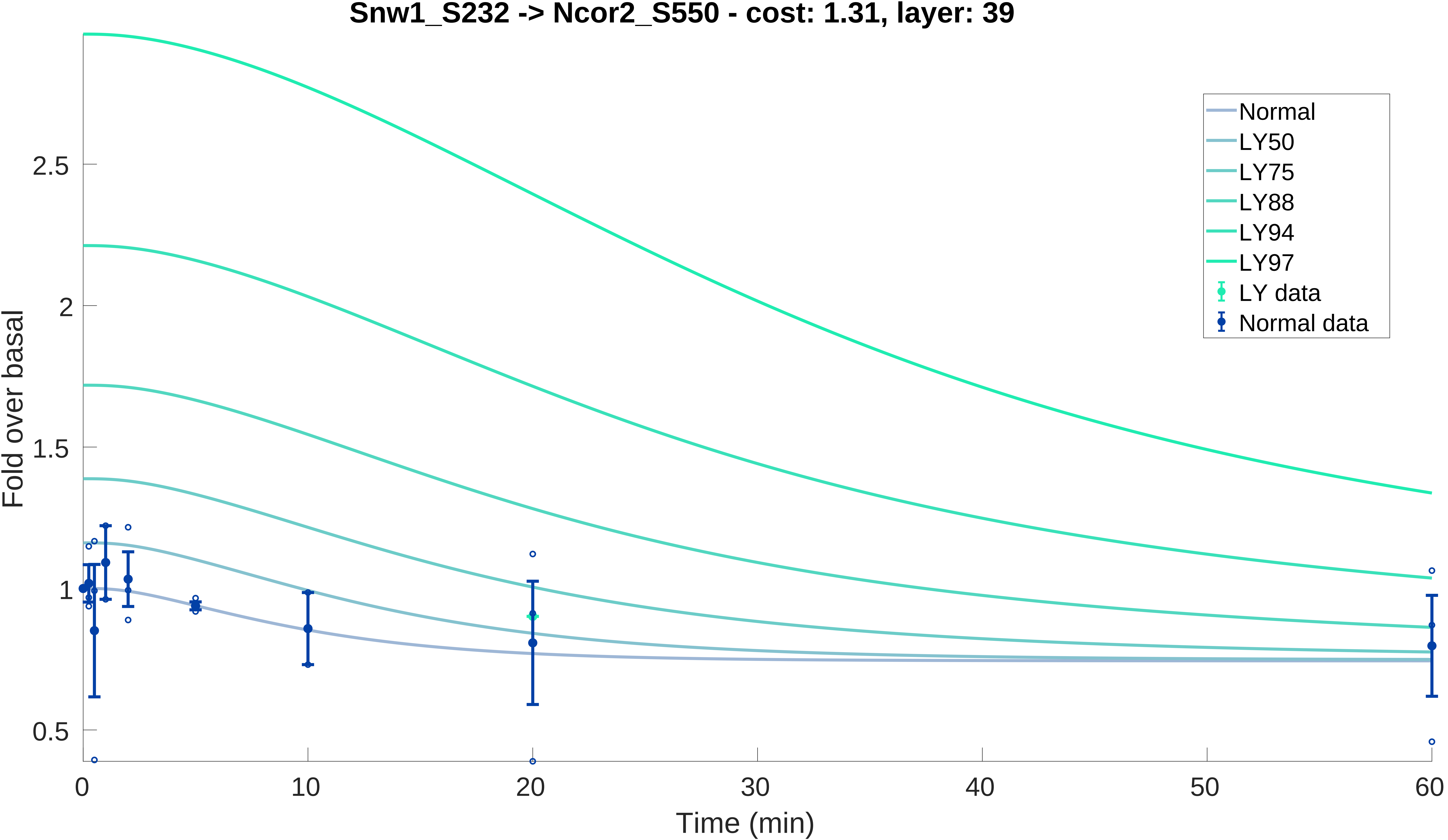
Snw1_S232 -> Ncor2_S550 - cost: 1.31, layer: 39
Fold over basal
Time (min)
0.5
1
1.5
2
2.5
0
10
20
30
40
50
60
Normal
LY50
LY75
LY88
LY94
LY97
LY data
Normal data

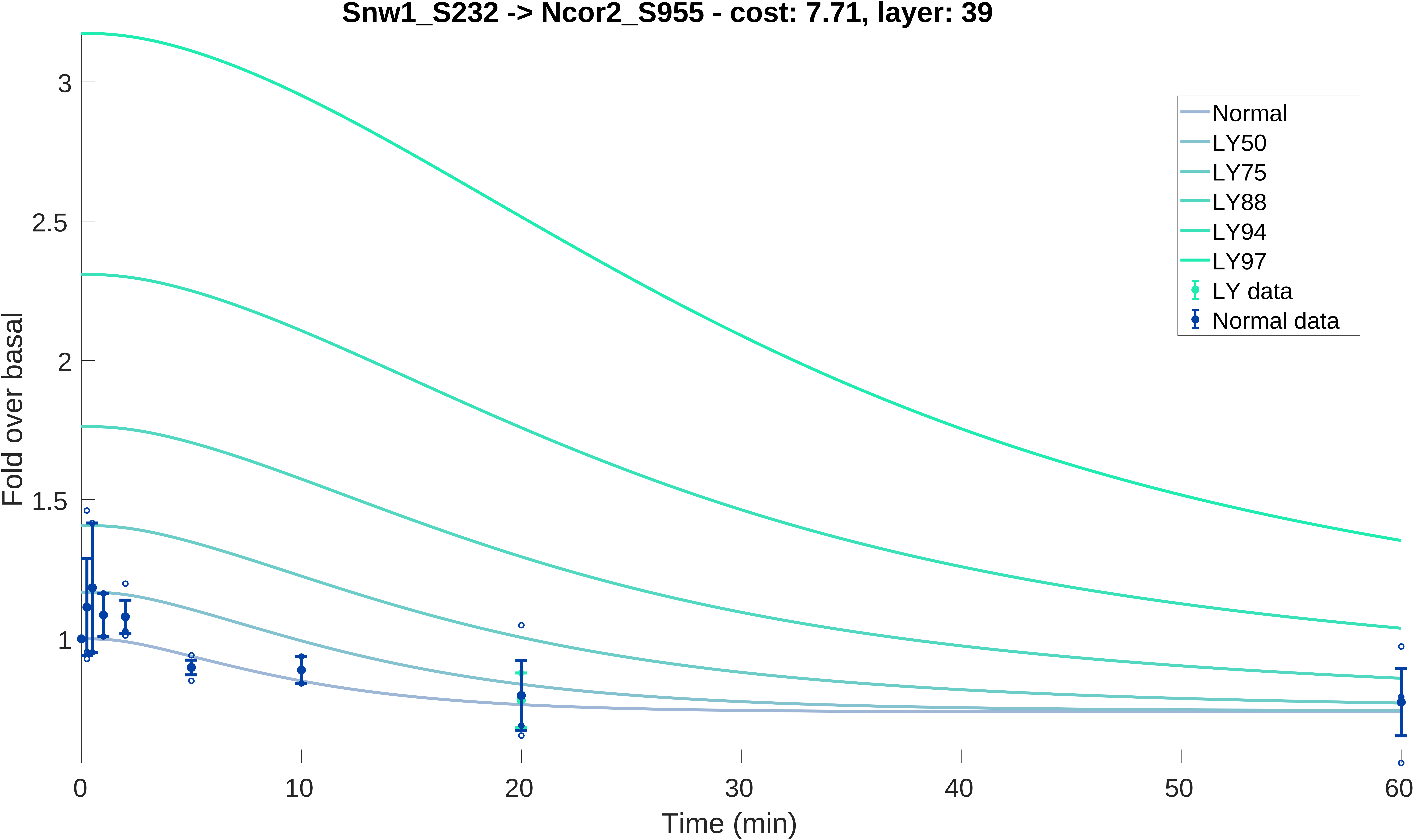

Snw1_S232 -> Ncor2_S955 - cost: 7.71, layer: 39
Fold over basal
Time (min)
Normal
LY50
LY75
LY88
LY94
LY97
LY data
Normal data
3
2.5
2
1.5
1
0
10
20
30
40
50
60

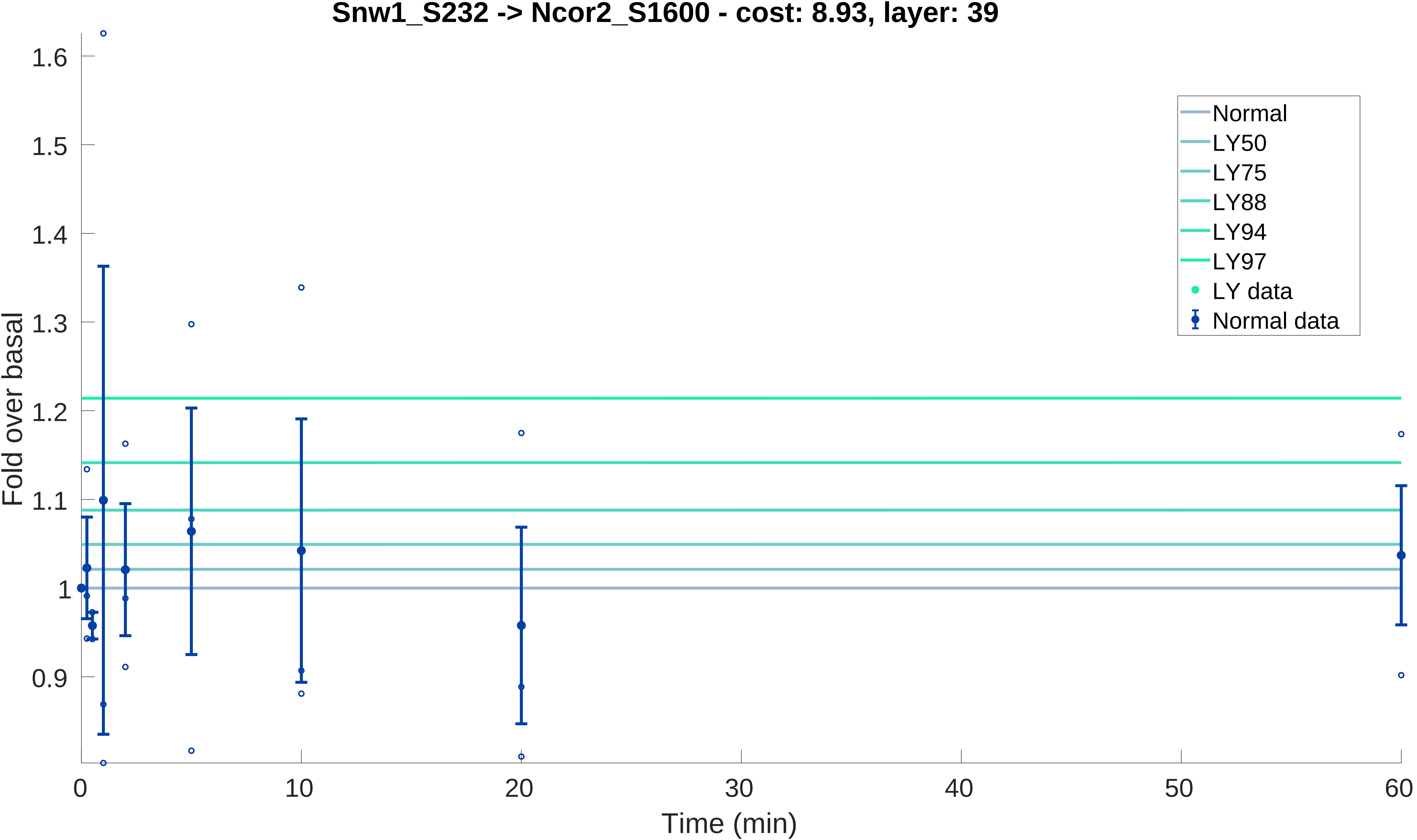
Snw1_S232 -> Ncor2_S1600 - cost: 8.93, layer: 39
Fold over basal
Time (min)
1.6
1.5
1.4
1.3
1.2
1.1
1
0.9
0
10
20
30
40
50
60
Normal
LY50
LY75
LY88
LY94
LY97
LY data
Normal data

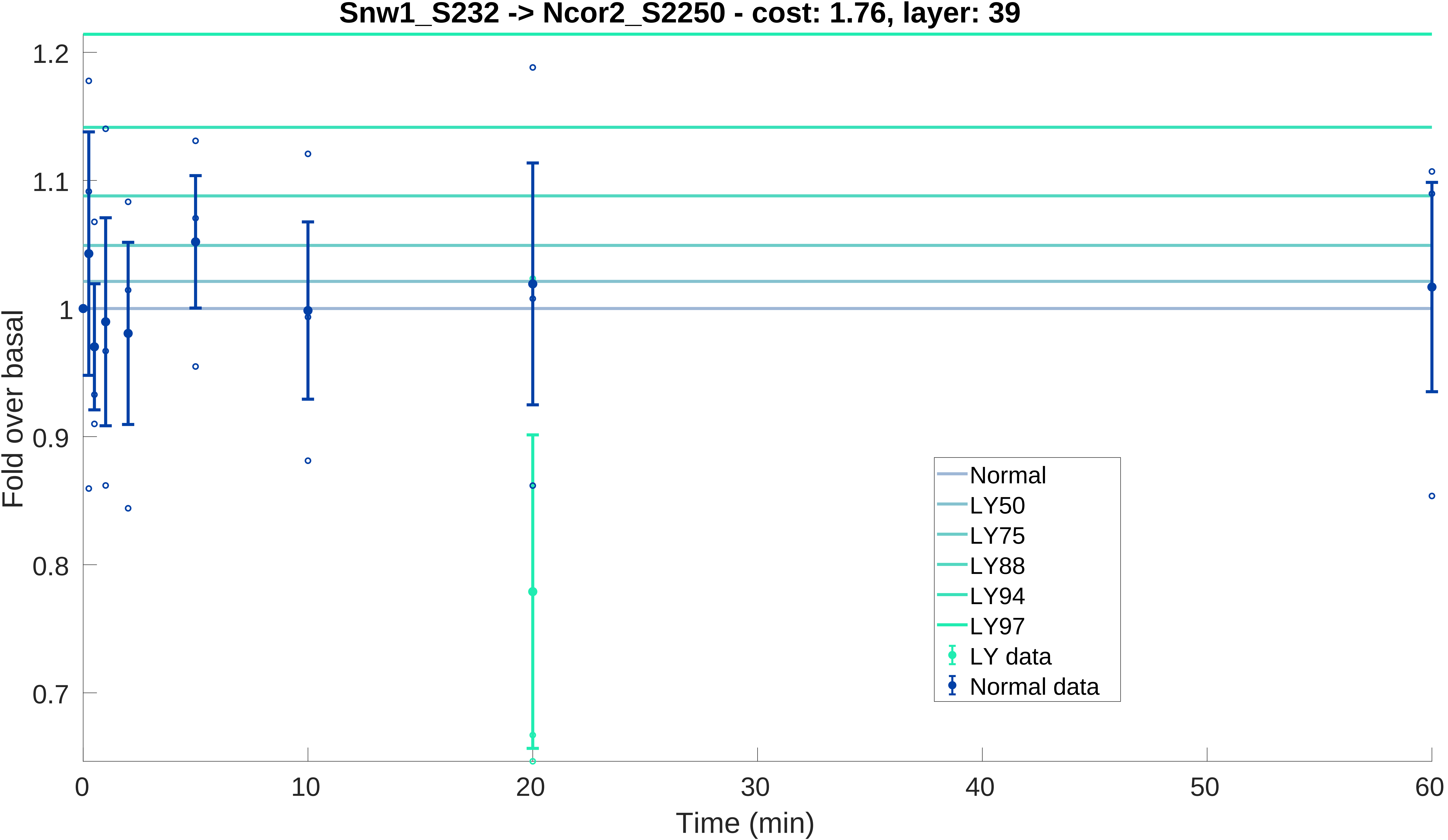

Snw1_S232 -> Ncor2_S2250 - cost: 1.76, layer: 39
Fold over basal
Time (min)
1.2
1.1
1
0.9
0.8
0.7
0
10
20
30
40
50
60
Normal
LY50
LY75
LY88
LY94
LY97
LY data
Normal data

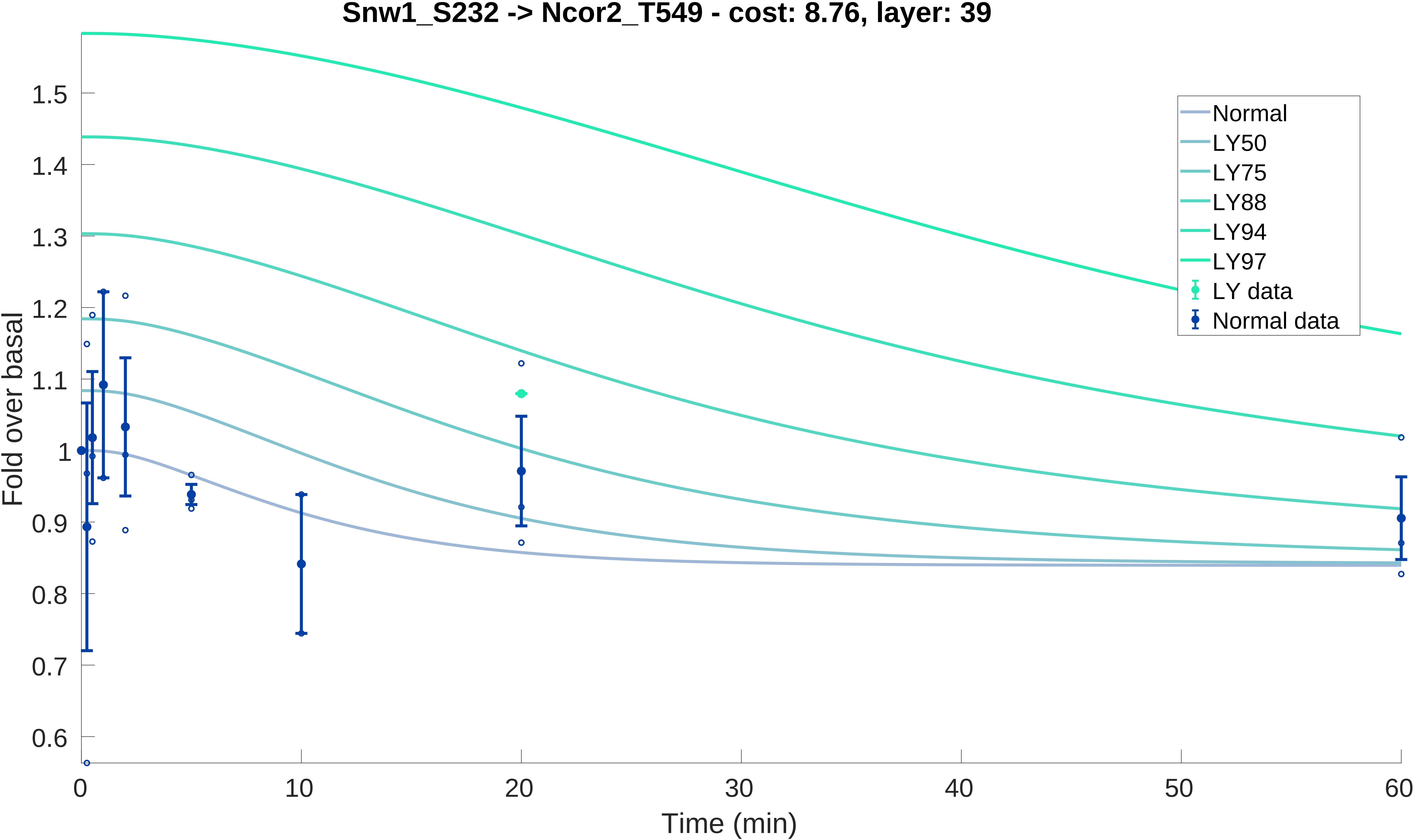

Snw1_S232 -> Ncor2_T549 - cost: 8.76, layer: 39
Fold over basal
Time (min)
1.5
1.4
1.3
1.2
1.1
1
0.9
0.8
0.7
0.6
0
10
20
30
40
50
60
Normal
LY50
LY75
LY88
LY94
LY97
LY data
Normal data

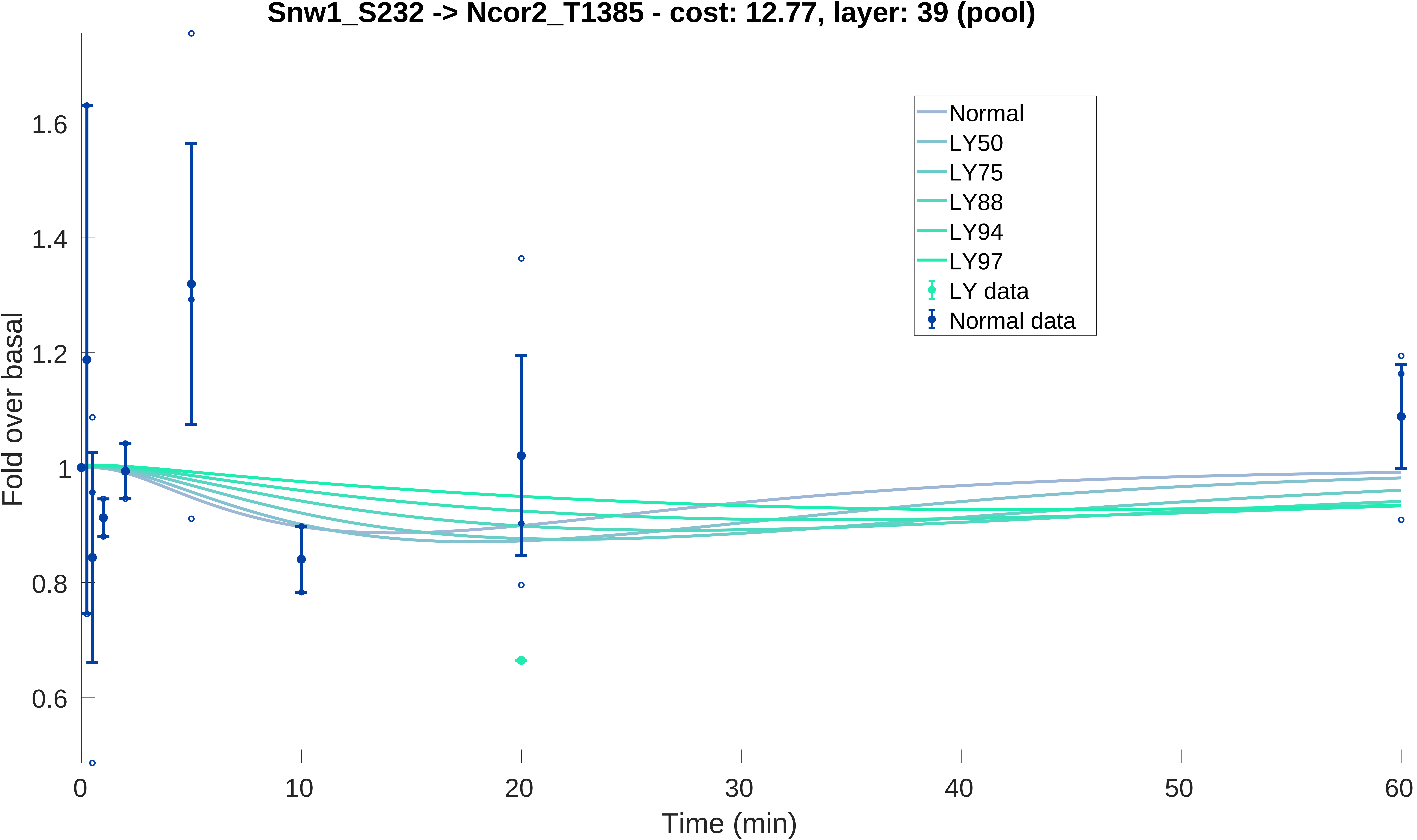
Snw1_S232 -> Ncor2_T1385 - cost: 12.77, layer: 39 (pool)
Fold over basal
Time (min)
0.6
0.8
1
1.2
1.4
1.6
0
10
20
30
40
50
60
Normal
LY50
LY75
LY88
LY94
LY97
LY data
Normal data

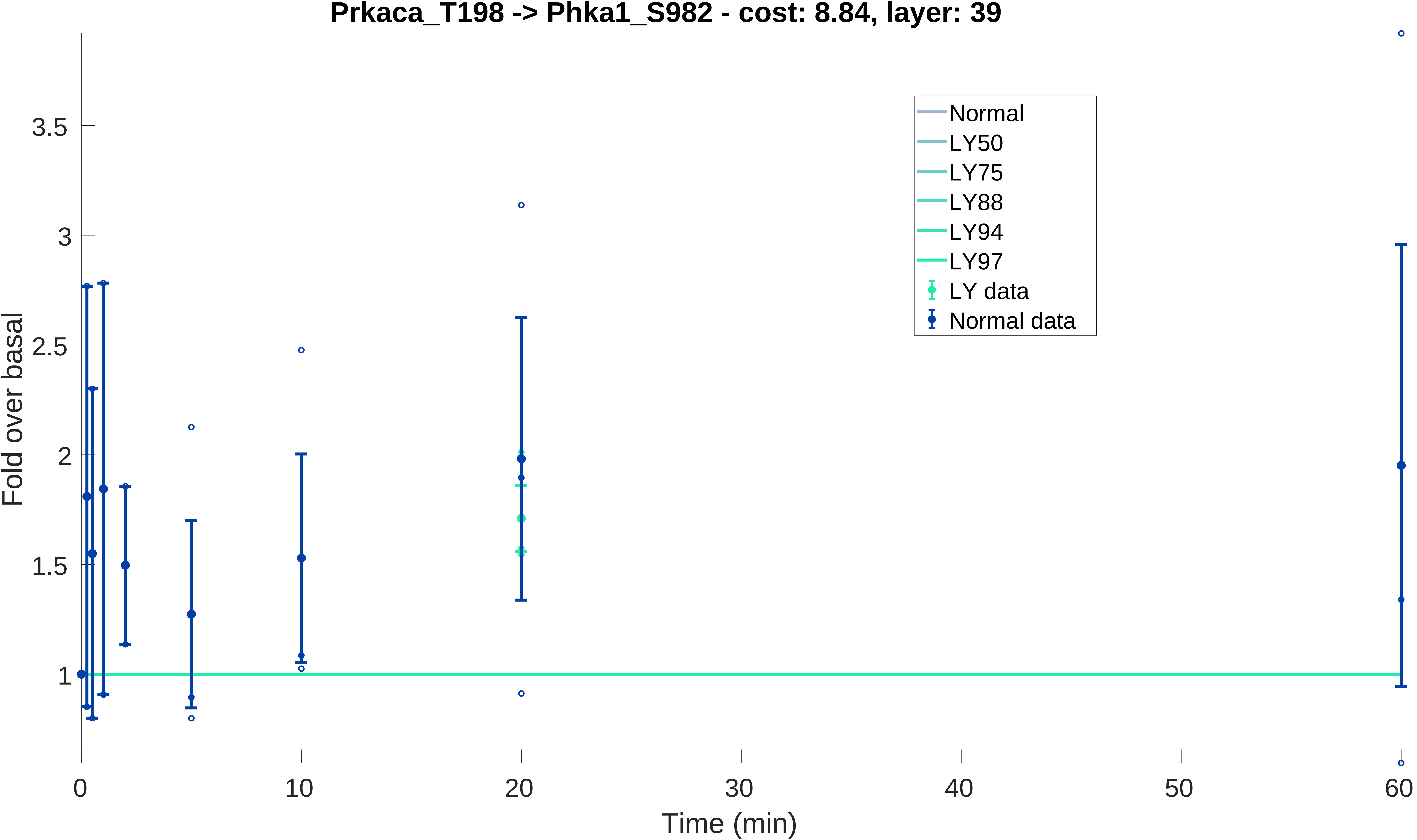

Prkaca_T198 -> Phka1_S982 - cost: 8.84, layer: 39
Fold over basal
Time (min)
0
10
20
30
40
50
60
1
1.5
2
2.5
3
3.5
Normal
LY50
LY75
LY88
LY94
LY97
LY data
Normal data

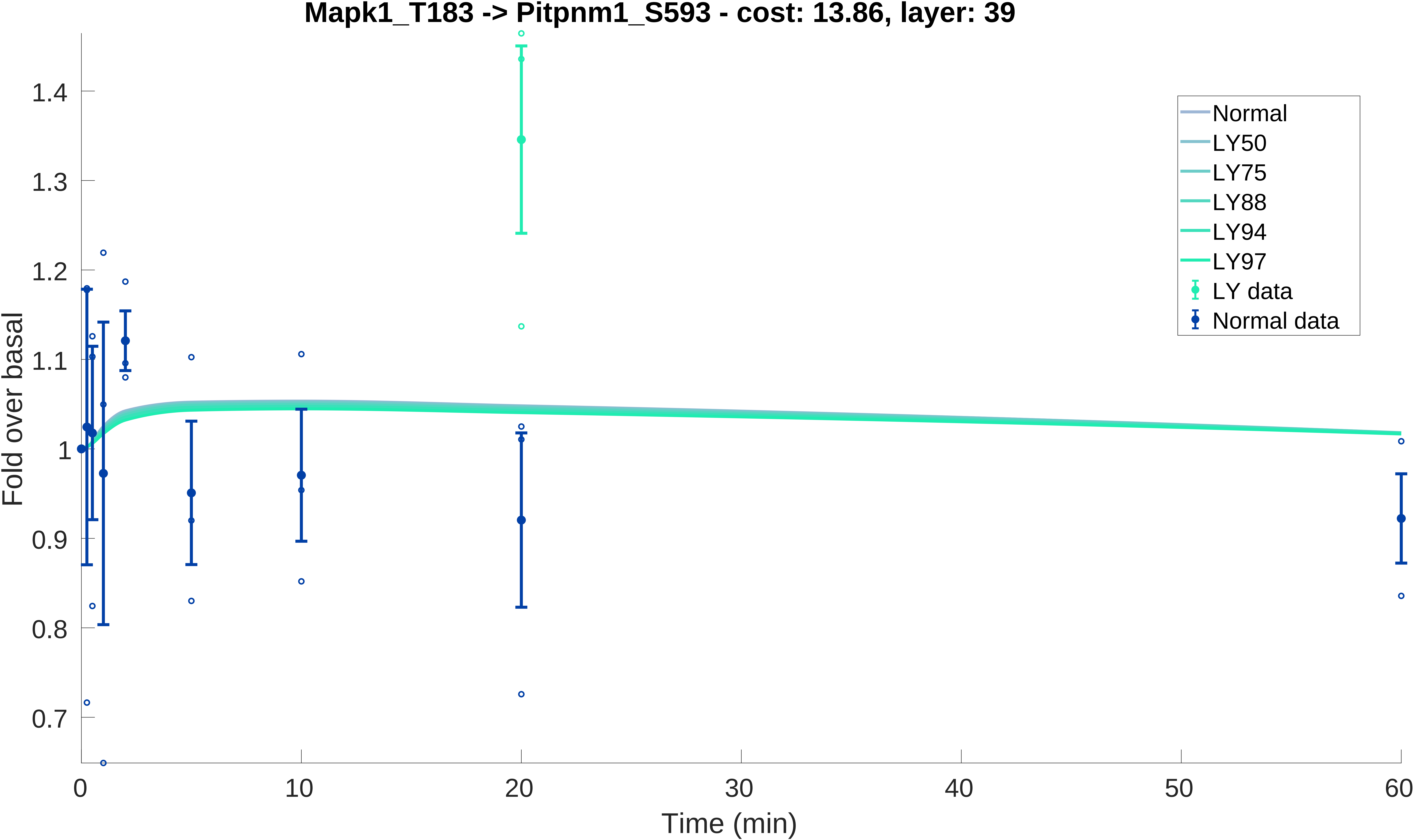

Mapk1_T183 -> Pitpnm1_S593 - cost: 13.86, layer: 39
Fold over basal
Time (min)
1.4
1.3
1.2
1.1
1
0.9
0.8
0.7
0
10
20
30
40
50
60
Normal
LY50
LY75
LY88
LY94
LY97
LY data
Normal data

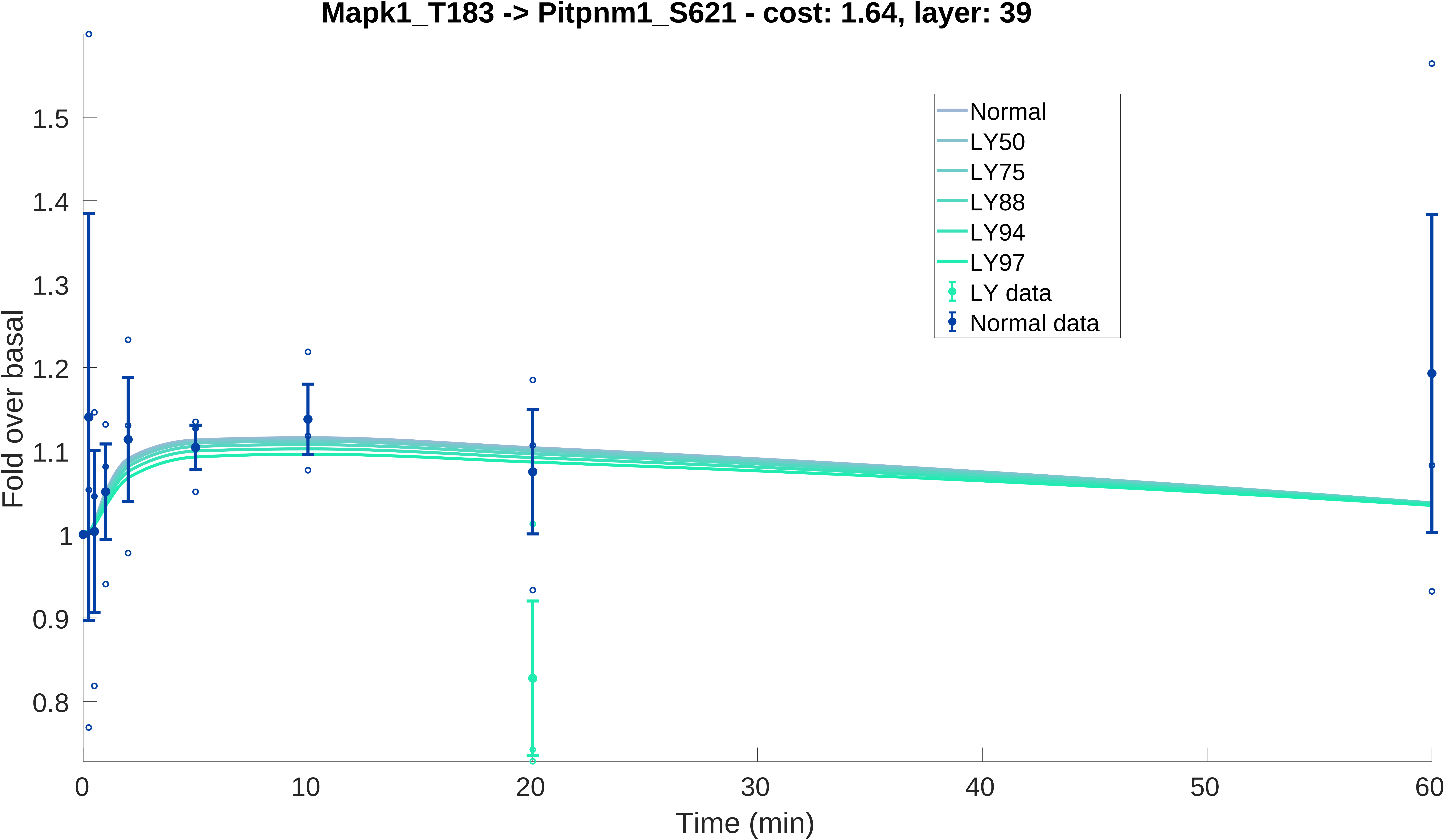

Mapk1_T183 -> Pitpnm1_S621 - cost: 1.64, layer: 39
Fold over basal
Time (min)
0.8
0.9
1
1.1
1.2
1.3
1.4
1.5
0
10
20
30
40
50
60
Normal
LY50
LY75
LY88
LY94
LY97
LY data
Normal data

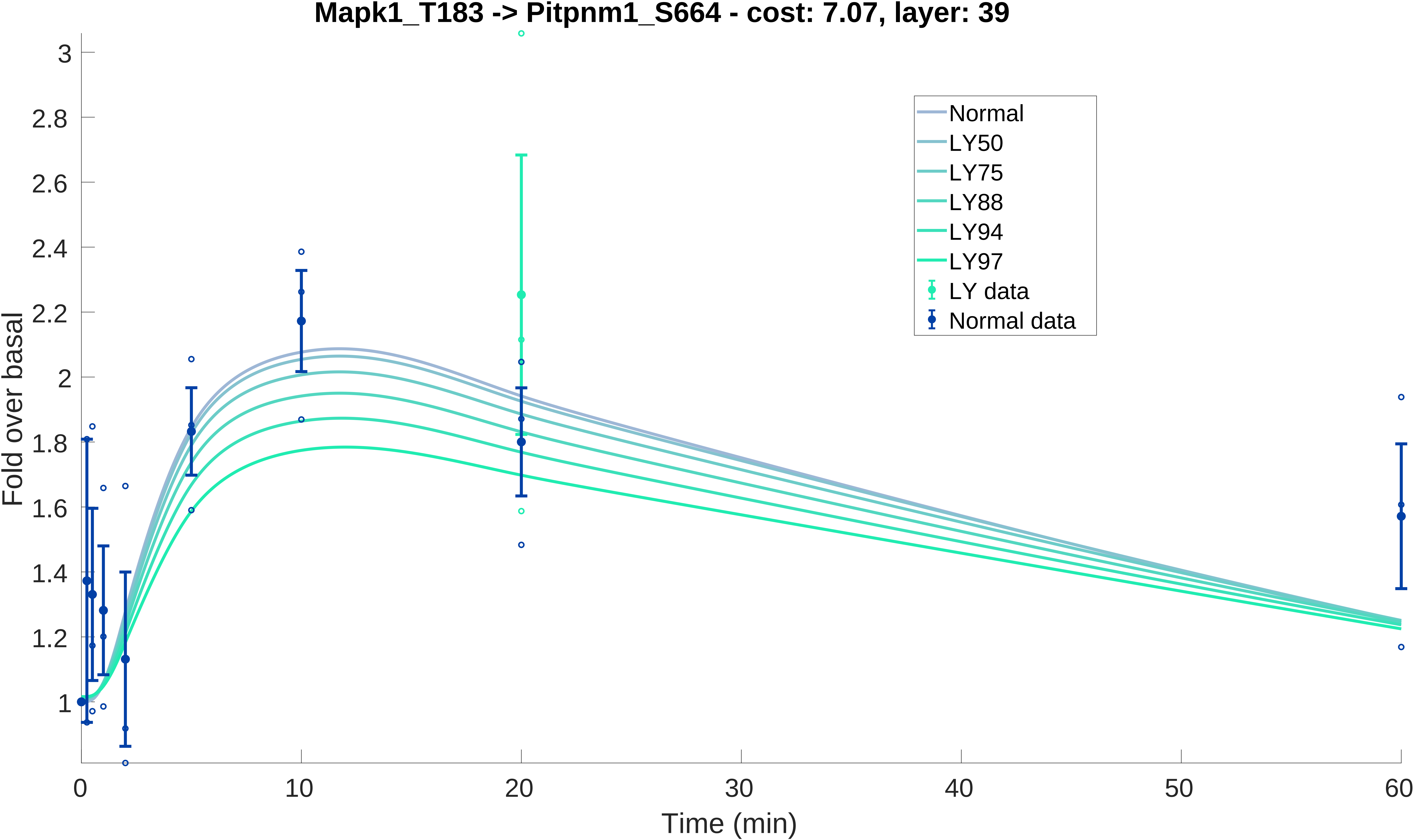
Mapk1_T183 -> Pitpnm1_S664 - cost: 7.07, layer: 39
Fold over basal
Time (min)
Normal
LY50
LY75
LY88
LY94
LY97
LY data
Normal data
0
10
20
30
40
50
60
1
1.2
1.4
1.6
1.8
2
2.2
2.4
2.6
2.8
3

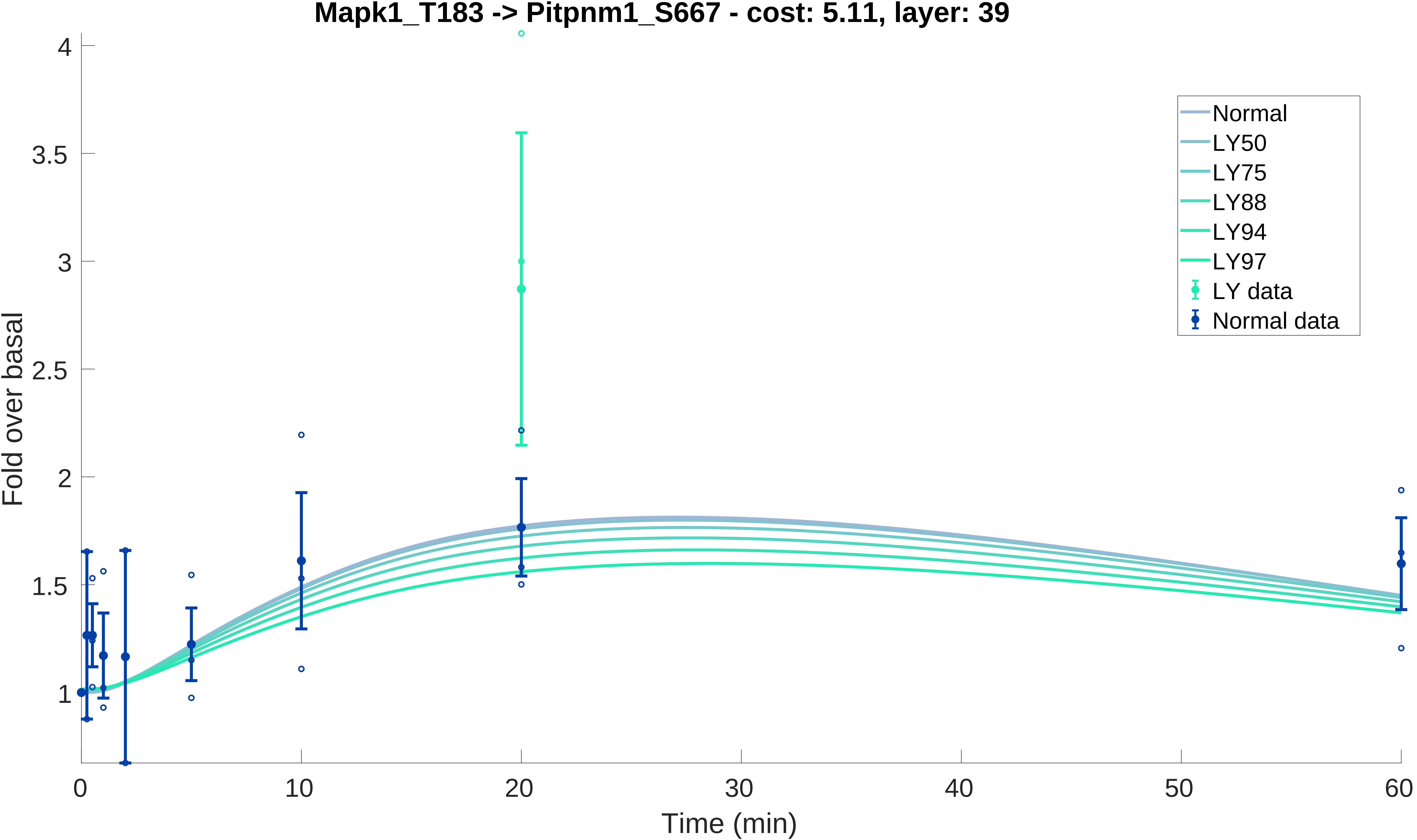

Mapk1_T183 -> Pitpnm1_S667 - cost: 5.11, layer: 39
Fold over basal
Time (min)
4
3.5
3
2.5
2
1.5
1
0
10
20
30
40
50
60
Normal
LY50
LY75
LY88
LY94
LY97
LY data
Normal data

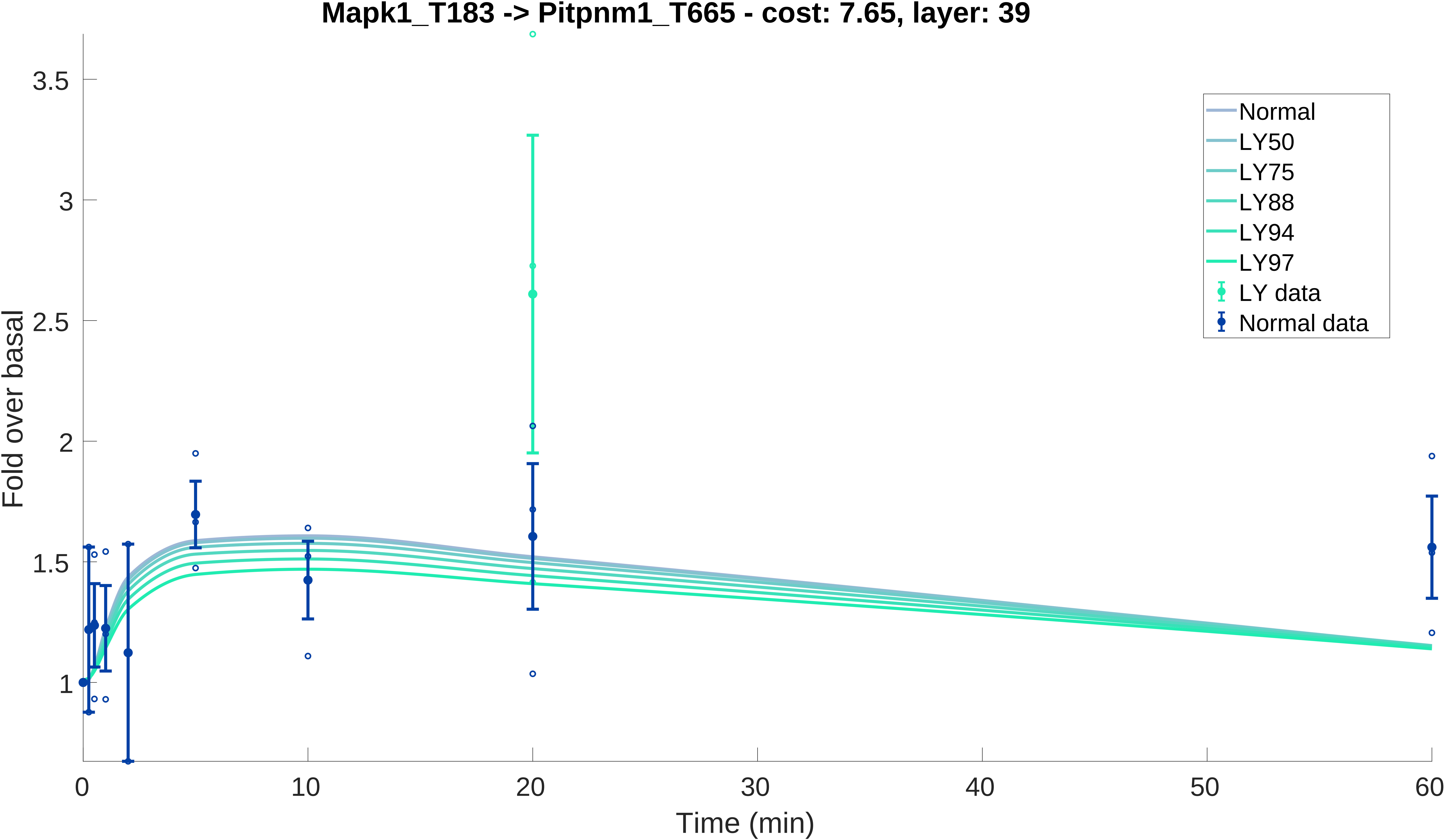

Mapk1_T183 -> Pitpnm1_T665 - cost: 7.65, layer: 39
Fold over basal
Time (min)
3.5
3
2.5
2
1.5
1
0
10
20
30
40
50
60
Normal
LY50
LY75
LY88
LY94
LY97
LY data
Normal data

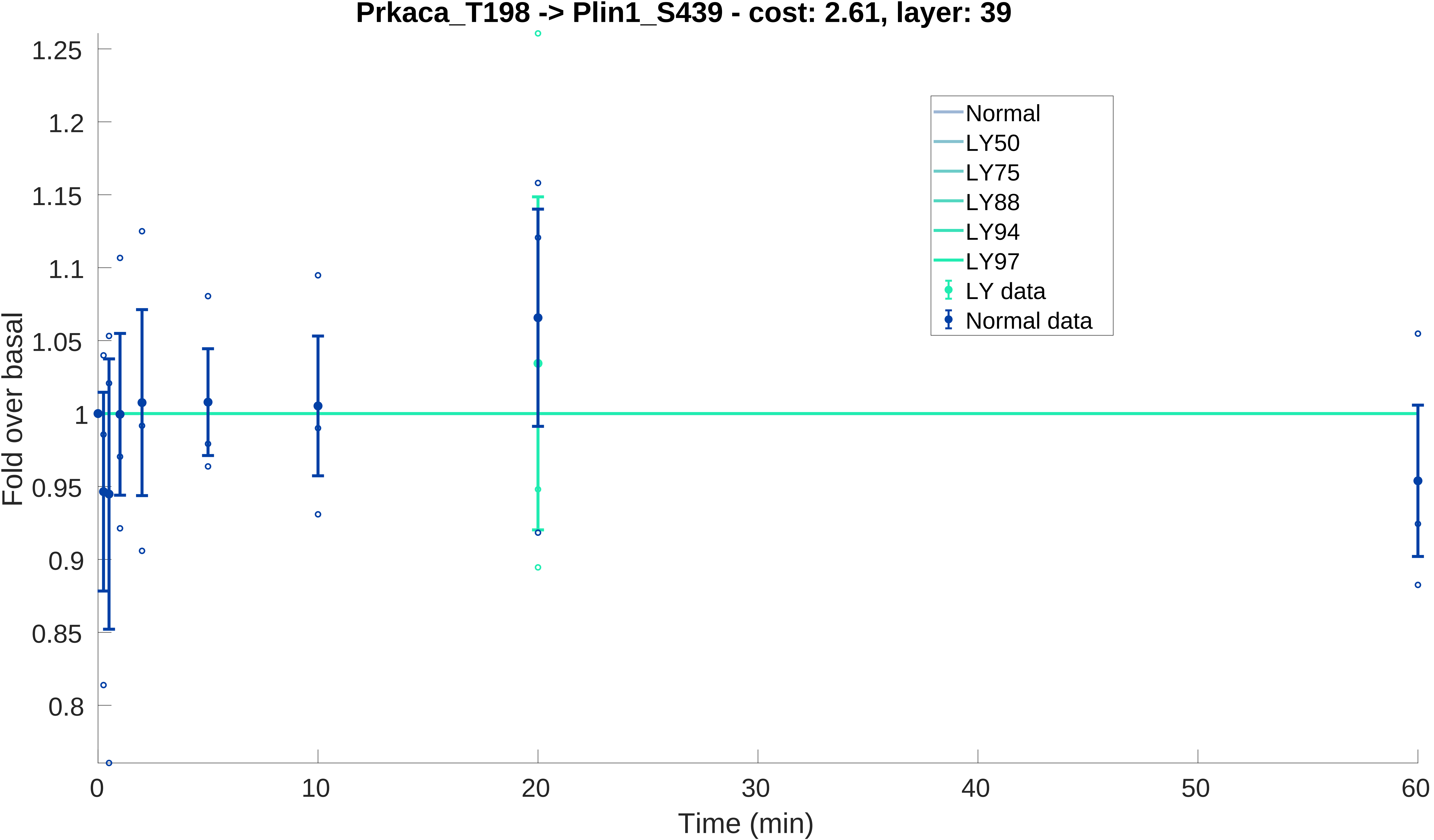

Prkaca_T198 -> Plin1_S439 - cost: 2.61, layer: 39
Fold over basal
1.25
1.2
1.15
1.1
1.05
1
0.95
0.9
0.85
0.8
0
10
20
30
40
50
60
Time (min)
Normal
LY50
LY75
LY88
LY94
LY97
LY data
Normal data

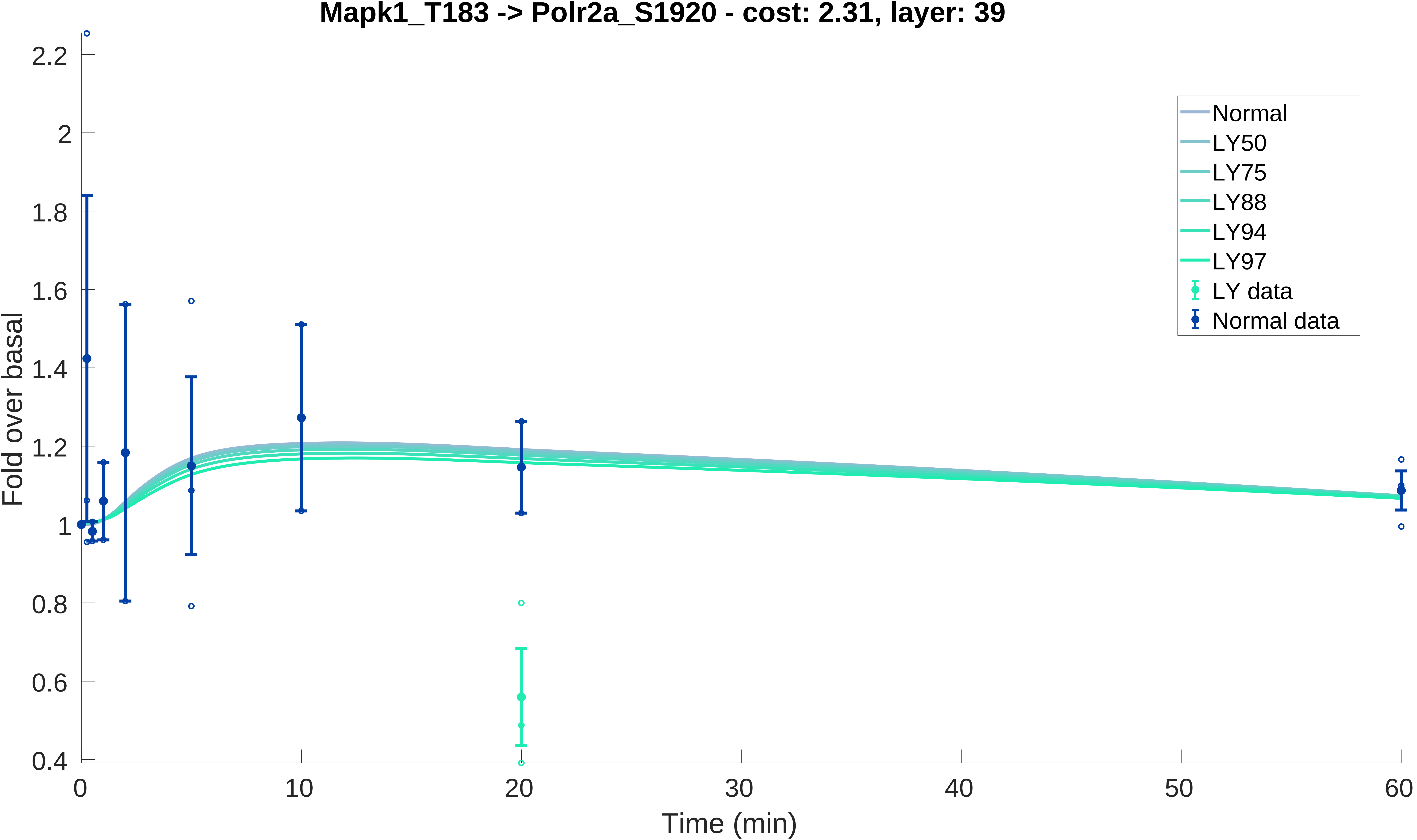

Mapk1_T183 -> Polr2a_S1920 - cost: 2.31, layer: 39
Fold over basal
Time (min)
0
10
20
30
40
50
60
0.4
0.6
0.8
1
1.2
1.4
1.6
1.8
2
2.2
Normal
LY50
LY75
LY88
LY94
LY97
LY data
Normal data

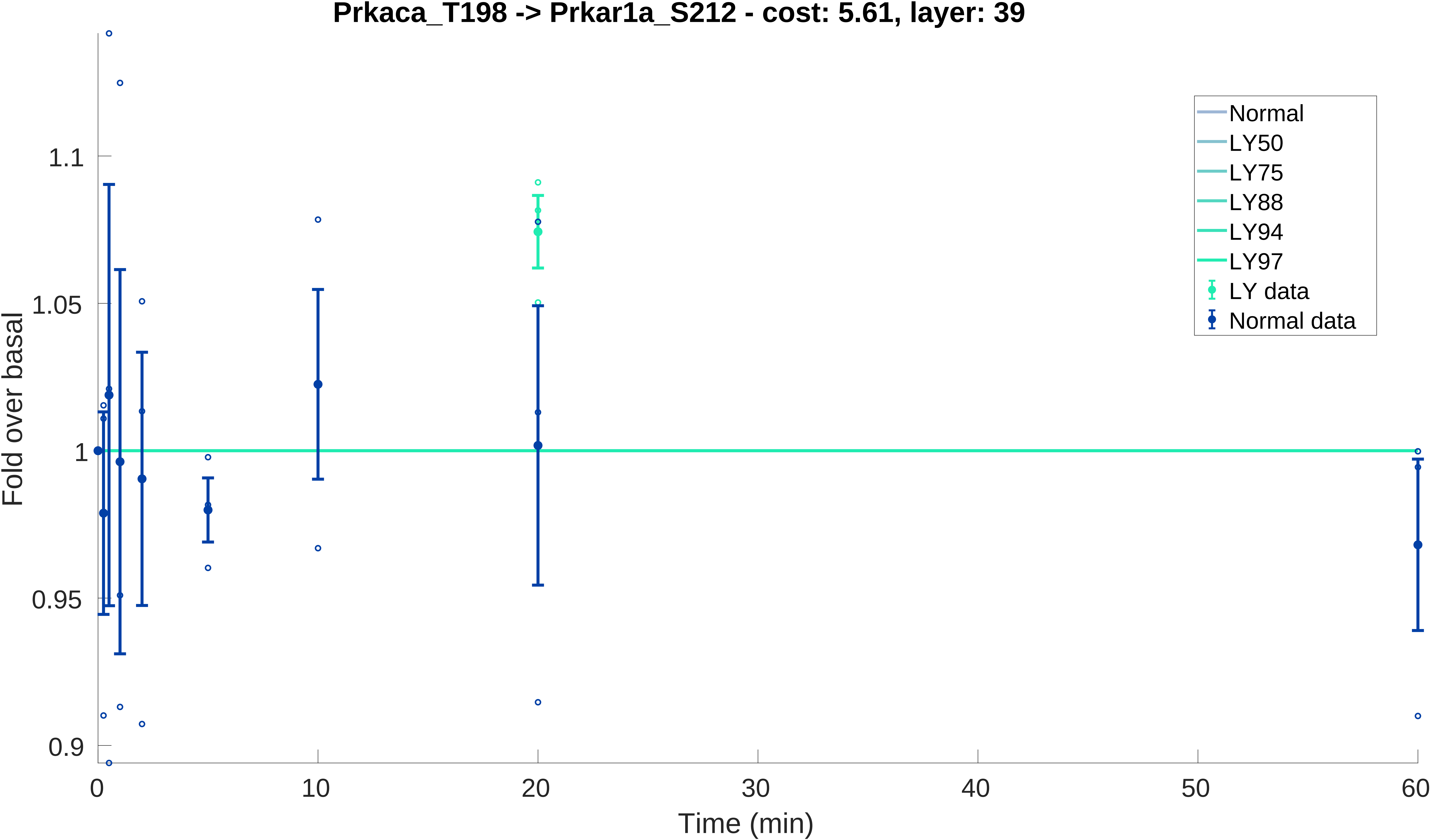

Prkaca_T198 -> Prkar1a_S212 - cost: 5.61, layer: 39
Fold over basal
Time (min)
1.1
1.05
1
0.95
0.9
0
10
20
30
40
50
60
Normal
LY50
LY75
LY88
LY94
LY97
LY data
Normal data

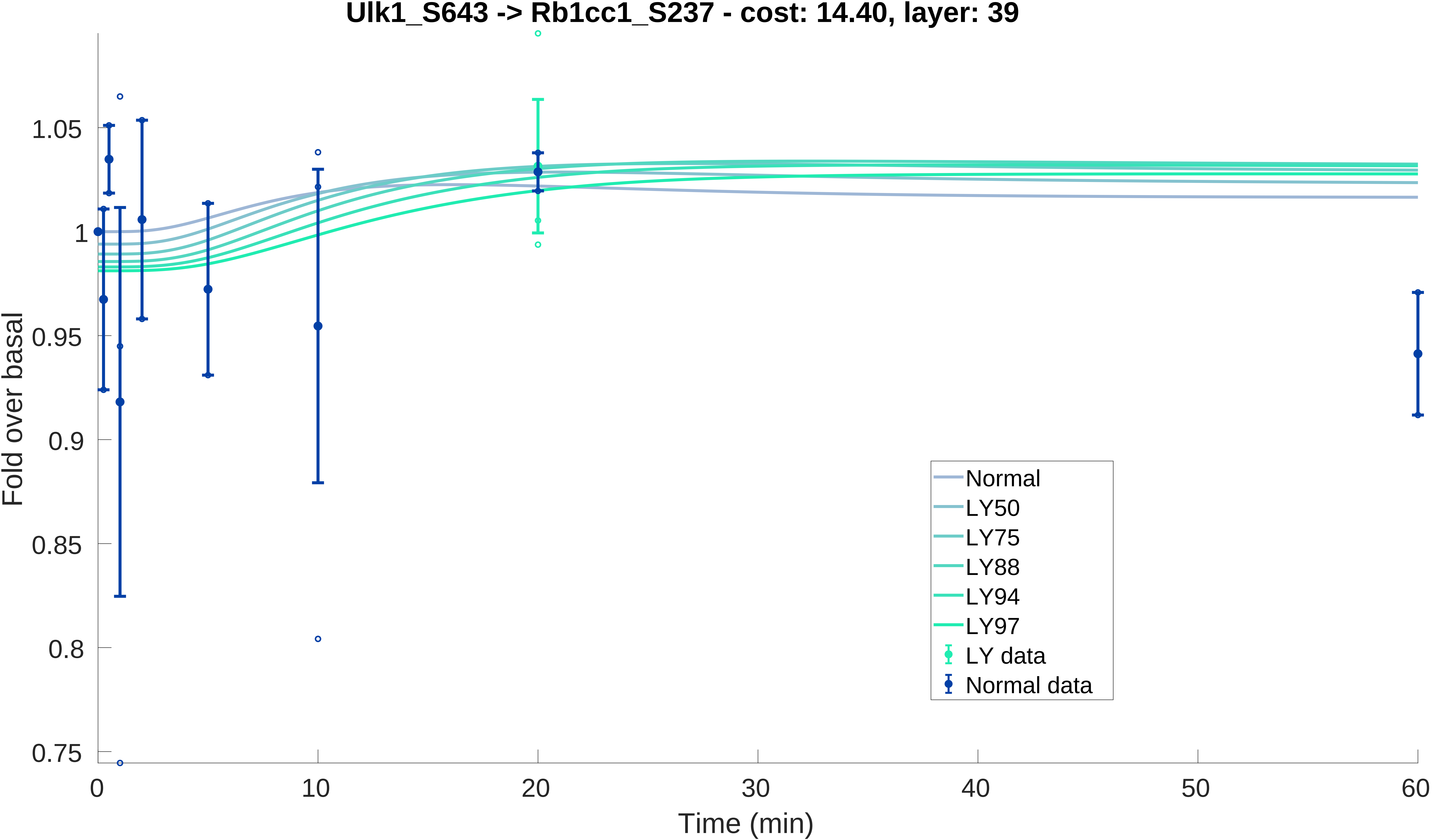
Ulk1_S643 -> Rb1cc1_S237 - cost: 14.40, layer: 39
Fold over basal
1.05
1
0.95
0.9
0.85
0.8
0.75
0
10
20
30
40
50
60
Time (min)
Normal
LY50
LY75
LY88
LY94
LY97
LY data
Normal data

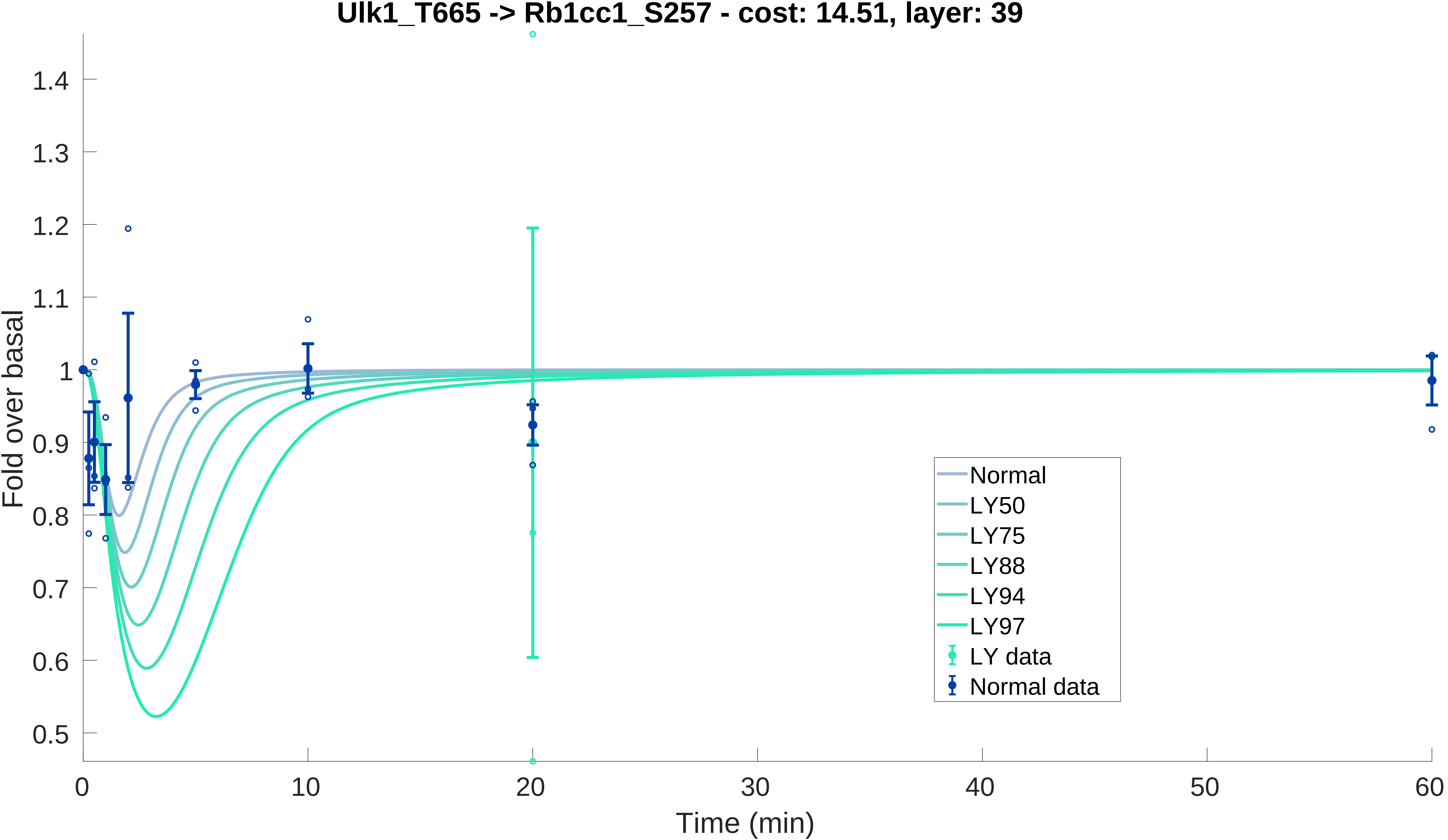

Ulk1_T665 -> Rb1cc1_S257 - cost: 14.51, layer: 39
Fold over basal
Time (min)
1.4
1.3
1.2
1.1
1
0.9
0.8
0.7
0.6
0.5
0
10
20
30
40
50
60
Normal
LY50
LY75
LY88
LY94
LY97
LY data
Normal data

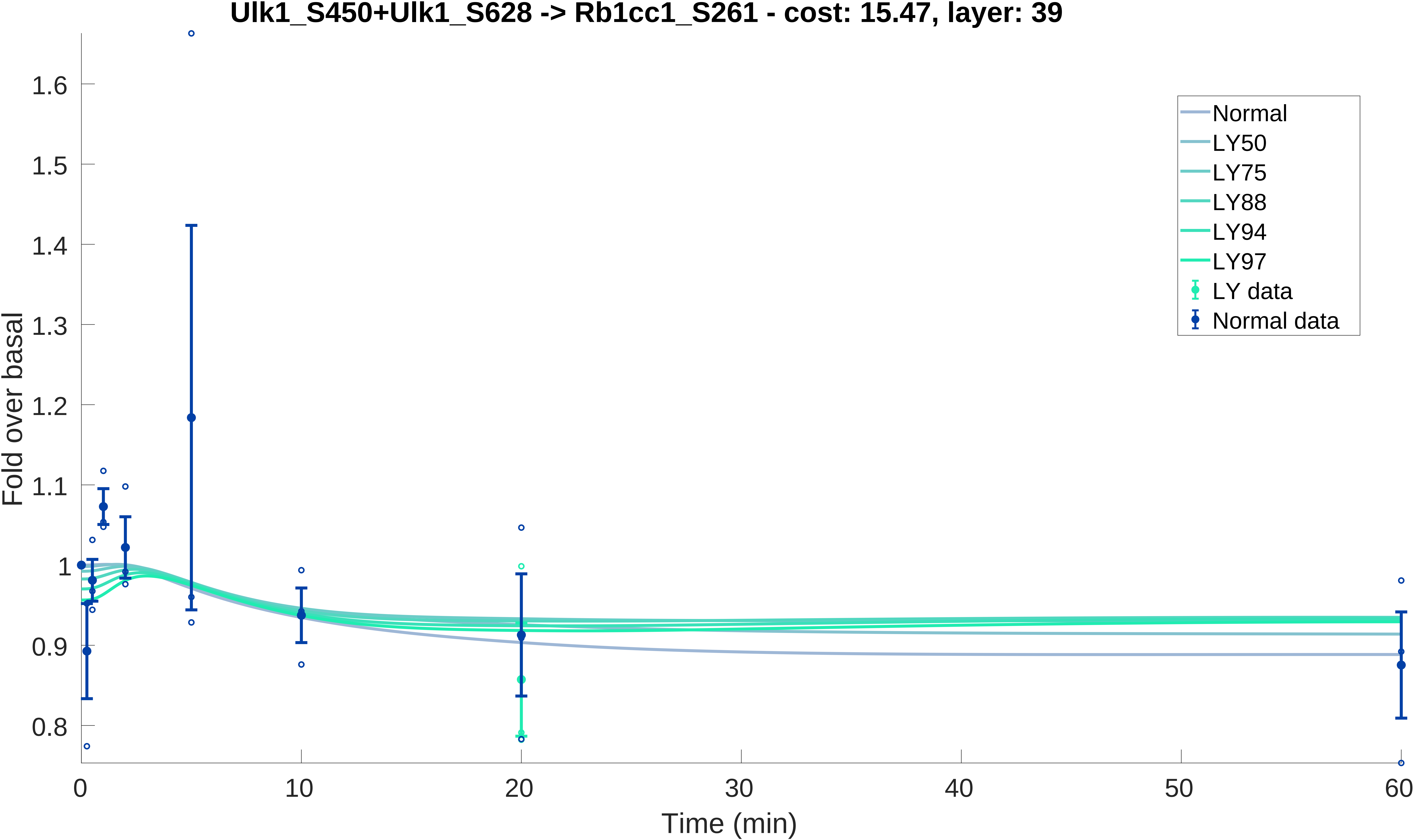

Ulk1_S450+Ulk1_S628 -> Rb1cc1_S261 - cost: 15.47, layer: 39
Fold over basal
Time (min)
0
10
20
30
40
50
60
0.8
0.9
1
1.1
1.2
1.3
1.4
1.5
1.6
Normal
LY50
LY75
LY88
LY94
LY97
LY data
Normal data

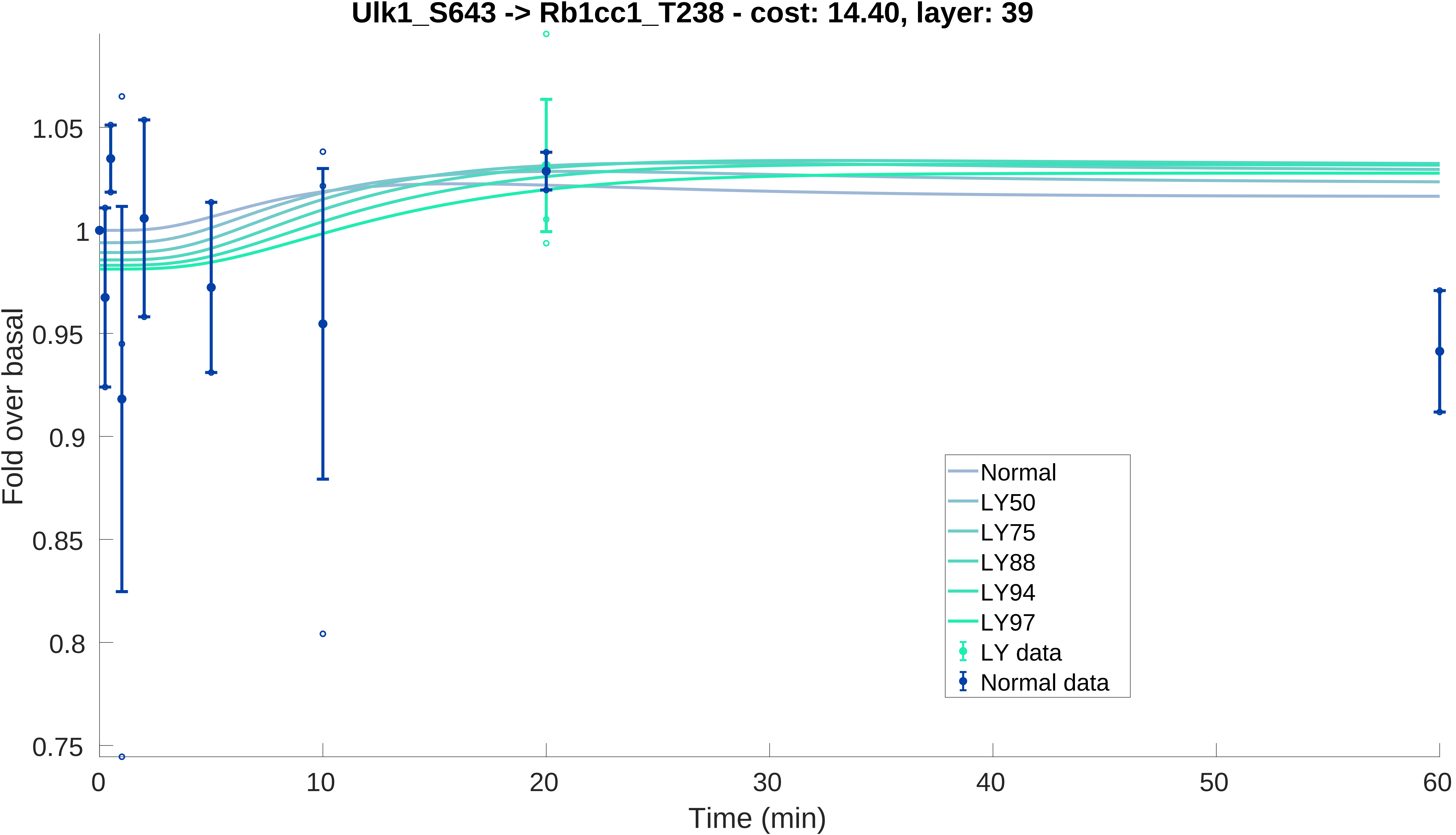

Ulk1_S643 -> Rb1cc1_T238 - cost: 14.40, layer: 39
Fold over basal
1.05
1
0.95
0.9
0.85
0.8
0.75
0
10
20
30
40
50
60
Time (min)
Normal
LY50
LY75
LY88
LY94
LY97
LY data
Normal data

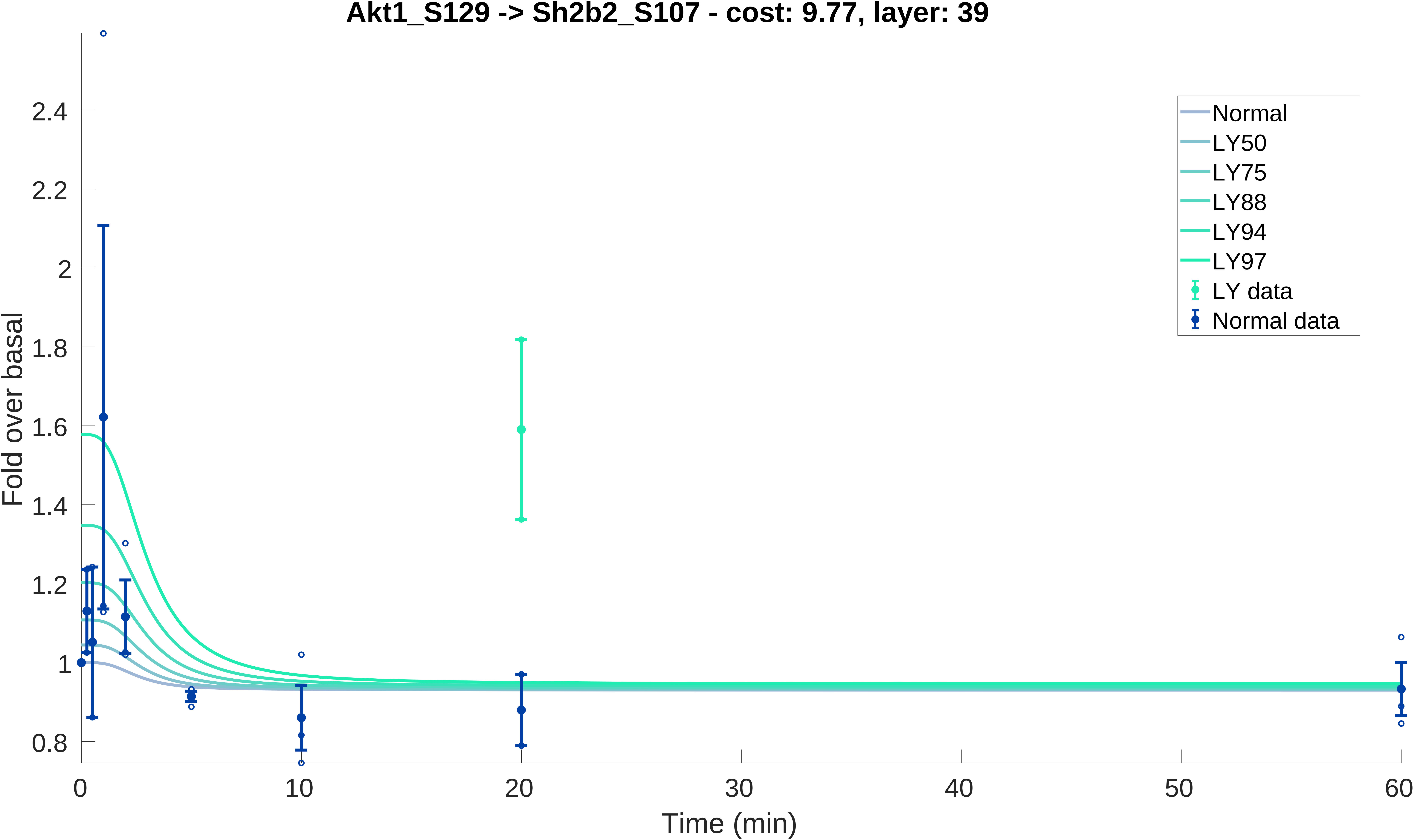

Akt1_S129 -> Sh2b2_S107 - cost: 9.77, layer: 39
Fold over basal
Time (min)
0.8
1
1.2
1.4
1.6
1.8
2
2.2
2.4
0
10
20
30
40
50
60
Normal
LY50
LY75
LY88
LY94
LY97
LY data
Normal data

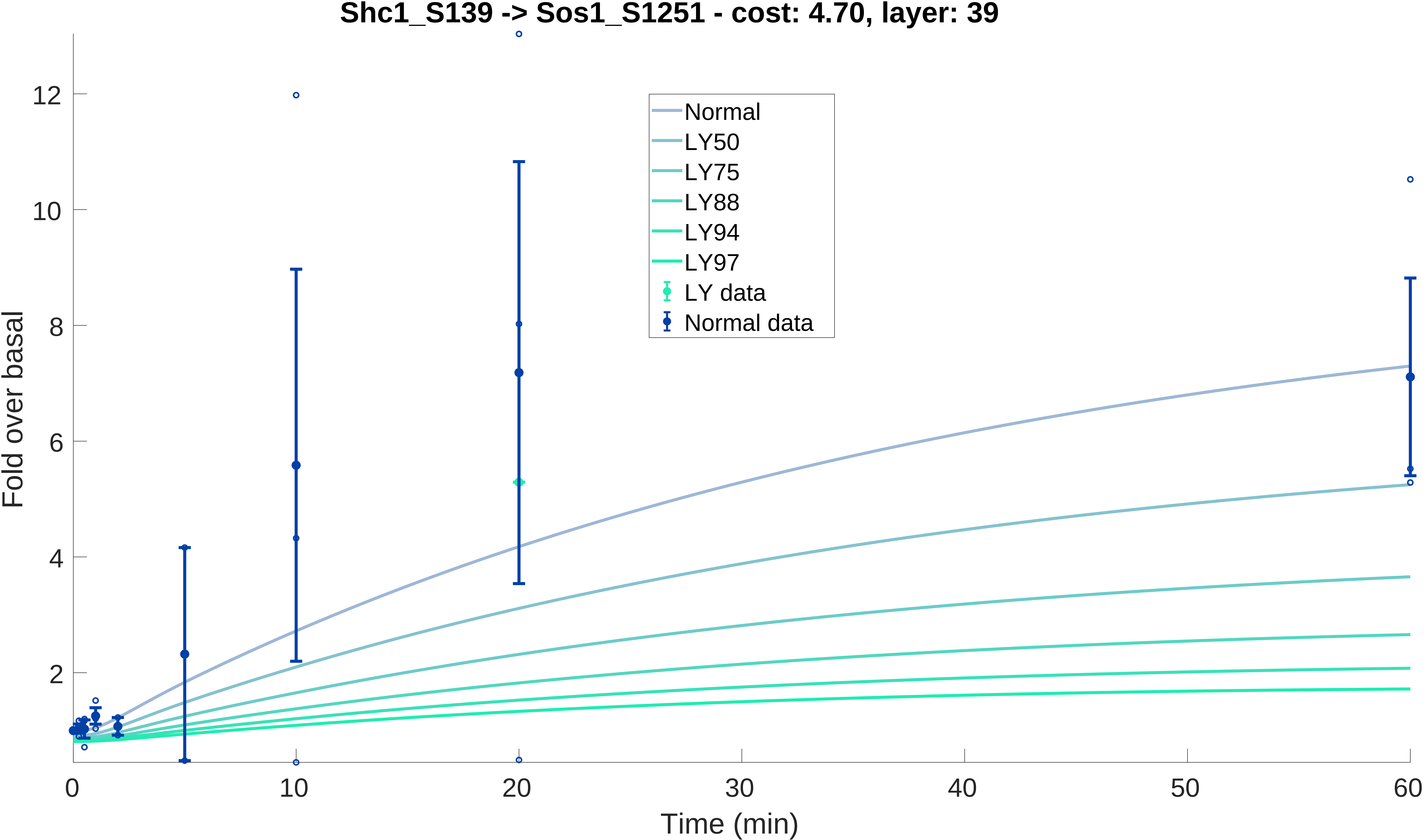

Shc1_S139 -> Sos1_S1251 - cost: 4.70, layer: 39
Normal
LY50
LY75
LY88
LY94
LY97
LY data
Normal data
Fold over basal
Time (min)
0
2
4
6
8
10
12
0
10
20
30
40
50
60

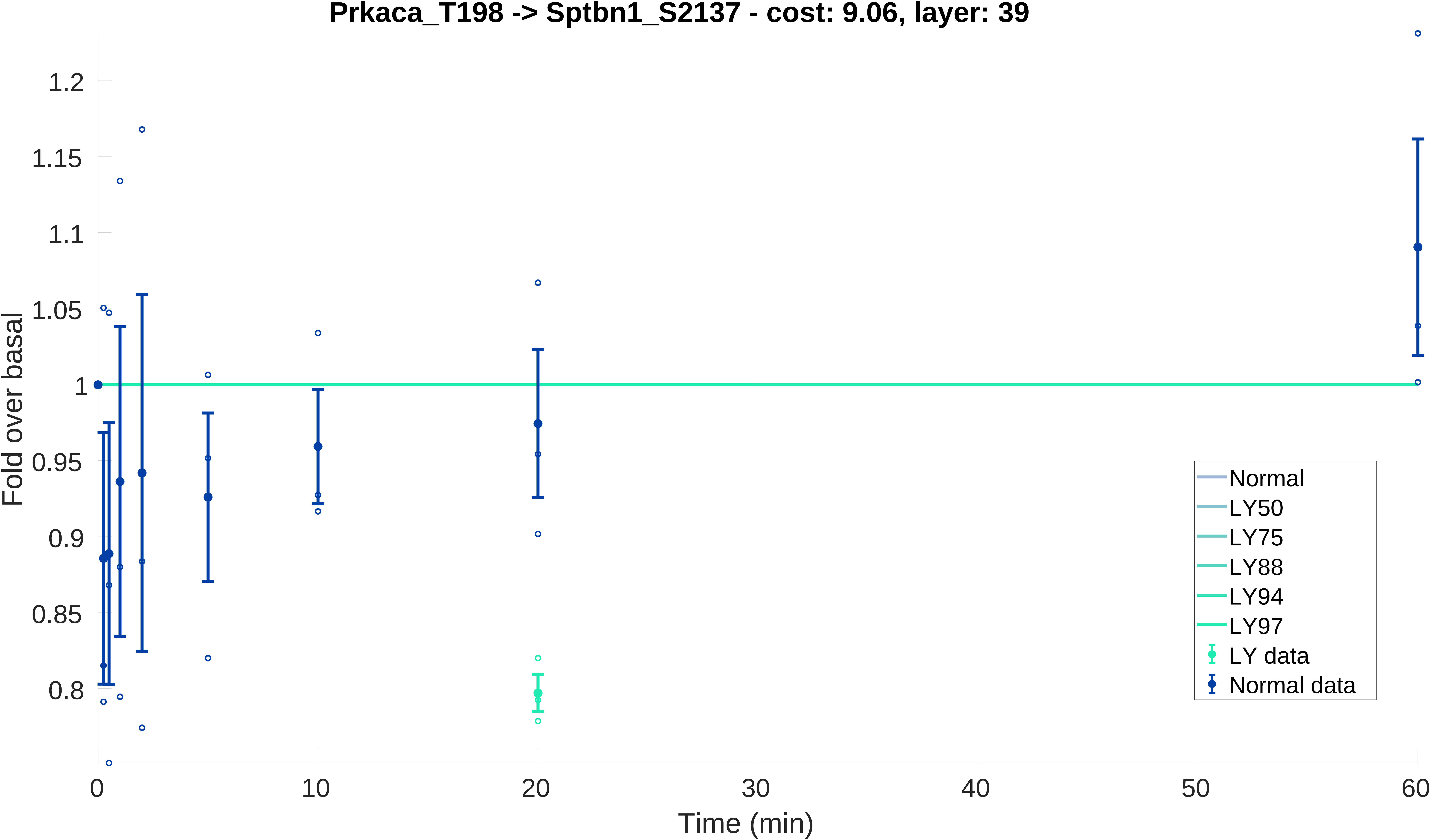

Prkaca_T198 -> Sptbn1_S2137 - cost: 9.06, layer: 39
Fold over basal
1.2
1.15
1.1
1.05
1
0.95
0.9
0.85
0.8
0
10
20
30
40
50
60
Time (min)
Normal
LY50
LY75
LY88
LY94
LY97
LY data
Normal data

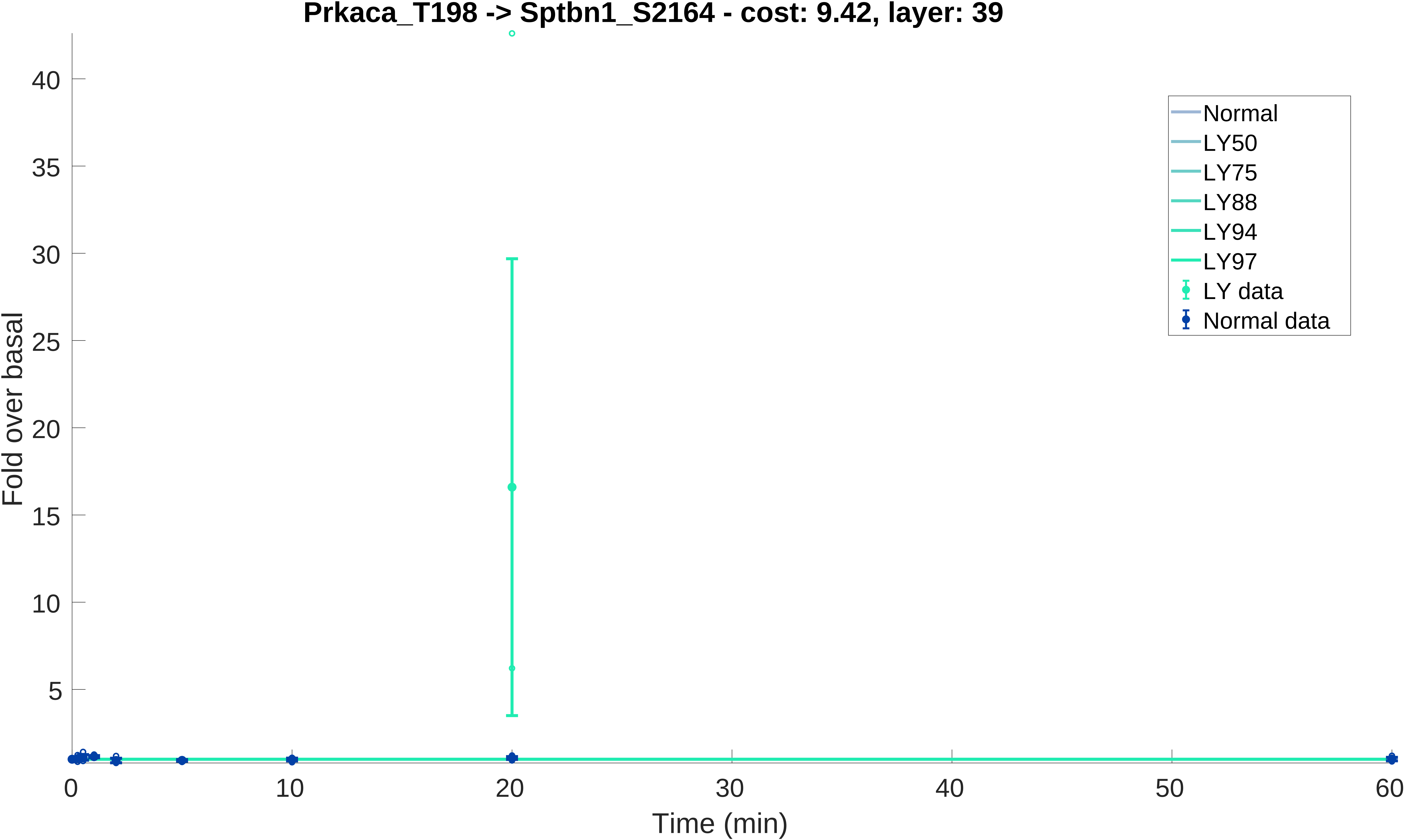

Prkaca_T198 -> Sptbn1_S2164 - cost: 9.42, layer: 39
Fold over basal
Time (min)
40
35
30
25
20
15
10
5
0
10
20
30
40
50
60
Normal
LY50
LY75
LY88
LY94
LY97
LY data
Normal data

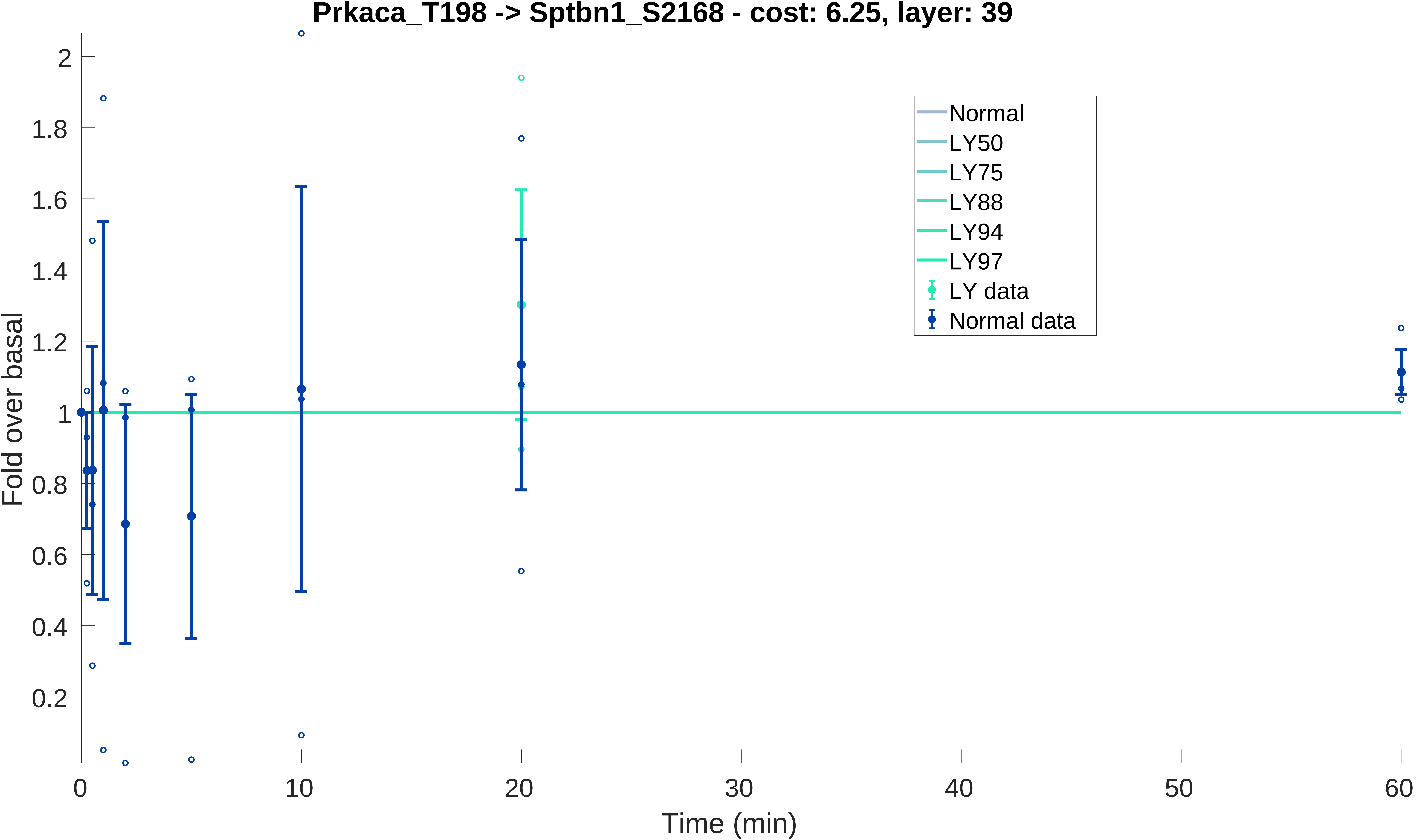

Prkaca_T198 -> Sptbn1_S2168 - cost: 6.25, layer: 39
Fold over basal
Time (min)
Normal
LY50
LY75
LY88
LY94
LY97
LY data
Normal data
0
10
20
30
40
50
60
0.2
0.4
0.6
0.8
1
1.2
1.4
1.6
1.8
2

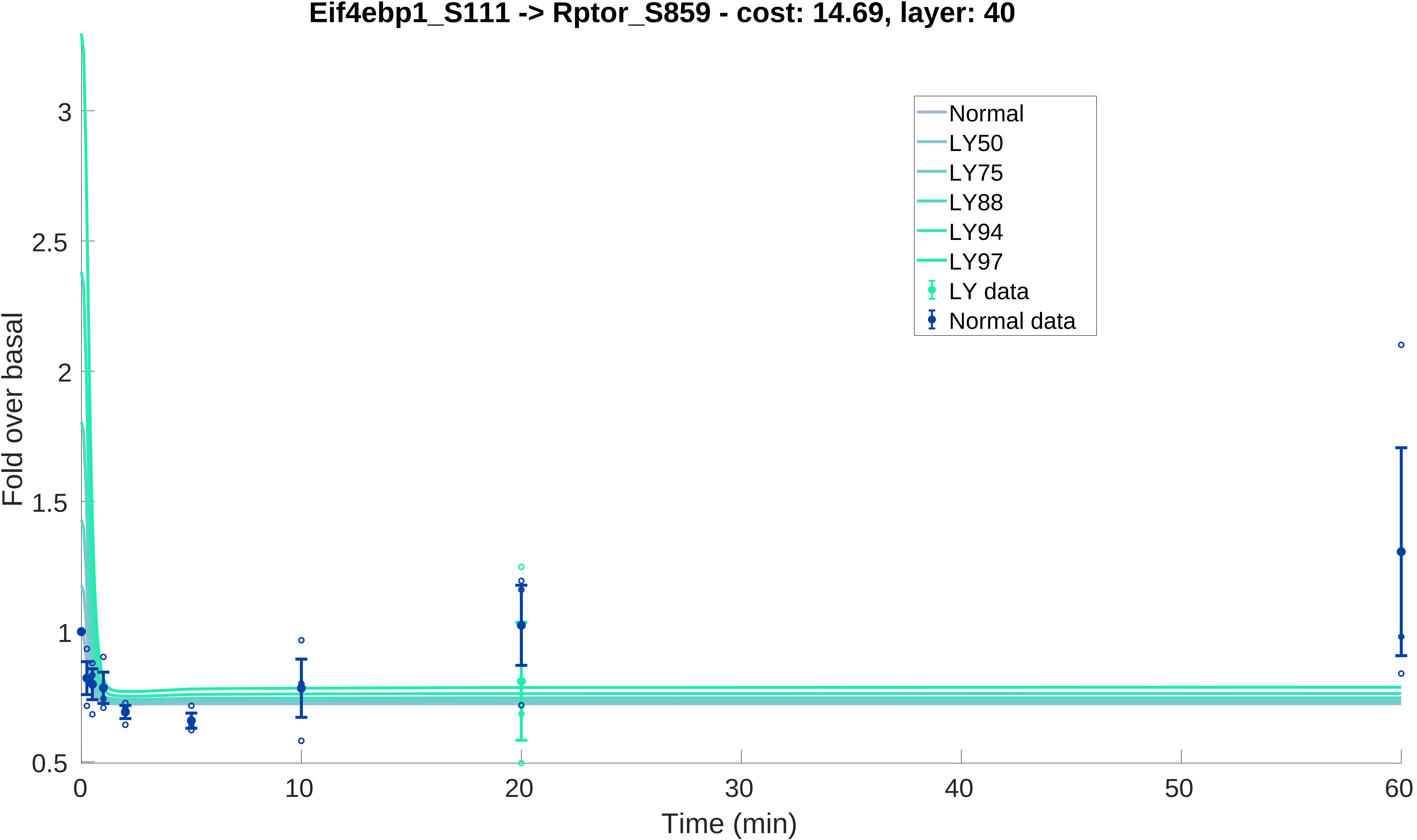

Eif4ebp1_S111 -> Rptor_S859 - cost: 14.69, layer: 40
Normal
LY50
LY75
LY88
LY94
LY97
LY data
Normal data
Fold over basal
Time (min)
0.5
1
1.5
2
2.5
3
0
10
20
30
40
50
60

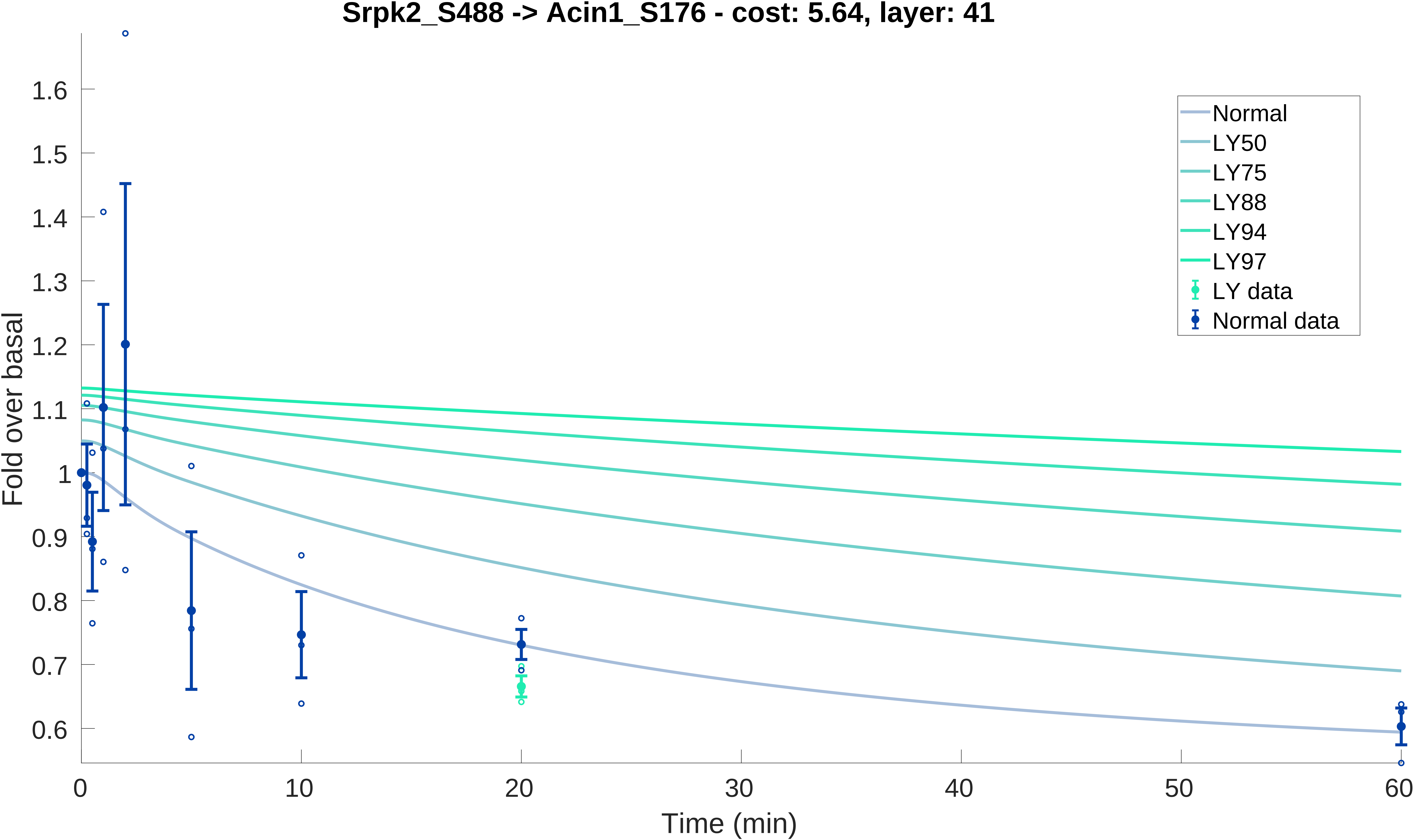

Srpk2_S488 -> Acin1_S176 - cost: 5.64, layer: 41
Fold over basal
Time (min)
1.6
1.5
1.4
1.3
1.2
1.1
1
0.9
0.8
0.7
0.6
0
10
20
30
40
50
60
Normal
LY50
LY75
LY88
LY94
LY97
LY data
Normal data

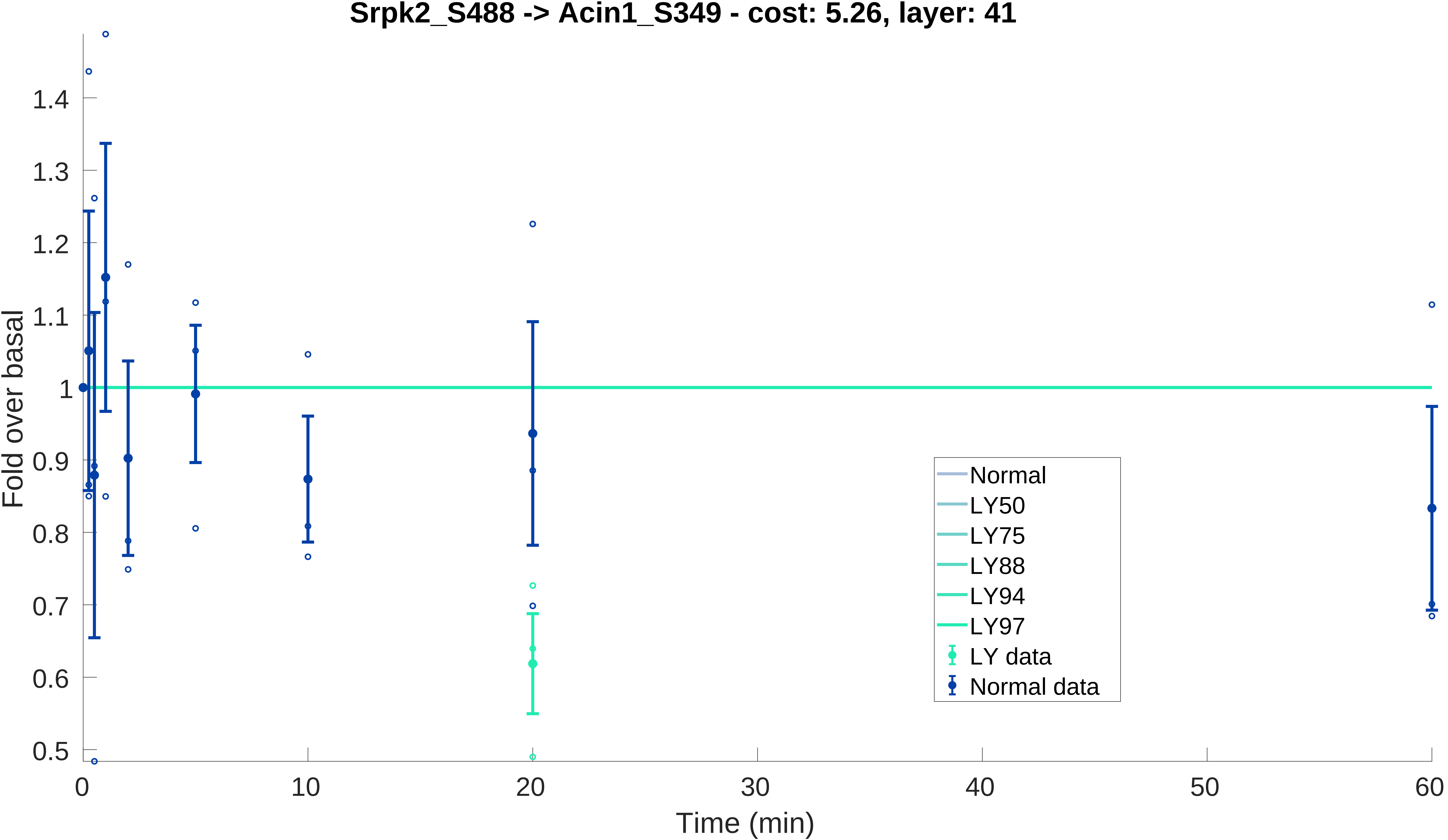

Srpk2_S488 -> Acin1_S349 - cost: 5.26, layer: 41
Fold over basal
Time (min)
0
10
20
30
40
50
60
0.5
0.6
0.7
0.8
0.9
1
1.1
1.2
1.3
1.4
Normal
LY50
LY75
LY88
LY94
LY97
LY data
Normal data

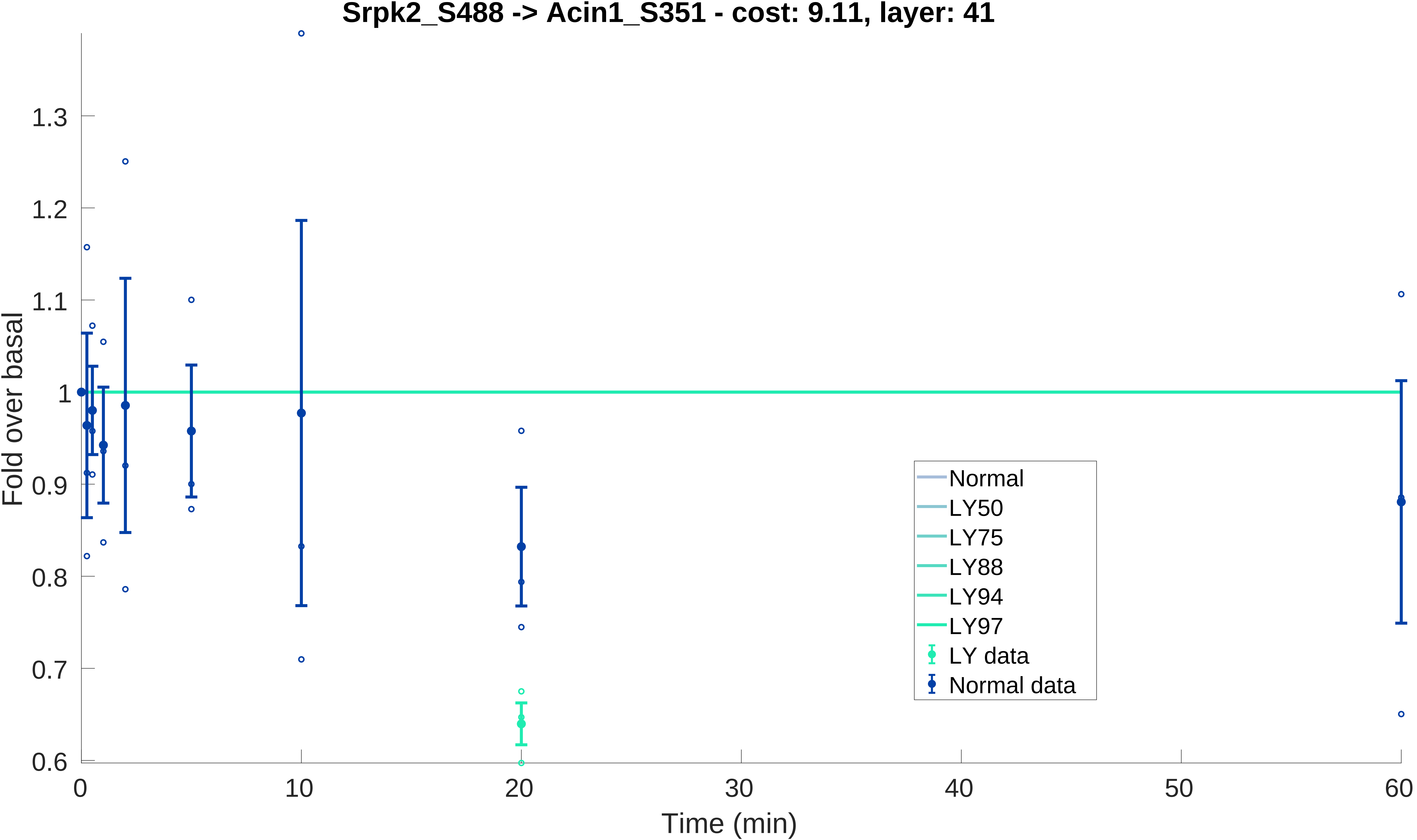

Srpk2_S488 -> Acin1_S351 - cost: 9.11, layer: 41
Fold over basal
Time (min)
0.6
0.7
0.8
0.9
1
1.1
1.2
1.3
0
10
20
30
40
50
60
Normal
LY50
LY75
LY88
LY94
LY97
LY data
Normal data

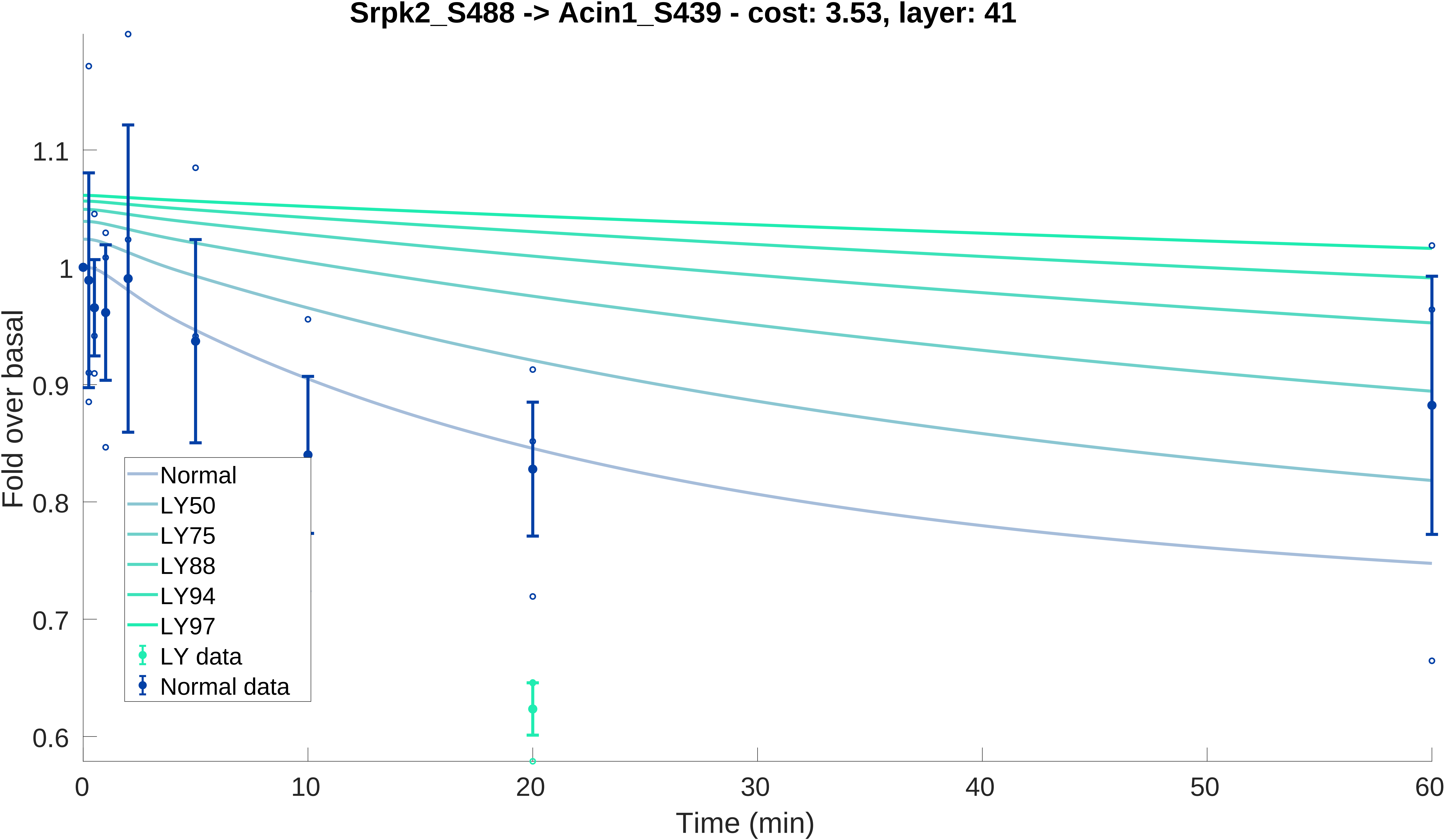

Srpk2_S488 -> Acin1_S439 - cost: 3.53, layer: 41
Fold over basal
1.1
1
0.9
0.8
0.7
0.6
0
10
20
30
40
50
60
Time (min)
Normal
LY50
LY75
LY88
LY94
LY97
LY data
Normal data

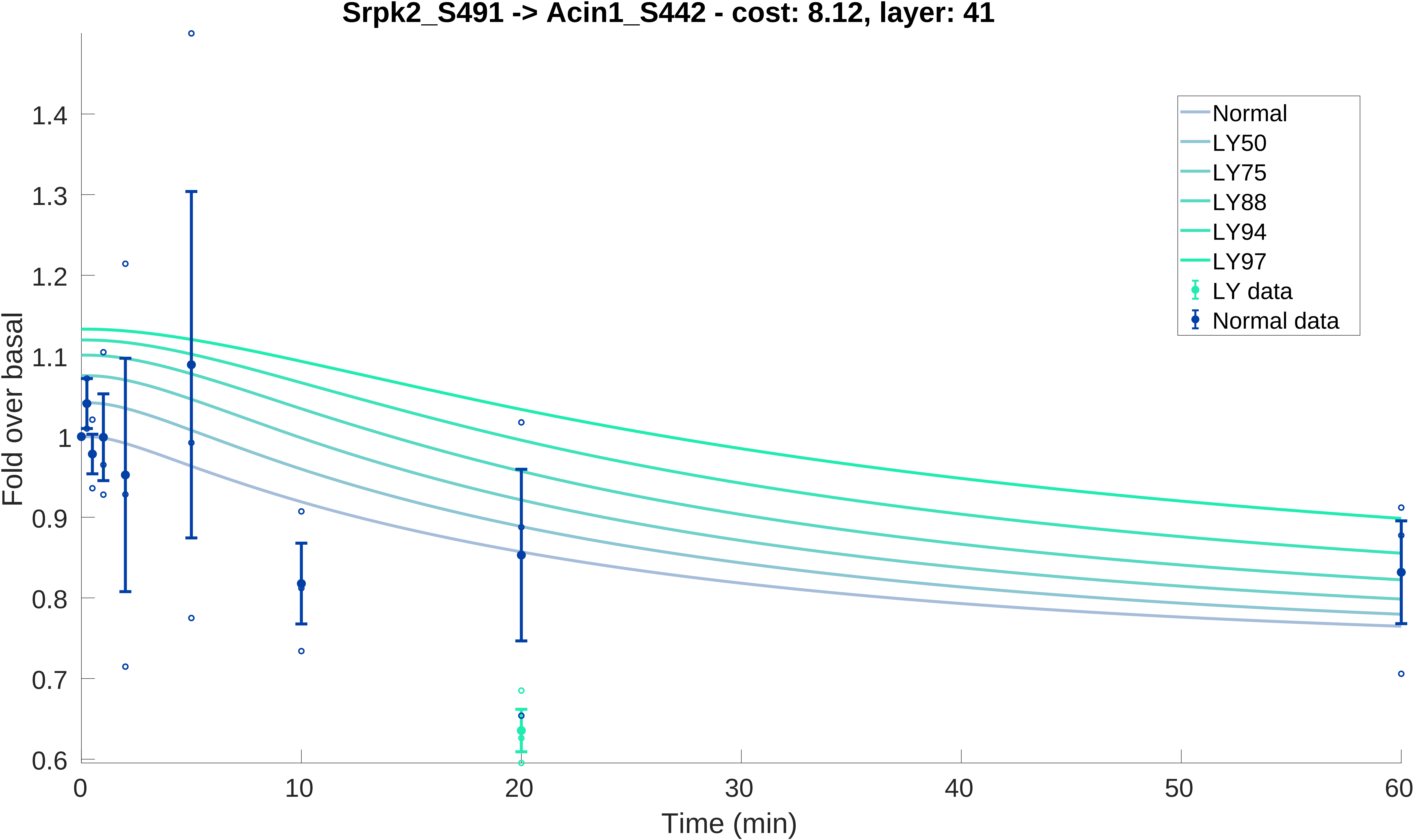

Srpk2_S491 -> Acin1_S442 - cost: 8.12, layer: 41
Fold over basal
Time (min)
Normal
LY50
LY75
LY88
LY94
LY97
LY data
Normal data
1.4
1.3
1.2
1.1
1
0.9
0.8
0.7
0.6
0
10
20
30
40
50
60

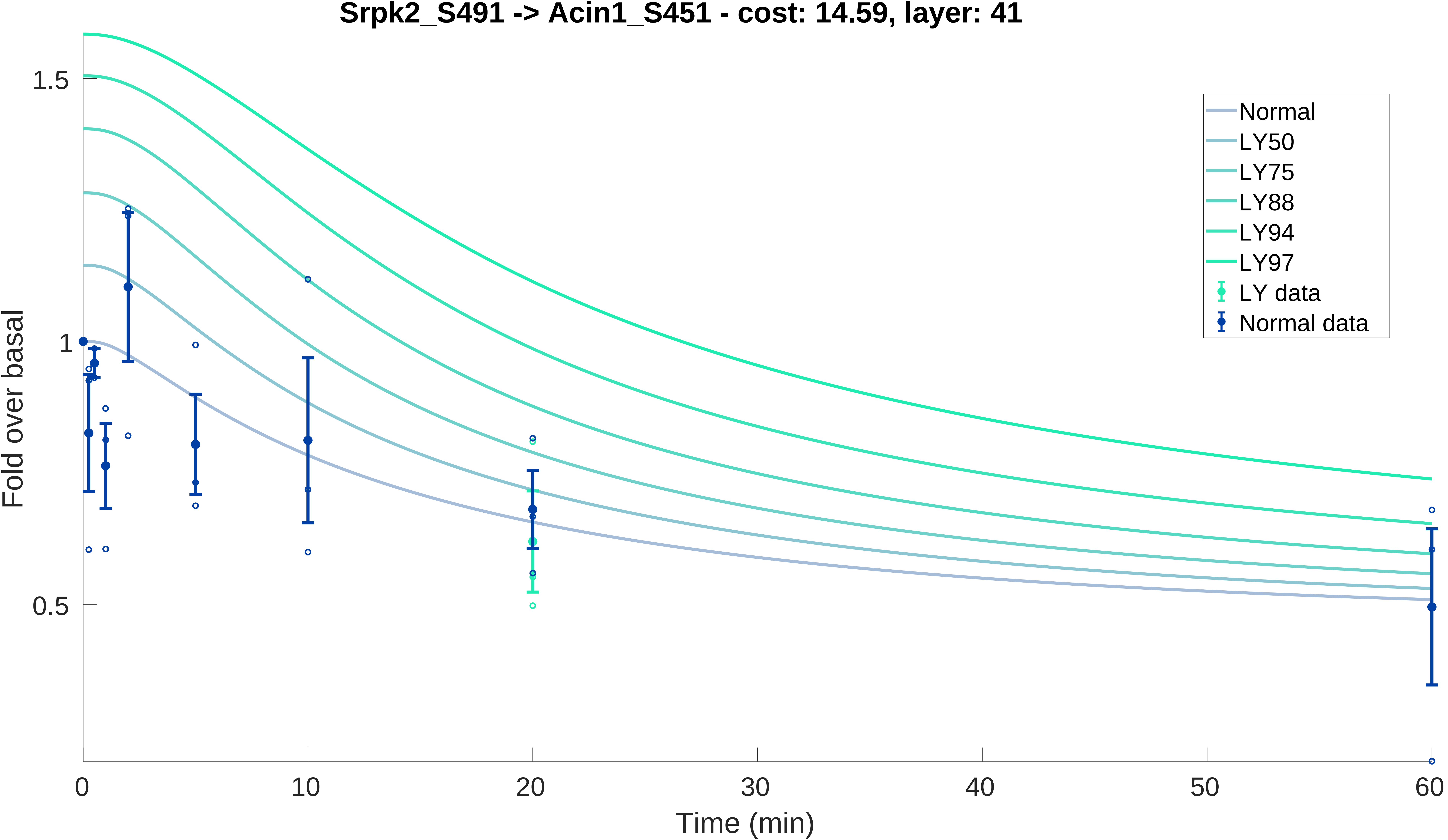

Srpk2_S491 -> Acin1_S451 - cost: 14.59, layer: 41
Fold over basal
1.5
1
0.5
0
10
20
30
40
50
60
Time (min)
Normal
LY50
LY75
LY88
LY94
LY97
LY data
Normal data

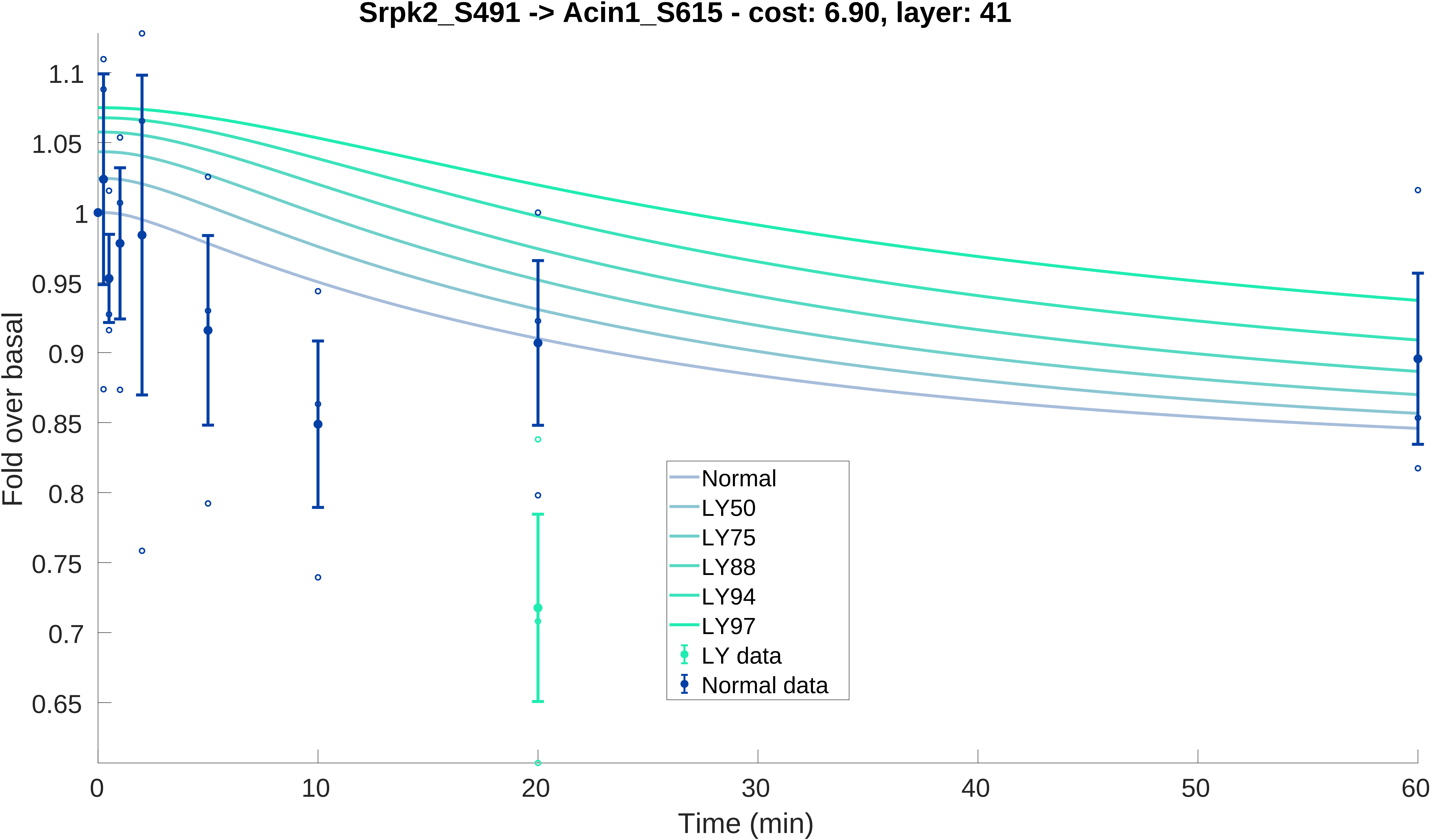

Srpk2_S491 -> Acin1_S615 - cost: 6.90, layer: 41
Fold over basal
Time (min)
1.1
1.05
1
0.95
0.9
0.85
0.8
0.75
0.7
0.65
0
10
20
30
40
50
60
Normal
LY50
LY75
LY88
LY94
LY97
LY data
Normal data

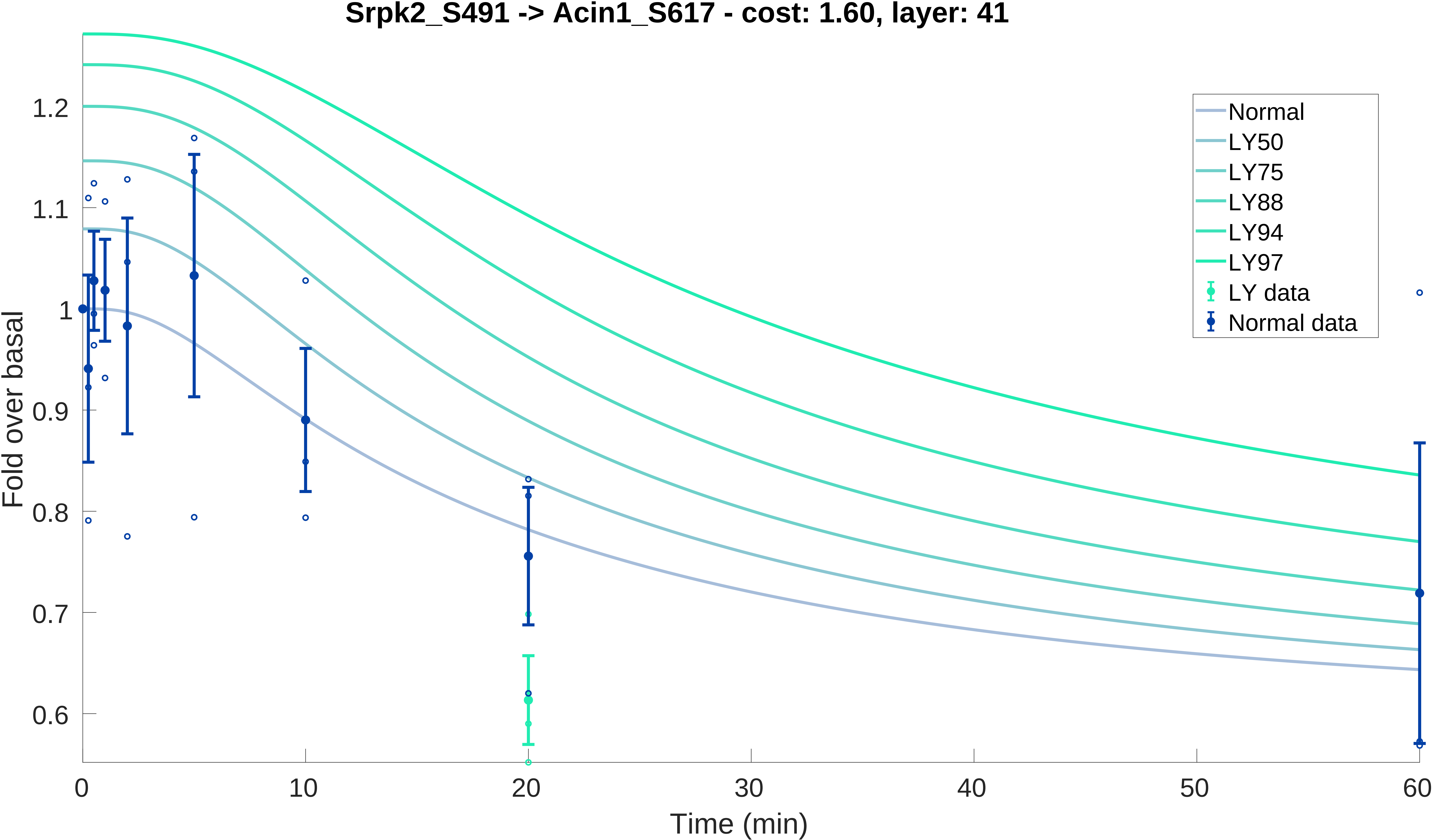
Srpk2_S491 -> Acin1_S617 - cost: 1.60, layer: 41
Fold over basal
1.2
1.1
1
0.9
0.8
0.7
0.6
0
10
20
30
40
50
60
Time (min)
Normal
LY50
LY75
LY88
LY94
LY97
LY data
Normal data

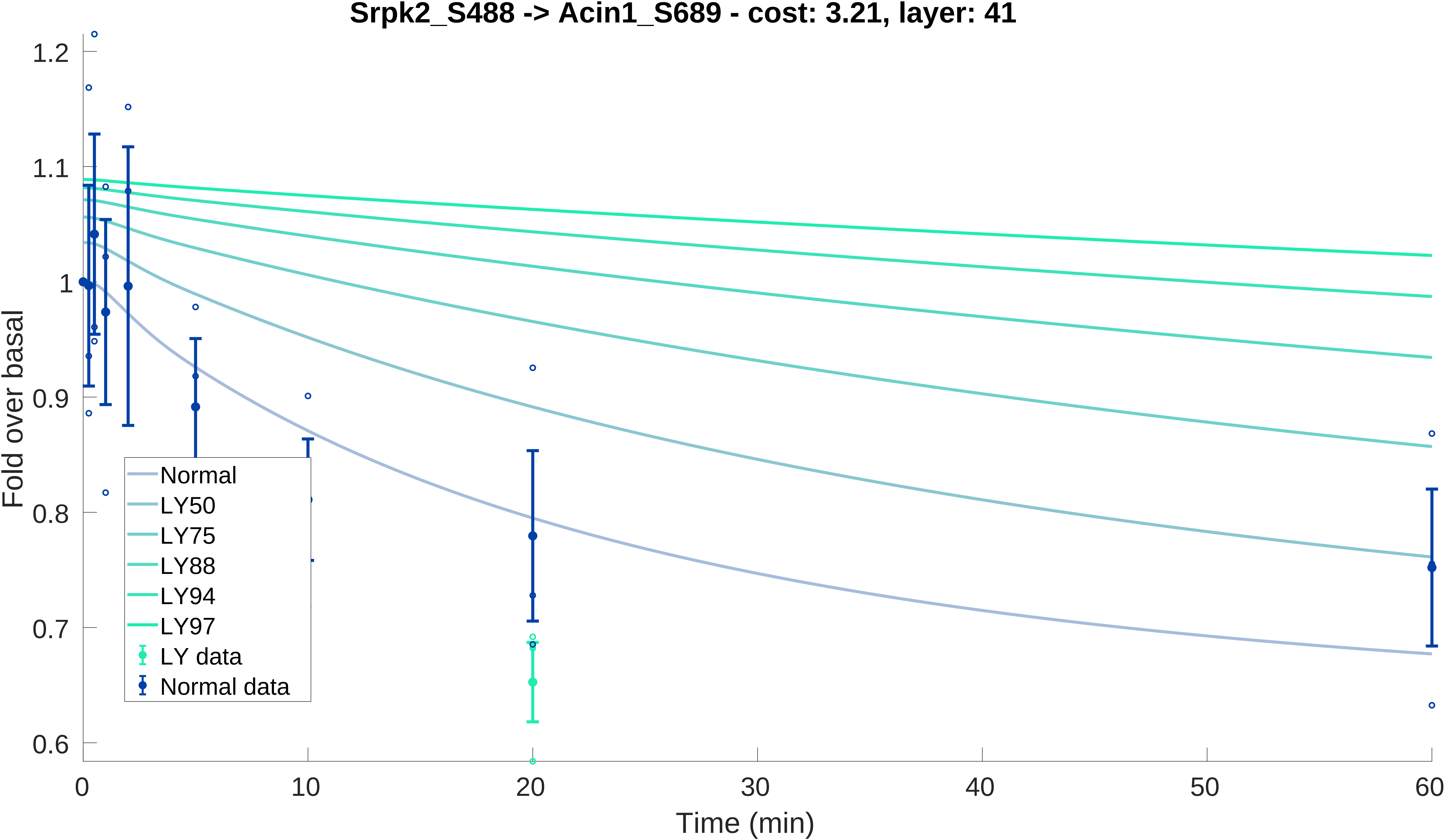
Srpk2_S488 -> Acin1_S689 - cost: 3.21, layer: 41
Fold over basal
Time (min)
0.6
0.7
0.8
0.9
1
1.1
1.2
0
10
20
30
40
50
60
Normal
LY50
LY75
LY88
LY94
LY97
LY data
Normal data

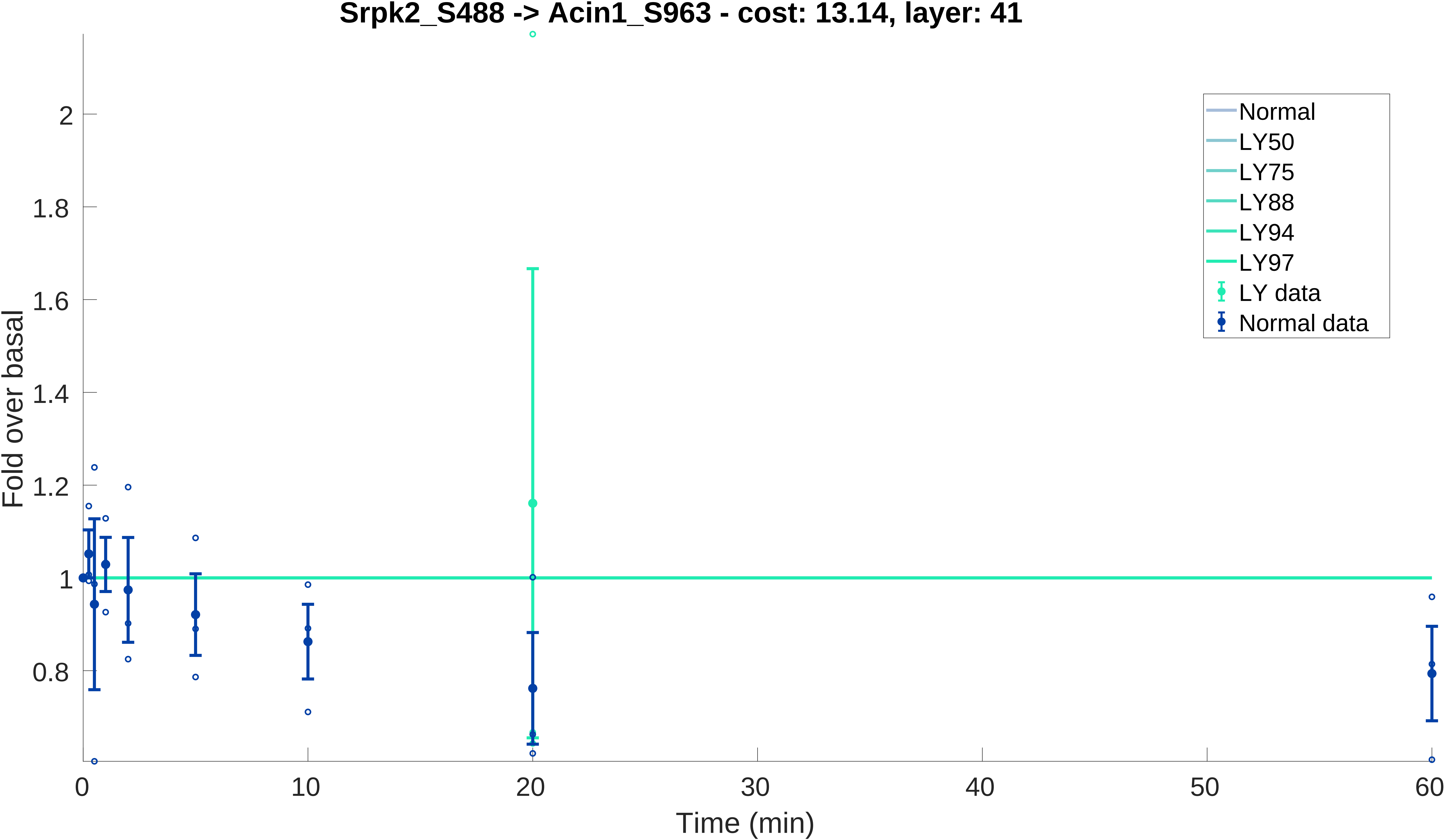

Srpk2_S488 -> Acin1_S963 - cost: 13.14, layer: 41
Fold over basal
Time (min)
Normal
LY50
LY75
LY88
LY94
LY97
LY data
Normal data
0
10
20
30
40
50
60
0.8
1
1.2
1.4
1.6
1.8
2

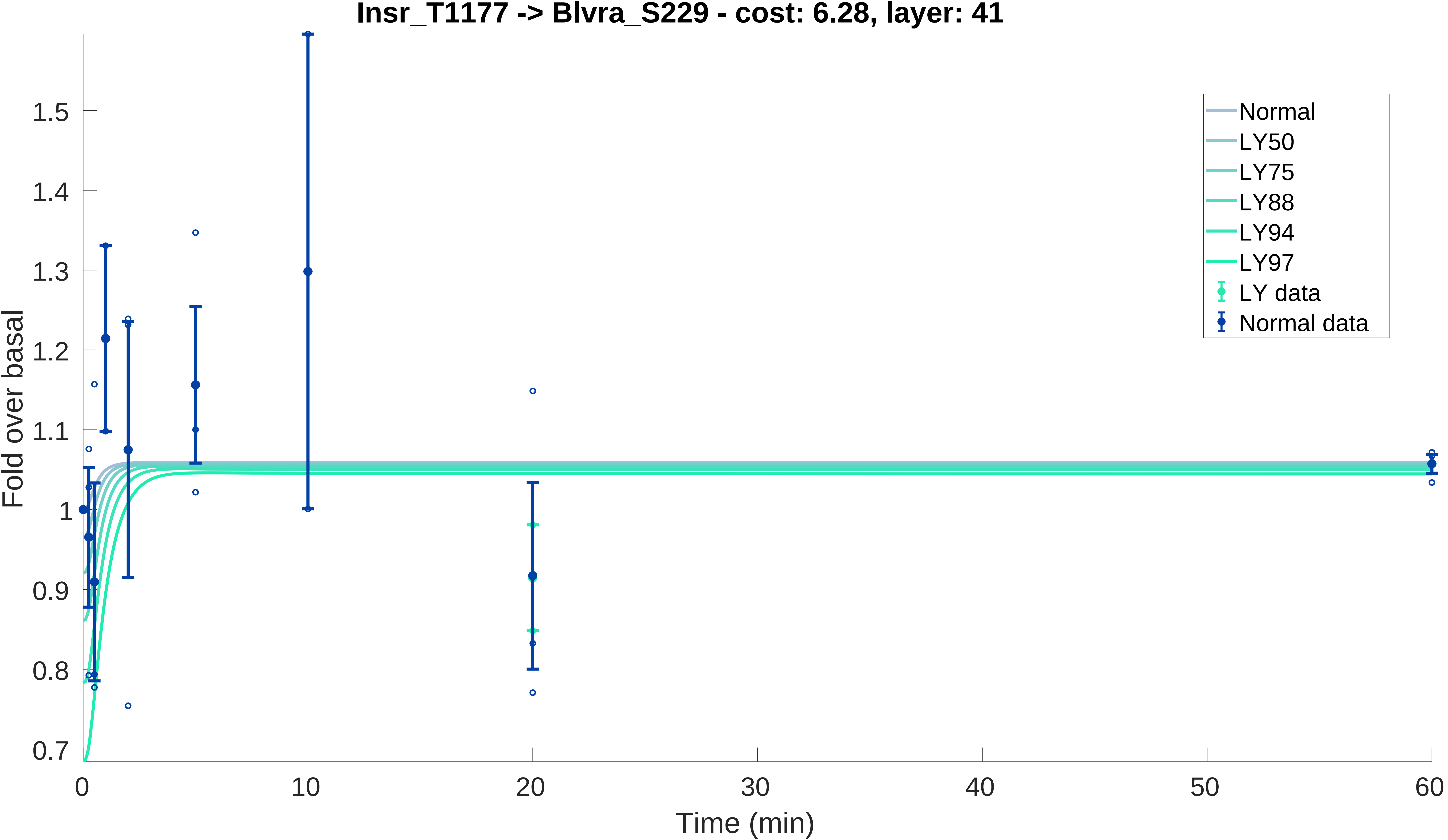

Insr_T1177 -> Blvra_S229 - cost: 6.28, layer: 41
Fold over basal
Time (min)
1.5
1.4
1.3
1.2
1.1
1
0.9
0.8
0.7
0
10
20
30
40
50
60
Normal
LY50
LY75
LY88
LY94
LY97
LY data
Normal data

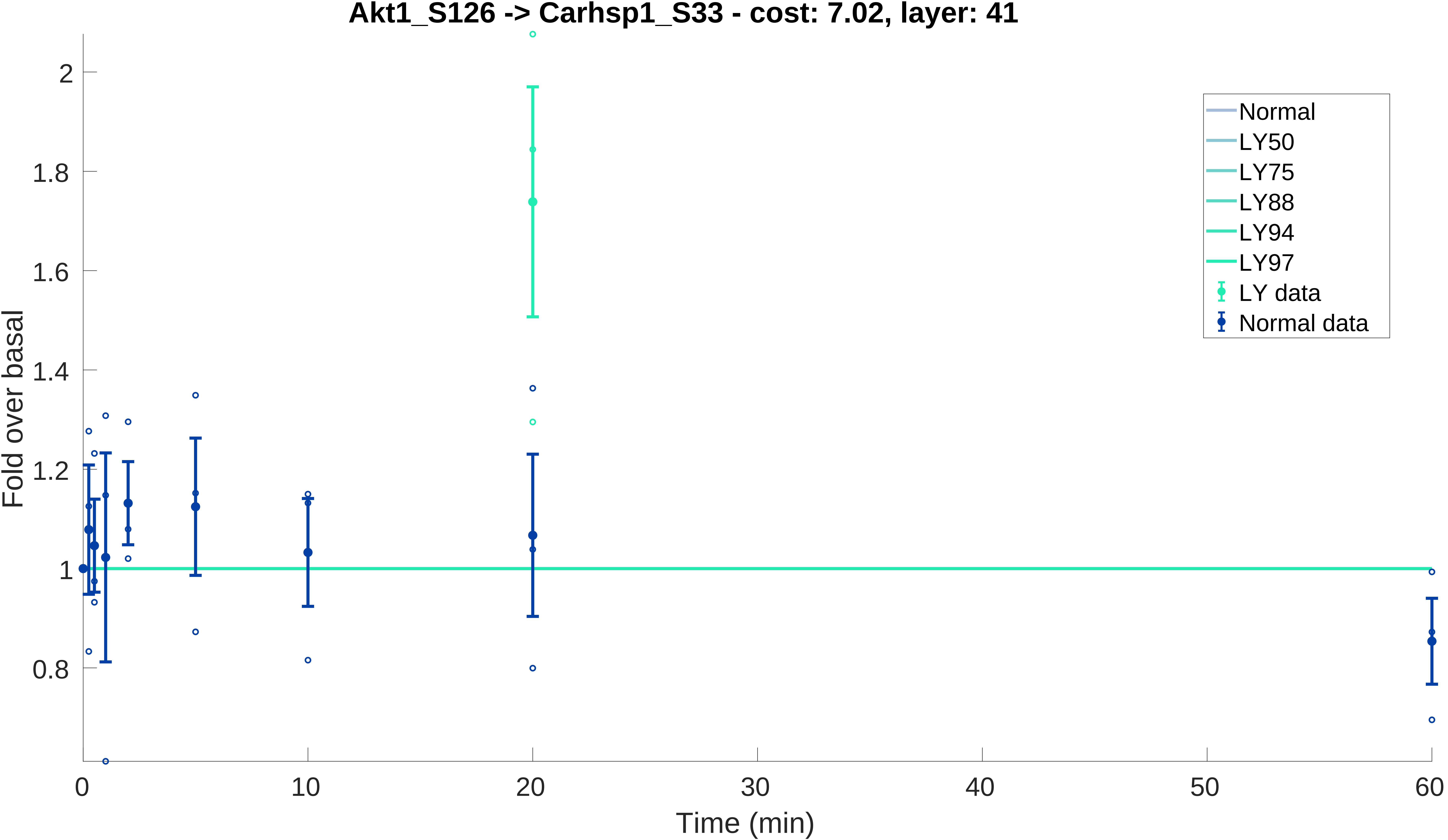
Akt1_S126 -> Carhsp1_S33 - cost: 7.02, layer: 41
Fold over basal
2
1.8
1.6
1.4
1.2
1
0.8
0
10
20
30
40
50
60
Time (min)
Normal
LY50
LY75
LY88
LY94
LY97
LY data
Normal data

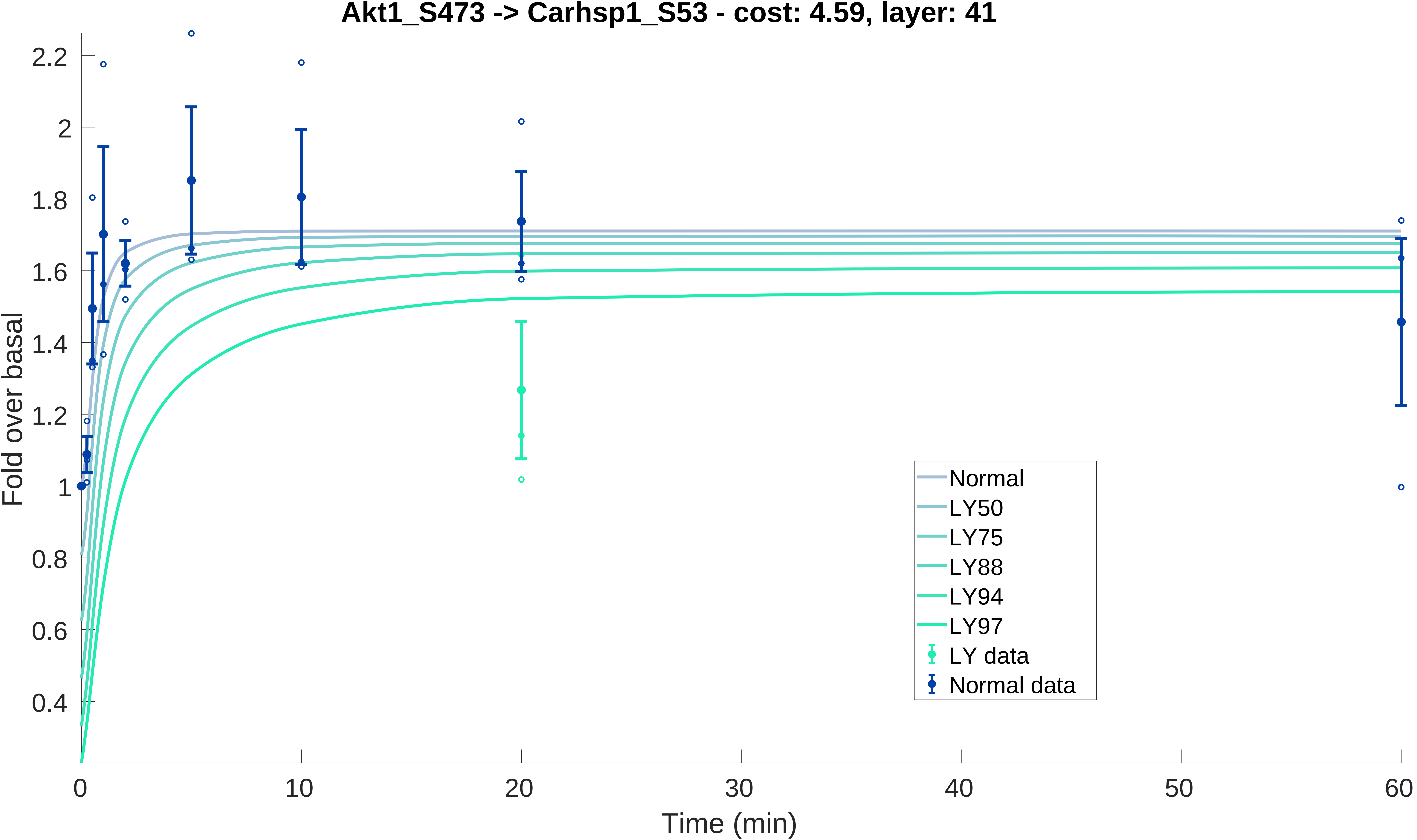

Akt1_S473 -> Carhsp1_S53 - cost: 4.59, layer: 41
2.2
2
1.8
1.6
1.4
1.2
1
0.8
0.6
0.4
Fold over basal
0
10
20
30
40
50
60
Time (min)
Normal
LY50
LY75
LY88
LY94
LY97
LY data
Normal data

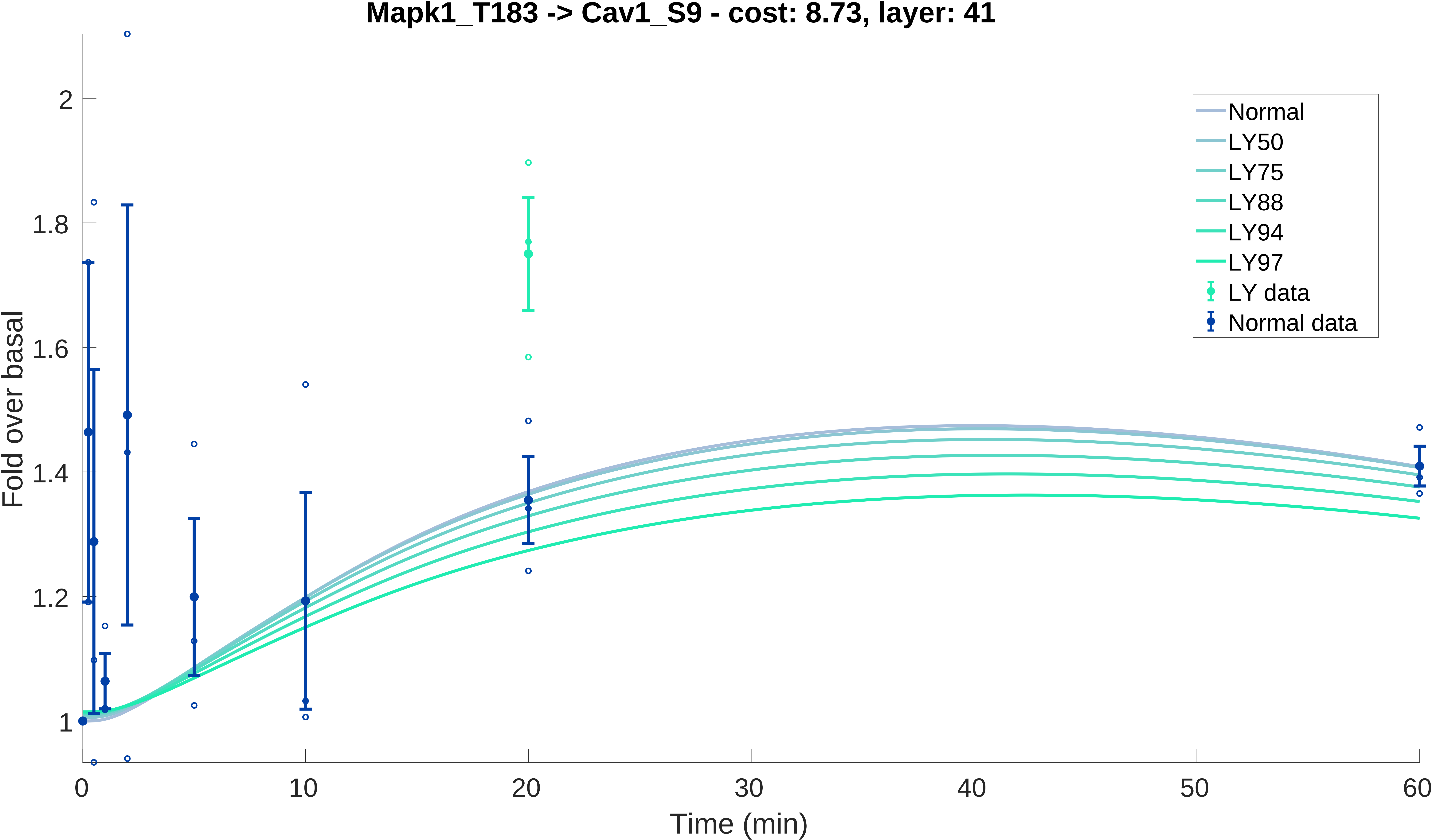

Mapk1_T183 -> Cav1_S9 - cost: 8.73, layer: 41
Fold over basal
Time (min)
0
10
20
30
40
50
60
1
1.2
1.4
1.6
1.8
2
Normal
LY50
LY75
LY88
LY94
LY97
LY data
Normal data

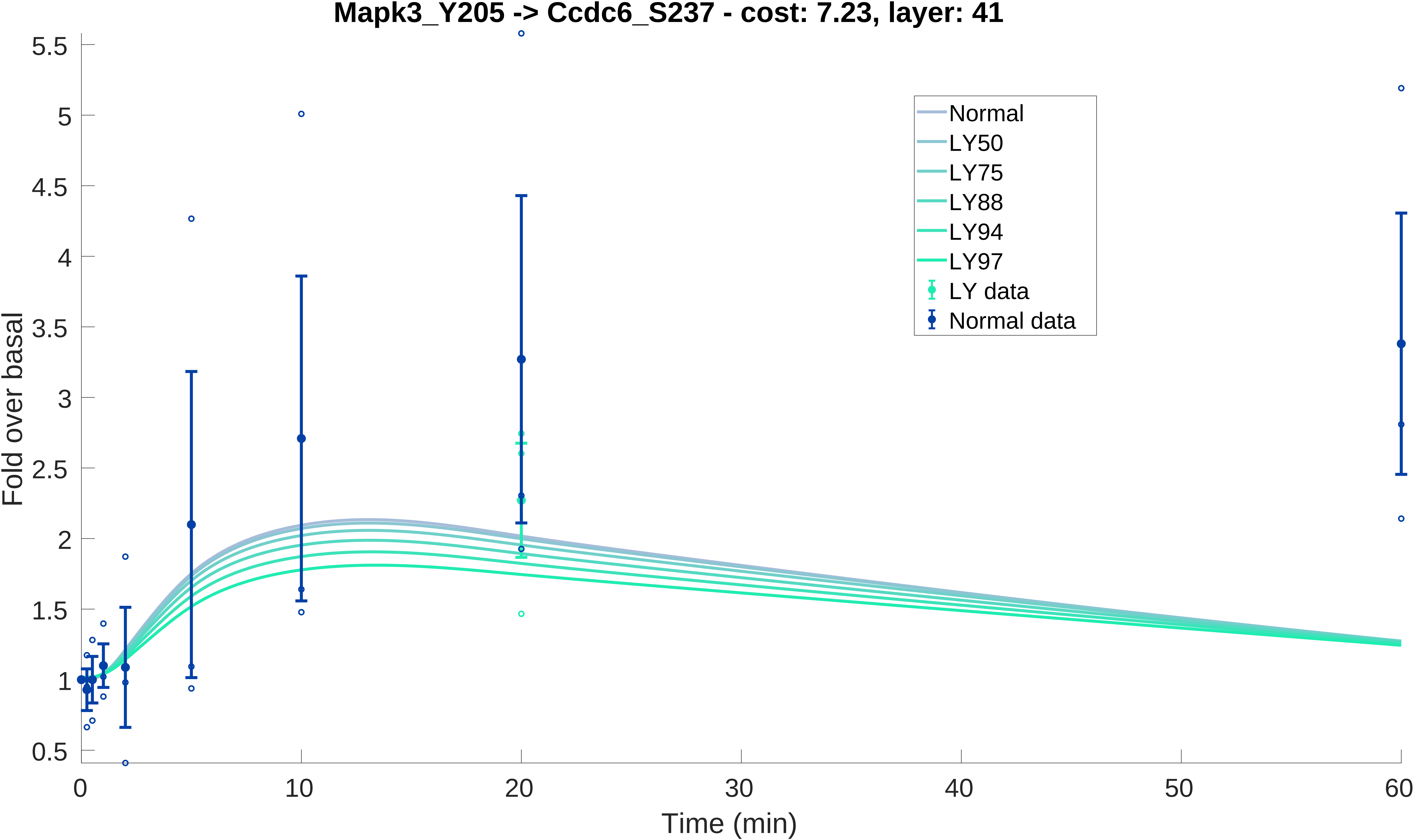

Mapk3_Y205 -> Ccdc6_S237 - cost: 7.23, layer: 41
Fold over basal
5.5
5
4.5
4
3.5
3
2.5
2
1.5
1
0.5
0
10
20
30
40
50
60
Time (min)
Normal
LY50
LY75
LY88
LY94
LY97
LY data
Normal data

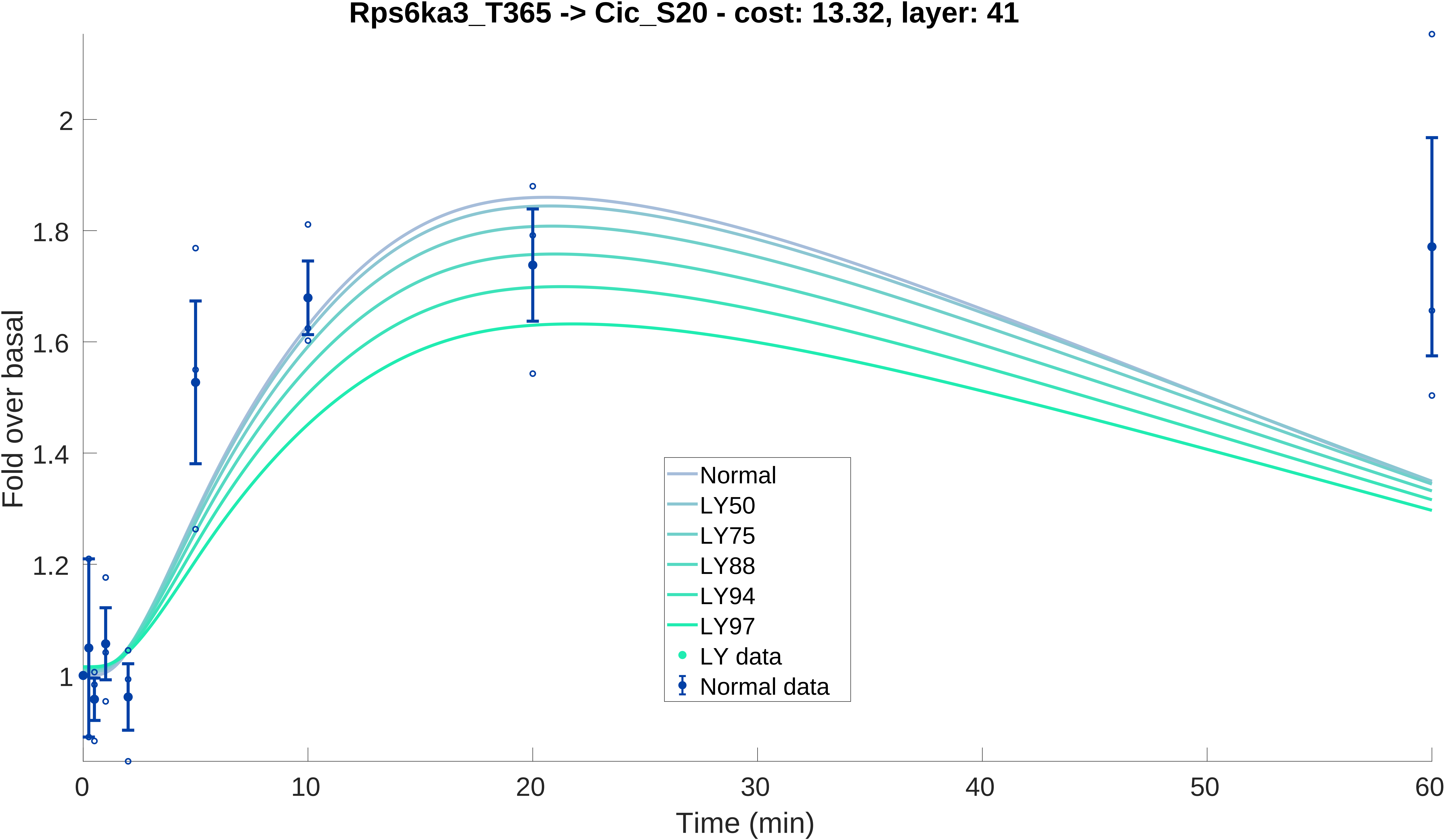

Rpps6ka3_T365 -> Cic_S20 - cost: 13.32, layer: 41
Fold over basal
Time (min)
Normal
LY50
LY75
LY88
LY94
LY97
LY data
Normal data

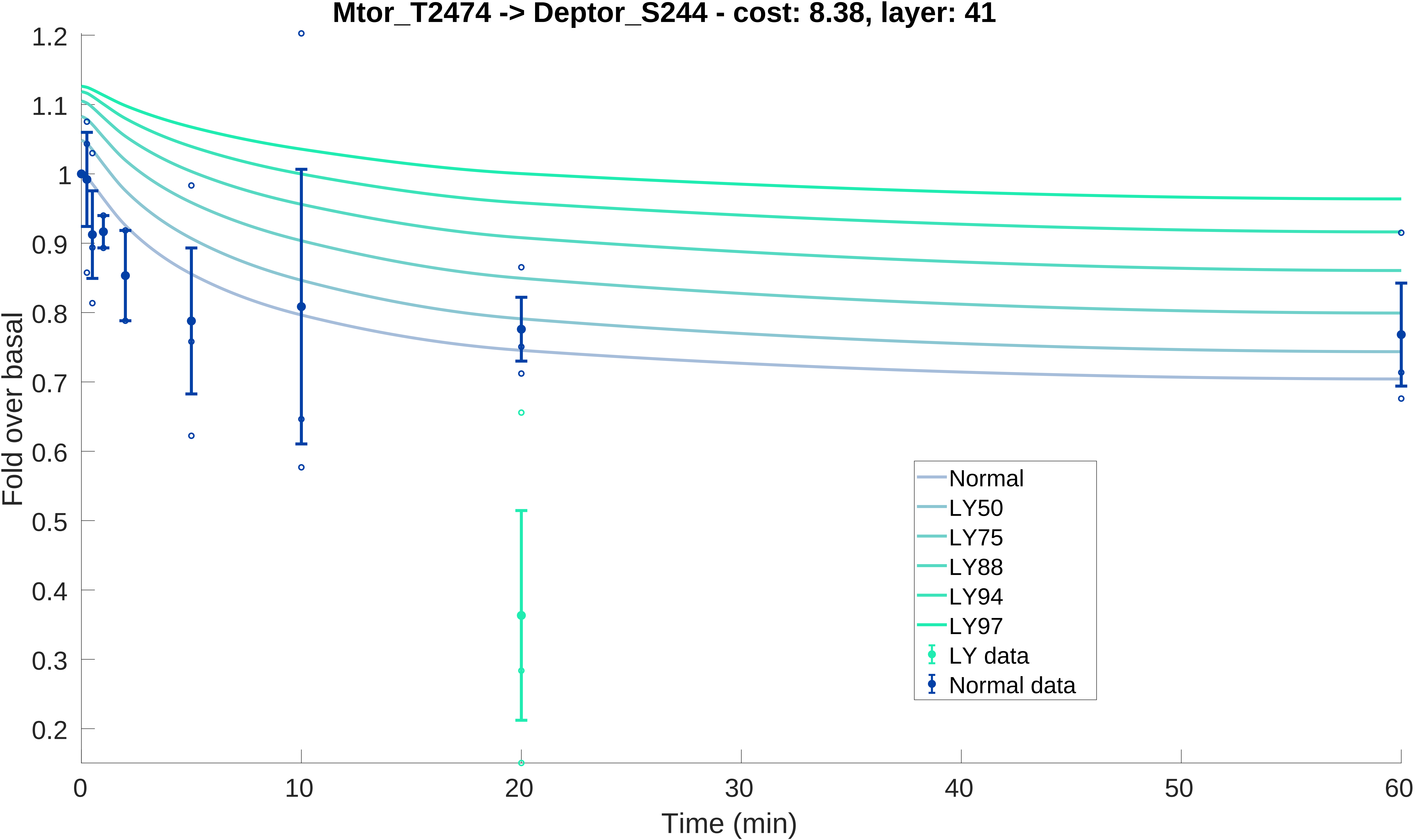
Mtor_T2474 -> Deptor_S244 - cost: 8.38, layer: 41
Fold over basal
Time (min)
1.2
1.1
1
0.9
0.8
0.7
0.6
0.5
0.4
0.3
0.2
0
10
20
30
40
50
60
Normal
LY50
LY75
LY88
LY94
LY97
LY data
Normal data

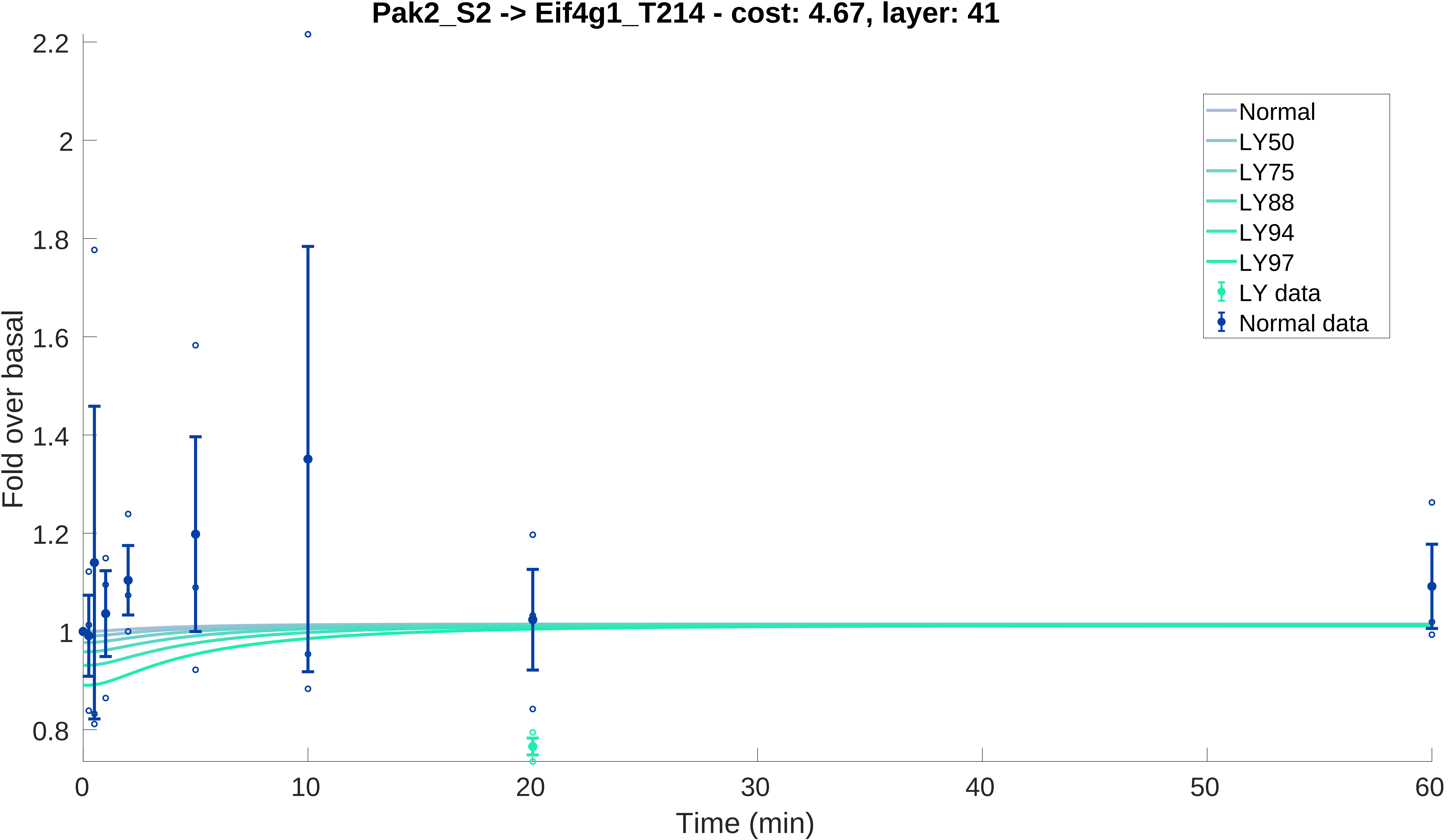

Pak2_S2 -> Eif4g1_T214 - cost: 4.67, layer: 41
Fold over basal
Time (min)
0.8
1
1.2
1.4
1.6
1.8
2
2.2
0
10
20
30
40
50
60
Normal
LY50
LY75
LY88
LY94
LY97
LY data
Normal data

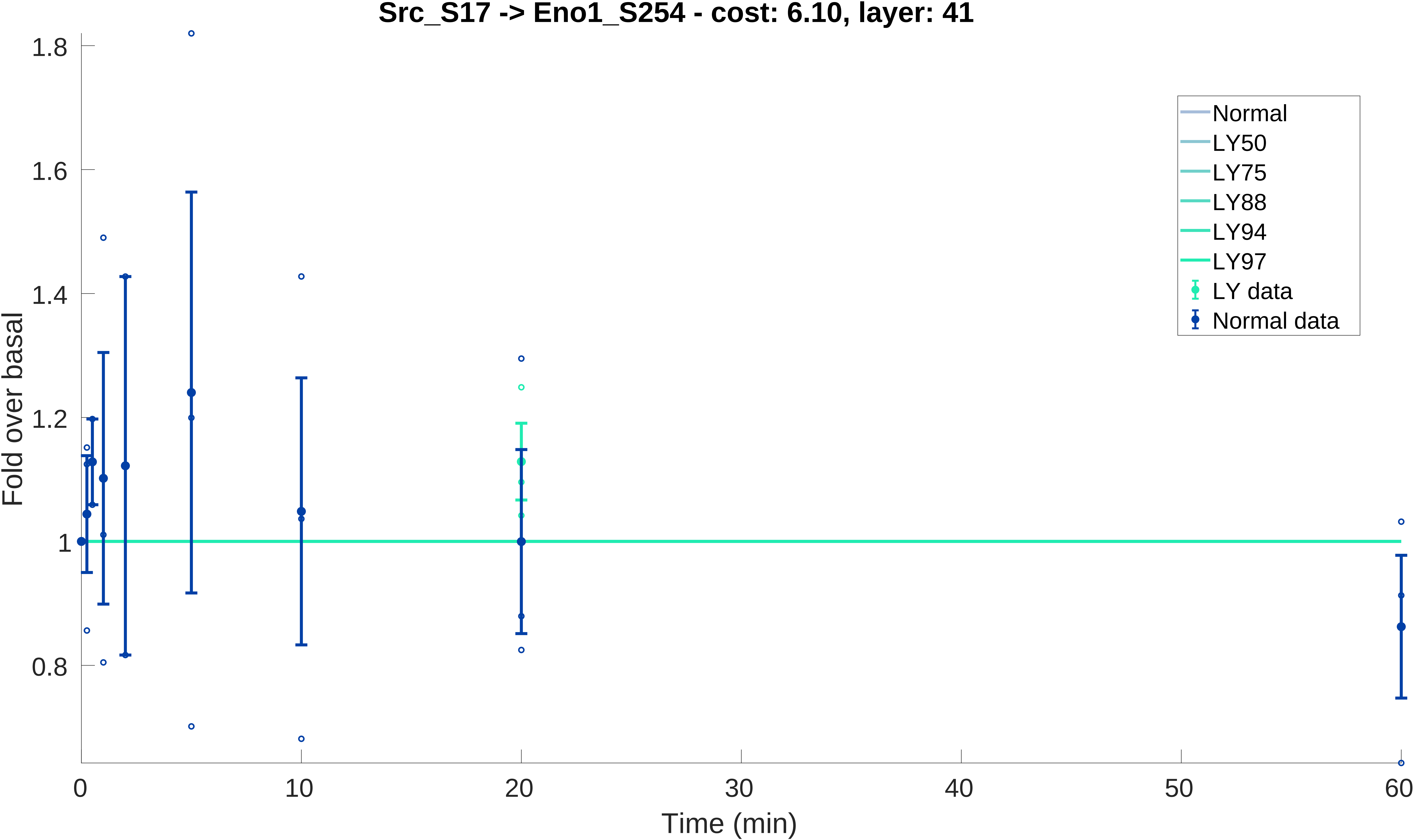

Src_S17 -> Eno1_S254 - cost: 6.10, layer: 41
Fold over basal
1.8
1.6
1.4
1.2
1
0.8
Time (min)
0
10
20
30
40
50
60
Normal
LY50
LY75
LY88
LY94
LY97
LY data
Normal data

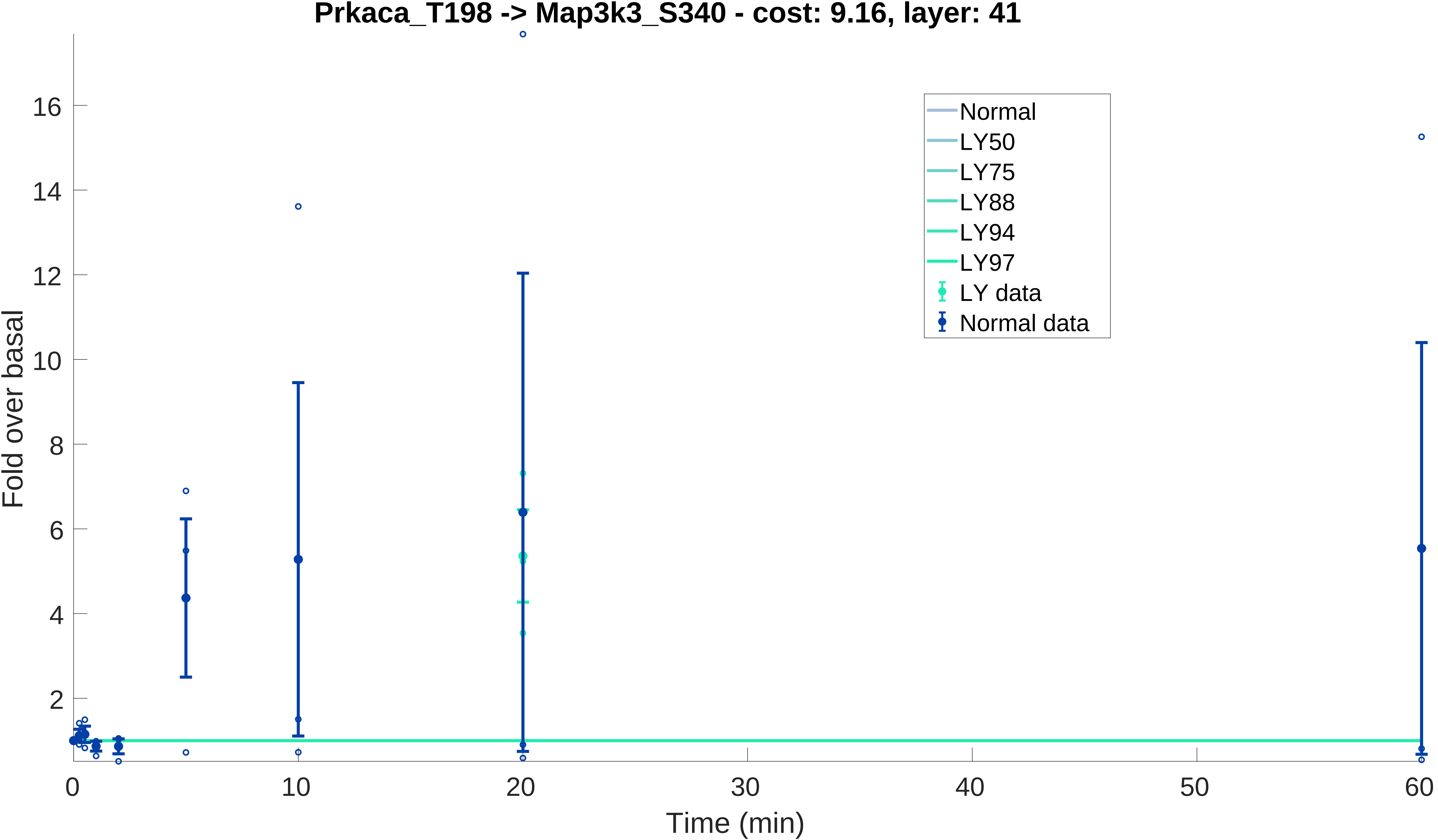
Prkaca_T198 -> Map3k3_S340 - cost: 9.16, layer: 41
Fold over basal
Time (min)
Normal
LY50
LY75
LY88
LY94
LY97
LY data
Normal data
0
10
20
30
40
50
60
2
4
6
8
10
12
14
16

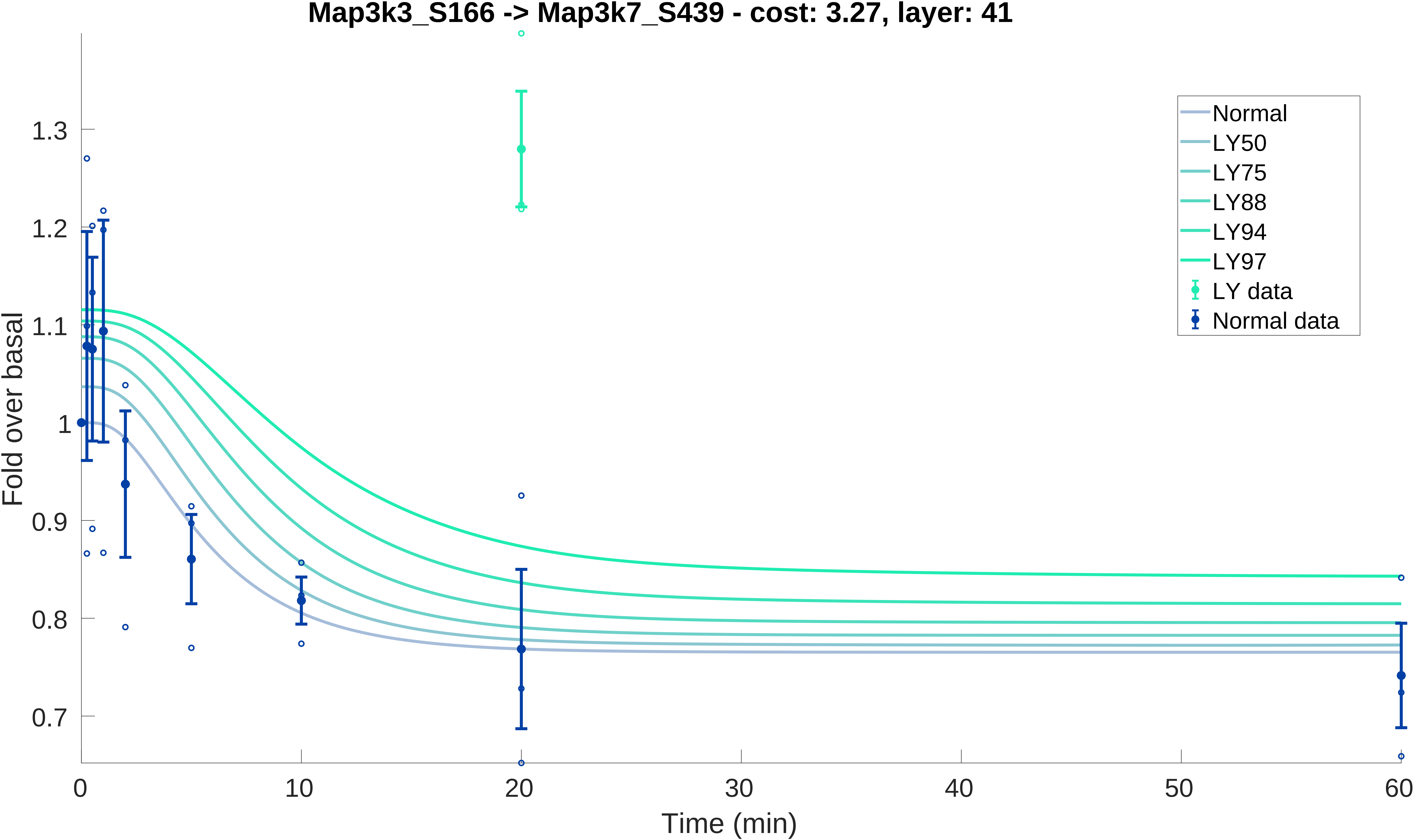
Map3k3_S166 -> Map3k7_S439 - cost: 3.27, layer: 41
Fold over basal
Time (min)
0.7
0.8
0.9
1
1.1
1.2
1.3
0
10
20
30
40
50
60
Normal
LY50
LY75
LY88
LY94
LY97
LY data
Normal data

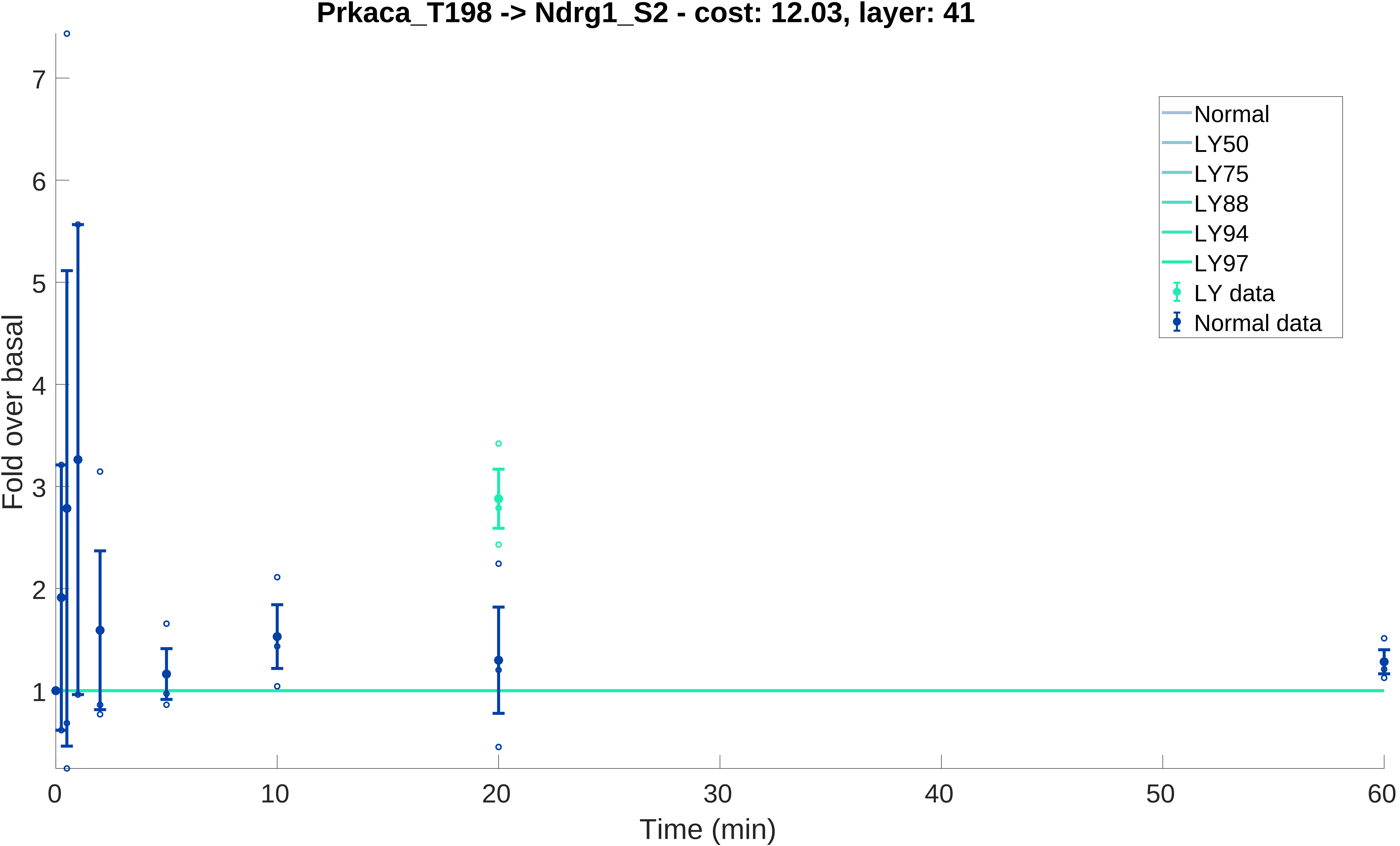

Prkaca_T198 -> Ndrg1_S2 - cost: 12.03, layer: 41
Fold over basal
Time (min)
0
10
20
30
40
50
60
1
2
3
4
5
6
7
Normal
LY50
LY75
LY88
LY94
LY97
LY data
Normal data

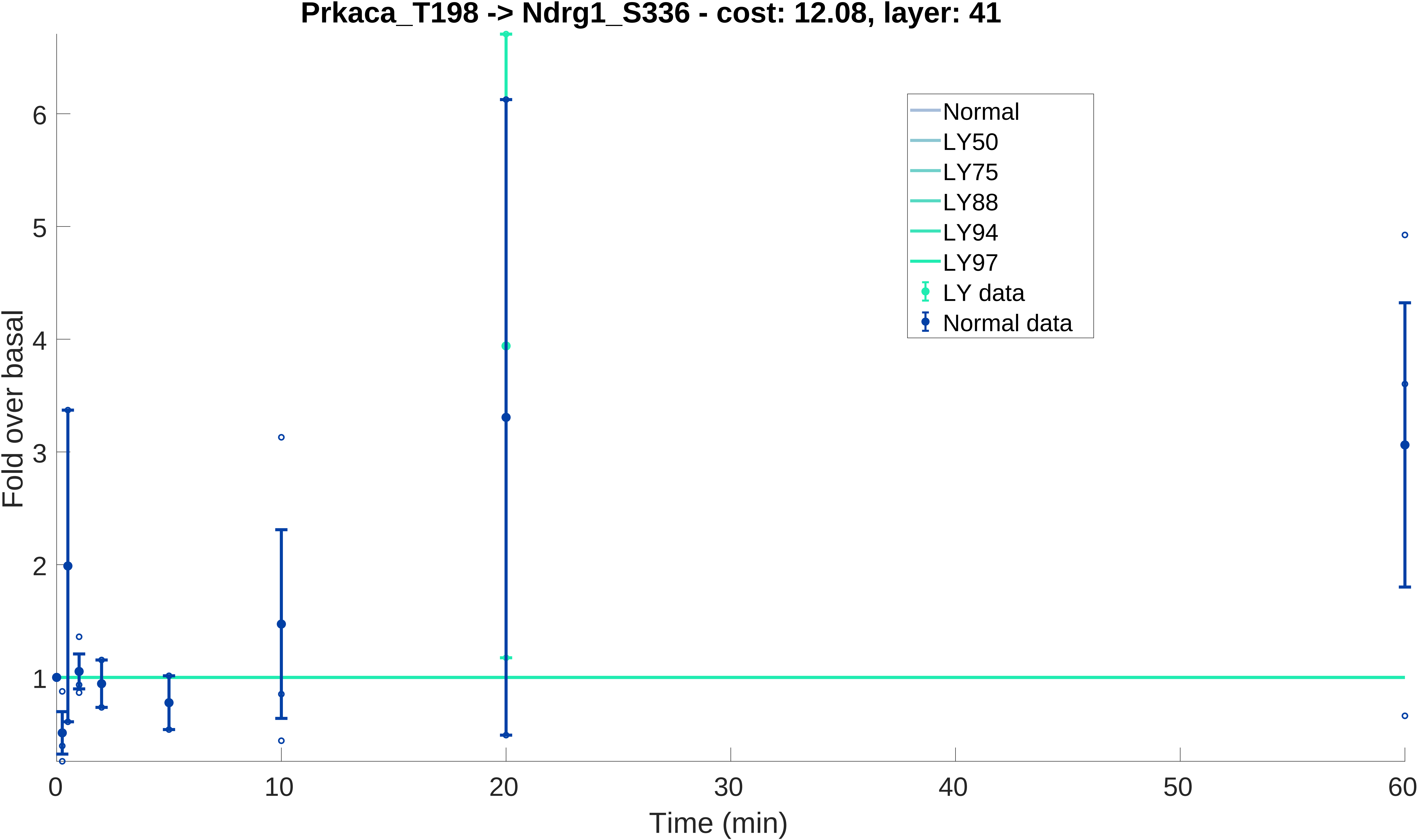

Prkaca_T198 -> Ndrg1_S336 - cost: 12.08, layer: 41
Fold over basal
Time (min)
0
10
20
30
40
50
60
1
2
3
4
5
6
Normal
LY50
LY75
LY88
LY94
LY97
LY data
Normal data

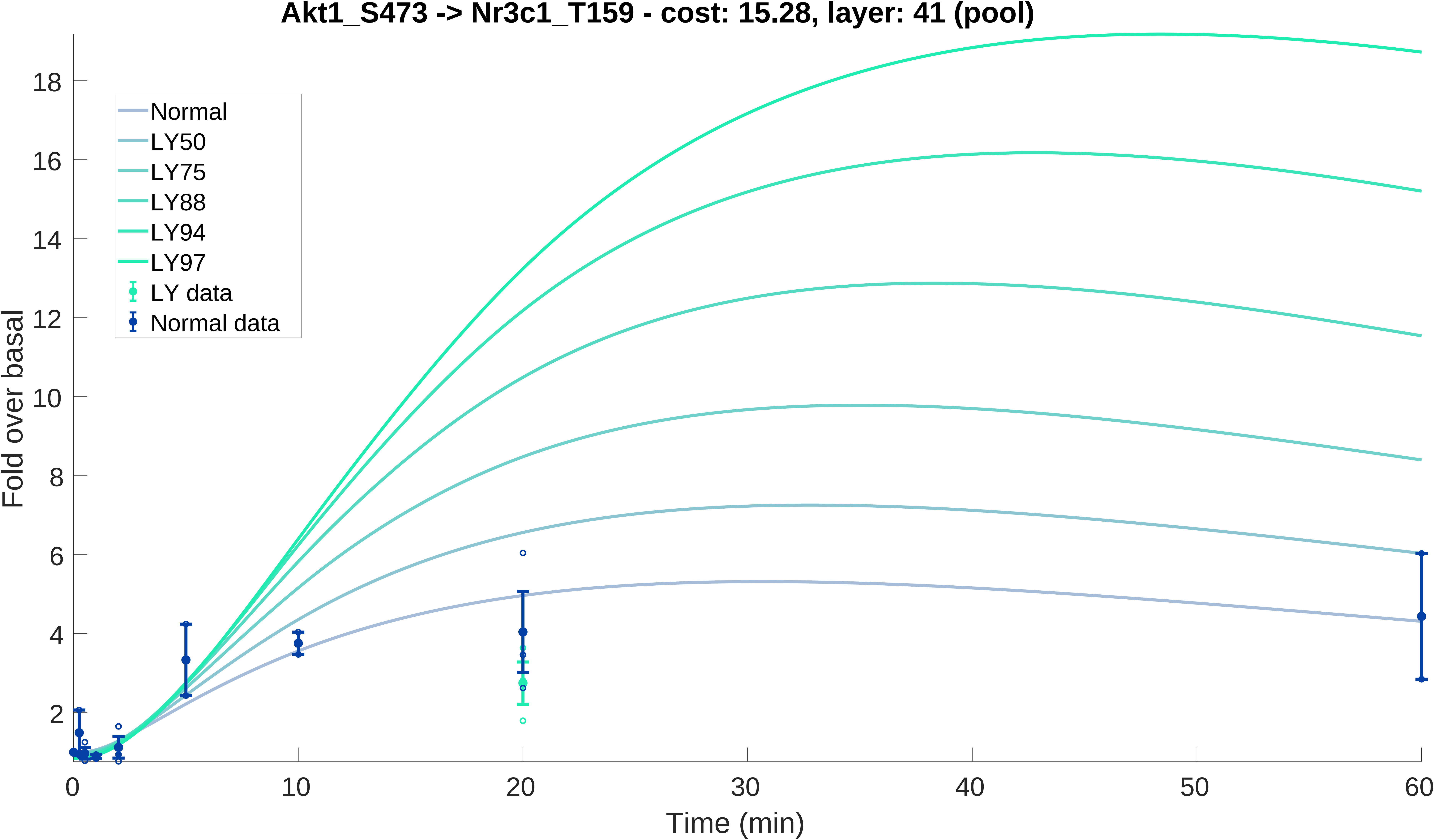

Akt1_S473 -> Nr3c1_T159 - cost: 15.28, layer: 41 (pool)
Normal
LY50
LY75
LY88
LY94
LY97
LY data
Normal data
Fold over basal
Time (min)
0
10
20
30
40
50
60
2
4
6
8
10
12
14
16
18

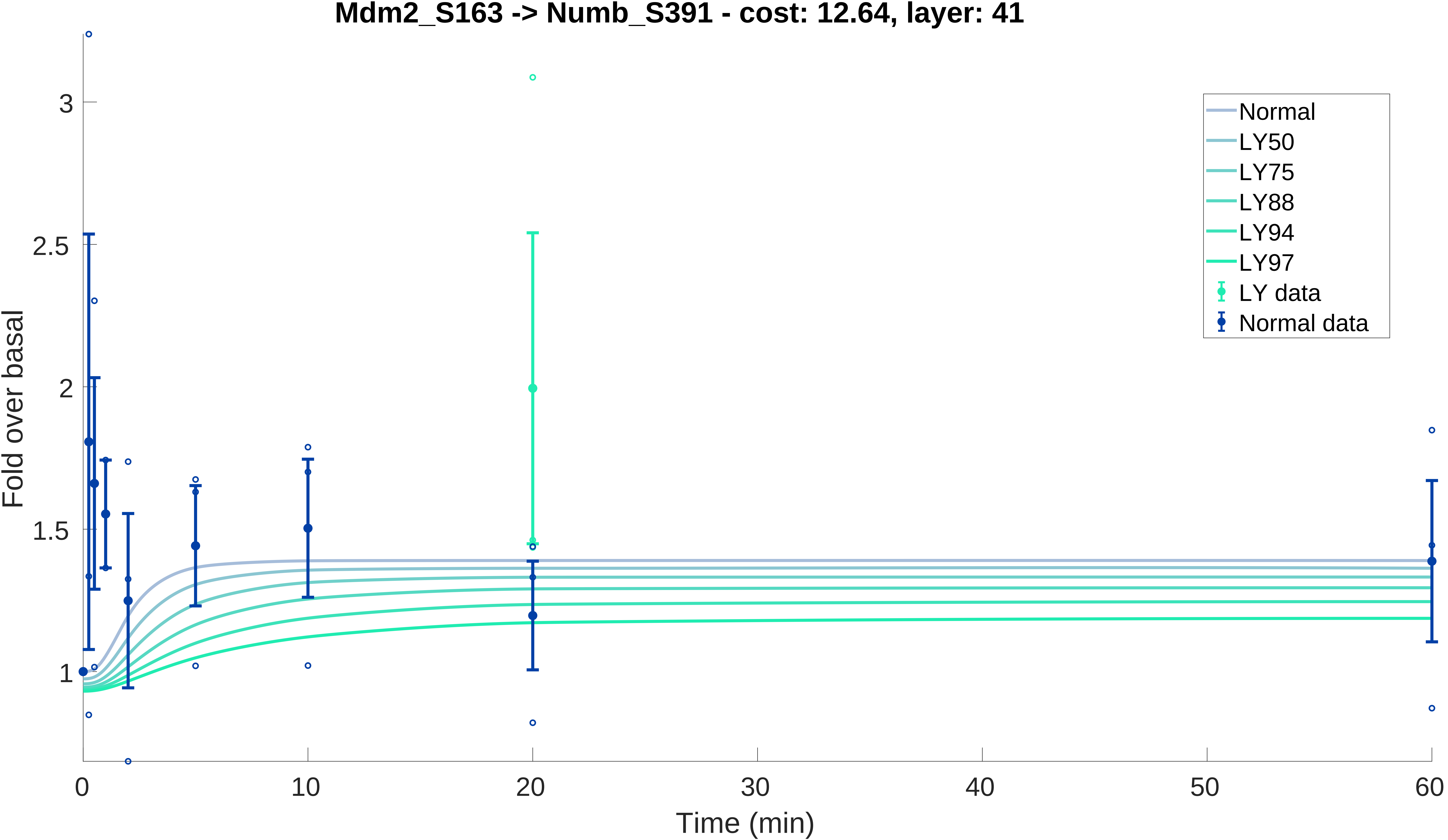
Mdm2_S163 -> Numb_S391 - cost: 12.64, layer: 41
Fold over basal
Time (min)
0
10
20
30
40
50
60
1
1.5
2
2.5
3
Normal
LY50
LY75
LY88
LY94
LY97
LY data
Normal data

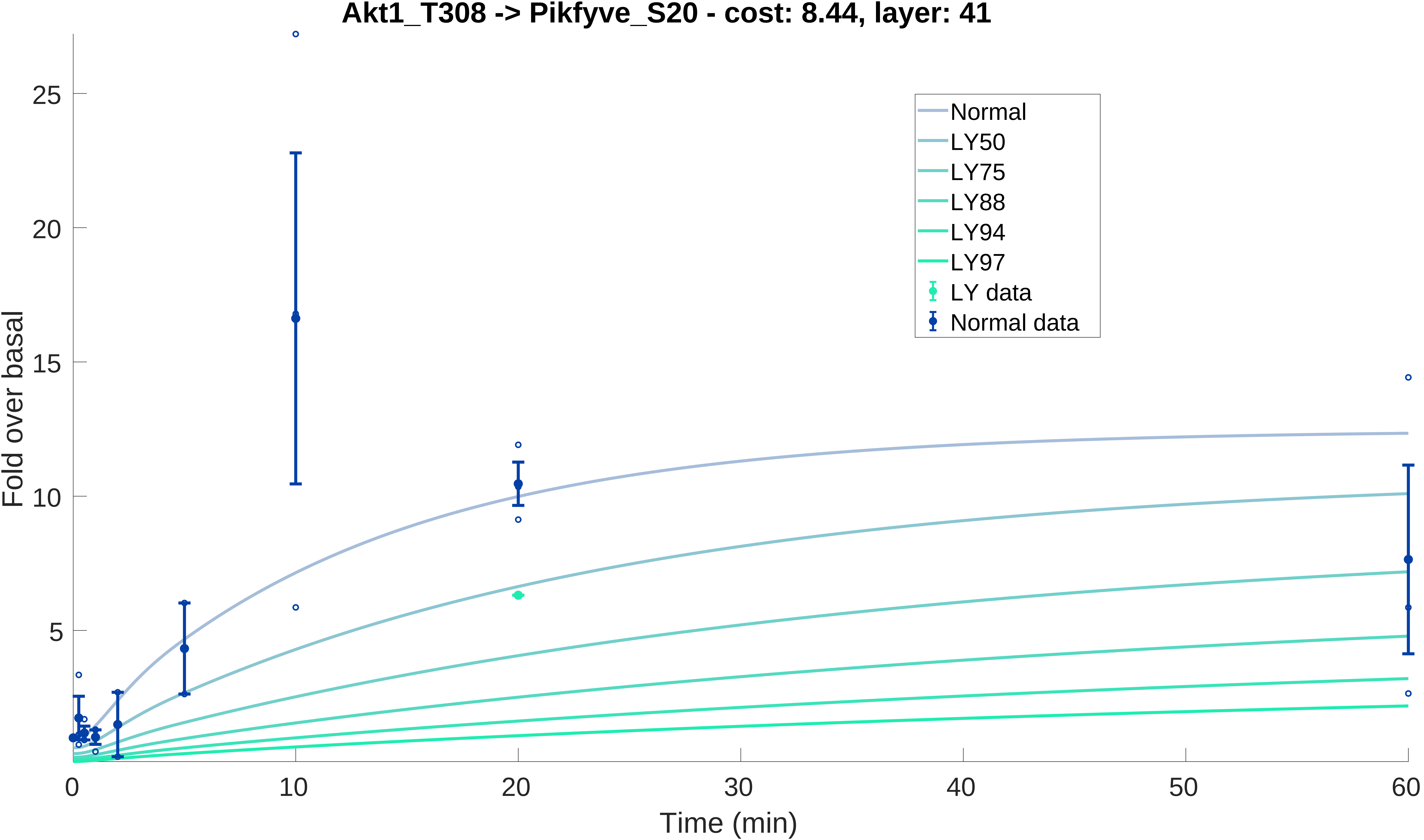
Akt1_T308 -> Pikfyve_S20 - cost: 8.44, layer: 41
Fold over basal
Time (min)
0
10
20
30
40
50
60
5
10
15
20
25
Normal
LY50
LY75
LY88
LY94
LY97
LY data
Normal data

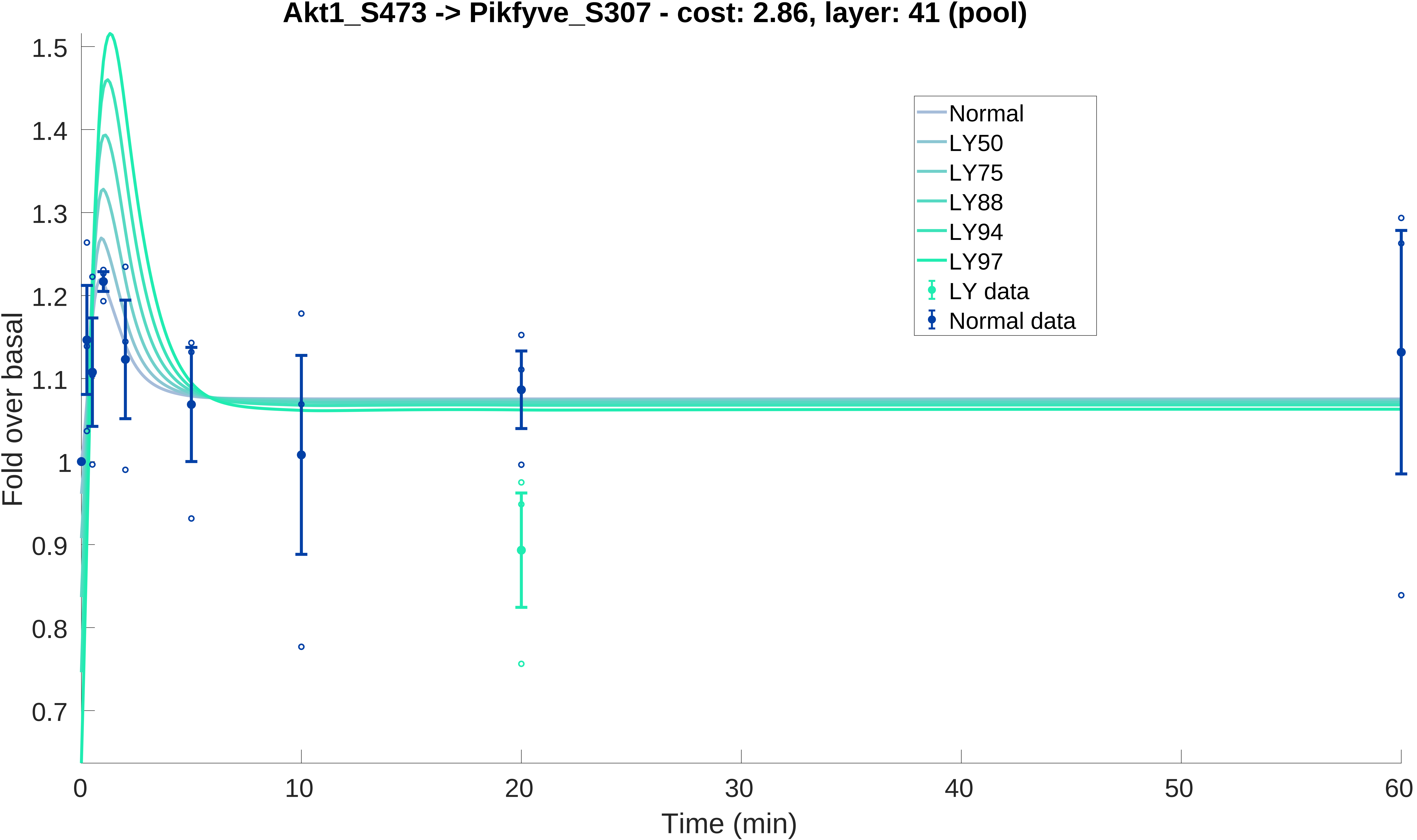

Akt1_S473 -> Pikfyve_S307 - cost: 2.86, layer: 41 (pool)
Fold over basal
Time (min)
Normal
LY50
LY75
LY88
LY94
LY97
LY data
Normal data
1.5
1.4
1.3
1.2
1.1
1
0.9
0.8
0.7
0
10
20
30
40
50
60

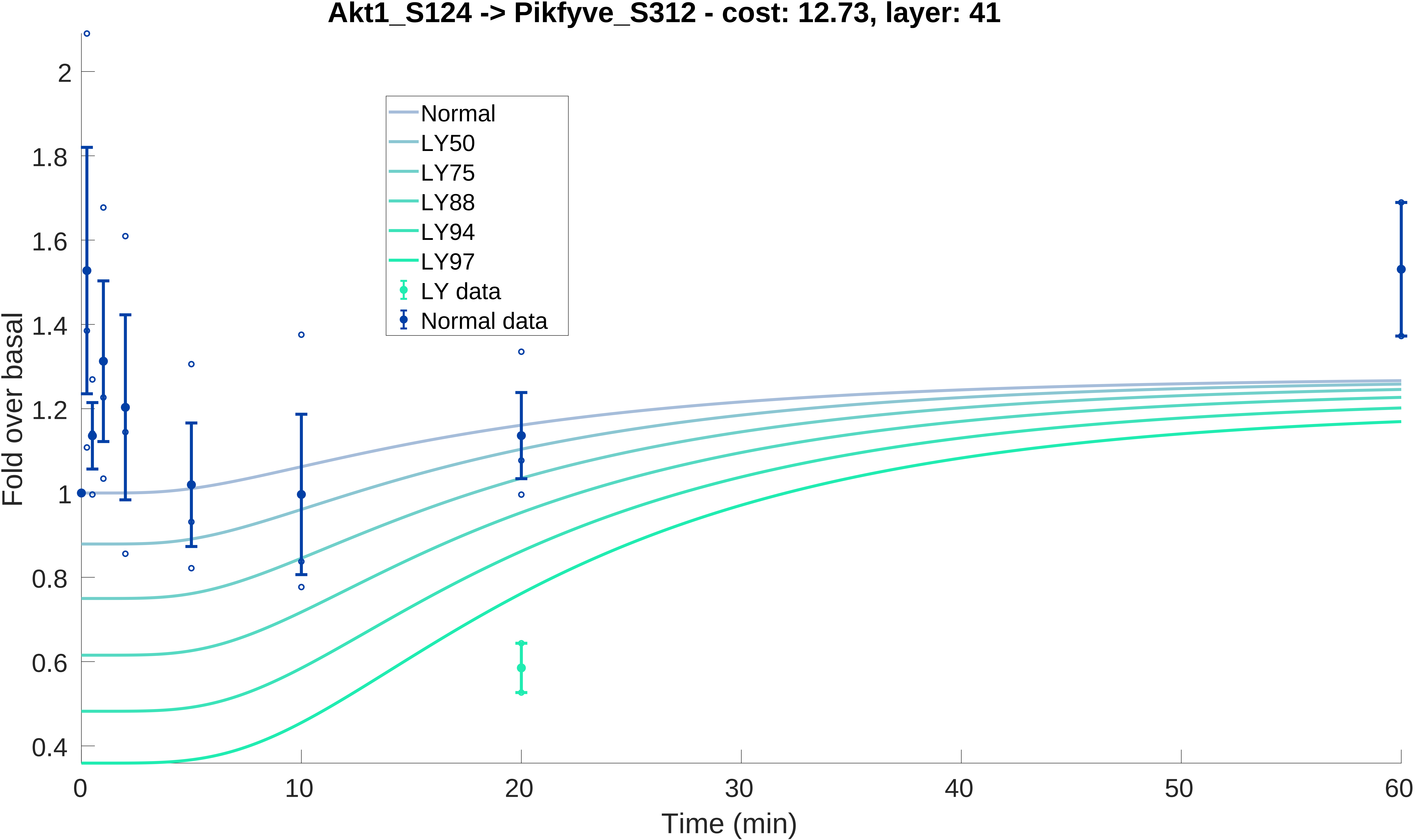

Akt1_S124 -> Pikfyve_S312 - cost: 12.73, layer: 41
Fold over basal
Time (min)
Normal
LY50
LY75
LY88
LY94
LY97
LY data
Normal data
0.4
0.6
0.8
1
1.2
1.4
1.6
1.8
2
0
10
20
30
40
50
60

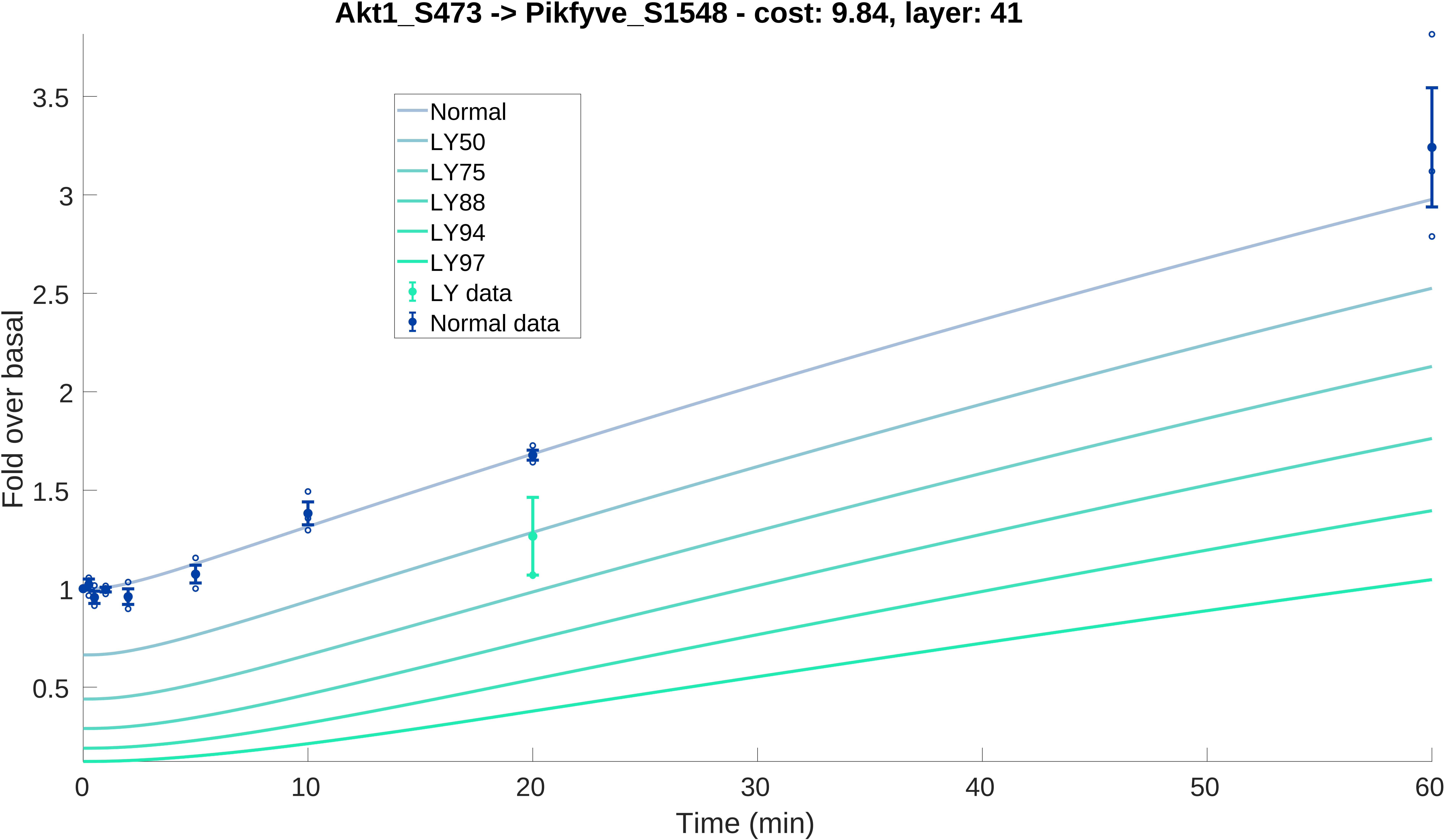
Akt1_S473 -> Pikfyve_S1548 - cost: 9.84, layer: 41
Normal
LY50
LY75
LY88
LY94
LY97
LY data
Normal data
Fold over basal
Time (min)
0.5
1
1.5
2
2.5
3
3.5
0
10
20
30
40
50
60

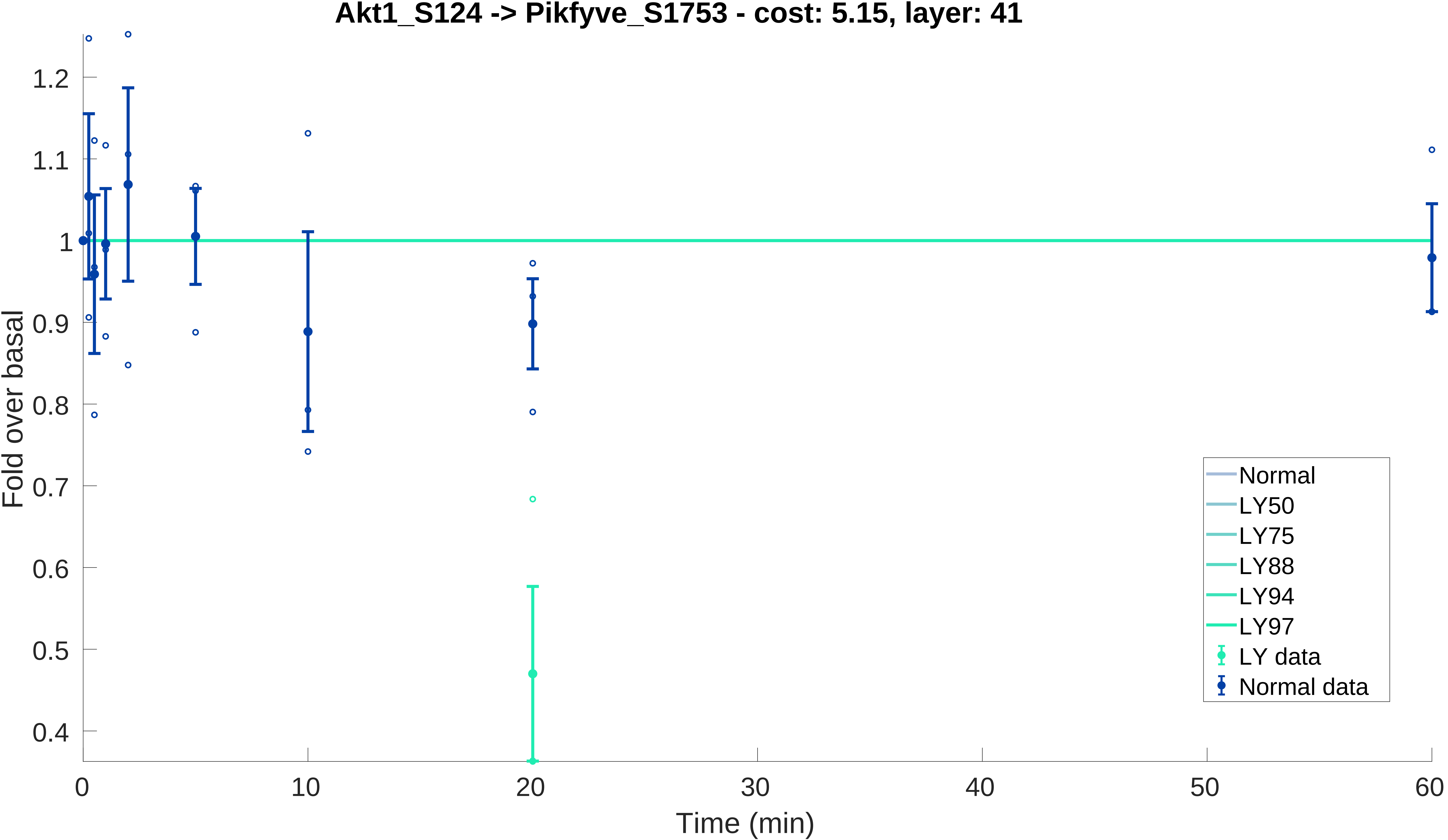

Akt1_S124 -> Pikfyve_S1753 - cost: 5.15, layer: 41
Fold over basal
1.2
1.1
1
0.9
0.8
0.7
0.6
0.5
0.4
0
10
20
30
40
50
60
Time (min)
Normal
LY50
LY75
LY88
LY94
LY97
LY data
Normal data

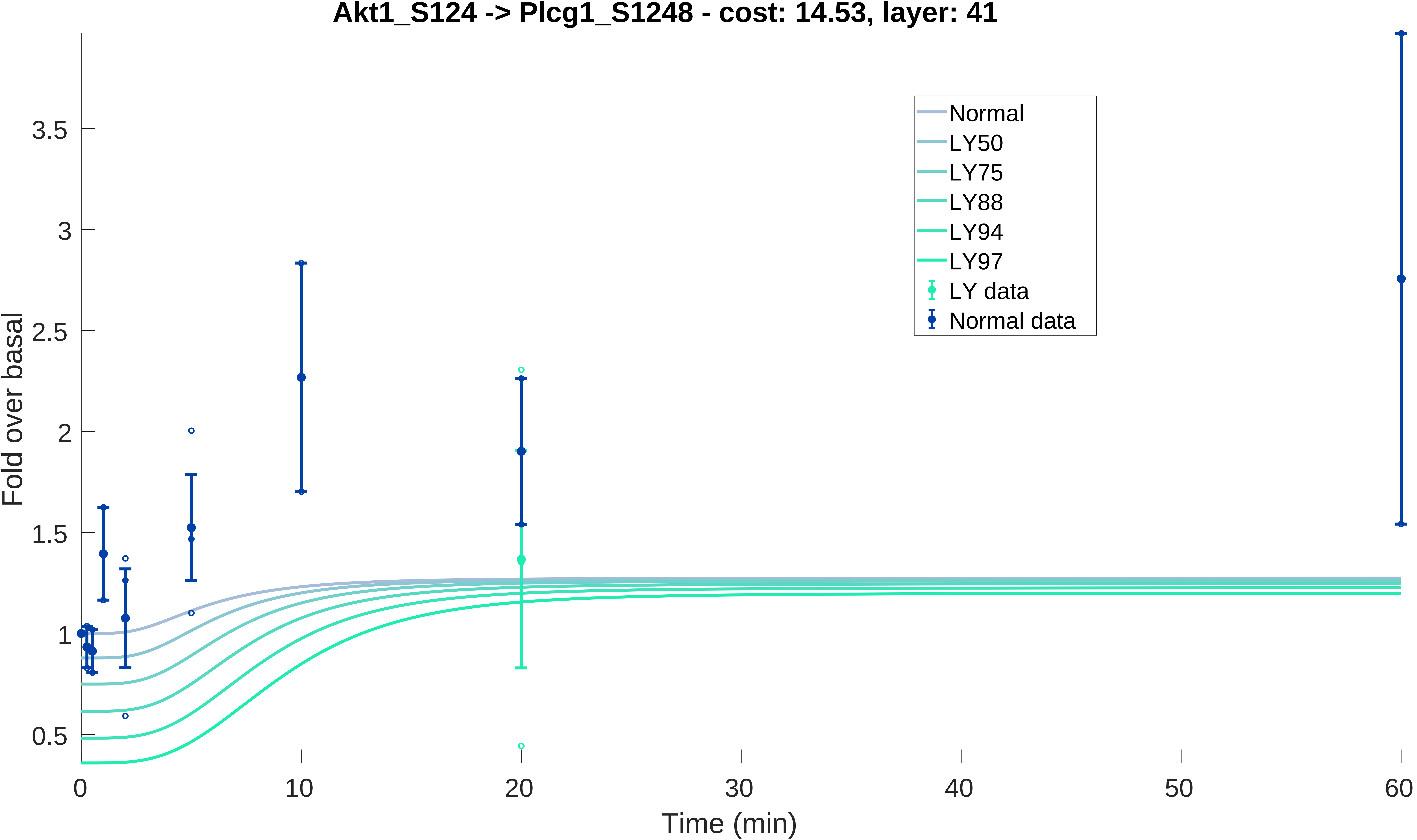

Akt1_S124 -> Plcg1_S1248 - cost: 14.53, layer: 41
Fold over basal
Time (min)
0
10
20
30
40
50
60
0.5
1
1.5
2
2.5
3
3.5
Normal
LY50
LY75
LY88
LY94
LY97
LY data
Normal data

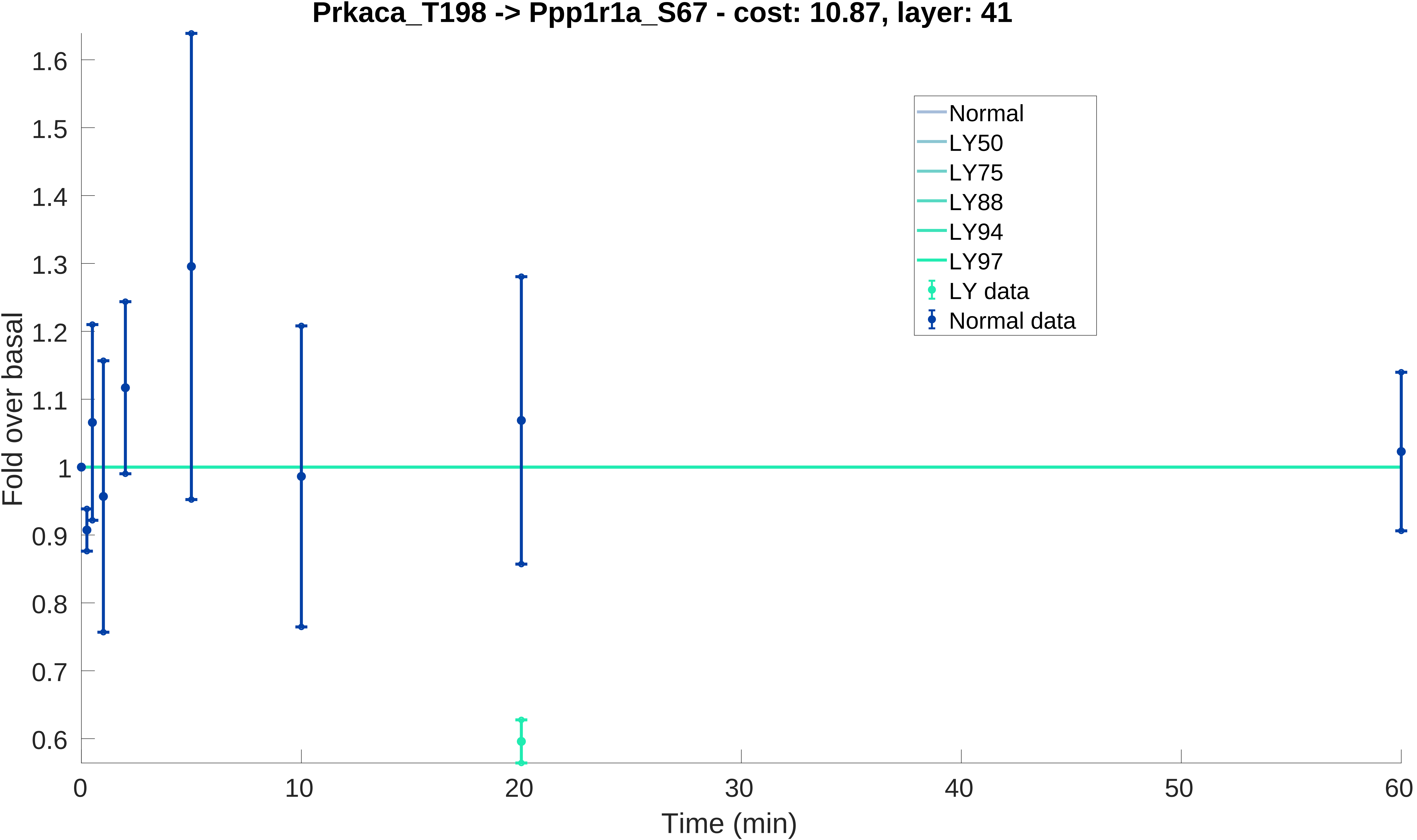
Prkaca_T198 -> Ppp1r1a_S67 - cost: 10.87, layer: 41
Fold over basal
1.6
1.5
1.4
1.3
1.2
1.1
1
0.9
0.8
0.7
0.6
0
10
20
30
40
50
60
Time (min)
Normal
LY50
LY75
LY88
LY94
LY97
LY data
Normal data

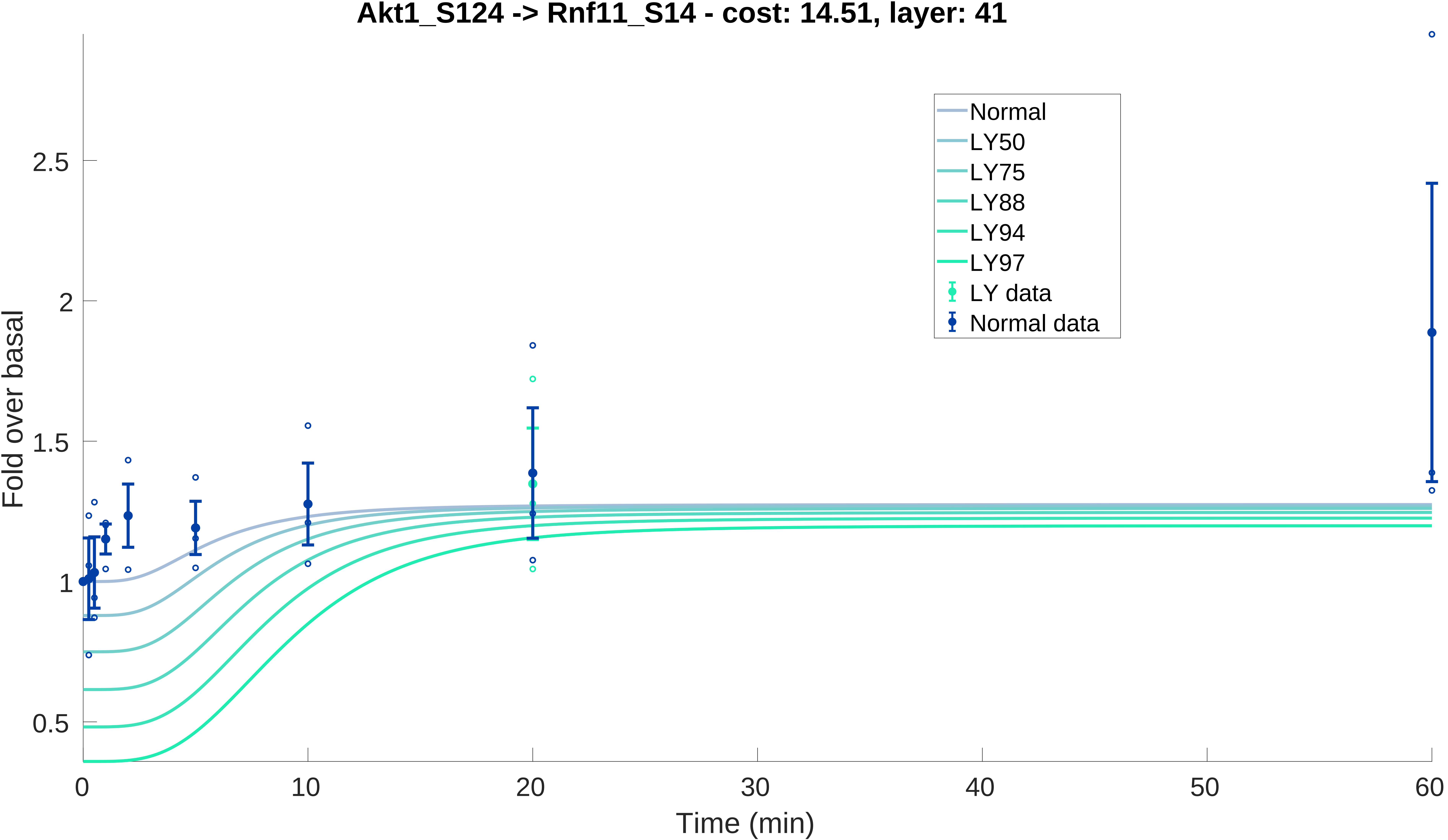

Akt1_S124 -> Rnf11_S14 - cost: 14.51, layer: 41
Fold over basal
Time (min)
0.5
1
1.5
2
2.5
0
10
20
30
40
50
60
Normal
LY50
LY75
LY88
LY94
LY97
LY data
Normal data

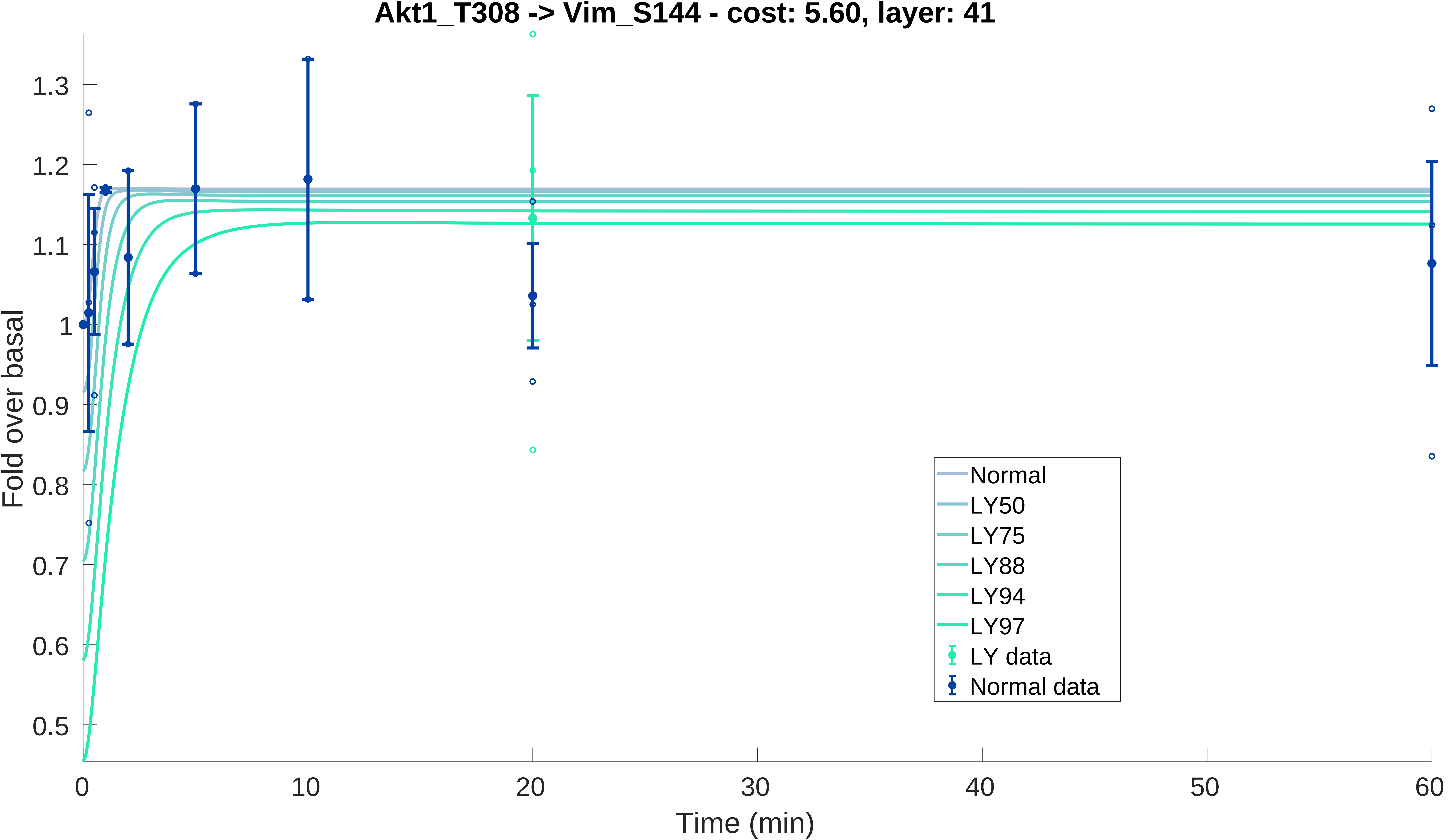

Akt1_T308 -> Vim_S144 - cost: 5.60, layer: 41
Fold over basal
1.3
1.2
1.1
1
0.9
0.8
0.7
0.6
0.5
0
10
20
30
40
50
60
Time (min)
Normal
LY50
LY75
LY88
LY94
LY97
LY data
Normal data

Akt1_T308 -> Vim_S214 - cost: 7.01, layer: 41

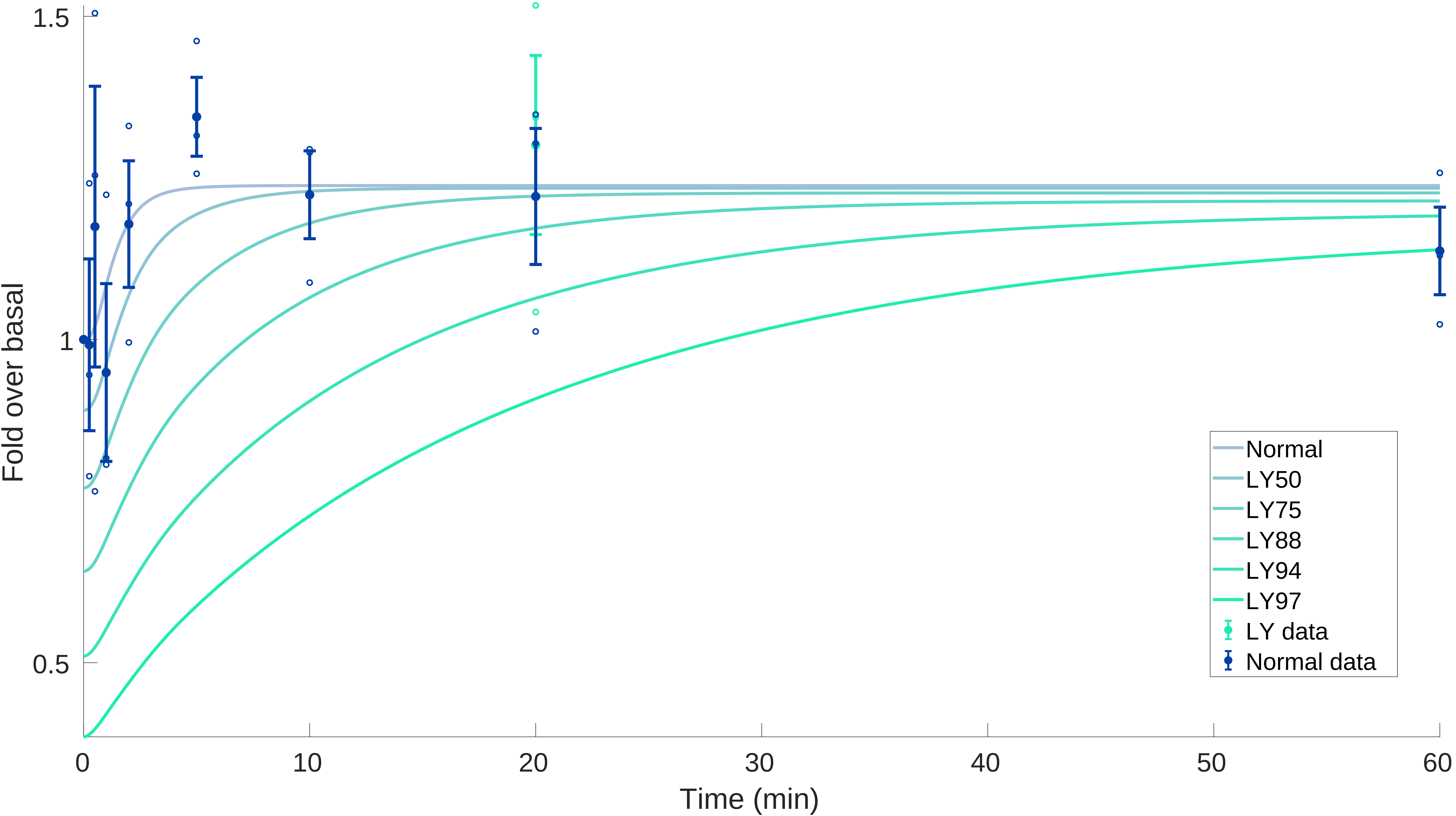

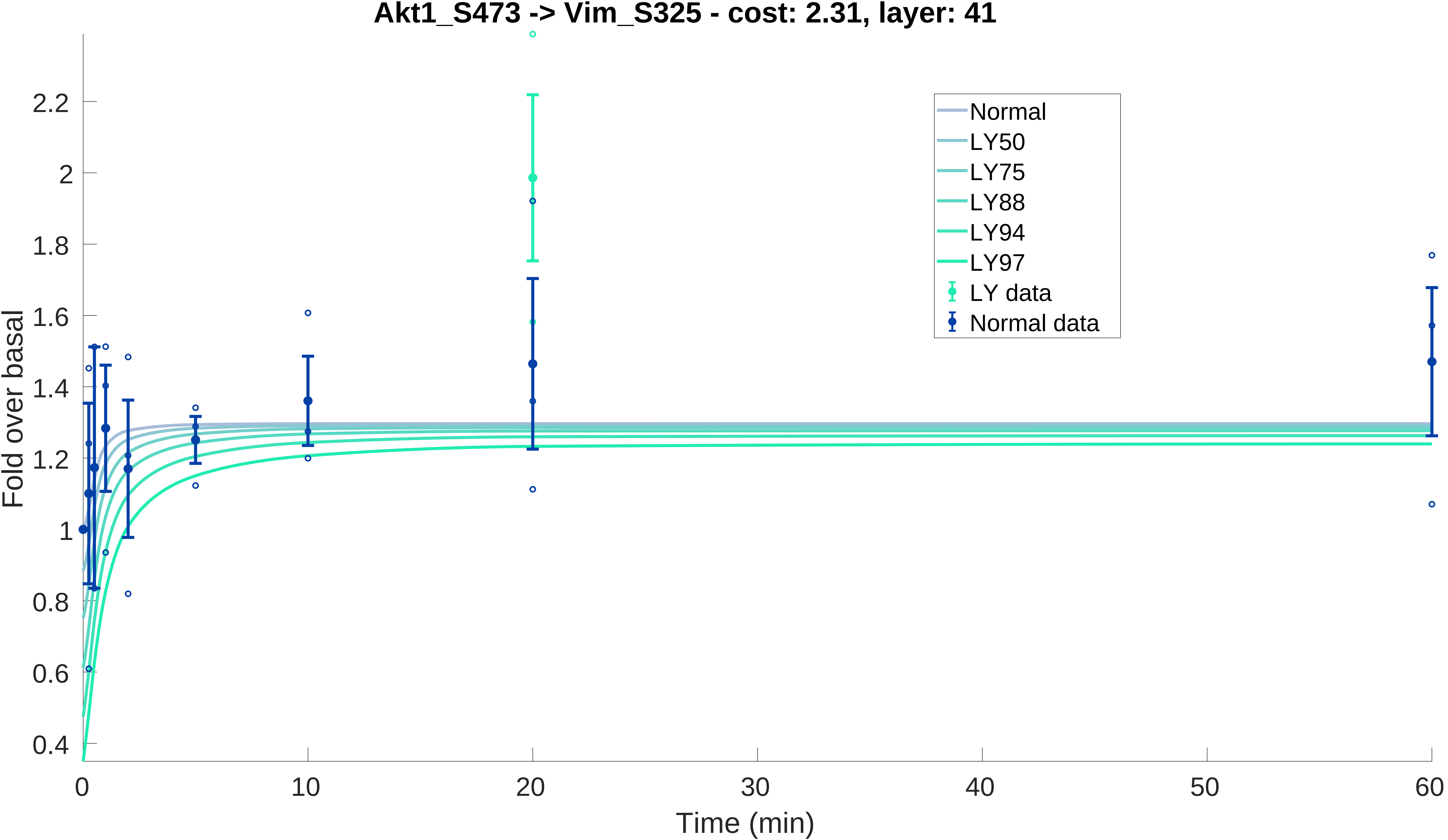

Akt1_S473 -> Vim_S325 - cost: 2.31, layer: 41
Fold over basal
Time (min)
0.4
0.6
0.8
1
1.2
1.4
1.6
1.8
2
2.2
0
10
20
30
40
50
60
Normal
LY50
LY75
LY88
LY94
LY97
LY data
Normal data

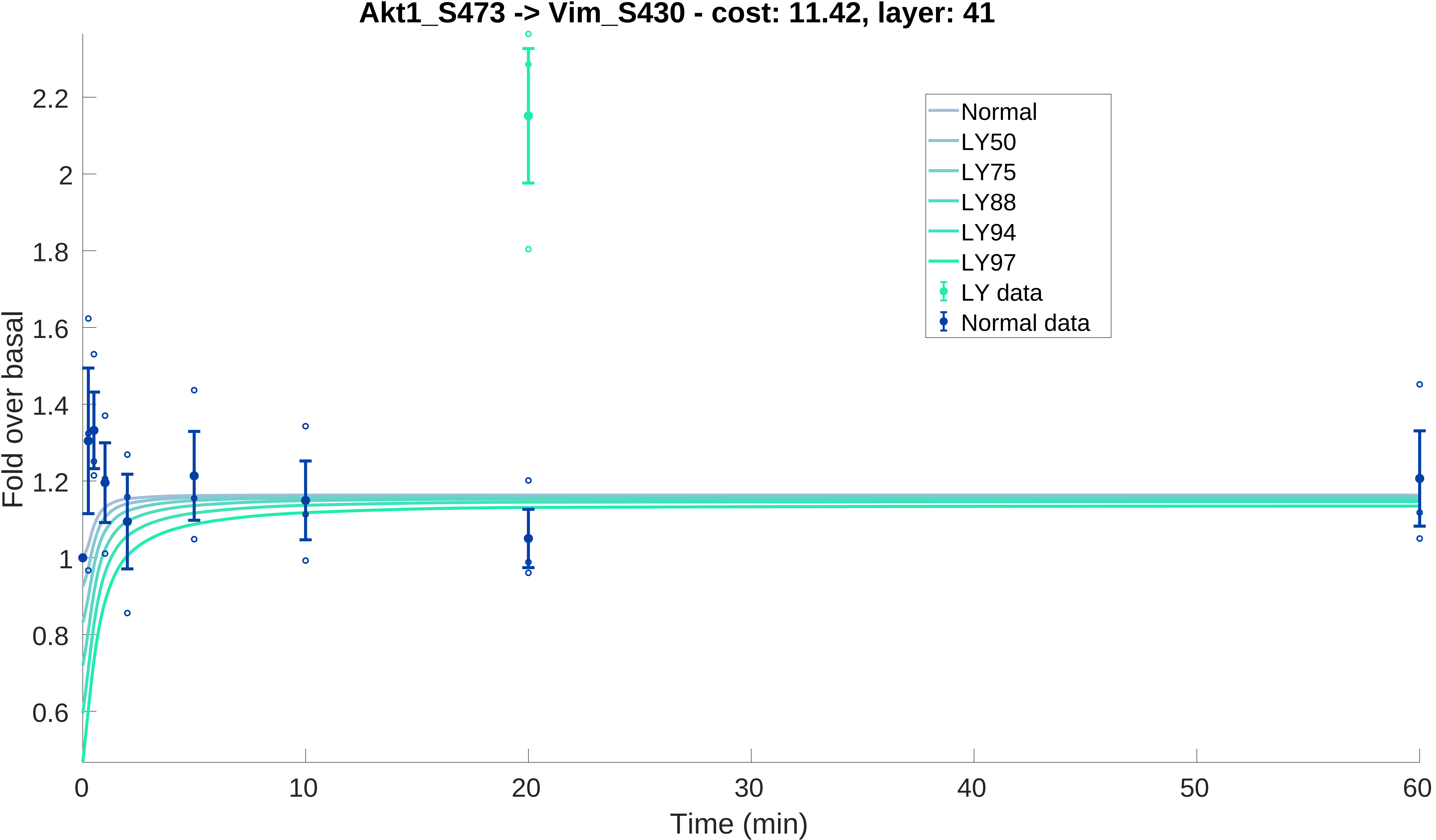
Akt1_S473 -> Vim_S430 - cost: 11.42, layer: 41
Fold over basal
Time (min)
Normal
LY50
LY75
LY88
LY94
LY97
LY data
Normal data
0.6
0.8
1
1.2
1.4
1.6
1.8
2
2.2
0
10
20
30
40
50
60

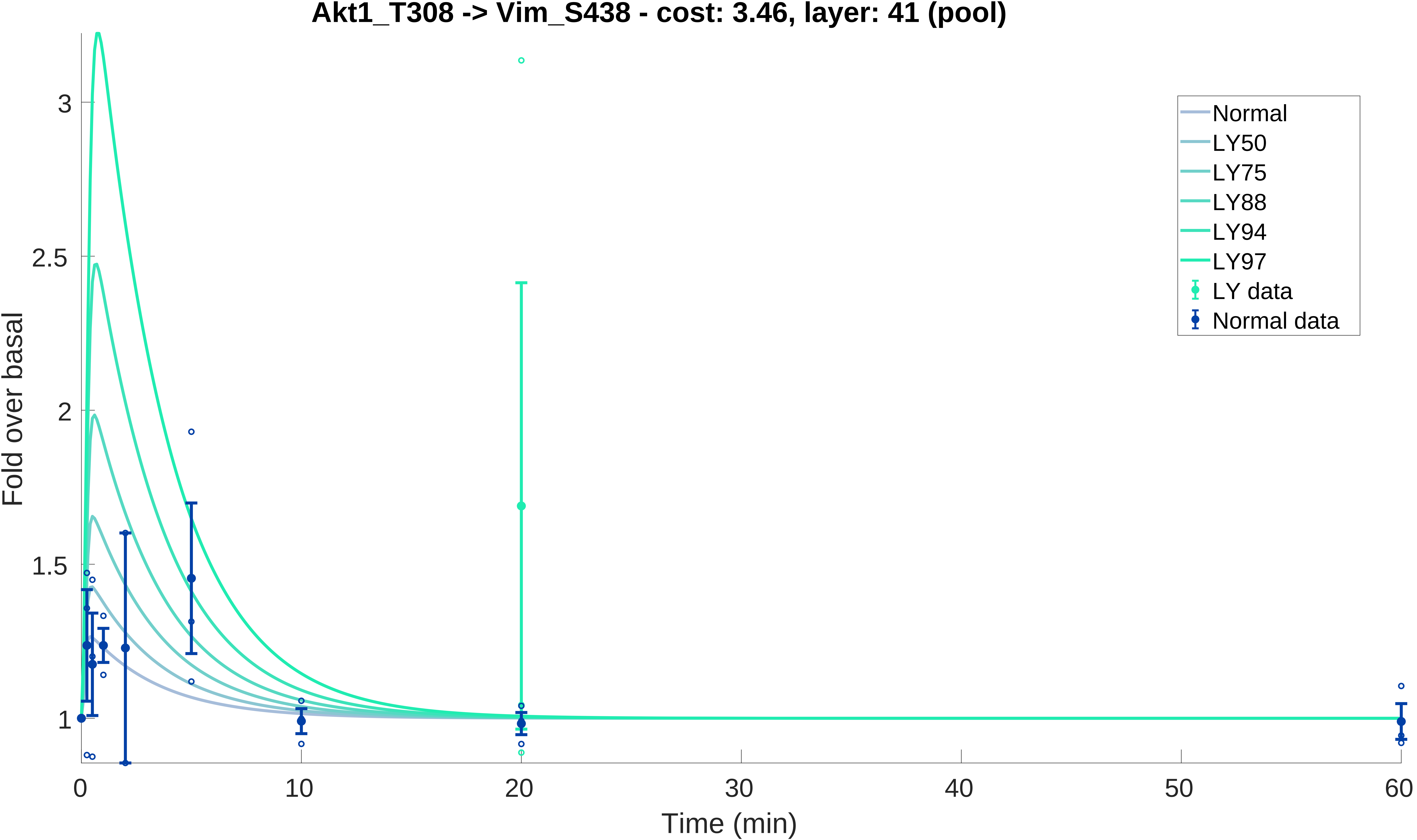
Akt1_T308 -> Vim_S438 - cost: 3.46, layer: 41 (pool)
Fold over basal
Time (min)
1
1.5
2
2.5
3
0
10
20
30
40
50
60
Normal
LY50
LY75
LY88
LY94
LY97
LY data
Normal data

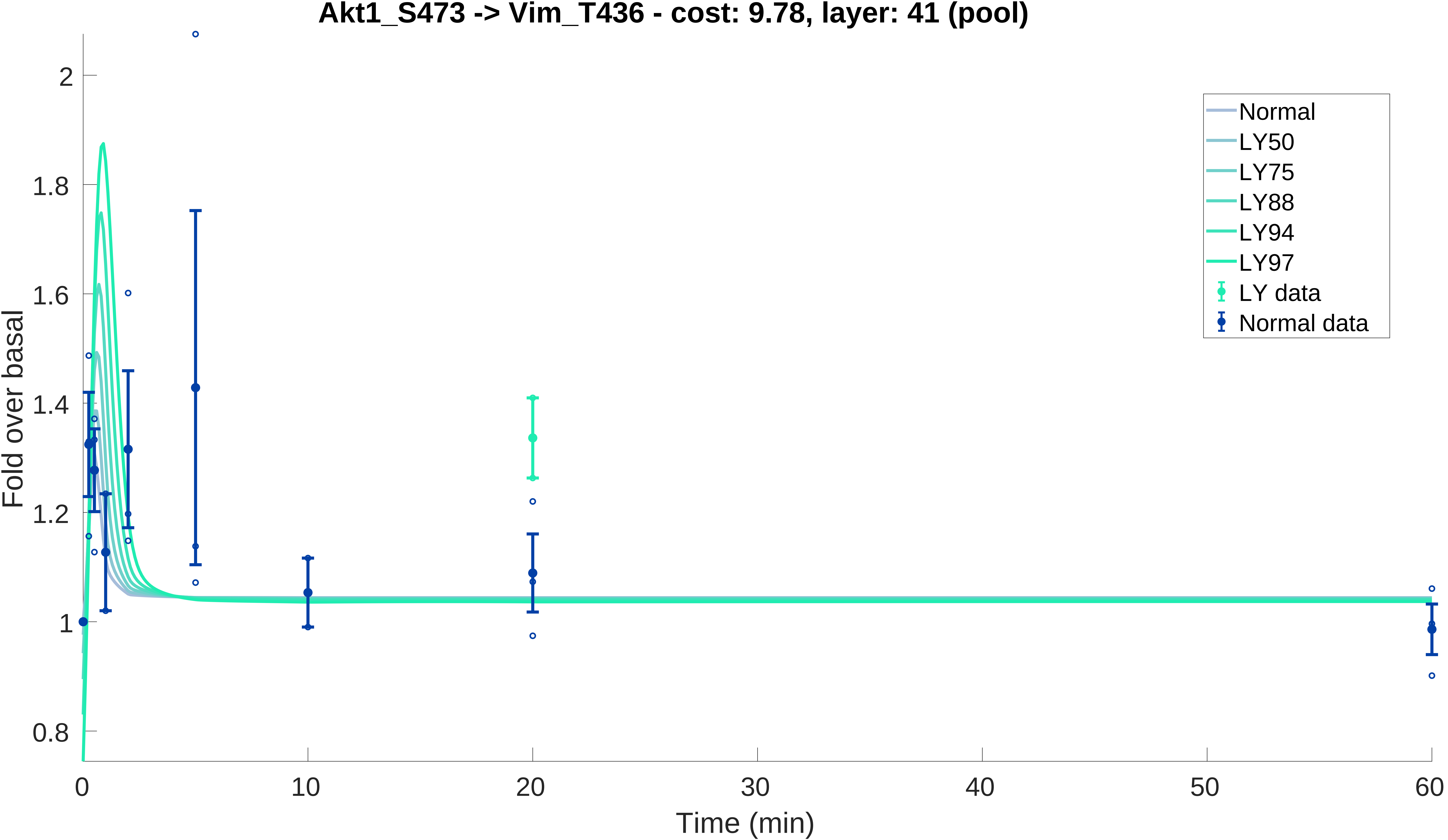

Akt1_S473 -> Vim_T436 - cost: 9.78, layer: 41 (pool)
Fold over basal
Time (min)
0.8
1
1.2
1.4
1.6
1.8
2
0
10
20
30
40
50
60
Normal
LY50
LY75
LY88
LY94
LY97
LY data
Normal data

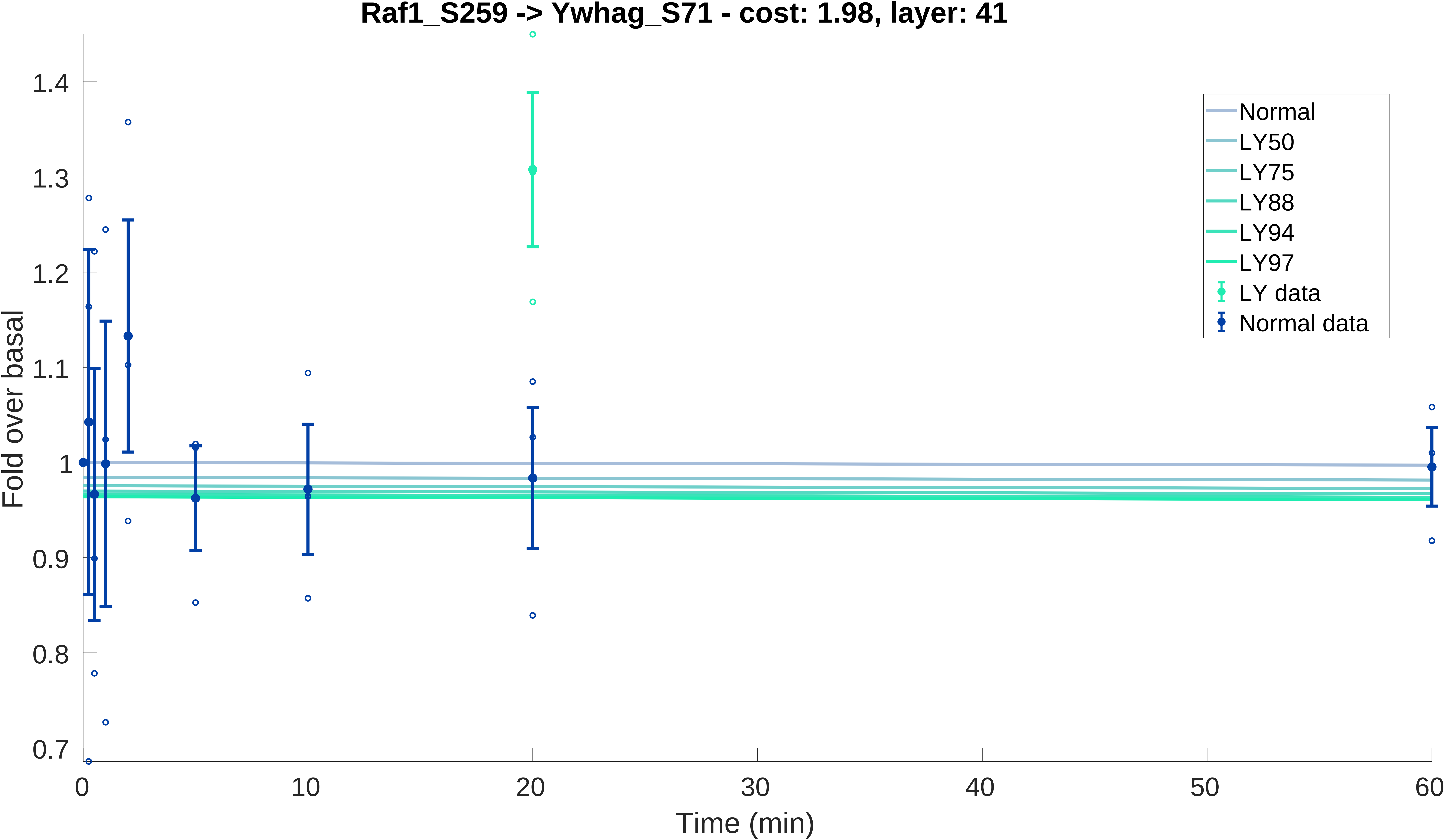
Raf1_S259 -> Ywhag_S71 - cost: 1.98, layer: 41
Fold over basal
1.4
1.3
1.2
1.1
1
0.9
0.8
0.7
0
10
20
30
40
50
60
Time (min)
Normal
LY50
LY75
LY88
LY94
LY97
LY data
Normal data

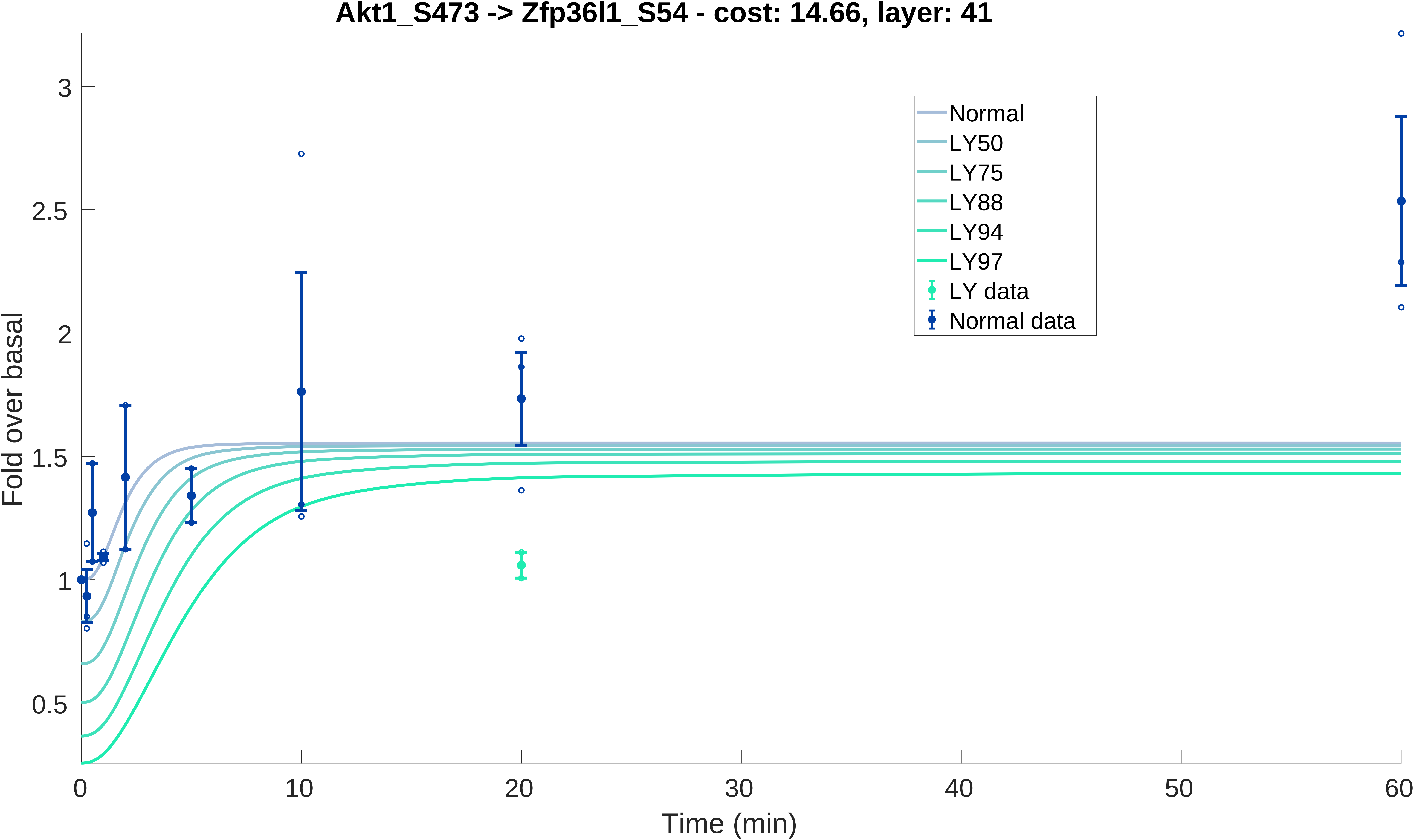
Akt1_S473 -> Zfp36l1_S54 - cost: 14.66, layer: 41
Fold over basal
Time (min)
0
10
20
30
40
50
60
0.5
1
1.5
2
2.5
3
Normal
LY50
LY75
LY88
LY94
LY97
LY data
Normal data

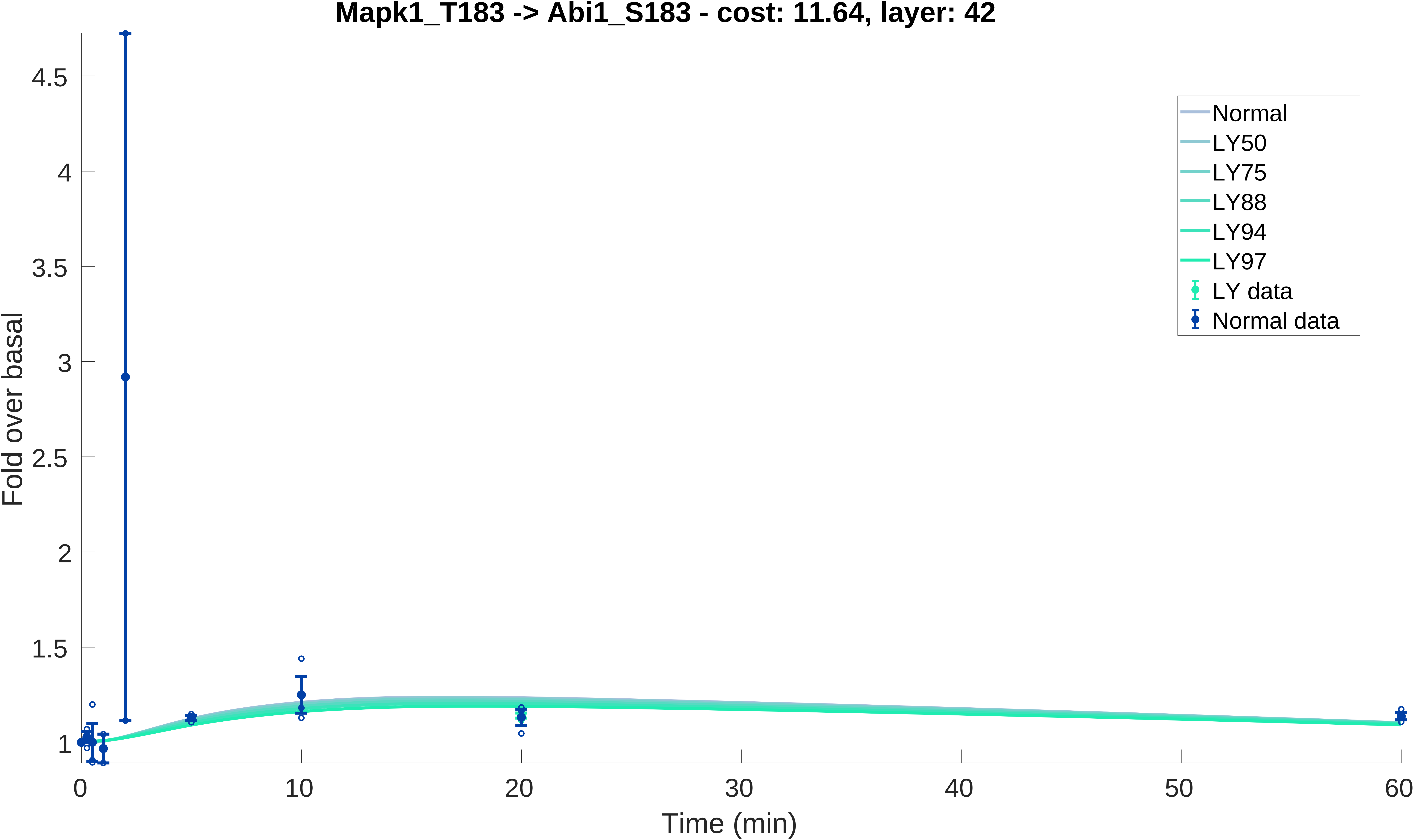

Mapk1_T183 -> Abi1_S183 - cost: 11.64, layer: 42
Fold over basal
Time (min)
Normal
LY50
LY75
LY88
LY94
LY97
LY data
Normal data
4.5
4
3.5
3
2.5
2
1.5
1
0
10
20
30
40
50
60

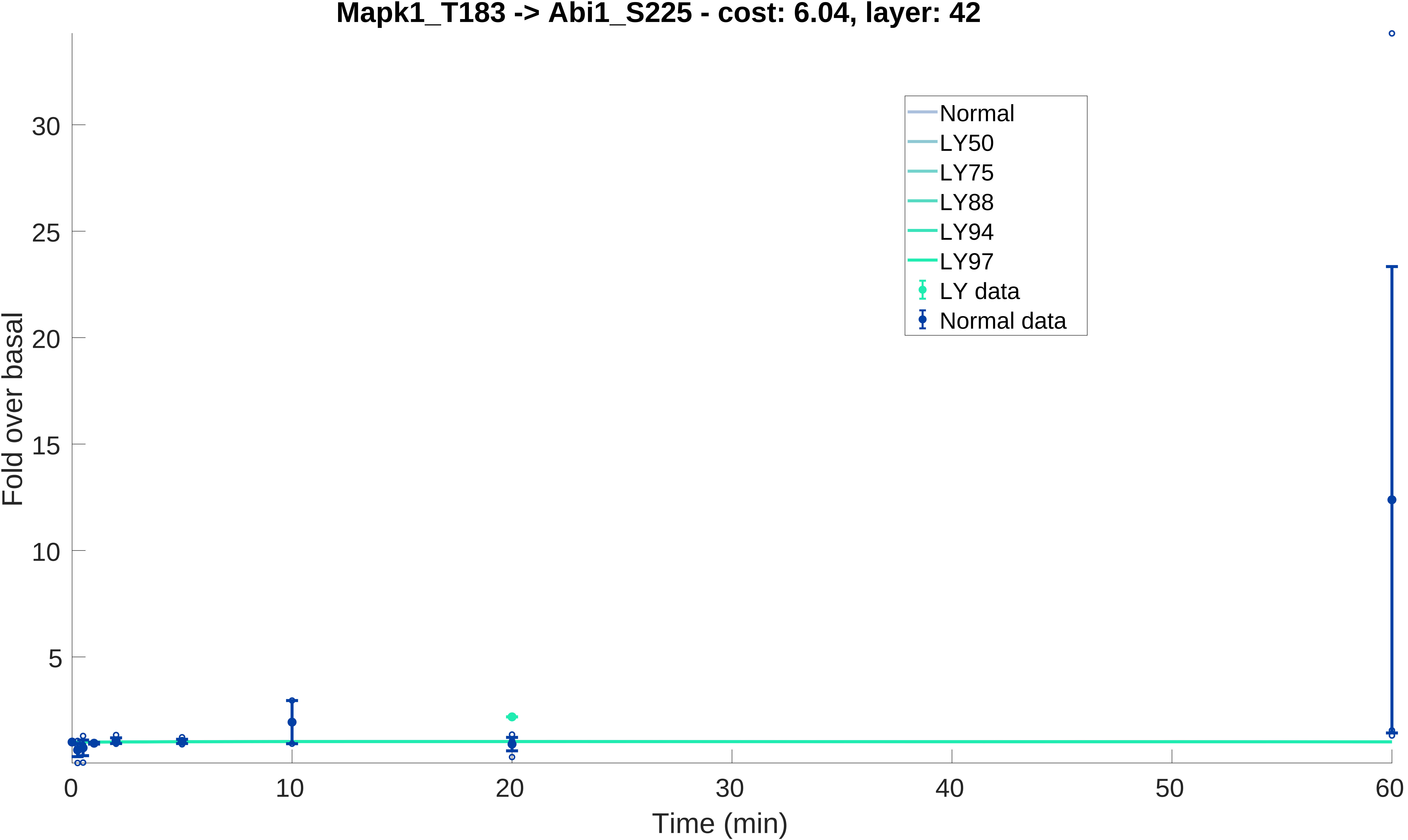

Mapk1_T183 -> Abi1_S225 - cost: 6.04, layer: 42
Normal
LY50
LY75
LY88
LY94
LY97
LY data
Normal data
Fold over basal
Time (min)
30
25
20
15
10
5
0
10
20
30
40
50
60

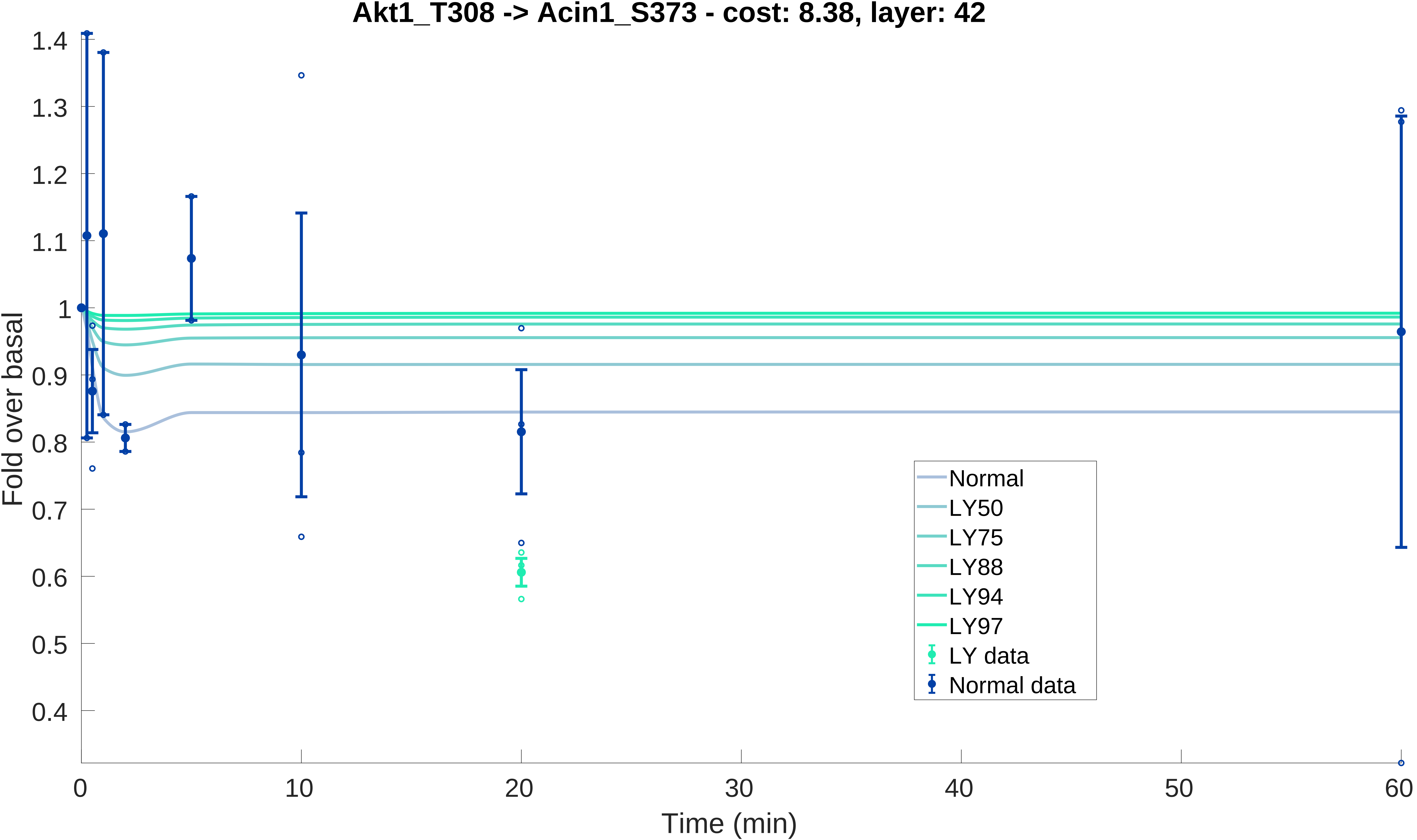

Akt1_T308 -> Acin1_S373 - cost: 8.38, layer: 42
Fold over basal
Time (min)
1.4
1.3
1.2
1.1
1
0.9
0.8
0.7
0.6
0.5
0.4
0
10
20
30
40
50
60
Normal
LY50
LY75
LY88
LY94
LY97
LY data
Normal data

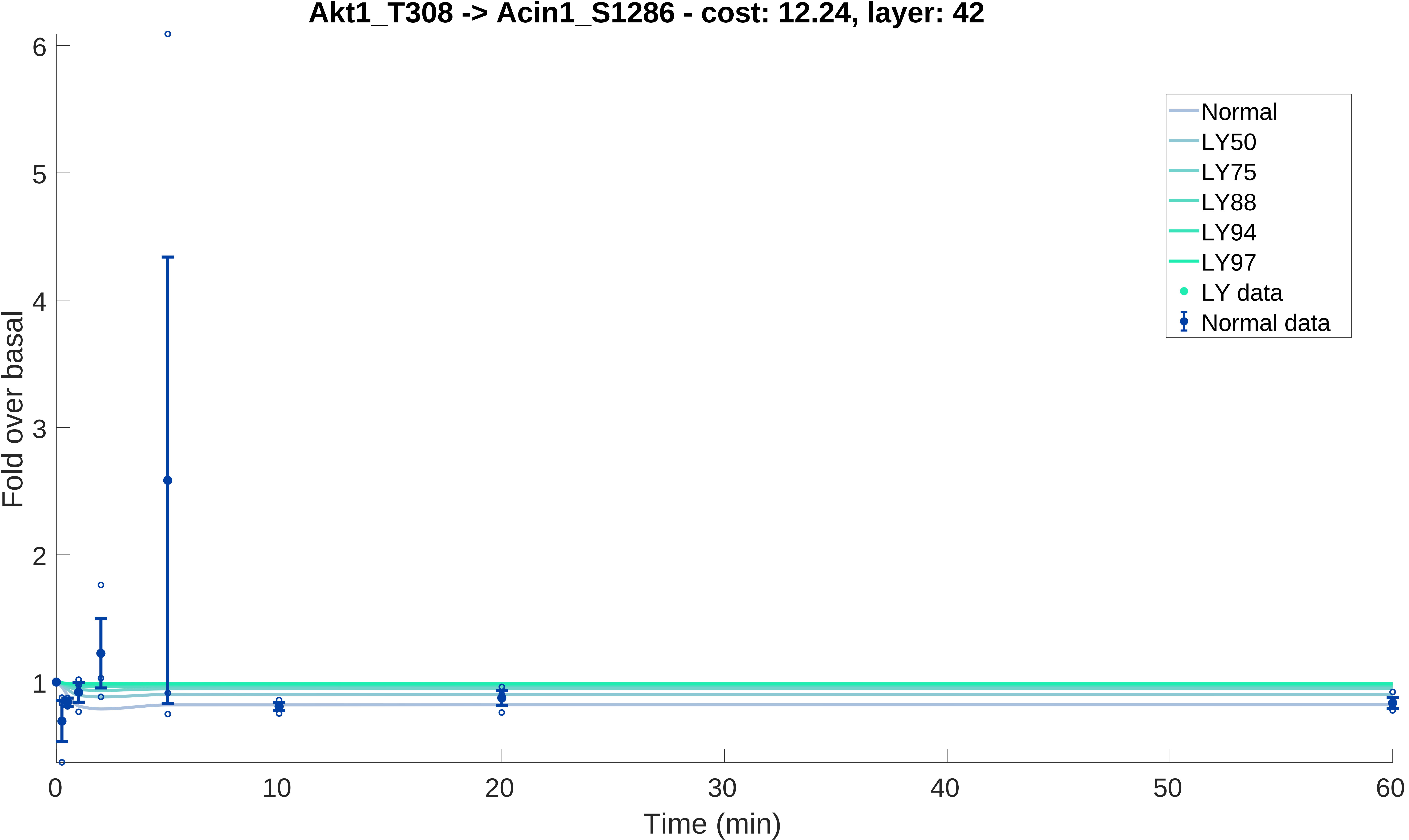

Akt1_T308 -> Acin1_S1286 - cost: 12.24, layer: 42
Fold over basal
Time (min)
0
10
20
30
40
50
60
1
2
3
4
5
6
Normal
LY50
LY75
LY88
LY94
LY97
LY data
Normal data

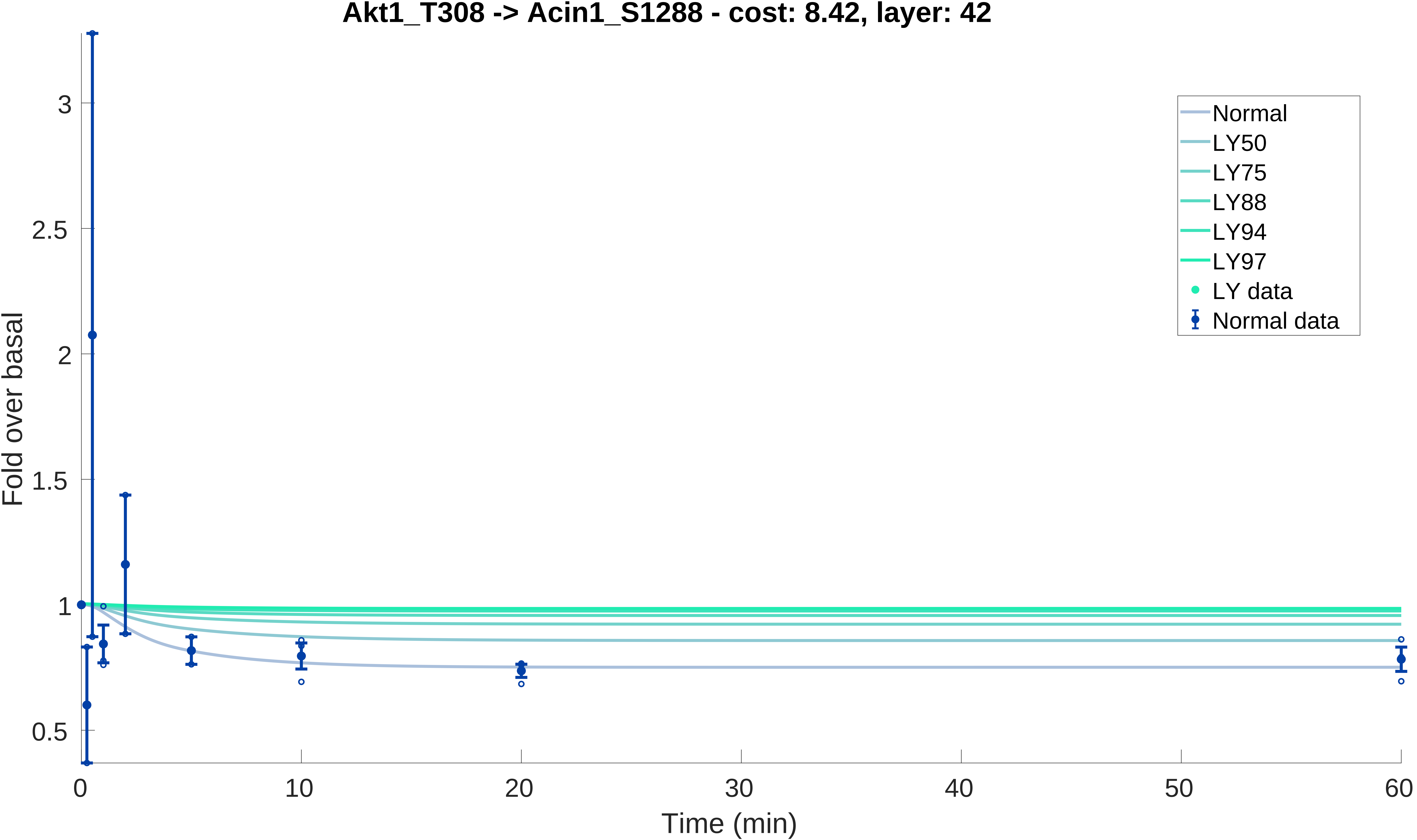
Akt1_T308 -> Acin1_S1288 - cost: 8.42, layer: 42
Fold over basal
Time (min)
0.5
1
1.5
2
2.5
3
0
10
20
30
40
50
60
Normal
LY50
LY75
LY88
LY94
LY97
LY data
Normal data

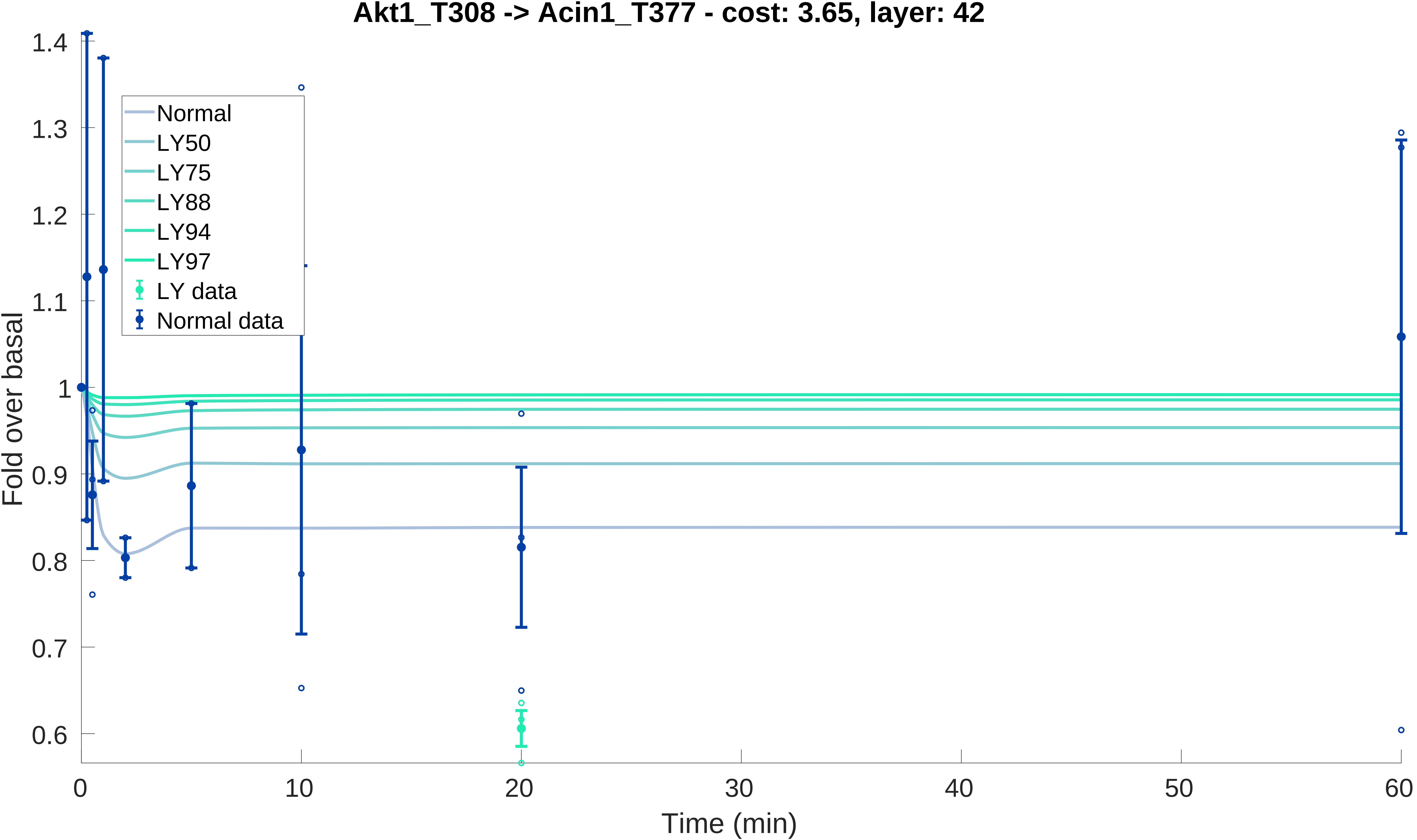

Akt1_T308 -> Acin1_T377 - cost: 3.65, layer: 42
Fold over basal
Time (min)
1.4
1.3
1.2
1.1
1
0.9
0.8
0.7
0.6
0
10
20
30
40
50
60
Normal
LY50
LY75
LY88
LY94
LY97
LY data
Normal data

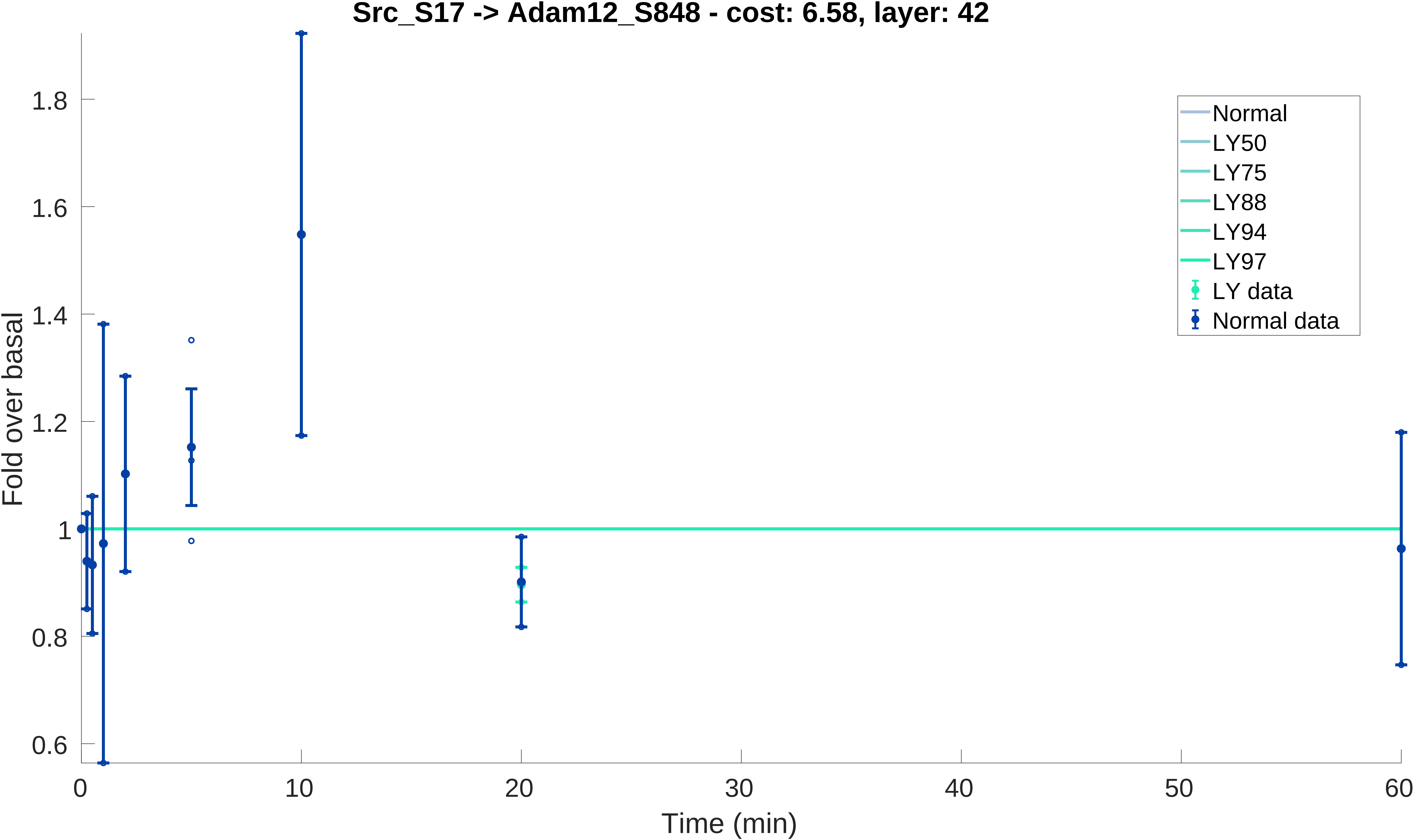

Src_S17 -> Adam12_S848 - cost: 6.58, layer: 42
Fold over basal
1.8
1.6
1.4
1.2
1
0.8
0.6
Time (min)
0
10
20
30
40
50
60
Normal
LY50
LY75
LY88
LY94
LY97
LY data
Normal data

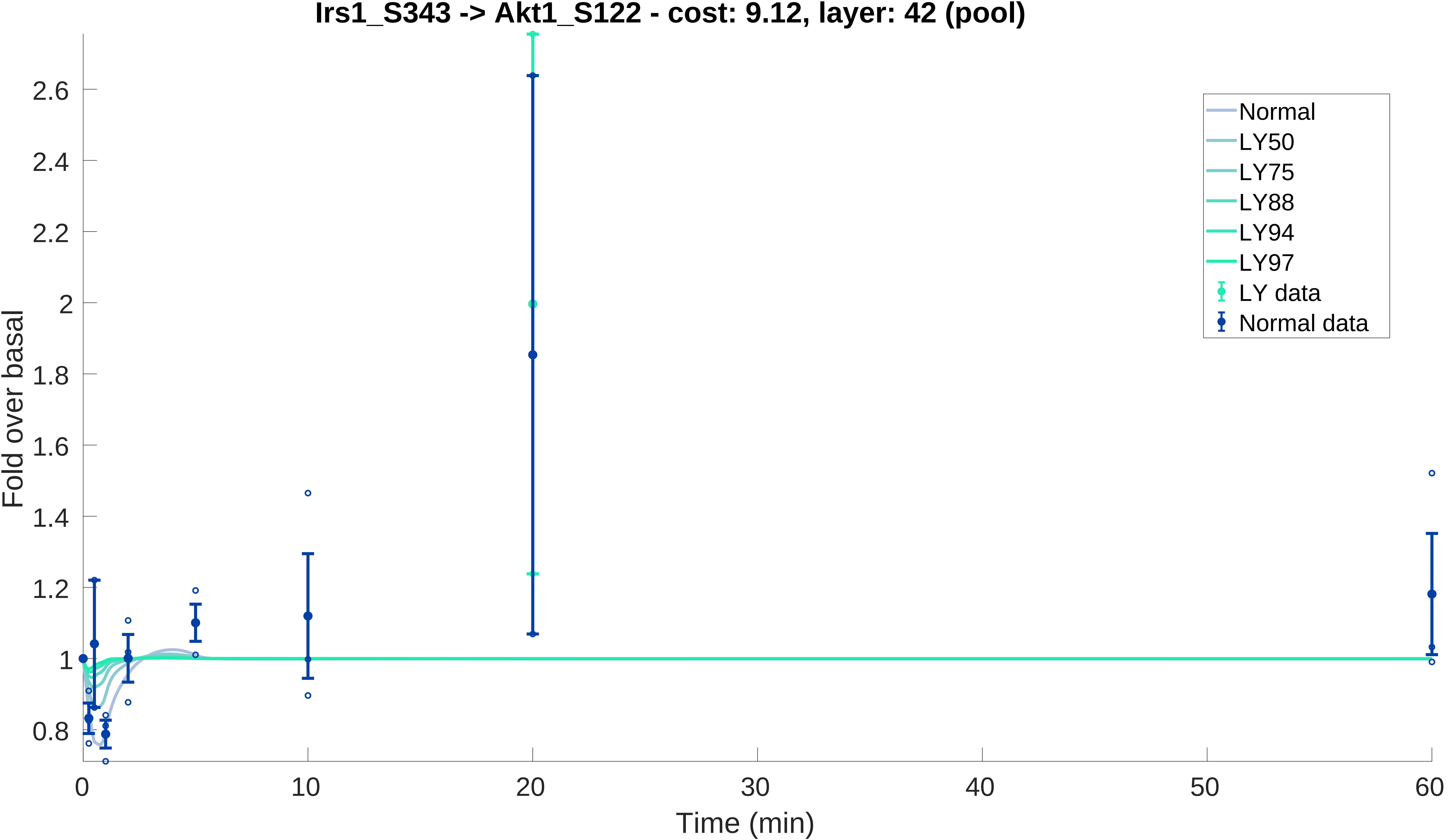

Irs1_S343 -> Akt1_S122 - cost: 9.12, layer: 42 (pool)
Fold over basal
Time (min)
0.8
1
1.2
1.4
1.6
1.8
2
2.2
2.4
2.6
0
10
20
30
40
50
60
Normal
LY50
LY75
LY88
LY94
LY97
LY data
Normal data

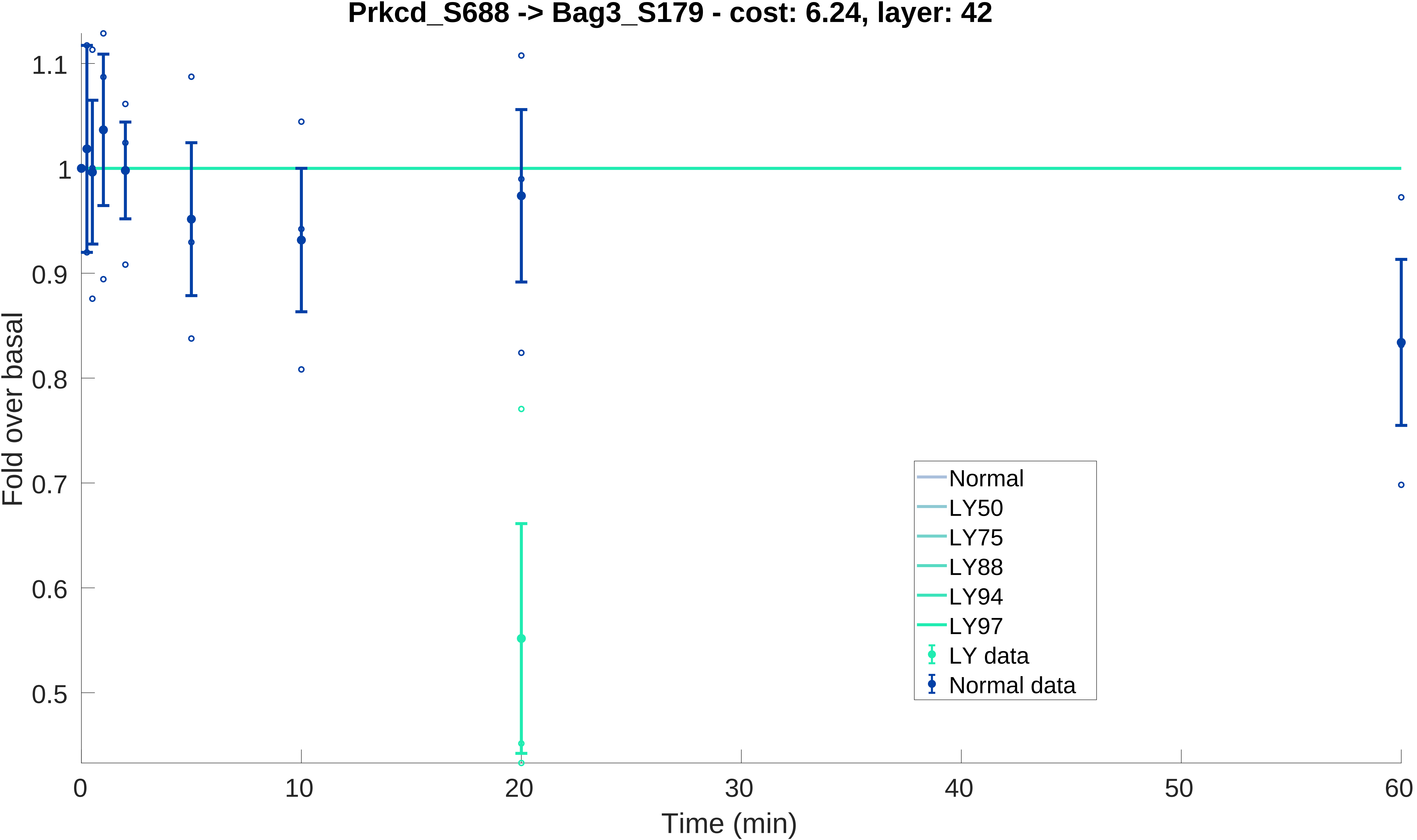
Prkcd_S688 -> Bag3_S179 - cost: 6.24, layer: 42
Fold over basal
Time (min)
0.5
0.6
0.7
0.8
0.9
1
1.1
0
10
20
30
40
50
60
Normal
LY50
LY75
LY88
LY94
LY97
LY data
Normal data

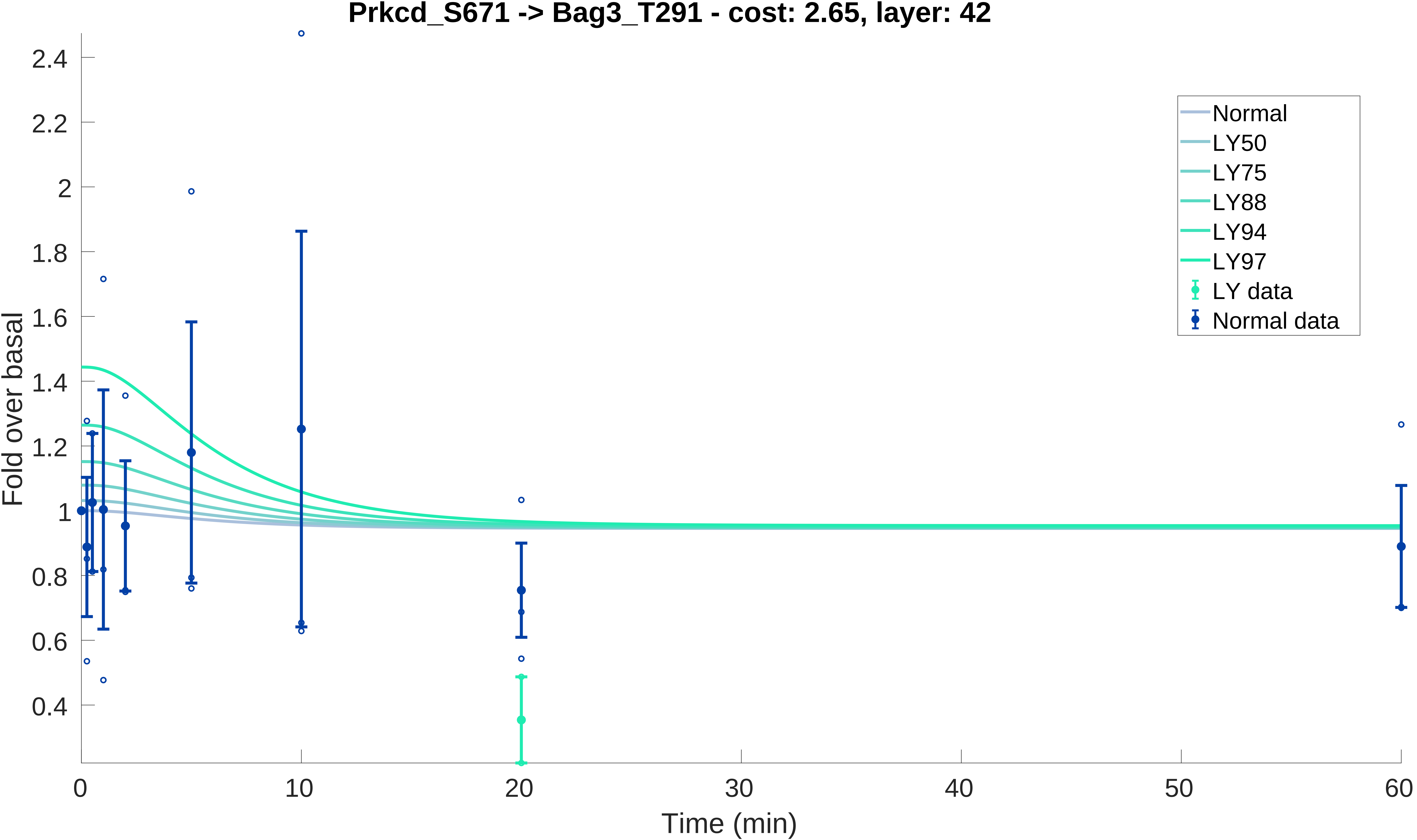

Prkcd_S671 -> Bag3_T291 - cost: 2.65, layer: 42
Fold over basal
Time (min)
0
10
20
30
40
50
60
0.4
0.6
0.8
1
1.2
1.4
1.6
1.8
2
2.2
2.4
Normal
LY50
LY75
LY88
LY94
LY97
LY data
Normal data

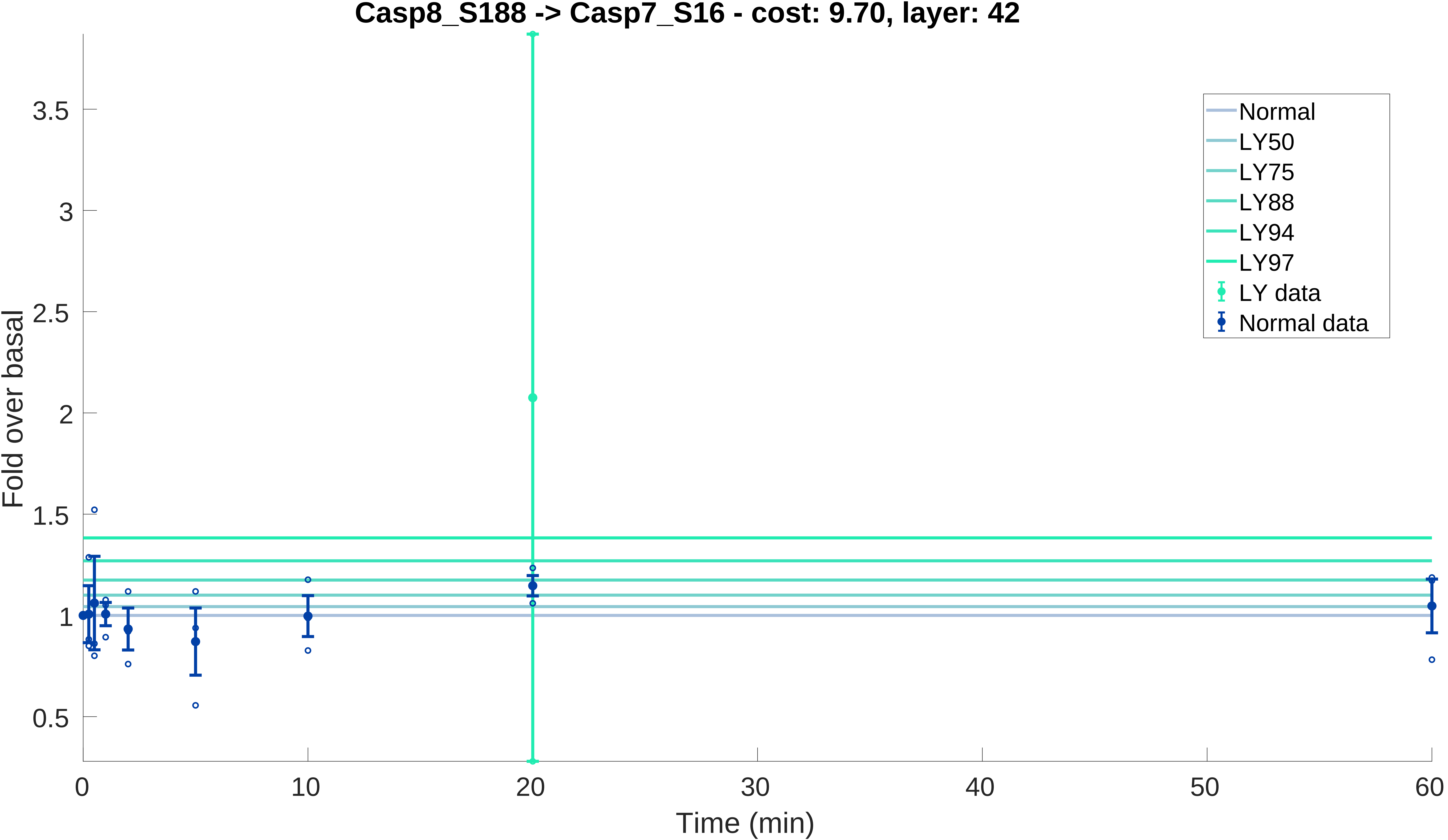

Casp8_S188 -> Casp7_S16 - cost: 9.70, layer: 42
Fold over basal
Time (min)
0.5
1
1.5
2
2.5
3
3.5
0
10
20
30
40
50
60
Normal
LY50
LY75
LY88
LY94
LY97
LY data
Normal data

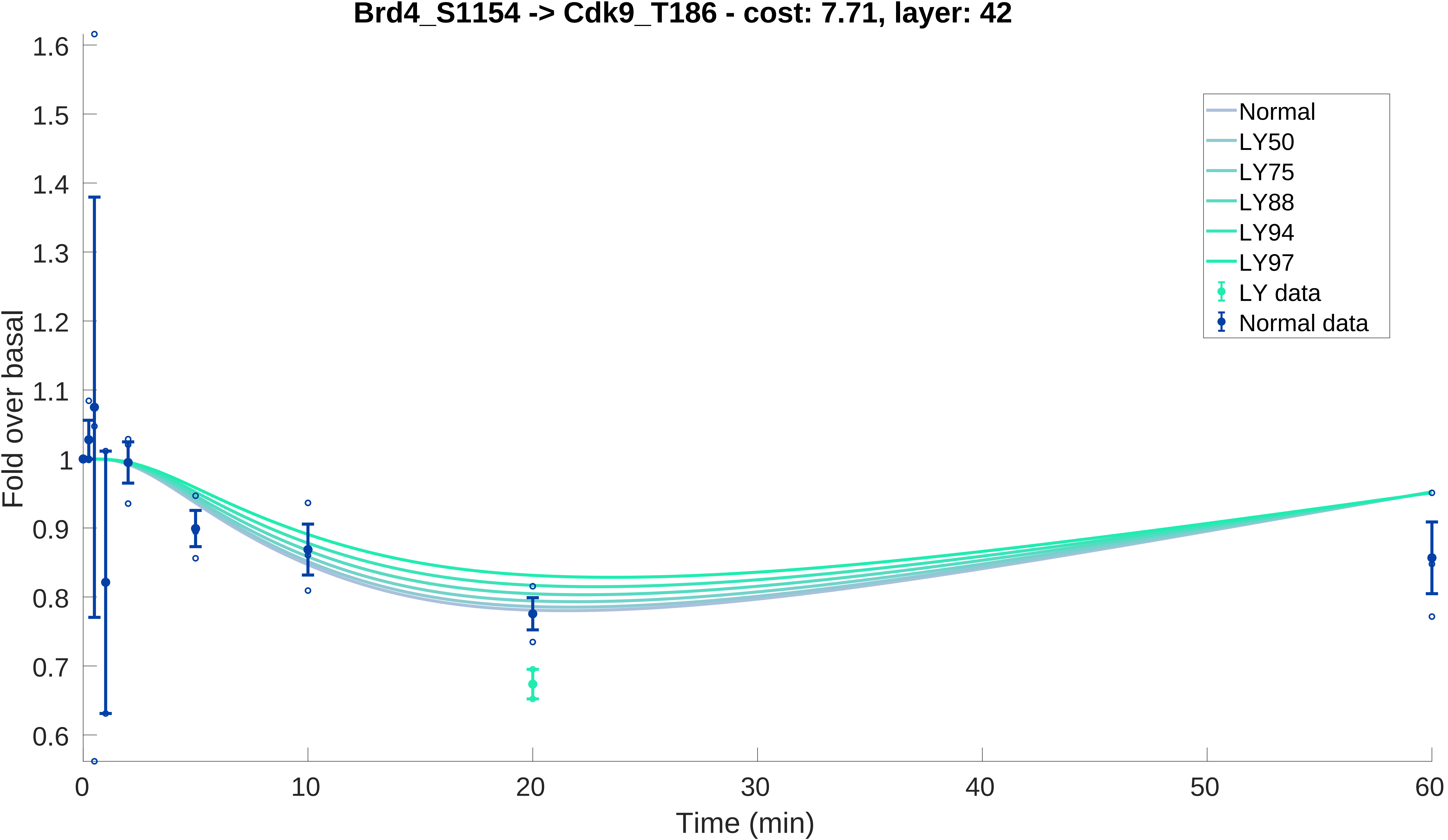
Brd4_S1154 -> Cdk9_T186 - cost: 7.71, layer: 42
Fold over basal
Time (min)
Normal
LY50
LY75
LY88
LY94
LY97
LY data
Normal data
1.6
1.5
1.4
1.3
1.2
1.1
1
0.9
0.8
0.7
0.6
0
10
20
30
40
50
60

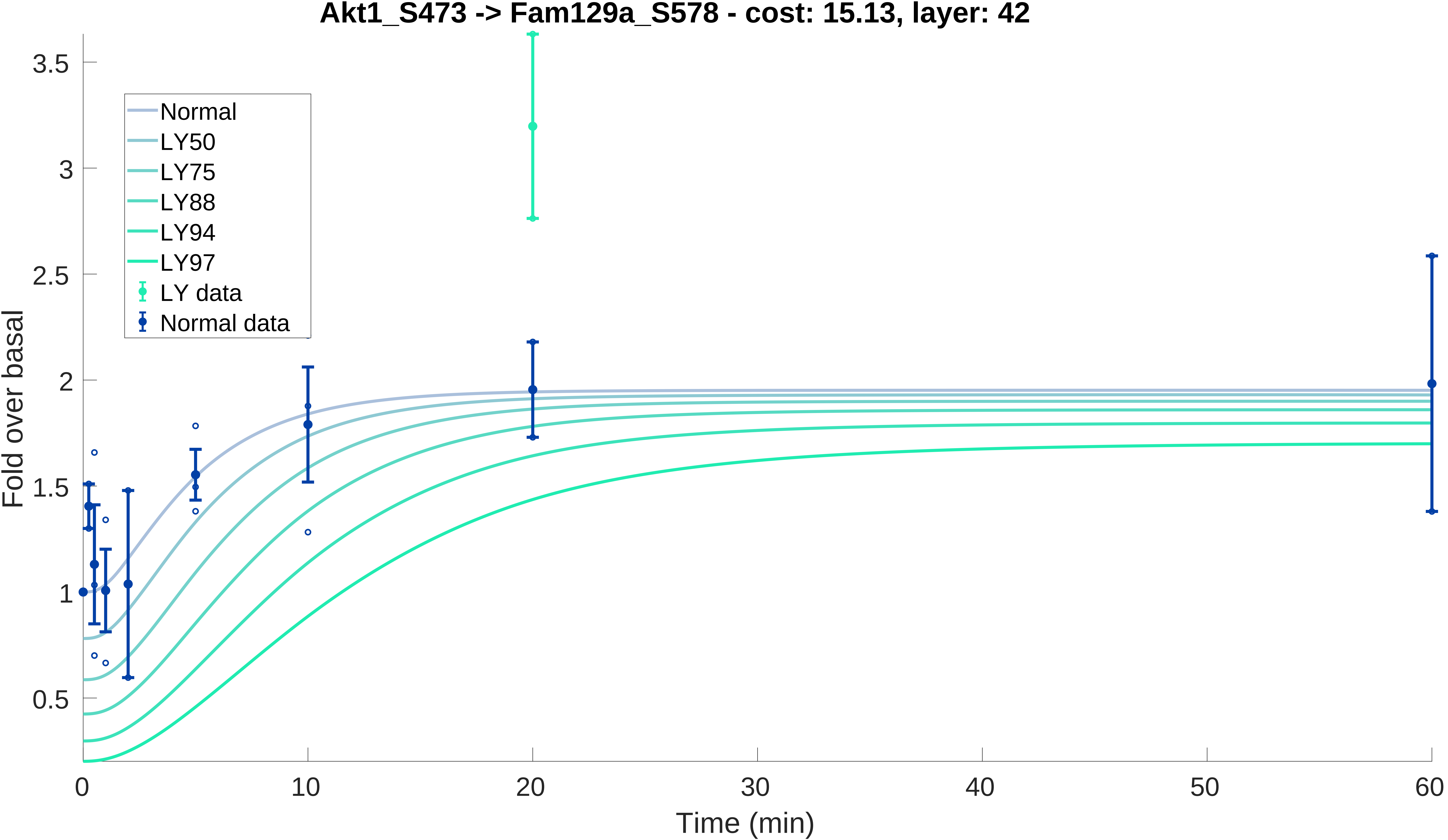
Akt1_S473 -> Fam129a_S578 - cost: 15.13, layer: 42
Fold over basal
Time (min)
Normal
LY50
LY75
LY88
LY94
LY97
LY data
Normal data
0.5
1
1.5
2
2.5
3
3.5
0
10
20
30
40
50
60

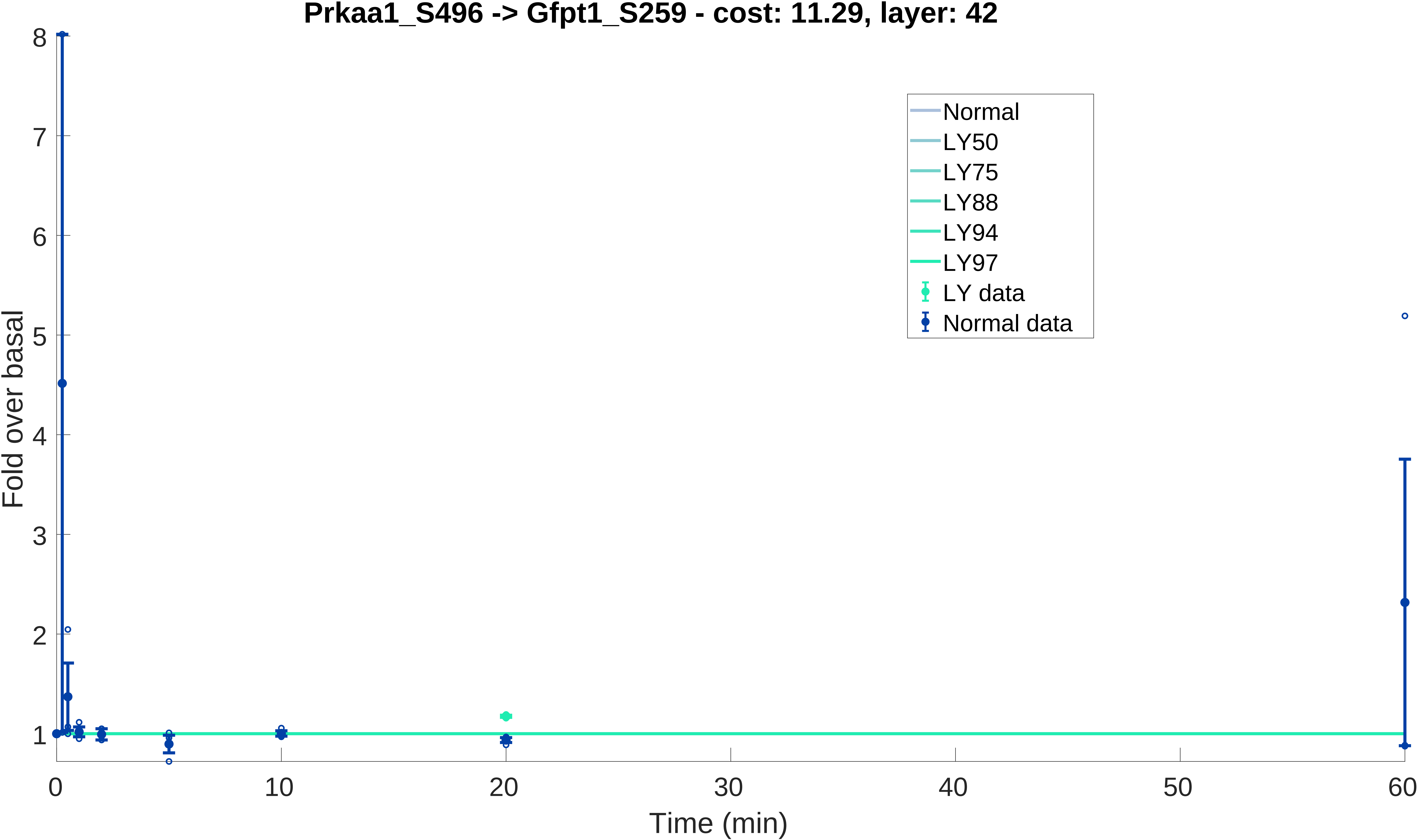

Prkaa1_S496 -> Gfpt1_S259 - cost: 11.29, layer: 42
Fold over basal
Time (min)
Normal
LY50
LY75
LY88
LY94
LY97
LY data
Normal data

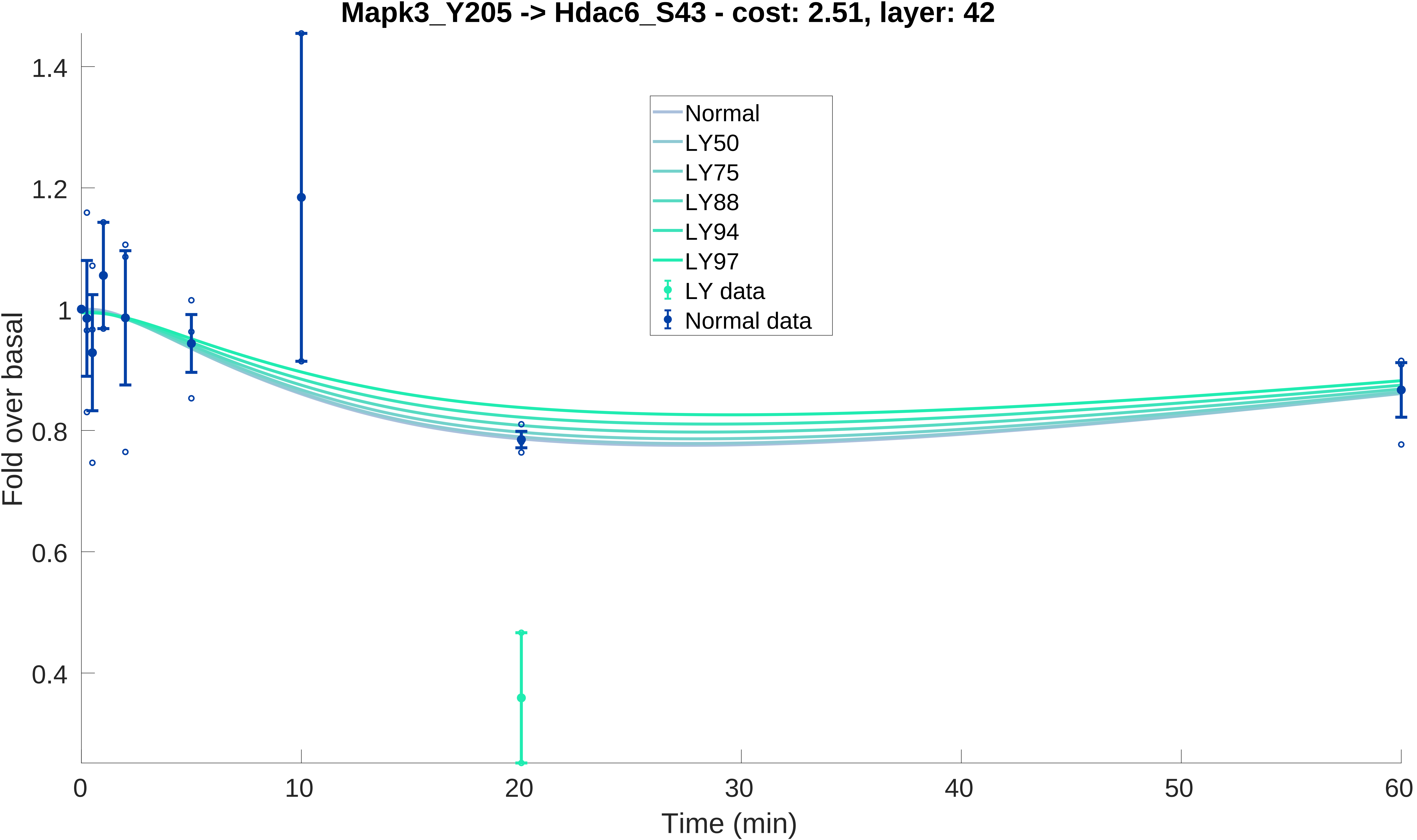
Mapk3_Y205 -> Hdac6_S43 - cost: 2.51, layer: 42
Fold over basal
1.4
1.2
1
0.8
0.6
0.4
Time (min)
0
10
20
30
40
50
60
Normal
LY50
LY75
LY88
LY94
LY97
LY data
Normal data

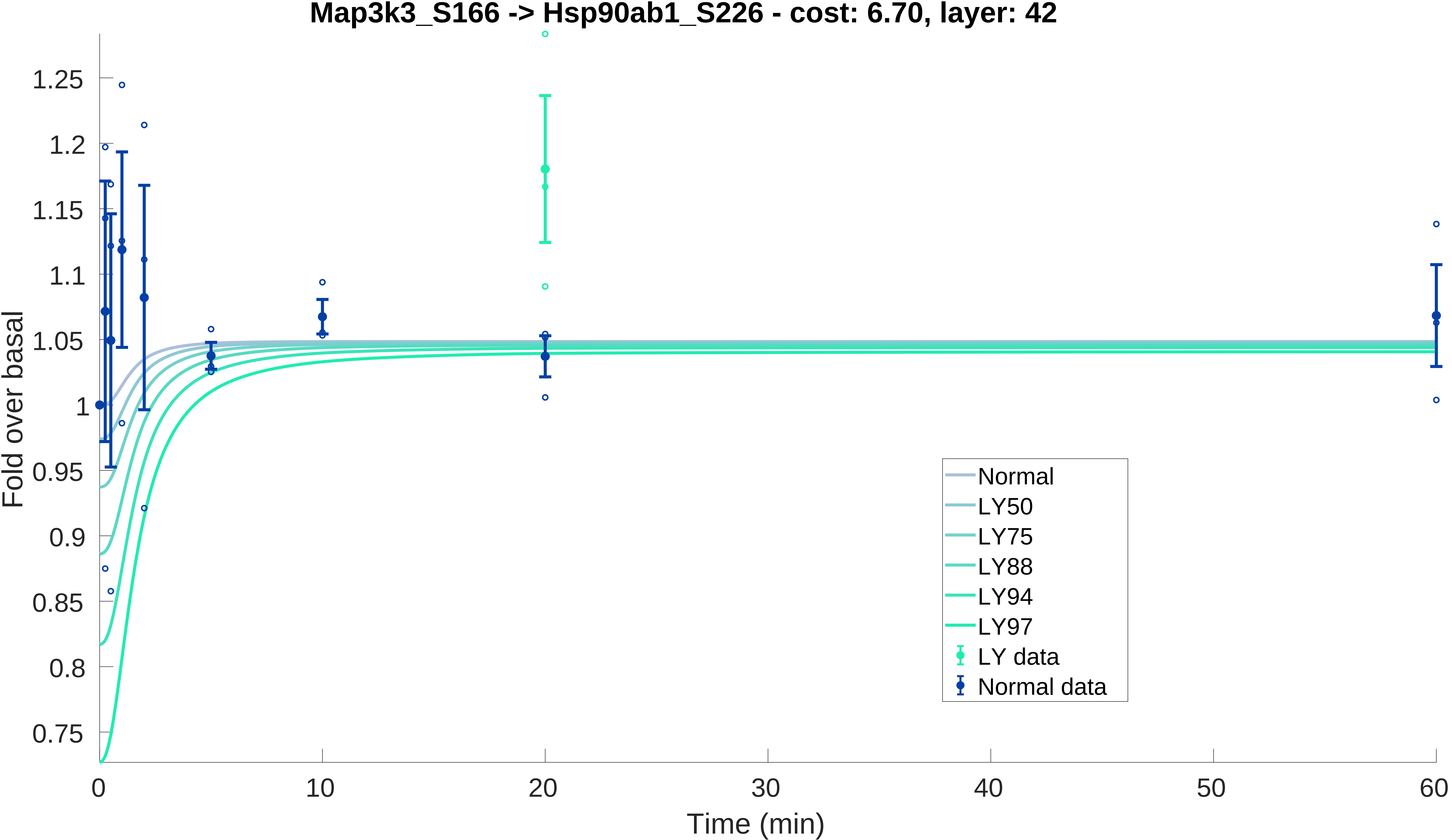

Map3k3_S166 -> Hsp90ab1_S226 - cost: 6.70, layer: 42
Fold over basal
1.25
1.2
1.15
1.1
1.05
1
0.95
0.9
0.85
0.8
0.75
0
10
20
30
40
50
60
Time (min)
Normal
LY50
LY75
LY88
LY94
LY97
LY data
Normal data

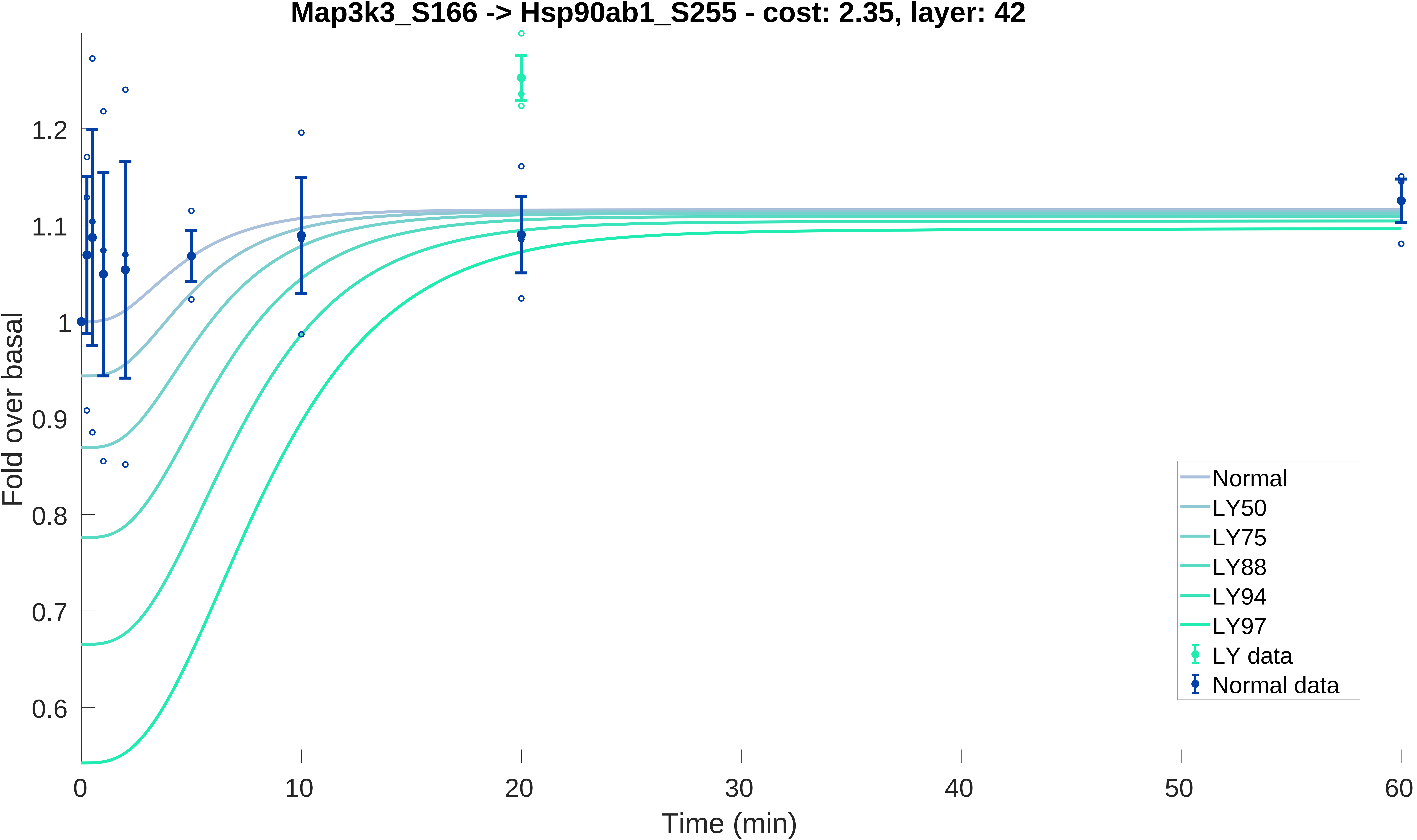
Map3k3_S166 -> Hsp90ab1_S255 - cost: 2.35, layer: 42
Fold over basal
1.2
1.1
1
0.9
0.8
0.7
0.6
0
10
20
30
40
50
60
Time (min)
Normal
LY50
LY75
LY88
LY94
LY97
LY data
Normal data

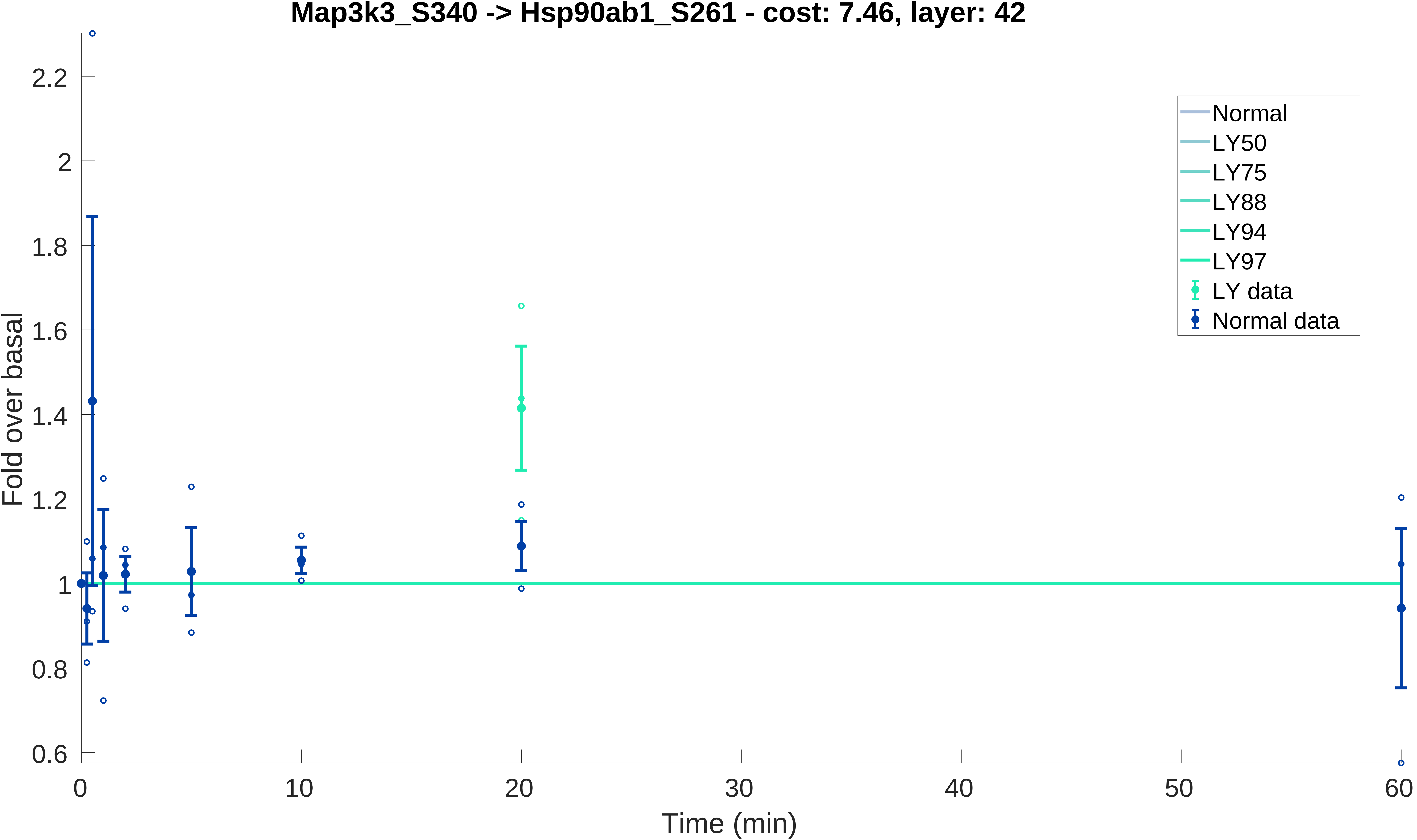

Map3k3_S340 -> Hsp90ab1_S261 - cost: 7.46, layer: 42
Fold over basal
Time (min)
Normal
LY50
LY75
LY88
LY94
LY97
LY data
Normal data
0.6
0.8
1
1.2
1.4
1.6
1.8
2
2.2
0
10
20
30
40
50
60

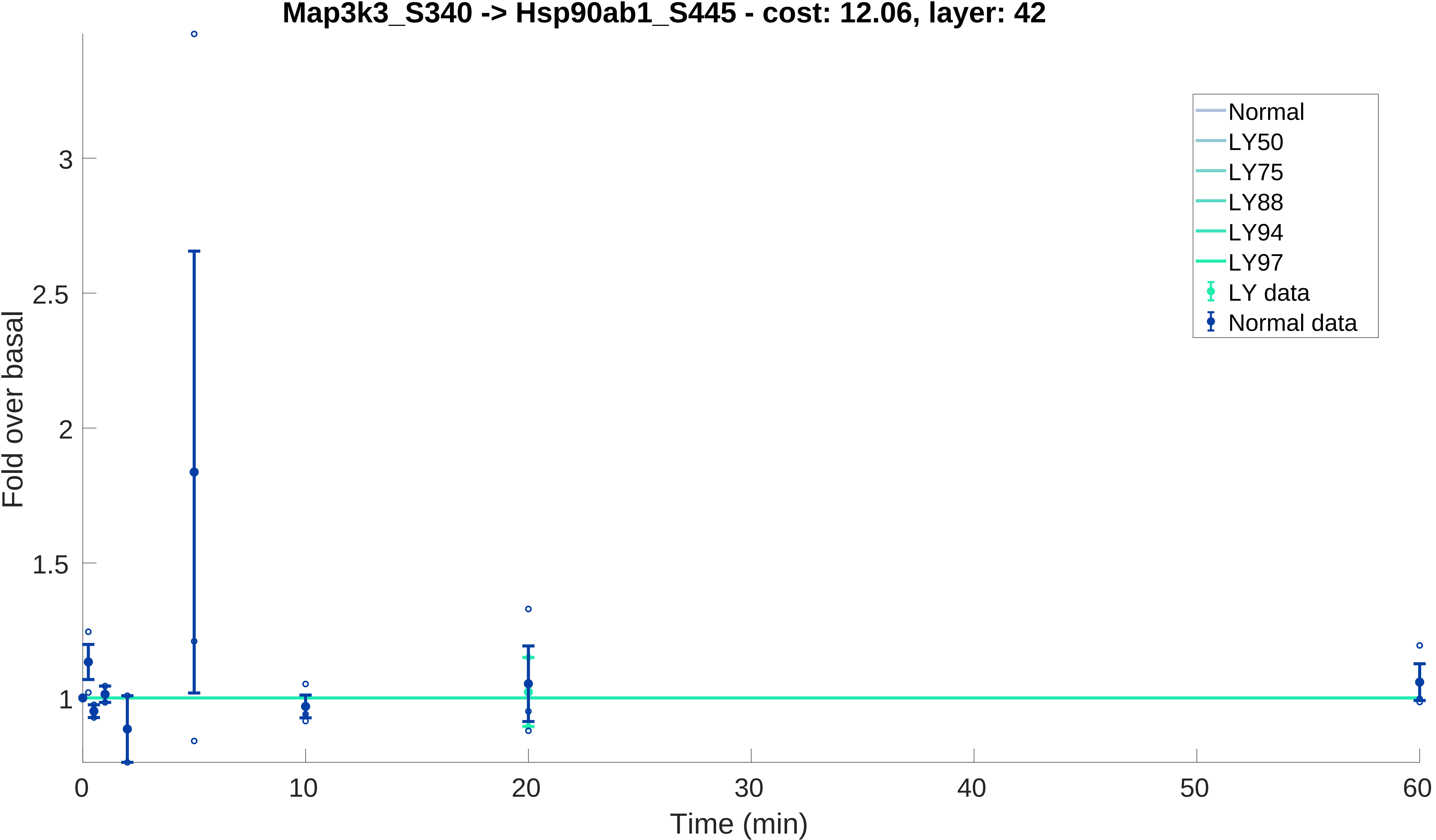

Map3k3_S340 -> Hsp90ab1_S445 - cost: 12.06, layer: 42
Fold over basal
3
2.5
2
1.5
1
0
10
20
30
40
50
60
Time (min)
Normal
LY50
LY75
LY88
LY94
LY97
LY data
Normal data

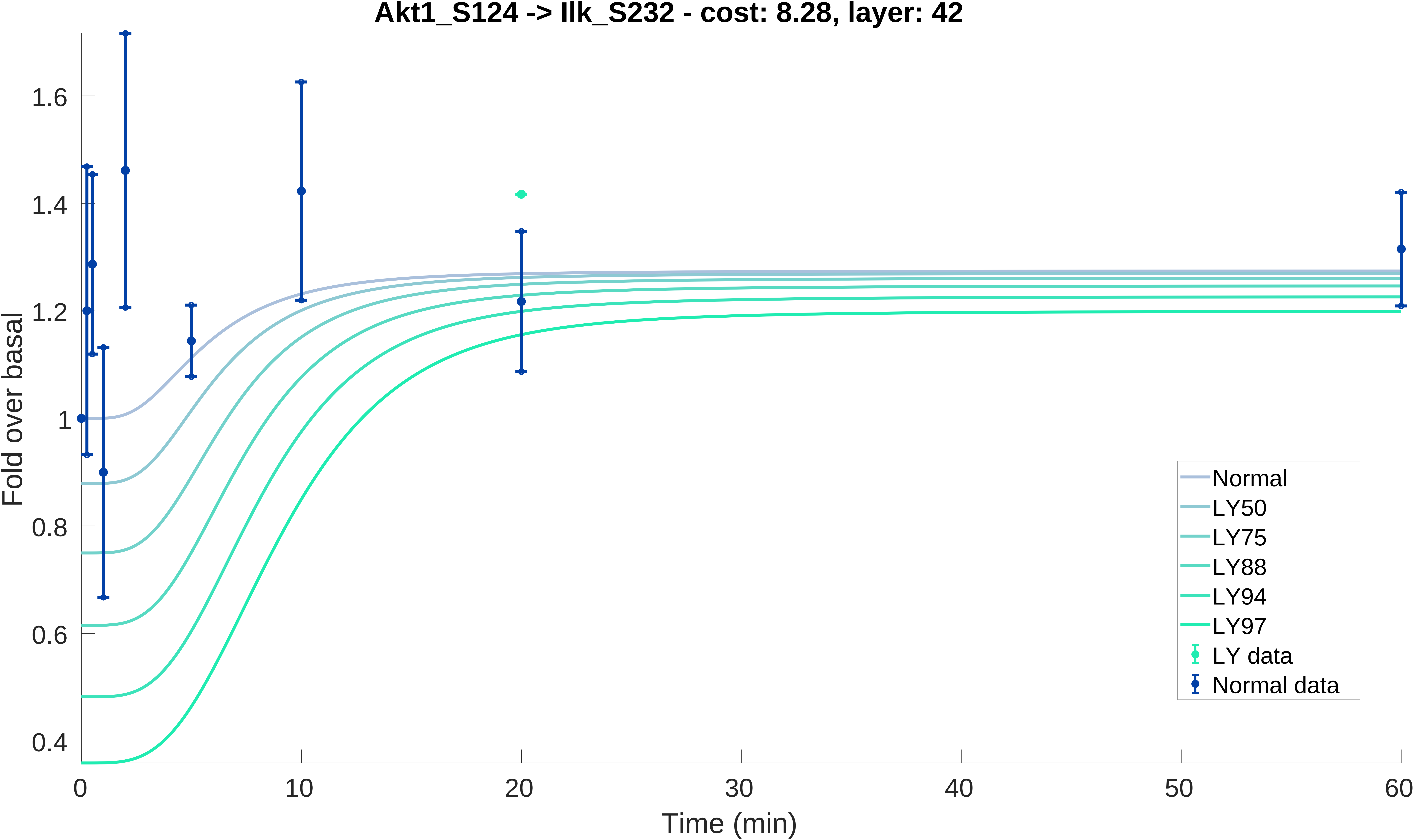

Akt1_S124 -> Ilk_S232 - cost: 8.28, layer: 42
Fold over basal
Time (min)
0
10
20
30
40
50
60
0.4
0.6
0.8
1
1.2
1.4
1.6
Normal
LY50
LY75
LY88
LY94
LY97
LY data
Normal data

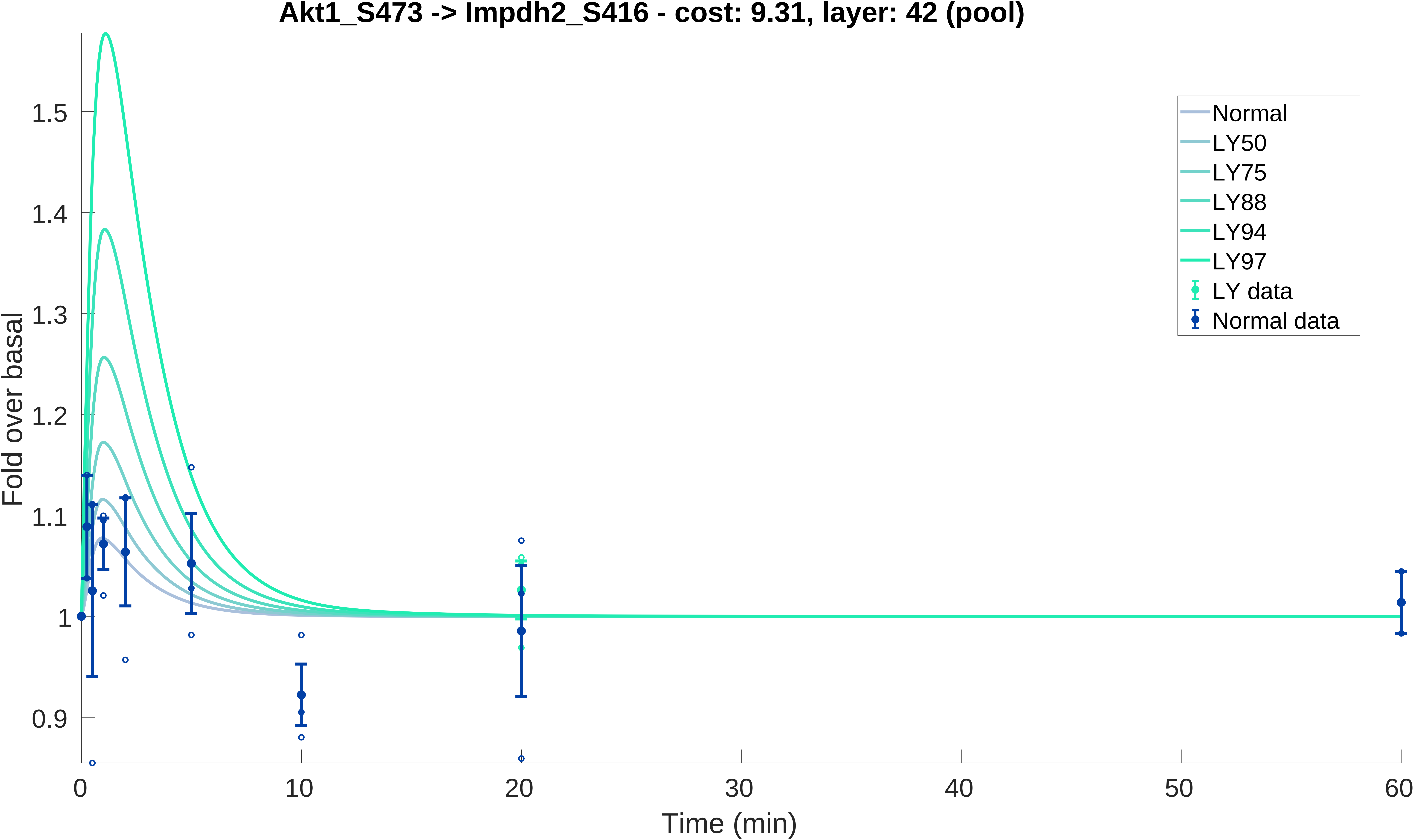
Akt1_S473 -> Impdh2_S416 - cost: 9.31, layer: 42 (pool)
Fold over basal
Time (min)
0.9
1
1.1
1.2
1.3
1.4
1.5
0
10
20
30
40
50
60
Normal
LY50
LY75
LY88
LY94
LY97
LY data
Normal data

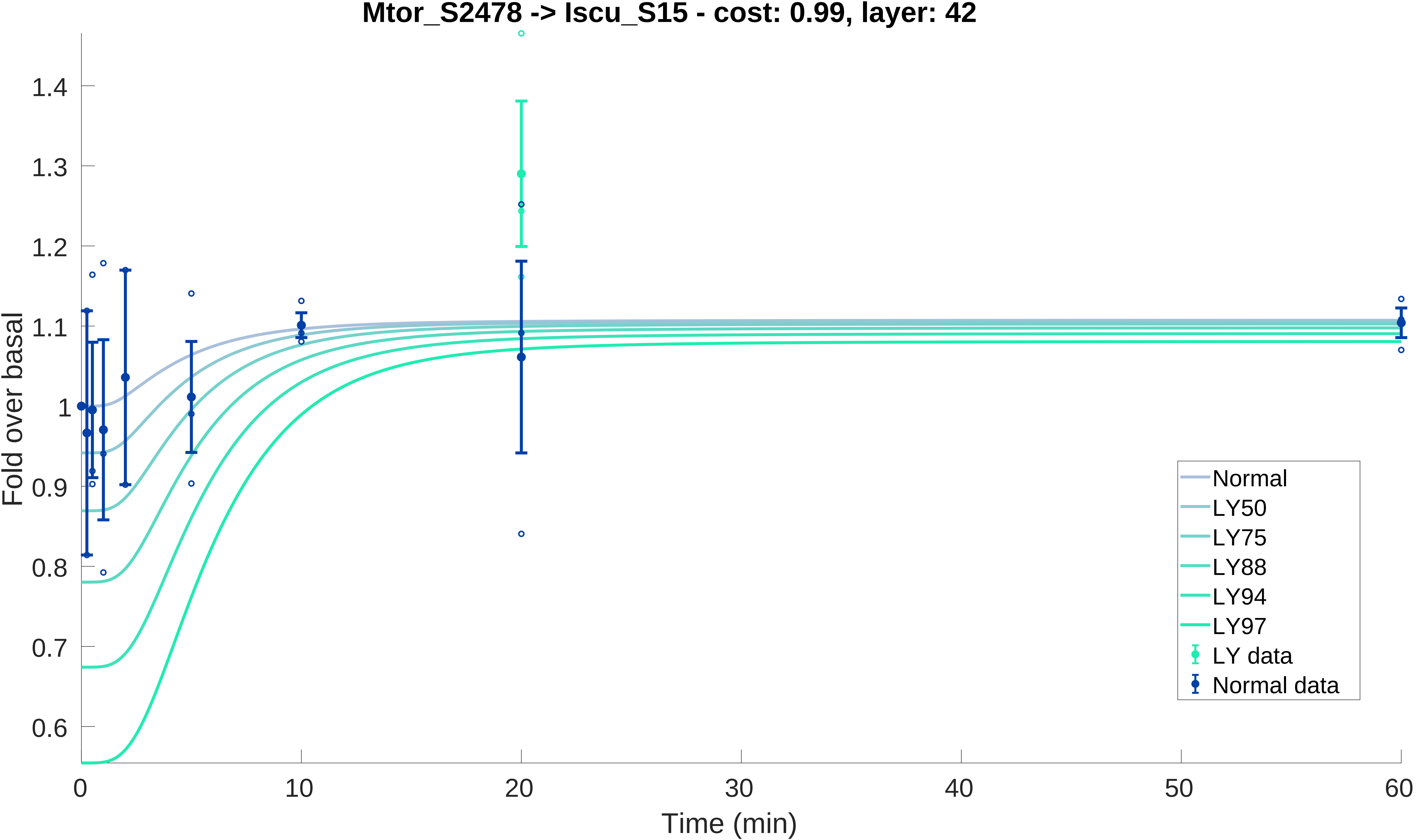

Mtor_S2478 -> Iscu_S15 - cost: 0.99, layer: 42
Fold over basal
1.4
1.3
1.2
1.1
1
0.9
0.8
0.7
0.6
0
10
20
30
40
50
60
Time (min)
Normal
LY50
LY75
LY88
LY94
LY97
LY data
Normal data

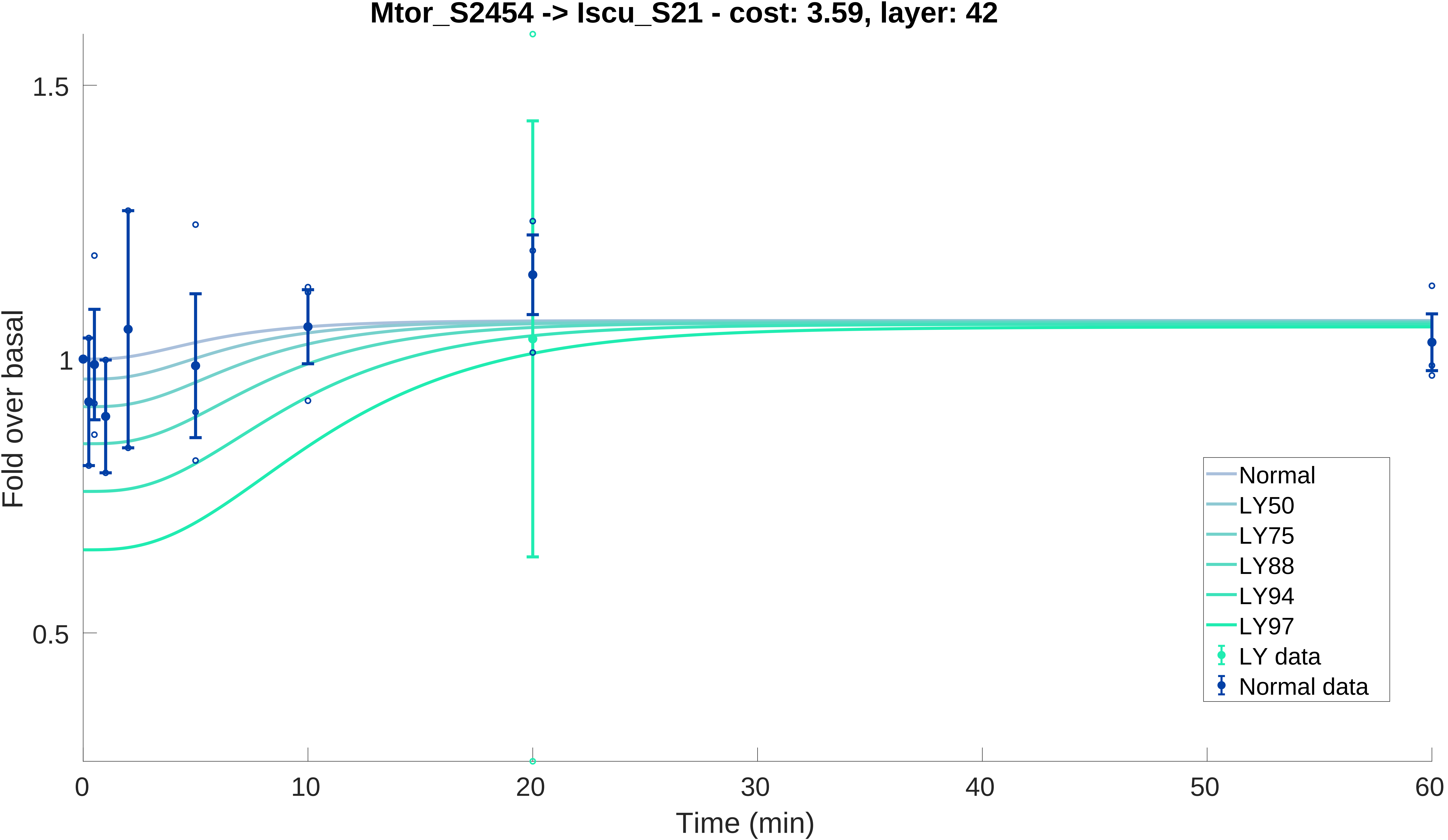

Mtor_S2454 -> Iscu_S21 - cost: 3.59, layer: 42
Fold over basal
1.5
1
0.5
0
10
20
30
40
50
60
Time (min)
Normal
LY50
LY75
LY88
LY94
LY97
LY data
Normal data

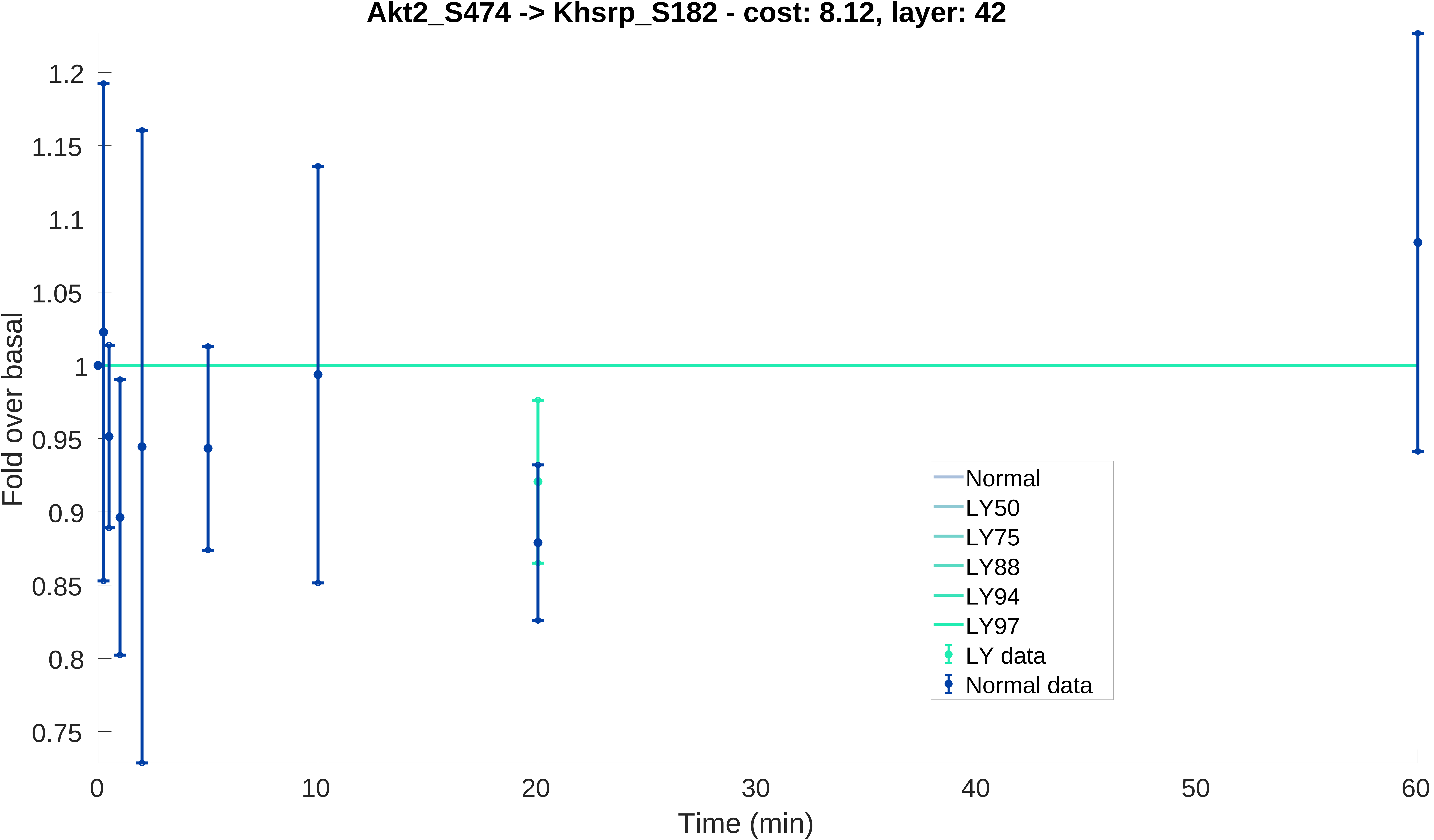

Akt2_S474 -> Khsrp_S182 - cost: 8.12, layer: 42
Fold over basal
1.2
1.15
1.1
1.05
1
0.95
0.9
0.85
0.8
0.75
0
10
20
30
40
50
60
Time (min)
Normal
LY50
LY75
LY88
LY94
LY97
LY data
Normal data

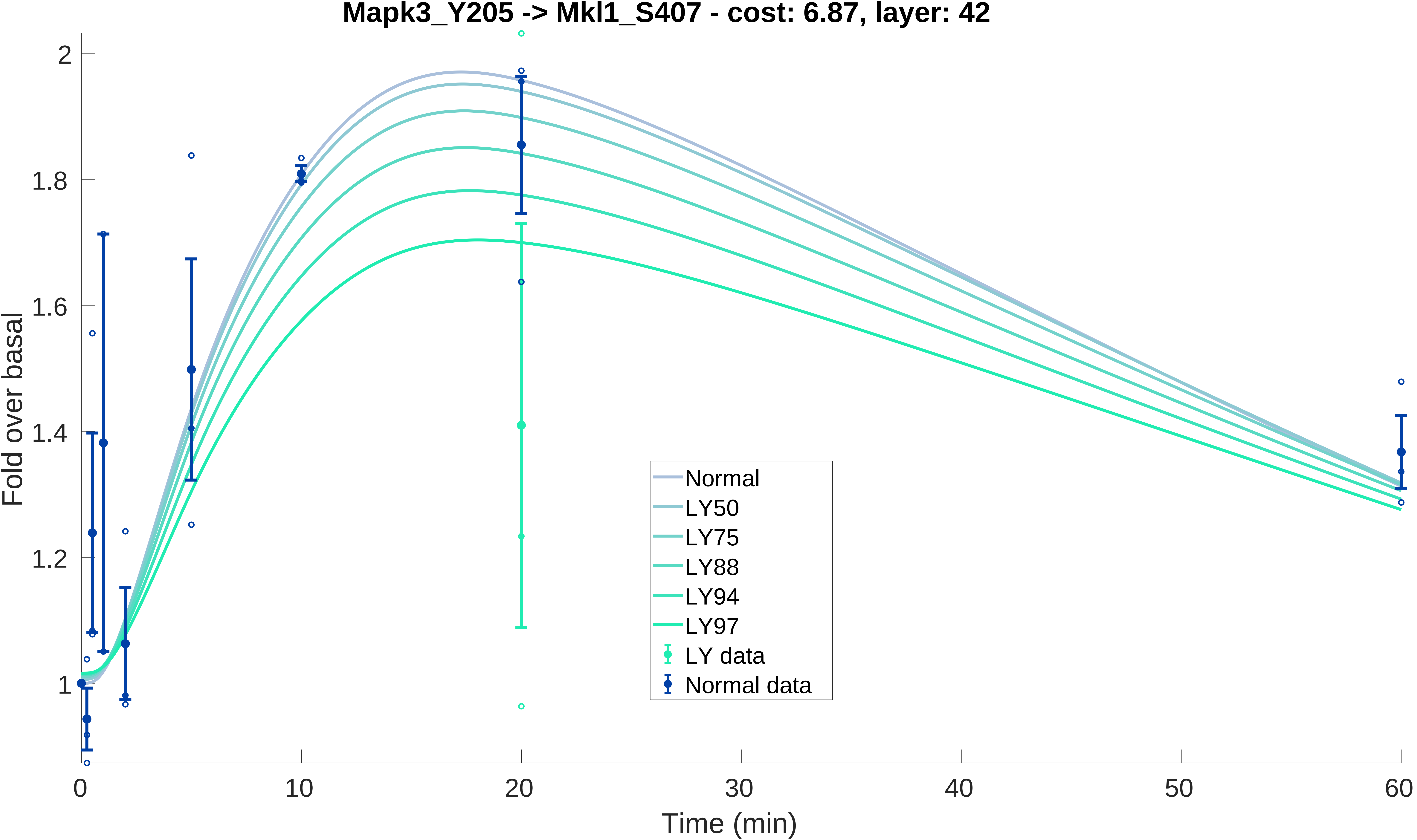

Mapk3_Y205 -> Mkl1_S407 - cost: 6.87, layer: 42
Fold over basal
Time (min)
0
10
20
30
40
50
60
1
1.2
1.4
1.6
1.8
2
Normal
LY50
LY75
LY88
LY94
LY97
LY data
Normal data

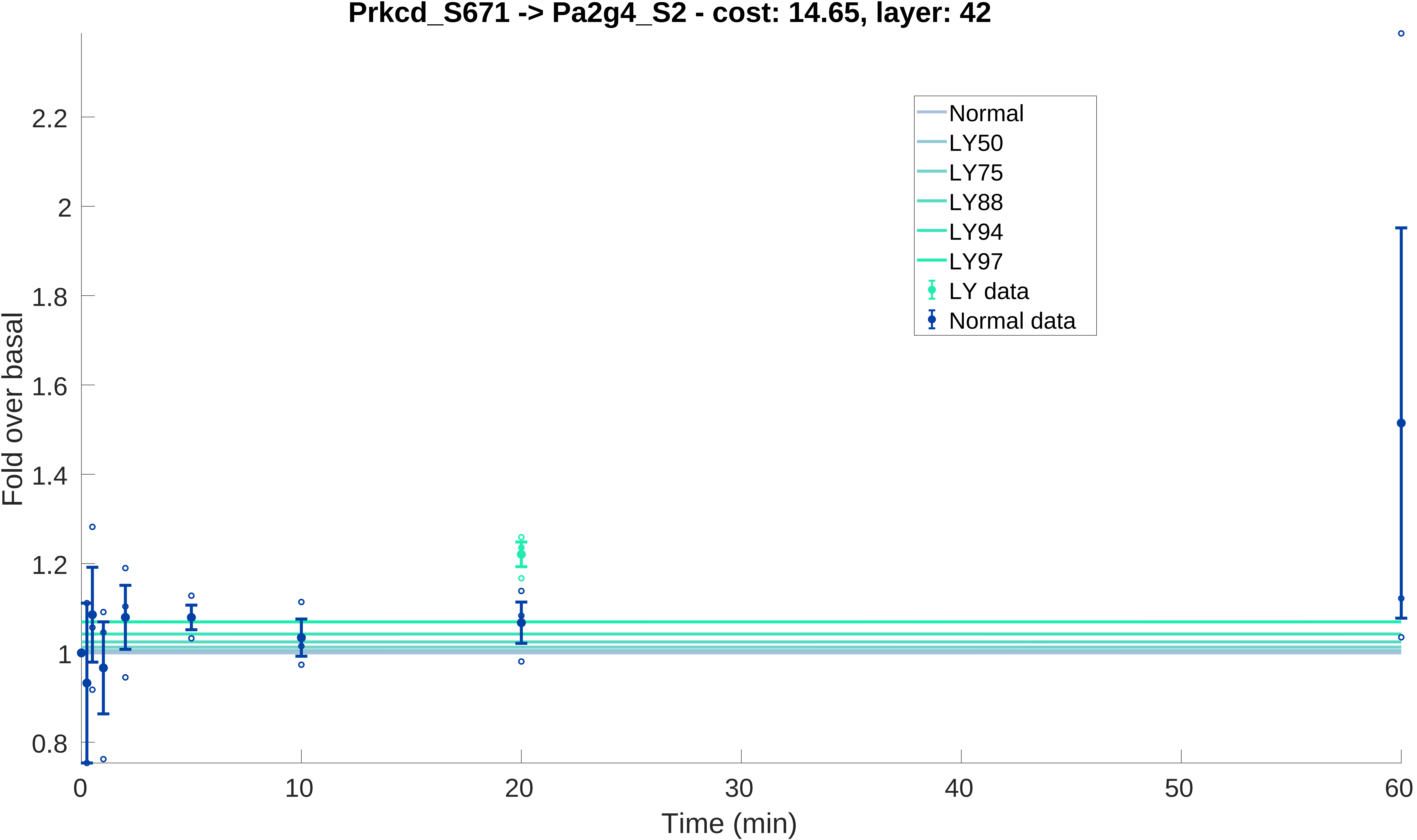

Prrkcd_S671 -> Pa2g4_S2 - cost: 14.65, layer: 42
Normal
LY50
LY75
LY88
LY94
LY97
LY data
Normal data
Fold over basal
Time (min)
0.8
1
1.2
1.4
1.6
1.8
2
2.2
0
10
20
30
40
50
60

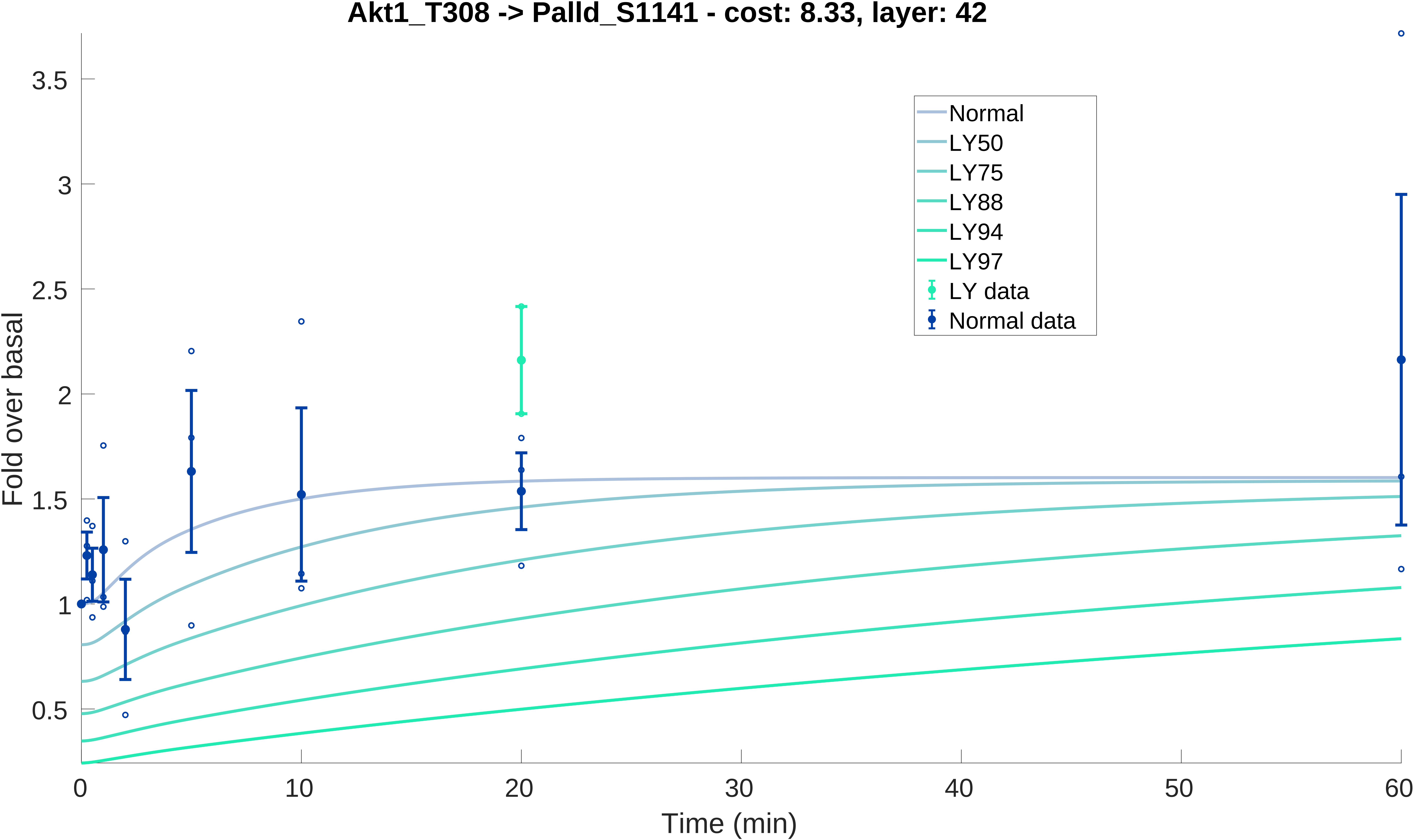

Akt1_T308 -> Palld_S1141 - cost: 8.33, layer: 42
Fold over basal
Time (min)
0.5
1
1.5
2
2.5
3
3.5
0
10
20
30
40
50
60
Normal
LY50
LY75
LY88
LY94
LY97
LY data
Normal data

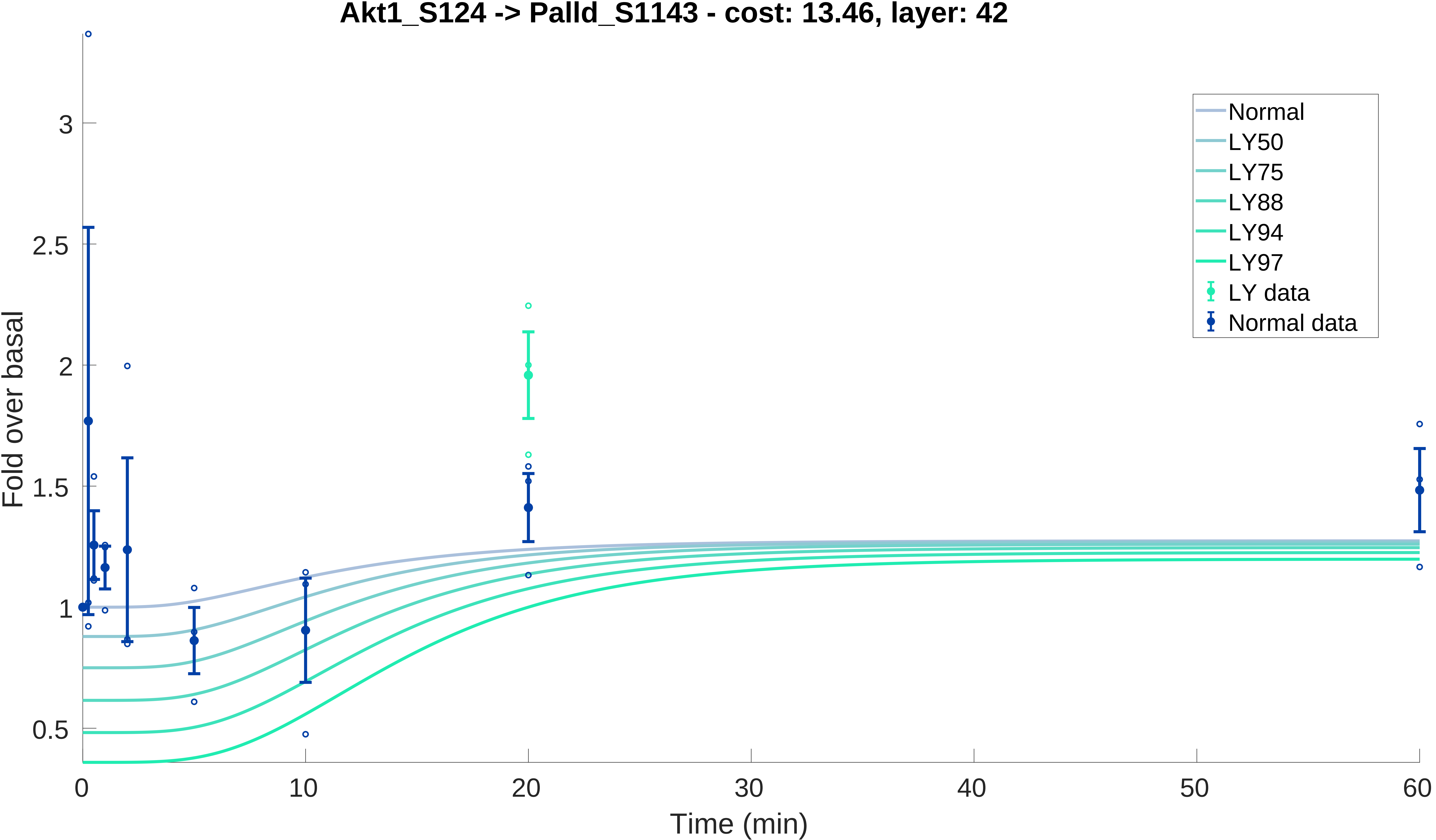
Akt1_S124 -> Palld_S1143 - cost: 13.46, layer: 42
Fold over basal
Time (min)
0
10
20
30
40
50
60
0.5
1
1.5
2
2.5
3
Normal
LY50
LY75
LY88
LY94
LY97
LY data
Normal data

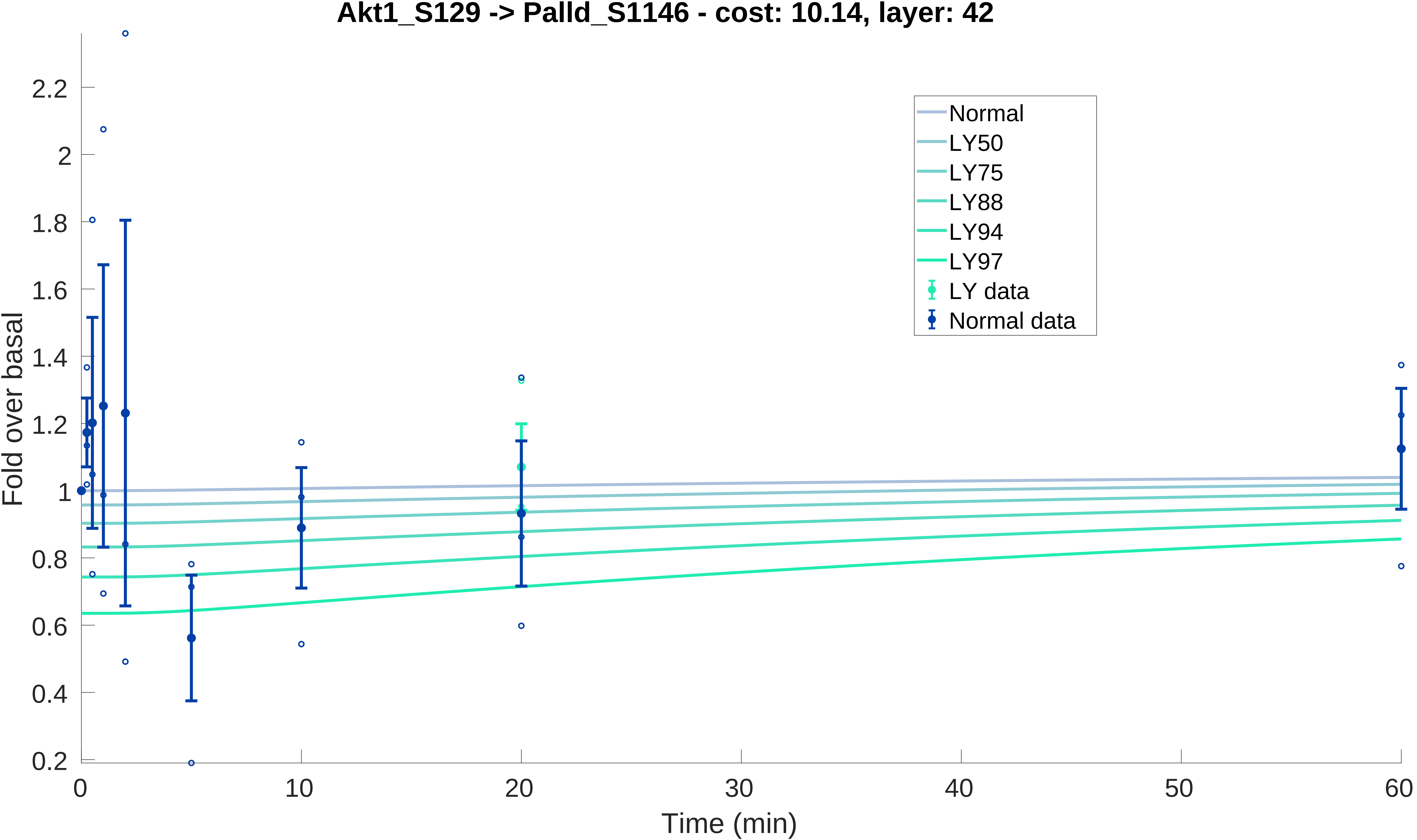

Akt1_S129 -> Palld_S1146 - cost: 10.14, layer: 42
Fold over basal
Time (min)
0.2
0.4
0.6
0.8
1
1.2
1.4
1.6
1.8
2
2.2
0
10
20
30
40
50
60
Normal
LY50
LY75
LY88
LY94
LY97
LY data
Normal data

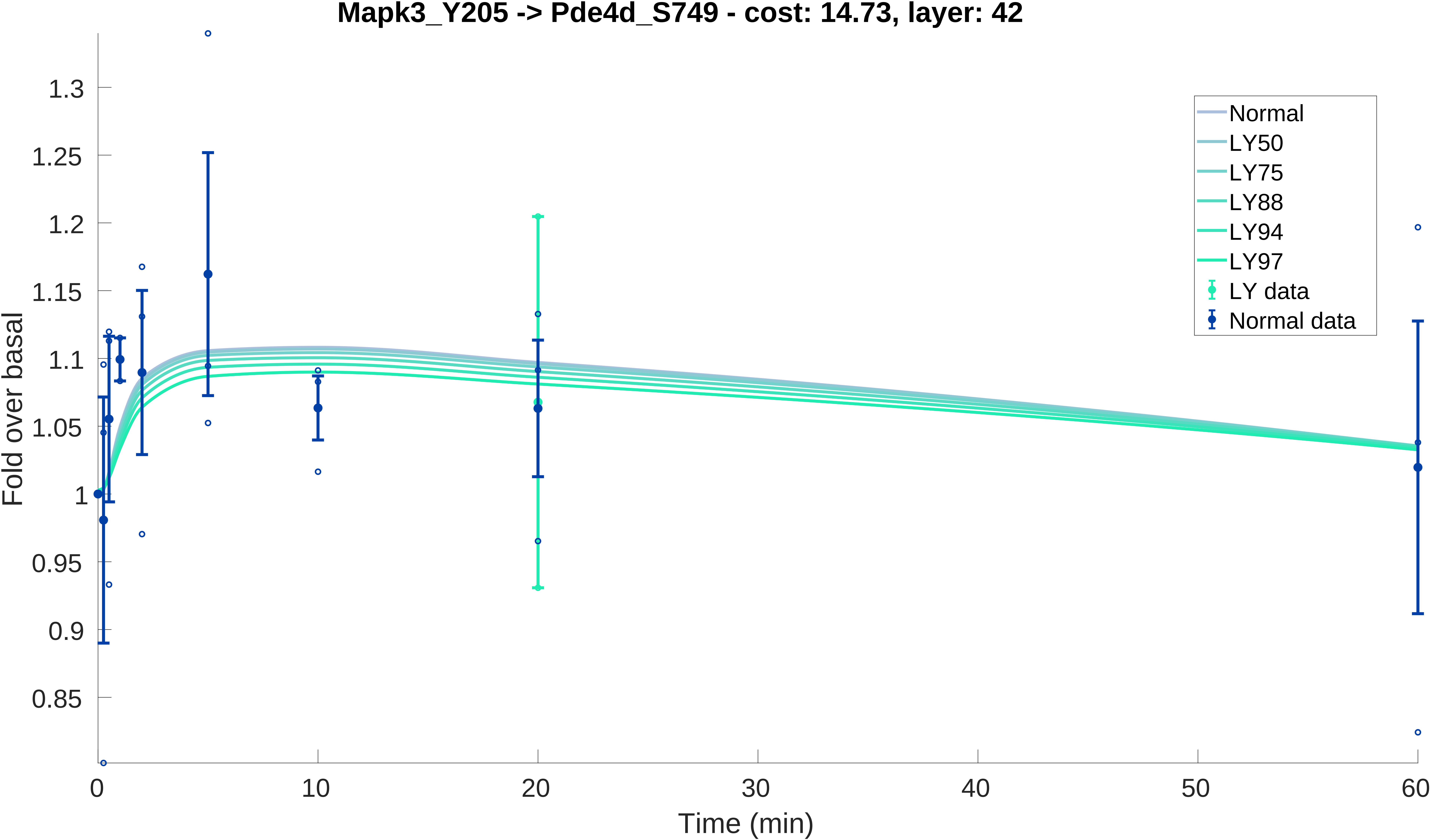

Mapk3_Y205 -> Pde4d_S749 - cost: 14.73, layer: 42
Fold over basal
Time (min)
1.3
1.25
1.2
1.15
1.1
1.05
1
0.95
0.9
0.85
0
10
20
30
40
50
60
Normal
LY50
LY75
LY88
LY94
LY97
LY data
Normal data

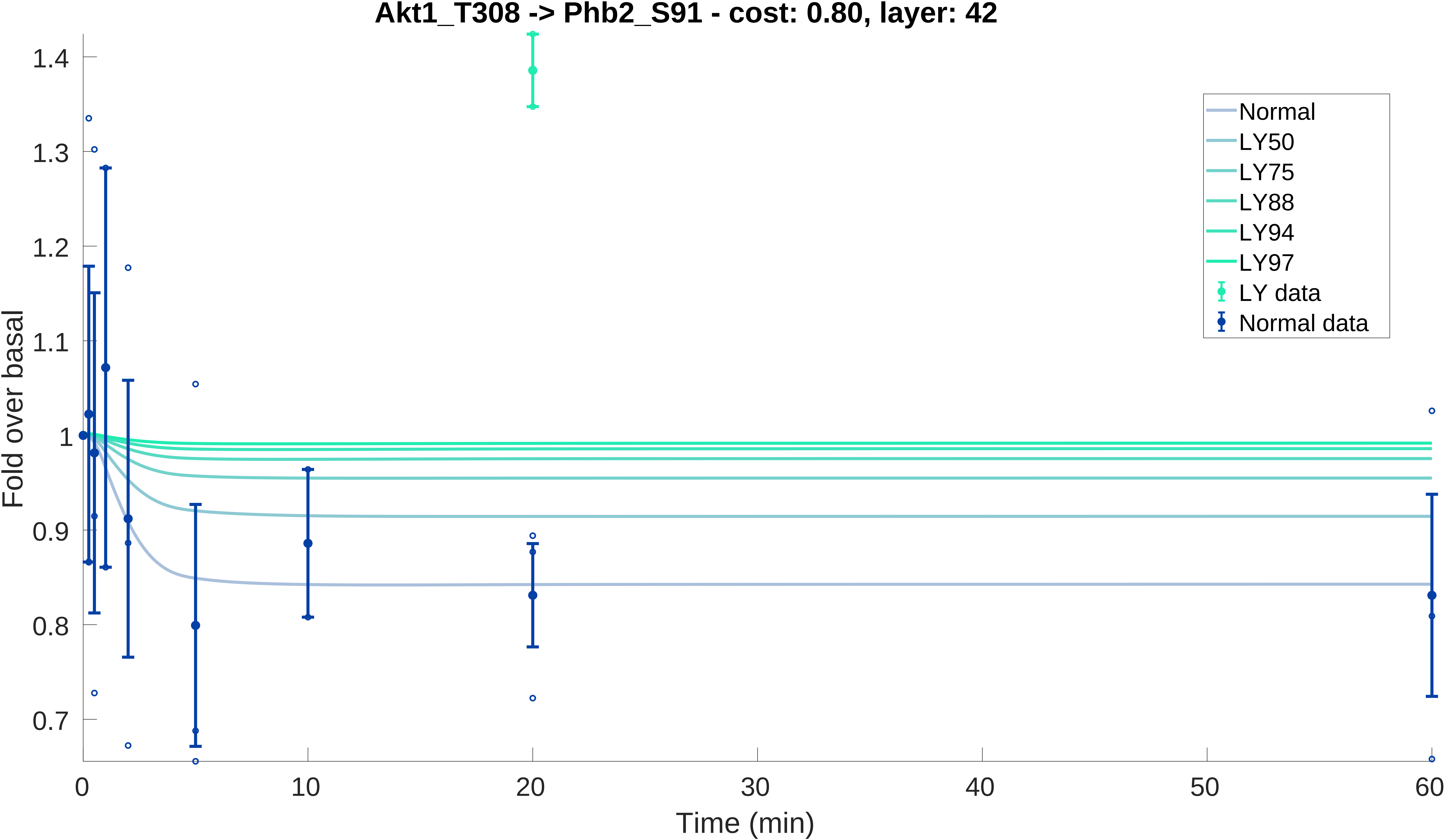

Akt1_T308 -> Phb2_S91 - cost: 0.80, layer: 42
Fold over basal
1.4
1.3
1.2
1.1
1
0.9
0.8
0.7
0
10
20
30
40
50
60
Time (min)
Normal
LY50
LY75
LY88
LY94
LY97
LY data
Normal data

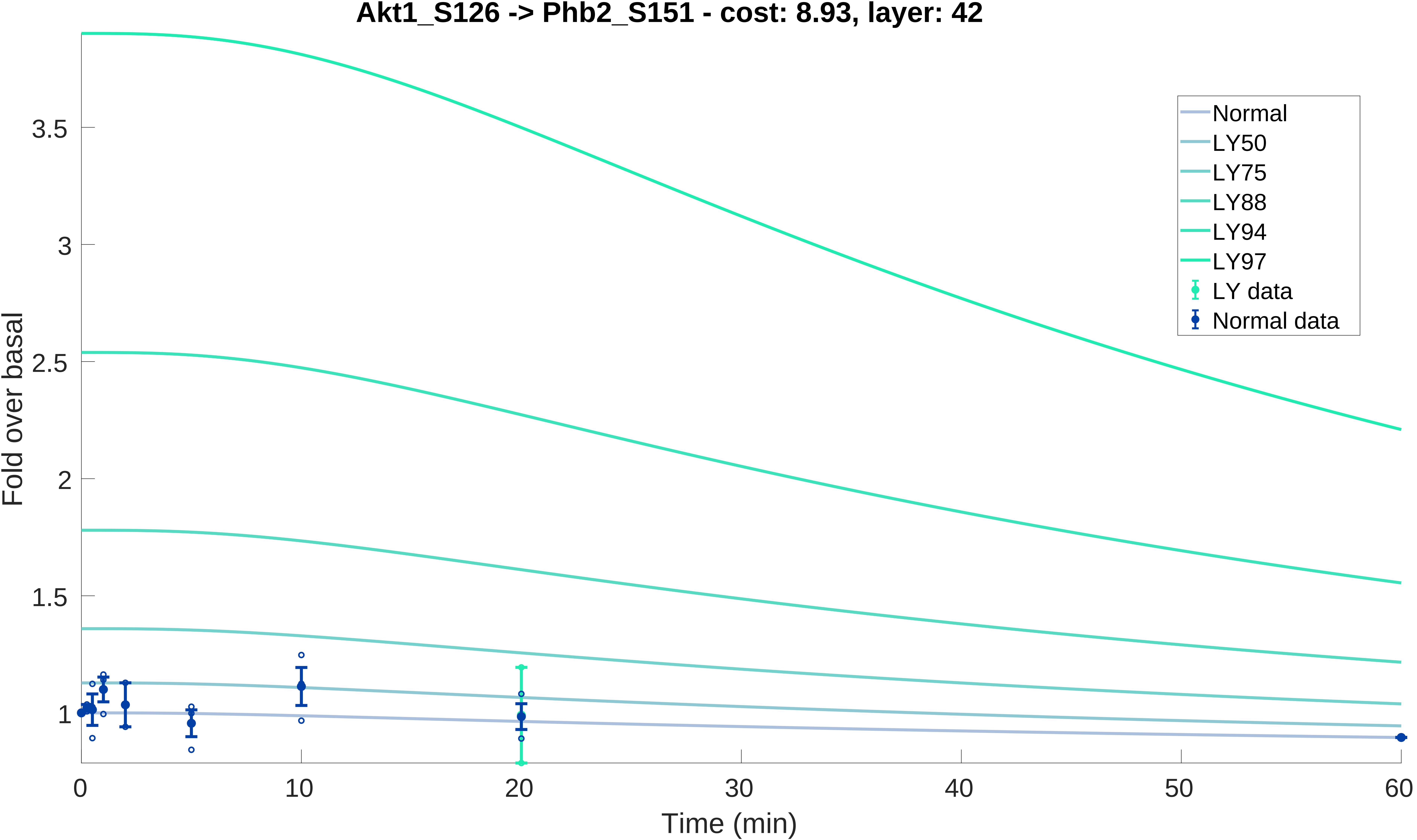
Akt1_S126 -> Phb2_S151 - cost: 8.93, layer: 42
Fold over basal
Time (min)
Normal
LY50
LY75
LY88
LY94
LY97
LY data
Normal data
3.5
3
2.5
2
1.5
1
0
10
20
30
40
50
60

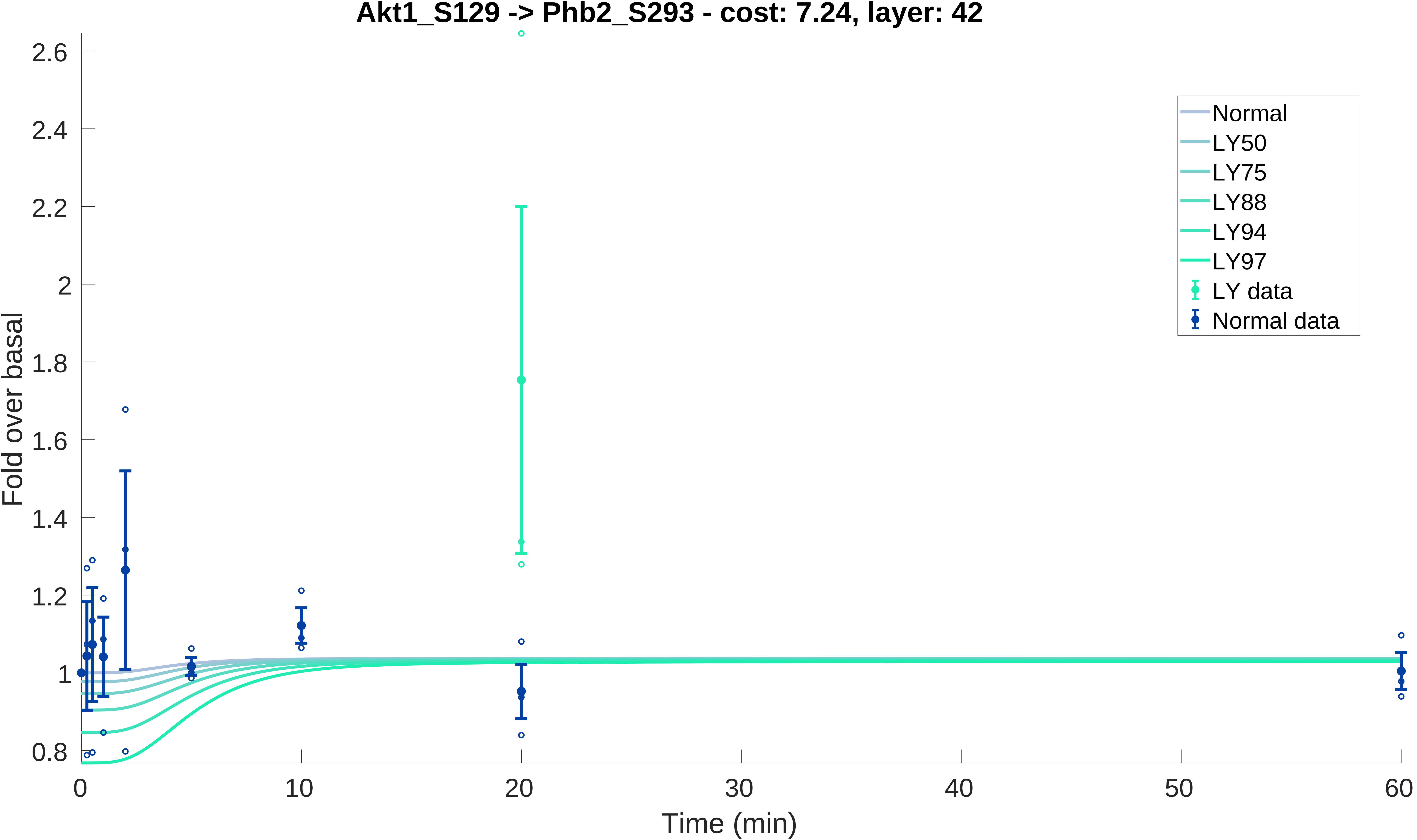

Akt1_S129 -> Phb2_S293 - cost: 7.24, layer: 42
Fold over basal
Time (min)
2.6
2.4
2.2
2
1.8
1.6
1.4
1.2
1
0.8
0
10
20
30
40
50
60
Normal
LY50
LY75
LY88
LY94
LY97
LY data
Normal data

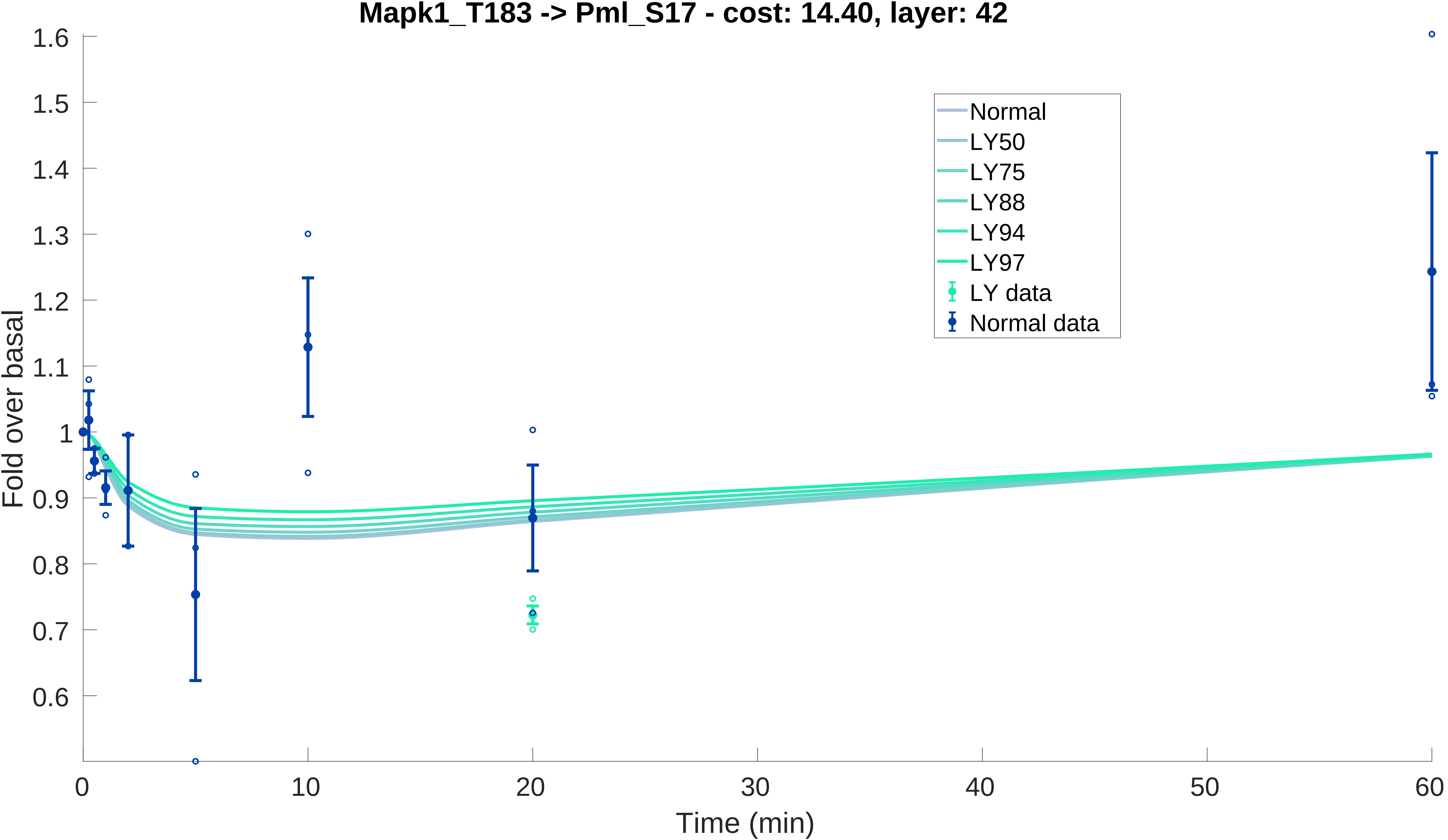

Mapk1_T183 -> Pml_S17 - cost: 14.40, layer: 42
Fold over basal
Time (min)
Normal
LY50
LY75
LY88
LY94
LY97
LY data
Normal data
1.6
1.5
1.4
1.3
1.2
1.1
1
0.9
0.8
0.7
0.6
0
10
20
30
40
50
60

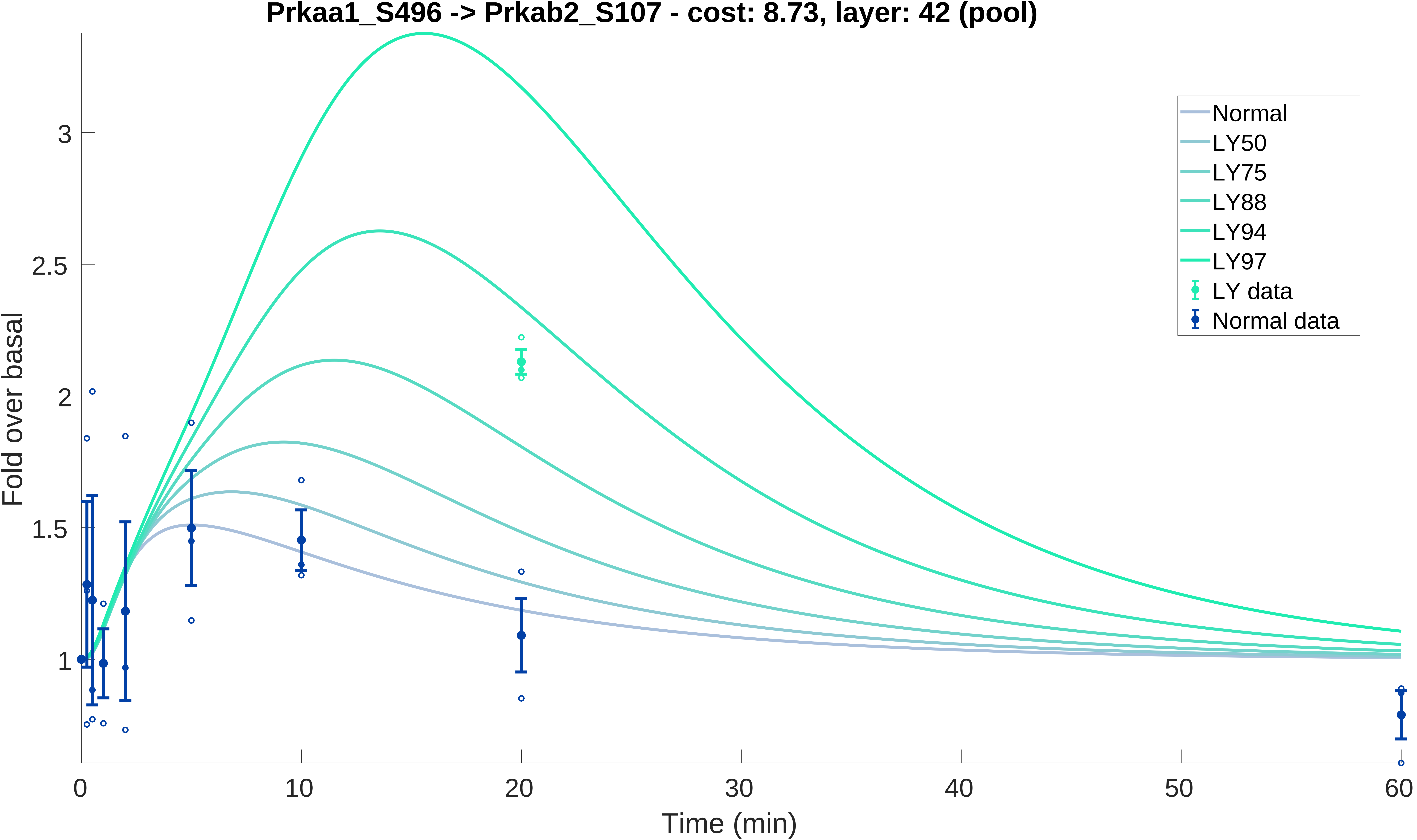

Prrkaa1_S496 -> Prkab2_S107 - cost: 8.73, layer: 42 (pool)
Fold over basal
Time (min)
3
2.5
2
1.5
1
0
10
20
30
40
50
60
Normal
LY50
LY75
LY88
LY94
LY97
LY data
Normal data

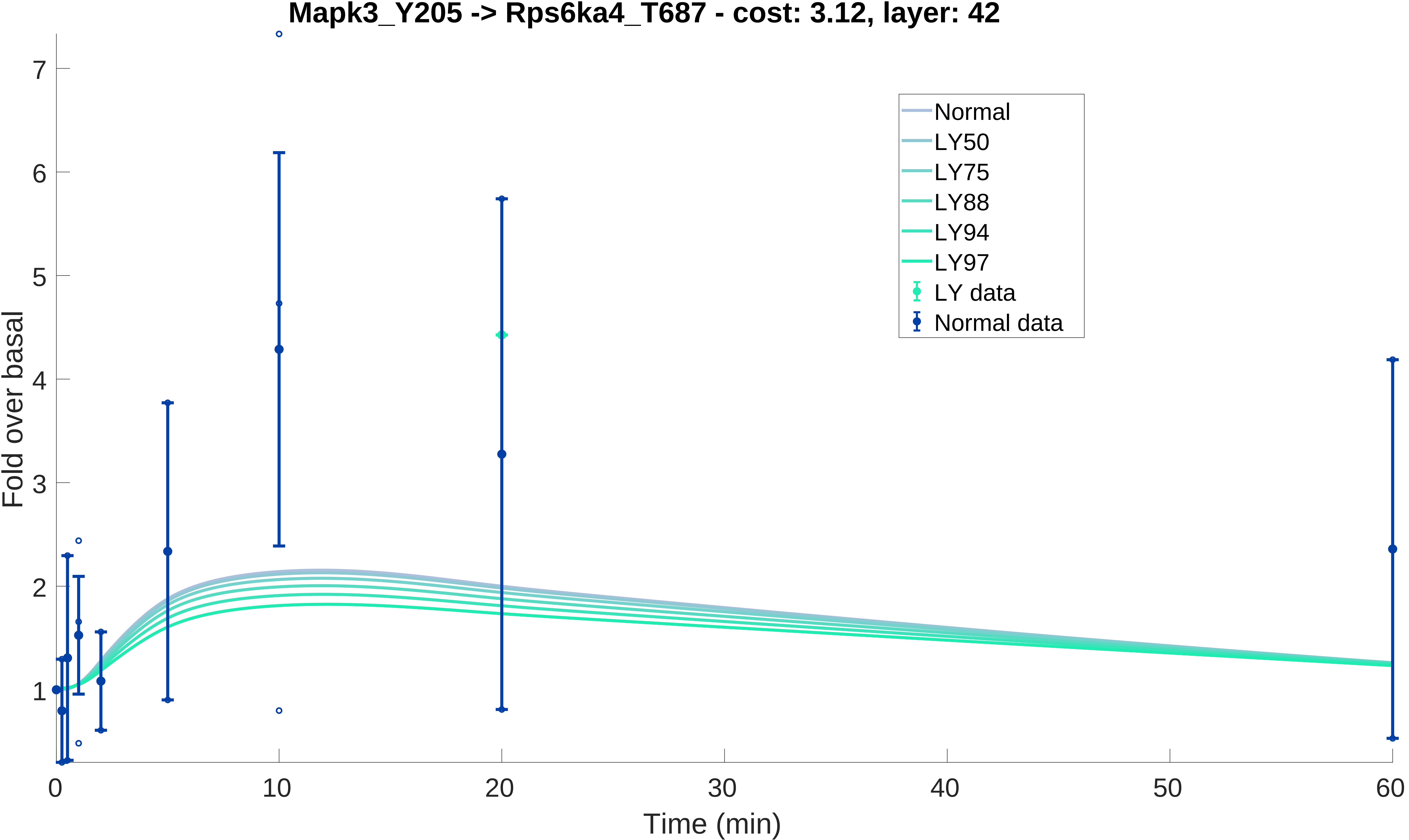
Mapk3_Y205 -> Rps6ka4_T687 - cost: 3.12, layer: 42
Fold over basal
Time (min)
0
10
20
30
40
50
60
1
2
3
4
5
6
7
Normal
LY50
LY75
LY88
LY94
LY97
LY data
Normal data

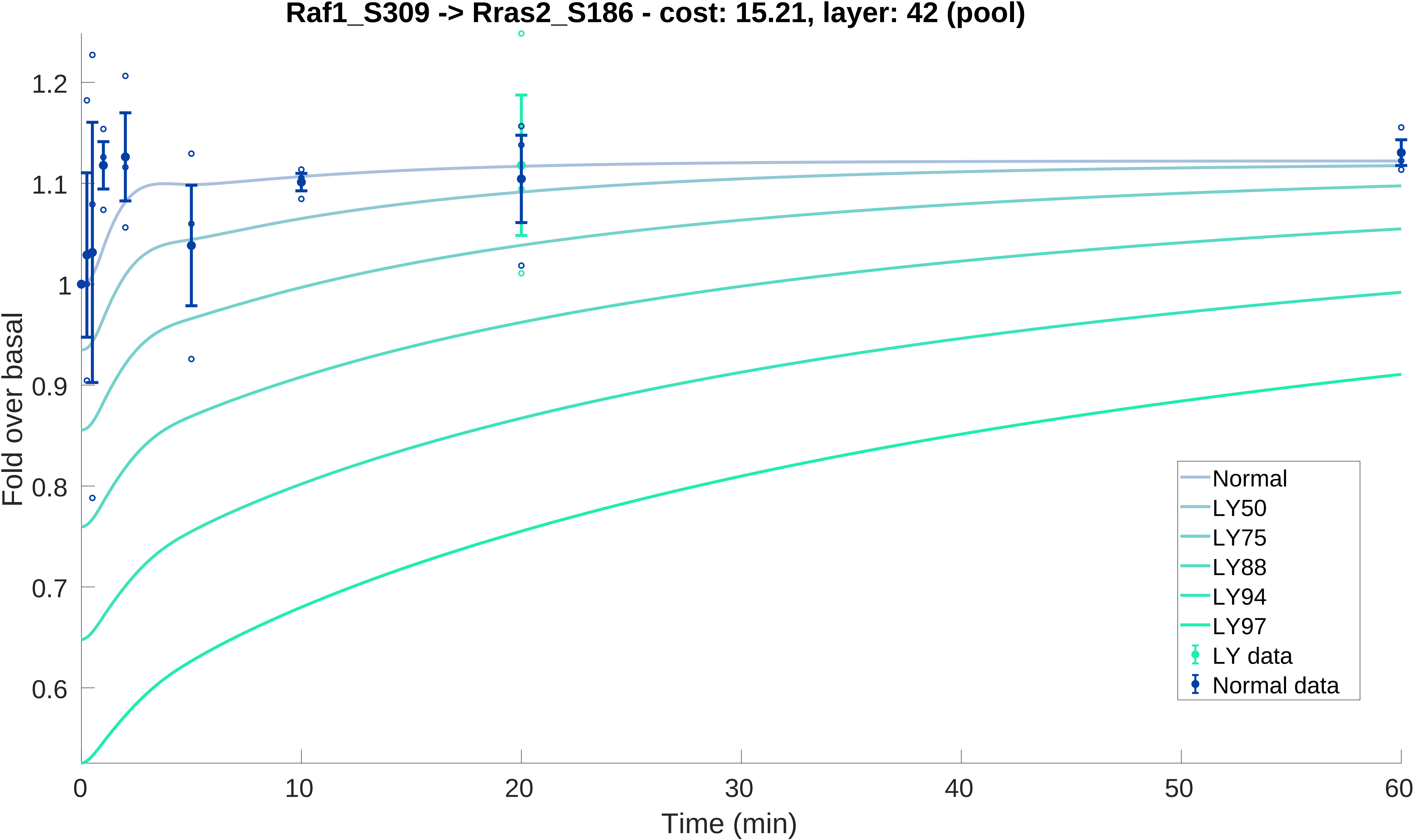

Raf1_S309 -> Rras2_S186 - cost: 15.21, layer: 42 (pool)
Fold over basal
Time (min)
0
10
20
30
40
50
60
0.6
0.7
0.8
0.9
1
1.1
1.2
Normal
LY50
LY75
LY88
LY94
LY97
LY data
Normal data

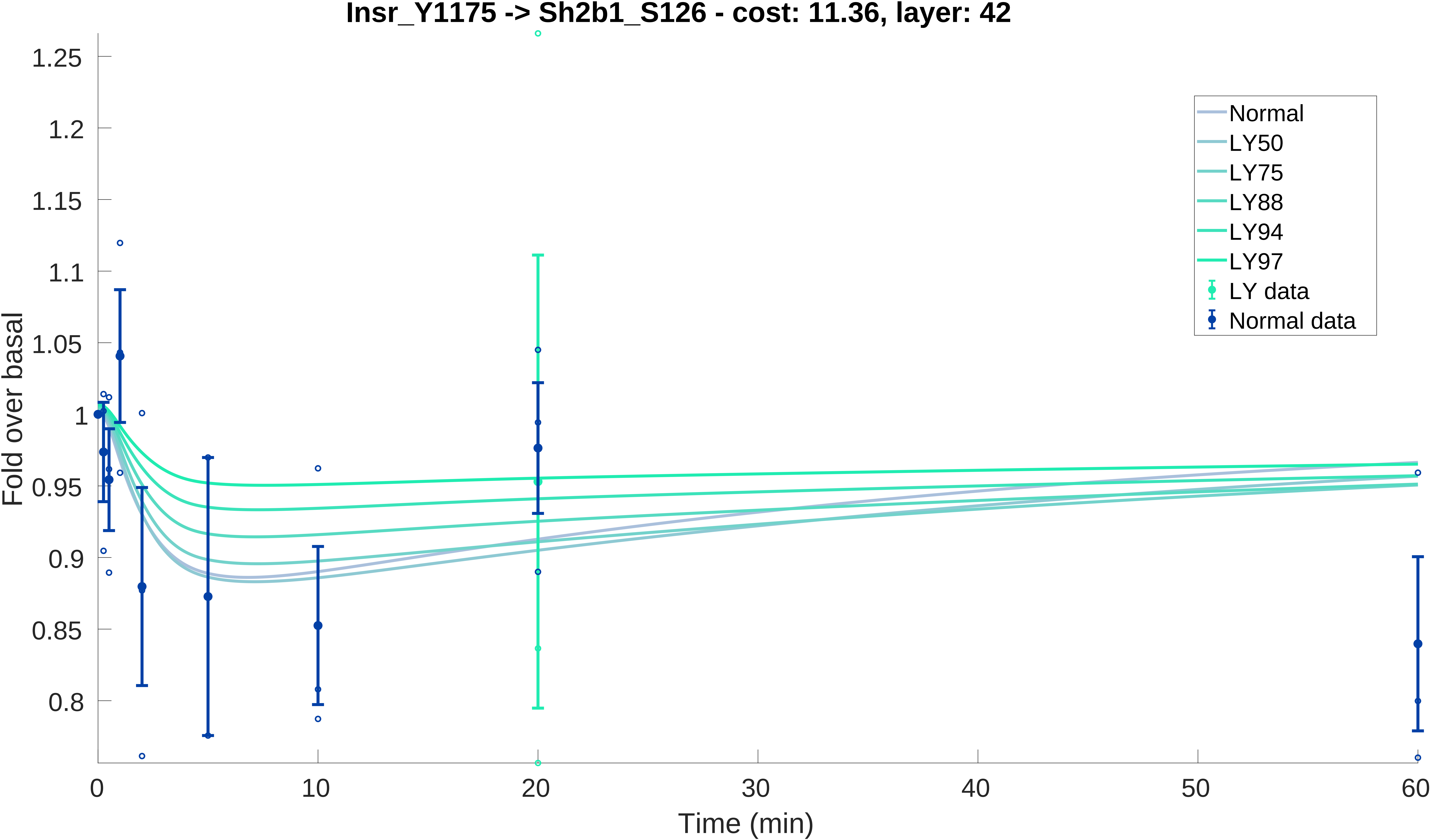
Insr_Y1175 -> Sh2b1_S126 - cost: 11.36, layer: 42
Fold over basal
Time (min)
Normal
LY50
LY75
LY88
LY94
LY97
LY data
Normal data
1.25
1.2
1.15
1.1
1.05
1
0.95
0.9
0.85
0.8
0
10
20
30
40
50
60

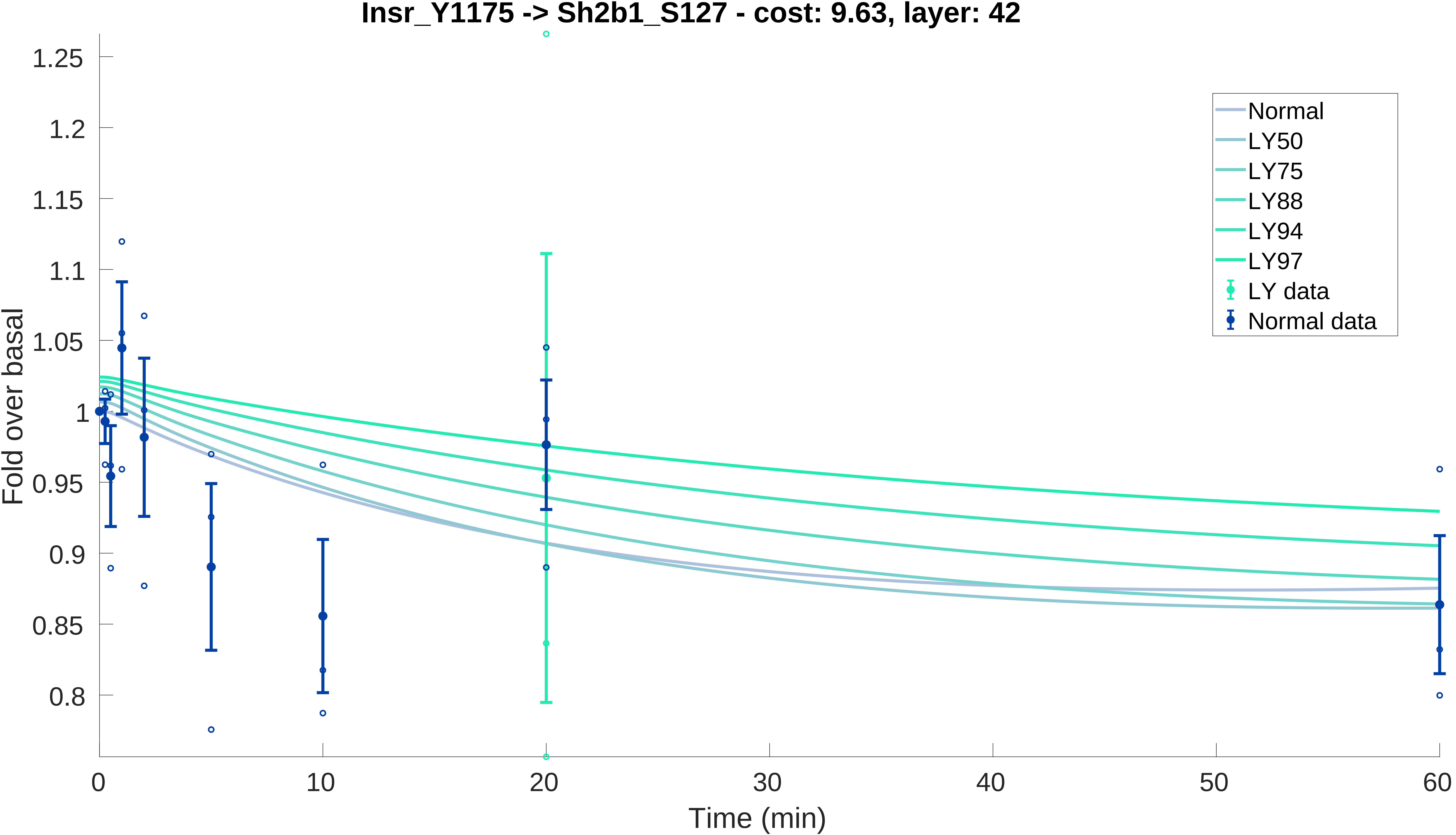
Insr_Y1175 -> Sh2b1_S127 - cost: 9.63, layer: 42
Fold over basal
Time (min)
1.25
1.2
1.15
1.1
1.05
1
0.95
0.9
0.85
0.8
0
10
20
30
40
50
60
Normal
LY50
LY75
LY88
LY94
LY97
LY data
Normal data

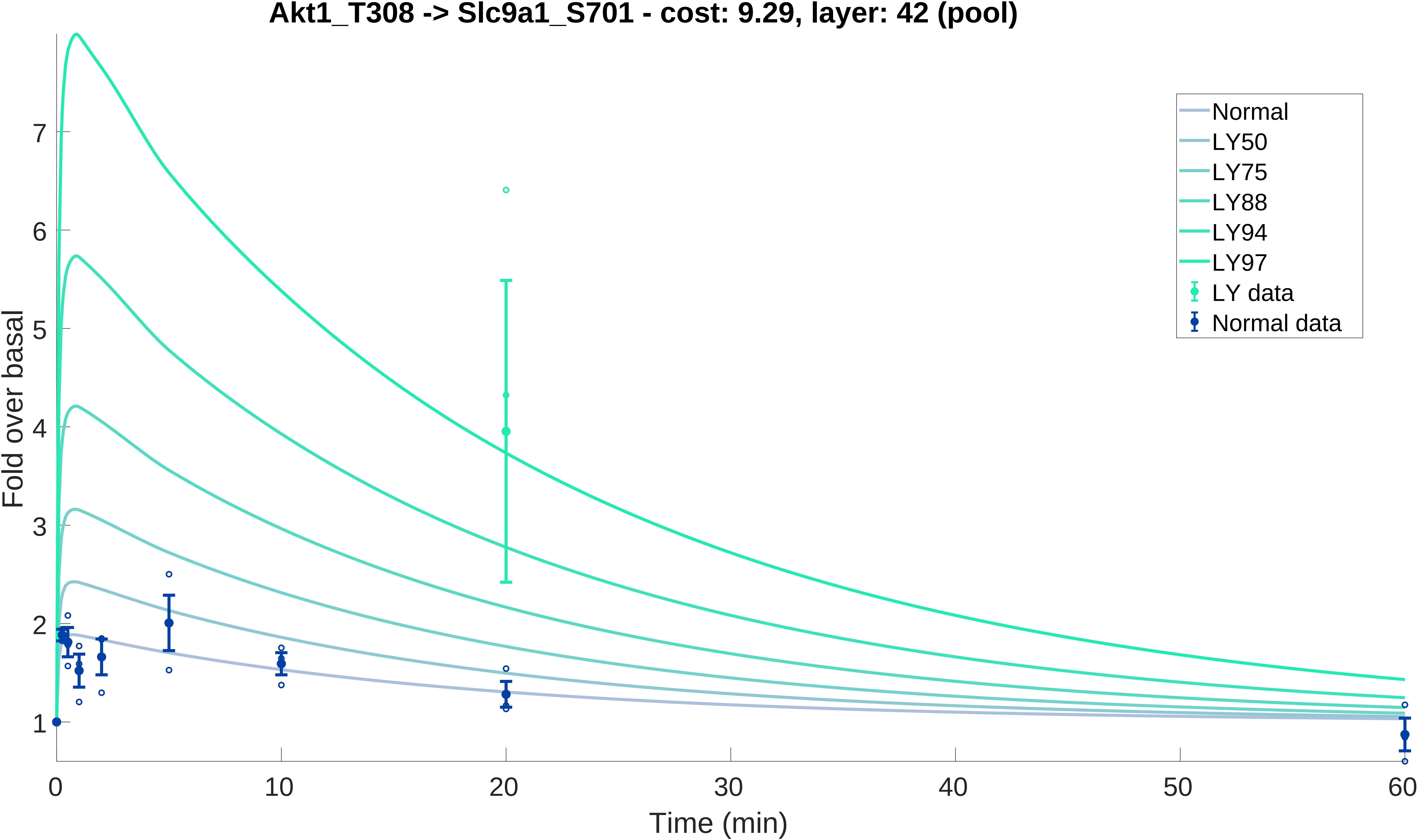

Akt1_T308 -> Slc9a1_S701 - cost: 9.29, layer: 42 (pool)
Fold over basal
Time (min)
0
10
20
30
40
50
60
1
2
3
4
5
6
7
Normal
LY50
LY75
LY88
LY94
LY97
LY data
Normal data

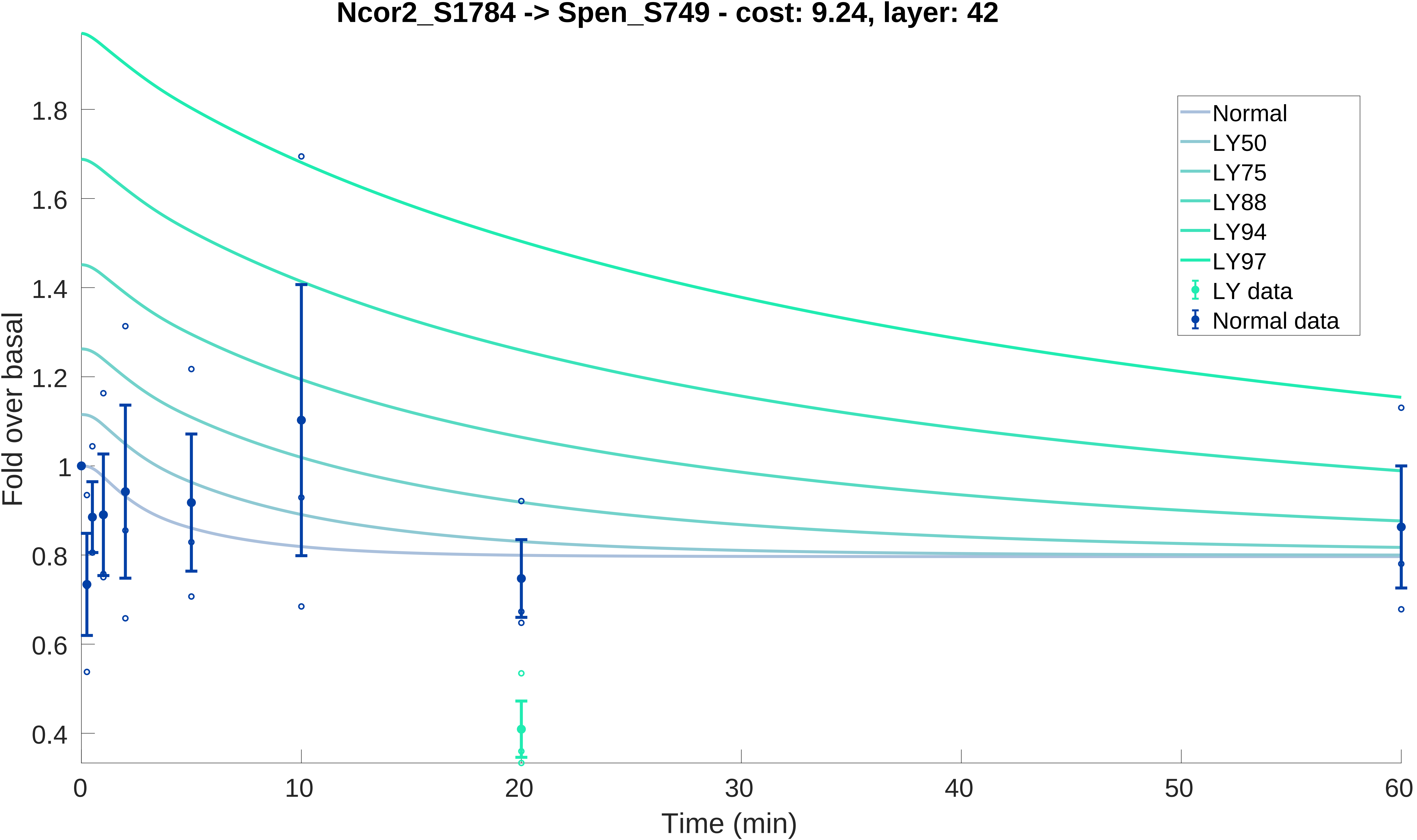
Ncor2_S1784 -> Spen_S749 - cost: 9.24, layer: 42
Fold over basal
Time (min)
Normal
LY50
LY75
LY88
LY94
LY97
LY data
Normal data
0.4
0.6
0.8
1
1.2
1.4
1.6
1.8
0
10
20
30
40
50
60

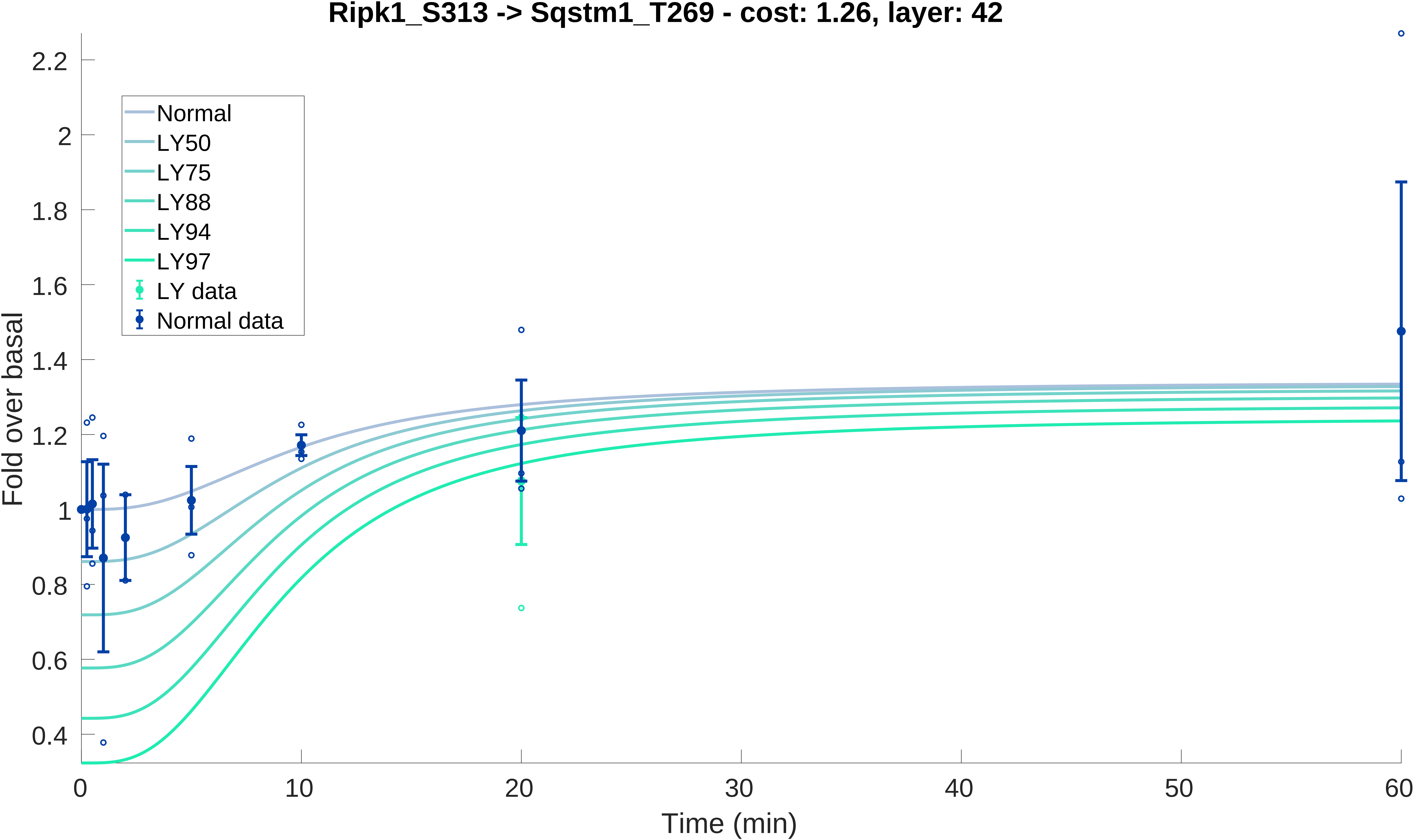

Ripk1_S313 -> Sqstm1_T269 - cost: 1.26, layer: 42
Normal
LY50
LY75
LY88
LY94
LY97
LY data
Normal data
Fold over basal
Time (min)
0.4
0.6
0.8
1
1.2
1.4
1.6
1.8
2
2.2
0
10
20
30
40
50
60

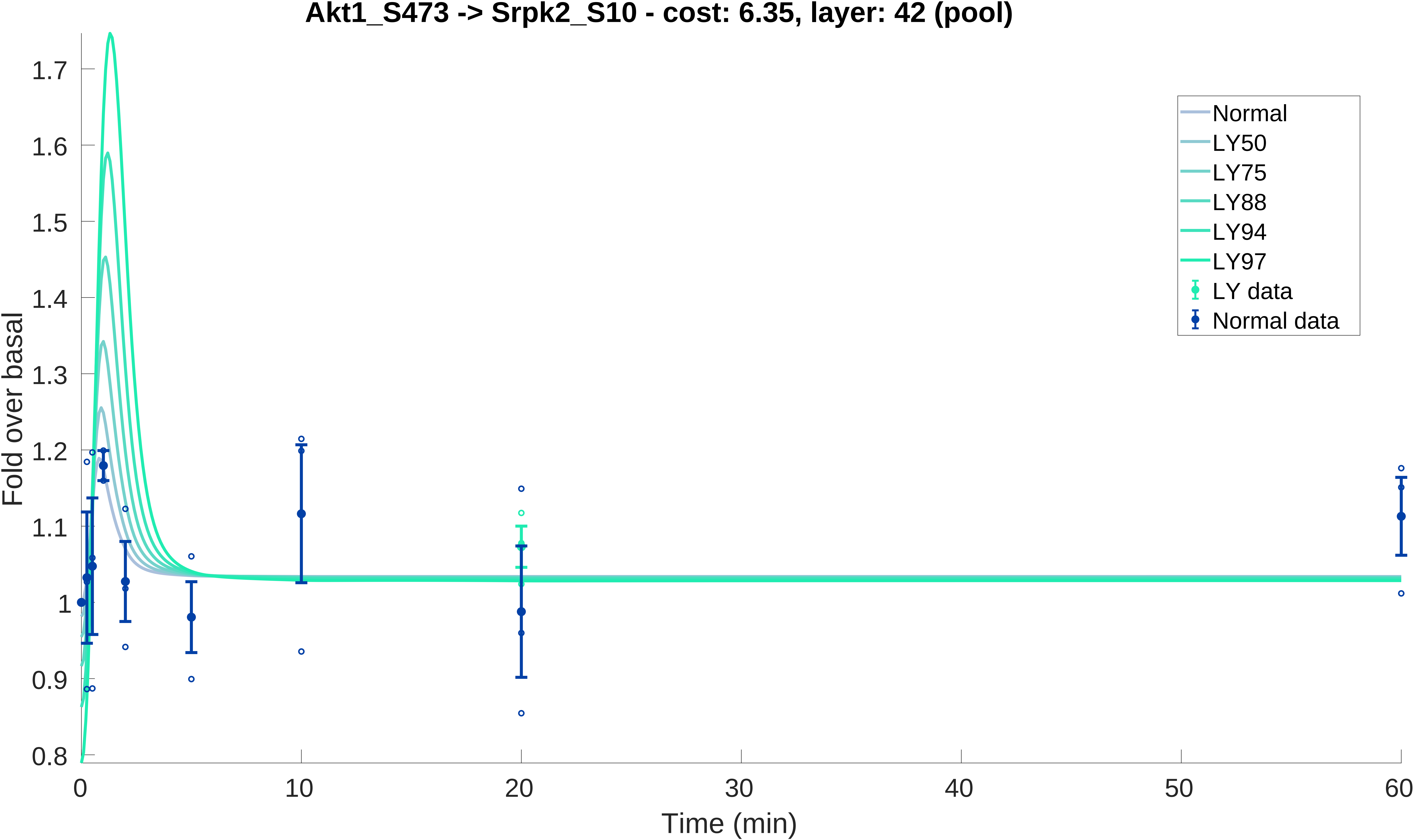

Akt1_S473 -> Srpk2_S10 - cost: 6.35, layer: 42 (pool)
Fold over basal
Time (min)
0.8
0.9
1
1.1
1.2
1.3
1.4
1.5
1.6
1.7
0
10
20
30
40
50
60
Normal
LY50
LY75
LY88
LY94
LY97
LY data
Normal data

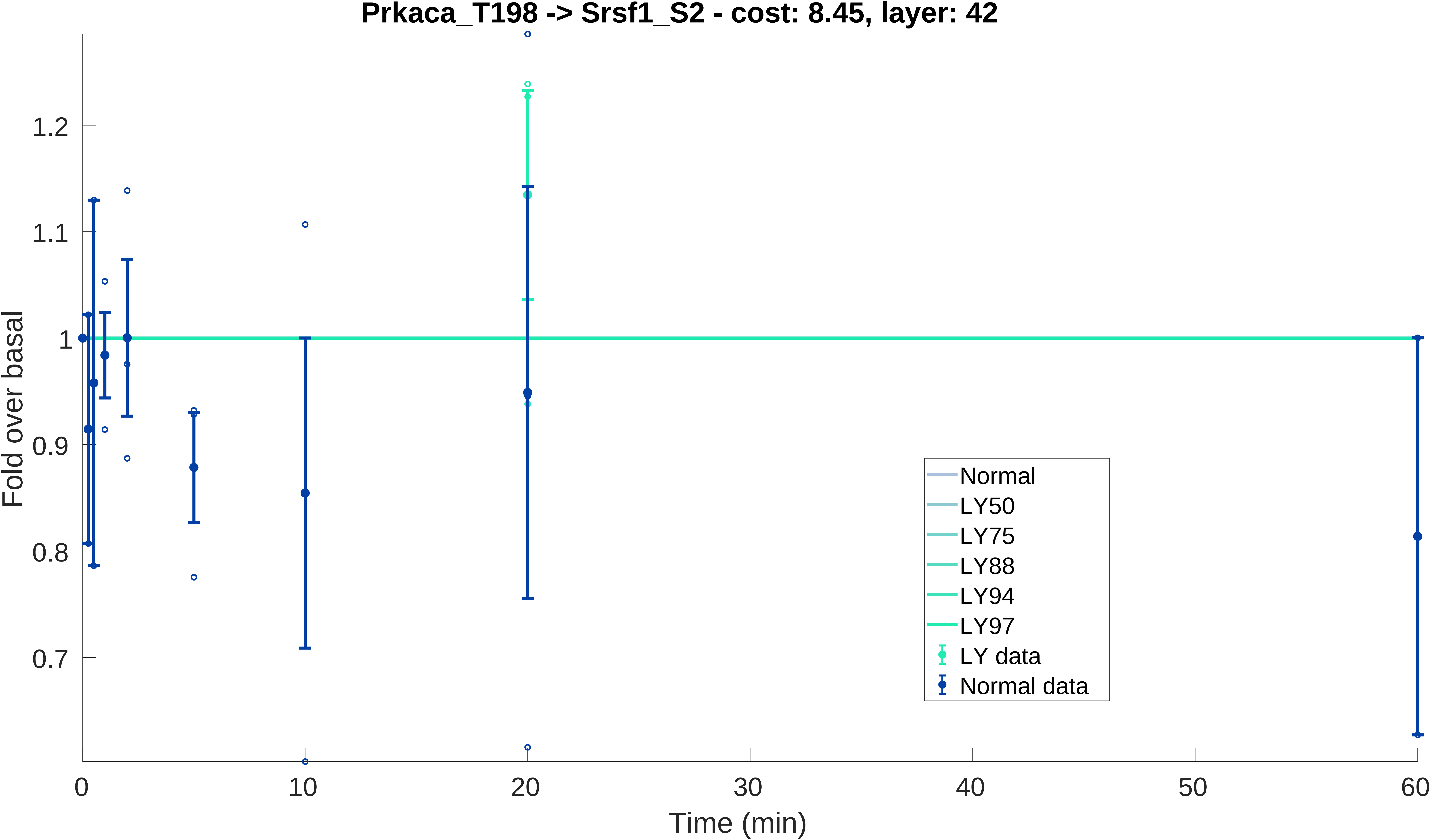

Prkaca_T198 -> Srsf1_S2 - cost: 8.45, layer: 42
Fold over basal
Time (min)
1.2
1.1
1
0.9
0.8
0.7
0
10
20
30
40
50
60
Normal
LY50
LY75
LY88
LY94
LY97
LY data
Normal data

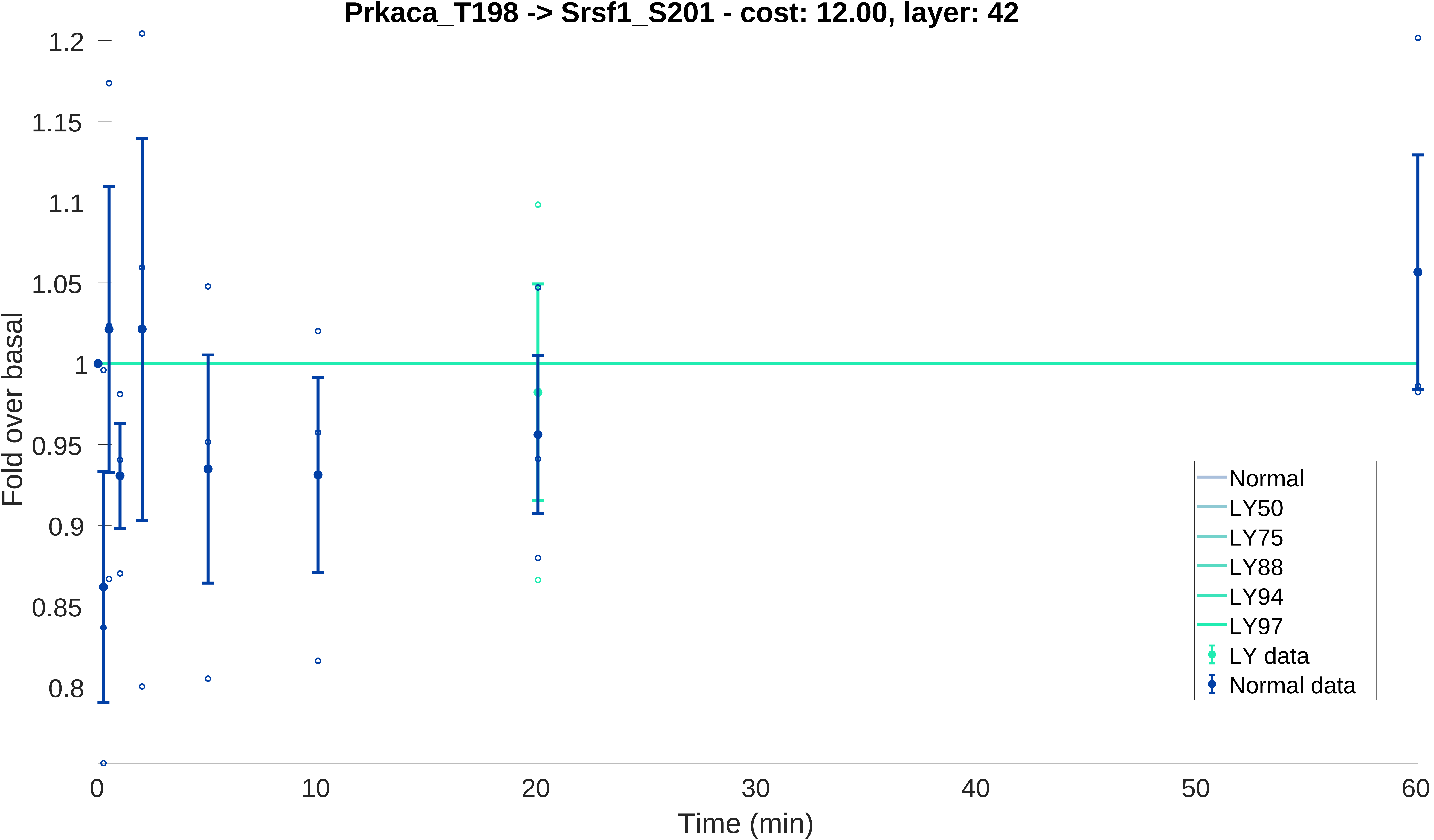

Prkaca_T198 -> Srsf1_S201 - cost: 12.00, layer: 42
Fold over basal
Time (min)
1.2
1.15
1.1
1.05
1
0.95
0.9
0.85
0.8
0
10
20
30
40
50
60
Normal
LY50
LY75
LY88
LY94
LY97
LY data
Normal data

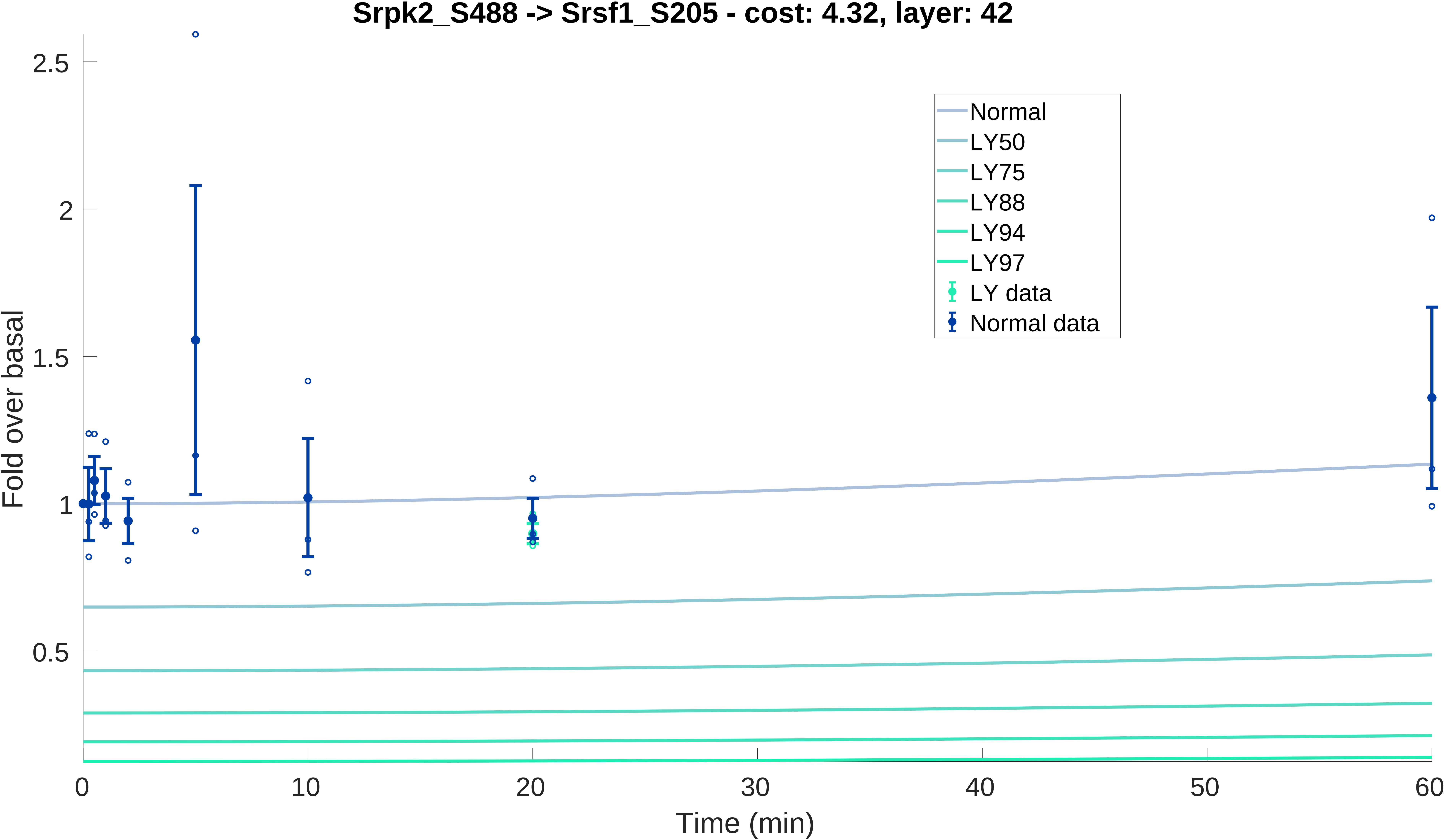

Srpk2_S488 -> Srsf1_S205 - cost: 4.32, layer: 42
Fold over basal
Time (min)
Normal
LY50
LY75
LY88
LY94
LY97
LY data
Normal data
0.5
1
1.5
2
2.5
0
10
20
30
40
50
60

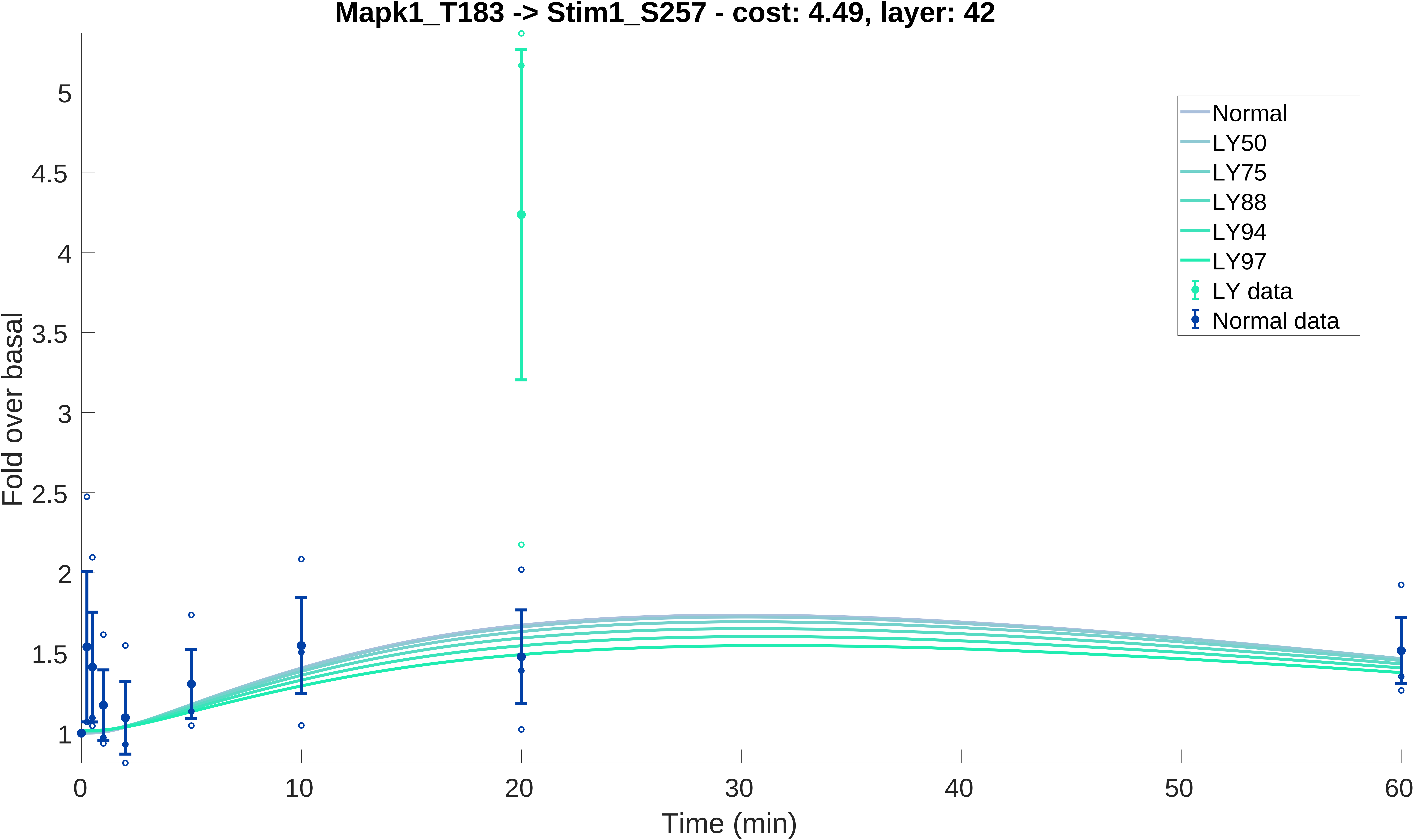
Mapk1_T183 -> Stim1_S257 - cost: 4.49, layer: 42
Fold over basal
Time (min)
0
10
20
30
40
50
60
1
1.5
2
2.5
3
3.5
4
4.5
5
Normal
LY50
LY75
LY88
LY94
LY97
LY data
Normal data

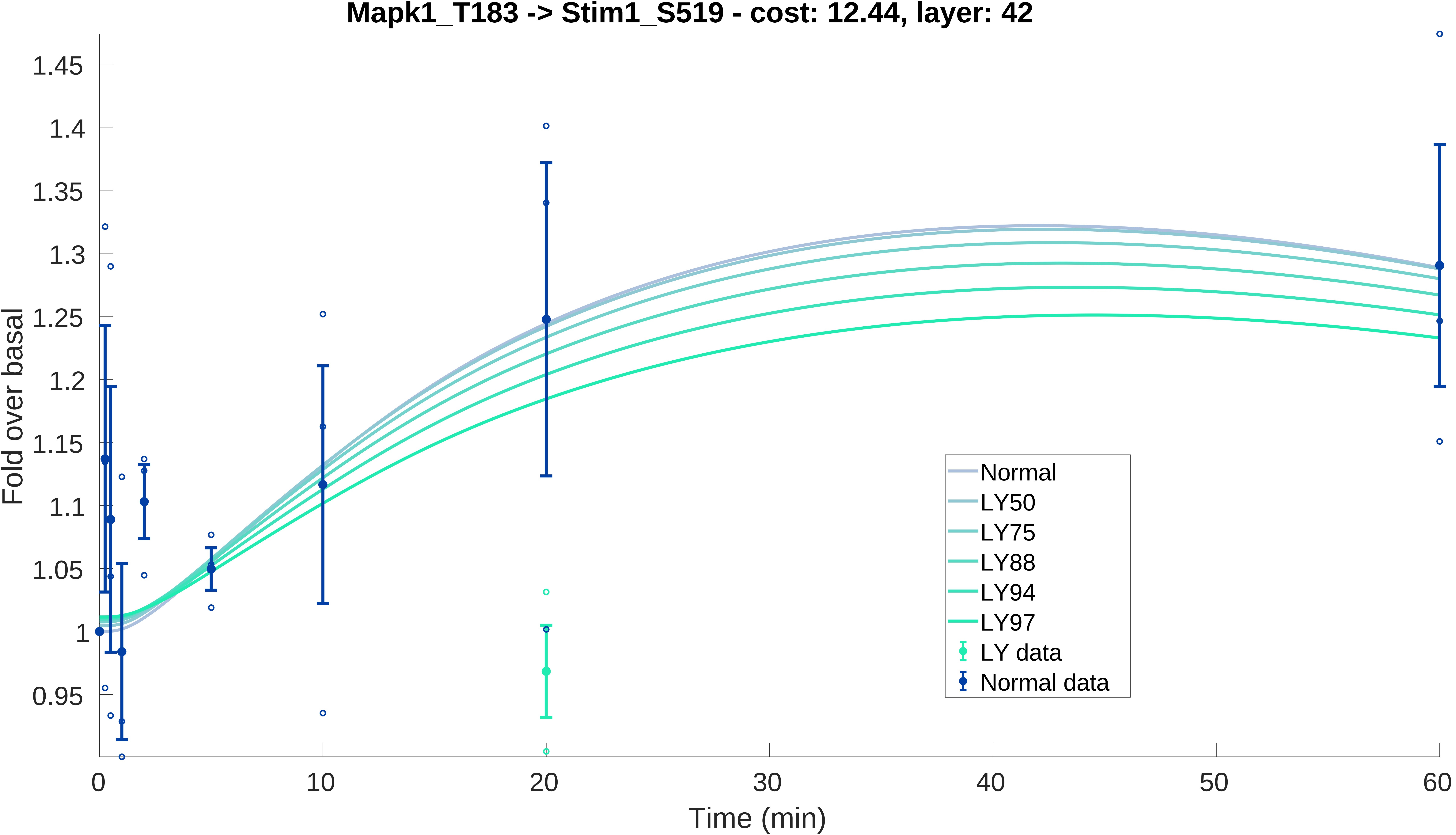

Mapk1_T183 -> Stim1_S519 - cost: 12.44, layer: 42
Fold over basal
Time (min)
1.45
1.4
1.35
1.3
1.25
1.2
1.15
1.1
1.05
1
0.95
0
10
20
30
40
50
60
Normal
LY50
LY75
LY88
LY94
LY97
LY data
Normal data

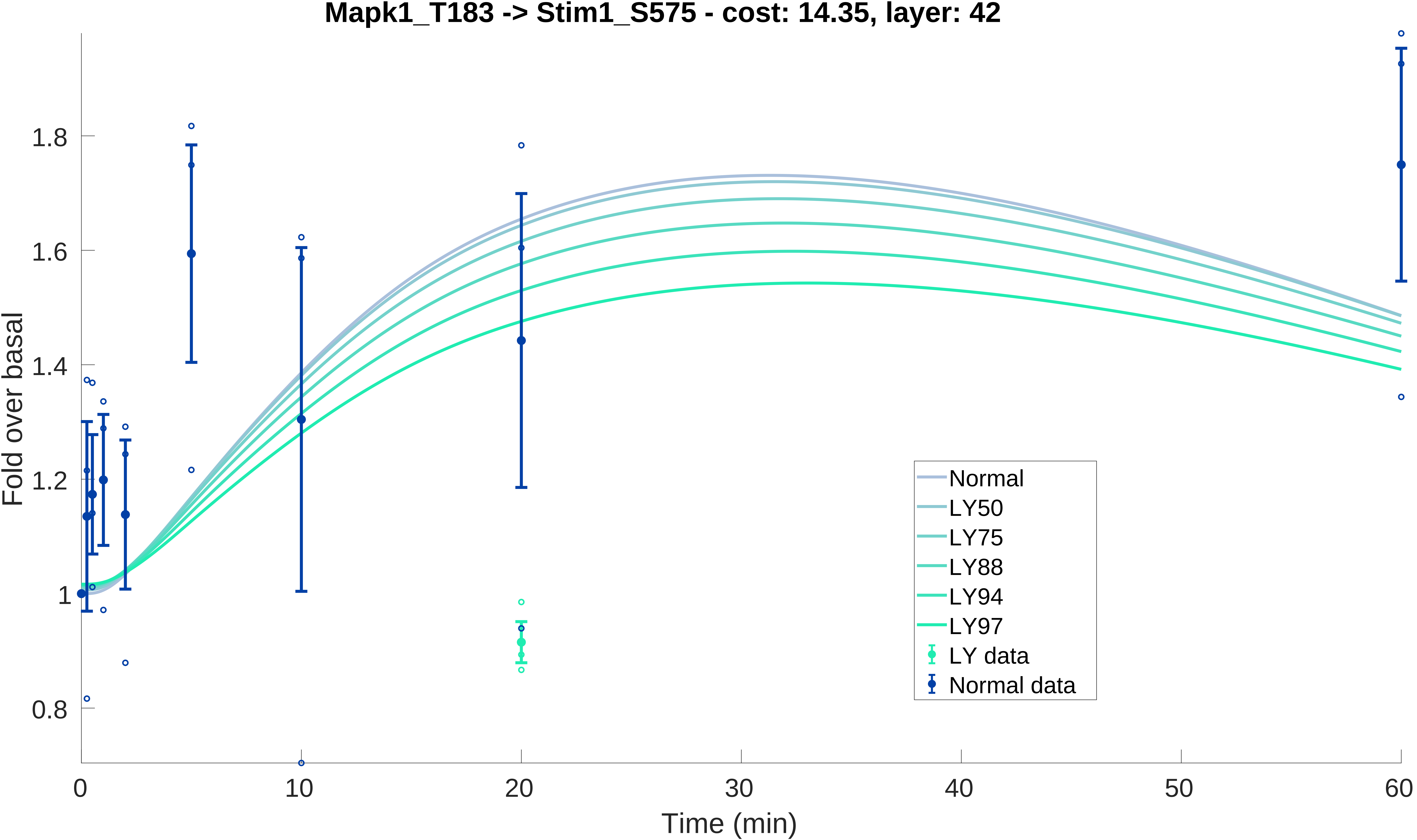

Mapk1_T183 -> Stim1_S575 - cost: 14.35, layer: 42
Fold over basal
1.8
1.6
1.4
1.2
1
0.8
0
10
20
30
40
50
60
Time (min)
Normal
LY50
LY75
LY88
LY94
LY97
LY data
Normal data

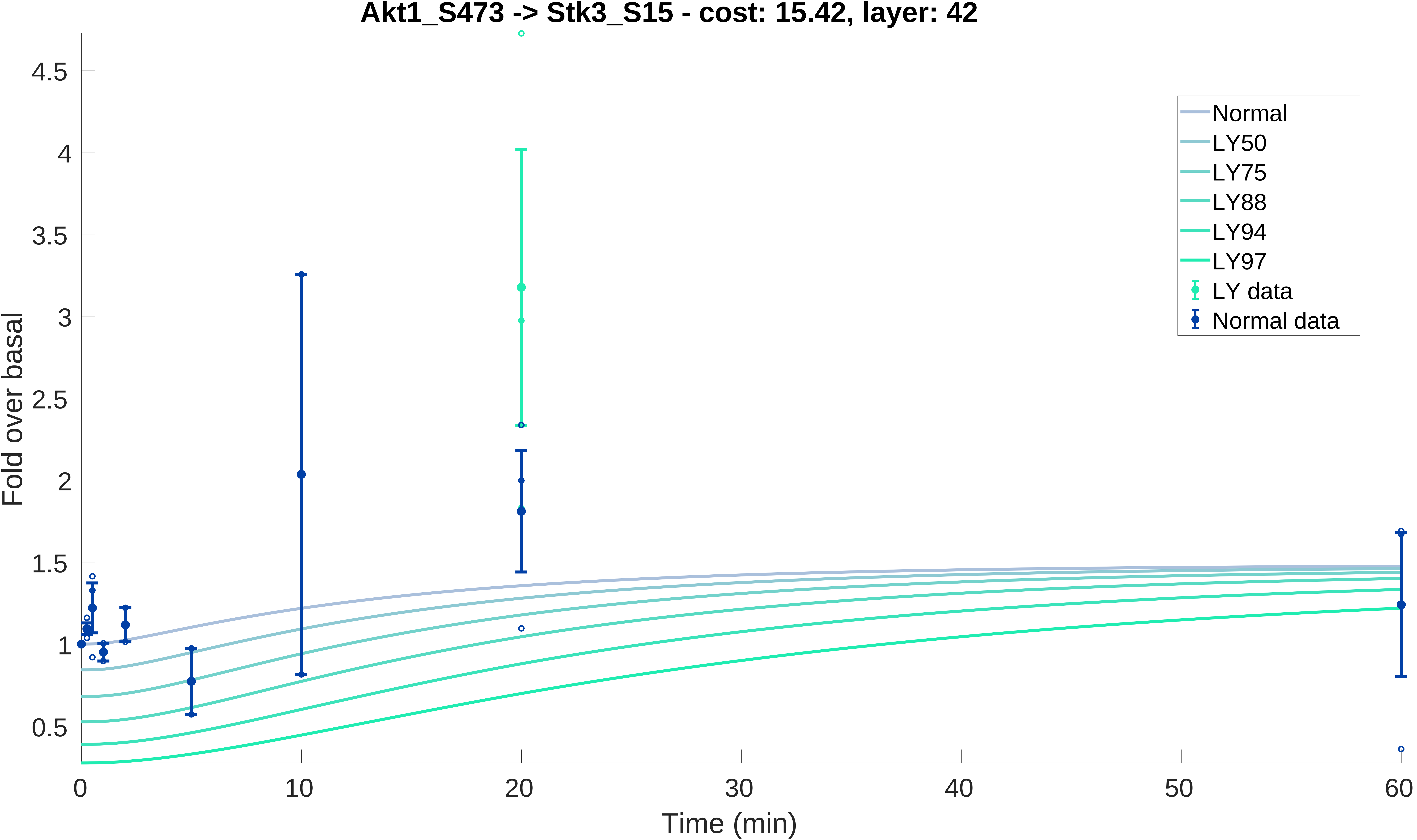

Akt1_S473 -> Stk3_S15 - cost: 15.42, layer: 42
Fold over basal
Time (min)
0.5
1
1.5
2
2.5
3
3.5
4
4.5
0
10
20
30
40
50
60
Normal
LY50
LY75
LY88
LY94
LY97
LY data
Normal data

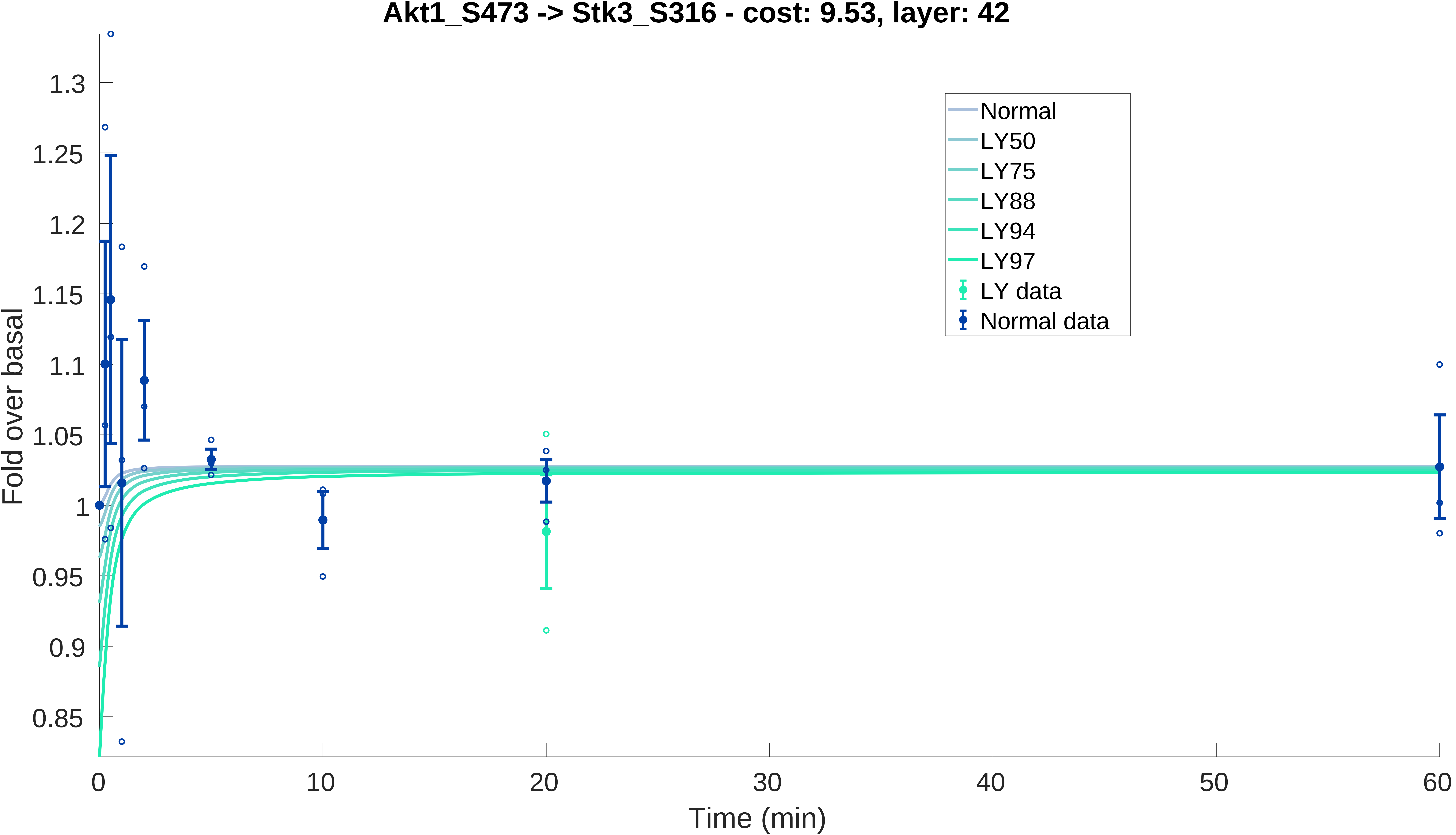

Akt1_S473 -> Stk3_S316 - cost: 9.53, layer: 42
Fold over basal
Time (min)
1.3
1.25
1.2
1.15
1.1
1.05
1
0.95
0.9
0.85
0
10
20
30
40
50
60
Normal
LY50
LY75
LY88
LY94
LY97
LY data
Normal data

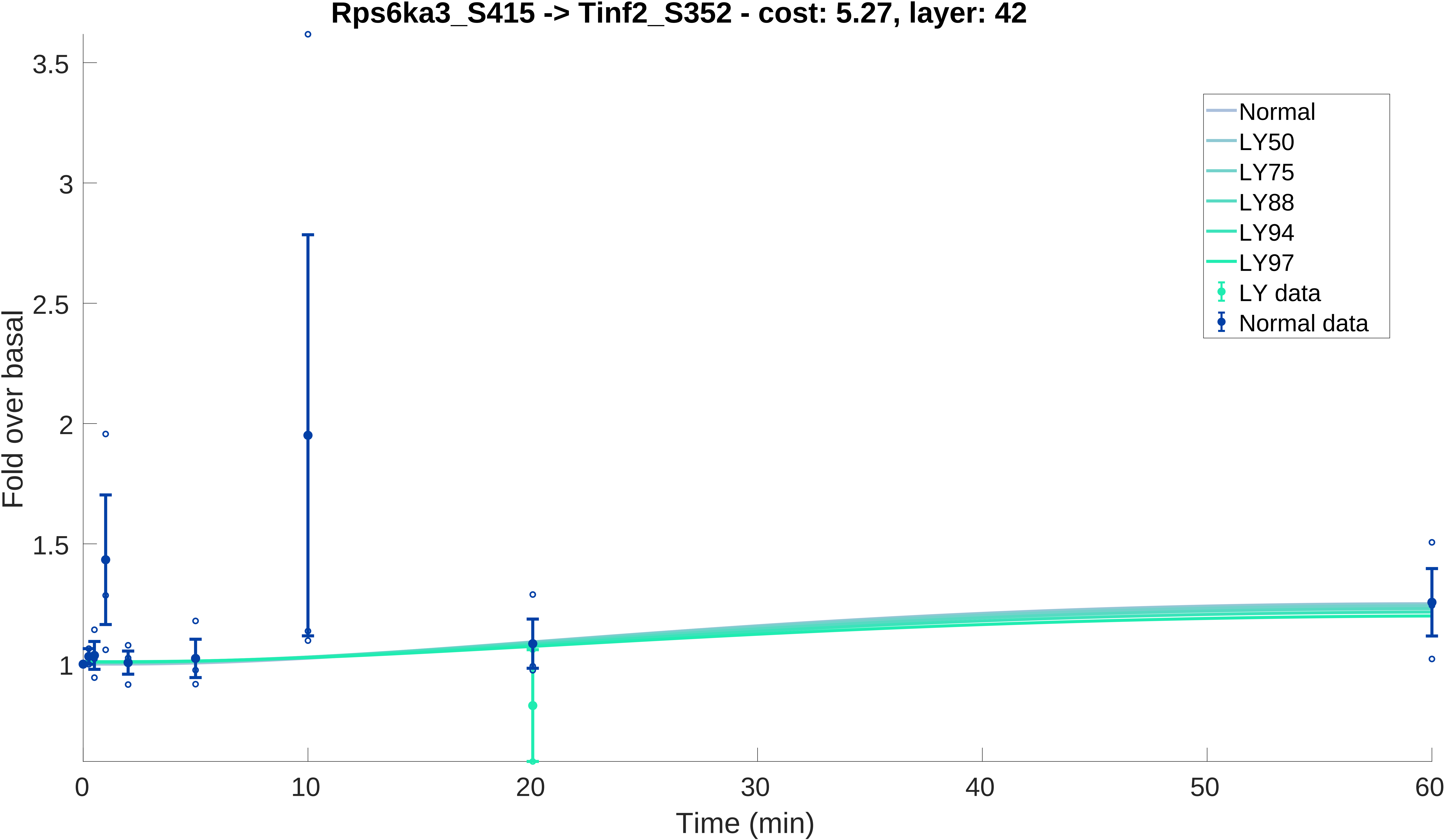

Rps6ka3_S415 -> Tinf2_S352 - cost: 5.27, layer: 42
Fold over basal
Time (min)
3.5
3
2.5
2
1.5
1
0
10
20
30
40
50
60
Normal
LY50
LY75
LY88
LY94
LY97
LY data
Normal data

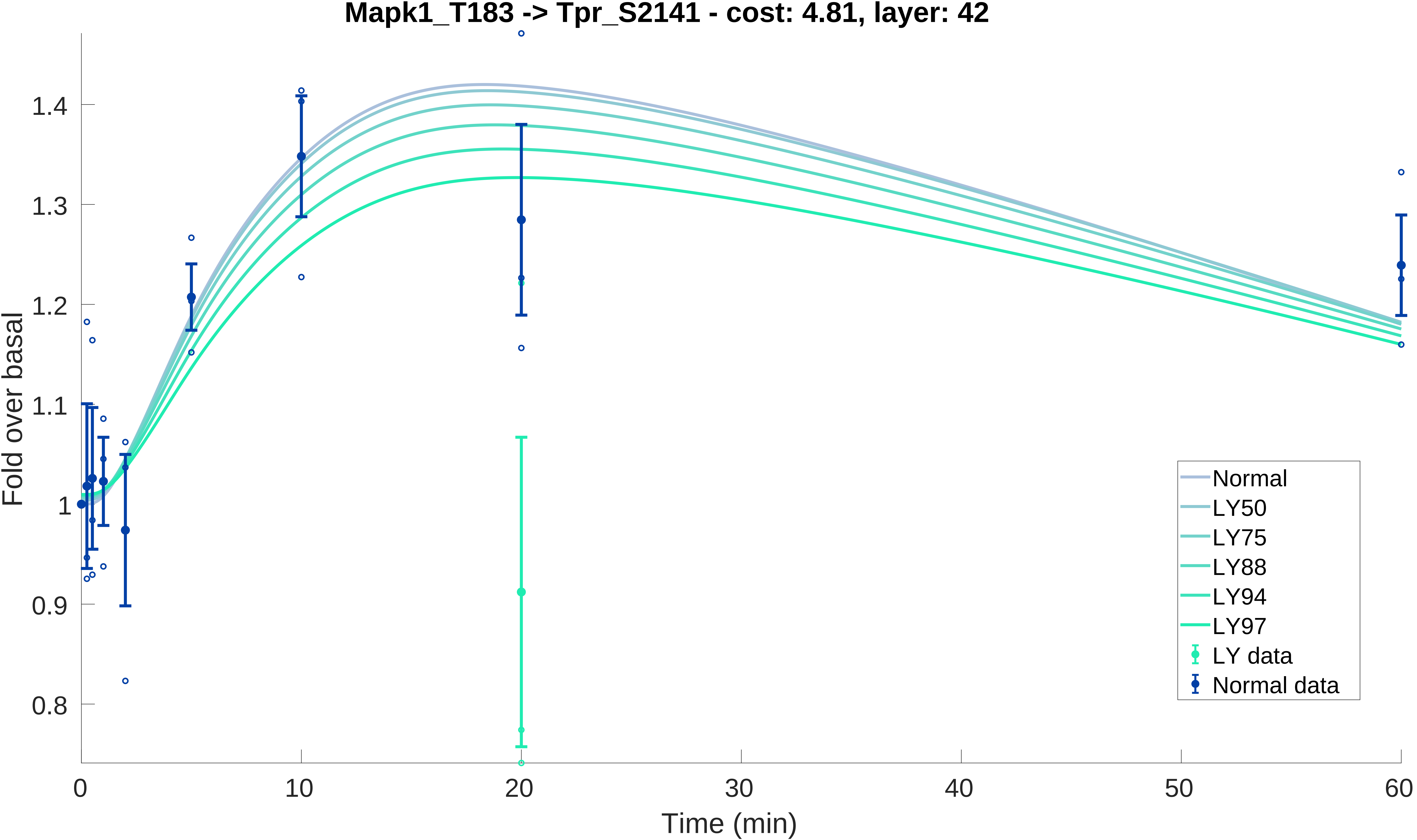

Mapk1_T183 -> Tpr_S2141 - cost: 4.81, layer: 42
Fold over basal
1.4
1.3
1.2
1.1
1
0.9
0.8
0
10
20
30
40
50
60
Time (min)
Normal
LY50
LY75
LY88
LY94
LY97
LY data
Normal data

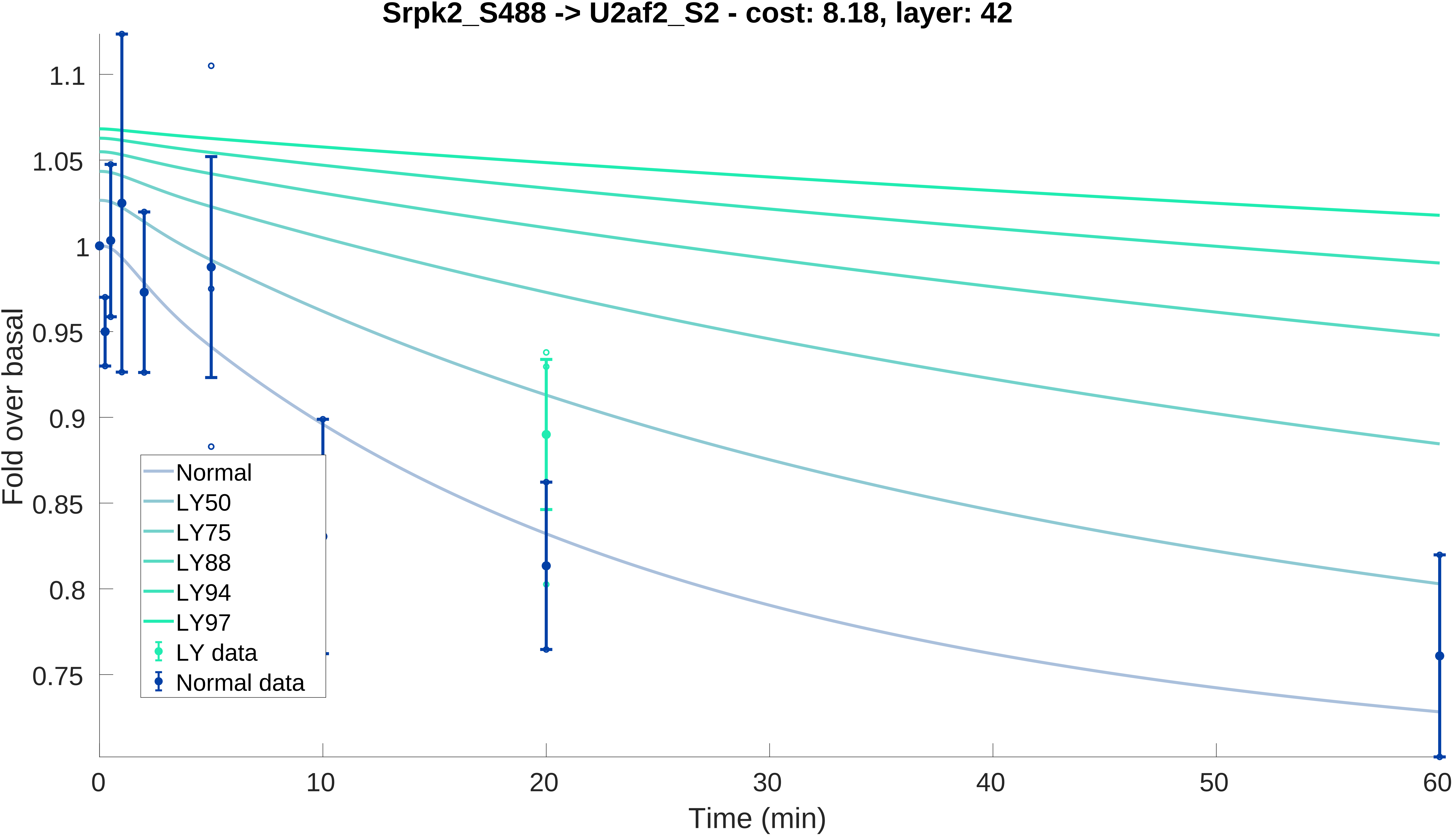

Srpk2_S488 -> U2af2_S2 - cost: 8.18, layer: 42
Fold over basal
Time (min)
1.1
1.05
1
0.95
0.9
0.85
0.8
0.75
0
10
20
30
40
50
60
Normal
LY50
LY75
LY88
LY94
LY97
LY data
Normal data

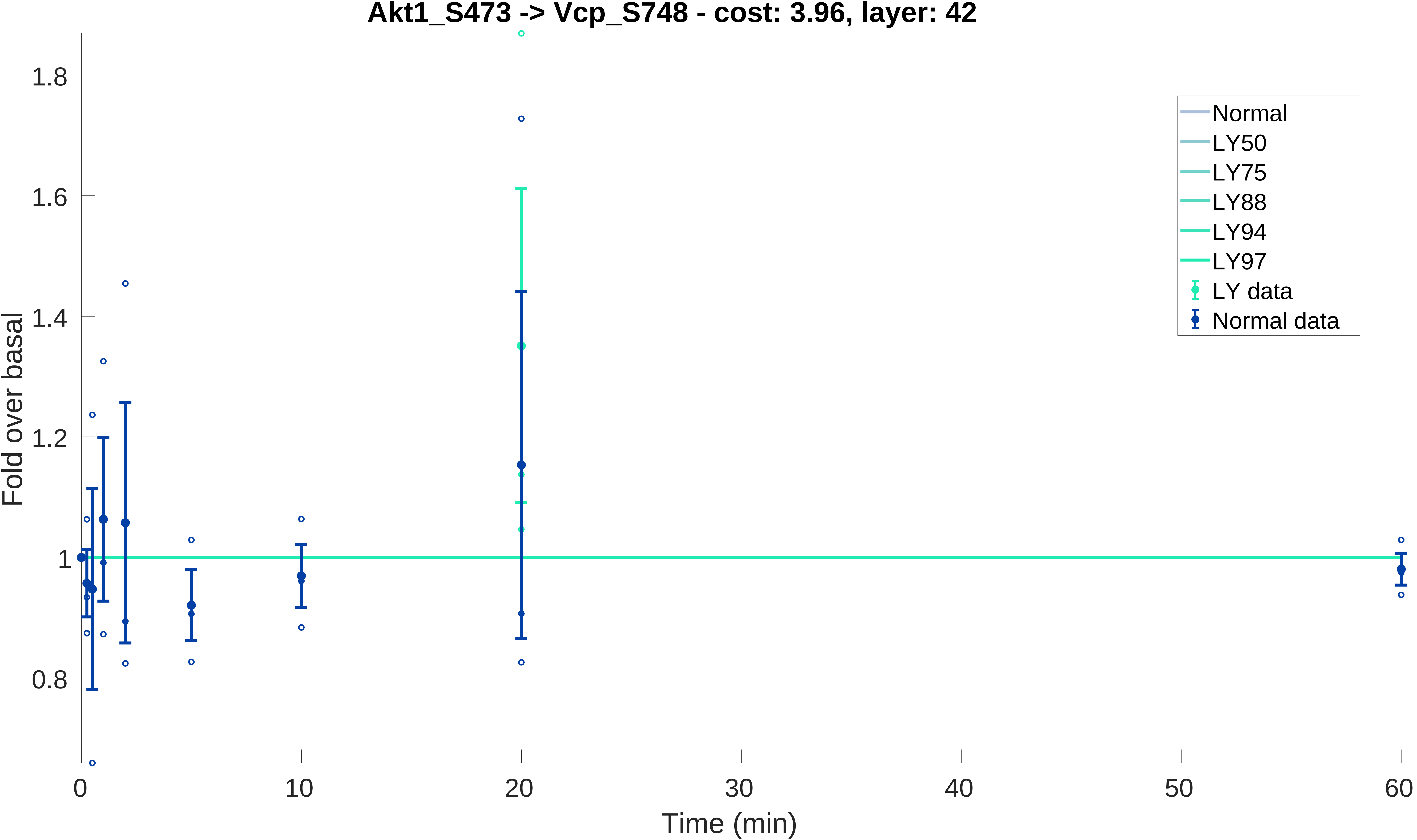

Akt1_S473 -> Vcp_S748 - cost: 3.96, layer: 42
Fold over basal
Time (min)
Normal
LY50
LY75
LY88
LY94
LY97
LY data
Normal data
1.8
1.6
1.4
1.2
1
0.8
0
10
20
30
40
50
60

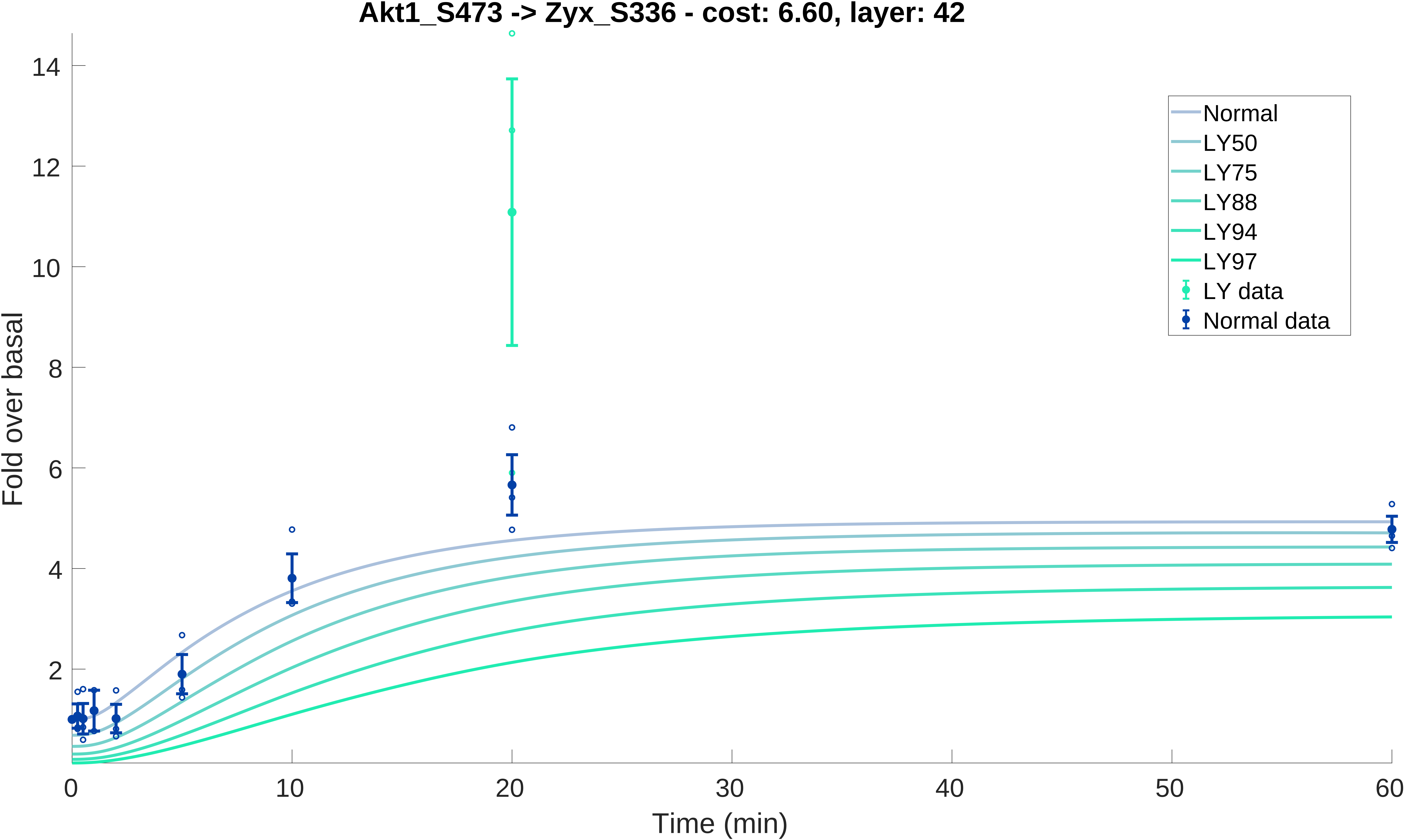

Akt1_S473 -> Zyx_S336 - cost: 6.60, layer: 42
Fold over basal
Time (min)
0
10
20
30
40
50
60
2
4
6
8
10
12
14
Normal
LY50
LY75
LY88
LY94
LY97
LY data
Normal data

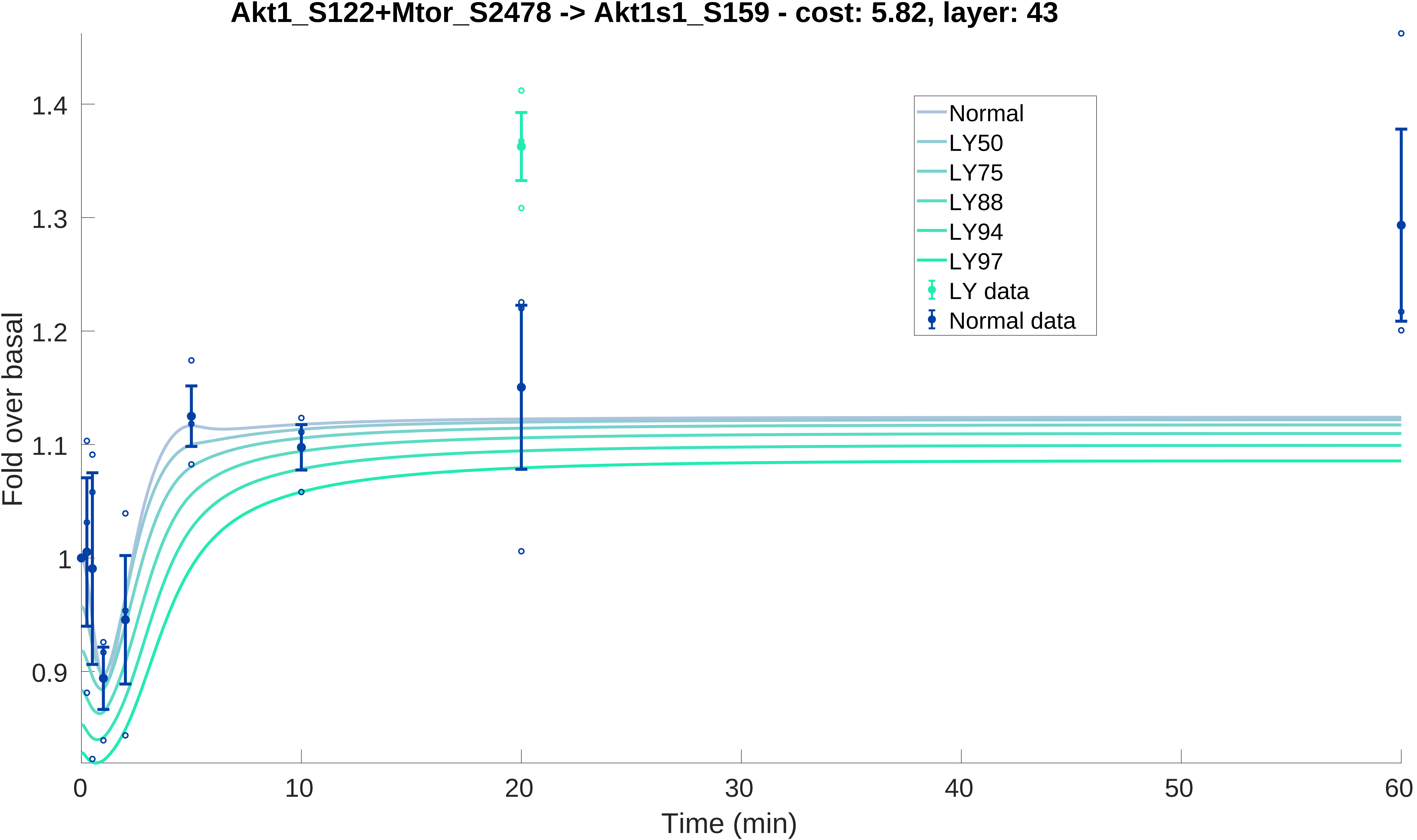
Akt1_S122+Mtor_S2478 -> Akt1s1_S159 - cost: 5.82, layer: 43
Fold over basal
1.4
1.3
1.2
1.1
1
0.9
0
10
20
30
40
50
60
Time (min)
Normal
LY50
LY75
LY88
LY94
LY97
LY data
Normal data

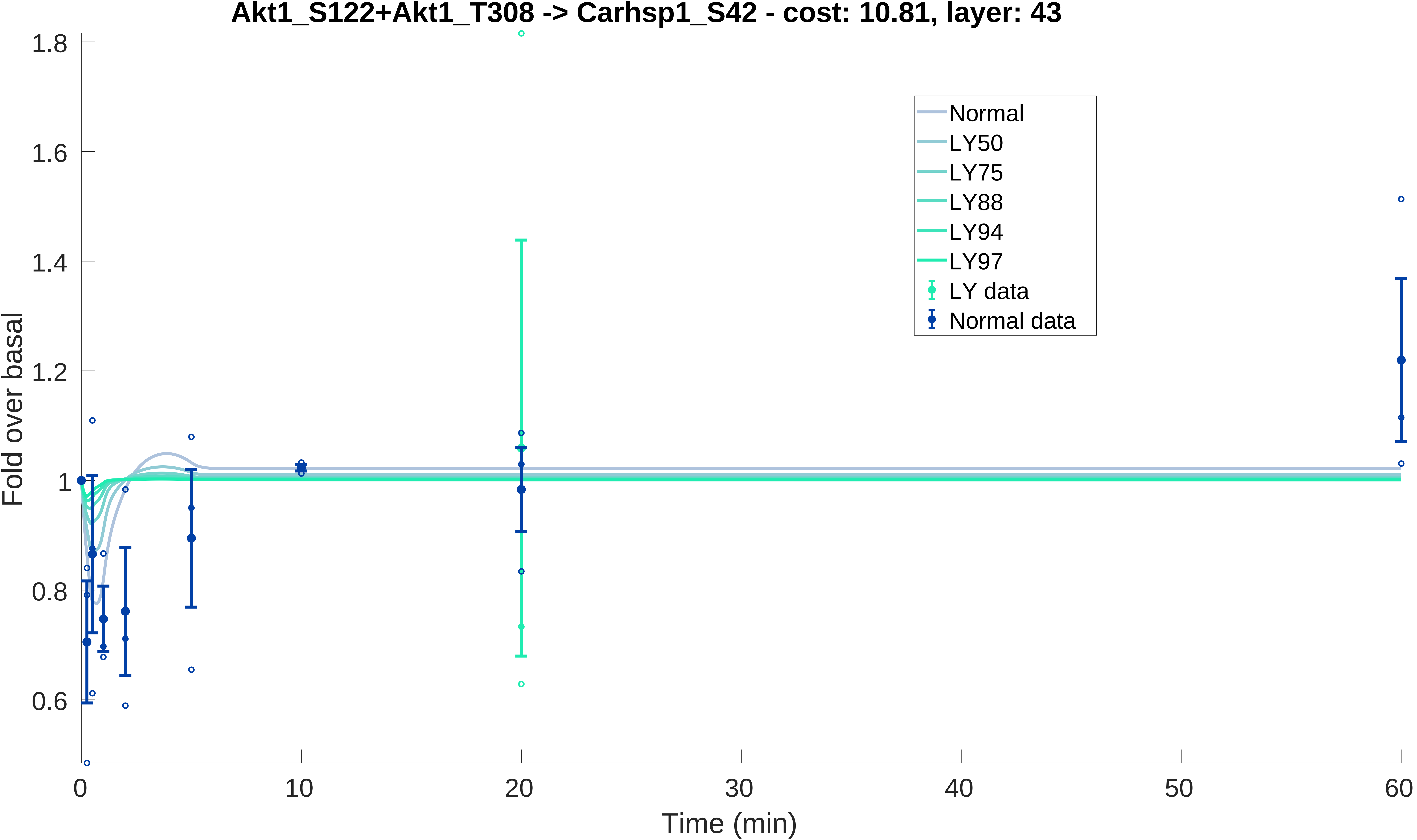

Akt1_S122+Akt1_T308 -> Carhsp1_S42 - cost: 10.81, layer: 43
Fold over basal
Time (min)
0.6
0.8
1
1.2
1.4
1.6
1.8
0
10
20
30
40
50
60
Normal
LY50
LY75
LY88
LY94
LY97
LY data
Normal data

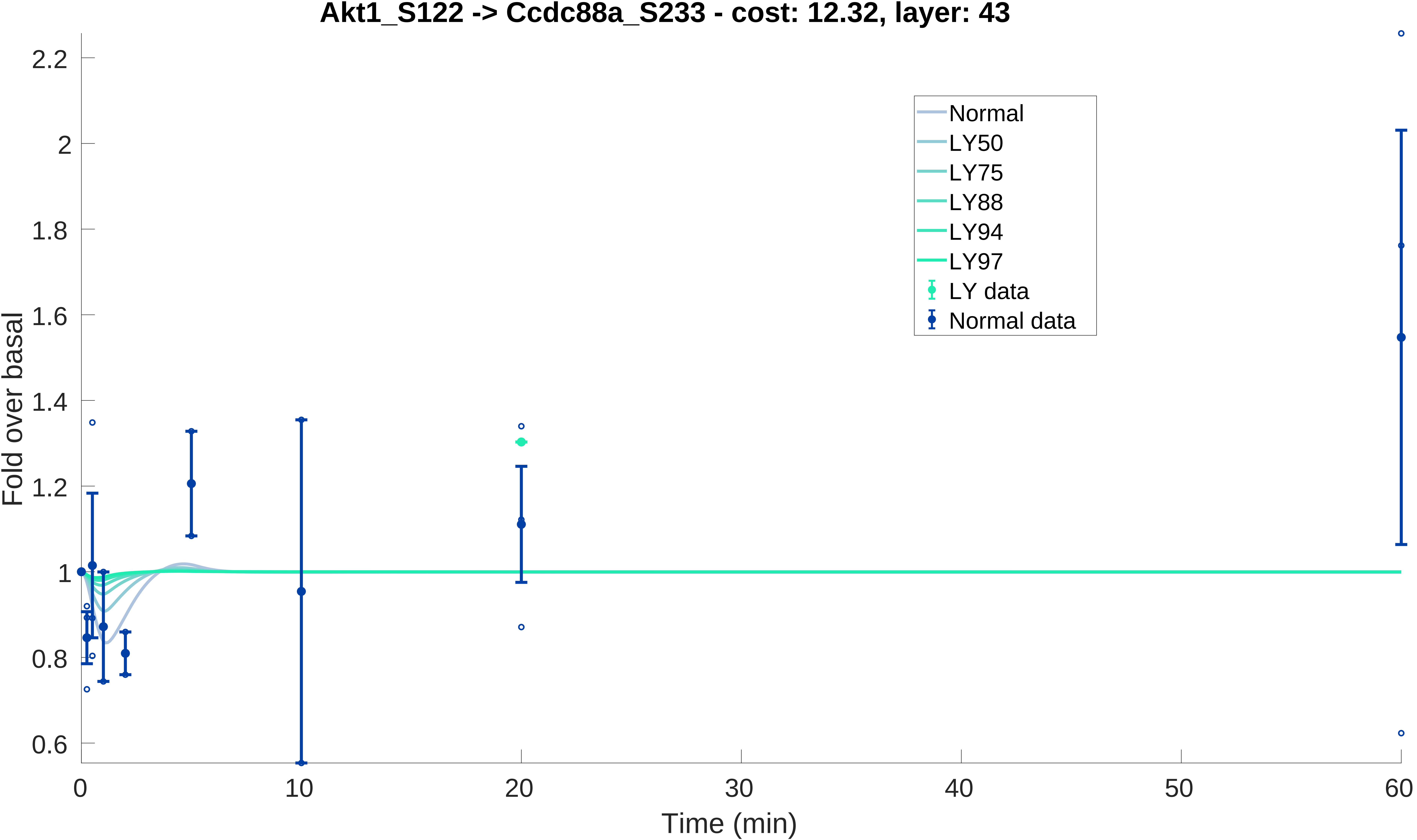
Akt1_S122 -> Ccdc88a_S233 - cost: 12.32, layer: 43
Fold over basal
Time (min)
0.6
0.8
1
1.2
1.4
1.6
1.8
2
2.2
0
10
20
30
40
50
60
Normal
LY50
LY75
LY88
LY94
LY97
LY data
Normal data

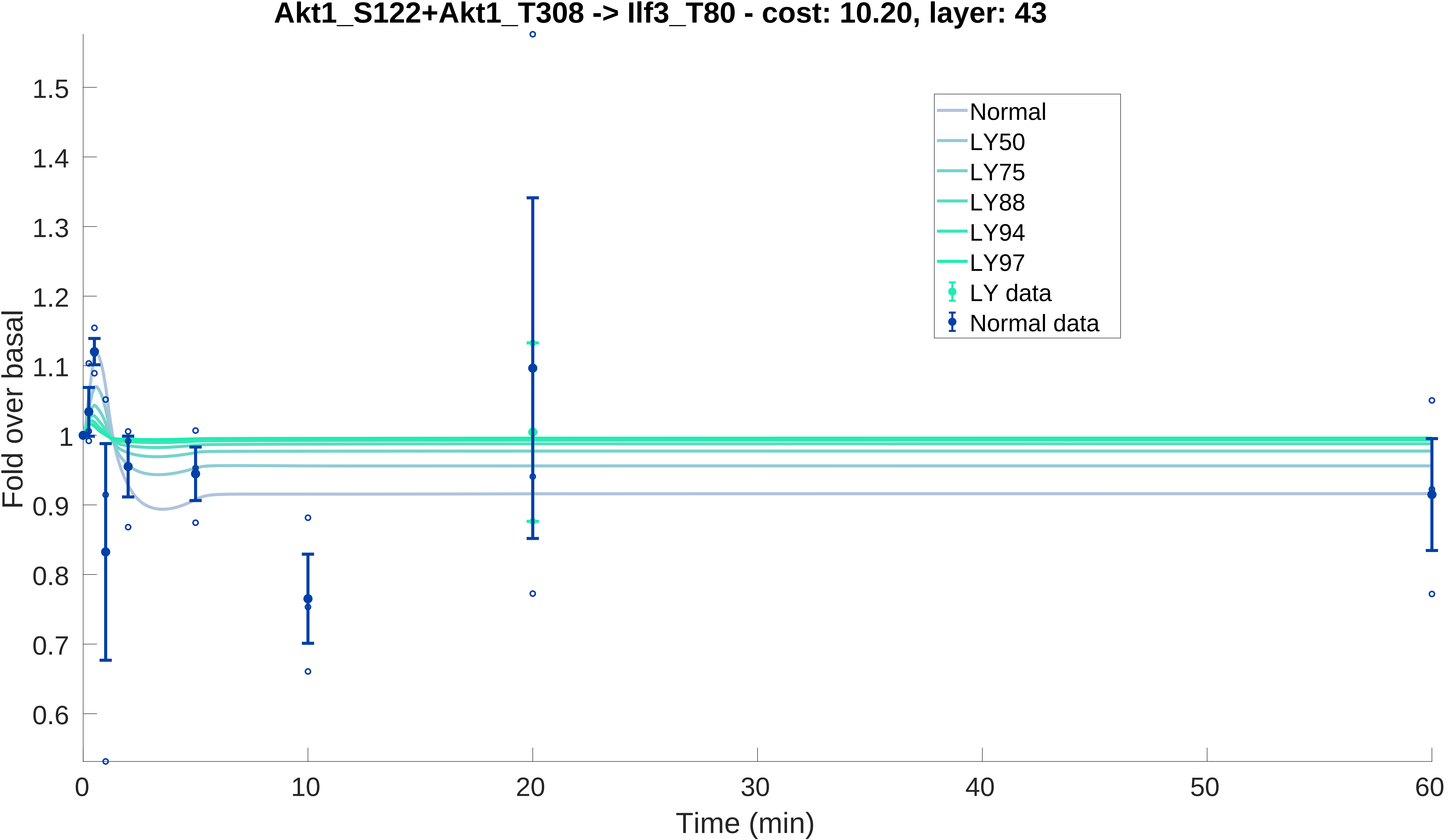

Akt1_S122+Akt1_T308 -> Ilf3_T80 - cost: 10.20, layer: 43
Fold over basal
Time (min)
Normal
LY50
LY75
LY88
LY94
LY97
LY data
Normal data
1.5
1.4
1.3
1.2
1.1
1
0.9
0.8
0.7
0.6
0
10
20
30
40
50
60

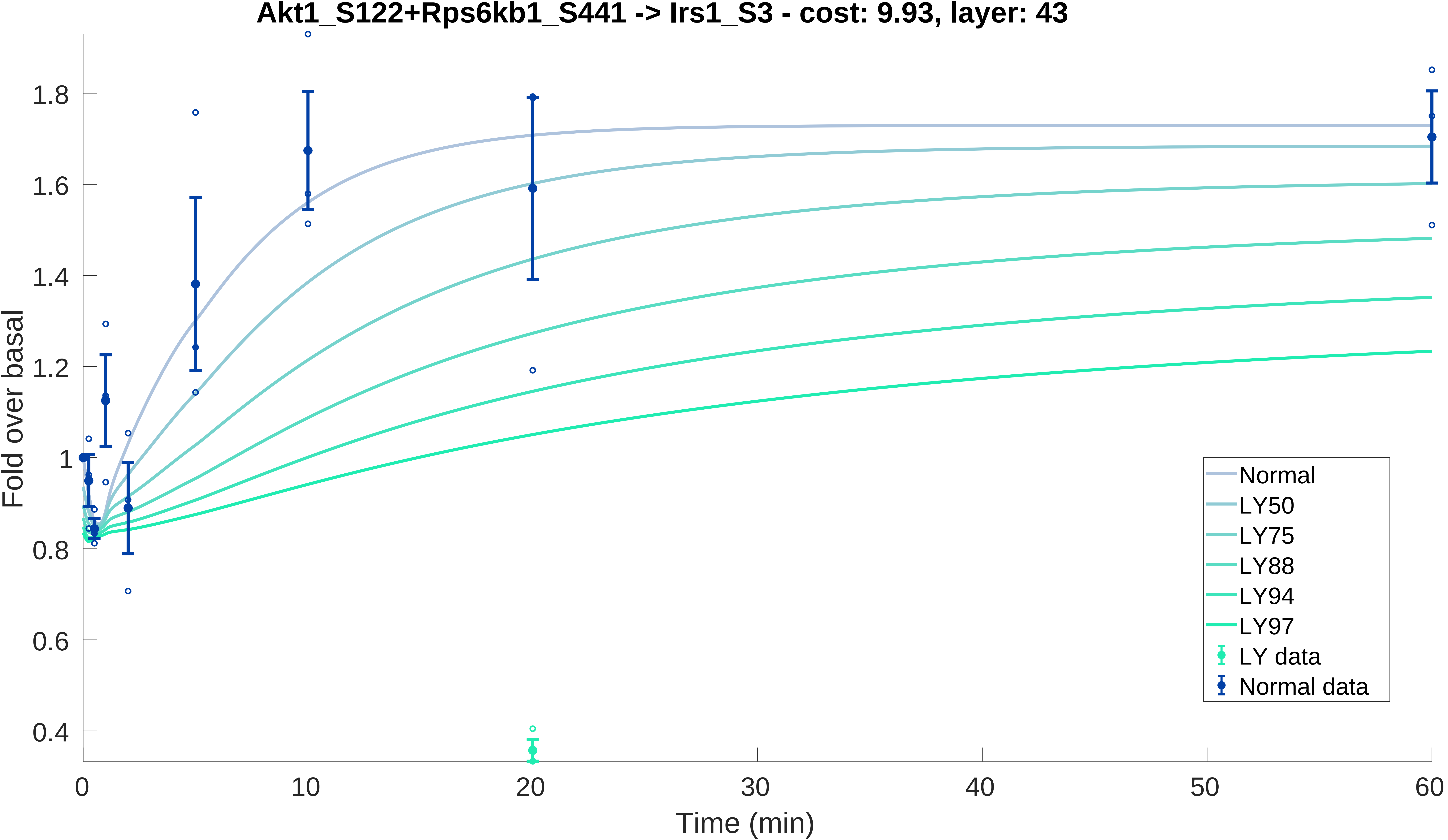

Akt1_S122+Rps6kb1_S441 -> Irs1_S3 - cost: 9.93, layer: 43
Fold over basal
Time (min)
0.4
0.6
0.8
1
1.2
1.4
1.6
1.8
0
10
20
30
40
50
60
Normal
LY50
LY75
LY88
LY94
LY97
LY data
Normal data

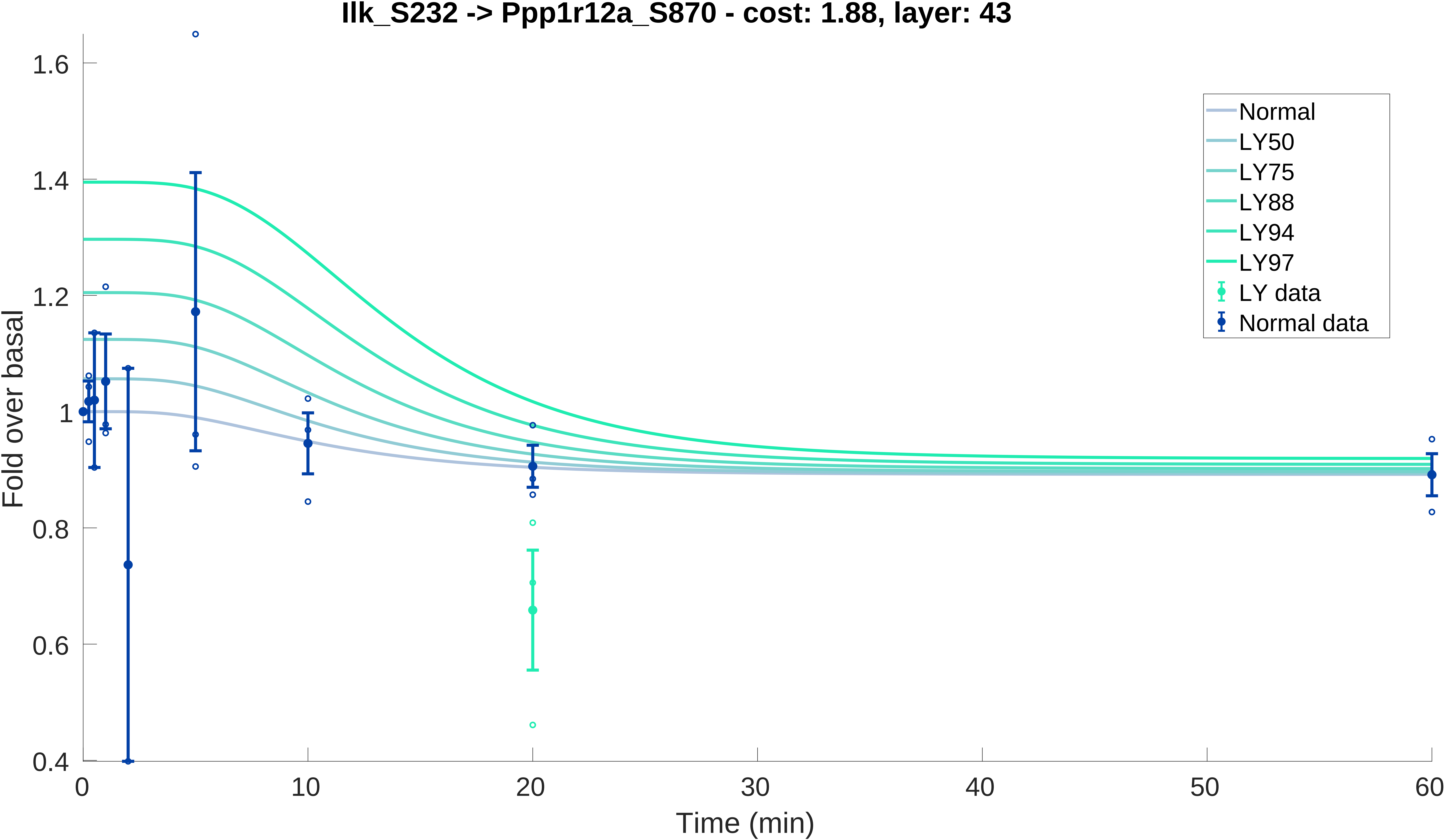

Ilk_S232 -> Ppp1r12a_S870 - cost: 1.88, layer: 43
Fold over basal
Time (min)
Normal
LY50
LY75
LY88
LY94
LY97
LY data
Normal data
0.4
0.6
0.8
1
1.2
1.4
1.6
0
10
20
30
40
50
60

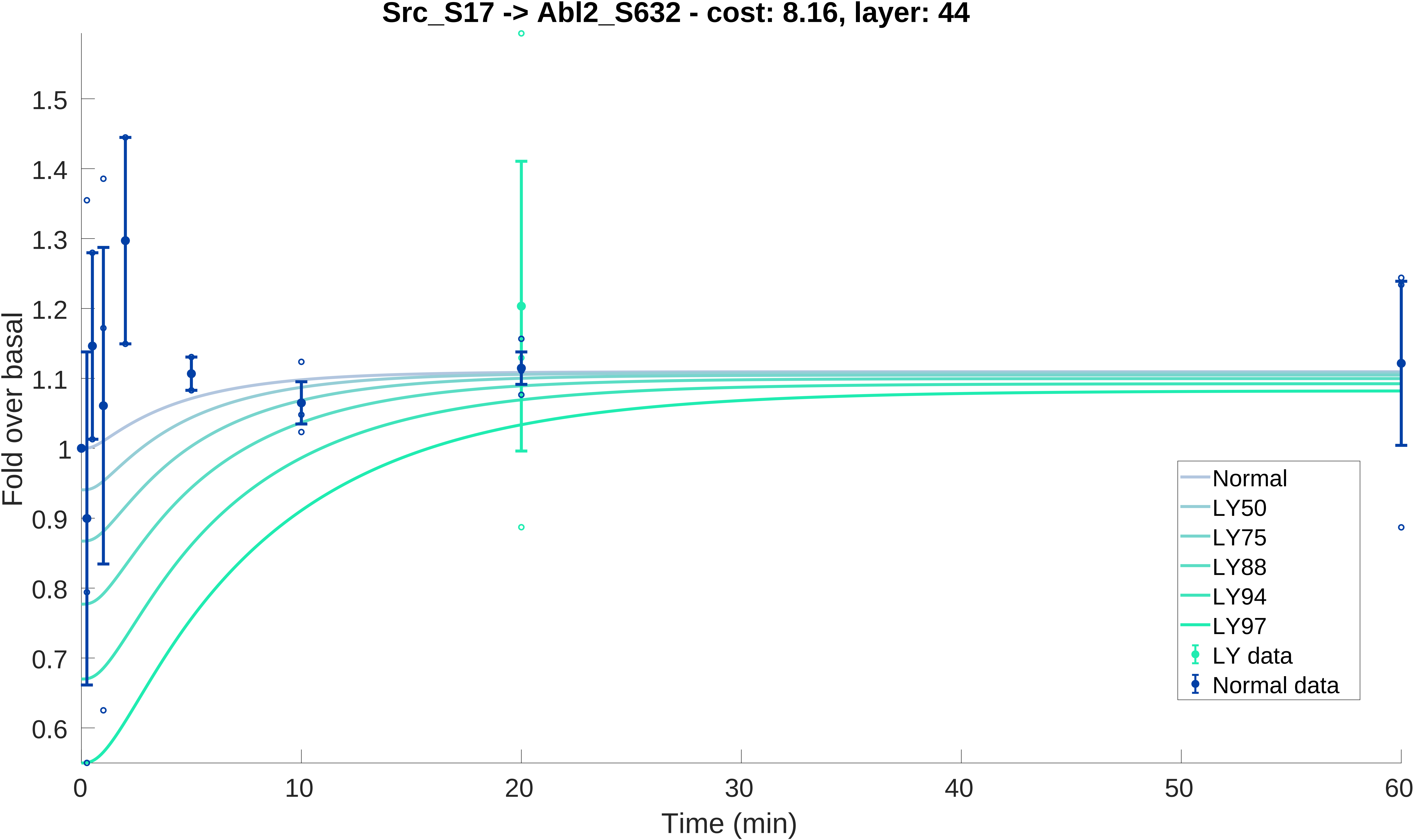

Src_S17 -> Abl2_S632 - cost: 8.16, layer: 44
Fold over basal
Time (min)
1.5
1.4
1.3
1.2
1.1
1
0.9
0.8
0.7
0.6
0
10
20
30
40
50
60
Normal
LY50
LY75
LY88
LY94
LY97
LY data
Normal data

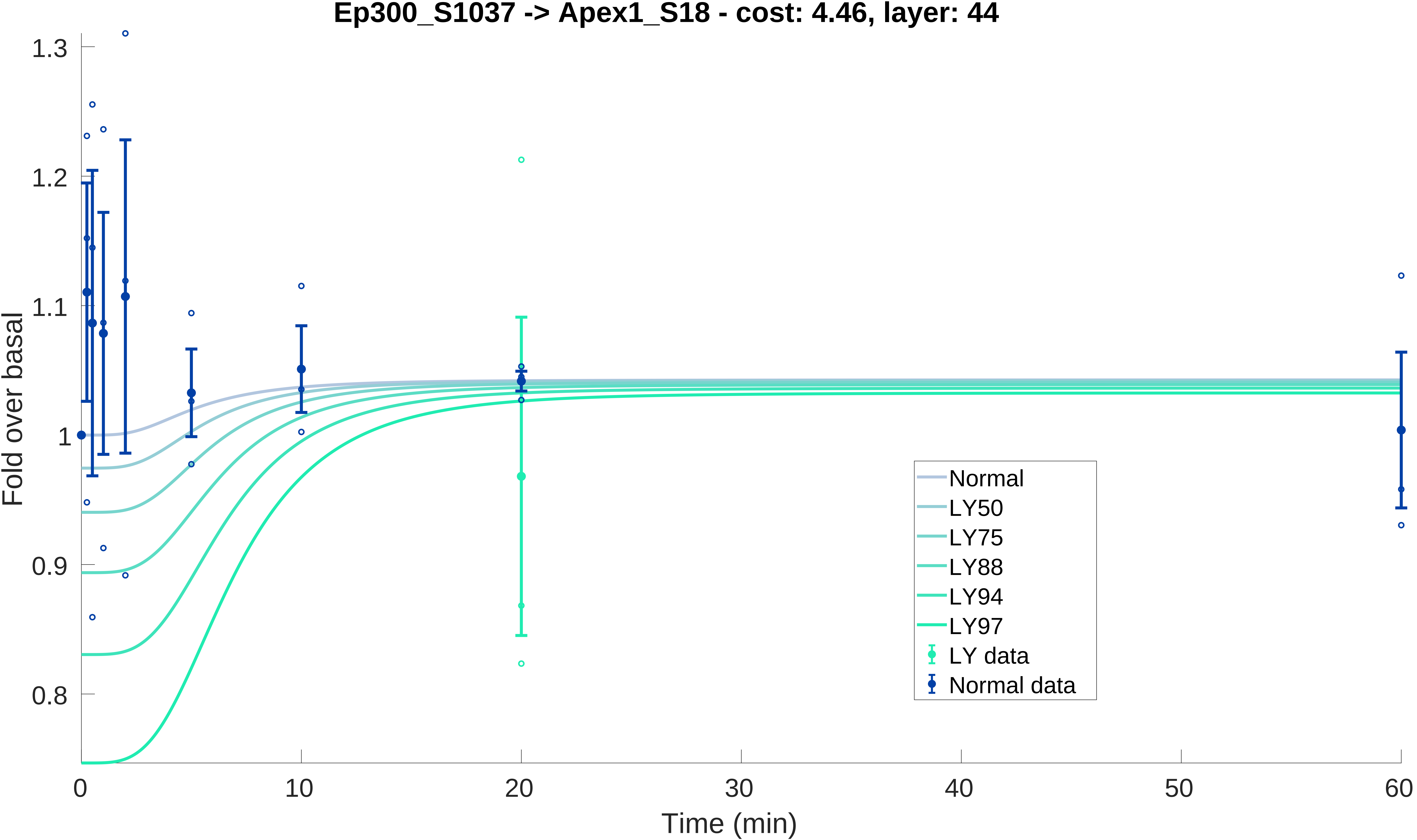
Ep300_S1037 -> Apex1_S18 - cost: 4.46, layer: 44
Fold over basal
Time (min)
0.8
0.9
1
1.1
1.2
1.3
0
10
20
30
40
50
60
Normal
LY50
LY75
LY88
LY94
LY97
LY data
Normal data

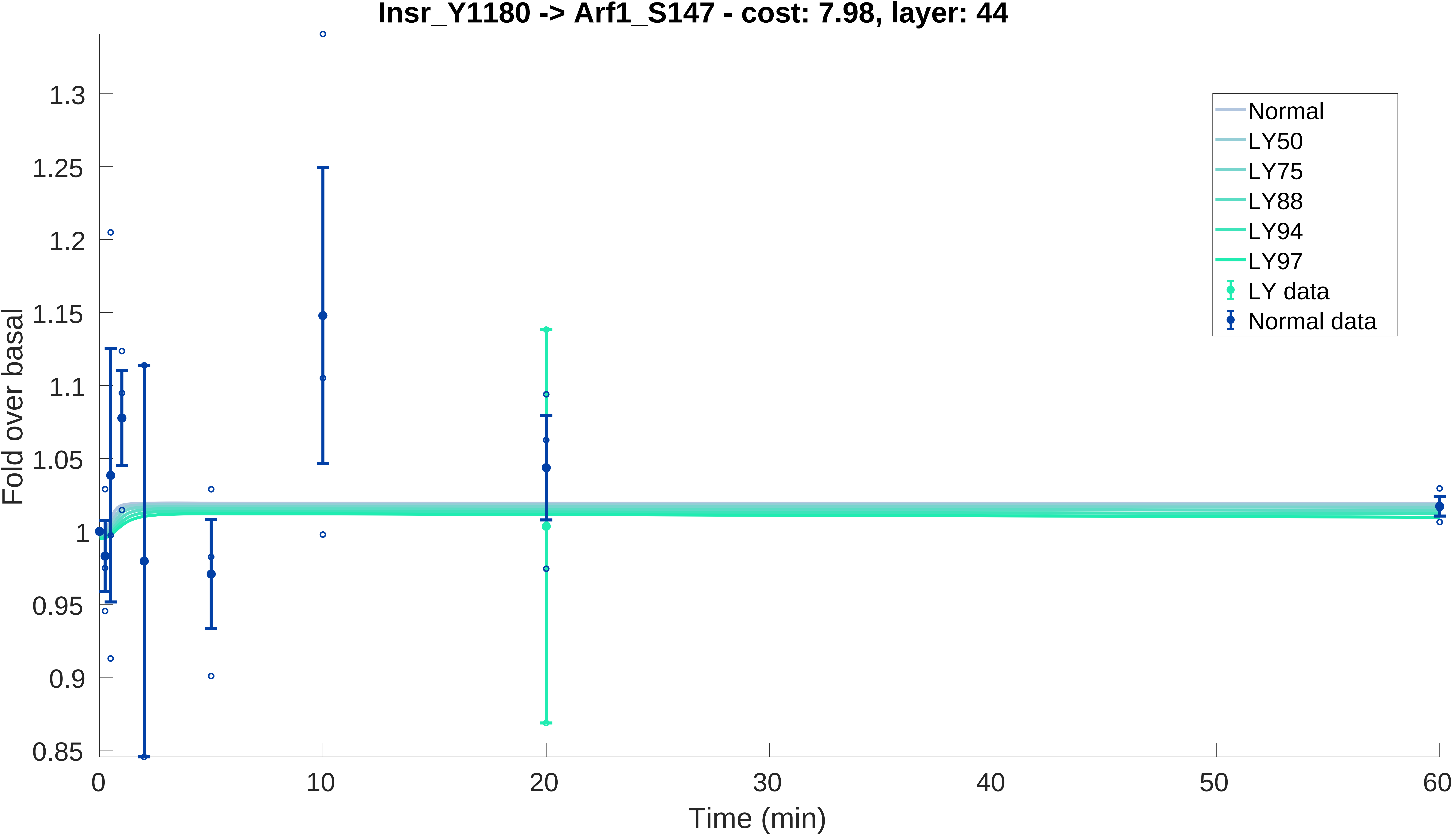
Insr_Y1180 -> Arf1_S147 - cost: 7.98, layer: 44
Fold over basal
Time (min)
1.3
1.25
1.2
1.15
1.1
1.05
1
0.95
0.9
0.85
0
10
20
30
40
50
60
Normal
LY50
LY75
LY88
LY94
LY97
LY data
Normal data

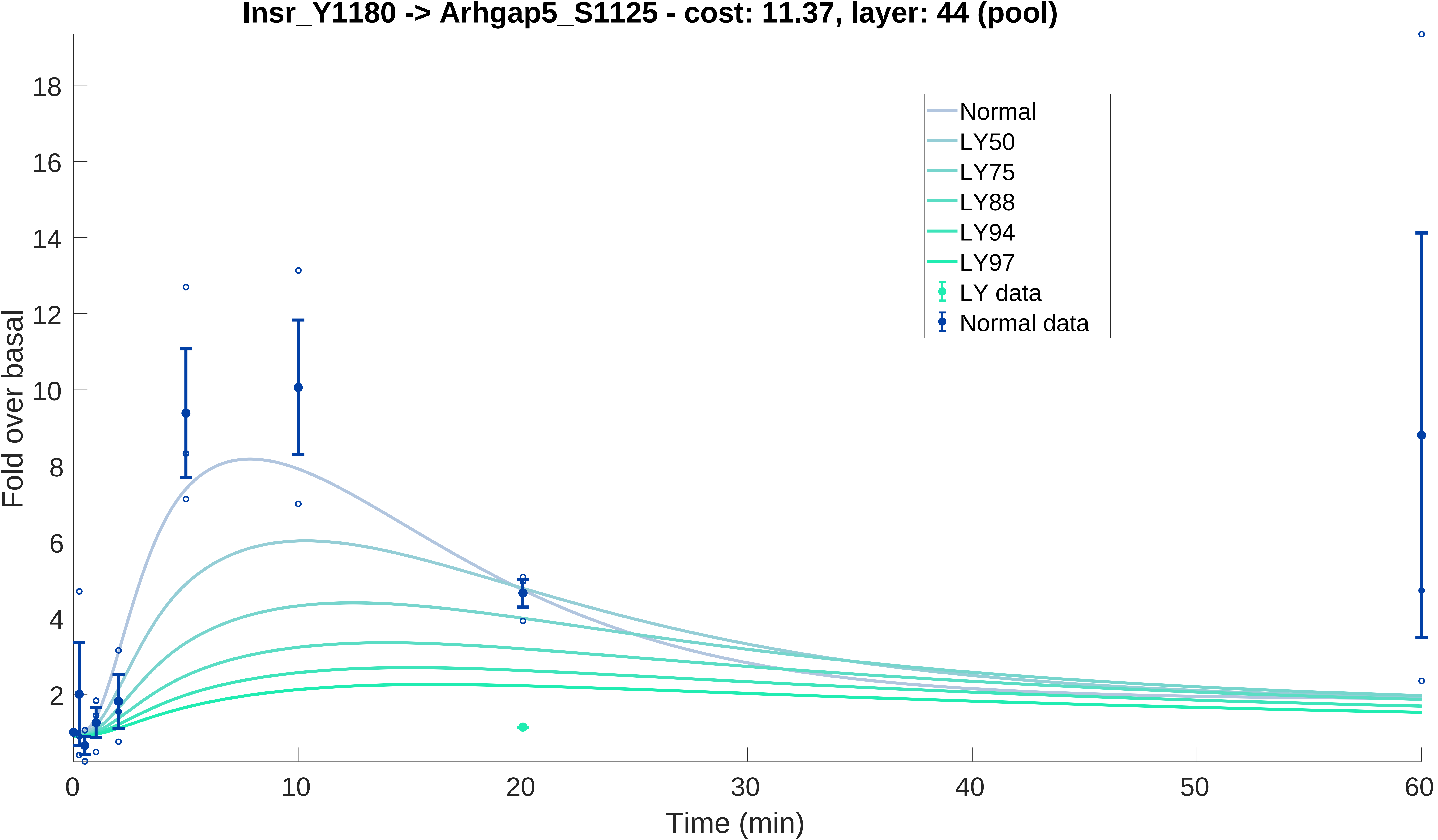

Insr_Y1180 -> Arhgap5_S1125 - cost: 11.37, layer: 44 (pool)
Fold over basal
Time (min)
Normal
LY50
LY75
LY88
LY94
LY97
LY data
Normal data
0
10
20
30
40
50
60
2
4
6
8
10
12
14
16
18

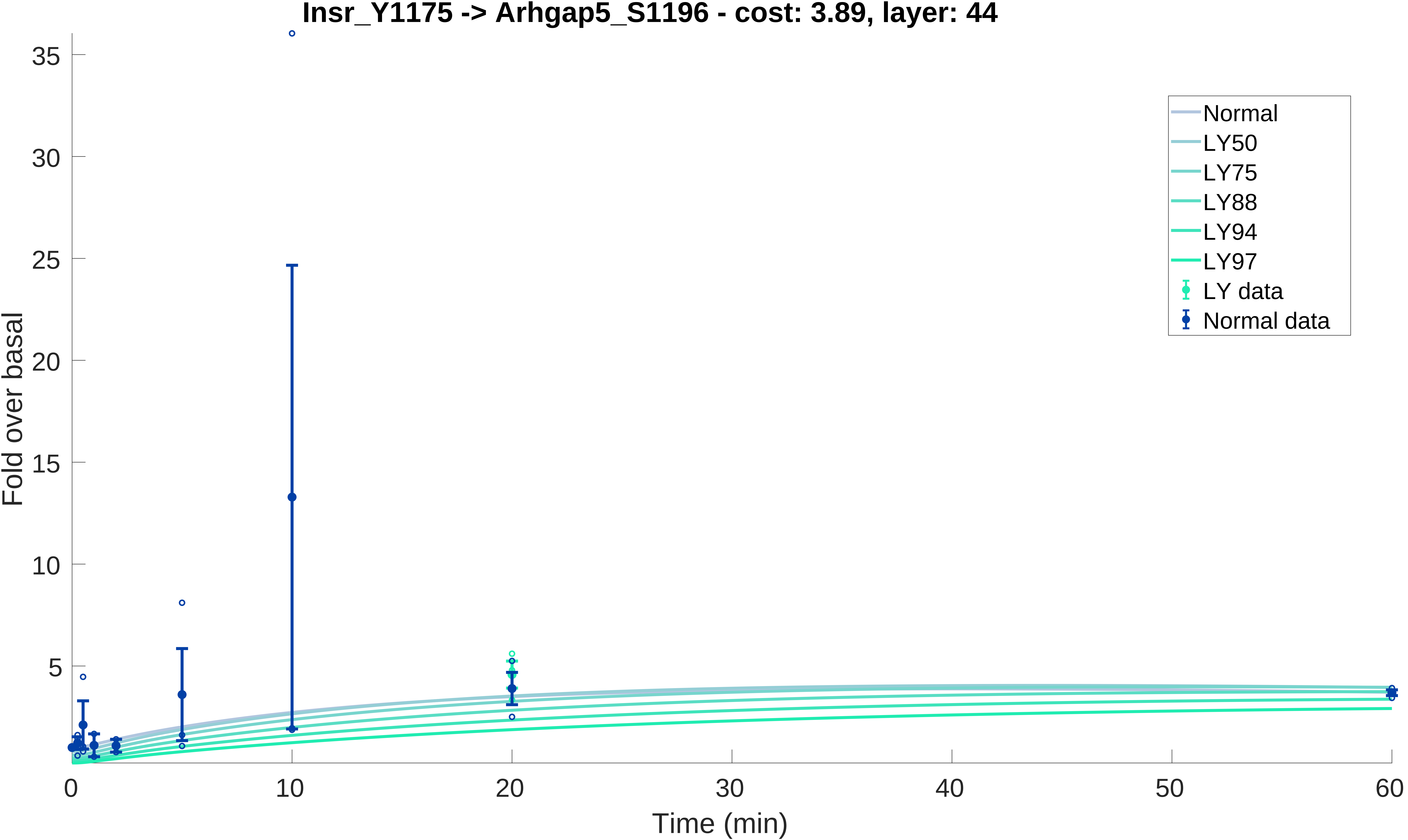

Insr_Y1175 -> Arhgap5_S1196 - cost: 3.89, layer: 44
Fold over basal
Time (min)
0
5
10
15
20
25
30
35
40
50
60
Normal
LY50
LY75
LY88
LY94
LY97
LY data
Normal data

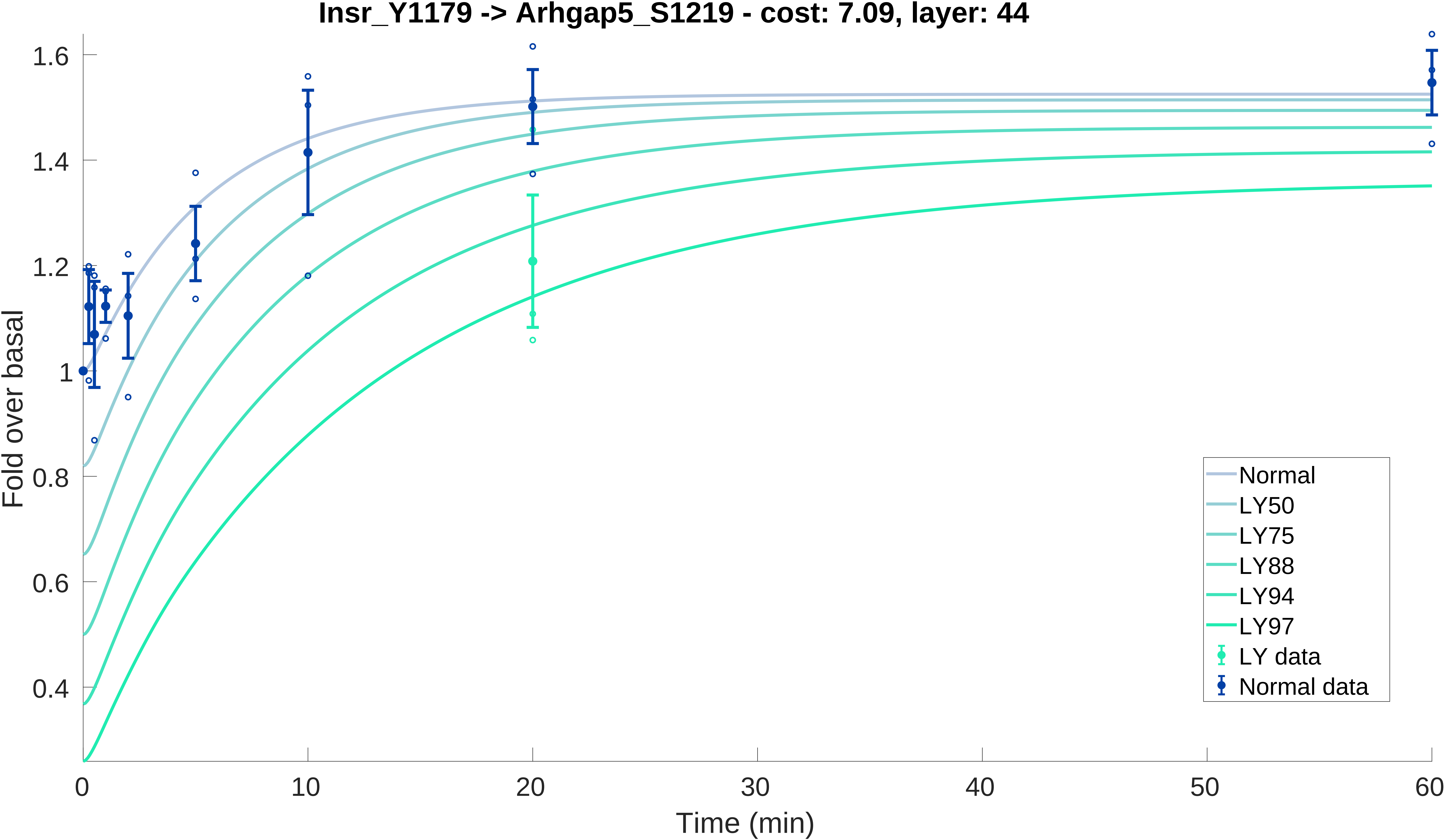
Insr_Y1179 -> Arhgap5_S1219 - cost: 7.09, layer: 44
Fold over basal
1.6
1.4
1.2
1
0.8
0.6
0.4
0
10
20
30
40
50
60
Time (min)
Normal
LY50
LY75
LY88
LY94
LY97
LY data
Normal data

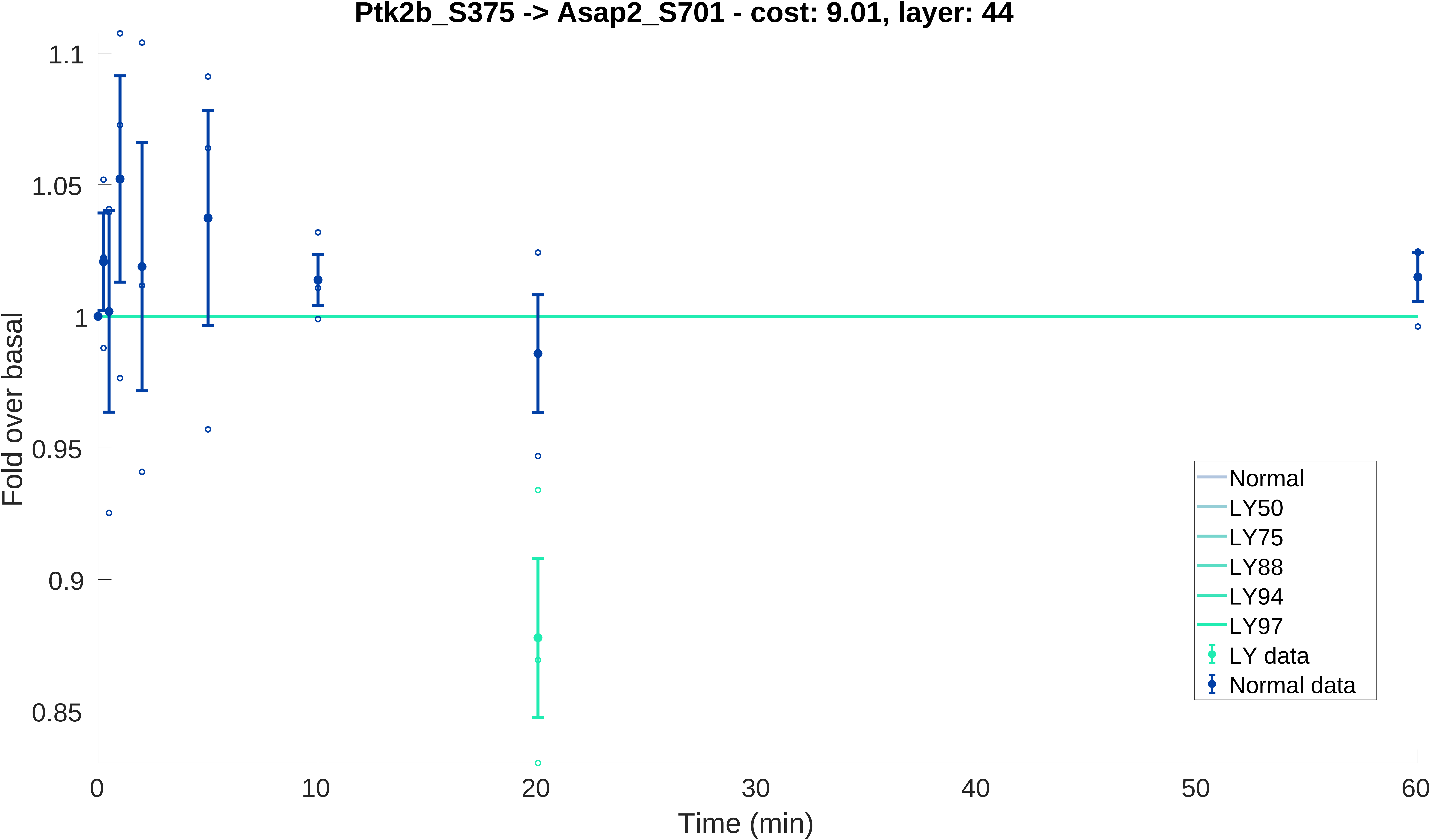

Ptk2b_S375 -> Asap2_S701 - cost: 9.01, layer: 44
Fold over basal
1.1
1.05
1
0.95
0.9
0.85
0
10
20
30
40
50
60
Time (min)
Normal
LY50
LY75
LY88
LY94
LY97
LY data
Normal data

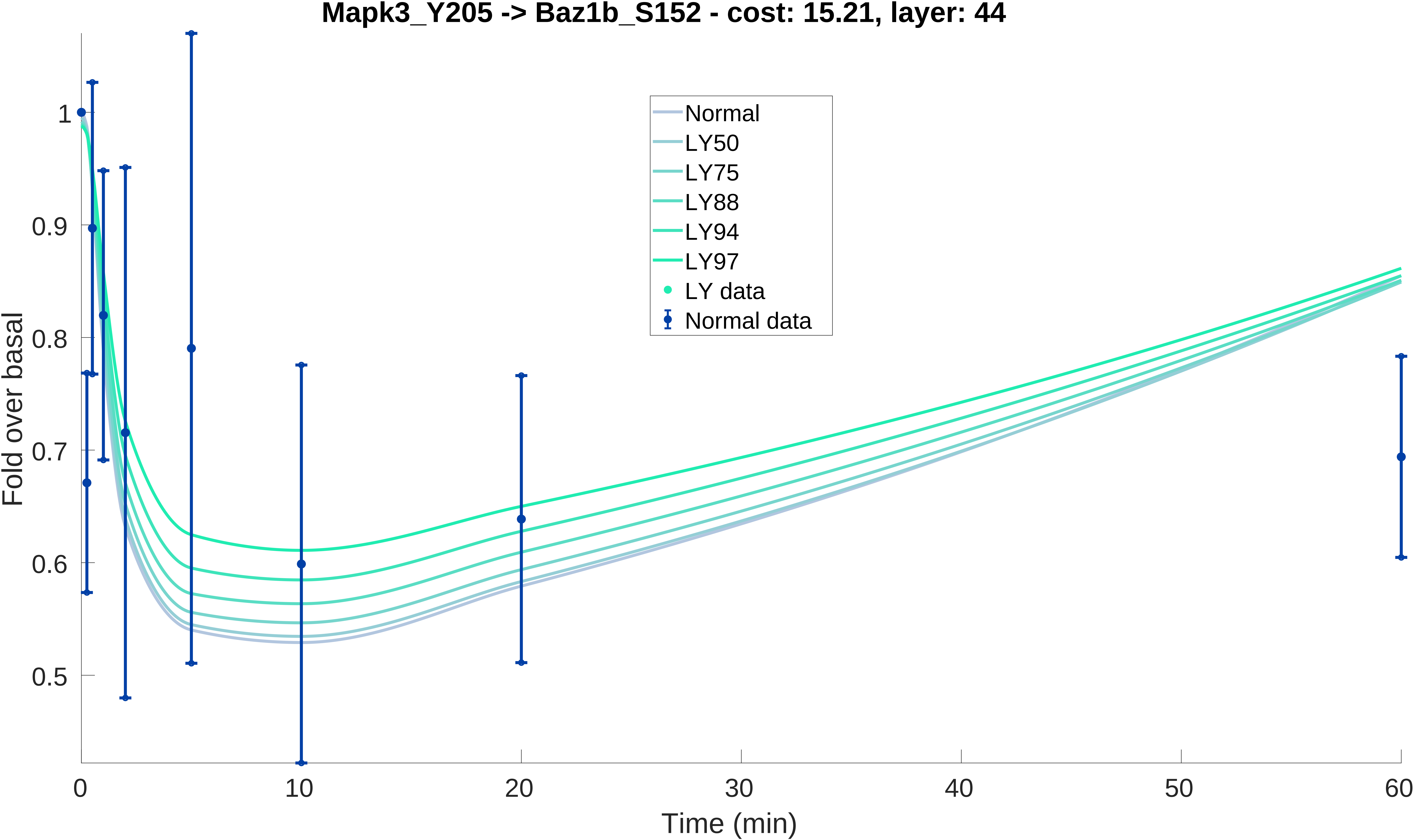

Mapk3_Y205 -> Baz1b_S152 - cost: 15.21, layer: 44
Fold over basal
Time (min)
Normal
LY50
LY75
LY88
LY94
LY97
LY data
Normal data
1
0.9
0.8
0.7
0.6
0.5
0
10
20
30
40
50
60

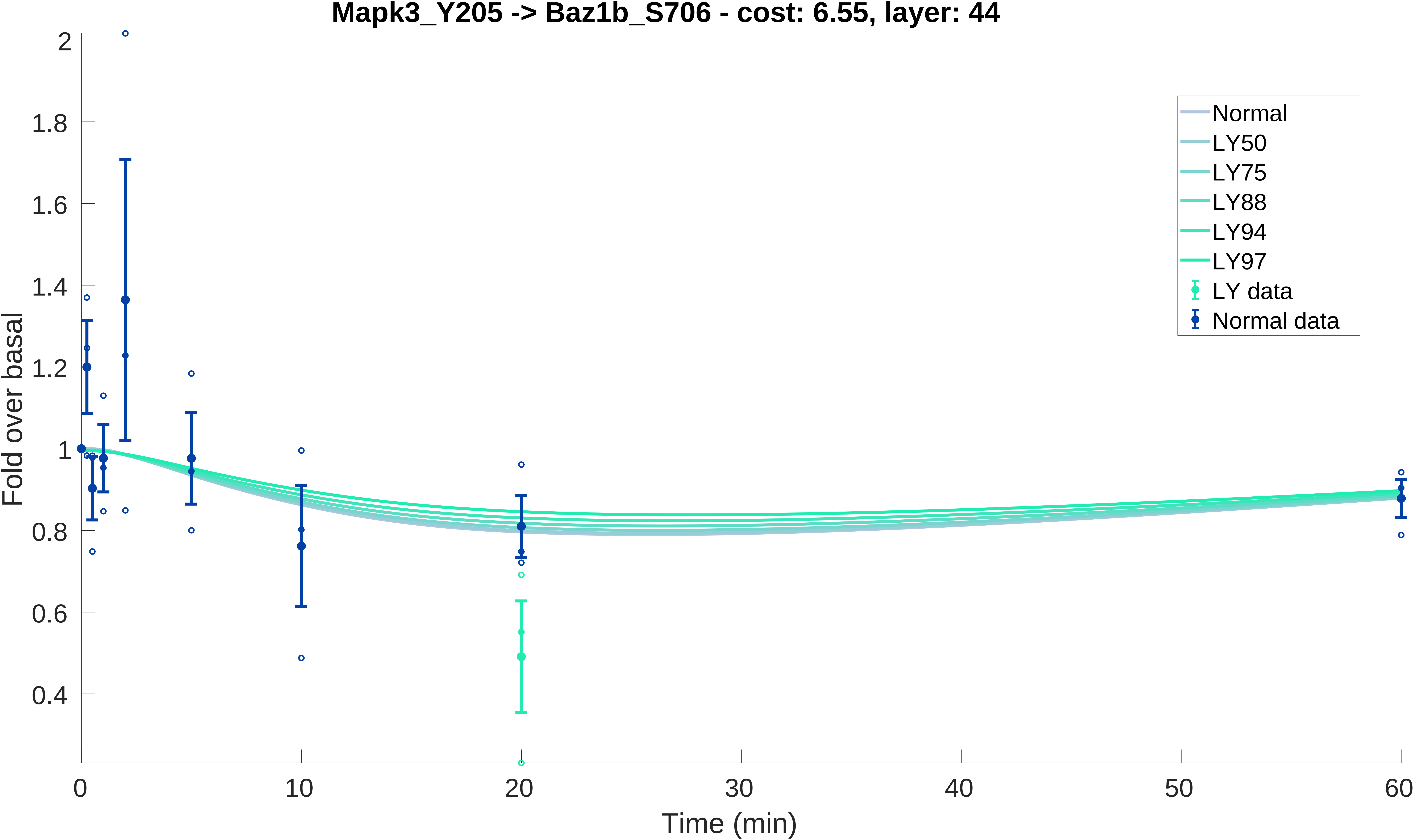

Mapk3_Y205 -> Baz1b_S706 - cost: 6.55, layer: 44
Fold over basal
Time (min)
Normal
LY50
LY75
LY88
LY94
LY97
LY data
Normal data
2
1.8
1.6
1.4
1.2
1
0.8
0.6
0.4
0
10
20
30
40
50
60

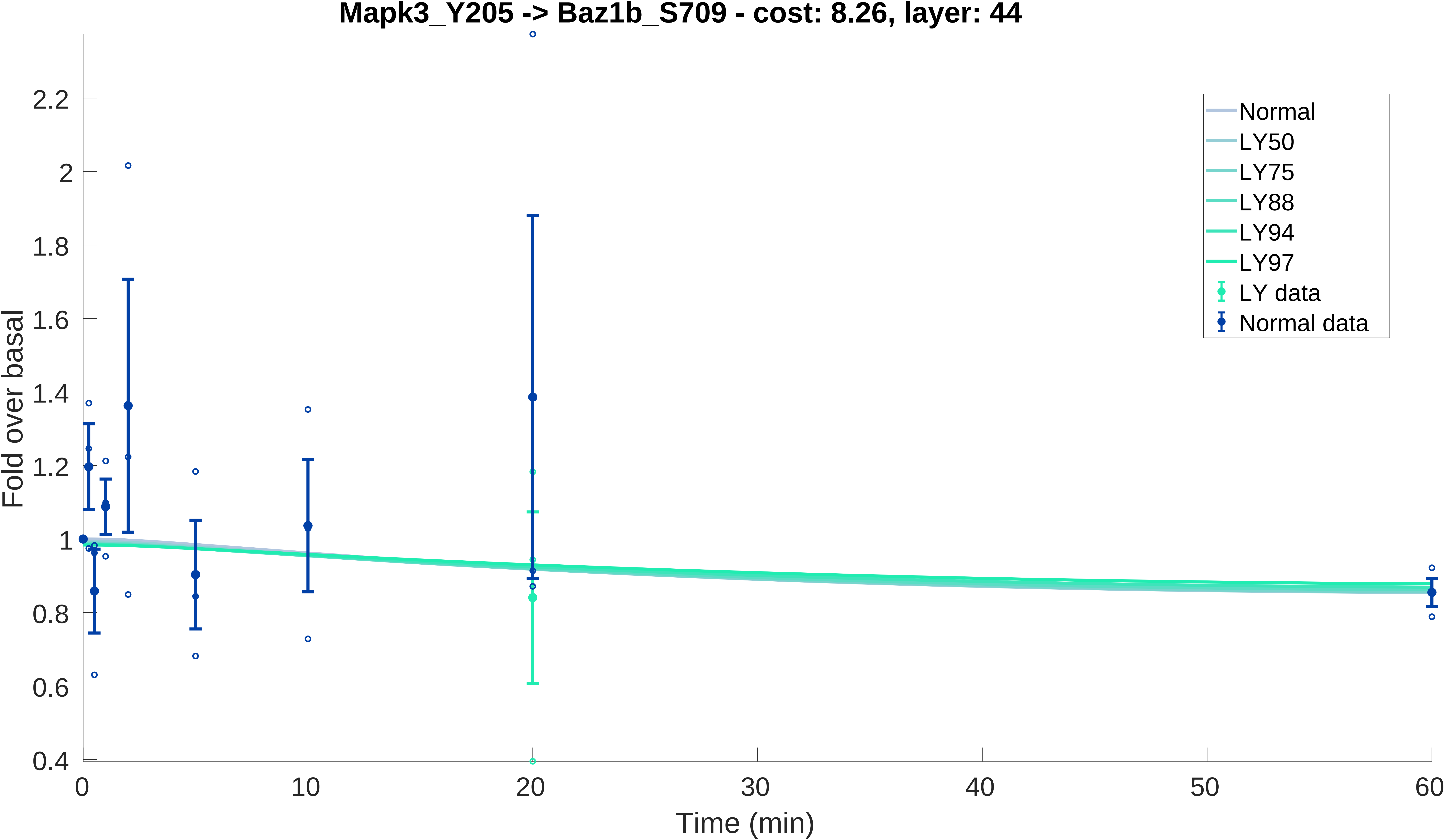

Mapk3_Y205 -> Baz1b_S709 - cost: 8.26, layer: 44
Fold over basal
Time (min)
0.4
0.6
0.8
1
1.2
1.4
1.6
1.8
2
2.2
0
10
20
30
40
50
60
Normal
LY50
LY75
LY88
LY94
LY97
LY data
Normal data

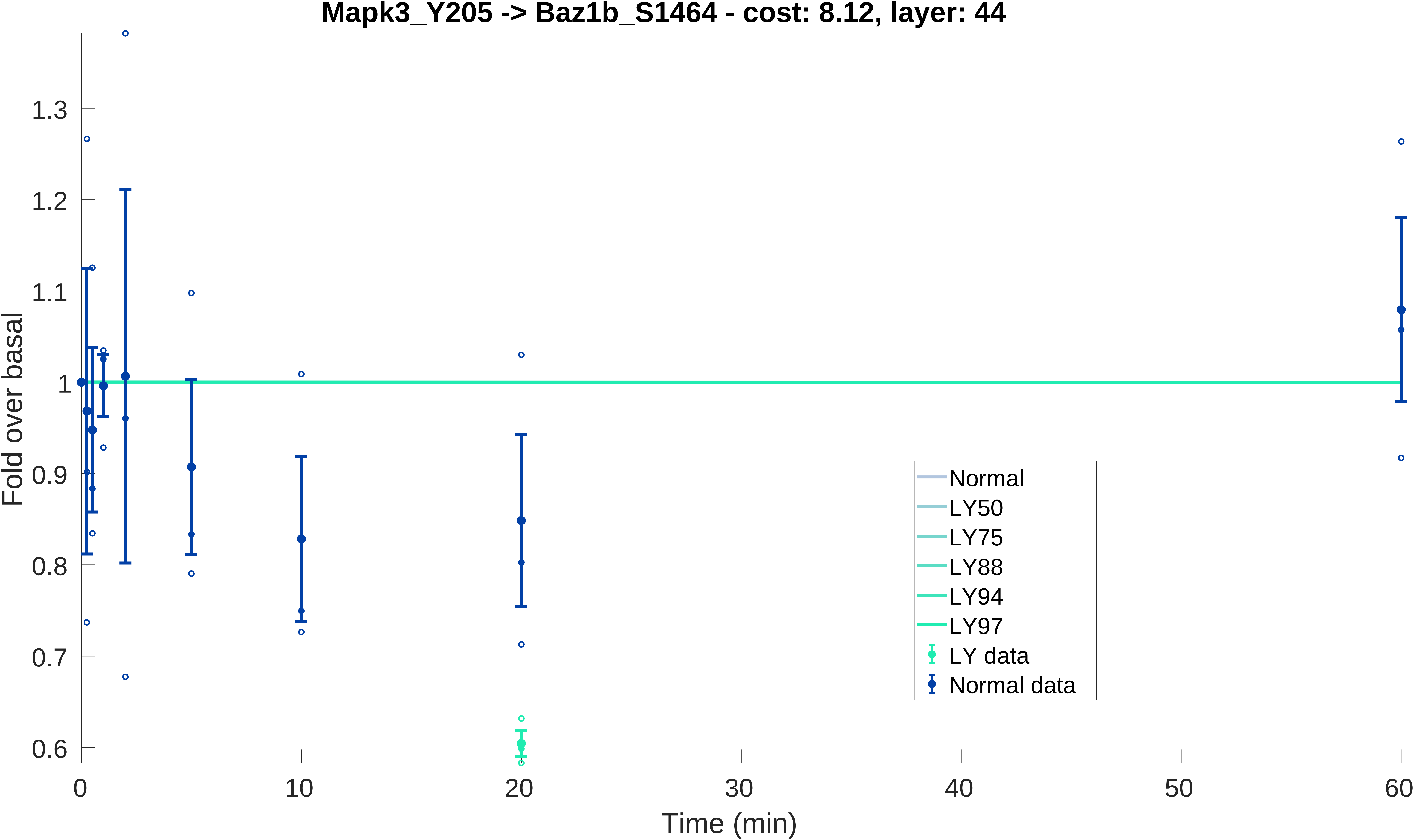

Mapk3_Y205 -> Baz1b_S1464 - cost: 8.12, layer: 44
Fold over basal
1.3
1.2
1.1
1
0.9
0.8
0.7
0.6
0
10
20
30
40
50
60
Time (min)
Normal
LY50
LY75
LY88
LY94
LY97
LY data
Normal data

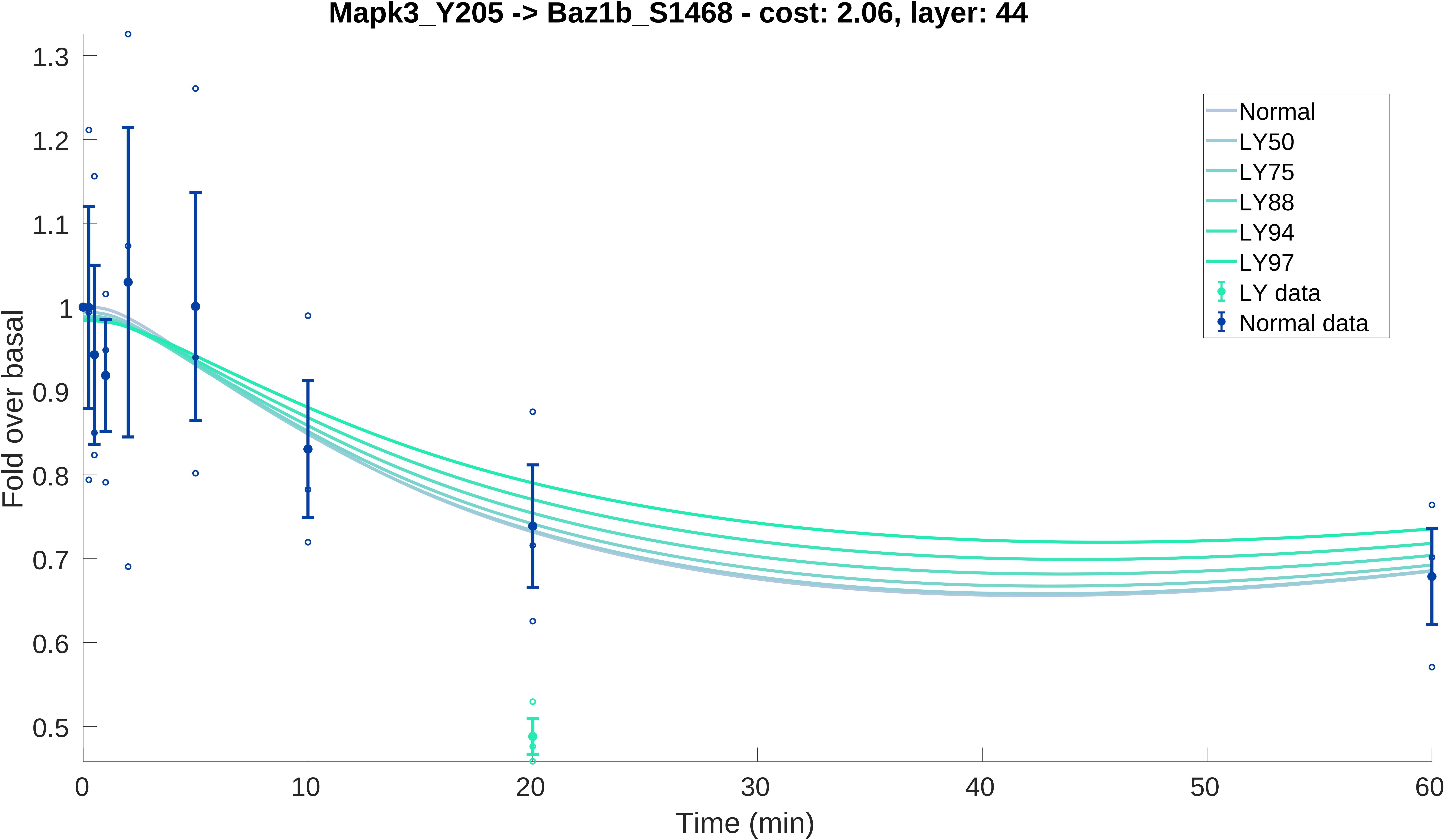

Mapk3_Y205 -> Baz1b_S1468 - cost: 2.06, layer: 44
Fold over basal
Time (min)
1.3
1.2
1.1
1
0.9
0.8
0.7
0.6
0.5
0
10
20
30
40
50
60
Normal
LY50
LY75
LY88
LY94
LY97
LY data
Normal data

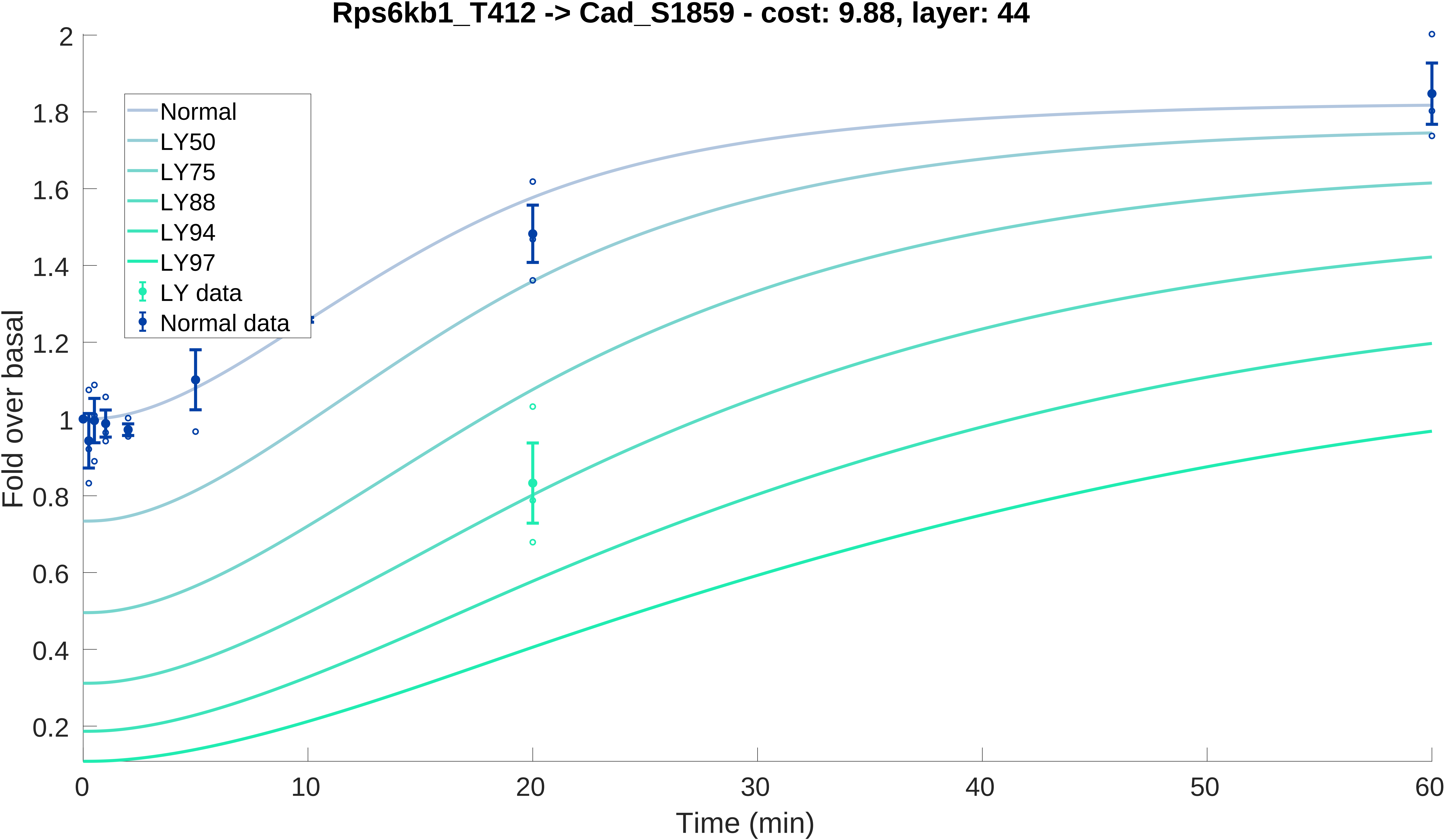

Rps6kb1_T412 -> Cad_S1859 - cost: 9.88, layer: 44
Fold over basal
Time (min)
Normal
LY50
LY75
LY88
LY94
LY97
LY data
Normal data
0
10
20
30
40
50
60
0.2
0.4
0.6
0.8
1
1.2
1.4
1.6
1.8
2

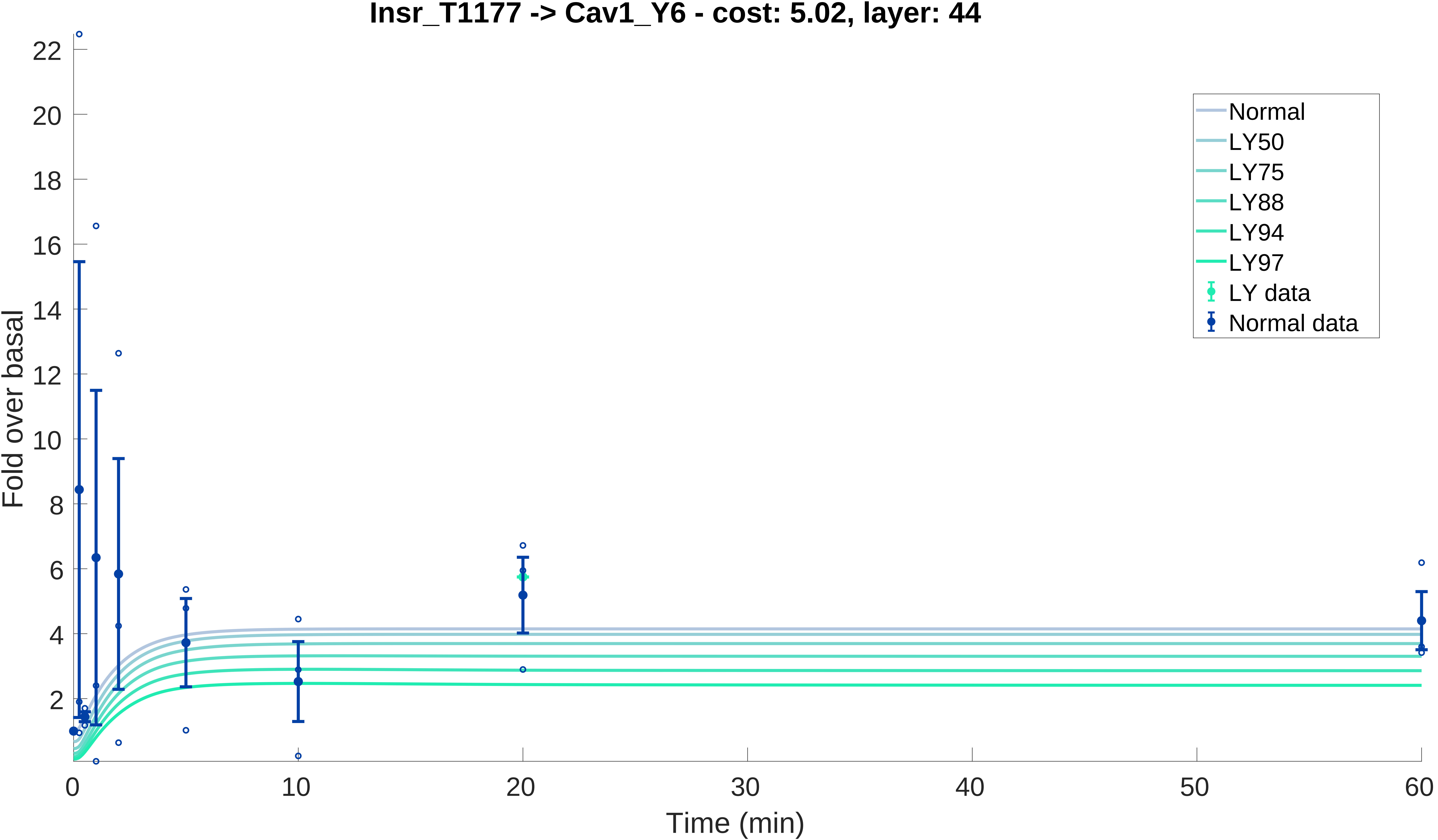
Insr_T1177 -> Cav1_Y6 - cost: 5.02, layer: 44
Fold over basal
Time (min)
0
10
20
30
40
50
60
2
4
6
8
10
12
14
16
18
20
22
Normal
LY50
LY75
LY88
LY94
LY97
LY data
Normal data

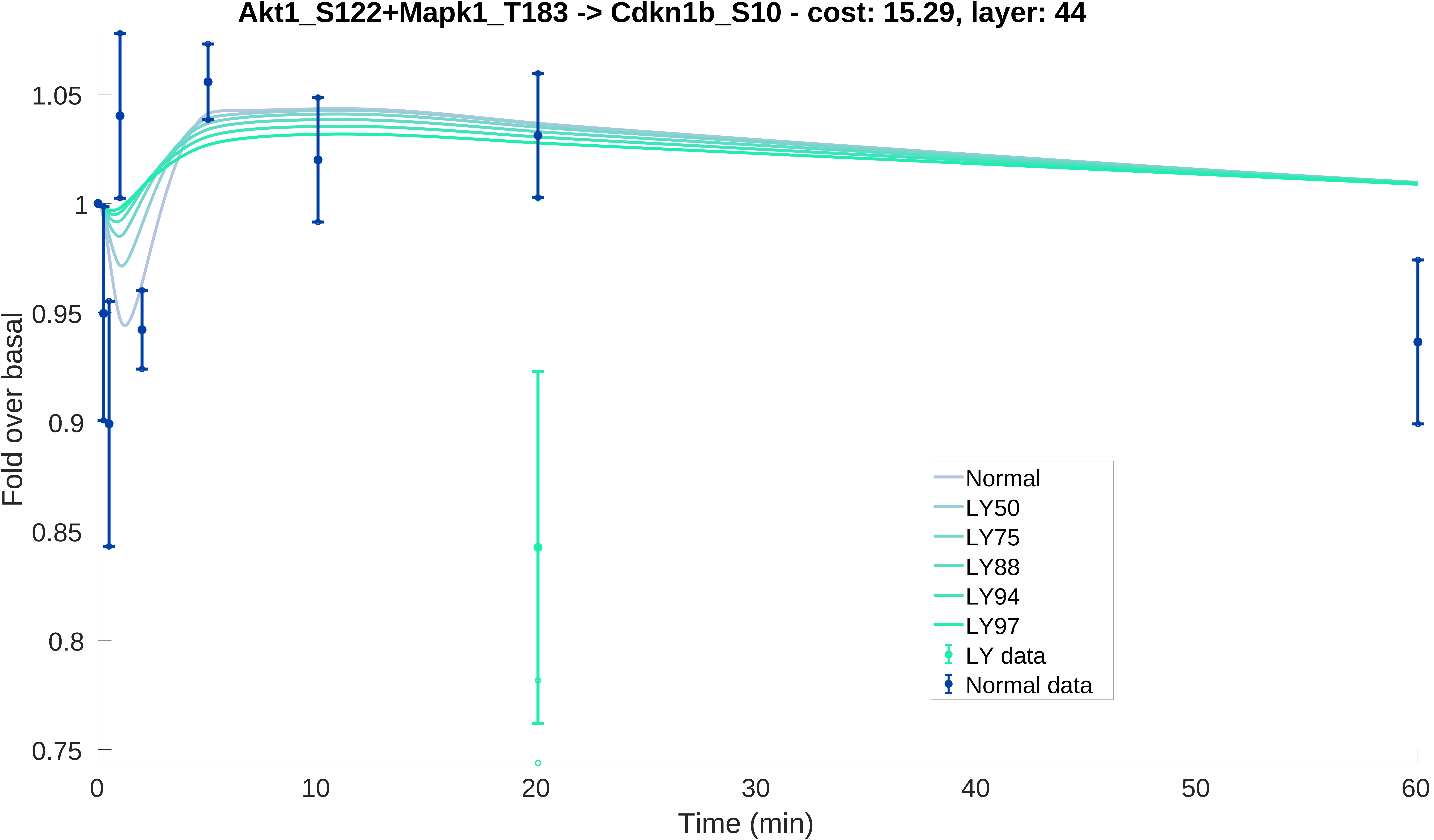

Akt1_S122+Mapk1_T183 -> Cdkn1b_S10 - cost: 15.29, layer: 44
1.05
1
0.95
0.9
0.85
0.8
0.75
Fold over basal
0
10
20
30
40
50
60
Time (min)
Normal
LY50
LY75
LY88
LY94
LY97
LY data
Normal data

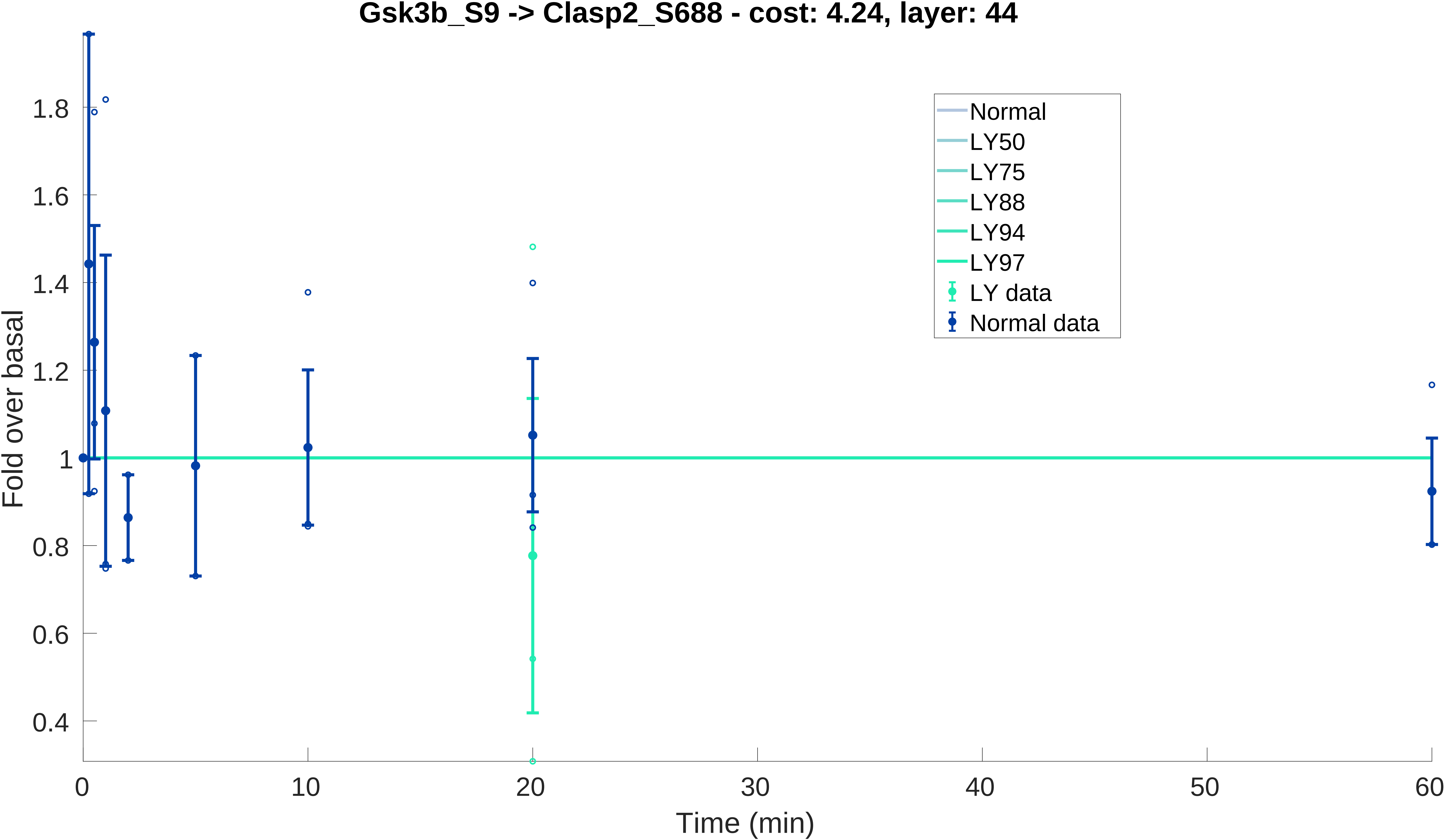

Gsk3b_S9 -> Clasp2_S688 - cost: 4.24, layer: 44
Fold over basal
Time (min)
0.4
0.6
0.8
1
1.2
1.4
1.6
1.8
0
10
20
30
40
50
60
Normal
LY50
LY75
LY88
LY94
LY97
LY data
Normal data

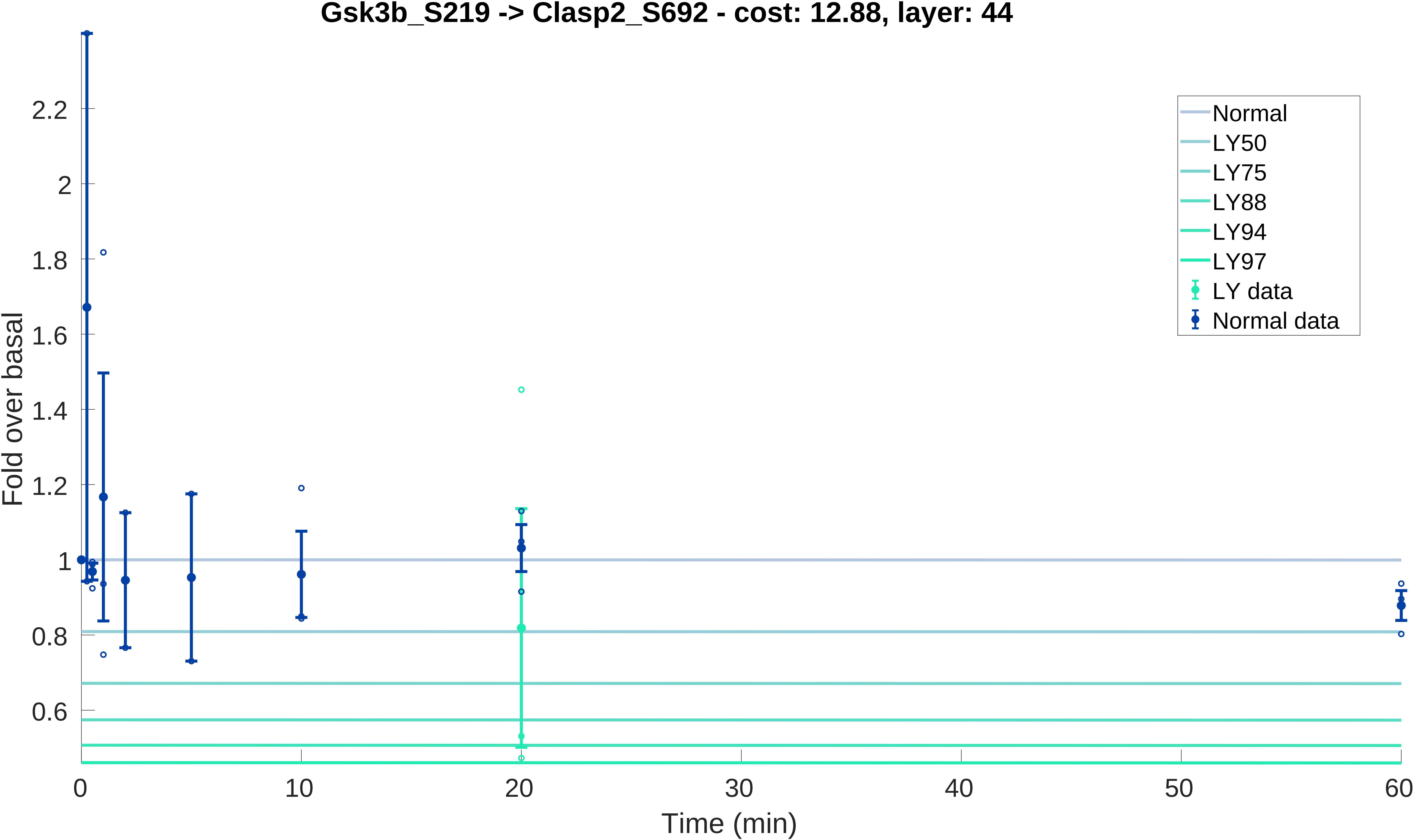

Gsk3b_S219 -> Clasp2_S692 - cost: 12.88, layer: 44
Fold over basal
Time (min)
2.2
2
1.8
1.6
1.4
1.2
1
0.8
0.6
0
10
20
30
40
50
60
Normal
LY50
LY75
LY88
LY94
LY97
LY data
Normal data

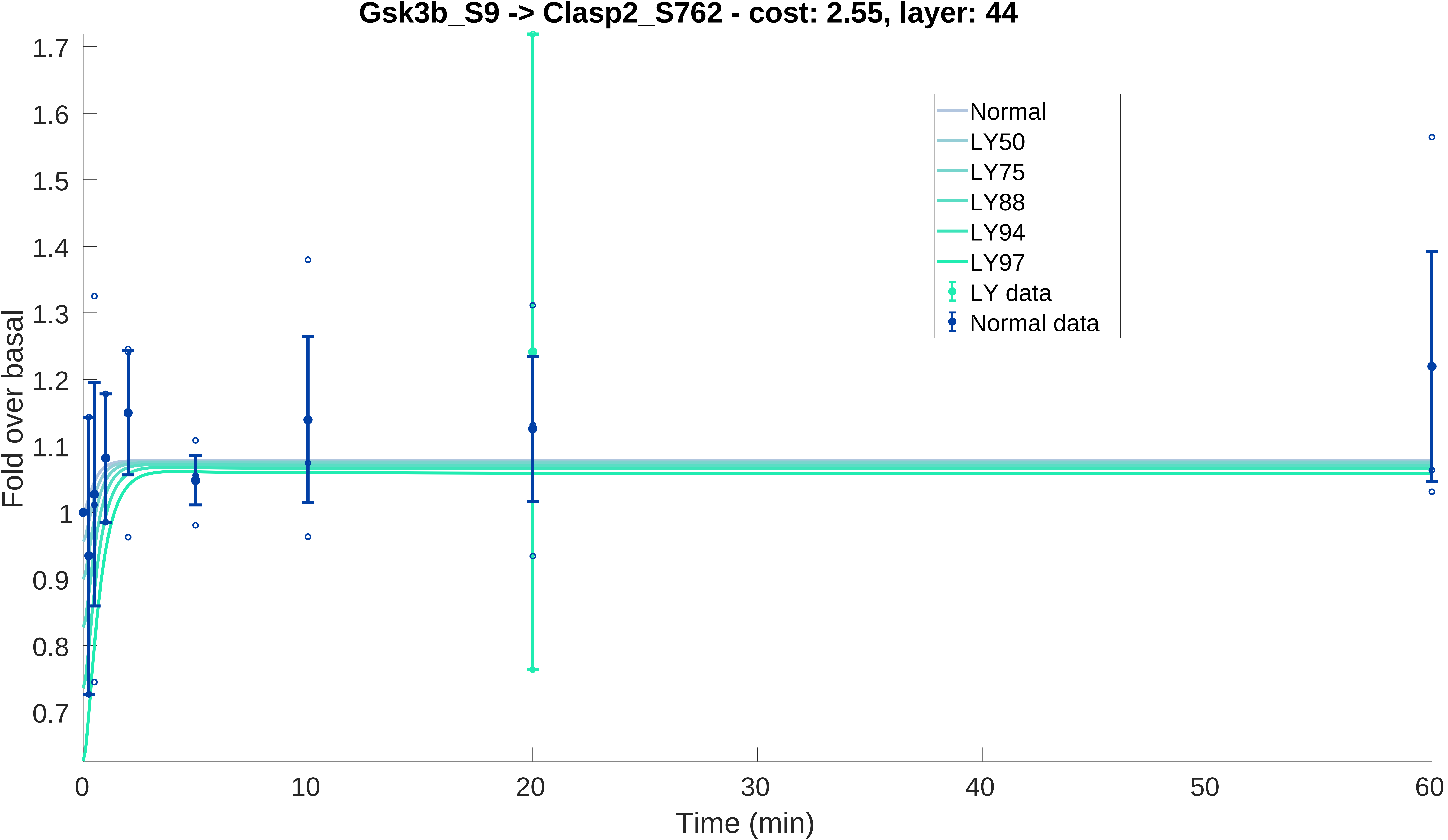

Gsk3b_S9 -> Clasp2_S762 - cost: 2.55, layer: 44
Fold over basal
Time (min)
0
10
20
30
40
50
60
0.7
0.8
0.9
1
1.1
1.2
1.3
1.4
1.5
1.6
1.7
Normal
LY50
LY75
LY88
LY94
LY97
LY data
Normal data

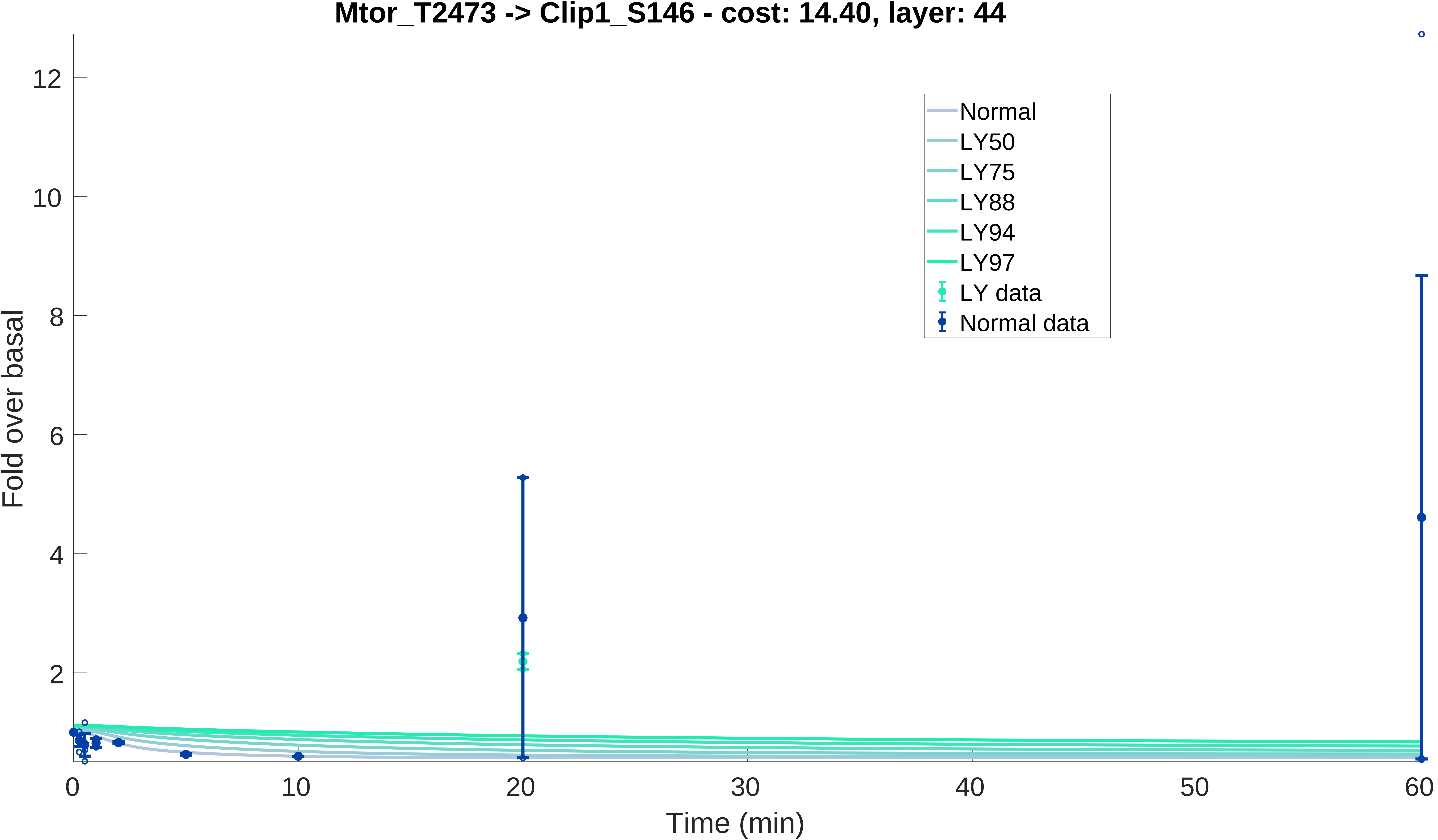

Mtor_T2473 -> Clip1_S146 - cost: 14.40, layer: 44
Fold over basal
Time (min)
0
10
20
30
40
50
60
2
4
6
8
10
12
Normal
LY50
LY75
LY88
LY94
LY97
LY data
Normal data

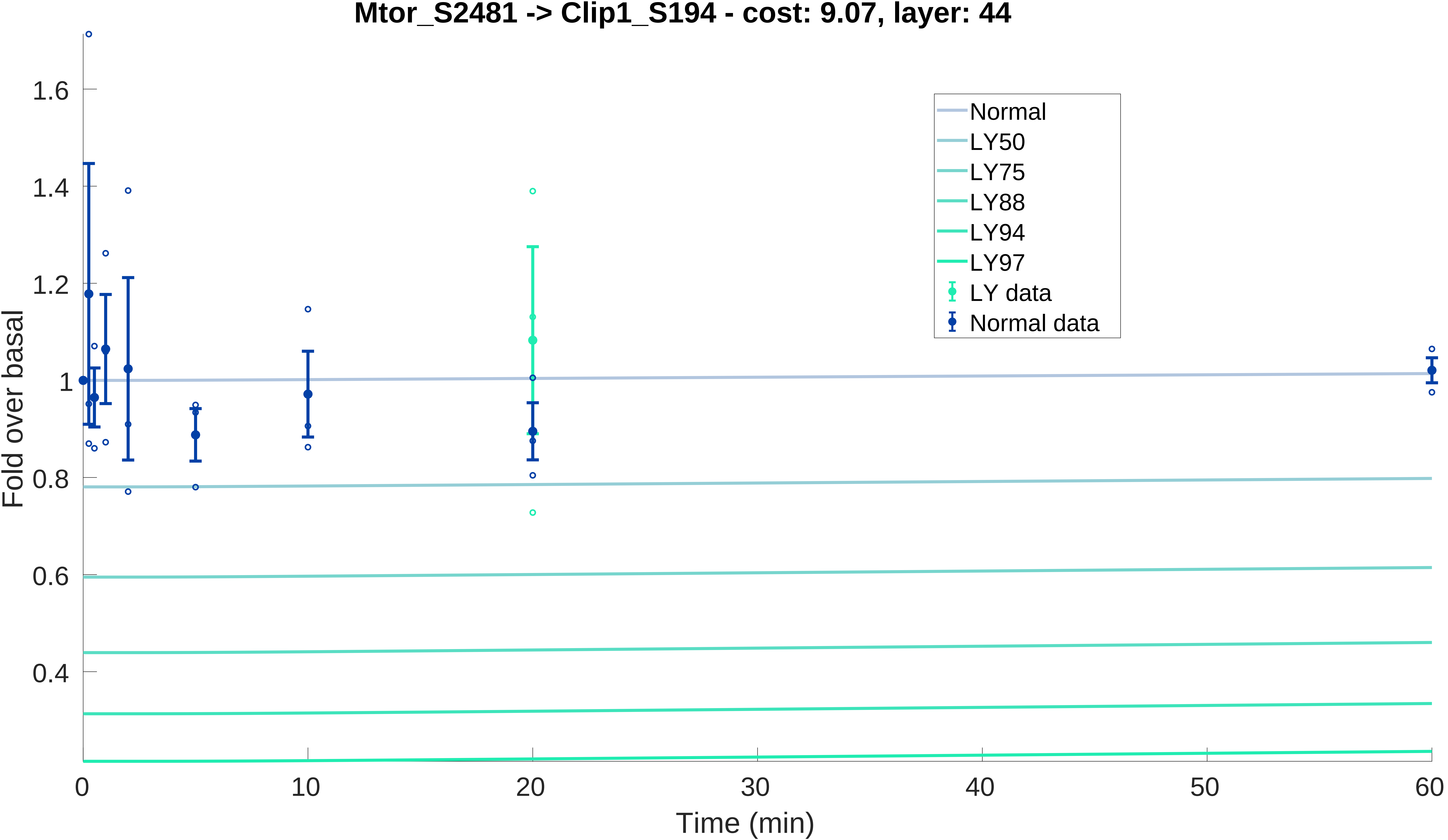

Mtor_S2481 -> Clip1_S194 - cost: 9.07, layer: 44
Fold over basal
Time (min)
Normal
LY50
LY75
LY88
LY94
LY97
LY data
Normal data
1.6
1.4
1.2
1
0.8
0.6
0.4
0
10
20
30
40
50
60

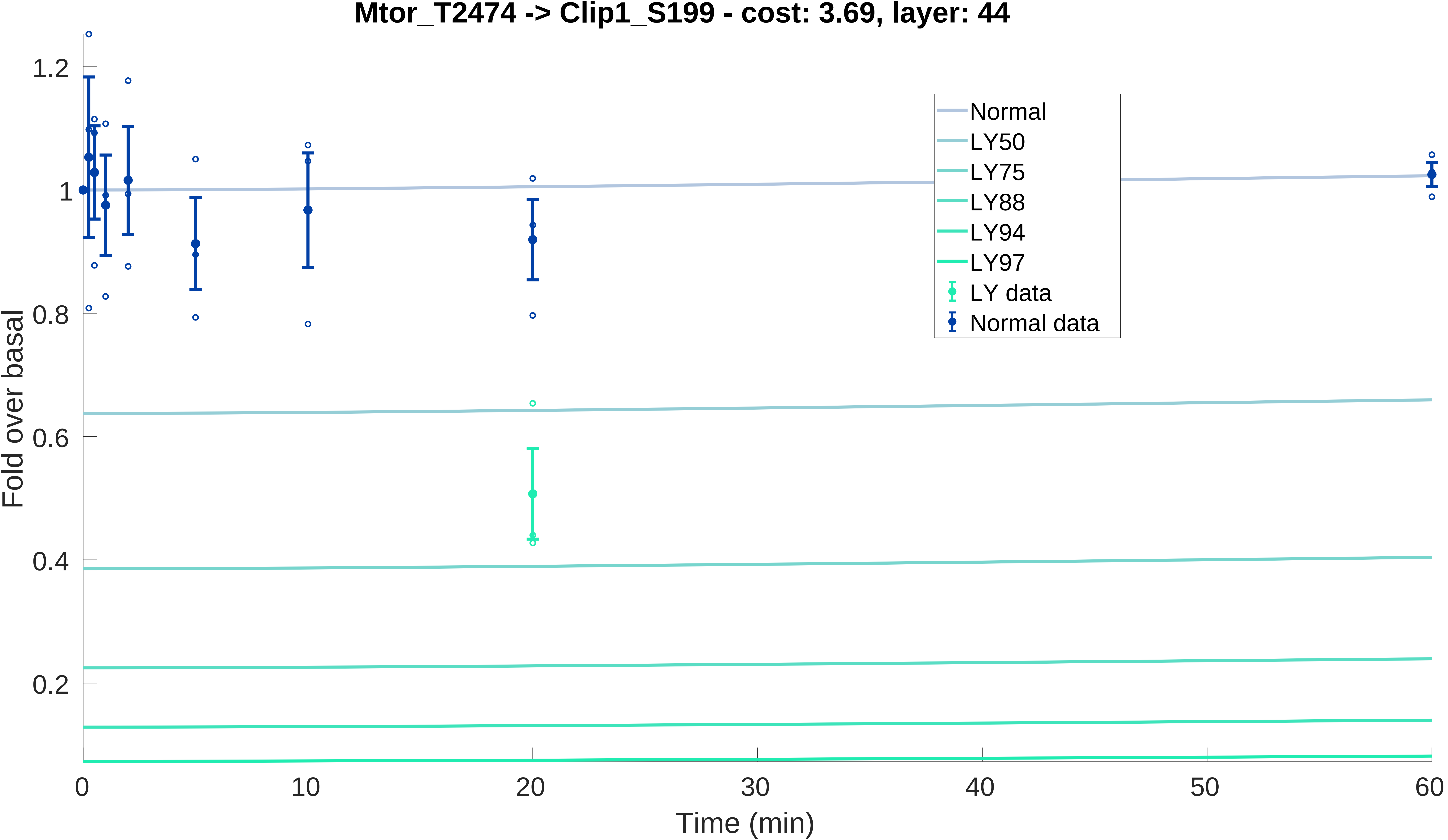

Mtor_T2474 -> Clip1_S199 - cost: 3.69, layer: 44
Fold over basal
Time (min)
1.2
1
0.8
0.6
0.4
0.2
0
10
20
30
40
50
60
Normal
LY50
LY75
LY88
LY94
LY97
LY data
Normal data

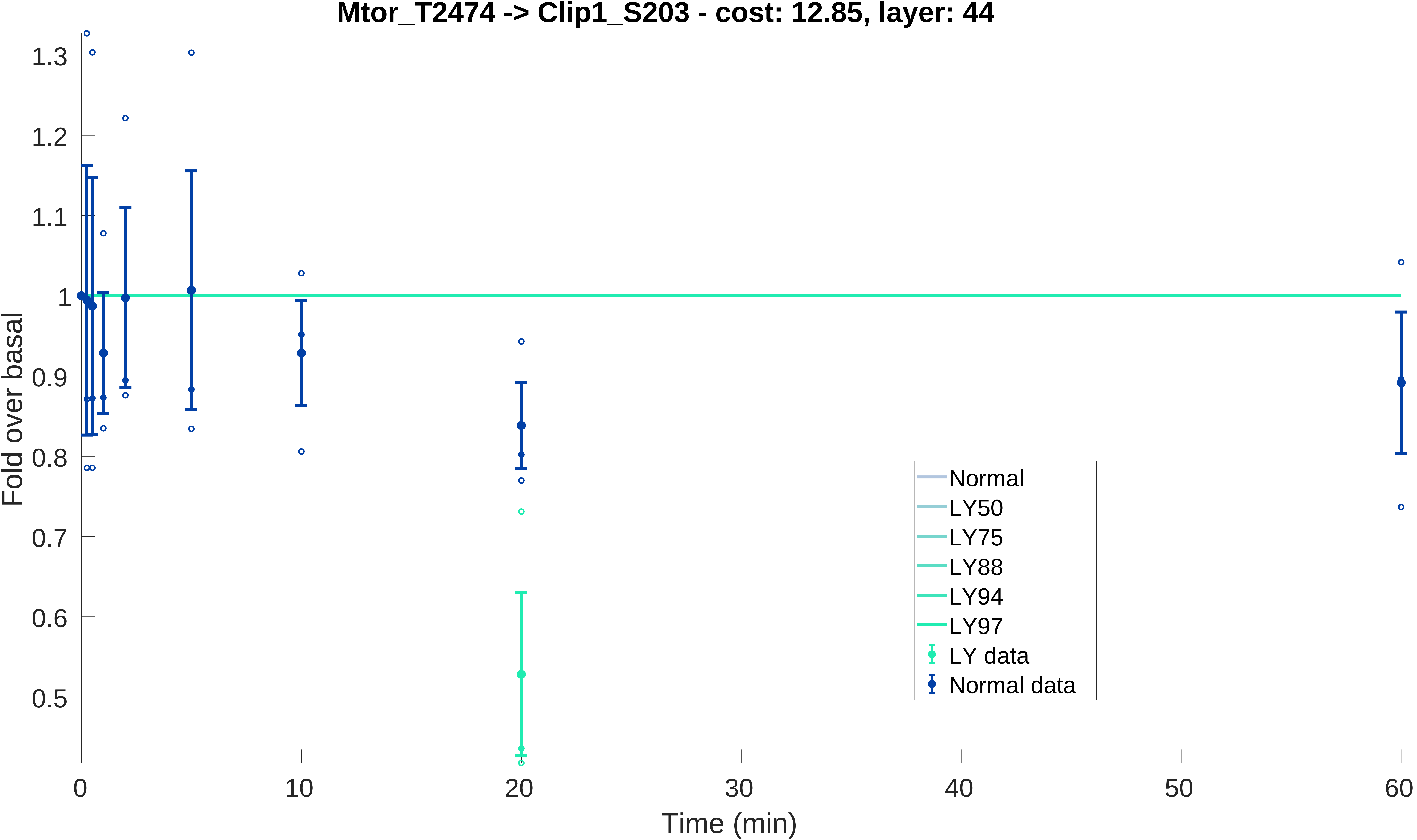

Mtor_T2474 -> Clip1_S203 - cost: 12.85, layer: 44
Fold over basal
Time (min)
1.3
1.2
1.1
1
0.9
0.8
0.7
0.6
0.5
0
10
20
30
40
50
60
Normal
LY50
LY75
LY88
LY94
LY97
LY data
Normal data

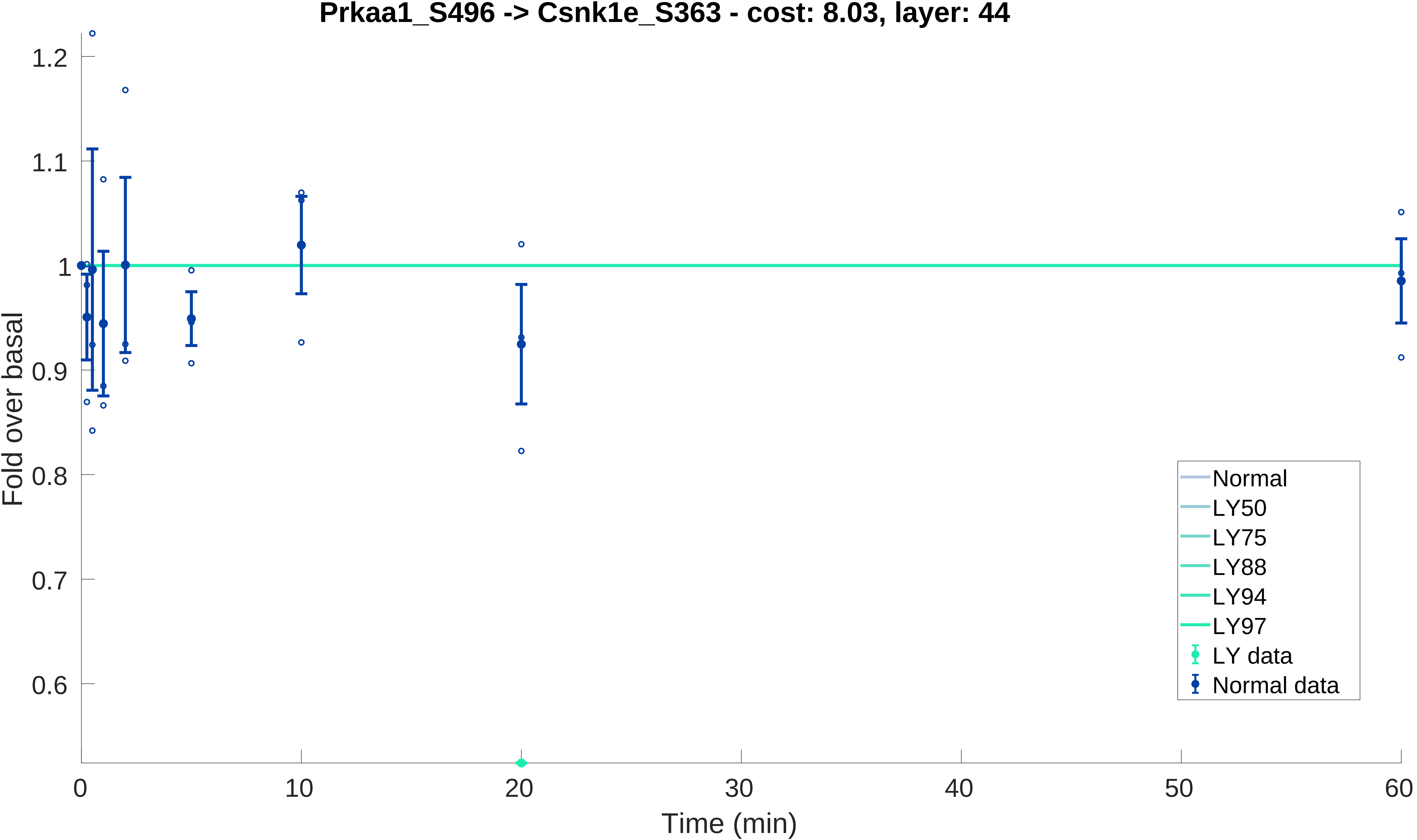

Prkaa1_S496 -> Csnk1e_S363 - cost: 8.03, layer: 44
Fold over basal
1.2
1.1
1
0.9
0.8
0.7
0.6
Time (min)
0
10
20
30
40
50
60
Normal
LY50
LY75
LY88
LY94
LY97
LY data
Normal data

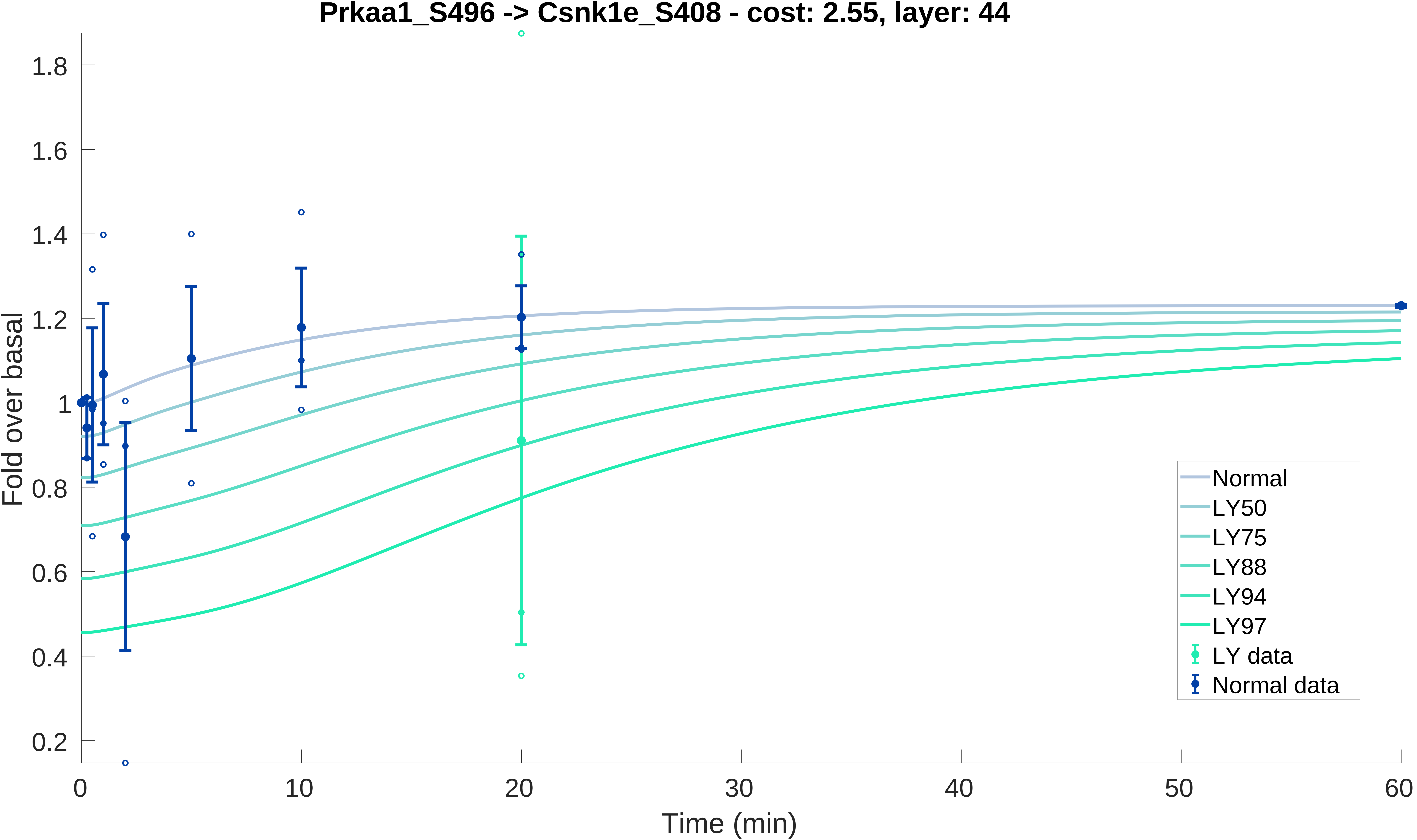

Prkaa1_S496 -> Csnk1e_S408 - cost: 2.55, layer: 44
Fold over basal
1.8
1.6
1.4
1.2
1
0.8
0.6
0.4
0.2
0
10
20
30
40
50
60
Time (min)
Normal
LY50
LY75
LY88
LY94
LY97
LY data
Normal data

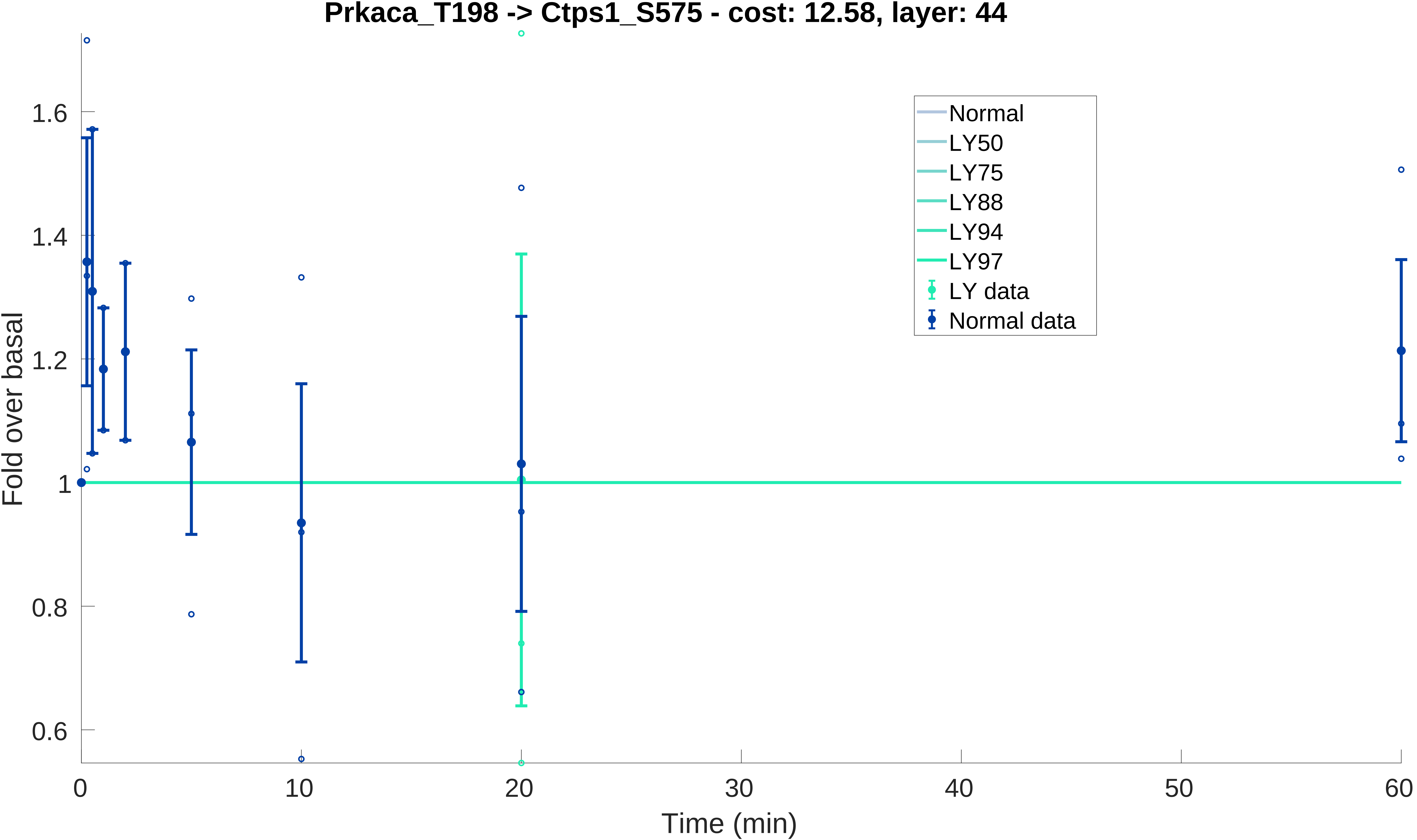

Prkaca_T198 -> Ctps1_S575 - cost: 12.58, layer: 44
Fold over basal
1.6
1.4
1.2
1
0.8
0.6
0
10
20
30
40
50
60
Time (min)
Normal
LY50
LY75
LY88
LY94
LY97
LY data
Normal data

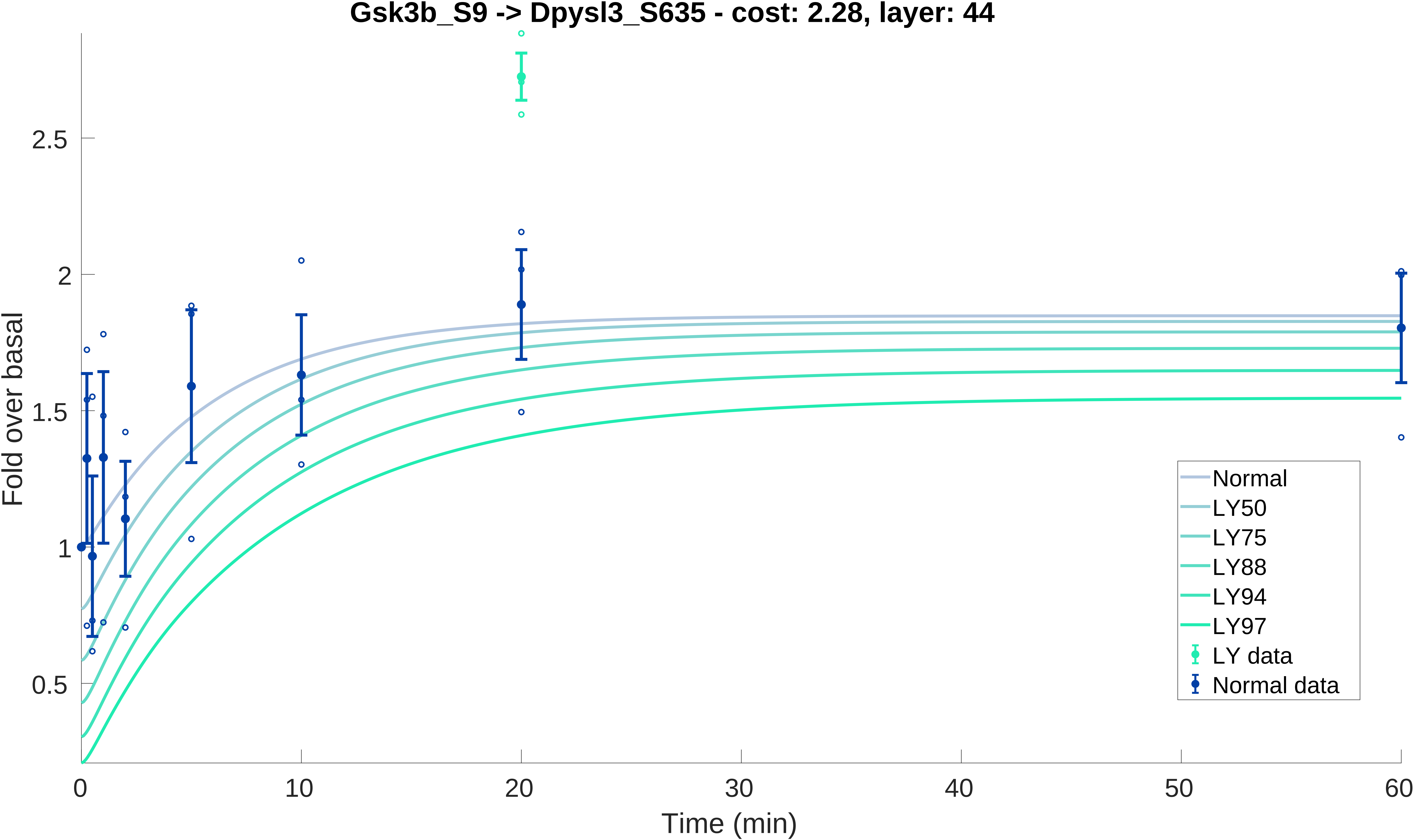
Gsk3b_S9 -> Dpysl3_S635 - cost: 2.28, layer: 44
Fold over basal
Time (min)
0
10
20
30
40
50
60
0.5
1
1.5
2
2.5
Normal
LY50
LY75
LY88
LY94
LY97
LY data
Normal data

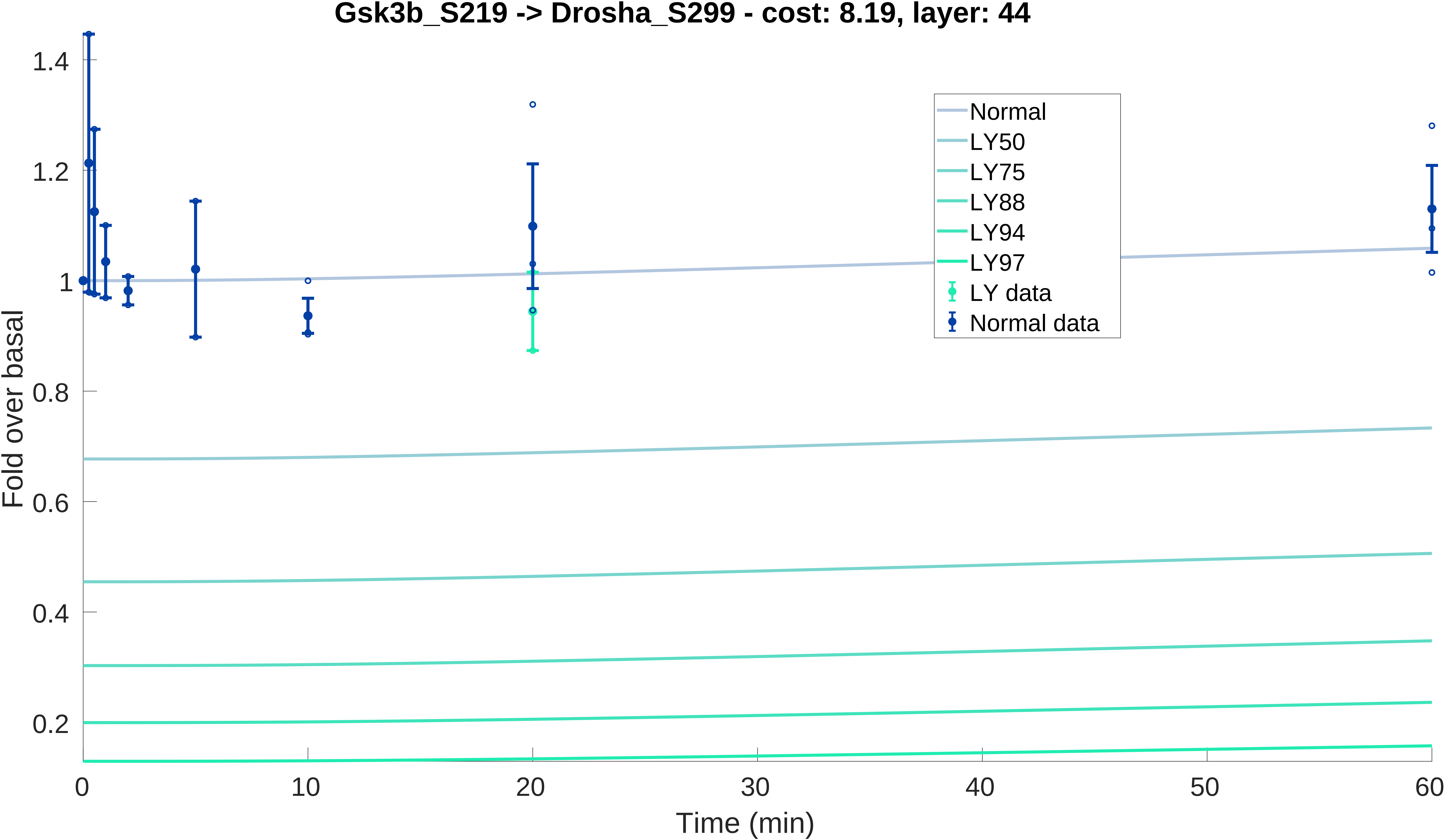
Gsk3b_S219 -> Drosha_S299 - cost: 8.19, layer: 44
Fold over basal
1.4
1.2
1
0.8
0.6
0.4
0.2
0
10
20
30
40
50
60
Time (min)
Normal
LY50
LY75
LY88
LY94
LY97
LY data
Normal data

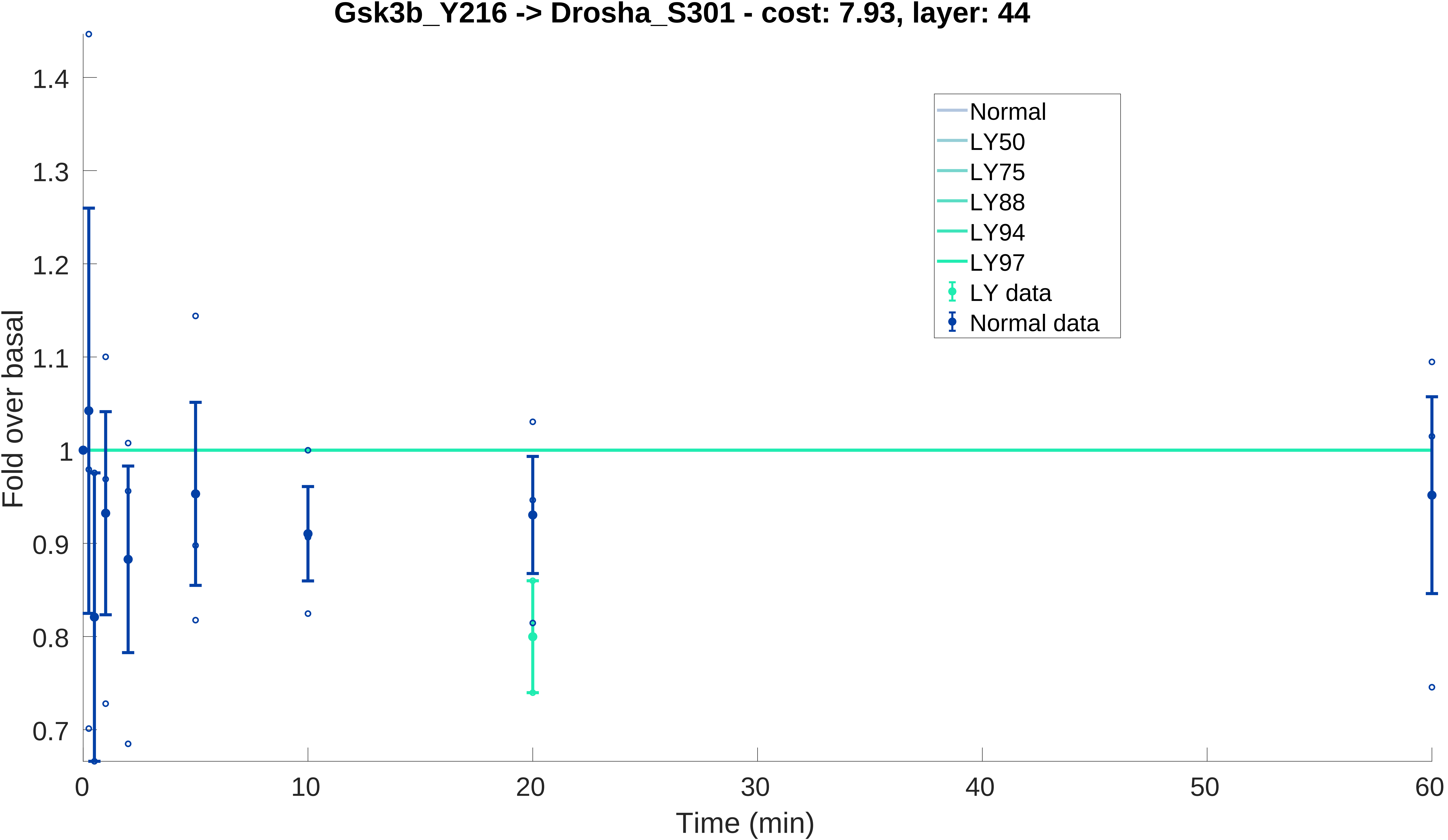

Gsk3b_Y216 -> Drosha_S301 - cost: 7.93, layer: 44
Fold over basal
Time (min)
Normal
LY50
LY75
LY88
LY94
LY97
LY data
Normal data
1.4
1.3
1.2
1.1
1
0.9
0.8
0.7
0
10
20
30
40
50
60

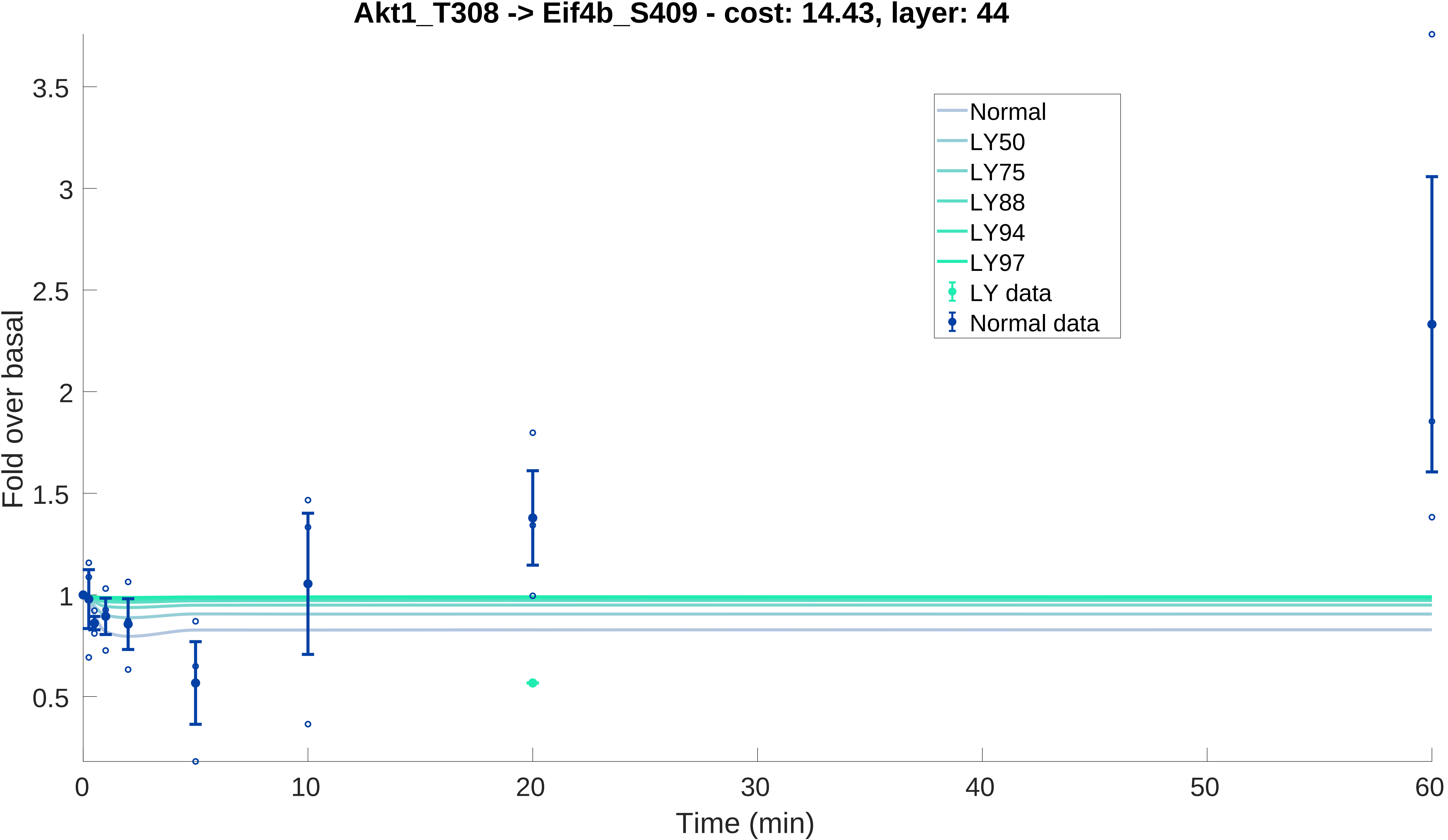

Akt1_T308 -> Eif4b_S409 - cost: 14.43, layer: 44
Fold over basal
Time (min)
Normal
LY50
LY75
LY88
LY94
LY97
LY data
Normal data
3.5
3
2.5
2
1.5
1
0.5
0
10
20
30
40
50
60

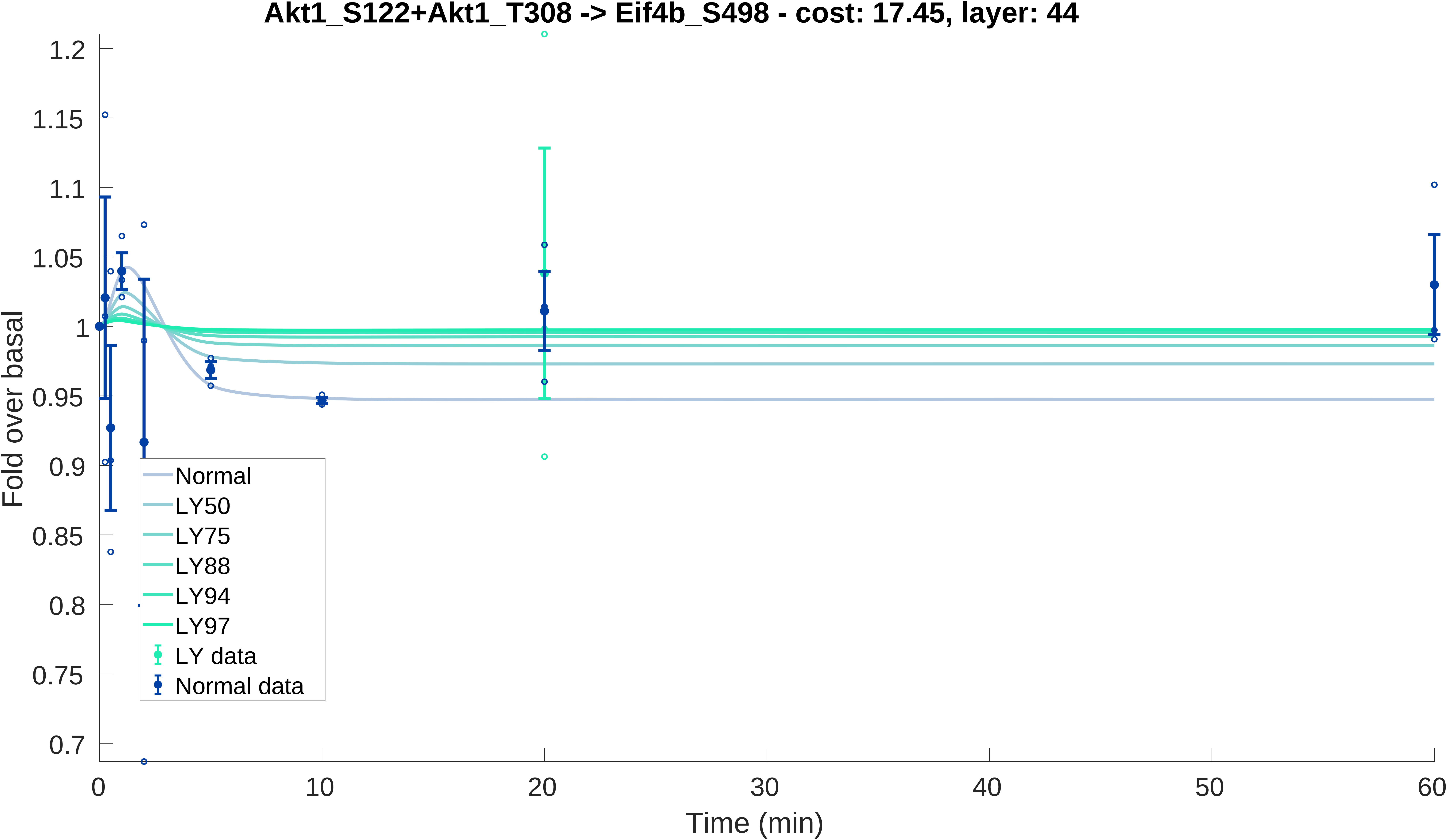
Akt1_S122+Akt1_T308 -> Eif4b_S498 - cost: 17.45, layer: 44
Fold over basal
Time (min)
1.2
1.15
1.1
1.05
1
0.95
0.9
0.85
0.8
0.75
0.7
0
10
20
30
40
50
60
Normal
LY50
LY75
LY88
LY94
LY97
LY data
Normal data

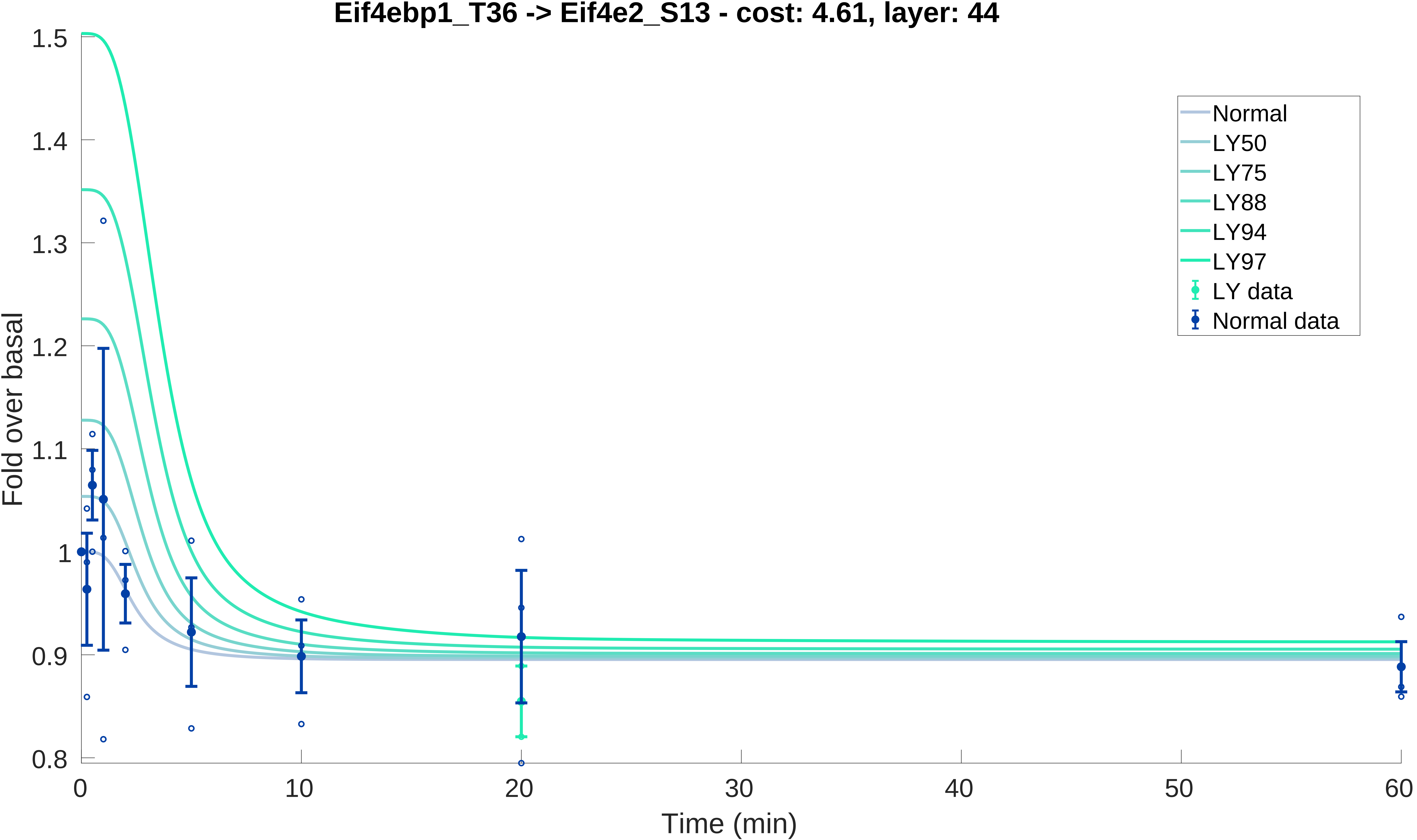

Eif4ebp1_T36 -> Eif4e2_S13 - cost: 4.61, layer: 44
Fold over basal
Time (min)
0.8
0.9
1
1.1
1.2
1.3
1.4
1.5
0
10
20
30
40
50
60
Normal
LY50
LY75
LY88
LY94
LY97
LY data
Normal data

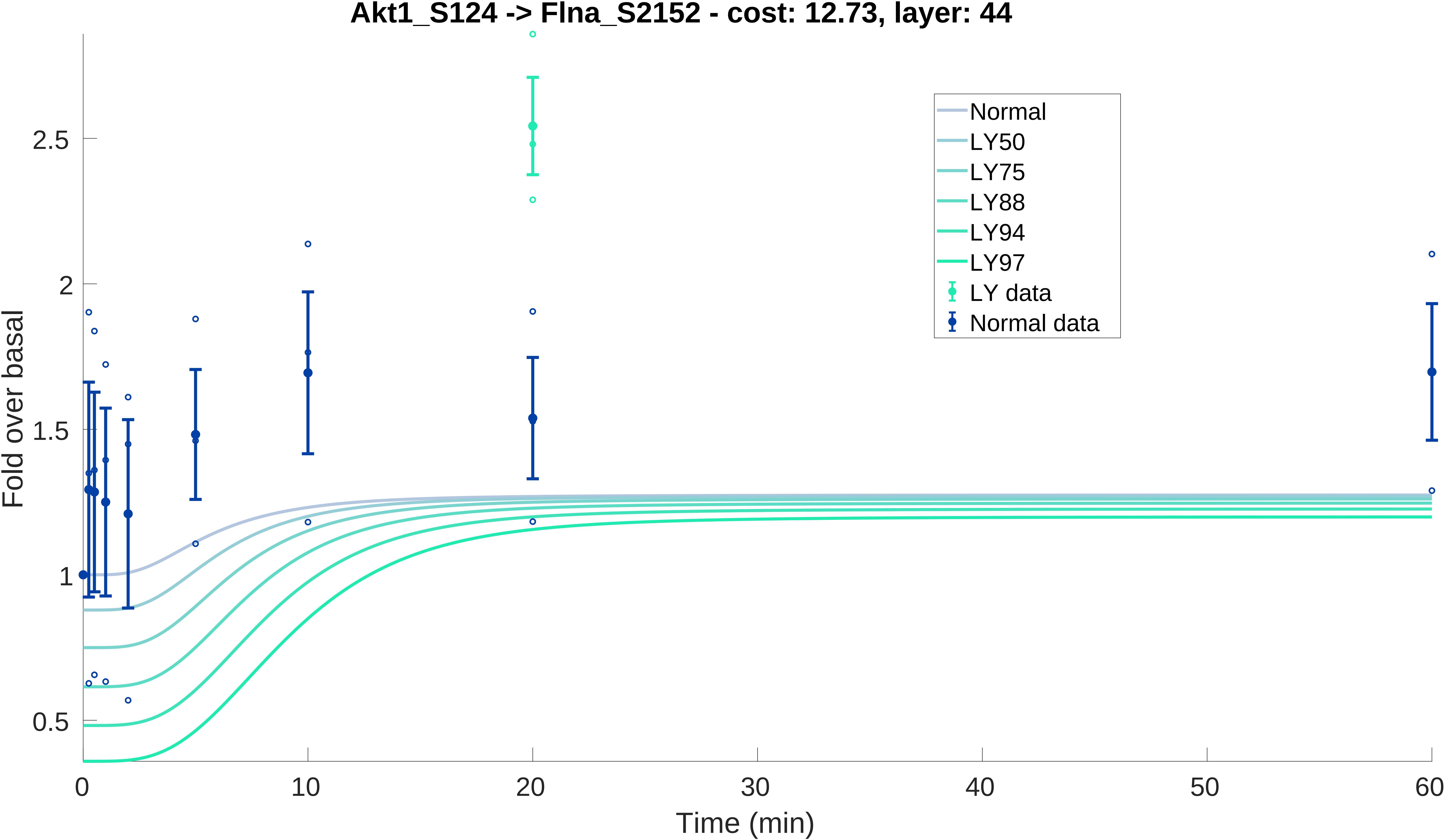
Akt1_S124 -> Flna_S2152 - cost: 12.73, layer: 44
Normal
LY50
LY75
LY88
LY94
LY97
LY data
Normal data
Fold over basal
2.5
2
1.5
1
0.5
0
10
20
30
40
50
60
Time (min)

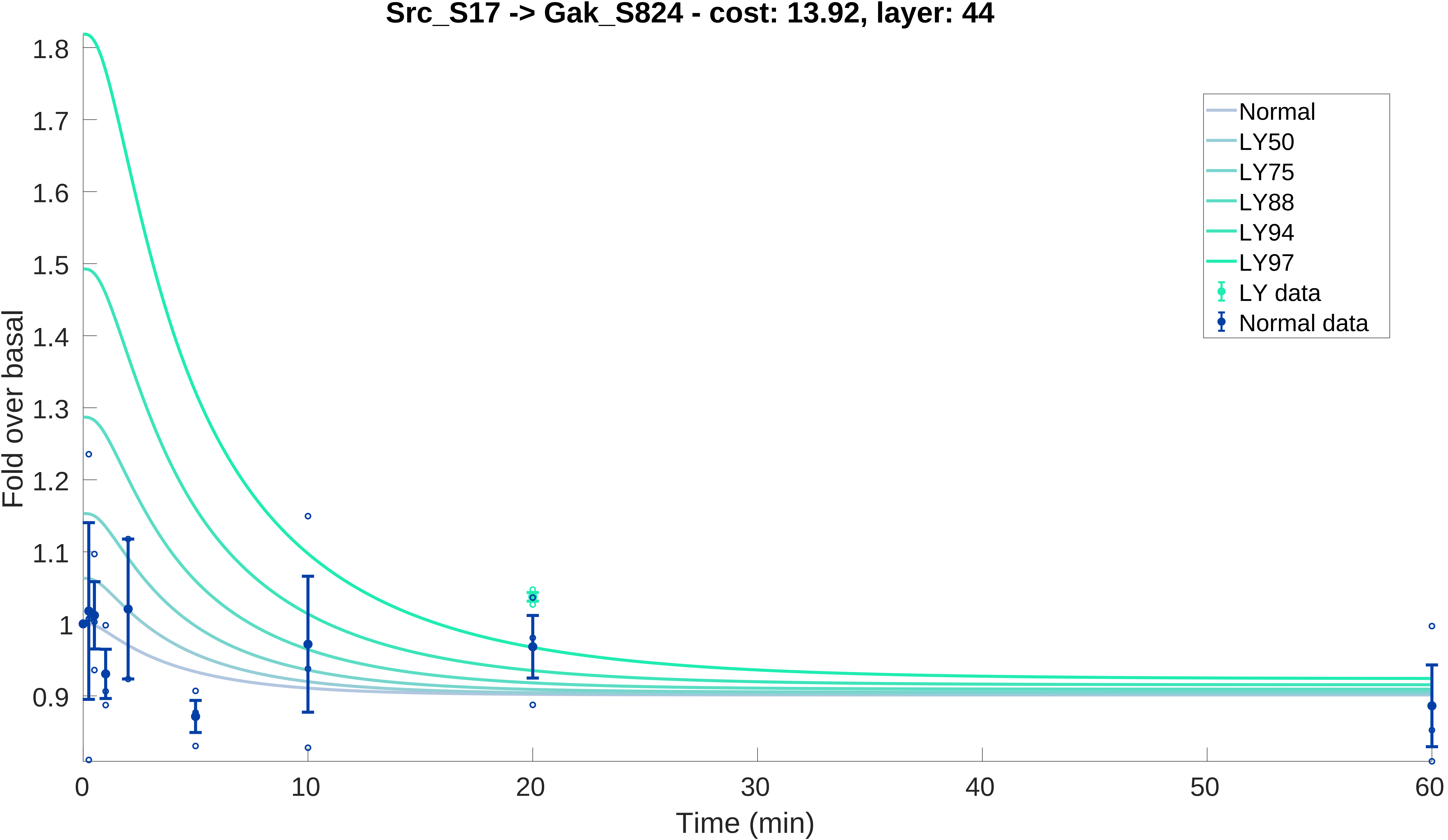
Src_S17 -> Gak_S824 - cost: 13.92, layer: 44
Fold over basal
1.8
1.7
1.6
1.5
1.4
1.3
1.2
1.1
1
0.9
0
10
20
30
40
50
60
Time (min)
Normal
LY50
LY75
LY88
LY94
LY97
LY data
Normal data

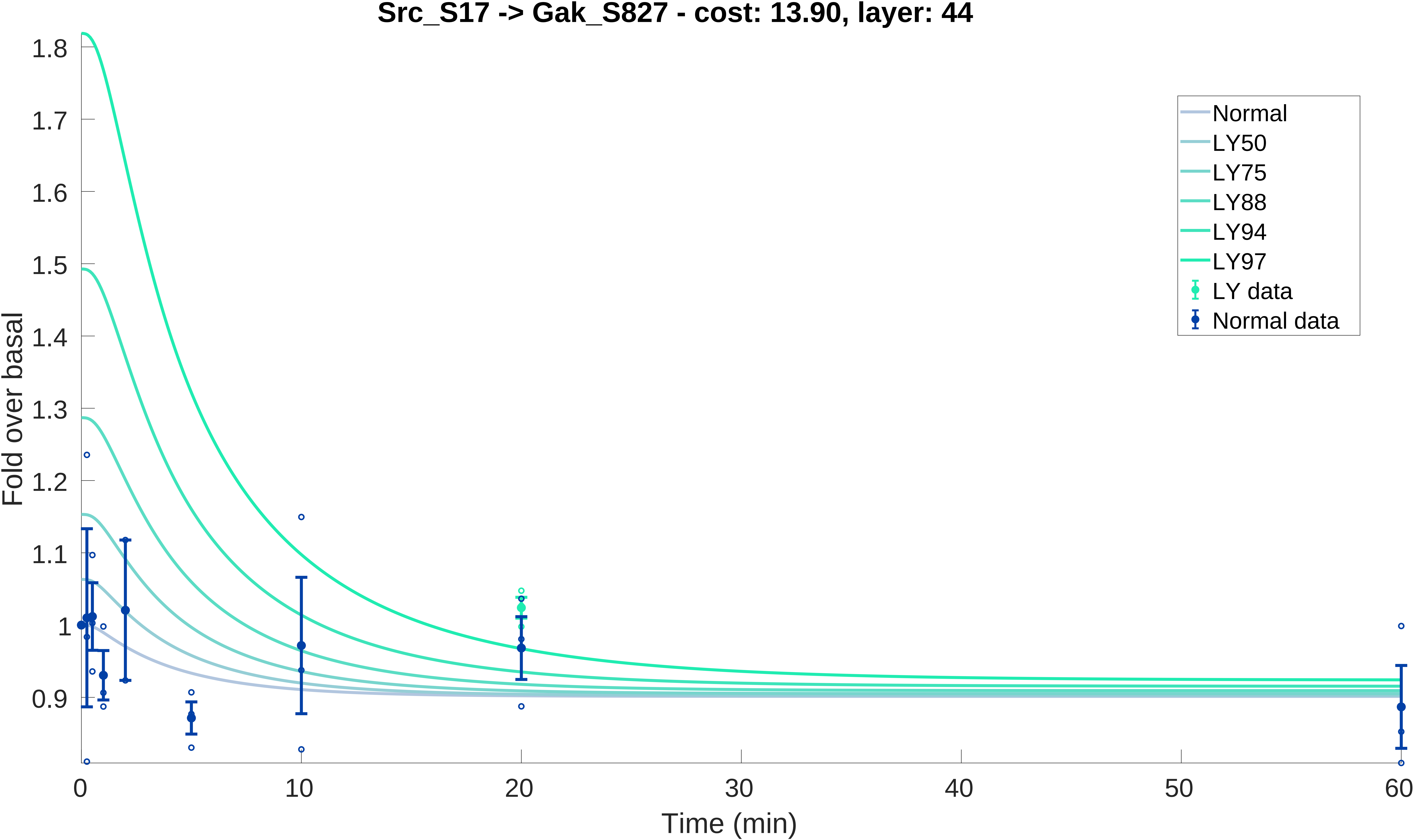
Src_S17 -> Gak_S827 - cost: 13.90, layer: 44
Fold over basal
1.8
1.7
1.6
1.5
1.4
1.3
1.2
1.1
1
0.9
Time (min)
0
10
20
30
40
50
60
Normal
LY50
LY75
LY88
LY94
LY97
LY data
Normal data

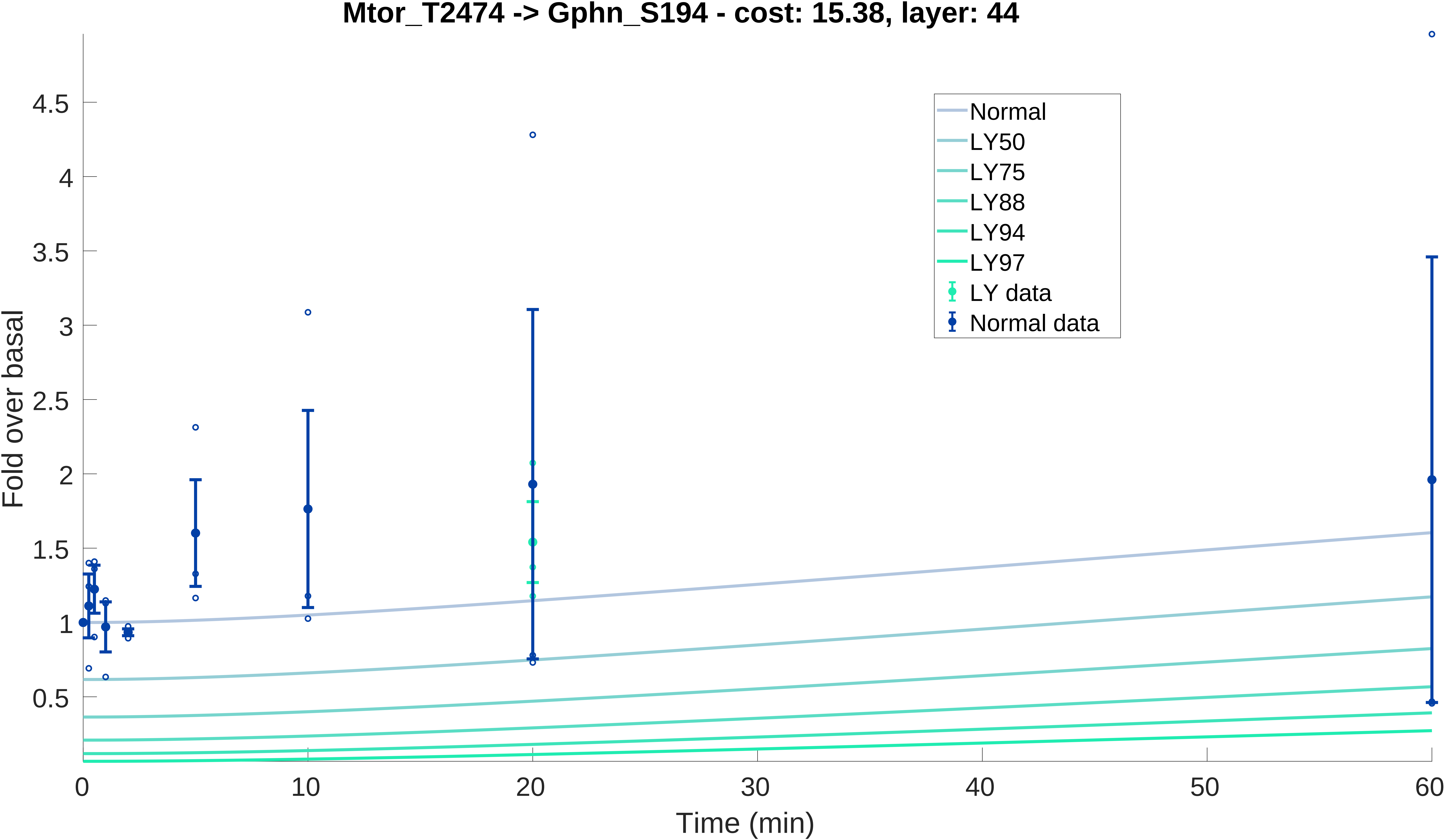

Mtor_T2474 -> Gphn_S194 - cost: 15.38, layer: 44
Normal
LY50
LY75
LY88
LY94
LY97
LY data
Normal data
Fold over basal
Time (min)
4.5
4
3.5
3
2.5
2
1.5
1
0.5
0
10
20
30
40
50
60

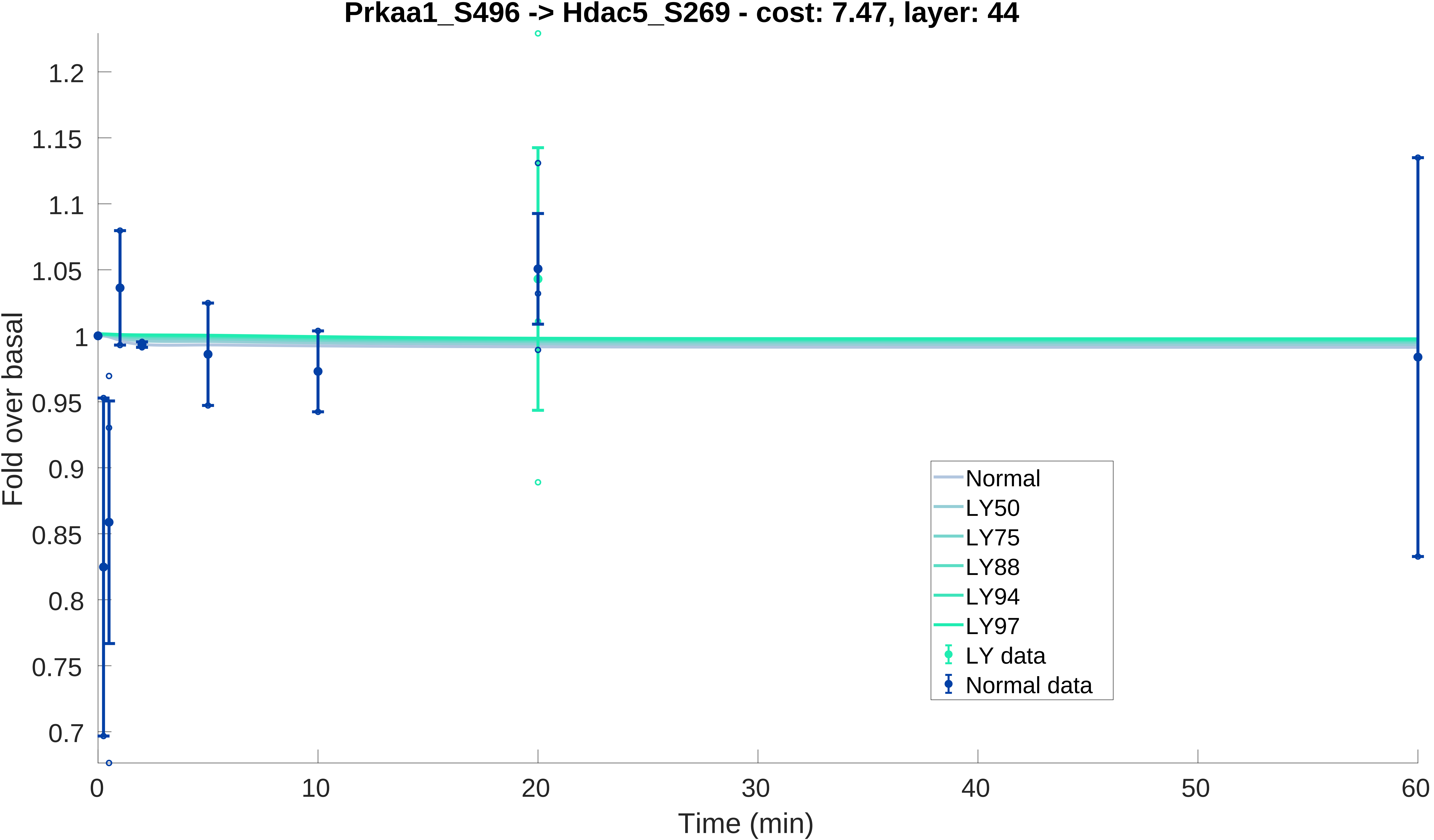

Prkaa1_S496 -> Hdac5_S269 - cost: 7.47, layer: 44
Fold over basal
Time (min)
1.2
1.15
1.1
1.05
1
0.95
0.9
0.85
0.8
0.75
0.7
0
10
20
30
40
50
60
Normal
LY50
LY75
LY88
LY94
LY97
LY data
Normal data

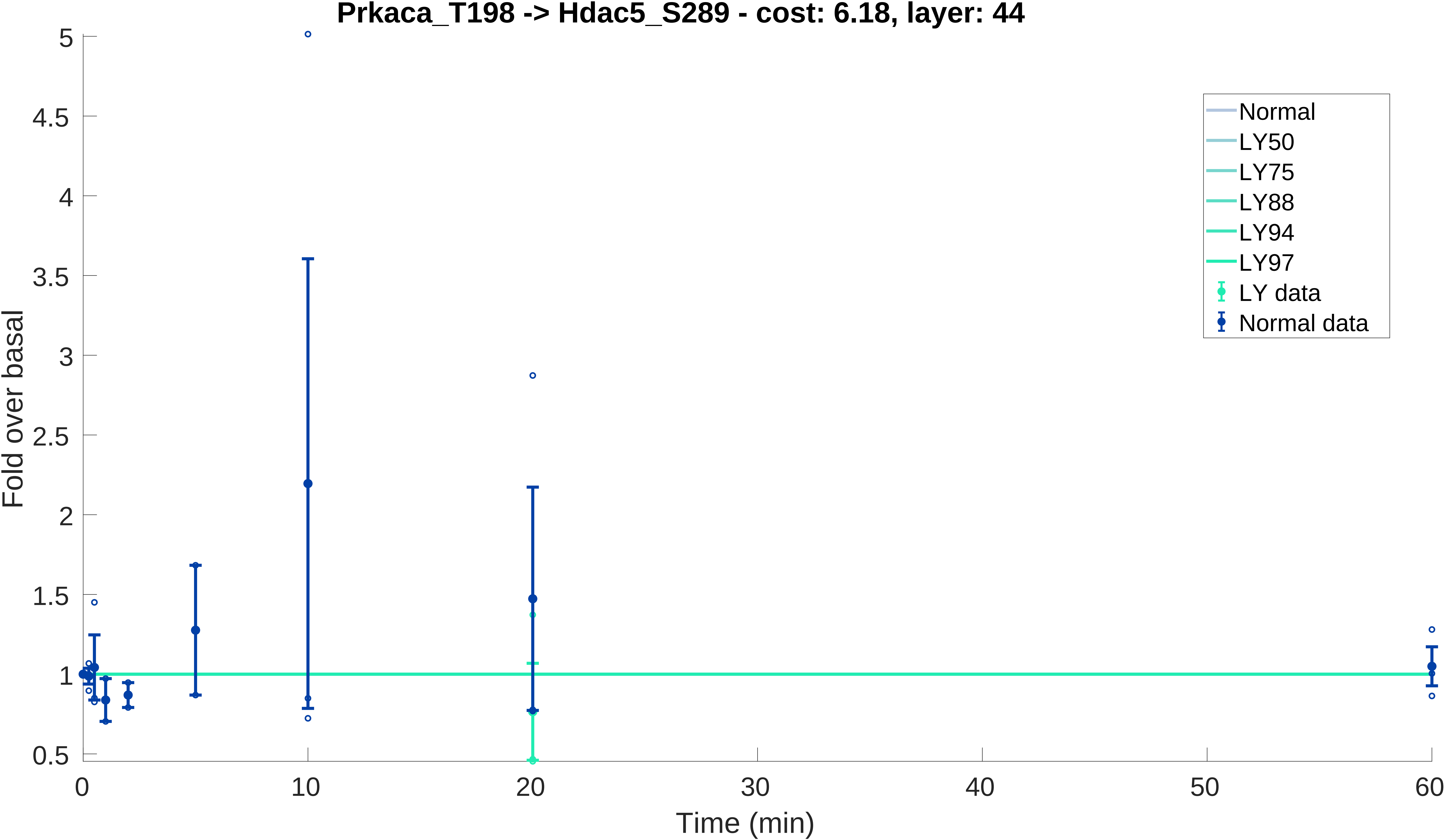

Prrkaca_T198 -> Hdac5_S289 - cost: 6.18, layer: 44
Fold over basal
Time (min)
0.5
1
1.5
2
2.5
3
3.5
4
4.5
5
0
10
20
30
40
50
60
Normal
LY50
LY75
LY88
LY94
LY97
LY data
Normal data

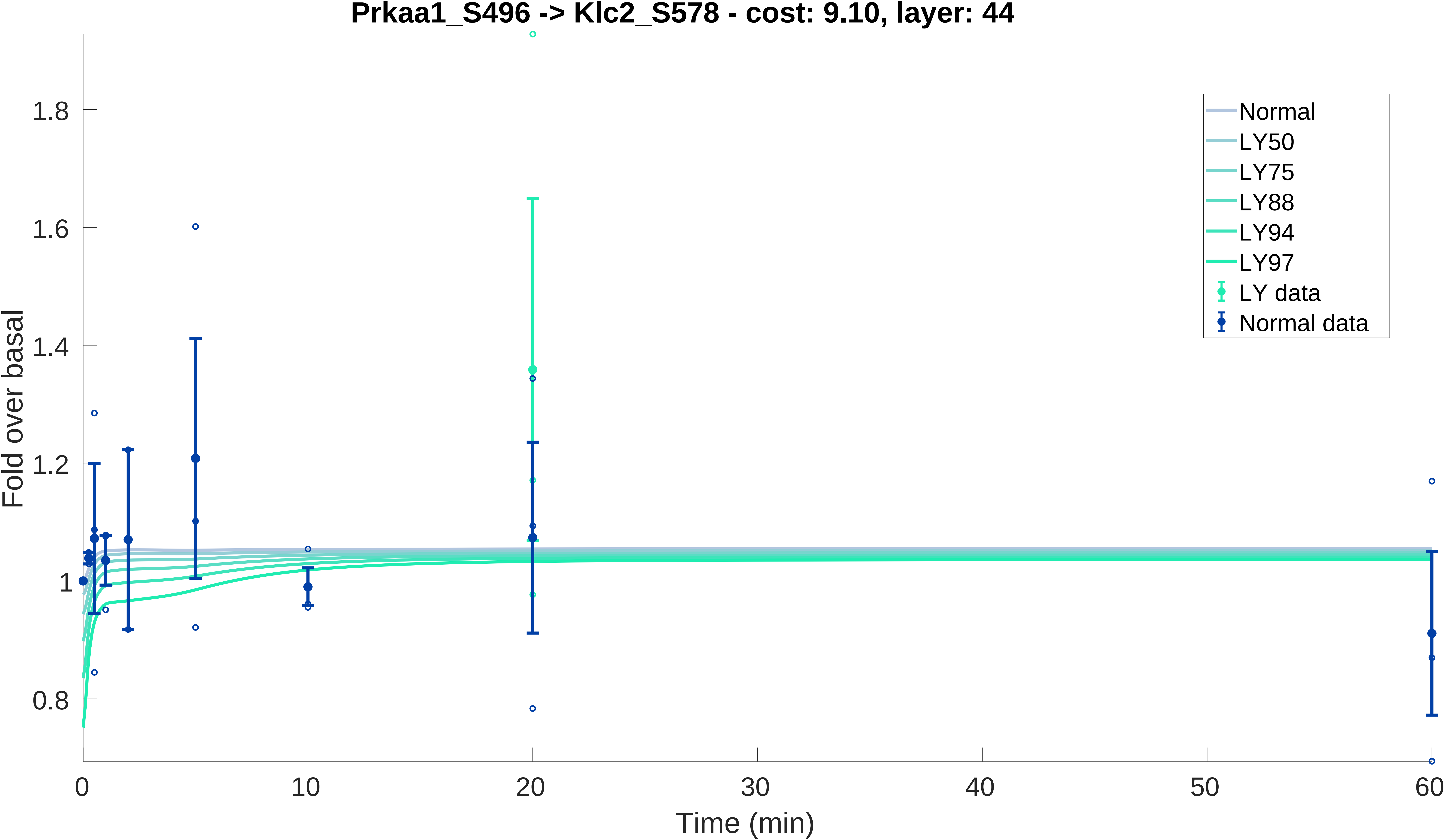

Prkaa1_S496 -> Klc2_S578 - cost: 9.10, layer: 44
Fold over basal
1.8
1.6
1.4
1.2
1
0.8
0
10
20
30
40
50
60
Time (min)
Normal
LY50
LY75
LY88
LY94
LY97
LY data
Normal data

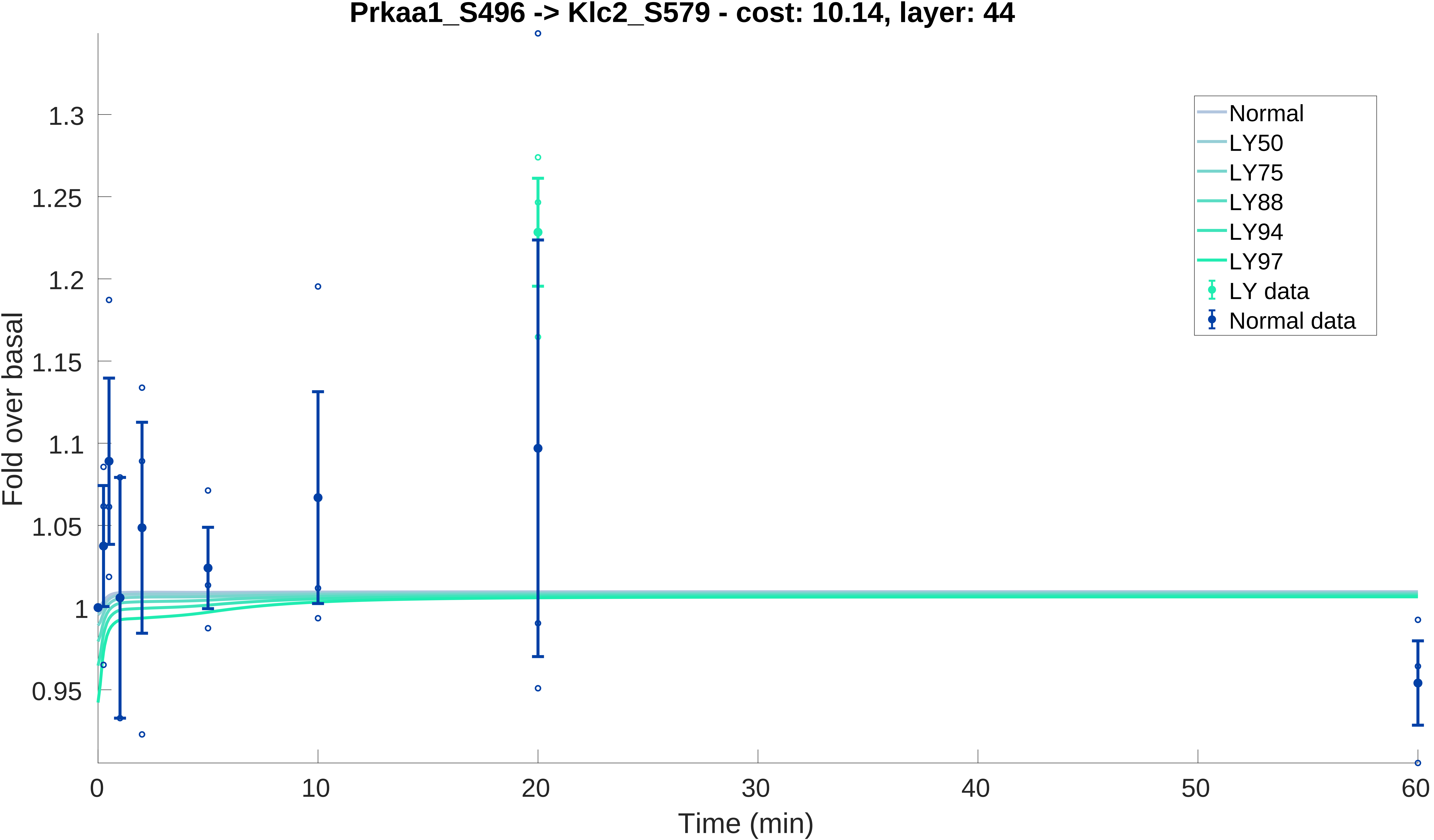
Prrkaa1_S496 -> Klc2_S579 - cost: 10.14, layer: 44
Fold over basal
Time (min)
Normal
LY50
LY75
LY88
LY94
LY97
LY data
Normal data
1.3
1.25
1.2
1.15
1.1
1.05
1
0.95
0
10
20
30
40
50
60

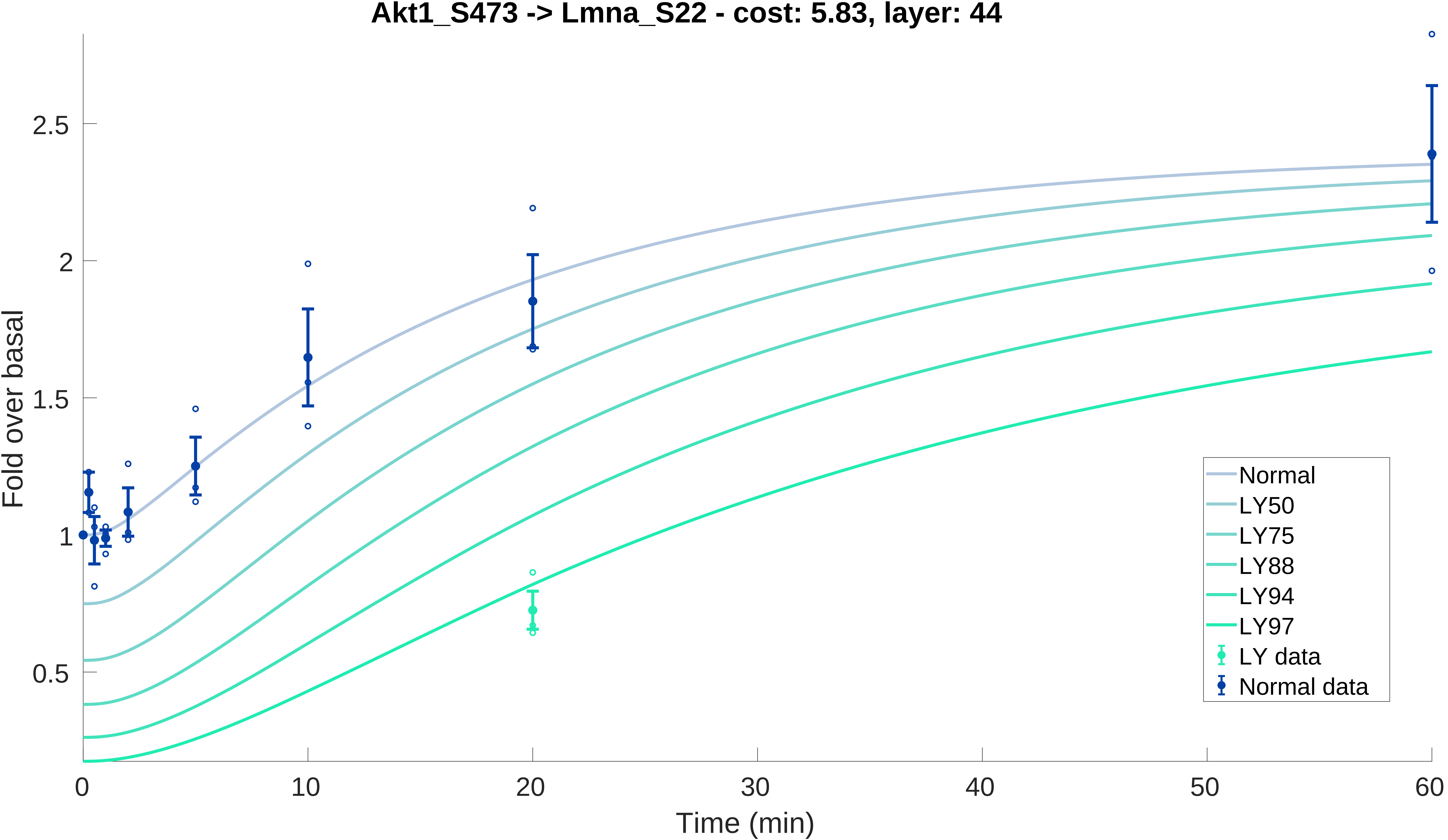
Akt1_S473 -> Lmna_S22 - cost: 5.83, layer: 44
Fold over basal
2.5
2
1.5
1
0.5
0
10
20
30
40
50
60
Time (min)
Normal
LY50
LY75
LY88
LY94
LY97
LY data
Normal data

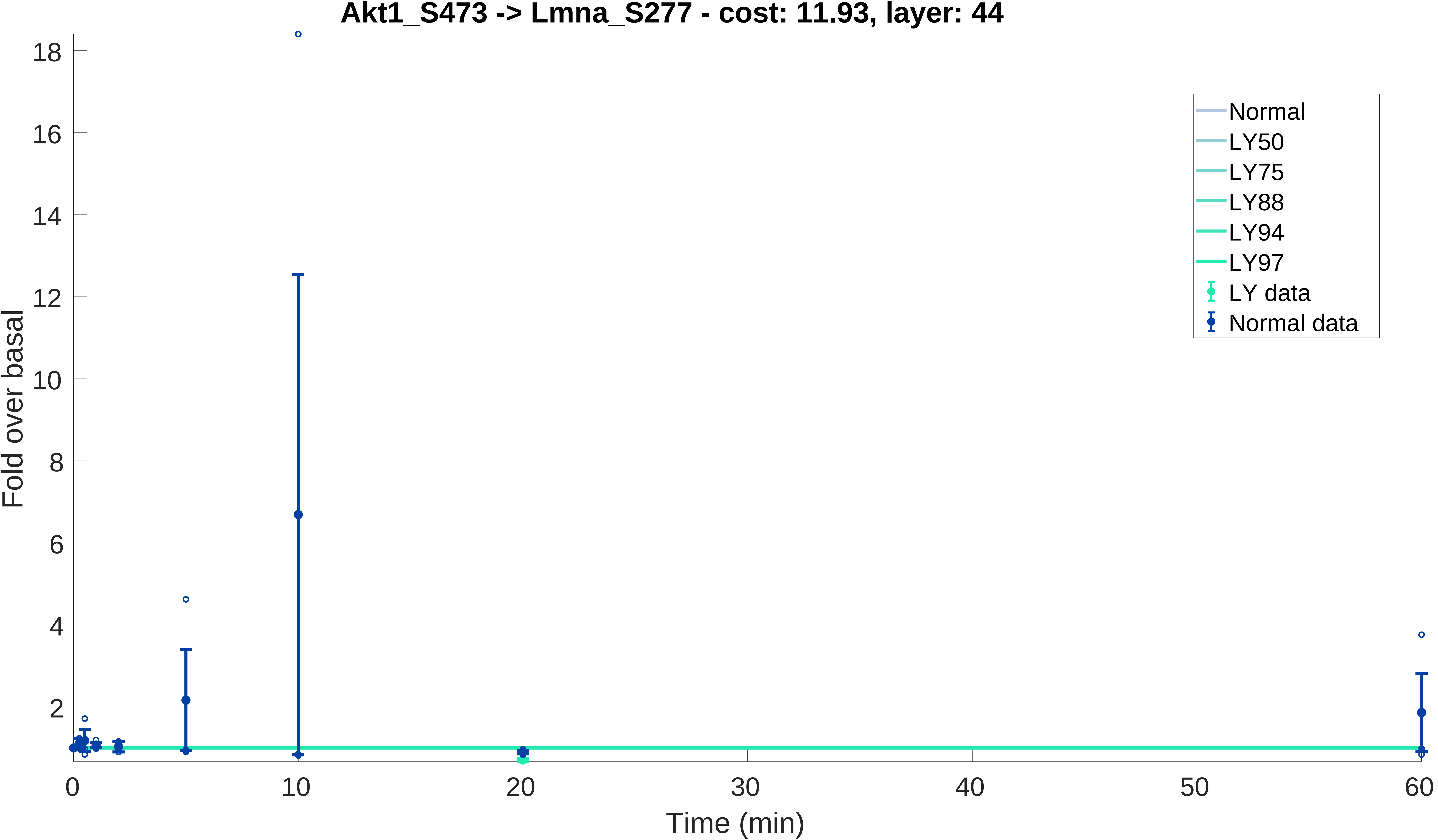
Akt1_S473 -> Lmna_S277 - cost: 11.93, layer: 44
Fold over basal
Time (min)
0
10
20
30
40
50
60
2
4
6
8
10
12
14
16
18
Normal
LY50
LY75
LY88
LY94
LY97
LY data
Normal data

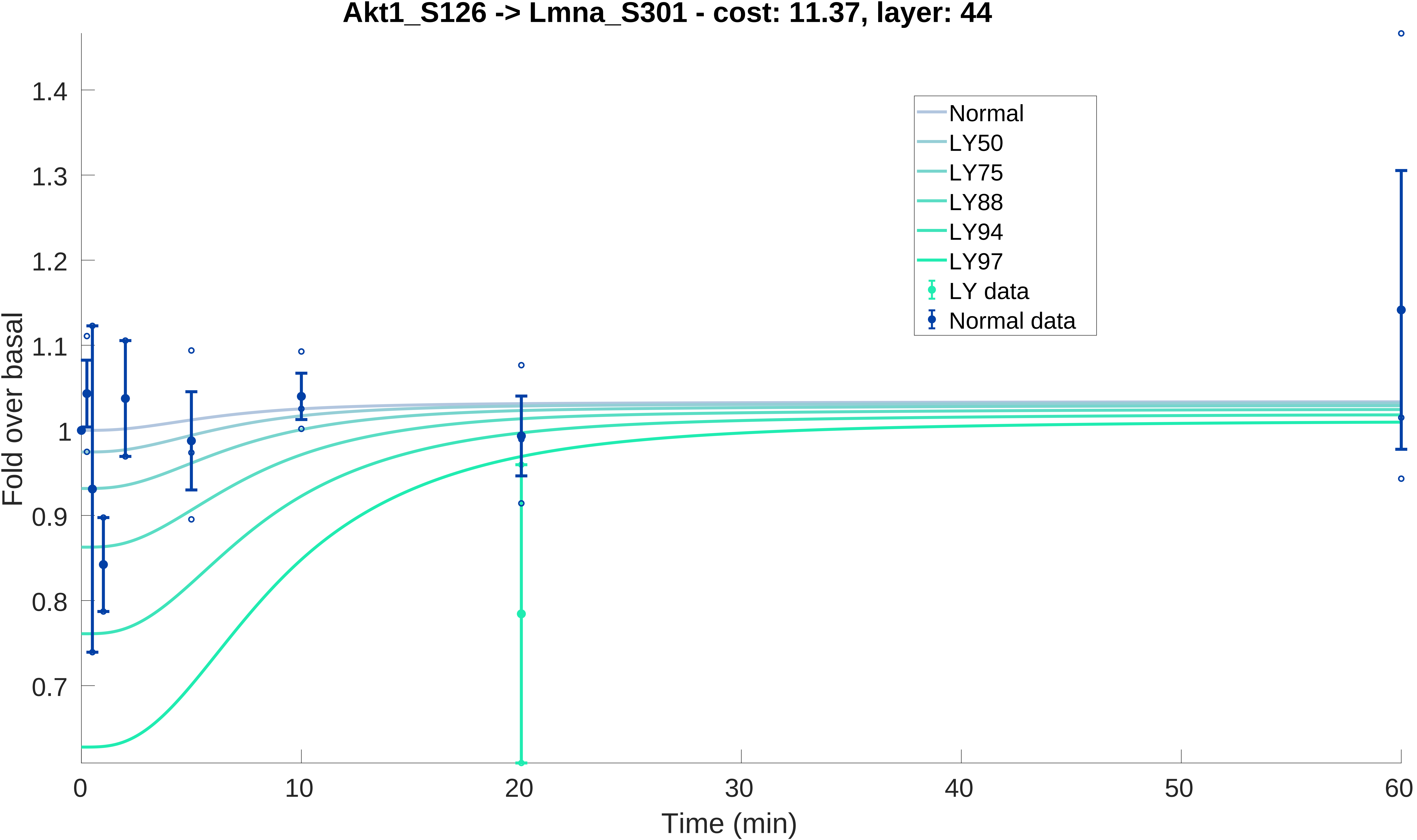

Akt1_S126 -> Lmna_S301 - cost: 11.37, layer: 44
Fold over basal
Time (min)
1.4
1.3
1.2
1.1
1
0.9
0.8
0.7
0
10
20
30
40
50
60
Normal
LY50
LY75
LY88
LY94
LY97
LY data
Normal data

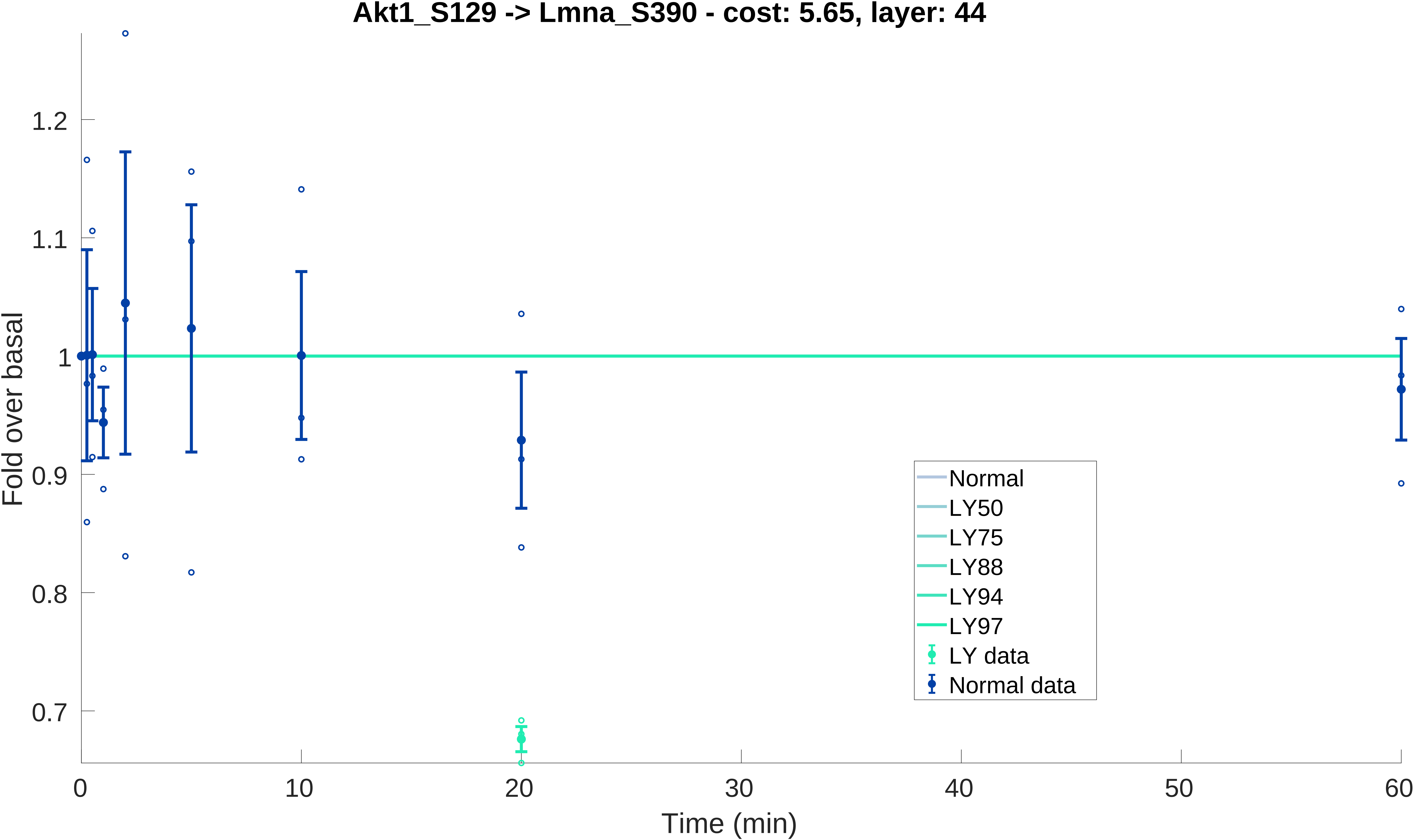

Akt1_S129 -> Lmna_S390 - cost: 5.65, layer: 44
Fold over basal
Time (min)
1.2
1.1
1
0.9
0.8
0.7
0
10
20
30
40
50
60
Normal
LY50
LY75
LY88
LY94
LY97
LY data
Normal data

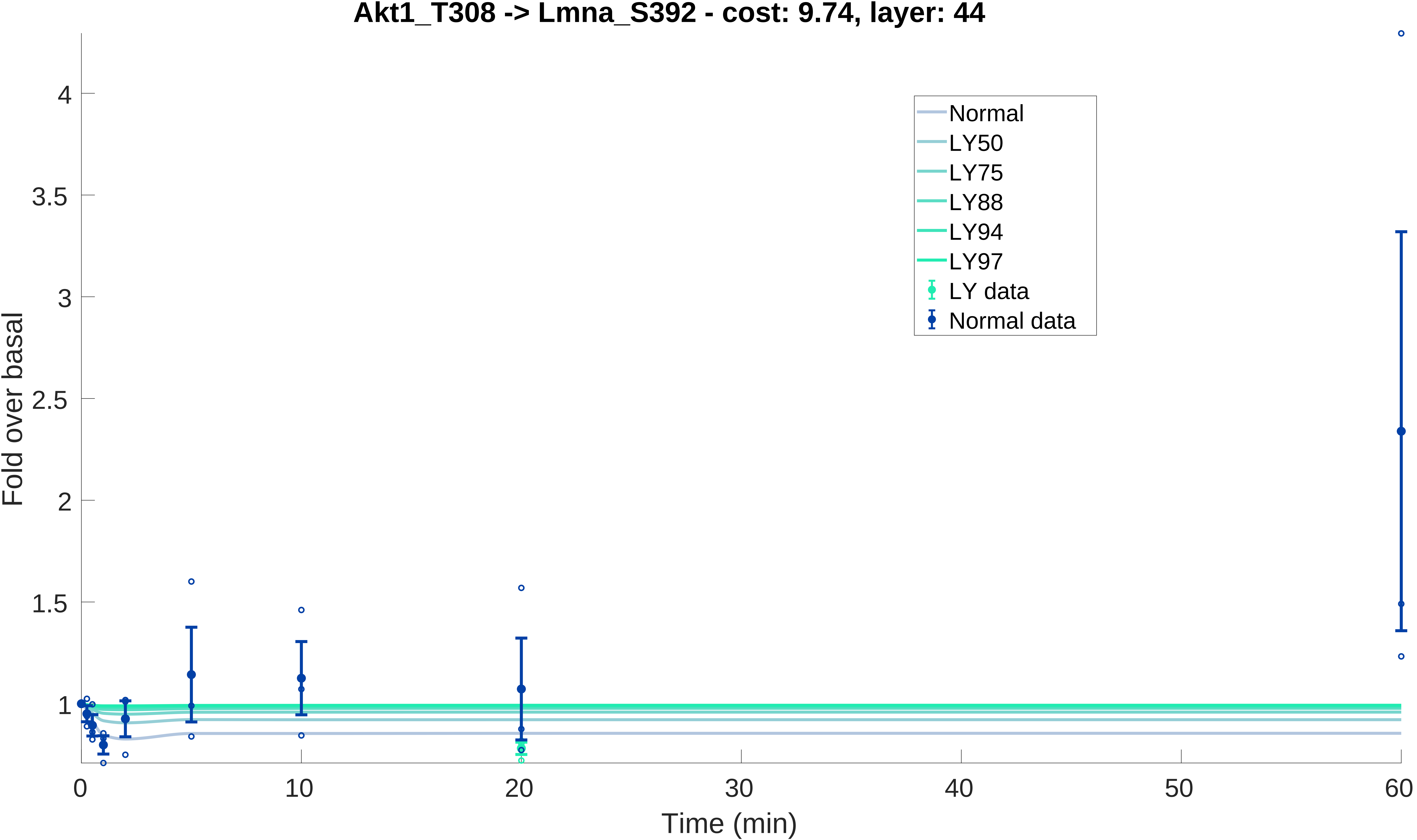
Akt1_T308 -> Lmna_S392 - cost: 9.74, layer: 44
Fold over basal
Time (min)
Normal
LY50
LY75
LY88
LY94
LY97
LY data
Normal data
0
10
20
30
40
50
60
1
1.5
2
2.5
3
3.5
4

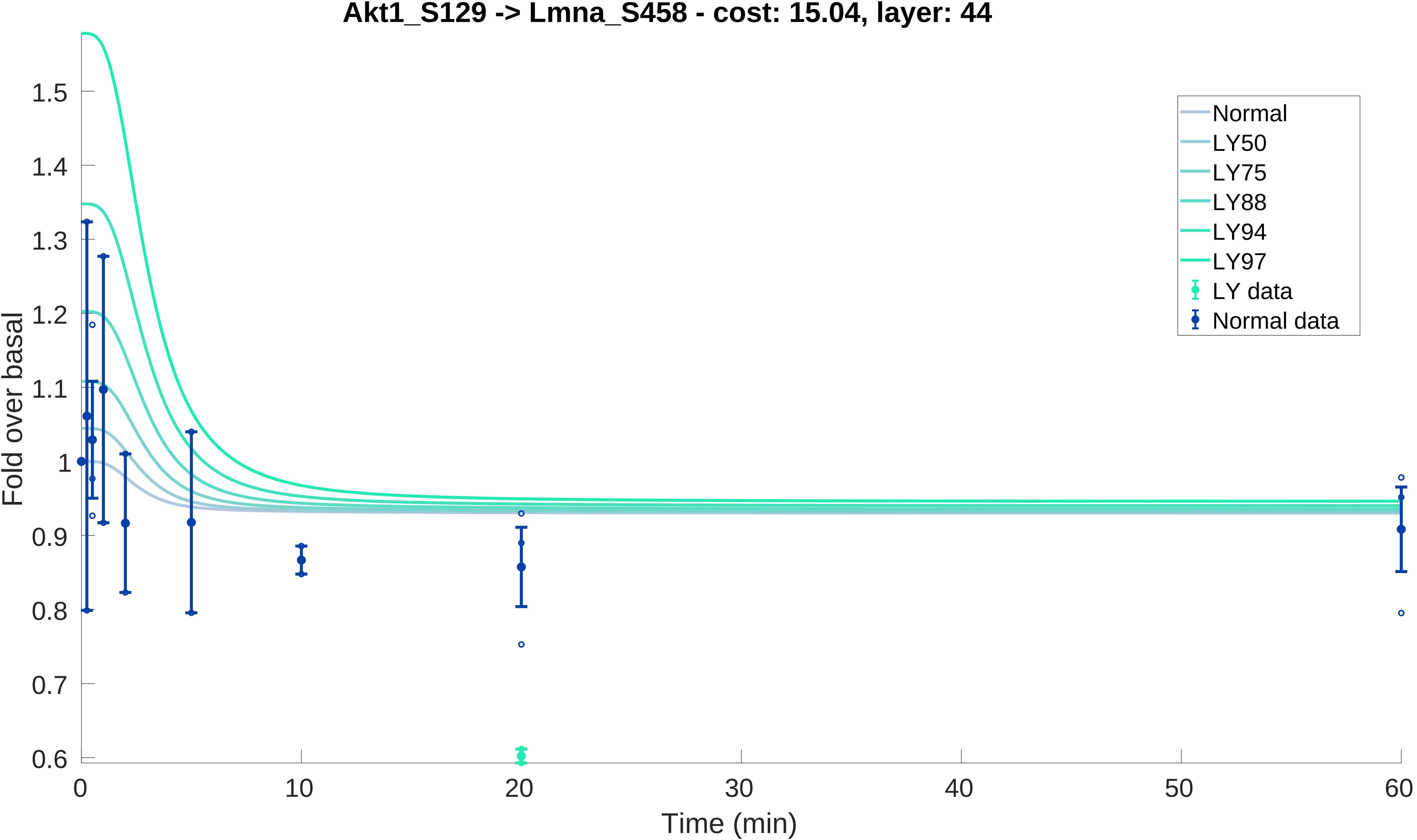
Akt1_S129 -> Lmna_S458 - cost: 15.04, layer: 44
Fold over basal
Time (min)
1.5
1.4
1.3
1.2
1.1
1
0.9
0.8
0.7
0.6
0
10
20
30
40
50
60
Normal
LY50
LY75
LY88
LY94
LY97
LY data
Normal data

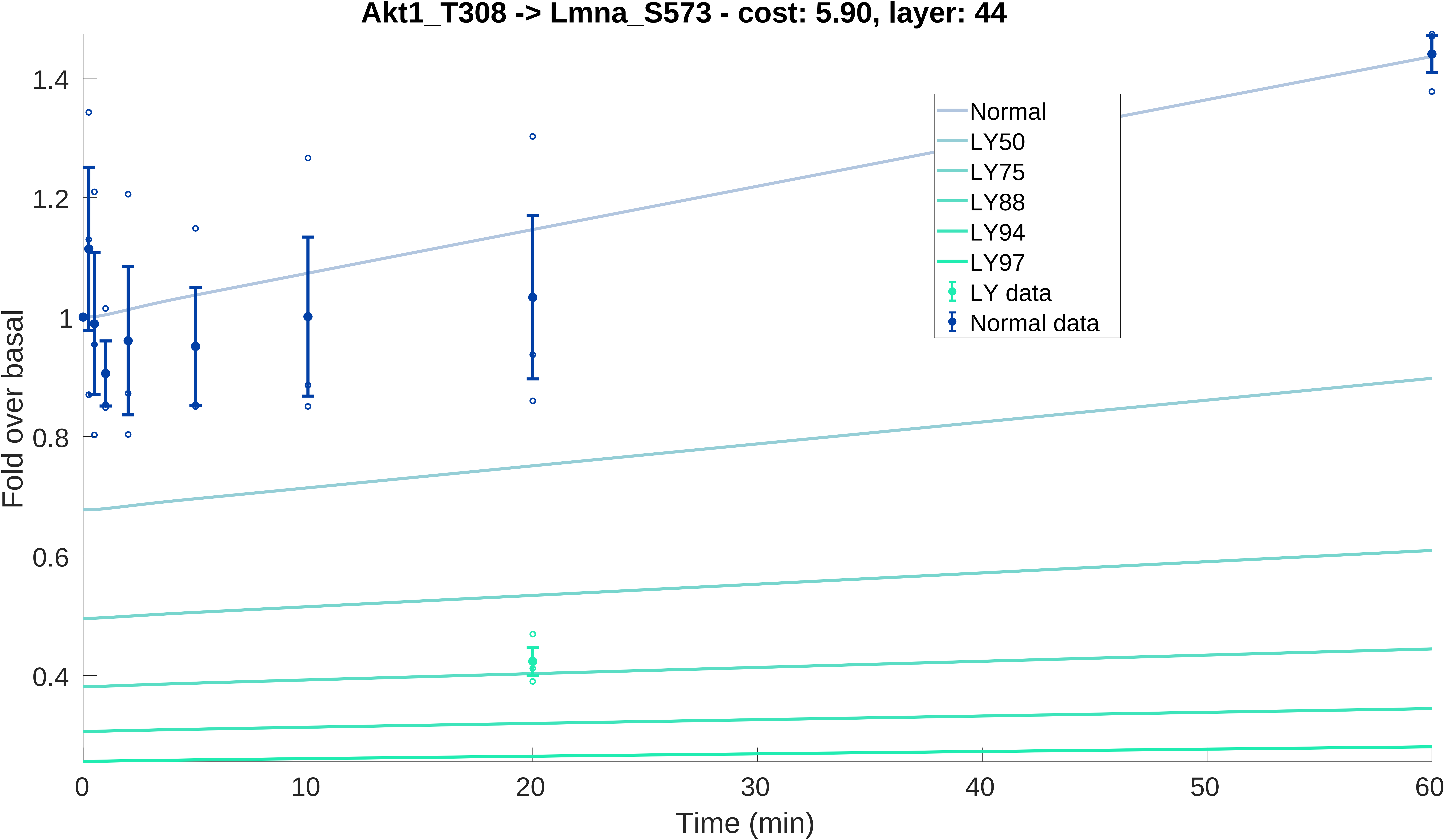
Akt1_T308 -> Lmna_S573 - cost: 5.90, layer: 44
Fold over basal
Time (min)
1.4
1.2
1
0.8
0.6
0.4
0
10
20
30
40
50
60
Normal
LY50
LY75
LY88
LY94
LY97
LY data
Normal data

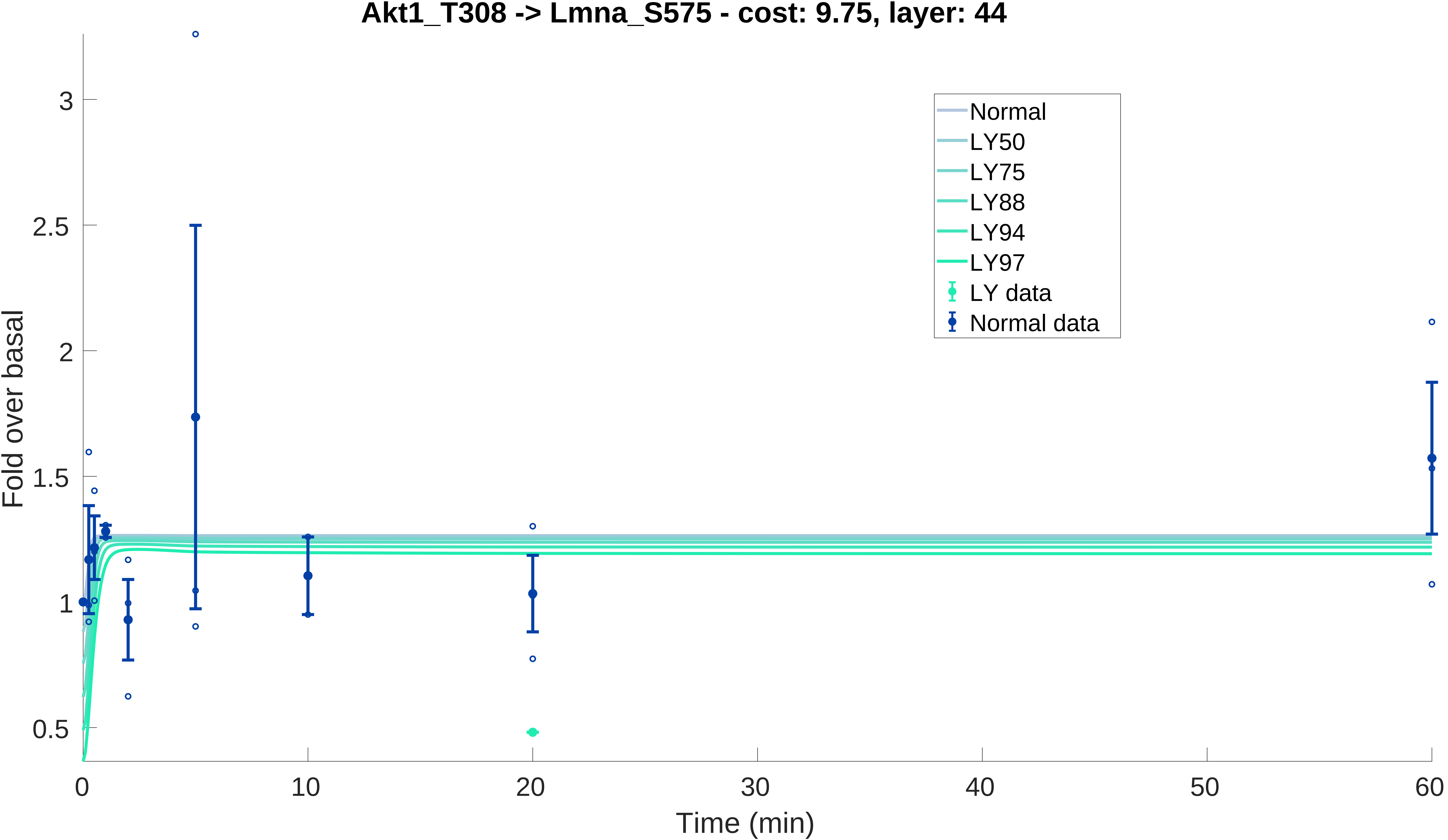

Akt1_T308 -> Lmna_S575 - cost: 9.75, layer: 44
Fold over basal
Time (min)
Normal
LY50
LY75
LY88
LY94
LY97
LY data
Normal data
0.5
1
1.5
2
2.5
3
0
10
20
30
40
50
60

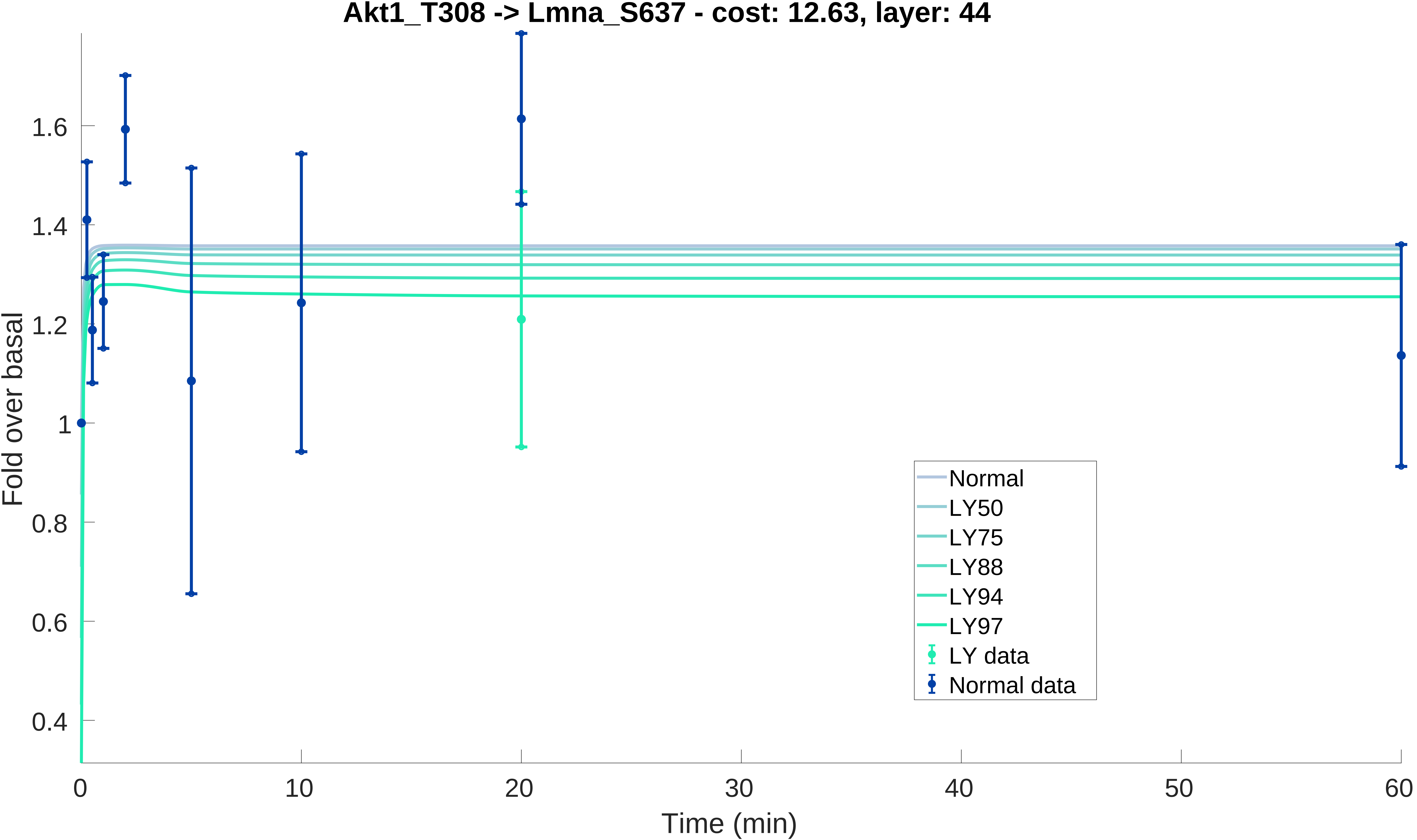

Akt1_T308 -> Lmna_S637 - cost: 12.63, layer: 44
Fold over basal
Time (min)
0
10
20
30
40
50
60
0.4
0.6
0.8
1
1.2
1.4
1.6
Normal
LY50
LY75
LY88
LY94
LY97
LY data
Normal data

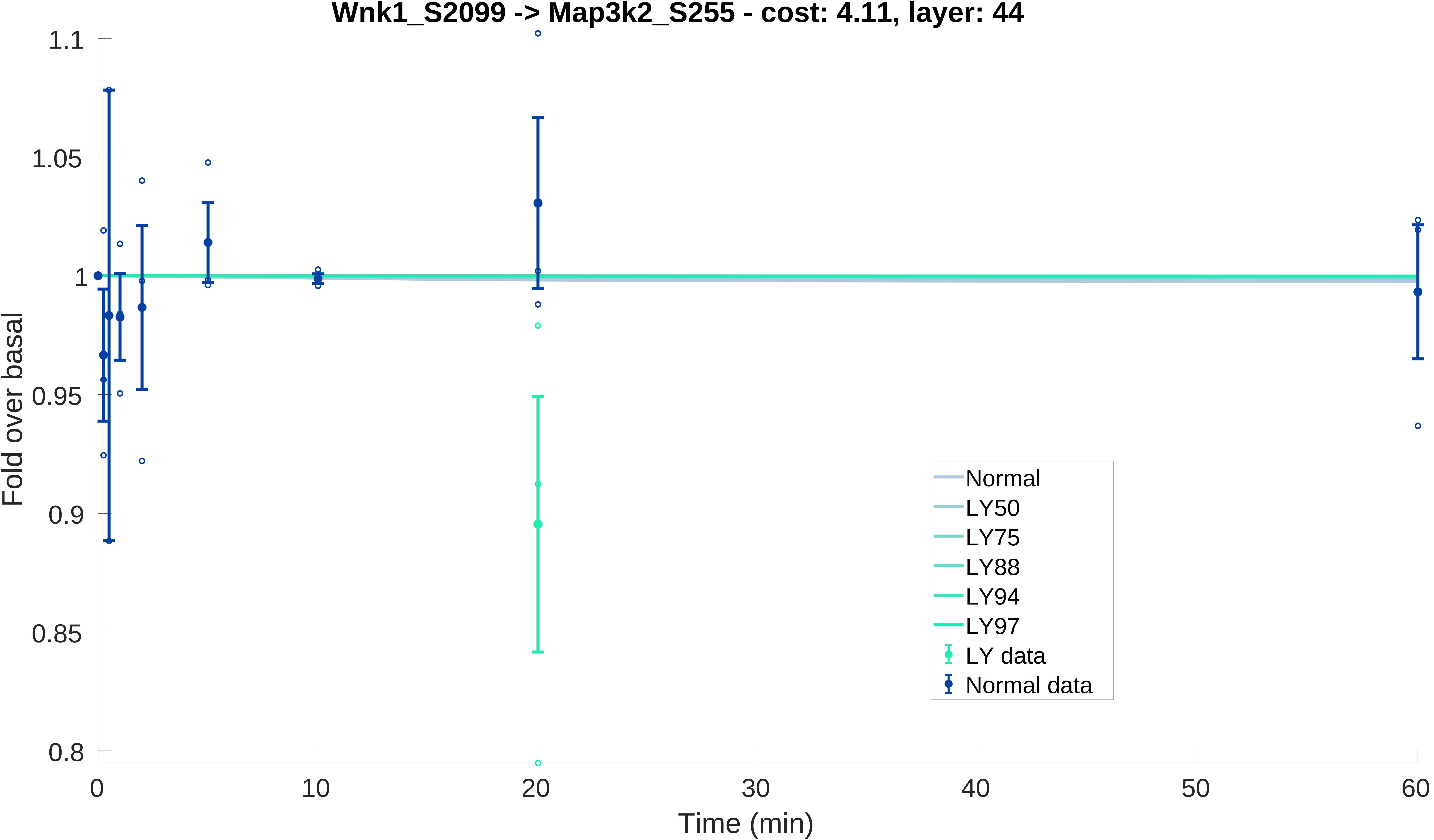

Wnk1_S2099 -> Map3k2_S255 - cost: 4.11, layer: 44
Fold over basal
Time (min)
1.1
1.05
1
0.95
0.9
0.85
0.8
0
10
20
30
40
50
60
Normal
LY50
LY75
LY88
LY94
LY97
LY data
Normal data

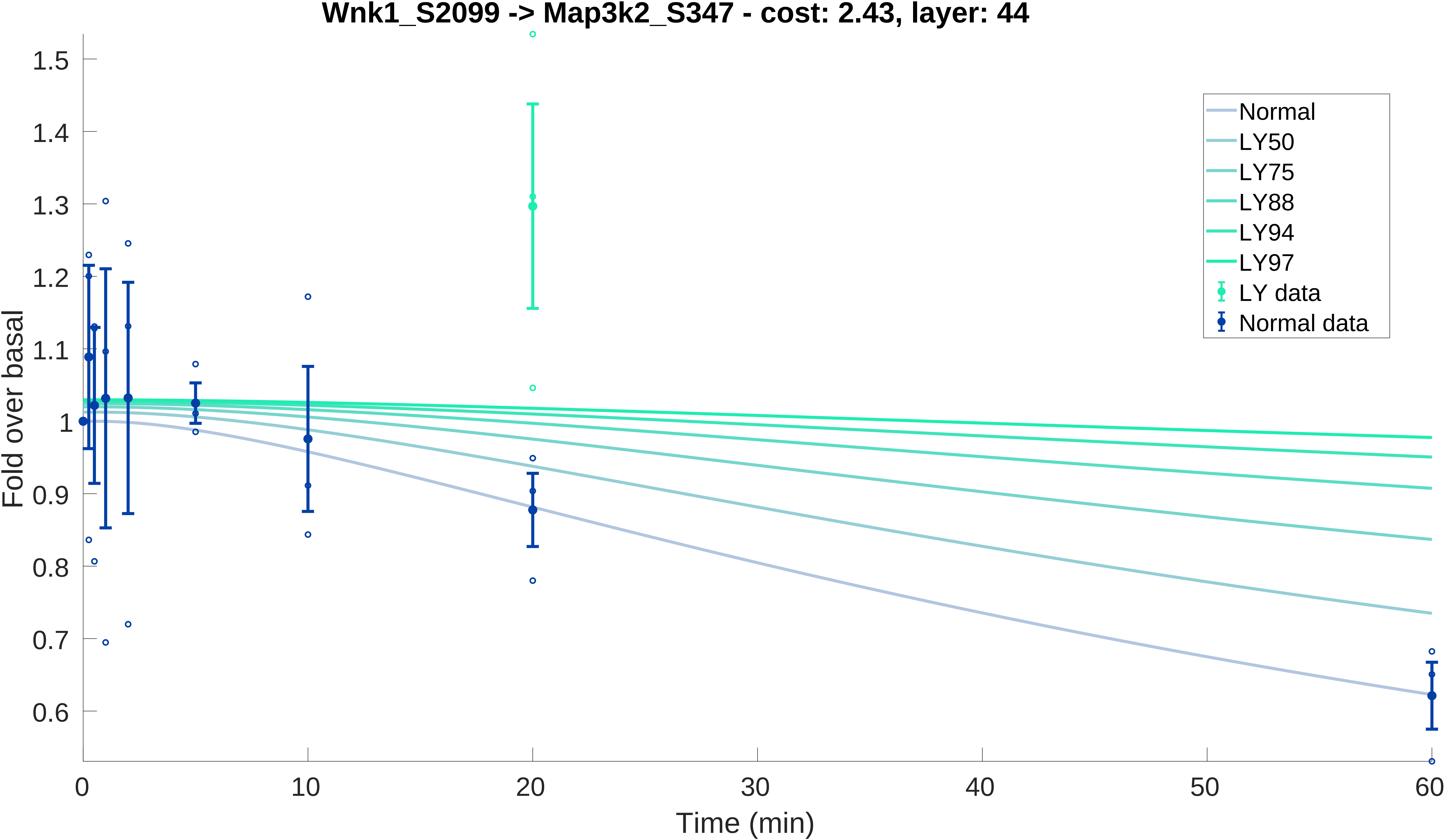

Wnk1_S2099 -> Map3k2_S347 - cost: 2.43, layer: 44
Fold over basal
1.5
1.4
1.3
1.2
1.1
1
0.9
0.8
0.7
0.6
0
10
20
30
40
50
60
Time (min)
Normal
LY50
LY75
LY88
LY94
LY97
LY data
Normal data

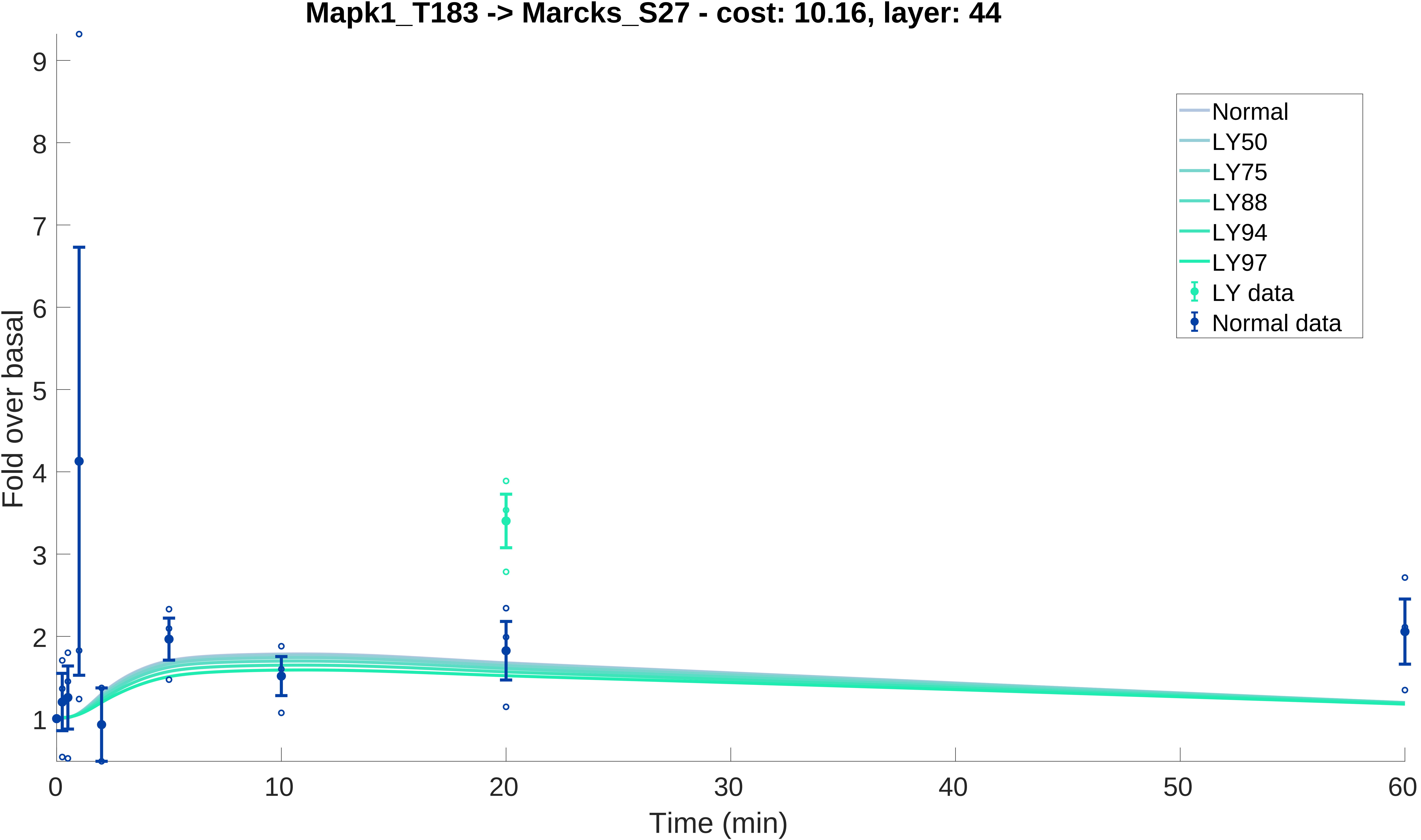

Mapk1_T183 -> Marcks_S27 - cost: 10.16, layer: 44
Fold over basal
Time (min)
0
10
20
30
40
50
60
1
2
3
4
5
6
7
8
9
Normal
LY50
LY75
LY88
LY94
LY97
LY data
Normal data

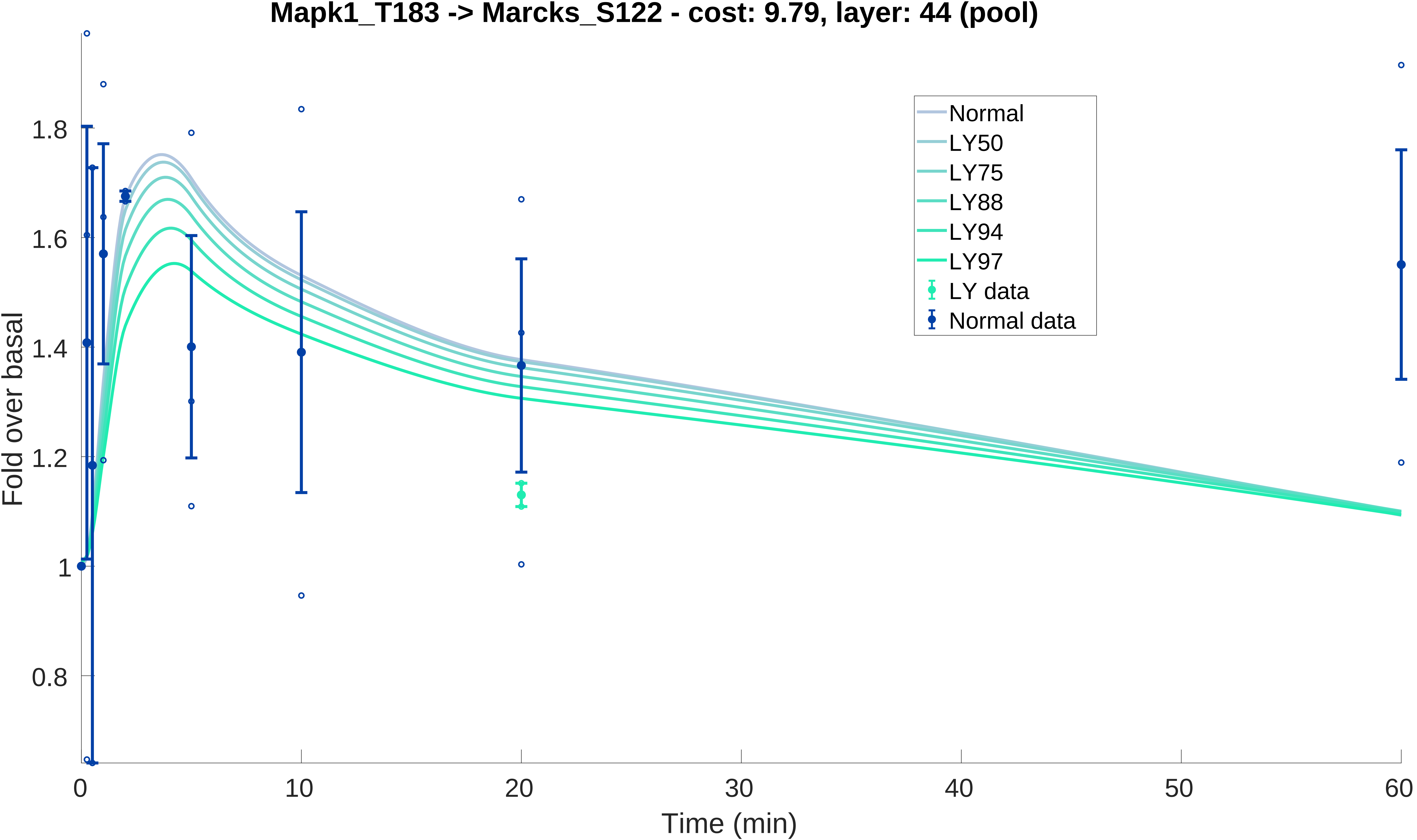

Mapk1_T183 -> Marcks_S122 - cost: 9.79, layer: 44 (pool)
Normal
LY50
LY75
LY88
LY94
LY97
LY data
Normal data
Fold over basal
0.8
1
1.2
1.4
1.6
1.8
0
10
20
30
40
50
60
Time (min)

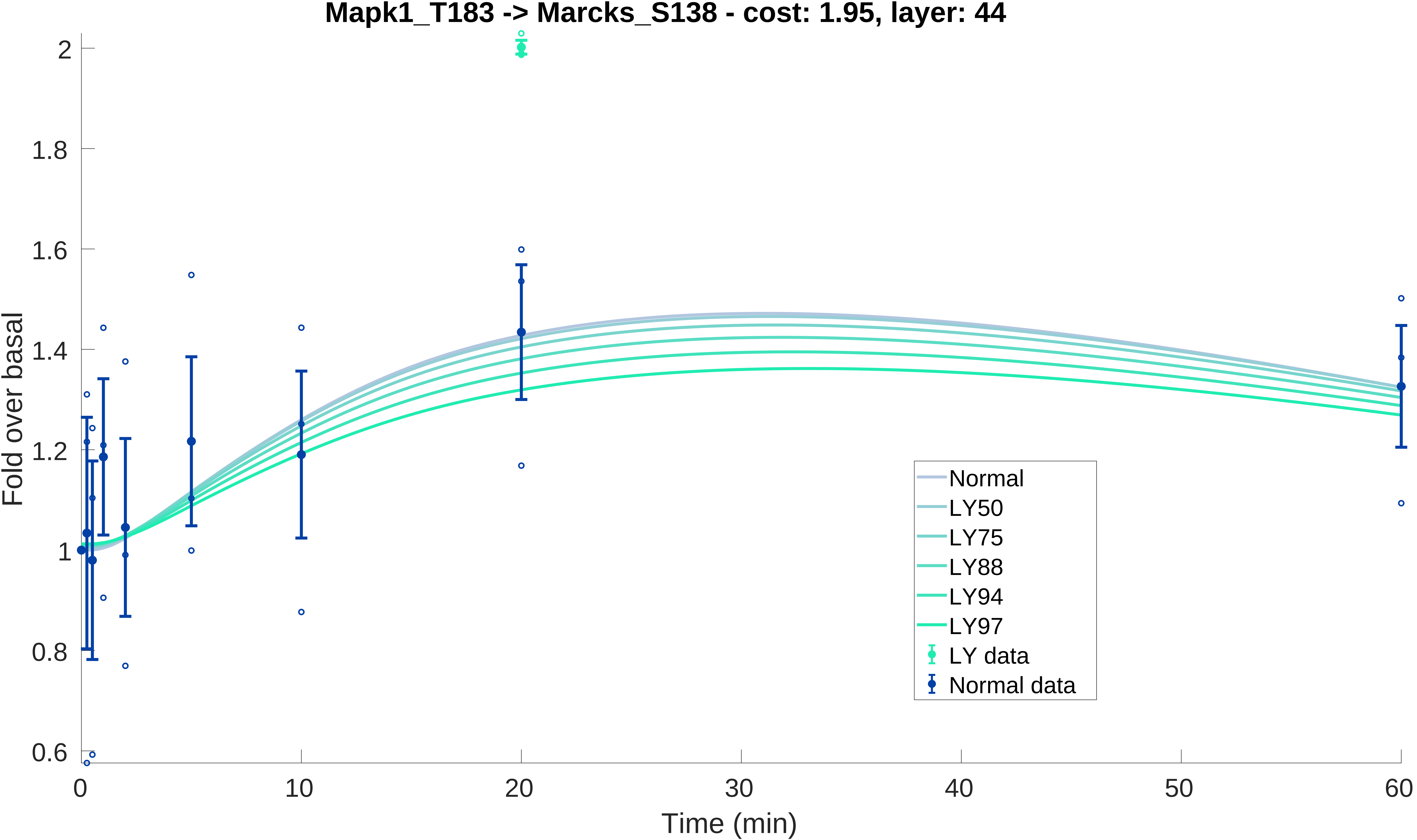
Mapk1_T183 -> Marcks_S138 - cost: 1.95, layer: 44
Fold over basal
Time (min)
0
10
20
30
40
50
60
0.6
0.8
1
1.2
1.4
1.6
1.8
2
Normal
LY50
LY75
LY88
LY94
LY97
LY data
Normal data

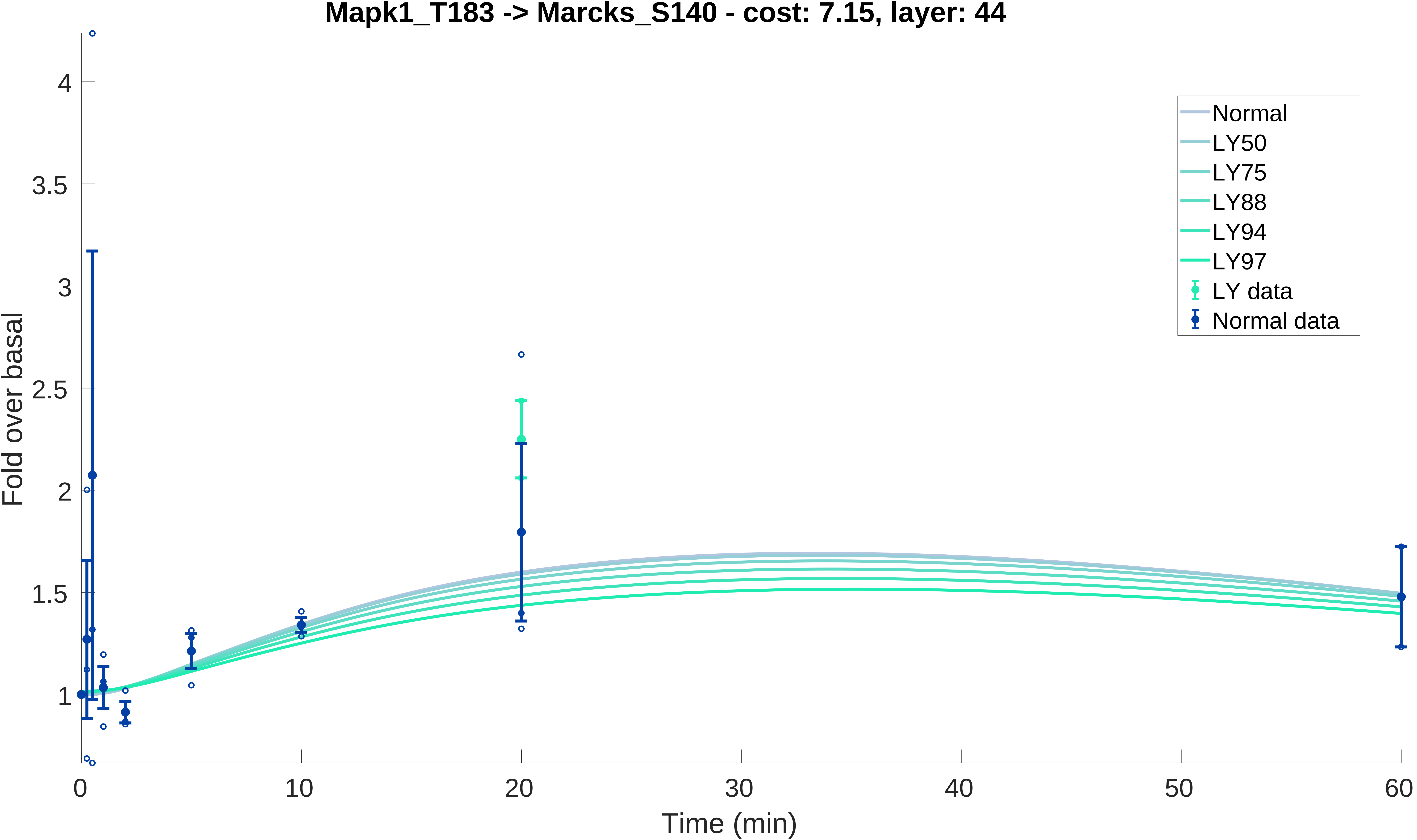
Mapk1_T183 -> Marcks_S140 - cost: 7.15, layer: 44
Fold over basal
Time (min)
0
10
20
30
40
50
60
1
1.5
2
2.5
3
3.5
4
Normal
LY50
LY75
LY88
LY94
LY97
LY data
Normal data

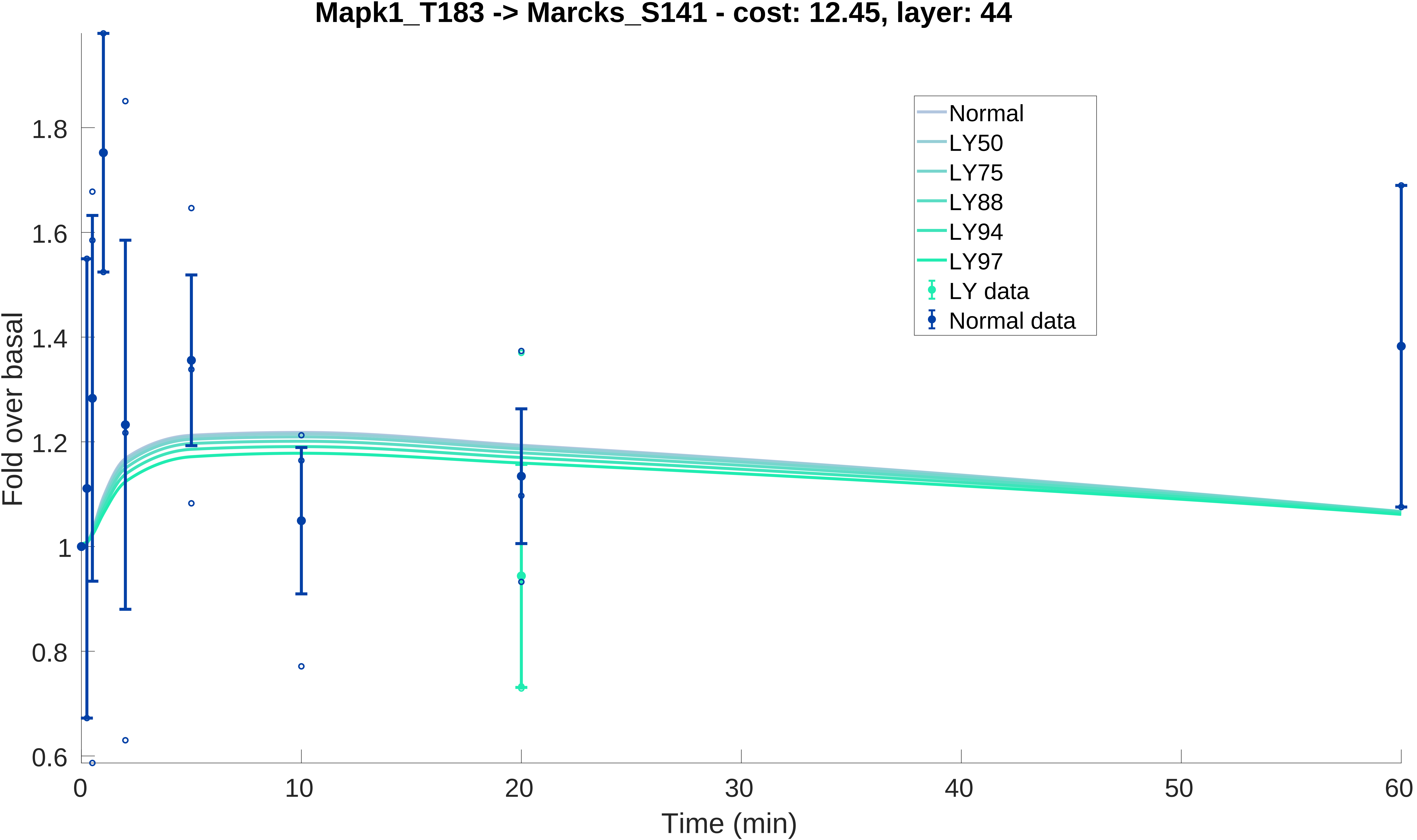

Mapk1_T183 -> Marcks_S141 - cost: 12.45, layer: 44
Normal
LY50
LY75
LY88
LY94
LY97
LY data
Normal data
Fold over basal
Time (min)
0.6
0.8
1
1.2
1.4
1.6
1.8
0
10
20
30
40
50
60

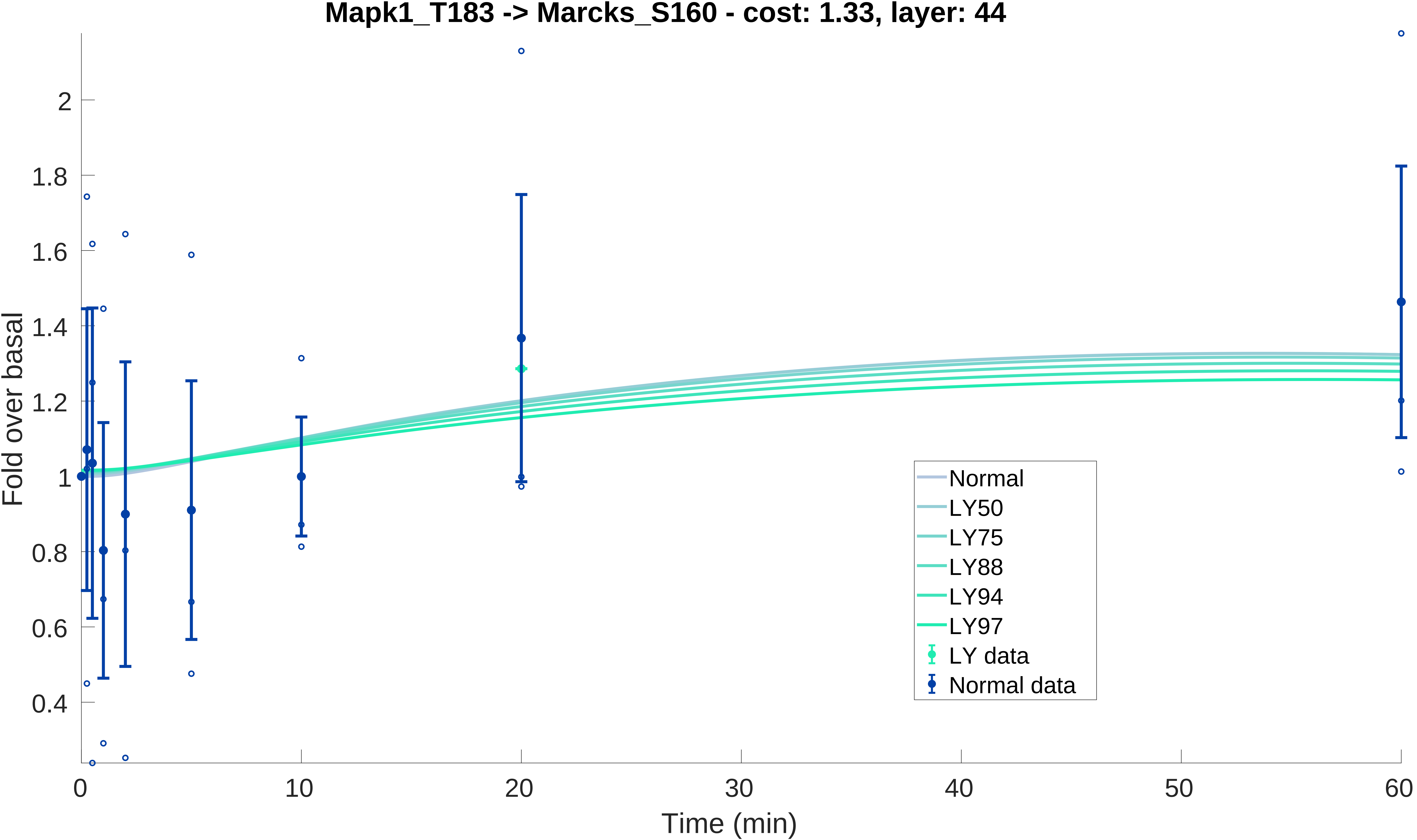

Mapk1_T183 -> Marcks_S160 - cost: 1.33, layer: 44
Fold over basal
Time (min)
0.4
0.6
0.8
1
1.2
1.4
1.6
1.8
2
0
10
20
30
40
50
60
Normal
LY50
LY75
LY88
LY94
LY97
LY data
Normal data

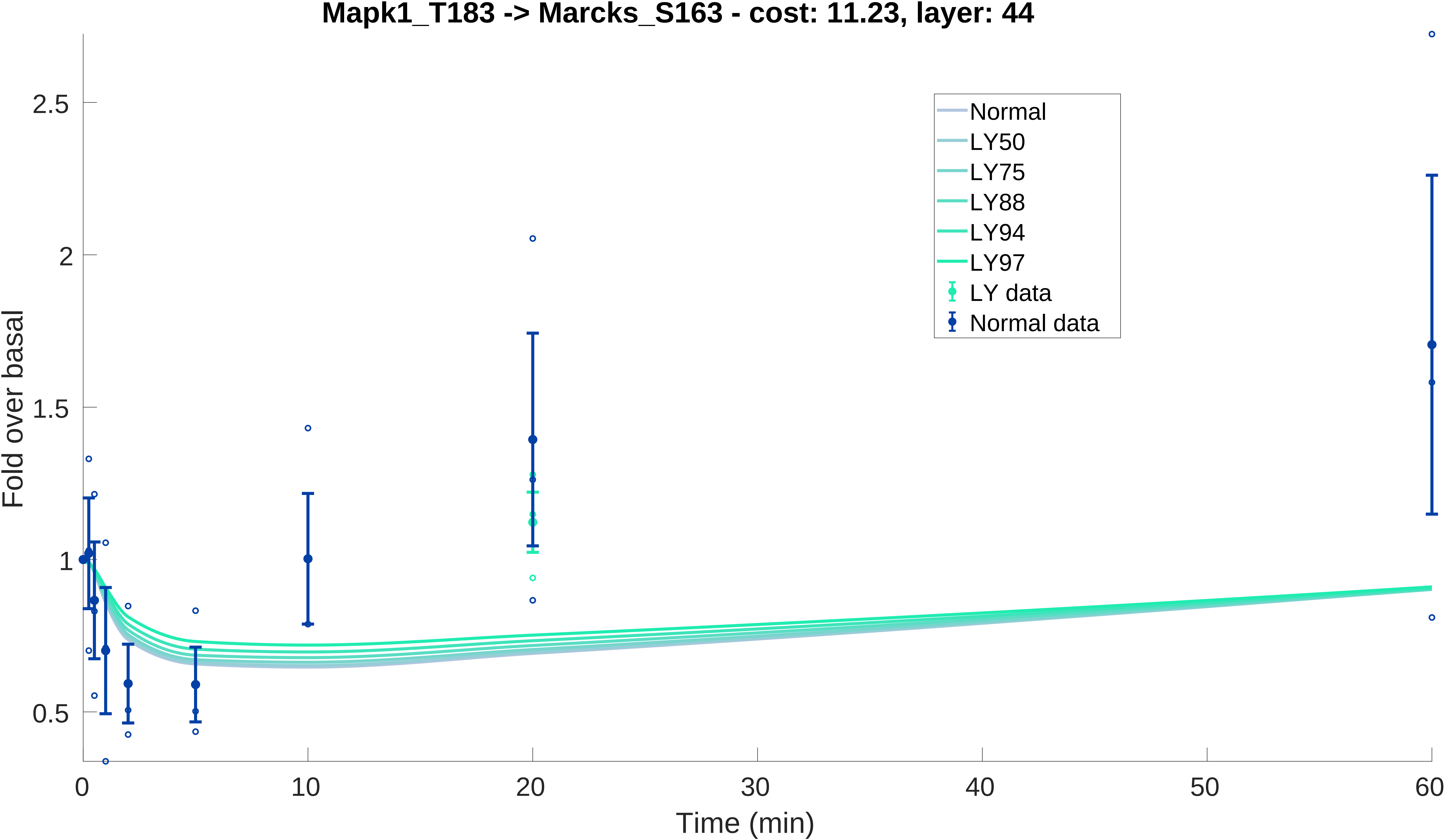
Mapk1_T183 -> Marcks_S163 - cost: 11.23, layer: 44
Fold over basal
Time (min)
0.5
1
1.5
2
2.5
0
10
20
30
40
50
60
Normal
LY50
LY75
LY88
LY94
LY97
LY data
Normal data

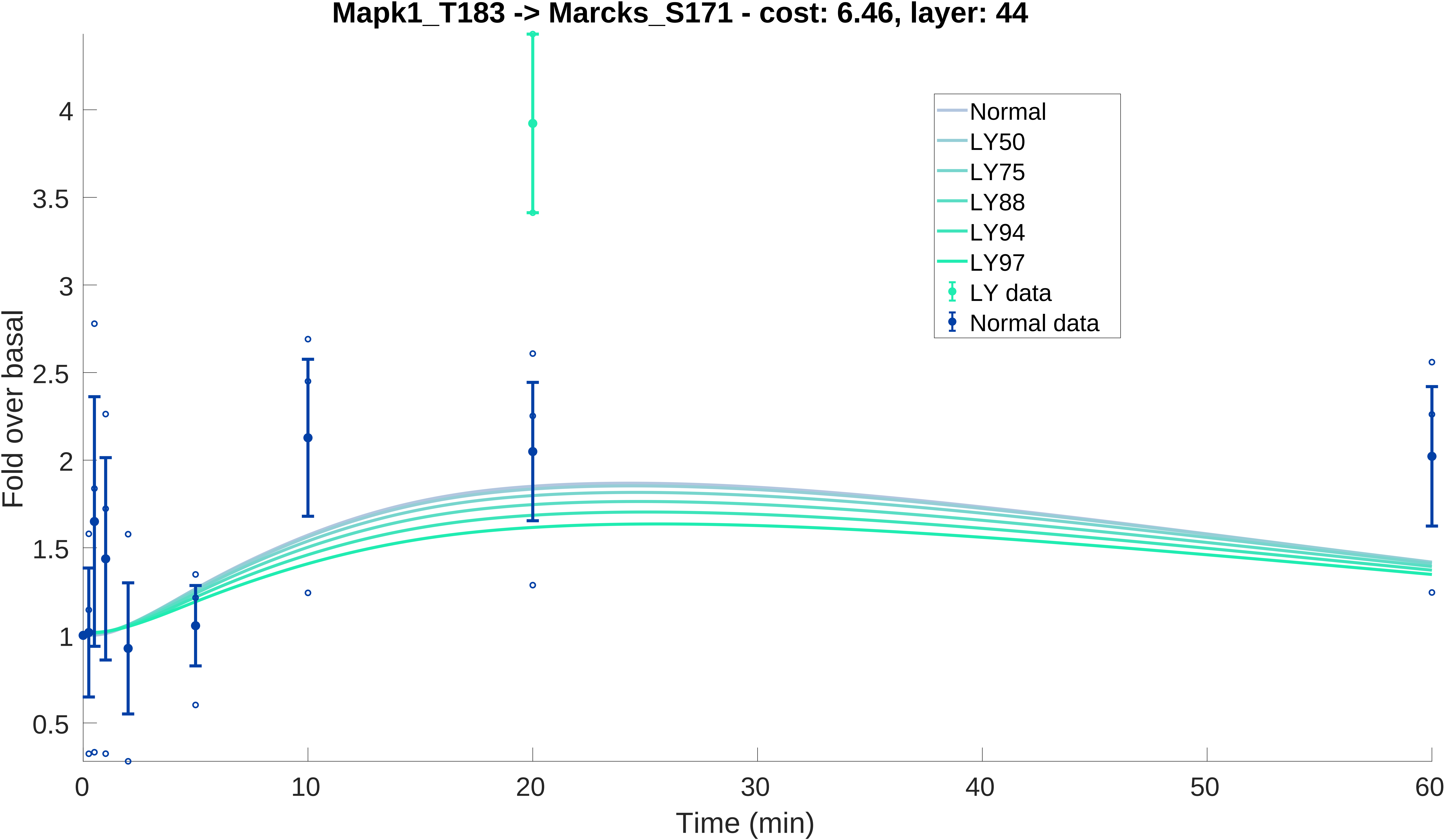

Mapk1_T183 -> Marcks_S171 - cost: 6.46, layer: 44
Fold over basal
Time (min)
0.5
1
1.5
2
2.5
3
3.5
4
0
10
20
30
40
50
60
Normal
LY50
LY75
LY88
LY94
LY97
LY data
Normal data

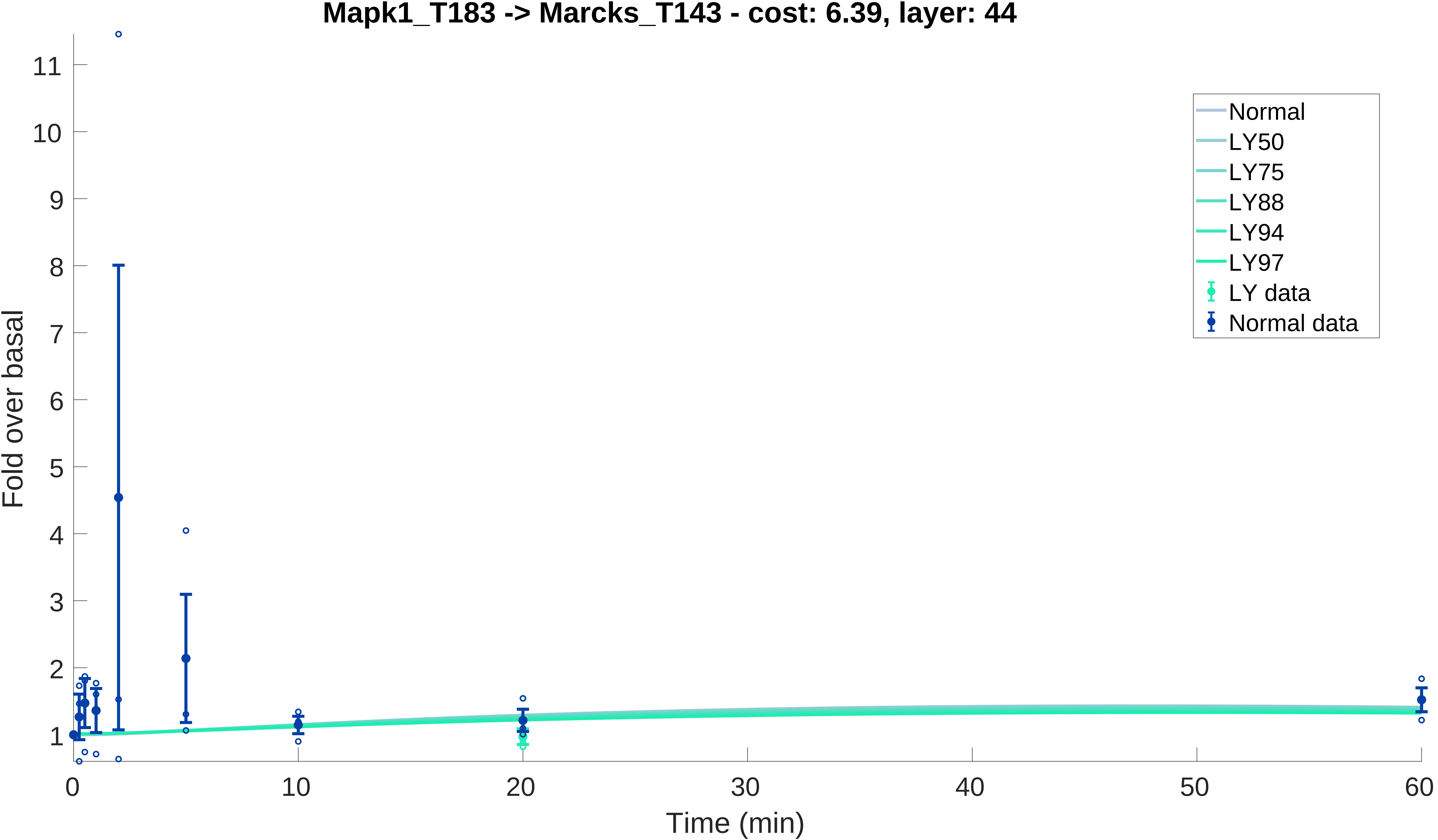

Mapk1_T183 -> Marcks_T143 - cost: 6.39, layer: 44
Fold over basal
Time (min)
1
2
3
4
5
6
7
8
9
10
11
0
10
20
30
40
50
60
Normal
LY50
LY75
LY88
LY94
LY97
LY data
Normal data

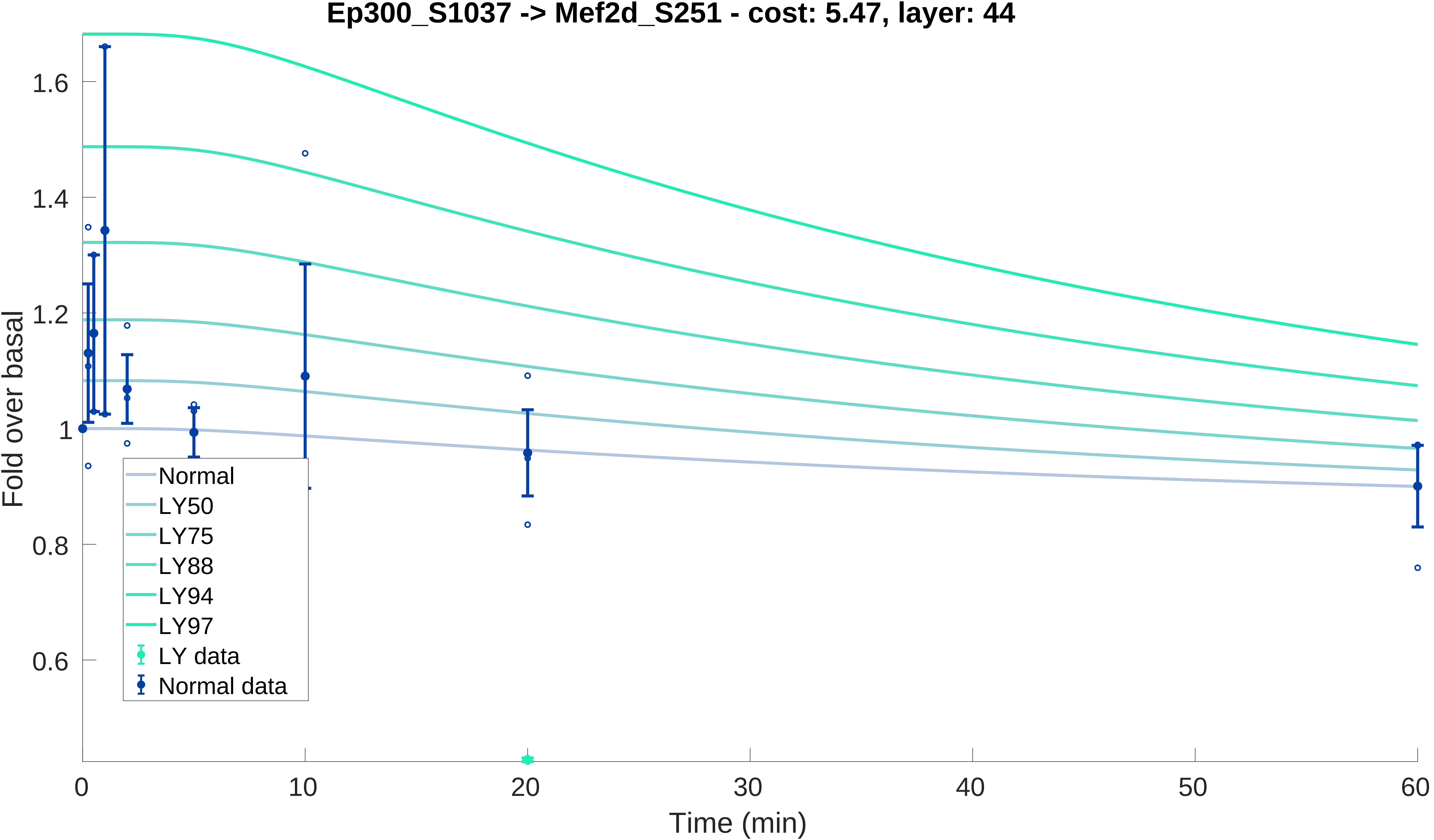

Ep300_S1037 -> Mef2d_S251 - cost: 5.47, layer: 44
Fold over basal
1.6
1.4
1.2
1
0.8
0.6
0
10
20
30
40
50
60
Time (min)
Normal
LY50
LY75
LY88
LY94
LY97
LY data
Normal data

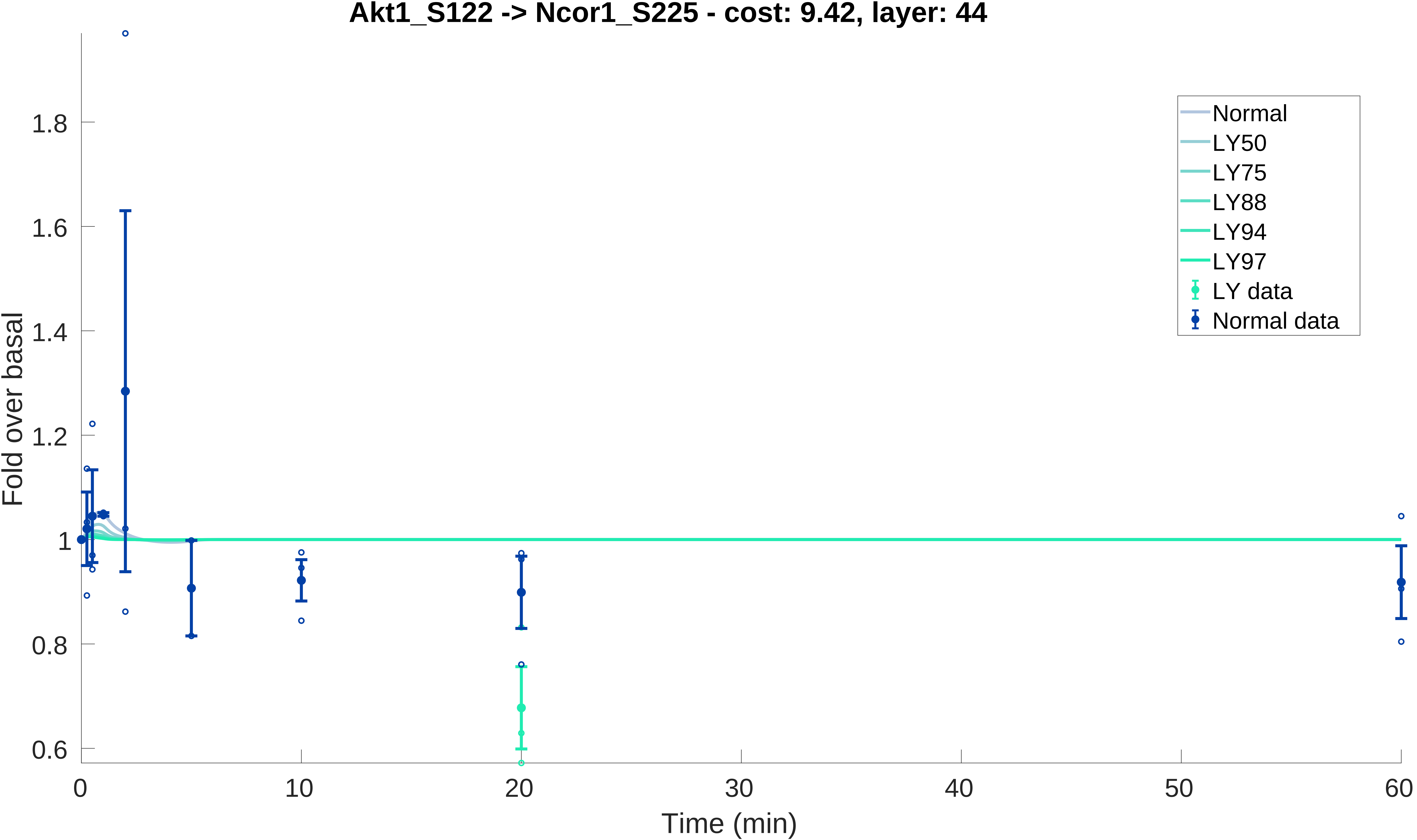

Akt1_S122 -> Ncor1_S225 - cost: 9.42, layer: 44
Fold over basal
Time (min)
0.6
0.8
1
1.2
1.4
1.6
1.8
0
10
20
30
40
50
60
Normal
LY50
LY75
LY88
LY94
LY97
LY data
Normal data

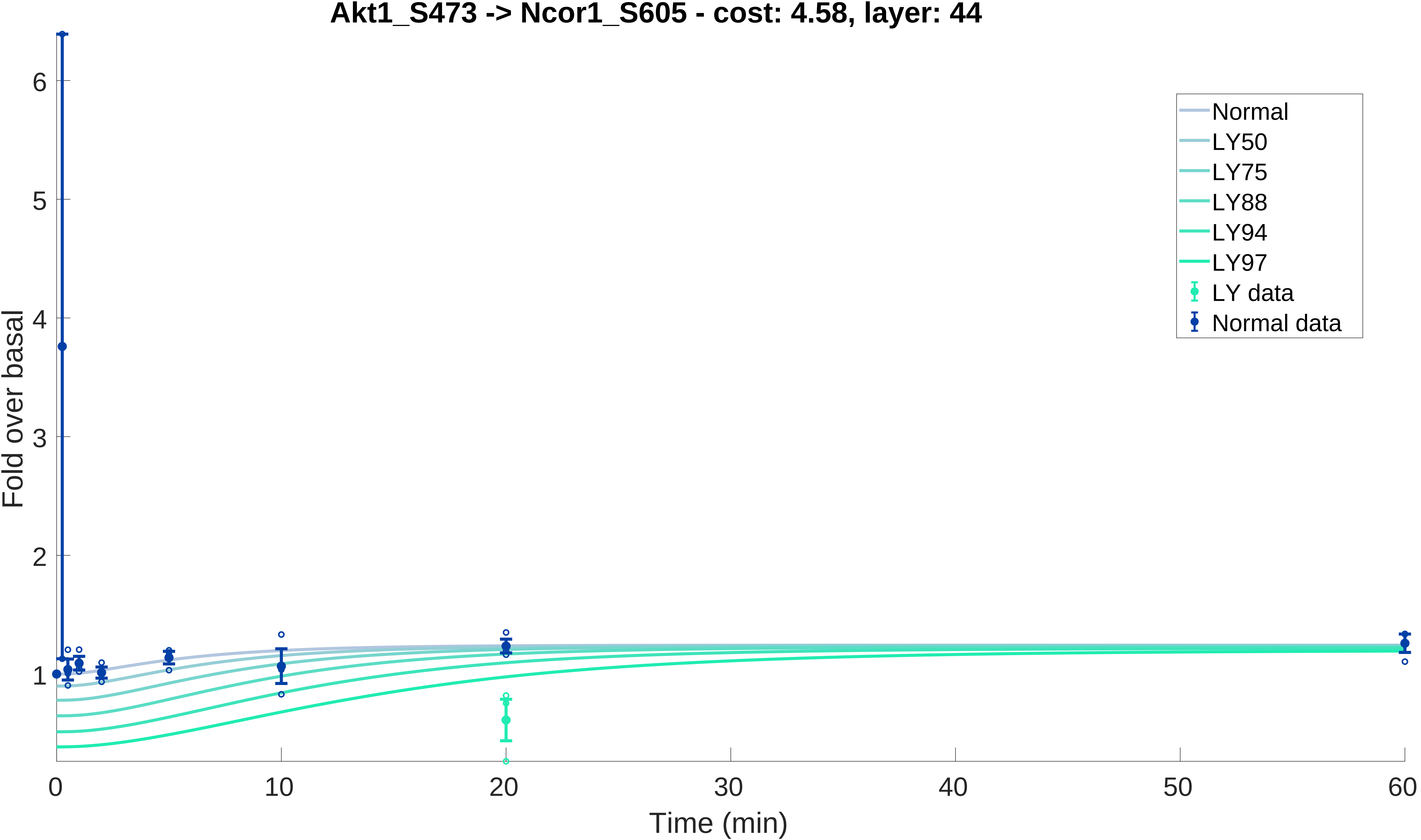

Akt1_S473 -> Ncor1_S605 - cost: 4.58, layer: 44
Fold over basal
Time (min)
1
2
3
4
5
6
0
10
20
30
40
50
60
Normal
LY50
LY75
LY88
LY94
LY97
LY data
Normal data

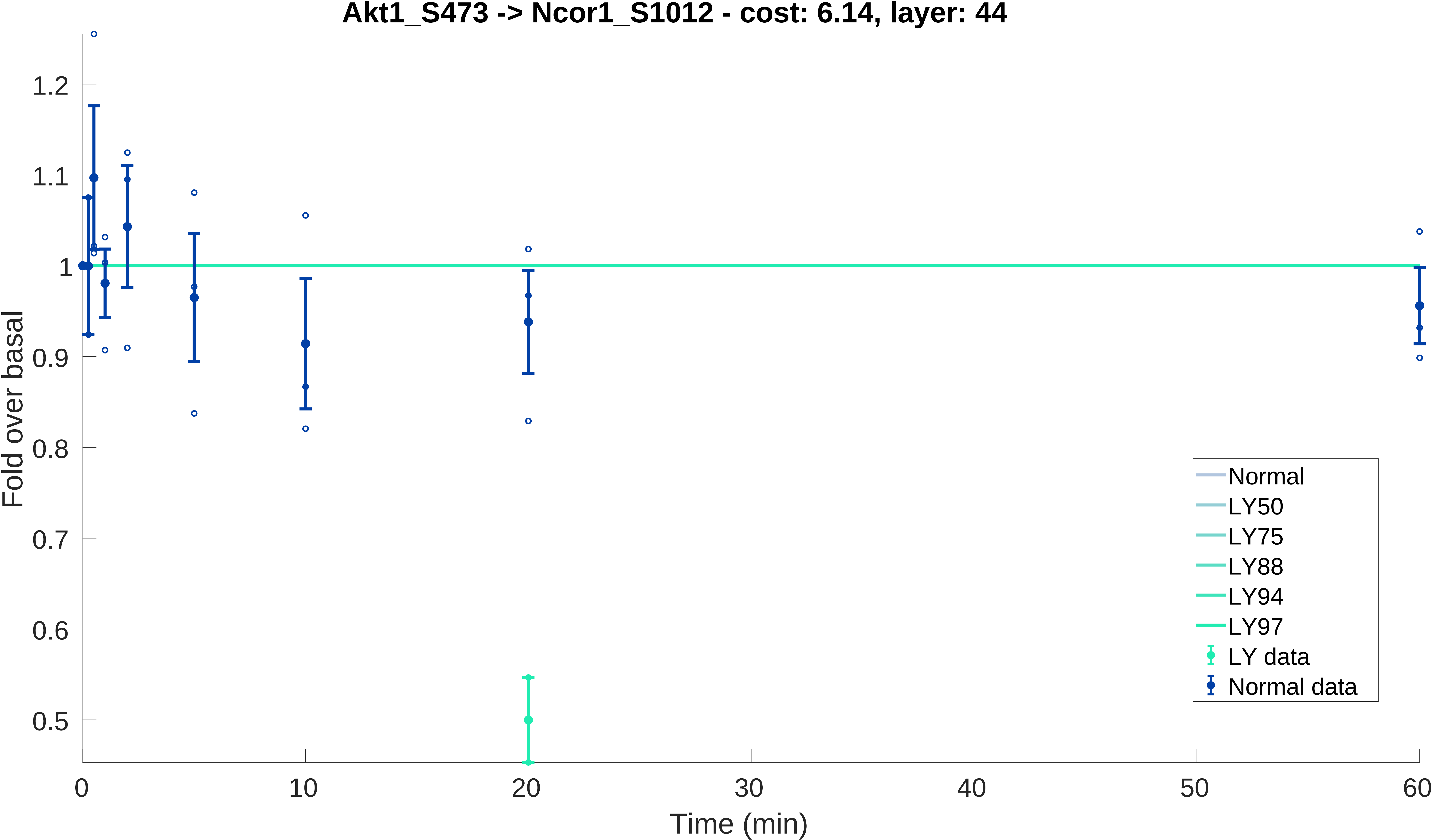
Akt1_S473 -> Ncor1_S1012 - cost: 6.14, layer: 44
Fold over basal
Time (min)
1.2
1.1
1
0.9
0.8
0.7
0.6
0.5
0
10
20
30
40
50
60
Normal
LY50
LY75
LY88
LY94
LY97
LY data
Normal data

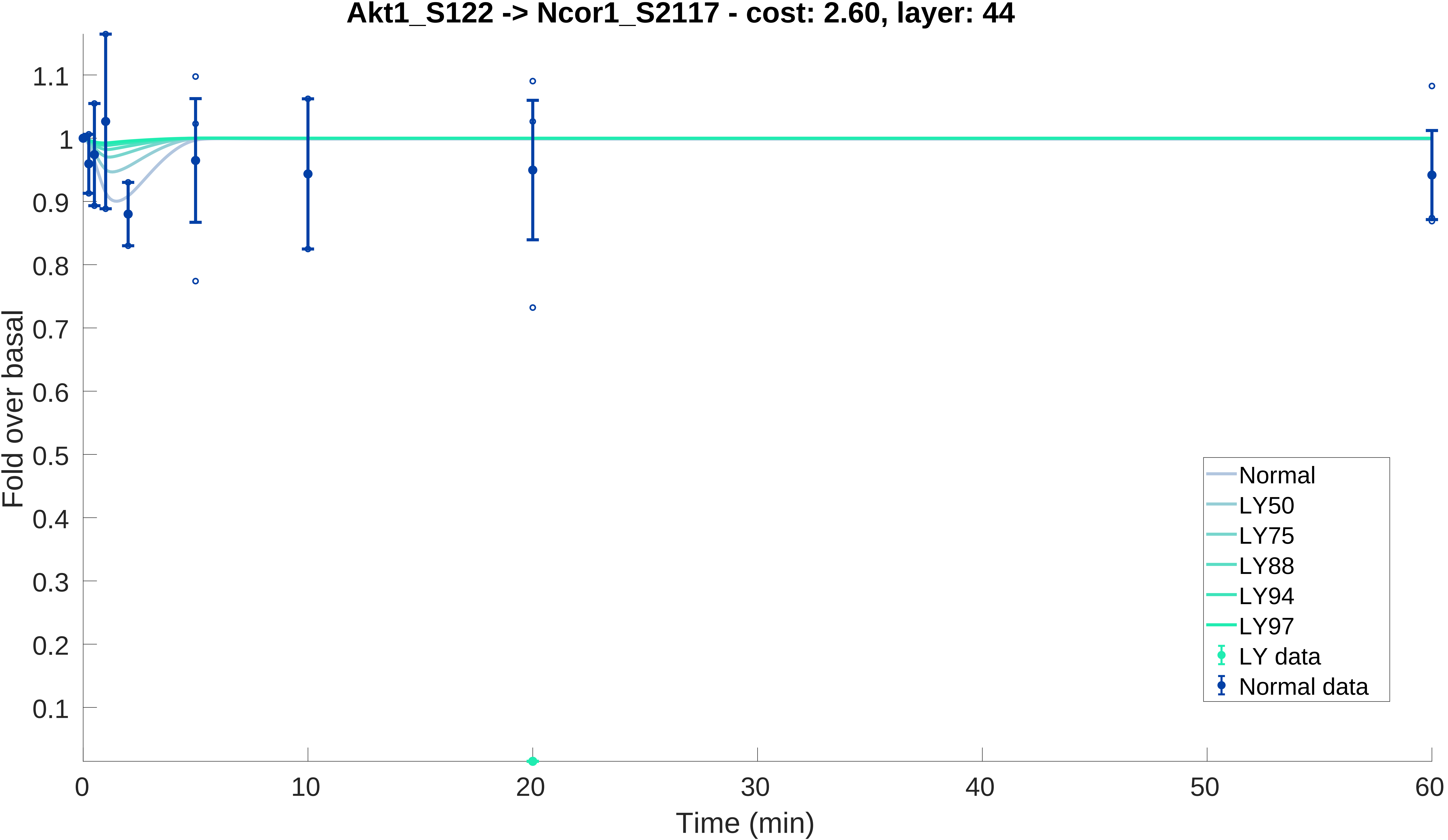

Akt1_S122 -> Ncor1_S2117 - cost: 2.60, layer: 44
Fold over basal
Time (min)
1.1
1
0.9
0.8
0.7
0.6
0.5
0.4
0.3
0.2
0.1
0
10
20
30
40
50
60
Normal
LY50
LY75
LY88
LY94
LY97
LY data
Normal data

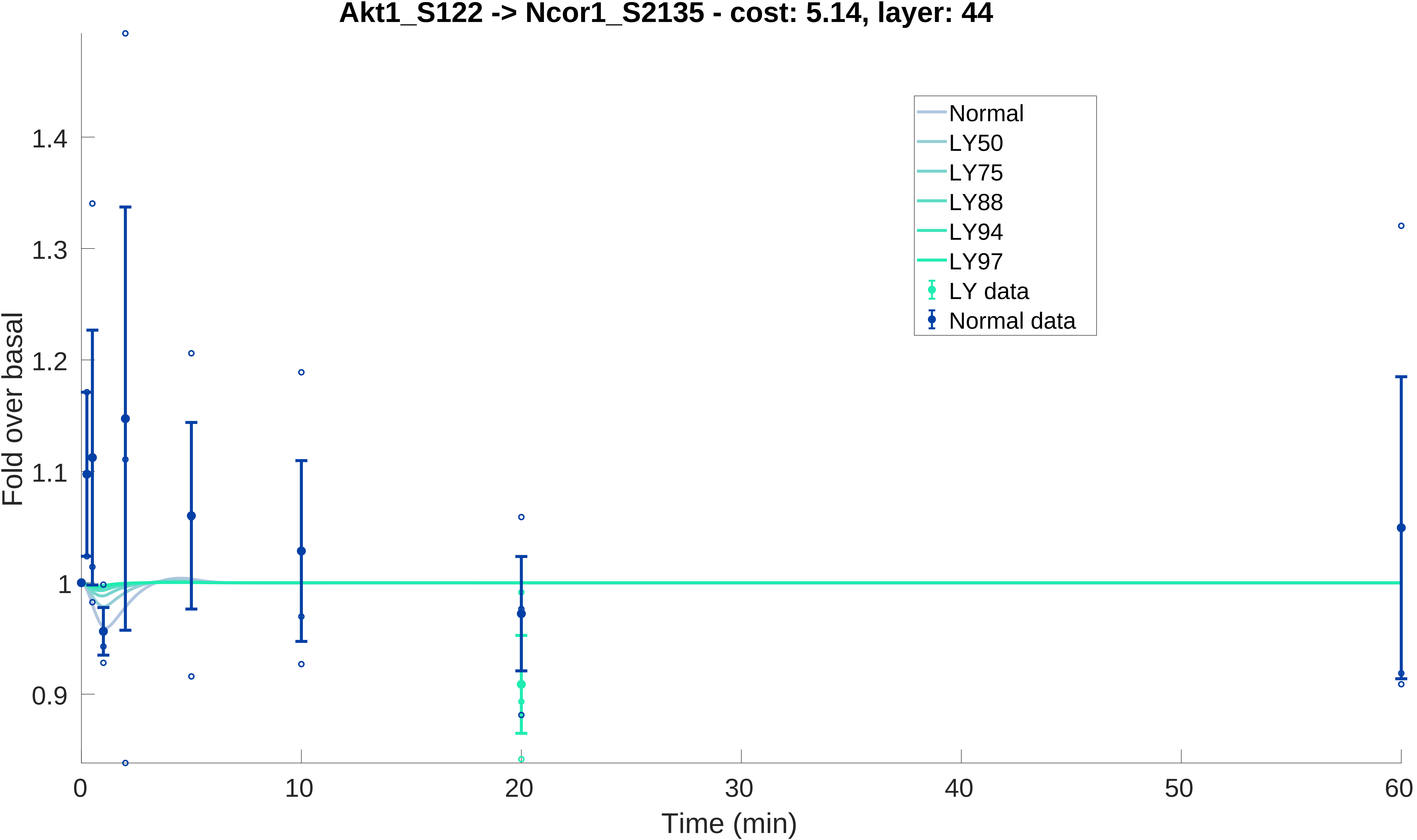
Akt1_S122 -> Ncor1_S2135 - cost: 5.14, layer: 44
Normal
LY50
LY75
LY88
LY94
LY97
LY data
Normal data
Fold over basal
1.4
1.3
1.2
1.1
1
0.9
0
10
20
30
40
50
60
Time (min)

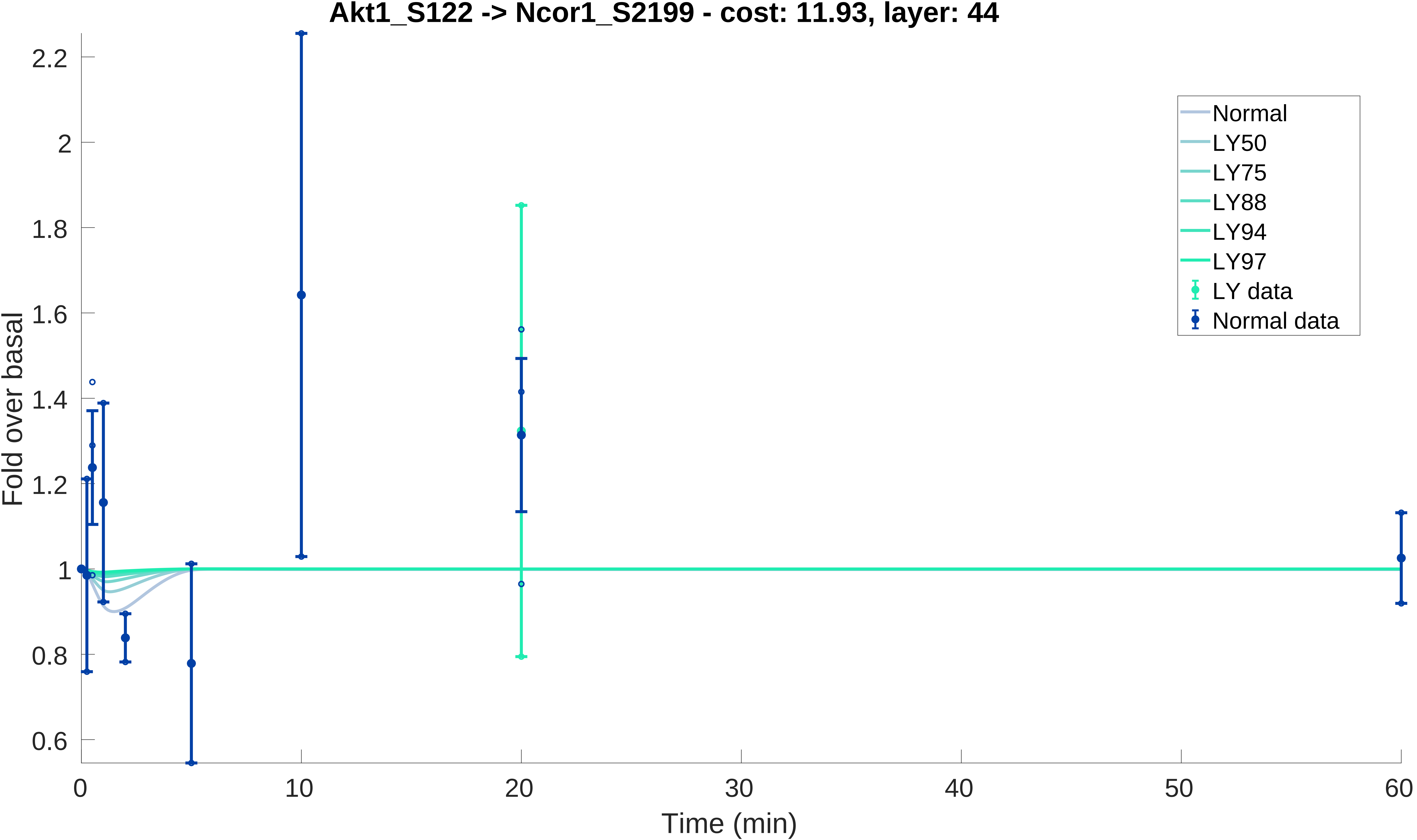

Akt1_S122 -> Ncor1_S2199 - cost: 11.93, layer: 44
Fold over basal
Time (min)
0.6
0.8
1
1.2
1.4
1.6
1.8
2
2.2
0
10
20
30
40
50
60
Normal
LY50
LY75
LY88
LY94
LY97
LY data
Normal data

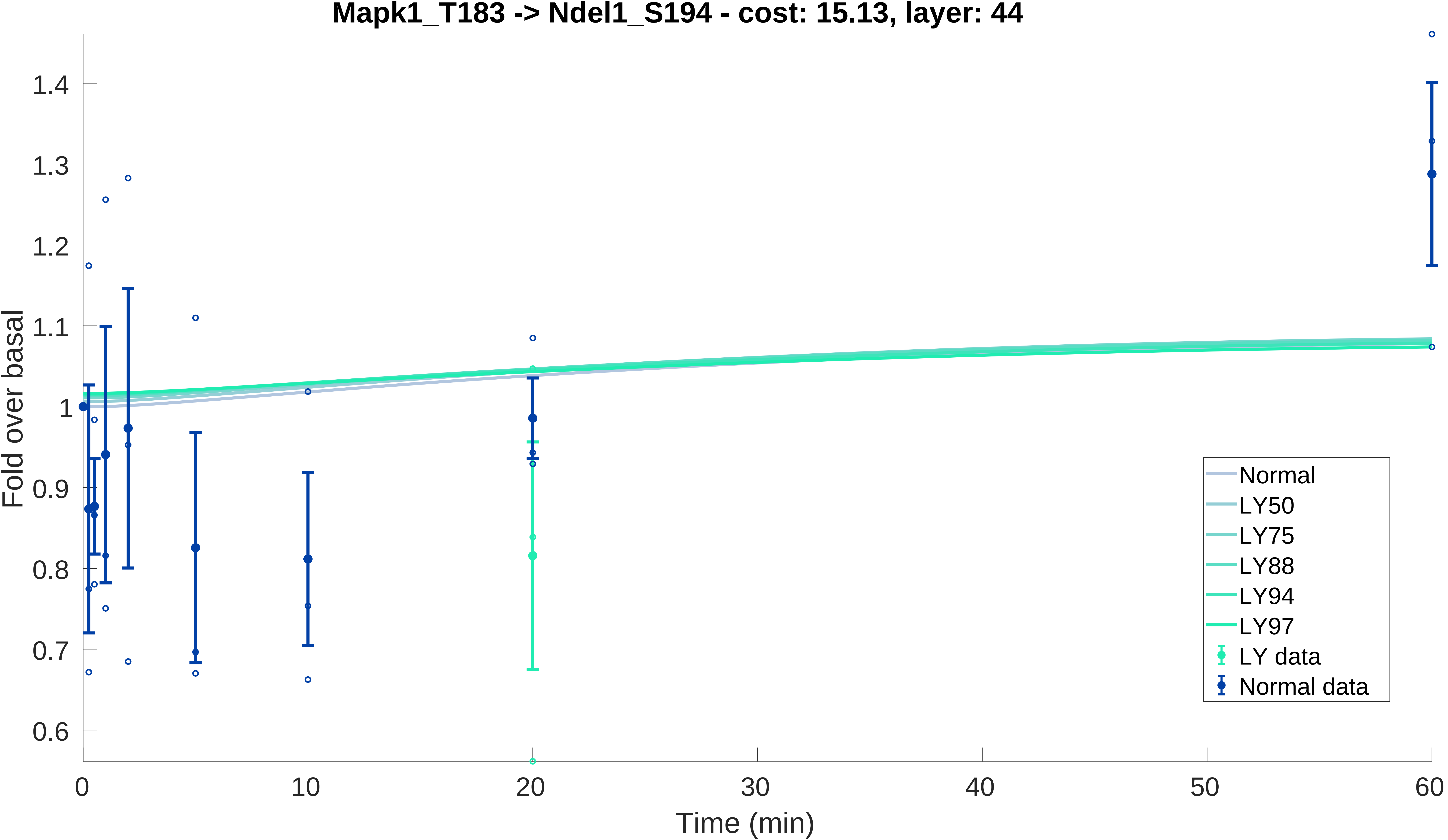
Mapk1_T183 -> Ndel1_S194 - cost: 15.13, layer: 44
Fold over basal
Time (min)
0
10
20
30
40
50
60
0.6
0.7
0.8
0.9
1
1.1
1.2
1.3
1.4
Normal
LY50
LY75
LY88
LY94
LY97
LY data
Normal data

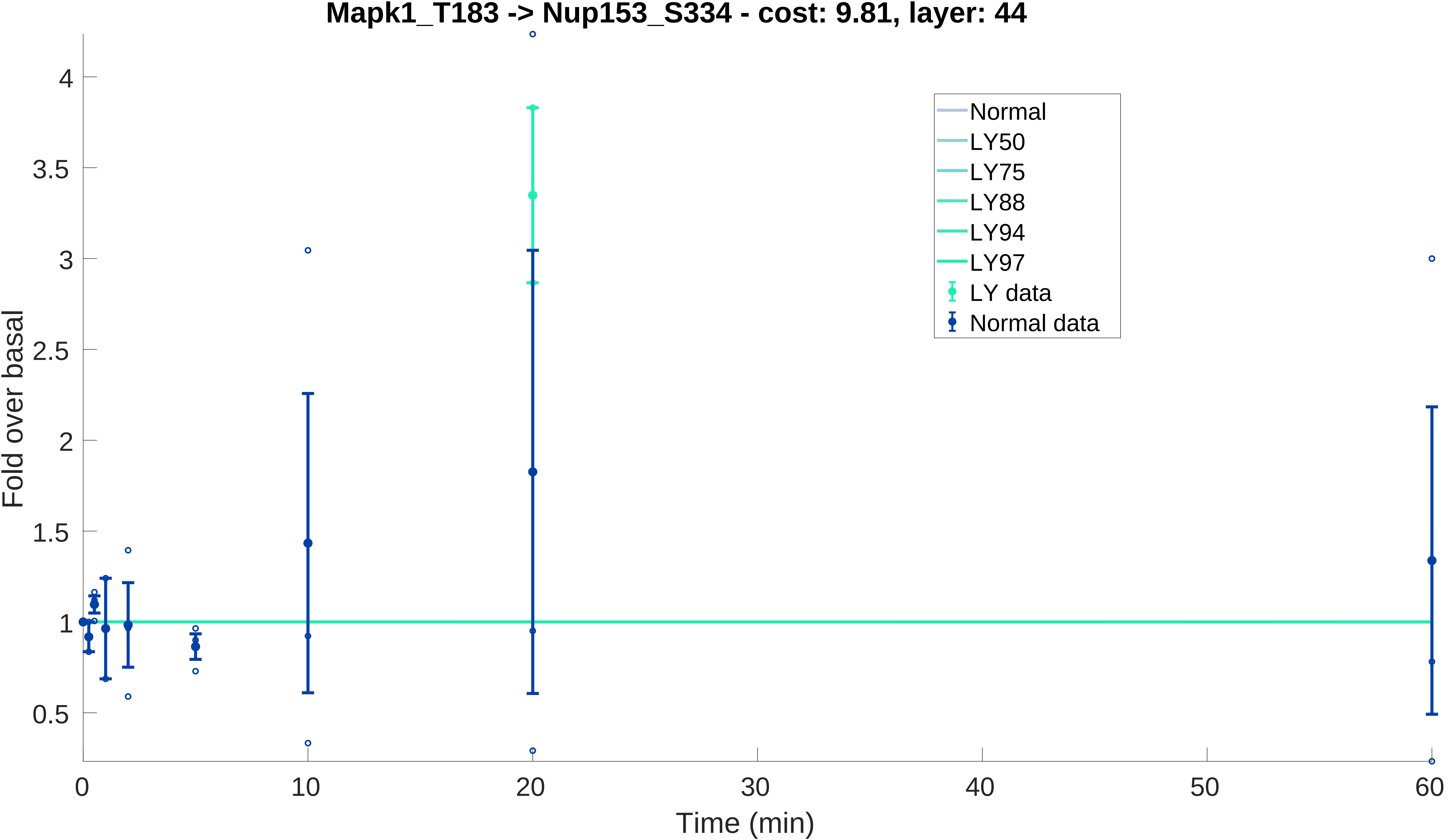
Mapk1_T183 -> Nup153_S334 - cost: 9.81, layer: 44
Fold over basal
Time (min)
0.5
1
1.5
2
2.5
3
3.5
4
0
10
20
30
40
50
60
Normal
LY50
LY75
LY88
LY94
LY97
LY data
Normal data

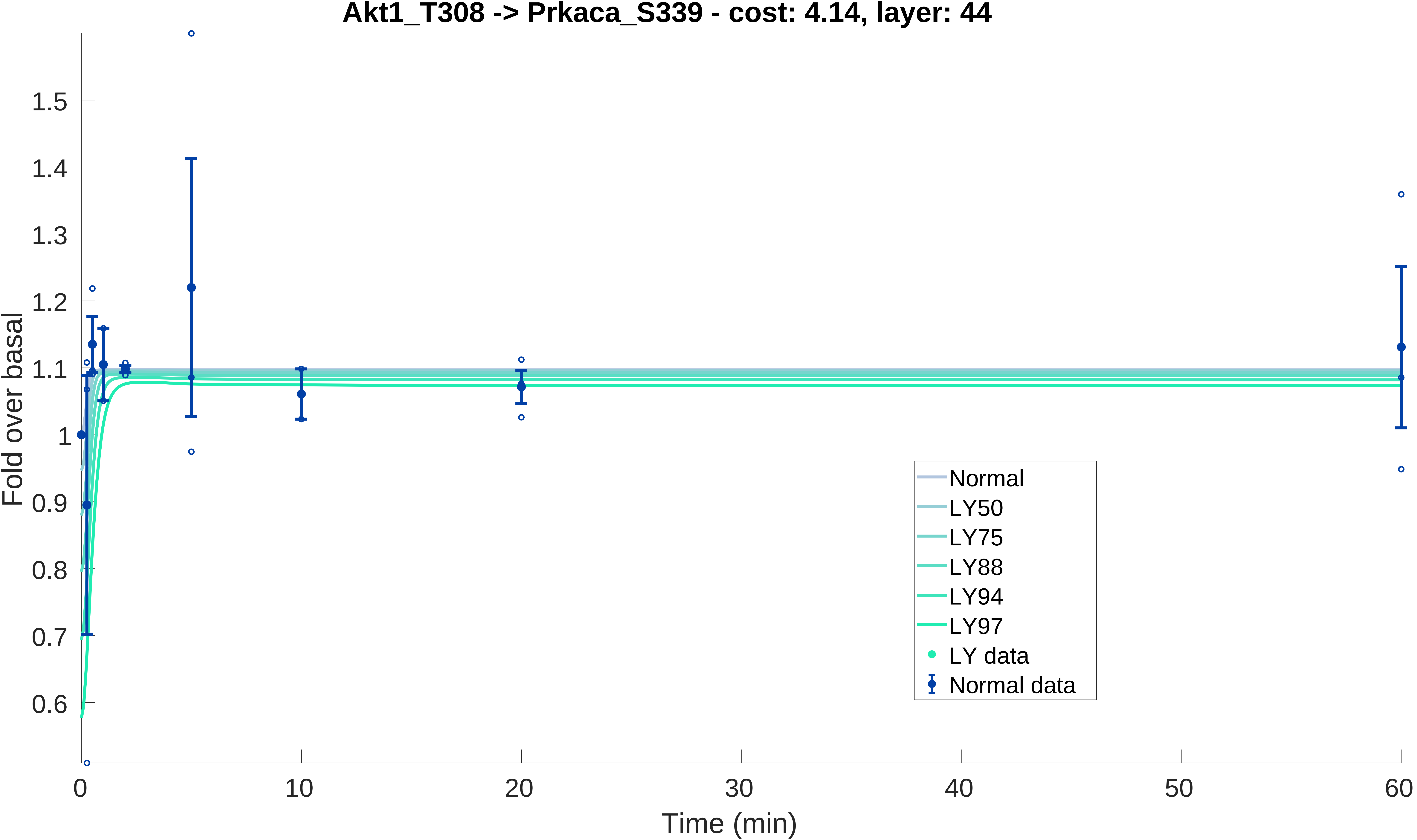

Akt1_T308 -> Prkaca_S339 - cost: 4.14, layer: 44
Fold over basal
Time (min)
1.5
1.4
1.3
1.2
1.1
1
0.9
0.8
0.7
0.6
0
10
20
30
40
50
60
Normal
LY50
LY75
LY88
LY94
LY97
LY data
Normal data

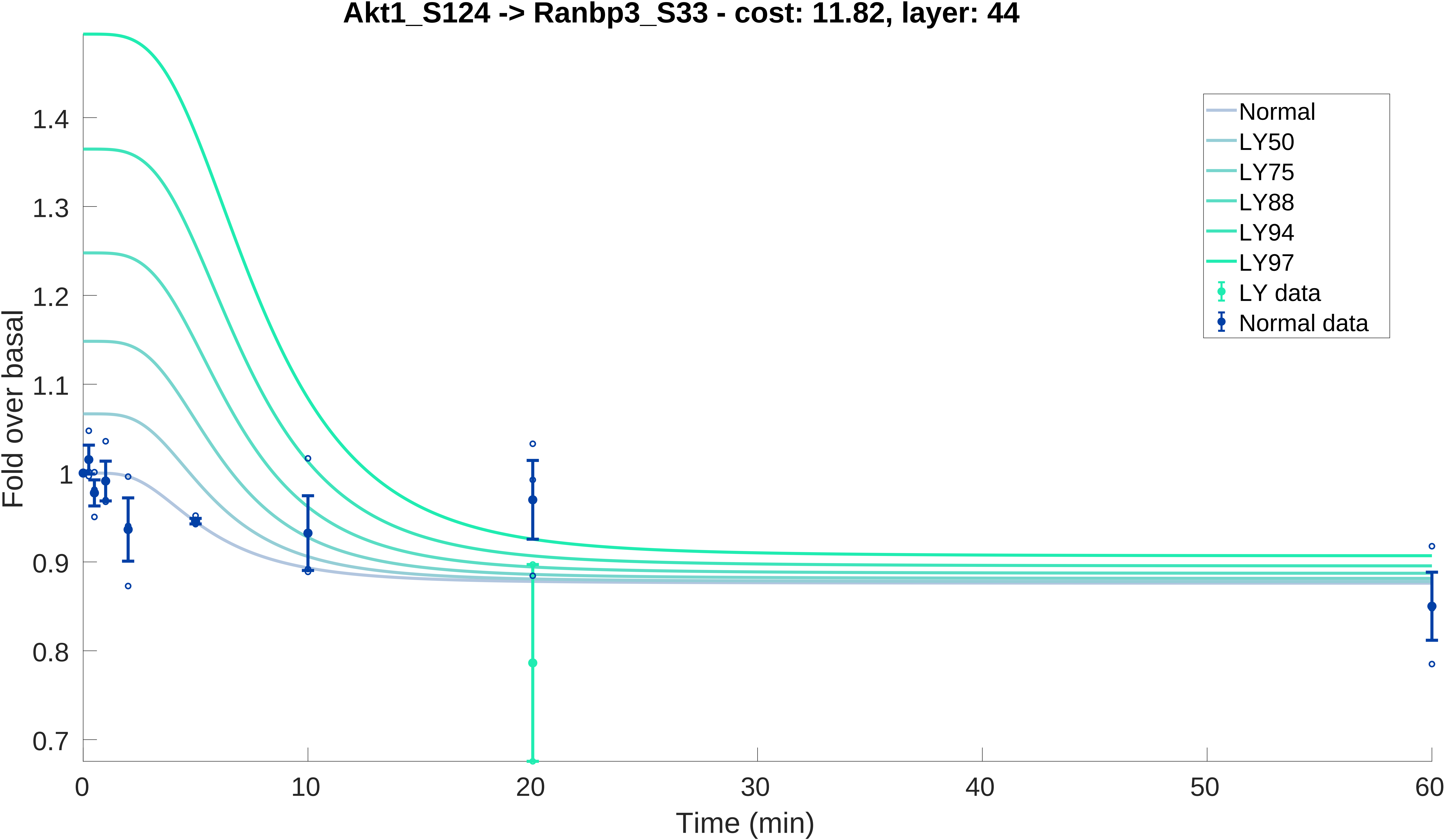

Akt1_S124 -> Ranbp3_S33 - cost: 11.82, layer: 44
Fold over basal
Time (min)
1.4
1.3
1.2
1.1
1
0.9
0.8
0.7
0
10
20
30
40
50
60
Normal
LY50
LY75
LY88
LY94
LY97
LY data
Normal data

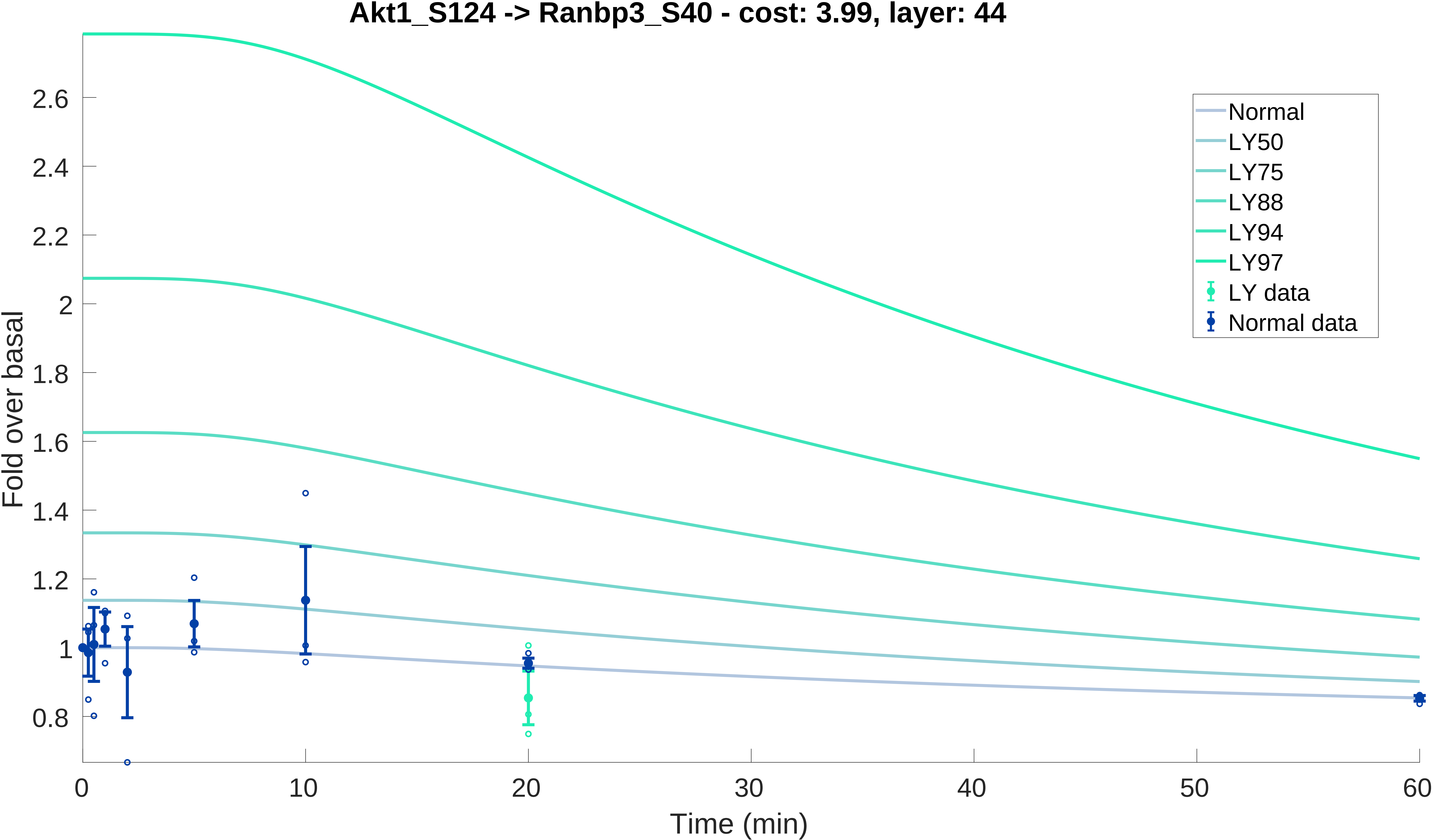

Akt1_S124 -> Ranbp3_S40 - cost: 3.99, layer: 44
Fold over basal
Time (min)
Normal
LY50
LY75
LY88
LY94
LY97
LY data
Normal data
0.8
1
1.2
1.4
1.6
1.8
2
2.2
2.4
2.6
0
10
20
30
40
50
60

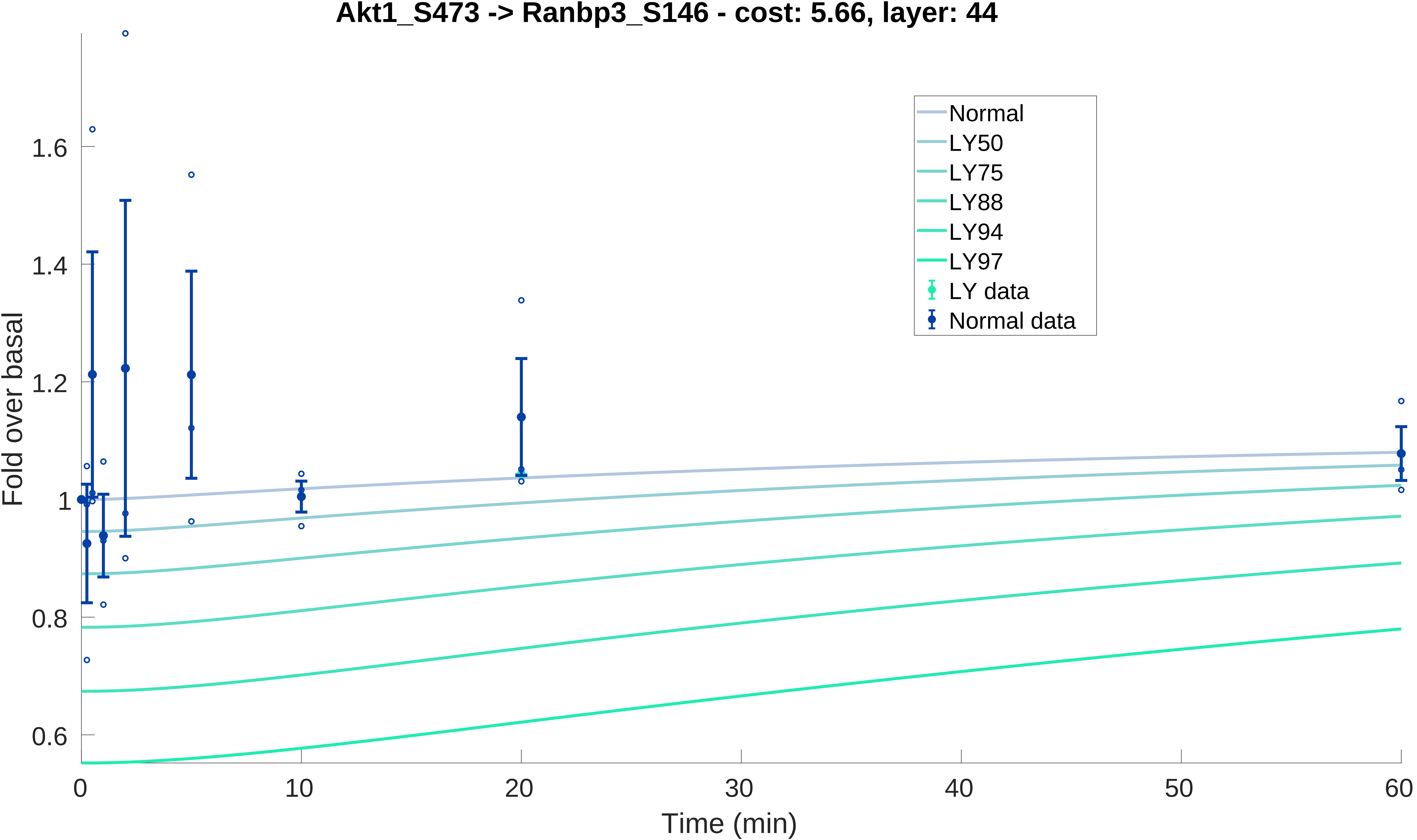

Akt1_S473 -> Ranbp3_S146 - cost: 5.66, layer: 44
Fold over basal
0.6
0.8
1
1.2
1.4
1.6
0
10
20
30
40
50
60
Time (min)
Normal
LY50
LY75
LY88
LY94
LY97
LY data
Normal data

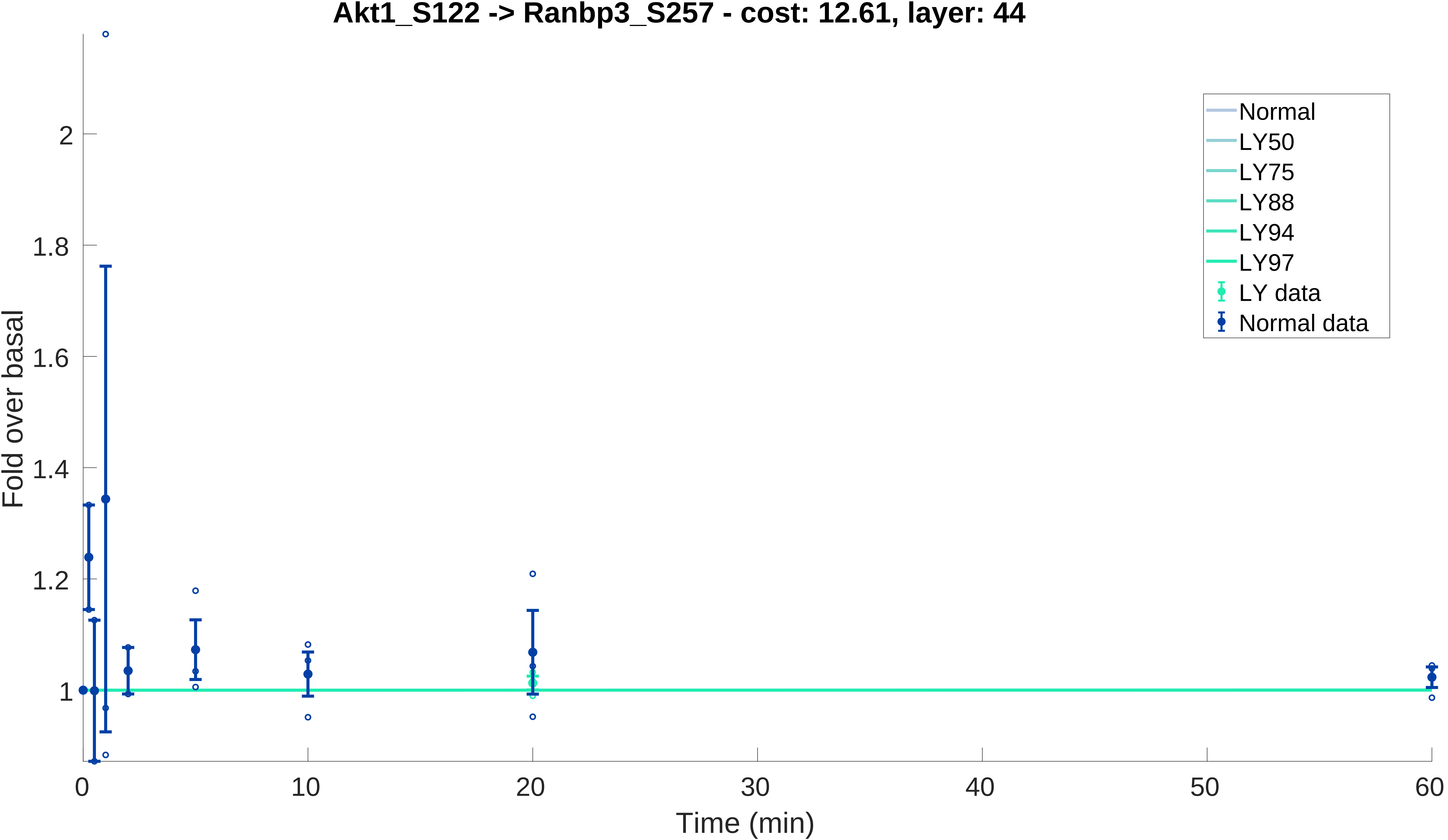

Akt1_S122 -> Ranbp3_S257 - cost: 12.61, layer: 44
Fold over basal
Time (min)
2
1.8
1.6
1.4
1.2
1
0
10
20
30
40
50
60
Normal
LY50
LY75
LY88
LY94
LY97
LY data
Normal data

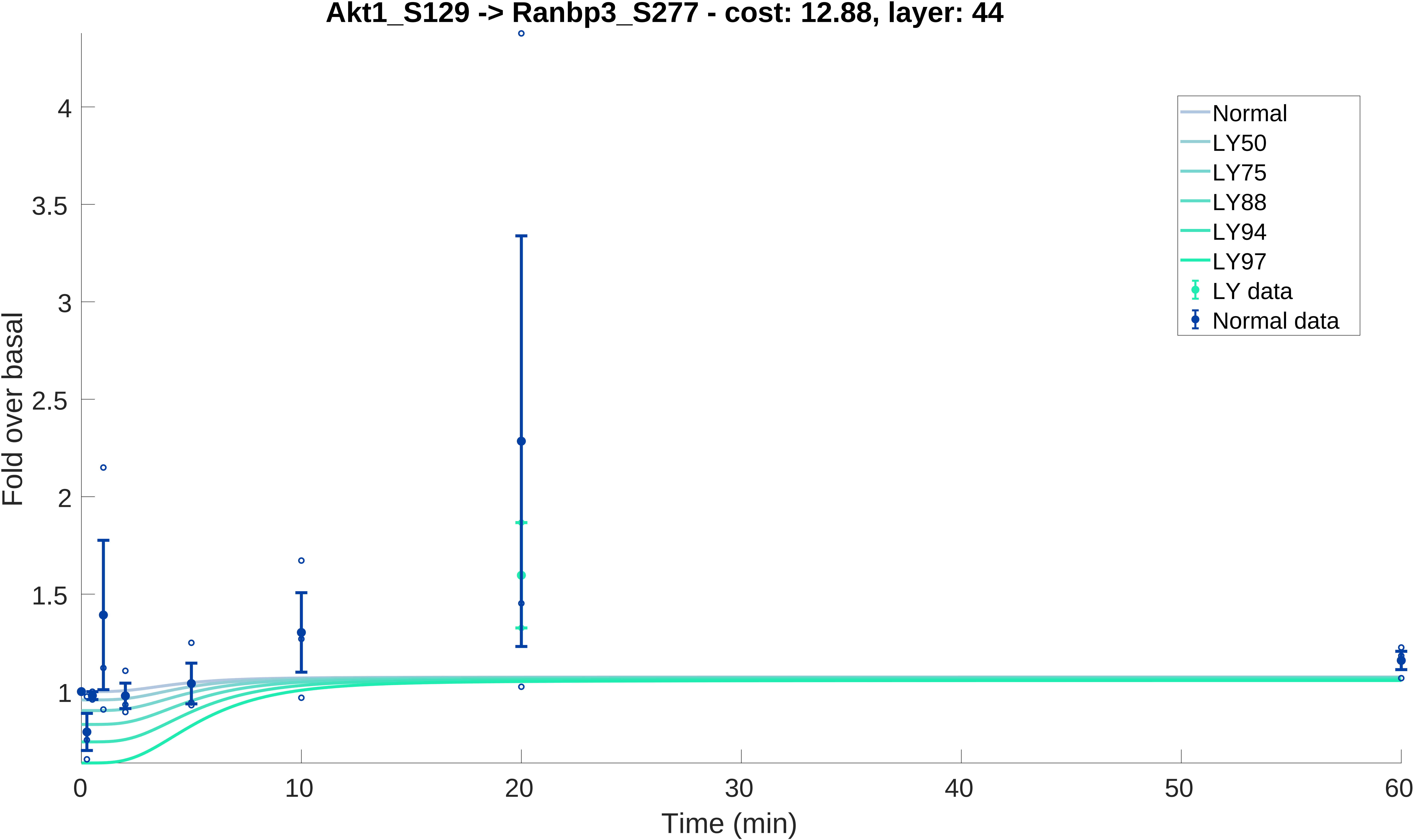

Akt1_S129 -> Ranbp3_S277 - cost: 12.88, layer: 44
Fold over basal
Time (min)
4
3.5
3
2.5
2
1.5
1
0
10
20
30
40
50
60
Normal
LY50
LY75
LY88
LY94
LY97
LY data
Normal data

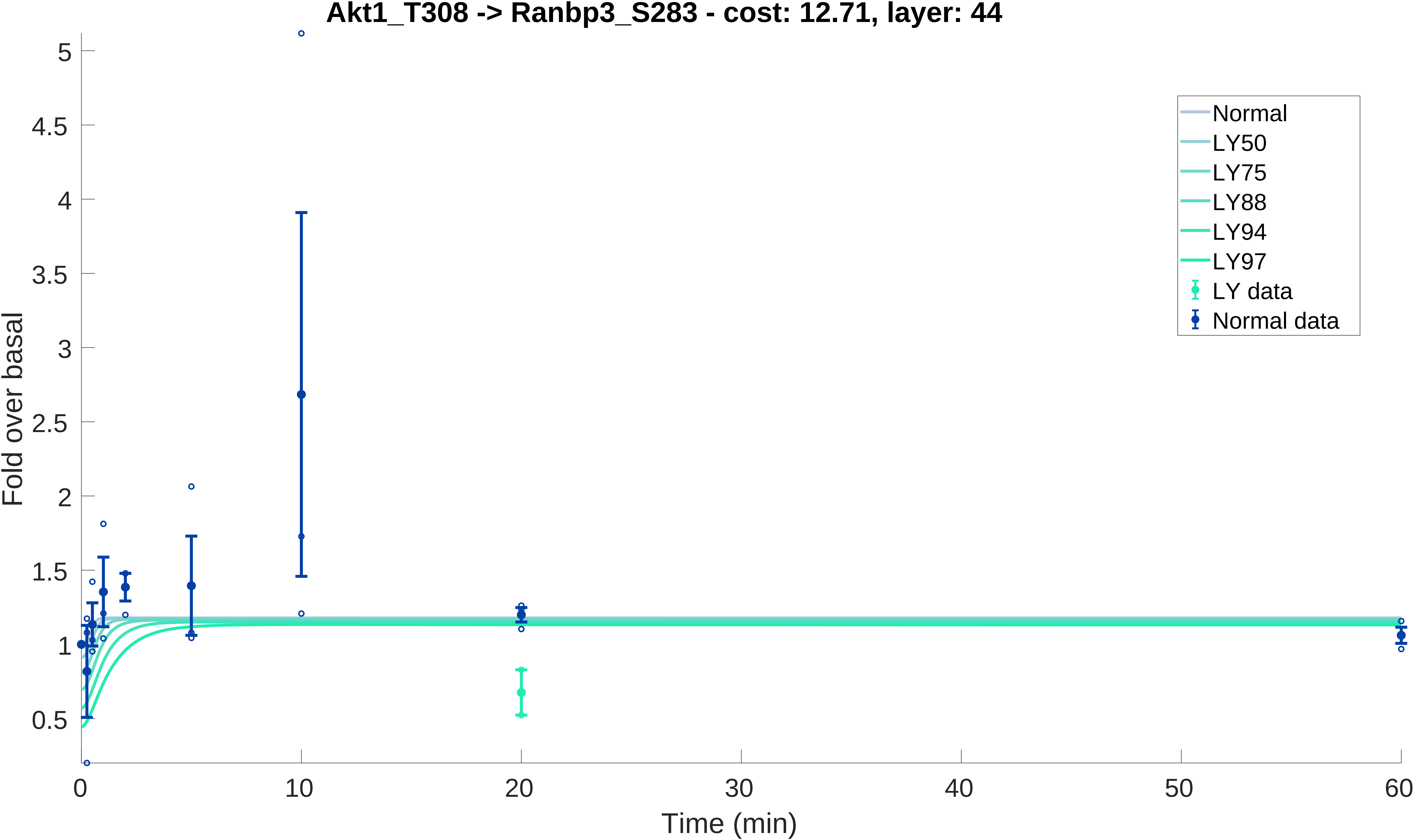

Akt1_T308 -> Ranbp3_S283 - cost: 12.71, layer: 44
Fold over basal
Time (min)
Normal
LY50
LY75
LY88
LY94
LY97
LY data
Normal data
0
10
20
30
40
50
60
0.5
1
1.5
2
2.5
3
3.5
4
4.5
5

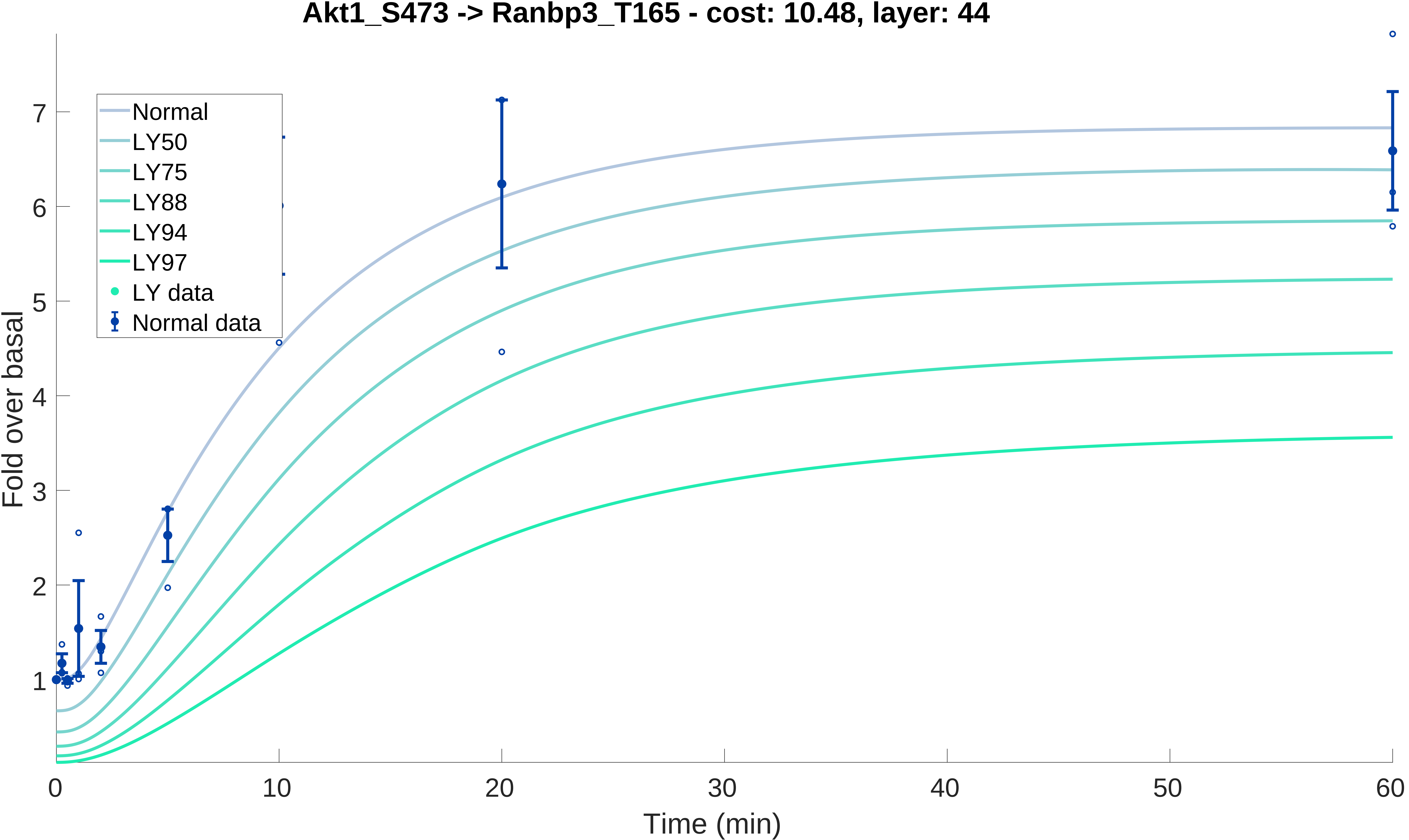

Akt1_S473 -> Ranbp3_T165 - cost: 10.48, layer: 44
Fold over basal
Time (min)
Normal
LY50
LY75
LY88
LY94
LY97
LY data
Normal data
0
10
20
30
40
50
60
1
2
3
4
5
6
7

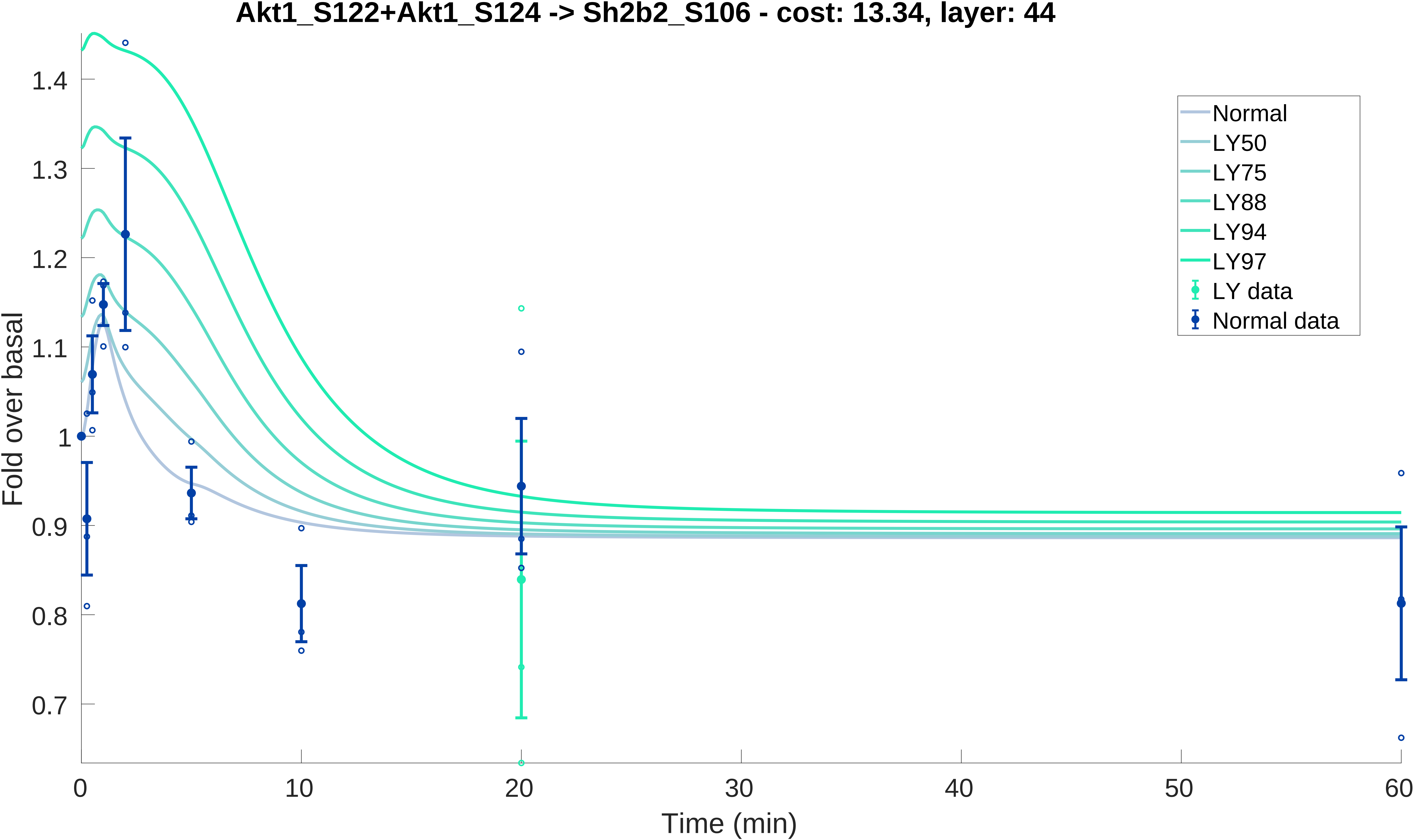

Akt1_S122+Akt1_S124 -> Sh2b2_S106 - cost: 13.34, layer: 44
Fold over basal
1.4
1.3
1.2
1.1
1
0.9
0.8
0.7
0
10
20
30
40
50
60
Time (min)
Normal
LY50
LY75
LY88
LY94
LY97
LY data
Normal data

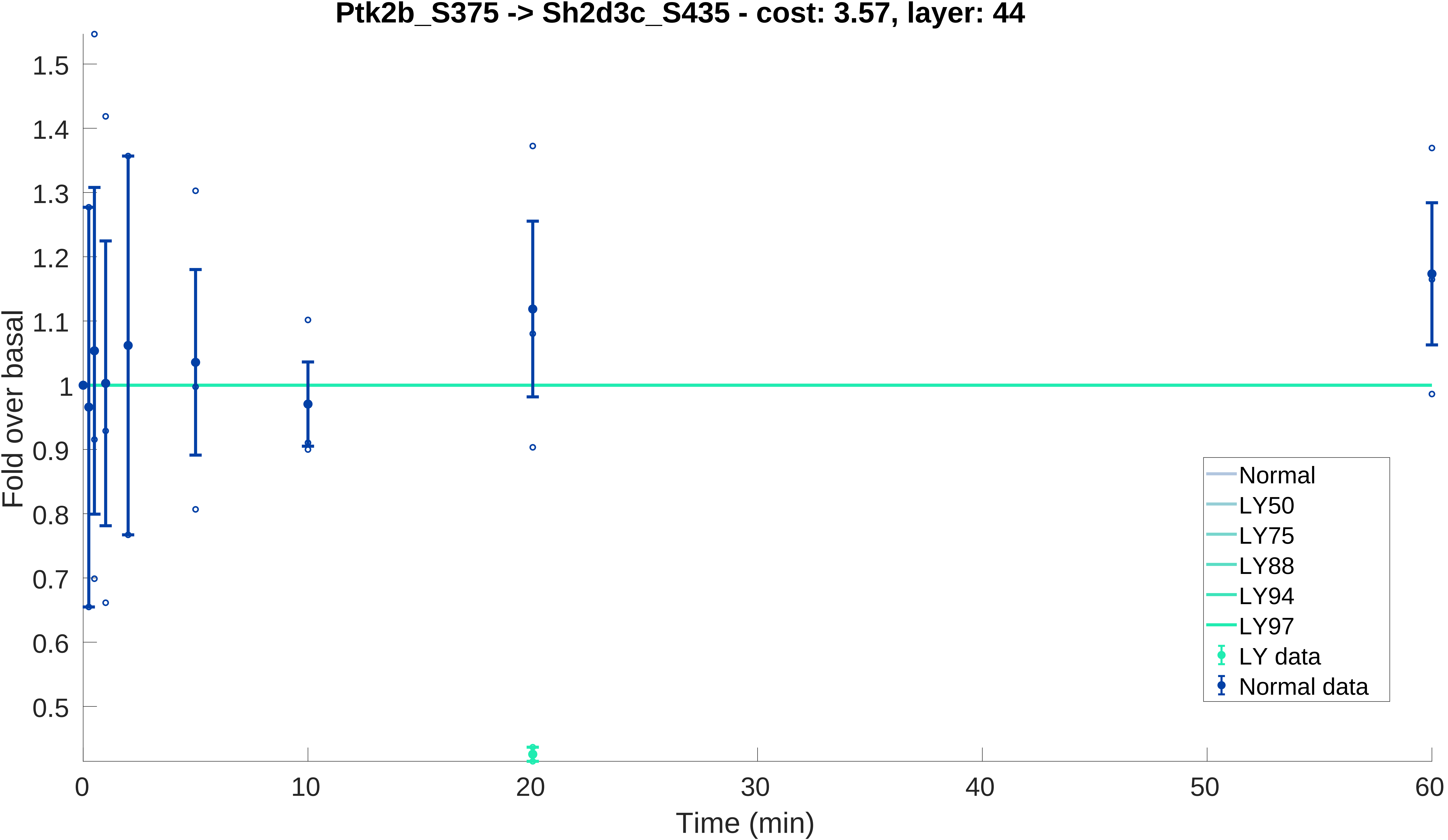

Ptk2b_S375 -> Sh2d3c_S435 - cost: 3.57, layer: 44
Fold over basal
Time (min)
0
10
20
30
40
50
60
0.5
0.6
0.7
0.8
0.9
1
1.1
1.2
1.3
1.4
1.5
Normal
LY50
LY75
LY88
LY94
LY97
LY data
Normal data

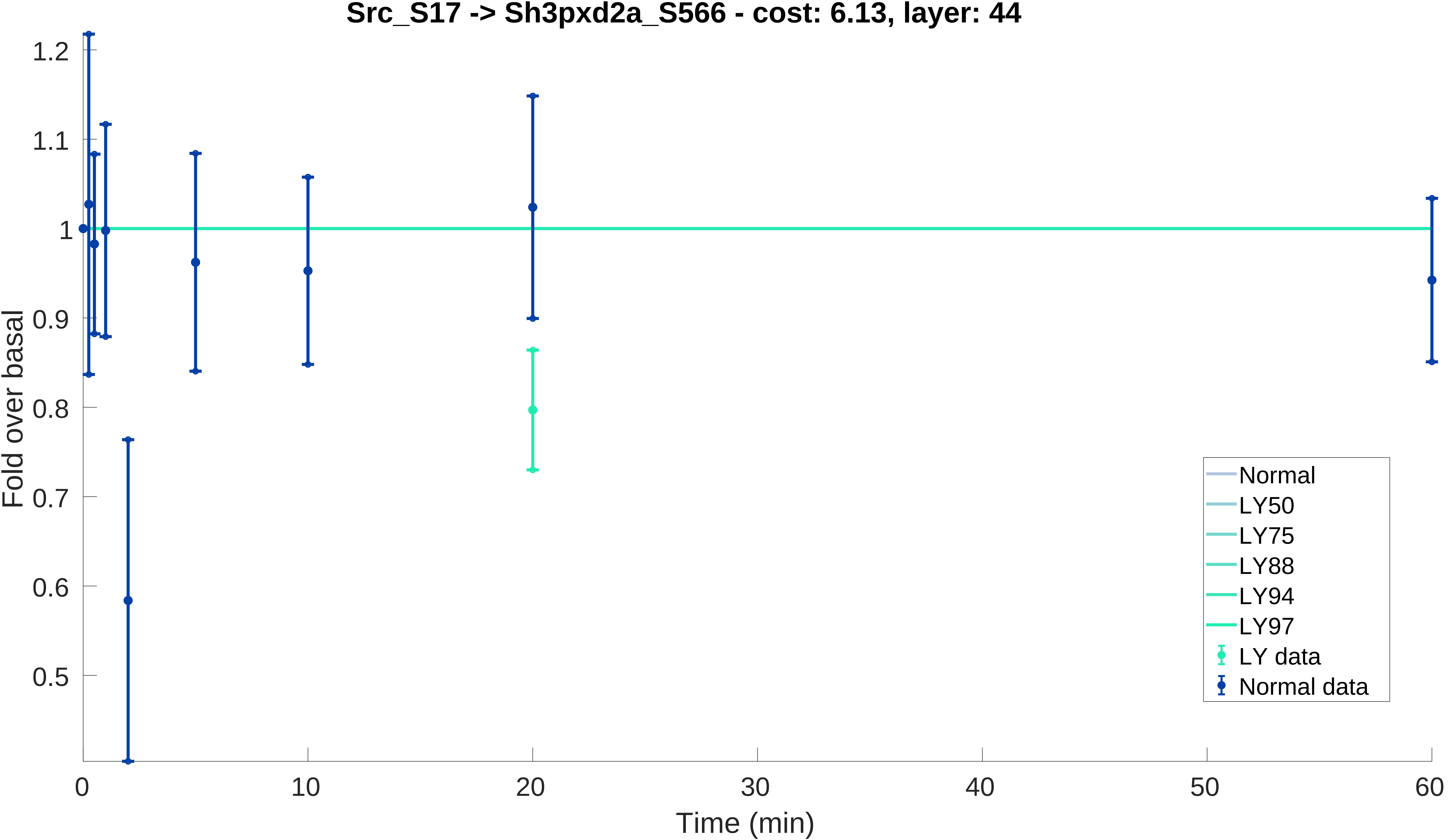

Src_S17 -> Sh3pxd2a_S566 - cost: 6.13, layer: 44
Fold over basal
1.2
1.1
1
0.9
0.8
0.7
0.6
0.5
0
10
20
30
40
50
60
Time (min)
Normal
LY50
LY75
LY88
LY94
LY97
LY data
Normal data

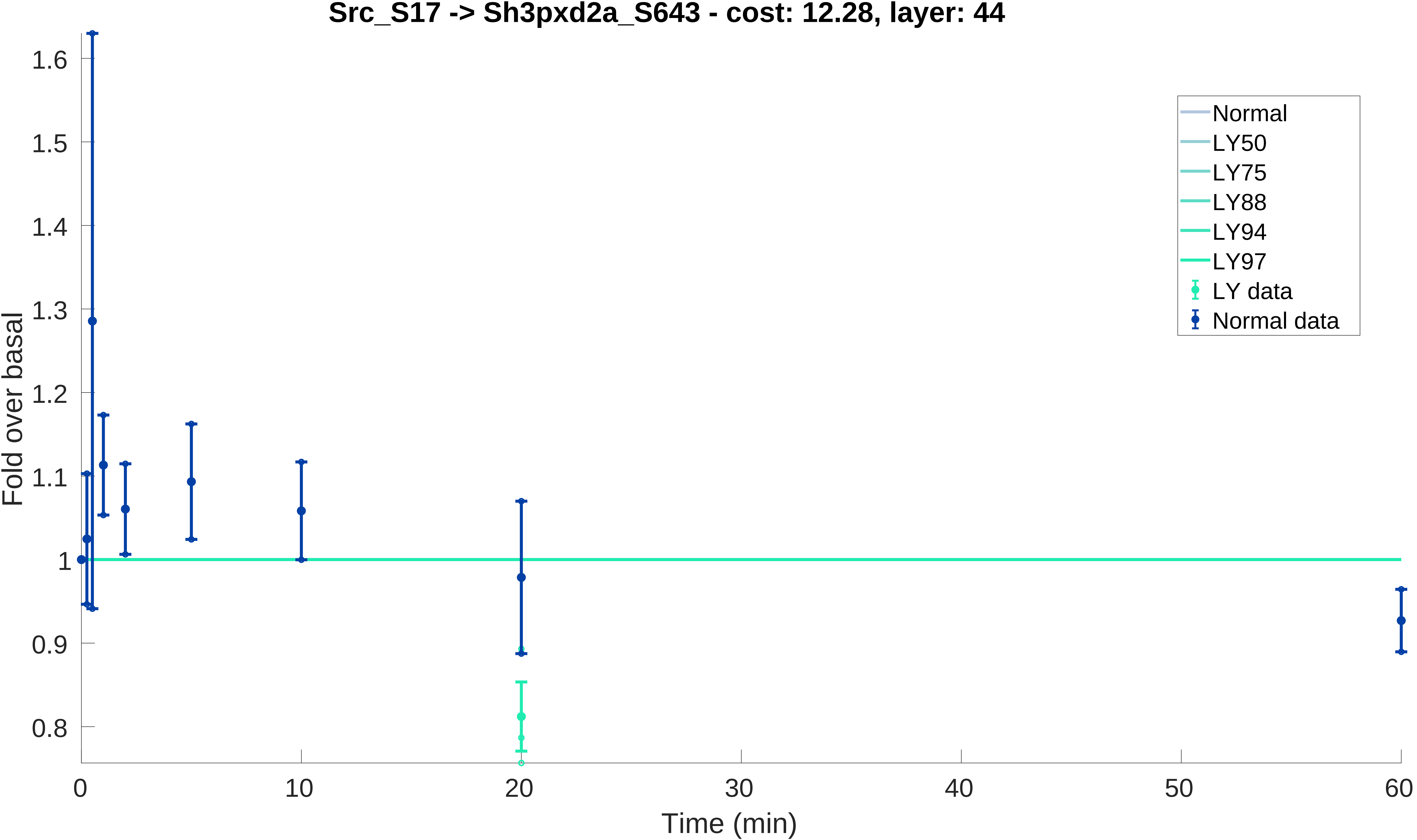

Src_S17 -> Sh3pxd2a_S643 - cost: 12.28, layer: 44
Fold over basal
1.6
1.5
1.4
1.3
1.2
1.1
1
0.9
0.8
0
10
20
30
40
50
60
Time (min)
Normal
LY50
LY75
LY88
LY94
LY97
LY data
Normal data

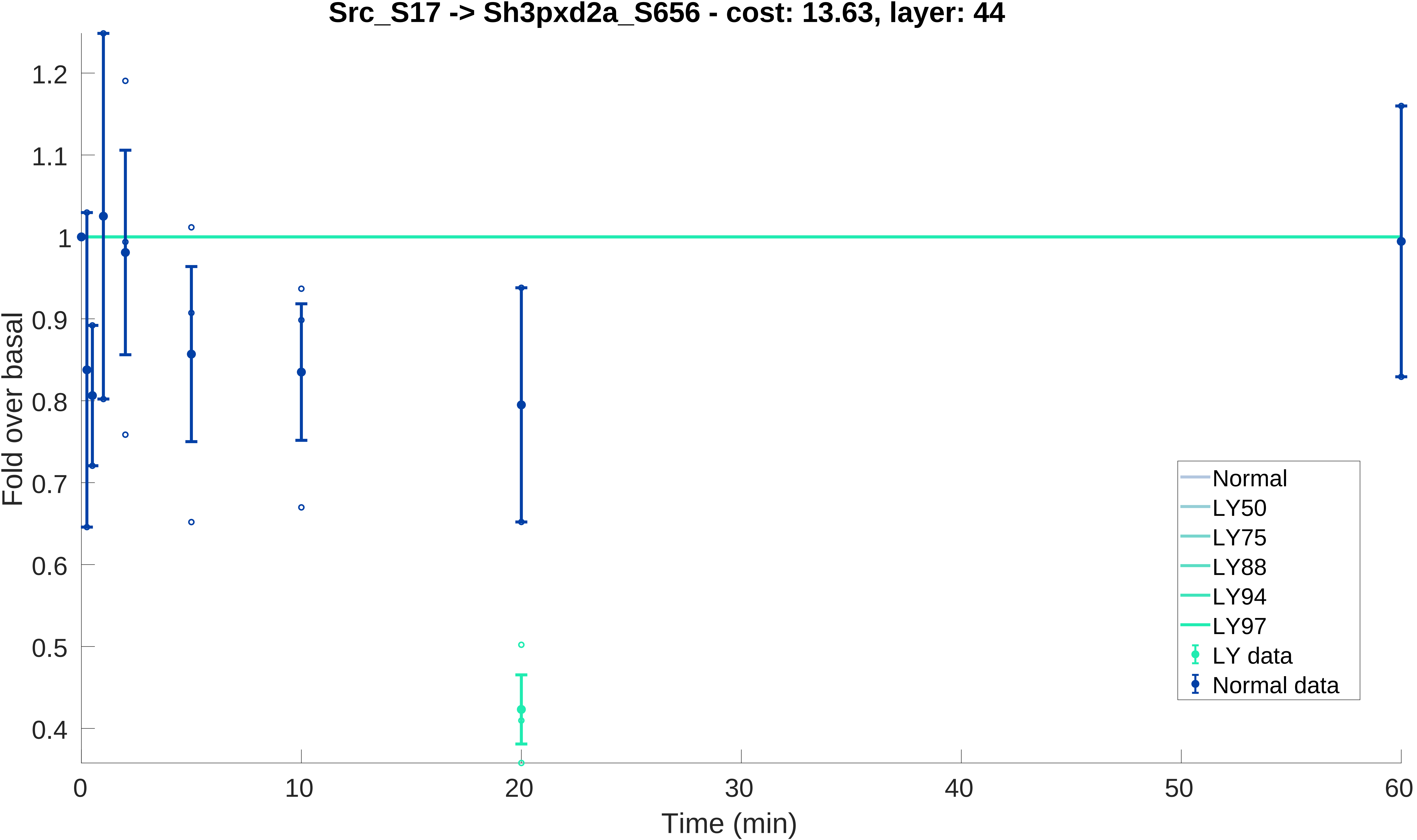

Src_S17 -> Sh3pxd2a_S656 - cost: 13.63, layer: 44
Fold over basal
1.2
1.1
1
0.9
0.8
0.7
0.6
0.5
0.4
0
10
20
30
40
50
60
Time (min)
Normal
LY50
LY75
LY88
LY94
LY97
LY data
Normal data

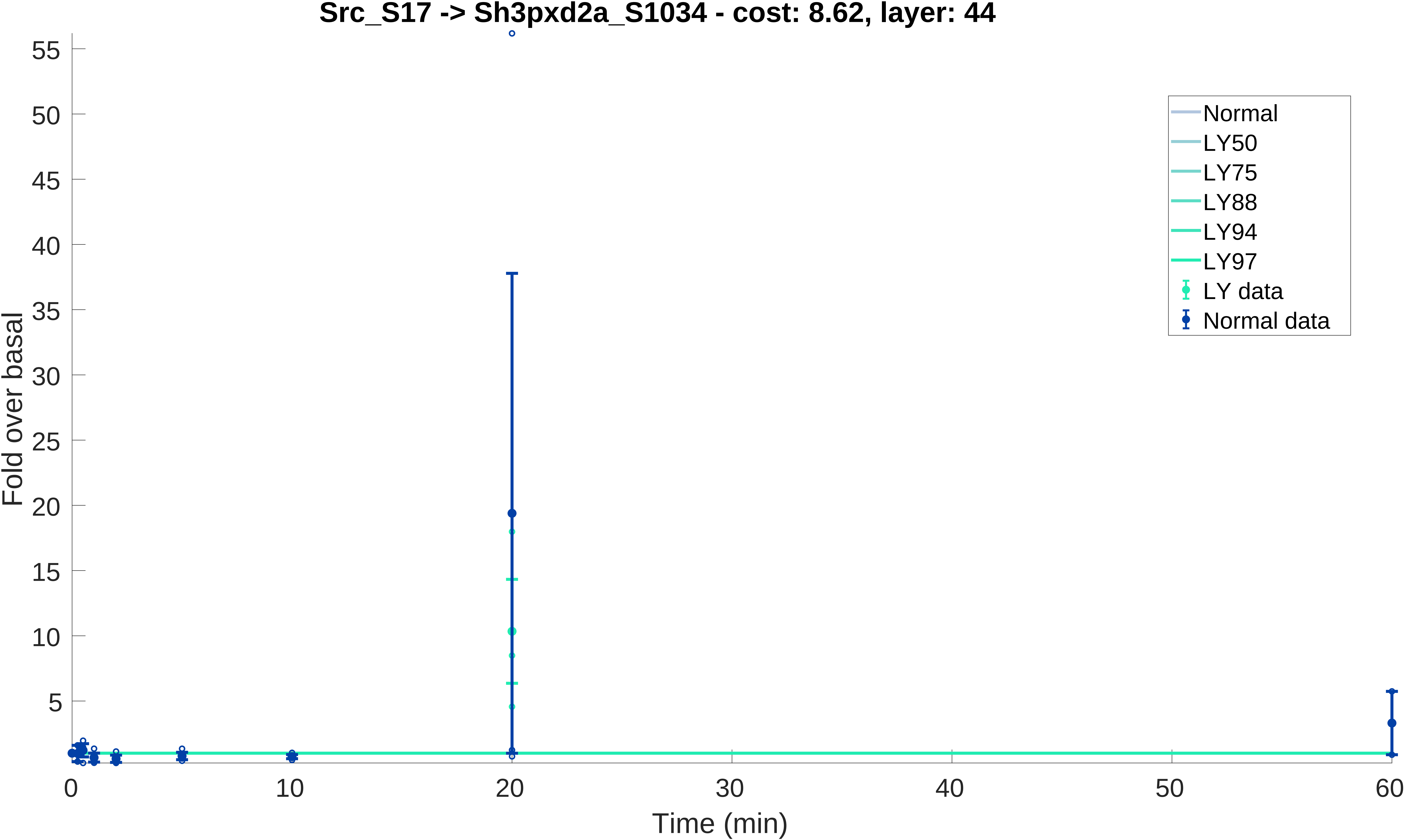
Src_S17 -> Sh3pxd2a_S1034 - cost: 8.62, layer: 44
Fold over basal
Time (min)
0
10
20
30
40
50
60
5
10
15
20
25
30
35
40
45
50
55
Normal
LY50
LY75
LY88
LY94
LY97
LY data
Normal data

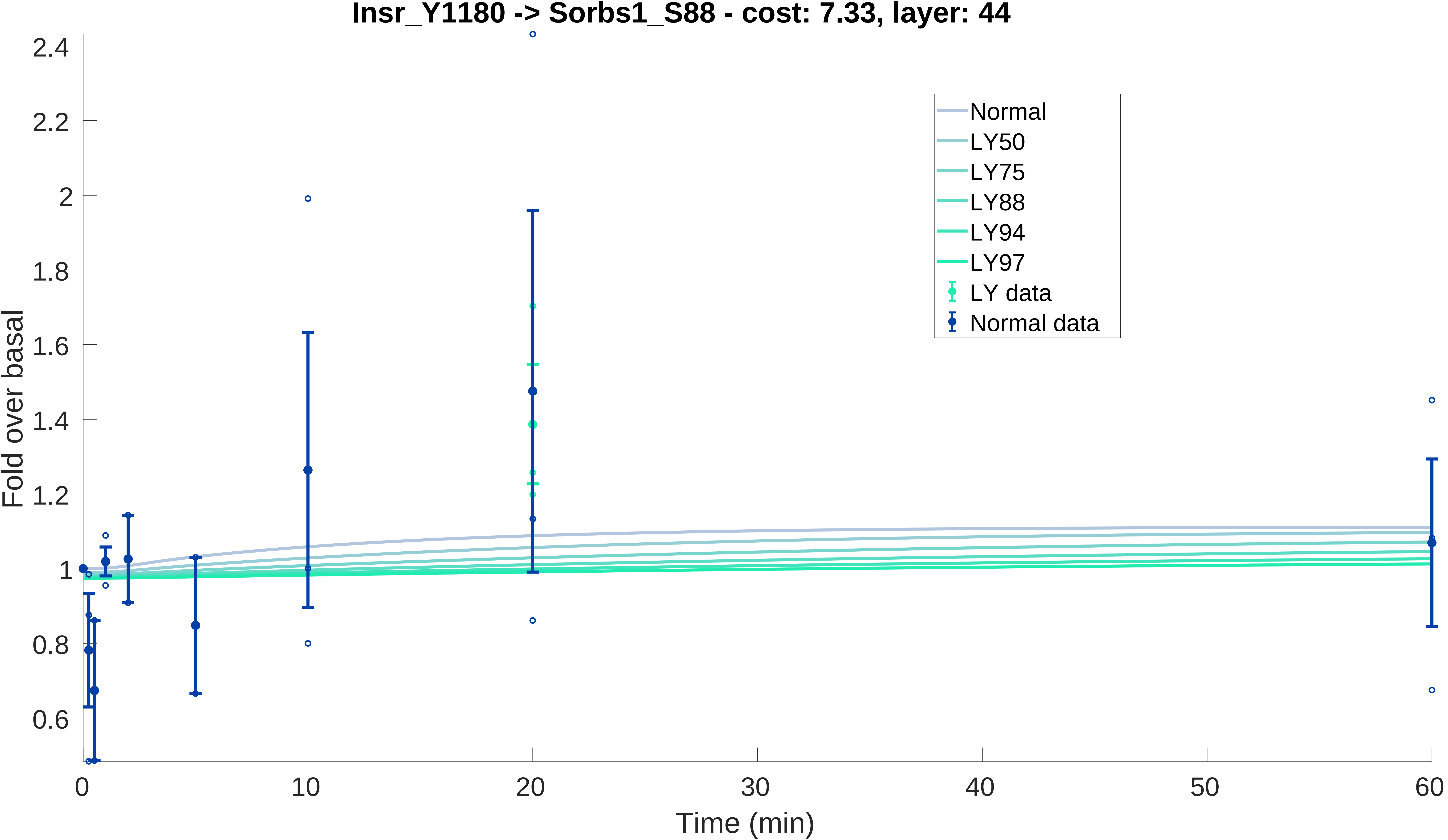

Insr_Y1180 -> Sorbs1_S88 - cost: 7.33, layer: 44
Fold over basal
Time (min)
Normal
LY50
LY75
LY88
LY94
LY97
LY data
Normal data
2.4
2.2
2
1.8
1.6
1.4
1.2
1
0.8
0.6
0
10
20
30
40
50
60

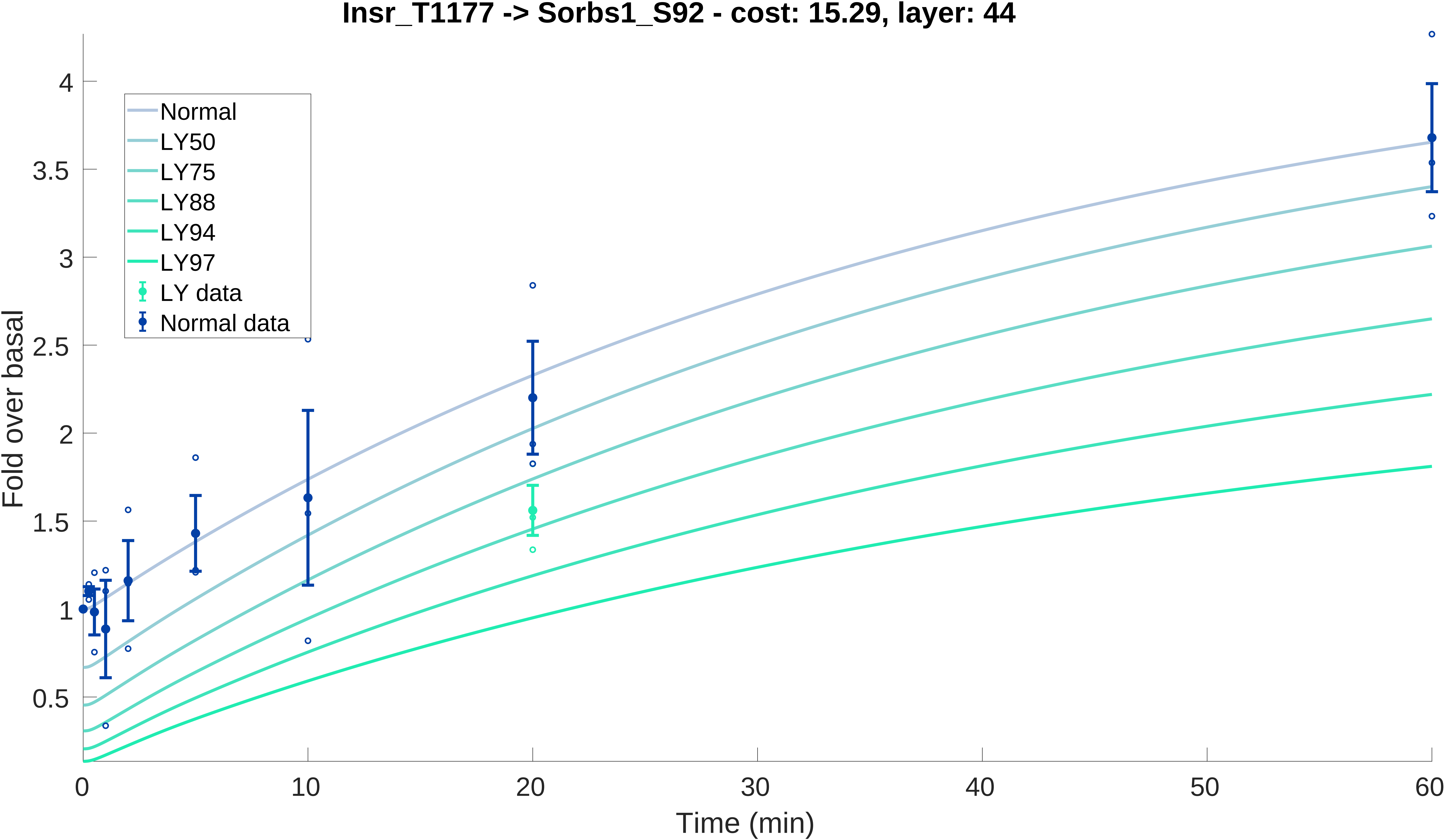
Insr_T1177 -> Sorbs1_S92 - cost: 15.29, layer: 44
Normal
LY50
LY75
LY88
LY94
LY97
LY data
Normal data
Fold over basal
Time (min)
0
10
20
30
40
50
60
0.5
1
1.5
2
2.5
3
3.5
4

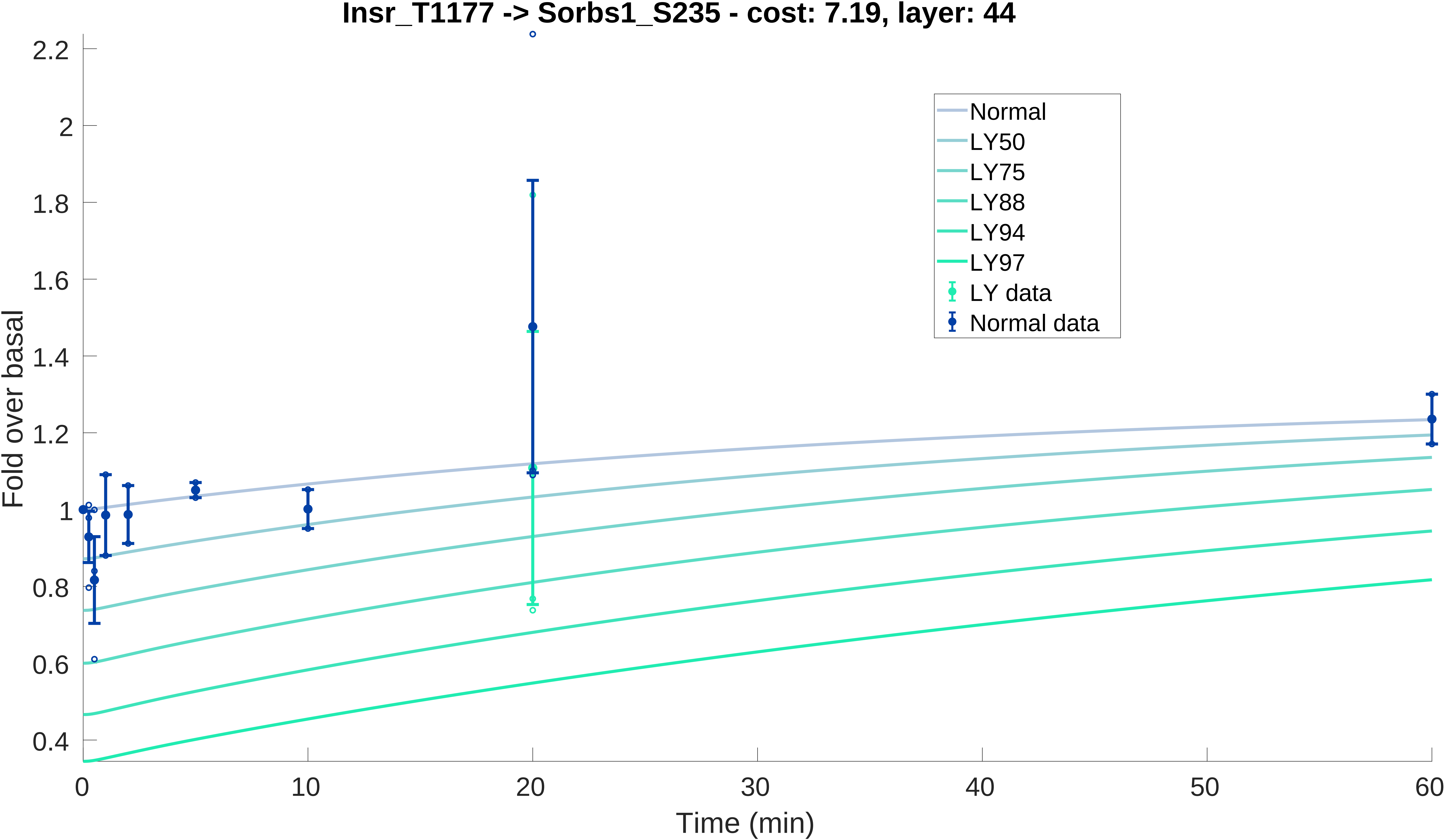
Insr_T1177 -> Sorbs1_S235 - cost: 7.19, layer: 44
Fold over basal
Time (min)
2.2
2
1.8
1.6
1.4
1.2
1
0.8
0.6
0.4
0
10
20
30
40
50
60
Normal
LY50
LY75
LY88
LY94
LY97
LY data
Normal data

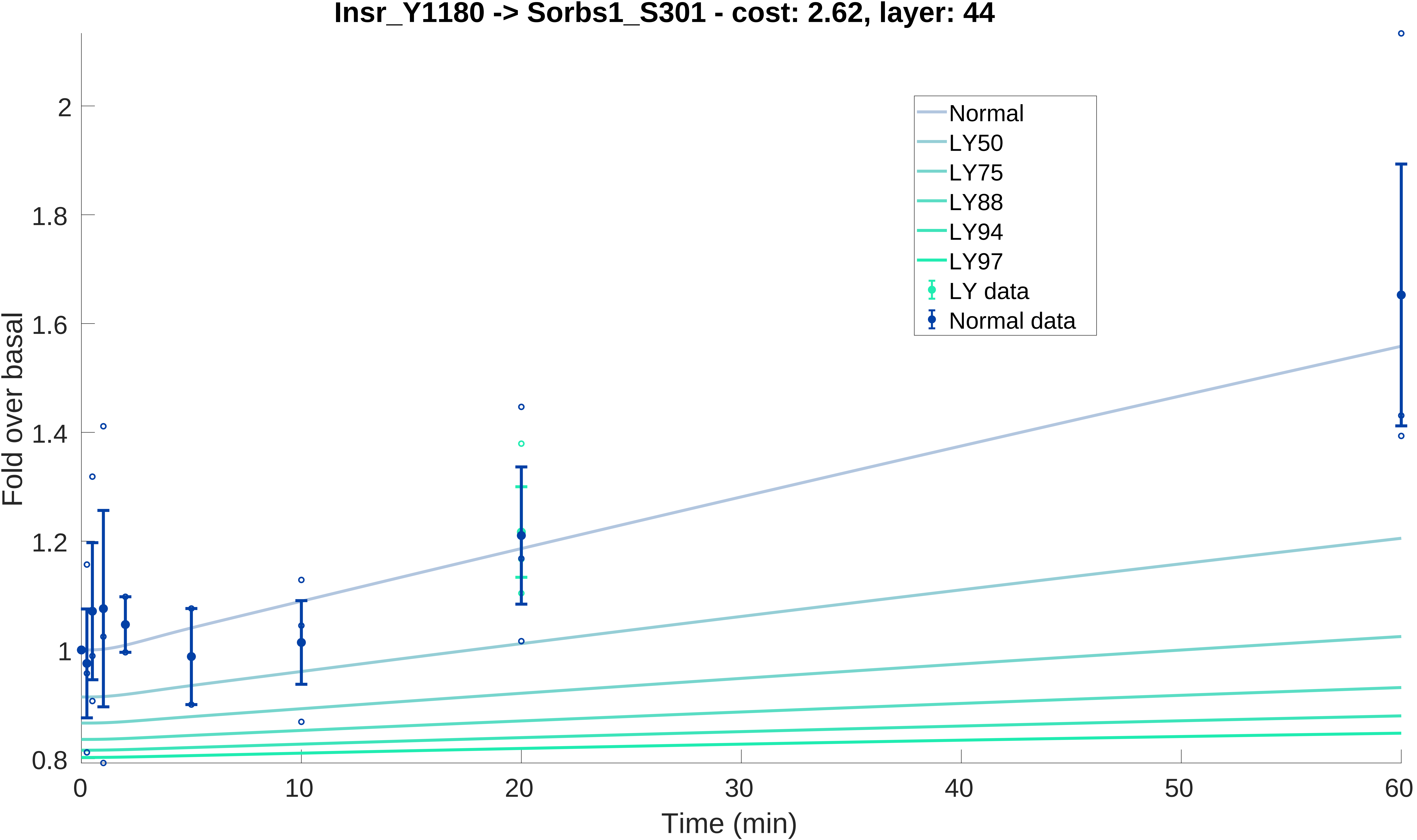
Insr_Y1180 -> Sorbs1_S301 - cost: 2.62, layer: 44
Normal
LY50
LY75
LY88
LY94
LY97
LY data
Normal data
Fold over basal
Time (min)
0.8
1
1.2
1.4
1.6
1.8
2
0
10
20
30
40
50
60

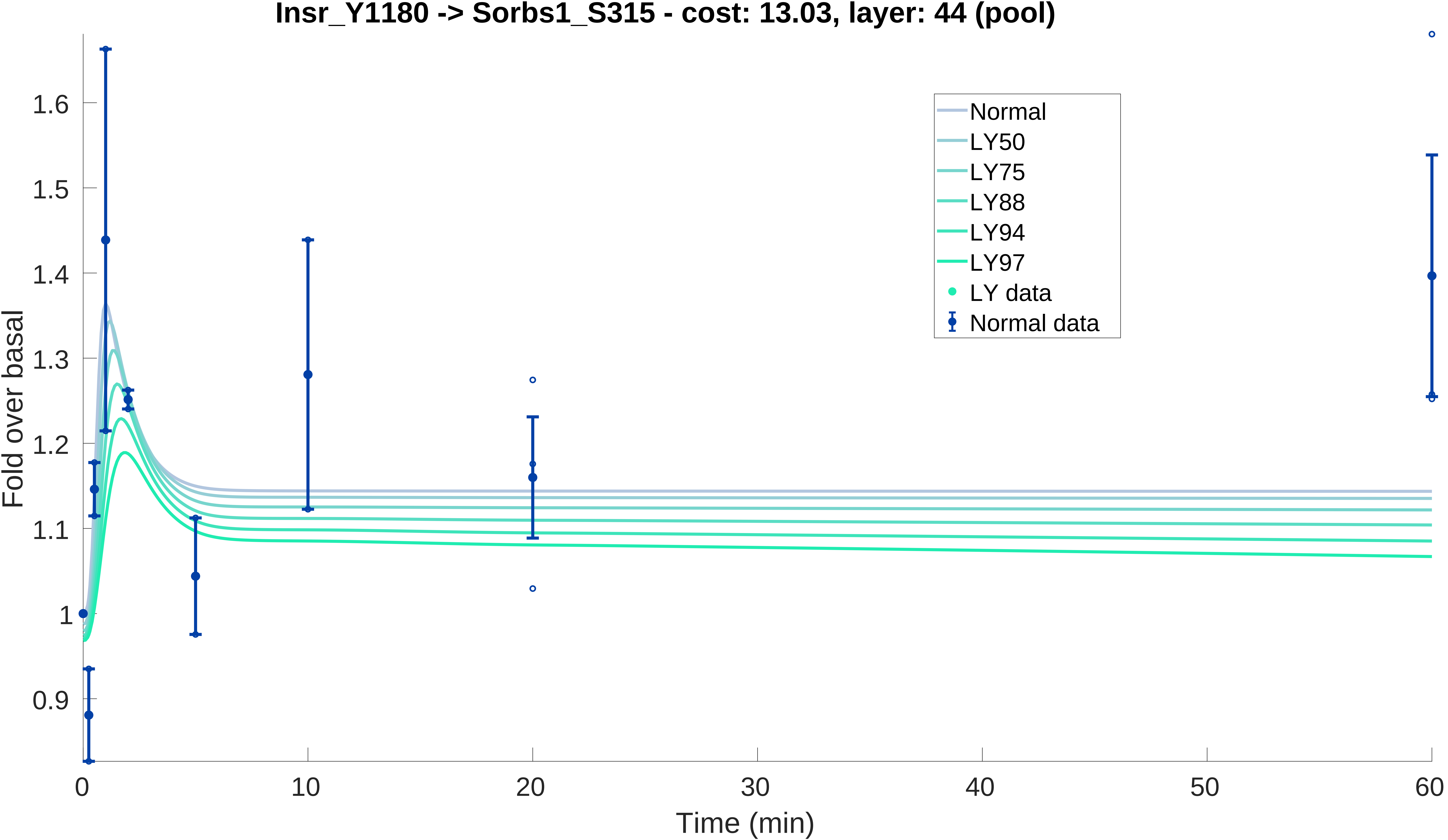
Insr_Y1180 -> Sorbs1_S315 - cost: 13.03, layer: 44 (pool)
Normal
LY50
LY75
LY88
LY94
LY97
LY data
Normal data
Fold over basal
Time (min)
0
10
20
30
40
50
60
0.9
1
1.1
1.2
1.3
1.4
1.5
1.6

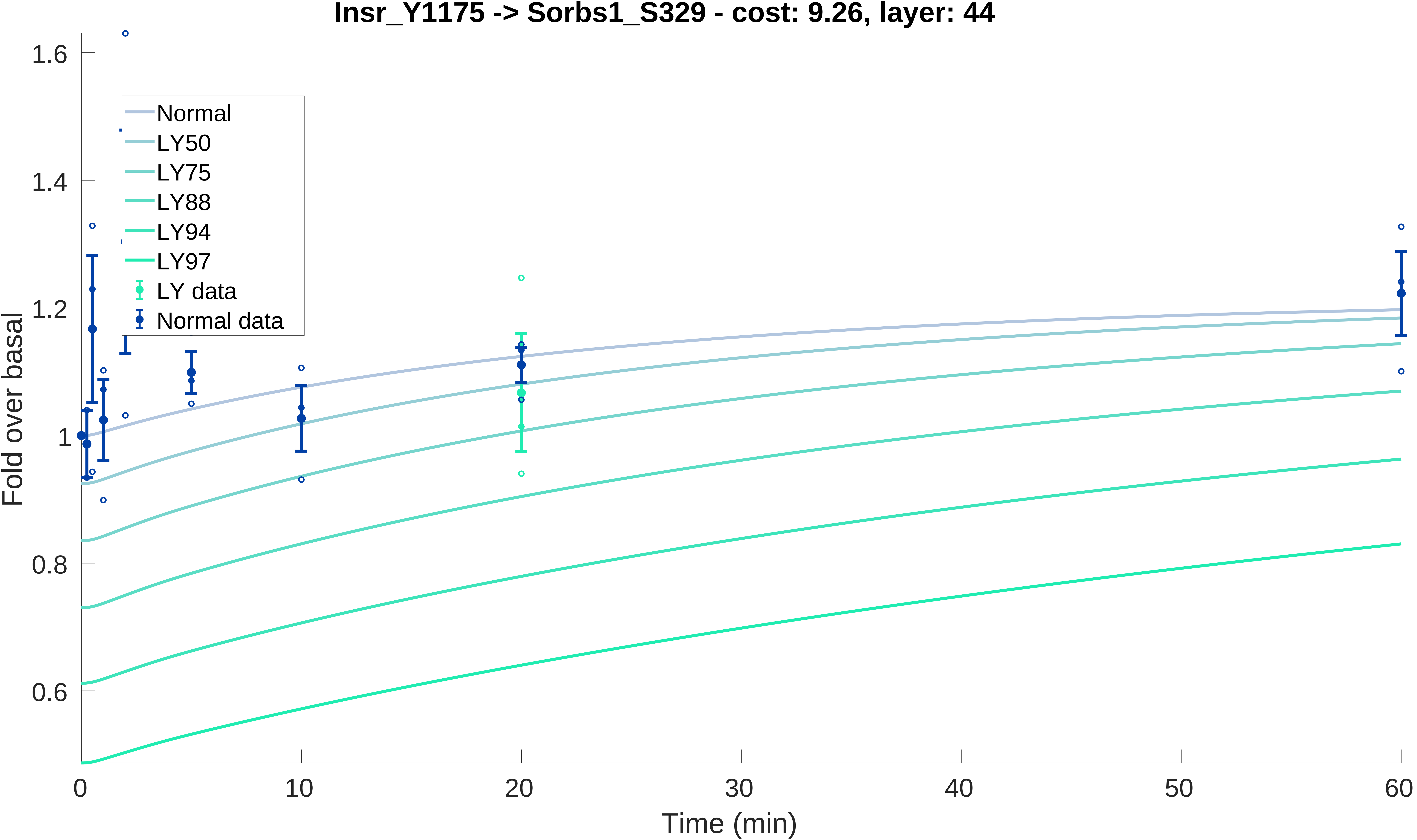

Insr_Y1175 -> Sorbs1_S329 - cost: 9.26, layer: 44
Fold over basal
Time (min)
Normal
LY50
LY75
LY88
LY94
LY97
LY data
Normal data
1.6
1.4
1.2
1
0.8
0.6
0
10
20
30
40
50
60

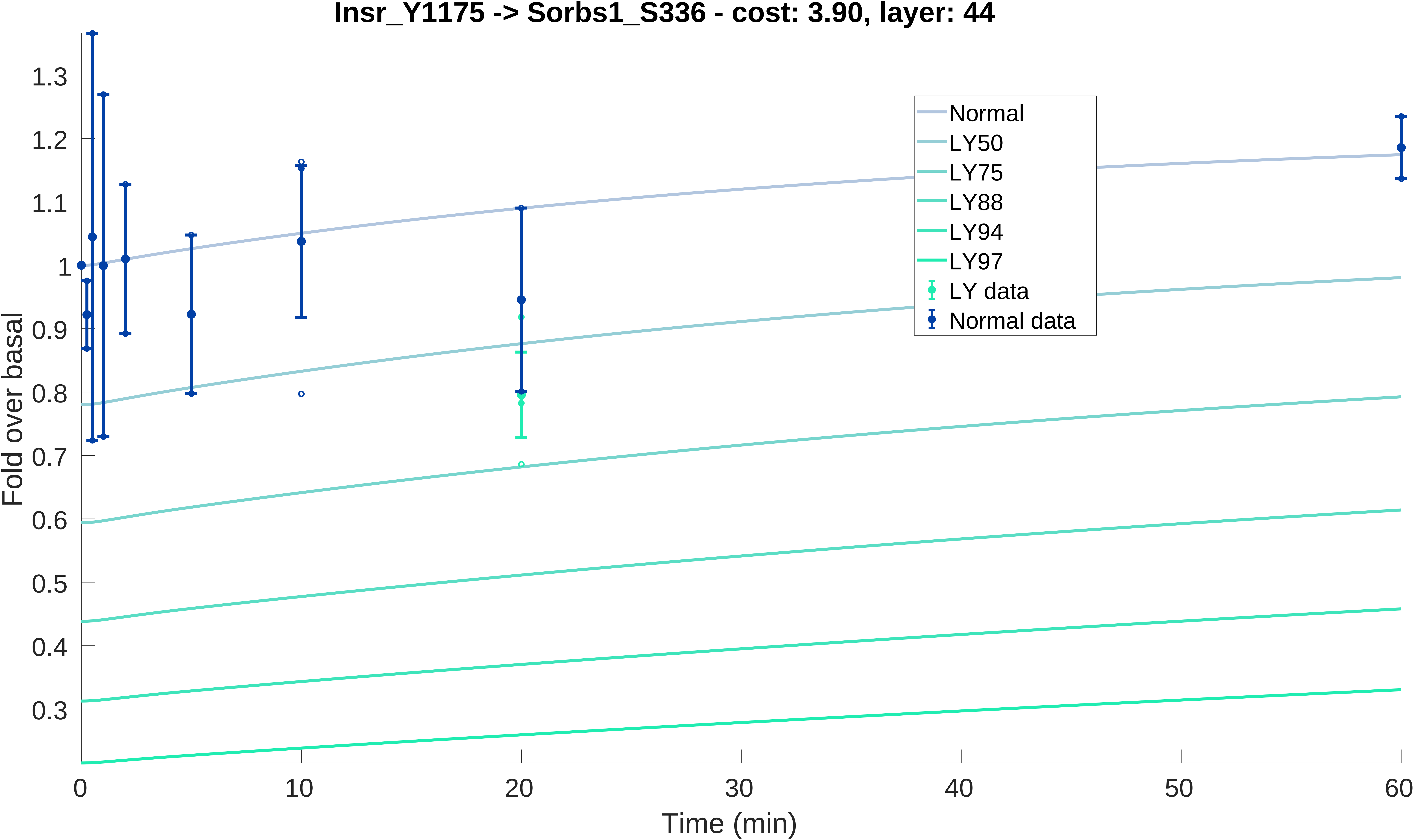

Insr_Y1175 -> Sorbs1_S336 - cost: 3.90, layer: 44
Fold over basal
Time (min)
1.3
1.2
1.1
1
0.9
0.8
0.7
0.6
0.5
0.4
0.3
0
10
20
30
40
50
60
Normal
LY50
LY75
LY88
LY94
LY97
LY data
Normal data

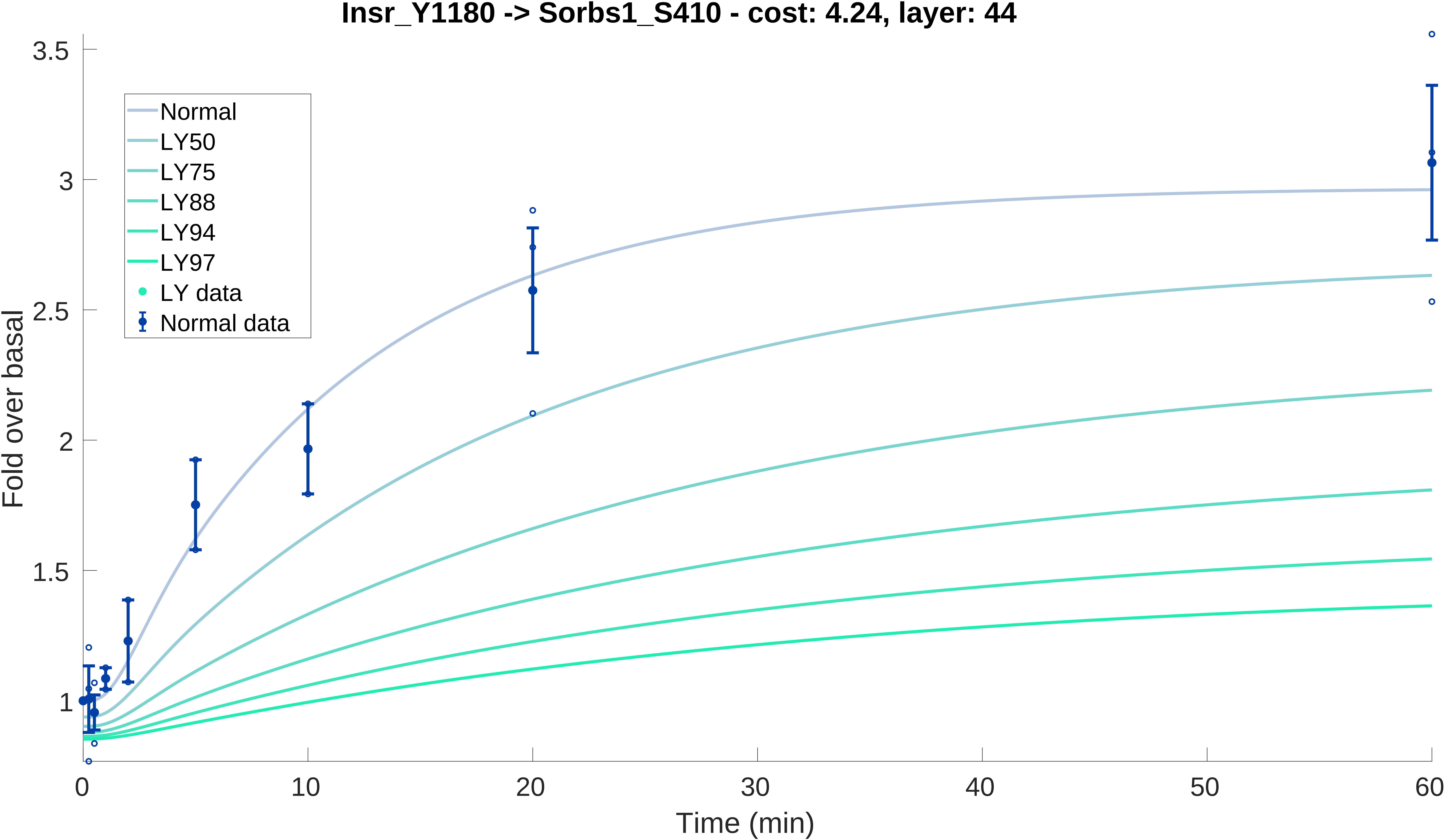
Insr_Y1180 -> Sorbs1_S410 - cost: 4.24, layer: 44
Fold over basal
Time (min)
0
10
20
30
40
50
60
1
1.5
2
2.5
3
3.5
Normal
LY50
LY75
LY88
LY94
LY97
LY data
Normal data

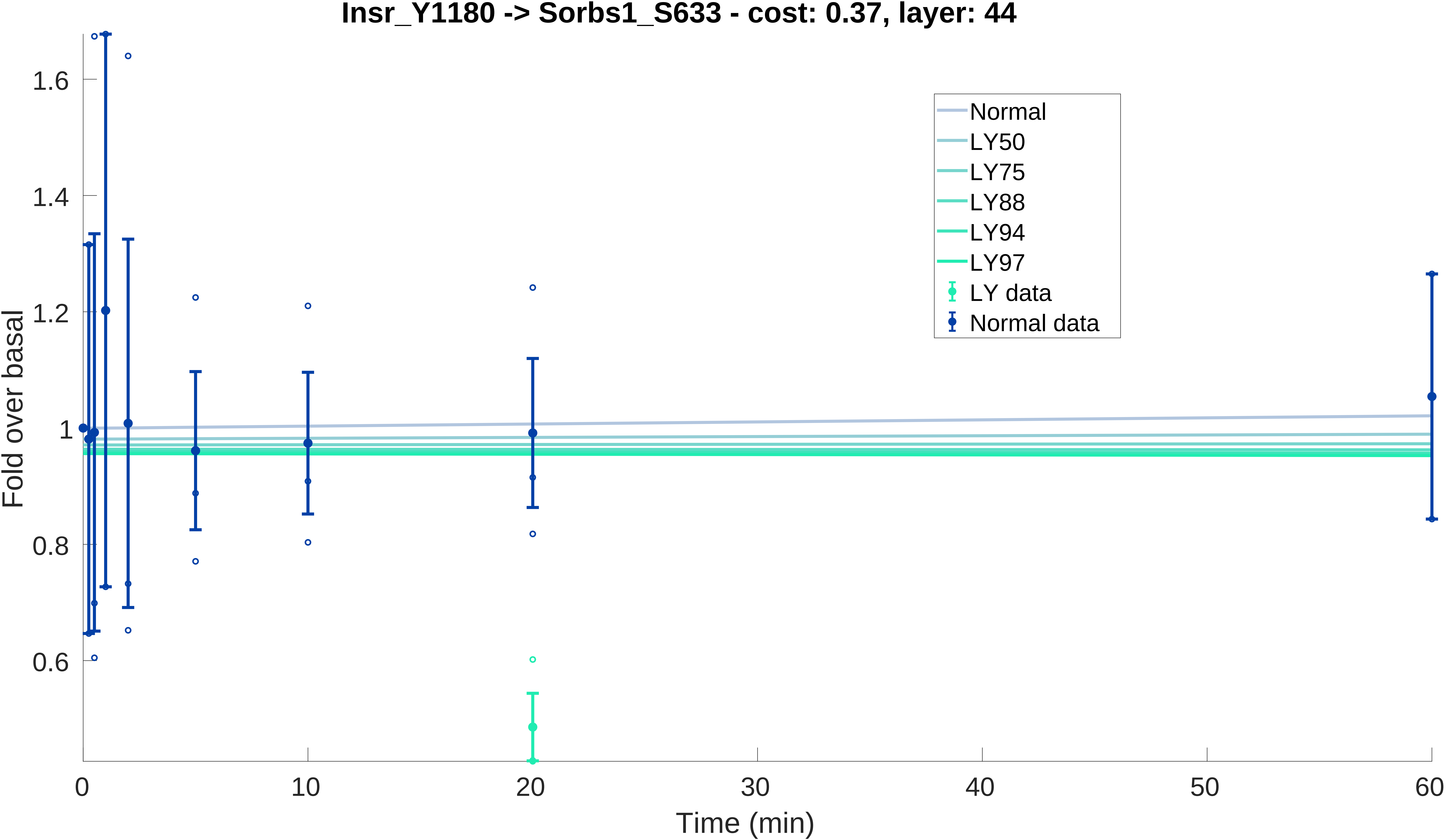

Insr_Y1180 -> Sorbs1_S633 - cost: 0.37, layer: 44
Fold over basal
Time (min)
1.6
1.4
1.2
1
0.8
0.6
0
10
20
30
40
50
60
Normal
LY50
LY75
LY88
LY94
LY97
LY data
Normal data

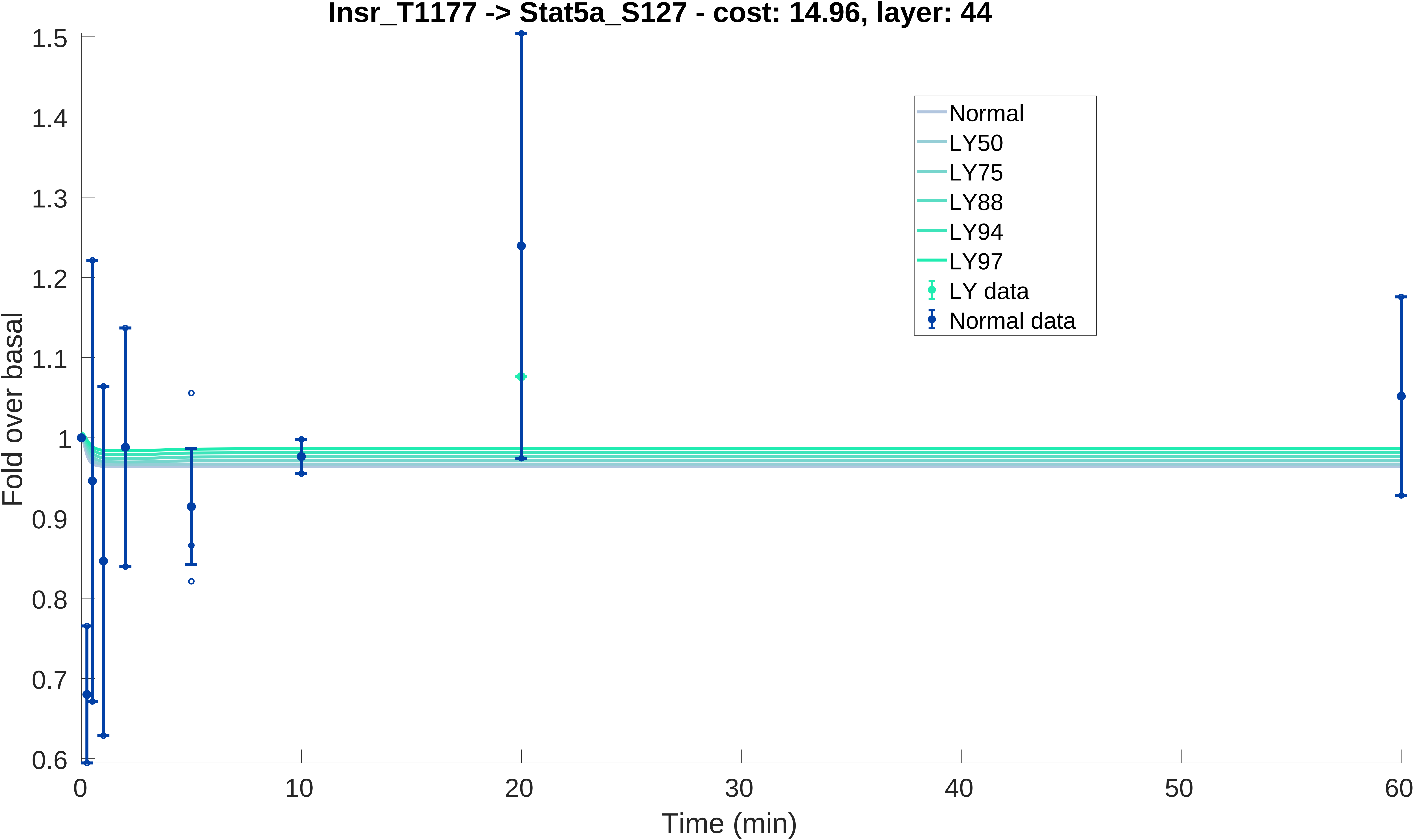

Insr_T1177 -> Stat5a_S127 - cost: 14.96, layer: 44
Fold over basal
Time (min)
1.5
1.4
1.3
1.2
1.1
1
0.9
0.8
0.7
0.6
0
10
20
30
40
50
60
Normal
LY50
LY75
LY88
LY94
LY97
LY data
Normal data

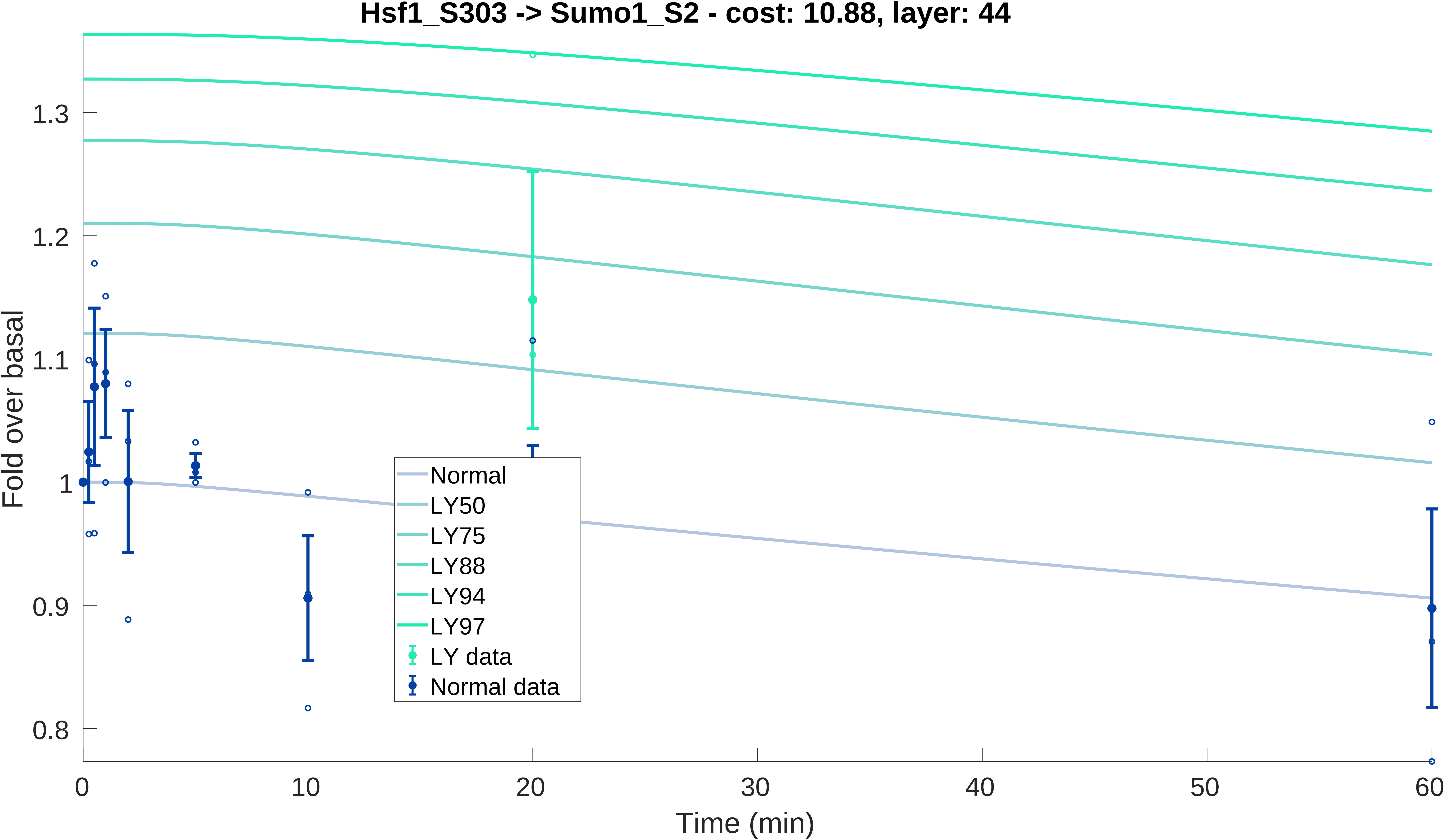

Hsf1_S303 -> Sumo1_S2 - cost: 10.88, layer: 44
Fold over basal
Time (min)
1.3
1.2
1.1
1
0.9
0.8
0
10
20
30
40
50
60
Normal
LY50
LY75
LY88
LY94
LY97
LY data
Normal data

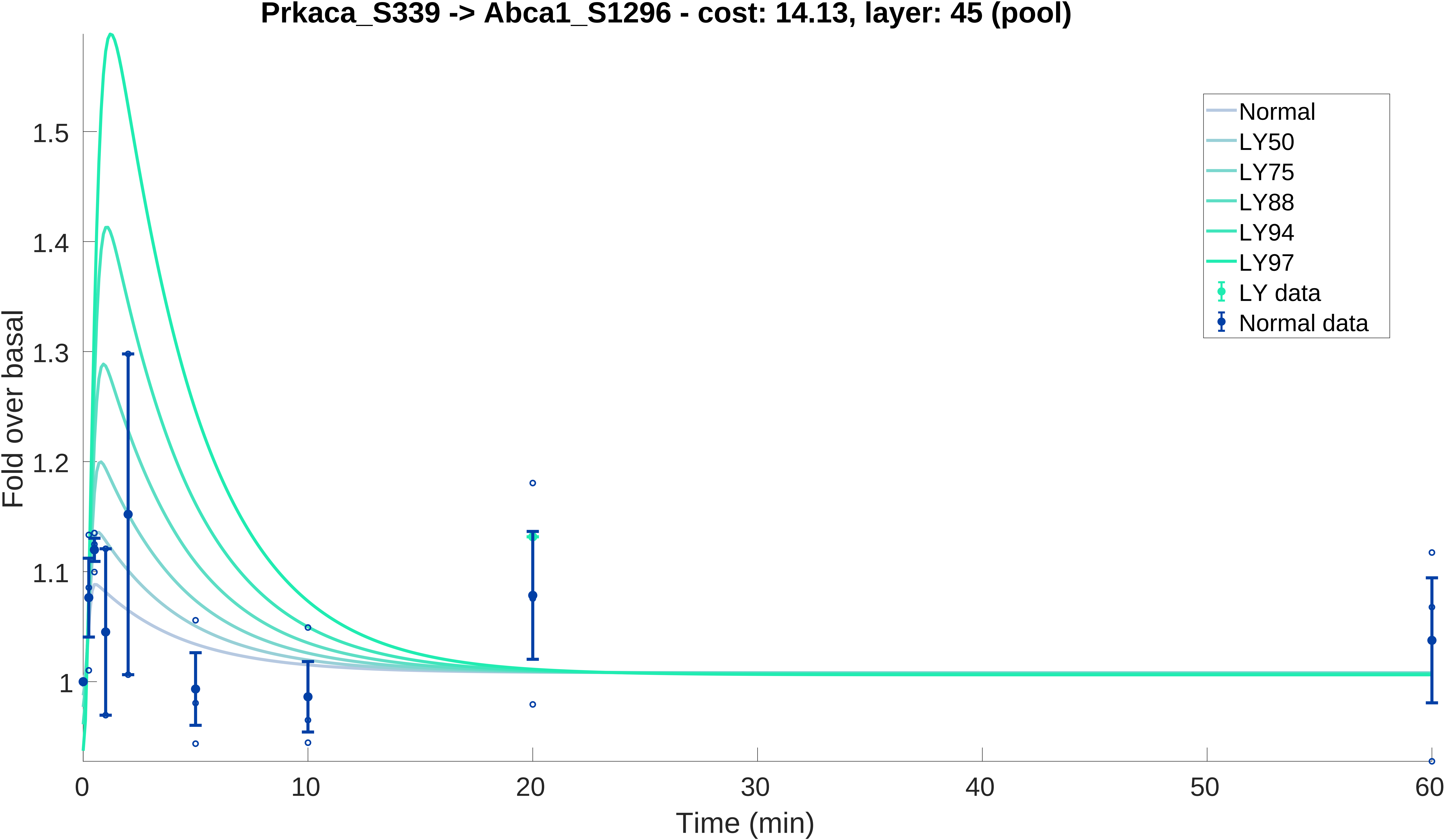
Prkaca_S339 -> Abca1_S1296 - cost: 14.13, layer: 45 (pool)
Fold over basal
Time (min)
1.5
1.4
1.3
1.2
1.1
1
0
10
20
30
40
50
60
Normal
LY50
LY75
LY88
LY94
LY97
LY data
Normal data

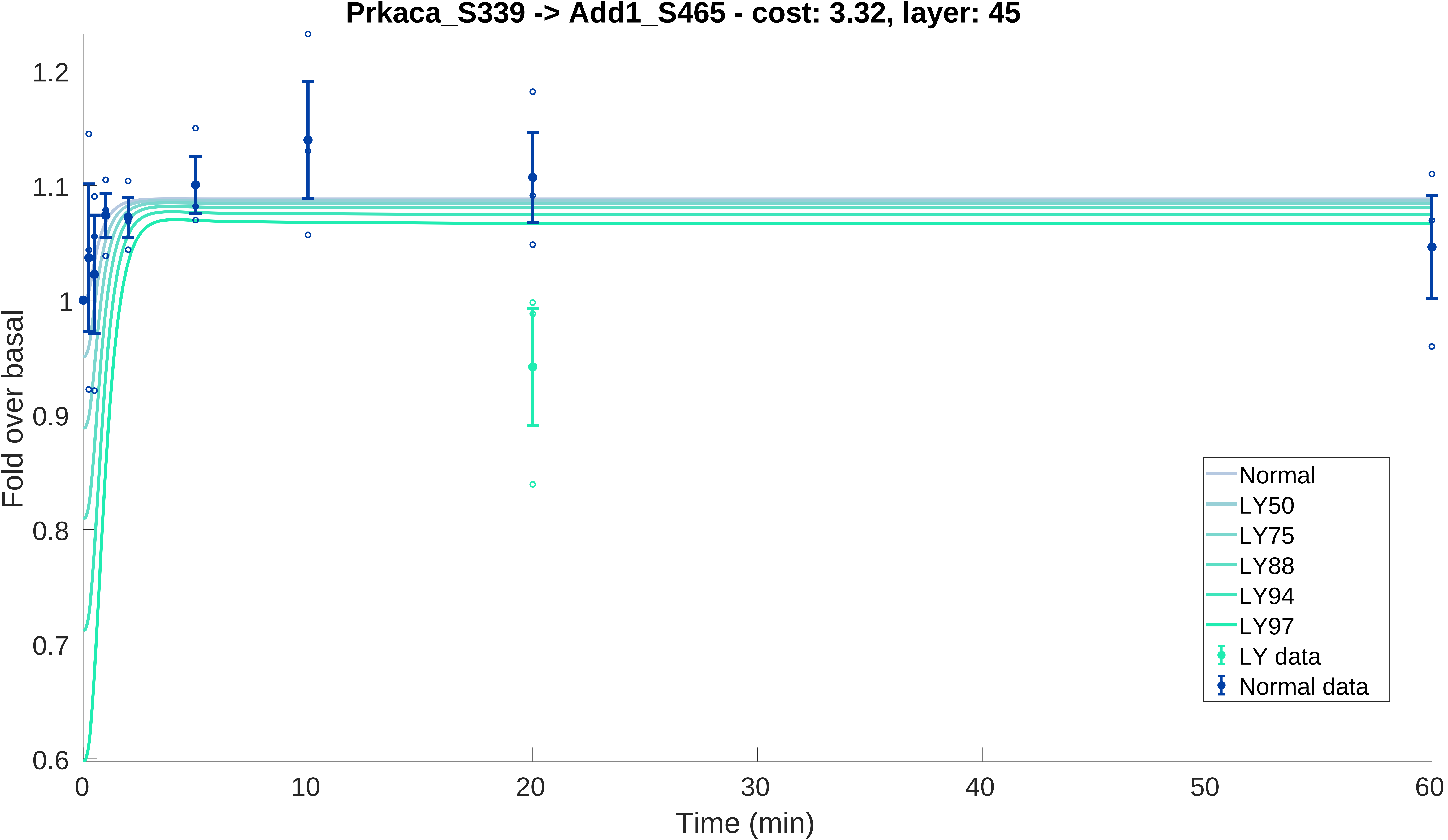

Prkaca_S339 -> Add1_S465 - cost: 3.32, layer: 45
Fold over basal
Time (min)
1.2
1.1
1
0.9
0.8
0.7
0.6
0
10
20
30
40
50
60
Normal
LY50
LY75
LY88
LY94
LY97
LY data
Normal data

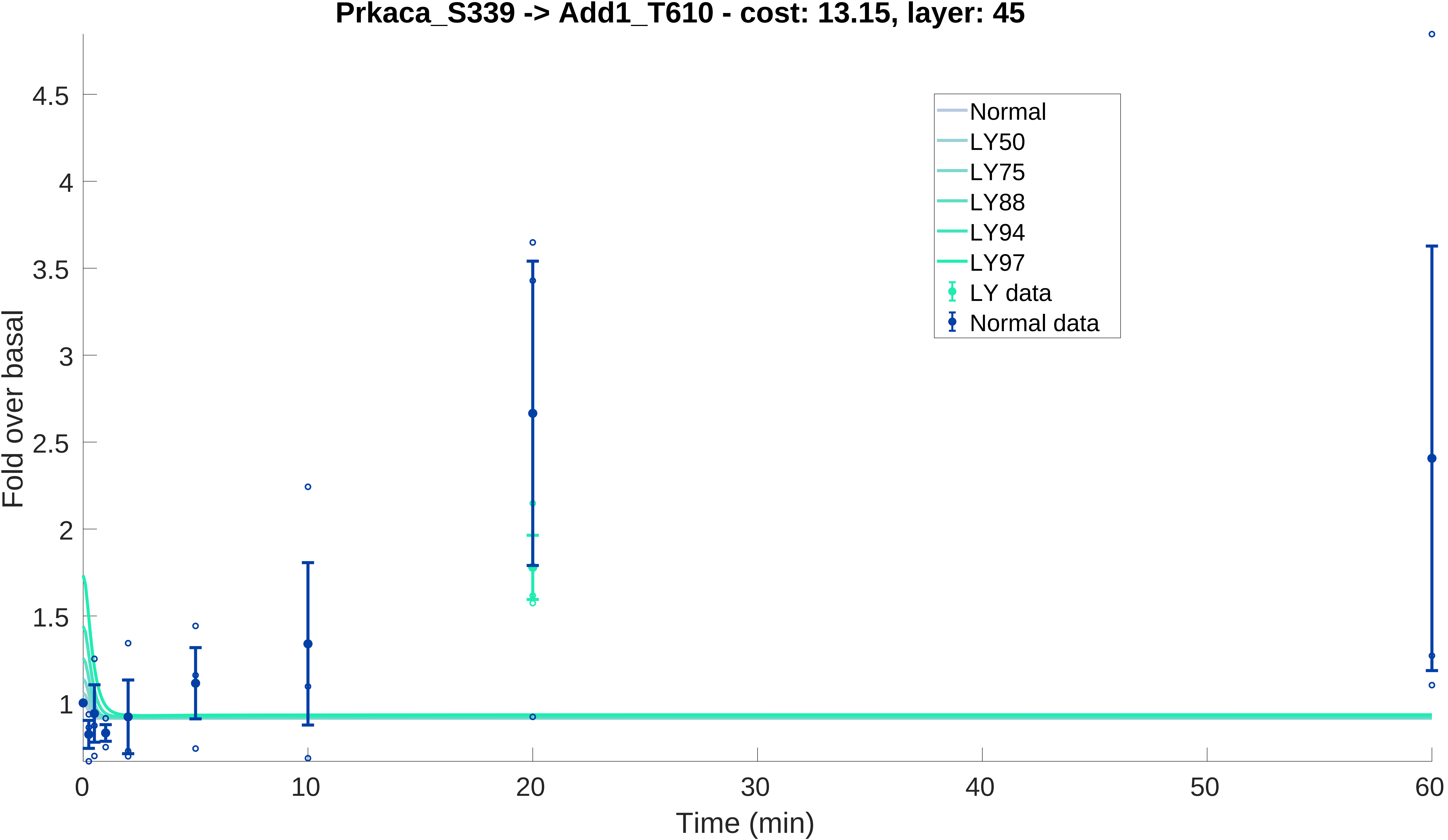

Prkaca_S339 -> Add1_T610 - cost: 13.15, layer: 45
Fold over basal
Time (min)
0
10
20
30
40
50
60
1
1.5
2
2.5
3
3.5
4
4.5
Normal
LY50
LY75
LY88
LY94
LY97
LY data
Normal data

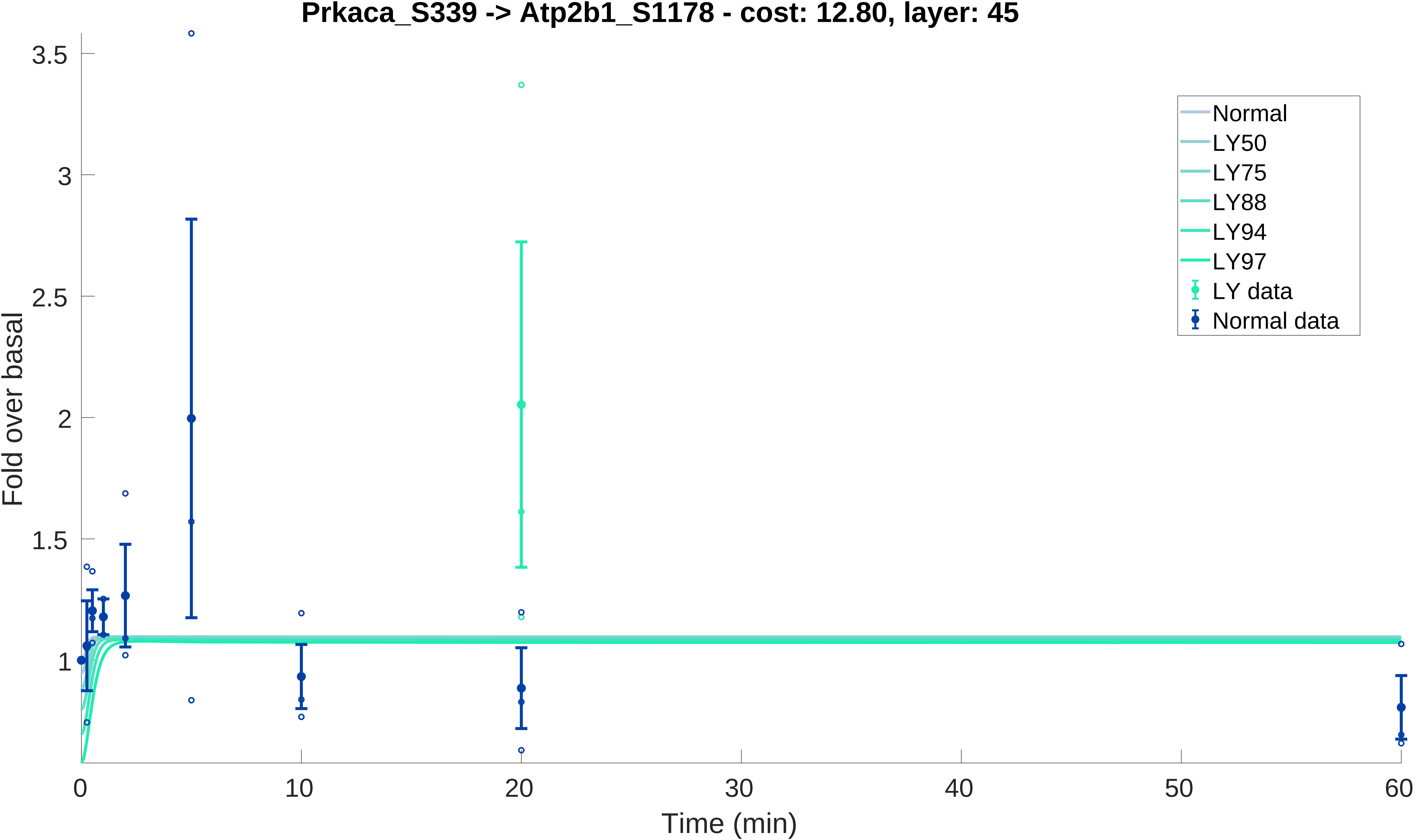

Prkaca_S339 -> Atp2b1_S1178 - cost: 12.80, layer: 45
Fold over basal
Time (min)
3.5
3
2.5
2
1.5
1
0
10
20
30
40
50
60
Normal
LY50
LY75
LY88
LY94
LY97
LY data
Normal data

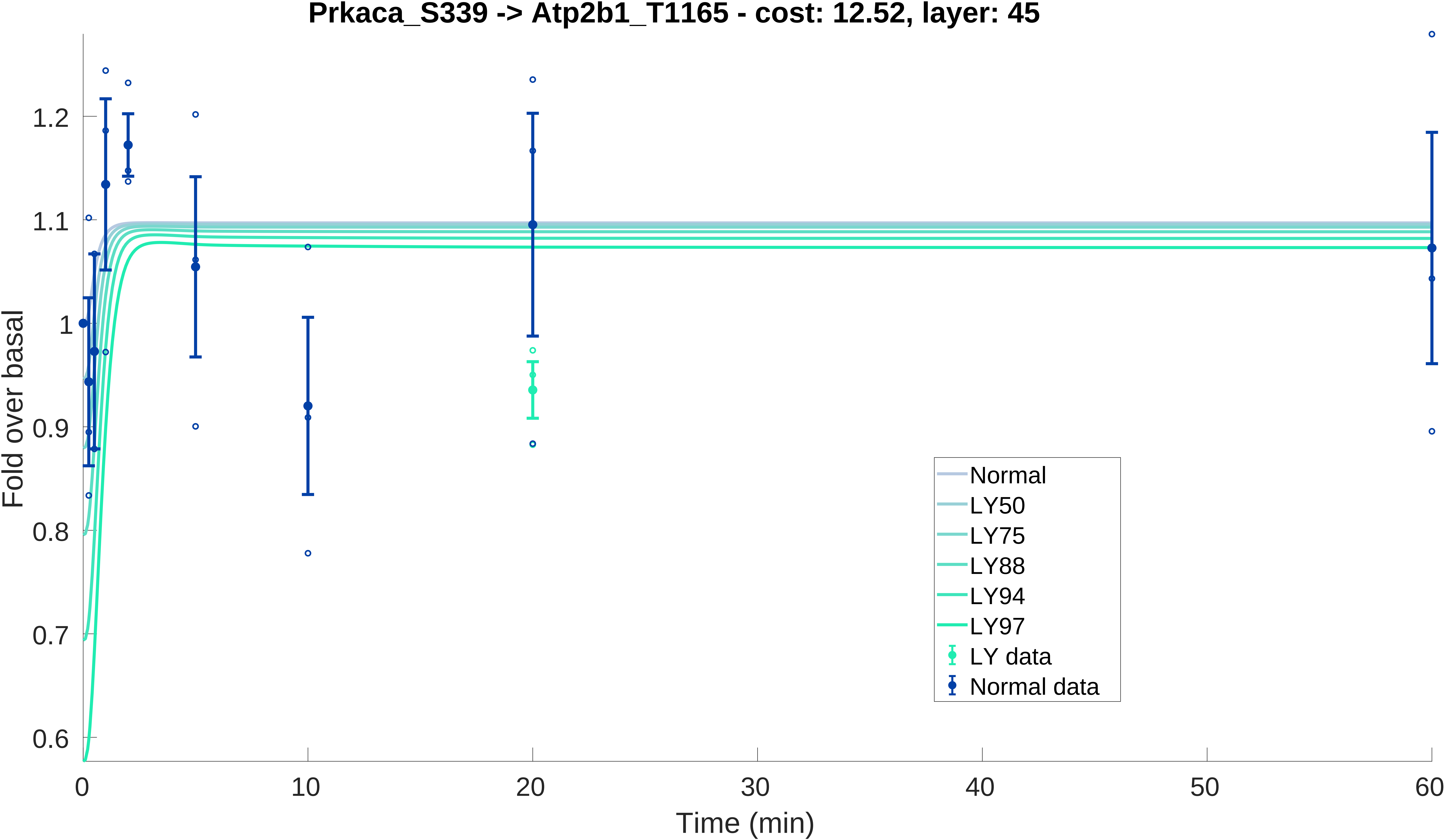
Prkaca_S339 -> Atp2b1_T1165 - cost: 12.52, layer: 45
Fold over basal
Time (min)
0.6
0.7
0.8
0.9
1
1.1
1.2
0
10
20
30
40
50
60
Normal
LY50
LY75
LY88
LY94
LY97
LY data
Normal data

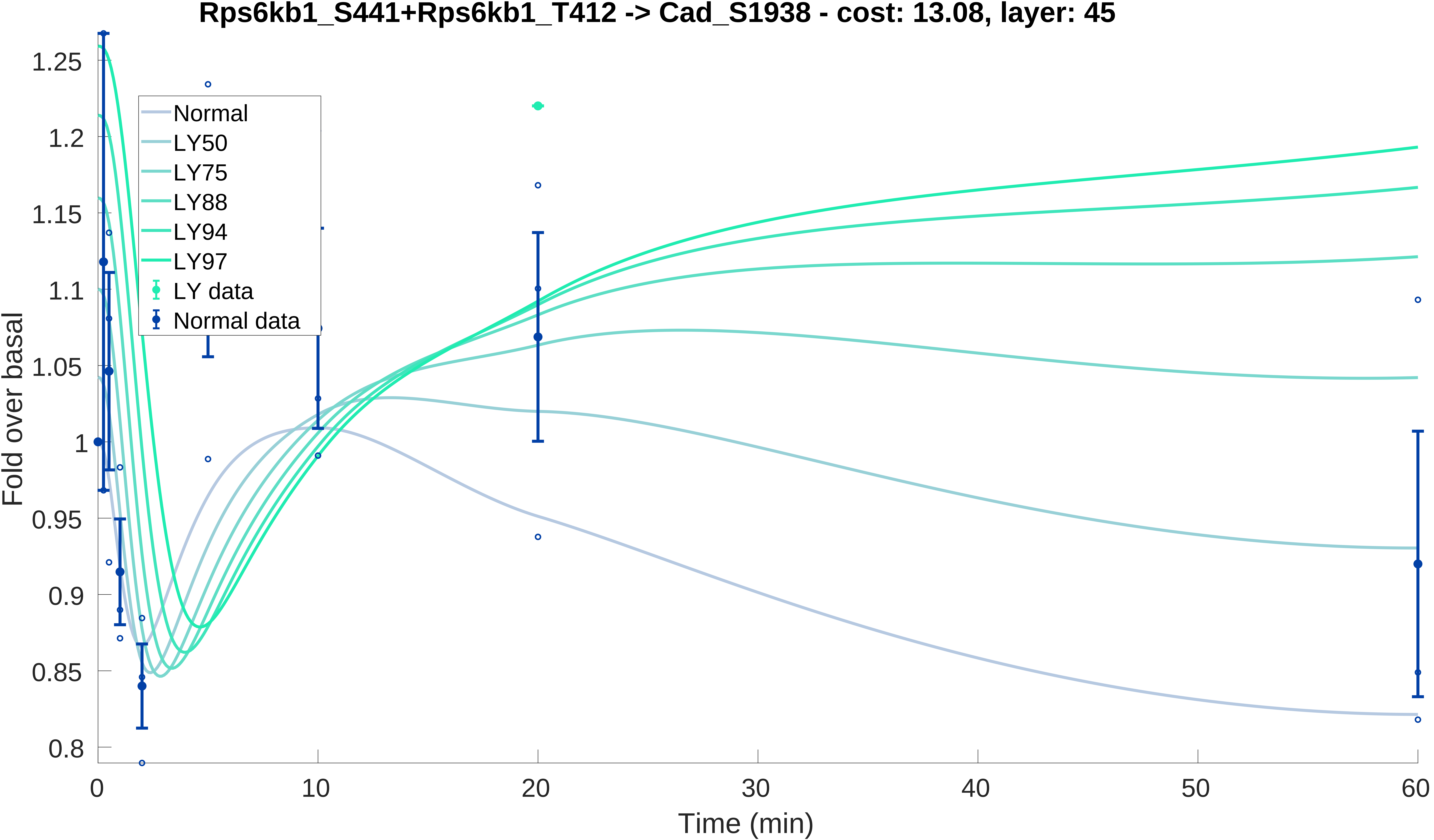

Rps6kb1_S441+Rps6kb1_T412 -> Cad_S1938 - cost: 13.08, layer: 45
Normal
LY50
LY75
LY88
LY94
LY97
LY data
Normal data
Fold over basal
Time (min)
1.25
1.2
1.15
1.1
1.05
1
0.95
0.9
0.85
0.8
0
10
20
30
40
50
60

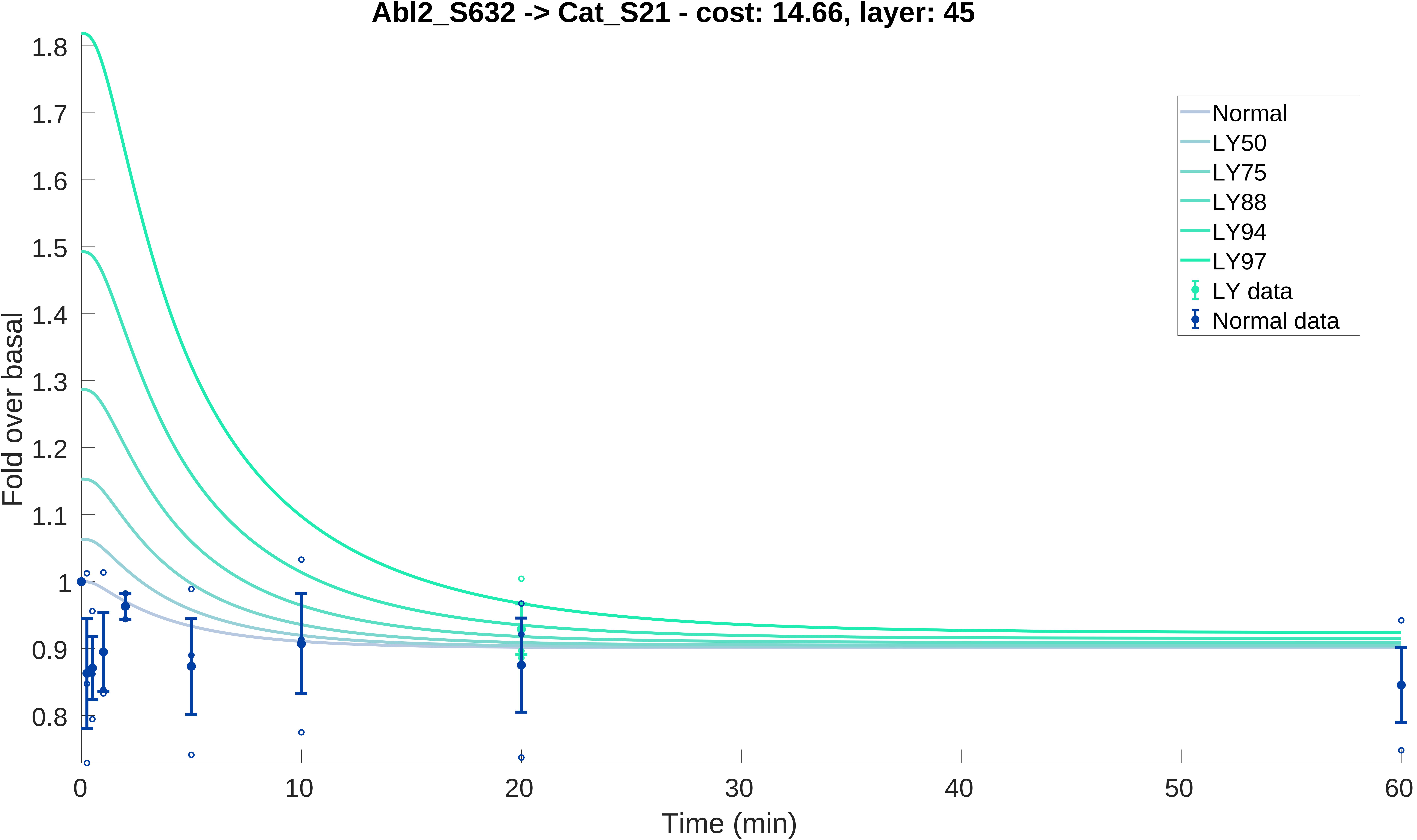

Abl2_S632 -> Cat_S21 - cost: 14.66, layer: 45
Fold over basal
1.8
1.7
1.6
1.5
1.4
1.3
1.2
1.1
1
0.9
0.8
Time (min)
0
10
20
30
40
50
60
Normal
LY50
LY75
LY88
LY94
LY97
LY data
Normal data

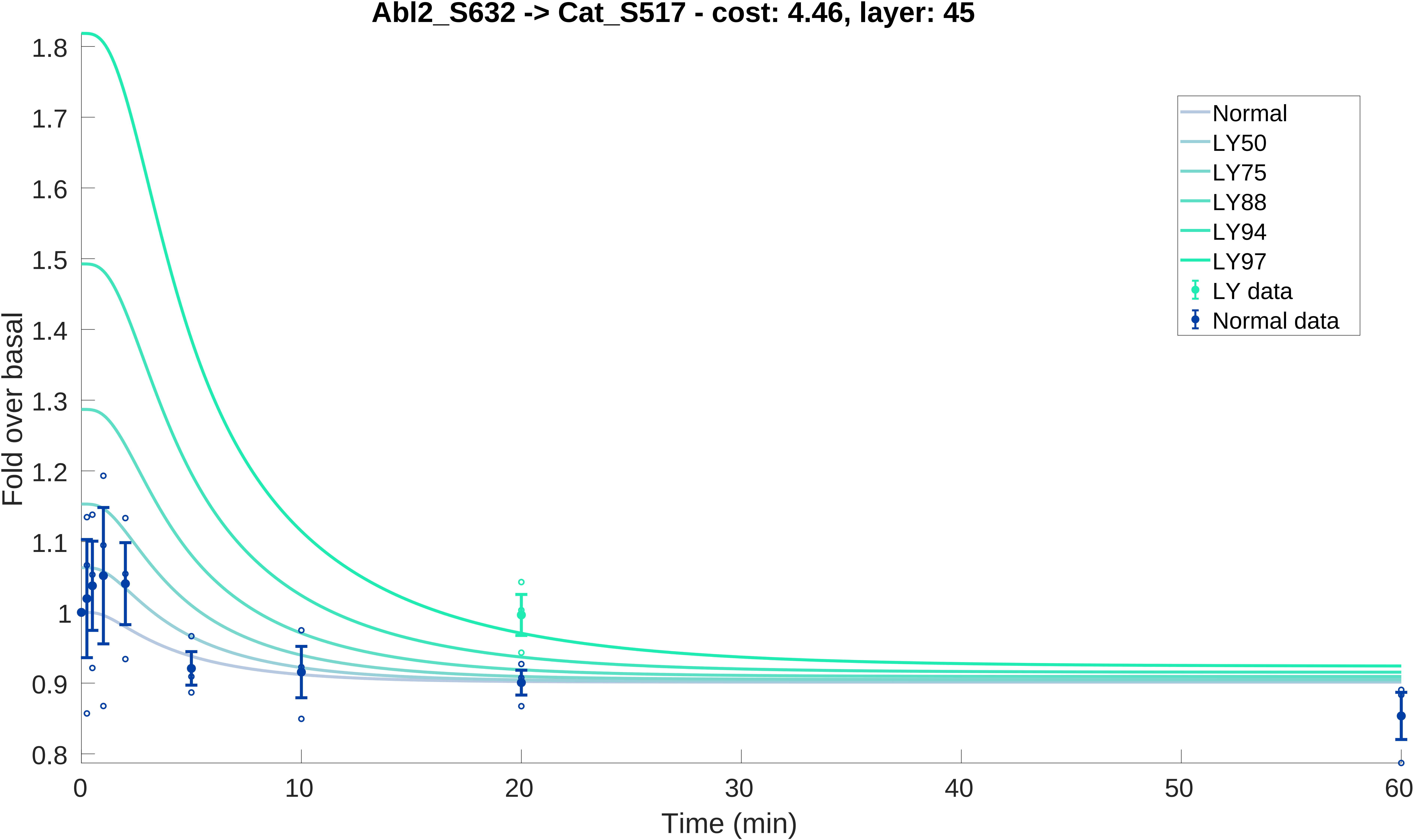
Abl2_S632 -> Cat_S517 - cost: 4.46, layer: 45
Fold over basal
1.8
1.7
1.6
1.5
1.4
1.3
1.2
1.1
1
0.9
0.8
0
10
20
30
40
50
60
Time (min)
Normal
LY50
LY75
LY88
LY94
LY97
LY data
Normal data

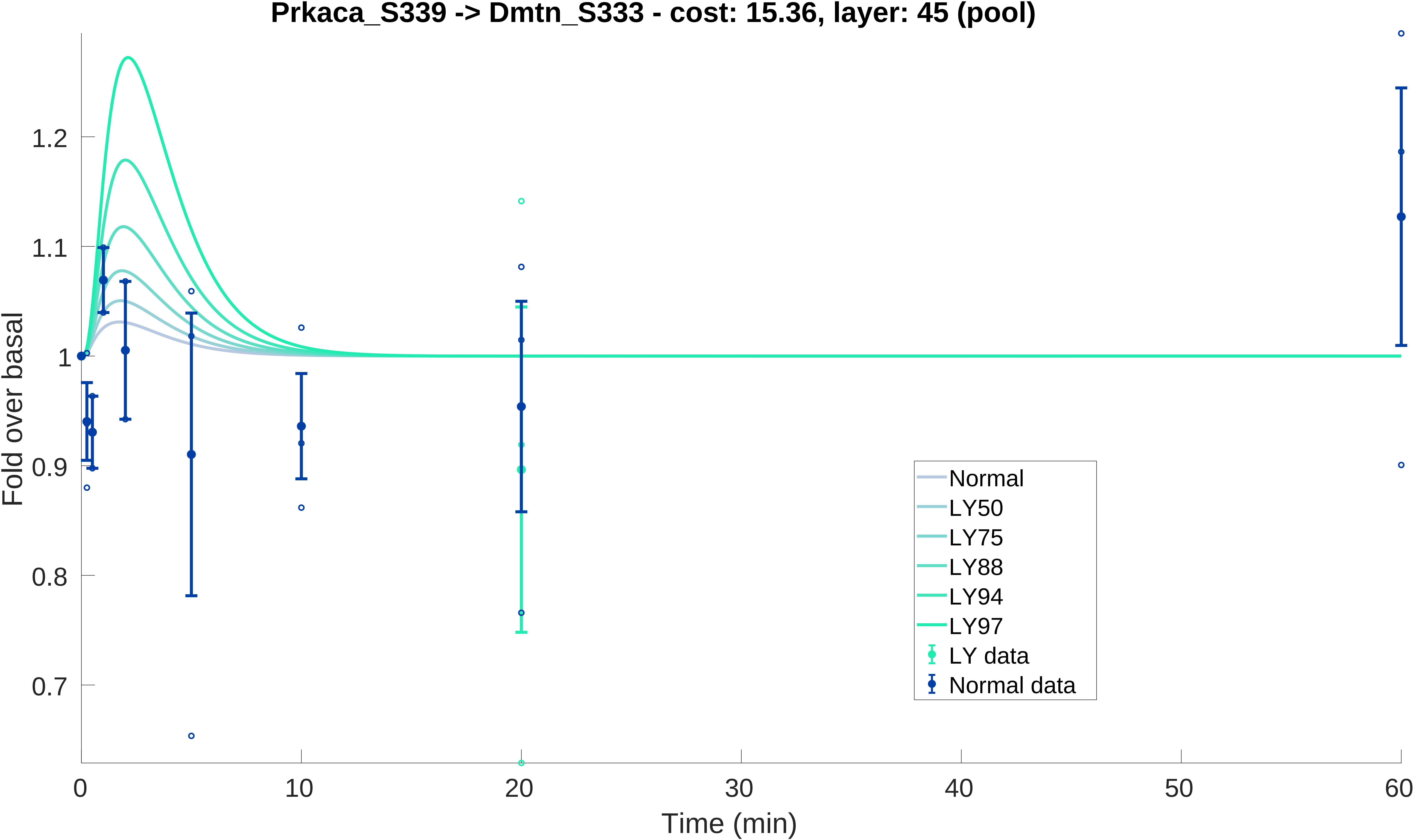

Prkaca_S339 -> Dmtn_S333 - cost: 15.36, layer: 45 (pool)
Fold over basal
Time (min)
0.7
0.8
0.9
1
1.1
1.2
0
10
20
30
40
50
60
Normal
LY50
LY75
LY88
LY94
LY97
LY data
Normal data

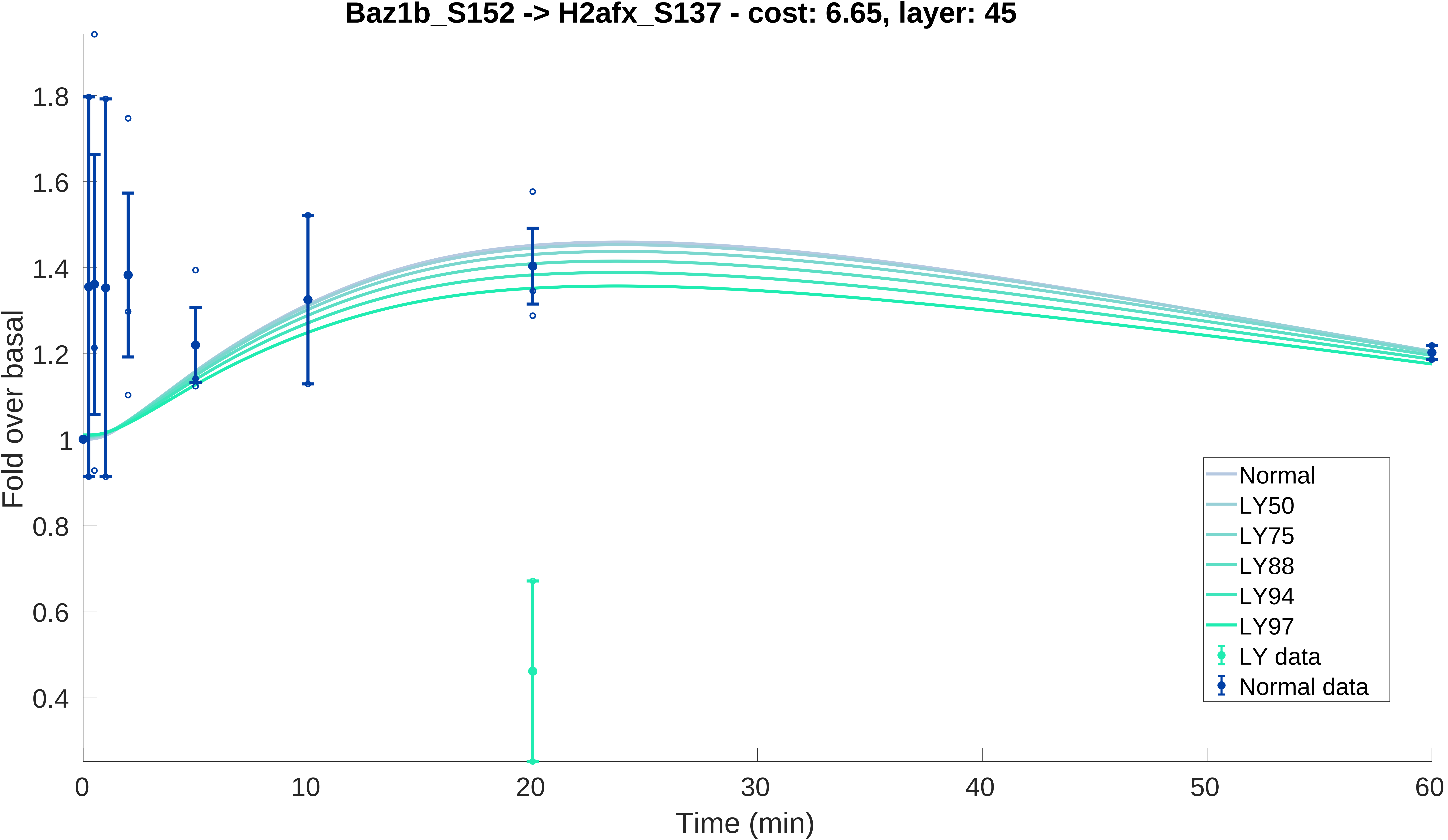
Baz1b_S152 -> H2afx_S137 - cost: 6.65, layer: 45
Fold over basal
Time (min)
1.8
1.6
1.4
1.2
1
0.8
0.6
0.4
0
10
20
30
40
50
60
Normal
LY50
LY75
LY88
LY94
LY97
LY data
Normal data

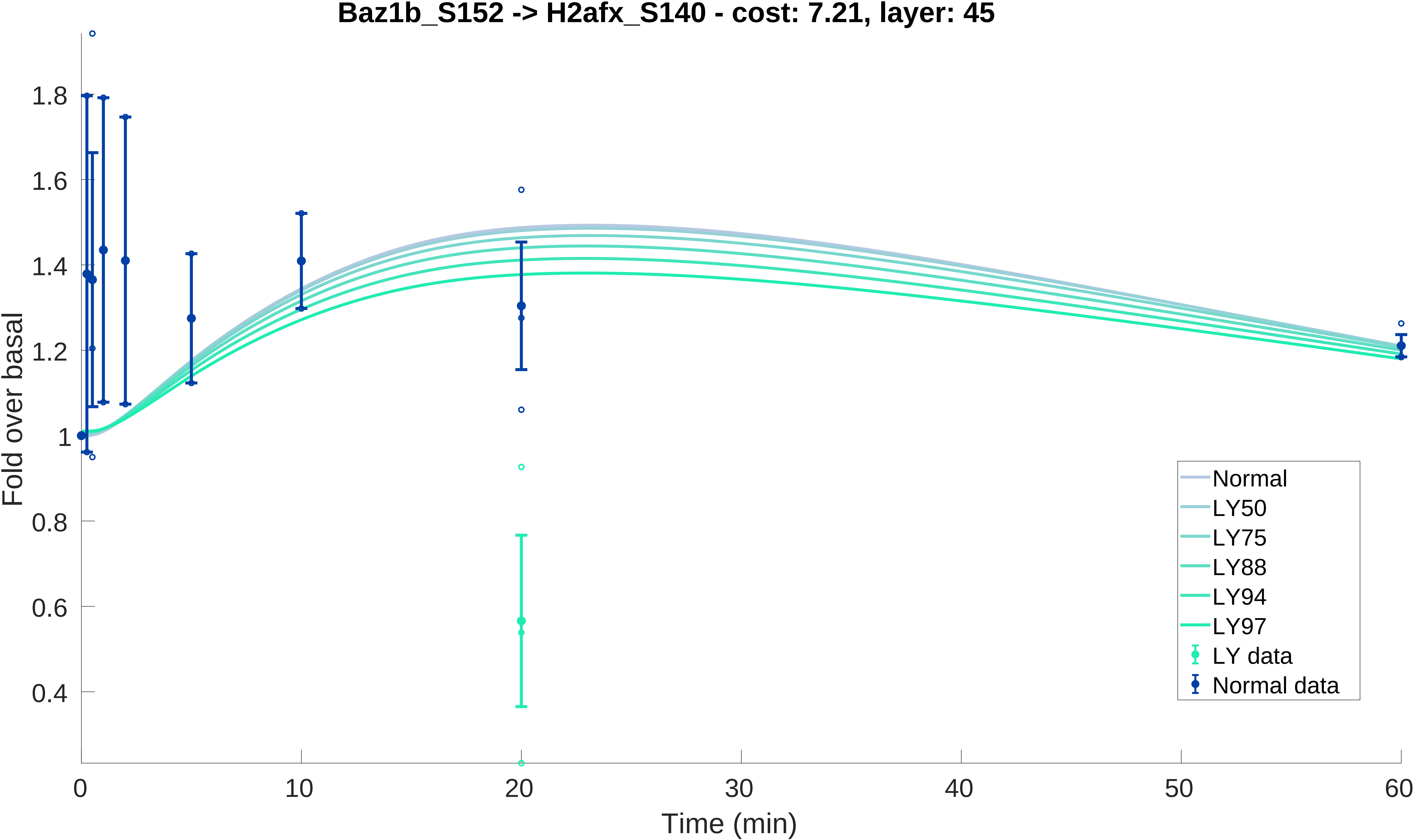

Baz1b_S152 -> H2afx_S140 - cost: 7.21, layer: 45
Fold over basal
1.8
1.6
1.4
1.2
1
0.8
0.6
0.4
Time (min)
0
10
20
30
40
50
60
Normal
LY50
LY75
LY88
LY94
LY97
LY data
Normal data

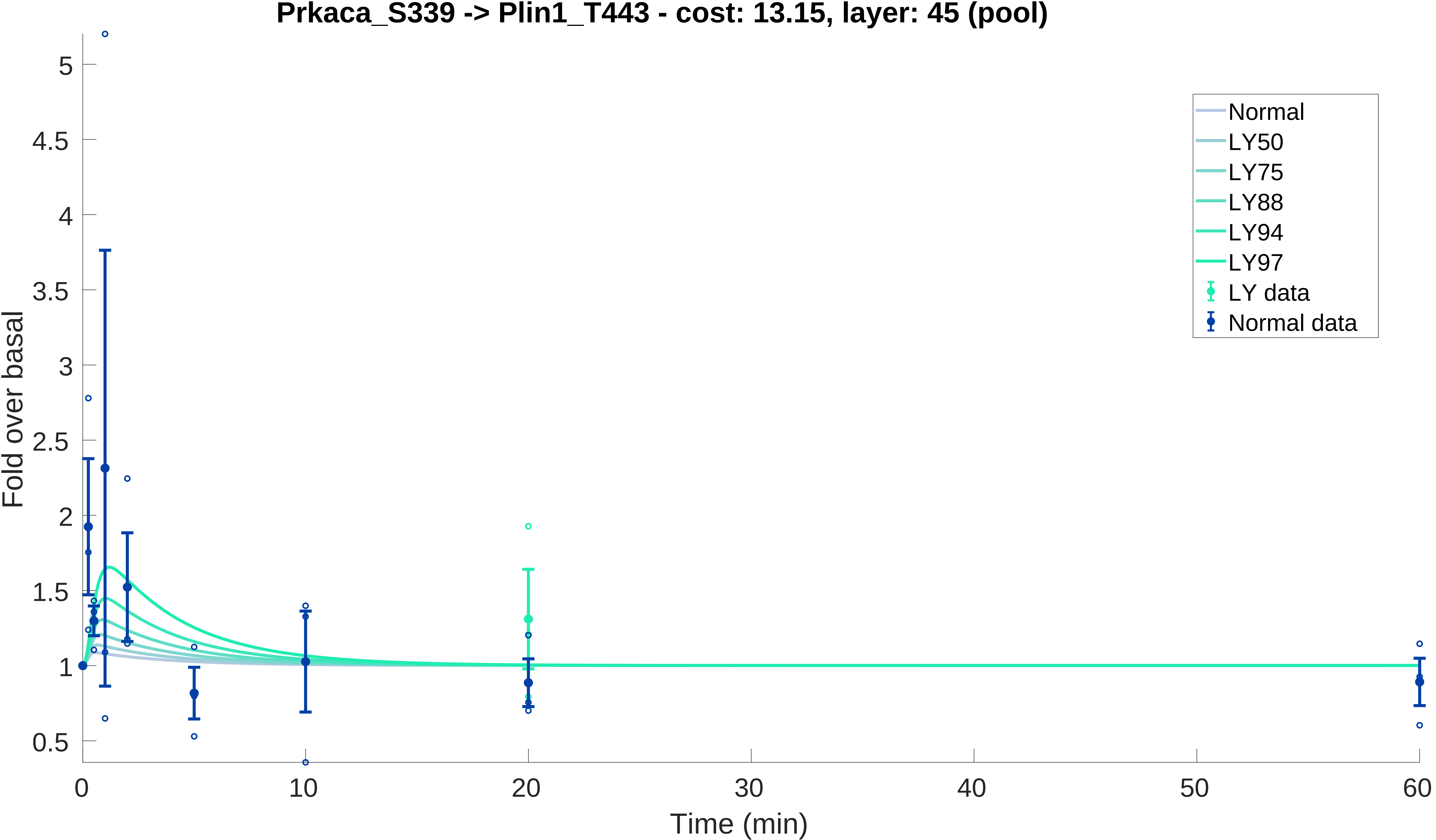

Prrkaca_S339 -> Plin1_T443 - cost: 13.15, layer: 45 (pool)
Fold over basal
Time (min)
Normal
LY50
LY75
LY88
LY94
LY97
LY data
Normal data
0.5
1
1.5
2
2.5
3
3.5
4
4.5
5
0
10
20
30
40
50
60

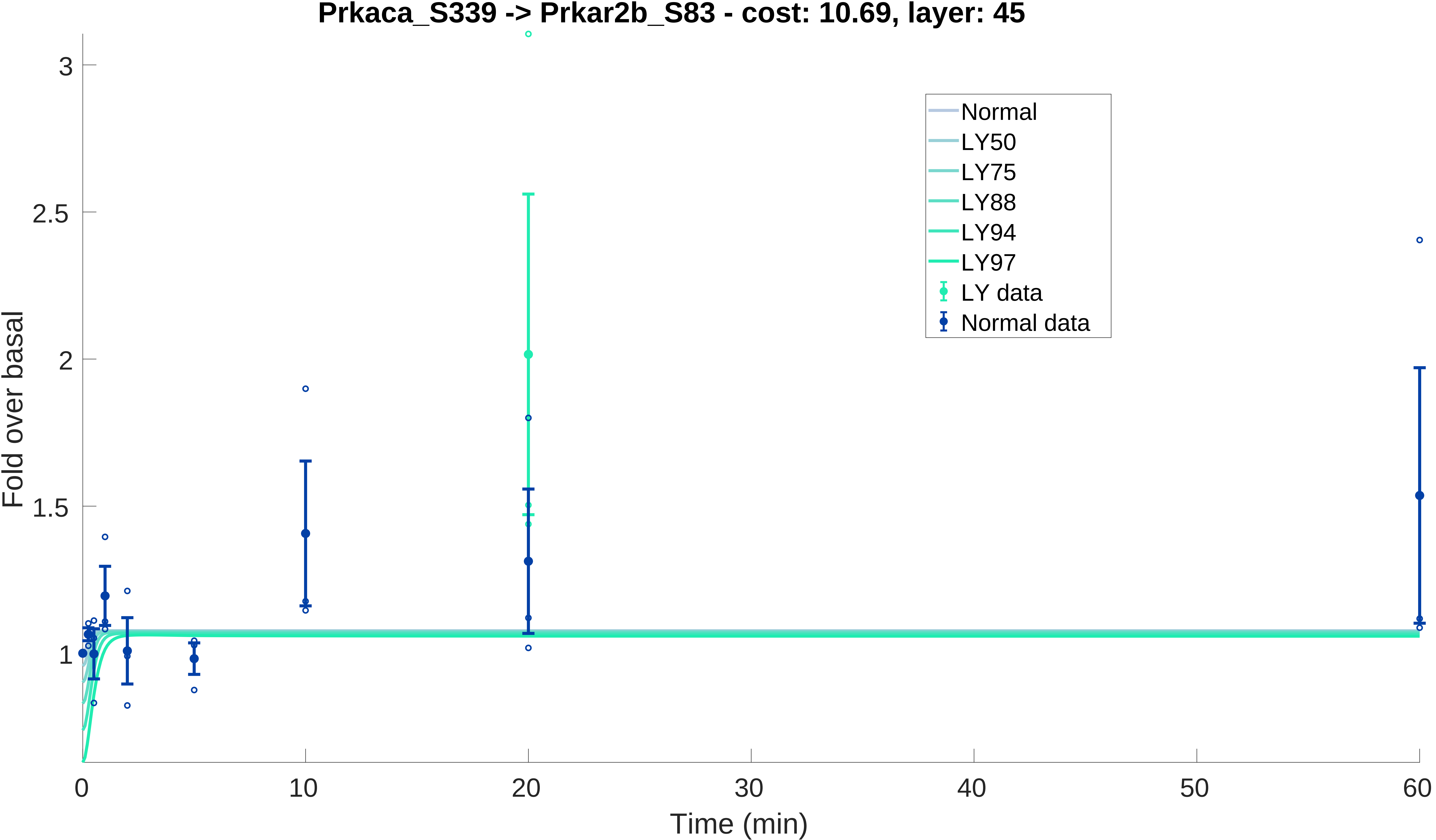

Prkaca_S339 -> Prkar2b_S83 - cost: 10.69, layer: 45
Fold over basal
Time (min)
0
10
20
30
40
50
60
1
1.5
2
2.5
3
Normal
LY50
LY75
LY88
LY94
LY97
LY data
Normal data

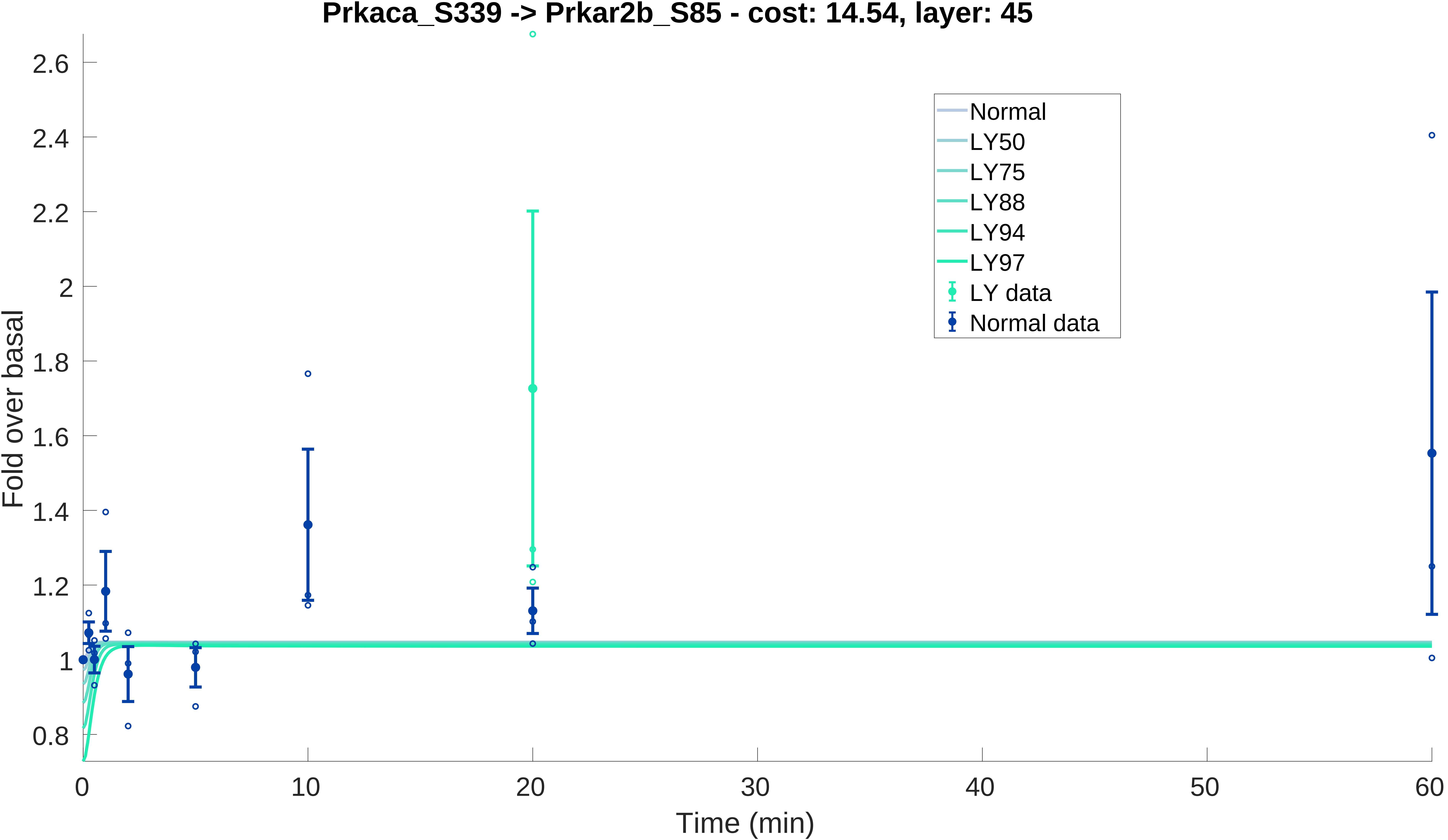

Prkaca_S339 -> Prkar2b_S85 - cost: 14.54, layer: 45
Fold over basal
Time (min)
0.8
1
1.2
1.4
1.6
1.8
2
2.2
2.4
2.6
0
10
20
30
40
50
60
Normal
LY50
LY75
LY88
LY94
LY97
LY data
Normal data

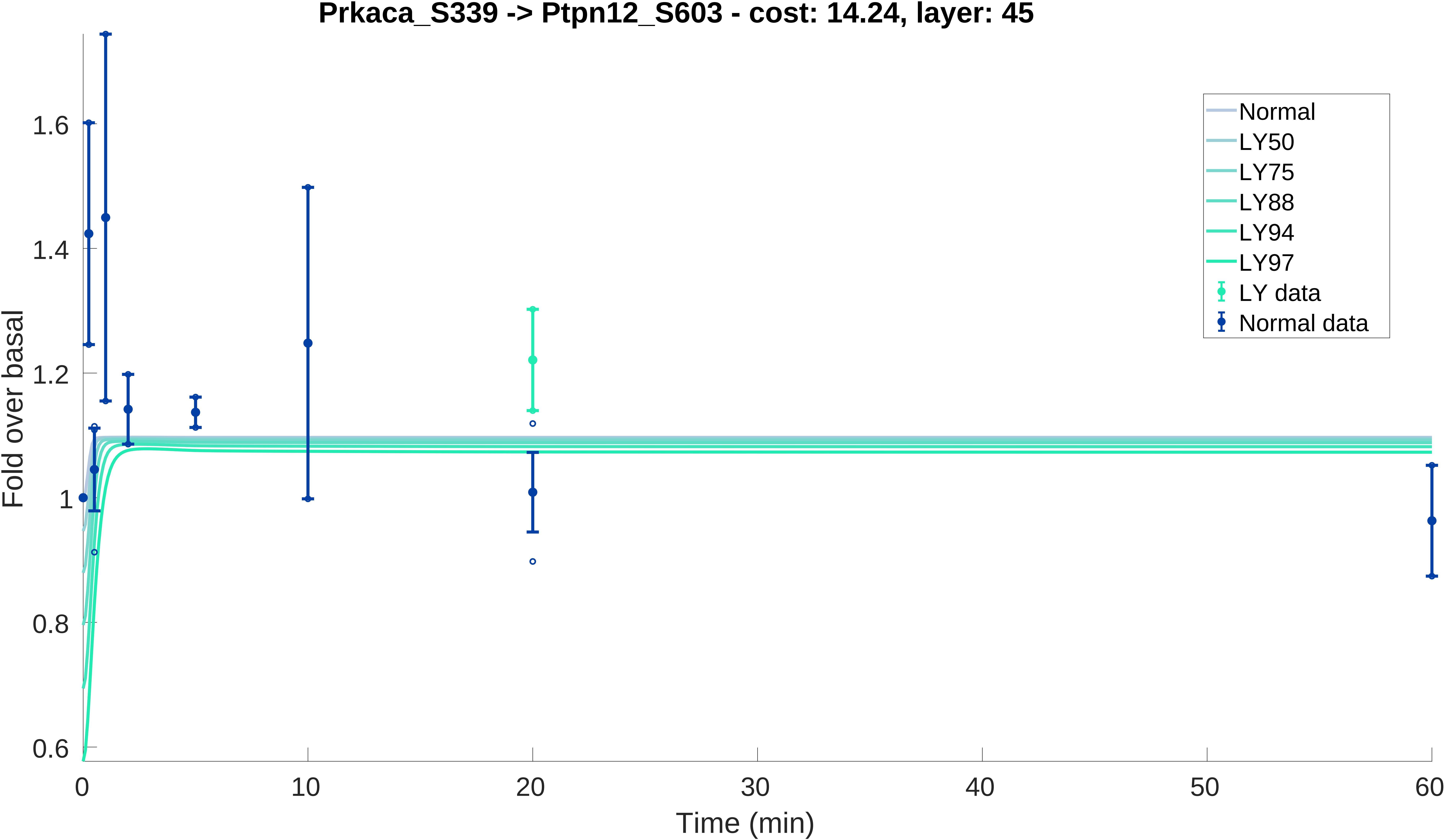
Prrkaca_S339 -> Ptpn12_S603 - cost: 14.24, layer: 45
Fold over basal
Time (min)
1.6
1.4
1.2
1
0.8
0.6
0
10
20
30
40
50
60
Normal
LY50
LY75
LY88
LY94
LY97
LY data
Normal data

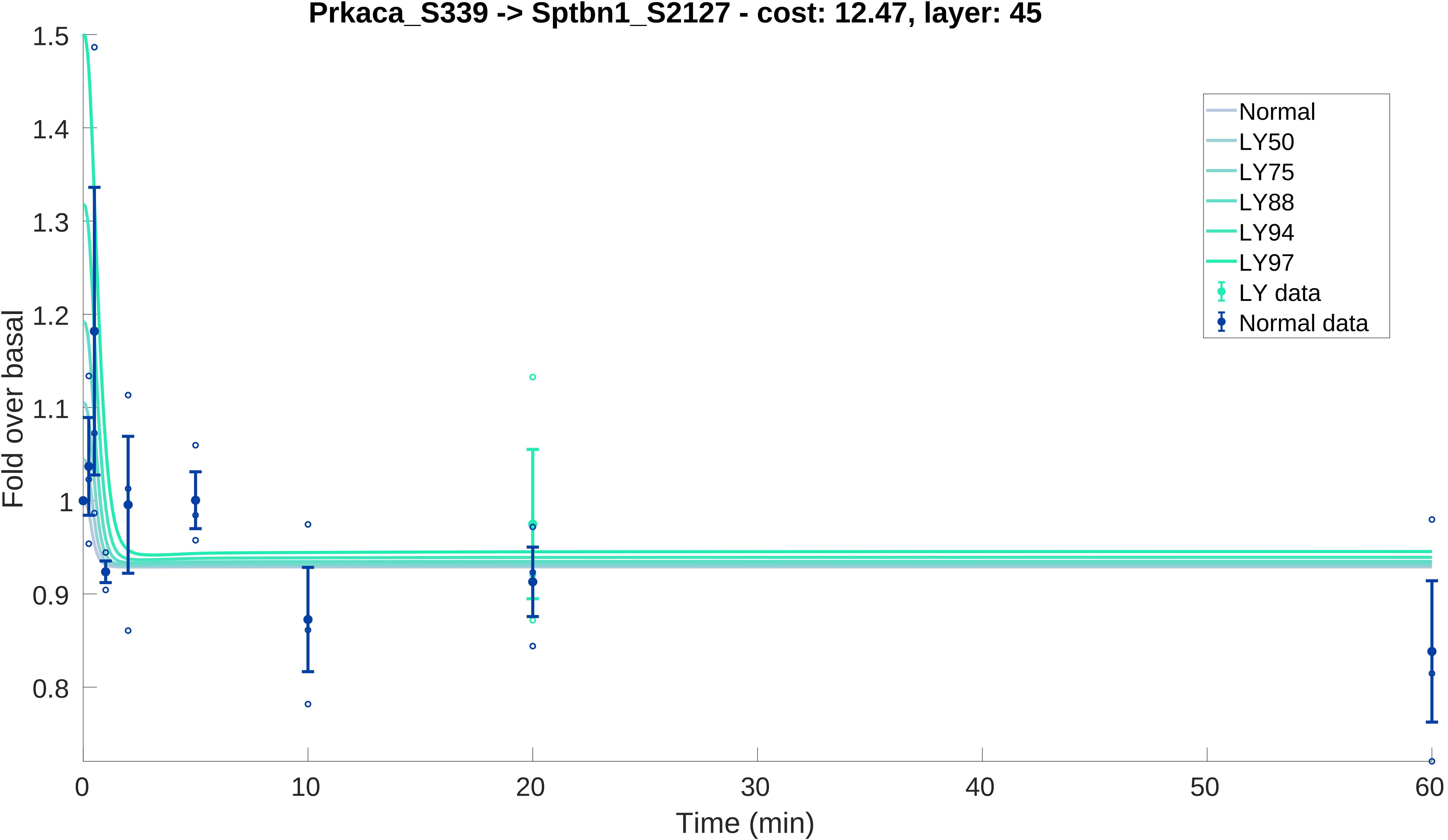

Prkaca_S339 -> Sptbn1_S2127 - cost: 12.47, layer: 45
Fold over basal
1.5
1.4
1.3
1.2
1.1
1
0.9
0.8
0
10
20
30
40
50
60
Time (min)
Normal
LY50
LY75
LY88
LY94
LY97
LY data
Normal data

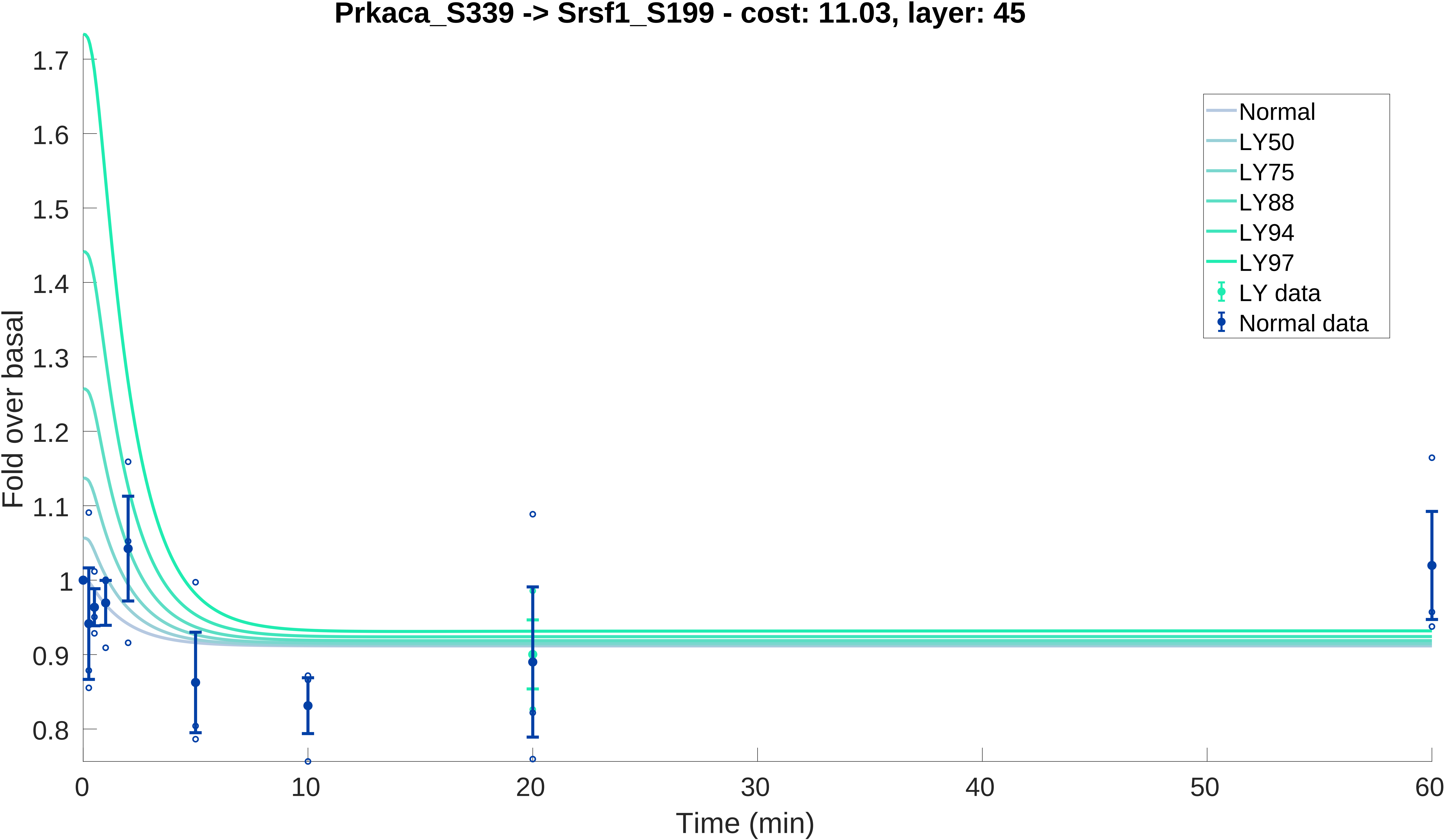

Prkaca_S339 -> Srsf1_S199 - cost: 11.03, layer: 45
Fold over basal
Time (min)
1.7
1.6
1.5
1.4
1.3
1.2
1.1
1
0.9
0.8
0
10
20
30
40
50
60
Normal
LY50
LY75
LY88
LY94
LY97
LY data
Normal data

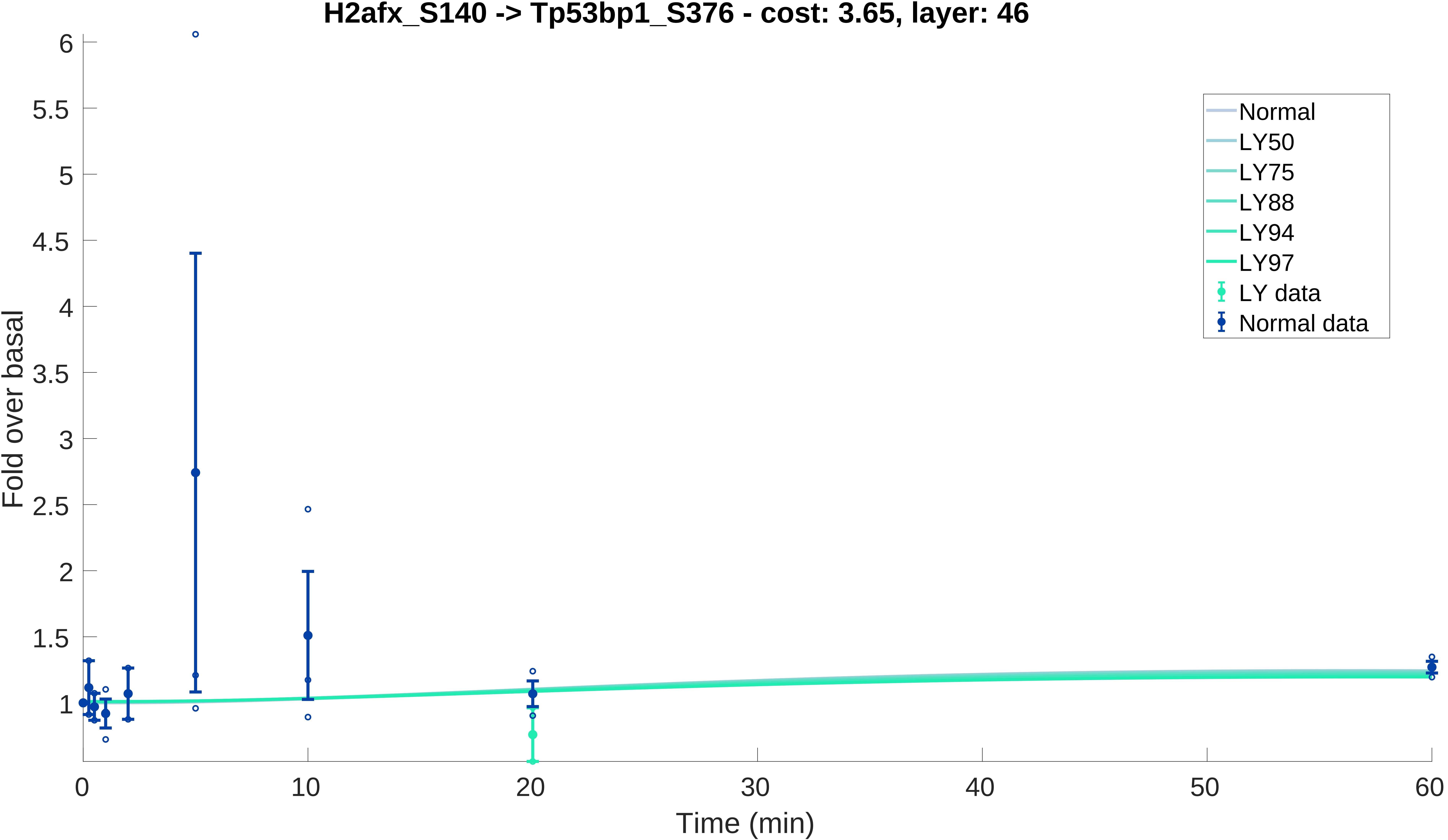
H2afx_S140 -> Tp53bp1_S376 - cost: 3.65, layer: 46
Fold over basal
6
5.5
5
4.5
4
3.5
3
2.5
2
1.5
1
0
10
20
30
40
50
60
Time (min)
Normal
LY50
LY75
LY88
LY94
LY97
LY data
Normal data

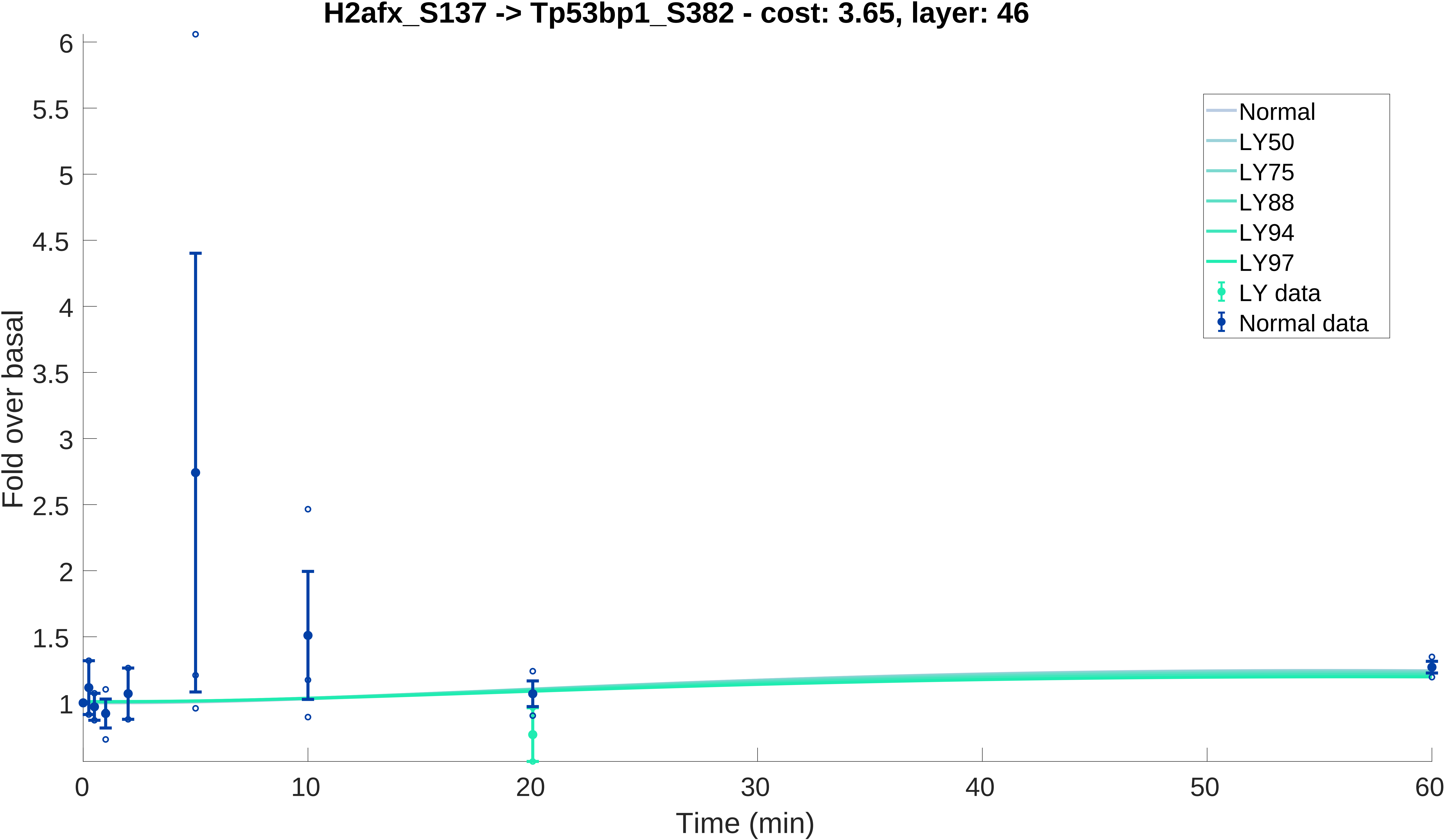

H2afx_S137 -> Tp53bp1_S382 - cost: 3.65, layer: 46
Fold over basal
Time (min)
6
5.5
5
4.5
4
3.5
3
2.5
2
1.5
1
0
10
20
30
40
50
60
Normal
LY50
LY75
LY88
LY94
LY97
LY data
Normal data

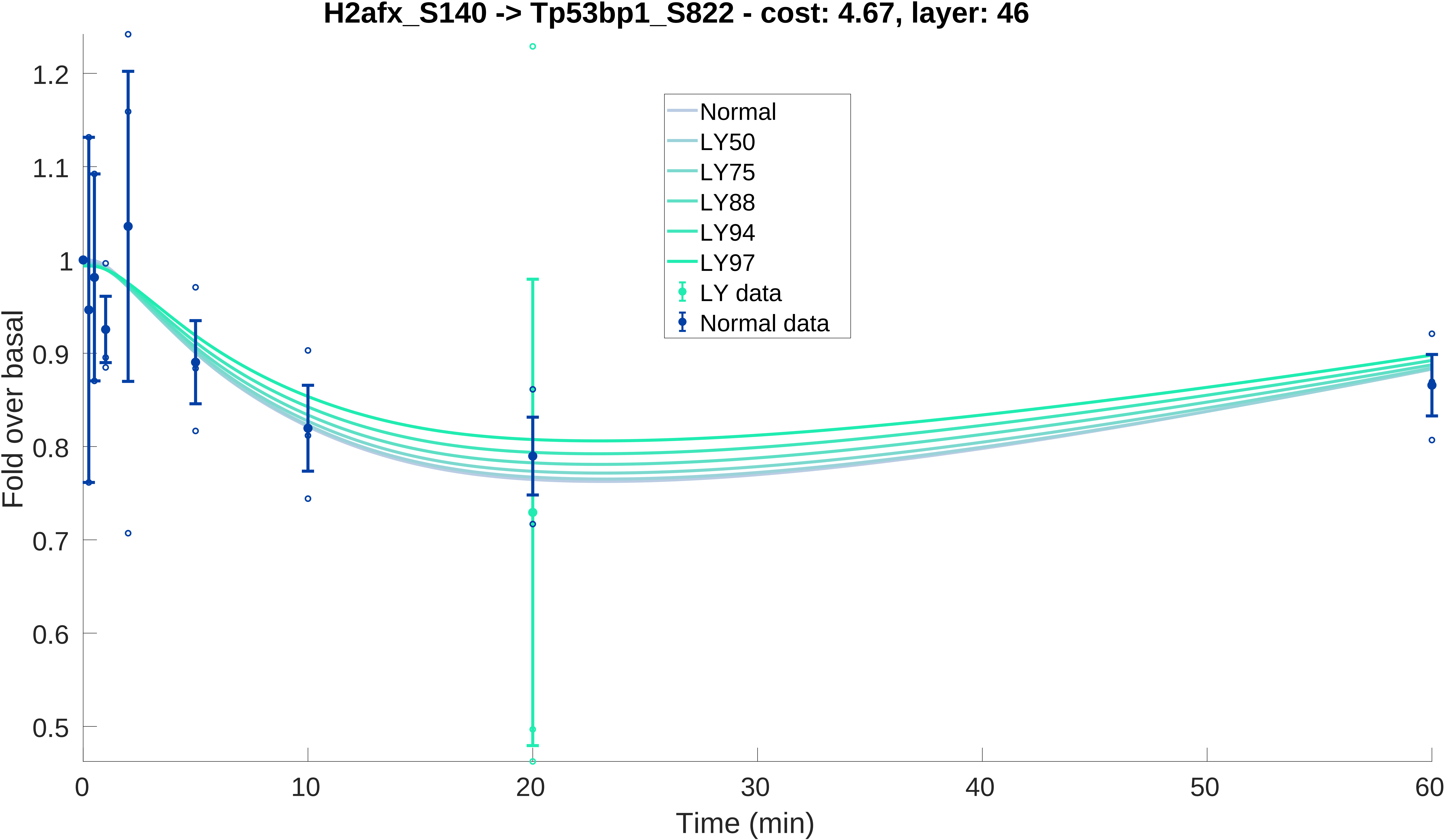

H2afx_S140 -> Tp53bp1_S822 - cost: 4.67, layer: 46
Normal
LY50
LY75
LY88
LY94
LY97
LY data
Normal data
Fold over basal
Time (min)
1.2
1.1
1
0.9
0.8
0.7
0.6
0.5
0
10
20
30
40
50
60

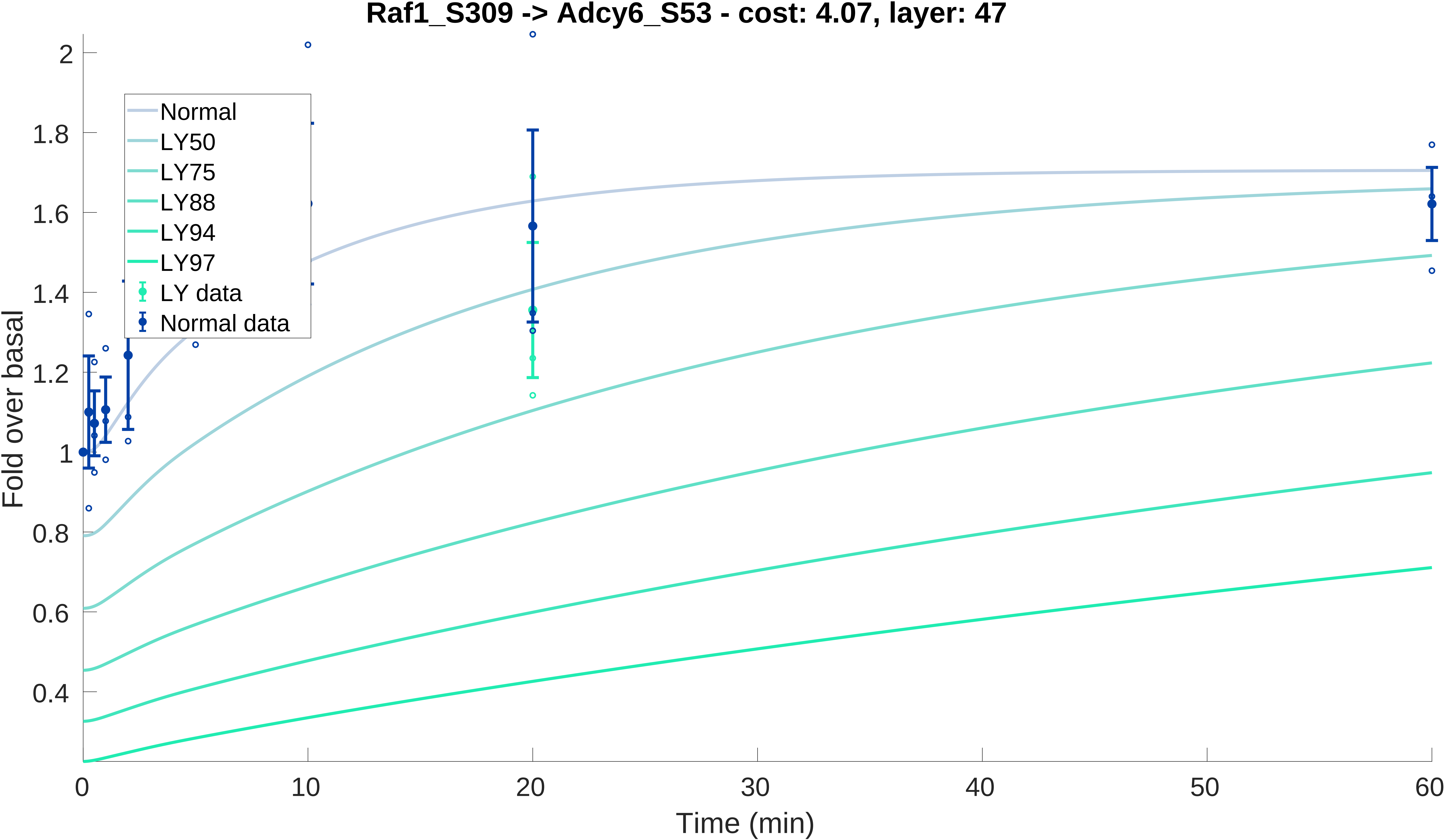

Raf1_S309 -> Adcy6_S53 - cost: 4.07, layer: 47
Normal
LY50
LY75
LY88
LY94
LY97
LY data
Normal data
Fold over basal
Time (min)
0
10
20
30
40
50
60
0.4
0.6
0.8
1
1.2
1.4
1.6
1.8
2

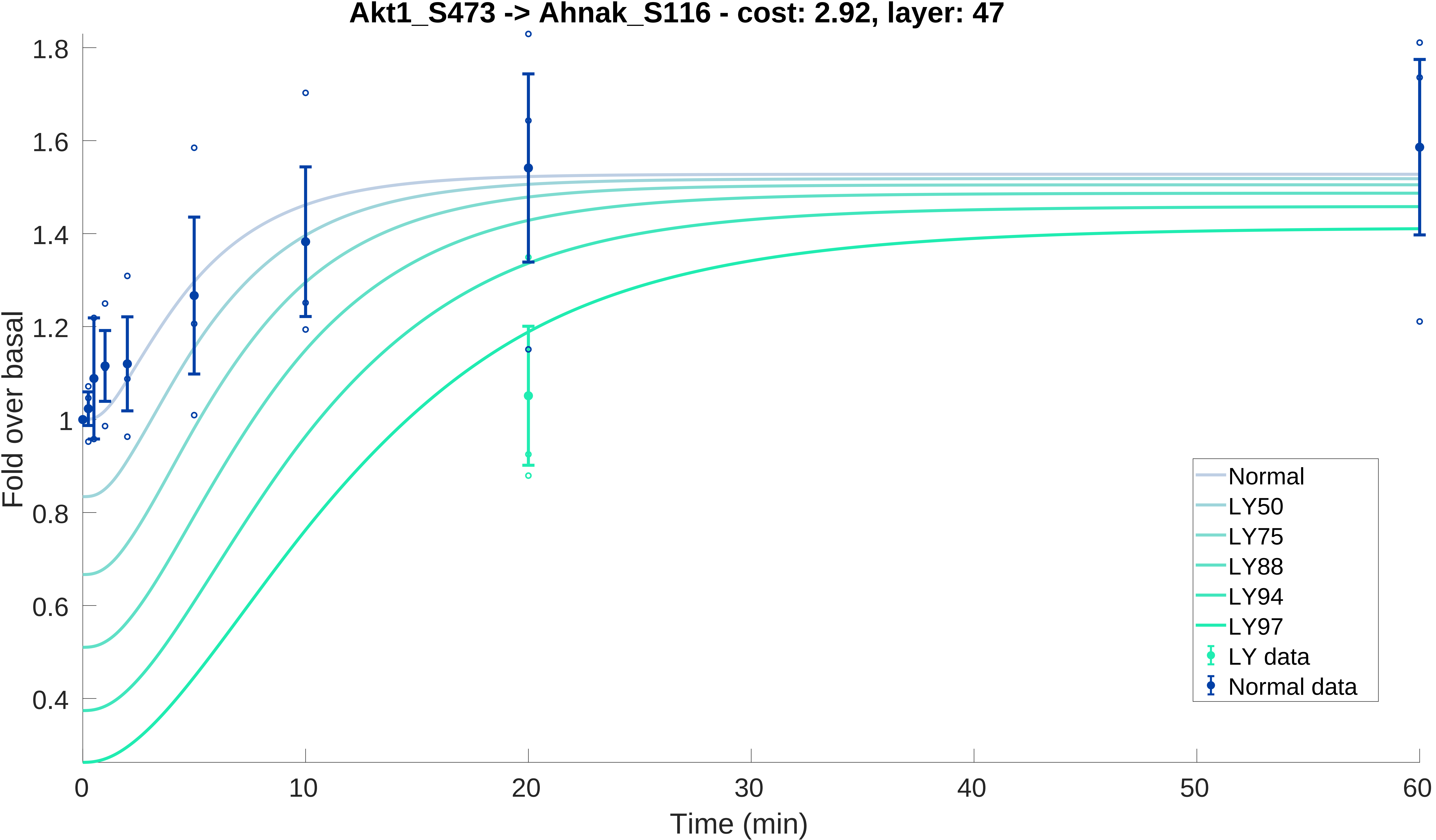

Akt1_S473 -> Ahnak_S116 - cost: 2.92, layer: 47
Fold over basal
Time (min)
0
10
20
30
40
50
60
0.4
0.6
0.8
1
1.2
1.4
1.6
1.8
Normal
LY50
LY75
LY88
LY94
LY97
LY data
Normal data

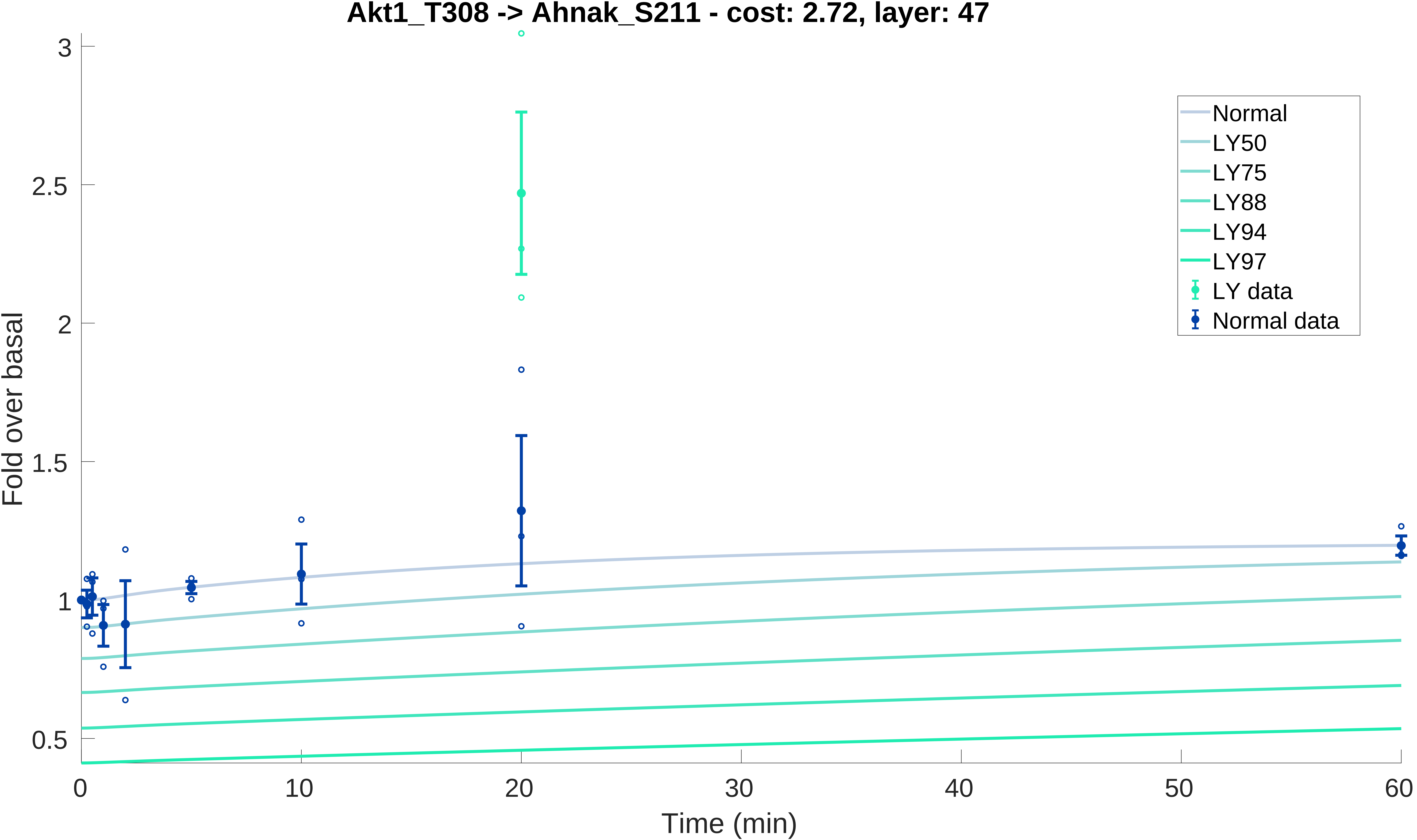

Akt1_T308 -> Ahnak_S211 - cost: 2.72, layer: 47
Fold over basal
Time (min)
0
10
20
30
40
50
60
0.5
1
1.5
2
2.5
3
Normal
LY50
LY75
LY88
LY94
LY97
LY data
Normal data

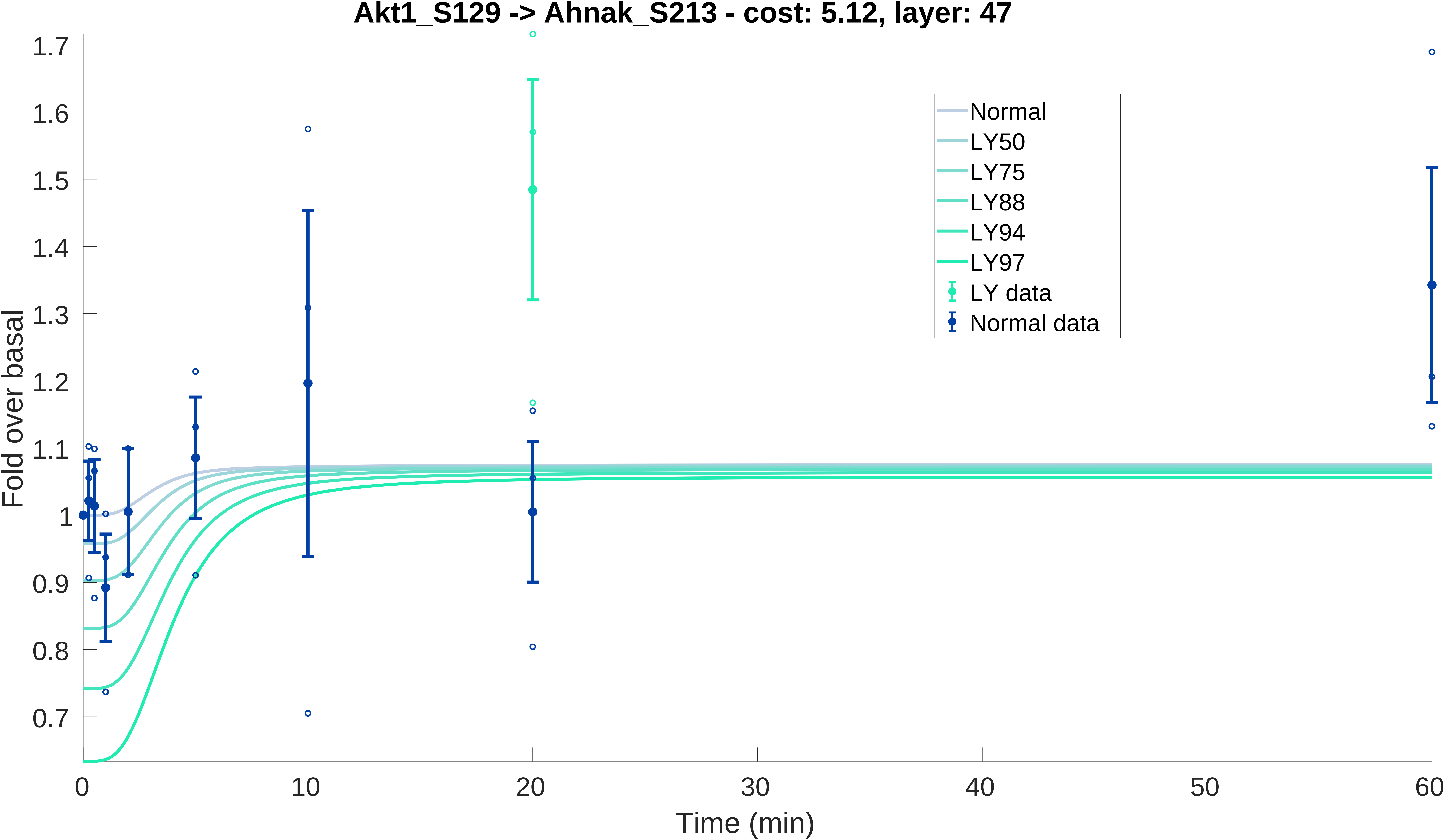

Akt1_S129 -> Ahnak_S213 - cost: 5.12, layer: 47
Fold over basal
Time (min)
0
10
20
30
40
50
60
0.7
0.8
0.9
1
1.1
1.2
1.3
1.4
1.5
1.6
1.7
Normal
LY50
LY75
LY88
LY94
LY97
LY data
Normal data

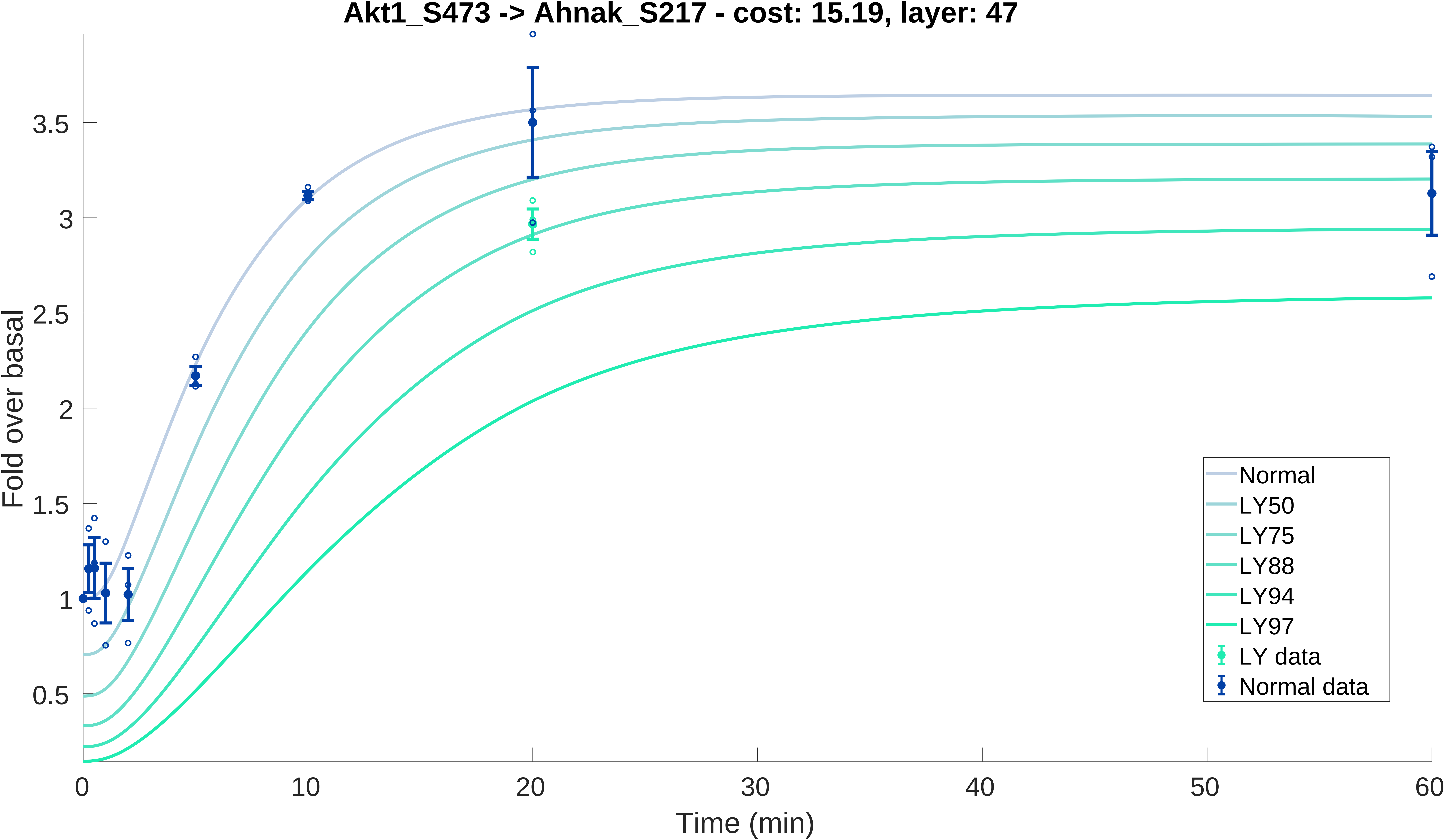
Akt1_S473 -> Ahnak_S217 - cost: 15.19, layer: 47
Fold over basal
3.5
3
2.5
2
1.5
1
0.5
0
10
20
30
40
50
60
Time (min)
Normal
LY50
LY75
LY88
LY94
LY97
LY data
Normal data

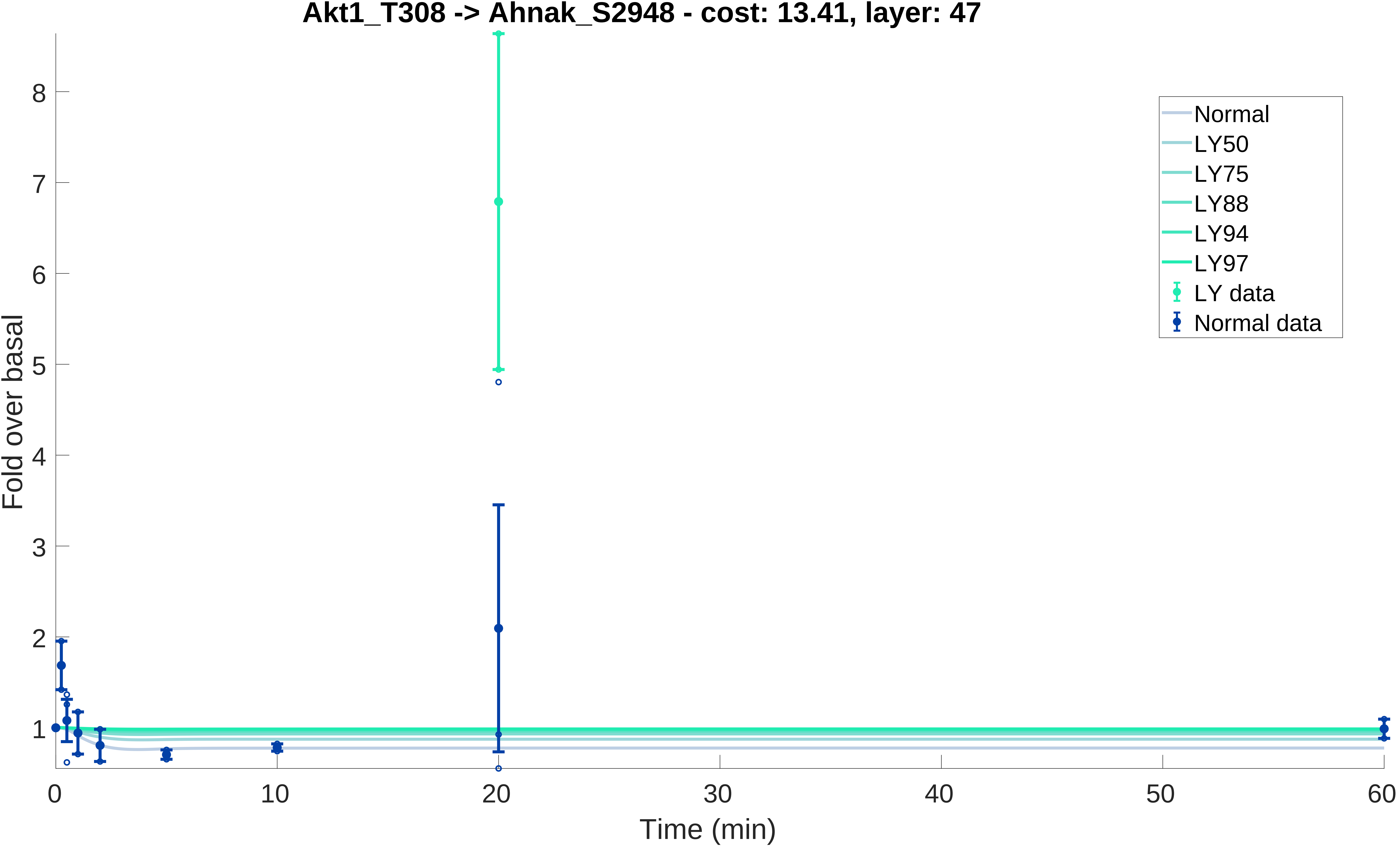

Akt1_T308 -> Ahnak_S2948 - cost: 13.41, layer: 47
Fold over basal
Time (min)
0
10
20
30
40
50
60
1
2
3
4
5
6
7
8
Normal
LY50
LY75
LY88
LY94
LY97
LY data
Normal data

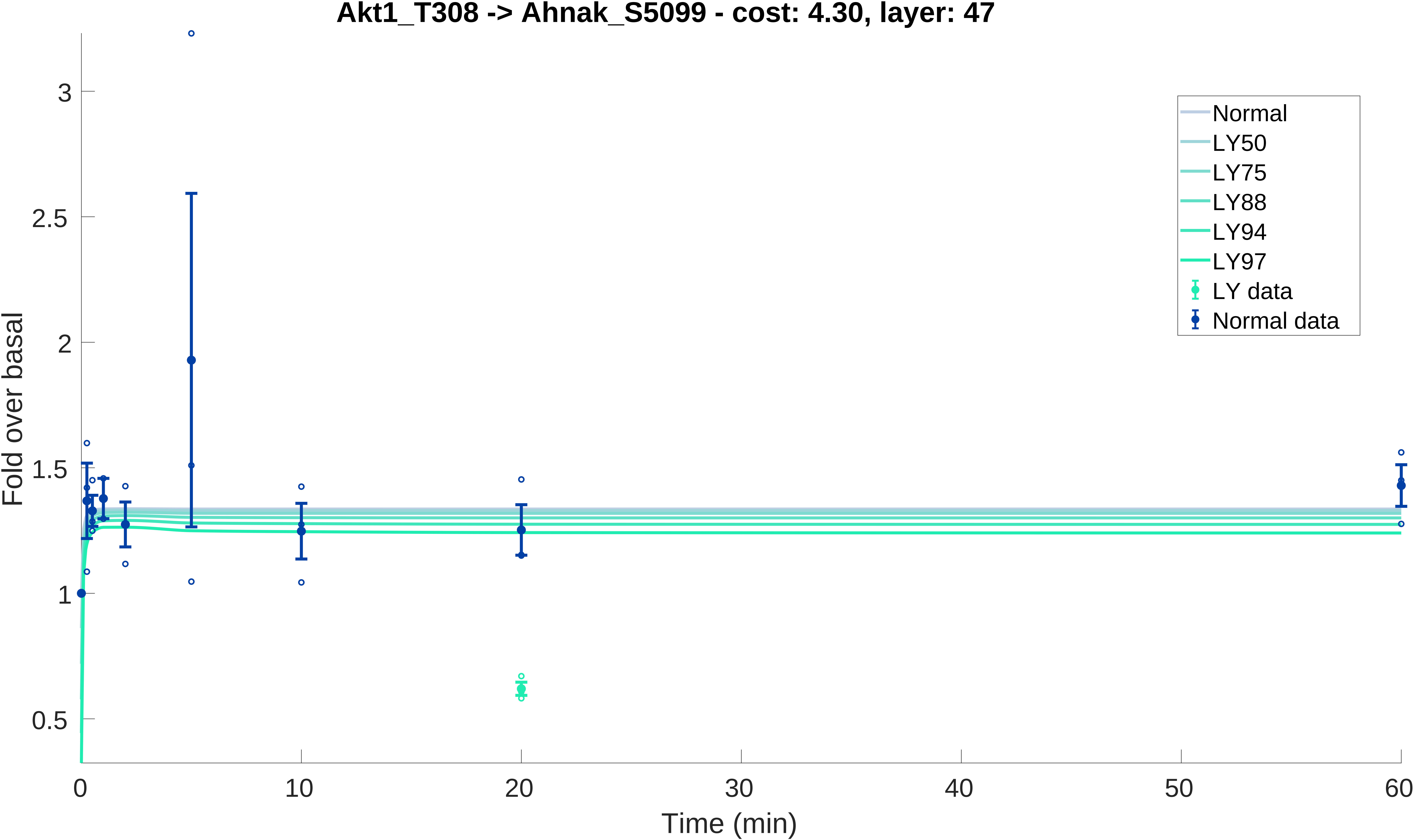

Akt1_T308 -> Ahnak_S5099 - cost: 4.30, layer: 47
Normal
LY50
LY75
LY88
LY94
LY97
LY data
Normal data
Fold over basal
0.5
1
1.5
2
2.5
3
Time (min)
0
10
20
30
40
50
60

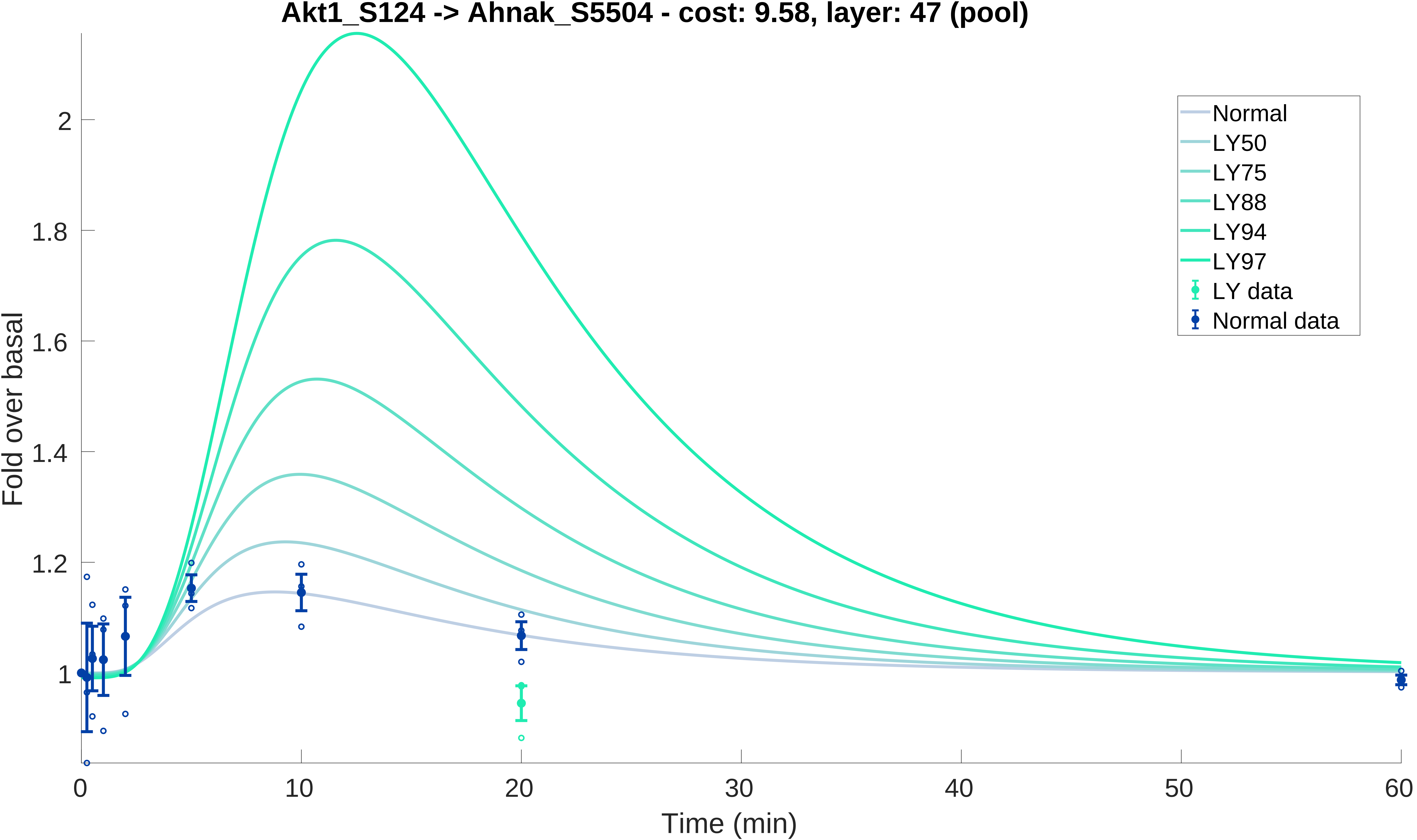

Akt1_S124 -> Ahnak_S5504 - cost: 9.58, layer: 47 (pool)
Fold over basal
Time (min)
2
1.8
1.6
1.4
1.2
1
0
10
20
30
40
50
60
Normal
LY50
LY75
LY88
LY94
LY97
LY data
Normal data

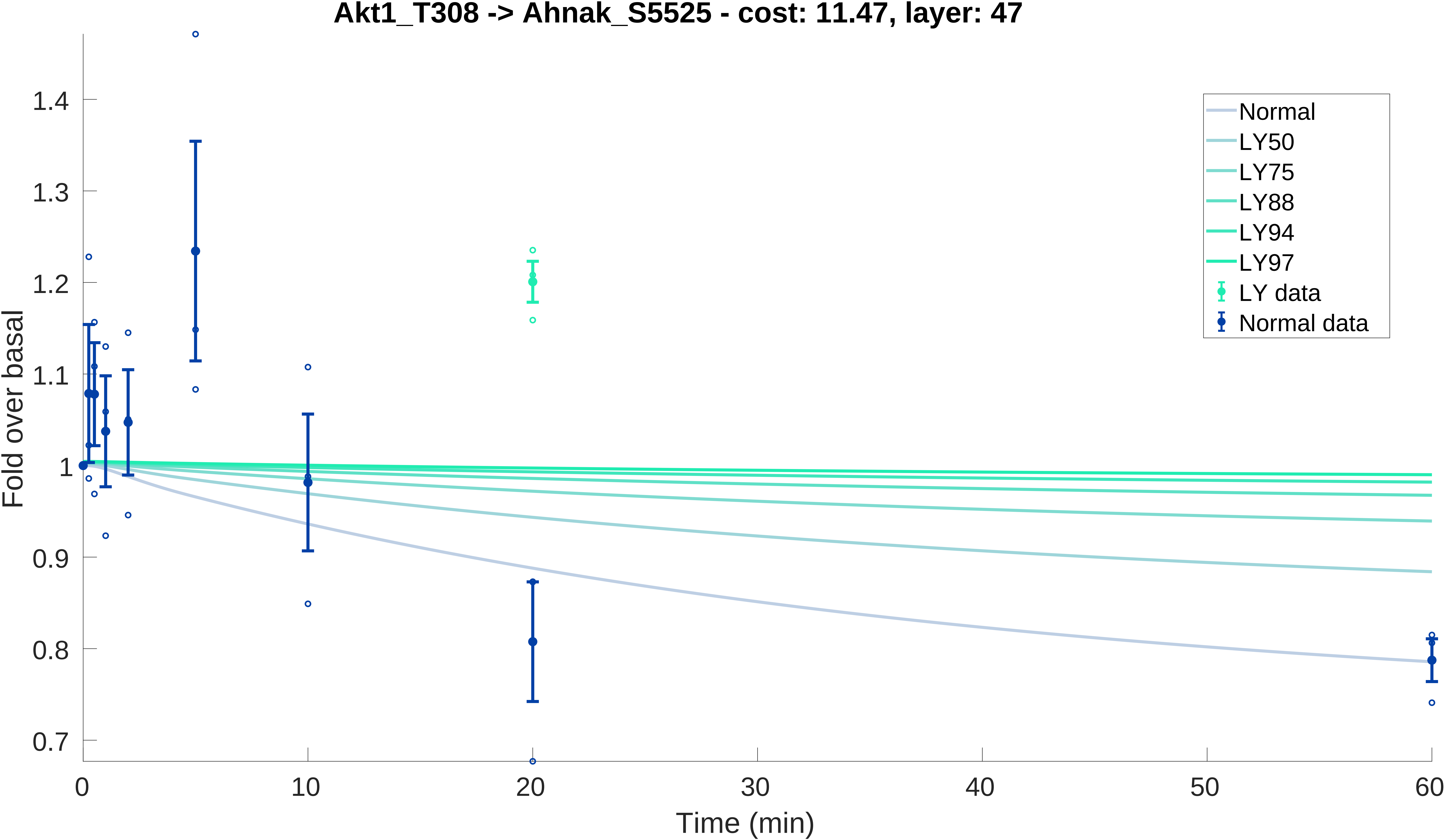

Akt1_T308 -> Ahnak_S5525 - cost: 11.47, layer: 47
Fold over basal
1.4
1.3
1.2
1.1
1
0.9
0.8
0.7
0
10
20
30
40
50
60
Time (min)
Normal
LY50
LY75
LY88
LY94
LY97
LY data
Normal data

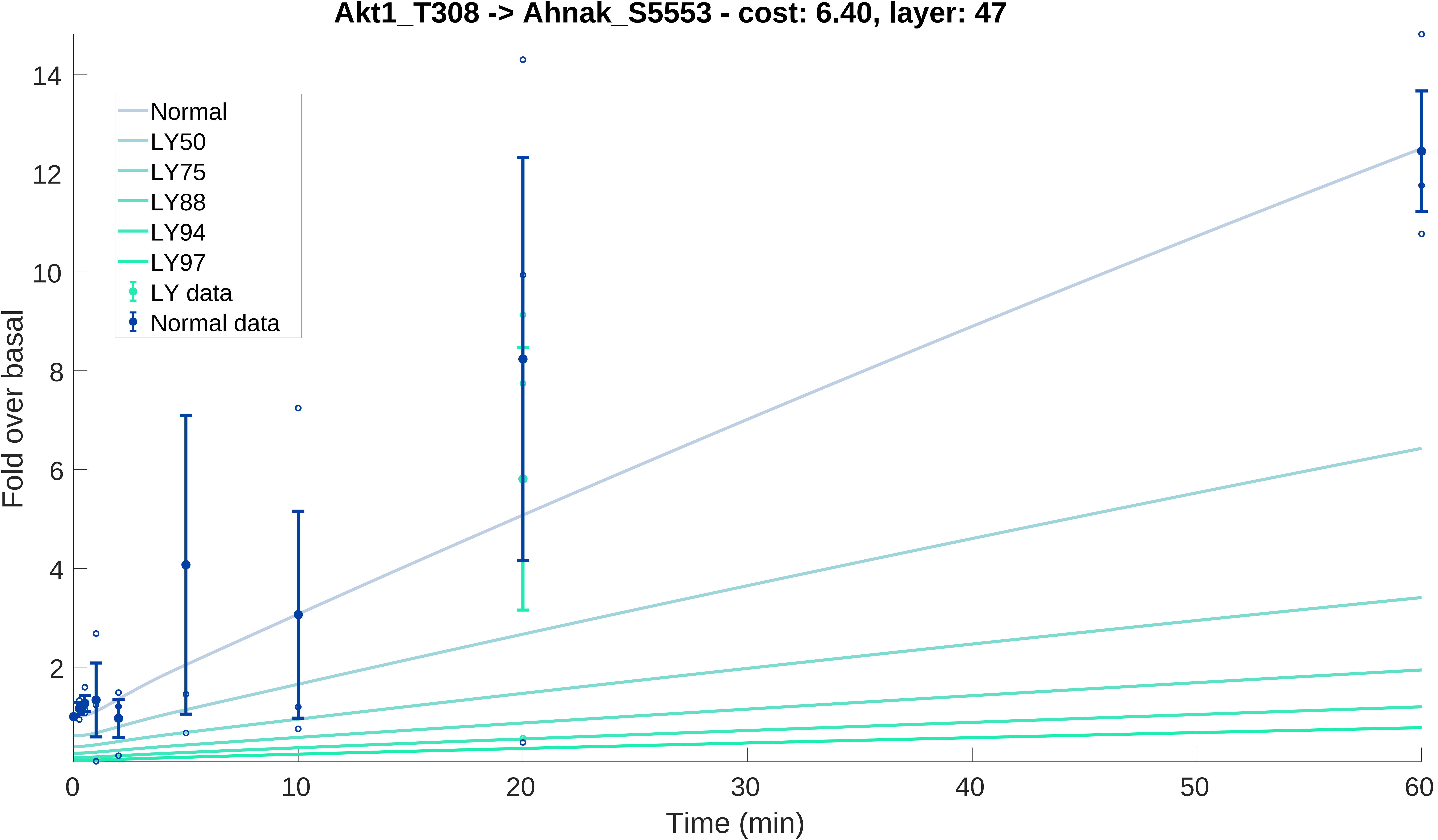

Akt1_T308 -> Ahnak_S5553 - cost: 6.40, layer: 47
Fold over basal
Time (min)
Normal
LY50
LY75
LY88
LY94
LY97
LY data
Normal data
0
2
4
6
8
10
12
14
0
10
20
30
40
50
60

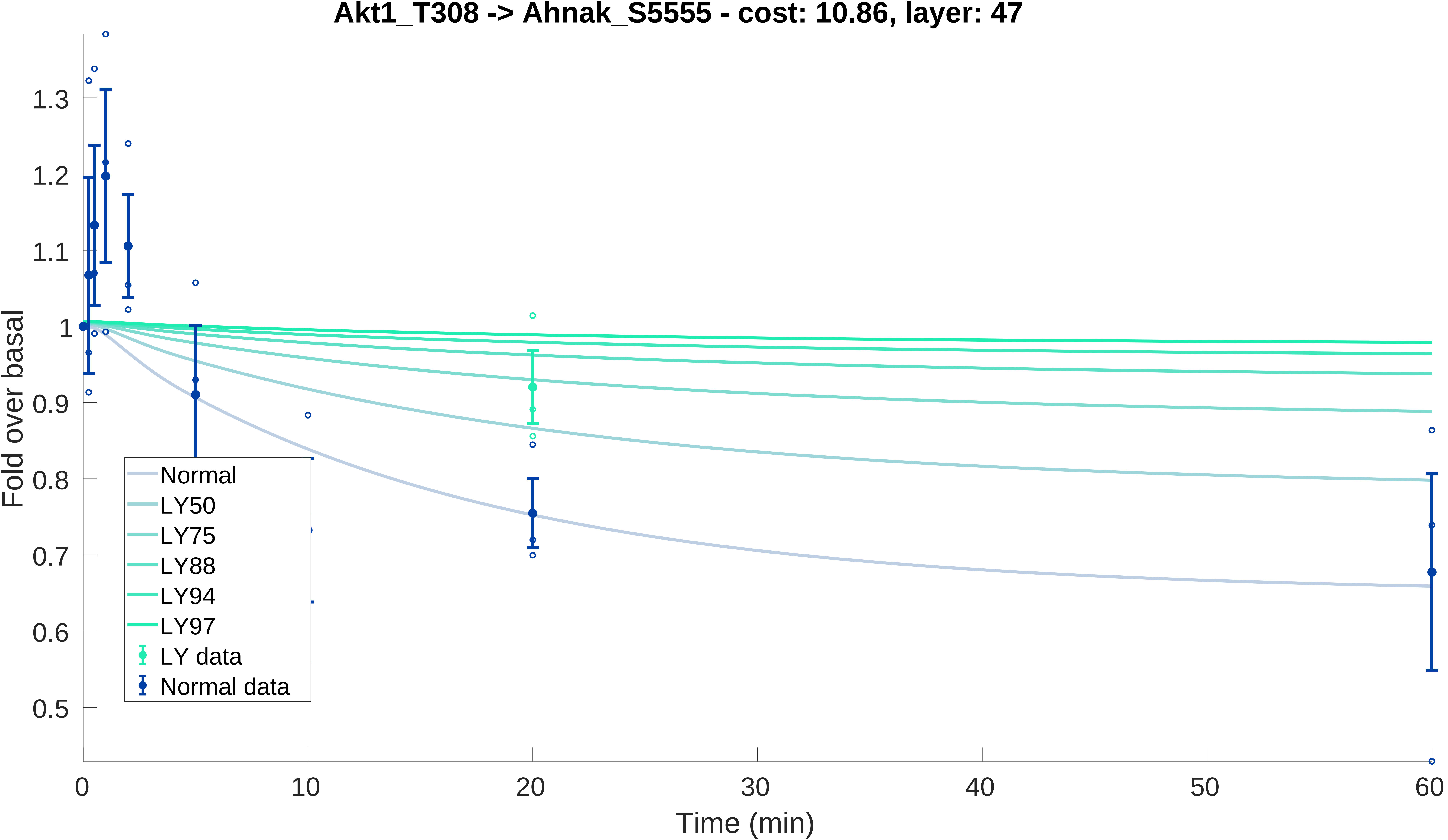
Akt1_T308 -> Ahnak_S5555 - cost: 10.86, layer: 47
Fold over basal
Time (min)
0
10
20
30
40
50
60
0.5
0.6
0.7
0.8
0.9
1
1.1
1.2
1.3
Normal
LY50
LY75
LY88
LY94
LY97
LY data
Normal data

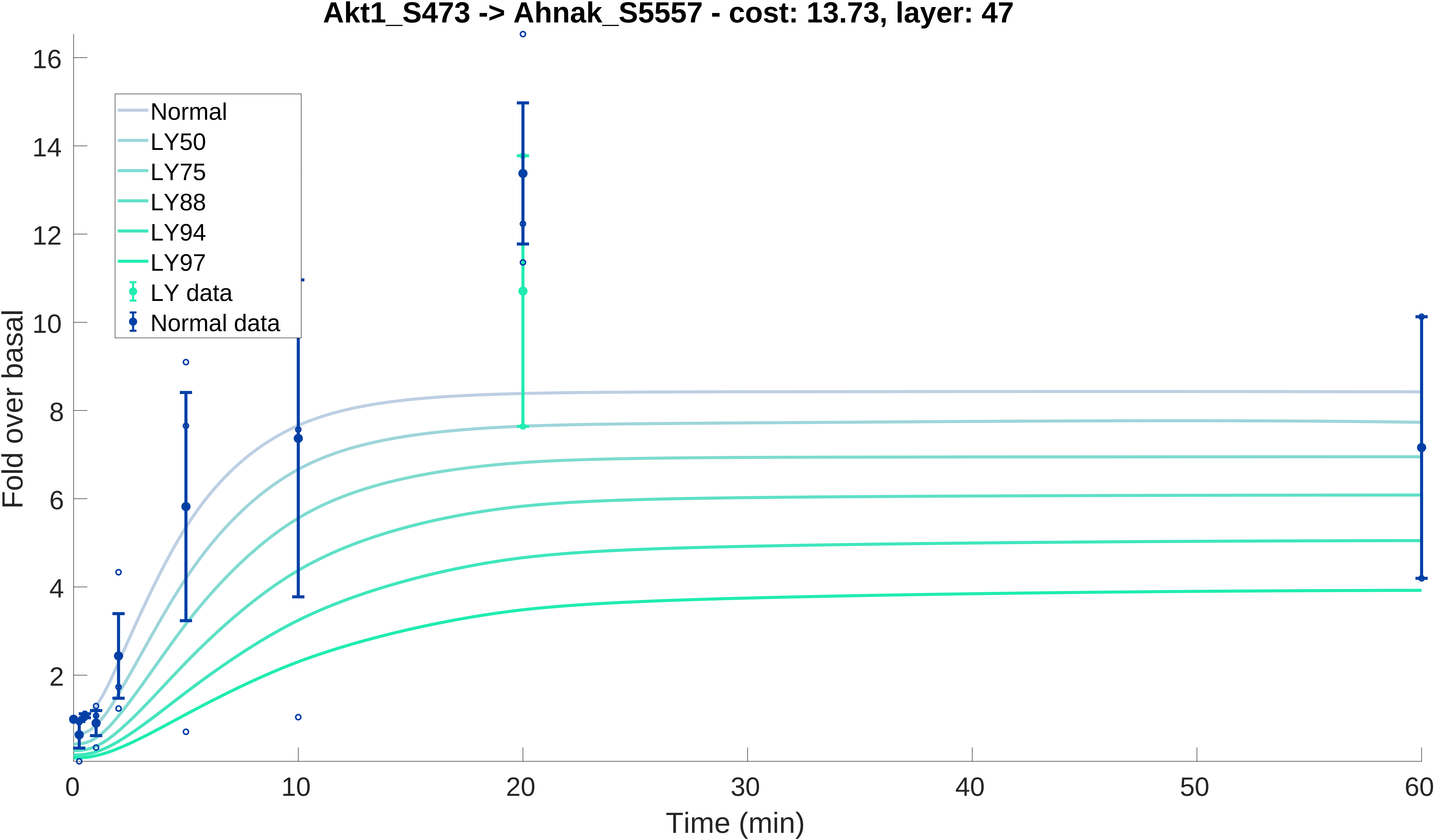
Akt1_S473 -> Ahnak_S5557 - cost: 13.73, layer: 47
Normal
LY50
LY75
LY88
LY94
LY97
LY data
Normal data
Fold over basal
Time (min)
0
10
20
30
40
50
60
2
4
6
8
10
12
14
16

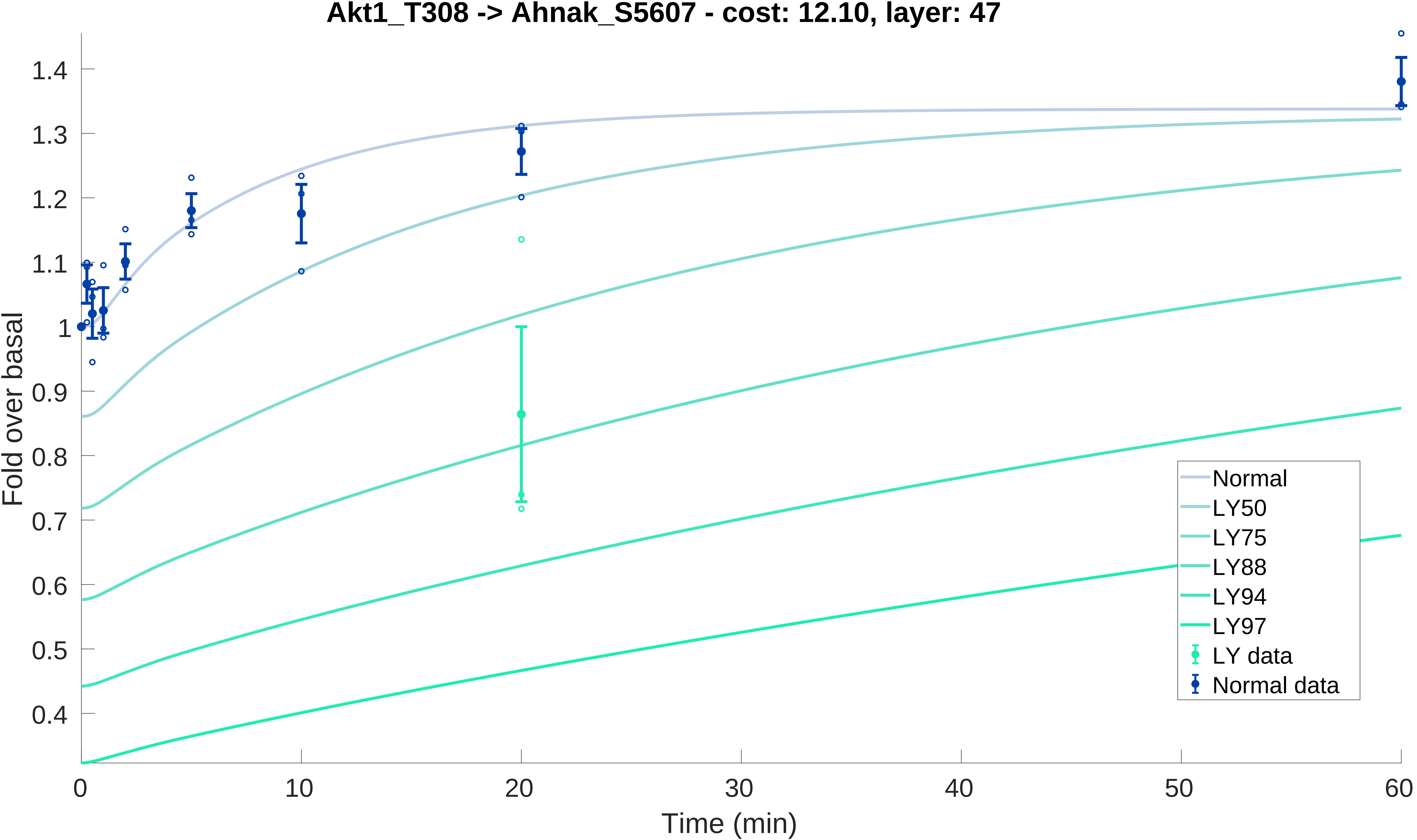
Akt1_T308 -> Ahnak_S5607 - cost: 12.10, layer: 47
Fold over basal
Time (min)
1.4
1.3
1.2
1.1
1
0.9
0.8
0.7
0.6
0.5
0.4
0
10
20
30
40
50
60
Normal
LY50
LY75
LY88
LY94
LY97
LY data
Normal data

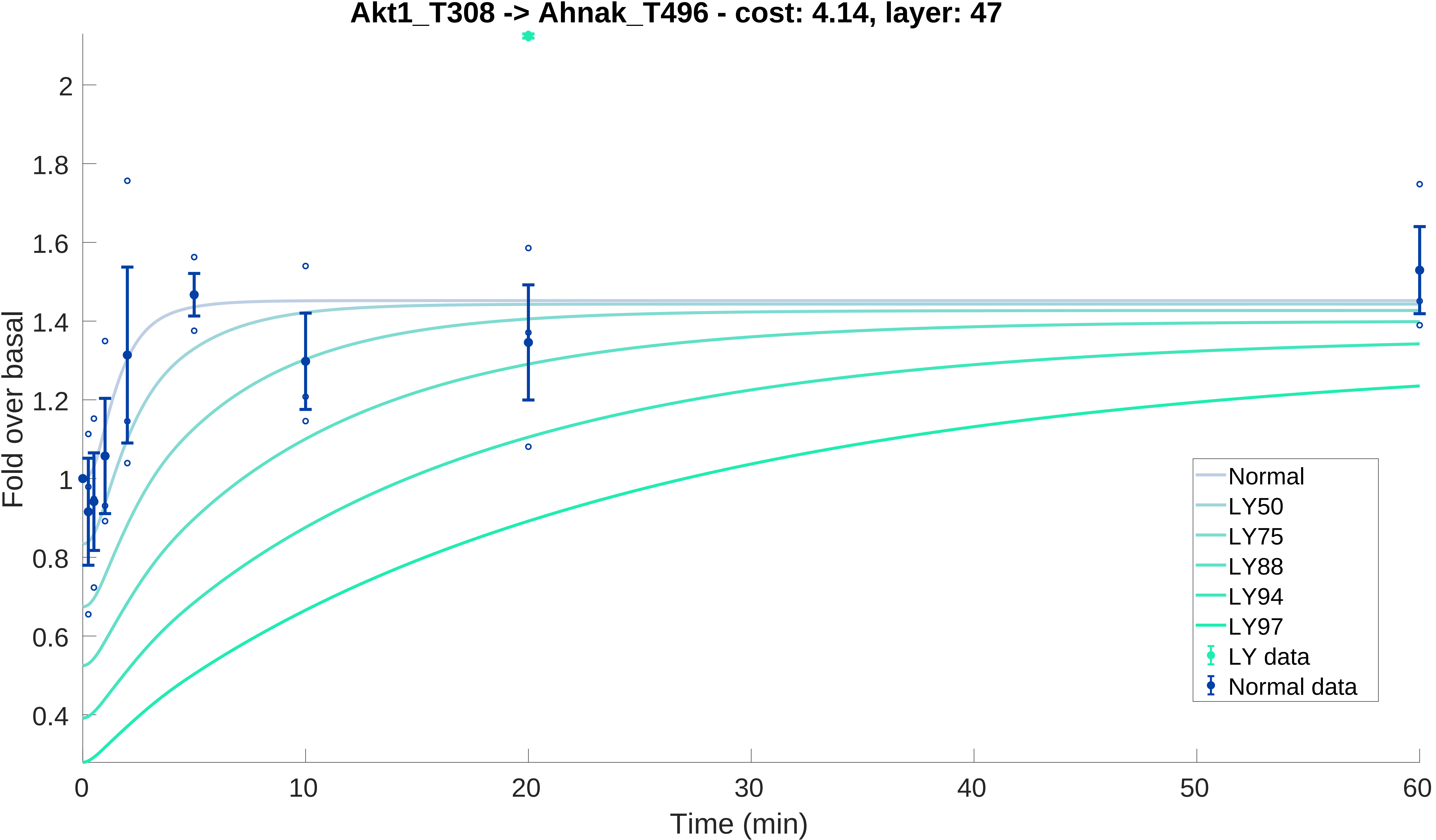

Akt1_T308 -> Ahnak_T496 - cost: 4.14, layer: 47
Fold over basal
2
1.8
1.6
1.4
1.2
1
0.8
0.6
0.4
0
10
20
30
40
50
60
Time (min)
Normal
LY50
LY75
LY88
LY94
LY97
LY data
Normal data

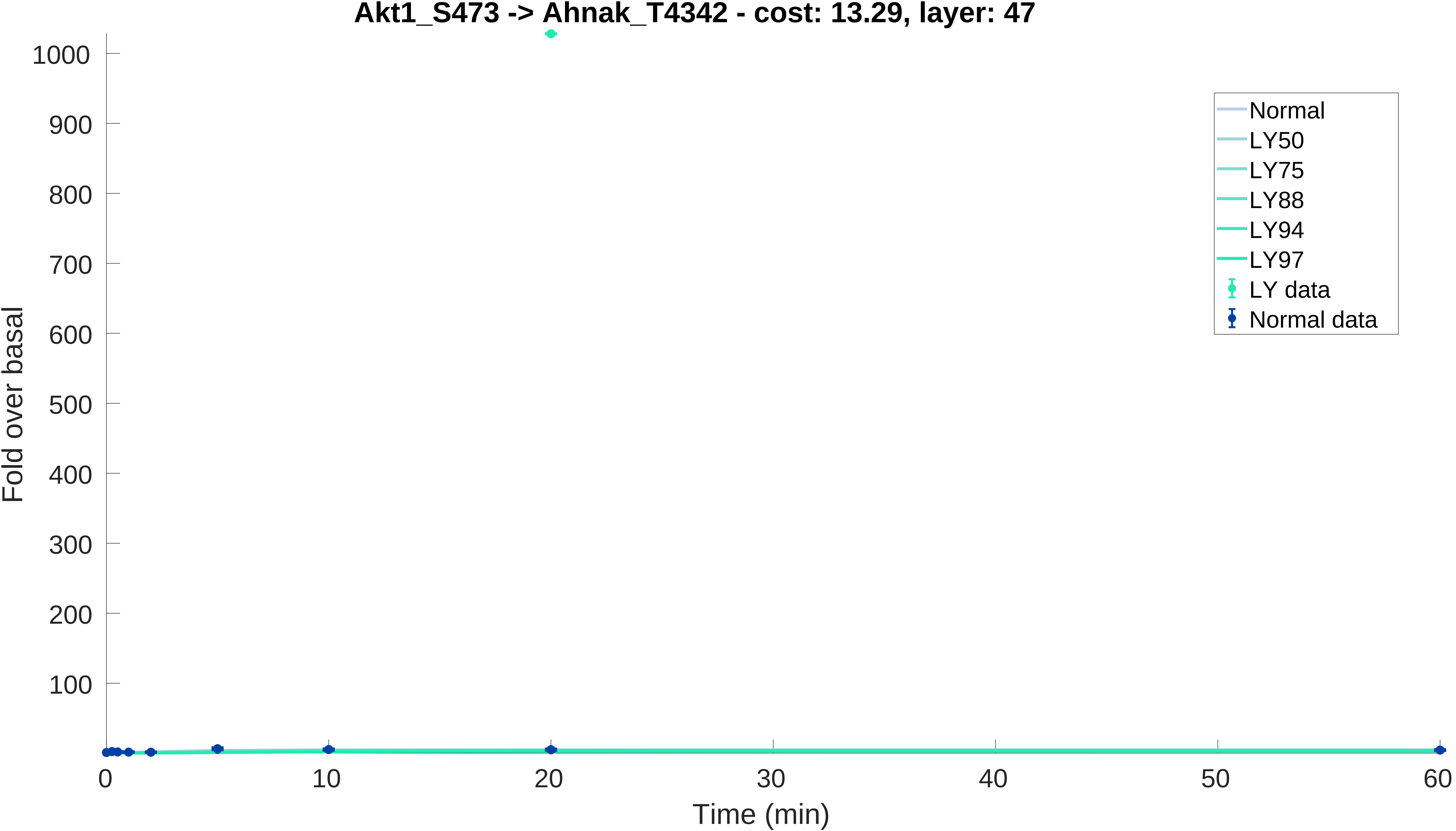

Akt1_S473 -> Ahnak_T4342 - cost: 13.29, layer: 47
Fold over basal
1000
900
800
700
600
500
400
300
200
100
0
10
20
30
40
50
60
Time (min)
Normal
LY50
LY75
LY88
LY94
LY97
LY data
Normal data

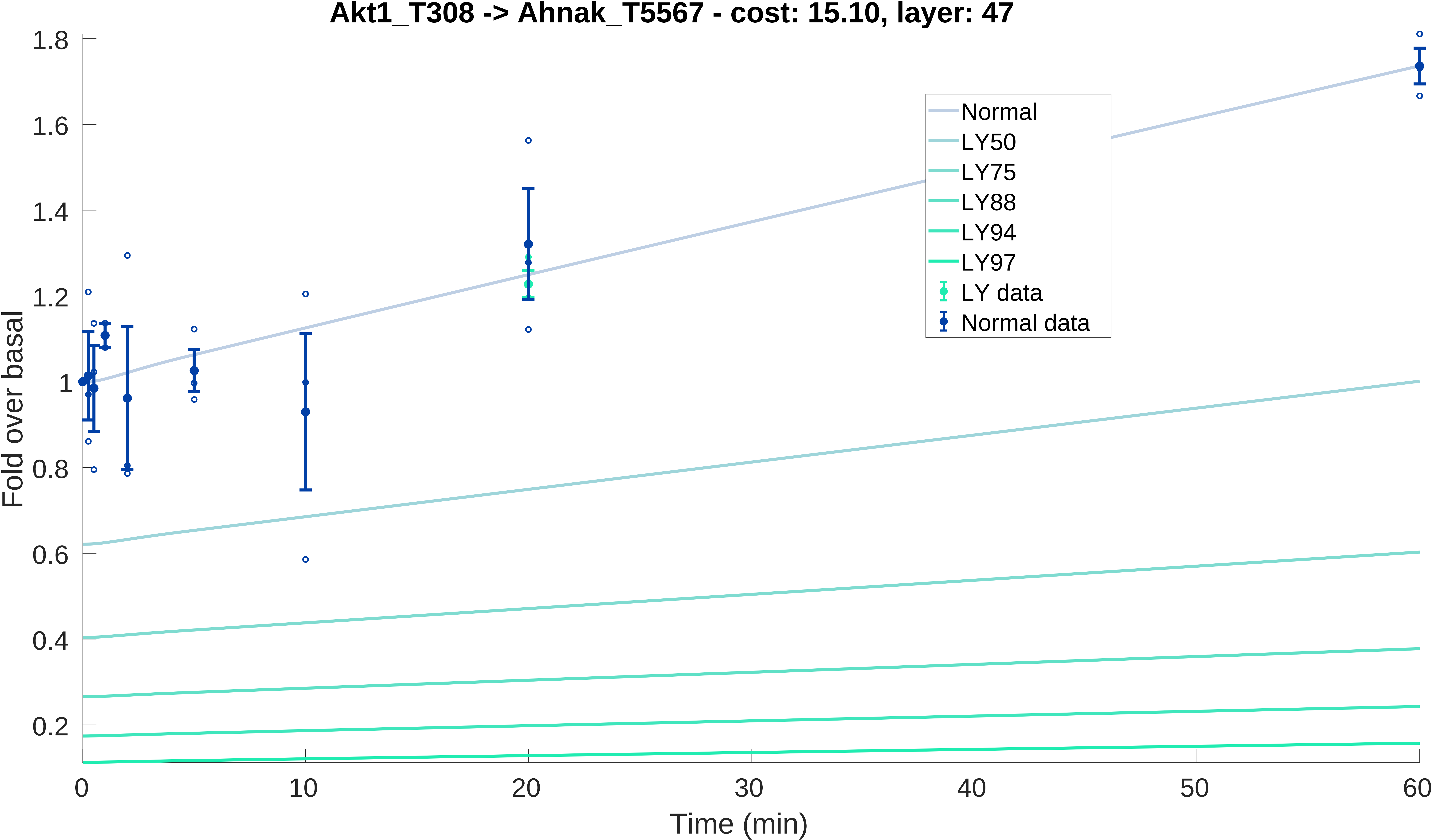

Akt1_T308 -> Ahnak_T5567 - cost: 15.10, layer: 47
Fold over basal
Time (min)
0
10
20
30
40
50
60
0.2
0.4
0.6
0.8
1
1.2
1.4
1.6
1.8
Normal
LY50
LY75
LY88
LY94
LY97
LY data
Normal data

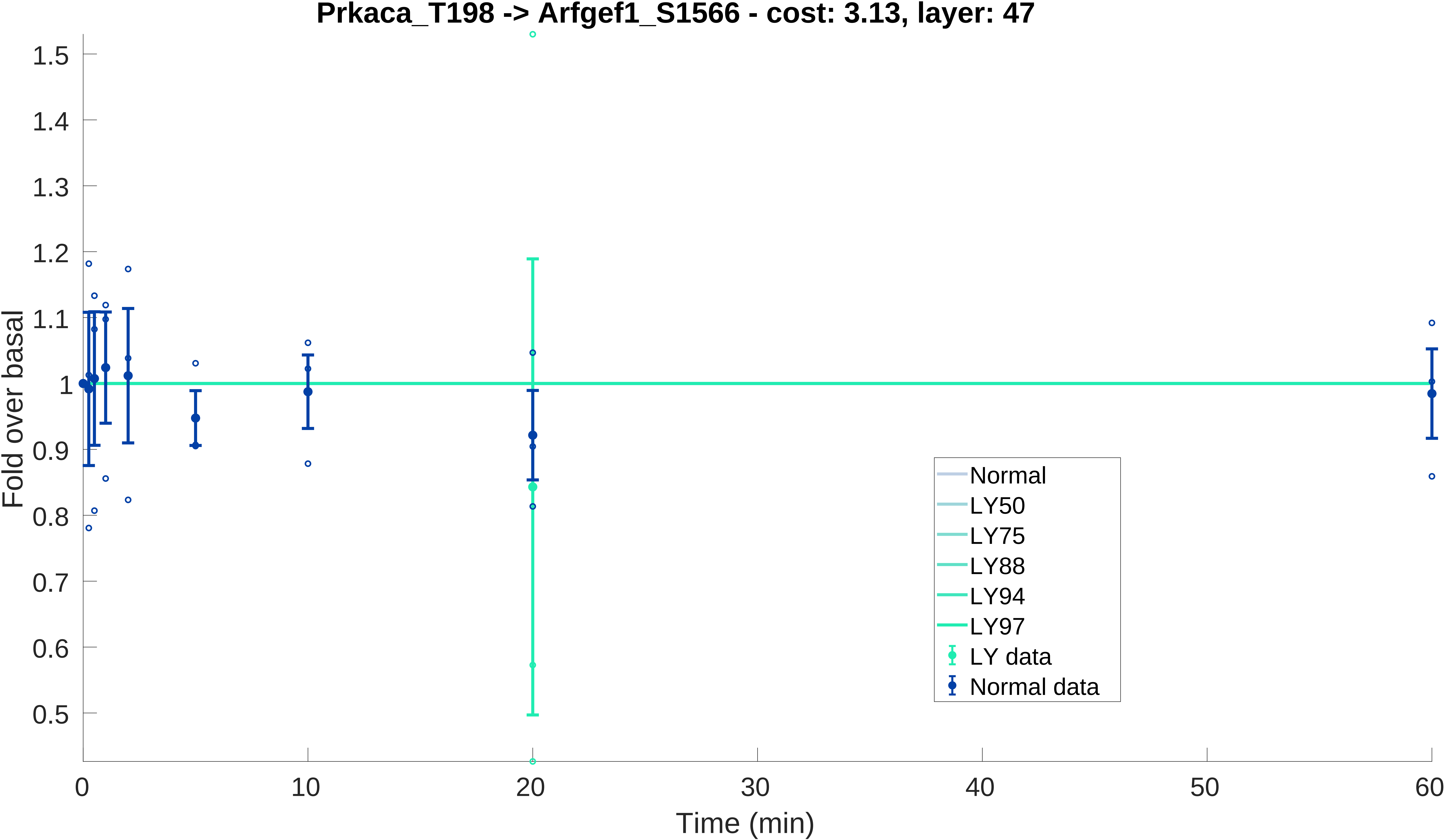

Prkaca_T198 -> Arfgef1_S1566 - cost: 3.13, layer: 47
Fold over basal
1.5
1.4
1.3
1.2
1.1
1
0.9
0.8
0.7
0.6
0.5
0
10
20
30
40
50
60
Time (min)
Normal
LY50
LY75
LY88
LY94
LY97
LY data
Normal data

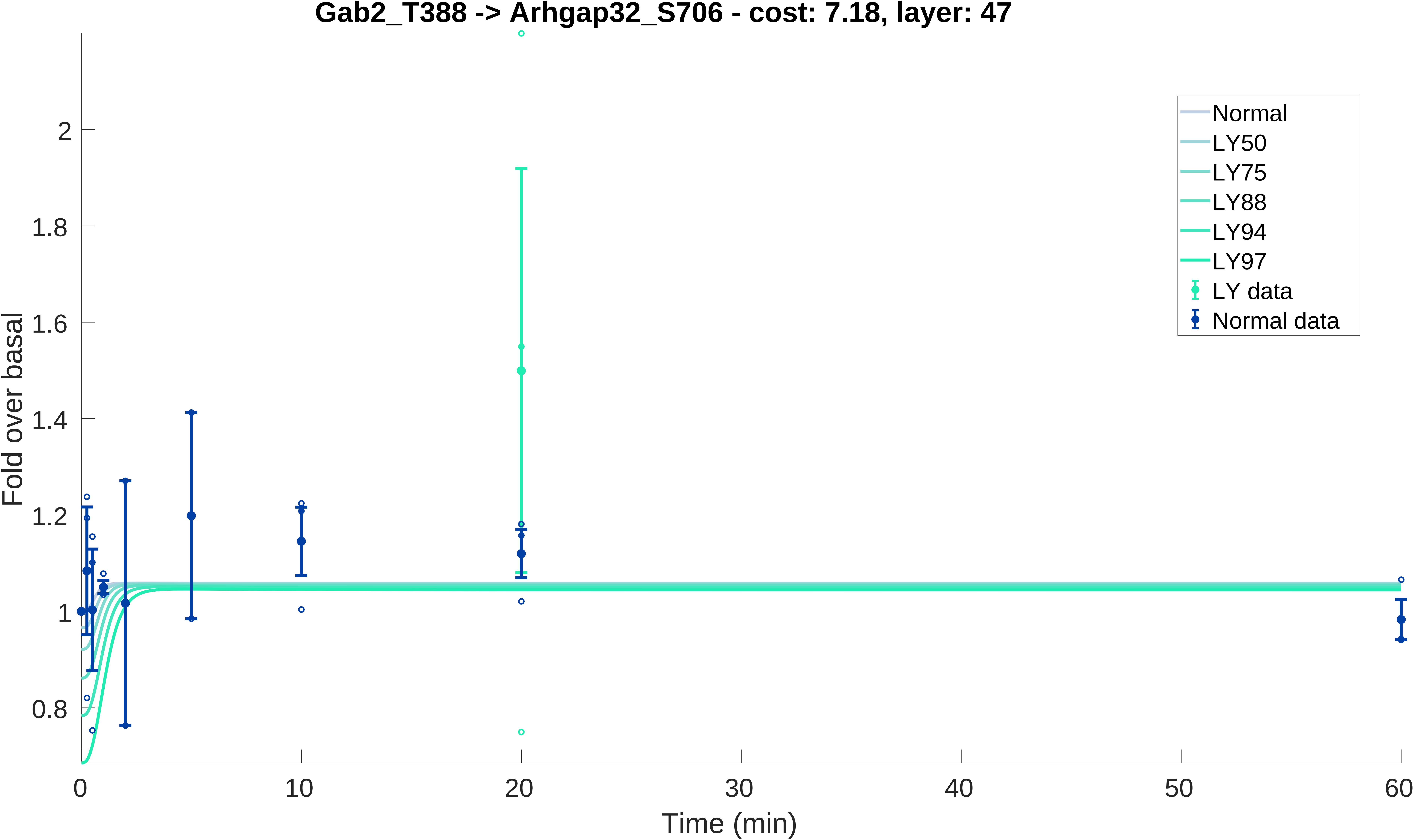

Gab2_T388 -> Arhgap32_S706 - cost: 7.18, layer: 47
Fold over basal
Time (min)
0
10
20
30
40
50
60
0.8
1
1.2
1.4
1.6
1.8
2
Normal
LY50
LY75
LY88
LY94
LY97
LY data
Normal data

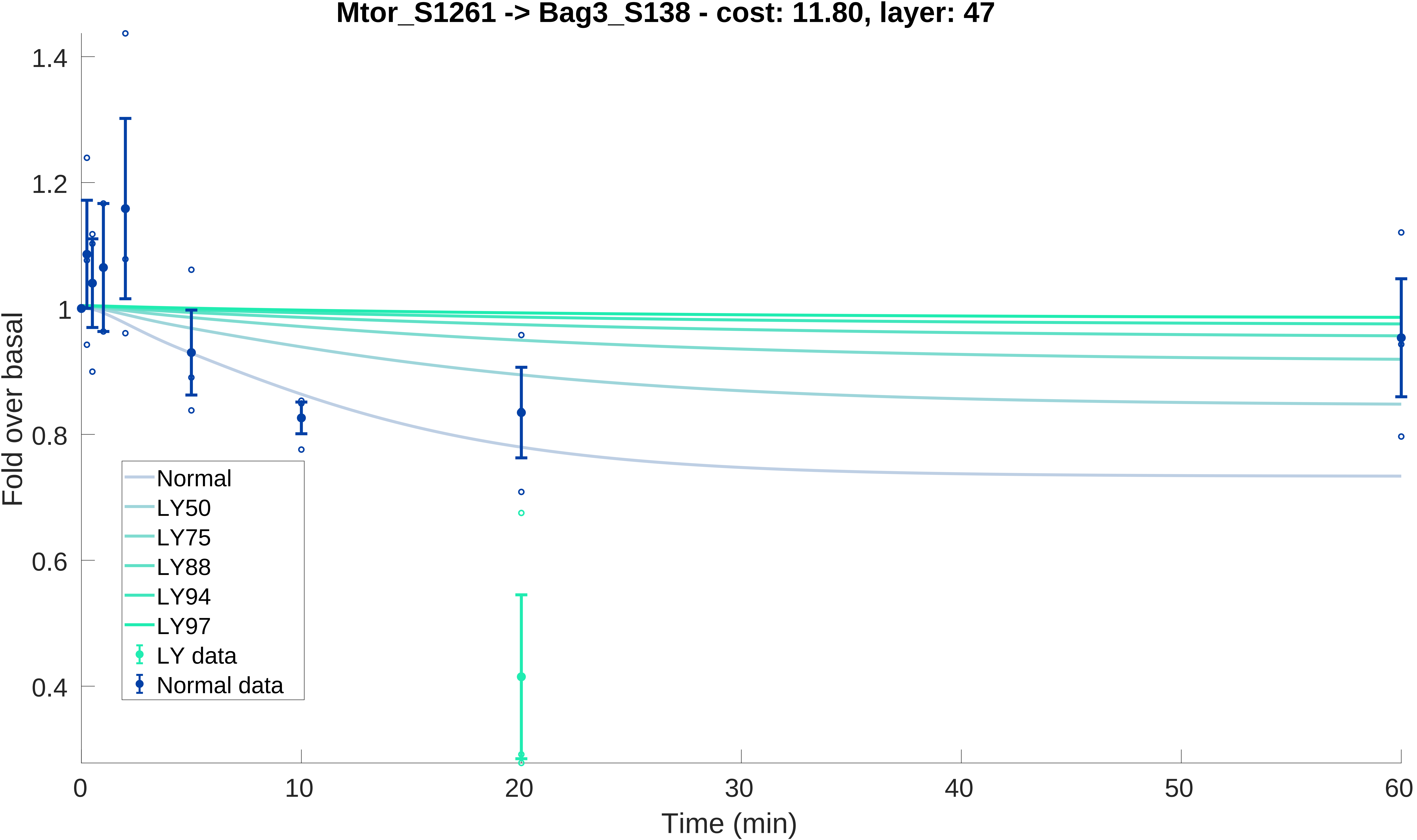
Mtor_S1261 -> Bag3_S138 - cost: 11.80, layer: 47
1.4
1.2
1
0.8
0.6
0.4
Fold over basal
0
10
20
30
40
50
60
Time (min)
Normal
LY50
LY75
LY88
LY94
LY97
LY data
Normal data

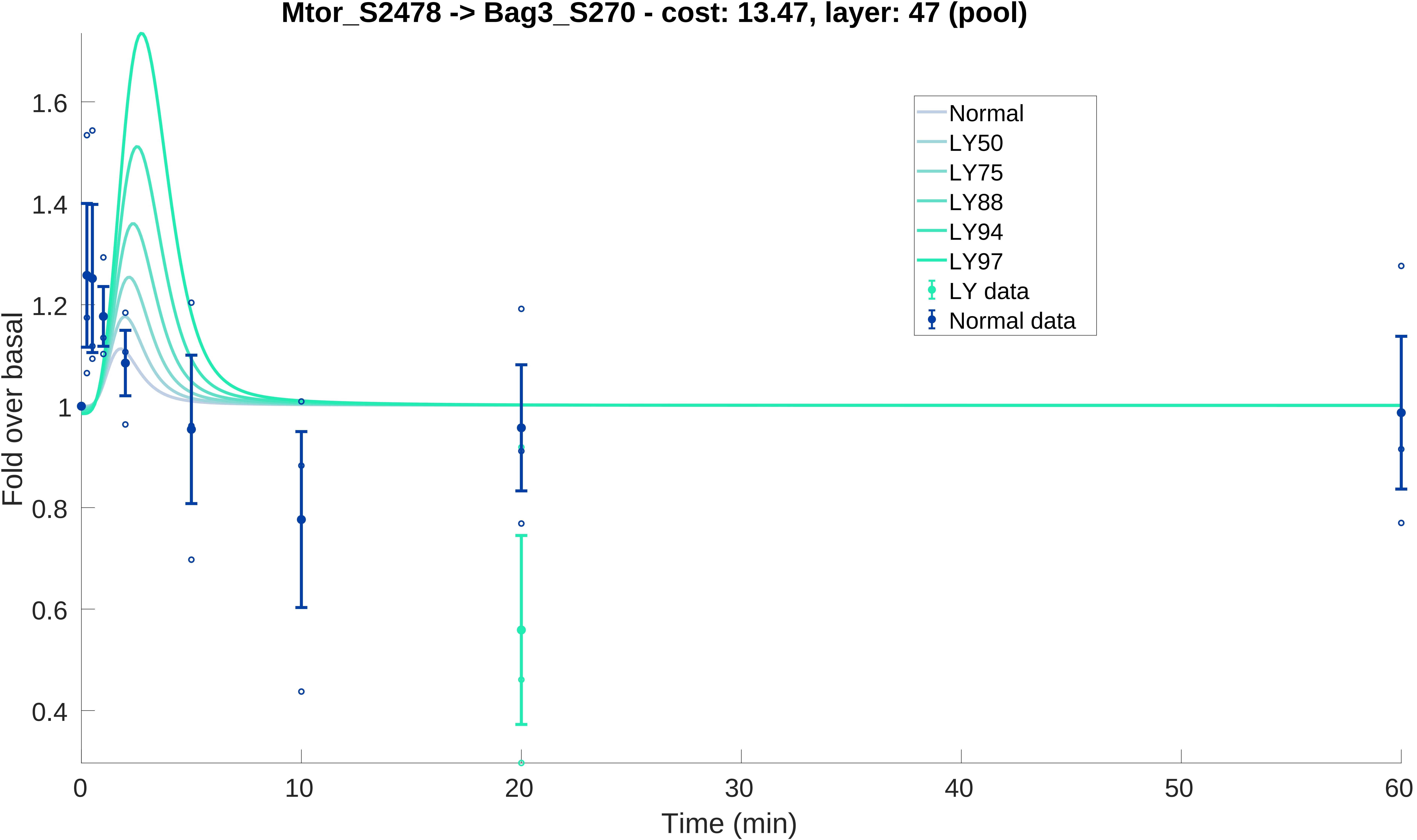

Mtor_S2478 -> Bag3_S270 - cost: 13.47, layer: 47 (pool)
Fold over basal
Time (min)
Normal
LY50
LY75
LY88
LY94
LY97
LY data
Normal data
0.4
0.6
0.8
1
1.2
1.4
1.6
0
10
20
30
40
50
60

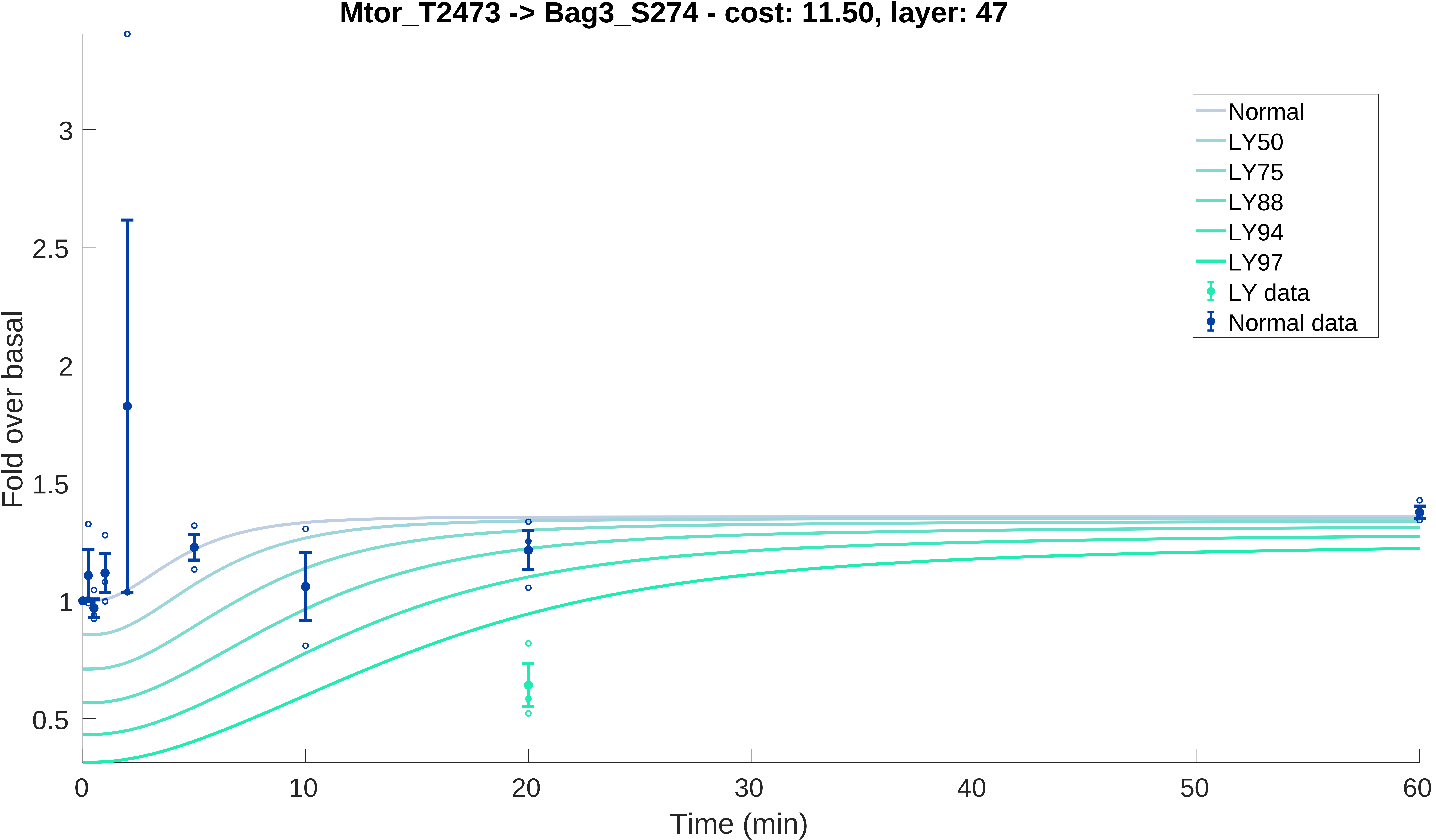
Mtor_T2473 -> Bag3_S274 - cost: 11.50, layer: 47
Fold over basal
Time (min)
Normal
LY50
LY75
LY88
LY94
LY97
LY data
Normal data
0
10
20
30
40
50
60
0.5
1
1.5
2
2.5
3

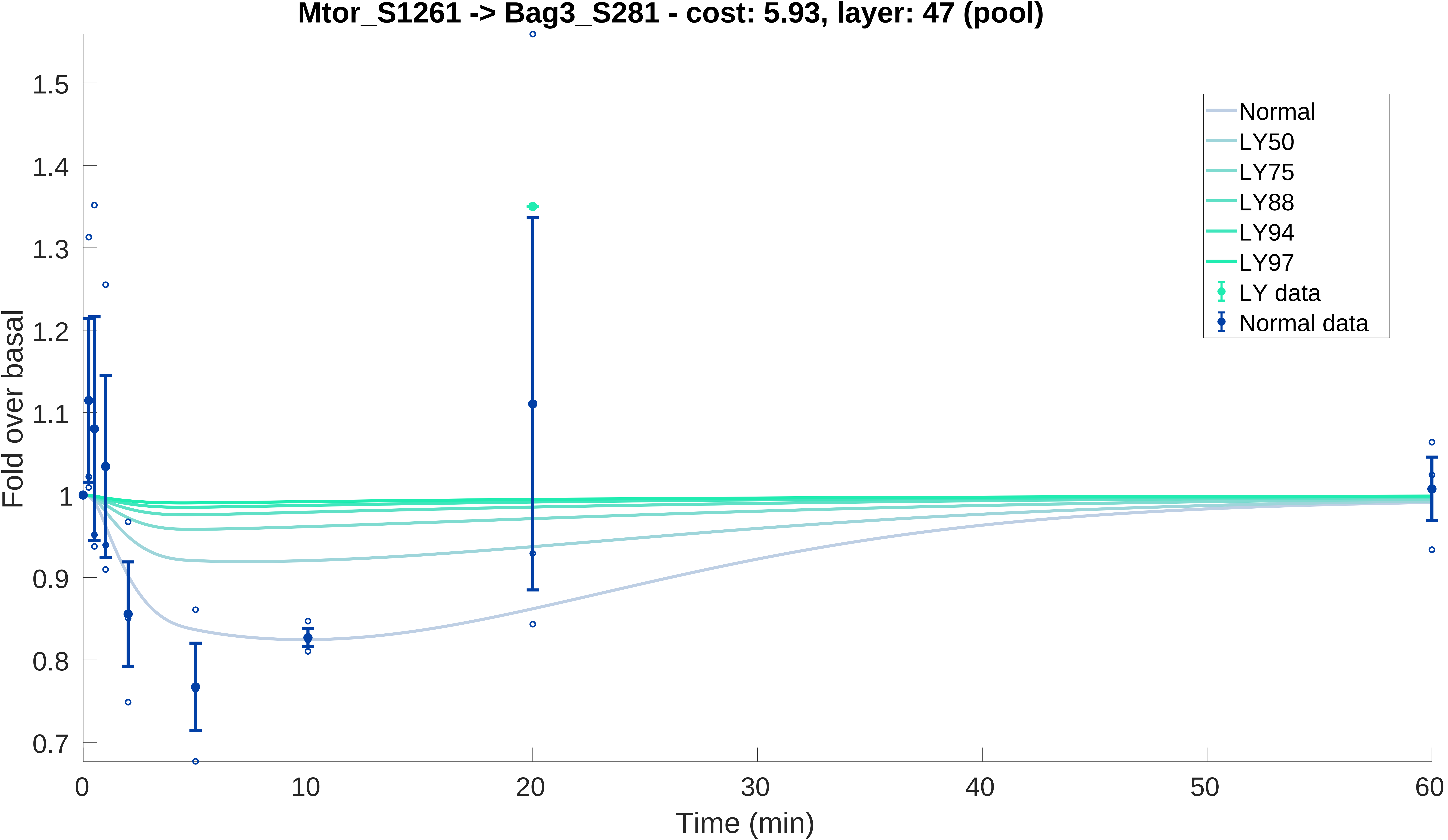

Mtor_S1261 -> Bag3_S281 - cost: 5.93, layer: 47 (pool)
Fold over basal
1.5
1.4
1.3
1.2
1.1
1
0.9
0.8
0.7
0
10
20
30
40
50
60
Time (min)
Normal
LY50
LY75
LY88
LY94
LY97
LY data
Normal data

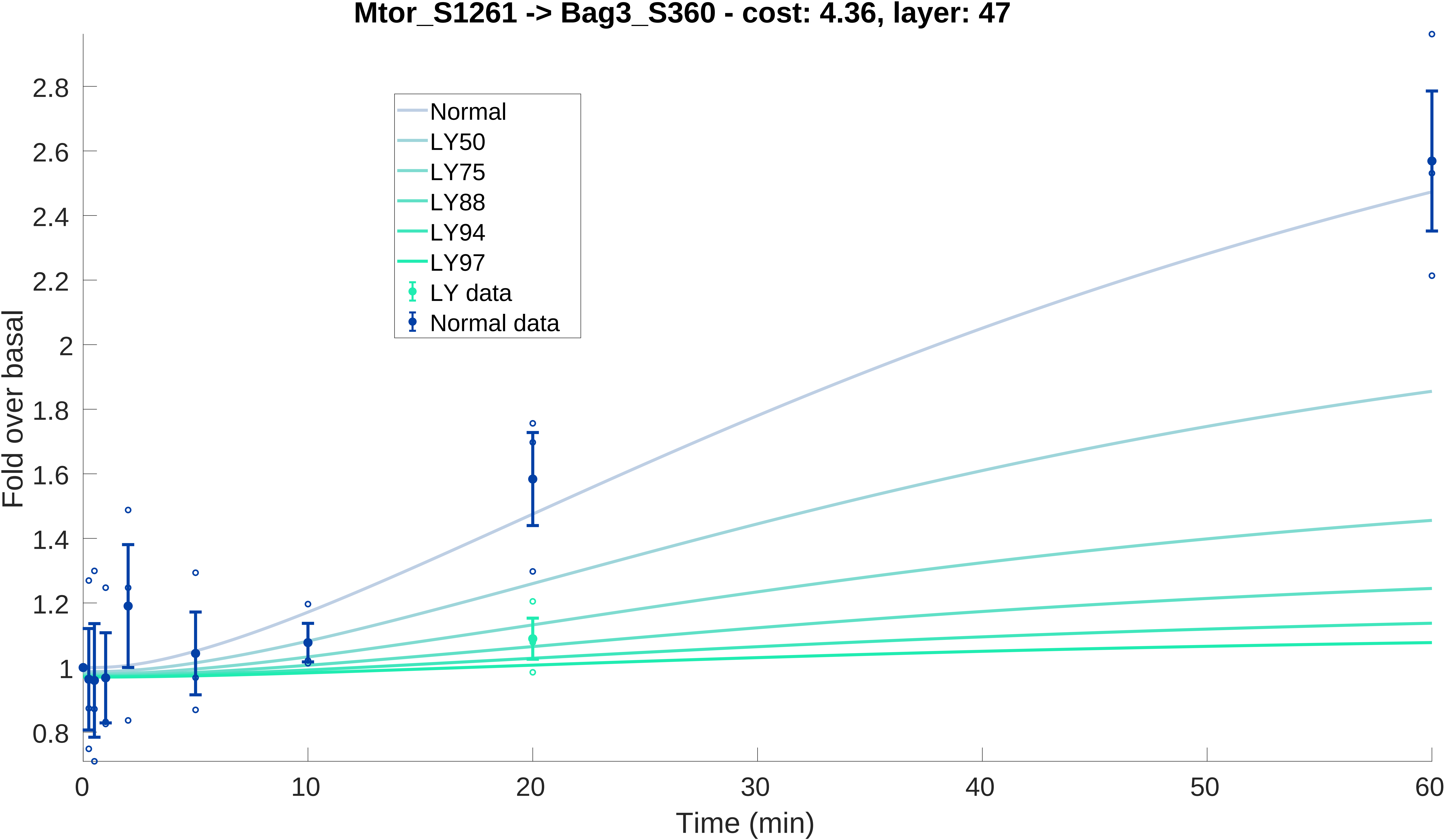
Mtor_S1261 -> Bag3_S360 - cost: 4.36, layer: 47
Normal
LY50
LY75
LY88
LY94
LY97
LY data
Normal data
Fold over basal
Time (min)
0.8
1
1.2
1.4
1.6
1.8
2
2.2
2.4
2.6
2.8
0
10
20
30
40
50
60

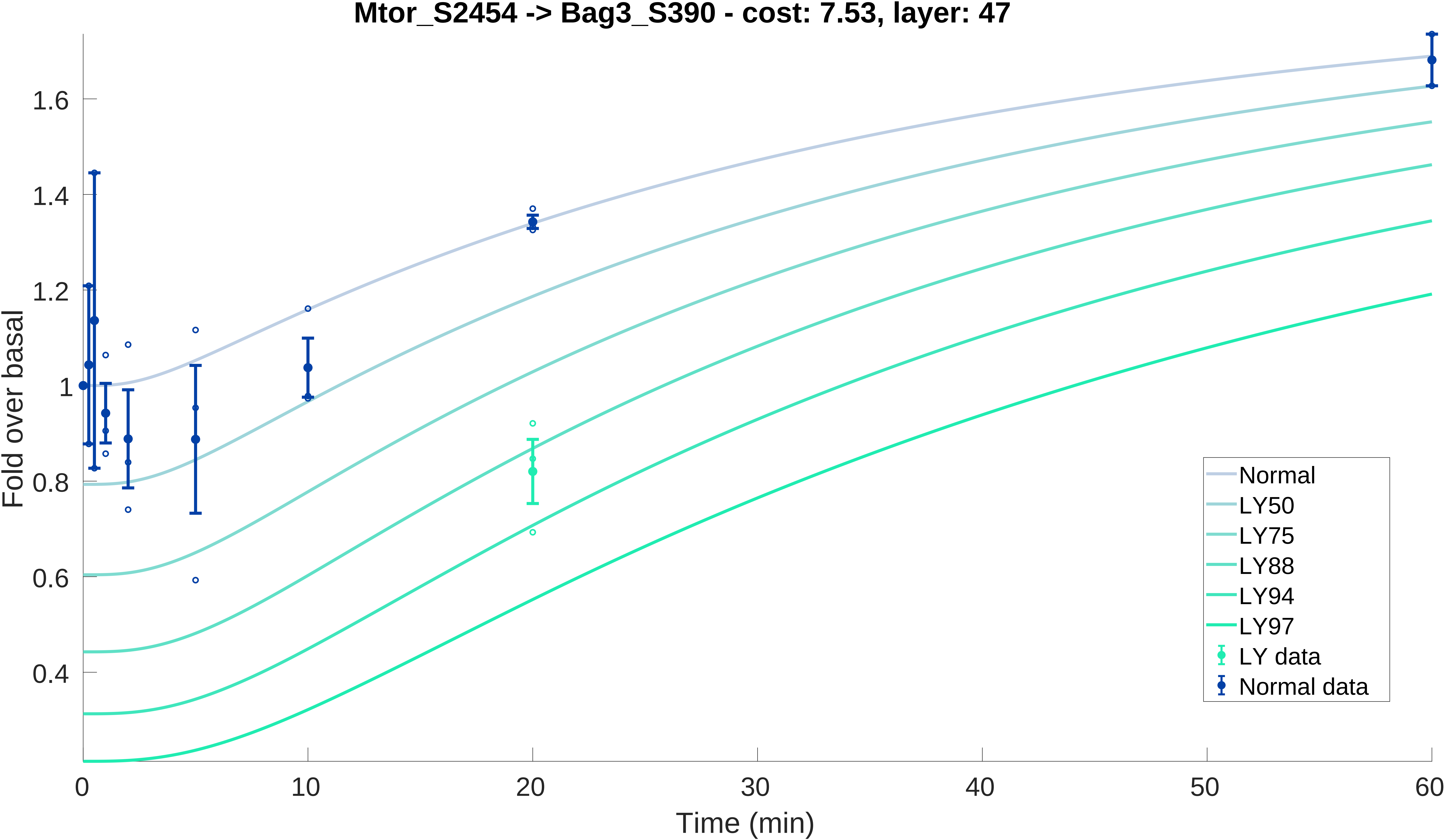

Mtor_S2454 -> Bag3_S390 - cost: 7.53, layer: 47
Fold over basal
Time (min)
1.6
1.4
1.2
1
0.8
0.6
0.4
0
10
20
30
40
50
60
Normal
LY50
LY75
LY88
LY94
LY97
LY data
Normal data

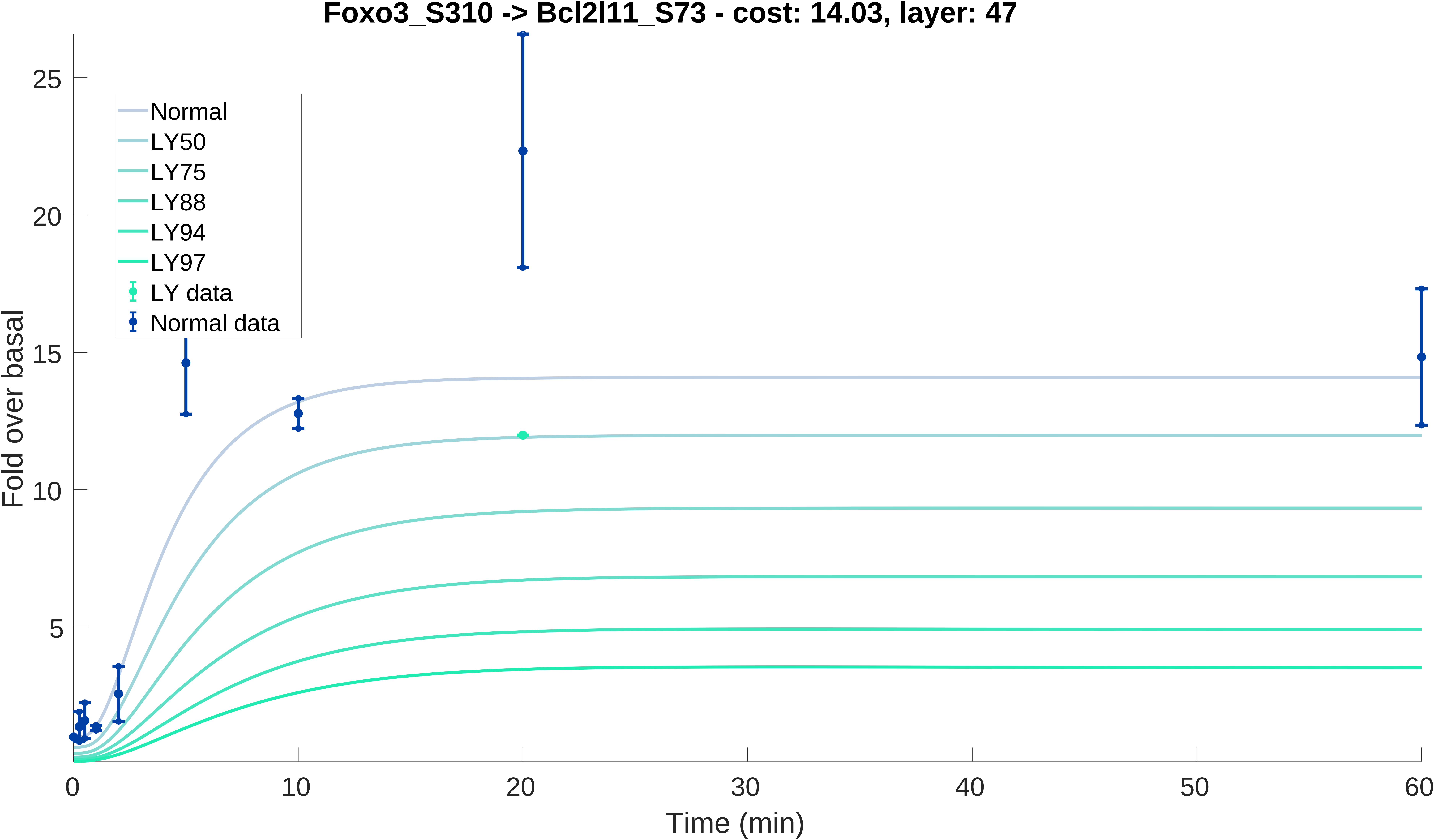

Foxo3_S310 -> Bcl2l11_S73 - cost: 14.03, layer: 47
Fold over basal
Time (min)
0
10
20
30
40
50
60
5
10
15
20
25
Normal
LY50
LY75
LY88
LY94
LY97
LY data
Normal data

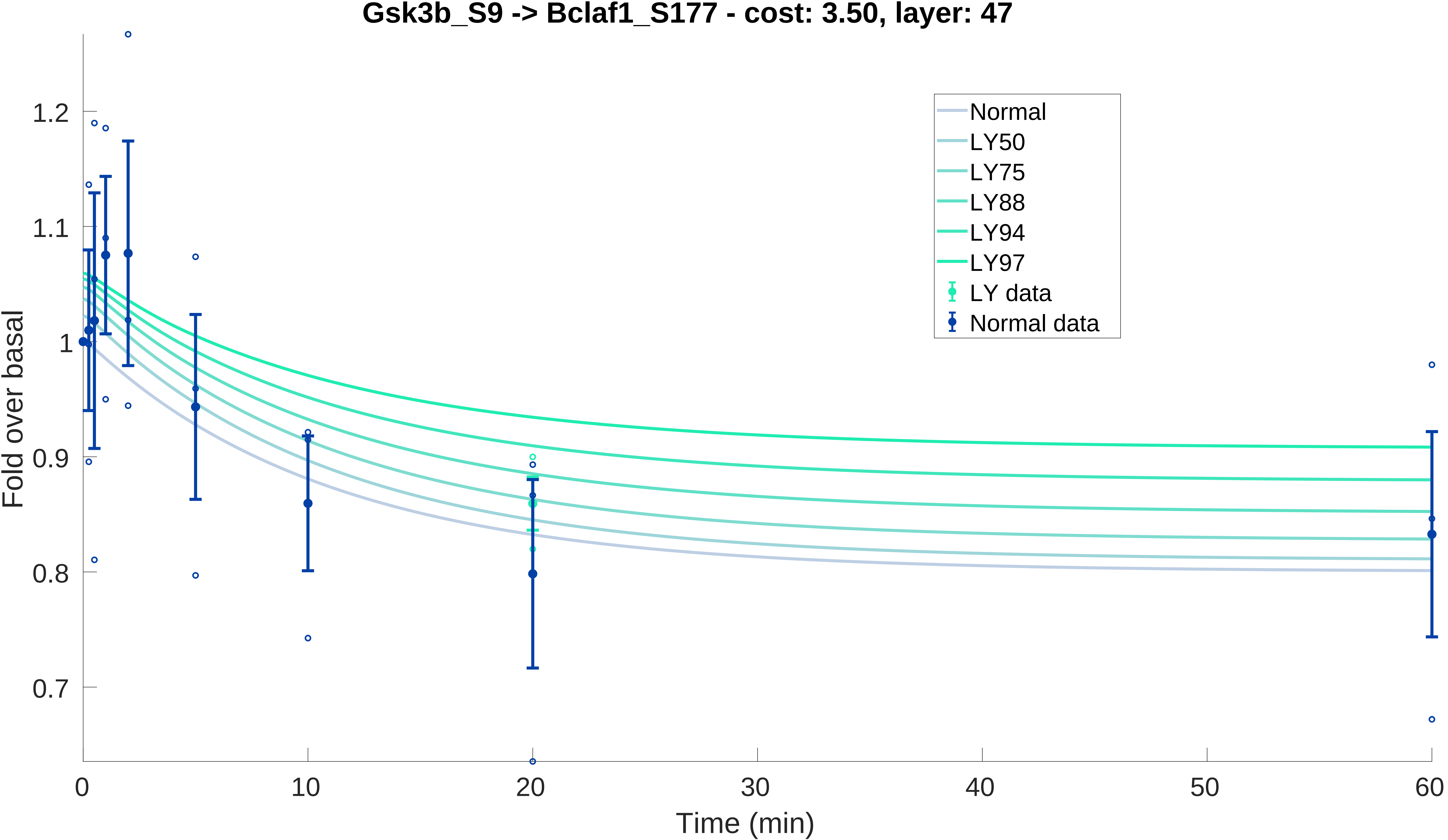
Gsk3b_S9 -> Bclaf1_S177 - cost: 3.50, layer: 47
Normal
LY50
LY75
LY88
LY94
LY97
LY data
Normal data
Fold over basal
Time (min)
0.7
0.8
0.9
1
1.1
1.2
0
10
20
30
40
50
60

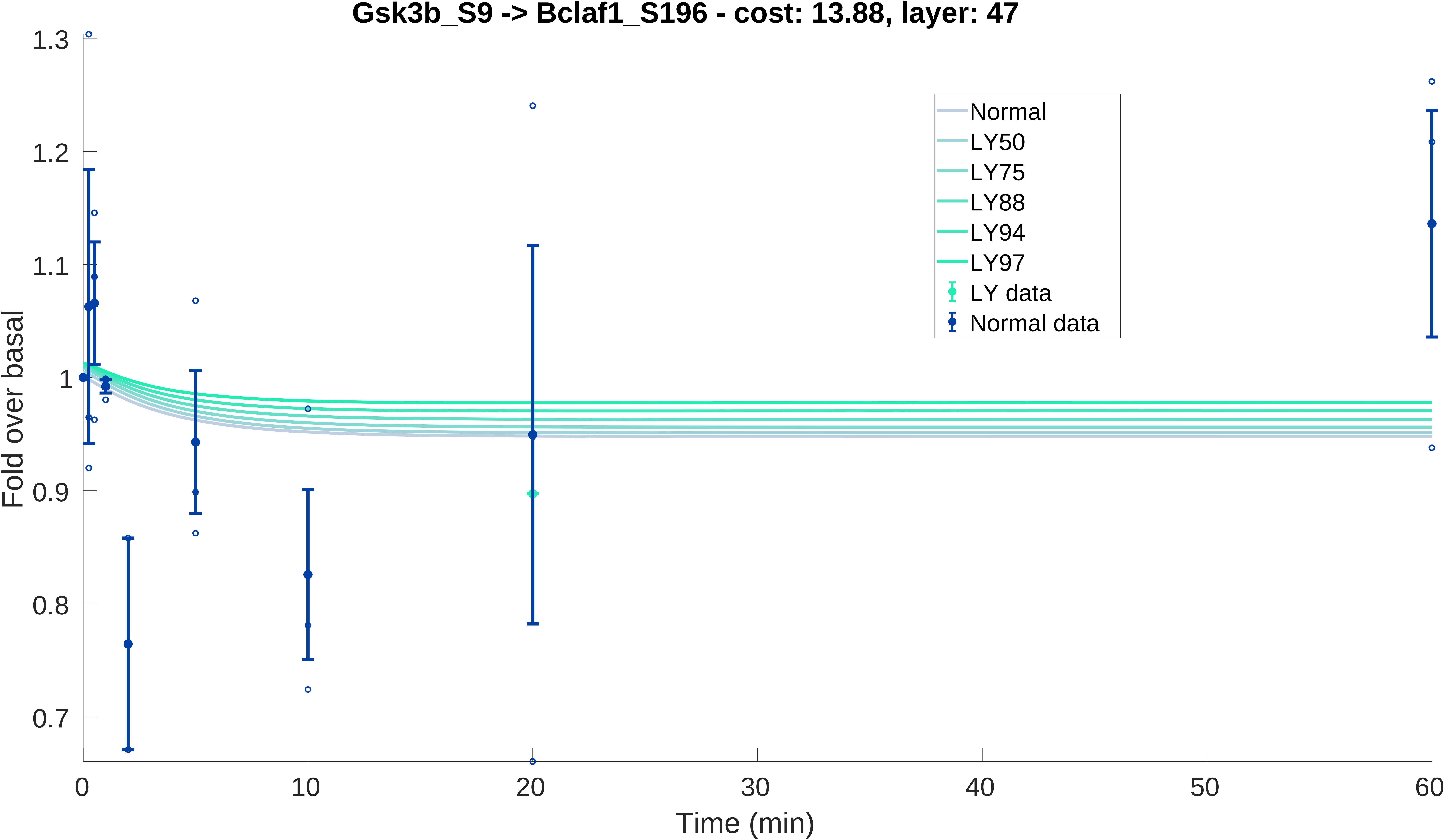

Gsk3b_S9 -> Bclaf1_S196 - cost: 13.88, layer: 47
Fold over basal
Time (min)
1.3
1.2
1.1
1
0.9
0.8
0.7
0
10
20
30
40
50
60
Normal
LY50
LY75
LY88
LY94
LY97
LY data
Normal data

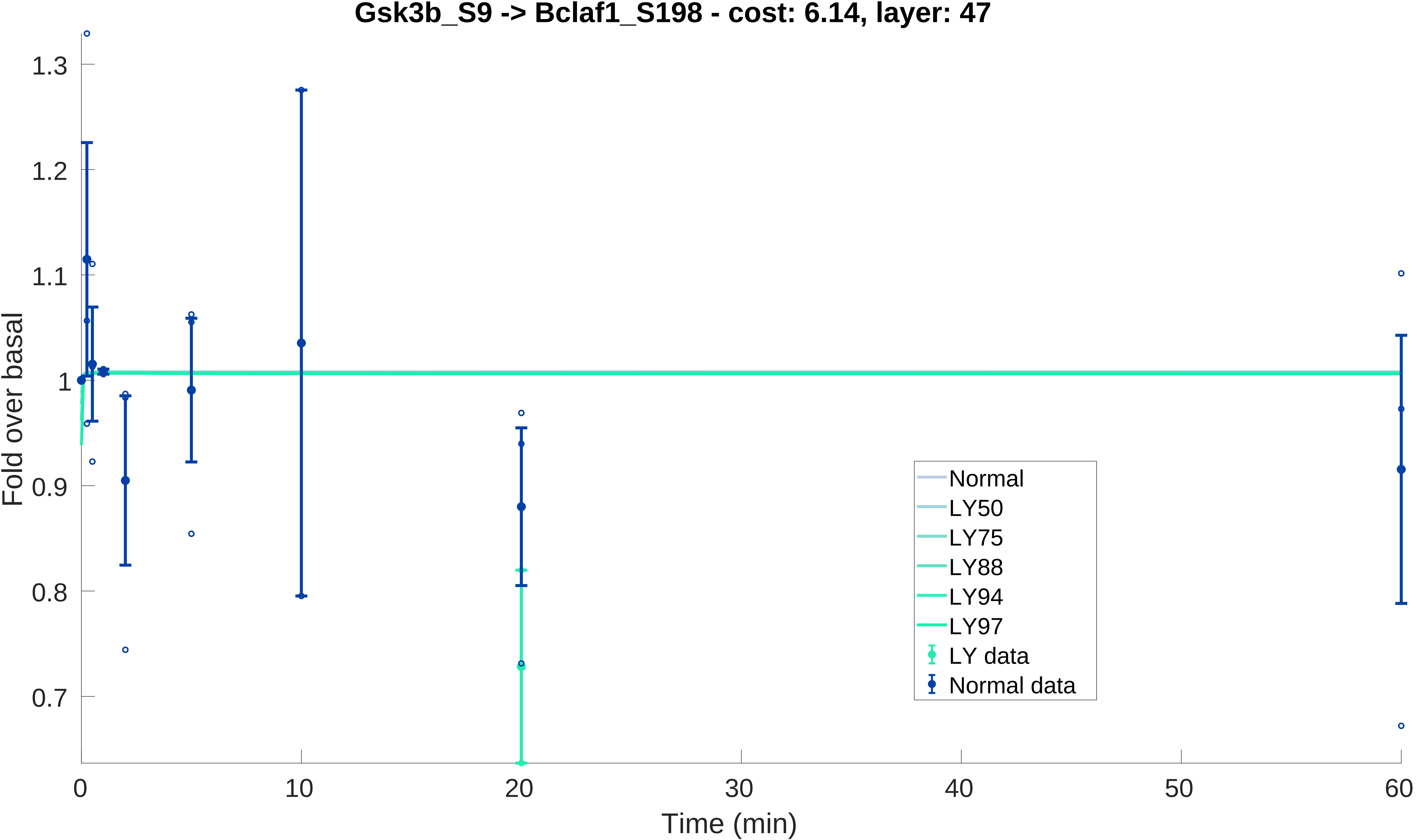

Gsk3b_S9 -> Bclaf1_S198 - cost: 6.14, layer: 47
Fold over basal
Time (min)
1.3
1.2
1.1
1
0.9
0.8
0.7
0
10
20
30
40
50
60
Normal
LY50
LY75
LY88
LY94
LY97
LY data
Normal data

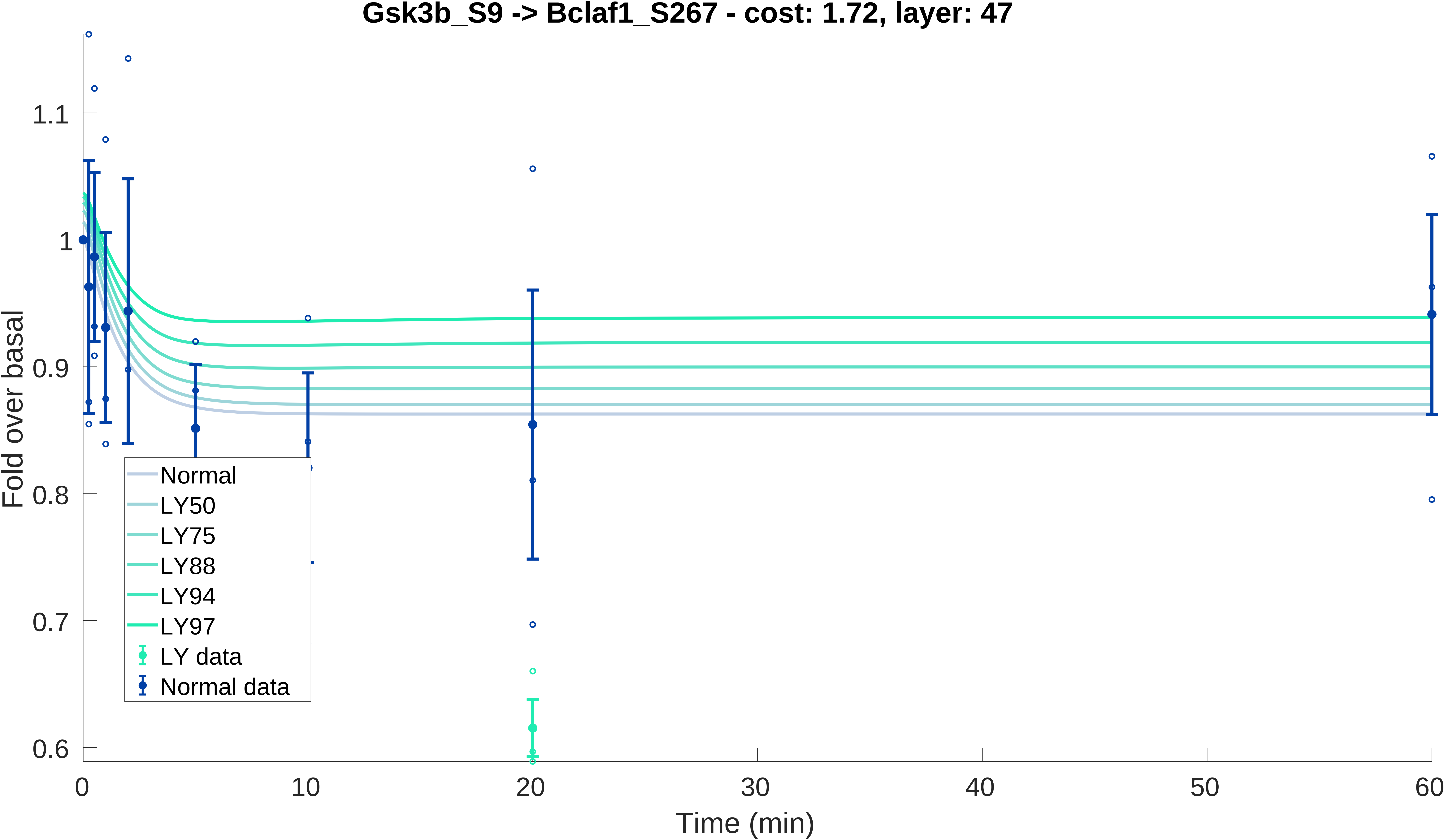

Gsk3b_S9 -> Bclaf1_S267 - cost: 1.72, layer: 47
Fold over basal
Time (min)
1.1
1
0.9
0.8
0.7
0.6
0
10
20
30
40
50
60
Normal
LY50
LY75
LY88
LY94
LY97
LY data
Normal data

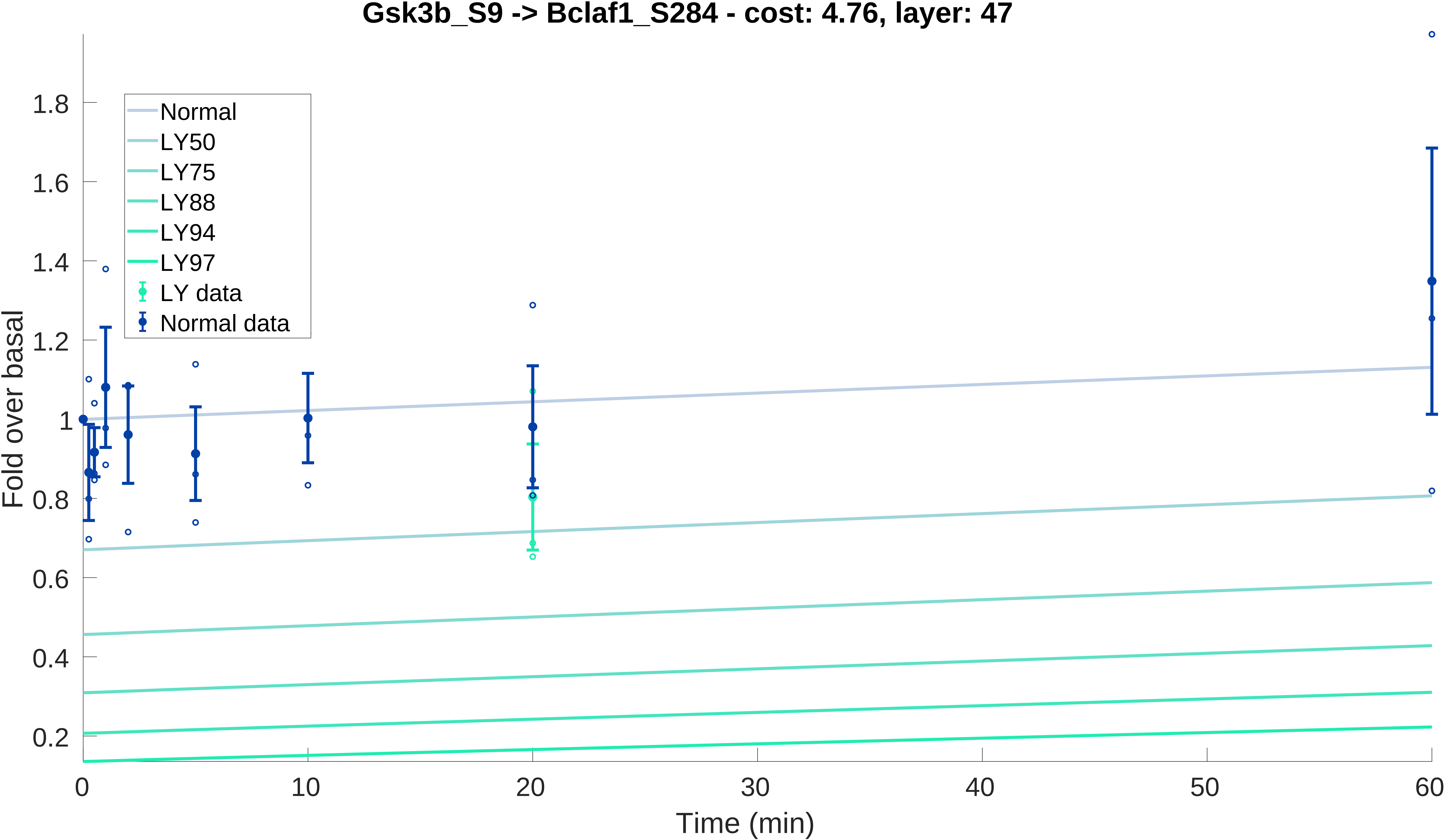
Gsk3b_S9 -> Bclaf1_S284 - cost: 4.76, layer: 47
Normal
LY50
LY75
LY88
LY94
LY97
LY data
Normal data
Fold over basal
Time (min)
0.2
0.4
0.6
0.8
1
1.2
1.4
1.6
1.8
0
10
20
30
40
50
60

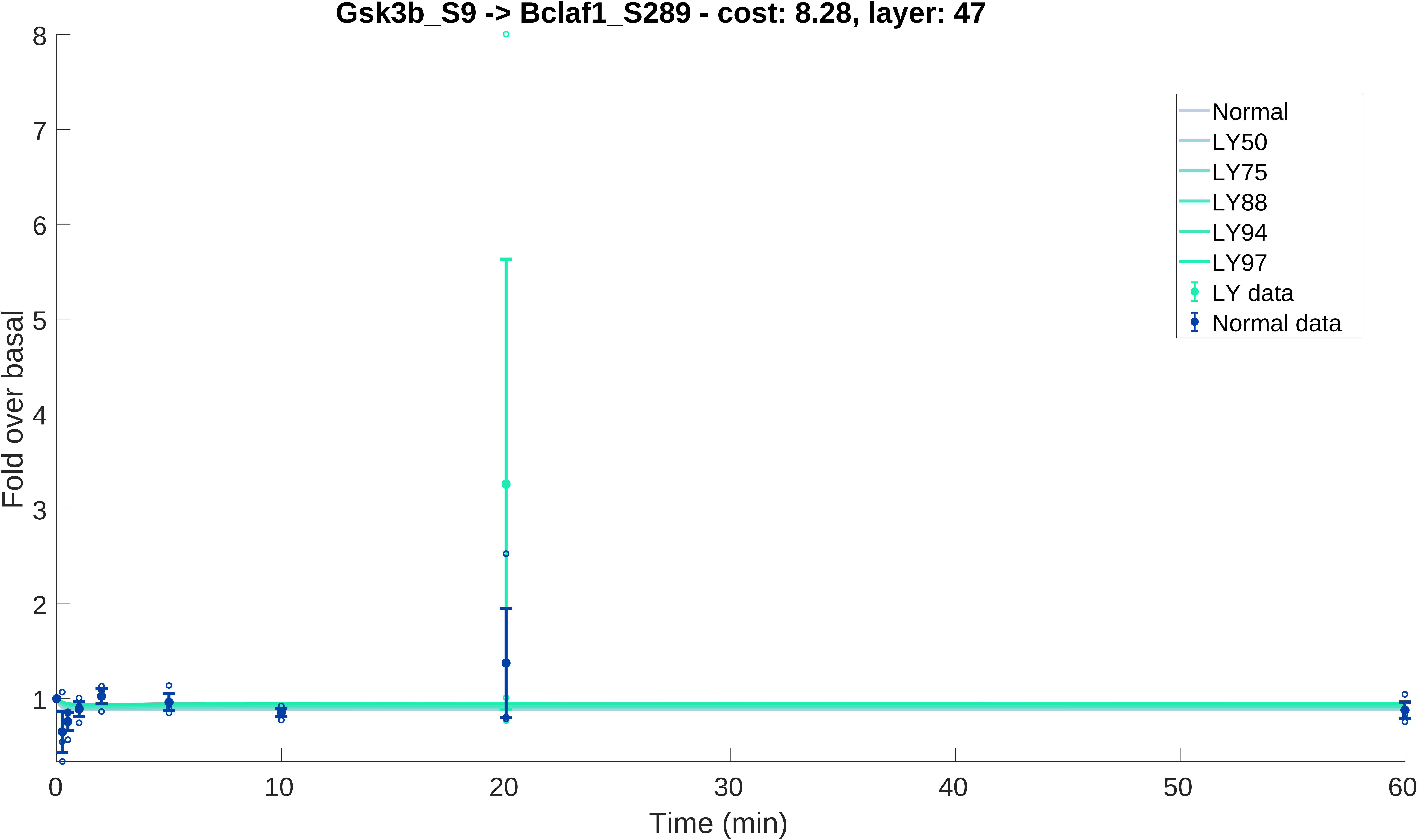

Gsk3b_S9 -> Bclaf1_S289 - cost: 8.28, layer: 47
Fold over basal
Time (min)
0
10
20
30
40
50
60
1
2
3
4
5
6
7
8
Normal
LY50
LY75
LY88
LY94
LY97
LY data
Normal data

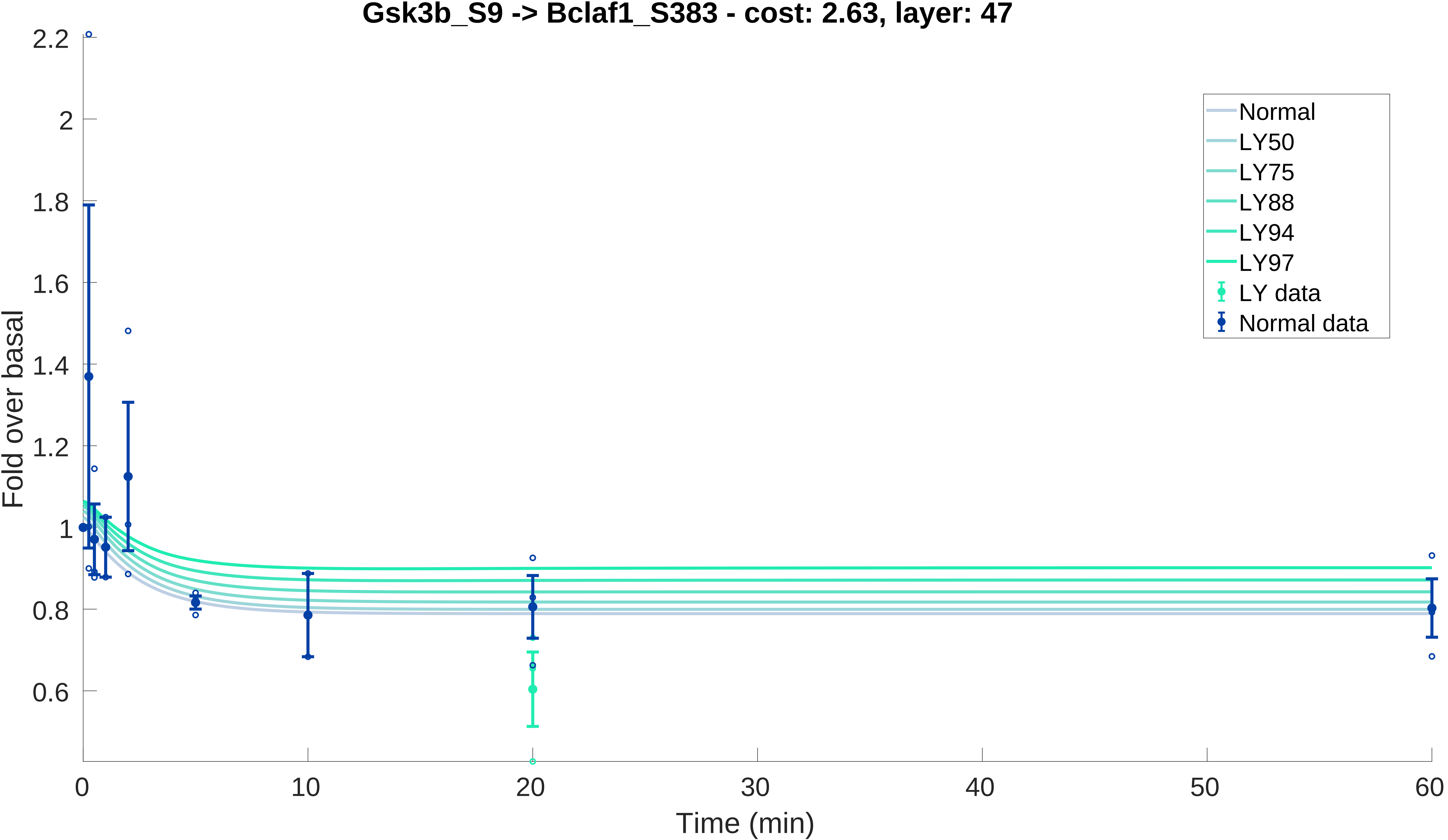

Gsk3b_S9 -> Bclaf1_S383 - cost: 2.63, layer: 47
Fold over basal
Time (min)
2.2
2
1.8
1.6
1.4
1.2
1
0.8
0.6
0
10
20
30
40
50
60
Normal
LY50
LY75
LY88
LY94
LY97
LY data
Normal data

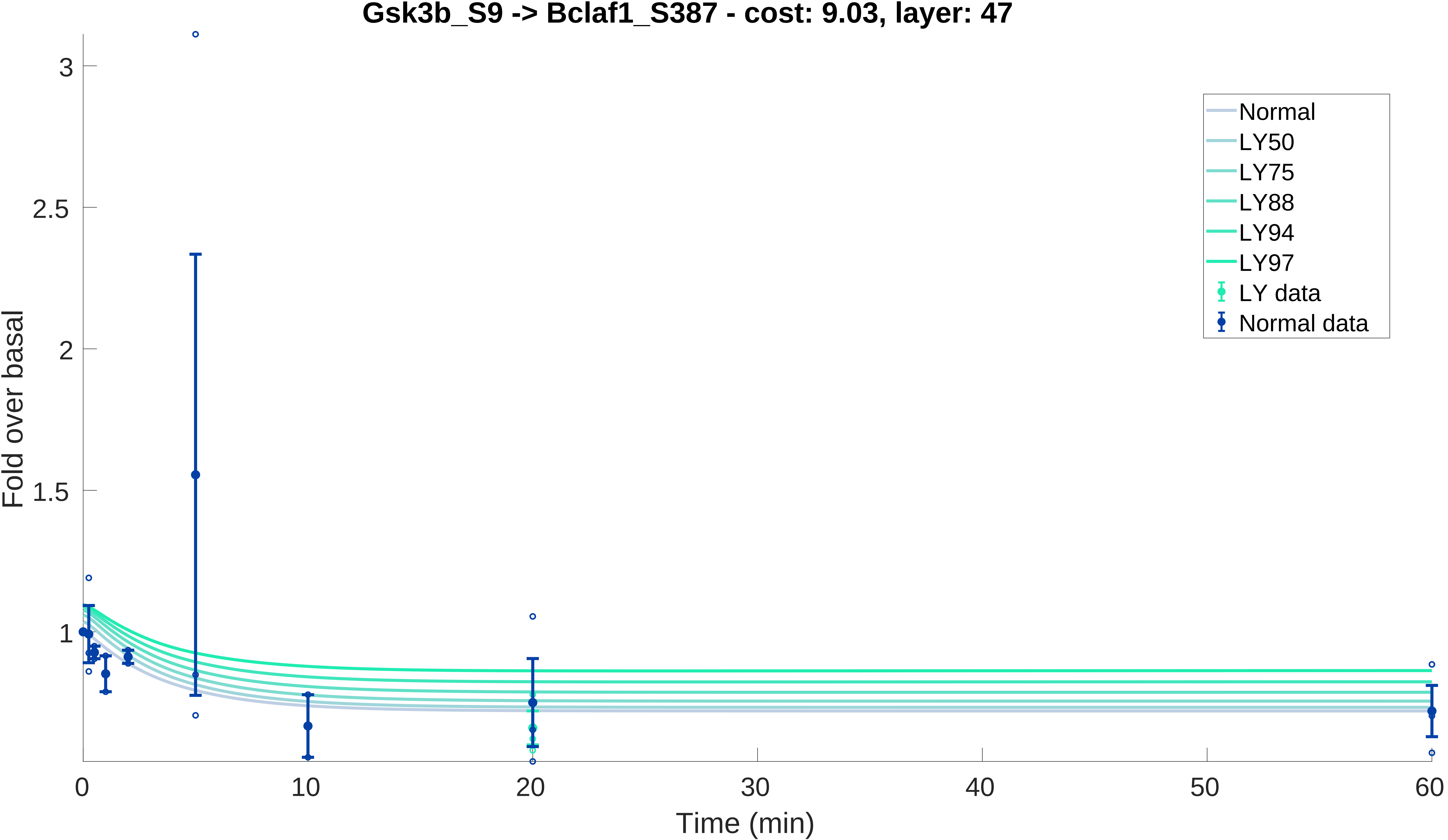

Gsk3b_S9 -> Bclaf1_S387 - cost: 9.03, layer: 47
Fold over basal
Time (min)
Normal
LY50
LY75
LY88
LY94
LY97
LY data
Normal data
0
10
20
30
40
50
60
1
1.5
2
2.5
3

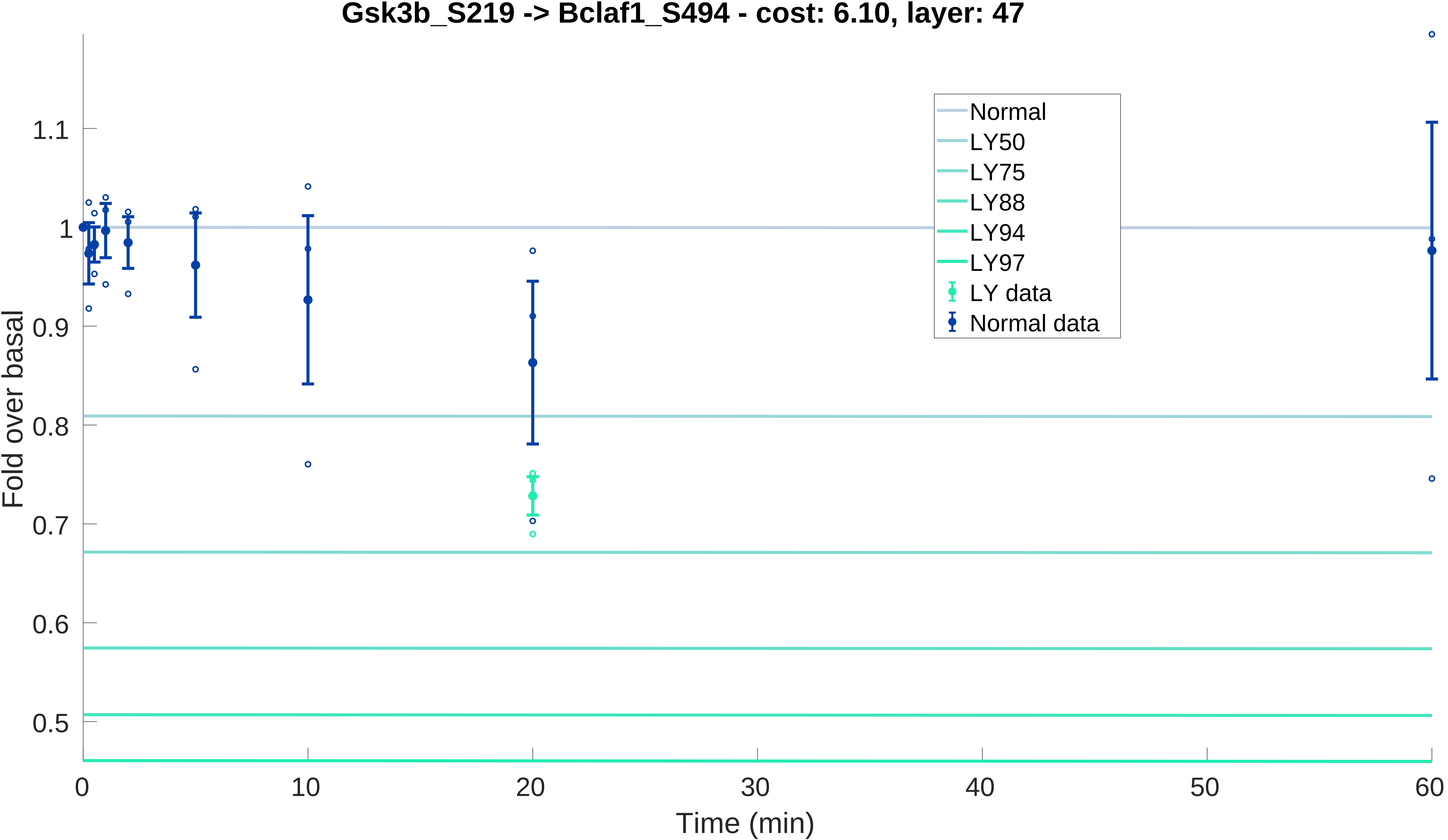

Gsk3b_S219 -> Bclaf1_S494 - cost: 6.10, layer: 47
Fold over basal
Time (min)
0.5
0.6
0.7
0.8
0.9
1
1.1
0
10
20
30
40
50
60
Normal
LY50
LY75
LY88
LY94
LY97
LY data
Normal data

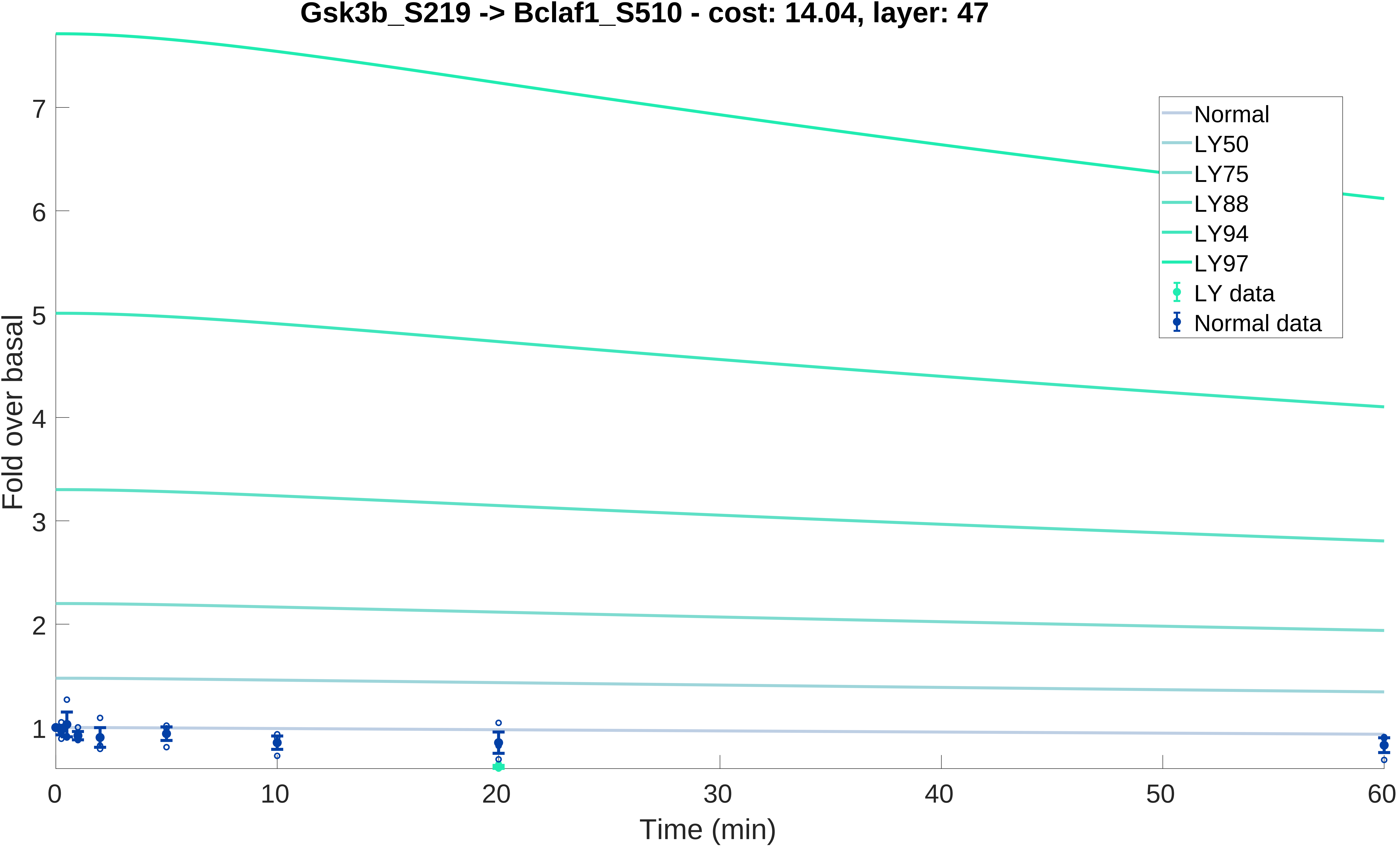
Gsk3b_S219 -> Bclaf1_S510 - cost: 14.04, layer: 47
Fold over basal
Time (min)
Normal
LY50
LY75
LY88
LY94
LY97
LY data
Normal data
0
10
20
30
40
50
60
1
2
3
4
5
6
7

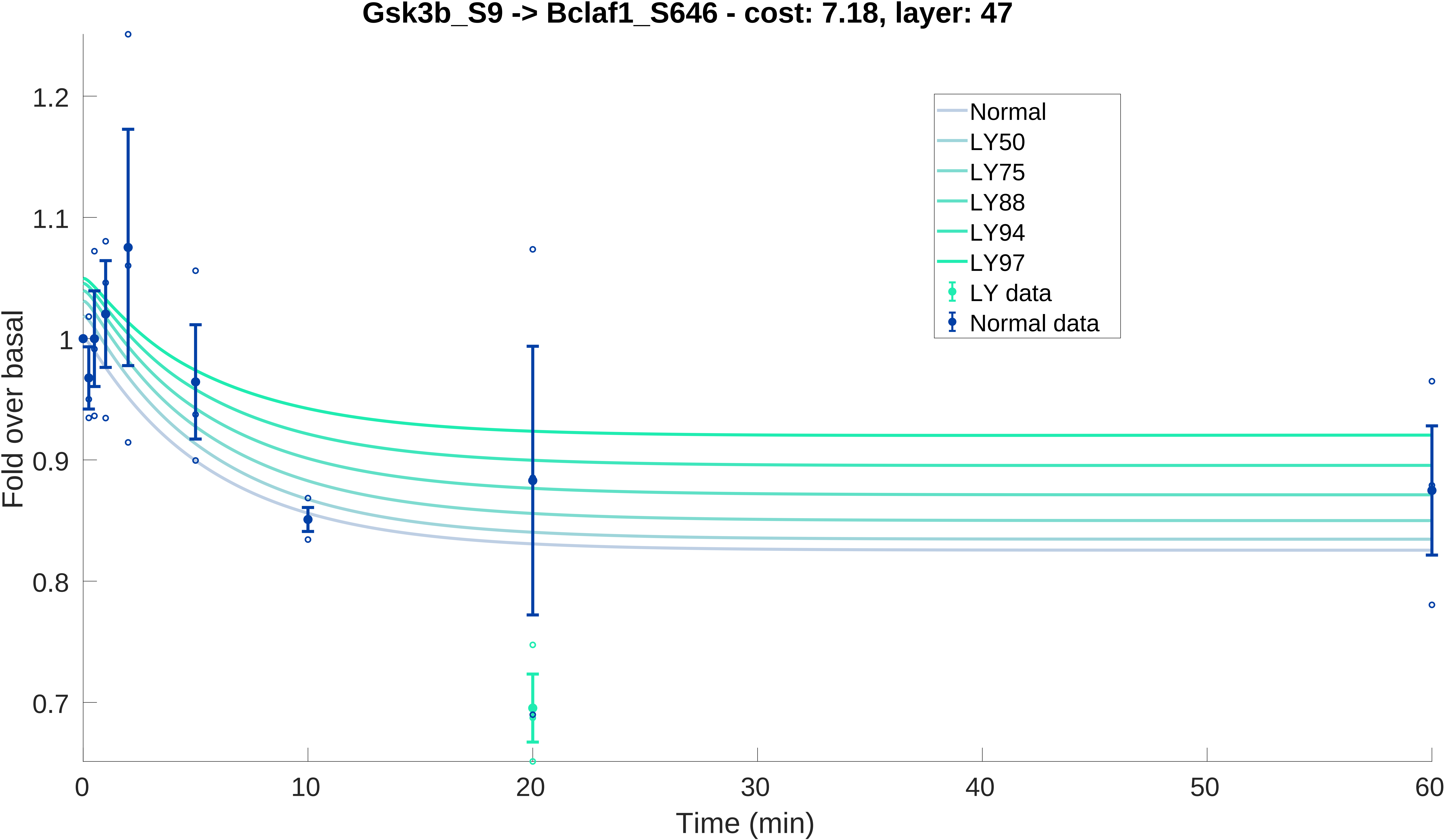

Gsk3b_S9 -> Bclaf1_S646 - cost: 7.18, layer: 47
Fold over basal
Time (min)
1.2
1.1
1
0.9
0.8
0.7
0
10
20
30
40
50
60
Normal
LY50
LY75
LY88
LY94
LY97
LY data
Normal data

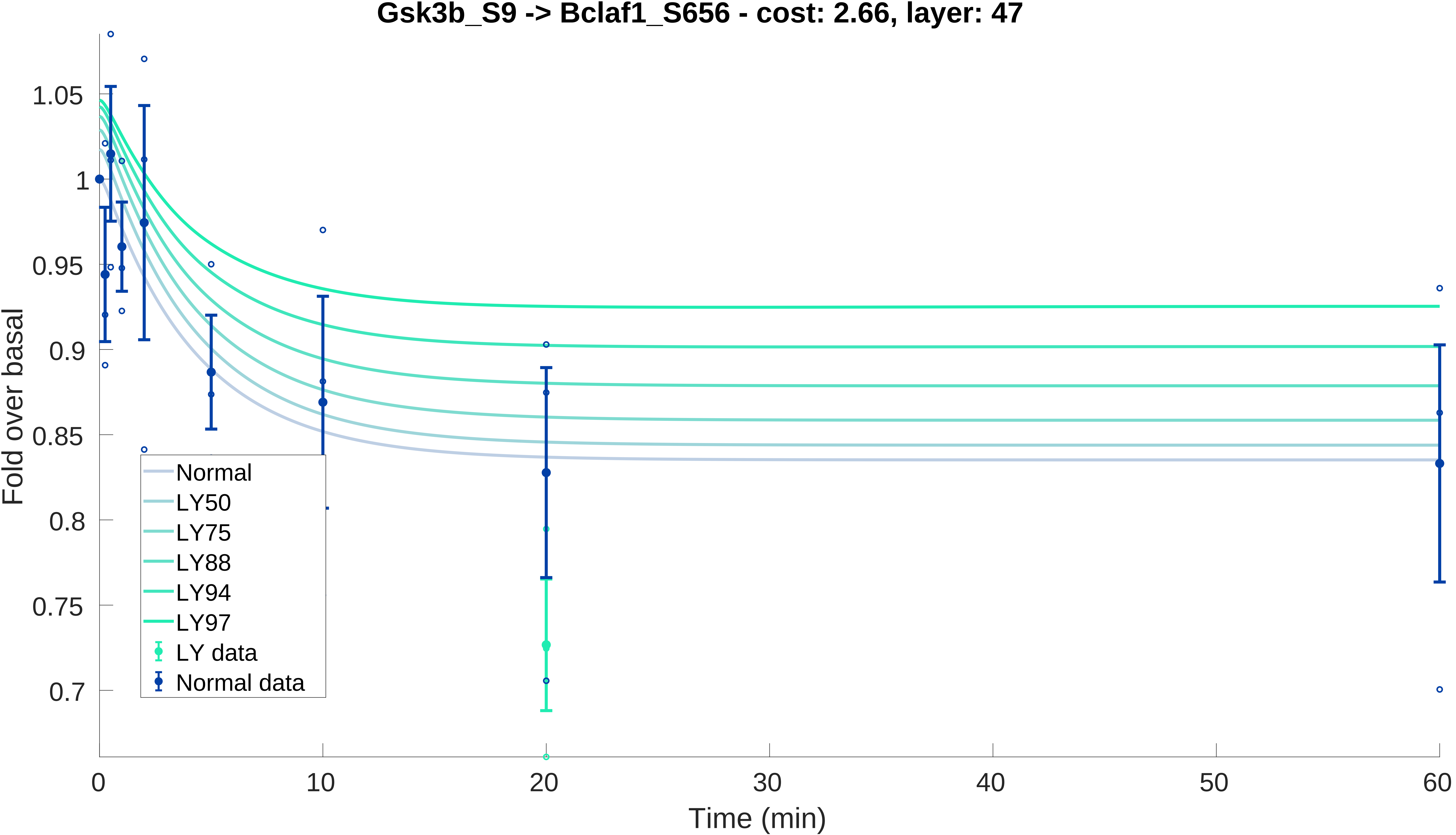

Gsk3b_S9 -> Bclaf1_S656 - cost: 2.66, layer: 47
Fold over basal
Time (min)
1.05
1
0.95
0.9
0.85
0.8
0.75
0.7
0
10
20
30
40
50
60
Normal
LY50
LY75
LY88
LY94
LY97
LY data
Normal data

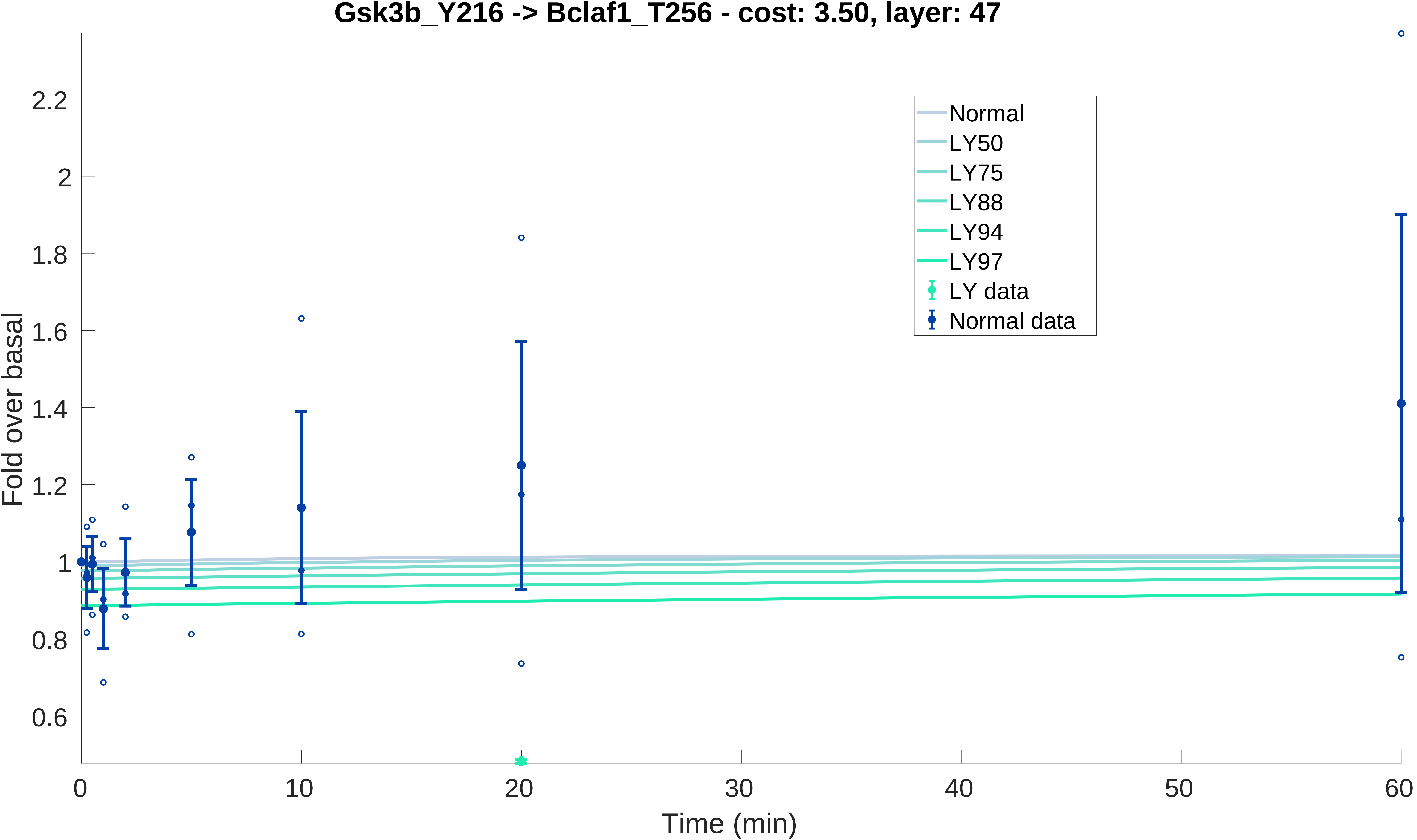

Gsk3b_Y216 -> Bclaf1_T256 - cost: 3.50, layer: 47
Normal
LY50
LY75
LY88
LY94
LY97
LY data
Normal data
Fold over basal
Time (min)
0.6
0.8
1
1.2
1.4
1.6
1.8
2
2.2
0
10
20
30
40
50
60

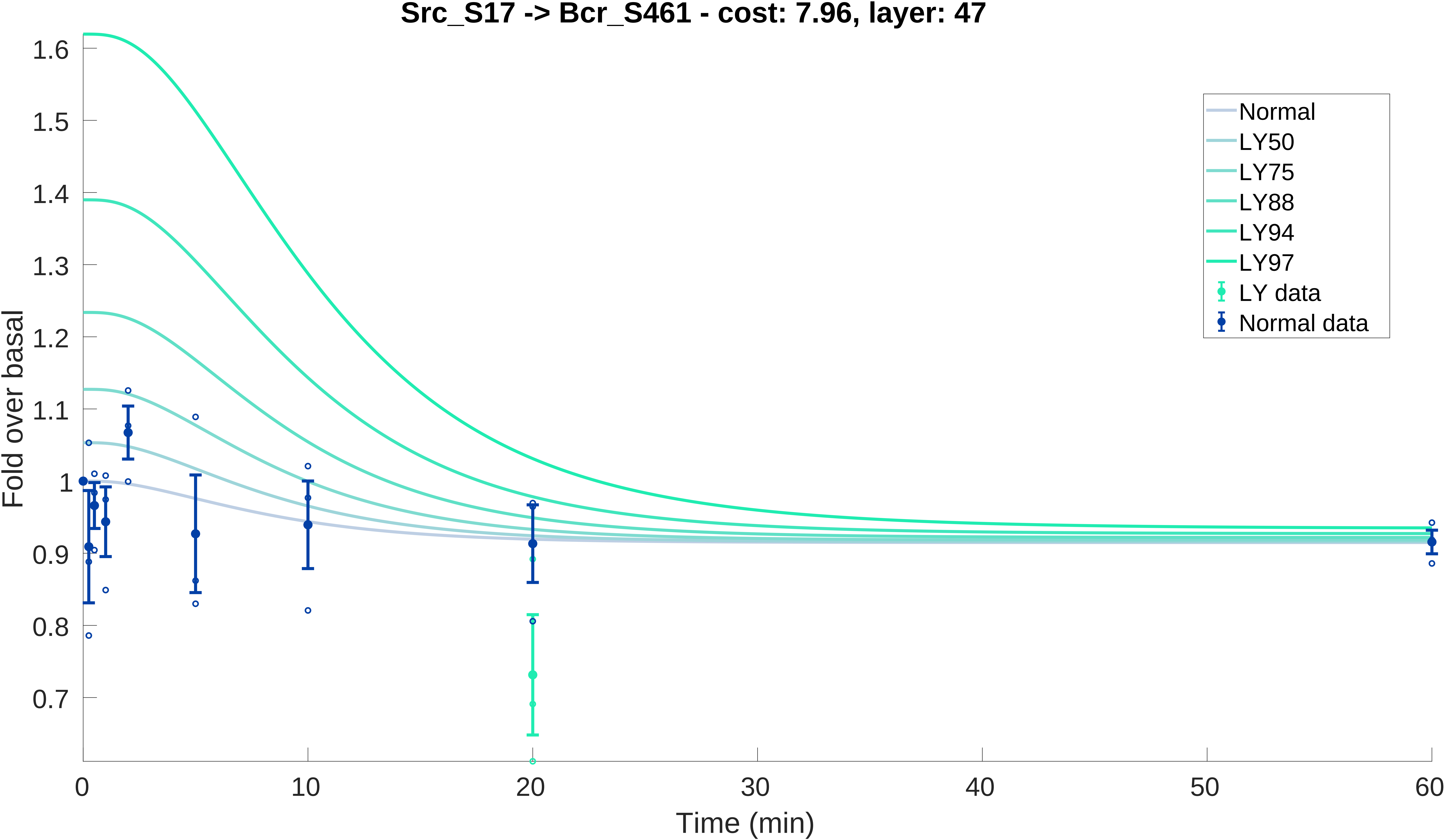

Src_S17 -> Bcr_S461 - cost: 7.96, layer: 47
Fold over basal
Time (min)
1.6
1.5
1.4
1.3
1.2
1.1
1
0.9
0.8
0.7
0
10
20
30
40
50
60
Normal
LY50
LY75
LY88
LY94
LY97
LY data
Normal data

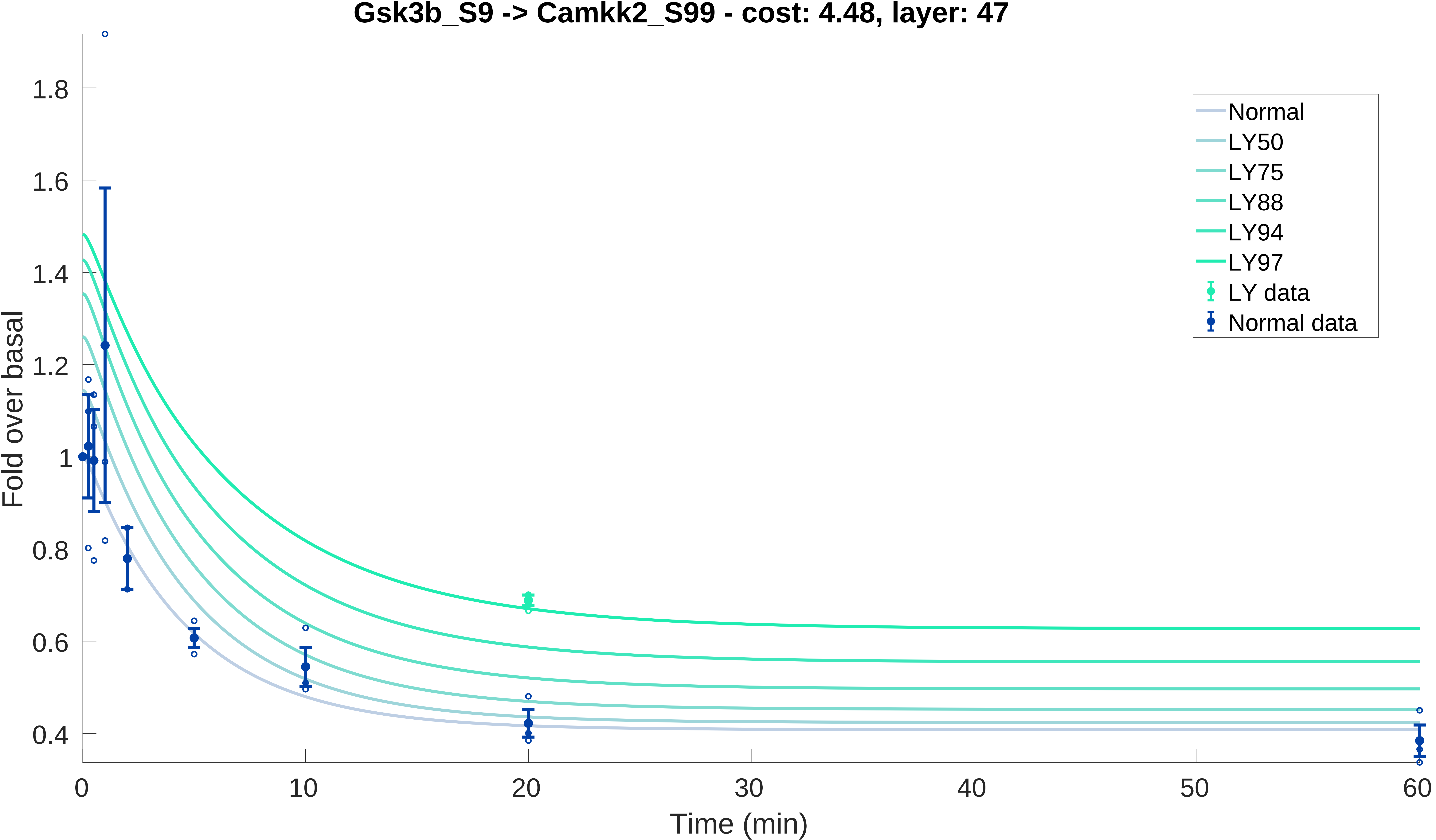
Gsk3b_S9 -> Camkk2_S99 - cost: 4.48, layer: 47
Fold over basal
Time (min)
Normal
LY50
LY75
LY88
LY94
LY97
LY data
Normal data
1.8
1.6
1.4
1.2
1
0.8
0.6
0.4
0
10
20
30
40
50
60

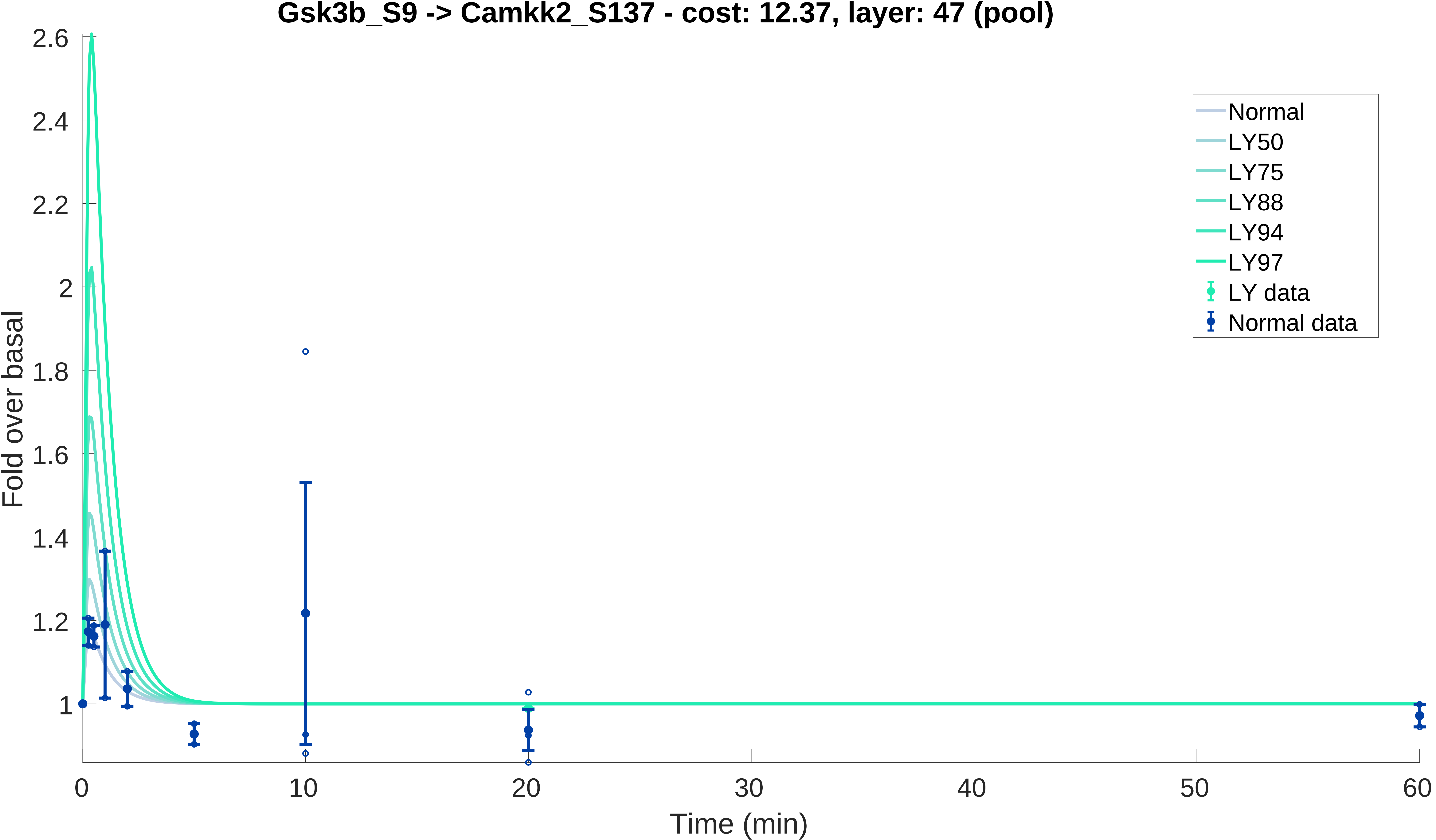
Gsk3b_S9 -> Camkk2_S137 - cost: 12.37, layer: 47 (pool)
Fold over basal
Time (min)
2.6
2.4
2.2
2
1.8
1.6
1.4
1.2
1
0
10
20
30
40
50
60
Normal
LY50
LY75
LY88
LY94
LY97
LY data
Normal data

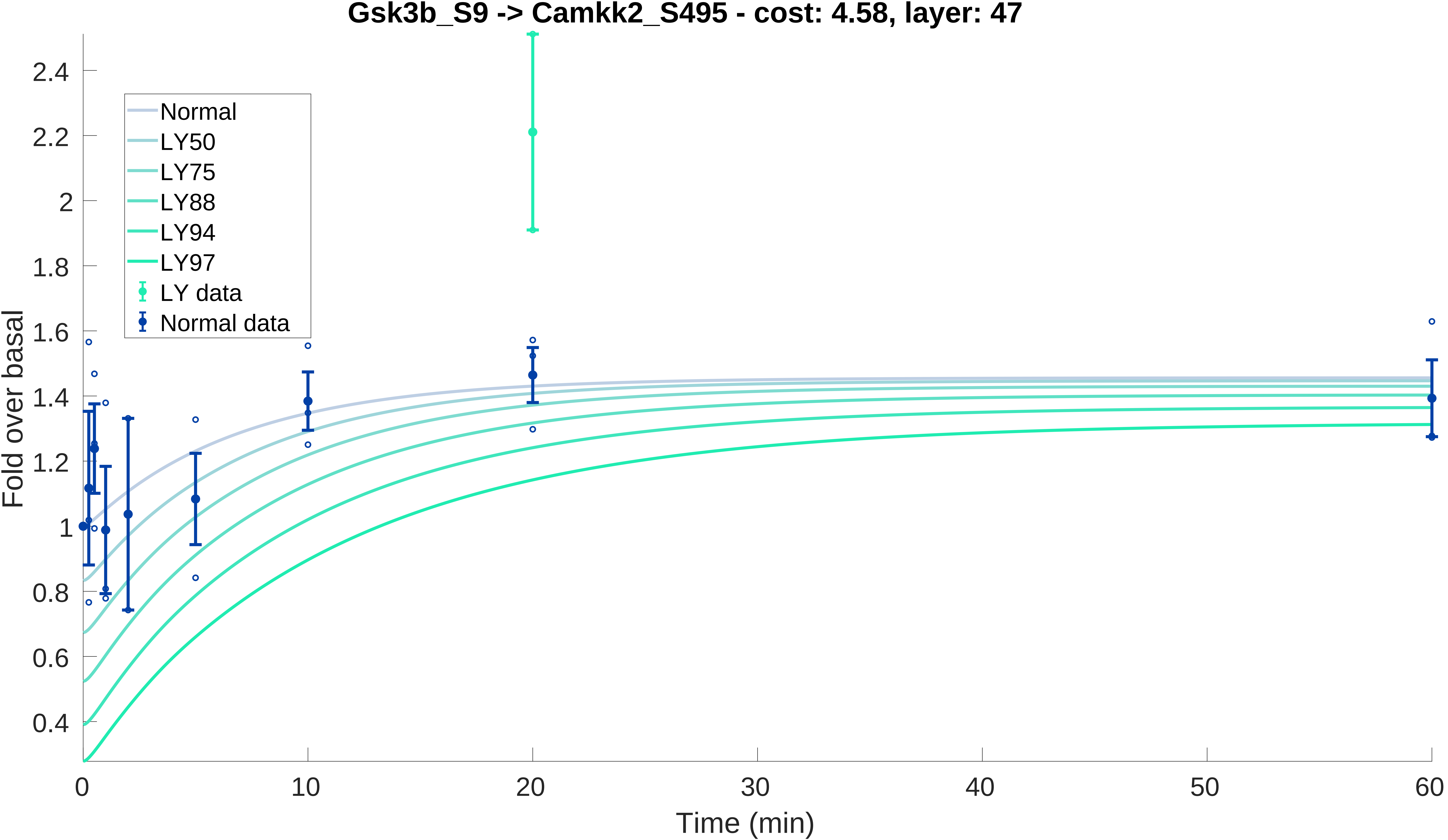

Gsk3b_S9 -> Camkk2_S495 - cost: 4.58, layer: 47
Normal
LY50
LY75
LY88
LY94
LY97
LY data
Normal data
Fold over basal
2.4
2.2
2
1.8
1.6
1.4
1.2
1
0.8
0.6
0.4
0
10
20
30
40
50
60
Time (min)

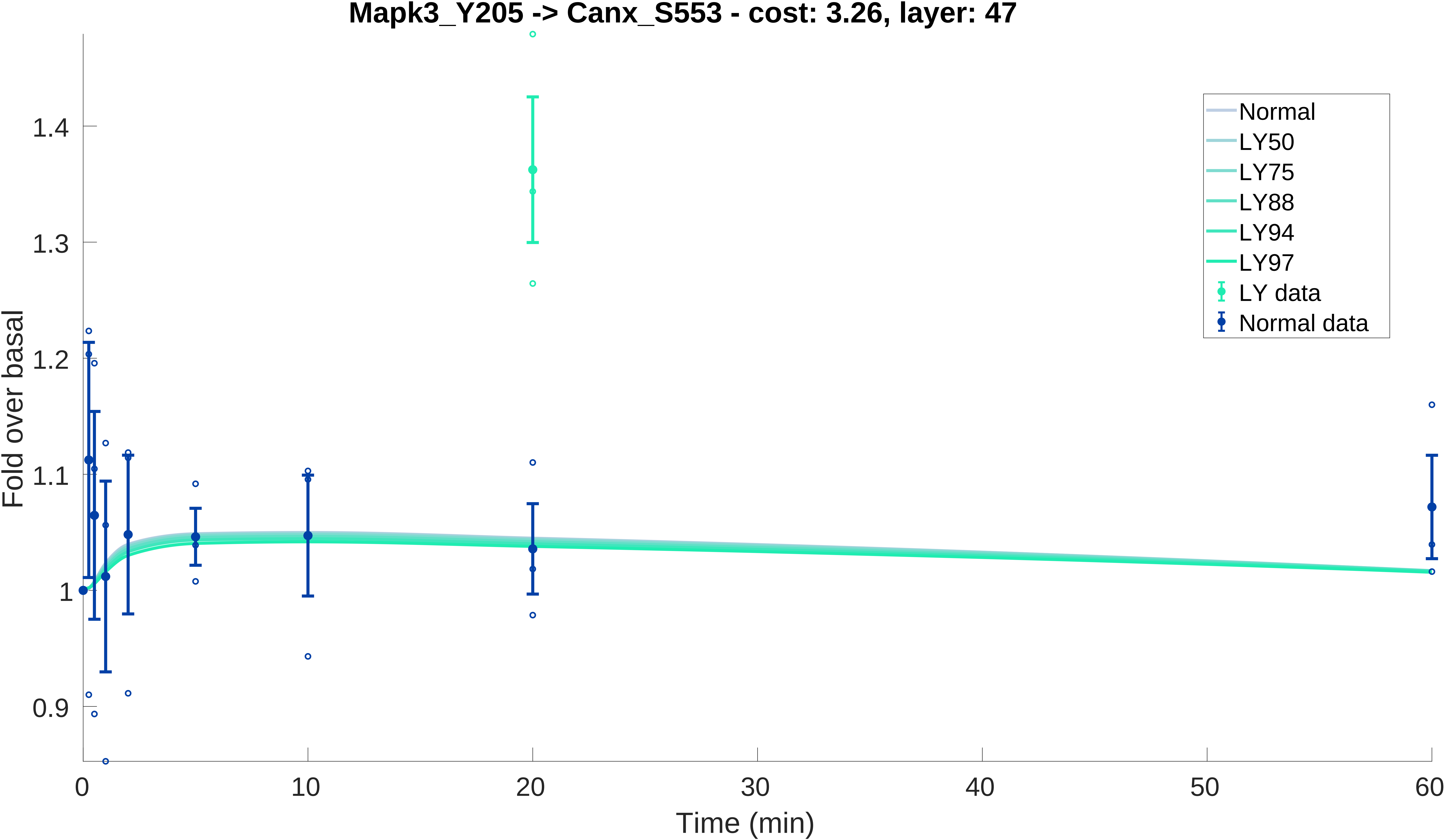
Mapk3_Y205 -> Canx_S553 - cost: 3.26, layer: 47
Fold over basal
Time (min)
0.9
1
1.1
1.2
1.3
1.4
0
10
20
30
40
50
60
Normal
LY50
LY75
LY88
LY94
LY97
LY data
Normal data

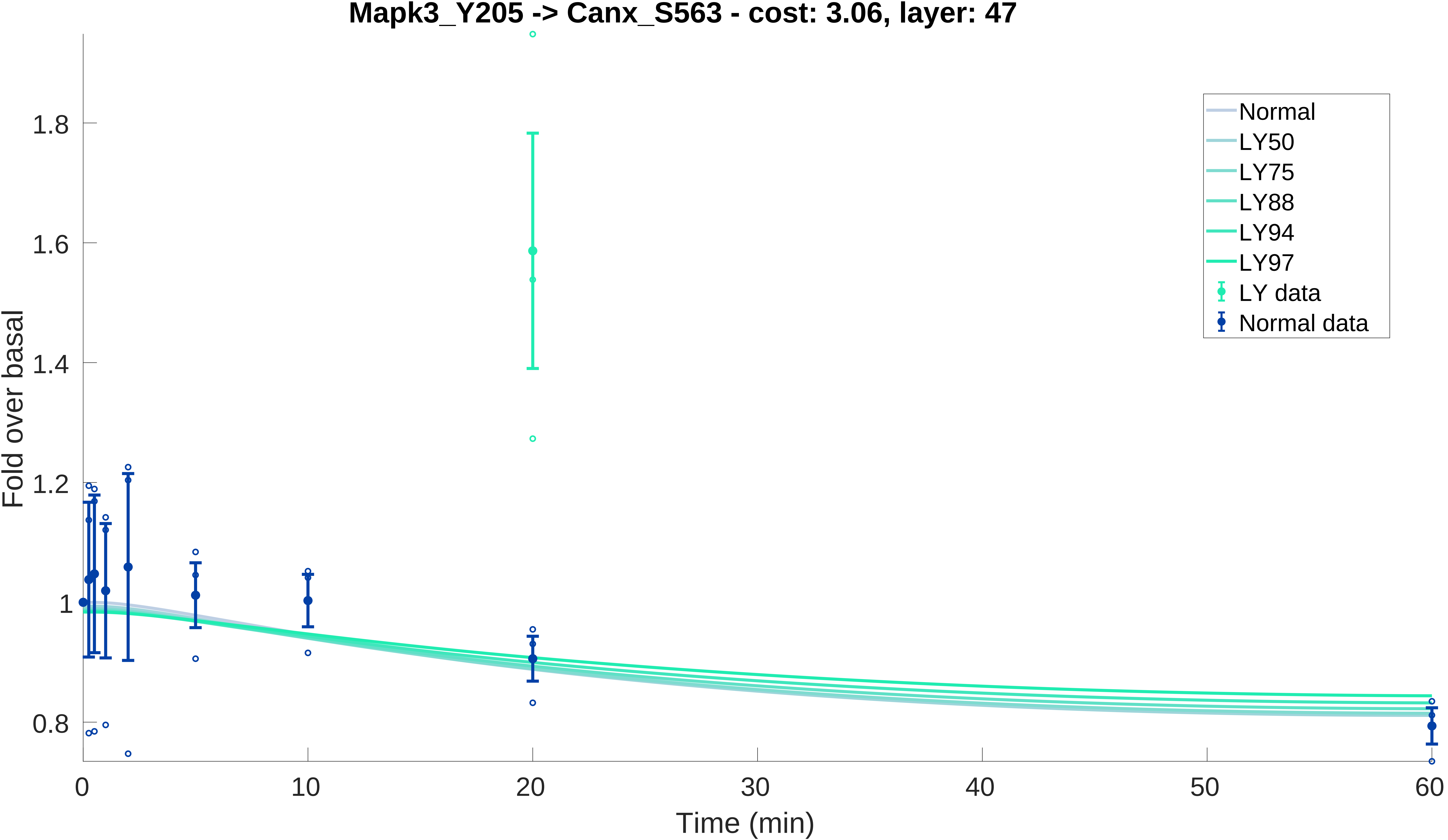
Mapk3_Y205 -> Canx_S563 - cost: 3.06, layer: 47
Fold over basal
Time (min)
0.8
1
1.2
1.4
1.6
1.8
0
10
20
30
40
50
60
Normal
LY50
LY75
LY88
LY94
LY97
LY data
Normal data

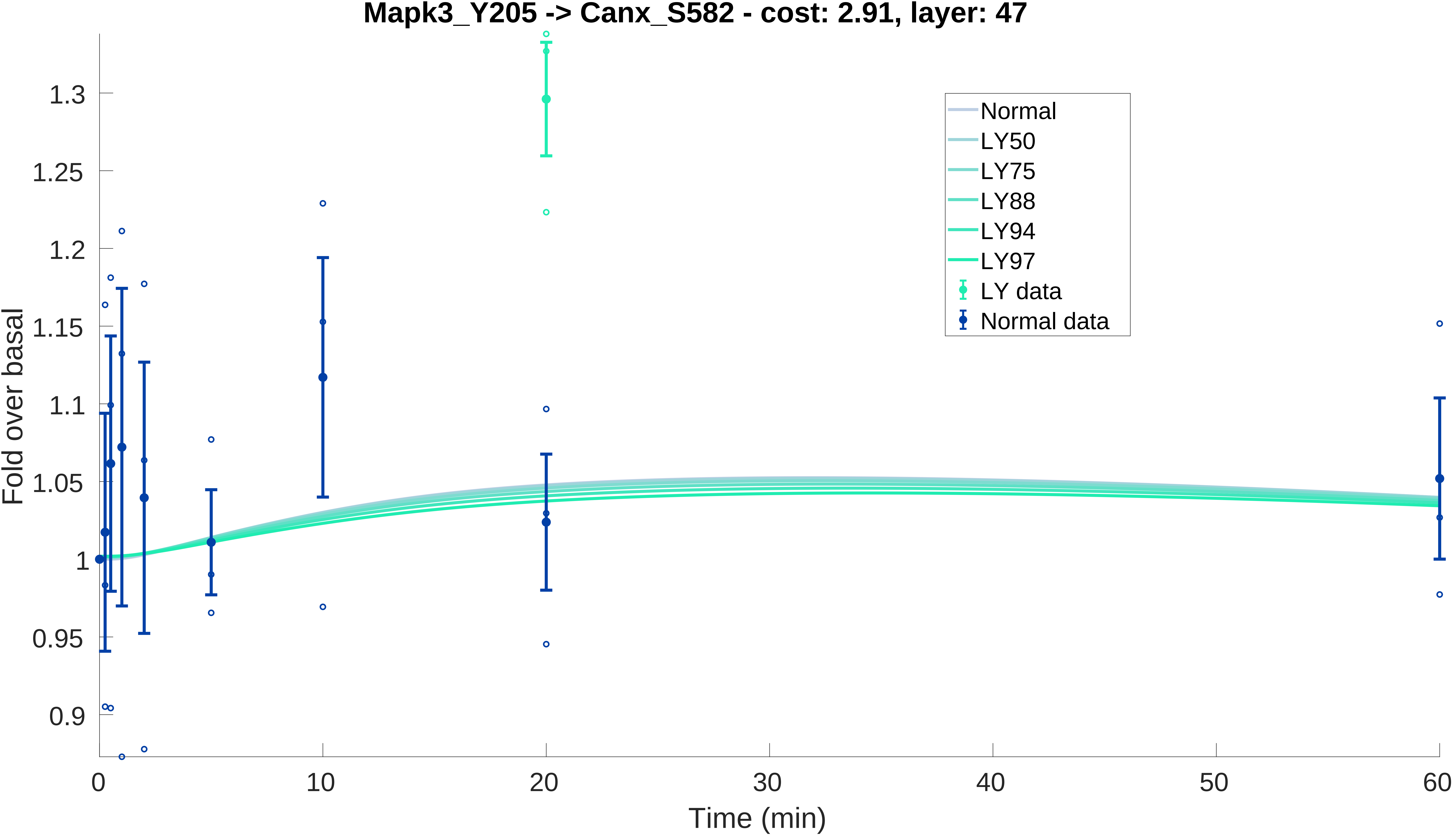

Mapk3_Y205 -> Canx_S582 - cost: 2.91, layer: 47
Fold over basal
1.3
1.25
1.2
1.15
1.1
1.05
1
0.95
0.9
0
10
20
30
40
50
60
Time (min)
Normal
LY50
LY75
LY88
LY94
LY97
LY data
Normal data

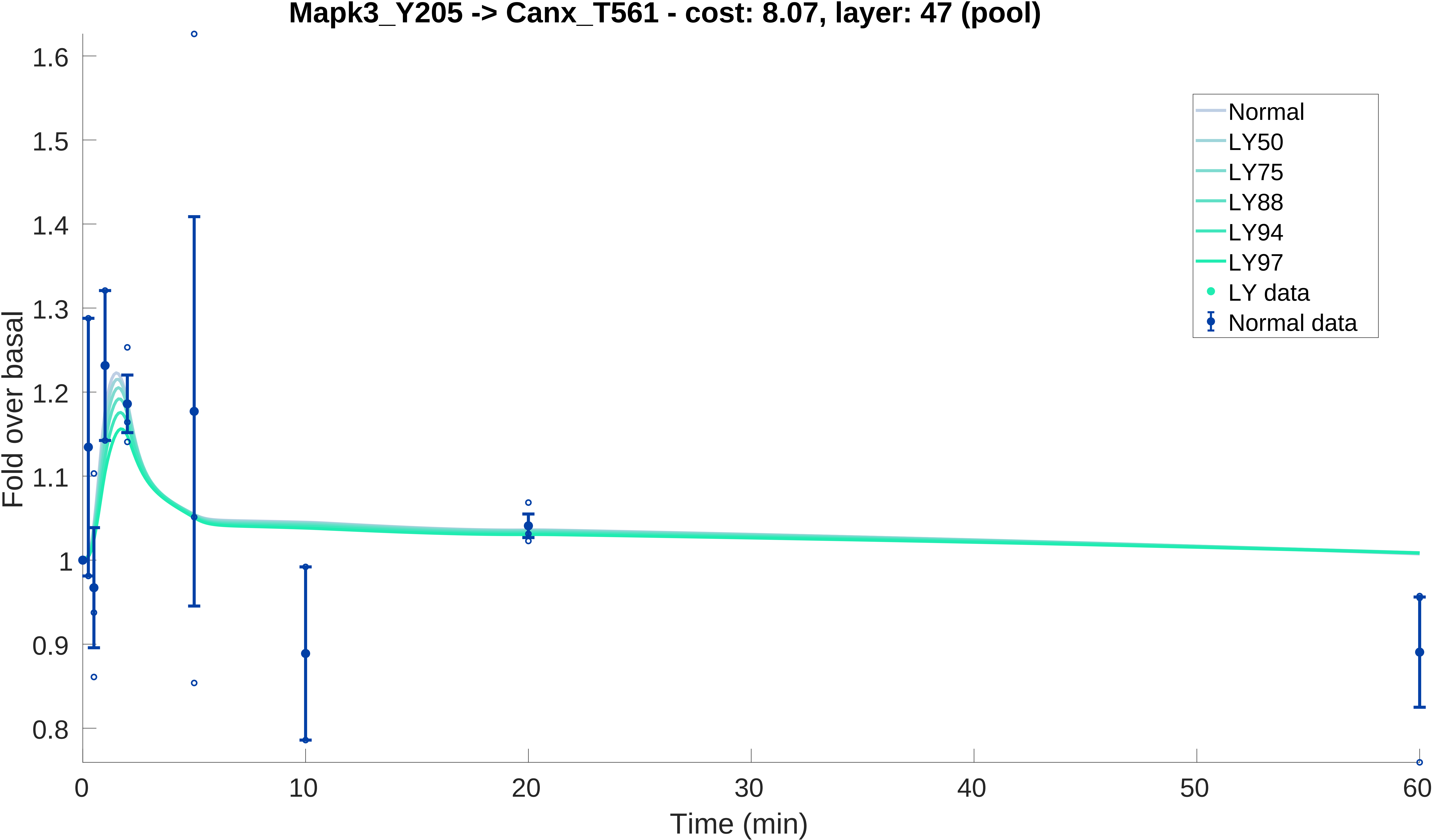

Mapk3_Y205 -> Canx_T561 - cost: 8.07, layer: 47 (pool)
Fold over basal
Time (min)
1.6
1.5
1.4
1.3
1.2
1.1
1
0.9
0.8
0
10
20
30
40
50
60
Normal
LY50
LY75
LY88
LY94
LY97
LY data
Normal data

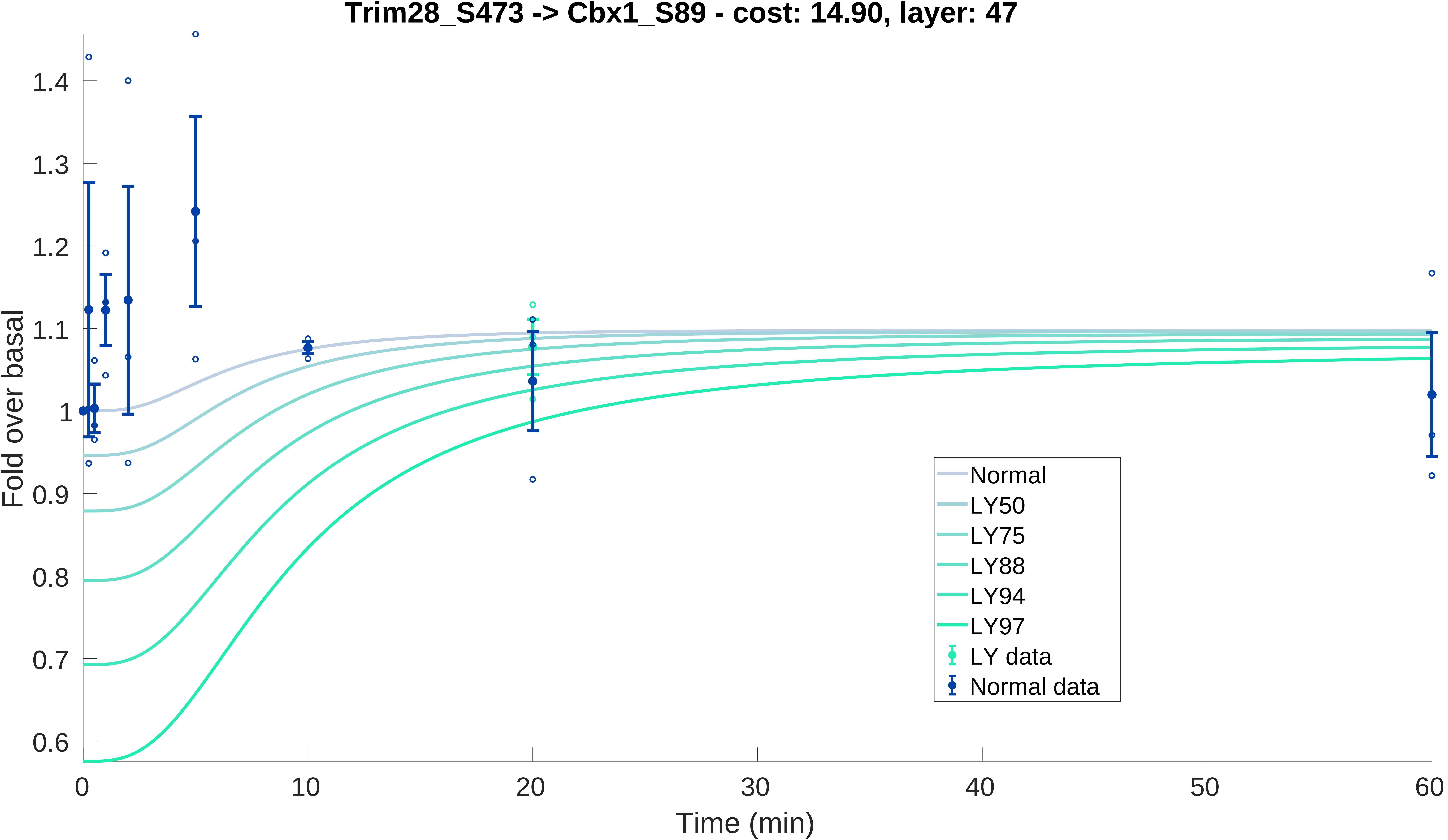

Trim28_S473 -> Cbx1_S89 - cost: 14.90, layer: 47
Fold over basal
1.4
1.3
1.2
1.1
1
0.9
0.8
0.7
0.6
0
10
20
30
40
50
60
Time (min)
Normal
LY50
LY75
LY88
LY94
LY97
LY data
Normal data

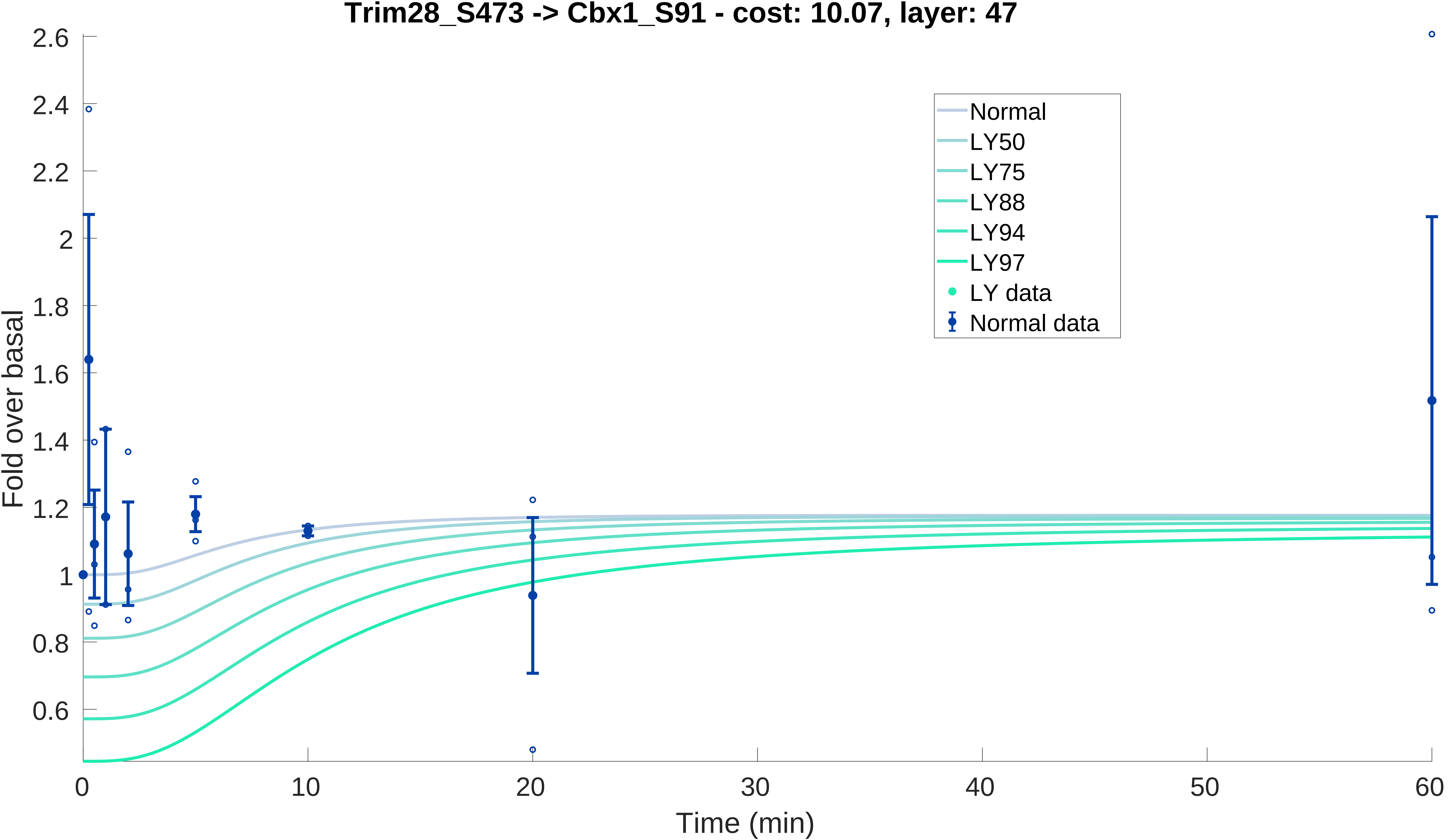

Trim28_S473 -> Cbx1_S91 - cost: 10.07, layer: 47
Fold over basal
2.6
2.4
2.2
2
1.8
1.6
1.4
1.2
1
0.8
0.6
0
10
20
30
40
50
60
Time (min)
Normal
LY50
LY75
LY88
LY94
LY97
LY data
Normal data

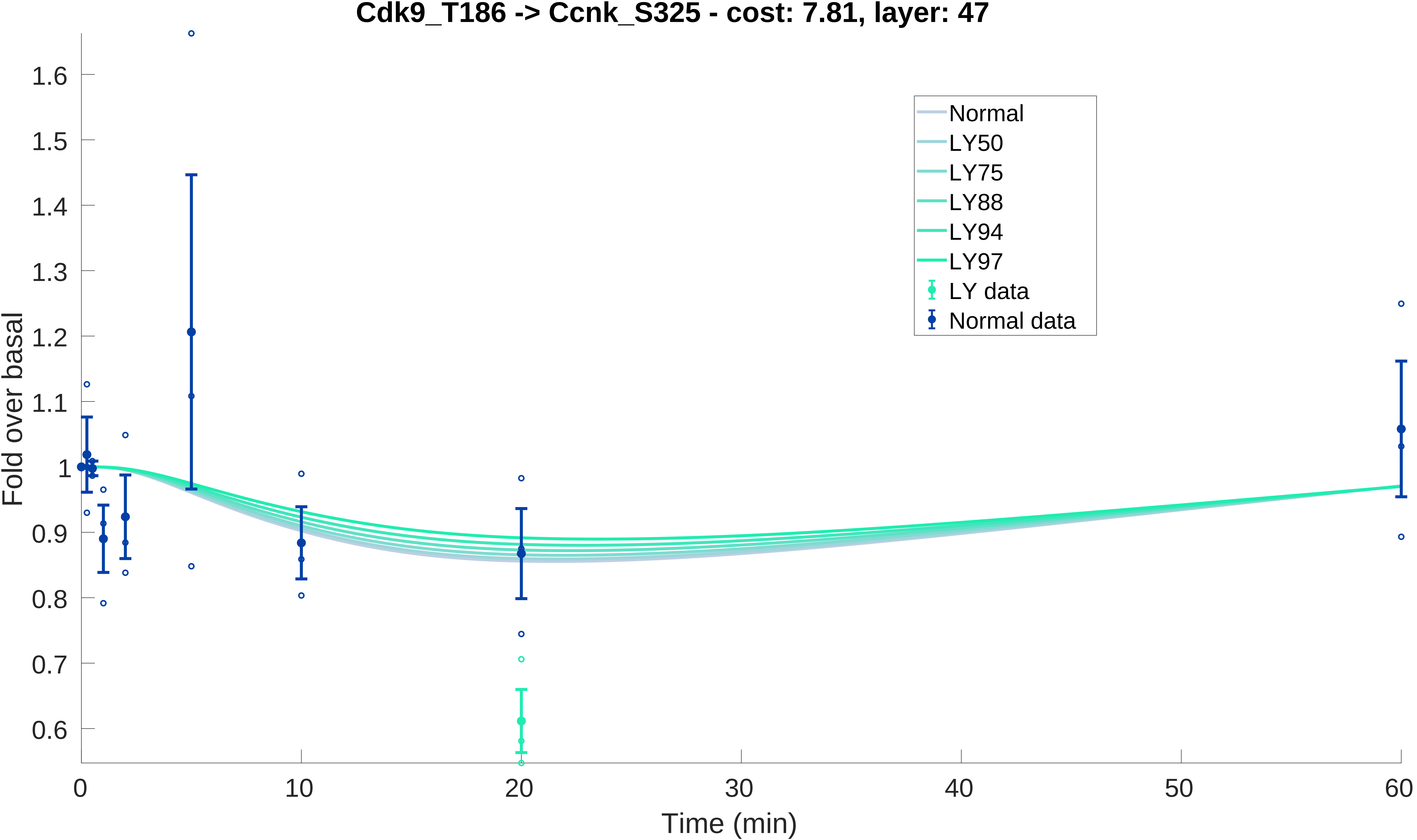

Cdk9_T186 -> Ccnk_S325 - cost: 7.81, layer: 47
Fold over basal
Time (min)
1.6
1.5
1.4
1.3
1.2
1.1
1
0.9
0.8
0.7
0.6
0
10
20
30
40
50
60
Normal
LY50
LY75
LY88
LY94
LY97
LY data
Normal data

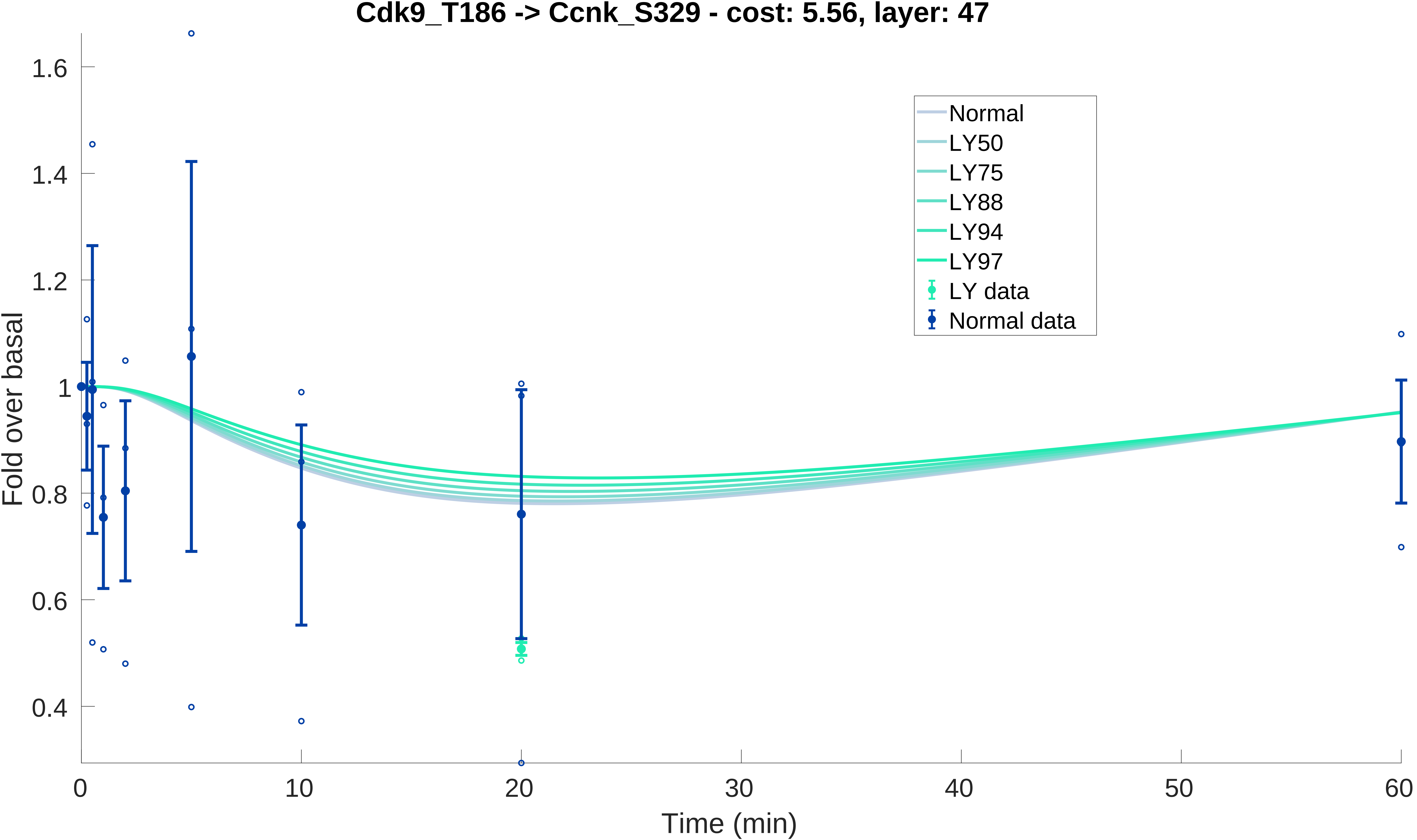
Cdk9_T186 -> Ccnk_S329 - cost: 5.56, layer: 47
Fold over basal
Time (min)
1.6
1.4
1.2
1
0.8
0.6
0.4
0
10
20
30
40
50
60
Normal
LY50
LY75
LY88
LY94
LY97
LY data
Normal data

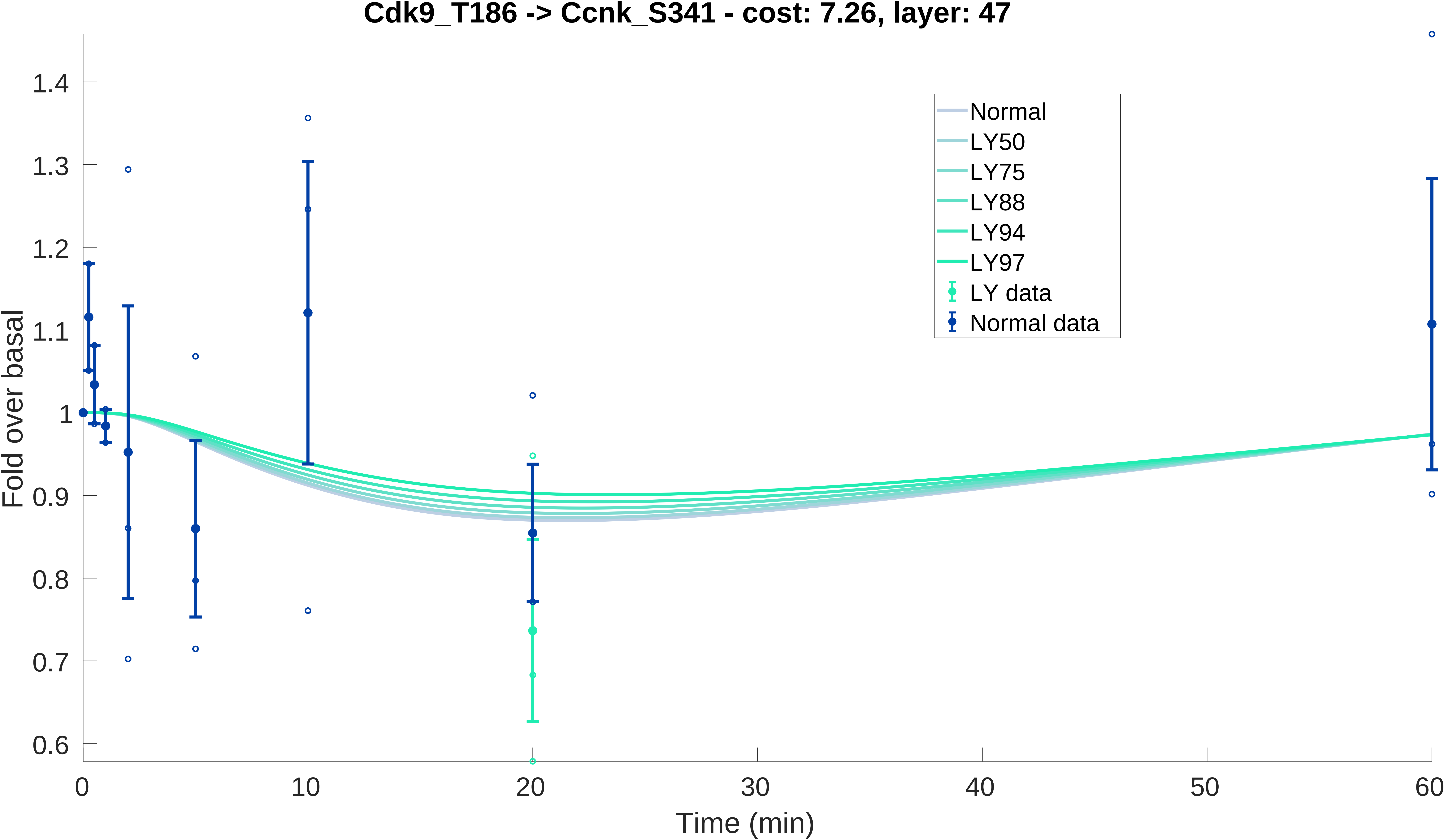

Cdk9_T186 -> Ccnk_S341 - cost: 7.26, layer: 47
Fold over basal
Time (min)
Normal
LY50
LY75
LY88
LY94
LY97
LY data
Normal data
1.4
1.3
1.2
1.1
1
0.9
0.8
0.7
0.6
0
10
20
30
40
50
60

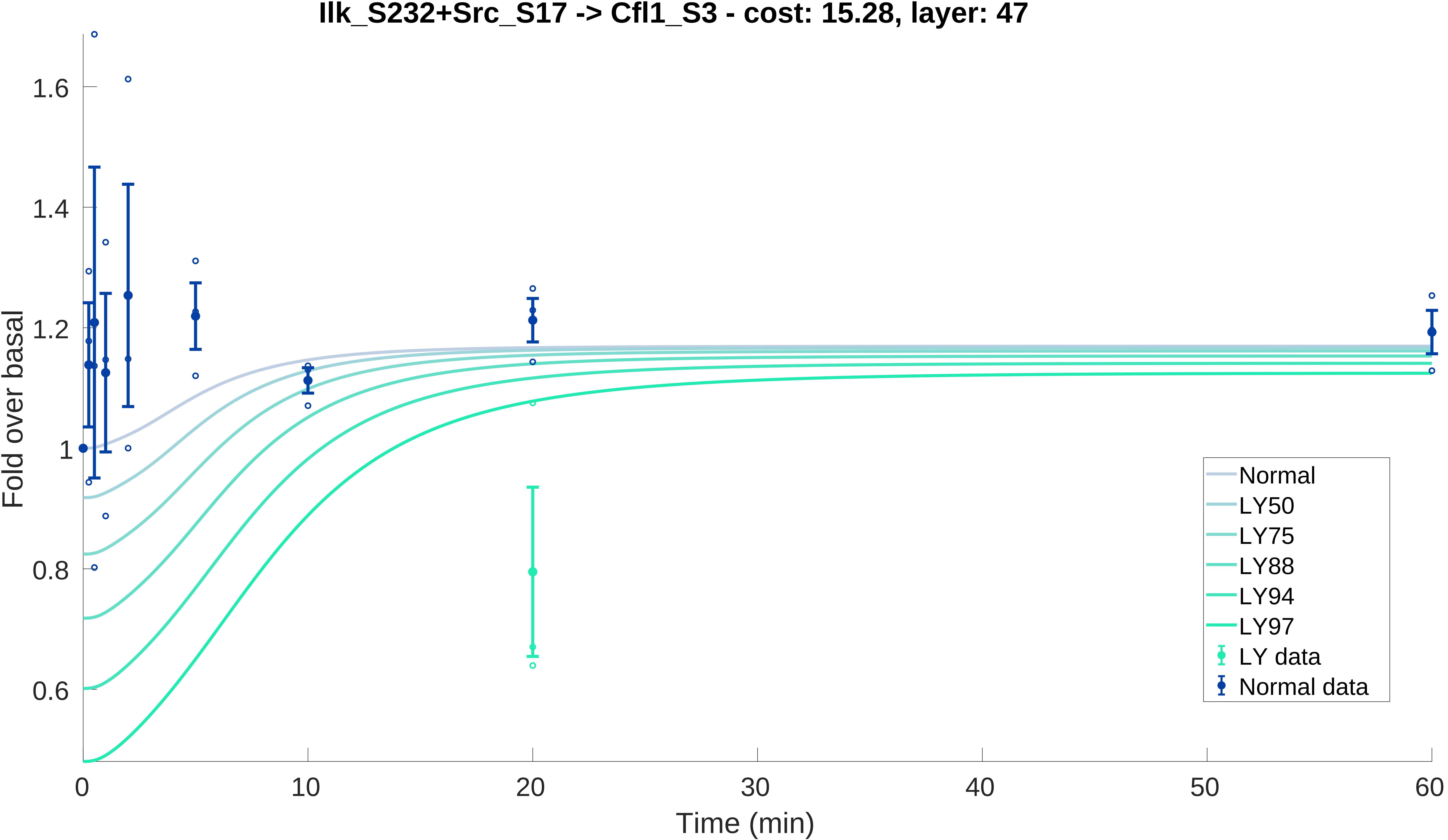

Ilk_S232+Src_S17 -> Cfl1_S3 - cost: 15.28, layer: 47
Fold over basal
Time (min)
0
10
20
30
40
50
60
0.6
0.8
1
1.2
1.4
1.6
Normal
LY50
LY75
LY88
LY94
LY97
LY data
Normal data

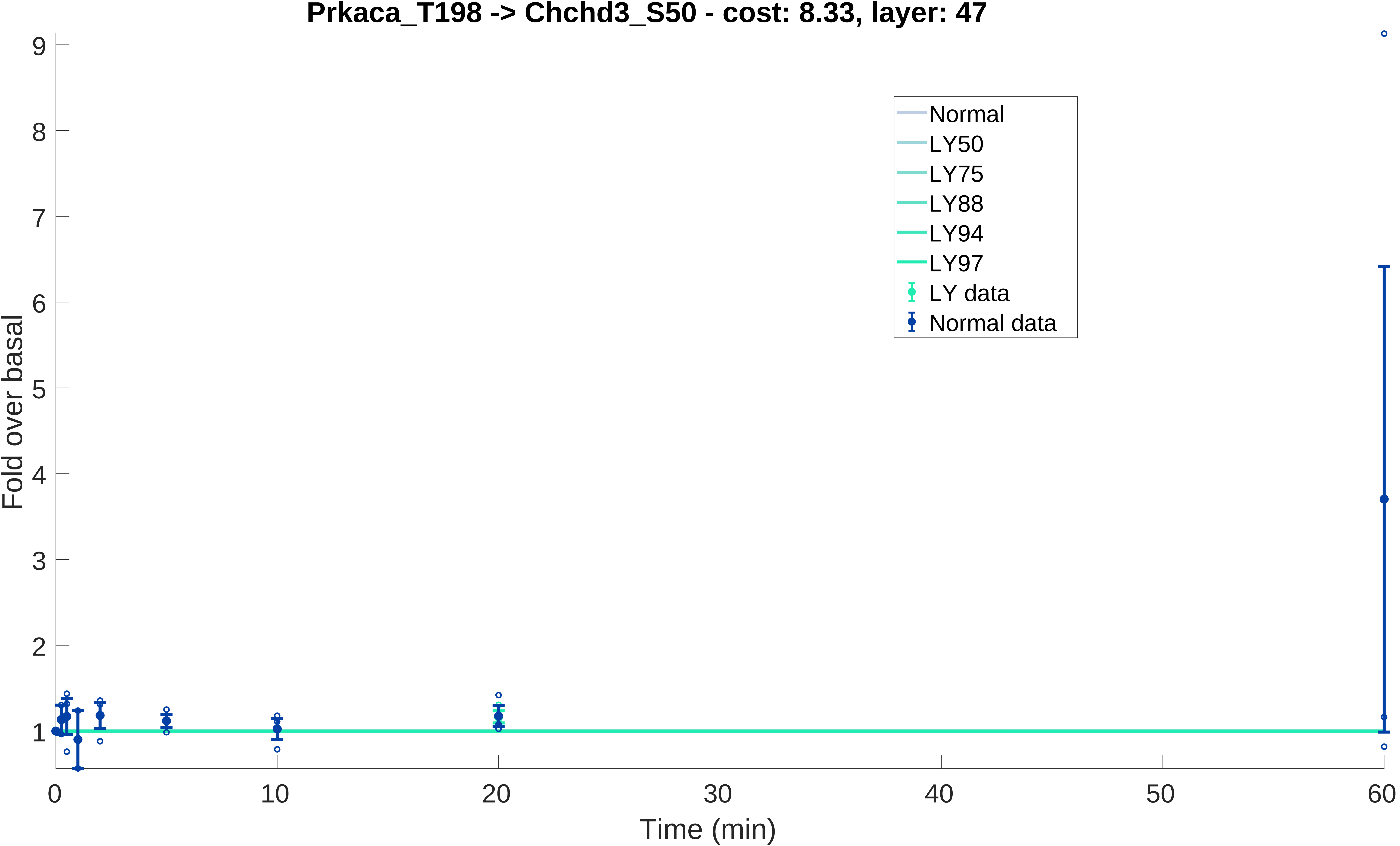

Prkaca_T198 -> Chchd3_S50 - cost: 8.33, layer: 47
Fold over basal
Time (min)
Normal
LY50
LY75
LY88
LY94
LY97
LY data
Normal data

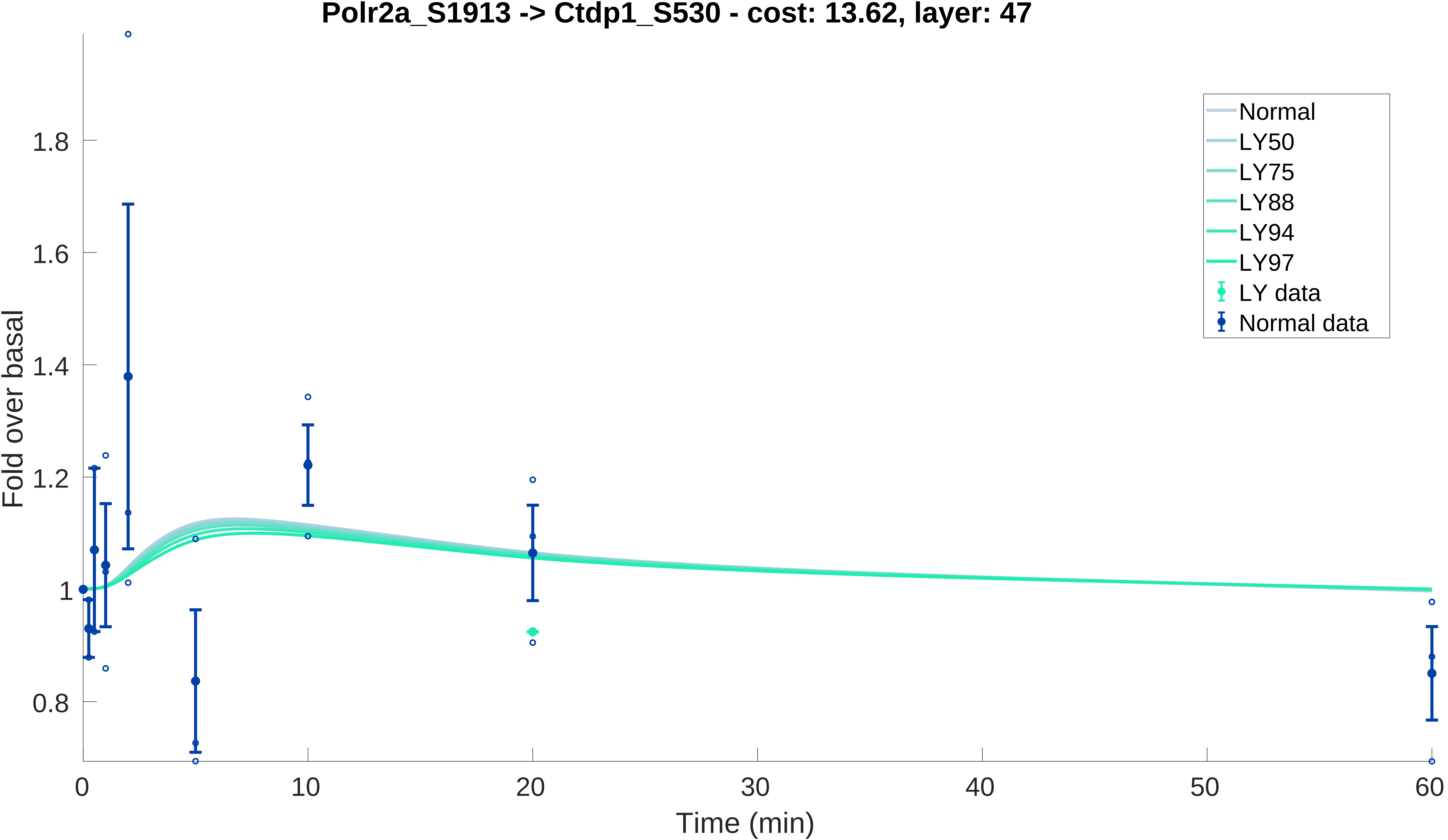

Polr2a_S1913 -> Ctdp1_S530 - cost: 13.62, layer: 47
Fold over basal
1.8
1.6
1.4
1.2
1
0.8
0
10
20
30
40
50
60
Time (min)
Normal
LY50
LY75
LY88
LY94
LY97
LY data
Normal data

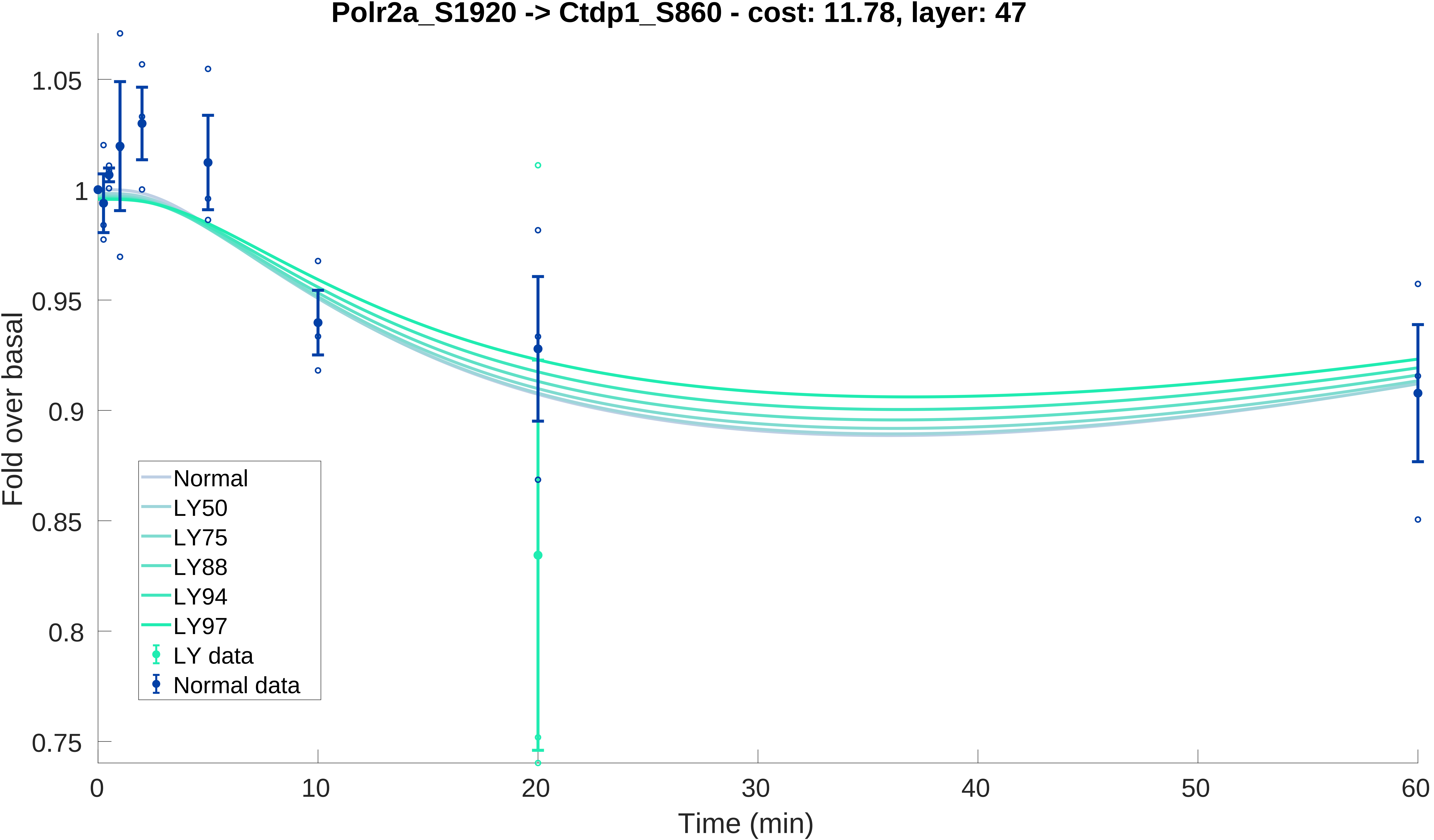

Polr2a_S1920 -> Ctdp1_S860 - cost: 11.78, layer: 47
Fold over basal
Time (min)
1.05
1
0.95
0.9
0.85
0.8
0.75
0
10
20
30
40
50
60
Normal
LY50
LY75
LY88
LY94
LY97
LY data
Normal data

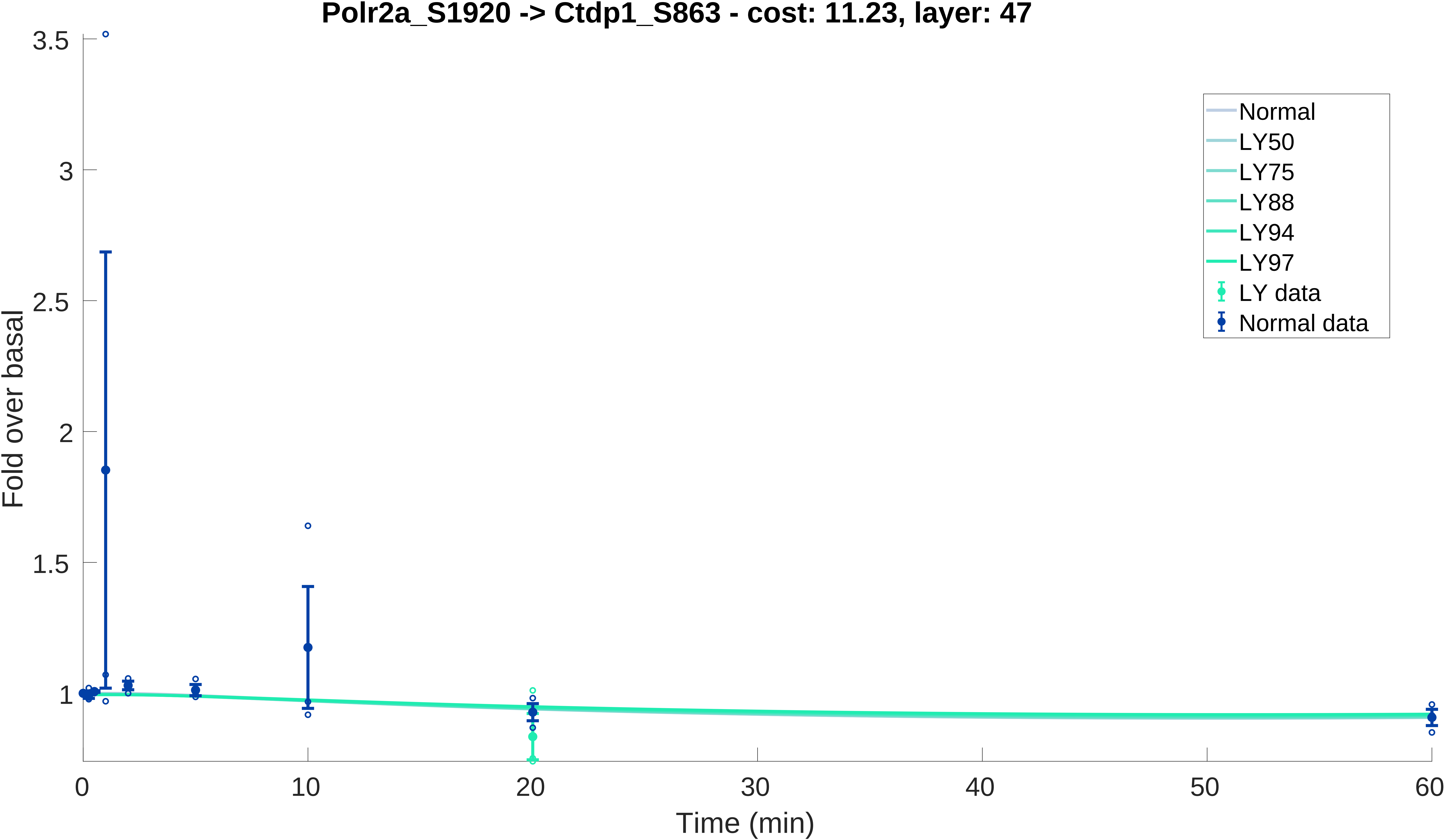

Polr2a_S1920 -> Ctdp1_S863 - cost: 11.23, layer: 47
Fold over basal
3.5
3
2.5
2
1.5
1
0
10
20
30
40
50
60
Time (min)
Normal
LY50
LY75
LY88
LY94
LY97
LY data
Normal data

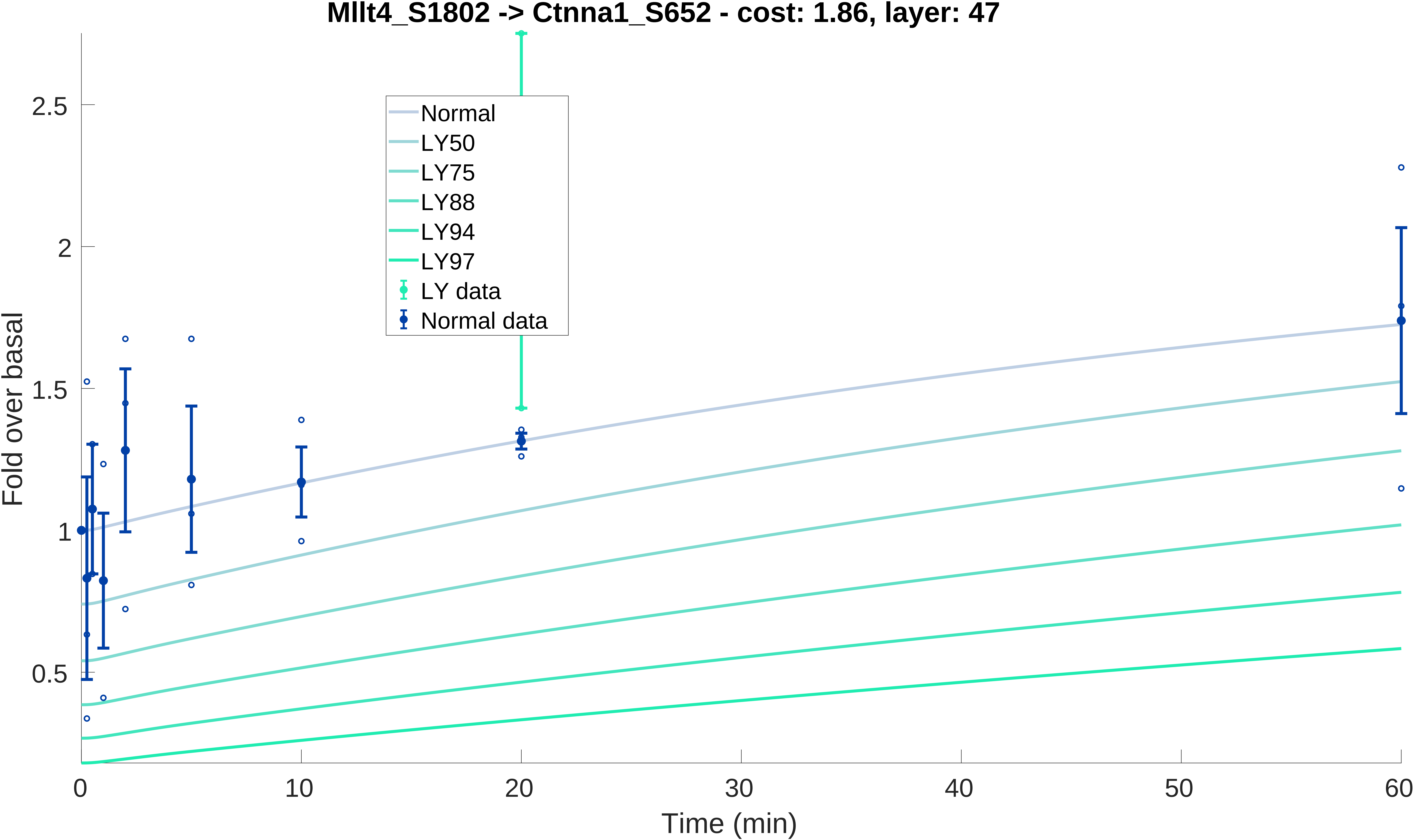

Mllt4_S1802 -> Ctnna1_S652 - cost: 1.86, layer: 47
Normal
LY50
LY75
LY88
LY94
LY97
LY data
Normal data
Fold over basal
Time (min)
0.5
1
1.5
2
2.5
0
10
20
30
40
50
60

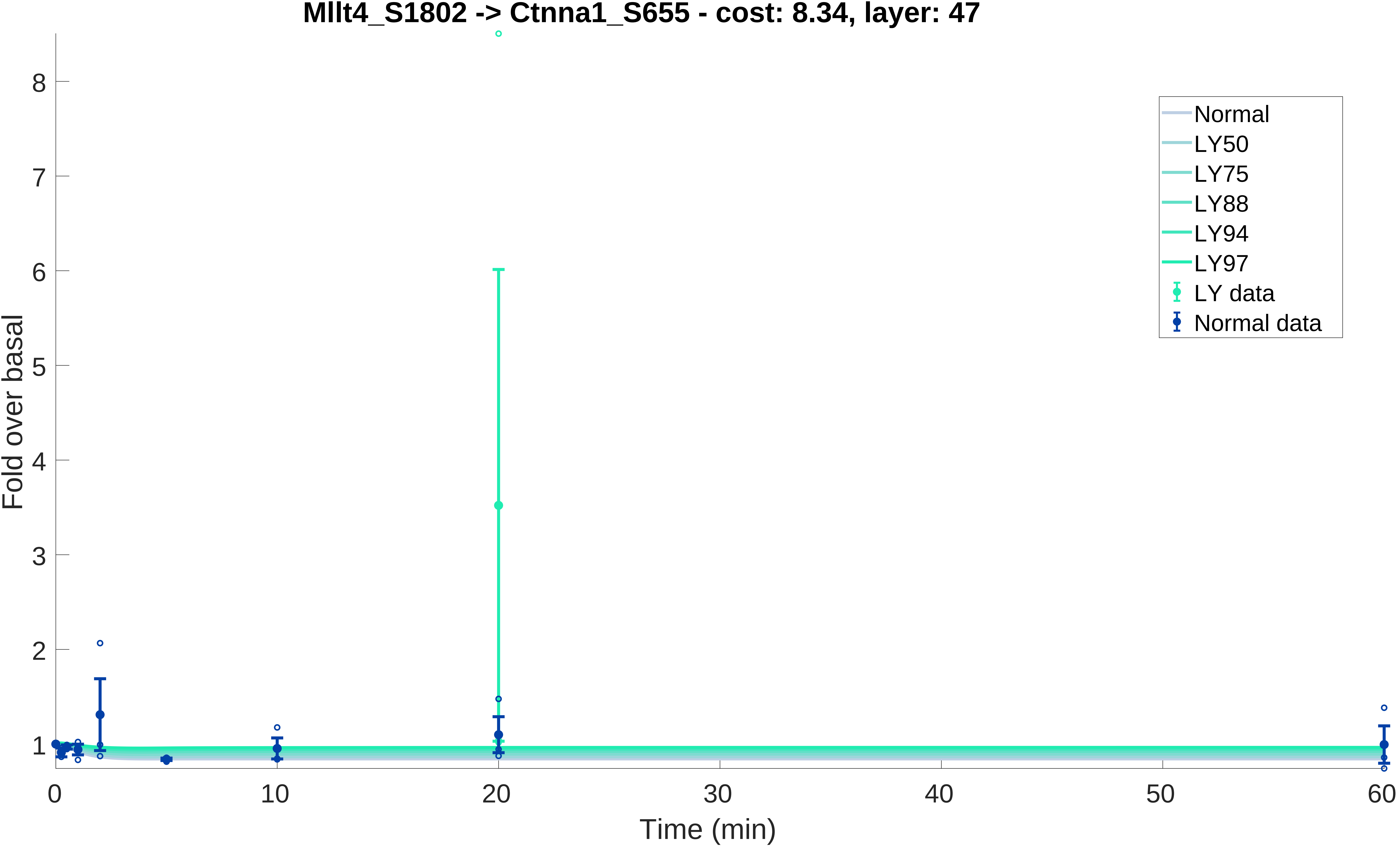

Mllt4_S1802 -> Ctnna1_S655 - cost: 8.34, layer: 47
Fold over basal
1
2
3
4
5
6
7
8
0
10
20
30
40
50
60
Time (min)
Normal
LY50
LY75
LY88
LY94
LY97
LY data
Normal data

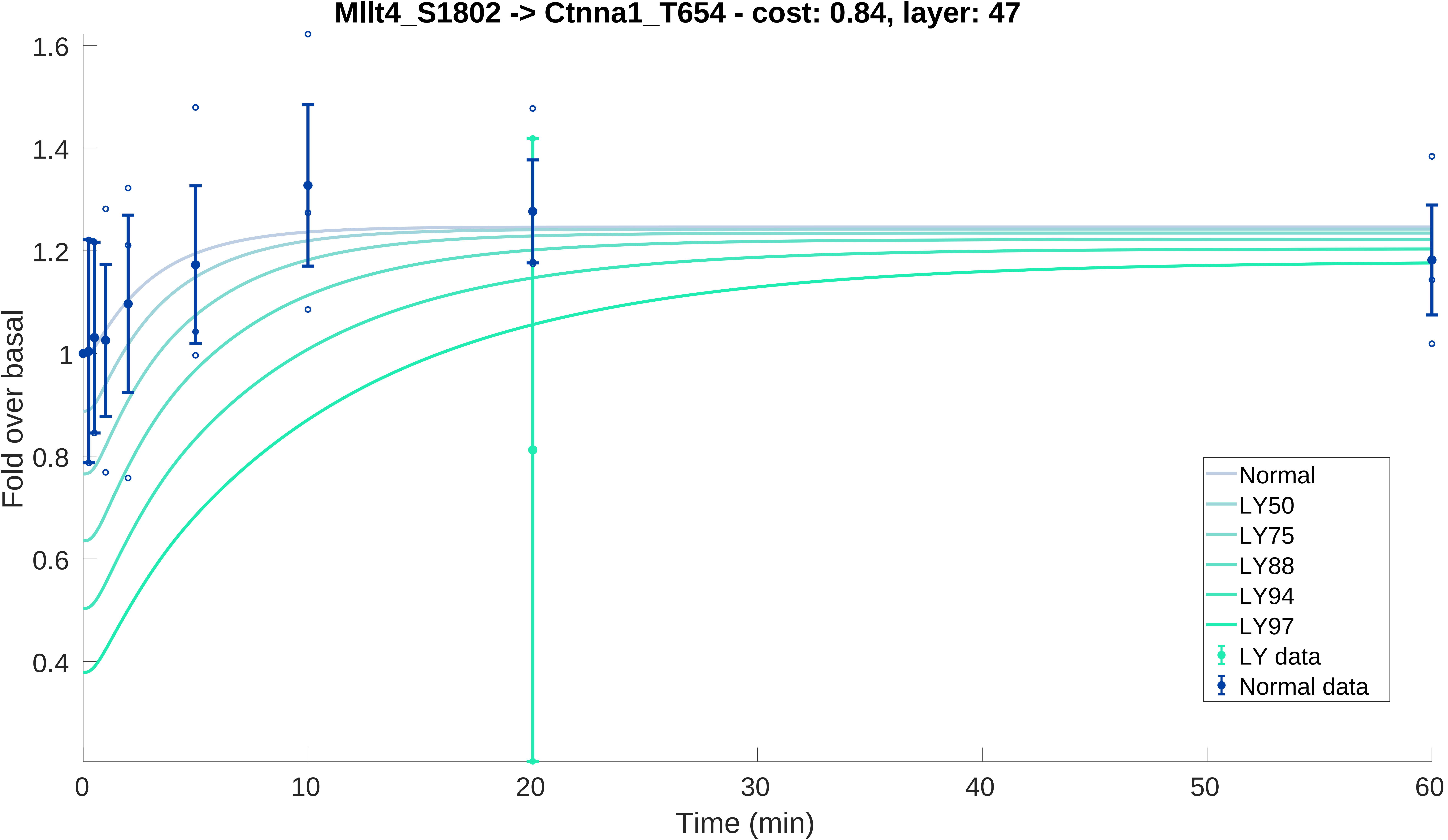

Mllt4_S1802 -> Ctnna1_T654 - cost: 0.84, layer: 47
Fold over basal
1.6
1.4
1.2
1
0.8
0.6
0.4
0
10
20
30
40
50
60
Time (min)
Normal
LY50
LY75
LY88
LY94
LY97
LY data
Normal data

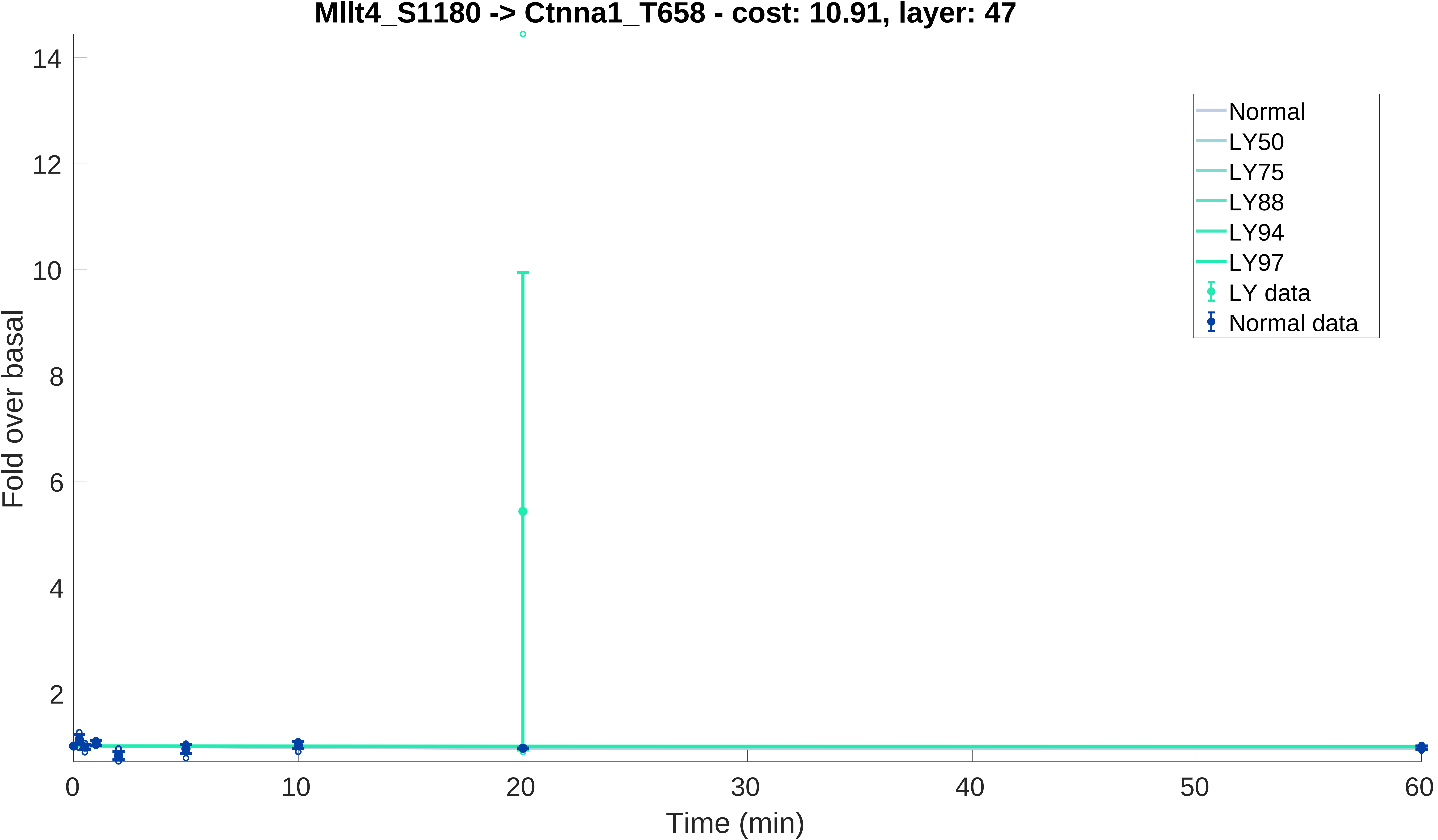

Mllt4_S1180 -> Ctnna1_T658 - cost: 10.91, layer: 47
Fold over basal
Time (min)
Normal
LY50
LY75
LY88
LY94
LY97
LY data
Normal data
0
2
4
6
8
10
12
14
0
10
20
30
40
50
60

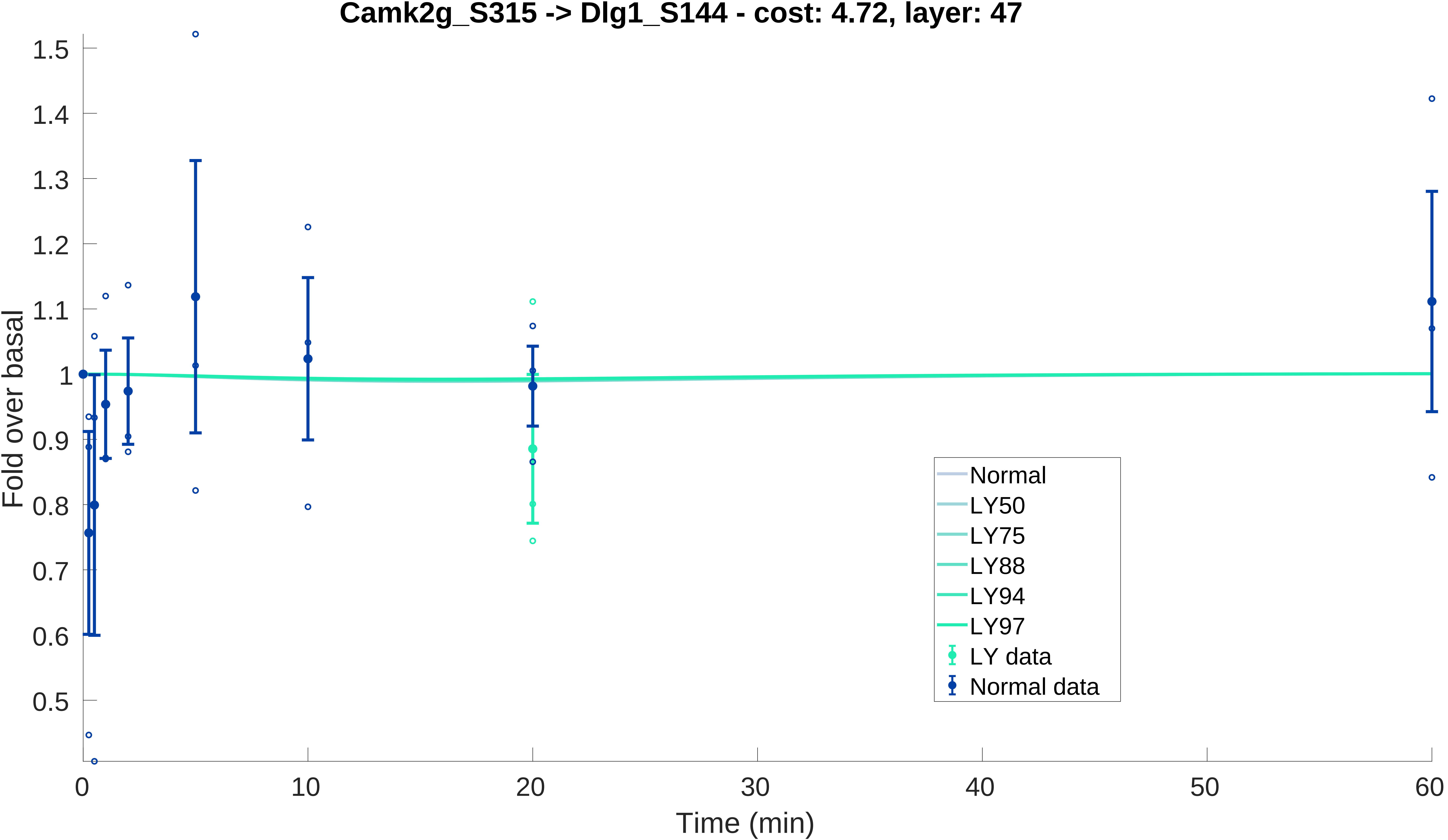

Camk2g_S315 -> Dlg1_S144 - cost: 4.72, layer: 47
Fold over basal
Time (min)
1.5
1.4
1.3
1.2
1.1
1
0.9
0.8
0.7
0.6
0.5
0
10
20
30
40
50
60
Normal
LY50
LY75
LY88
LY94
LY97
LY data
Normal data

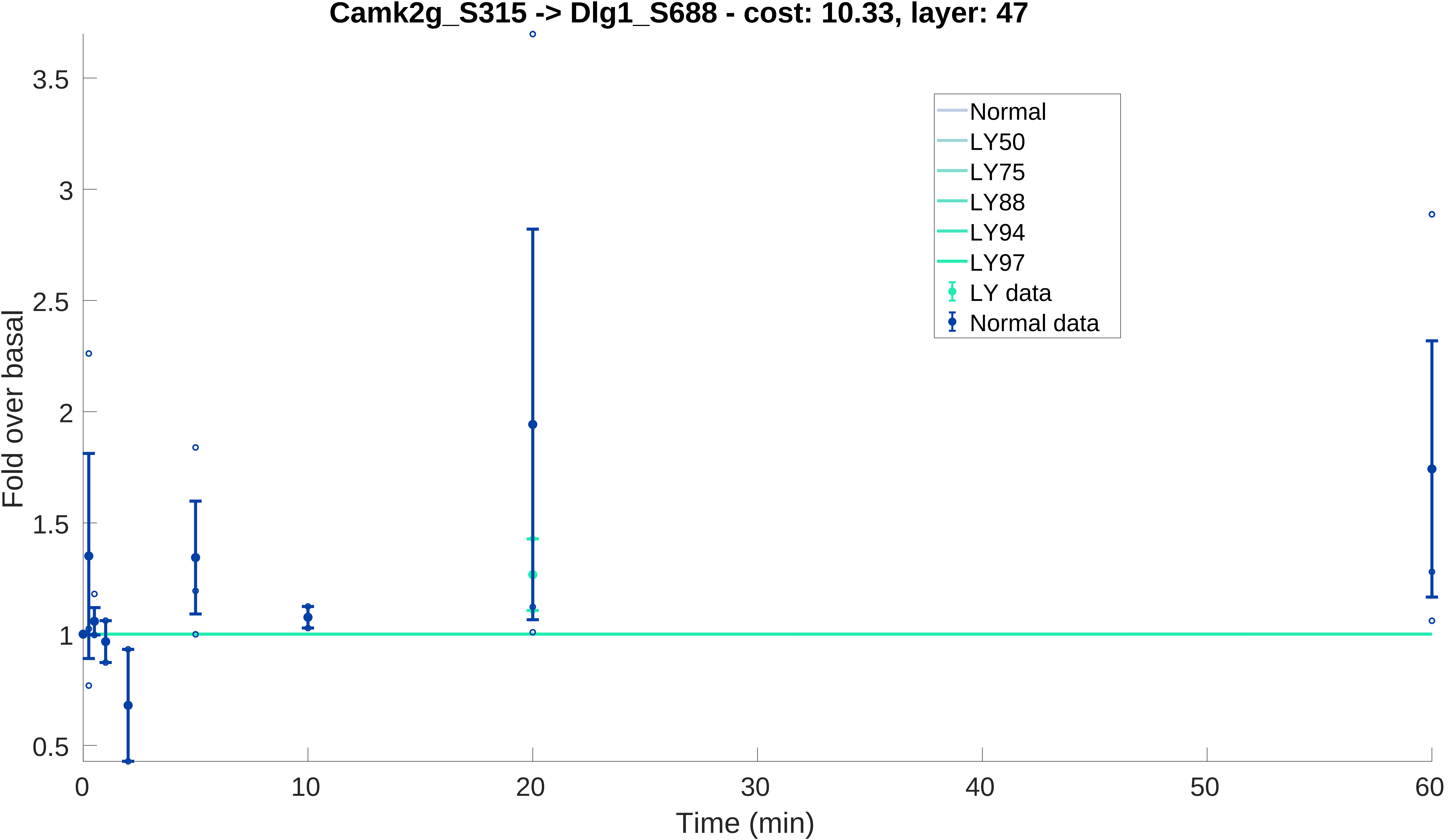

Camk2g_S315 -> Dlg1_S688 - cost: 10.33, layer: 47
Fold over basal
3.5
3
2.5
2
1.5
1
0.5
0
10
20
30
40
50
60
Time (min)
Normal
LY50
LY75
LY88
LY94
LY97
LY data
Normal data

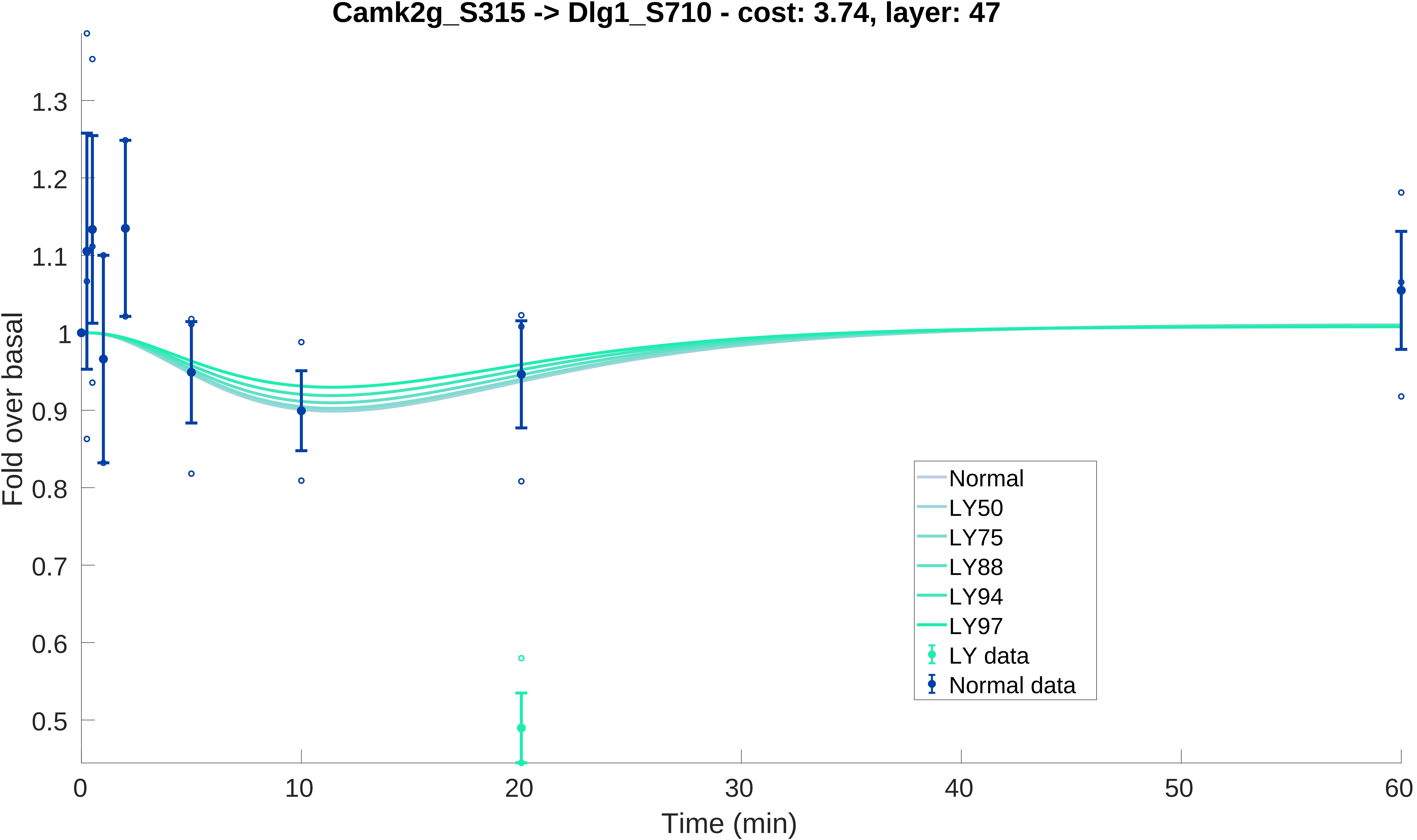

Camk2g_S315 -> Dlg1_S710 - cost: 3.74, layer: 47
Fold over basal
Time (min)
1.3
1.2
1.1
1
0.9
0.8
0.7
0.6
0.5
0
10
20
30
40
50
60
Normal
LY50
LY75
LY88
LY94
LY97
LY data
Normal data

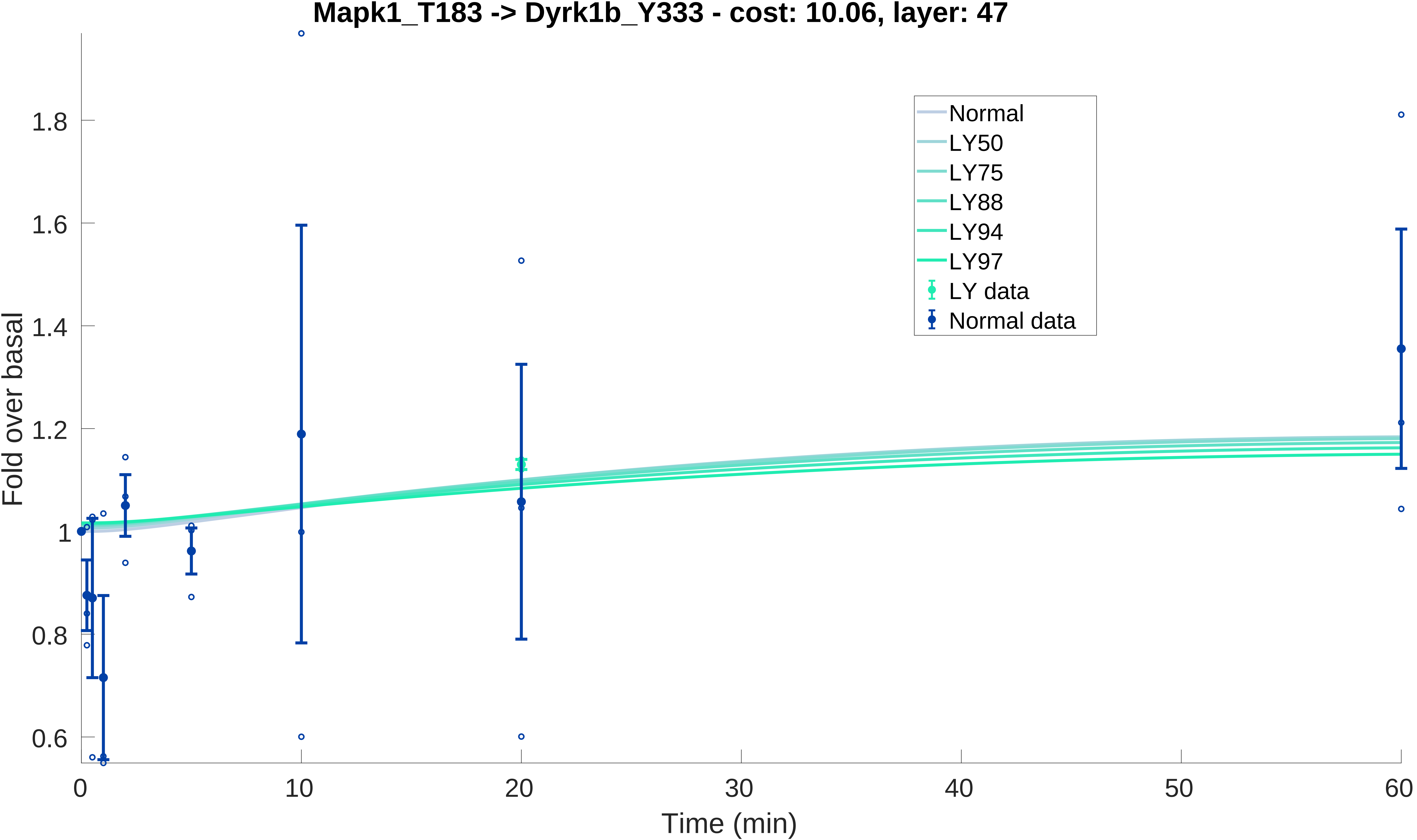
Mapk1_T183 -> Dyrk1b_Y333 - cost: 10.06, layer: 47
Fold over basal
Time (min)
0.6
0.8
1
1.2
1.4
1.6
1.8
0
10
20
30
40
50
60
Normal
LY50
LY75
LY88
LY94
LY97
LY data
Normal data

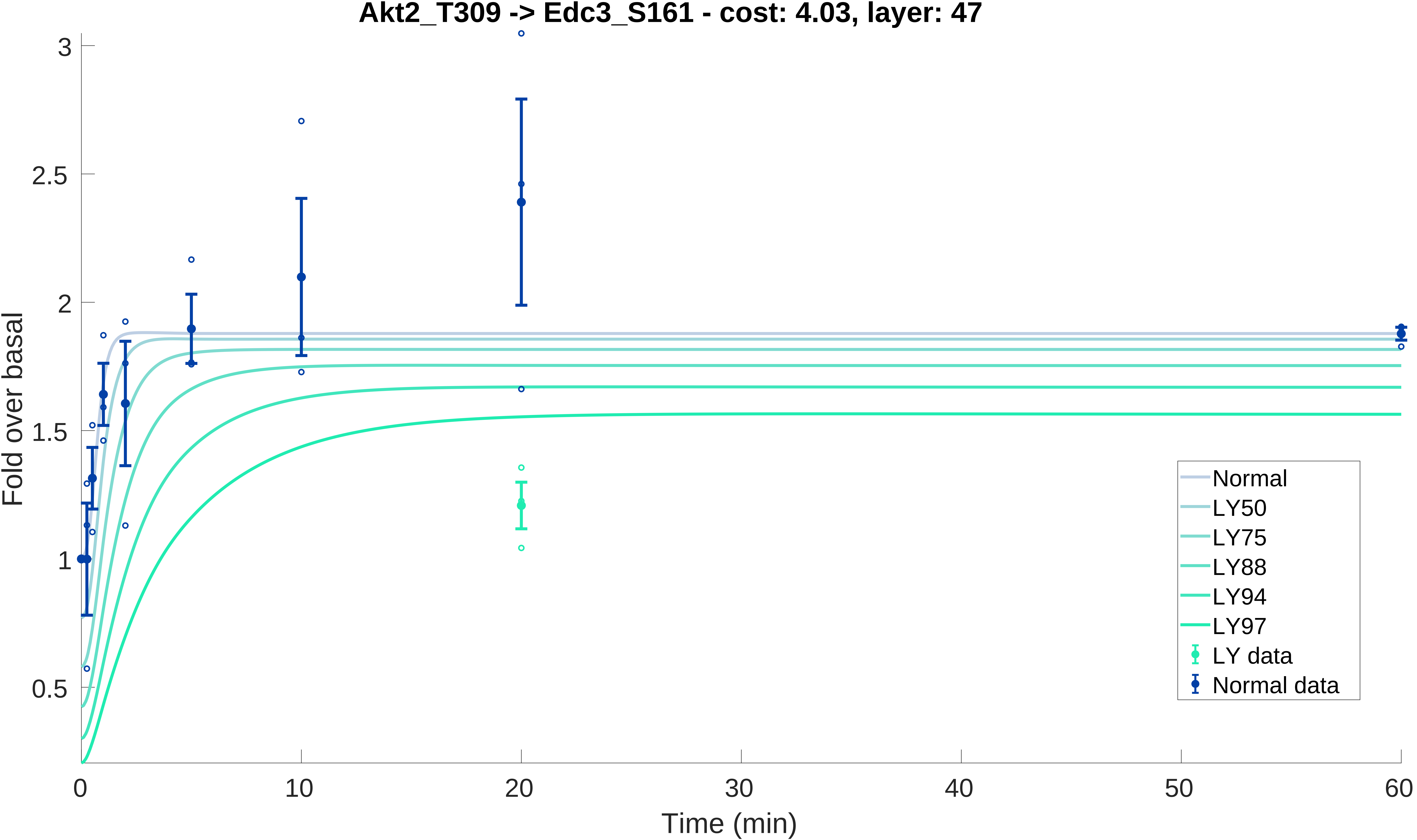

Akt2_T309 -> Edc3_S161 - cost: 4.03, layer: 47
Fold over basal
Time (min)
0
10
20
30
40
50
60
0.5
1
1.5
2
2.5
3
Normal
LY50
LY75
LY88
LY94
LY97
LY data
Normal data

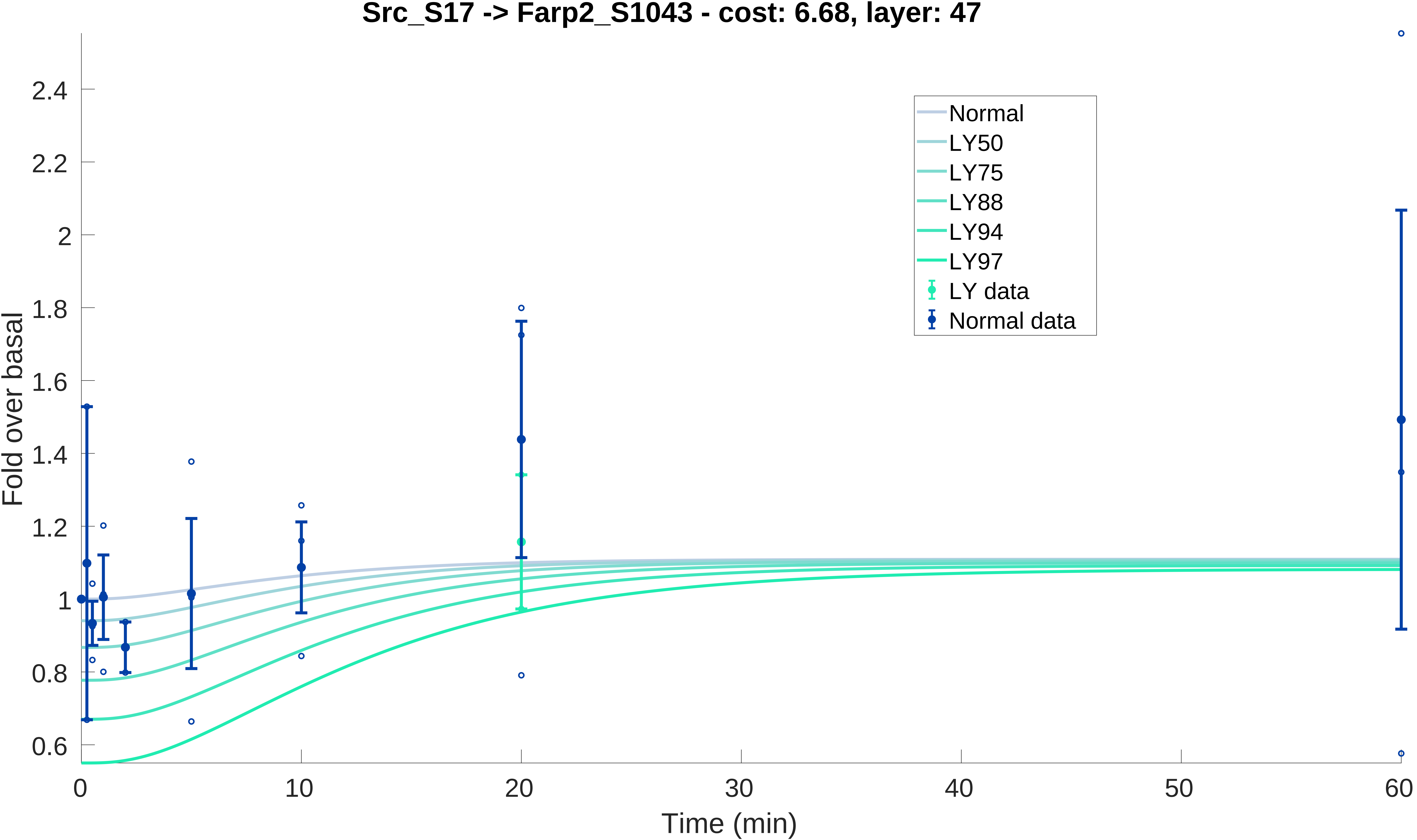

Src_S17 -> Farp2_S1043 - cost: 6.68, layer: 47
Fold over basal
Time (min)
Normal
LY50
LY75
LY88
LY94
LY97
LY data
Normal data
0.6
0.8
1
1.2
1.4
1.6
1.8
2
2.2
2.4
0
10
20
30
40
50
60

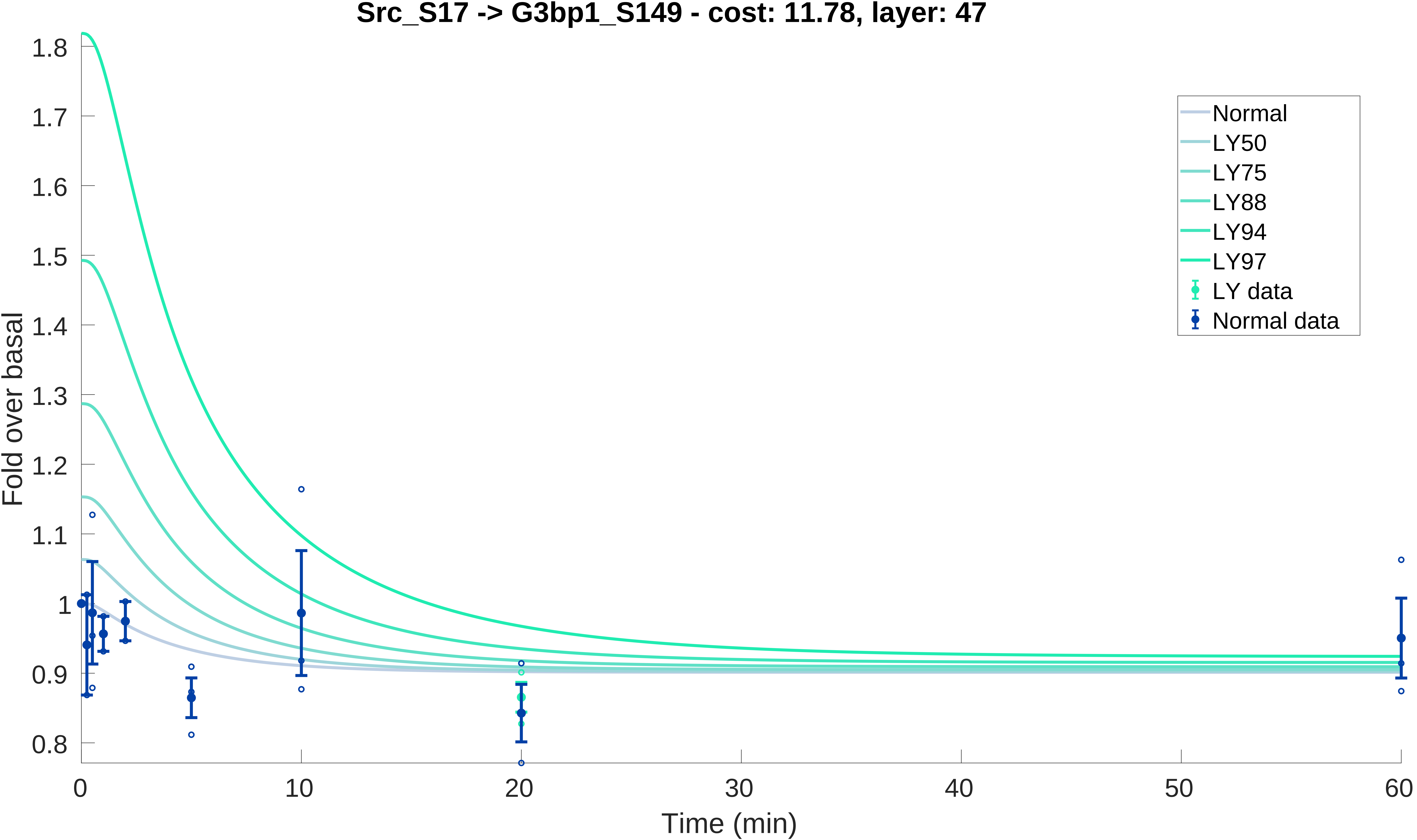

Src_S17 -> G3bp1_S149 - cost: 11.78, layer: 47
Fold over basal
Time (min)
Normal
LY50
LY75
LY88
LY94
LY97
LY data
Normal data
1.8
1.7
1.6
1.5
1.4
1.3
1.2
1.1
1
0.9
0.8
0
10
20
30
40
50
60

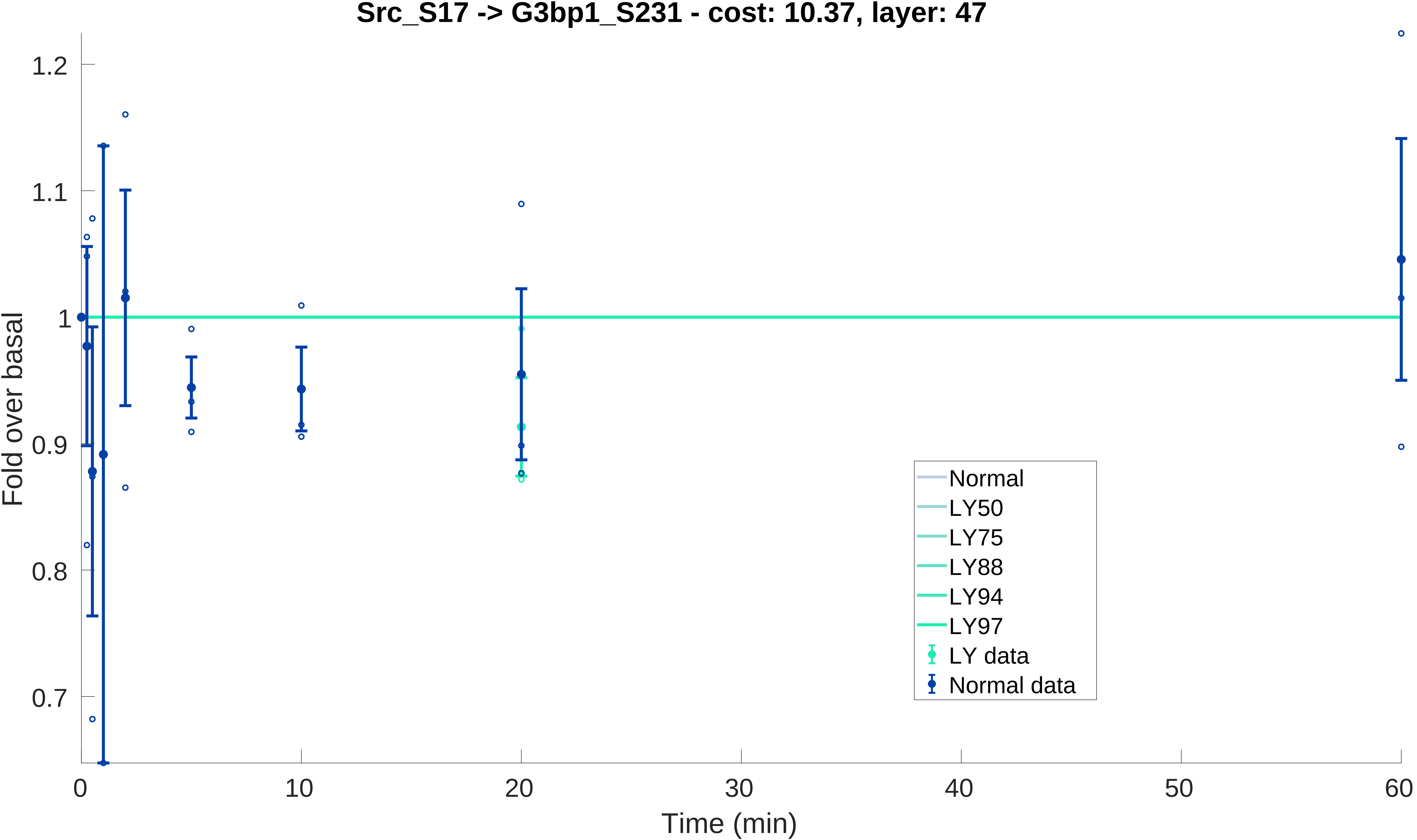

Src_S17 -> G3bp1_S231 - cost: 10.37, layer: 47
Fold over basal
1.2
1.1
1
0.9
0.8
0.7
0
10
20
30
40
50
60
Time (min)
Normal
LY50
LY75
LY88
LY94
LY97
LY data
Normal data

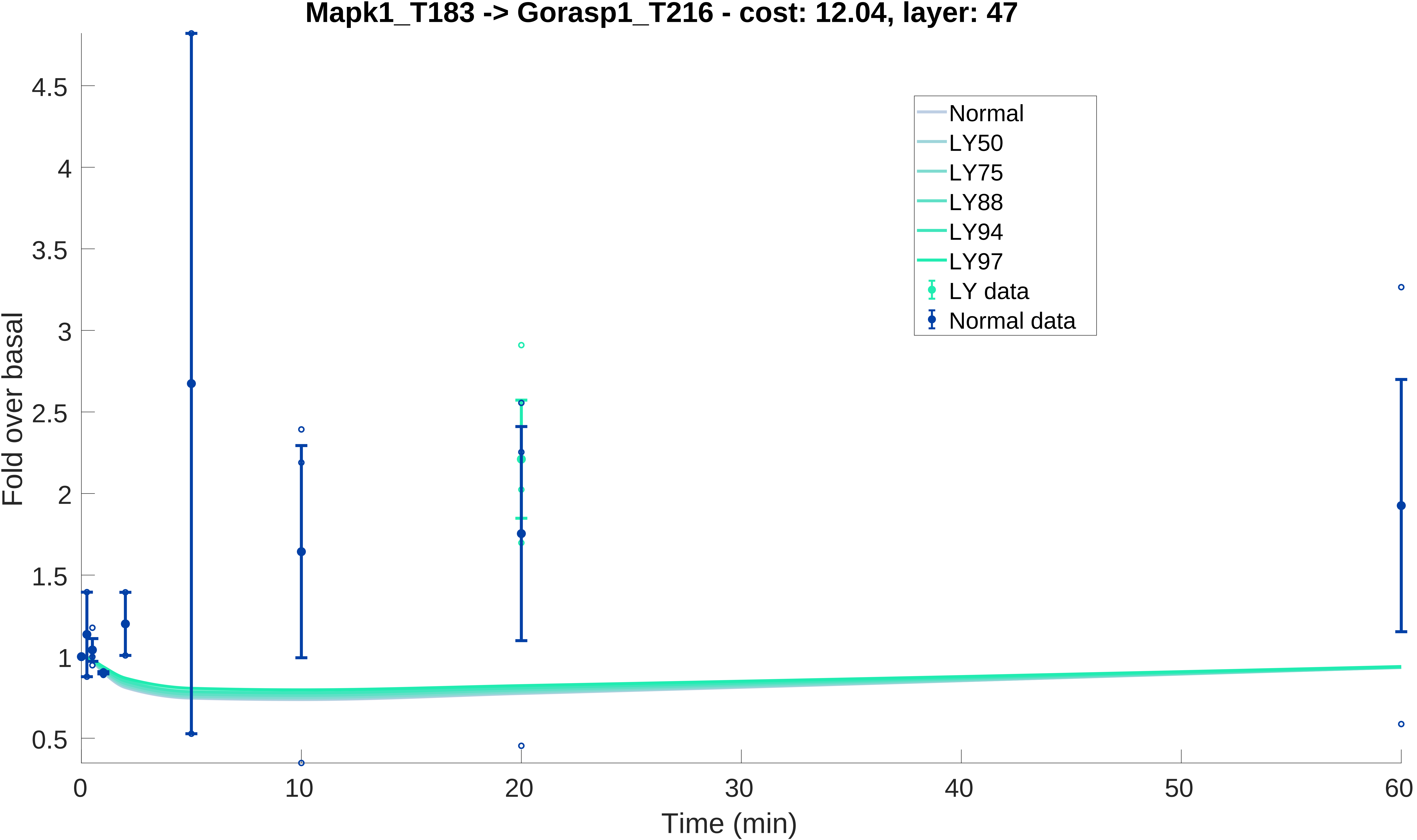
Mapk1_T183 -> Gorasp1_T216 - cost: 12.04, layer: 47
Fold over basal
Time (min)
0.5
1
1.5
2
2.5
3
3.5
4
4.5
0
10
20
30
40
50
60
Normal
LY50
LY75
LY88
LY94
LY97
LY data
Normal data

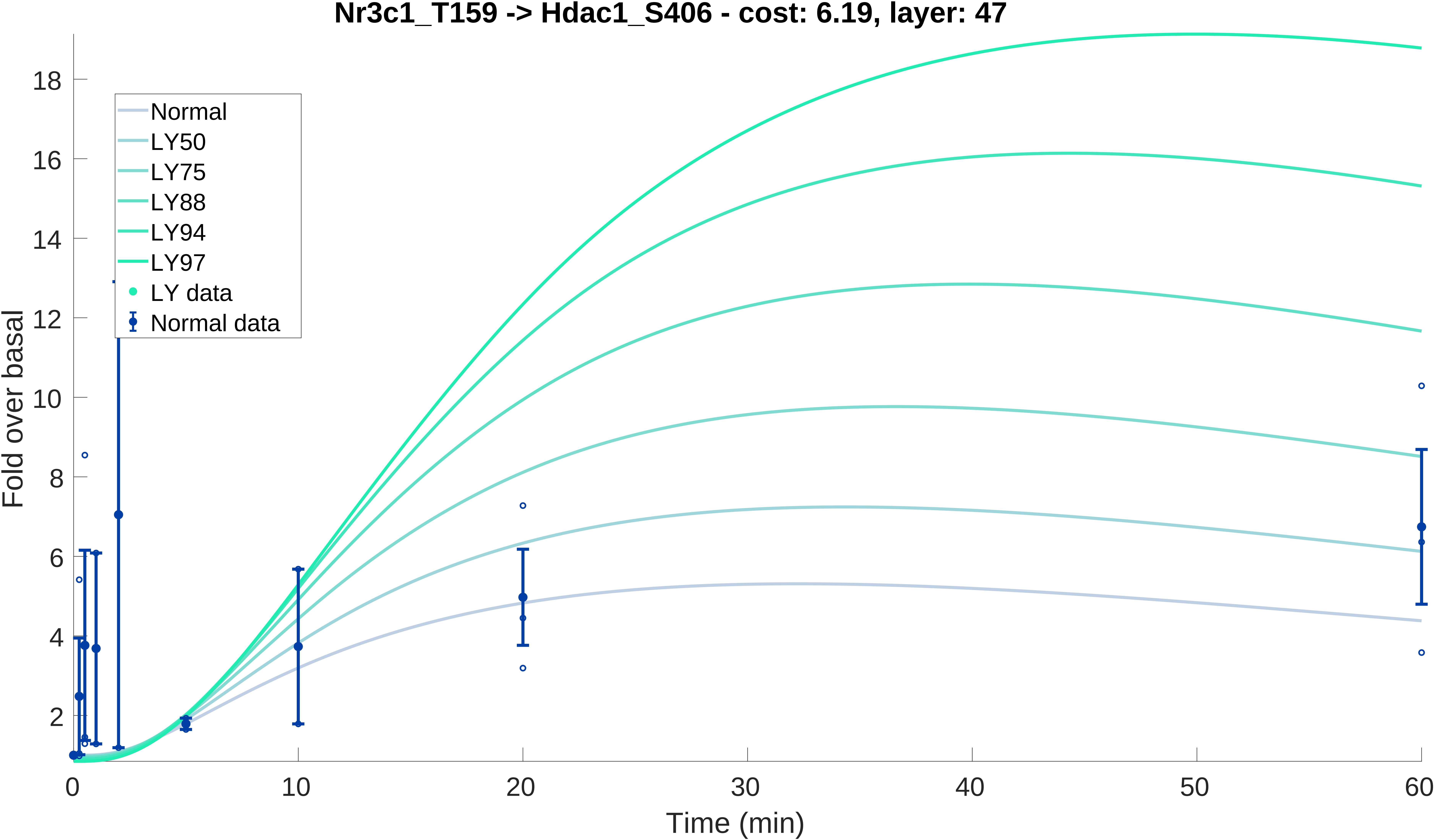

Nr3c1_T159 -> Hdac1_S406 - cost: 6.19, layer: 47
Normal
LY50
LY75
LY88
LY94
LY97
LY data
Normal data
Fold over basal
Time (min)
18
16
14
12
10
8
6
4
2
0
10
20
30
40
50
60

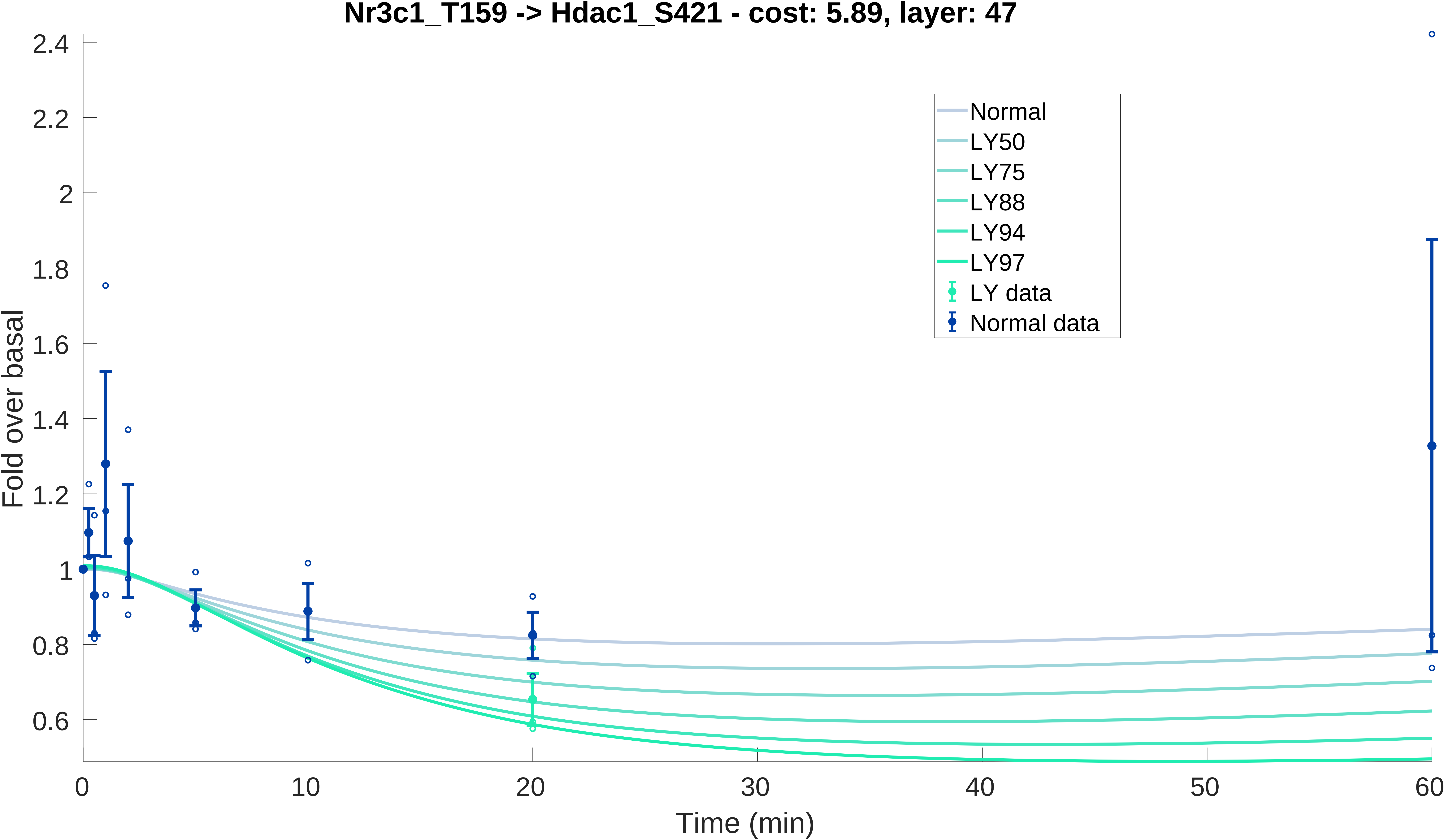

Nr3c1_T159 -> Hdac1_S421 - cost: 5.89, layer: 47
Fold over basal
2.4
2.2
2
1.8
1.6
1.4
1.2
1
0.8
0.6
Time (min)
0
10
20
30
40
50
60
Normal
LY50
LY75
LY88
LY94
LY97
LY data
Normal data

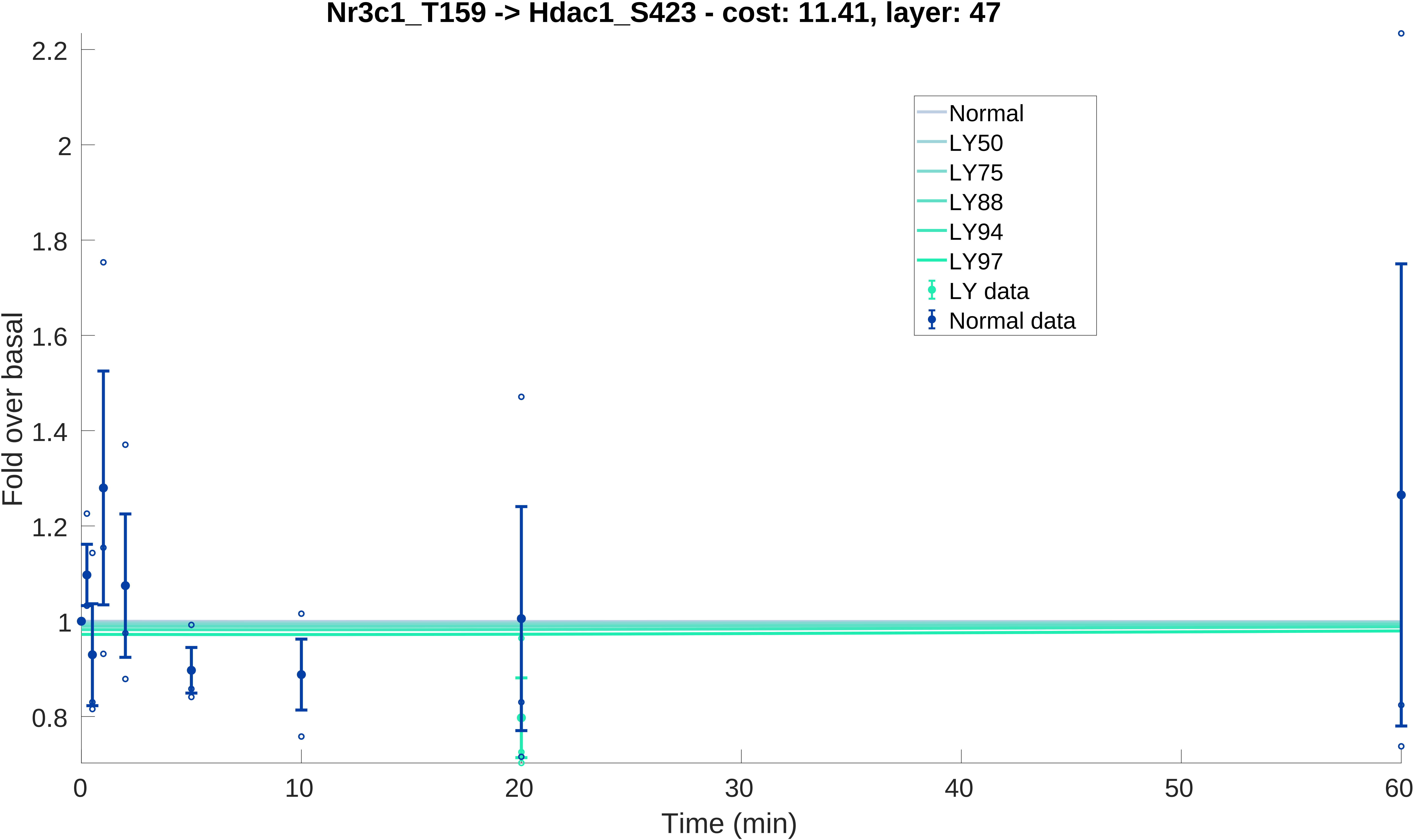
Nr3c1_T159 -> Hdac1_S423 - cost: 11.41, layer: 47
Fold over basal
Time (min)
0.8
1
1.2
1.4
1.6
1.8
2
2.2
0
10
20
30
40
50
60
Normal
LY50
LY75
LY88
LY94
LY97
LY data
Normal data

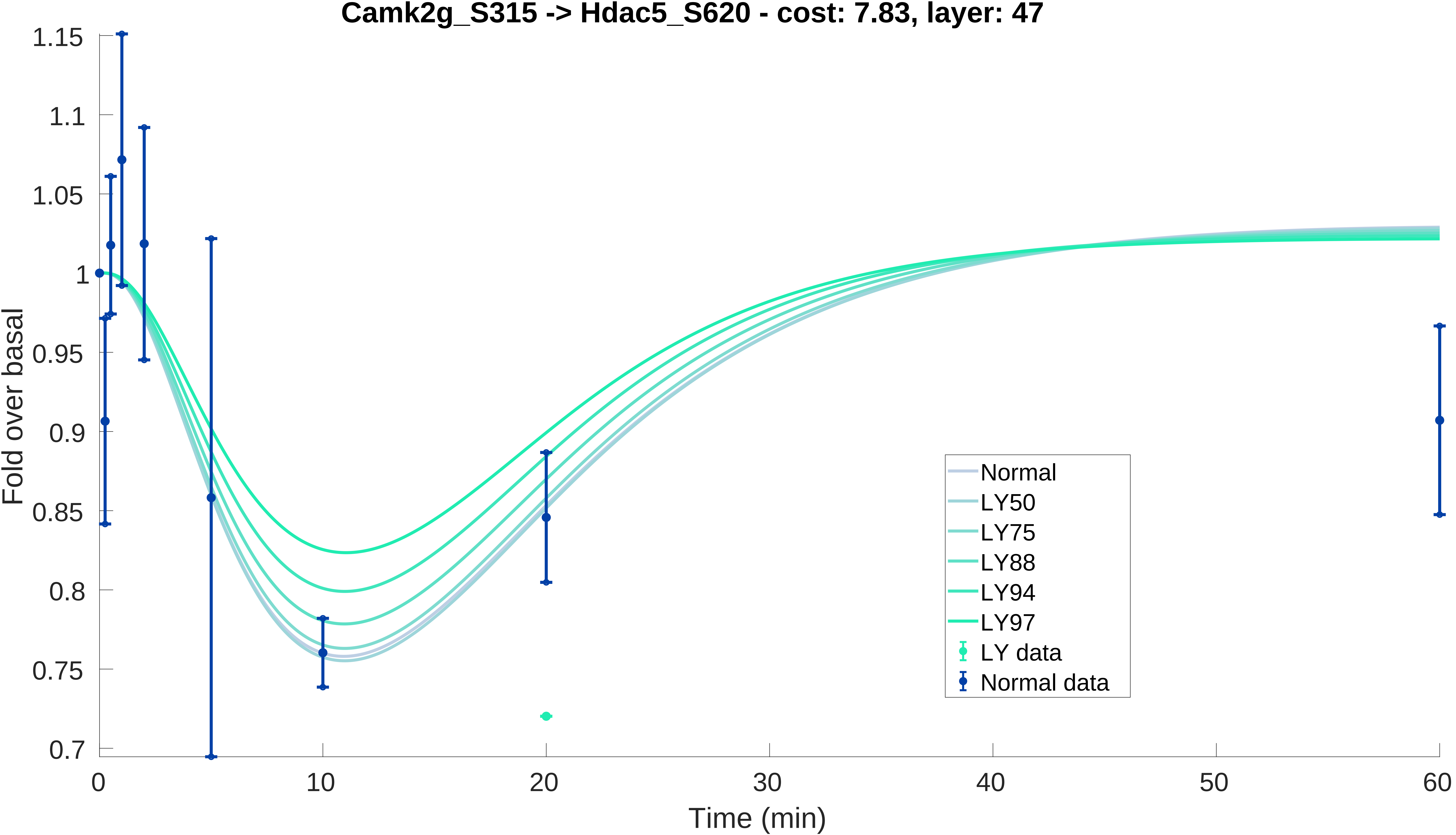

Camk2g_S315 -> Hdac5_S620 - cost: 7.83, layer: 47
Fold over basal
Time (min)
1.15
1.1
1.05
1
0.95
0.9
0.85
0.8
0.75
0.7
0
10
20
30
40
50
60
Normal
LY50
LY75
LY88
LY94
LY97
LY data
Normal data

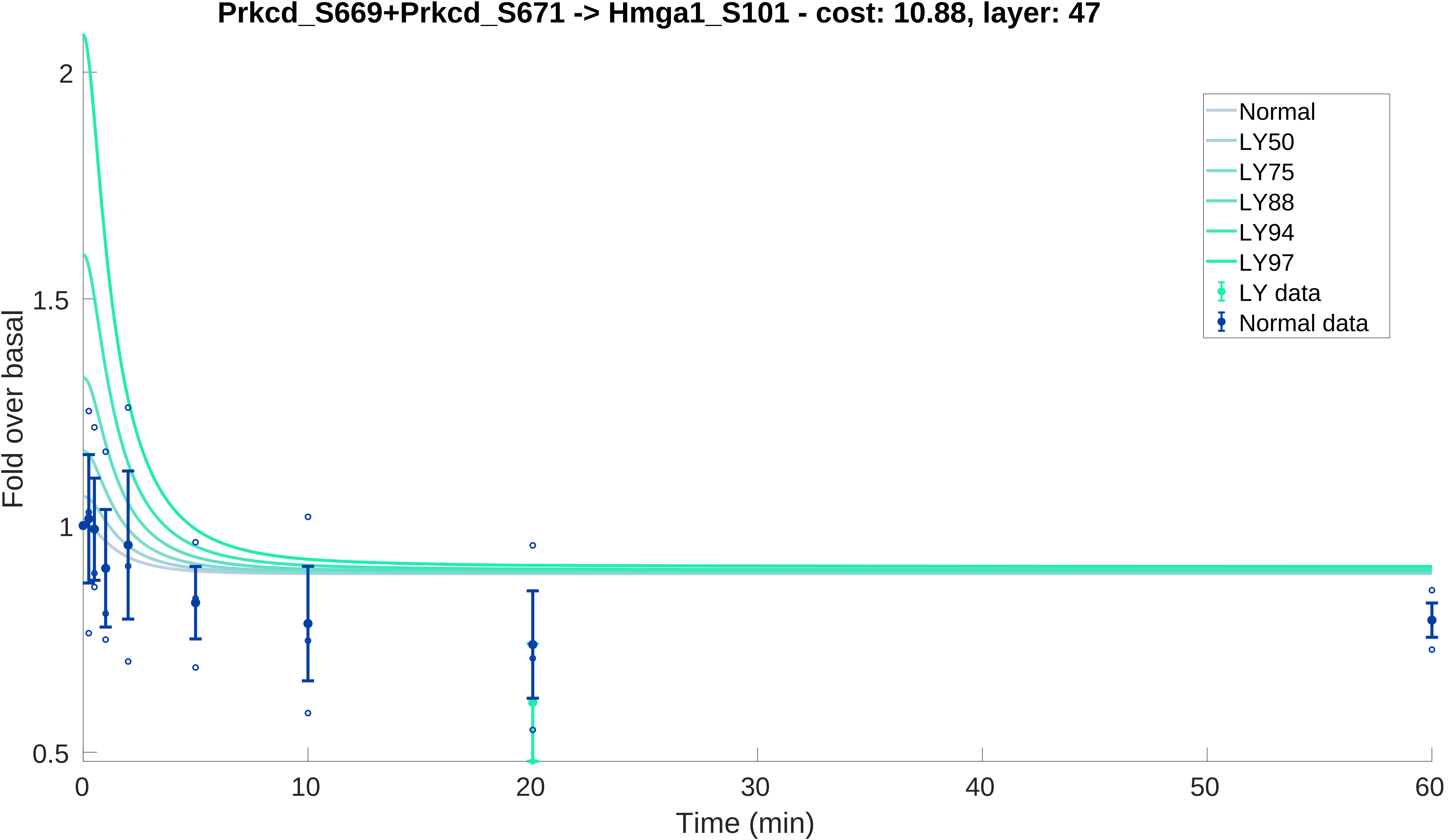

Prkcd_S669+Prkcd_S671 -> Hmga1_S101 - cost: 10.88, layer: 47
Fold over basal
Time (min)
0.5
1
1.5
2
0
10
20
30
40
50
60
Normal
LY50
LY75
LY88
LY94
LY97
LY data
Normal data

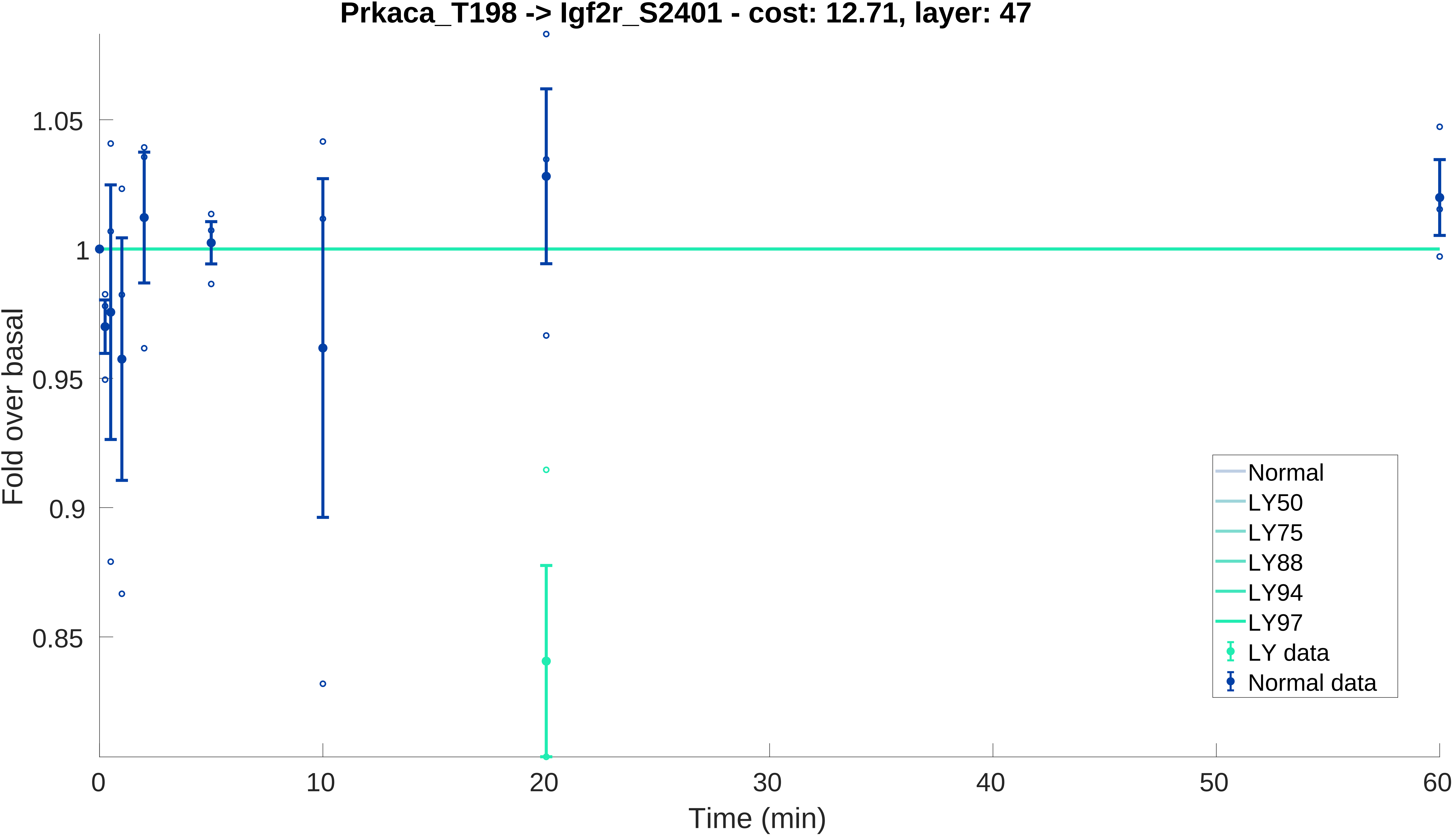
Prkaca_T198 -> Igf2r_S2401 - cost: 12.71, layer: 47
Fold over basal
1.05
1
0.95
0.9
0.85
0
10
20
30
40
50
60
Time (min)
Normal
LY50
LY75
LY88
LY94
LY97
LY data
Normal data

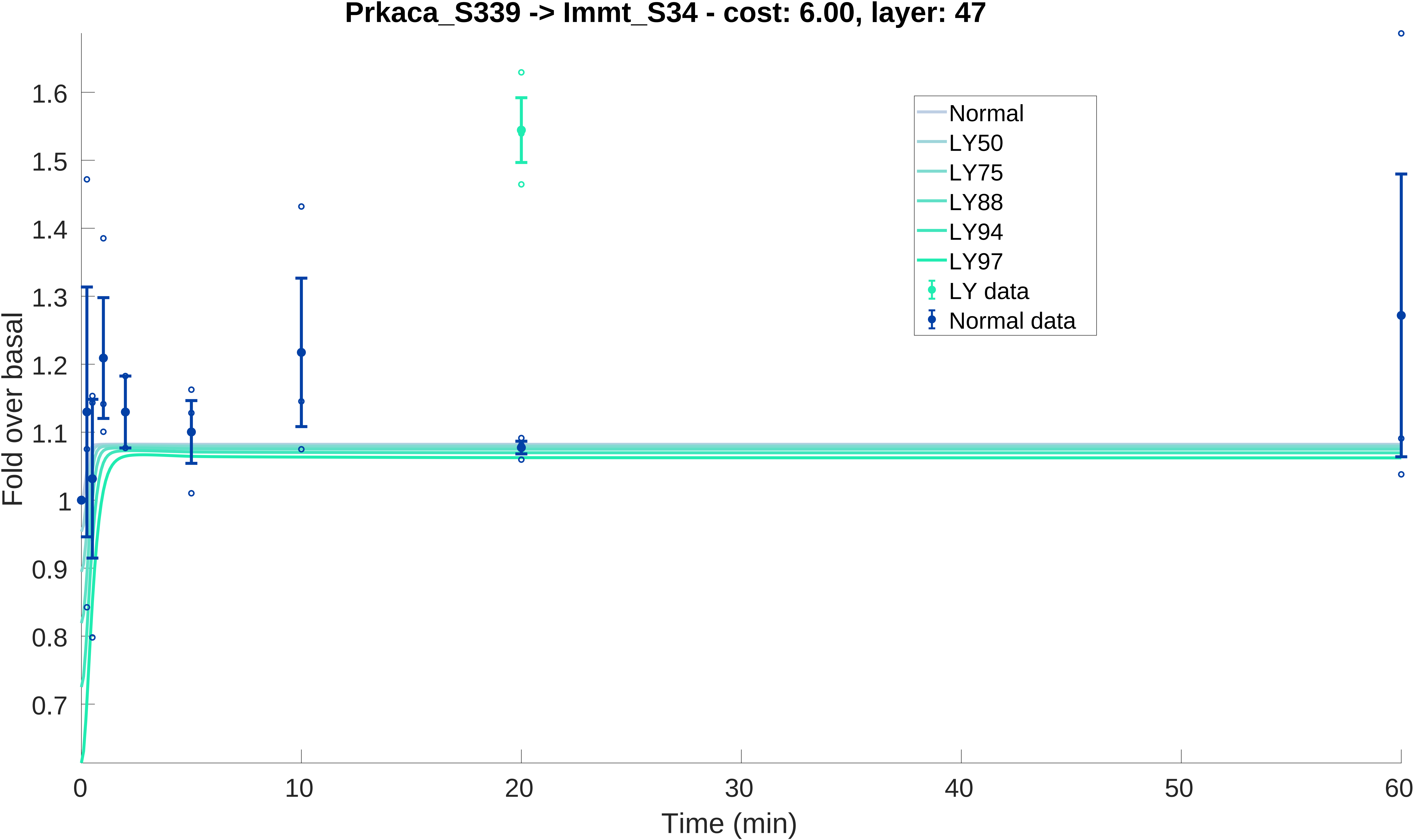

Prkaca_S339 -> Immt_S34 - cost: 6.00, layer: 47
Normal
LY50
LY75
LY88
LY94
LY97
LY data
Normal data
Fold over basal
Time (min)
0.7
0.8
0.9
1
1.1
1.2
1.3
1.4
1.5
1.6
0
10
20
30
40
50
60

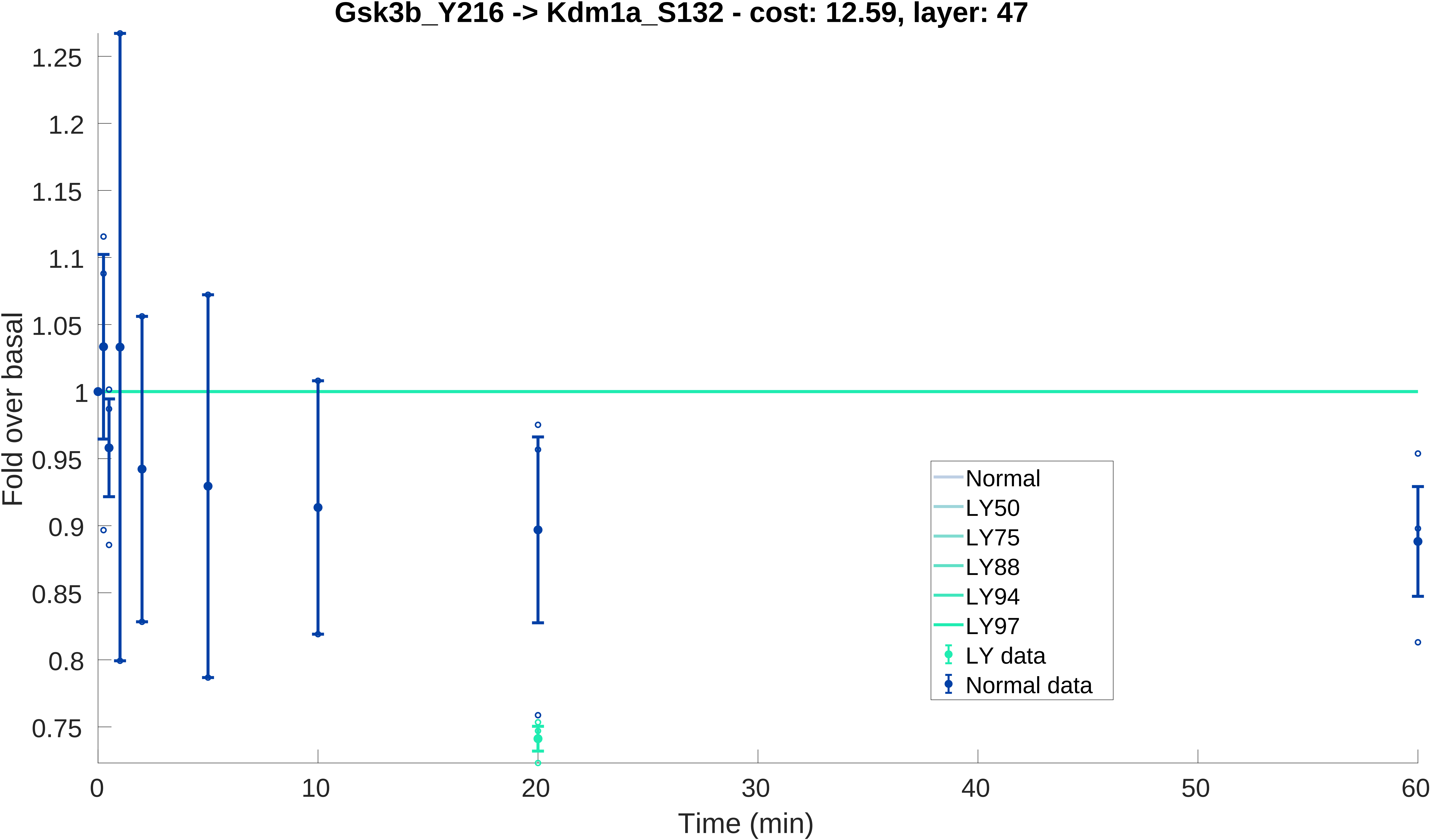

Gsk3b_Y216 -> Kdm1a_S132 - cost: 12.59, layer: 47
Fold over basal
Time (min)
1.25
1.2
1.15
1.1
1.05
1
0.95
0.9
0.85
0.8
0.75
0
10
20
30
40
50
60
Normal
LY50
LY75
LY88
LY94
LY97
LY data
Normal data

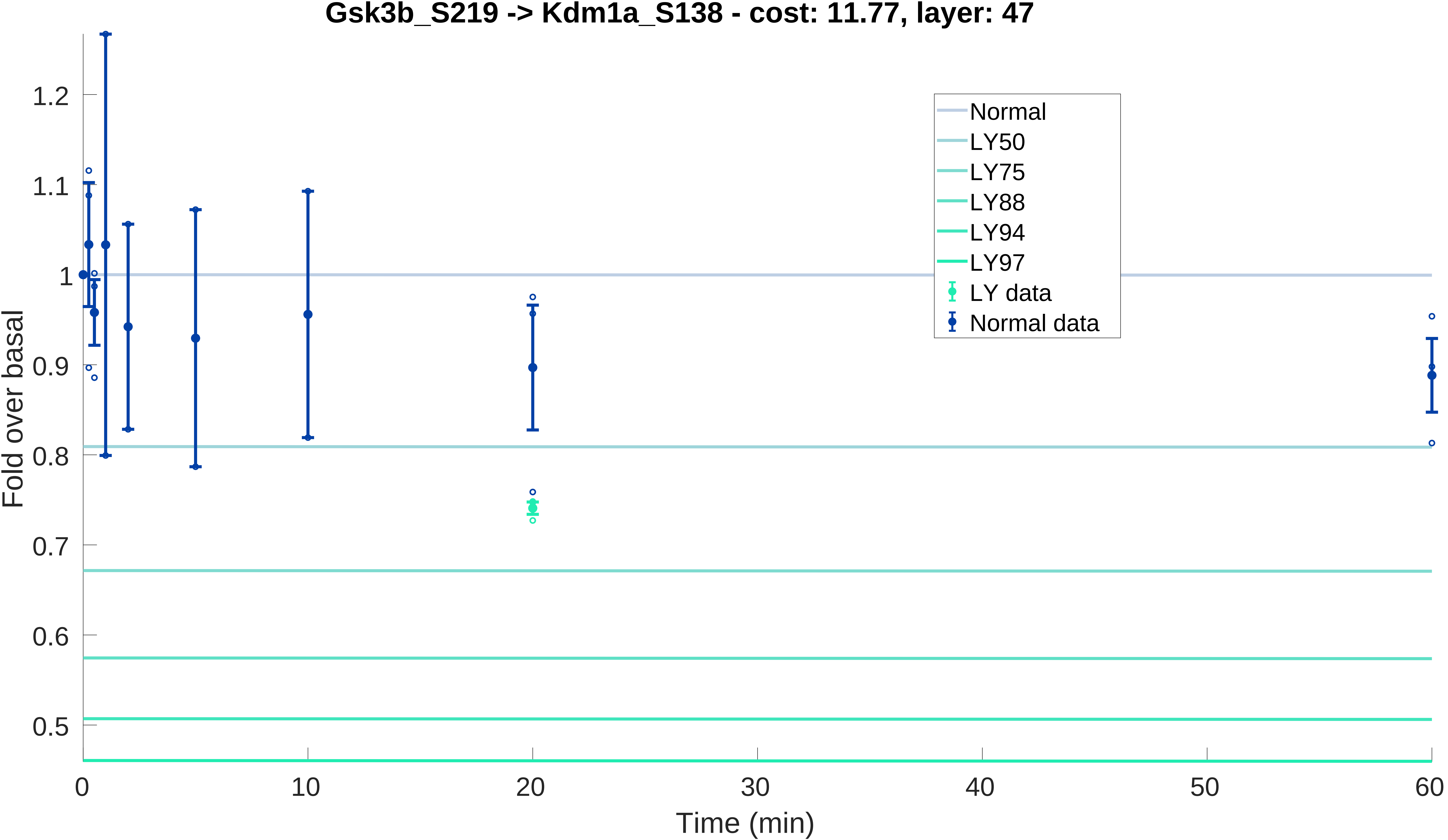

Gsk3b_S219 -> Kdm1a_S138 - cost: 11.77, layer: 47
Fold over basal
Time (min)
0
10
20
30
40
50
60
0.5
0.6
0.7
0.8
0.9
1
1.1
1.2
Normal
LY50
LY75
LY88
LY94
LY97
LY data
Normal data

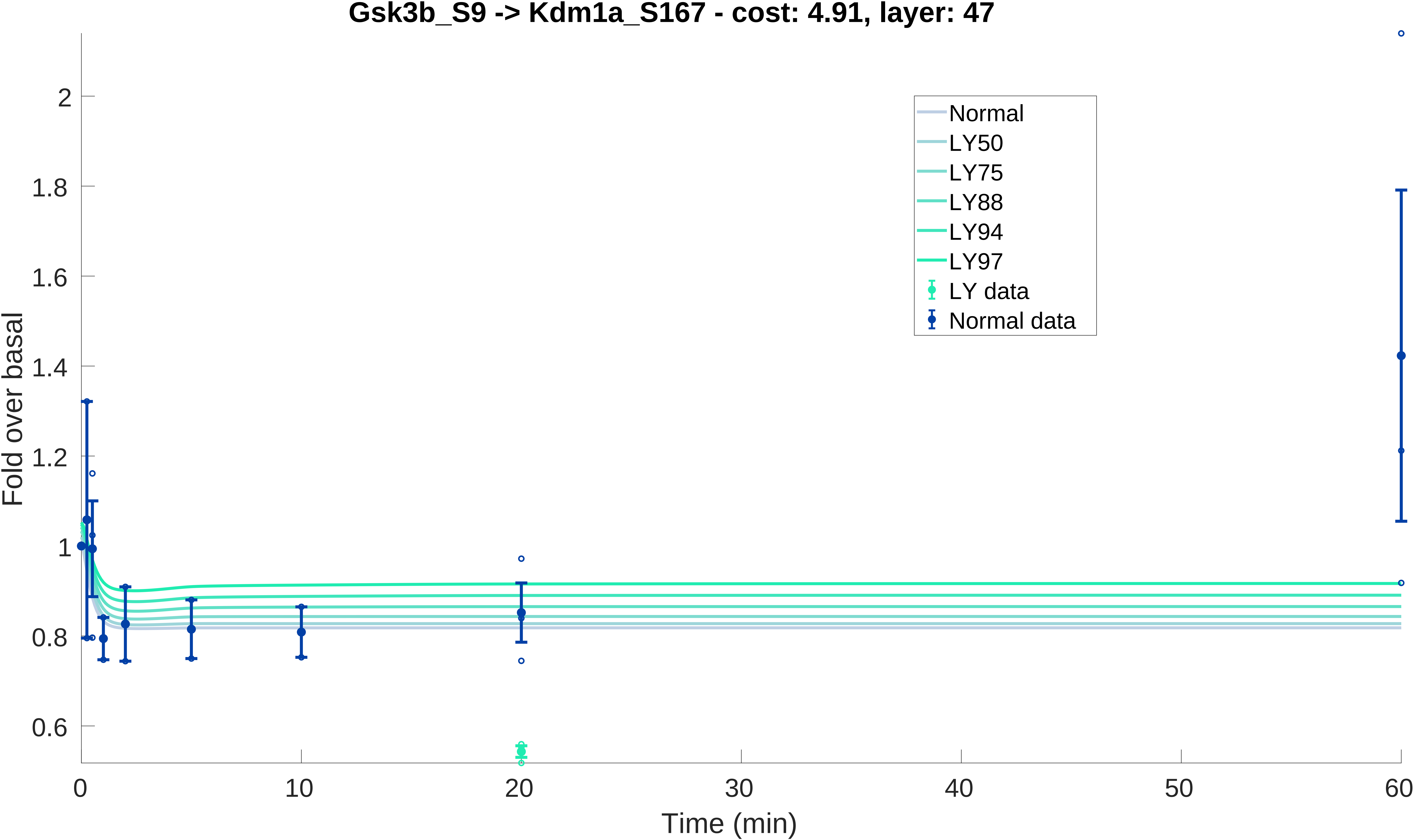

Gsk3b_S9 -> Kdm1a_S167 - cost: 4.91, layer: 47
Fold over basal
Time (min)
0.6
0.8
1
1.2
1.4
1.6
1.8
2
0
10
20
30
40
50
60
Normal
LY50
LY75
LY88
LY94
LY97
LY data
Normal data

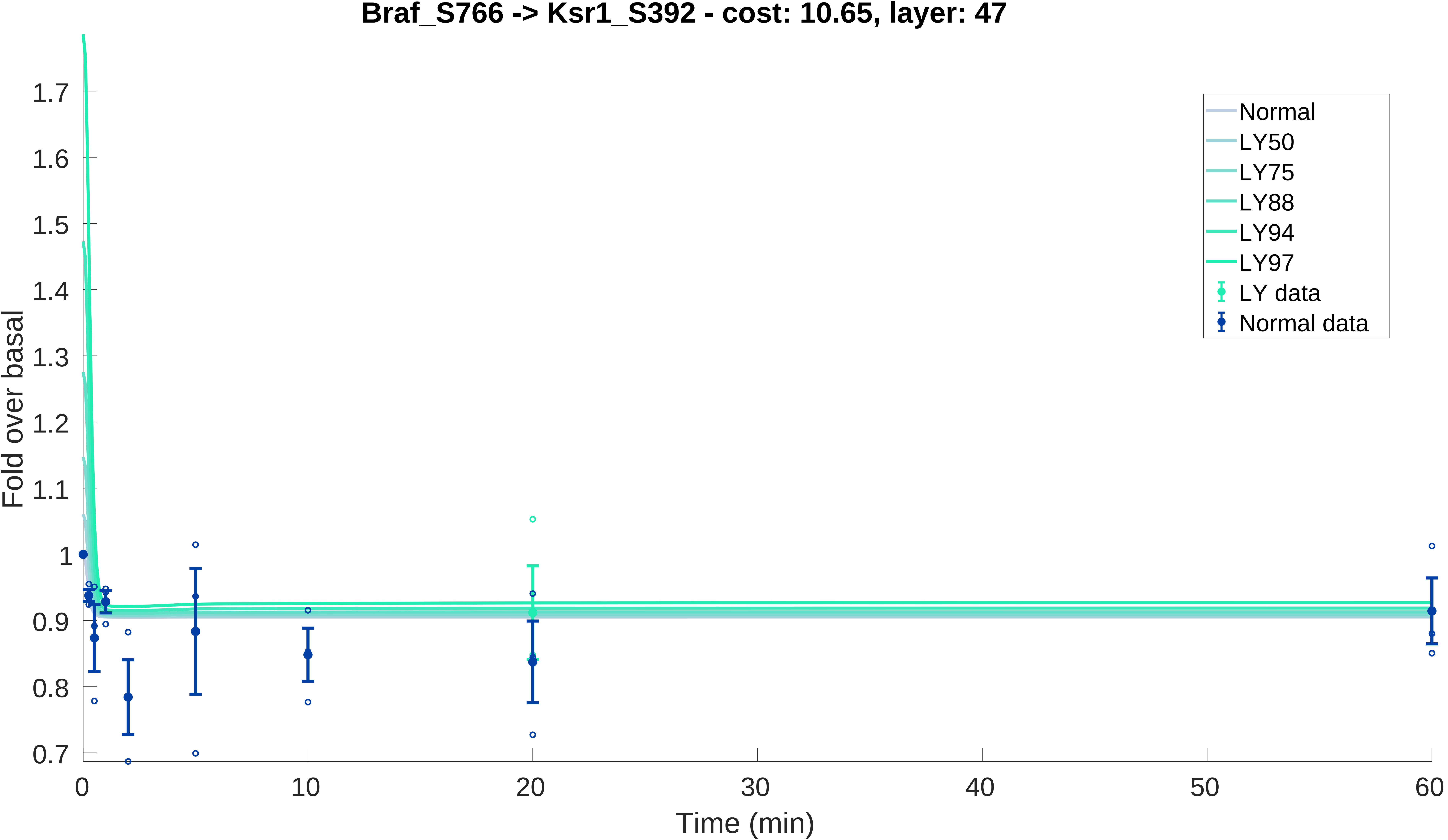
Braf_S766 -> Ksr1_S392 - cost: 10.65, layer: 47
Fold over basal
Time (min)
Normal
LY50
LY75
LY88
LY94
LY97
LY data
Normal data
1.7
1.6
1.5
1.4
1.3
1.2
1.1
1
0.9
0.8
0.7
0
10
20
30
40
50
60

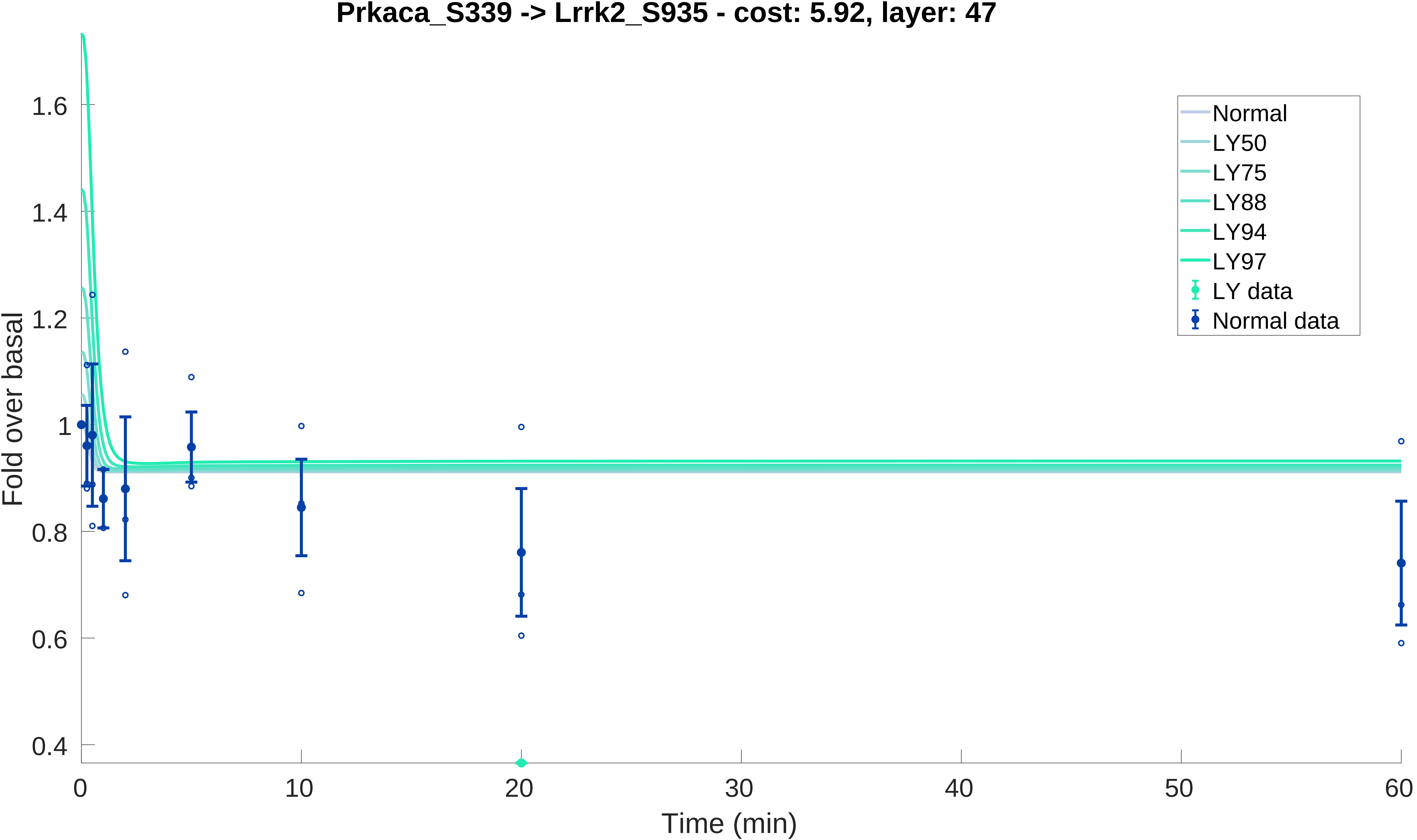

Prkaca_S339 -> Lrrk2_S935 - cost: 5.92, layer: 47
Normal
LY50
LY75
LY88
LY94
LY97
LY data
Normal data
Fold over basal
Time (min)
1.6
1.4
1.2
1
0.8
0.6
0.4
0
10
20
30
40
50
60

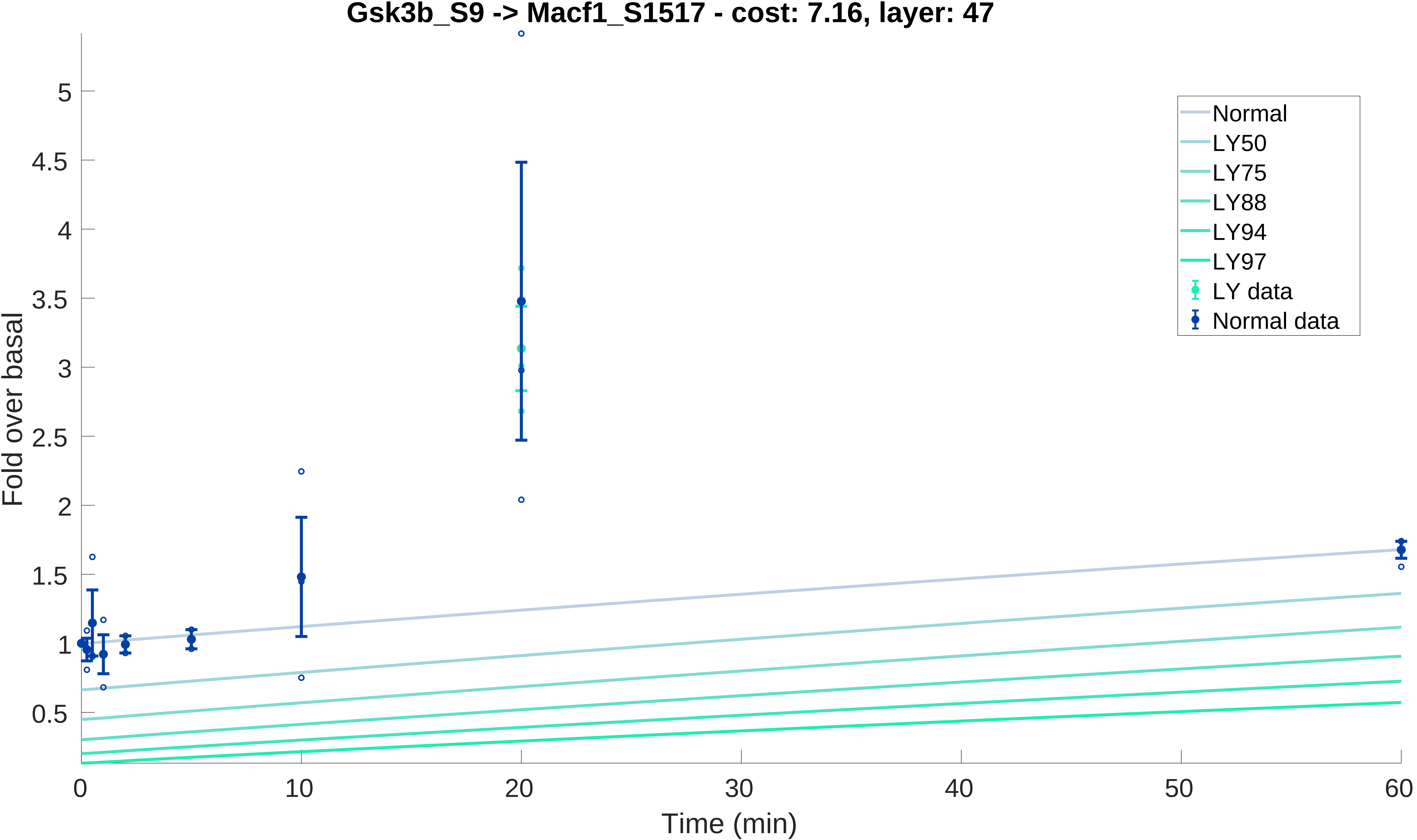
Gsk3b_S9 -> Macf1_S1517 - cost: 7.16, layer: 47
Fold over basal
Time (min)
Normal
LY50
LY75
LY88
LY94
LY97
LY data
Normal data
0
10
20
30
40
50
60
0.5
1
1.5
2
2.5
3
3.5
4
4.5
5

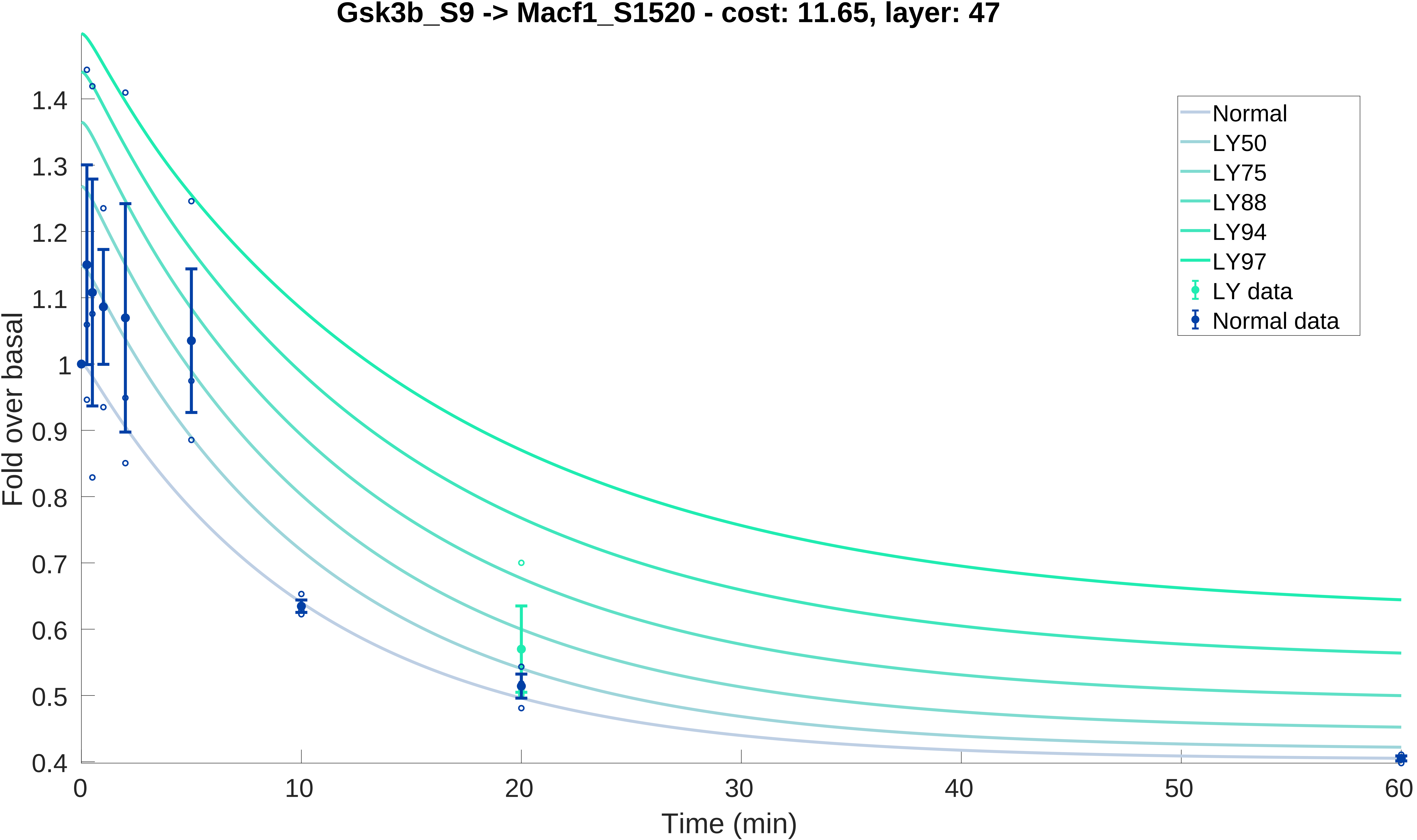

Gsk3b_S9 -> Macf1_S1520 - cost: 11.65, layer: 47
Fold over basal
Time (min)
1.4
1.3
1.2
1.1
1
0.9
0.8
0.7
0.6
0.5
0.4
0
10
20
30
40
50
60
Normal
LY50
LY75
LY88
LY94
LY97
LY data
Normal data

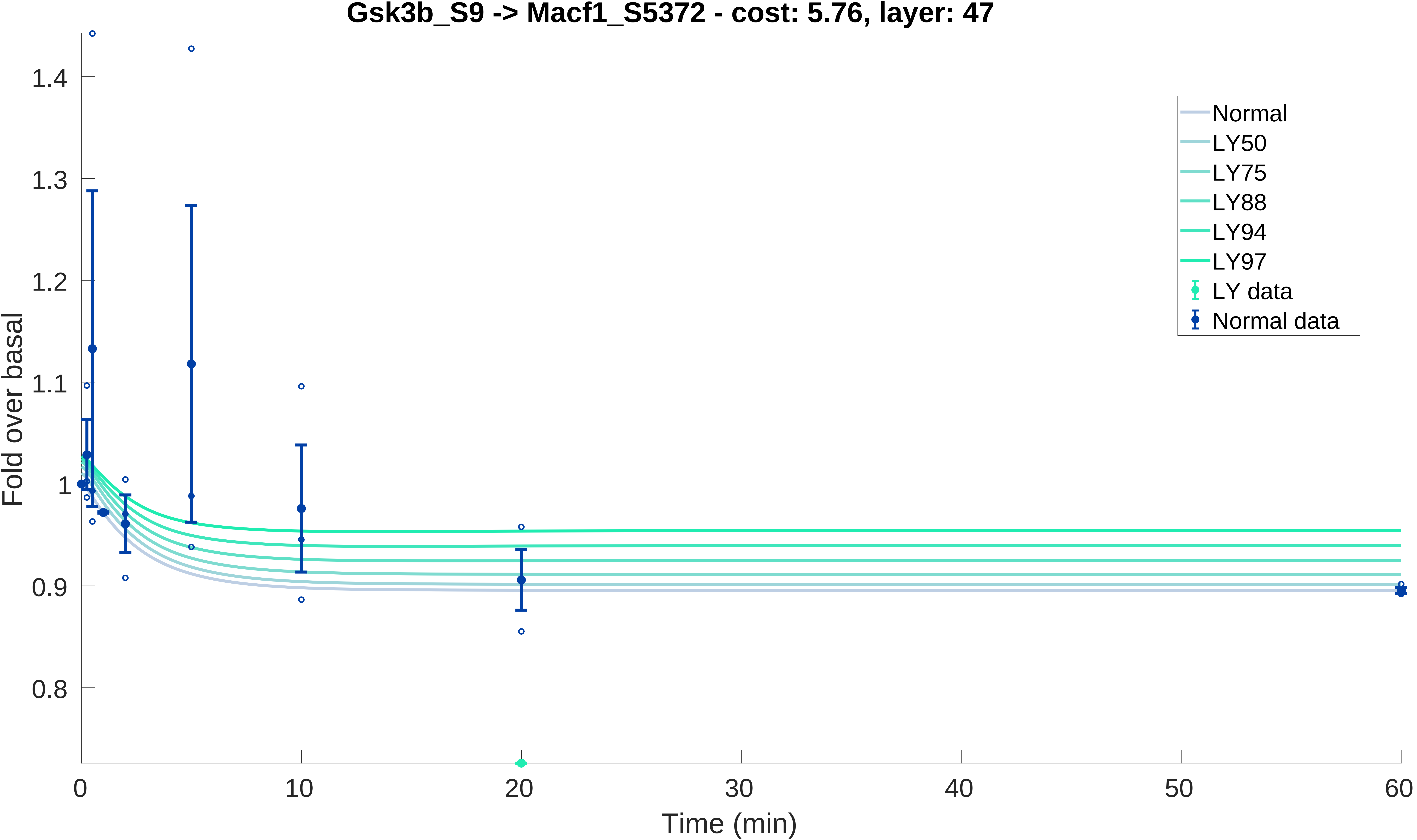

Gsk3b_S9 -> Macf1_S5372 - cost: 5.76, layer: 47
Fold over basal
1.4
1.3
1.2
1.1
1
0.9
0.8
0
10
20
30
40
50
60
Time (min)
Normal
LY50
LY75
LY88
LY94
LY97
LY data
Normal data

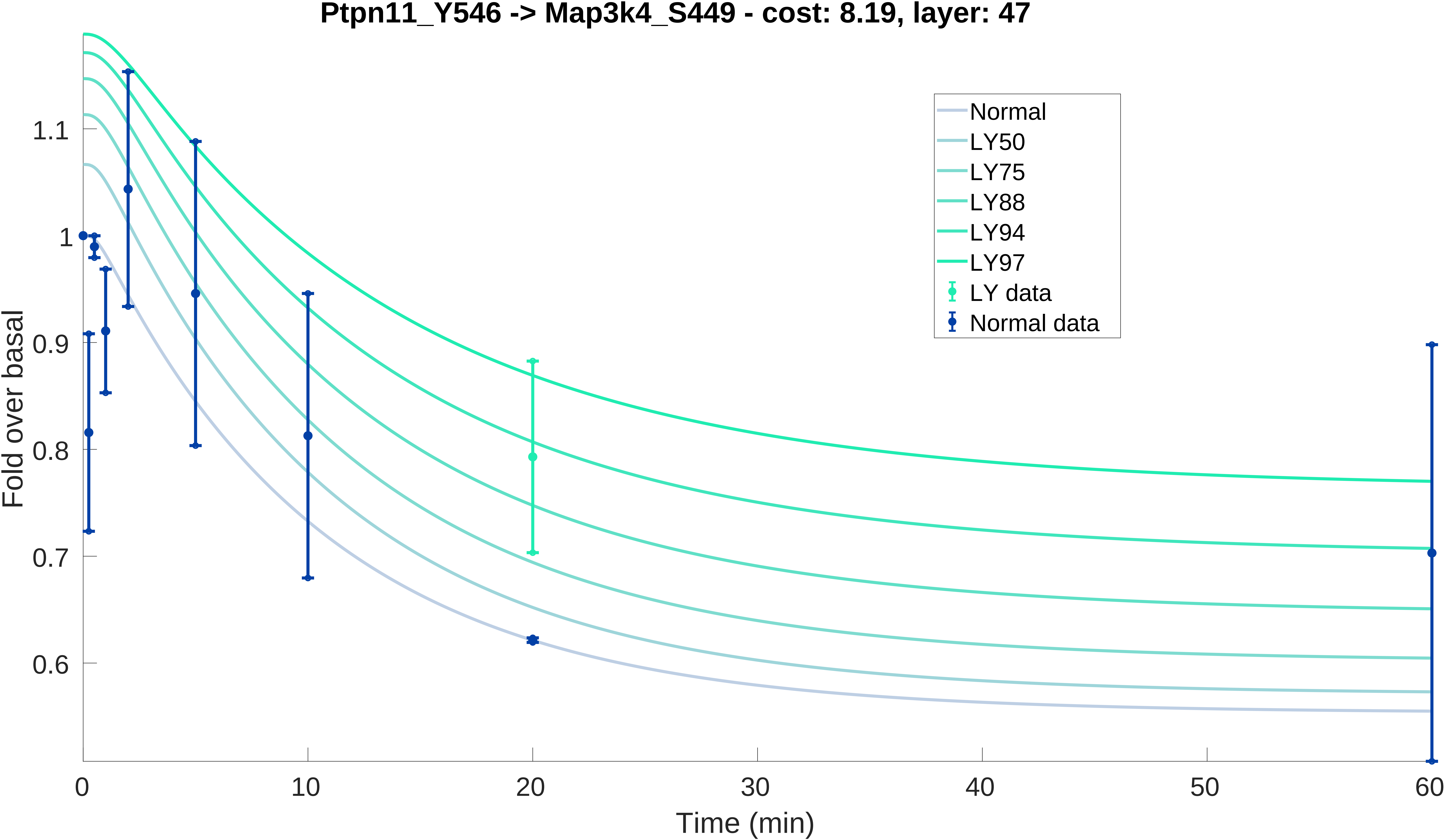

Ptpn11_Y546 -> Map3k4_S449 - cost: 8.19, layer: 47
Normal
LY50
LY75
LY88
LY94
LY97
LY data
Normal data
1.1
1
0.9
0.8
0.7
0.6
Fold over basal
0
10
20
30
40
50
60
Time (min)

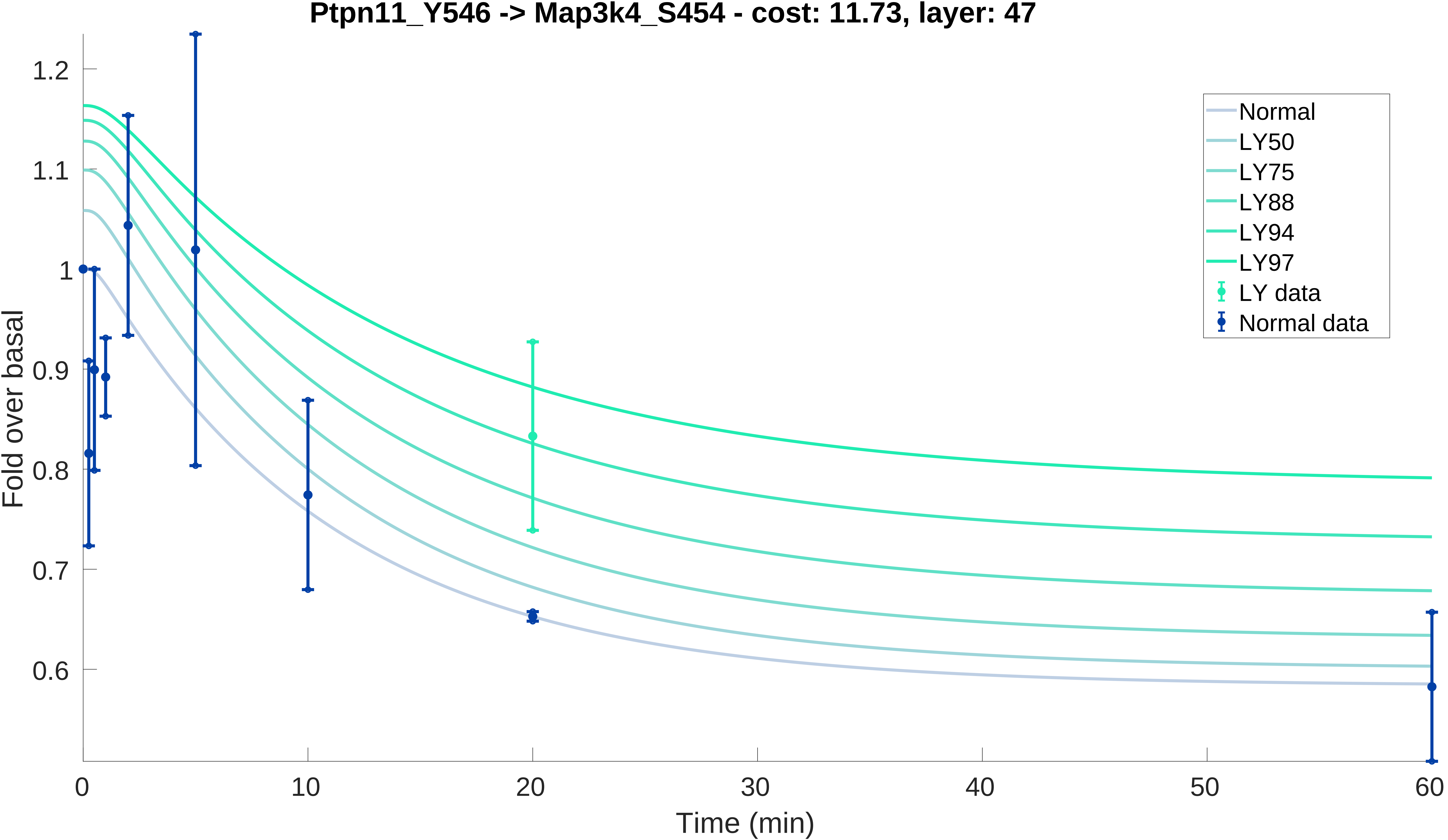
Ptpn11_Y546 -> Map3k4_S454 - cost: 11.73, layer: 47
Fold over basal
Time (min)
Normal
LY50
LY75
LY88
LY94
LY97
LY data
Normal data
0
10
20
30
40
50
60
0.6
0.7
0.8
0.9
1
1.1
1.2

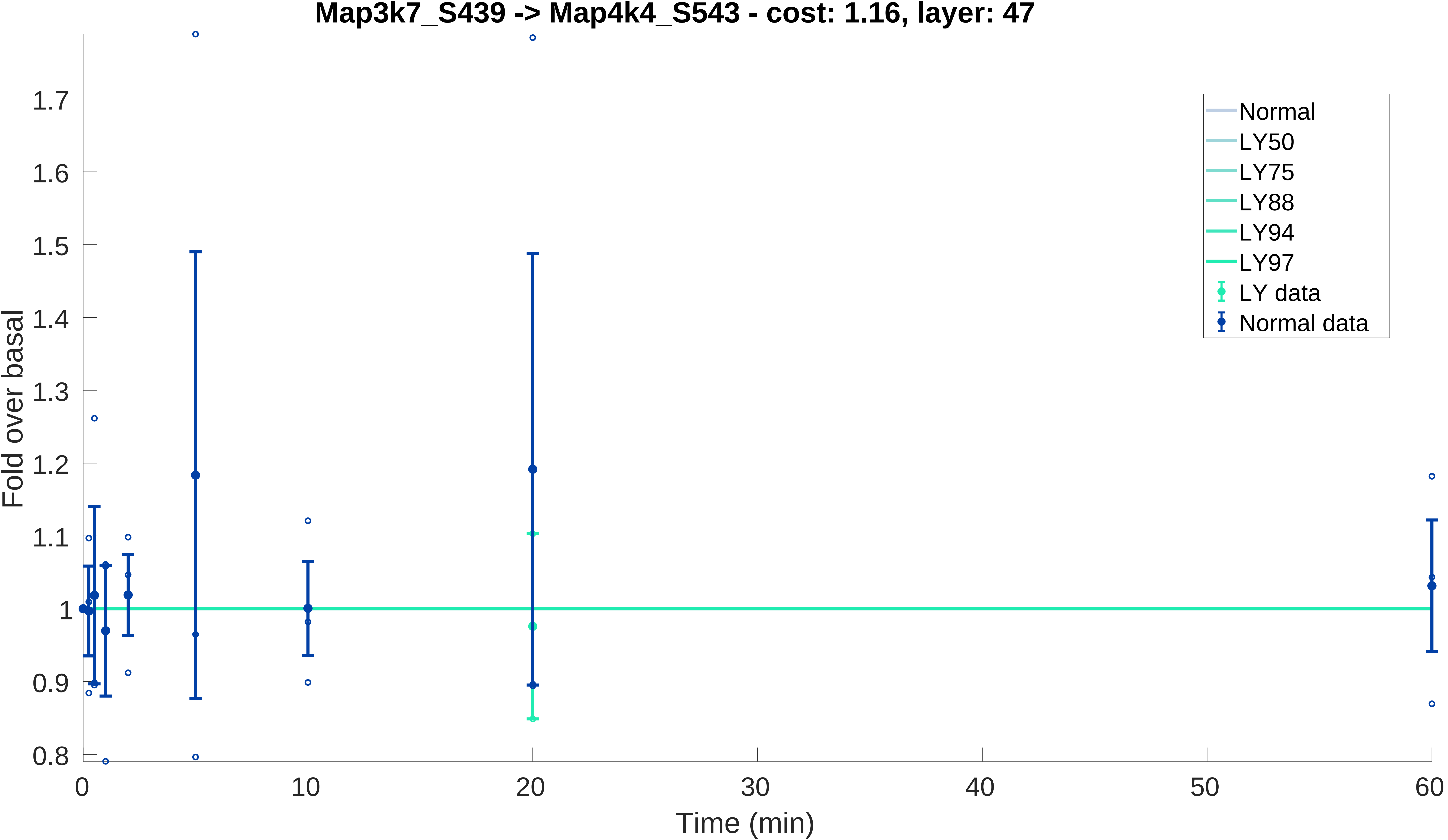

Map3k7_S439 -> Map4k4_S543 - cost: 1.16, layer: 47
Fold over basal
Time (min)
0.8
0.9
1
1.1
1.2
1.3
1.4
1.5
1.6
1.7
0
10
20
30
40
50
60
Normal
LY50
LY75
LY88
LY94
LY97
LY data
Normal data

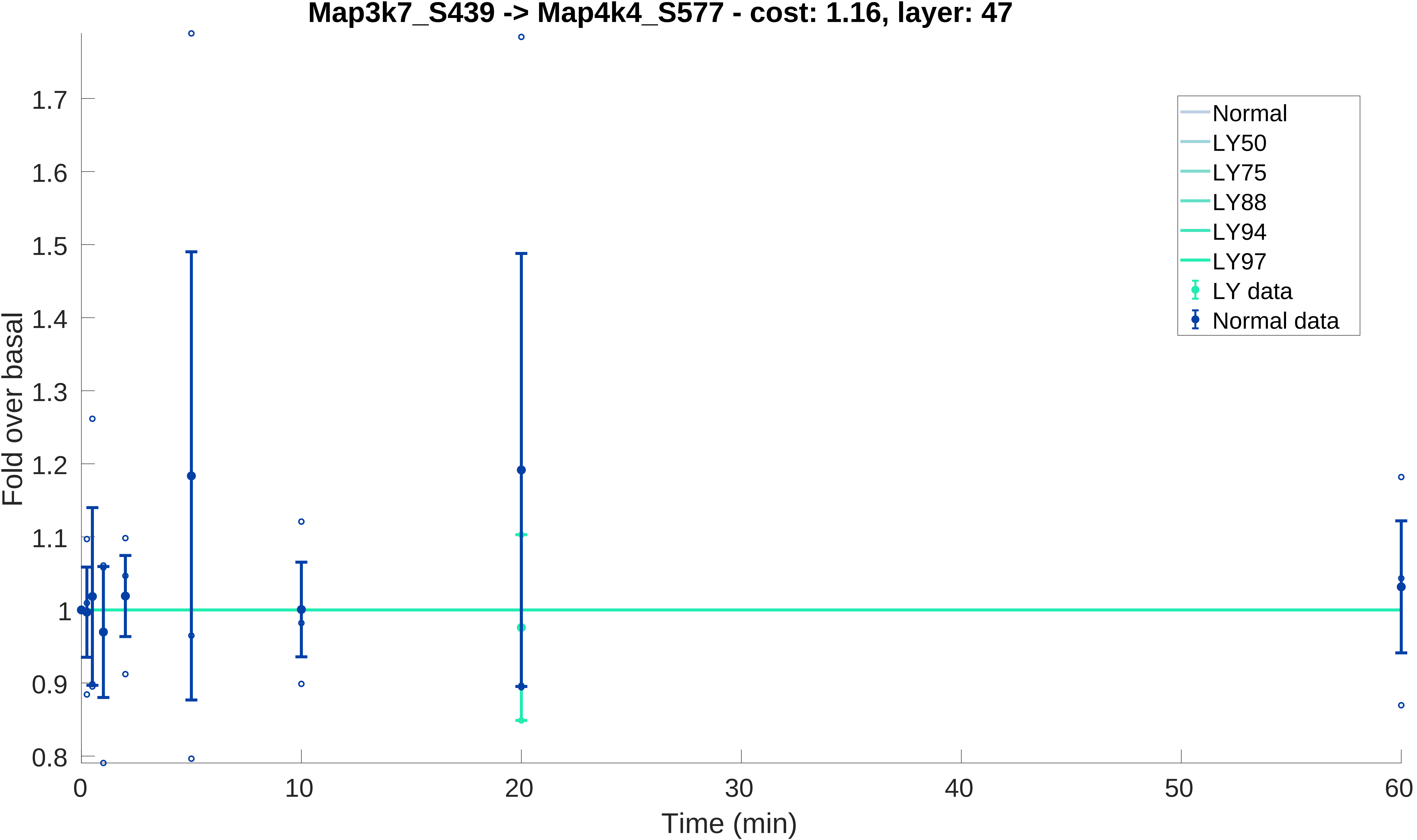

Map3k7_S439 -> Map4k4_S577 - cost: 1.16, layer: 47
Fold over basal
Time (min)
Normal
LY50
LY75
LY88
LY94
LY97
LY data
Normal data
0.8
0.9
1
1.1
1.2
1.3
1.4
1.5
1.6
1.7
0
10
20
30
40
50
60

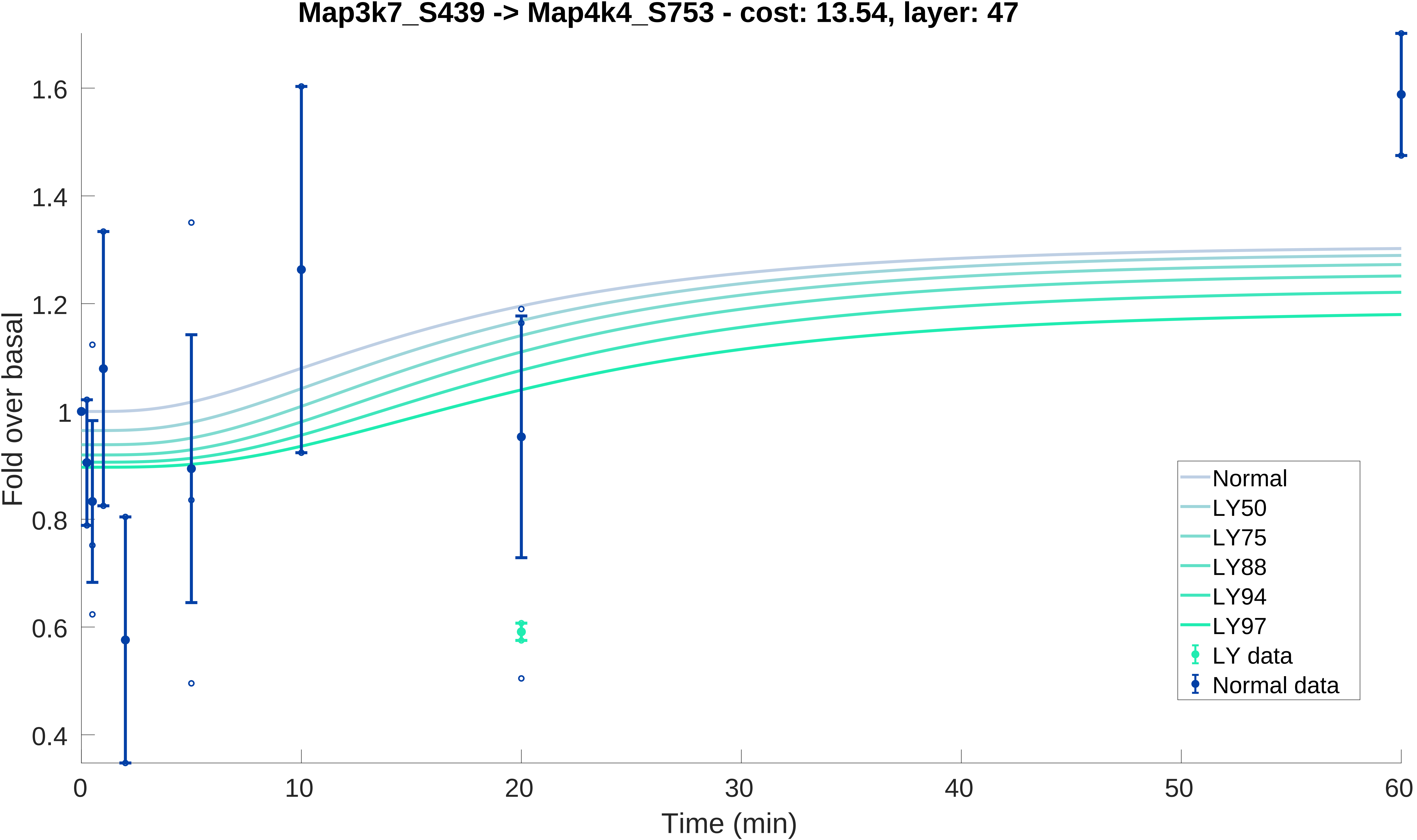
Map3k7_S439 -> Map4k4_S753 - cost: 13.54, layer: 47
Fold over basal
Time (min)
0.4
0.6
0.8
1
1.2
1.4
1.6
0
10
20
30
40
50
60
Normal
LY50
LY75
LY88
LY94
LY97
LY data
Normal data

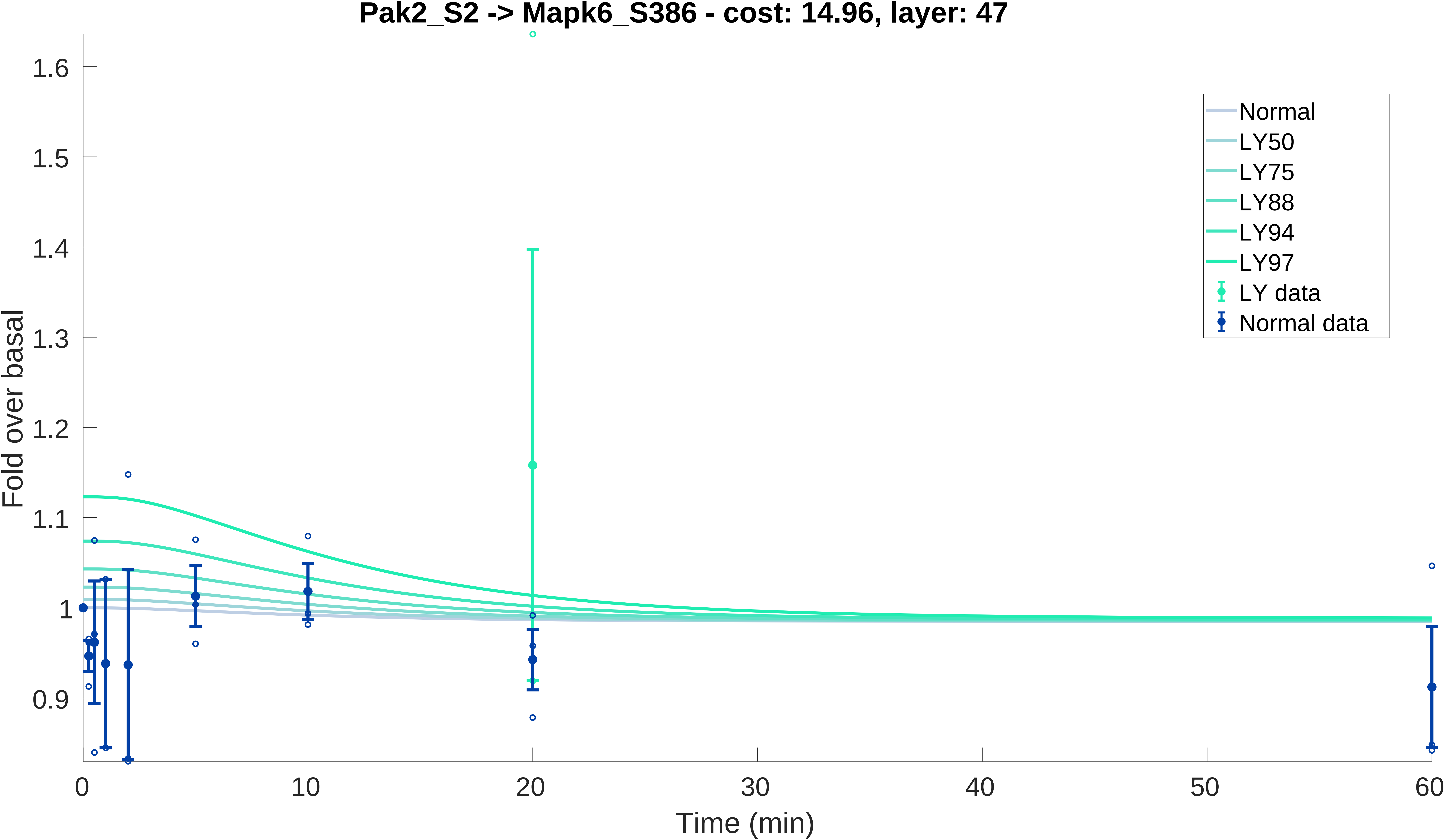
Pak2_S2 -> Mapk6_S386 - cost: 14.96, layer: 47
Fold over basal
Time (min)
1.6
1.5
1.4
1.3
1.2
1.1
1
0.9
0
10
20
30
40
50
60
Normal
LY50
LY75
LY88
LY94
LY97
LY data
Normal data

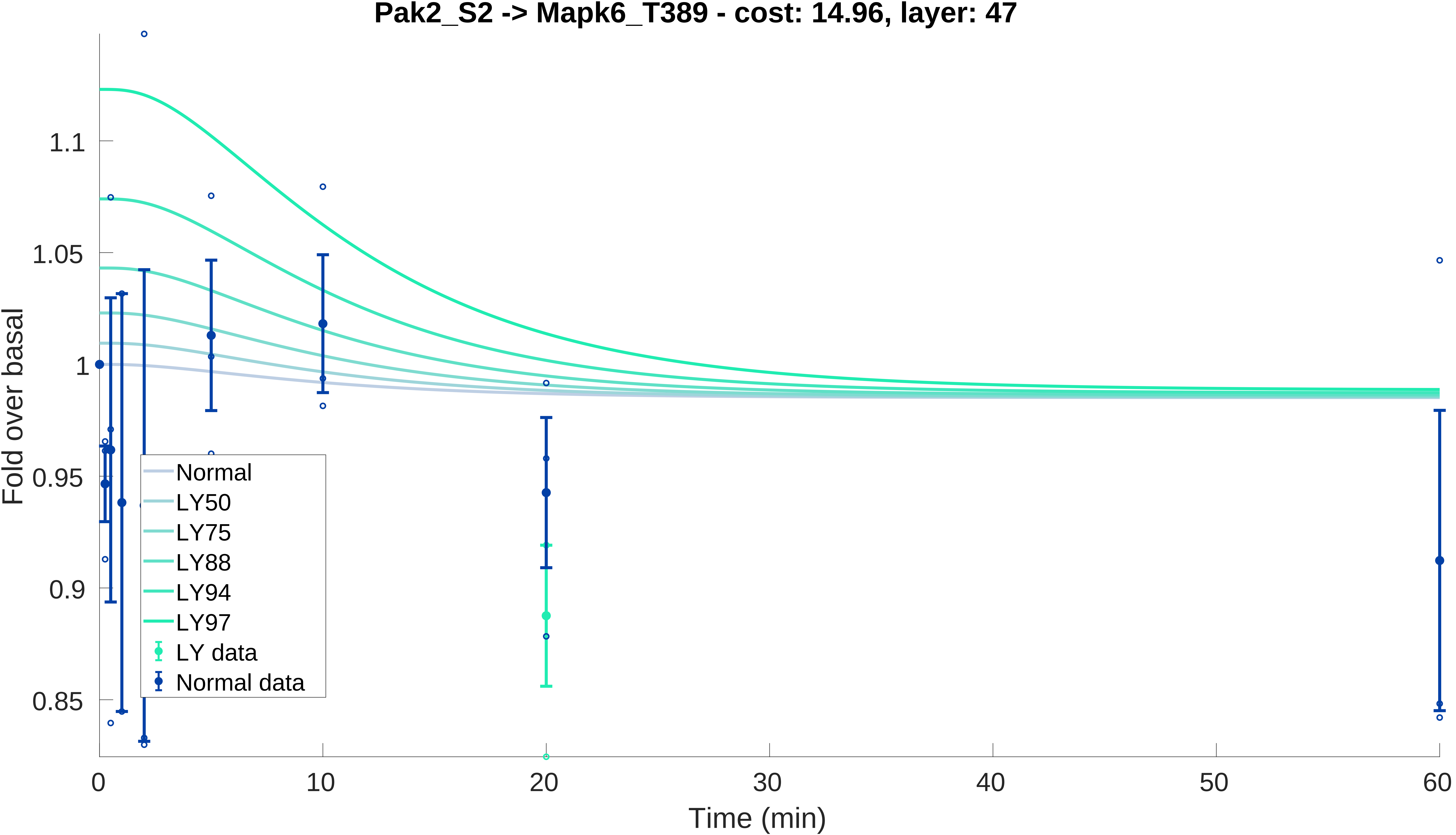

Pak2_S2 -> Mapk6_T389 - cost: 14.96, layer: 47
Fold over basal
Time (min)
1.1
1.05
1
0.95
0.9
0.85
0
10
20
30
40
50
60
Normal
LY50
LY75
LY88
LY94
LY97
LY data
Normal data

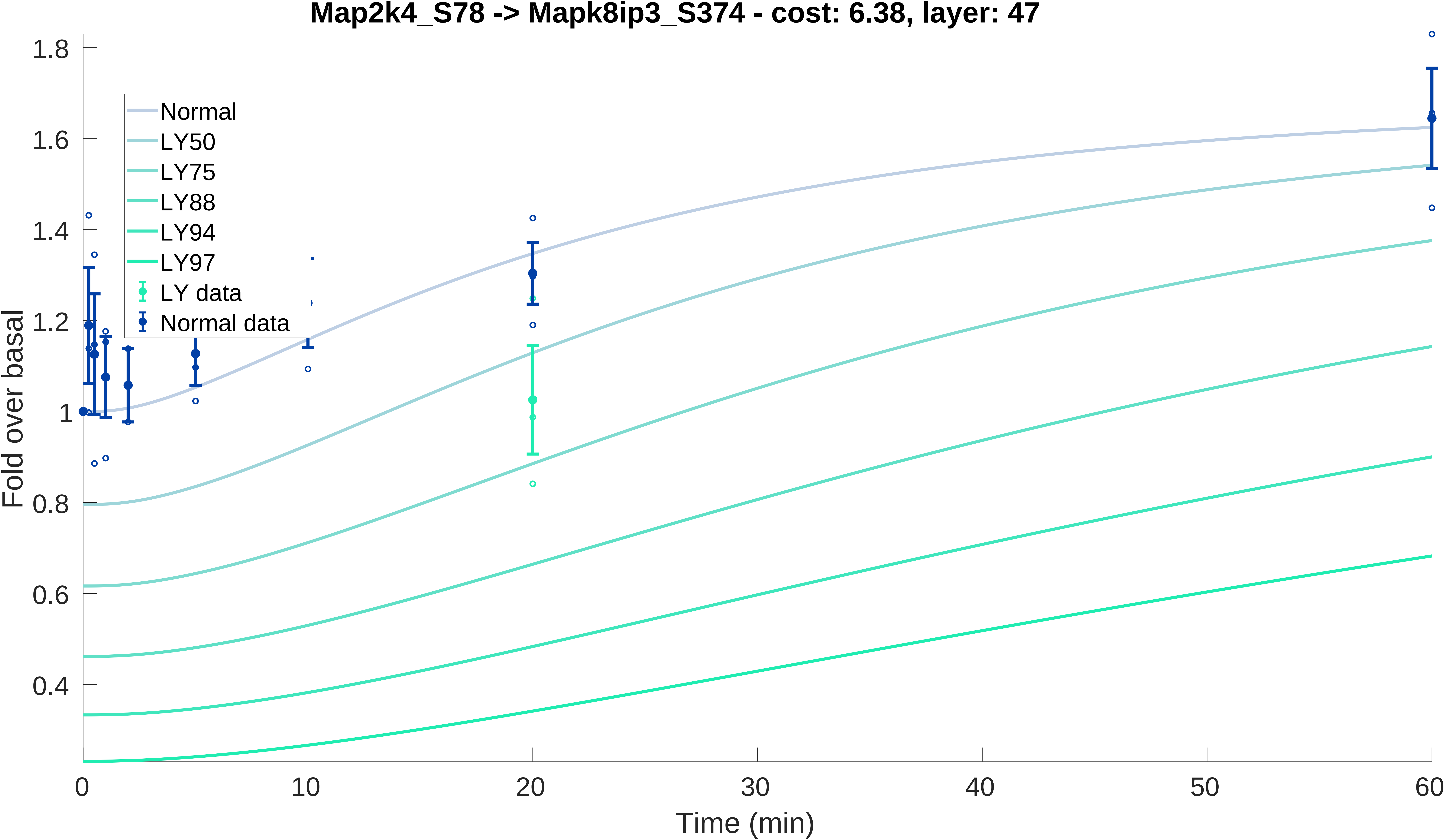

Map2k4_S78 -> Mapk8ip3_S374 - cost: 6.38, layer: 47
Normal
LY50
LY75
LY88
LY94
LY97
LY data
Normal data
Fold over basal
Time (min)
0.4
0.6
0.8
1
1.2
1.4
1.6
1.8
0
10
20
30
40
50
60

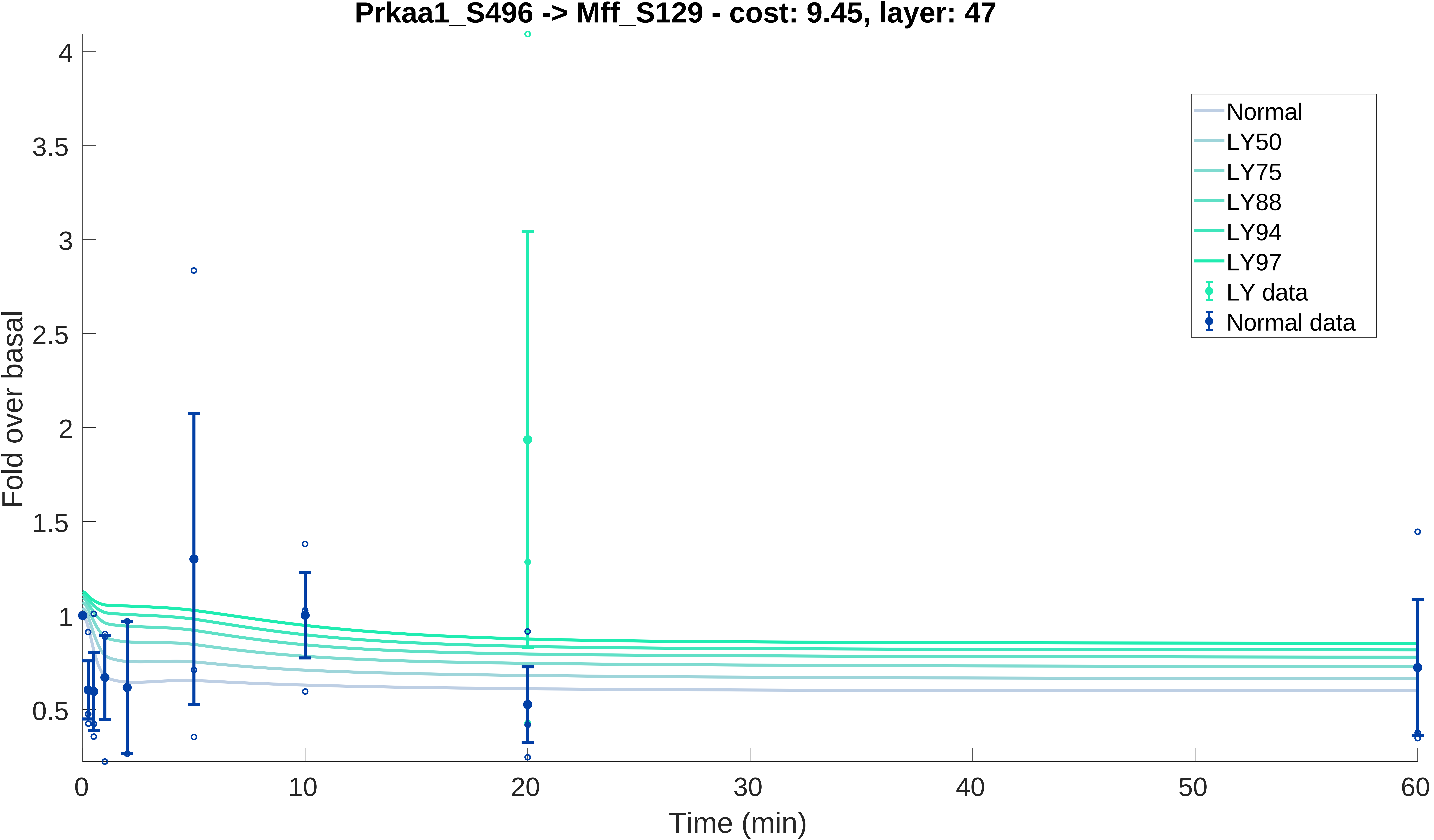
Prkaa1_S496 -> Mff_S129 - cost: 9.45, layer: 47
Fold over basal
4
3.5
3
2.5
2
1.5
1
0.5
0
10
20
30
40
50
60
Time (min)
Normal
LY50
LY75
LY88
LY94
LY97
LY data
Normal data

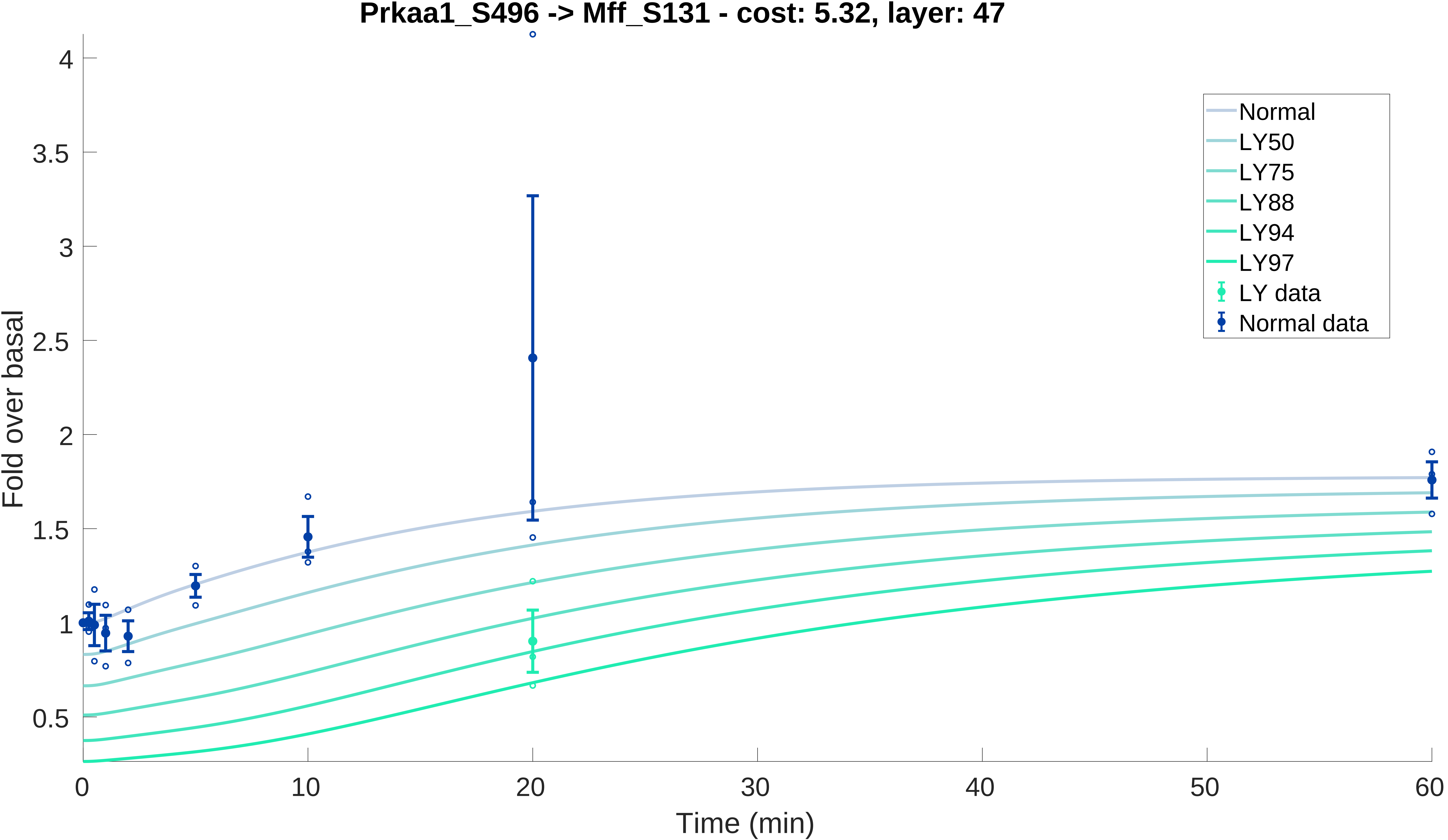

Prkaa1_S496 -> Mff_S131 - cost: 5.32, layer: 47
Fold over basal
4
3.5
3
2.5
2
1.5
1
0.5
0
10
20
30
40
50
60
Time (min)
Normal
LY50
LY75
LY88
LY94
LY97
LY data
Normal data

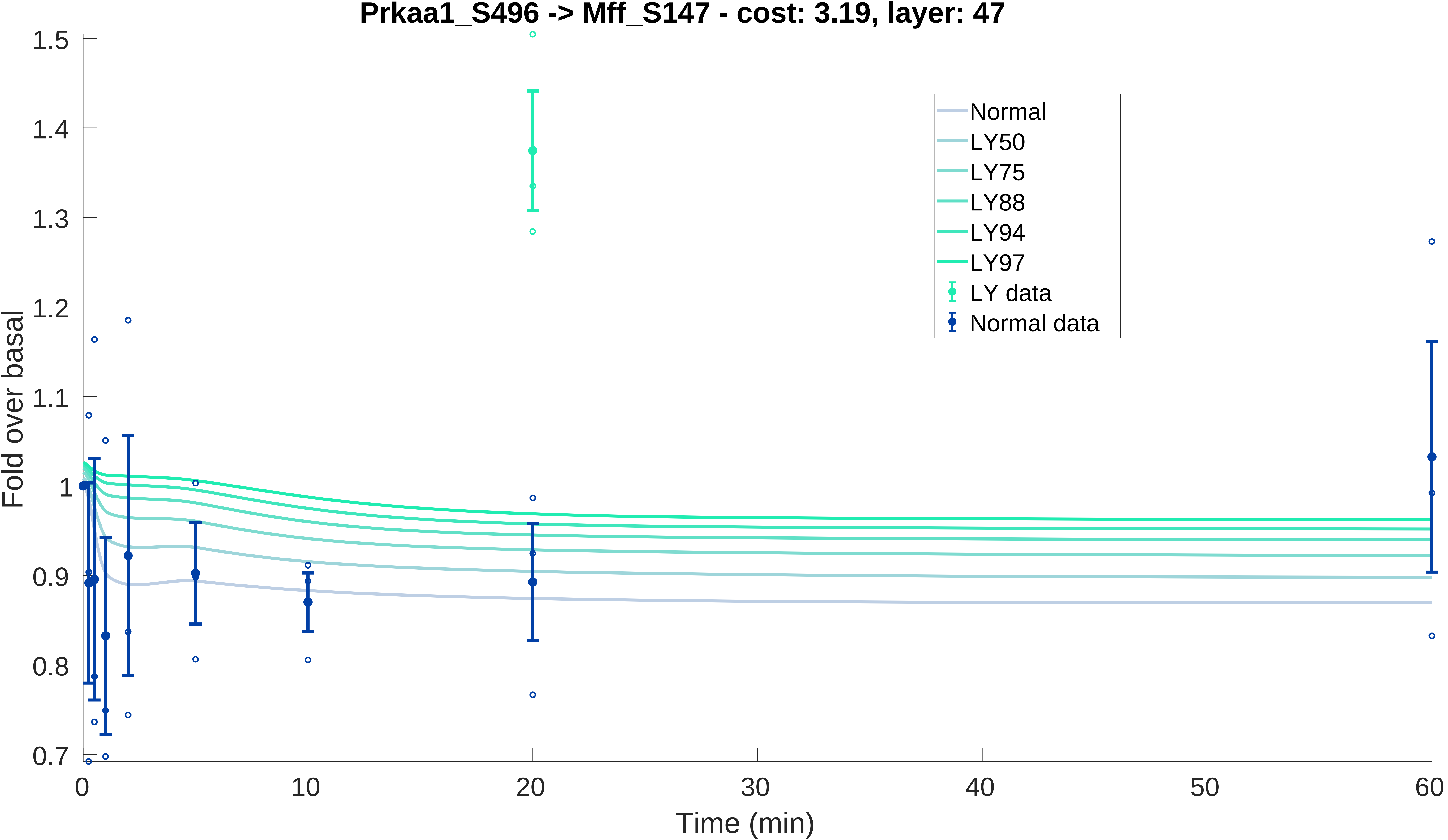

Prkaa1_S496 -> Mff_S147 - cost: 3.19, layer: 47
1.5
1.4
1.3
1.2
1.1
1
0.9
0.8
0.7
Fold over basal
0
10
20
30
40
50
60
Time (min)
Normal
LY50
LY75
LY88
LY94
LY97
LY data
Normal data

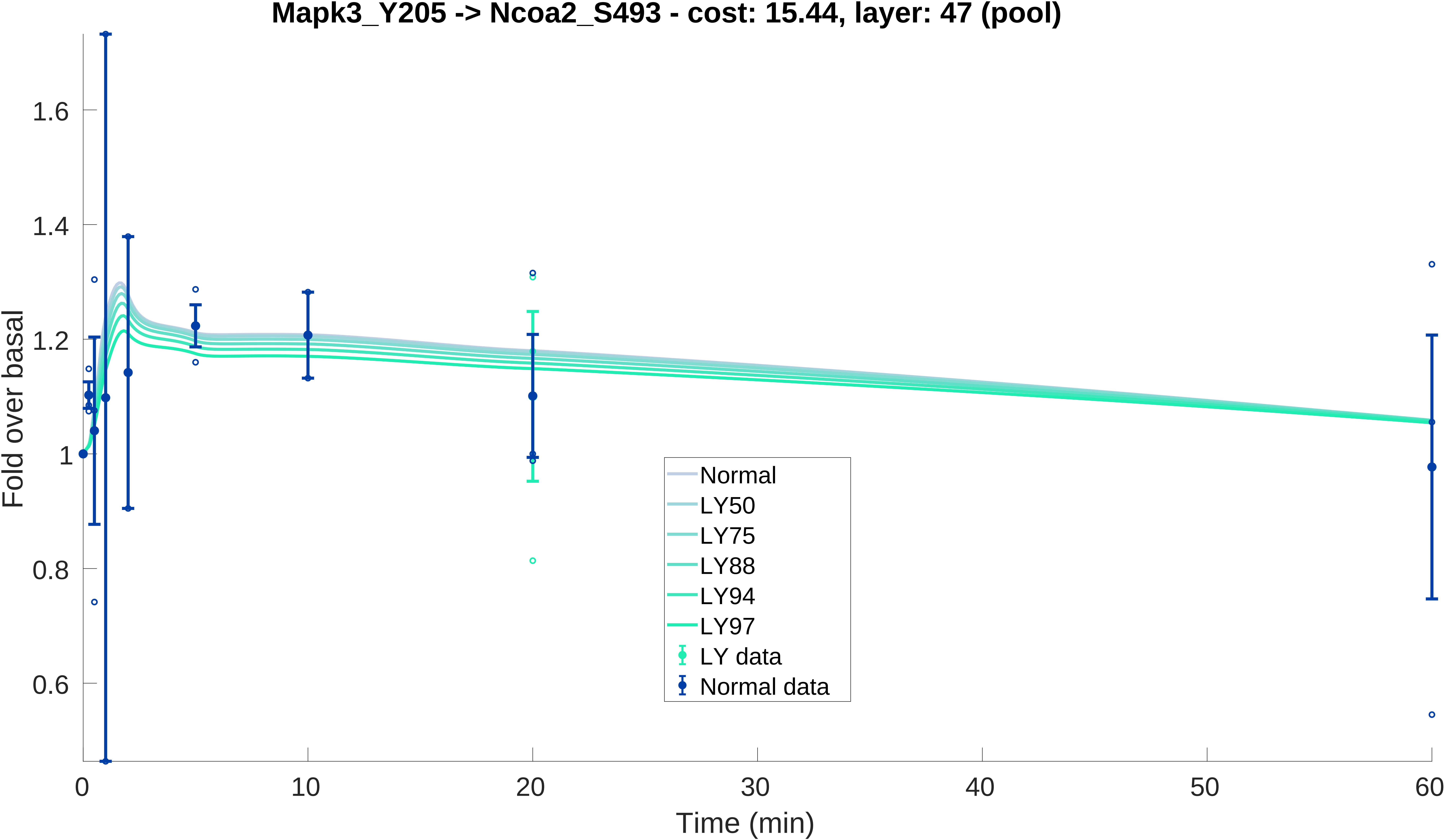
Mapk3_Y205 -> Ncoa2_S493 - cost: 15.44, layer: 47 (pool)
Fold over basal
Time (min)
0.6
0.8
1
1.2
1.4
1.6
0
10
20
30
40
50
60
Normal
LY50
LY75
LY88
LY94
LY97
LY data
Normal data

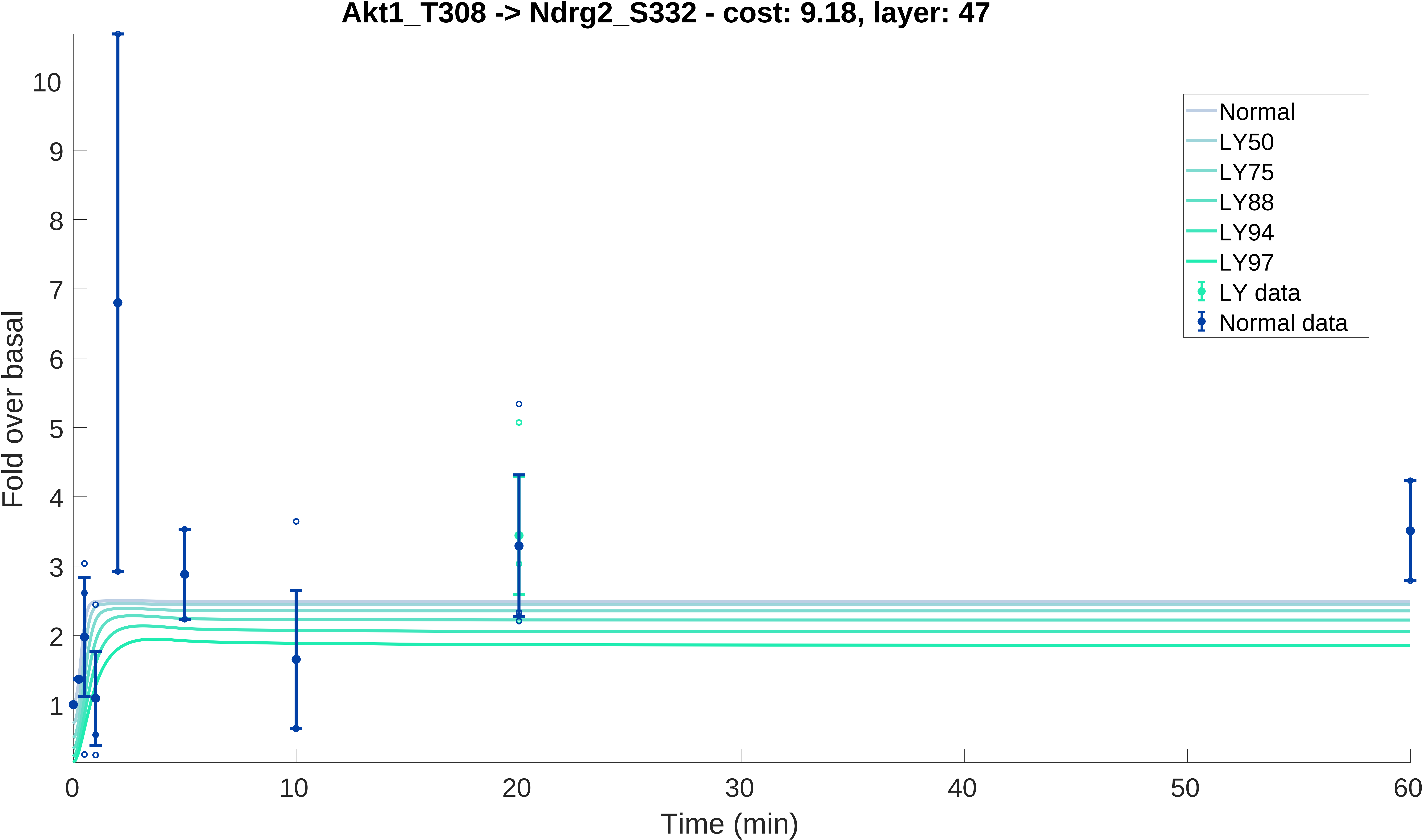

Akt1_T308 -> Ndrg2_S332 - cost: 9.18, layer: 47
Fold over basal
Time (min)
0
10
20
30
40
50
60
1
2
3
4
5
6
7
8
9
10
Normal
LY50
LY75
LY88
LY94
LY97
LY data
Normal data

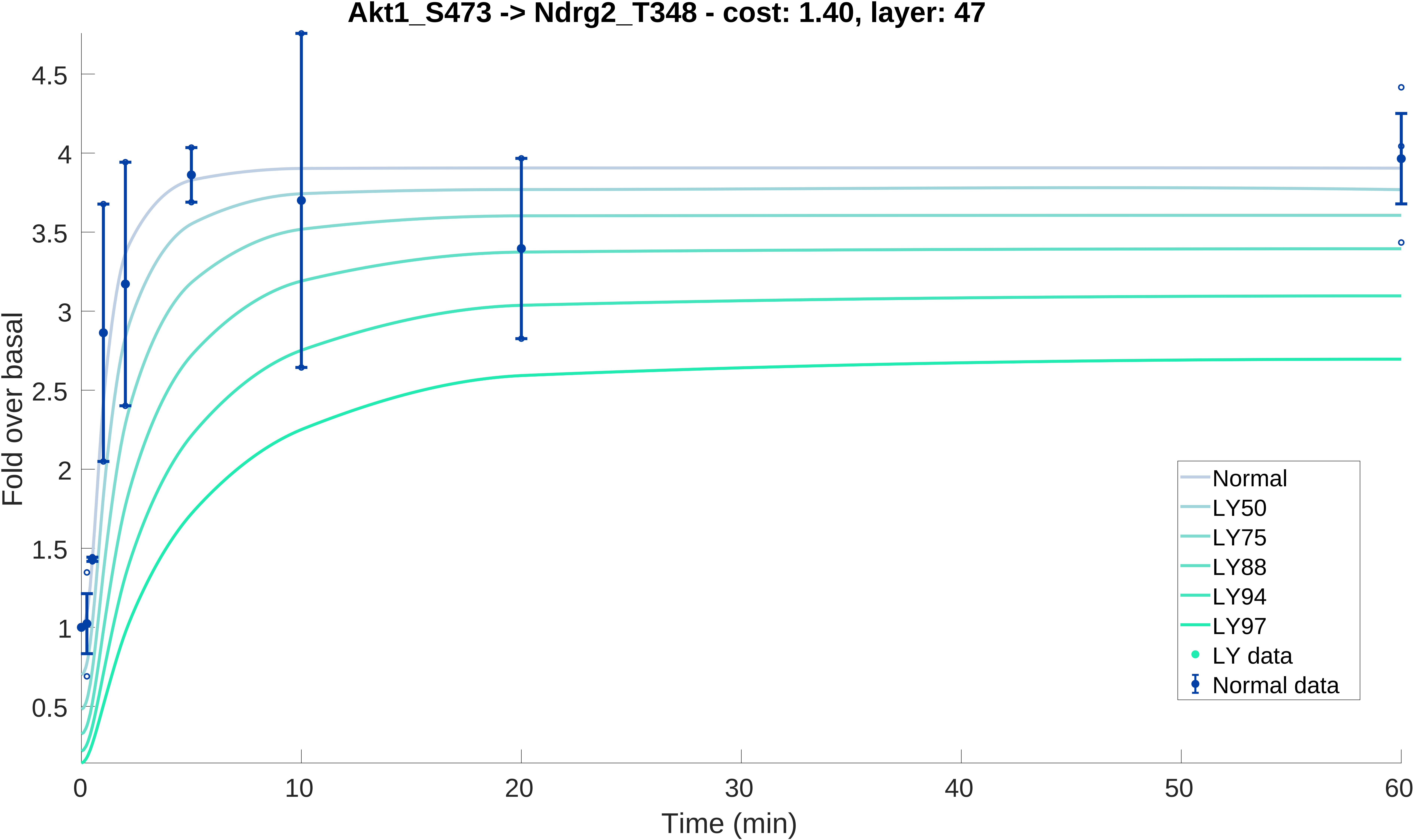
Akt1_S473 -> Ndrg2_T348 - cost: 1.40, layer: 47
Fold over basal
Time (min)
0.5
1
1.5
2
2.5
3
3.5
4
4.5
0
10
20
30
40
50
60
Normal
LY50
LY75
LY88
LY94
LY97
LY data
Normal data

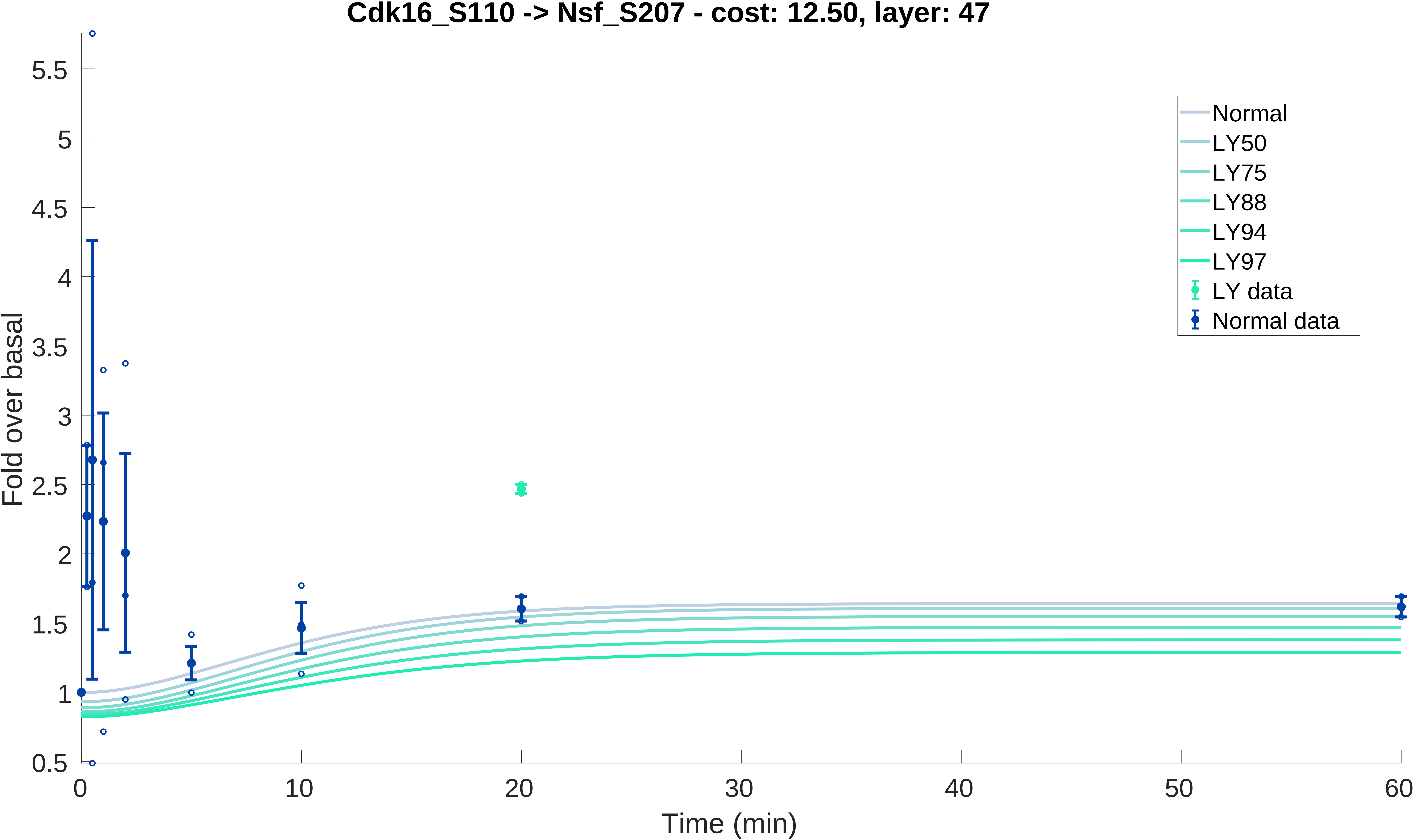
Cdk16_S110 -> Nsf_S207 - cost: 12.50, layer: 47
Fold over basal
Time (min)
Normal
LY50
LY75
LY88
LY94
LY97
LY data
Normal data
0.5
1
1.5
2
2.5
3
3.5
4
4.5
5
5.5
0
10
20
30
40
50
60

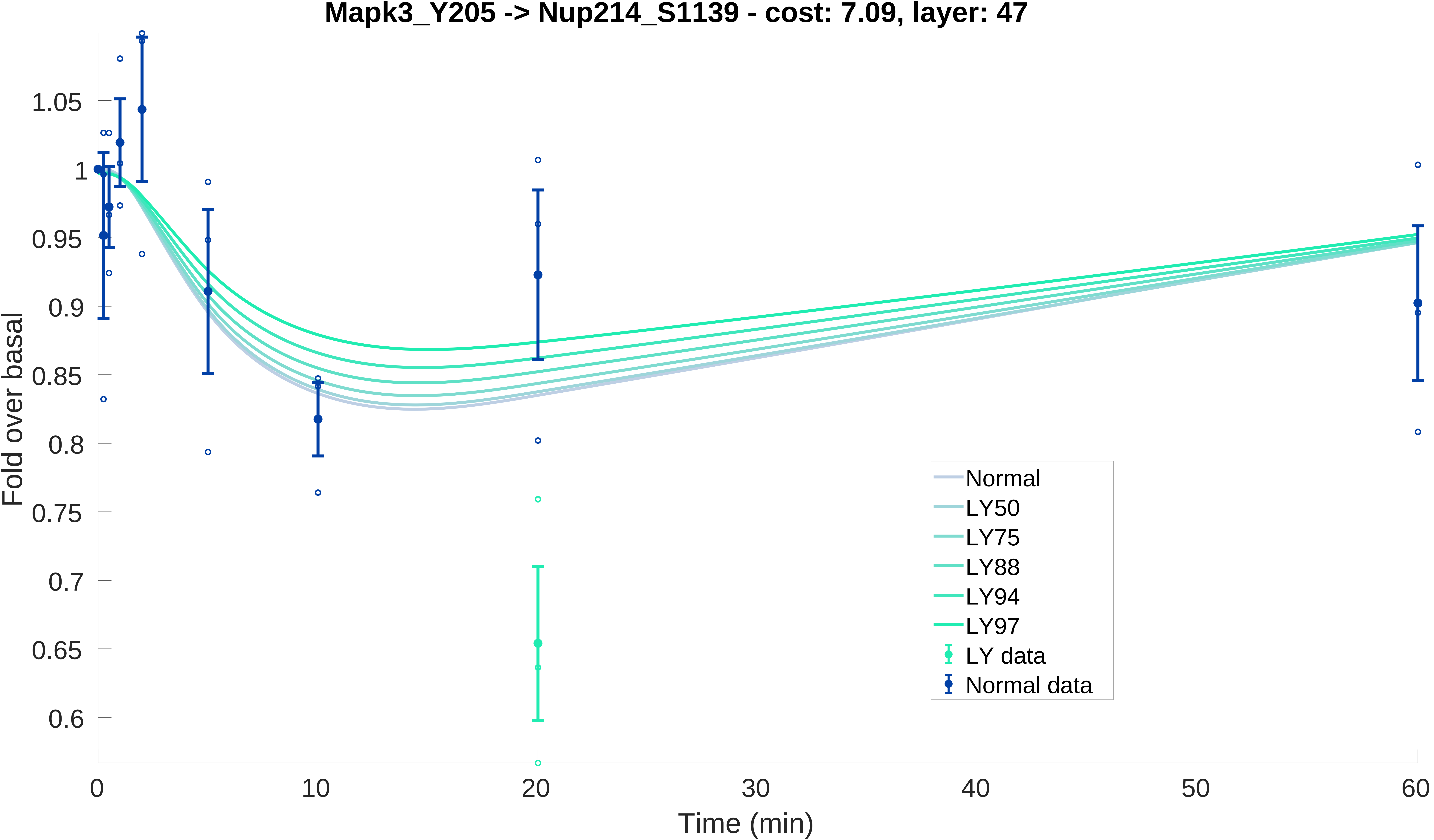

Mapk3_Y205 -> Nup214_S1139 - cost: 7.09, layer: 47
Fold over basal
1.05
1
0.95
0.9
0.85
0.8
0.75
0.7
0.65
0.6
0
10
20
30
40
50
60
Time (min)
Normal
LY50
LY75
LY88
LY94
LY97
LY data
Normal data

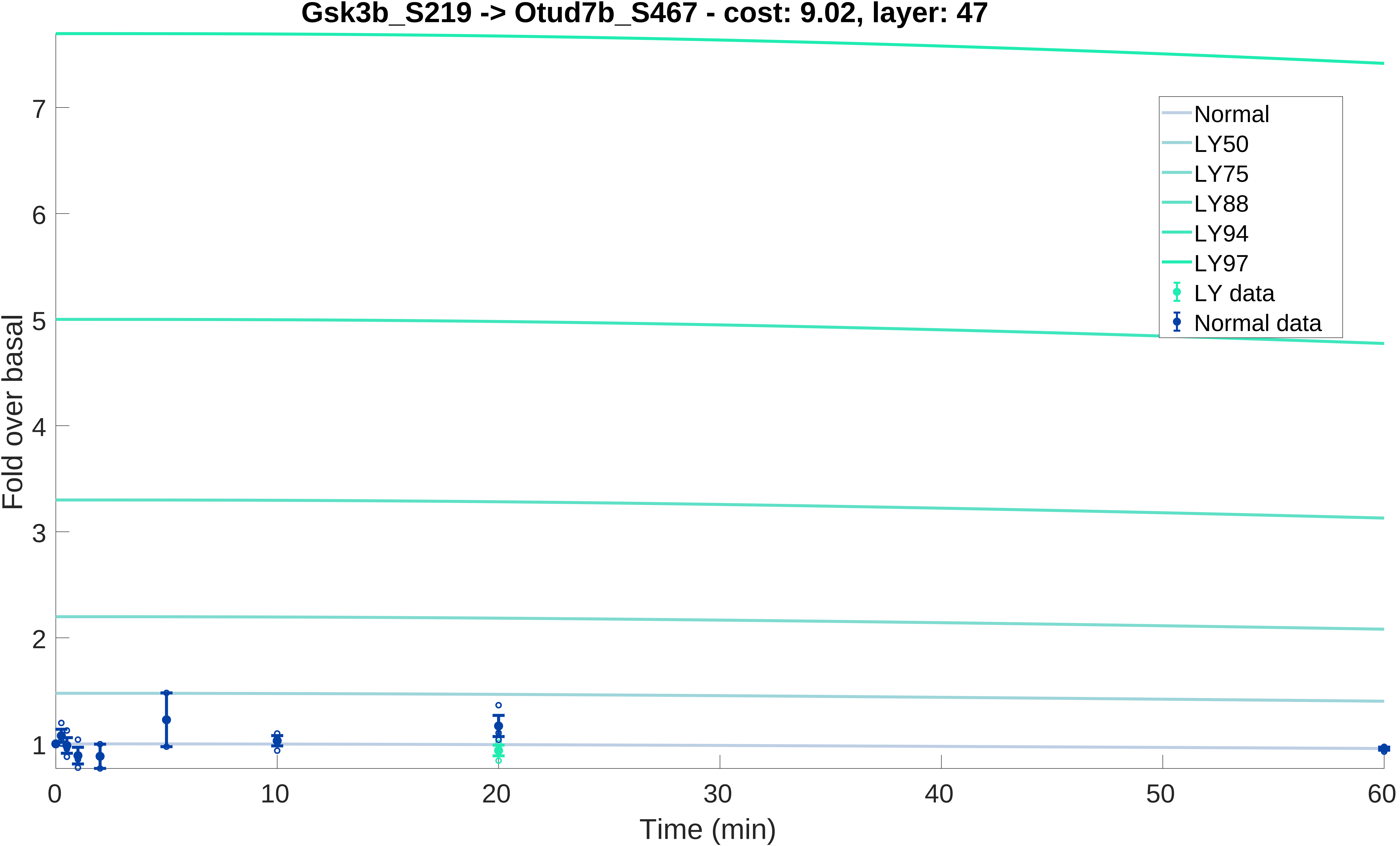
Gsk3b_S219 -> Otud7b_S467 - cost: 9.02, layer: 47
Fold over basal
Time (min)
0
10
20
30
40
50
60
1
2
3
4
5
6
7
Normal
LY50
LY75
LY88
LY94
LY97
LY data
Normal data

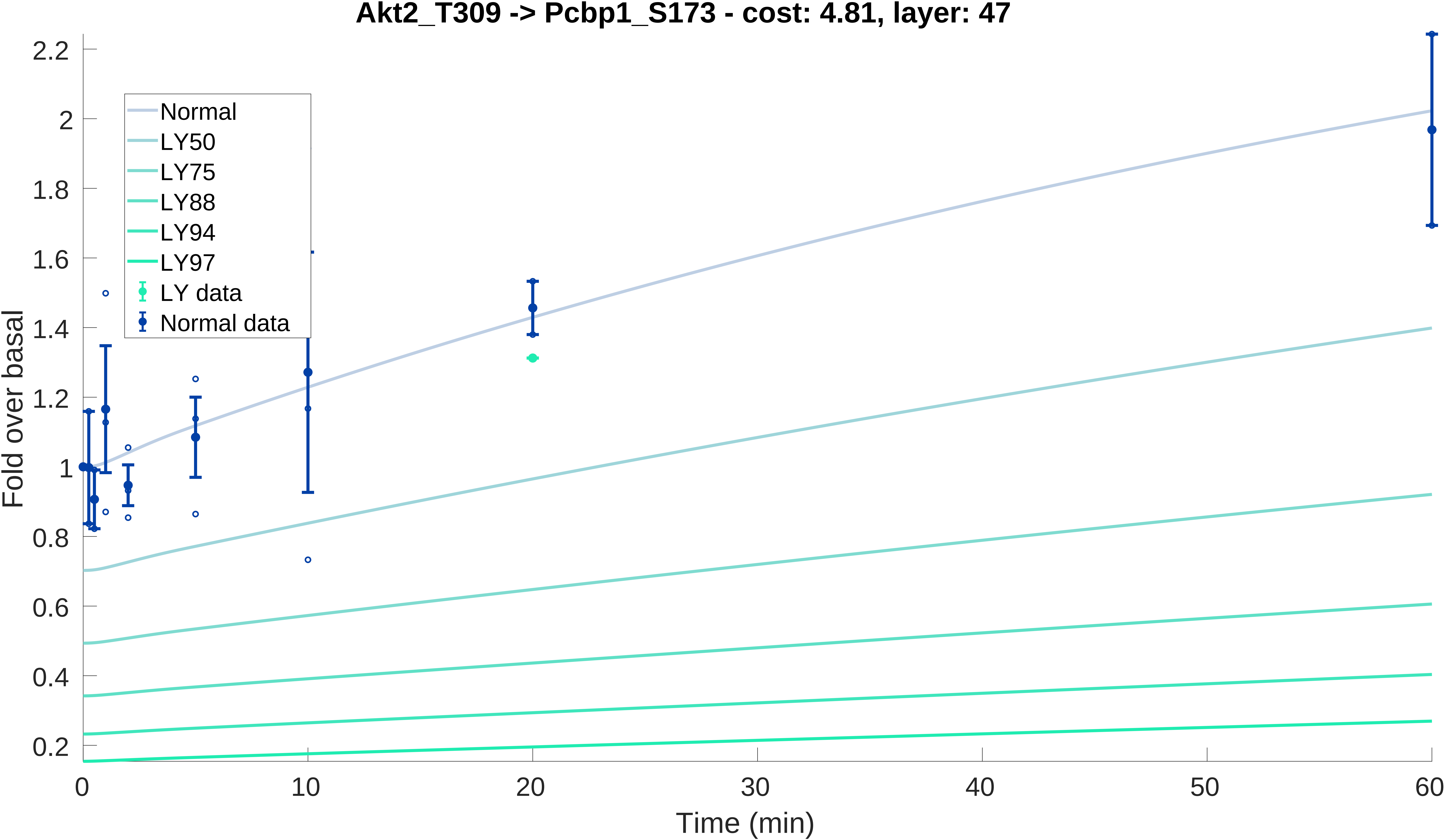

Akt2_T309 -> Pcbp1_S173 - cost: 4.81, layer: 47
Fold over basal
Time (min)
Normal
LY50
LY75
LY88
LY94
LY97
LY data
Normal data
0.2
0.4
0.6
0.8
1
1.2
1.4
1.6
1.8
2
2.2
0
10
20
30
40
50
60

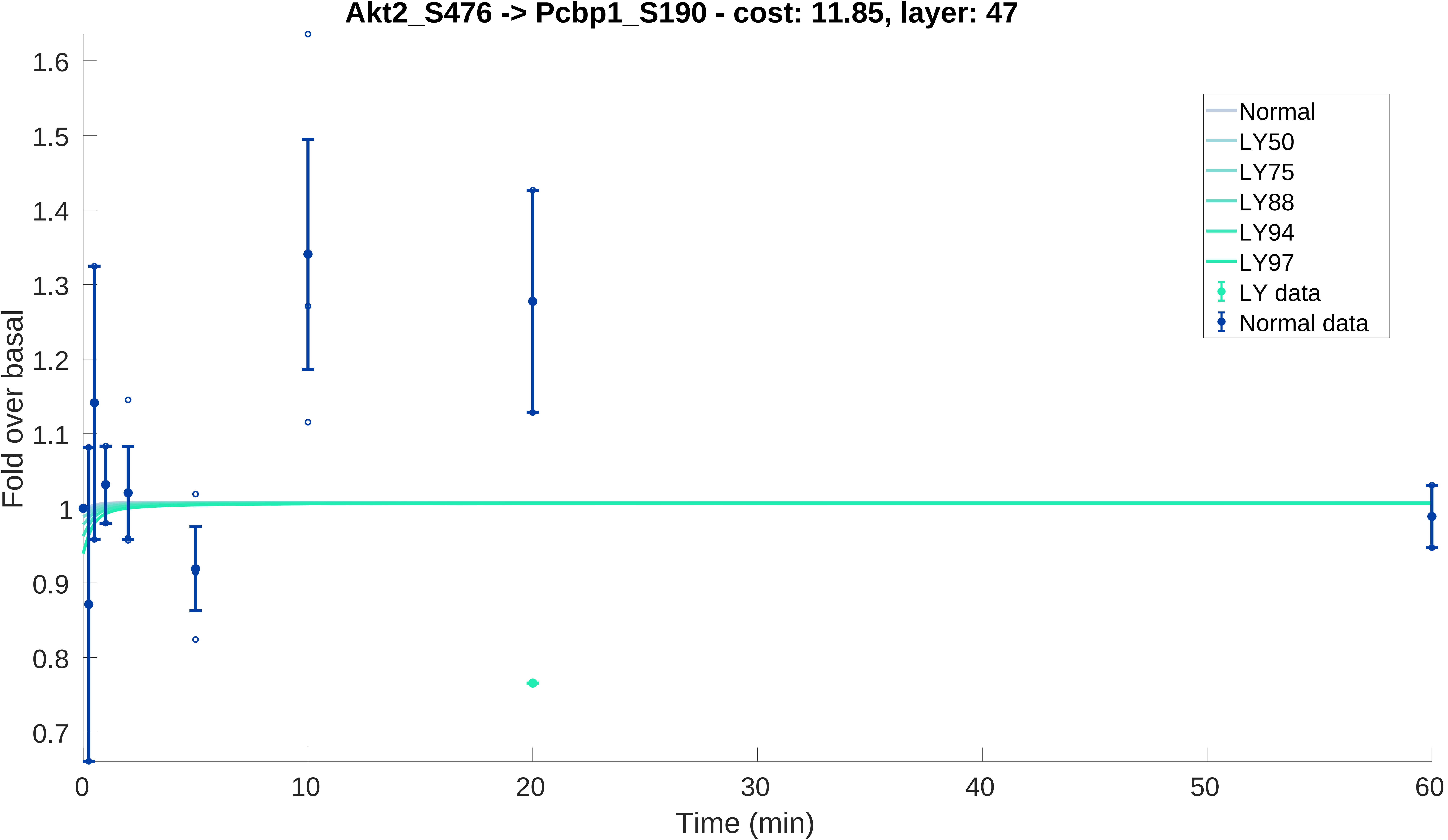

Akt2_S476 -> Pcbp1_S190 - cost: 11.85, layer: 47
Fold over basal
Time (min)
1.6
1.5
1.4
1.3
1.2
1.1
1
0.9
0.8
0.7
0
10
20
30
40
50
60
Normal
LY50
LY75
LY88
LY94
LY97
LY data
Normal data

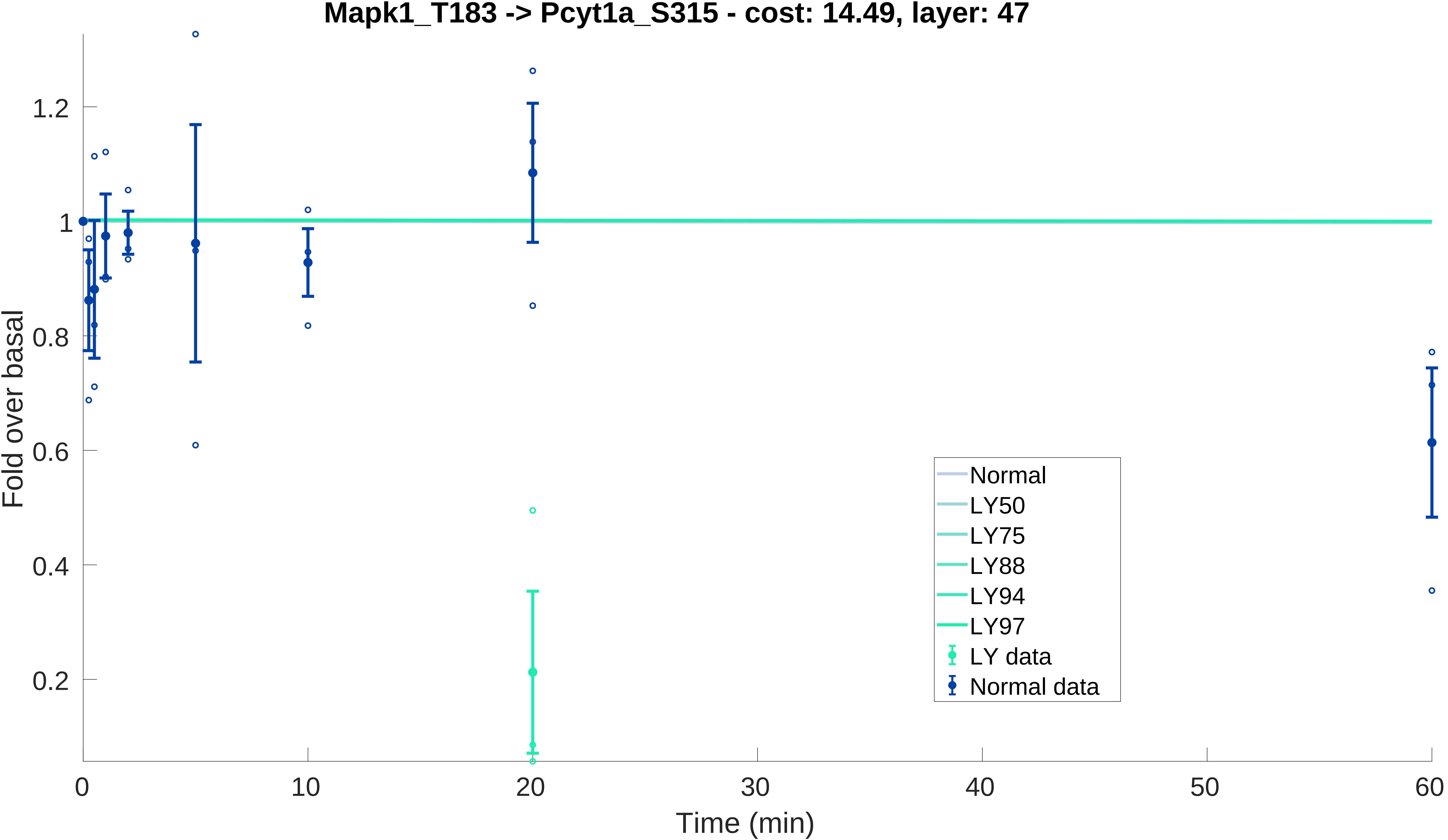

Mapk1_T183 -> Pcyt1a_S315 - cost: 14.49, layer: 47
Fold over basal
1.2
1
0.8
0.6
0.4
0.2
0
10
20
30
40
50
60
Time (min)
Normal
LY50
LY75
LY88
LY94
LY97
LY data
Normal data

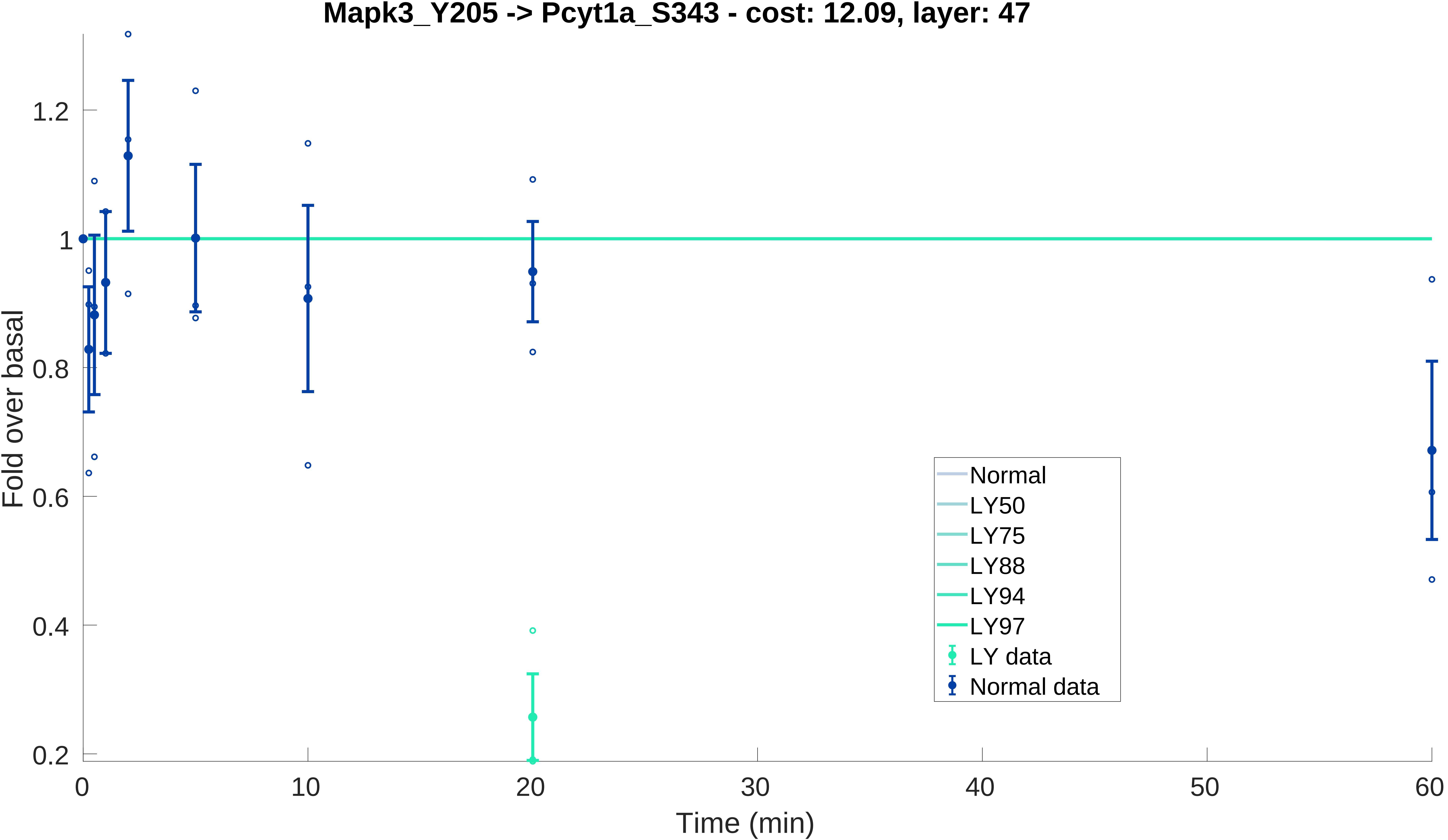

Mapk3_Y205 -> Pcyt1a_S343 - cost: 12.09, layer: 47
Fold over basal
1.2
1
0.8
0.6
0.4
0.2
0
10
20
30
40
50
60
Time (min)
Normal
LY50
LY75
LY88
LY94
LY97
LY data
Normal data

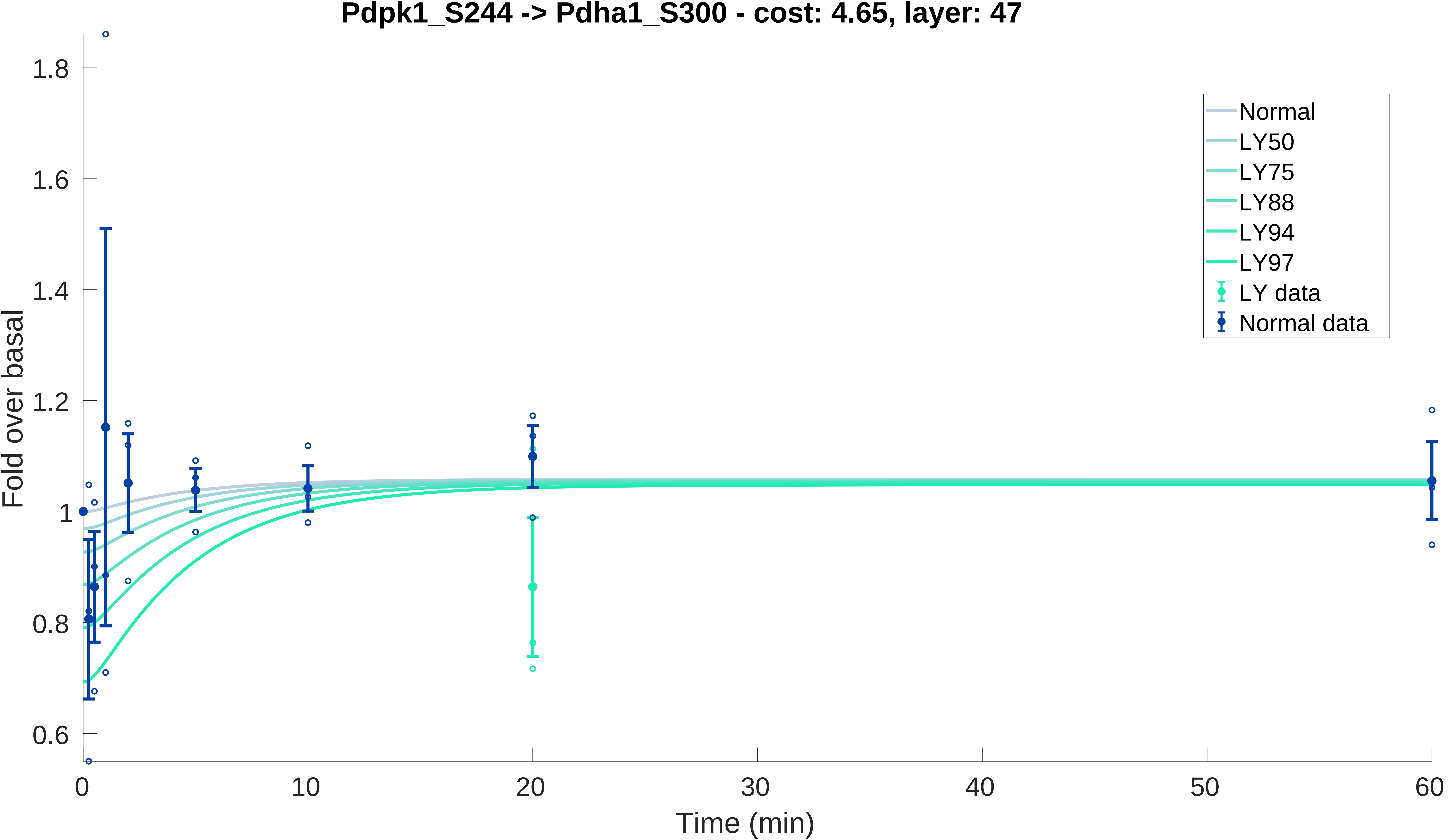

Pdpk1_S244 -> Pdha1_S300 - cost: 4.65, layer: 47
Fold over basal
1.8
1.6
1.4
1.2
1
0.8
0.6
0
10
20
30
40
50
60
Time (min)
Normal
LY50
LY75
LY88
LY94
LY97
LY data
Normal data

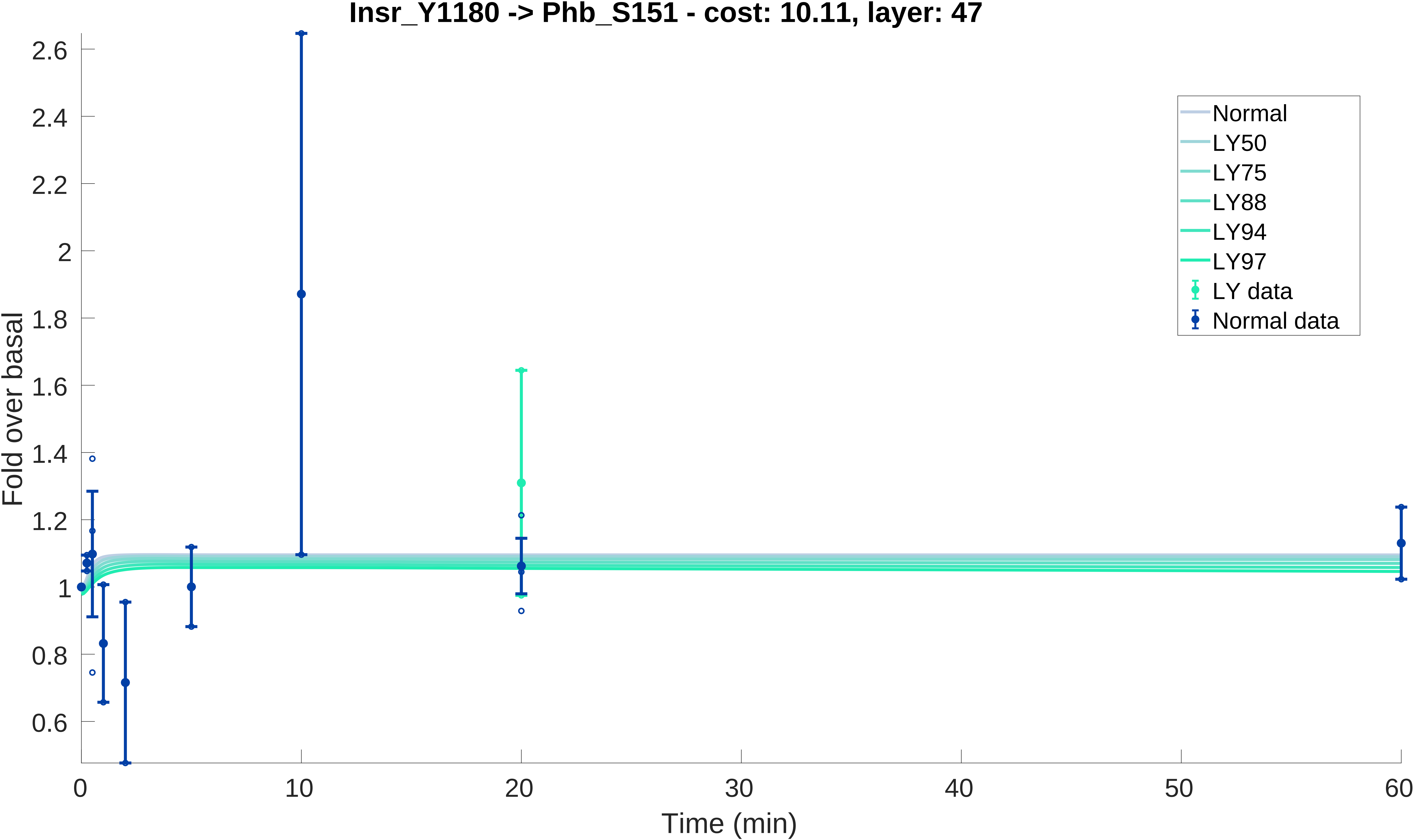

Insr_Y1180 -> Phb_S151 - cost: 10.11, layer: 47
Fold over basal
Time (min)
2.6
2.4
2.2
2
1.8
1.6
1.4
1.2
1
0.8
0.6
0
10
20
30
40
50
60
Normal
LY50
LY75
LY88
LY94
LY97
LY data
Normal data

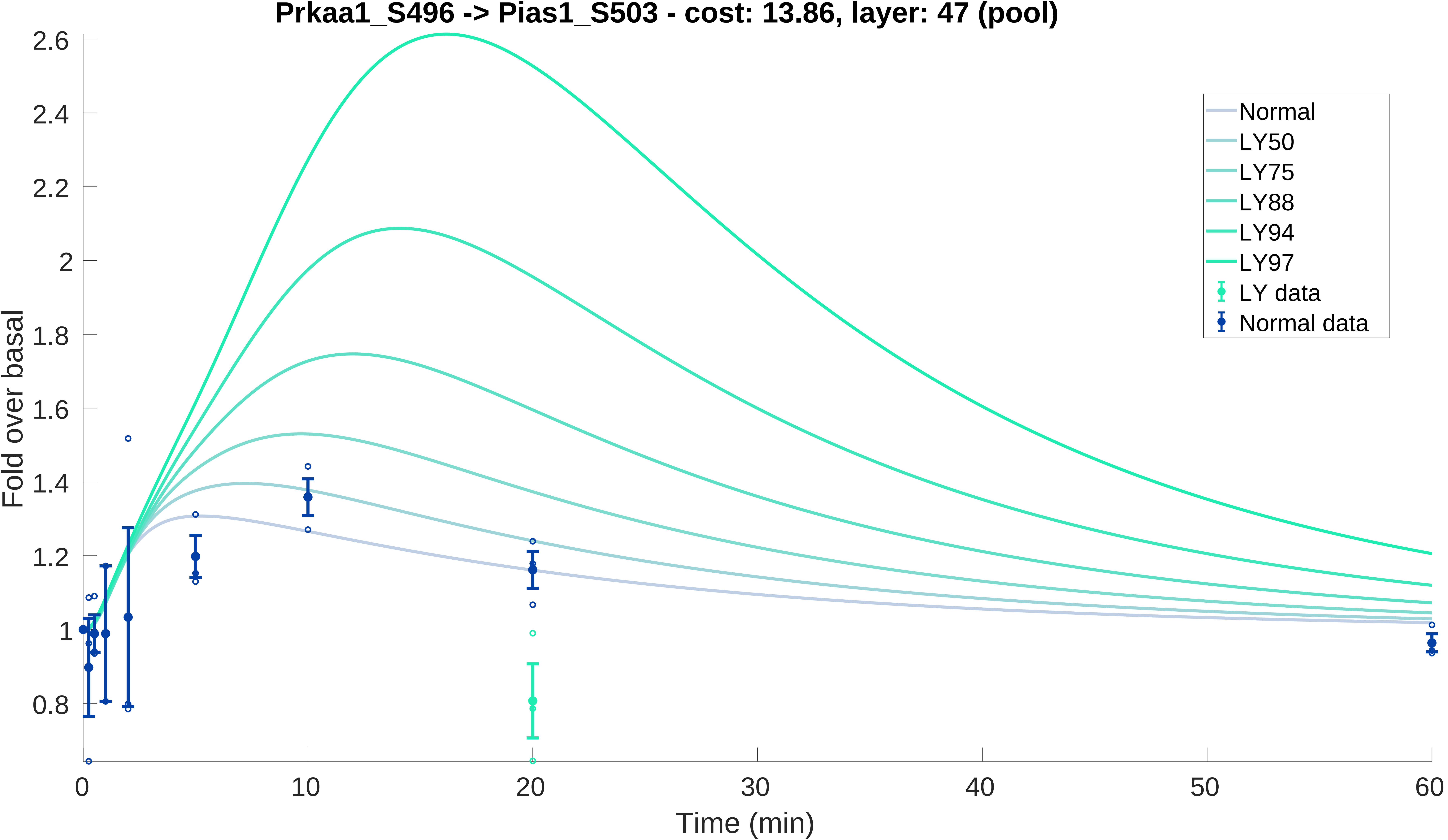

Prkaa1_S496 -> Pias1_S503 - cost: 13.86, layer: 47 (pool)
Fold over basal
Time (min)
0.8
1
1.2
1.4
1.6
1.8
2
2.2
2.4
2.6
0
10
20
30
40
50
60
Normal
LY50
LY75
LY88
LY94
LY97
LY data
Normal data

Ptk2b_S375 -> Pitpnm2_S316 - cost: 9.80, layer: 47

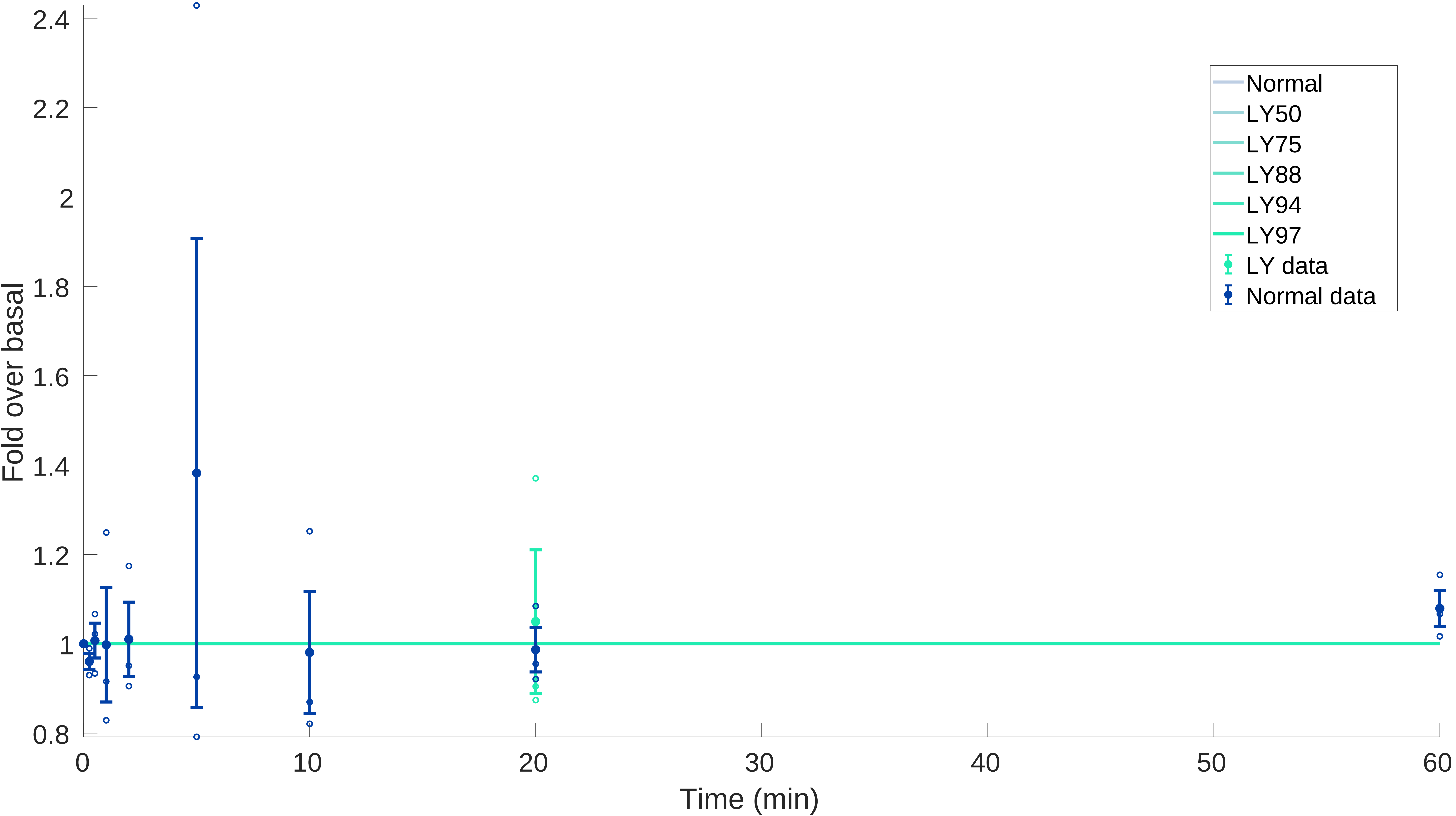

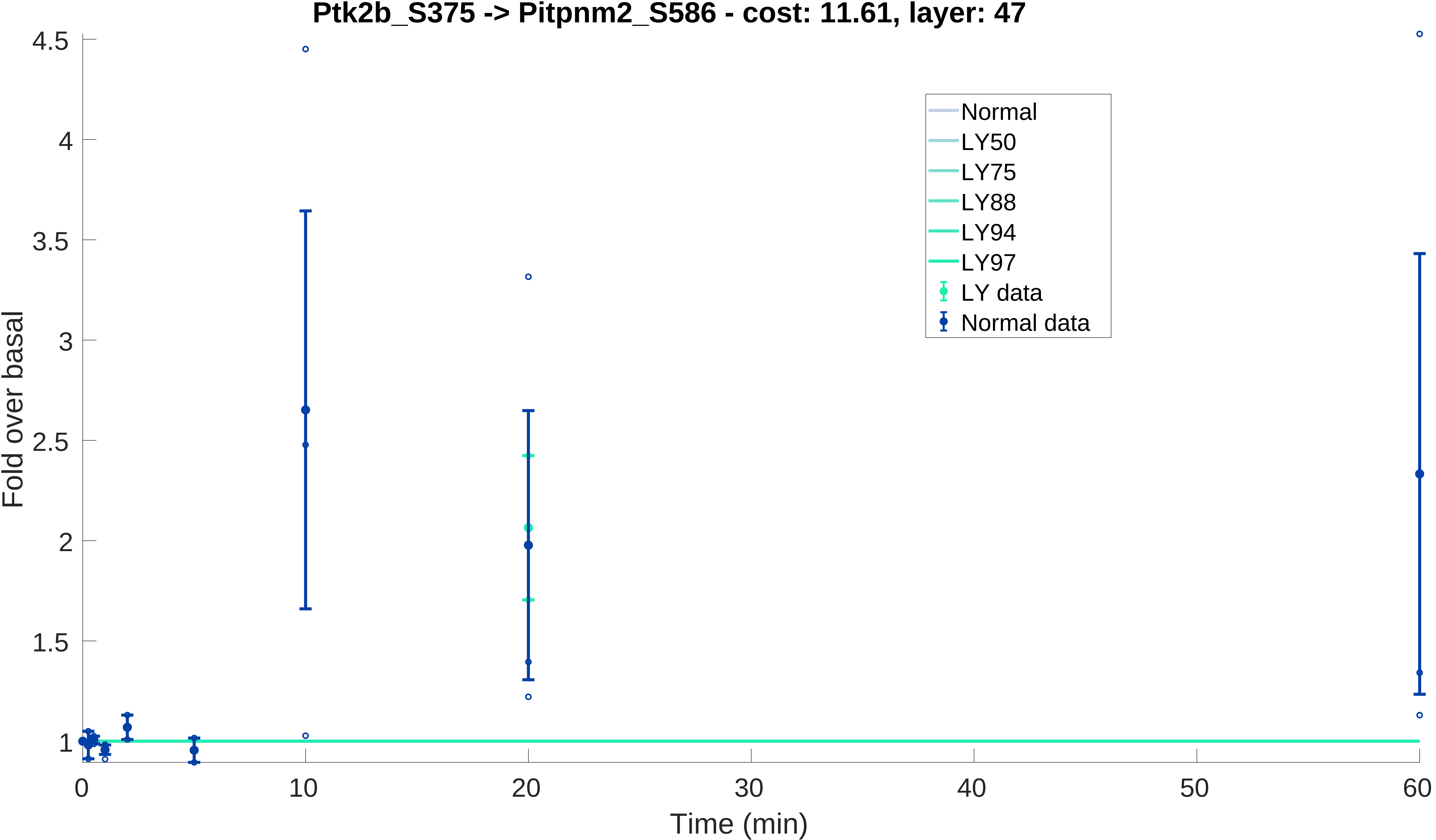
Ptk2b_S375 -> Pitpnm2_S586 - cost: 11.61, layer: 47
Fold over basal
Time (min)
4.5
4
3.5
3
2.5
2
1.5
1
0
10
20
30
40
50
60
Normal
LY50
LY75
LY88
LY94
LY97
LY data
Normal data

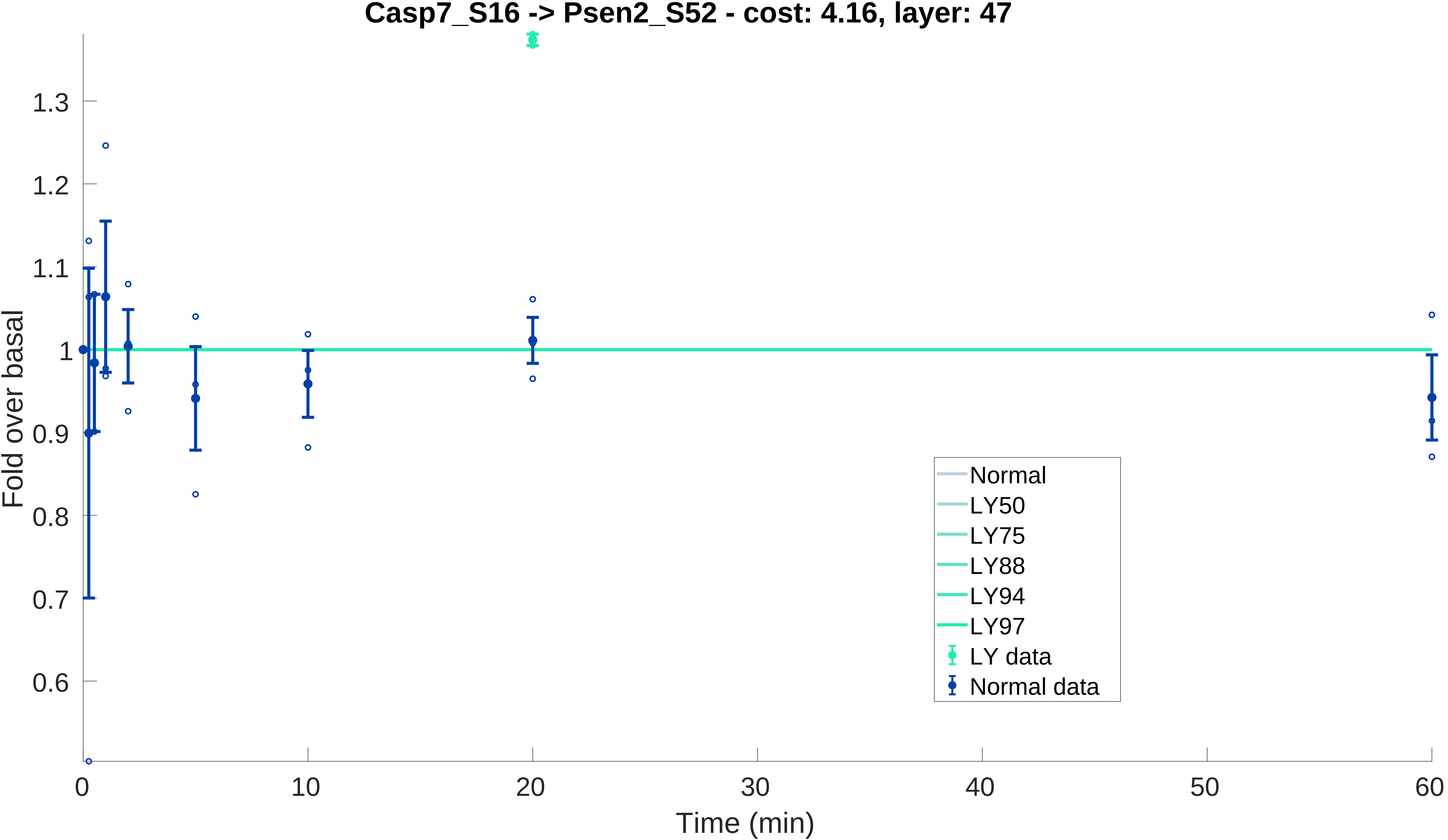
Casp7_S16 -> Psen2_S52 - cost: 4.16, layer: 47
Fold over basal
Time (min)
1.3
1.2
1.1
1
0.9
0.8
0.7
0.6
0
10
20
30
40
50
60
Normal
LY50
LY75
LY88
LY94
LY97
LY data
Normal data

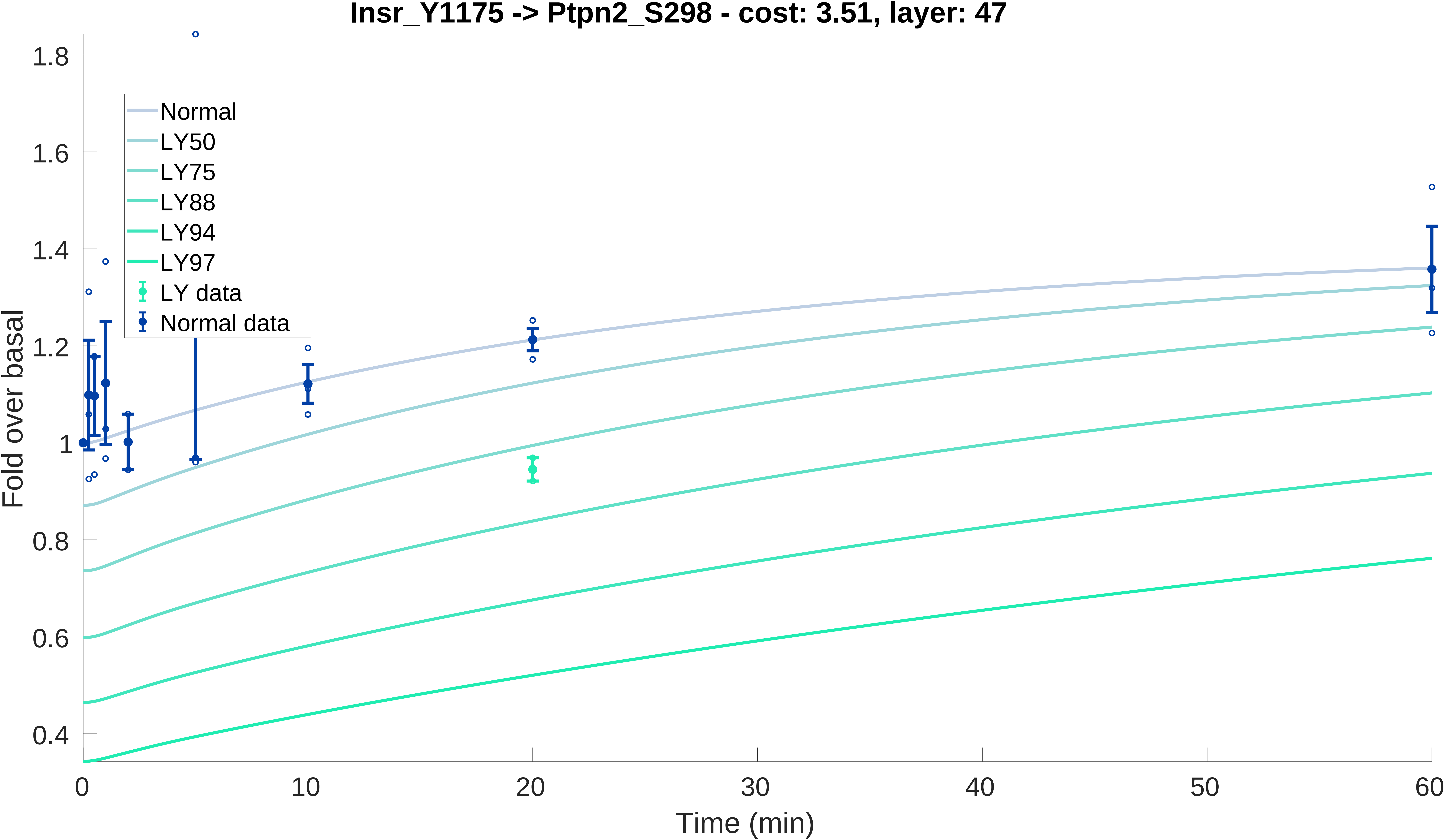
Insr_Y1175 -> Ptpn2_S298 - cost: 3.51, layer: 47
Normal
LY50
LY75
LY88
LY94
LY97
LY data
Normal data
Fold over basal
Time (min)
0.4
0.6
0.8
1
1.2
1.4
1.6
1.8
0
10
20
30
40
50
60

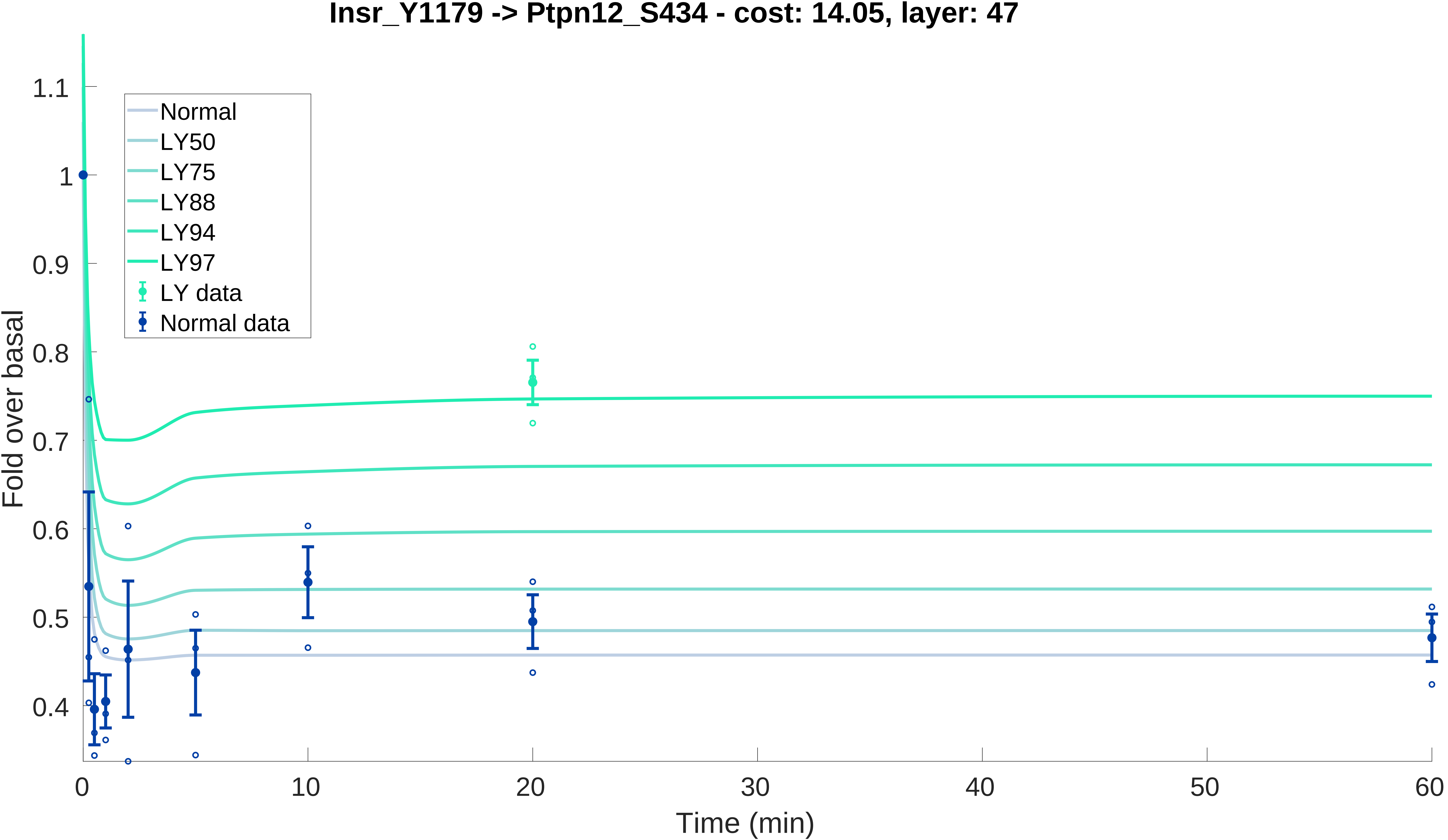

Insr_Y1179 -> Ptpn12_S434 - cost: 14.05, layer: 47
Fold over basal
Time (min)
Normal
LY50
LY75
LY88
LY94
LY97
LY data
Normal data
1.1
1
0.9
0.8
0.7
0.6
0.5
0.4
0
10
20
30
40
50
60

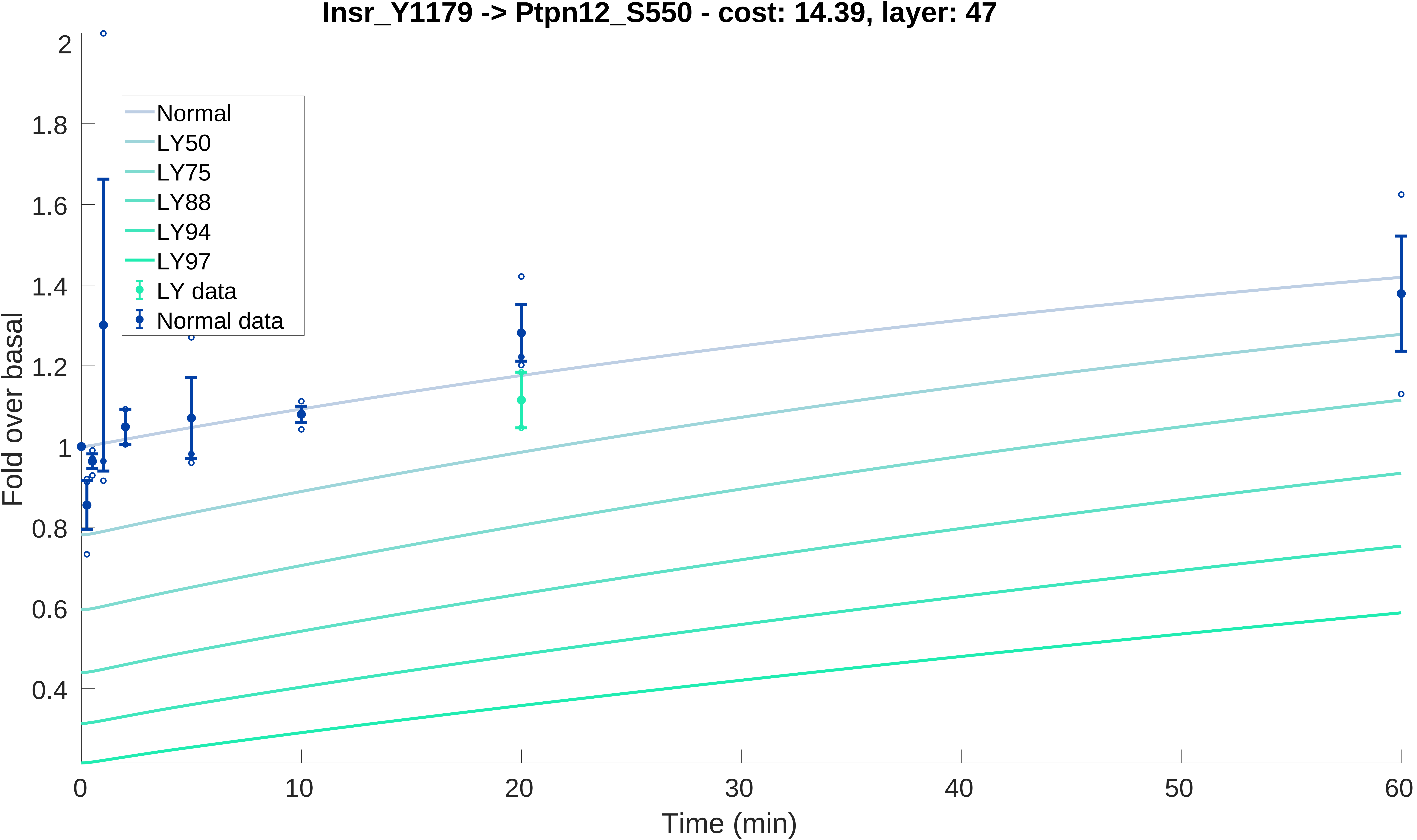

Insr_Y1179 -> Ptpn12_S550 - cost: 14.39, layer: 47
Normal
LY50
LY75
LY88
LY94
LY97
LY data
Normal data
Fold over basal
Time (min)
0
10
20
30
40
50
60
0.4
0.6
0.8
1
1.2
1.4
1.6
1.8
2

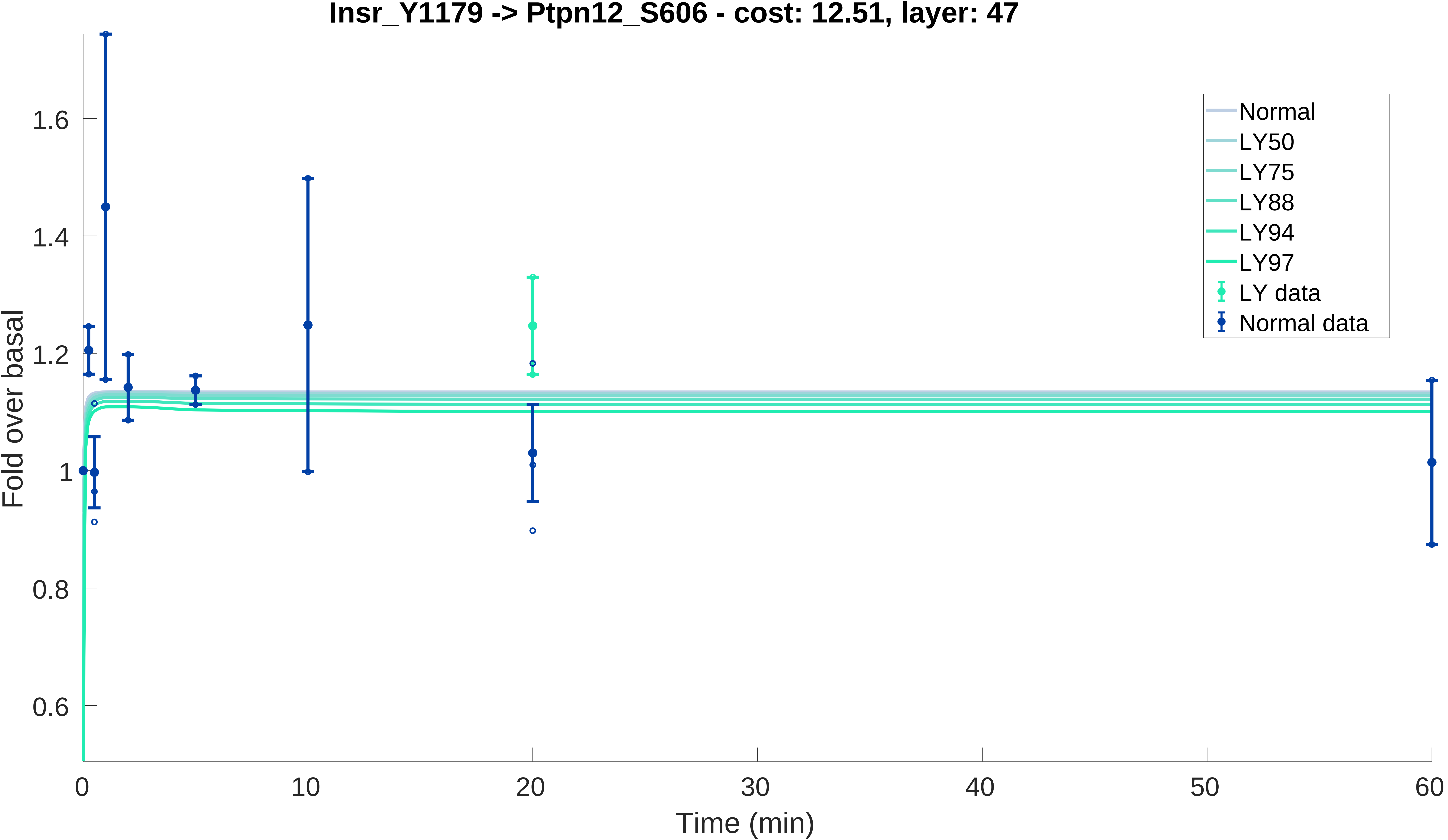
Insr_Y1179 -> Ptpn12_S606 - cost: 12.51, layer: 47
Fold over basal
1.6
1.4
1.2
1
0.8
0.6
0
10
20
30
40
50
60
Time (min)
Normal
LY50
LY75
LY88
LY94
LY97
LY data
Normal data

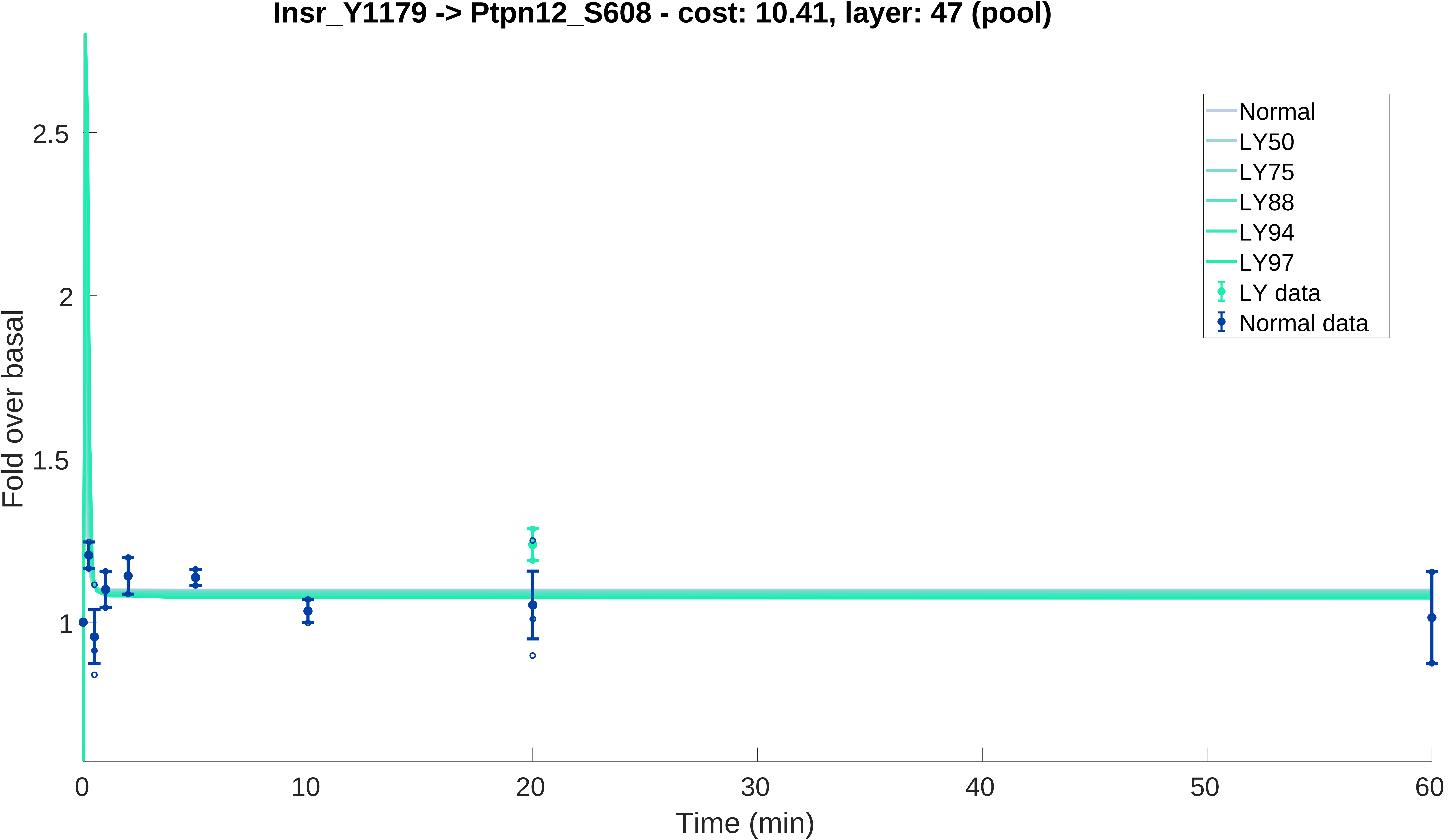

Insr_Y1179 -> Ptpn12_S608 - cost: 10.41, layer: 47 (pool)
Fold over basal
Time (min)
2.5
2
1.5
1
0
10
20
30
40
50
60
Normal
LY50
LY75
LY88
LY94
LY97
LY data
Normal data

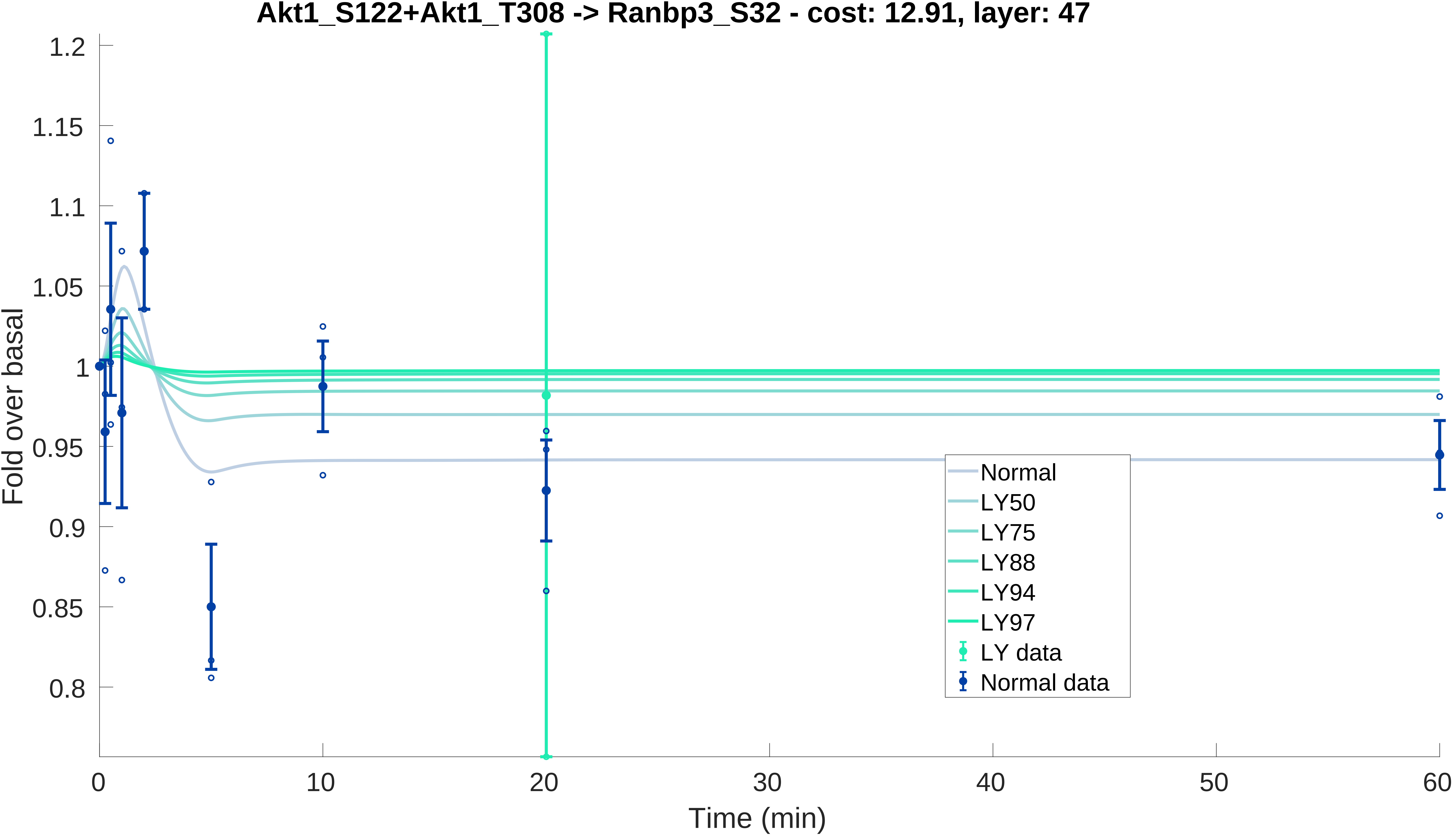

Akt1_S122+Akt1_T308 -> Ranbp3_S32 - cost: 12.91, layer: 47
Fold over basal
1.2
1.15
1.1
1.05
1
0.95
0.9
0.85
0.8
0
10
20
30
40
50
60
Time (min)
Normal
LY50
LY75
LY88
LY94
LY97
LY data
Normal data

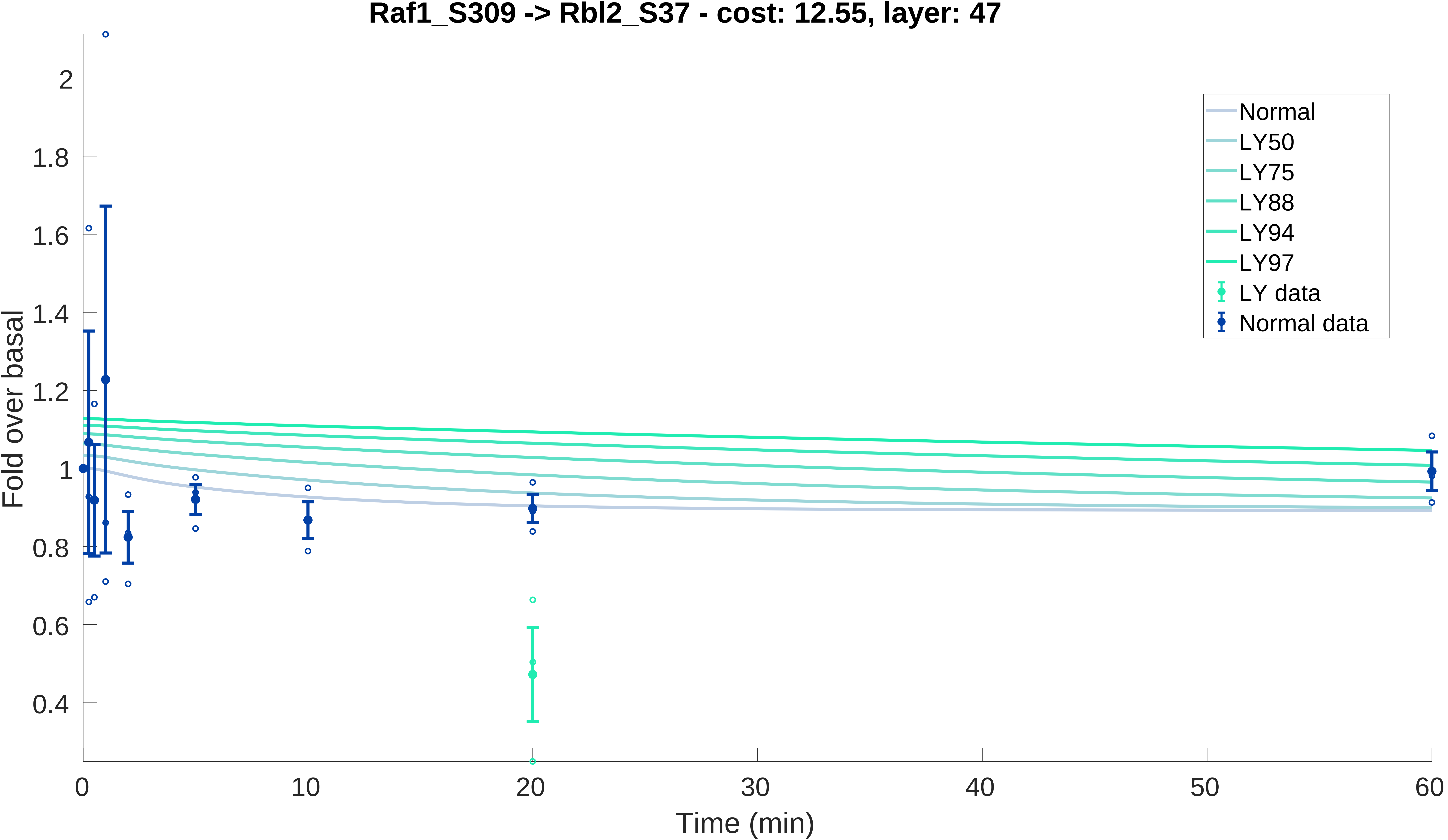

Raf1_S309 -> Rbl2_S37 - cost: 12.55, layer: 47
Fold over basal
Time (min)
0
10
20
30
40
50
60
0.4
0.6
0.8
1
1.2
1.4
1.6
1.8
2
Normal
LY50
LY75
LY88
LY94
LY97
LY data
Normal data

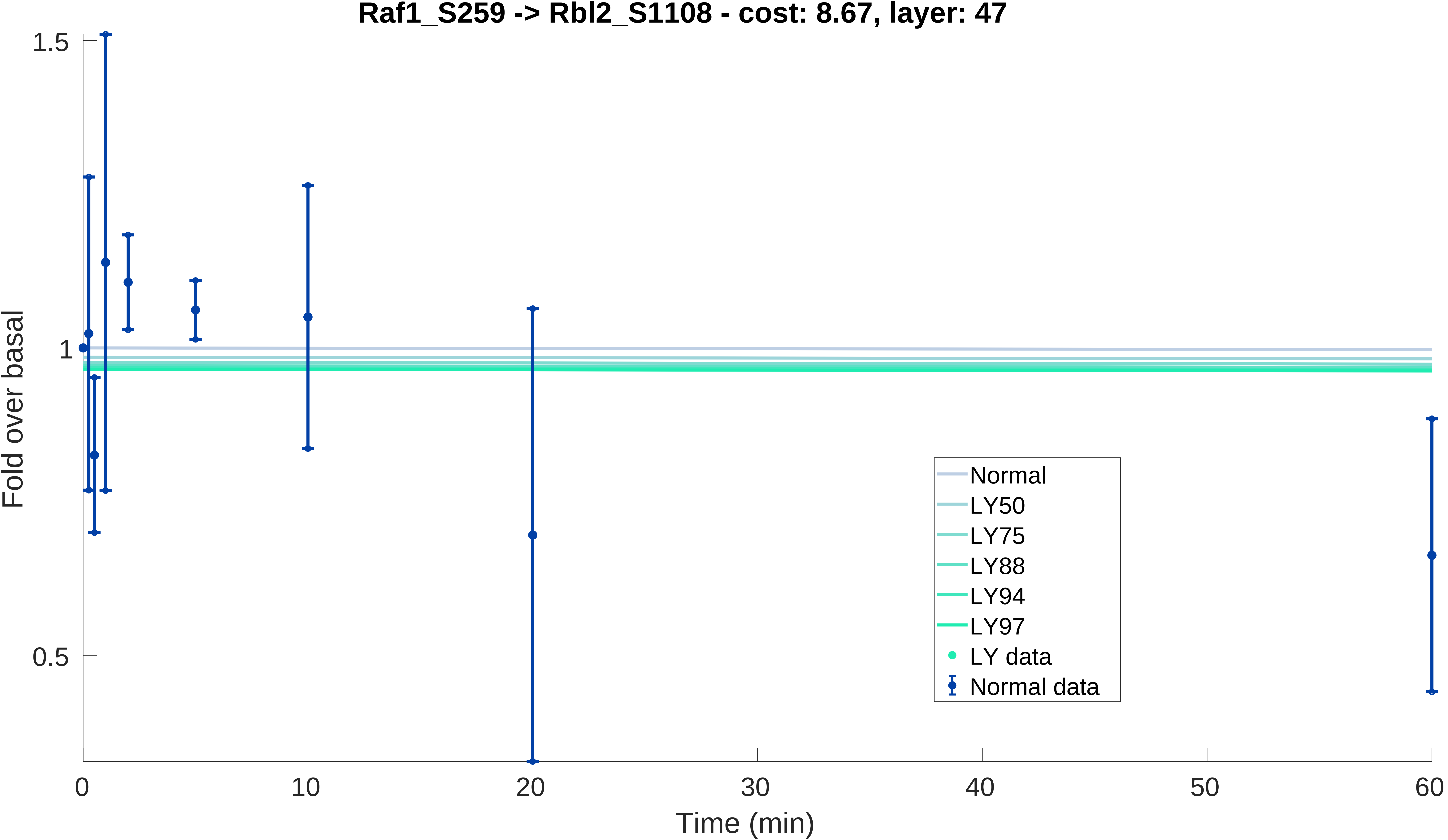
Raf1_S259 -> Rbl2_S1108 - cost: 8.67, layer: 47
Fold over basal
1.5
1
0.5
0
10
20
30
40
50
60
Time (min)
Normal
LY50
LY75
LY88
LY94
LY97
LY data
Normal data

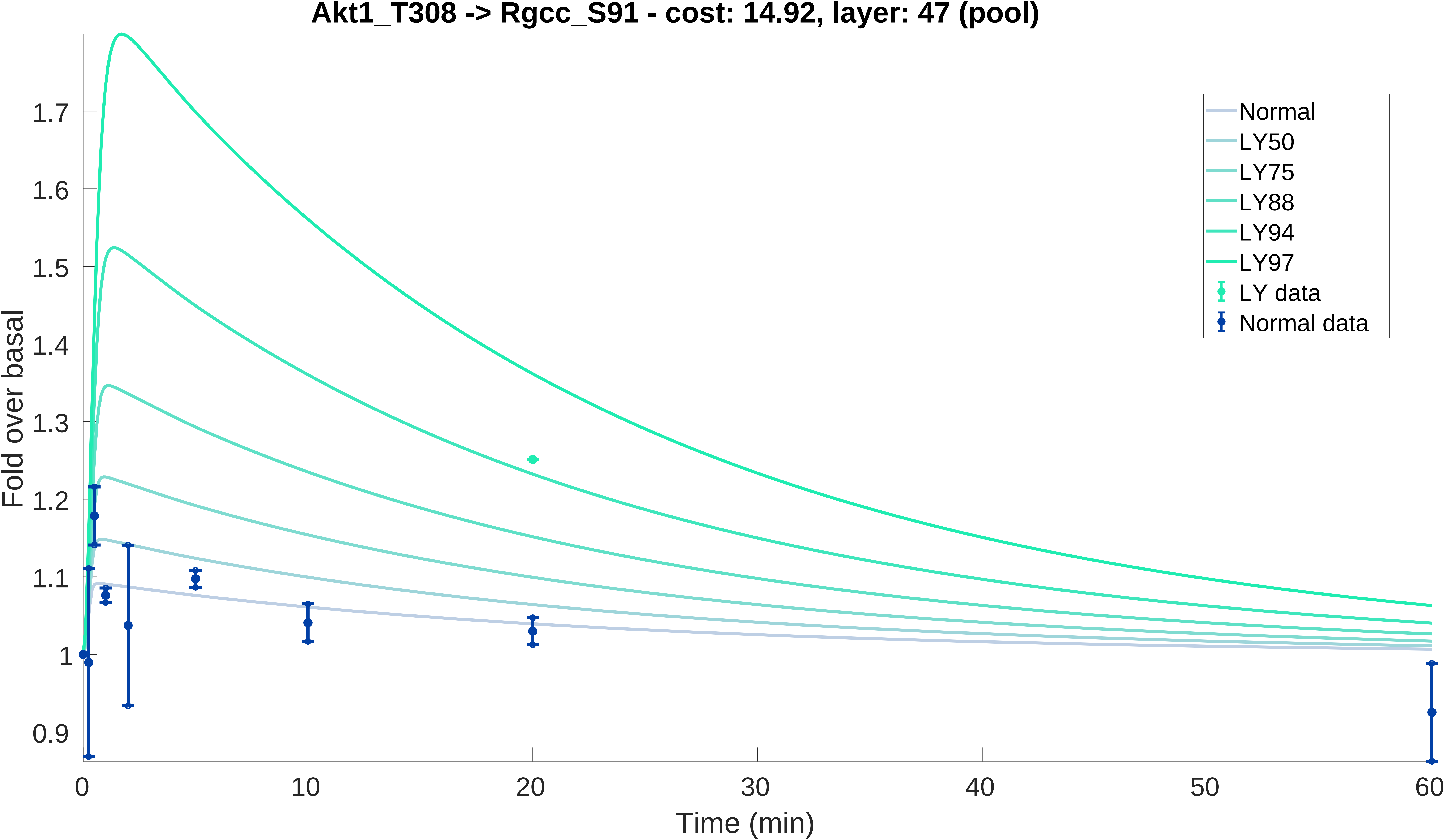

Akt1_T308 -> Rgcc_S91 - cost: 14.92, layer: 47 (pool)
Fold over basal
Time (min)
0.9
1
1.1
1.2
1.3
1.4
1.5
1.6
1.7
0
10
20
30
40
50
60
Normal
LY50
LY75
LY88
LY94
LY97
LY data
Normal data

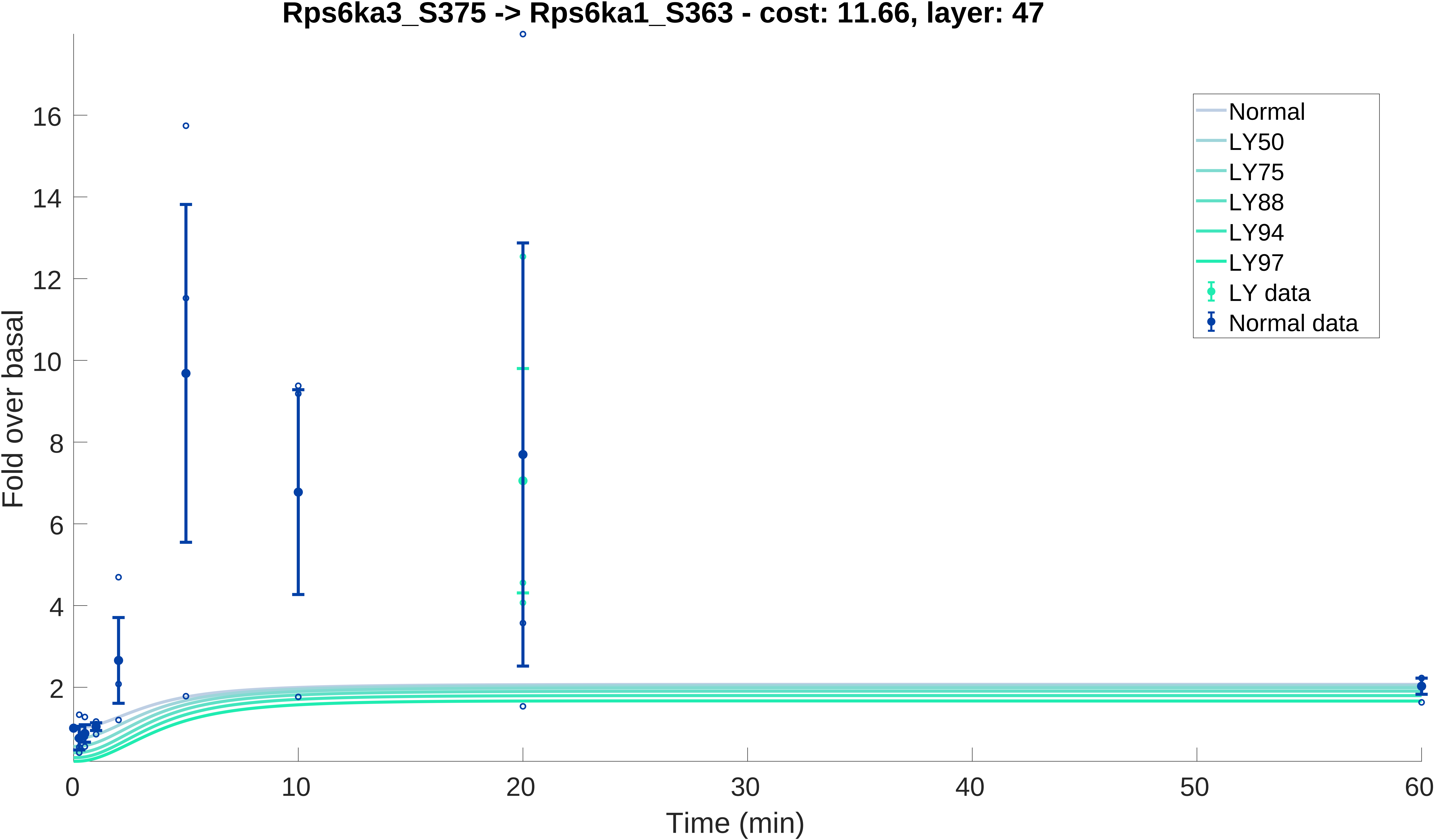

Rps6ka3_S375 -> Rps6ka1_S363 - cost: 11.66, layer: 47
Fold over basal
Time (min)
0
10
20
30
40
50
60
2
4
6
8
10
12
14
16
Normal
LY50
LY75
LY88
LY94
LY97
LY data
Normal data

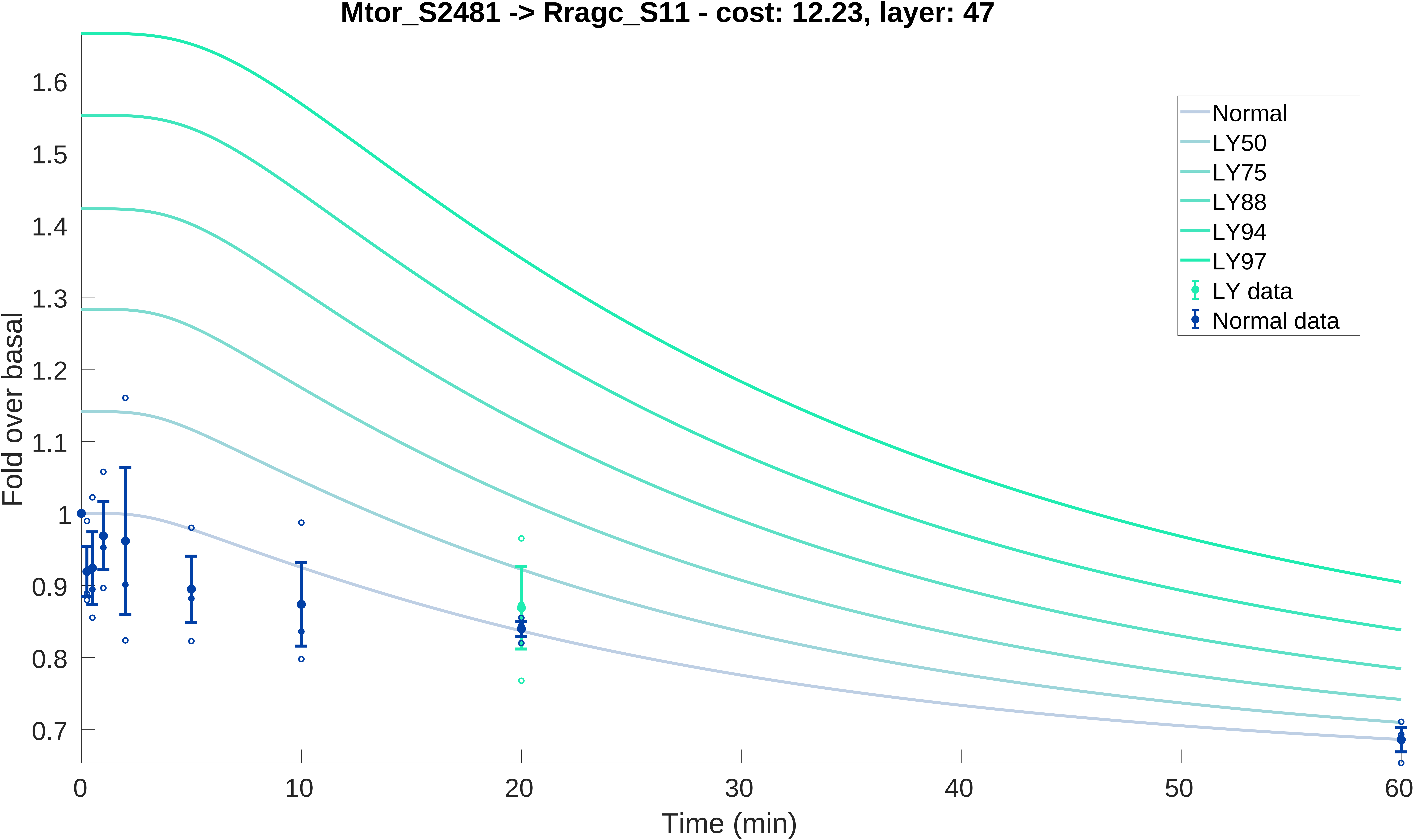
Mtor_S2481 -> Rragc_S11 - cost: 12.23, layer: 47
Normal
LY50
LY75
LY88
LY94
LY97
LY data
Normal data
Fold over basal
Time (min)
0
10
20
30
40
50
60
0.7
0.8
0.9
1
1.1
1.2
1.3
1.4
1.5
1.6

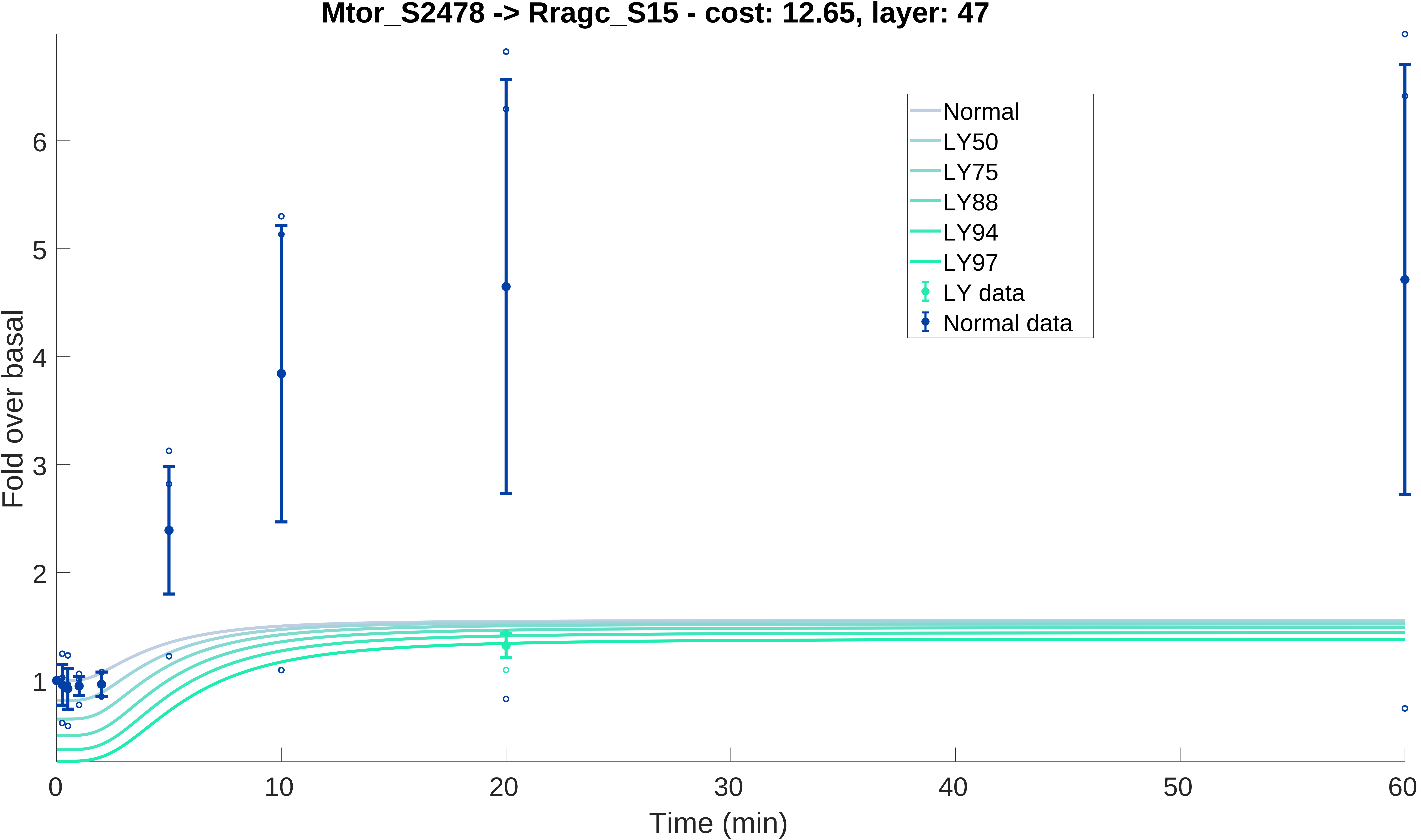

Mtor_S2478 -> Rragc_S15 - cost: 12.65, layer: 47
Fold over basal
1
2
3
4
5
6
0
10
20
30
40
50
60
Time (min)
Normal
LY50
LY75
LY88
LY94
LY97
LY data
Normal data

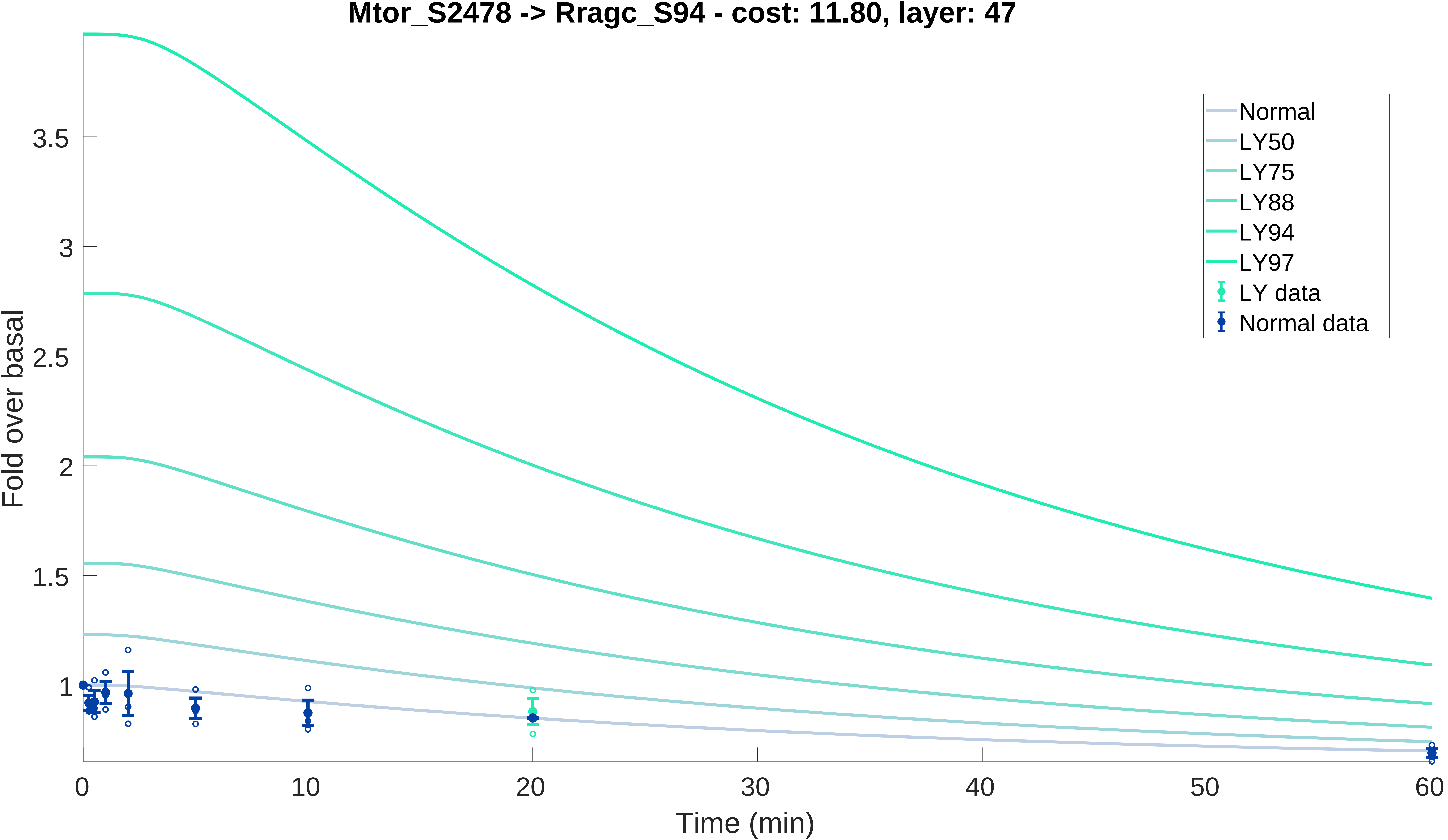

Mtor_S2478 -> Rragc_S94 - cost: 11.80, layer: 47
Fold over basal
Time (min)
0
10
20
30
40
50
60
1
1.5
2
2.5
3
3.5
Normal
LY50
LY75
LY88
LY94
LY97
LY data
Normal data

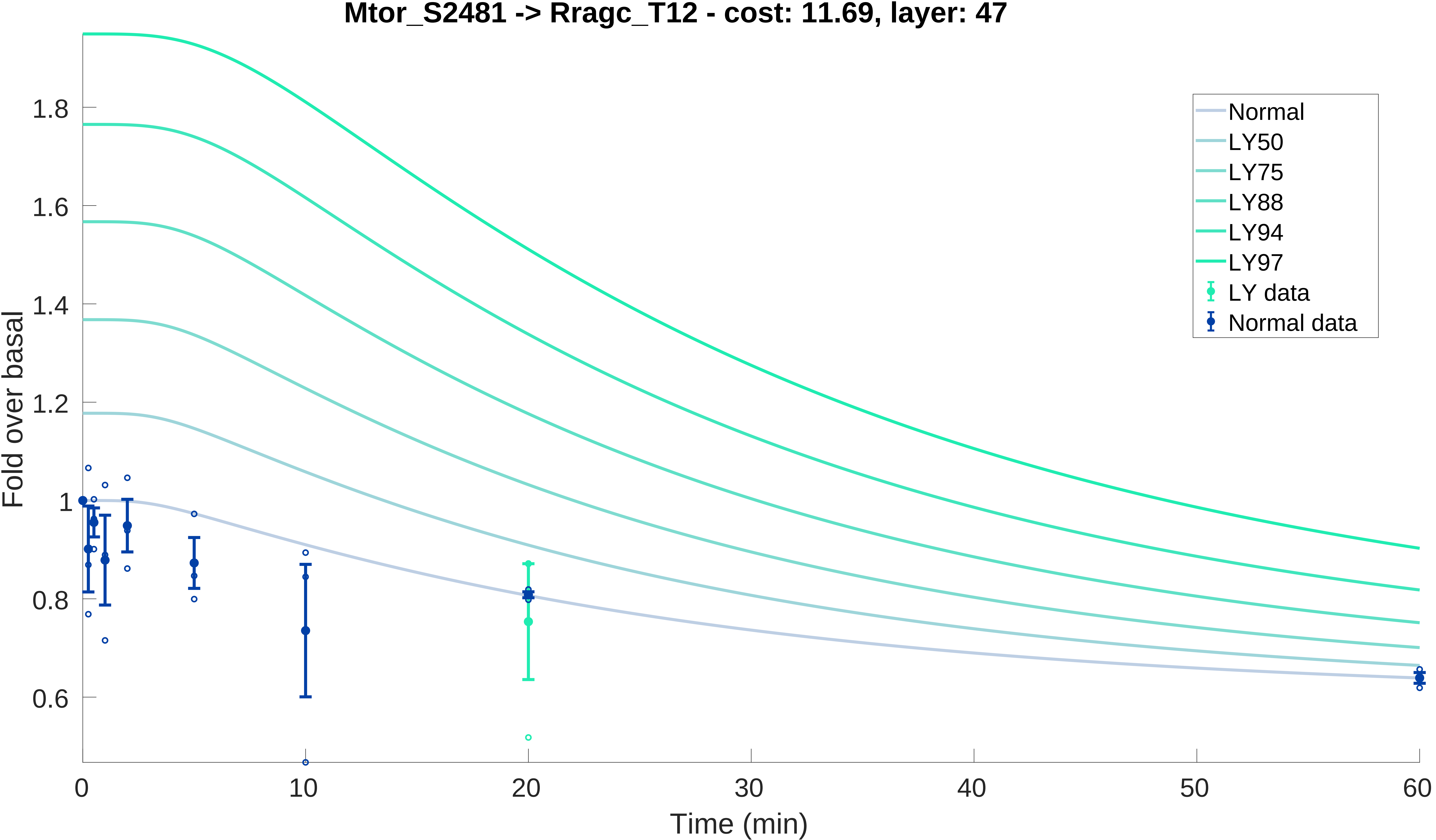

Mtor_S2481 -> Rragc_T12 - cost: 11.69, layer: 47
Fold over basal
Time (min)
1.8
1.6
1.4
1.2
1
0.8
0.6
0
10
20
30
40
50
60
Normal
LY50
LY75
LY88
LY94
LY97
LY data
Normal data

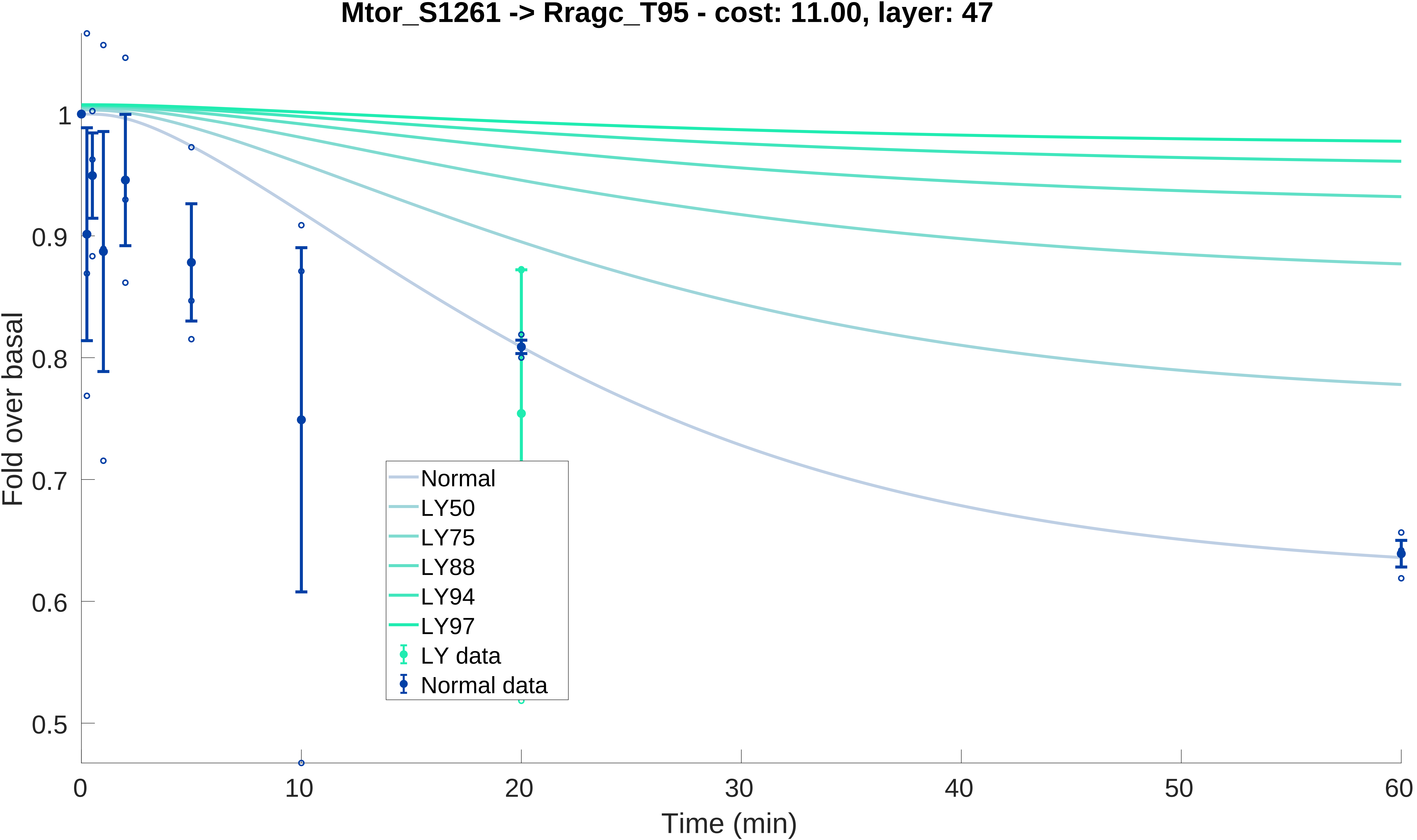

Mtor_S1261 -> Rragc_T95 - cost: 11.00, layer: 47
Fold over basal
Time (min)
1
0.9
0.8
0.7
0.6
0.5
0
10
20
30
40
50
60
Normal
LY50
LY75
LY88
LY94
LY97
LY data
Normal data

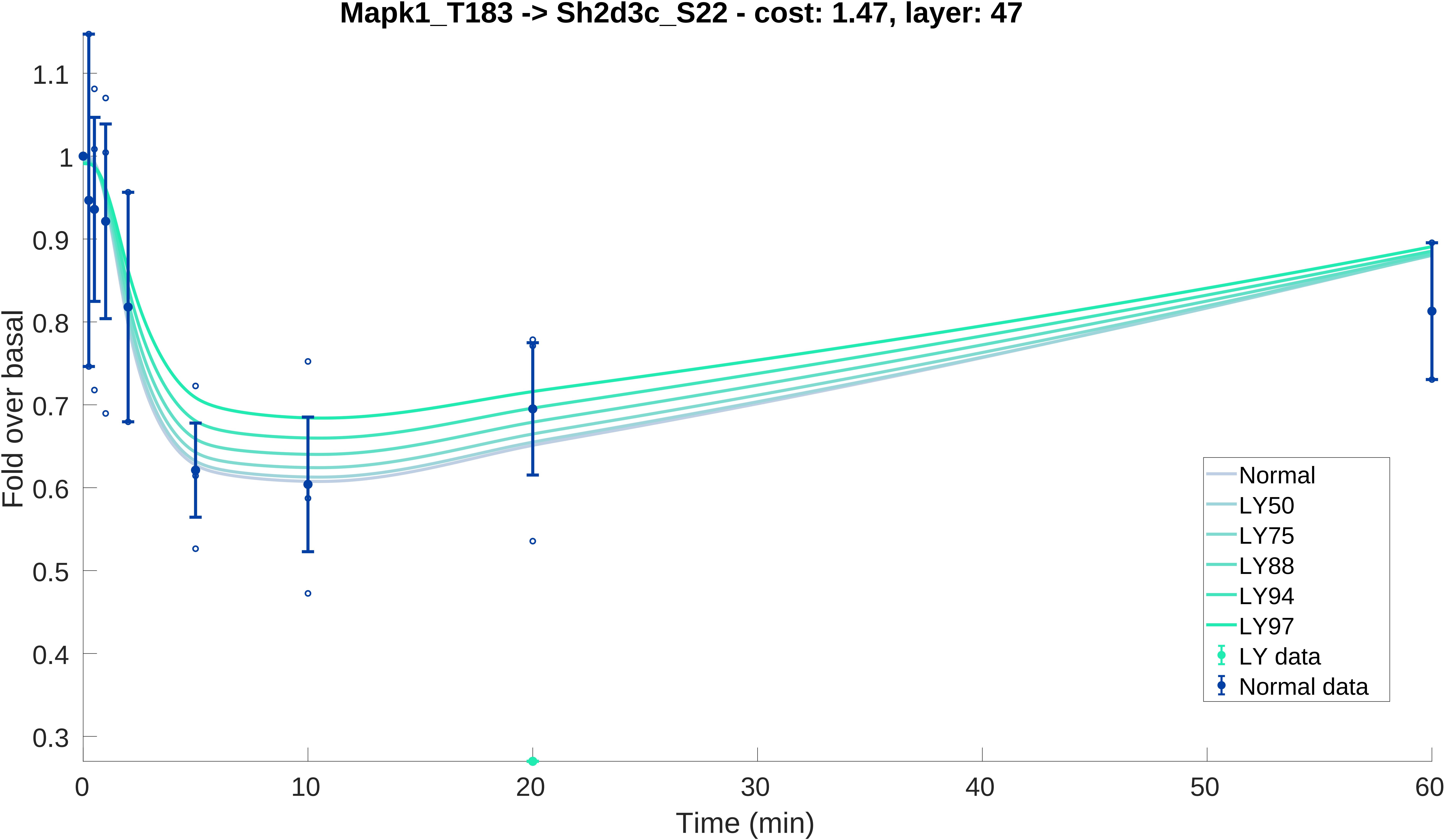

Mapk1_T183 -> Sh2d3c_S22 - cost: 1.47, layer: 47
Fold over basal
Time (min)
1.1
1
0.9
0.8
0.7
0.6
0.5
0.4
0.3
0
10
20
30
40
50
60
Normal
LY50
LY75
LY88
LY94
LY97
LY data
Normal data

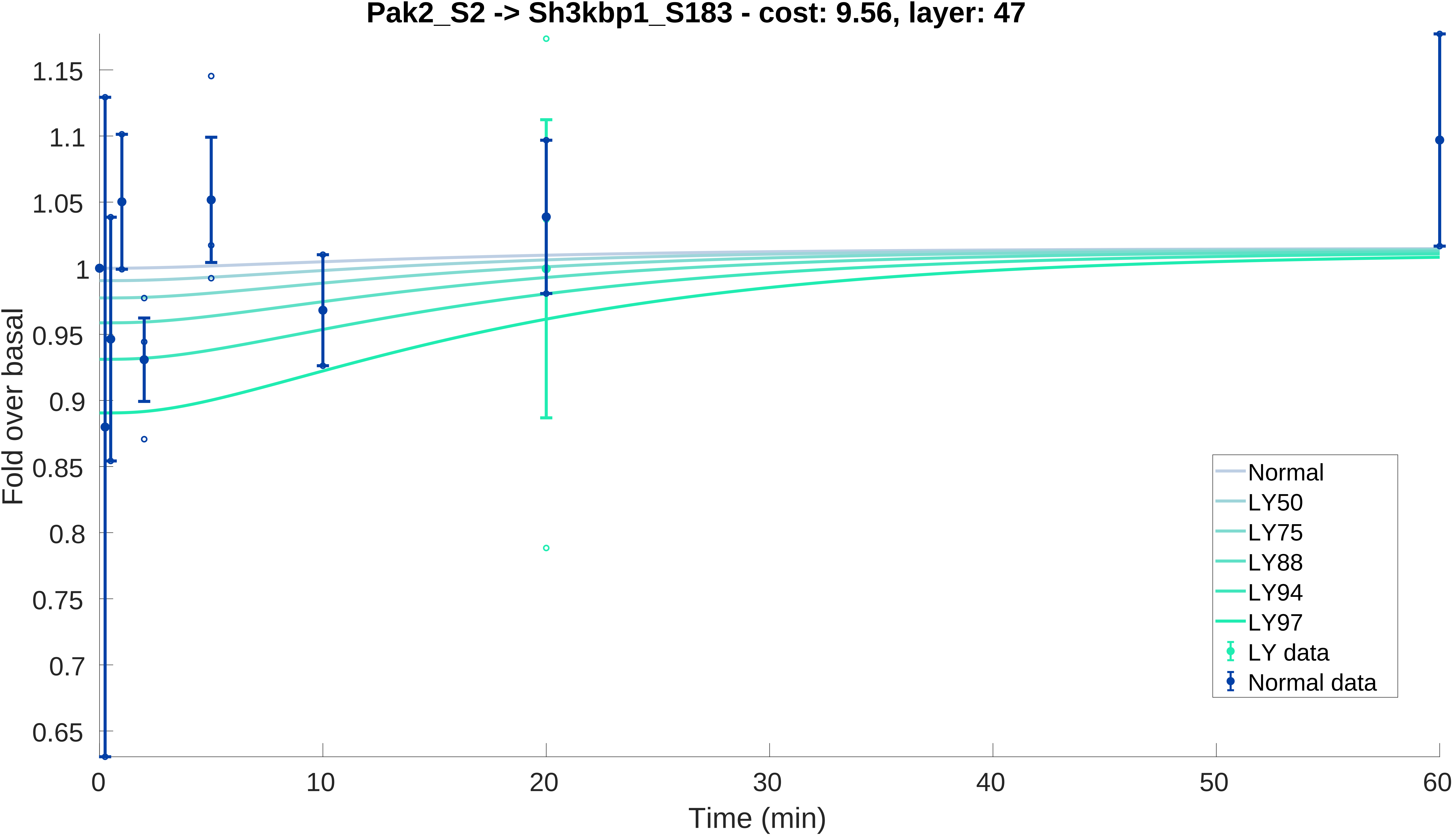

Pak2_S2 -> Sh3kbp1_S183 - cost: 9.56, layer: 47
Fold over basal
Time (min)
1.15
1.1
1.05
1
0.95
0.9
0.85
0.8
0.75
0.7
0.65
0
10
20
30
40
50
60
Normal
LY50
LY75
LY88
LY94
LY97
LY data
Normal data

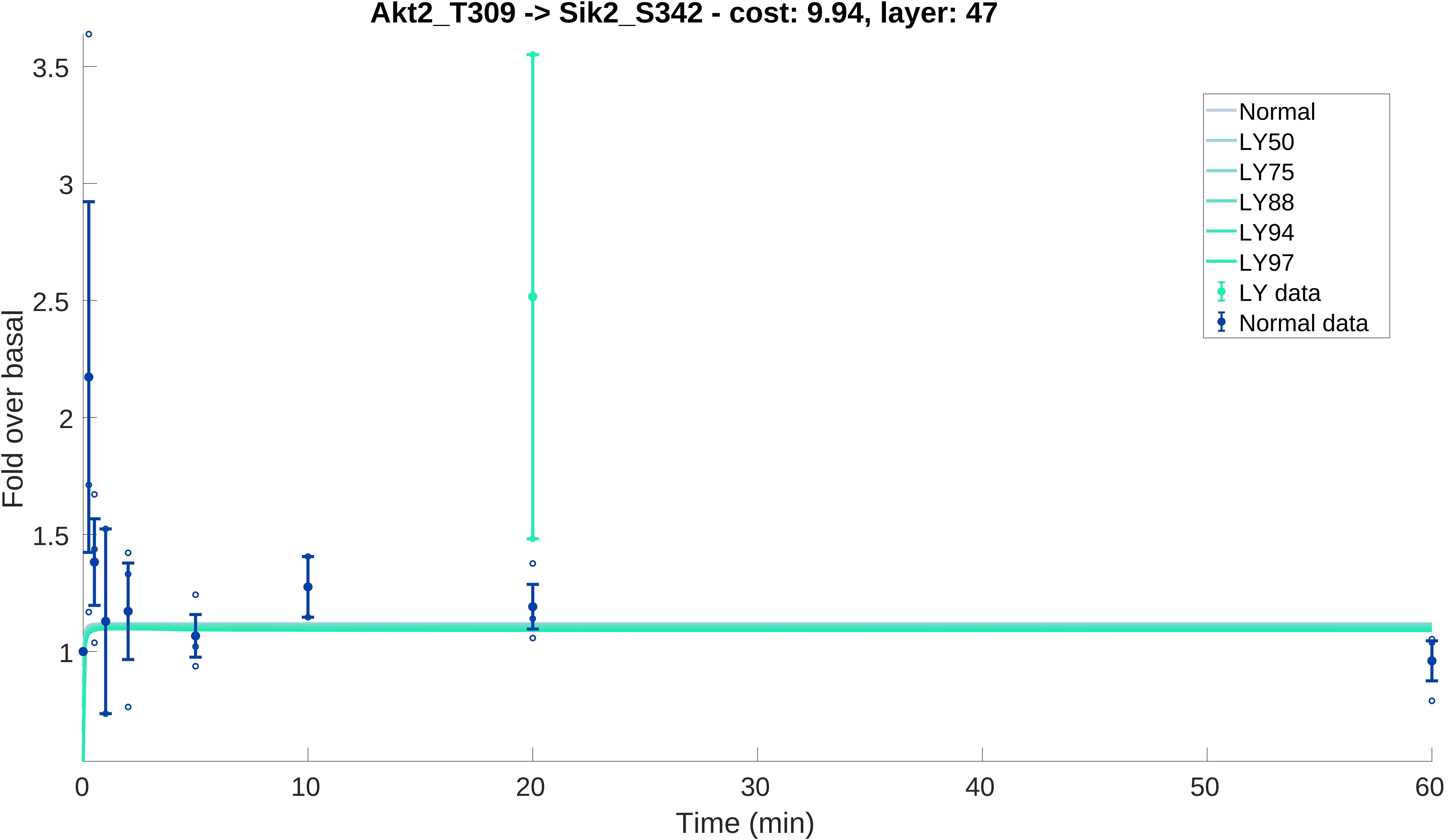

Akt2_T309 -> Sik2_S342 - cost: 9.94, layer: 47
Fold over basal
3.5
3
2.5
2
1.5
1
0
10
20
30
40
50
60
Time (min)
Normal
LY50
LY75
LY88
LY94
LY97
LY data
Normal data

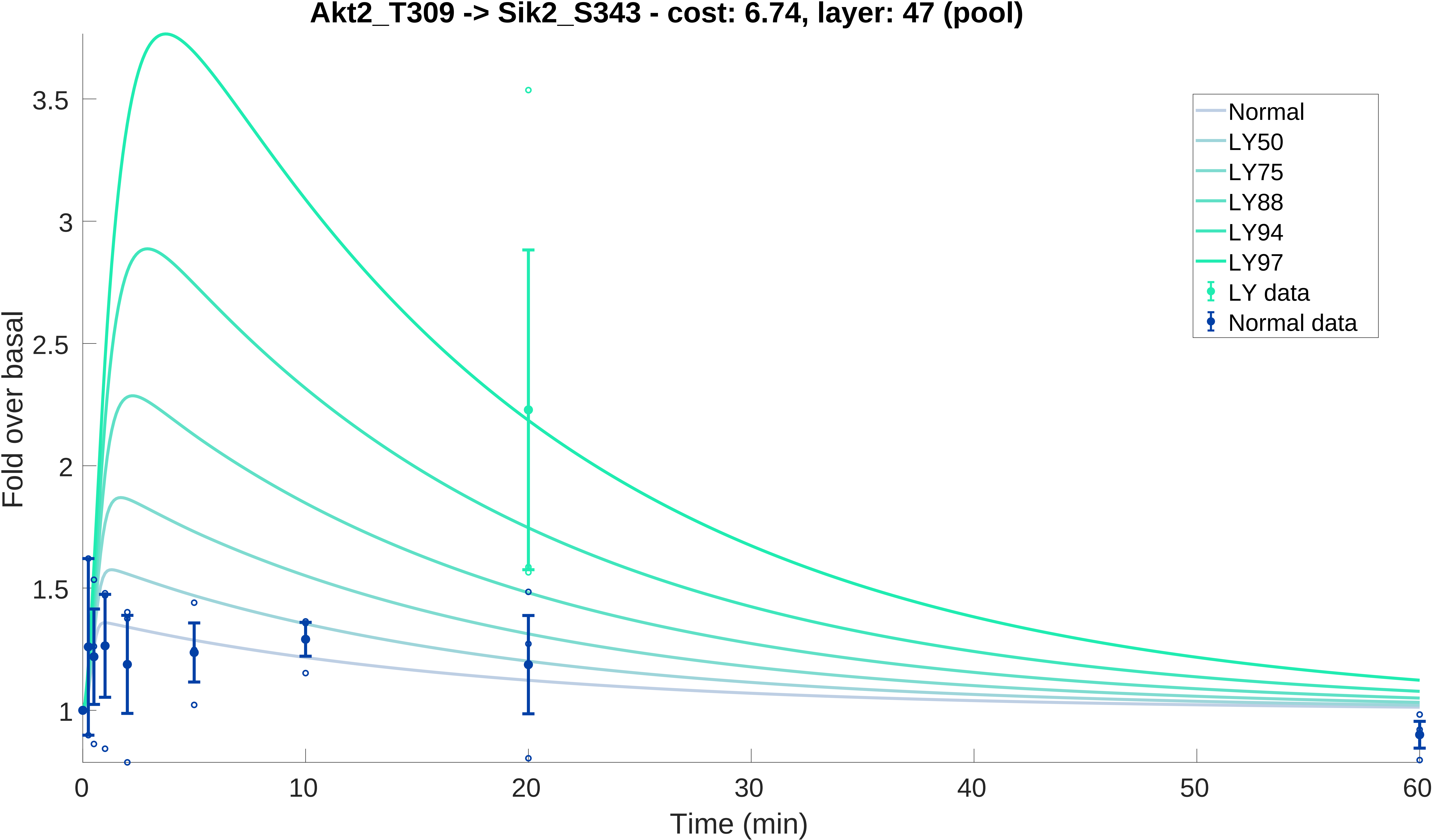
Akt2_T309 -> Sik2_S343 - cost: 6.74, layer: 47 (pool)
Fold over basal
Time (min)
3.5
3
2.5
2
1.5
1
0
10
20
30
40
50
60
Normal
LY50
LY75
LY88
LY94
LY97
LY data
Normal data

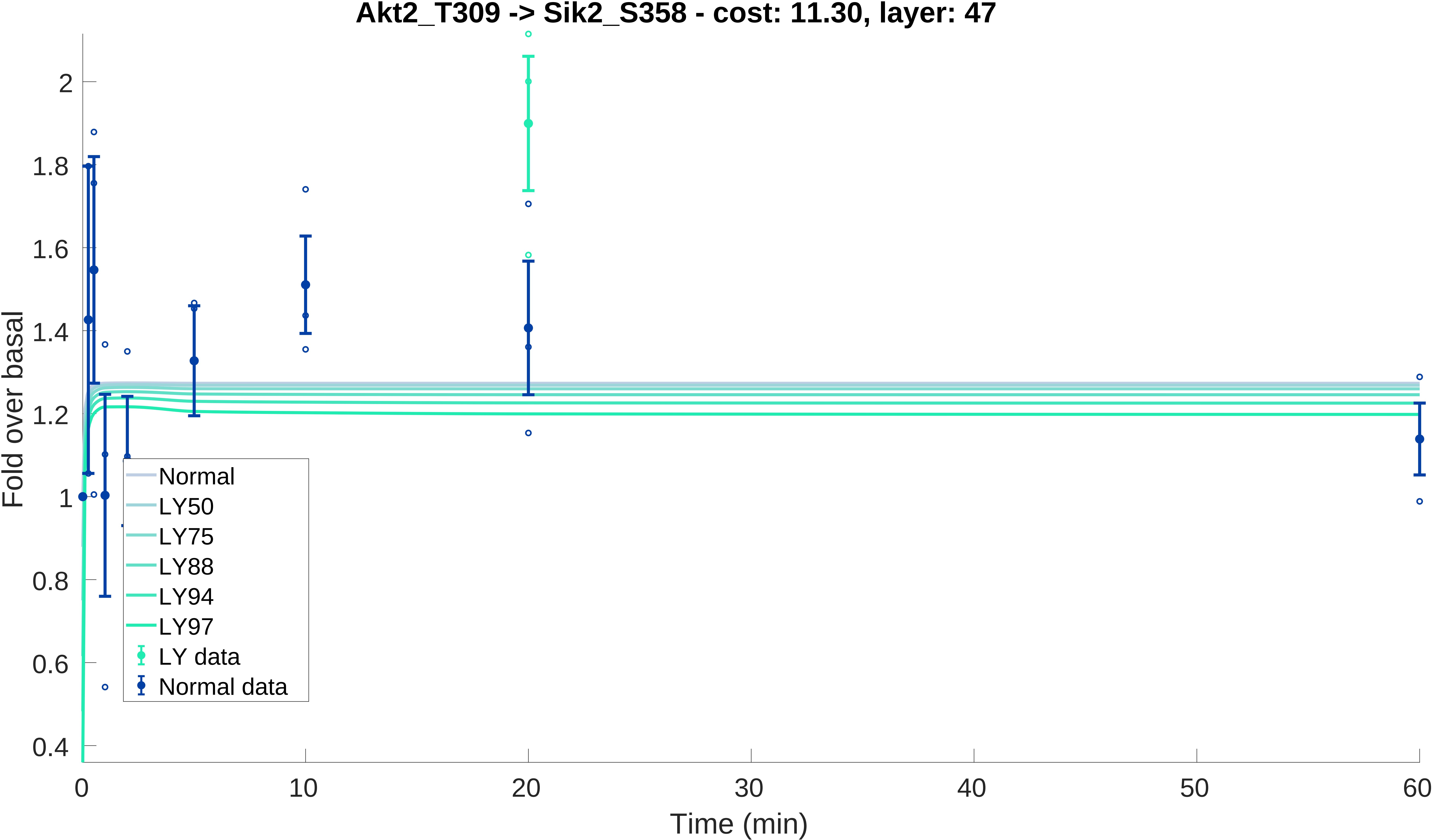

Akt2_T309 -> Sik2_S358 - cost: 11.30, layer: 47
Fold over basal
Time (min)
0.4
0.6
0.8
1
1.2
1.4
1.6
1.8
2
0
10
20
30
40
50
60
Normal
LY50
LY75
LY88
LY94
LY97
LY data
Normal data

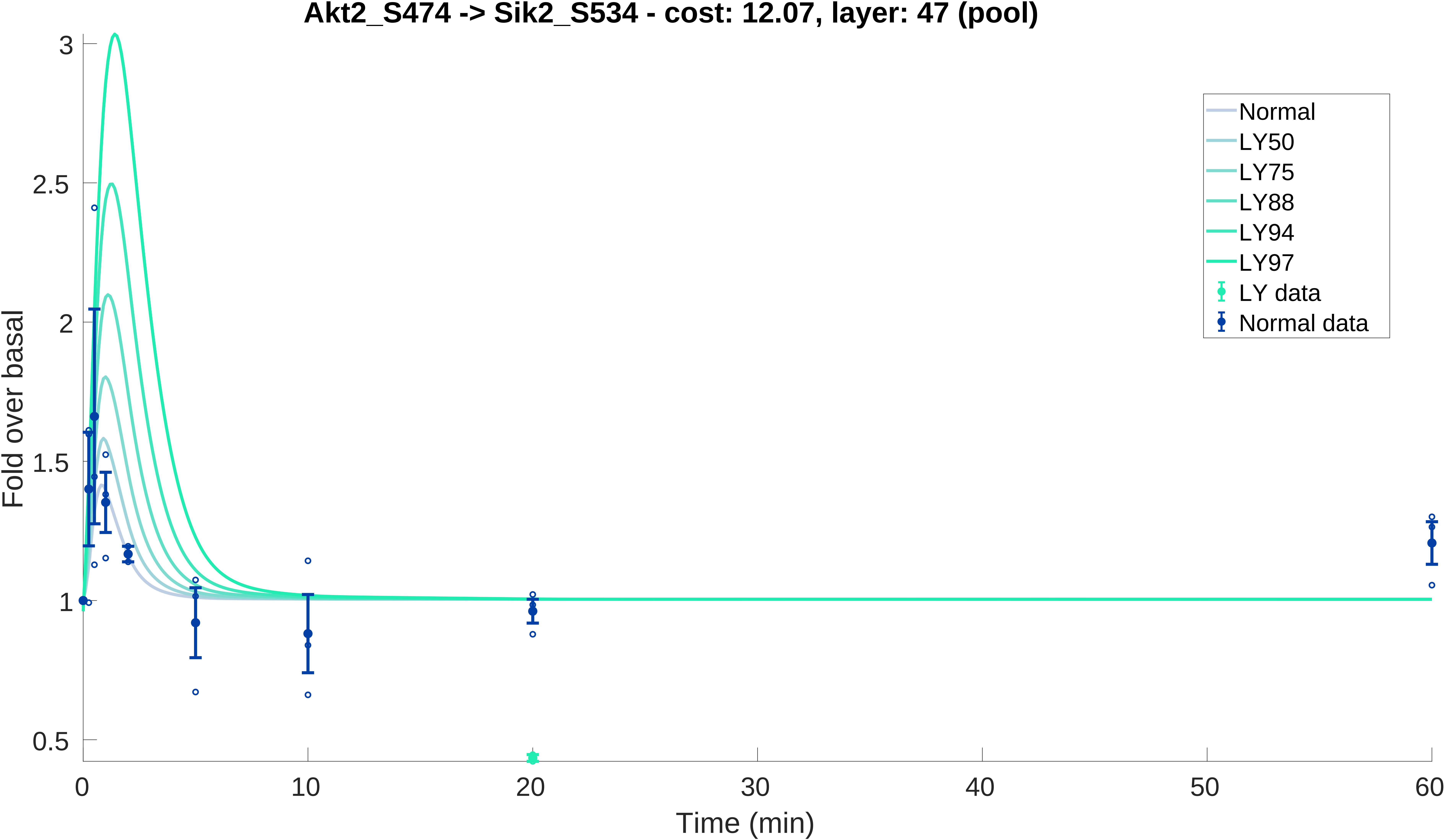
Akt2_S474 -> Sik2_S534 - cost: 12.07, layer: 47 (pool)
Fold over basal
3
2.5
2
1.5
1
0.5
0
10
20
30
40
50
60
Time (min)
Normal
LY50
LY75
LY88
LY94
LY97
LY data
Normal data

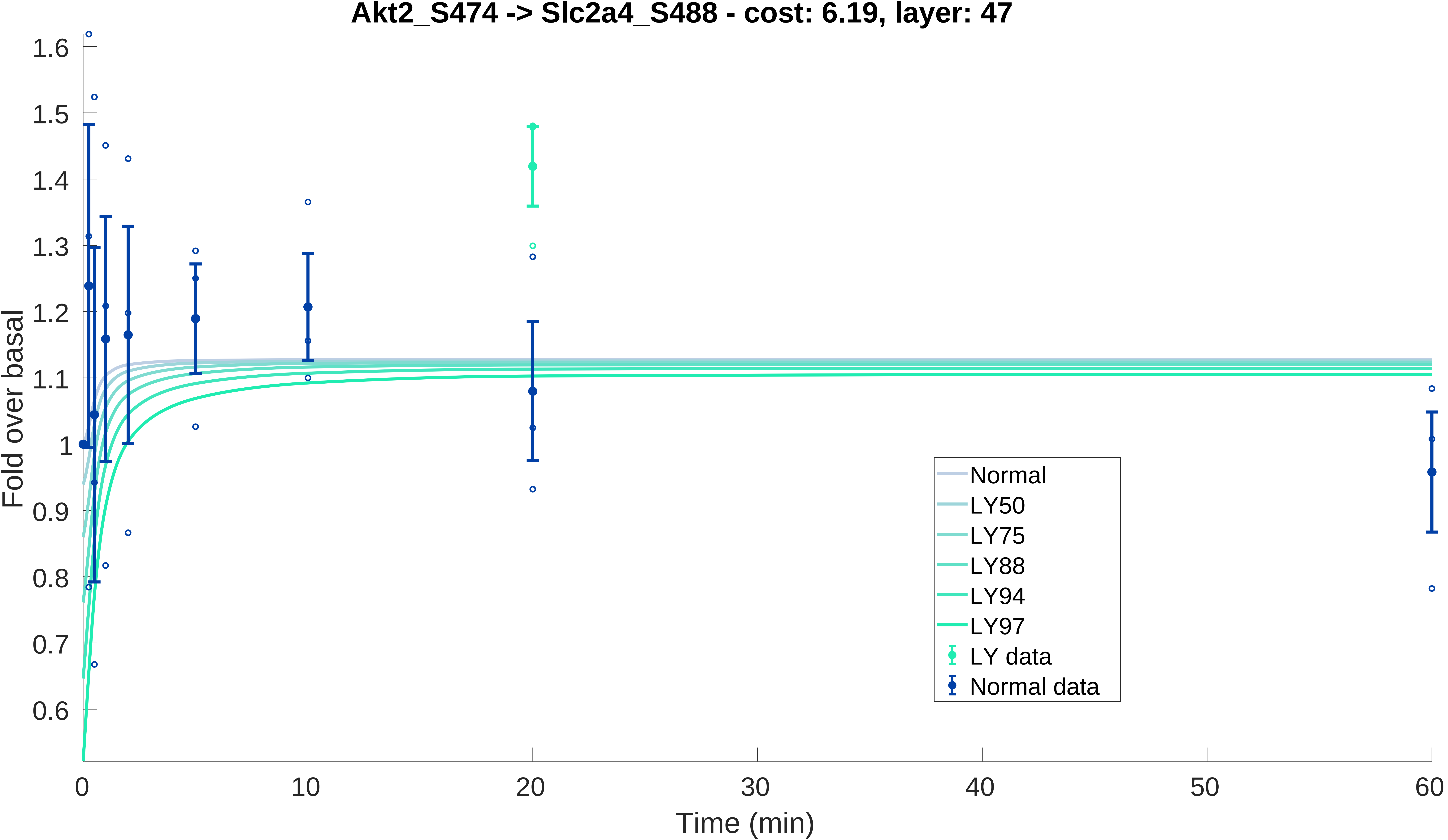

Akt2_S474 -> Slc2a4_S488 - cost: 6.19, layer: 47
Fold over basal
Time (min)
1.6
1.5
1.4
1.3
1.2
1.1
1
0.9
0.8
0.7
0.6
0
10
20
30
40
50
60
Normal
LY50
LY75
LY88
LY94
LY97
LY data
Normal data

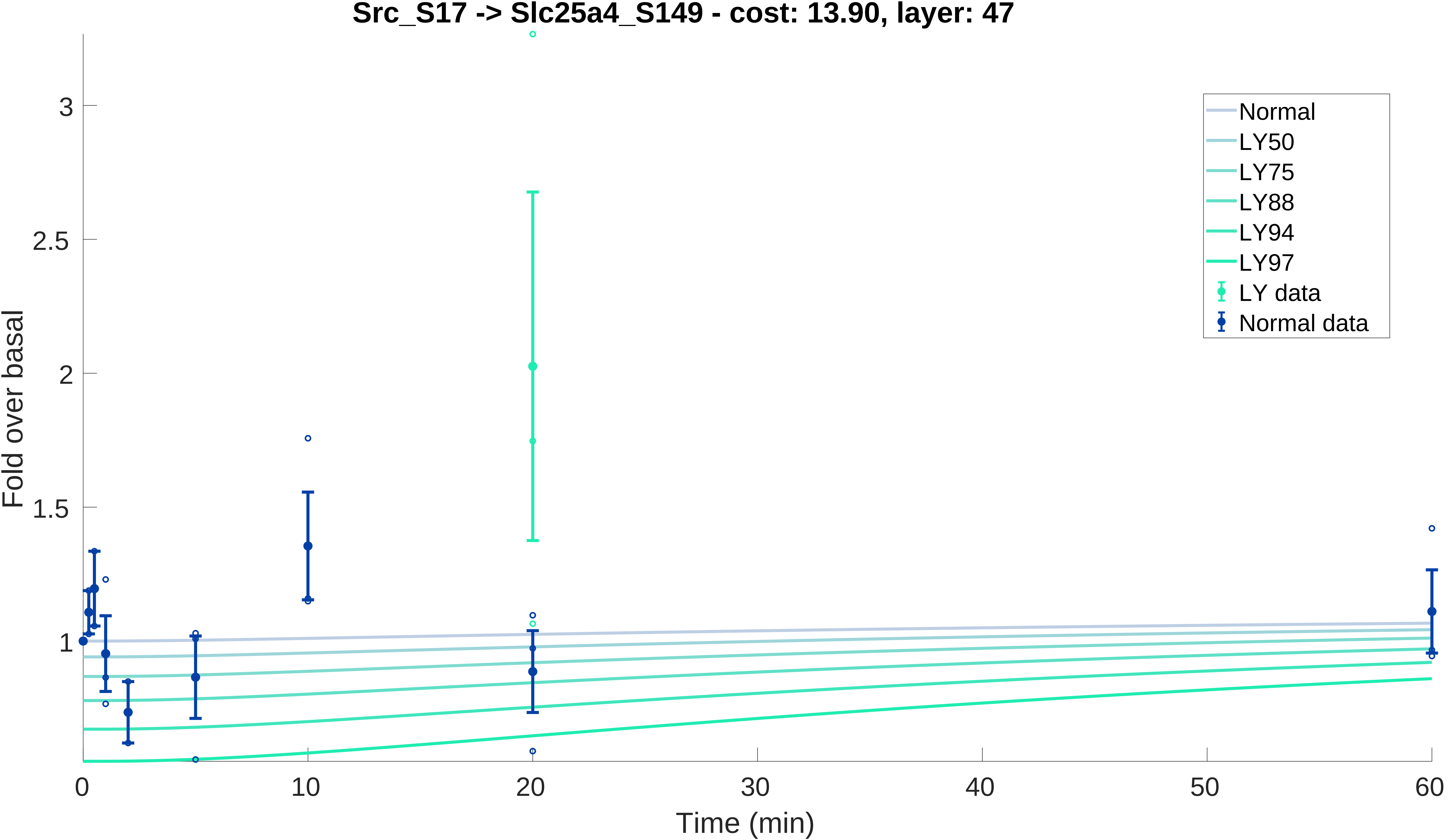

Src_S17 -> Slc25a4_S149 - cost: 13.90, layer: 47
Fold over basal
Time (min)
Normal
LY50
LY75
LY88
LY94
LY97
LY data
Normal data
3
2.5
2
1.5
1
0
10
20
30
40
50
60

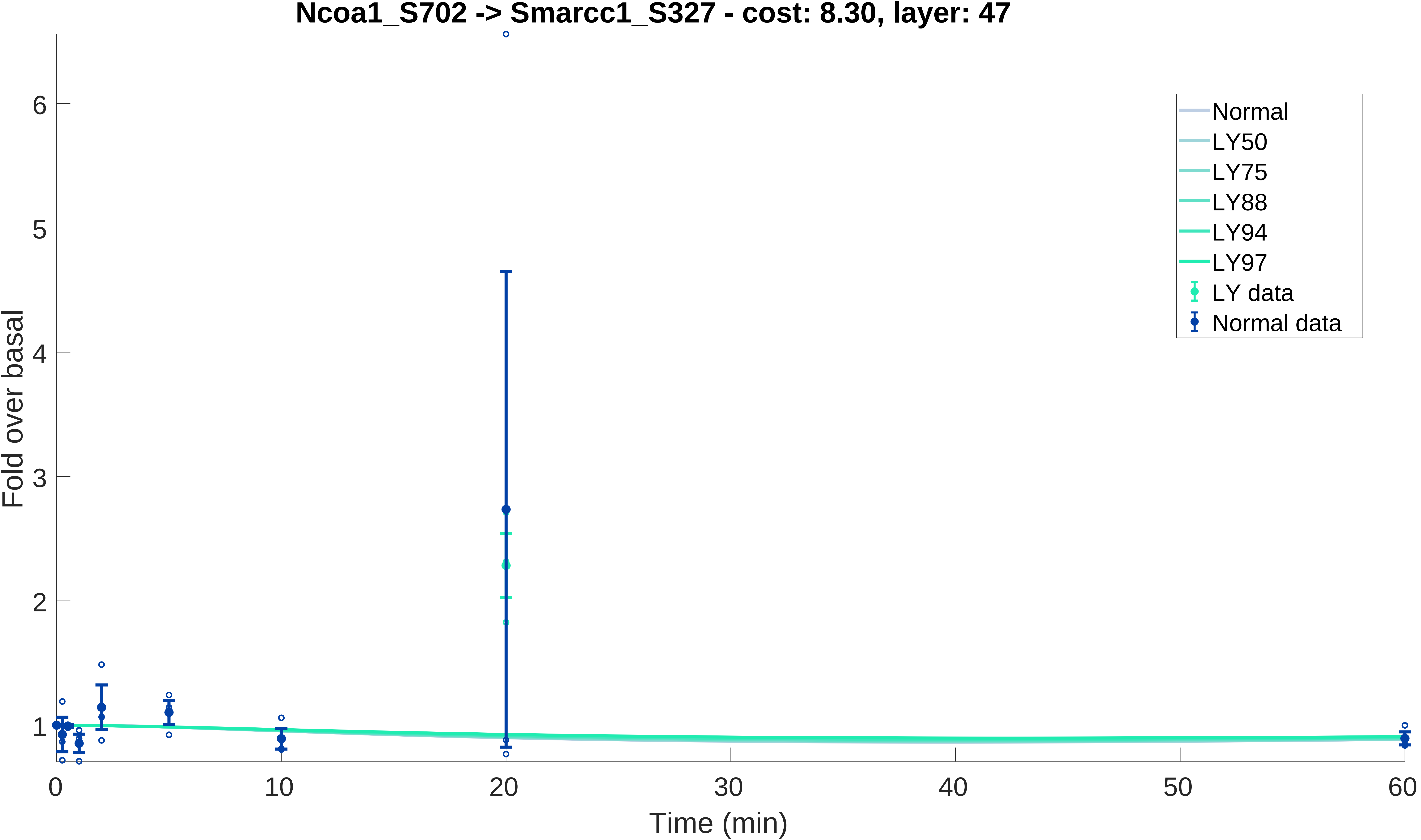

Ncoa1_S702 -> Smarcc1_S327 - cost: 8.30, layer: 47
Fold over basal
Time (min)
0
10
20
30
40
50
60
1
2
3
4
5
6
Normal
LY50
LY75
LY88
LY94
LY97
LY data
Normal data

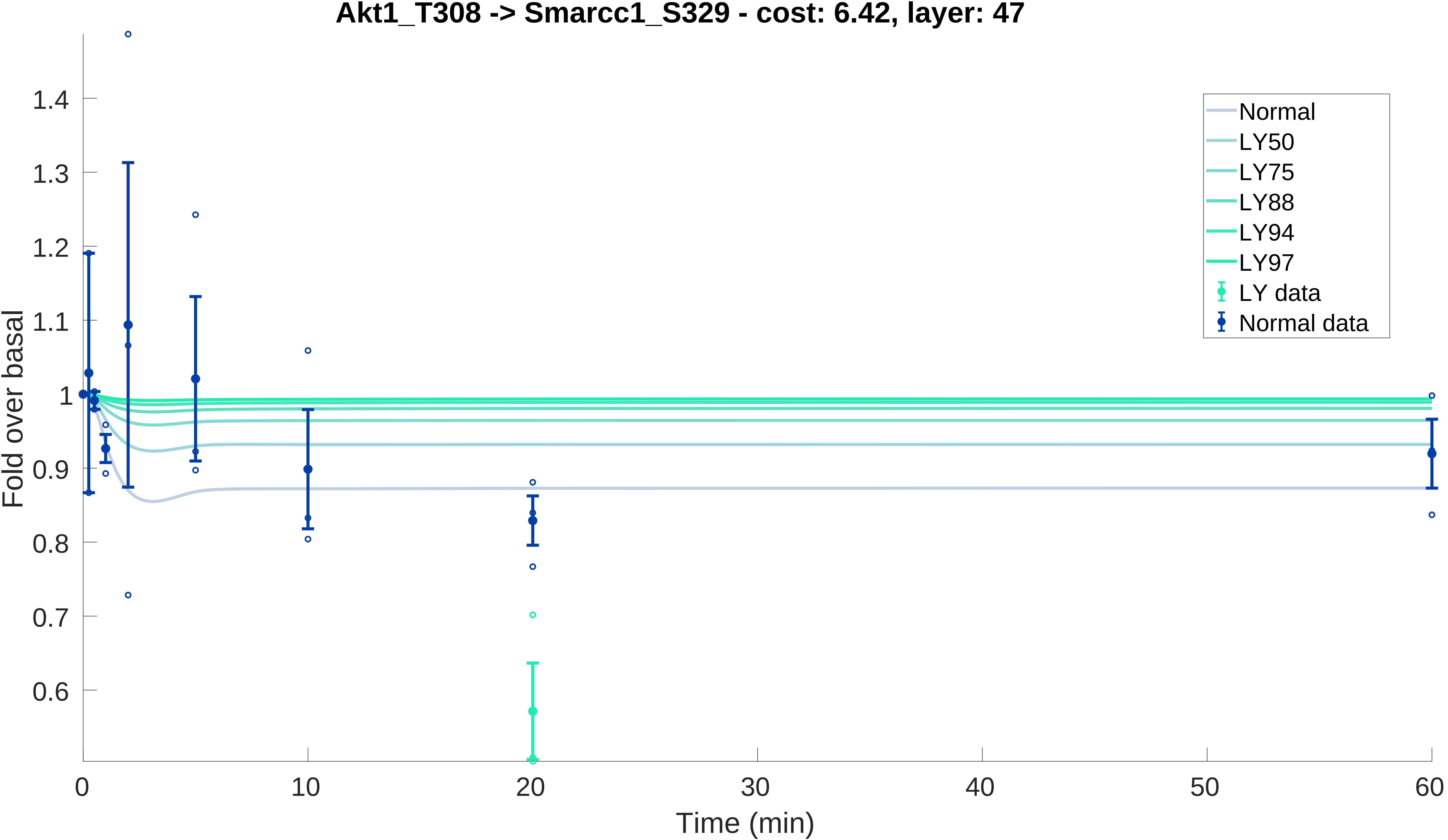
Akt1_T308 -> Smarcc1_S329 - cost: 6.42, layer: 47
Fold over basal
Time (min)
0.6
0.7
0.8
0.9
1
1.1
1.2
1.3
1.4
0
10
20
30
40
50
60
Normal
LY50
LY75
LY88
LY94
LY97
LY data
Normal data

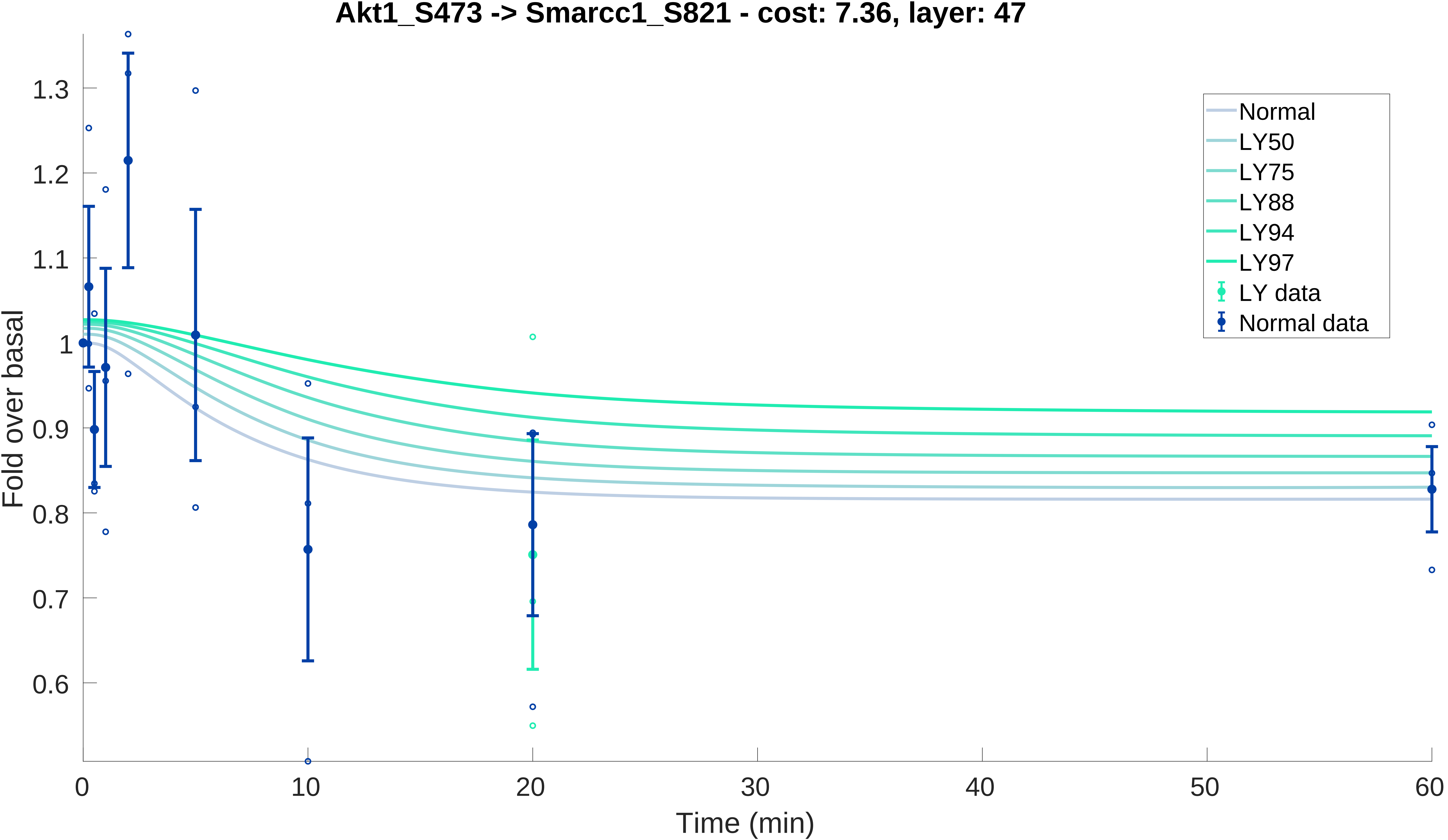

Akt1_S473 -> Smarcc1_S821 - cost: 7.36, layer: 47
Fold over basal
Time (min)
Normal
LY50
LY75
LY88
LY94
LY97
LY data
Normal data
1.3
1.2
1.1
1
0.9
0.8
0.7
0.6
0
10
20
30
40
50
60

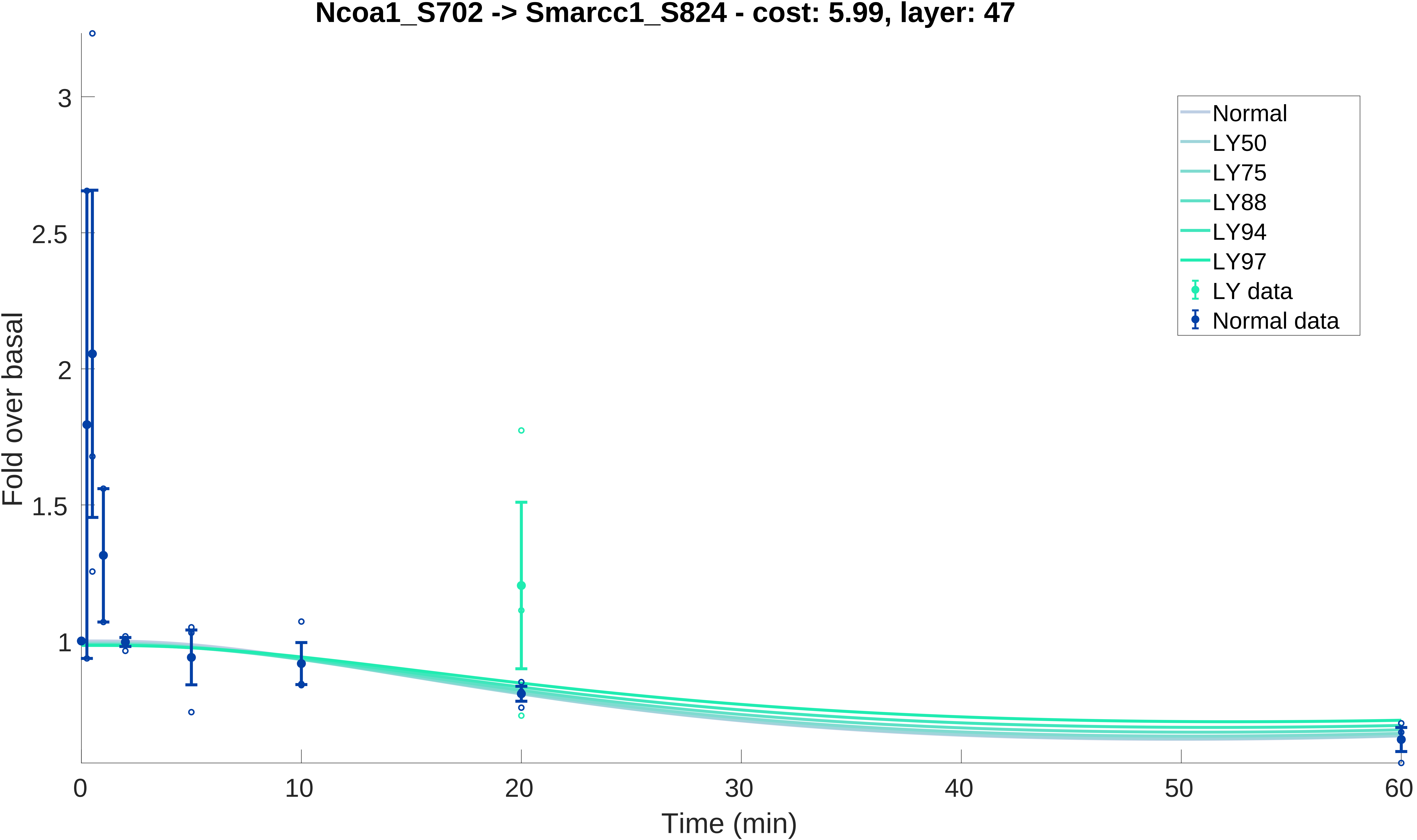

Ncoa1_S702 -> Smarcc1_S824 - cost: 5.99, layer: 47
Fold over basal
Time (min)
0
10
20
30
40
50
60
1
1.5
2
2.5
3
Normal
LY50
LY75
LY88
LY94
LY97
LY data
Normal data

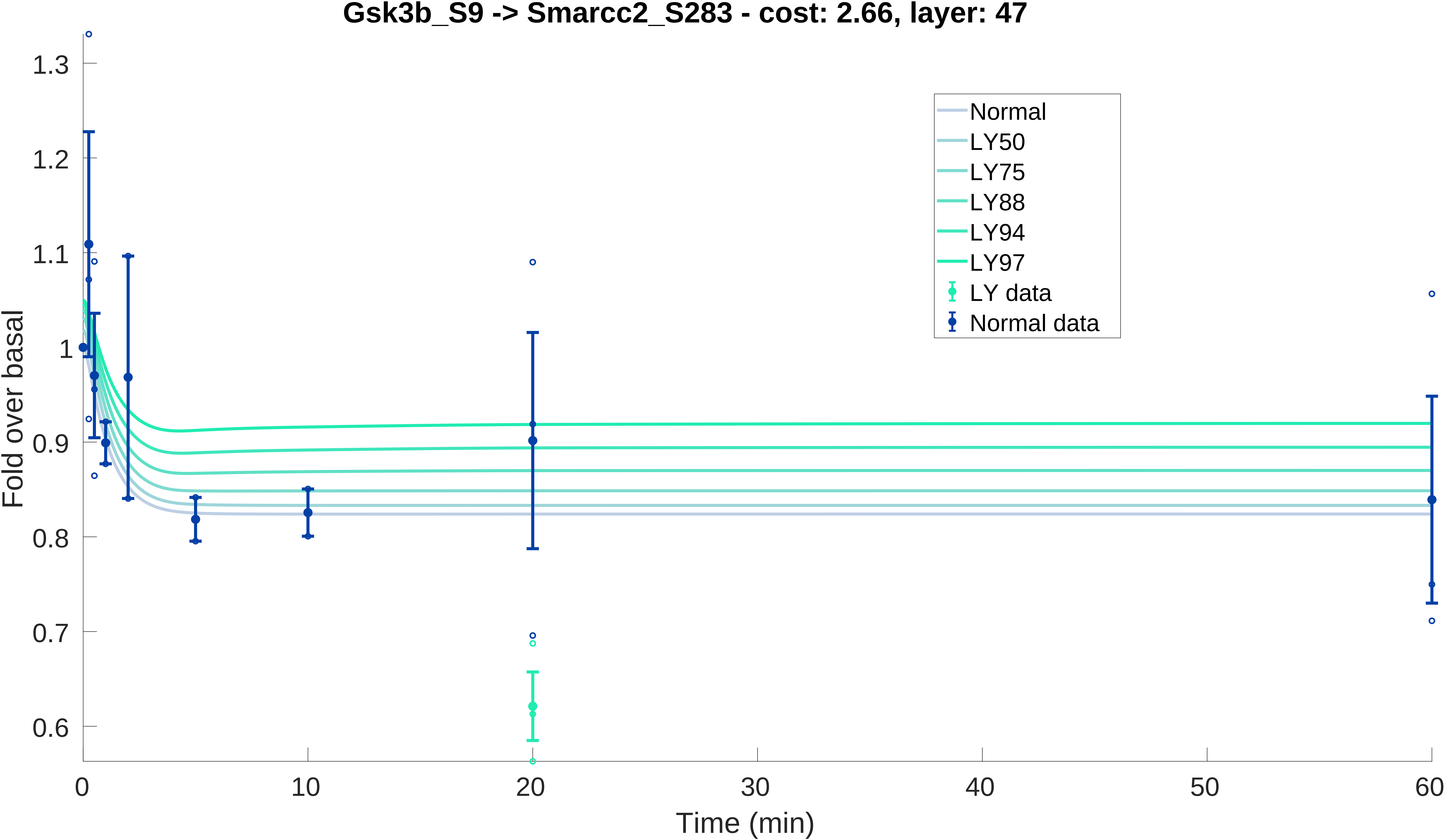
Gsk3b_S9 -> Smarcc2_S283 - cost: 2.66, layer: 47
Fold over basal
Time (min)
1.3
1.2
1.1
1
0.9
0.8
0.7
0.6
0
10
20
30
40
50
60
Normal
LY50
LY75
LY88
LY94
LY97
LY data
Normal data

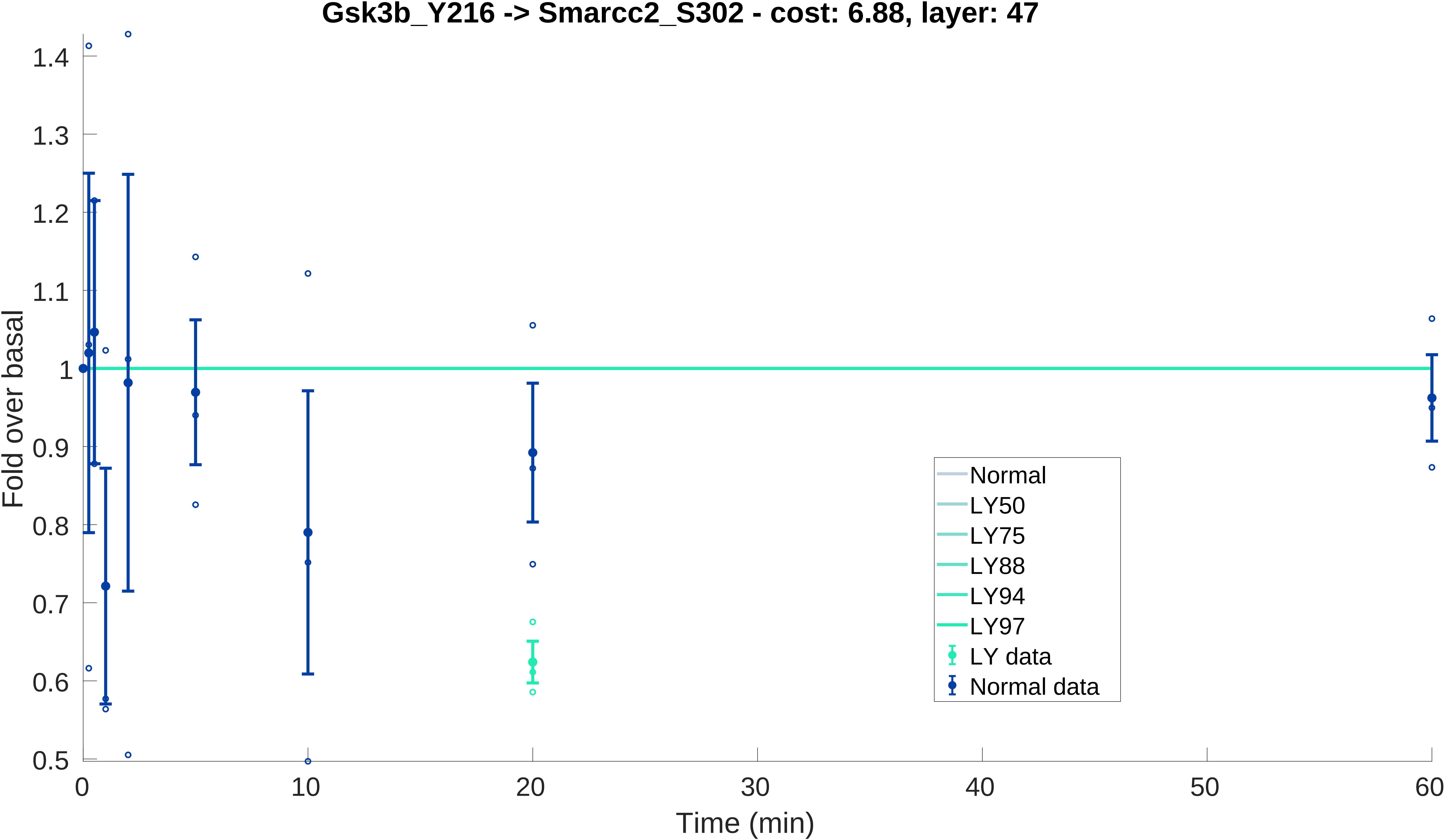

Gsk3b_Y216 -> Smarcc2_S302 - cost: 6.88, layer: 47
Fold over basal
Time (min)
1.4
1.3
1.2
1.1
1
0.9
0.8
0.7
0.6
0.5
0
10
20
30
40
50
60
Normal
LY50
LY75
LY88
LY94
LY97
LY data
Normal data

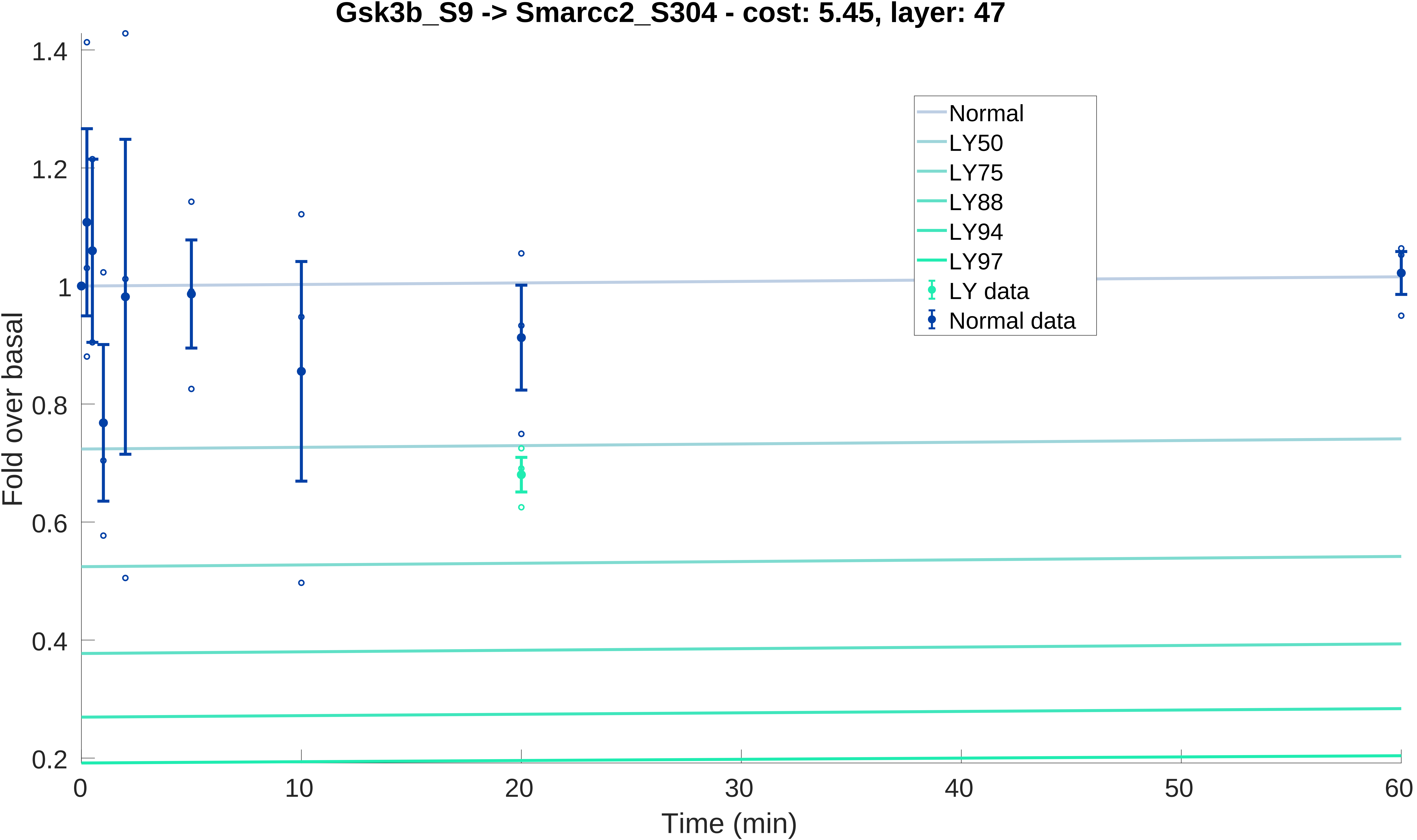

Gsk3b_S9 -> Smarcc2_S304 - cost: 5.45, layer: 47
Fold over basal
Time (min)
0.2
0.4
0.6
0.8
1
1.2
1.4
0
10
20
30
40
50
60
Normal
LY50
LY75
LY88
LY94
LY97
LY data
Normal data

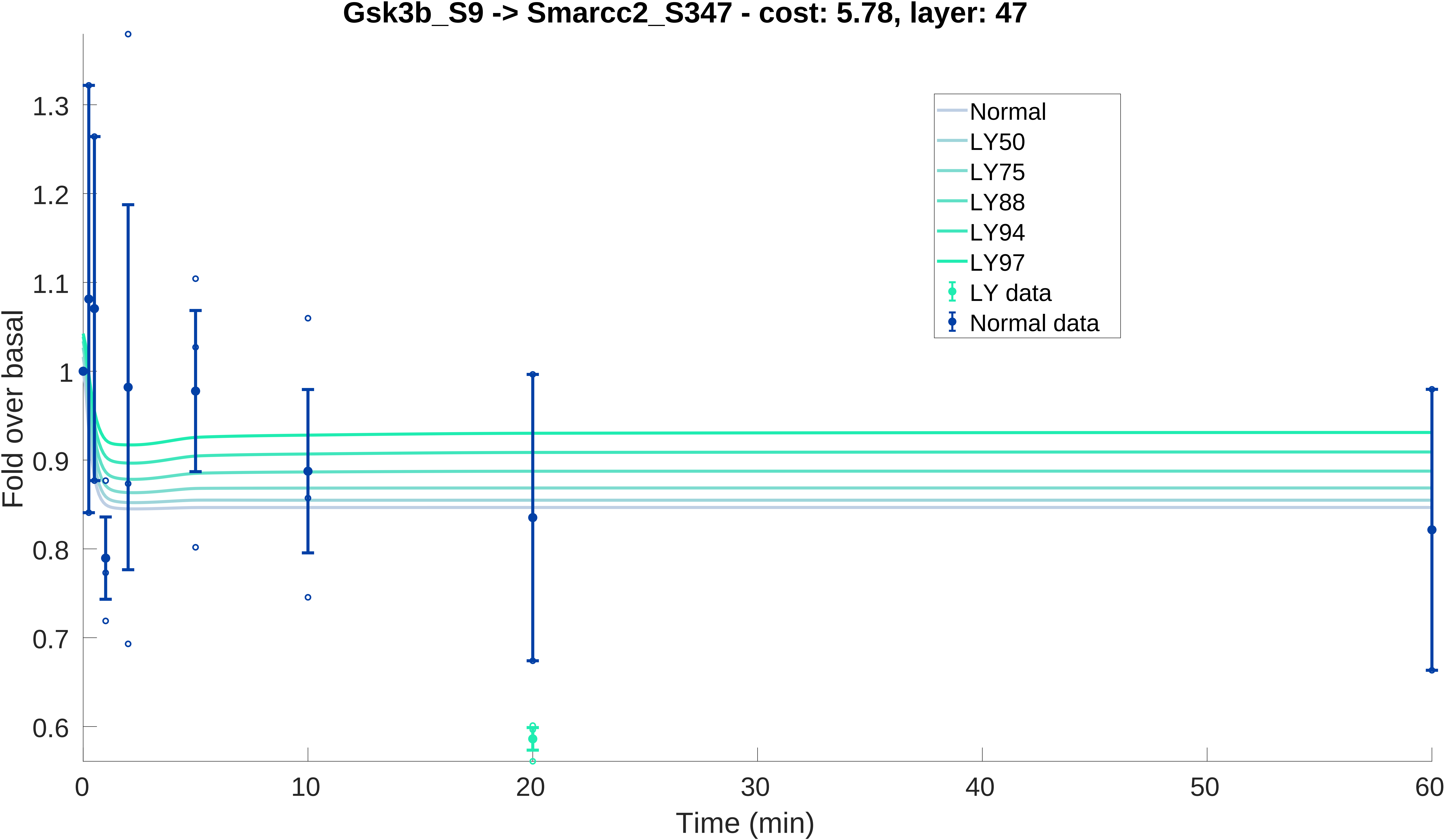

Gsk3b_S9 -> Smarcc2_S347 - cost: 5.78, layer: 47
Fold over basal
Time (min)
0.6
0.7
0.8
0.9
1
1.1
1.2
1.3
0
10
20
30
40
50
60
Normal
LY50
LY75
LY88
LY94
LY97
LY data
Normal data

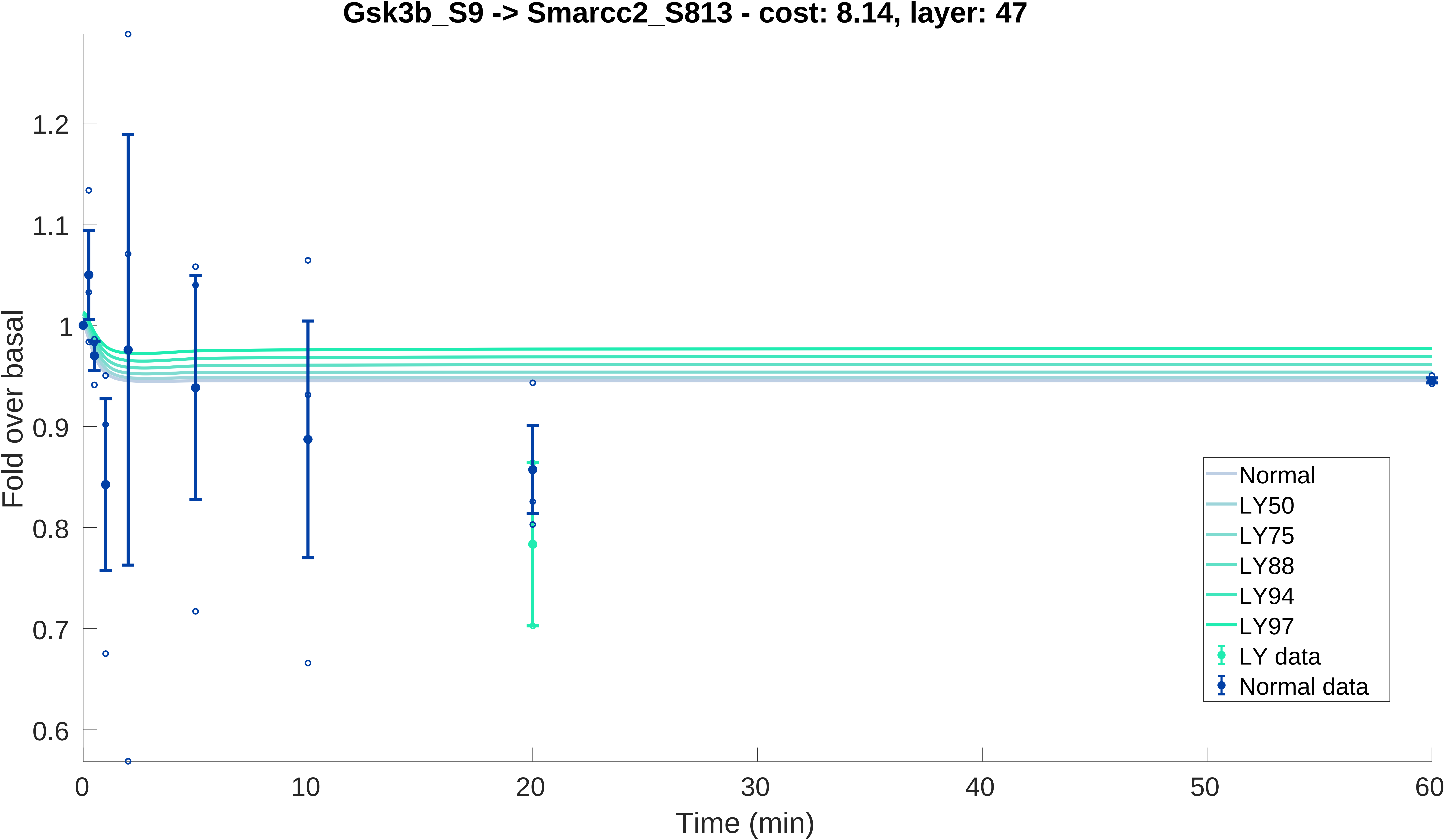

Gsk3b_S9 -> Smarcc2_S813 - cost: 8.14, layer: 47
Fold over basal
1.2
1.1
1
0.9
0.8
0.7
0.6
0
10
20
30
40
50
60
Time (min)
Normal
LY50
LY75
LY88
LY94
LY97
LY data
Normal data

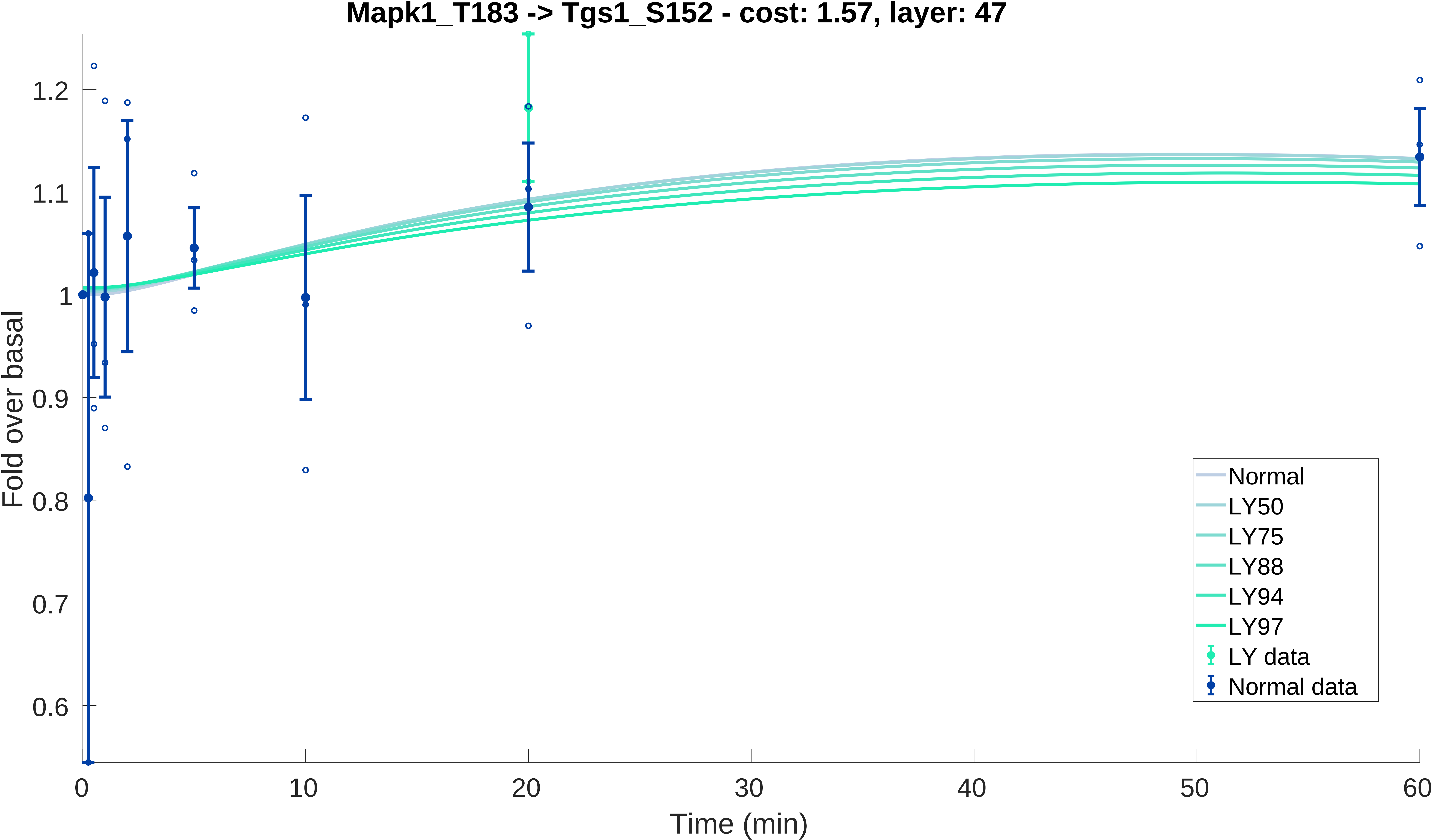

Mapk1_T183 -> Tgs1_S152 - cost: 1.57, layer: 47
Fold over basal
Time (min)
0
10
20
30
40
50
60
0.6
0.7
0.8
0.9
1
1.1
1.2
Normal
LY50
LY75
LY88
LY94
LY97
LY data
Normal data

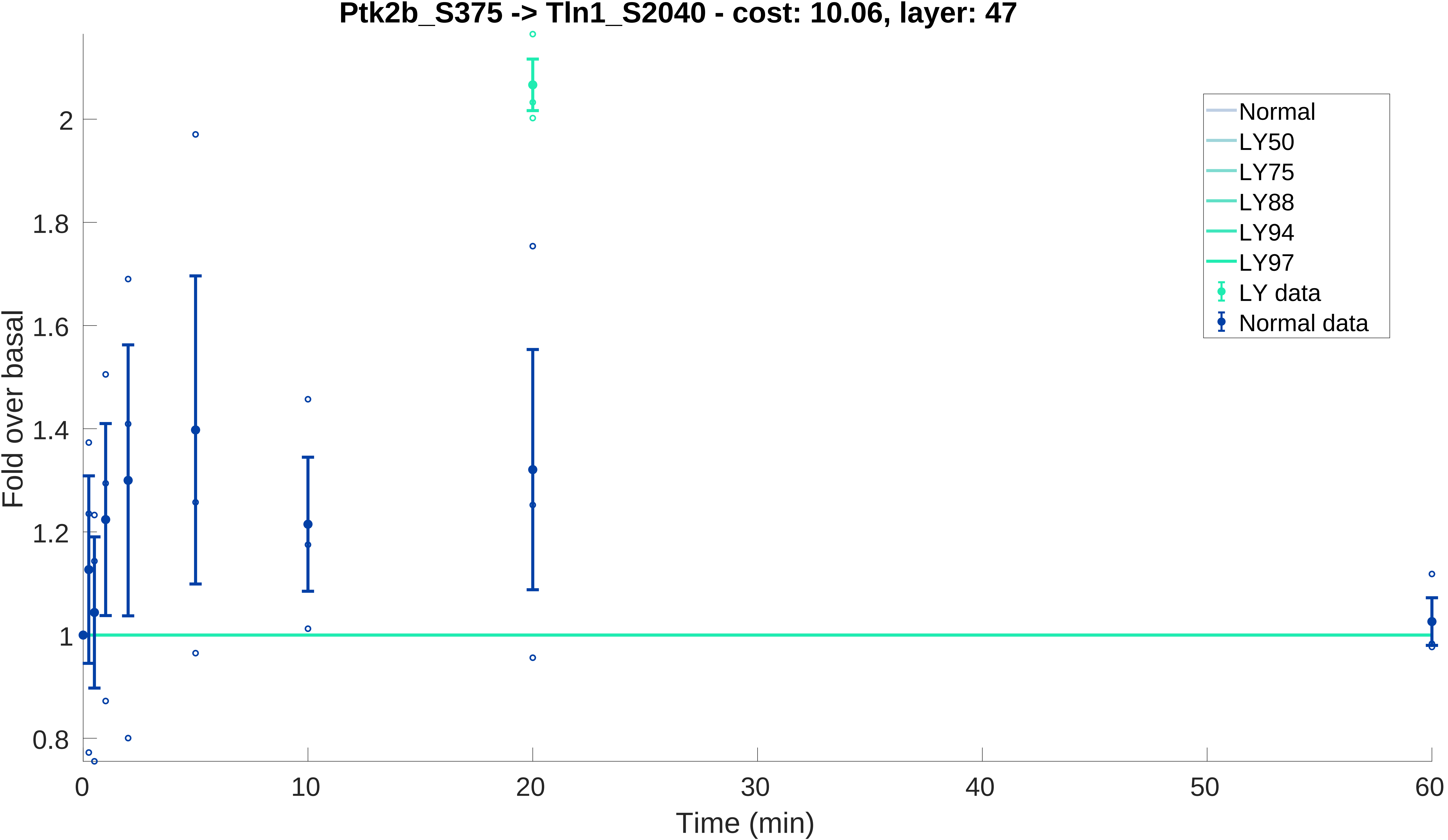

Ptk2b_S375 -> Tln1_S2040 - cost: 10.06, layer: 47
Fold over basal
Time (min)
0.8
1
1.2
1.4
1.6
1.8
2
0
10
20
30
40
50
60
Normal
LY50
LY75
LY88
LY94
LY97
LY data
Normal data

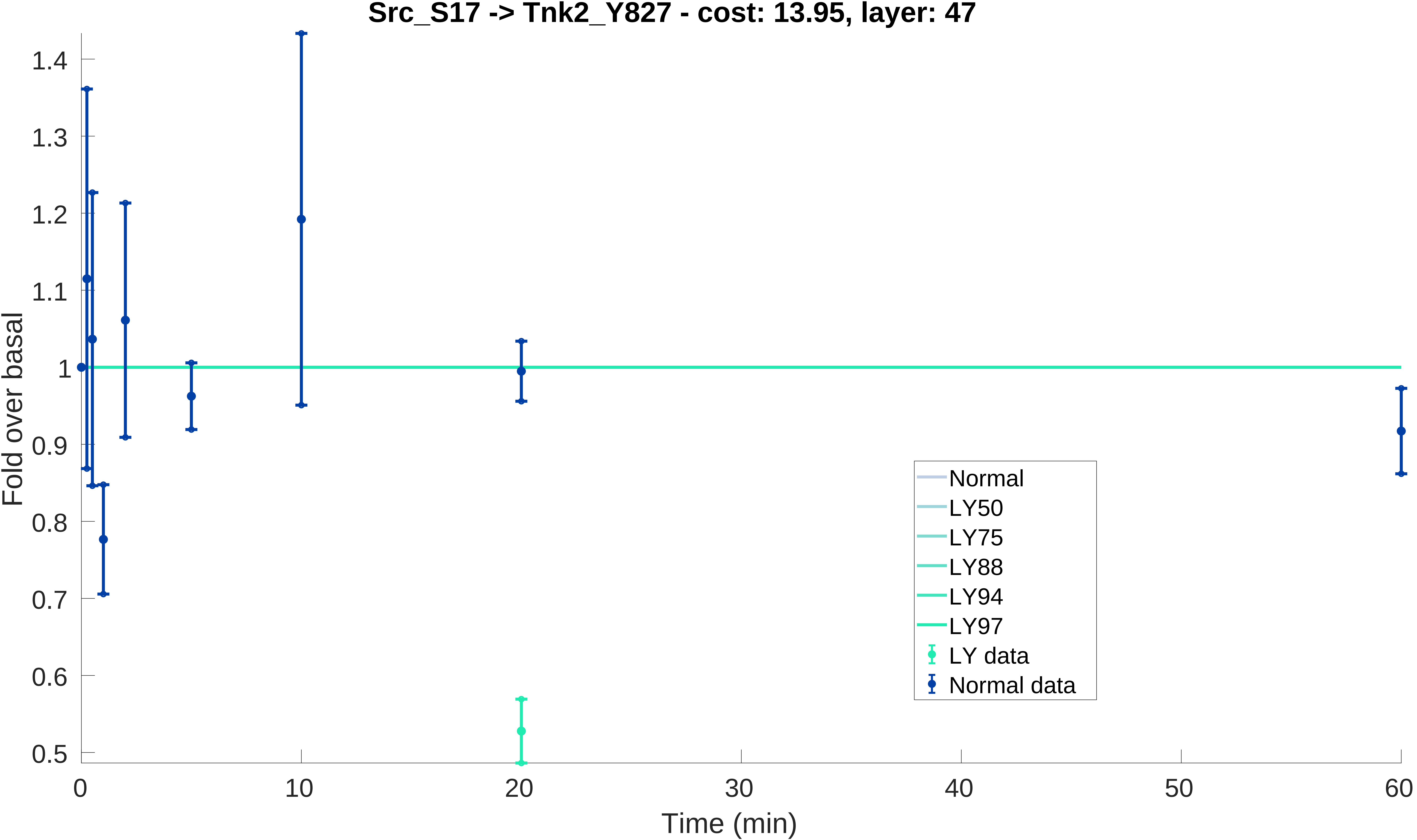

Src_S17 -> Tnk2_Y827 - cost: 13.95, layer: 47
Fold over basal
1.4
1.3
1.2
1.1
1
0.9
0.8
0.7
0.6
0.5
0
10
20
30
40
50
60
Time (min)
Normal
LY50
LY75
LY88
LY94
LY97
LY data
Normal data

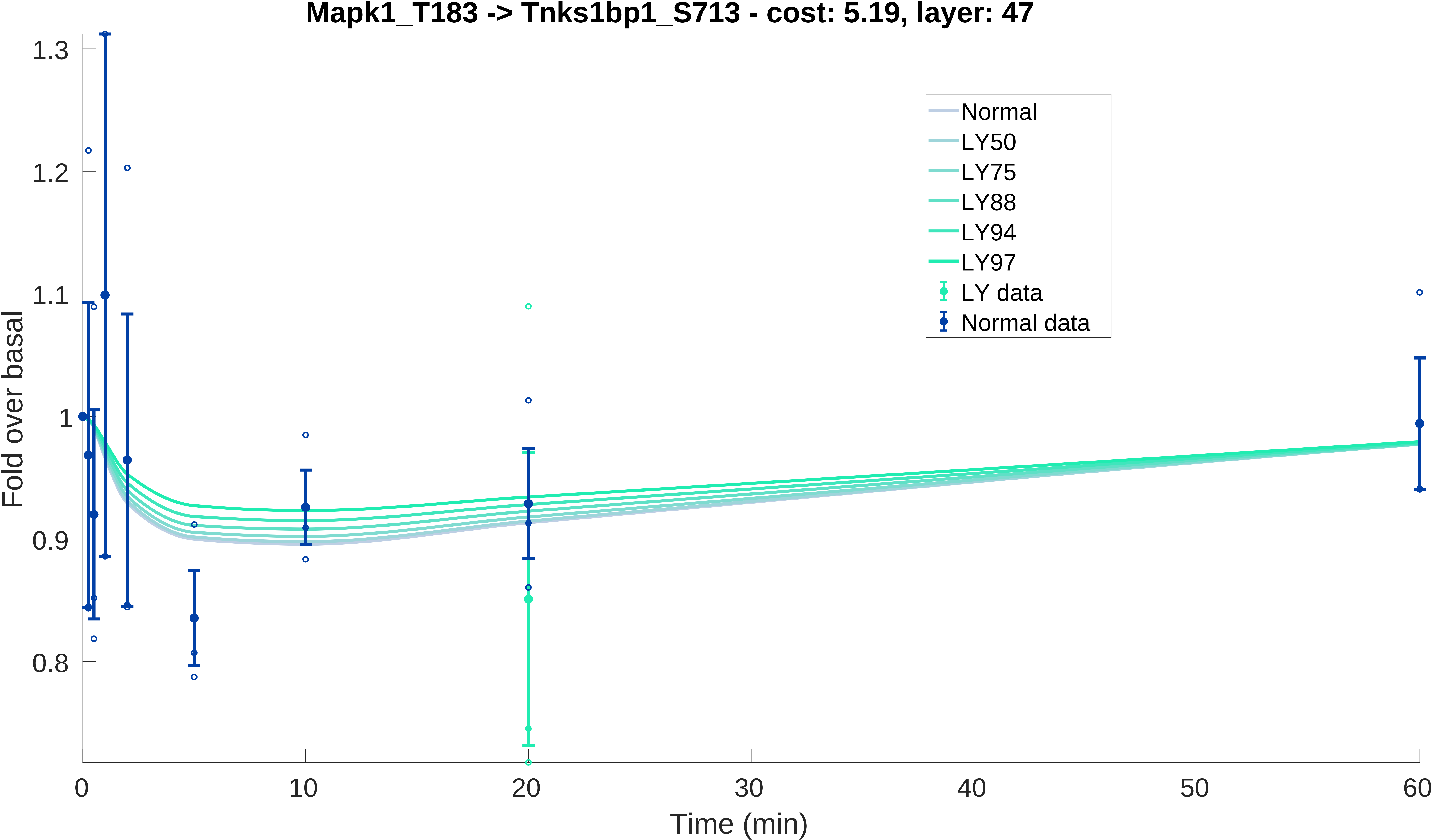

Mapk1_T183 -> Tnks1bp1_S713 - cost: 5.19, layer: 47
Fold over basal
Time (min)
Normal
LY50
LY75
LY88
LY94
LY97
LY data
Normal data
1.3
1.2
1.1
1
0.9
0.8
0
10
20
30
40
50
60

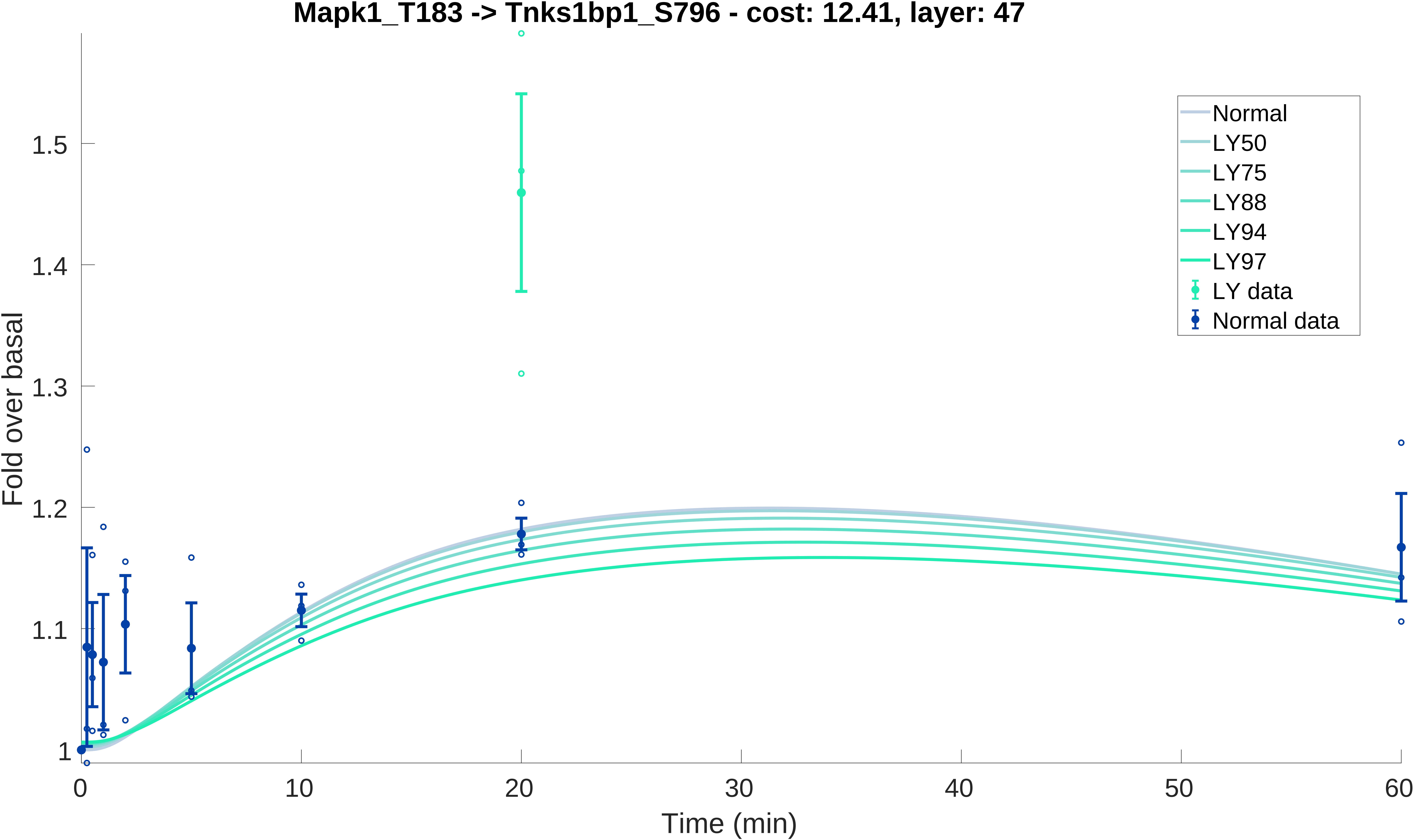

Mapk1_T183 -> Tnks1bp1_S796 - cost: 12.41, layer: 47
Normal
LY50
LY75
LY88
LY94
LY97
LY data
Normal data
Fold over basal
1
1.1
1.2
1.3
1.4
1.5
0
10
20
30
40
50
60
Time (min)

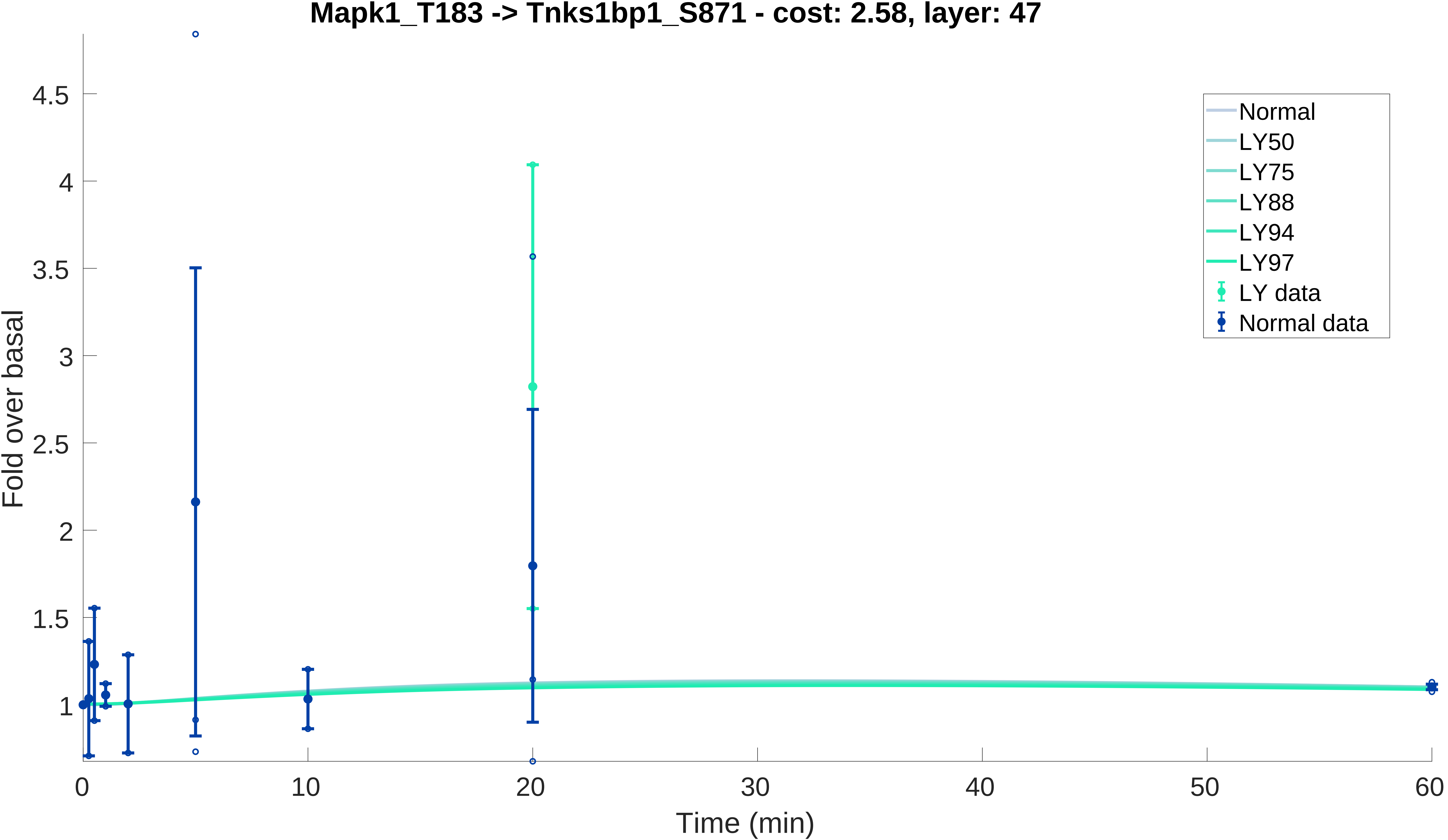

Mapk1_T183 -> Tnks1bp1_S871 - cost: 2.58, layer: 47
Fold over basal
Time (min)
4.5
4
3.5
3
2.5
2
1.5
1
0
10
20
30
40
50
60
Normal
LY50
LY75
LY88
LY94
LY97
LY data
Normal data

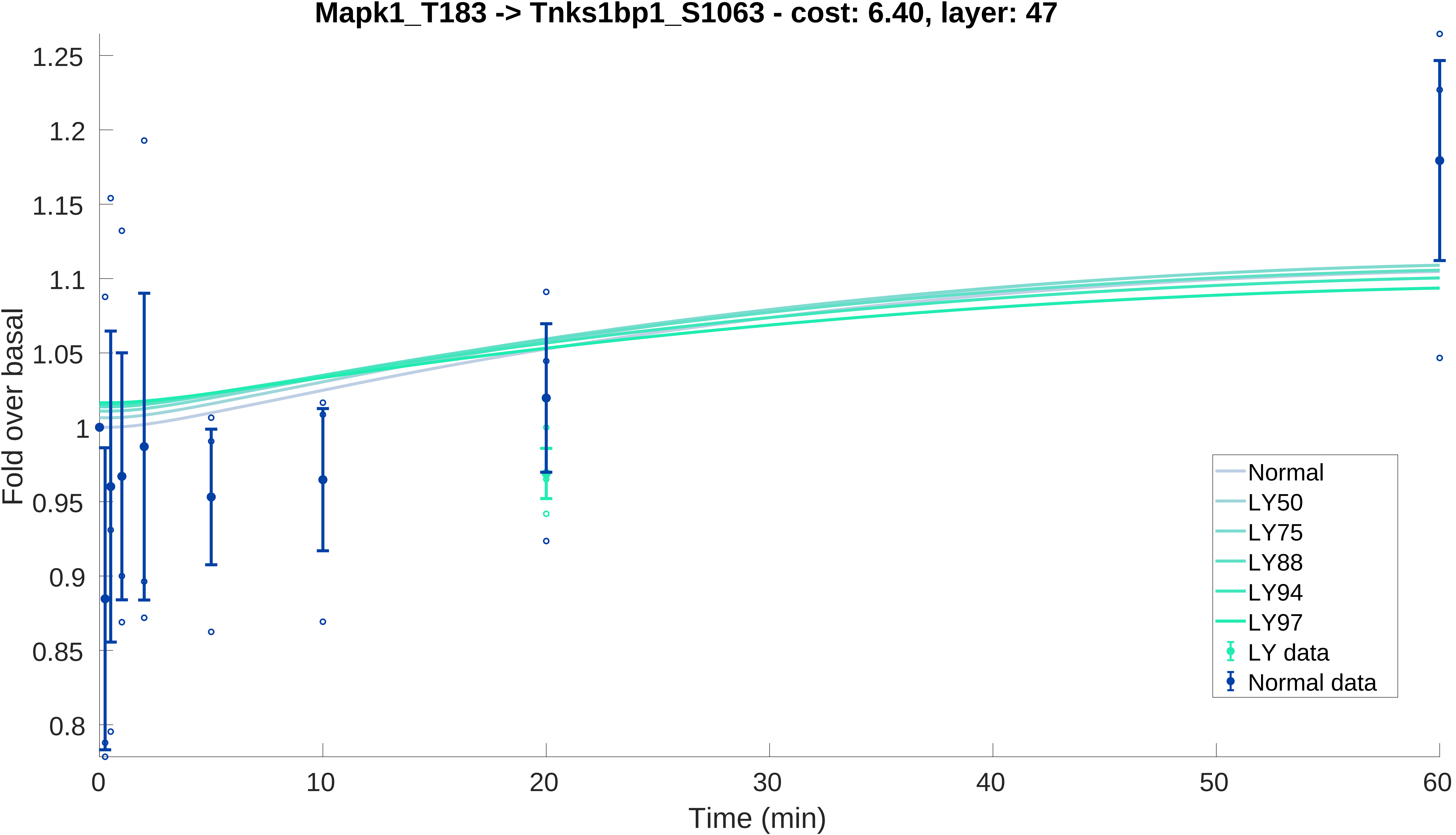

Mapk1_T183 -> Tnks1bp1_S1063 - cost: 6.40, layer: 47
Fold over basal
Time (min)
1.25
1.2
1.15
1.1
1.05
1
0.95
0.9
0.85
0.8
0
10
20
30
40
50
60
Normal
LY50
LY75
LY88
LY94
LY97
LY data
Normal data

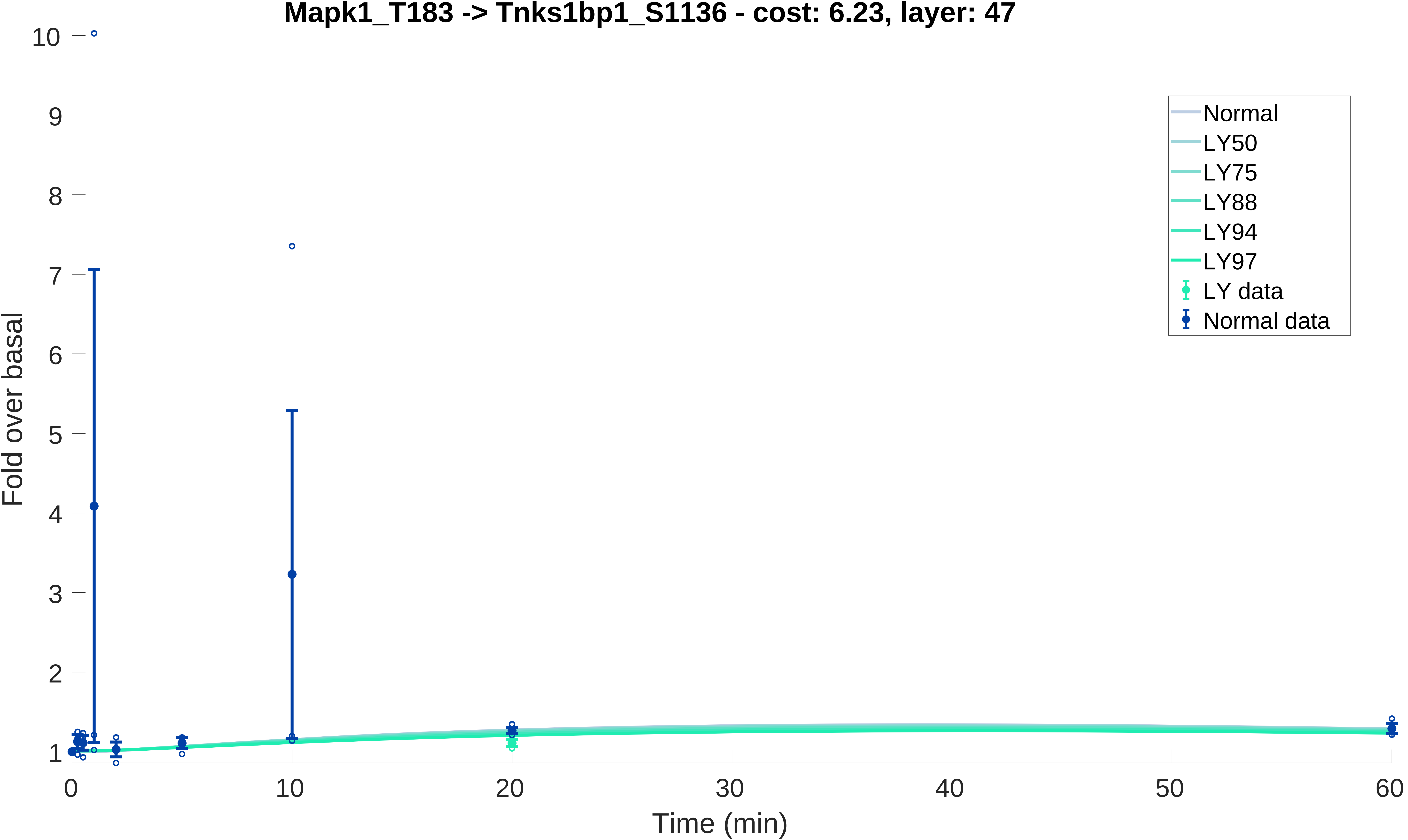

Mapk1_T183 -> Tnks1bp1_S1136 - cost: 6.23, layer: 47
Fold over basal
Time (min)
Normal
LY50
LY75
LY88
LY94
LY97
LY data
Normal data

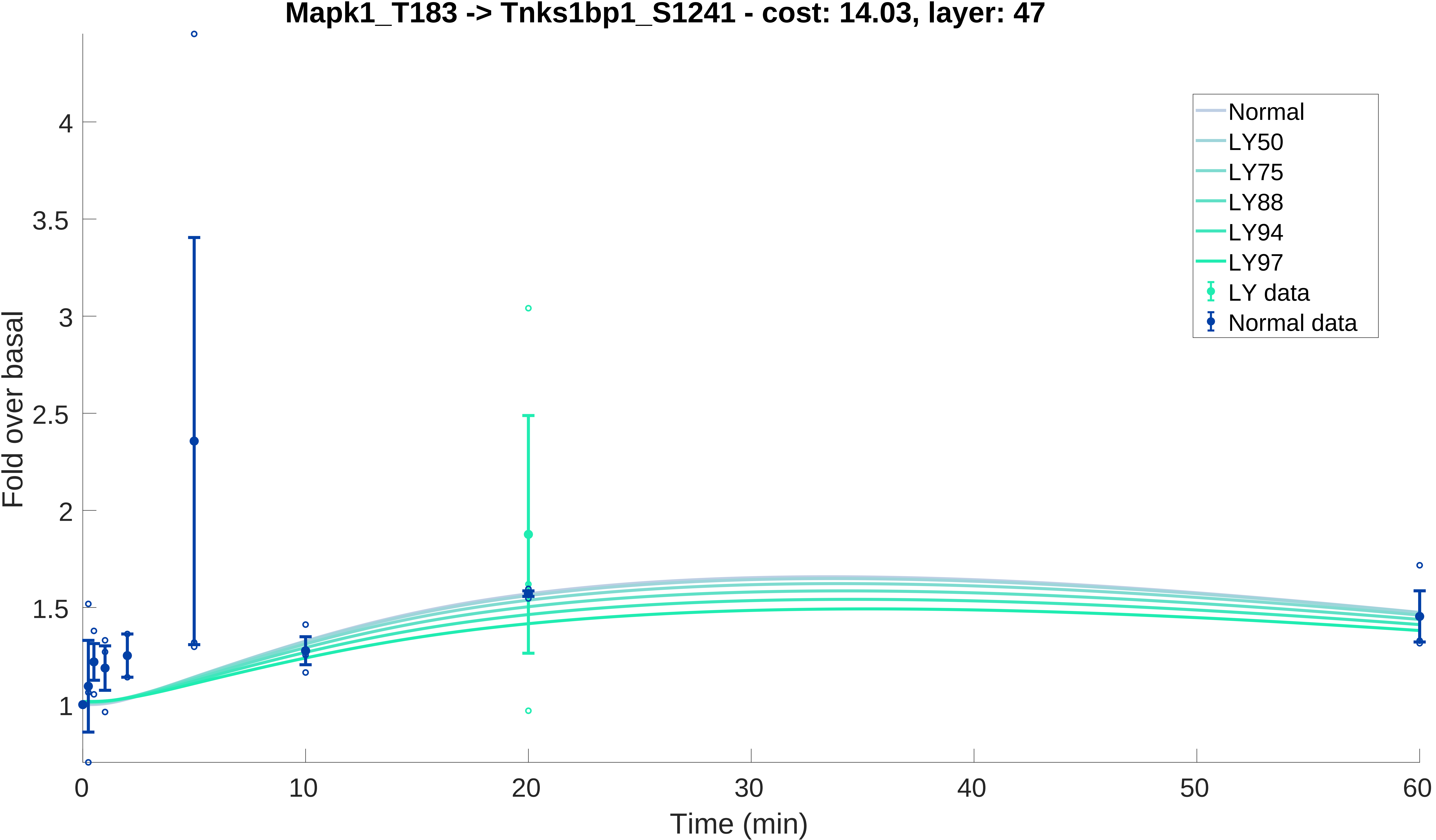
Mapk1_T183 -> Tnks1bp1_S1241 - cost: 14.03, layer: 47
Fold over basal
Time (min)
Normal
LY50
LY75
LY88
LY94
LY97
LY data
Normal data
0
10
20
30
40
50
60
1
1.5
2
2.5
3
3.5
4

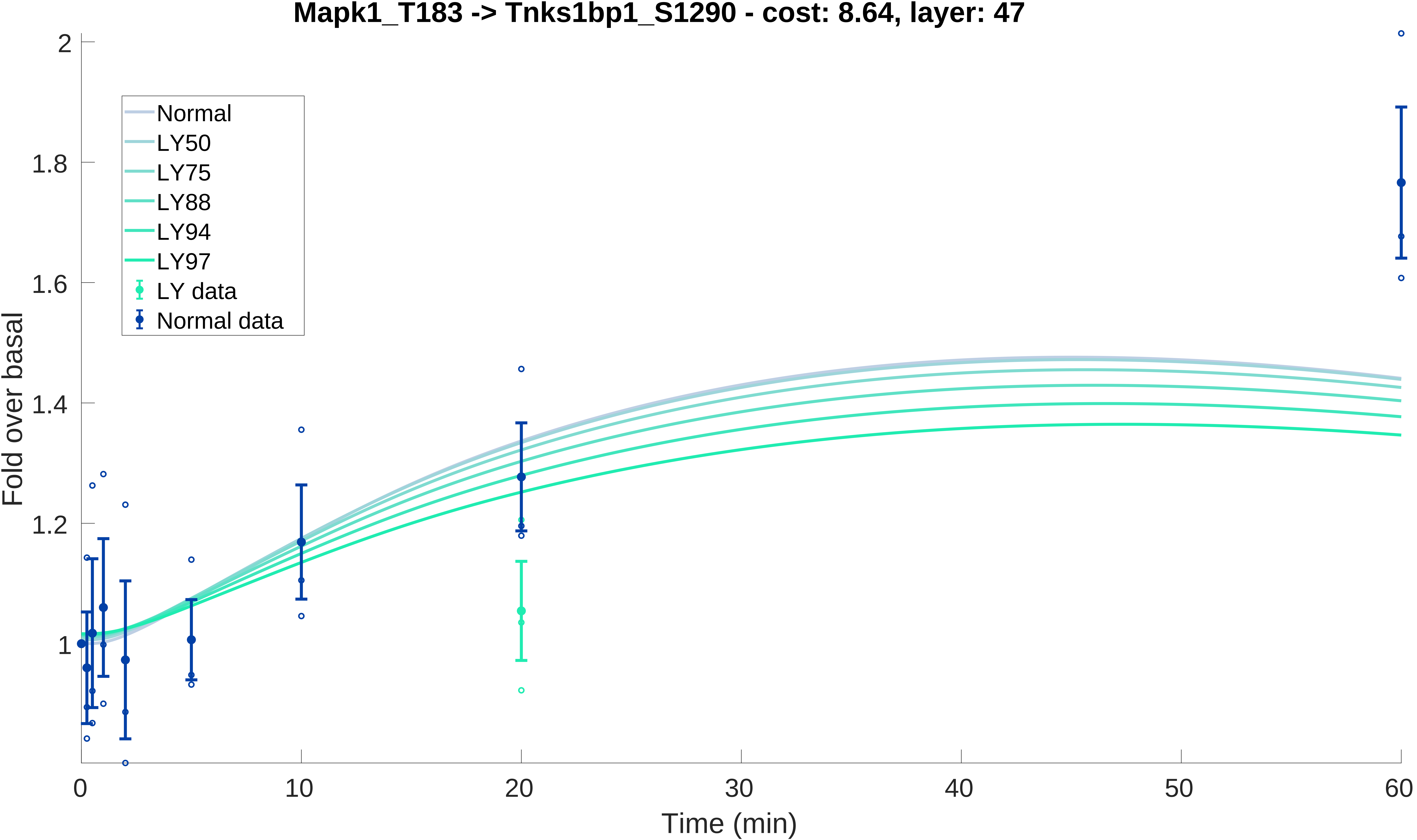

Mapk1_T183 -> Tnks1bp1_S1290 - cost: 8.64, layer: 47
Normal
LY50
LY75
LY88
LY94
LY97
LY data
Normal data
Fold over basal
Time (min)
2
1.8
1.6
1.4
1.2
1
0
10
20
30
40
50
60

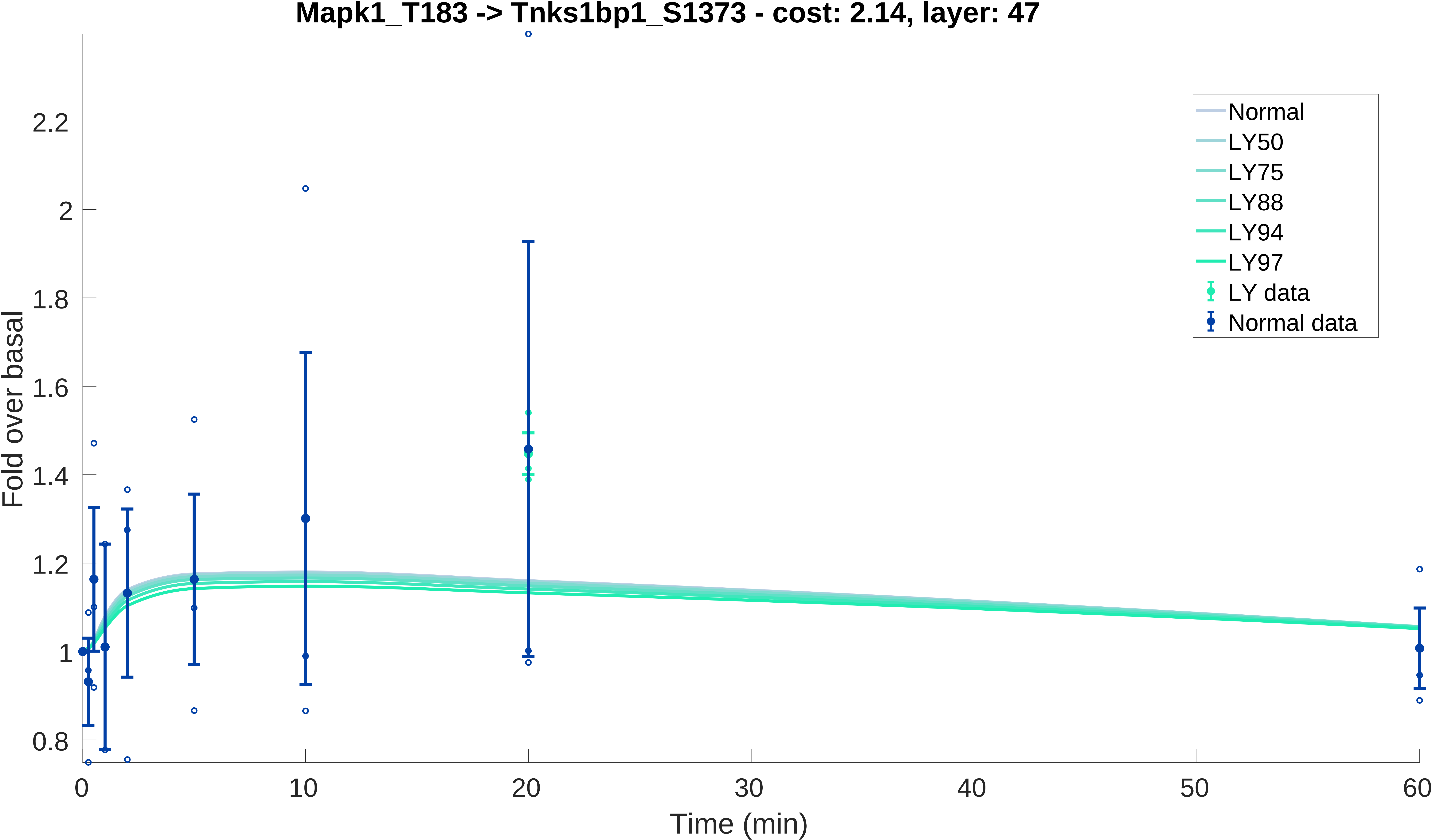
Mapk1_T183 -> Tnks1bp1_S1373 - cost: 2.14, layer: 47
Fold over basal
Time (min)
0.8
1
1.2
1.4
1.6
1.8
2
2.2
0
10
20
30
40
50
60
Normal
LY50
LY75
LY88
LY94
LY97
LY data
Normal data

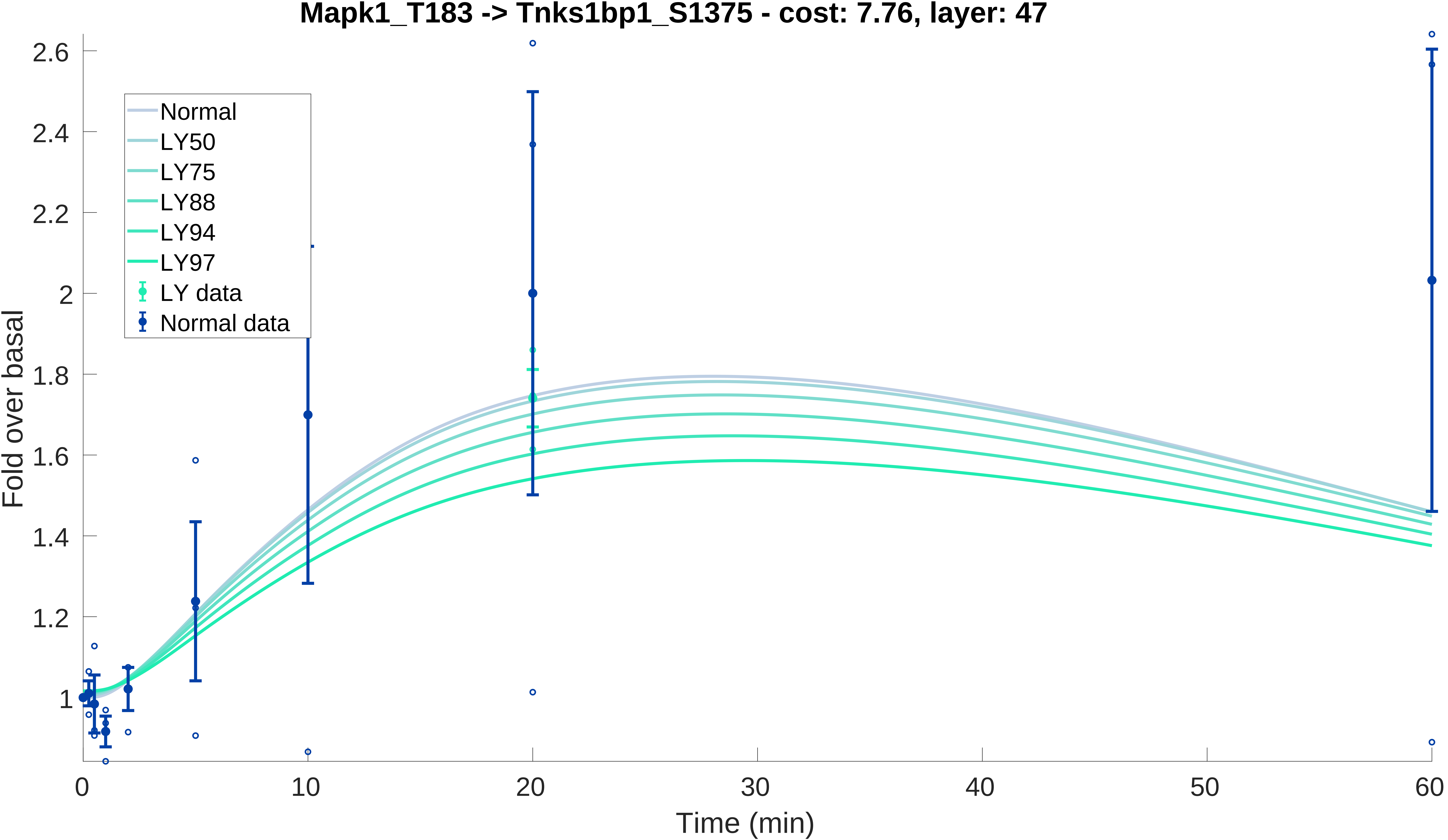

Mapk1_T183 -> Tnks1bp1_S1375 - cost: 7.76, layer: 47
Fold over basal
Time (min)
Normal
LY50
LY75
LY88
LY94
LY97
LY data
Normal data
2.6
2.4
2.2
2
1.8
1.6
1.4
1.2
1
0
10
20
30
40
50
60

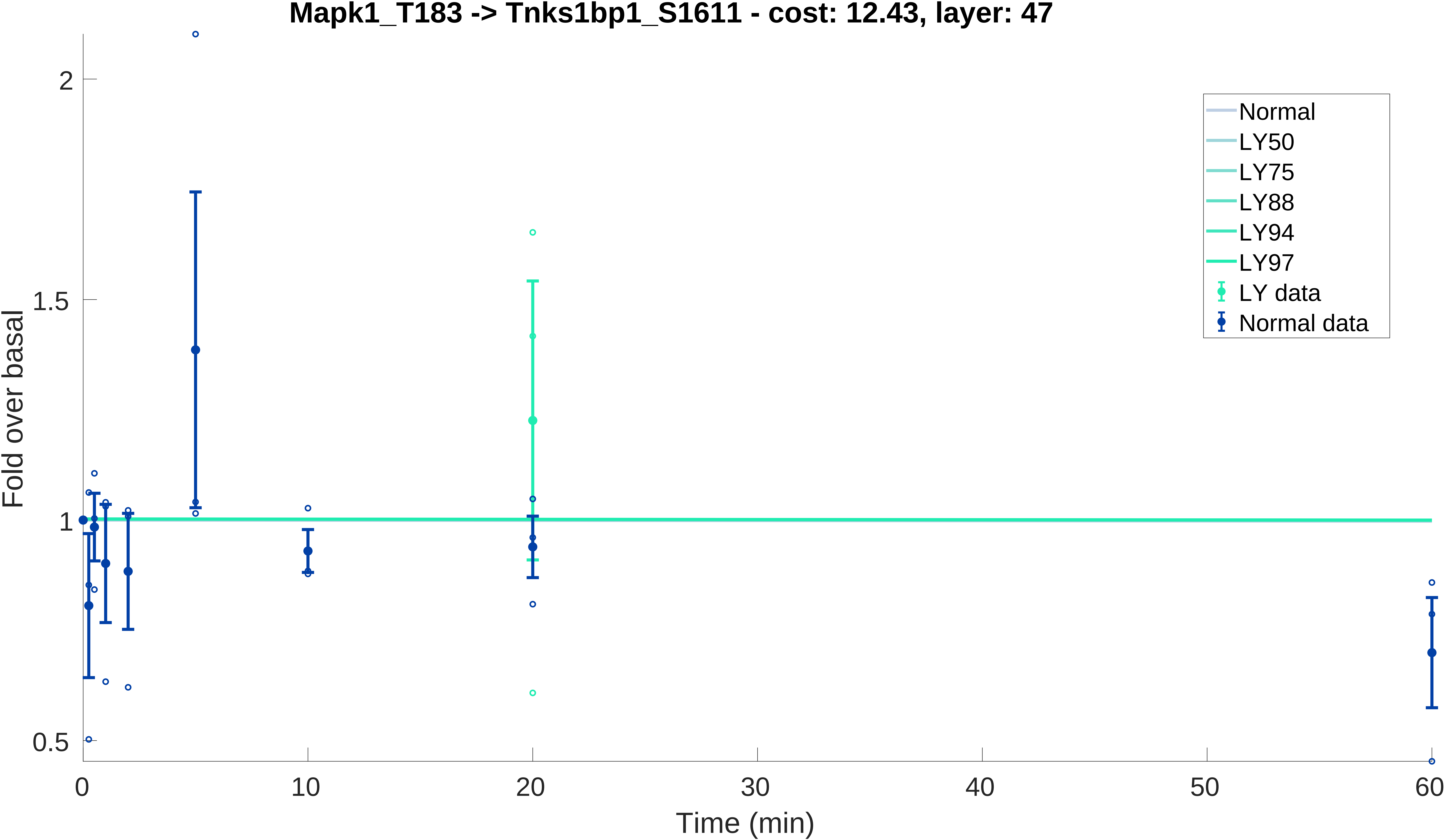
Mapk1_T183 -> Tnks1bp1_S1611 - cost: 12.43, layer: 47
Fold over basal
Time (min)
0.5
1
1.5
2
0
10
20
30
40
50
60
Normal
LY50
LY75
LY88
LY94
LY97
LY data
Normal data

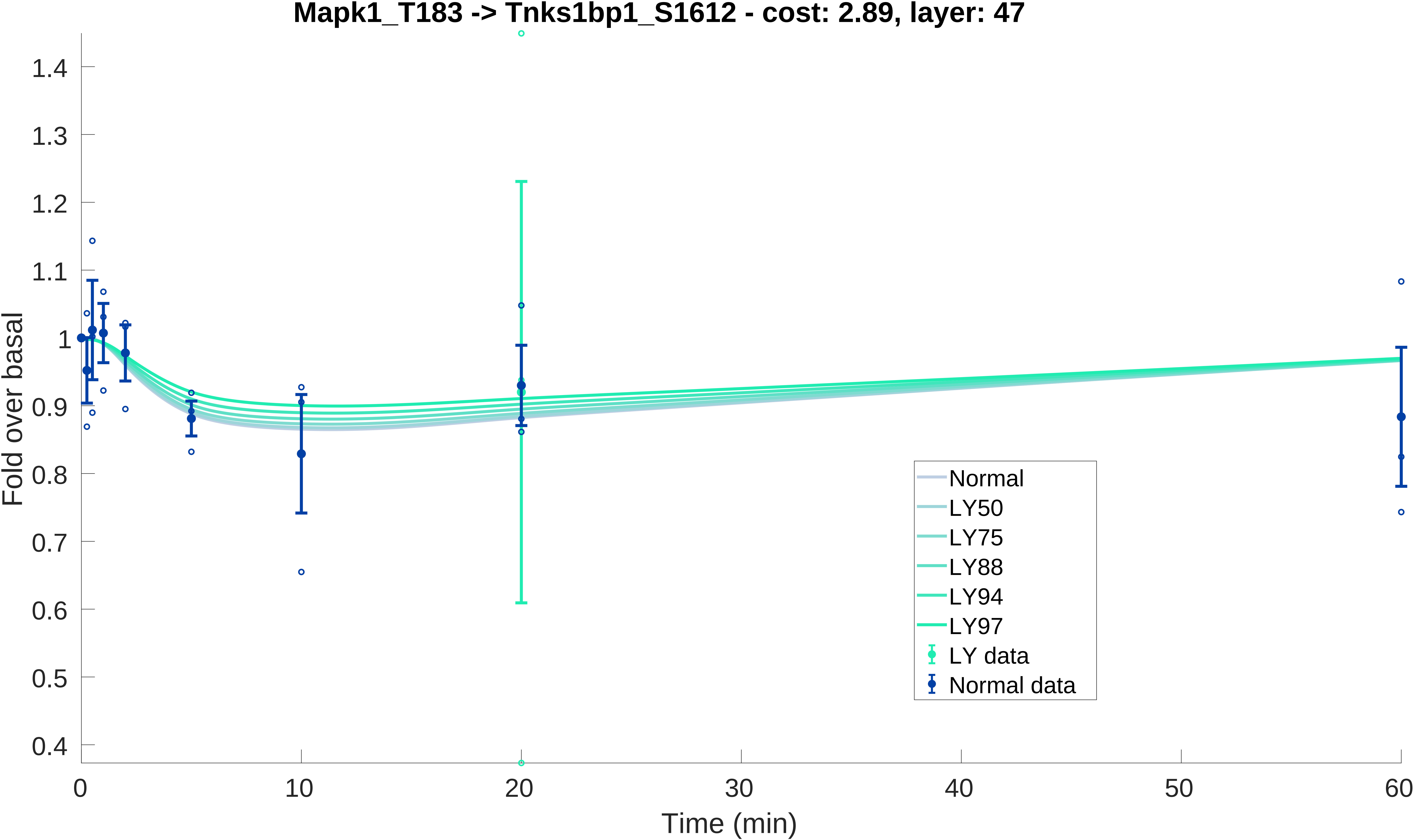

Mapk1_T183 -> Tnks1bp1_S1612 - cost: 2.89, layer: 47
Fold over basal
Time (min)
1.4
1.3
1.2
1.1
1
0.9
0.8
0.7
0.6
0.5
0.4
0
10
20
30
40
50
60
Normal
LY50
LY75
LY88
LY94
LY97
LY data
Normal data

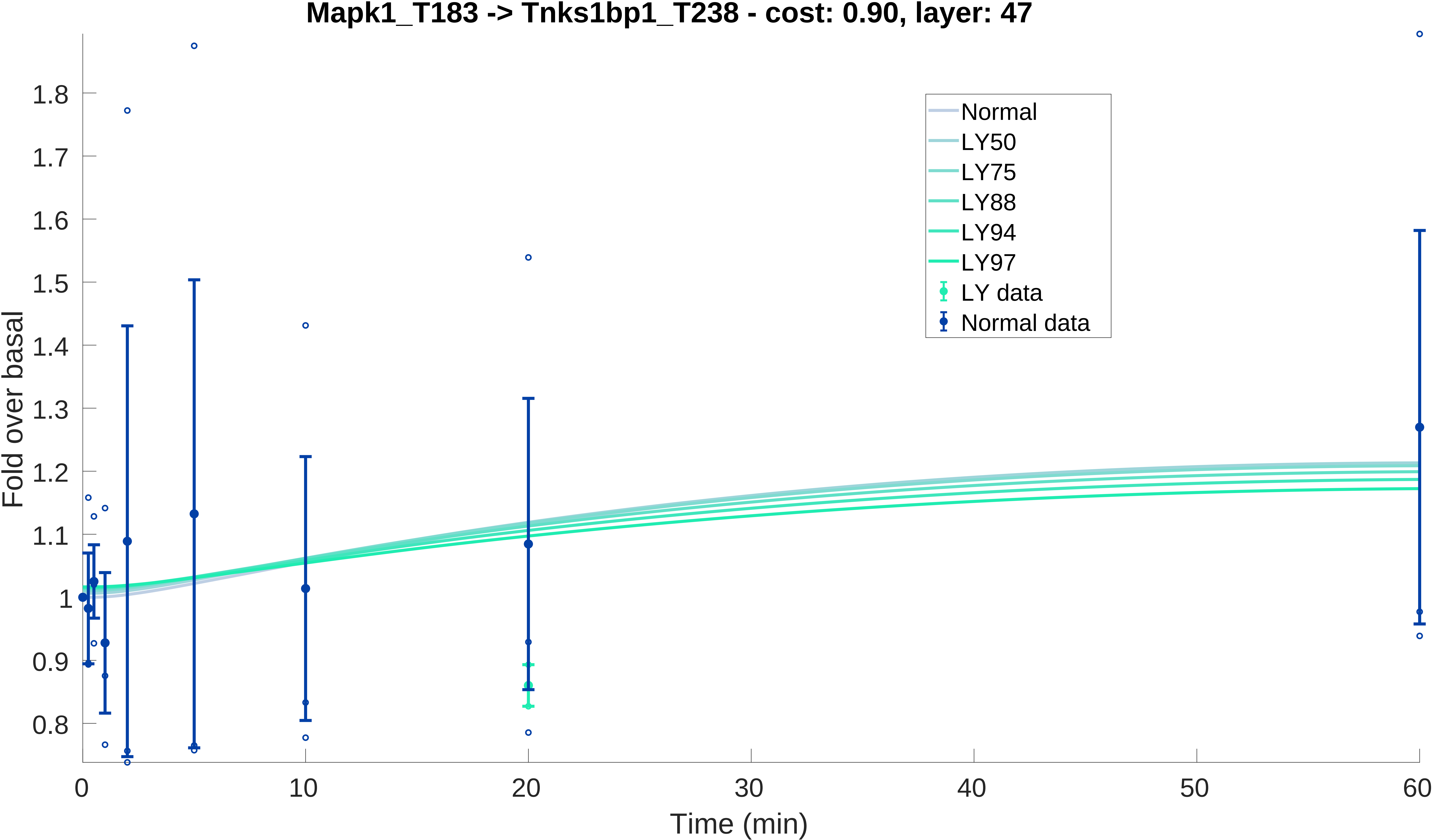

Mapk1_T183 -> Tnks1bp1_T238 - cost: 0.90, layer: 47
Fold over basal
Time (min)
Normal
LY50
LY75
LY88
LY94
LY97
LY data
Normal data
0.8
0.9
1
1.1
1.2
1.3
1.4
1.5
1.6
1.7
1.8
0
10
20
30
40
50
60

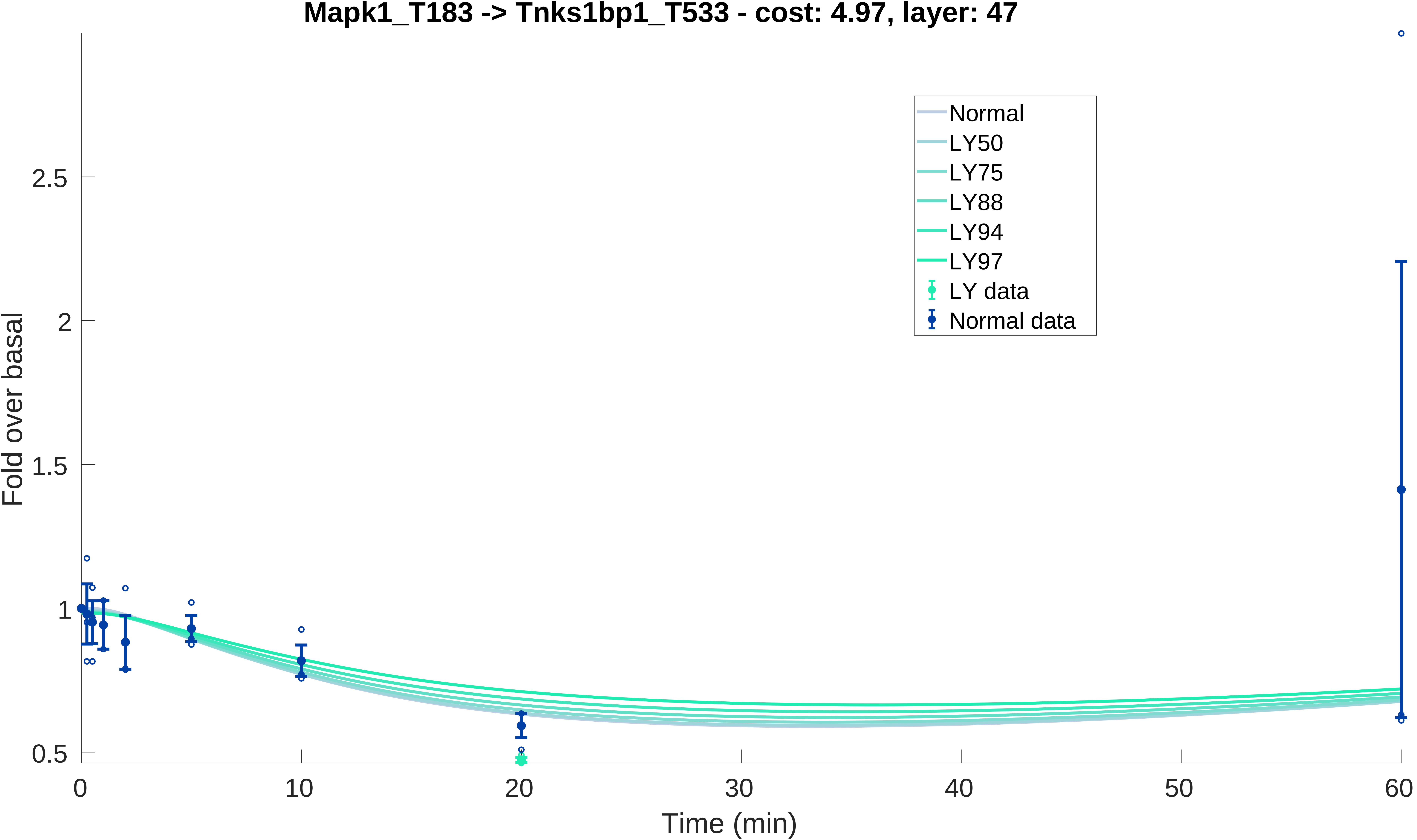
Mapk1_T183 -> Tnks1bp1_T533 - cost: 4.97, layer: 47
Fold over basal
Time (min)
0.5
1
1.5
2
2.5
0
10
20
30
40
50
60
Normal
LY50
LY75
LY88
LY94
LY97
LY data
Normal data

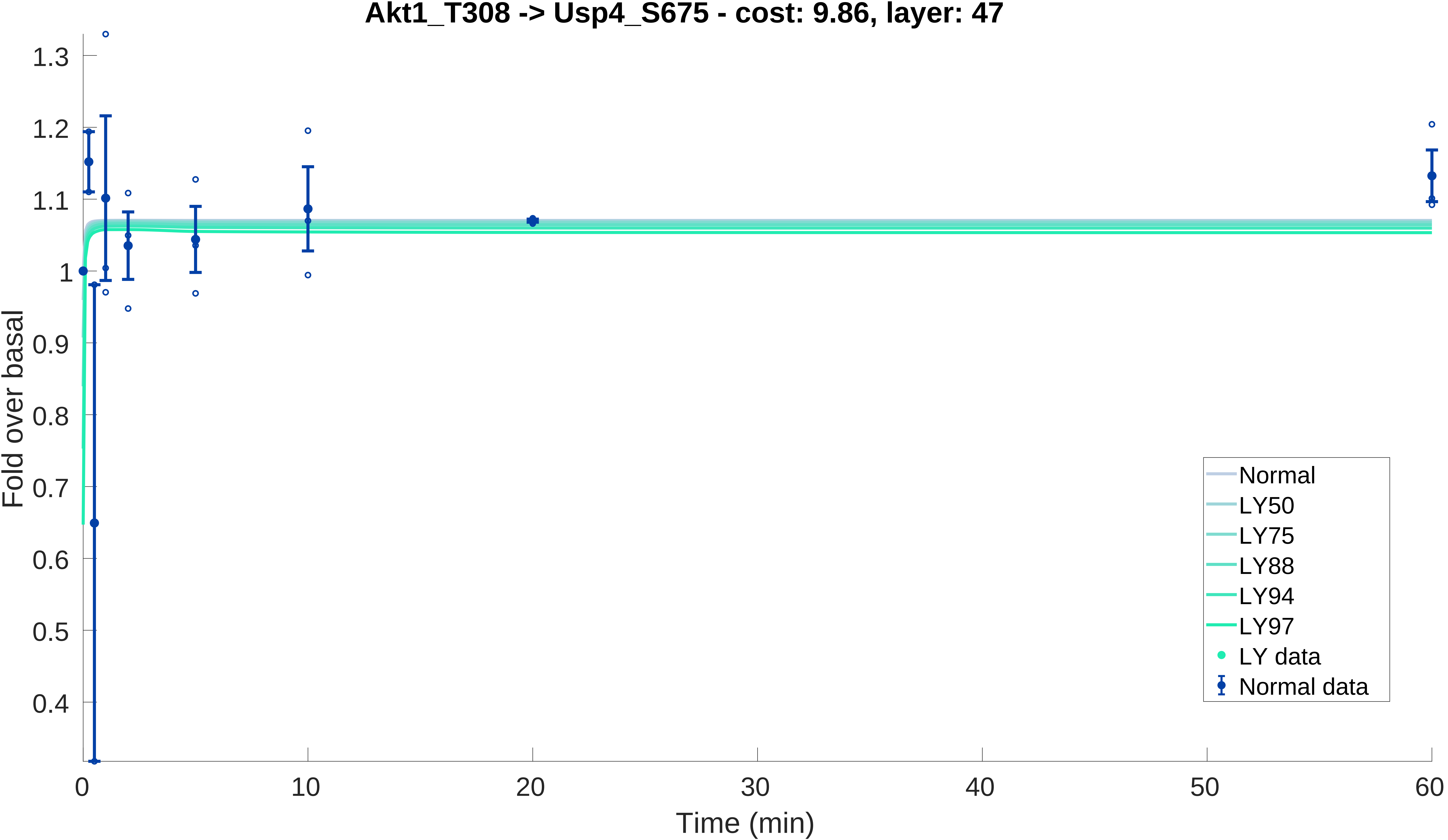

Akt1_T308 -> Usp4_S675 - cost: 9.86, layer: 47
Fold over basal
1.3
1.2
1.1
1
0.9
0.8
0.7
0.6
0.5
0.4
0
10
20
30
40
50
60
Time (min)
Normal
LY50
LY75
LY88
LY94
LY97
LY data
Normal data

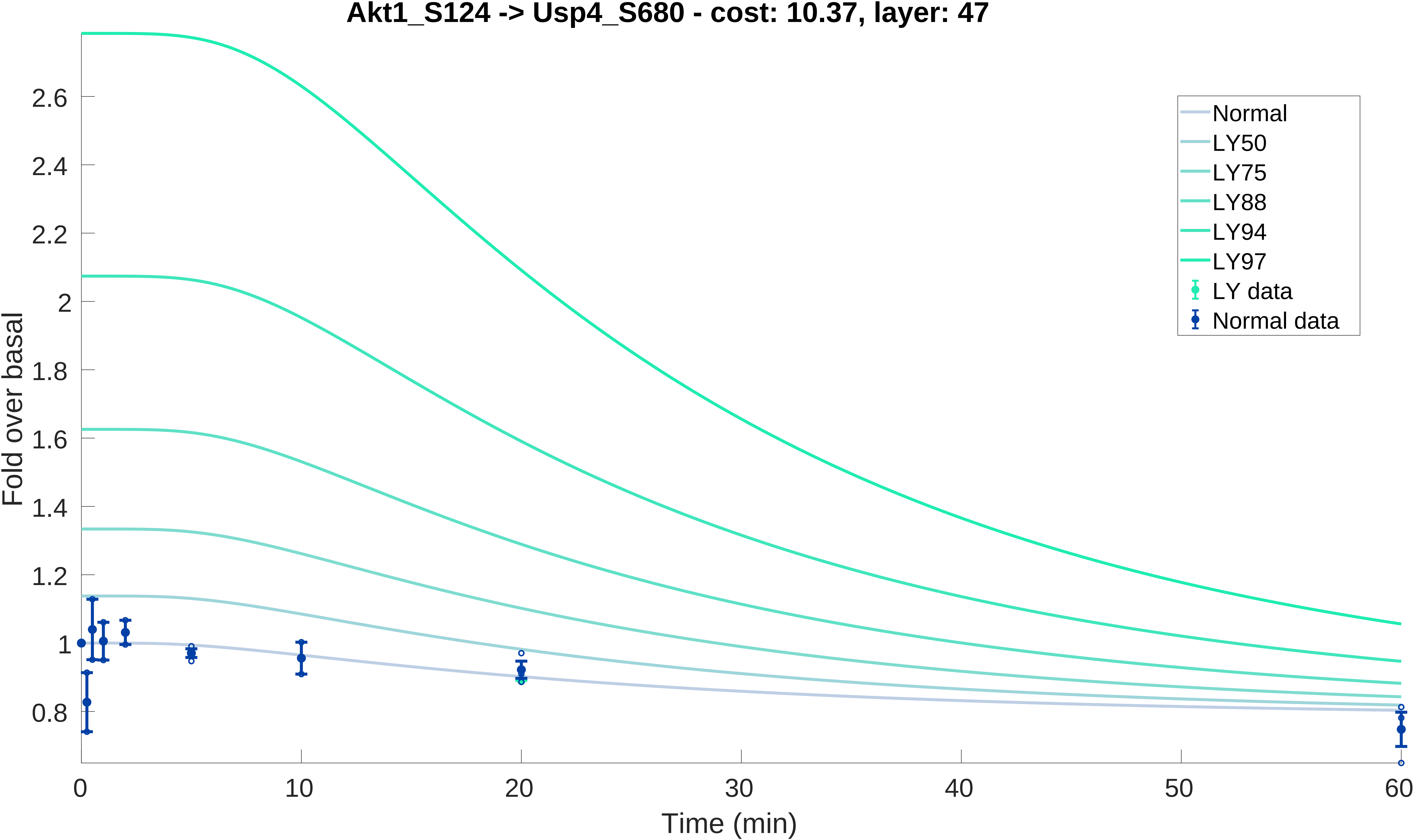

Akt1_S124 -> Usp4_S680 - cost: 10.37, layer: 47
Fold over basal
Time (min)
Normal
LY50
LY75
LY88
LY94
LY97
LY data
Normal data
2.6
2.4
2.2
2
1.8
1.6
1.4
1.2
1
0.8
0
10
20
30
40
50
60

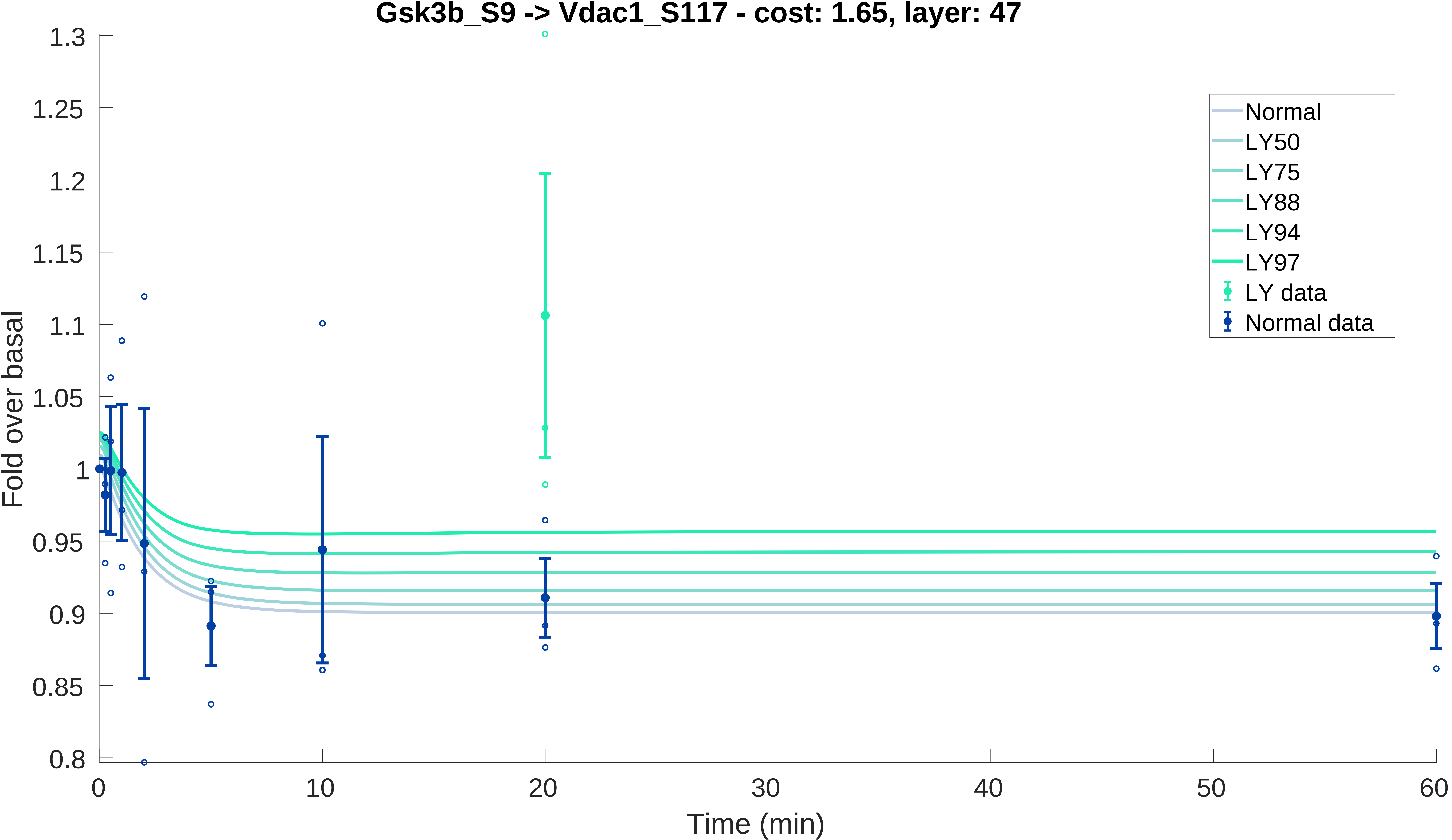
Gsk3b_S9 -> Vdac1_S117 - cost: 1.65, layer: 47
Fold over basal
Time (min)
1.3
1.25
1.2
1.15
1.1
1.05
1
0.95
0.9
0.85
0.8
0
10
20
30
40
50
60
Normal
LY50
LY75
LY88
LY94
LY97
LY data
Normal data

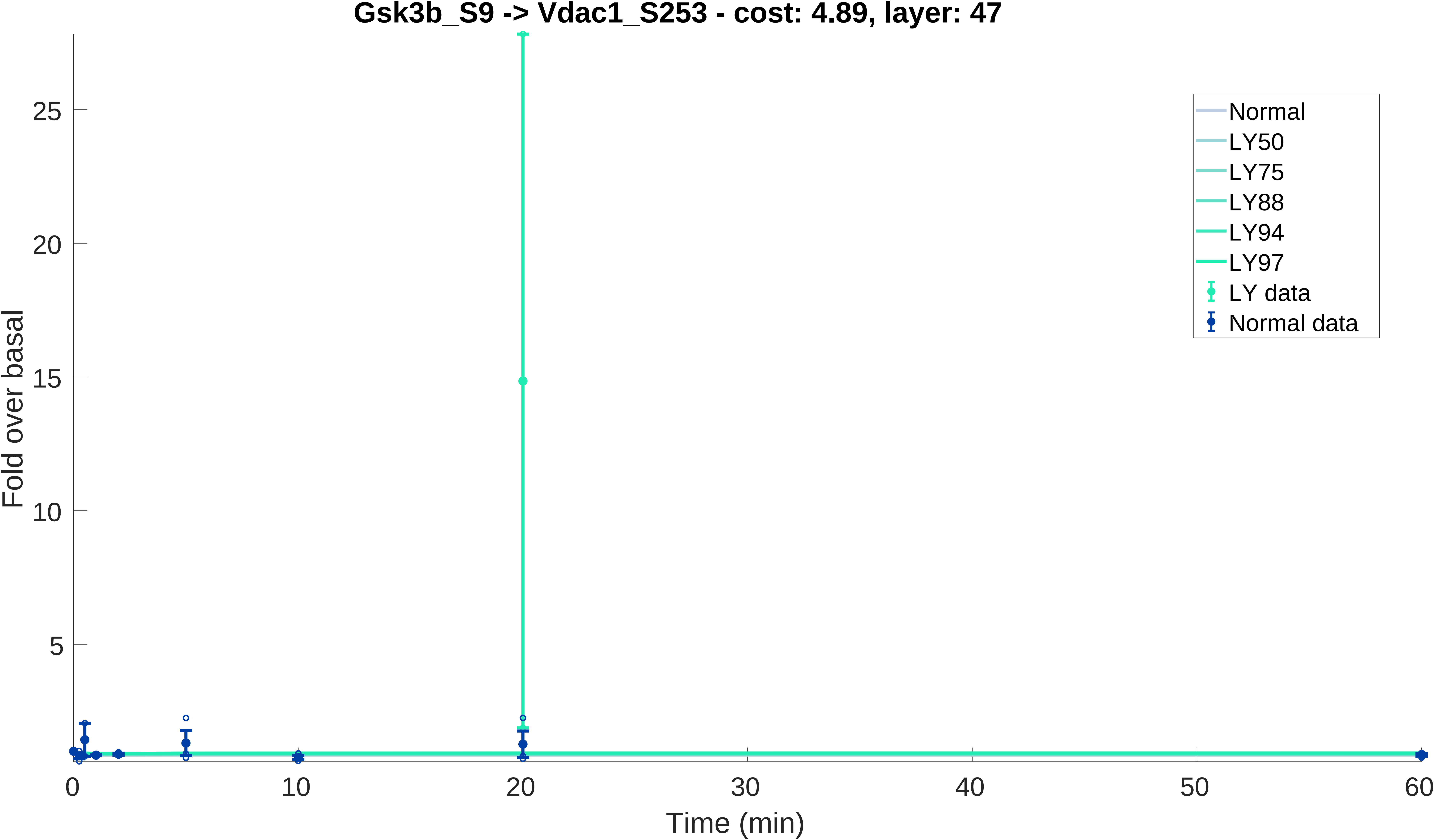
Gsk3b_S9 -> Vdac1_S253 - cost: 4.89, layer: 47
Fold over basal
Time (min)
0
5
10
15
20
25
0
10
20
30
40
50
60
Normal
LY50
LY75
LY88
LY94
LY97
LY data
Normal data

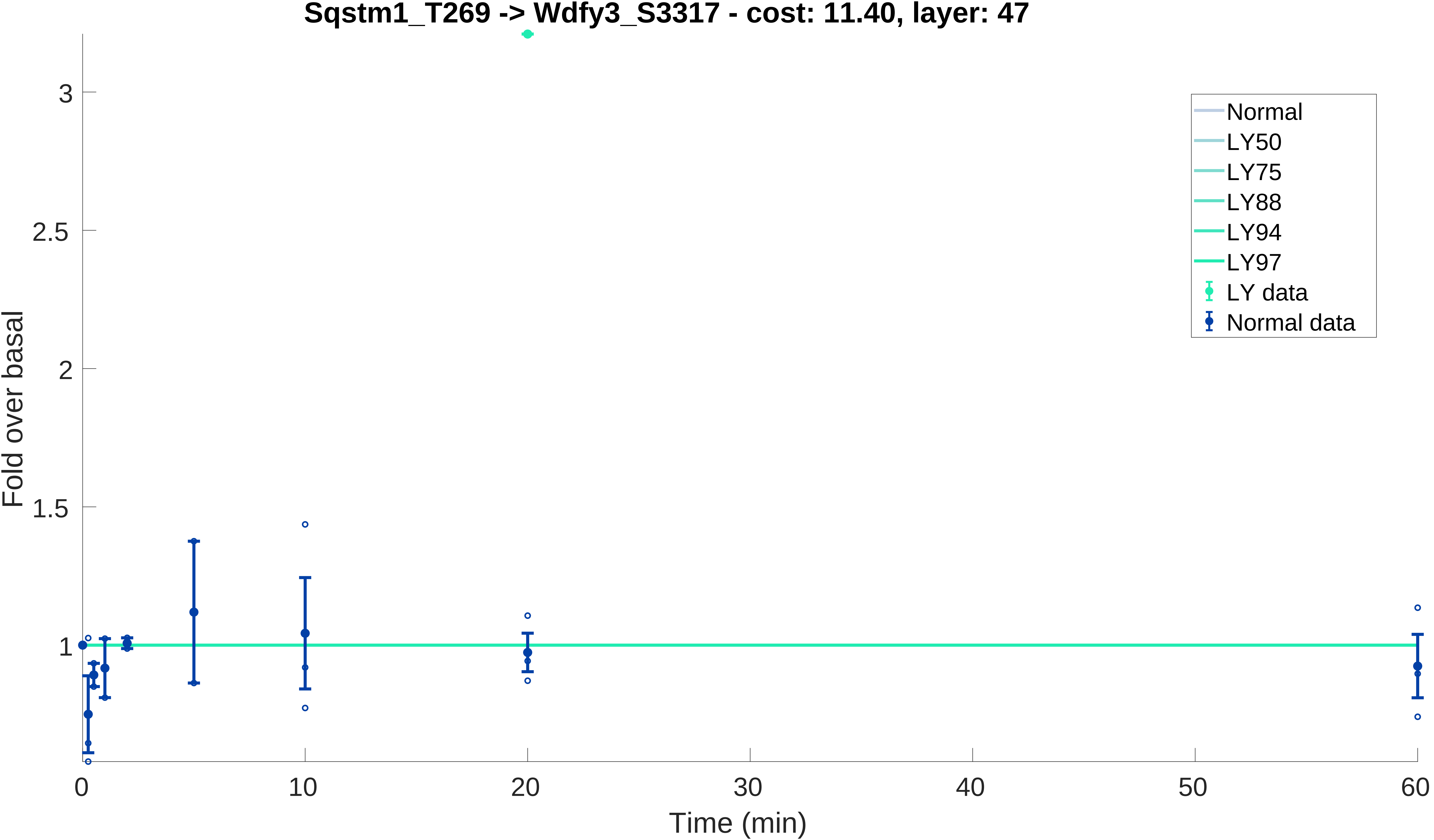

Sqstm1_T269 -> Wdfy3_S3317 - cost: 11.40, layer: 47
Fold over basal
Time (min)
Normal
LY50
LY75
LY88
LY94
LY97
LY data
Normal data
3
2.5
2
1.5
1
0
10
20
30
40
50
60

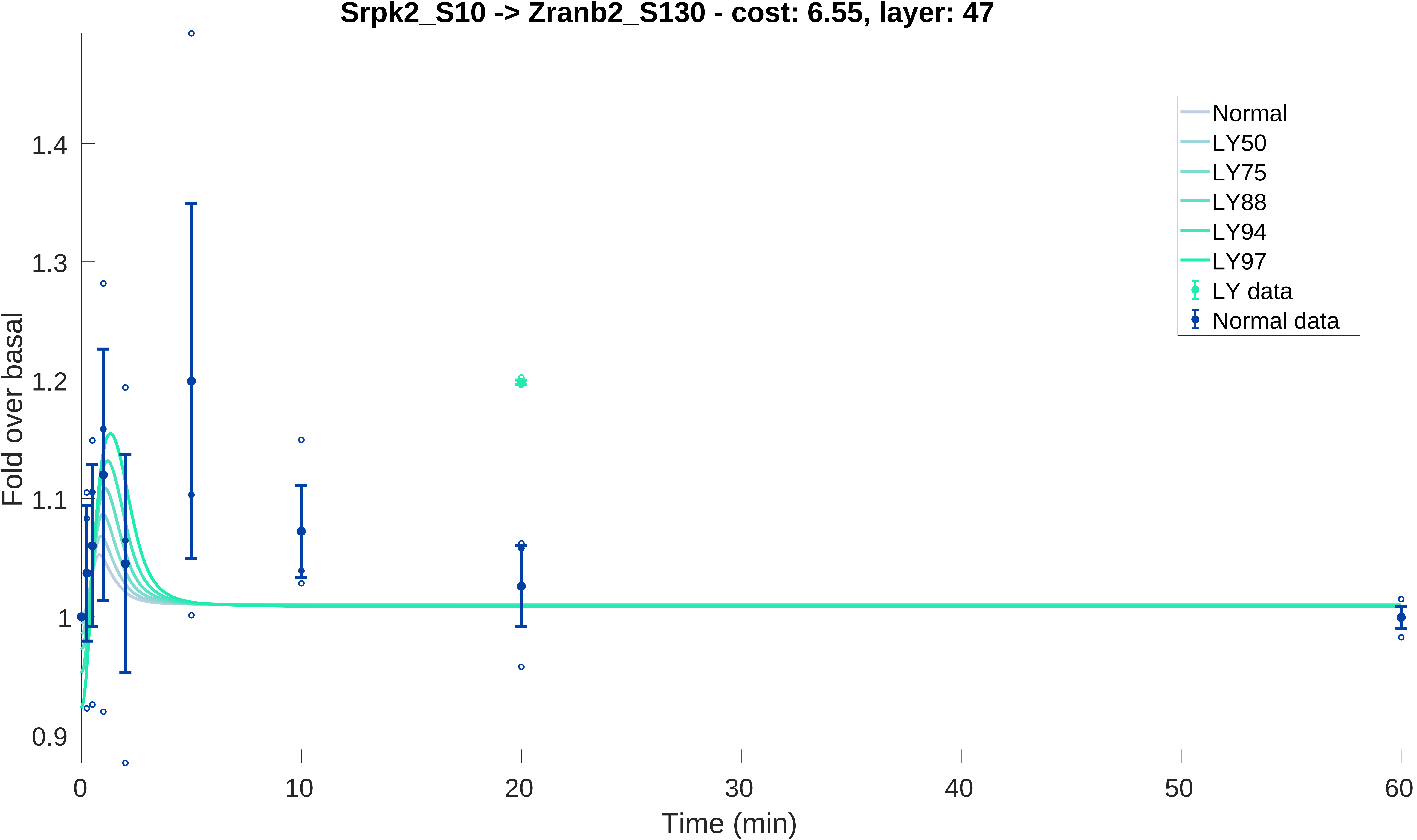
Srpk2_S10 -> Zranb2_S130 - cost: 6.55, layer: 47
Fold over basal
Time (min)
Normal
LY50
LY75
LY88
LY94
LY97
LY data
Normal data
0.9
1
1.1
1.2
1.3
1.4
0
10
20
30
40
50
60

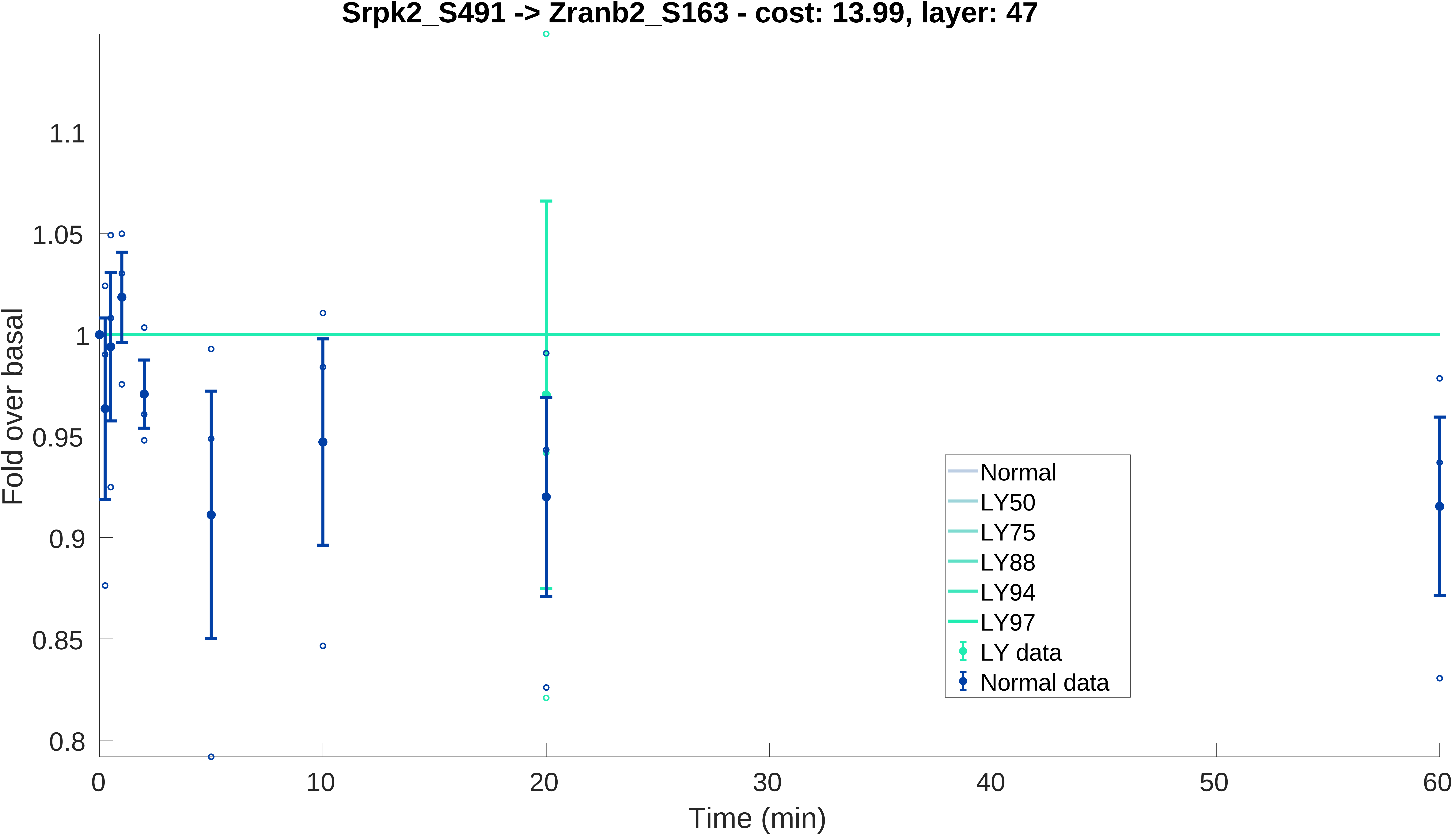

Srpk2_S491 -> Zranb2_S163 - cost: 13.99, layer: 47
Fold over basal
1.1
1.05
1
0.95
0.9
0.85
0.8
0
10
20
30
40
50
60
Time (min)
Normal
LY50
LY75
LY88
LY94
LY97
LY data
Normal data

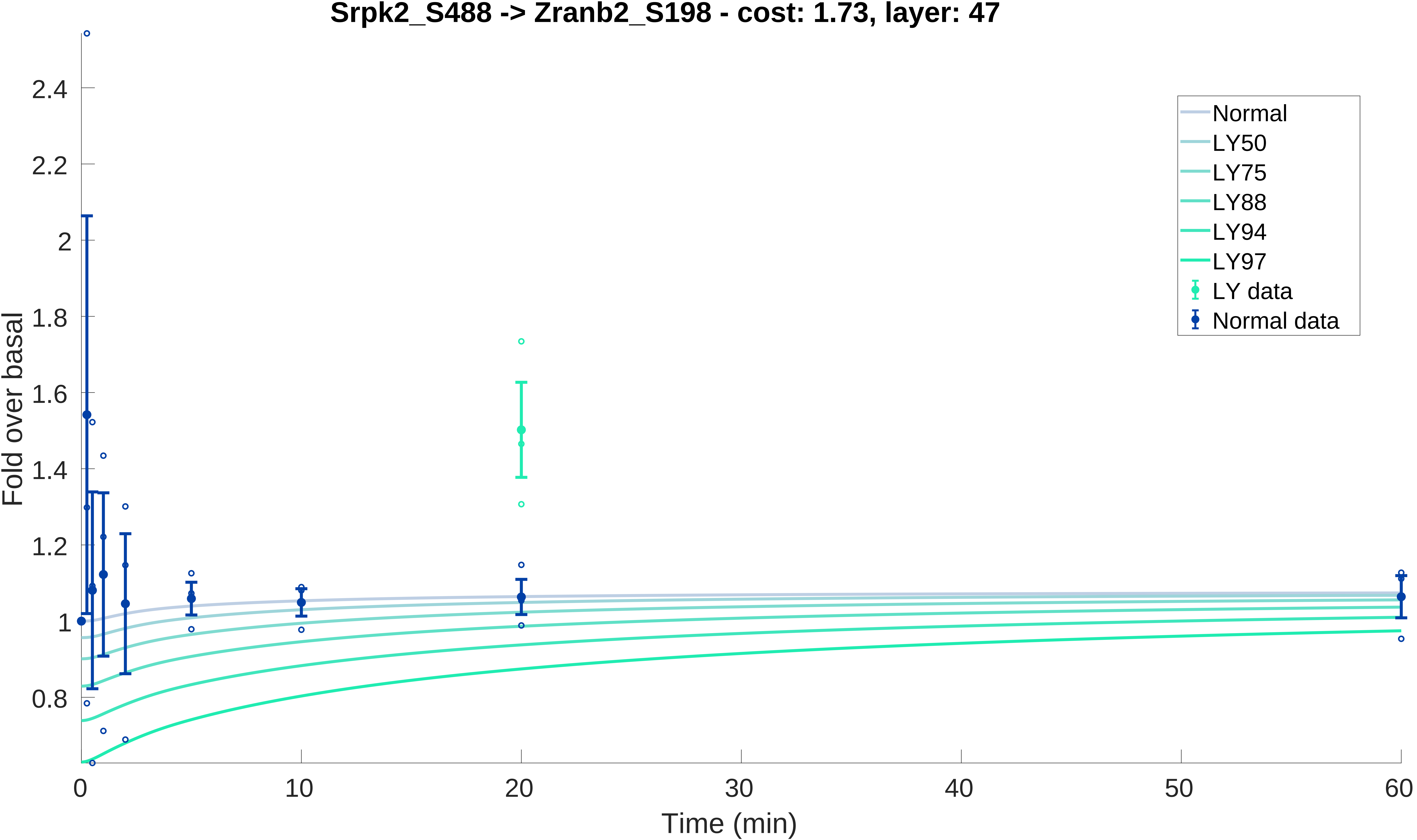
Srpk2_S488 -> Zranb2_S198 - cost: 1.73, layer: 47
Fold over basal
Time (min)
2.4
2.2
2
1.8
1.6
1.4
1.2
1
0.8
0
10
20
30
40
50
60
Normal
LY50
LY75
LY88
LY94
LY97
LY data
Normal data

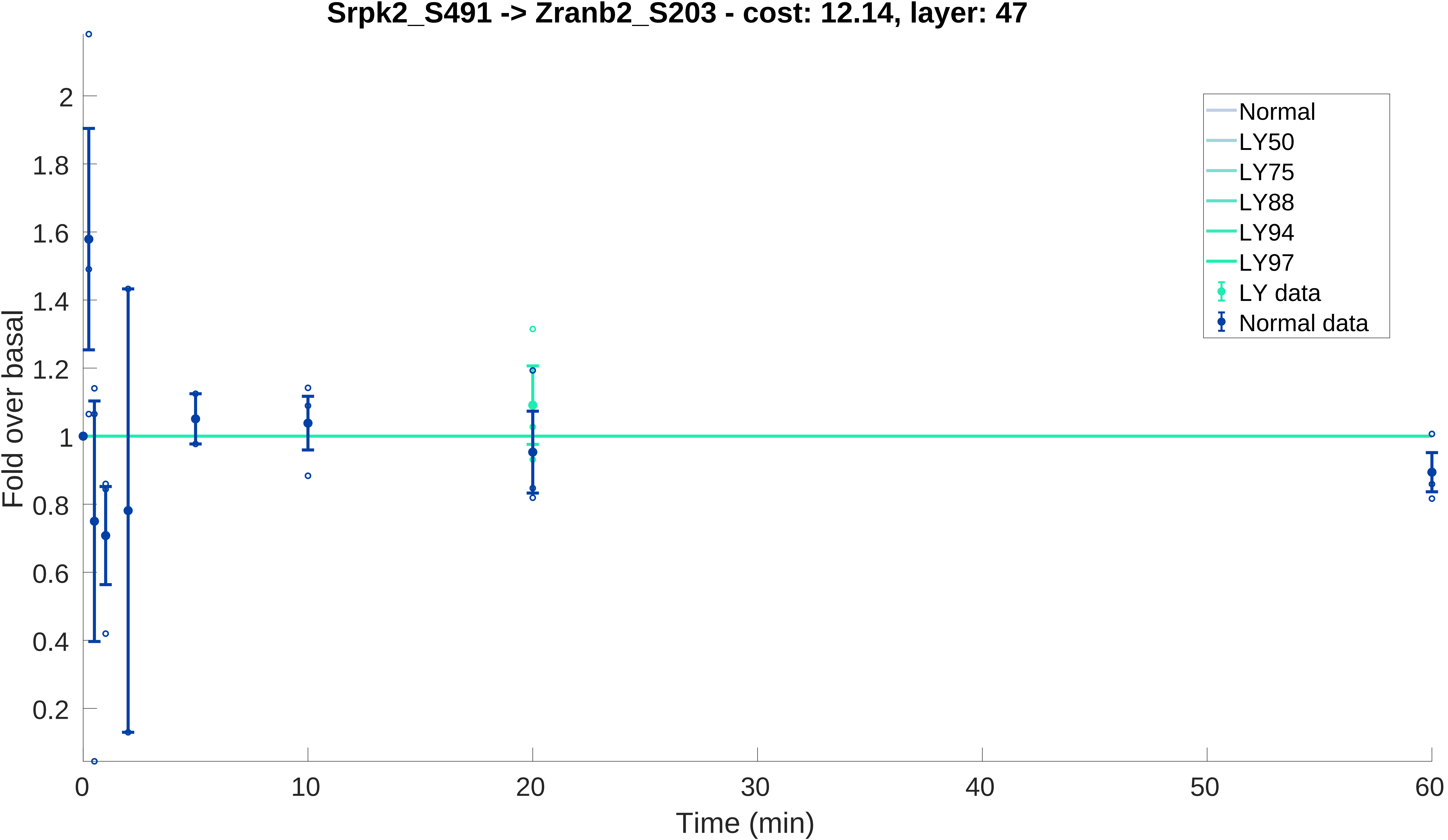

Srpk2_S491 -> Zranb2_S203 - cost: 12.14, layer: 47
Fold over basal
Time (min)
0.2
0.4
0.6
0.8
1
1.2
1.4
1.6
1.8
2
0
10
20
30
40
50
60
Normal
LY50
LY75
LY88
LY94
LY97
LY data
Normal data

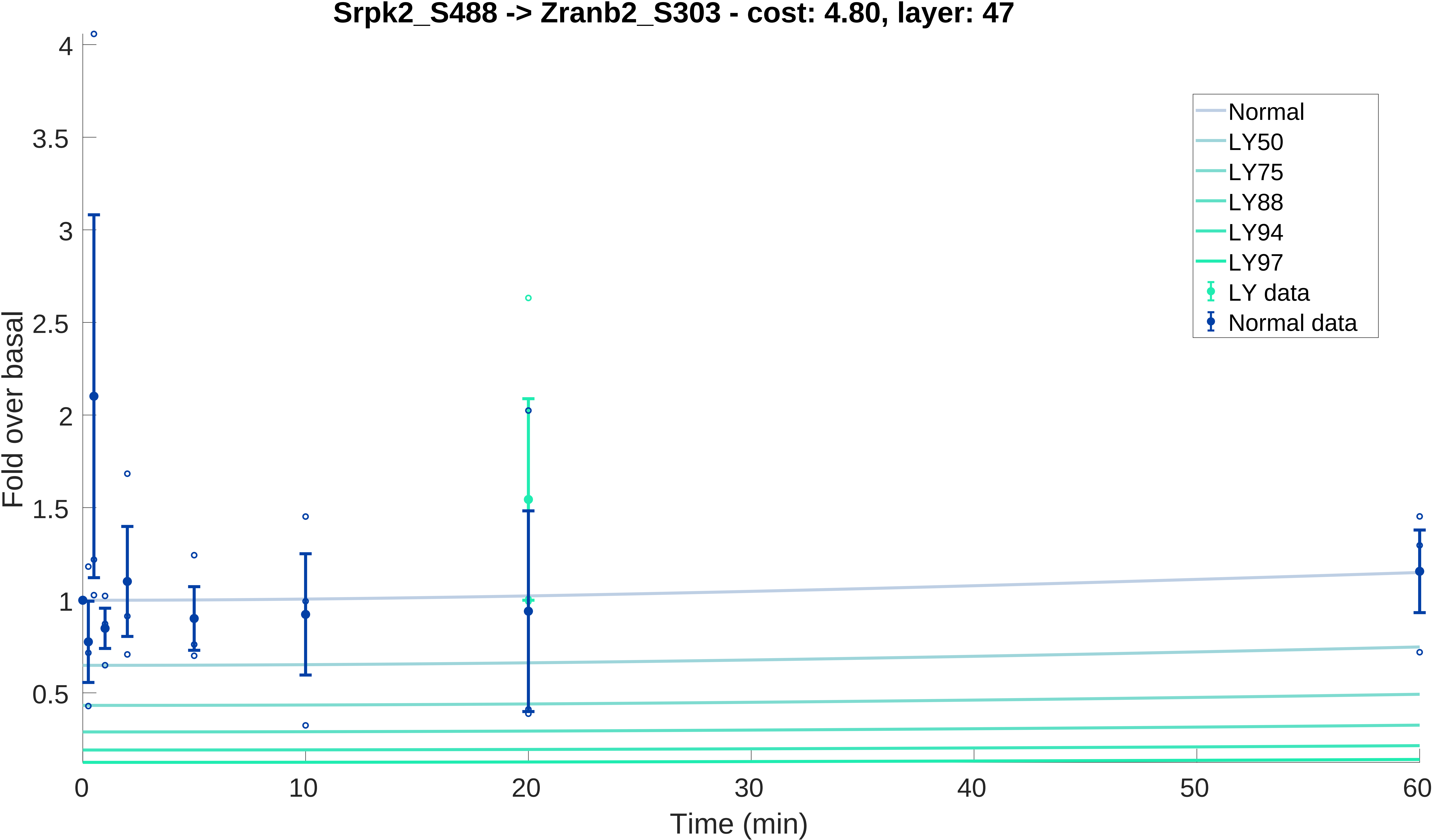
Srpk2_S488 -> Zranb2_S303 - cost: 4.80, layer: 47
Fold over basal
Time (min)
0
10
20
30
40
50
60
0.5
1
1.5
2
2.5
3
3.5
4
Normal
LY50
LY75
LY88
LY94
LY97
LY data
Normal data

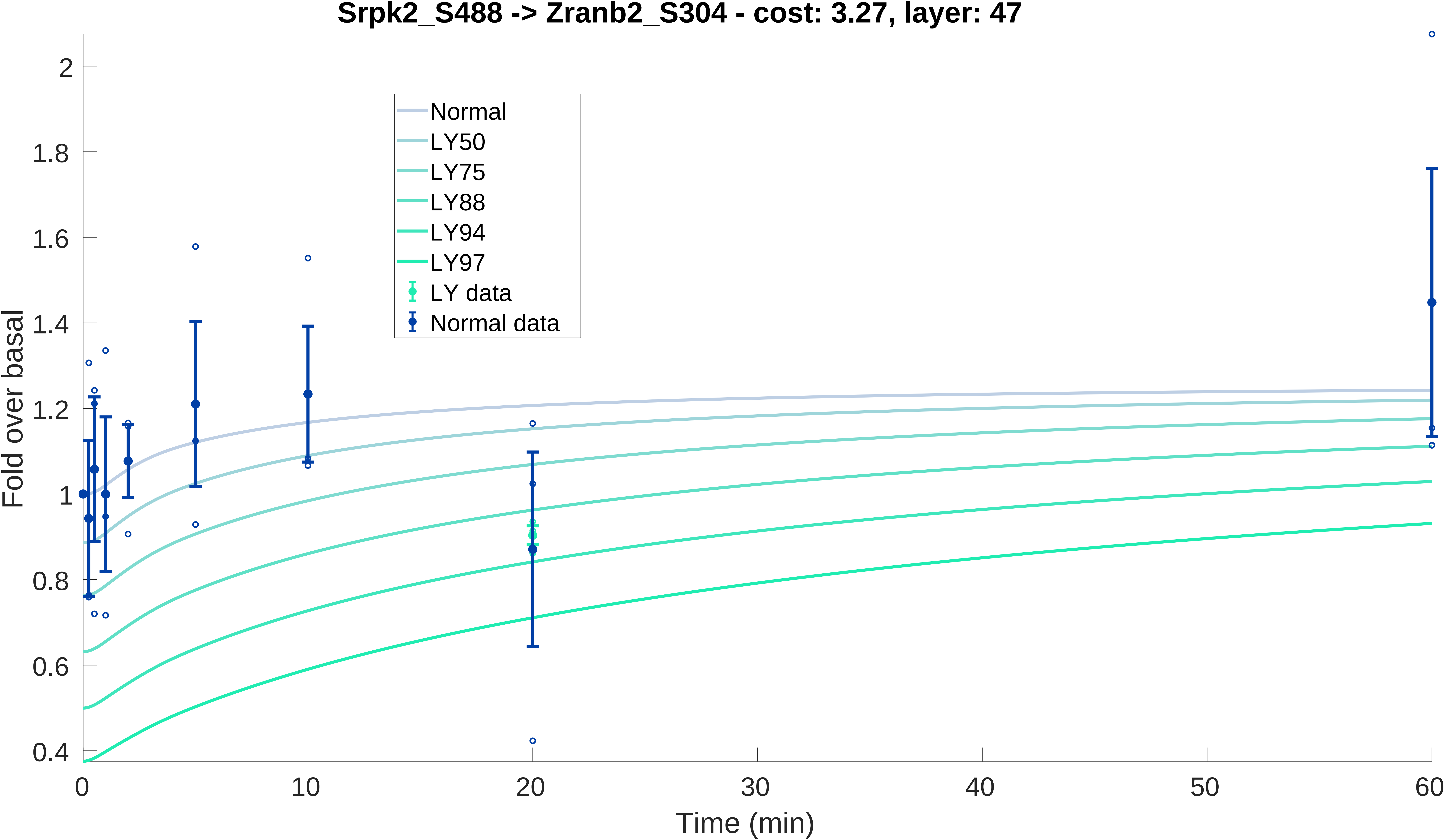
Srpk2_S488 -> Zranb2_S304 - cost: 3.27, layer: 47
Normal
LY50
LY75
LY88
LY94
LY97
LY data
Normal data
Fold over basal
Time (min)
0.4
0.6
0.8
1
1.2
1.4
1.6
1.8
2
0
10
20
30
40
50
60

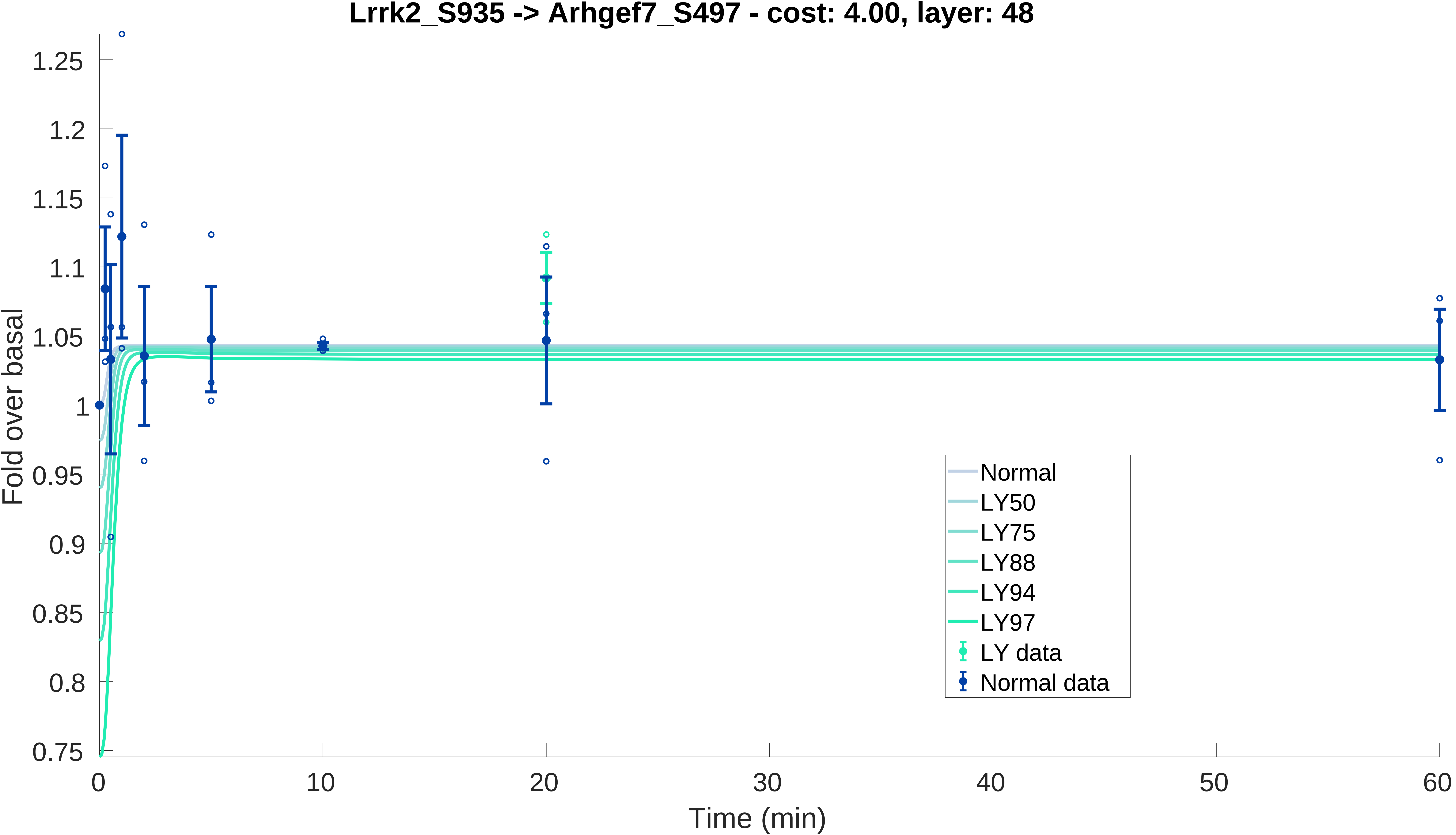

Lrrk2_S935 -> Arhgef7_S497 - cost: 4.00, layer: 48
Fold over basal
Time (min)
0
10
20
30
40
50
60
0.75
0.8
0.85
0.9
0.95
1
1.05
1.1
1.15
1.2
1.25
Normal
LY50
LY75
LY88
LY94
LY97
LY data
Normal data

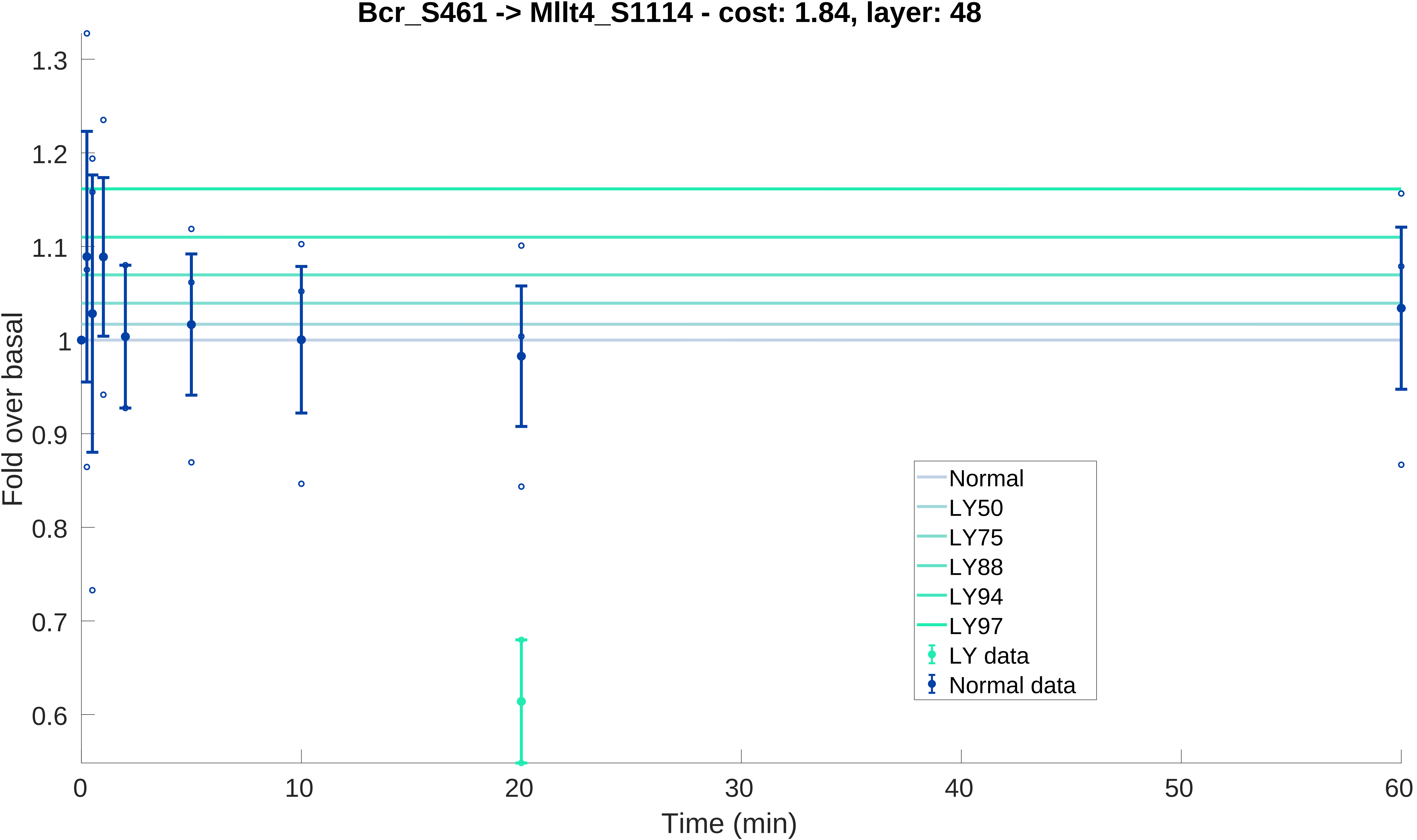
Bcr_S461 -> Mllt4_S1114 - cost: 1.84, layer: 48
Fold over basal
Time (min)
1.3
1.2
1.1
1
0.9
0.8
0.7
0.6
0
10
20
30
40
50
60
Normal
LY50
LY75
LY88
LY94
LY97
LY data
Normal data

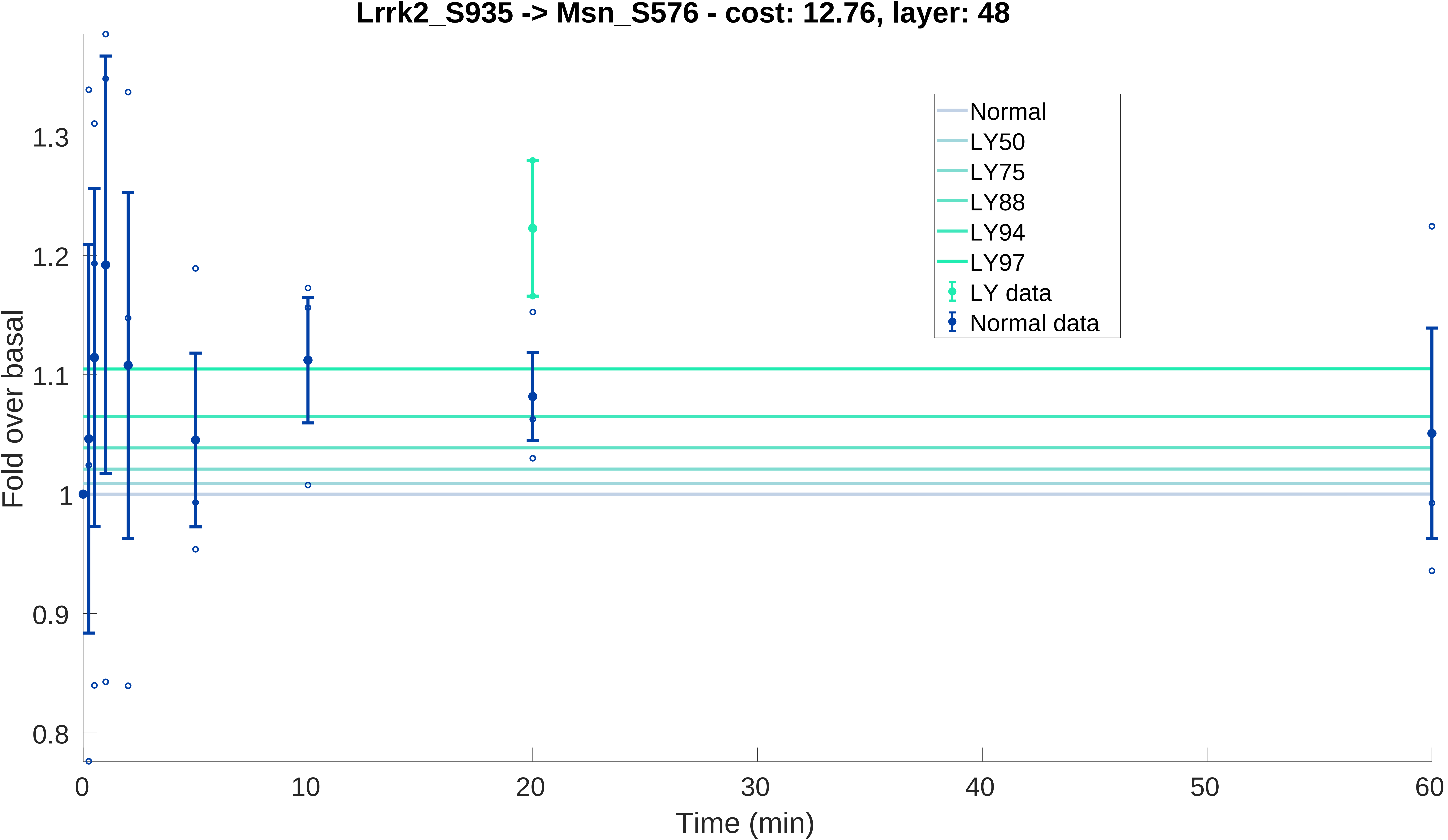
Lrrk2_S935 -> Msn_S576 - cost: 12.76, layer: 48
Normal
LY50
LY75
LY88
LY94
LY97
LY data
Normal data
Fold over basal
1.3
1.2
1.1
1
0.9
0.8
0
10
20
30
40
50
60
Time (min)

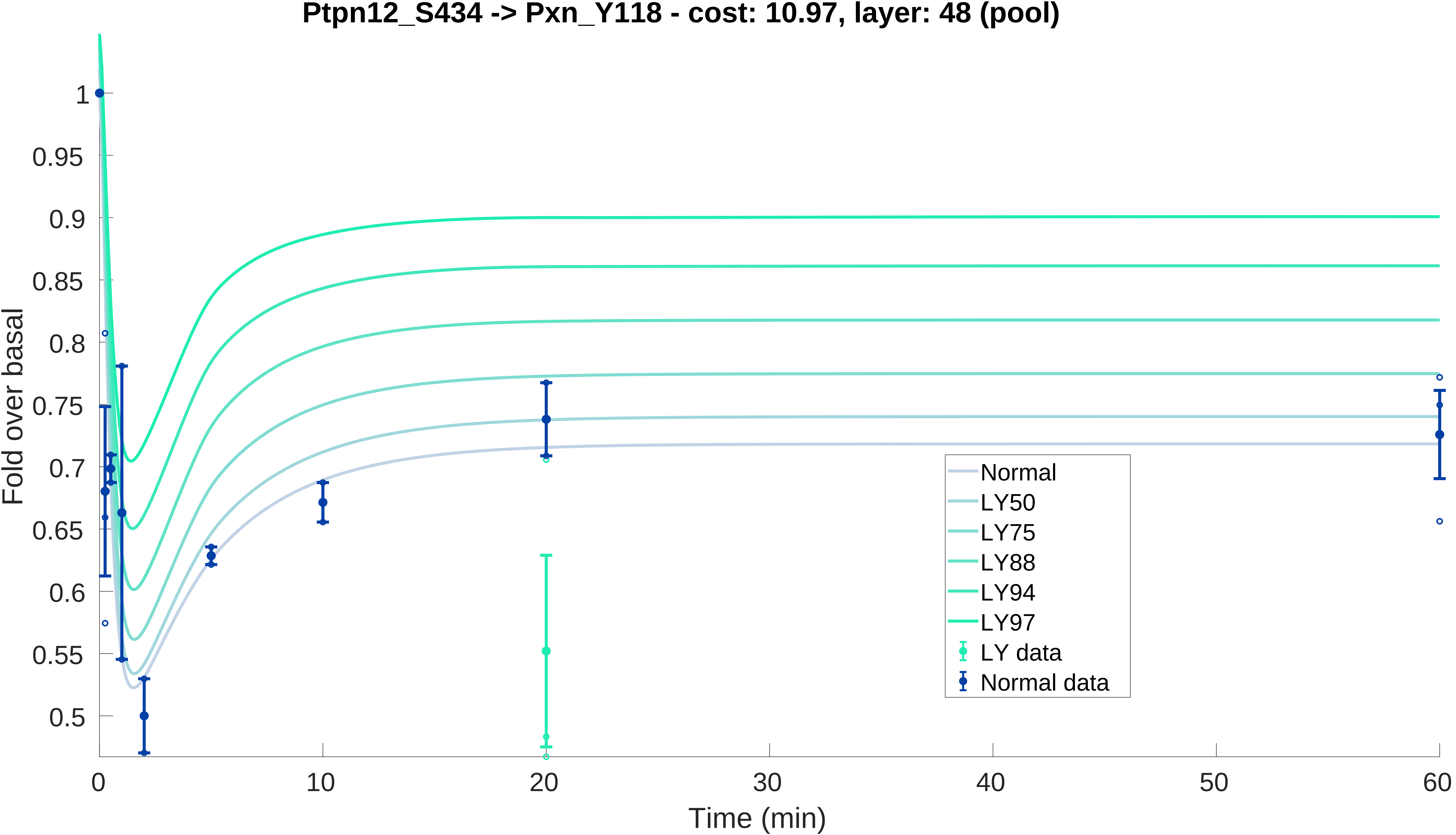

Ptpn12_S434 -> Pxn_Y118 - cost: 10.97, layer: 48 (pool)
Fold over basal
1
0.95
0.9
0.85
0.8
0.75
0.7
0.65
0.6
0.55
0.5
0
10
20
30
40
50
60
Time (min)
Normal
LY50
LY75
LY88
LY94
LY97
LY data
Normal data

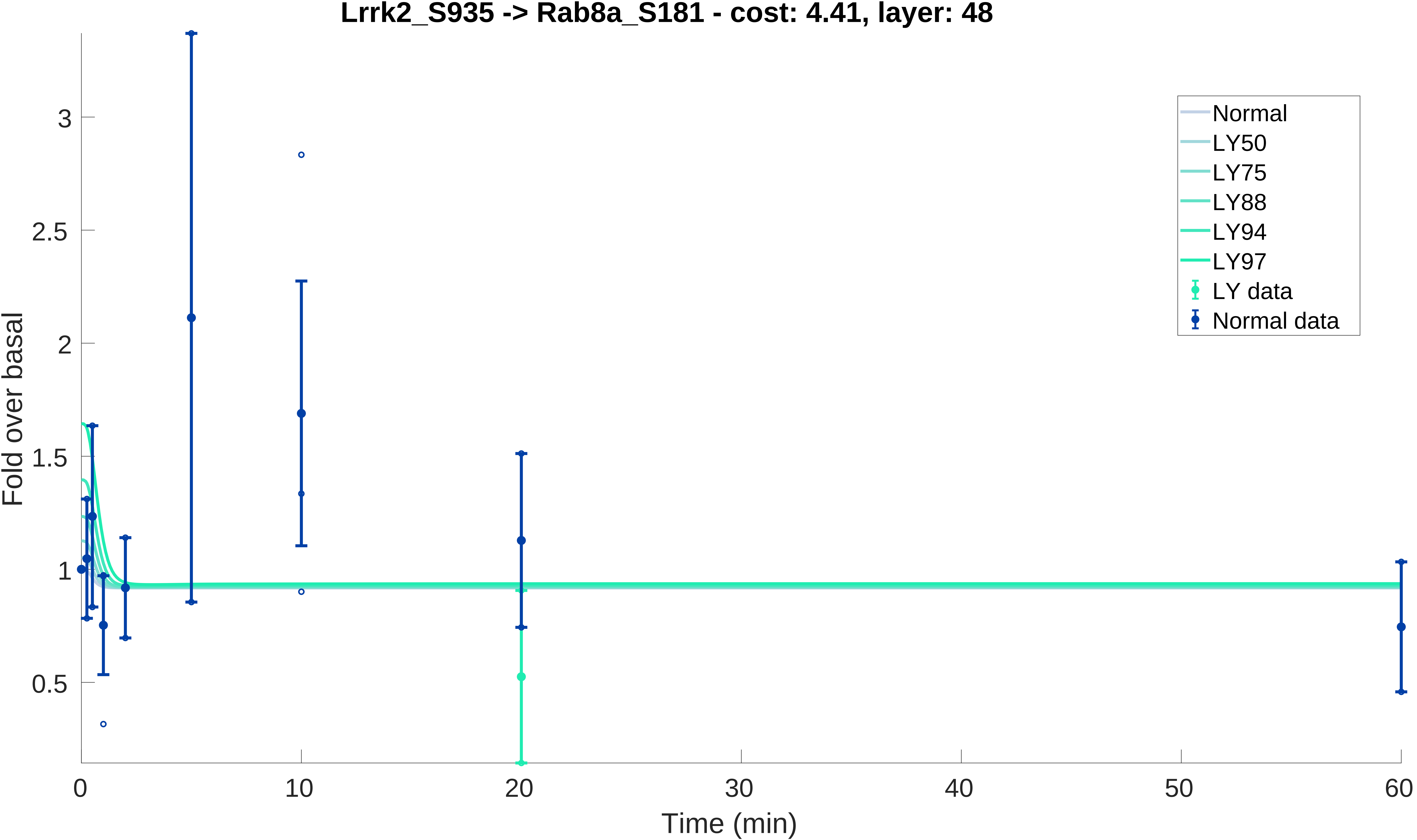

Lrrk2_S935 -> Rab8a_S181 - cost: 4.41, layer: 48
Fold over basal
Time (min)
Normal
LY50
LY75
LY88
LY94
LY97
LY data
Normal data
0.5
1
1.5
2
2.5
3
0
10
20
30
40
50
60

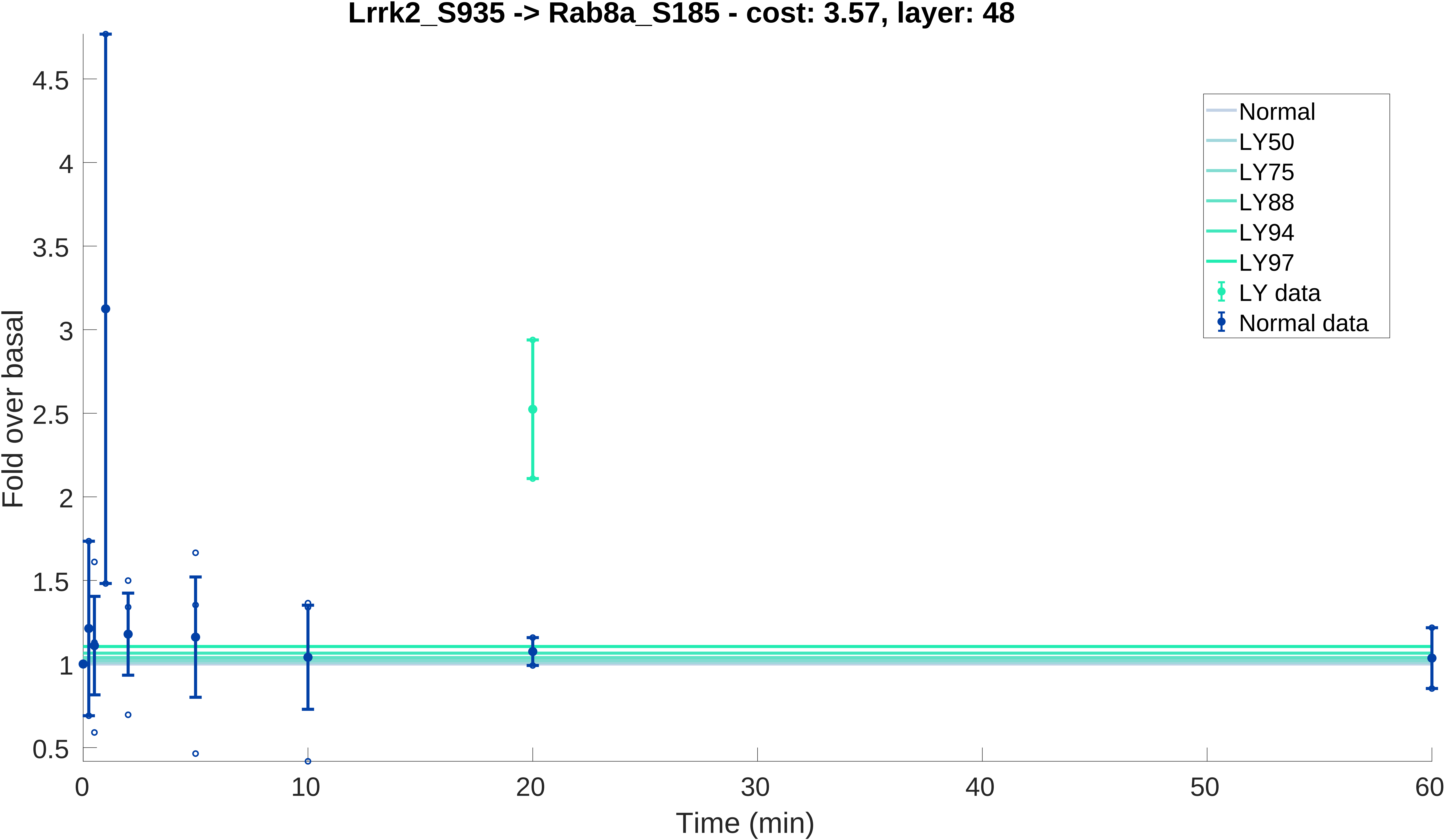

Lrrk2_S935 -> Rab8a_S185 - cost: 3.57, layer: 48
Fold over basal
Time (min)
0.5
1
1.5
2
2.5
3
3.5
4
4.5
0
10
20
30
40
50
60
Normal
LY50
LY75
LY88
LY94
LY97
LY data
Normal data

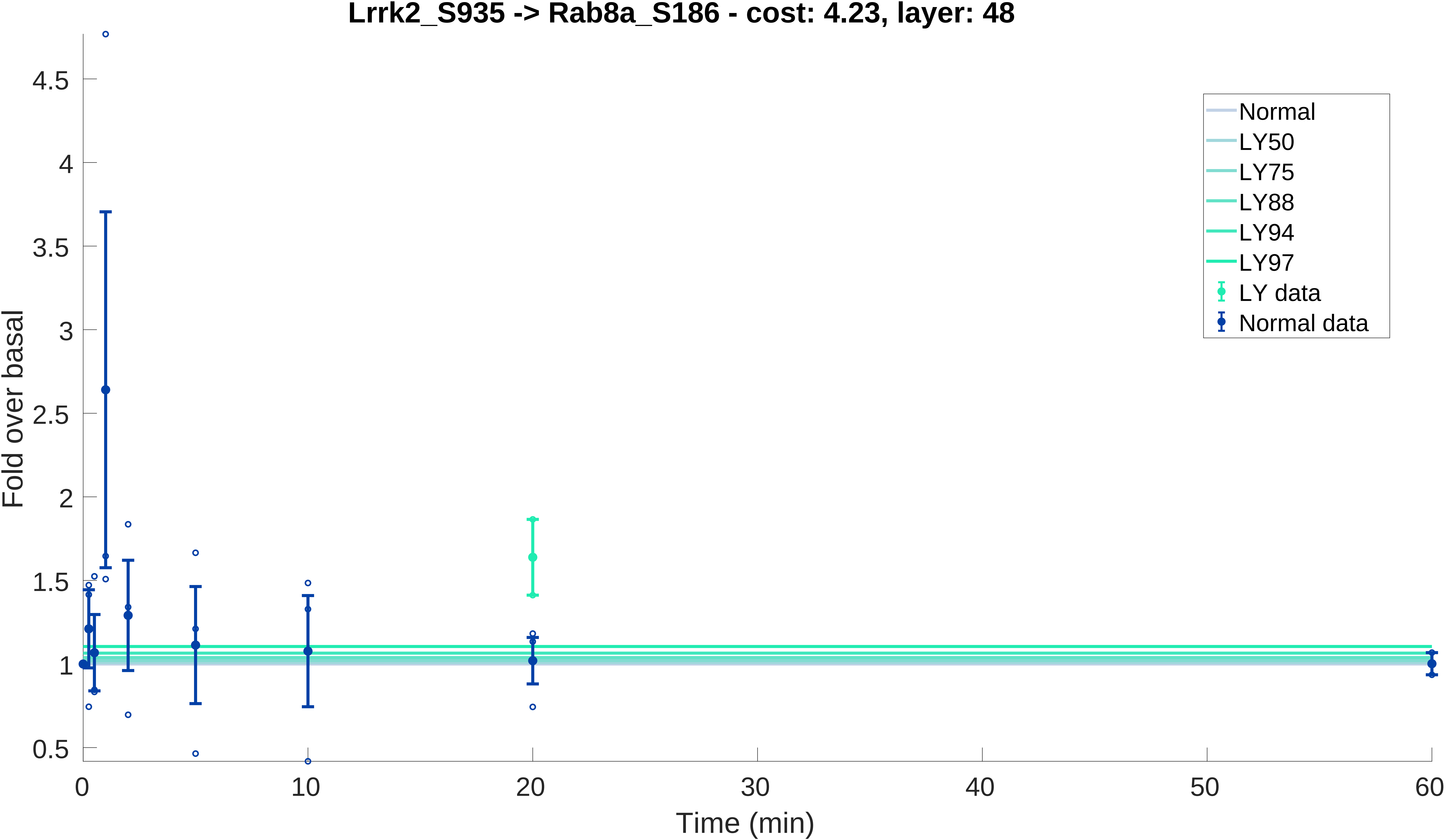

Lrrk2_S935 -> Rab8a_S186 - cost: 4.23, layer: 48
Fold over basal
4.5
4
3.5
3
2.5
2
1.5
1
0.5
0
10
20
30
40
50
60
Time (min)
Normal
LY50
LY75
LY88
LY94
LY97
LY data
Normal data

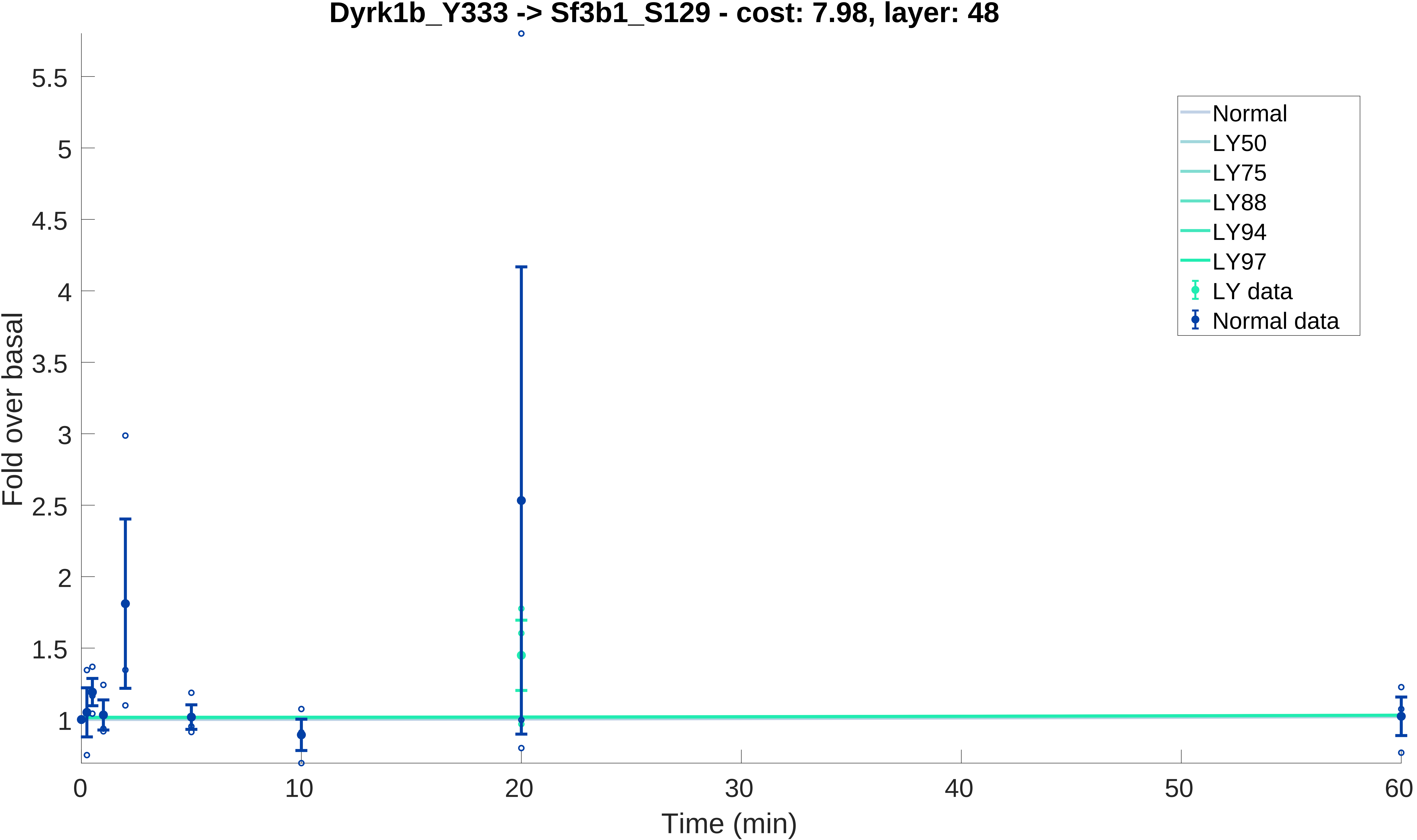

Dyrk1b_Y333 -> Sf3b1_S129 - cost: 7.98, layer: 48
Fold over basal
Time (min)
Normal
LY50
LY75
LY88
LY94
LY97
LY data
Normal data
5.5
5
4.5
4
3.5
3
2.5
2
1.5
1
0
10
20
30
40
50
60

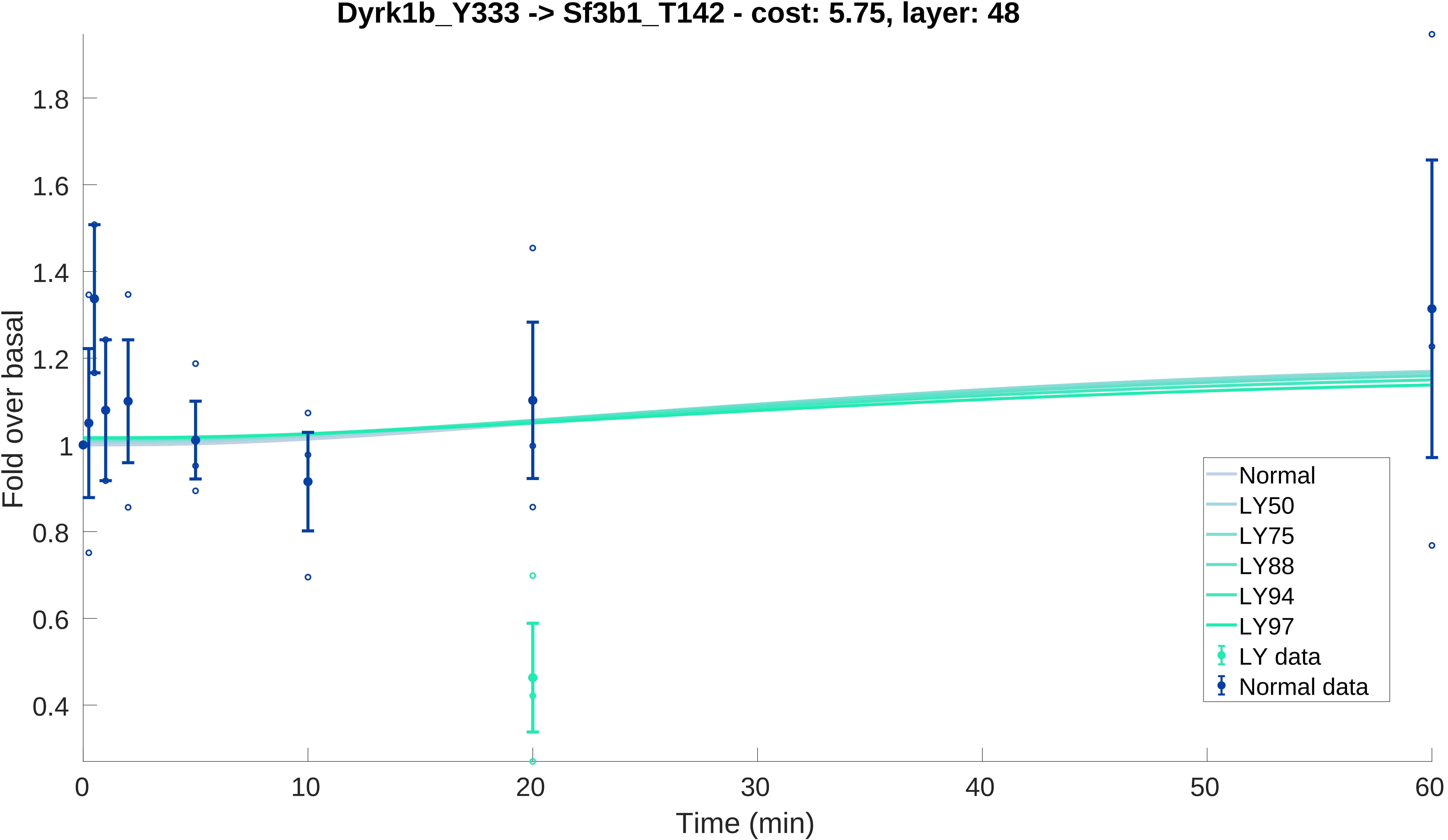

Dyrk1b_Y333 -> Sf3b1_T142 - cost: 5.75, layer: 48
Fold over basal
Time (min)
0.4
0.6
0.8
1
1.2
1.4
1.6
1.8
0
10
20
30
40
50
60
Normal
LY50
LY75
LY88
LY94
LY97
LY data
Normal data

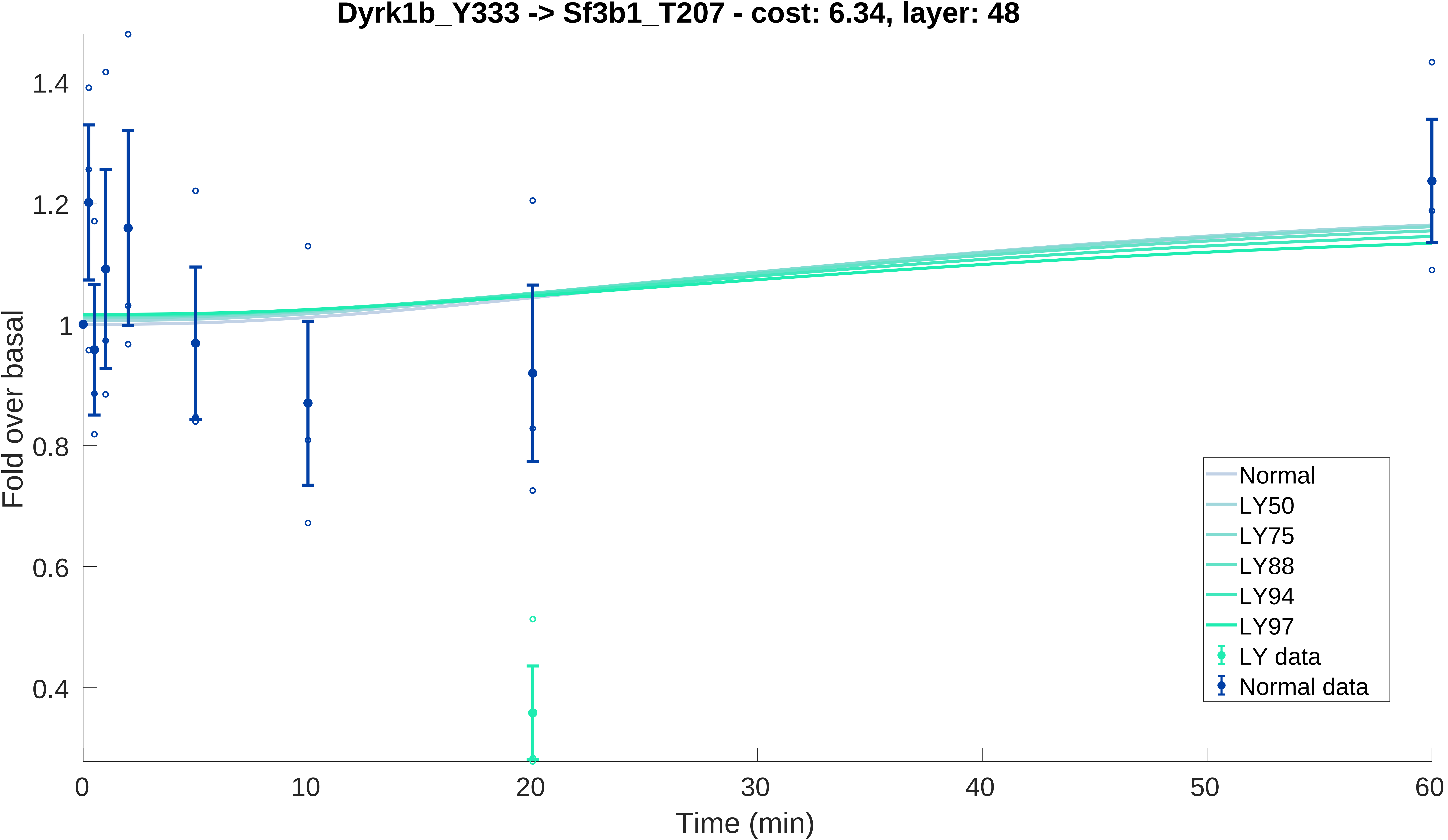

Dyrk1b_Y333 -> Sf3b1_T207 - cost: 6.34, layer: 48
Fold over basal
1.4
1.2
1
0.8
0.6
0.4
0
10
20
30
40
50
60
Time (min)
Normal
LY50
LY75
LY88
LY94
LY97
LY data
Normal data

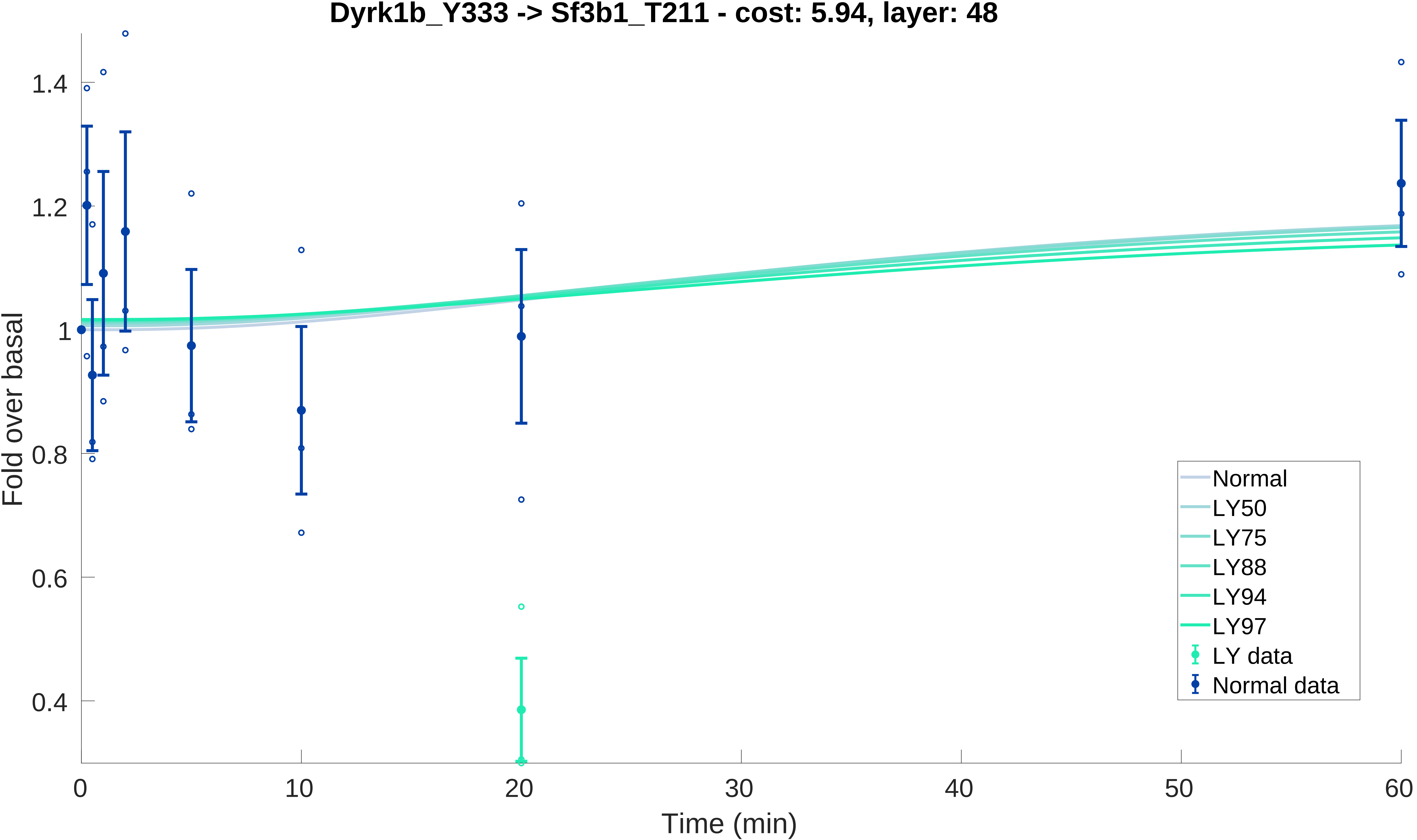

Dyrk1b_Y333 -> Sf3b1_T211 - cost: 5.94, layer: 48
Fold over basal
1.4
1.2
1
0.8
0.6
0.4
0
10
20
30
40
50
60
Time (min)
Normal
LY50
LY75
LY88
LY94
LY97
LY data
Normal data

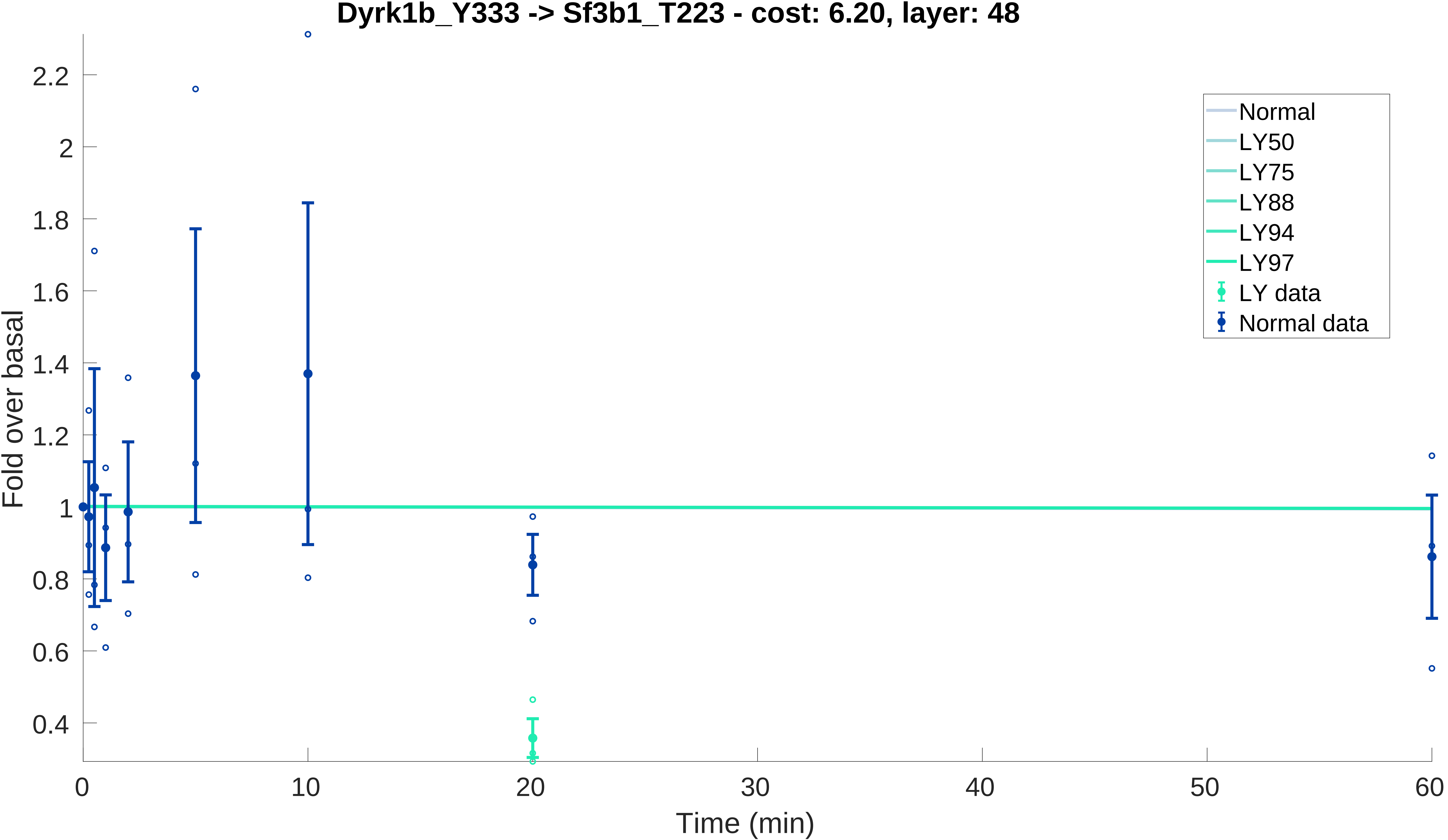
Dyrk1b_Y333 -> Sf3b1_T223 - cost: 6.20, layer: 48
Fold over basal
Time (min)
0
10
20
30
40
50
60
0.4
0.6
0.8
1
1.2
1.4
1.6
1.8
2
2.2
Normal
LY50
LY75
LY88
LY94
LY97
LY data
Normal data

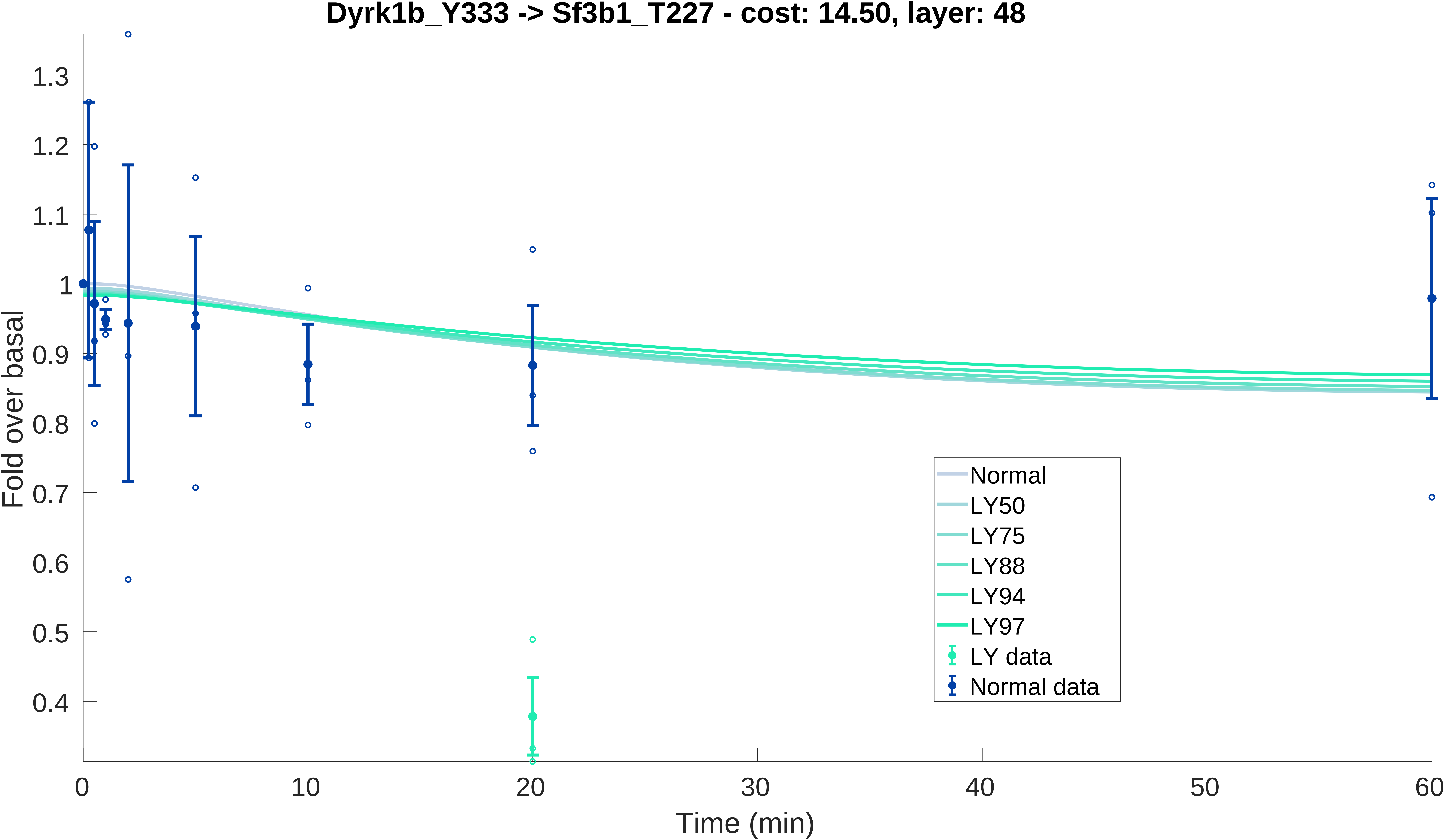

Dyrk1b_Y333 -> Sf3b1_T227 - cost: 14.50, layer: 48
Fold over basal
Time (min)
1.3
1.2
1.1
1
0.9
0.8
0.7
0.6
0.5
0.4
0
10
20
30
40
50
60
Normal
LY50
LY75
LY88
LY94
LY97
LY data
Normal data

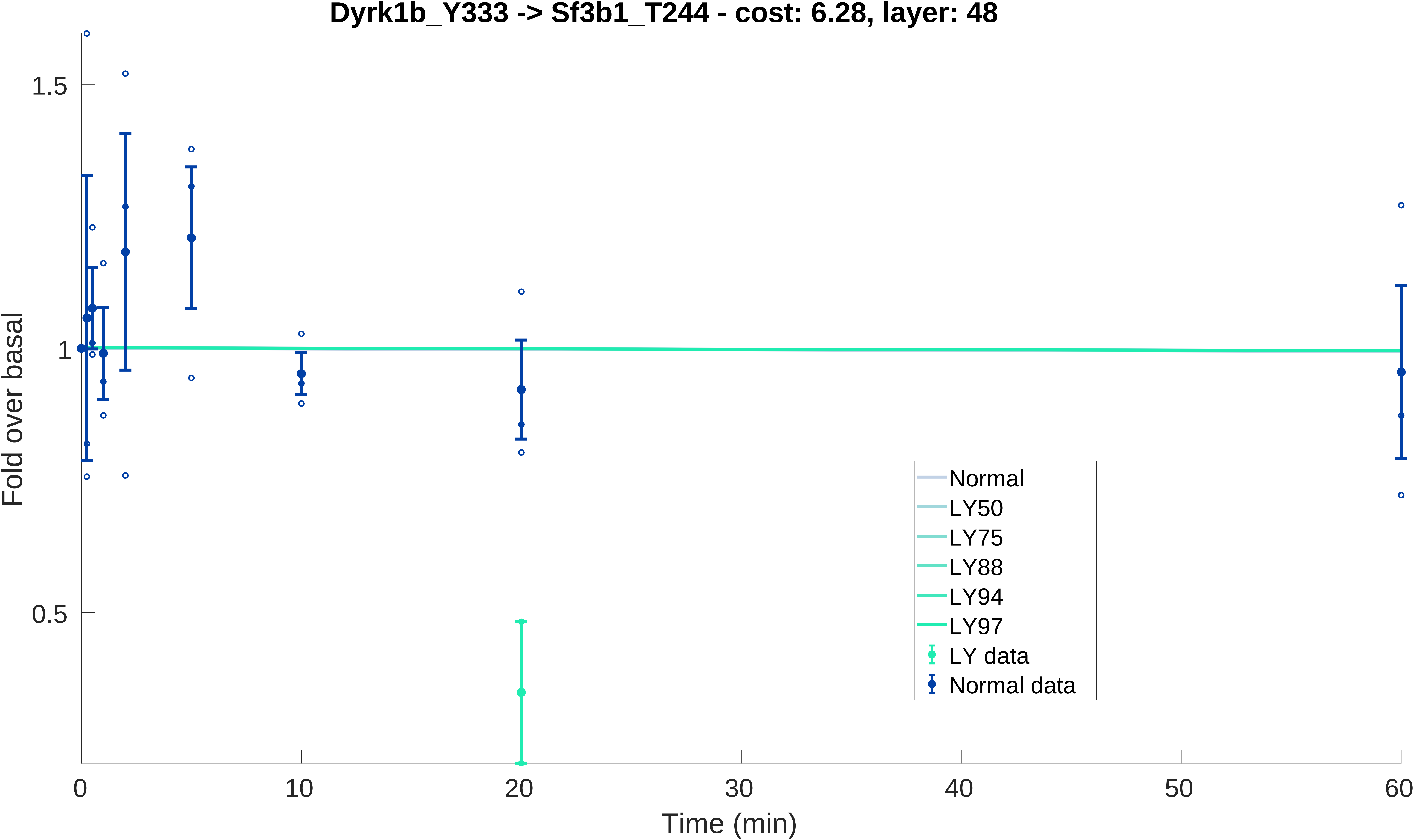

Dyrk1b_Y333 -> Sf3b1_T244 - cost: 6.28, layer: 48
Fold over basal
1.5
1
0.5
0
10
20
30
40
50
60
Time (min)
Normal
LY50
LY75
LY88
LY94
LY97
LY data
Normal data

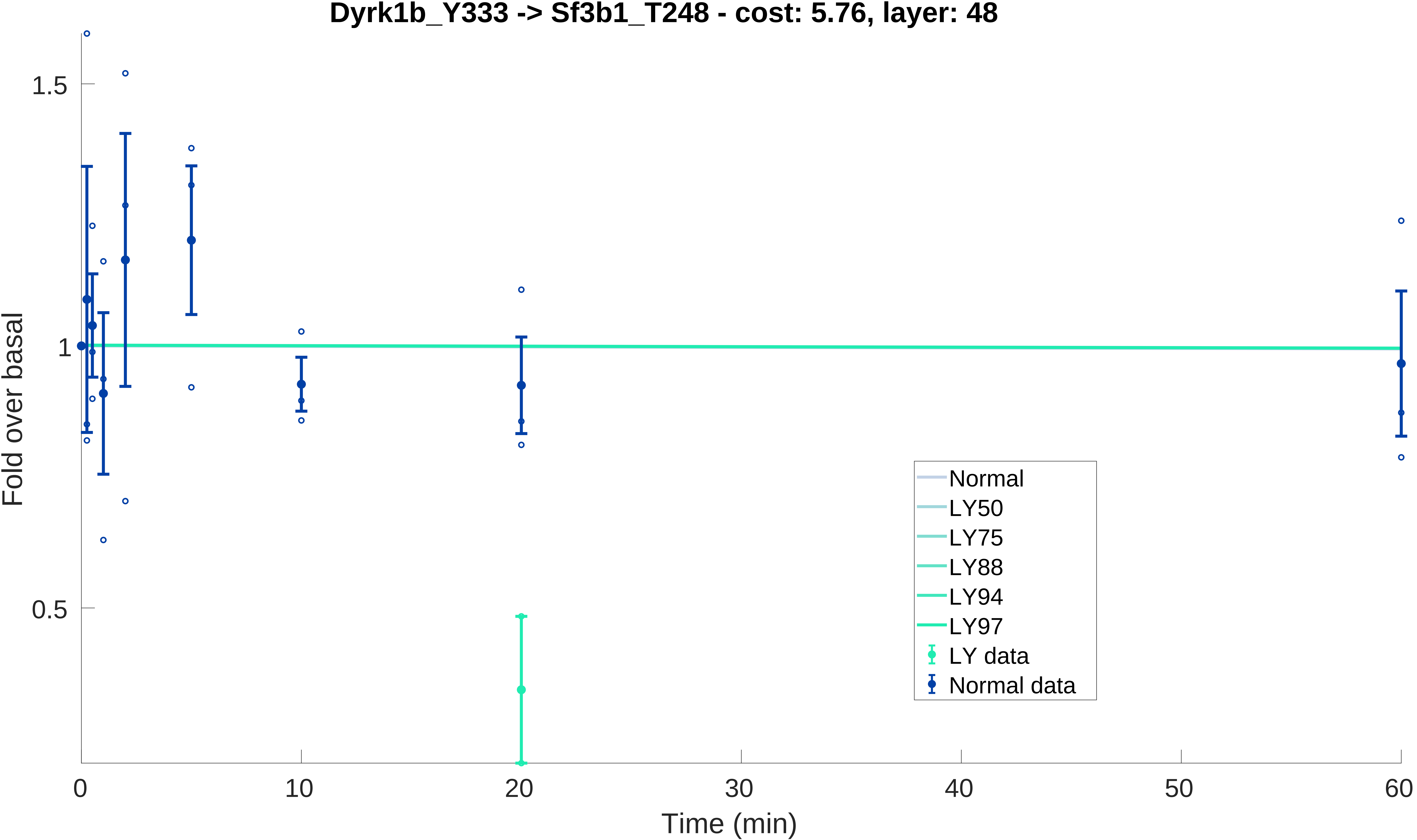

Dyrk1b_Y333 -> Sf3b1_T248 - cost: 5.76, layer: 48
Fold over basal
1.5
1
0.5
0
10
20
30
40
50
60
Time (min)
Normal
LY50
LY75
LY88
LY94
LY97
LY data
Normal data

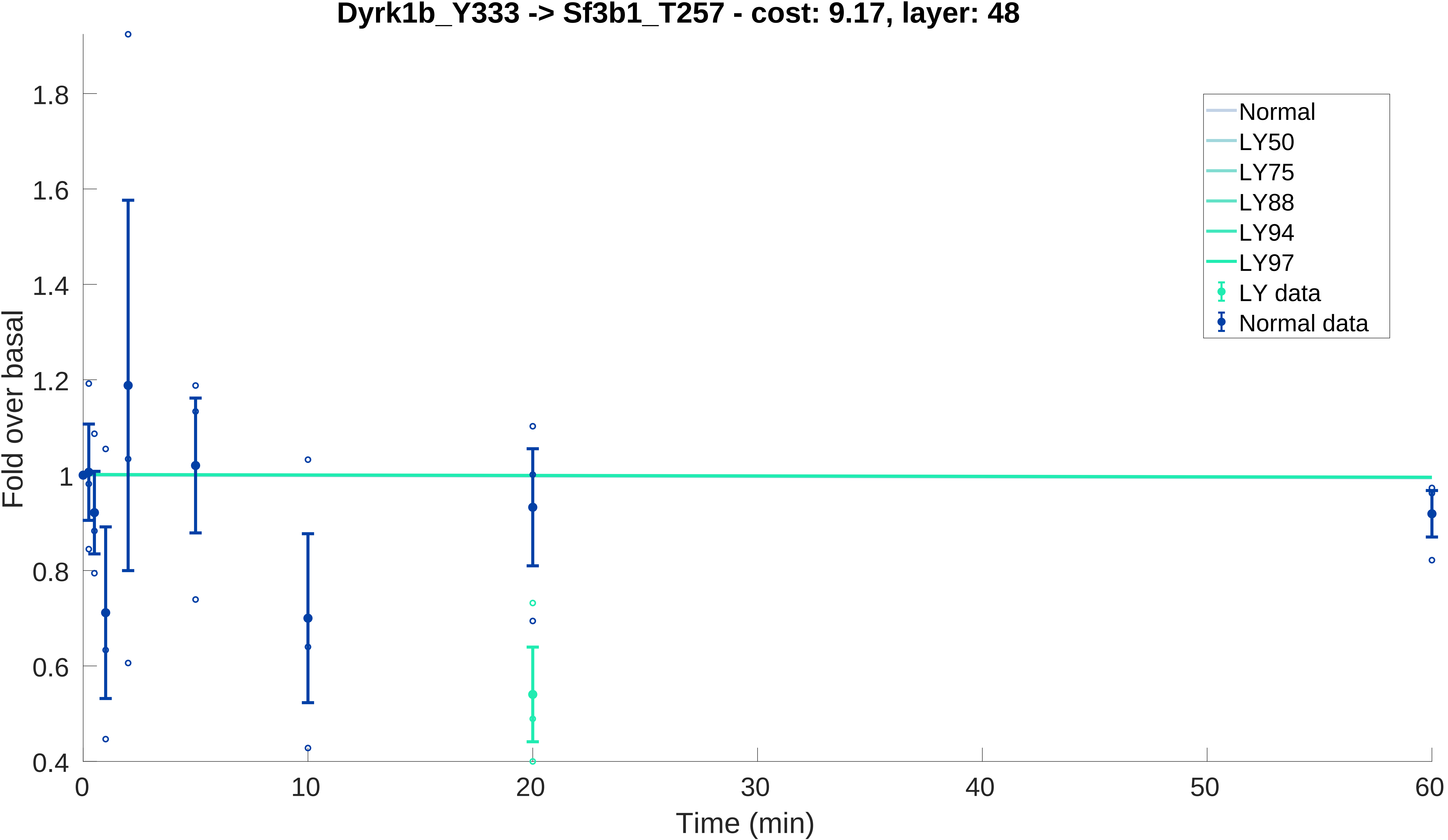
Dyrk1b_Y333 -> Sf3b1_T257 - cost: 9.17, layer: 48
Fold over basal
Time (min)
1.8
1.6
1.4
1.2
1
0.8
0.6
0.4
0
10
20
30
40
50
60
Normal
LY50
LY75
LY88
LY94
LY97
LY data
Normal data

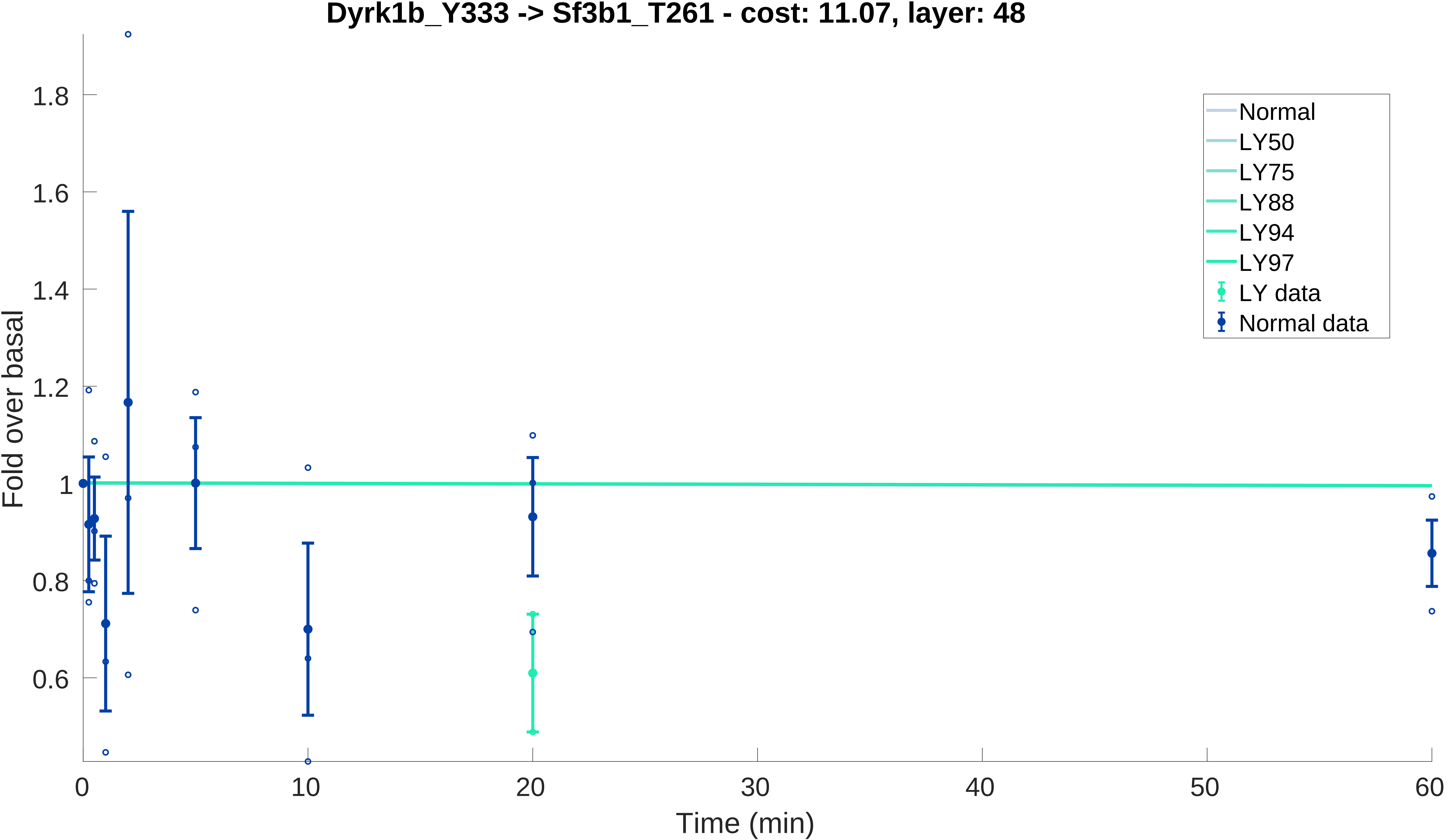

Dyrk1b_Y333 -> Sf3b1_T261 - cost: 11.07, layer: 48
Fold over basal
1.8
1.6
1.4
1.2
1
0.8
0.6
0
10
20
30
40
50
60
Time (min)
Normal
LY50
LY75
LY88
LY94
LY97
LY data
Normal data

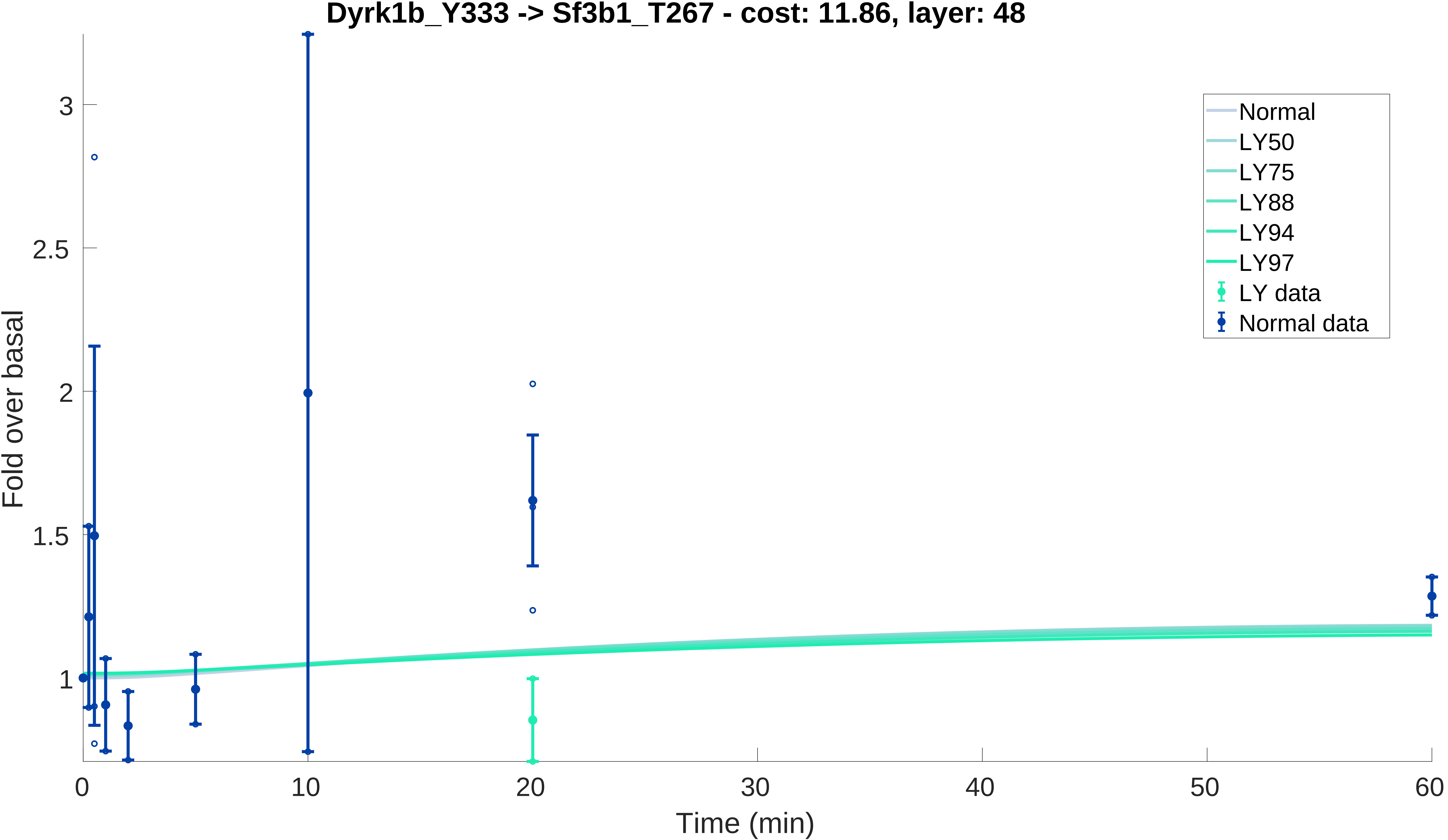
Dyrk1b_Y333 -> Sf3b1_T267 - cost: 11.86, layer: 48
Fold over basal
Time (min)
0
10
20
30
40
50
60
1
1.5
2
2.5
3
Normal
LY50
LY75
LY88
LY94
LY97
LY data
Normal data

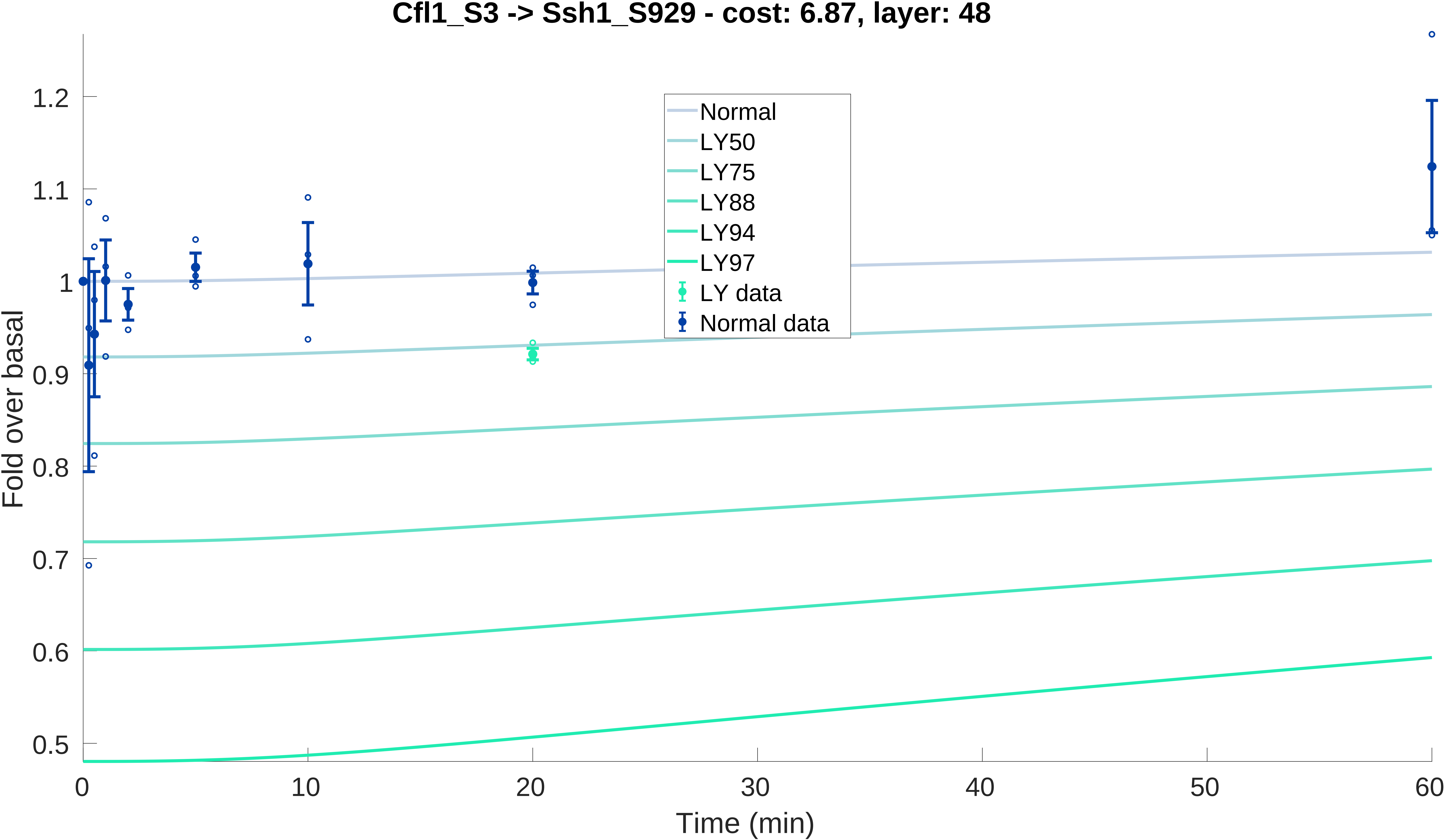
Cfl1_S3 -> Ssh1_S929 - cost: 6.87, layer: 48
Normal
LY50
LY75
LY88
LY94
LY97
LY data
Normal data
Fold over basal
Time (min)
0
10
20
30
40
50
60
0.5
0.6
0.7
0.8
0.9
1
1.1
1.2

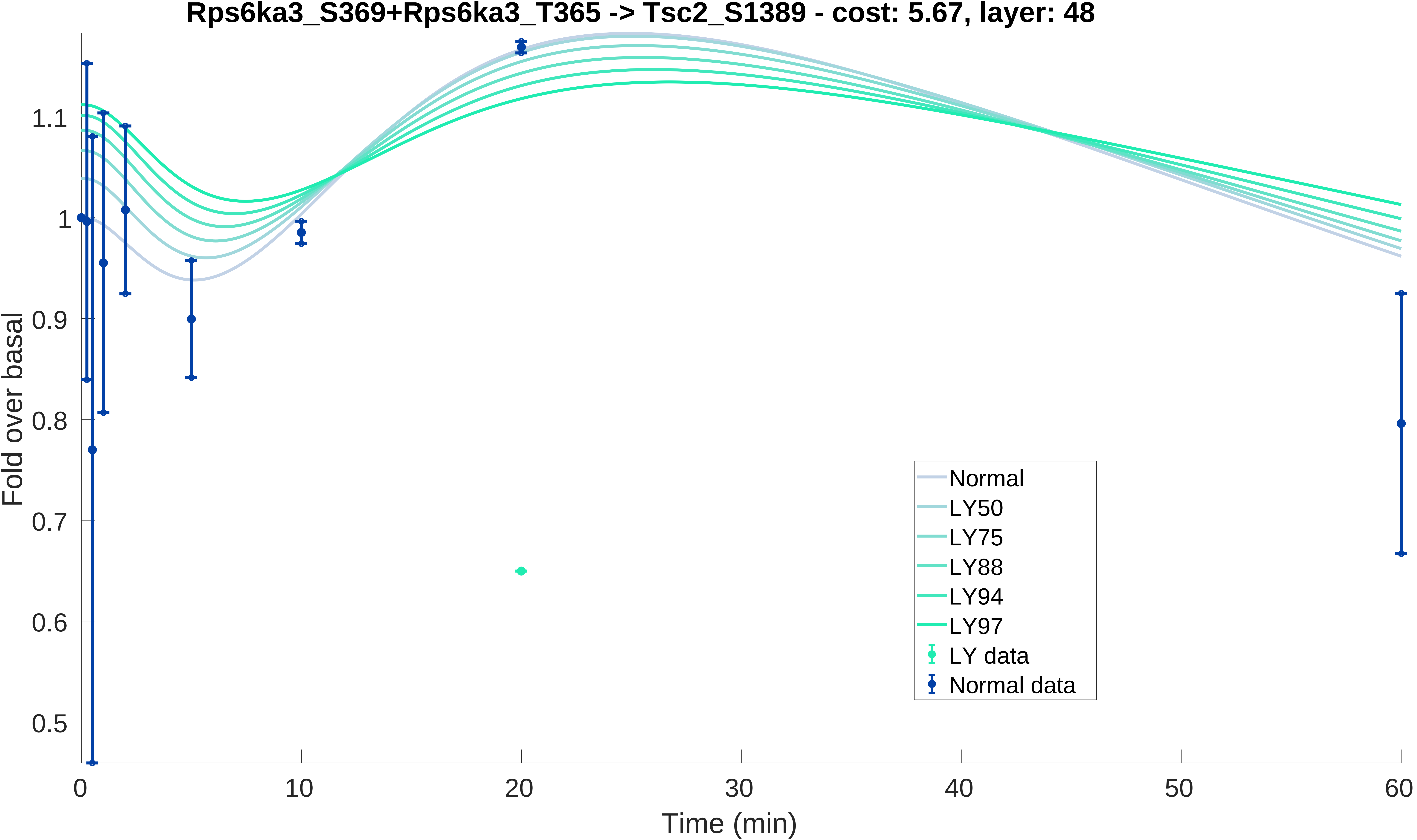

Rps6ka3_S369+Rps6ka3_T365 -> Tsc2_S1389 - cost: 5.67, layer: 48
Fold over basal
Time (min)
0.5
0.6
0.7
0.8
0.9
1
1.1
0
10
20
30
40
50
60
Normal
LY50
LY75
LY88
LY94
LY97
LY data
Normal data

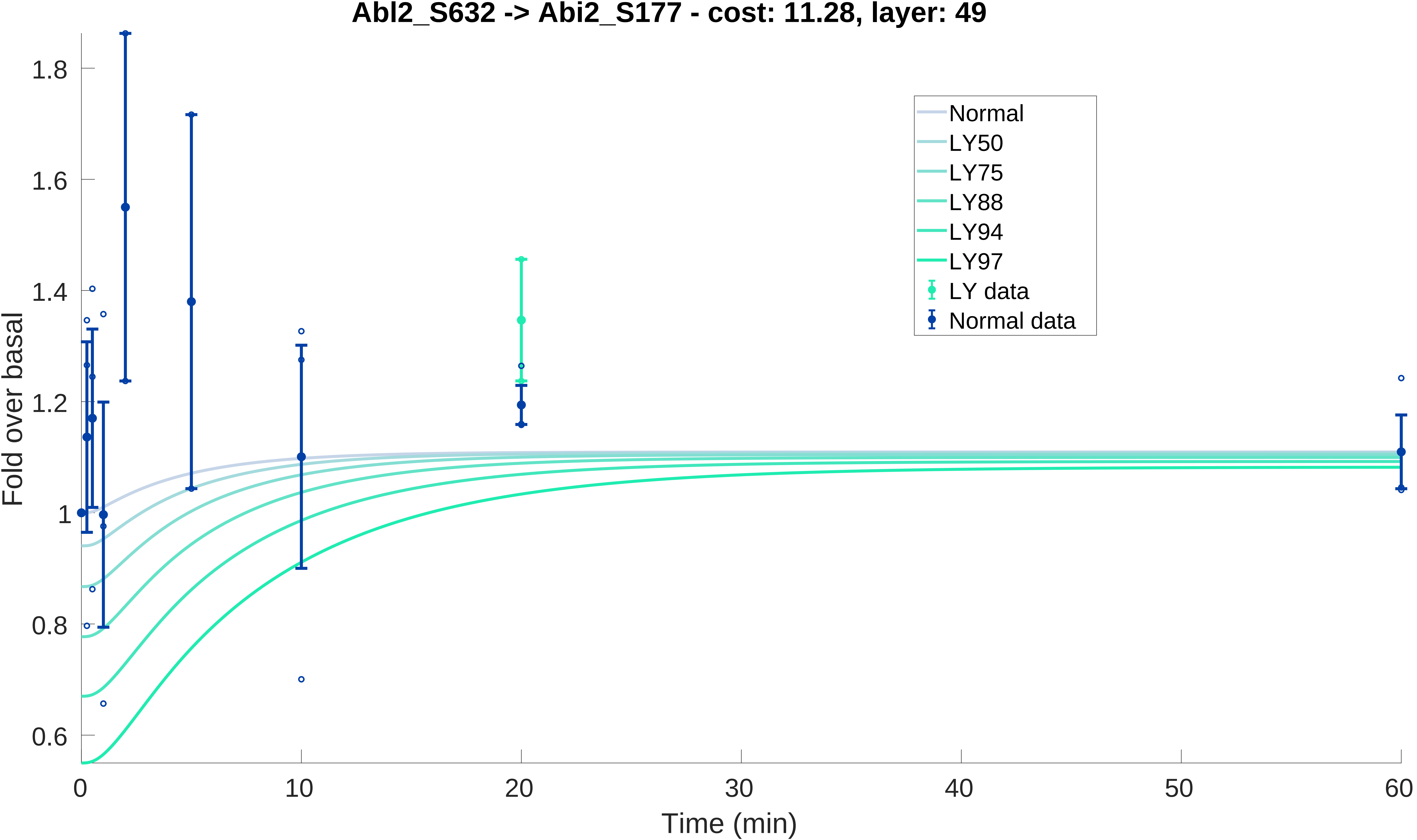

Abl2_S632 -> Abi2_S177 - cost: 11.28, layer: 49
Fold over basal
Time (min)
1.8
1.6
1.4
1.2
1
0.8
0.6
0
10
20
30
40
50
60
Normal
LY50
LY75
LY88
LY94
LY97
LY data
Normal data

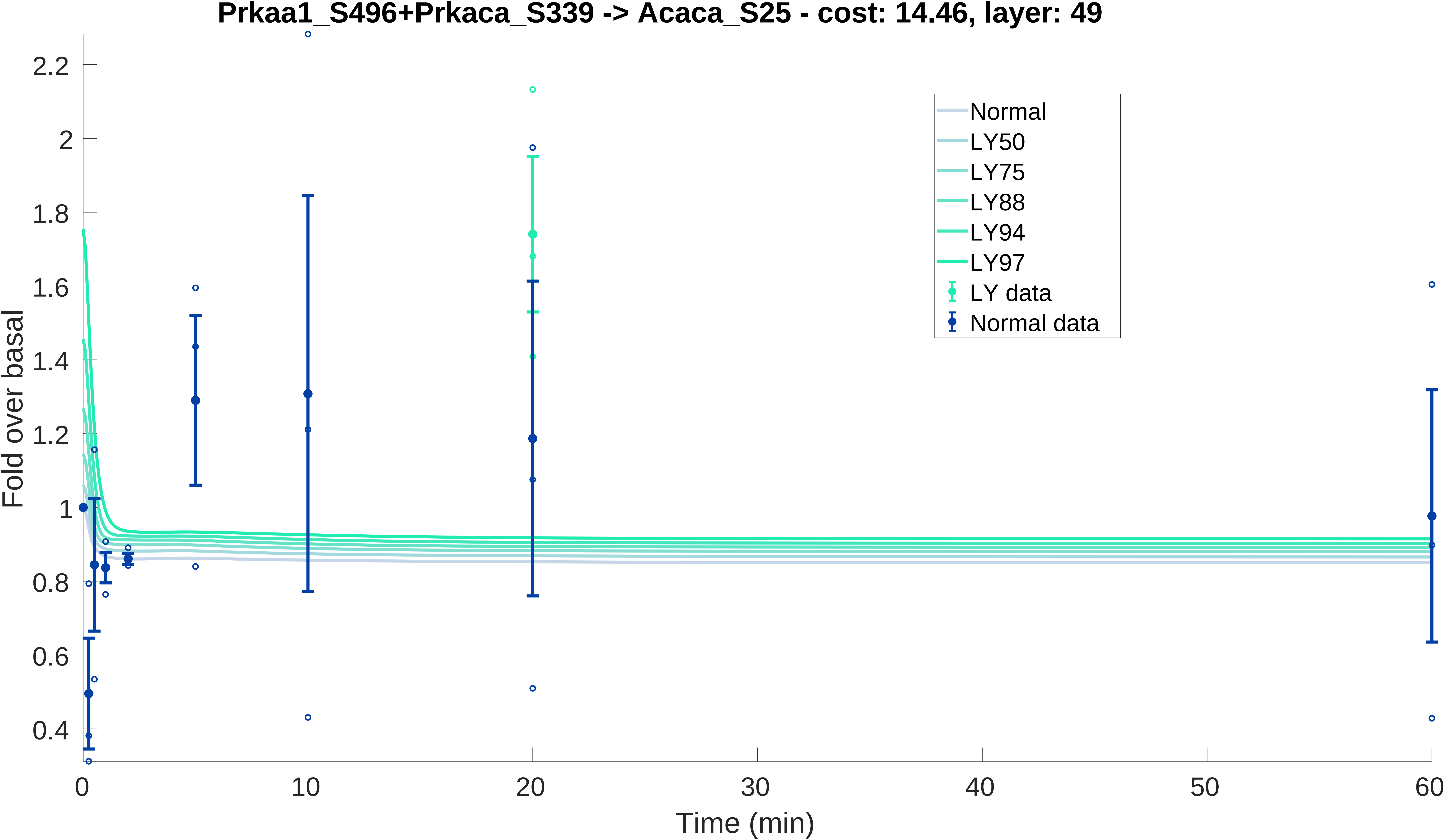
Prrkaa1_S496+Prkaca_S339 -> Acaca_S25 - cost: 14.46, layer: 49
Fold over basal
Time (min)
0
10
20
30
40
50
60
0.4
0.6
0.8
1
1.2
1.4
1.6
1.8
2
2.2
Normal
LY50
LY75
LY88
LY94
LY97
LY data
Normal data

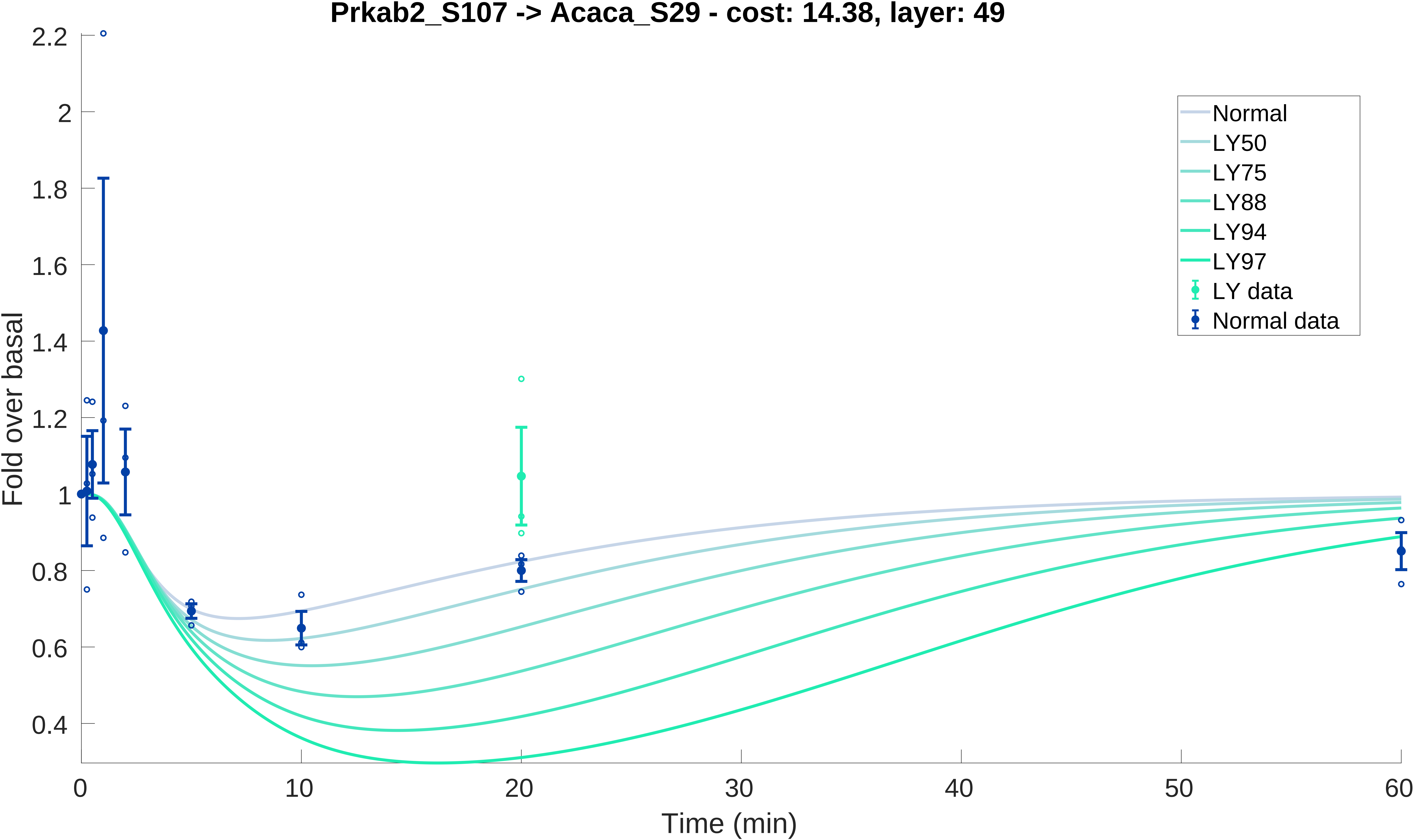
Prkab2_S107 -> Acaca_S29 - cost: 14.38, layer: 49
Fold over basal
Time (min)
2.2
2
1.8
1.6
1.4
1.2
1
0.8
0.6
0.4
0
10
20
30
40
50
60
Normal
LY50
LY75
LY88
LY94
LY97
LY data
Normal data

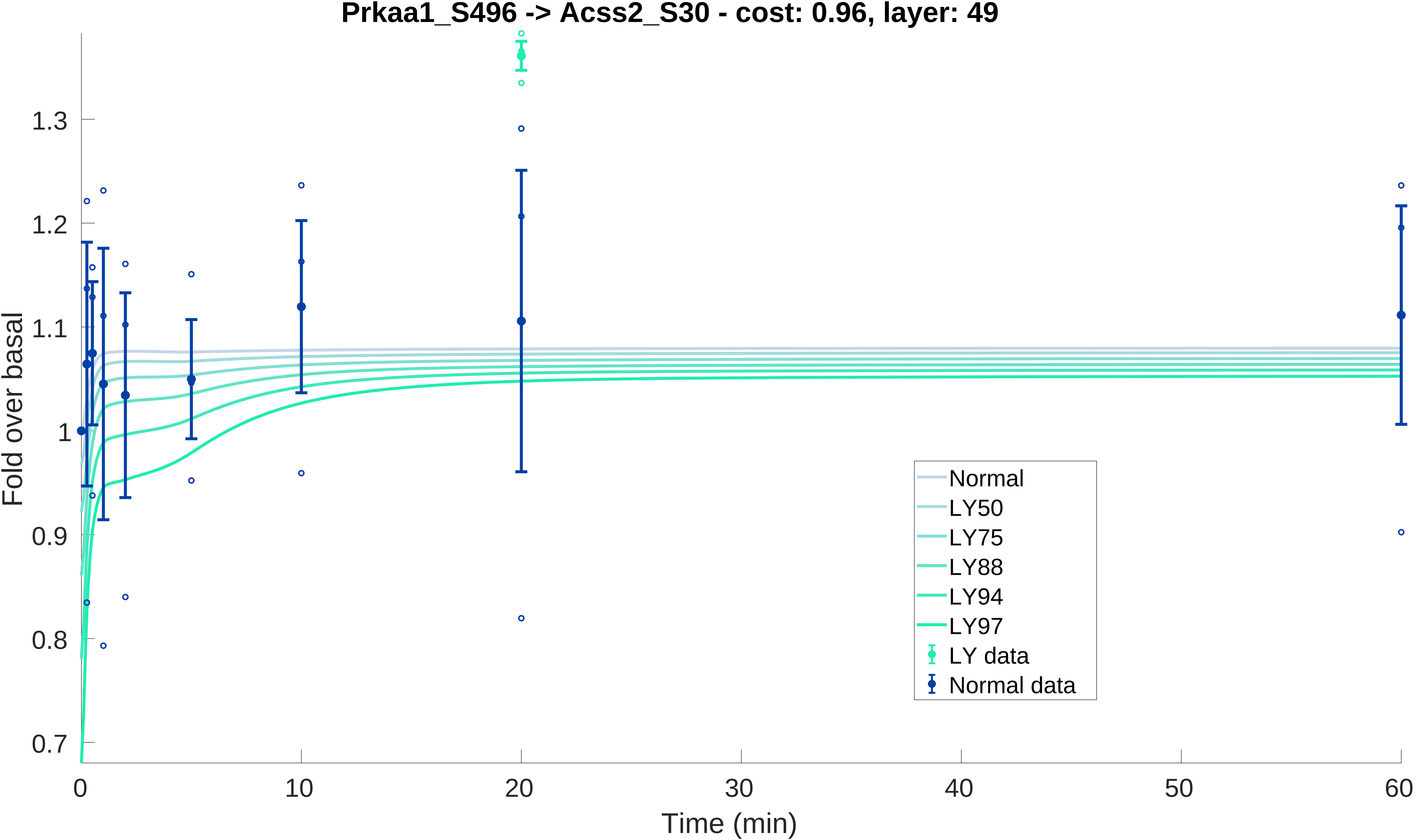

Prrkaa1_S496 -> Acss2_S30 - cost: 0.96, layer: 49
Fold over basal
Time (min)
0.7
0.8
0.9
1
1.1
1.2
1.3
0
10
20
30
40
50
60
Normal
LY50
LY75
LY88
LY94
LY97
LY data
Normal data

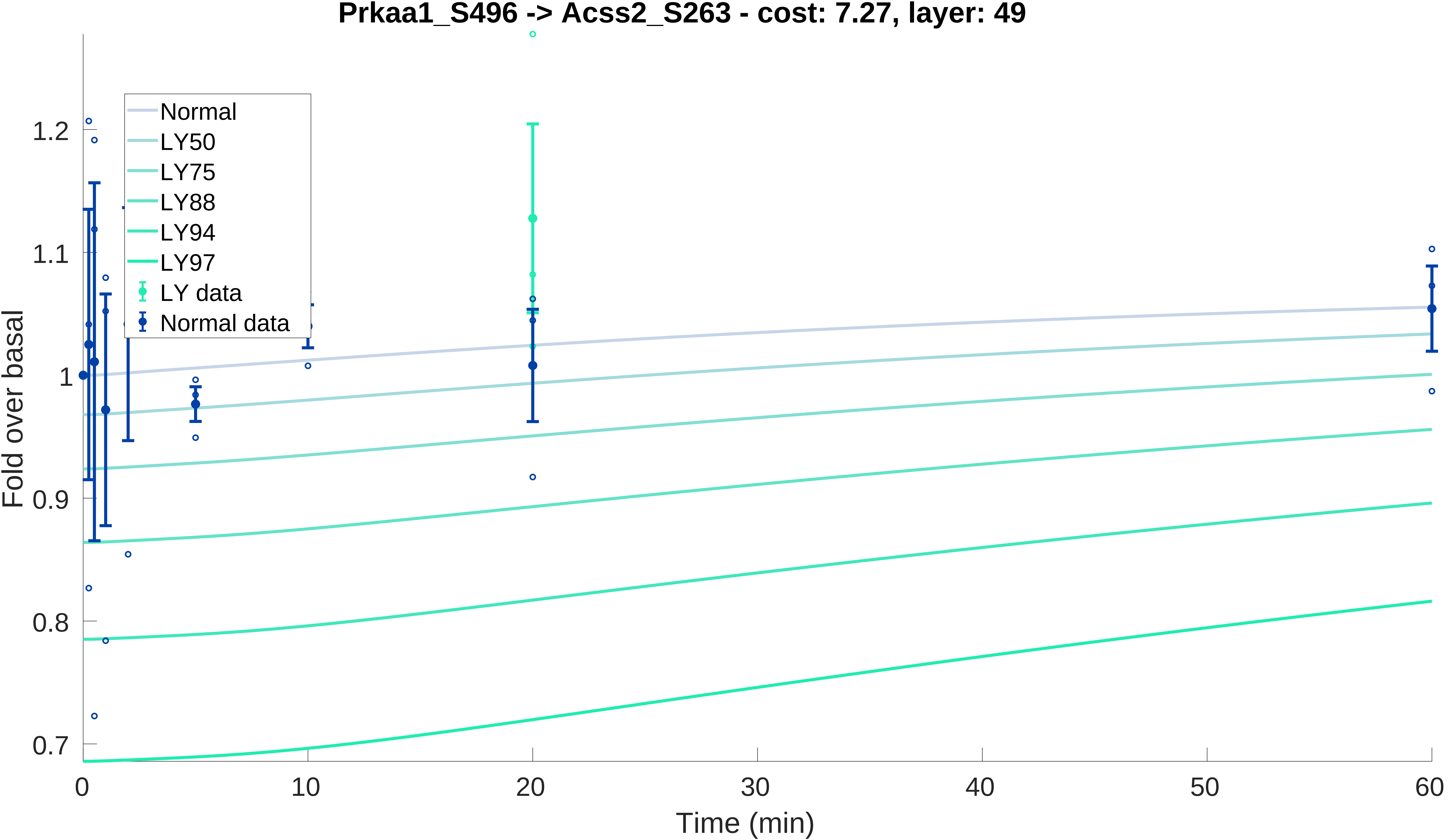

Prkaa1_S496 -> Acss2_S263 - cost: 7.27, layer: 49
Normal
LY50
LY75
LY88
LY94
LY97
LY data
Normal data
Fold over basal
1.2
1.1
1
0.9
0.8
0.7
0
10
20
30
40
50
60
Time (min)

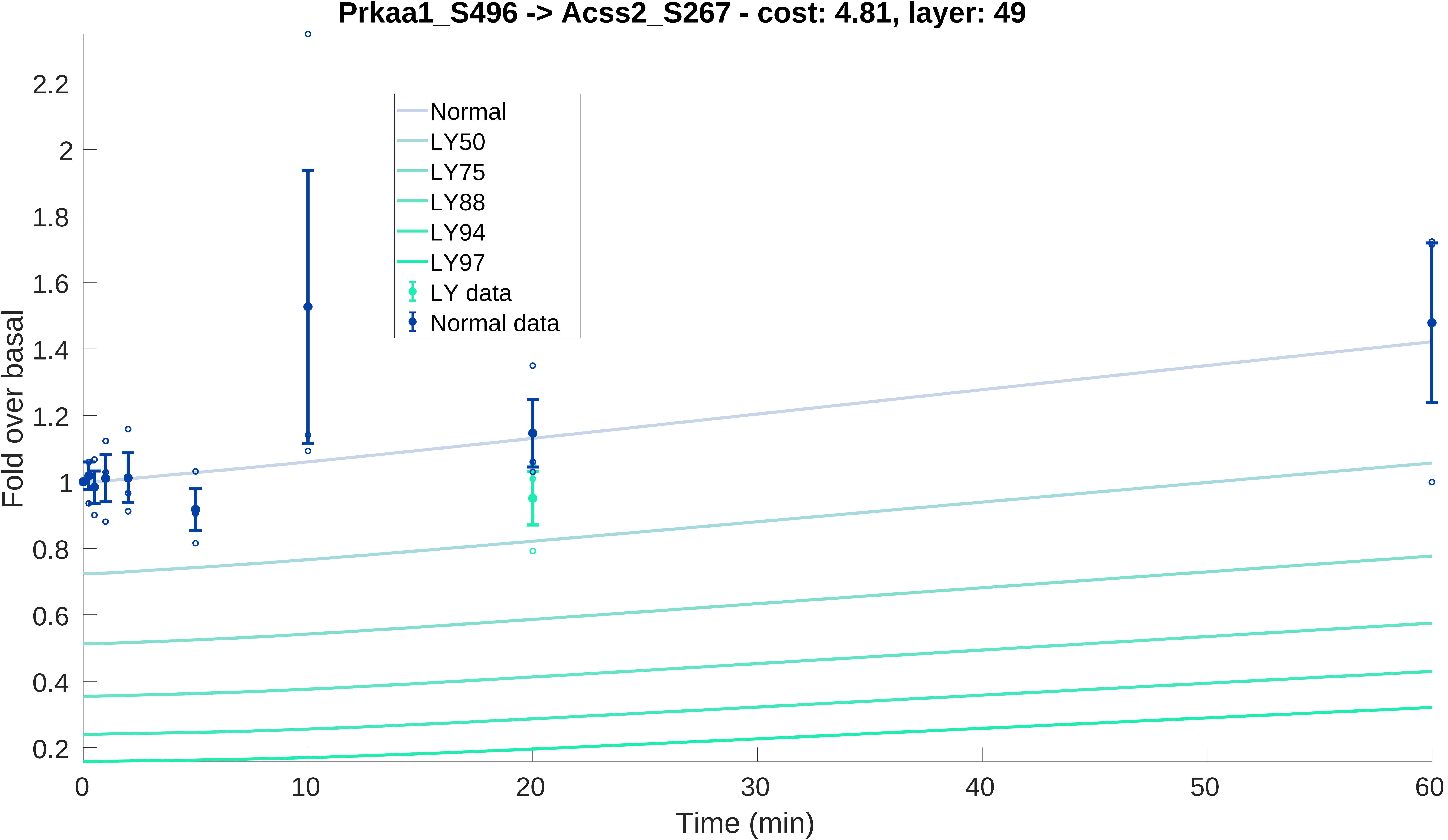

Prkaa1_S496 -> Acss2_S267 - cost: 4.81, layer: 49
Normal
LY50
LY75
LY88
LY94
LY97
LY data
Normal data
Fold over basal
Time (min)
0.2
0.4
0.6
0.8
1
1.2
1.4
1.6
1.8
2
2.2
0
10
20
30
40
50
60

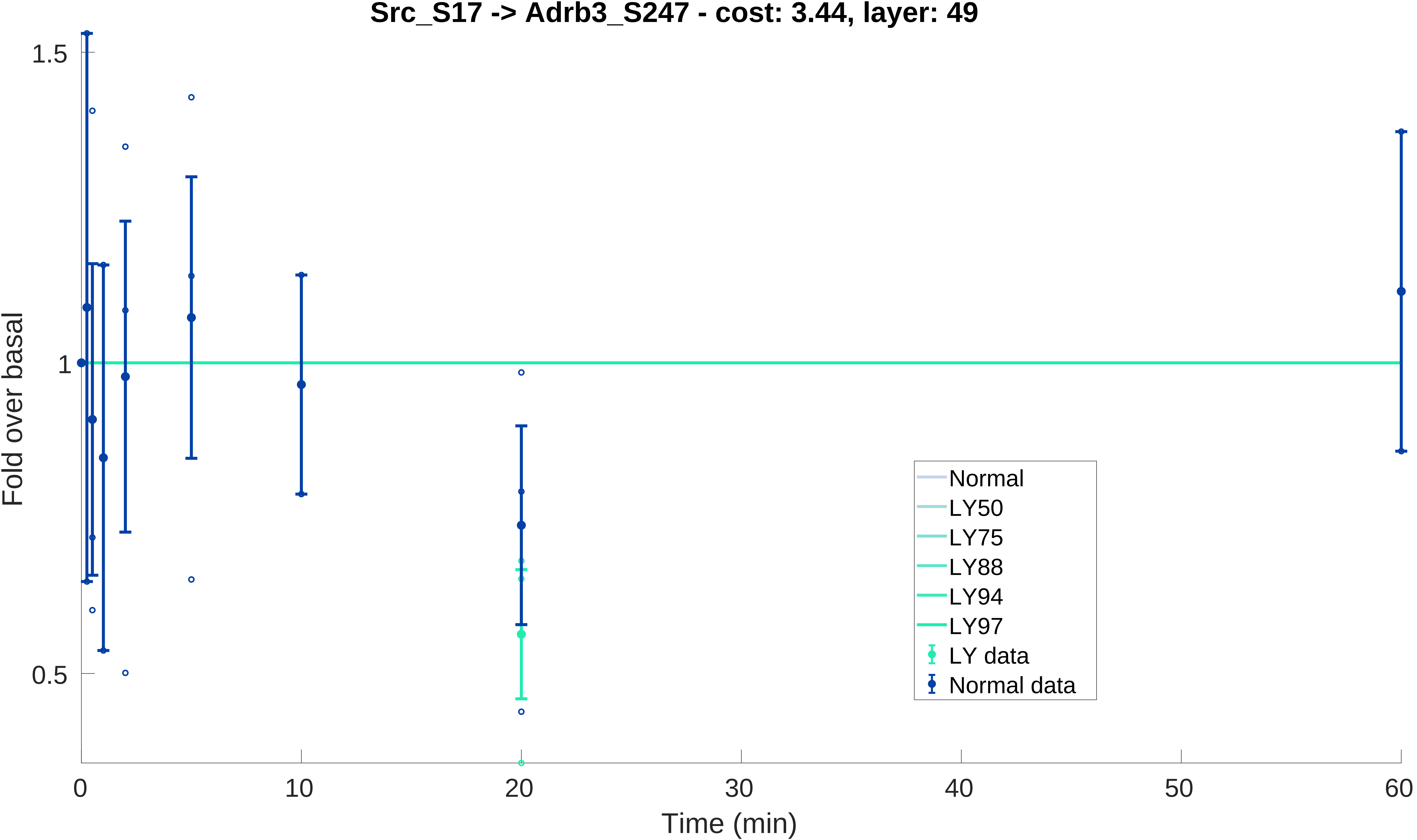

Src_S17 -> Adrb3_S247 - cost: 3.44, layer: 49
Fold over basal
1.5
1
0.5
Time (min)
0
10
20
30
40
50
60
Normal
LY50
LY75
LY88
LY94
LY97
LY data
Normal data

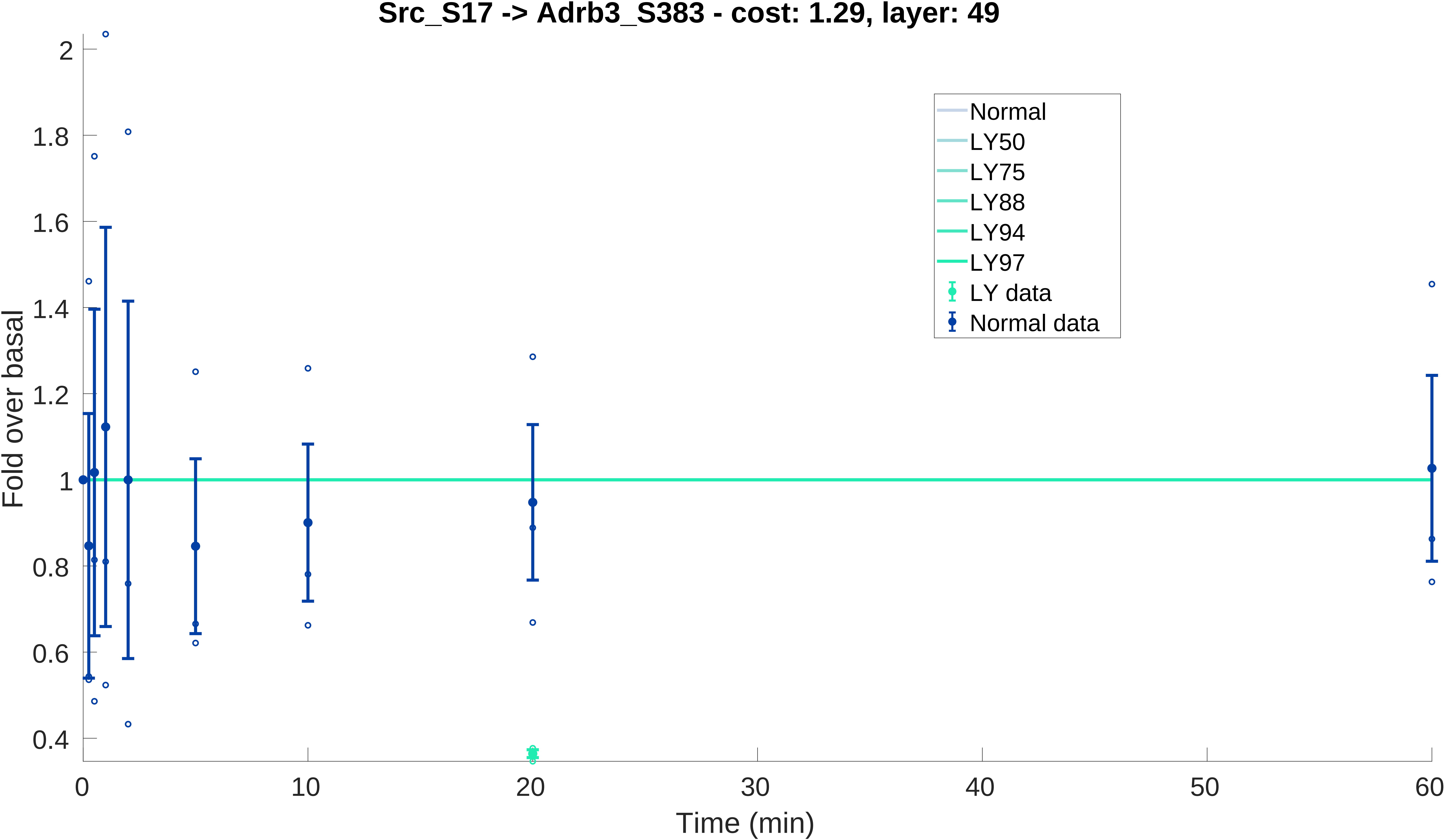

Src_S17 -> Adrb3_S383 - cost: 1.29, layer: 49
Fold over basal
Time (min)
0
10
20
30
40
50
60
0.4
0.6
0.8
1
1.2
1.4
1.6
1.8
2
Normal
LY50
LY75
LY88
LY94
LY97
LY data
Normal data

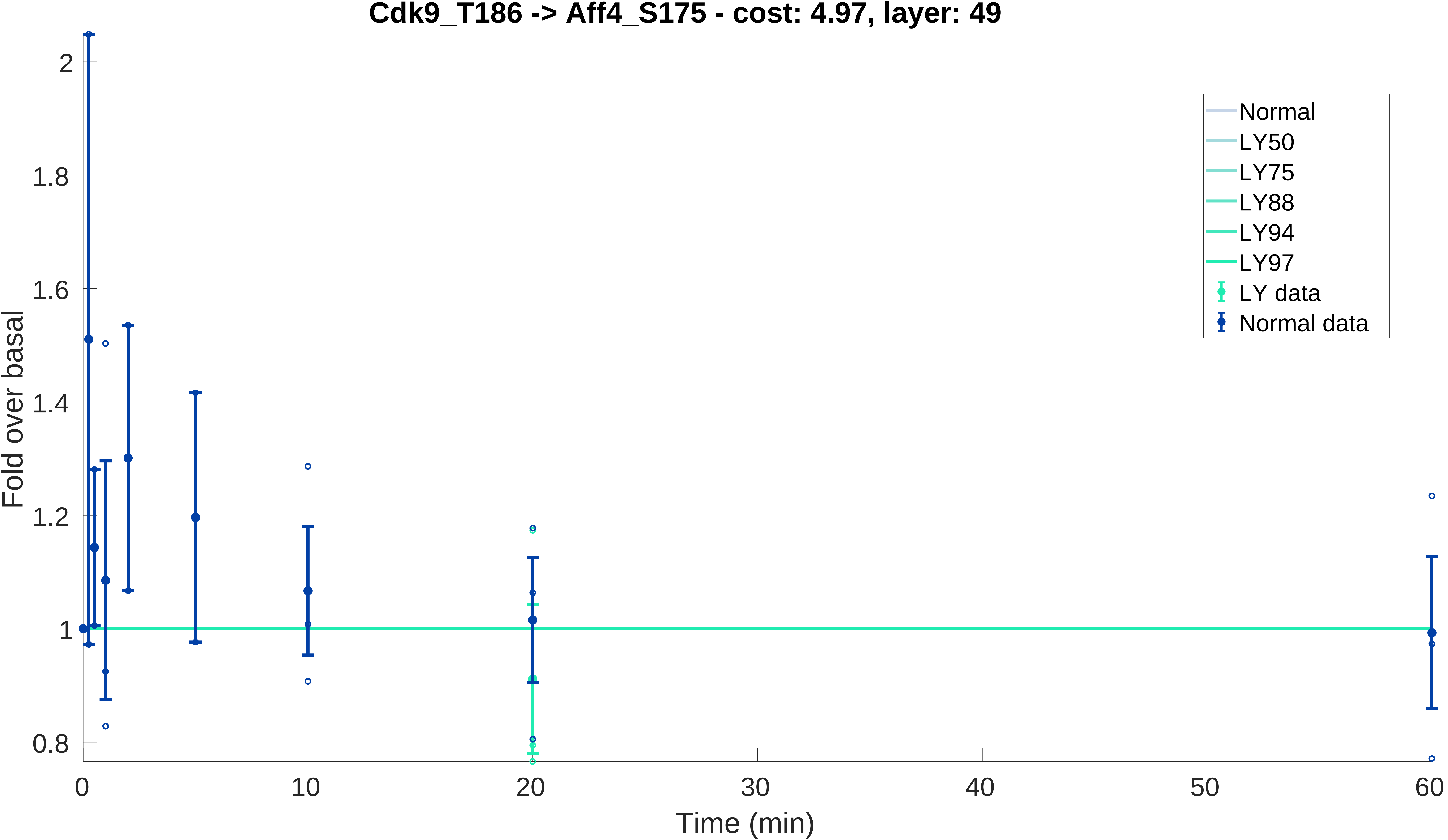

Cdk9_T186 -> Aff4_S175 - cost: 4.97, layer: 49
Fold over basal
Time (min)
0.8
1
1.2
1.4
1.6
1.8
2
0
10
20
30
40
50
60
Normal
LY50
LY75
LY88
LY94
LY97
LY data
Normal data

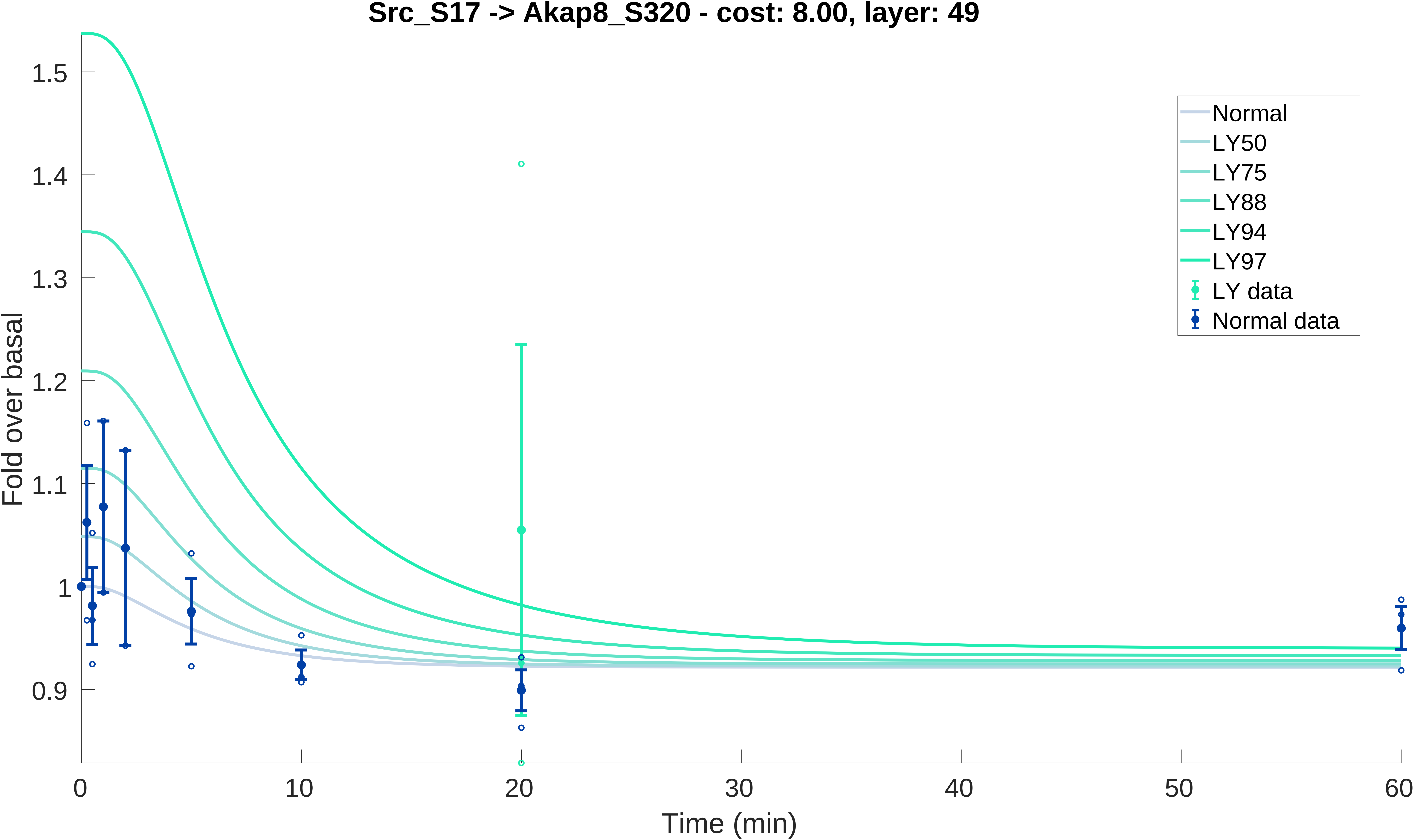
Src_S17 -> Akap8_S320 - cost: 8.00, layer: 49
Fold over basal
Time (min)
0.9
1
1.1
1.2
1.3
1.4
1.5
0
10
20
30
40
50
60
Normal
LY50
LY75
LY88
LY94
LY97
LY data
Normal data

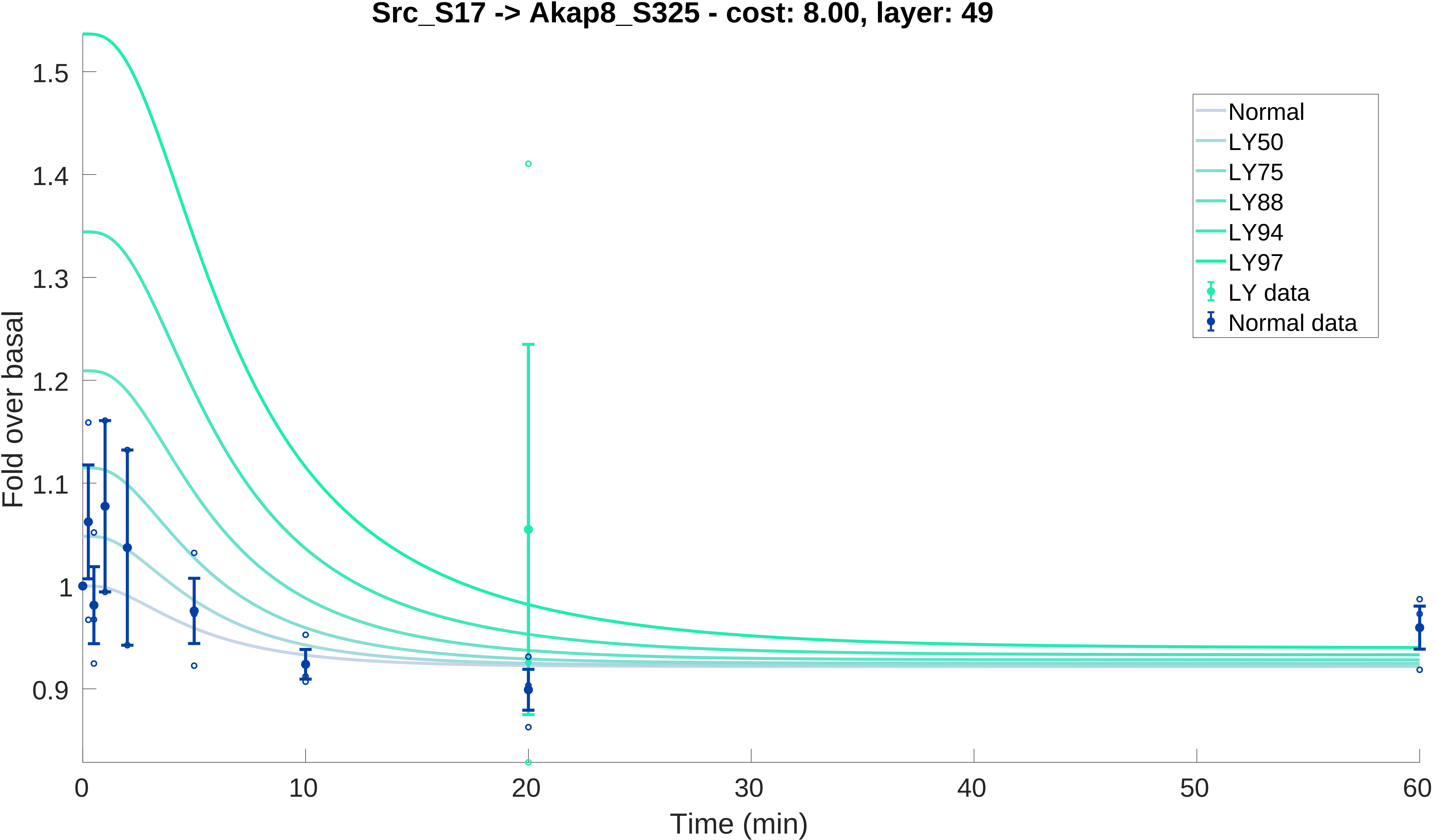

Src_S17 -> Akap8_S325 - cost: 8.00, layer: 49
Fold over basal
Time (min)
1.5
1.4
1.3
1.2
1.1
1
0.9
0
10
20
30
40
50
60
Normal
LY50
LY75
LY88
LY94
LY97
LY data
Normal data

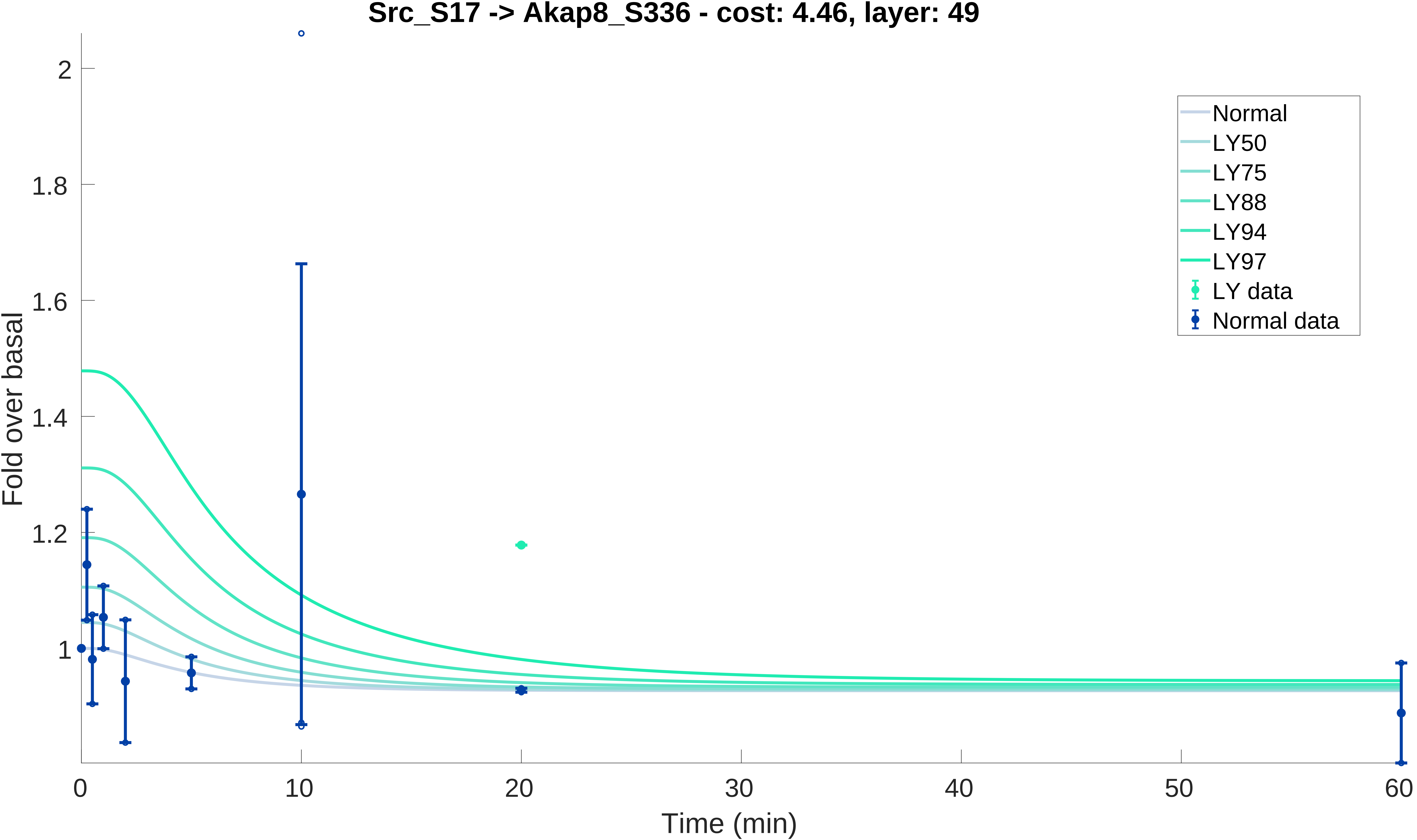

Src_S17 -> Akap8_S336 - cost: 4.46, layer: 49
Fold over basal
Time (min)
Normal
LY50
LY75
LY88
LY94
LY97
LY data
Normal data
2
1.8
1.6
1.4
1.2
1
0
10
20
30
40
50
60

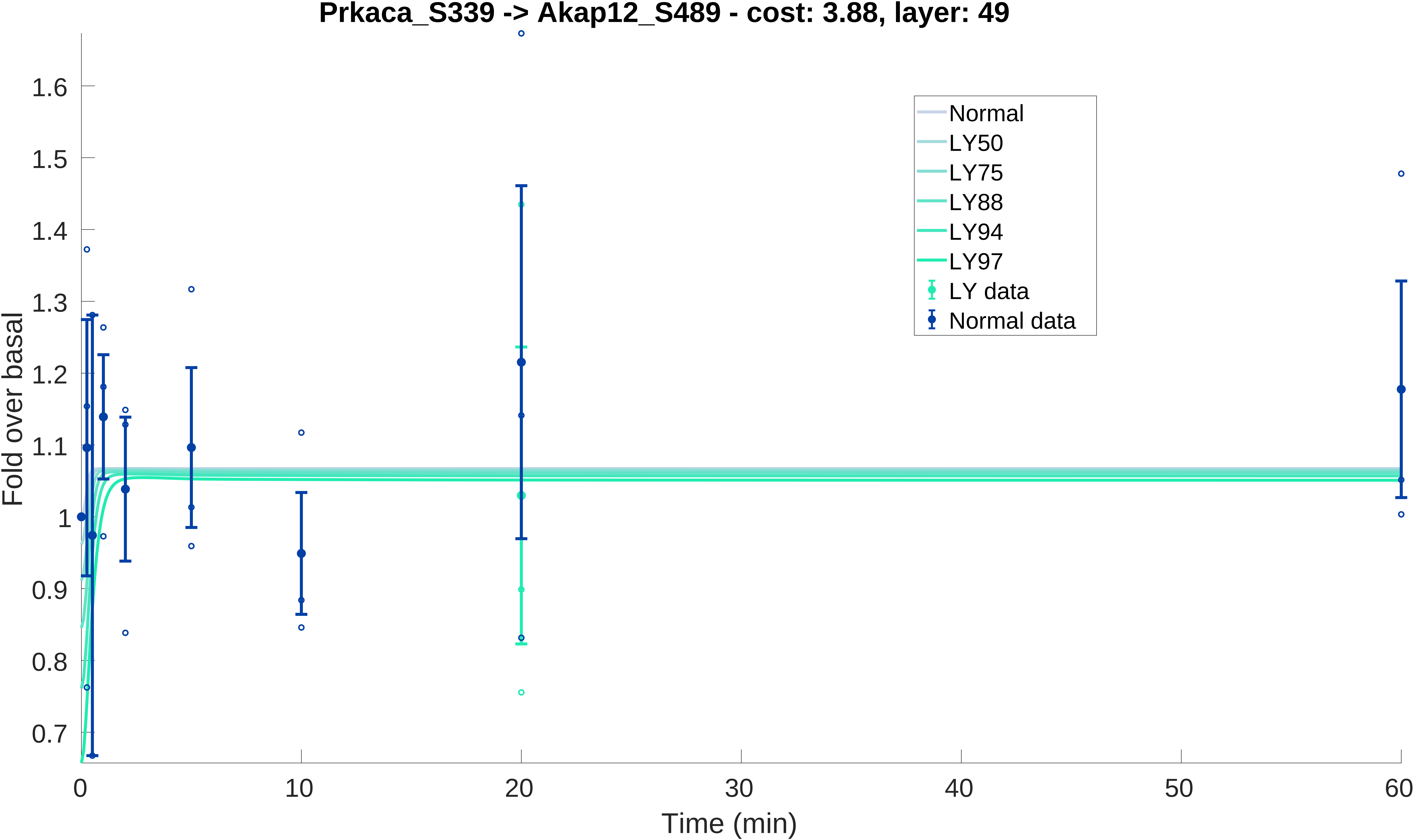

Prkaca_S339 -> Akap12_S489 - cost: 3.88, layer: 49
Fold over basal
1.6
1.5
1.4
1.3
1.2
1.1
1
0.9
0.8
0.7
0
10
20
30
40
50
60
Time (min)
Normal
LY50
LY75
LY88
LY94
LY97
LY data
Normal data

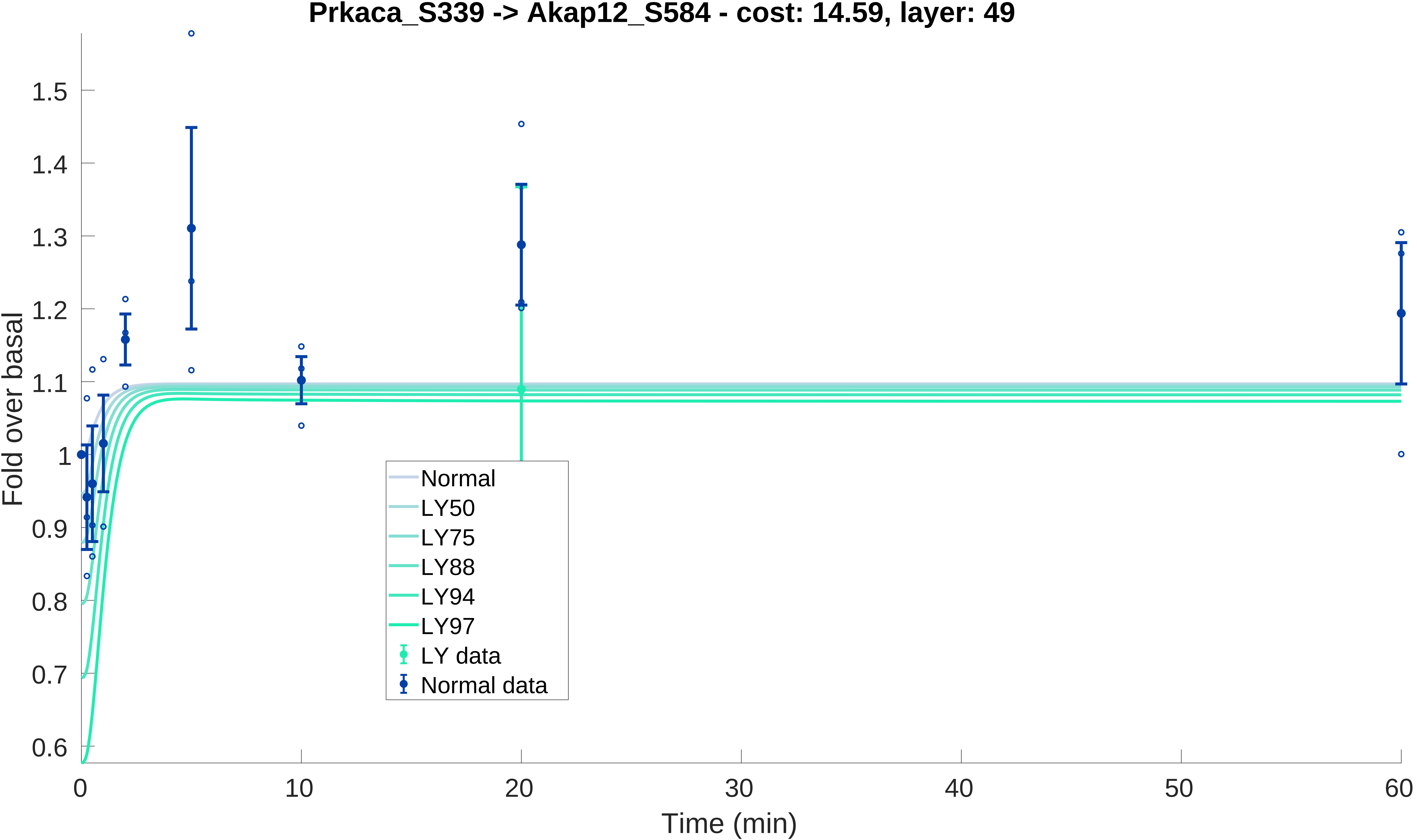

Prkaca_S339 -> Akap12_S584 - cost: 14.59, layer: 49
Fold over basal
Time (min)
0.6
0.7
0.8
0.9
1
1.1
1.2
1.3
1.4
1.5
0
10
20
30
40
50
60
Normal
LY50
LY75
LY88
LY94
LY97
LY data
Normal data

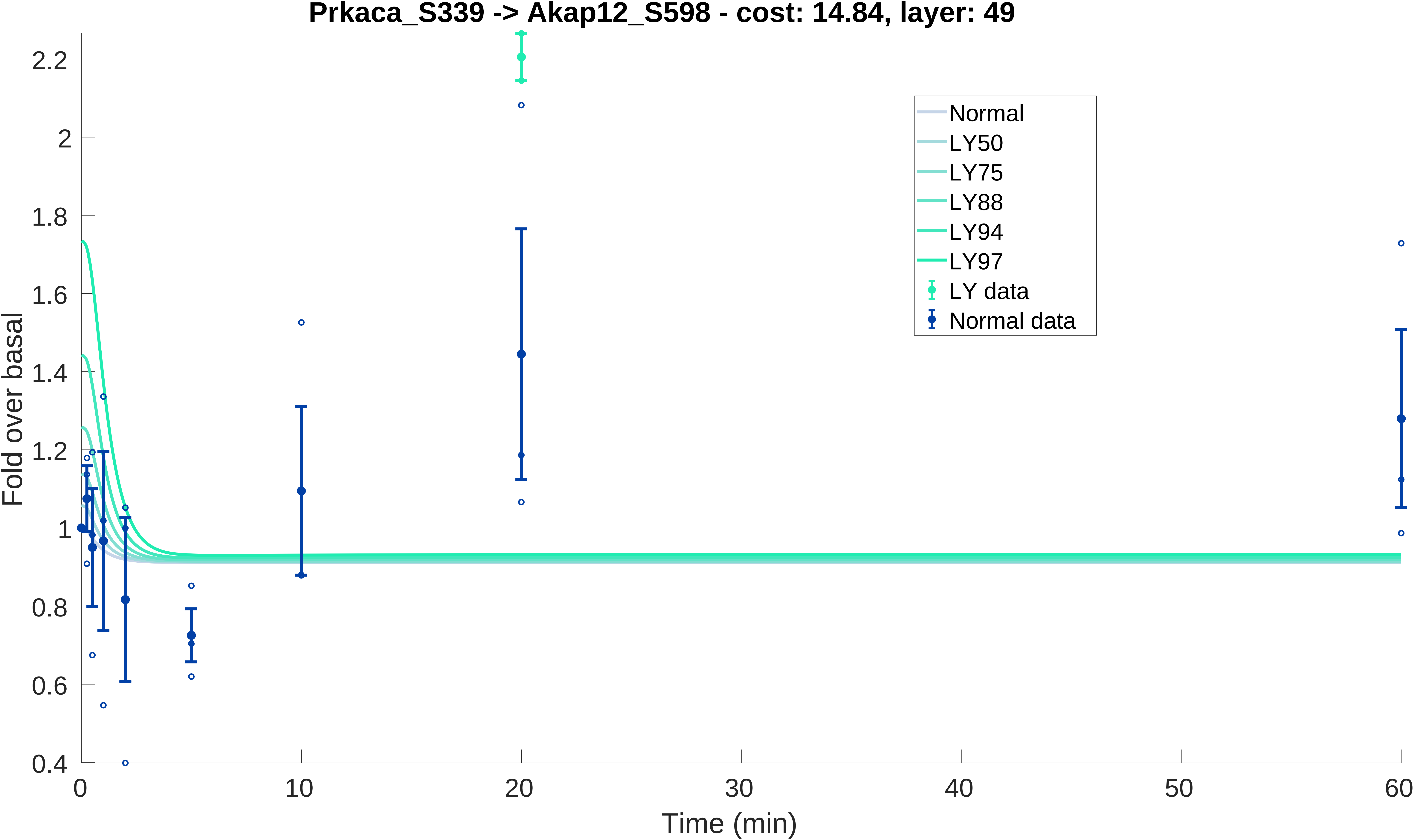
Prkaca_S339 -> Akap12_S598 - cost: 14.84, layer: 49
Fold over basal
Time (min)
0
10
20
30
40
50
60
0.4
0.6
0.8
1
1.2
1.4
1.6
1.8
2
2.2
Normal
LY50
LY75
LY88
LY94
LY97
LY data
Normal data

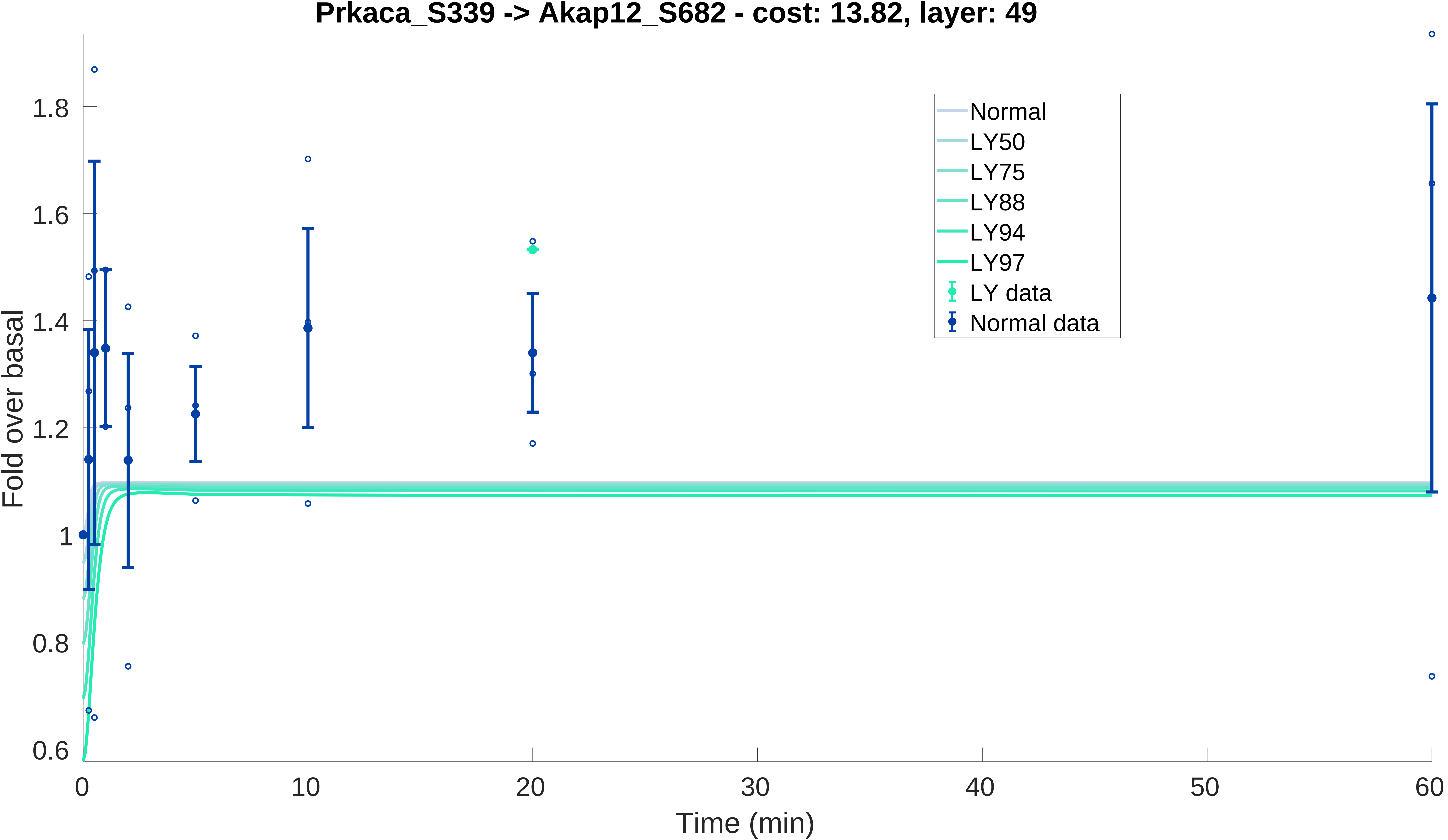

Prkaca_S339 -> Akap12_S682 - cost: 13.82, layer: 49
Fold over basal
Time (min)
0.6
0.8
1
1.2
1.4
1.6
1.8
0
10
20
30
40
50
60
Normal
LY50
LY75
LY88
LY94
LY97
LY data
Normal data

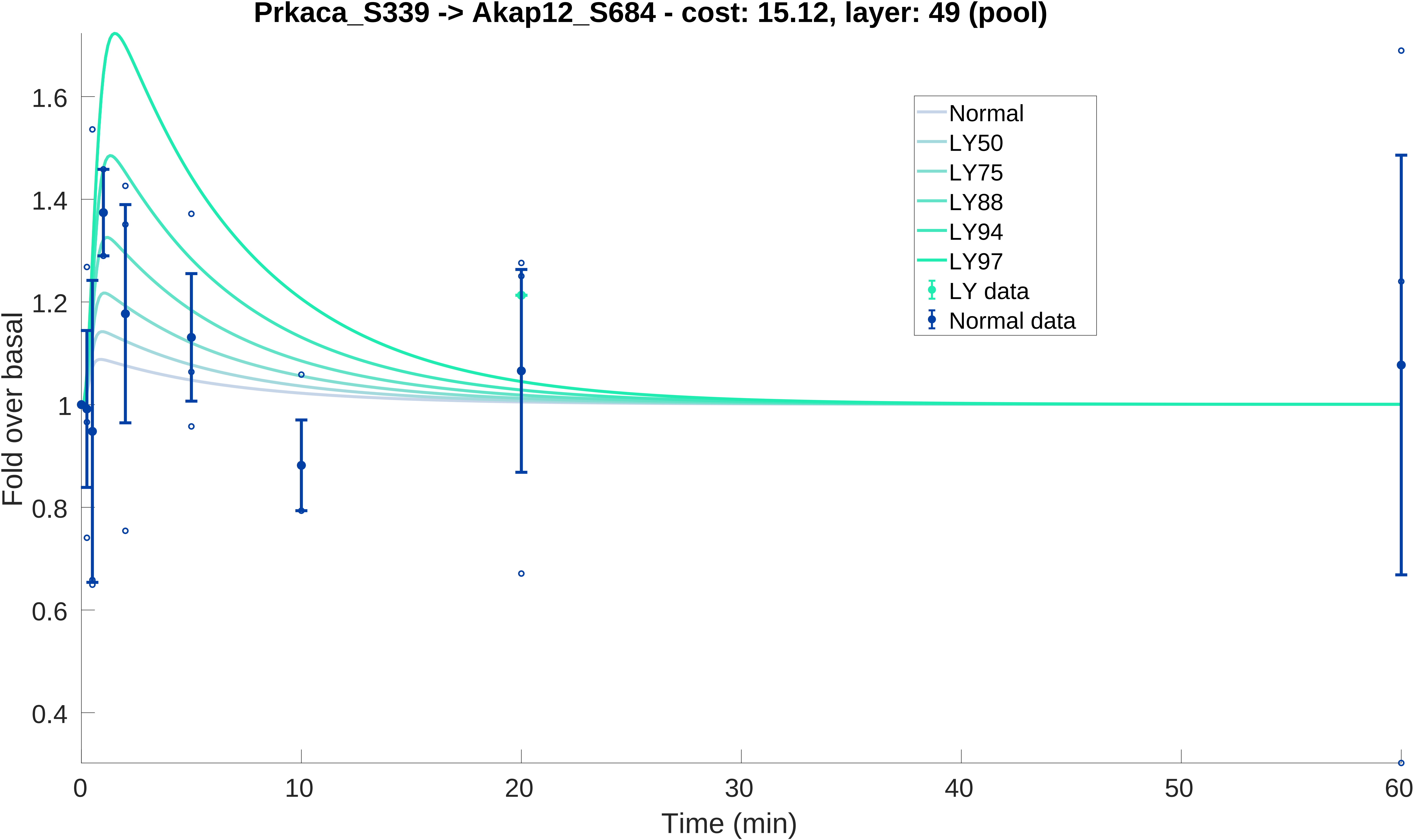
Prkaca_S339 -> Akap12_S684 - cost: 15.12, layer: 49 (pool)
Normal
LY50
LY75
LY88
LY94
LY97
LY data
Normal data
Fold over basal
Time (min)
1.6
1.4
1.2
1
0.8
0.6
0.4
0
10
20
30
40
50
60

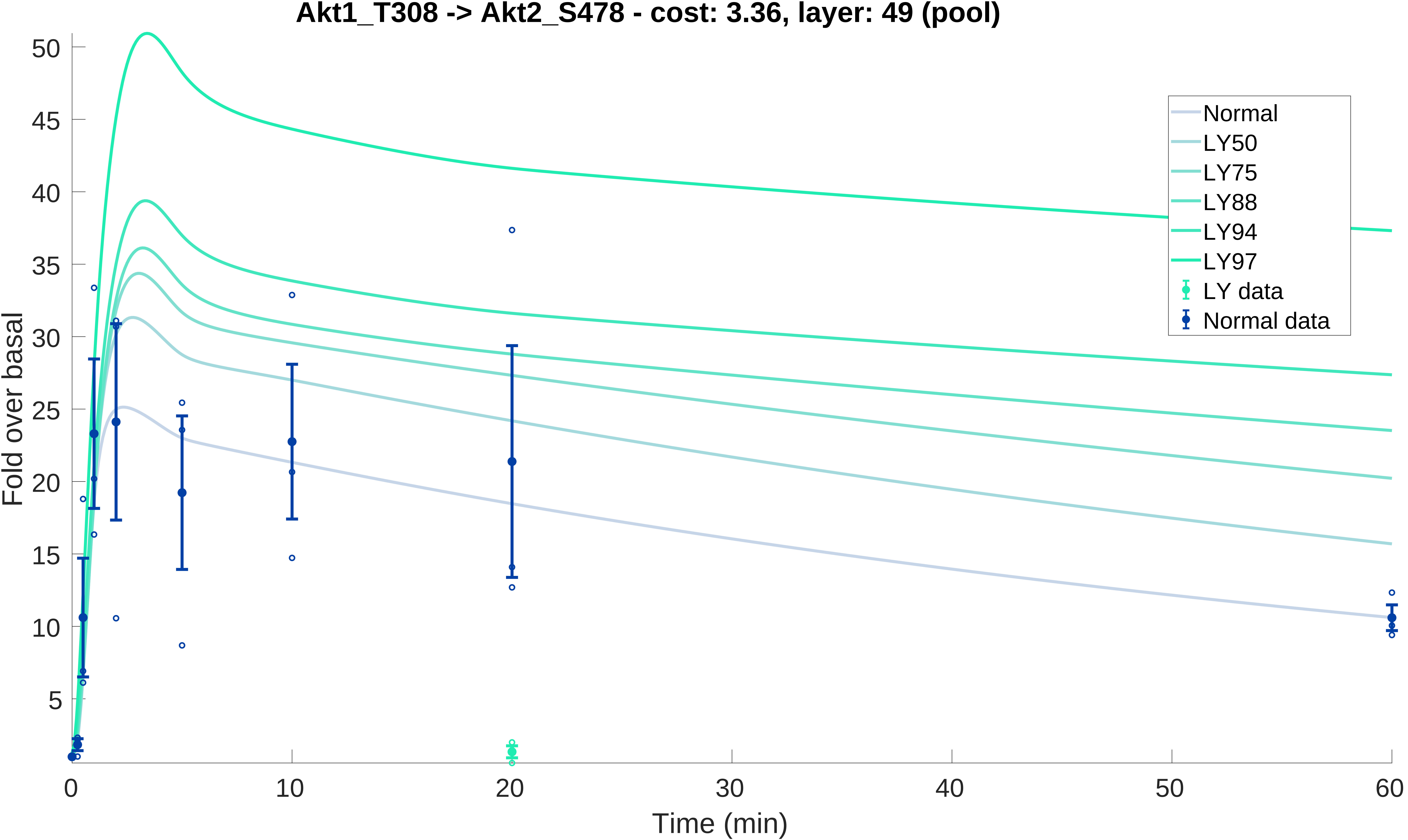

Akt1_T308 -> Akt2_S478 - cost: 3.36, layer: 49 (pool)
Fold over basal
Time (min)
Normal
LY50
LY75
LY88
LY94
LY97
LY data
Normal data
0
10
20
30
40
50
60
5
10
15
20
25
30
35
40
45
50

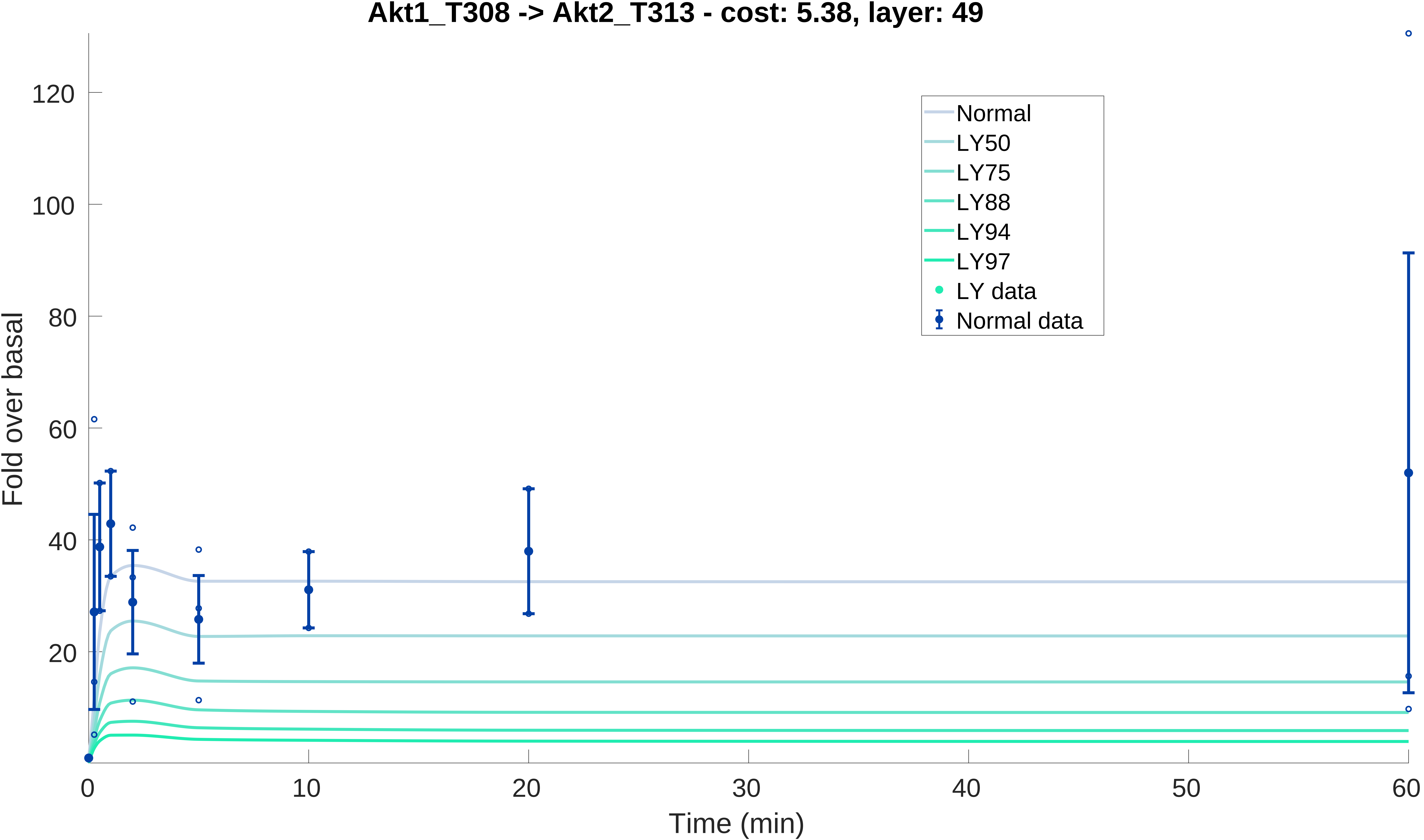

Akt1_T308 -> Akt2_T313 - cost: 5.38, layer: 49
Fold over basal
Time (min)
0
10
20
30
40
50
60
20
40
60
80
100
120
Normal
LY50
LY75
LY88
LY94
LY97
LY data
Normal data

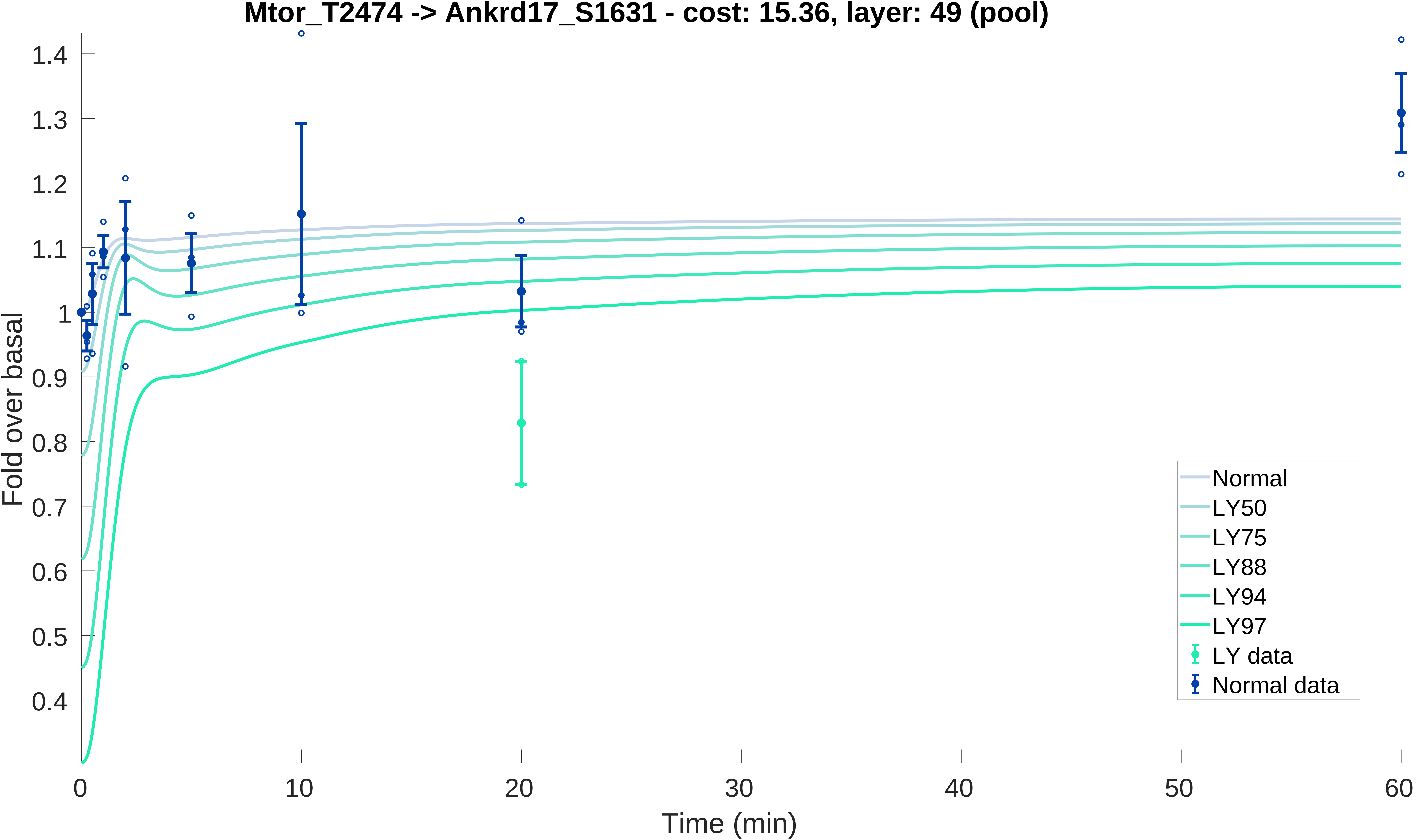
Mtor_T2474 -> Ankrd17_S1631 - cost: 15.36, layer: 49 (pool)
Fold over basal
Time (min)
0
10
20
30
40
50
60
0.4
0.5
0.6
0.7
0.8
0.9
1
1.1
1.2
1.3
1.4
Normal
LY50
LY75
LY88
LY94
LY97
LY data
Normal data

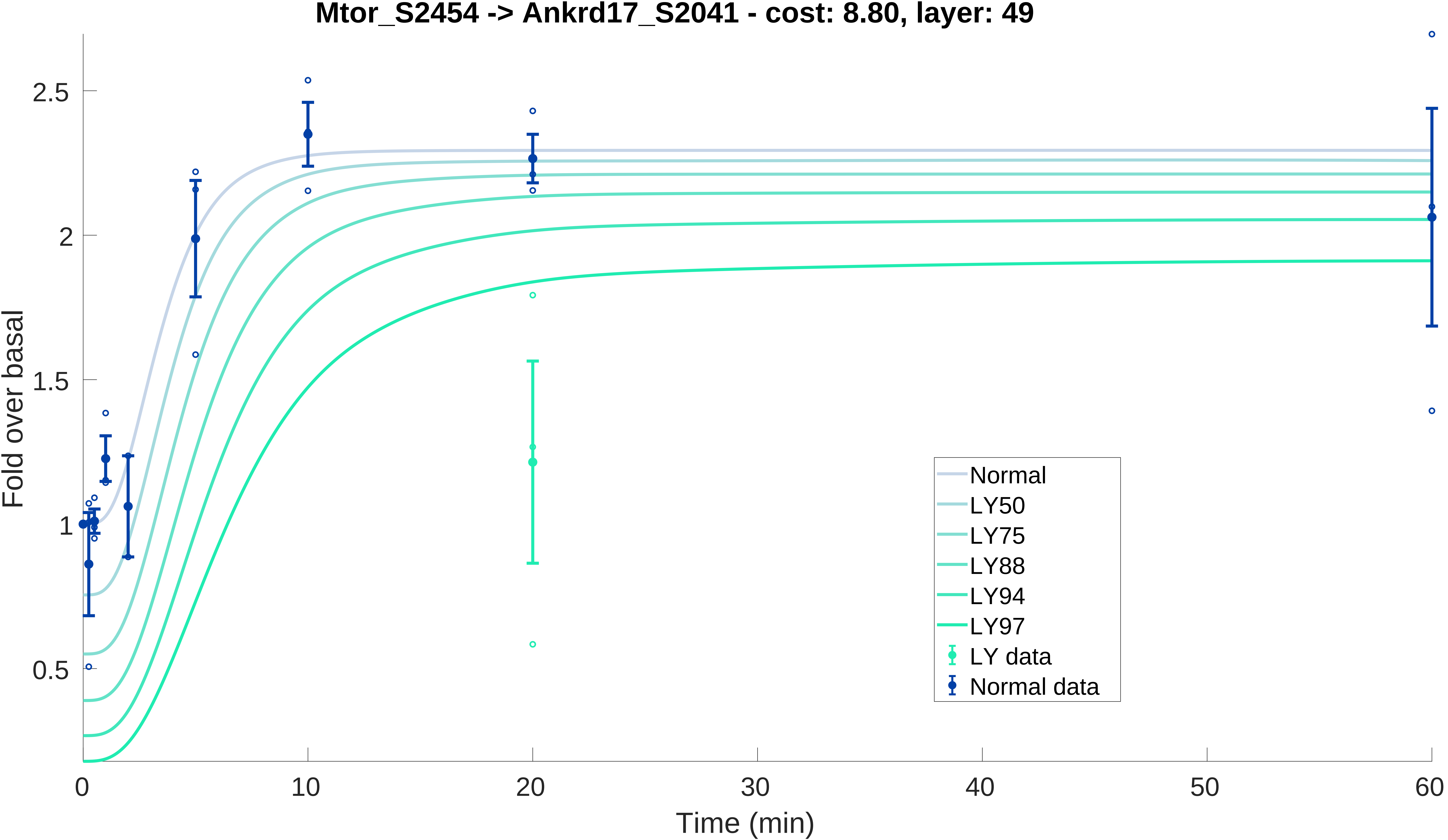

Mtor_S2454 -> Ankrd17_S2041 - cost: 8.80, layer: 49
Fold over basal
Time (min)
0.5
1
1.5
2
2.5
0
10
20
30
40
50
60
Normal
LY50
LY75
LY88
LY94
LY97
LY data
Normal data

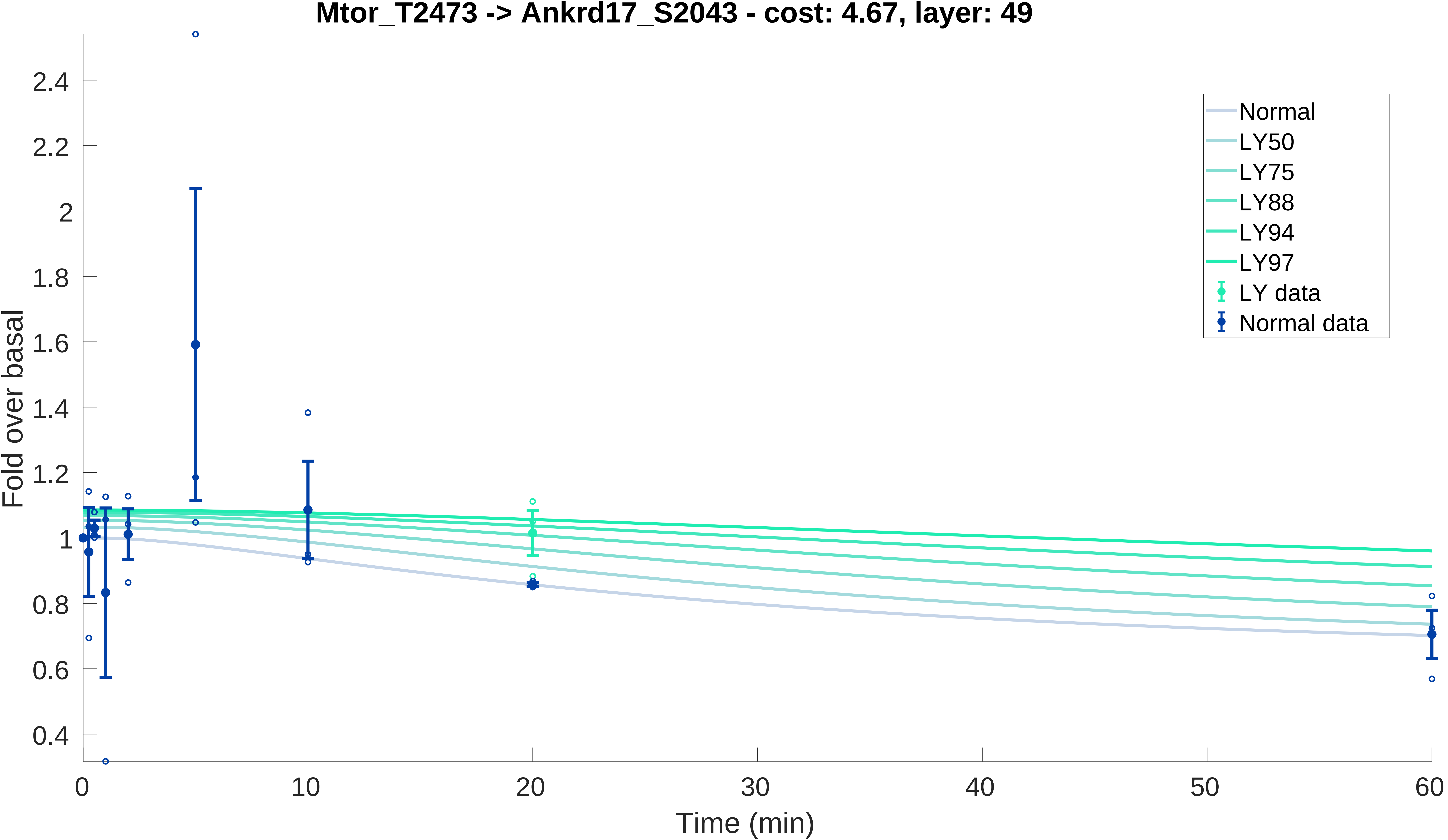

Mtor_T2473 -> Ankrd17_S2043 - cost: 4.67, layer: 49
Fold over basal
Time (min)
0.4
0.6
0.8
1
1.2
1.4
1.6
1.8
2
2.2
2.4
0
10
20
30
40
50
60
Normal
LY50
LY75
LY88
LY94
LY97
LY data
Normal data

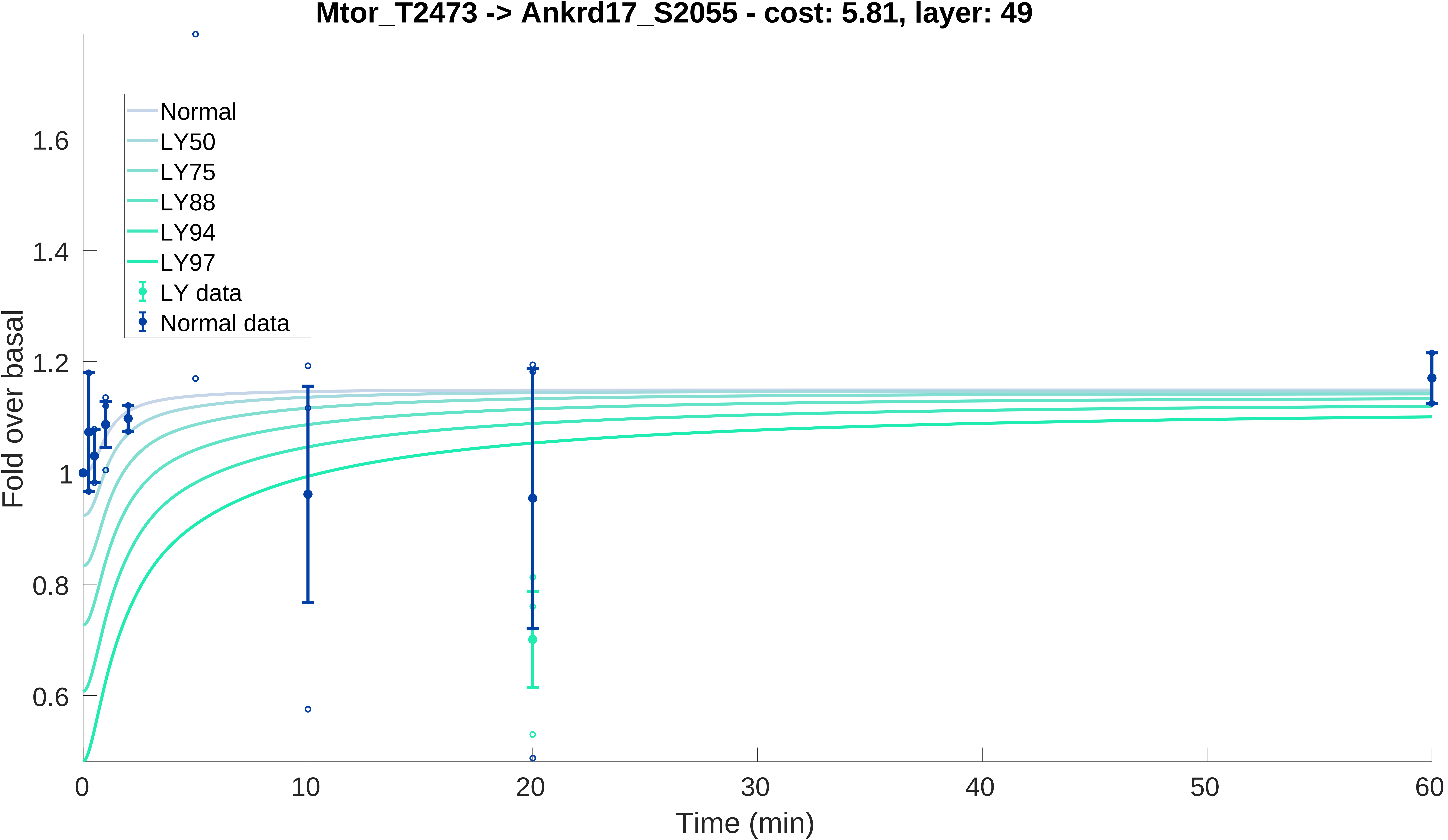

Mtor_T2473 -> Ankrd17_S2055 - cost: 5.81, layer: 49
Normal
LY50
LY75
LY88
LY94
LY97
LY data
Normal data
Fold over basal
1.6
1.4
1.2
1
0.8
0.6
0
10
20
30
40
50
60
Time (min)

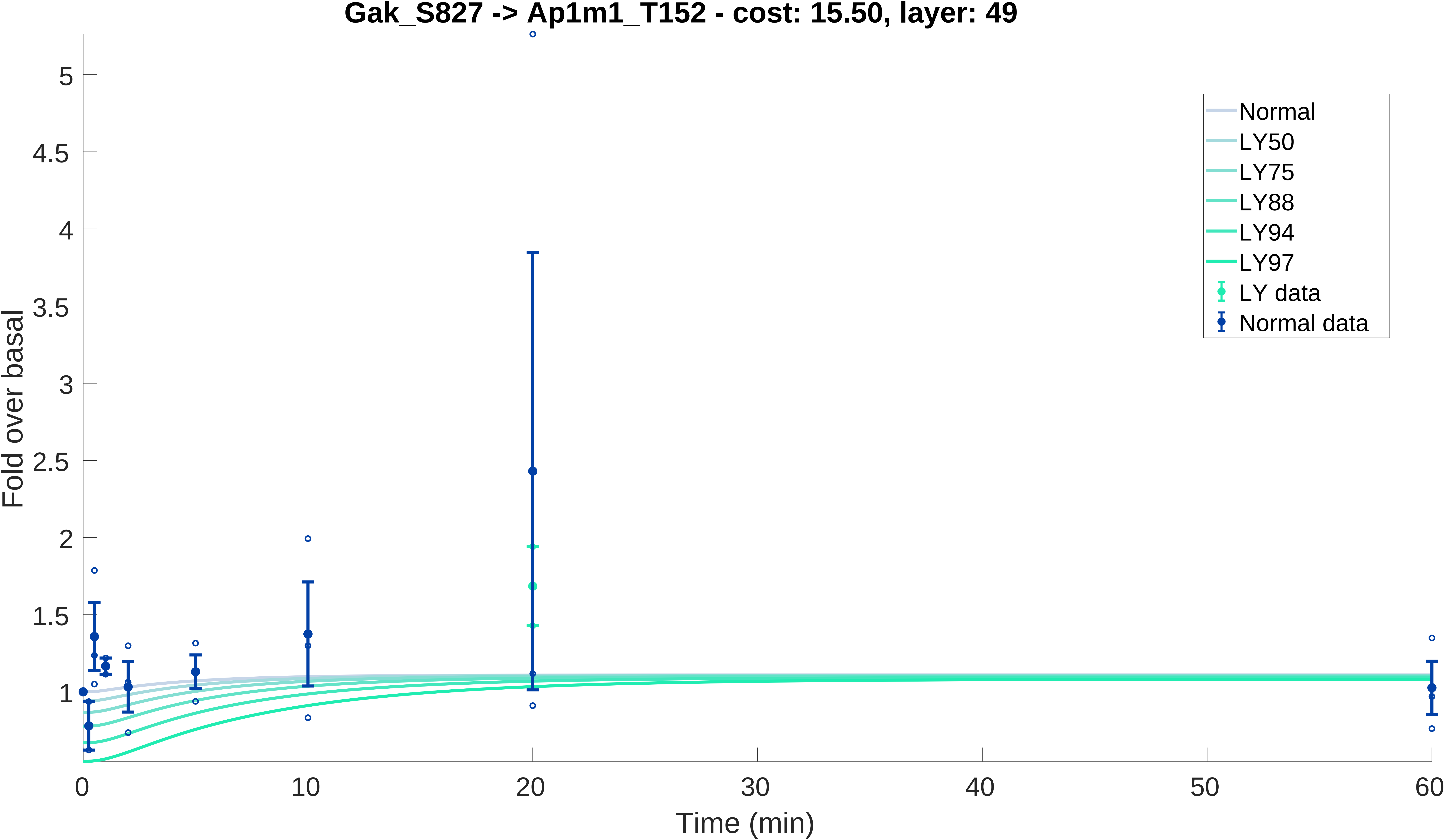

Gak_S827 -> Ap1m1_T152 - cost: 15.50, layer: 49
Fold over basal
5
4.5
4
3.5
3
2.5
2
1.5
1
0
10
20
30
40
50
60
Time (min)
Normal
LY50
LY75
LY88
LY94
LY97
LY data
Normal data

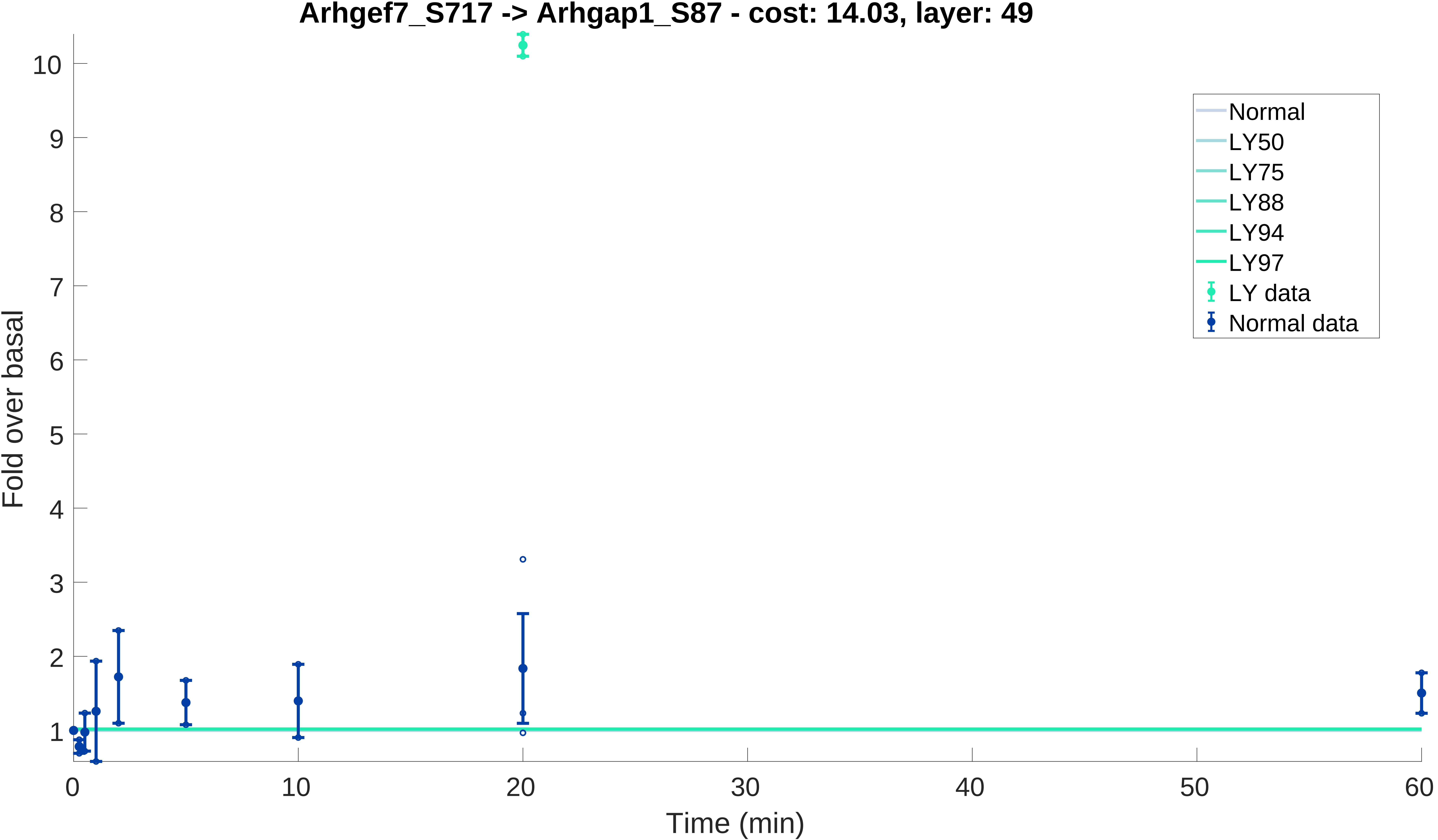

Arhgef7_S717 -> Arhgap1_S87 - cost: 14.03, layer: 49
Fold over basal
Time (min)
Normal
LY50
LY75
LY88
LY94
LY97
LY data
Normal data
0
10
20
30
40
50
60
1
2
3
4
5
6
7
8
9
10

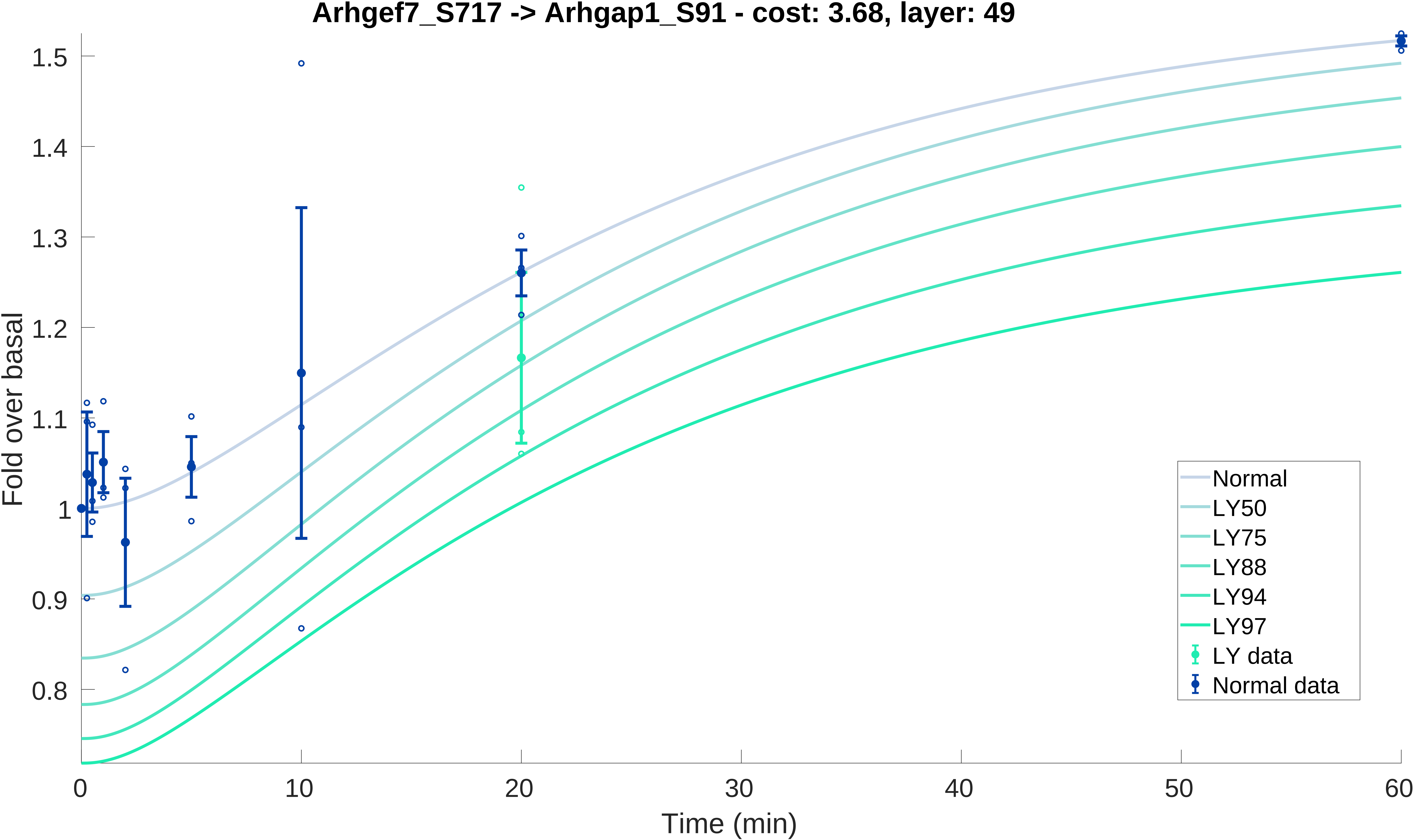

Arhgef7_S717 -> Arhgap1_S91 - cost: 3.68, layer: 49
Fold over basal
1.5
1.4
1.3
1.2
1.1
1
0.9
0.8
0
10
20
30
40
50
60
Time (min)
Normal
LY50
LY75
LY88
LY94
LY97
LY data
Normal data

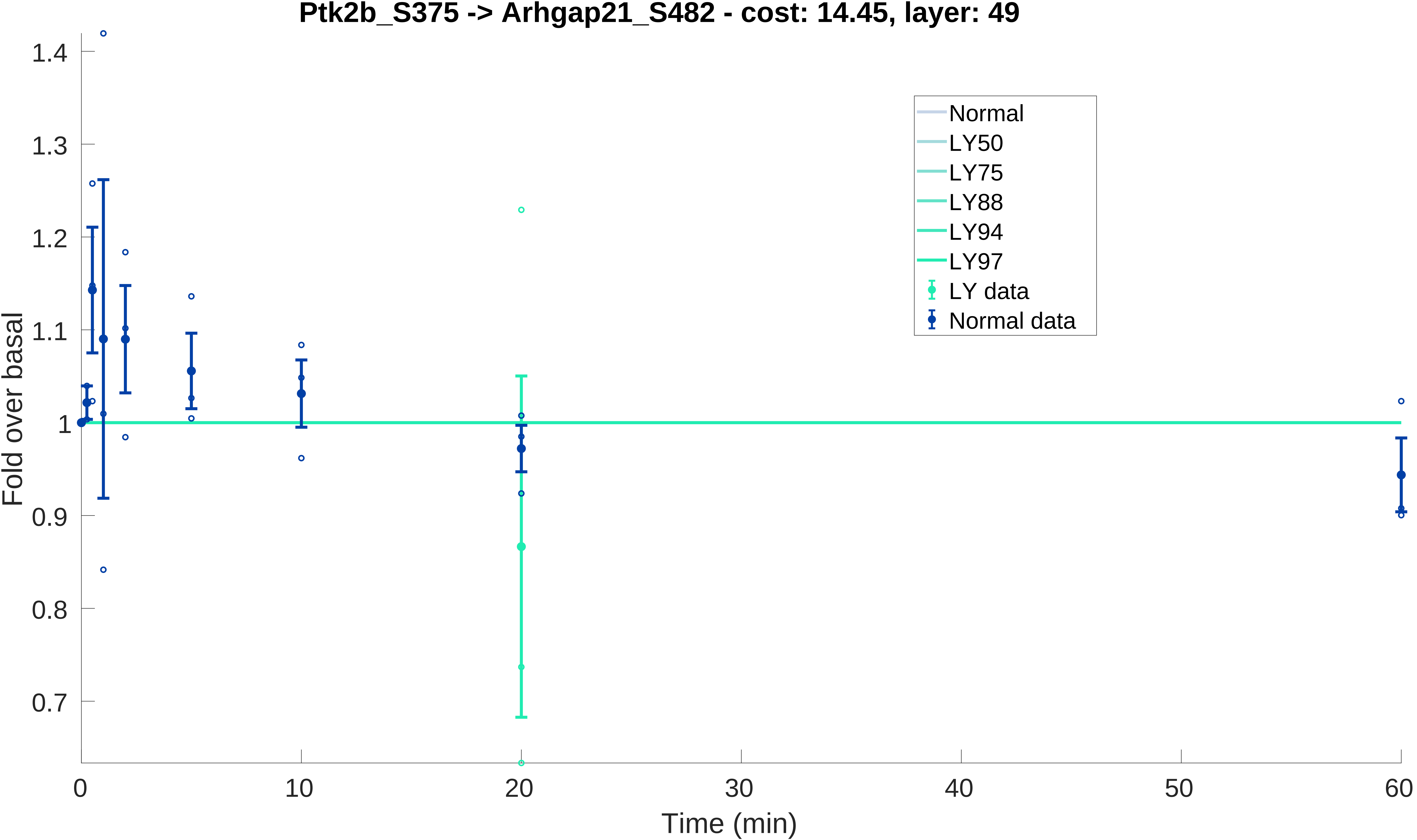

Ptk2b_S375 -> Arhgap21_S482 - cost: 14.45, layer: 49
Fold over basal
1.4
1.3
1.2
1.1
1
0.9
0.8
0.7
Time (min)
0
10
20
30
40
50
60
Normal
LY50
LY75
LY88
LY94
LY97
LY data
Normal data

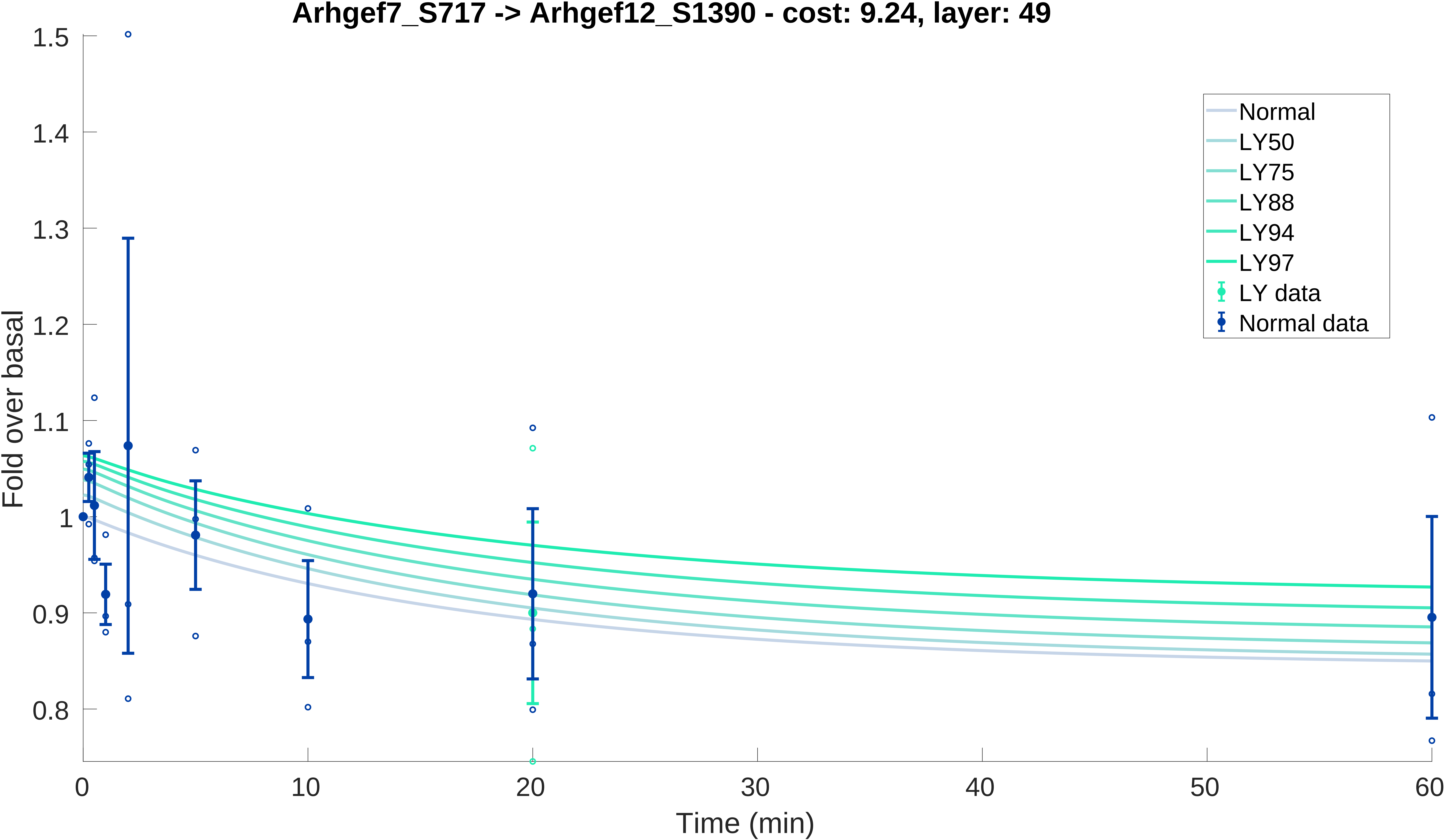
Arhgef7_S717 -> Arhgef12_S1390 - cost: 9.24, layer: 49
Fold over basal
Time (min)
1.5
1.4
1.3
1.2
1.1
1
0.9
0.8
0
10
20
30
40
50
60
Normal
LY50
LY75
LY88
LY94
LY97
LY data
Normal data

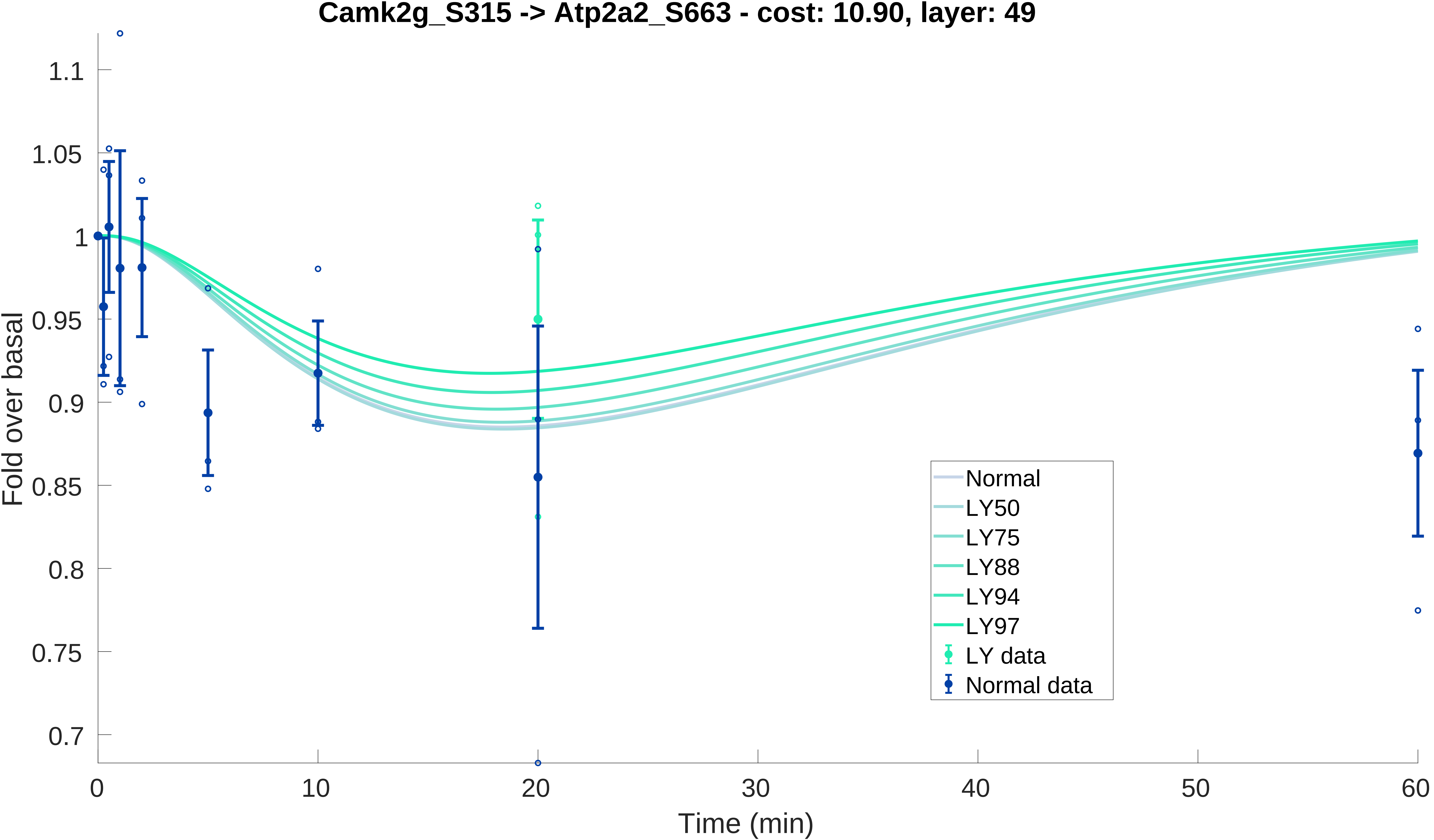
Camk2g_S315 -> Atp2a2_S663 - cost: 10.90, layer: 49
Fold over basal
1.1
1.05
1
0.95
0.9
0.85
0.8
0.75
0.7
0
10
20
30
40
50
60
Time (min)
Normal
LY50
LY75
LY88
LY94
LY97
LY data
Normal data

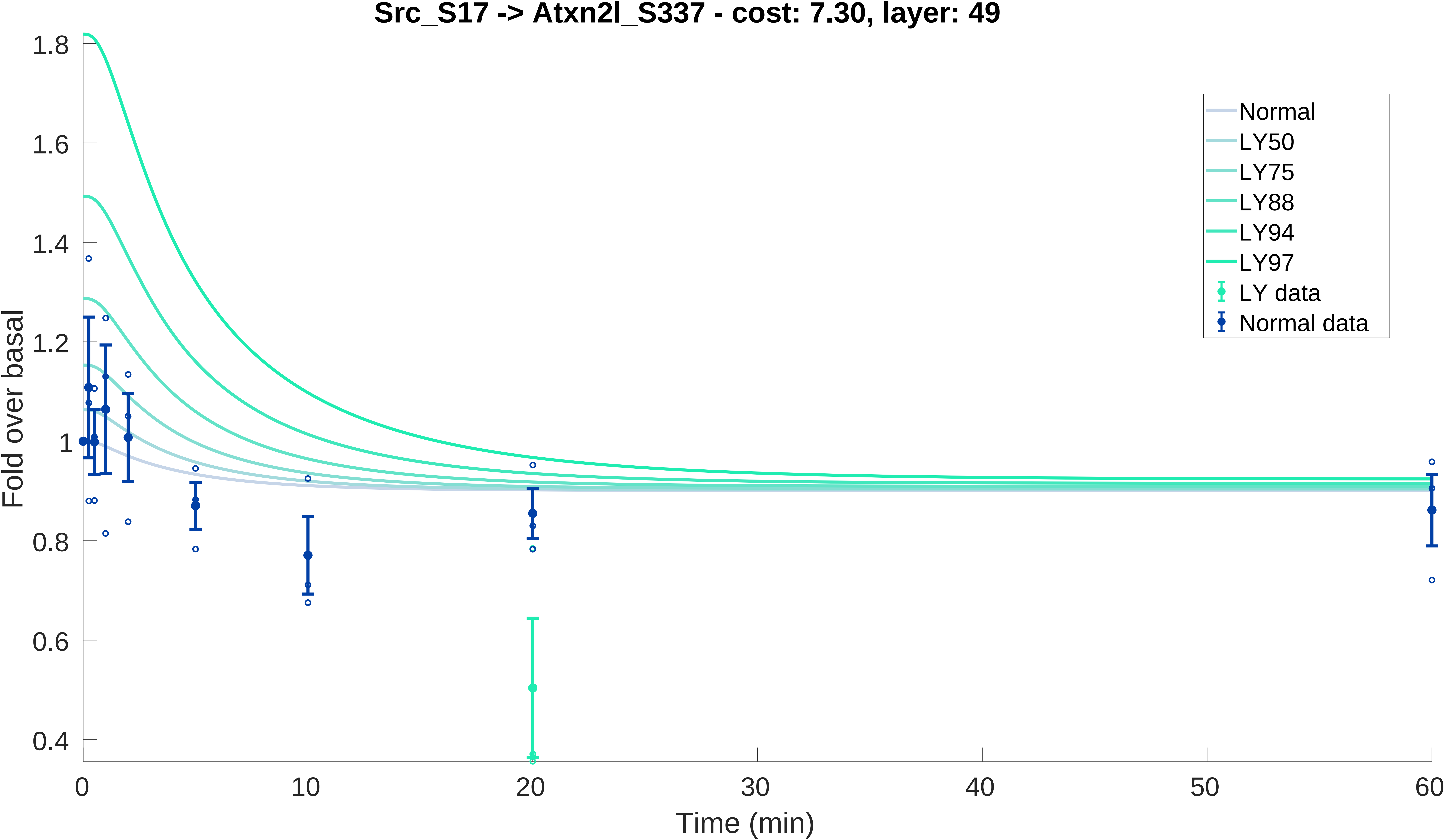

Src_S17 -> Atxn2l_S337 - cost: 7.30, layer: 49
Fold over basal
1.8
1.6
1.4
1.2
1
0.8
0.6
0.4
0
10
20
30
40
50
60
Time (min)
Normal
LY50
LY75
LY88
LY94
LY97
LY data
Normal data

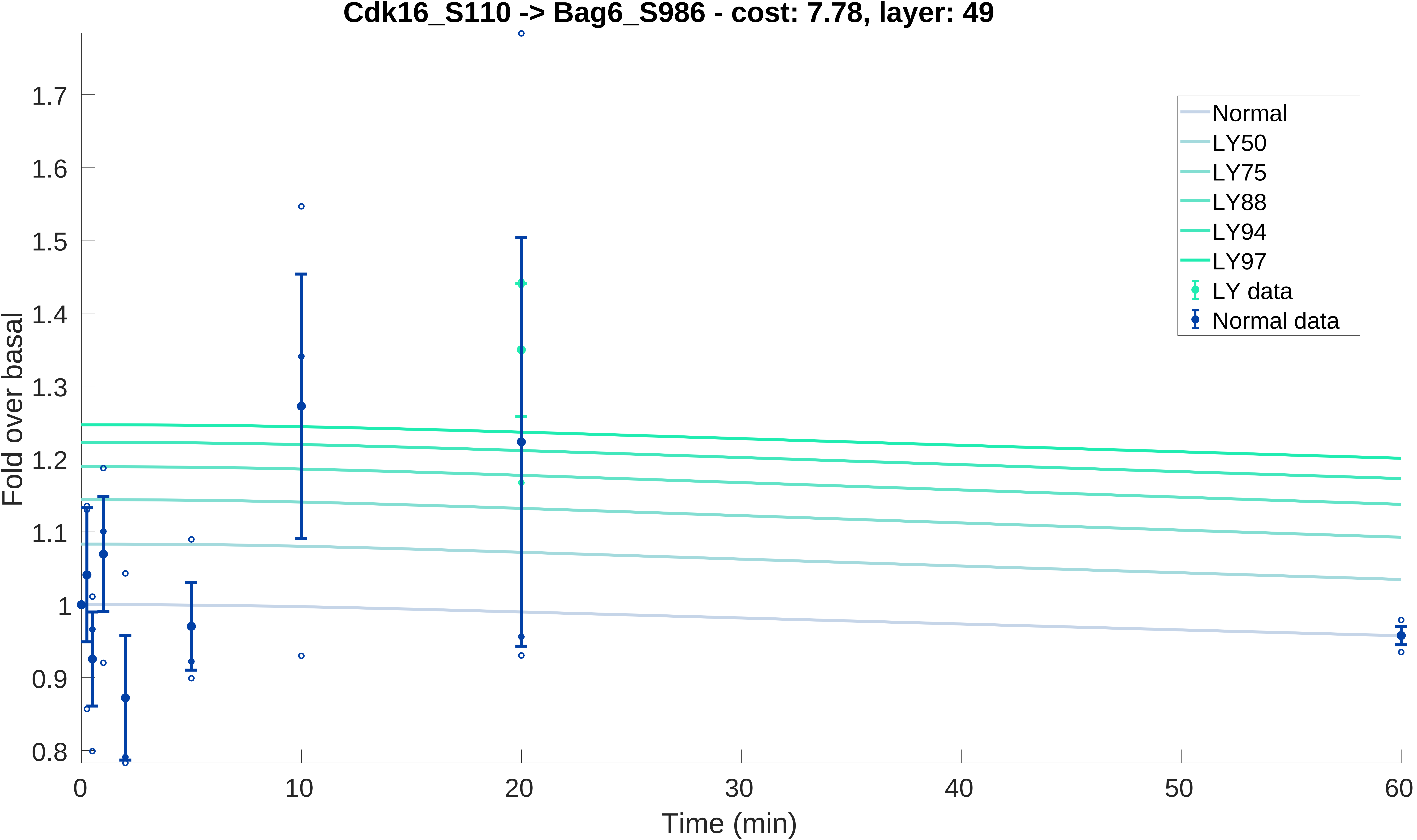

Cdk16_S110 -> Bag6_S986 - cost: 7.78, layer: 49
Fold over basal
Time (min)
0.8
0.9
1
1.1
1.2
1.3
1.4
1.5
1.6
1.7
0
10
20
30
40
50
60
Normal
LY50
LY75
LY88
LY94
LY97
LY data
Normal data

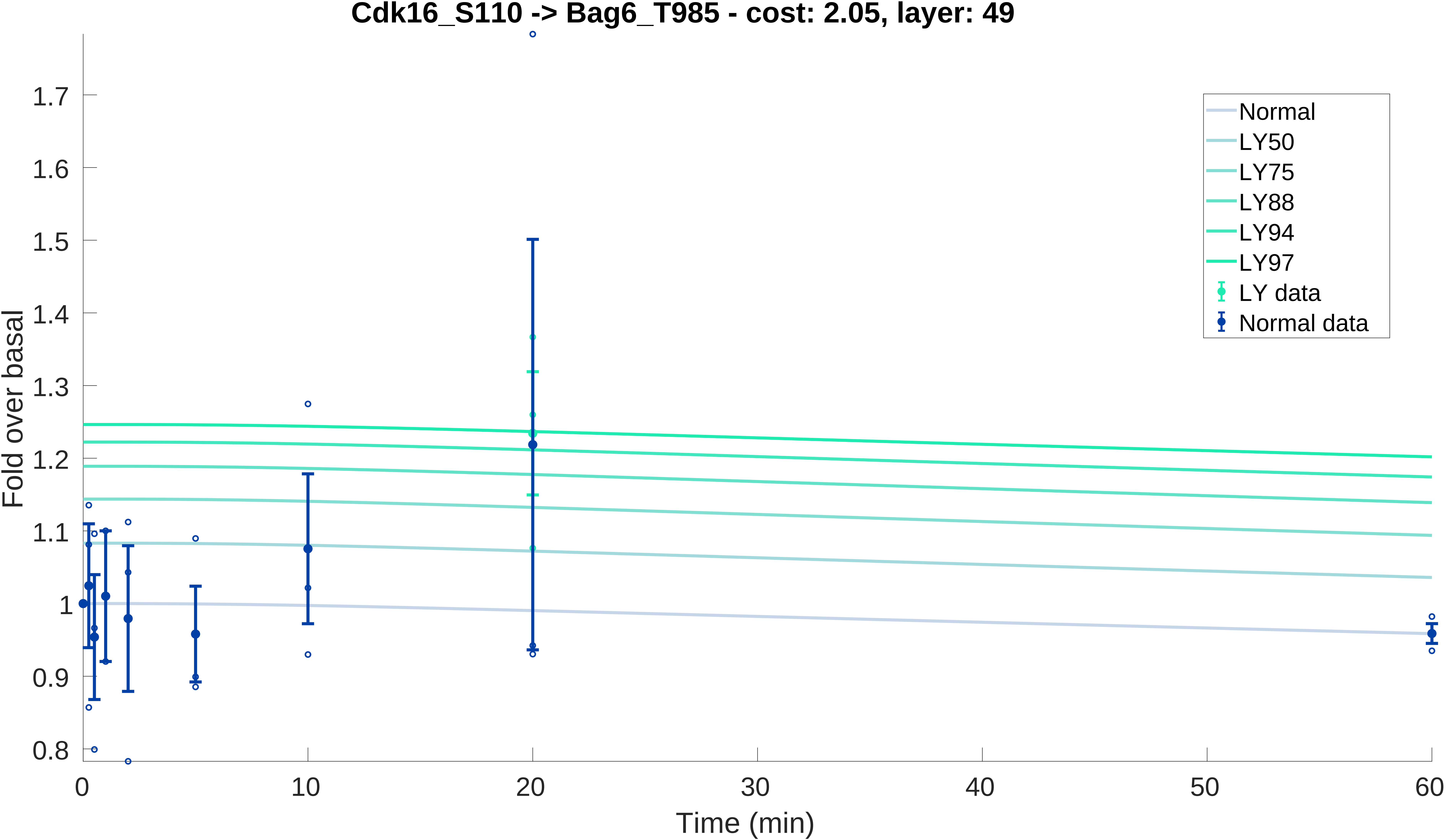

Cdk16_S110 -> Bag6_T985 - cost: 2.05, layer: 49
Fold over basal
Time (min)
1.7
1.6
1.5
1.4
1.3
1.2
1.1
1
0.9
0.8
0
10
20
30
40
50
60
Normal
LY50
LY75
LY88
LY94
LY97
LY data
Normal data

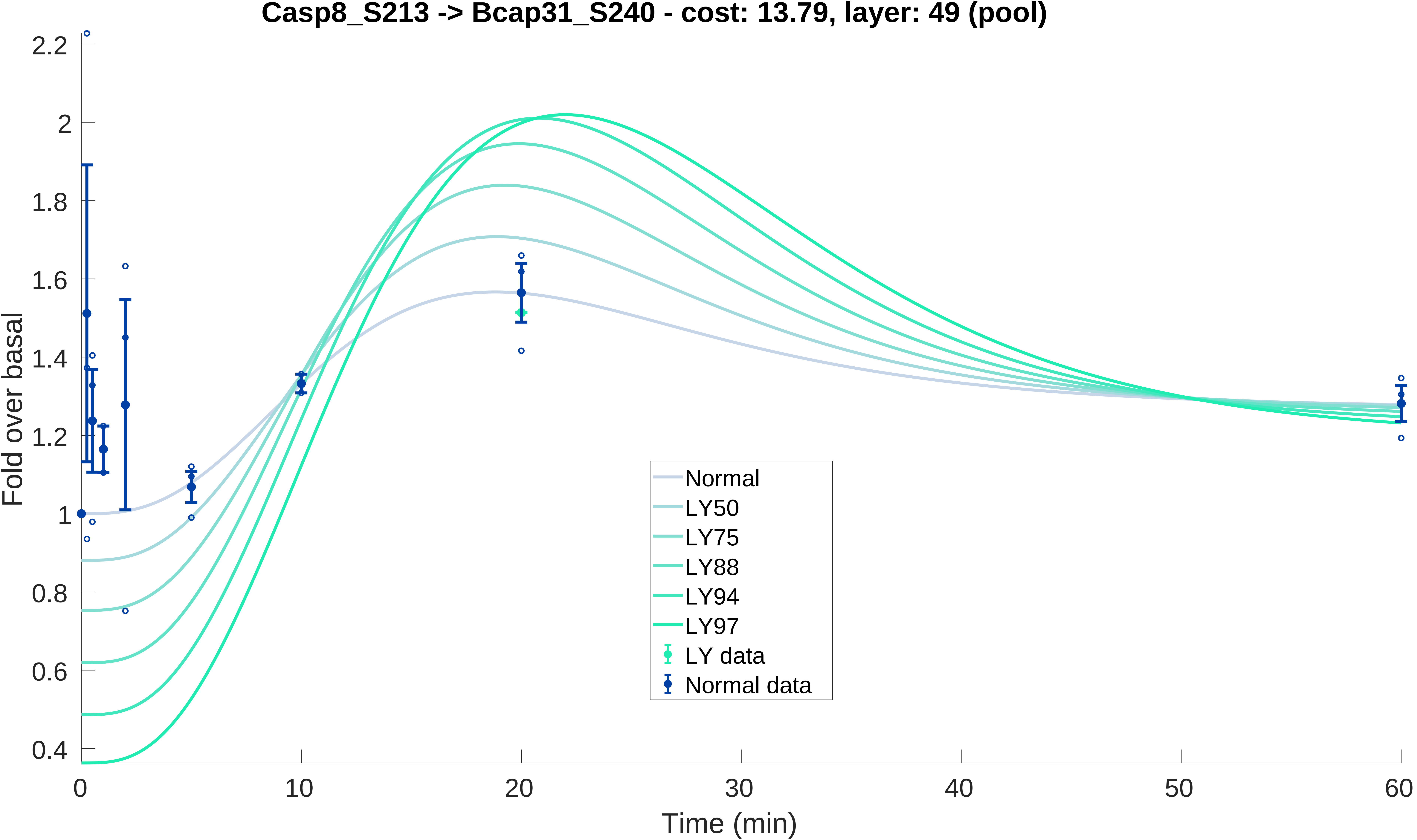

Casp8_S213 -> Bcap31_S240 - cost: 13.79, layer: 49 (pool)
Fold over basal
Time (min)
2.2
2
1.8
1.6
1.4
1.2
1
0.8
0.6
0.4
0
10
20
30
40
50
60
Normal
LY50
LY75
LY88
LY94
LY97
LY data
Normal data

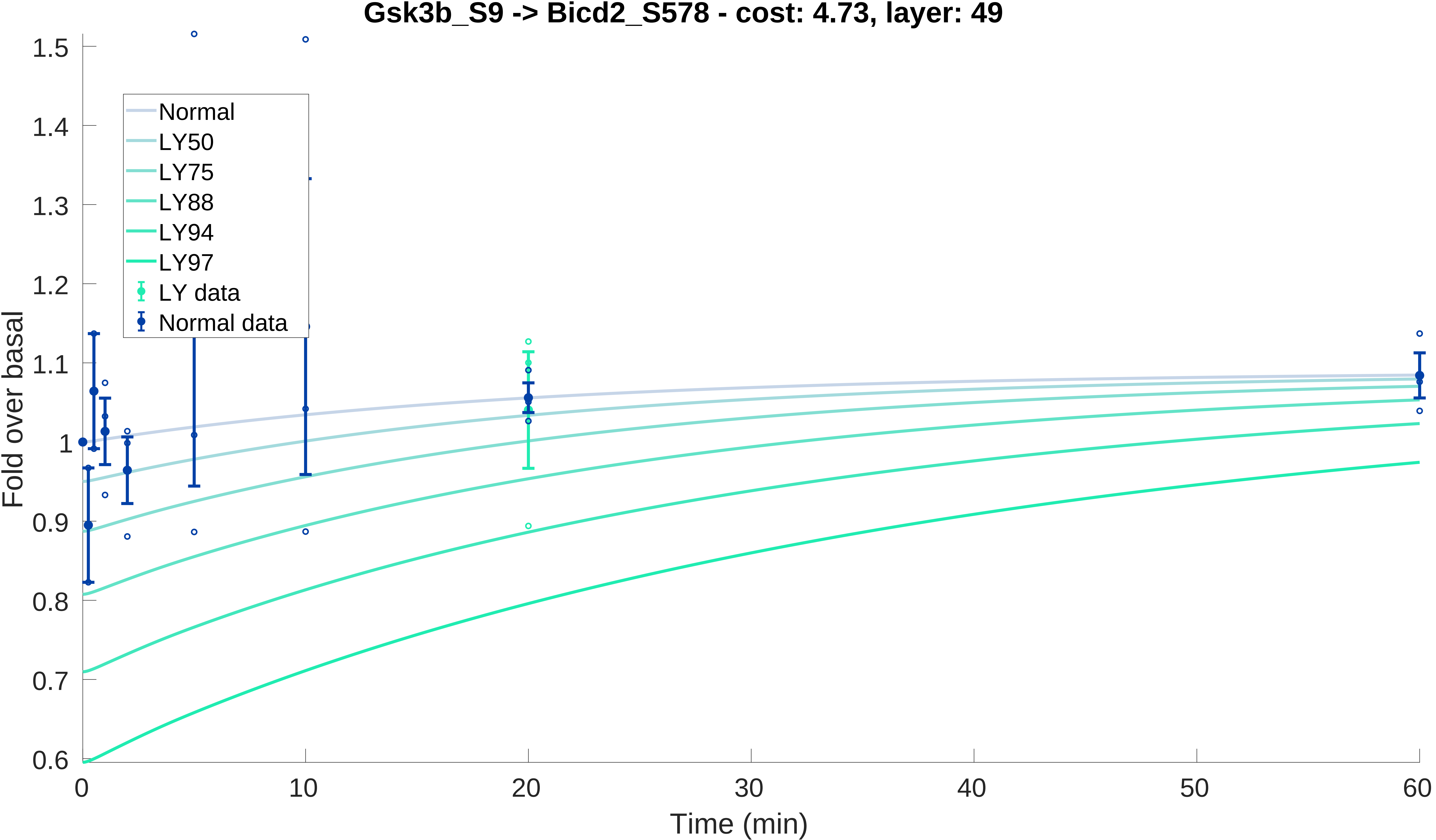
Gsk3b_S9 -> Bicd2_S578 - cost: 4.73, layer: 49
Normal
LY50
LY75
LY88
LY94
LY97
LY data
Normal data
Fold over basal
Time (min)
0.6
0.7
0.8
0.9
1
1.1
1.2
1.3
1.4
1.5
0
10
20
30
40
50
60

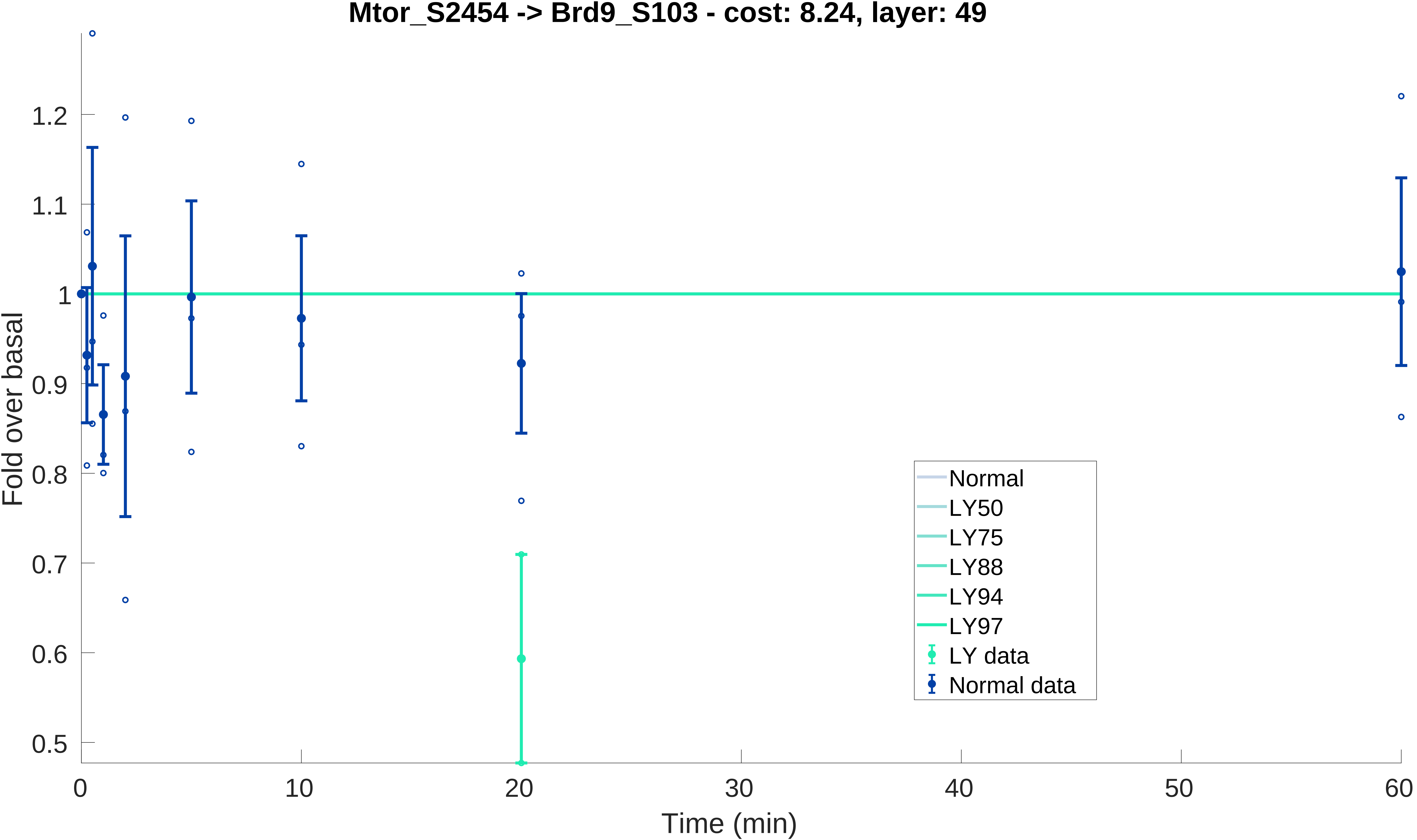

Mtor_S2454 -> Brd9_S103 - cost: 8.24, layer: 49
Fold over basal
1.2
1.1
1
0.9
0.8
0.7
0.6
0.5
0
10
20
30
40
50
60
Time (min)
Normal
LY50
LY75
LY88
LY94
LY97
LY data
Normal data

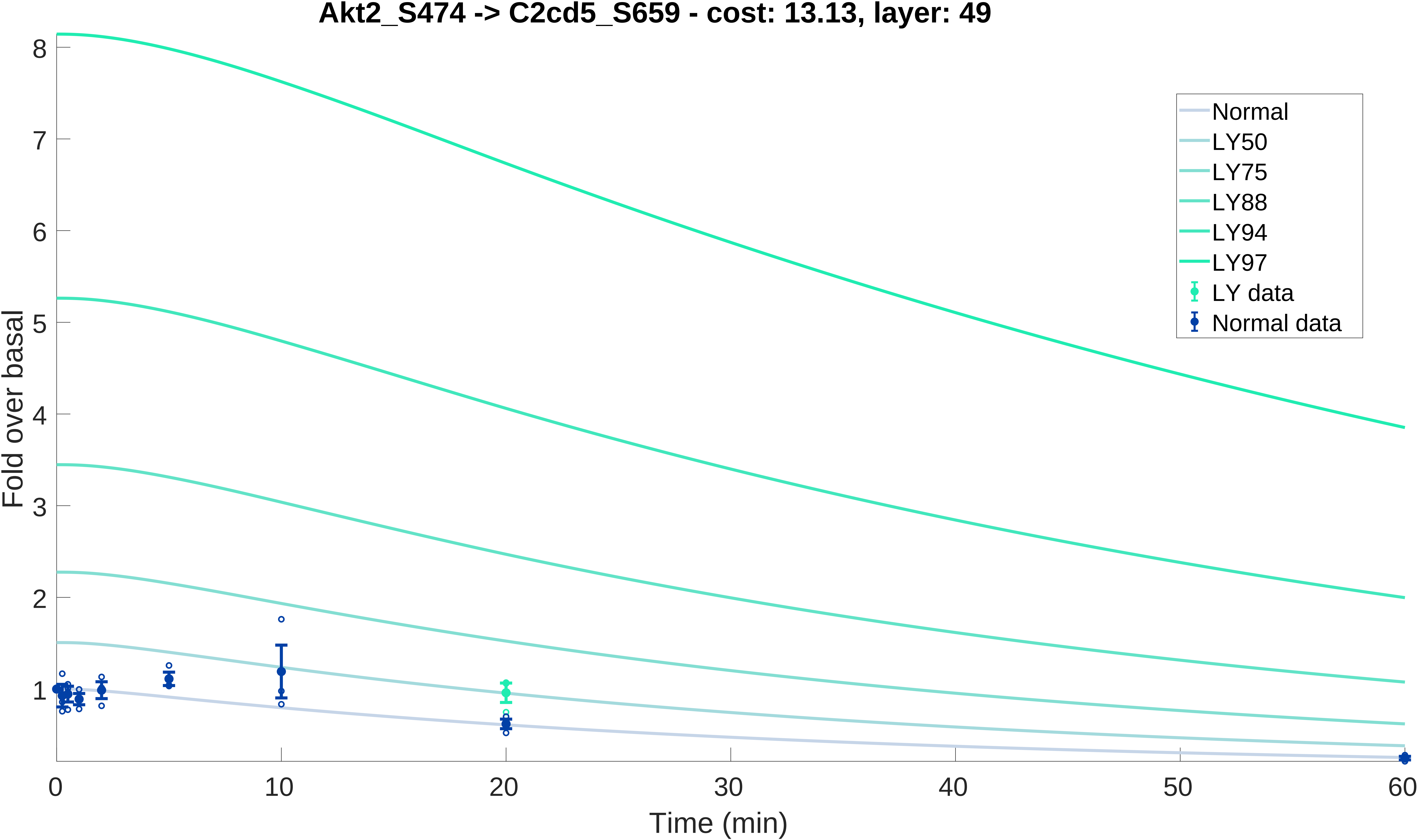

Akt2_S474 -> C2cd5_S659 - cost: 13.13, layer: 49
Fold over basal
Time (min)
0
10
20
30
40
50
60
1
2
3
4
5
6
7
8
Normal
LY50
LY75
LY88
LY94
LY97
LY data
Normal data

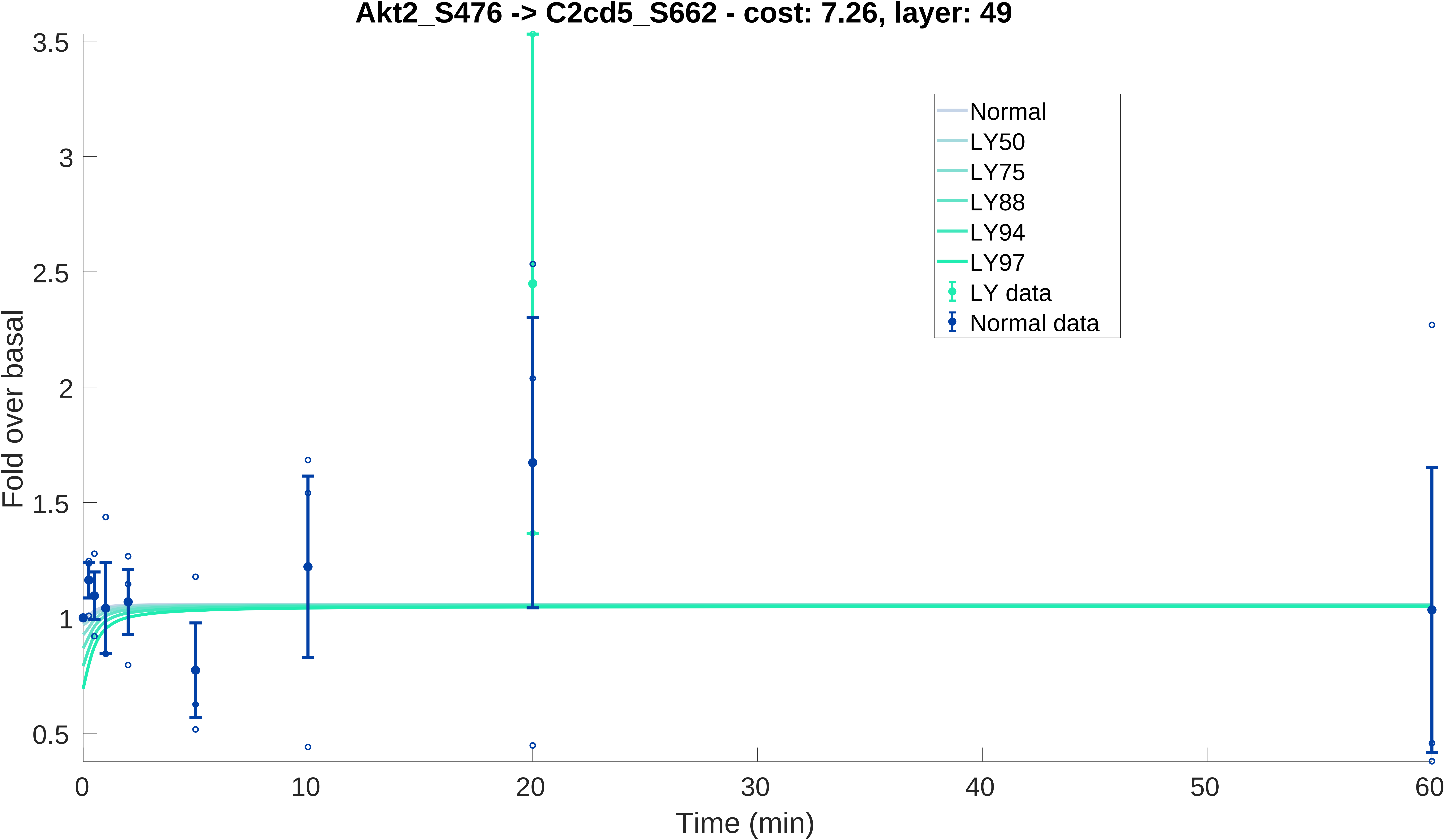

Akt2_S476 -> C2cd5_S662 - cost: 7.26, layer: 49
Fold over basal
3.5
3
2.5
2
1.5
1
0.5
0
10
20
30
40
50
60
Time (min)
Normal
LY50
LY75
LY88
LY94
LY97
LY data
Normal data

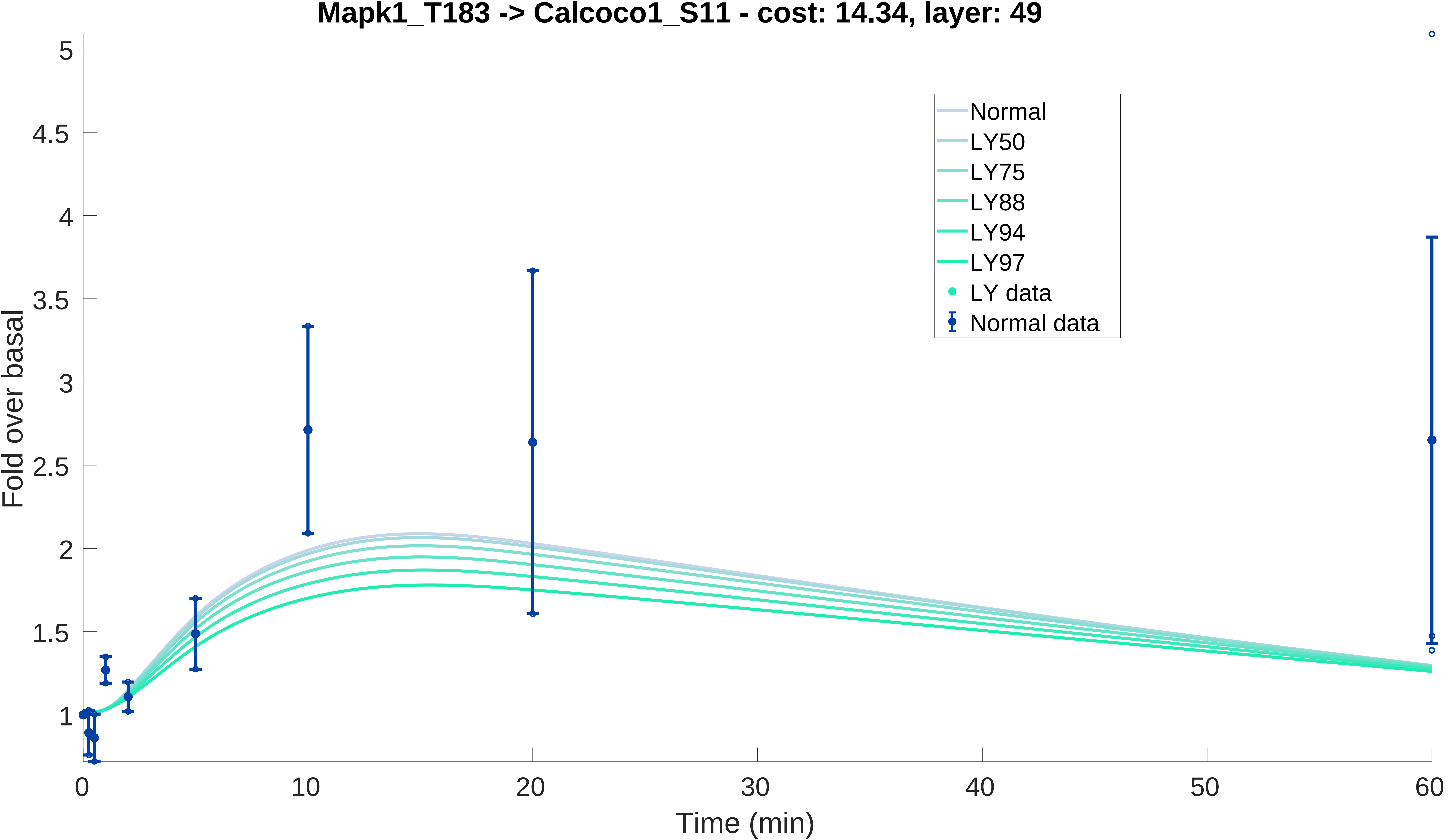

Mapk1_T183 -> Calcoco1_S11 - cost: 14.34, layer: 49
Fold over basal
Time (min)
Normal
LY50
LY75
LY88
LY94
LY97
LY data
Normal data
5
4.5
4
3.5
3
2.5
2
1.5
1
0
10
20
30
40
50
60

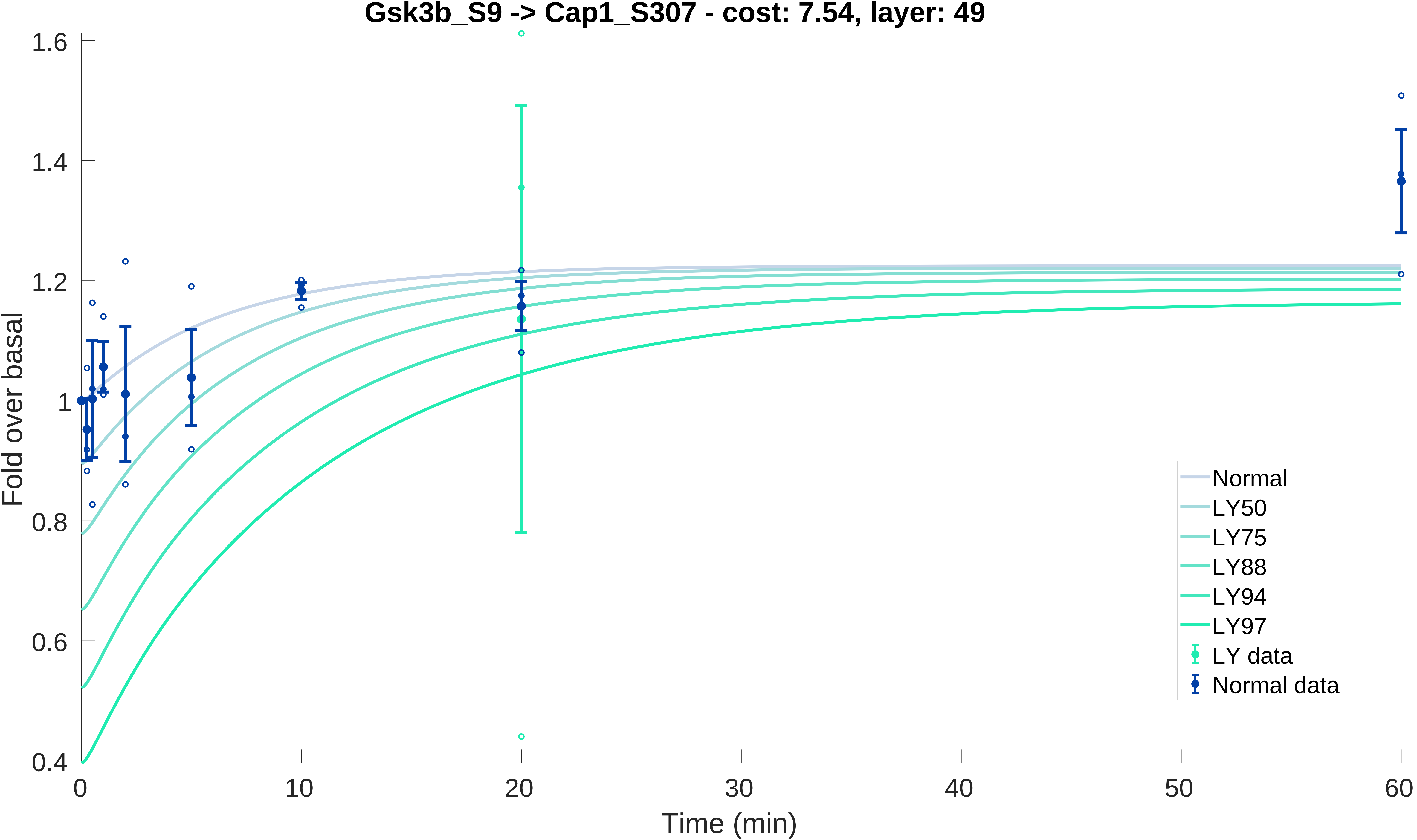

Gsk3b_S9 -> Cap1_S307 - cost: 7.54, layer: 49
1.6
1.4
1.2
1
0.8
0.6
0.4
Fold over basal
0
10
20
30
40
50
60
Time (min)
Normal
LY50
LY75
LY88
LY94
LY97
LY data
Normal data

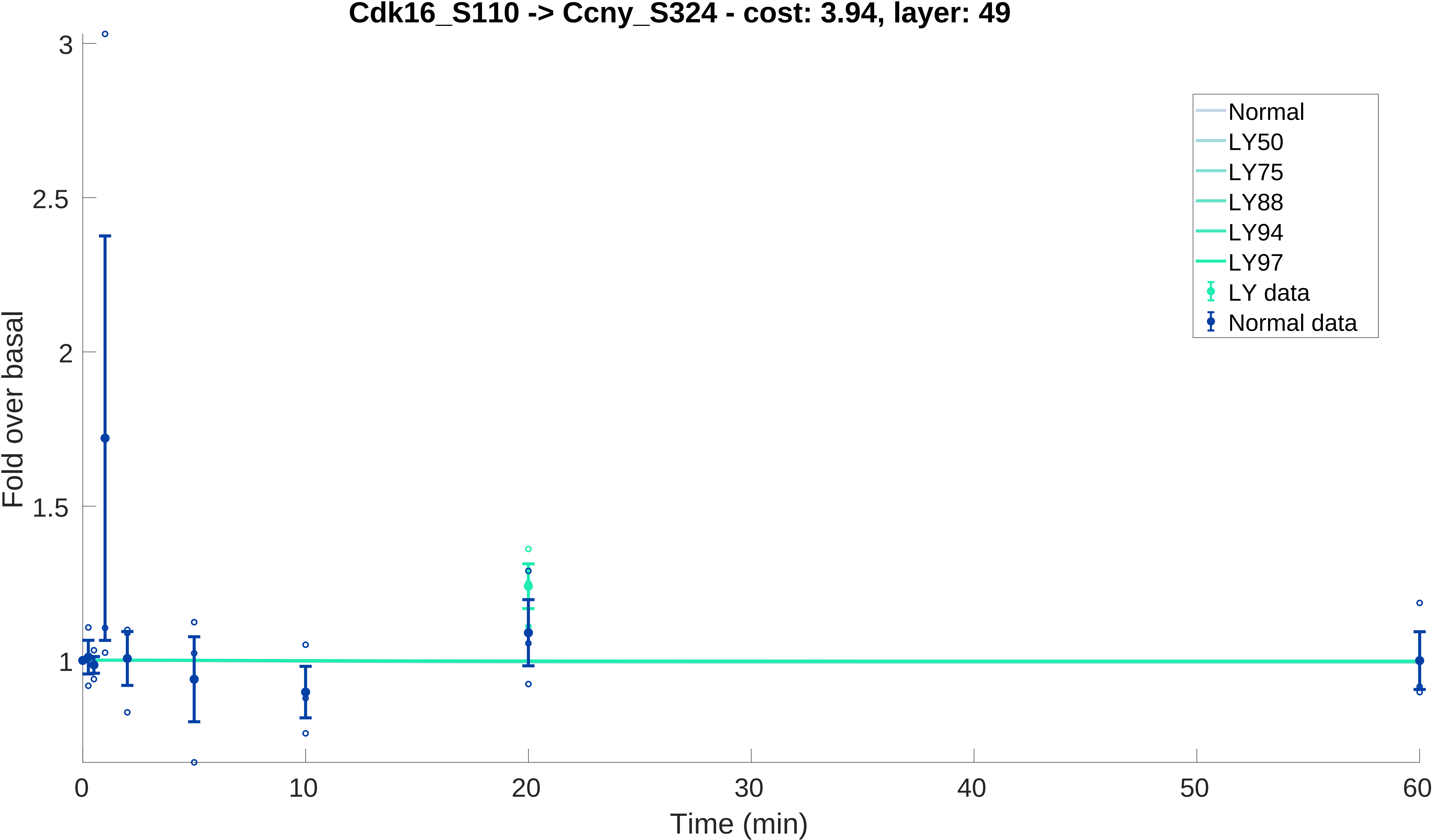

Cdk16_S110 -> Ccny_S324 - cost: 3.94, layer: 49
Fold over basal
Time (min)
3
2.5
2
1.5
1
0
10
20
30
40
50
60
Normal
LY50
LY75
LY88
LY94
LY97
LY data
Normal data

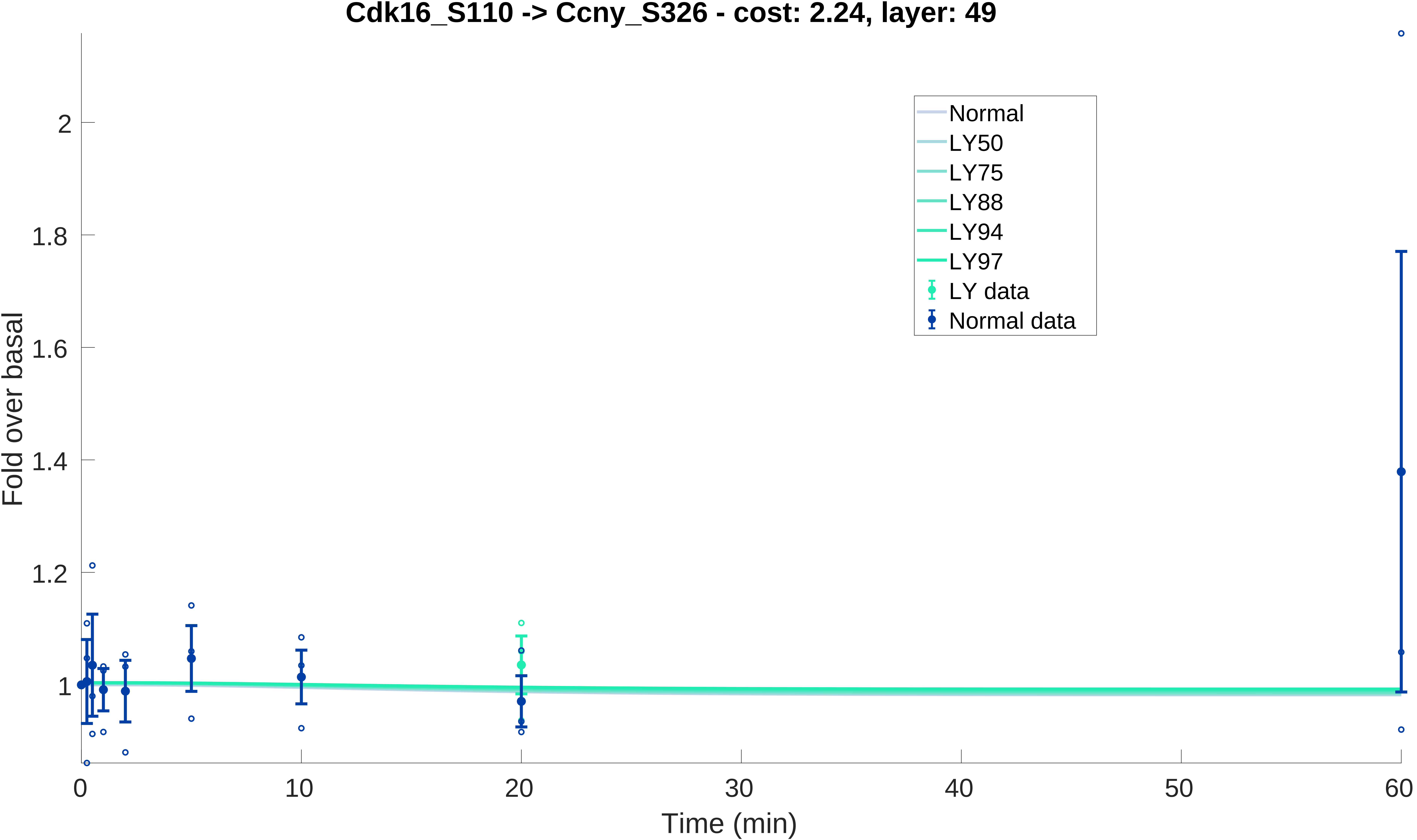
Cdk16_S110 -> Ccny_S326 - cost: 2.24, layer: 49
Fold over basal
Time (min)
Normal
LY50
LY75
LY88
LY94
LY97
LY data
Normal data
2
1.8
1.6
1.4
1.2
1
0
10
20
30
40
50
60

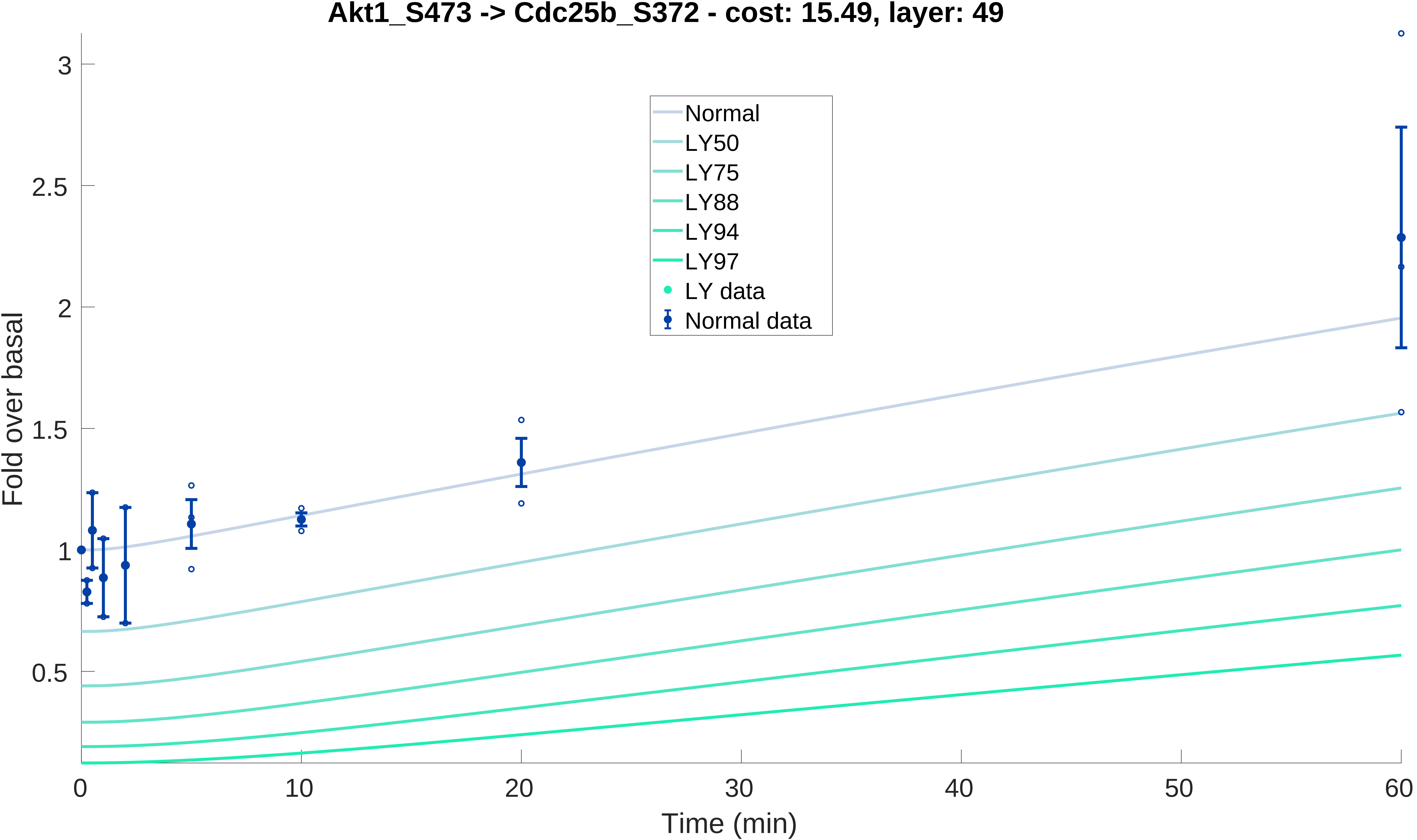
Akt1_S473 -> Cdc25b_S372 - cost: 15.49, layer: 49
Fold over basal
Time (min)
Normal
LY50
LY75
LY88
LY94
LY97
LY data
Normal data
0
10
20
30
40
50
60
0.5
1
1.5
2
2.5
3

Mapk1_T183 -> Cdc42ep1_S121 - cost: 8.21, layer: 49

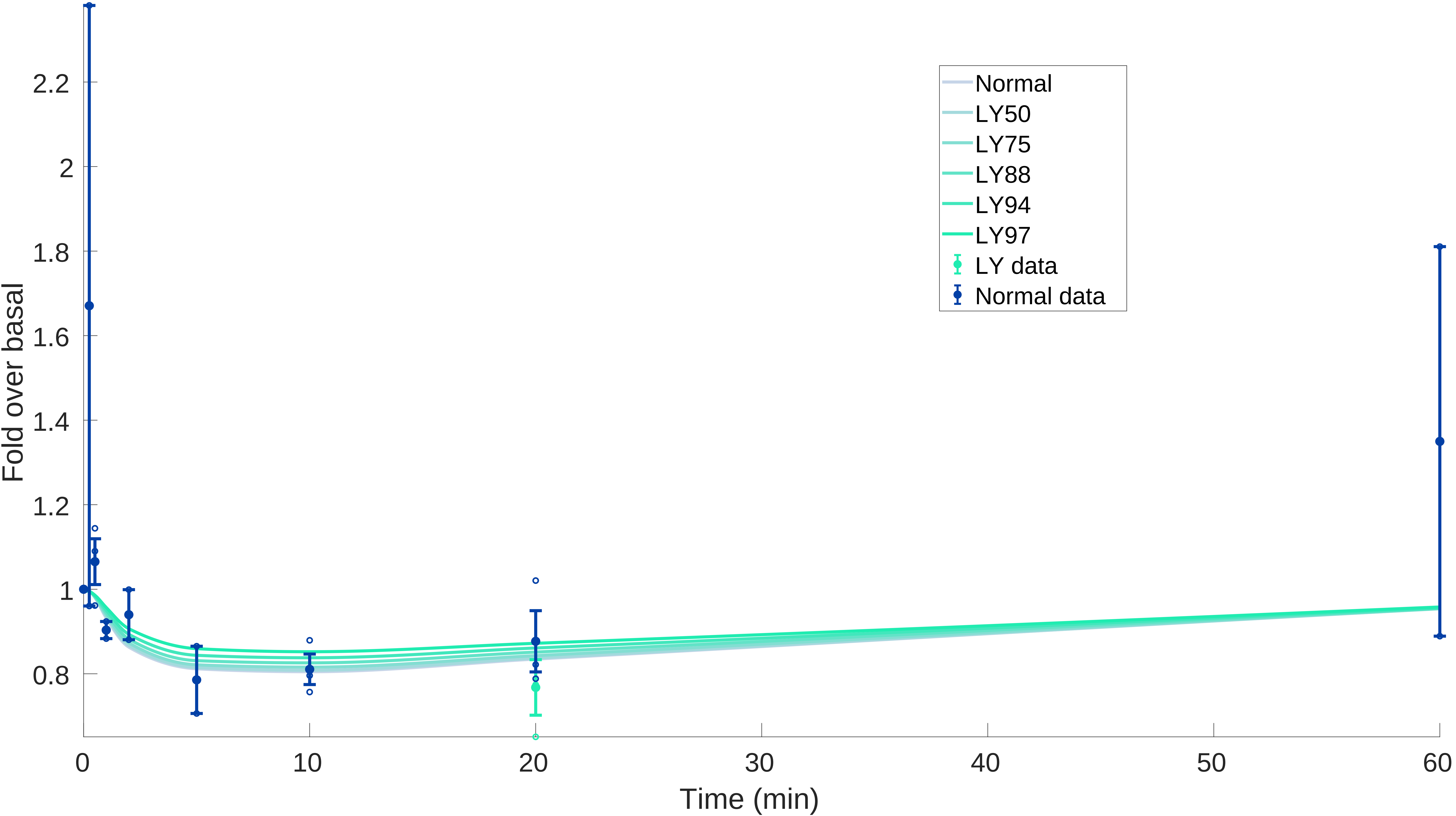

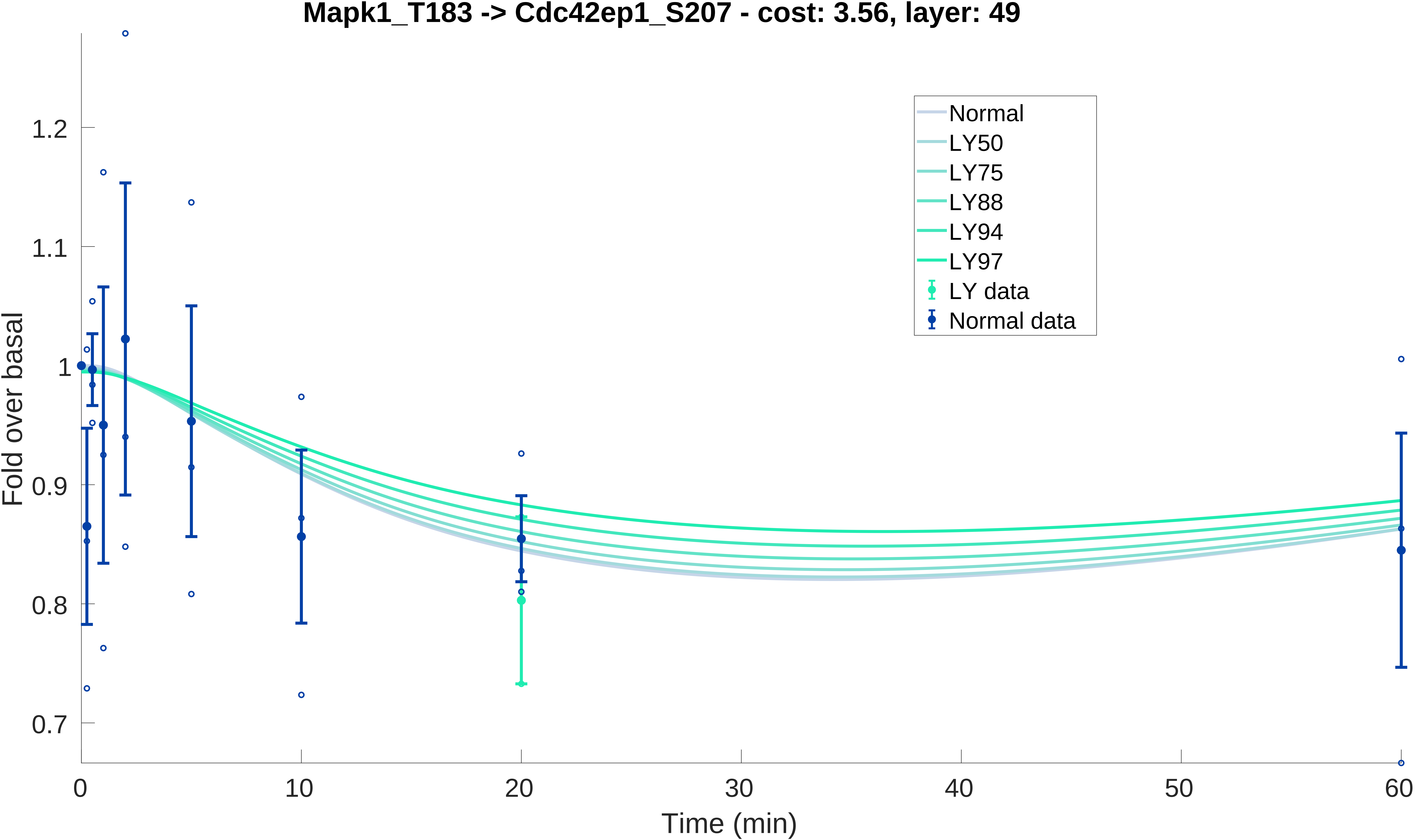

Mapk1_T183 -> Cdc42ep1_S207 - cost: 3.56, layer: 49
Normal
LY50
LY75
LY88
LY94
LY97
LY data
Normal data
Fold over basal
Time (min)
0.7
0.8
0.9
1
1.1
1.2
0
10
20
30
40
50
60

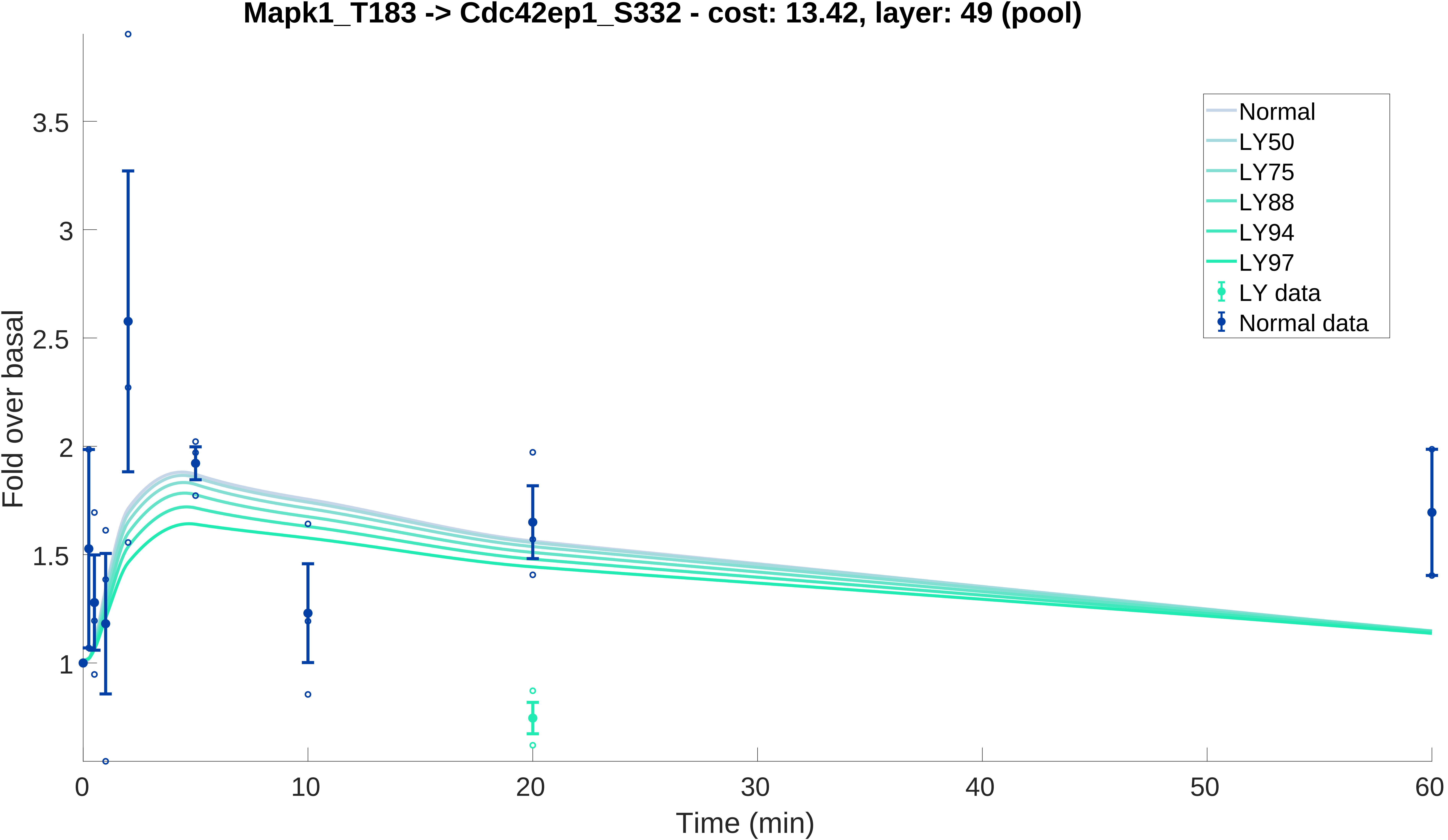

Mapk1_T183 -> Cdc42ep1_S332 - cost: 13.42, layer: 49 (pool)
Fold over basal
Time (min)
3.5
3
2.5
2
1.5
1
0
10
20
30
40
50
60
Normal
LY50
LY75
LY88
LY94
LY97
LY data
Normal data

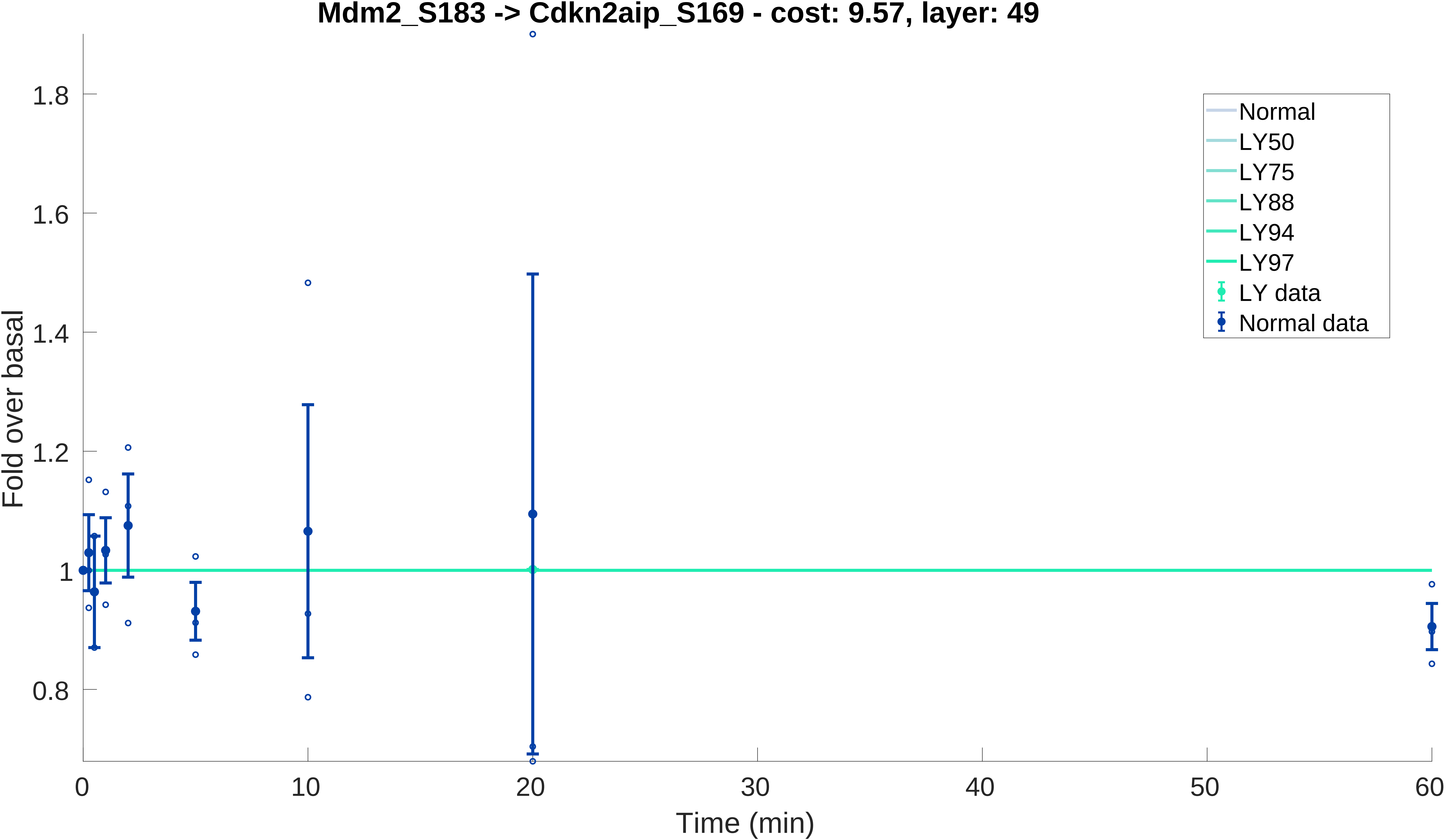

Mdm2_S183 -> Cdkn2aip_S169 - cost: 9.57, layer: 49
Fold over basal
1.8
1.6
1.4
1.2
1
0.8
0
10
20
30
40
50
60
Time (min)
Normal
LY50
LY75
LY88
LY94
LY97
LY data
Normal data

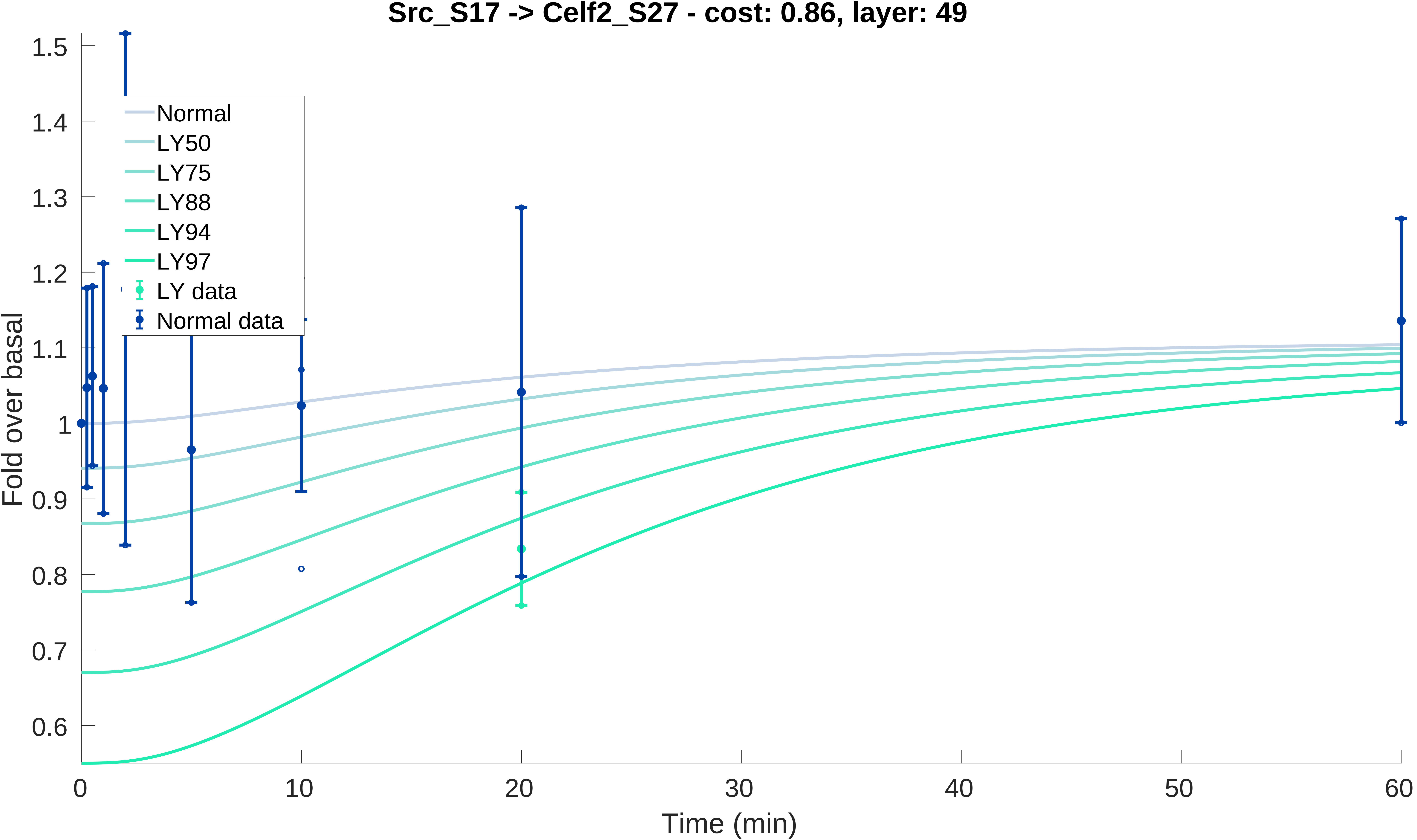

Src_S17 -> Celf2_S27 - cost: 0.86, layer: 49
Normal
LY50
LY75
LY88
LY94
LY97
LY data
Normal data
Fold over basal
1.5
1.4
1.3
1.2
1.1
1
0.9
0.8
0.7
0.6
0
10
20
30
40
50
60
Time (min)

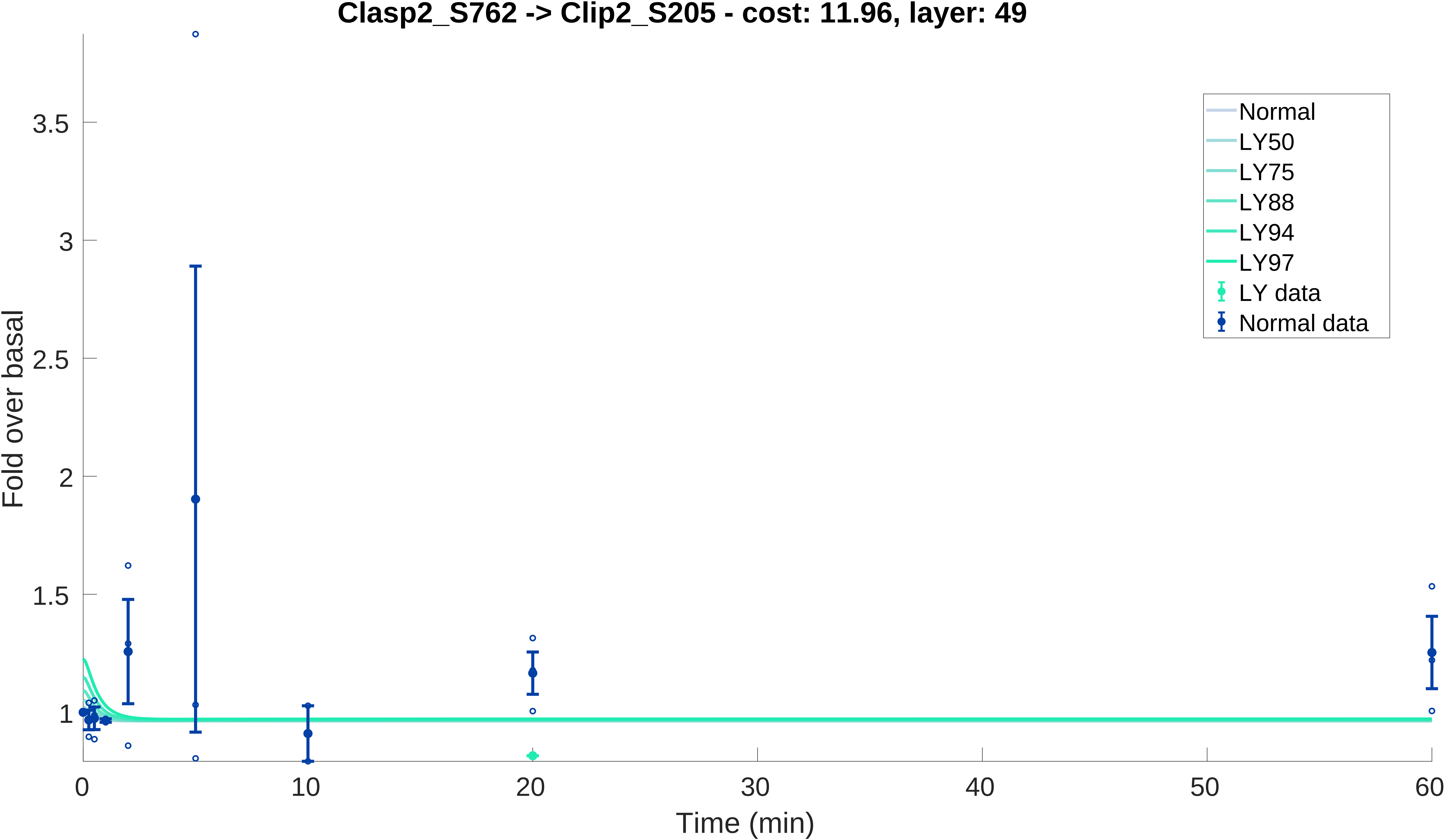

Clasp2_S762 -> Clip2_S205 - cost: 11.96, layer: 49
Fold over basal
Time (min)
3.5
3
2.5
2
1.5
1
0
10
20
30
40
50
60
Normal
LY50
LY75
LY88
LY94
LY97
LY data
Normal data

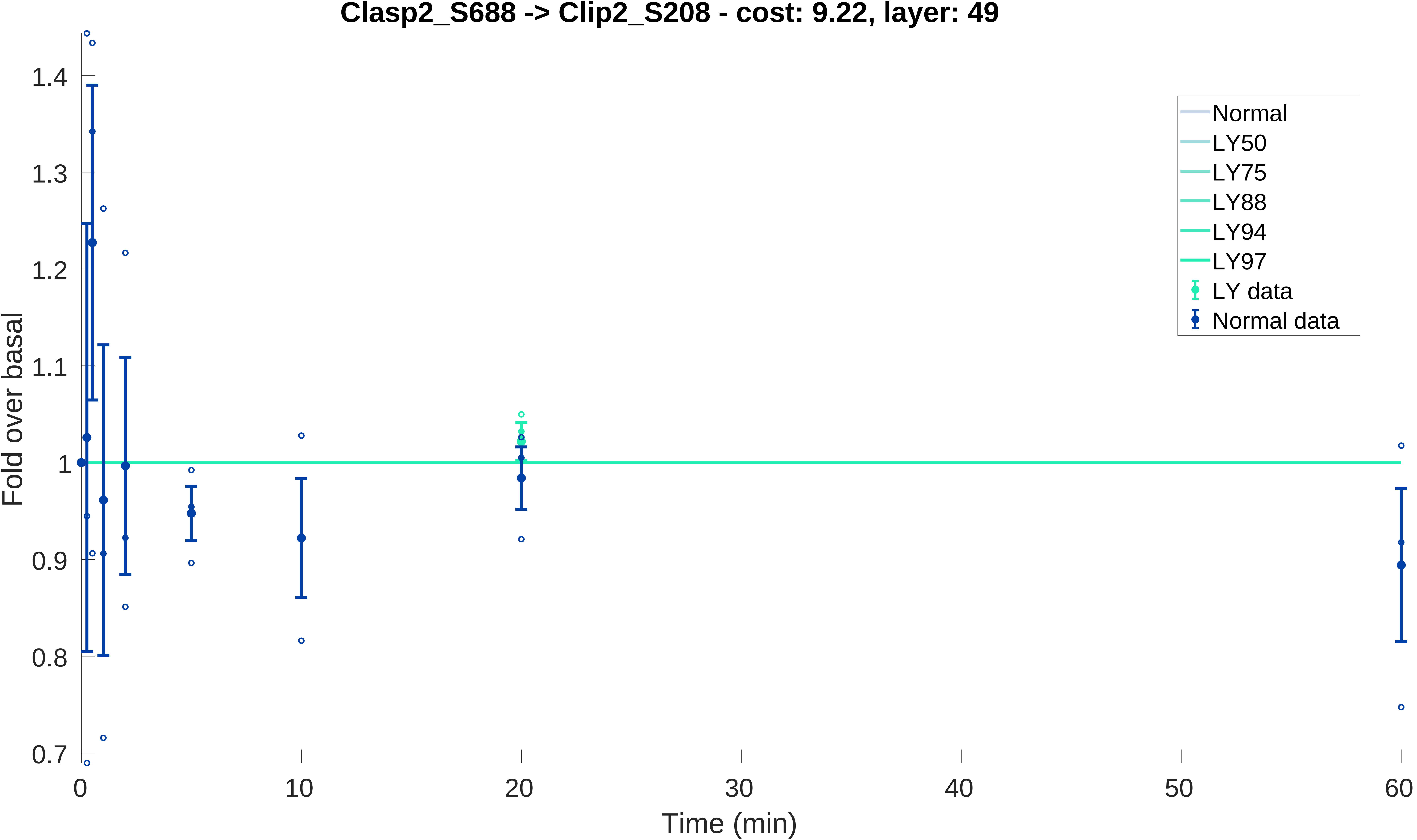
Clasp2_S688 -> Clip2_S208 - cost: 9.22, layer: 49
Fold over basal
Time (min)
Normal
LY50
LY75
LY88
LY94
LY97
LY data
Normal data
1.4
1.3
1.2
1.1
1
0.9
0.8
0.7
0
10
20
30
40
50
60

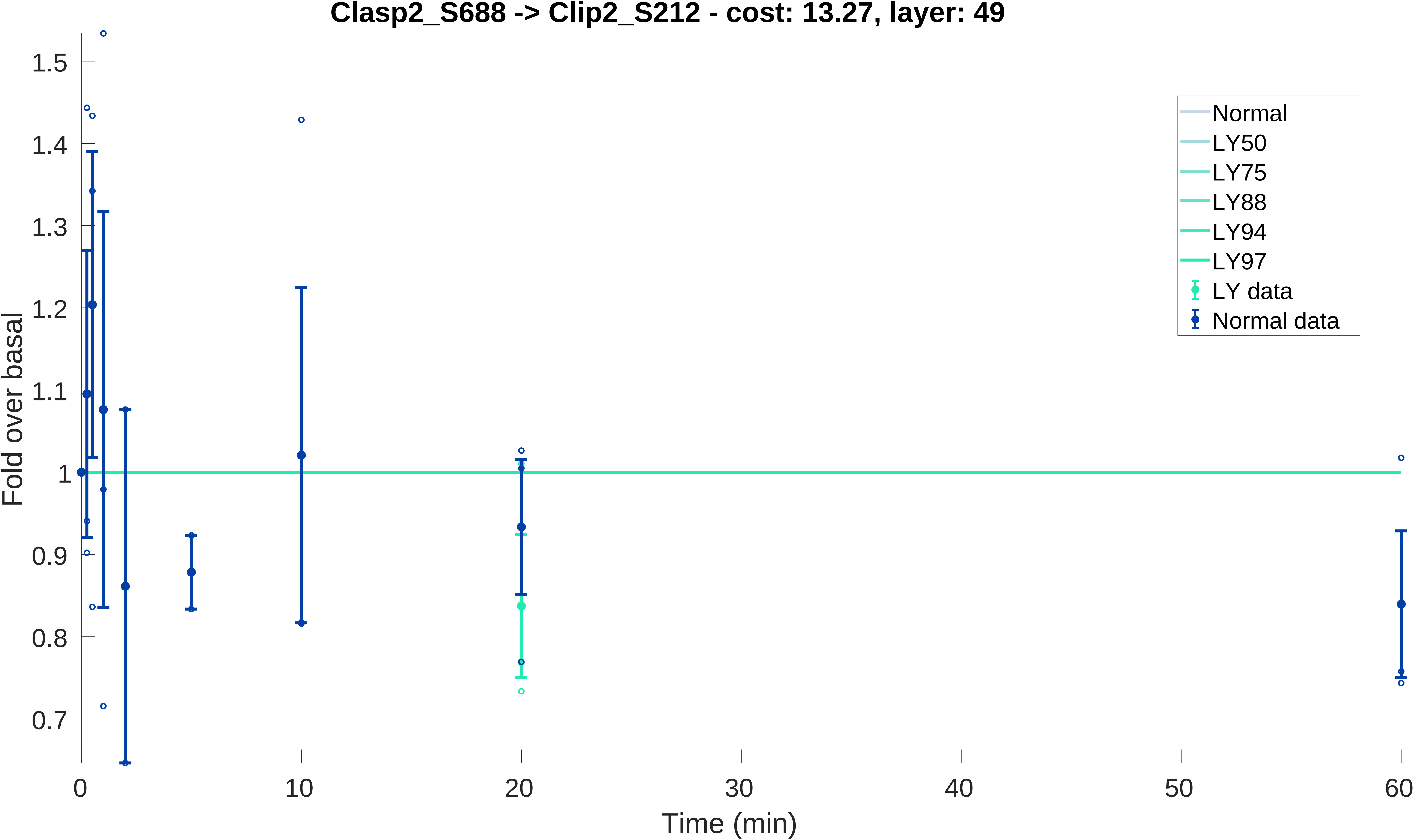

Clasp2_S688 -> Clip2_S212 - cost: 13.27, layer: 49
Fold over basal
Time (min)
1.5
1.4
1.3
1.2
1.1
1
0.9
0.8
0.7
0
10
20
30
40
50
60
Normal
LY50
LY75
LY88
LY94
LY97
LY data
Normal data

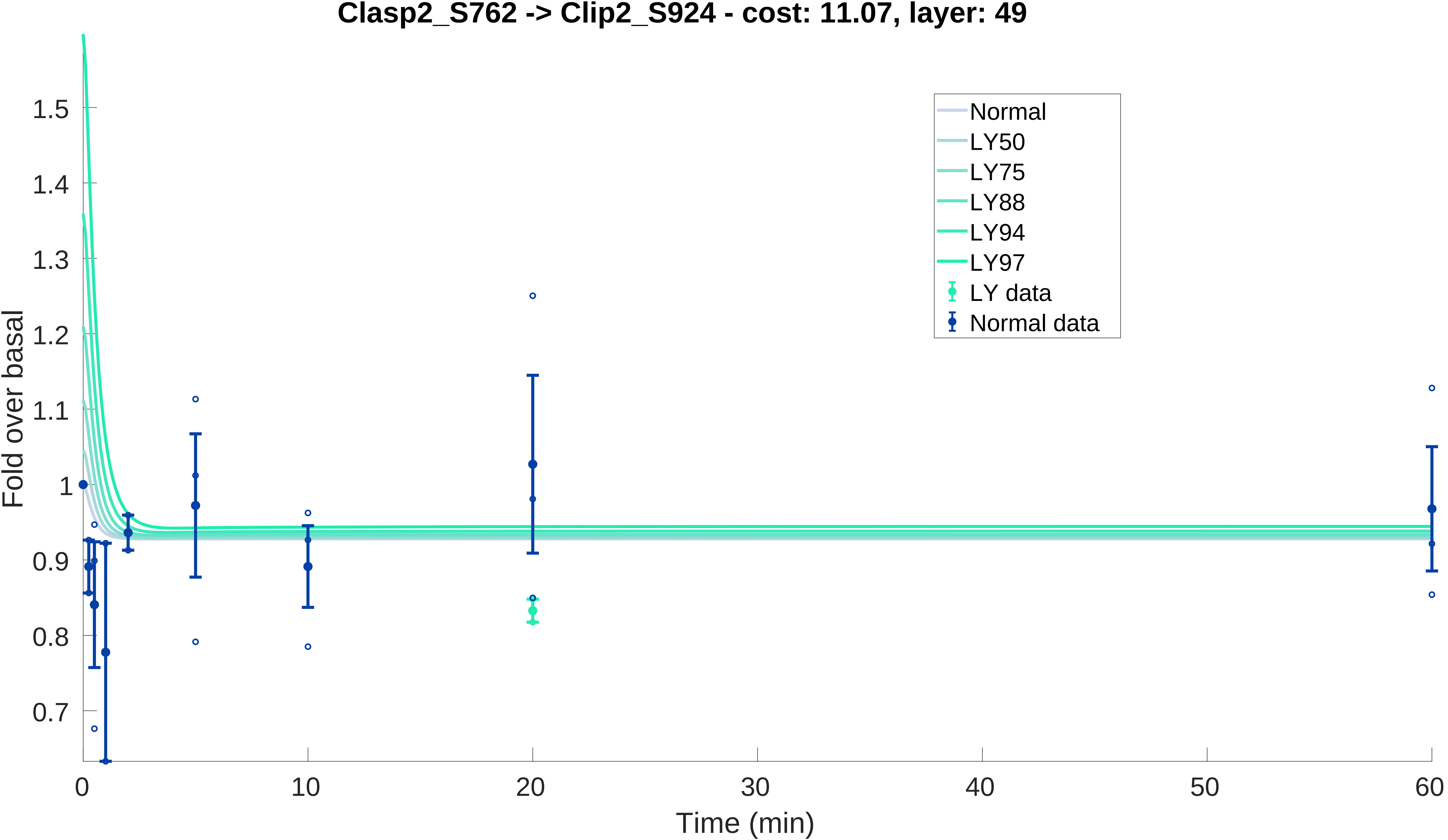

Clasp2_S762 -> Clip2_S924 - cost: 11.07, layer: 49
Fold over basal
Time (min)
0
10
20
30
40
50
60
0.7
0.8
0.9
1
1.1
1.2
1.3
1.4
1.5
Normal
LY50
LY75
LY88
LY94
LY97
LY data
Normal data

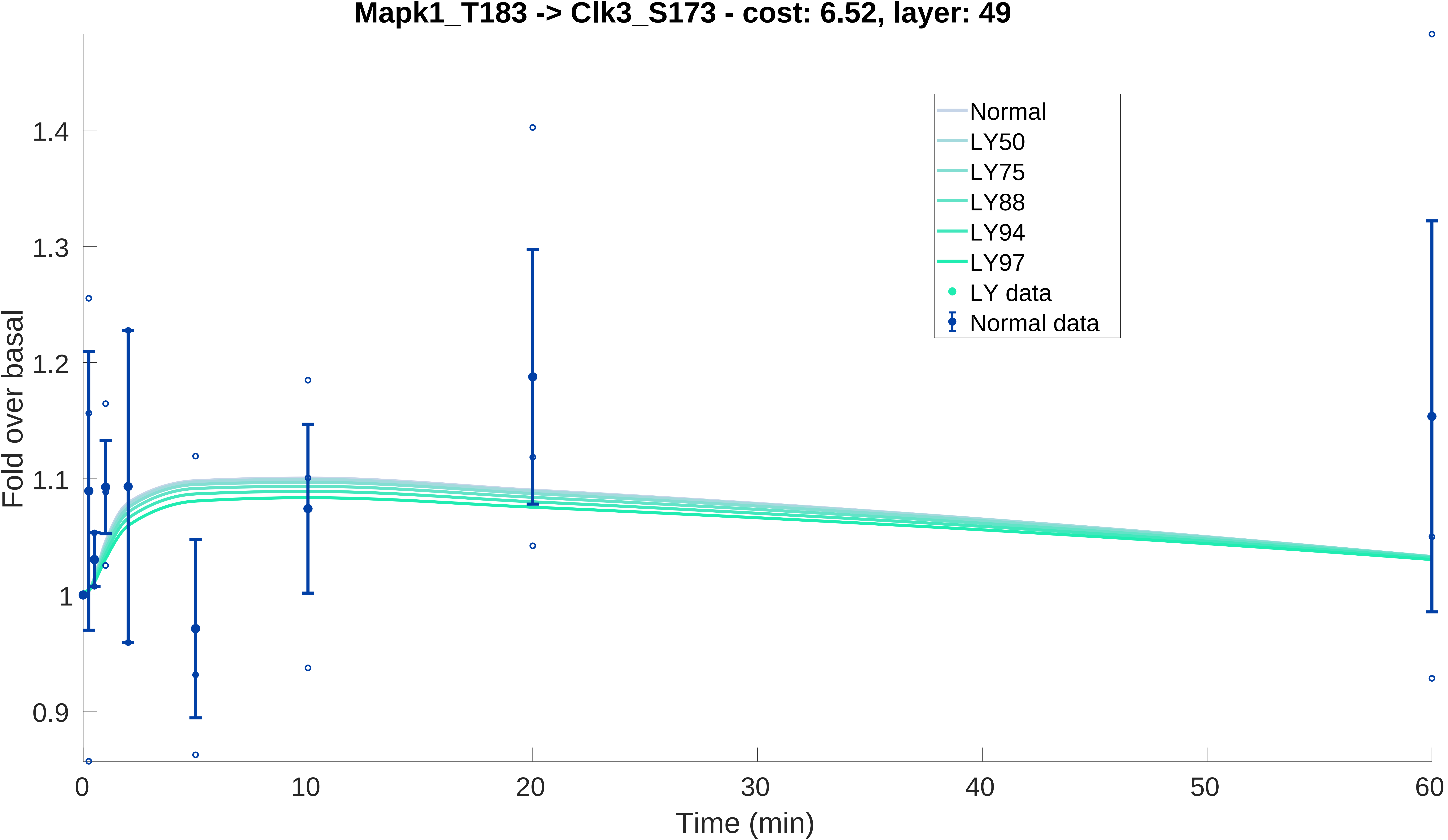

Mapk1_T183 -> Clk3_S173 - cost: 6.52, layer: 49
Normal
LY50
LY75
LY88
LY94
LY97
LY data
Normal data
Fold over basal
Time (min)
0
10
20
30
40
50
60
0.9
1
1.1
1.2
1.3
1.4

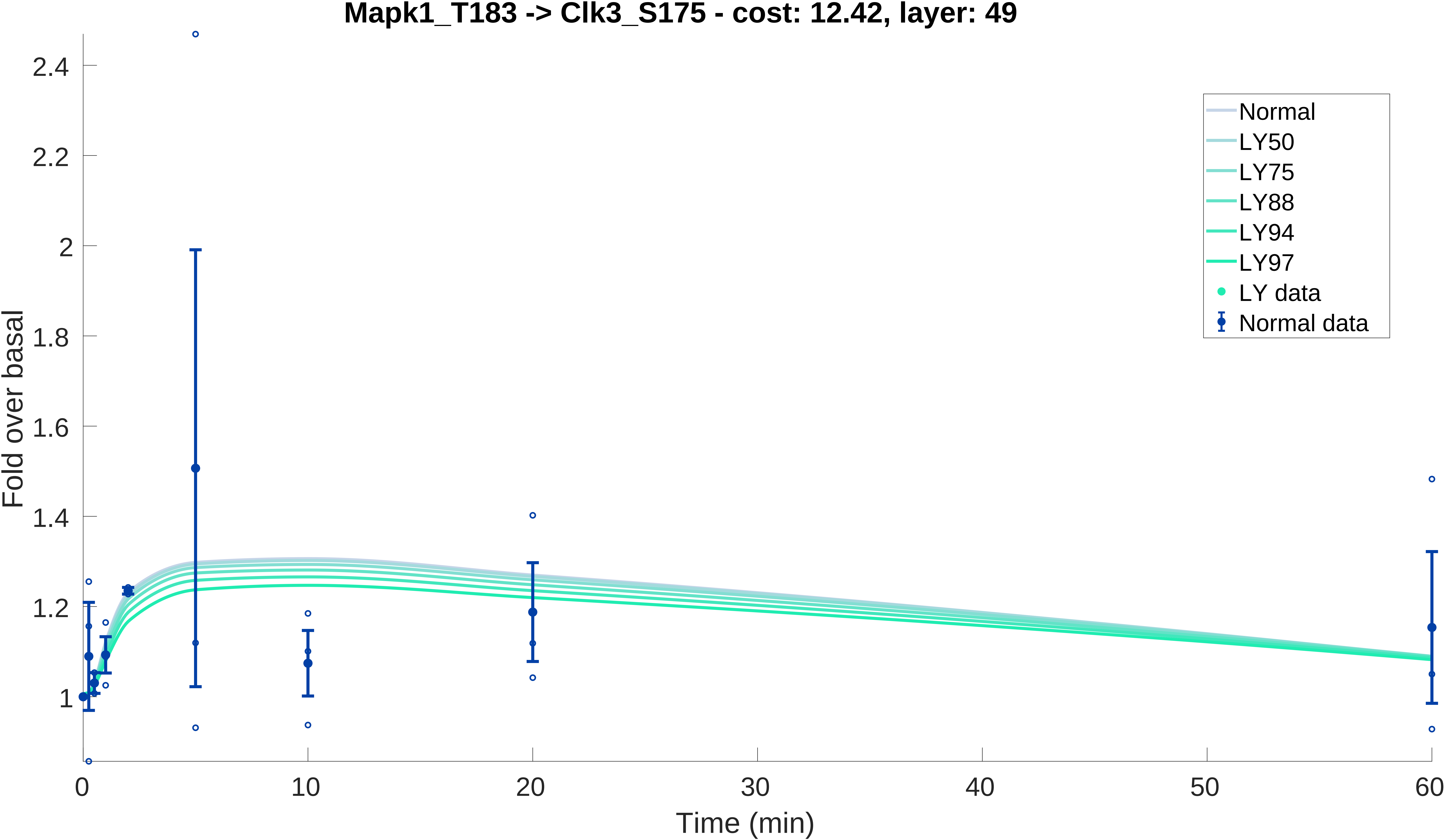

Mapk1_T183 -> Clk3_S175 - cost: 12.42, layer: 49
Fold over basal
2.4
2.2
2
1.8
1.6
1.4
1.2
1
0
10
20
30
40
50
60
Time (min)
Normal
LY50
LY75
LY88
LY94
LY97
LY data
Normal data

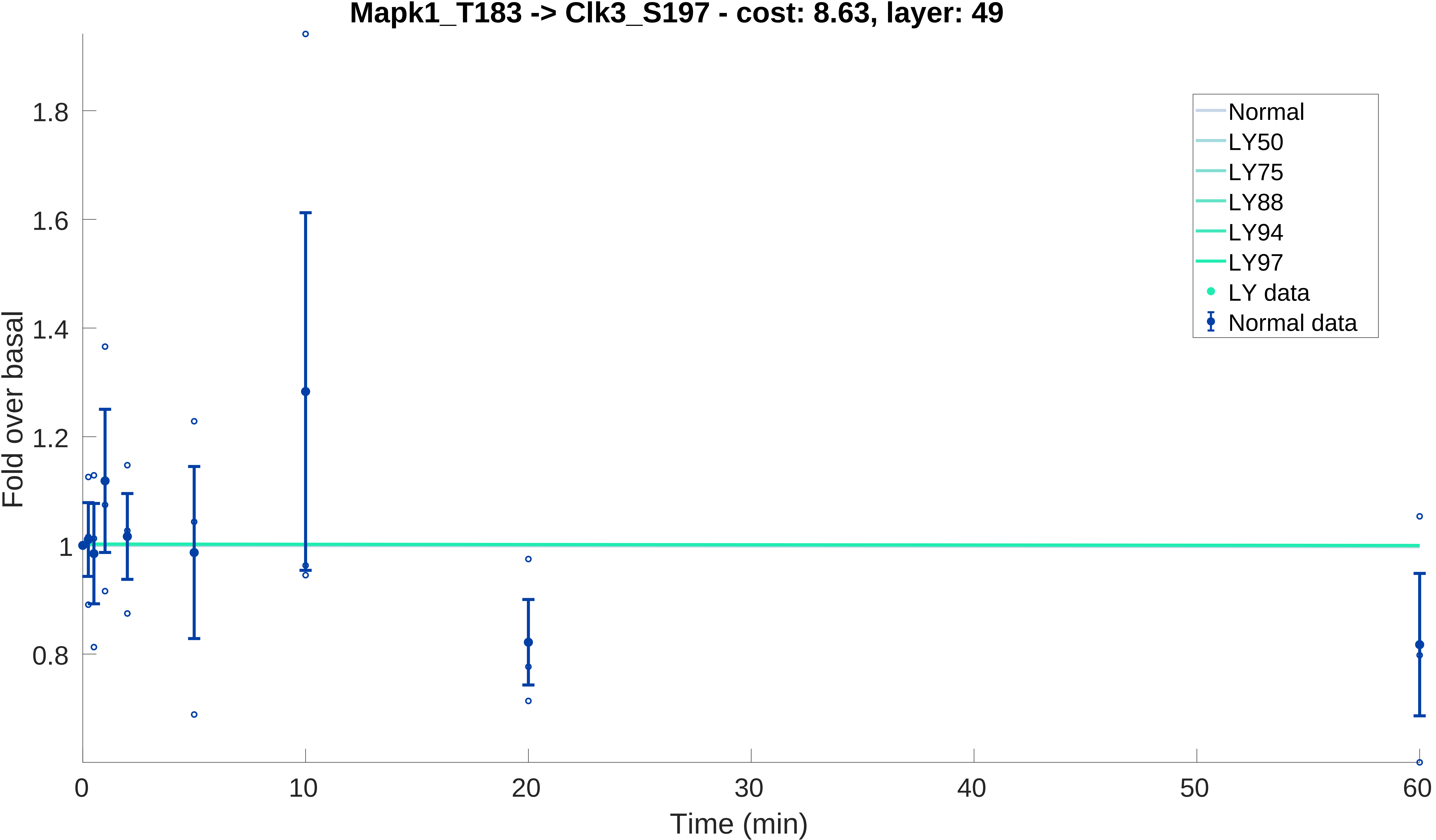

Mapk1_T183 -> Clk3_S197 - cost: 8.63, layer: 49
Fold over basal
Time (min)
1.8
1.6
1.4
1.2
1
0.8
0
10
20
30
40
50
60
Normal
LY50
LY75
LY88
LY94
LY97
LY data
Normal data

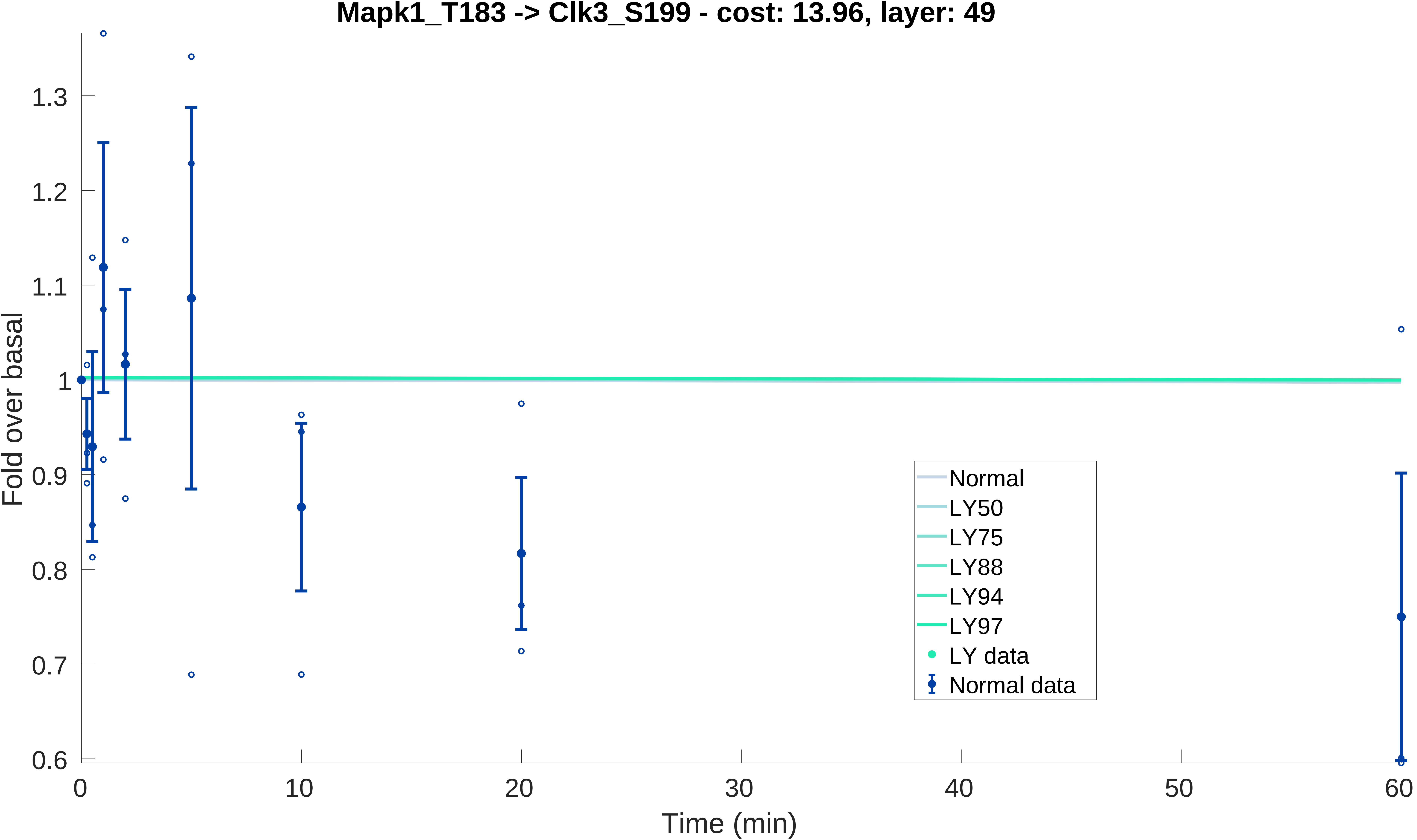

Mapk1_T183 -> Clk3_S199 - cost: 13.96, layer: 49
Fold over basal
1.3
1.2
1.1
1
0.9
0.8
0.7
0.6
0
10
20
30
40
50
60
Time (min)
Normal
LY50
LY75
LY88
LY94
LY97
LY data
Normal data

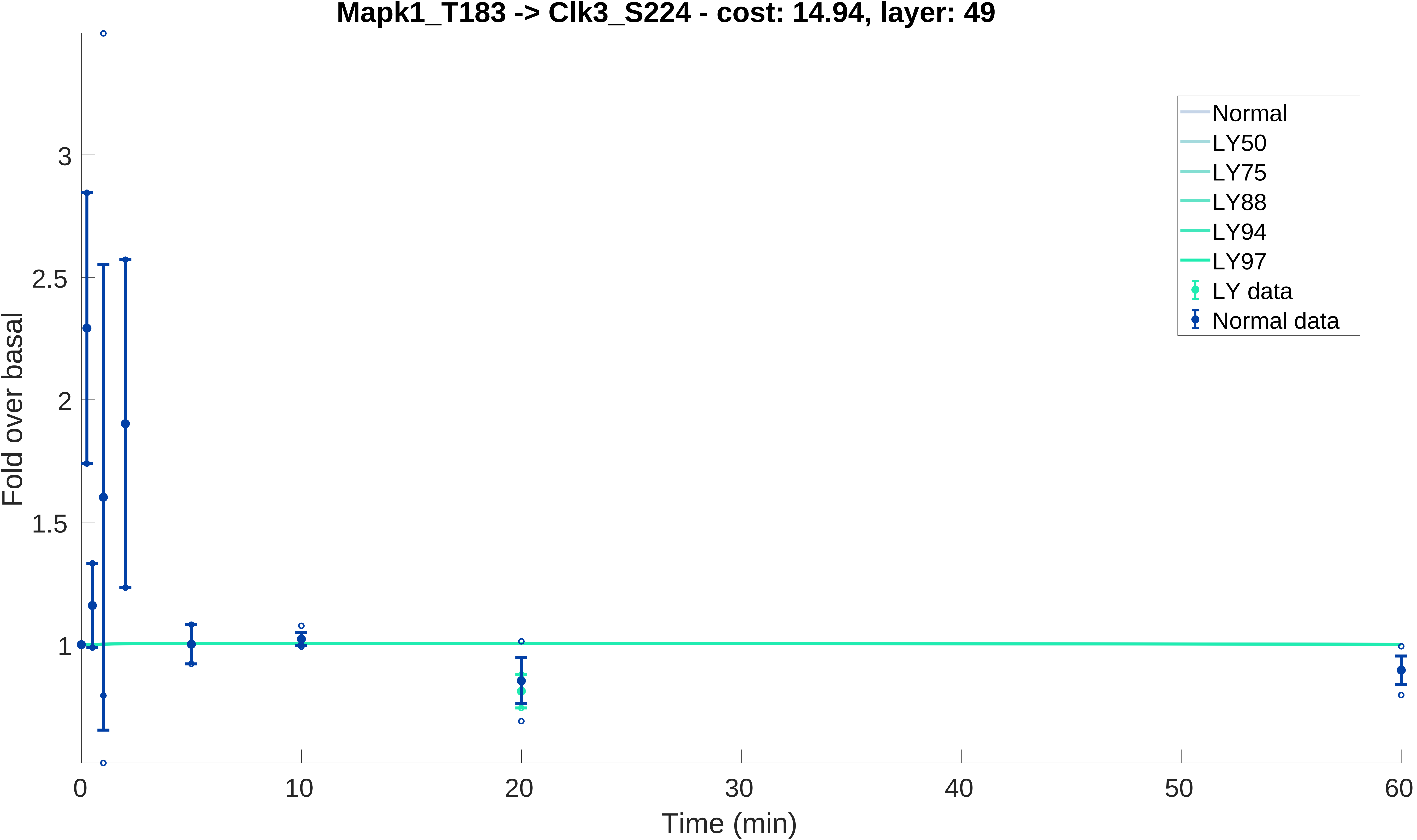

Mapk1_T183 -> Clk3_S224 - cost: 14.94, layer: 49
Fold over basal
Time (min)
0
10
20
30
40
50
60
1
1.5
2
2.5
3
Normal
LY50
LY75
LY88
LY94
LY97
LY data
Normal data

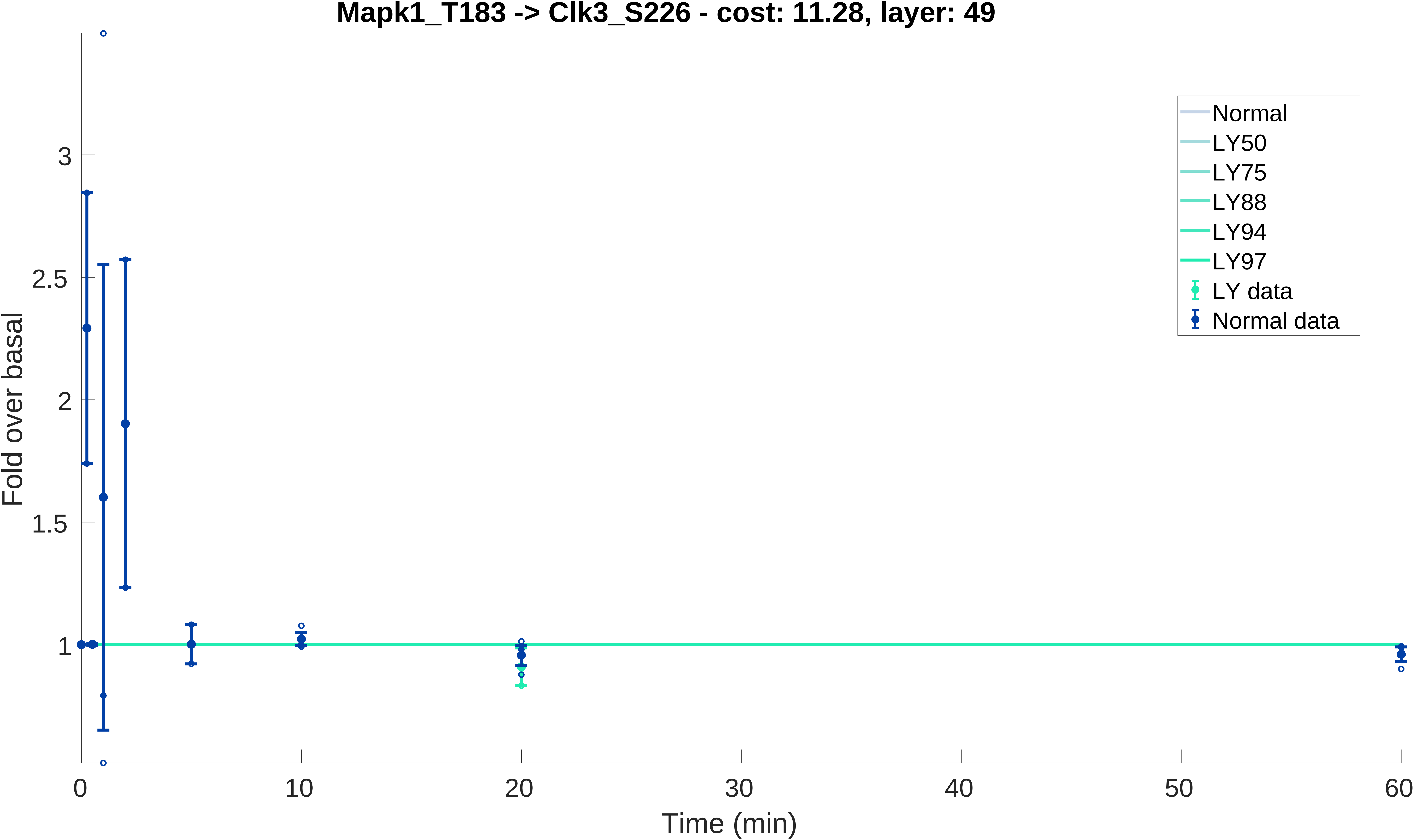

Mapk1_T183 -> Clk3_S226 - cost: 11.28, layer: 49
Fold over basal
Time (min)
Normal
LY50
LY75
LY88
LY94
LY97
LY data
Normal data
3
2.5
2
1.5
1
0
10
20
30
40
50
60

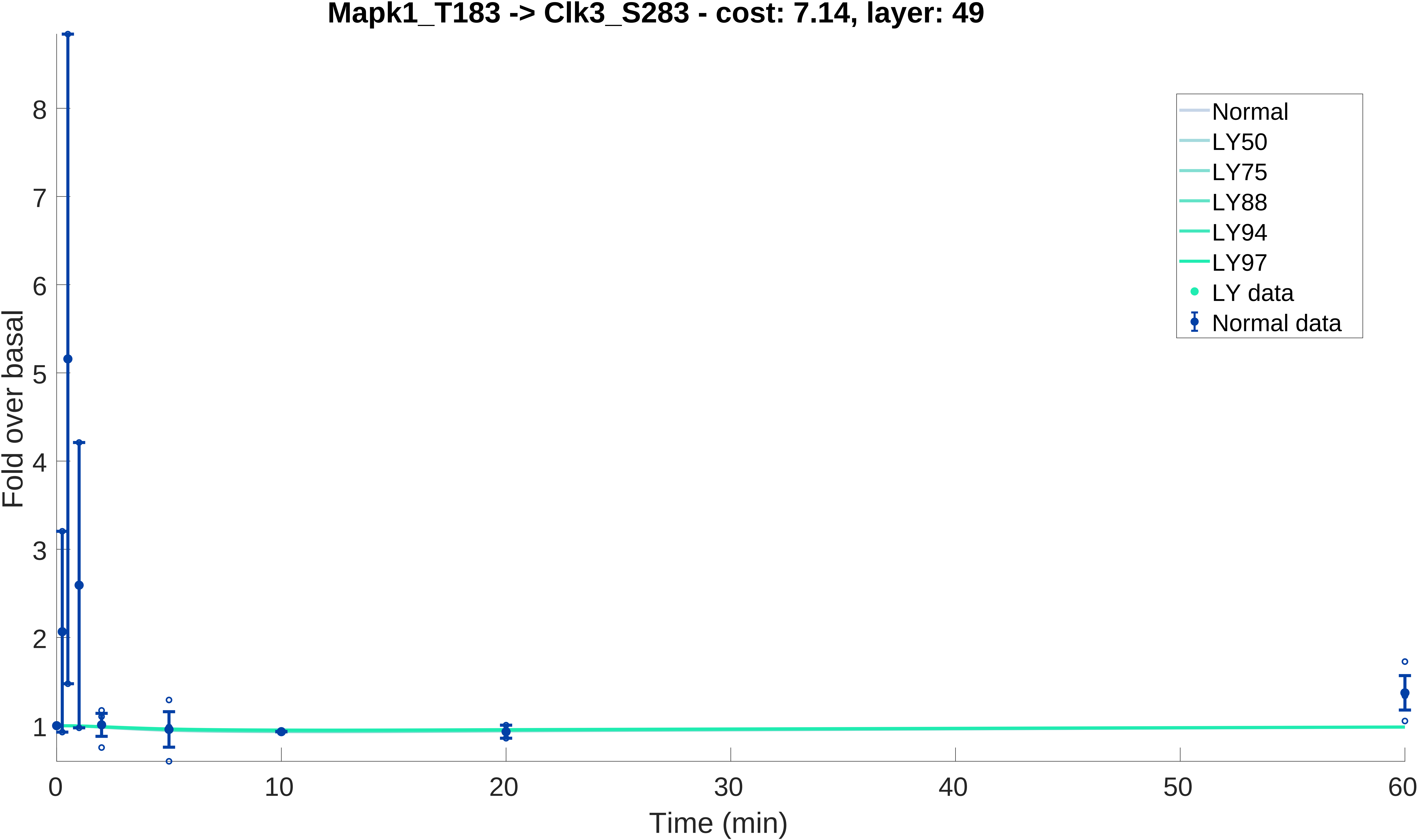

Mapk1_T183 -> Clk3_S283 - cost: 7.14, layer: 49
Fold over basal
Time (min)
0
10
20
30
40
50
60
1
2
3
4
5
6
7
8
Normal
LY50
LY75
LY88
LY94
LY97
LY data
Normal data

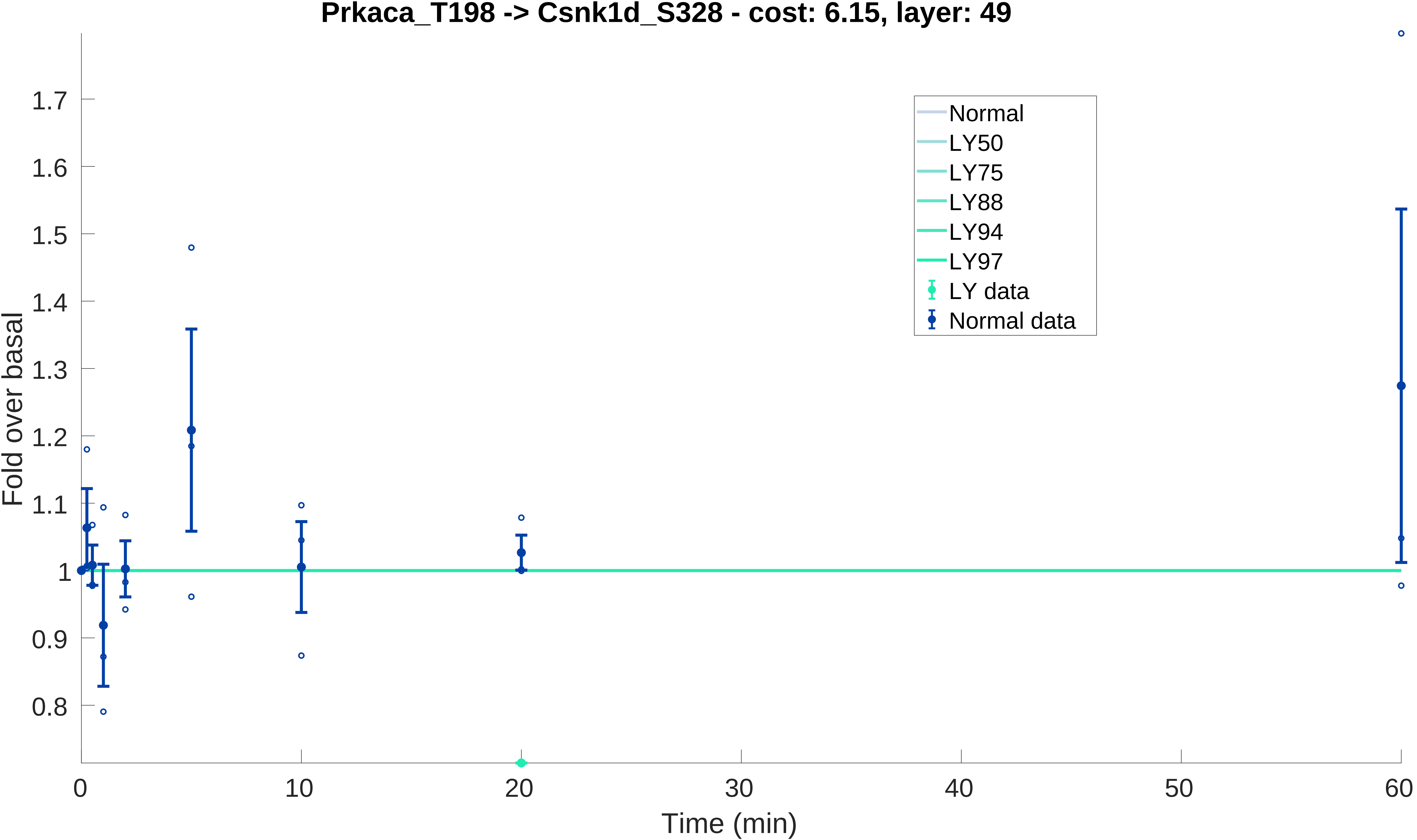

Prkaca_T198 -> Csnk1d_S328 - cost: 6.15, layer: 49
Fold over basal
Time (min)
0.8
0.9
1
1.1
1.2
1.3
1.4
1.5
1.6
1.7
0
10
20
30
40
50
60
Normal
LY50
LY75
LY88
LY94
LY97
LY data
Normal data

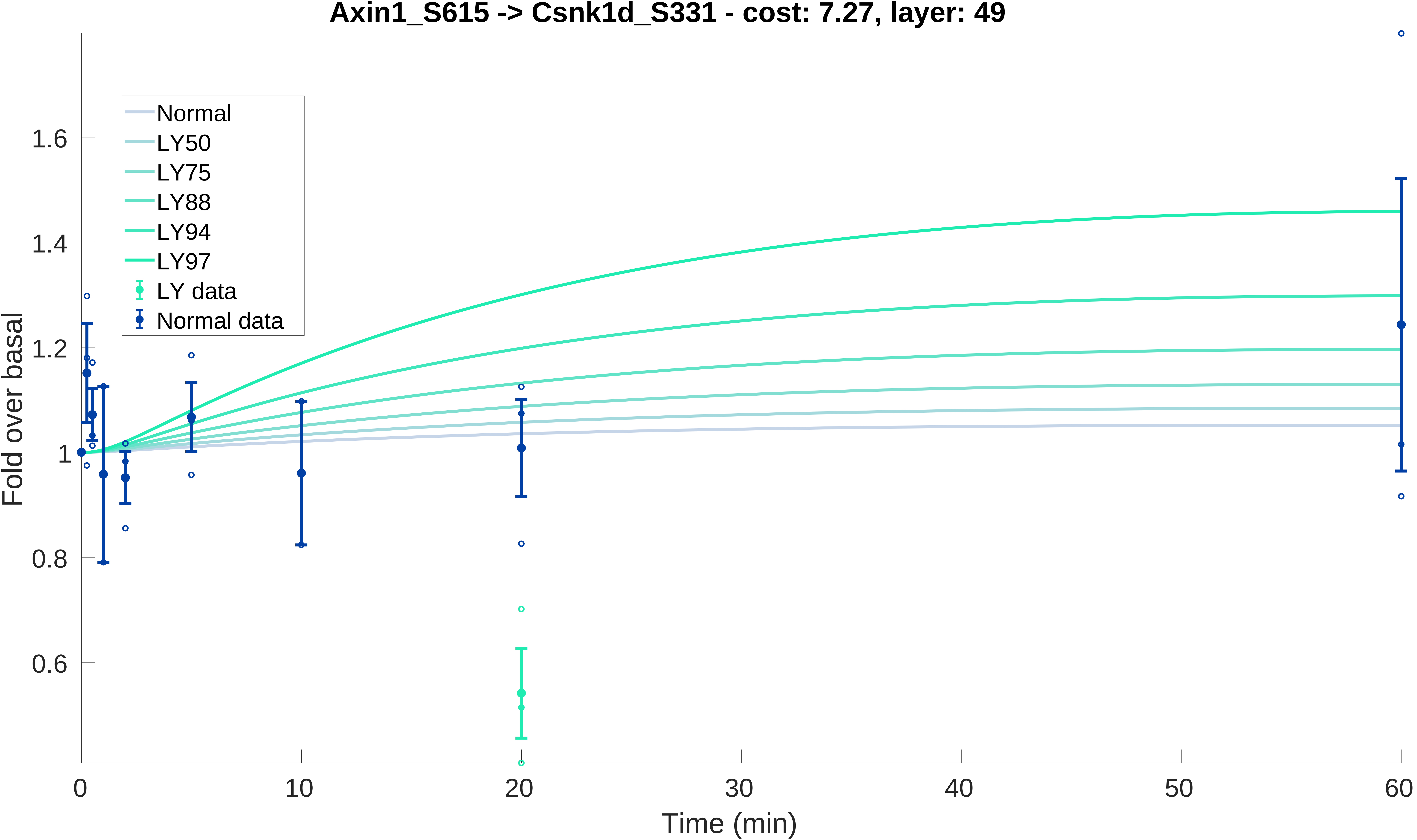

Axin1_S615 -> Csnk1d_S331 - cost: 7.27, layer: 49
Normal
LY50
LY75
LY88
LY94
LY97
LY data
Normal data
Fold over basal
Time (min)
1.6
1.4
1.2
1
0.8
0.6
0
10
20
30
40
50
60

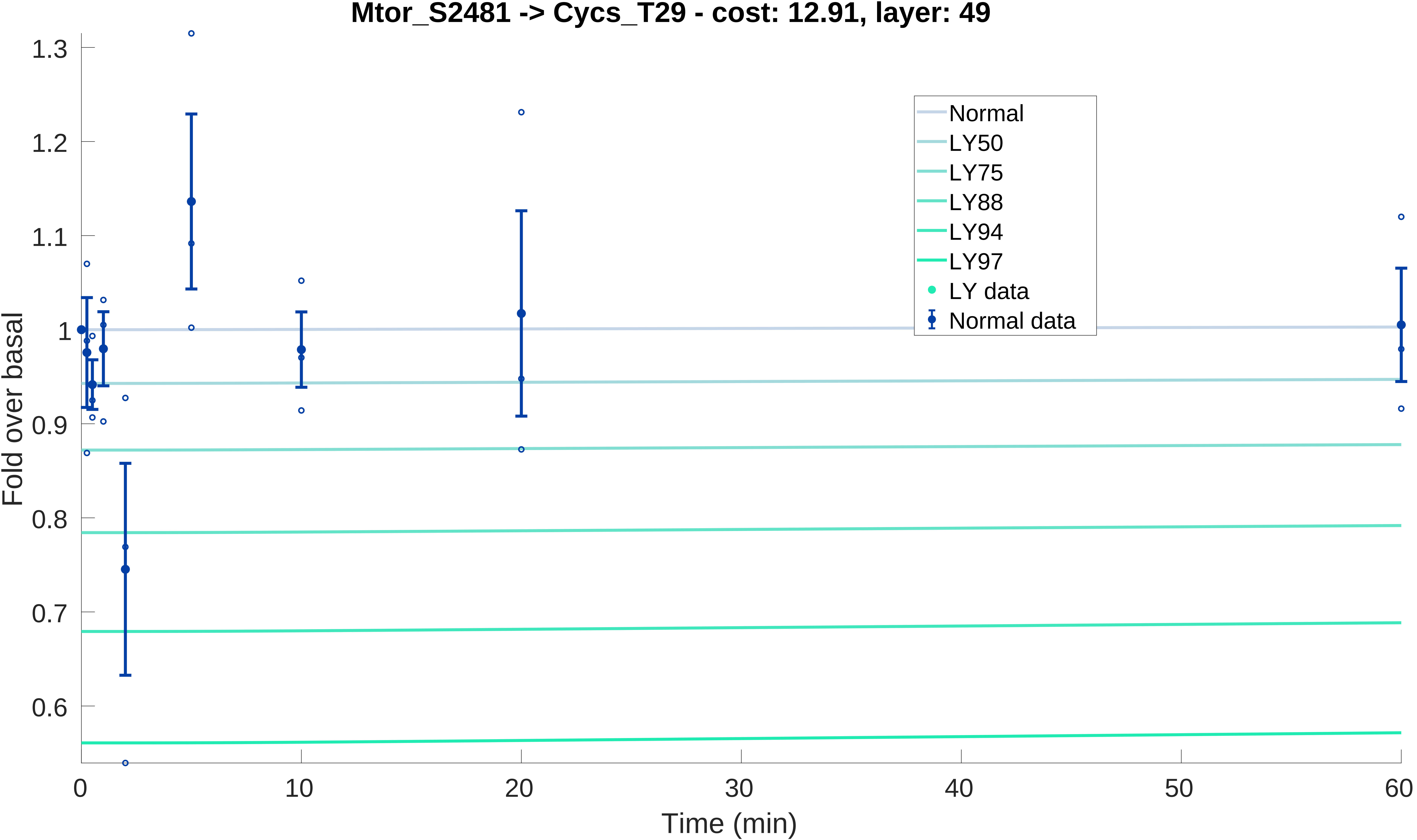

Mtor_S2481 -> Cycs_T29 - cost: 12.91, layer: 49
Fold over basal
Time (min)
1.3
1.2
1.1
1
0.9
0.8
0.7
0.6
0
10
20
30
40
50
60
Normal
LY50
LY75
LY88
LY94
LY97
LY data
Normal data

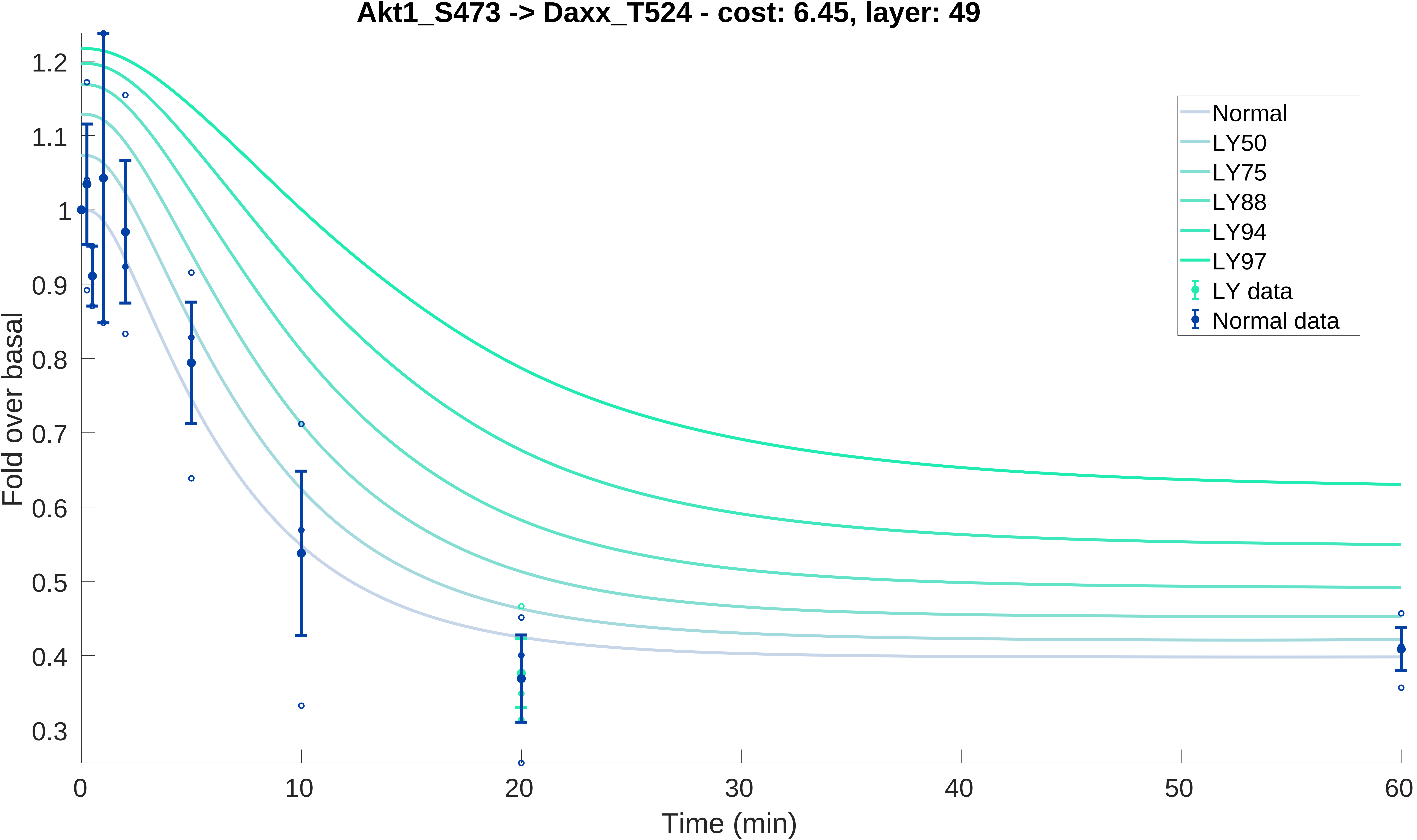
Akt1_S473 -> Daxx_T524 - cost: 6.45, layer: 49
Fold over basal
Time (min)
Normal
LY50
LY75
LY88
LY94
LY97
LY data
Normal data
0
10
20
30
40
50
60
0.3
0.4
0.5
0.6
0.7
0.8
0.9
1
1.1
1.2

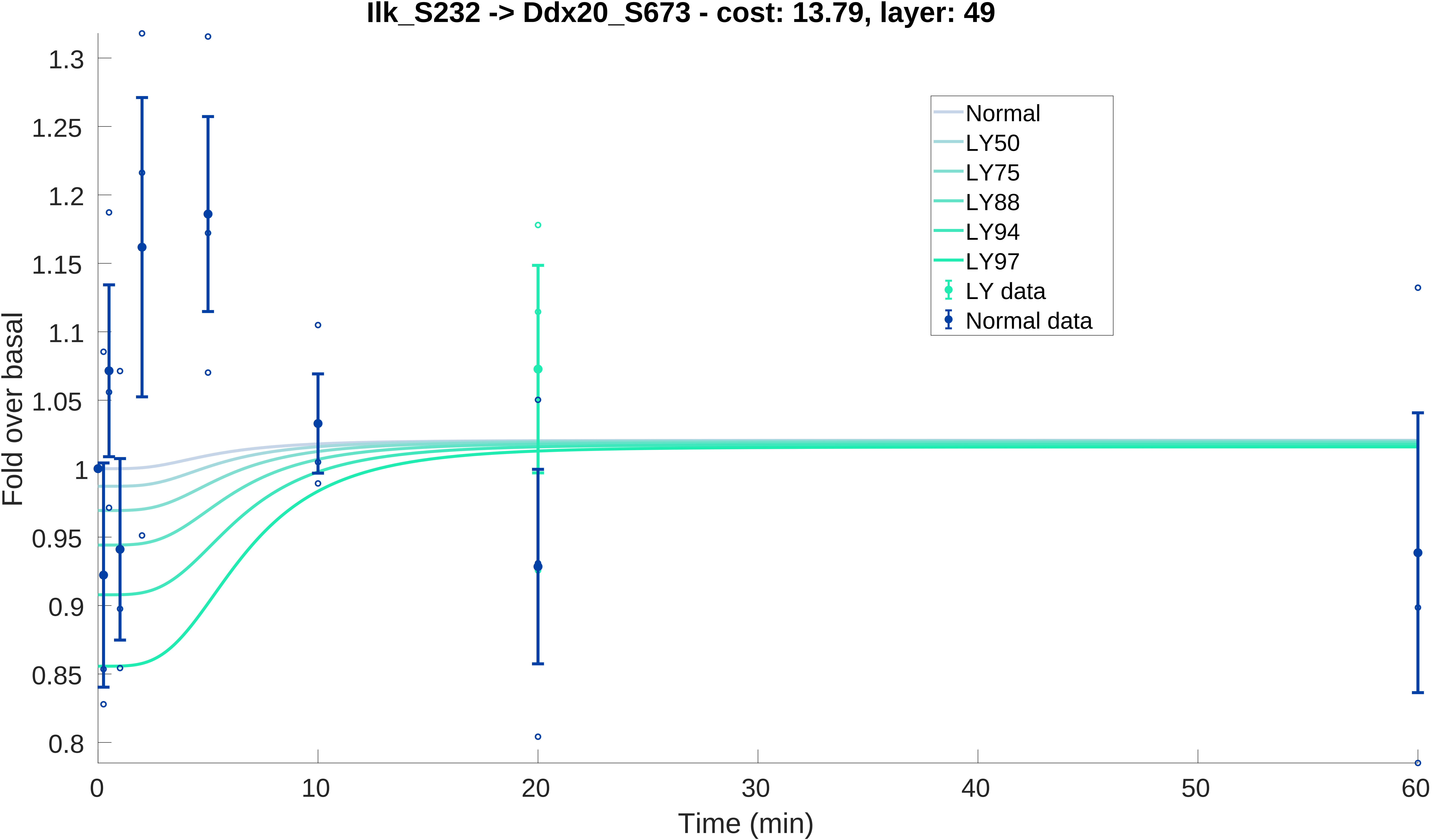

Ilk_S232 -> Ddx20_S673 - cost: 13.79, layer: 49
Fold over basal
1.3
1.25
1.2
1.15
1.1
1.05
1
0.95
0.9
0.85
0.8
0
10
20
30
40
50
60
Time (min)
Normal
LY50
LY75
LY88
LY94
LY97
LY data
Normal data

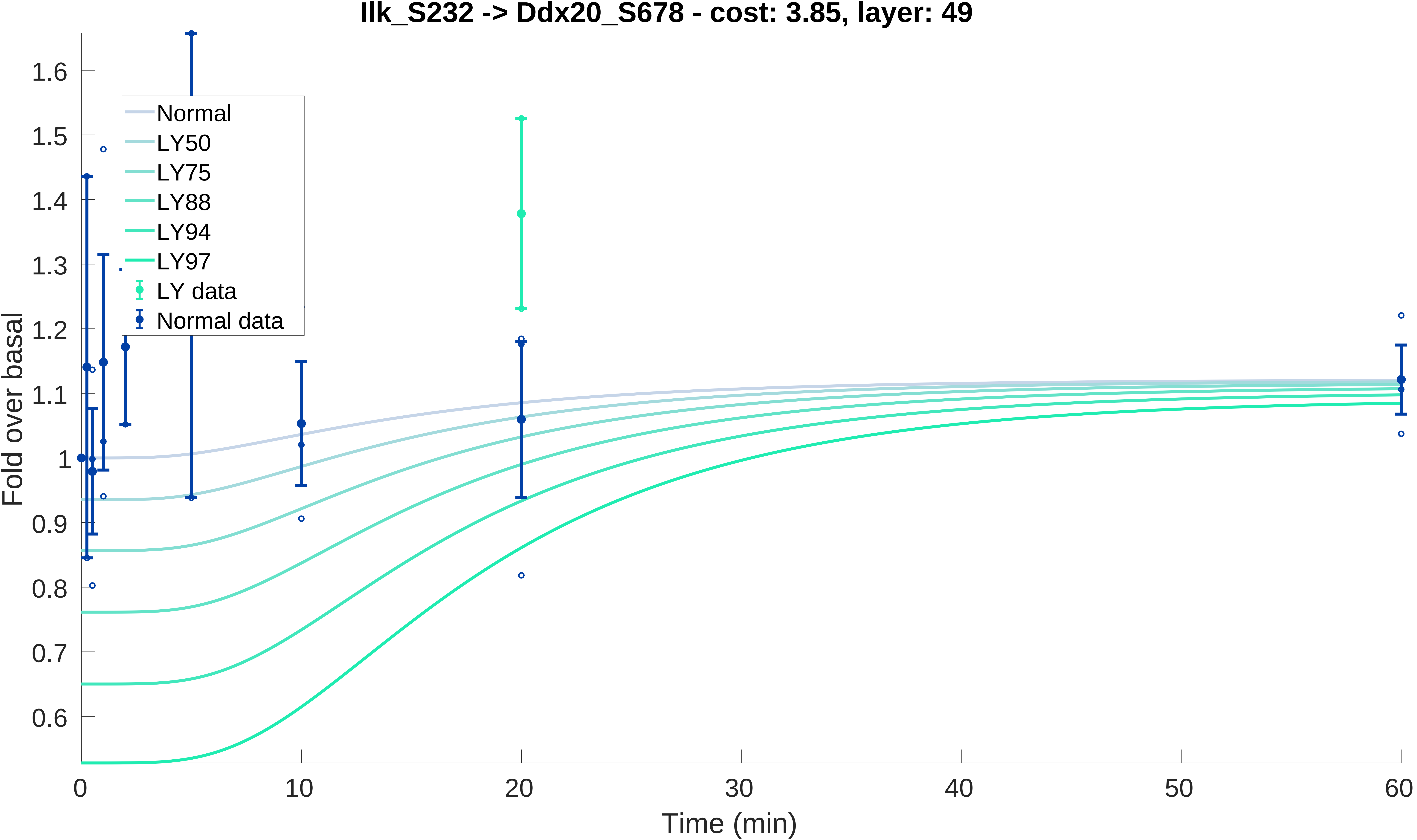

Ilk_S232 -> Ddx20_S678 - cost: 3.85, layer: 49
Normal
LY50
LY75
LY88
LY94
LY97
LY data
Normal data
Fold over basal
Time (min)
1.6
1.5
1.4
1.3
1.2
1.1
1
0.9
0.8
0.7
0.6
0
10
20
30
40
50
60

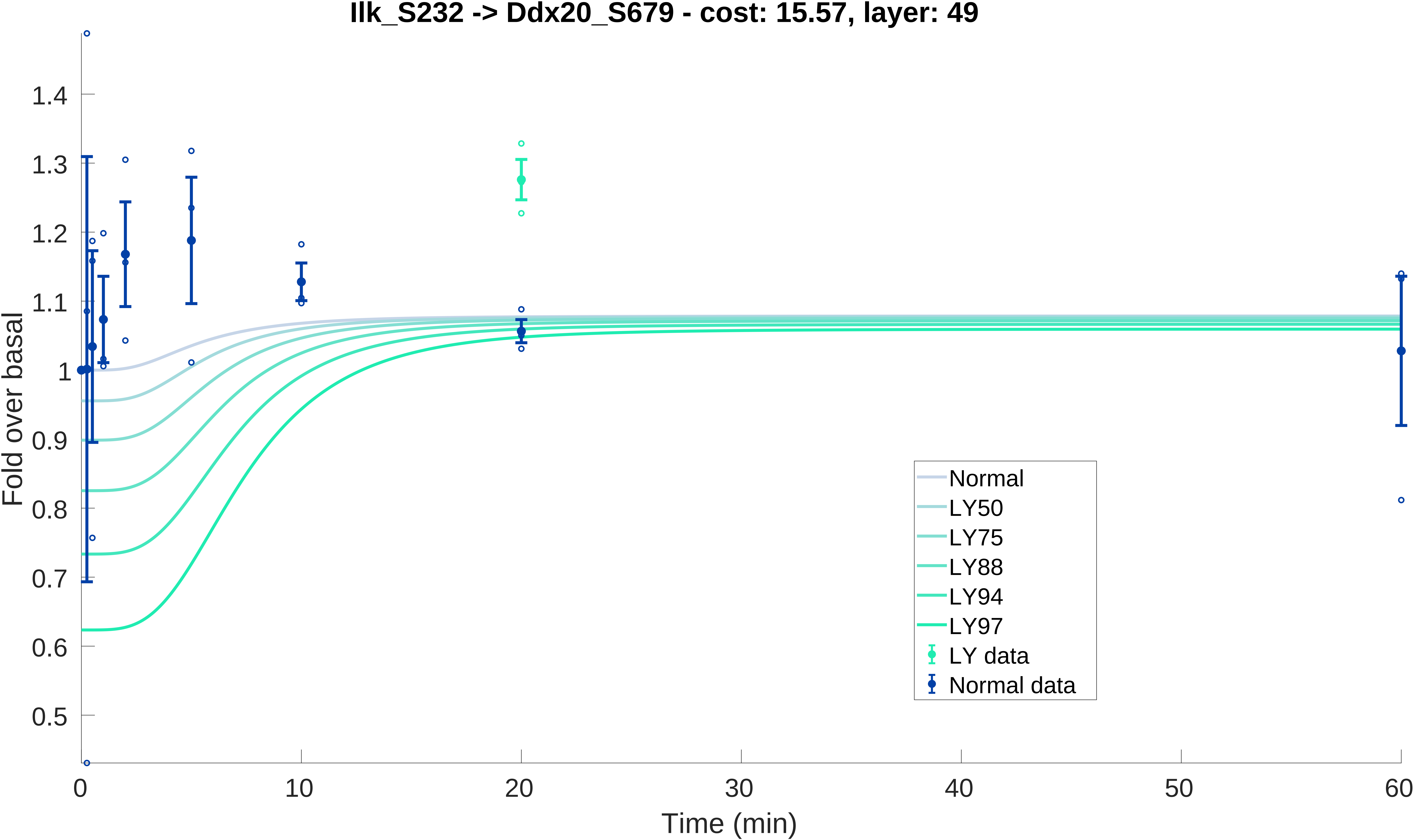

Ilk_S232 -> Ddx20_S679 - cost: 15.57, layer: 49
Fold over basal
Time (min)
1.4
1.3
1.2
1.1
1
0.9
0.8
0.7
0.6
0.5
0
10
20
30
40
50
60
Normal
LY50
LY75
LY88
LY94
LY97
LY data
Normal data

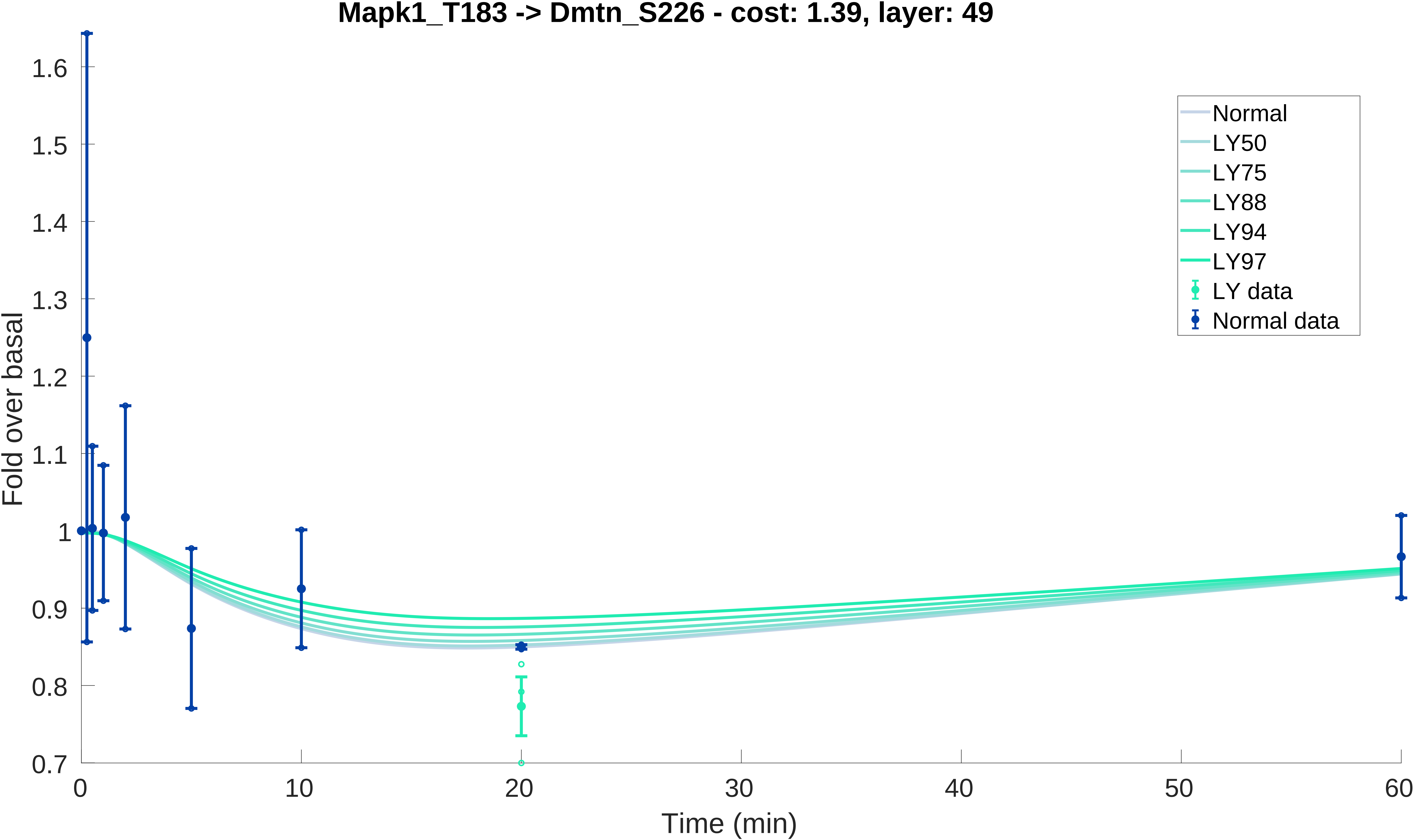
Mapk1_T183 -> Dmtn_S226 - cost: 1.39, layer: 49
Fold over basal
Time (min)
Normal
LY50
LY75
LY88
LY94
LY97
LY data
Normal data
1.6
1.5
1.4
1.3
1.2
1.1
1
0.9
0.8
0.7
0
10
20
30
40
50
60

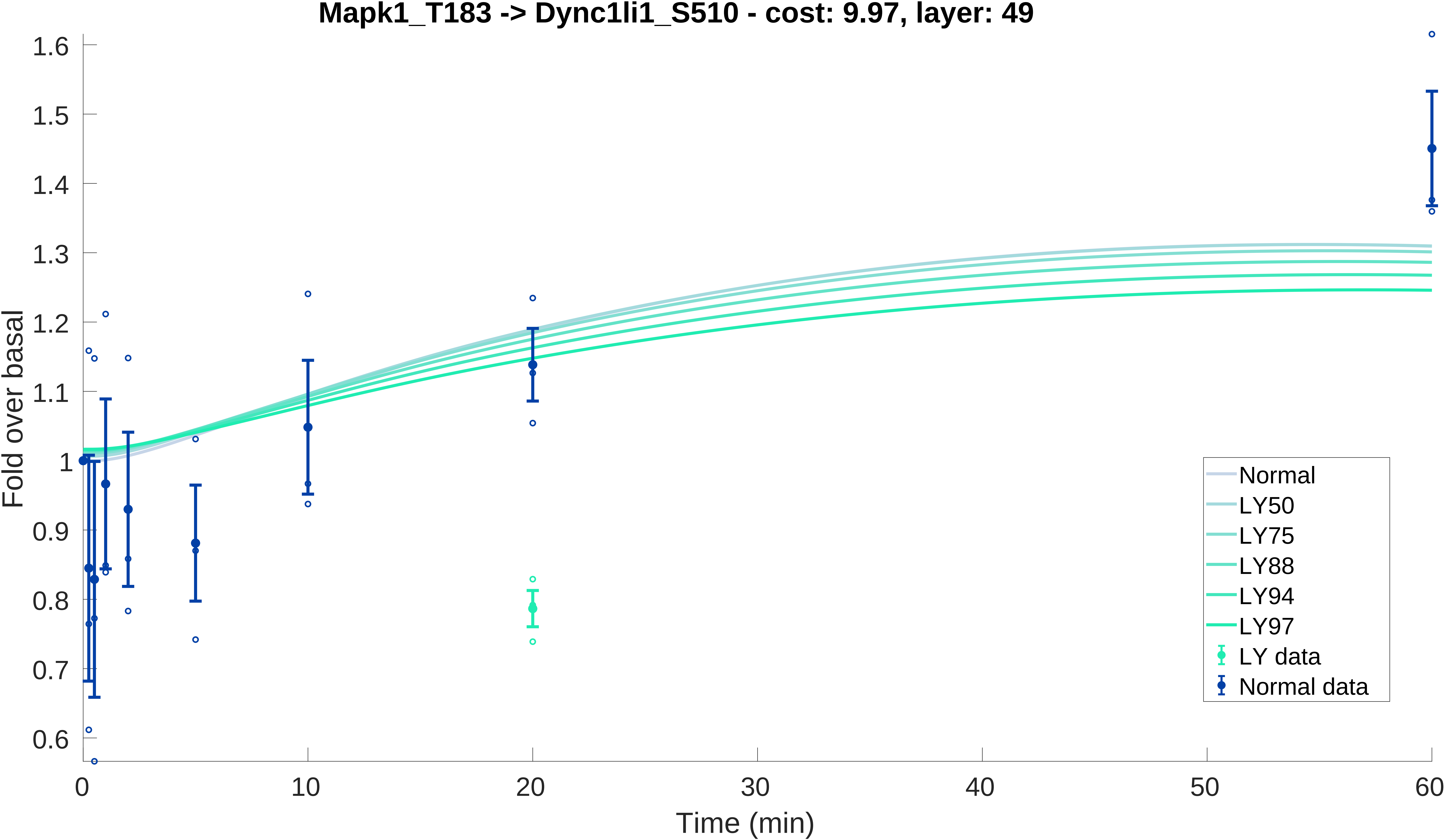

Mapk1_T183 -> Dync1li1_S510 - cost: 9.97, layer: 49
Fold over basal
Time (min)
1.6
1.5
1.4
1.3
1.2
1.1
1
0.9
0.8
0.7
0.6
0
10
20
30
40
50
60
Normal
LY50
LY75
LY88
LY94
LY97
LY data
Normal data

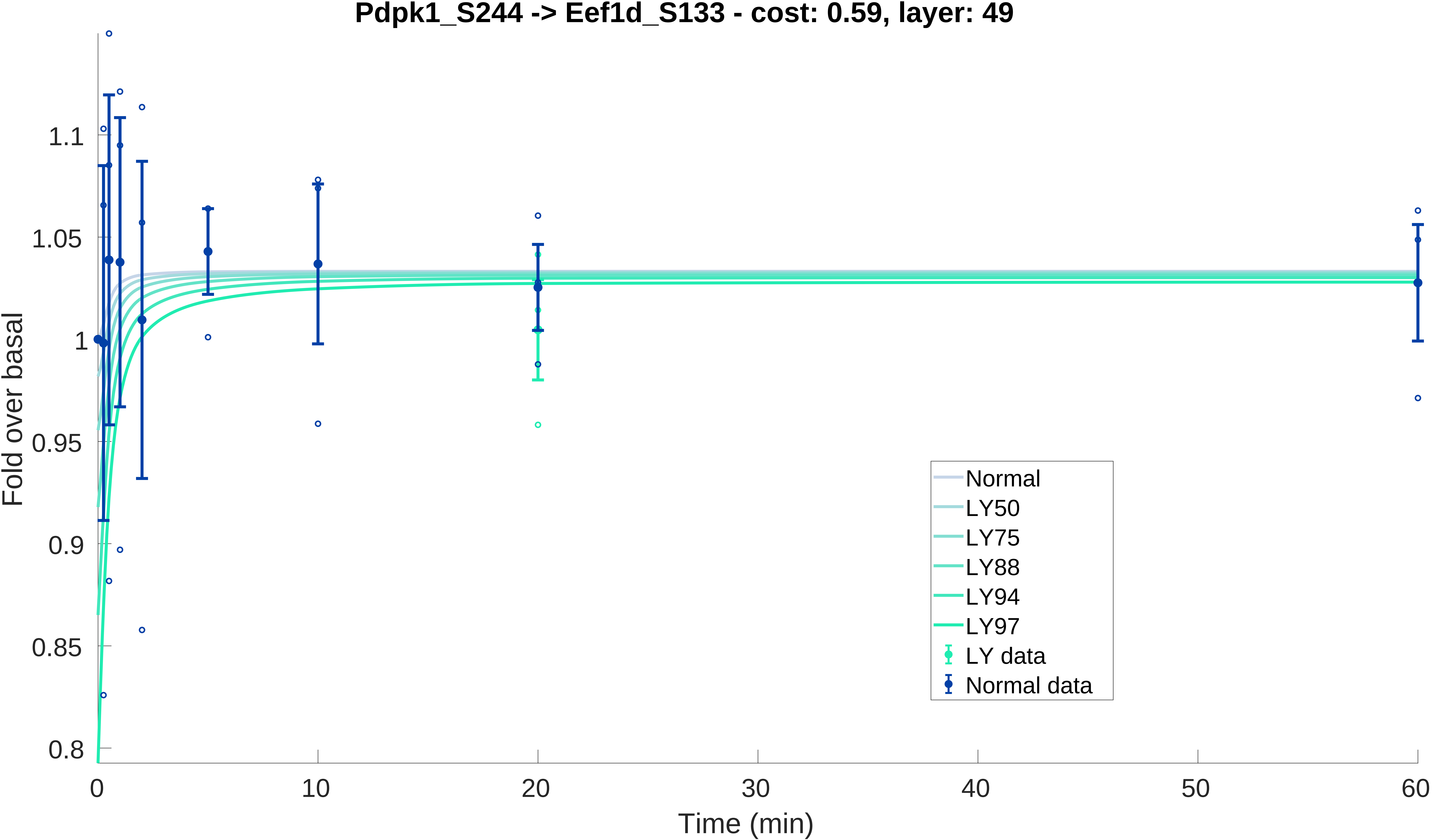
Pdpk1_S244 -> Eef1d_S133 - cost: 0.59, layer: 49
Fold over basal
Time (min)
0
10
20
30
40
50
60
0.8
0.85
0.9
0.95
1
1.05
1.1
Normal
LY50
LY75
LY88
LY94
LY97
LY data
Normal data

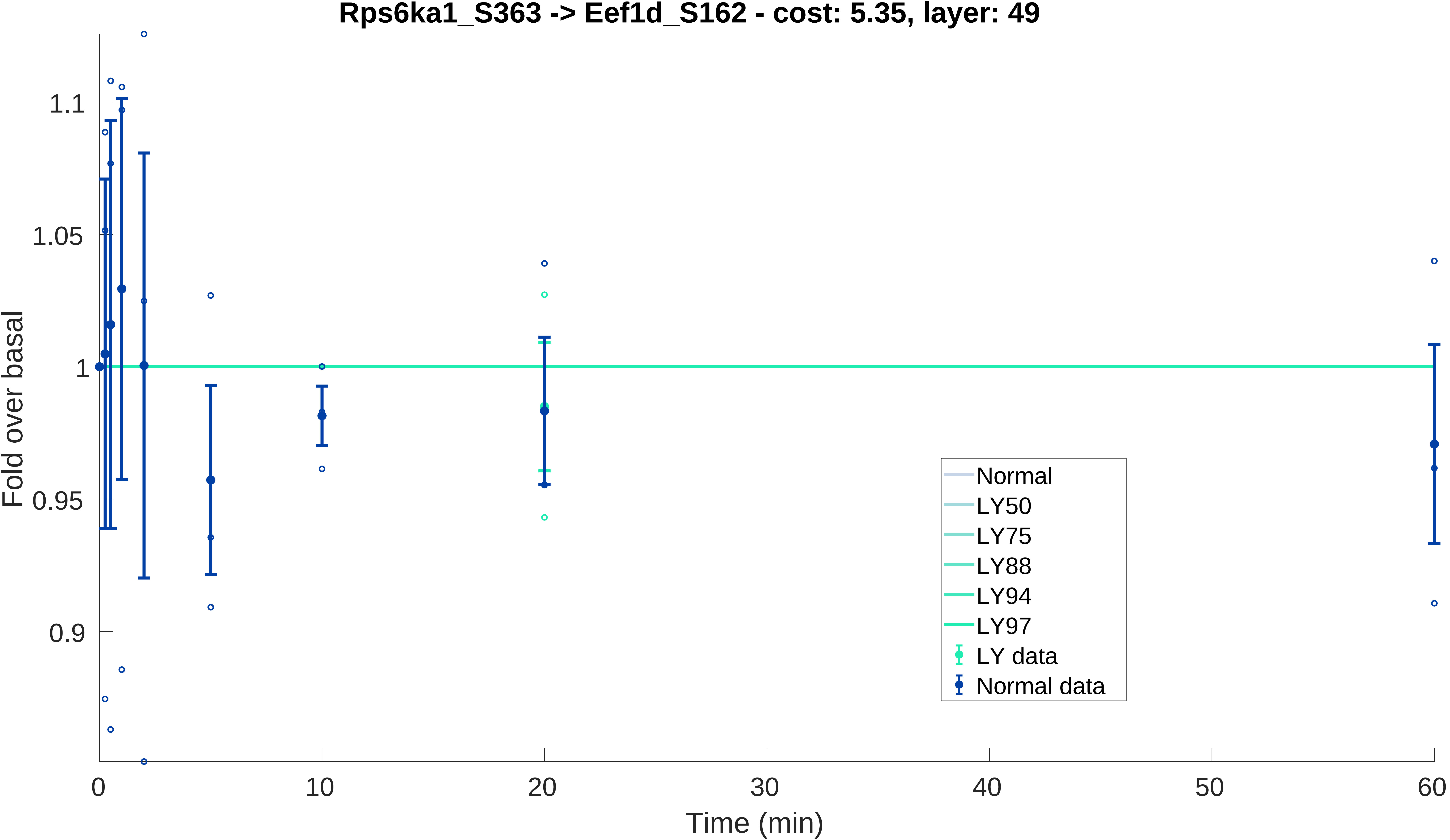
Rps6ka1_S363 -> Eef1d_S162 - cost: 5.35, layer: 49
Fold over basal
1.1
1.05
1
0.95
0.9
0
10
20
30
40
50
60
Time (min)
Normal
LY50
LY75
LY88
LY94
LY97
LY data
Normal data

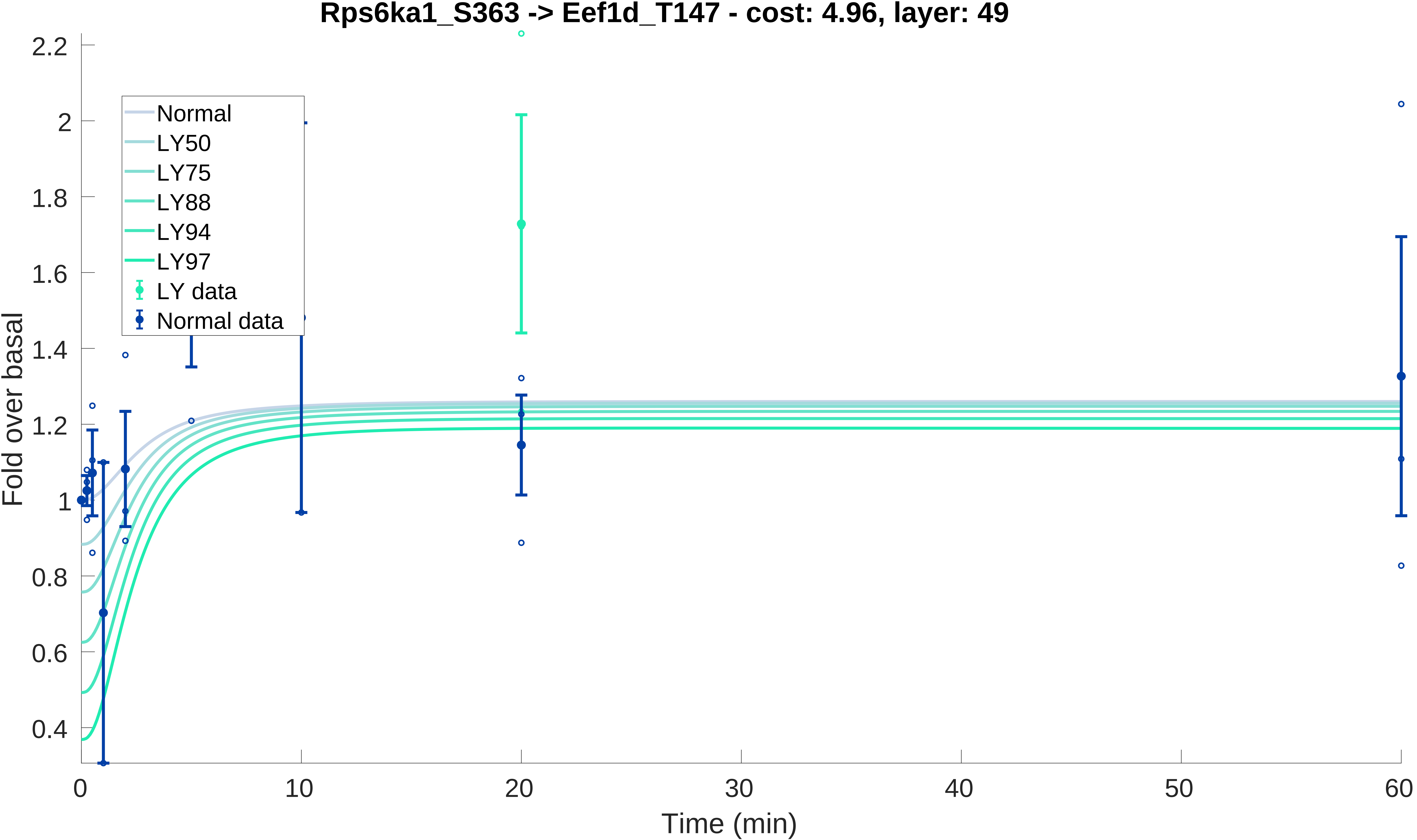
Rps6ka1_S363 -> Eef1d_T147 - cost: 4.96, layer: 49
Normal
LY50
LY75
LY88
LY94
LY97
LY data
Normal data
Fold over basal
2.2
2
1.8
1.6
1.4
1.2
1
0.8
0.6
0.4
0
10
20
30
40
50
60
Time (min)

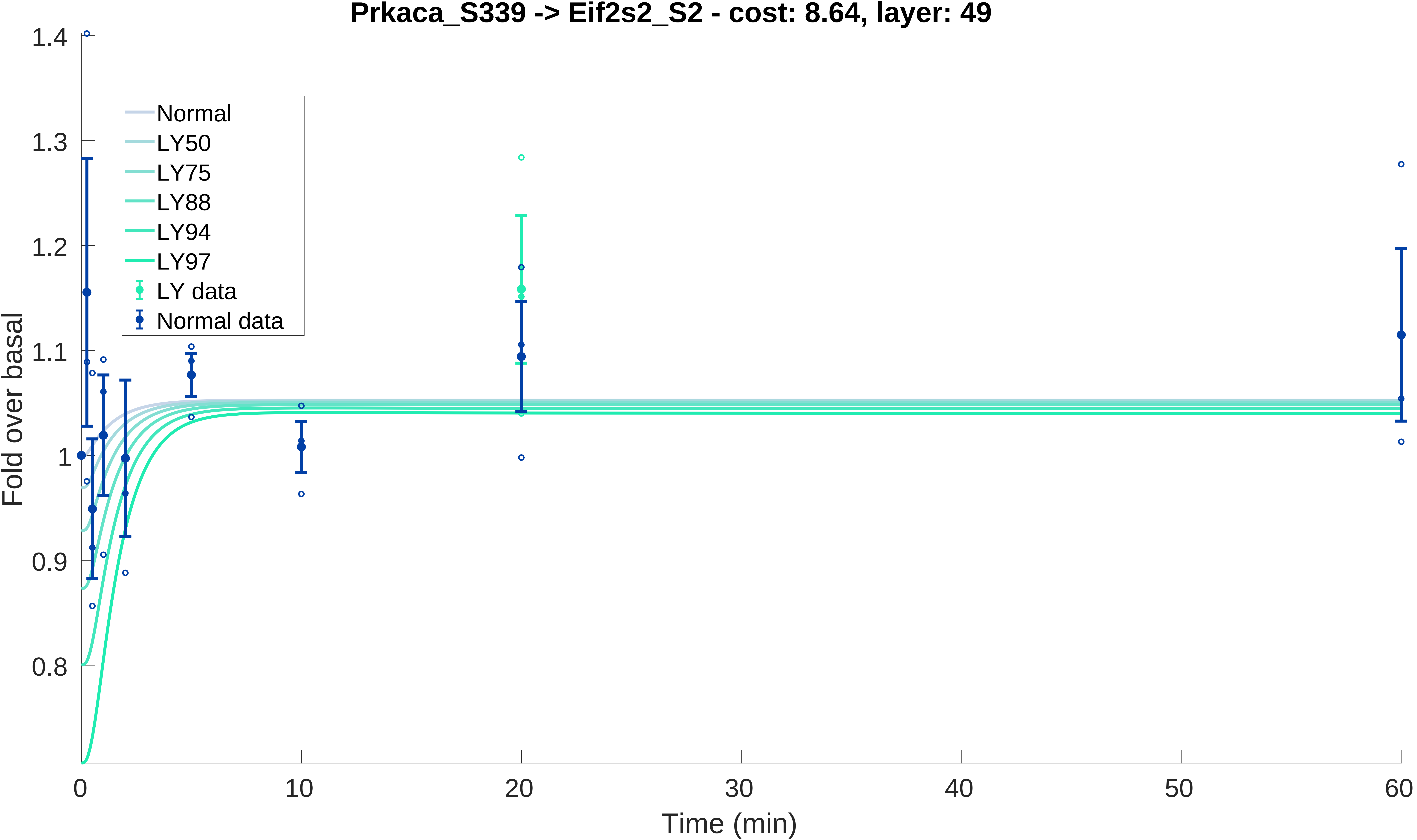

Prkaca_S339 -> Eif2s2_S2 - cost: 8.64, layer: 49
Fold over basal
Time (min)
Normal
LY50
LY75
LY88
LY94
LY97
LY data
Normal data
1.4
1.3
1.2
1.1
1
0.9
0.8
0
10
20
30
40
50
60

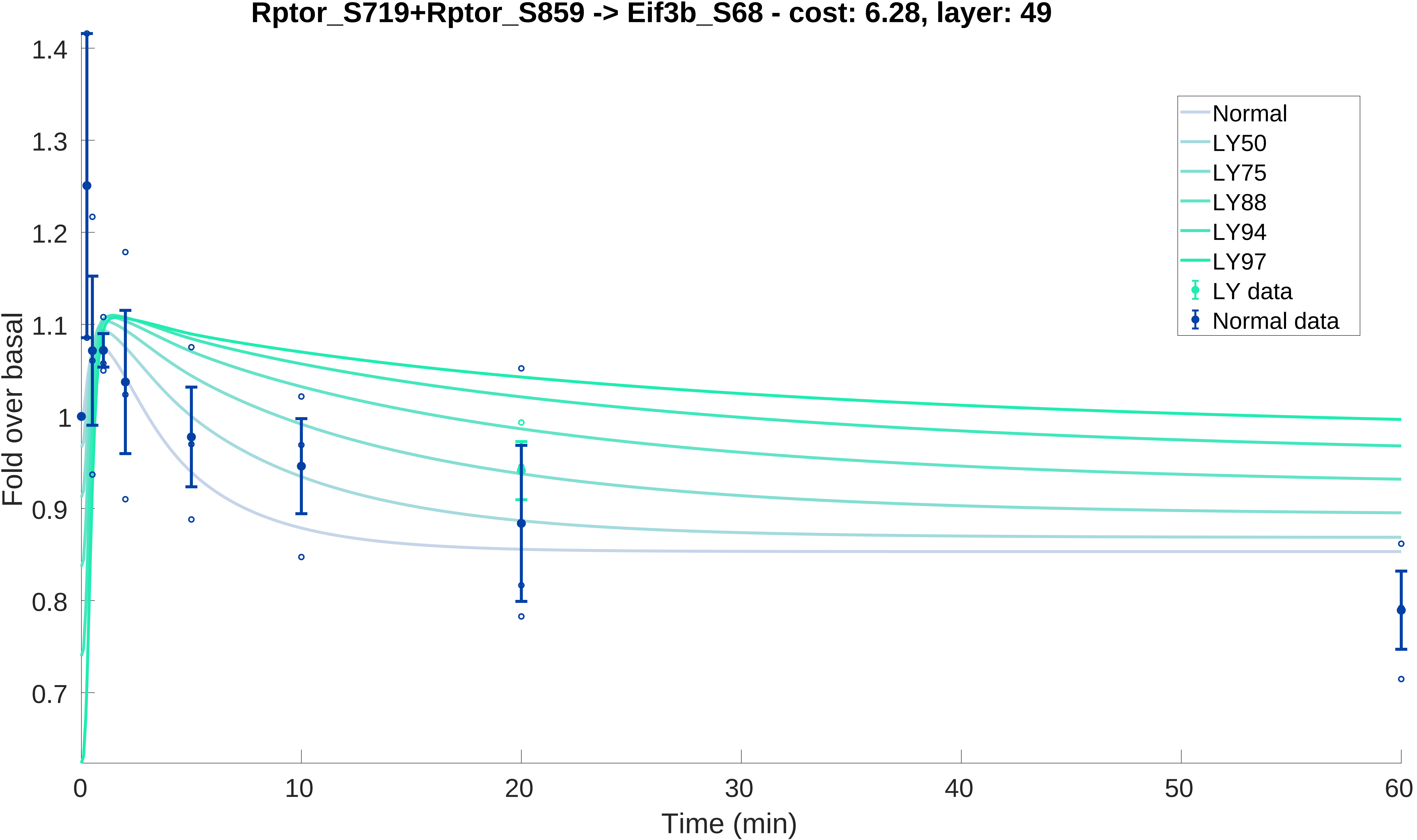
Rptor_S719+Rptor_S859 -> Eif3b_S68 - cost: 6.28, layer: 49
Fold over basal
Time (min)
0.7
0.8
0.9
1
1.1
1.2
1.3
1.4
0
10
20
30
40
50
60
Normal
LY50
LY75
LY88
LY94
LY97
LY data
Normal data

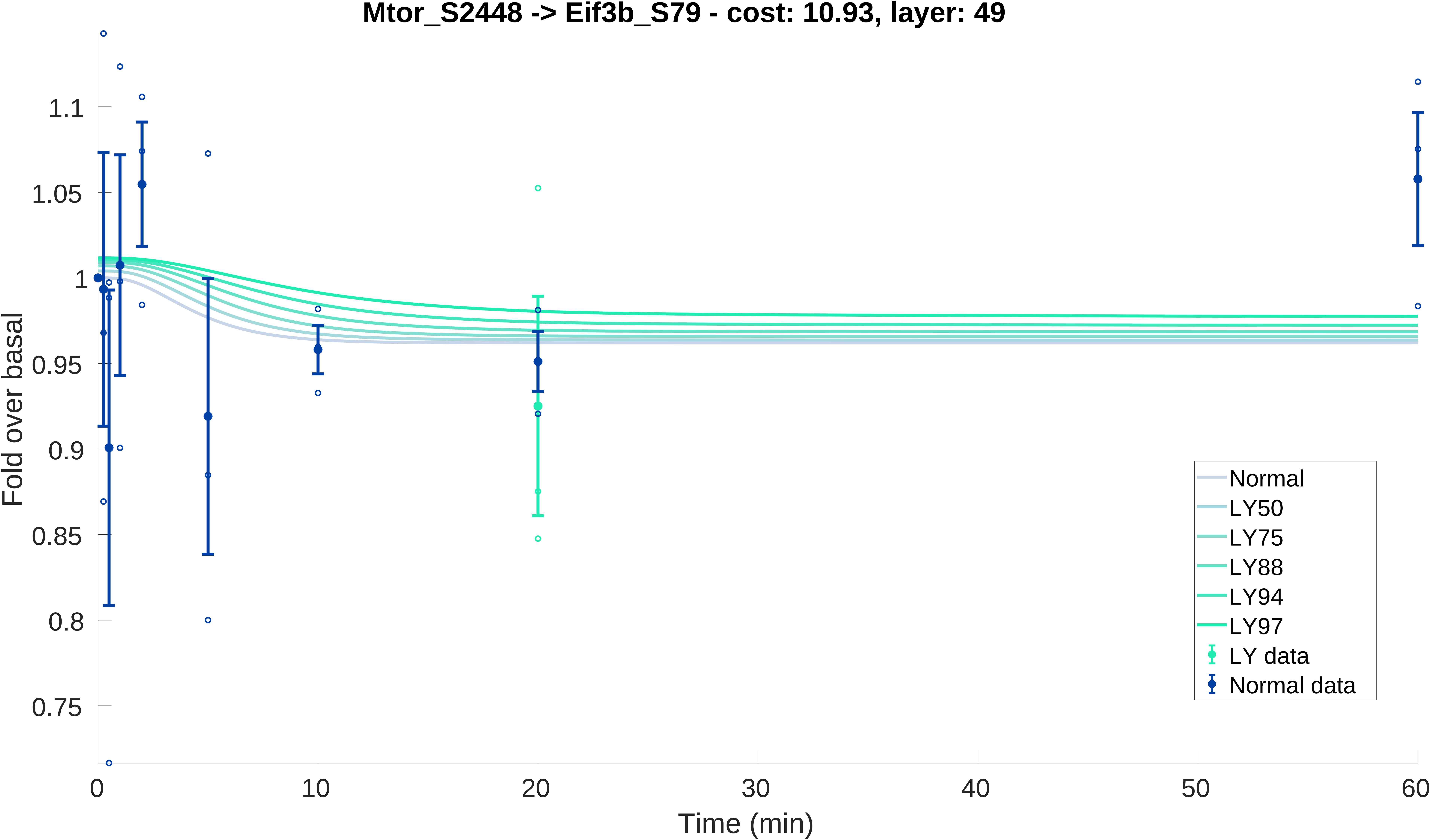

Mtor_S2448 -> Eif3b_S79 - cost: 10.93, layer: 49
Fold over basal
1.1
1.05
1
0.95
0.9
0.85
0.8
0.75
0
10
20
30
40
50
60
Time (min)
Normal
LY50
LY75
LY88
LY94
LY97
LY data
Normal data

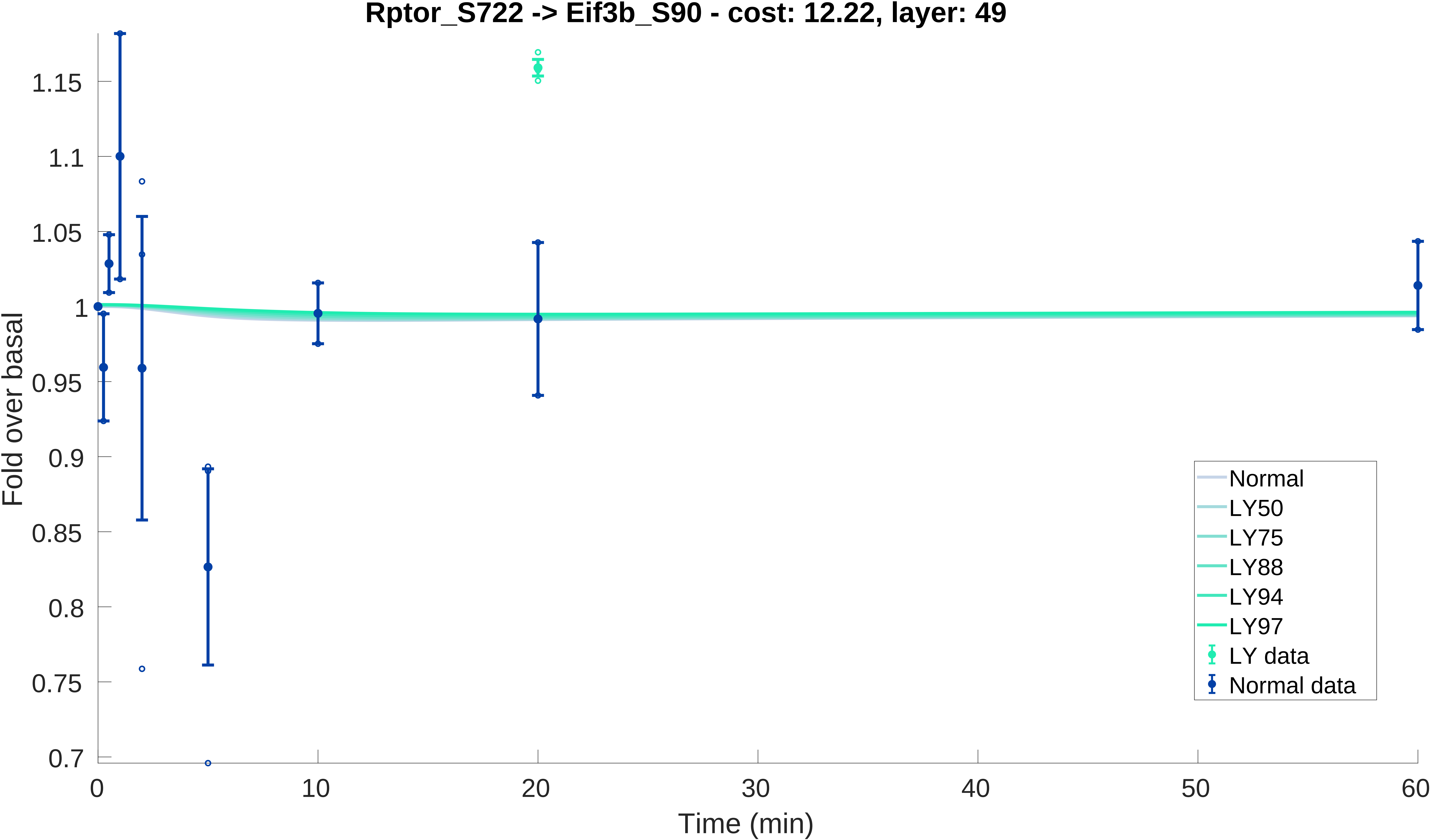

Rptor_S722 -> Eif3b_S90 - cost: 12.22, layer: 49
Fold over basal
1.15
1.1
1.05
1
0.95
0.9
0.85
0.8
0.75
0.7
0
10
20
30
40
50
60
Time (min)
Normal
LY50
LY75
LY88
LY94
LY97
LY data
Normal data

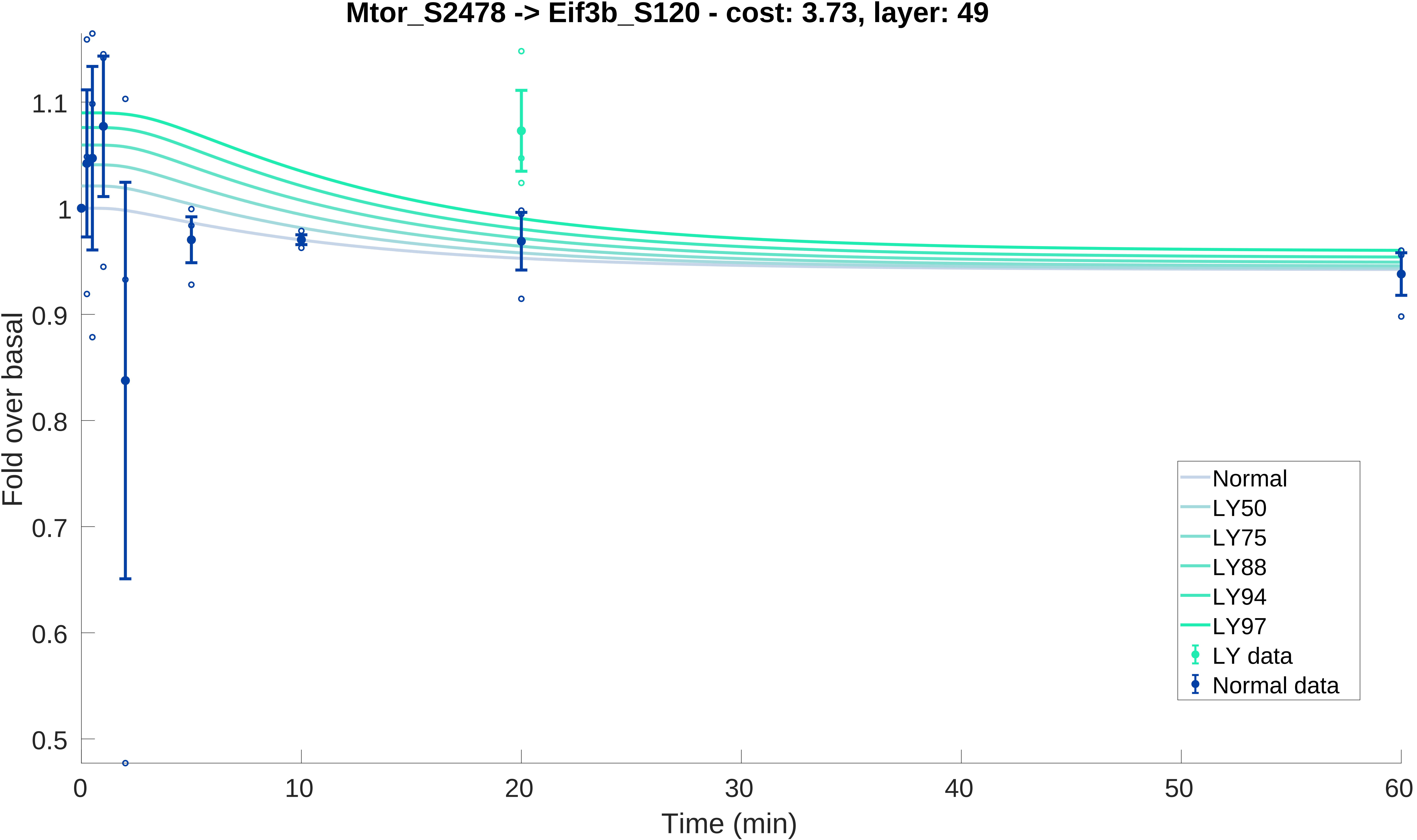

Mtor_S2478 -> Eif3b_S120 - cost: 3.73, layer: 49
Fold over basal
1.1
1
0.9
0.8
0.7
0.6
0.5
0
10
20
30
40
50
60
Time (min)
Normal
LY50
LY75
LY88
LY94
LY97
LY data
Normal data

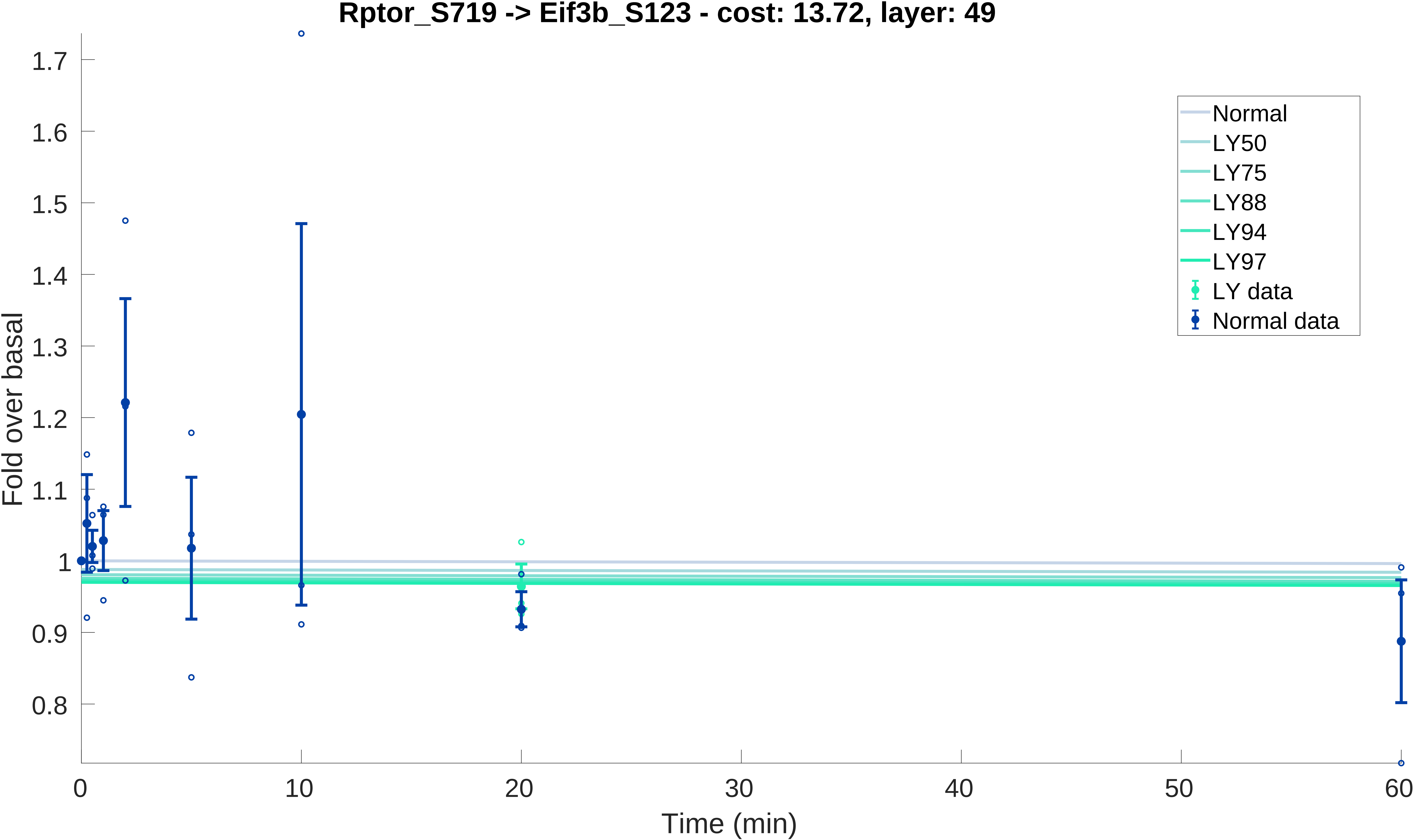
Rptor_S719 -> Eif3b_S123 - cost: 13.72, layer: 49
Fold over basal
Time (min)
0
10
20
30
40
50
60
0.8
0.9
1
1.1
1.2
1.3
1.4
1.5
1.6
1.7
Normal
LY50
LY75
LY88
LY94
LY97
LY data
Normal data

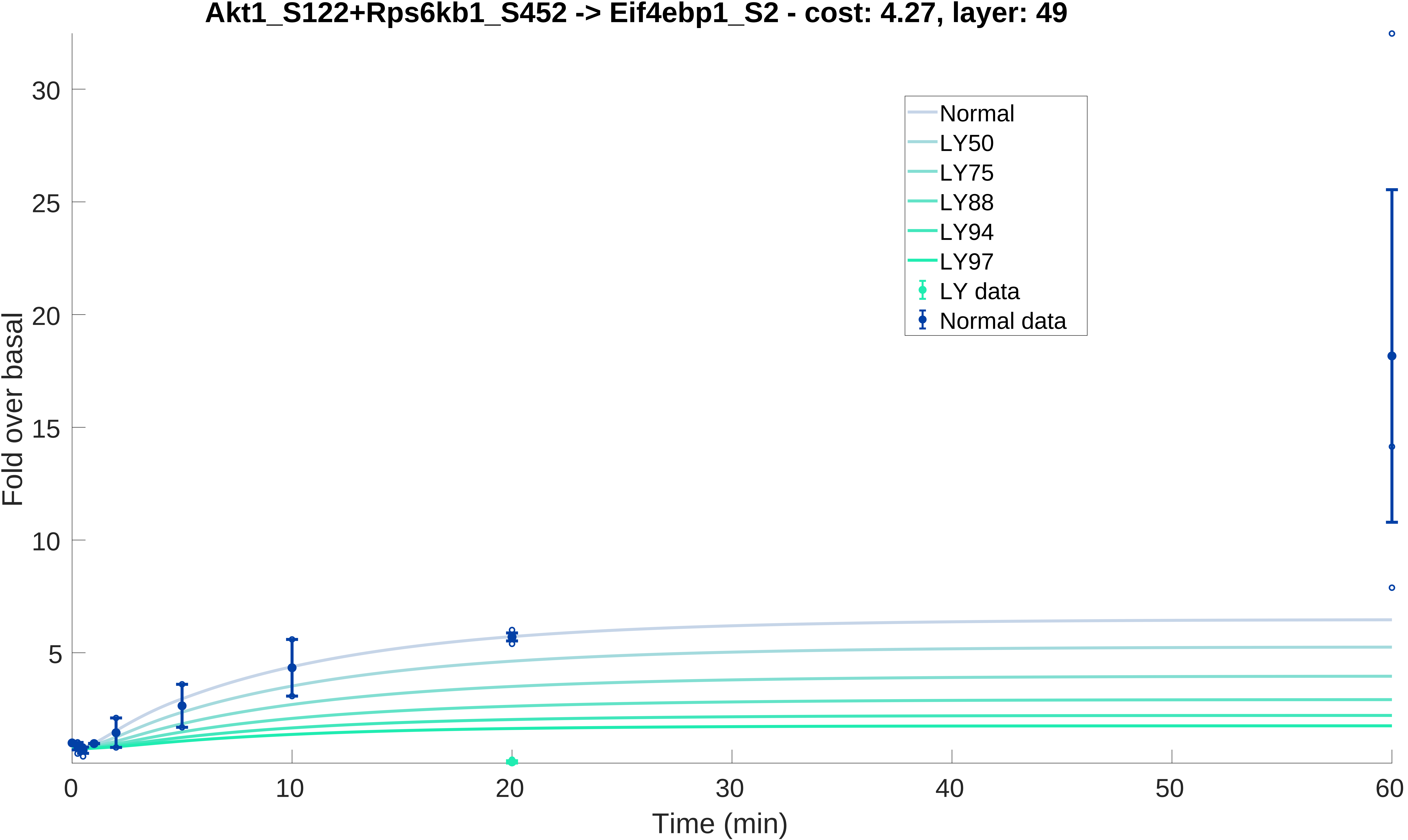

Akt1_S122+Rps6kb1_S452 -> Eif4ebp1_S2 - cost: 4.27, layer: 49
Fold over basal
Time (min)
Normal
LY50
LY75
LY88
LY94
LY97
LY data
Normal data

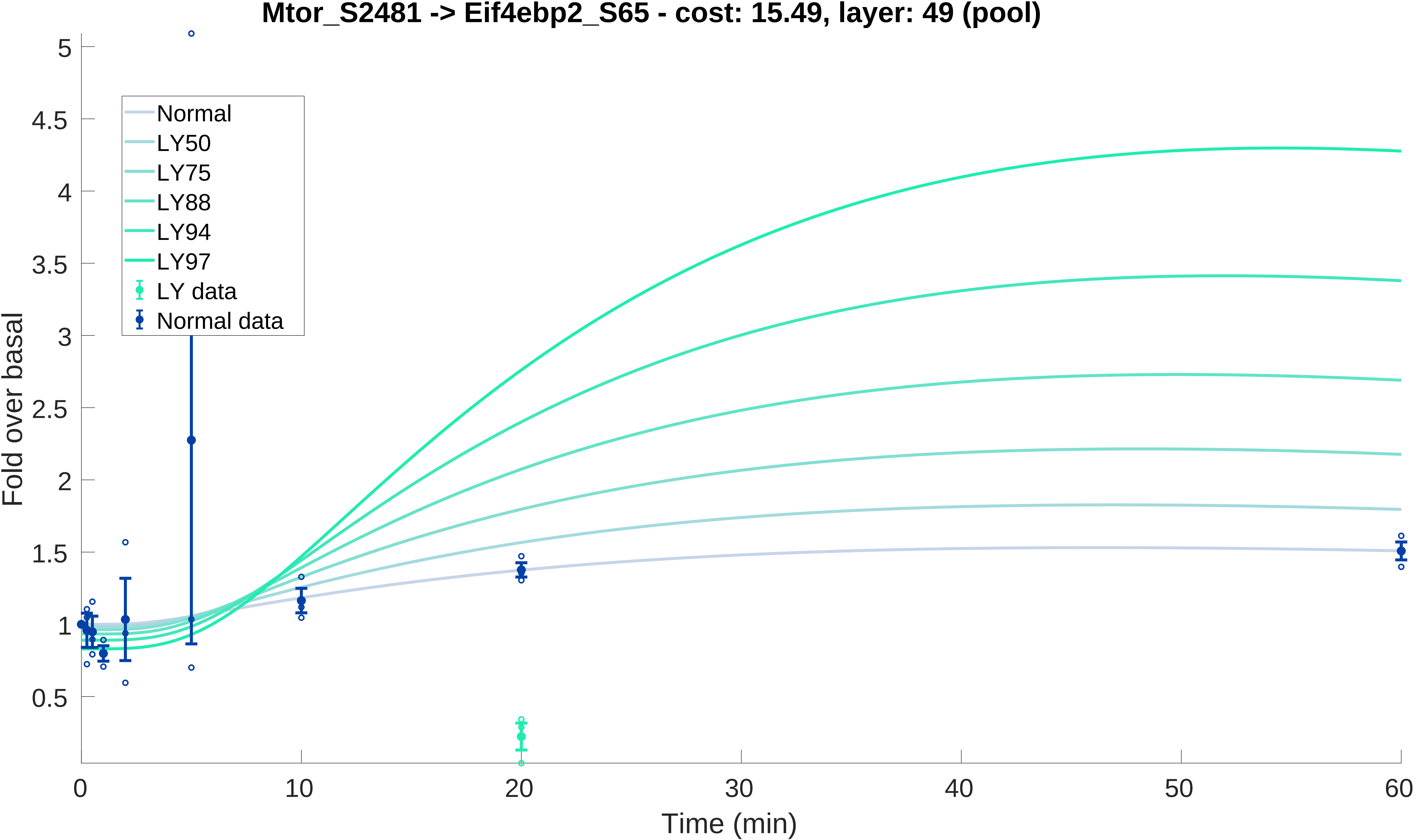

Mtor_S2481 -> Eif4ebp2_S65 - cost: 15.49, layer: 49 (pool)
Fold over basal
Time (min)
0
10
20
30
40
50
60
0.5
1
1.5
2
2.5
3
3.5
4
4.5
5
Normal
LY50
LY75
LY88
LY94
LY97
LY data
Normal data

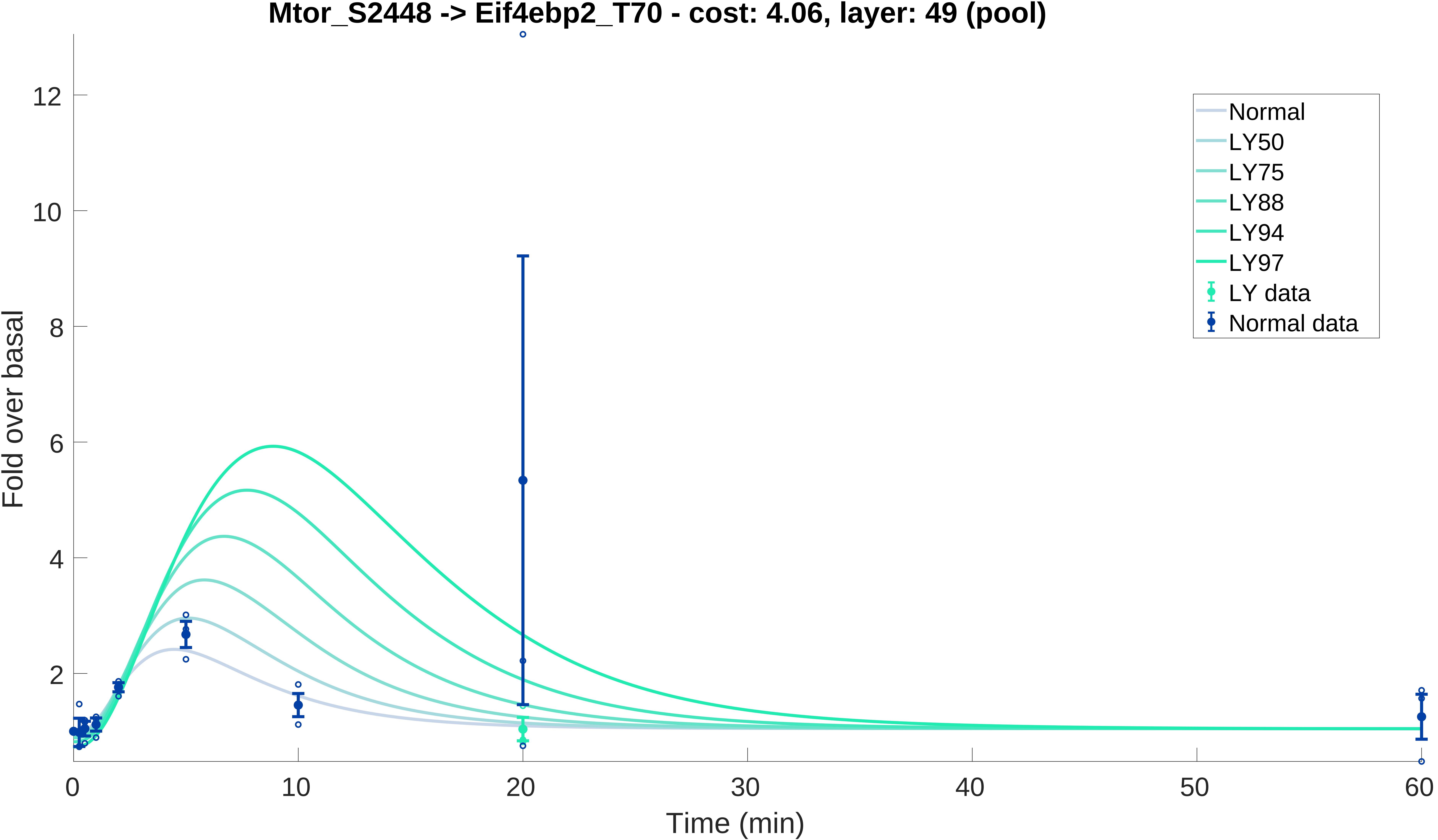
Mtor_S2448 -> Eif4ebp2_T70 - cost: 4.06, layer: 49 (pool)
Fold over basal
Time (min)
0
10
20
30
40
50
60
2
4
6
8
10
12
Normal
LY50
LY75
LY88
LY94
LY97
LY data
Normal data

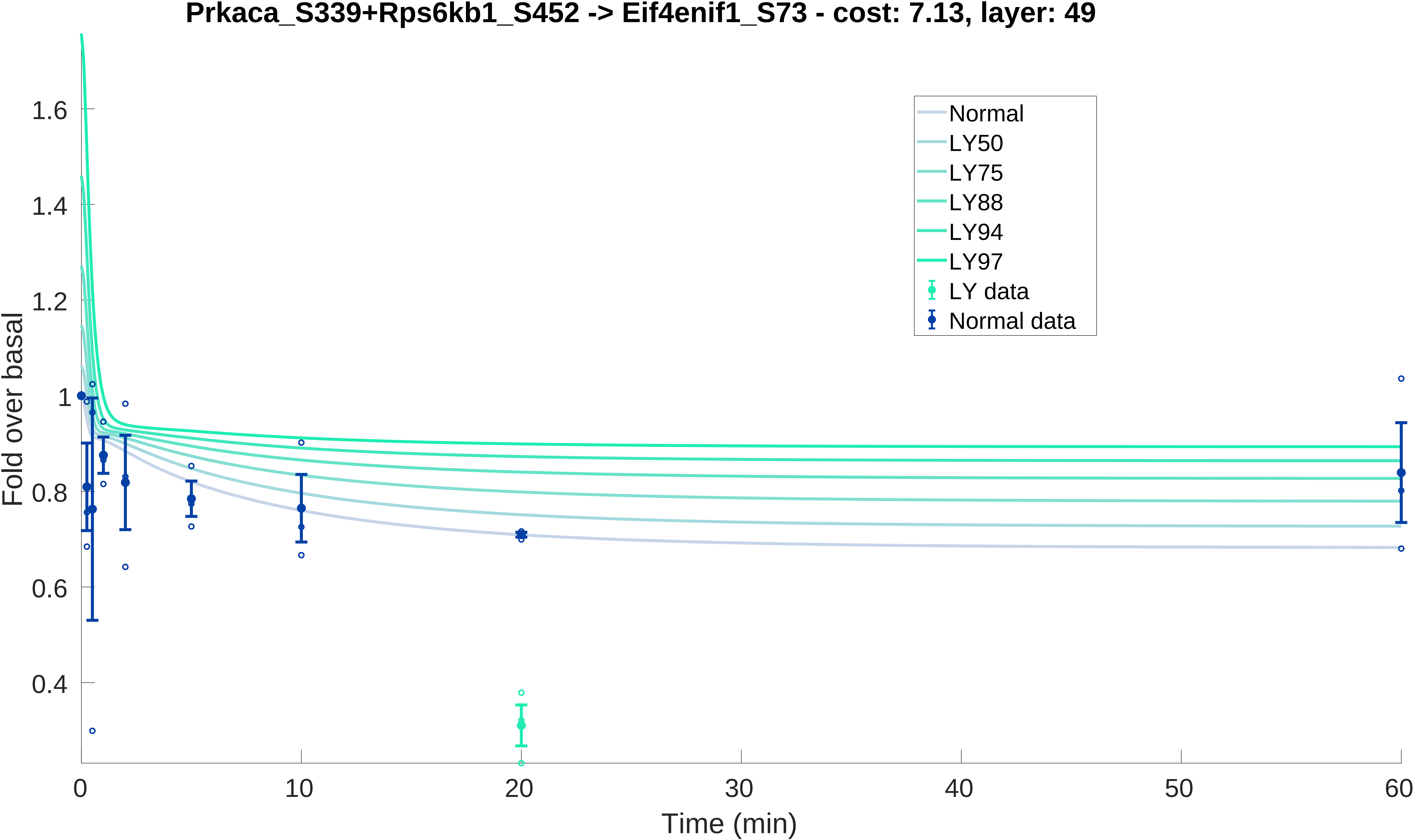

Prkaca_S339+Rps6kb1_S452 -> Eif4enif1_S73 - cost: 7.13, layer: 49
Fold over basal
1.6
1.4
1.2
1
0.8
0.6
0.4
0
10
20
30
40
50
60
Time (min)
Normal
LY50
LY75
LY88
LY94
LY97
LY data
Normal data

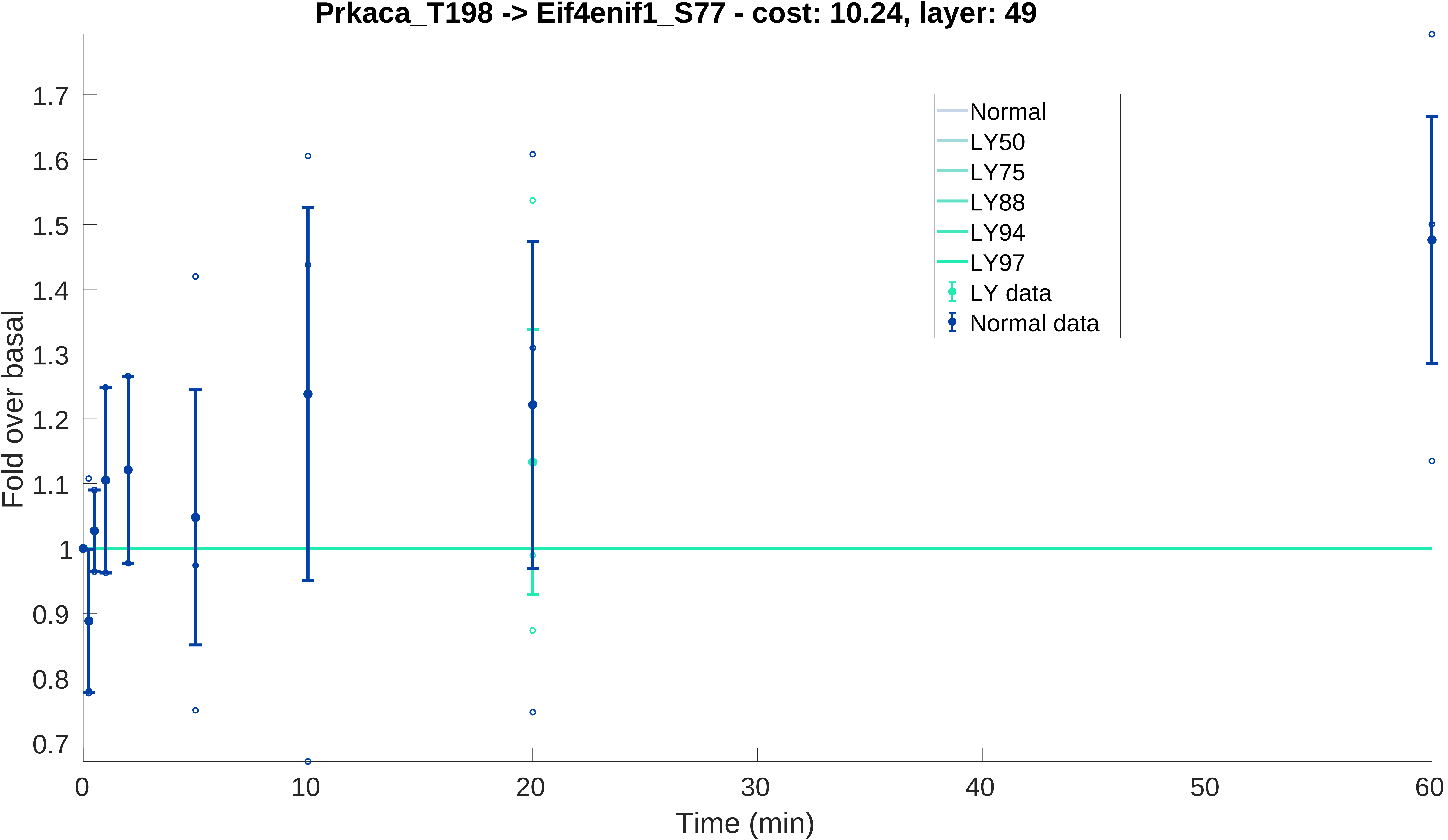

Prkaca_T198 -> Eif4enif1_S77 - cost: 10.24, layer: 49
Fold over basal
Time (min)
0.7
0.8
0.9
1
1.1
1.2
1.3
1.4
1.5
1.6
1.7
0
10
20
30
40
50
60
Normal
LY50
LY75
LY88
LY94
LY97
LY data
Normal data

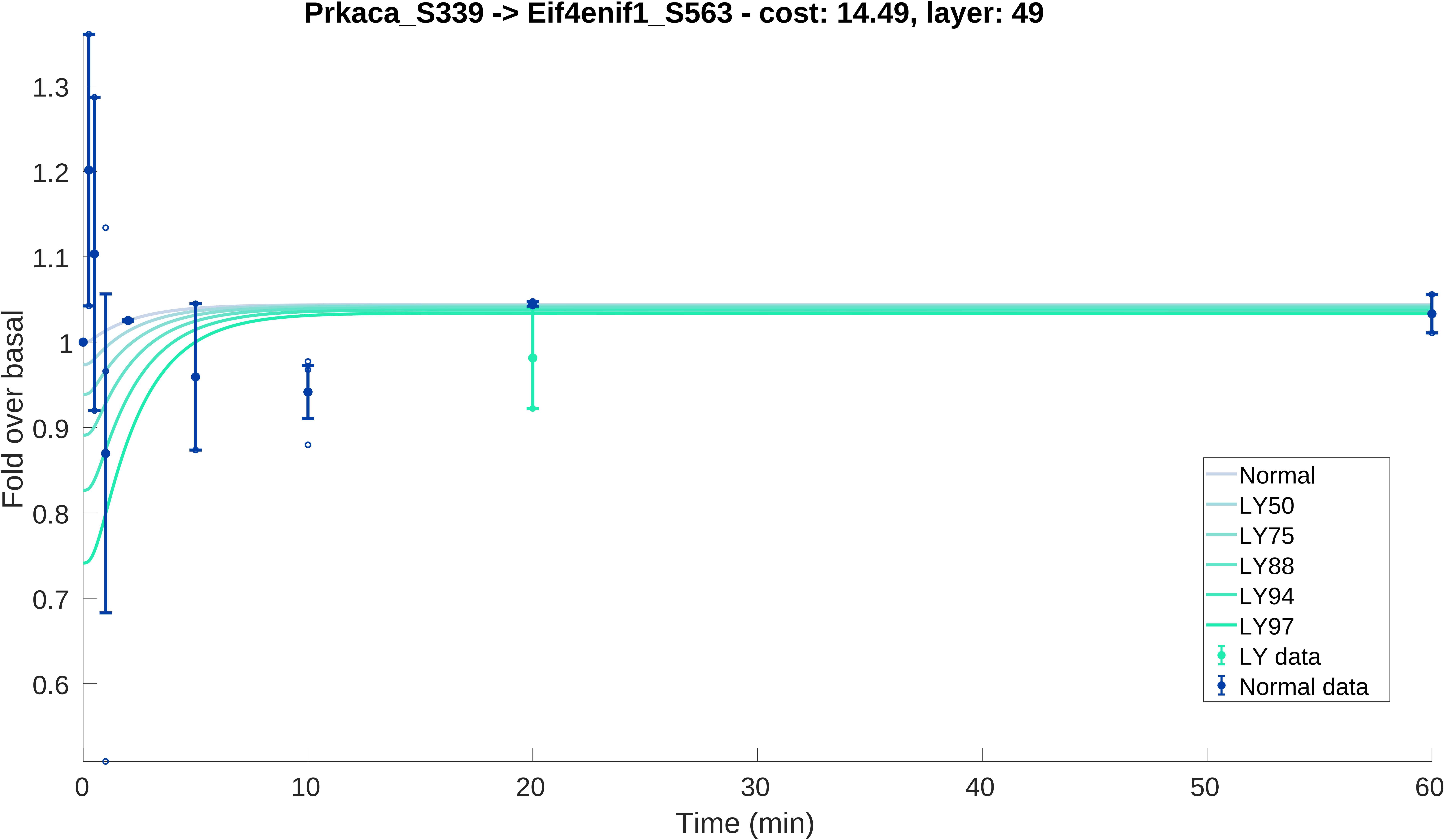

Prkaca_S339 -> Eif4enif1_S563 - cost: 14.49, layer: 49
Fold over basal
1.3
1.2
1.1
1
0.9
0.8
0.7
0.6
0
10
20
30
40
50
60
Time (min)
Normal
LY50
LY75
LY88
LY94
LY97
LY data
Normal data

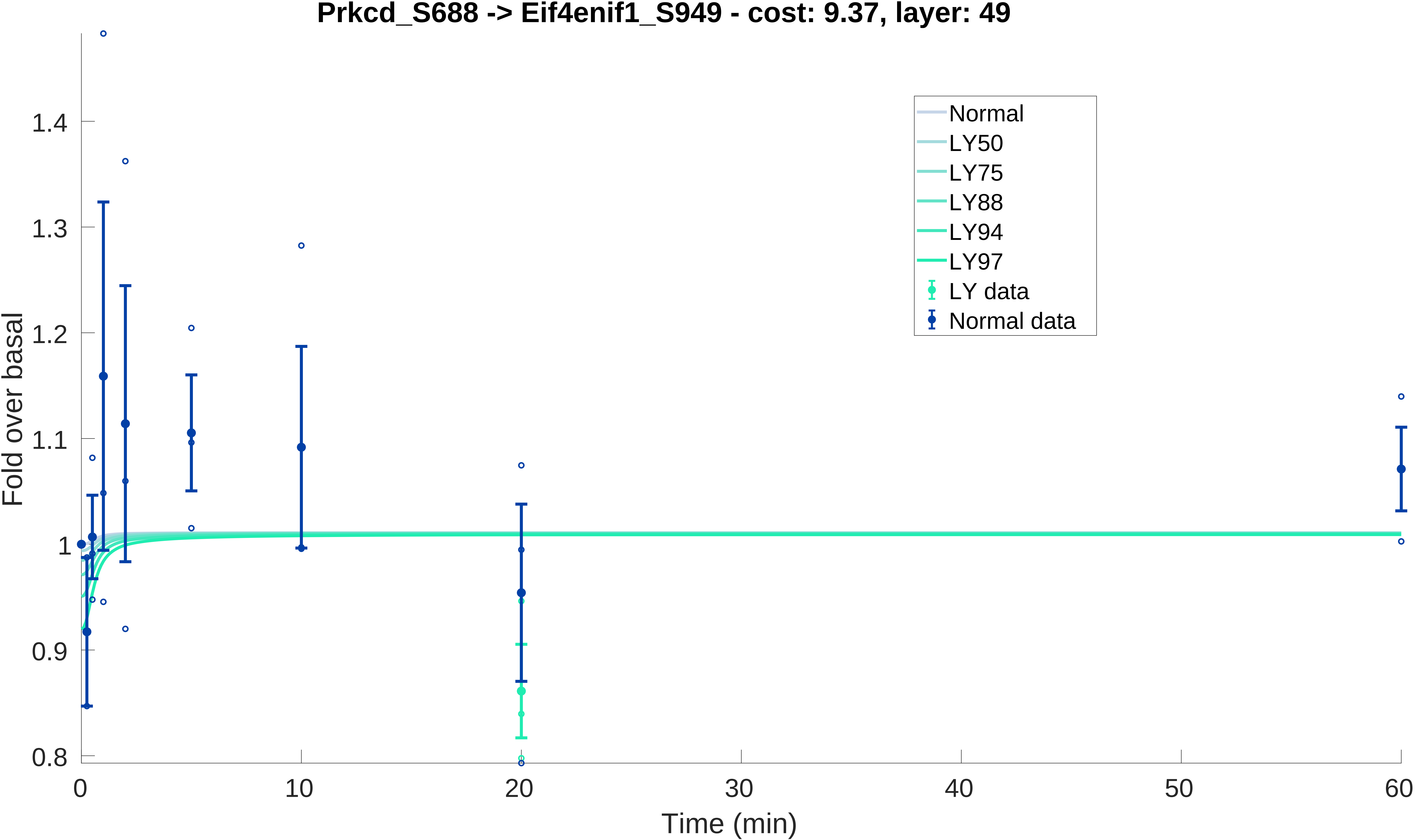

Prkcd_S688 -> Eif4enif1_S949 - cost: 9.37, layer: 49
Normal
LY50
LY75
LY88
LY94
LY97
LY data
Normal data
Fold over basal
Time (min)
0.8
0.9
1
1.1
1.2
1.3
1.4
0
10
20
30
40
50
60

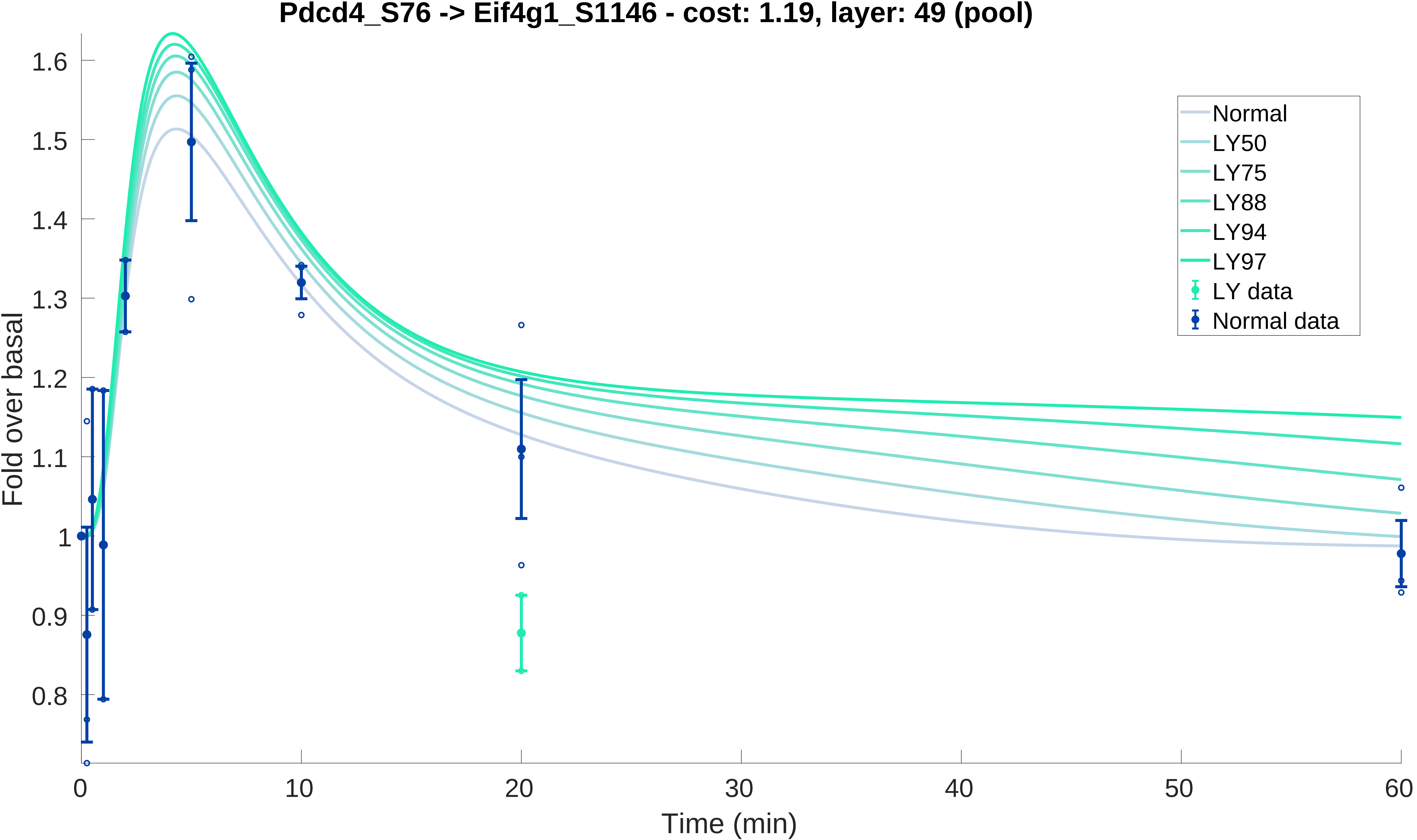
Pdcd4_S76 -> Eif4g1_S1146 - cost: 1.19, layer: 49 (pool)
Fold over basal
Time (min)
0.8
0.9
1
1.1
1.2
1.3
1.4
1.5
1.6
0
10
20
30
40
50
60
Normal
LY50
LY75
LY88
LY94
LY97
LY data
Normal data

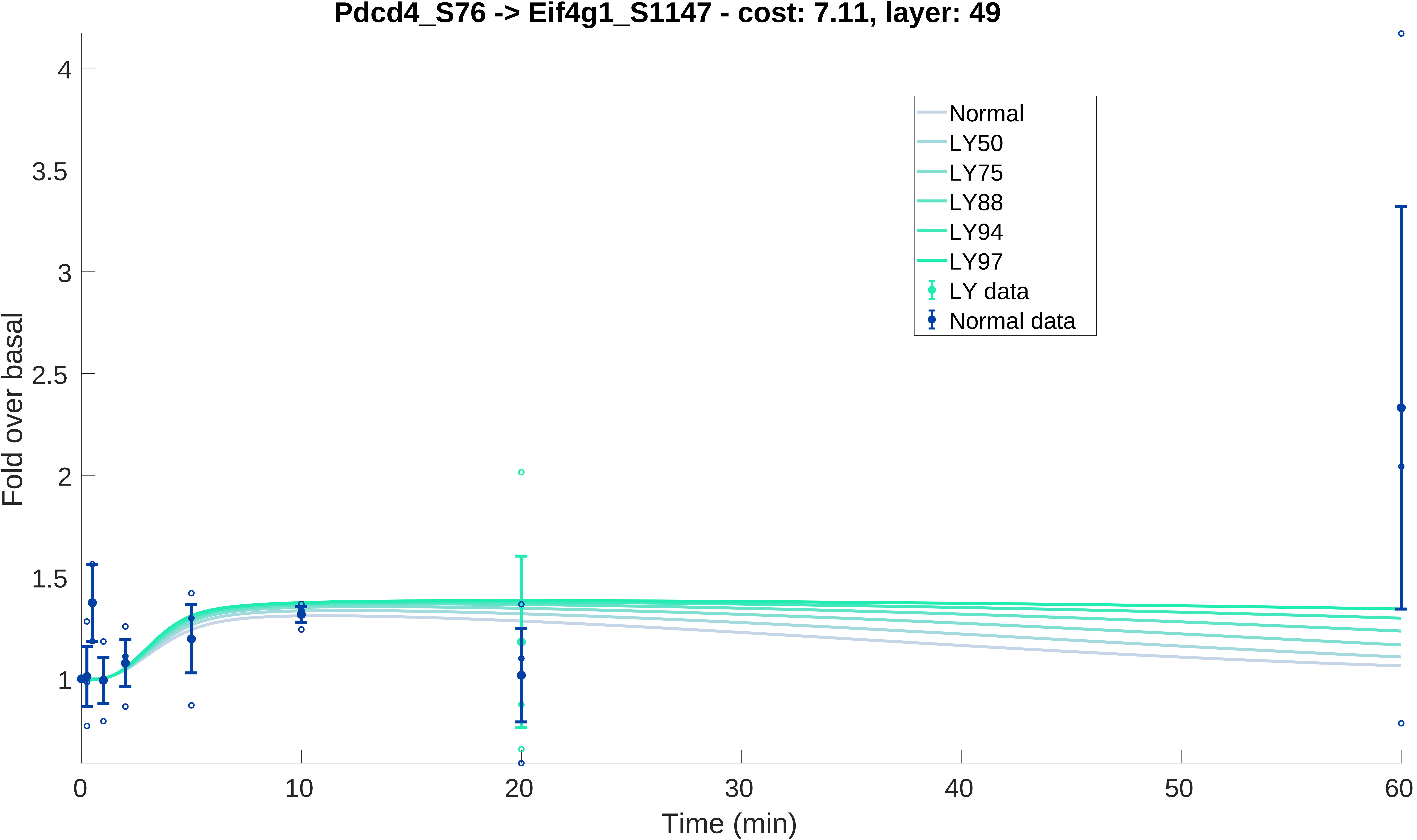

Pdcd4_S76 -> Eif4g1_S1147 - cost: 7.11, layer: 49
Fold over basal
Time (min)
0
10
20
30
40
50
60
1
1.5
2
2.5
3
3.5
4
Normal
LY50
LY75
LY88
LY94
LY97
LY data
Normal data

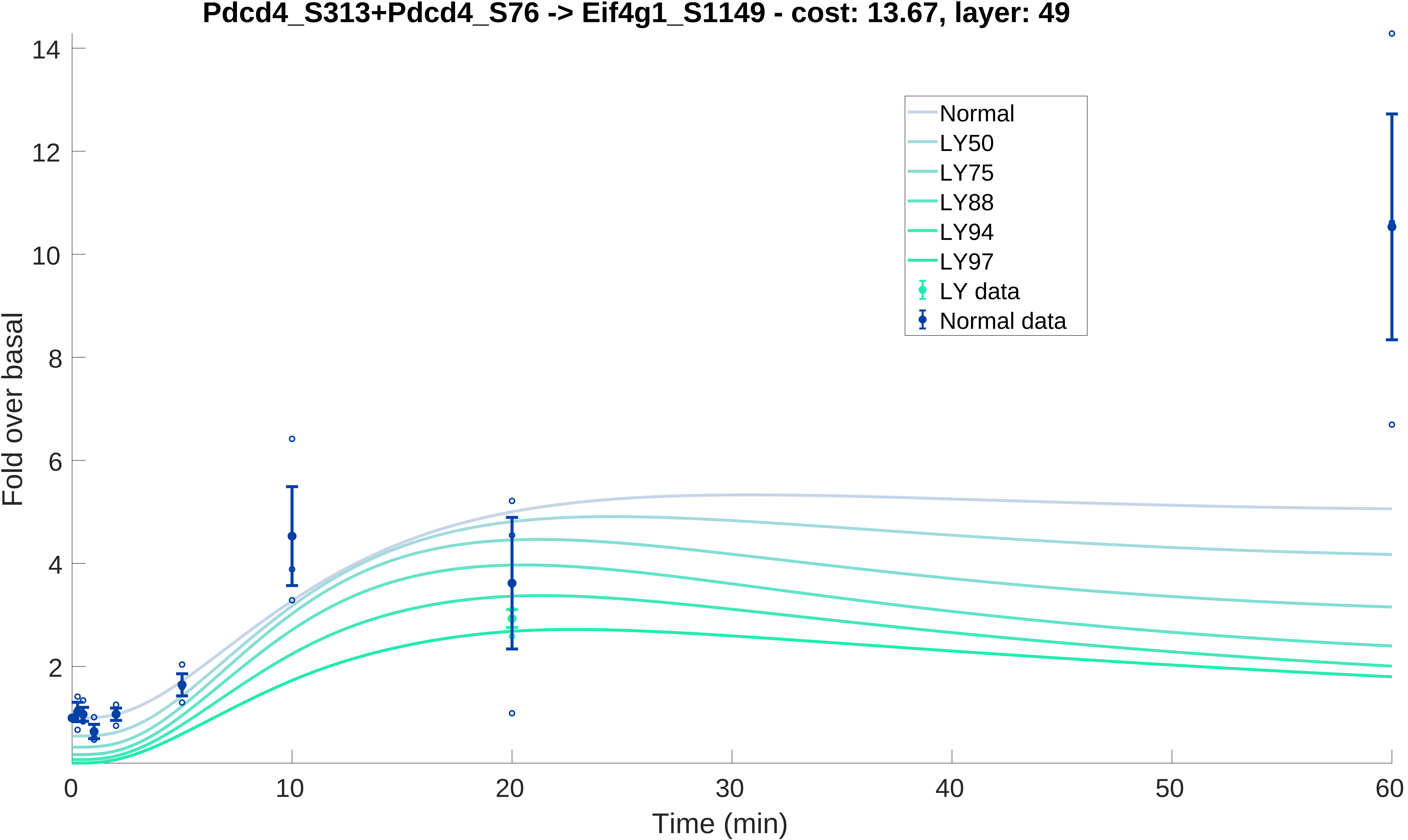

Pdcd4_S313+Pdcd4_S76 -> Eif4g1_S1149 - cost: 13.67, layer: 49
Fold over basal
Time (min)
0
10
20
30
40
50
60
2
4
6
8
10
12
14
Normal
LY50
LY75
LY88
LY94
LY97
LY data
Normal data

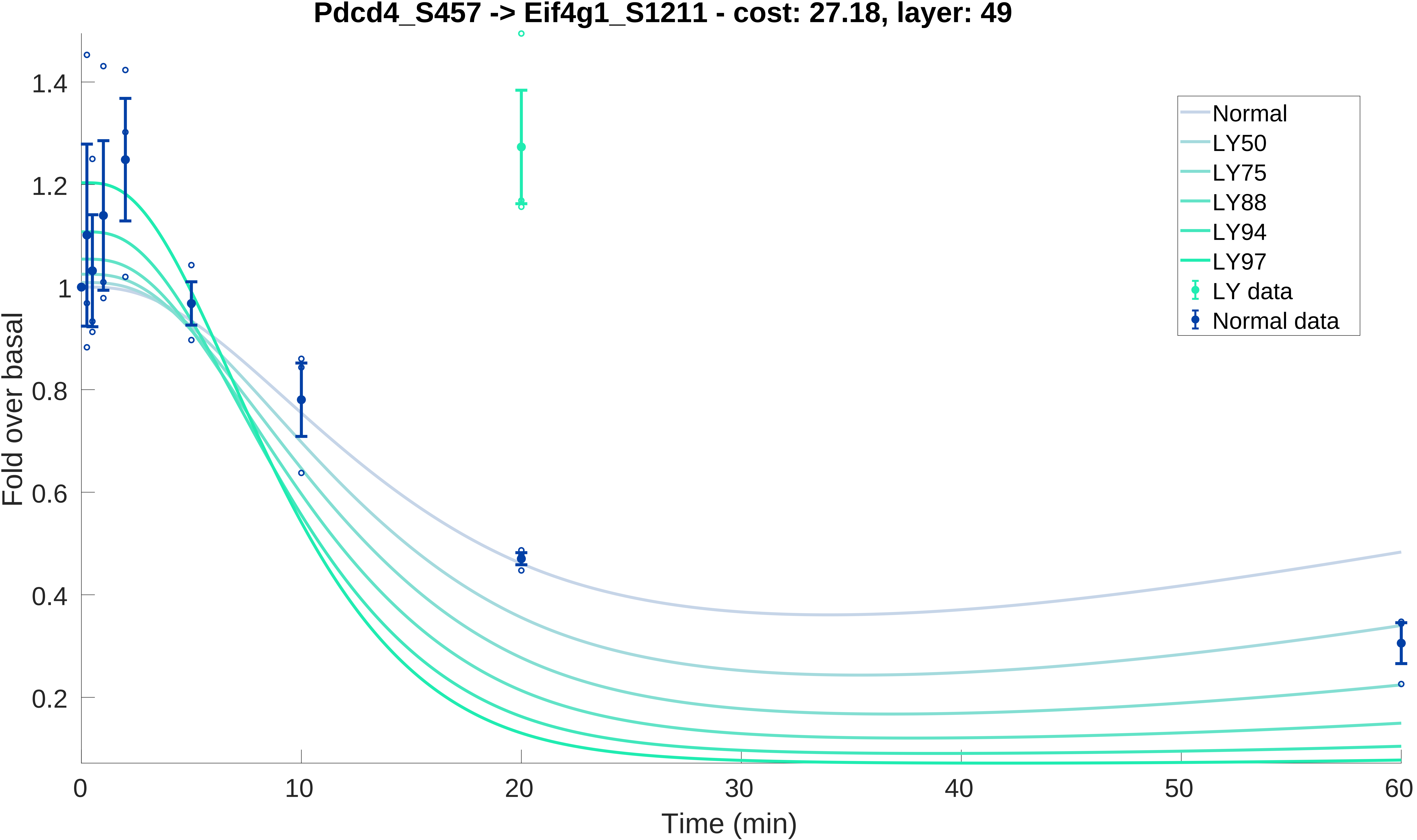

Pdcd4_S457 -> Eif4g1_S1211 - cost: 27.18, layer: 49
Normal
LY50
LY75
LY88
LY94
LY97
LY data
Normal data
Fold over basal
Time (min)
0
10
20
30
40
50
60
0.2
0.4
0.6
0.8
1
1.2
1.4

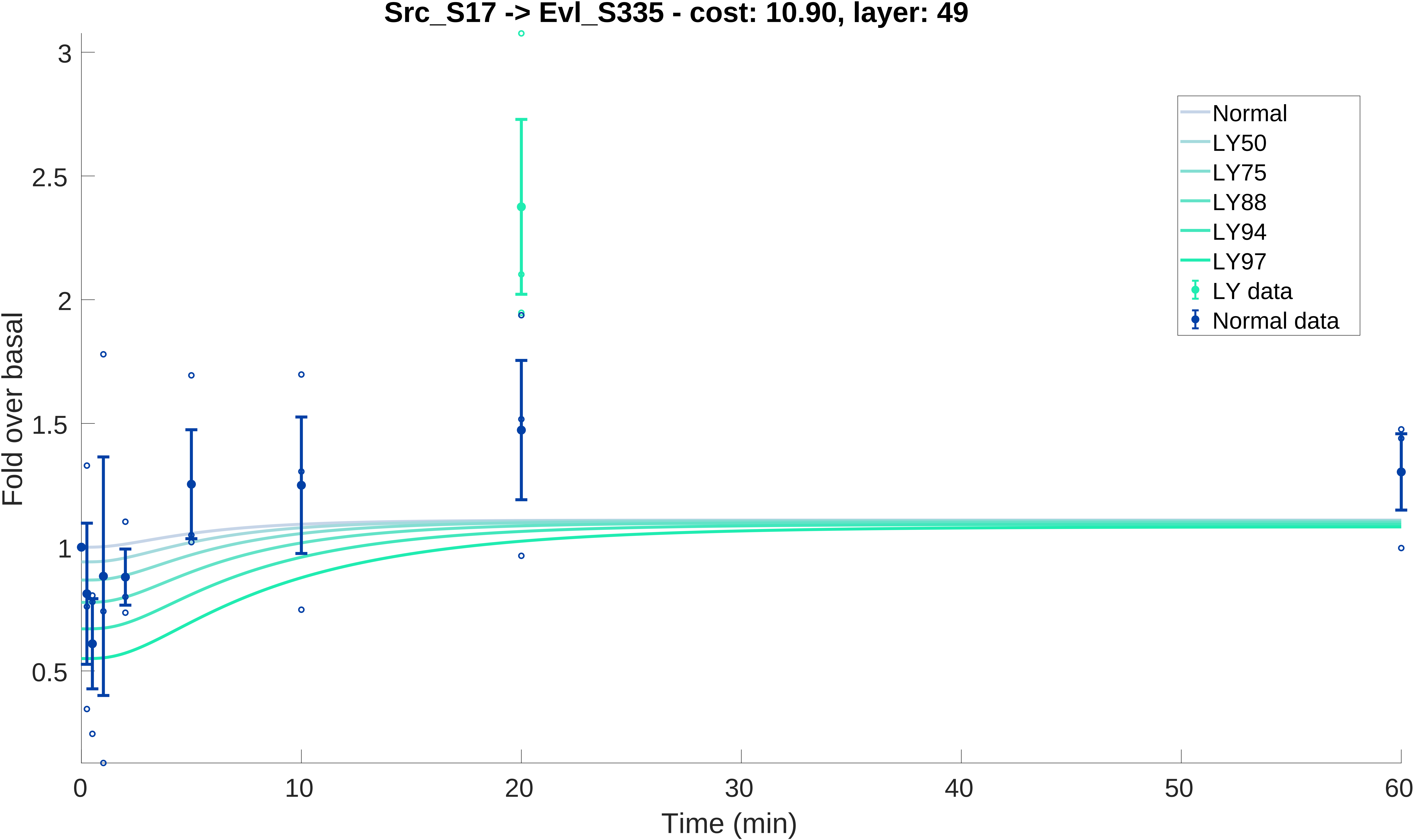

Src_S17 -> Evl_S335 - cost: 10.90, layer: 49
Fold over basal
Time (min)
0.5
1
1.5
2
2.5
3
0
10
20
30
40
50
60
Normal
LY50
LY75
LY88
LY94
LY97
LY data
Normal data

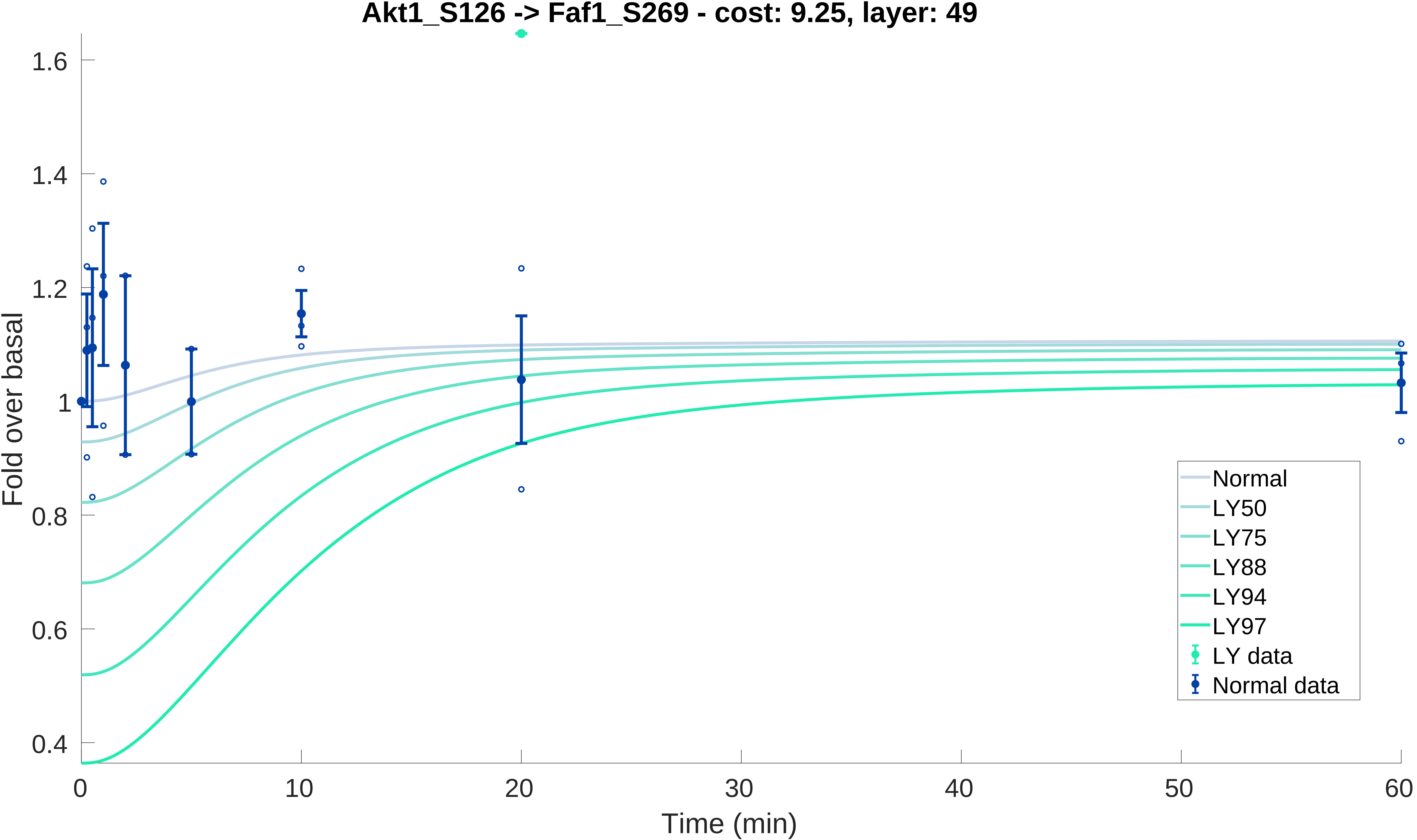

Akt1_S126 -> Faf1_S269 - cost: 9.25, layer: 49
Fold over basal
1.6
1.4
1.2
1
0.8
0.6
0.4
0
10
20
30
40
50
60
Time (min)
Normal
LY50
LY75
LY88
LY94
LY97
LY data
Normal data

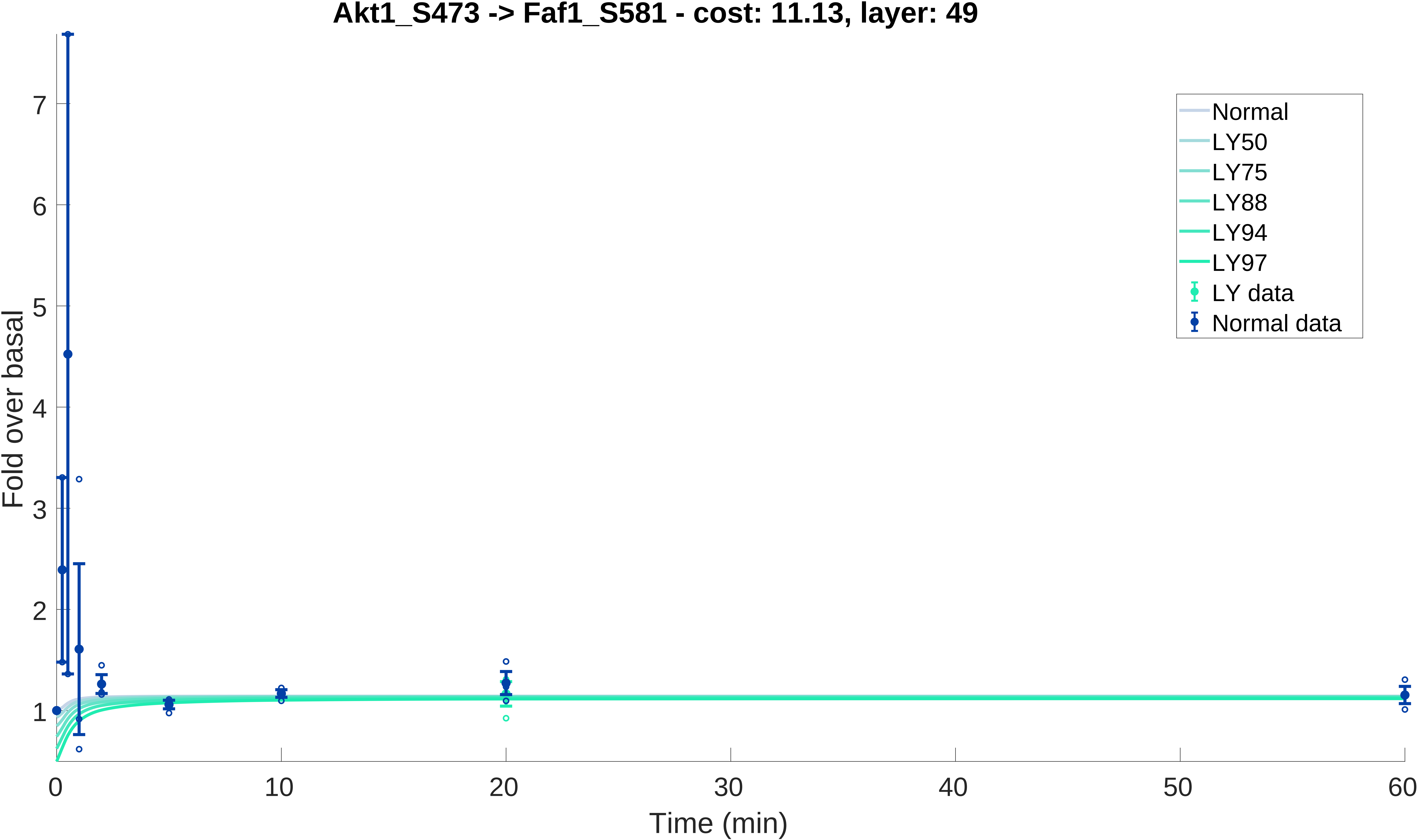
Akt1_S473 -> Faf1_S581 - cost: 11.13, layer: 49
Fold over basal
Time (min)
1
2
3
4
5
6
7
0
10
20
30
40
50
60
Normal
LY50
LY75
LY88
LY94
LY97
LY data
Normal data

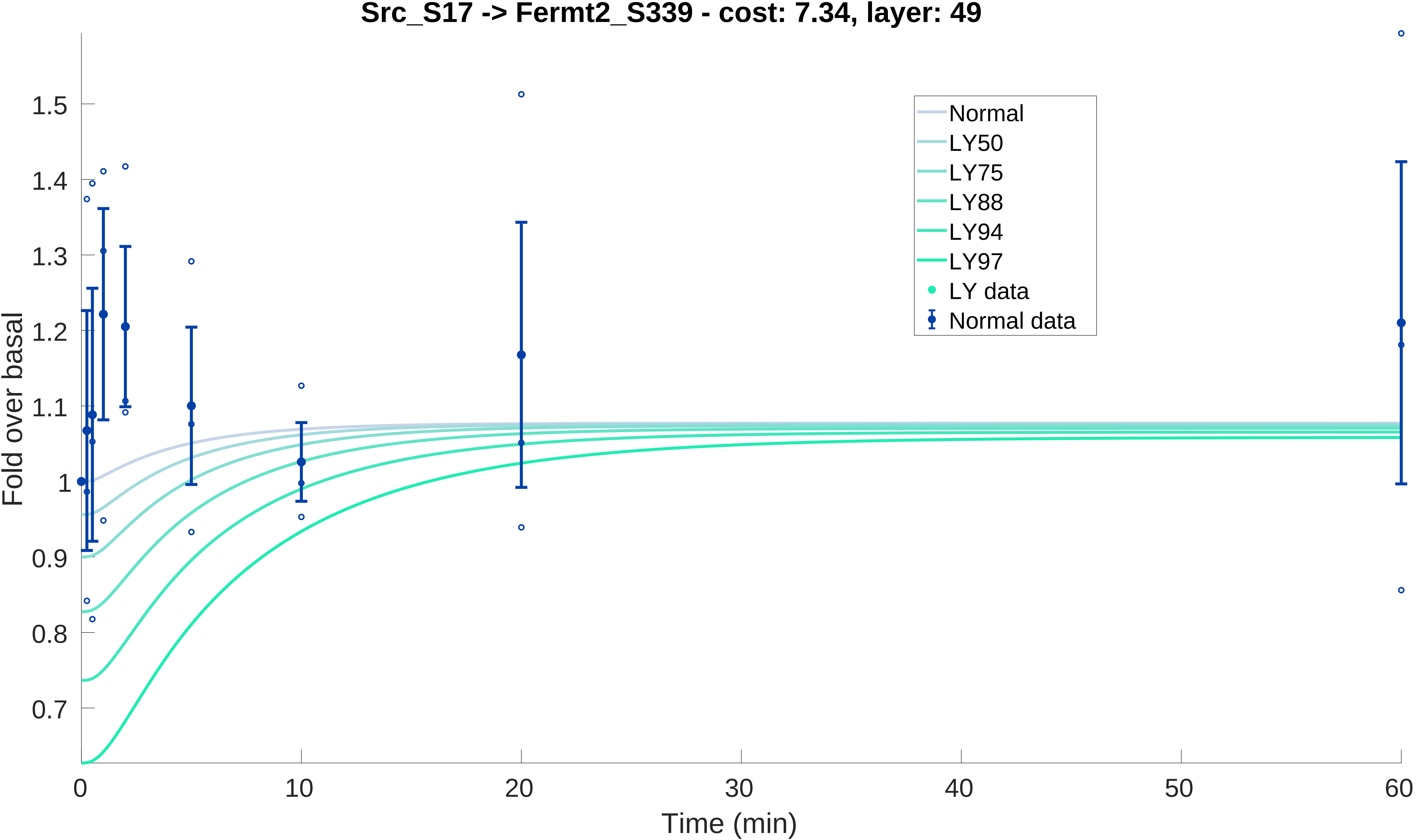

Src_S17 -> Fermt2_S339 - cost: 7.34, layer: 49
Fold over basal
Time (min)
Normal
LY50
LY75
LY88
LY94
LY97
LY data
Normal data
1.5
1.4
1.3
1.2
1.1
1
0.9
0.8
0.7
0
10
20
30
40
50
60

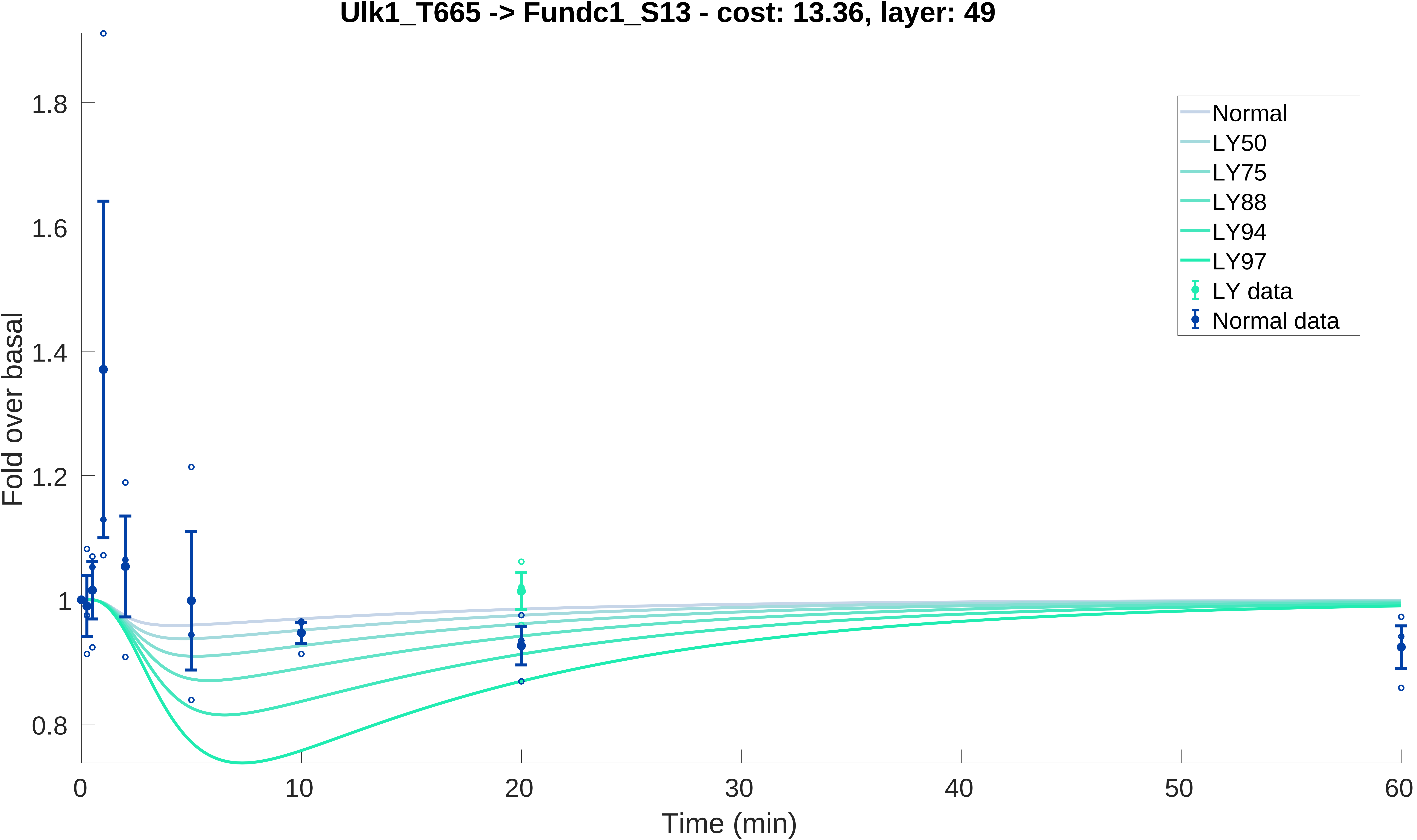
Ulk1_T665 -> Fundc1_S13 - cost: 13.36, layer: 49
Fold over basal
1.8
1.6
1.4
1.2
1
0.8
0
10
20
30
40
50
60
Time (min)
Normal
LY50
LY75
LY88
LY94
LY97
LY data
Normal data

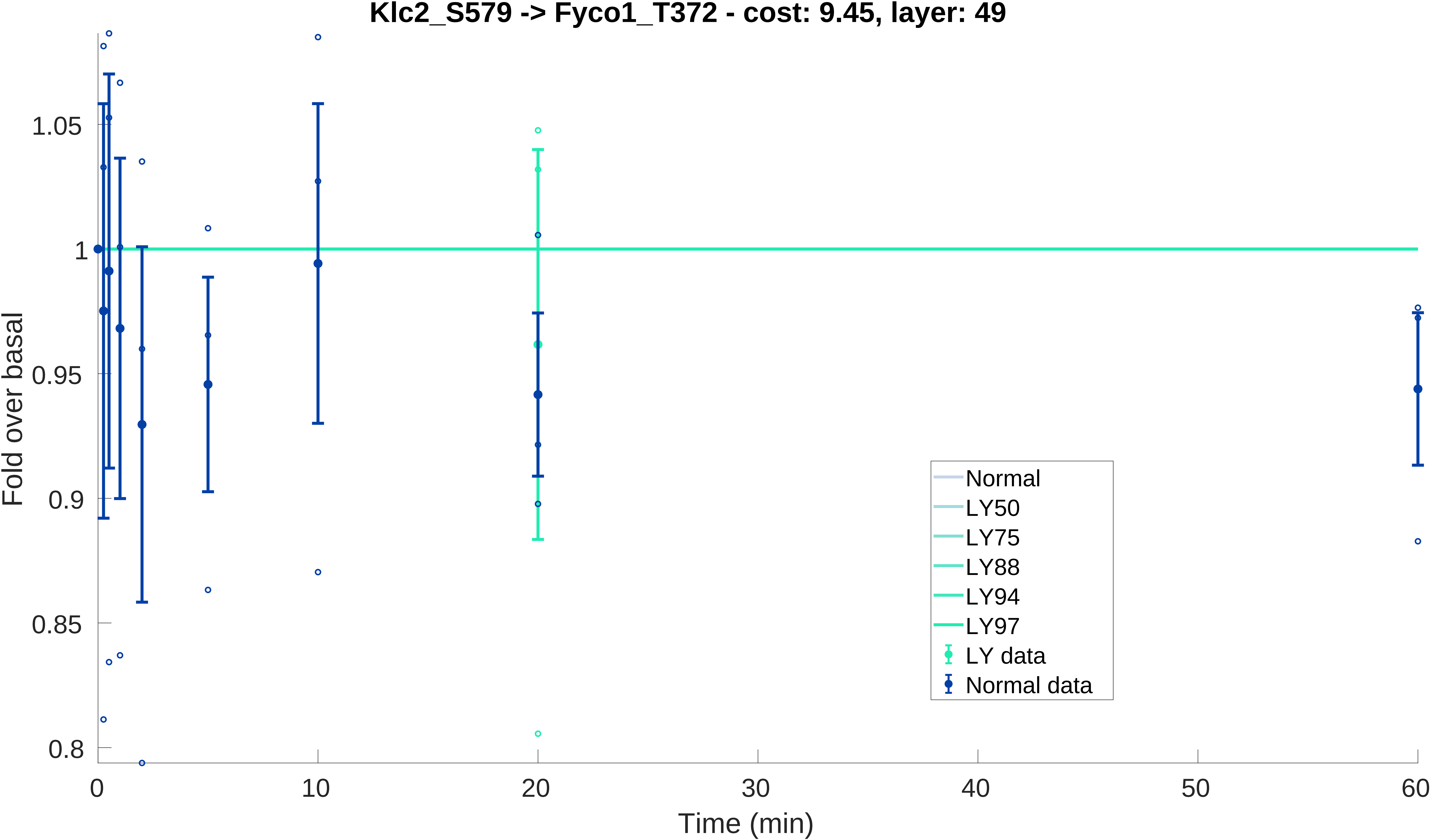

Klc2_S579 -> Fyco1_T372 - cost: 9.45, layer: 49
Fold over basal
1.05
1
0.95
0.9
0.85
0.8
0
10
20
30
40
50
60
Time (min)
Normal
LY50
LY75
LY88
LY94
LY97
LY data
Normal data

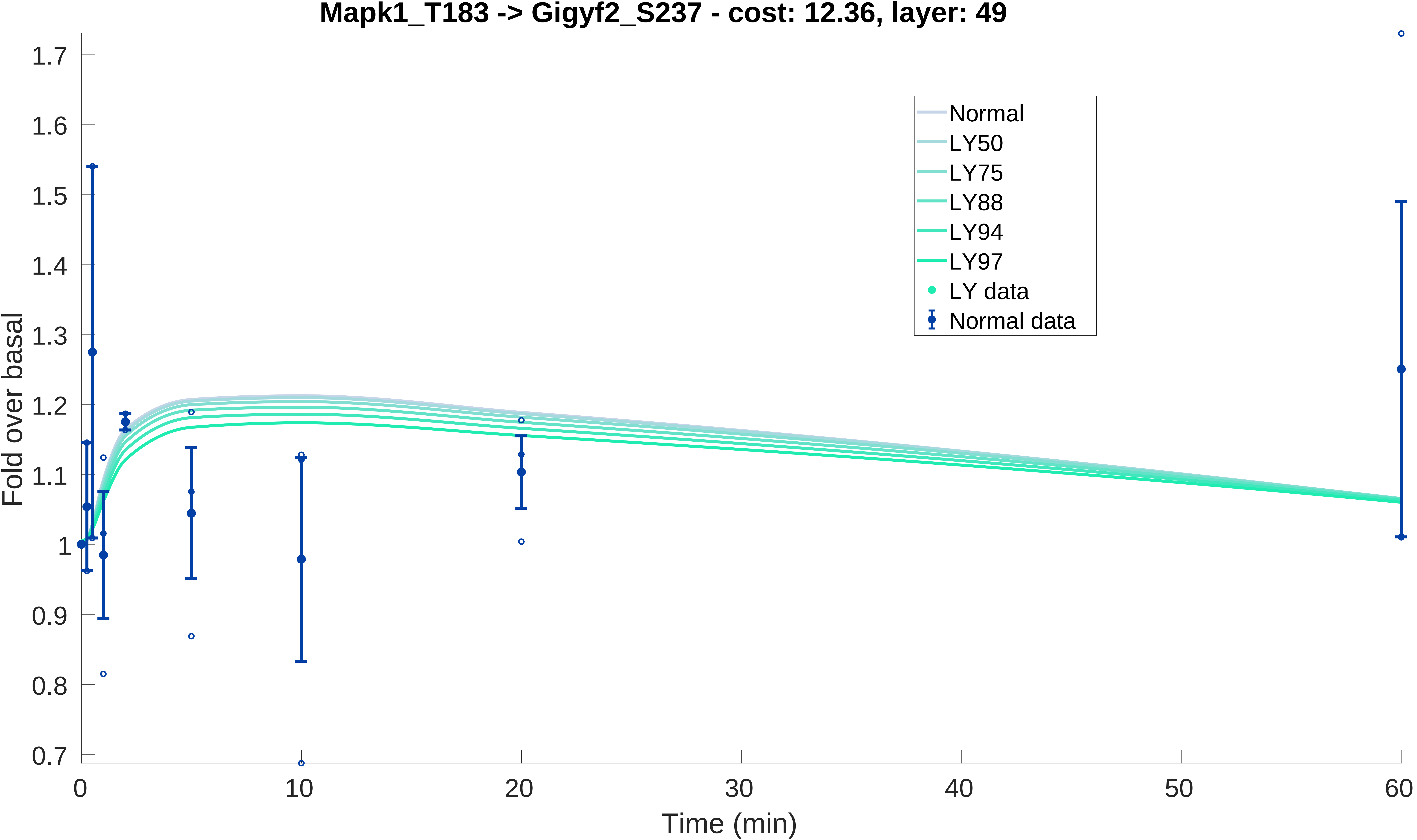

Mapk1_T183 -> Gigyf2_S237 - cost: 12.36, layer: 49
Fold over basal
Time (min)
0.7
0.8
0.9
1
1.1
1.2
1.3
1.4
1.5
1.6
1.7
0
10
20
30
40
50
60
Normal
LY50
LY75
LY88
LY94
LY97
LY data
Normal data

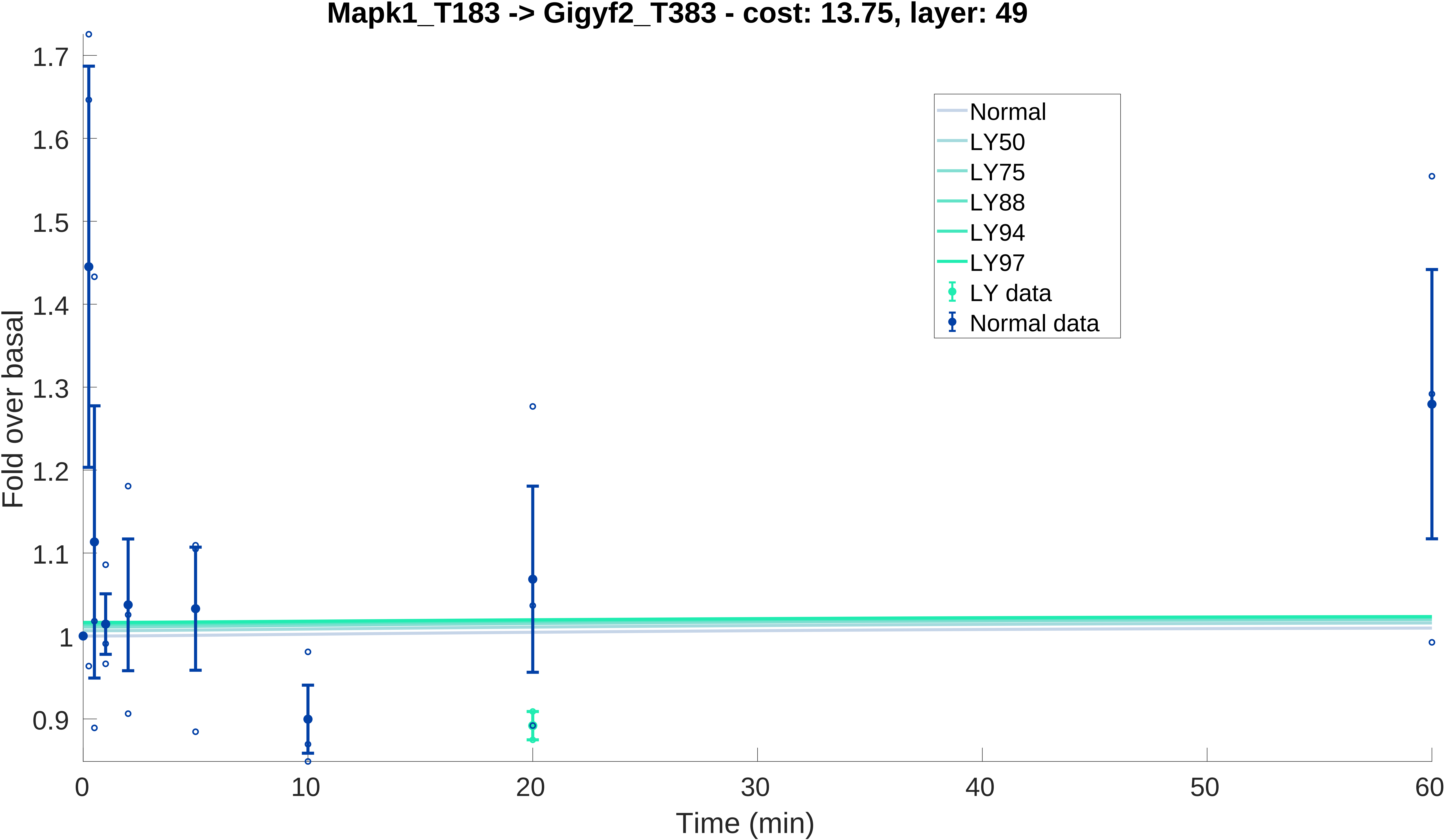
Mapk1_T183 -> Gigyf2_T383 - cost: 13.75, layer: 49
Fold over basal
Time (min)
0
10
20
30
40
50
60
0.9
1
1.1
1.2
1.3
1.4
1.5
1.6
1.7
Normal
LY50
LY75
LY88
LY94
LY97
LY data
Normal data

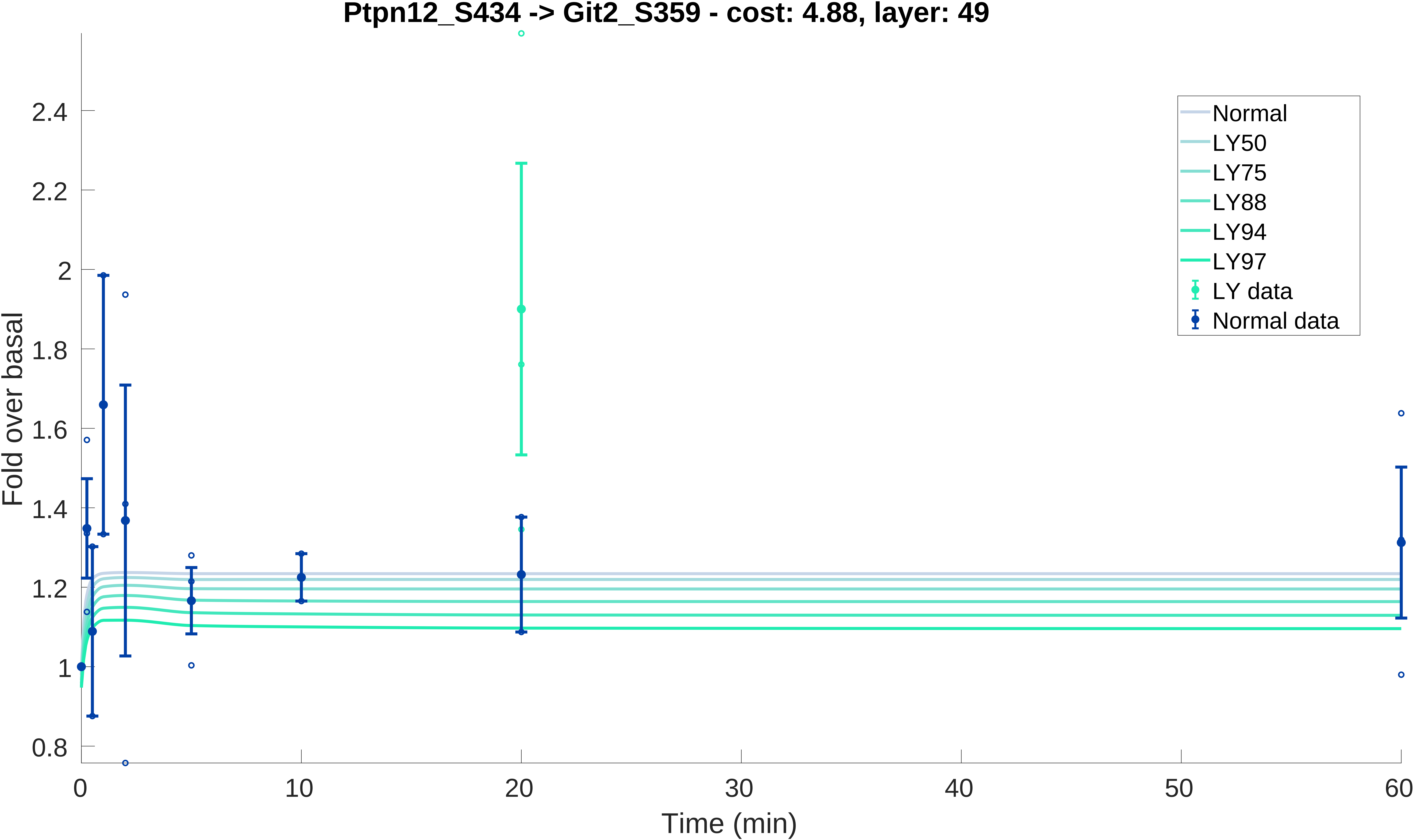

Ptpn12_S434 -> Git2_S359 - cost: 4.88, layer: 49
Fold over basal
Time (min)
Normal
LY50
LY75
LY88
LY94
LY97
LY data
Normal data
0.8
1
1.2
1.4
1.6
1.8
2
2.2
2.4
0
10
20
30
40
50
60

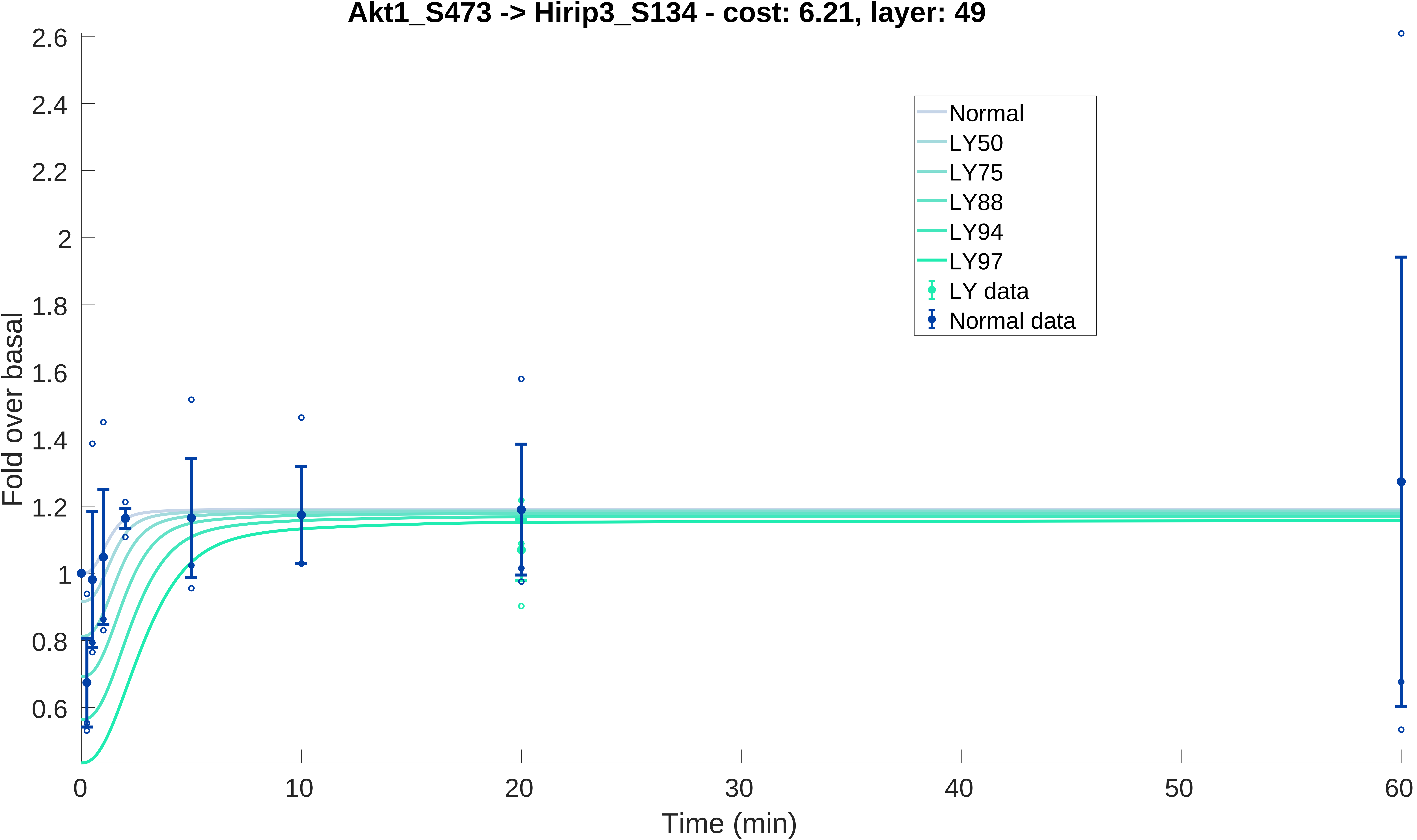

Akt1_S473 -> Hirip3_S134 - cost: 6.21, layer: 49
Fold over basal
Time (min)
2.6
2.4
2.2
2
1.8
1.6
1.4
1.2
1
0.8
0.6
0
10
20
30
40
50
60
Normal
LY50
LY75
LY88
LY94
LY97
LY data
Normal data

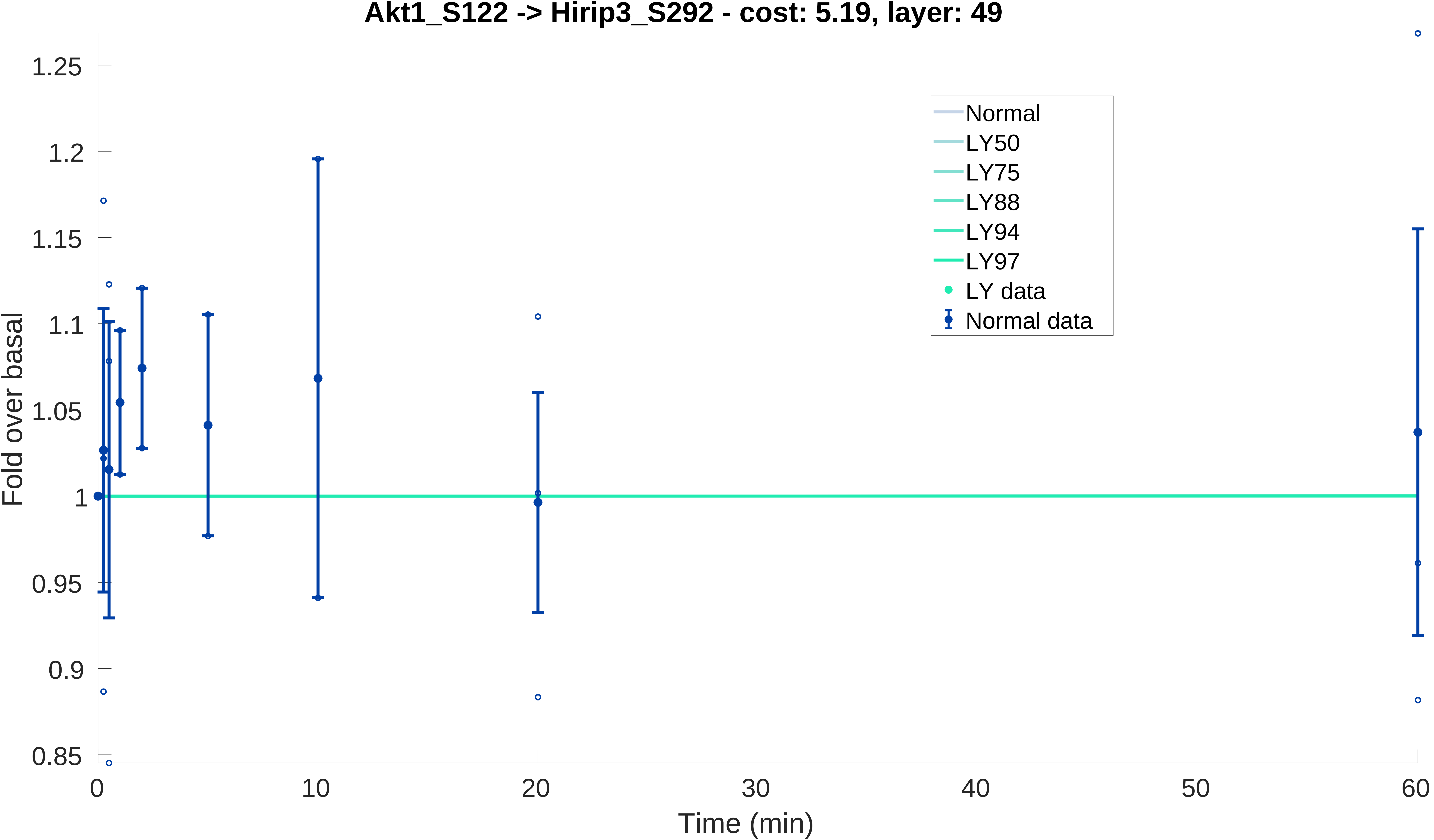

Akt1_S122 -> Hirip3_S292 - cost: 5.19, layer: 49
Fold over basal
Time (min)
1.25
1.2
1.15
1.1
1.05
1
0.95
0.9
0.85
0
10
20
30
40
50
60
Normal
LY50
LY75
LY88
LY94
LY97
LY data
Normal data

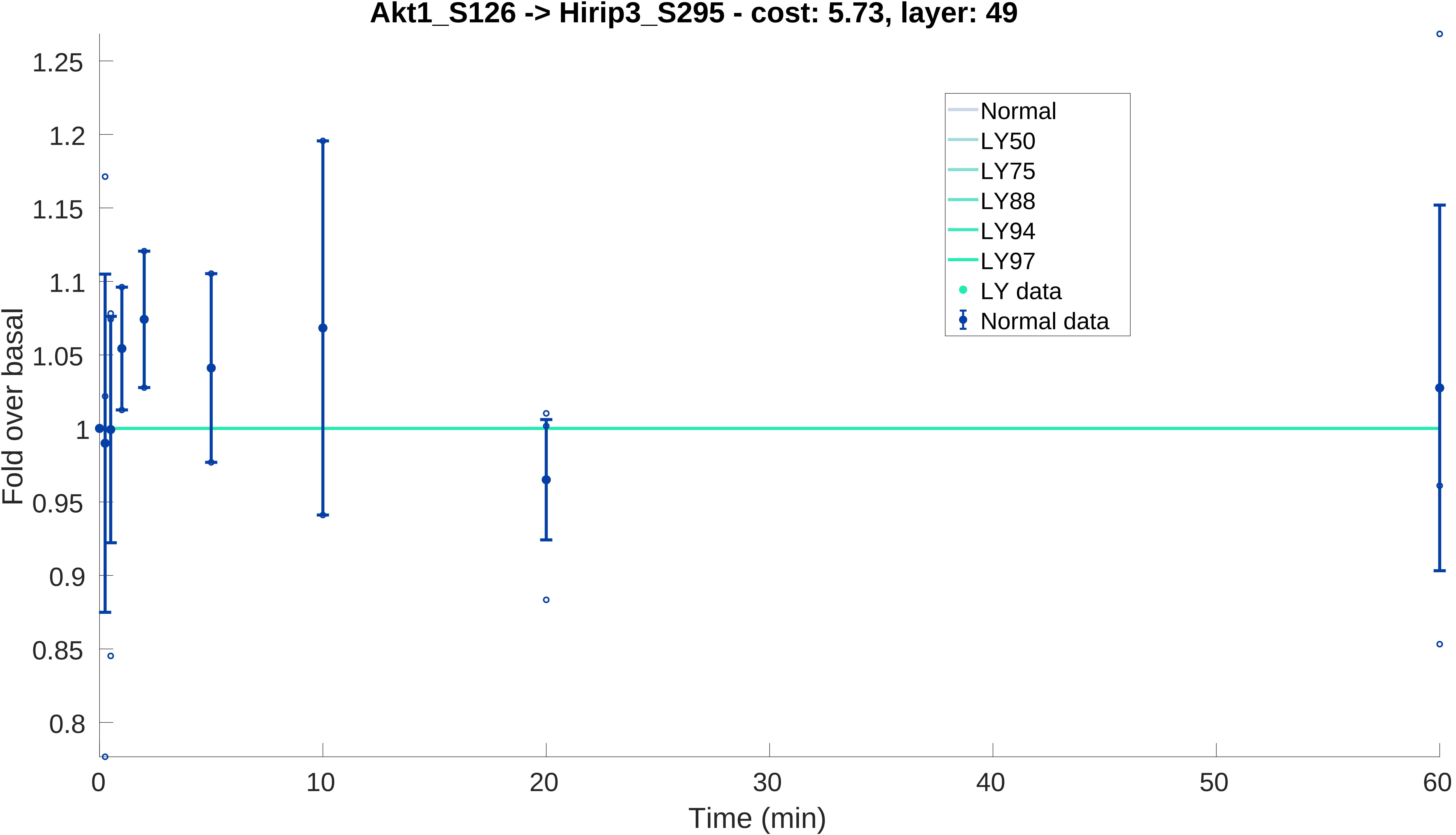

Akt1_S126 -> Hirip3_S295 - cost: 5.73, layer: 49
Fold over basal
Time (min)
Normal
LY50
LY75
LY88
LY94
LY97
LY data
Normal data
1.25
1.2
1.15
1.1
1.05
1
0.95
0.9
0.85
0.8
0
10
20
30
40
50
60

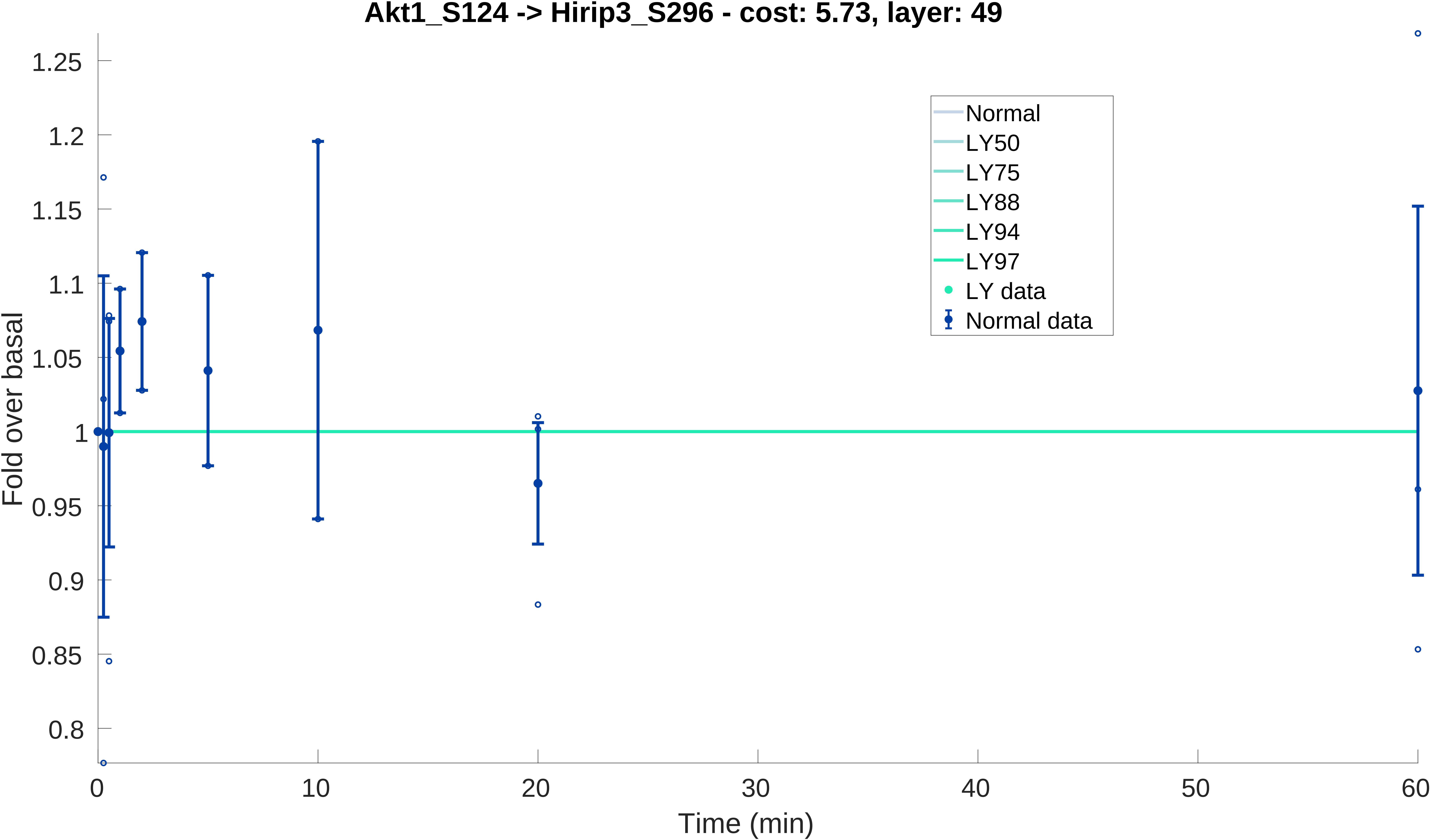

Akt1_S124 -> Hirip3_S296 - cost: 5.73, layer: 49
Fold over basal
Time (min)
1.25
1.2
1.15
1.1
1.05
1
0.95
0.9
0.85
0.8
0
10
20
30
40
50
60
Normal
LY50
LY75
LY88
LY94
LY97
LY data
Normal data

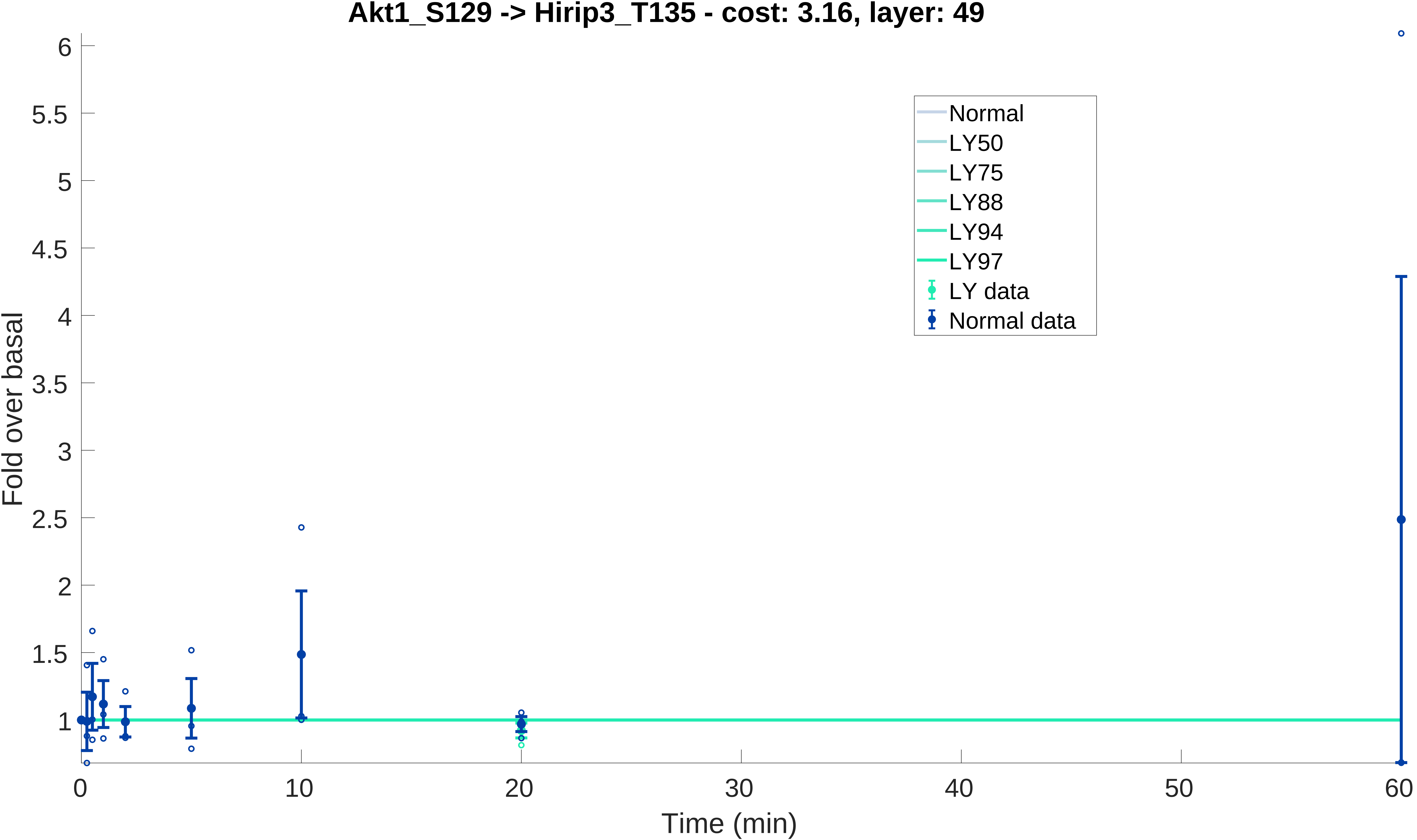

Akt1_S129 -> Hirip3_T135 - cost: 3.16, layer: 49
Fold over basal
Time (min)
Normal
LY50
LY75
LY88
LY94
LY97
LY data
Normal data
6
5.5
5
4.5
4
3.5
3
2.5
2
1.5
1
0
10
20
30
40
50
60

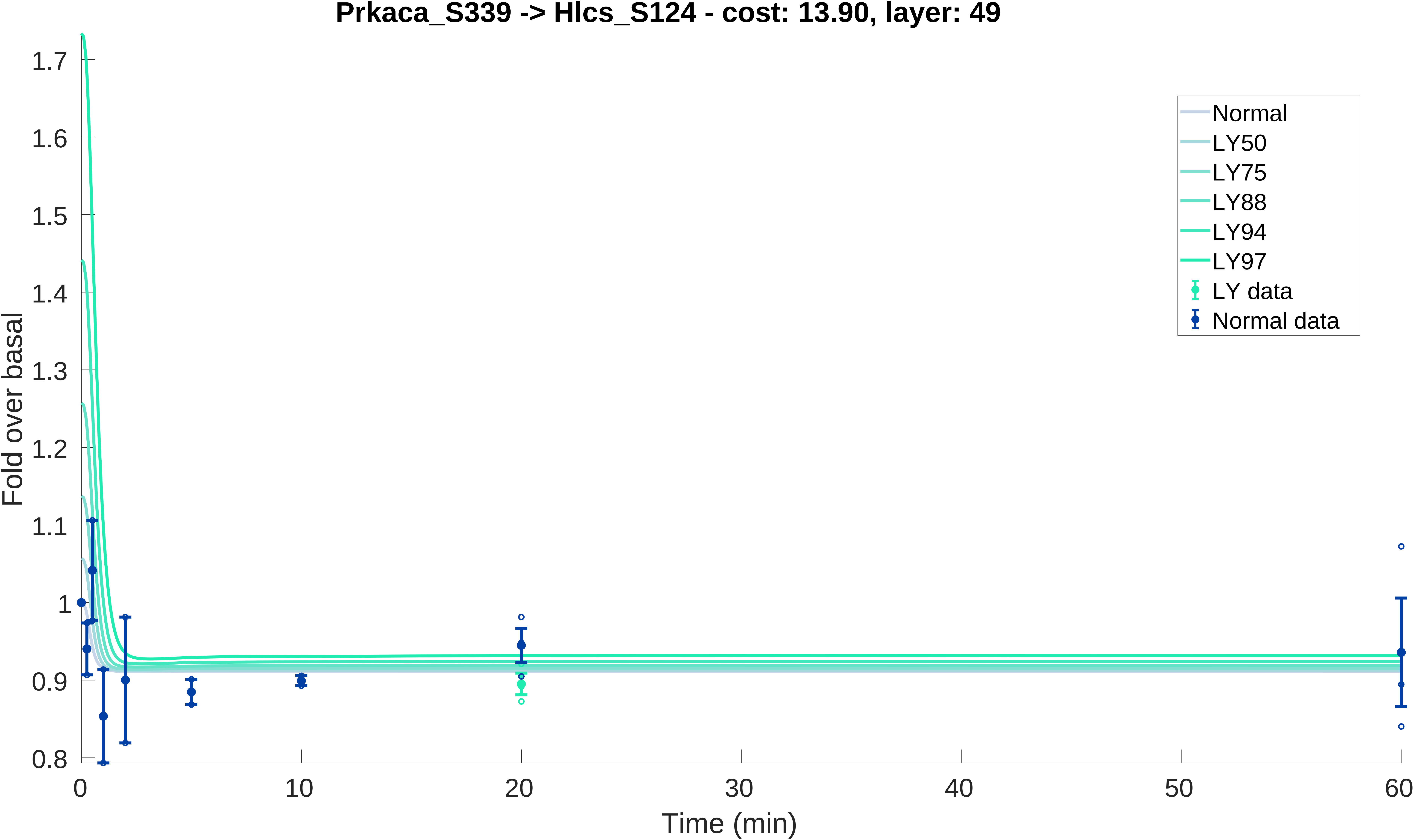
Prkaca_S339 -> Hlcs_S124 - cost: 13.90, layer: 49
Fold over basal
Time (min)
Normal
LY50
LY75
LY88
LY94
LY97
LY data
Normal data
0.8
0.9
1
1.1
1.2
1.3
1.4
1.5
1.6
1.7
0
10
20
30
40
50
60

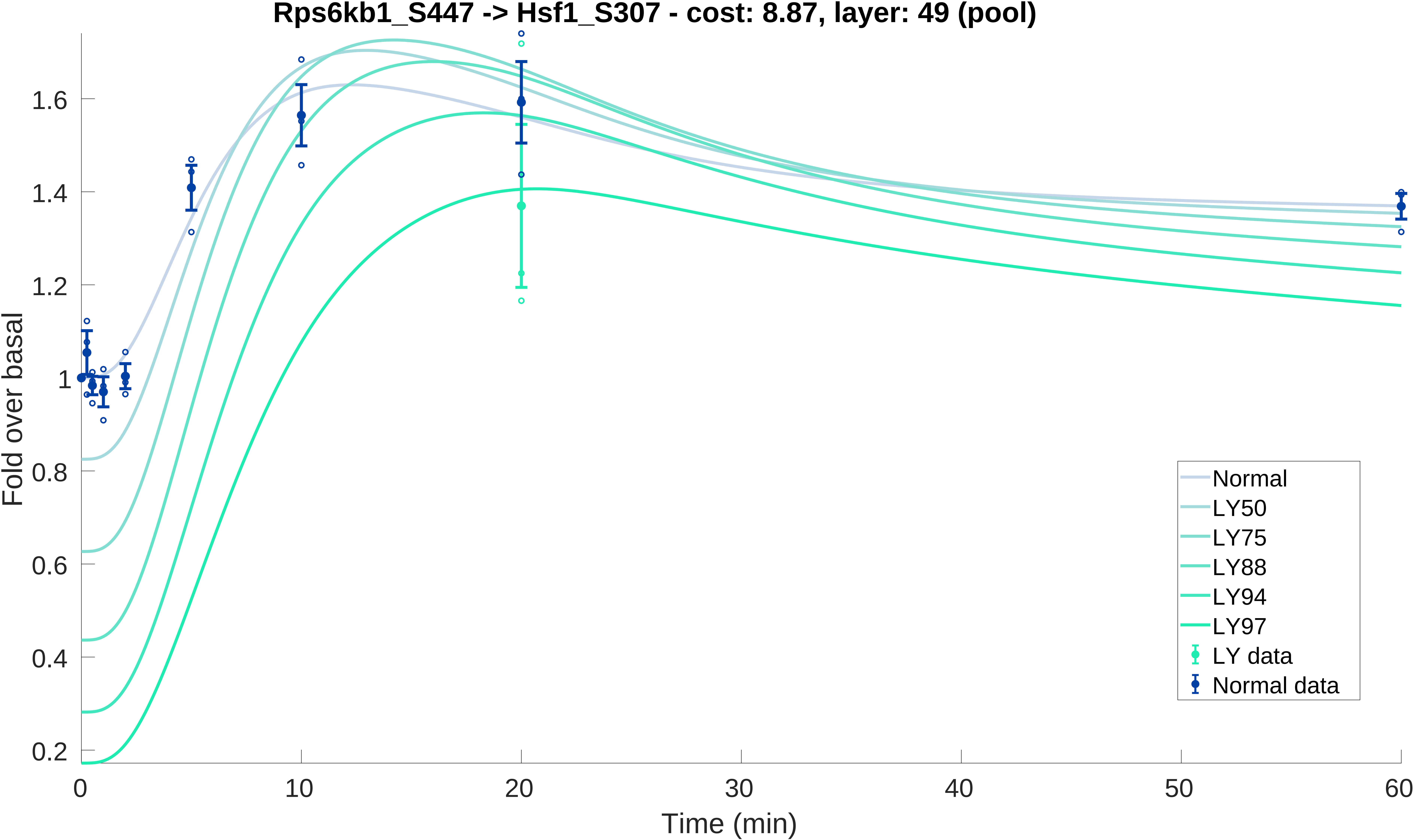

Rps6kb1_S447 -> Hsf1_S307 - cost: 8.87, layer: 49 (pool)
Fold over basal
Time (min)
1.6
1.4
1.2
1
0.8
0.6
0.4
0.2
0
10
20
30
40
50
60
Normal
LY50
LY75
LY88
LY94
LY97
LY data
Normal data

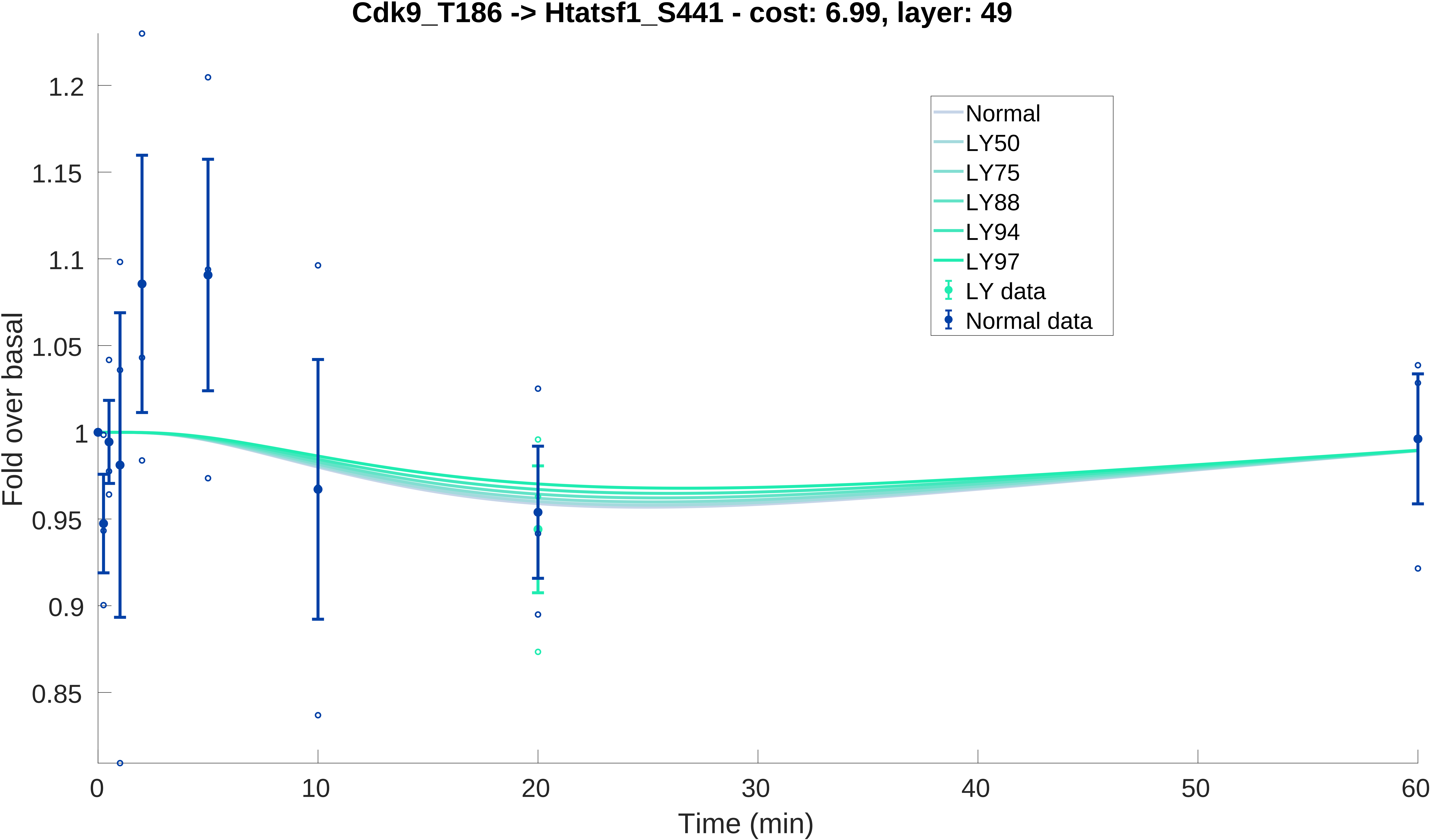

Cdk9_T186 -> Htatsf1_S441 - cost: 6.99, layer: 49
Fold over basal
Time (min)
1.2
1.15
1.1
1.05
1
0.95
0.9
0.85
0
10
20
30
40
50
60
Normal
LY50
LY75
LY88
LY94
LY97
LY data
Normal data

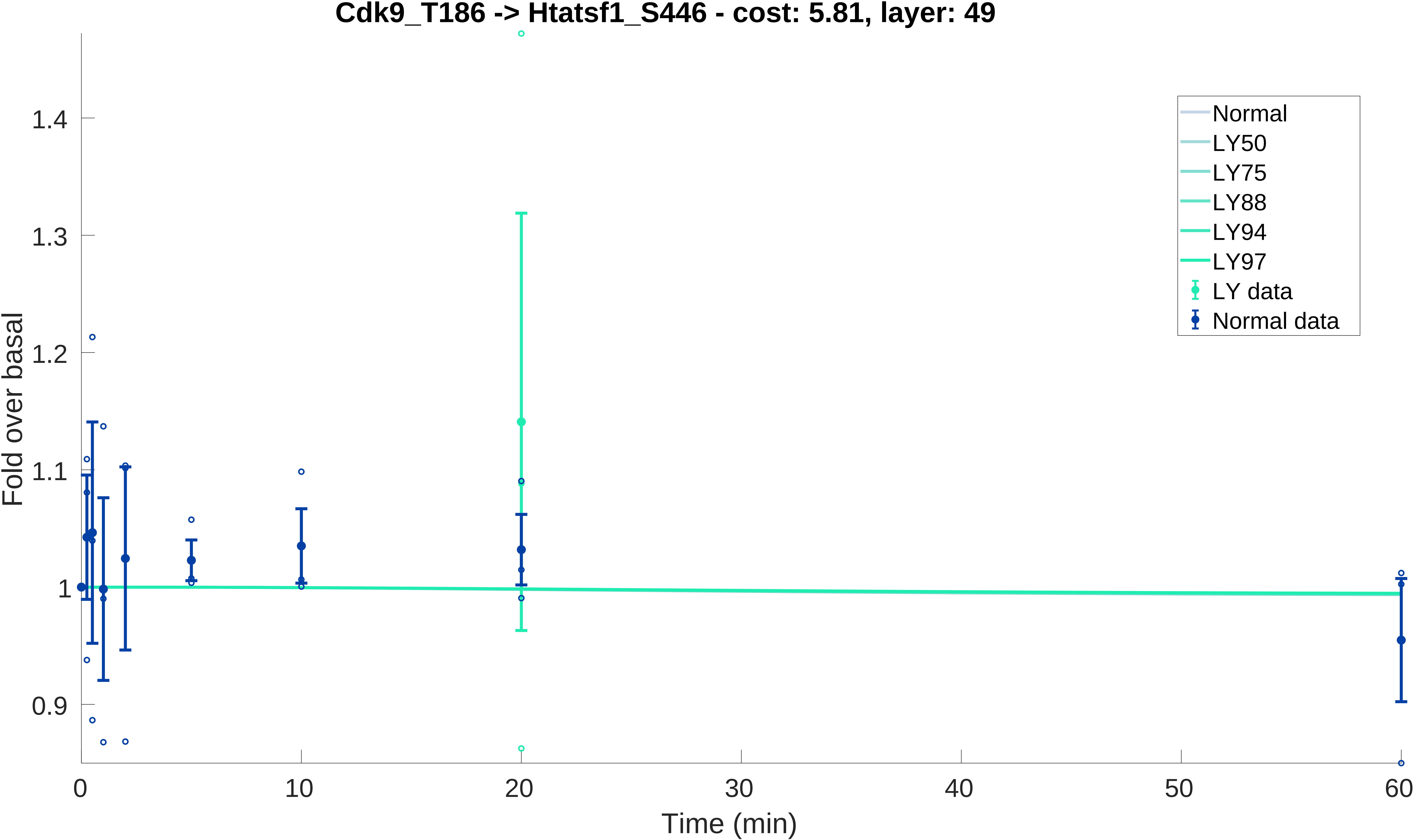

Cdk9_T186 -> Htatsf1_S446 - cost: 5.81, layer: 49
Fold over basal
Time (min)
Normal
LY50
LY75
LY88
LY94
LY97
LY data
Normal data
1.4
1.3
1.2
1.1
1
0.9
0
10
20
30
40
50
60

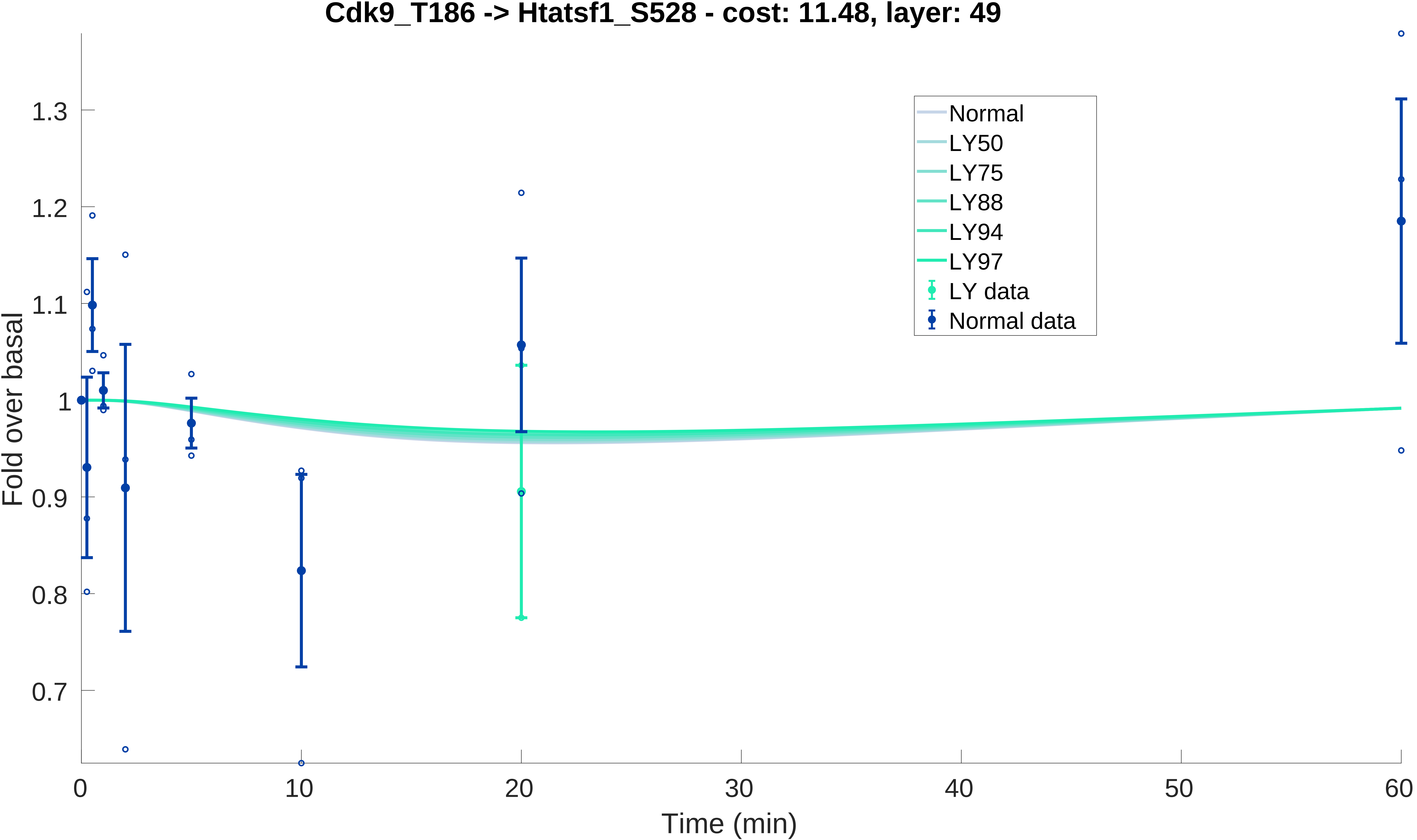

Cdk9_T186 -> Htatsf1_S528 - cost: 11.48, layer: 49
Normal
LY50
LY75
LY88
LY94
LY97
LY data
Normal data
Fold over basal
Time (min)
1.3
1.2
1.1
1
0.9
0.8
0.7
0
10
20
30
40
50
60

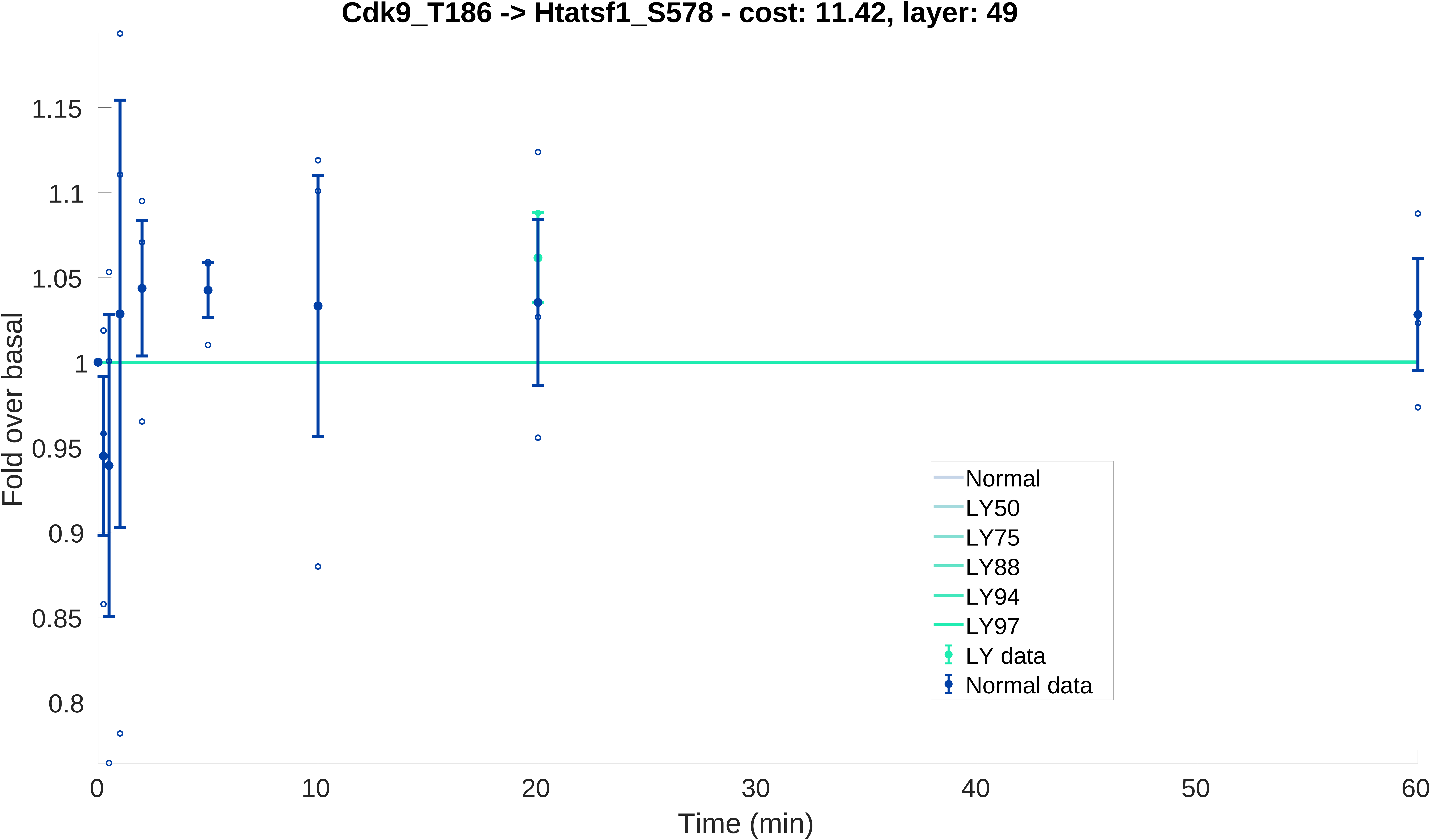

Cdk9_T186 -> Htatsf1_S578 - cost: 11.42, layer: 49
Fold over basal
1.15
1.1
1.05
1
0.95
0.9
0.85
0.8
0
10
20
30
40
50
60
Time (min)
Normal
LY50
LY75
LY88
LY94
LY97
LY data
Normal data

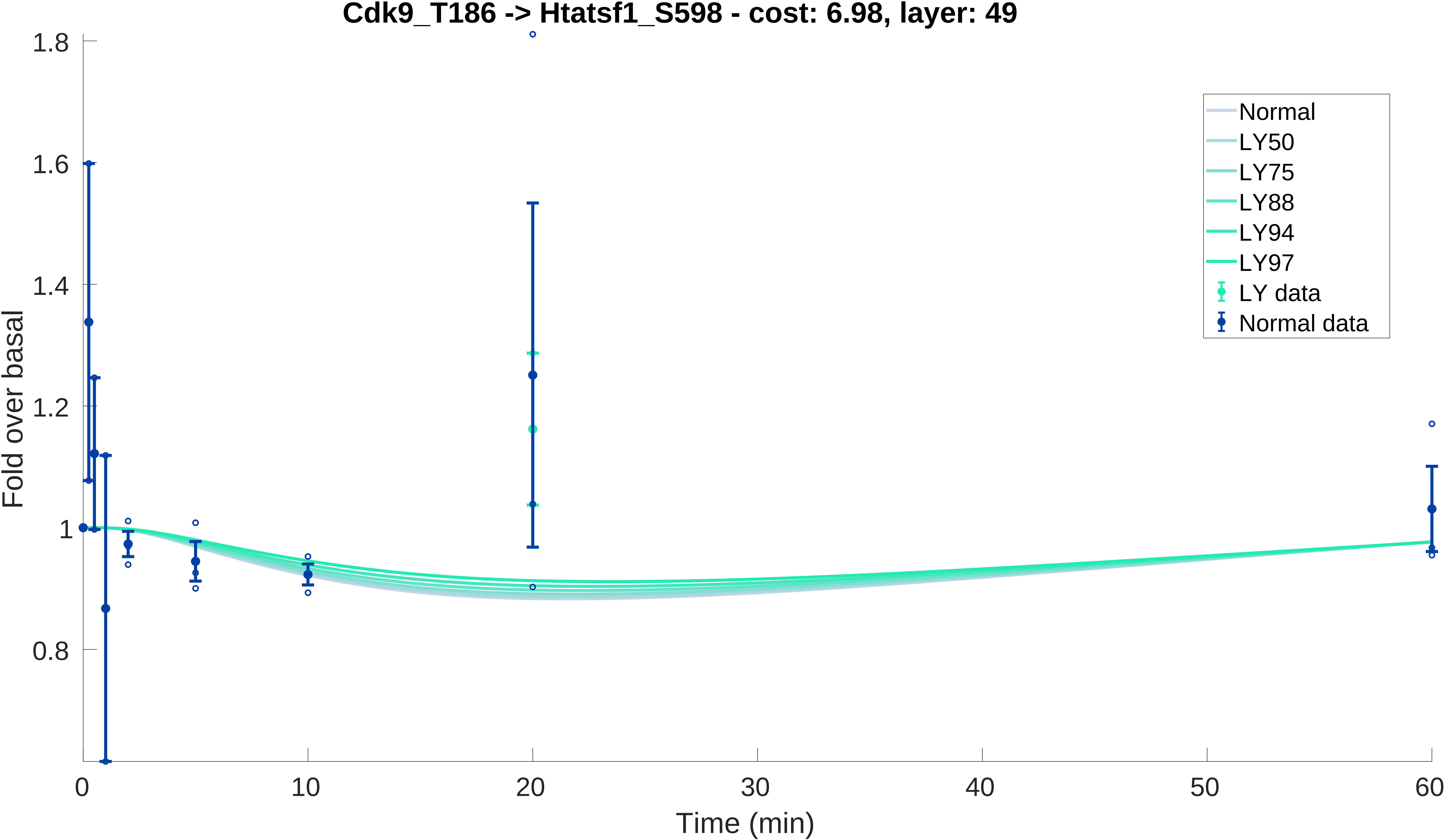

Cdk9_T186 -> Htatsf1_S598 - cost: 6.98, layer: 49
Fold over basal
Time (min)
0.8
1
1.2
1.4
1.6
1.8
0
10
20
30
40
50
60
Normal
LY50
LY75
LY88
LY94
LY97
LY data
Normal data

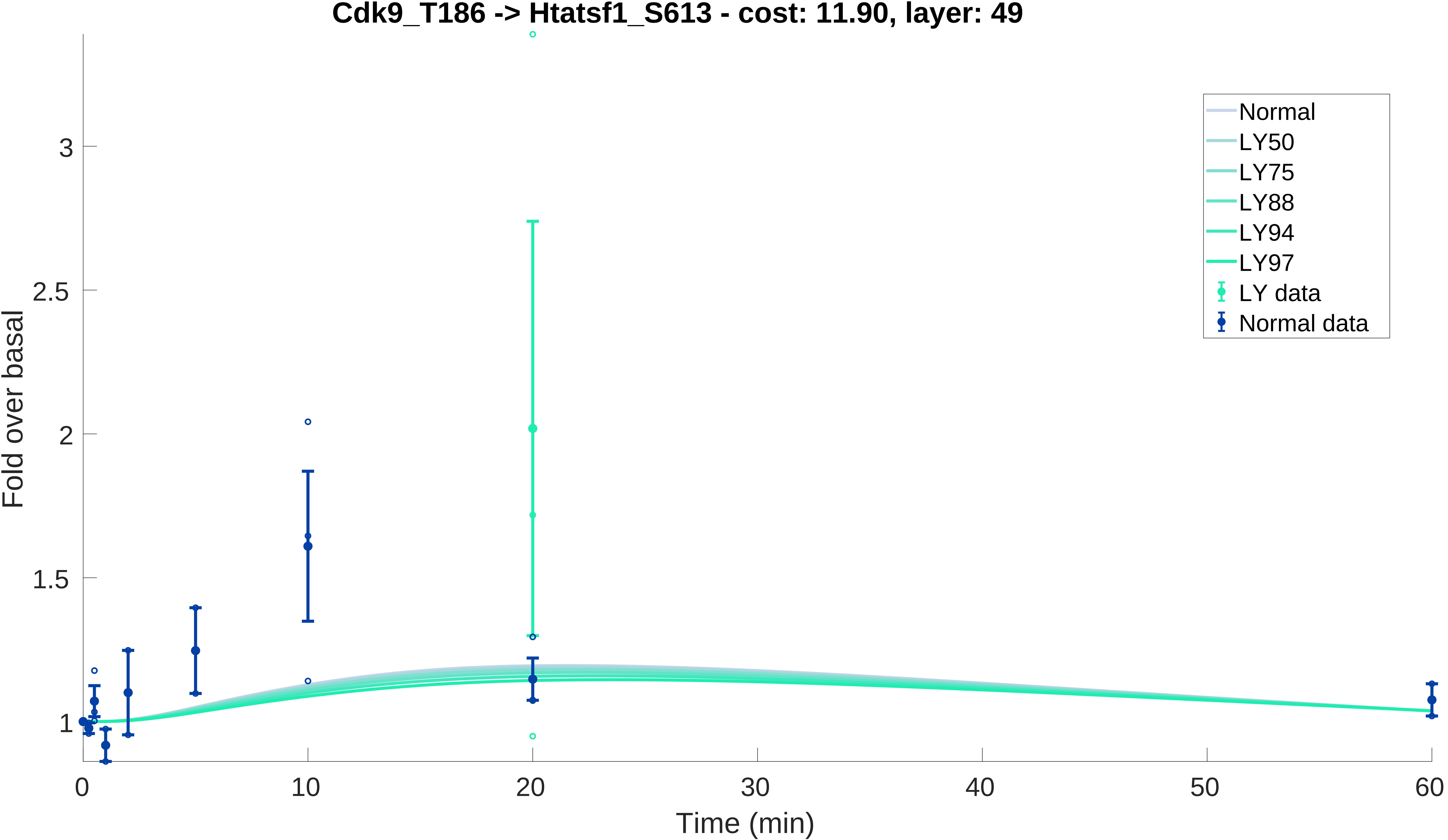

Cdk9_T186 -> Htatsf1_S613 - cost: 11.90, layer: 49
Fold over basal
Time (min)
3
2.5
2
1.5
1
0
10
20
30
40
50
60
Normal
LY50
LY75
LY88
LY94
LY97
LY data
Normal data

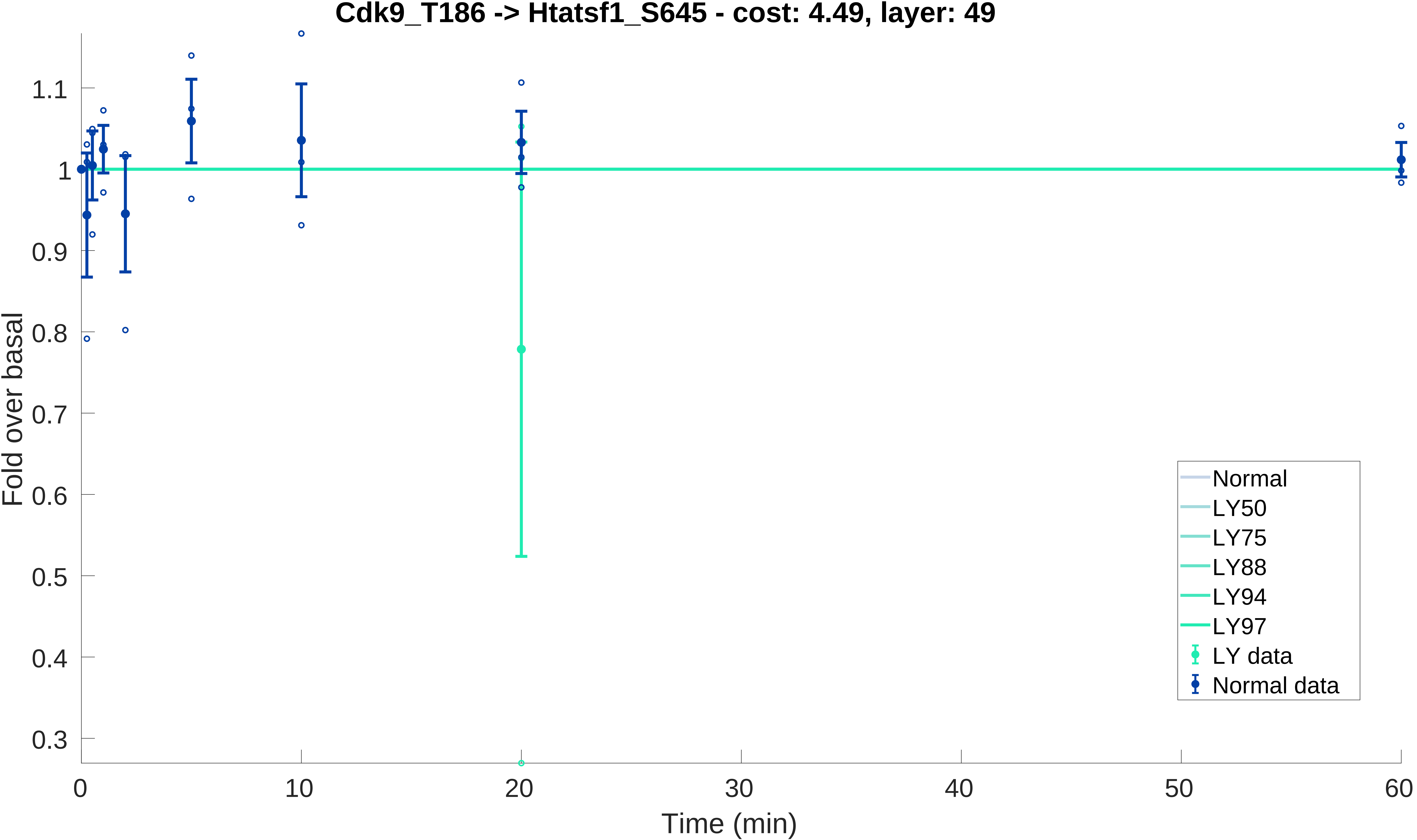

Cdk9_T186 -> Htatsf1_S645 - cost: 4.49, layer: 49
Fold over basal
Time (min)
1.1
1
0.9
0.8
0.7
0.6
0.5
0.4
0.3
0
10
20
30
40
50
60
Normal
LY50
LY75
LY88
LY94
LY97
LY data
Normal data

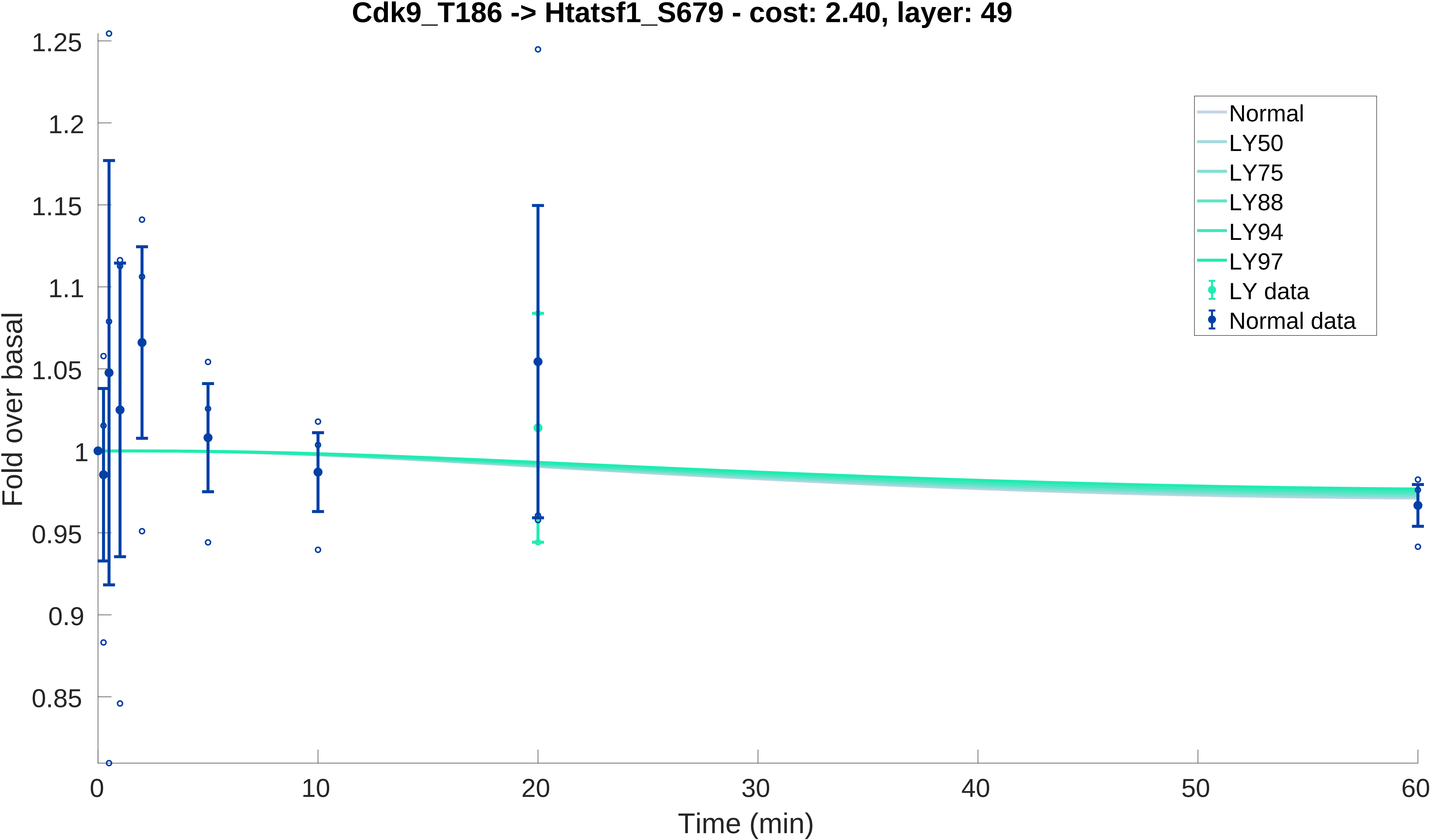

Cdk9_T186 -> Htatsf1_S679 - cost: 2.40, layer: 49
Fold over basal
Time (min)
1.25
1.2
1.15
1.1
1.05
1
0.95
0.9
0.85
0
10
20
30
40
50
60
Normal
LY50
LY75
LY88
LY94
LY97
LY data
Normal data

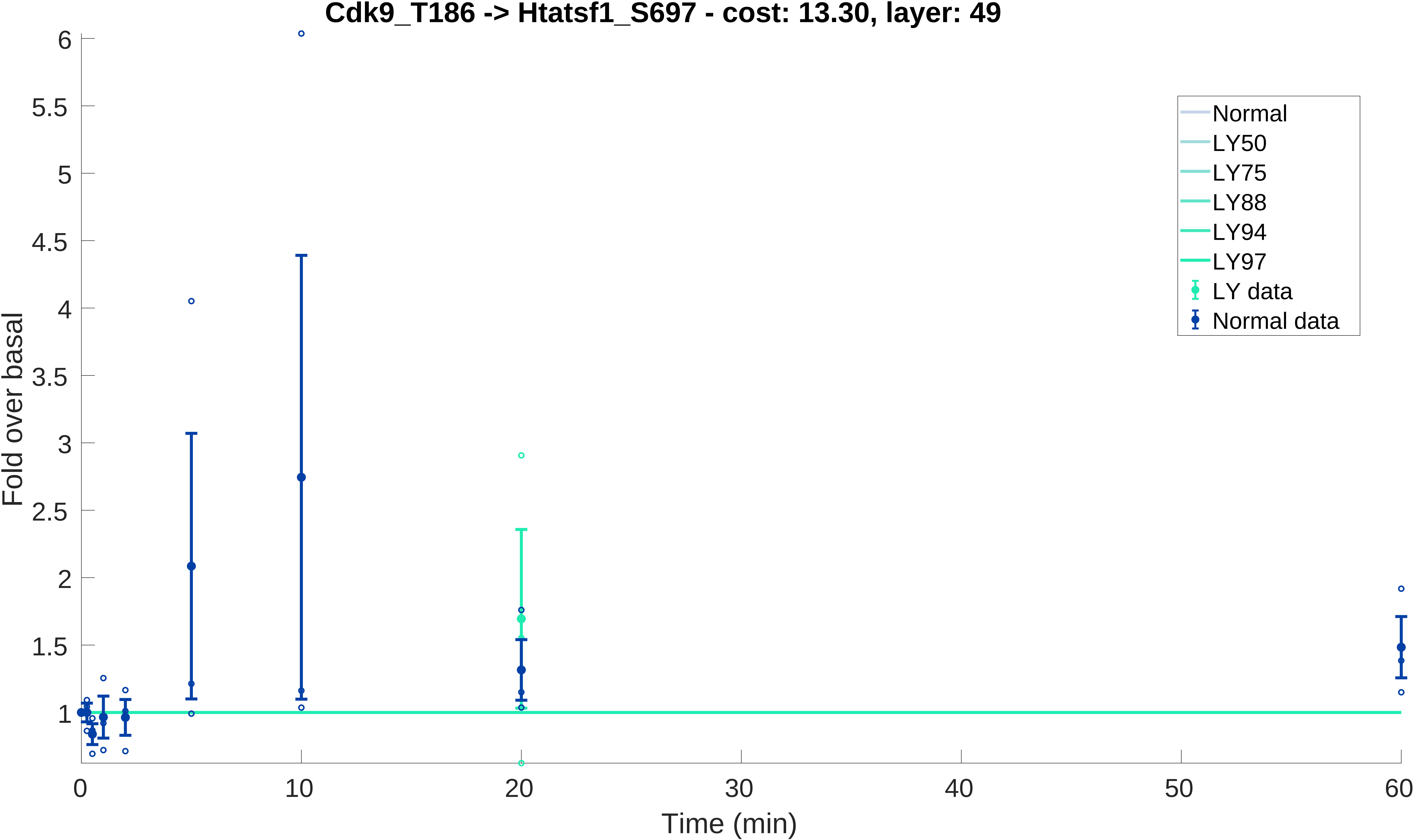

Cdk9_T186 -> Htatsf1_S697 - cost: 13.30, layer: 49
Fold over basal
Time (min)
6
5.5
5
4.5
4
3.5
3
2.5
2
1.5
1
0
10
20
30
40
50
60
Normal
LY50
LY75
LY88
LY94
LY97
LY data
Normal data

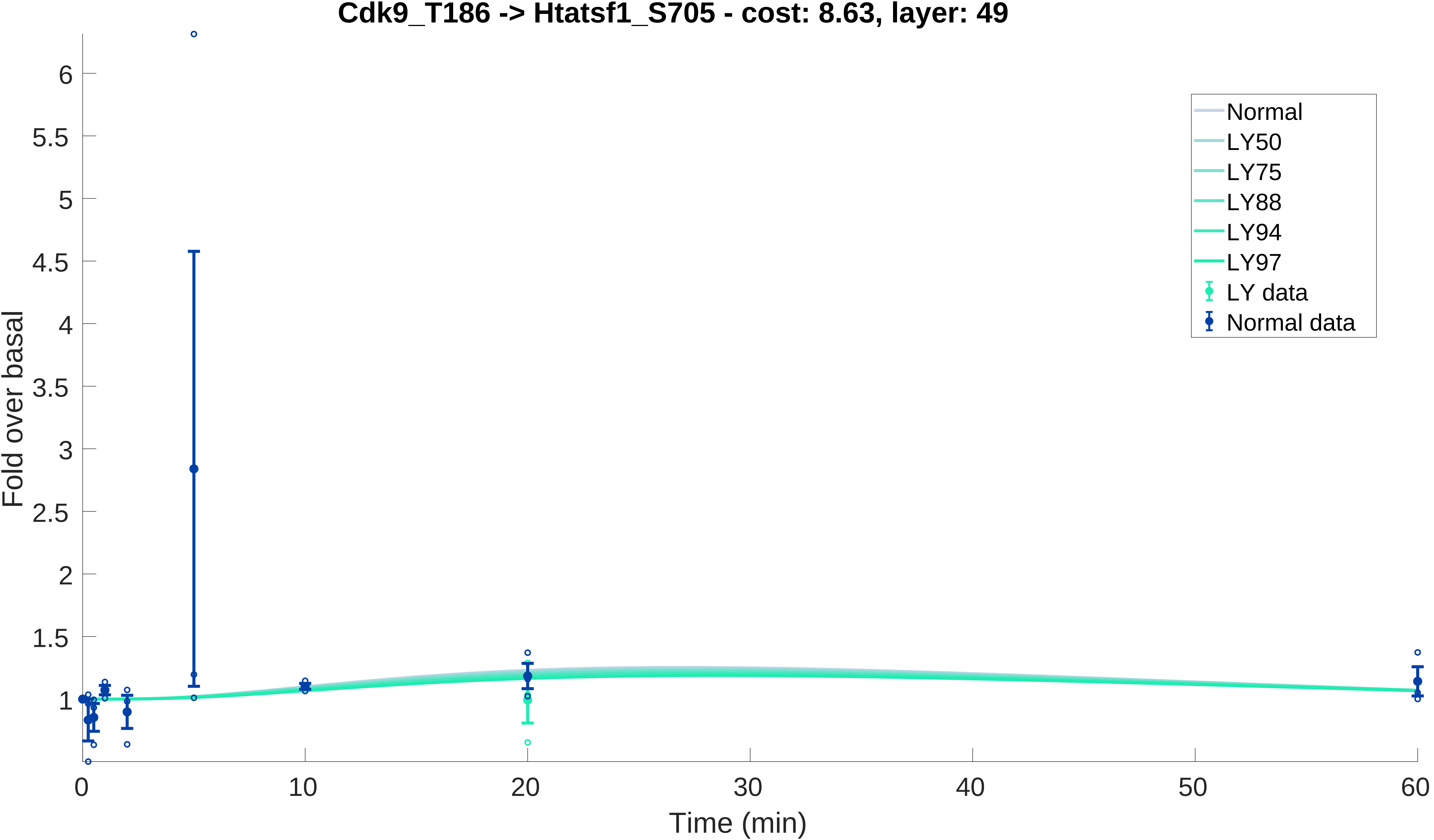

Cdk9_T186 -> Htatsf1_S705 - cost: 8.63, layer: 49
Fold over basal
Time (min)
Normal
LY50
LY75
LY88
LY94
LY97
LY data
Normal data

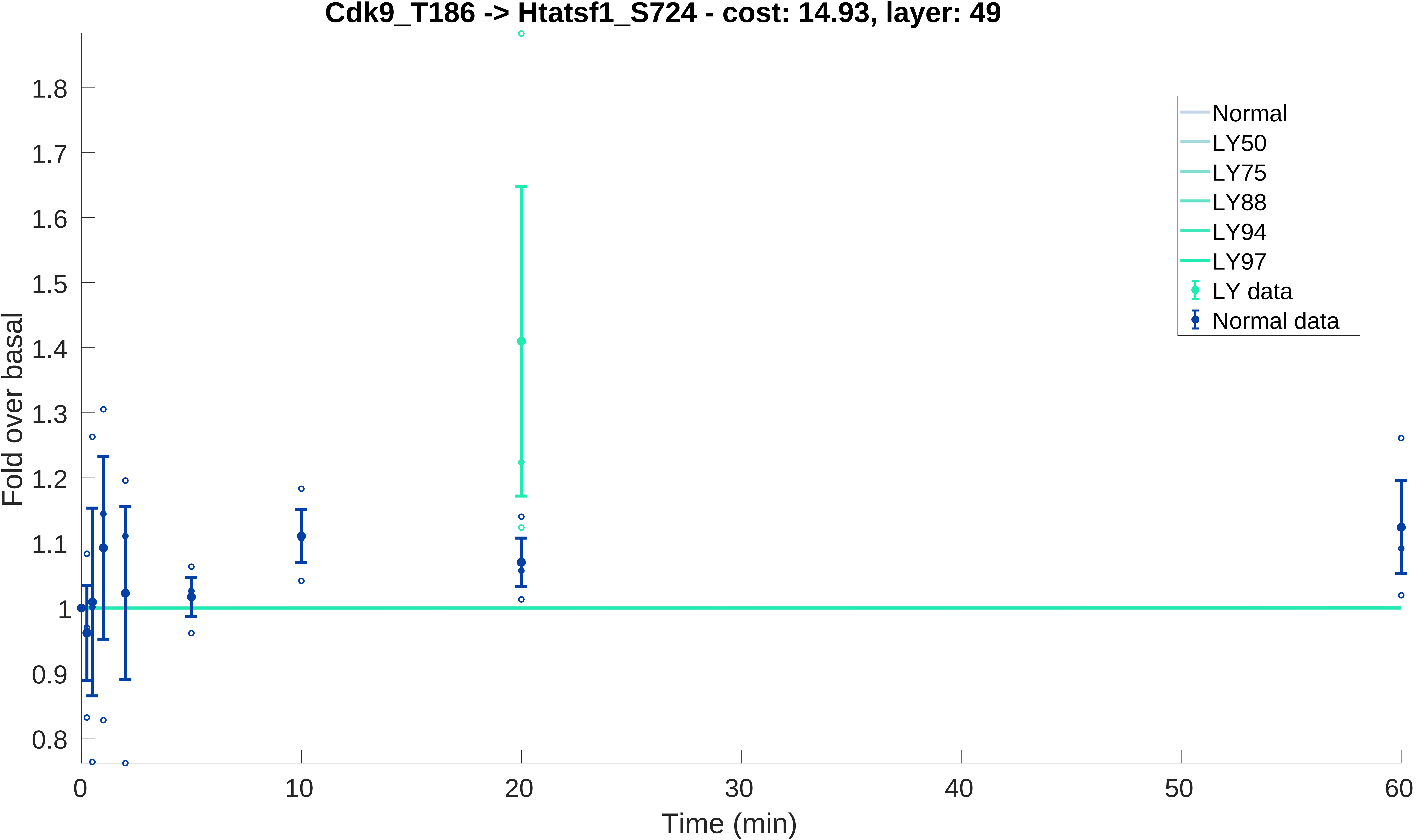

Cdk9_T186 -> Htatsf1_S724 - cost: 14.93, layer: 49
Fold over basal
Time (min)
Normal
LY50
LY75
LY88
LY94
LY97
LY data
Normal data
0.8
0.9
1
1.1
1.2
1.3
1.4
1.5
1.6
1.7
1.8
0
10
20
30
40
50
60

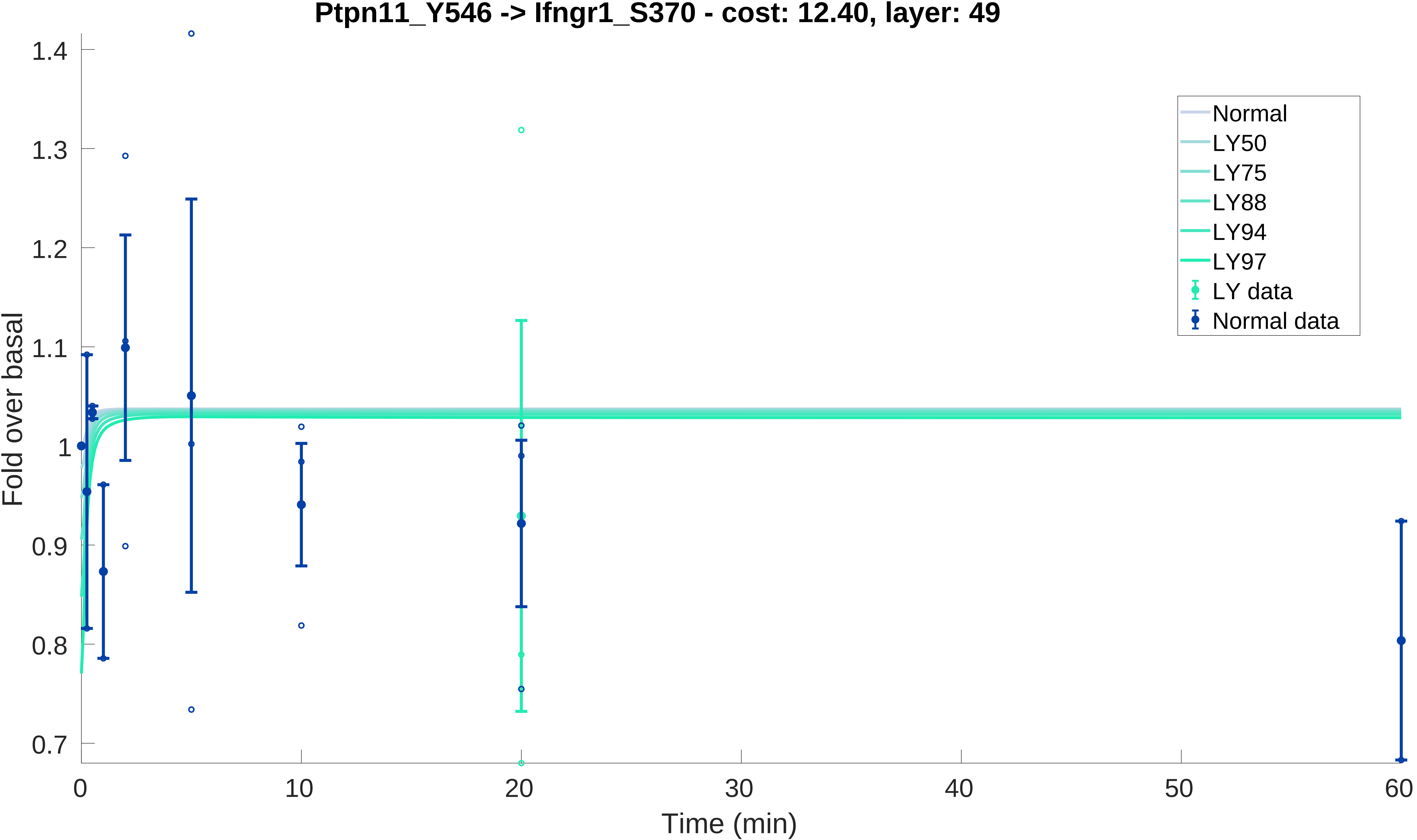

Ptpn11_Y546 -> Ifngr1_S370 - cost: 12.40, layer: 49
Fold over basal
Time (min)
1.4
1.3
1.2
1.1
1
0.9
0.8
0.7
0
10
20
30
40
50
60
Normal
LY50
LY75
LY88
LY94
LY97
LY data
Normal data

Ptpn11_Y546 -> Ifngr1_T302 - cost: 2.56, layer: 49

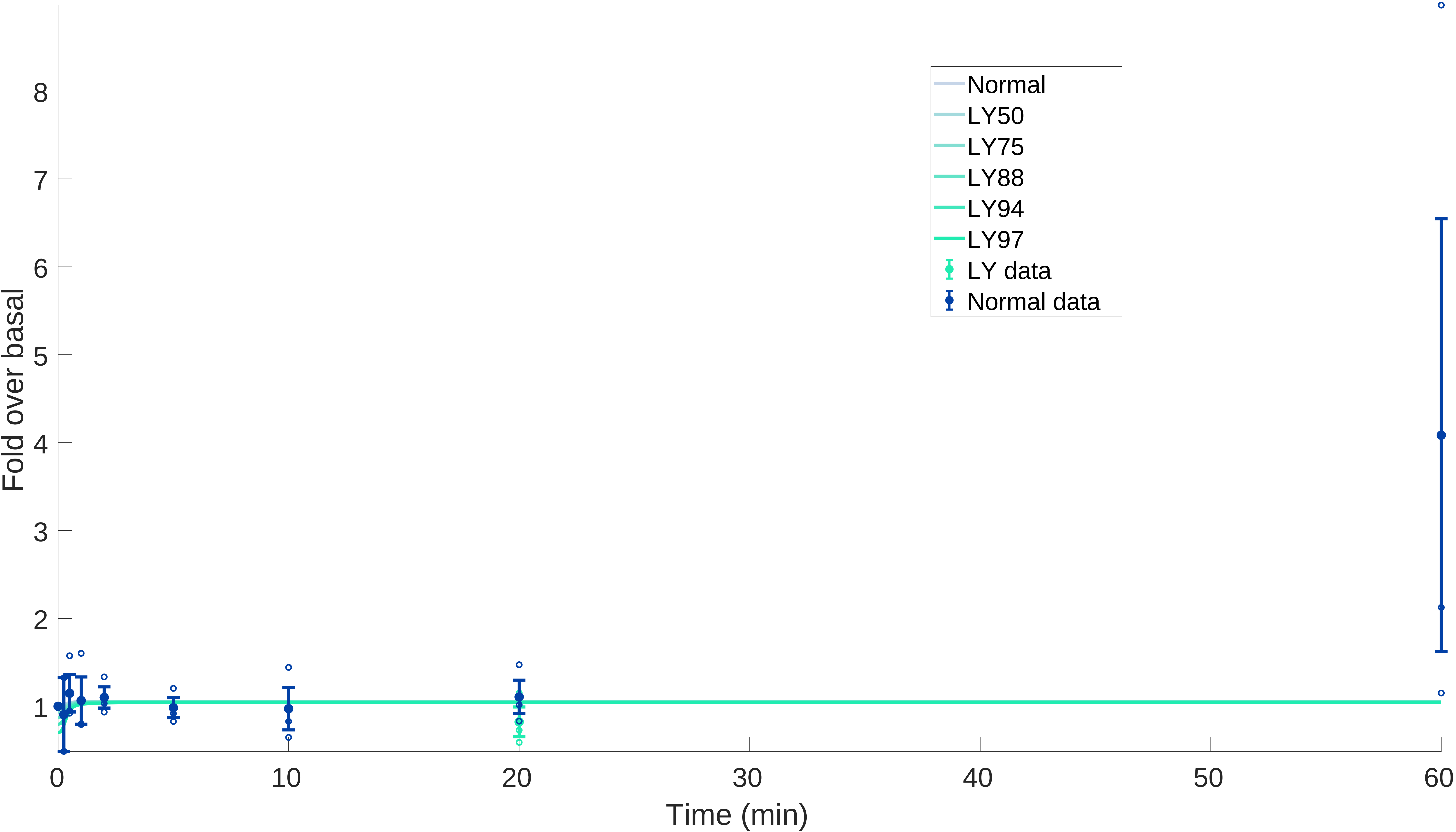

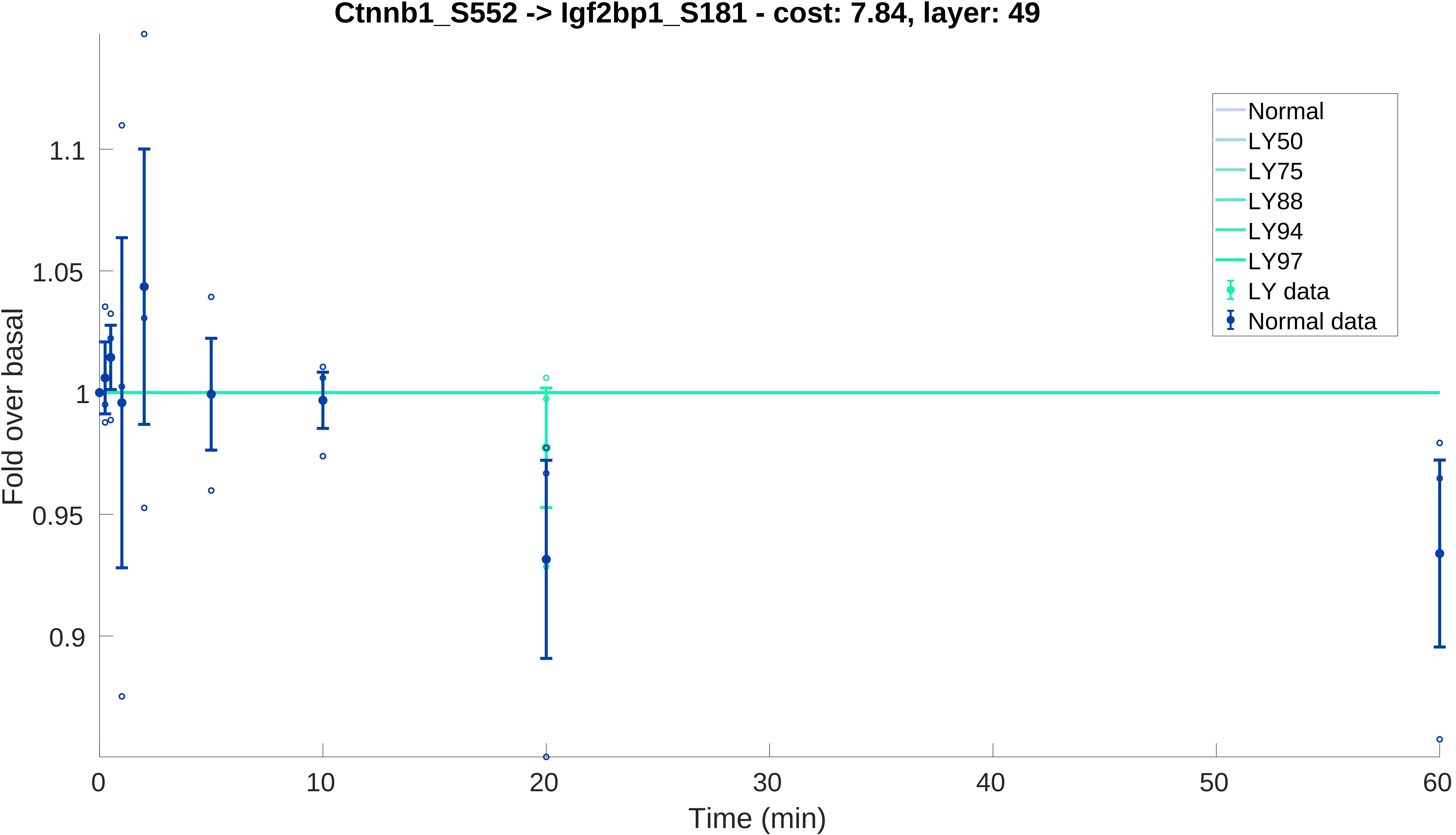

Ctnnb1_S552 -> Igf2bp1_S181 - cost: 7.84, layer: 49
Fold over basal
1.1
1.05
1
0.95
0.9
0
10
20
30
40
50
60
Time (min)
Normal
LY50
LY75
LY88
LY94
LY97
LY data
Normal data

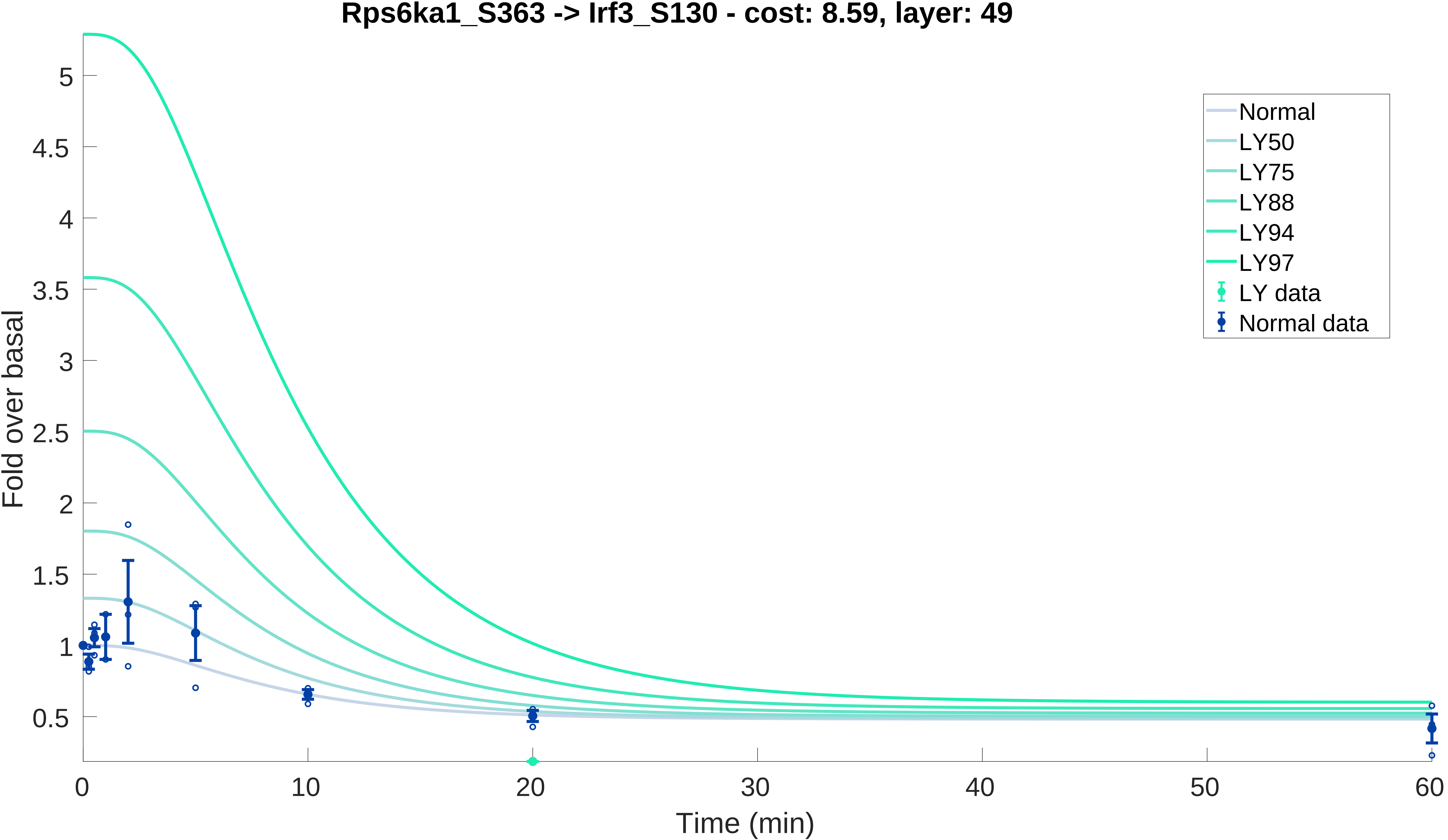

Rps6ka1_S363 -> Irf3_S130 - cost: 8.59, layer: 49
Fold over basal
Time (min)
0.5
1
1.5
2
2.5
3
3.5
4
4.5
5
0
10
20
30
40
50
60
Normal
LY50
LY75
LY88
LY94
LY97
LY data
Normal data

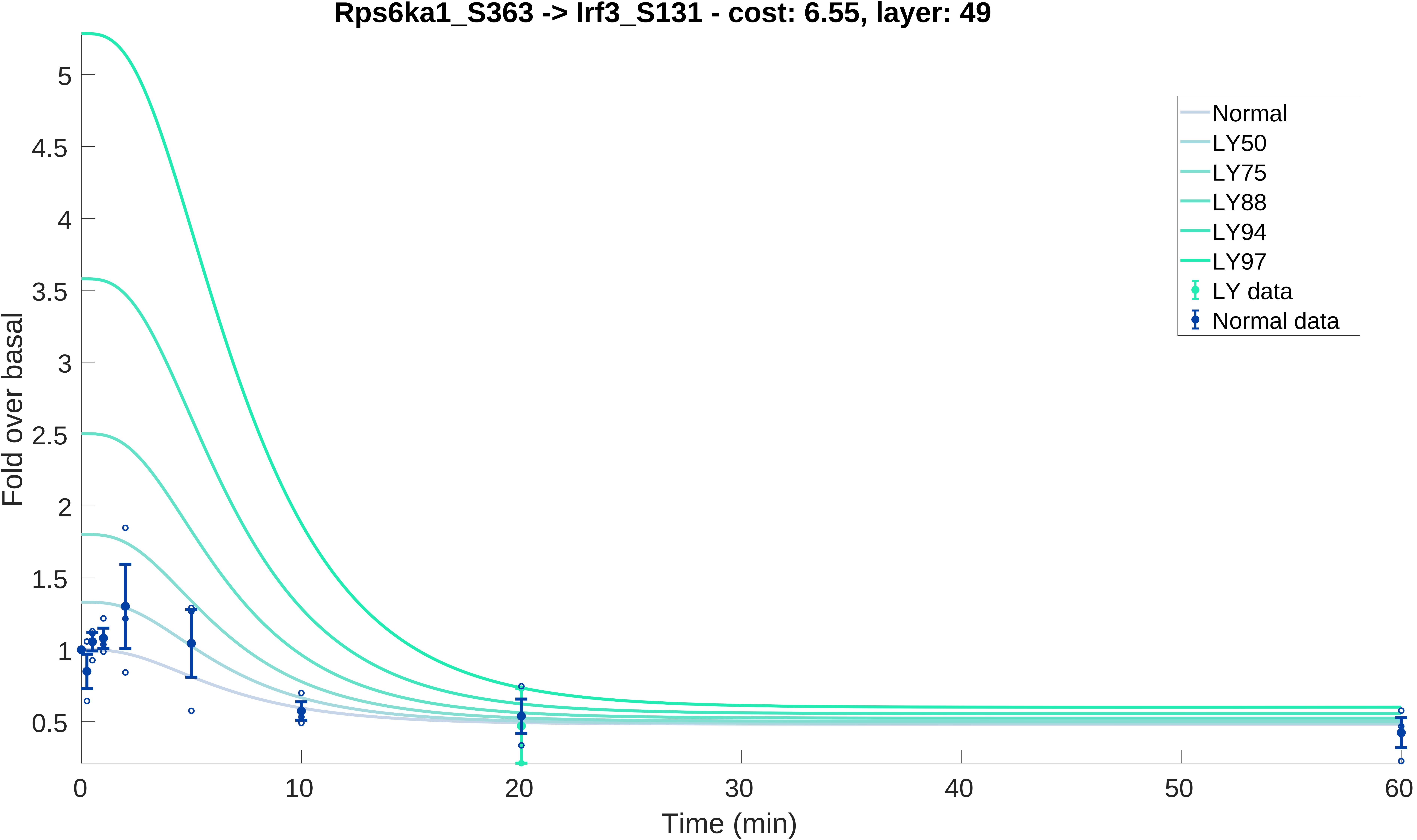

Rps6ka1_S363 -> Irf3_S131 - cost: 6.55, layer: 49
Fold over basal
Time (min)
0.5
1
1.5
2
2.5
3
3.5
4
4.5
5
0
10
20
30
40
50
60
Normal
LY50
LY75
LY88
LY94
LY97
LY data
Normal data

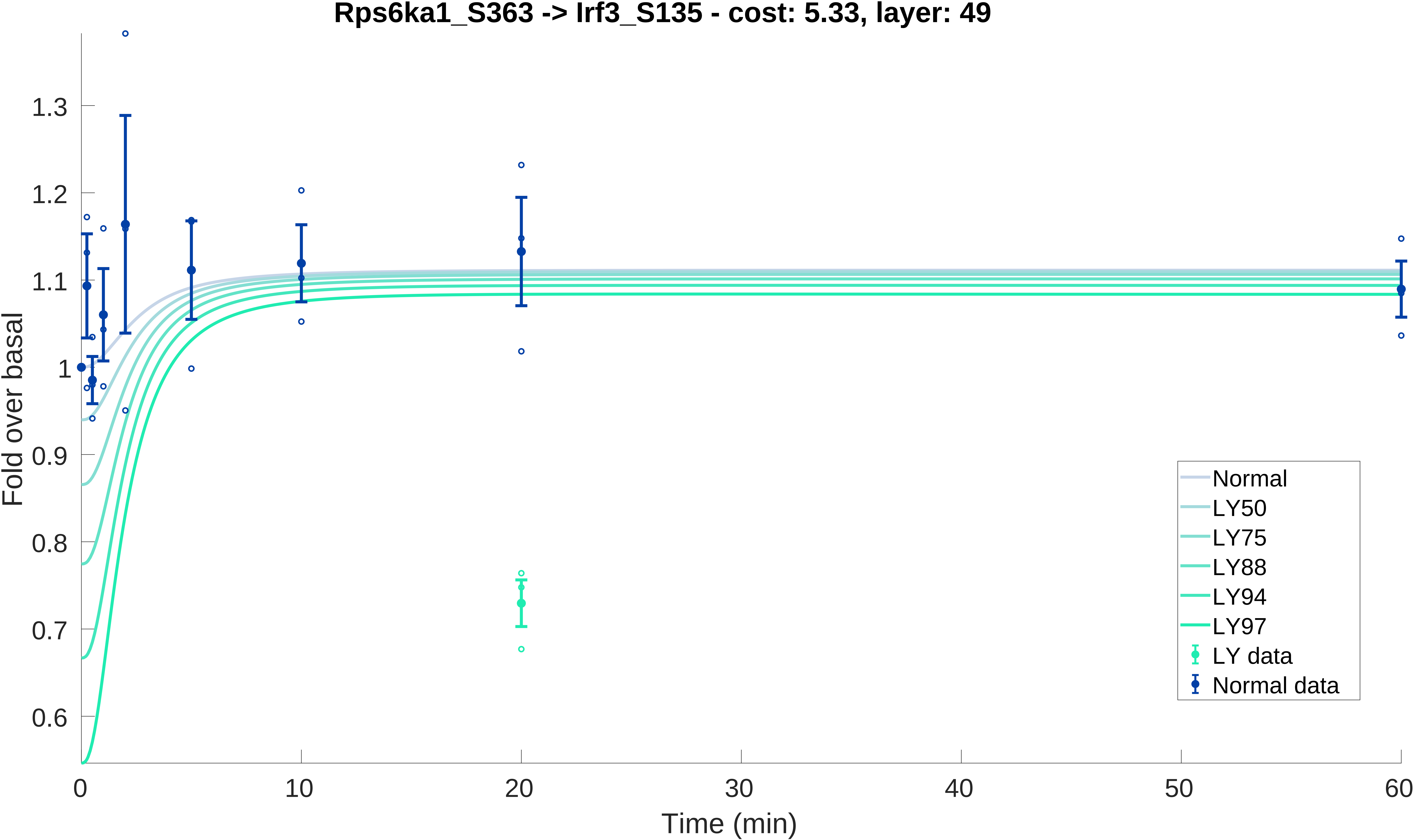

Rps6ka1_S363 -> Irf3_S135 - cost: 5.33, layer: 49
Fold over basal
Time (min)
0
10
20
30
40
50
60
0.6
0.7
0.8
0.9
1
1.1
1.2
1.3
Normal
LY50
LY75
LY88
LY94
LY97
LY data
Normal data

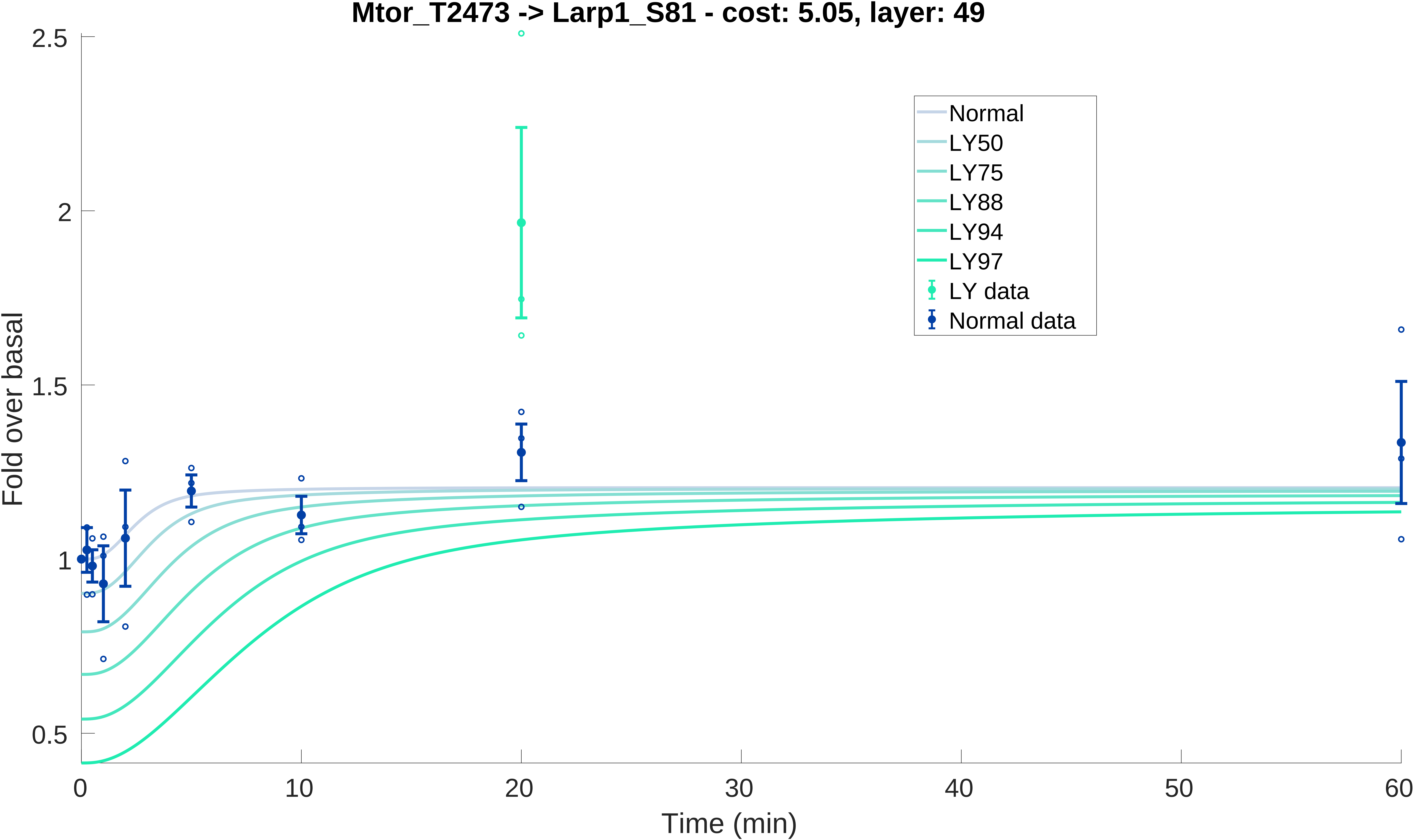
Mtor_T2473 -> Larp1_S81 - cost: 5.05, layer: 49
Fold over basal
Time (min)
2.5
2
1.5
1
0.5
0
10
20
30
40
50
60
Normal
LY50
LY75
LY88
LY94
LY97
LY data
Normal data

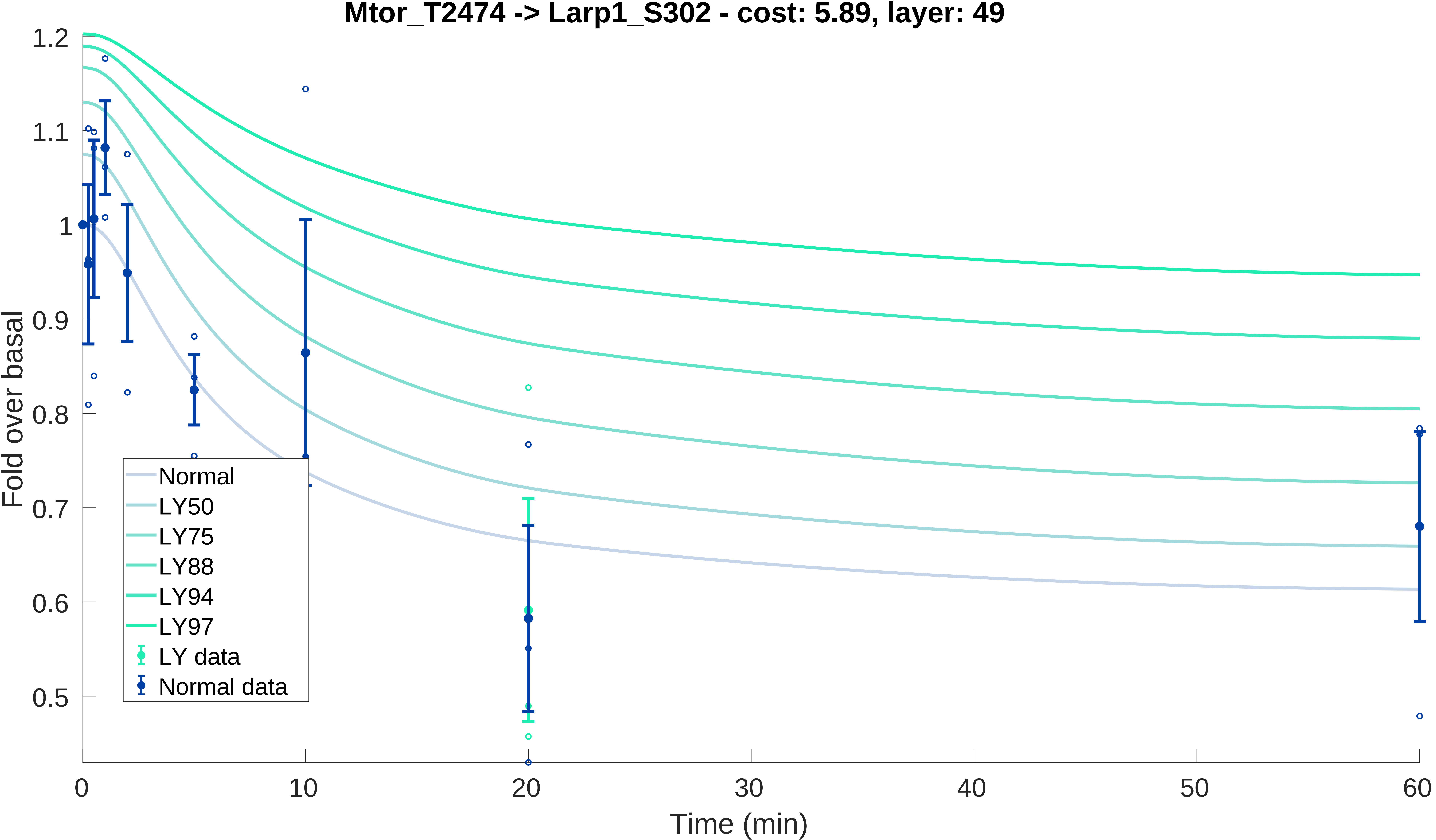

Mtor_T2474 -> Larp1_S302 - cost: 5.89, layer: 49
Fold over basal
Time (min)
1.2
1.1
1
0.9
0.8
0.7
0.6
0.5
0
10
20
30
40
50
60
Normal
LY50
LY75
LY88
LY94
LY97
LY data
Normal data

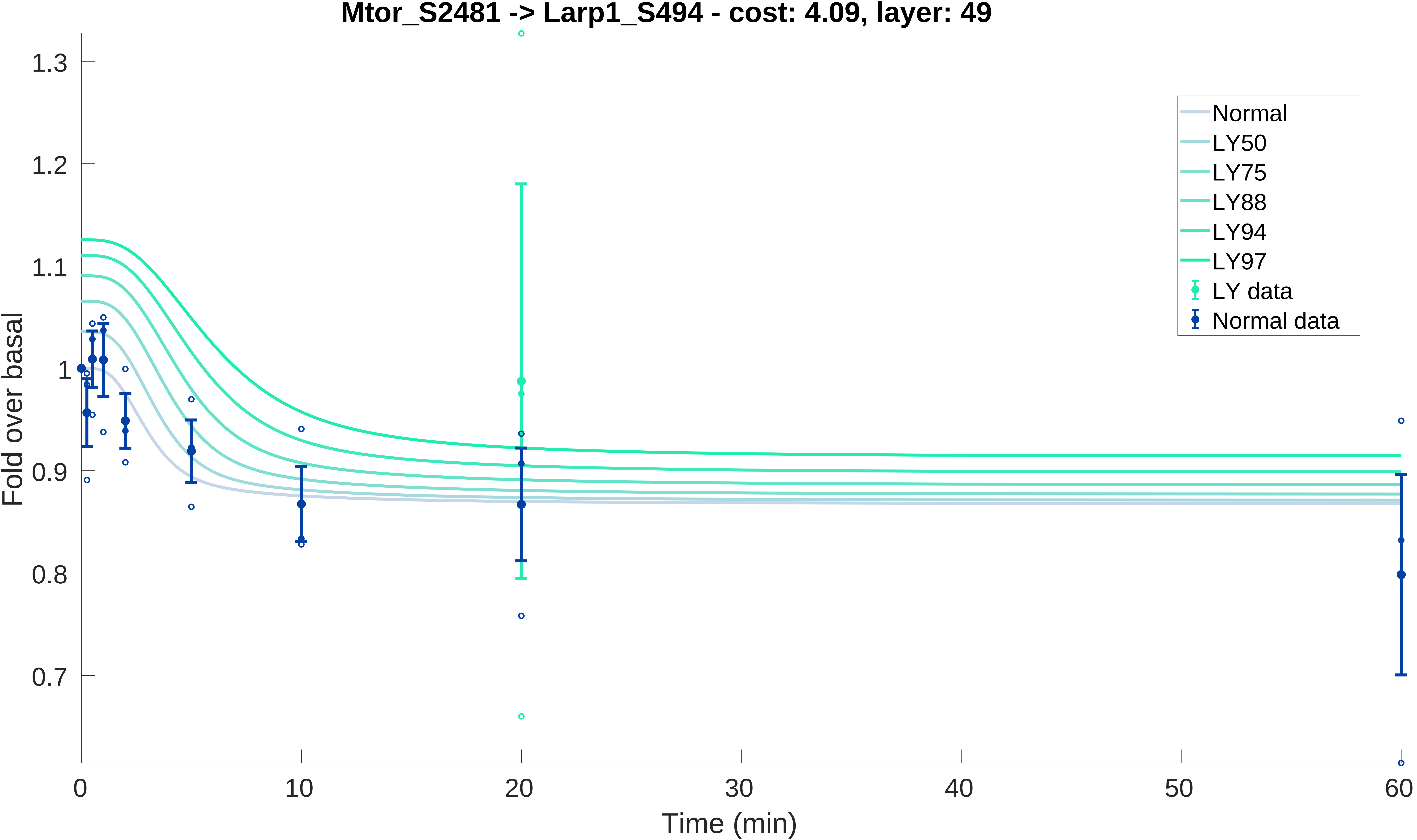

Mtor_S2481 -> Larp1_S494 - cost: 4.09, layer: 49
Fold over basal
Time (min)
Normal
LY50
LY75
LY88
LY94
LY97
LY data
Normal data
1.3
1.2
1.1
1
0.9
0.8
0.7
0
10
20
30
40
50
60

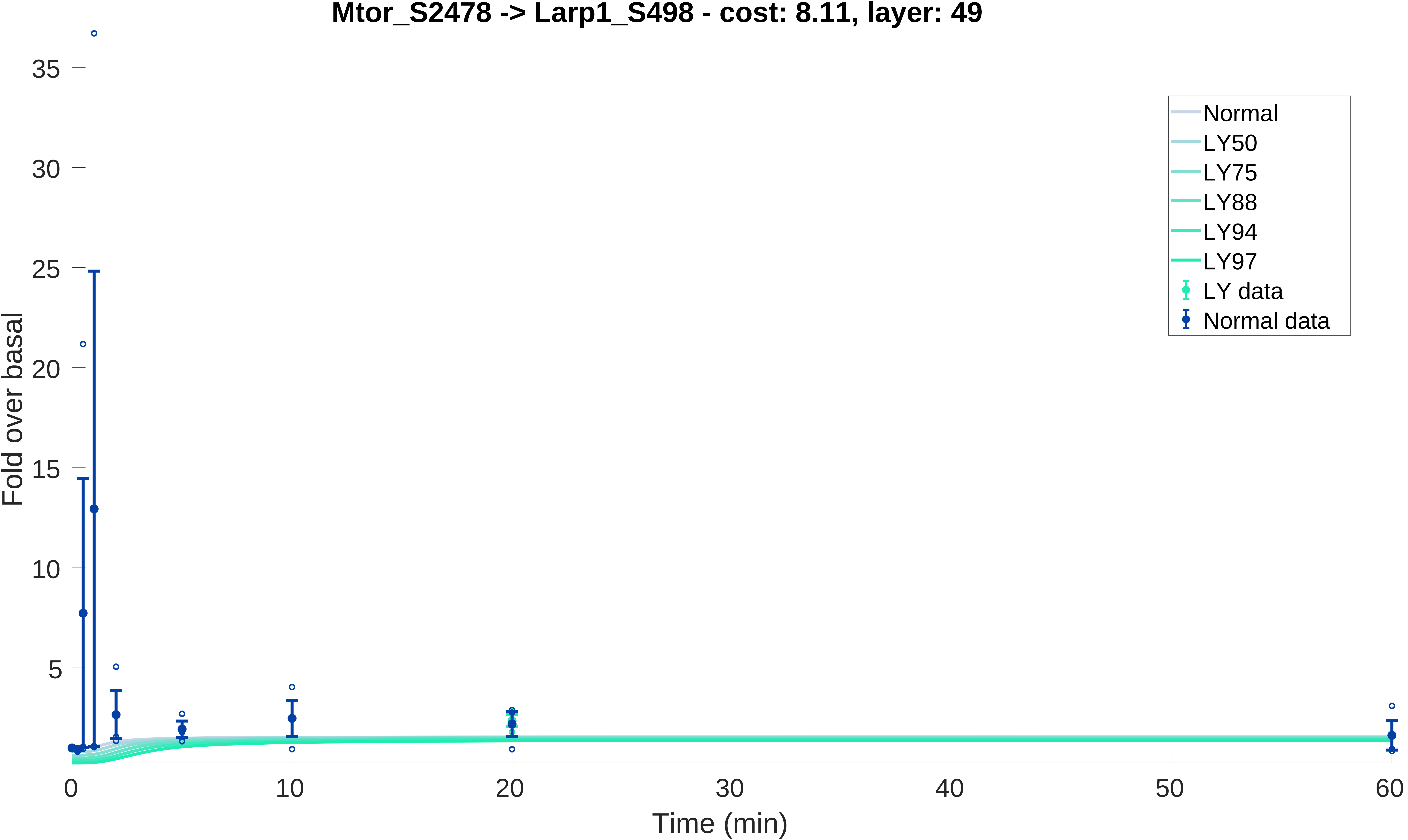

Mtor_S2478 -> Larp1_S498 - cost: 8.11, layer: 49
Fold over basal
Time (min)
0
5
10
15
20
25
30
35
40
50
60
Normal
LY50
LY75
LY88
LY94
LY97
LY data
Normal data

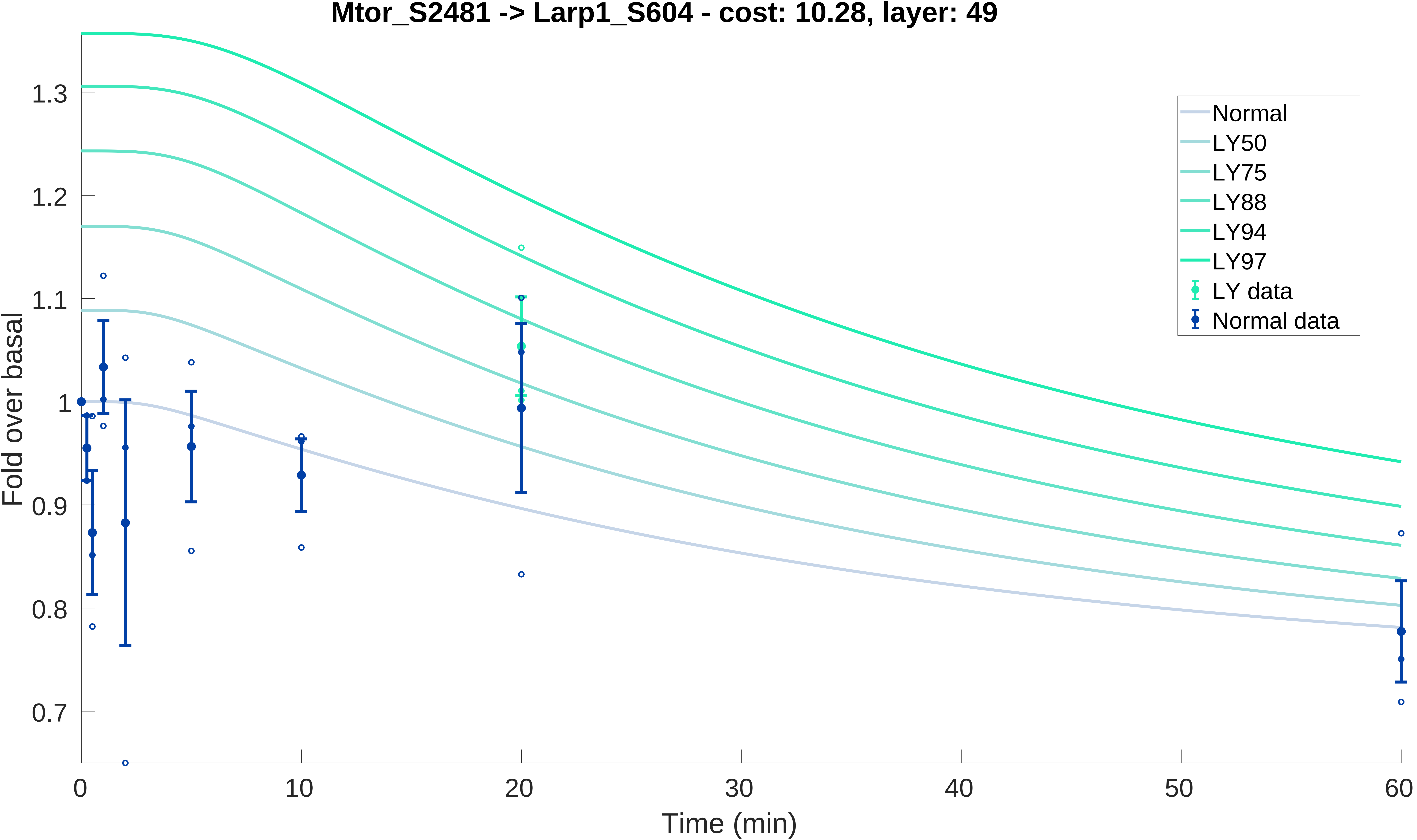
Mtor_S2481 -> Larp1_S604 - cost: 10.28, layer: 49
Fold over basal
Time (min)
1.3
1.2
1.1
1
0.9
0.8
0.7
0
10
20
30
40
50
60
Normal
LY50
LY75
LY88
LY94
LY97
LY data
Normal data

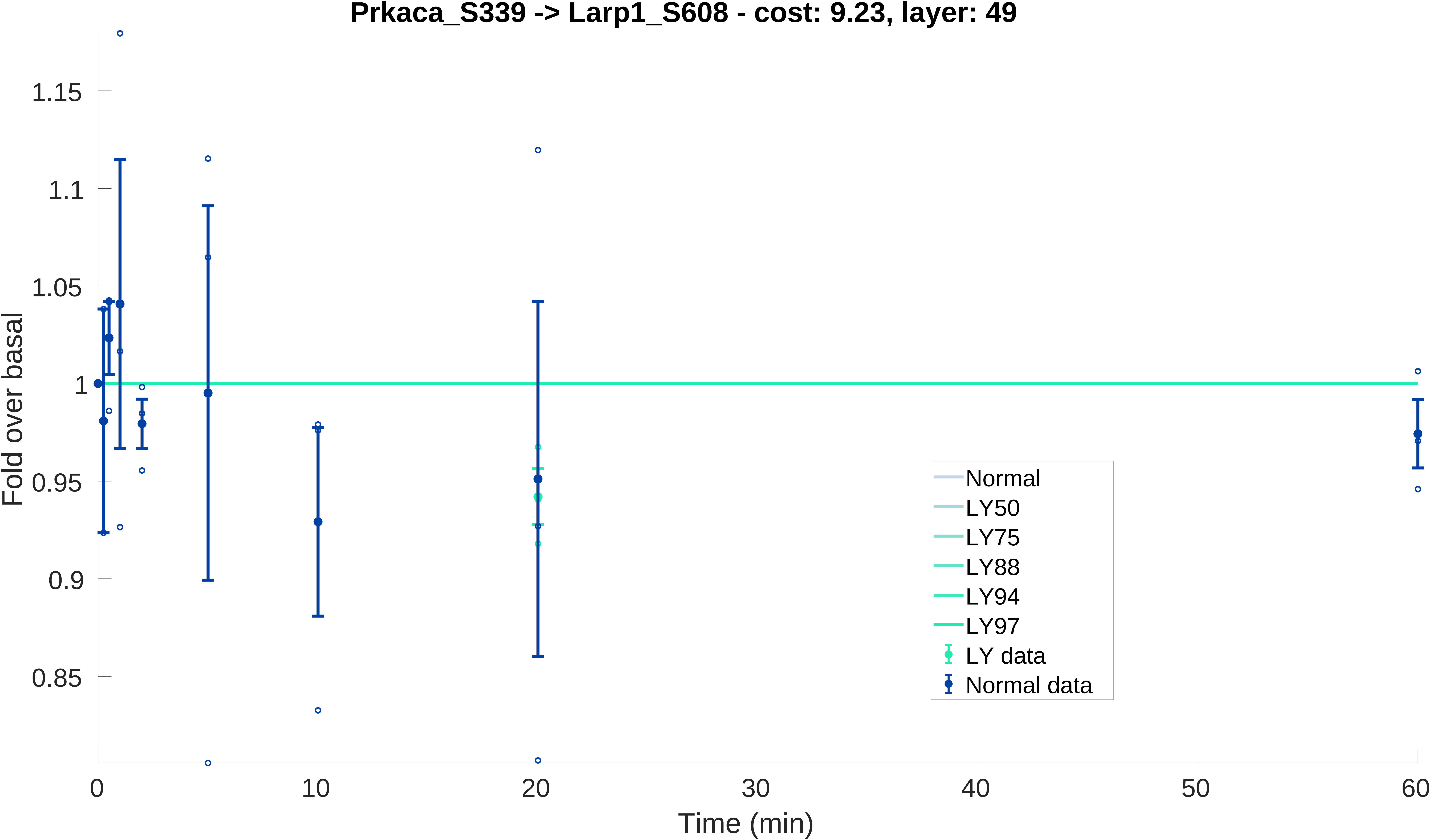
Prkaca_S339 -> Larp1_S608 - cost: 9.23, layer: 49
Fold over basal
1.15
1.1
1.05
1
0.95
0.9
0.85
0
10
20
30
40
50
60
Time (min)
Normal
LY50
LY75
LY88
LY94
LY97
LY data
Normal data

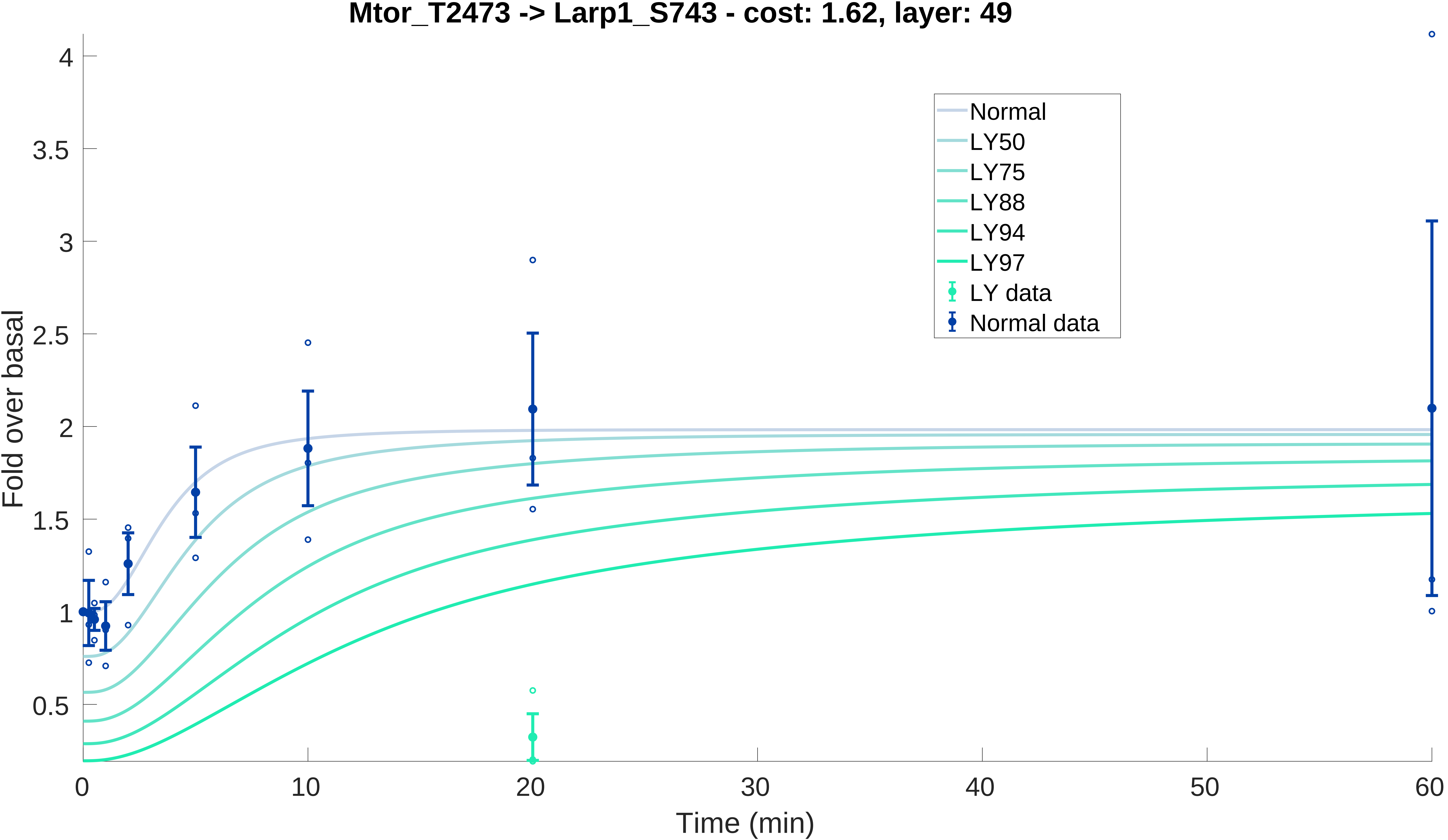

Mtor_T2473 -> Larp1_S743 - cost: 1.62, layer: 49
Fold over basal
Time (min)
0
10
20
30
40
50
60
0.5
1
1.5
2
2.5
3
3.5
4
Normal
LY50
LY75
LY88
LY94
LY97
LY data
Normal data

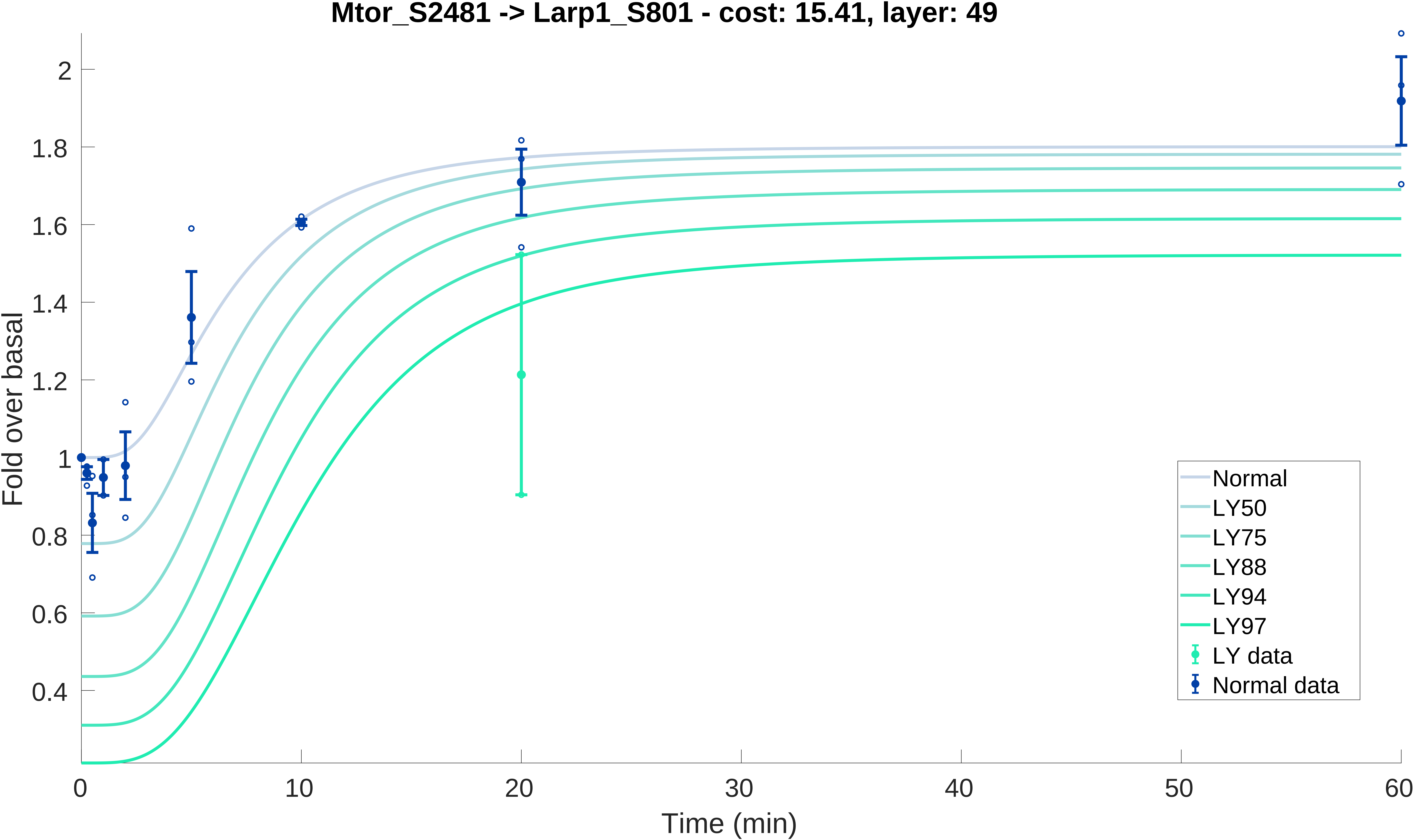
Mtor_S2481 -> Larp1_S801 - cost: 15.41, layer: 49
Fold over basal
2
1.8
1.6
1.4
1.2
1
0.8
0.6
0.4
0
10
20
30
40
50
60
Time (min)
Normal
LY50
LY75
LY88
LY94
LY97
LY data
Normal data

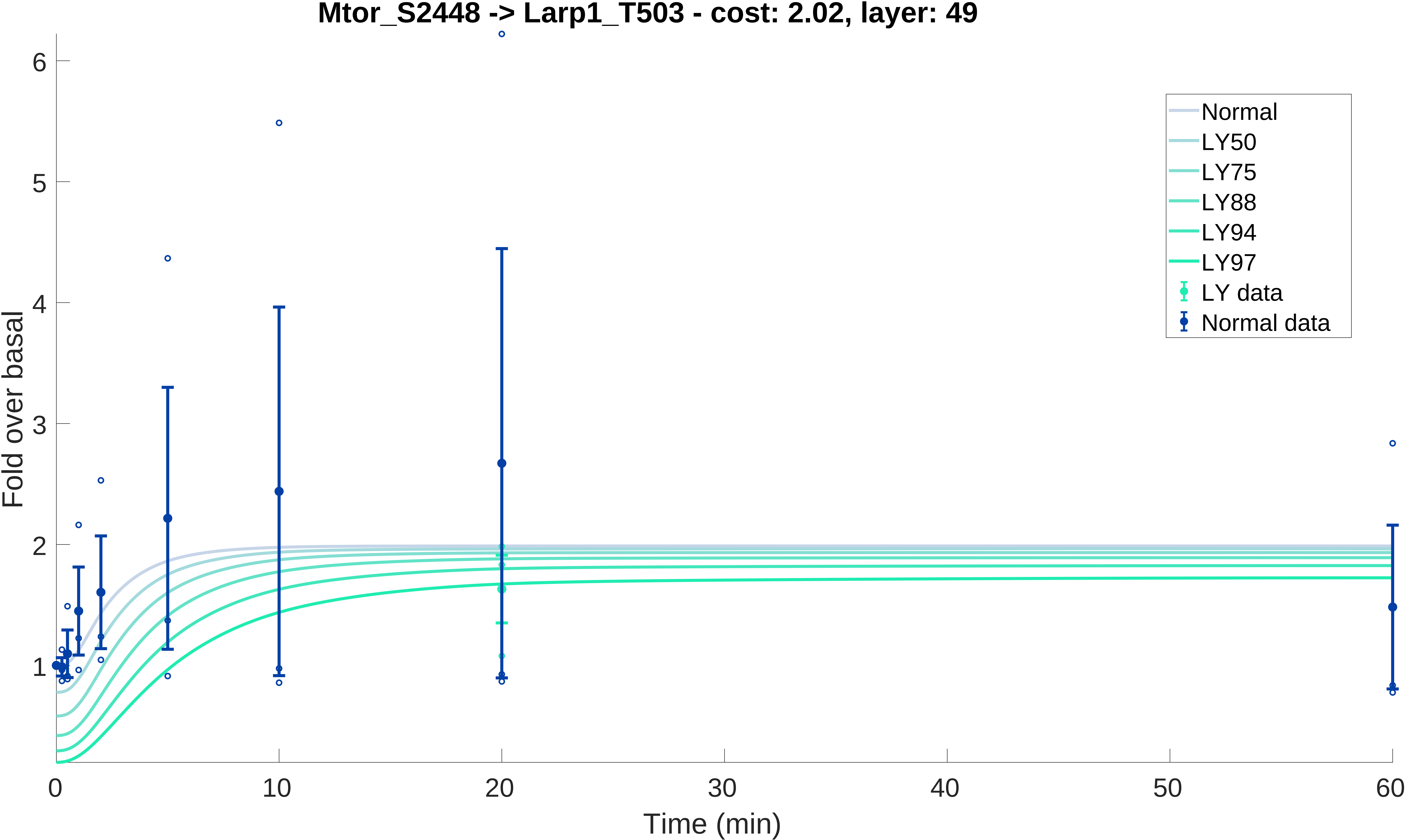

Mtor_S2448 -> Larp1_T503 - cost: 2.02, layer: 49
Fold over basal
Time (min)
Normal
LY50
LY75
LY88
LY94
LY97
LY data
Normal data

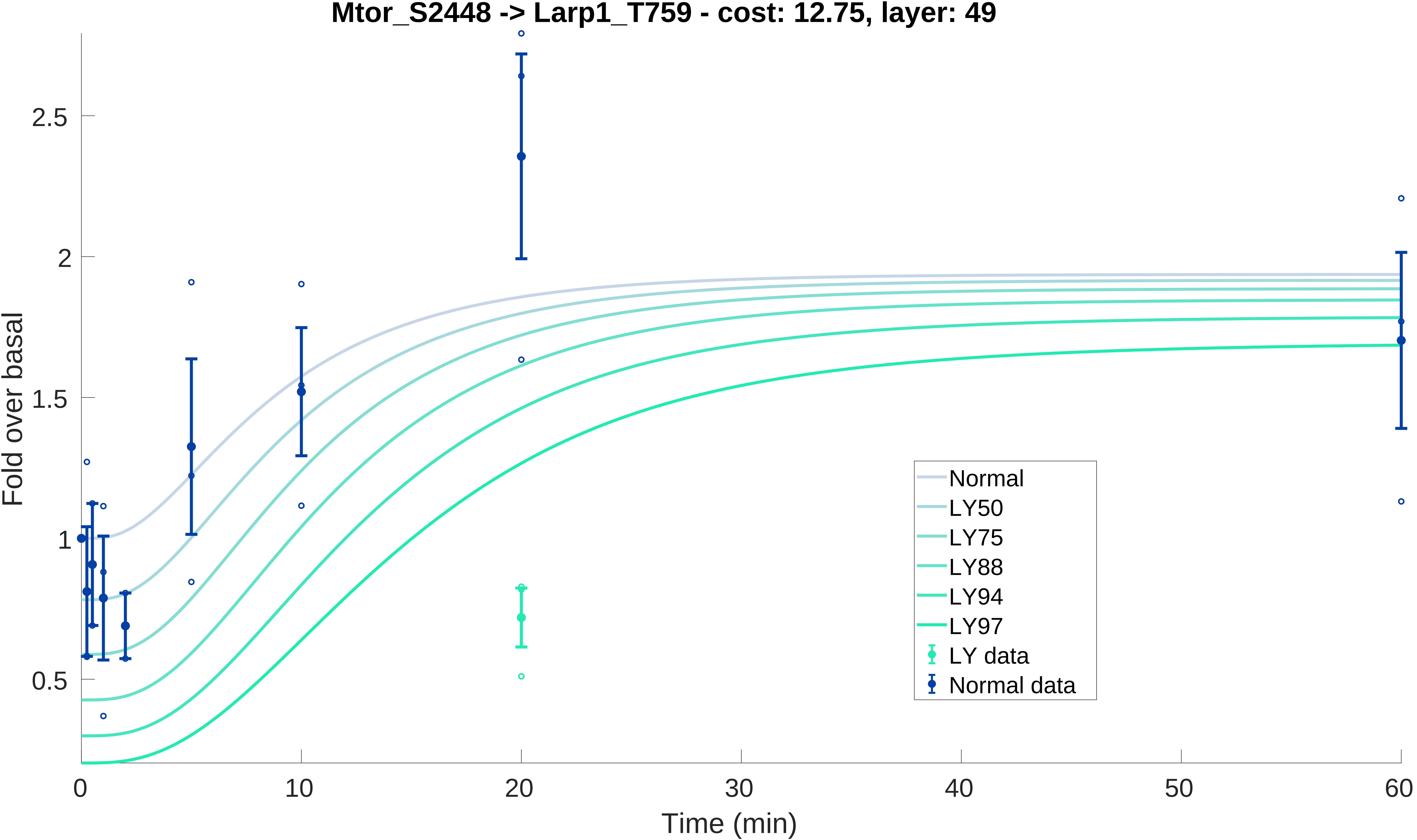

Mtor_S2448 -> Larp1_T759 - cost: 12.75, layer: 49
Fold over basal
Time (min)
0.5
1
1.5
2
2.5
0
10
20
30
40
50
60
Normal
LY50
LY75
LY88
LY94
LY97
LY data
Normal data

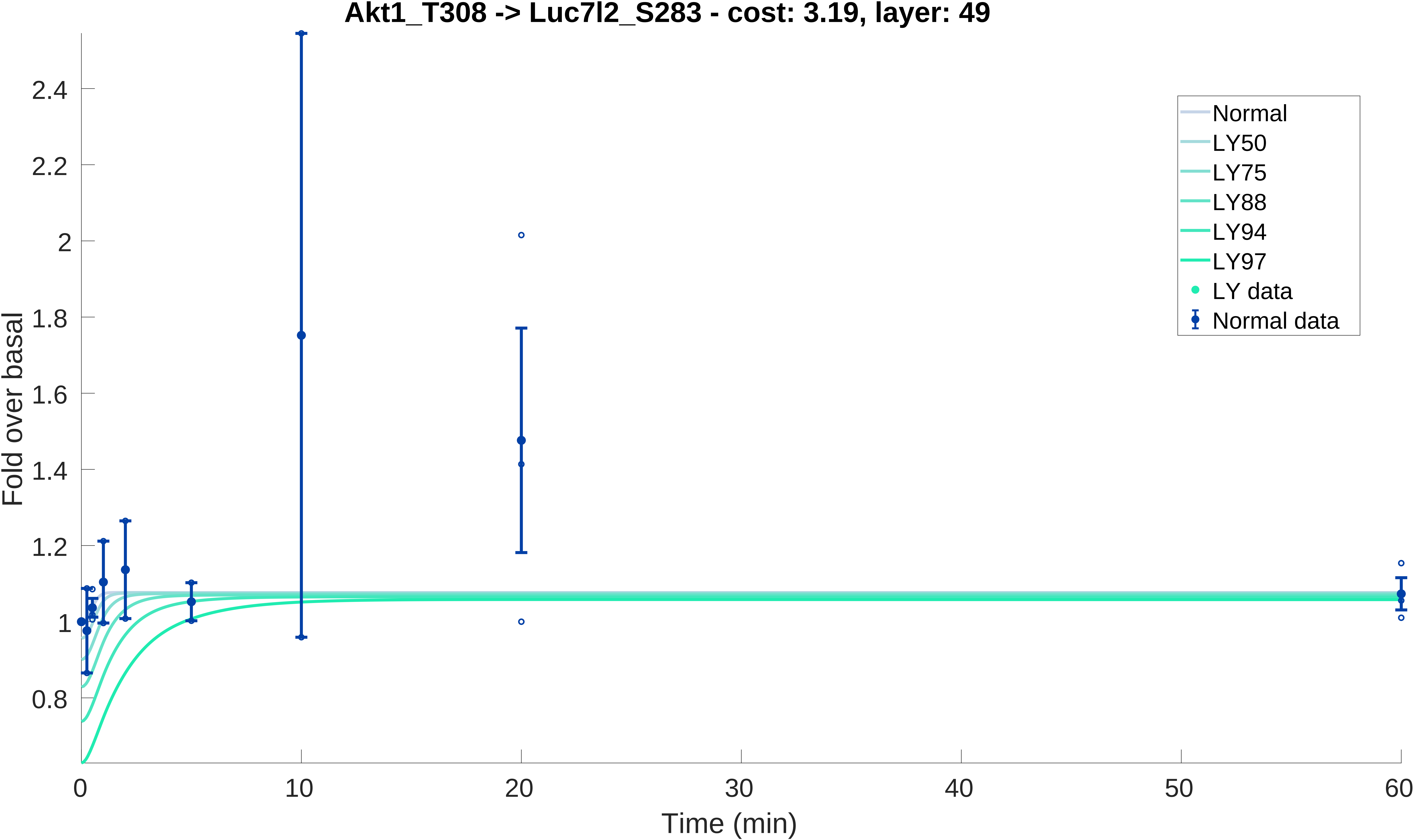

Akt1_T308 -> Luc7l2_S283 - cost: 3.19, layer: 49
Fold over basal
Time (min)
2.4
2.2
2
1.8
1.6
1.4
1.2
1
0.8
0
10
20
30
40
50
60
Normal
LY50
LY75
LY88
LY94
LY97
LY data
Normal data

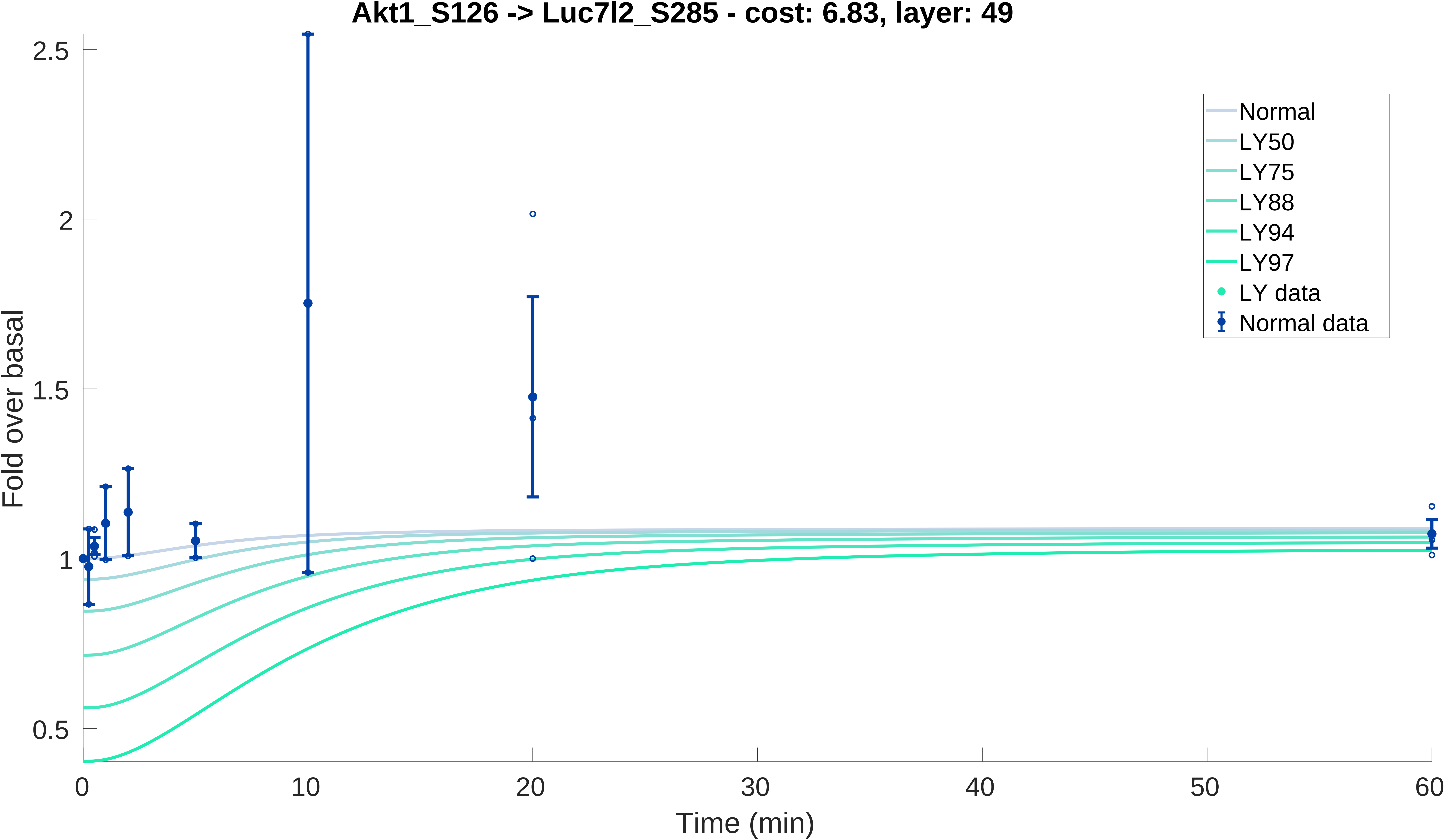

Akt1_S126 -> Luc7l2_S285 - cost: 6.83, layer: 49
Fold over basal
Time (min)
0
10
20
30
40
50
60
0.5
1
1.5
2
2.5
Normal
LY50
LY75
LY88
LY94
LY97
LY data
Normal data

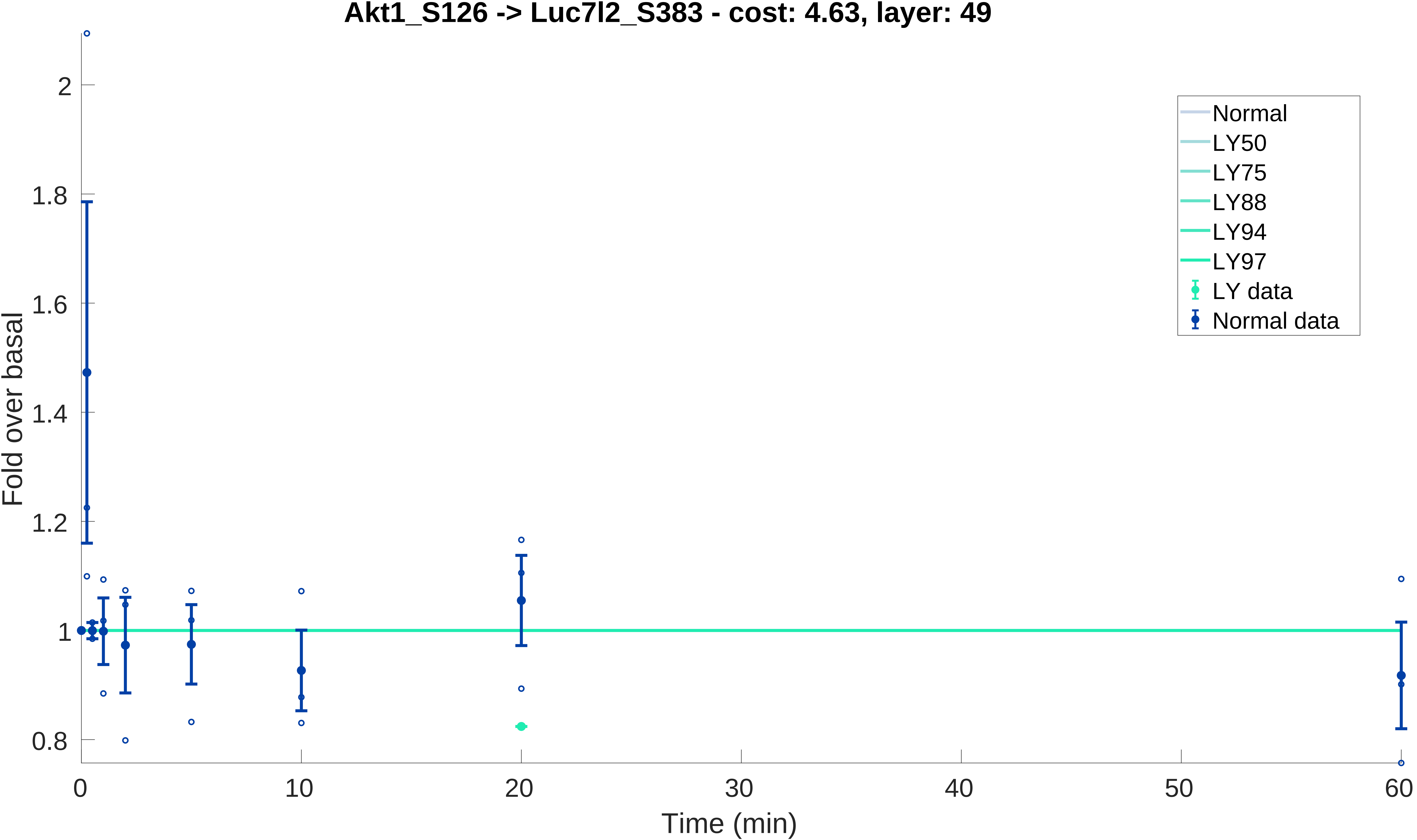

Akt1_S126 -> Luc7l2_S383 - cost: 4.63, layer: 49
Fold over basal
2
1.8
1.6
1.4
1.2
1
0.8
0
10
20
30
40
50
60
Time (min)
Normal
LY50
LY75
LY88
LY94
LY97
LY data
Normal data

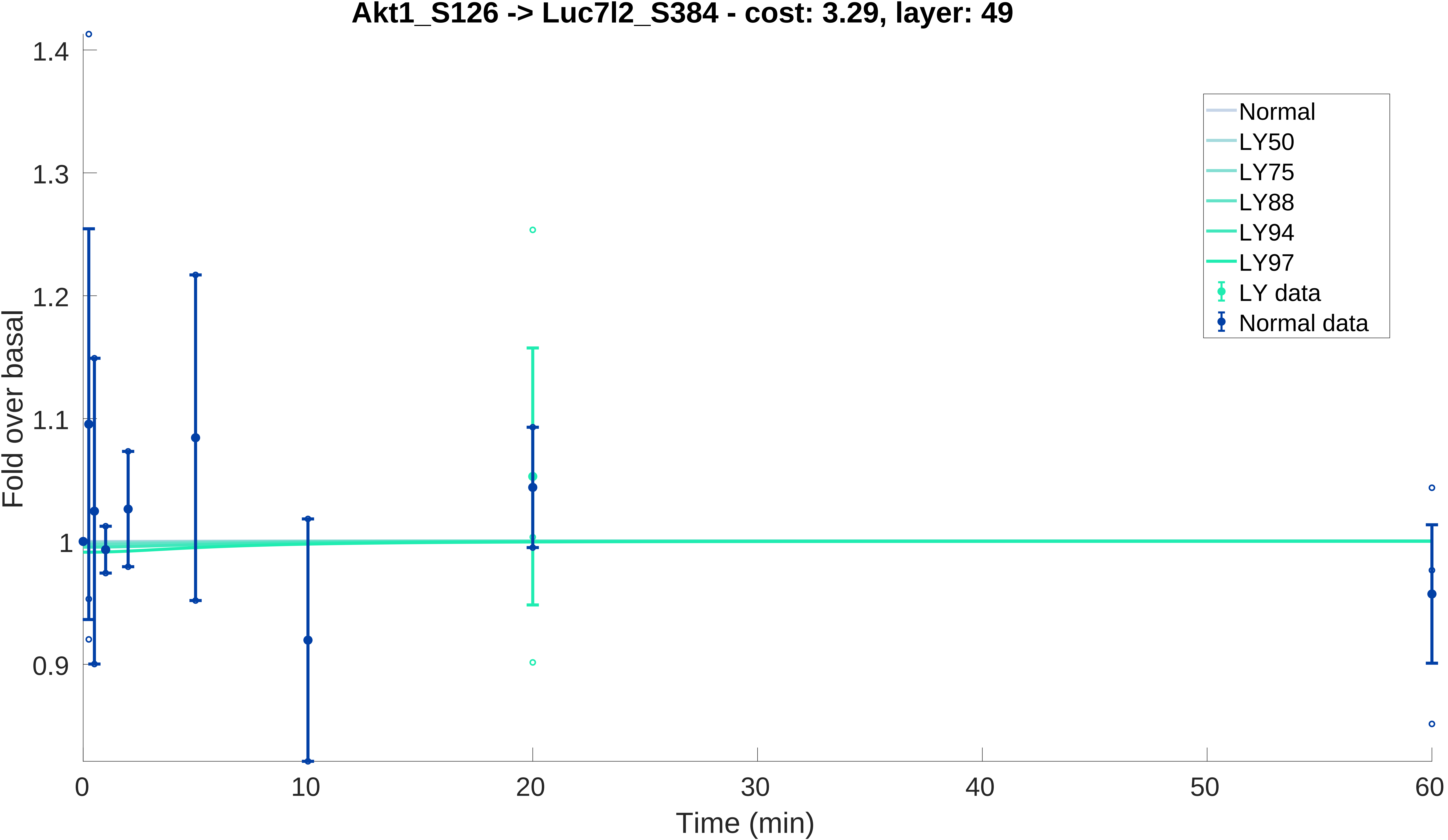

Akt1_S126 -> Luc7l2_S384 - cost: 3.29, layer: 49
Fold over basal
Time (min)
1.4
1.3
1.2
1.1
1
0.9
0
10
20
30
40
50
60
Normal
LY50
LY75
LY88
LY94
LY97
LY data
Normal data

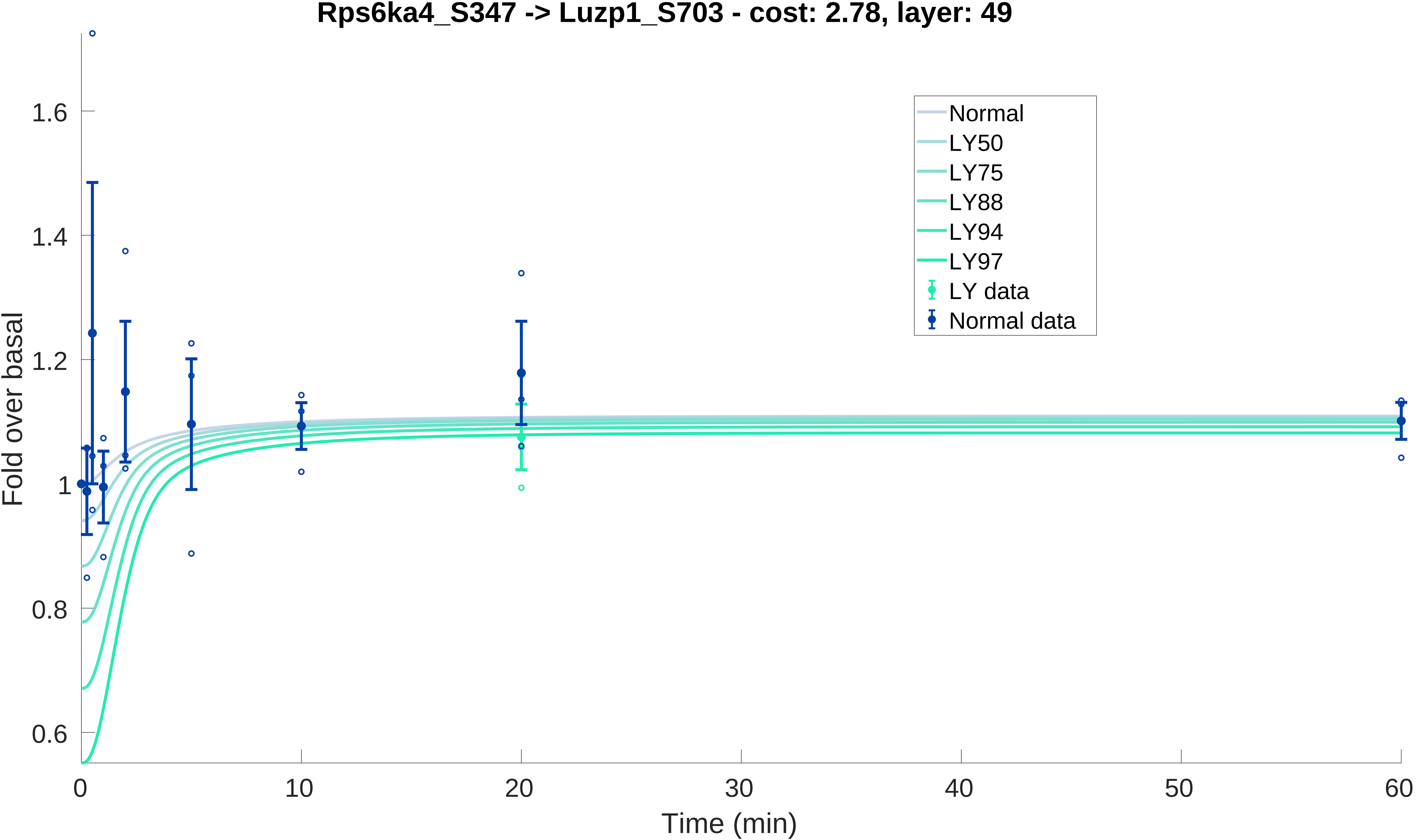

Rps6ka4_S347 -> Luzp1_S703 - cost: 2.78, layer: 49
Fold over basal
Time (min)
0.6
0.8
1
1.2
1.4
1.6
0
10
20
30
40
50
60
Normal
LY50
LY75
LY88
LY94
LY97
LY data
Normal data

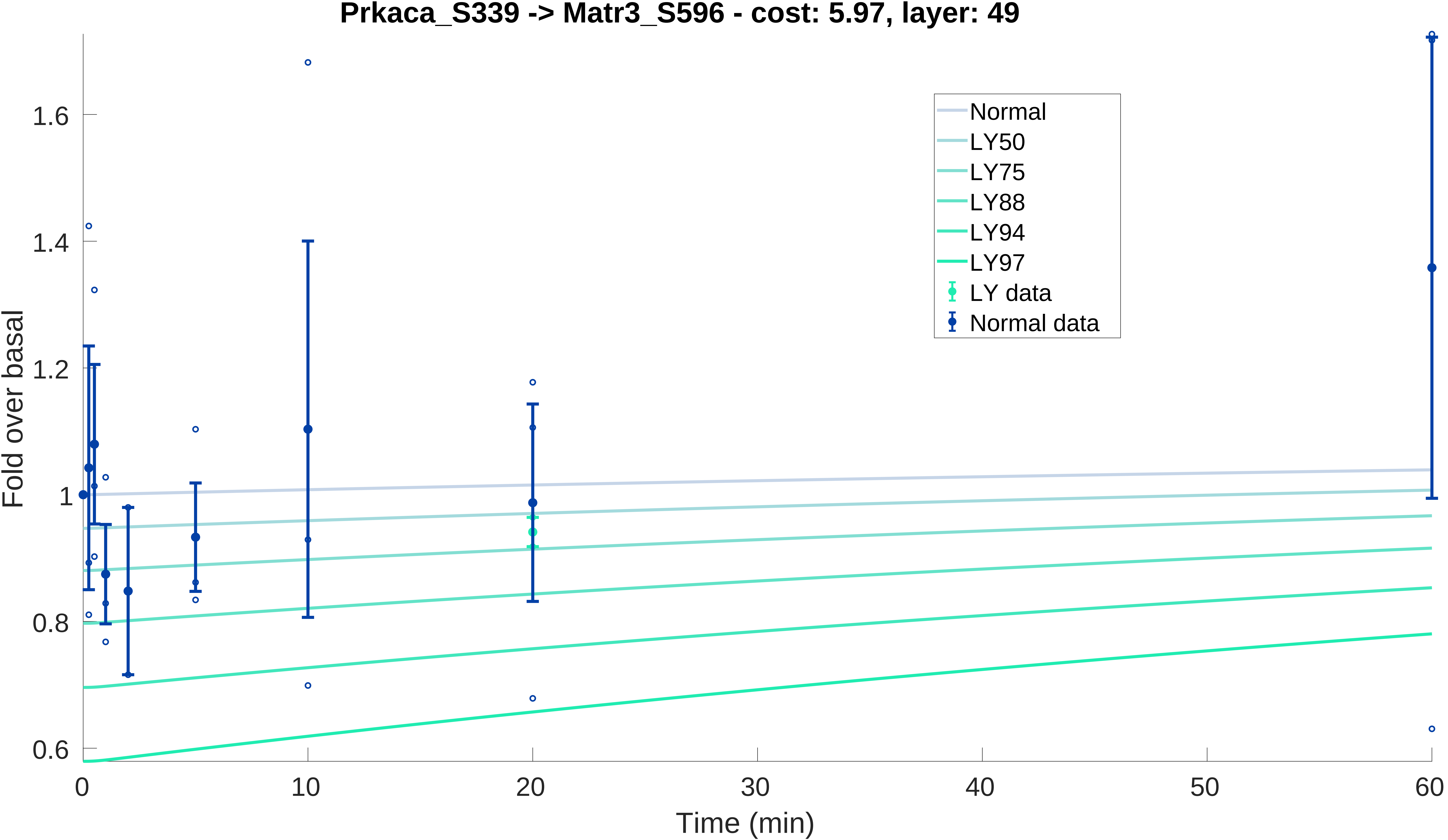

Prkaca_S339 -> Matr3_S596 - cost: 5.97, layer: 49
Fold over basal
Time (min)
Normal
LY50
LY75
LY88
LY94
LY97
LY data
Normal data
0.6
0.8
1
1.2
1.4
1.6
0
10
20
30
40
50
60

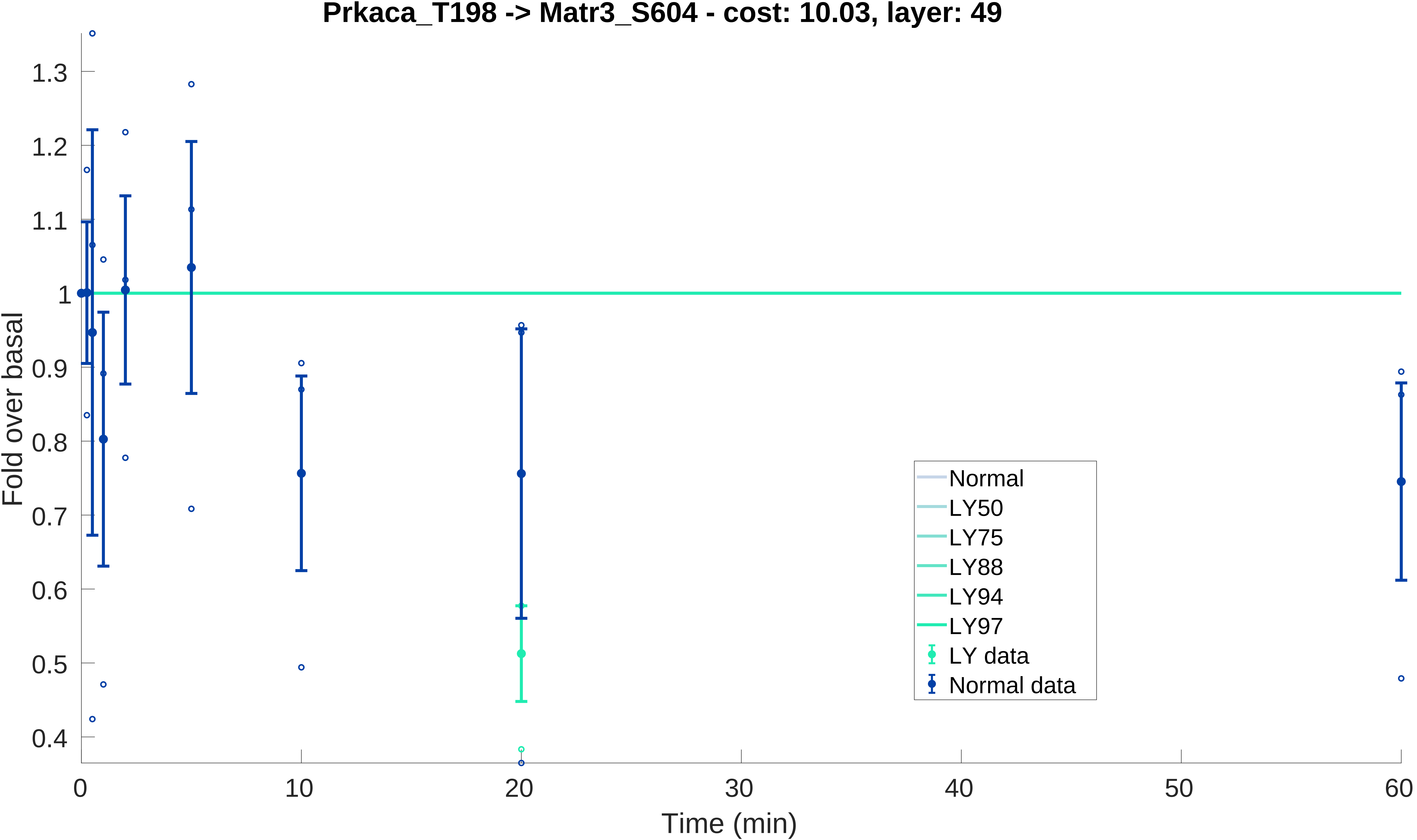

Prkaca_T198 -> Matr3_S604 - cost: 10.03, layer: 49
Fold over basal
1.3
1.2
1.1
1
0.9
0.8
0.7
0.6
0.5
0.4
0
10
20
30
40
50
60
Time (min)
Normal
LY50
LY75
LY88
LY94
LY97
LY data
Normal data

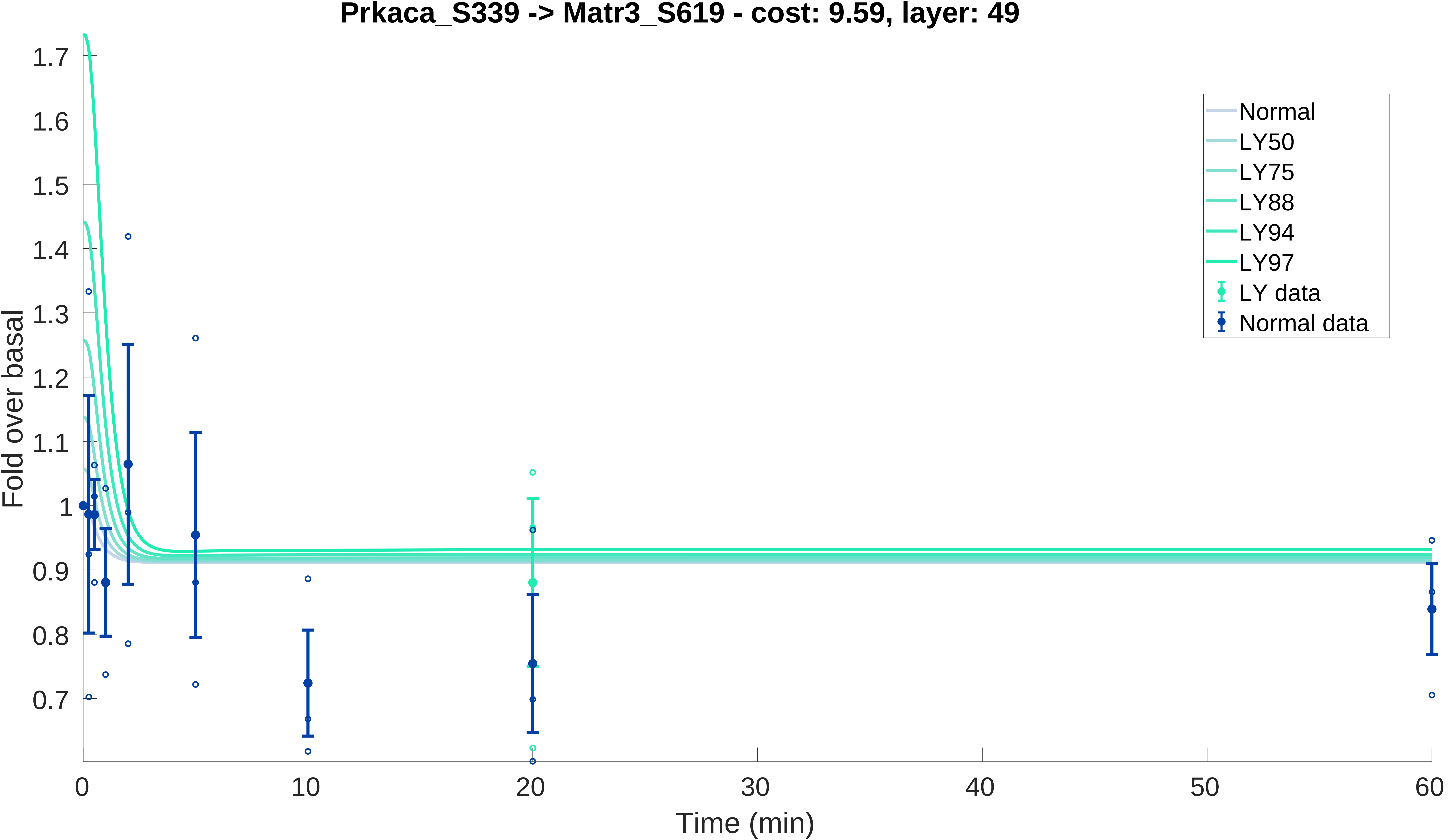
Prkaca_S339 -> Matr3_S619 - cost: 9.59, layer: 49
Fold over basal
1.7
1.6
1.5
1.4
1.3
1.2
1.1
1
0.9
0.8
0.7
0
10
20
30
40
50
60
Time (min)
Normal
LY50
LY75
LY88
LY94
LY97
LY data
Normal data

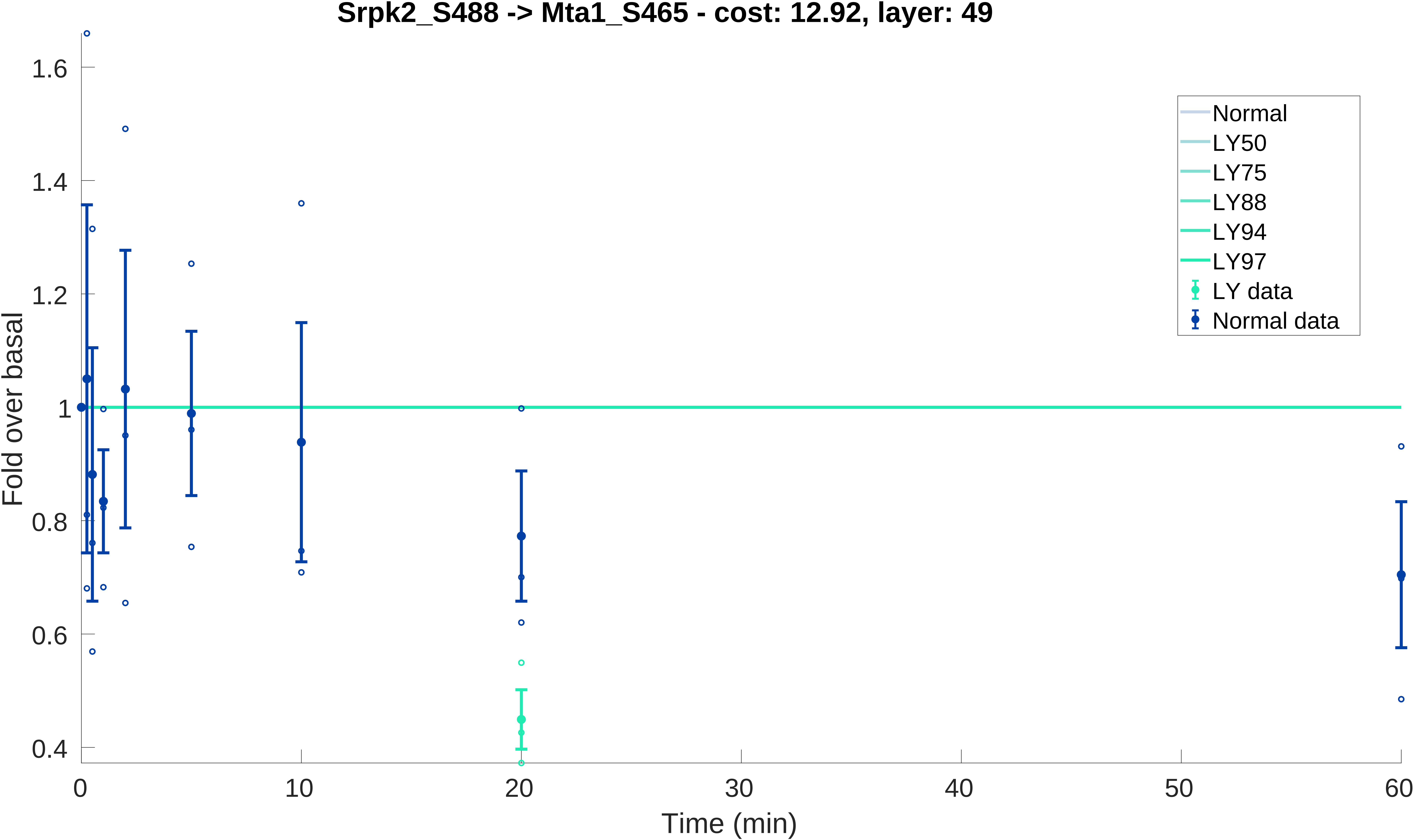

Srpk2_S488 -> Mta1_S465 - cost: 12.92, layer: 49
Fold over basal
Time (min)
1.6
1.4
1.2
1
0.8
0.6
0.4
0
10
20
30
40
50
60
Normal
LY50
LY75
LY88
LY94
LY97
LY data
Normal data

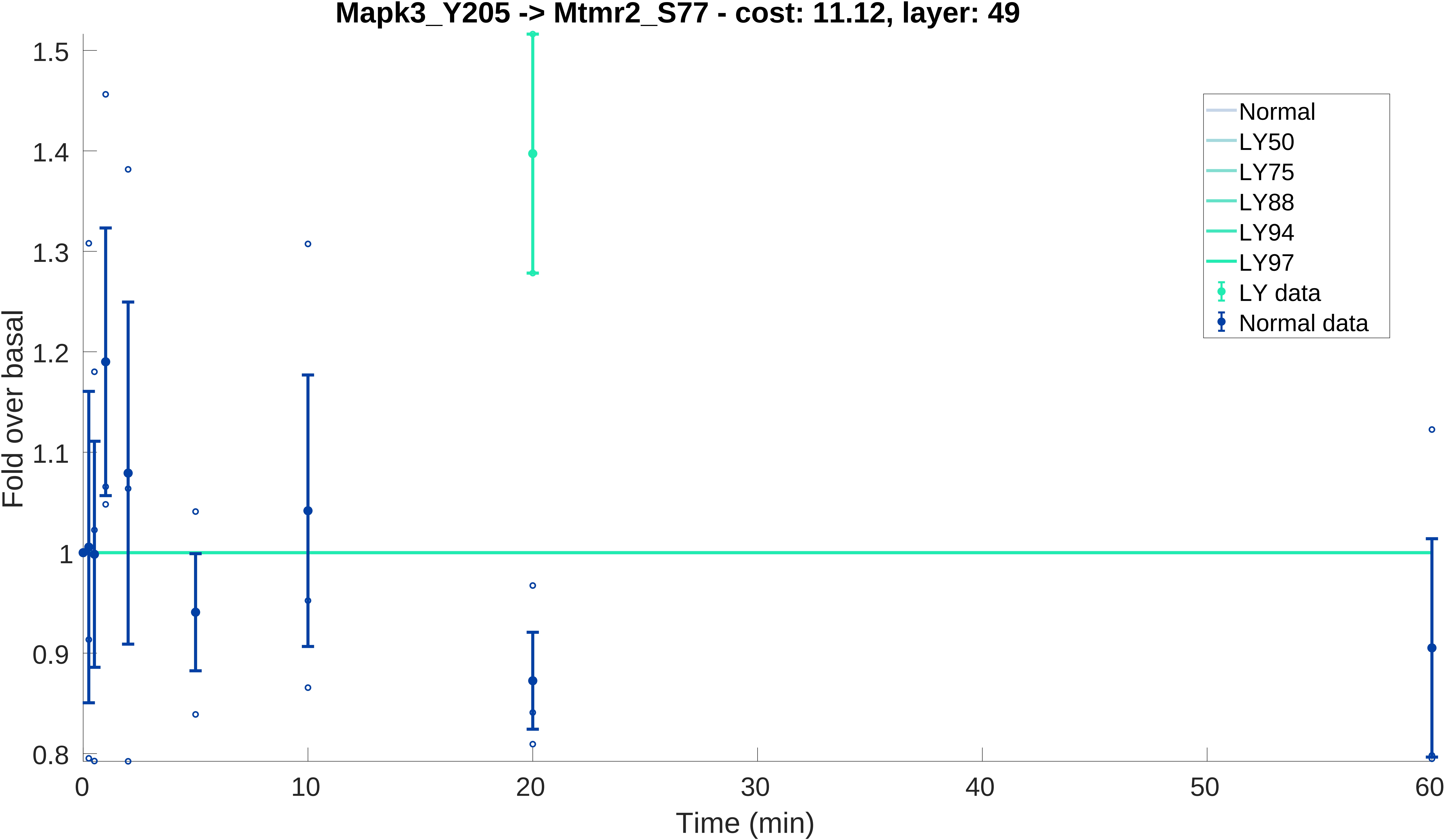

Mapk3_Y205 -> Mtmr2_S77 - cost: 11.12, layer: 49
Fold over basal
Time (min)
0
10
20
30
40
50
60
0.8
0.9
1
1.1
1.2
1.3
1.4
1.5
Normal
LY50
LY75
LY88
LY94
LY97
LY data
Normal data

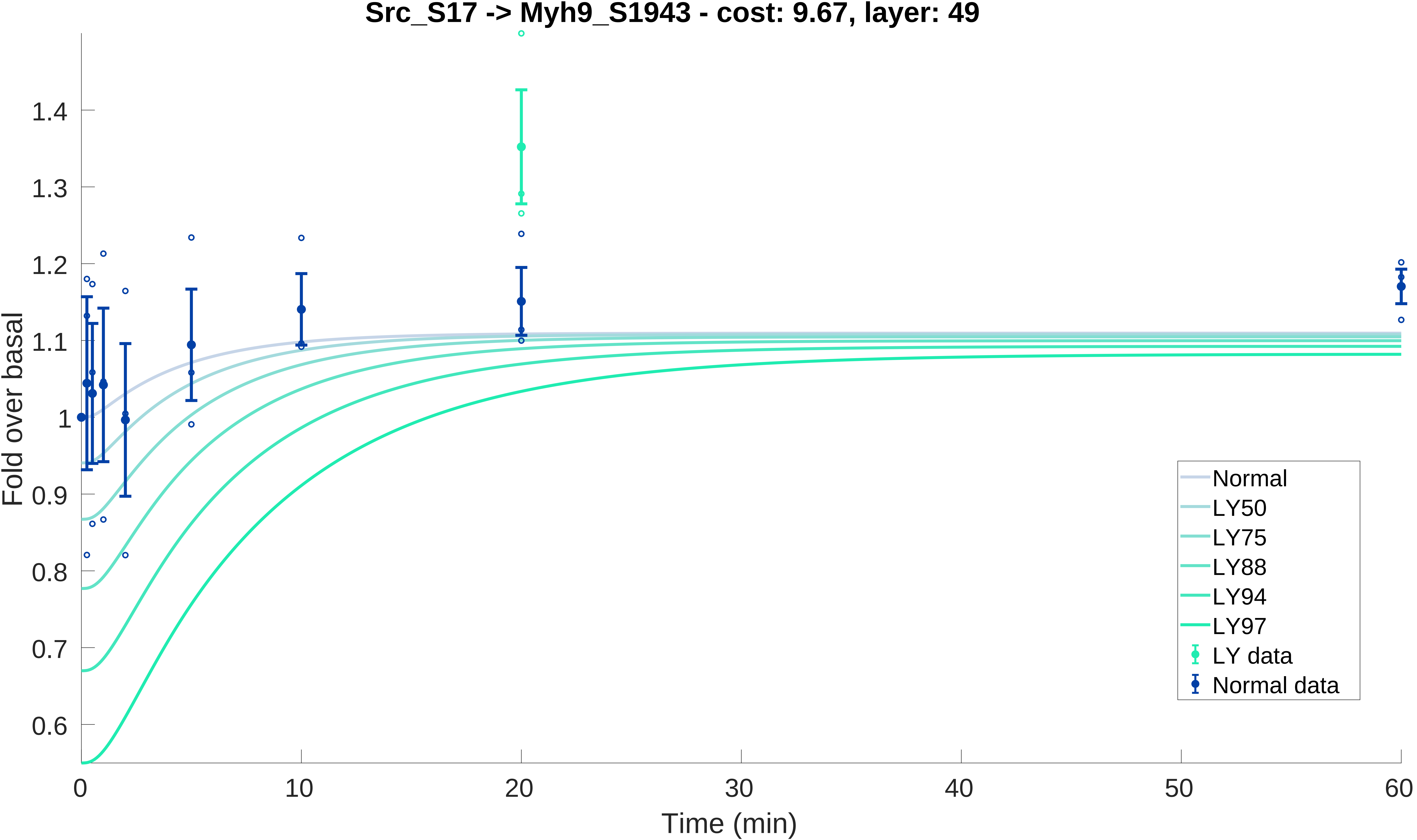
Src_S17 -> Myh9_S1943 - cost: 9.67, layer: 49
Fold over basal
Time (min)
1.4
1.3
1.2
1.1
1
0.9
0.8
0.7
0.6
0
10
20
30
40
50
60
Normal
LY50
LY75
LY88
LY94
LY97
LY data
Normal data

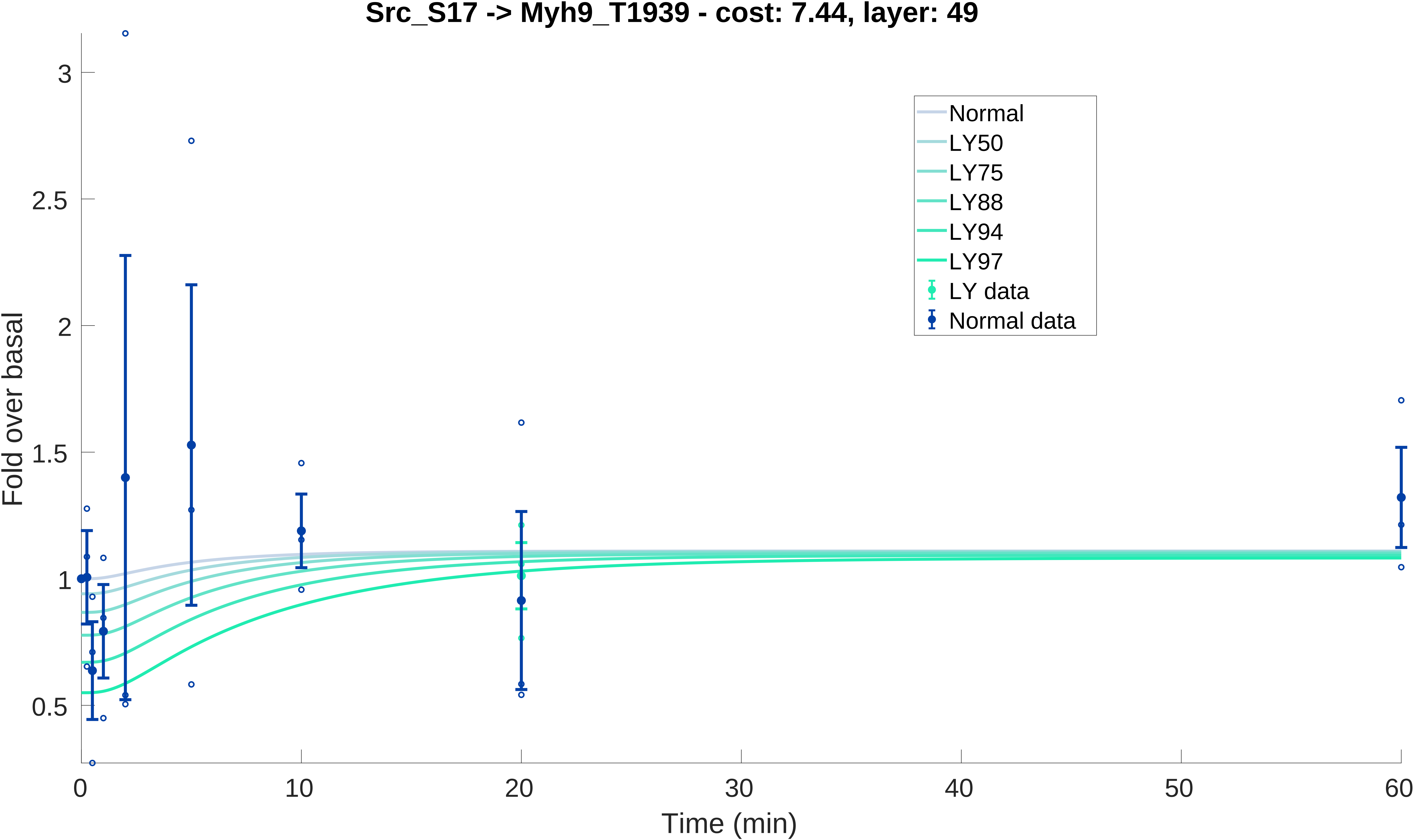

Src_S17 -> Myh9_T1939 - cost: 7.44, layer: 49
Fold over basal
Time (min)
Normal
LY50
LY75
LY88
LY94
LY97
LY data
Normal data
0.5
1
1.5
2
2.5
3
0
10
20
30
40
50
60

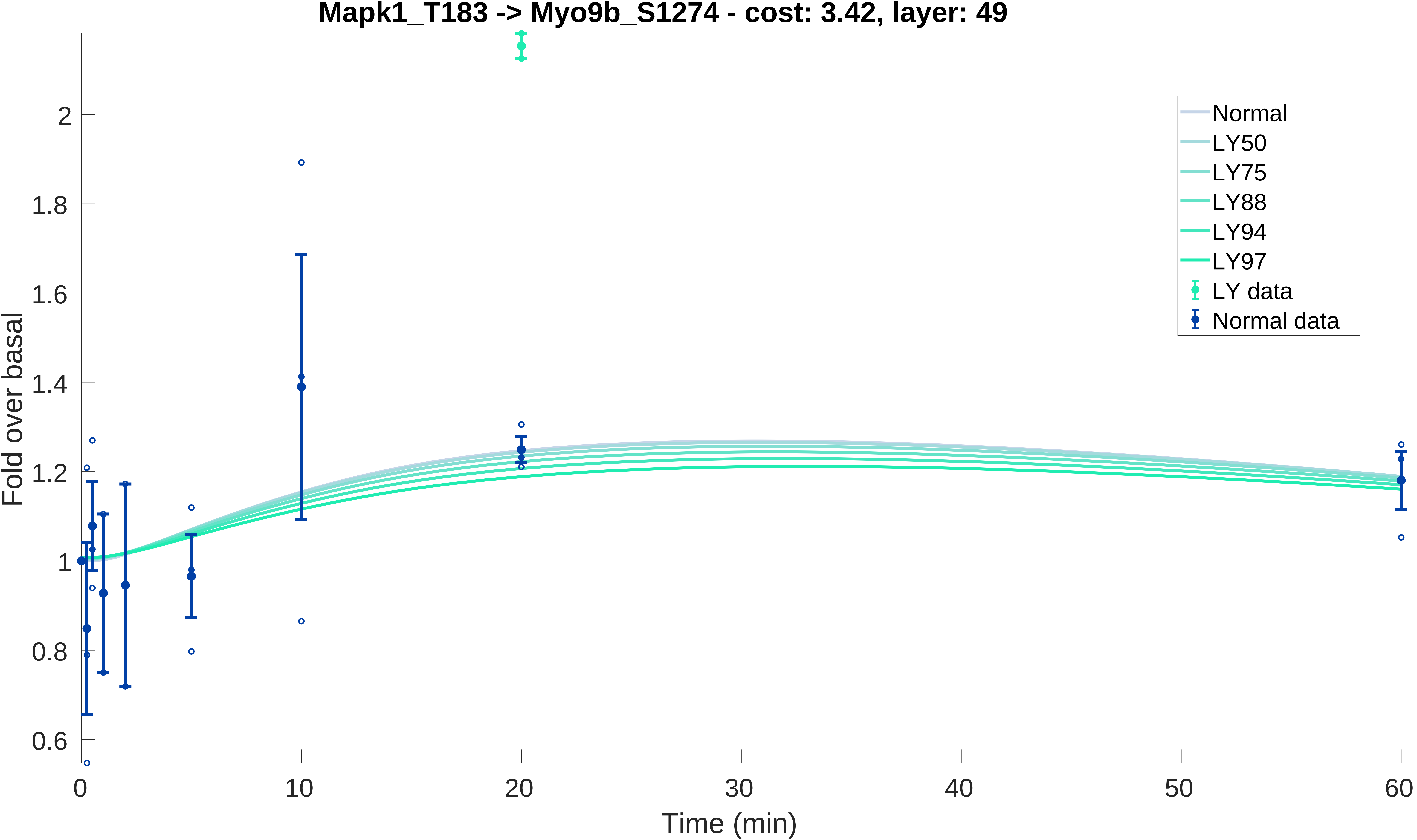

Mapk1_T183 -> Myo9b_S1274 - cost: 3.42, layer: 49
Fold over basal
Time (min)
0.6
0.8
1
1.2
1.4
1.6
1.8
2
0
10
20
30
40
50
60
Normal
LY50
LY75
LY88
LY94
LY97
LY data
Normal data

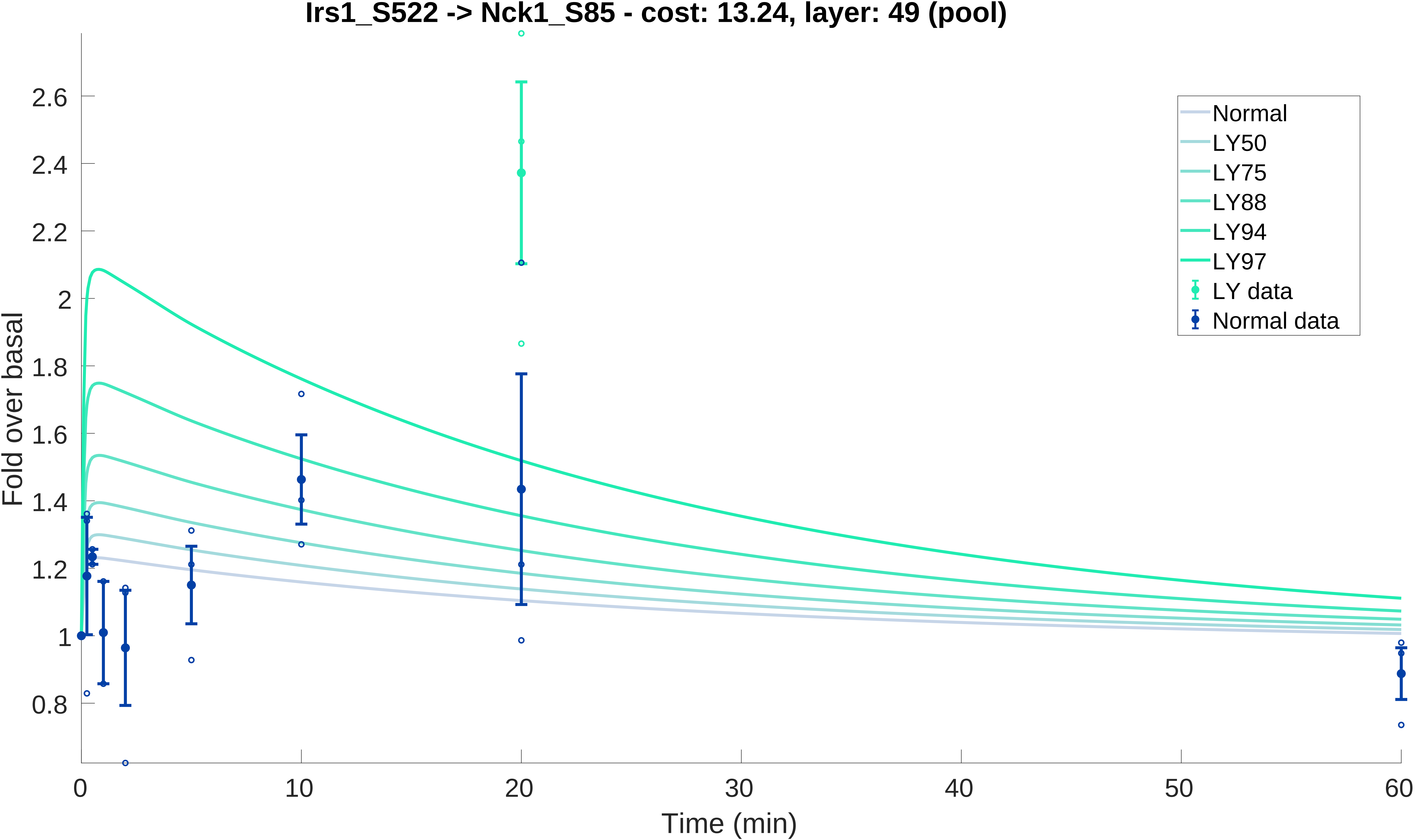

Irs1_S522 -> Nck1_S85 - cost: 13.24, layer: 49 (pool)
Fold over basal
Time (min)
0.8
1
1.2
1.4
1.6
1.8
2
2.2
2.4
2.6
0
10
20
30
40
50
60
Normal
LY50
LY75
LY88
LY94
LY97
LY data
Normal data

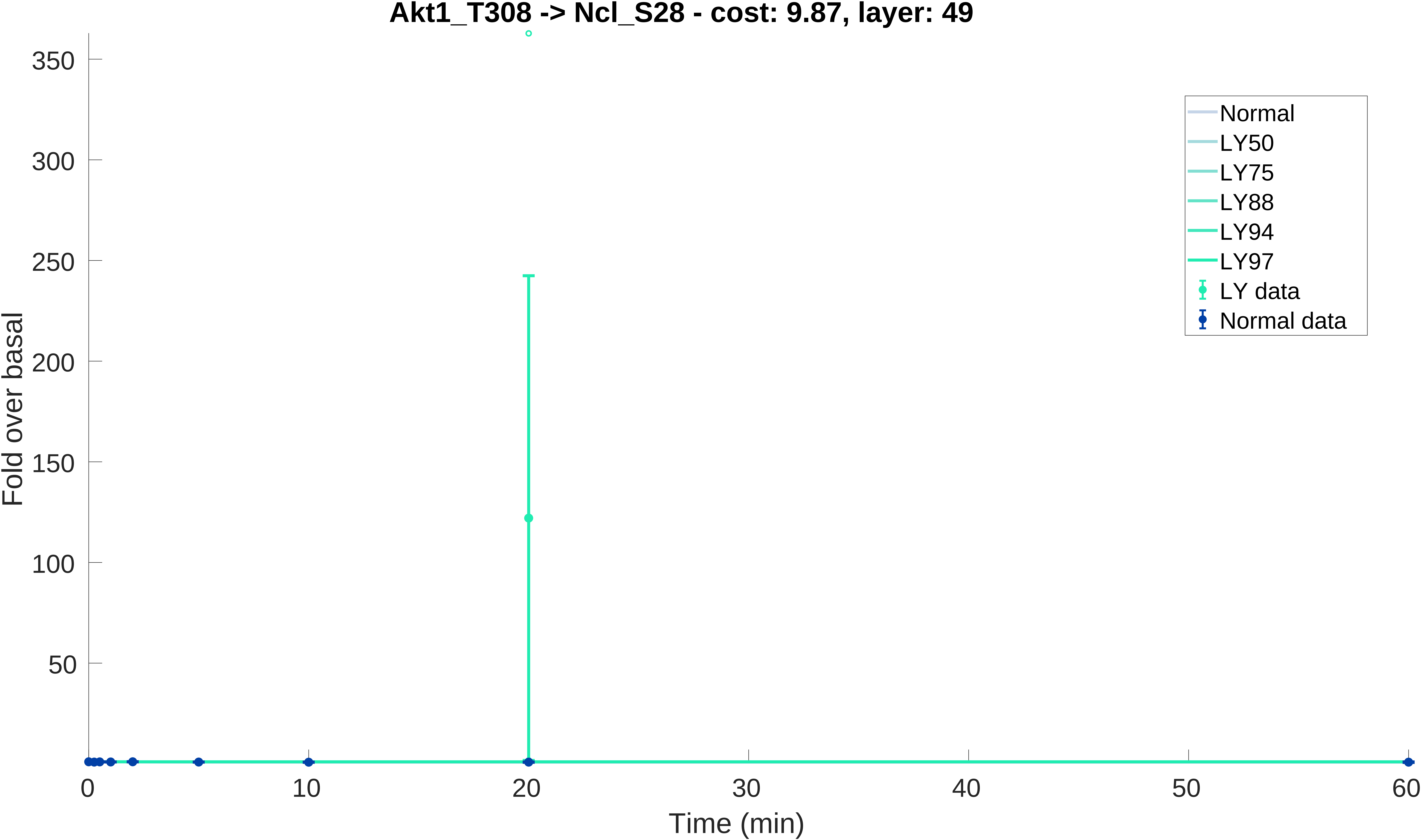

Akt1_T308 -> Ncl_S28 - cost: 9.87, layer: 49
Fold over basal
Time (min)
350
300
250
200
150
100
50
0
10
20
30
40
50
60
Normal
LY50
LY75
LY88
LY94
LY97
LY data
Normal data

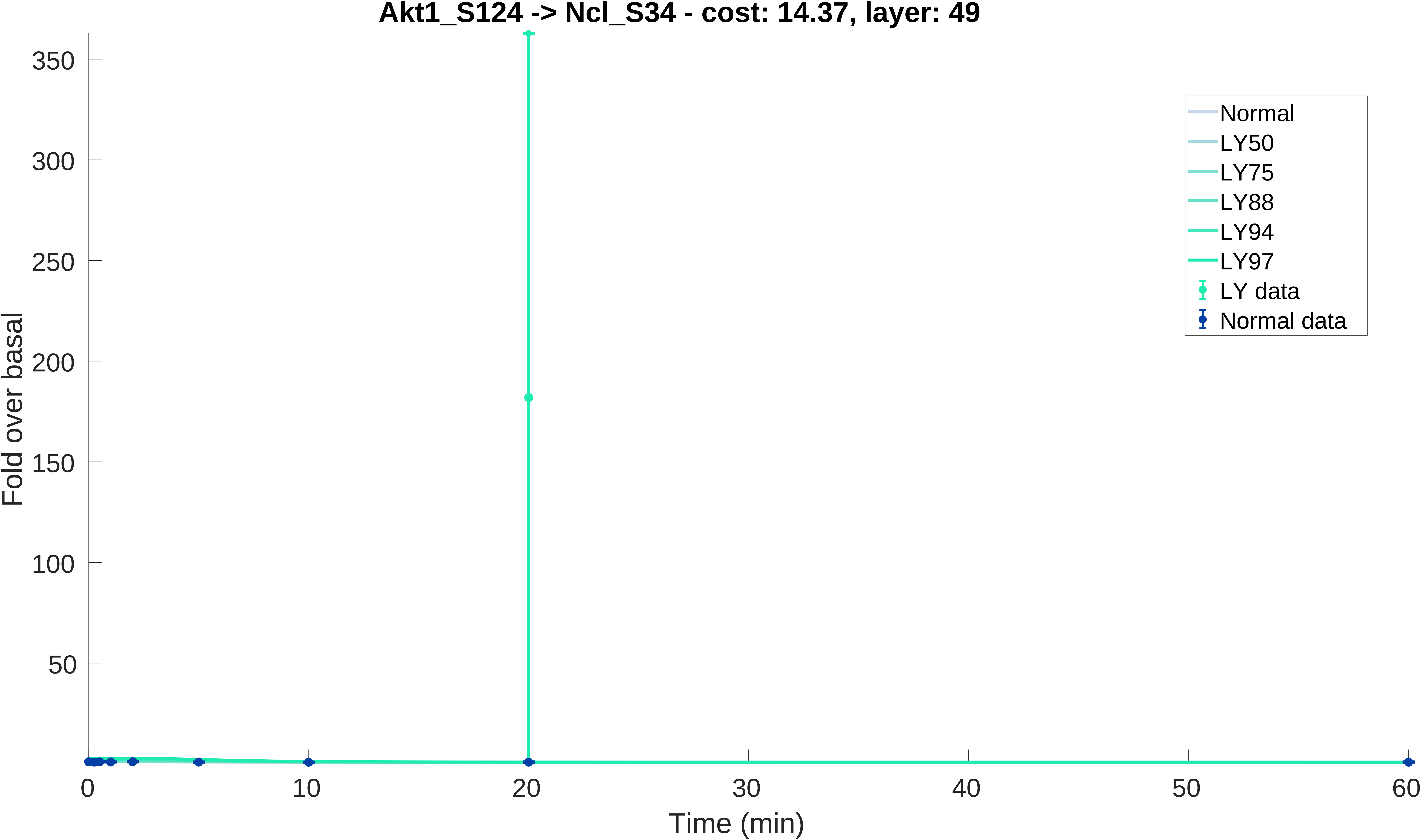

Akt1_S124 -> Ncl_S34 - cost: 14.37, layer: 49
Fold over basal
350
300
250
200
150
100
50
0
10
20
30
40
50
60
Time (min)
Normal
LY50
LY75
LY88
LY94
LY97
LY data
Normal data

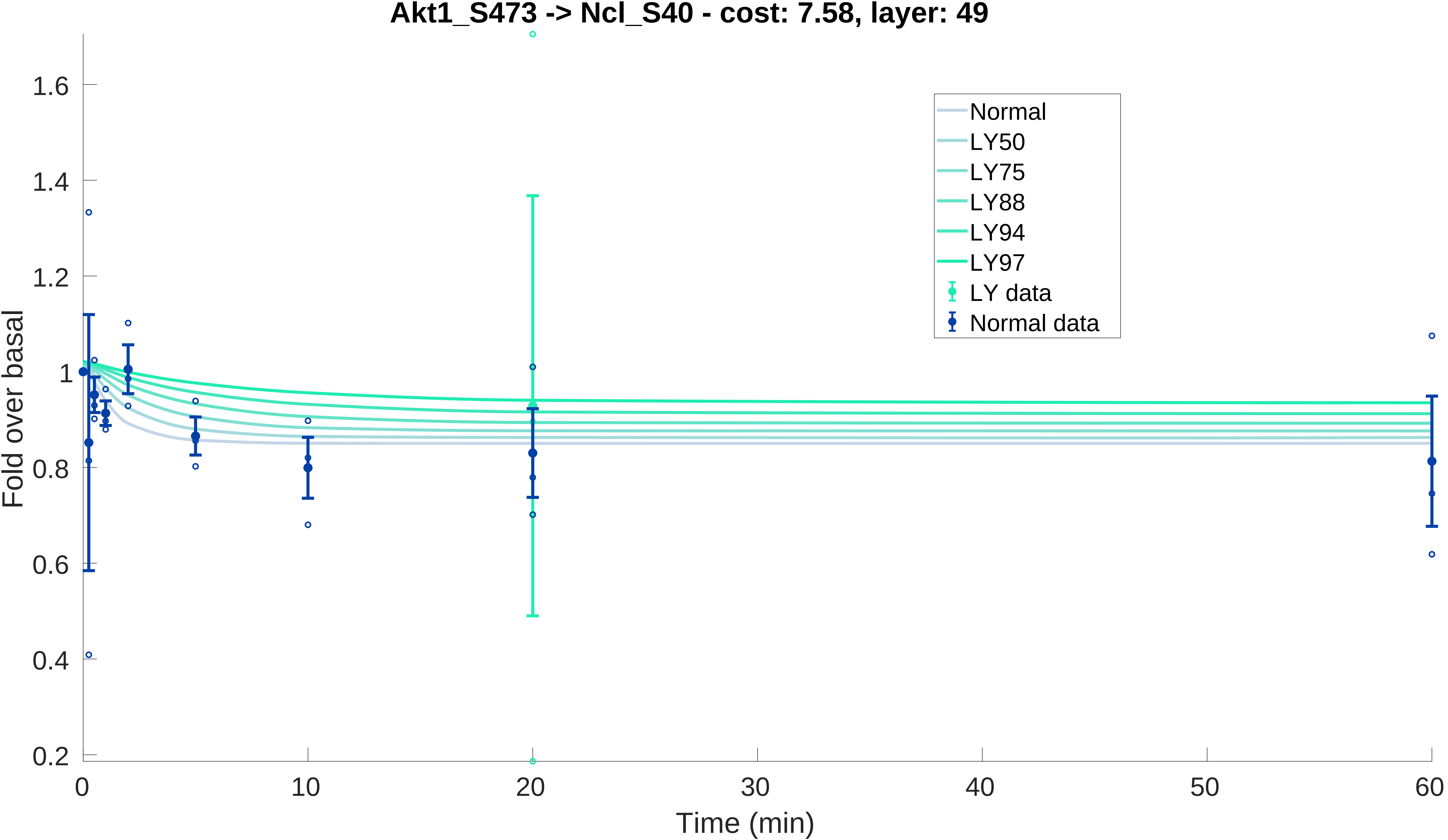

Akt1_S473 -> Ncl_S40 - cost: 7.58, layer: 49
Fold over basal
1.6
1.4
1.2
1
0.8
0.6
0.4
0.2
0
10
20
30
40
50
60
Time (min)
Normal
LY50
LY75
LY88
LY94
LY97
LY data
Normal data

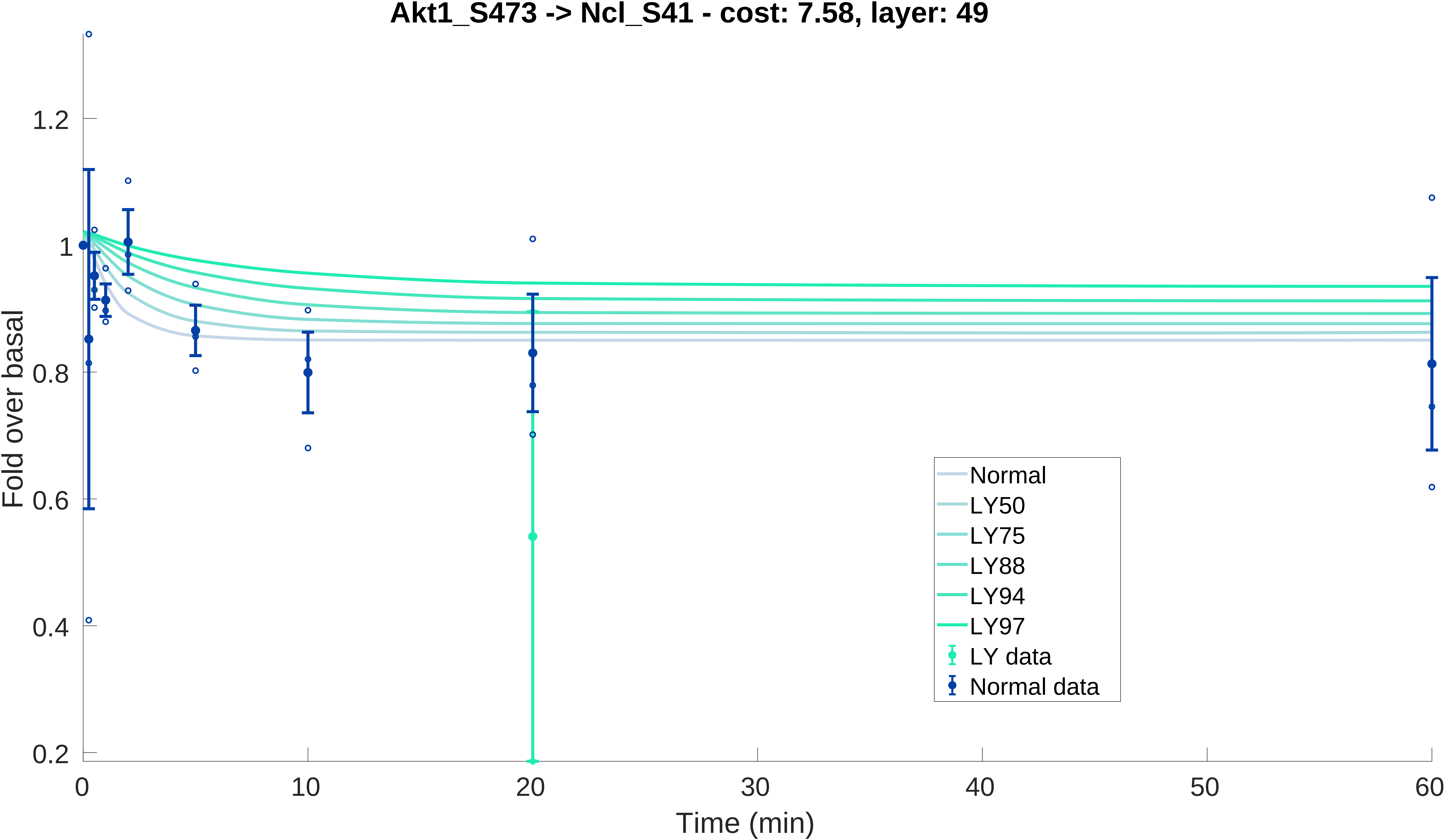
Akt1_S473 -> Ncl_S41 - cost: 7.58, layer: 49
Fold over basal
Time (min)
0
10
20
30
40
50
60
0.2
0.4
0.6
0.8
1
1.2
Normal
LY50
LY75
LY88
LY94
LY97
LY data
Normal data

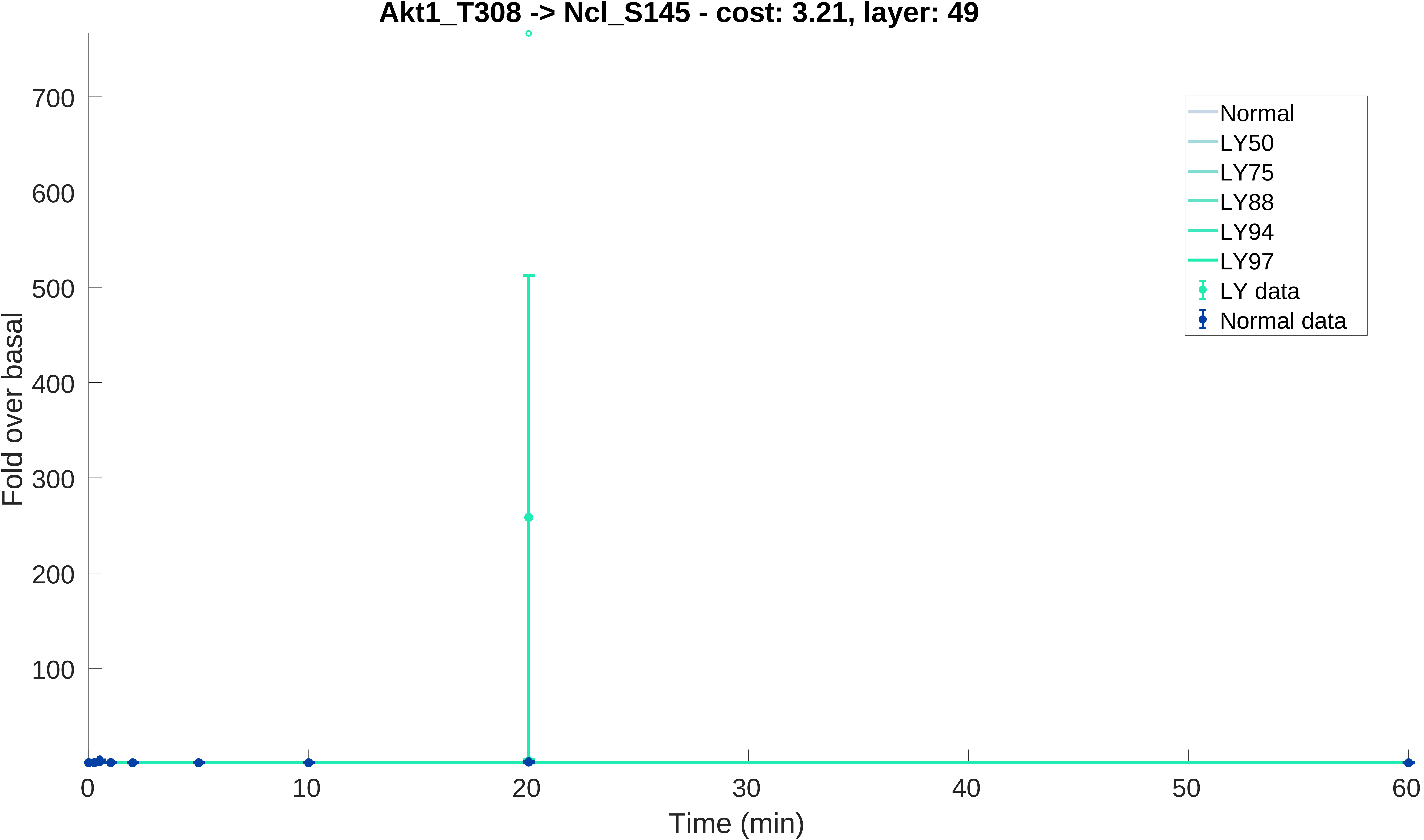

Akt1_T308 -> Ncl_S145 - cost: 3.21, layer: 49
Fold over basal
700
600
500
400
300
200
100
0
10
20
30
40
50
60
Time (min)
Normal
LY50
LY75
LY88
LY94
LY97
LY data
Normal data

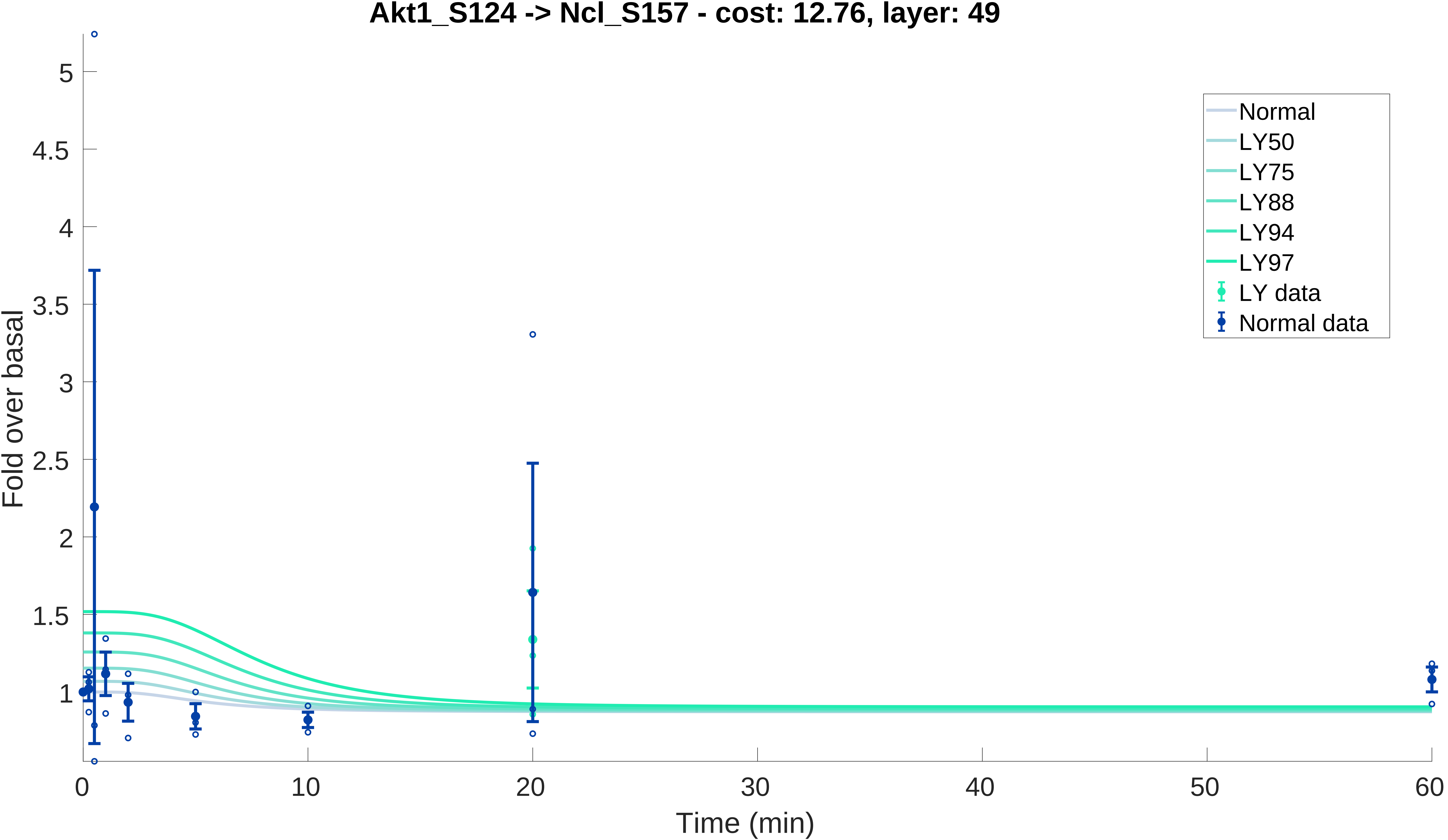

Akt1_S124 -> Ncl_S157 - cost: 12.76, layer: 49
Fold over basal
5
4.5
4
3.5
3
2.5
2
1.5
1
0
10
20
30
40
50
60
Time (min)
Normal
LY50
LY75
LY88
LY94
LY97
LY data
Normal data

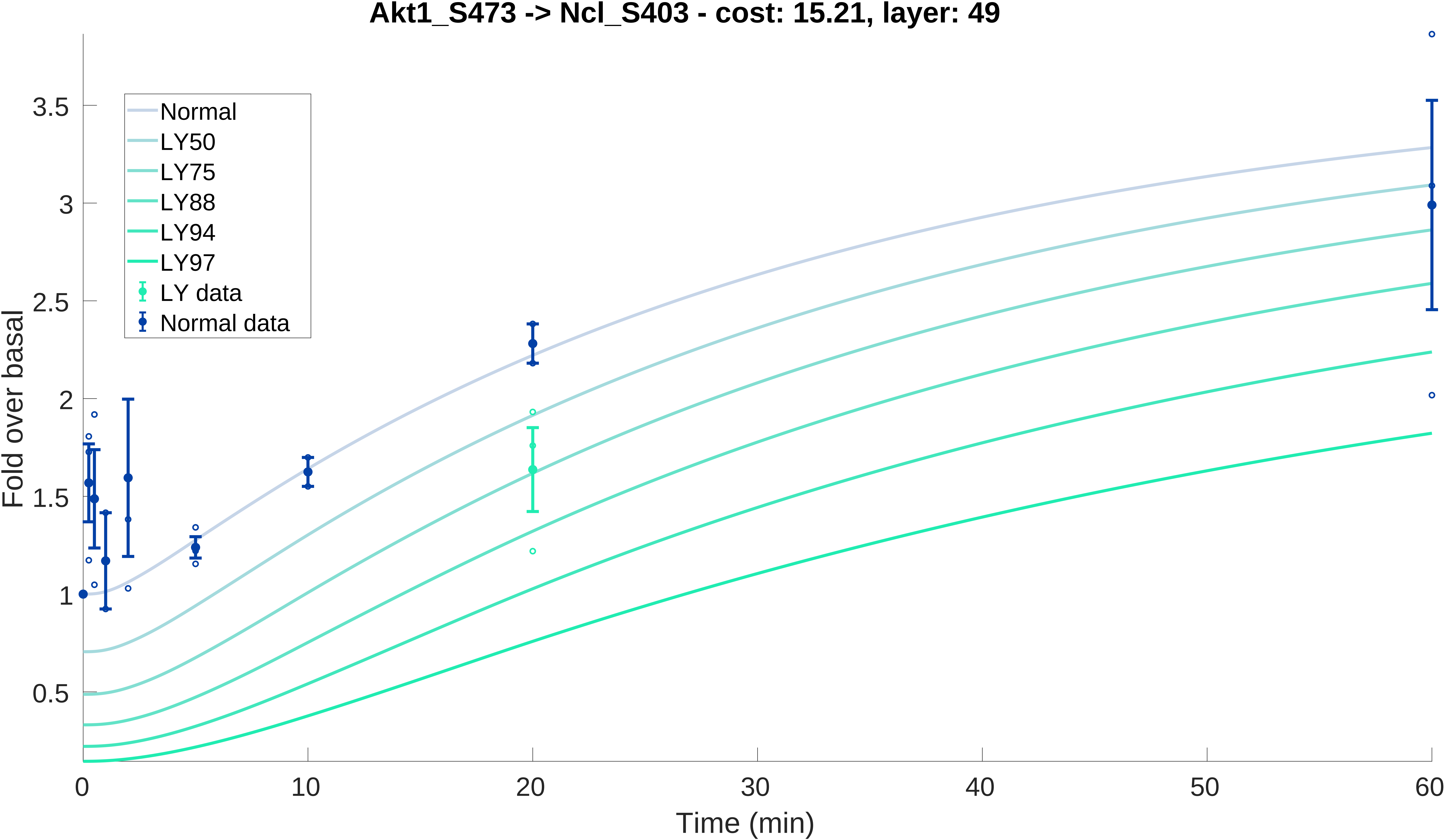

Akt1_S473 -> Ncl_S403 - cost: 15.21, layer: 49
Normal
LY50
LY75
LY88
LY94
LY97
LY data
Normal data
Fold over basal
Time (min)
0.5
1
1.5
2
2.5
3
3.5
0
10
20
30
40
50
60

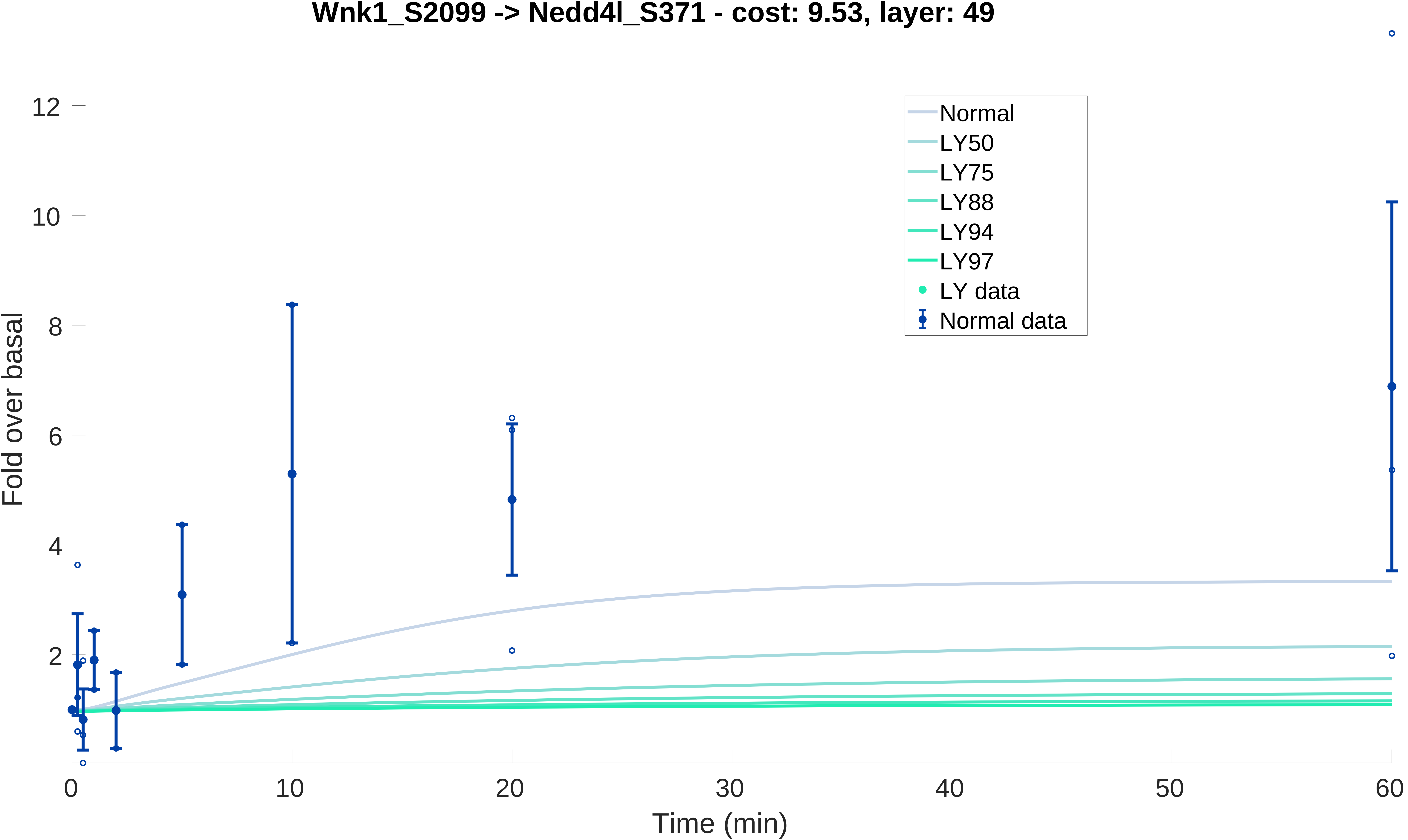

Wnk1_S2099 -> Nedd4l_S371 - cost: 9.53, layer: 49
Normal
LY50
LY75
LY88
LY94
LY97
LY data
Normal data
Fold over basal
Time (min)
0
10
20
30
40
50
60
2
4
6
8
10
12

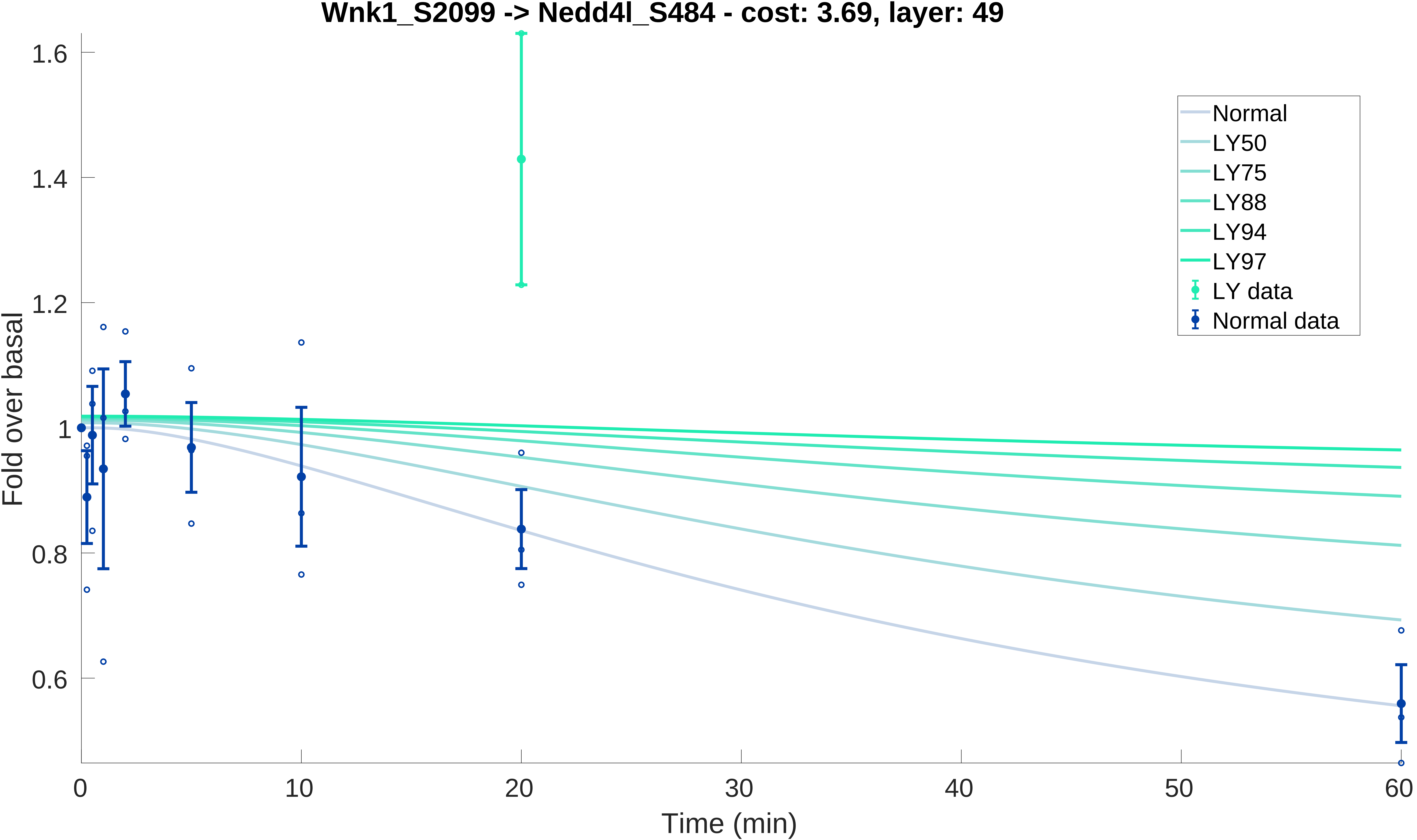

Wnk1_S2099 -> Nedd4l_S484 - cost: 3.69, layer: 49
Fold over basal
Time (min)
0
10
20
30
40
50
60
0.6
0.8
1
1.2
1.4
1.6
Normal
LY50
LY75
LY88
LY94
LY97
LY data
Normal data

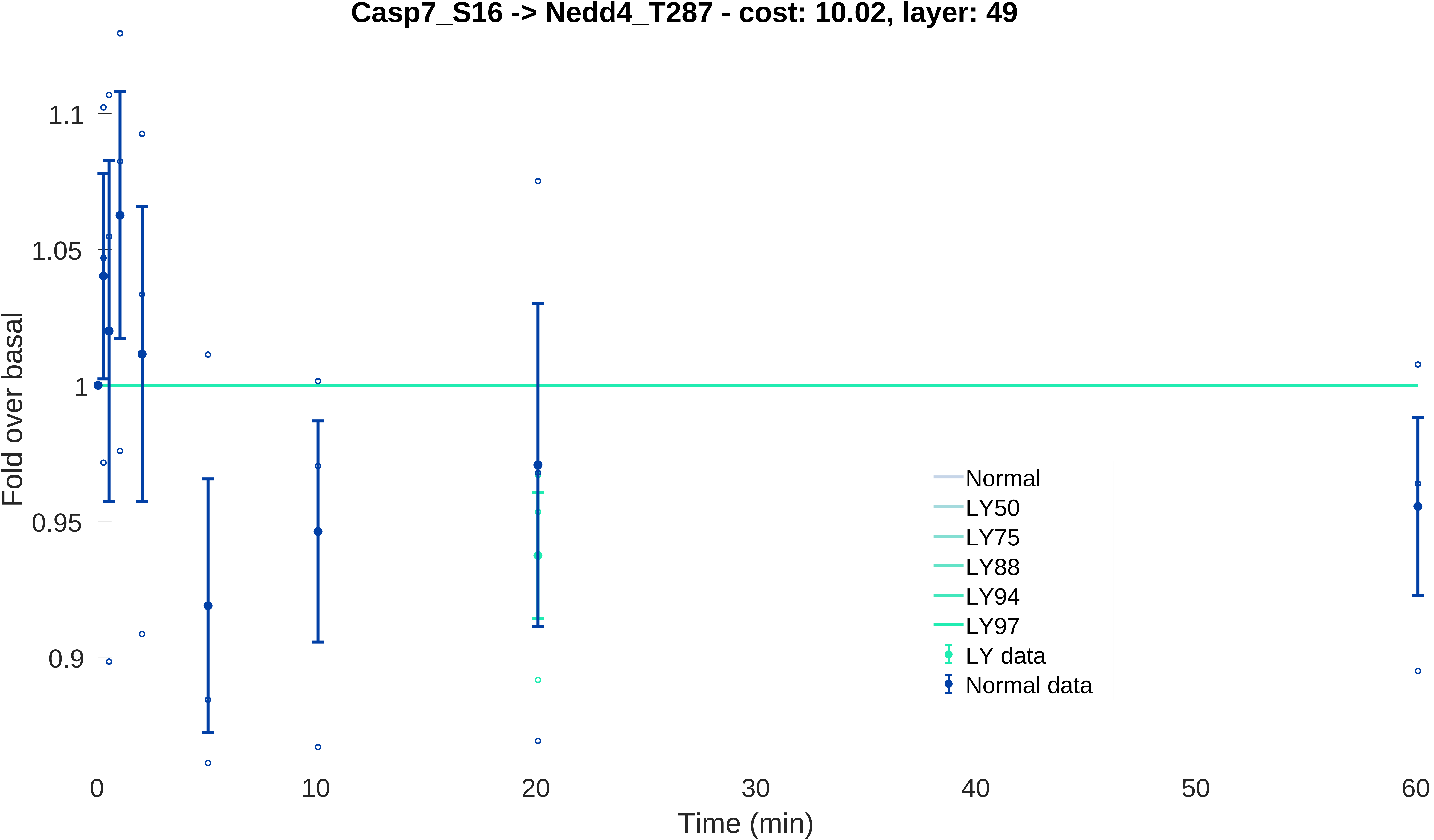

Casp7_S16 -> Nedd4_T287 - cost: 10.02, layer: 49
Fold over basal
1.1
1.05
1
0.95
0.9
0
10
20
30
40
50
60
Time (min)
Normal
LY50
LY75
LY88
LY94
LY97
LY data
Normal data

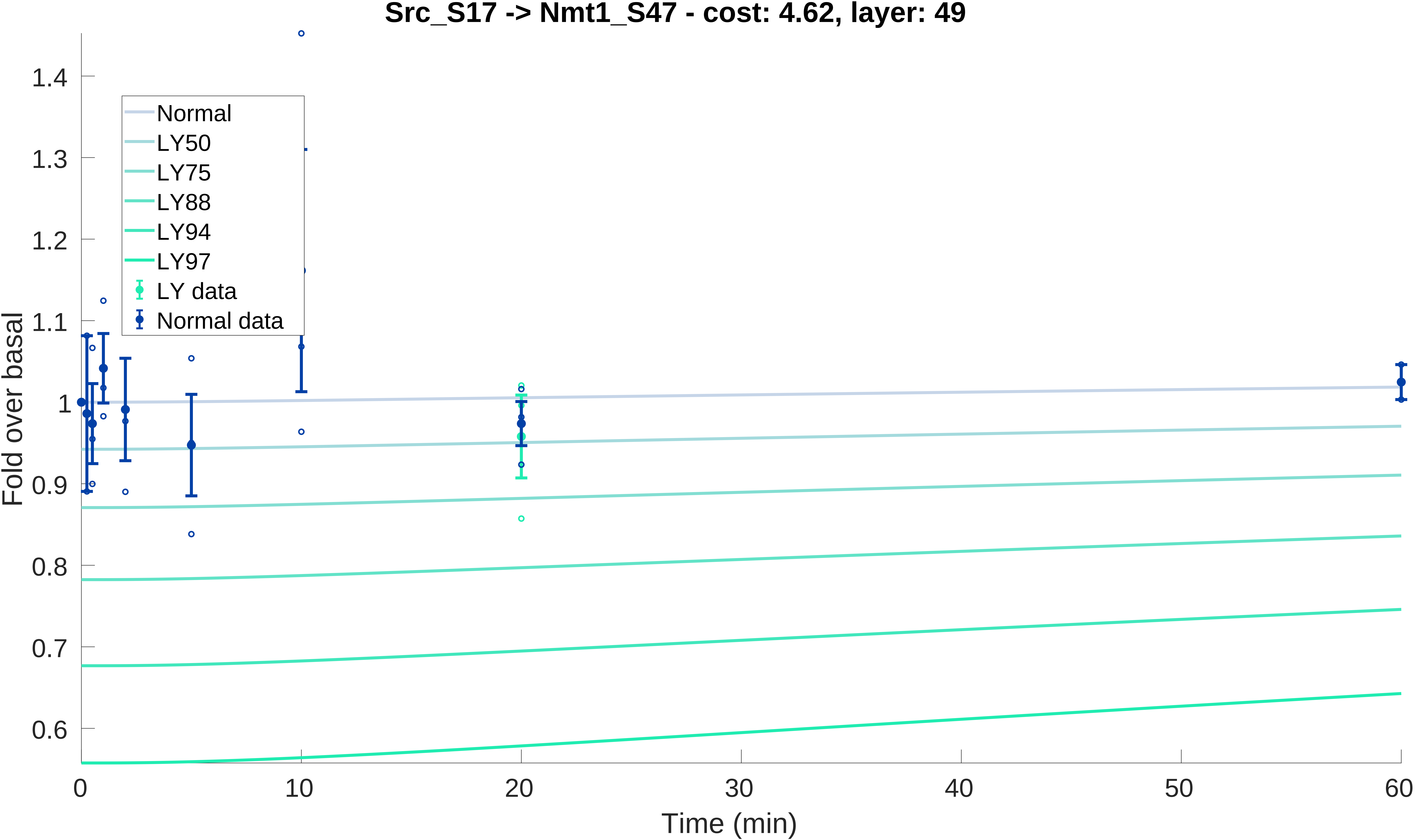

Src_S17 -> Nmt1_S47 - cost: 4.62, layer: 49
Normal
LY50
LY75
LY88
LY94
LY97
LY data
Normal data
Fold over basal
Time (min)
1.4
1.3
1.2
1.1
1
0.9
0.8
0.7
0.6
0
10
20
30
40
50
60

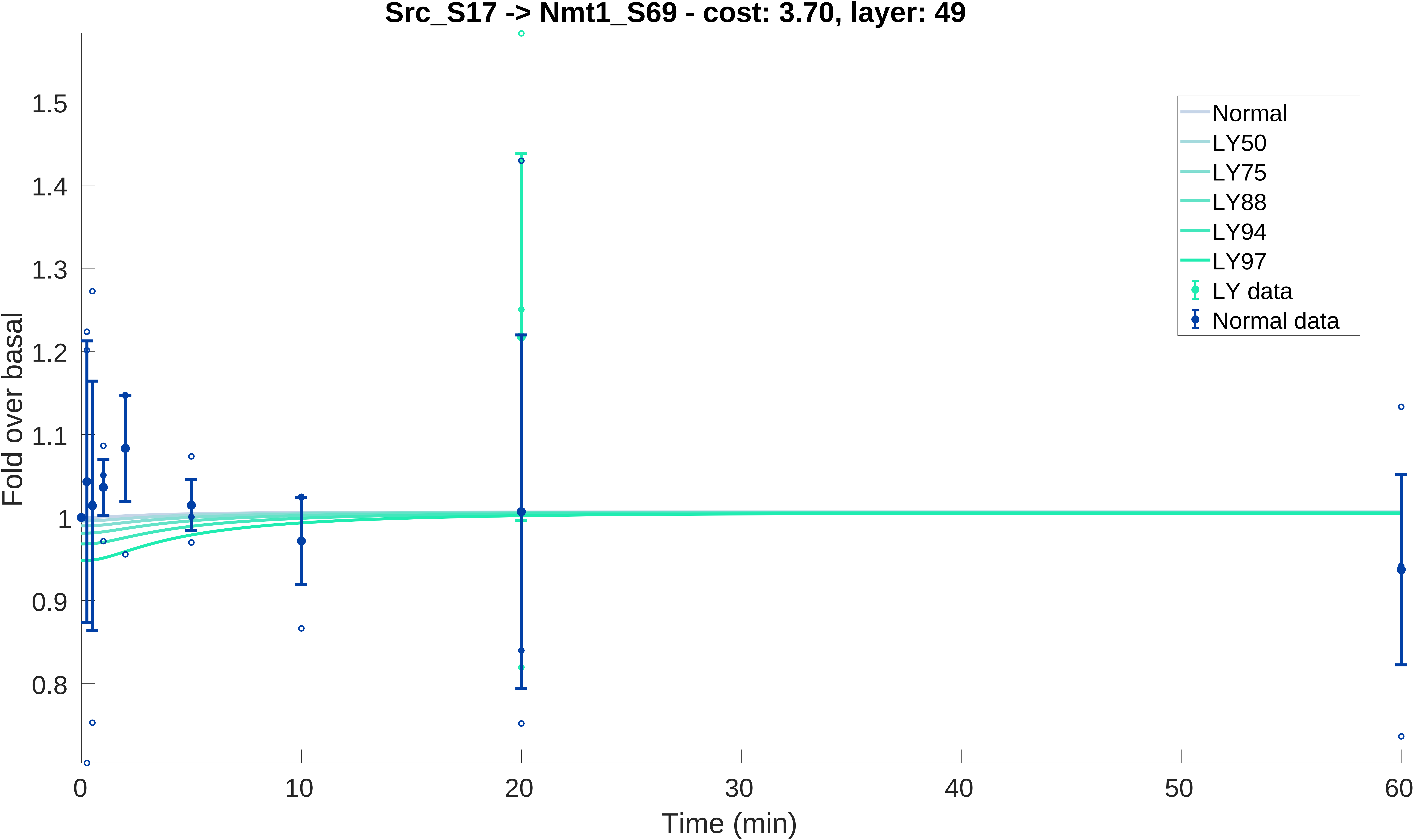

Src_S17 -> Nmt1_S69 - cost: 3.70, layer: 49
Fold over basal
1.5
1.4
1.3
1.2
1.1
1
0.9
0.8
Time (min)
0
10
20
30
40
50
60
Normal
LY50
LY75
LY88
LY94
LY97
LY data
Normal data

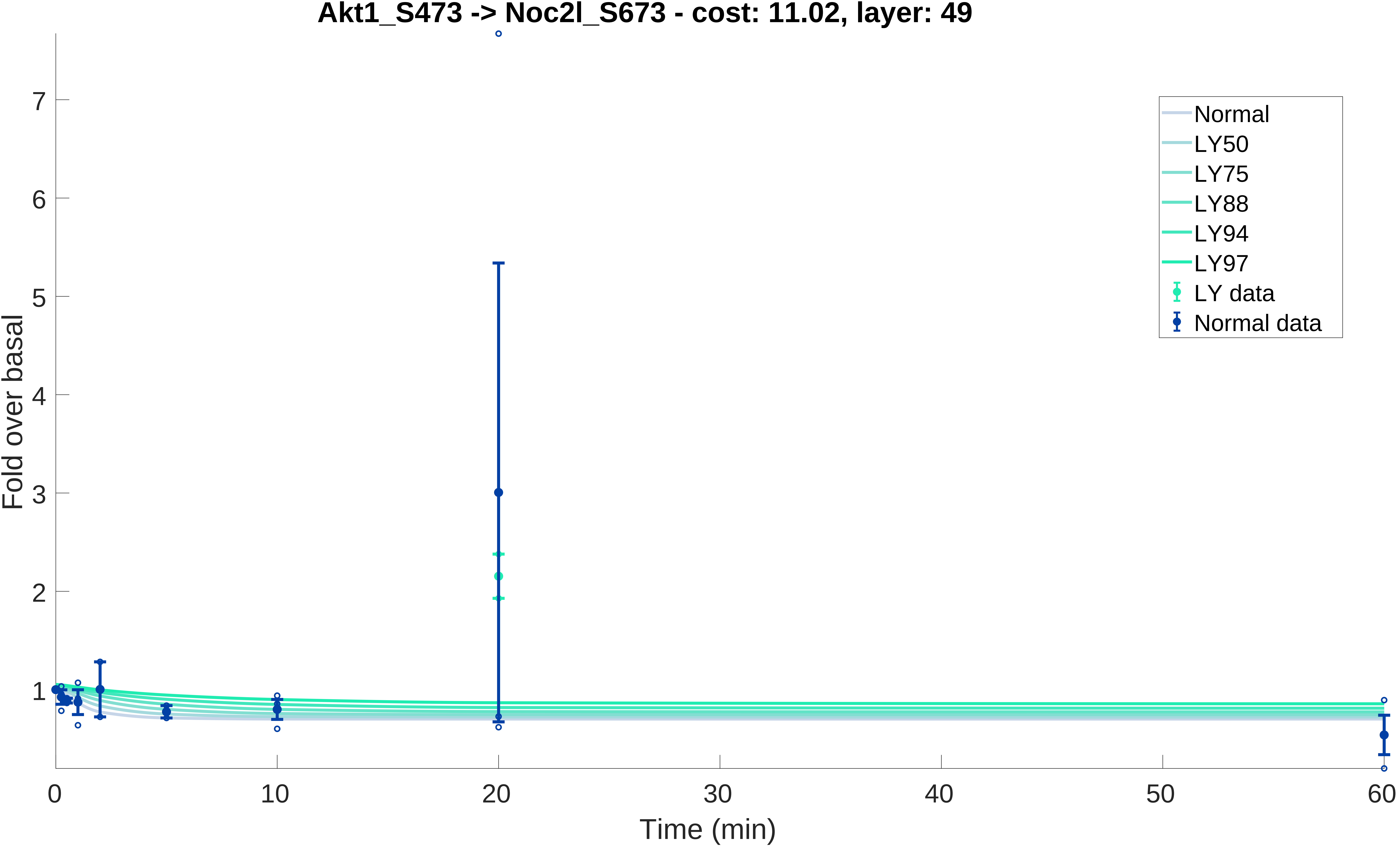

Akt1_S473 -> Noc2l_S673 - cost: 11.02, layer: 49
Fold over basal
Time (min)
0
10
20
30
40
50
60
1
2
3
4
5
6
7
Normal
LY50
LY75
LY88
LY94
LY97
LY data
Normal data

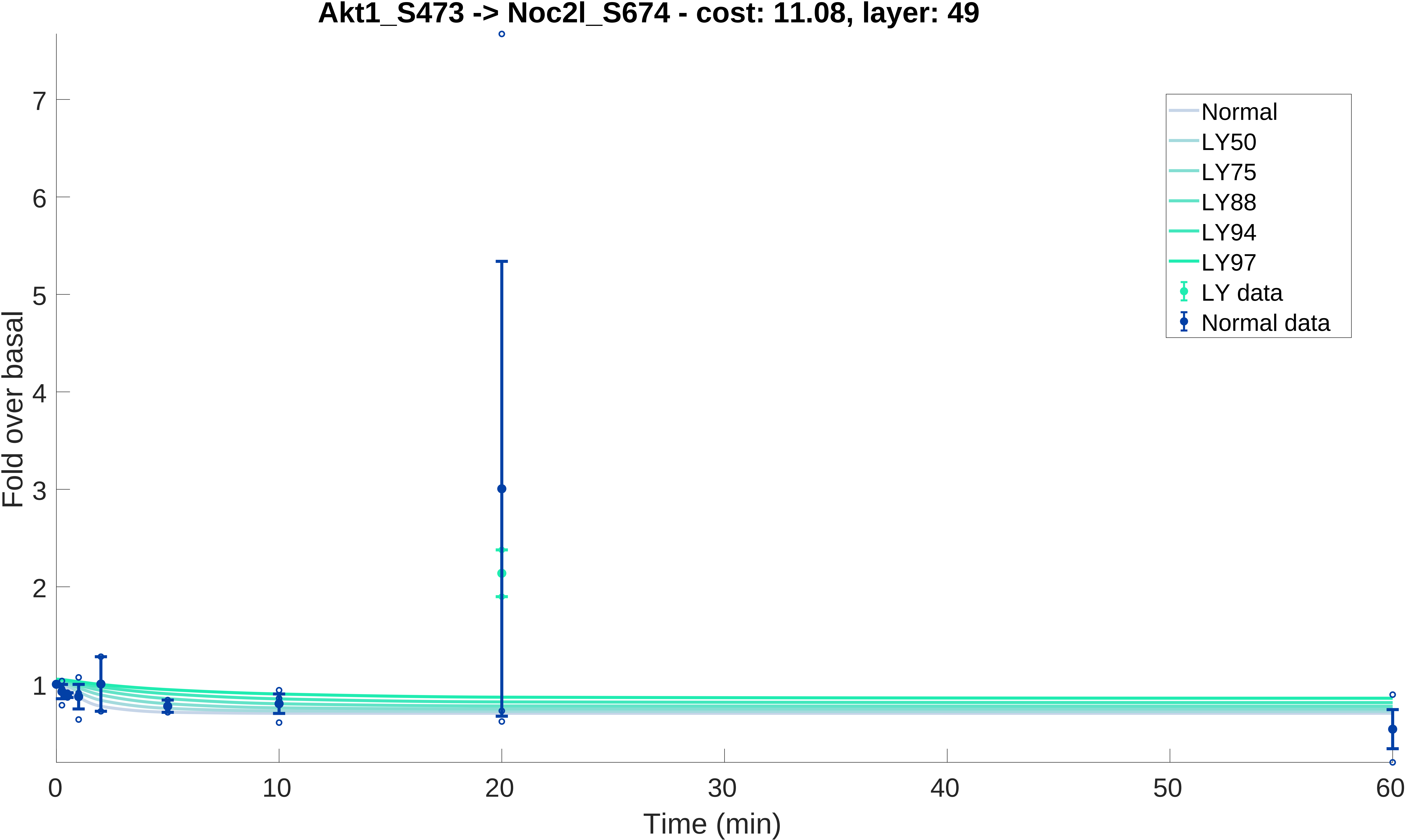

Akt1_S473 -> Noc2l_S674 - cost: 11.08, layer: 49
Fold over basal
Time (min)
0
10
20
30
40
50
60
1
2
3
4
5
6
7
Normal
LY50
LY75
LY88
LY94
LY97
LY data
Normal data

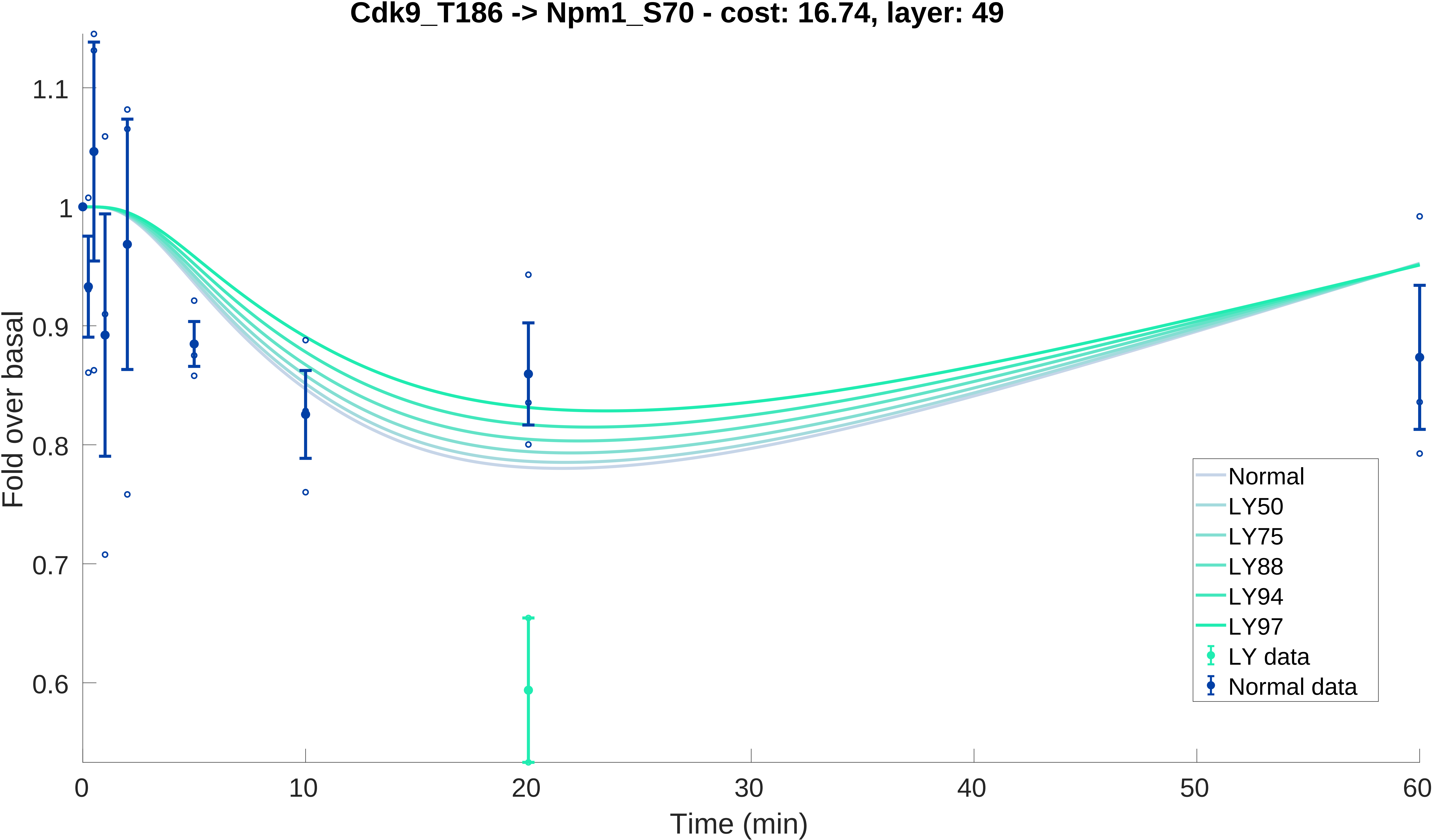

Cdk9_T186 -> Npm1_S70 - cost: 16.74, layer: 49
Fold over basal
Time (min)
0.6
0.7
0.8
0.9
1
1.1
0
10
20
30
40
50
60
Normal
LY50
LY75
LY88
LY94
LY97
LY data
Normal data

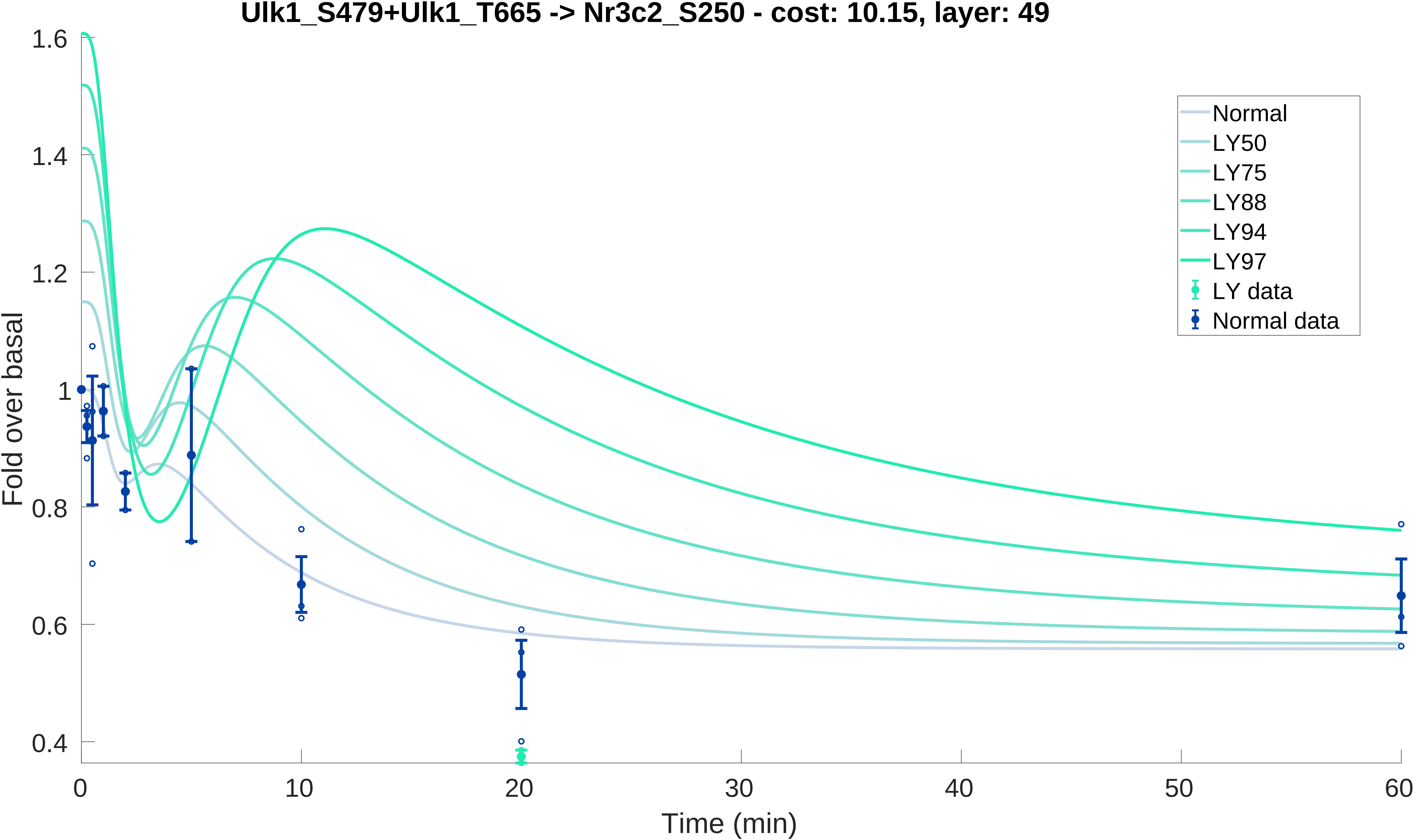

Ulk1_S479+Ulk1_T665 -> Nr3c2_S250 - cost: 10.15, layer: 49
Fold over basal
Time (min)
0
10
20
30
40
50
60
0.4
0.6
0.8
1
1.2
1.4
1.6
Normal
LY50
LY75
LY88
LY94
LY97
LY data
Normal data

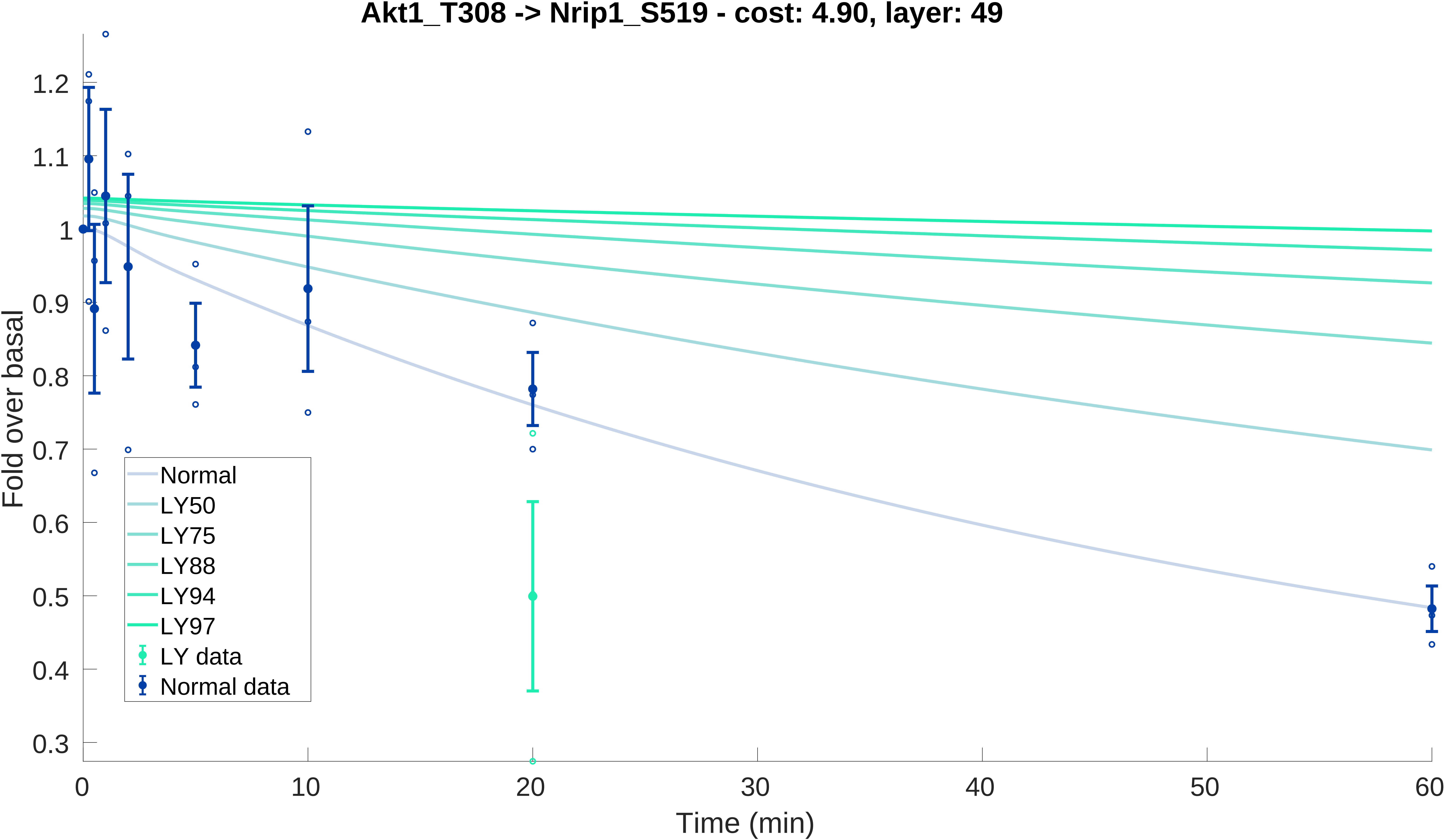
Akt1_T308 -> Nrip1_S519 - cost: 4.90, layer: 49
Fold over basal
1.2
1.1
1
0.9
0.8
0.7
0.6
0.5
0.4
0.3
0
10
20
30
40
50
60
Time (min)
Normal
LY50
LY75
LY88
LY94
LY97
LY data
Normal data

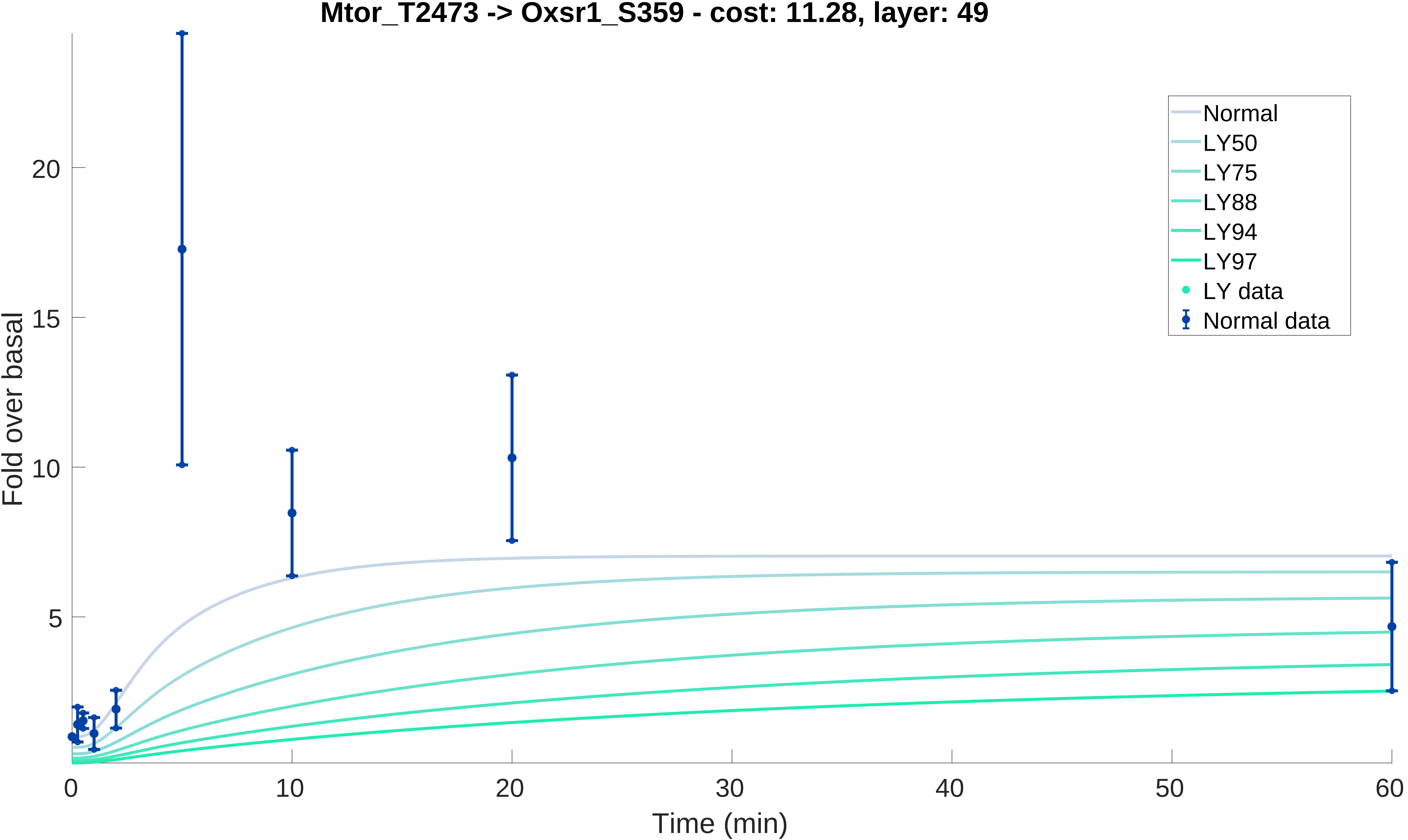

Mtor_T2473 -> Oxsr1_S359 - cost: 11.28, layer: 49
Fold over basal
Time (min)
0
10
20
30
40
50
60
5
10
15
20
Normal
LY50
LY75
LY88
LY94
LY97
LY data
Normal data

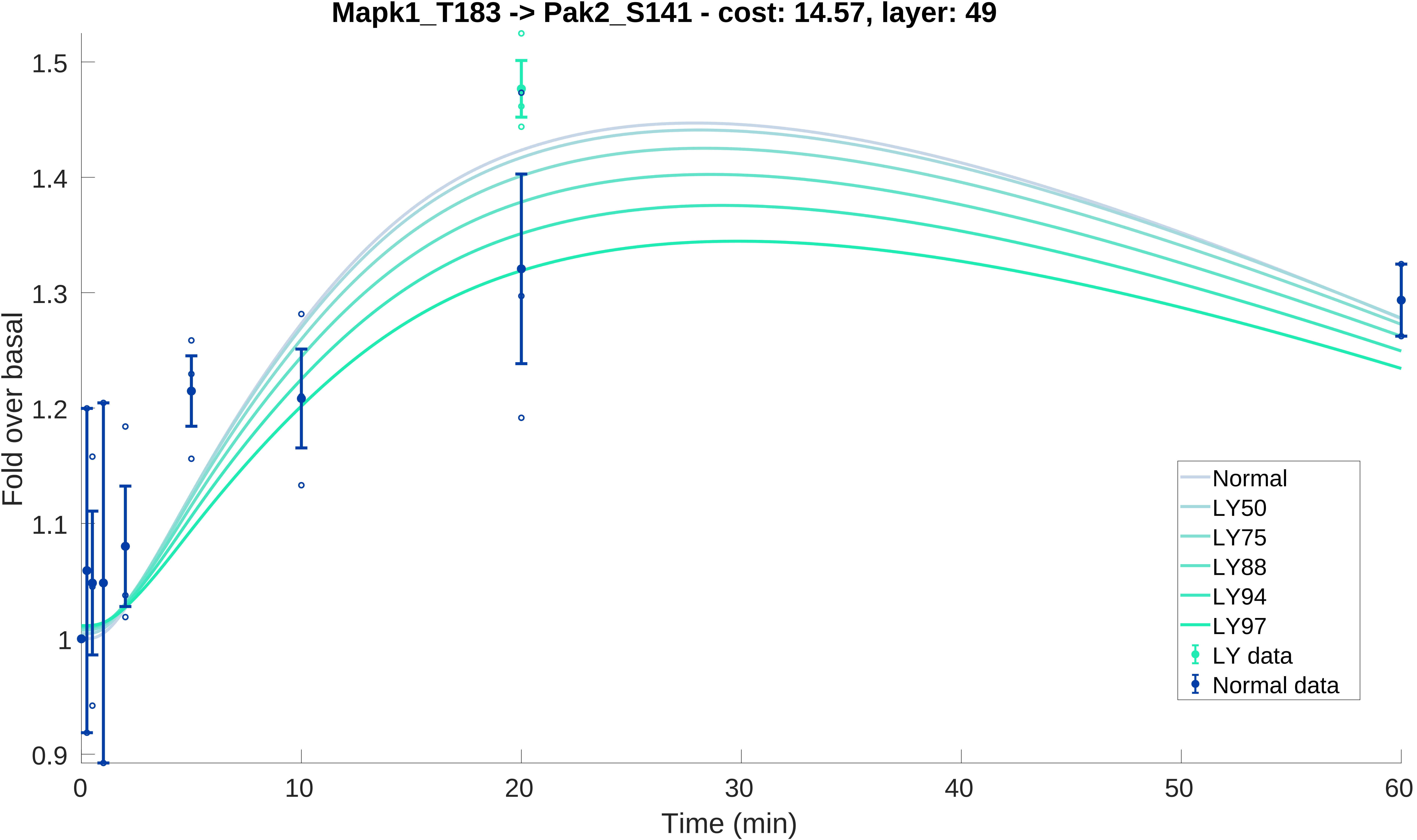
Mapk1_T183 -> Pak2_S141 - cost: 14.57, layer: 49
Fold over basal
1.5
1.4
1.3
1.2
1.1
1
0.9
0
10
20
30
40
50
60
Time (min)
Normal
LY50
LY75
LY88
LY94
LY97
LY data
Normal data

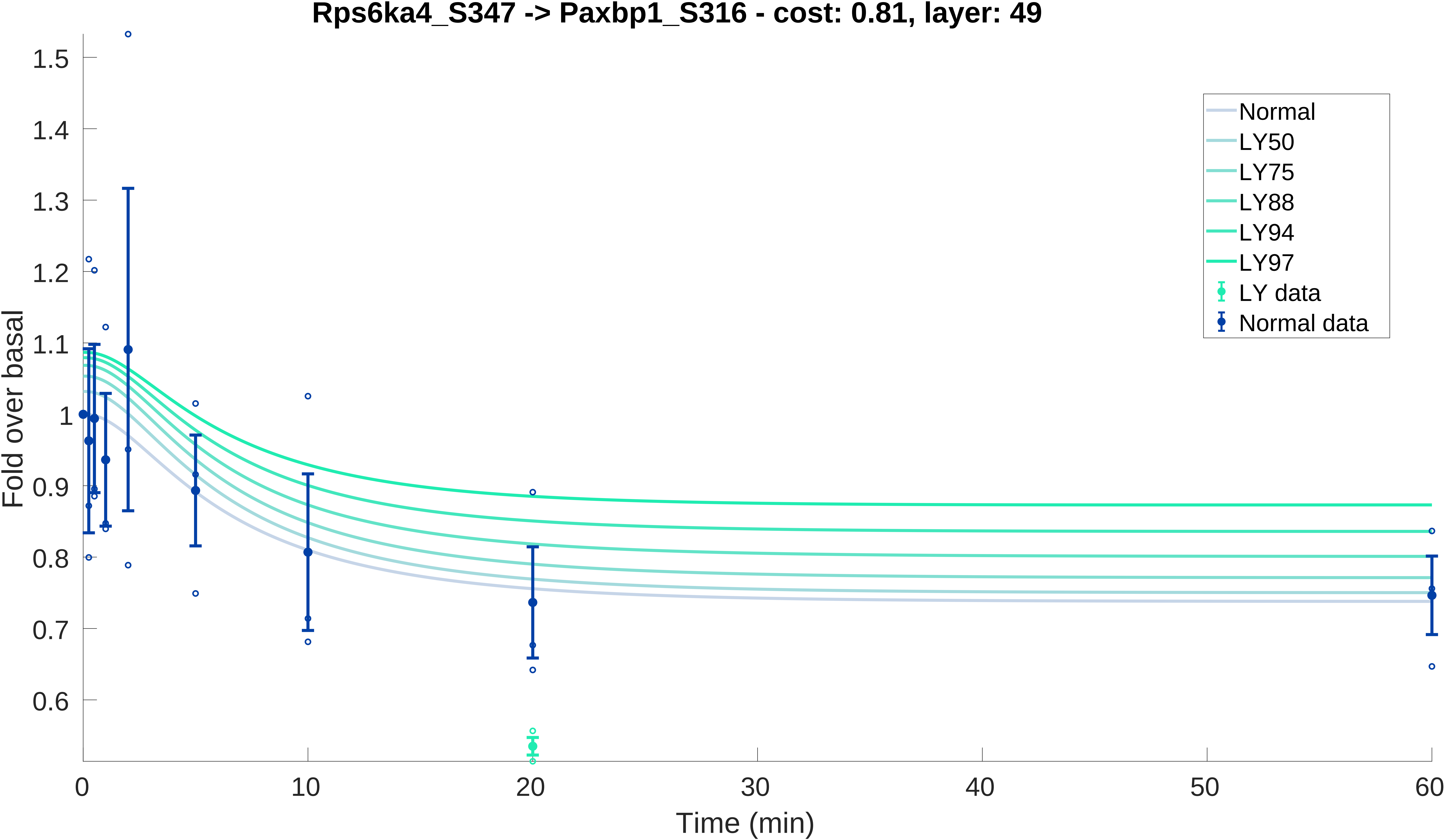

Rps6ka4_S347 -> Paxbp1_S316 - cost: 0.81, layer: 49
Fold over basal
Time (min)
Normal
LY50
LY75
LY88
LY94
LY97
LY data
Normal data
1.5
1.4
1.3
1.2
1.1
1
0.9
0.8
0.7
0.6
0
10
20
30
40
50
60

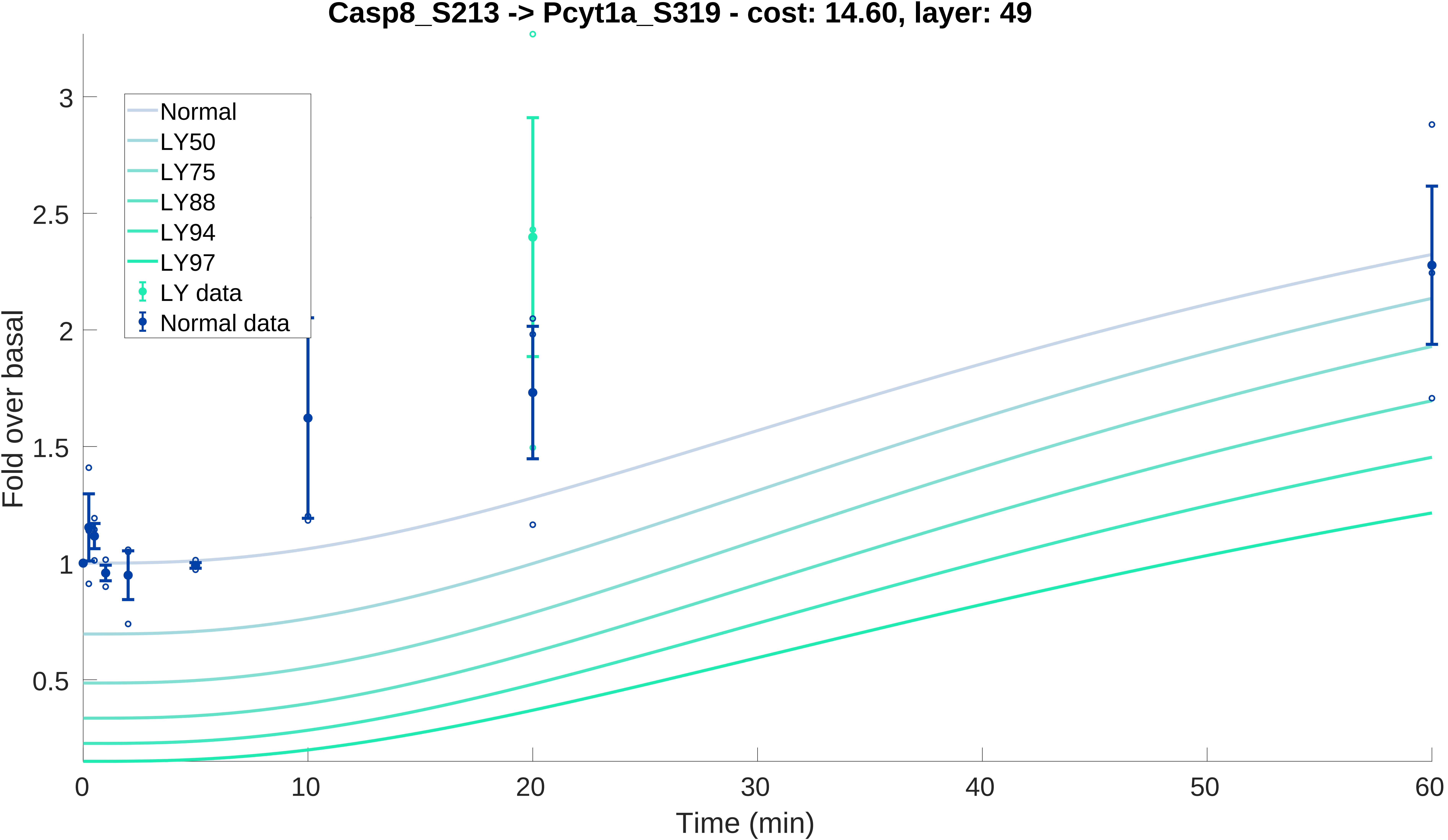
Casp8_S213 -> Pcyt1a_S319 - cost: 14.60, layer: 49
Normal
LY50
LY75
LY88
LY94
LY97
LY data
Normal data
Fold over basal
0.5
1
1.5
2
2.5
3
0
10
20
30
40
50
60
Time (min)

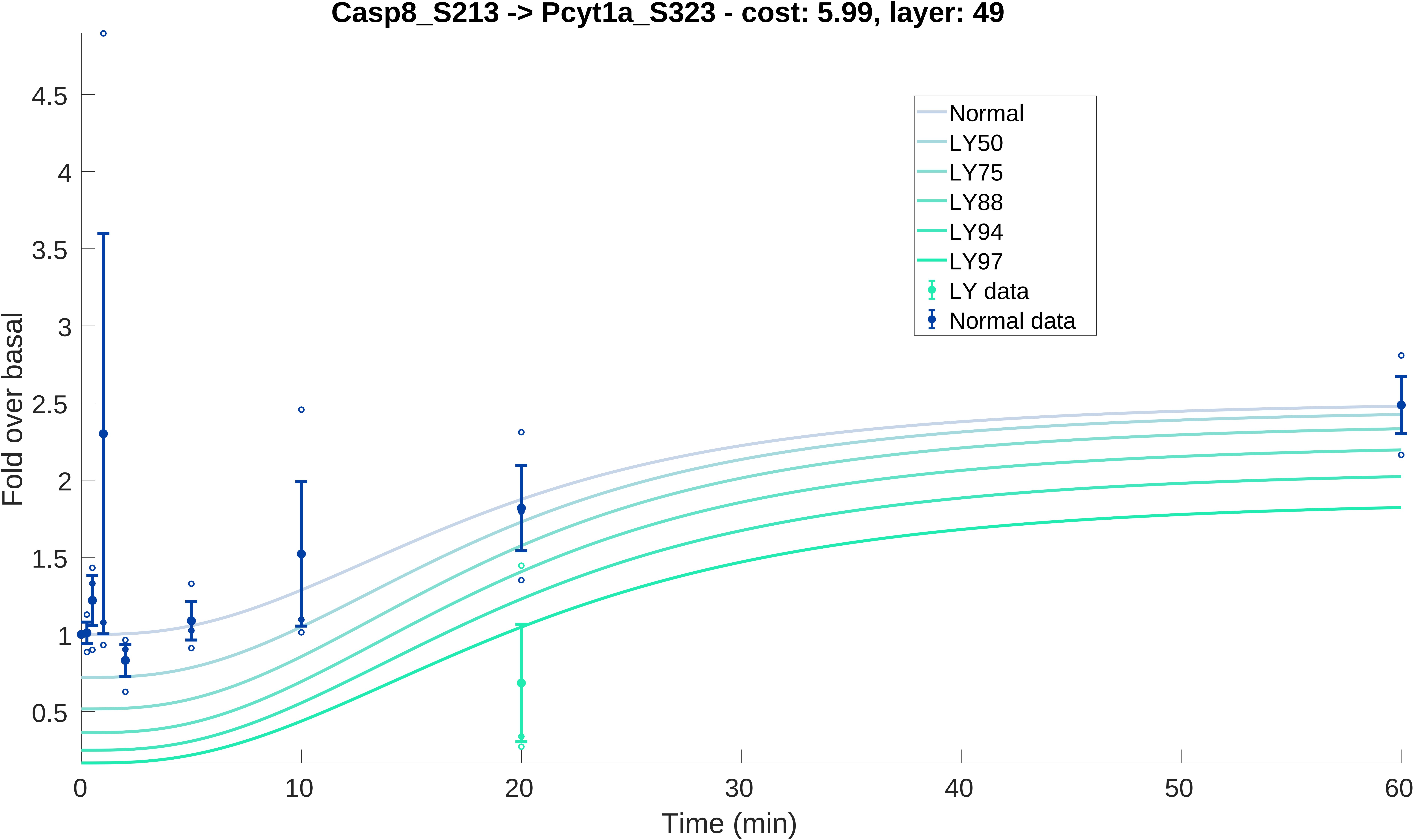

Casp8_S213 -> Pcyt1a_S323 - cost: 5.99, layer: 49
Fold over basal
Time (min)
0
10
20
30
40
50
60
0.5
1
1.5
2
2.5
3
3.5
4
4.5
Normal
LY50
LY75
LY88
LY94
LY97
LY data
Normal data

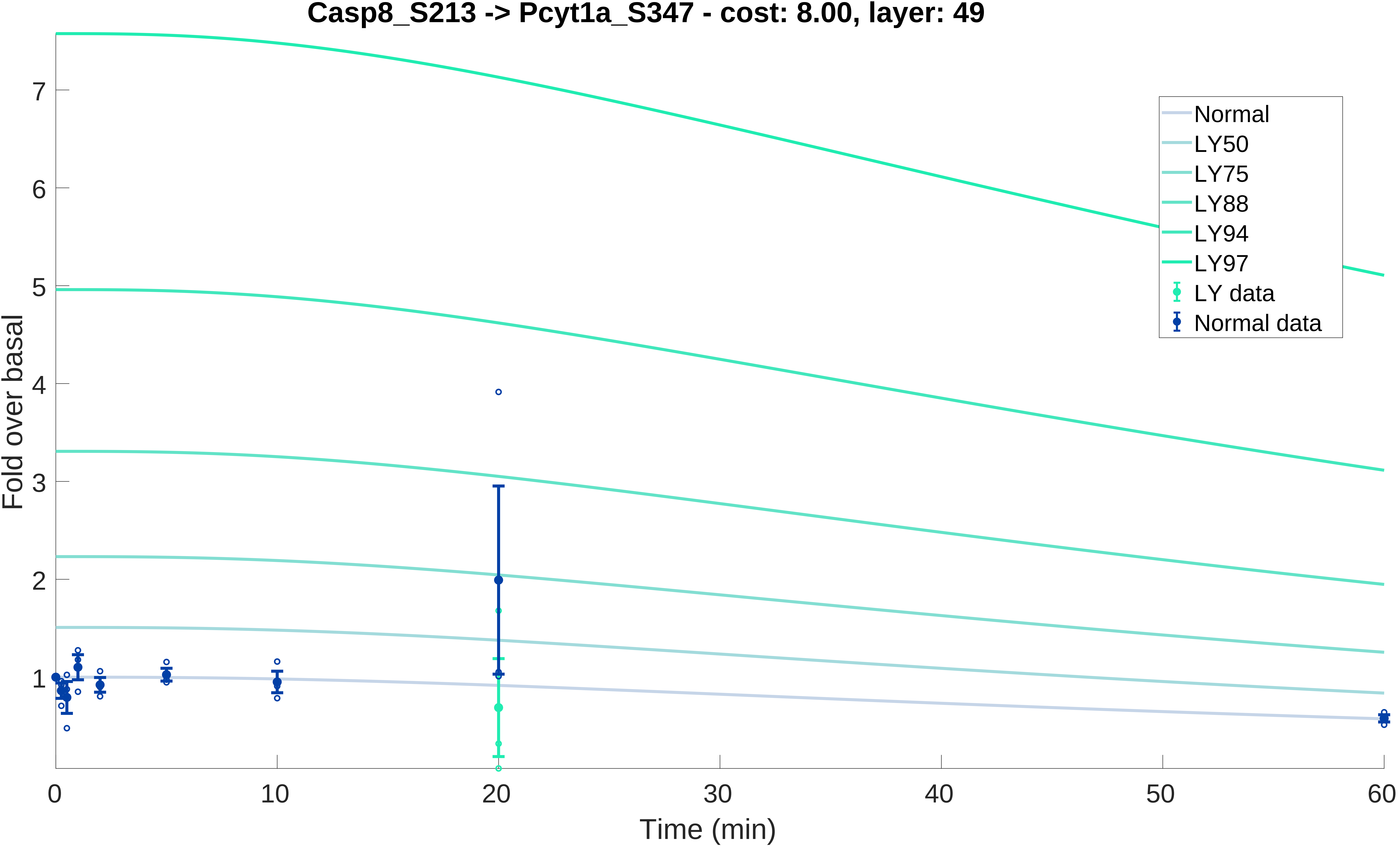

Casp8_S213 -> Pcyt1a_S347 - cost: 8.00, layer: 49
Fold over basal
Time (min)
0
10
20
30
40
50
60
1
2
3
4
5
6
7
Normal
LY50
LY75
LY88
LY94
LY97
LY data
Normal data

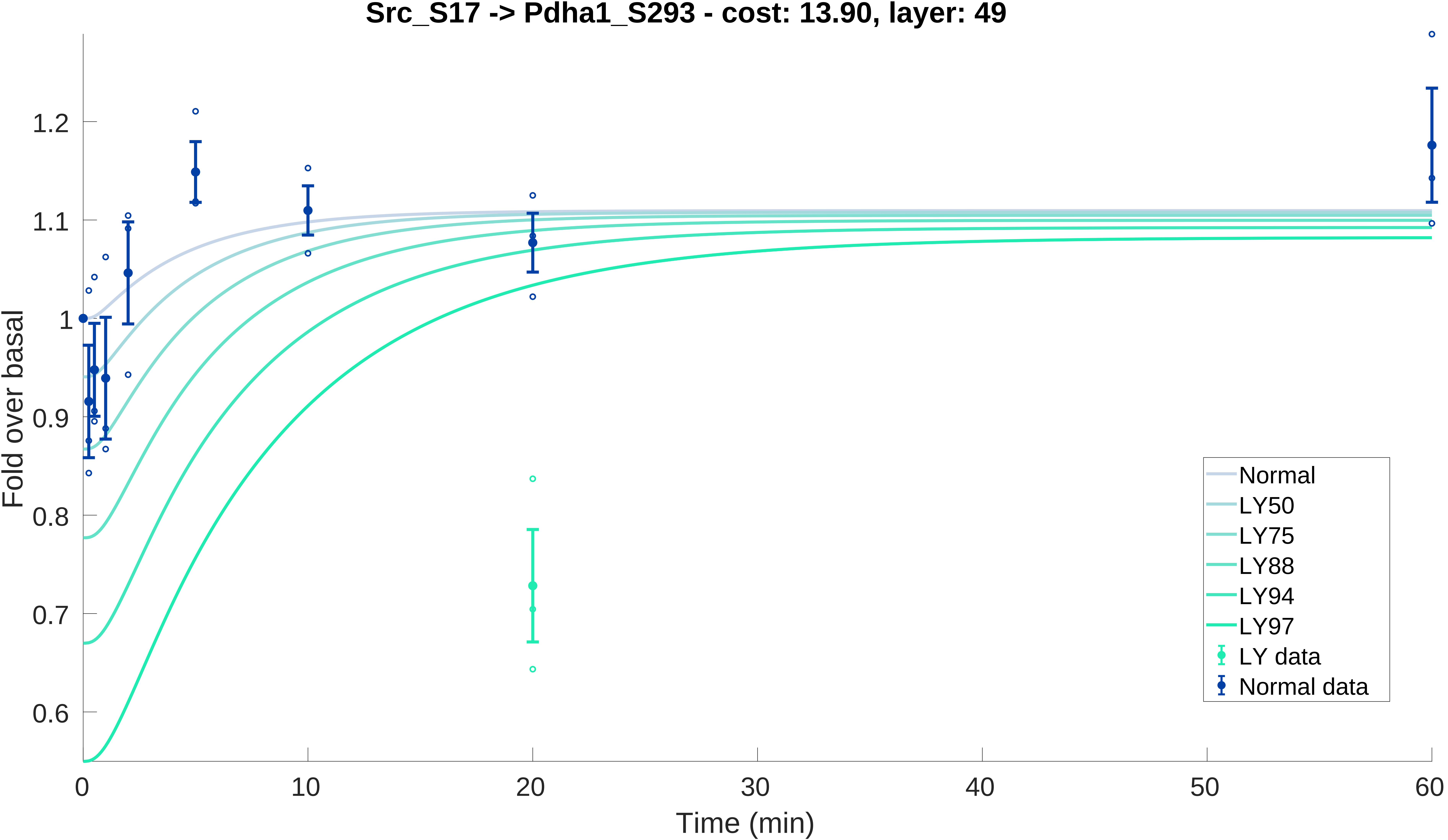

Src_S17 -> Pdha1_S293 - cost: 13.90, layer: 49
Fold over basal
Time (min)
0.6
0.7
0.8
0.9
1
1.1
1.2
0
10
20
30
40
50
60
Normal
LY50
LY75
LY88
LY94
LY97
LY data
Normal data

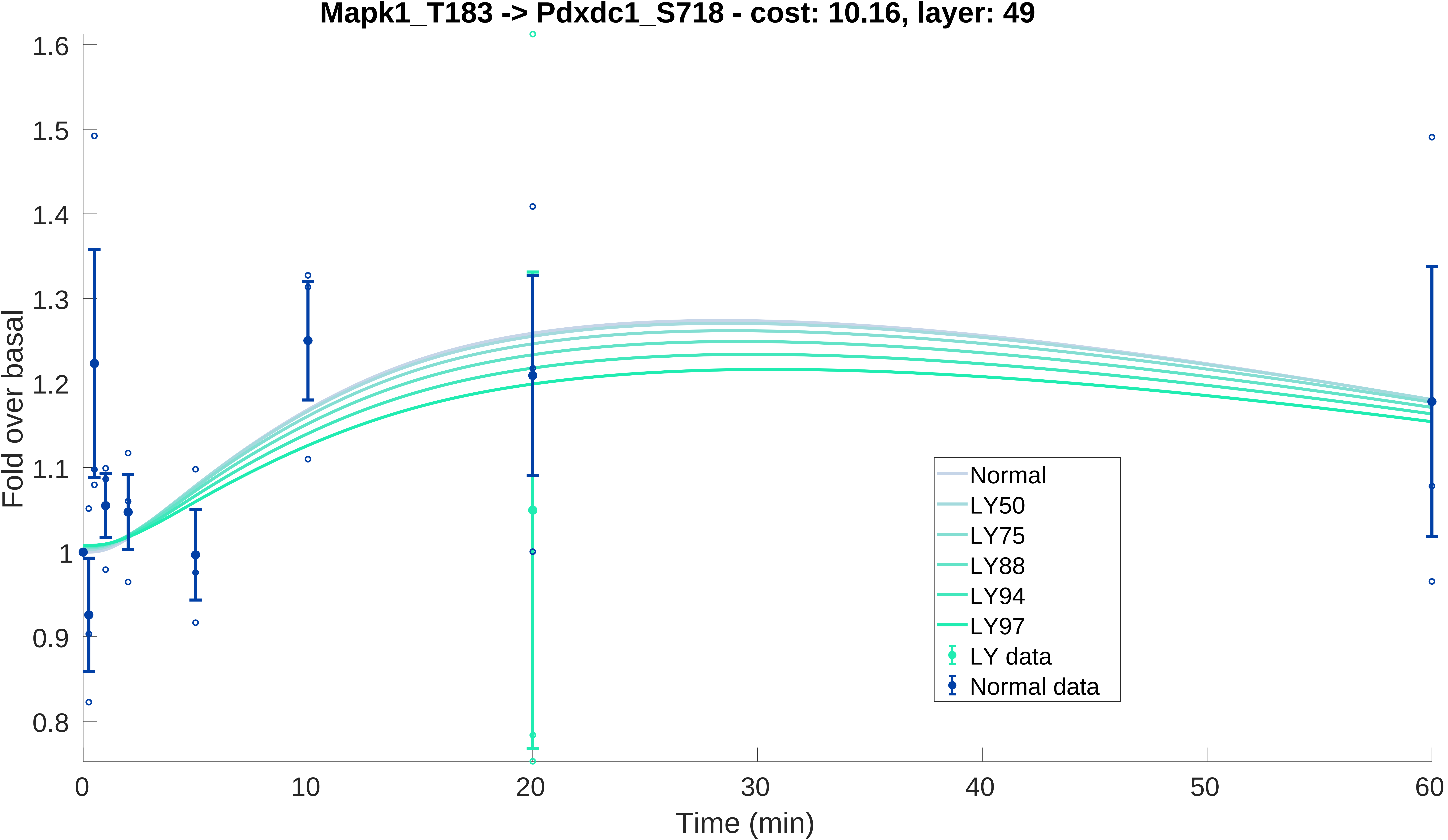
Mapk1_T183 -> Pdxdc1_S718 - cost: 10.16, layer: 49
Fold over basal
Time (min)
0.8
0.9
1
1.1
1.2
1.3
1.4
1.5
1.6
0
10
20
30
40
50
60
Normal
LY50
LY75
LY88
LY94
LY97
LY data
Normal data

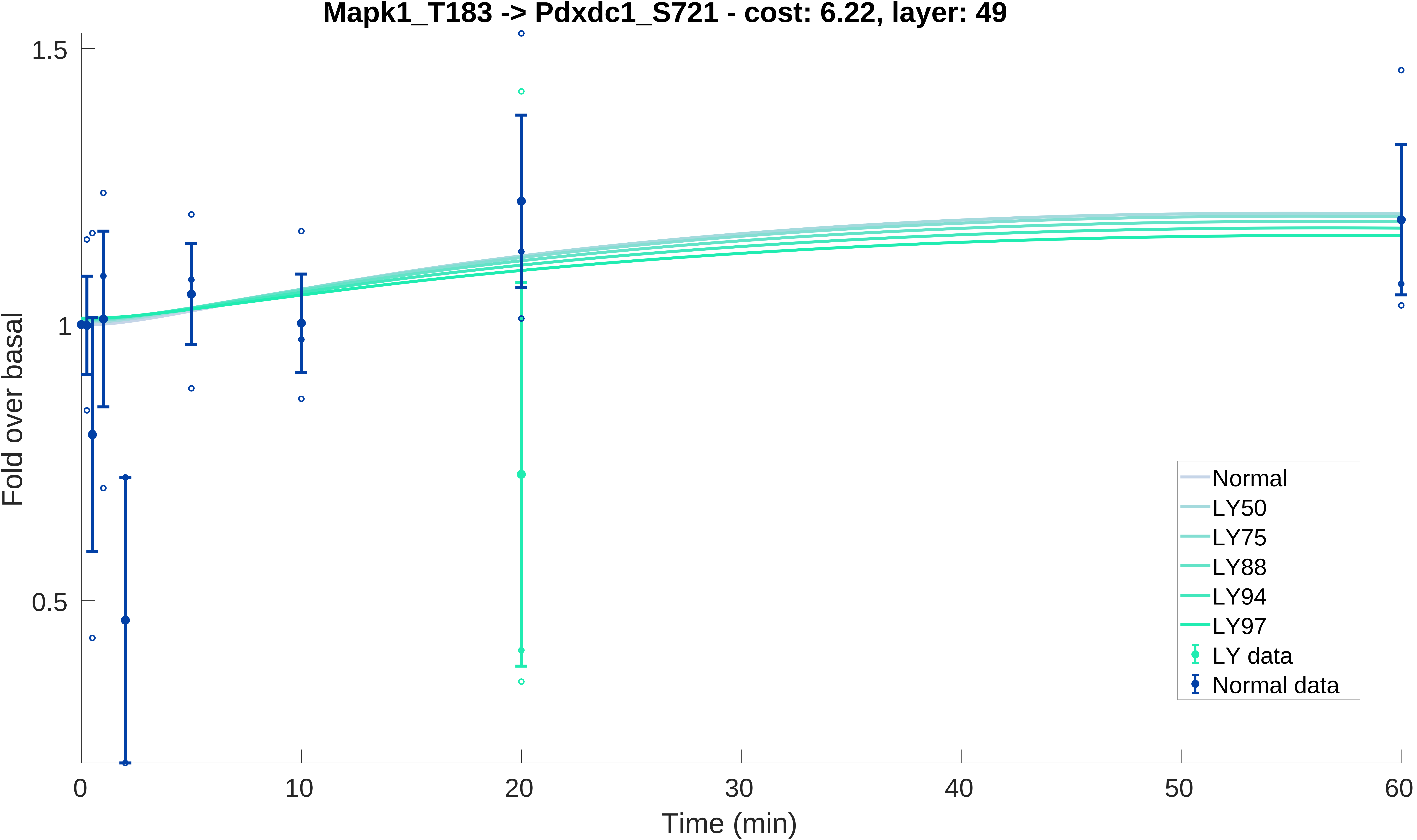

Mapk1_T183 -> Pdxdc1_S721 - cost: 6.22, layer: 49
Fold over basal
1.5
1
0.5
0
10
20
30
40
50
60
Time (min)
Normal
LY50
LY75
LY88
LY94
LY97
LY data
Normal data

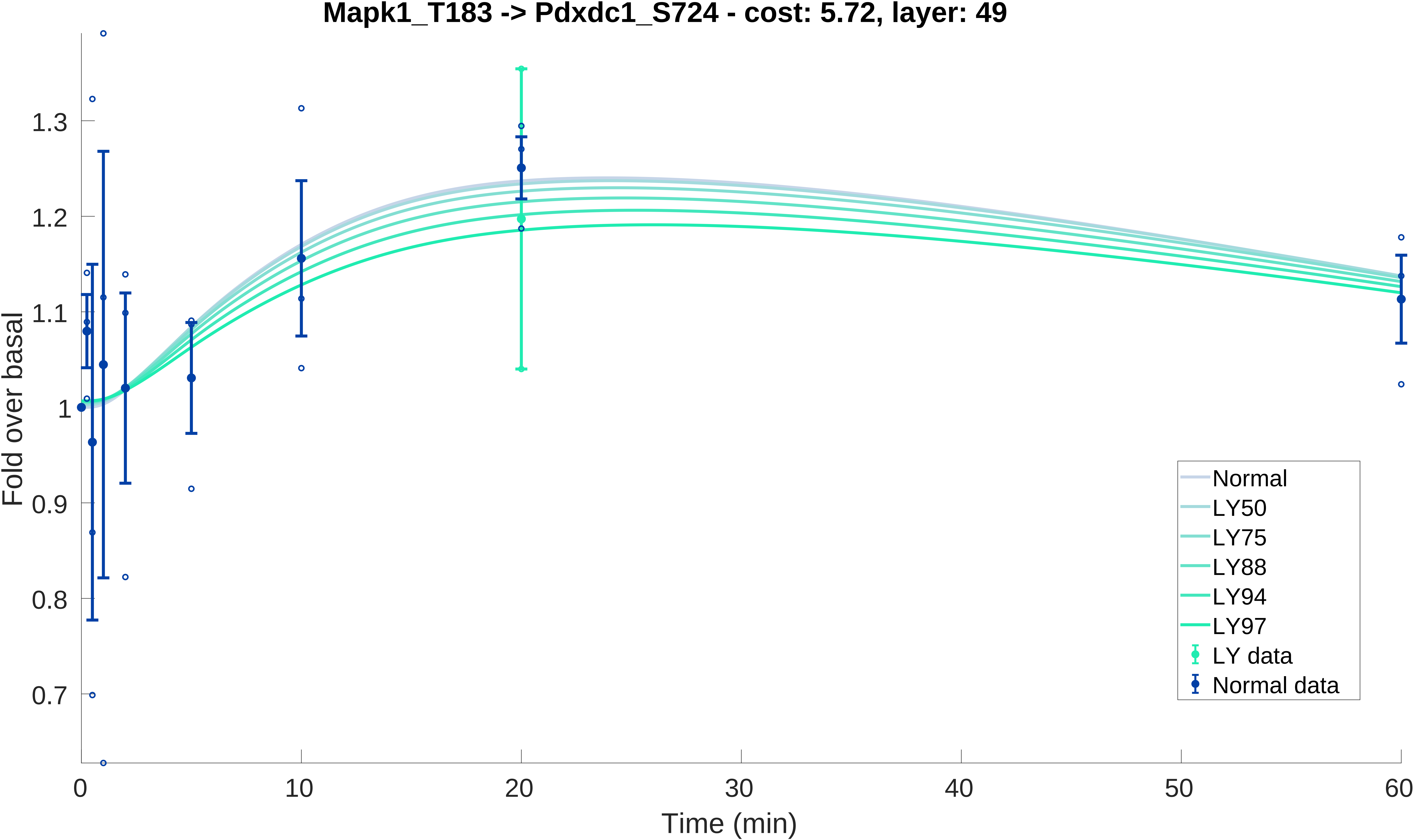

Mapk1_T183 -> Pdxdc1_S724 - cost: 5.72, layer: 49
Fold over basal
1.3
1.2
1.1
1
0.9
0.8
0.7
0
10
20
30
40
50
60
Time (min)
Normal
LY50
LY75
LY88
LY94
LY97
LY data
Normal data

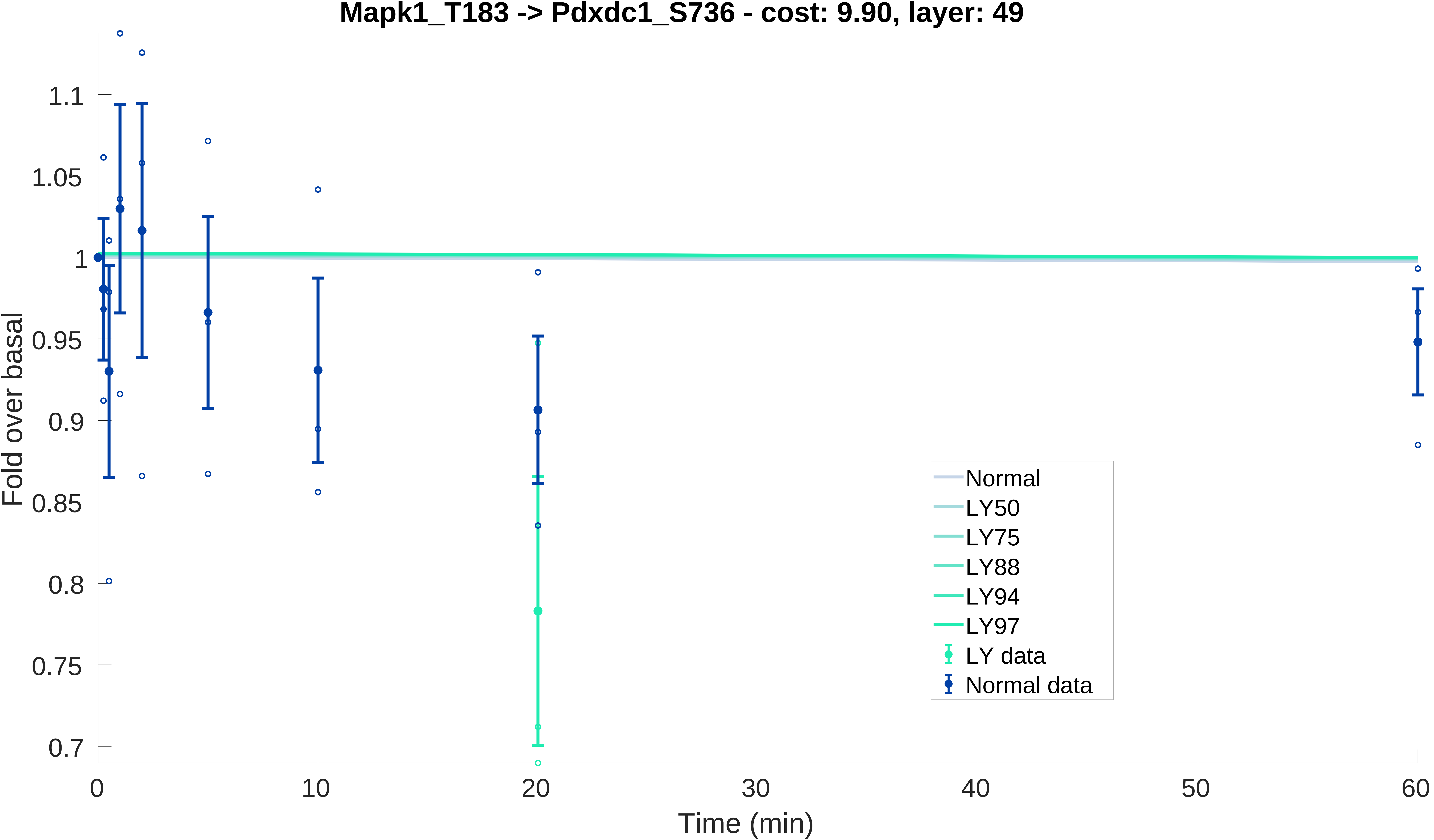
Mapk1_T183 -> Pdxdc1_S736 - cost: 9.90, layer: 49
Fold over basal
Time (min)
1.1
1.05
1
0.95
0.9
0.85
0.8
0.75
0.7
0
10
20
30
40
50
60
Normal
LY50
LY75
LY88
LY94
LY97
LY data
Normal data

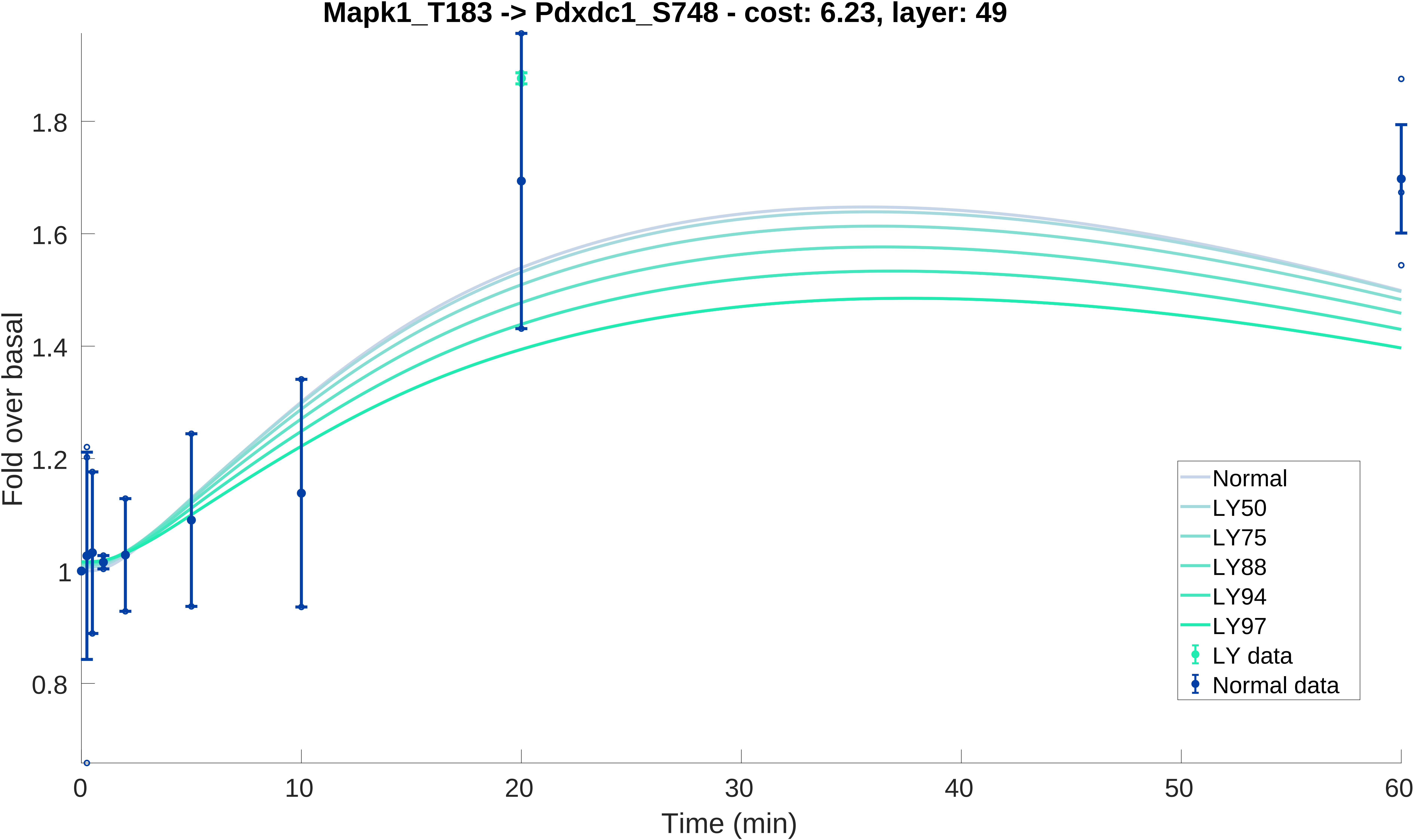

Mapk1_T183 -> Pdxdc1_S748 - cost: 6.23, layer: 49
Fold over basal
Time (min)
0.8
1
1.2
1.4
1.6
1.8
0
10
20
30
40
50
60
Normal
LY50
LY75
LY88
LY94
LY97
LY data
Normal data

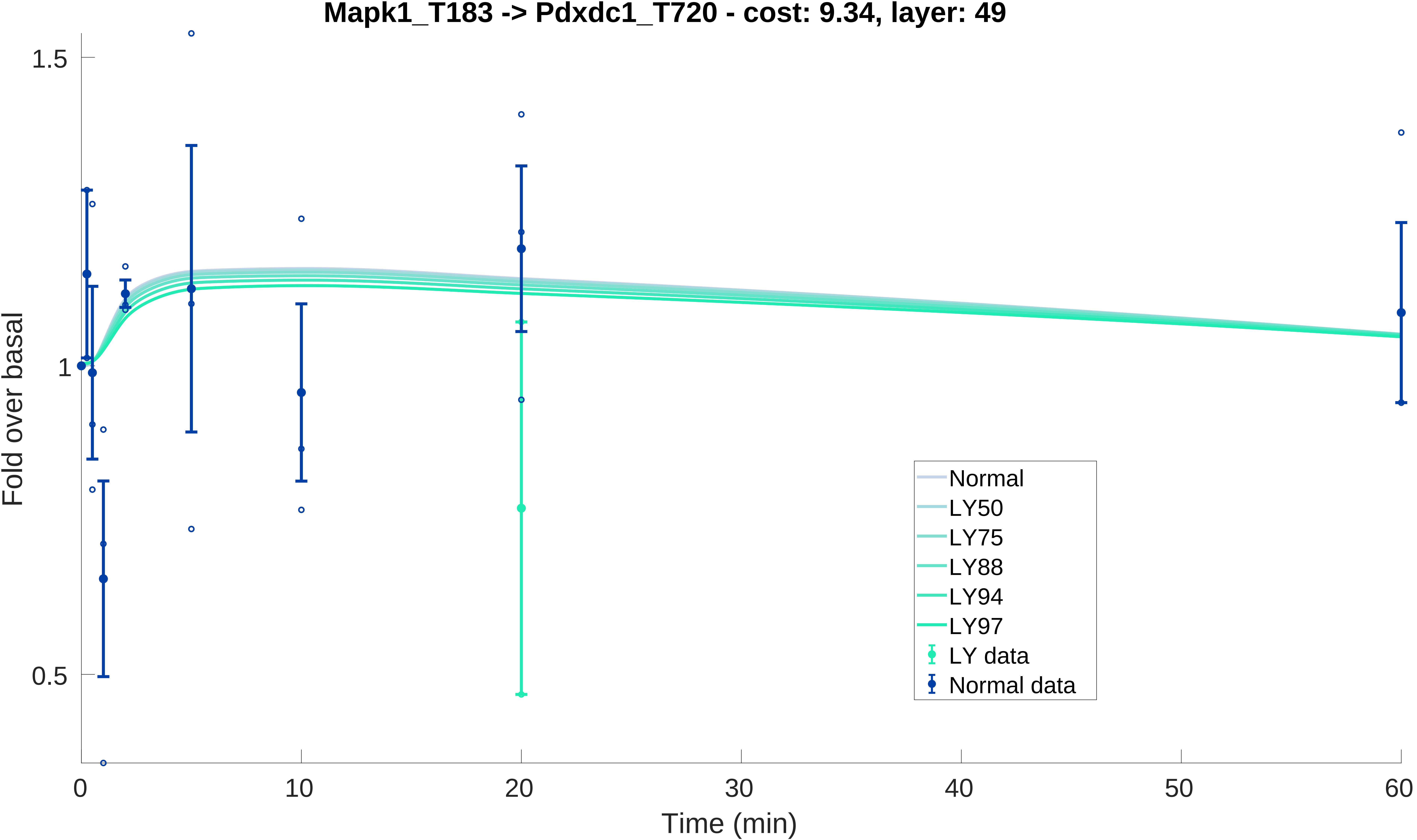

Mapk1_T183 -> Pdxdc1_T720 - cost: 9.34, layer: 49
Fold over basal
1.5
1
0.5
0
10
20
30
40
50
60
Time (min)
Normal
LY50
LY75
LY88
LY94
LY97
LY data
Normal data

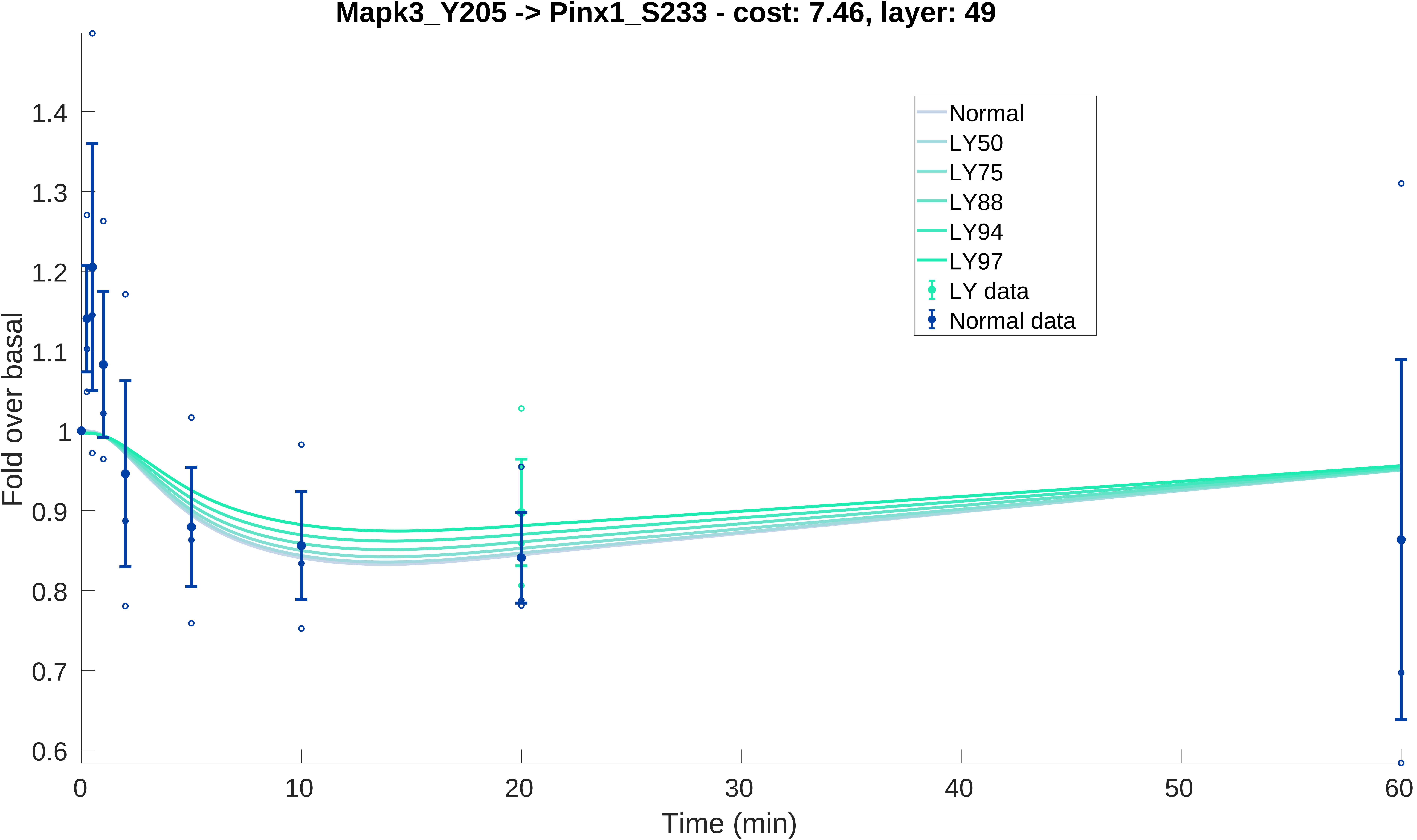

Mapk3_Y205 -> Pinx1_S233 - cost: 7.46, layer: 49
Normal
LY50
LY75
LY88
LY94
LY97
LY data
Normal data
Fold over basal
1.4
1.3
1.2
1.1
1
0.9
0.8
0.7
0.6
0
10
20
30
40
50
60
Time (min)

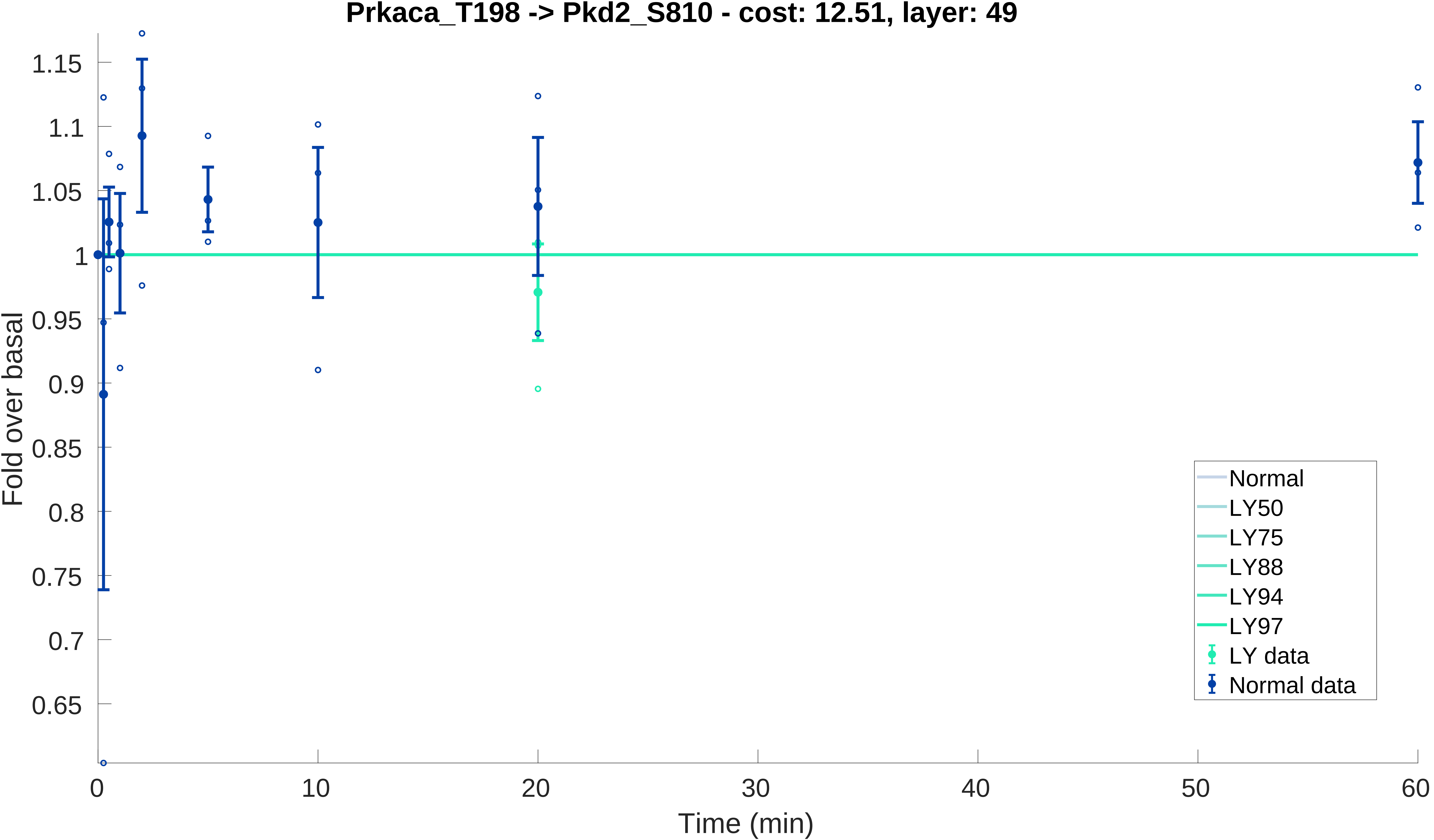

Prrkaca_T198 -> Pkd2_S810 - cost: 12.51, layer: 49
Fold over basal
Time (min)
1.15
1.1
1.05
1
0.95
0.9
0.85
0.8
0.75
0.7
0.65
0
10
20
30
40
50
60
Normal
LY50
LY75
LY88
LY94
LY97
LY data
Normal data

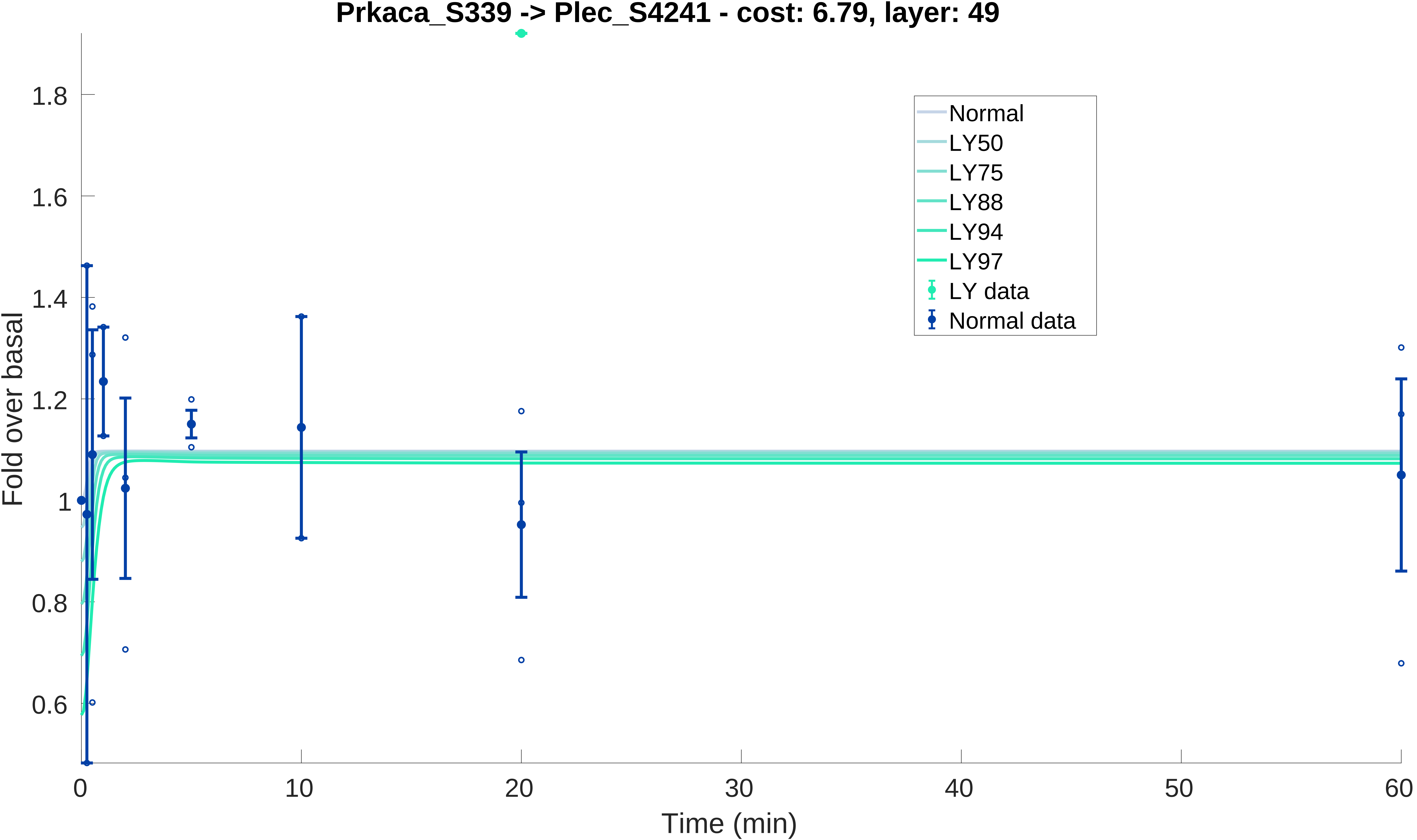

Prkaca_S339 -> Plec_S4241 - cost: 6.79, layer: 49
Fold over basal
Time (min)
0.6
0.8
1
1.2
1.4
1.6
1.8
0
10
20
30
40
50
60
Normal
LY50
LY75
LY88
LY94
LY97
LY data
Normal data

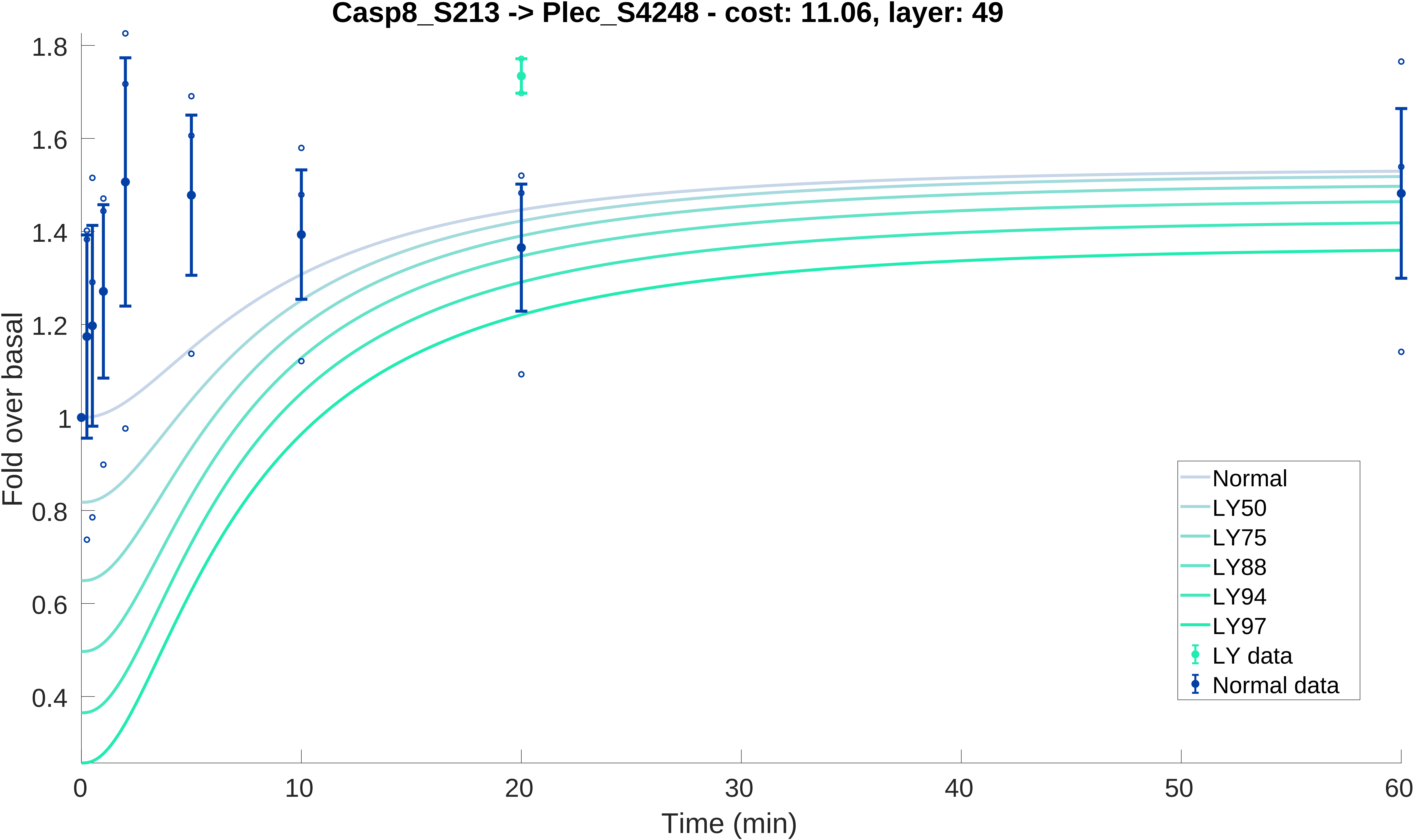
Casp8_S213 -> Plec_S4248 - cost: 11.06, layer: 49
Fold over basal
1.8
1.6
1.4
1.2
1
0.8
0.6
0.4
0
10
20
30
40
50
60
Time (min)
Normal
LY50
LY75
LY88
LY94
LY97
LY data
Normal data

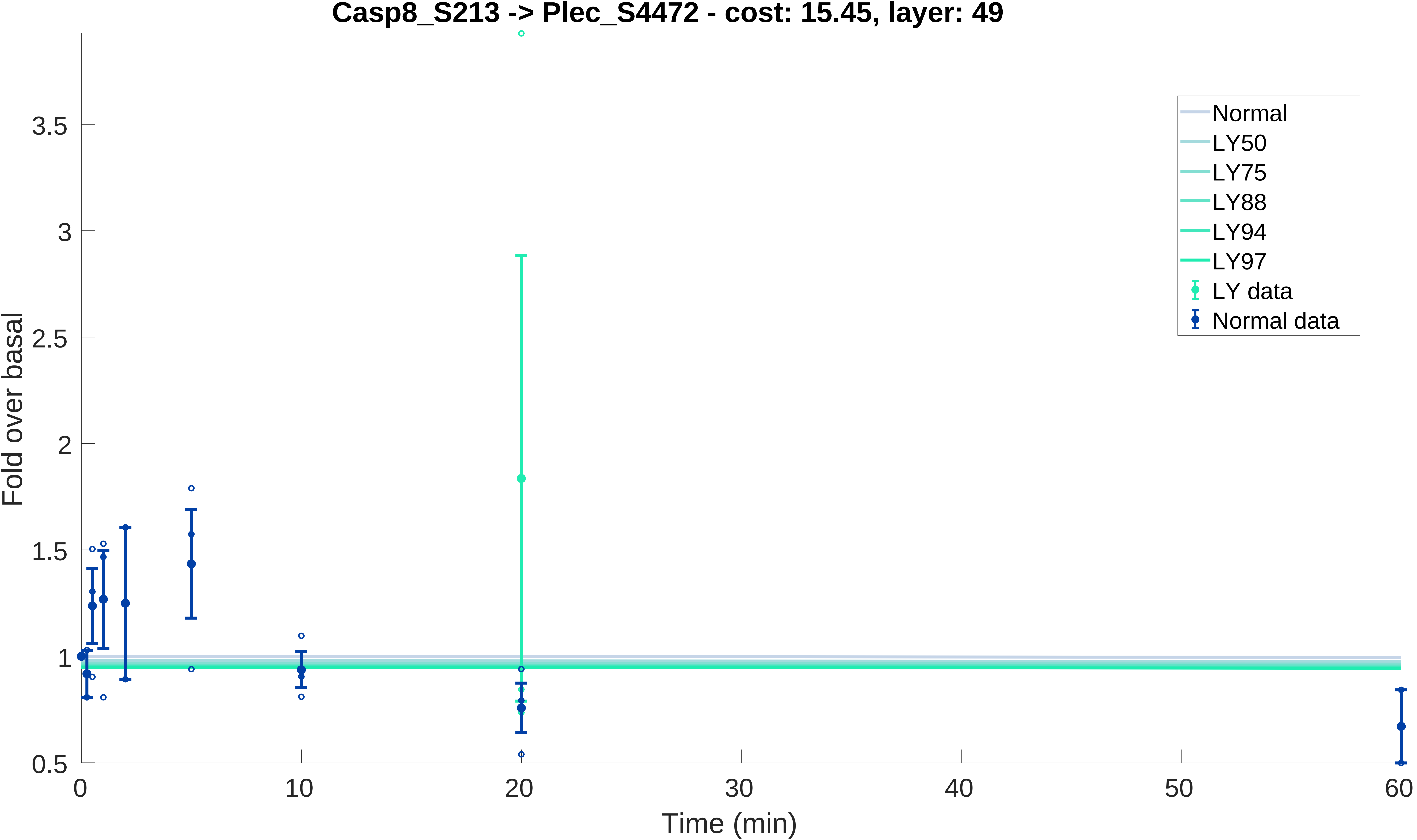

Casp8_S213 -> Plec_S4472 - cost: 15.45, layer: 49
Fold over basal
Time (min)
0.5
1
1.5
2
2.5
3
3.5
0
10
20
30
40
50
60
Normal
LY50
LY75
LY88
LY94
LY97
LY data
Normal data

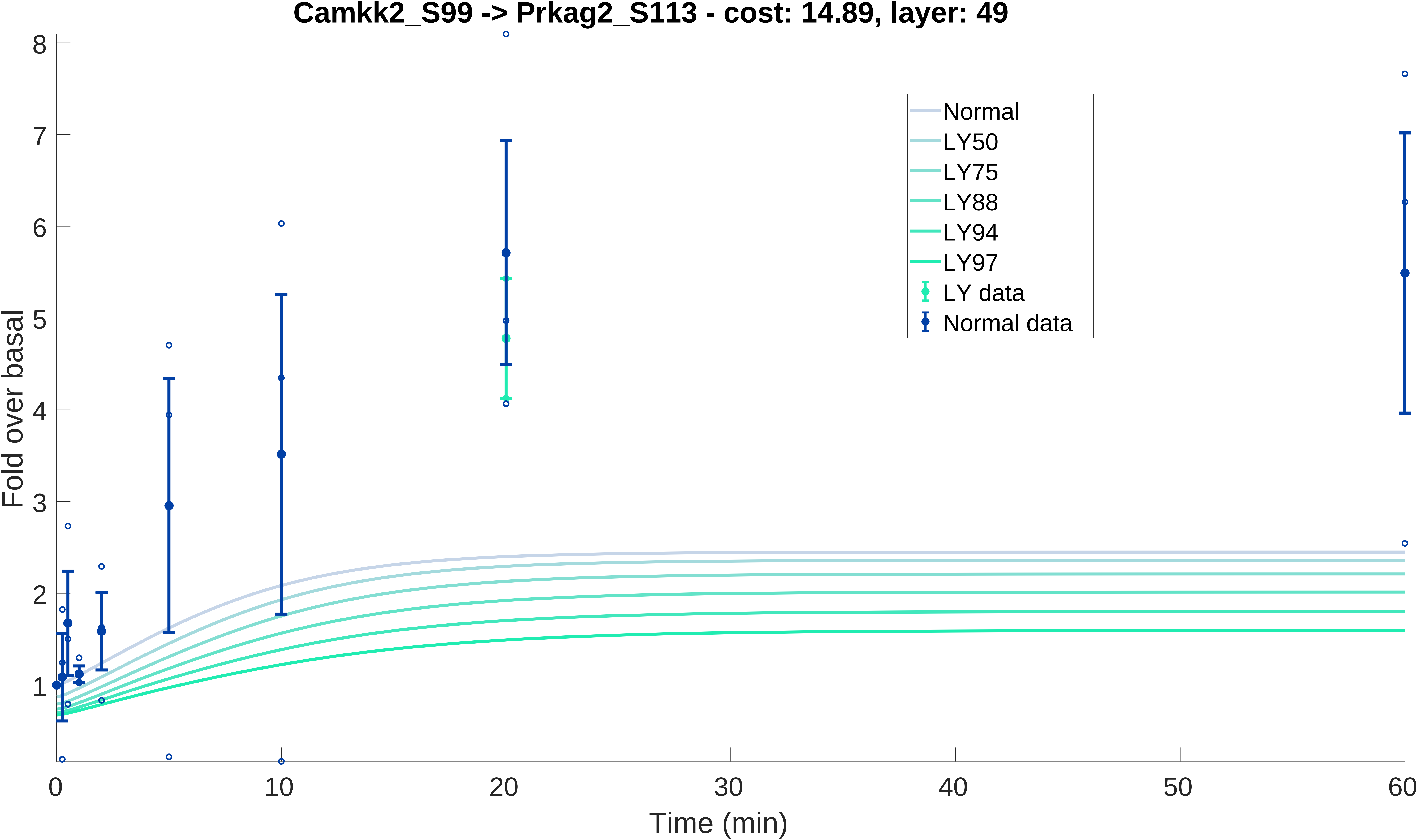
Camkk2_S99 -> Prkag2_S113 - cost: 14.89, layer: 49
Fold over basal
Time (min)
Normal
LY50
LY75
LY88
LY94
LY97
LY data
Normal data
0
10
20
30
40
50
60
1
2
3
4
5
6
7
8

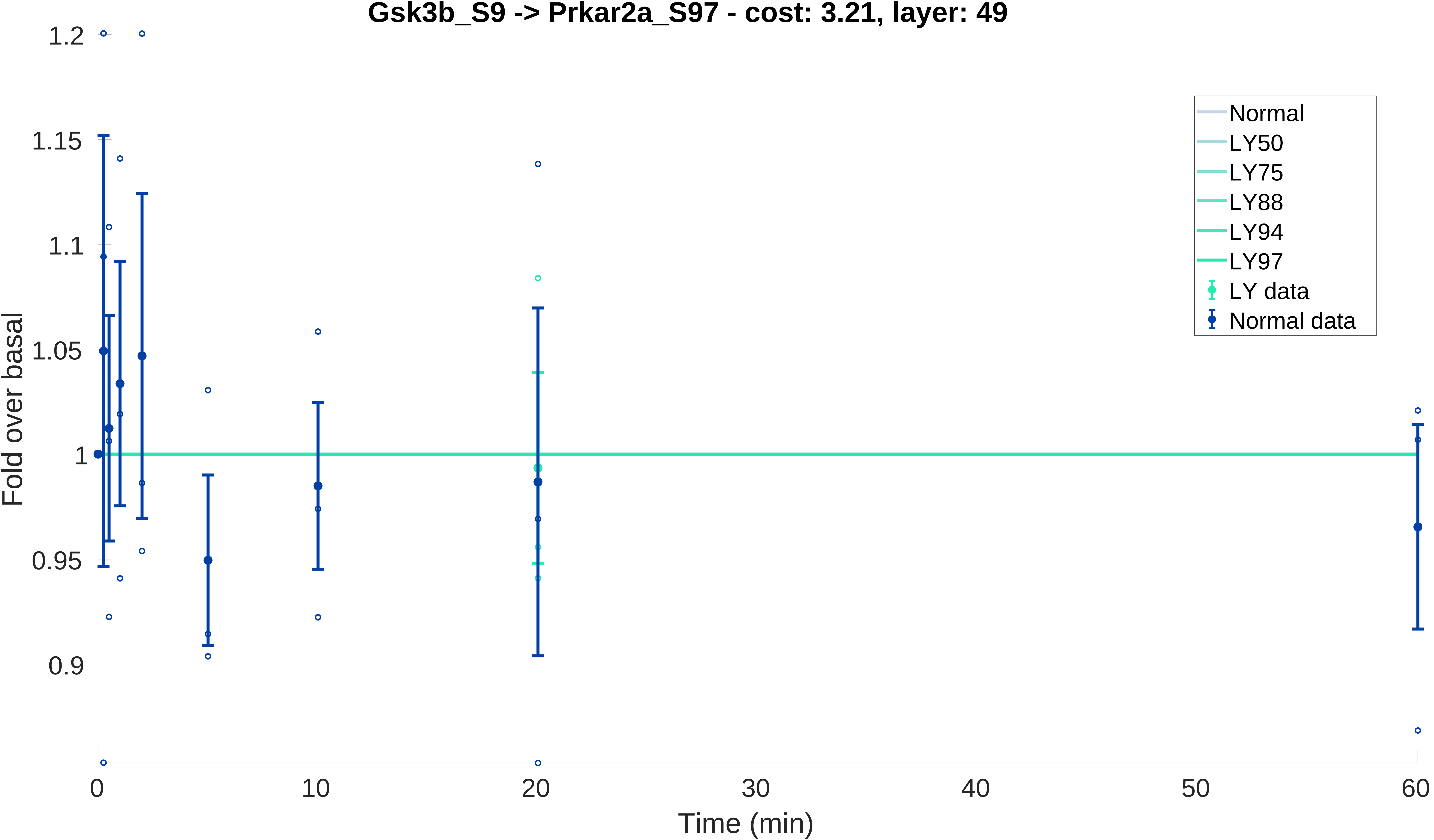

Gsk3b_S9 -> Prkar2a_S97 - cost: 3.21, layer: 49
1.2
1.15
1.1
1.05
1
0.95
0.9
Fold over basal
0
10
20
30
40
50
60
Time (min)
Normal
LY50
LY75
LY88
LY94
LY97
LY data
Normal data

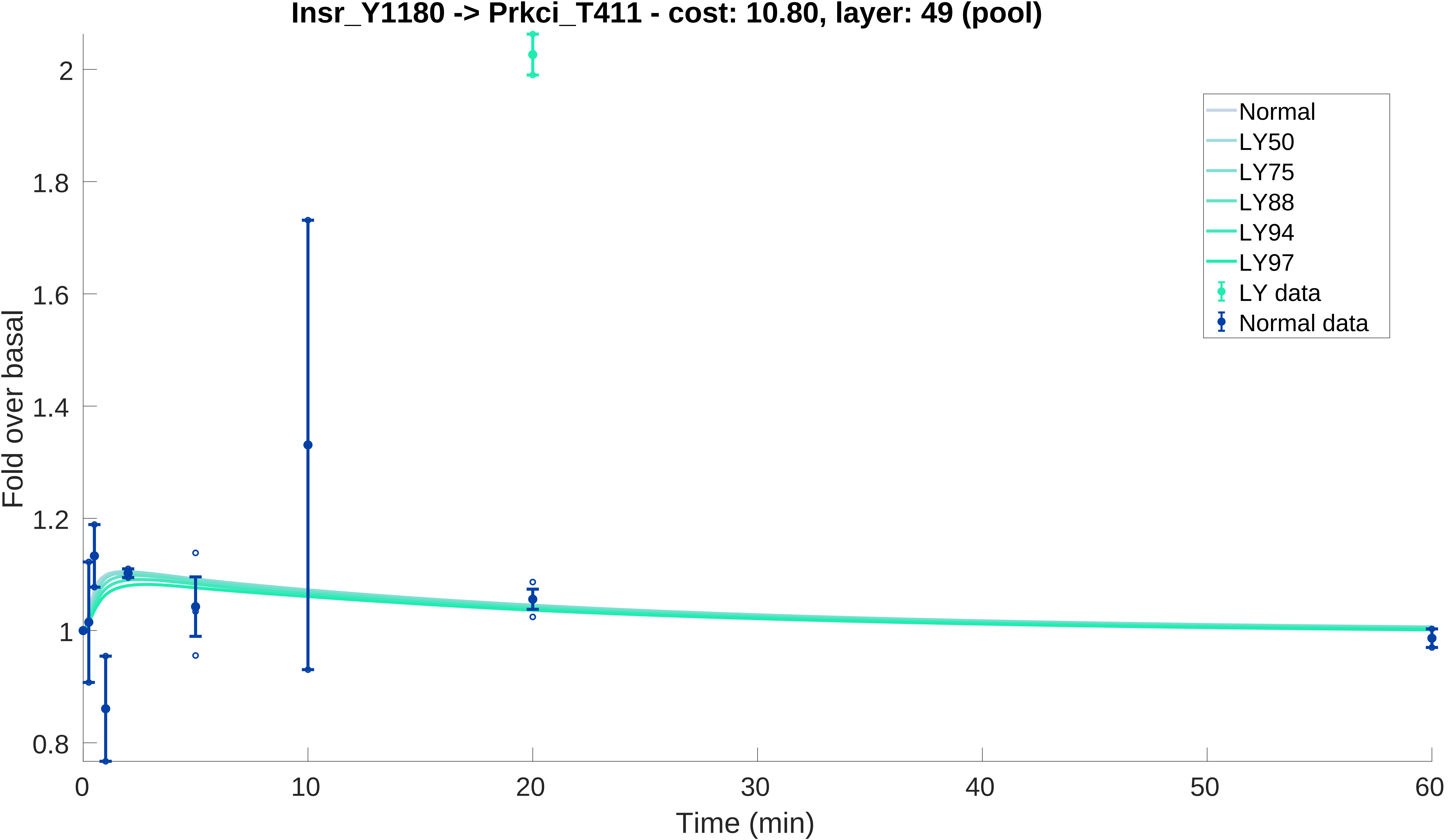
Insr_Y1180 -> Prkci_T411 - cost: 10.80, layer: 49 (pool)
Fold over basal
Time (min)
0.8
1
1.2
1.4
1.6
1.8
2
0
10
20
30
40
50
60
Normal
LY50
LY75
LY88
LY94
LY97
LY data
Normal data

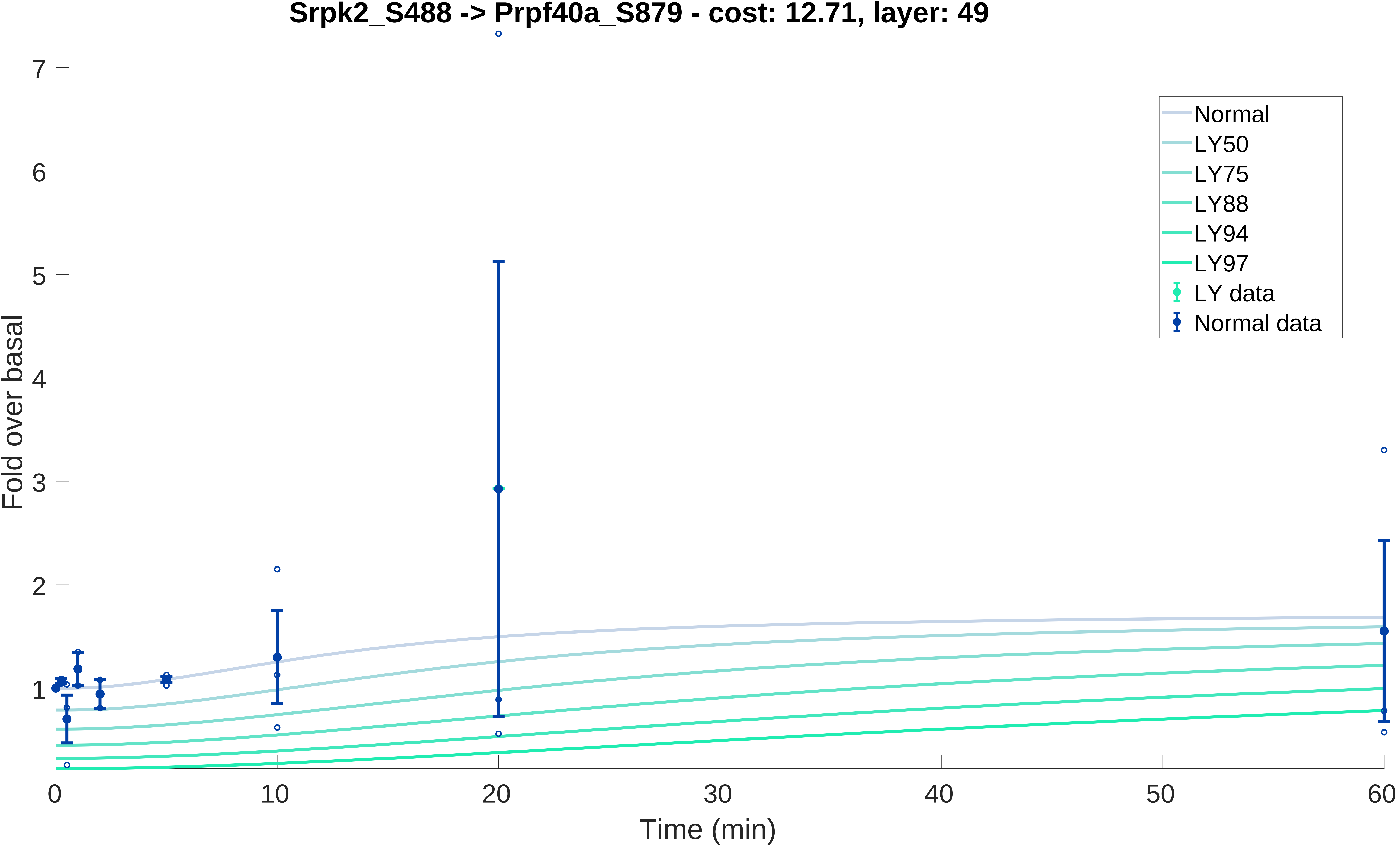

Srpk2_S488 -> Prpf40a_S879 - cost: 12.71, layer: 49
Fold over basal
Time (min)
1
2
3
4
5
6
7
0
10
20
30
40
50
60
Normal
LY50
LY75
LY88
LY94
LY97
LY data
Normal data

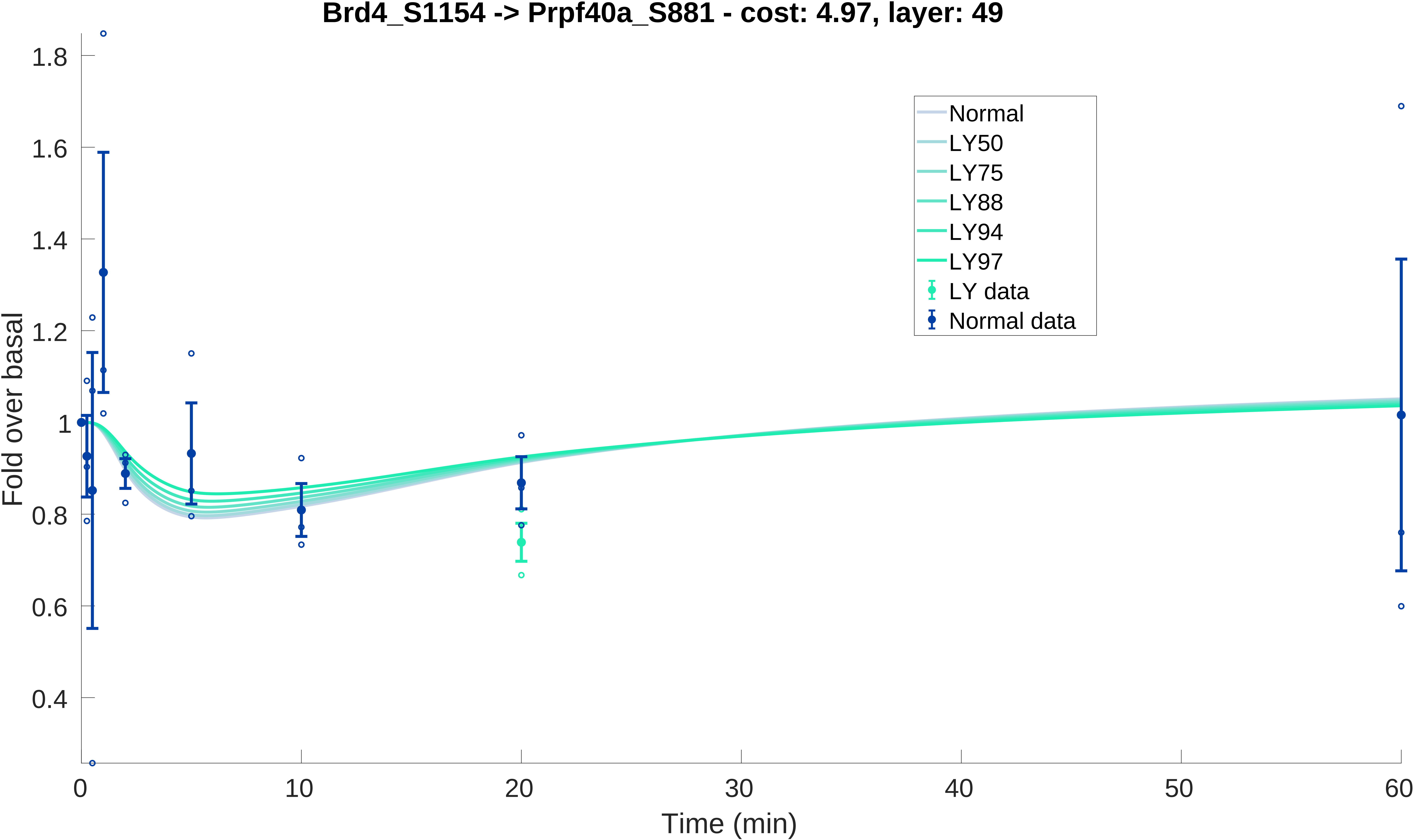

Brd4_S1154 -> Prpf40a_S881 - cost: 4.97, layer: 49
Fold over basal
Time (min)
Normal
LY50
LY75
LY88
LY94
LY97
LY data
Normal data
0.4
0.6
0.8
1
1.2
1.4
1.6
1.8
0
10
20
30
40
50
60

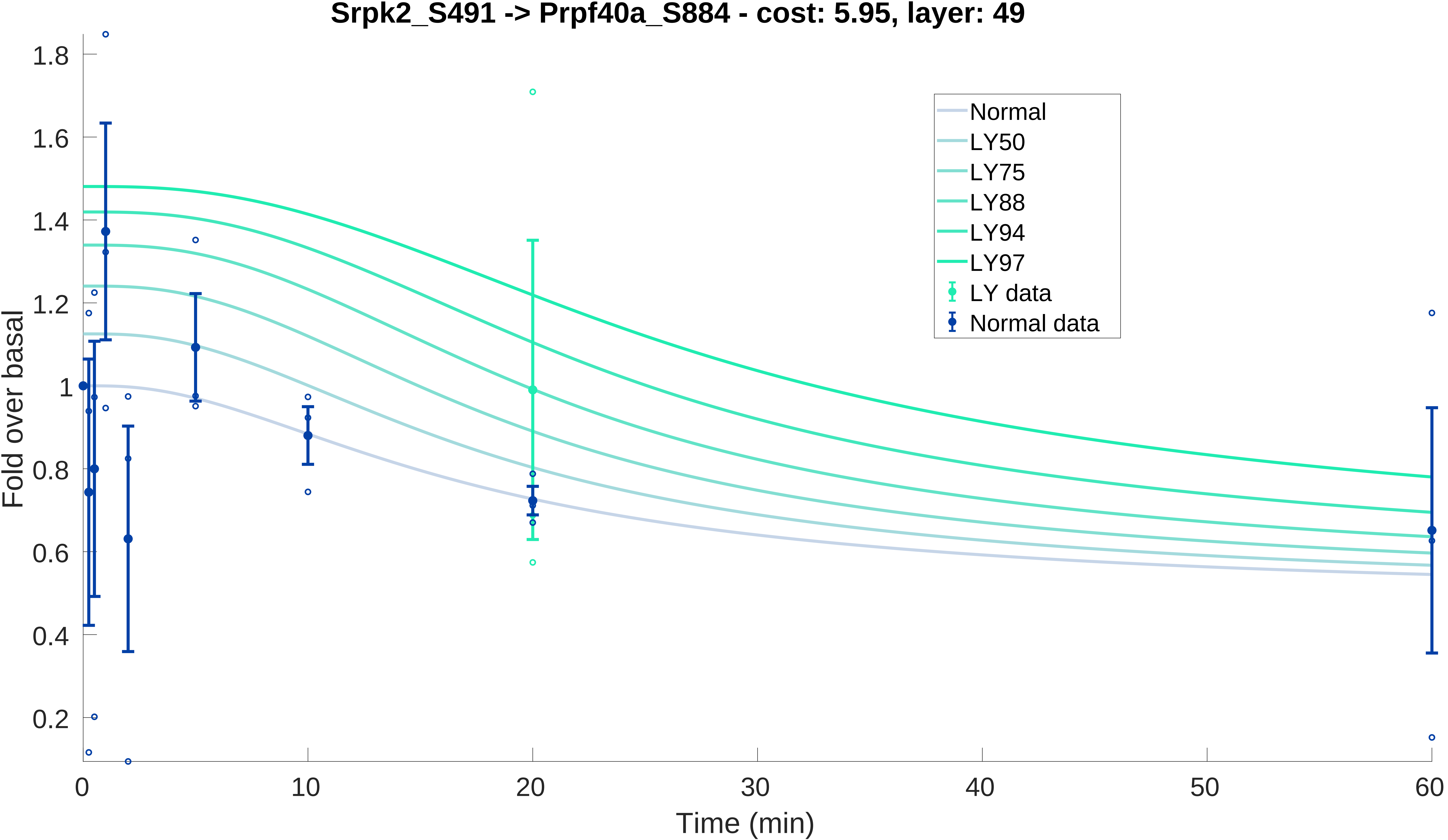

Srpk2_S491 -> Prpf40a_S884 - cost: 5.95, layer: 49
Fold over basal
Time (min)
0.2
0.4
0.6
0.8
1
1.2
1.4
1.6
1.8
0
10
20
30
40
50
60
Normal
LY50
LY75
LY88
LY94
LY97
LY data
Normal data

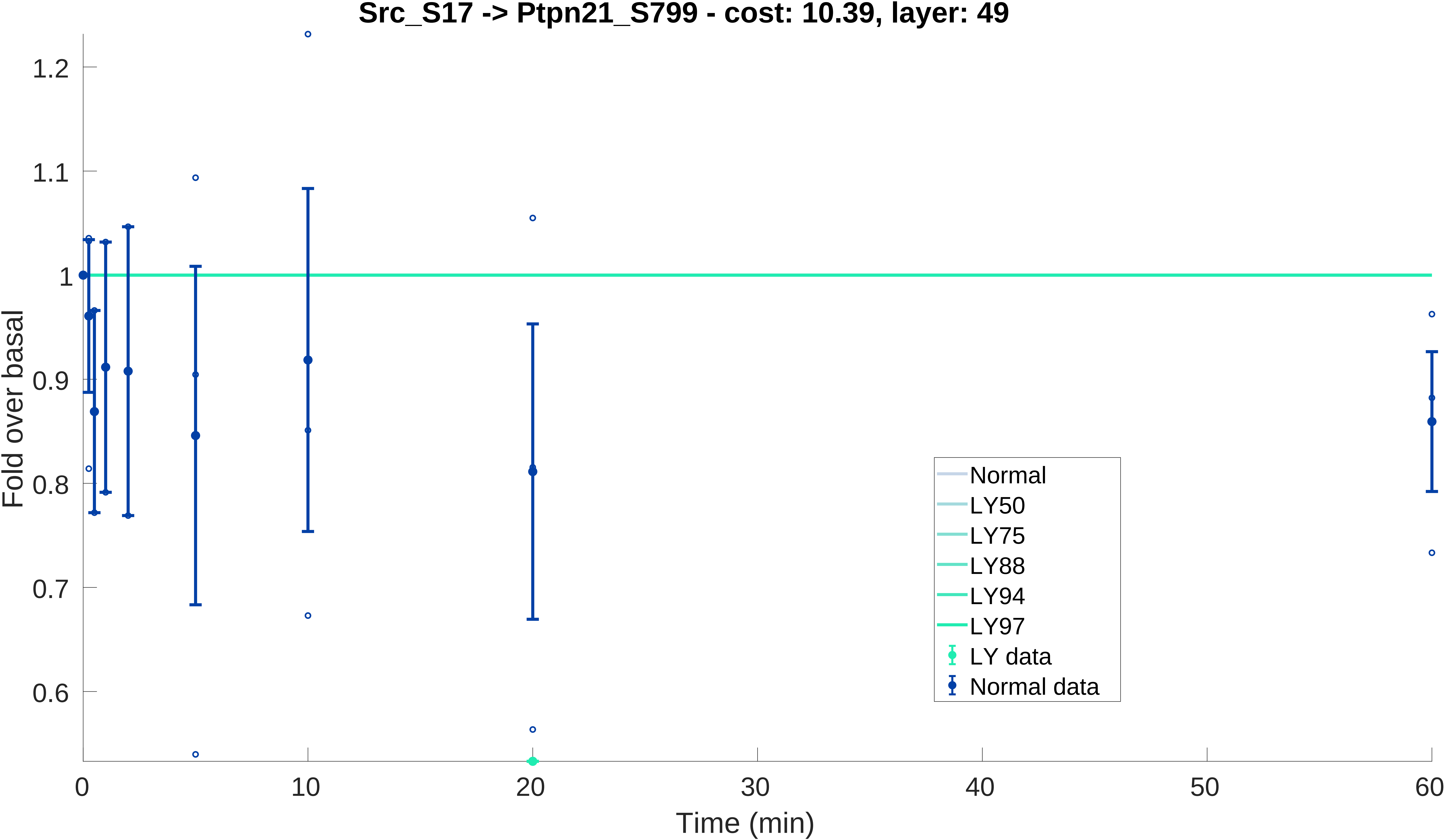

Src_S17 -> Ptpn21_S799 - cost: 10.39, layer: 49
Fold over basal
1.2
1.1
1
0.9
0.8
0.7
0.6
0
10
20
30
40
50
60
Time (min)
Normal
LY50
LY75
LY88
LY94
LY97
LY data
Normal data

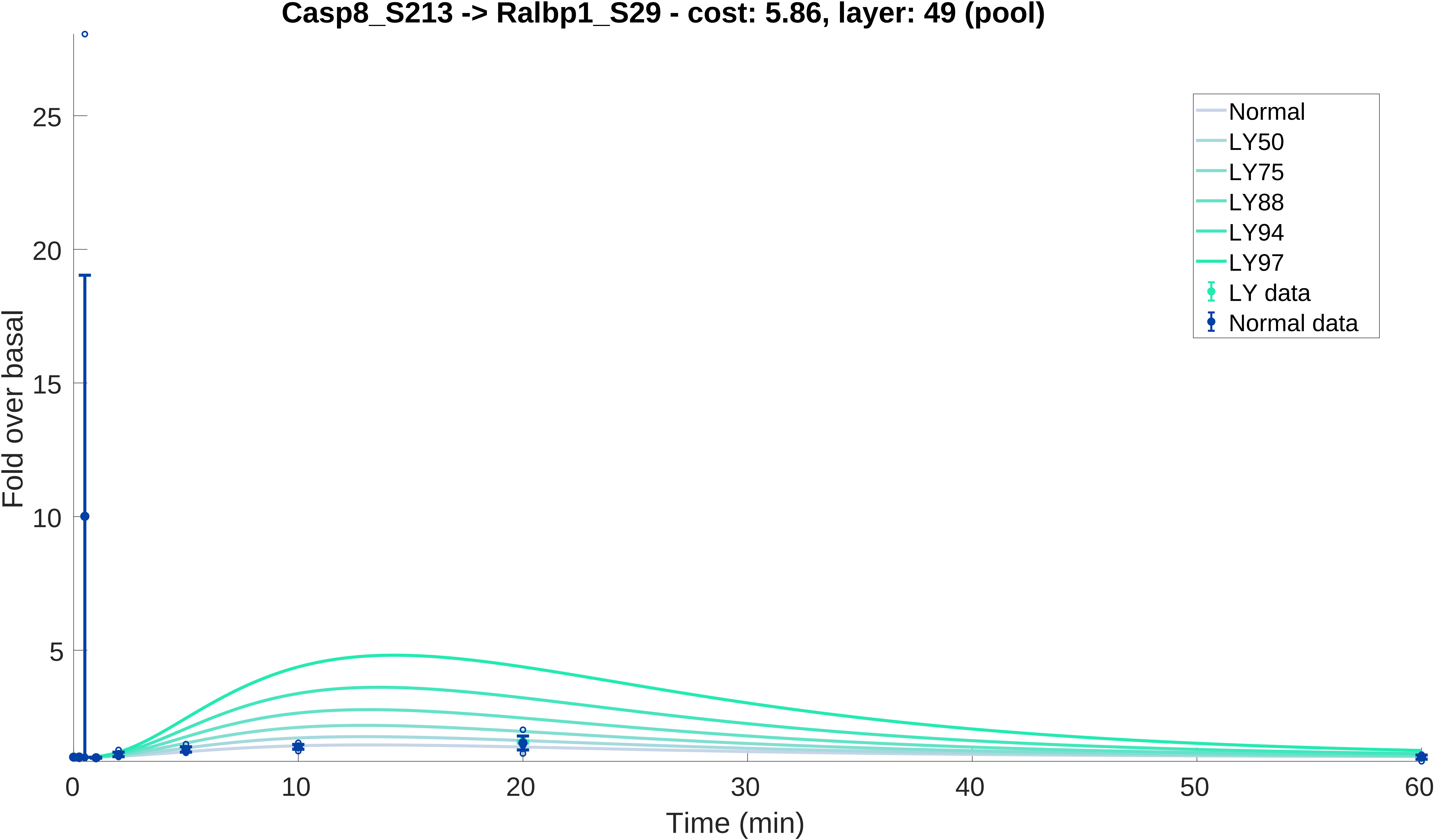
Casp8_S213 -> Ralbp1_S29 - cost: 5.86, layer: 49 (pool)
Fold over basal
Time (min)
0
5
10
15
20
25
0
10
20
30
40
50
60
Normal
LY50
LY75
LY88
LY94
LY97
LY data
Normal data

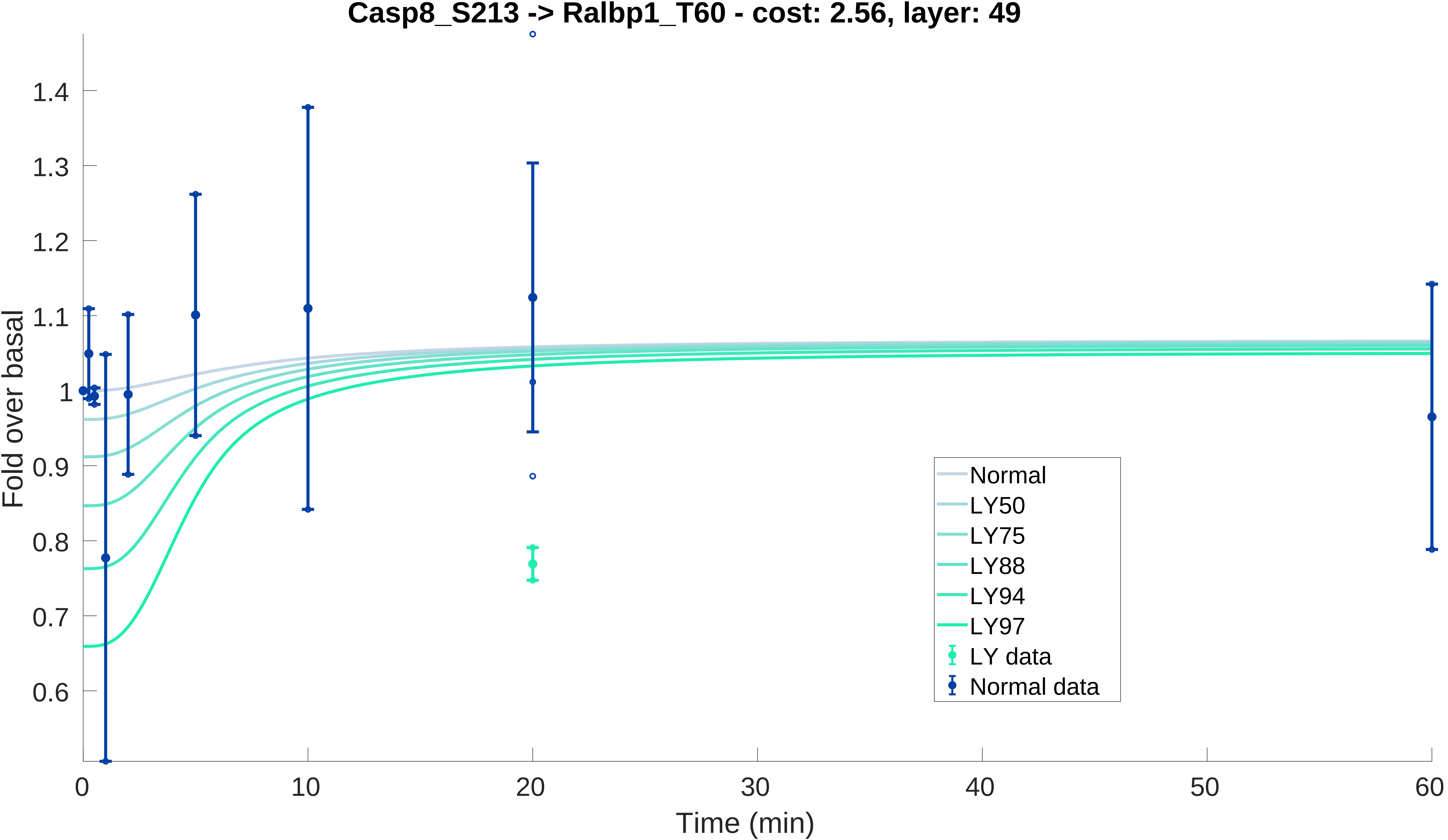
Casp8_S213 -> Ralbp1_T60 - cost: 2.56, layer: 49
Fold over basal
Time (min)
1.4
1.3
1.2
1.1
1
0.9
0.8
0.7
0.6
0
10
20
30
40
50
60
Normal
LY50
LY75
LY88
LY94
LY97
LY data
Normal data

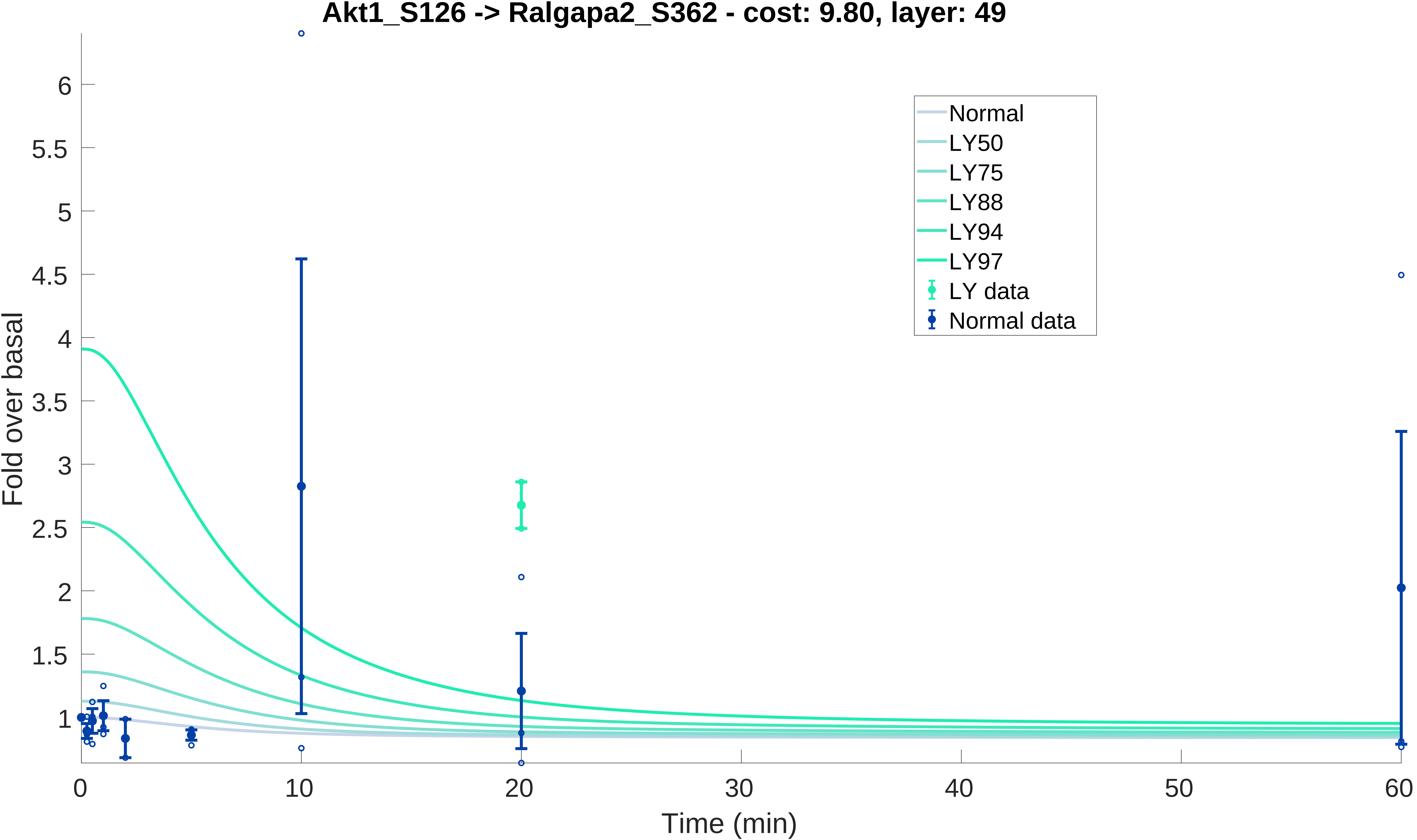
Akt1_S126 -> Ralgapa2_S362 - cost: 9.80, layer: 49
Fold over basal
Time (min)
0
10
20
30
40
50
60
1
1.5
2
2.5
3
3.5
4
4.5
5
5.5
6
Normal
LY50
LY75
LY88
LY94
LY97
LY data
Normal data

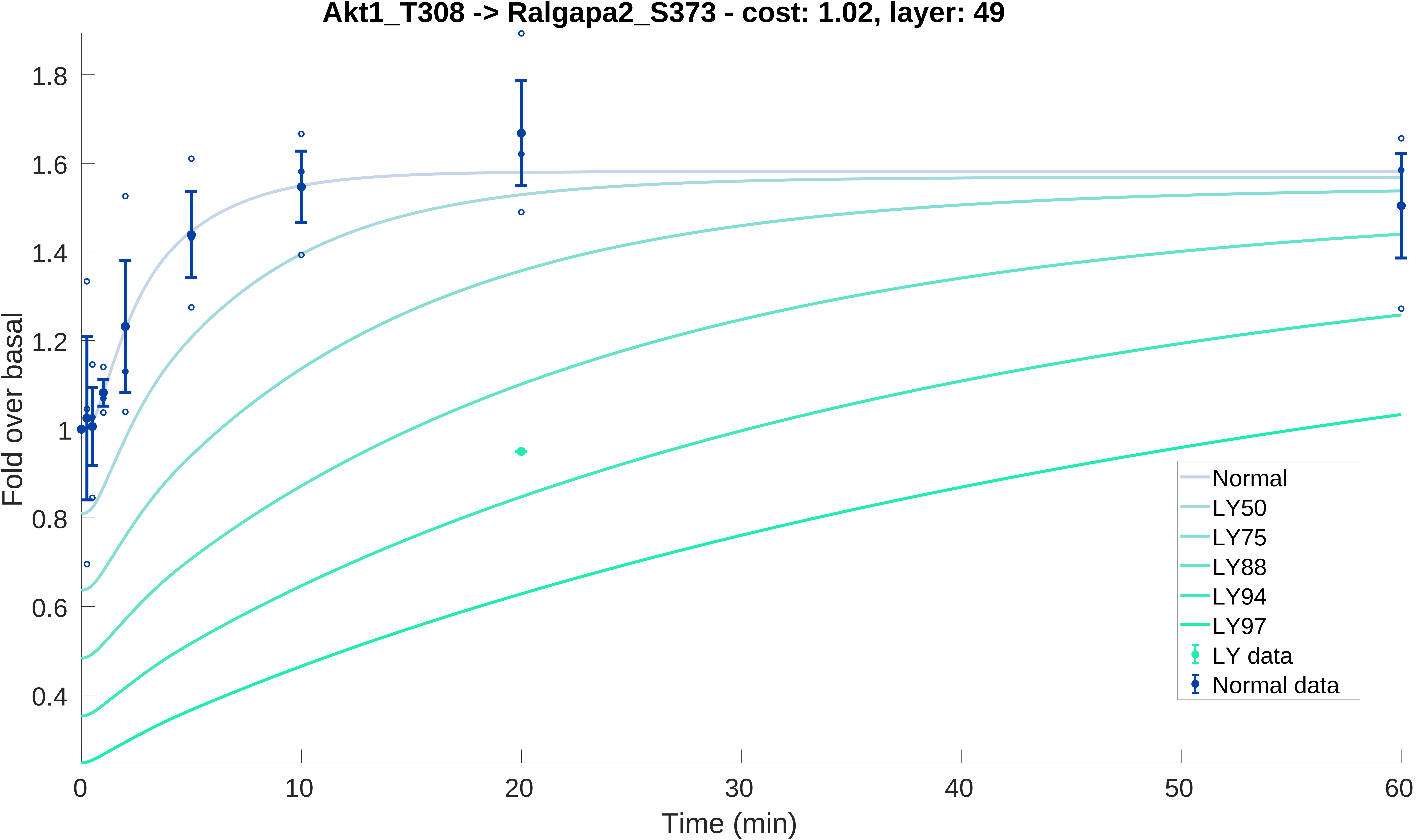

Akt1_T308 -> Ralgapa2_S373 - cost: 1.02, layer: 49
Fold over basal
1.8
1.6
1.4
1.2
1
0.8
0.6
0.4
0
10
20
30
40
50
60
Time (min)
Normal
LY50
LY75
LY88
LY94
LY97
LY data
Normal data

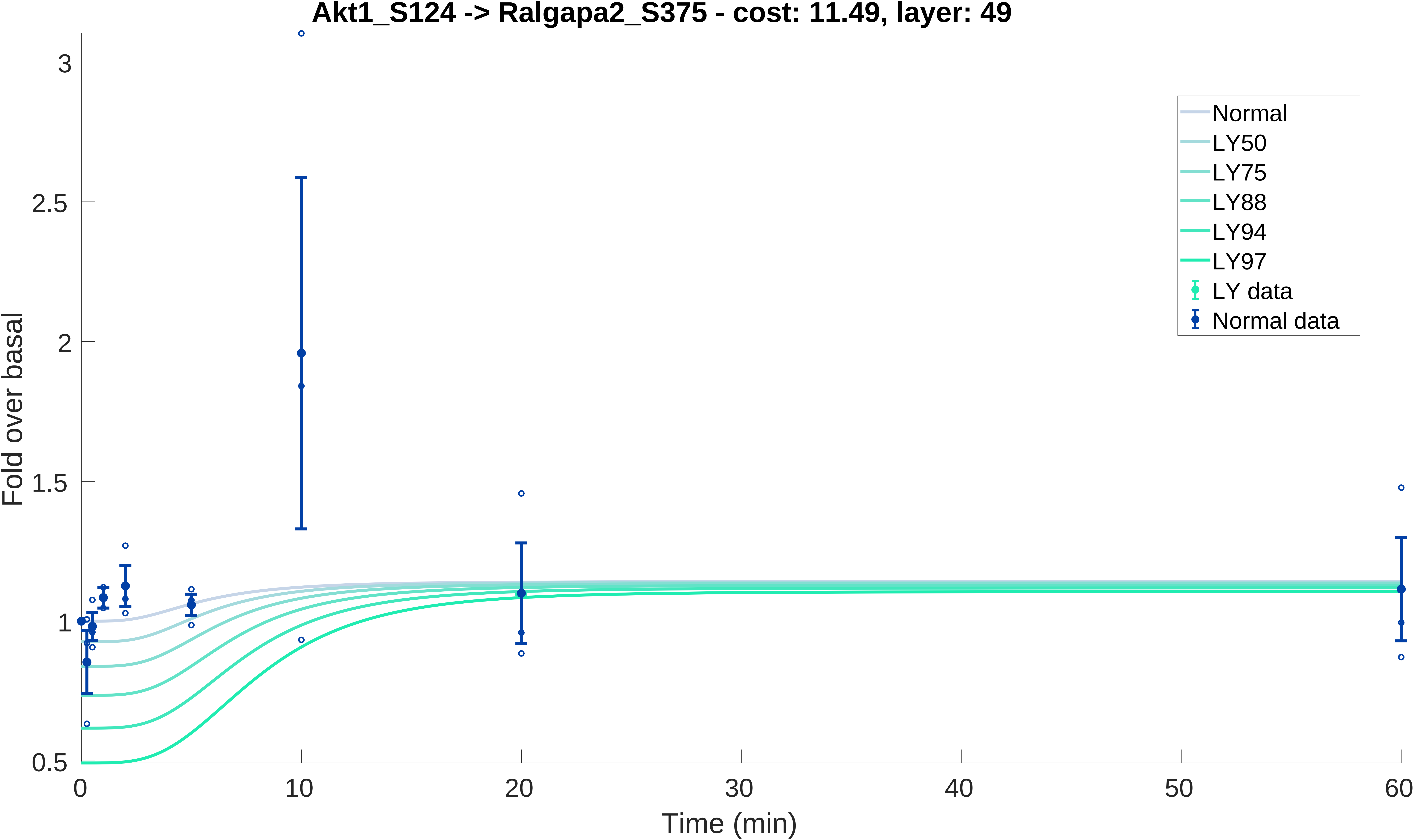

Akt1_S124 -> Ralgapa2_S375 - cost: 11.49, layer: 49
Fold over basal
Time (min)
0.5
1
1.5
2
2.5
3
0
10
20
30
40
50
60
Normal
LY50
LY75
LY88
LY94
LY97
LY data
Normal data

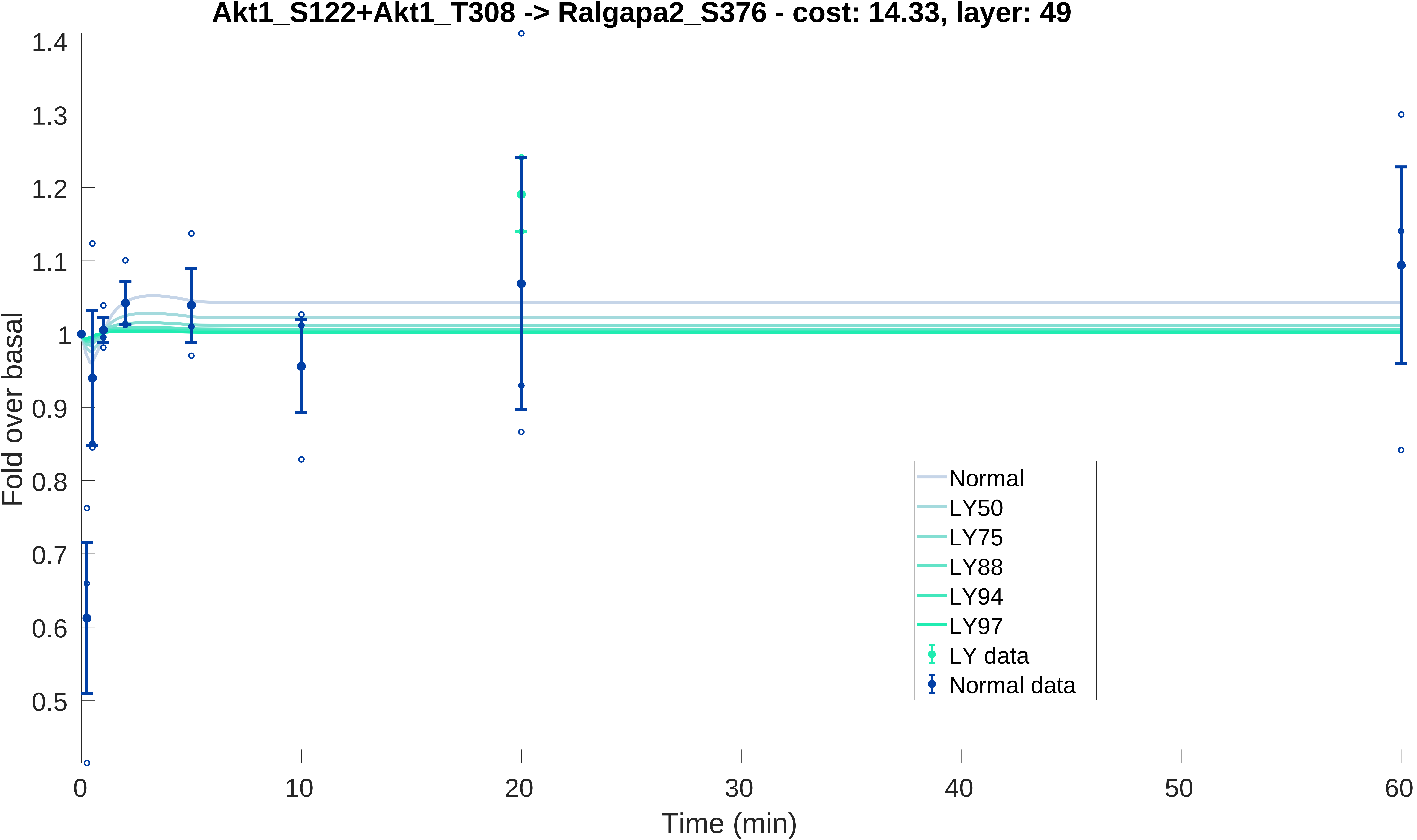
Akt1_S122+Akt1_T308 -> Ralgapa2_S376 - cost: 14.33, layer: 49
Fold over basal
Time (min)
1.4
1.3
1.2
1.1
1
0.9
0.8
0.7
0.6
0.5
0
10
20
30
40
50
60
Normal
LY50
LY75
LY88
LY94
LY97
LY data
Normal data

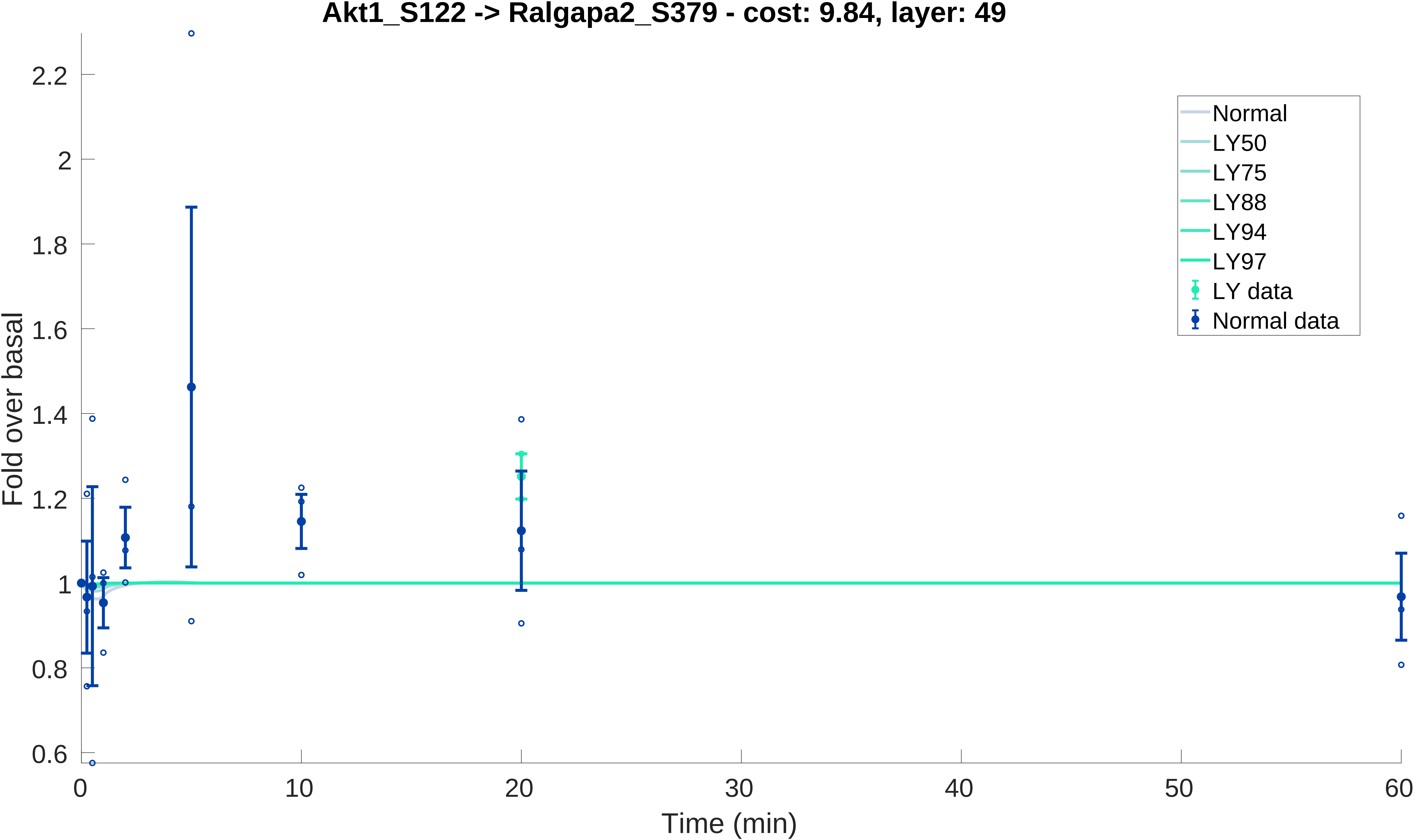
Akt1_S122 -> Ralgapa2_S379 - cost: 9.84, layer: 49
Fold over basal
Time (min)
0.6
0.8
1
1.2
1.4
1.6
1.8
2
2.2
0
10
20
30
40
50
60
Normal
LY50
LY75
LY88
LY94
LY97
LY data
Normal data

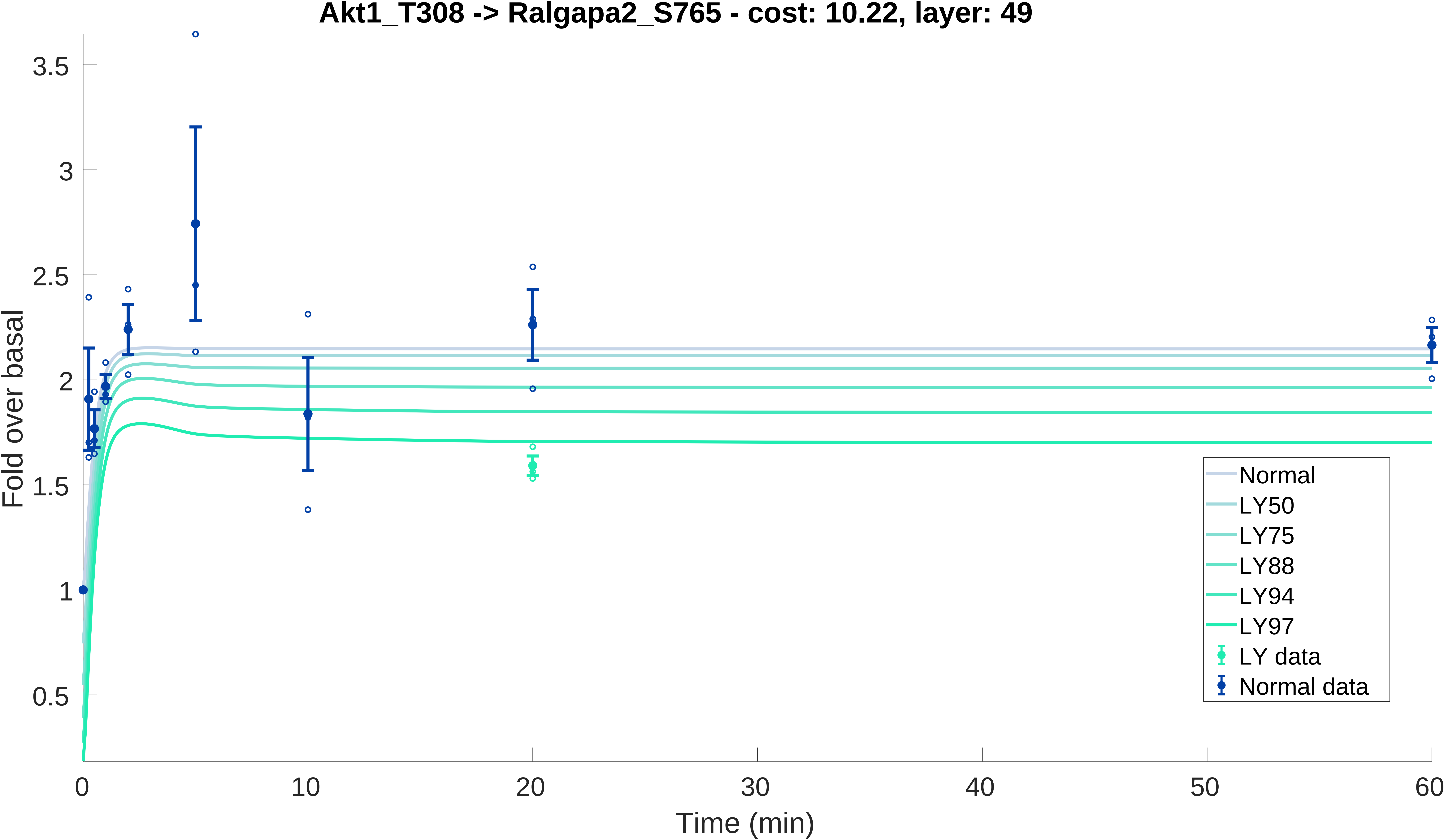
Akt1_T308 -> Ralgapa2_S765 - cost: 10.22, layer: 49
Fold over basal
Time (min)
0.5
1
1.5
2
2.5
3
3.5
0
10
20
30
40
50
60
Normal
LY50
LY75
LY88
LY94
LY97
LY data
Normal data

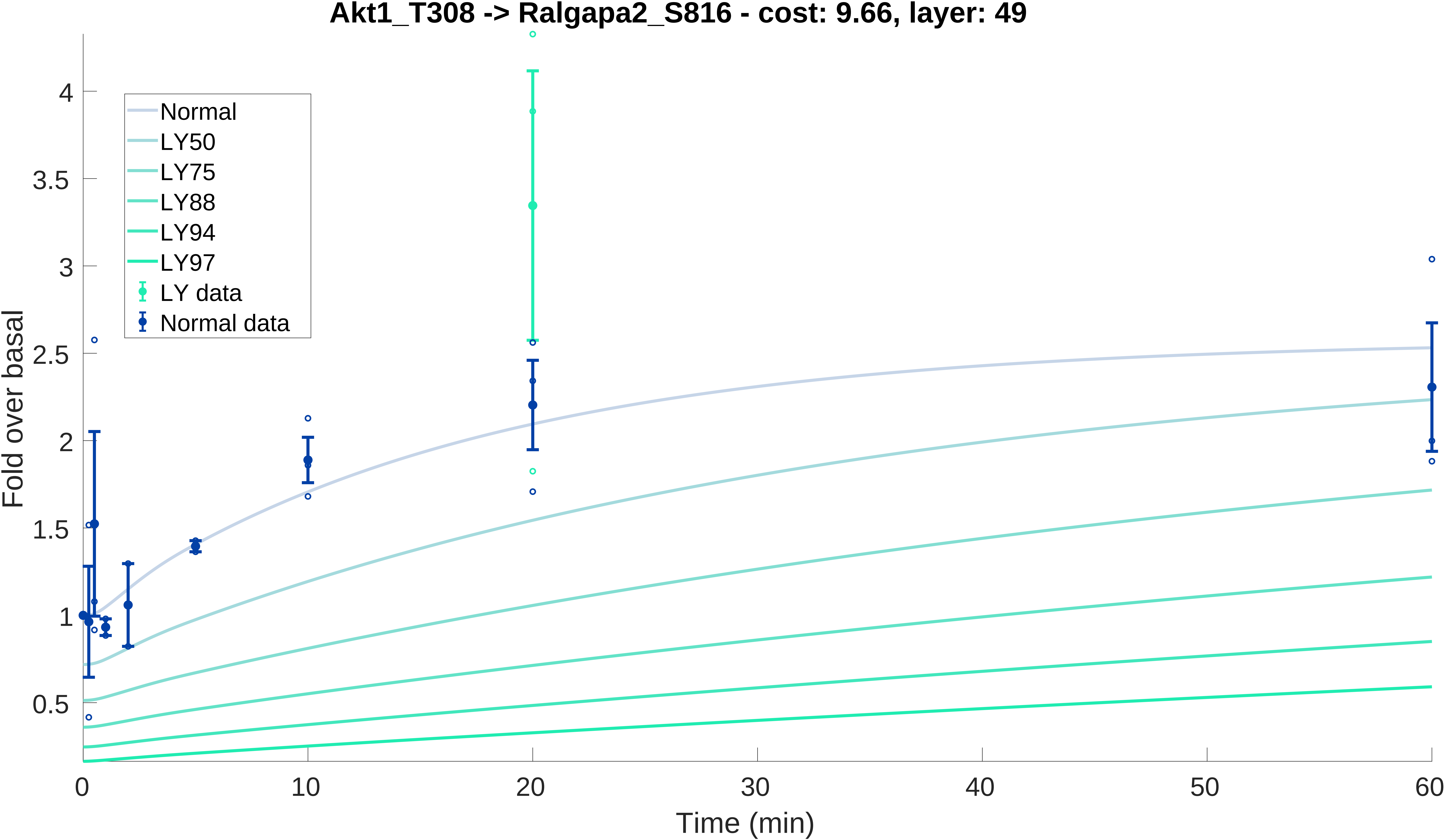

Akt1_T308 -> Ralgapa2_S816 - cost: 9.66, layer: 49
Normal
LY50
LY75
LY88
LY94
LY97
LY data
Normal data
Fold over basal
Time (min)
0
10
20
30
40
50
60
0.5
1
1.5
2
2.5
3
3.5
4

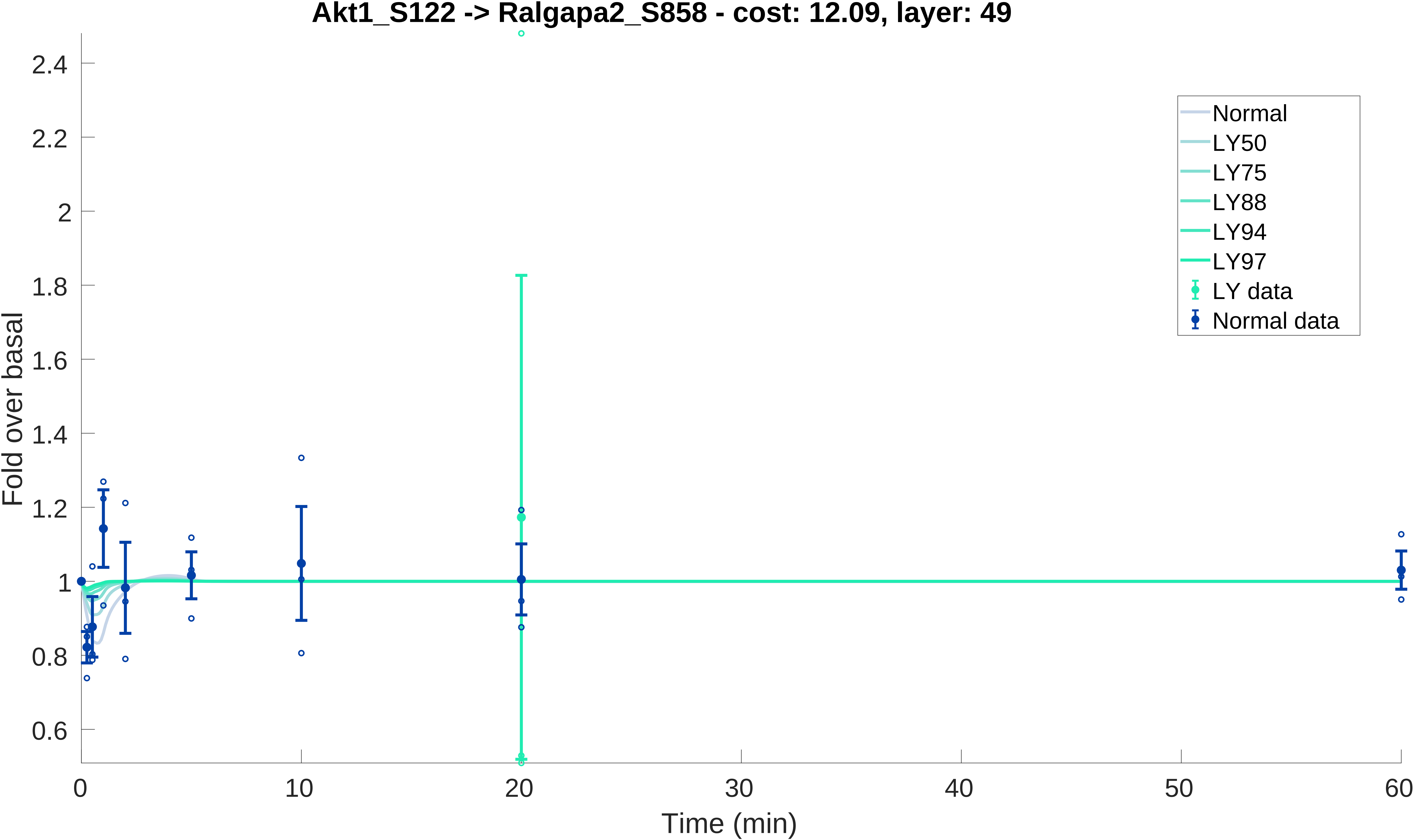

Akt1_S122 -> Ralgapa2_S858 - cost: 12.09, layer: 49
Fold over basal
Time (min)
0.6
0.8
1
1.2
1.4
1.6
1.8
2
2.2
2.4
0
10
20
30
40
50
60
Normal
LY50
LY75
LY88
LY94
LY97
LY data
Normal data

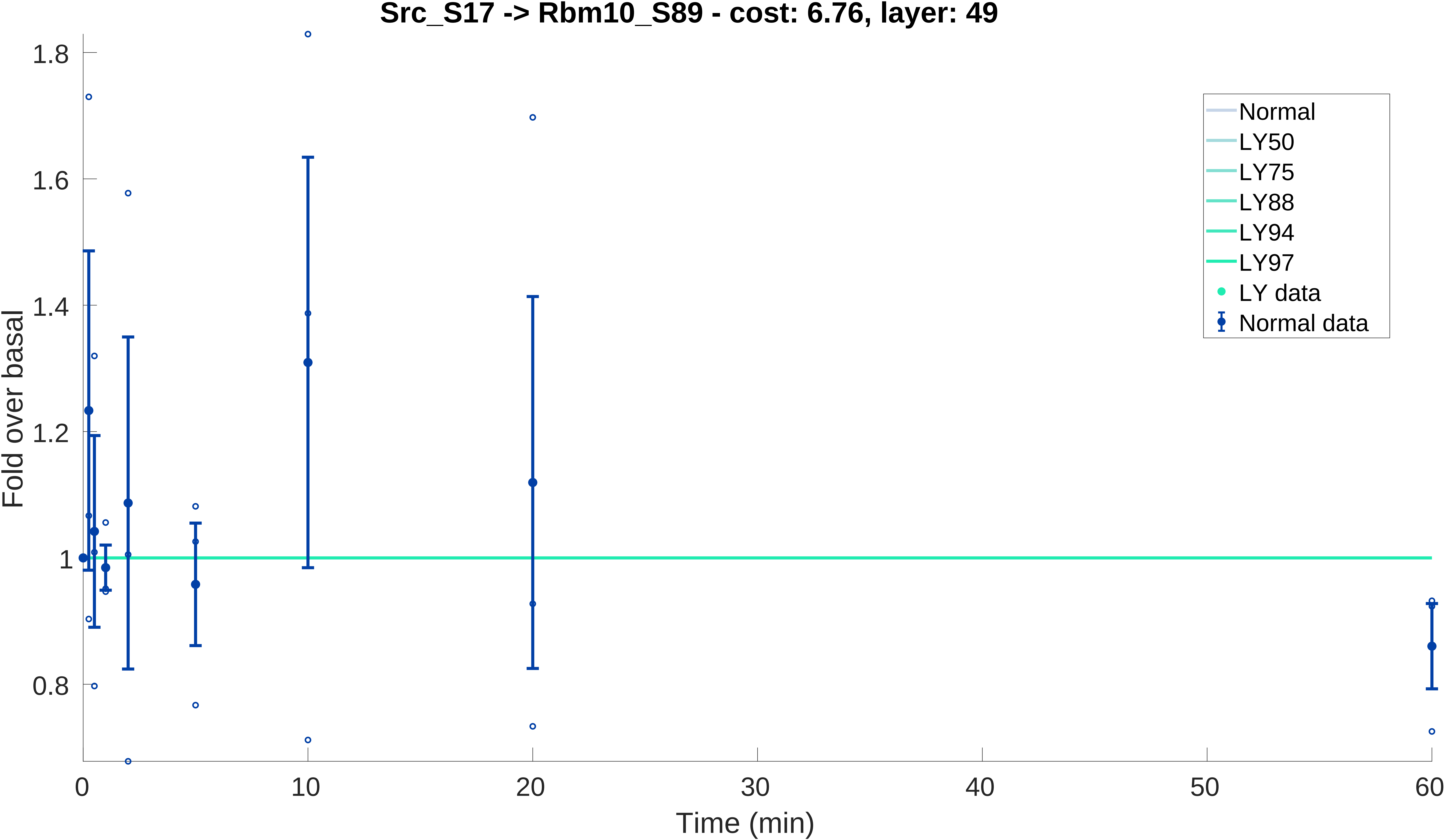

Src_S17 -> Rbm10_S89 - cost: 6.76, layer: 49
Fold over basal
1.8
1.6
1.4
1.2
1
0.8
0
10
20
30
40
50
60
Time (min)
Normal
LY50
LY75
LY88
LY94
LY97
LY data
Normal data

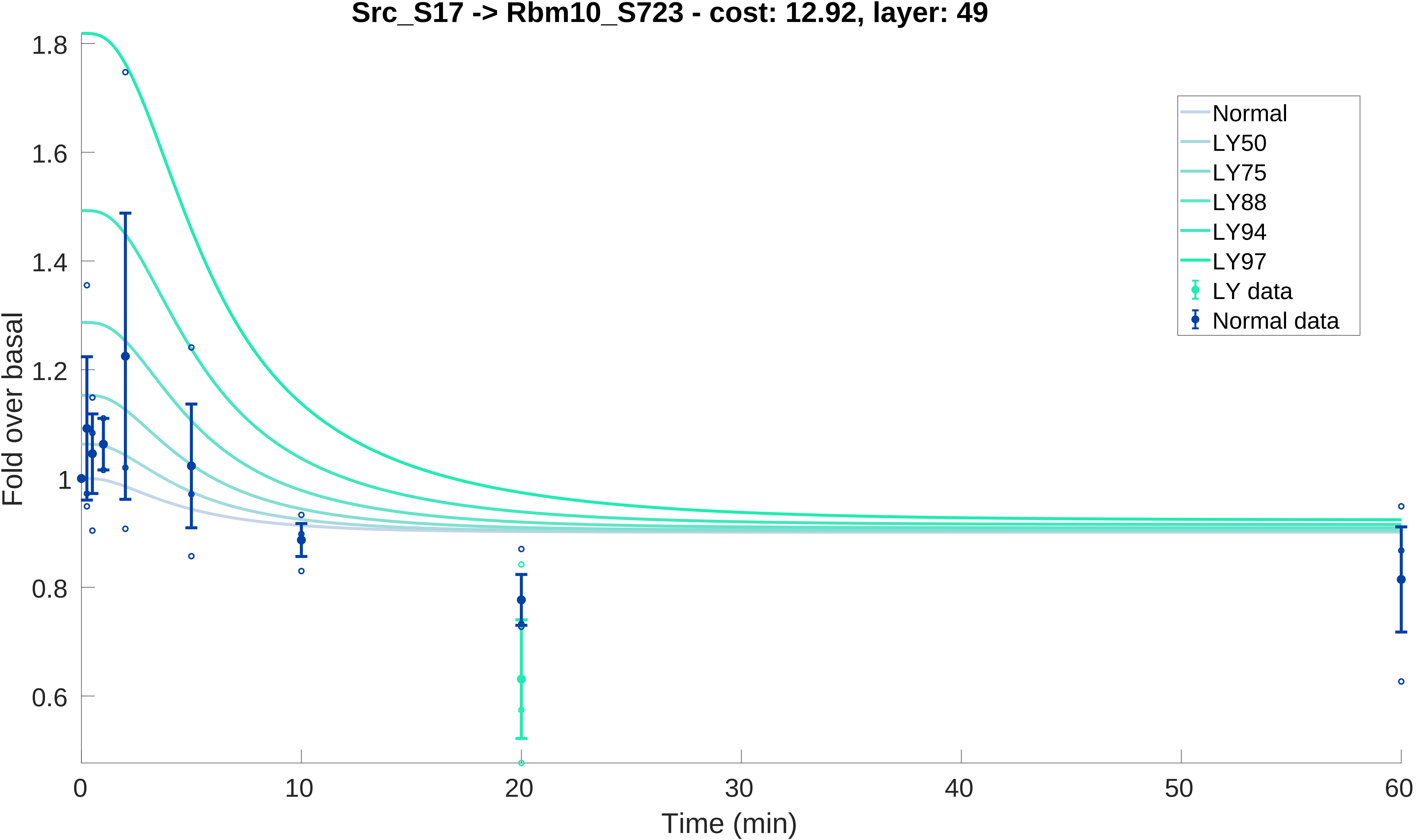
Src_S17 -> Rbm10_S723 - cost: 12.92, layer: 49
Fold over basal
Time (min)
1.8
1.6
1.4
1.2
1
0.8
0.6
0
10
20
30
40
50
60
Normal
LY50
LY75
LY88
LY94
LY97
LY data
Normal data

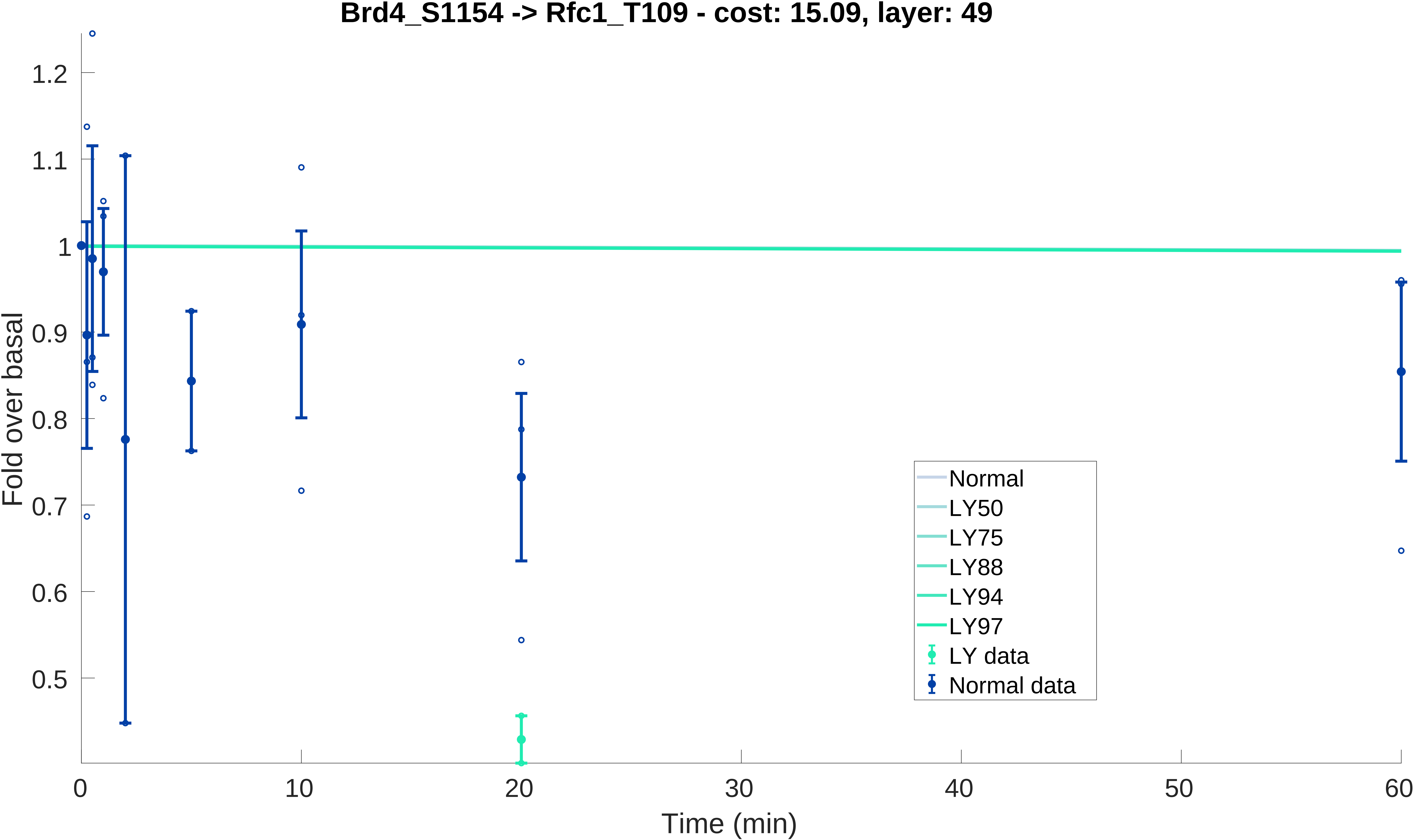

Brd4_S1154 -> Rfc1_T109 - cost: 15.09, layer: 49
Fold over basal
1.2
1.1
1
0.9
0.8
0.7
0.6
0.5
0
10
20
30
40
50
60
Time (min)
Normal
LY50
LY75
LY88
LY94
LY97
LY data
Normal data

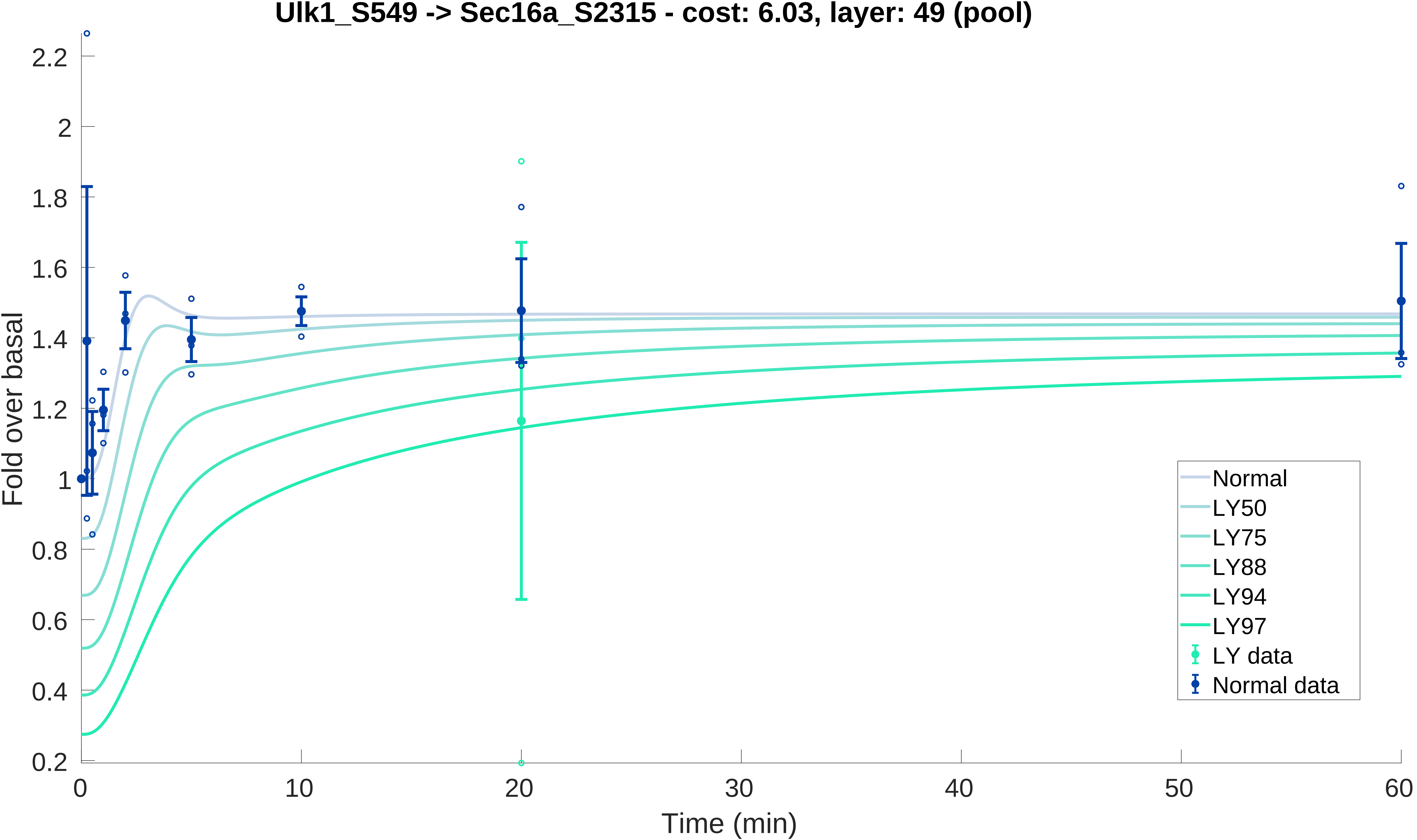

Ulk1_S549 -> Sec16a_S2315 - cost: 6.03, layer: 49 (pool)
Fold over basal
Time (min)
0.2
0.4
0.6
0.8
1
1.2
1.4
1.6
1.8
2
2.2
0
10
20
30
40
50
60
Normal
LY50
LY75
LY88
LY94
LY97
LY data
Normal data

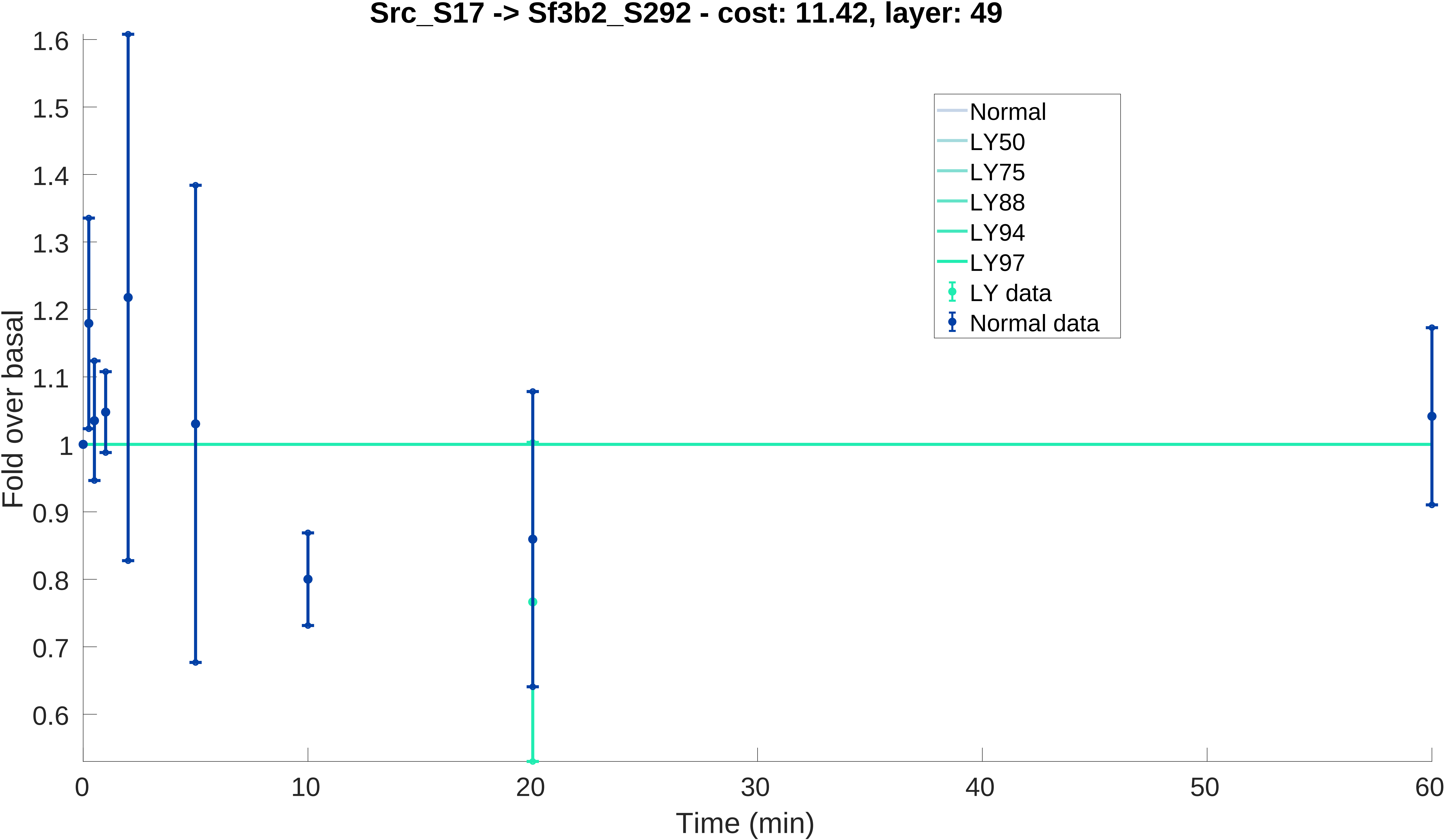

Src_S17 -> Sf3b2_S292 - cost: 11.42, layer: 49
Fold over basal
Time (min)
1.6
1.5
1.4
1.3
1.2
1.1
1
0.9
0.8
0.7
0.6
0
10
20
30
40
50
60
Normal
LY50
LY75
LY88
LY94
LY97
LY data
Normal data

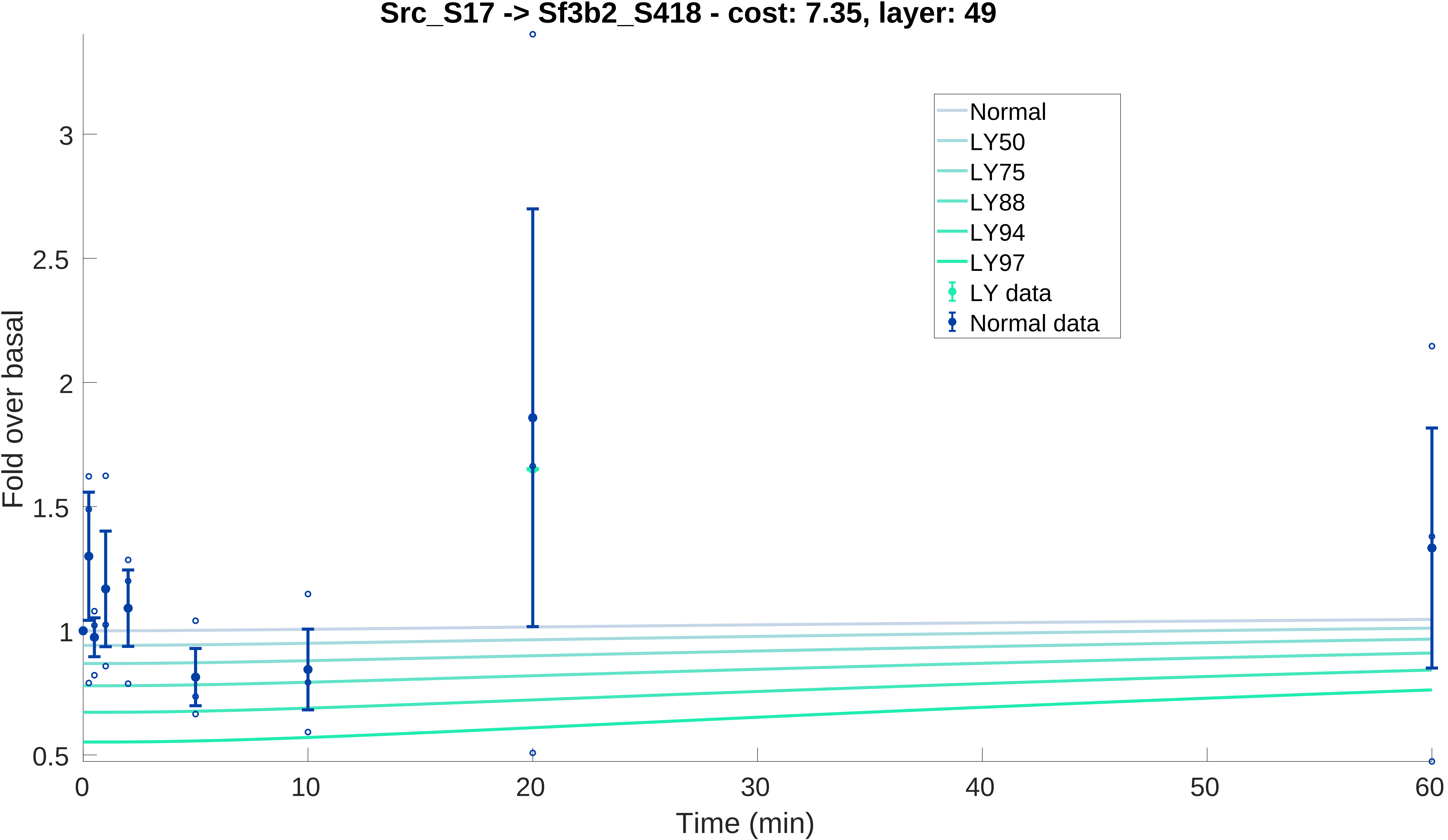

Src_S17 -> Sf3b2_S418 - cost: 7.35, layer: 49
Fold over basal
0.5
1
1.5
2
2.5
3
0
10
20
30
40
50
60
Time (min)
Normal
LY50
LY75
LY88
LY94
LY97
LY data
Normal data

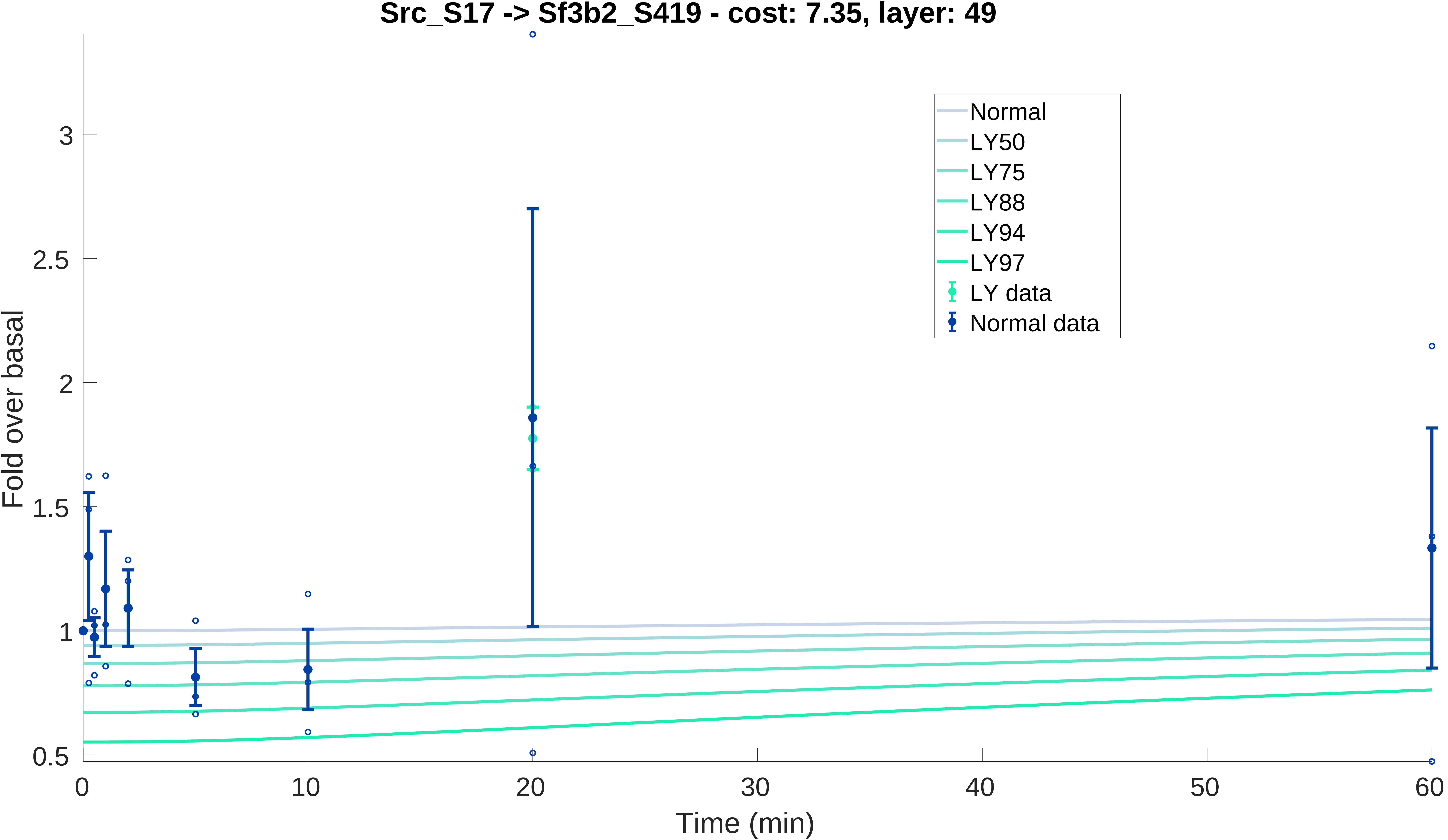

Src_S17 -> Sf3b2_S419 - cost: 7.35, layer: 49
Normal
LY50
LY75
LY88
LY94
LY97
LY data
Normal data
Fold over basal
Time (min)
0.5
1
1.5
2
2.5
3
0
10
20
30
40
50
60

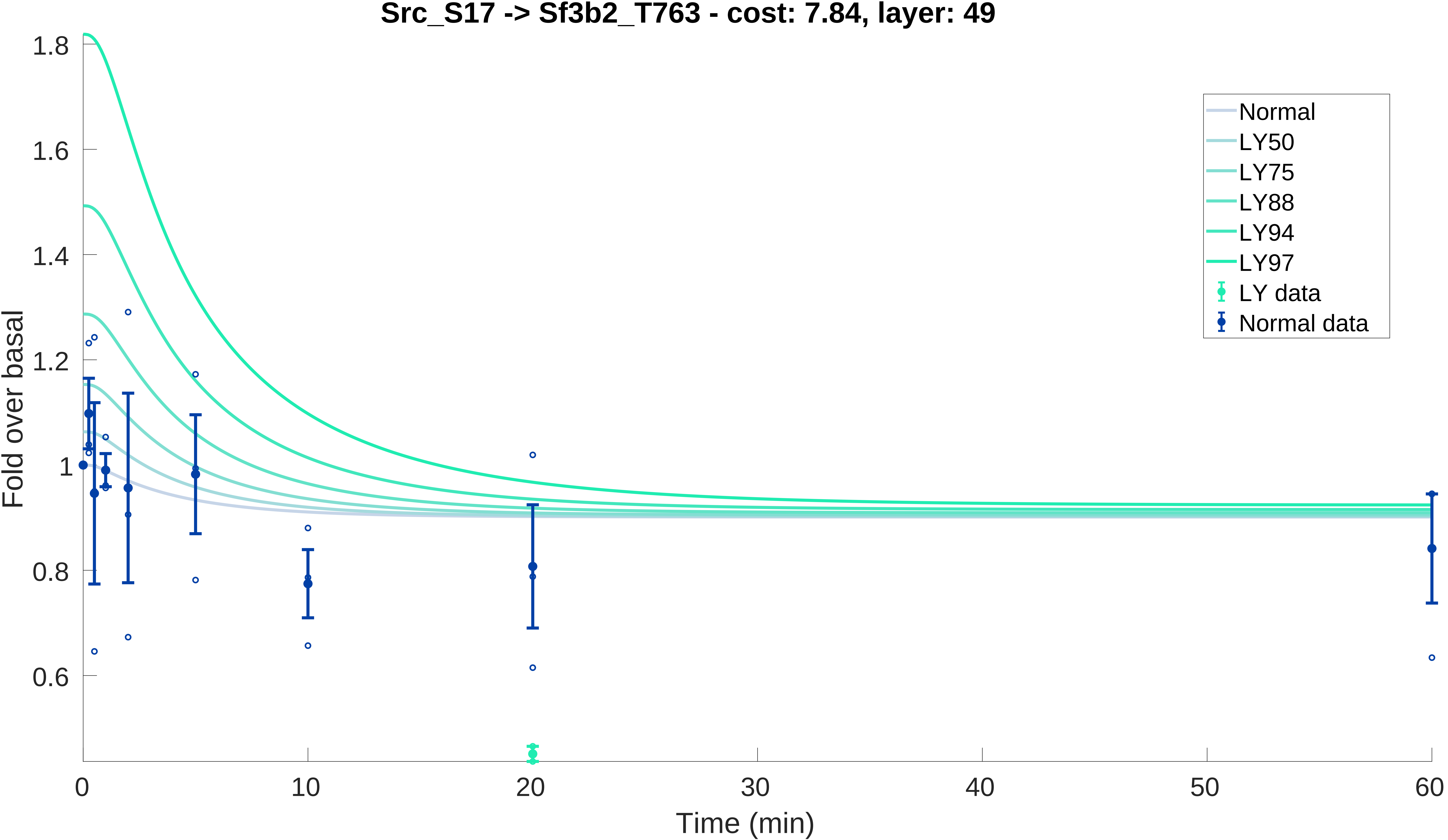

Src_S17 -> Sf3b2_T763 - cost: 7.84, layer: 49
1.8
1.6
1.4
1.2
1
0.8
0.6
Fold over basal
0
10
20
30
40
50
60
Time (min)
Normal
LY50
LY75
LY88
LY94
LY97
LY data
Normal data

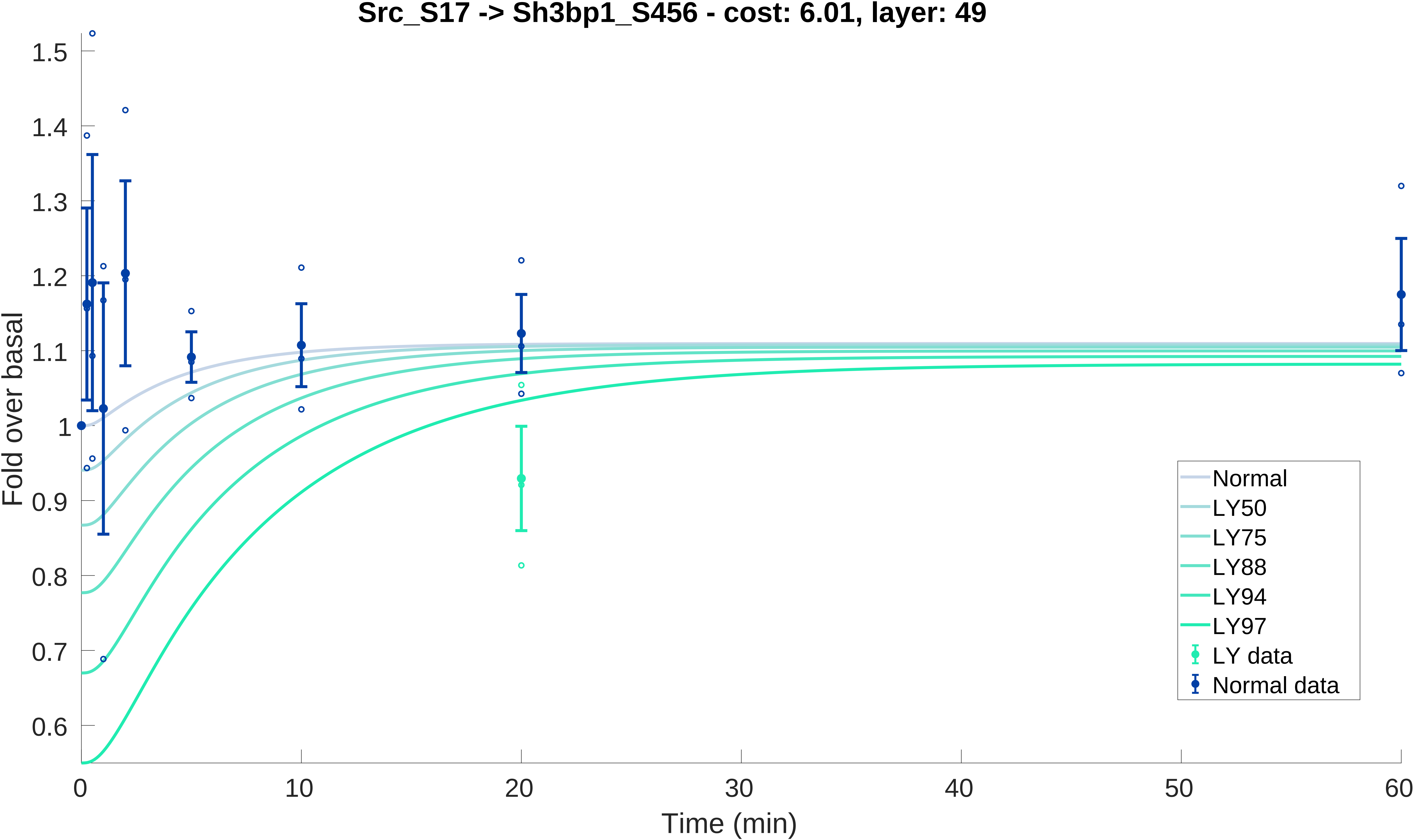

Src_S17 -> Sh3bp1_S456 - cost: 6.01, layer: 49
Fold over basal
Time (min)
0
10
20
30
40
50
60
0.6
0.7
0.8
0.9
1
1.1
1.2
1.3
1.4
1.5
Normal
LY50
LY75
LY88
LY94
LY97
LY data
Normal data

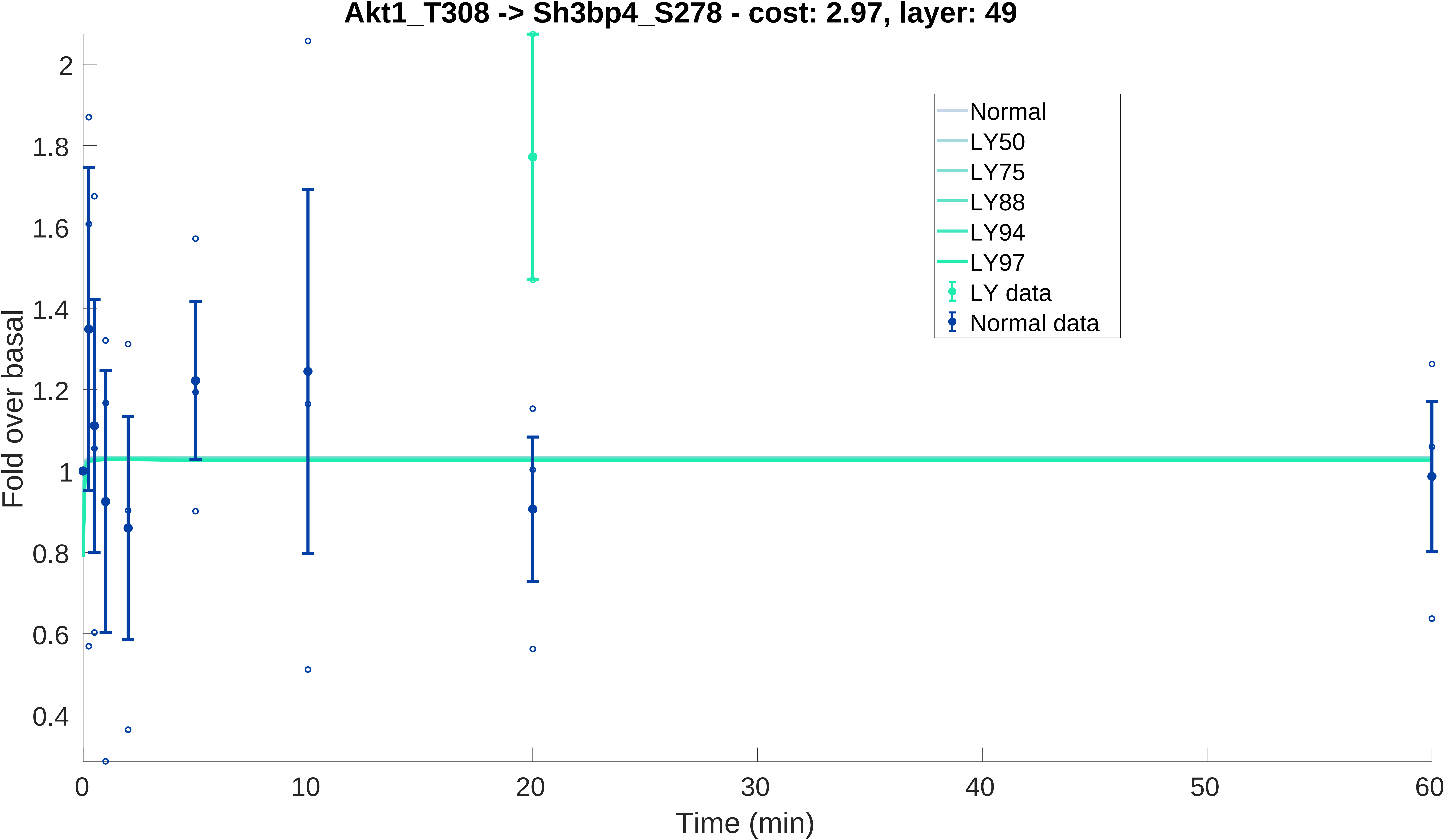
Akt1_T308 -> Sh3bp4_S278 - cost: 2.97, layer: 49
Fold over basal
Time (min)
0.4
0.6
0.8
1
1.2
1.4
1.6
1.8
2
0
10
20
30
40
50
60
Normal
LY50
LY75
LY88
LY94
LY97
LY data
Normal data

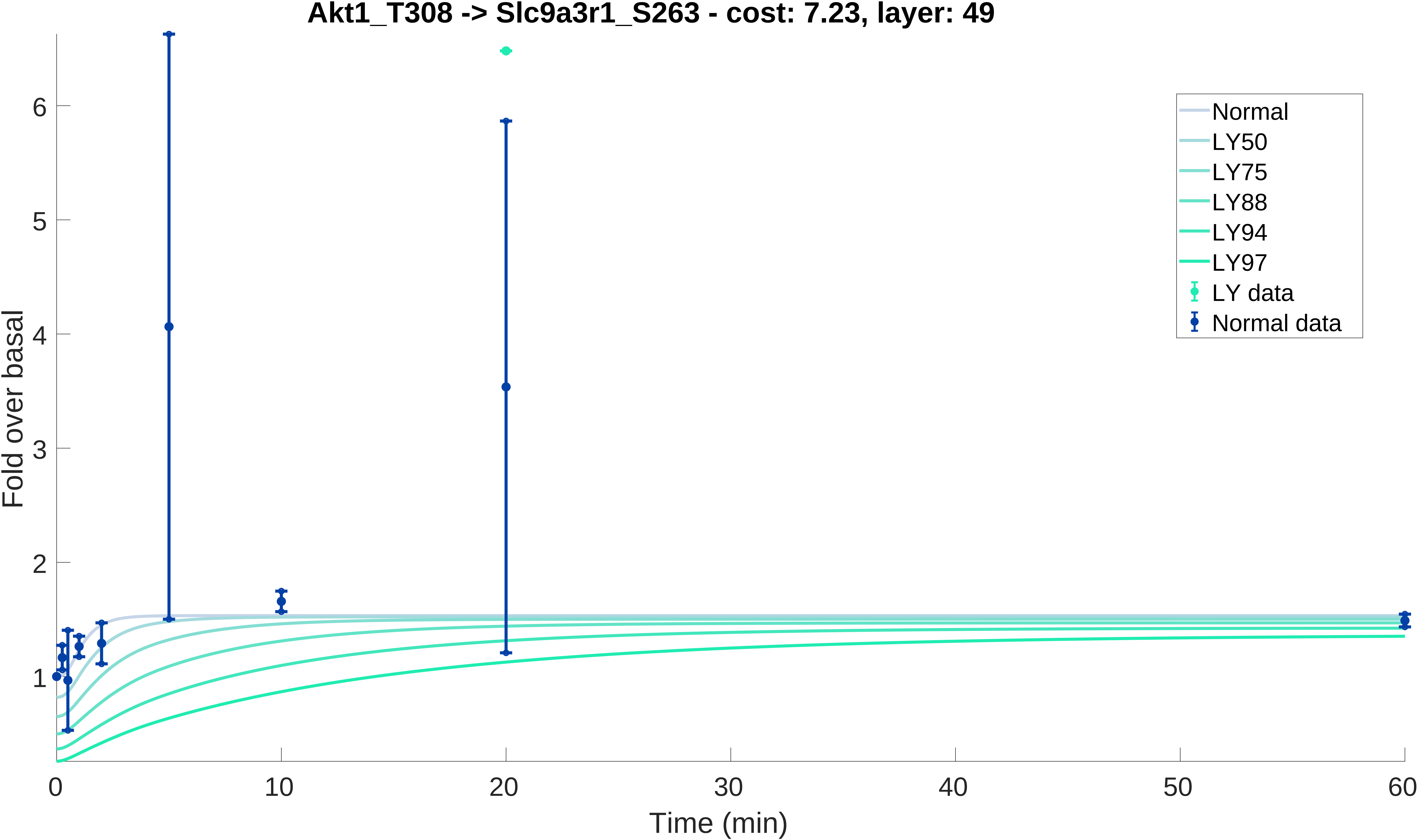

Akt1_T308 -> Slc9a3r1_S263 - cost: 7.23, layer: 49
Fold over basal
Time (min)
1
2
3
4
5
6
0
10
20
30
40
50
60
Normal
LY50
LY75
LY88
LY94
LY97
LY data
Normal data

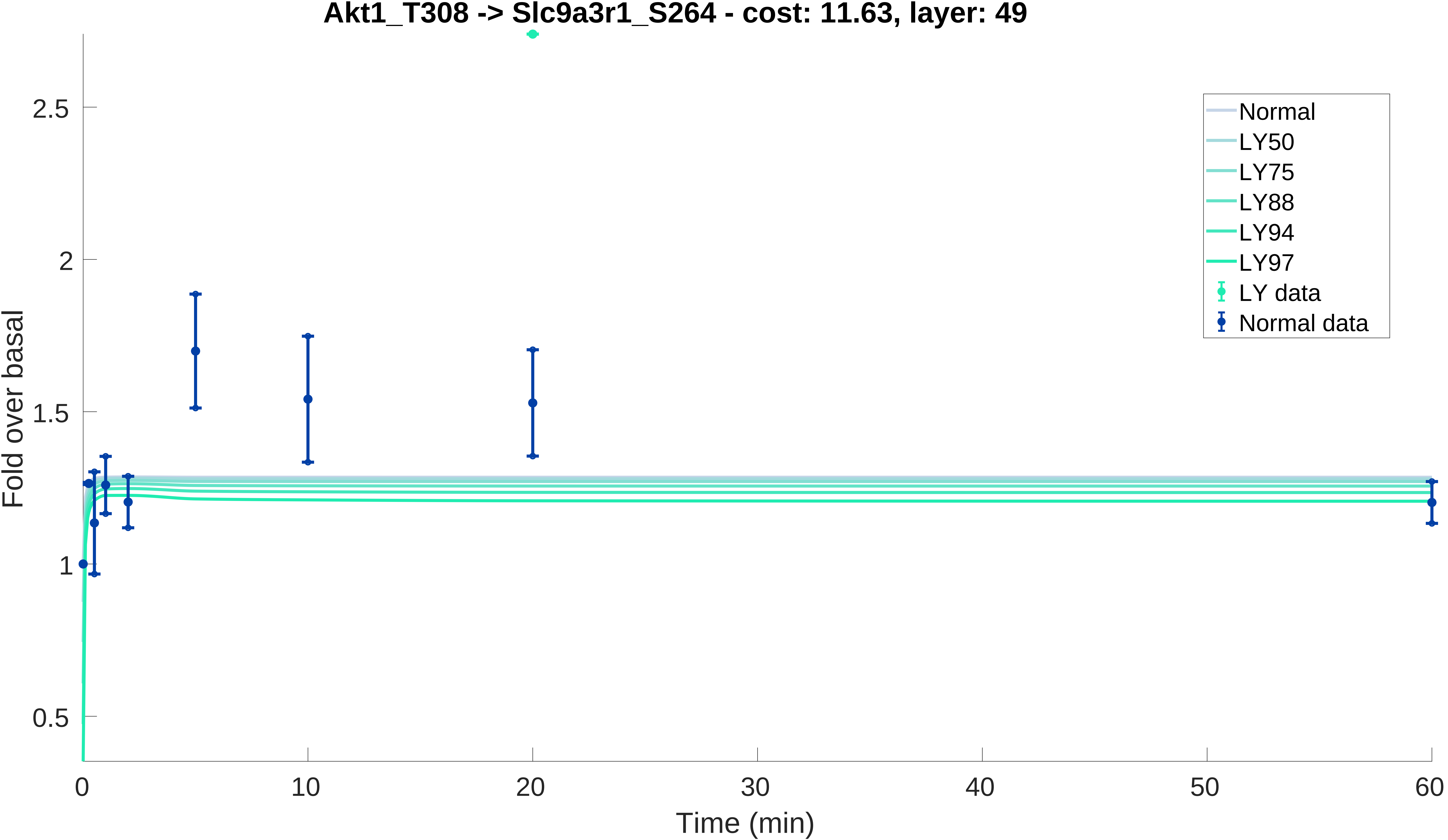

Akt1_T308 -> Slc9a3r1_S264 - cost: 11.63, layer: 49
Fold over basal
Time (min)
0.5
1
1.5
2
2.5
0
10
20
30
40
50
60
Normal
LY50
LY75
LY88
LY94
LY97
LY data
Normal data

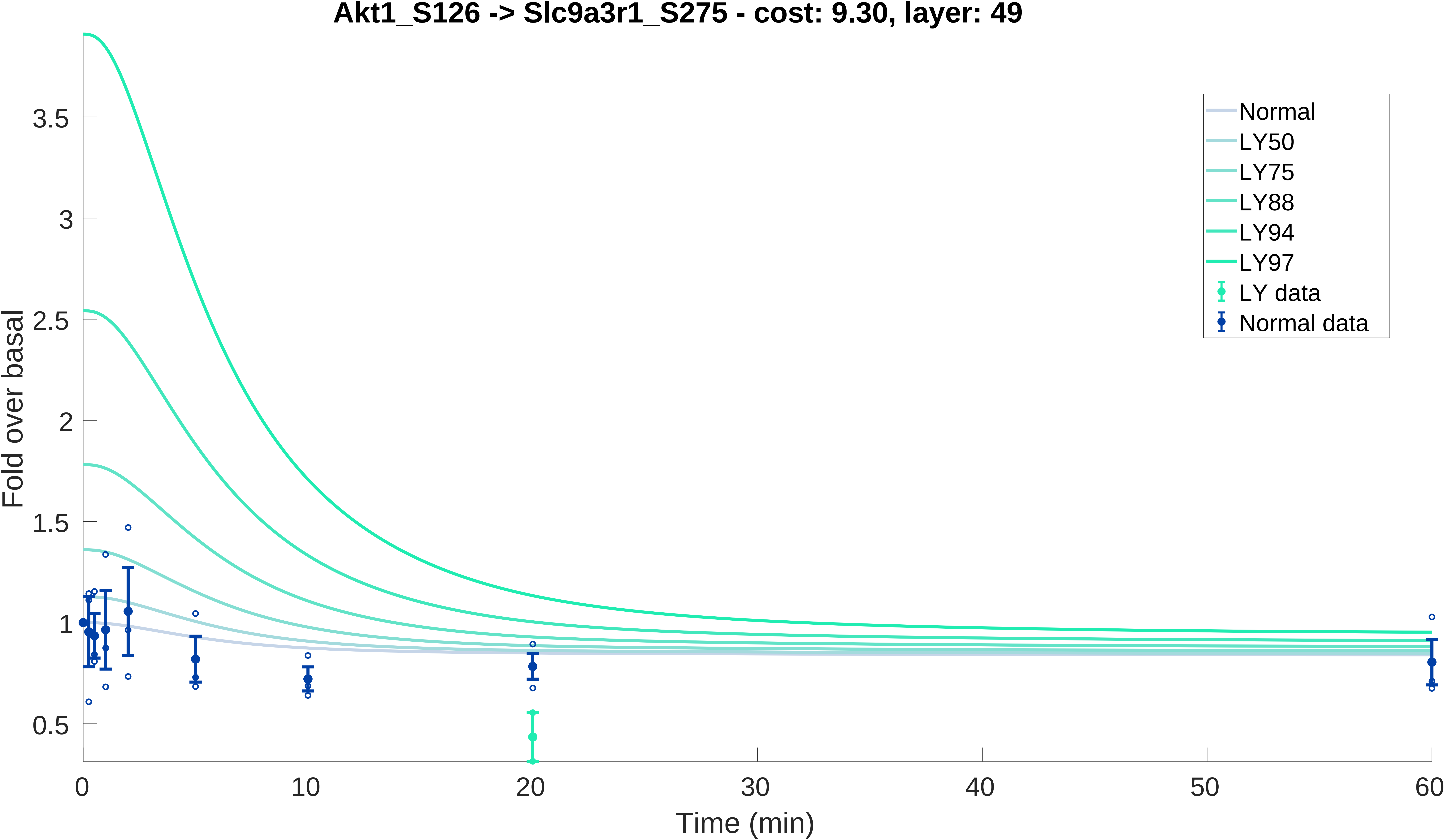
Akt1_S126 -> Slc9a3r1_S275 - cost: 9.30, layer: 49
Fold over basal
Time (min)
0.5
1
1.5
2
2.5
3
3.5
0
10
20
30
40
50
60
Normal
LY50
LY75
LY88
LY94
LY97
LY data
Normal data

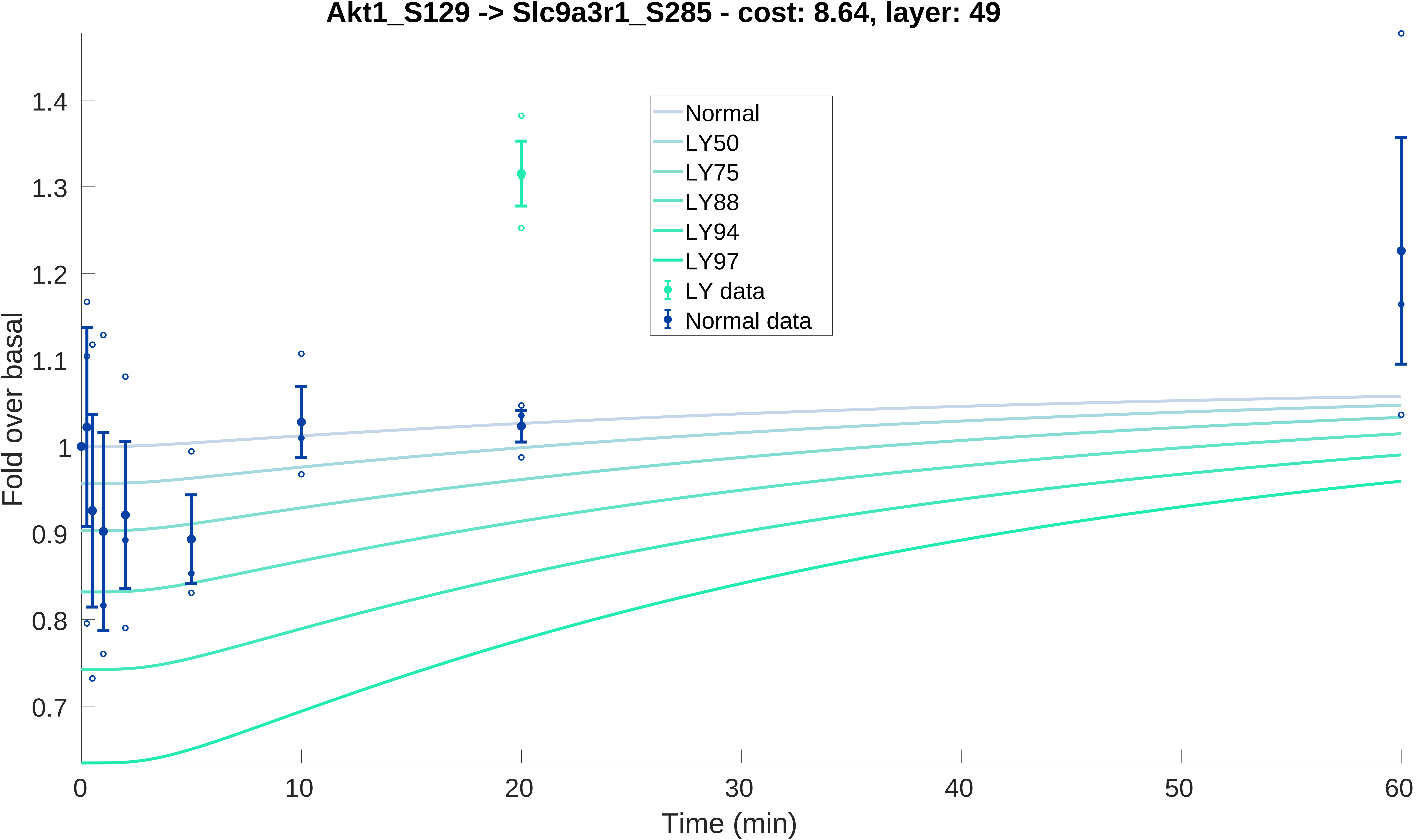

Akt1_S129 -> Slc9a3r1_S285 - cost: 8.64, layer: 49
Fold over basal
Time (min)
1.4
1.3
1.2
1.1
1
0.9
0.8
0.7
0
10
20
30
40
50
60
Normal
LY50
LY75
LY88
LY94
LY97
LY data
Normal data

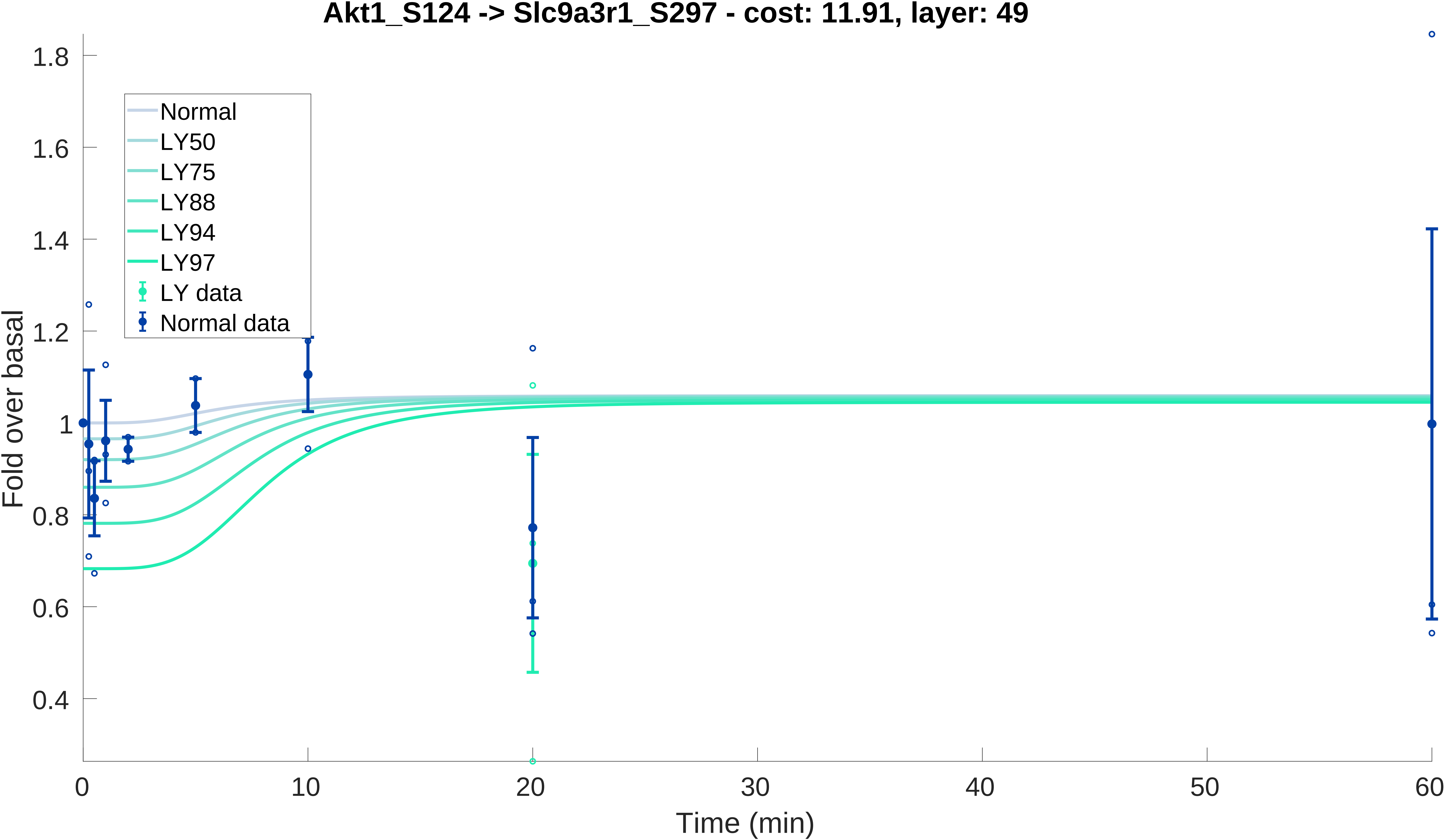

Akt1_S124 -> Slc9a3r1_S297 - cost: 11.91, layer: 49
Fold over basal
Time (min)
Normal
LY50
LY75
LY88
LY94
LY97
LY data
Normal data
1.8
1.6
1.4
1.2
1
0.8
0.6
0.4
0
10
20
30
40
50
60

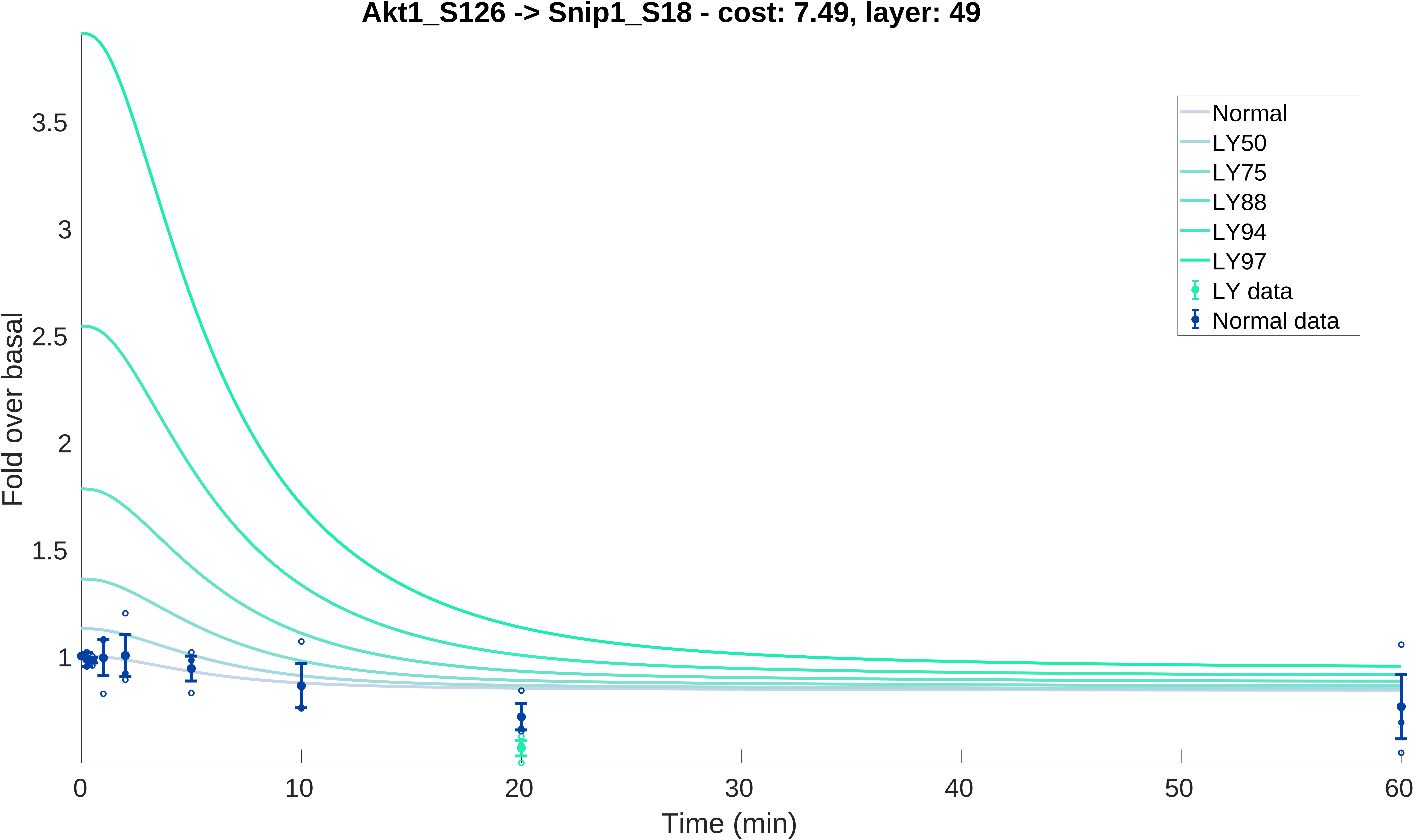

Akt1_S126 -> Snip1_S18 - cost: 7.49, layer: 49
Fold over basal
3.5
3
2.5
2
1.5
1
0
10
20
30
40
50
60
Time (min)
Normal
LY50
LY75
LY88
LY94
LY97
LY data
Normal data

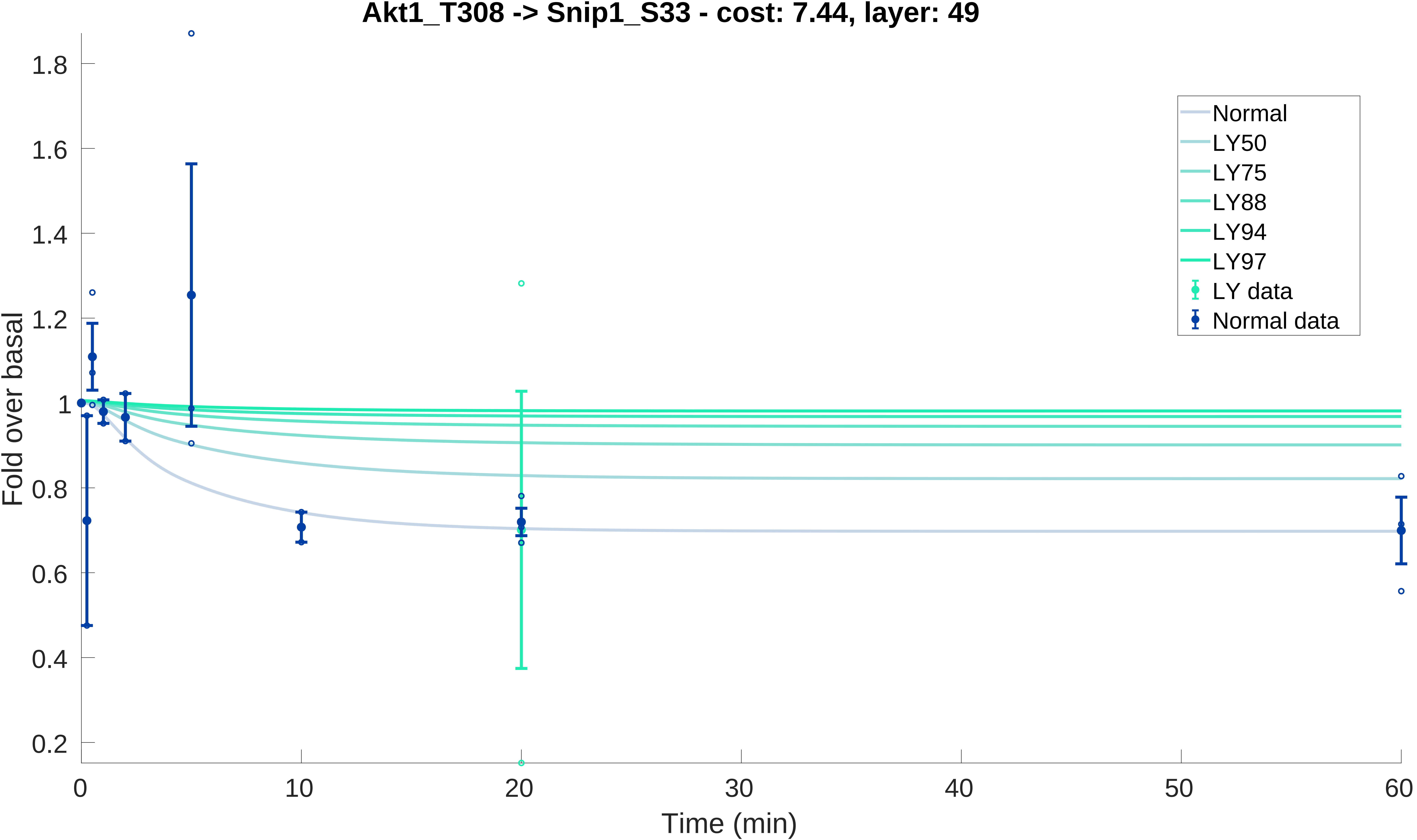

Akt1_T308 -> Snip1_S33 - cost: 7.44, layer: 49
Fold over basal
1.8
1.6
1.4
1.2
1
0.8
0.6
0.4
0.2
0
10
20
30
40
50
60
Time (min)
Normal
LY50
LY75
LY88
LY94
LY97
LY data
Normal data

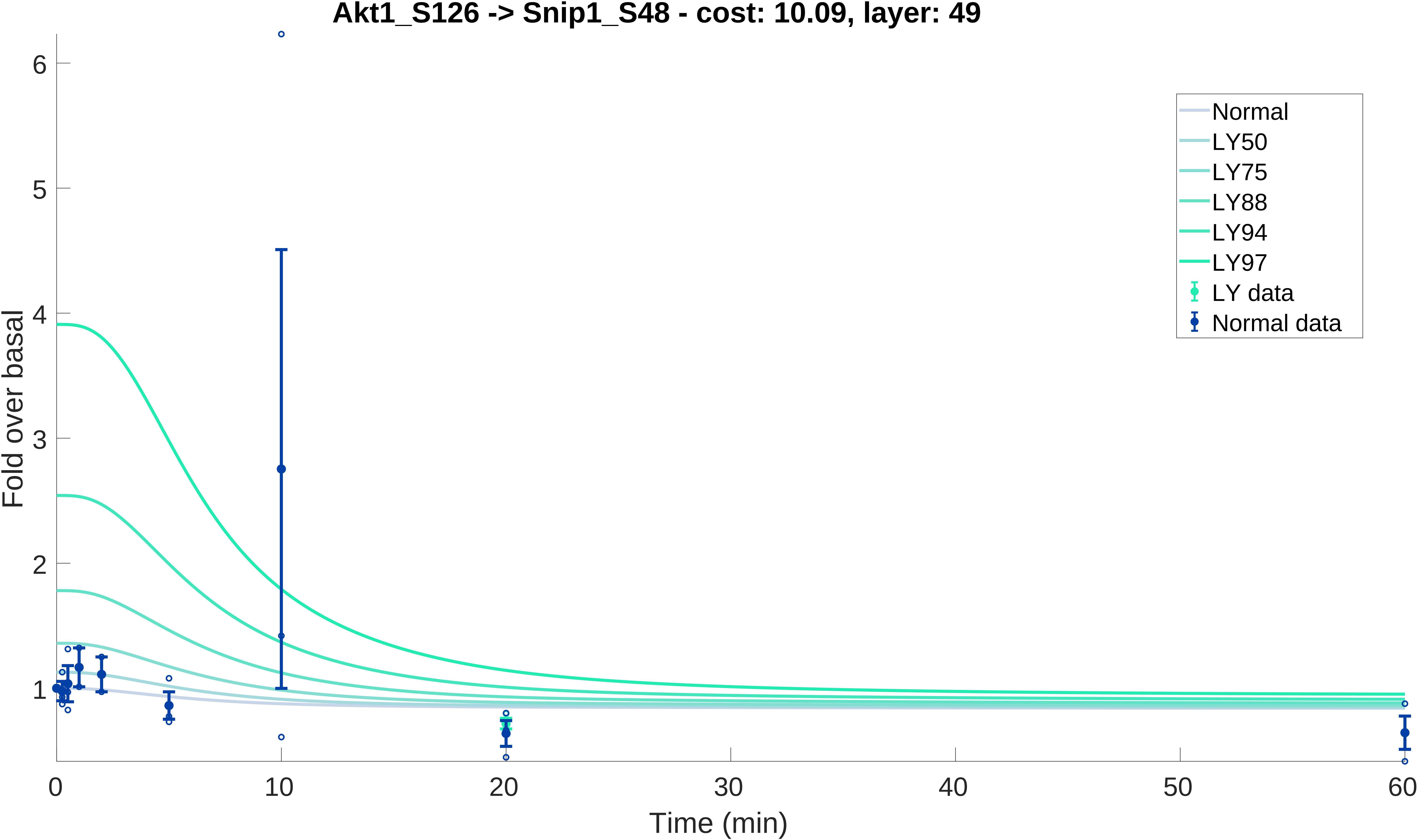
Akt1_S126 -> Snip1_S48 - cost: 10.09, layer: 49
Fold over basal
Time (min)
0
10
20
30
40
50
60
1
2
3
4
5
6
Normal
LY50
LY75
LY88
LY94
LY97
LY data
Normal data

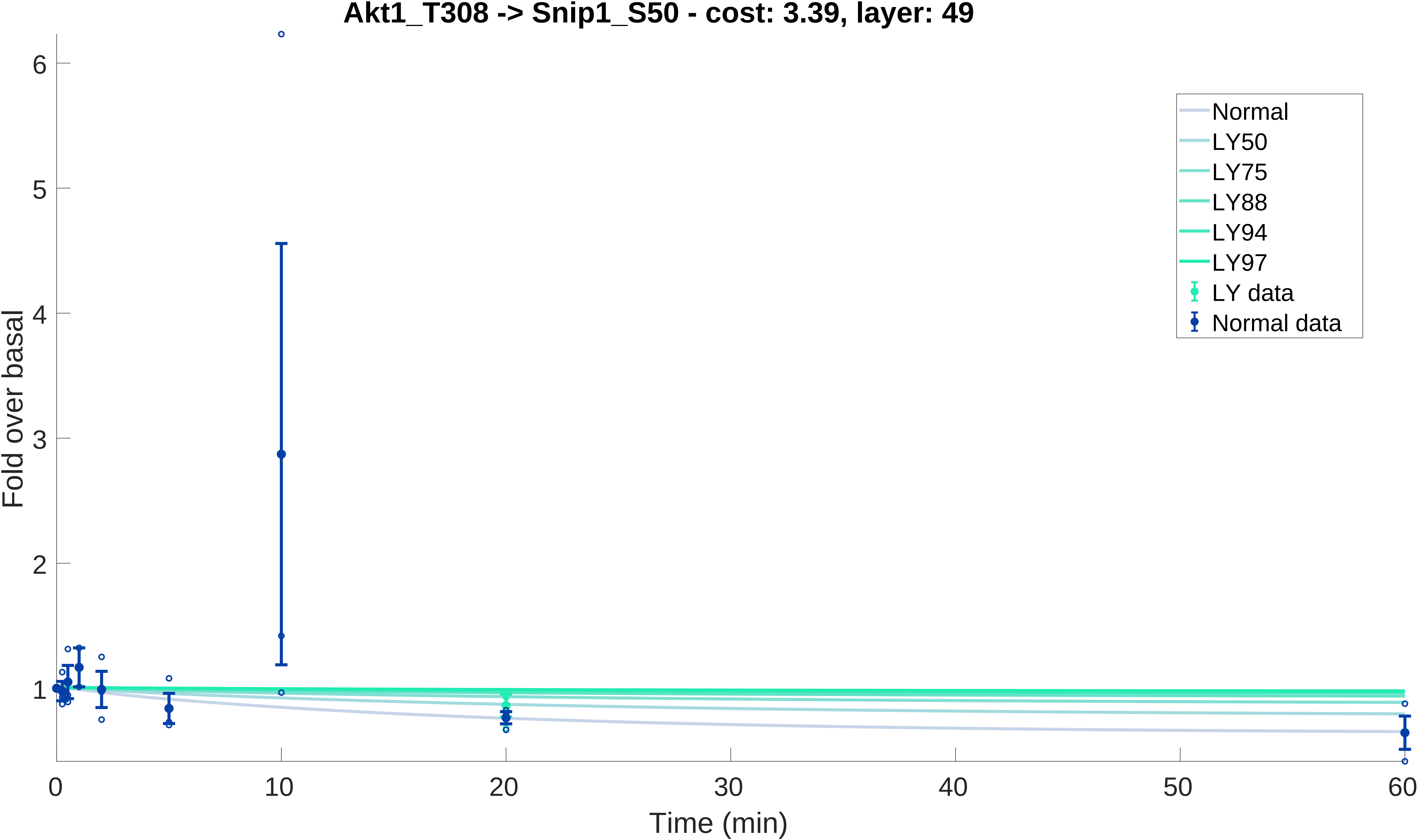

Akt1_T308 -> Snip1_S50 - cost: 3.39, layer: 49
Fold over basal
Time (min)
0
10
20
30
40
50
60
1
2
3
4
5
6
Normal
LY50
LY75
LY88
LY94
LY97
LY data
Normal data

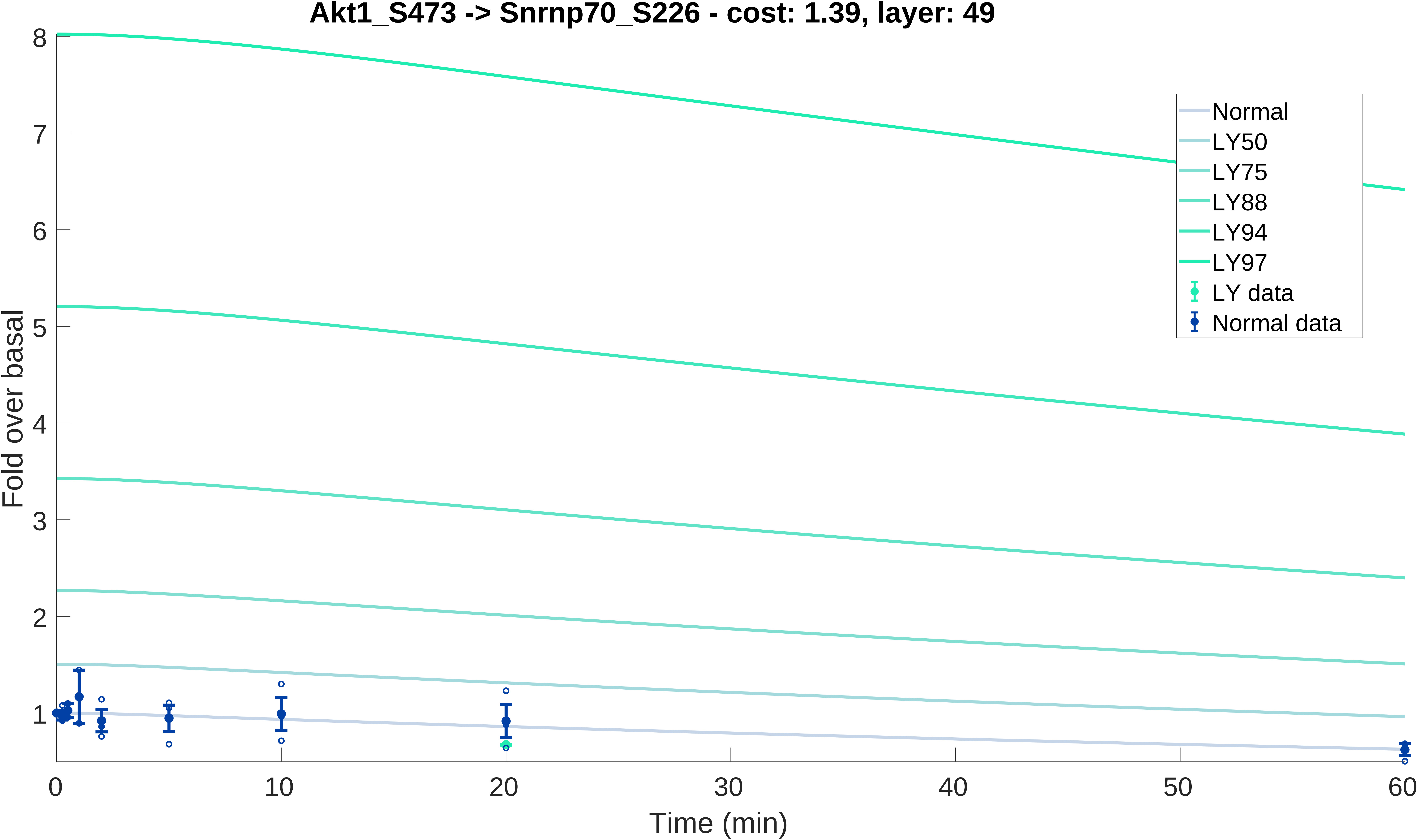

Akt1_S473 -> Snrnp70_S226 - cost: 1.39, layer: 49
Fold over basal
Time (min)
Normal
LY50
LY75
LY88
LY94
LY97
LY data
Normal data
0
10
20
30
40
50
60
1
2
3
4
5
6
7
8

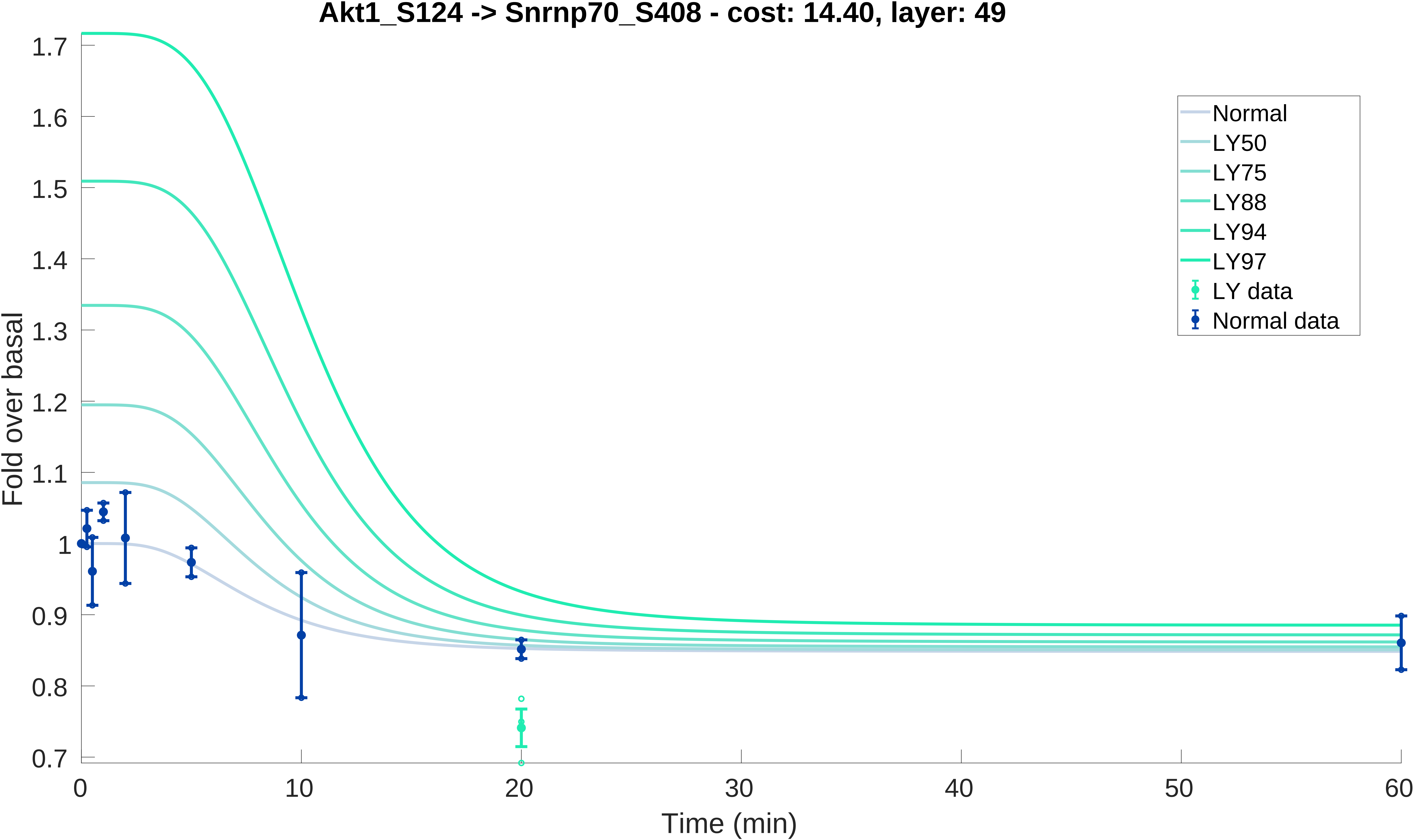

Akt1_S124 -> Snrnp70_S408 - cost: 14.40, layer: 49
Fold over basal
Time (min)
0
10
20
30
40
50
60
0.7
0.8
0.9
1
1.1
1.2
1.3
1.4
1.5
1.6
1.7
Normal
LY50
LY75
LY88
LY94
LY97
LY data
Normal data

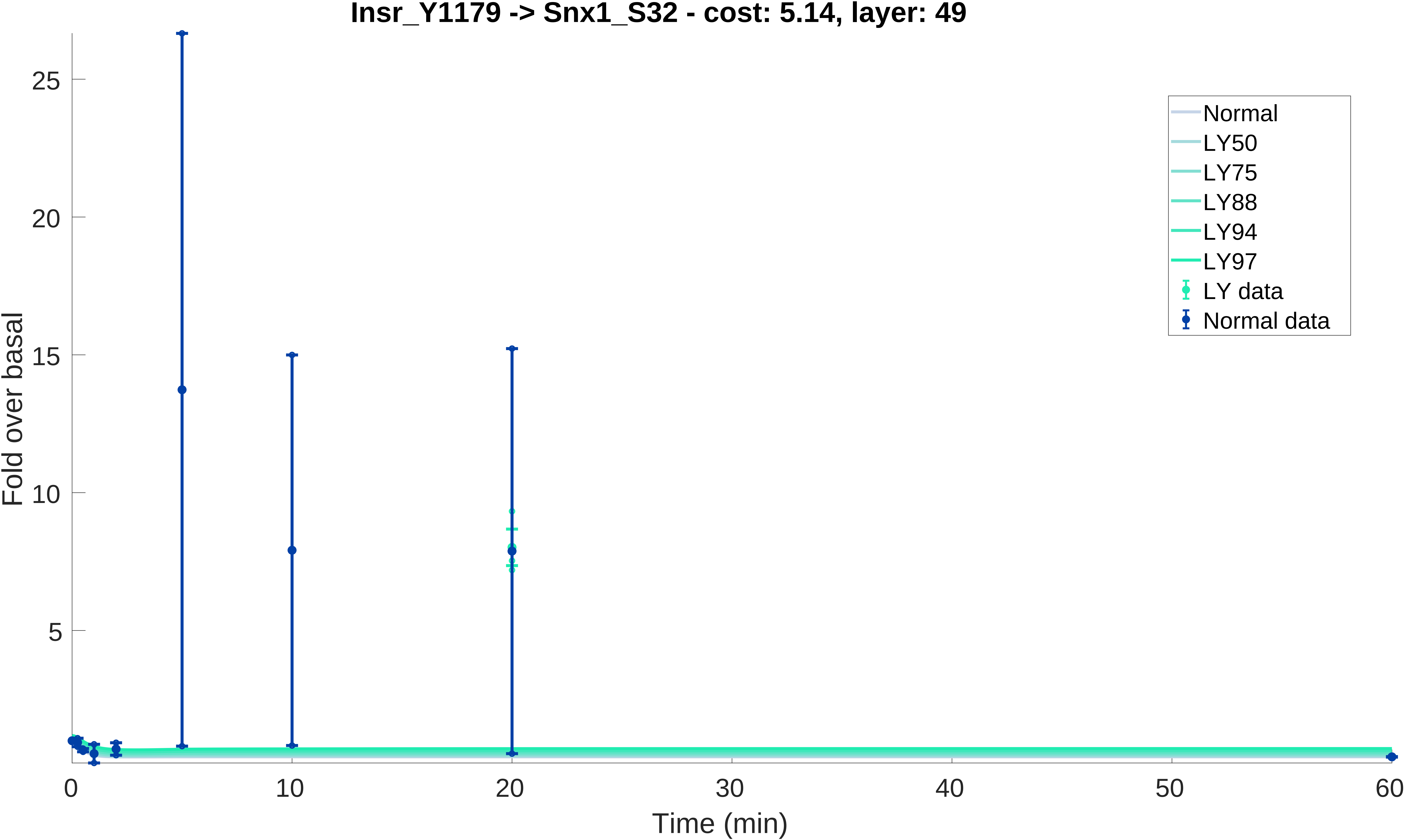

Insr_Y1179 -> Snx1_S32 - cost: 5.14, layer: 49
Fold over basal
Time (min)
0
10
20
30
40
50
60
5
10
15
20
25
Normal
LY50
LY75
LY88
LY94
LY97
LY data
Normal data

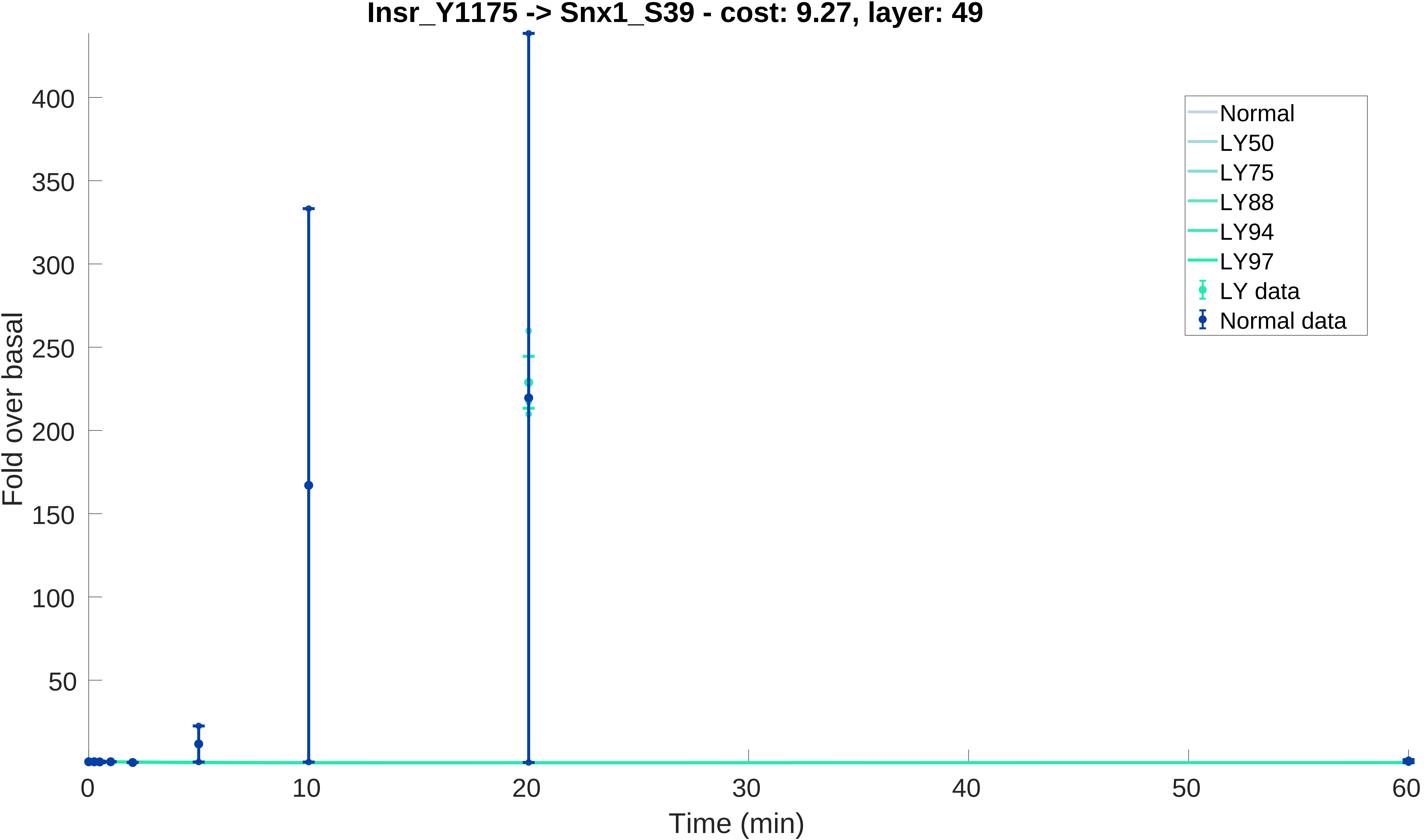

Insr_Y1175 -> Snx1_S39 - cost: 9.27, layer: 49
Fold over basal
Time (min)
400
350
300
250
200
150
100
50
0
10
20
30
40
50
60
Normal
LY50
LY75
LY88
LY94
LY97
LY data
Normal data

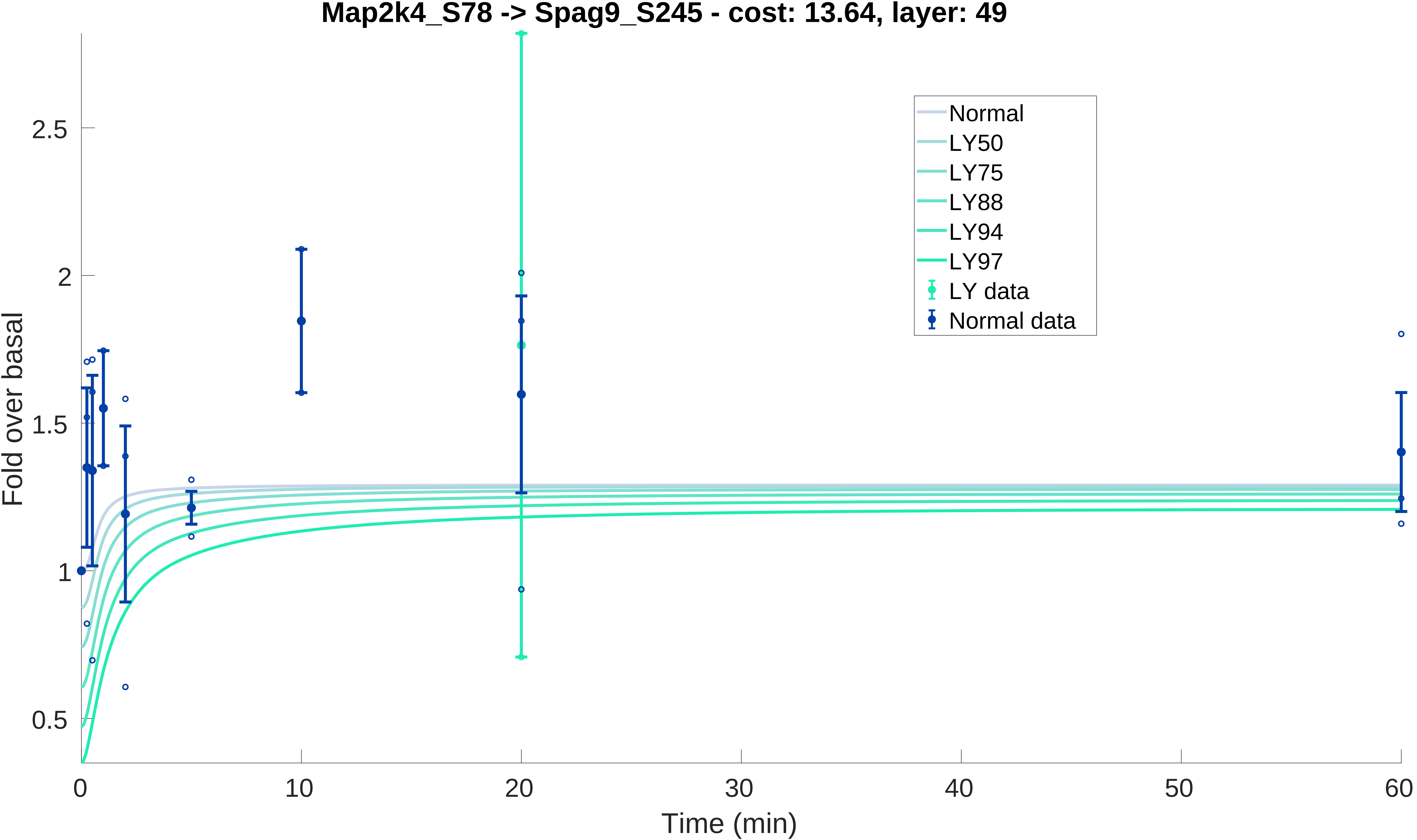

Map2k4_S78 -> Spag9_S245 - cost: 13.64, layer: 49
Fold over basal
Time (min)
0.5
1
1.5
2
2.5
0
10
20
30
40
50
60
Normal
LY50
LY75
LY88
LY94
LY97
LY data
Normal data

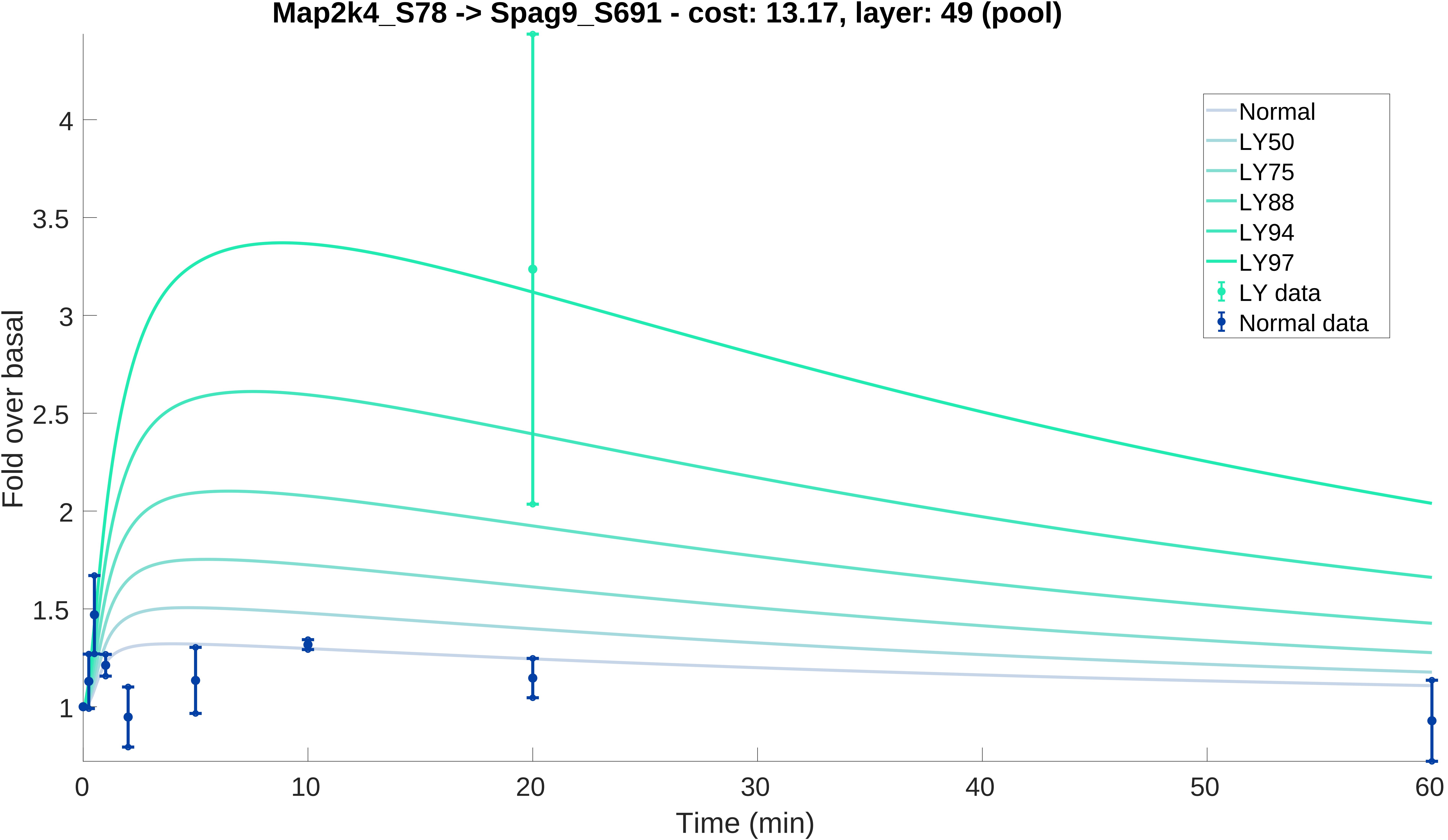

Map2k4_S78 -> Spag9_S691 - cost: 13.17, layer: 49 (pool)
Fold over basal
Time (min)
Normal
LY50
LY75
LY88
LY94
LY97
LY data
Normal data
0
10
20
30
40
50
60
1
1.5
2
2.5
3
3.5
4

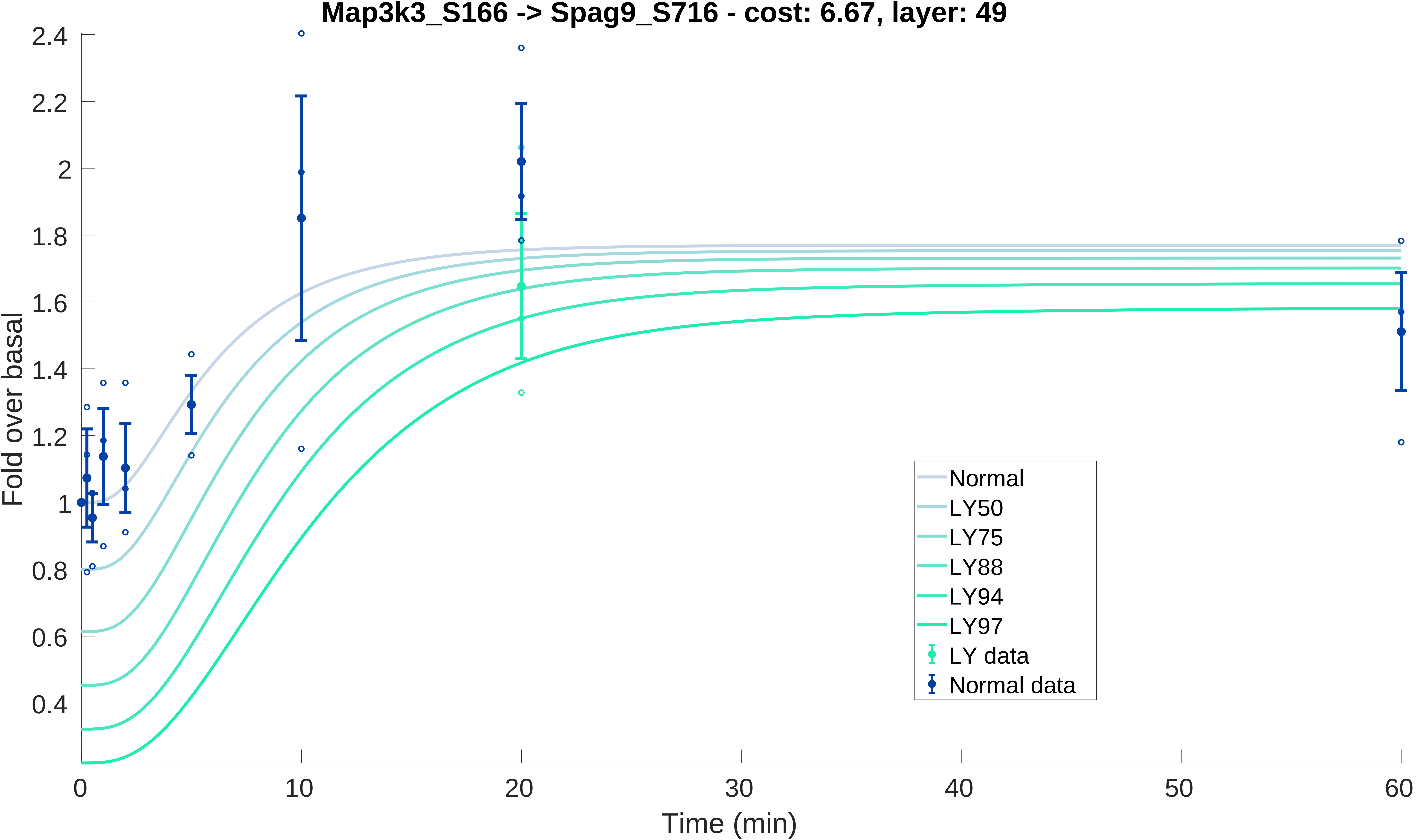

Map3k3_S166 -> Spag9_S716 - cost: 6.67, layer: 49
Fold over basal
Time (min)
0
10
20
30
40
50
60
0.4
0.6
0.8
1
1.2
1.4
1.6
1.8
2
2.2
2.4
Normal
LY50
LY75
LY88
LY94
LY97
LY data
Normal data

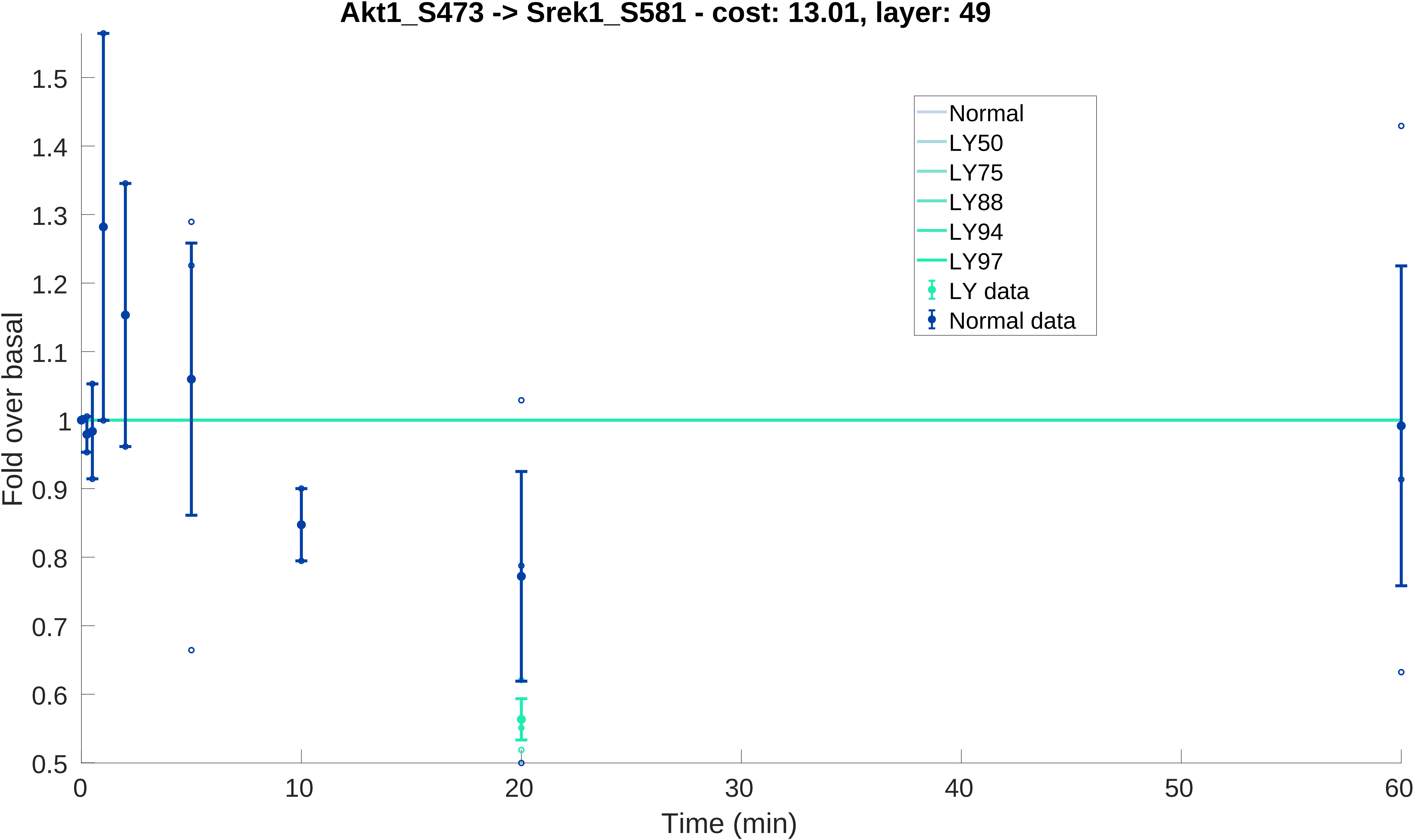

Akt1_S473 -> Srek1_S581 - cost: 13.01, layer: 49
Fold over basal
Time (min)
1.5
1.4
1.3
1.2
1.1
1
0.9
0.8
0.7
0.6
0.5
0
10
20
30
40
50
60
Normal
LY50
LY75
LY88
LY94
LY97
LY data
Normal data

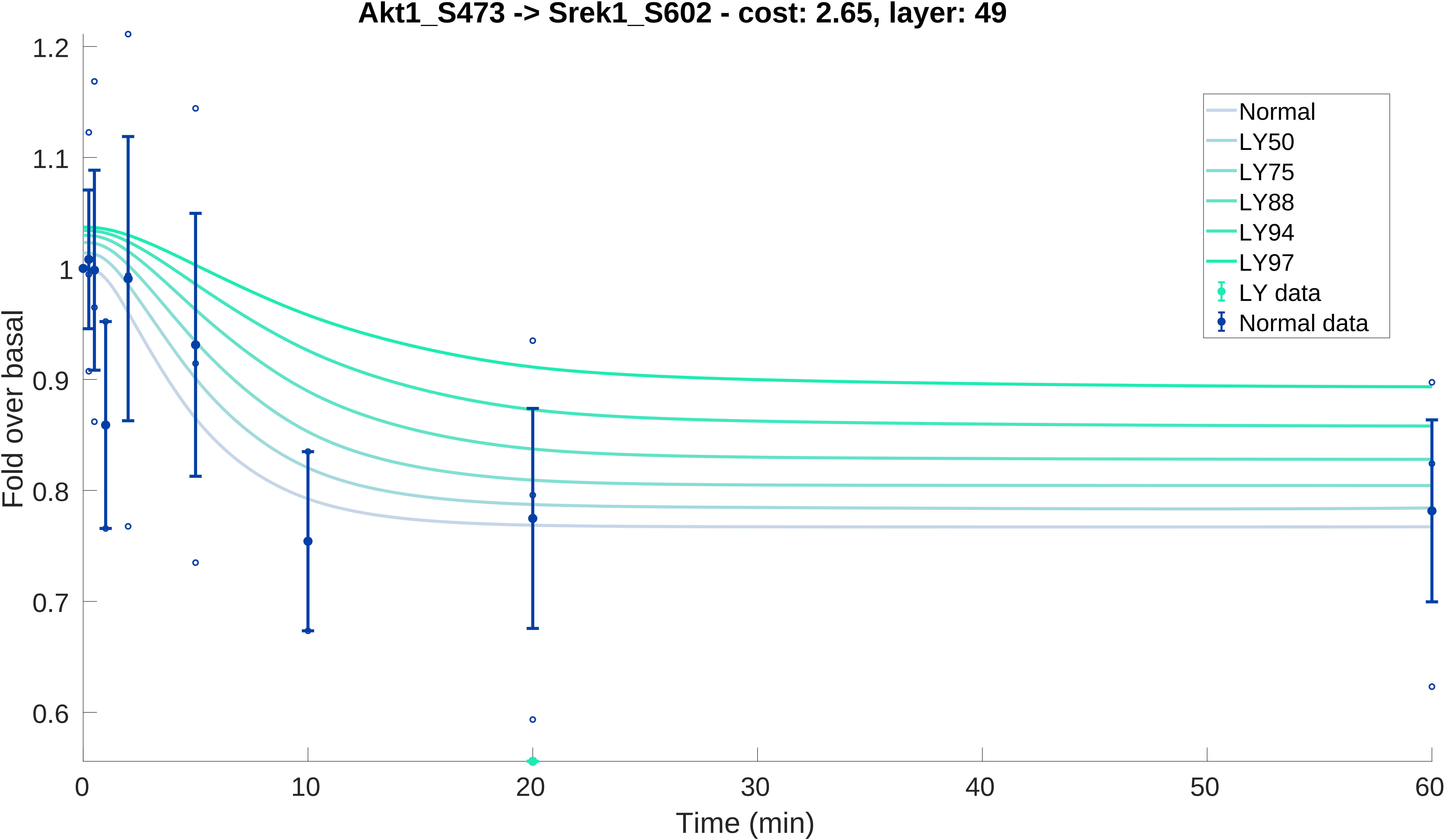
Akt1_S473 -> Srek1_S602 - cost: 2.65, layer: 49
Fold over basal
Time (min)
1.2
1.1
1
0.9
0.8
0.7
0.6
0
10
20
30
40
50
60
Normal
LY50
LY75
LY88
LY94
LY97
LY data
Normal data

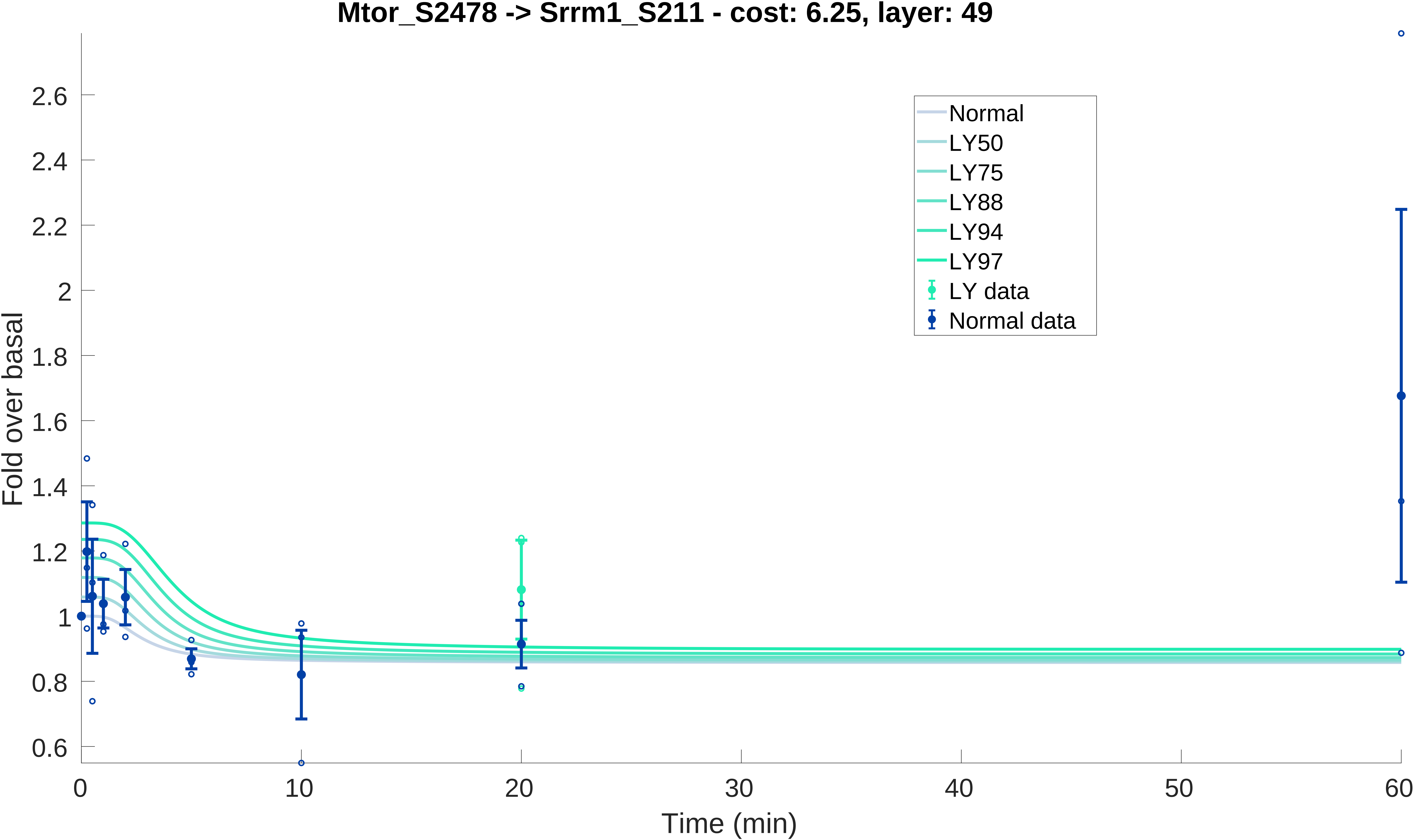

Mtor_S2478 -> Srrm1_S211 - cost: 6.25, layer: 49
Fold over basal
Time (min)
0.6
0.8
1
1.2
1.4
1.6
1.8
2
2.2
2.4
2.6
0
10
20
30
40
50
60
Normal
LY50
LY75
LY88
LY94
LY97
LY data
Normal data

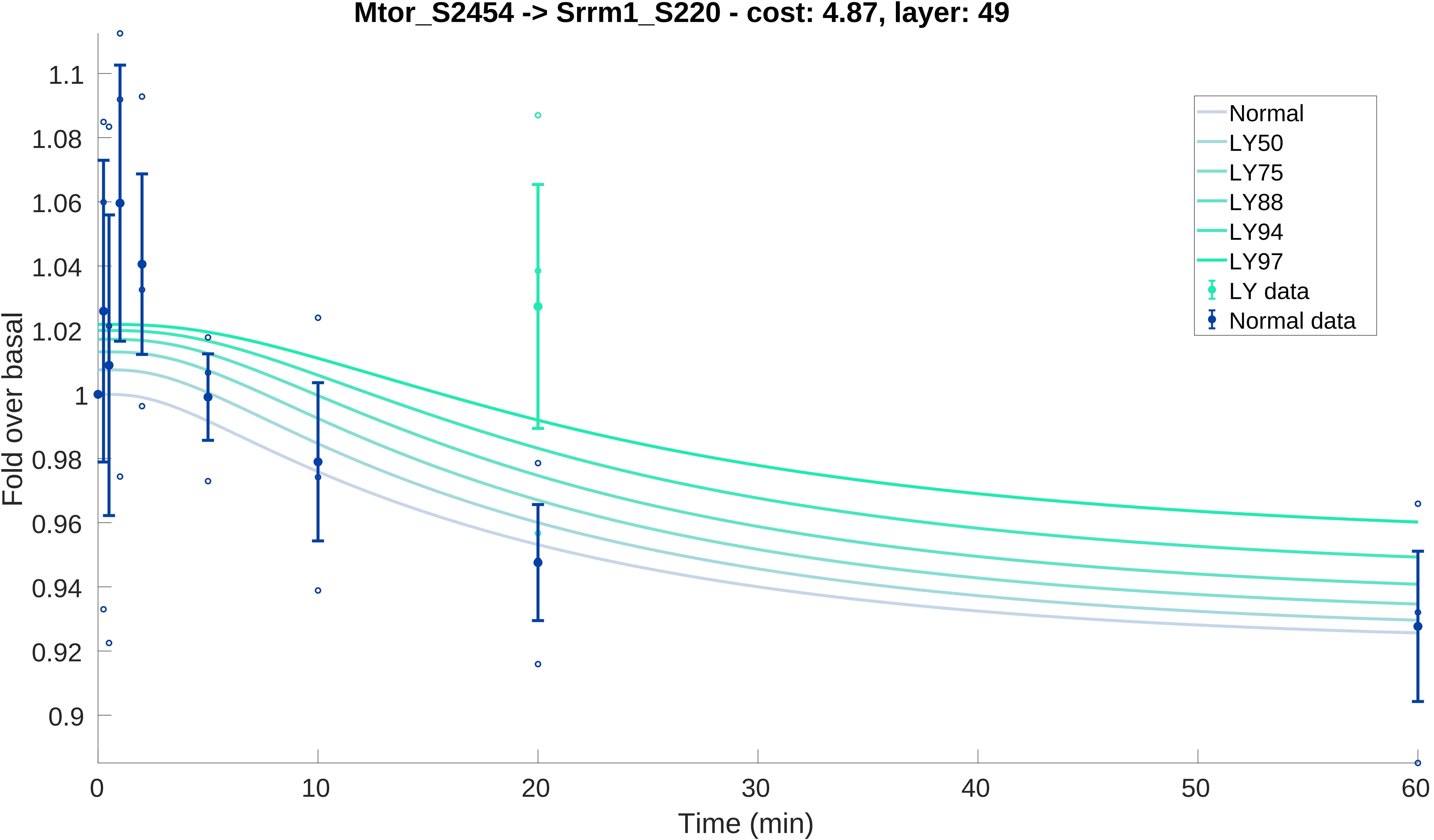
Mtor_S2454 -> Srrm1_S220 - cost: 4.87, layer: 49
Fold over basal
Time (min)
1.1
1.08
1.06
1.04
1.02
1
0.98
0.96
0.94
0.92
0.9
0
10
20
30
40
50
60
Normal
LY50
LY75
LY88
LY94
LY97
LY data
Normal data

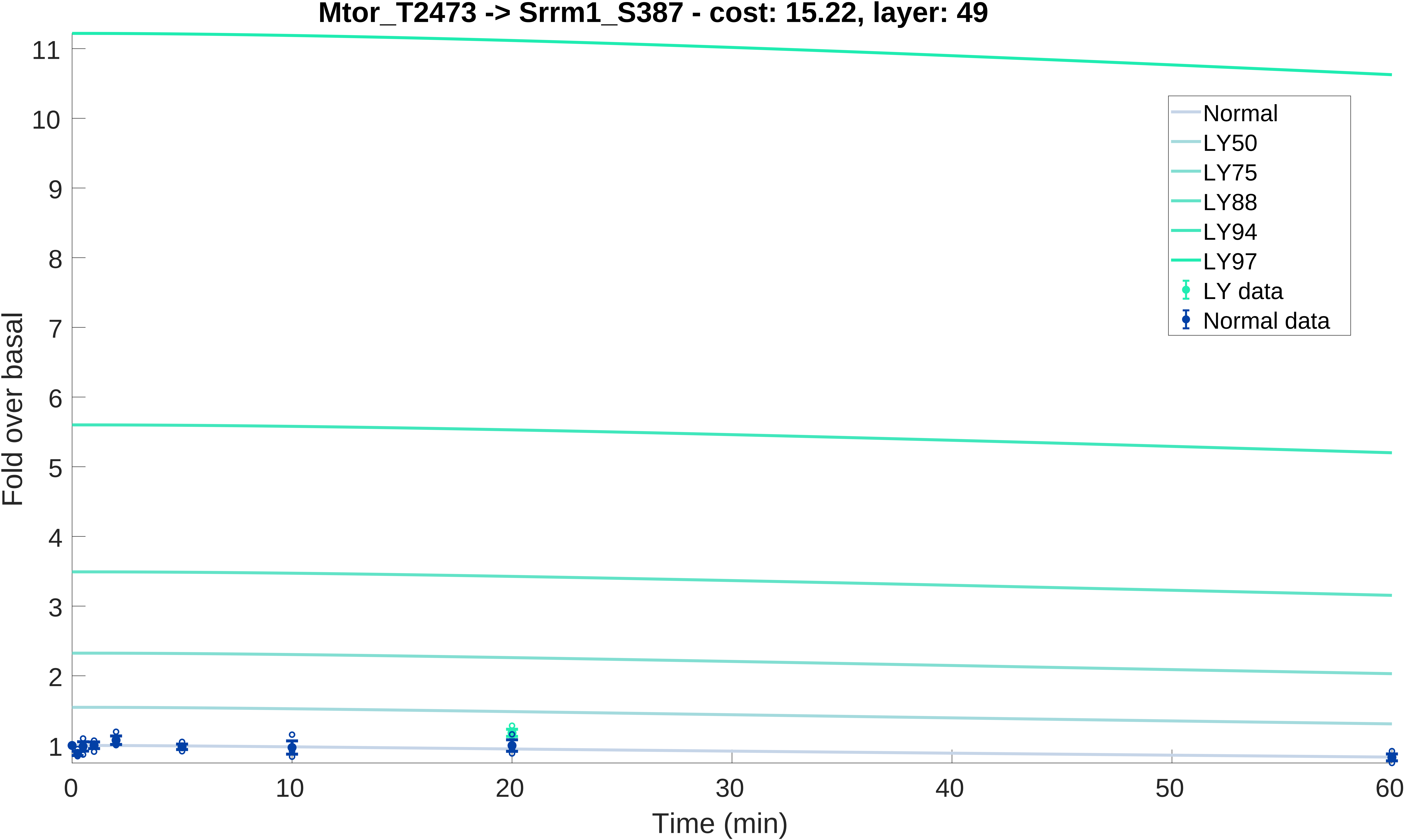

Mtor_T2473 -> Srrm1_S387 - cost: 15.22, layer: 49
Fold over basal
Time (min)
Normal
LY50
LY75
LY88
LY94
LY97
LY data
Normal data
11
10
9
8
7
6
5
4
3
2
1
0
10
20
30
40
50
60

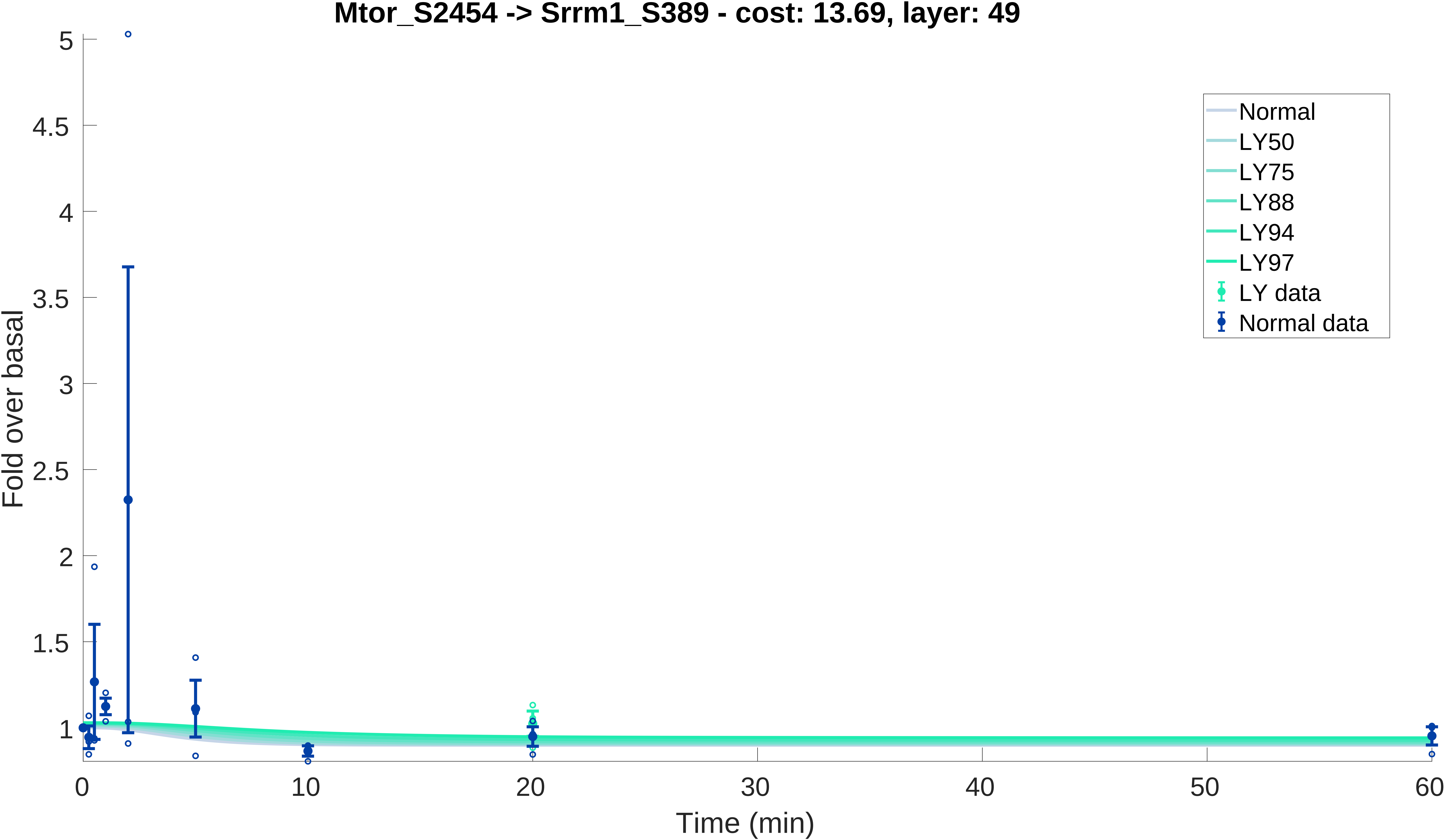

Mtor_S2454 -> Srrm1_S389 - cost: 13.69, layer: 49
Fold over basal
Time (min)
0
10
20
30
40
50
60
1
1.5
2
2.5
3
3.5
4
4.5
5
Normal
LY50
LY75
LY88
LY94
LY97
LY data
Normal data

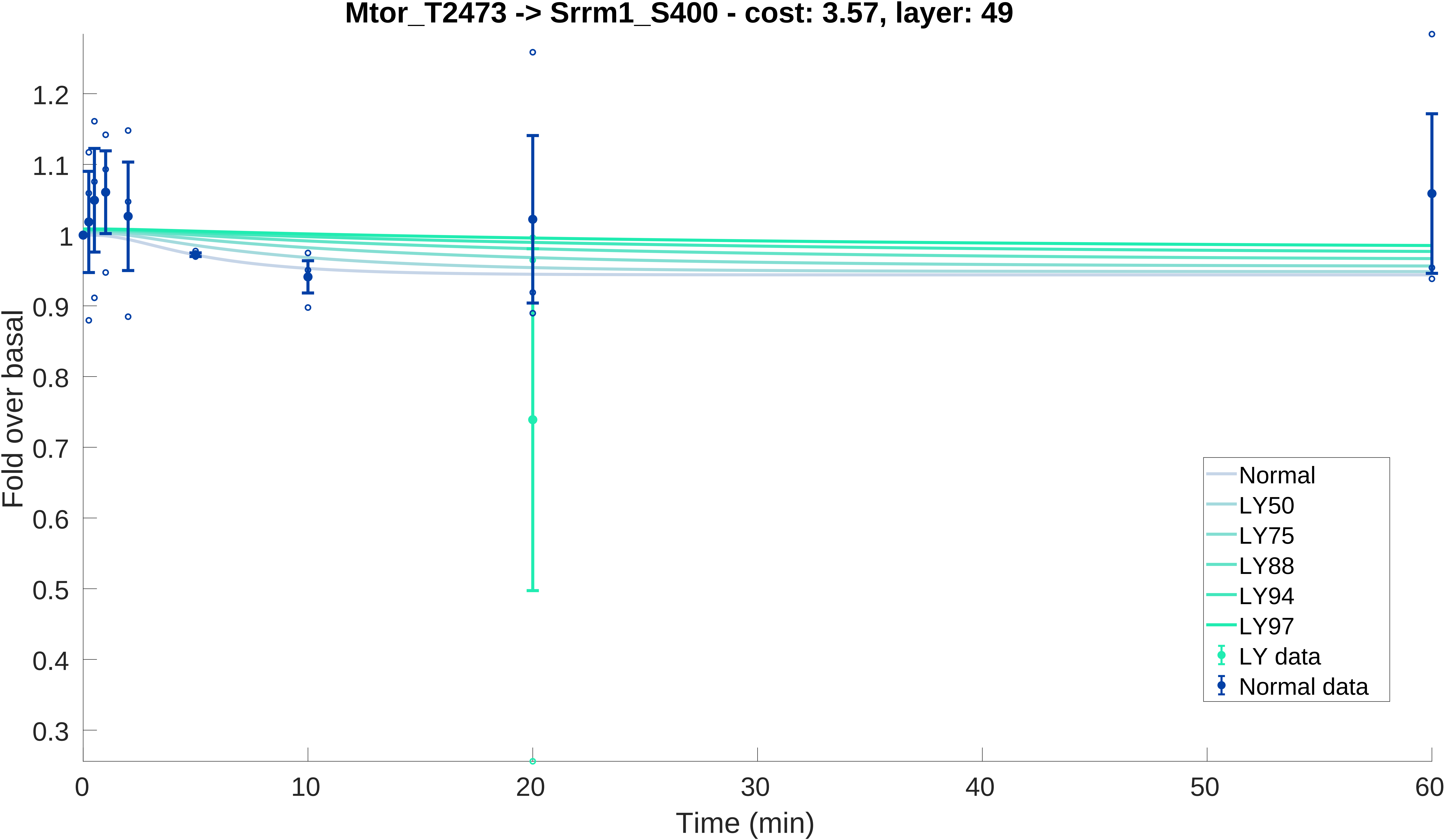

Mtor_T2473 -> Srrm1_S400 - cost: 3.57, layer: 49
Fold over basal
Time (min)
0.3
0.4
0.5
0.6
0.7
0.8
0.9
1
1.1
1.2
0
10
20
30
40
50
60
Normal
LY50
LY75
LY88
LY94
LY97
LY data
Normal data

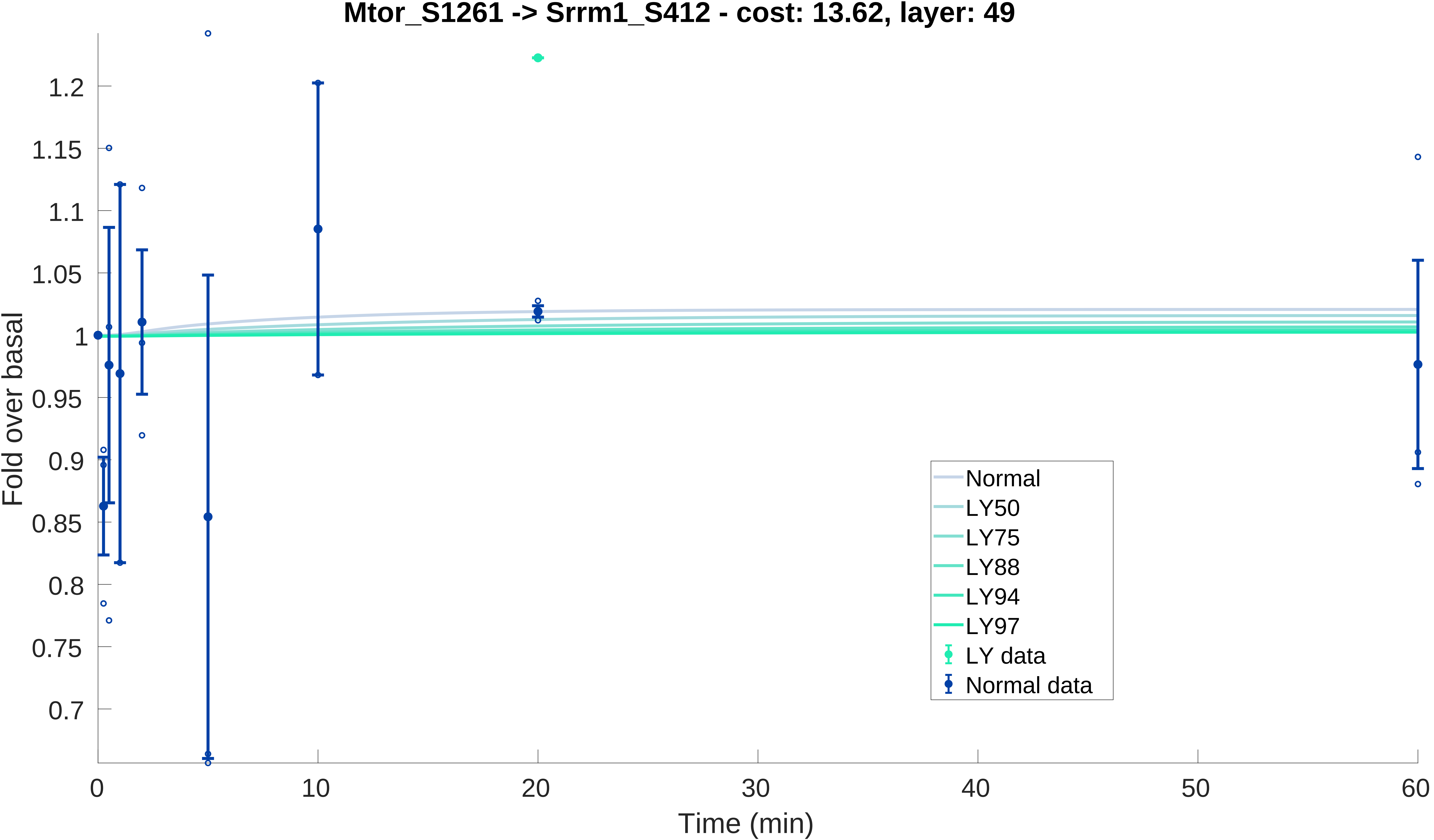
Mtor_S1261 -> Srrm1_S412 - cost: 13.62, layer: 49
Fold over basal
1.2
1.15
1.1
1.05
1
0.95
0.9
0.85
0.8
0.75
0.7
0
10
20
30
40
50
60
Time (min)
Normal
LY50
LY75
LY88
LY94
LY97
LY data
Normal data

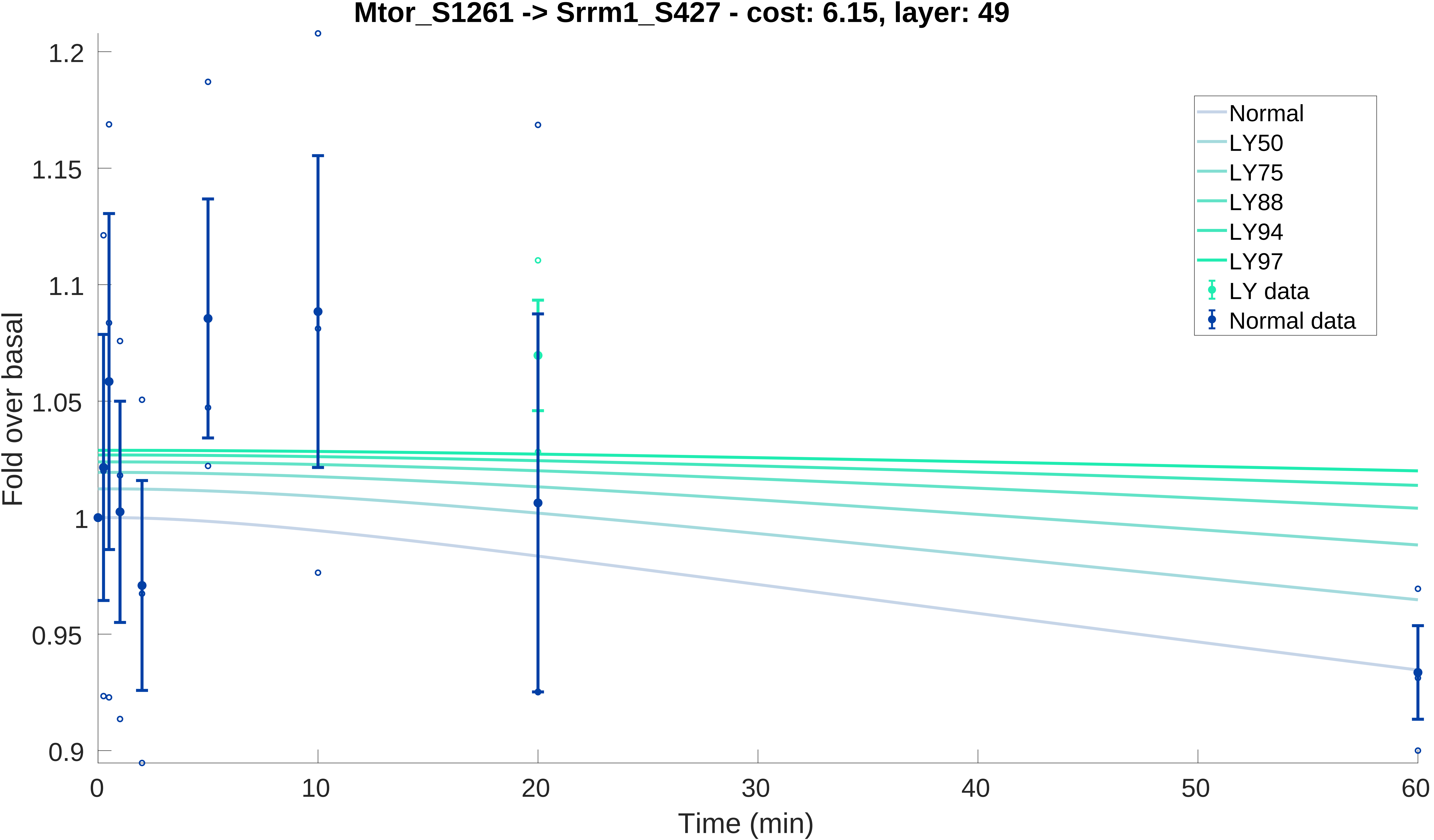

Mtor_S1261 -> Srrm1_S427 - cost: 6.15, layer: 49
Fold over basal
Time (min)
1.2
1.15
1.1
1.05
1
0.95
0.9
0
10
20
30
40
50
60
Normal
LY50
LY75
LY88
LY94
LY97
LY data
Normal data

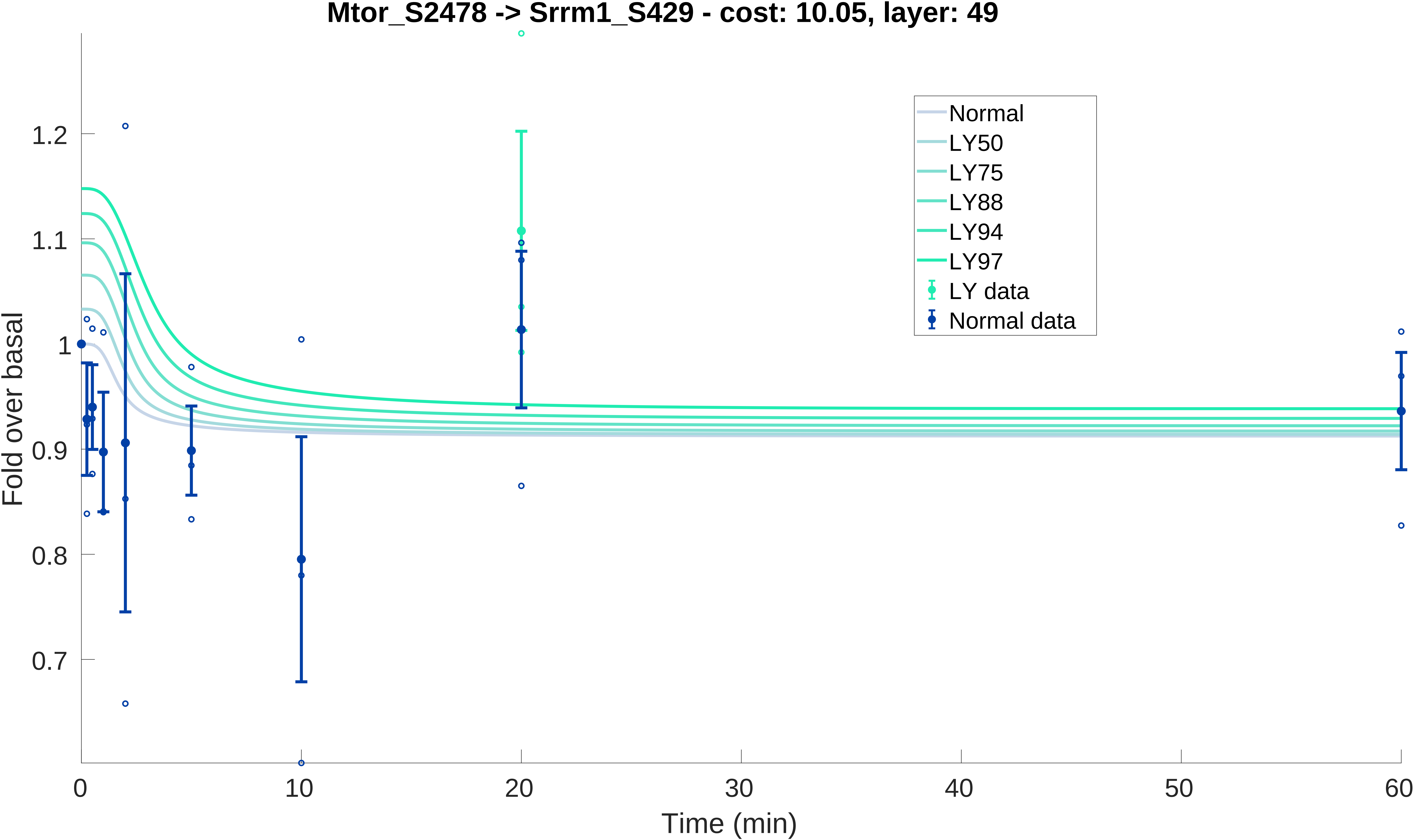

Mtor_S2478 -> Srrm1_S429 - cost: 10.05, layer: 49
Normal
LY50
LY75
LY88
LY94
LY97
LY data
Normal data
Fold over basal
1.2
1.1
1
0.9
0.8
0.7
0
10
20
30
40
50
60
Time (min)

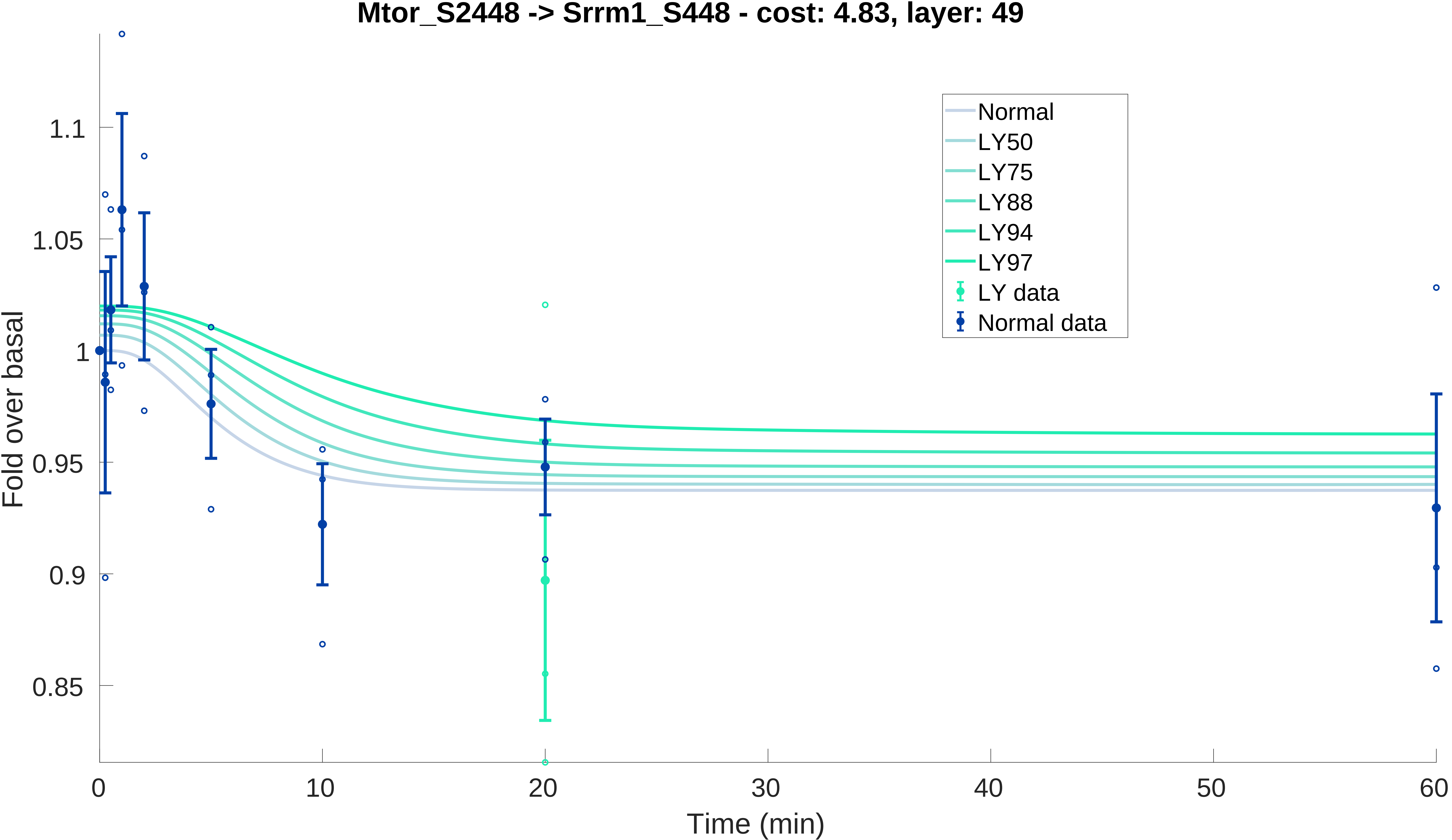

Mtor_S2448 -> Srrm1_S448 - cost: 4.83, layer: 49
Normal
LY50
LY75
LY88
LY94
LY97
LY data
Normal data
Fold over basal
1.1
1.05
1
0.95
0.9
0.85
0
10
20
30
40
50
60
Time (min)

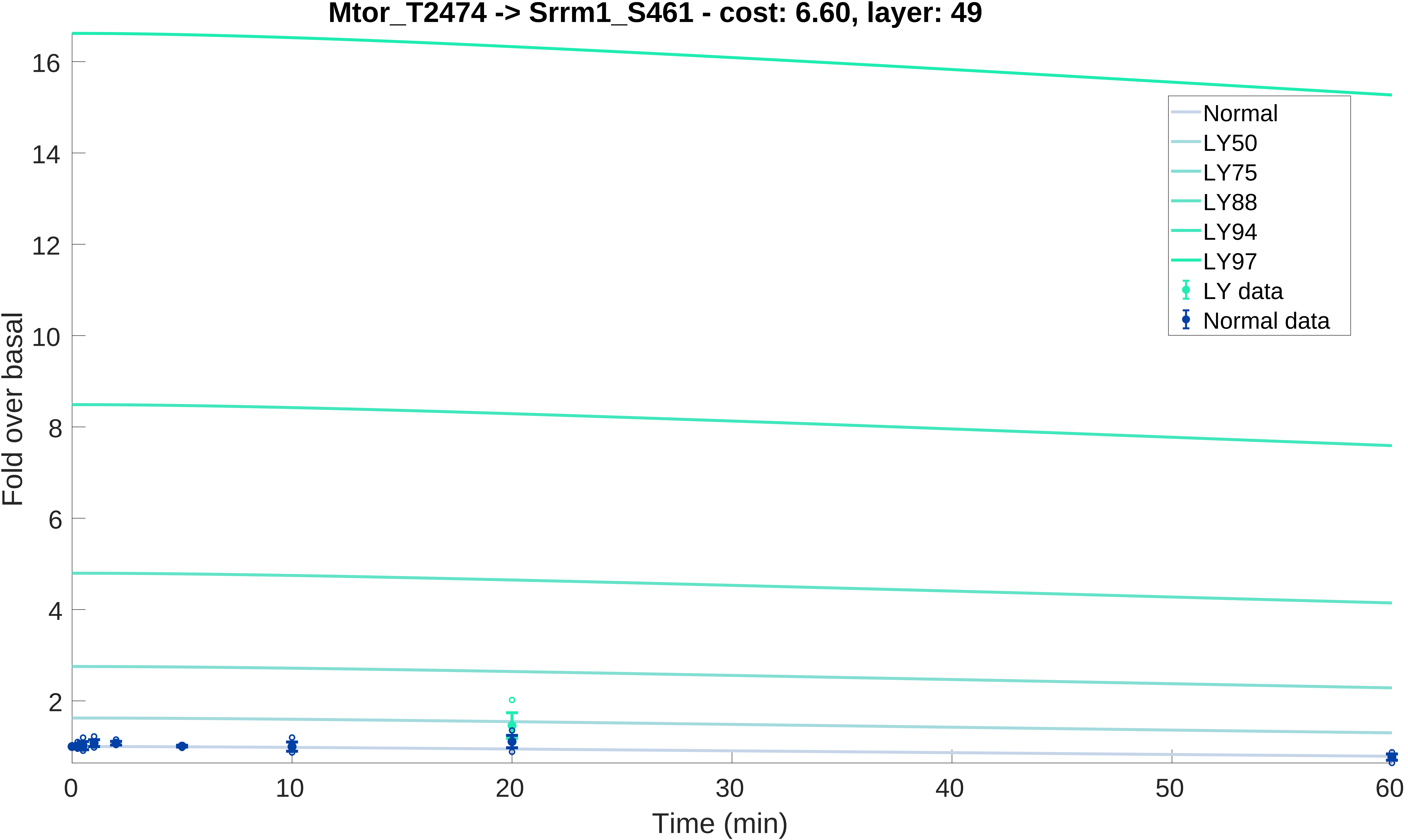

Mtor_T2474 -> Srrm1_S461 - cost: 6.60, layer: 49
Fold over basal
Time (min)
0
10
20
30
40
50
60
2
4
6
8
10
12
14
16
Normal
LY50
LY75
LY88
LY94
LY97
LY data
Normal data

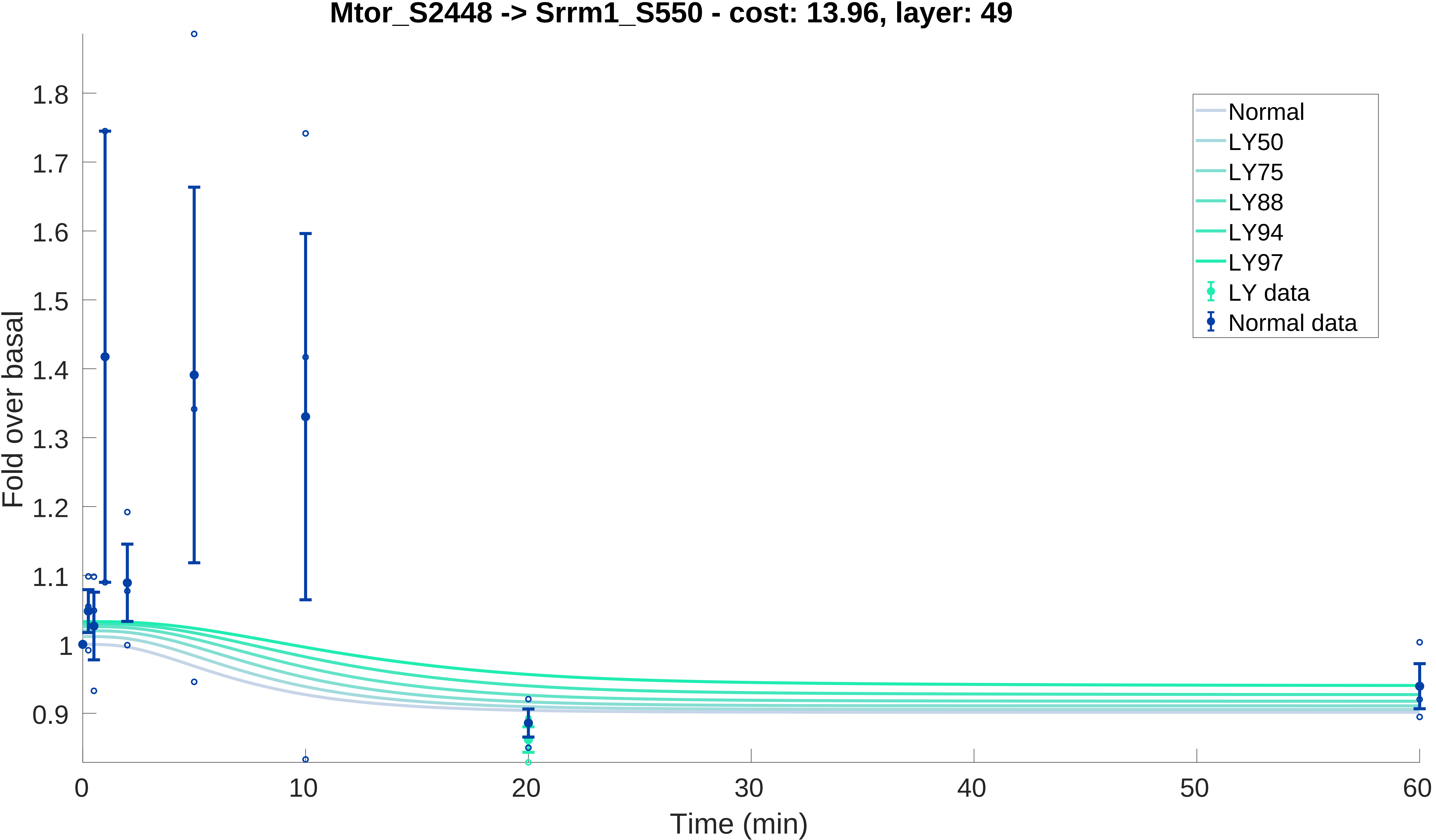

Mtor_S2448 -> Srrm1_S550 - cost: 13.96, layer: 49
Fold over basal
Time (min)
0.9
1
1.1
1.2
1.3
1.4
1.5
1.6
1.7
1.8
0
10
20
30
40
50
60
Normal
LY50
LY75
LY88
LY94
LY97
LY data
Normal data

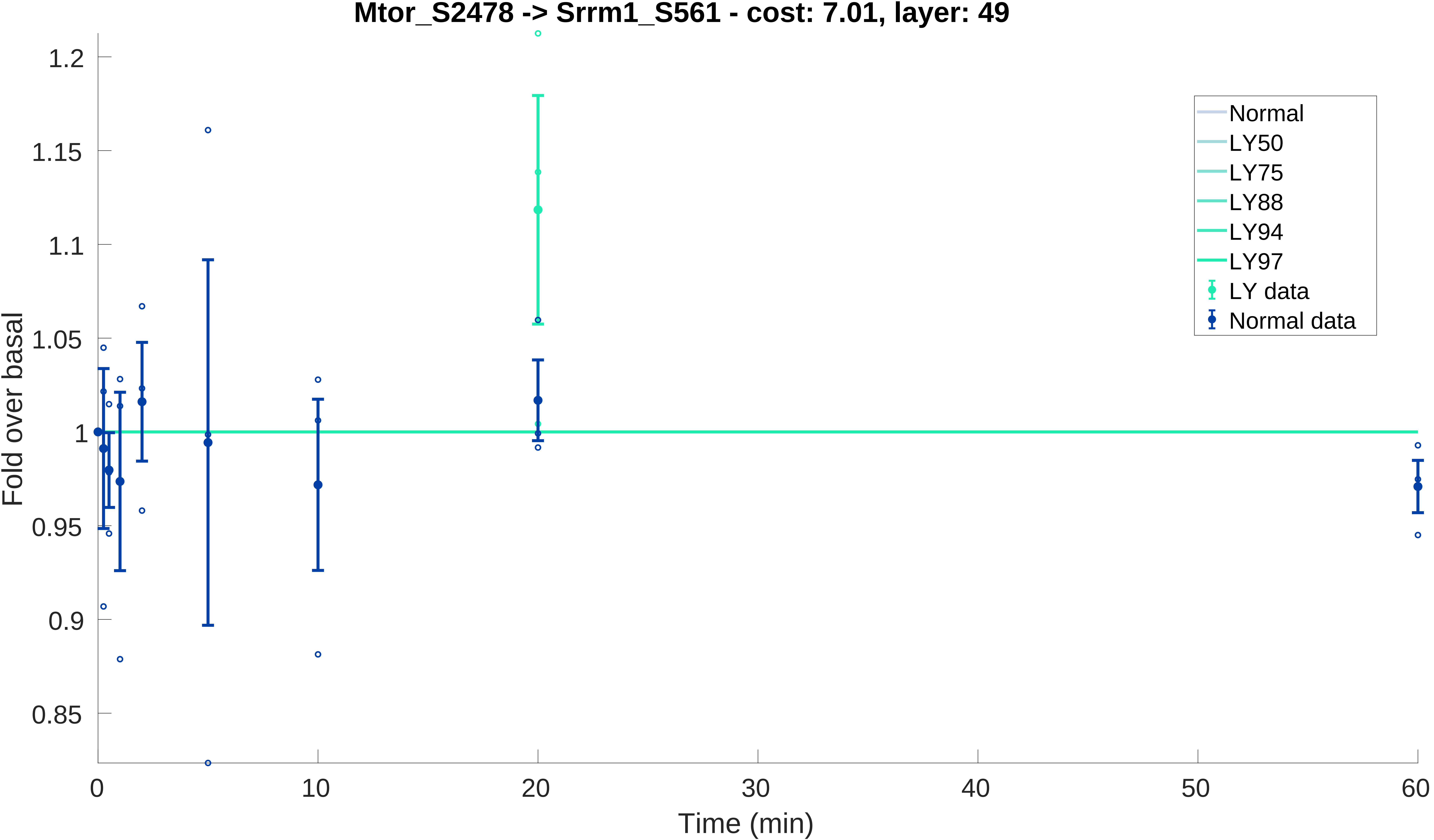
Mtor_S2478 -> Srrm1_S561 - cost: 7.01, layer: 49
Fold over basal
Time (min)
1.2
1.15
1.1
1.05
1
0.95
0.9
0.85
0
10
20
30
40
50
60
Normal
LY50
LY75
LY88
LY94
LY97
LY data
Normal data

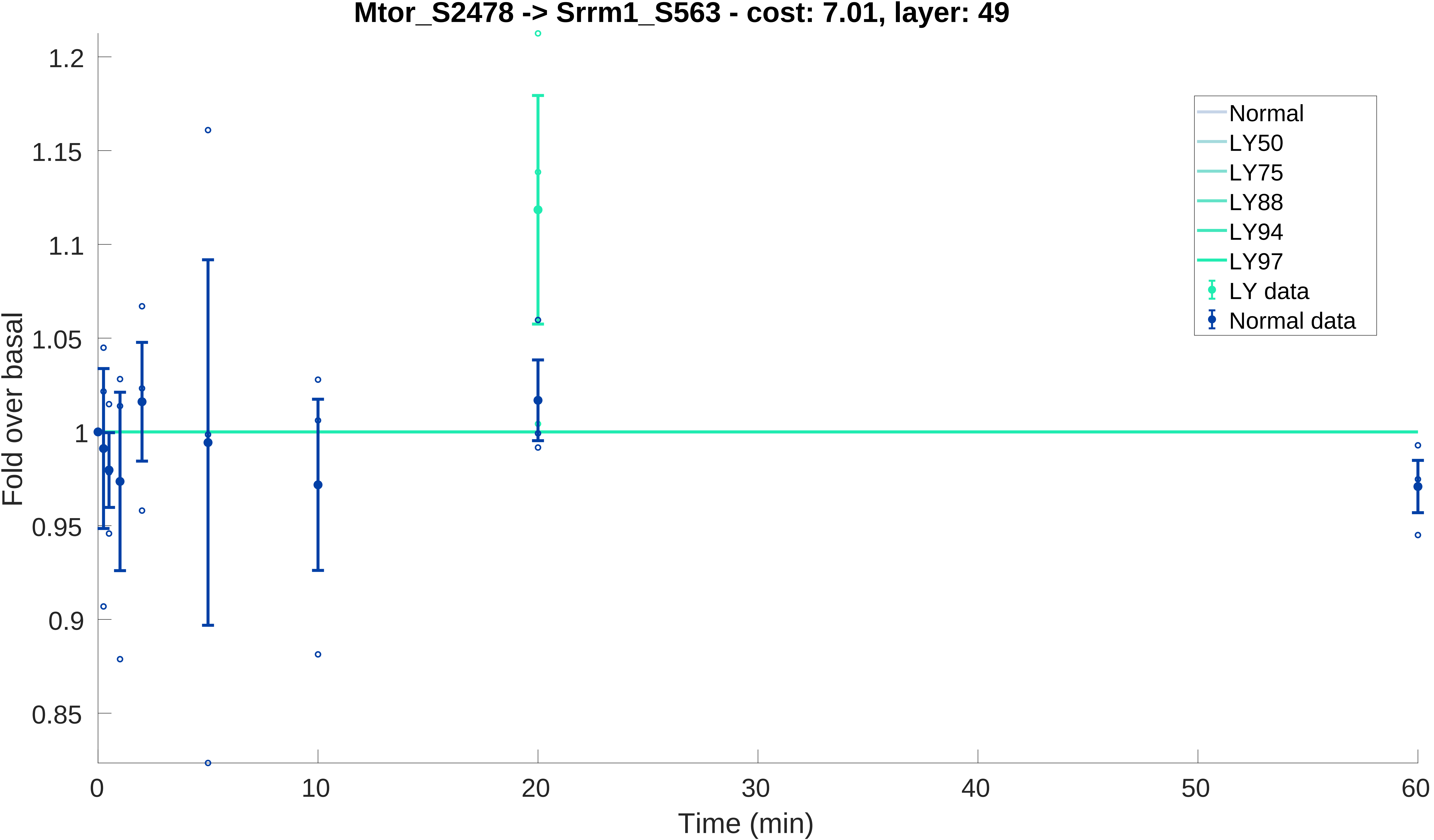

Mtor_S2478 -> Srrm1_S563 - cost: 7.01, layer: 49
Fold over basal
1.2
1.15
1.1
1.05
1
0.95
0.9
0.85
0
10
20
30
40
50
60
Time (min)
Normal
LY50
LY75
LY88
LY94
LY97
LY data
Normal data

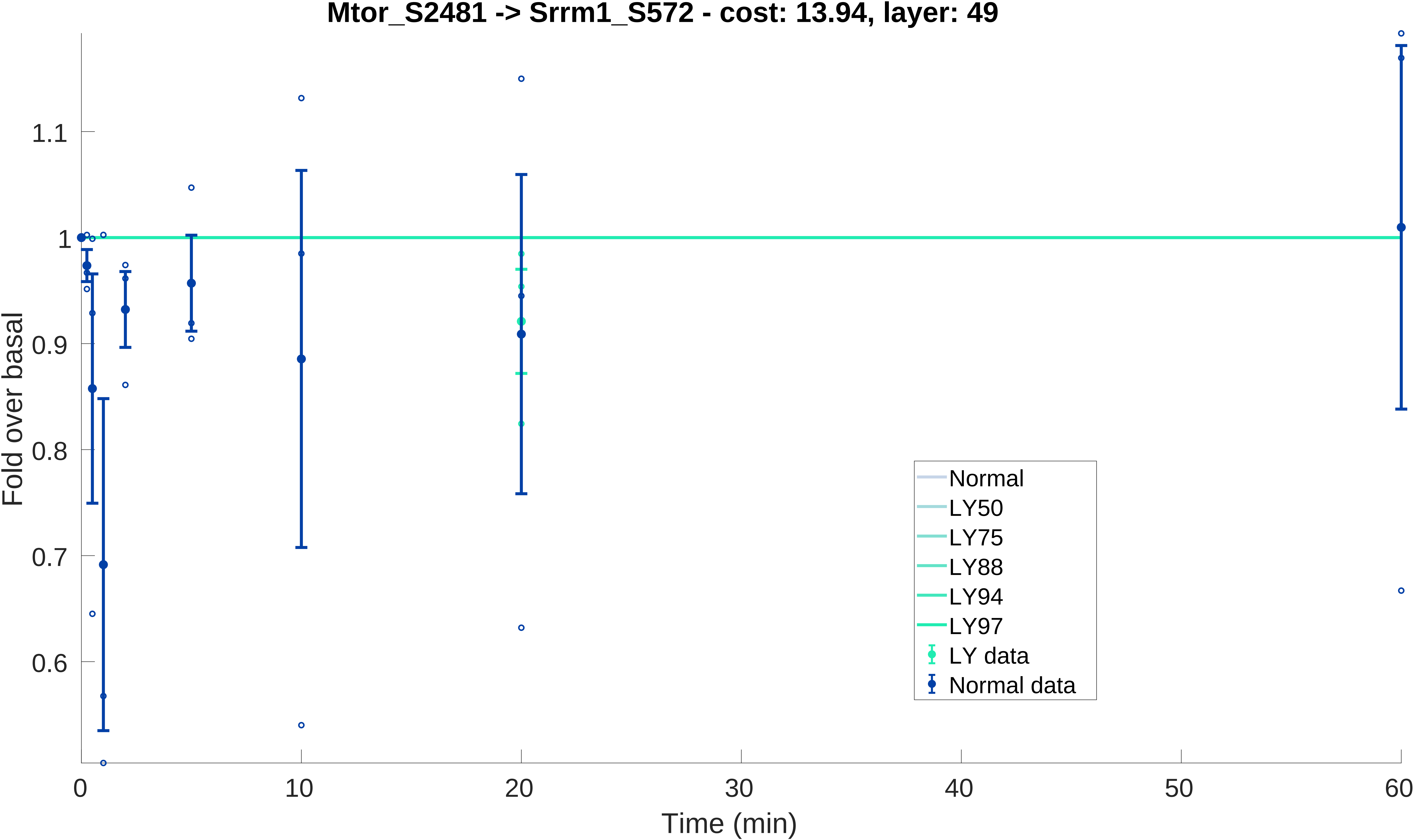

Mtor_S2481 -> Srrm1_S572 - cost: 13.94, layer: 49
Fold over basal
Time (min)
0.6
0.7
0.8
0.9
1
1.1
0
10
20
30
40
50
60
Normal
LY50
LY75
LY88
LY94
LY97
LY data
Normal data

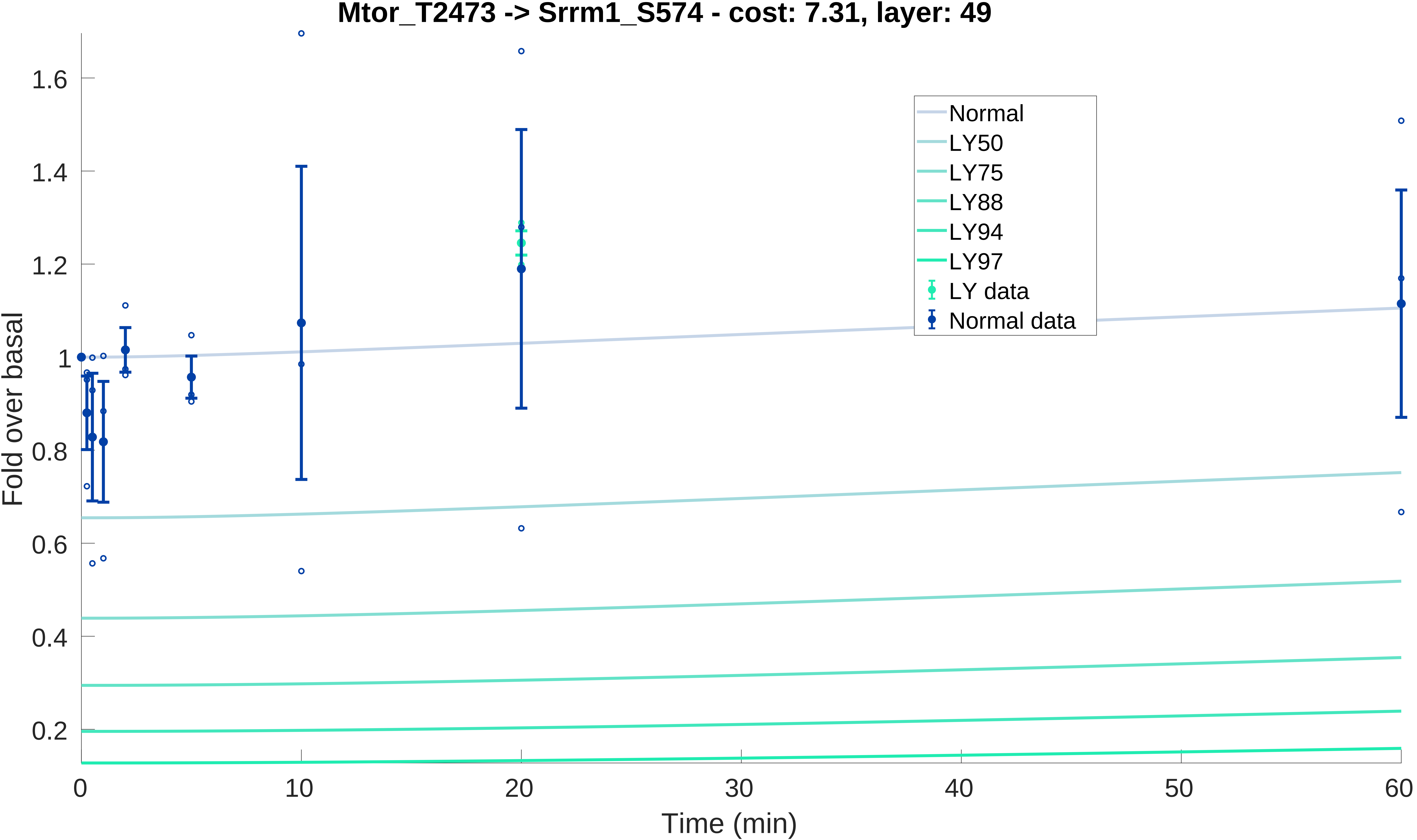

Mtor_T2473 -> Srrm1_S574 - cost: 7.31, layer: 49
Fold over basal
Time (min)
1.6
1.4
1.2
1
0.8
0.6
0.4
0.2
0
10
20
30
40
50
60
Normal
LY50
LY75
LY88
LY94
LY97
LY data
Normal data

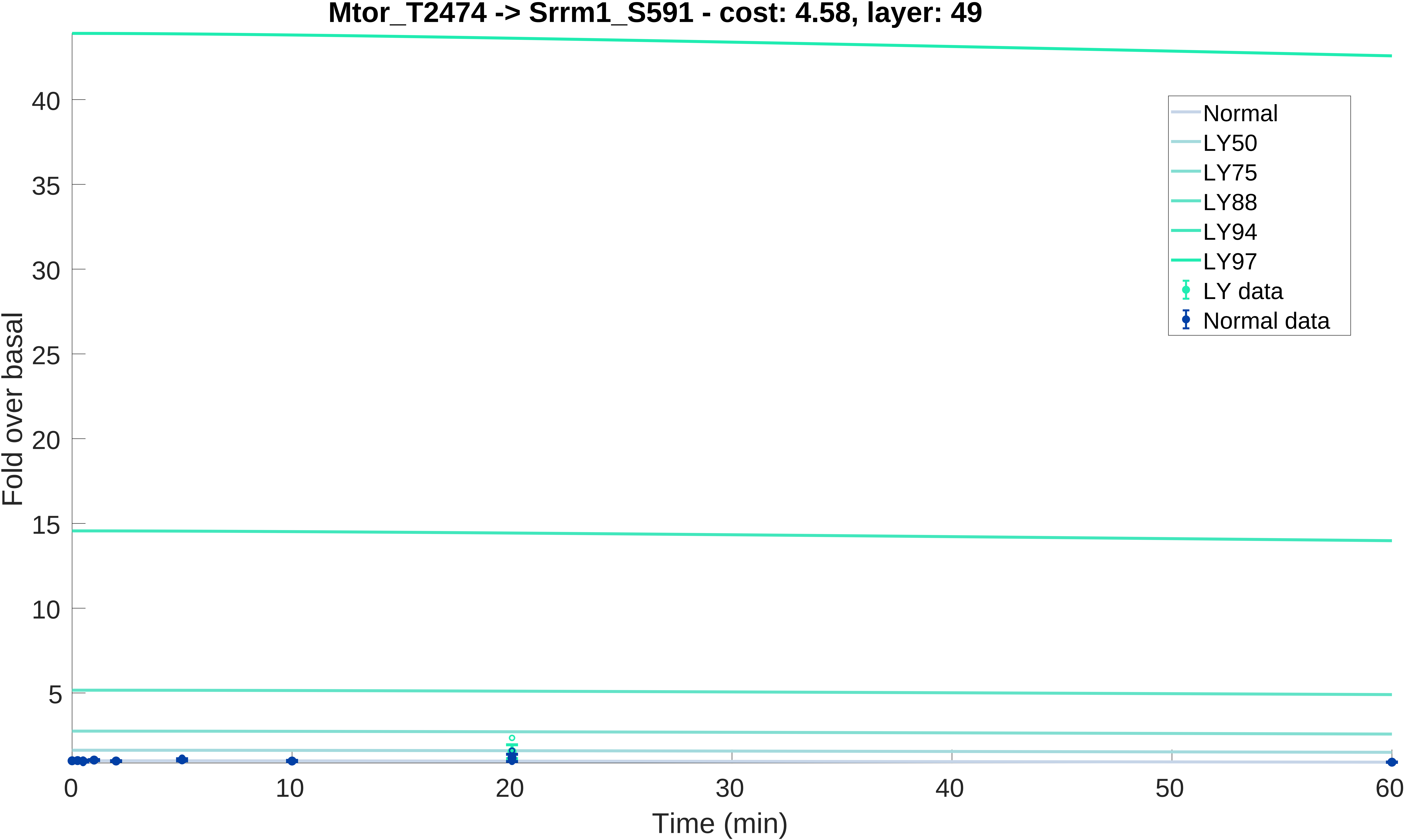

Mtor_T2474 -> Srrm1_S591 - cost: 4.58, layer: 49
Fold over basal
Time (min)
0
10
20
30
40
50
60
5
10
15
20
25
30
35
40
Normal
LY50
LY75
LY88
LY94
LY97
LY data
Normal data

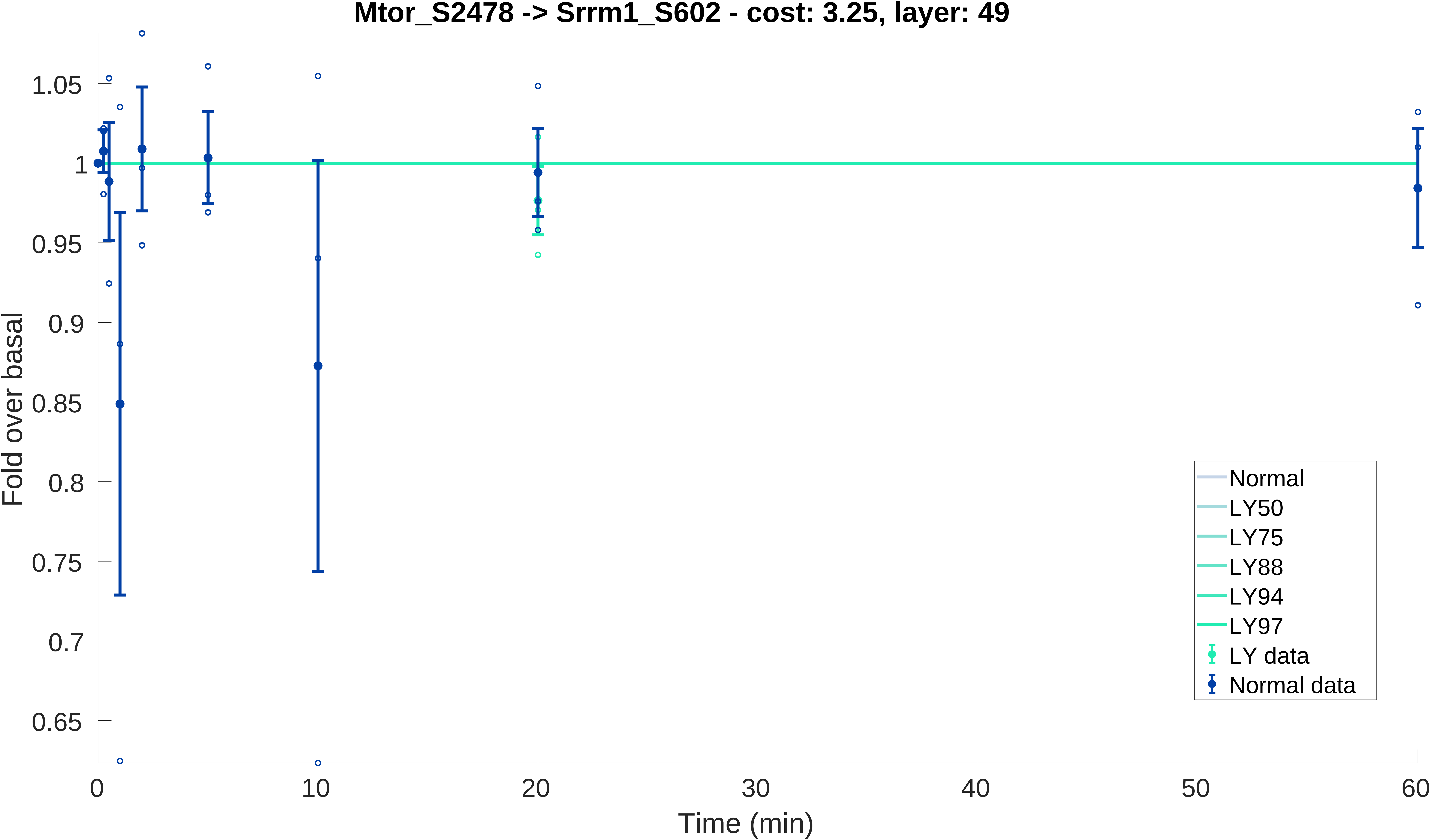

Mtor_S2478 -> Srrm1_S602 - cost: 3.25, layer: 49
Fold over basal
Time (min)
1.05
1
0.95
0.9
0.85
0.8
0.75
0.7
0.65
0
10
20
30
40
50
60
Normal
LY50
LY75
LY88
LY94
LY97
LY data
Normal data

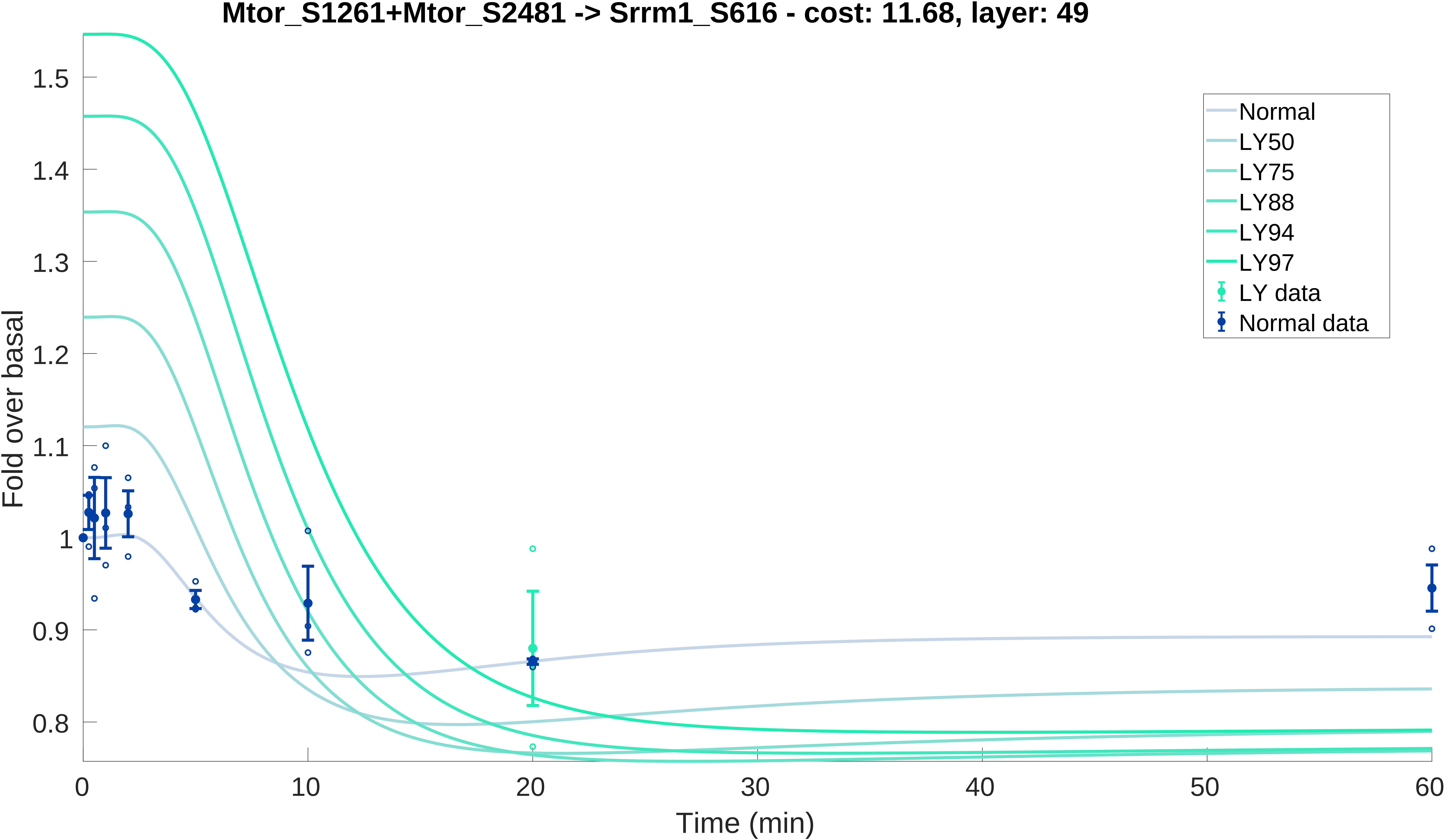
Mtor_S1261+Mtor_S2481 -> Srrm1_S616 - cost: 11.68, layer: 49
Fold over basal
Time (min)
1.5
1.4
1.3
1.2
1.1
1
0.9
0.8
0
10
20
30
40
50
60
Normal
LY50
LY75
LY88
LY94
LY97
LY data
Normal data

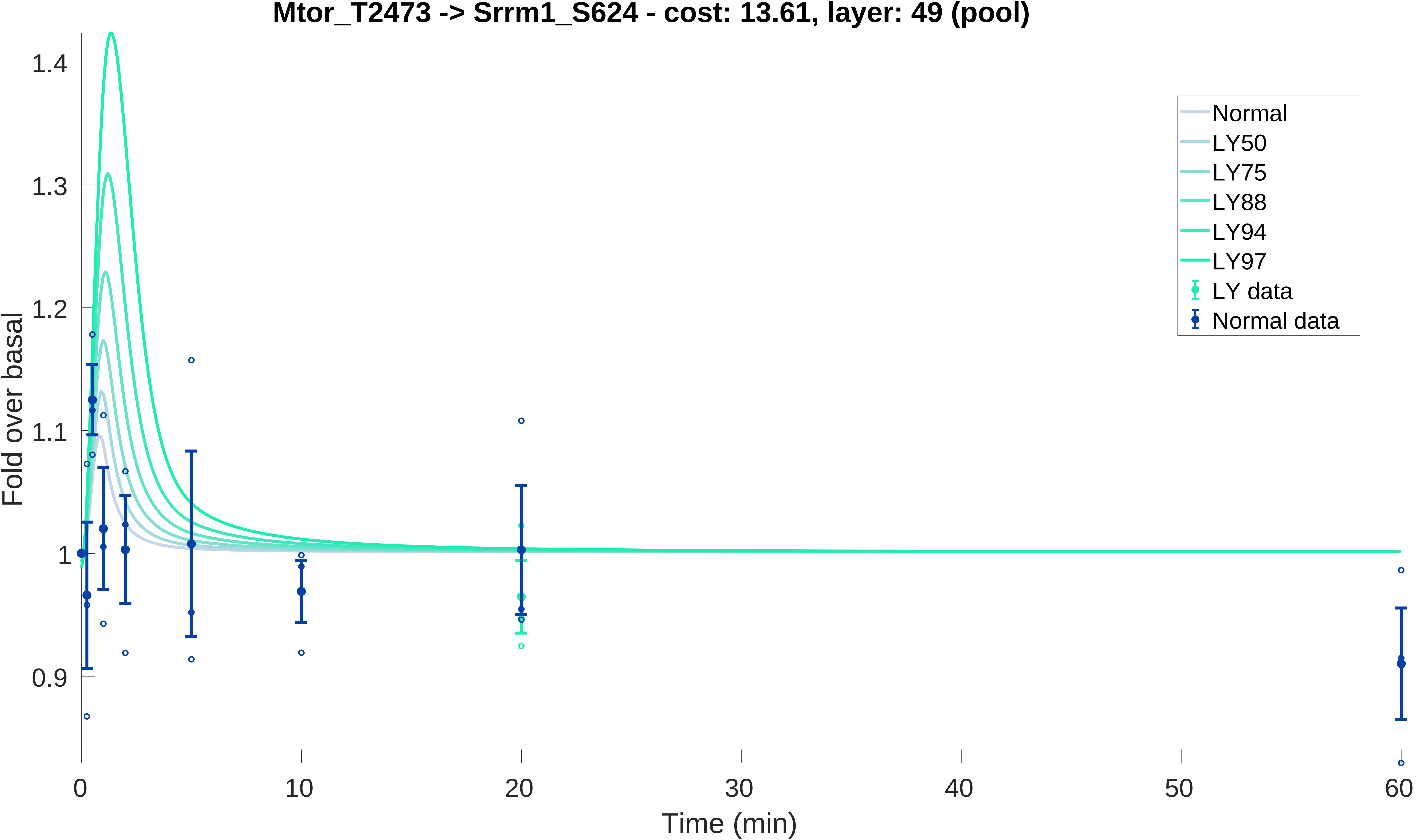

Mtor_T2473 -> Srrm1_S624 - cost: 13.61, layer: 49 (pool)
Fold over basal
Time (min)
1.4
1.3
1.2
1.1
1
0.9
0
10
20
30
40
50
60
Normal
LY50
LY75
LY88
LY94
LY97
LY data
Normal data

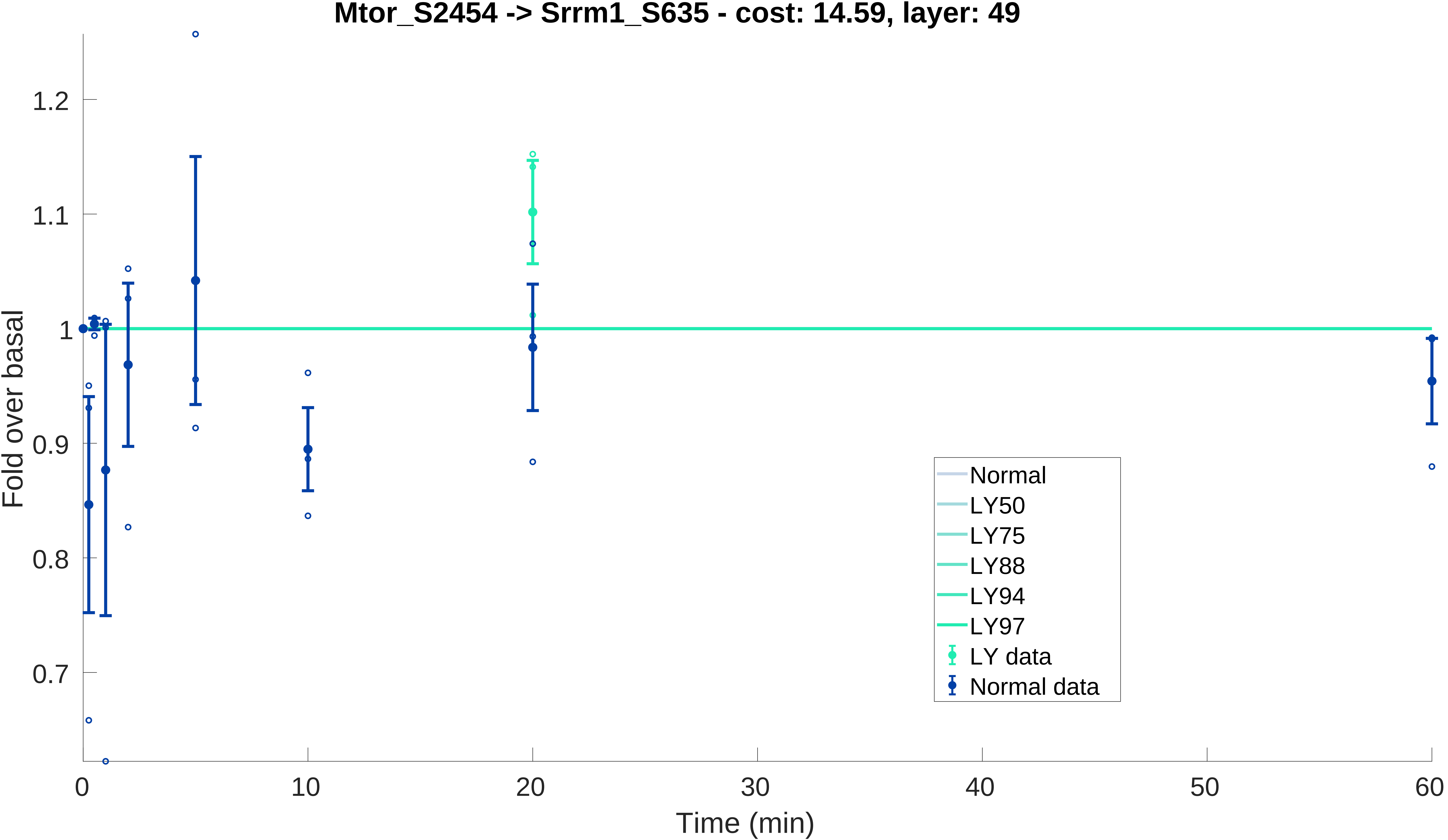

Mtor_S2454 -> Srrm1_S635 - cost: 14.59, layer: 49
Fold over basal
1.2
1.1
1
0.9
0.8
0.7
0
10
20
30
40
50
60
Time (min)
Normal
LY50
LY75
LY88
LY94
LY97
LY data
Normal data

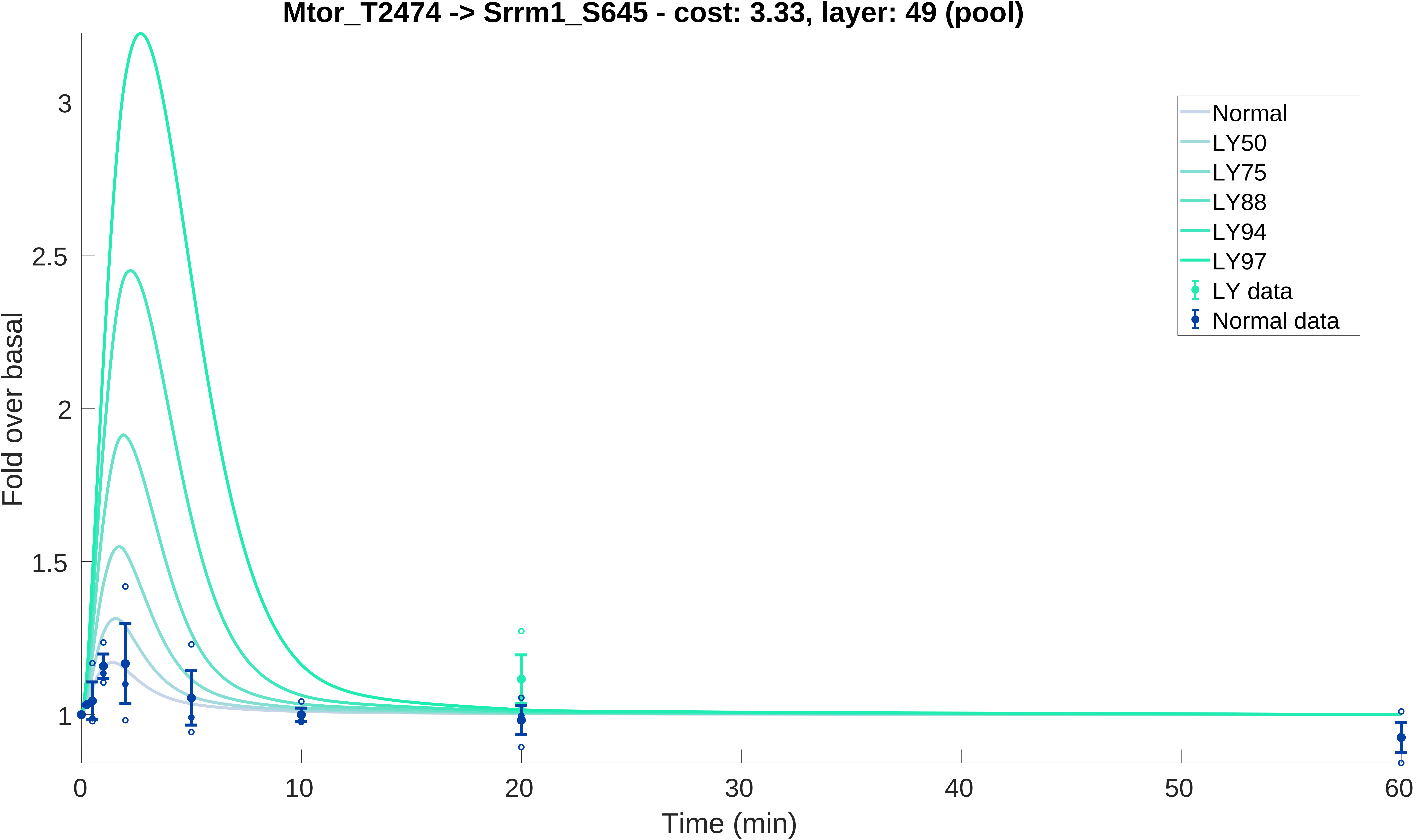

Mtor_T2474 -> Srrm1_S645 - cost: 3.33, layer: 49 (pool)
Fold over basal
Time (min)
0
10
20
30
40
50
60
1
1.5
2
2.5
3
Normal
LY50
LY75
LY88
LY94
LY97
LY data
Normal data

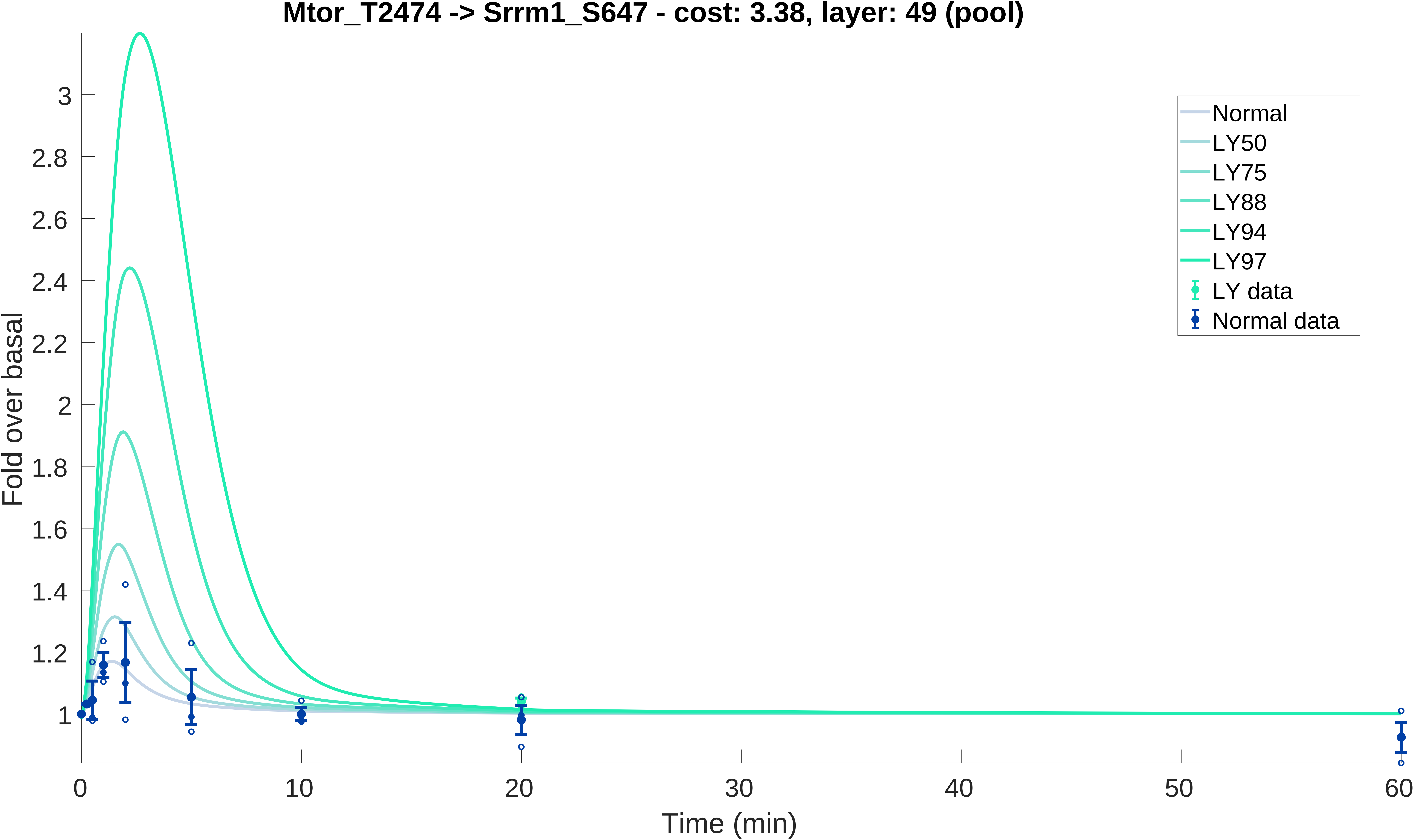

Mtor_T2474 -> Srrm1_S647 - cost: 3.38, layer: 49 (pool)
Fold over basal
Time (min)
0
10
20
30
40
50
60
1
1.2
1.4
1.6
1.8
2
2.2
2.4
2.6
2.8
3
Normal
LY50
LY75
LY88
LY94
LY97
LY data
Normal data

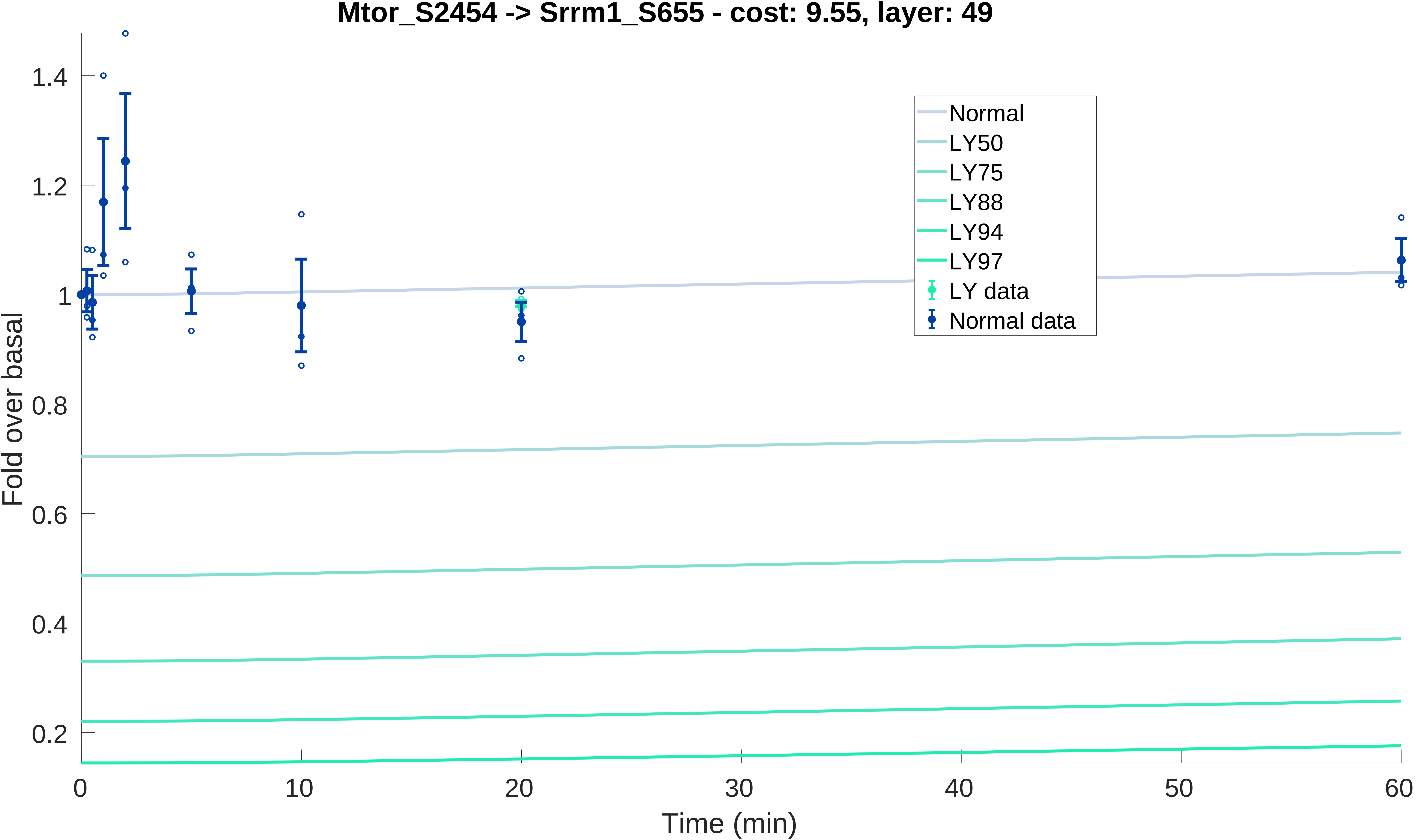

Mtor_S2454 -> Srrm1_S655 - cost: 9.55, layer: 49
Fold over basal
Time (min)
0.2
0.4
0.6
0.8
1
1.2
1.4
0
10
20
30
40
50
60
Normal
LY50
LY75
LY88
LY94
LY97
LY data
Normal data

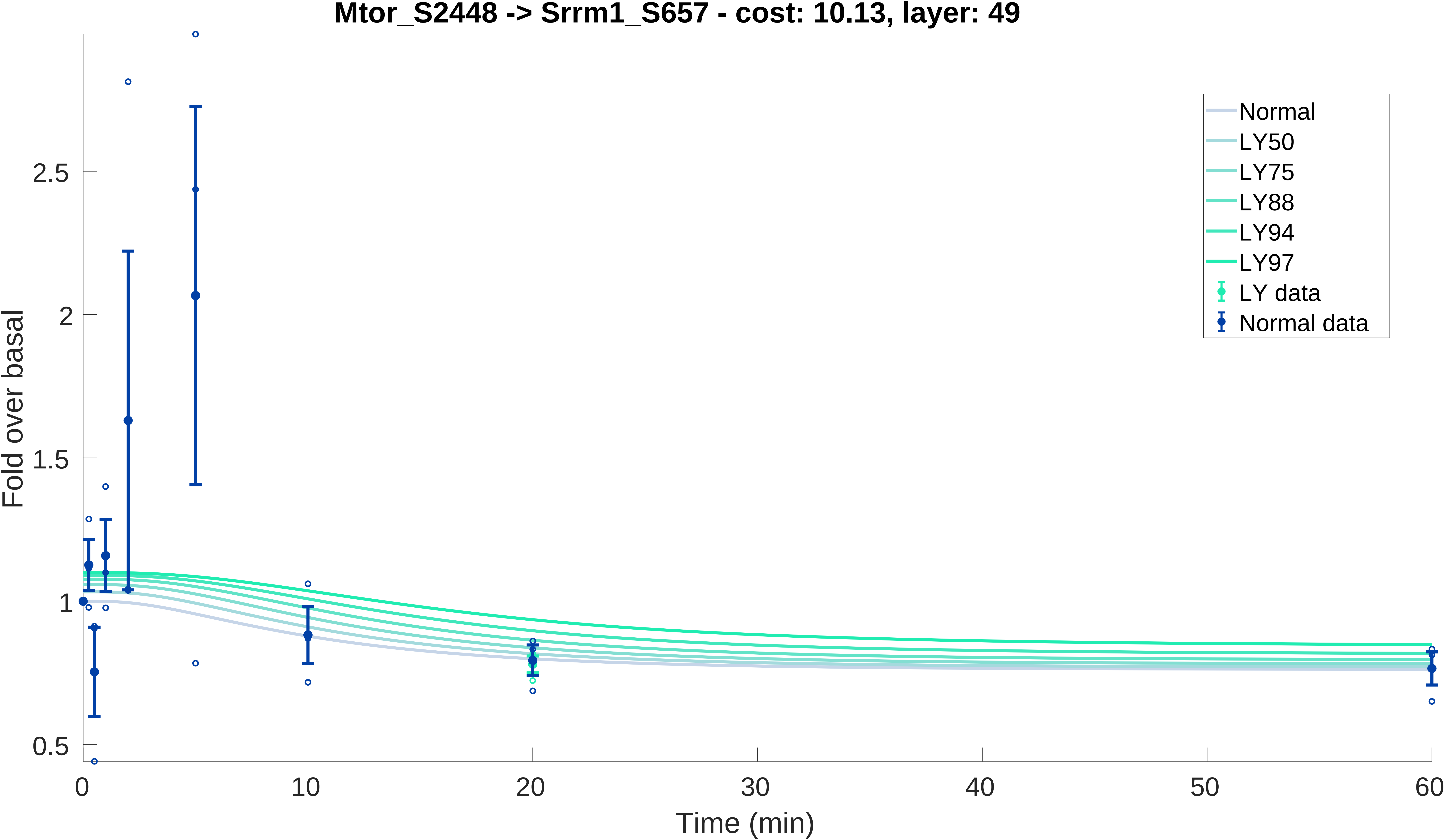

Mtor_S2448 -> Srrm1_S657 - cost: 10.13, layer: 49
Fold over basal
Time (min)
0.5
1
1.5
2
2.5
0
10
20
30
40
50
60
Normal
LY50
LY75
LY88
LY94
LY97
LY data
Normal data

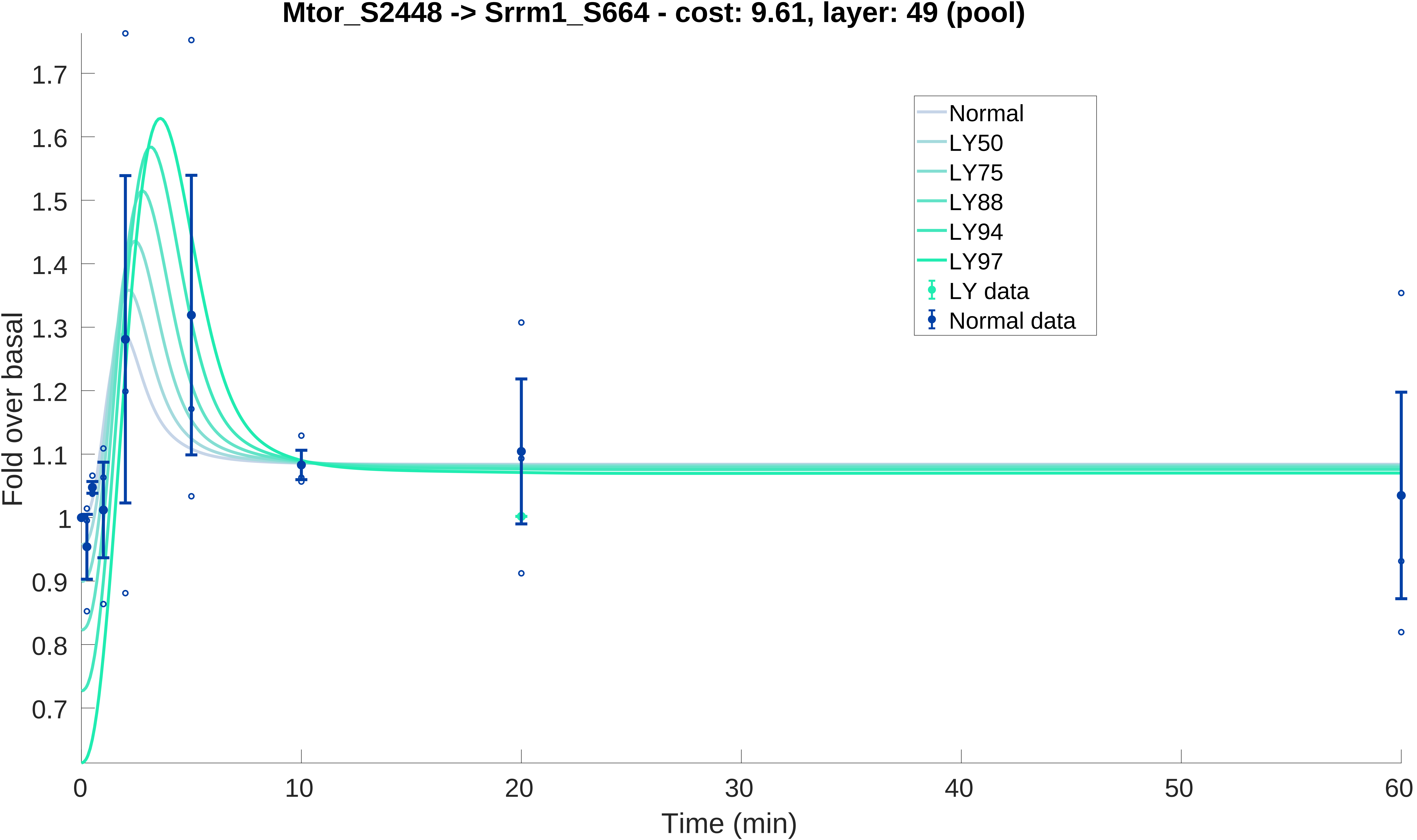
Mtor_S2448 -> Srrm1_S664 - cost: 9.61, layer: 49 (pool)
Fold over basal
Time (min)
Normal
LY50
LY75
LY88
LY94
LY97
LY data
Normal data
0.7
0.8
0.9
1
1.1
1.2
1.3
1.4
1.5
1.6
1.7
0
10
20
30
40
50
60

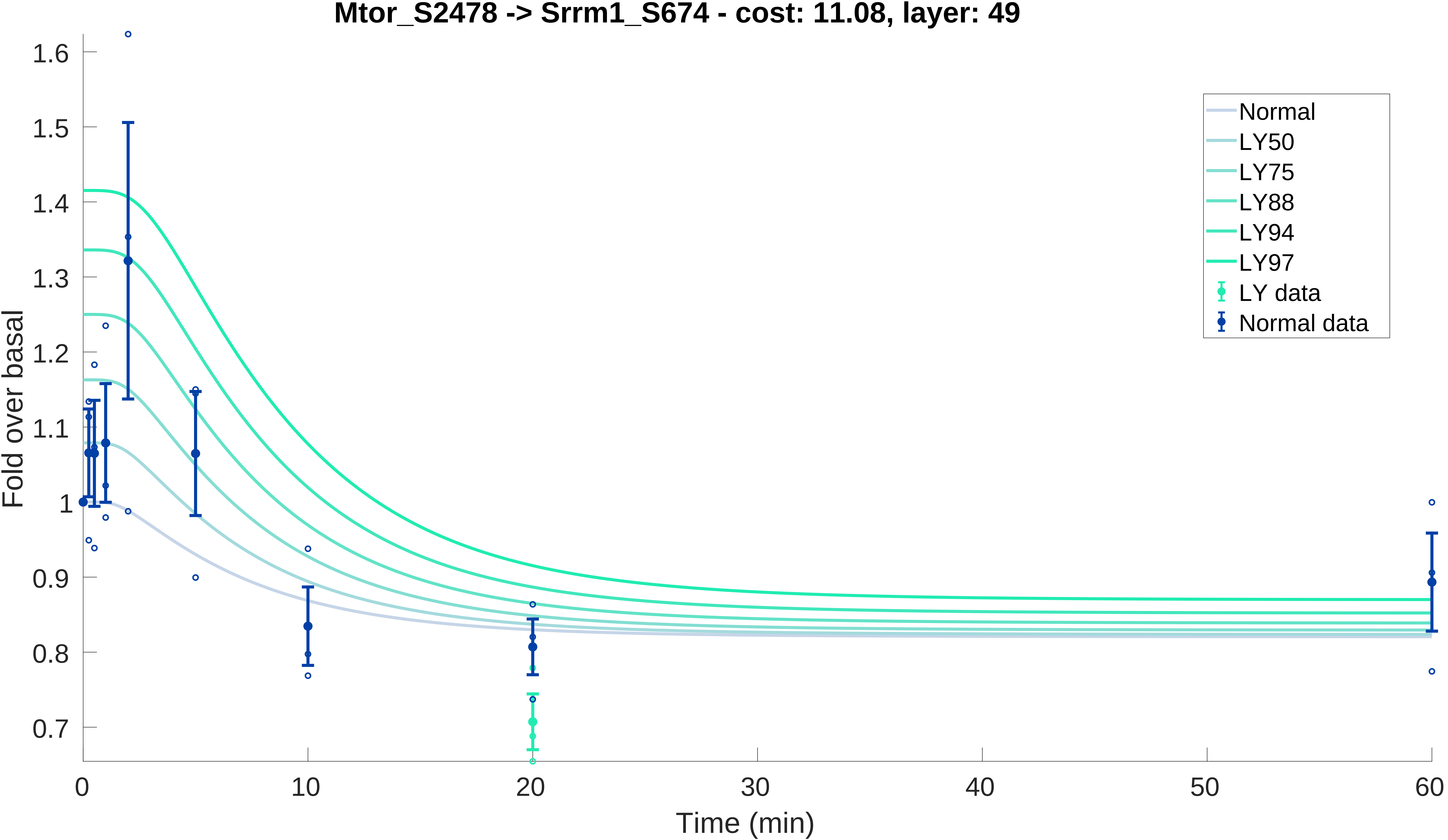

Mtor_S2478 -> Srrm1_S674 - cost: 11.08, layer: 49
Fold over basal
1.6
1.5
1.4
1.3
1.2
1.1
1
0.9
0.8
0.7
0
10
20
30
40
50
60
Time (min)
Normal
LY50
LY75
LY88
LY94
LY97
LY data
Normal data

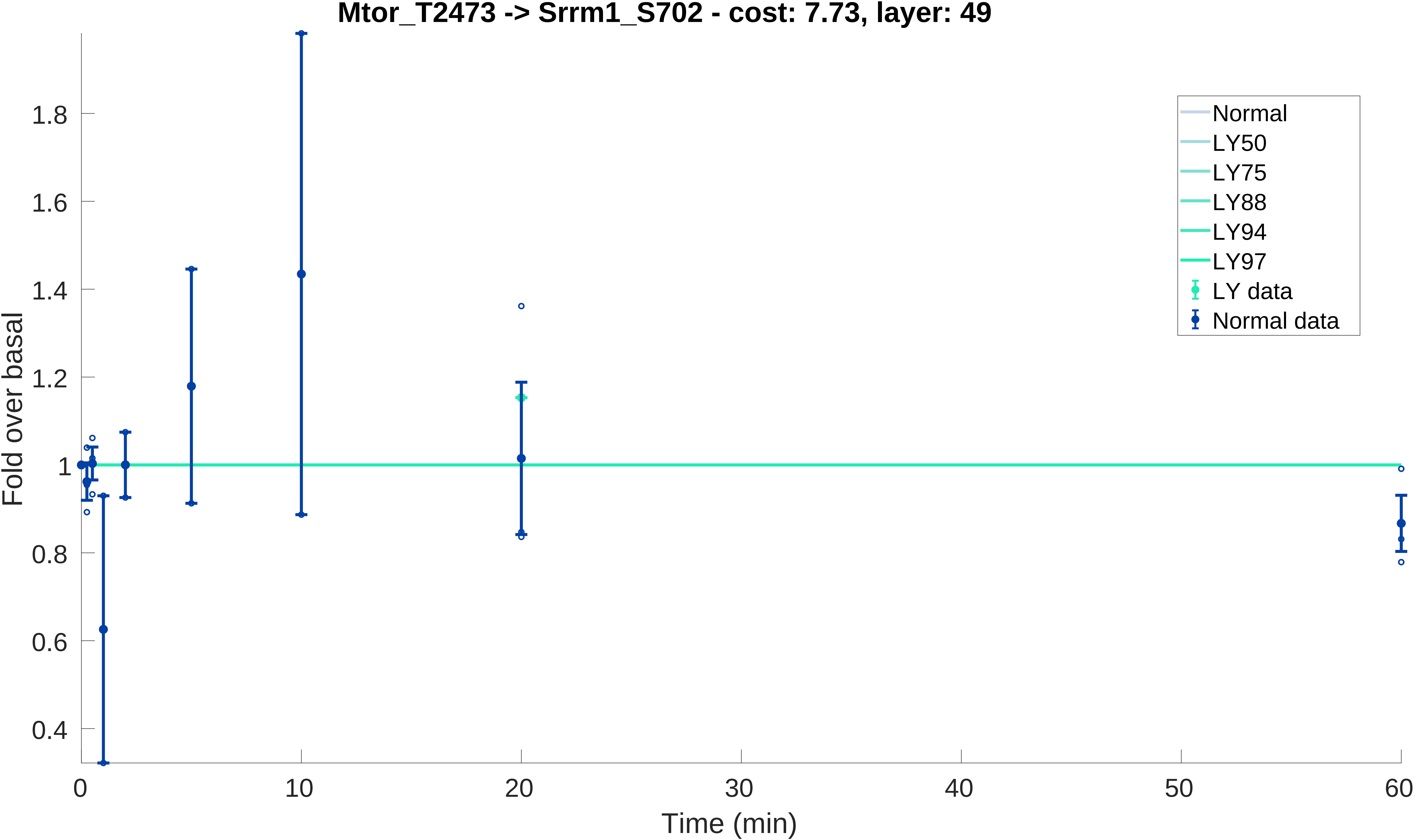

Mtor_T2473 -> Srrm1_S702 - cost: 7.73, layer: 49
Fold over basal
Time (min)
1.8
1.6
1.4
1.2
1
0.8
0.6
0.4
0
10
20
30
40
50
60
Normal
LY50
LY75
LY88
LY94
LY97
LY data
Normal data

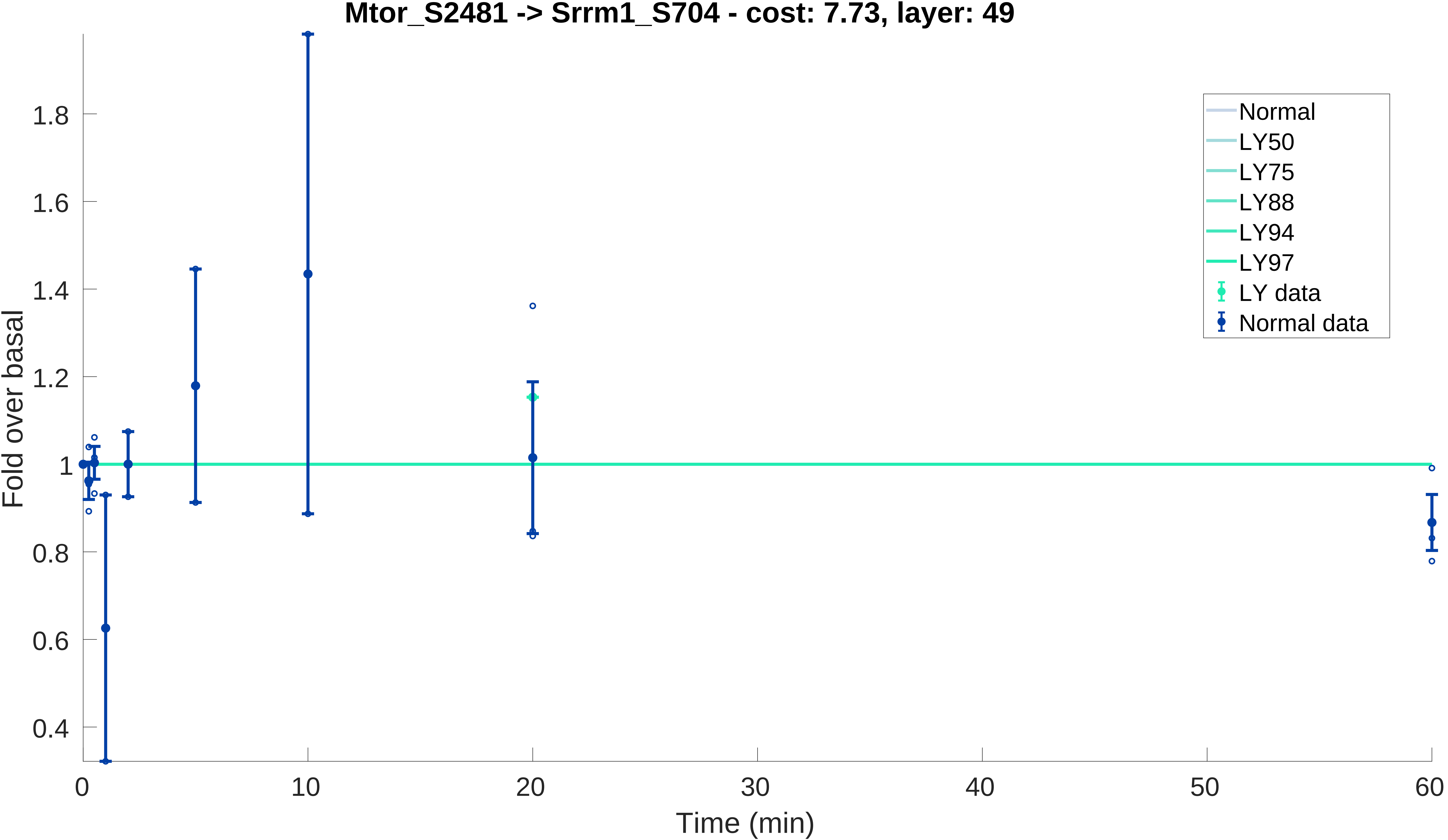

Mtor_S2481 -> Srrm1_S704 - cost: 7.73, layer: 49
Fold over basal
Time (min)
0
10
20
30
40
50
60
0.4
0.6
0.8
1
1.2
1.4
1.6
1.8
Normal
LY50
LY75
LY88
LY94
LY97
LY data
Normal data

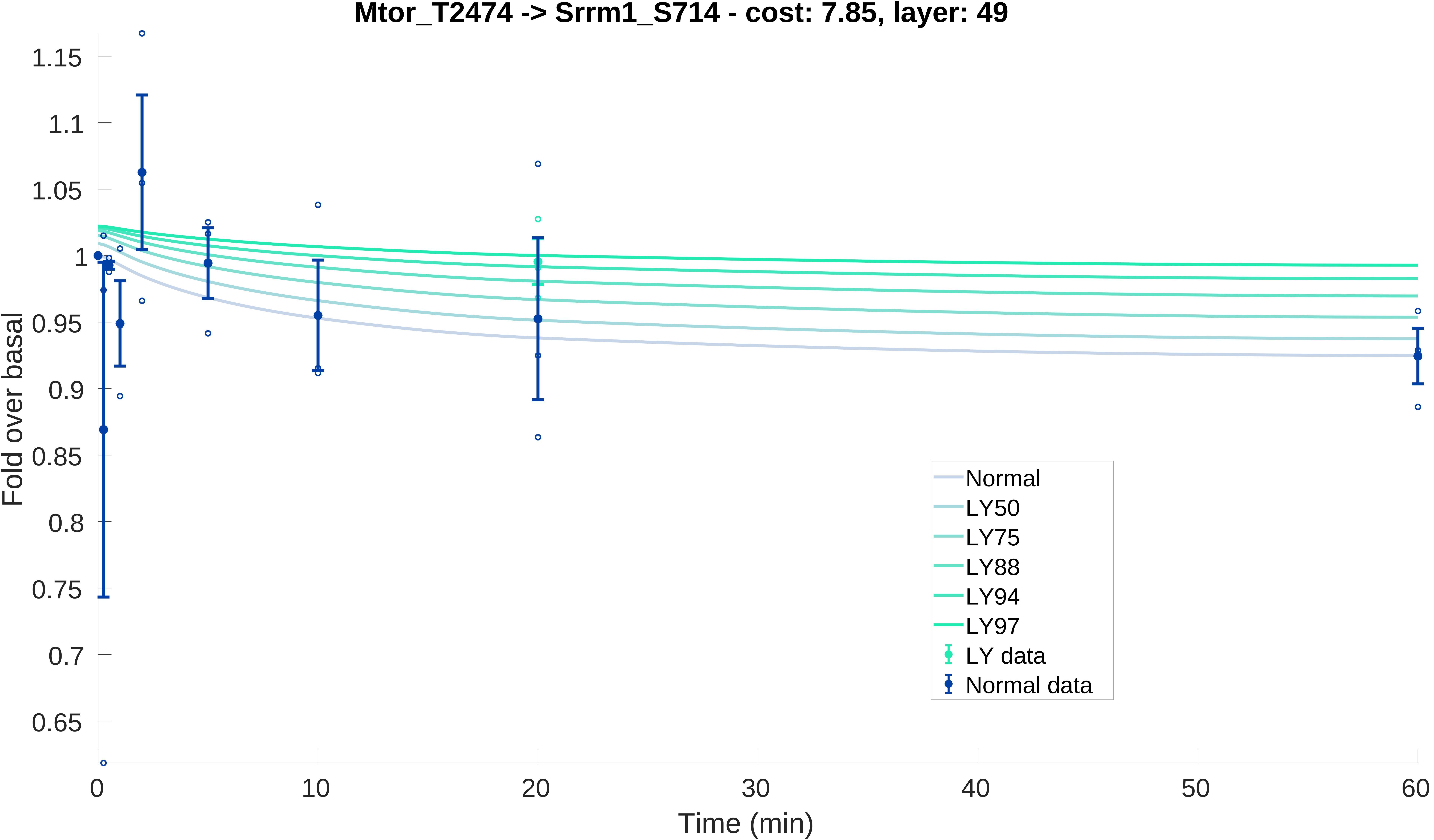
Mtor_T2474 -> Srrm1_S714 - cost: 7.85, layer: 49
Fold over basal
1.15
1.1
1.05
1
0.95
0.9
0.85
0.8
0.75
0.7
0.65
0
10
20
30
40
50
60
Time (min)
Normal
LY50
LY75
LY88
LY94
LY97
LY data
Normal data

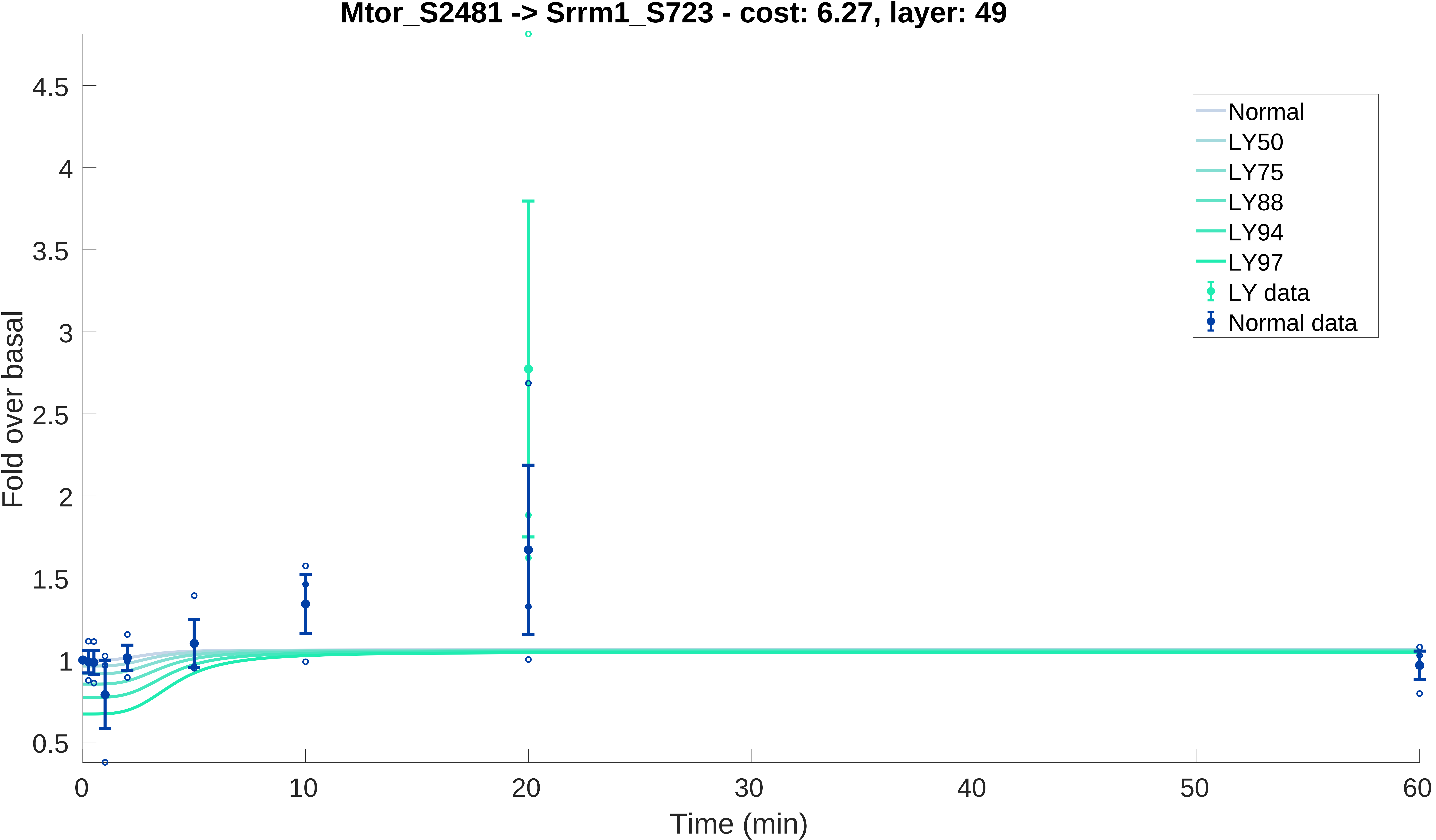

Mtor_S2481 -> Srrm1_S723 - cost: 6.27, layer: 49
Fold over basal
4.5
4
3.5
3
2.5
2
1.5
1
0.5
0
10
20
30
40
50
60
Time (min)
Normal
LY50
LY75
LY88
LY94
LY97
LY data
Normal data

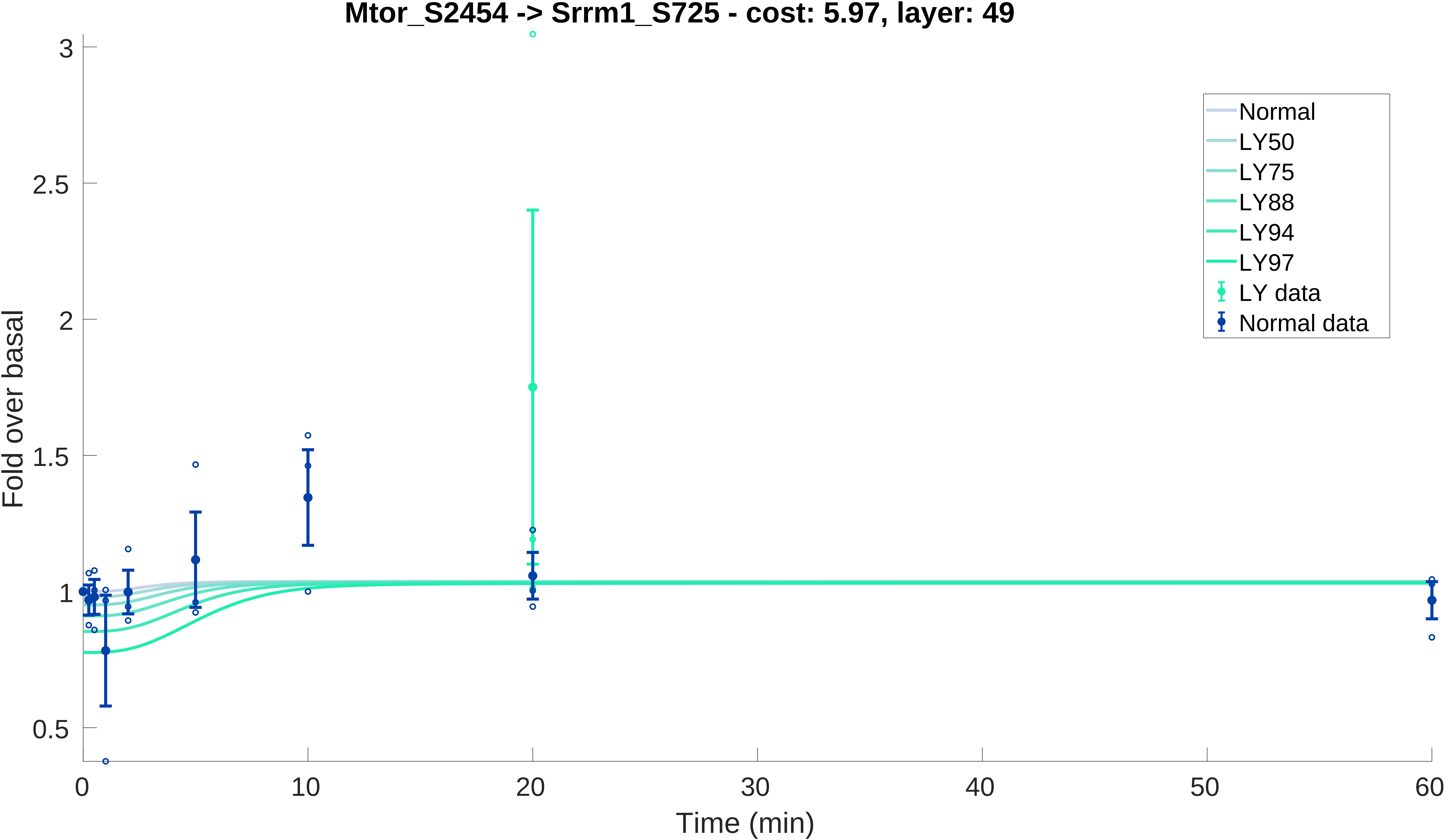

Mtor_S2454 -> Srrm1_S725 - cost: 5.97, layer: 49
Fold over basal
Time (min)
0.5
1
1.5
2
2.5
3
0
10
20
30
40
50
60
Normal
LY50
LY75
LY88
LY94
LY97
LY data
Normal data

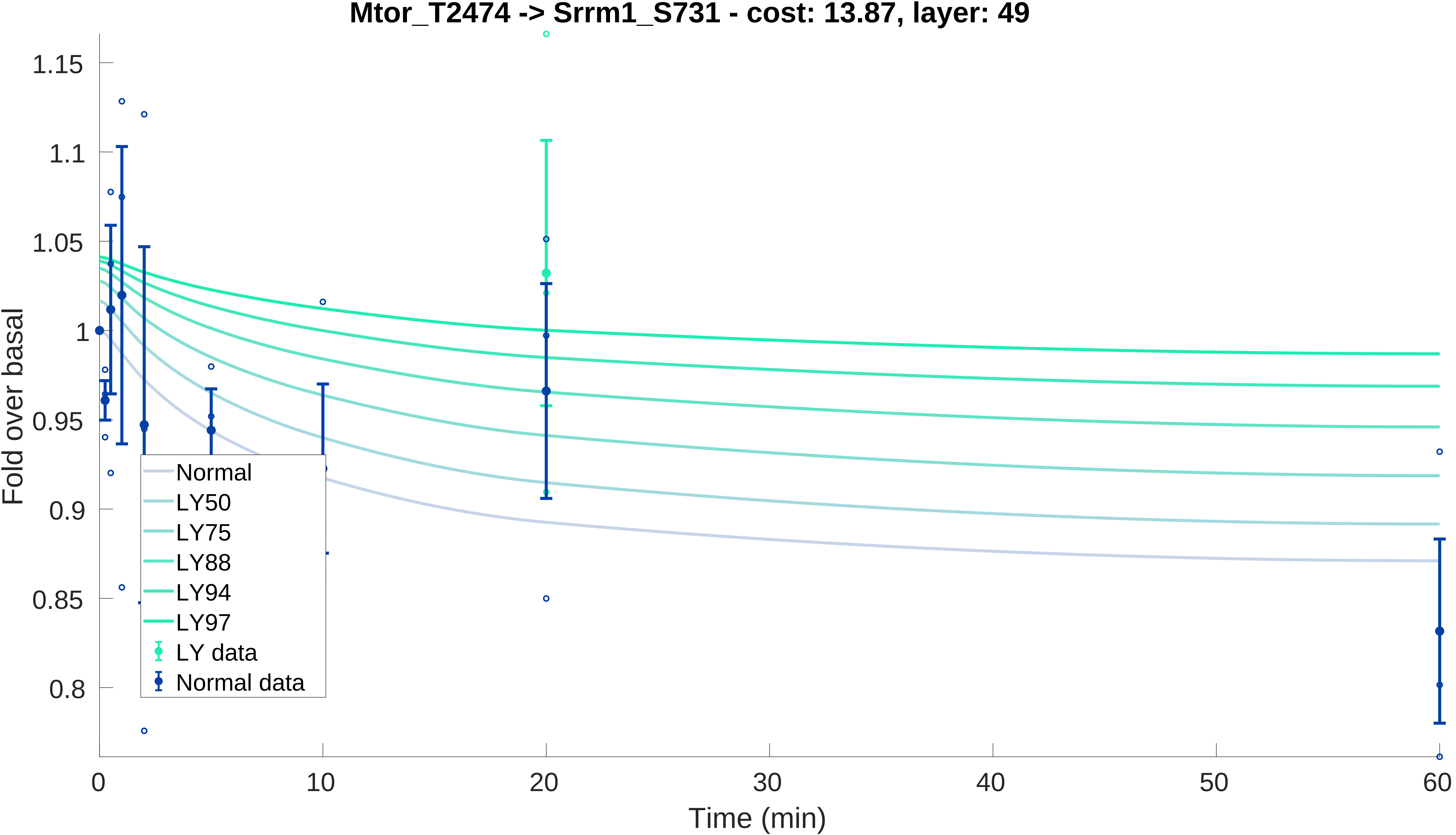
Mtor_T2474 -> Srrm1_S731 - cost: 13.87, layer: 49
Fold over basal
Time (min)
1.15
1.1
1.05
1
0.95
0.9
0.85
0.8
0
10
20
30
40
50
60
Normal
LY50
LY75
LY88
LY94
LY97
LY data
Normal data

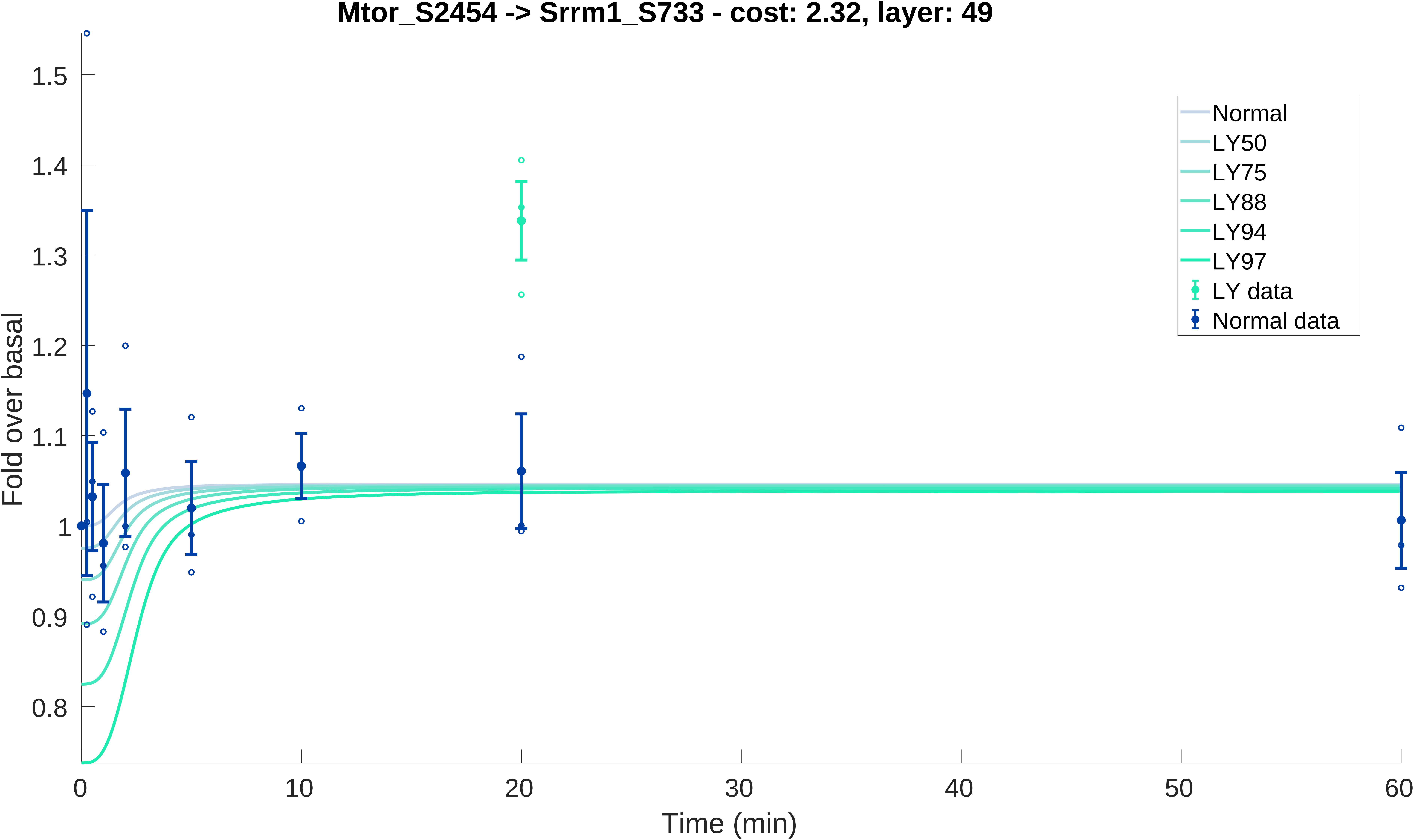

Mtor_S2454 -> Srrm1_S733 - cost: 2.32, layer: 49
Fold over basal
1.5
1.4
1.3
1.2
1.1
1
0.9
0.8
0
10
20
30
40
50
60
Time (min)
Normal
LY50
LY75
LY88
LY94
LY97
LY data
Normal data

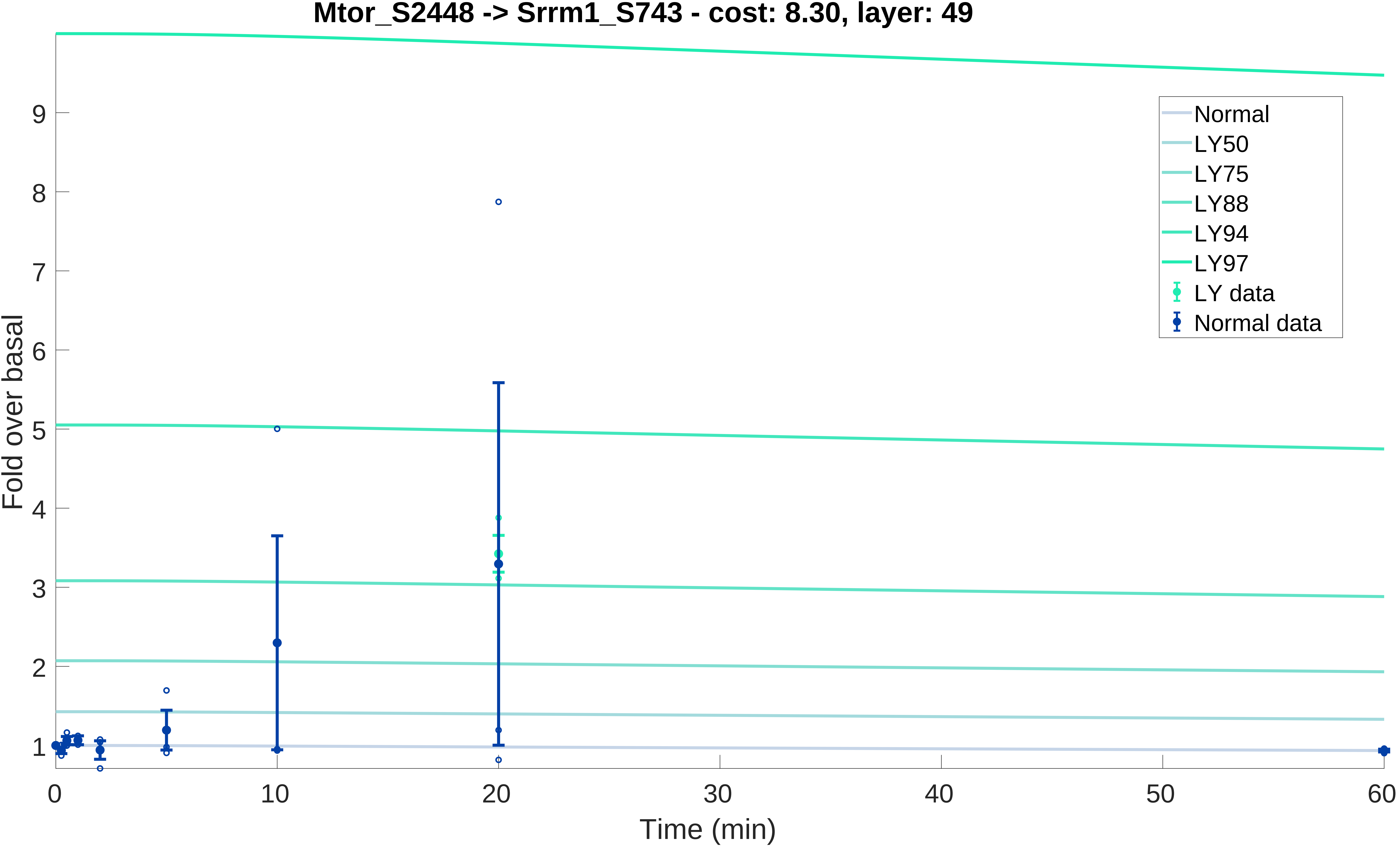

Mtor_S2448 -> Srrm1_S743 - cost: 8.30, layer: 49
Fold over basal
Time (min)
Normal
LY50
LY75
LY88
LY94
LY97
LY data
Normal data
1
2
3
4
5
6
7
8
9
0
10
20
30
40
50
60

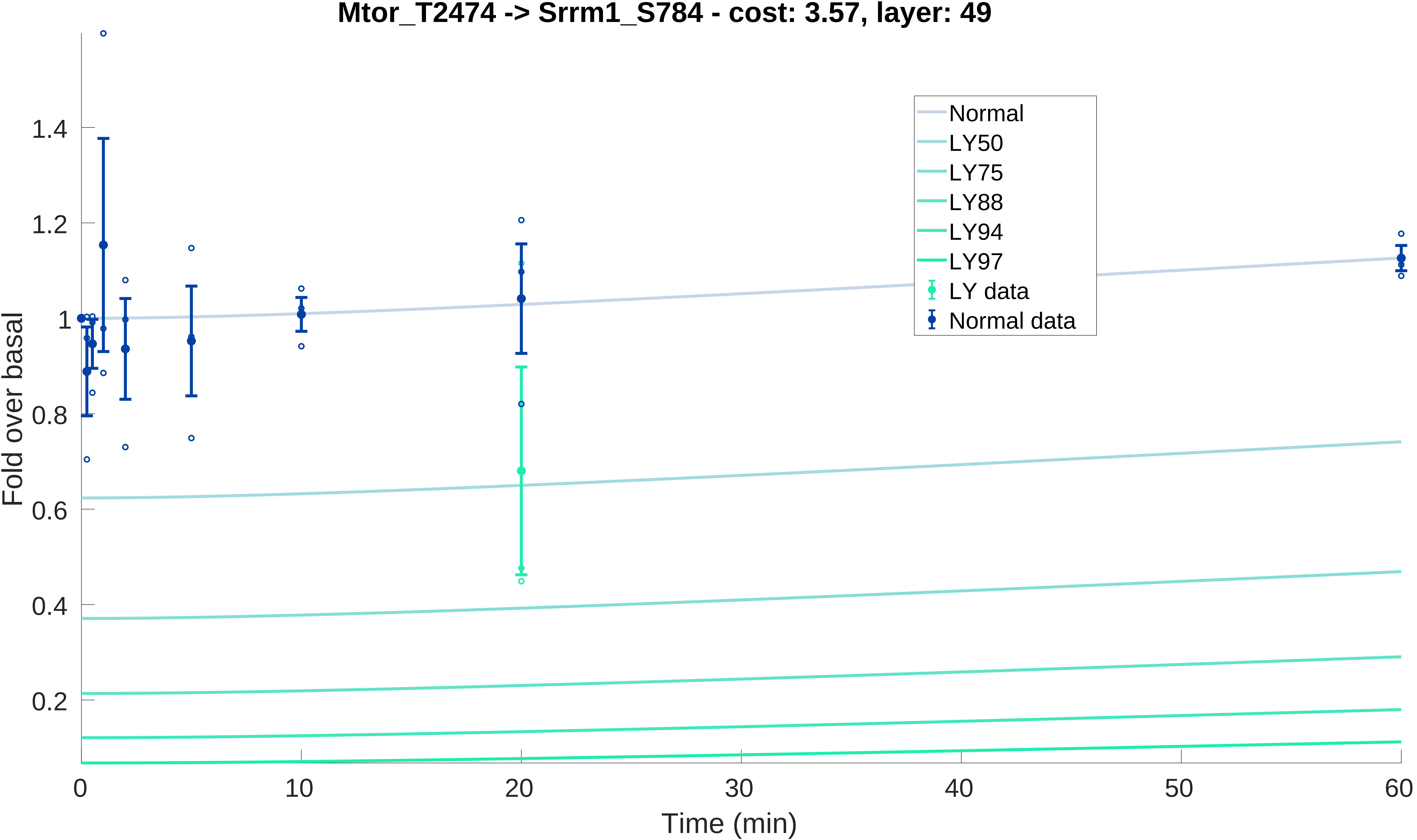
Mtor_T2474 -> Srrm1_S784 - cost: 3.57, layer: 49
Normal
LY50
LY75
LY88
LY94
LY97
LY data
Normal data
Fold over basal
0.2
0.4
0.6
0.8
1
1.2
1.4
0
10
20
30
40
50
60
Time (min)

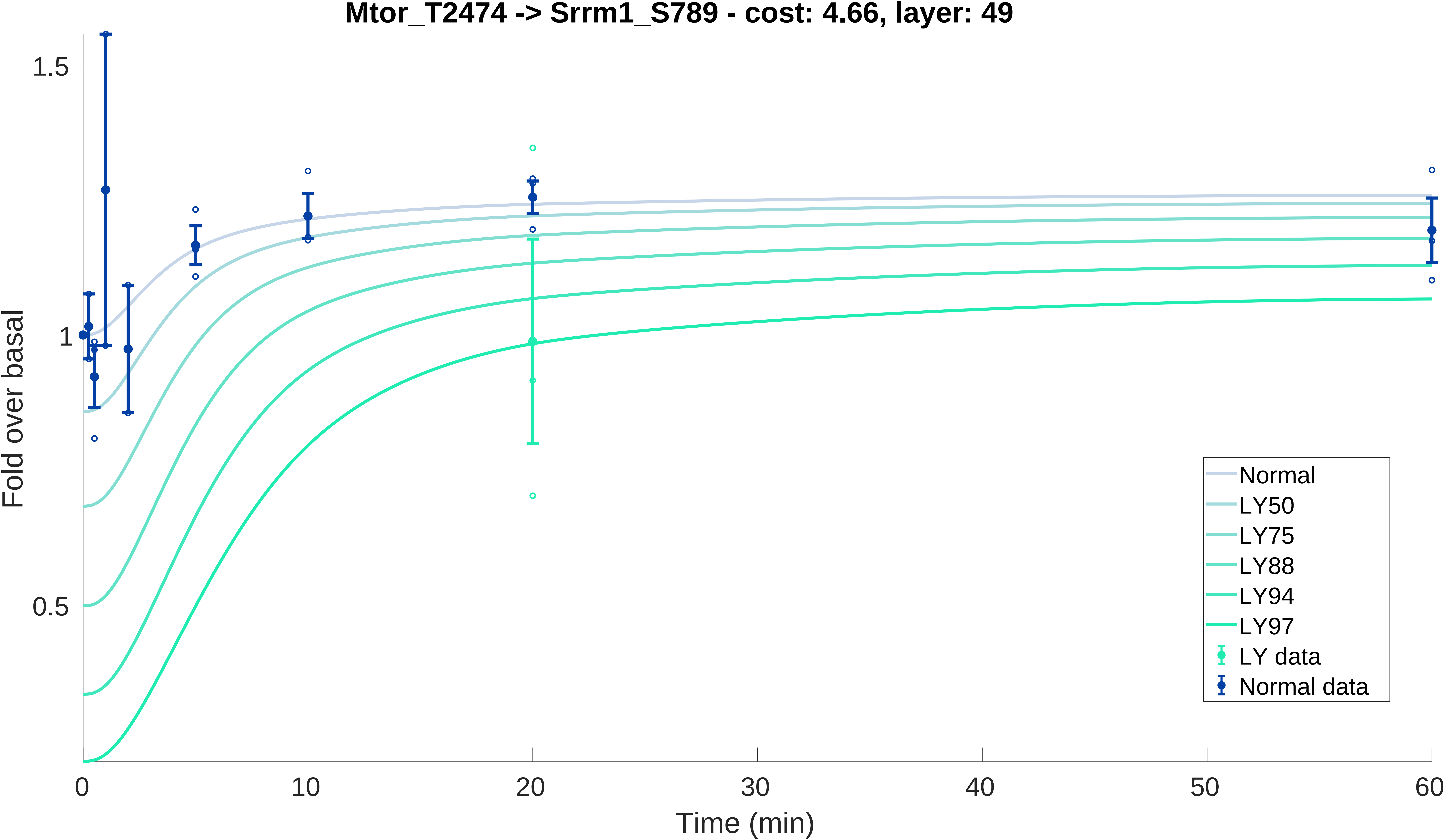

Mtor_T2474 -> Srrm1_S789 - cost: 4.66, layer: 49
Fold over basal
1.5
1
0.5
0
10
20
30
40
50
60
Time (min)
Normal
LY50
LY75
LY88
LY94
LY97
LY data
Normal data

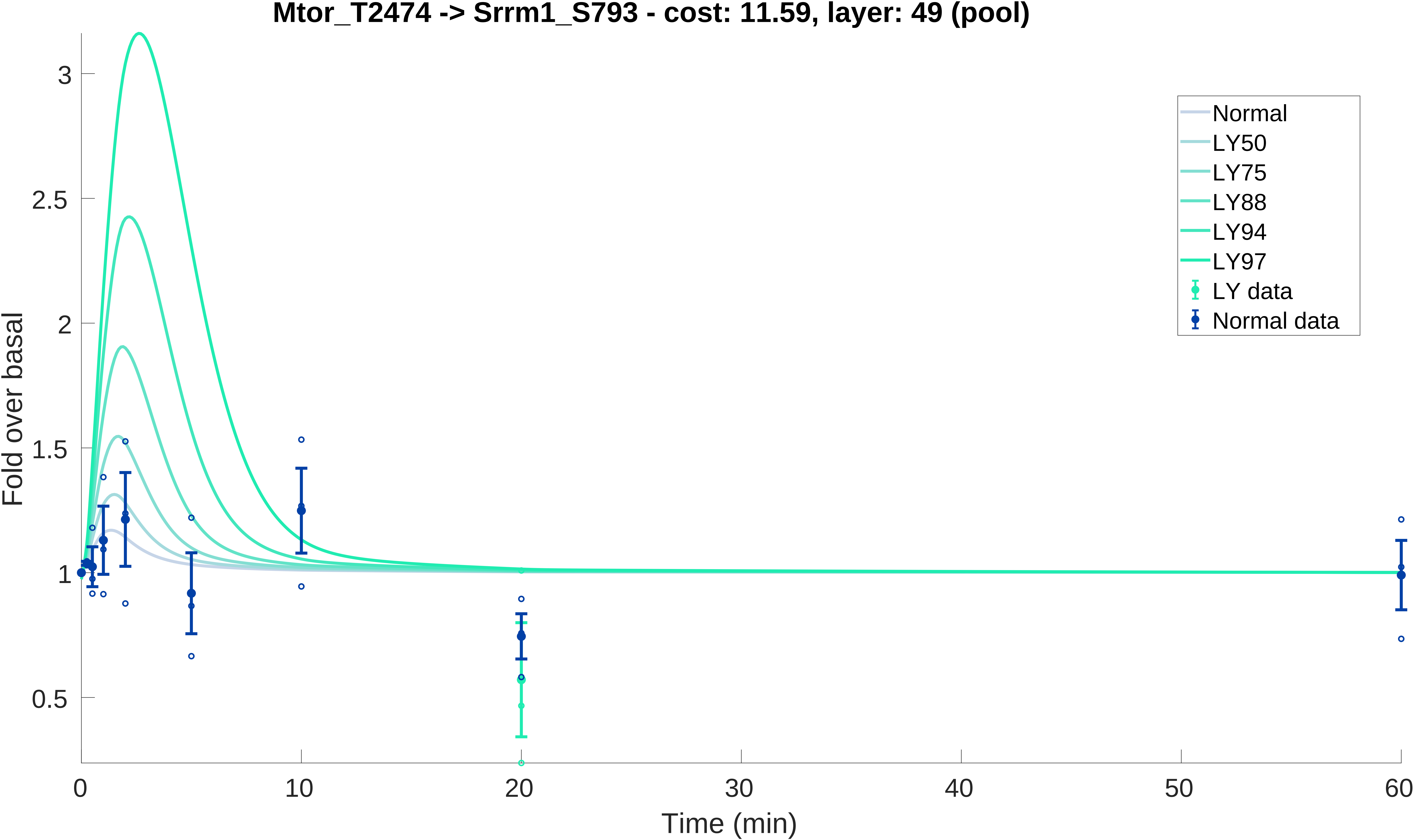

Mtor_T2474 -> Srrm1_S793 - cost: 11.59, layer: 49 (pool)
Fold over basal
Time (min)
0.5
1
1.5
2
2.5
3
0
10
20
30
40
50
60
Normal
LY50
LY75
LY88
LY94
LY97
LY data
Normal data

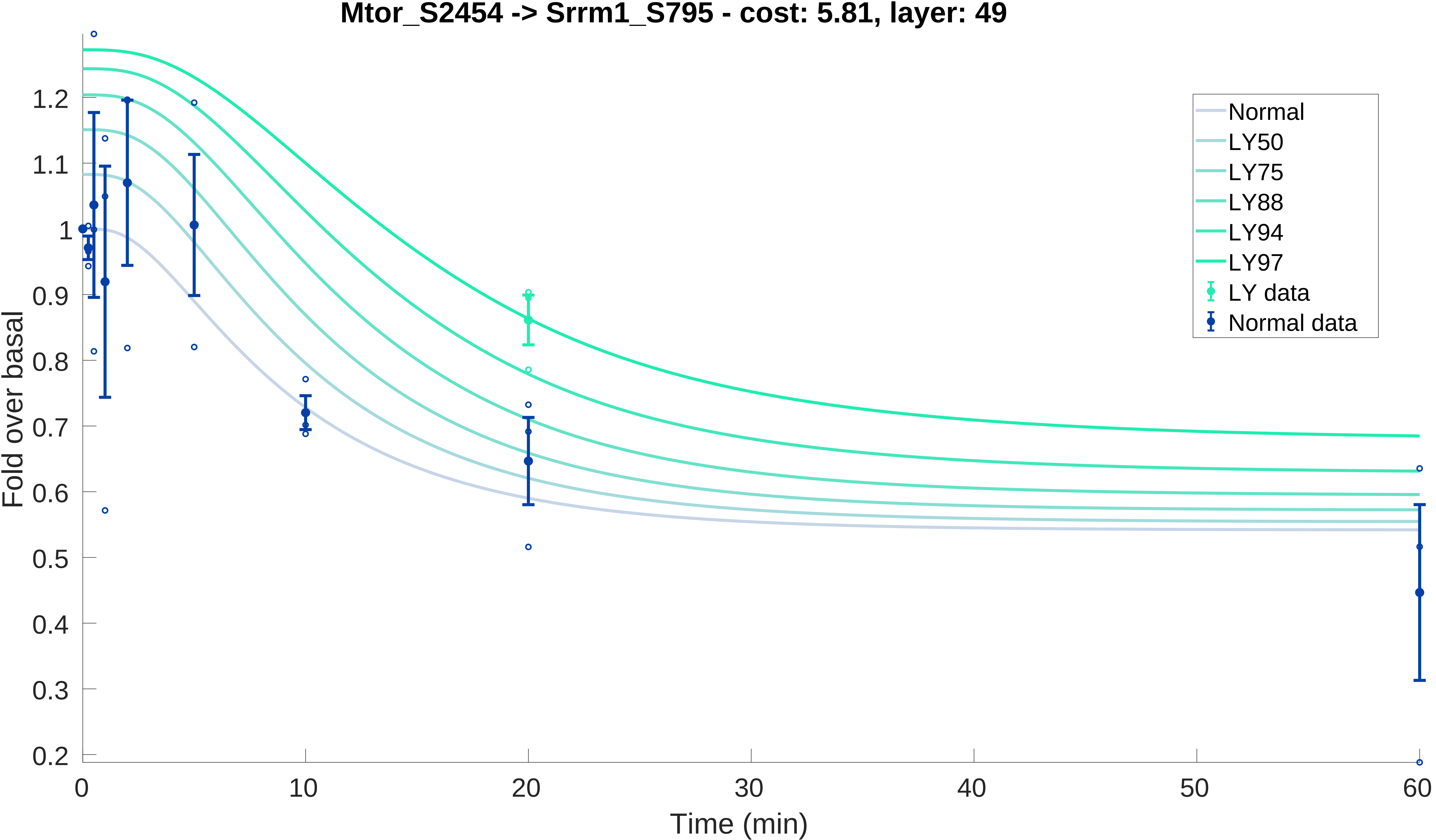

Mtor_S2454 -> Srrm1_S795 - cost: 5.81, layer: 49
Normal
LY50
LY75
LY88
LY94
LY97
LY data
Normal data
Fold over basal
Time (min)
0.2
0.3
0.4
0.5
0.6
0.7
0.8
0.9
1
1.1
1.2
0
10
20
30
40
50
60

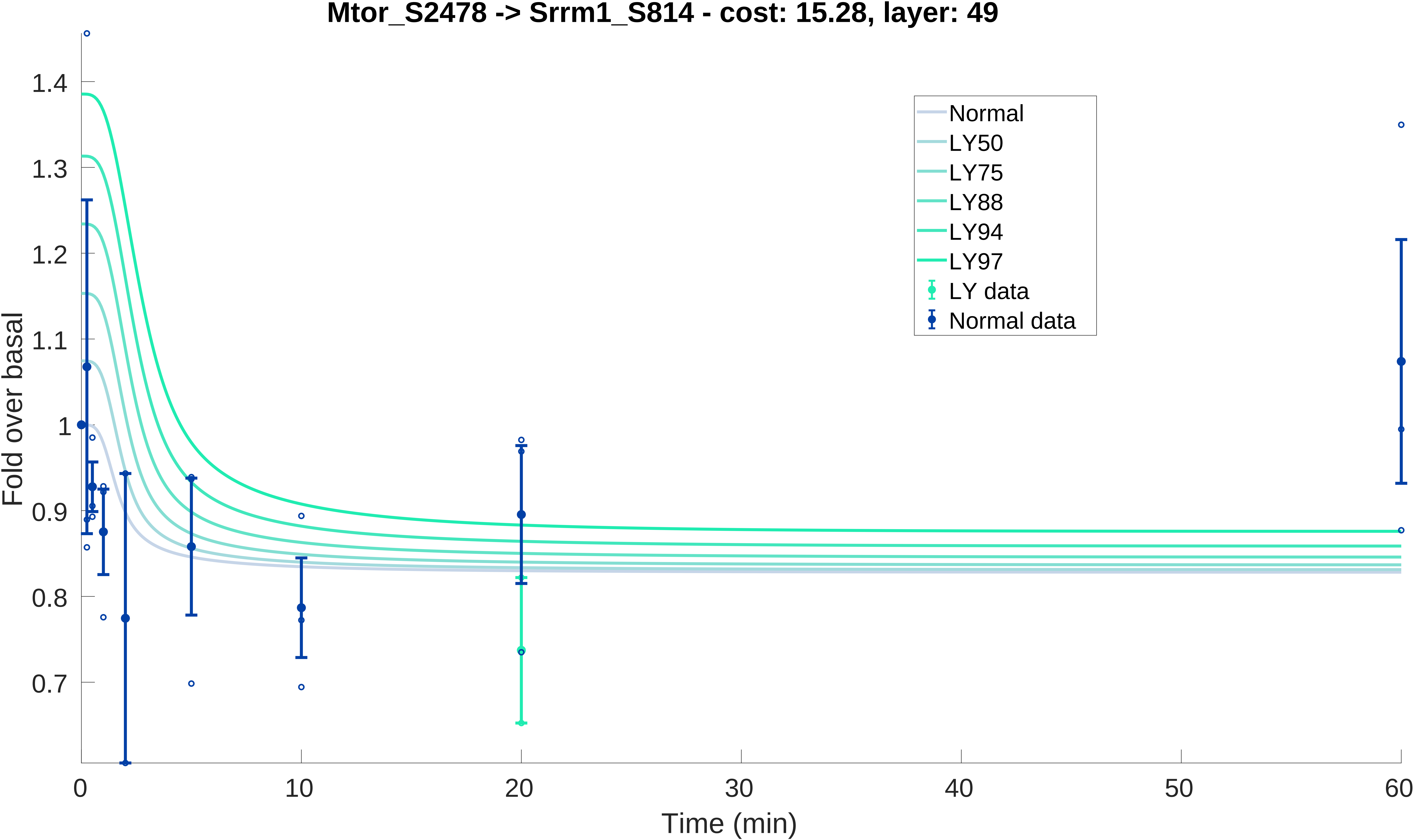
Mtor_S2478 -> Srrm1_S814 - cost: 15.28, layer: 49
Fold over basal
Time (min)
Normal
LY50
LY75
LY88
LY94
LY97
LY data
Normal data
1.4
1.3
1.2
1.1
1
0.9
0.8
0.7
0
10
20
30
40
50
60

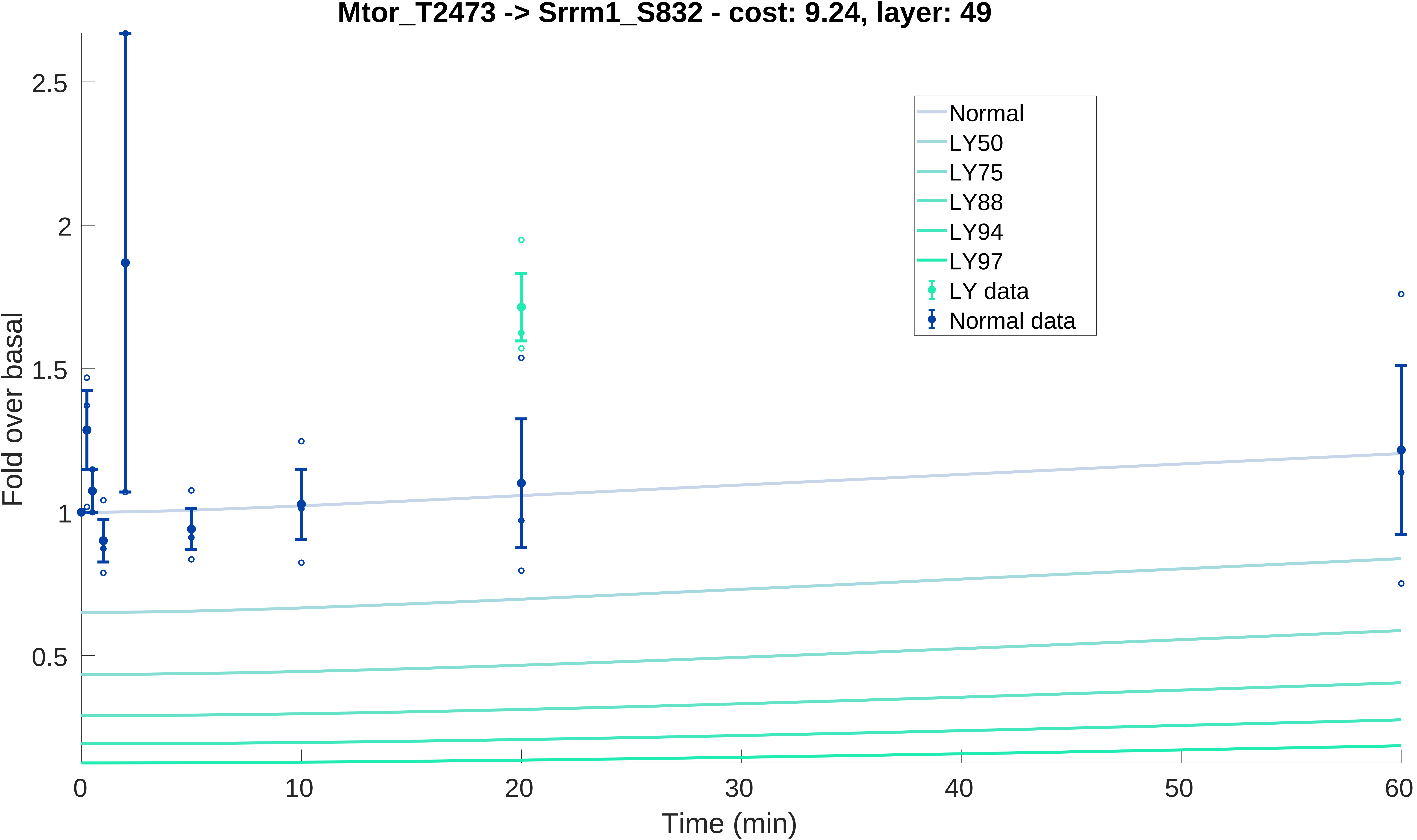

Mtor_T2473 -> Srrm1_S832 - cost: 9.24, layer: 49
Fold over basal
Time (min)
0.5
1
1.5
2
2.5
0
10
20
30
40
50
60
Normal
LY50
LY75
LY88
LY94
LY97
LY data
Normal data

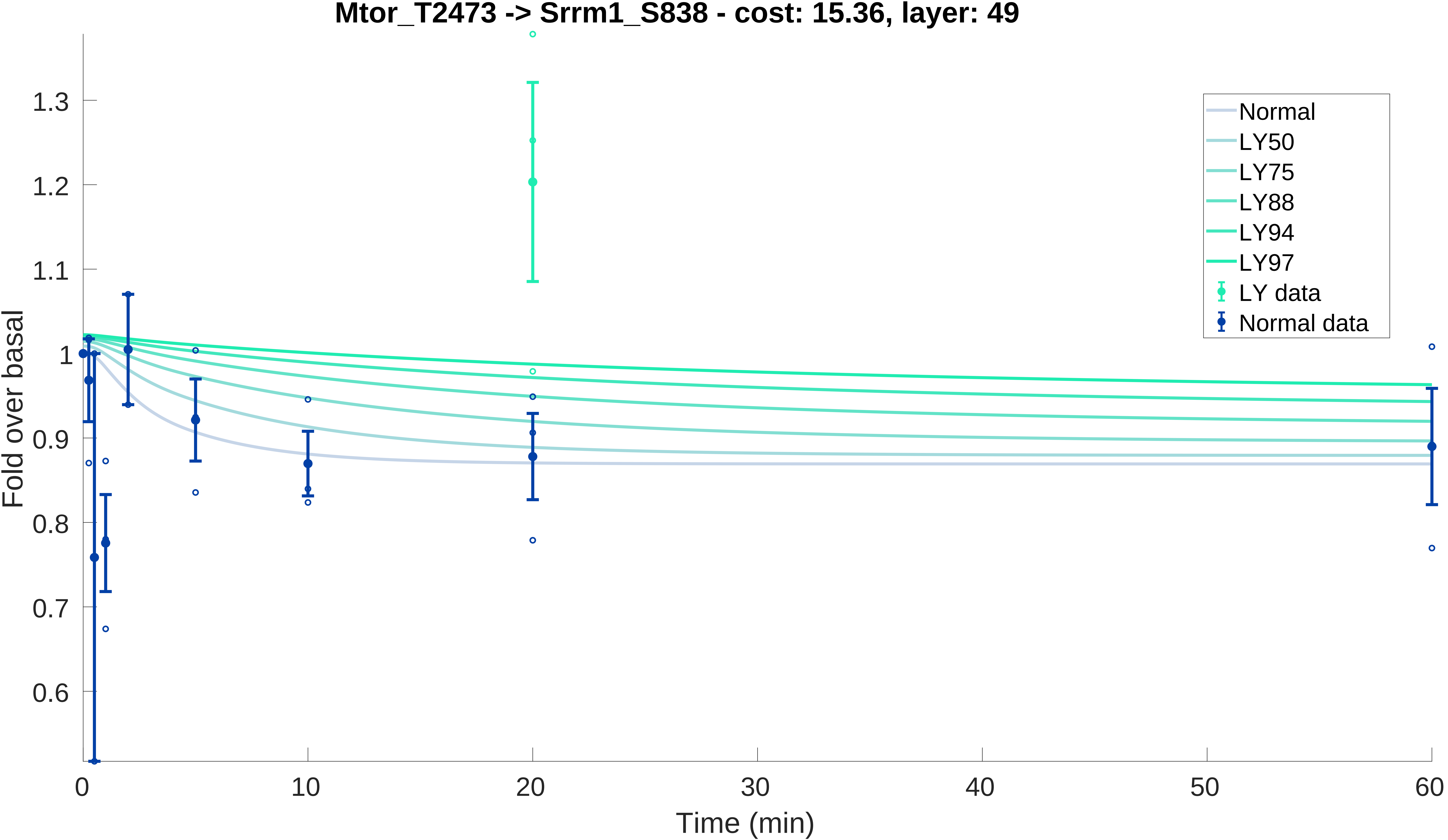

Mtor_T2473 -> Srrm1_S838 - cost: 15.36, layer: 49
Fold over basal
Time (min)
1.3
1.2
1.1
1
0.9
0.8
0.7
0.6
0
10
20
30
40
50
60
Normal
LY50
LY75
LY88
LY94
LY97
LY data
Normal data

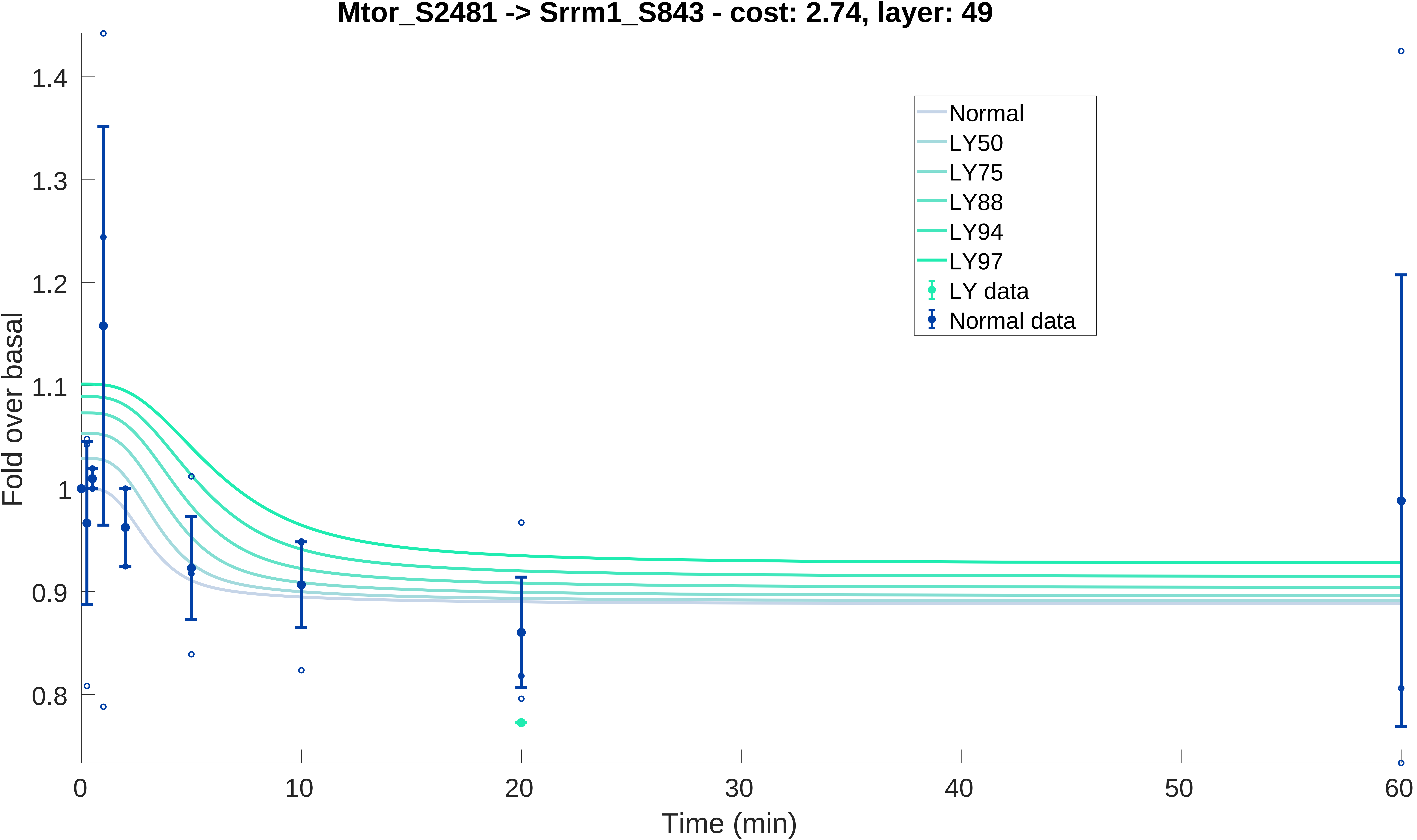
Mtor_S2481 -> Srrm1_S843 - cost: 2.74, layer: 49
Fold over basal
Time (min)
0
10
20
30
40
50
60
0.8
0.9
1
1.1
1.2
1.3
1.4
Normal
LY50
LY75
LY88
LY94
LY97
LY data
Normal data

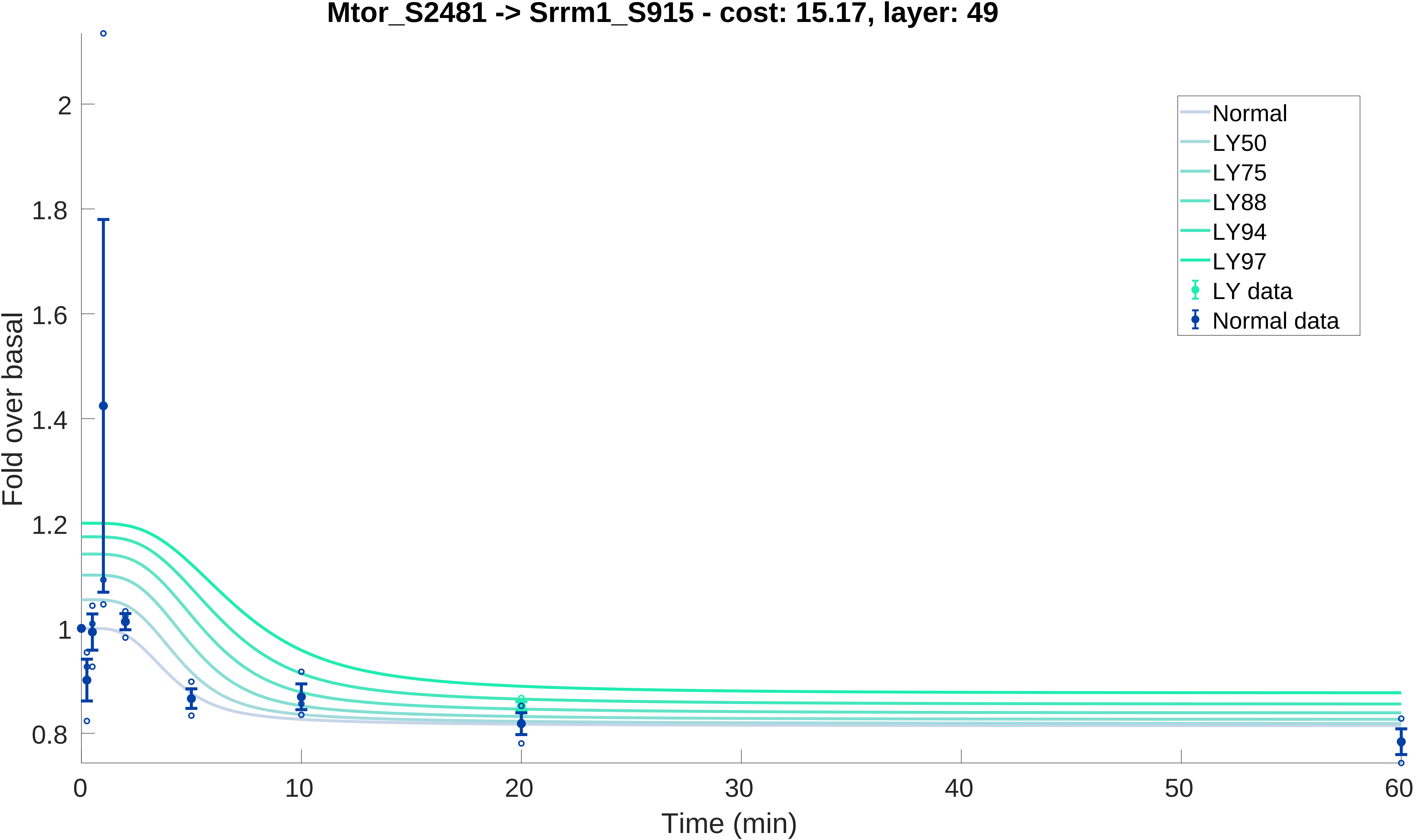

Mtor_S2481 -> Srrm1_S915 - cost: 15.17, layer: 49
Fold over basal
Time (min)
0
10
20
30
40
50
60
0.8
1
1.2
1.4
1.6
1.8
2
Normal
LY50
LY75
LY88
LY94
LY97
LY data
Normal data

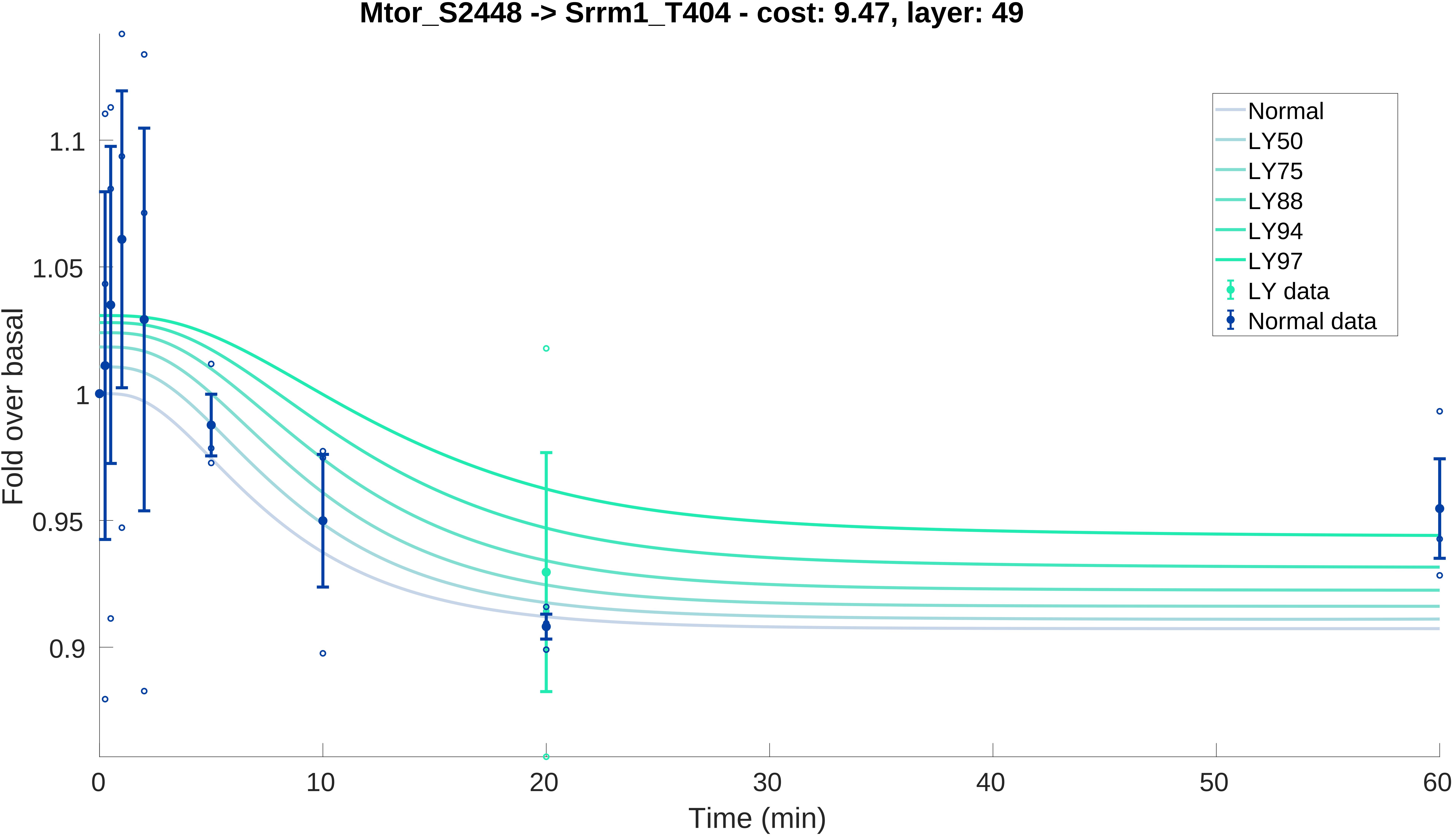

Mtor_S2448 -> Srrm1_T404 - cost: 9.47, layer: 49
Fold over basal
1.1
1.05
1
0.95
0.9
0
10
20
30
40
50
60
Time (min)
Normal
LY50
LY75
LY88
LY94
LY97
LY data
Normal data

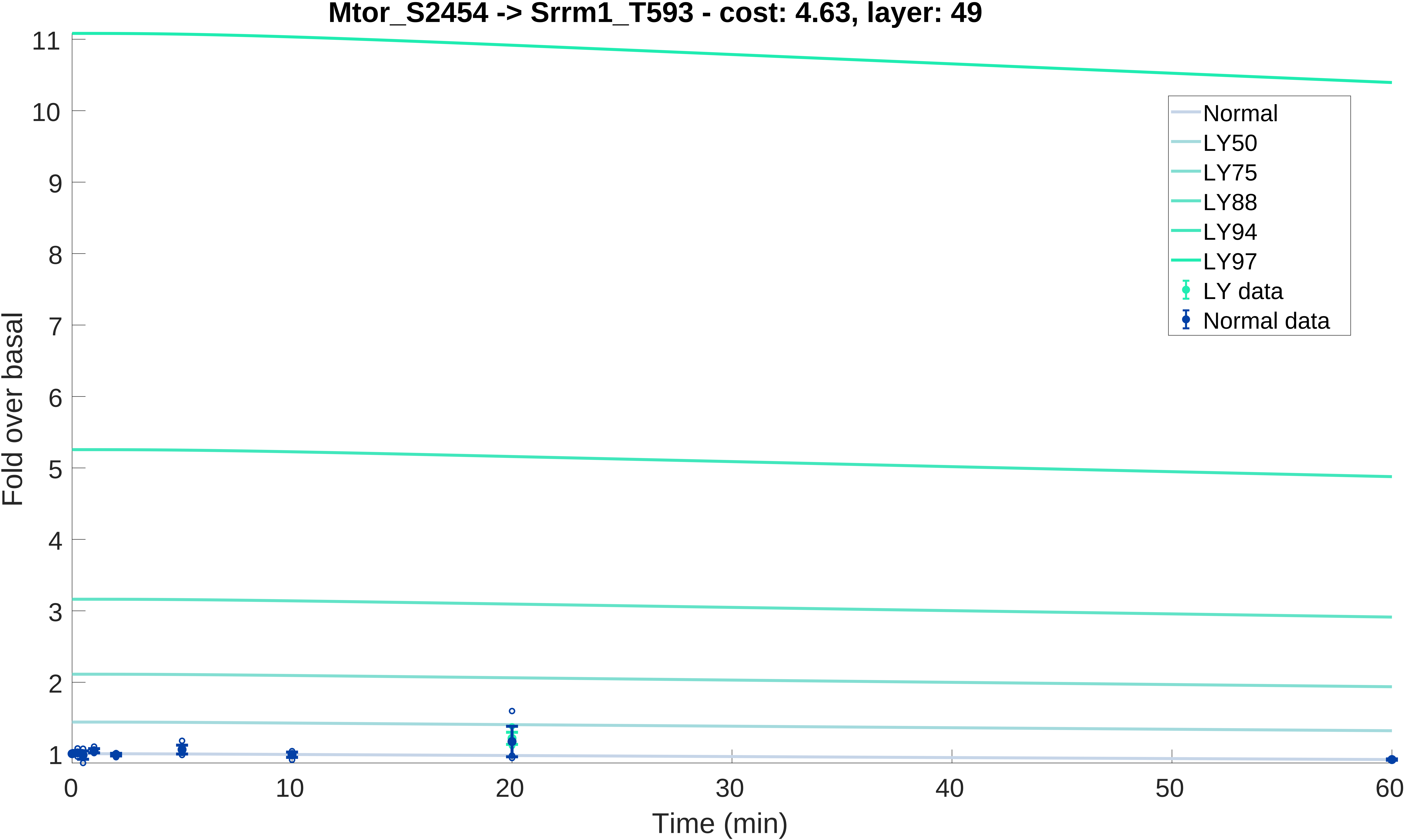

Mtor_S2454 -> Srrm1_T593 - cost: 4.63, layer: 49
Fold over basal
Time (min)
0
10
20
30
40
50
60
1
2
3
4
5
6
7
8
9
10
11
Normal
LY50
LY75
LY88
LY94
LY97
LY data
Normal data

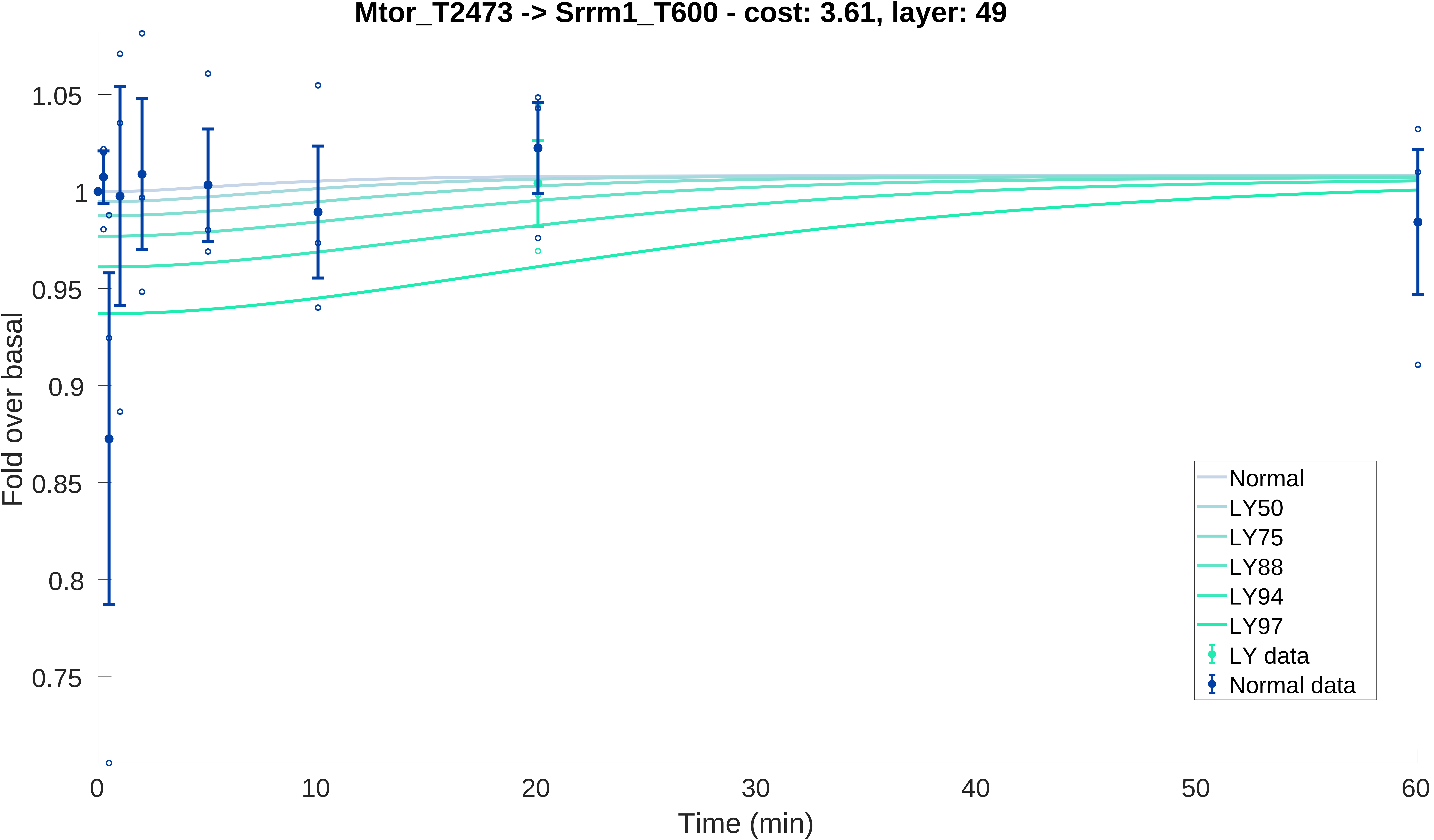

Mtor_T2473 -> Srrm1_T600 - cost: 3.61, layer: 49
Fold over basal
Time (min)
1.05
1
0.95
0.9
0.85
0.8
0.75
0
10
20
30
40
50
60
Normal
LY50
LY75
LY88
LY94
LY97
LY data
Normal data

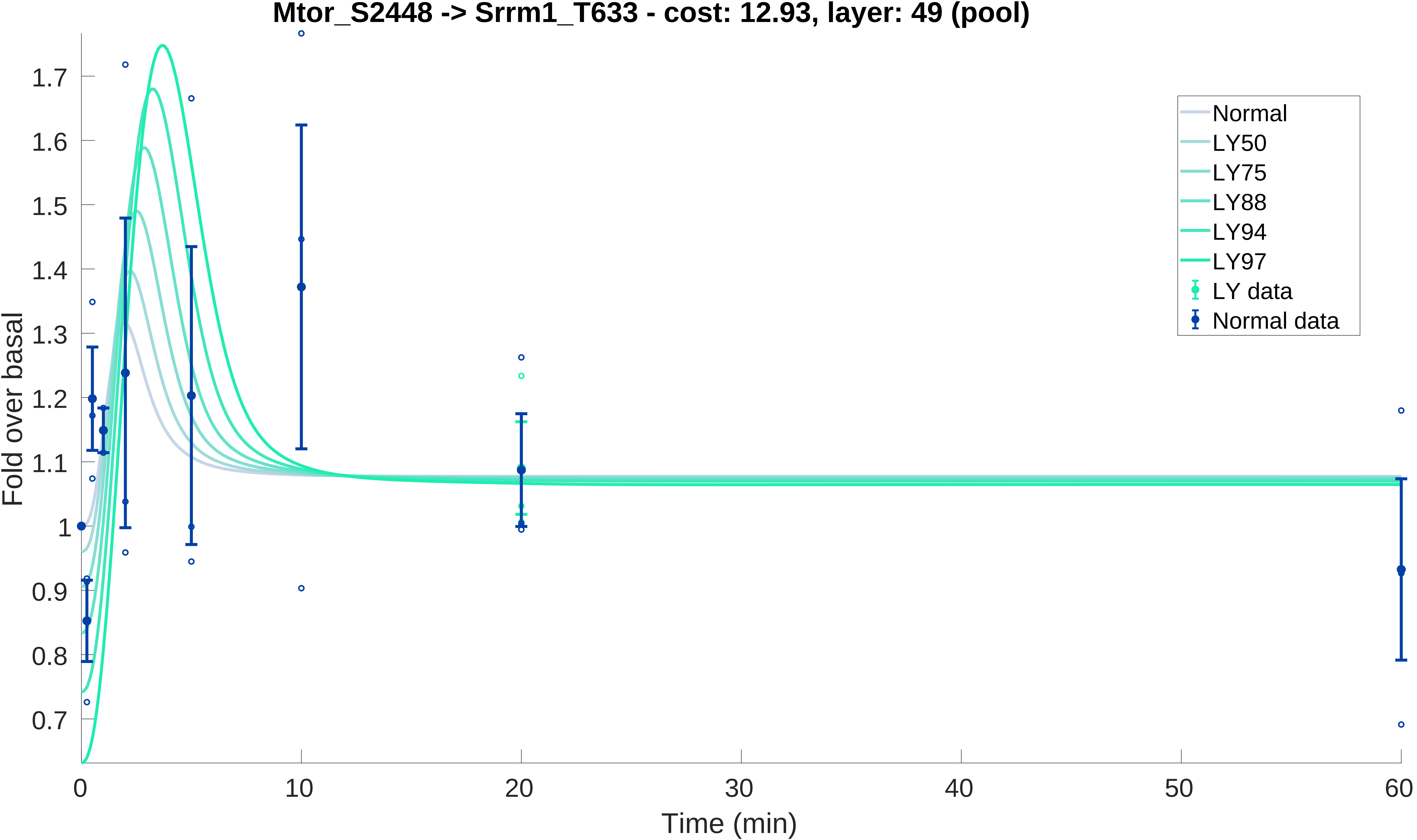

Mtor_S2448 -> Srrm1_T633 - cost: 12.93, layer: 49 (pool)
Fold over basal
Time (min)
1.7
1.6
1.5
1.4
1.3
1.2
1.1
1
0.9
0.8
0.7
0
10
20
30
40
50
60
Normal
LY50
LY75
LY88
LY94
LY97
LY data
Normal data

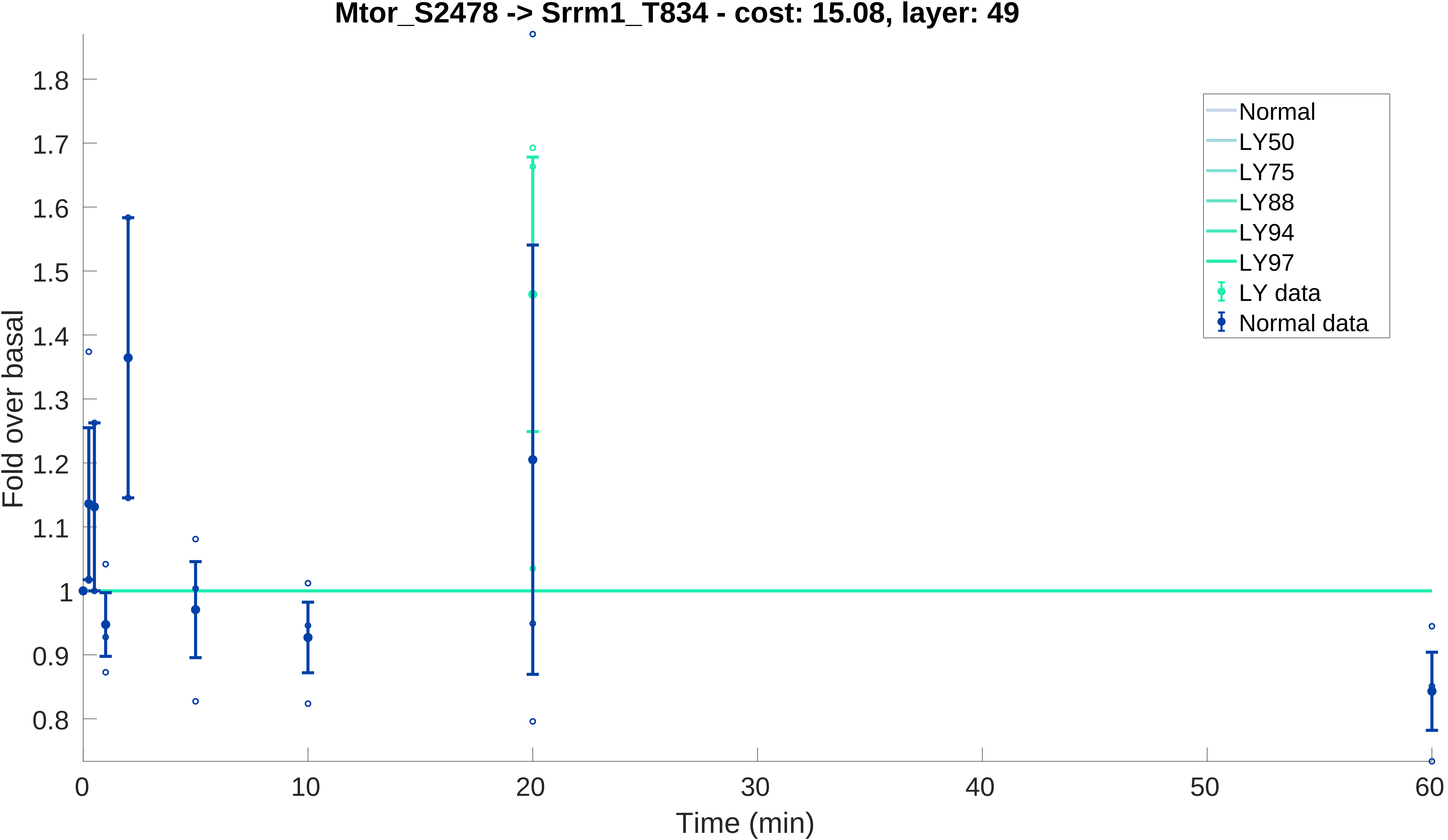

Mtor_S2478 -> Srrm1_T834 - cost: 15.08, layer: 49
Fold over basal
1.8
1.7
1.6
1.5
1.4
1.3
1.2
1.1
1
0.9
0.8
0
10
20
30
40
50
60
Time (min)
Normal
LY50
LY75
LY88
LY94
LY97
LY data
Normal data

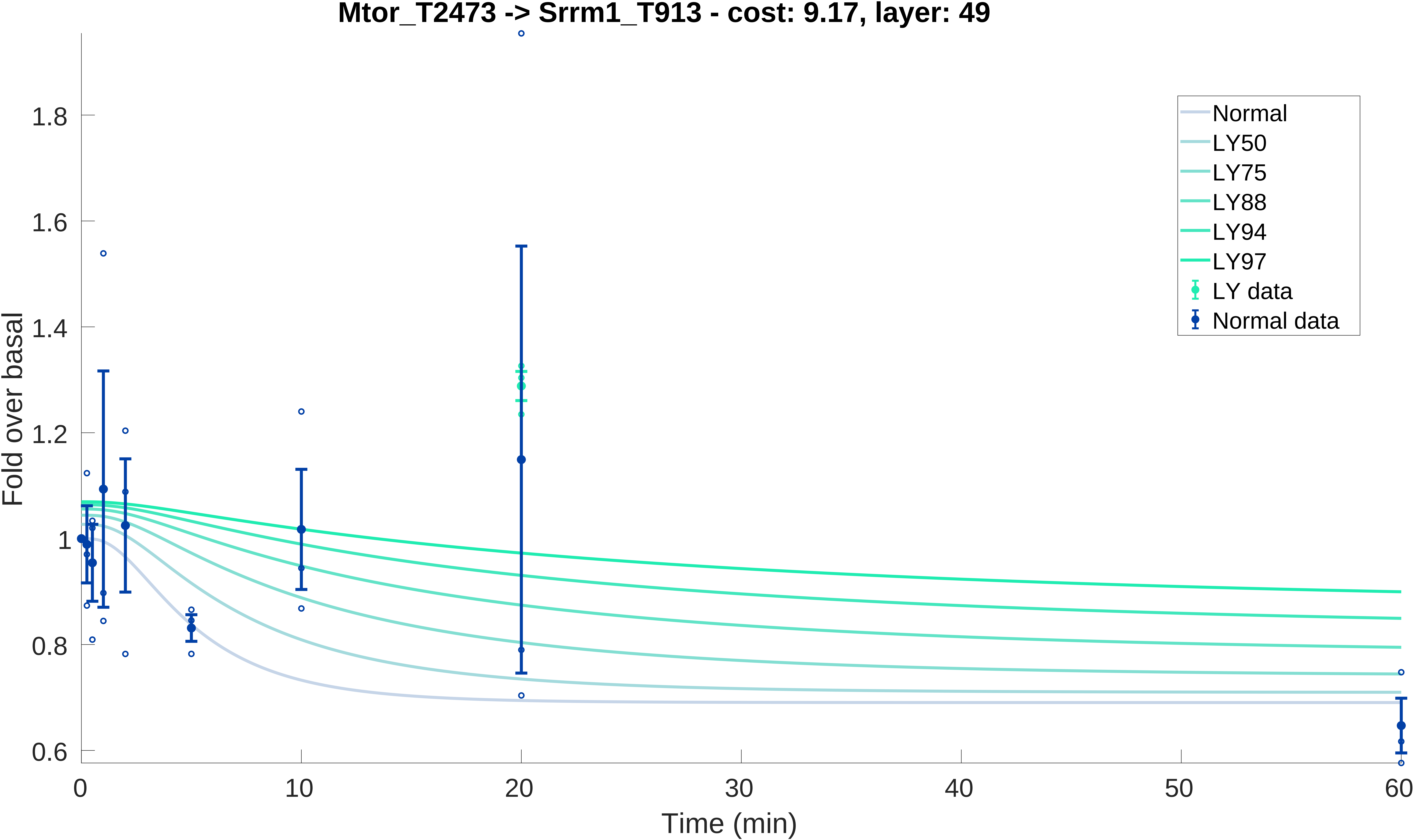

Mtor_T2473 -> Srrm1_T913 - cost: 9.17, layer: 49
Fold over basal
Time (min)
Normal
LY50
LY75
LY88
LY94
LY97
LY data
Normal data
0.6
0.8
1
1.2
1.4
1.6
1.8
0
10
20
30
40
50
60

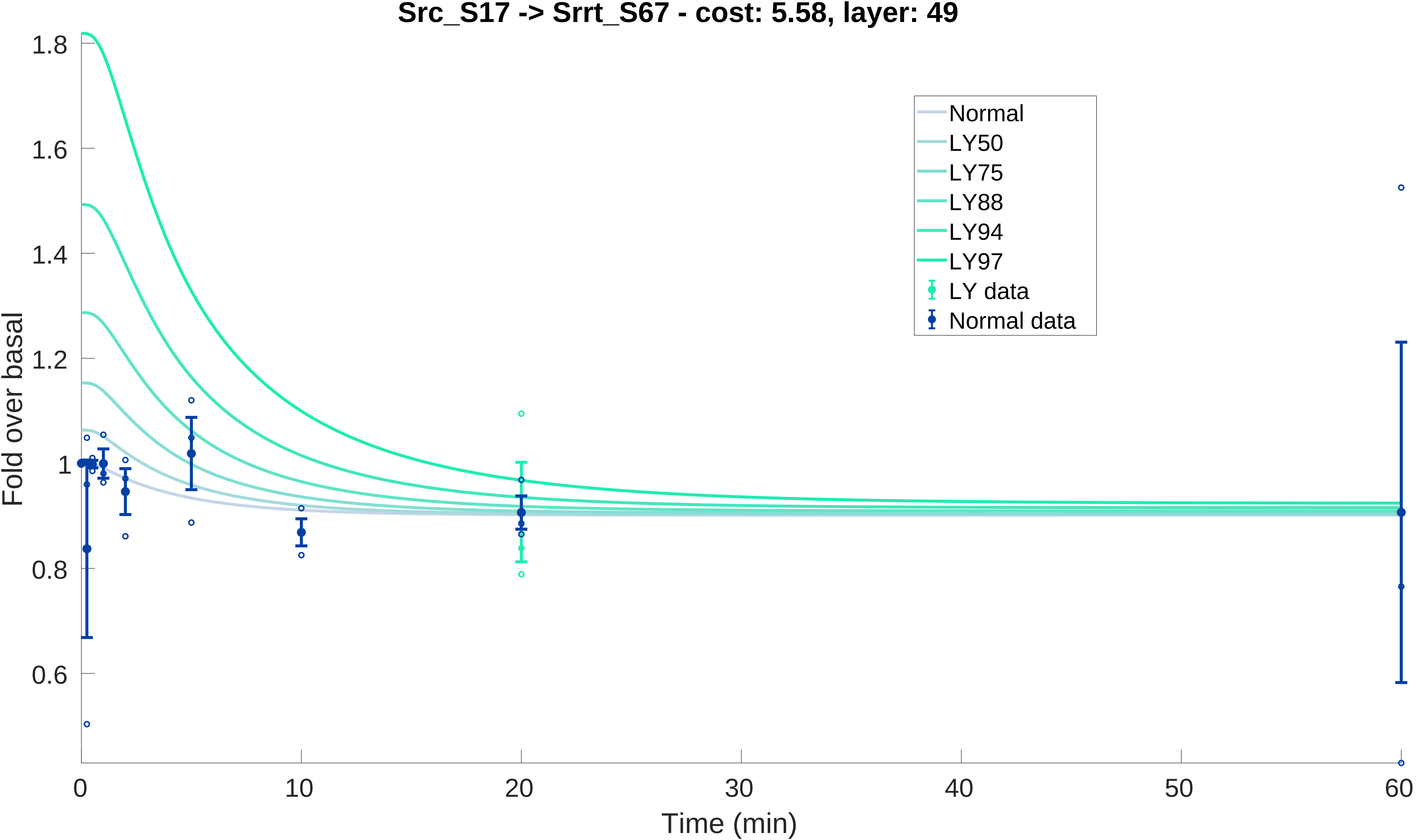
Src_S17 -> Srrt_S67 - cost: 5.58, layer: 49
Fold over basal
1.8
1.6
1.4
1.2
1
0.8
0.6
0
10
20
30
40
50
60
Time (min)
Normal
LY50
LY75
LY88
LY94
LY97
LY data
Normal data

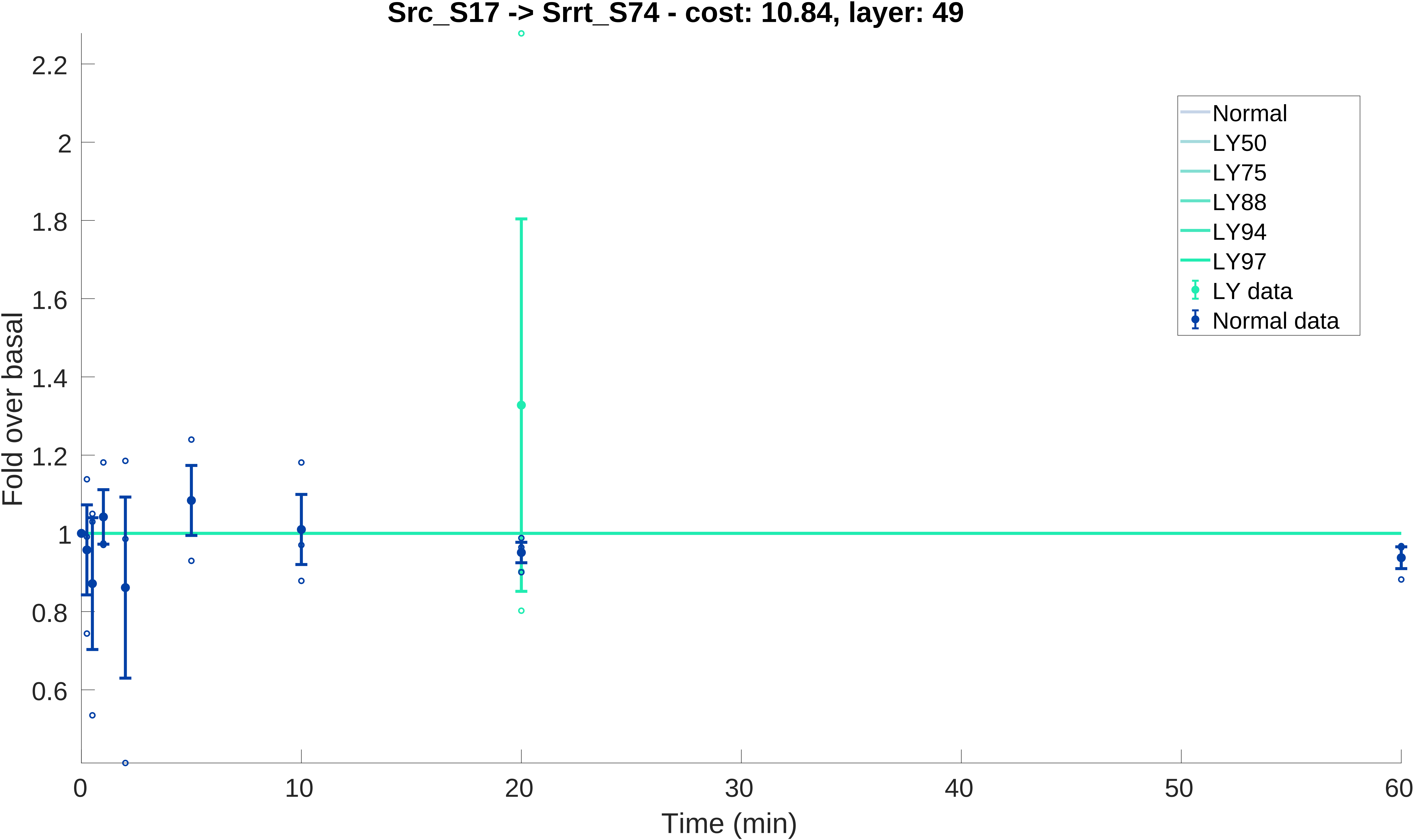

Src_S17 -> Srrt_S74 - cost: 10.84, layer: 49
Fold over basal
Time (min)
2.2
2
1.8
1.6
1.4
1.2
1
0.8
0.6
0
10
20
30
40
50
60
Normal
LY50
LY75
LY88
LY94
LY97
LY data
Normal data

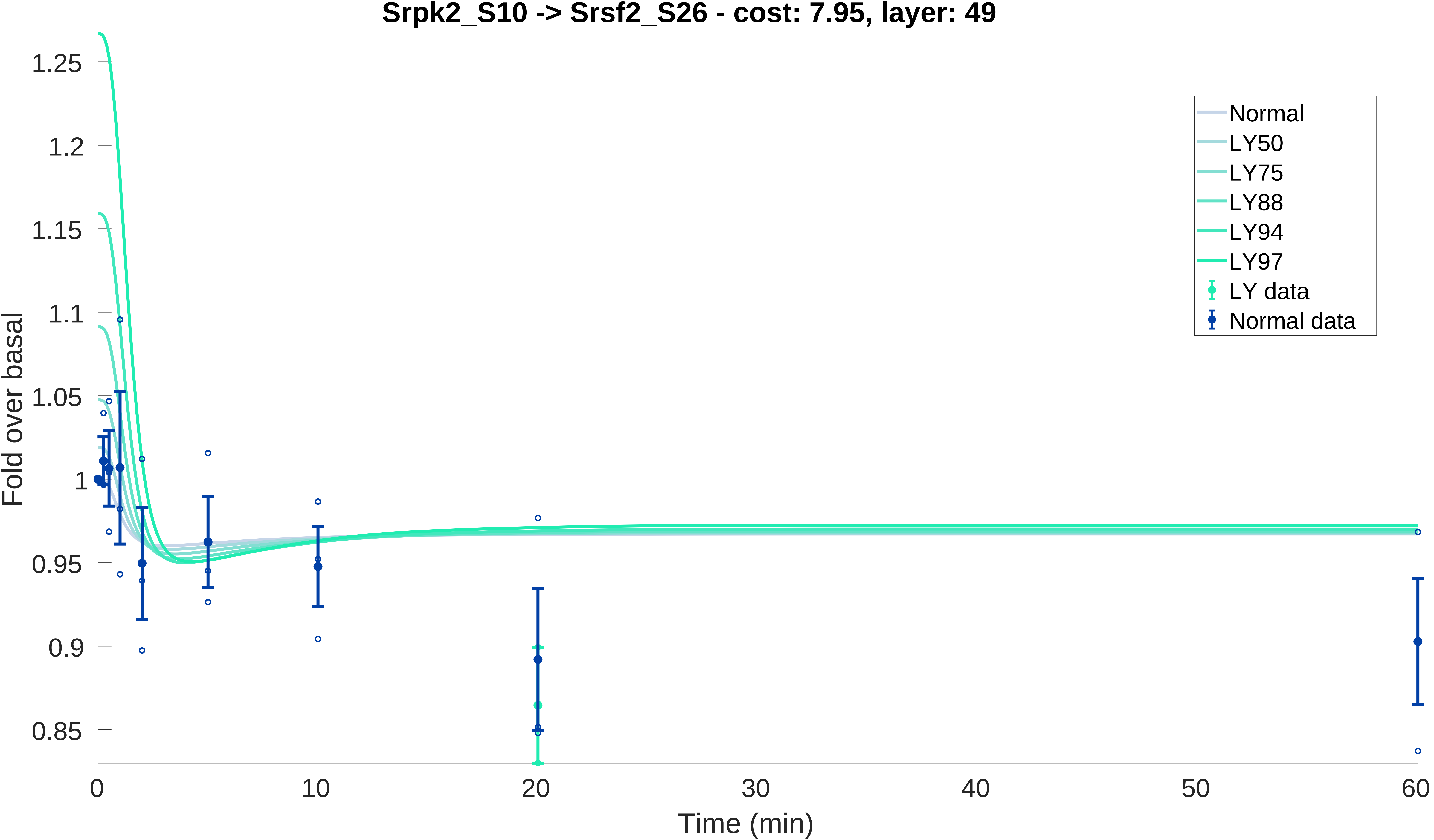

Srpk2_S10 -> Srsf2_S26 - cost: 7.95, layer: 49
Fold over basal
1.25
1.2
1.15
1.1
1.05
1
0.95
0.9
0.85
0
10
20
30
40
50
60
Time (min)
Normal
LY50
LY75
LY88
LY94
LY97
LY data
Normal data

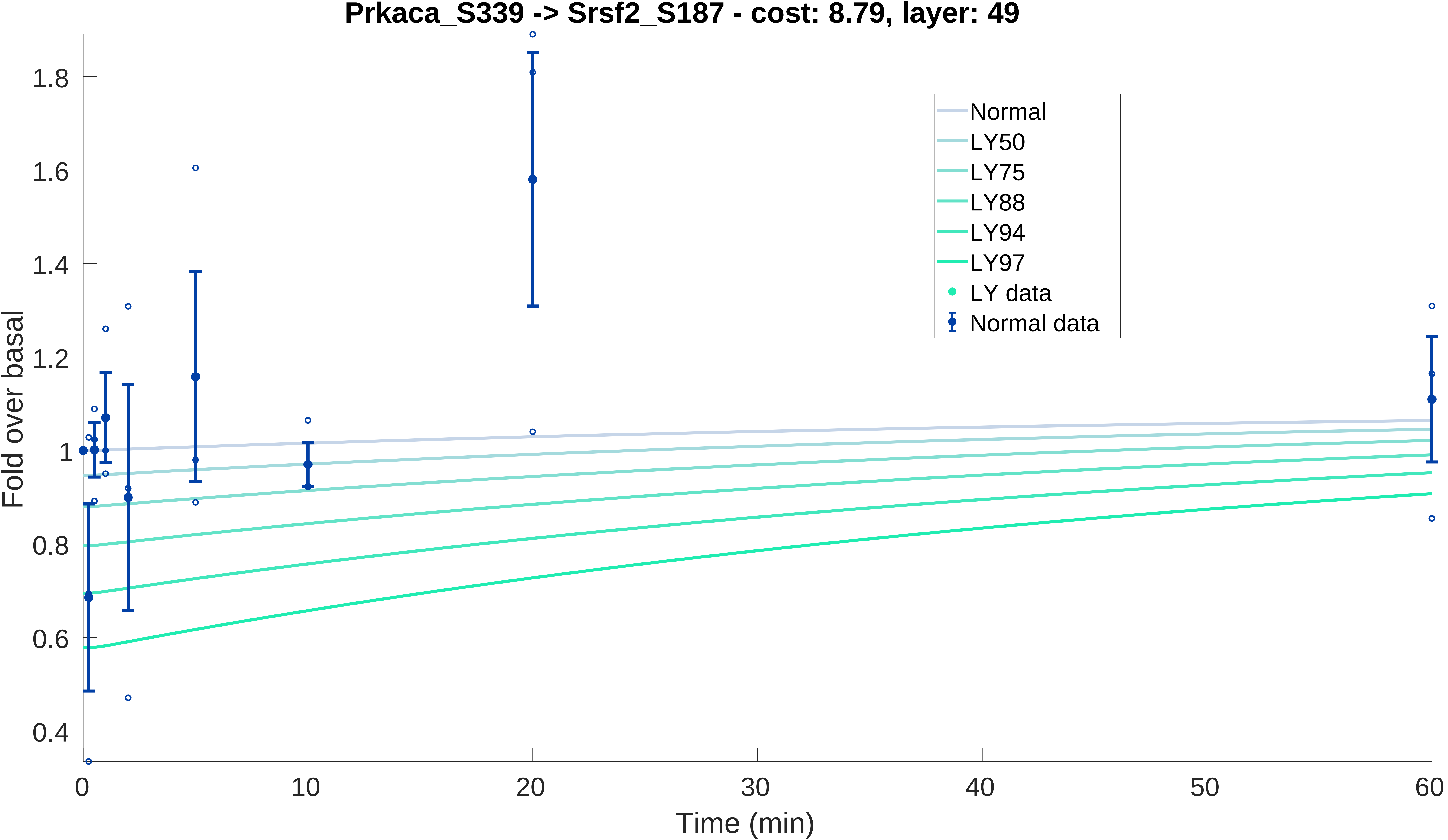

Prkaca_S339 -> Srsf2_S187 - cost: 8.79, layer: 49
Fold over basal
Time (min)
0.4
0.6
0.8
1
1.2
1.4
1.6
1.8
0
10
20
30
40
50
60
Normal
LY50
LY75
LY88
LY94
LY97
LY data
Normal data

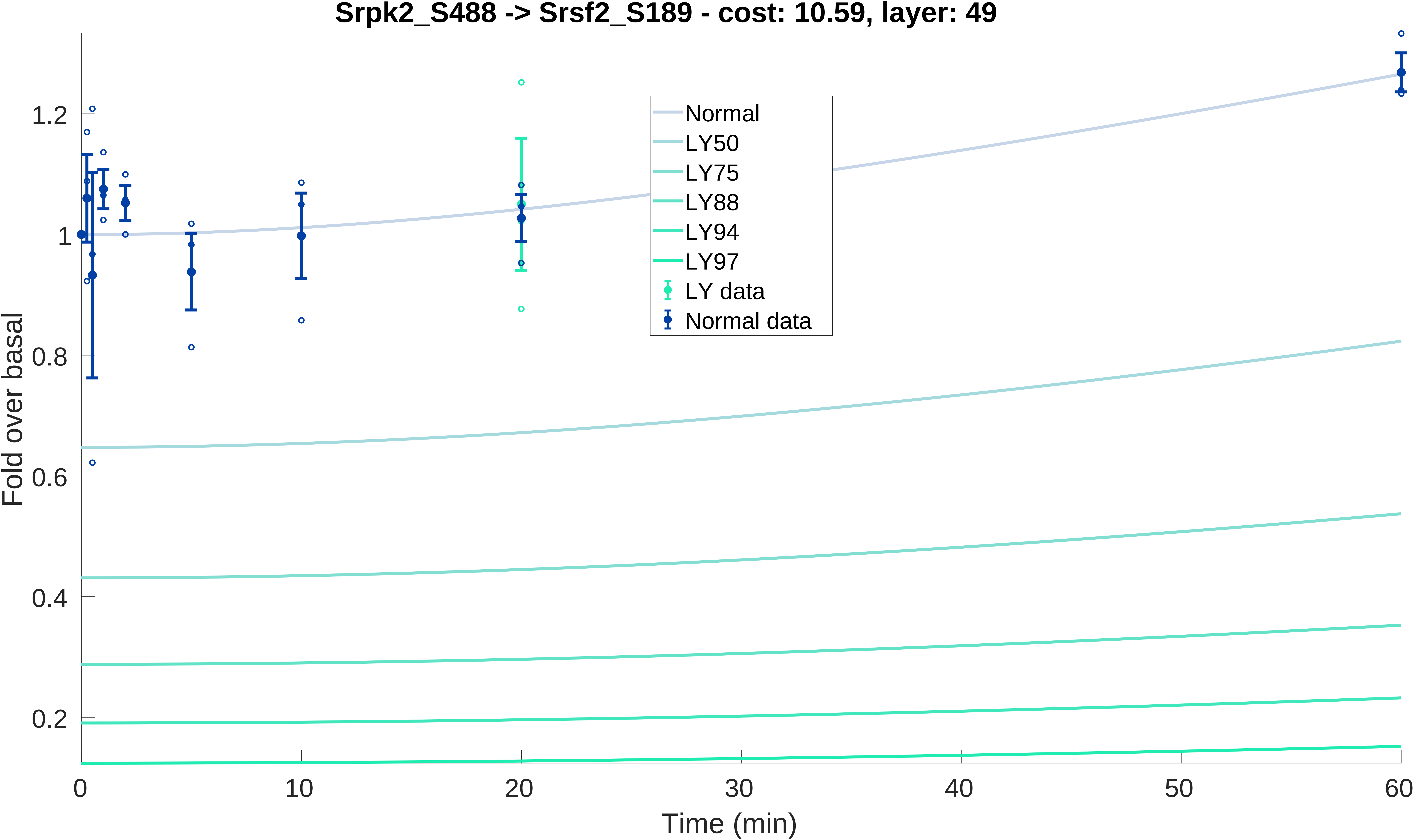
Srpk2_S488 -> Srsf2_S189 - cost: 10.59, layer: 49
Fold over basal
Time (min)
0
10
20
30
40
50
60
0.2
0.4
0.6
0.8
1
1.2
Normal
LY50
LY75
LY88
LY94
LY97
LY data
Normal data

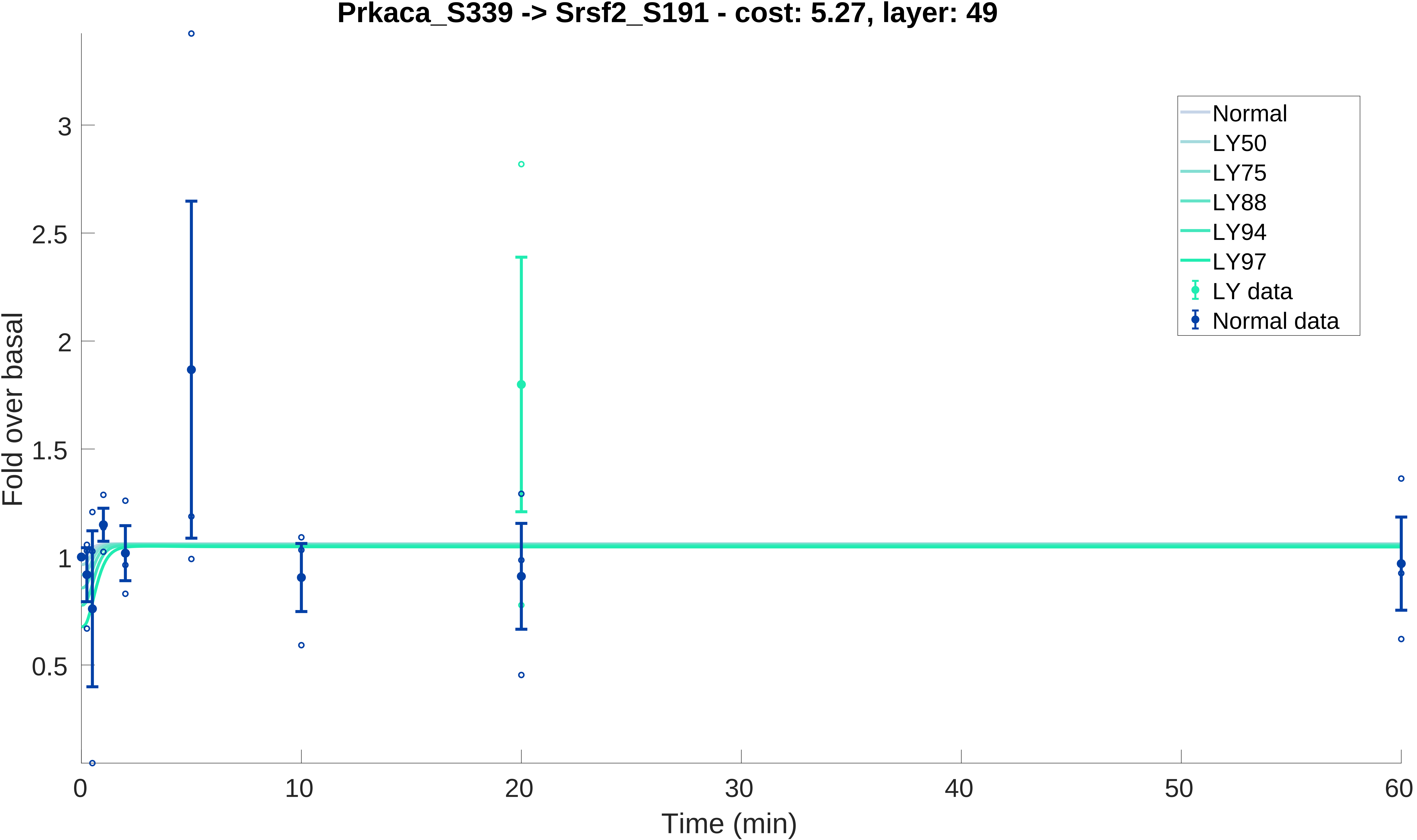

Prkaca_S339 -> Srsf2_S191 - cost: 5.27, layer: 49
Fold over basal
Time (min)
0.5
1
1.5
2
2.5
3
0
10
20
30
40
50
60
Normal
LY50
LY75
LY88
LY94
LY97
LY data
Normal data

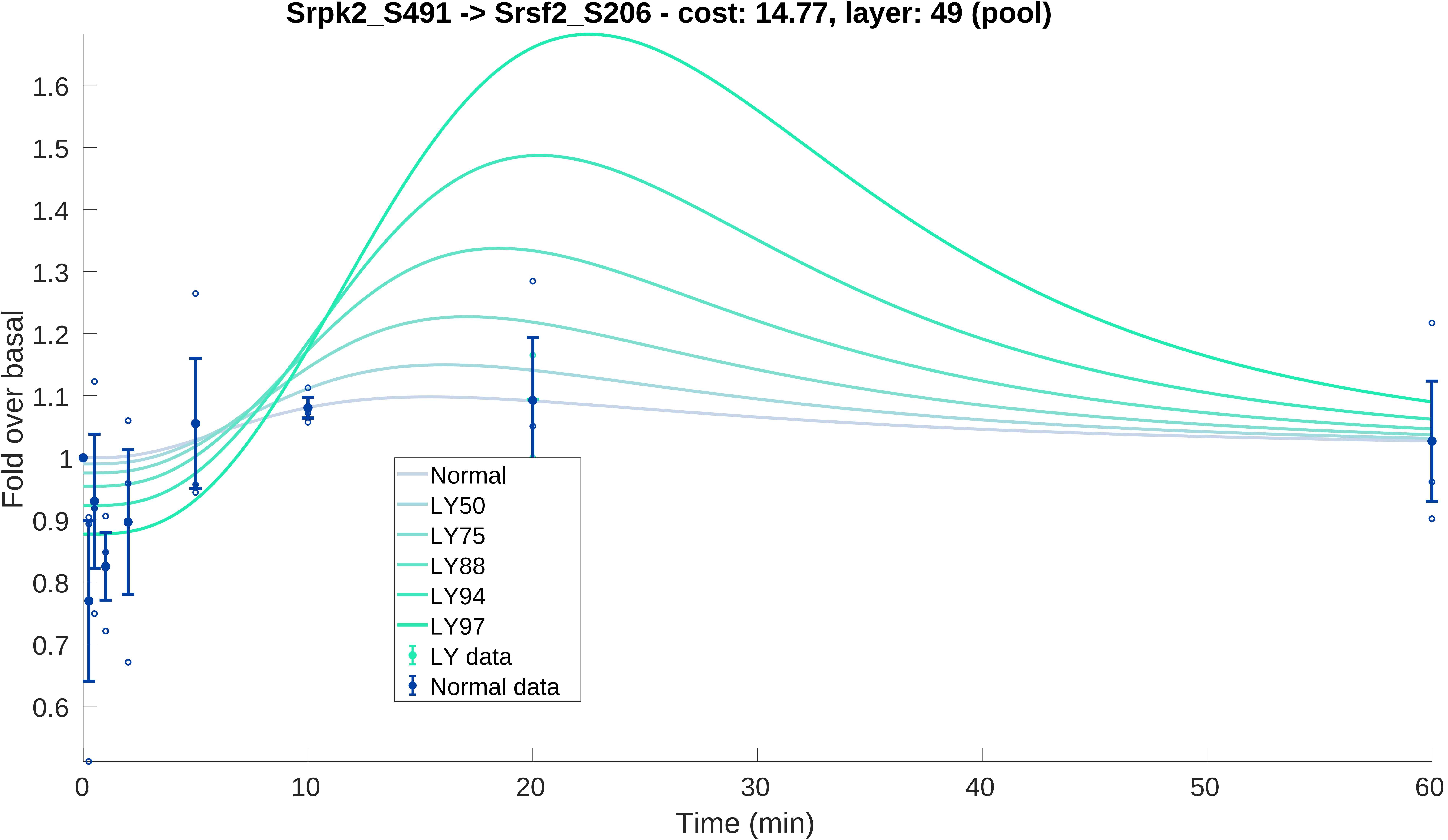

Srpk2_S491 -> Srsf2_S206 - cost: 14.77, layer: 49 (pool)
Fold over basal
Time (min)
1.6
1.5
1.4
1.3
1.2
1.1
1
0.9
0.8
0.7
0.6
0
10
20
30
40
50
60
Normal
LY50
LY75
LY88
LY94
LY97
LY data
Normal data

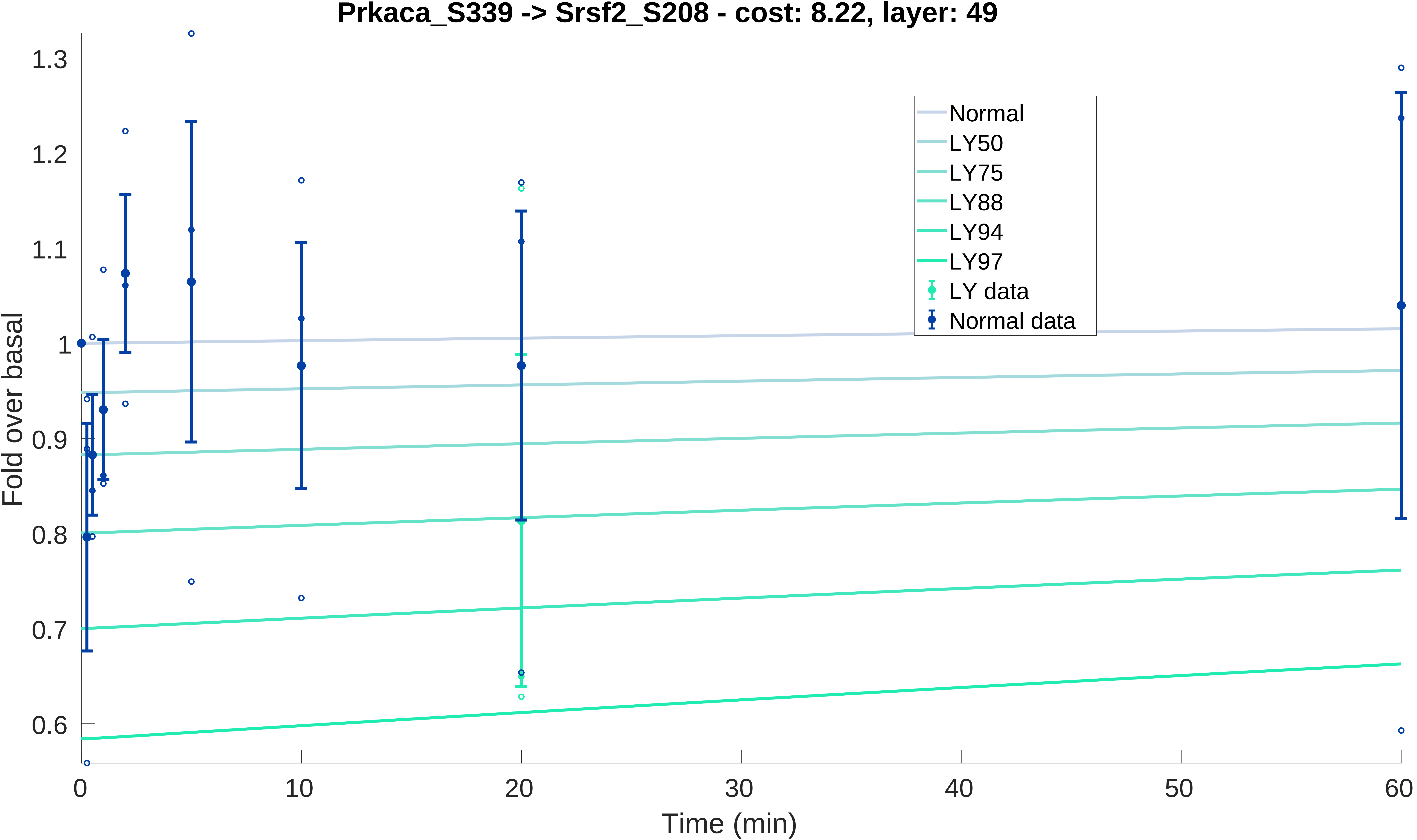
Prkaca_S339 -> Srsf2_S208 - cost: 8.22, layer: 49
Fold over basal
Time (min)
1.3
1.2
1.1
1
0.9
0.8
0.7
0.6
0
10
20
30
40
50
60
Normal
LY50
LY75
LY88
LY94
LY97
LY data
Normal data

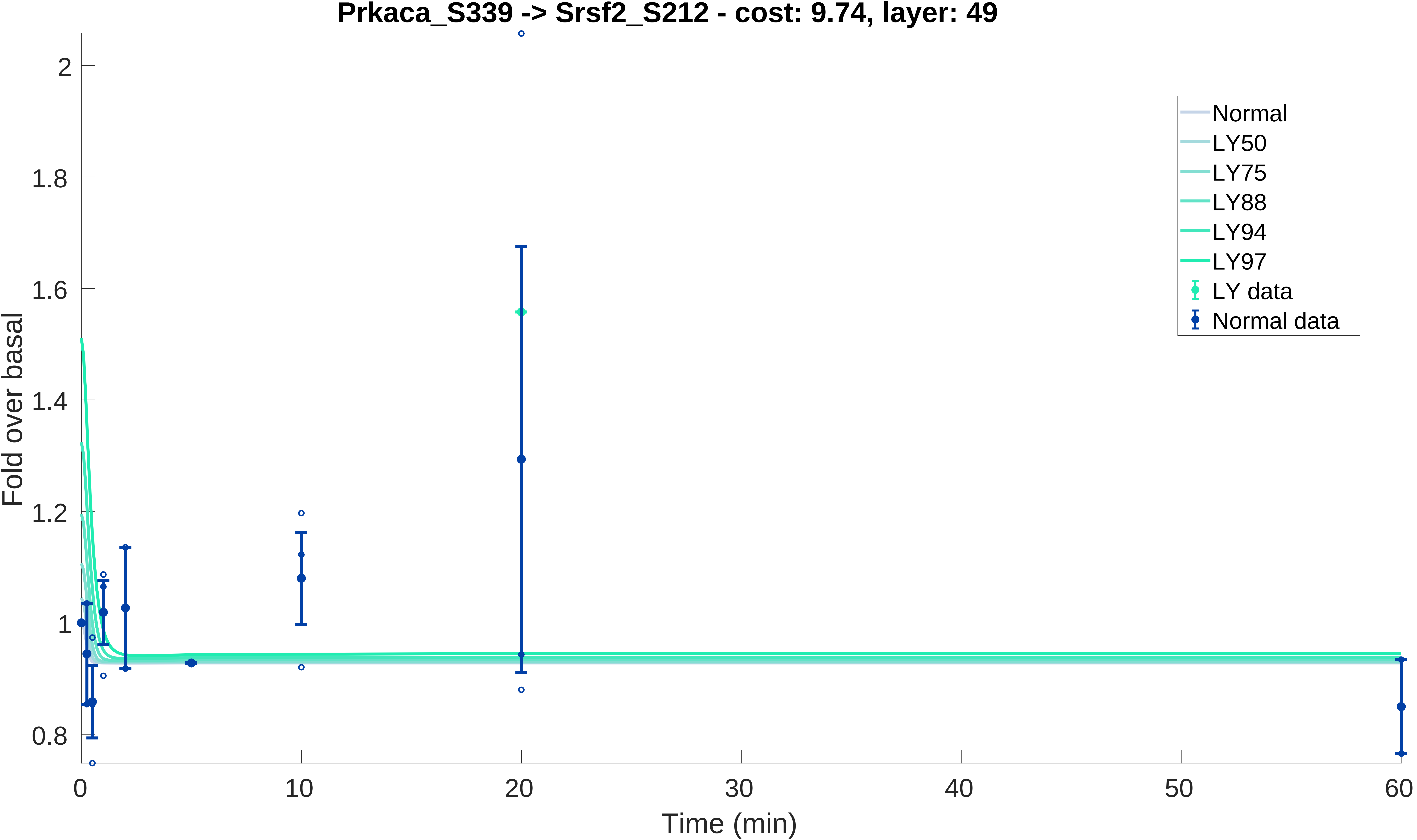

Prkaca_S339 -> Srsf2_S212 - cost: 9.74, layer: 49
Fold over basal
Time (min)
0.8
1
1.2
1.4
1.6
1.8
2
0
10
20
30
40
50
60
Normal
LY50
LY75
LY88
LY94
LY97
LY data
Normal data

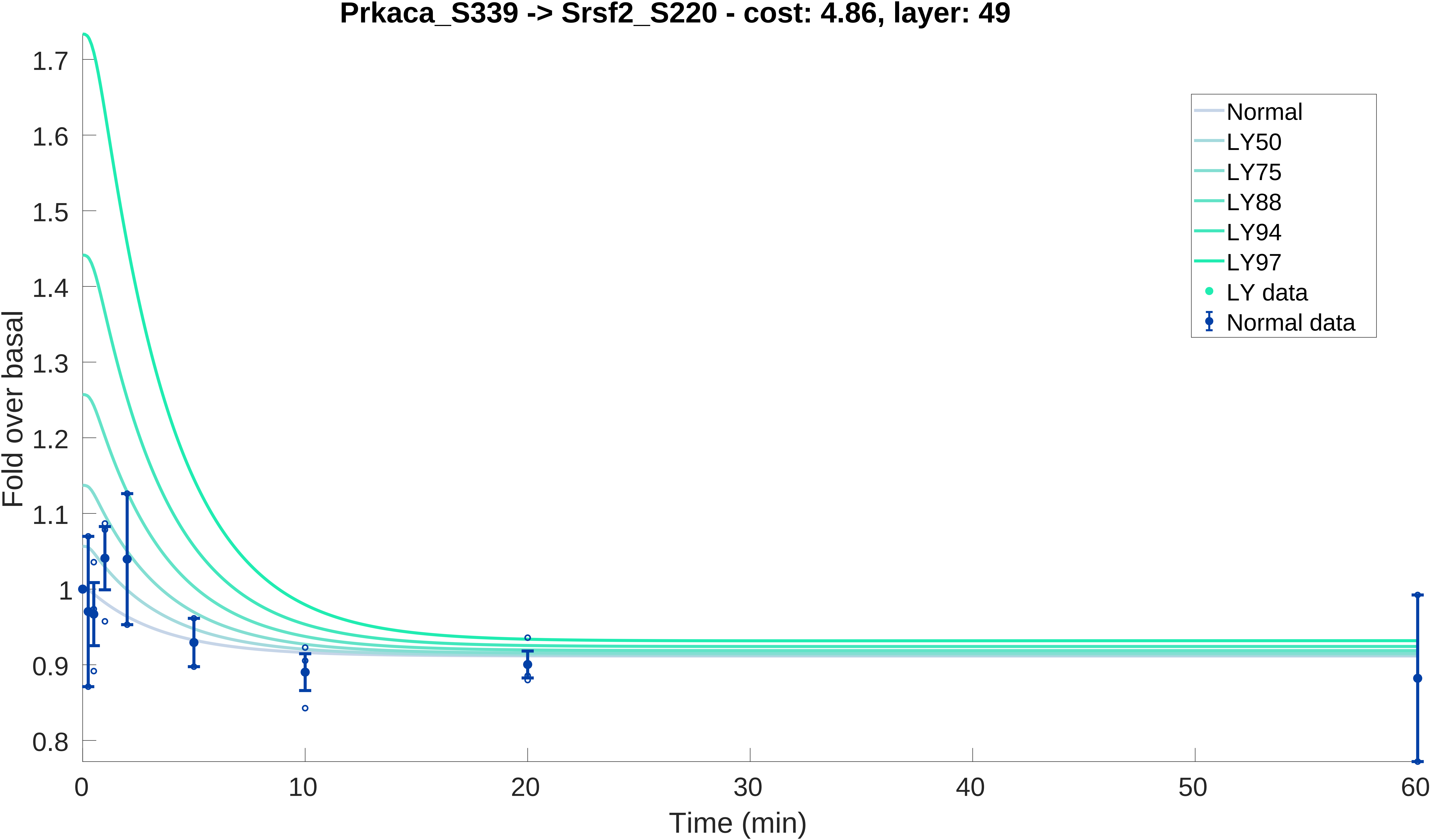

Prkaca_S339 -> Srsf2_S220 - cost: 4.86, layer: 49
Fold over basal
1.7
1.6
1.5
1.4
1.3
1.2
1.1
1
0.9
0.8
0
10
20
30
40
50
60
Time (min)
Normal
LY50
LY75
LY88
LY94
LY97
LY data
Normal data

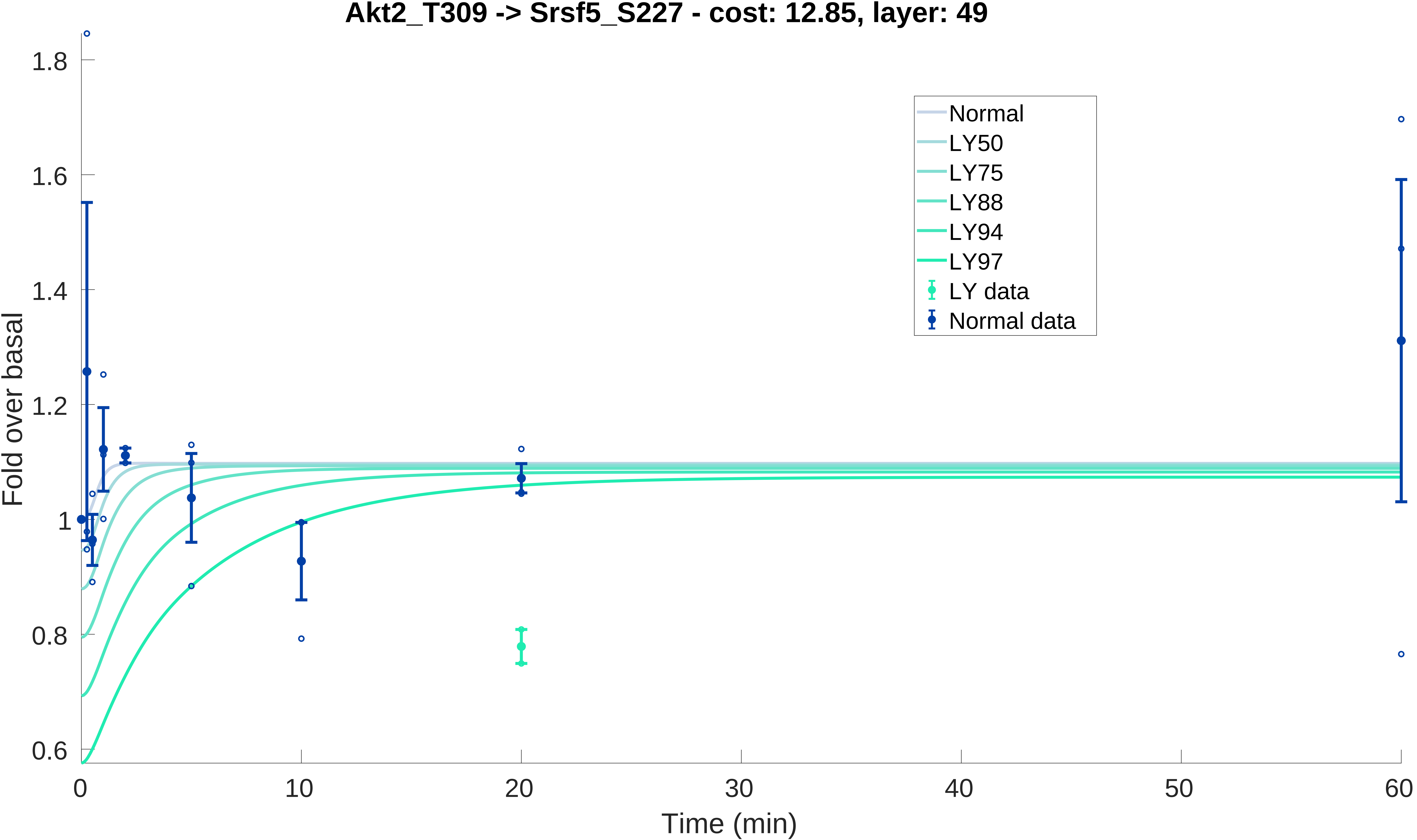

Akt2_T309 -> Srsf5_S227 - cost: 12.85, layer: 49
Fold over basal
Time (min)
Normal
LY50
LY75
LY88
LY94
LY97
LY data
Normal data
0.6
0.8
1
1.2
1.4
1.6
1.8
0
10
20
30
40
50
60

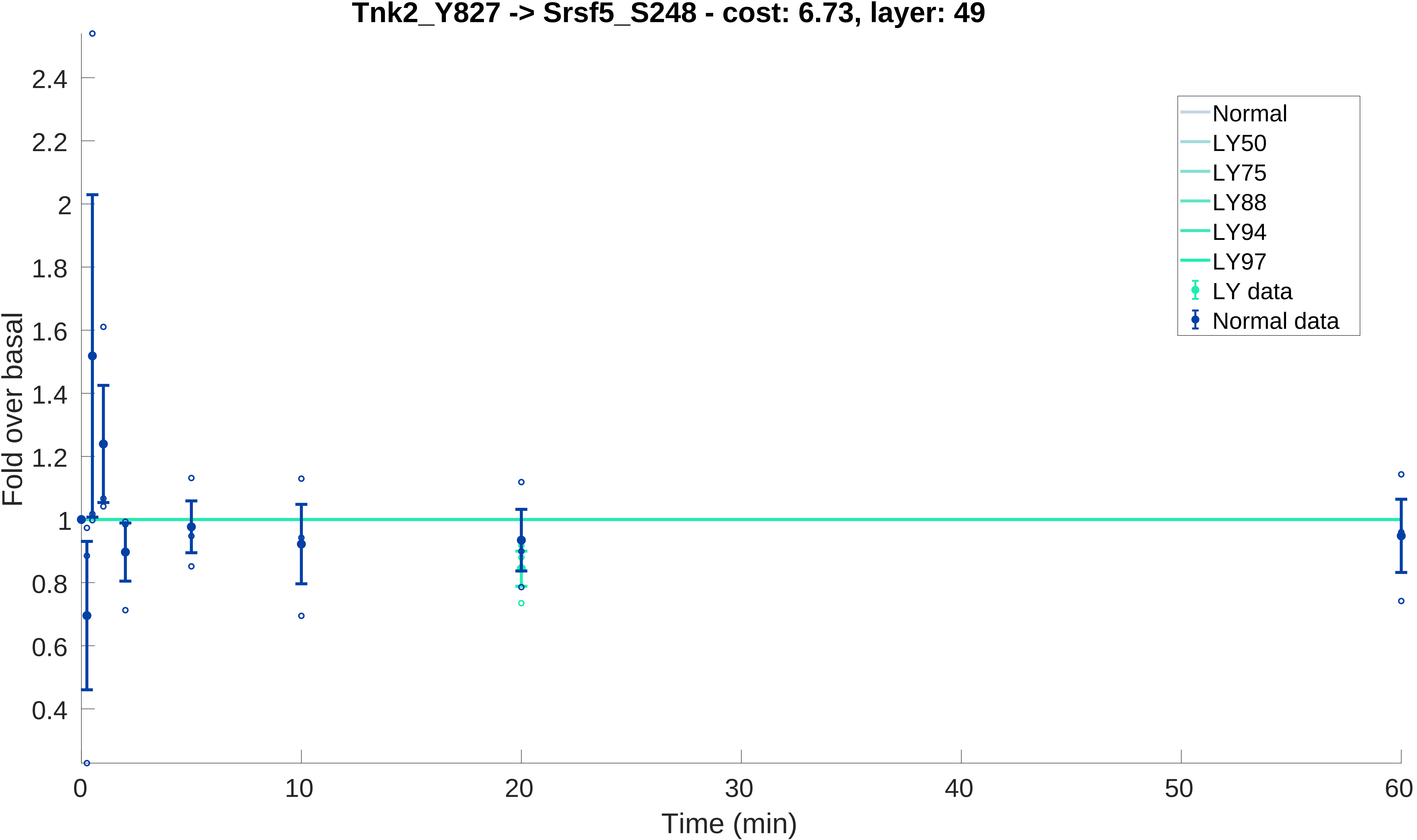
Tnk2_Y827 -> Srsf5_S248 - cost: 6.73, layer: 49
Fold over basal
Time (min)
Normal
LY50
LY75
LY88
LY94
LY97
LY data
Normal data
0.4
0.6
0.8
1
1.2
1.4
1.6
1.8
2
2.2
2.4
0
10
20
30
40
50
60

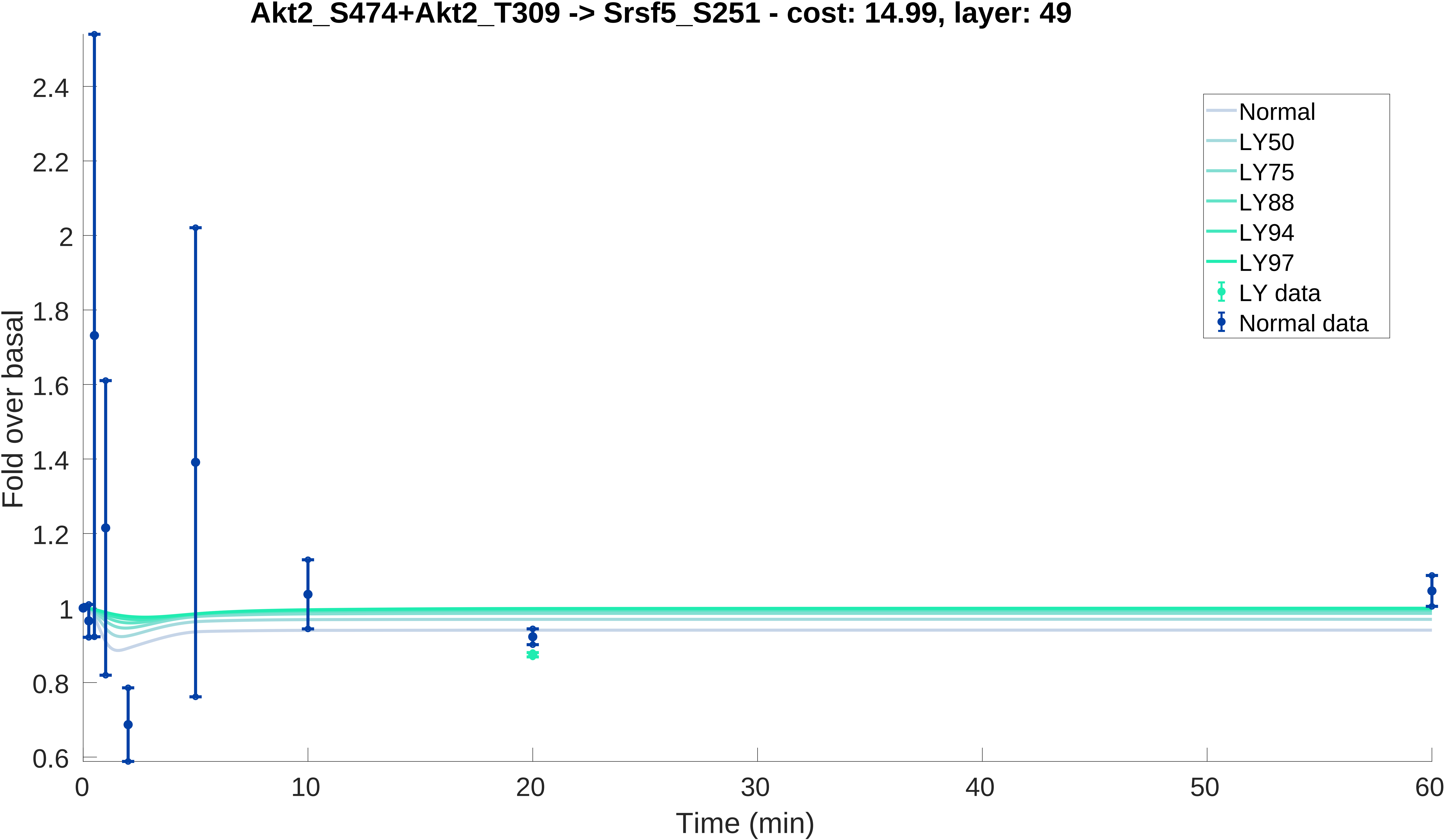
Akt2_S474+Akt2_T309 -> Srsf5_S251 - cost: 14.99, layer: 49
Fold over basal
Time (min)
0
10
20
30
40
50
60
0.6
0.8
1
1.2
1.4
1.6
1.8
2
2.2
2.4
Normal
LY50
LY75
LY88
LY94
LY97
LY data
Normal data

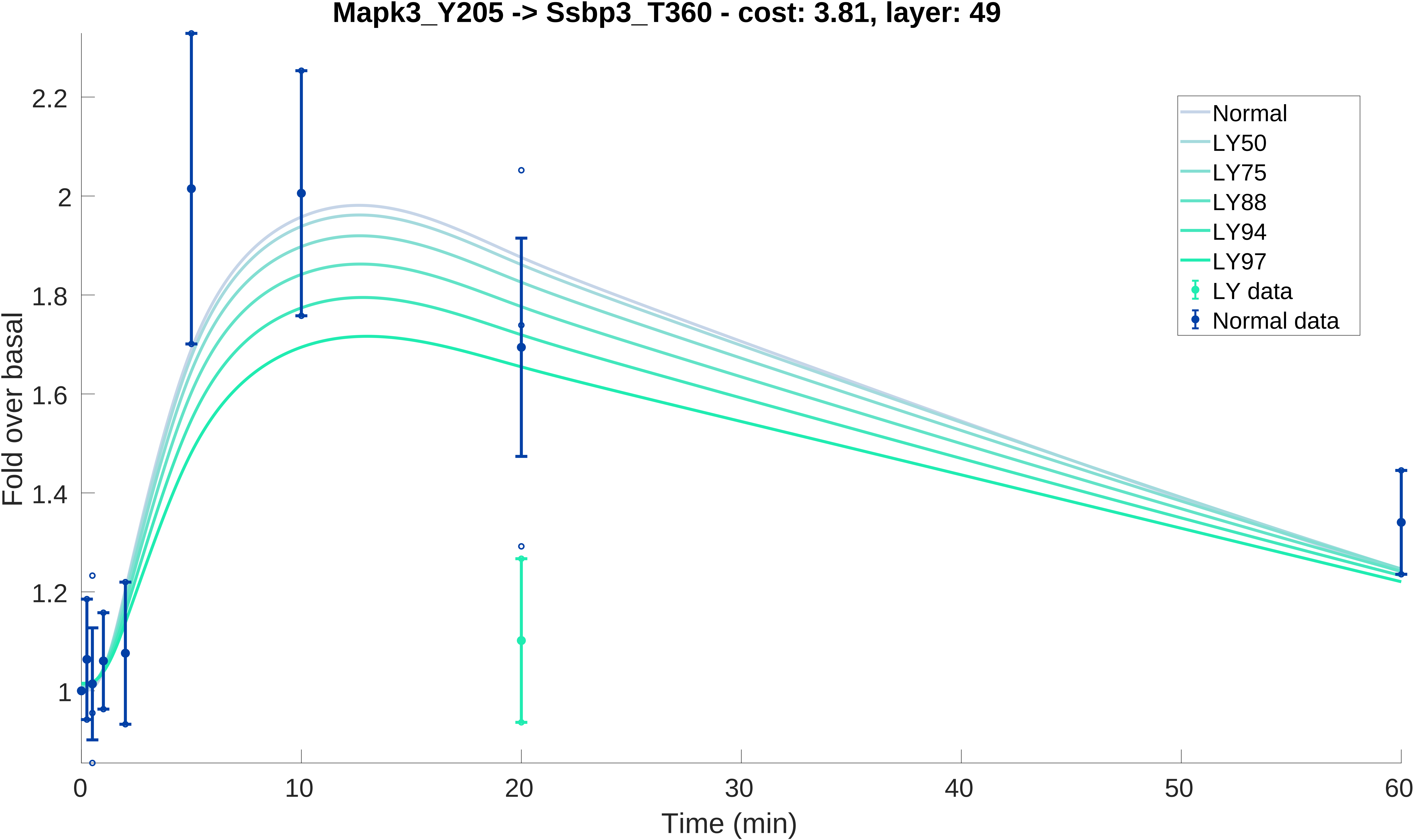

Mapk3_Y205 -> Ssbp3_T360 - cost: 3.81, layer: 49
Fold over basal
Time (min)
0
10
20
30
40
50
60
1
1.2
1.4
1.6
1.8
2
2.2
Normal
LY50
LY75
LY88
LY94
LY97
LY data
Normal data

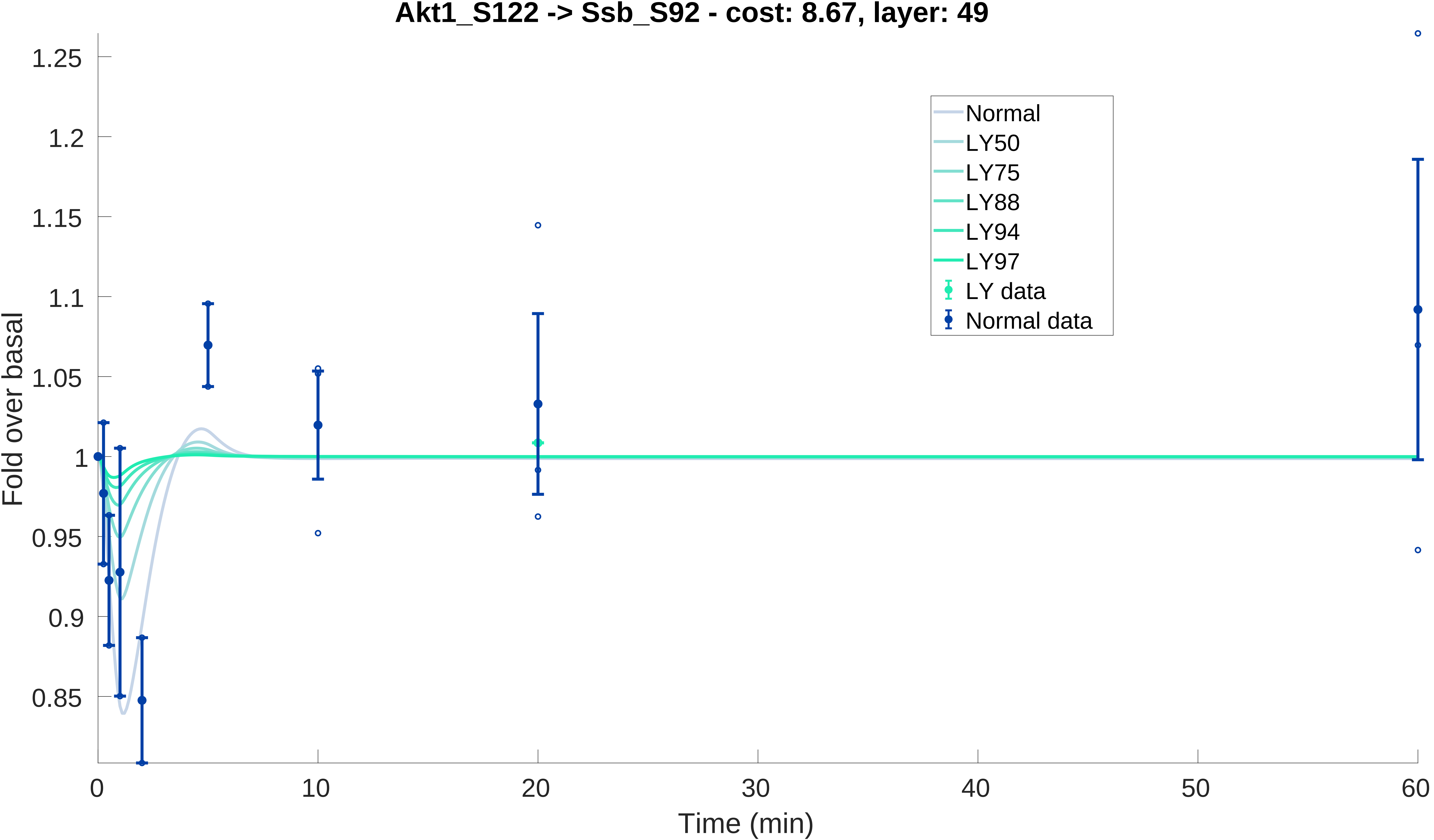
Akt1_S122 -> Ssb_S92 - cost: 8.67, layer: 49
Normal
LY50
LY75
LY88
LY94
LY97
LY data
Normal data
Fold over basal
Time (min)
1.25
1.2
1.15
1.1
1.05
1
0.95
0.9
0.85
0
10
20
30
40
50
60

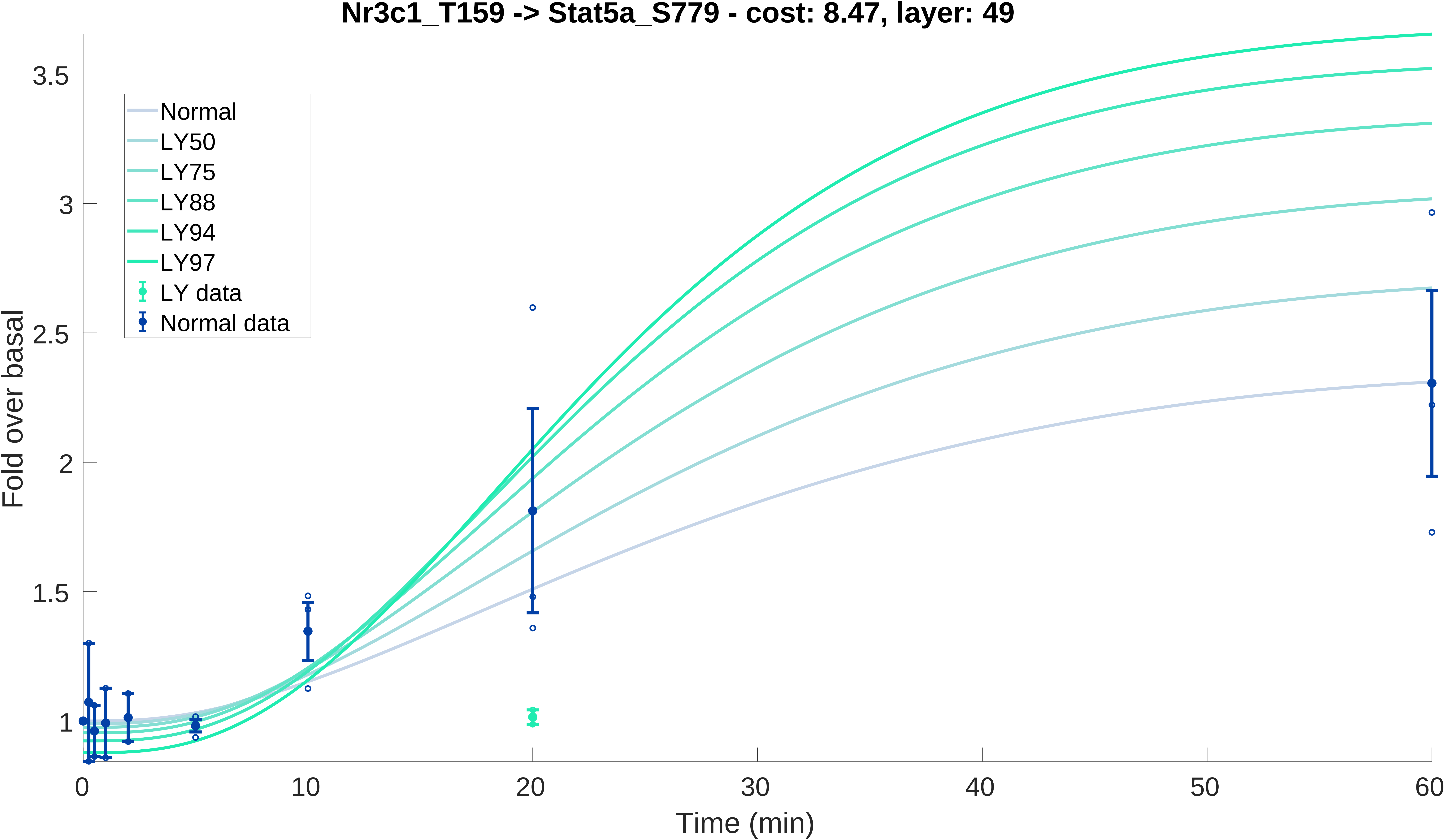
Nr3c1_T159 -> Stat5a_S779 - cost: 8.47, layer: 49
Normal
LY50
LY75
LY88
LY94
LY97
LY data
Normal data
Fold over basal
Time (min)
3.5
3
2.5
2
1.5
1
0
10
20
30
40
50
60

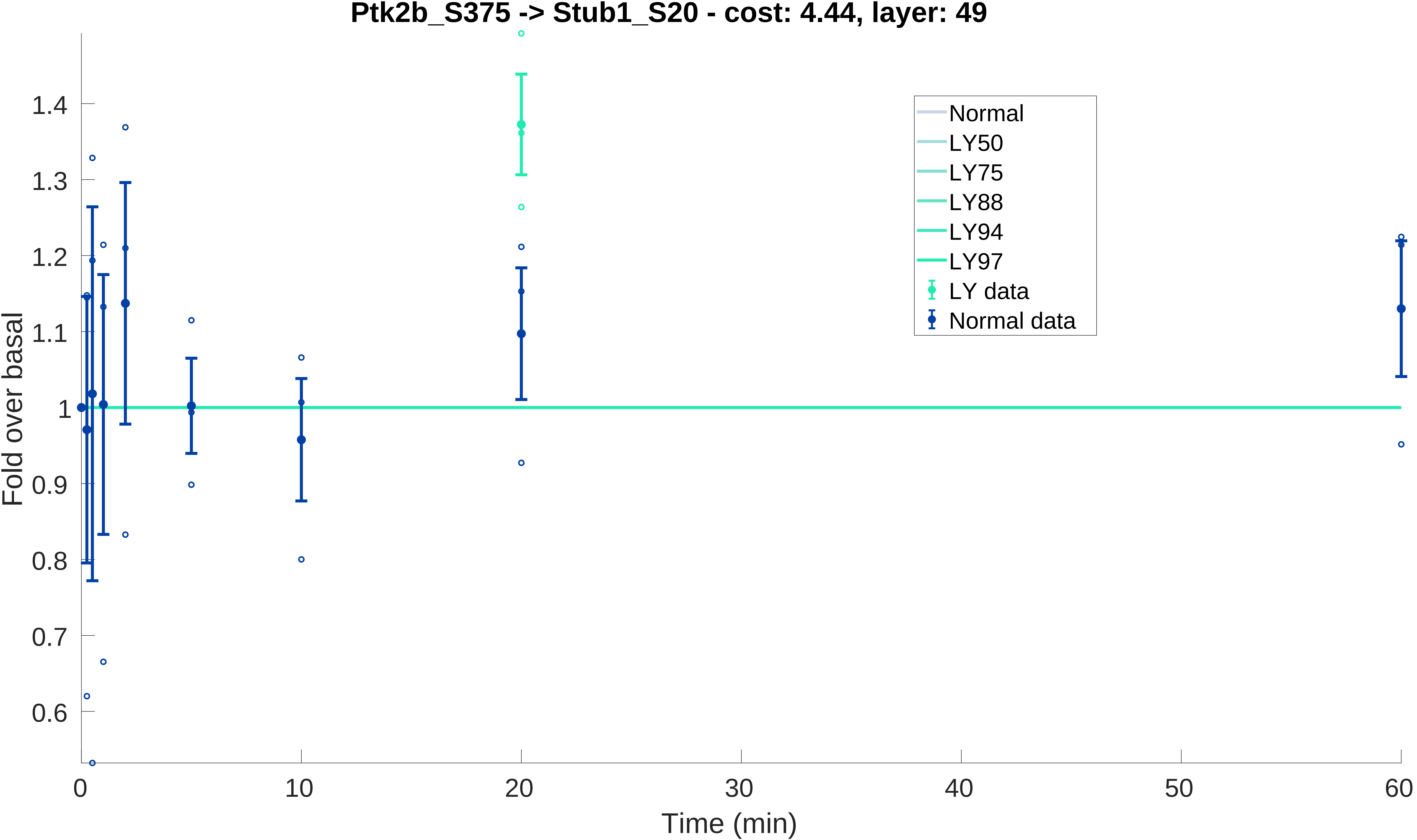

Ptk2b_S375 -> Stub1_S20 - cost: 4.44, layer: 49
Normal
LY50
LY75
LY88
LY94
LY97
LY data
Normal data
Fold over basal
Time (min)
1.4
1.3
1.2
1.1
1
0.9
0.8
0.7
0.6
0
10
20
30
40
50
60

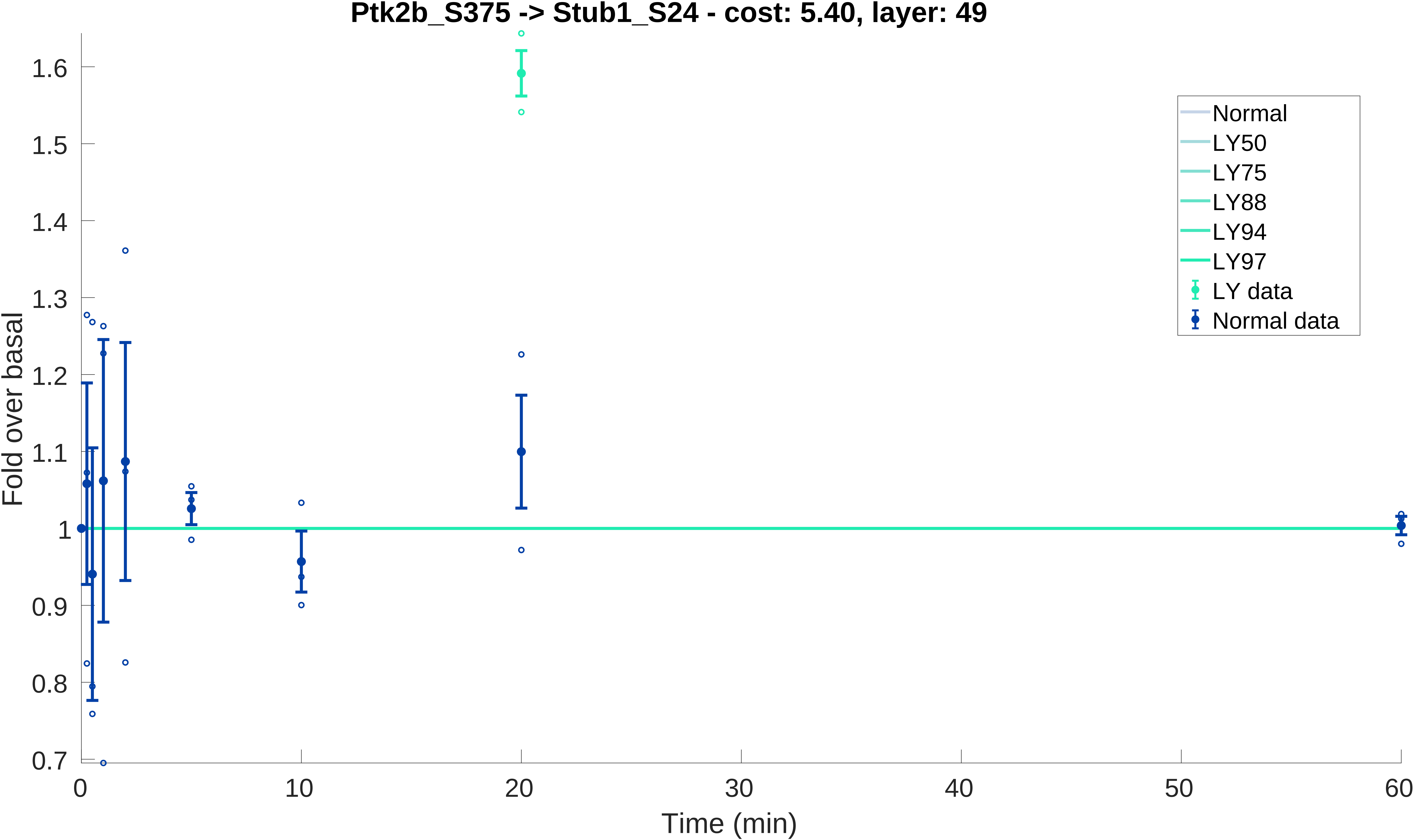

Ptk2b_S375 -> Stub1_S24 - cost: 5.40, layer: 49
Fold over basal
Time (min)
1.6
1.5
1.4
1.3
1.2
1.1
1
0.9
0.8
0.7
0
10
20
30
40
50
60
Normal
LY50
LY75
LY88
LY94
LY97
LY data
Normal data

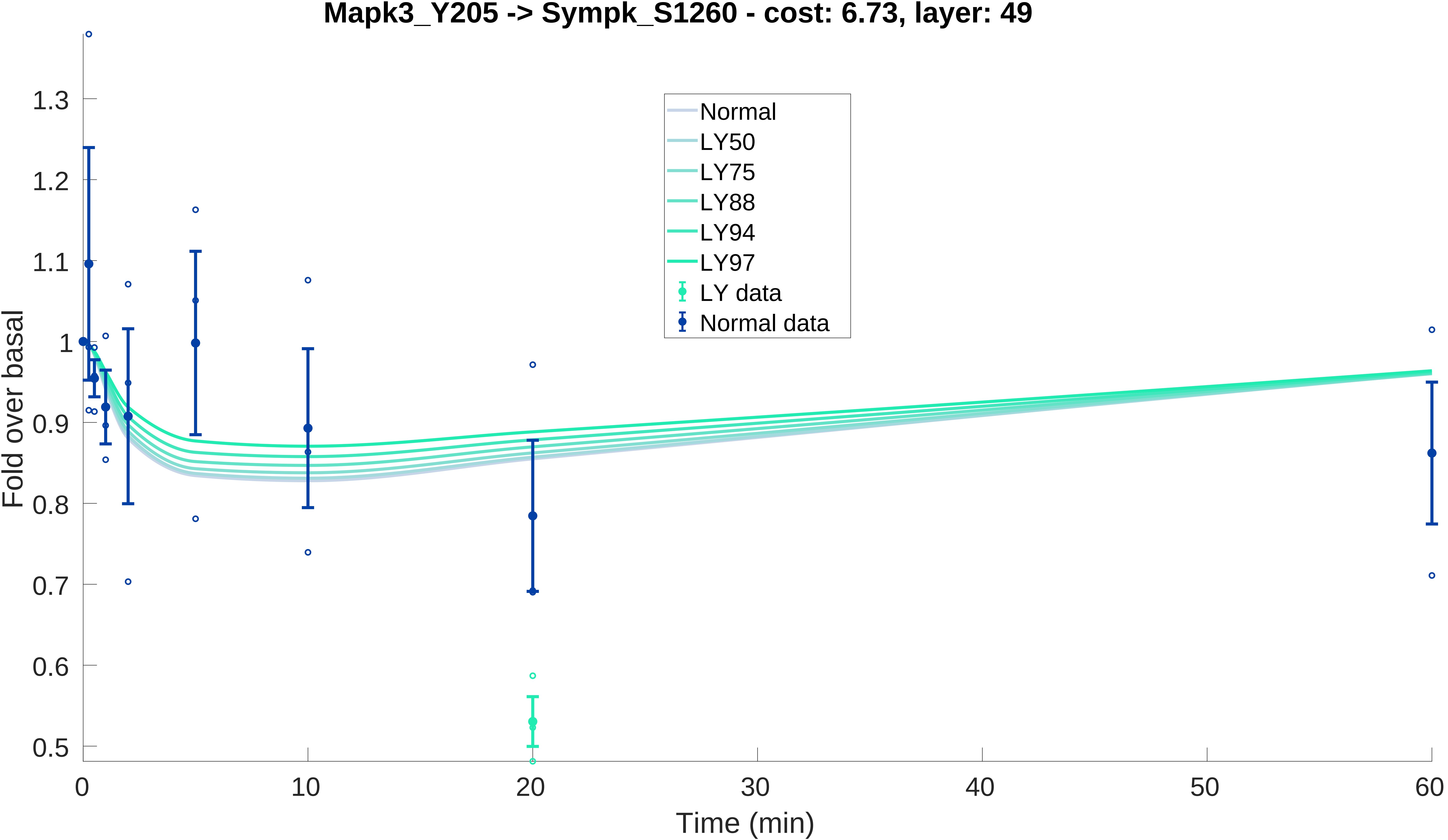

Mapk3_Y205 -> Sympk_S1260 - cost: 6.73, layer: 49
Normal
LY50
LY75
LY88
LY94
LY97
LY data
Normal data
Fold over basal
Time (min)
1.3
1.2
1.1
1
0.9
0.8
0.7
0.6
0.5
0
10
20
30
40
50
60

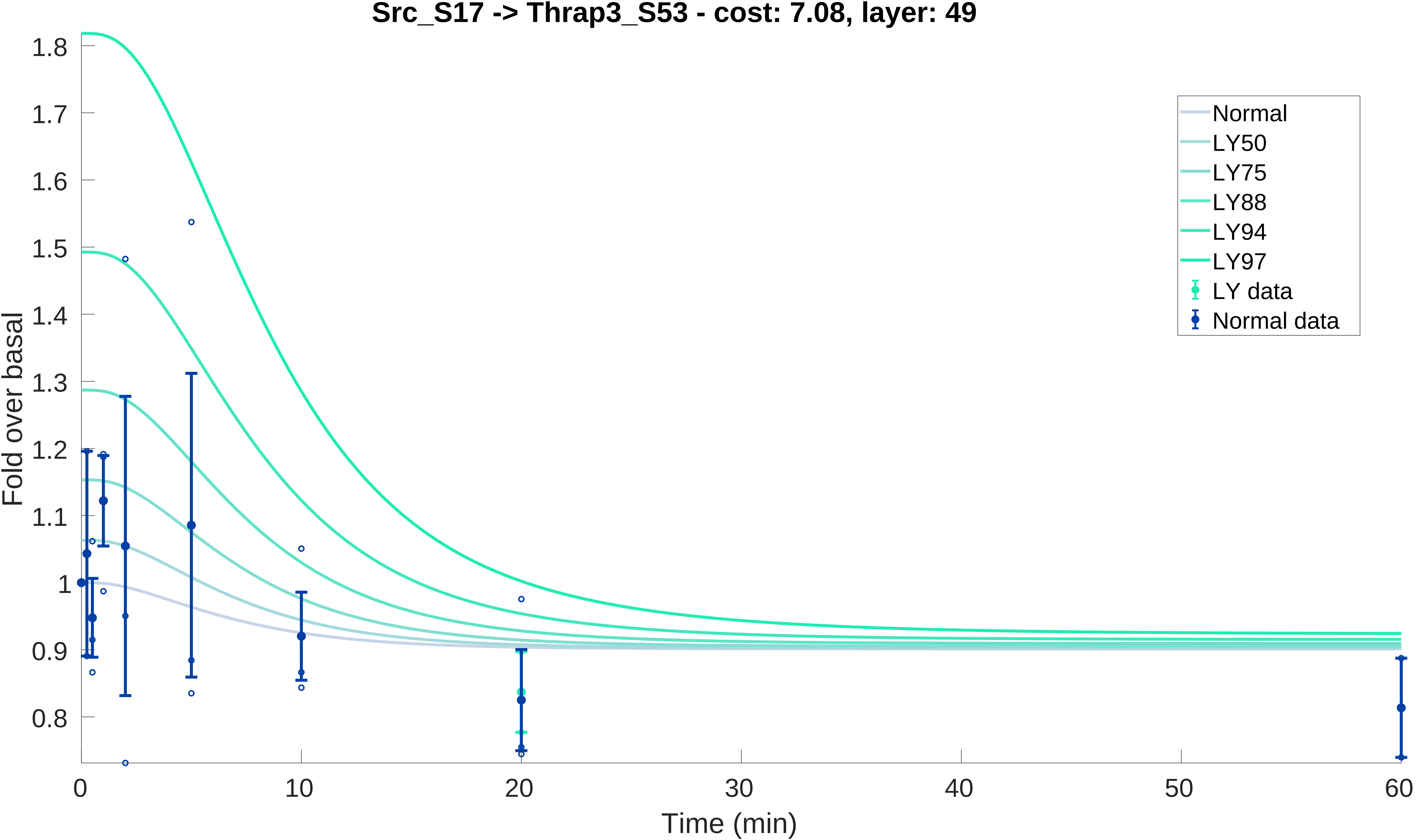

Src_S17 -> Thrap3_S53 - cost: 7.08, layer: 49
Fold over basal
Time (min)
Normal
LY50
LY75
LY88
LY94
LY97
LY data
Normal data
1.8
1.7
1.6
1.5
1.4
1.3
1.2
1.1
1
0.9
0.8
0
10
20
30
40
50
60

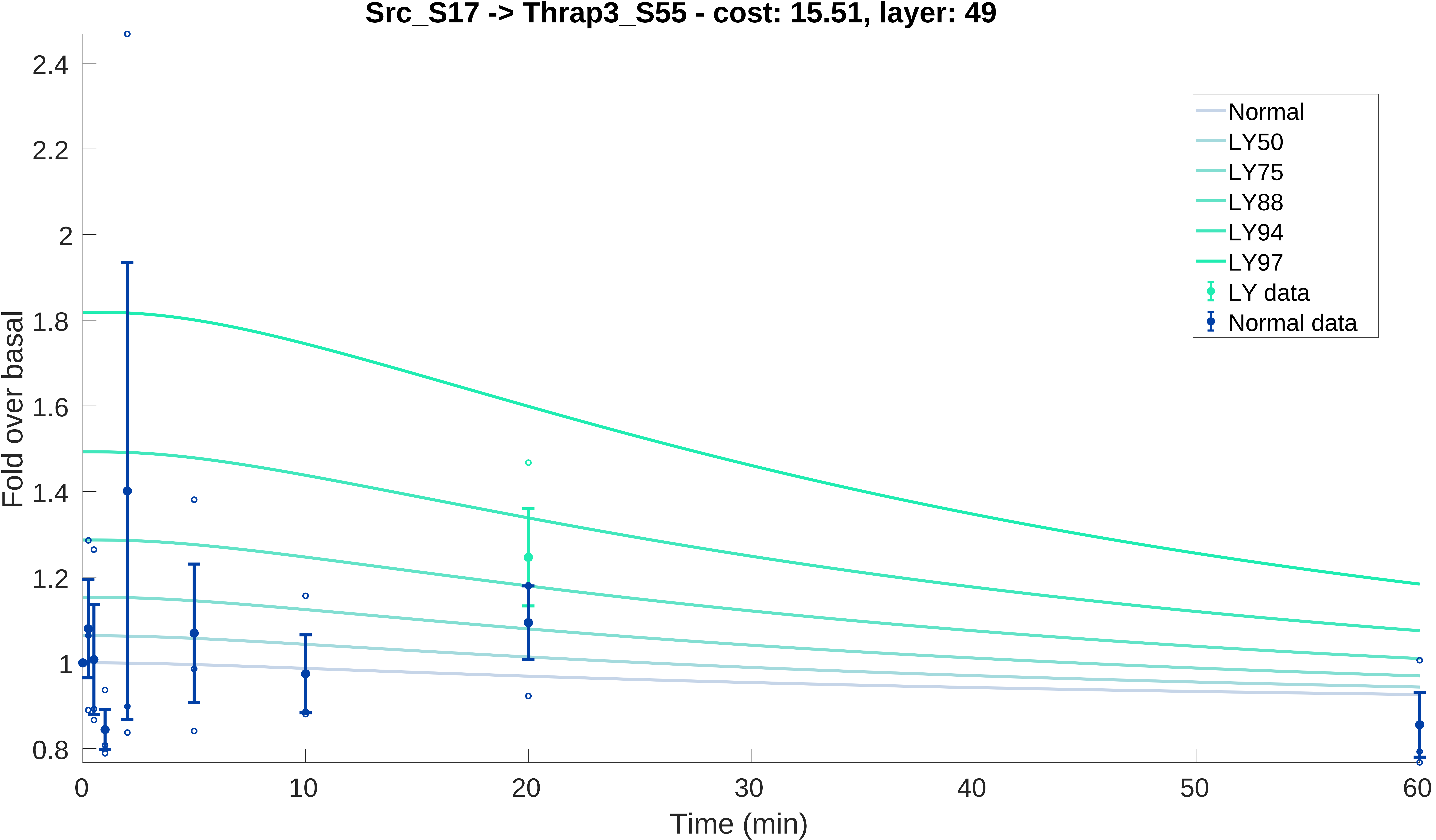

Src_S17 -> Thrap3_S55 - cost: 15.51, layer: 49
Fold over basal
Time (min)
Normal
LY50
LY75
LY88
LY94
LY97
LY data
Normal data
0.8
1
1.2
1.4
1.6
1.8
2
2.2
2.4
0
10
20
30
40
50
60

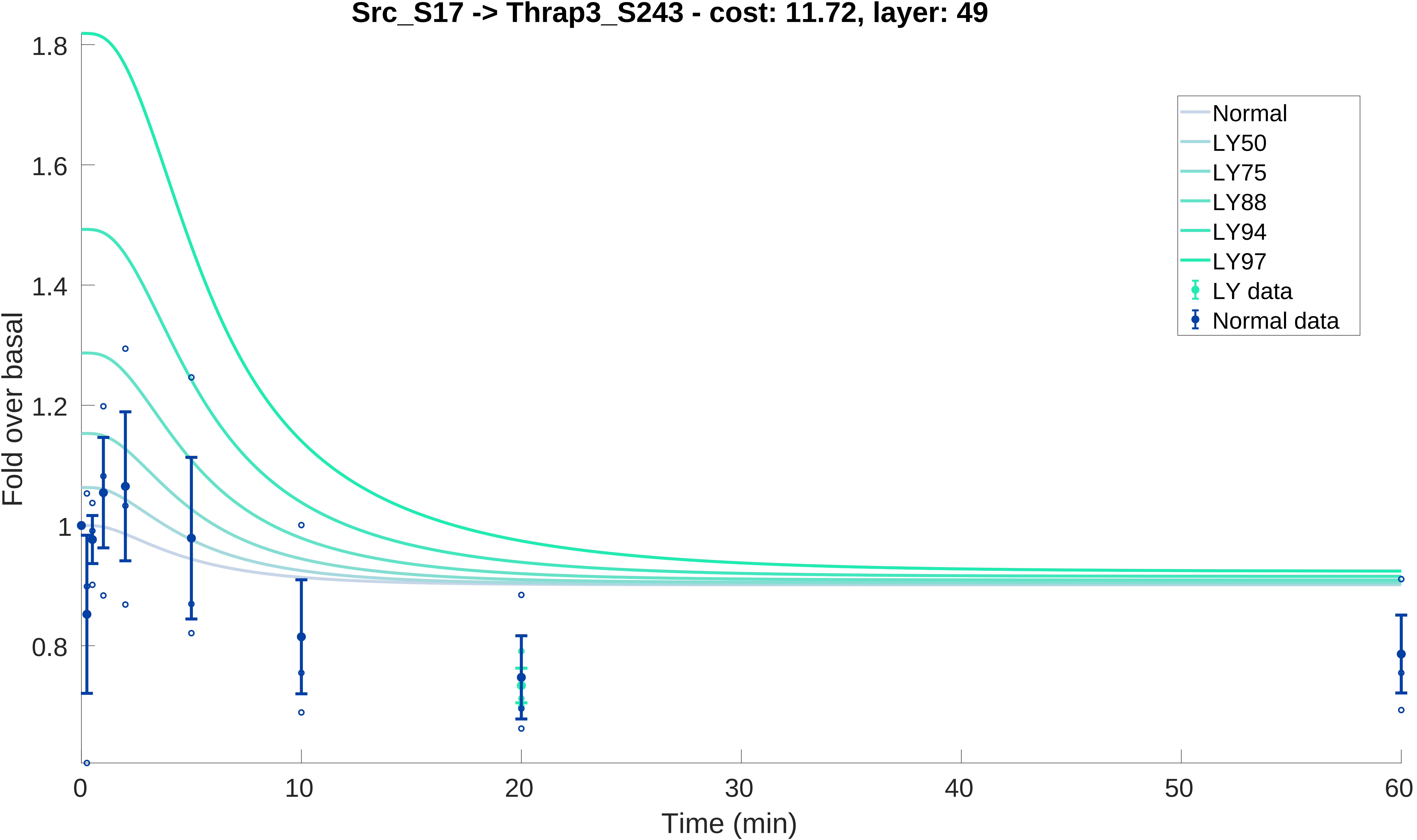

Src_S17 -> Thrap3_S243 - cost: 11.72, layer: 49
Fold over basal
1.8
1.6
1.4
1.2
1
0.8
0
10
20
30
40
50
60
Time (min)
Normal
LY50
LY75
LY88
LY94
LY97
LY data
Normal data

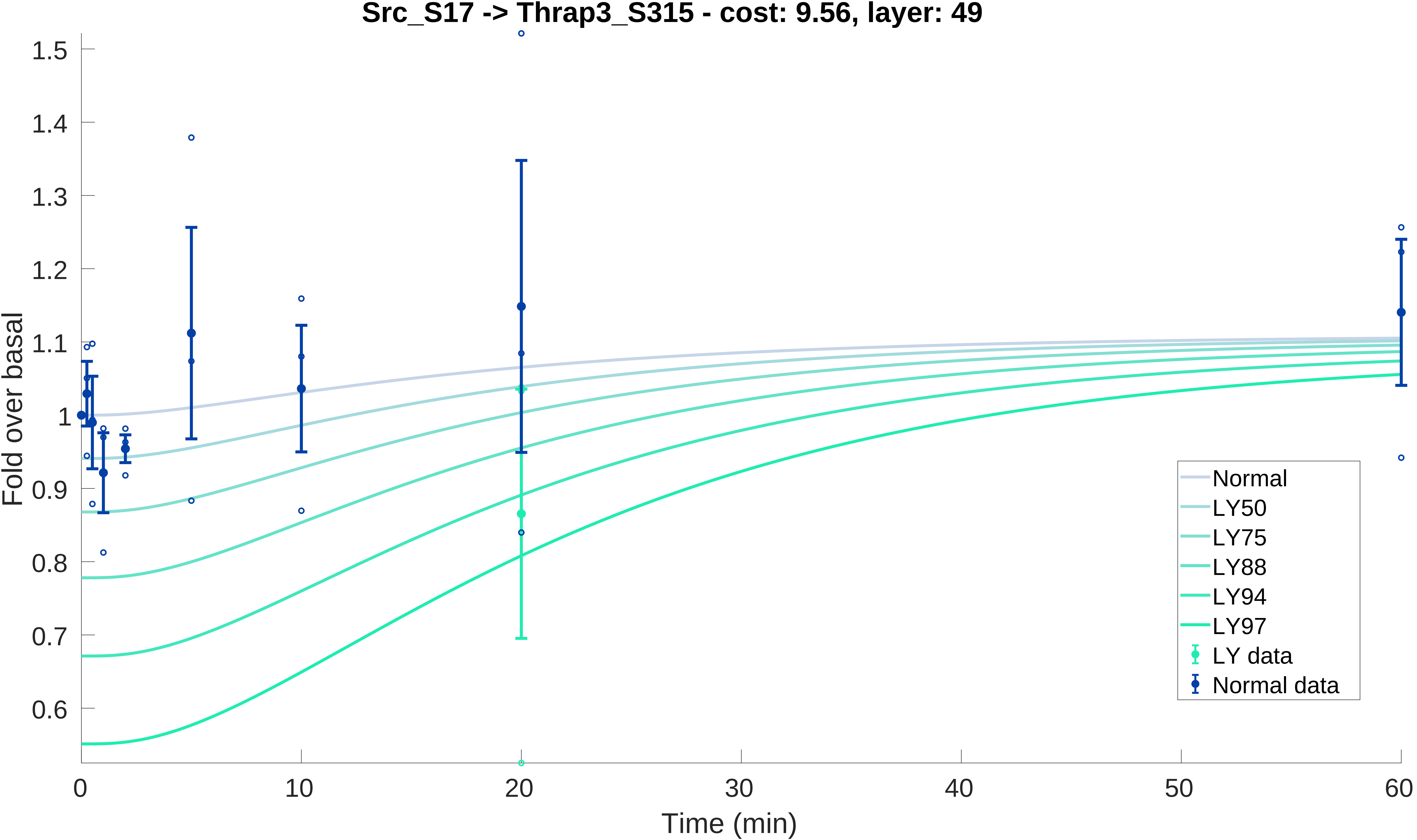
Src_S17 -> Thrap3_S315 - cost: 9.56, layer: 49
Fold over basal
Time (min)
1.5
1.4
1.3
1.2
1.1
1
0.9
0.8
0.7
0.6
0
10
20
30
40
50
60
Normal
LY50
LY75
LY88
LY94
LY97
LY data
Normal data

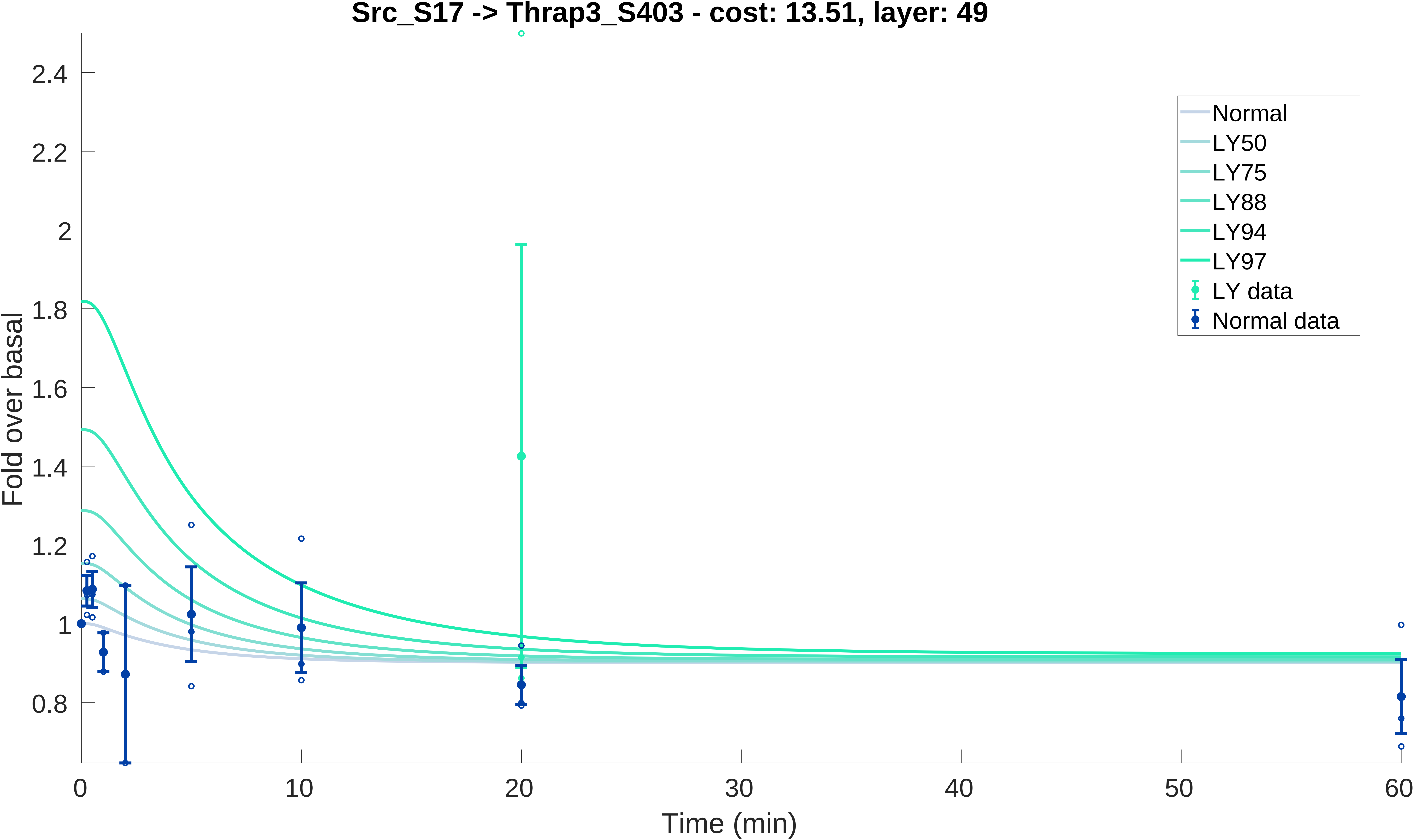

Src_S17 -> Thrap3_S403 - cost: 13.51, layer: 49
Fold over basal
Time (min)
0
10
20
30
40
50
60
0.8
1
1.2
1.4
1.6
1.8
2
2.2
2.4
Normal
LY50
LY75
LY88
LY94
LY97
LY data
Normal data

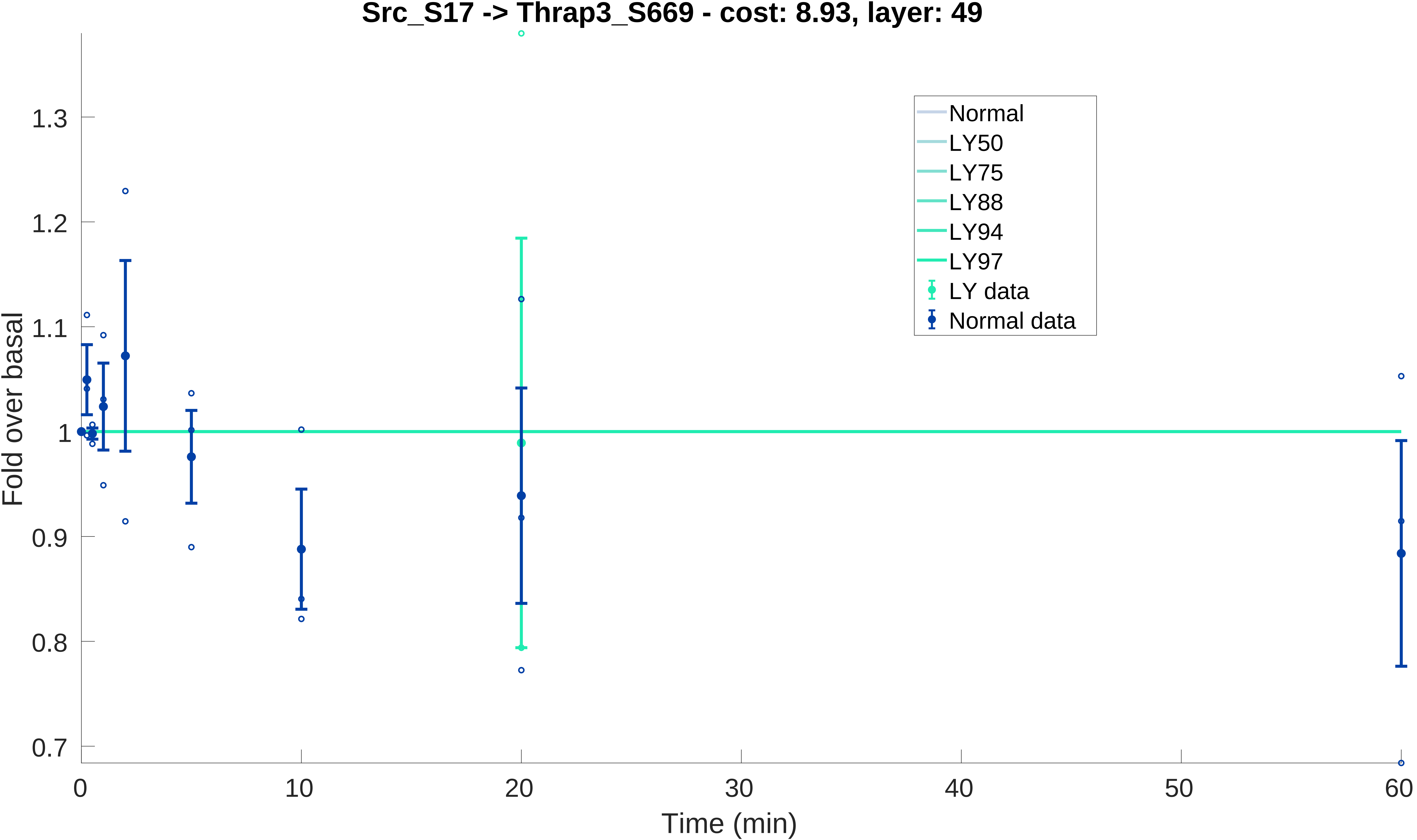

Src_S17 -> Thrap3_S669 - cost: 8.93, layer: 49
Normal
LY50
LY75
LY88
LY94
LY97
LY data
Normal data
Fold over basal
Time (min)
1.3
1.2
1.1
1
0.9
0.8
0.7
0
10
20
30
40
50
60

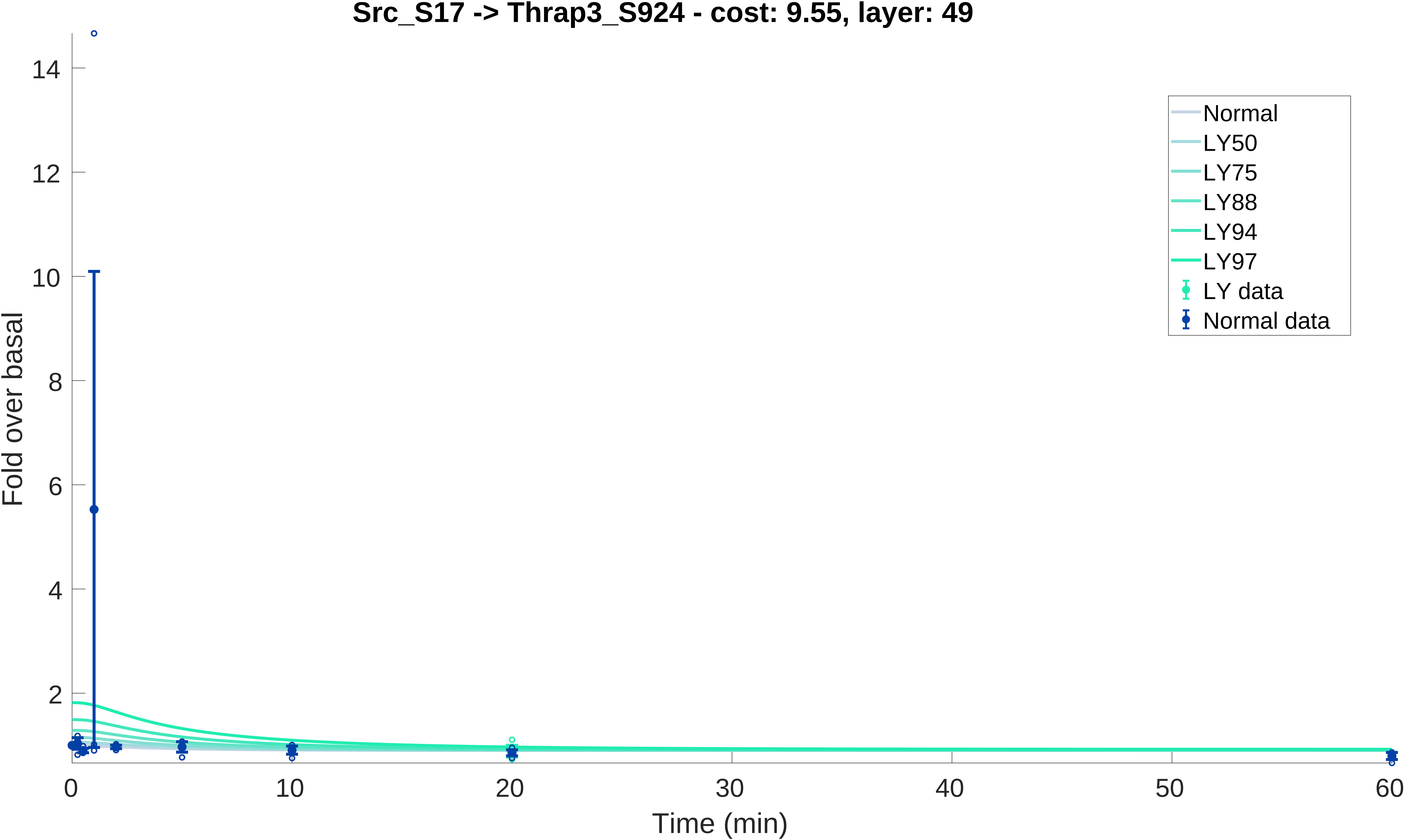

Src_S17 -> Thrap3_S924 - cost: 9.55, layer: 49
Fold over basal
Time (min)
0
10
20
30
40
50
60
2
4
6
8
10
12
14
Normal
LY50
LY75
LY88
LY94
LY97
LY data
Normal data

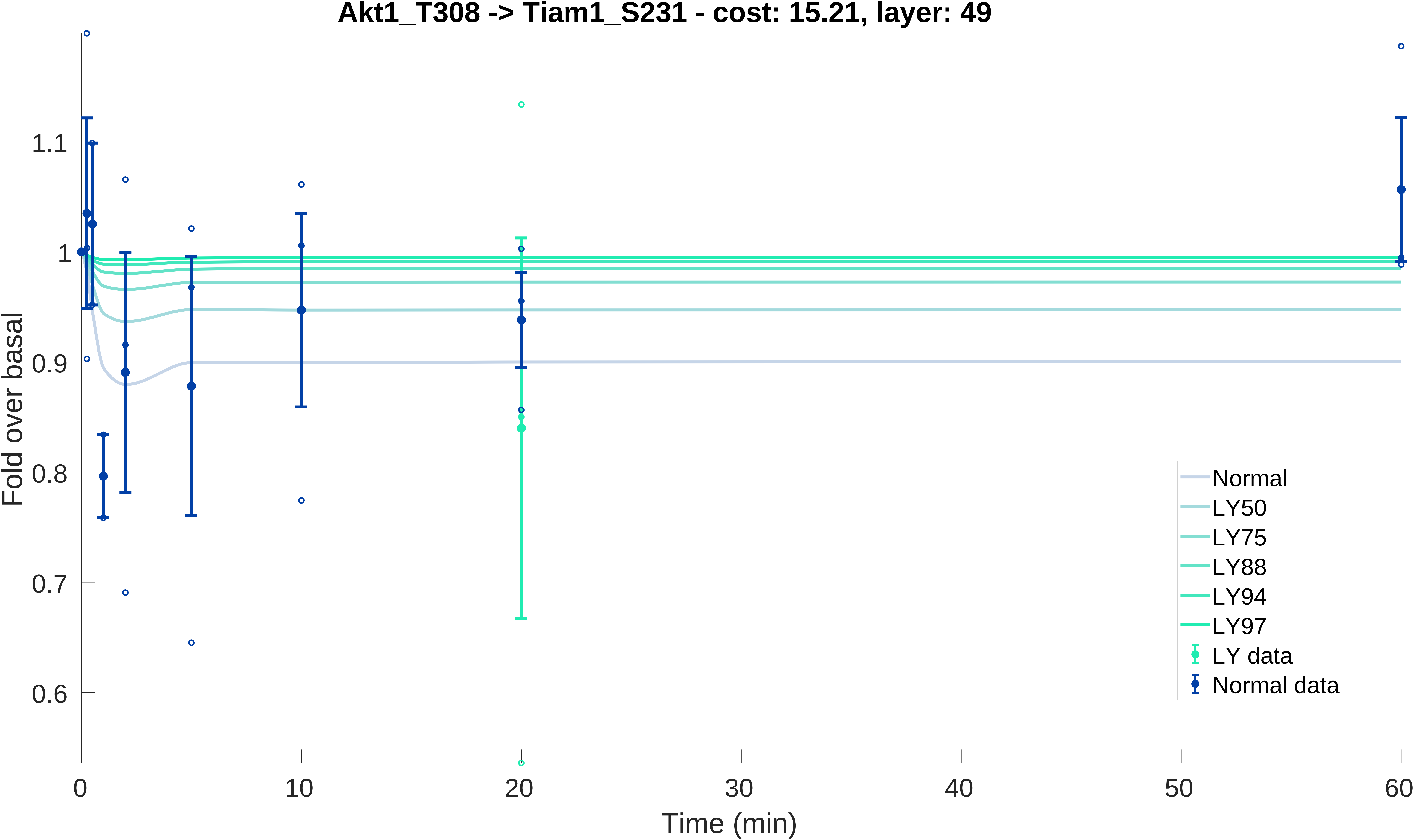

Akt1_T308 -> Tiam1_S231 - cost: 15.21, layer: 49
Fold over basal
Time (min)
0.6
0.7
0.8
0.9
1
1.1
0
10
20
30
40
50
60
Normal
LY50
LY75
LY88
LY94
LY97
LY data
Normal data

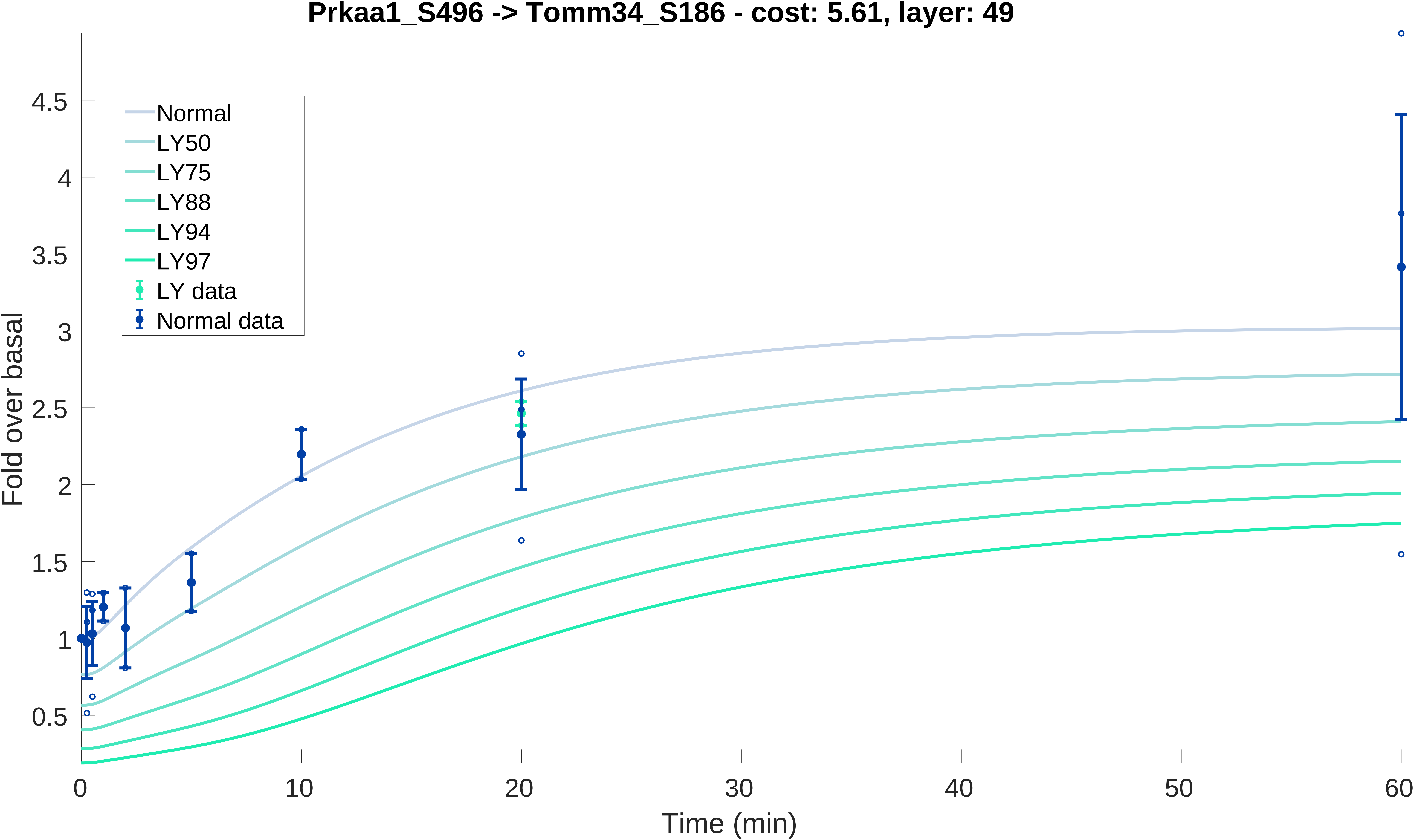
Prkaa1_S496 -> Tomm34_S186 - cost: 5.61, layer: 49
Normal
LY50
LY75
LY88
LY94
LY97
LY data
Normal data
Fold over basal
0.5
1
1.5
2
2.5
3
3.5
4
4.5
0
10
20
30
40
50
60
Time (min)

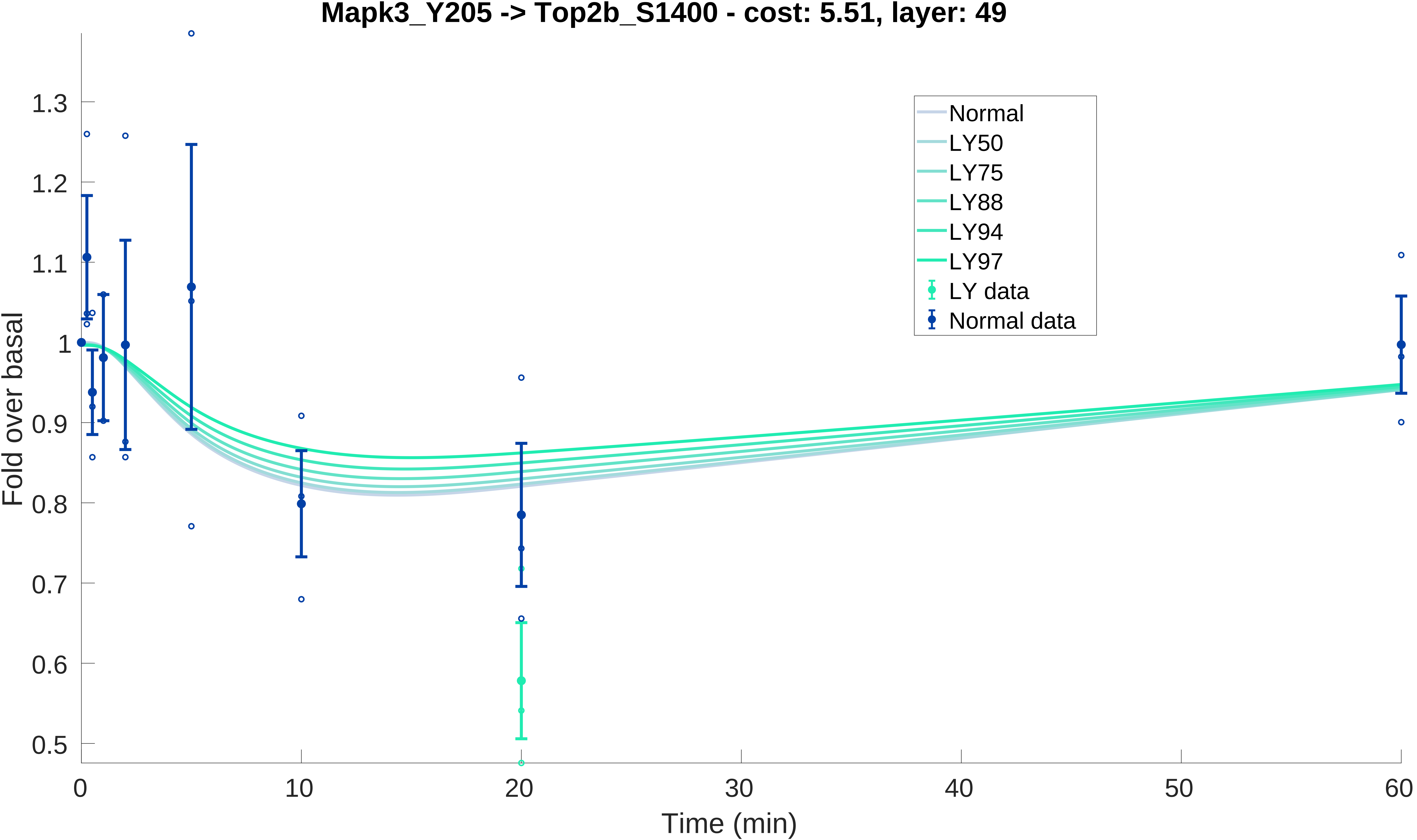

Mapk3_Y205 -> Top2b_S1400 - cost: 5.51, layer: 49
Fold over basal
Time (min)
1.3
1.2
1.1
1
0.9
0.8
0.7
0.6
0.5
0
10
20
30
40
50
60
Normal
LY50
LY75
LY88
LY94
LY97
LY data
Normal data

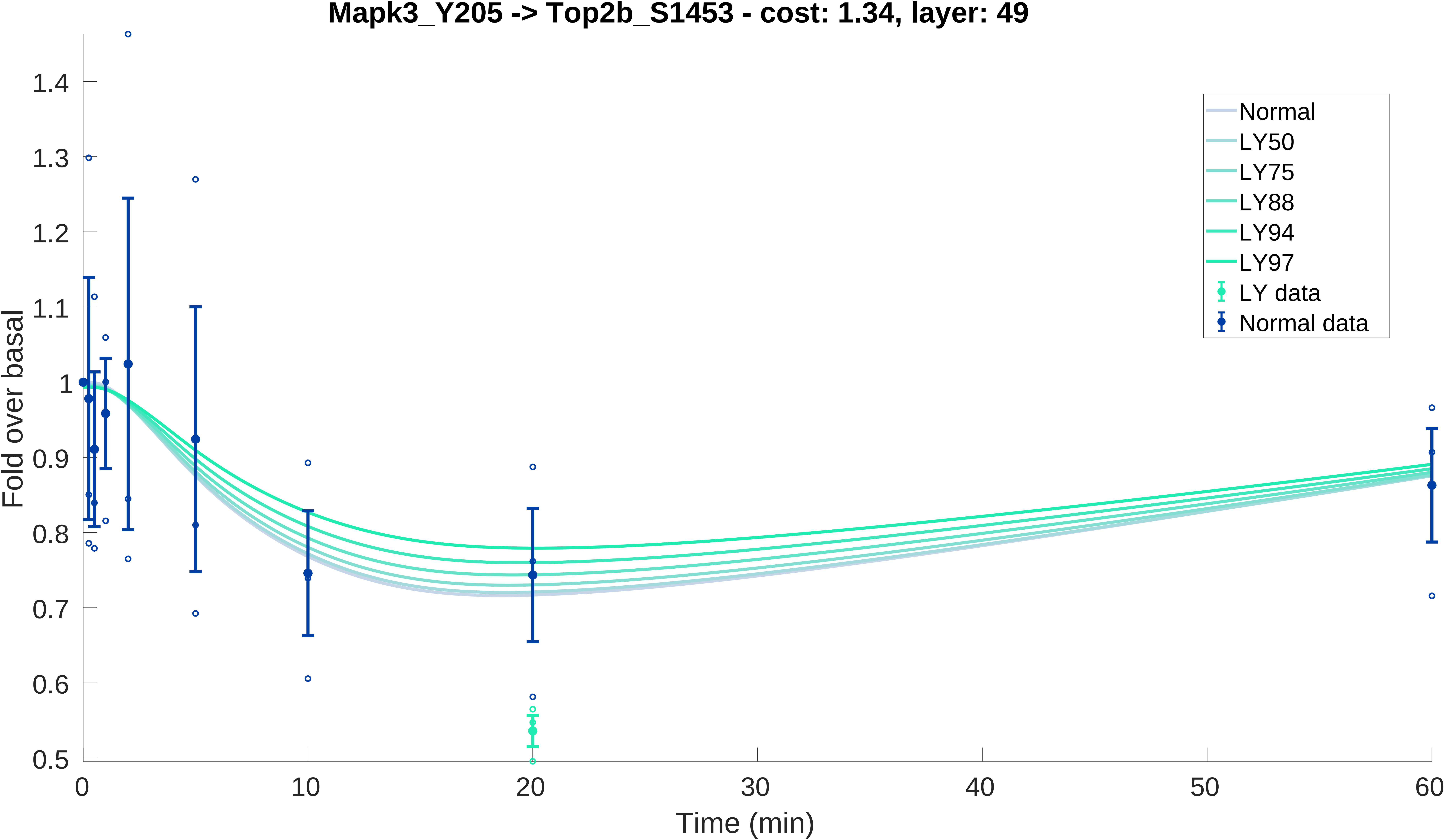

Mapk3_Y205 -> Top2b_S1453 - cost: 1.34, layer: 49
Fold over basal
Time (min)
1.4
1.3
1.2
1.1
1
0.9
0.8
0.7
0.6
0.5
0
10
20
30
40
50
60
Normal
LY50
LY75
LY88
LY94
LY97
LY data
Normal data

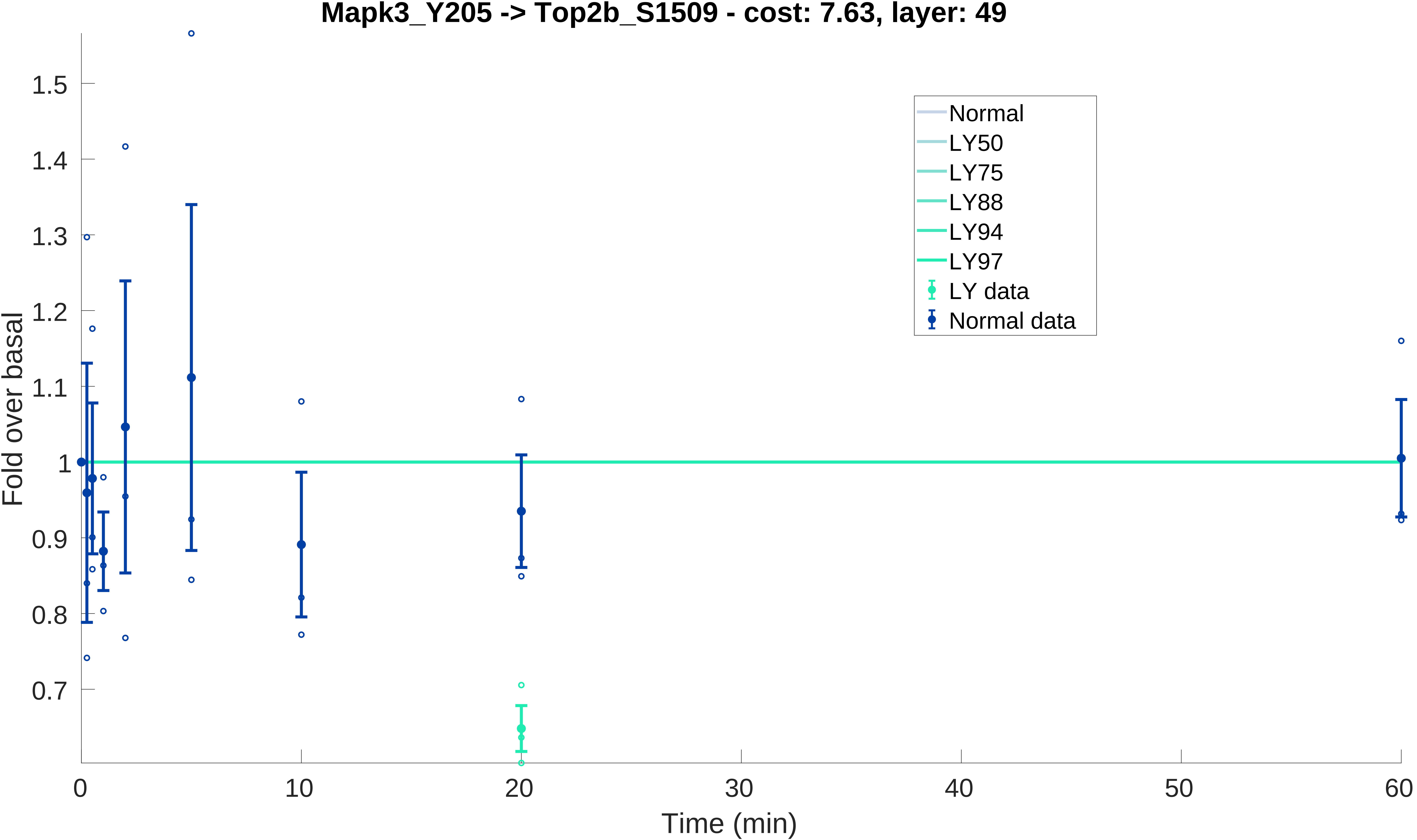

Mapk3_Y205 -> Top2b_S1509 - cost: 7.63, layer: 49
Fold over basal
1.5
1.4
1.3
1.2
1.1
1
0.9
0.8
0.7
0
10
20
30
40
50
60
Time (min)
Normal
LY50
LY75
LY88
LY94
LY97
LY data
Normal data

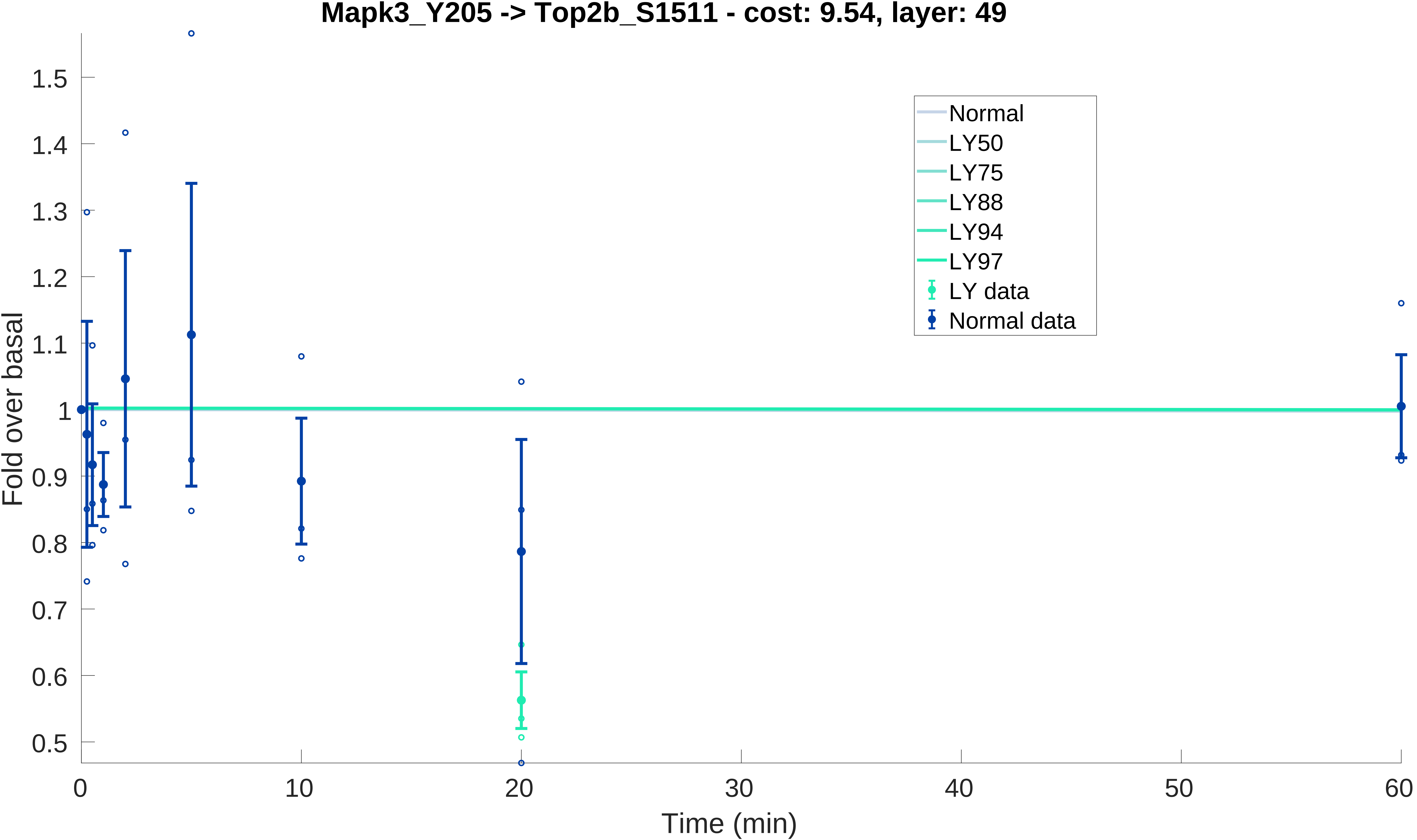

Mapk3_Y205 -> Top2b_S1511 - cost: 9.54, layer: 49
Fold over basal
Time (min)
1.5
1.4
1.3
1.2
1.1
1
0.9
0.8
0.7
0.6
0.5
0
10
20
30
40
50
60
Normal
LY50
LY75
LY88
LY94
LY97
LY data
Normal data

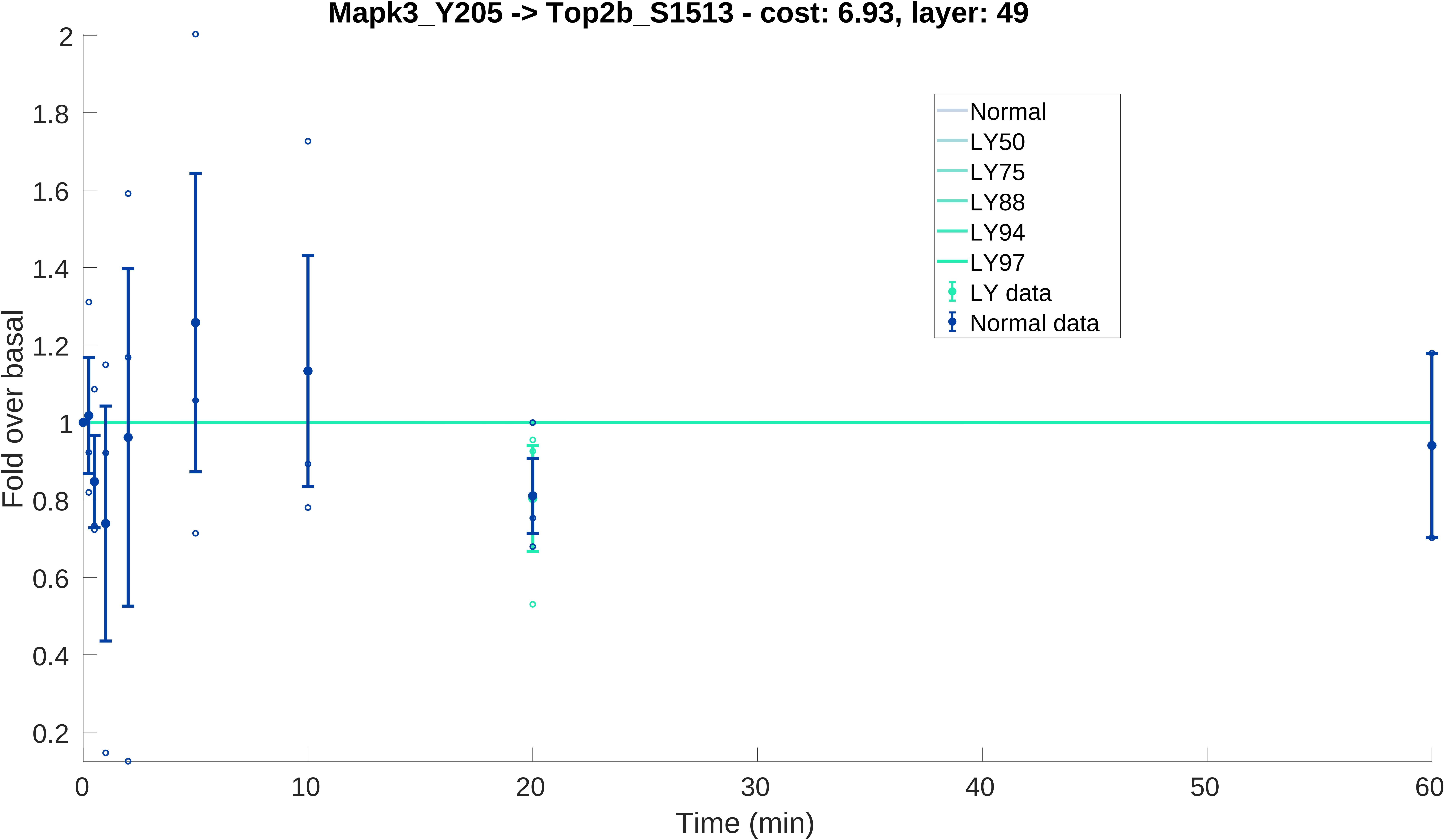

Mapk3_Y205 -> Top2b_S1513 - cost: 6.93, layer: 49
Fold over basal
Time (min)
0
10
20
30
40
50
60
0.2
0.4
0.6
0.8
1
1.2
1.4
1.6
1.8
2
Normal
LY50
LY75
LY88
LY94
LY97
LY data
Normal data

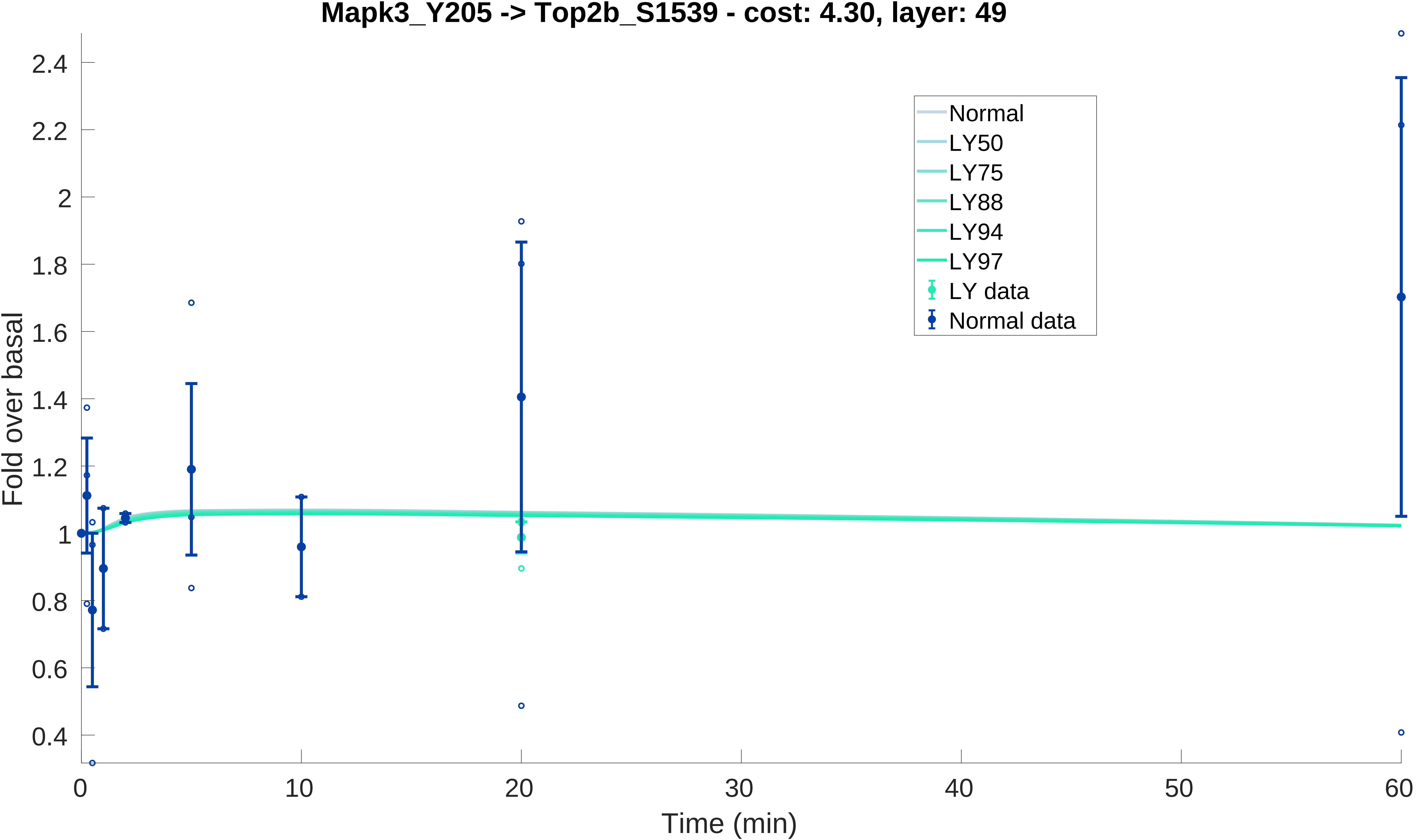
Mapk3_Y205 -> Top2b_S1539 - cost: 4.30, layer: 49
Fold over basal
Time (min)
Normal
LY50
LY75
LY88
LY94
LY97
LY data
Normal data
2.4
2.2
2
1.8
1.6
1.4
1.2
1
0.8
0.6
0.4
0
10
20
30
40
50
60

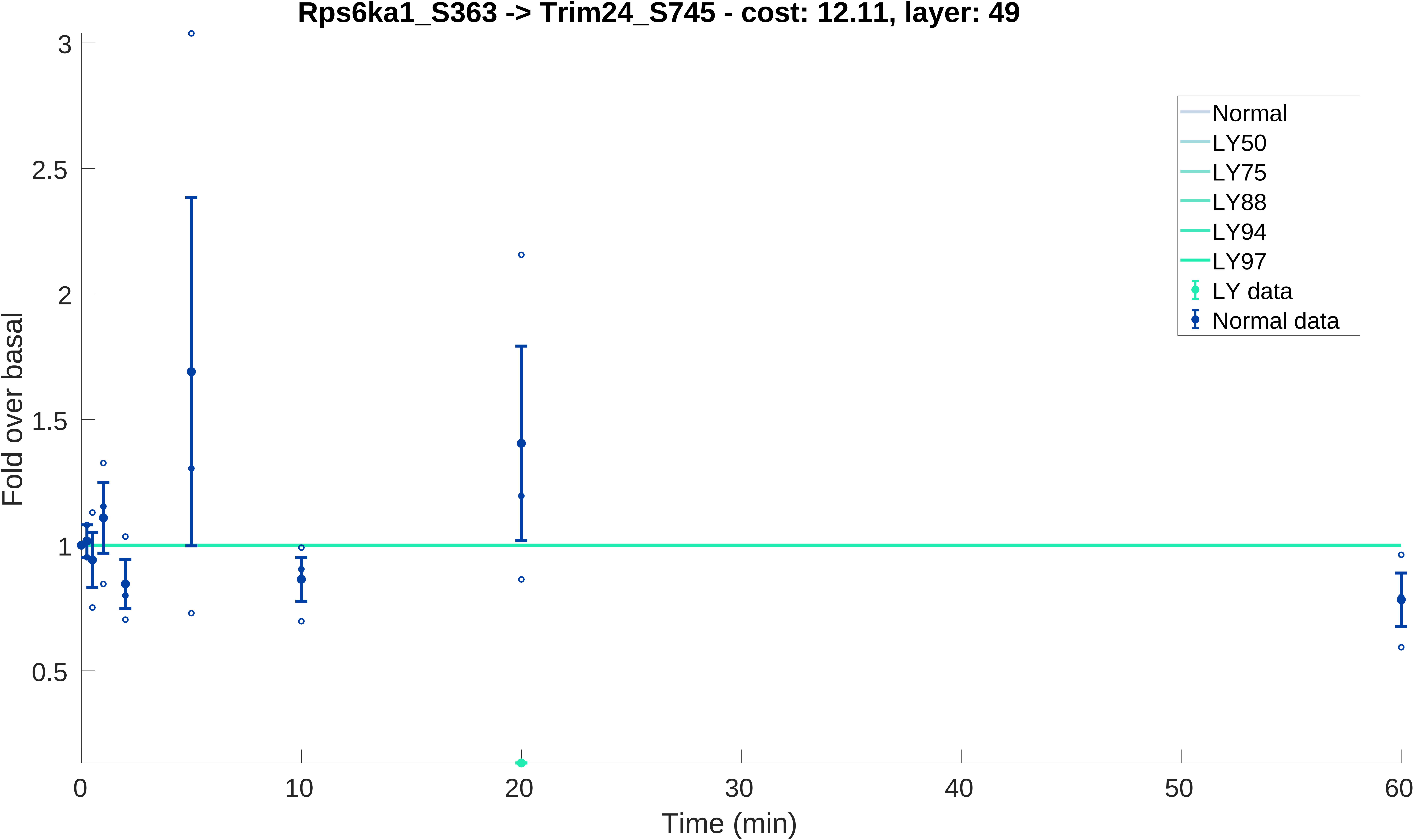

Rps6ka1_S363 -> Trim24_S745 - cost: 12.11, layer: 49
Fold over basal
3
2.5
2
1.5
1
0.5
0
10
20
30
40
50
60
Time (min)
Normal
LY50
LY75
LY88
LY94
LY97
LY data
Normal data

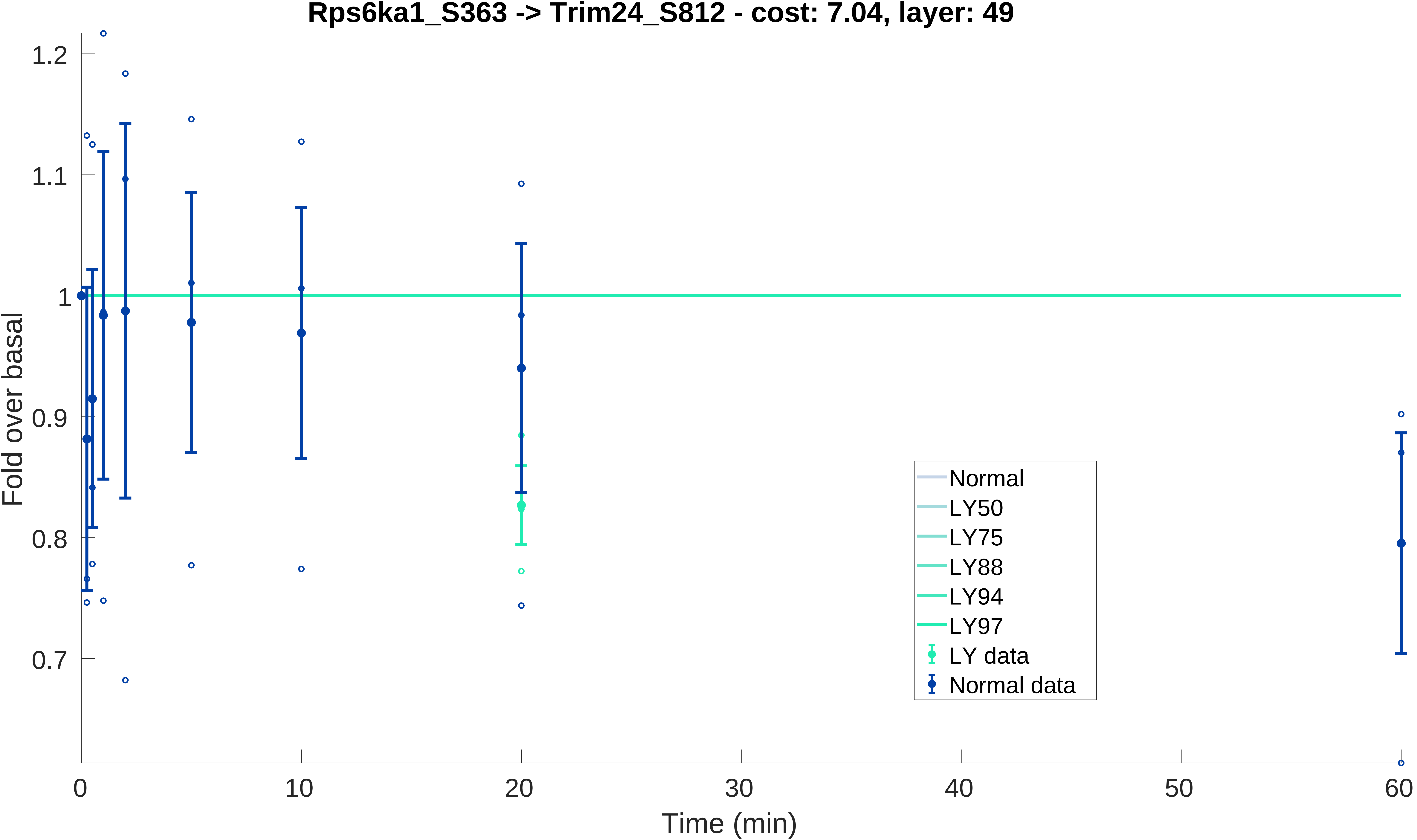

Rps6ka1_S363 -> Trim24_S812 - cost: 7.04, layer: 49
Fold over basal
1.2
1.1
1
0.9
0.8
0.7
0
10
20
30
40
50
60
Time (min)
Normal
LY50
LY75
LY88
LY94
LY97
LY data
Normal data

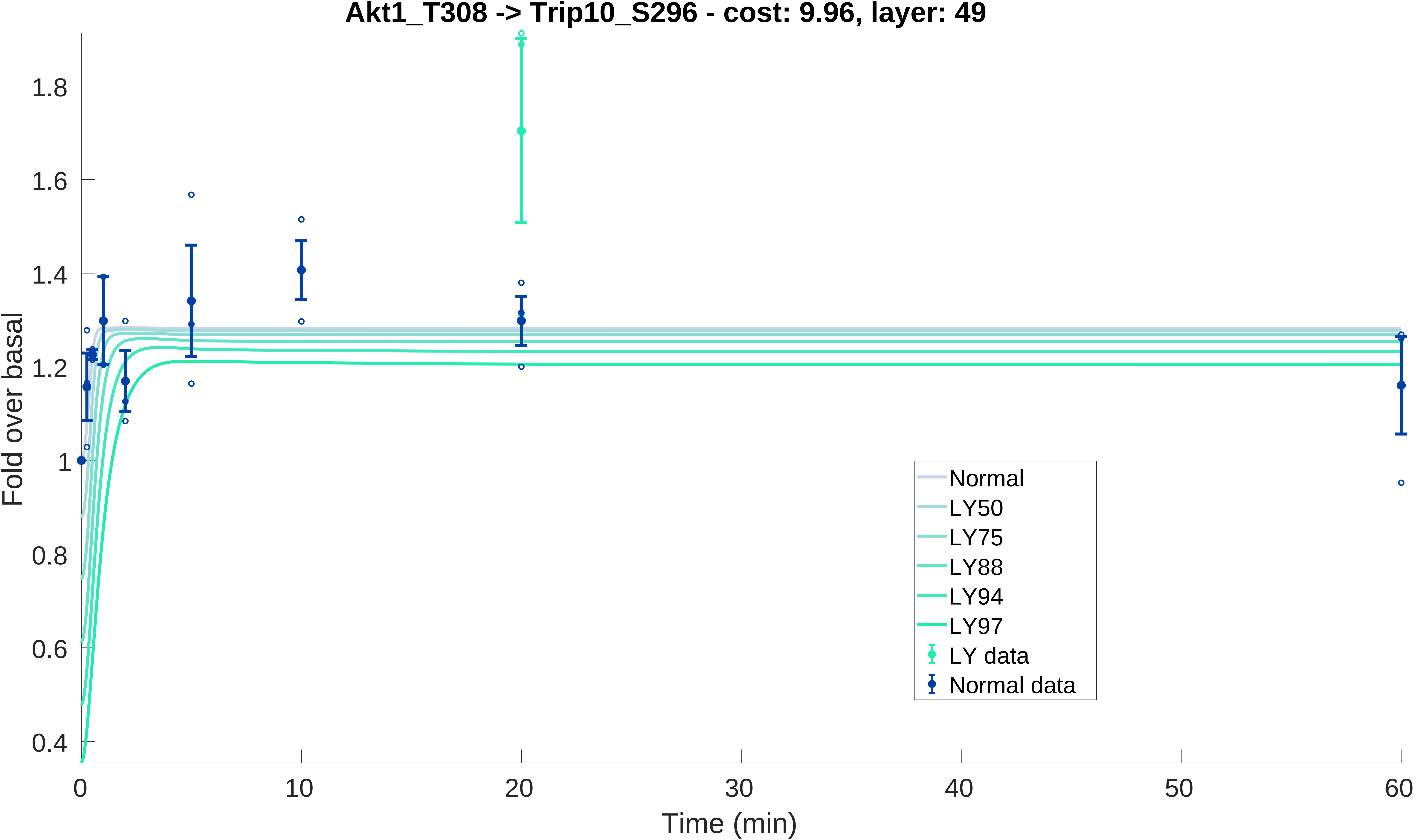

Akt1_T308 -> Trip10_S296 - cost: 9.96, layer: 49
Fold over basal
1.8
1.6
1.4
1.2
1
0.8
0.6
0.4
0
10
20
30
40
50
60
Time (min)
Normal
LY50
LY75
LY88
LY94
LY97
LY data
Normal data

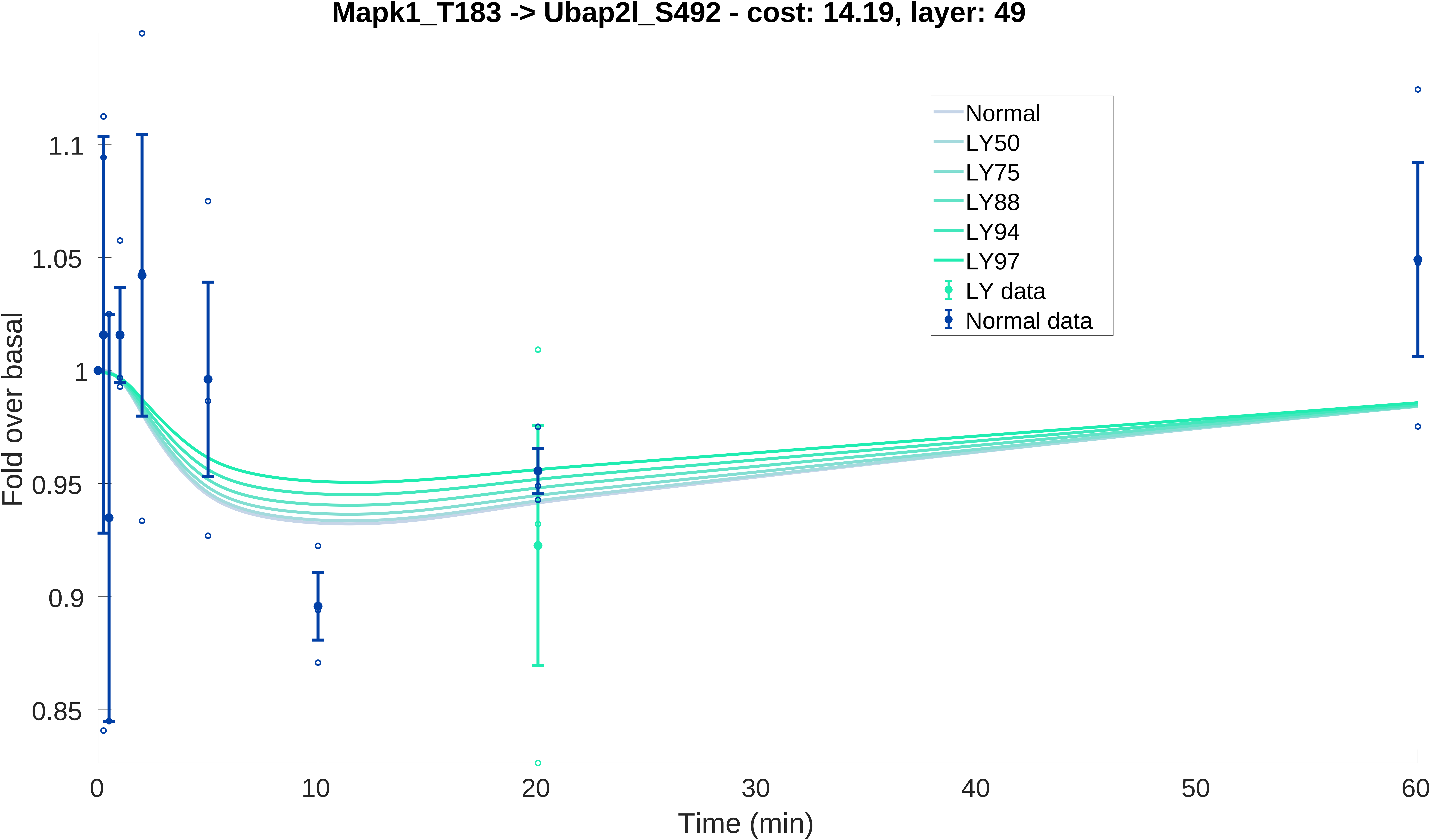
Mapk1_T183 -> Ubap2l_S492 - cost: 14.19, layer: 49
Fold over basal
1.1
1.05
1
0.95
0.9
0.85
0
10
20
30
40
50
60
Time (min)
Normal
LY50
LY75
LY88
LY94
LY97
LY data
Normal data

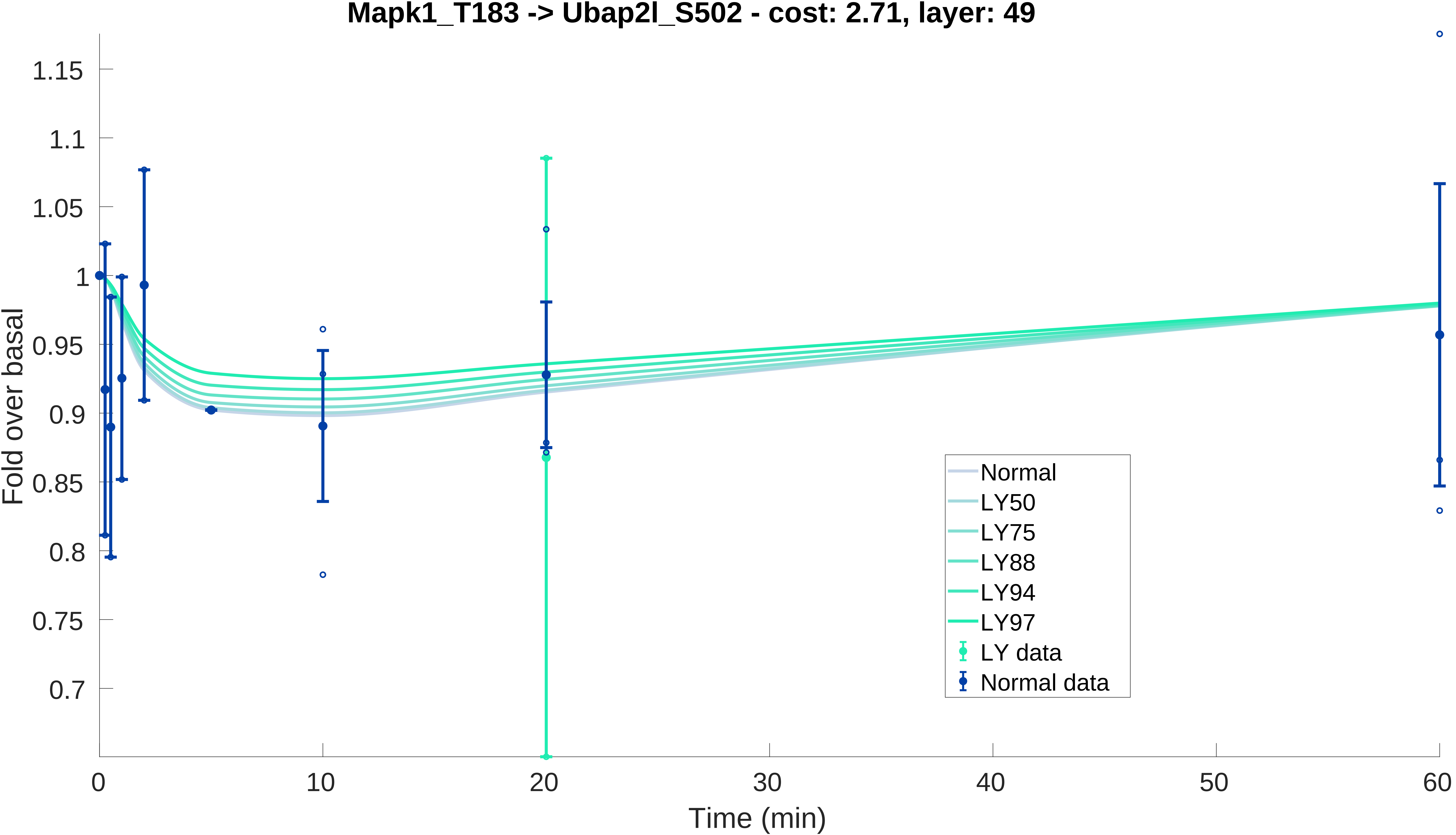

Mapk1_T183 -> Ubap2l_S502 - cost: 2.71, layer: 49
Fold over basal
1.15
1.1
1.05
1
0.95
0.9
0.85
0.8
0.75
0.7
0
10
20
30
40
50
60
Time (min)
Normal
LY50
LY75
LY88
LY94
LY97
LY data
Normal data

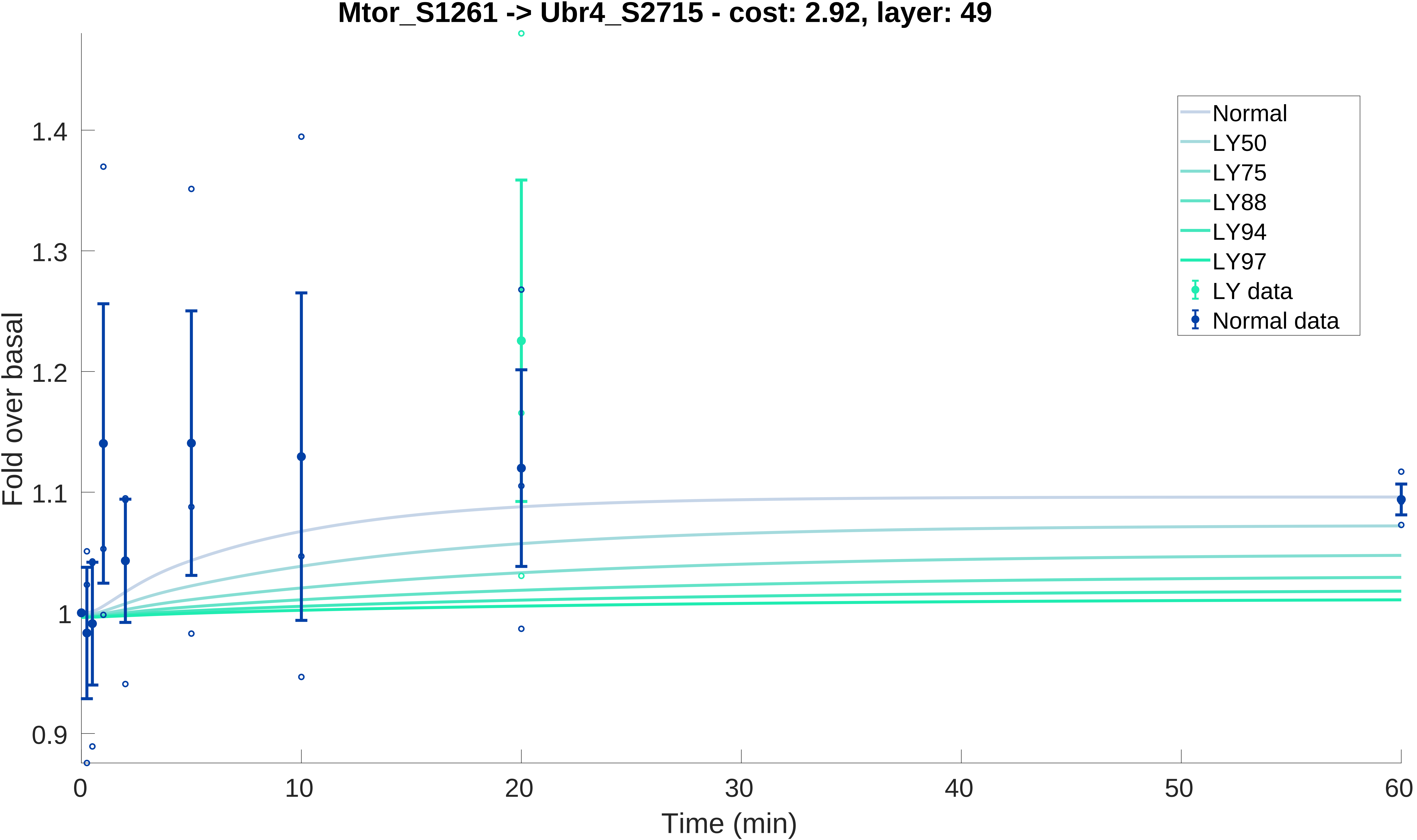

Mtor_S1261 -> Ubr4_S2715 - cost: 2.92, layer: 49
Fold over basal
0.9
1
1.1
1.2
1.3
1.4
Time (min)
0
10
20
30
40
50
60
Normal
LY50
LY75
LY88
LY94
LY97
LY data
Normal data

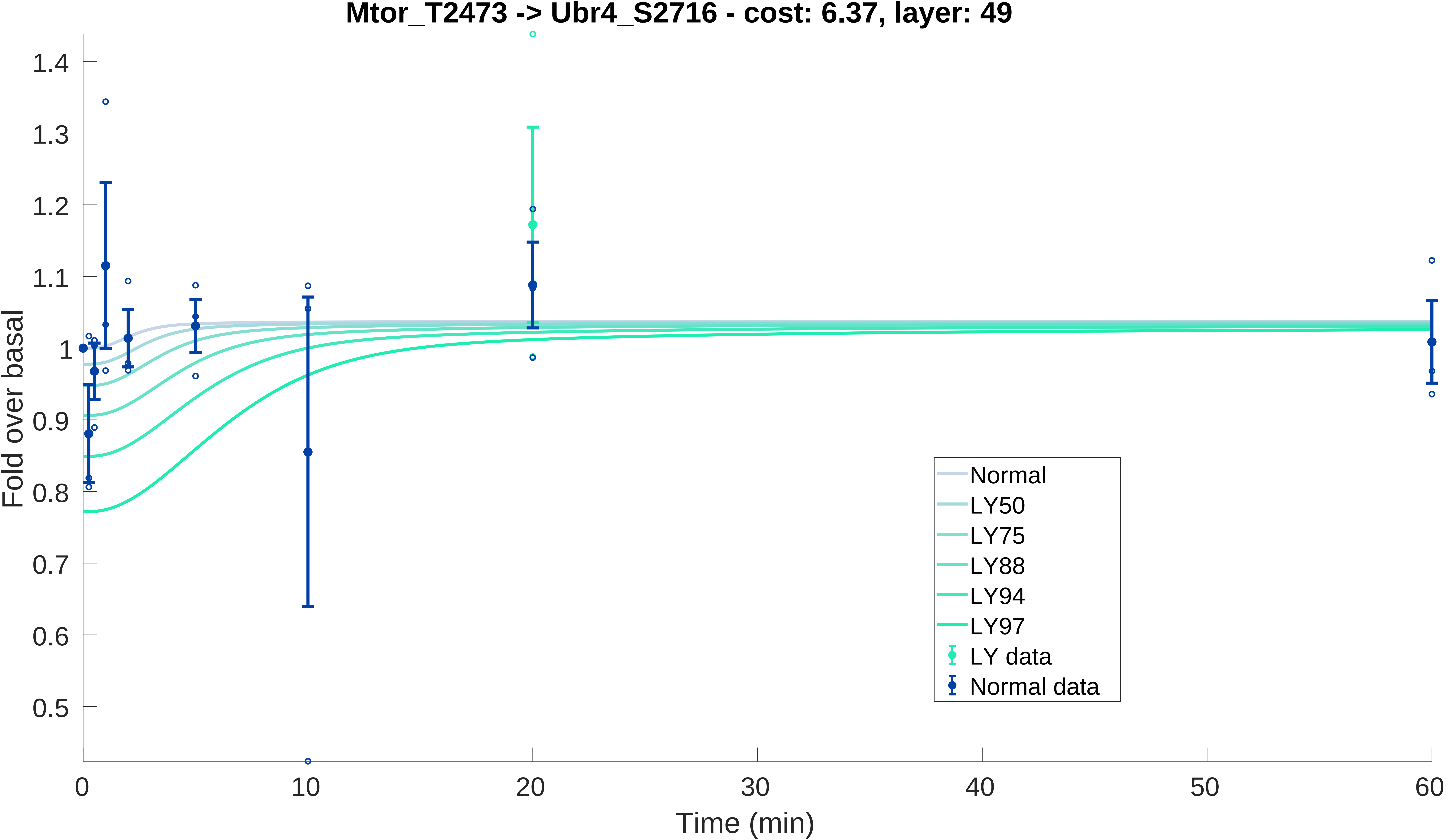
Mtor_T2473 -> Ubr4_S2716 - cost: 6.37, layer: 49
Fold over basal
Time (min)
1.4
1.3
1.2
1.1
1
0.9
0.8
0.7
0.6
0.5
0
10
20
30
40
50
60
Normal
LY50
LY75
LY88
LY94
LY97
LY data
Normal data

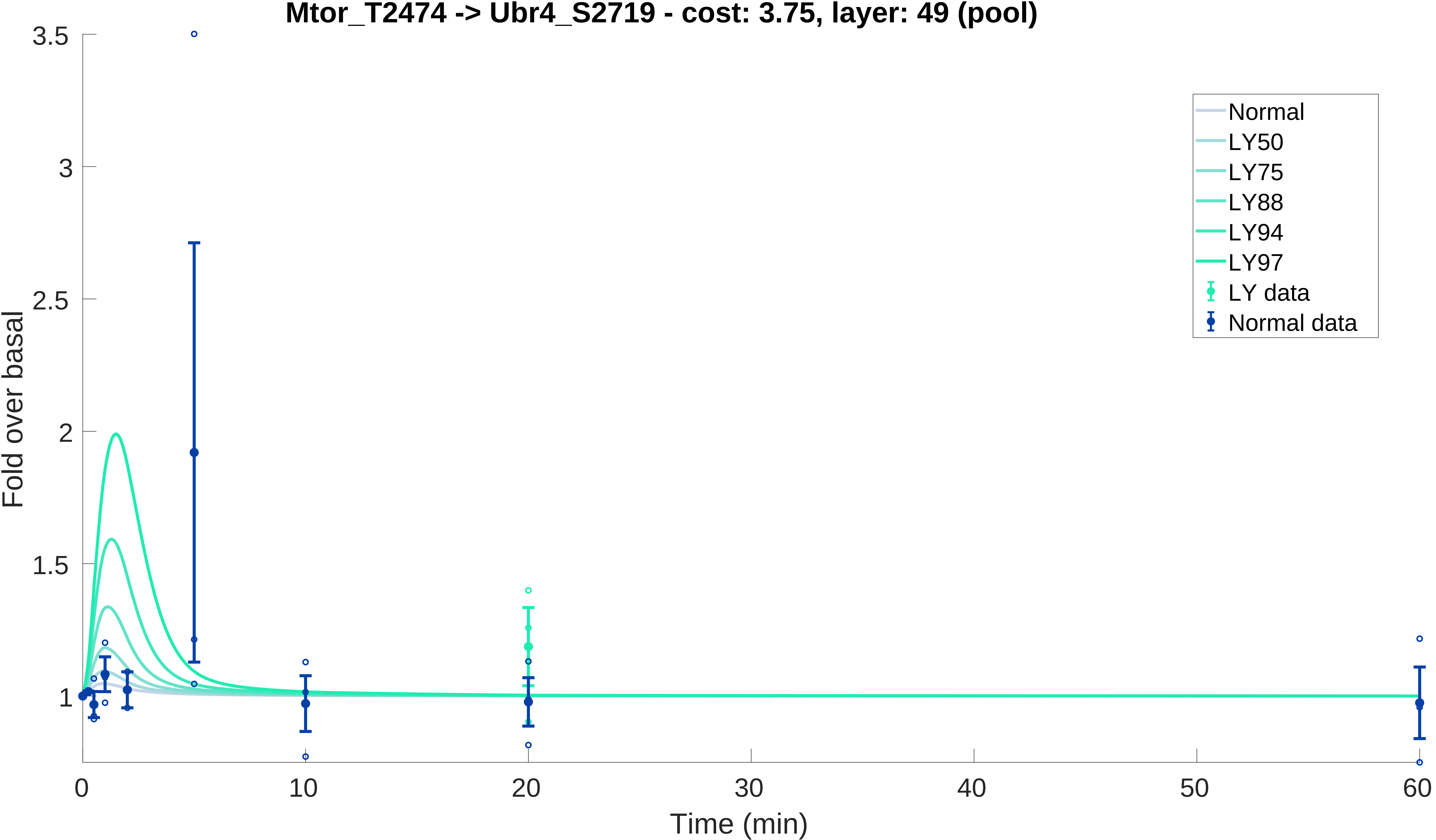

Mtor_T2474 -> Ubr4_S2719 - cost: 3.75, layer: 49 (pool)
Fold over basal
Time (min)
0
10
20
30
40
50
60
1
1.5
2
2.5
3
3.5
Normal
LY50
LY75
LY88
LY94
LY97
LY data
Normal data

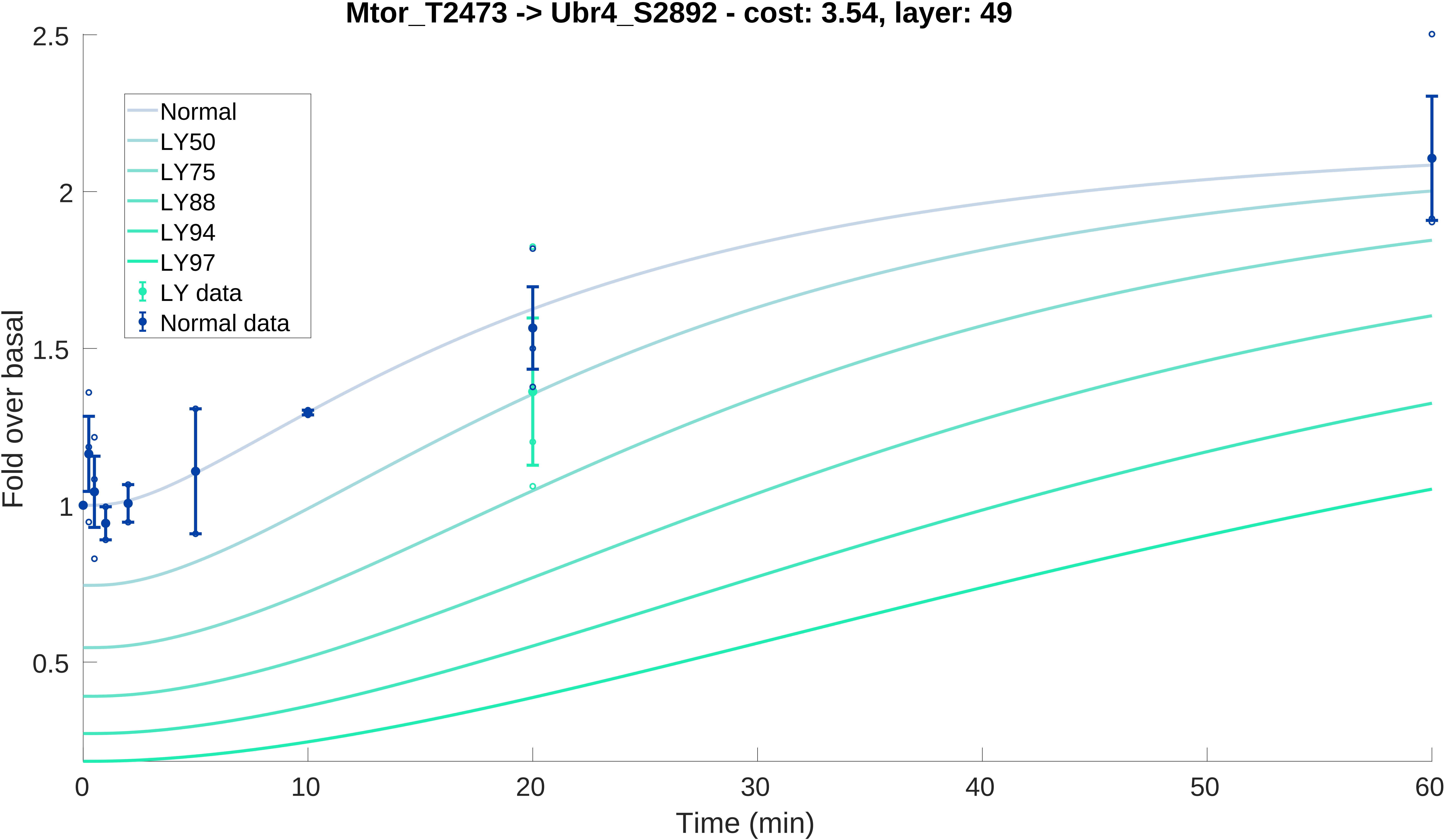

Mtor_T2473 -> Ubr4_S2892 - cost: 3.54, layer: 49
Normal
LY50
LY75
LY88
LY94
LY97
LY data
Normal data
Fold over basal
Time (min)
0.5
1
1.5
2
2.5
0
10
20
30
40
50
60

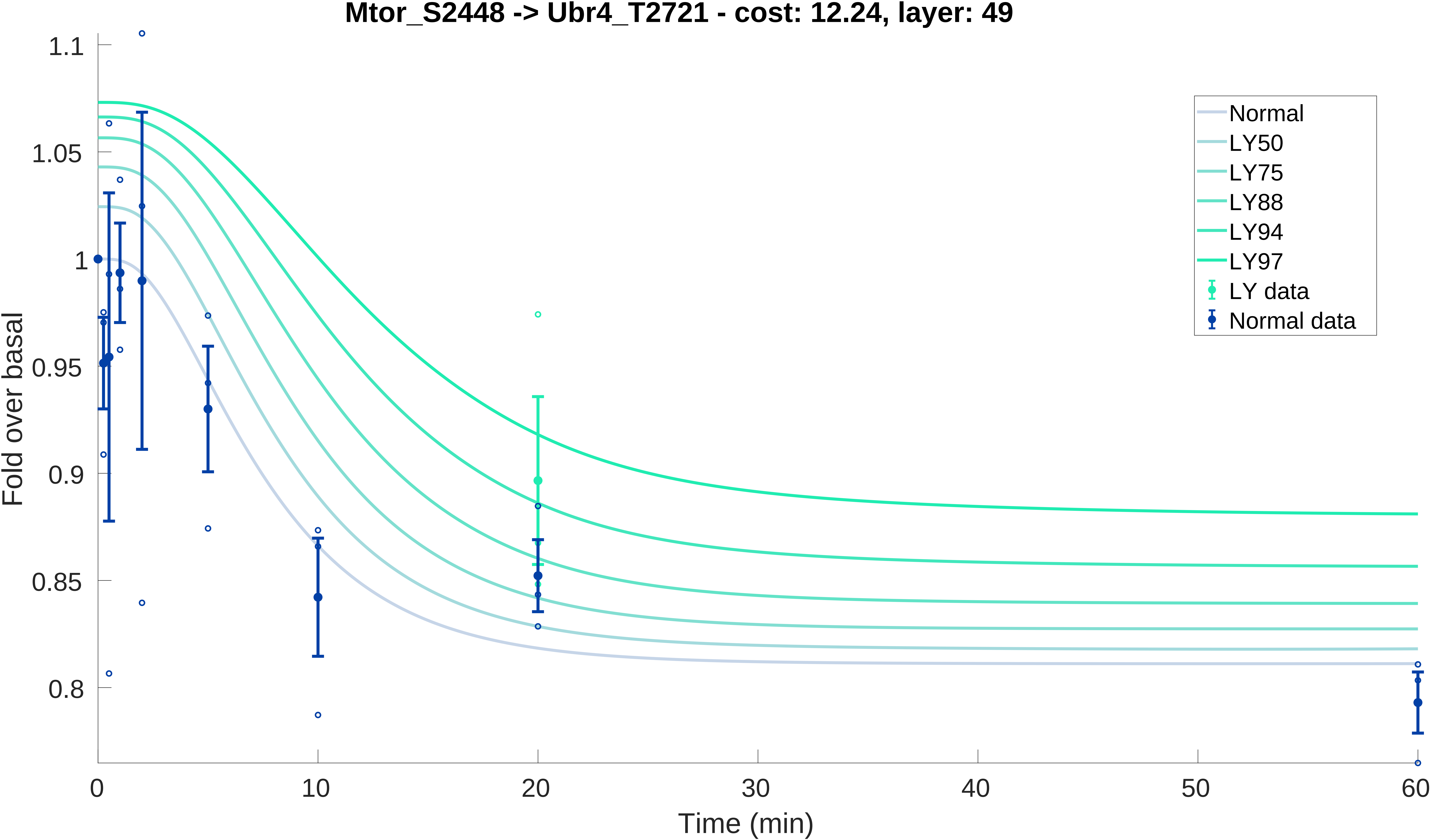

Mtor_S2448 -> Ubr4_T2721 - cost: 12.24, layer: 49
Fold over basal
1.1
1.05
1
0.95
0.9
0.85
0.8
0
10
20
30
40
50
60
Time (min)
Normal
LY50
LY75
LY88
LY94
LY97
LY data
Normal data

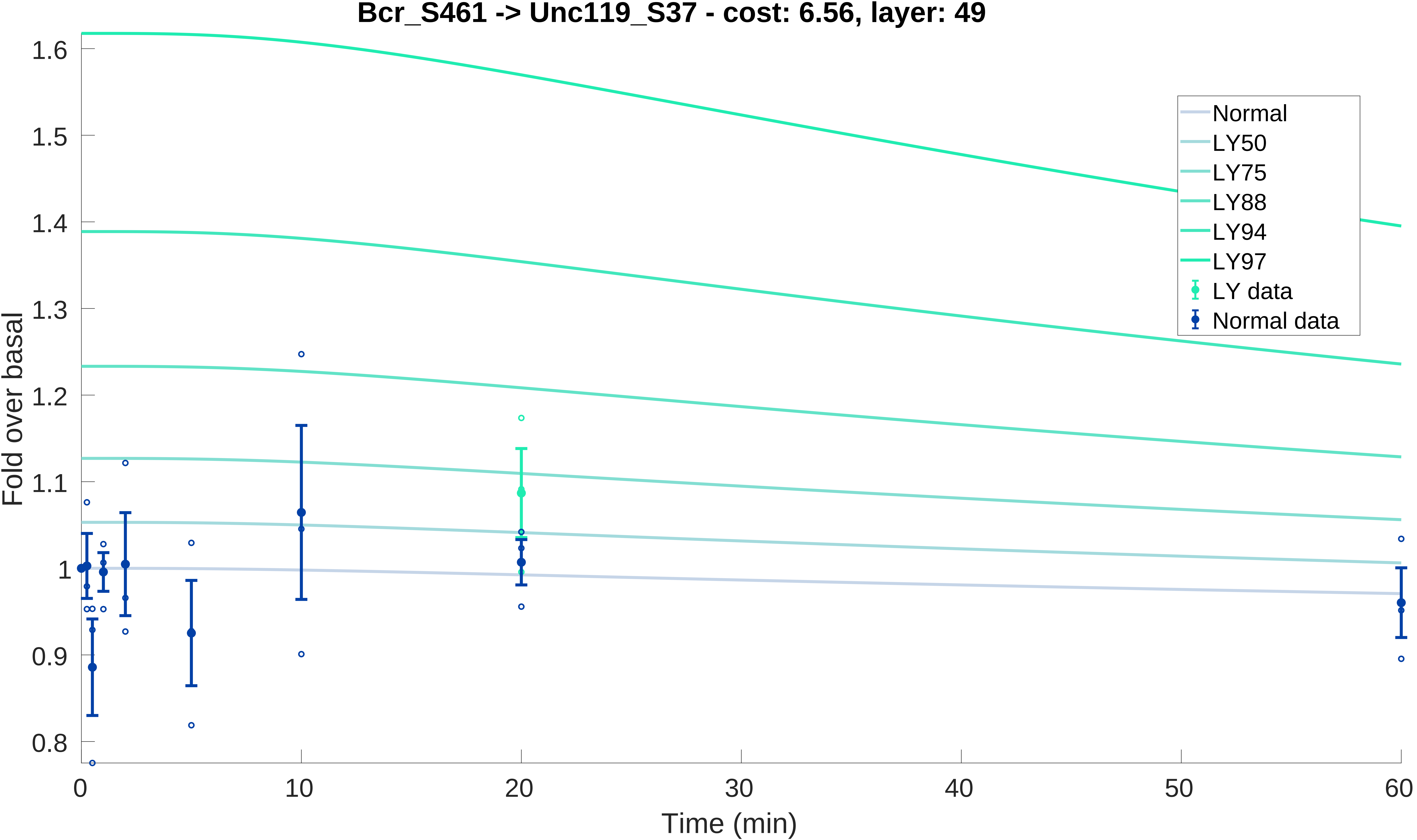

Bcr_S461 -> Unc119_S37 - cost: 6.56, layer: 49
Fold over basal
Time (min)
0.8
0.9
1
1.1
1.2
1.3
1.4
1.5
1.6
0
10
20
30
40
50
60
Normal
LY50
LY75
LY88
LY94
LY97
LY data
Normal data

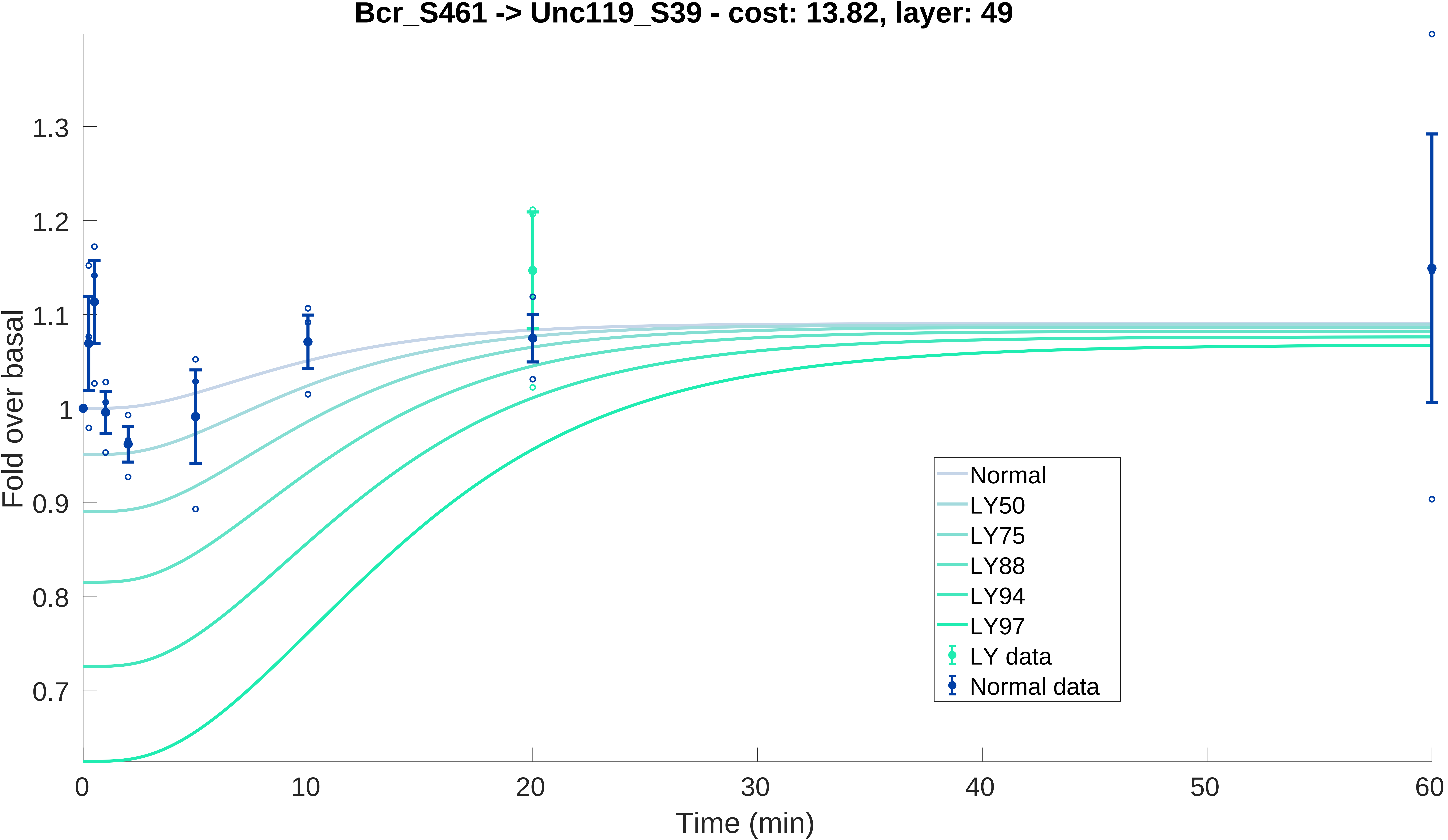

Bcr_S461 -> Unc119_S39 - cost: 13.82, layer: 49
Fold over basal
1.3
1.2
1.1
1
0.9
0.8
0.7
0
10
20
30
40
50
60
Time (min)
Normal
LY50
LY75
LY88
LY94
LY97
LY data
Normal data

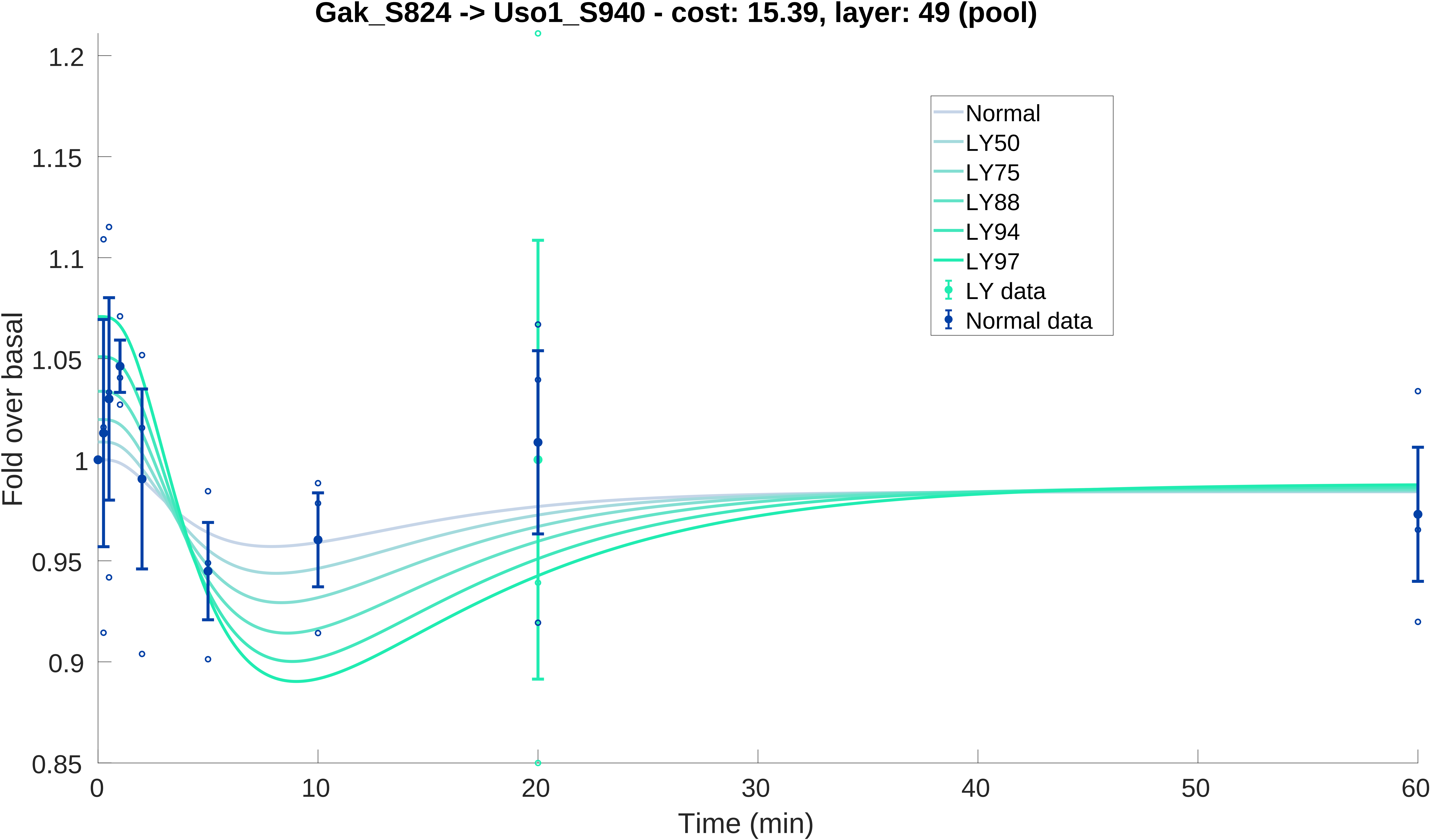

Gak_S824 -> Uso1_S940 - cost: 15.39, layer: 49 (pool)
Fold over basal
1.2
1.15
1.1
1.05
1
0.95
0.9
0.85
0
10
20
30
40
50
60
Time (min)
Normal
LY50
LY75
LY88
LY94
LY97
LY data
Normal data

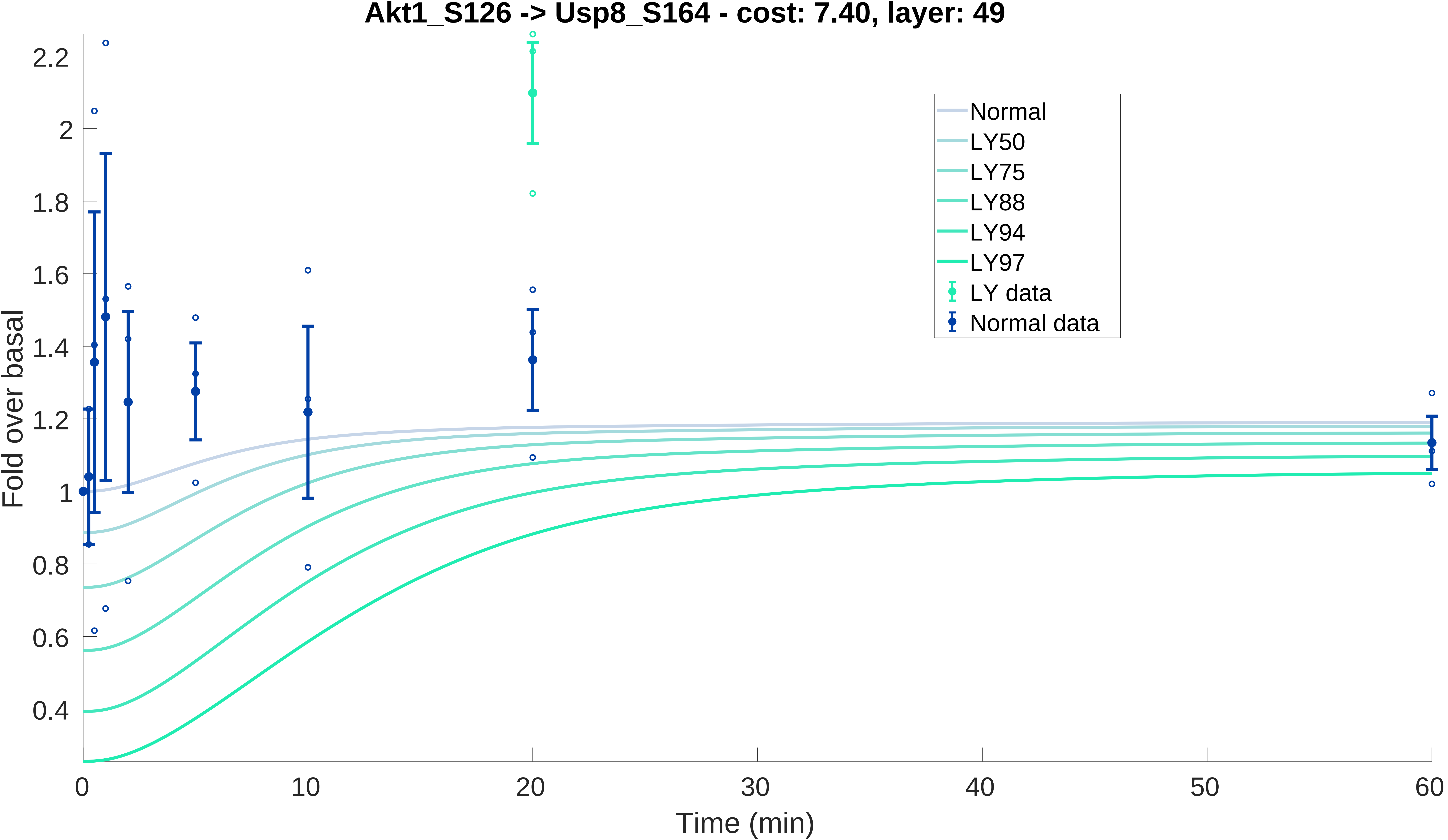

Akt1_S126 -> Usp8_S164 - cost: 7.40, layer: 49
Fold over basal
Time (min)
0
10
20
30
40
50
60
0.4
0.6
0.8
1
1.2
1.4
1.6
1.8
2
2.2
Normal
LY50
LY75
LY88
LY94
LY97
LY data
Normal data

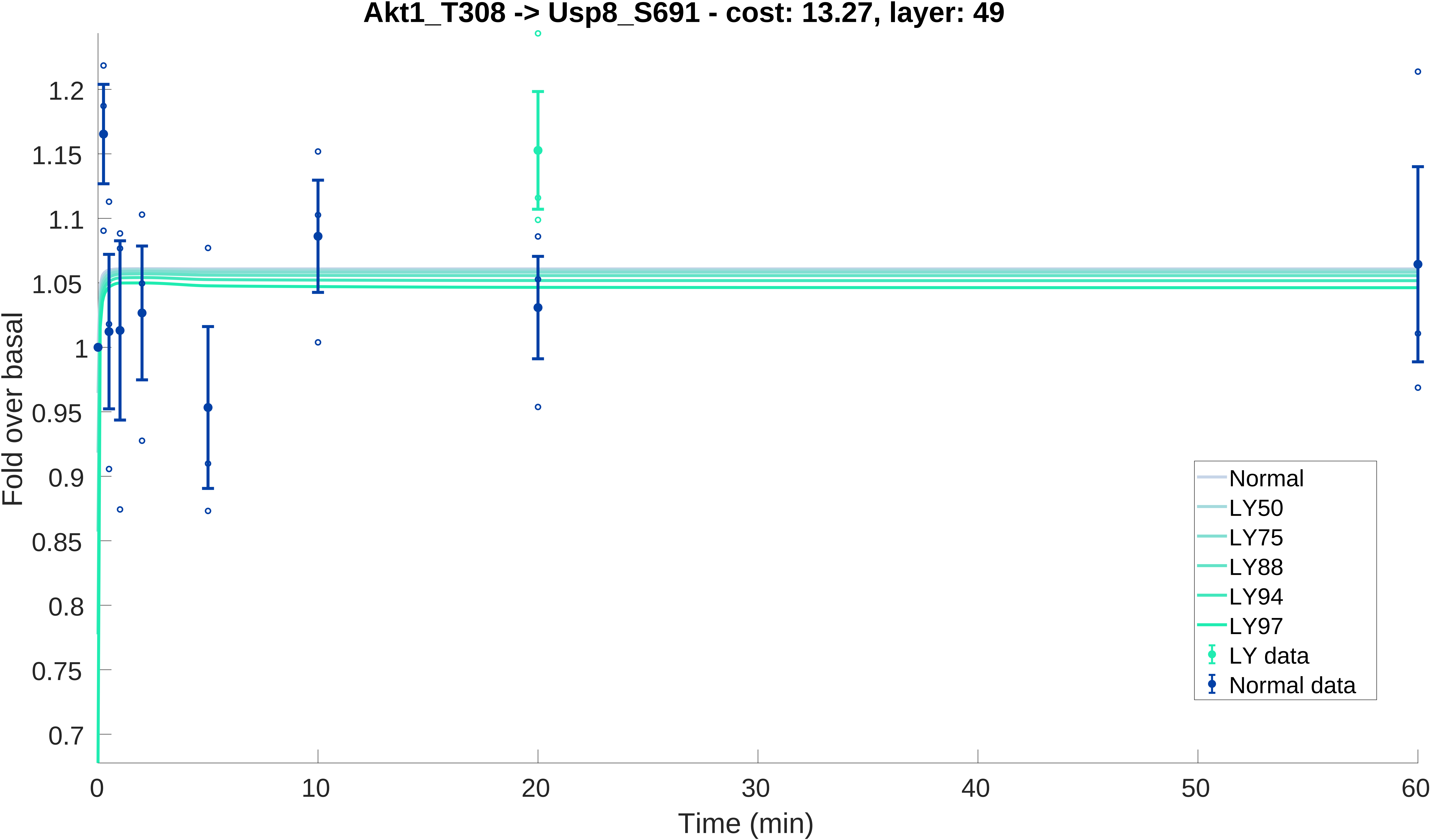

Akt1_T308 -> Usp8_S691 - cost: 13.27, layer: 49
Fold over basal
1.2
1.15
1.1
1.05
1
0.95
0.9
0.85
0.8
0.75
0.7
0
10
20
30
40
50
60
Time (min)
Normal
LY50
LY75
LY88
LY94
LY97
LY data
Normal data

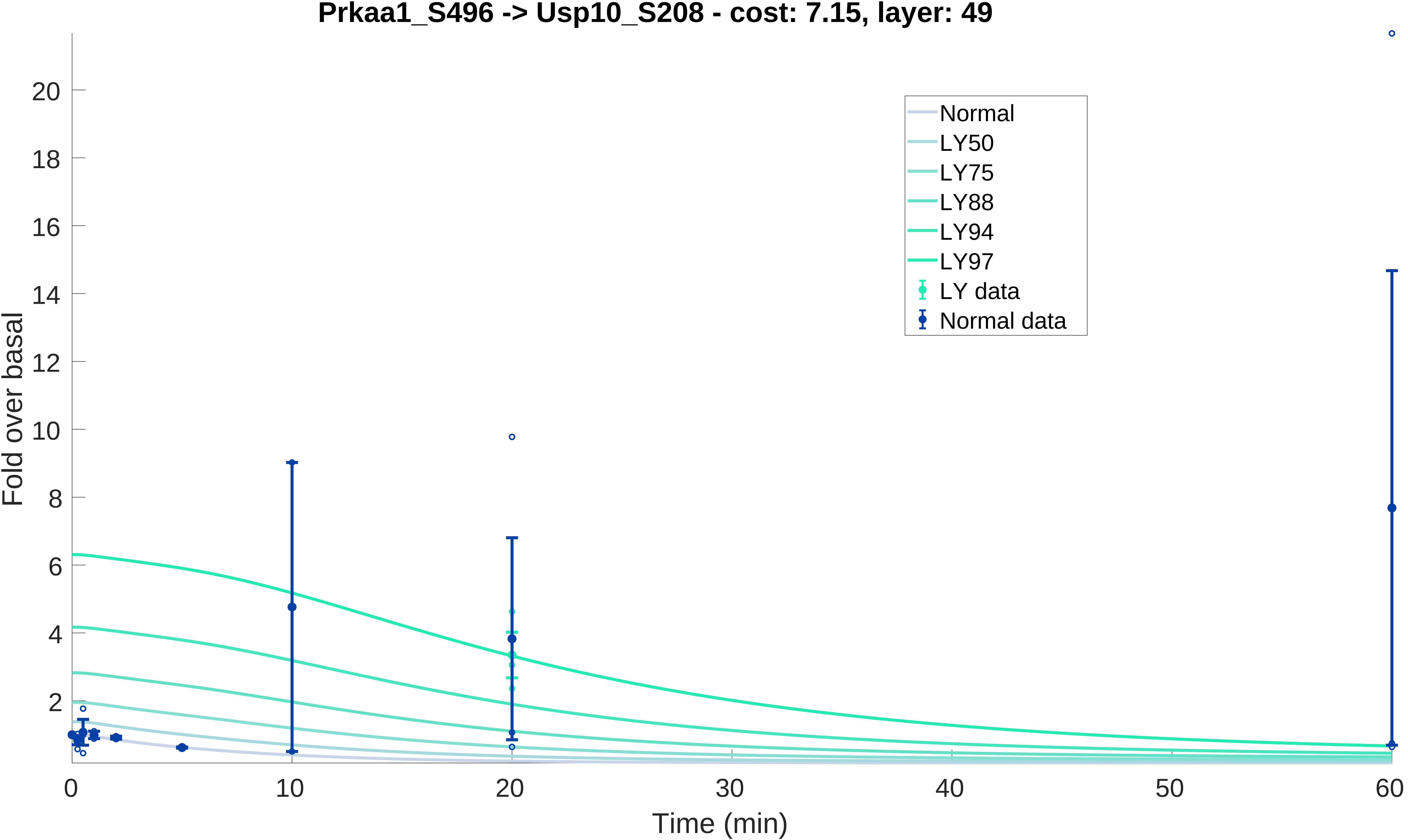

Prkaa1_S496 -> Usp10_S208 - cost: 7.15, layer: 49
Fold over basal
Time (min)
0
10
20
30
40
50
60
2
4
6
8
10
12
14
16
18
20
Normal
LY50
LY75
LY88
LY94
LY97
LY data
Normal data

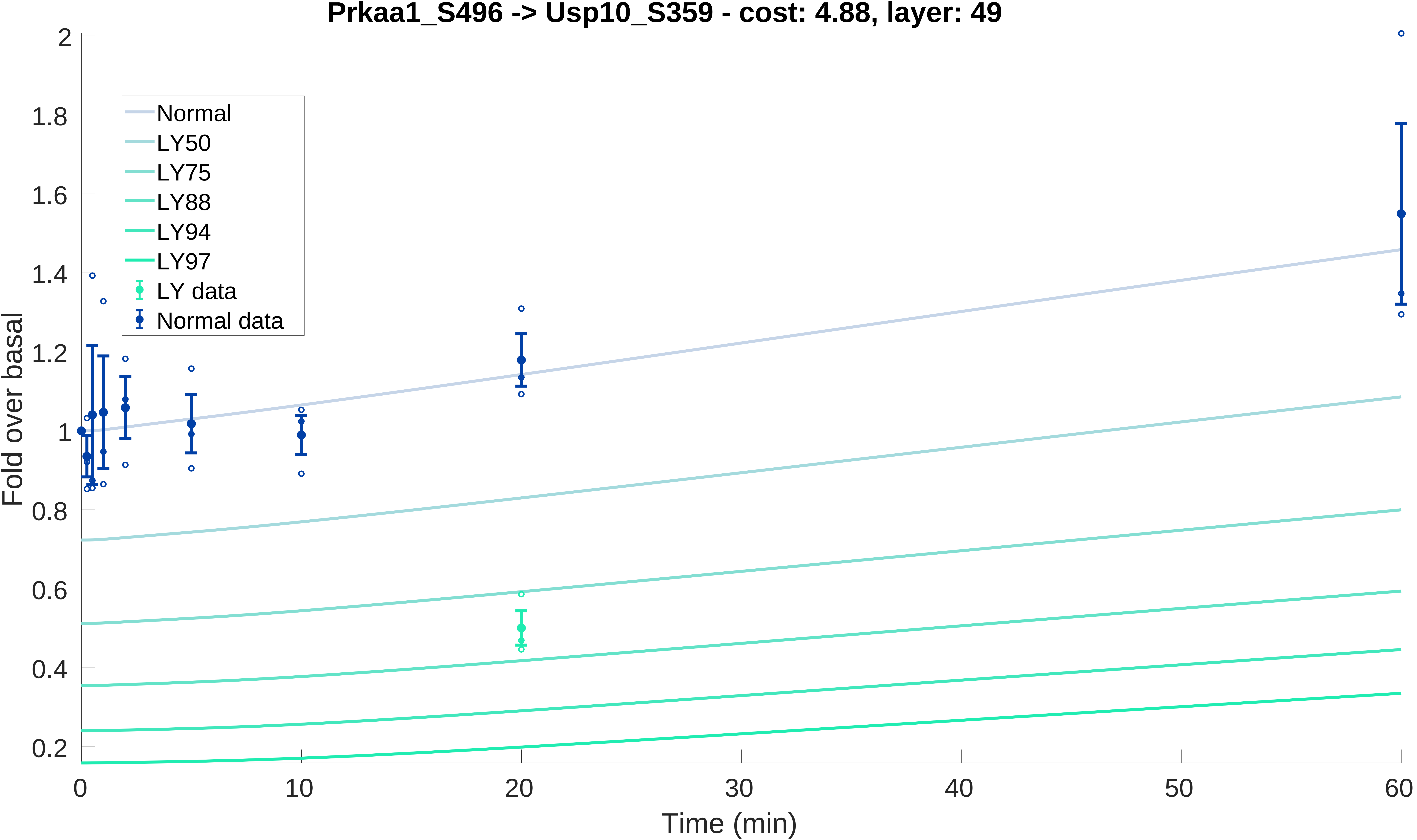

Prrkaa1_S496 -> Usp10_S359 - cost: 4.88, layer: 49
Fold over basal
Time (min)
0
10
20
30
40
50
60
0.2
0.4
0.6
0.8
1
1.2
1.4
1.6
1.8
2
Normal
LY50
LY75
LY88
LY94
LY97
LY data
Normal data

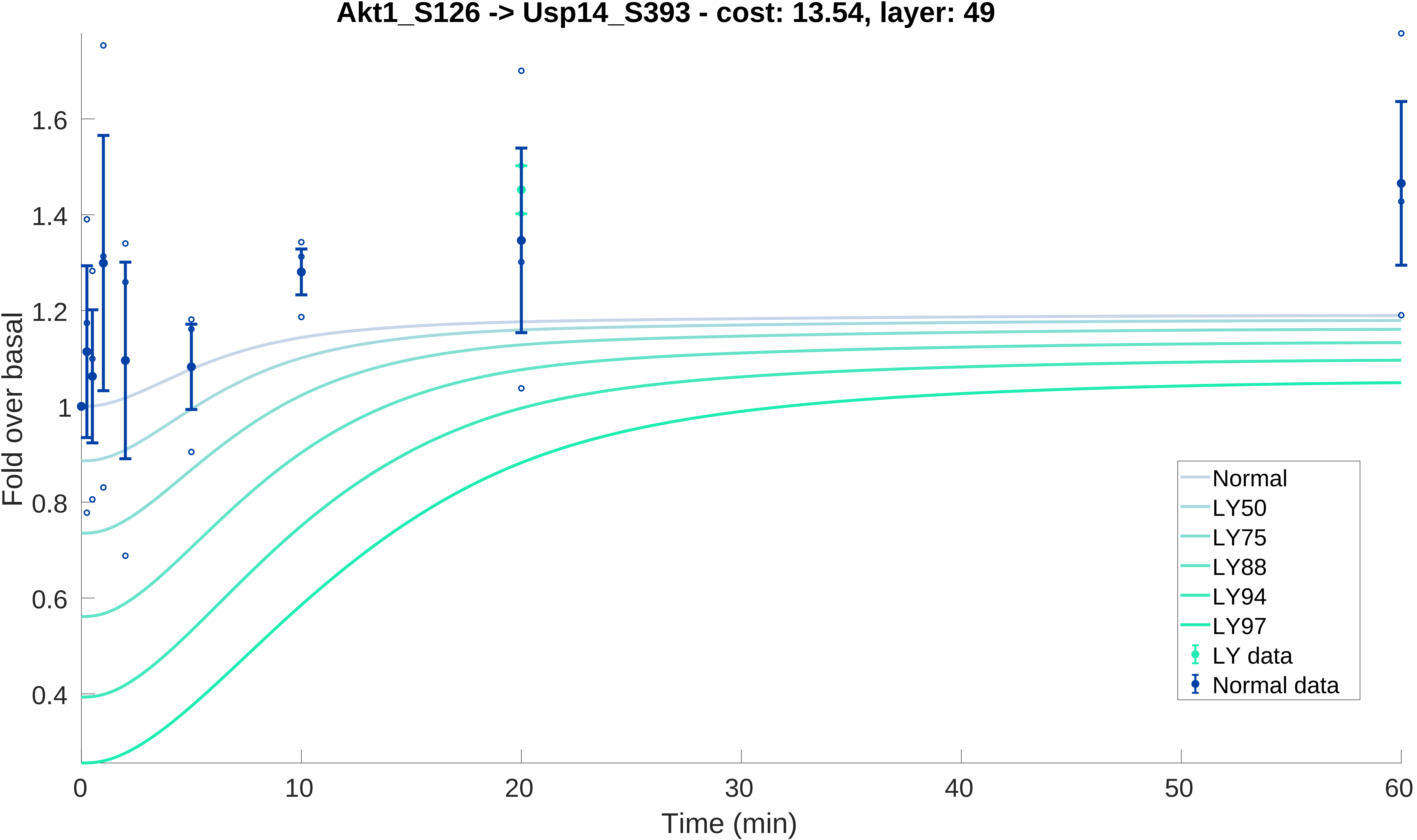

Akt1_S126 -> Usp14_S393 - cost: 13.54, layer: 49
Fold over basal
1.6
1.4
1.2
1
0.8
0.6
0.4
0
10
20
30
40
50
60
Time (min)
Normal
LY50
LY75
LY88
LY94
LY97
LY data
Normal data

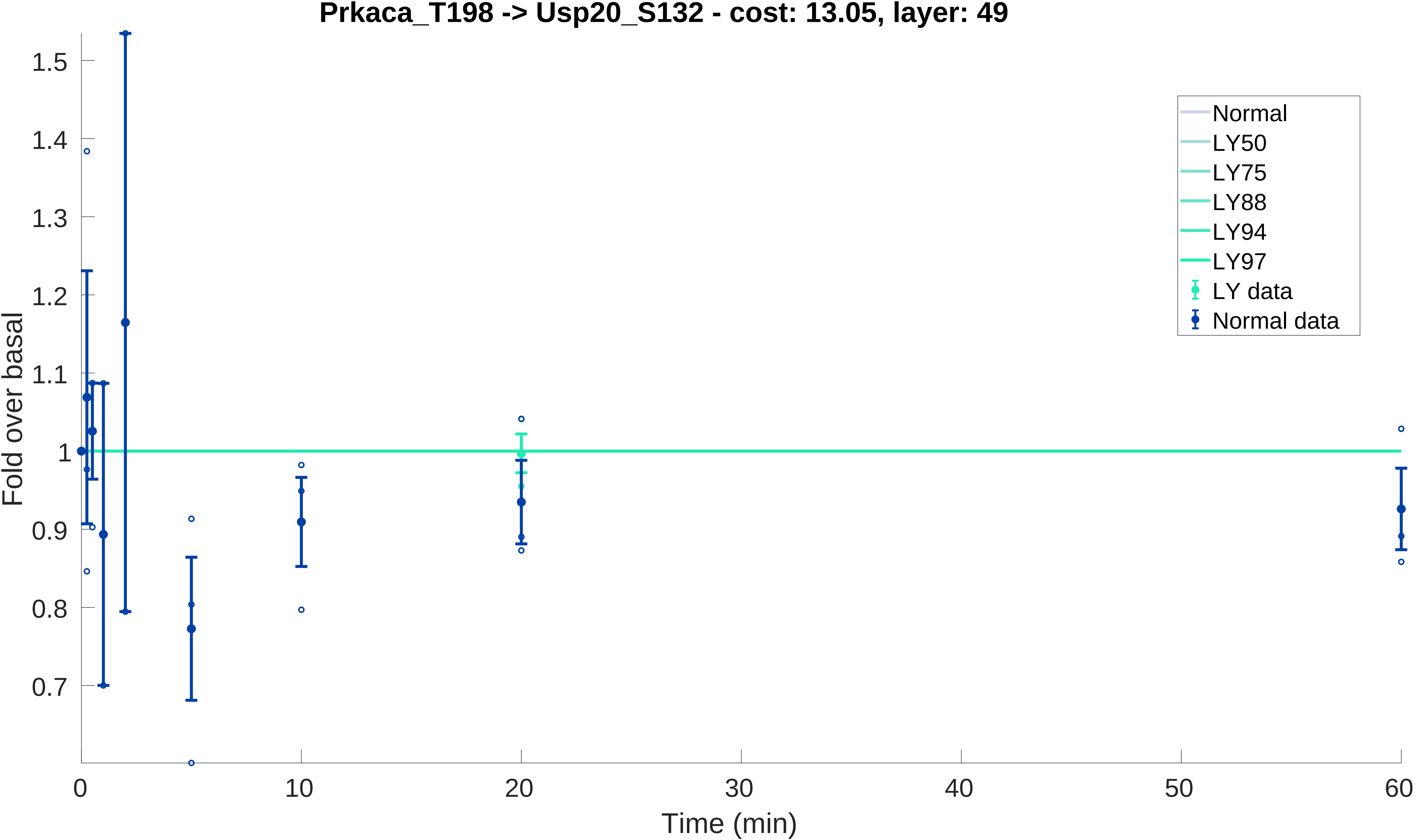

Prkaca_T198 -> Usp20_S132 - cost: 13.05, layer: 49
Fold over basal
Time (min)
1.5
1.4
1.3
1.2
1.1
1
0.9
0.8
0.7
0
10
20
30
40
50
60
Normal
LY50
LY75
LY88
LY94
LY97
LY data
Normal data

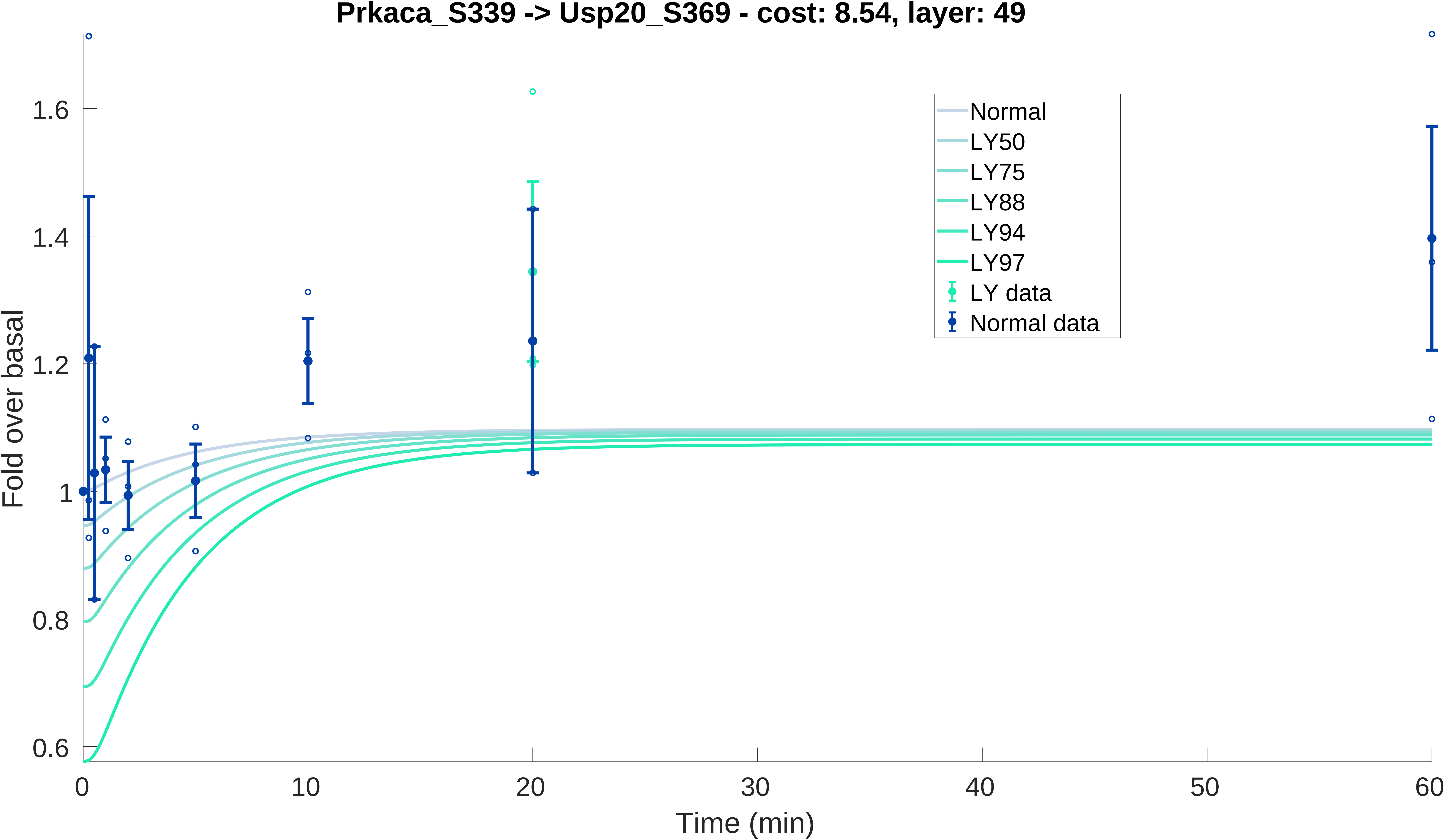

Prkaca_S339 -> Usp20_S369 - cost: 8.54, layer: 49
Normal
LY50
LY75
LY88
LY94
LY97
LY data
Normal data
Fold over basal
Time (min)
0.6
0.8
1
1.2
1.4
1.6
0
10
20
30
40
50
60

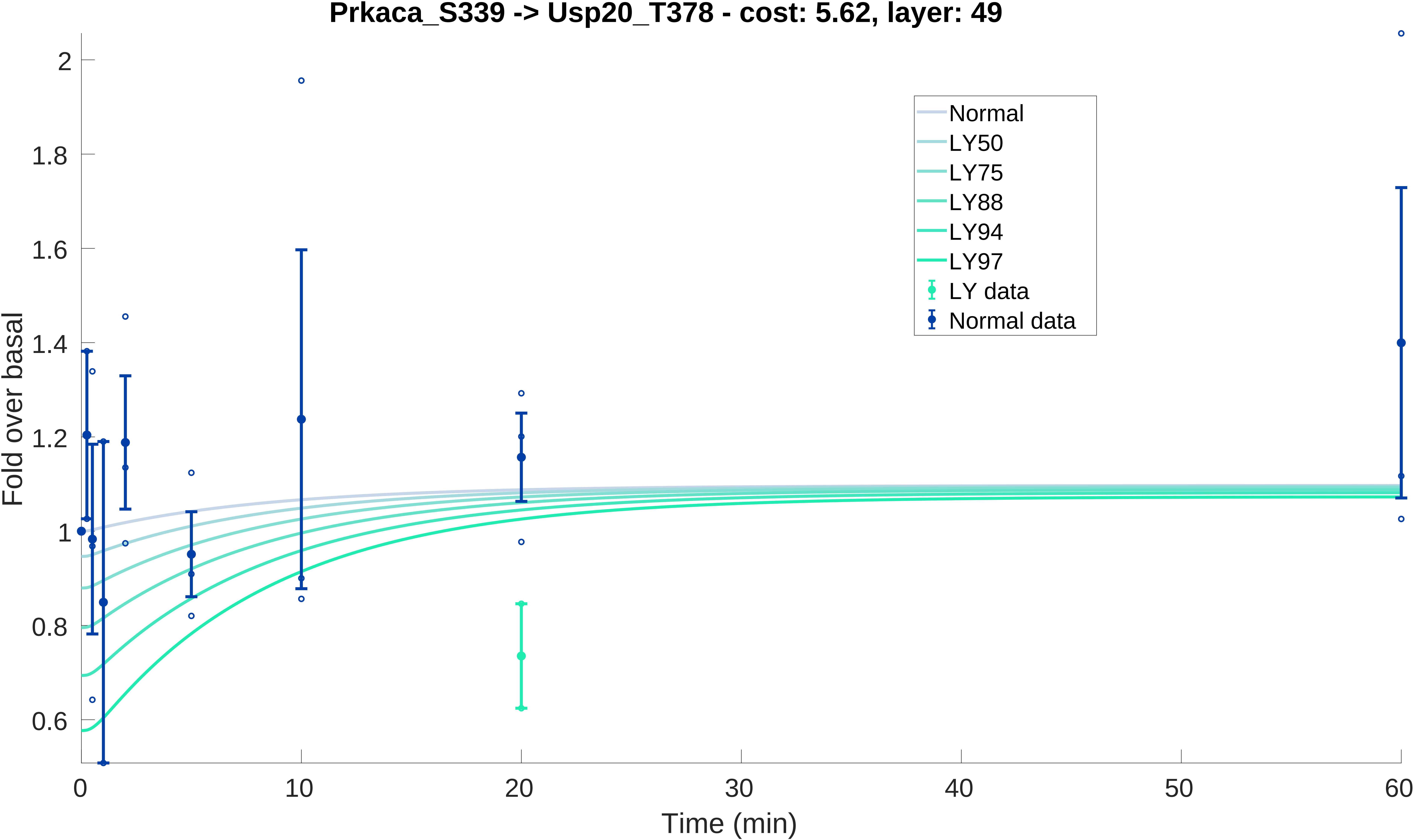

Prkaca_S339 -> Usp20_T378 - cost: 5.62, layer: 49
Fold over basal
Time (min)
Normal
LY50
LY75
LY88
LY94
LY97
LY data
Normal data
0.6
0.8
1
1.2
1.4
1.6
1.8
2
0
10
20
30
40
50
60

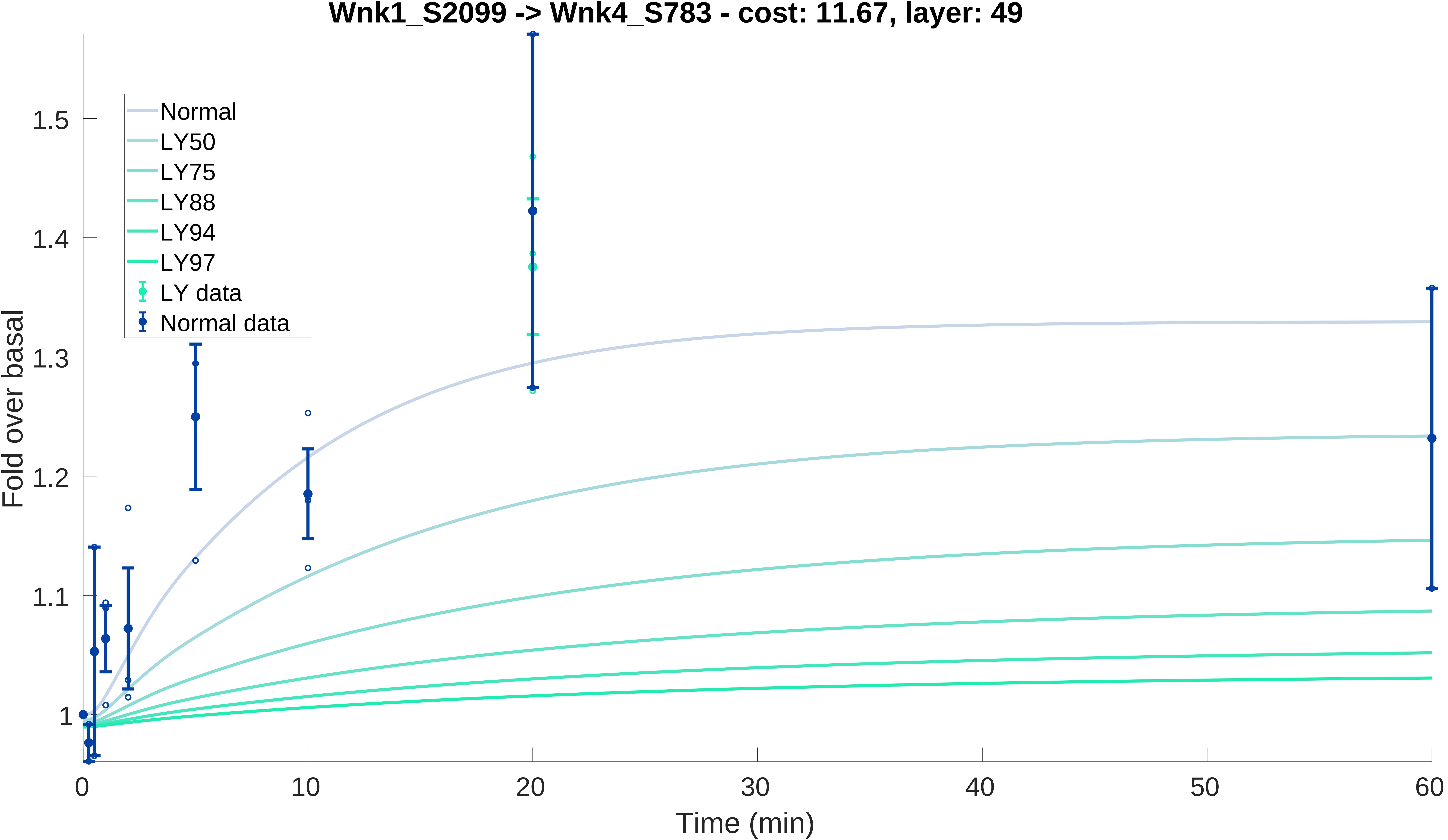

Wnk1_S2099 -> Wnk4_S783 - cost: 11.67, layer: 49
Normal
LY50
LY75
LY88
LY94
LY97
LY data
Normal data
Fold over basal
1.5
1.4
1.3
1.2
1.1
1
0
10
20
30
40
50
60
Time (min)

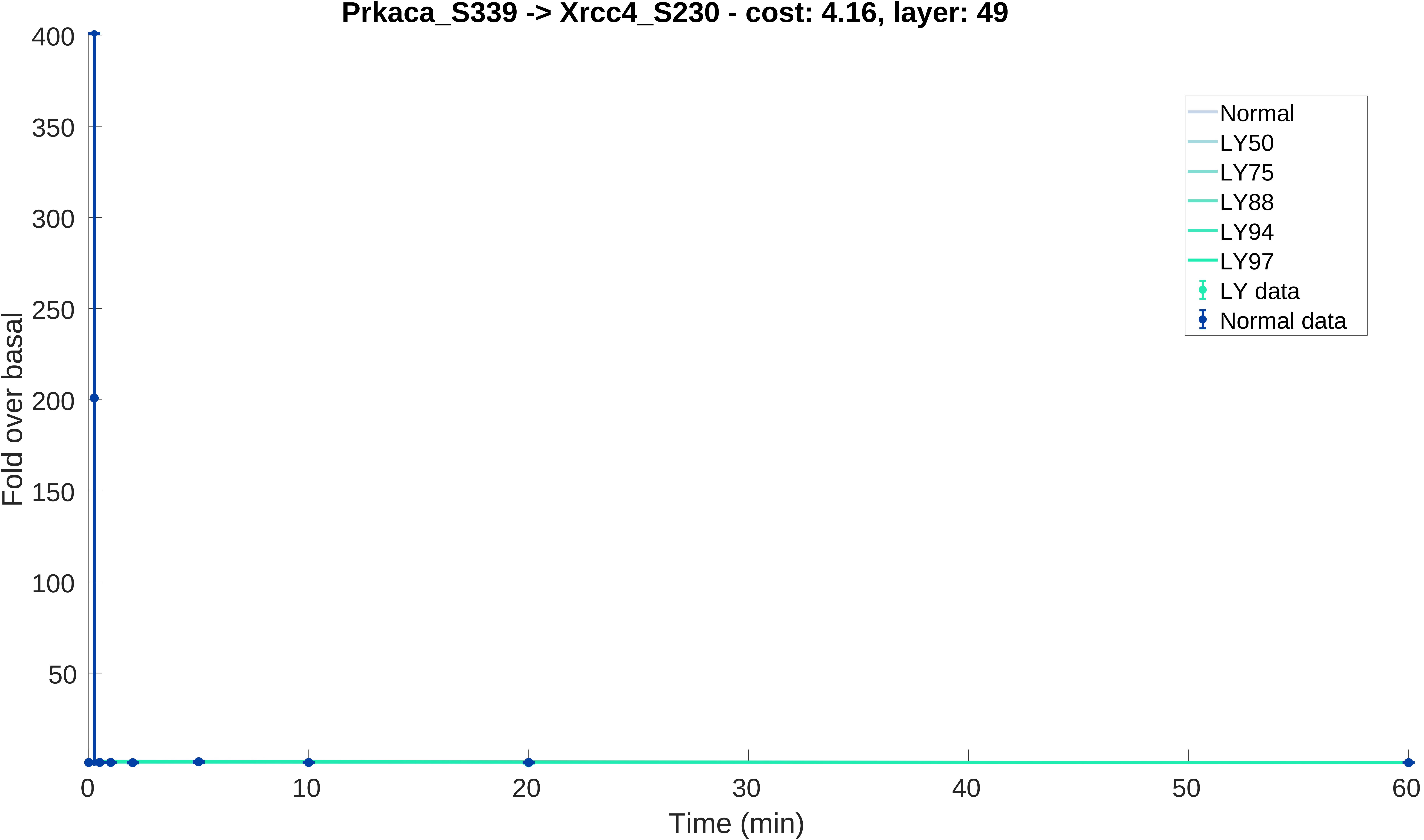

Prkaca_S339 -> Xrcc4_S230 - cost: 4.16, layer: 49
Fold over basal
400
350
300
250
200
150
100
50
0
10
20
30
40
50
60
Time (min)
Normal
LY50
LY75
LY88
LY94
LY97
LY data
Normal data

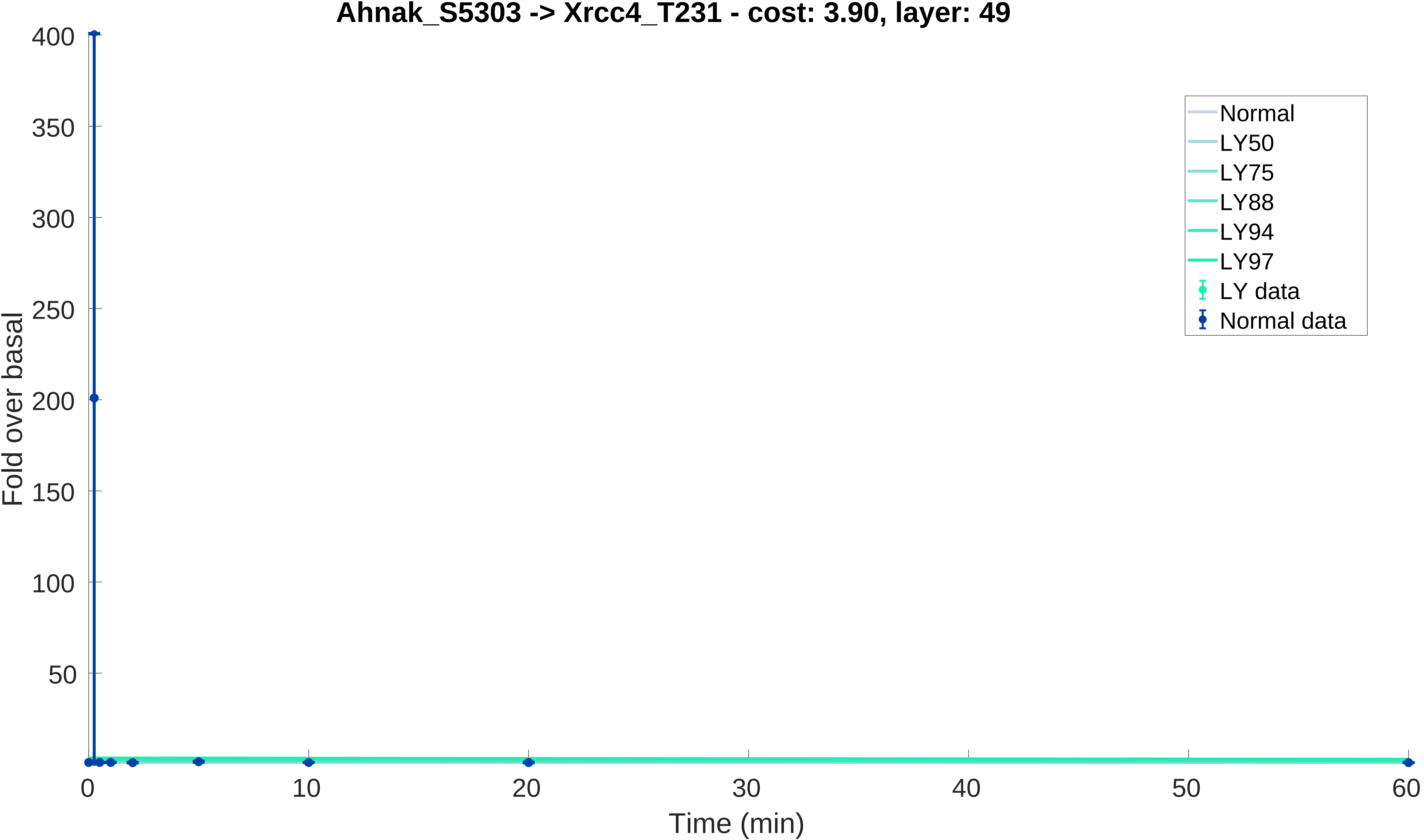

Ahnak_S5303 -> Xrcc4_T231 - cost: 3.90, layer: 49
Fold over basal
400
350
300
250
200
150
100
50
0
10
20
30
40
50
60
Time (min)
Normal
LY50
LY75
LY88
LY94
LY97
LY data
Normal data

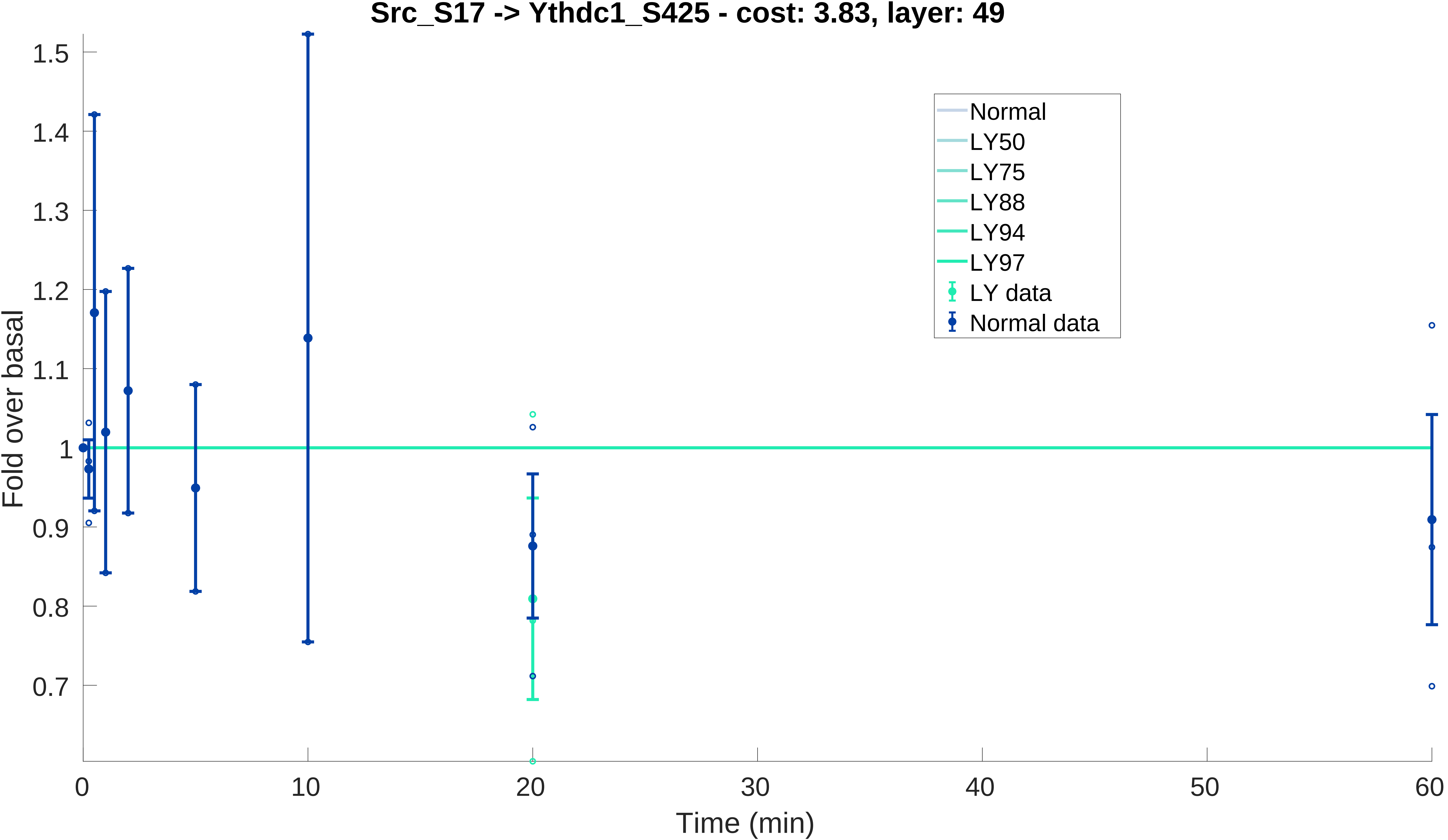

Src_S17 -> Ythdc1_S425 - cost: 3.83, layer: 49
Fold over basal
1.5
1.4
1.3
1.2
1.1
1
0.9
0.8
0.7
Time (min)
0
10
20
30
40
50
60
Normal
LY50
LY75
LY88
LY94
LY97
LY data
Normal data

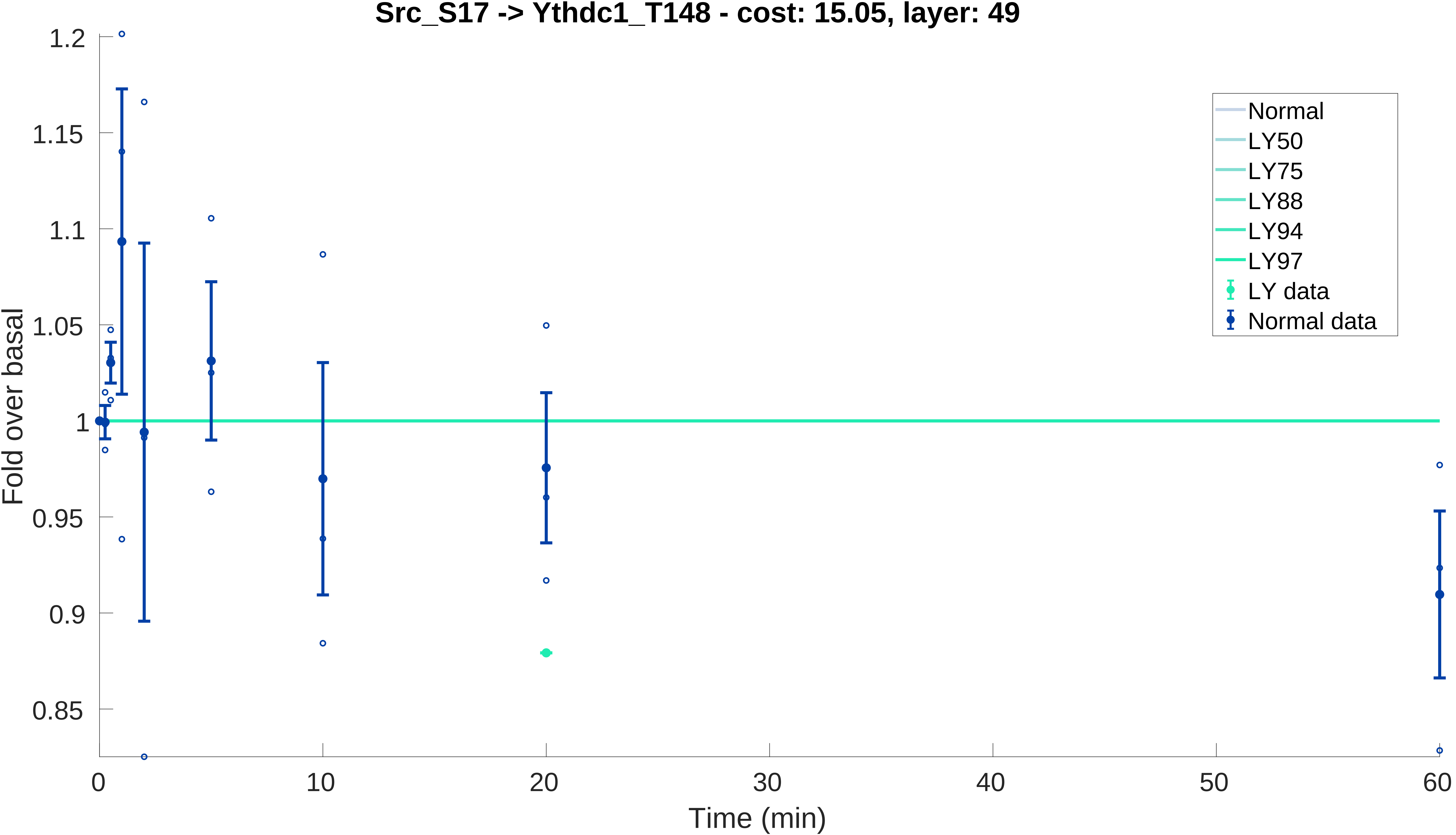
Src_S17 -> Ythdc1_T148 - cost: 15.05, layer: 49
Fold over basal
1.2
1.15
1.1
1.05
1
0.95
0.9
0.85
0
10
20
30
40
50
60
Time (min)
Normal
LY50
LY75
LY88
LY94
LY97
LY data
Normal data

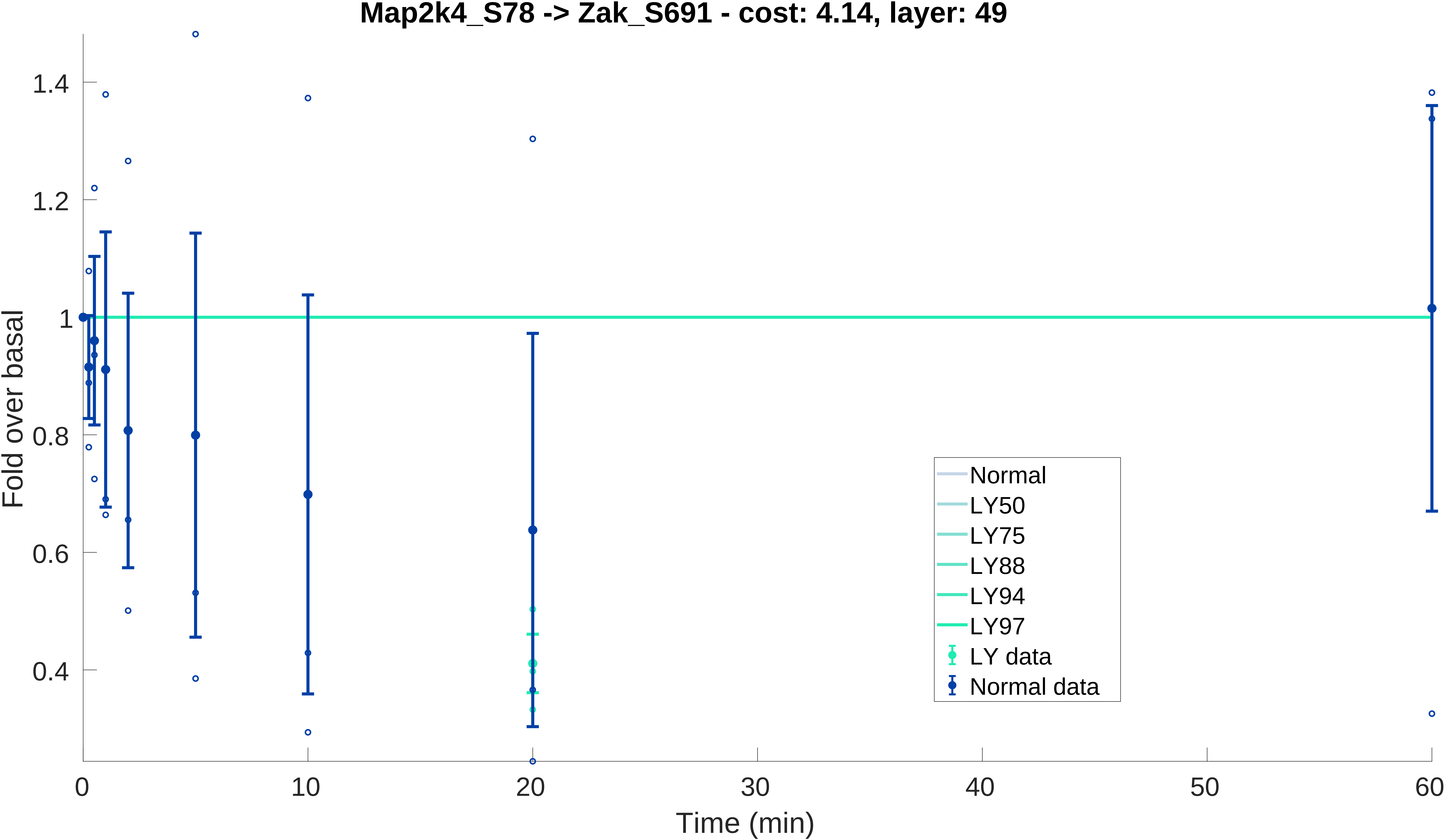

Map2k4_S78 -> Zak_S691 - cost: 4.14, layer: 49
Fold over basal
1.4
1.2
1
0.8
0.6
0.4
0
10
20
30
40
50
60
Time (min)
Normal
LY50
LY75
LY88
LY94
LY97
LY data
Normal data

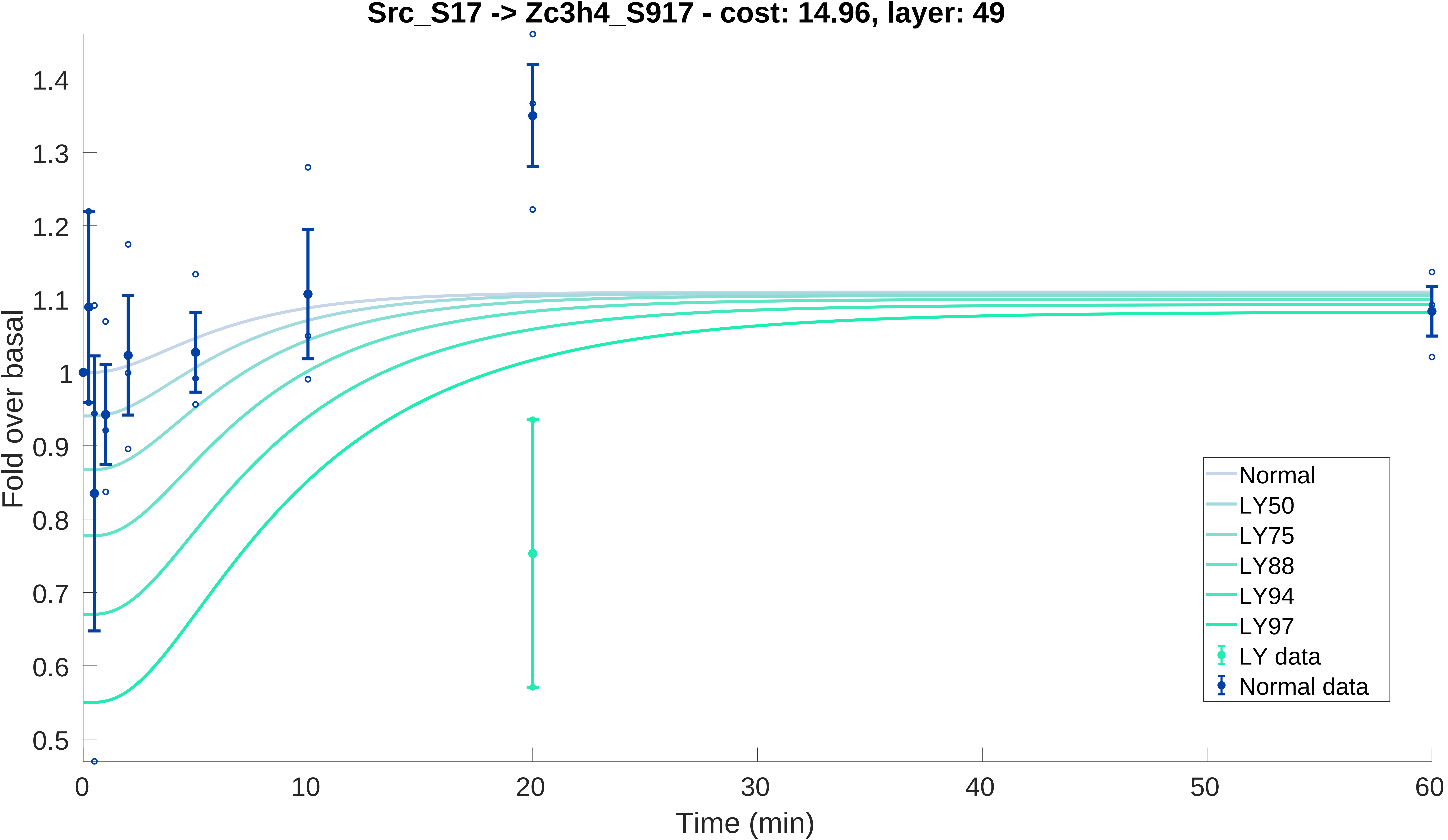
Src_S17 -> Zc3h4_S917 - cost: 14.96, layer: 49
Fold over basal
1.4
1.3
1.2
1.1
1
0.9
0.8
0.7
0.6
0.5
0
10
20
30
40
50
60
Time (min)
Normal
LY50
LY75
LY88
LY94
LY97
LY data
Normal data

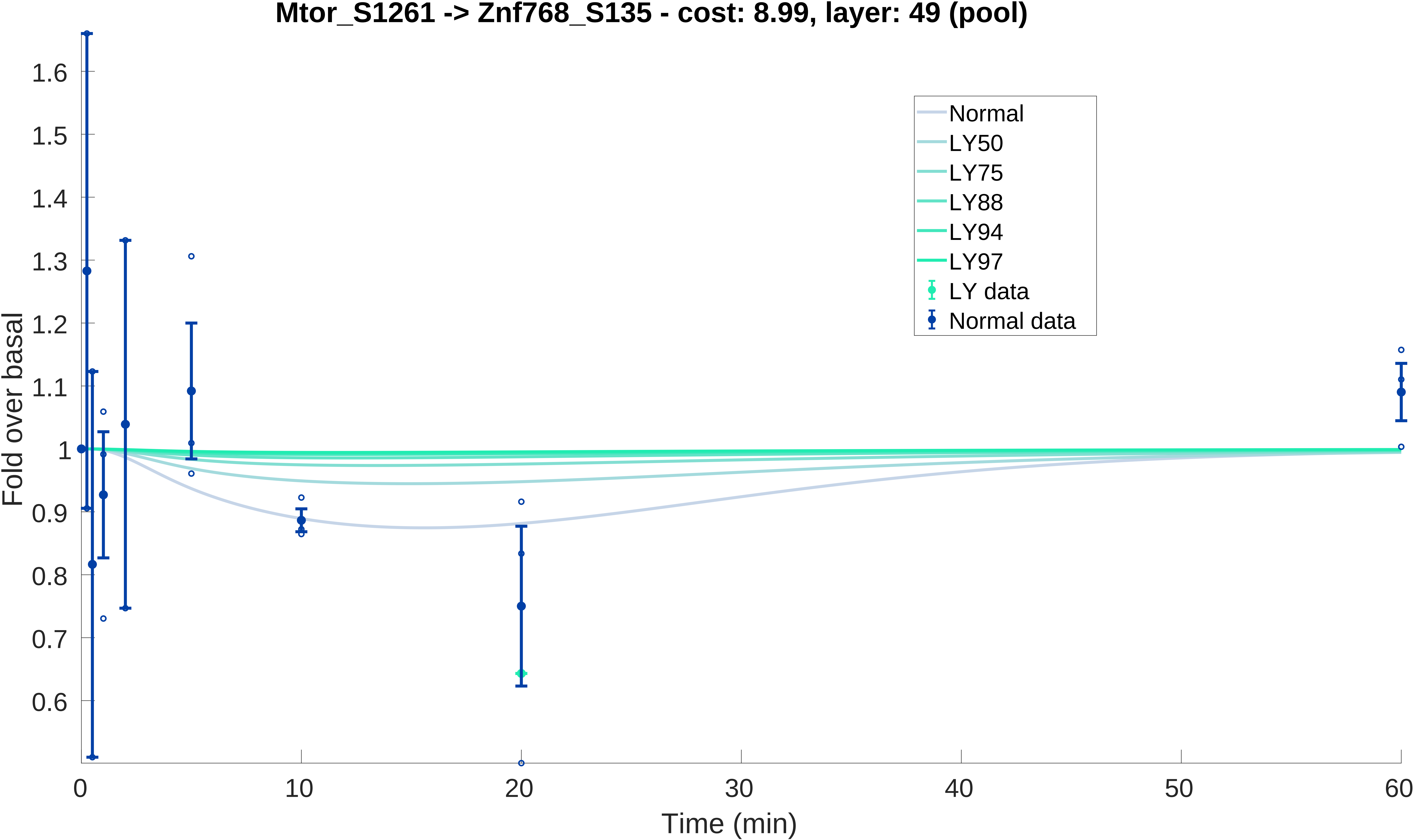

Mtor_S1261 -> Znf768_S135 - cost: 8.99, layer: 49 (pool)
Fold over basal
Time (min)
Normal
LY50
LY75
LY88
LY94
LY97
LY data
Normal data
0.6
0.7
0.8
0.9
1
1.1
1.2
1.3
1.4
1.5
1.6
0
10
20
30
40
50
60

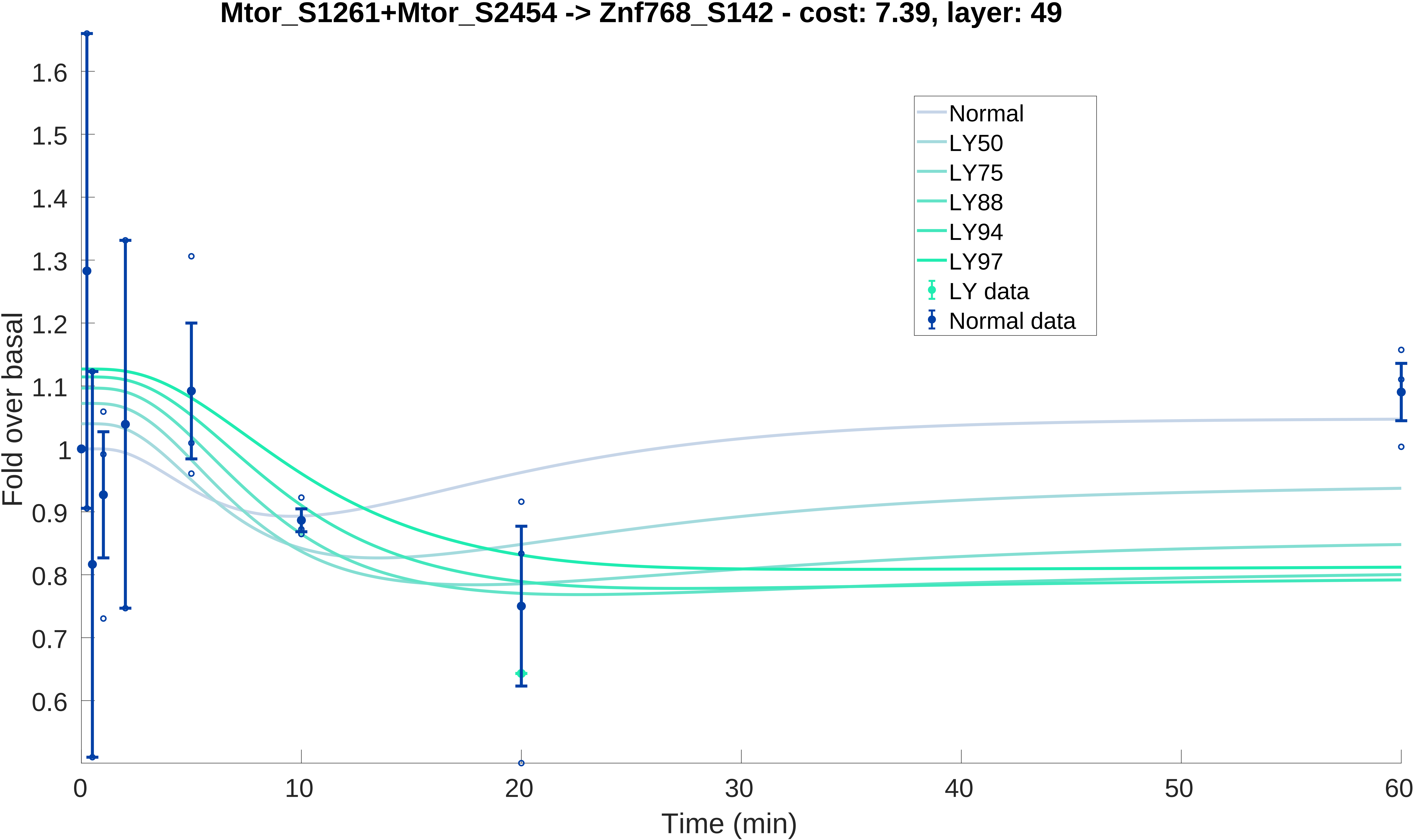
Mtor_S1261+Mtor_S2454 -> Znf768_S142 - cost: 7.39, layer: 49
Fold over basal
1.6
1.5
1.4
1.3
1.2
1.1
1
0.9
0.8
0.7
0.6
0
10
20
30
40
50
60
Time (min)
Normal
LY50
LY75
LY88
LY94
LY97
LY data
Normal data

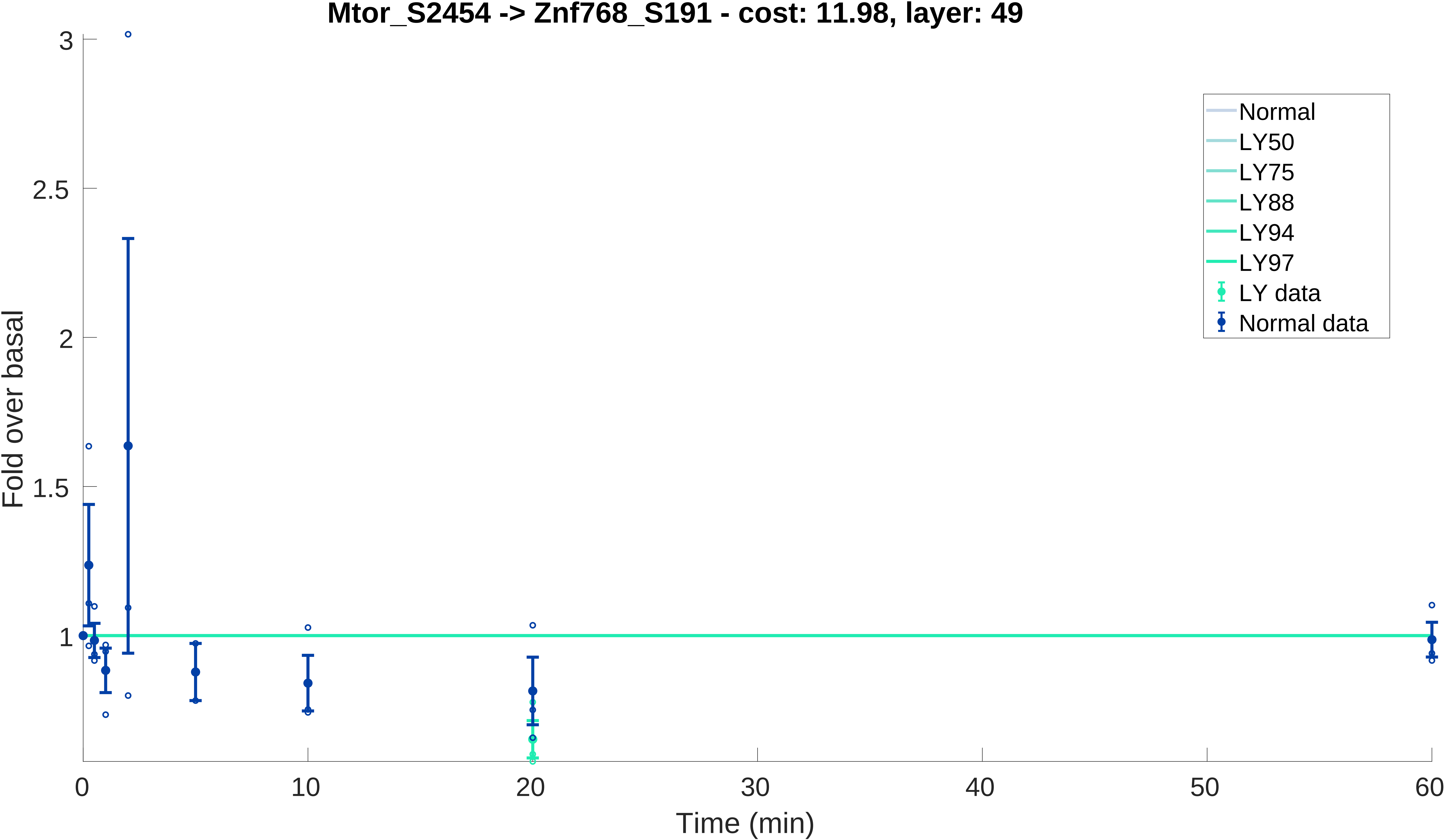

Mtor_S2454 -> Znf768_S191 - cost: 11.98, layer: 49
Fold over basal
Time (min)
3
2.5
2
1.5
1
0
10
20
30
40
50
60
Normal
LY50
LY75
LY88
LY94
LY97
LY data
Normal data

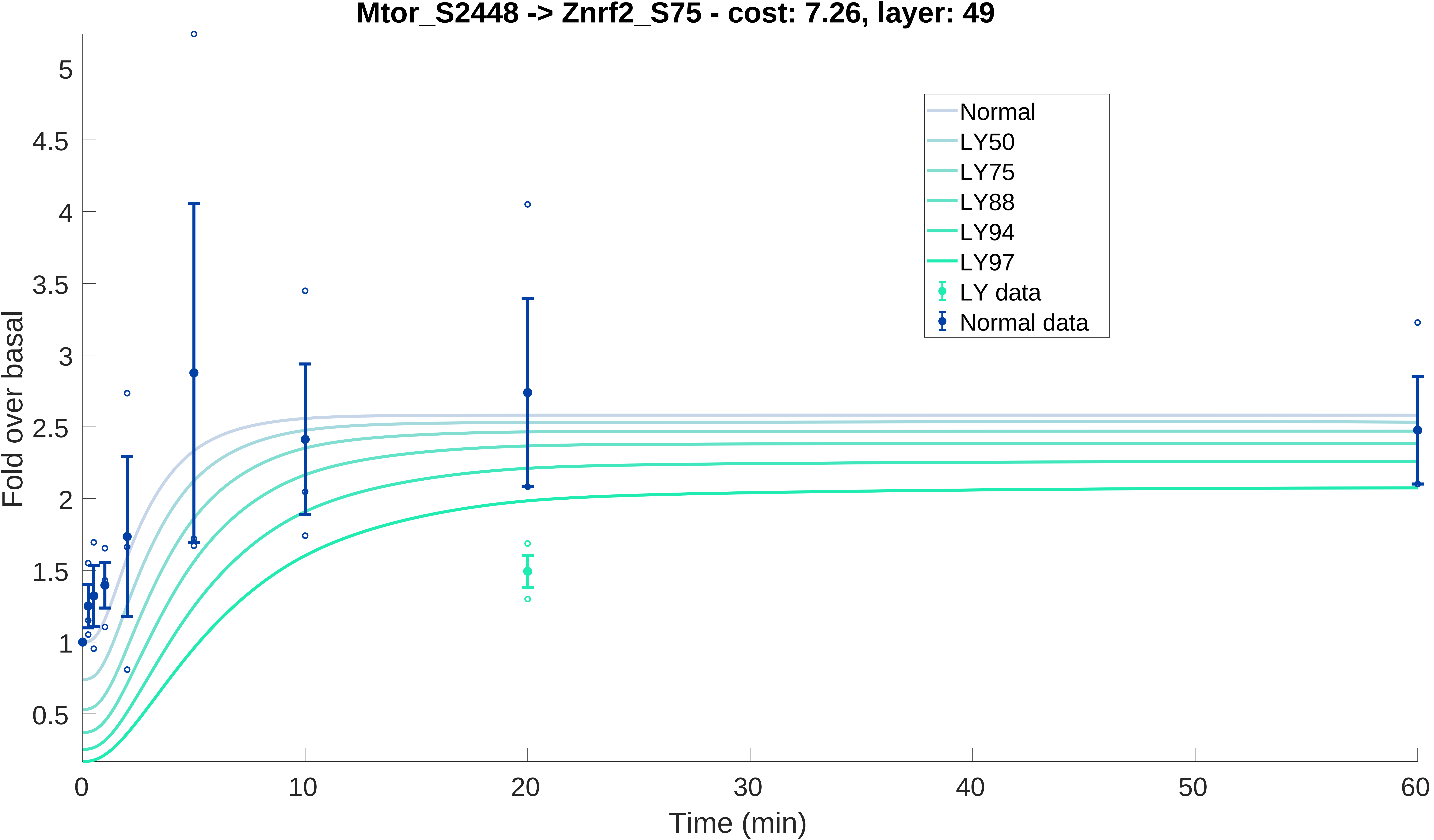

Mtor_S2448 -> Znrf2_S75 - cost: 7.26, layer: 49
Fold over basal
5
4.5
4
3.5
3
2.5
2
1.5
1
0.5
0
10
20
30
40
50
60
Time (min)
Normal
LY50
LY75
LY88
LY94
LY97
LY data
Normal data

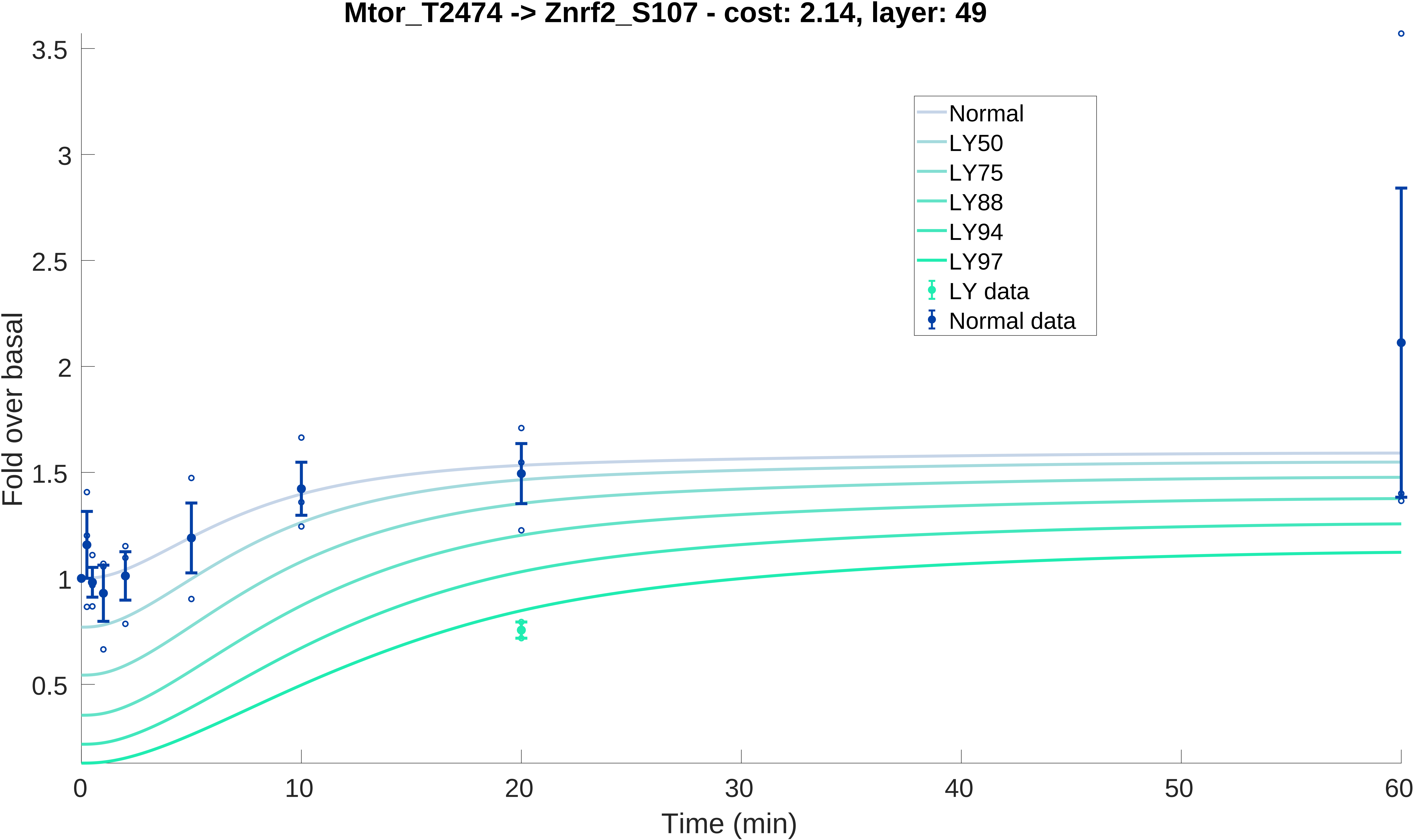

Mtor_T2474 -> Znrf2_S107 - cost: 2.14, layer: 49
Fold over basal
Time (min)
3.5
3
2.5
2
1.5
1
0.5
0
10
20
30
40
50
60
Normal
LY50
LY75
LY88
LY94
LY97
LY data
Normal data

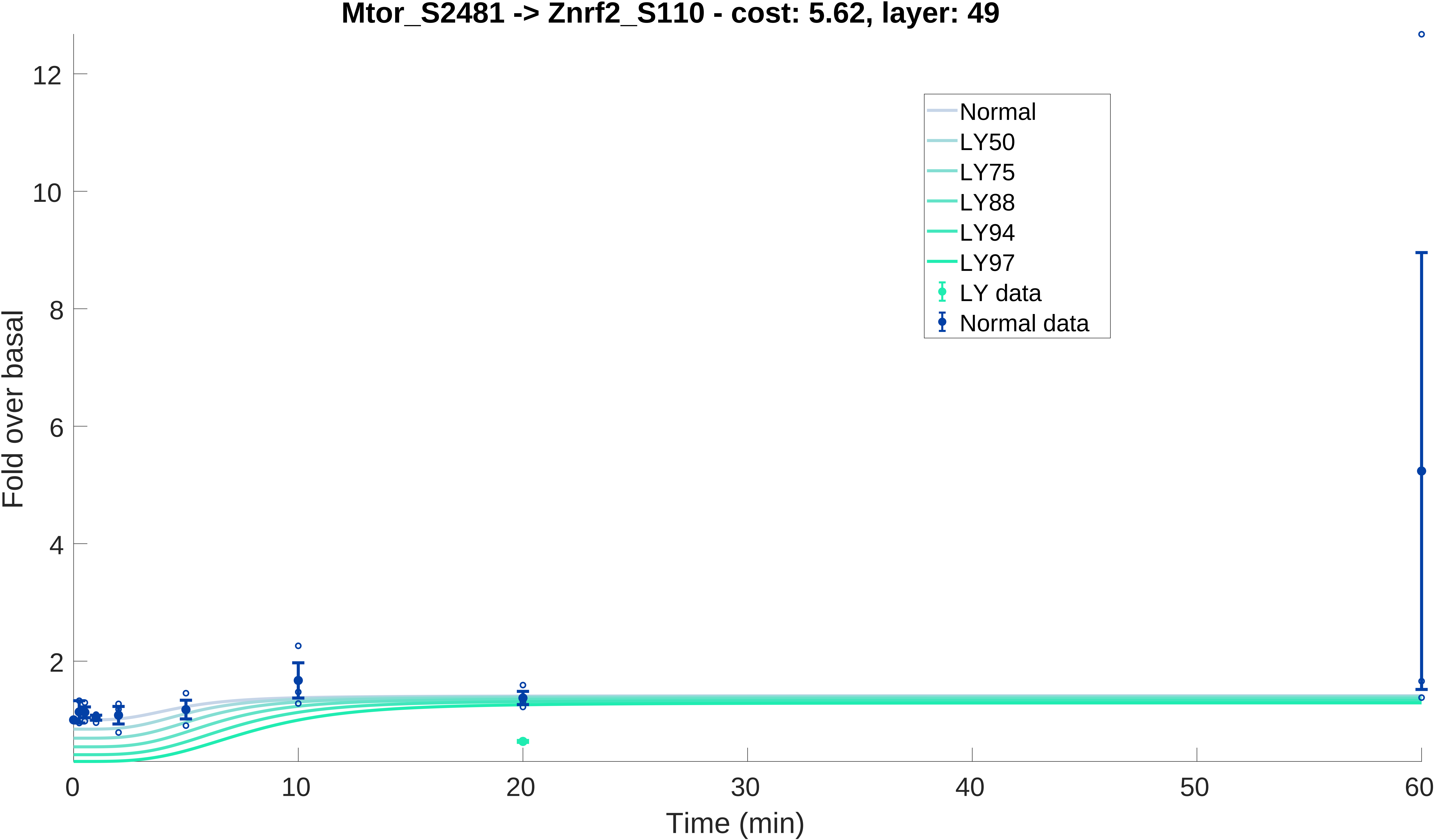

Mtor_S2481 -> Znrf2_S110 - cost: 5.62, layer: 49
Fold over basal
Time (min)
Normal
LY50
LY75
LY88
LY94
LY97
LY data
Normal data
0
10
20
30
40
50
60
2
4
6
8
10
12

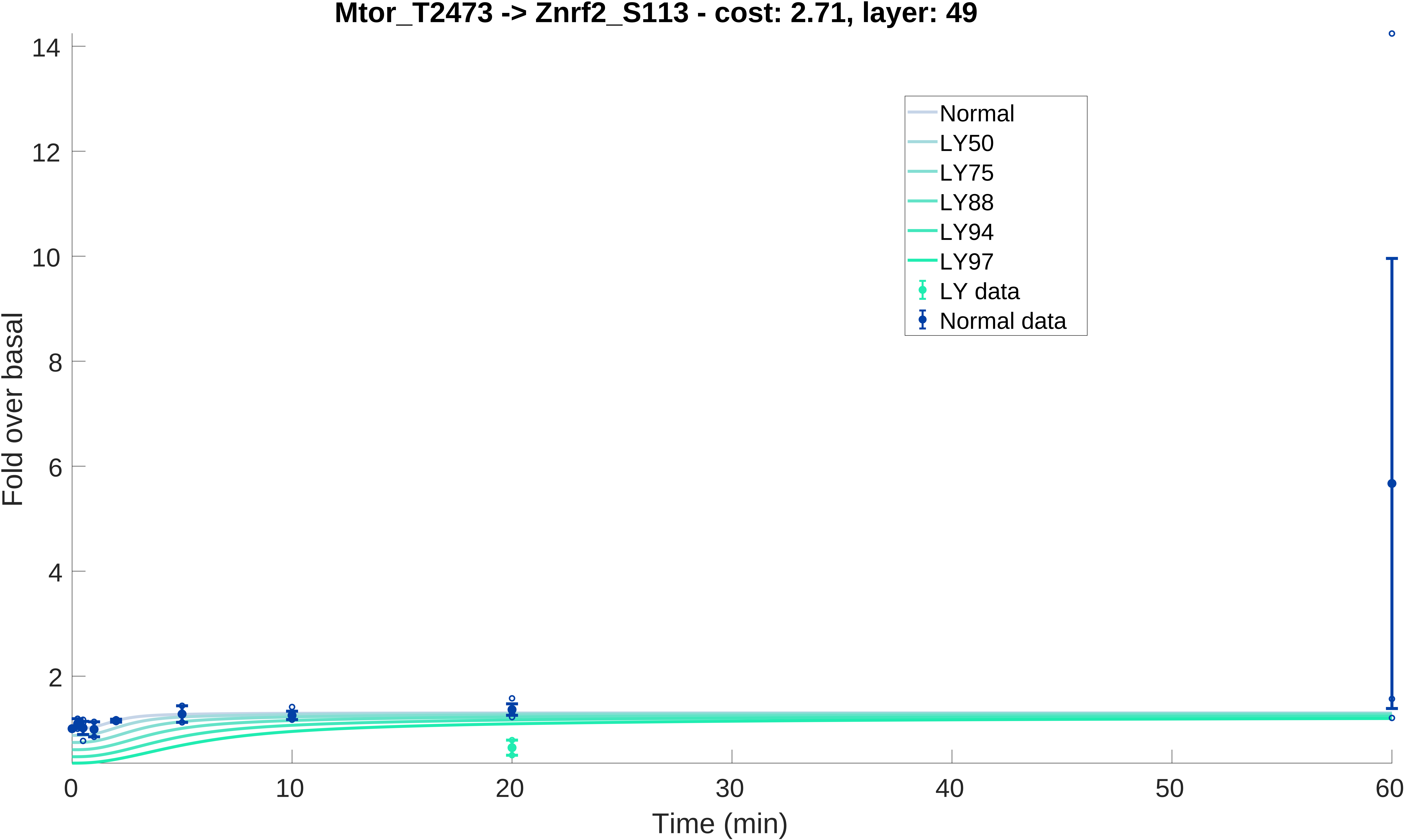

Mtor_T2473 -> Znrf2_S113 - cost: 2.71, layer: 49
Fold over basal
Time (min)
0
10
20
30
40
50
60
2
4
6
8
10
12
14
Normal
LY50
LY75
LY88
LY94
LY97
LY data
Normal data

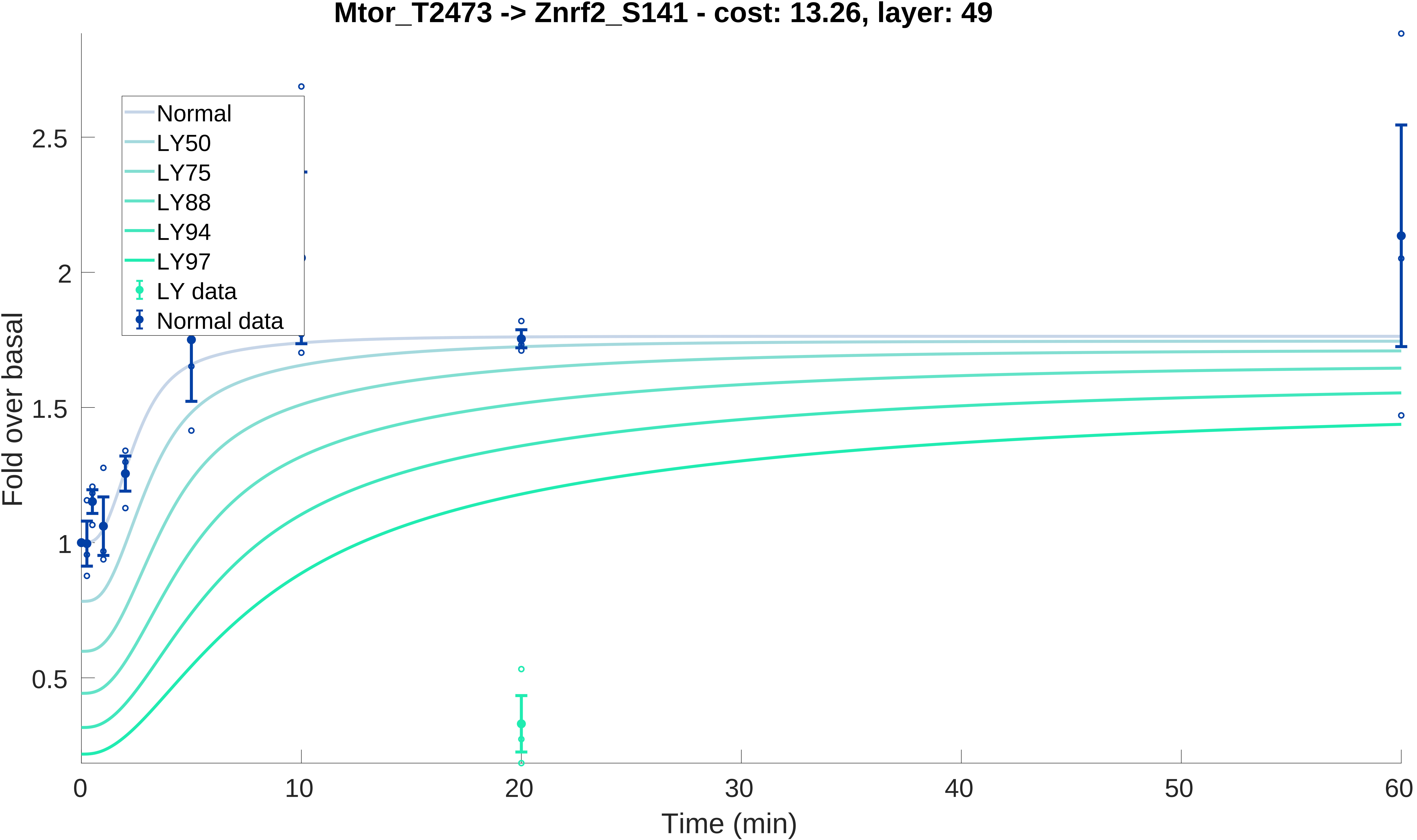
Mtor_T2473 -> Znrf2_S141 - cost: 13.26, layer: 49
Normal
LY50
LY75
LY88
LY94
LY97
LY data
Normal data
Fold over basal
Time (min)
0.5
1
1.5
2
2.5
0
10
20
30
40
50
60

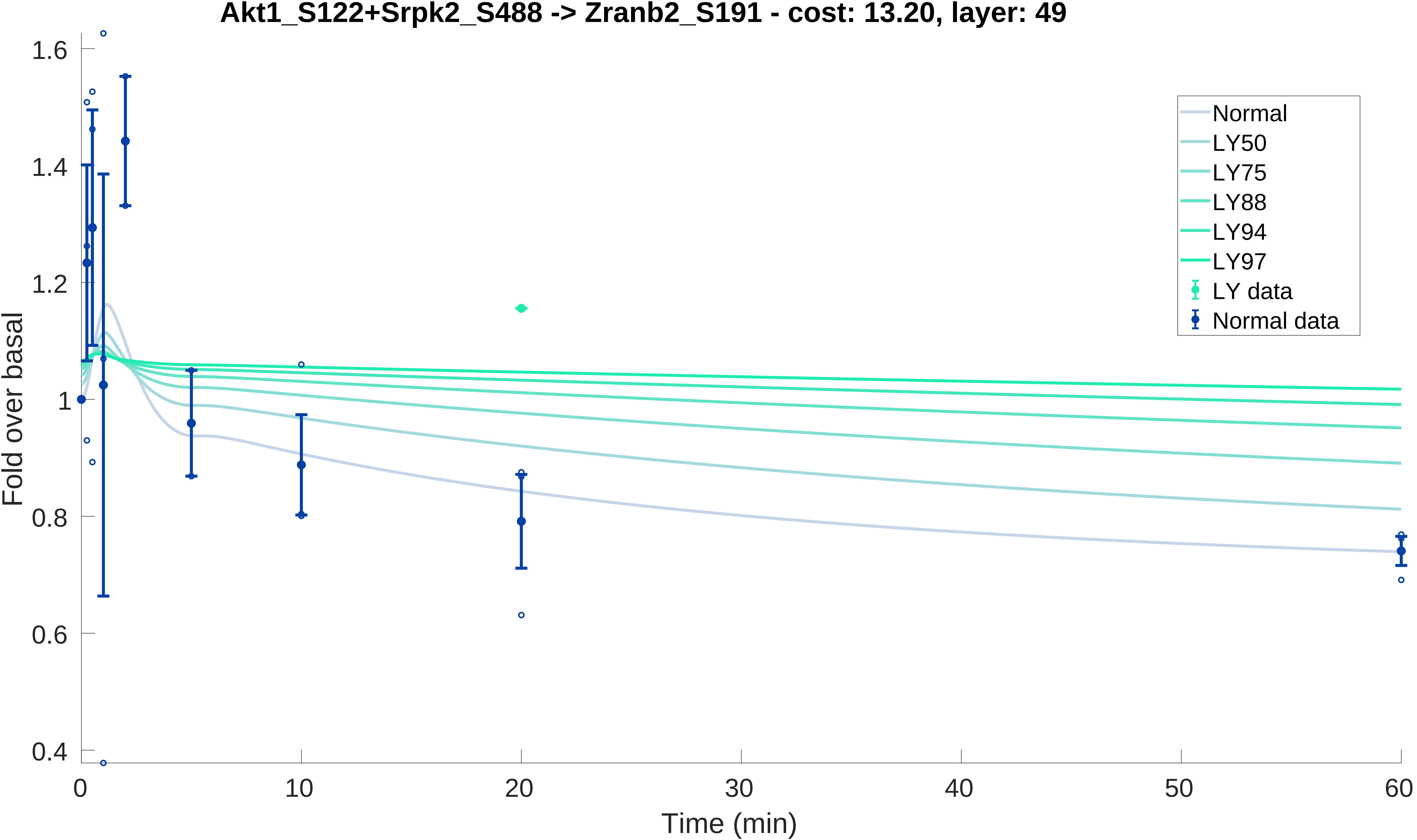
Akt1_S122+Srpk2_S488 -> Zranb2_S191 - cost: 13.20, layer: 49
Fold over basal
1.6
1.4
1.2
1
0.8
0.6
0.4
0
10
20
30
40
50
60
Time (min)
Normal
LY50
LY75
LY88
LY94
LY97
LY data
Normal data

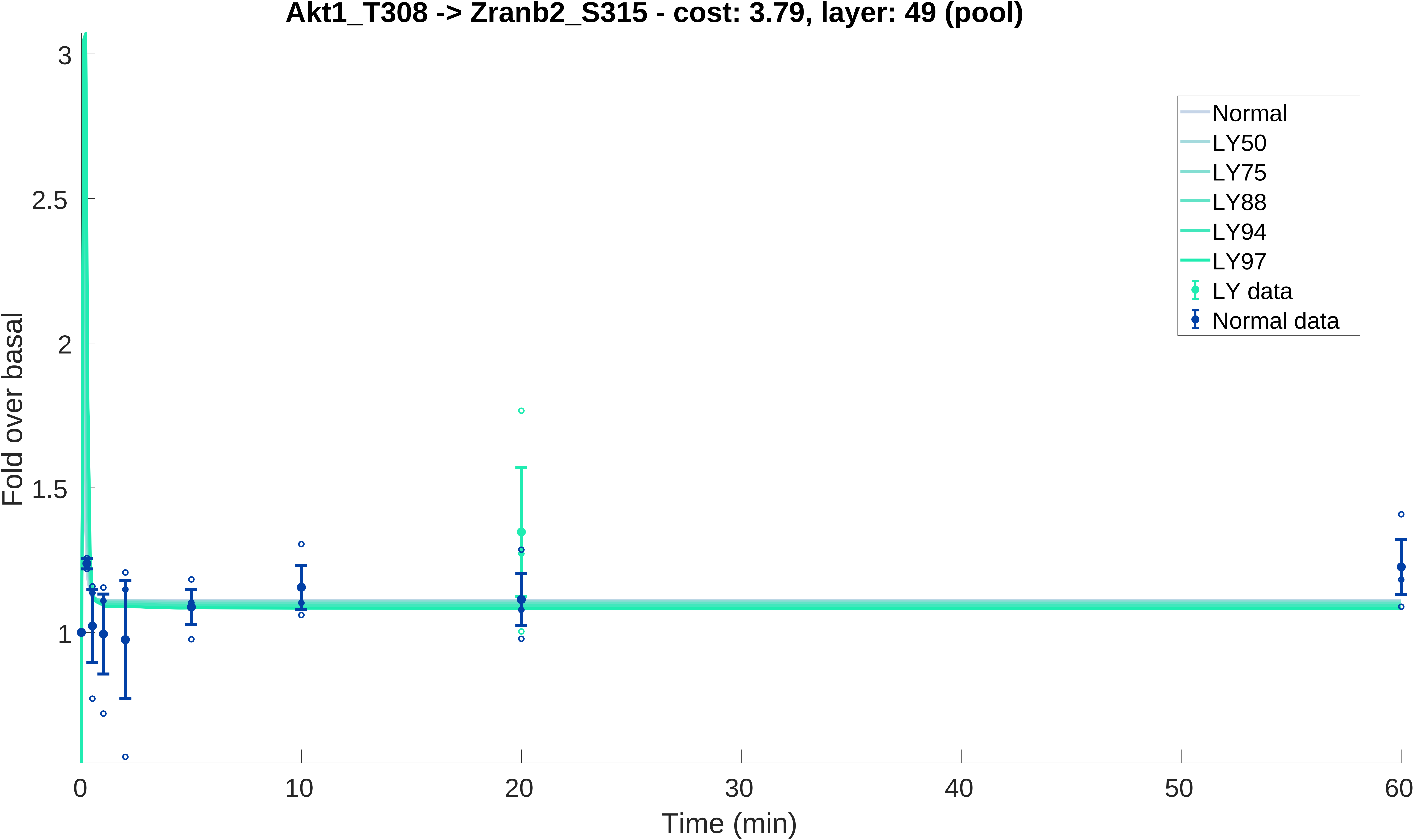
Akt1_T308 -> Zranb2_S315 - cost: 3.79, layer: 49 (pool)
Fold over basal
3
2.5
2
1.5
1
Time (min)
0
10
20
30
40
50
60
Normal
LY50
LY75
LY88
LY94
LY97
LY data
Normal data

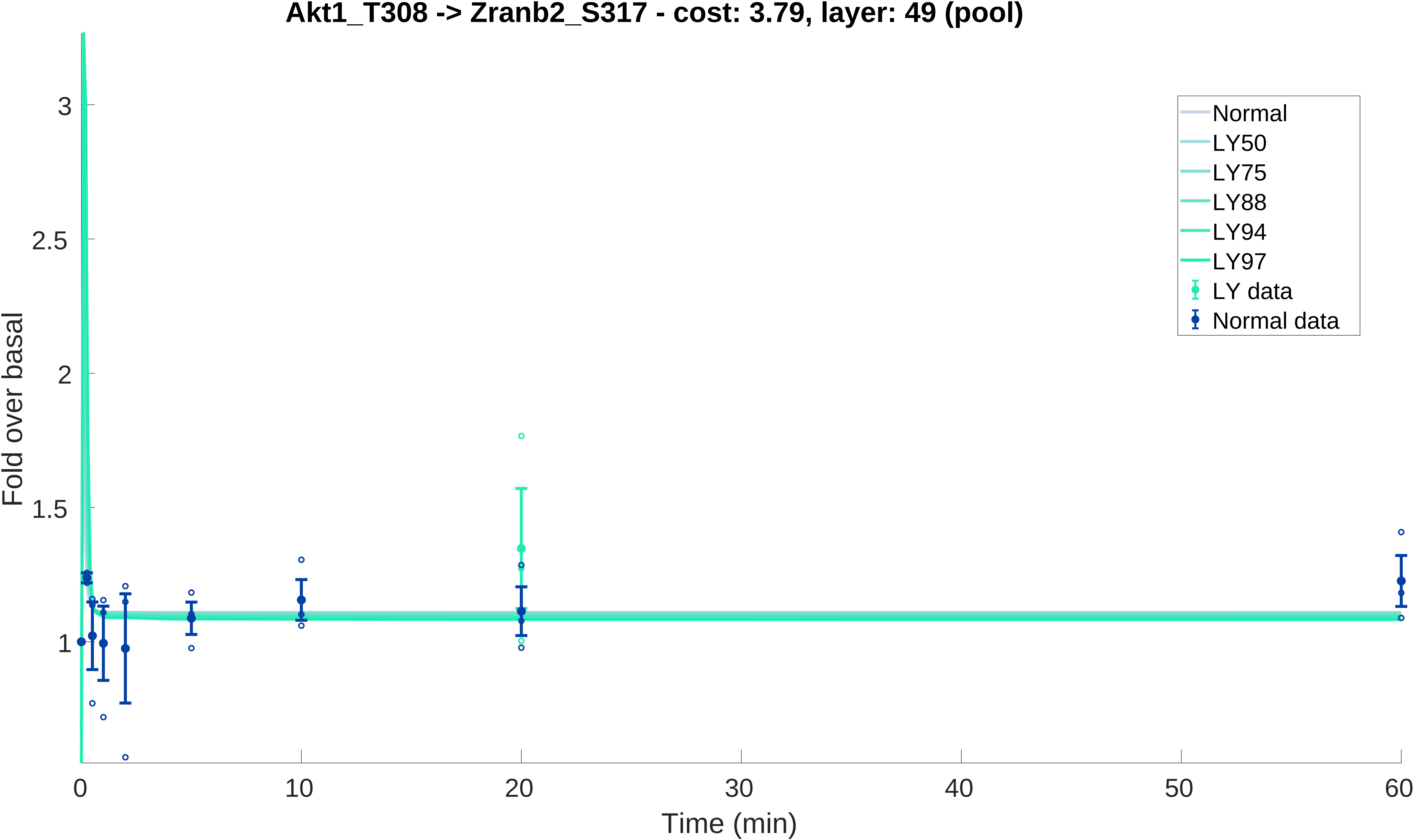

Akt1_T308 -> Zranb2_S317 - cost: 3.79, layer: 49 (pool)
Fold over basal
Time (min)
Normal
LY50
LY75
LY88
LY94
LY97
LY data
Normal data
3
2.5
2
1.5
1
0
10
20
30
40
50
60

Clk3_S224 -> Anks1a_S636 - cost: 11.42, layer: 50

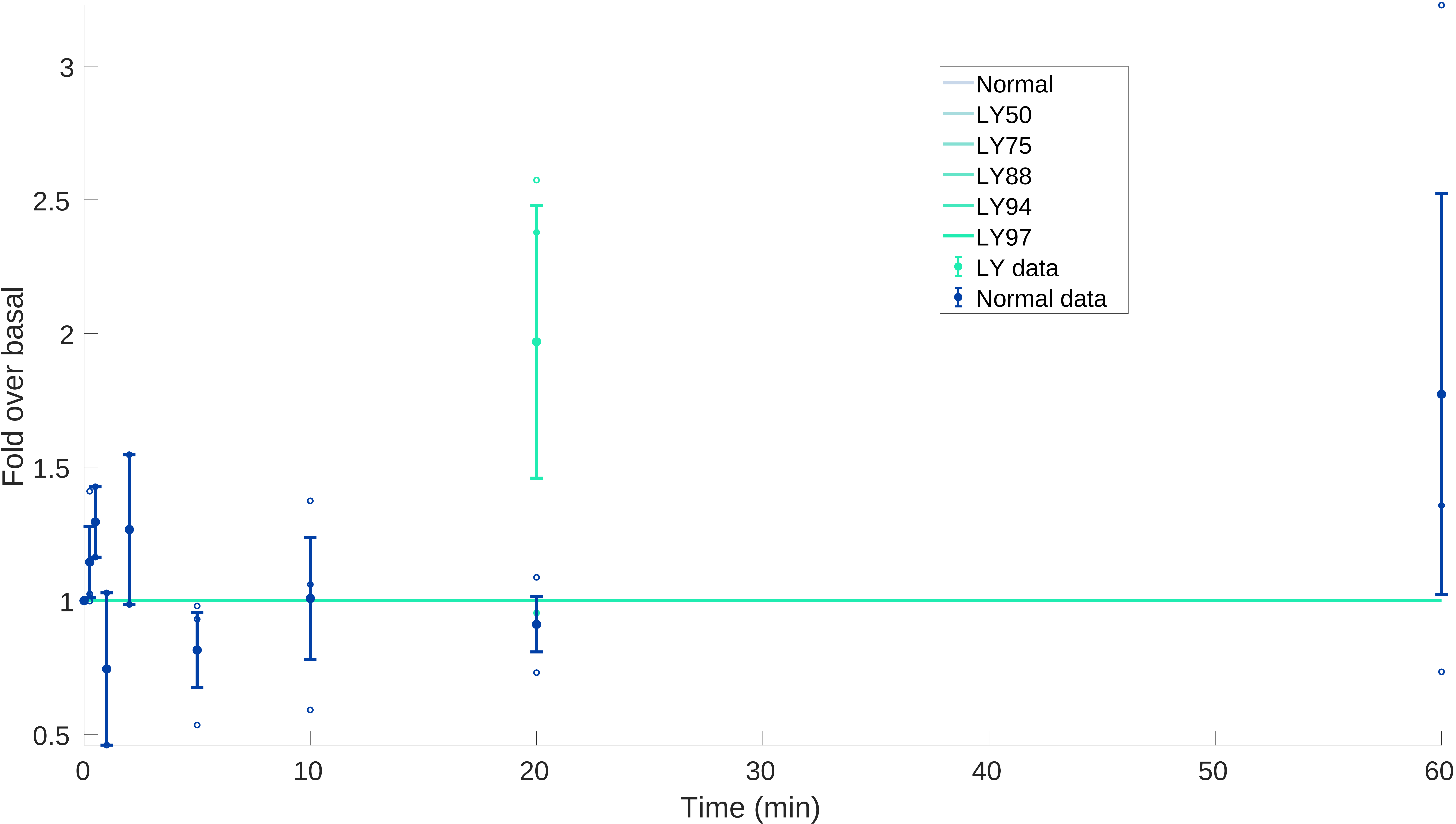

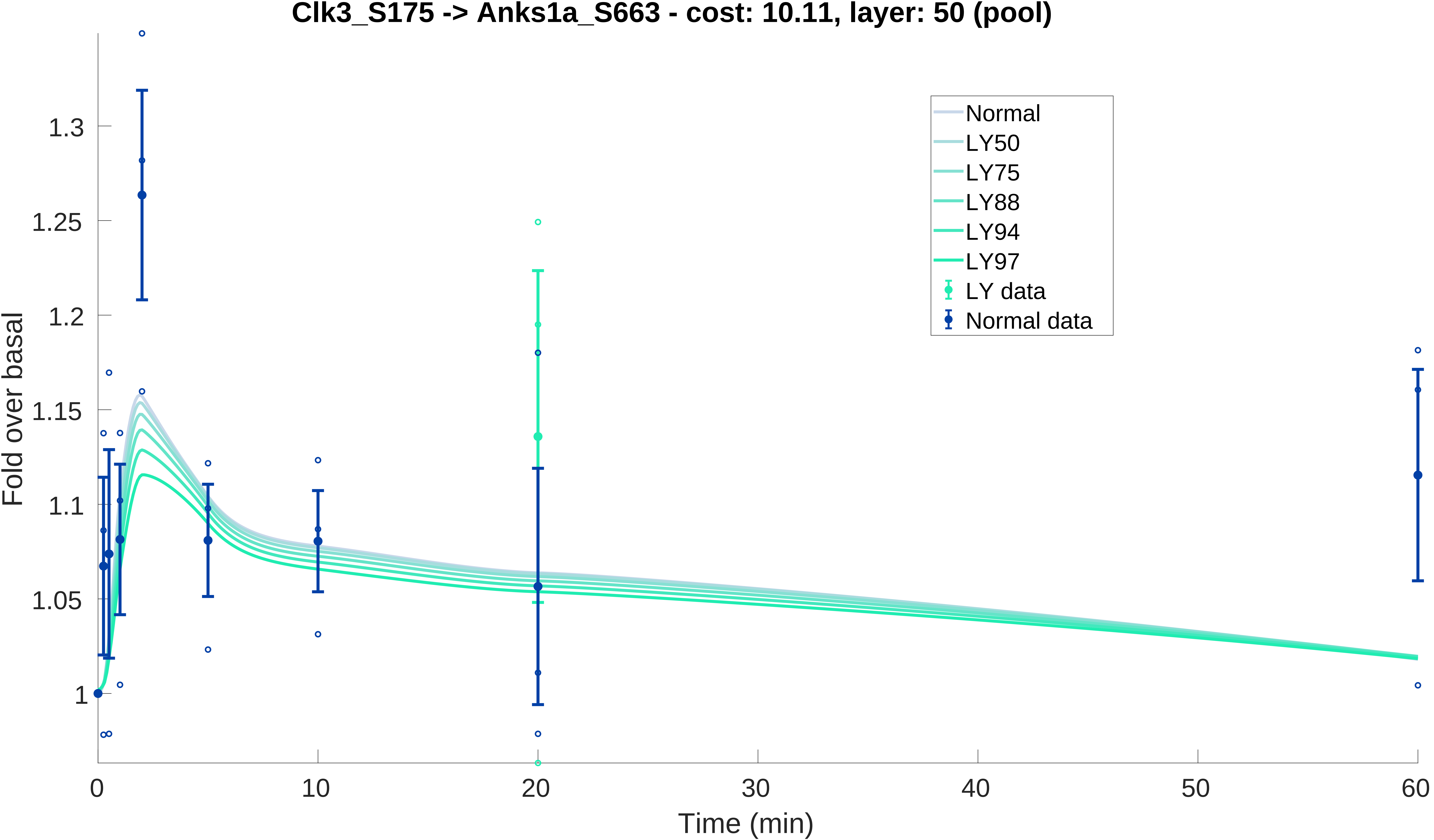

Clk3_S175 -> Anks1a_S663 - cost: 10.11, layer: 50 (pool)
Fold over basal
Time (min)
1.3
1.25
1.2
1.15
1.1
1.05
1
0
10
20
30
40
50
60
Normal
LY50
LY75
LY88
LY94
LY97
LY data
Normal data

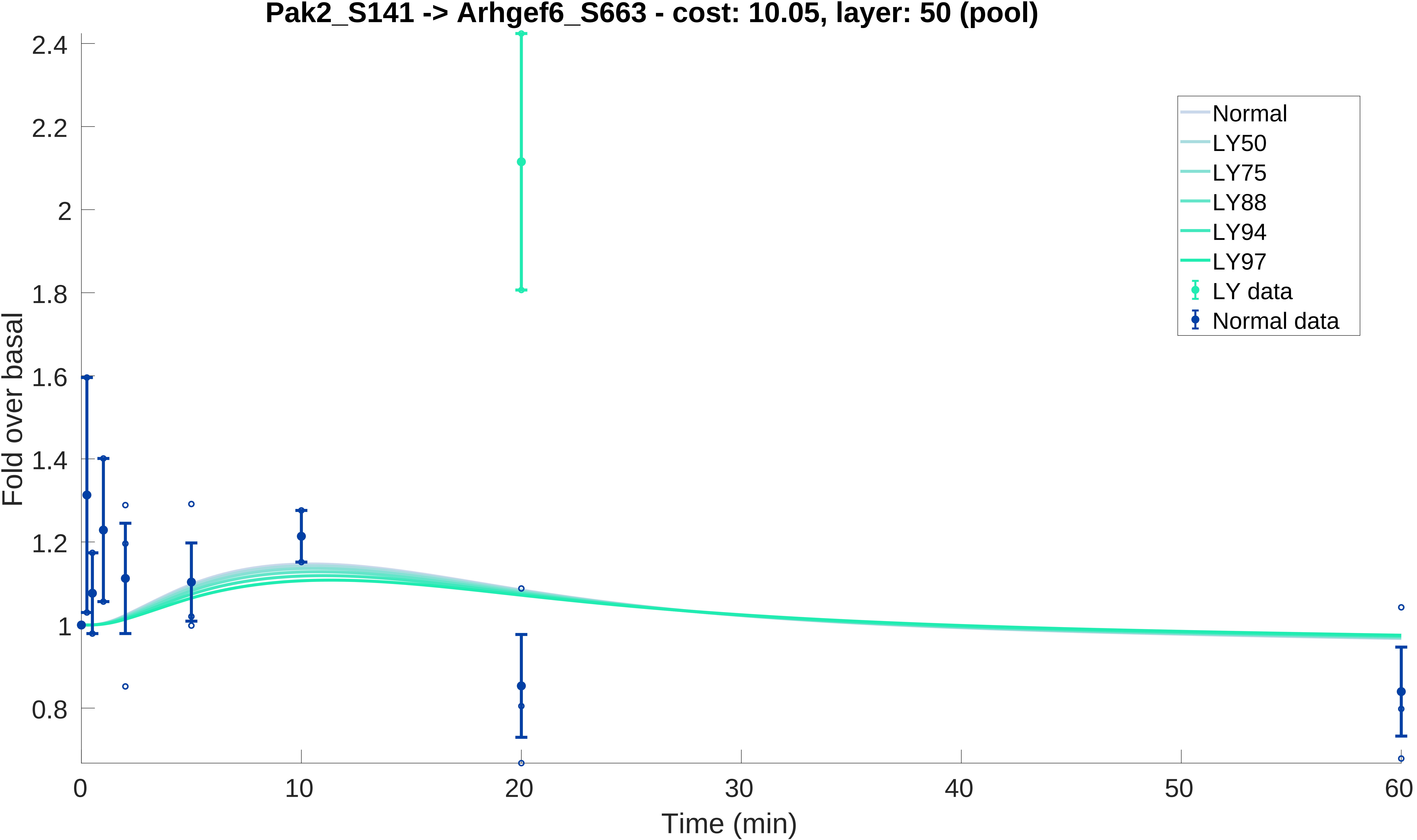

Pak2_S141 -> Arhgef6_S663 - cost: 10.05, layer: 50 (pool)
Fold over basal
Time (min)
Normal
LY50
LY75
LY88
LY94
LY97
LY data
Normal data
2.4
2.2
2
1.8
1.6
1.4
1.2
1
0.8
0
10
20
30
40
50
60

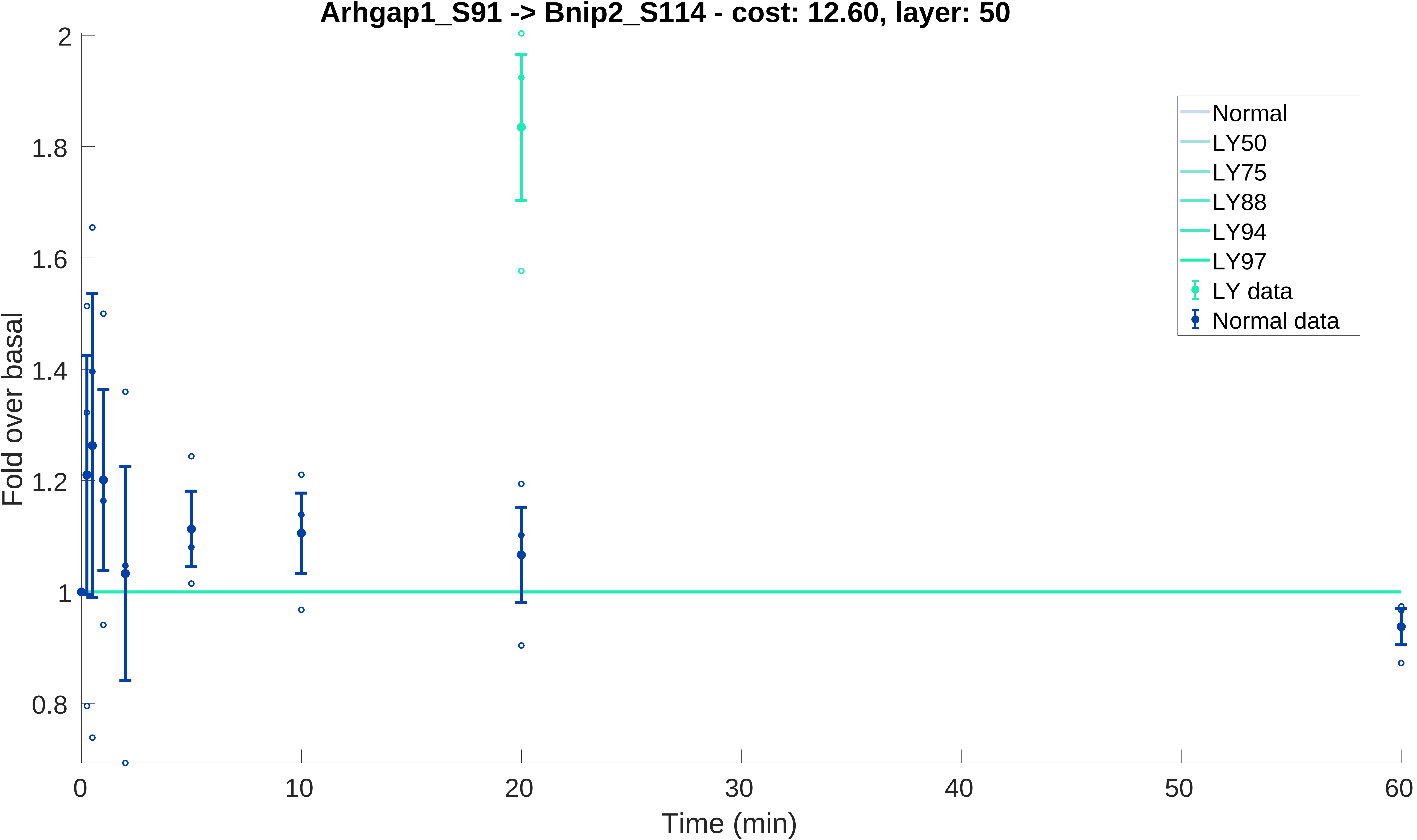

Arhgap1_S91 -> Bnip2_S114 - cost: 12.60, layer: 50
Fold over basal
Time (min)
2
1.8
1.6
1.4
1.2
1
0.8
0
10
20
30
40
50
60
Normal
LY50
LY75
LY88
LY94
LY97
LY data
Normal data

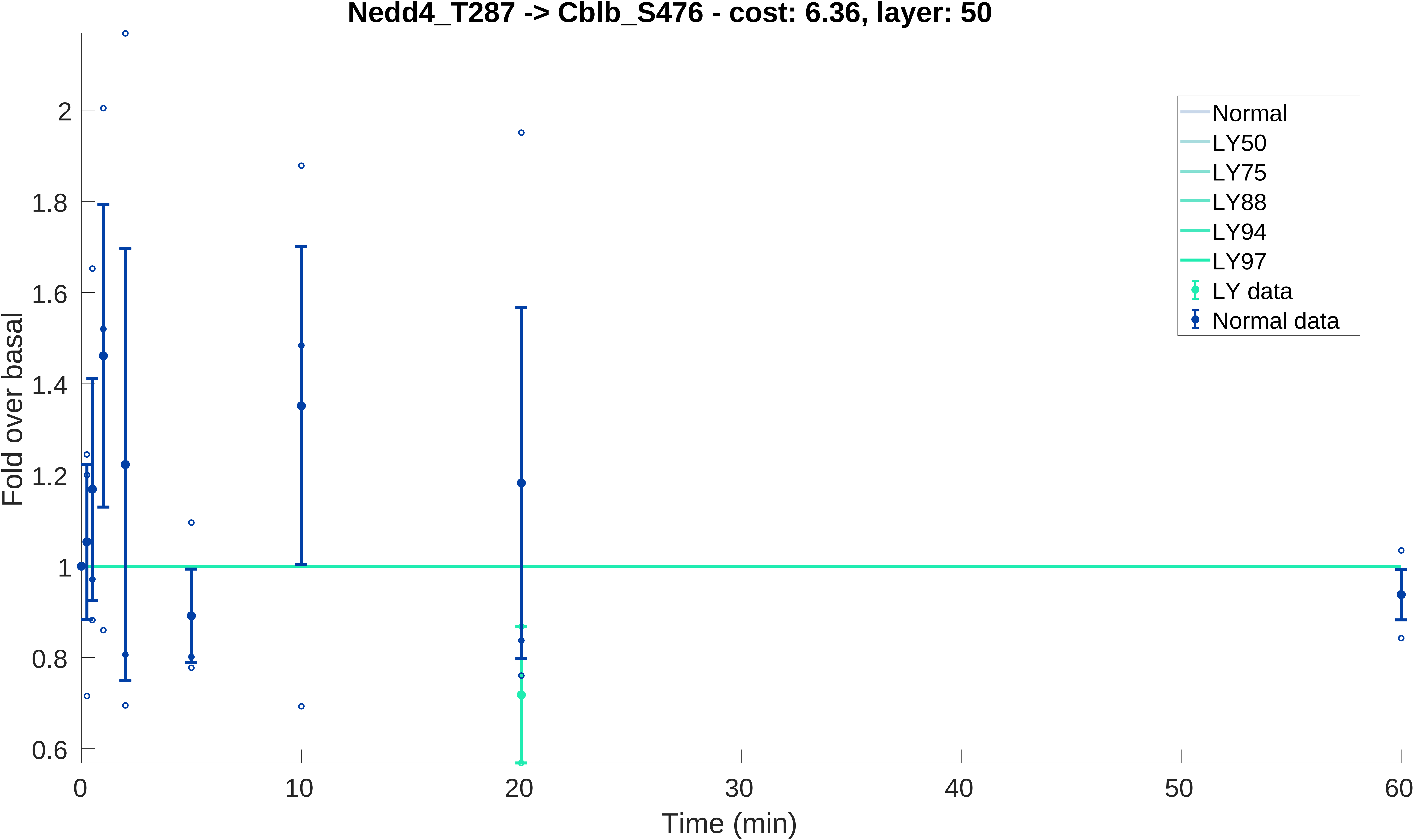

Nedd4_T287 -> Cblb_S476 - cost: 6.36, layer: 50
Fold over basal
Time (min)
Normal
LY50
LY75
LY88
LY94
LY97
LY data
Normal data
0.6
0.8
1
1.2
1.4
1.6
1.8
2
0
10
20
30
40
50
60

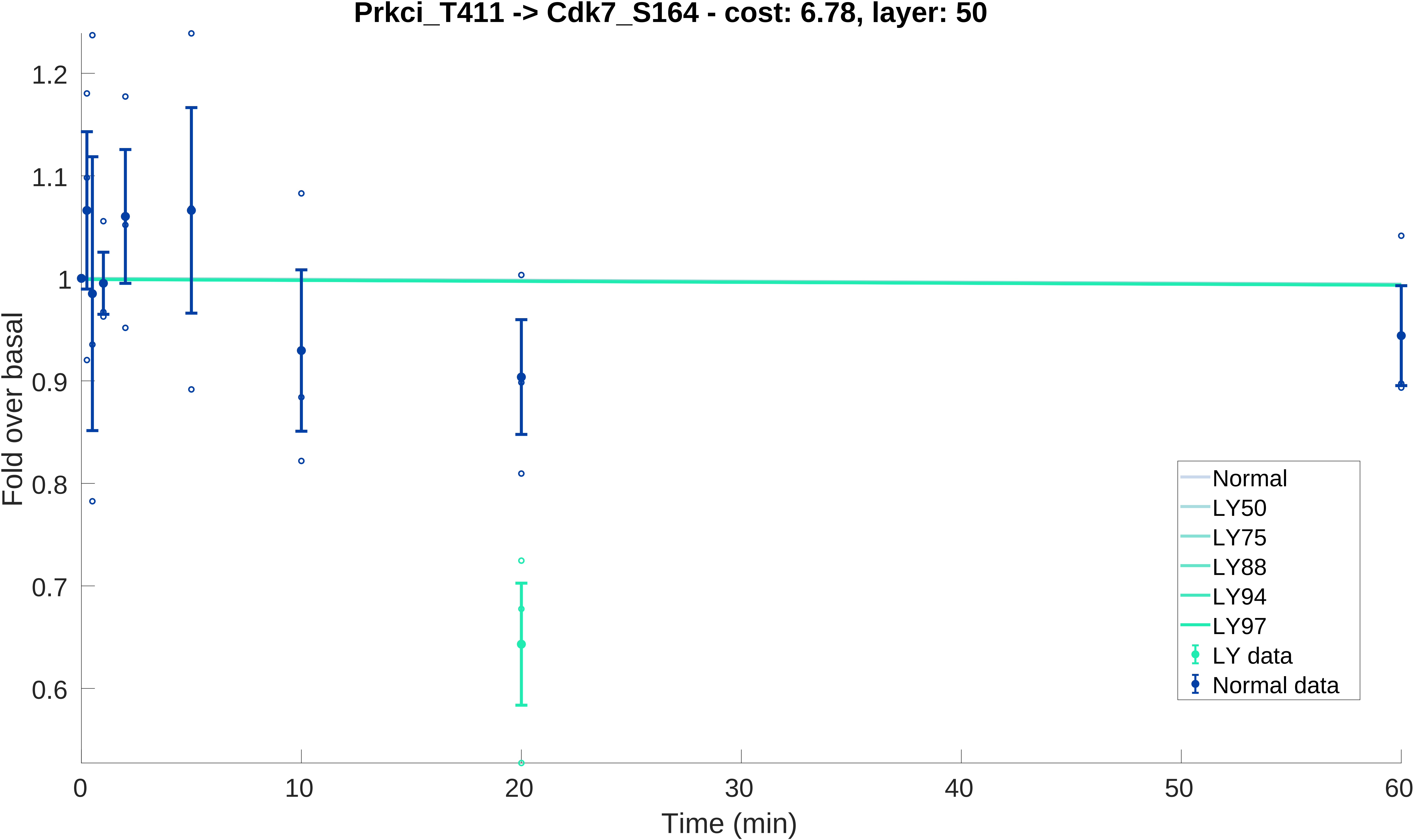

Prkci_T411 -> Cdk7_S164 - cost: 6.78, layer: 50
Fold over basal
1.2
1.1
1
0.9
0.8
0.7
0.6
0
10
20
30
40
50
60
Time (min)
Normal
LY50
LY75
LY88
LY94
LY97
LY data
Normal data

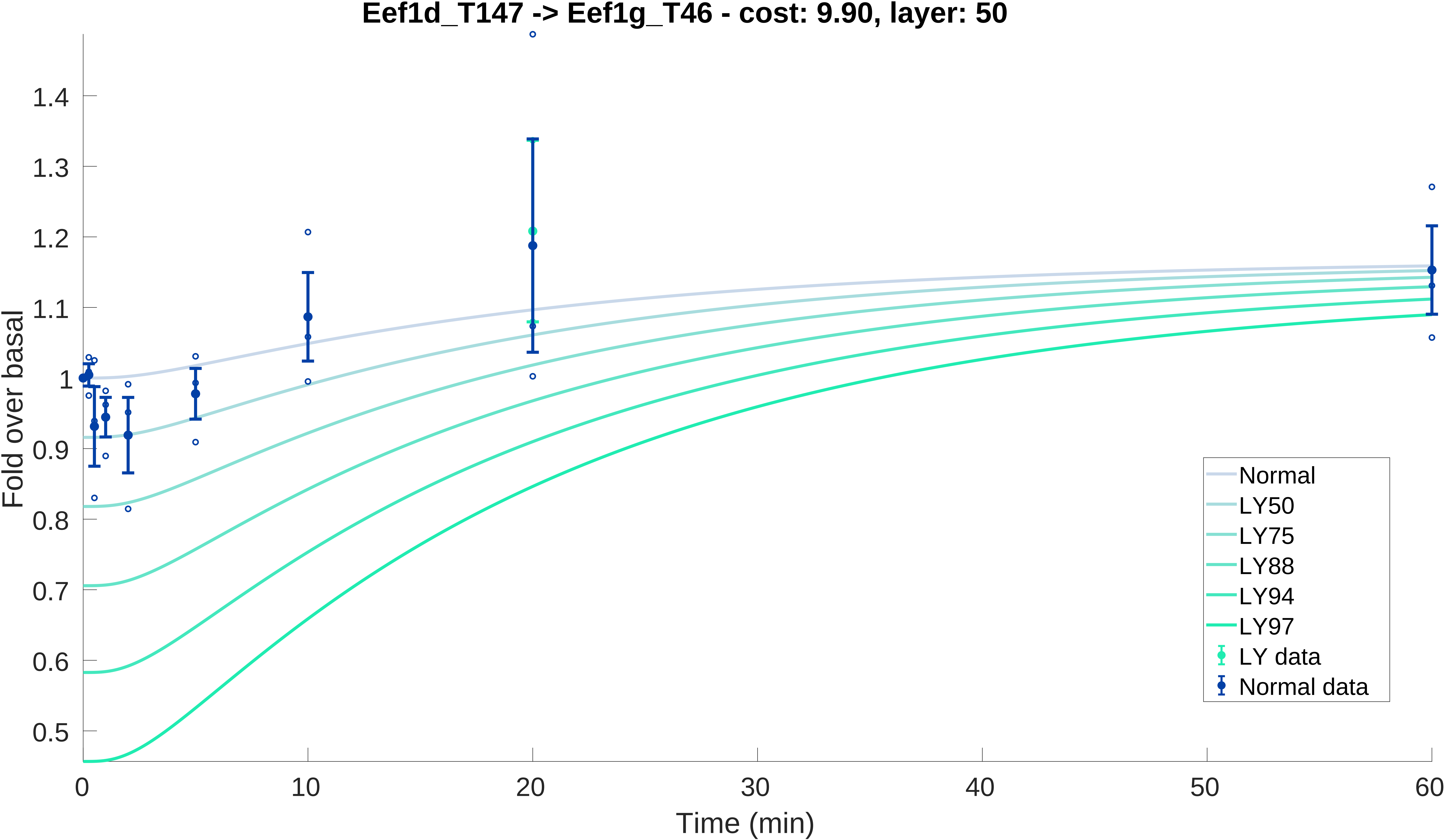

Eef1d_T147 -> Eef1g_T46 - cost: 9.90, layer: 50
Fold over basal
Time (min)
0.5
0.6
0.7
0.8
0.9
1
1.1
1.2
1.3
1.4
0
10
20
30
40
50
60
Normal
LY50
LY75
LY88
LY94
LY97
LY data
Normal data

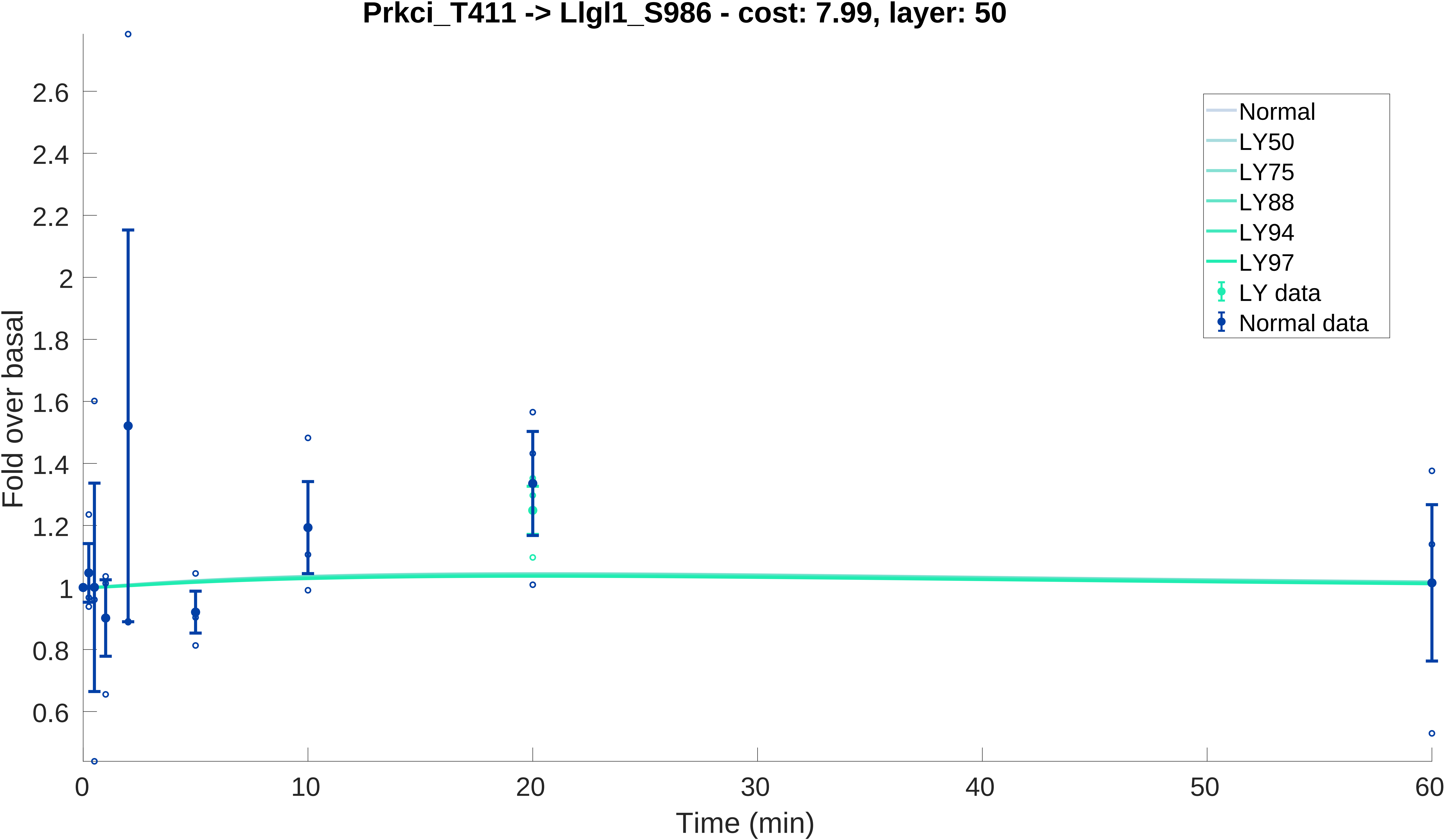

Prkci_T411 -> Llgl1_S986 - cost: 7.99, layer: 50
Fold over basal
Time (min)
0.6
0.8
1
1.2
1.4
1.6
1.8
2
2.2
2.4
2.6
0
10
20
30
40
50
60
Normal
LY50
LY75
LY88
LY94
LY97
LY data
Normal data

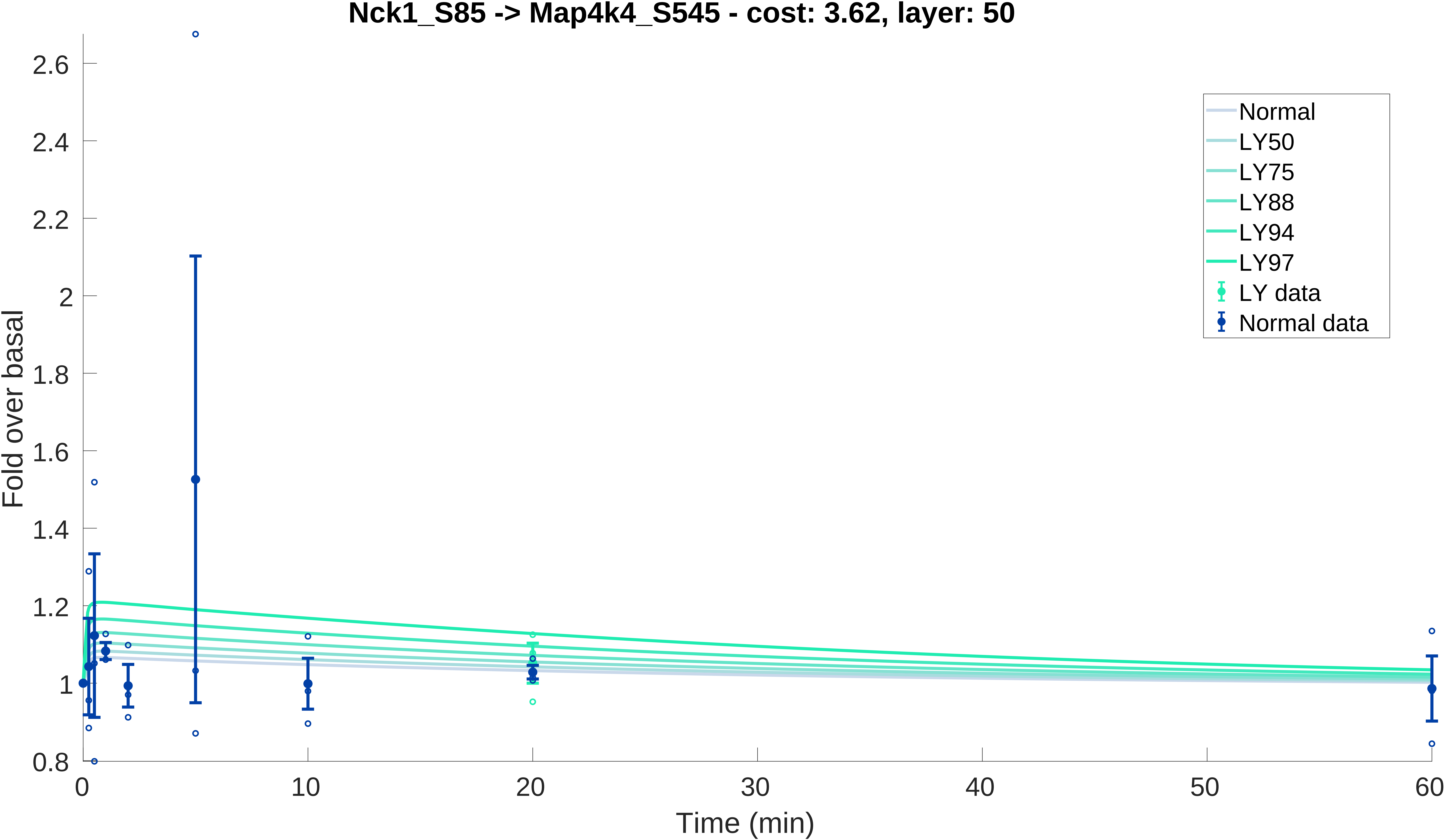
Nck1_S85 -> Map4k4_S545 - cost: 3.62, layer: 50
Fold over basal
Time (min)
0.8
1
1.2
1.4
1.6
1.8
2
2.2
2.4
2.6
0
10
20
30
40
50
60
Normal
LY50
LY75
LY88
LY94
LY97
LY data
Normal data

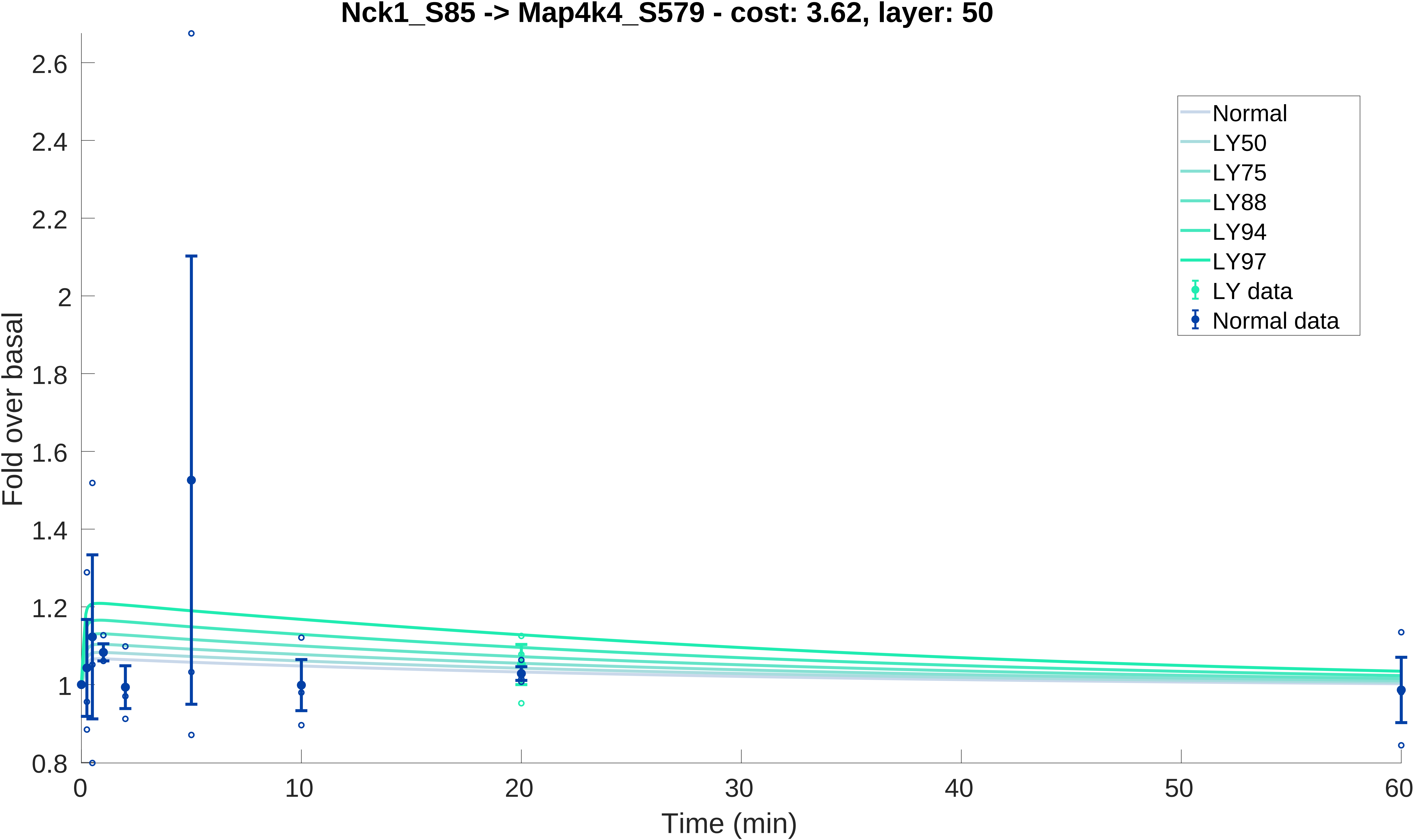
Nck1_S85 -> Map4k4_S579 - cost: 3.62, layer: 50
Fold over basal
Time (min)
0.8
1
1.2
1.4
1.6
1.8
2
2.2
2.4
2.6
0
10
20
30
40
50
60
Normal
LY50
LY75
LY88
LY94
LY97
LY data
Normal data

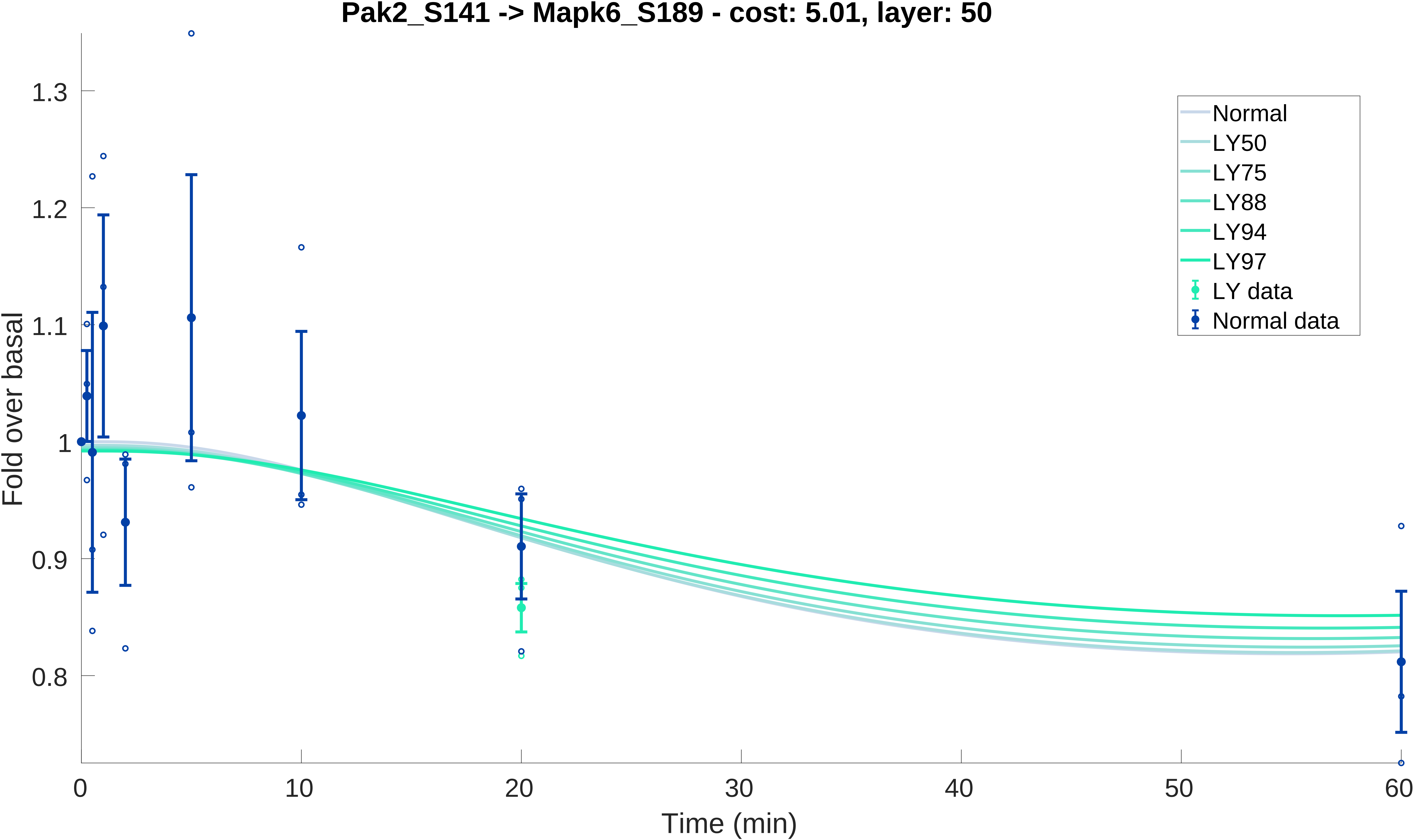

Pak2_S141 -> Mapk6_S189 - cost: 5.01, layer: 50
Fold over basal
Time (min)
1.3
1.2
1.1
1
0.9
0.8
0
10
20
30
40
50
60
Normal
LY50
LY75
LY88
LY94
LY97
LY data
Normal data

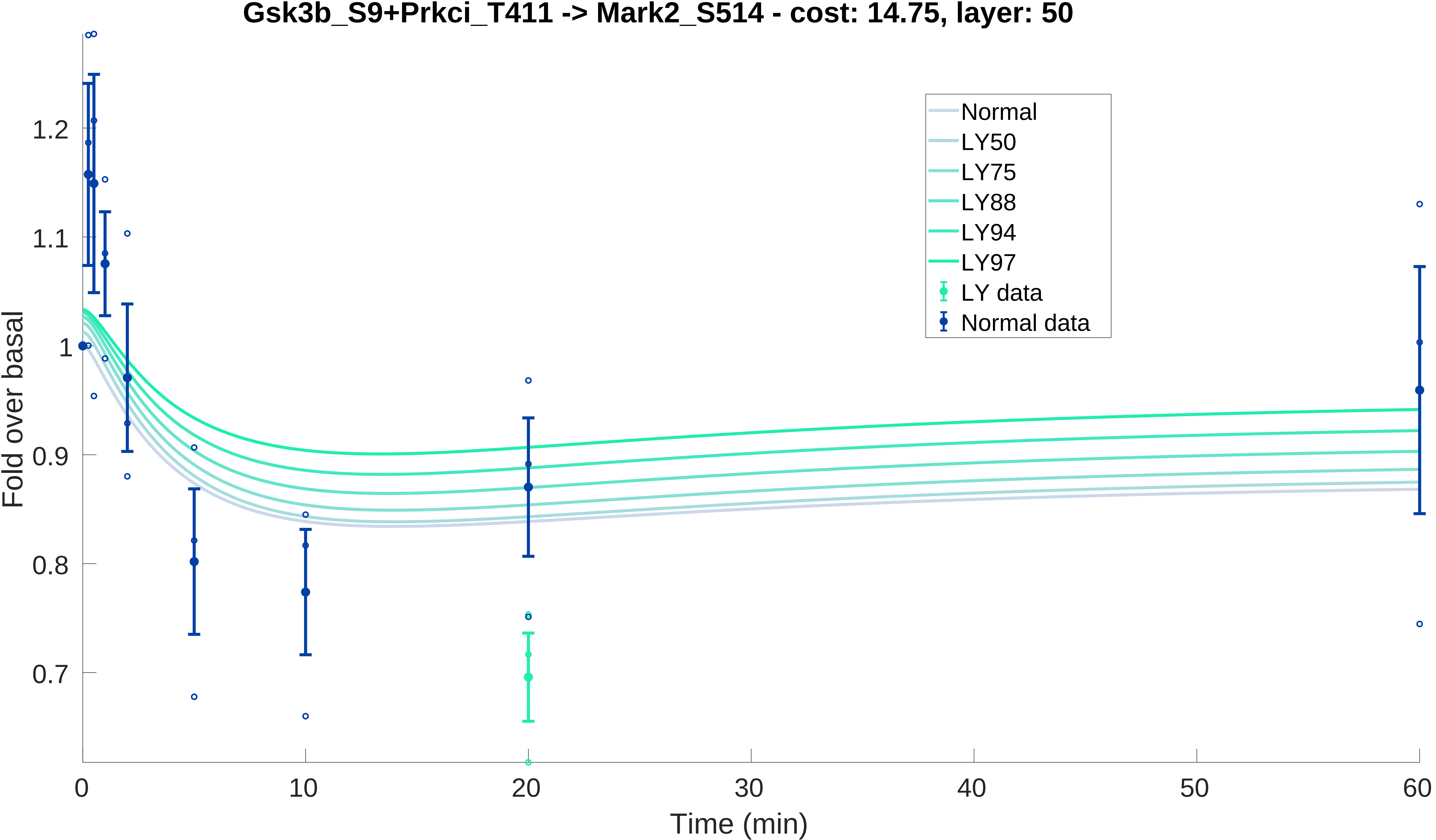
Gsk3b_S9+Prkci_T411 -> Mark2_S514 - cost: 14.75, layer: 50
Fold over basal
Time (min)
0.7
0.8
0.9
1
1.1
1.2
0
10
20
30
40
50
60
Normal
LY50
LY75
LY88
LY94
LY97
LY data
Normal data

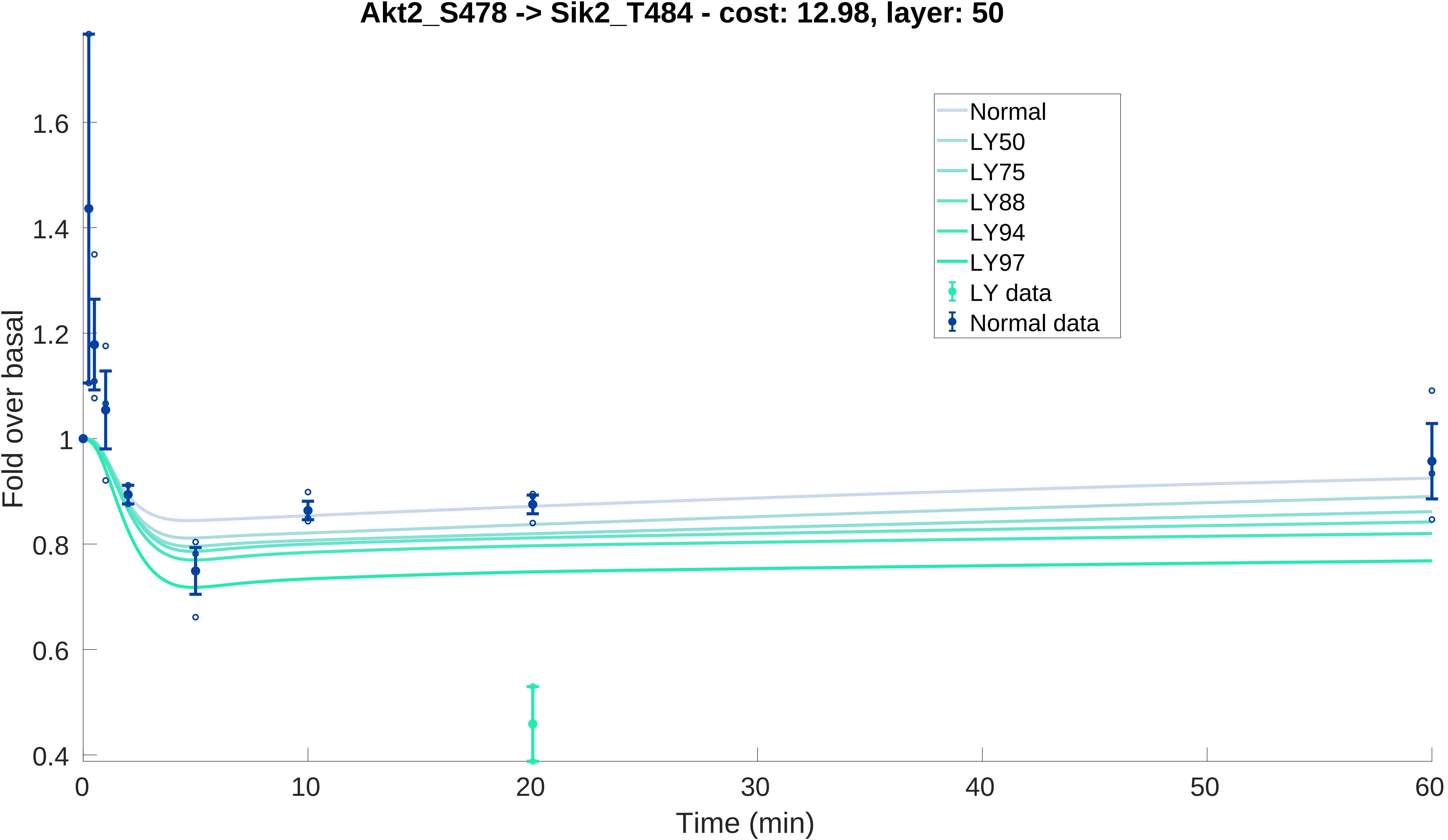

Akt2_S478 -> Sik2_T484 - cost: 12.98, layer: 50
Fold over basal
Time (min)
Normal
LY50
LY75
LY88
LY94
LY97
LY data
Normal data
0.4
0.6
0.8
1
1.2
1.4
1.6
0
10
20
30
40
50
60

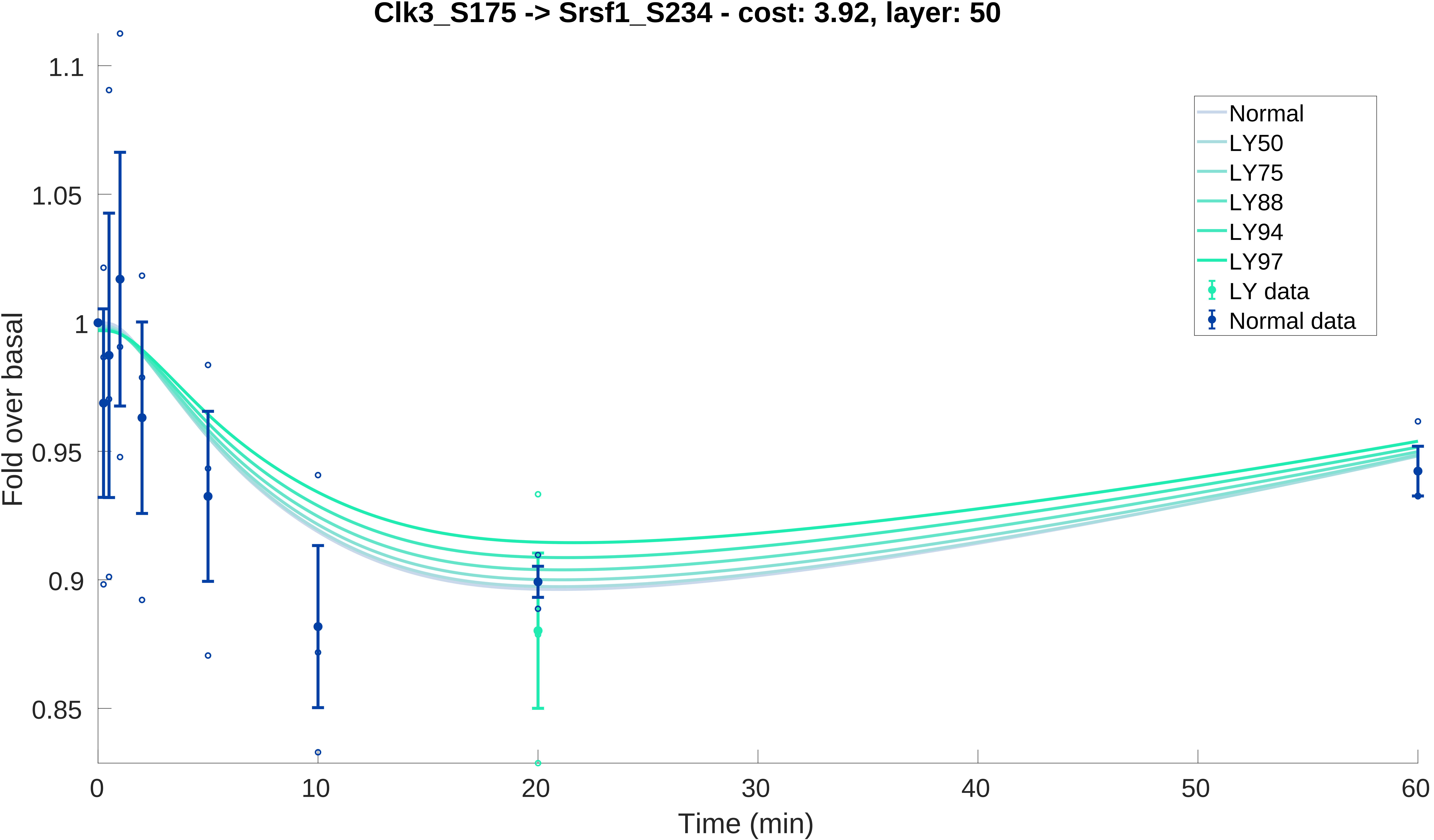

Clk3_S175 -> Srsf1_S234 - cost: 3.92, layer: 50
Fold over basal
Time (min)
1.1
1.05
1
0.95
0.9
0.85
0
10
20
30
40
50
60
Normal
LY50
LY75
LY88
LY94
LY97
LY data
Normal data

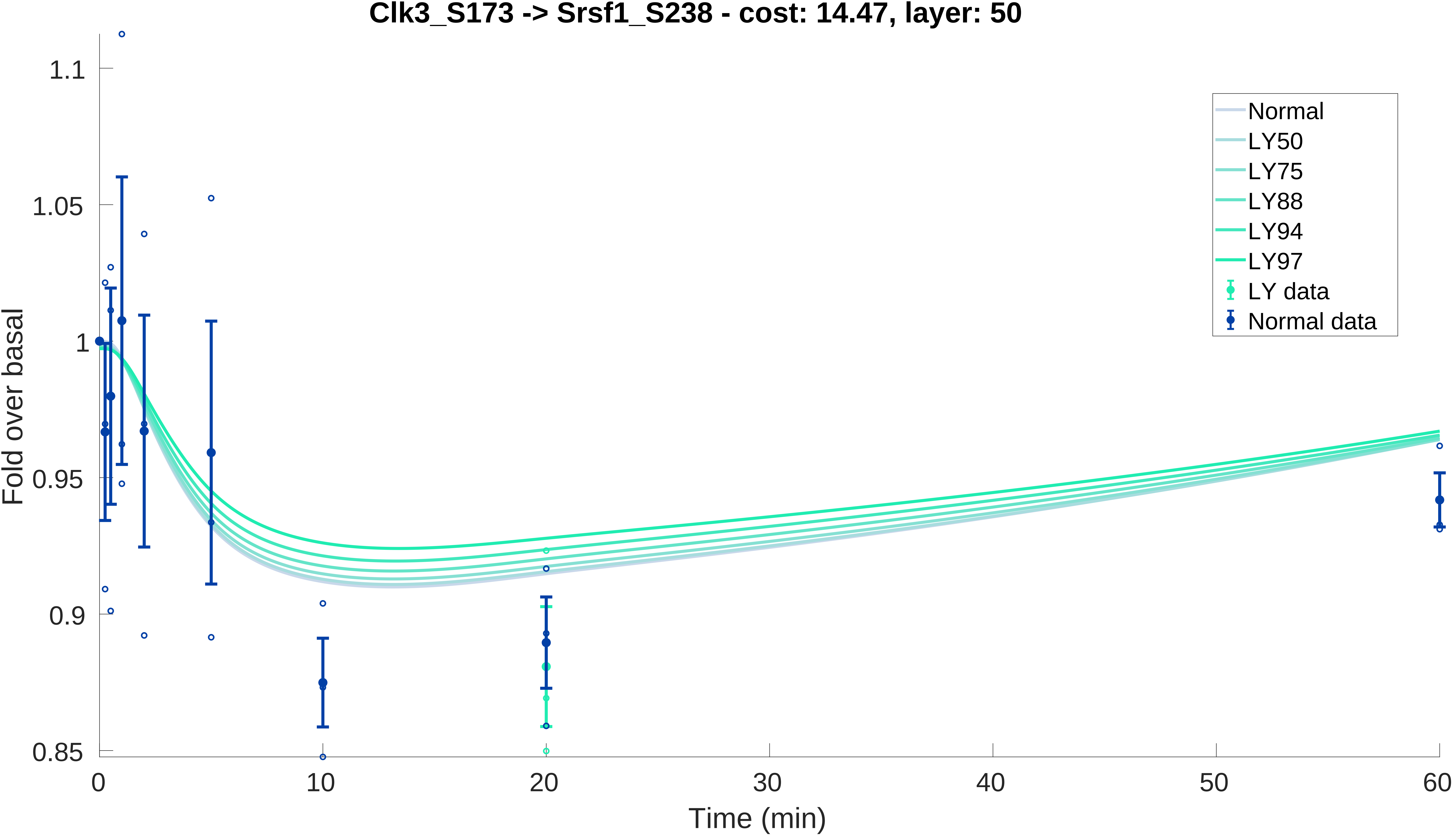

Clk3_S173 -> Srsf1_S238 - cost: 14.47, layer: 50
Fold over basal
Time (min)
1.1
1.05
1
0.95
0.9
0.85
0
10
20
30
40
50
60
Normal
LY50
LY75
LY88
LY94
LY97
LY data
Normal data

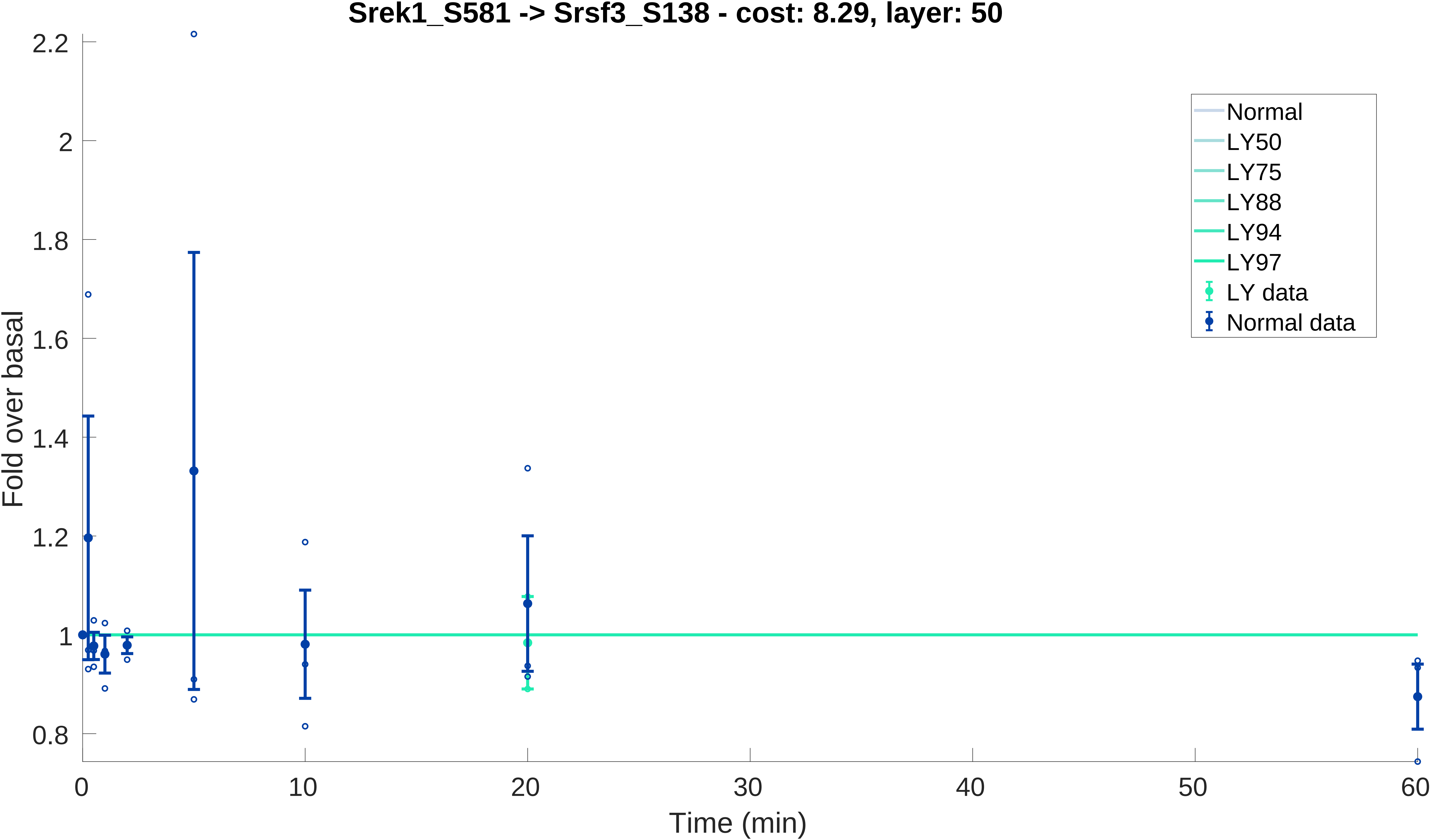

Srek1_S581 -> Srsf3_S138 - cost: 8.29, layer: 50
Fold over basal
Time (min)
Normal
LY50
LY75
LY88
LY94
LY97
LY data
Normal data
0.8
1
1.2
1.4
1.6
1.8
2
2.2
0
10
20
30
40
50
60

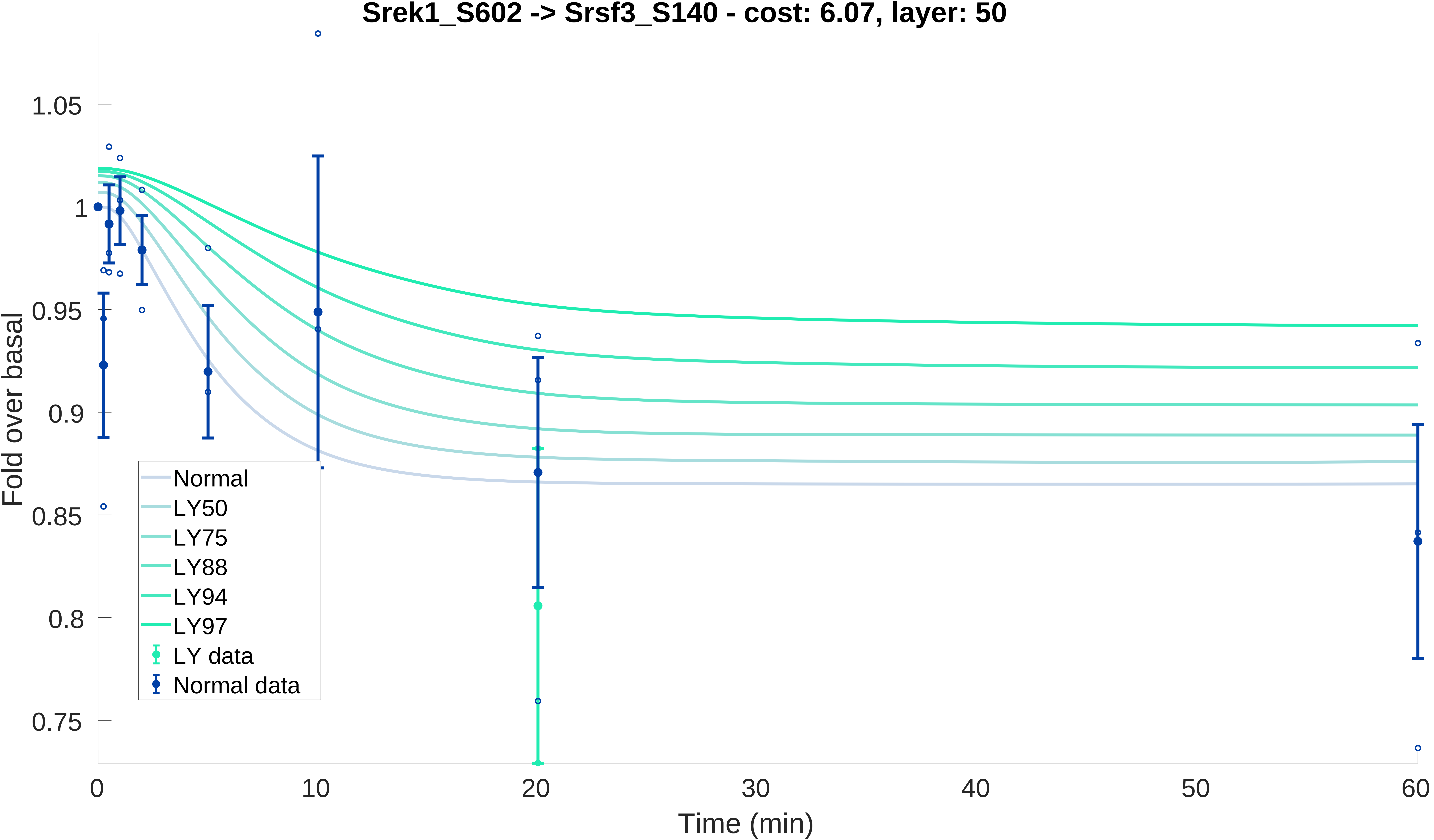
Srek1_S602 -> Srsf3_S140 - cost: 6.07, layer: 50
Fold over basal
Time (min)
1.05
1
0.95
0.9
0.85
0.8
0.75
0
10
20
30
40
50
60
Normal
LY50
LY75
LY88
LY94
LY97
LY data
Normal data

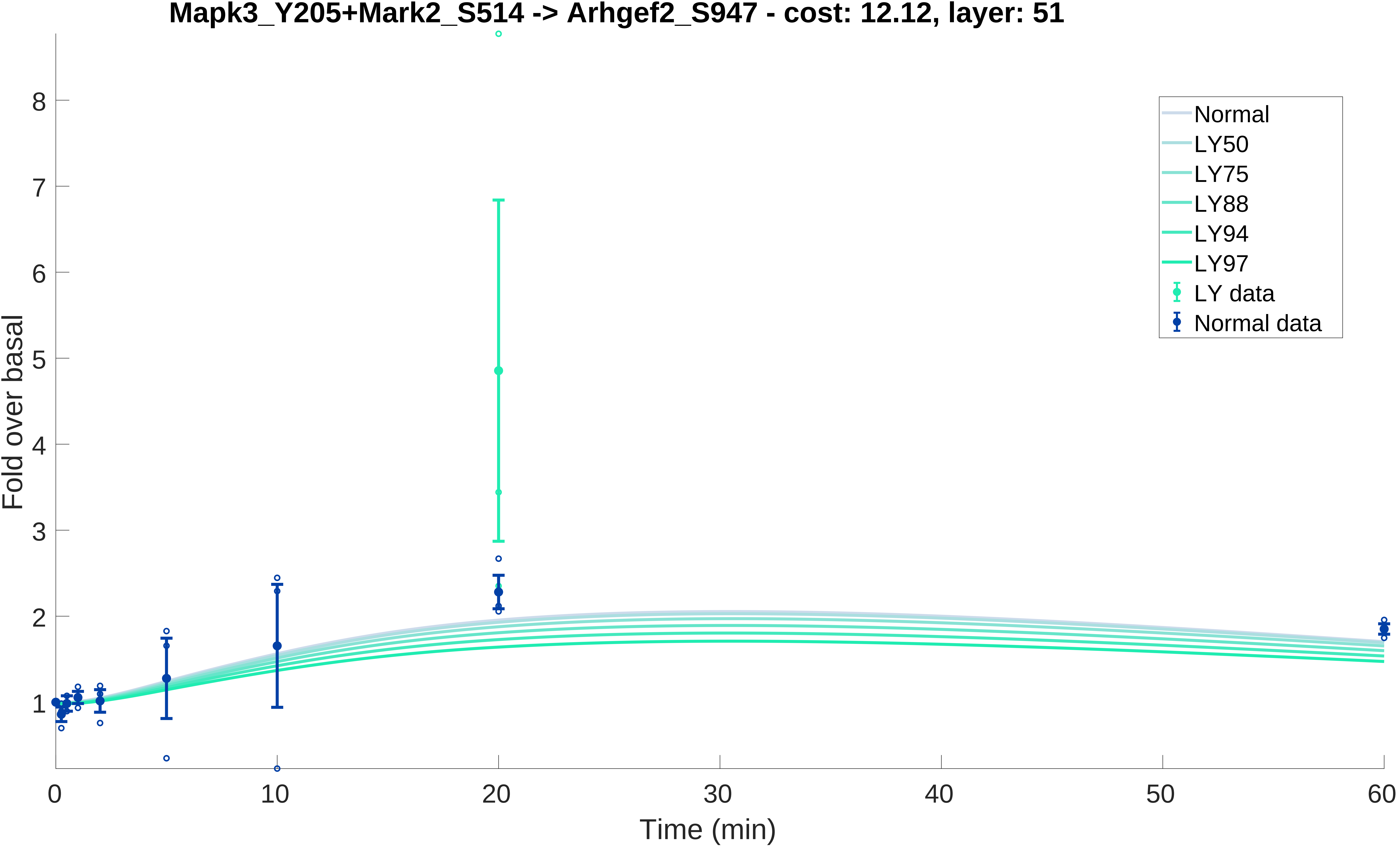

Mapk3_Y205+Mark2_S514 -> Arhgef2_S947 - cost: 12.12, layer: 51
Fold over basal
Time (min)
Normal
LY50
LY75
LY88
LY94
LY97
LY data
Normal data
1
2
3
4
5
6
7
8
0
10
20
30
40
50
60

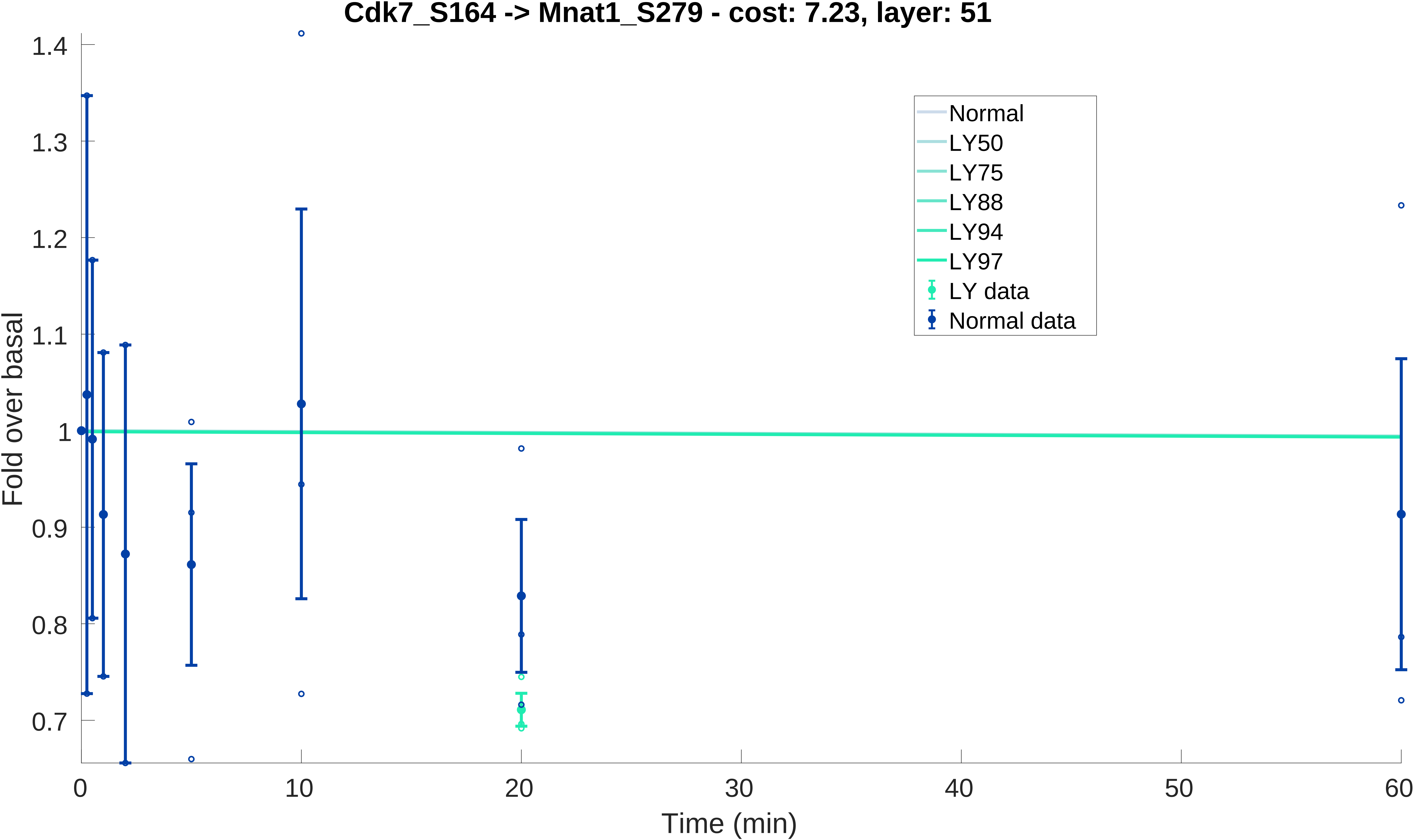
Cdk7_S164 -> Mnat1_S279 - cost: 7.23, layer: 51
Fold over basal
Time (min)
1.4
1.3
1.2
1.1
1
0.9
0.8
0.7
0
10
20
30
40
50
60
Normal
LY50
LY75
LY88
LY94
LY97
LY data
Normal data

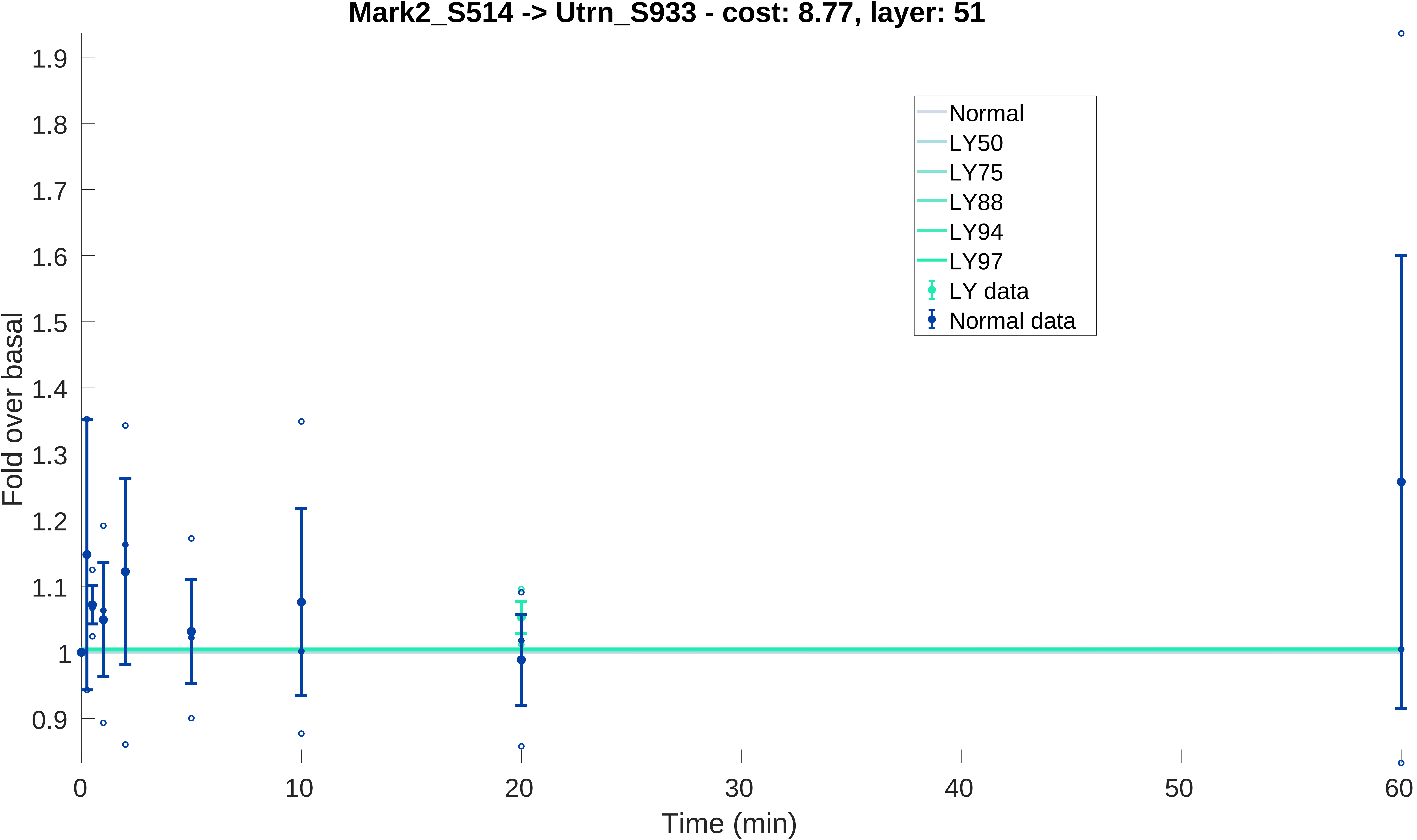

Mark2_S514 -> Utrn_S933 - cost: 8.77, layer: 51
Fold over basal
Time (min)
Normal
LY50
LY75
LY88
LY94
LY97
LY data
Normal data
0.9
1
1.1
1.2
1.3
1.4
1.5
1.6
1.7
1.8
1.9
0
10
20
30
40
50
60

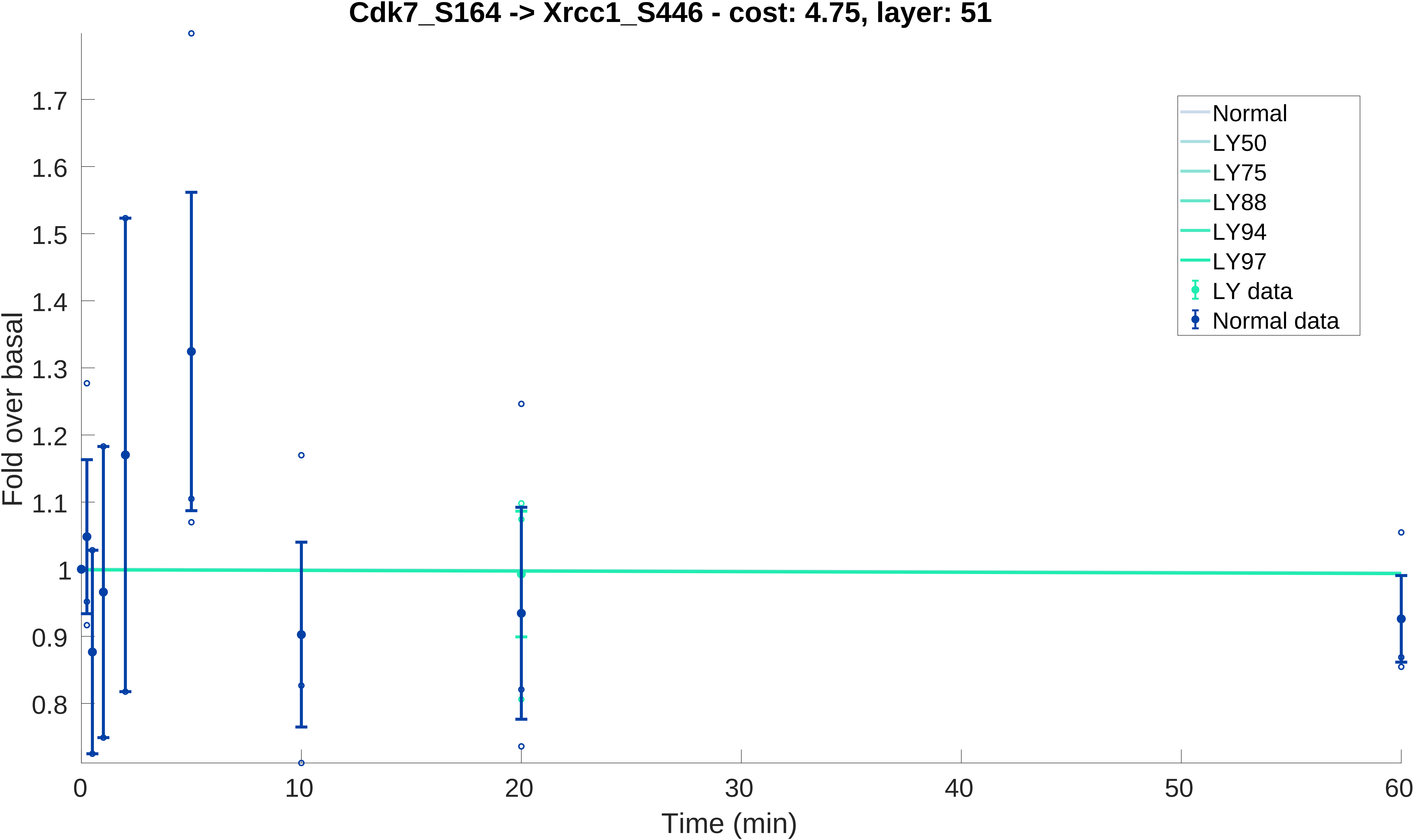

Cdk7_S164 -> Xrcc1_S446 - cost: 4.75, layer: 51
Fold over basal
Time (min)
0.8
0.9
1
1.1
1.2
1.3
1.4
1.5
1.6
1.7
0
10
20
30
40
50
60
Normal
LY50
LY75
LY88
LY94
LY97
LY data
Normal data

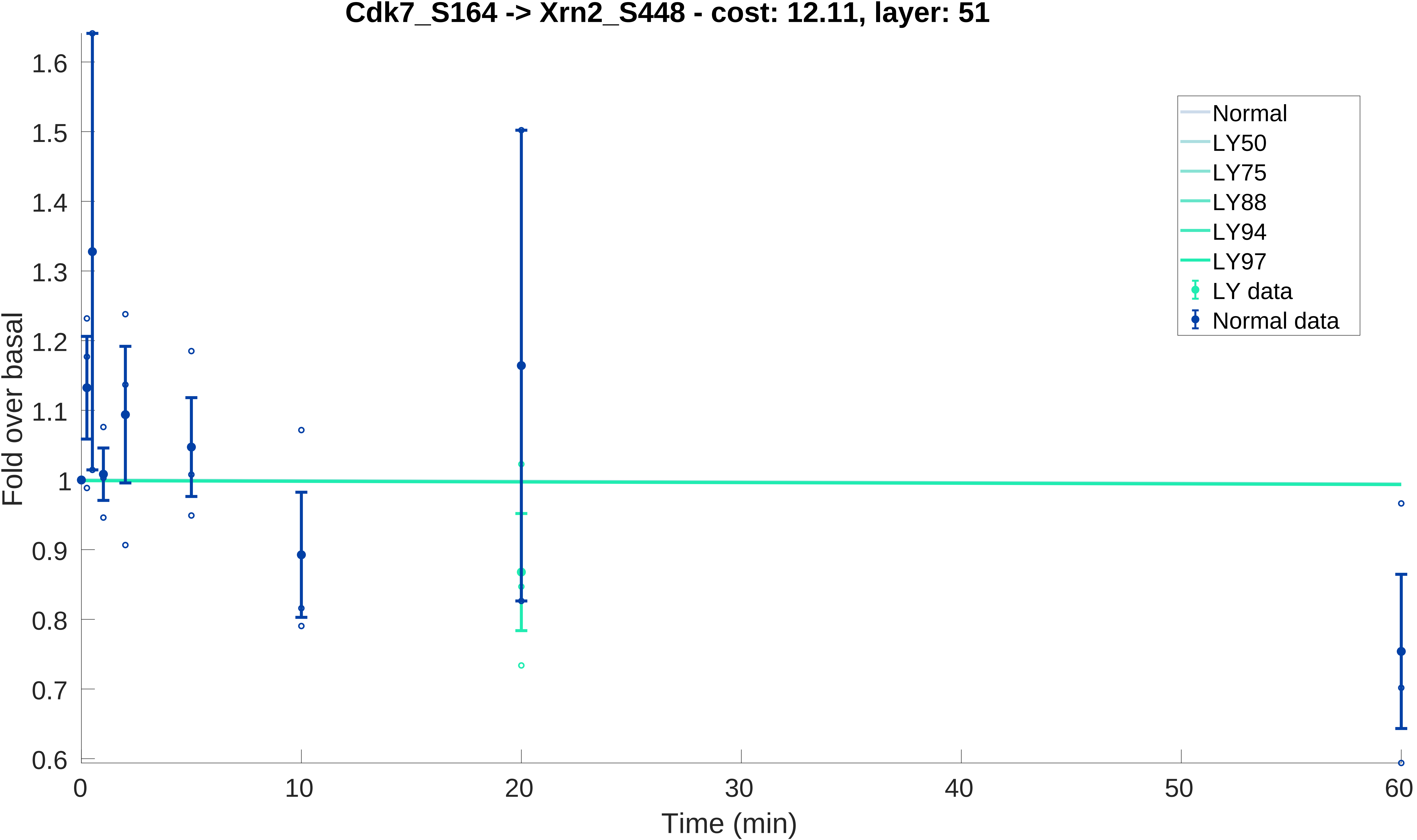

Cdk7_S164 -> Xrn2_S448 - cost: 12.11, layer: 51
Fold over basal
Time (min)
1.6
1.5
1.4
1.3
1.2
1.1
1
0.9
0.8
0.7
0.6
0
10
20
30
40
50
60
Normal
LY50
LY75
LY88
LY94
LY97
LY data
Normal data

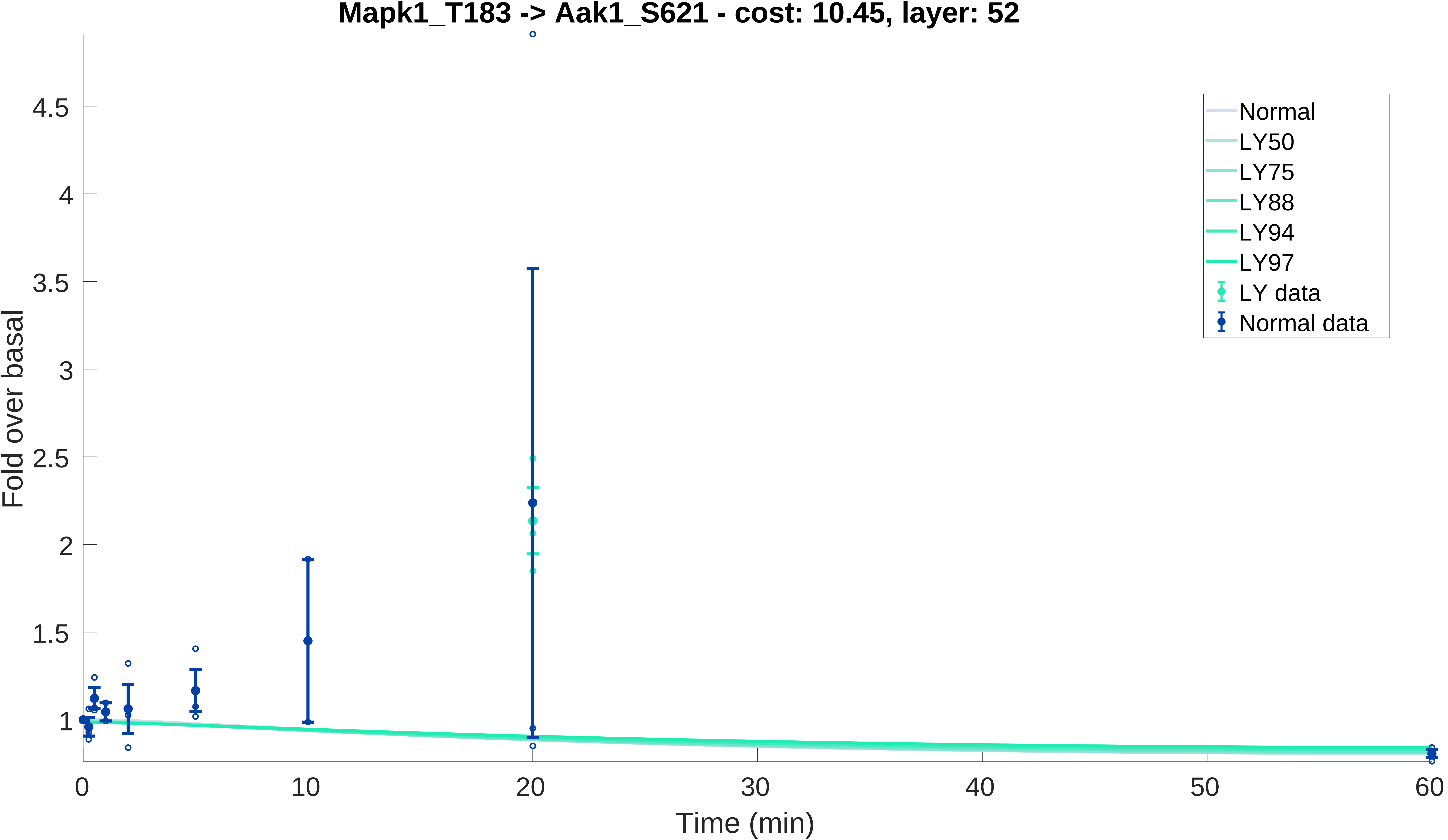

Mapk1_T183 -> Aak1_S621 - cost: 10.45, layer: 52
Fold over basal
Time (min)
4.5
4
3.5
3
2.5
2
1.5
1
0
10
20
30
40
50
60
Normal
LY50
LY75
LY88
LY94
LY97
LY data
Normal data

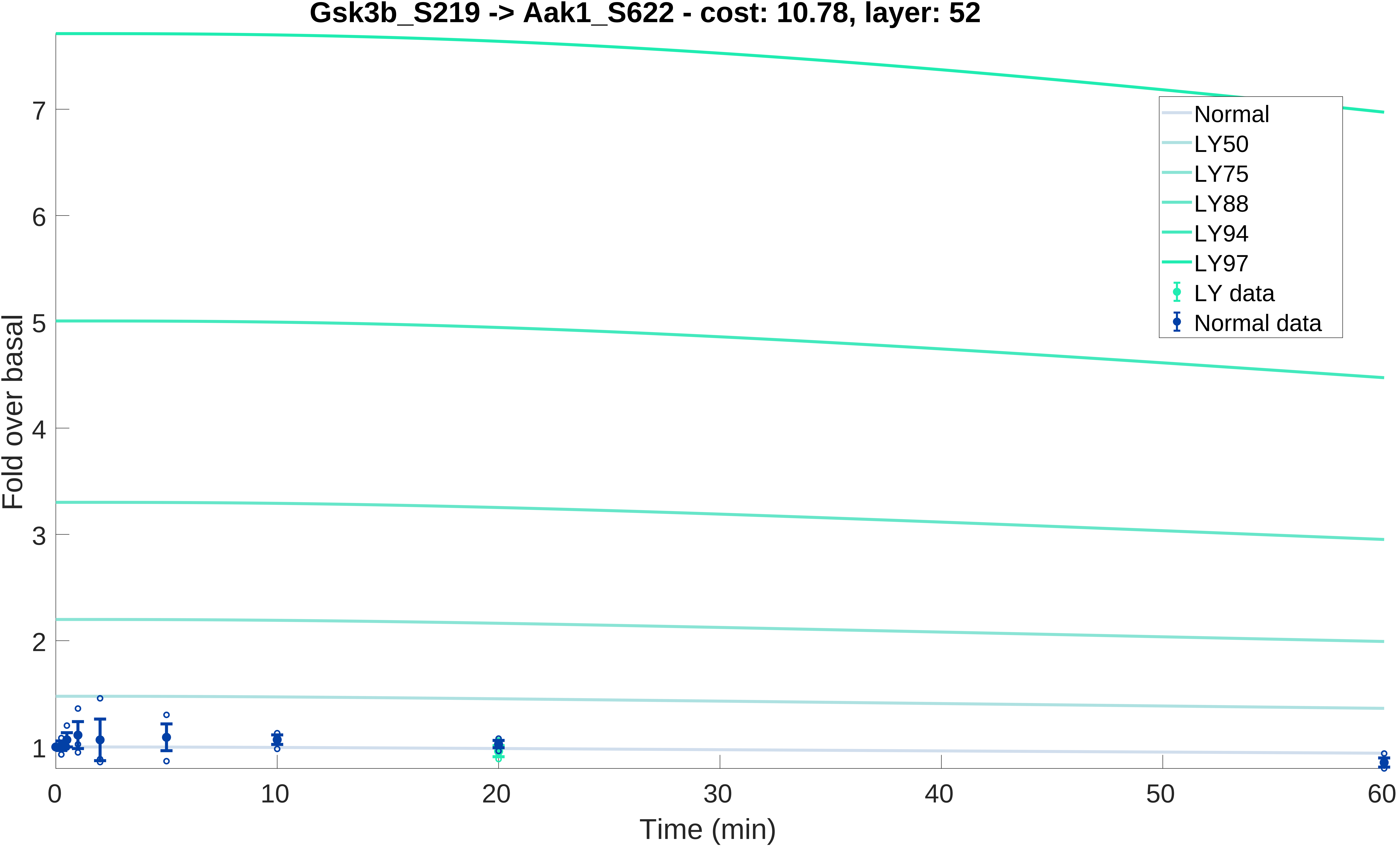

Gsk3b_S219 -> Aak1_S622 - cost: 10.78, layer: 52
Fold over basal
Time (min)
0
10
20
30
40
50
60
1
2
3
4
5
6
7
Normal
LY50
LY75
LY88
LY94
LY97
LY data
Normal data

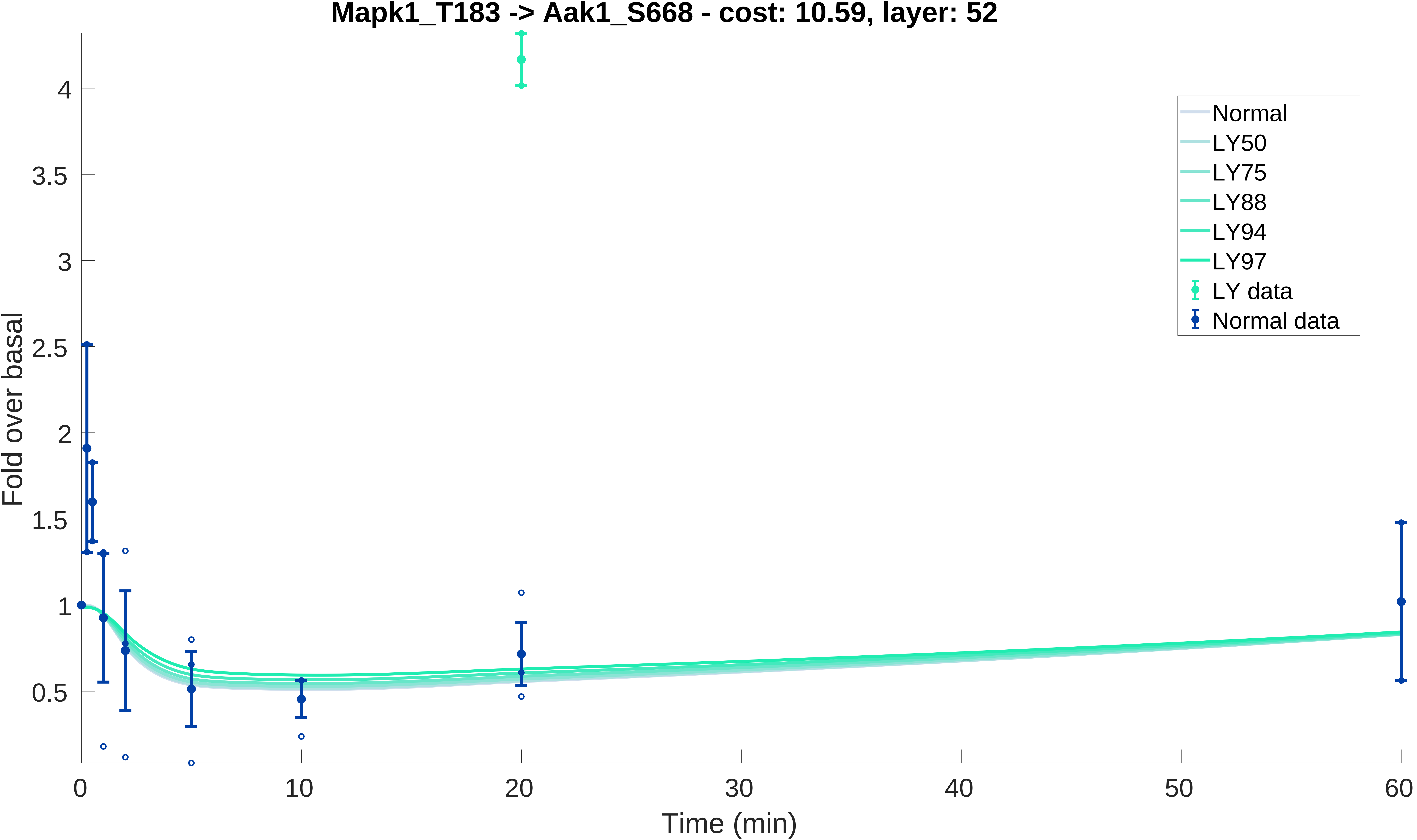

Mapk1_T183 -> Aak1_S668 - cost: 10.59, layer: 52
Fold over basal
Time (min)
0
10
20
30
40
50
60
0.5
1
1.5
2
2.5
3
3.5
4
Normal
LY50
LY75
LY88
LY94
LY97
LY data
Normal data

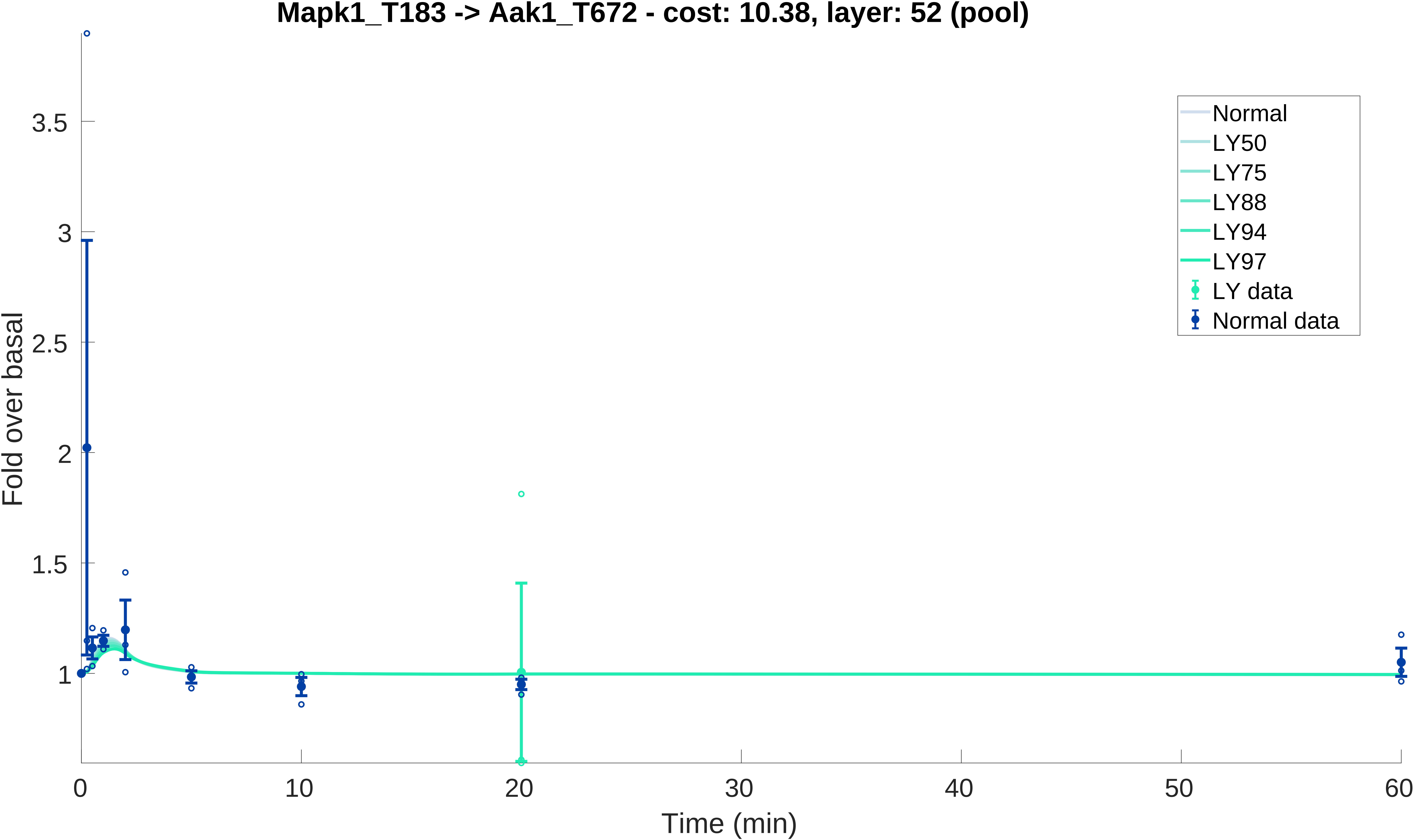
Mapk1_T183 -> Aak1_T672 - cost: 10.38, layer: 52 (pool)
Fold over basal
Time (min)
3.5
3
2.5
2
1.5
1
0
10
20
30
40
50
60
Normal
LY50
LY75
LY88
LY94
LY97
LY data
Normal data

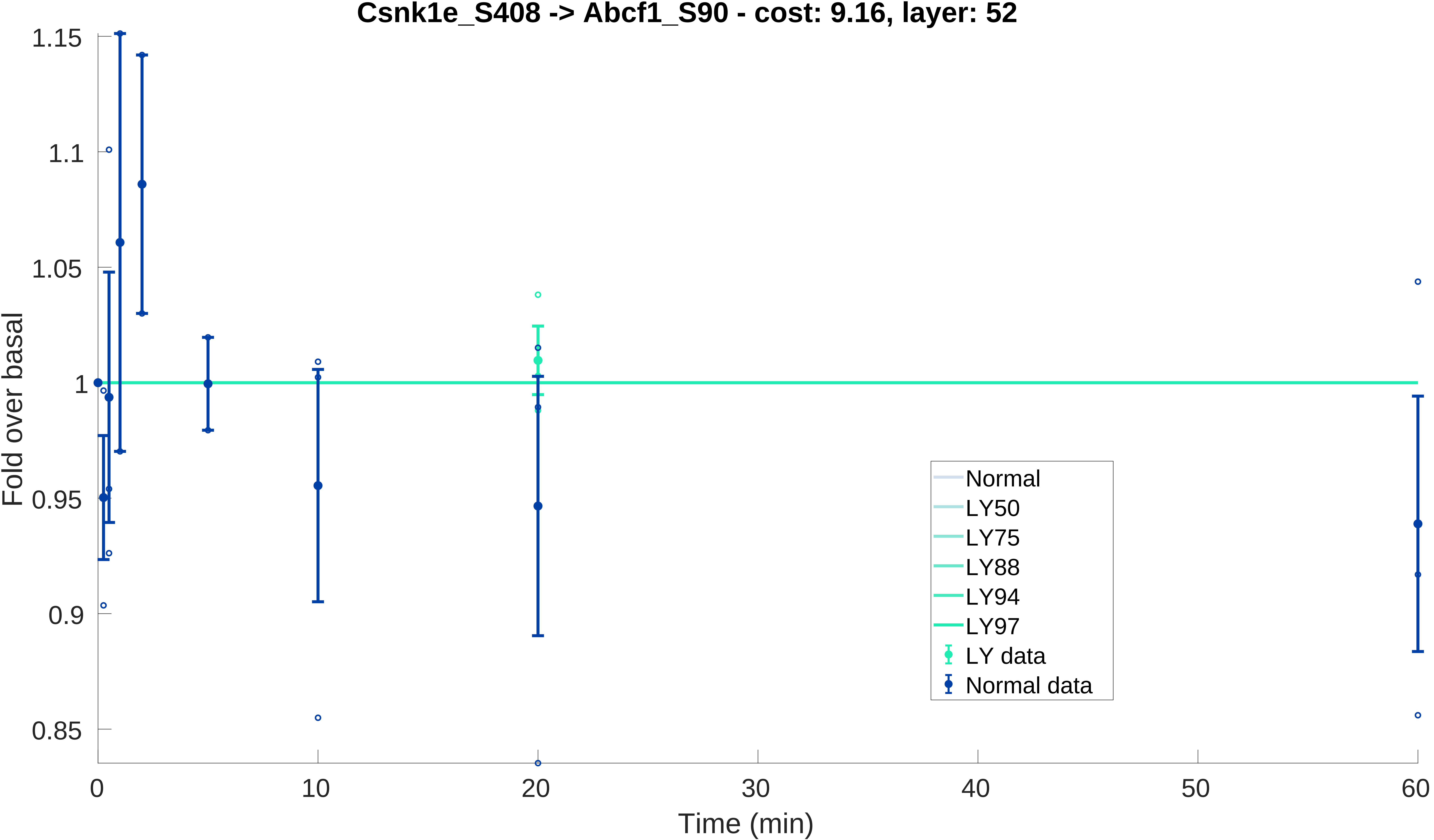

Csnk1e_S408 -> Abcf1_S90 - cost: 9.16, layer: 52
Fold over basal
Time (min)
1.15
1.1
1.05
1
0.95
0.9
0.85
0
10
20
30
40
50
60
Normal
LY50
LY75
LY88
LY94
LY97
LY data
Normal data

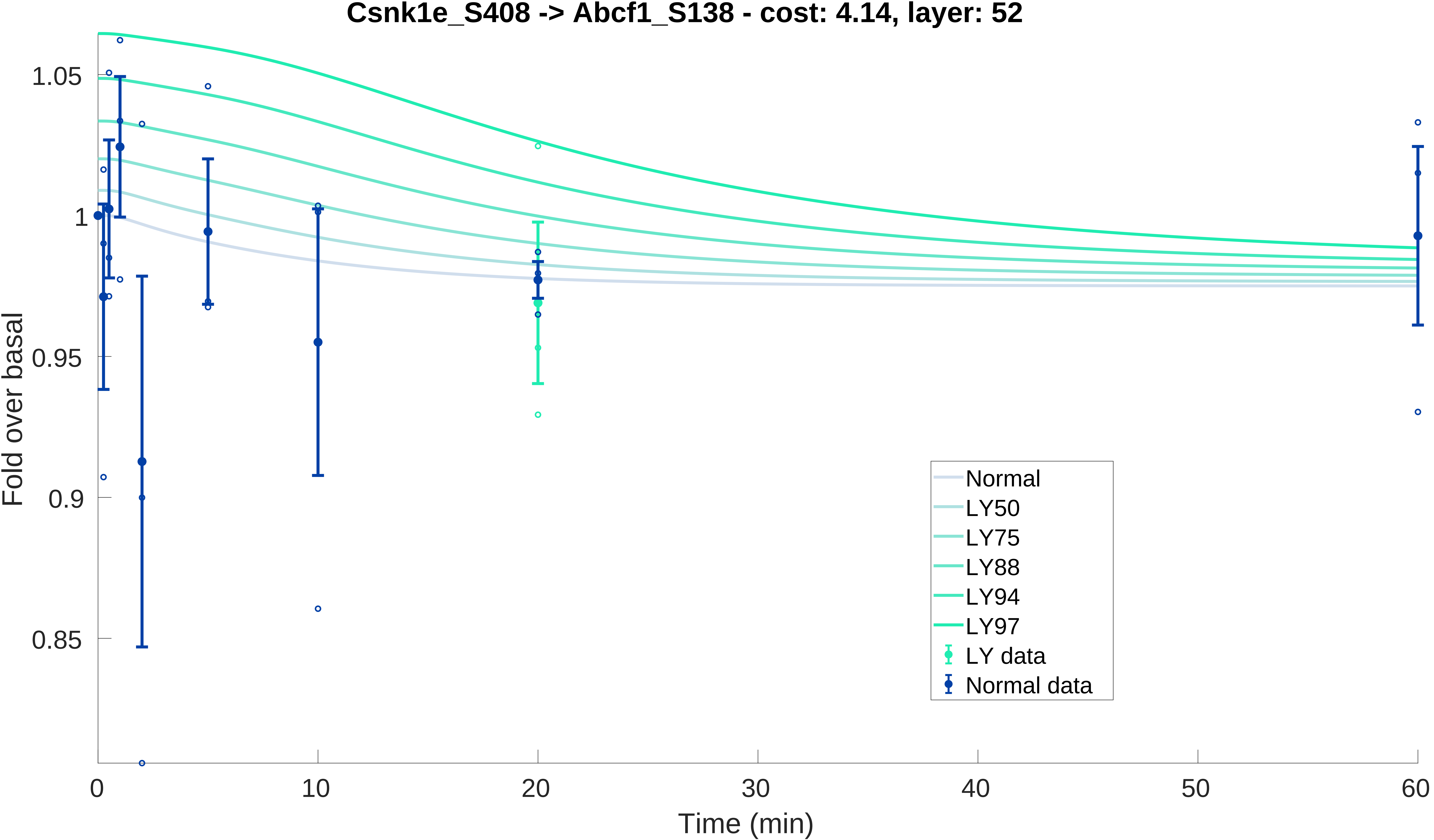
Csnk1e_S408 -> Abcf1_S138 - cost: 4.14, layer: 52
Fold over basal
1.05
1
0.95
0.9
0.85
0
10
20
30
40
50
60
Time (min)
Normal
LY50
LY75
LY88
LY94
LY97
LY data
Normal data

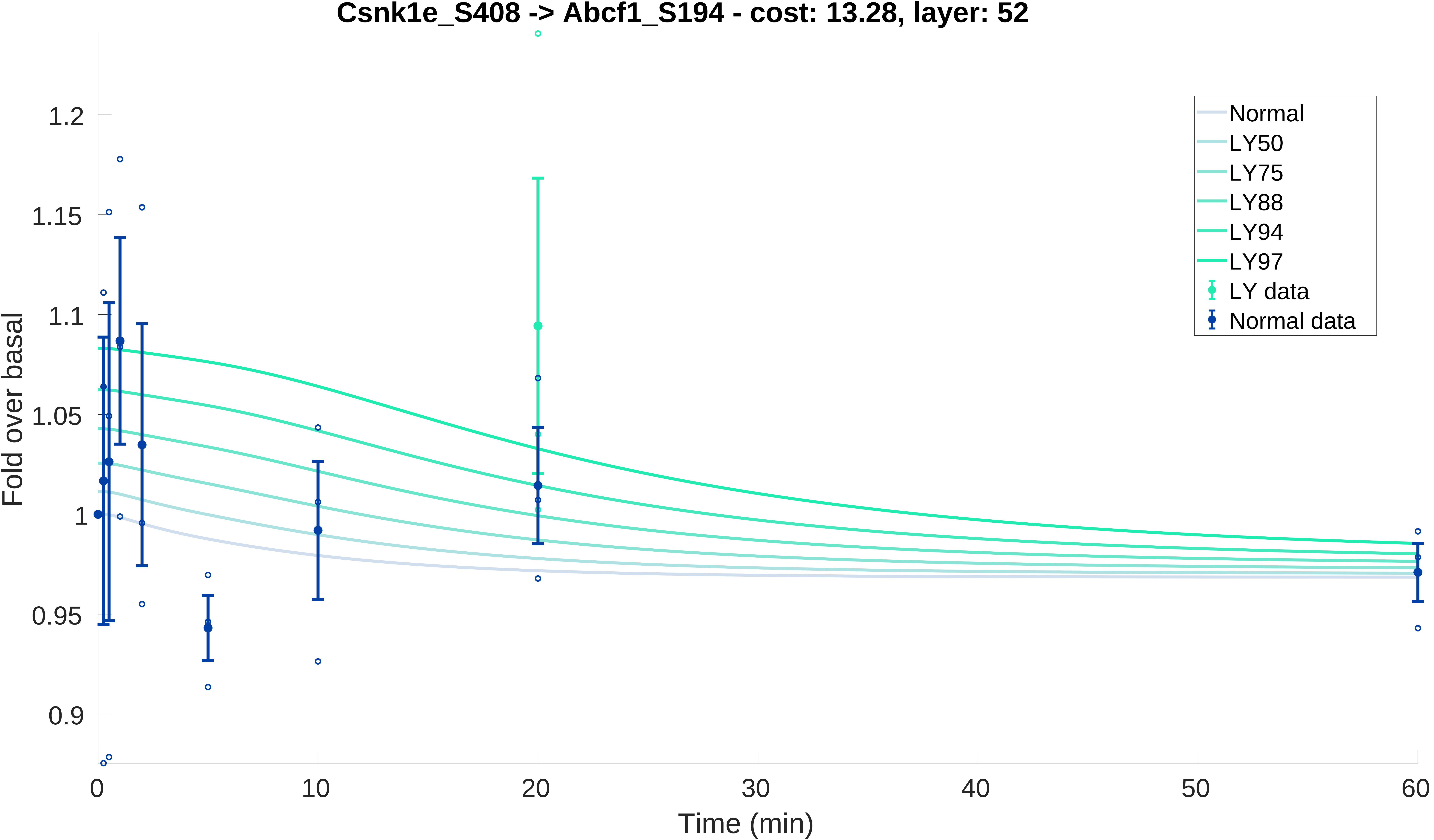

Csnk1e_S408 -> Abcf1_S194 - cost: 13.28, layer: 52
Fold over basal
Time (min)
1.2
1.15
1.1
1.05
1
0.95
0.9
0
10
20
30
40
50
60
Normal
LY50
LY75
LY88
LY94
LY97
LY data
Normal data

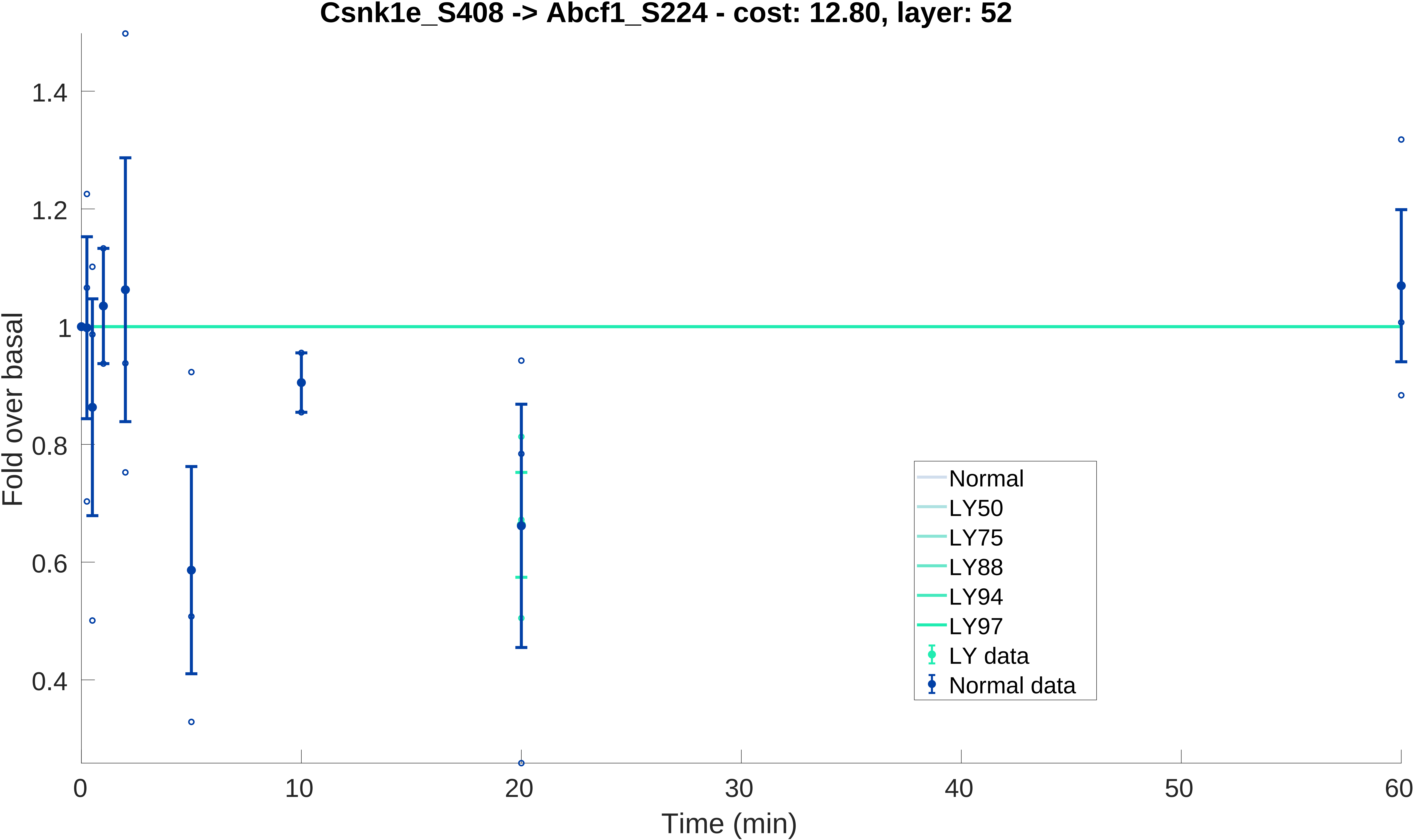

Csnk1e_S408 -> Abcf1_S224 - cost: 12.80, layer: 52
Fold over basal
1.4
1.2
1
0.8
0.6
0.4
0
10
20
30
40
50
60
Time (min)
Normal
LY50
LY75
LY88
LY94
LY97
LY data
Normal data

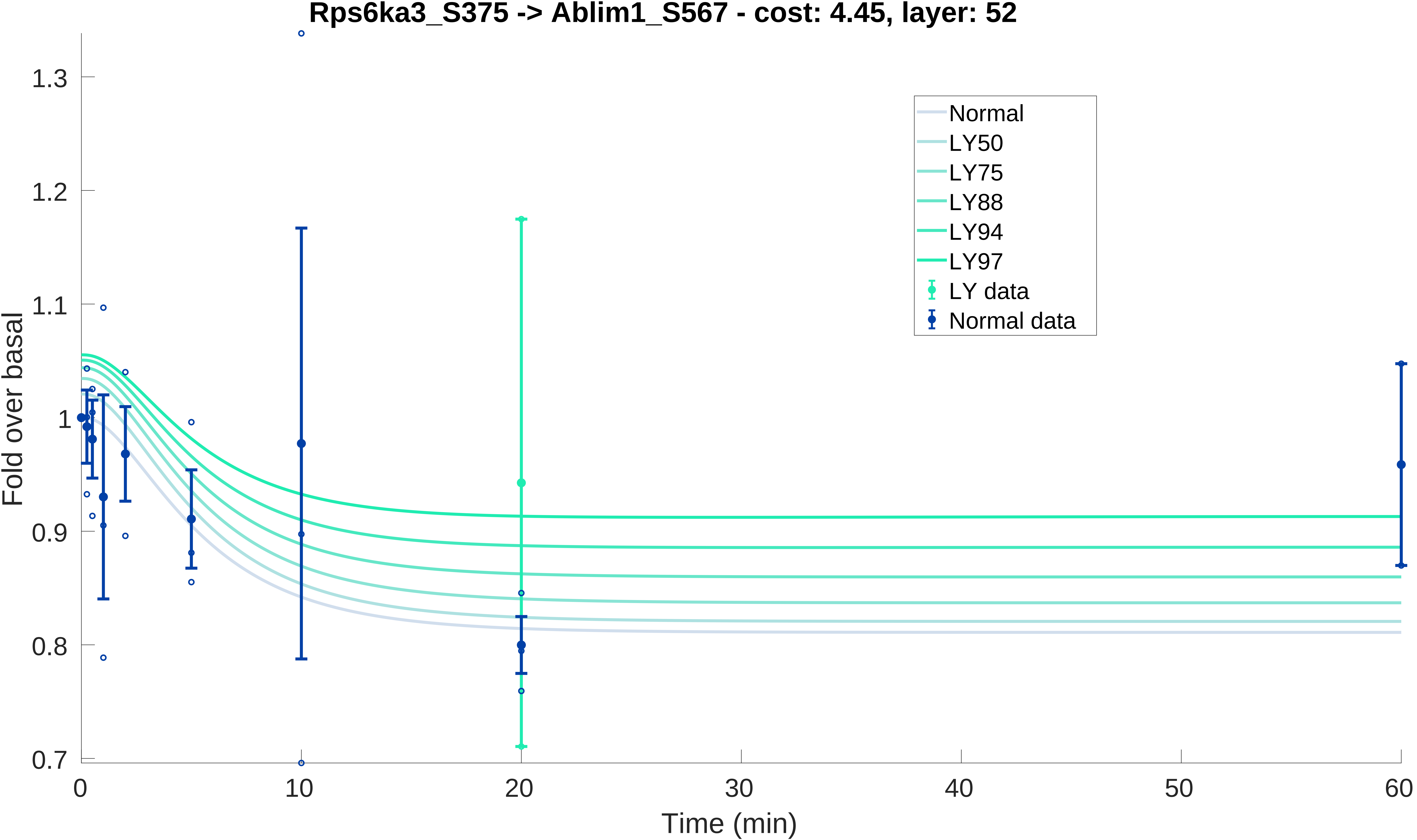
Rps6ka3_S375 -> Ablim1_S567 - cost: 4.45, layer: 52
Fold over basal
1.3
1.2
1.1
1
0.9
0.8
0.7
0
10
20
30
40
50
60
Time (min)
Normal
LY50
LY75
LY88
LY94
LY97
LY data
Normal data

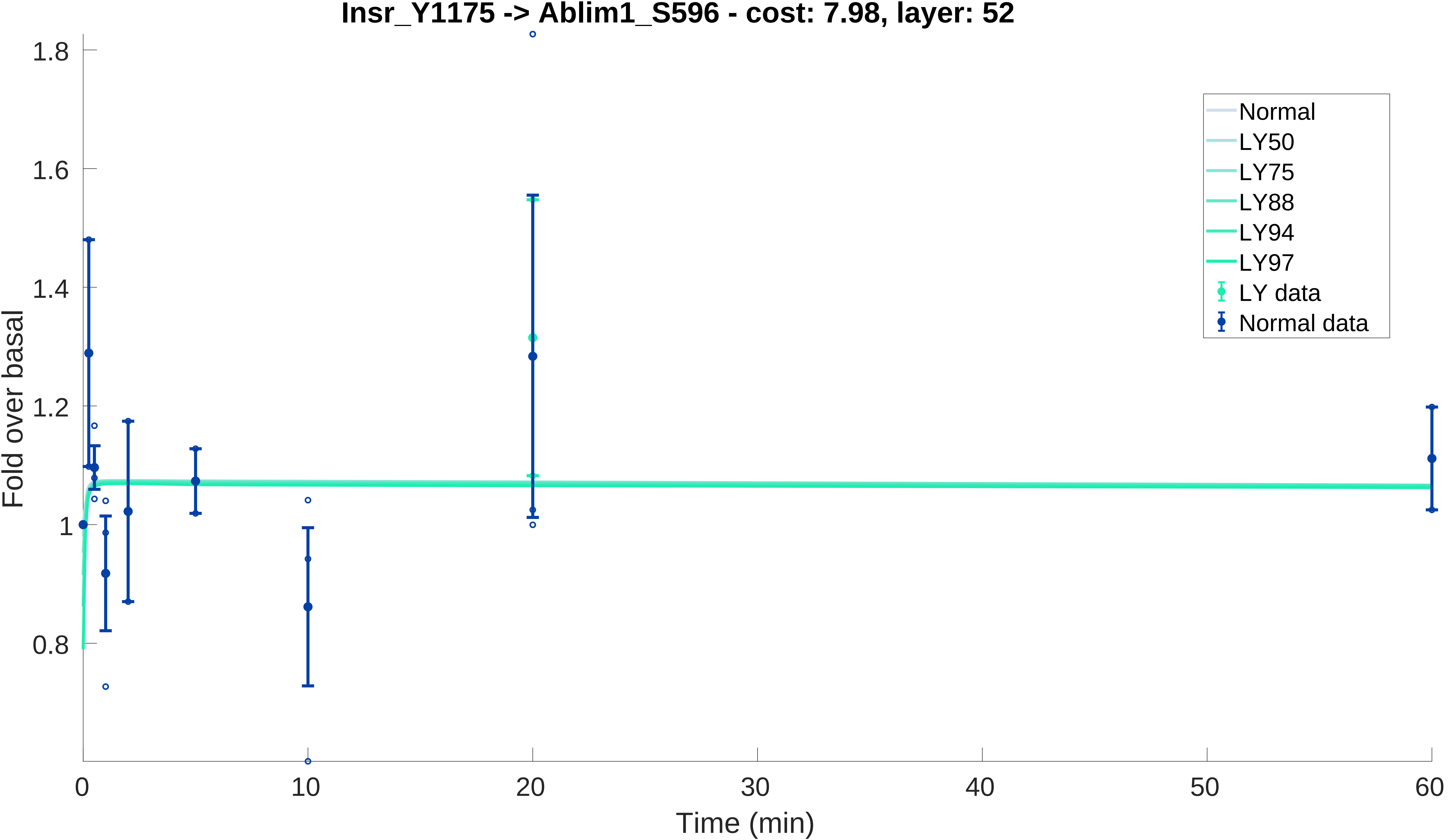

Insr_Y1175 -> Ablim1_S596 - cost: 7.98, layer: 52
Fold over basal
1.8
1.6
1.4
1.2
1
0.8
0
10
20
30
40
50
60
Time (min)
Normal
LY50
LY75
LY88
LY94
LY97
LY data
Normal data

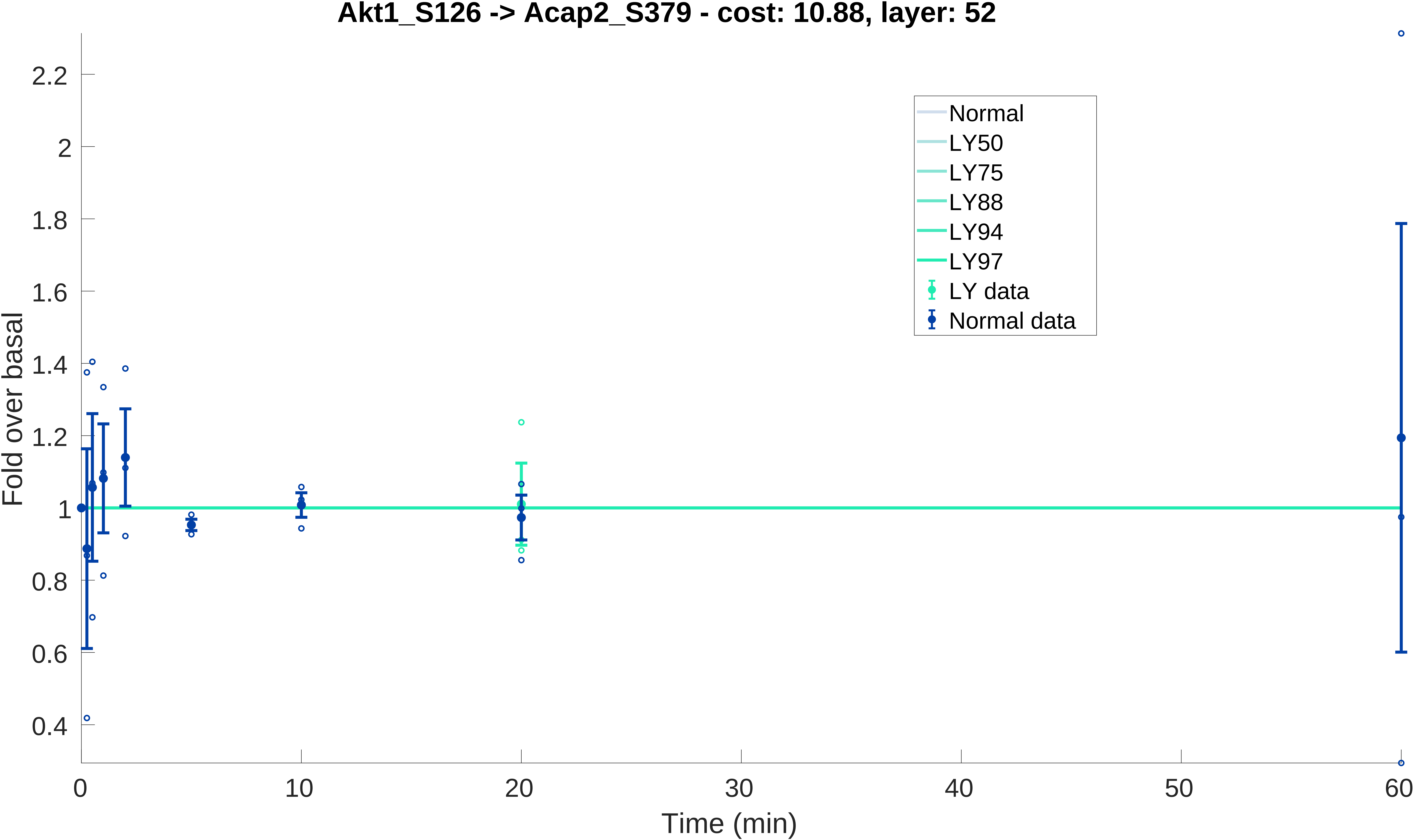

Akt1_S126 -> Acap2_S379 - cost: 10.88, layer: 52
Fold over basal
Time (min)
2.2
2
1.8
1.6
1.4
1.2
1
0.8
0.6
0.4
0
10
20
30
40
50
60
Normal
LY50
LY75
LY88
LY94
LY97
LY data
Normal data

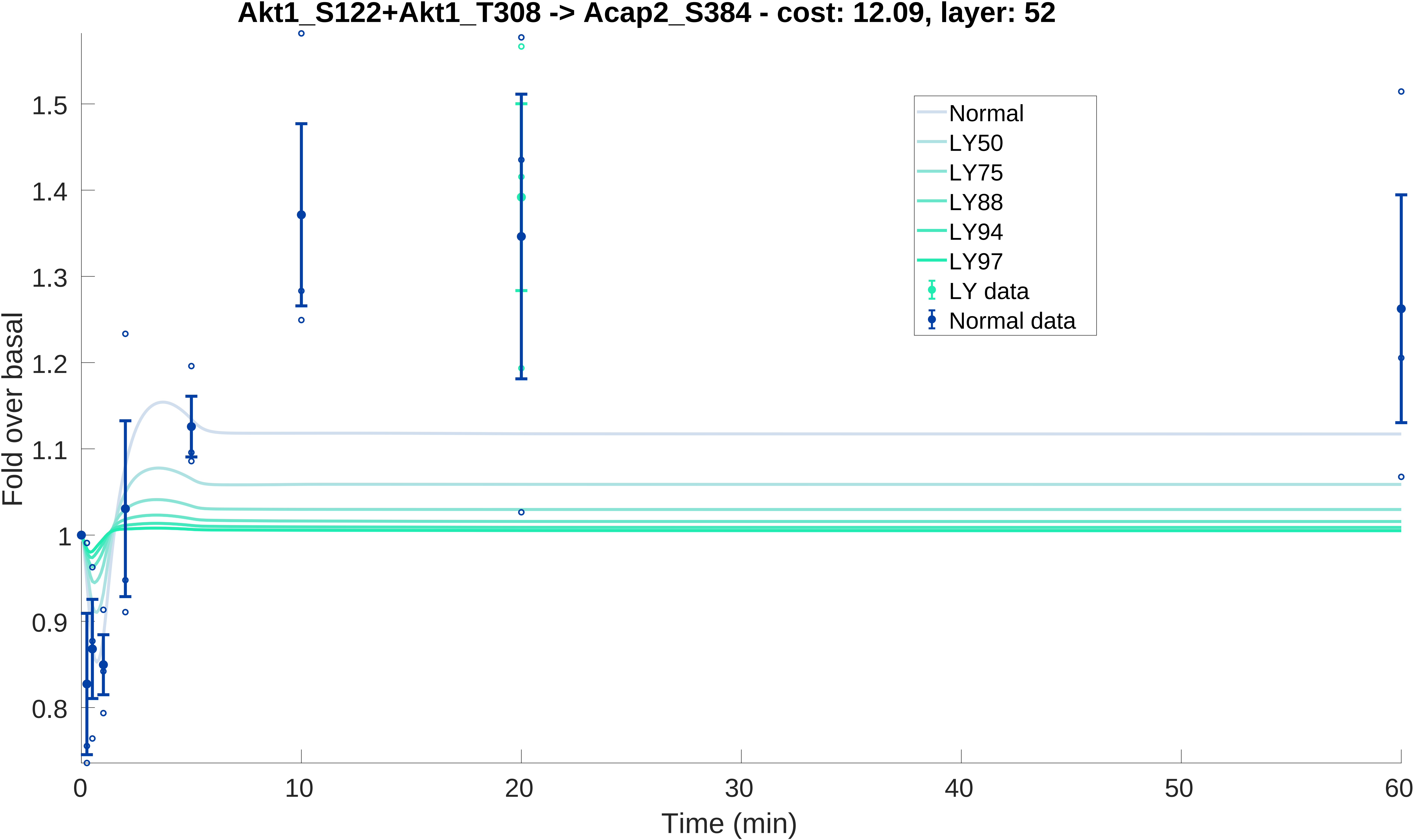

Akt1_S122+Akt1_T308 -> Acap2_S384 - cost: 12.09, layer: 52
Fold over basal
Time (min)
Normal
LY50
LY75
LY88
LY94
LY97
LY data
Normal data
1.5
1.4
1.3
1.2
1.1
1
0.9
0.8
0
10
20
30
40
50
60

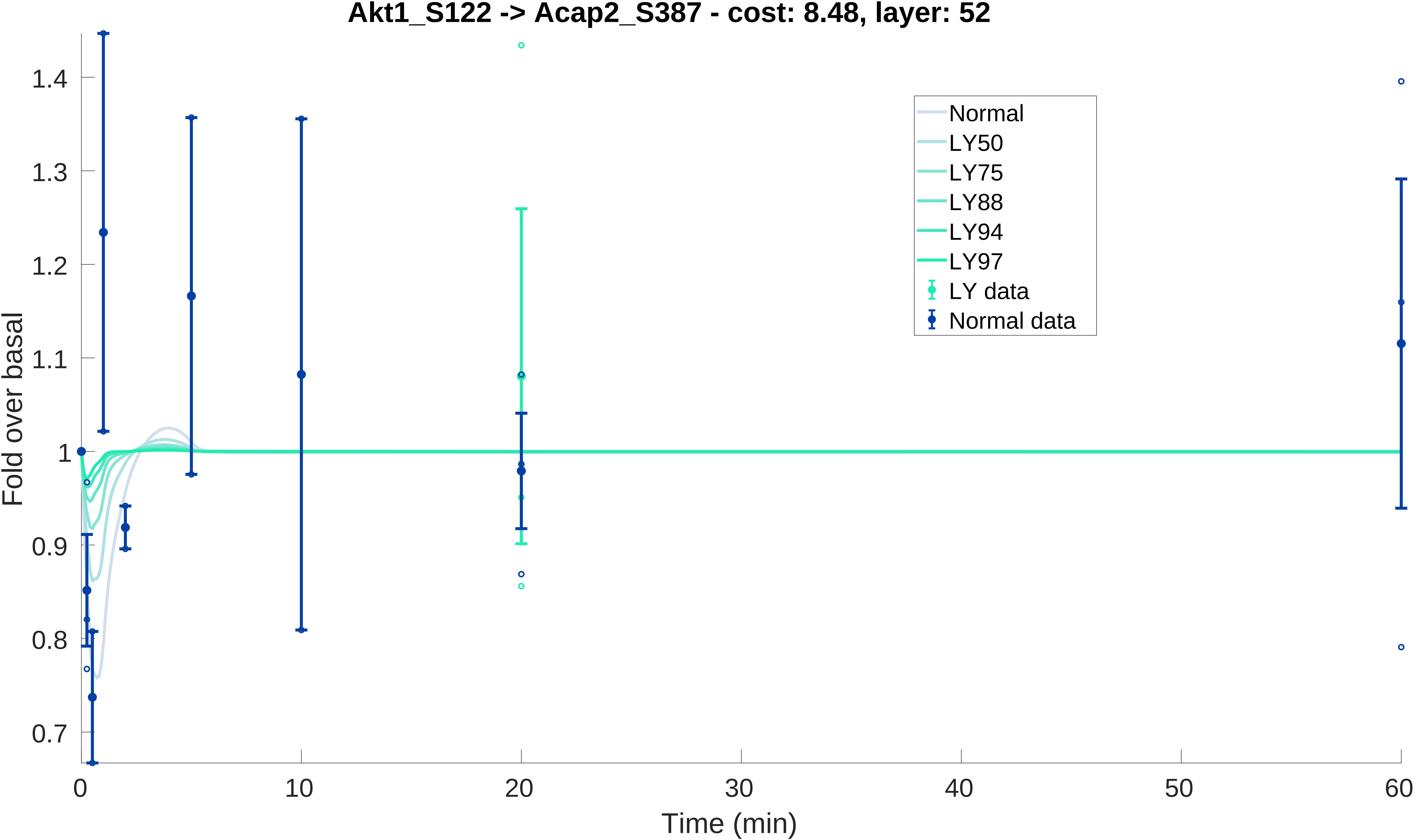
Akt1_S122 -> Acap2_S387 - cost: 8.48, layer: 52
Fold over basal
Time (min)
Normal
LY50
LY75
LY88
LY94
LY97
LY data
Normal data
0.7
0.8
0.9
1
1.1
1.2
1.3
1.4
0
10
20
30
40
50
60

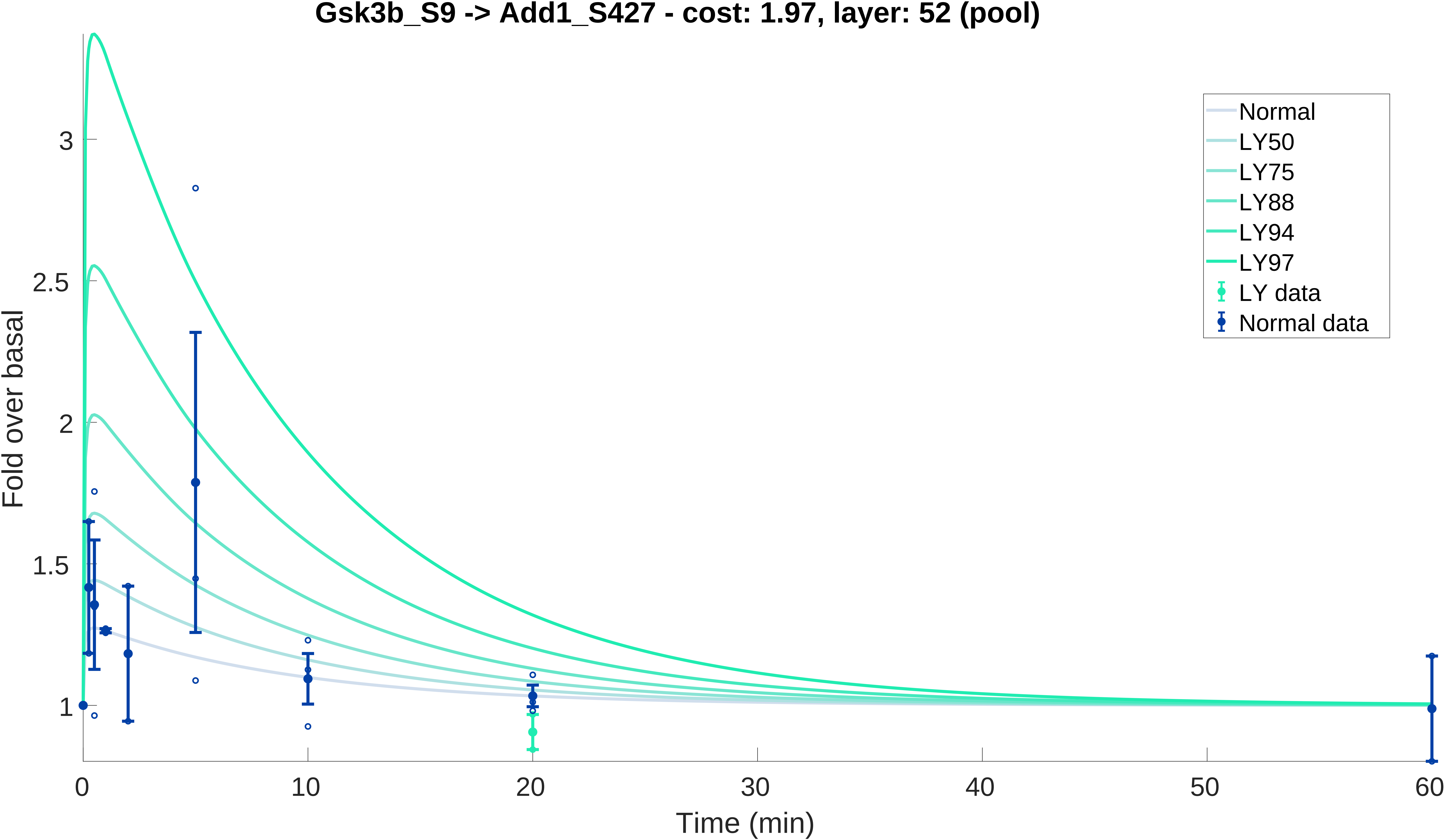
Gsk3b_S9 -> Add1_S427 - cost: 1.97, layer: 52 (pool)
Fold over basal
Time (min)
0
10
20
30
40
50
60
1
1.5
2
2.5
3
Normal
LY50
LY75
LY88
LY94
LY97
LY data
Normal data

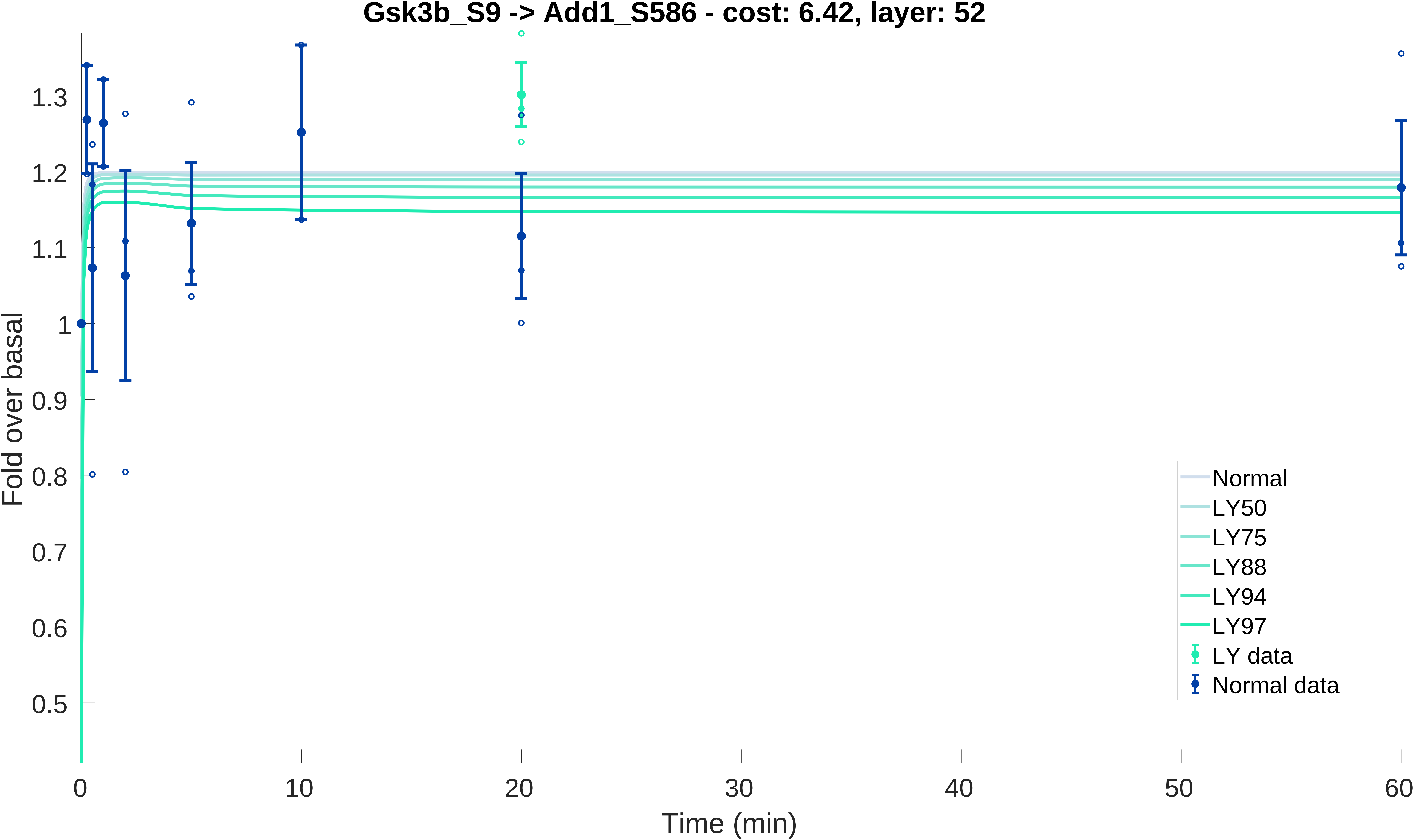

Gsk3b_S9 -> Add1_S586 - cost: 6.42, layer: 52
Fold over basal
1.3
1.2
1.1
1
0.9
0.8
0.7
0.6
0.5
0
10
20
30
40
50
60
Time (min)
Normal
LY50
LY75
LY88
LY94
LY97
LY data
Normal data

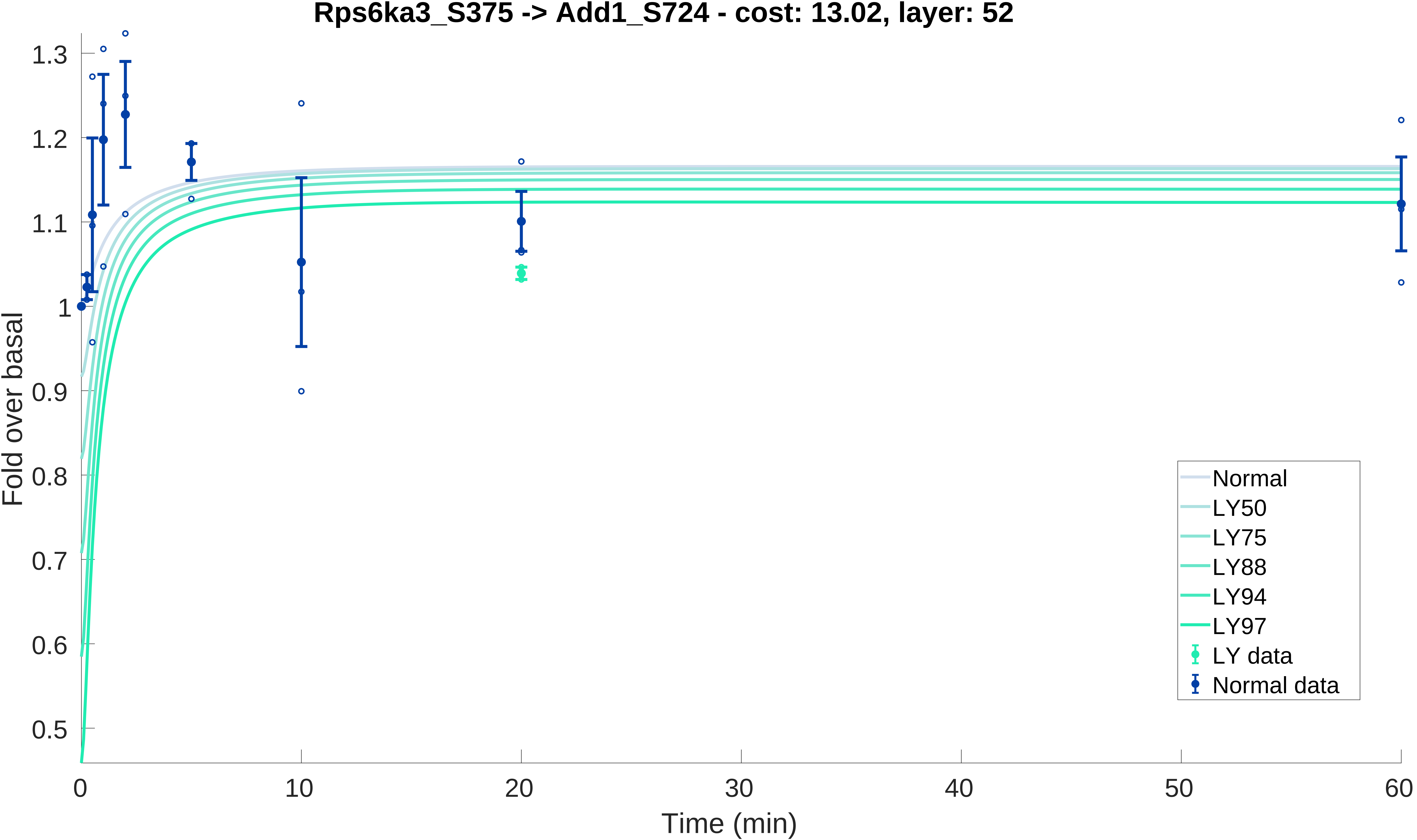
Rps6ka3_S375 -> Add1_S724 - cost: 13.02, layer: 52
Fold over basal
Time (min)
0
10
20
30
40
50
60
0.5
0.6
0.7
0.8
0.9
1
1.1
1.2
1.3
Normal
LY50
LY75
LY88
LY94
LY97
LY data
Normal data

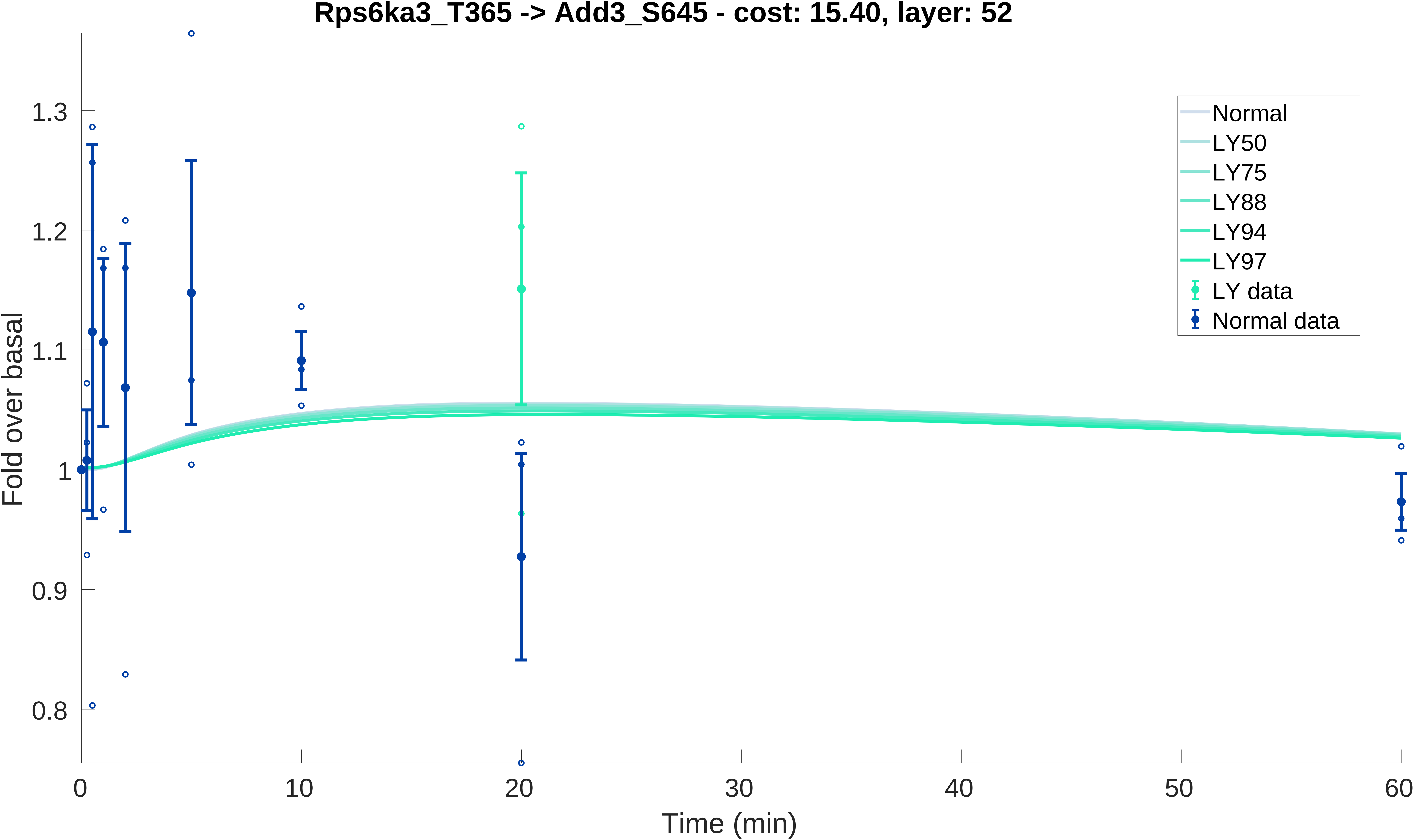
Rps6ka3_T365 -> Add3_S645 - cost: 15.40, layer: 52
Fold over basal
Time (min)
0.8
0.9
1
1.1
1.2
1.3
0
10
20
30
40
50
60
Normal
LY50
LY75
LY88
LY94
LY97
LY data
Normal data

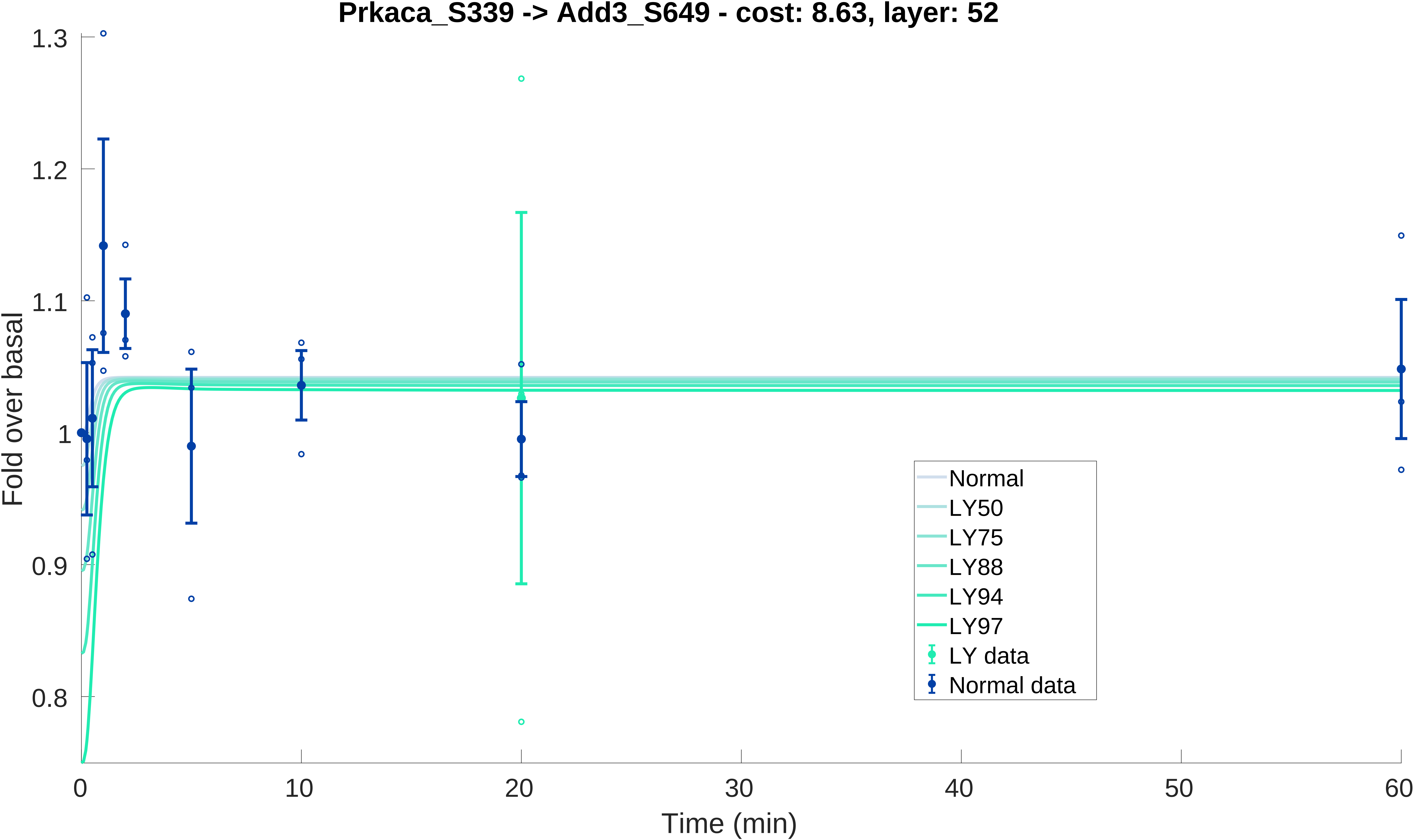

Prkaca_S339 -> Add3_S649 - cost: 8.63, layer: 52
Fold over basal
Time (min)
1.3
1.2
1.1
1
0.9
0.8
0
10
20
30
40
50
60
Normal
LY50
LY75
LY88
LY94
LY97
LY data
Normal data

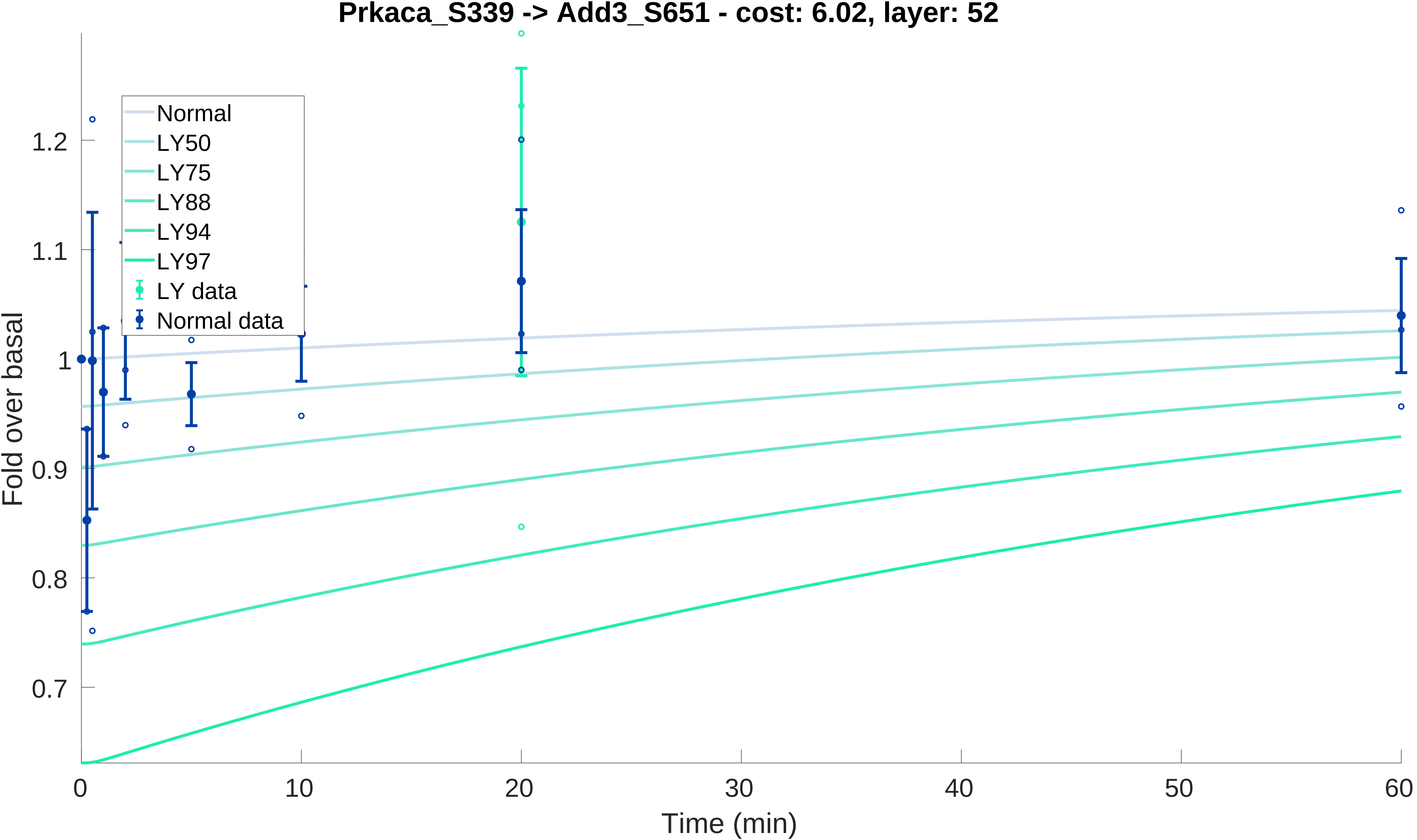
Prkaca_S339 -> Add3_S651 - cost: 6.02, layer: 52
Normal
LY50
LY75
LY88
LY94
LY97
LY data
Normal data
Fold over basal
Time (min)
0.7
0.8
0.9
1
1.1
1.2
0
10
20
30
40
50
60

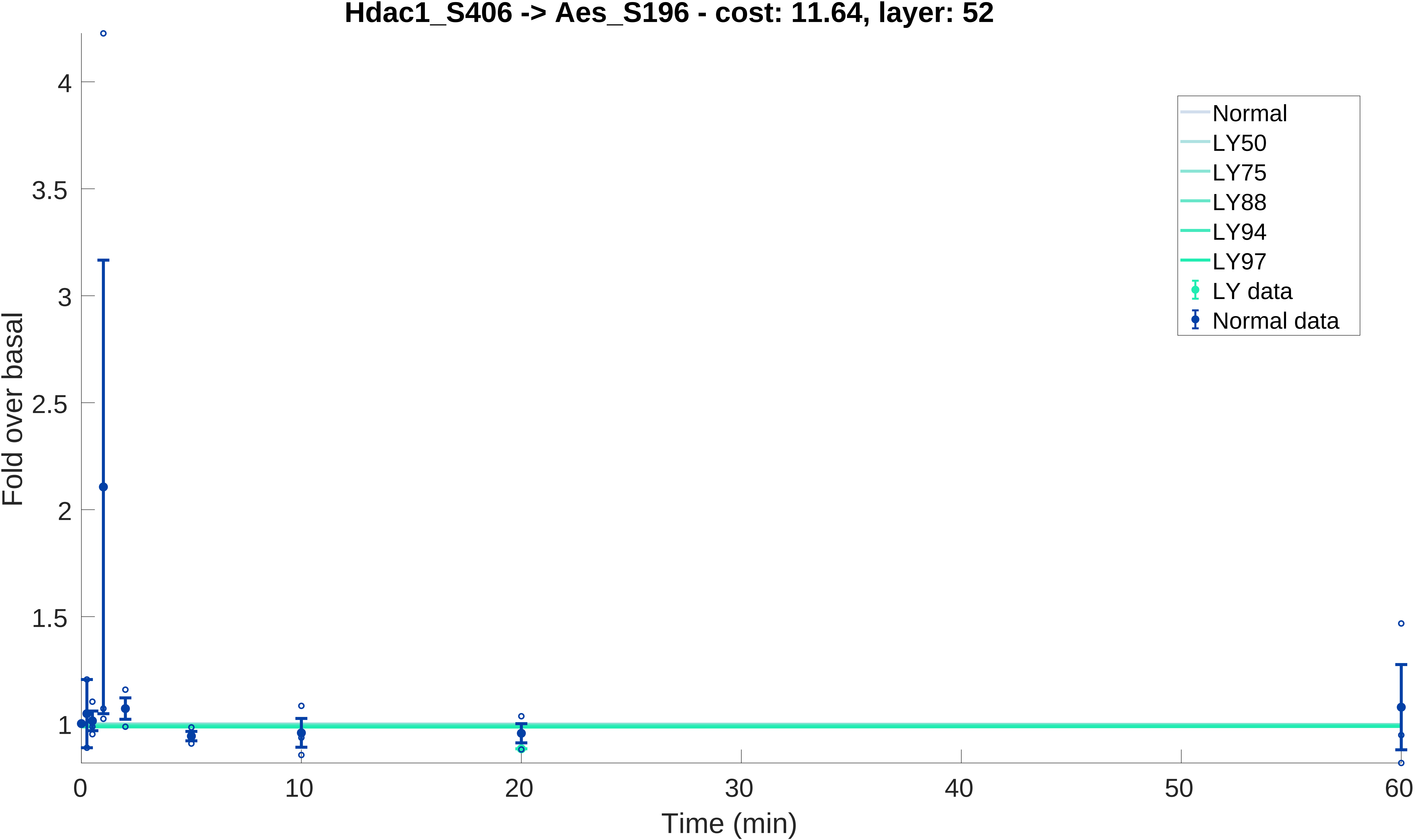
Hdac1_S406 -> Aes_S196 - cost: 11.64, layer: 52
Fold over basal
Time (min)
4
3.5
3
2.5
2
1.5
1
0
10
20
30
40
50
60
Normal
LY50
LY75
LY88
LY94
LY97
LY data
Normal data

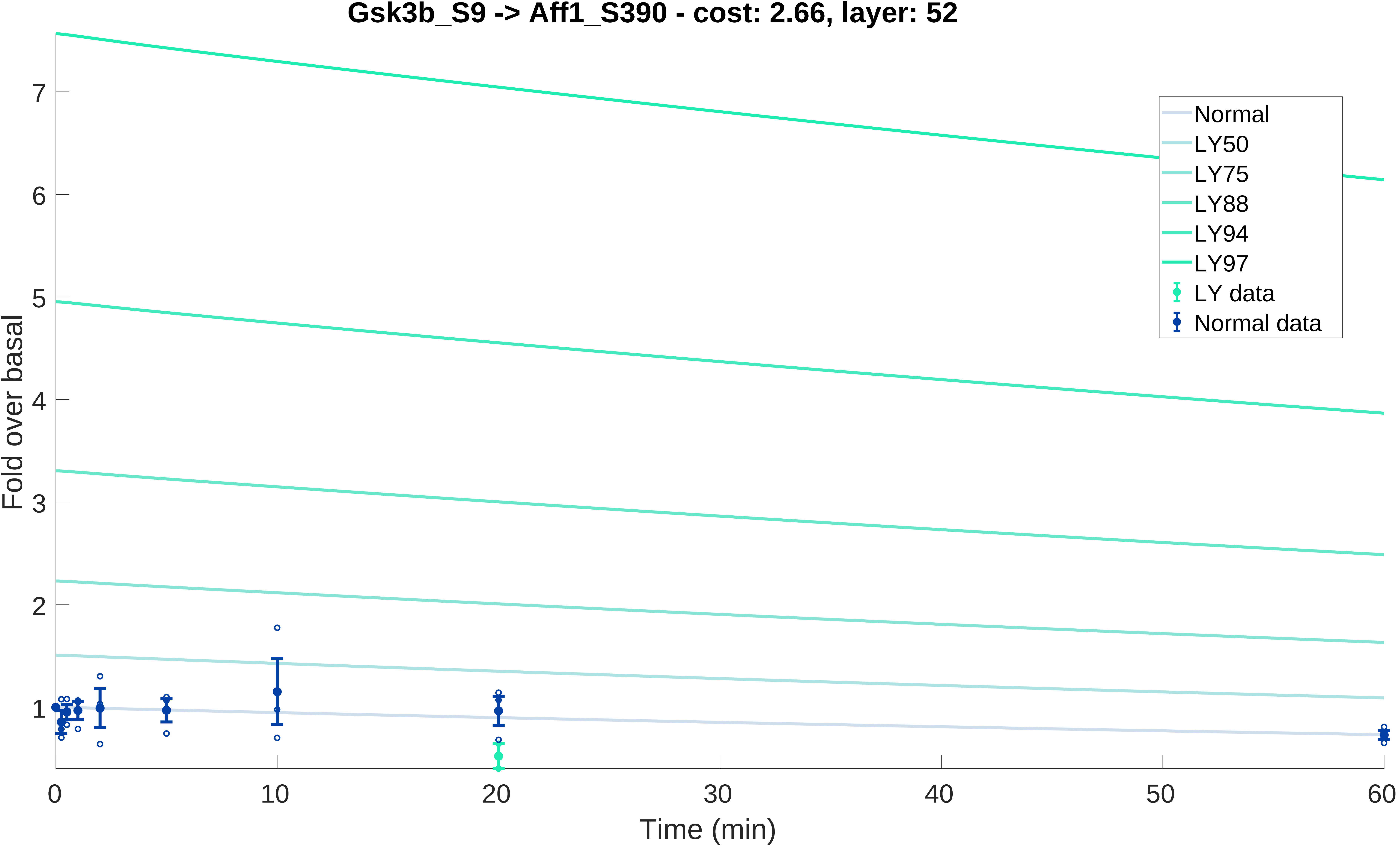

Gsk3b_S9 -> Aff1_S390 - cost: 2.66, layer: 52
Fold over basal
Time (min)
0
10
20
30
40
50
60
1
2
3
4
5
6
7
Normal
LY50
LY75
LY88
LY94
LY97
LY data
Normal data

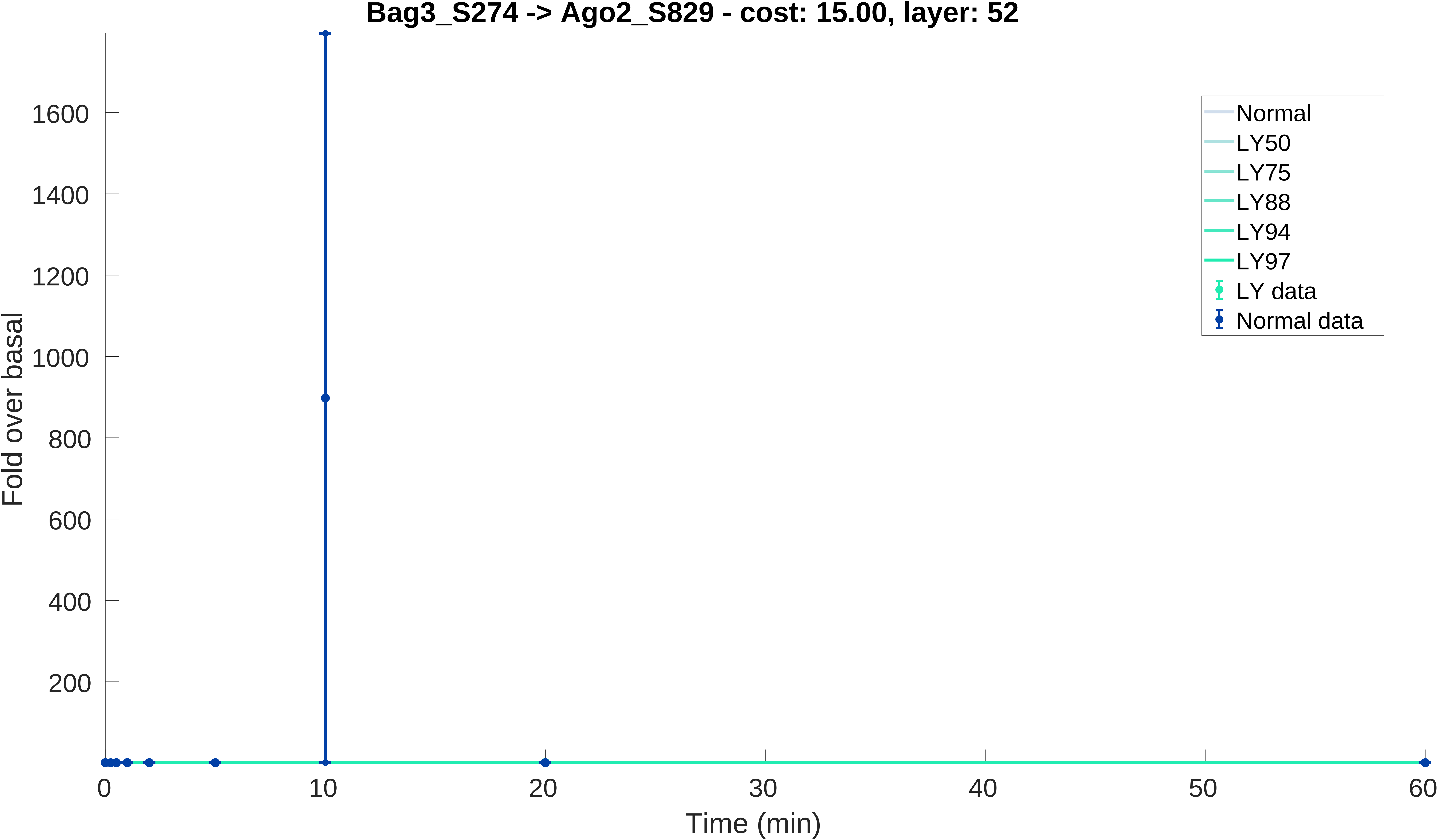

Bag3_S274 -> Ago2_S829 - cost: 15.00, layer: 52
Fold over basal
1600
1400
1200
1000
800
600
400
200
0
10
20
30
40
50
60
Time (min)
Normal
LY50
LY75
LY88
LY94
LY97
LY data
Normal data

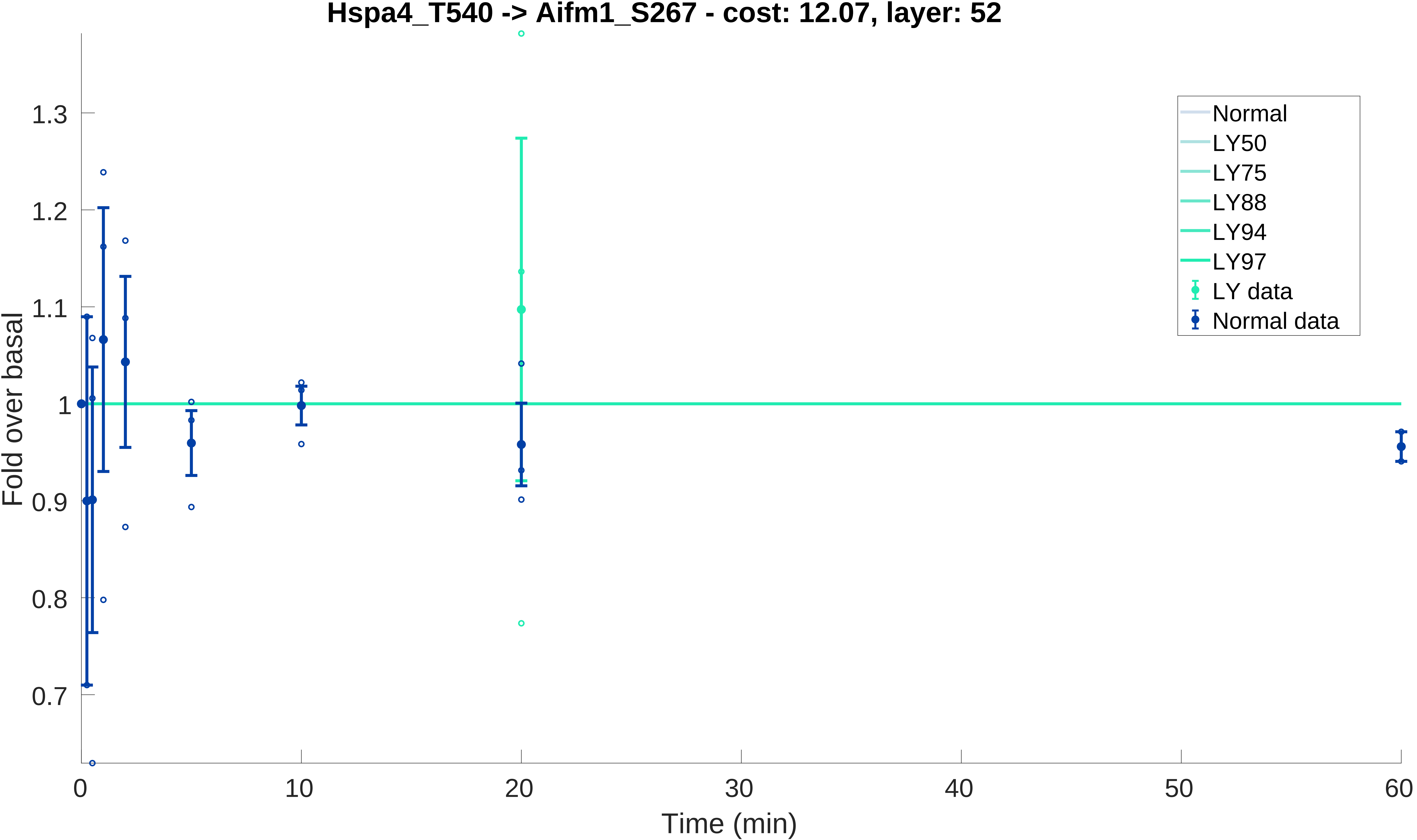

Hspa4_T540 -> Aifm1_S267 - cost: 12.07, layer: 52
Fold over basal
Time (min)
1.3
1.2
1.1
1
0.9
0.8
0.7
0
10
20
30
40
50
60
Normal
LY50
LY75
LY88
LY94
LY97
LY data
Normal data

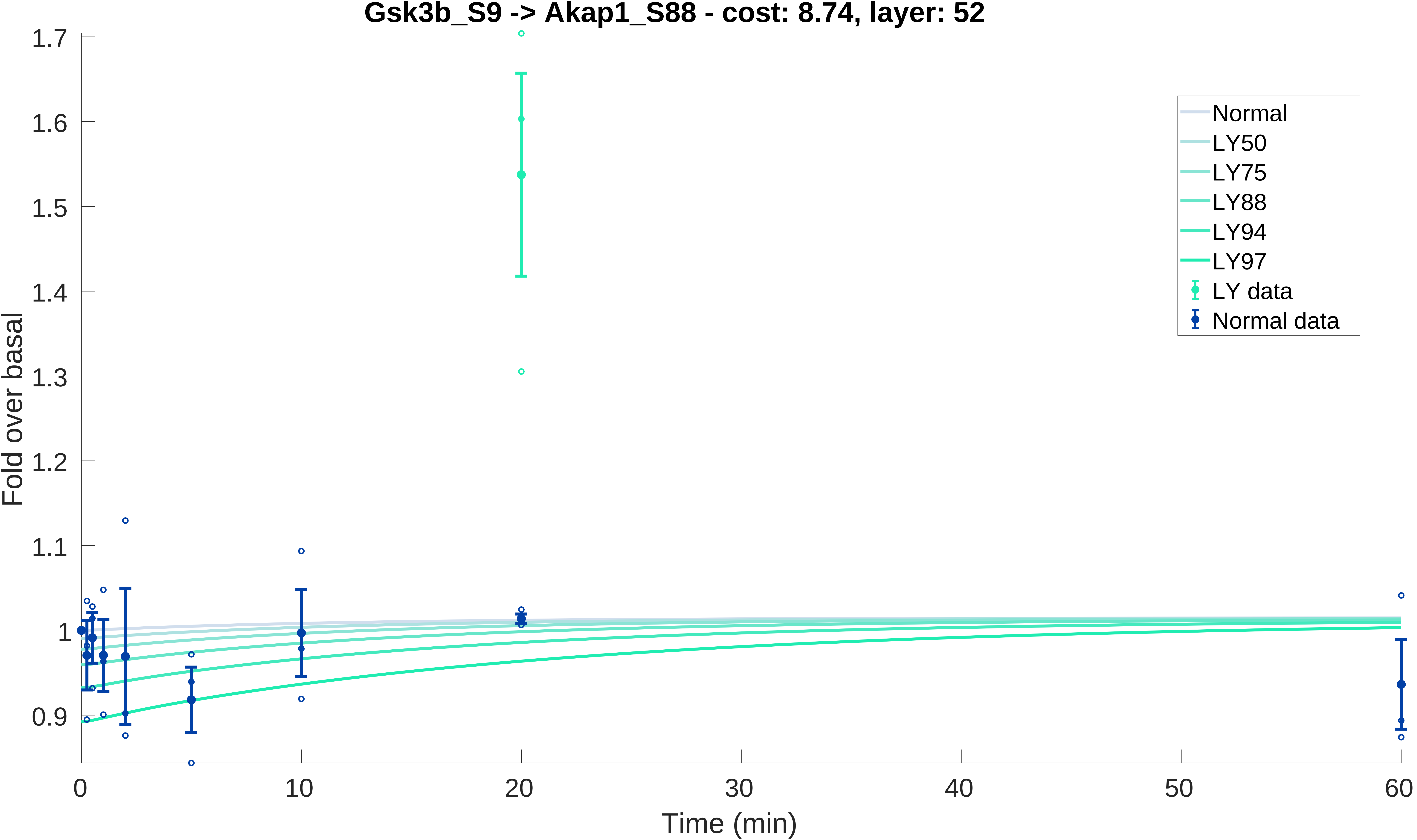

Gsk3b_S9 -> Akap1_S88 - cost: 8.74, layer: 52
Fold over basal
Time (min)
1.7
1.6
1.5
1.4
1.3
1.2
1.1
1
0.9
0
10
20
30
40
50
60
Normal
LY50
LY75
LY88
LY94
LY97
LY data
Normal data

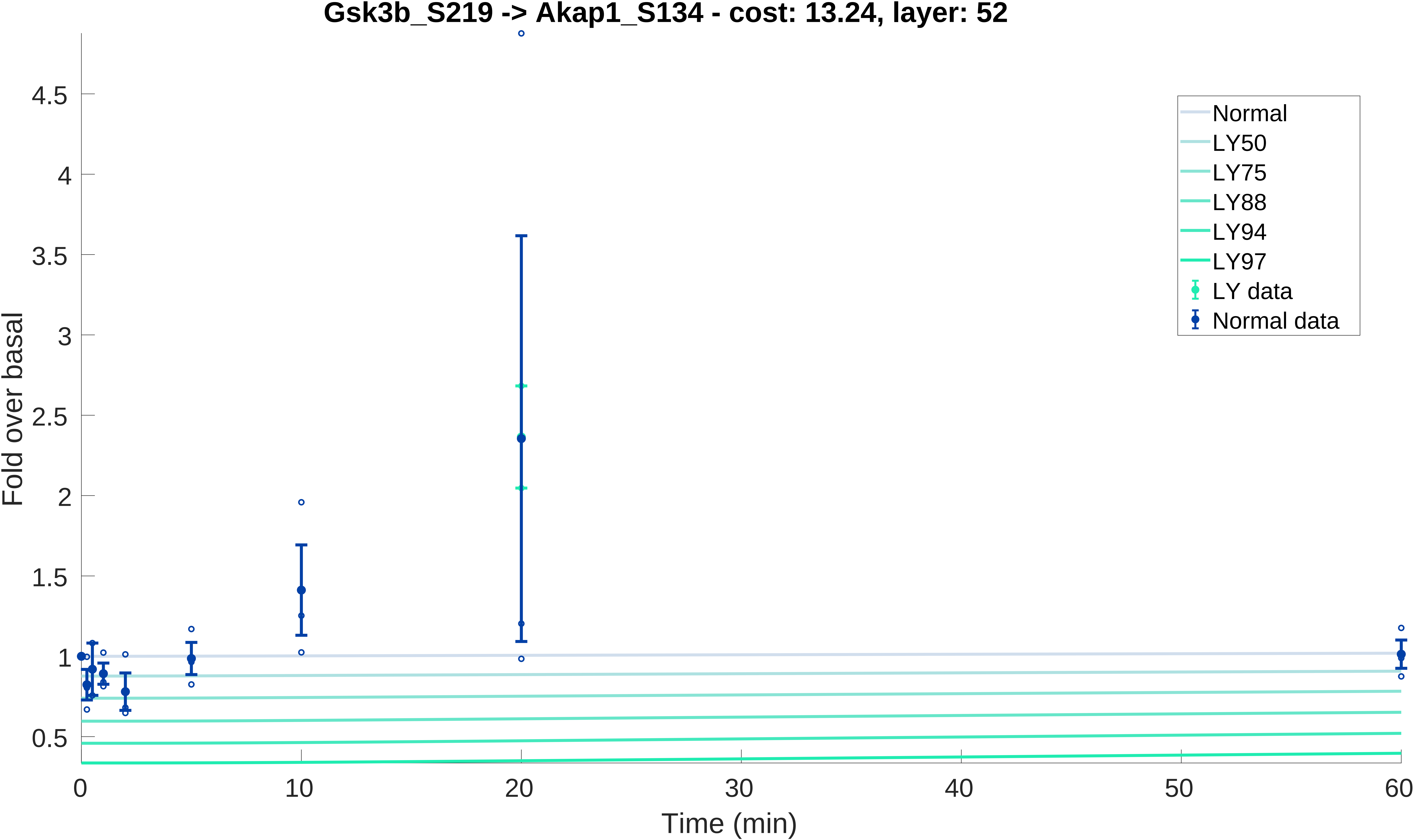
Gsk3b_S219 -> Akap1_S134 - cost: 13.24, layer: 52
Fold over basal
0.5
1
1.5
2
2.5
3
3.5
4
4.5
Time (min)
0
10
20
30
40
50
60
Normal
LY50
LY75
LY88
LY94
LY97
LY data
Normal data

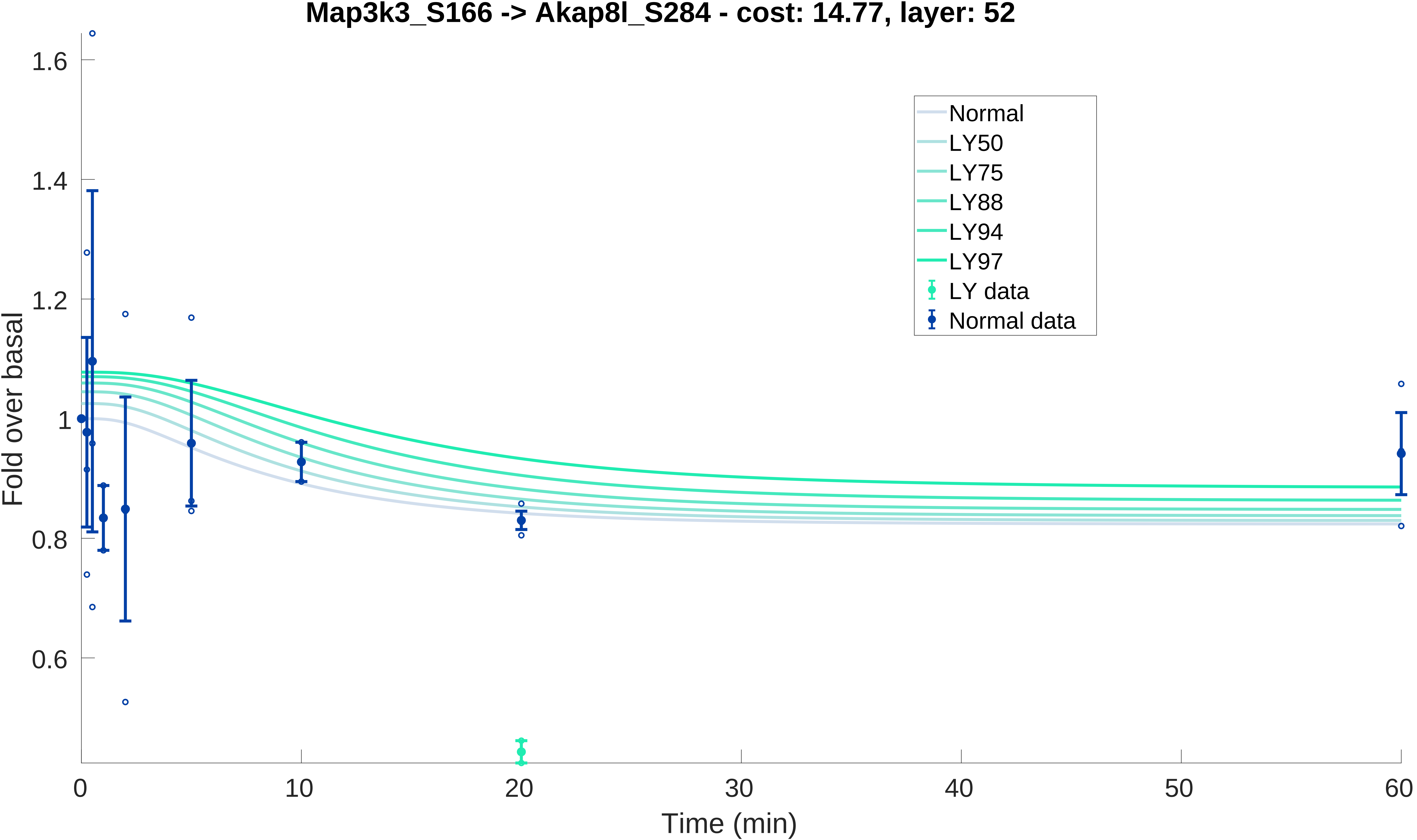

Map3k3_S166 -> Akap8l_S284 - cost: 14.77, layer: 52
Fold over basal
Time (min)
1.6
1.4
1.2
1
0.8
0.6
0
10
20
30
40
50
60
Normal
LY50
LY75
LY88
LY94
LY97
LY data
Normal data

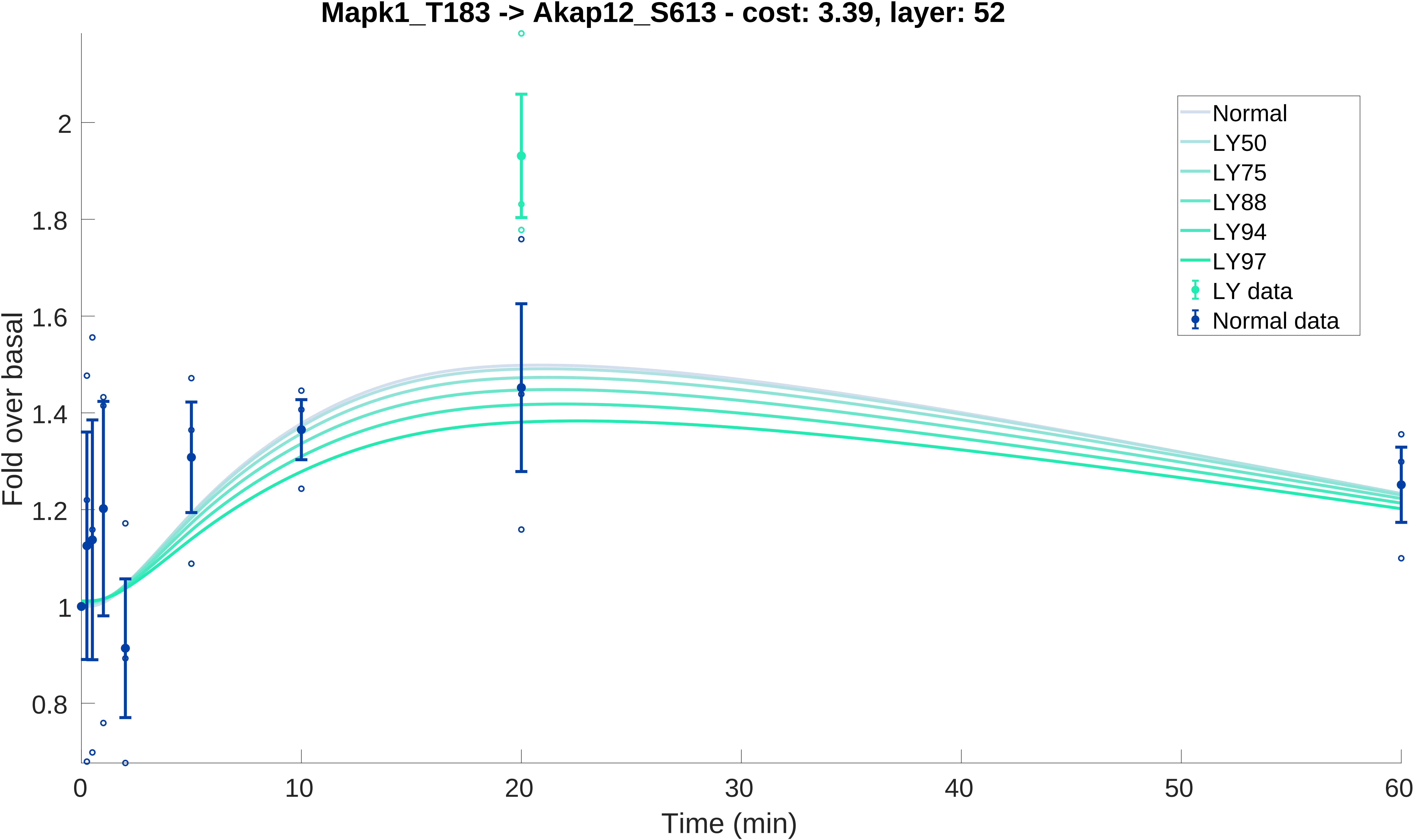
Mapk1_T183 -> Akap12_S613 - cost: 3.39, layer: 52
Fold over basal
Time (min)
Normal
LY50
LY75
LY88
LY94
LY97
LY data
Normal data
0.8
1
1.2
1.4
1.6
1.8
2
0
10
20
30
40
50
60

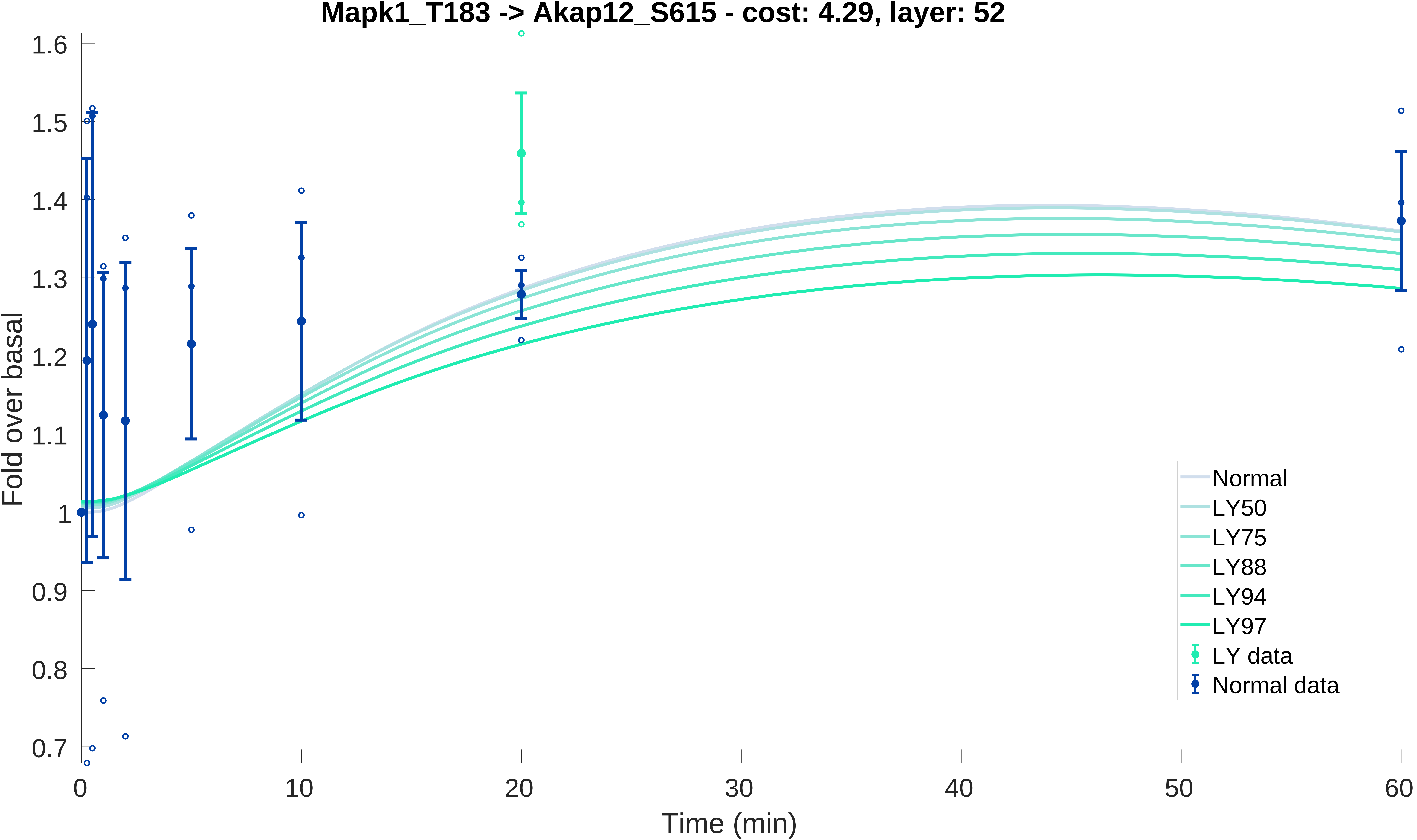

Mapk1_T183 -> Akap12_S615 - cost: 4.29, layer: 52
Fold over basal
1.6
1.5
1.4
1.3
1.2
1.1
1
0.9
0.8
0.7
0
10
20
30
40
50
60
Time (min)
Normal
LY50
LY75
LY88
LY94
LY97
LY data
Normal data

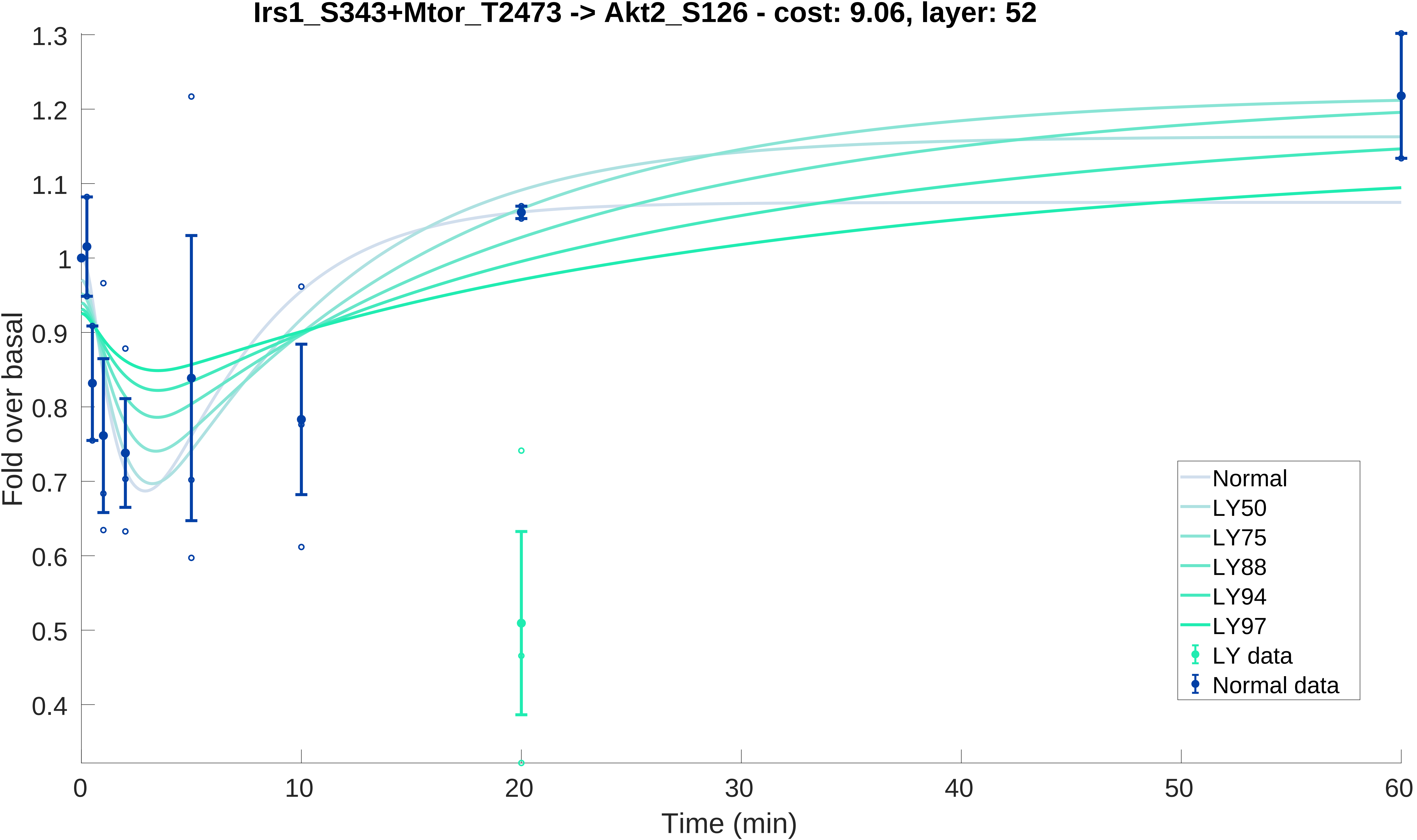
Irs1_S343+Mtor_T2473 -> Akt2_S126 - cost: 9.06, layer: 52
Fold over basal
Time (min)
1.3
1.2
1.1
1
0.9
0.8
0.7
0.6
0.5
0.4
0
10
20
30
40
50
60
Normal
LY50
LY75
LY88
LY94
LY97
LY data
Normal data

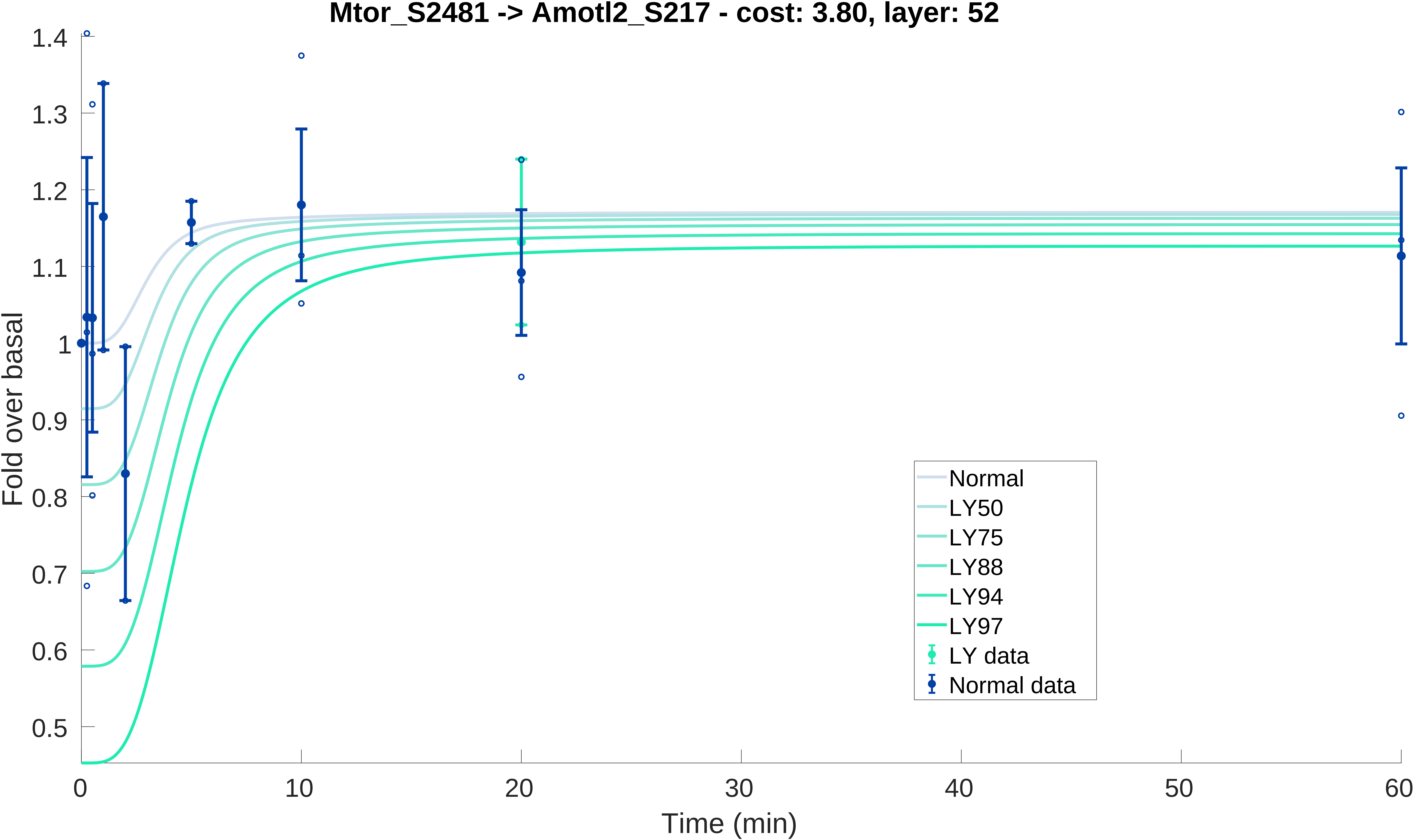

Mtor_S2481 -> Amotl2_S217 - cost: 3.80, layer: 52
Fold over basal
Time (min)
1.4
1.3
1.2
1.1
1
0.9
0.8
0.7
0.6
0.5
0
10
20
30
40
50
60
Normal
LY50
LY75
LY88
LY94
LY97
LY data
Normal data

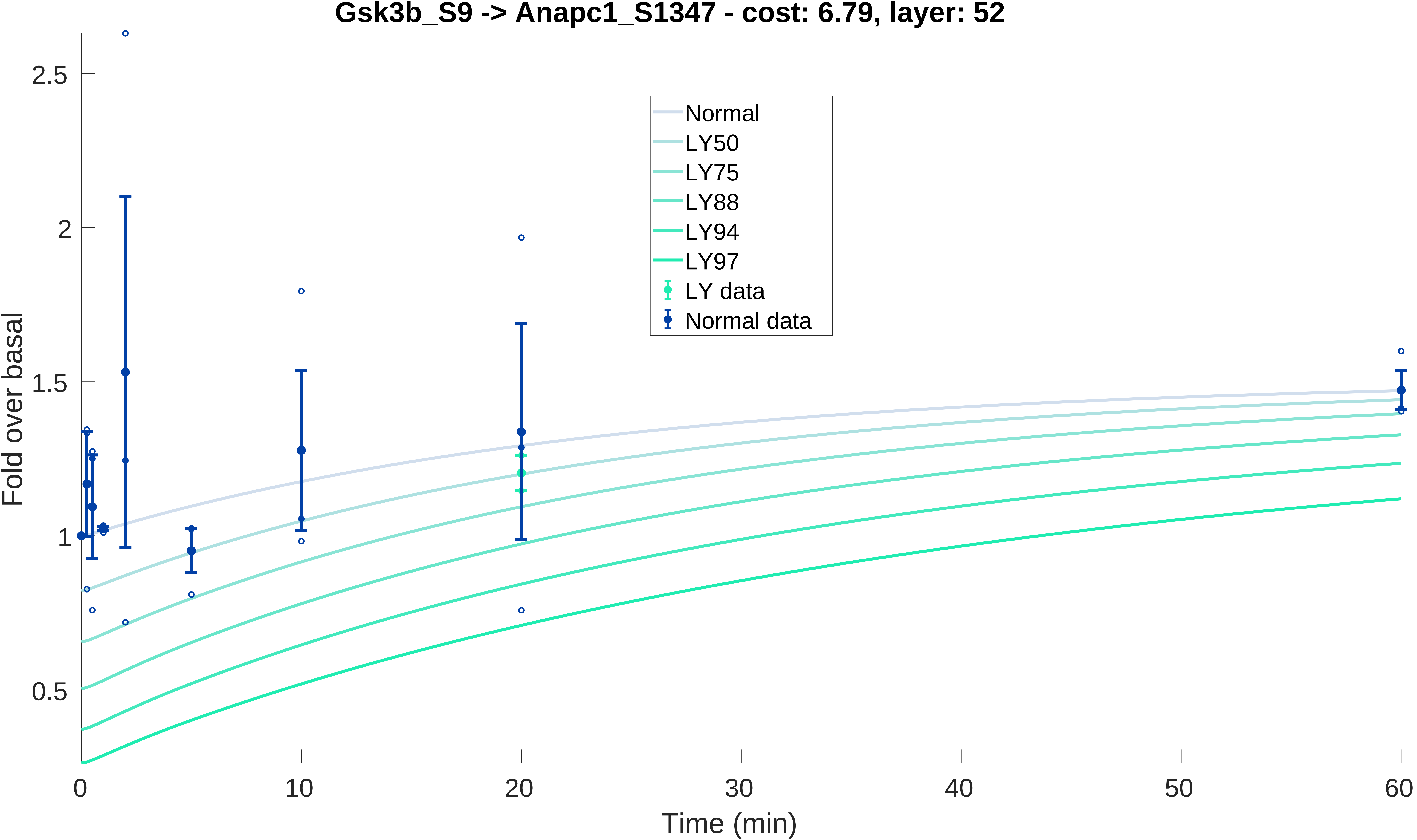

Gsk3b_S9 -> Anapc1_S1347 - cost: 6.79, layer: 52
Normal
LY50
LY75
LY88
LY94
LY97
LY data
Normal data
Fold over basal
Time (min)
0.5
1
1.5
2
2.5
0
10
20
30
40
50
60

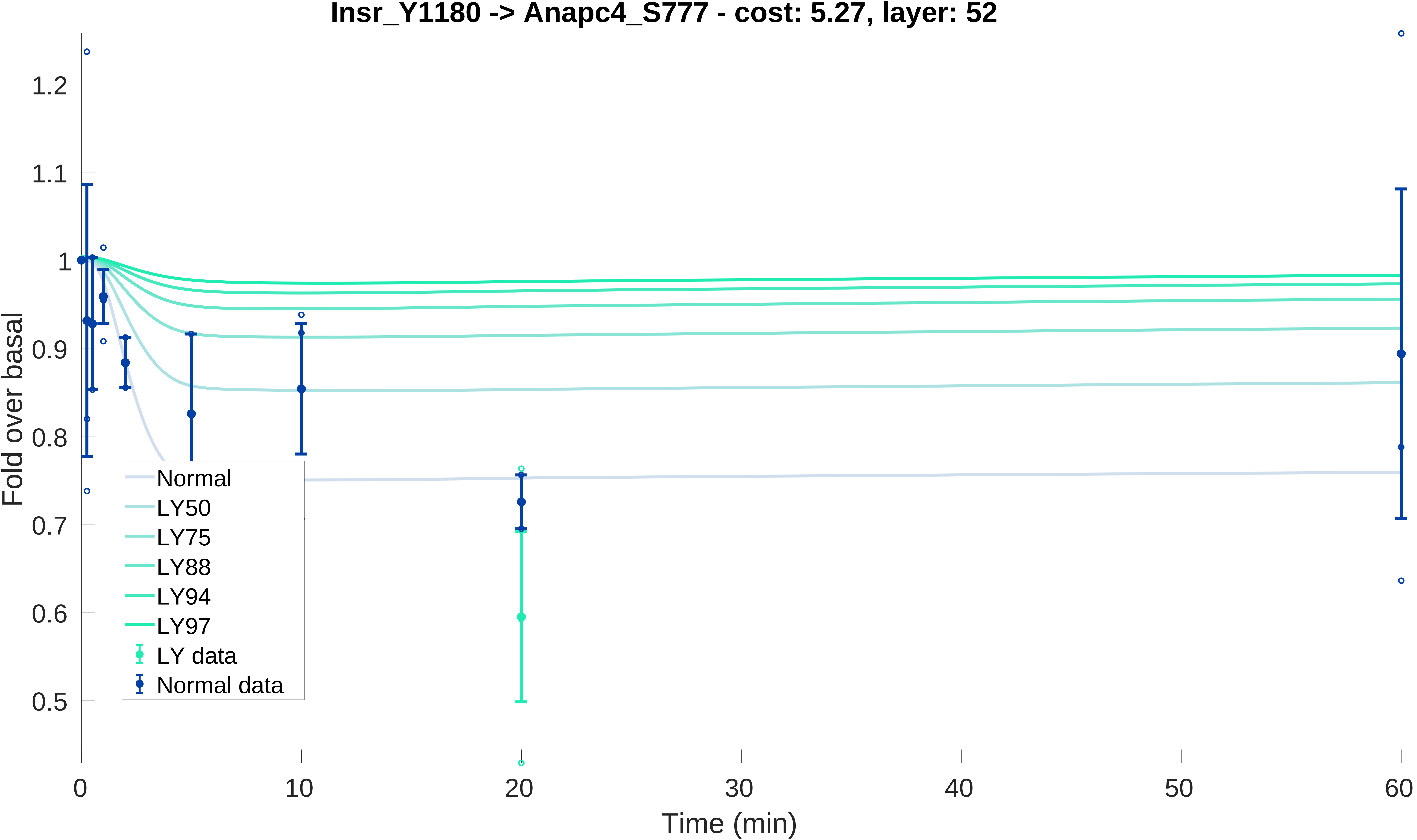

Insr_Y1180 -> Anapc4_S777 - cost: 5.27, layer: 52
Fold over basal
Time (min)
1.2
1.1
1
0.9
0.8
0.7
0.6
0.5
0
10
20
30
40
50
60
Normal
LY50
LY75
LY88
LY94
LY97
LY data
Normal data

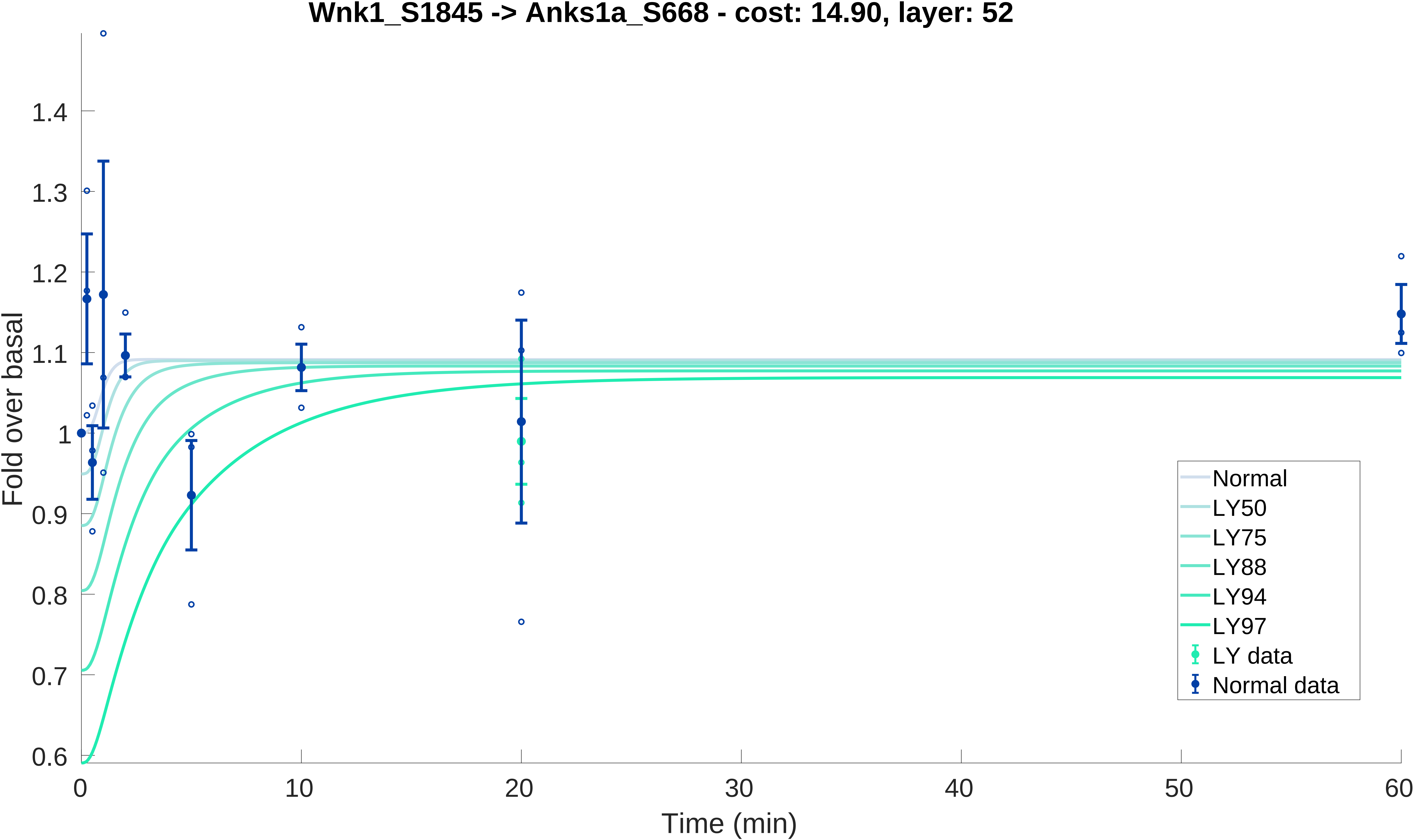
Wnk1_S1845 -> Anks1a_S668 - cost: 14.90, layer: 52
Fold over basal
Time (min)
0.6
0.7
0.8
0.9
1
1.1
1.2
1.3
1.4
0
10
20
30
40
50
60
Normal
LY50
LY75
LY88
LY94
LY97
LY data
Normal data

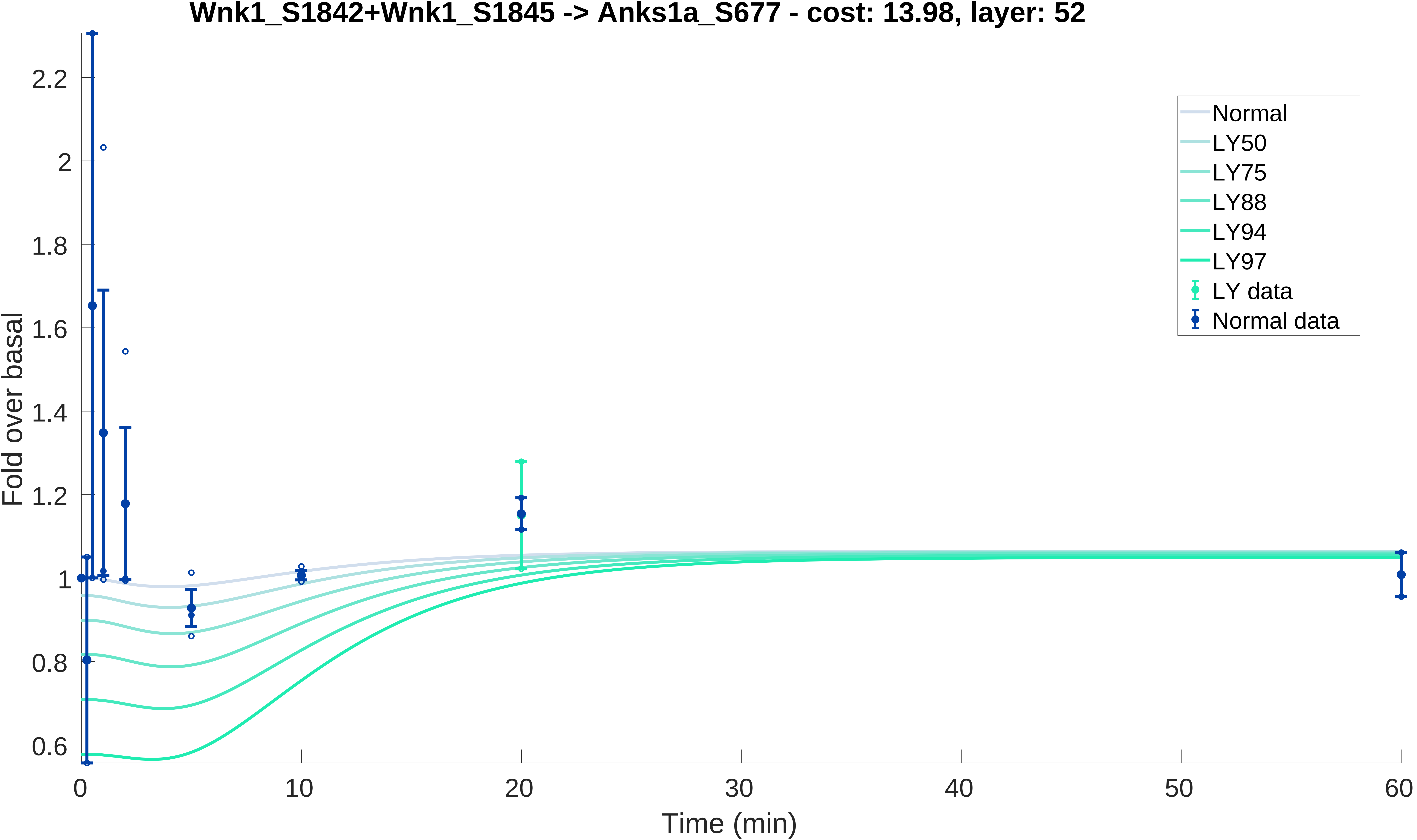

Wnk1_S1842+Wnk1_S1845 -> Anks1a_S677 - cost: 13.98, layer: 52
Fold over basal
Time (min)
0.6
0.8
1
1.2
1.4
1.6
1.8
2
2.2
0
10
20
30
40
50
60
Normal
LY50
LY75
LY88
LY94
LY97
LY data
Normal data

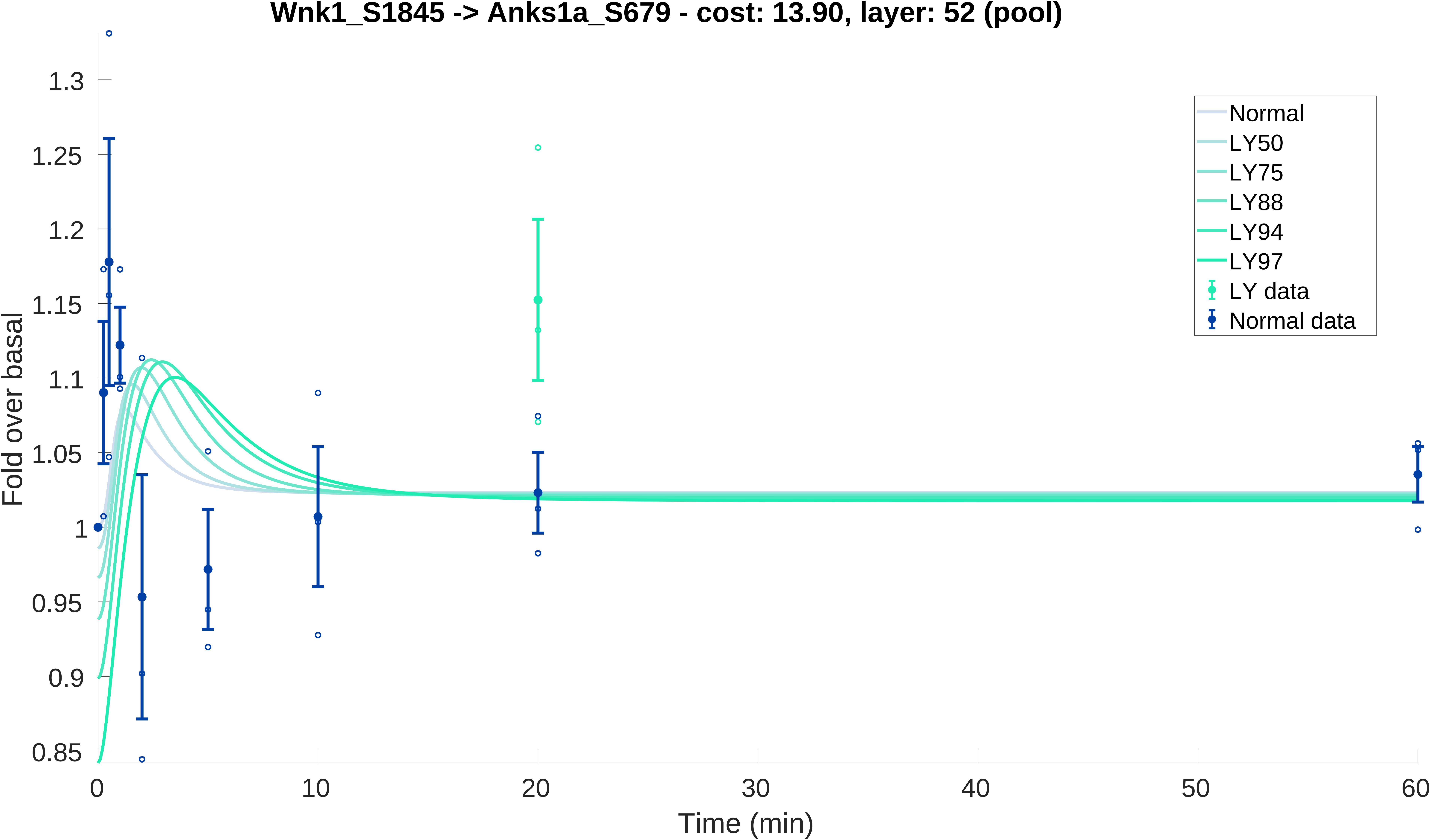

Wnk1_S1845 -> Anks1a_S679 - cost: 13.90, layer: 52 (pool)
Fold over basal
Time (min)
1.3
1.25
1.2
1.15
1.1
1.05
1
0.95
0.9
0.85
0
10
20
30
40
50
60
Normal
LY50
LY75
LY88
LY94
LY97
LY data
Normal data

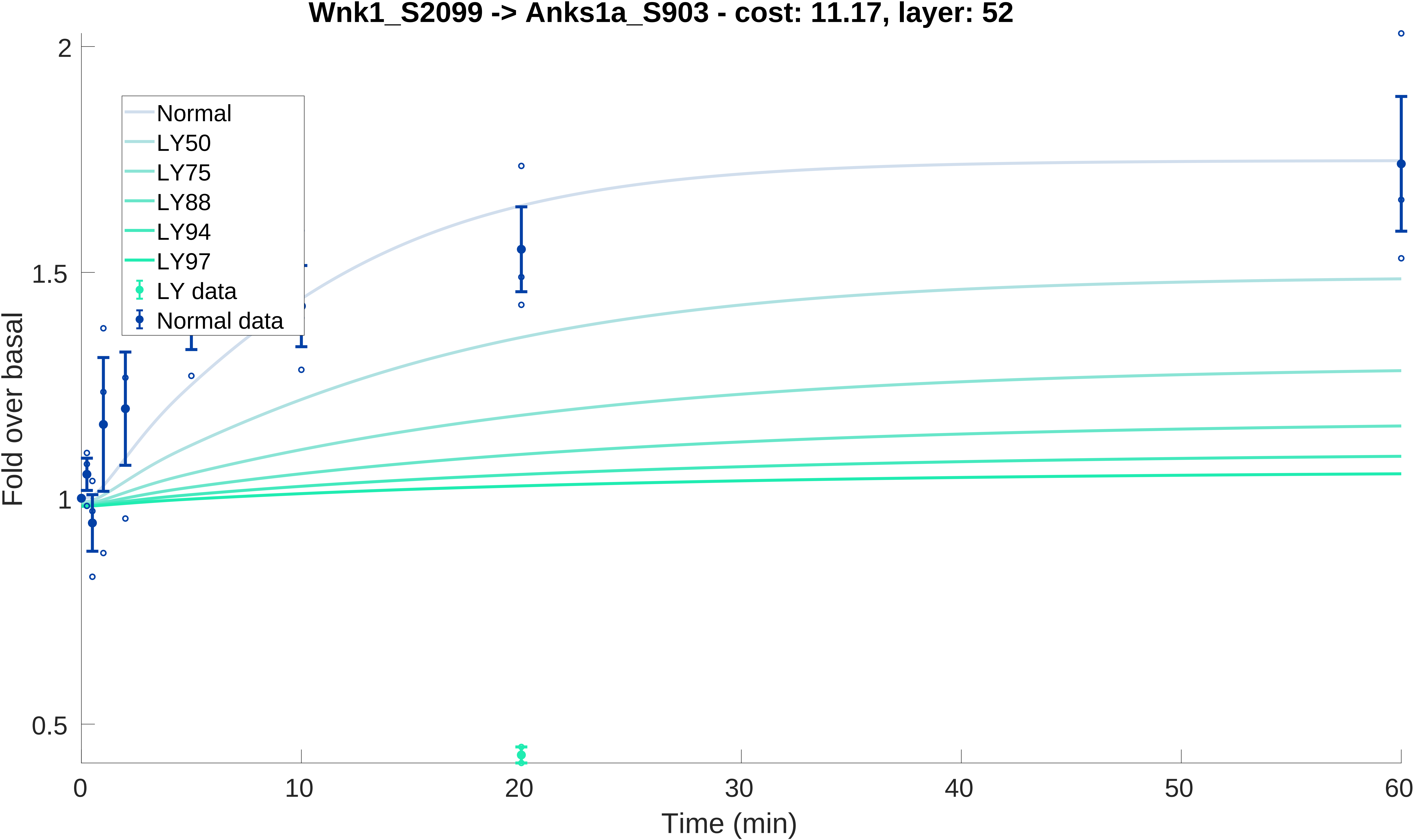

Wnk1_S2099 -> Anks1a_S903 - cost: 11.17, layer: 52
Normal
LY50
LY75
LY88
LY94
LY97
LY data
Normal data
Fold over basal
Time (min)
2
1.5
1
0.5
0
10
20
30
40
50
60

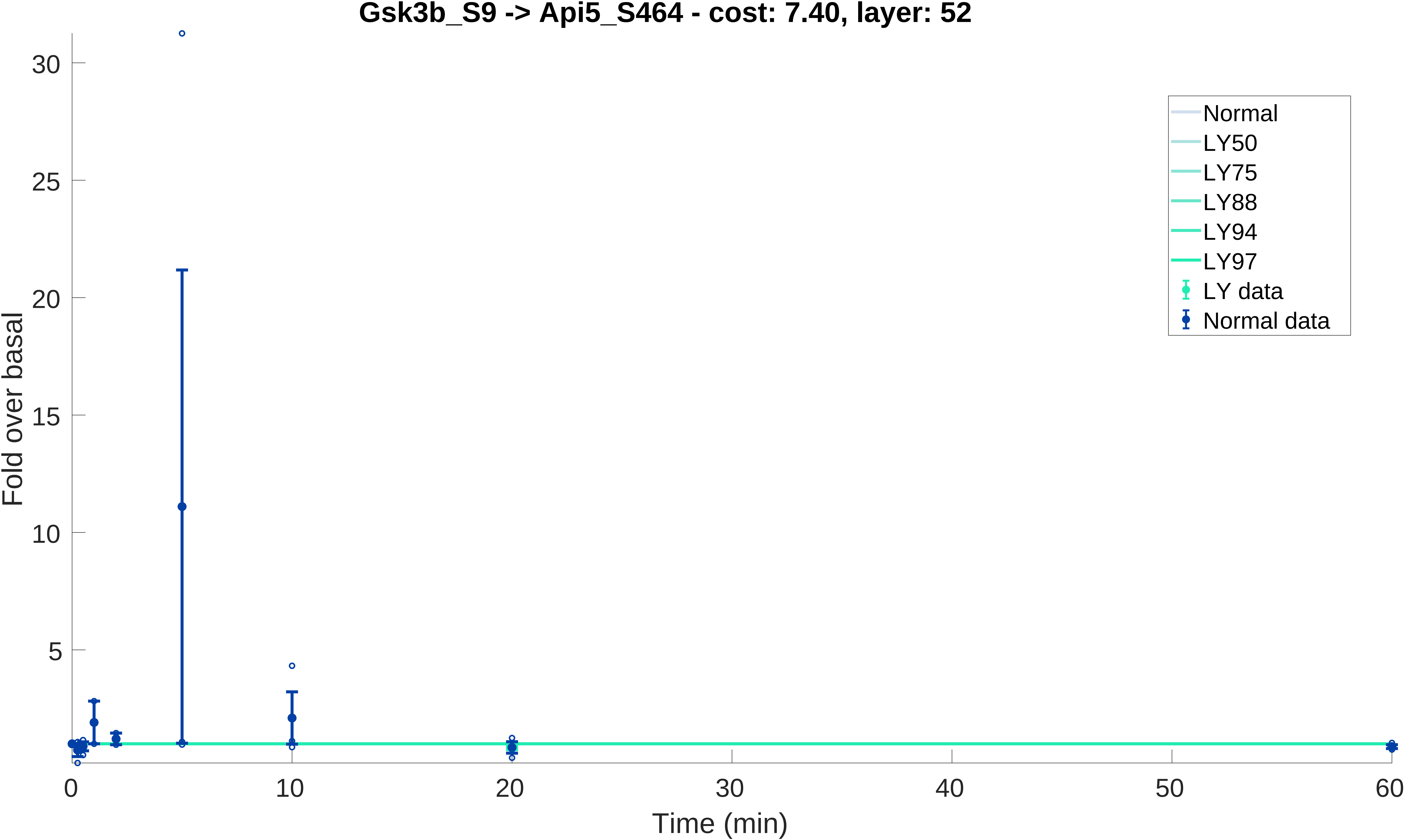

Gsk3b_S9 -> Api5_S464 - cost: 7.40, layer: 52
Fold over basal
Time (min)
0
5
10
15
20
25
30
40
50
60
Normal
LY50
LY75
LY88
LY94
LY97
LY data
Normal data

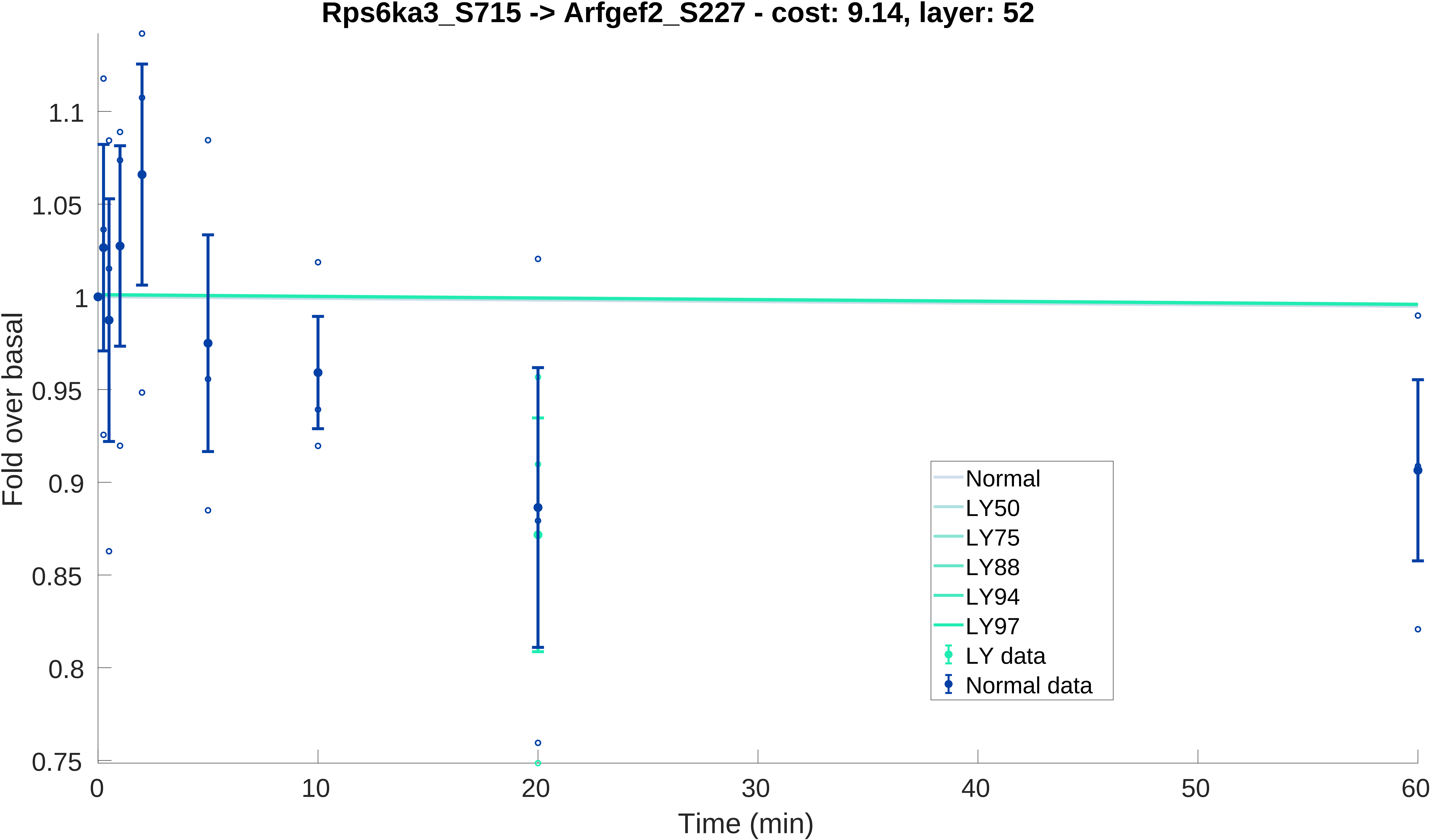

Rps6ka3_S715 -> Arfgef2_S227 - cost: 9.14, layer: 52
Fold over basal
Time (min)
1.1
1.05
1
0.95
0.9
0.85
0.8
0.75
0
10
20
30
40
50
60
Normal
LY50
LY75
LY88
LY94
LY97
LY data
Normal data

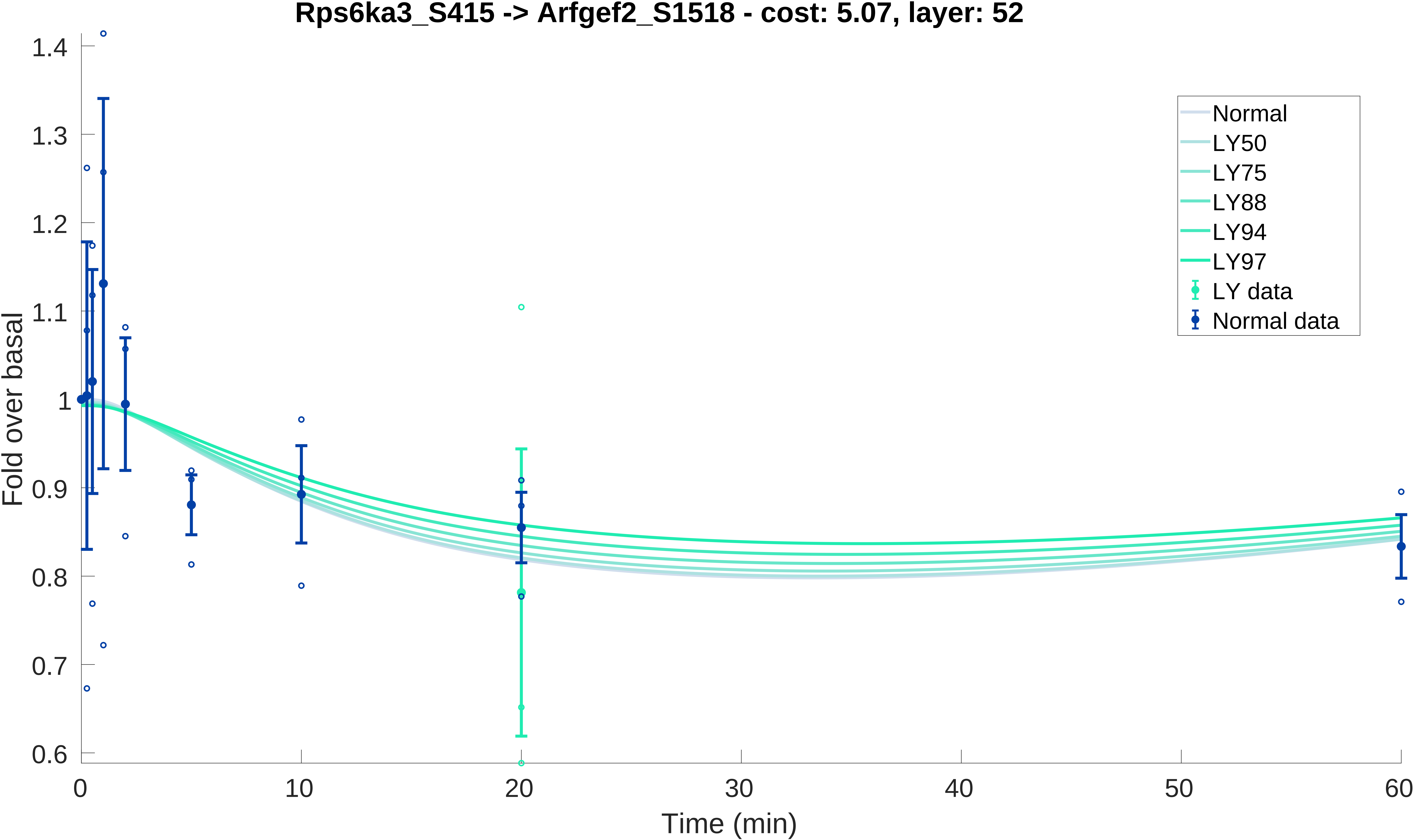

Rps6ka3_S415 -> Arfgef2_S1518 - cost: 5.07, layer: 52
Fold over basal
1.4
1.3
1.2
1.1
1
0.9
0.8
0.7
0.6
0
10
20
30
40
50
60
Time (min)
Normal
LY50
LY75
LY88
LY94
LY97
LY data
Normal data

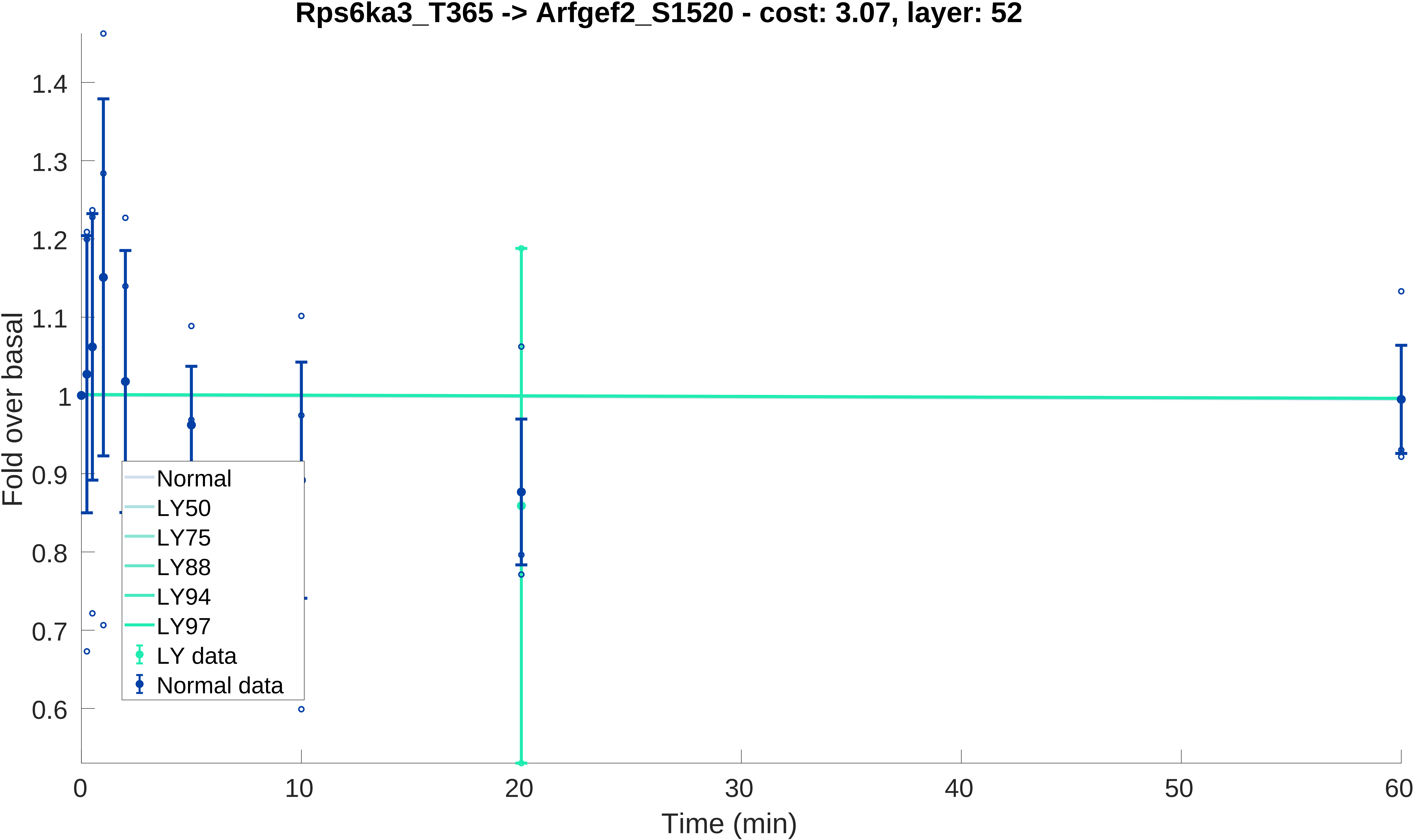

Rps6ka3_T365 -> Arfgef2_S1520 - cost: 3.07, layer: 52
Fold over basal
Time (min)
1.4
1.3
1.2
1.1
1
0.9
0.8
0.7
0.6
0
10
20
30
40
50
60
Normal
LY50
LY75
LY88
LY94
LY97
LY data
Normal data

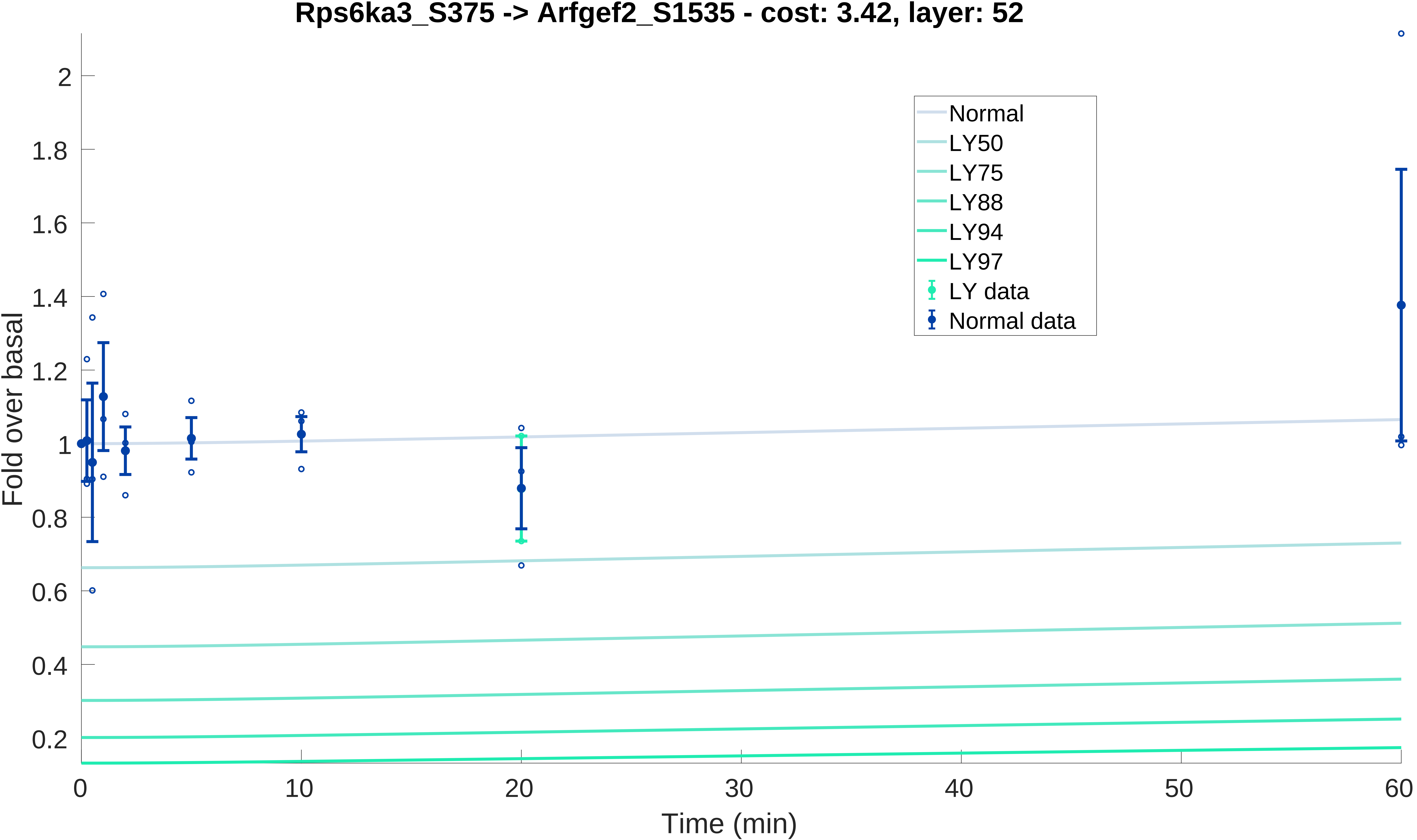

Rps6ka3_S375 -> Arfgef2_S1535 - cost: 3.42, layer: 52
Fold over basal
Time (min)
Normal
LY50
LY75
LY88
LY94
LY97
LY data
Normal data
0.2
0.4
0.6
0.8
1
1.2
1.4
1.6
1.8
2
0
10
20
30
40
50
60

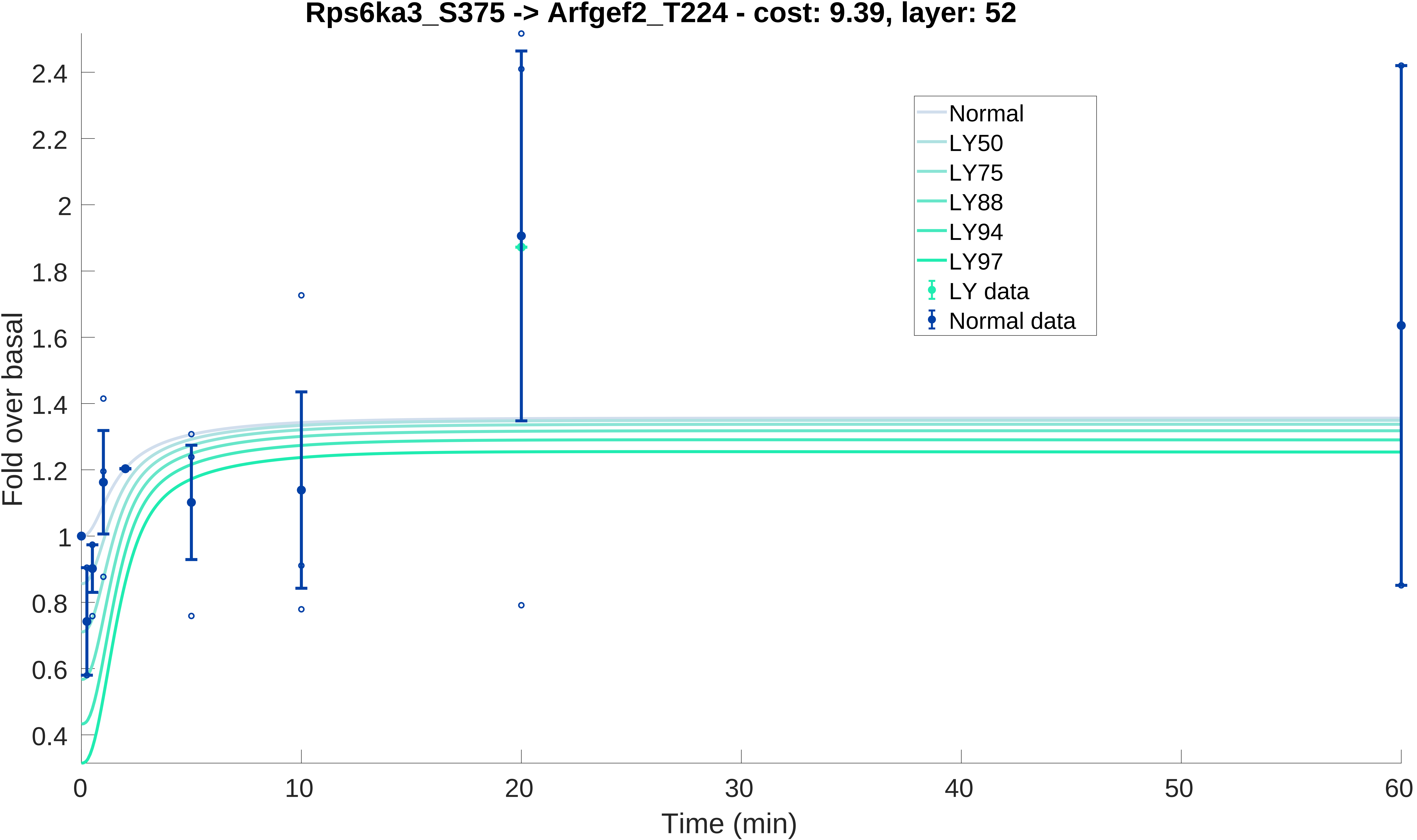

Rps6ka3_S375 -> Arfgef2_T224 - cost: 9.39, layer: 52
Fold over basal
Time (min)
0.4
0.6
0.8
1
1.2
1.4
1.6
1.8
2
2.2
2.4
0
10
20
30
40
50
60
Normal
LY50
LY75
LY88
LY94
LY97
LY data
Normal data

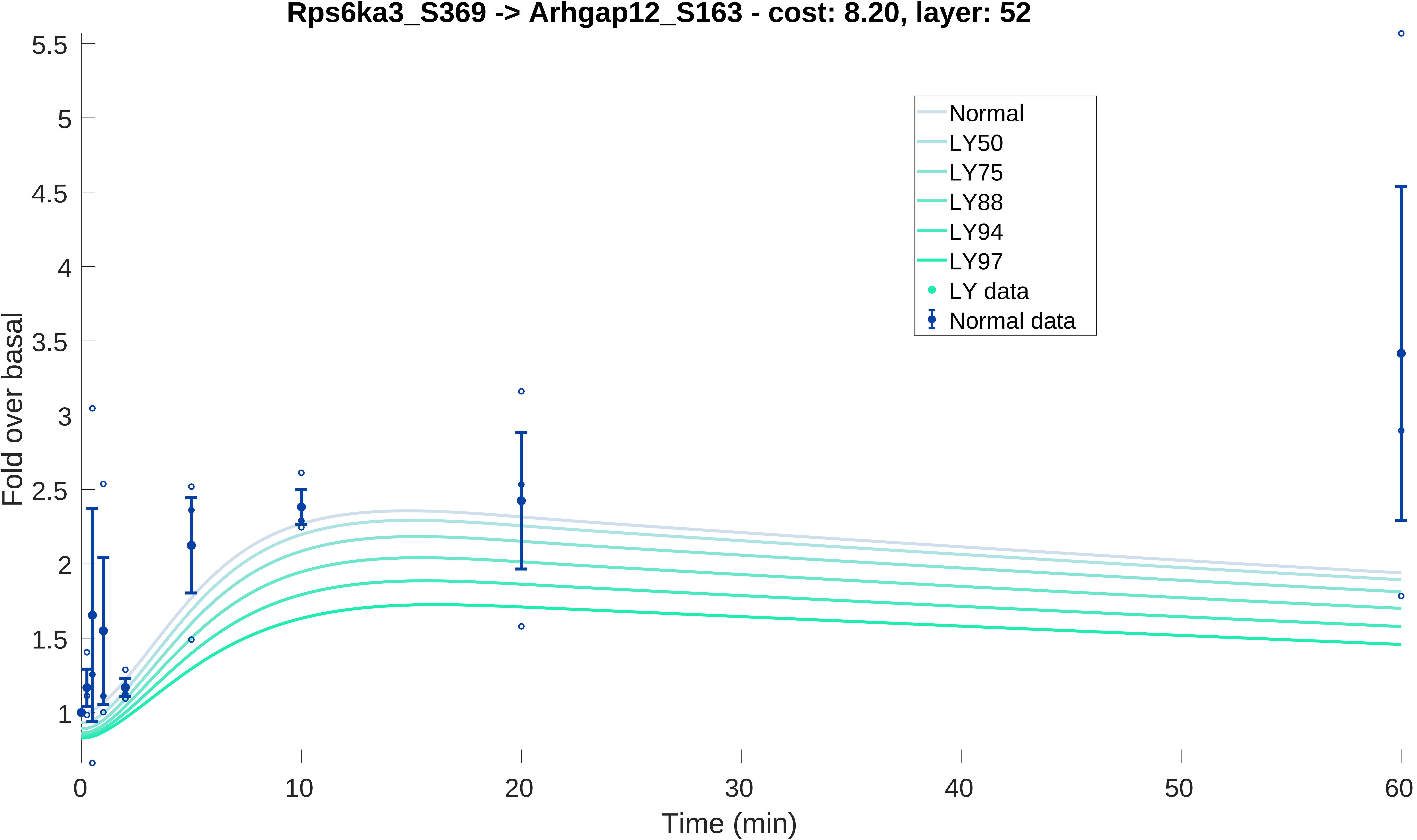

Rps6ka3_S369 -> Arhgap12_S163 - cost: 8.20, layer: 52
Fold over basal
Time (min)
0
10
20
30
40
50
60
1
1.5
2
2.5
3
3.5
4
4.5
5
5.5
Normal
LY50
LY75
LY88
LY94
LY97
LY data
Normal data

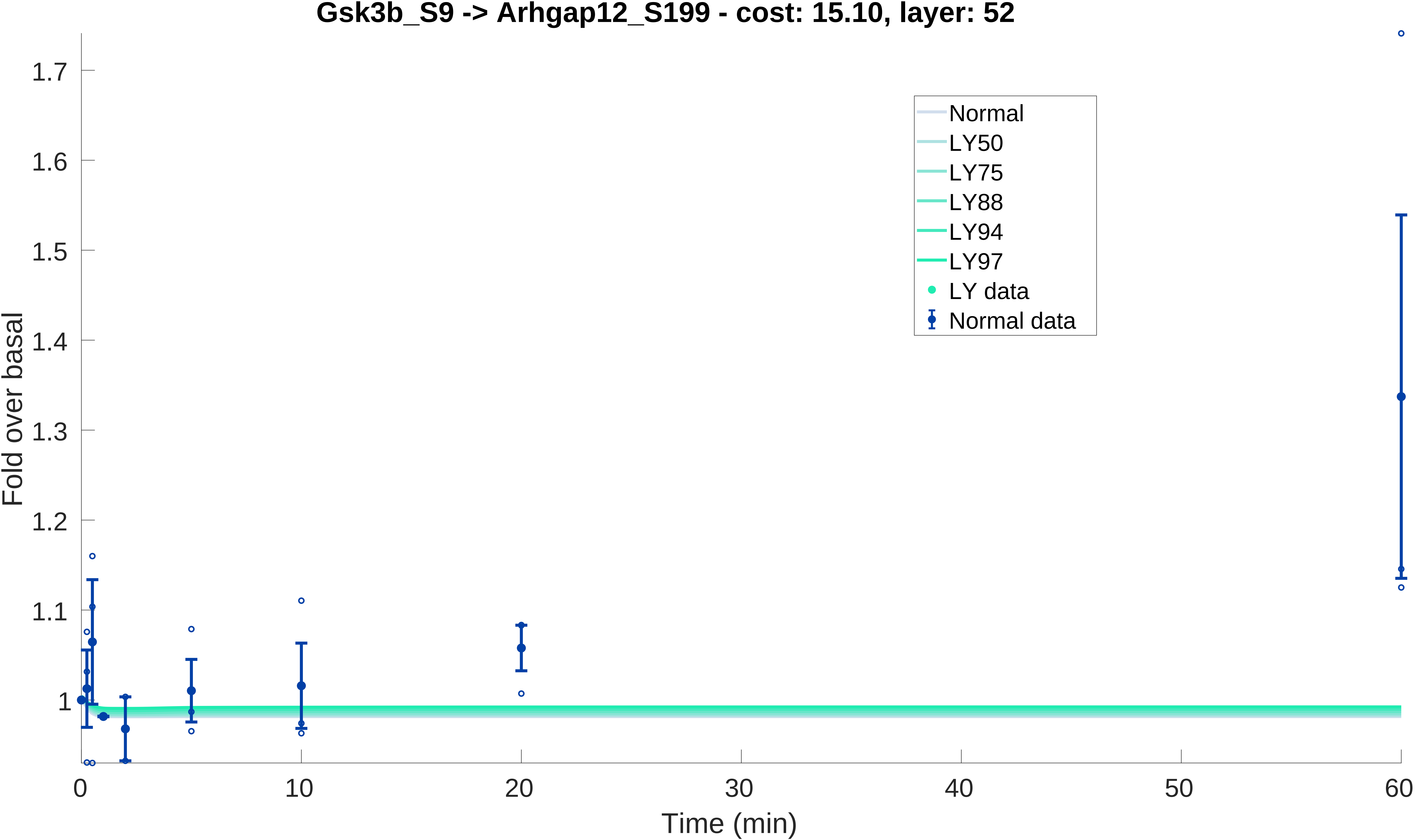
Gsk3b_S9 -> Arhgap12_S199 - cost: 15.10, layer: 52
Fold over basal
1.7
1.6
1.5
1.4
1.3
1.2
1.1
1
Time (min)
0
10
20
30
40
50
60
Normal
LY50
LY75
LY88
LY94
LY97
LY data
Normal data

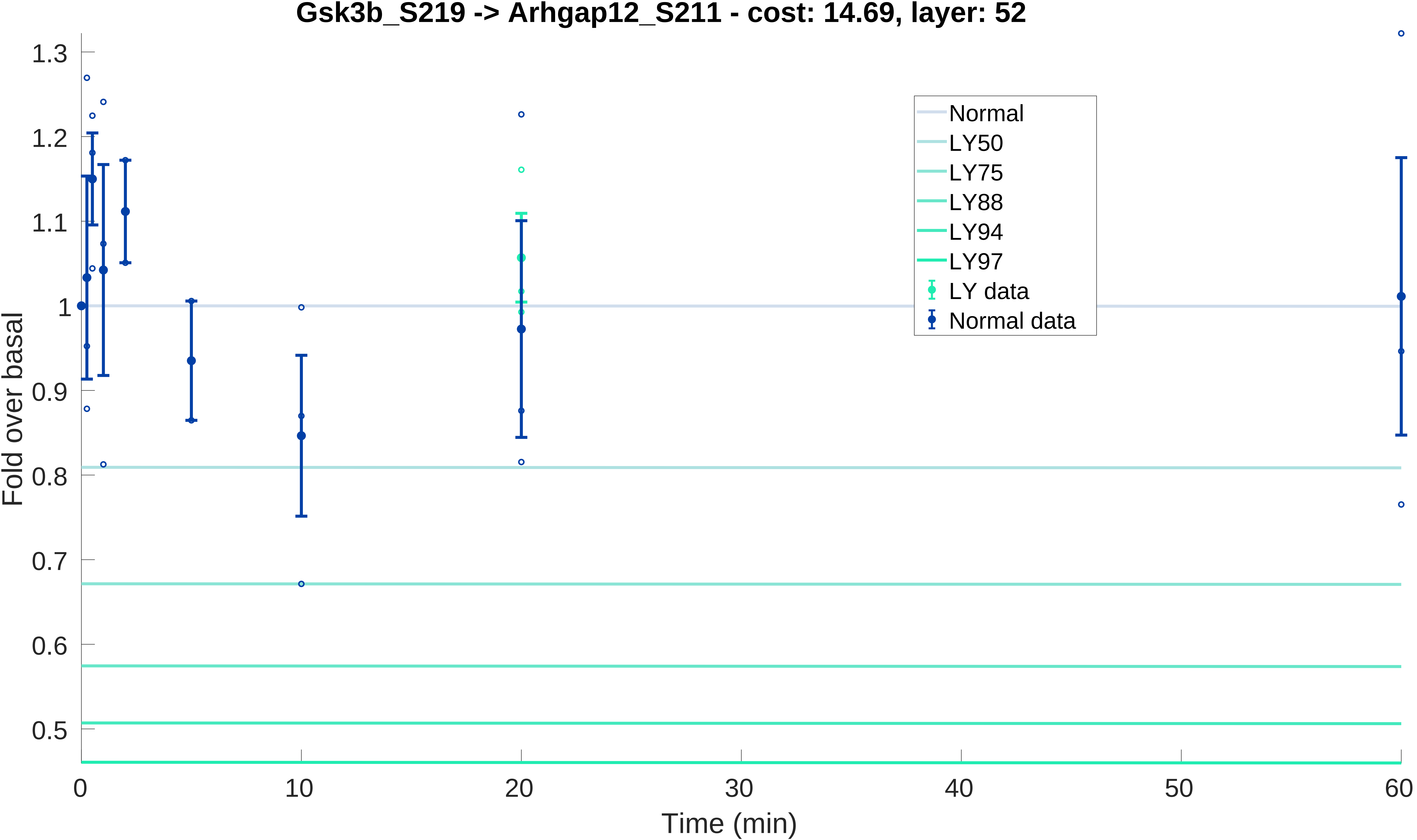

Gsk3b_S219 -> Arhgap12_S211 - cost: 14.69, layer: 52
Fold over basal
Time (min)
Normal
LY50
LY75
LY88
LY94
LY97
LY data
Normal data
1.3
1.2
1.1
1
0.9
0.8
0.7
0.6
0.5
0
10
20
30
40
50
60

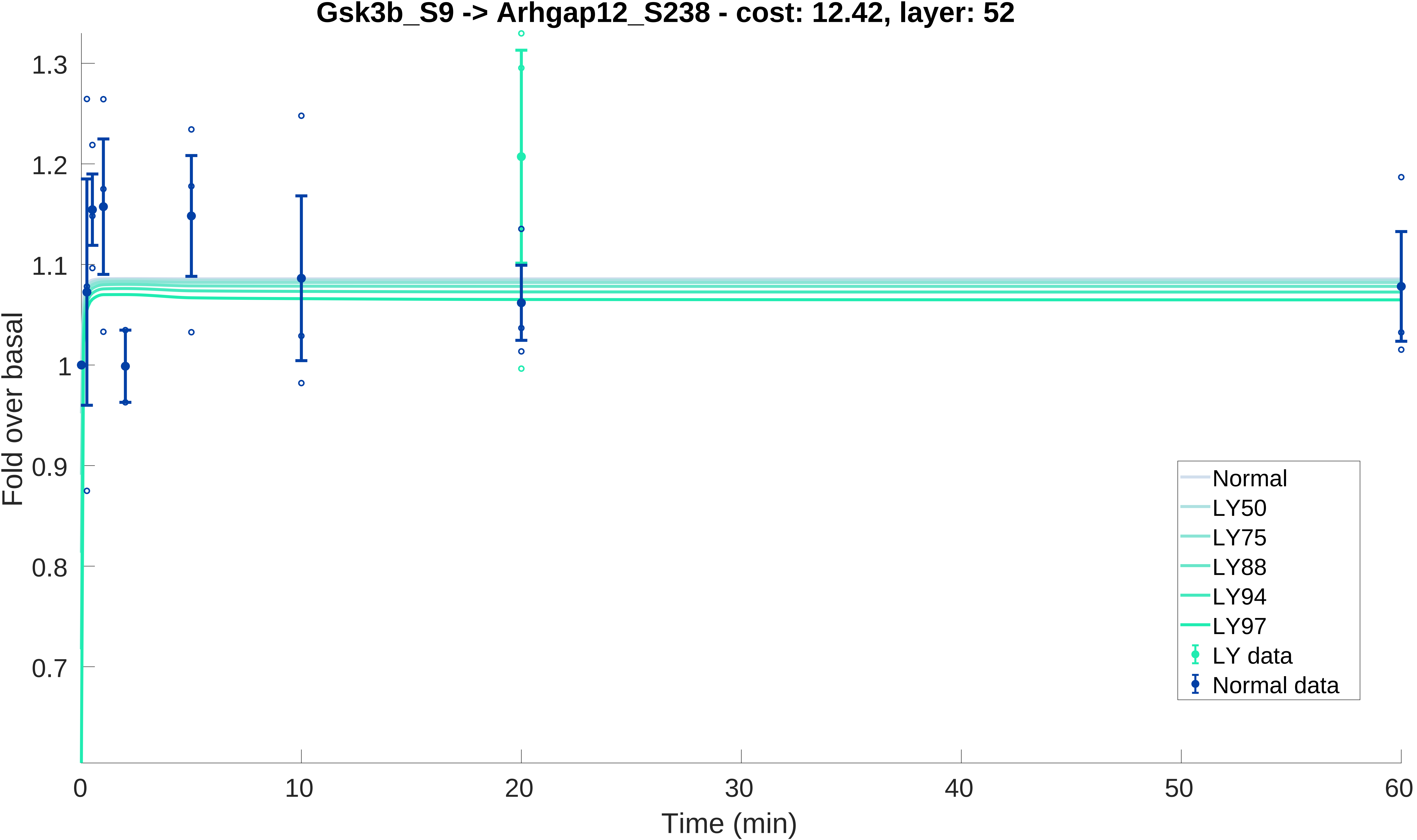

Gsk3b_S9 -> Arhgap12_S238 - cost: 12.42, layer: 52
Fold over basal
1.3
1.2
1.1
1
0.9
0.8
0.7
0
10
20
30
40
50
60
Time (min)
Normal
LY50
LY75
LY88
LY94
LY97
LY data
Normal data

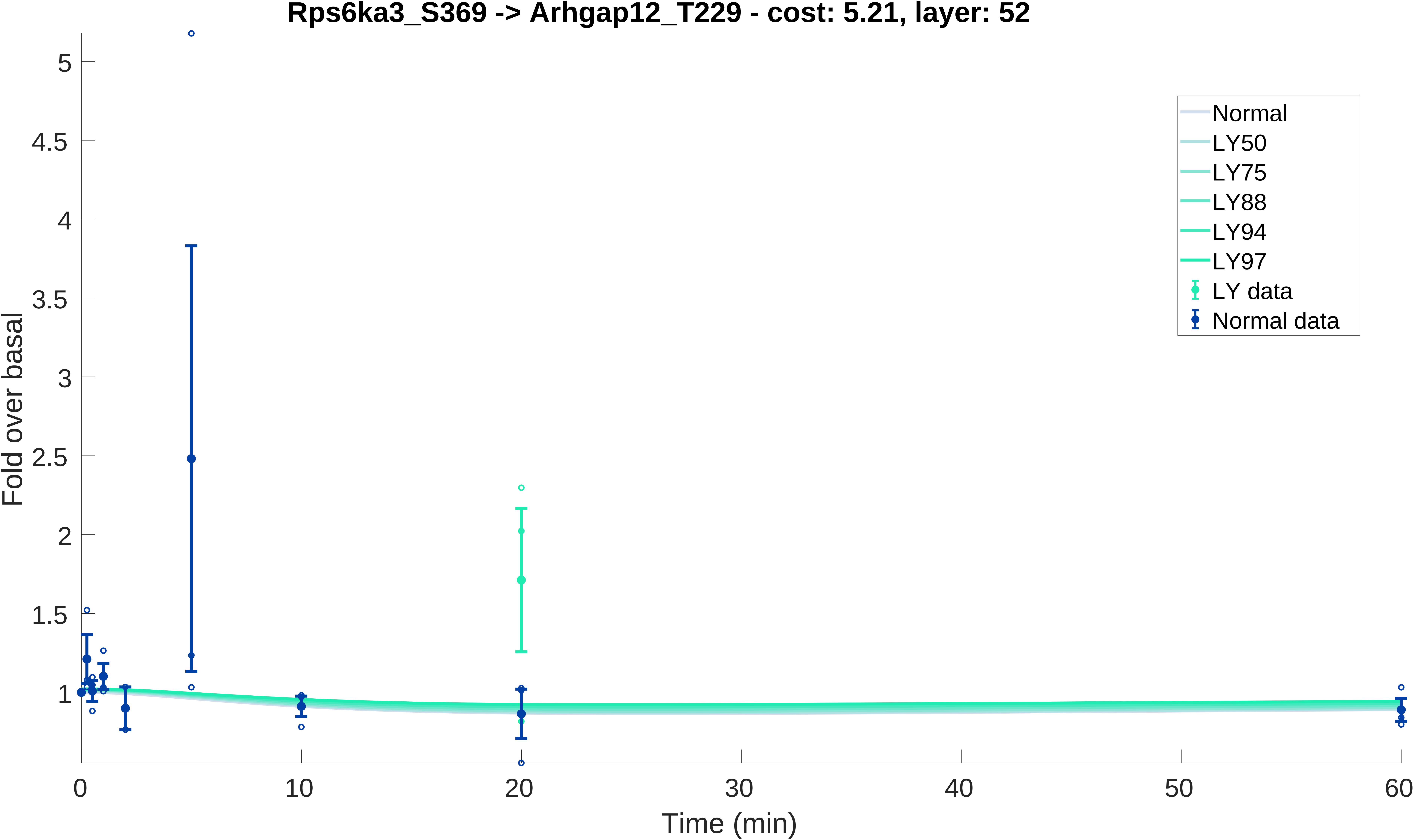

Rps6ka3_S369 -> Arhgap12_T229 - cost: 5.21, layer: 52
Fold over basal
Time (min)
0
10
20
30
40
50
60
1
1.5
2
2.5
3
3.5
4
4.5
5
Normal
LY50
LY75
LY88
LY94
LY97
LY data
Normal data

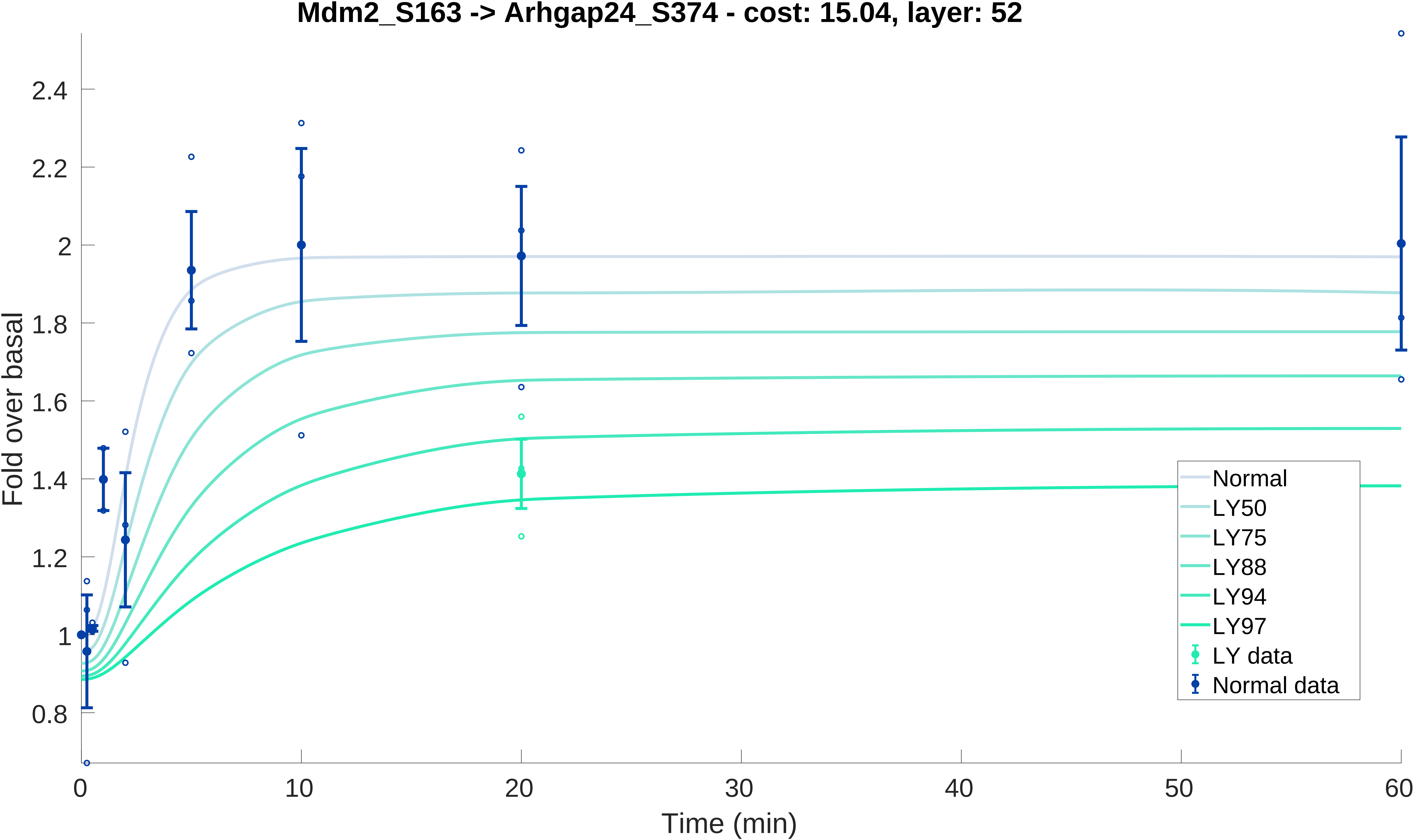

Mdm2_S163 -> Arhgap24_S374 - cost: 15.04, layer: 52
Fold over basal
Time (min)
0.8
1
1.2
1.4
1.6
1.8
2
2.2
2.4
0
10
20
30
40
50
60
Normal
LY50
LY75
LY88
LY94
LY97
LY data
Normal data

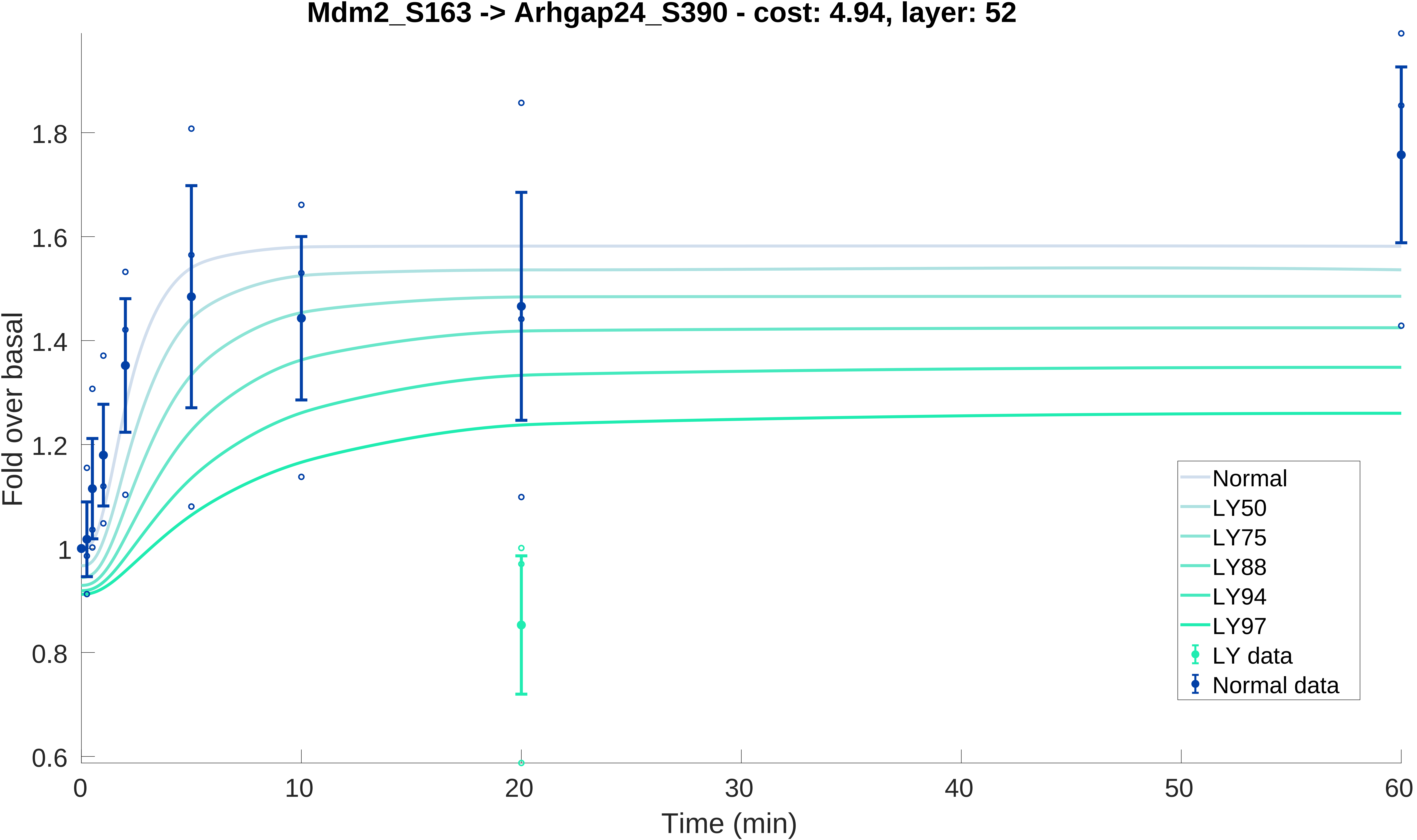

Mdm2_S163 -> Arhgap24_S390 - cost: 4.94, layer: 52
Fold over basal
Time (min)
0.6
0.8
1
1.2
1.4
1.6
1.8
0
10
20
30
40
50
60
Normal
LY50
LY75
LY88
LY94
LY97
LY data
Normal data

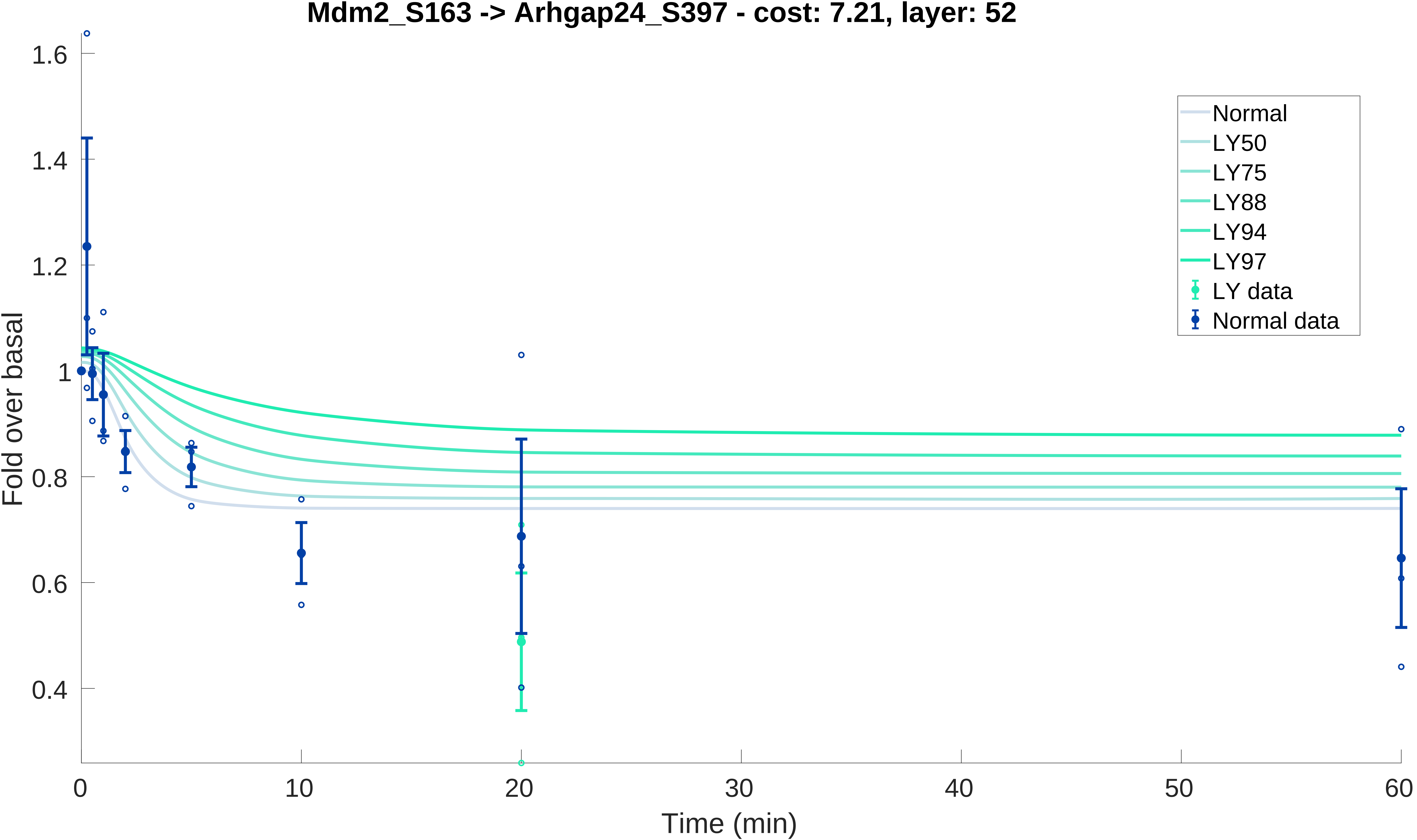
Mdm2_S163 -> Arhgap24_S397 - cost: 7.21, layer: 52
Fold over basal
Time (min)
1.6
1.4
1.2
1
0.8
0.6
0.4
0
10
20
30
40
50
60
Normal
LY50
LY75
LY88
LY94
LY97
LY data
Normal data

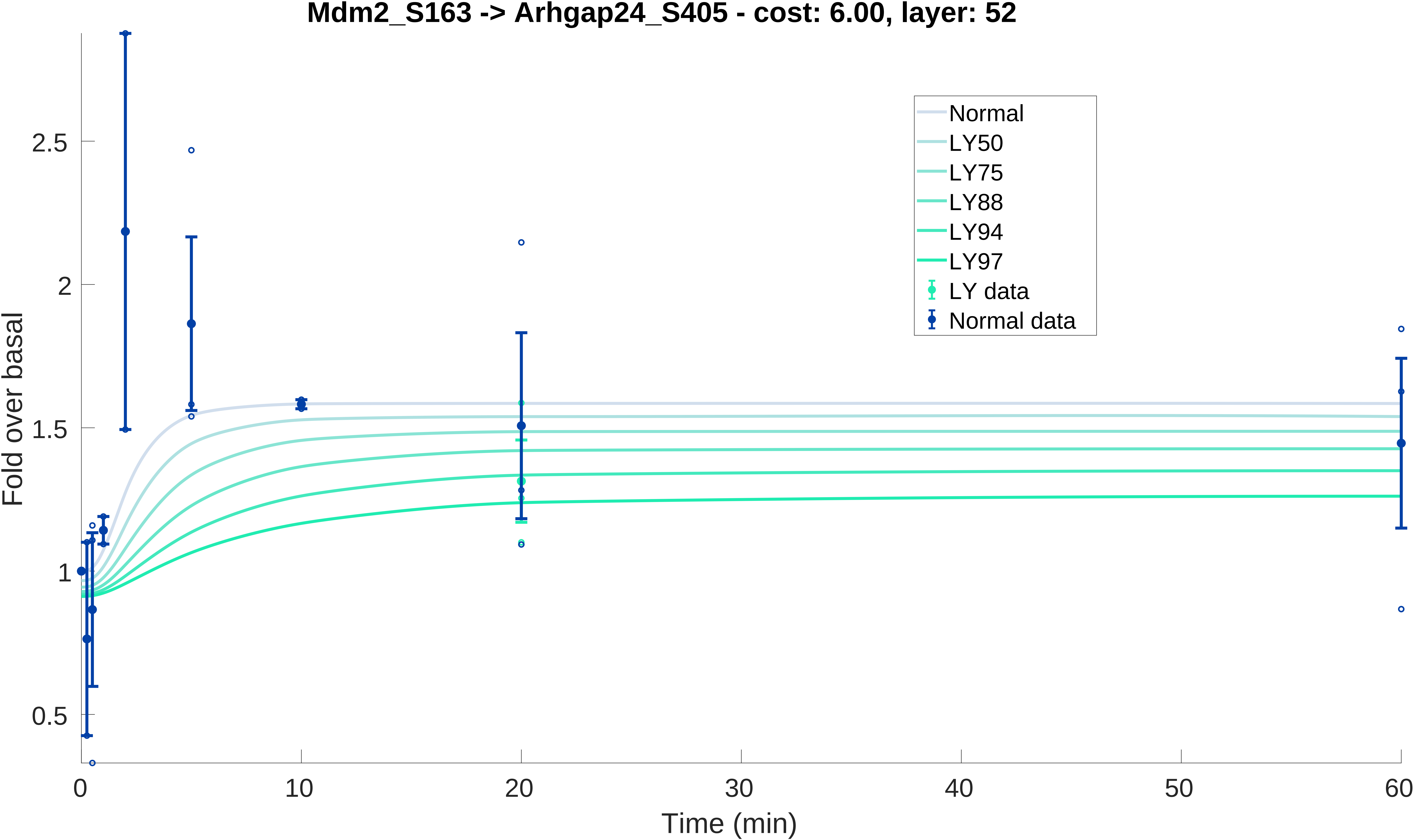

Mdm2_S163 -> Arhgap24_S405 - cost: 6.00, layer: 52
Normal
LY50
LY75
LY88
LY94
LY97
LY data
Normal data
Fold over basal
2.5
2
1.5
1
0.5
0
10
20
30
40
50
60
Time (min)

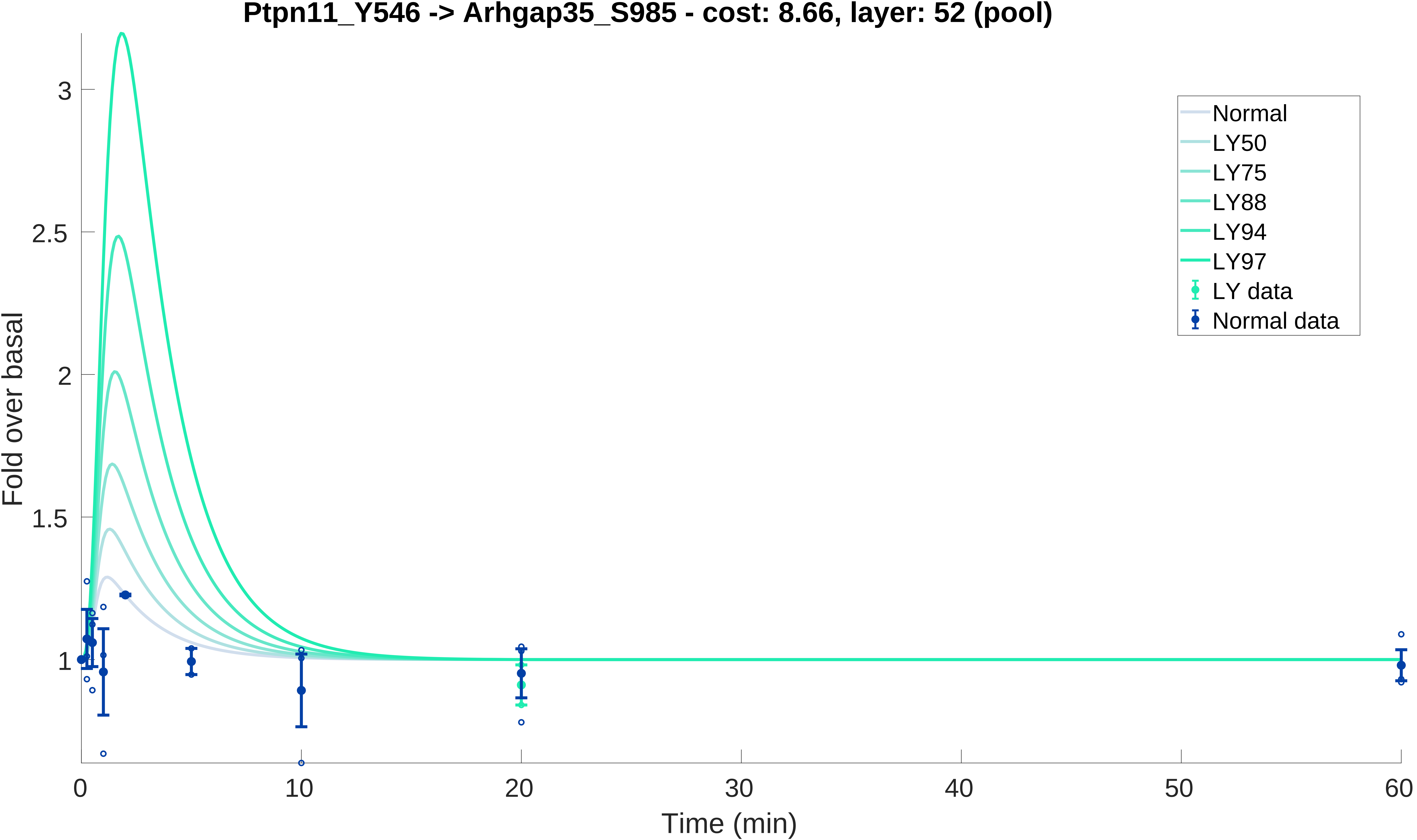

Ptpn11_Y546 -> Arhgap35_S985 - cost: 8.66, layer: 52 (pool)
Fold over basal
Time (min)
3
2.5
2
1.5
1
0
10
20
30
40
50
60
Normal
LY50
LY75
LY88
LY94
LY97
LY data
Normal data

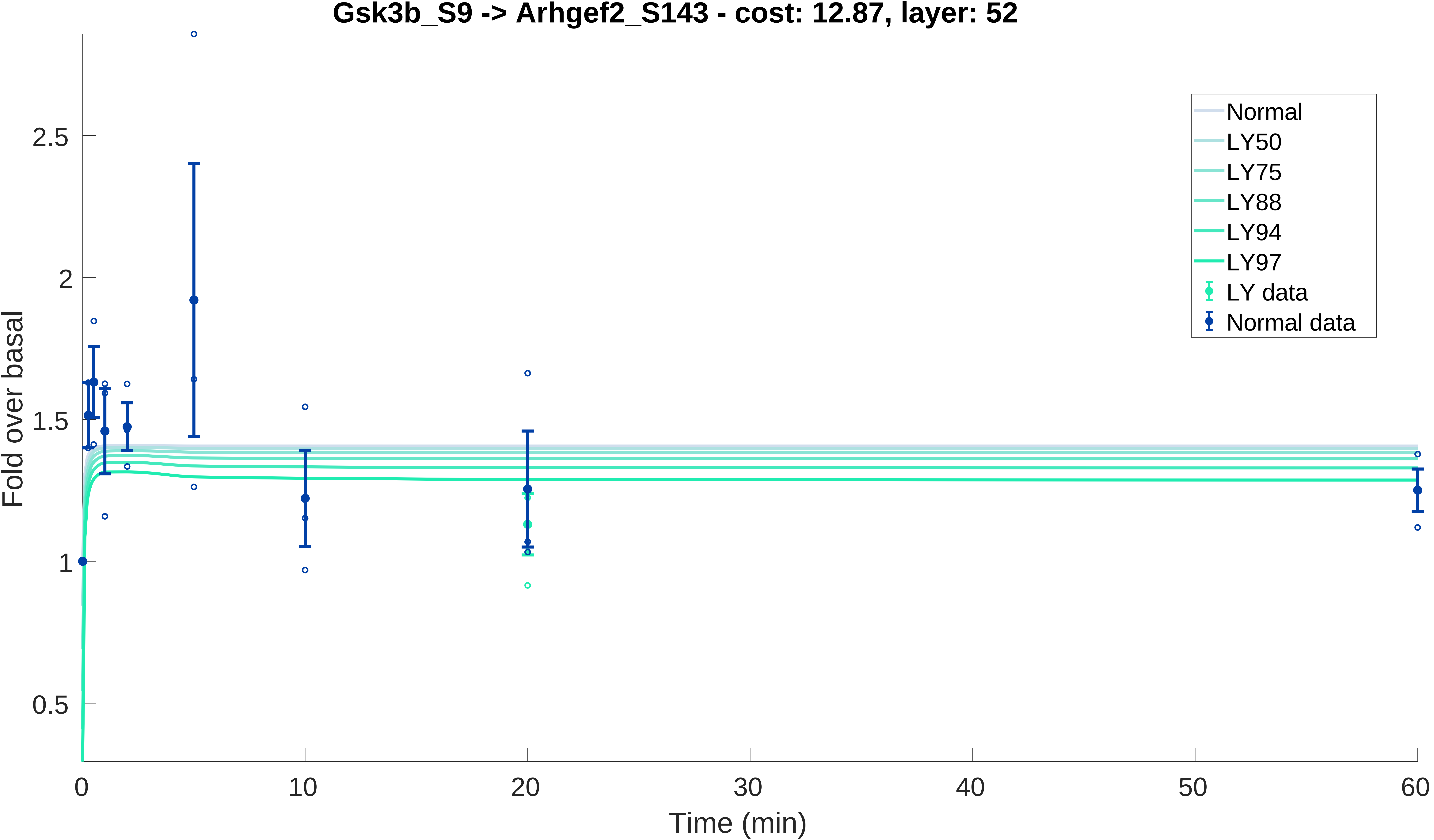
Gsk3b_S9 -> Arhgef2_S143 - cost: 12.87, layer: 52
Fold over basal
Time (min)
0.5
1
1.5
2
2.5
0
10
20
30
40
50
60
Normal
LY50
LY75
LY88
LY94
LY97
LY data
Normal data

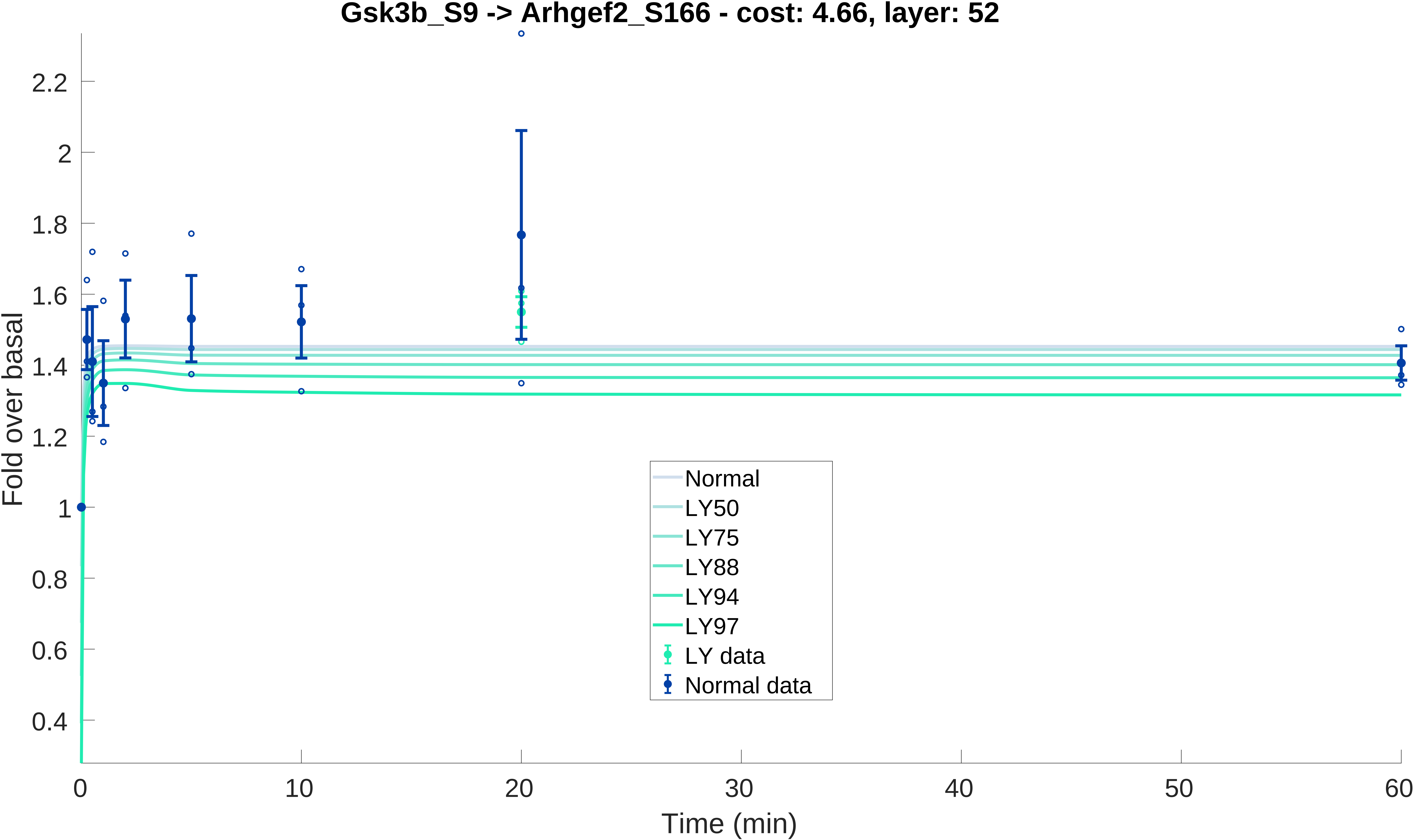

Gsk3b_S9 -> Arhgef2_S166 - cost: 4.66, layer: 52
Fold over basal
Time (min)
2.2
2
1.8
1.6
1.4
1.2
1
0.8
0.6
0.4
0
10
20
30
40
50
60
Normal
LY50
LY75
LY88
LY94
LY97
LY data
Normal data

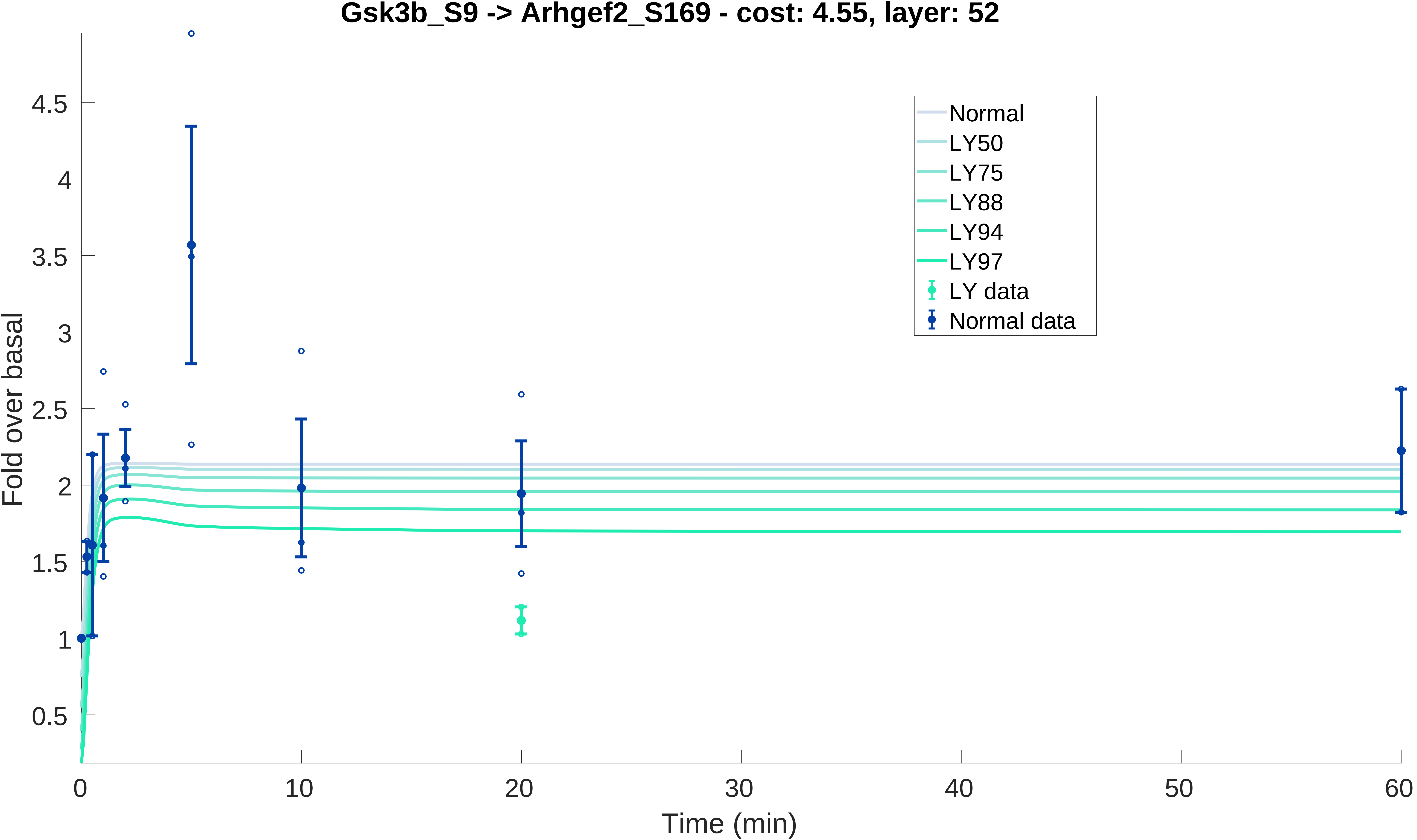

Gsk3b_S9 -> Arhgef2_S169 - cost: 4.55, layer: 52
Fold over basal
Time (min)
Normal
LY50
LY75
LY88
LY94
LY97
LY data
Normal data
0.5
1
1.5
2
2.5
3
3.5
4
4.5
0
10
20
30
40
50
60

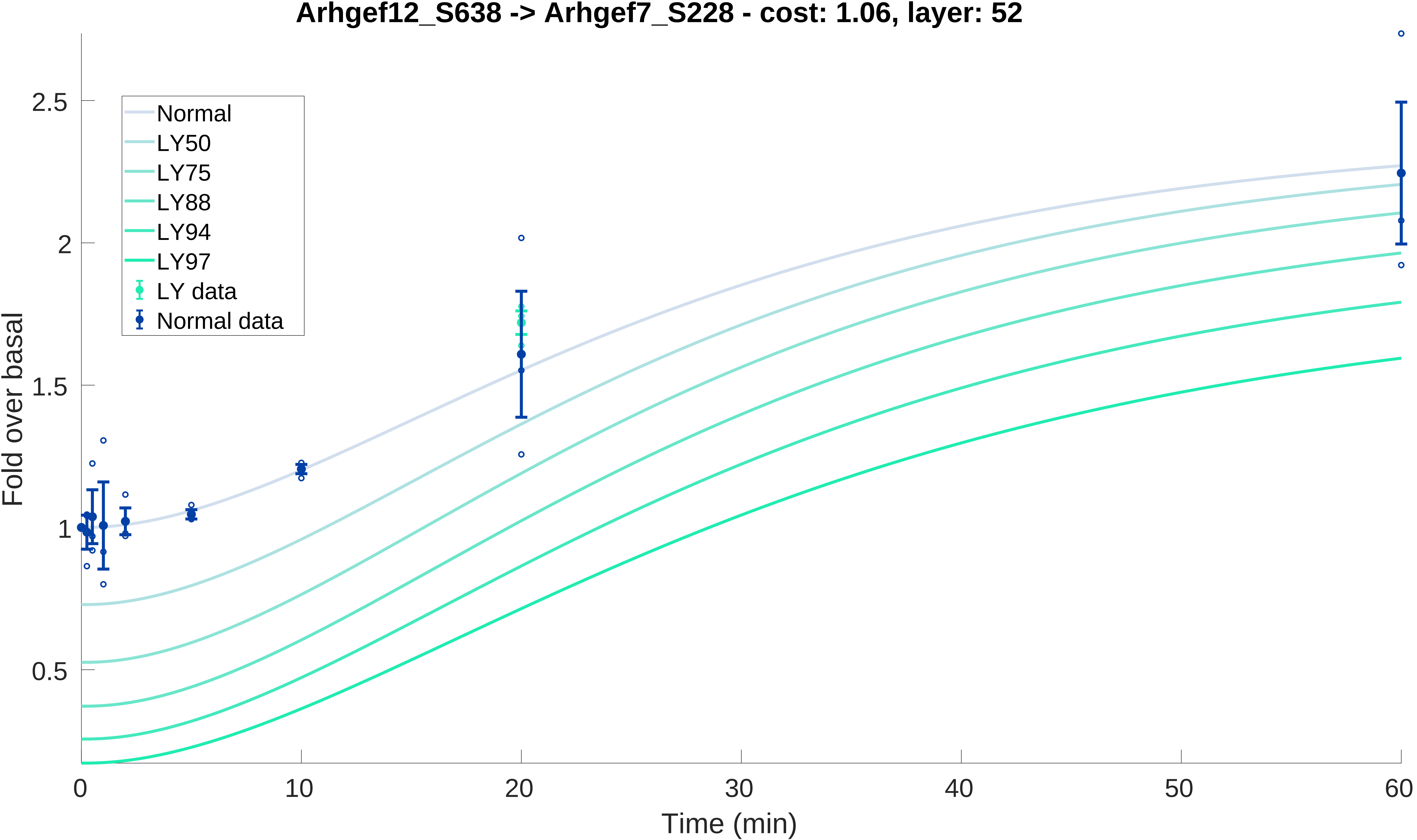

Arhgef12_S638 -> Arhgef7_S228 - cost: 1.06, layer: 52
Normal
LY50
LY75
LY88
LY94
LY97
LY data
Normal data
Fold over basal
2.5
2
1.5
1
0.5
0
10
20
30
40
50
60
Time (min)

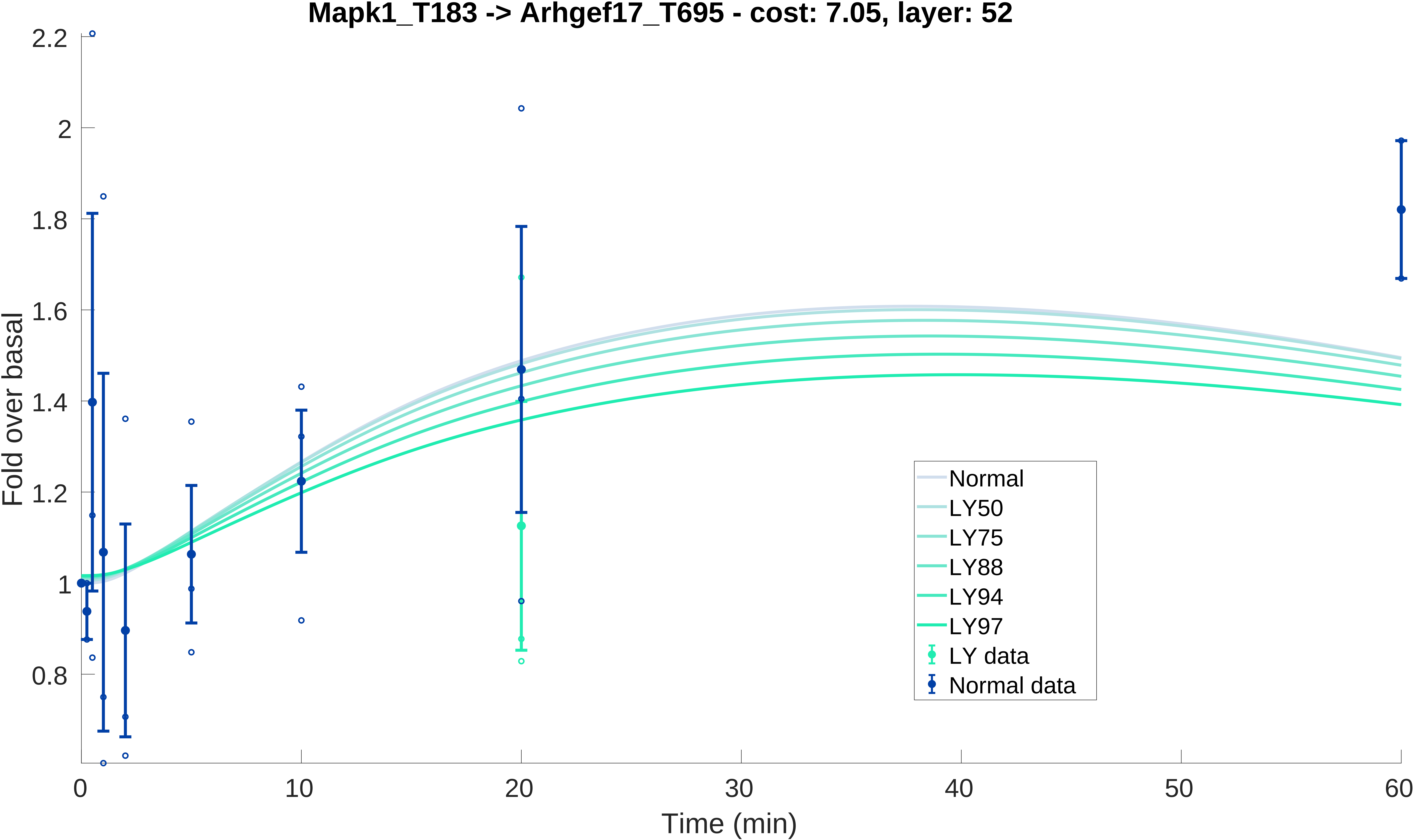

Mapk1_T183 -> Arhgef17_T695 - cost: 7.05, layer: 52
Fold over basal
2.2
2
1.8
1.6
1.4
1.2
1
0.8
0
10
20
30
40
50
60
Time (min)
Normal
LY50
LY75
LY88
LY94
LY97
LY data
Normal data

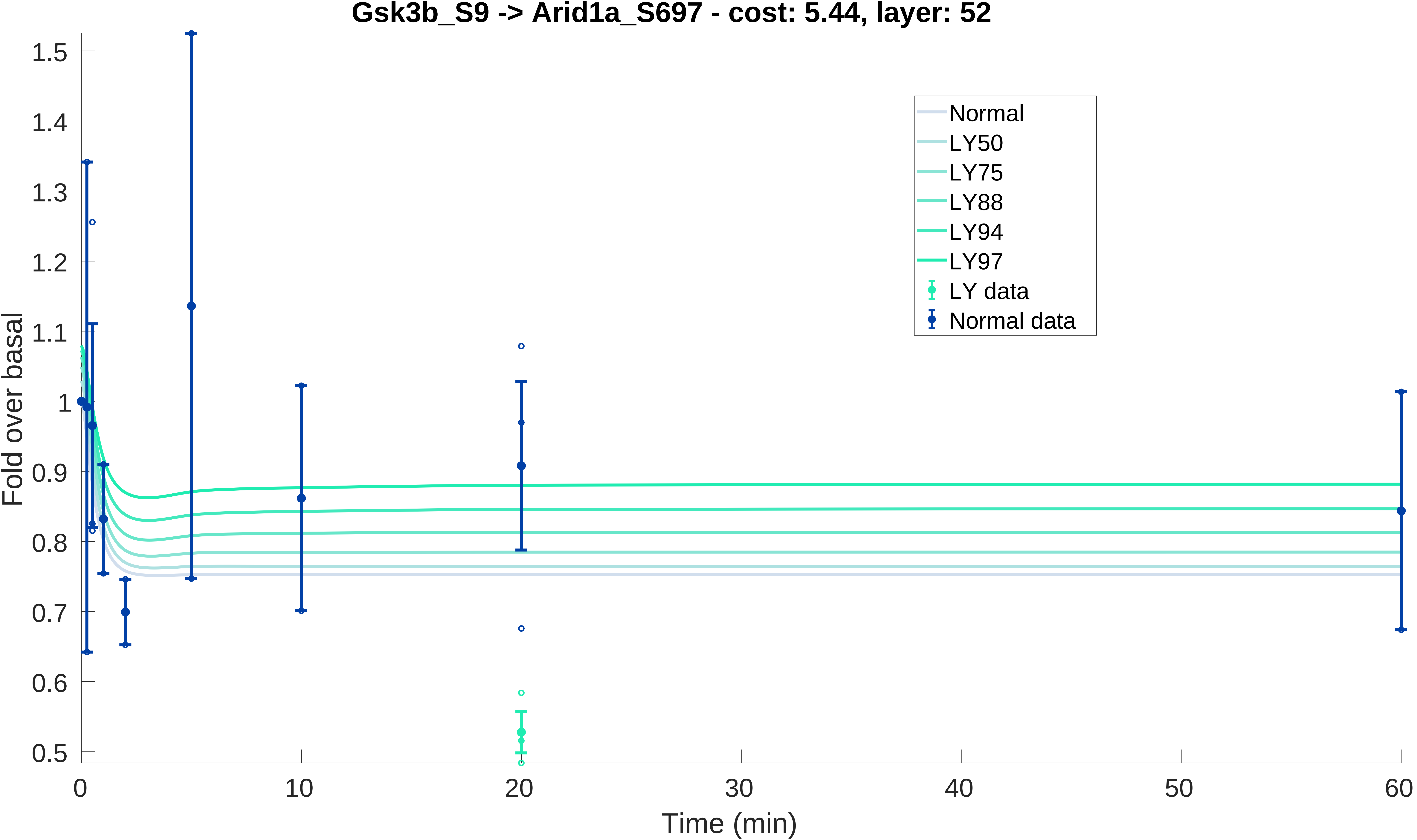
Gsk3b_S9 -> Arid1a_S697 - cost: 5.44, layer: 52
Fold over basal
Time (min)
Normal
LY50
LY75
LY88
LY94
LY97
LY data
Normal data
1.5
1.4
1.3
1.2
1.1
1
0.9
0.8
0.7
0.6
0.5
0
10
20
30
40
50
60

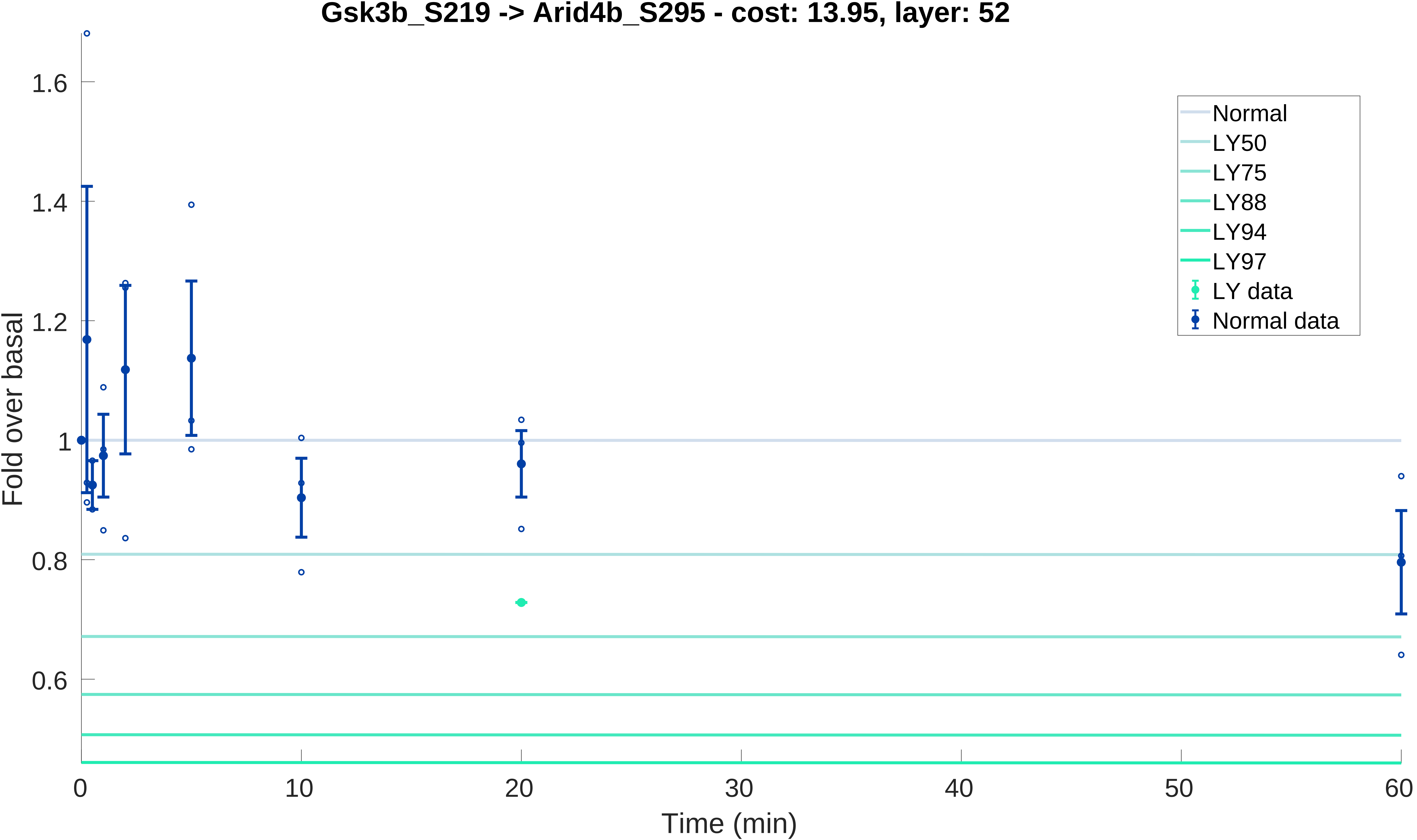

Gsk3b_S219 -> Arid4b_S295 - cost: 13.95, layer: 52
Fold over basal
1.6
1.4
1.2
1
0.8
0.6
0
10
20
30
40
50
60
Time (min)
Normal
LY50
LY75
LY88
LY94
LY97
LY data
Normal data

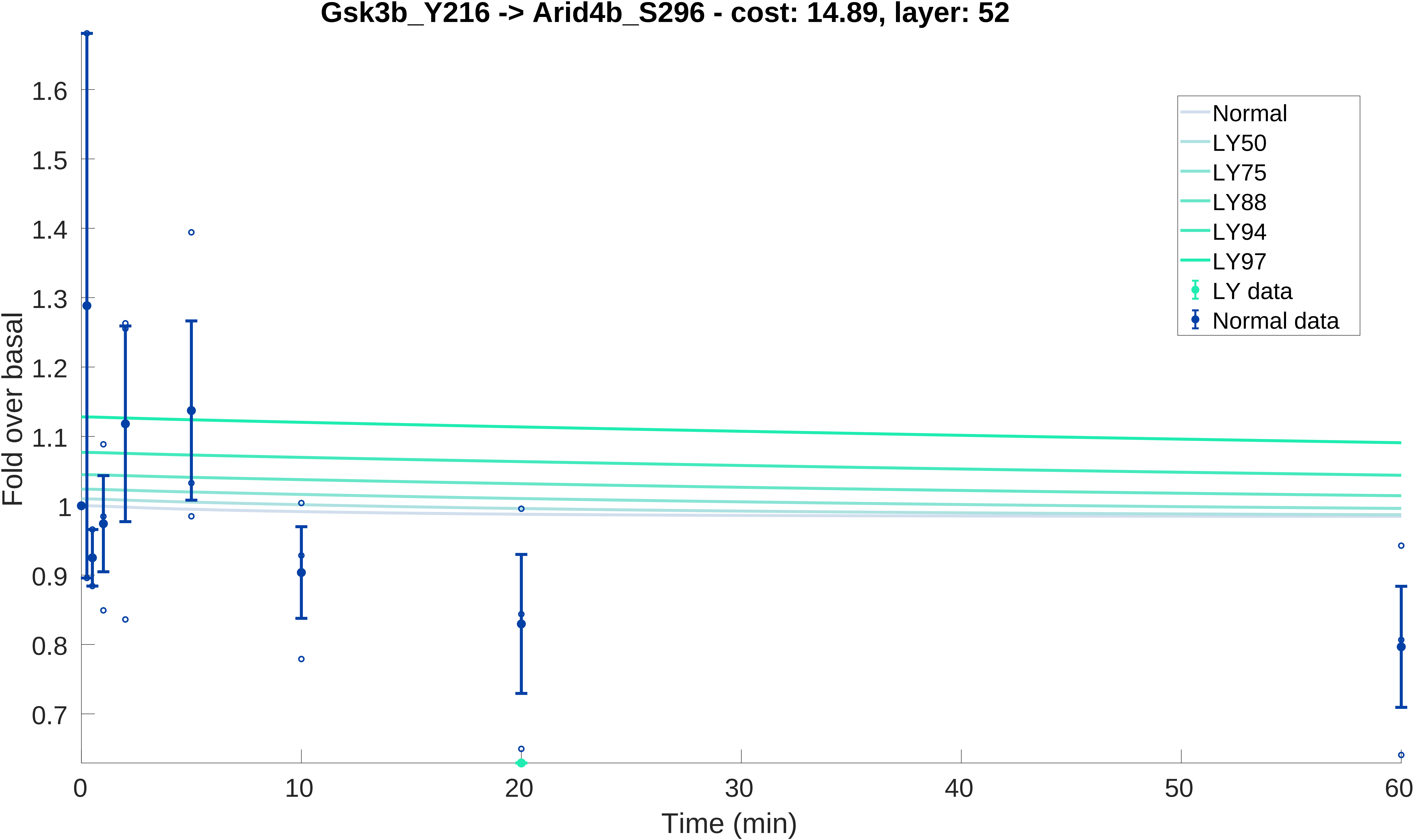

Gsk3b_Y216 -> Arid4b_S296 - cost: 14.89, layer: 52
Fold over basal
Time (min)
Normal
LY50
LY75
LY88
LY94
LY97
LY data
Normal data
0.7
0.8
0.9
1
1.1
1.2
1.3
1.4
1.5
1.6
0
10
20
30
40
50
60

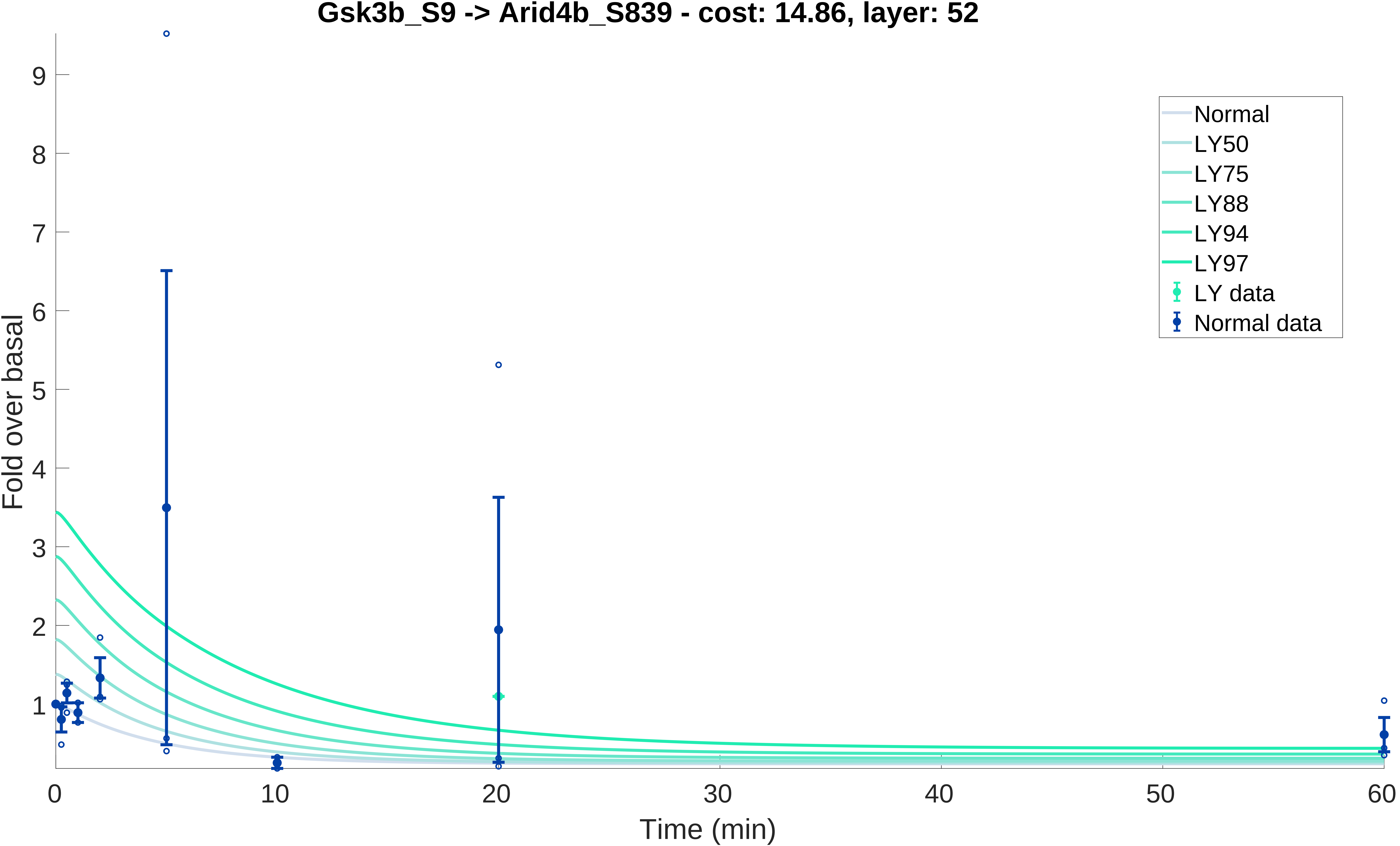

Gsk3b_S9 -> Arid4b_S839 - cost: 14.86, layer: 52
Fold over basal
Time (min)
Normal
LY50
LY75
LY88
LY94
LY97
LY data
Normal data
0
10
20
30
40
50
60
1
2
3
4
5
6
7
8
9

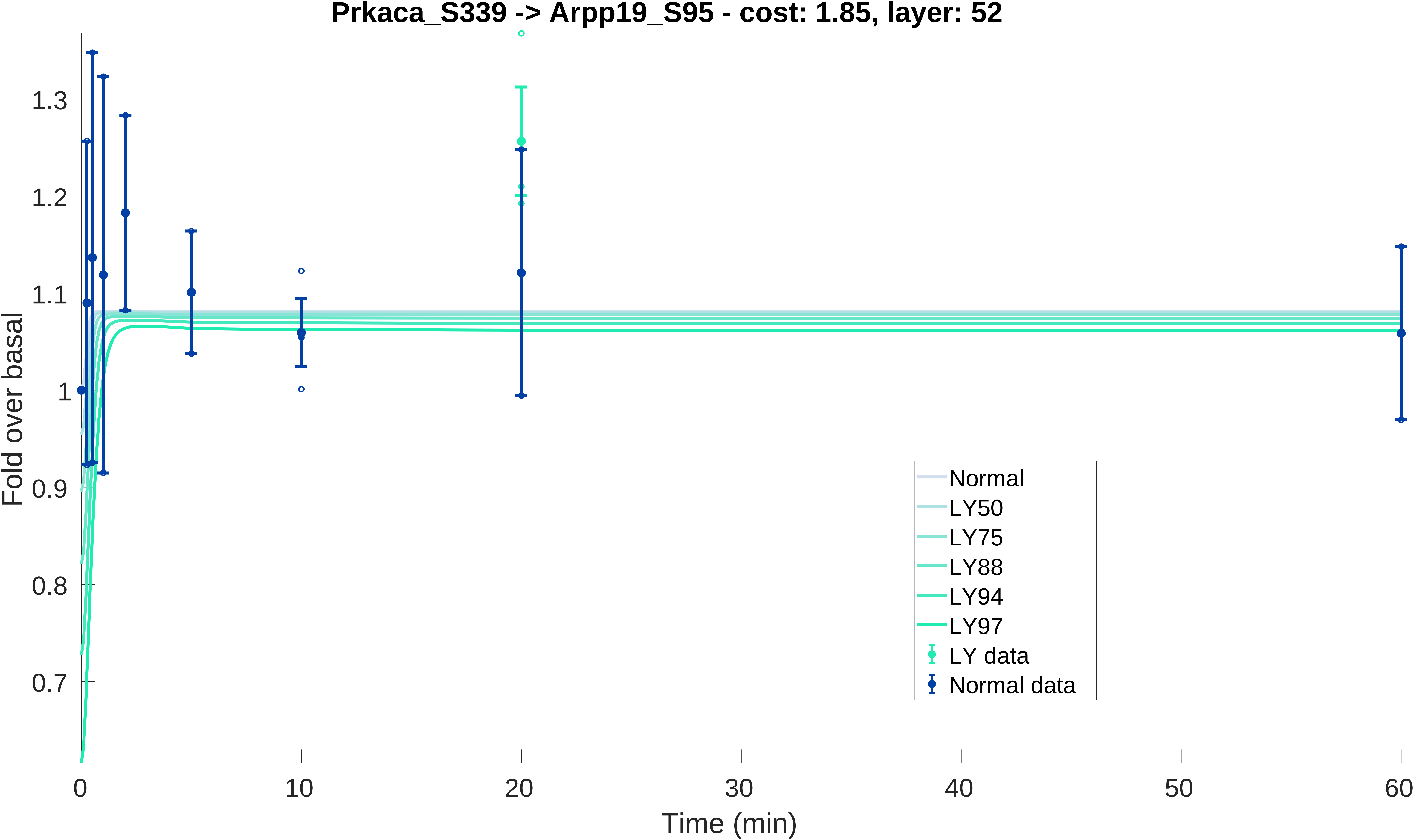
Prkaca_S339 -> Arpp19_S95 - cost: 1.85, layer: 52
Fold over basal
Time (min)
0.7
0.8
0.9
1
1.1
1.2
1.3
0
10
20
30
40
50
60
Normal
LY50
LY75
LY88
LY94
LY97
LY data
Normal data

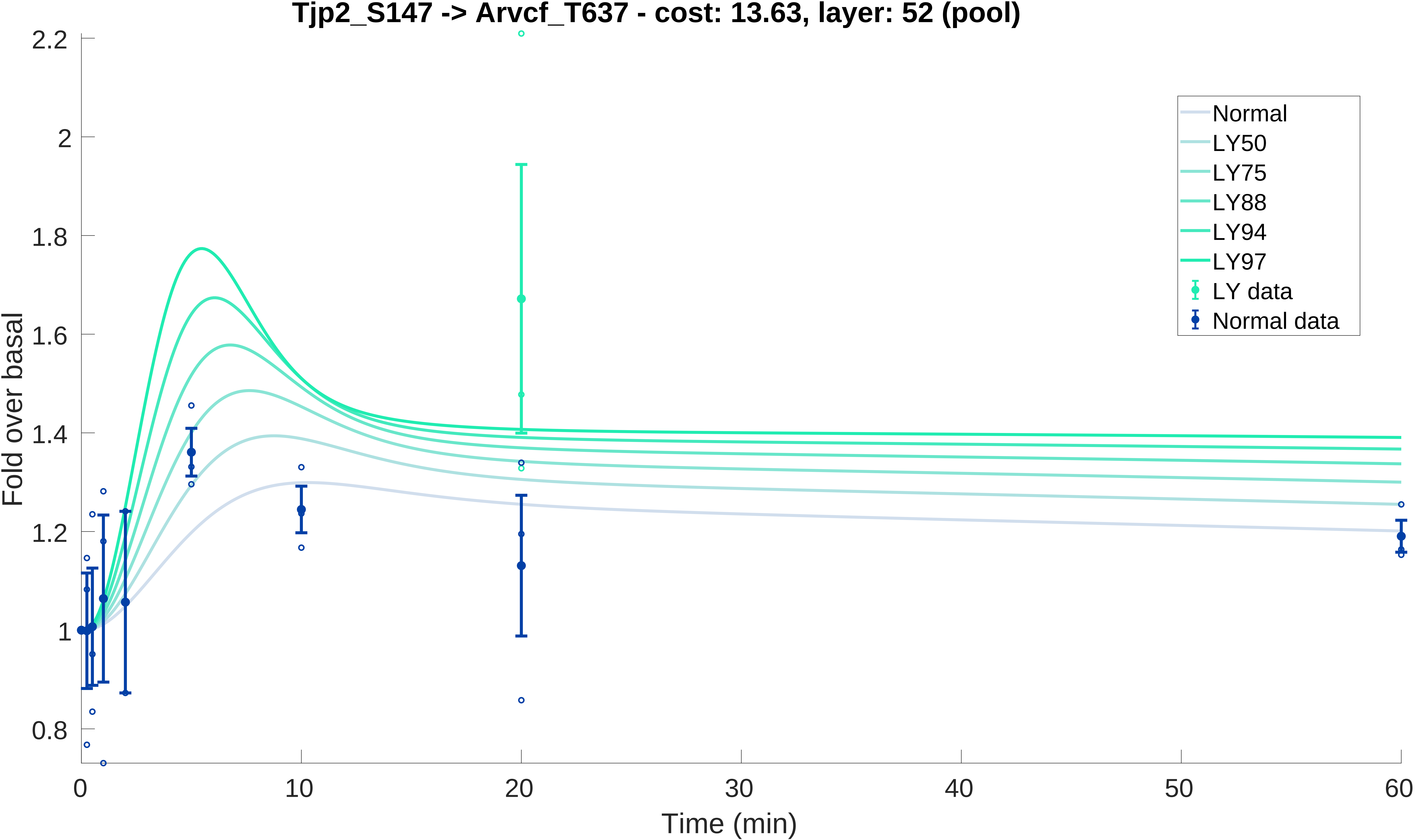
Tjp2_S147 -> Arvcf_T637 - cost: 13.63, layer: 52 (pool)
Fold over basal
Time (min)
Normal
LY50
LY75
LY88
LY94
LY97
LY data
Normal data
0.8
1
1.2
1.4
1.6
1.8
2
2.2
0
10
20
30
40
50
60

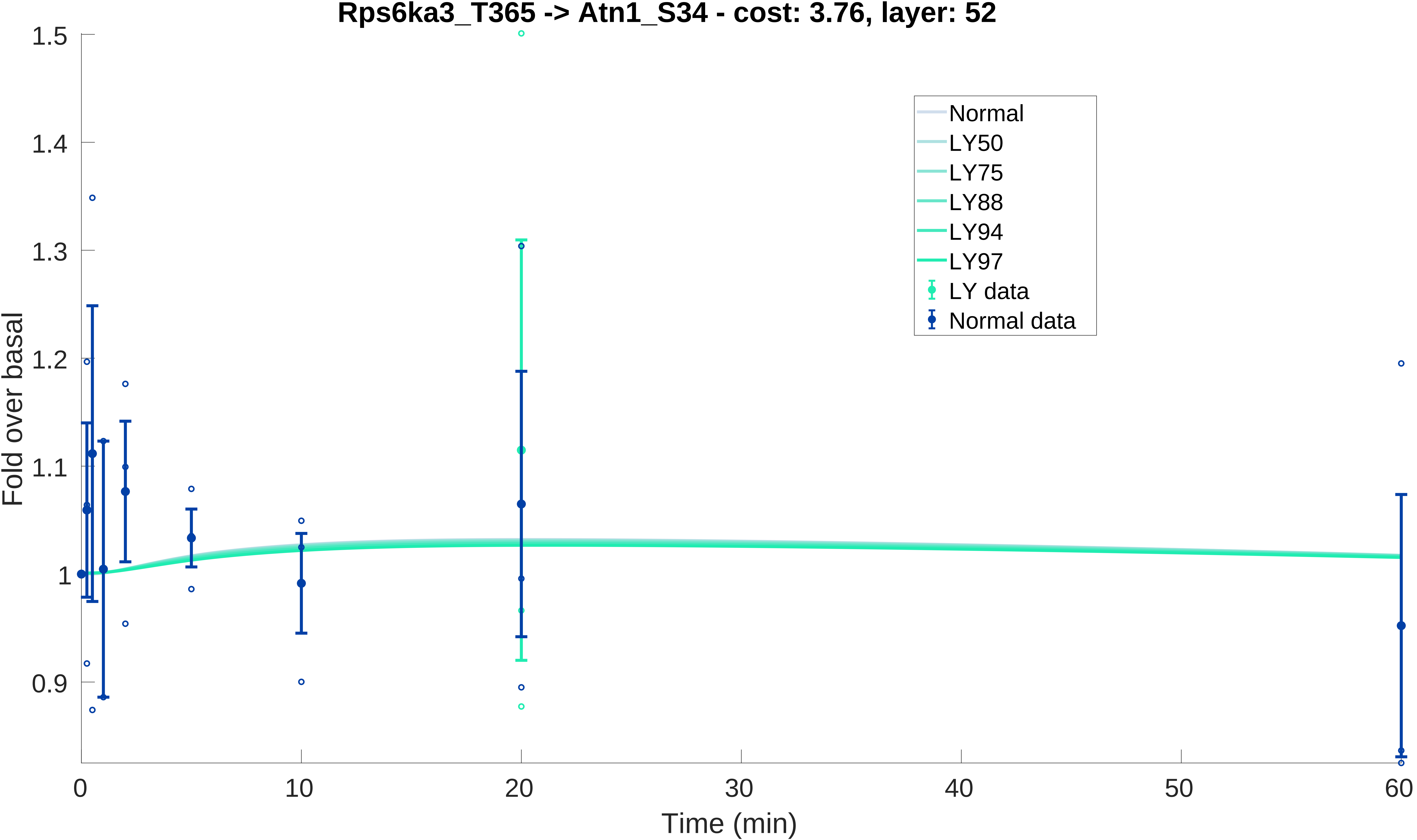

Rps6ka3_T365 -> Atn1_S34 - cost: 3.76, layer: 52
1.5
1.4
1.3
1.2
1.1
1
0.9
Fold over basal
0
10
20
30
40
50
60
Time (min)
Normal
LY50
LY75
LY88
LY94
LY97
LY data
Normal data

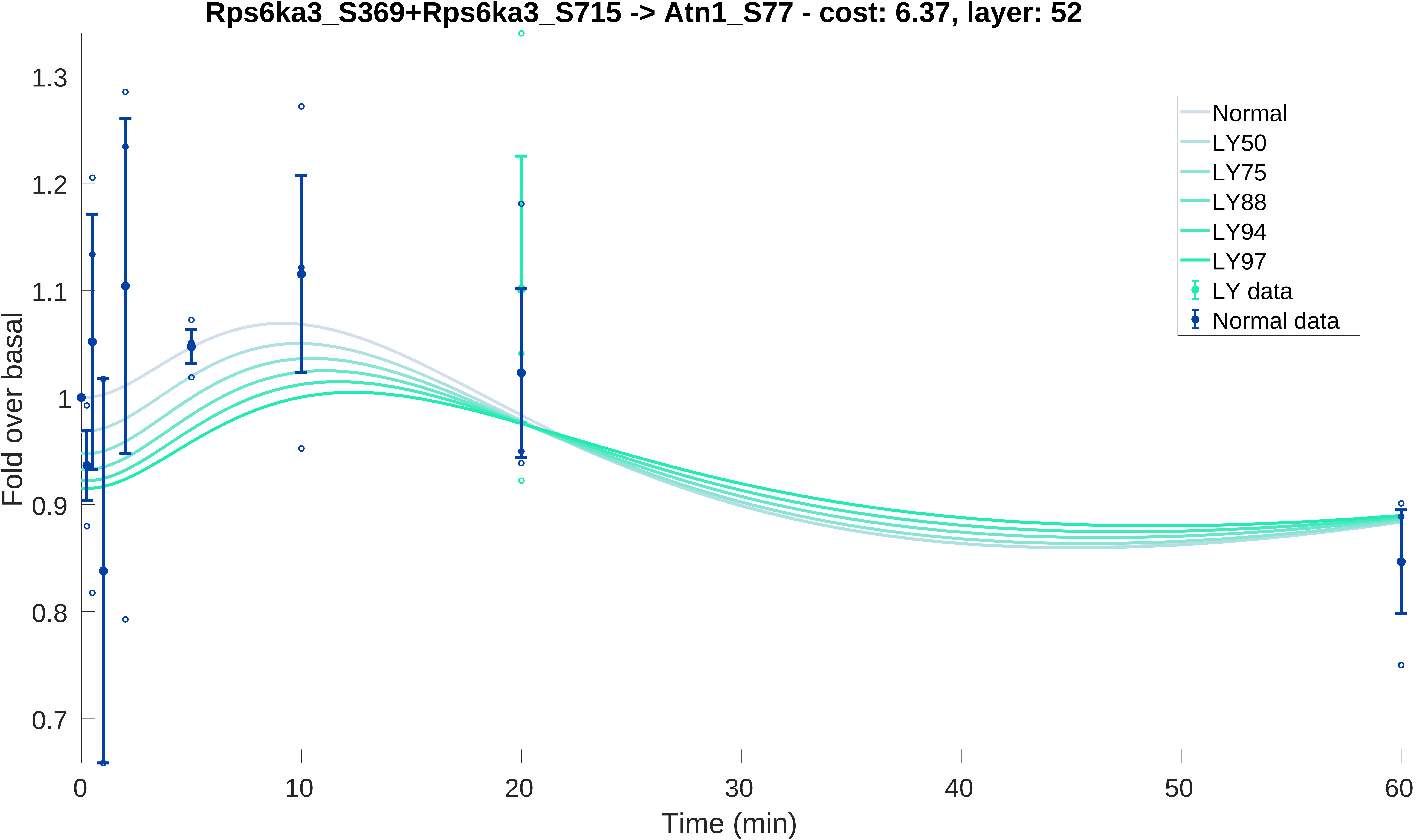

Rps6ka3_S369+Rps6ka3_S715 -> Atn1_S77 - cost: 6.37, layer: 52
Fold over basal
Time (min)
0.7
0.8
0.9
1
1.1
1.2
1.3
0
10
20
30
40
50
60
Normal
LY50
LY75
LY88
LY94
LY97
LY data
Normal data

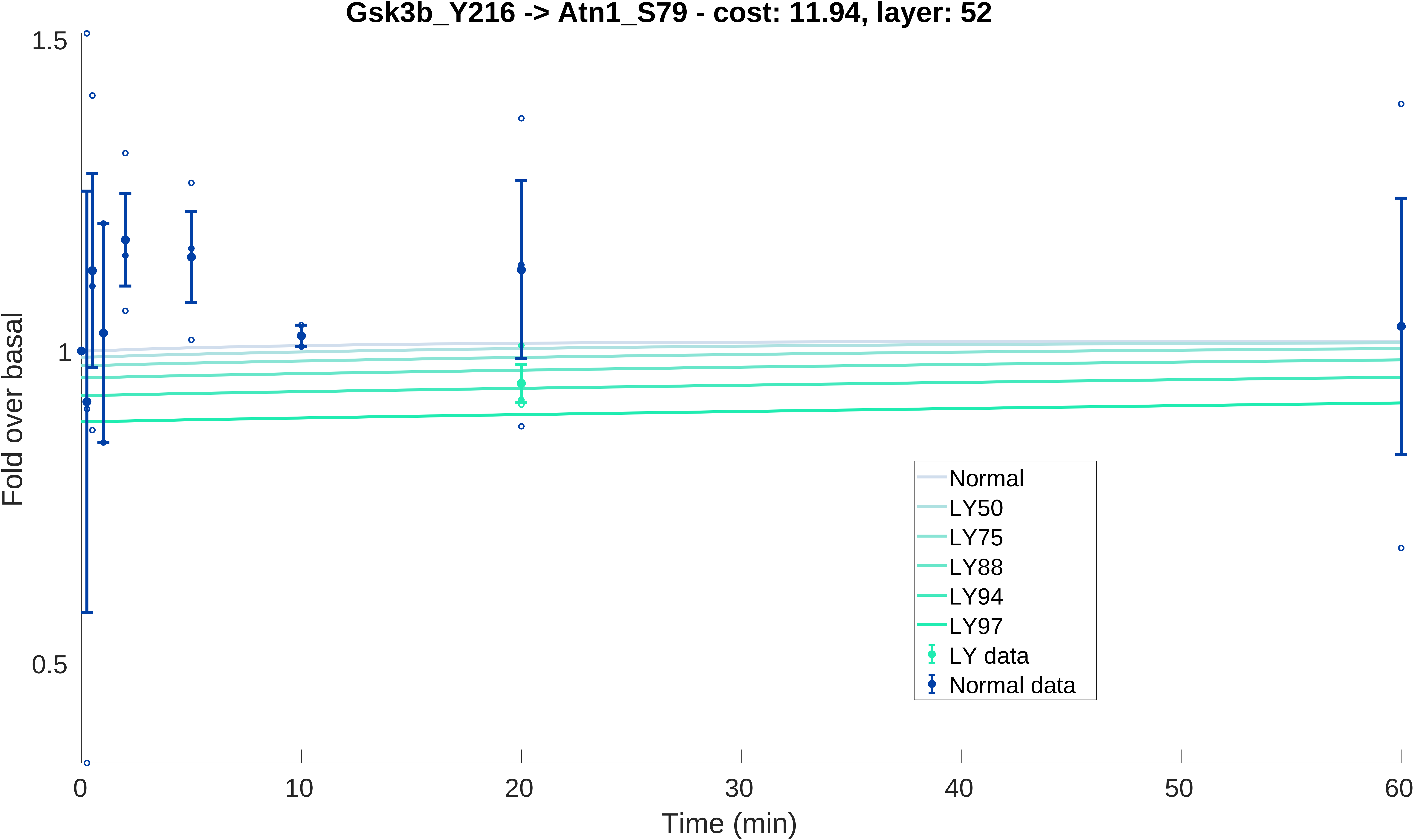

Gsk3b_Y216 -> Atn1_S79 - cost: 11.94, layer: 52
1.5
1
0.5
Fold over basal
0
10
20
30
40
50
60
Time (min)
Normal
LY50
LY75
LY88
LY94
LY97
LY data
Normal data

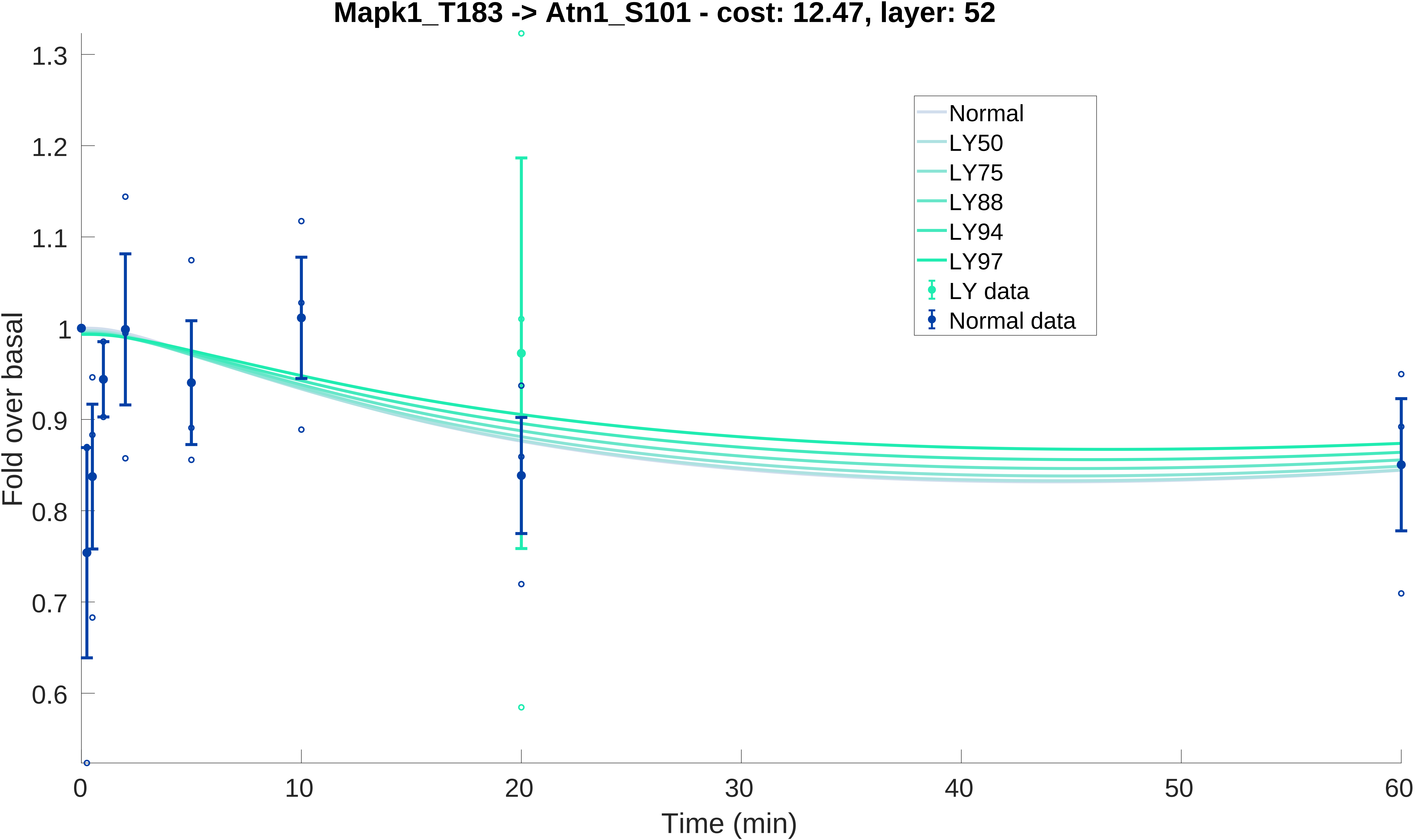
Mapk1_T183 -> Atn1_S101 - cost: 12.47, layer: 52
Fold over basal
Time (min)
Normal
LY50
LY75
LY88
LY94
LY97
LY data
Normal data
1.3
1.2
1.1
1
0.9
0.8
0.7
0.6
0
10
20
30
40
50
60

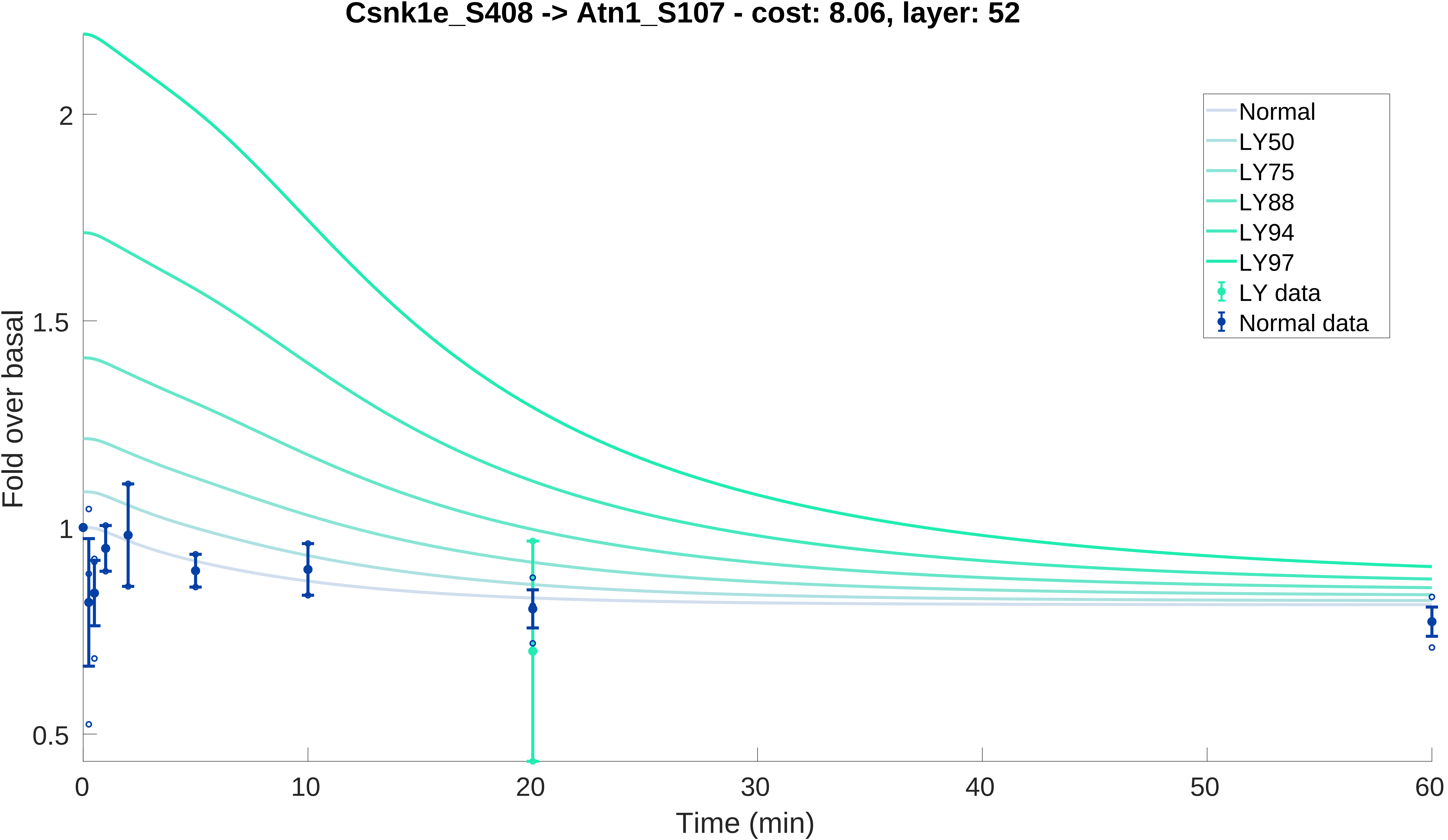

Csnk1e_S408 -> Atn1_S107 - cost: 8.06, layer: 52
Fold over basal
Time (min)
0
10
20
30
40
50
60
0.5
1
1.5
2
Normal
LY50
LY75
LY88
LY94
LY97
LY data
Normal data

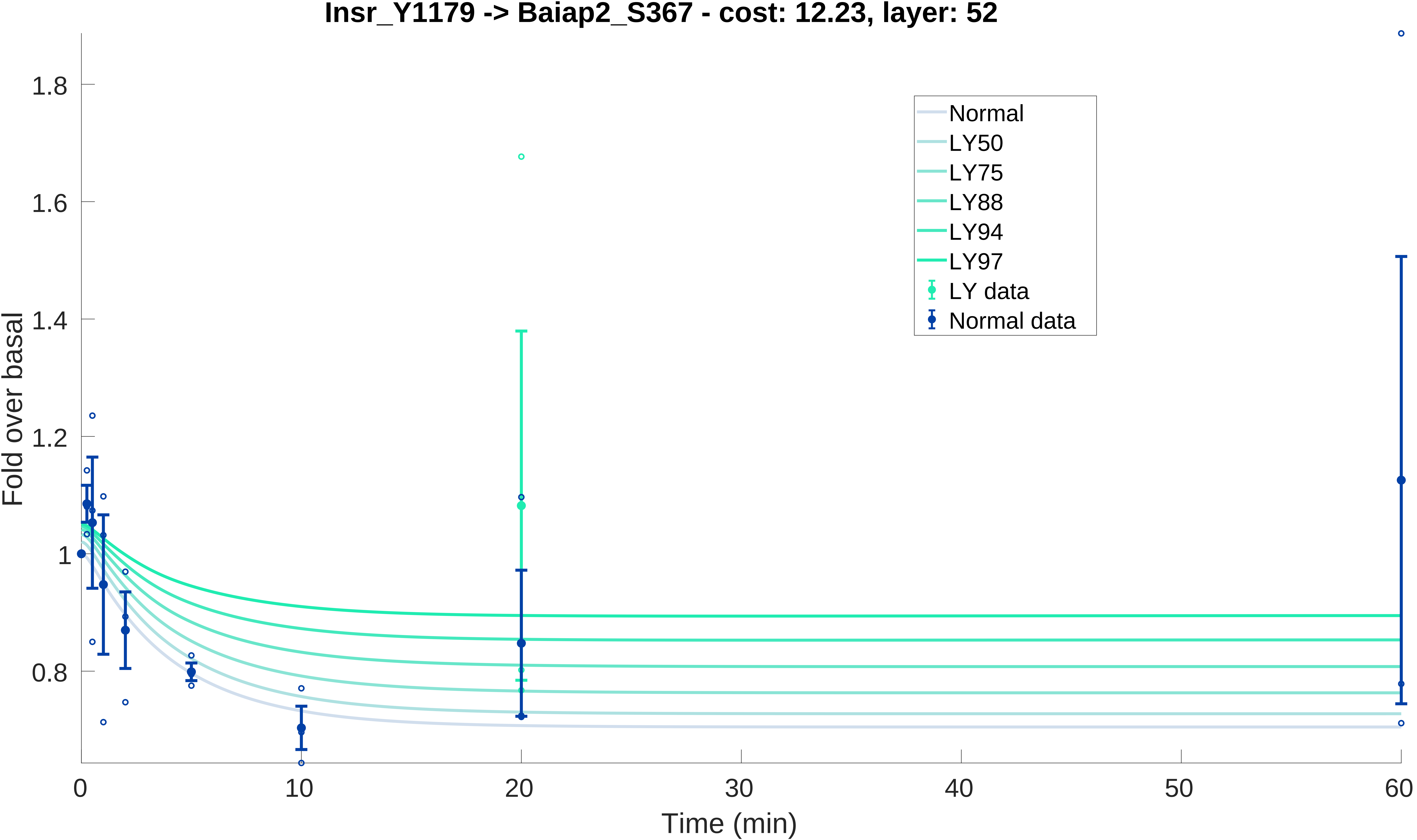
Insr_Y1179 -> Baiap2_S367 - cost: 12.23, layer: 52
Fold over basal
1.8
1.6
1.4
1.2
1
0.8
0
10
20
30
40
50
60
Time (min)
Normal
LY50
LY75
LY88
LY94
LY97
LY data
Normal data

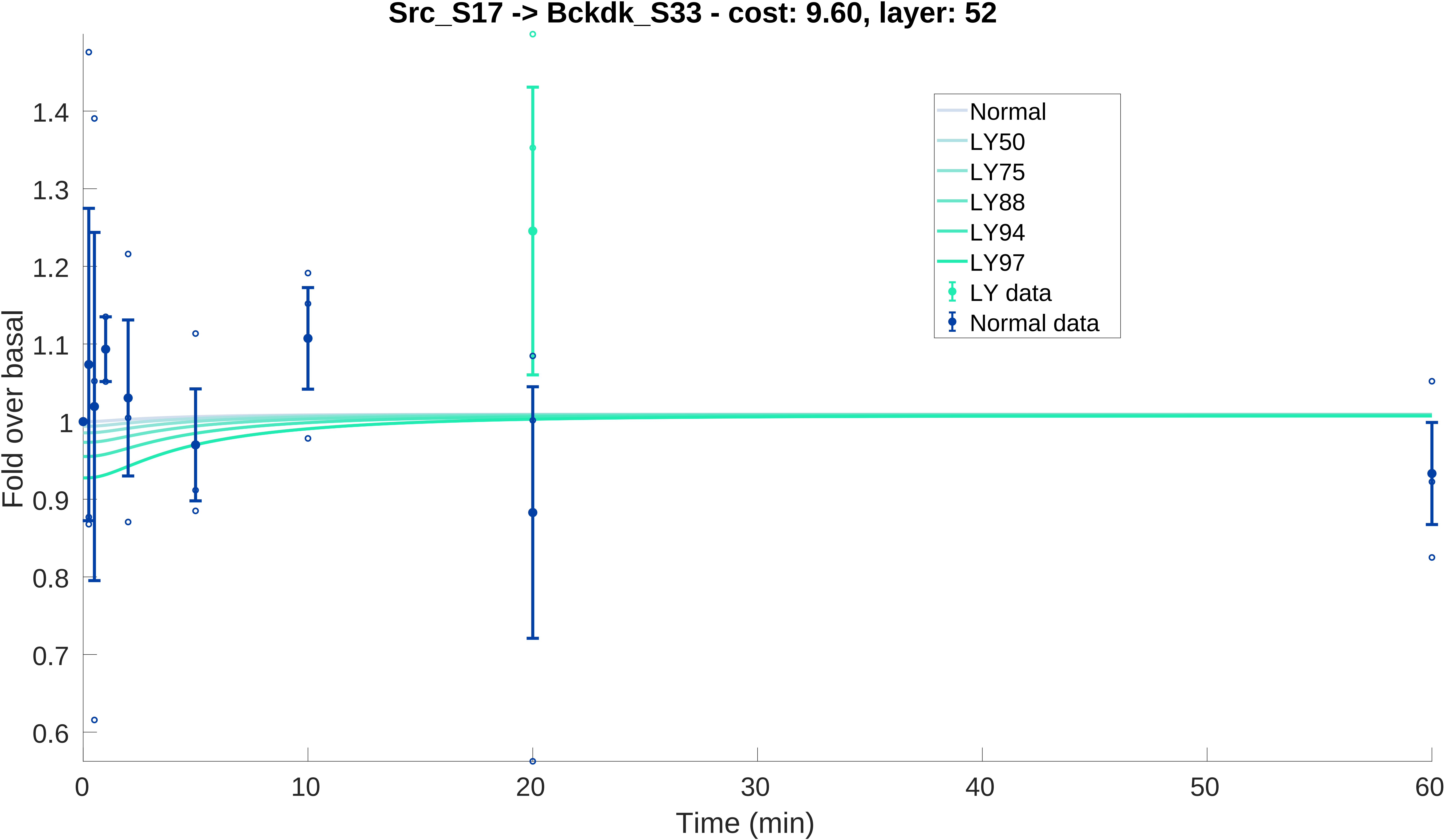

Src_S17 -> Bckdk_S33 - cost: 9.60, layer: 52
Fold over basal
Time (min)
0.6
0.7
0.8
0.9
1
1.1
1.2
1.3
1.4
0
10
20
30
40
50
60
Normal
LY50
LY75
LY88
LY94
LY97
LY data
Normal data

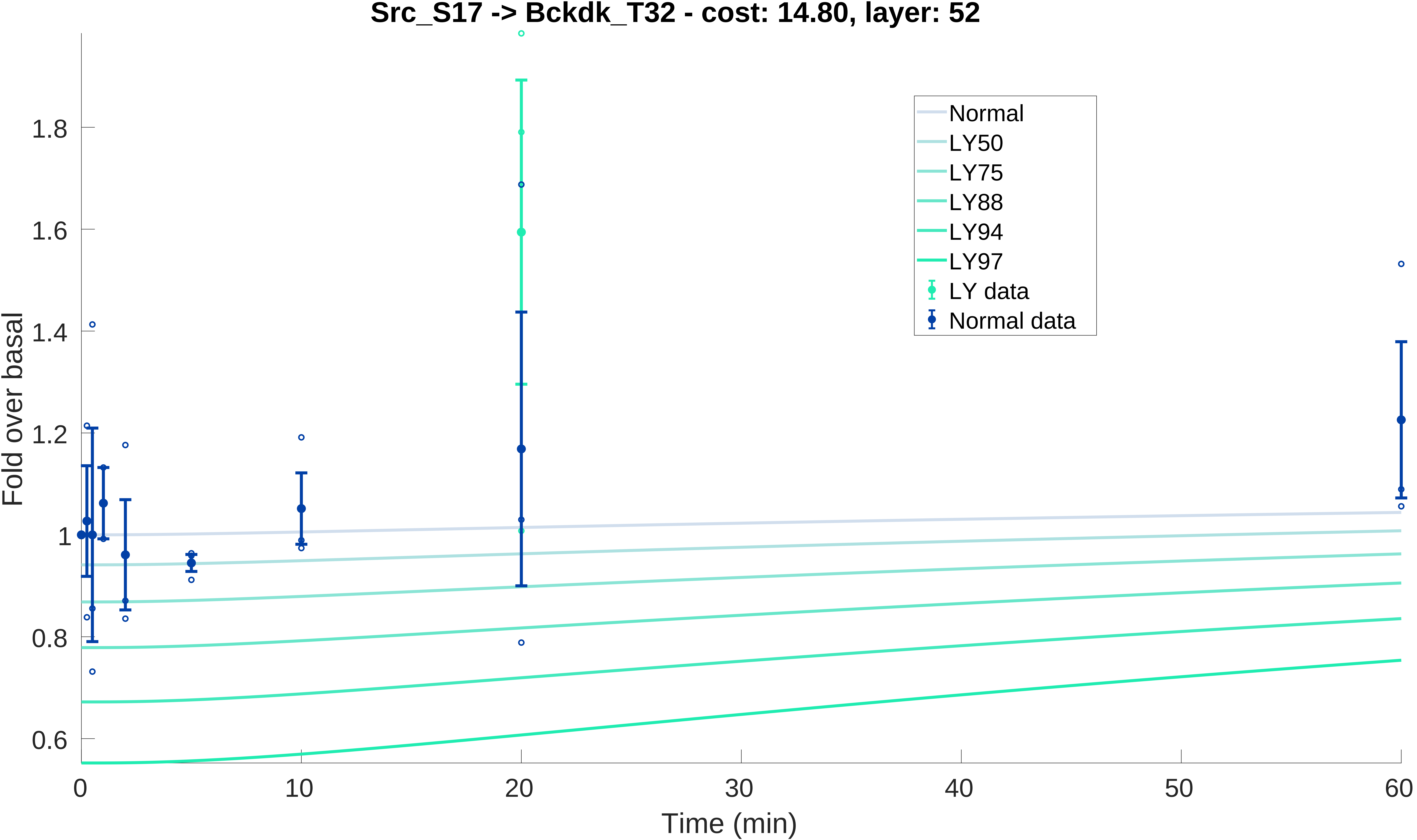

Src_S17 -> Bckdk_T32 - cost: 14.80, layer: 52
Fold over basal
Time (min)
Normal
LY50
LY75
LY88
LY94
LY97
LY data
Normal data
0.6
0.8
1
1.2
1.4
1.6
1.8
0
10
20
30
40
50
60

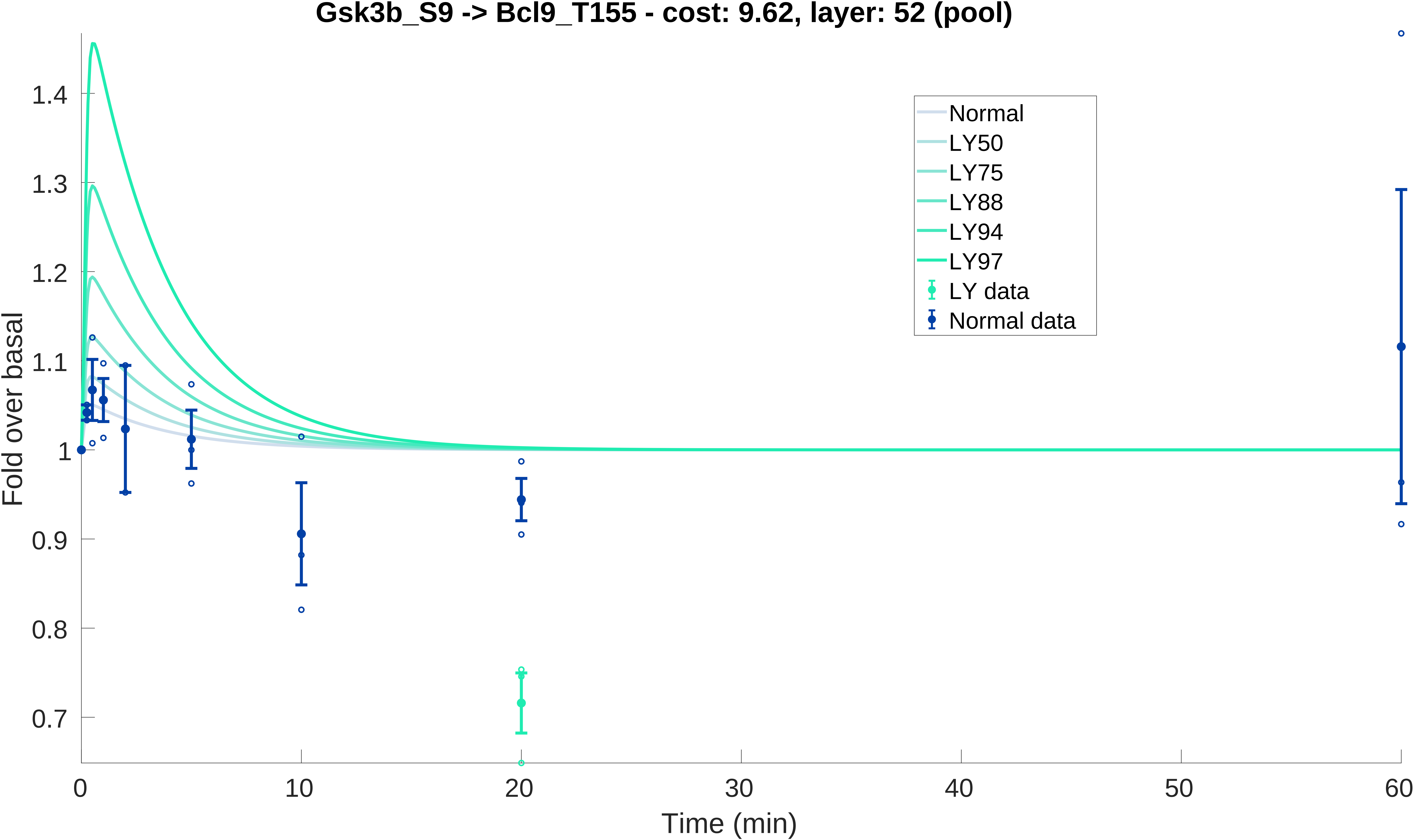
Gsk3b_S9 -> Bcl9_T155 - cost: 9.62, layer: 52 (pool)
Fold over basal
1.4
1.3
1.2
1.1
1
0.9
0.8
0.7
0
10
20
30
40
50
60
Time (min)
Normal
LY50
LY75
LY88
LY94
LY97
LY data
Normal data

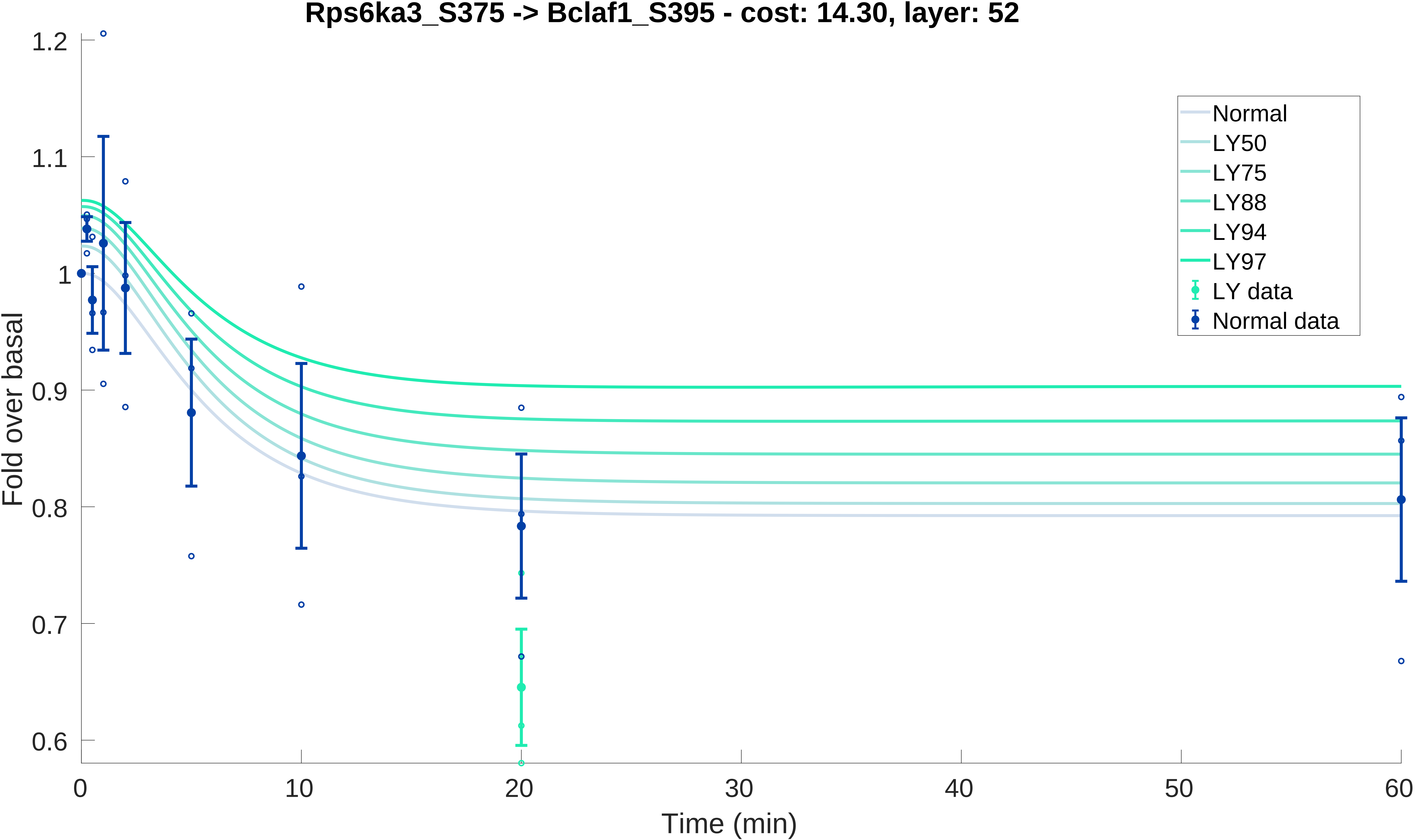
Rps6ka3_S375 -> Bclaf1_S395 - cost: 14.30, layer: 52
Fold over basal
1.2
1.1
1
0.9
0.8
0.7
0.6
0
10
20
30
40
50
60
Time (min)
Normal
LY50
LY75
LY88
LY94
LY97
LY data
Normal data

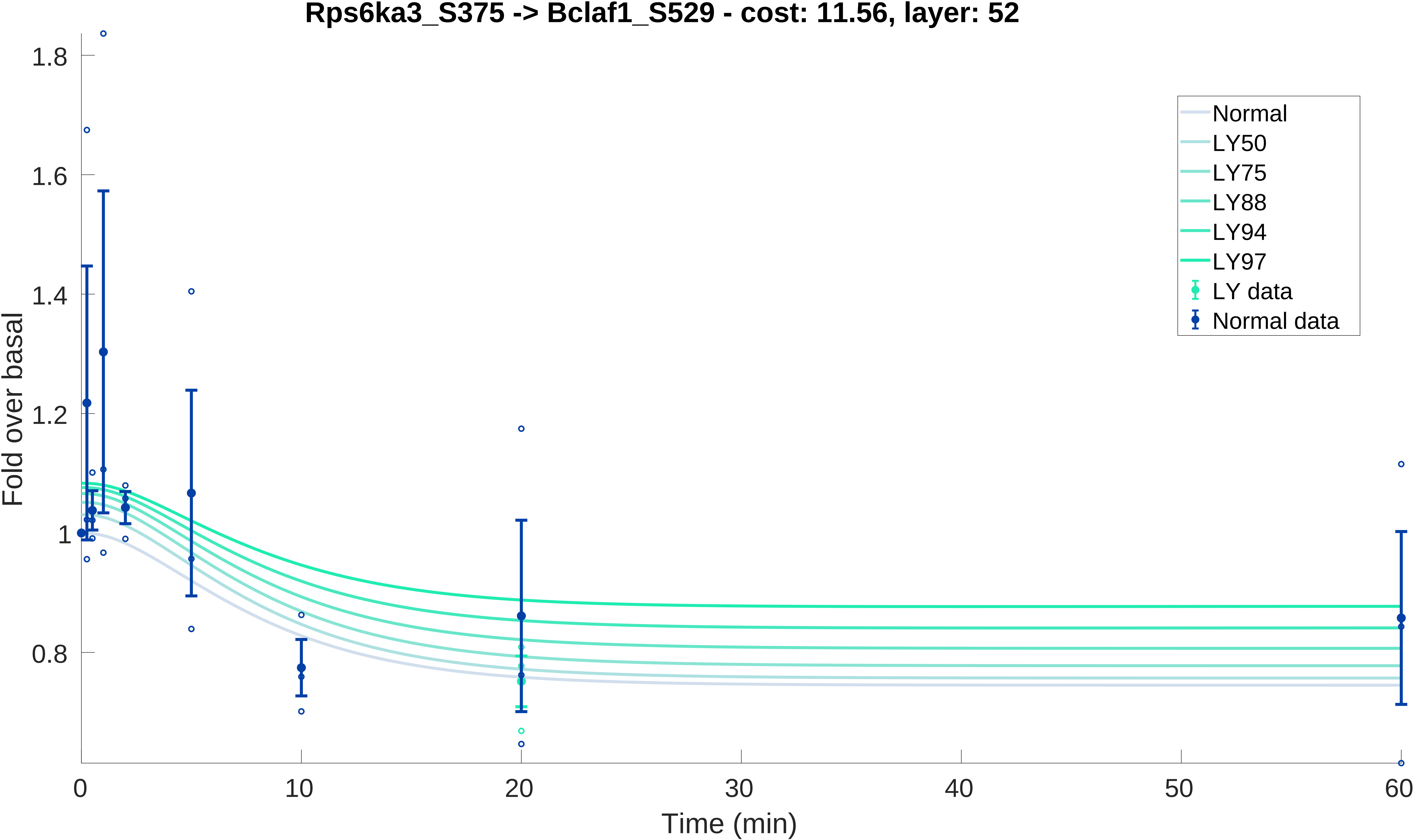

Rps6ka3_S375 -> Bclaf1_S529 - cost: 11.56, layer: 52
Fold over basal
Time (min)
0
10
20
30
40
50
60
0.8
1
1.2
1.4
1.6
1.8
Normal
LY50
LY75
LY88
LY94
LY97
LY data
Normal data

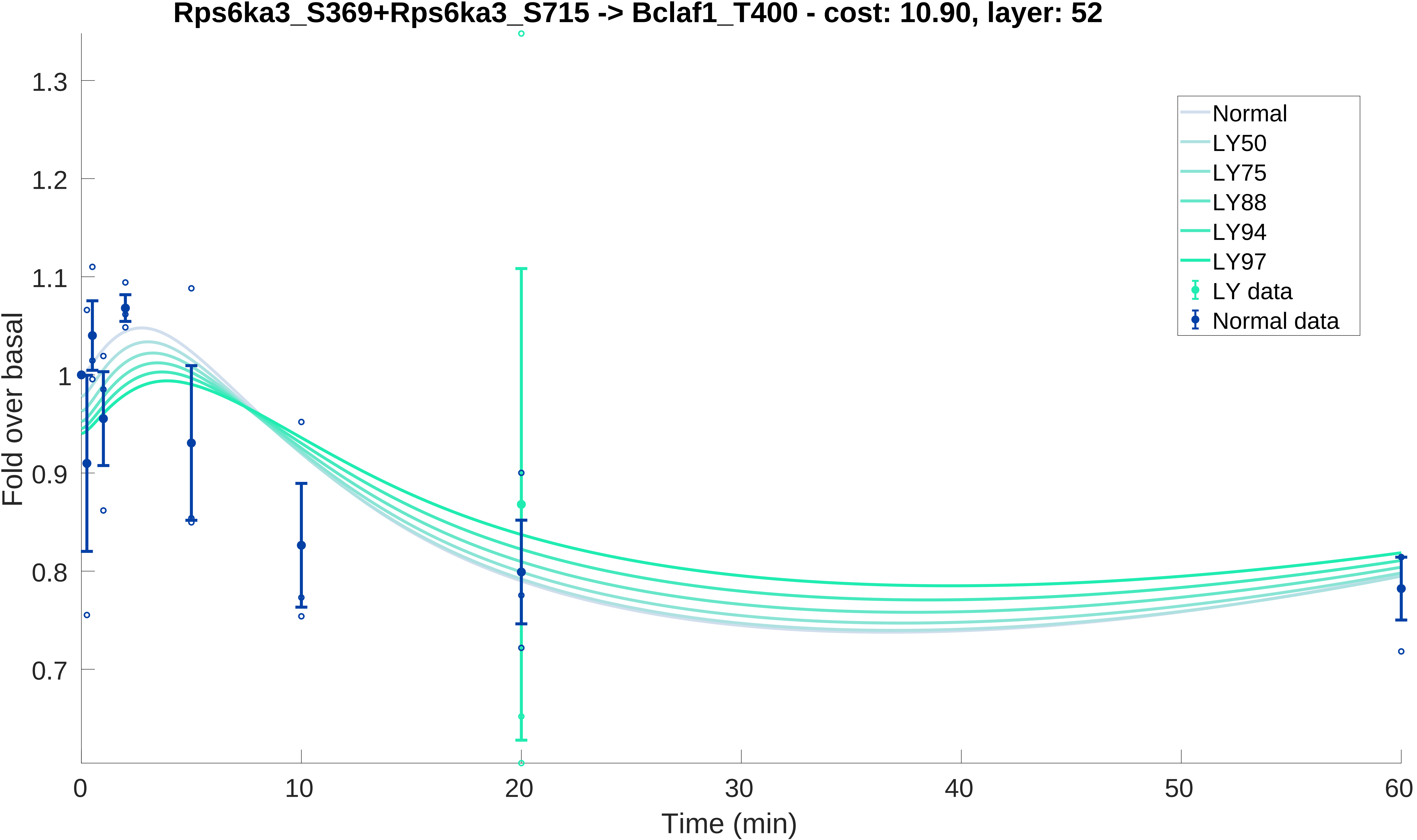

Rps6ka3_S369+Rps6ka3_S715 -> Bclaf1_T400 - cost: 10.90, layer: 52
Fold over basal
Time (min)
0
10
20
30
40
50
60
0.7
0.8
0.9
1
1.1
1.2
1.3
Normal
LY50
LY75
LY88
LY94
LY97
LY data
Normal data

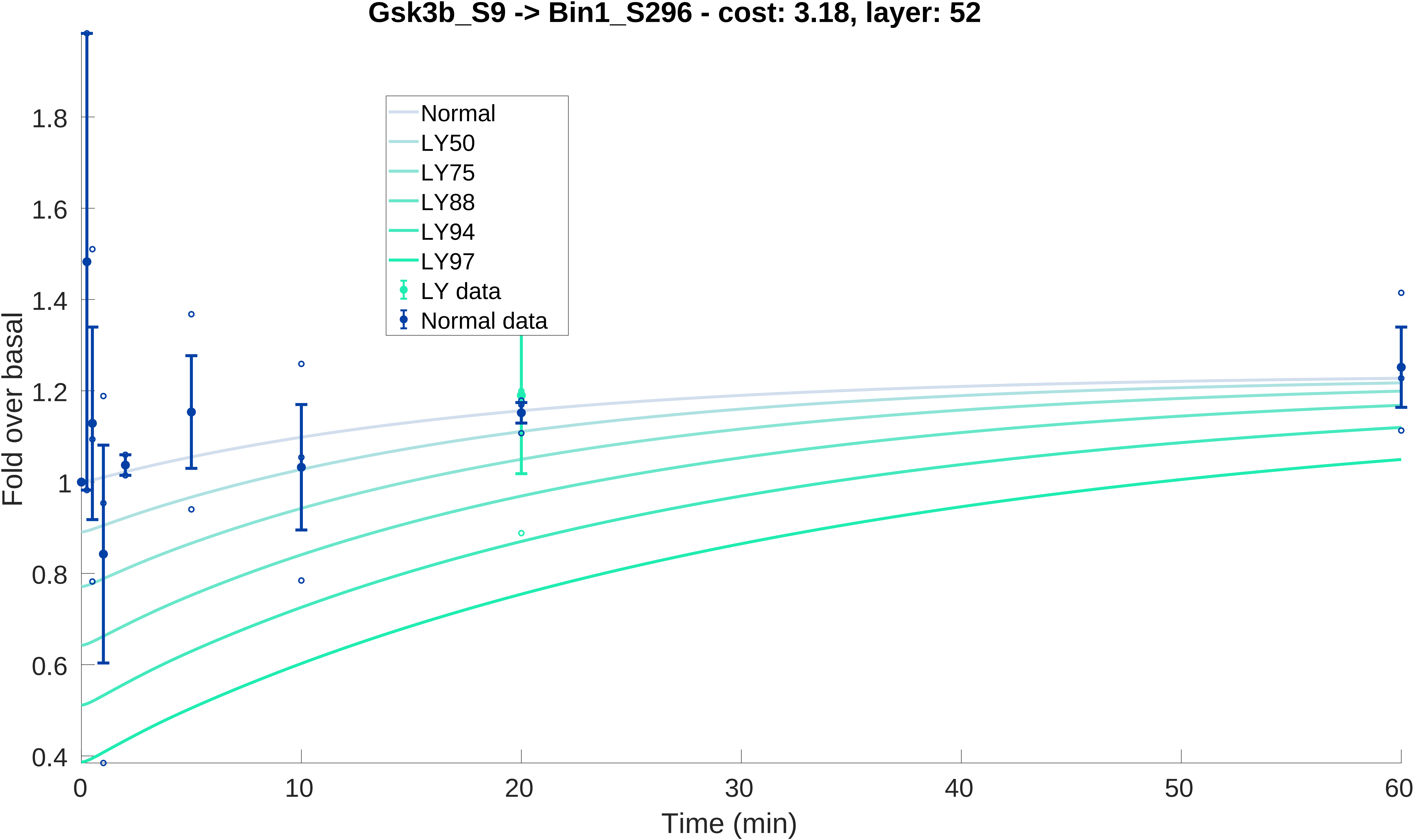

Gsk3b_S9 -> Bin1_S296 - cost: 3.18, layer: 52
Normal
LY50
LY75
LY88
LY94
LY97
LY data
Normal data
Fold over basal
Time (min)
1.8
1.6
1.4
1.2
1
0.8
0.6
0.4
0
10
20
30
40
50
60

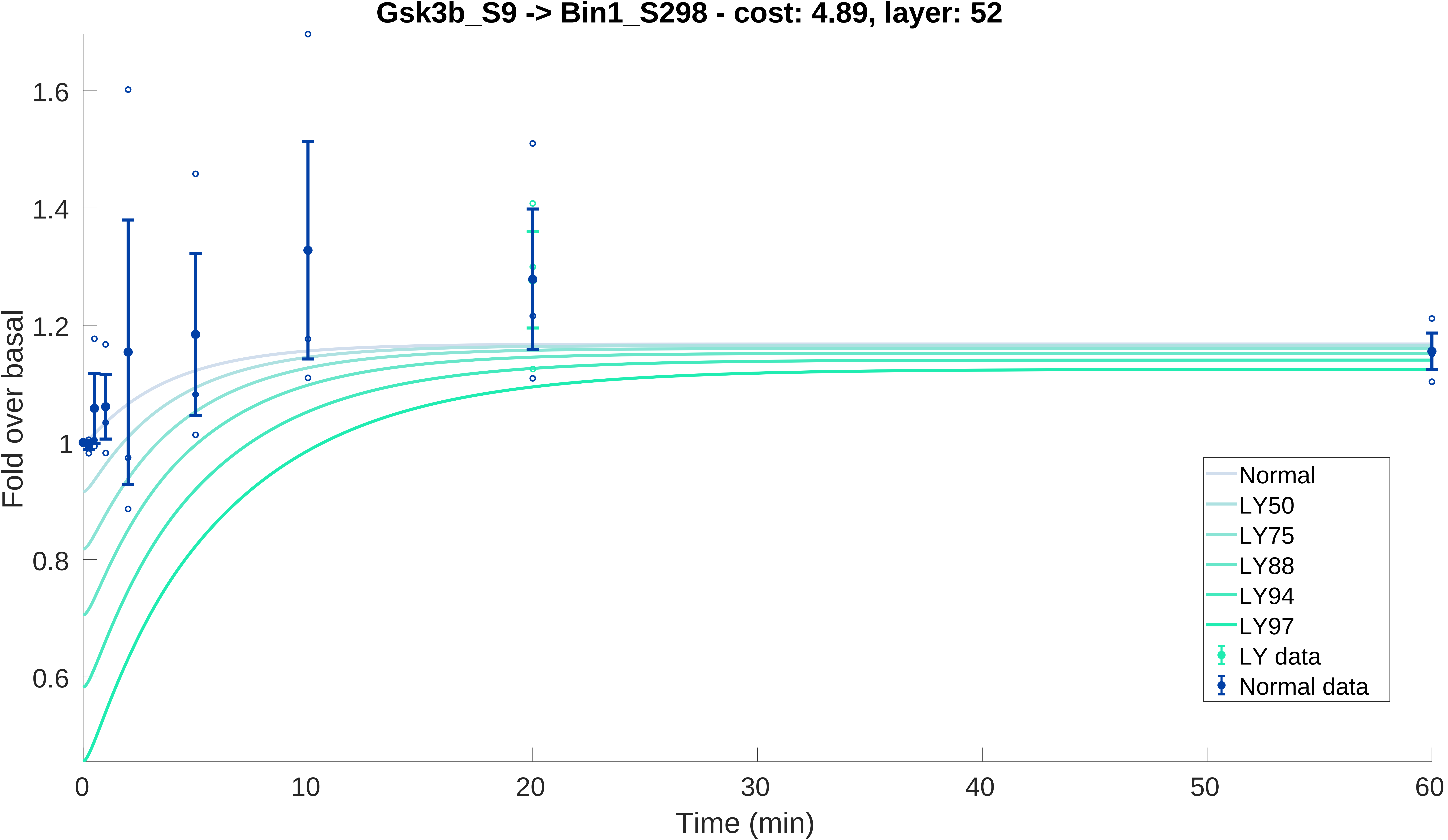

Gsk3b_S9 -> Bin1_S298 - cost: 4.89, layer: 52
Fold over basal
1.6
1.4
1.2
1
0.8
0.6
0
10
20
30
40
50
60
Time (min)
Normal
LY50
LY75
LY88
LY94
LY97
LY data
Normal data

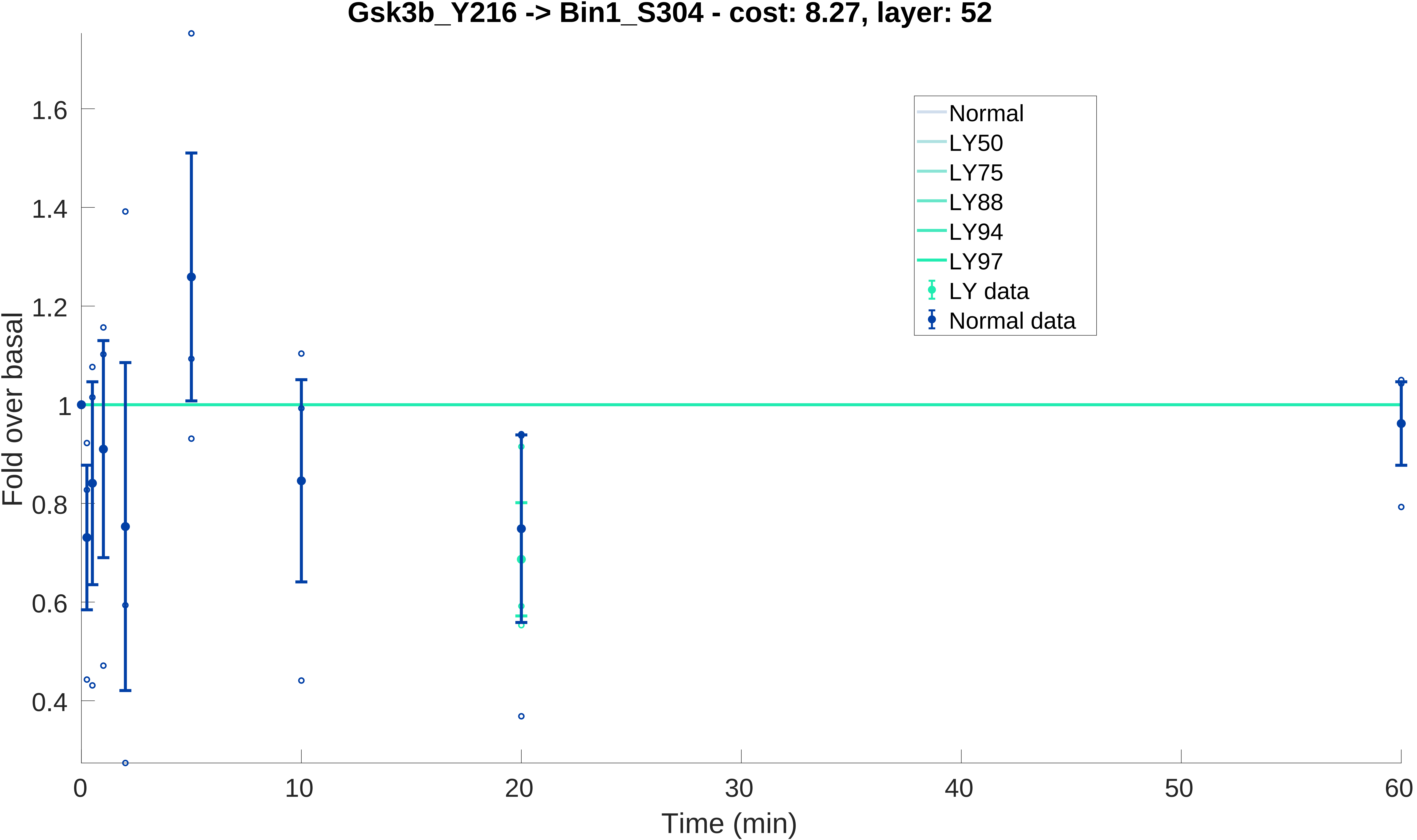

Gsk3b_Y216 -> Bin1_S304 - cost: 8.27, layer: 52
Fold over basal
Time (min)
0.4
0.6
0.8
1
1.2
1.4
1.6
0
10
20
30
40
50
60
Normal
LY50
LY75
LY88
LY94
LY97
LY data
Normal data

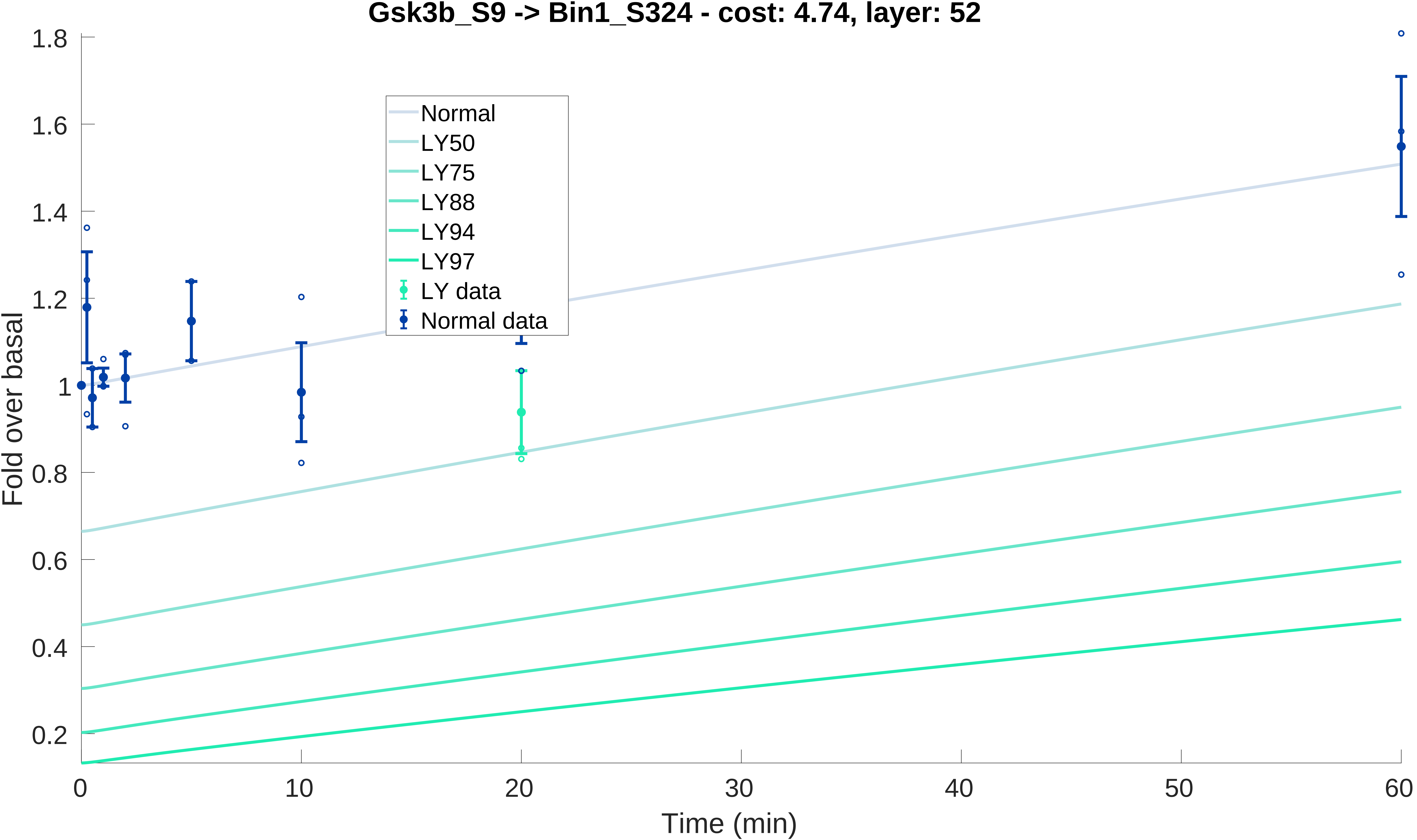

Gsk3b_S9 -> Bin1_S324 - cost: 4.74, layer: 52
Normal
LY50
LY75
LY88
LY94
LY97
LY data
Normal data
Fold over basal
Time (min)
1.8
1.6
1.4
1.2
1
0.8
0.6
0.4
0.2
0
10
20
30
40
50
60

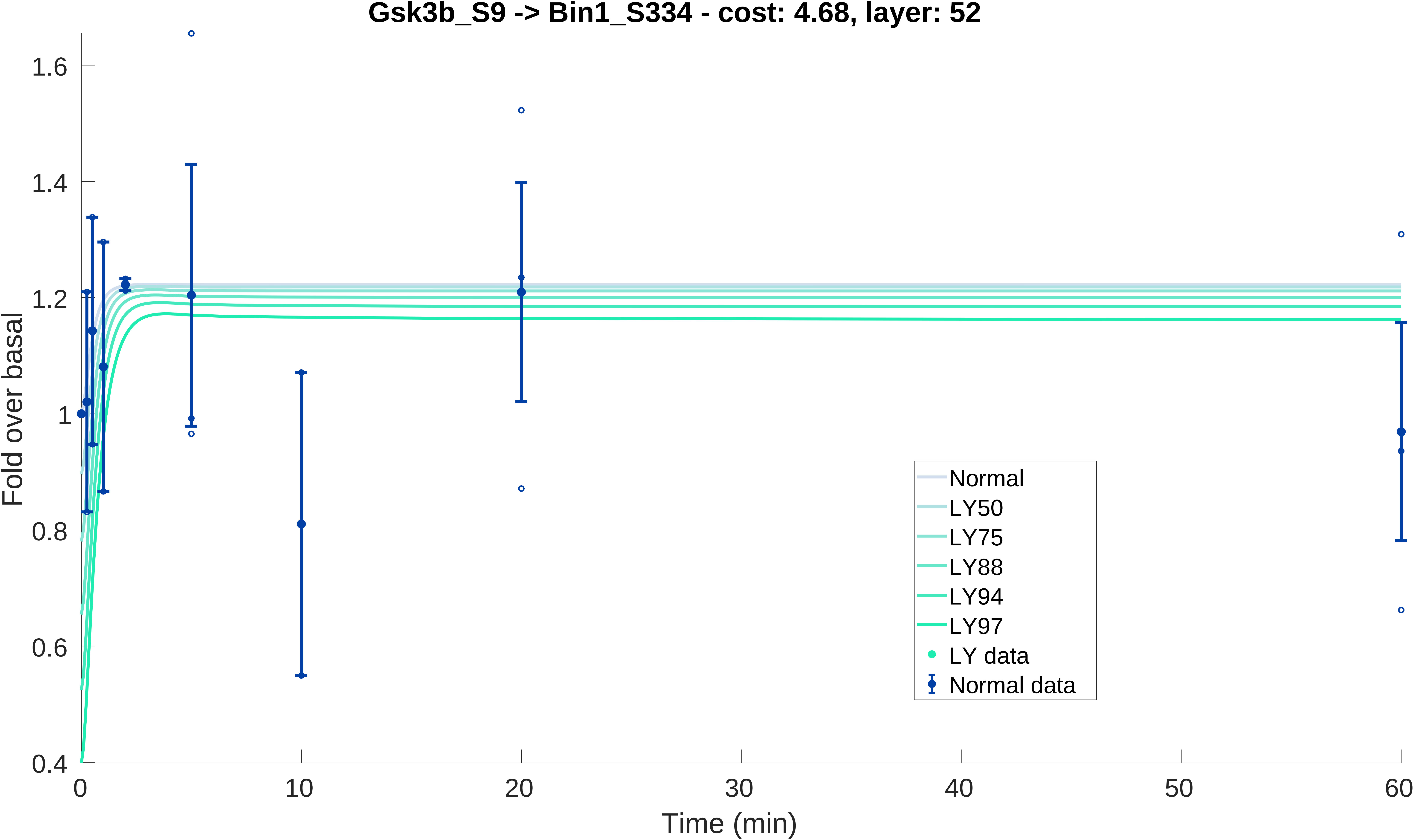
Gsk3b_S9 -> Bin1_S334 - cost: 4.68, layer: 52
Fold over basal
Time (min)
1.6
1.4
1.2
1
0.8
0.6
0.4
0
10
20
30
40
50
60
Normal
LY50
LY75
LY88
LY94
LY97
LY data
Normal data

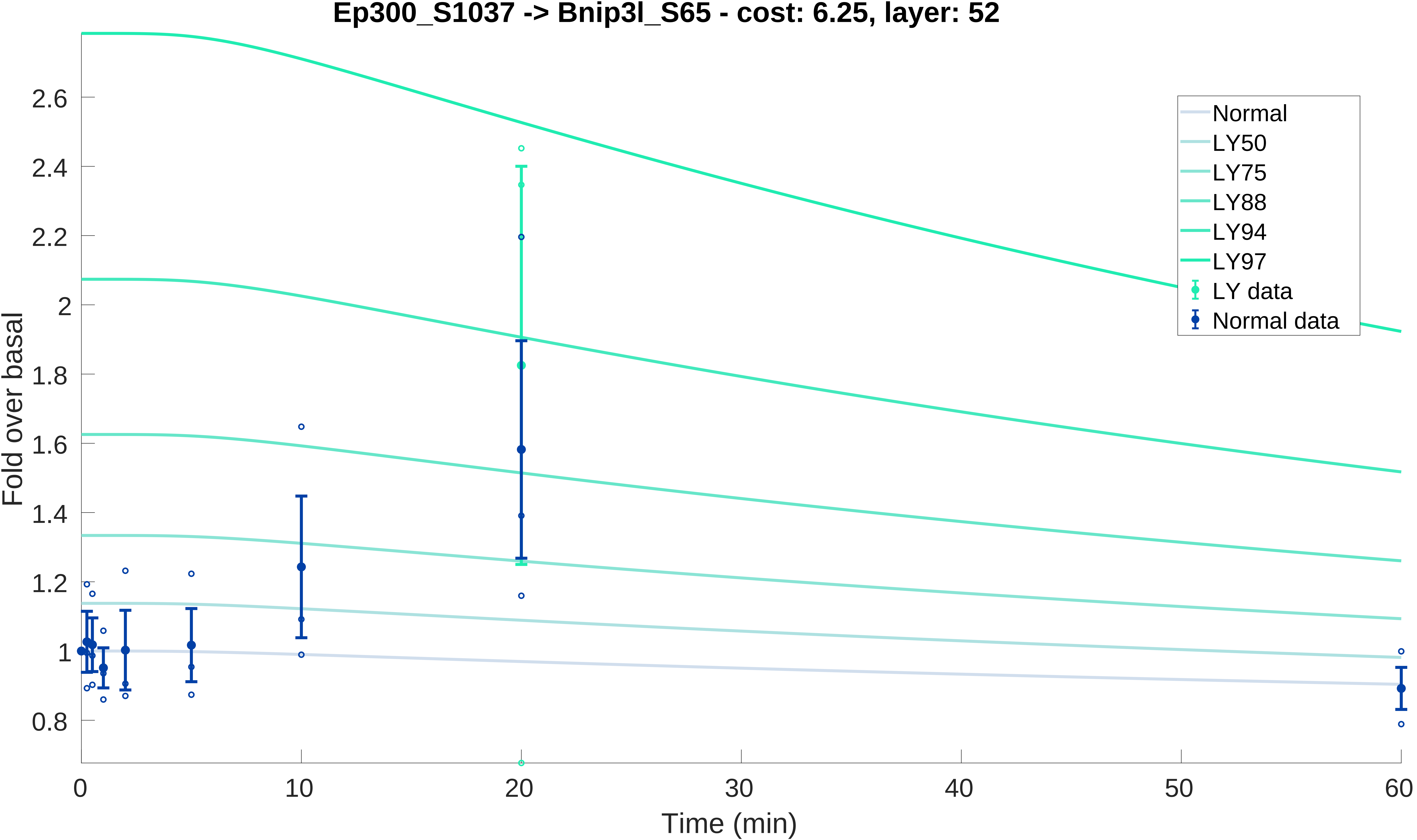
Ep300_S1037 -> Bnip3l_S65 - cost: 6.25, layer: 52
Fold over basal
Time (min)
0
10
20
30
40
50
60
0.8
1
1.2
1.4
1.6
1.8
2
2.2
2.4
2.6
Normal
LY50
LY75
LY88
LY94
LY97
LY data
Normal data

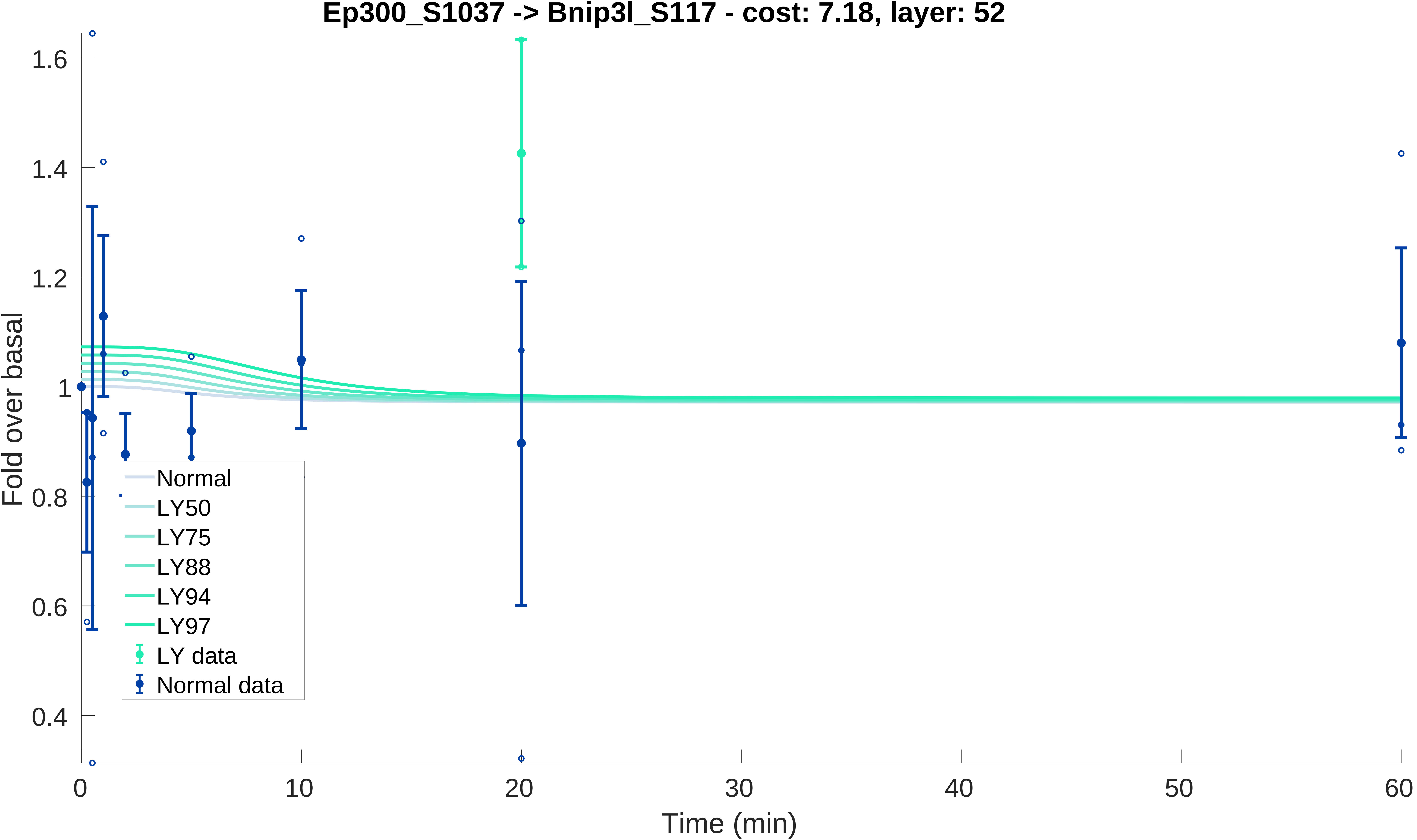

Ep300_S1037 -> Bnip3l_S117 - cost: 7.18, layer: 52
Fold over basal
1.6
1.4
1.2
1
0.8
0.6
0.4
0
10
20
30
40
50
60
Time (min)
Normal
LY50
LY75
LY88
LY94
LY97
LY data
Normal data

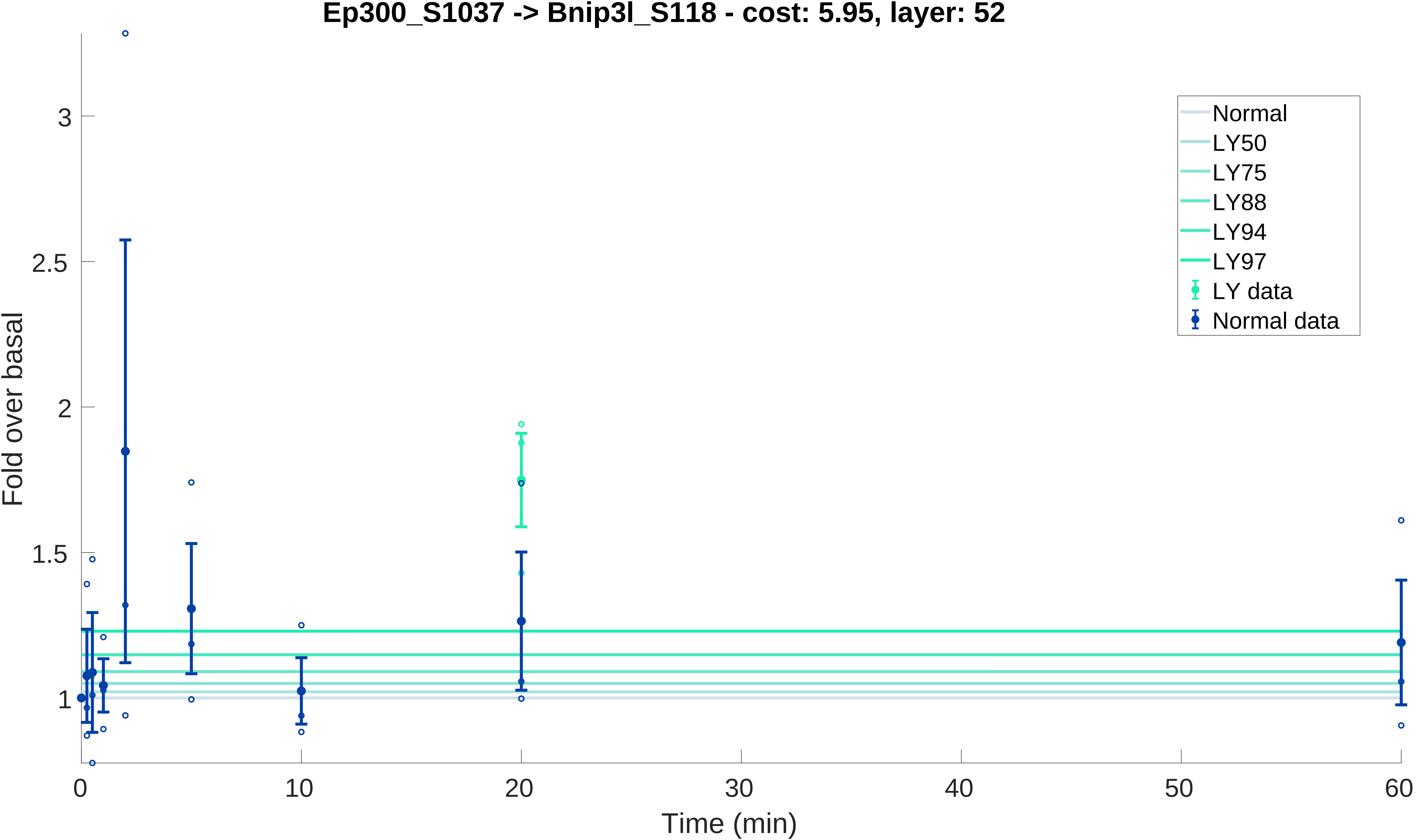

Ep300_S1037 -> Bnip3l_S118 - cost: 5.95, layer: 52
Fold over basal
Time (min)
0
10
20
30
40
50
60
1
1.5
2
2.5
3
Normal
LY50
LY75
LY88
LY94
LY97
LY data
Normal data

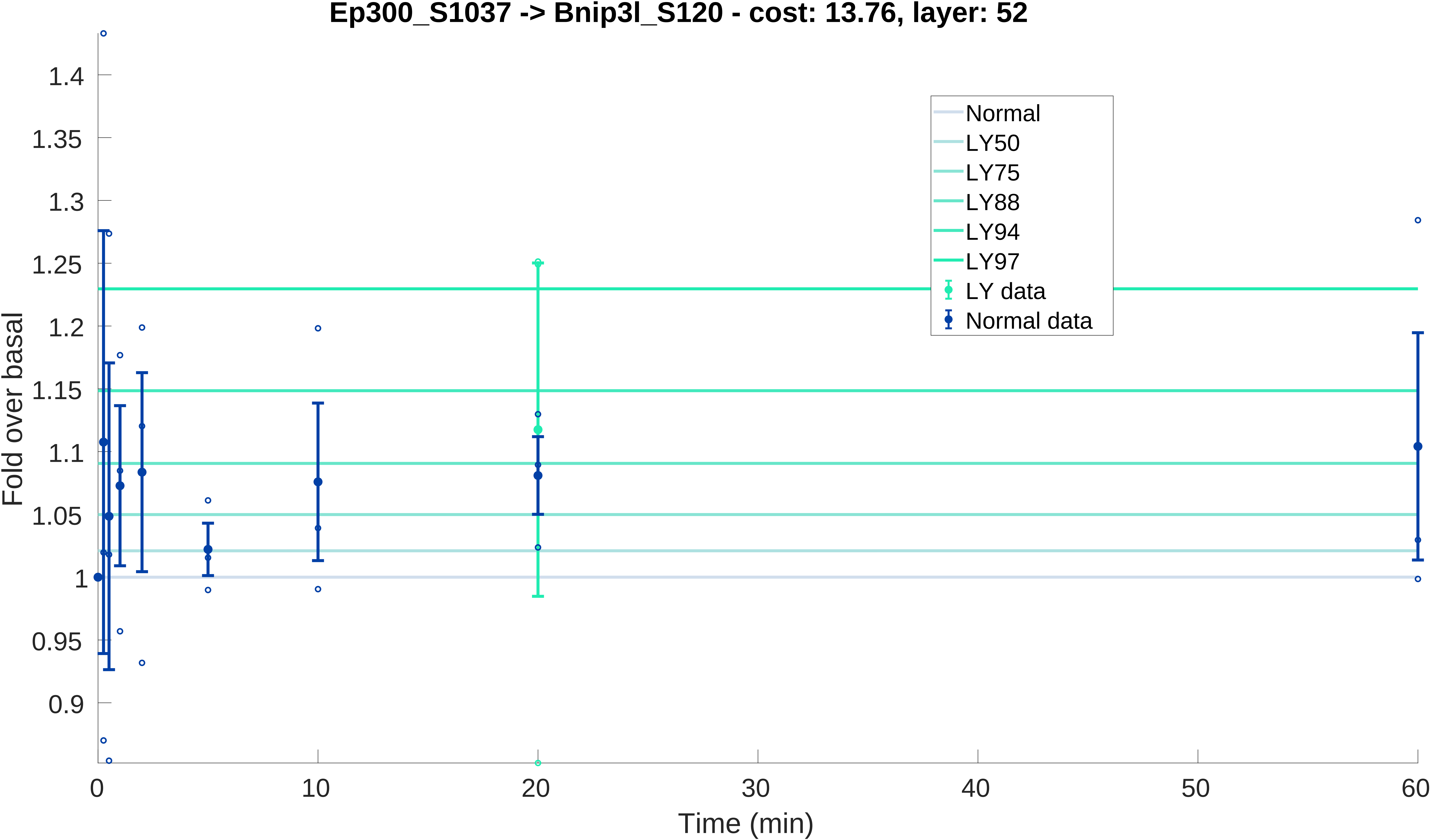
Ep300_S1037 -> Bnip3l_S120 - cost: 13.76, layer: 52
Fold over basal
1.4
1.35
1.3
1.25
1.2
1.15
1.1
1.05
1
0.95
0.9
0
10
20
30
40
50
60
Time (min)
Normal
LY50
LY75
LY88
LY94
LY97
LY data
Normal data

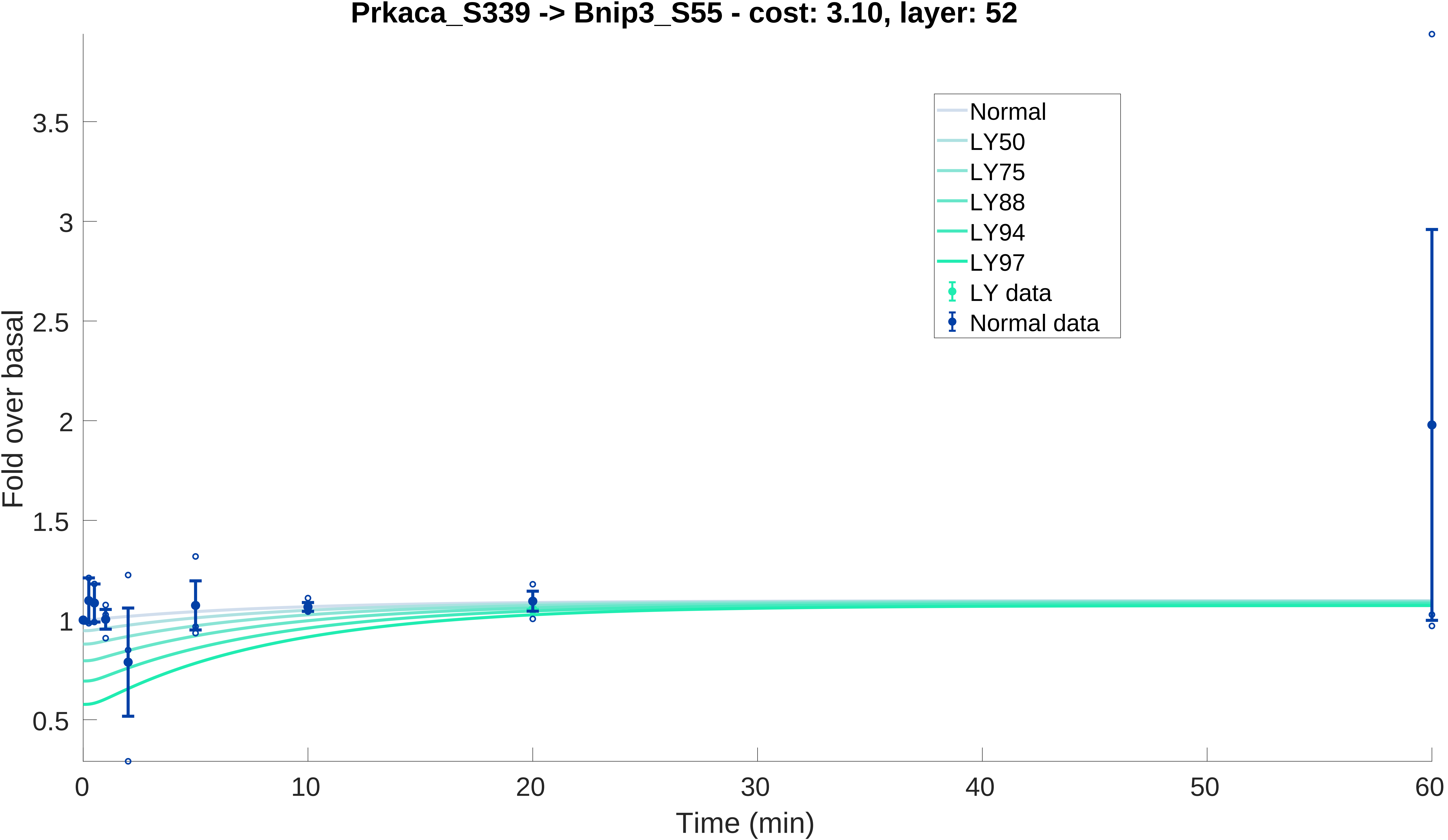

Prkaca_S339 -> Bnip3_S55 - cost: 3.10, layer: 52
Fold over basal
Time (min)
0.5
1
1.5
2
2.5
3
3.5
0
10
20
30
40
50
60
Normal
LY50
LY75
LY88
LY94
LY97
LY data
Normal data

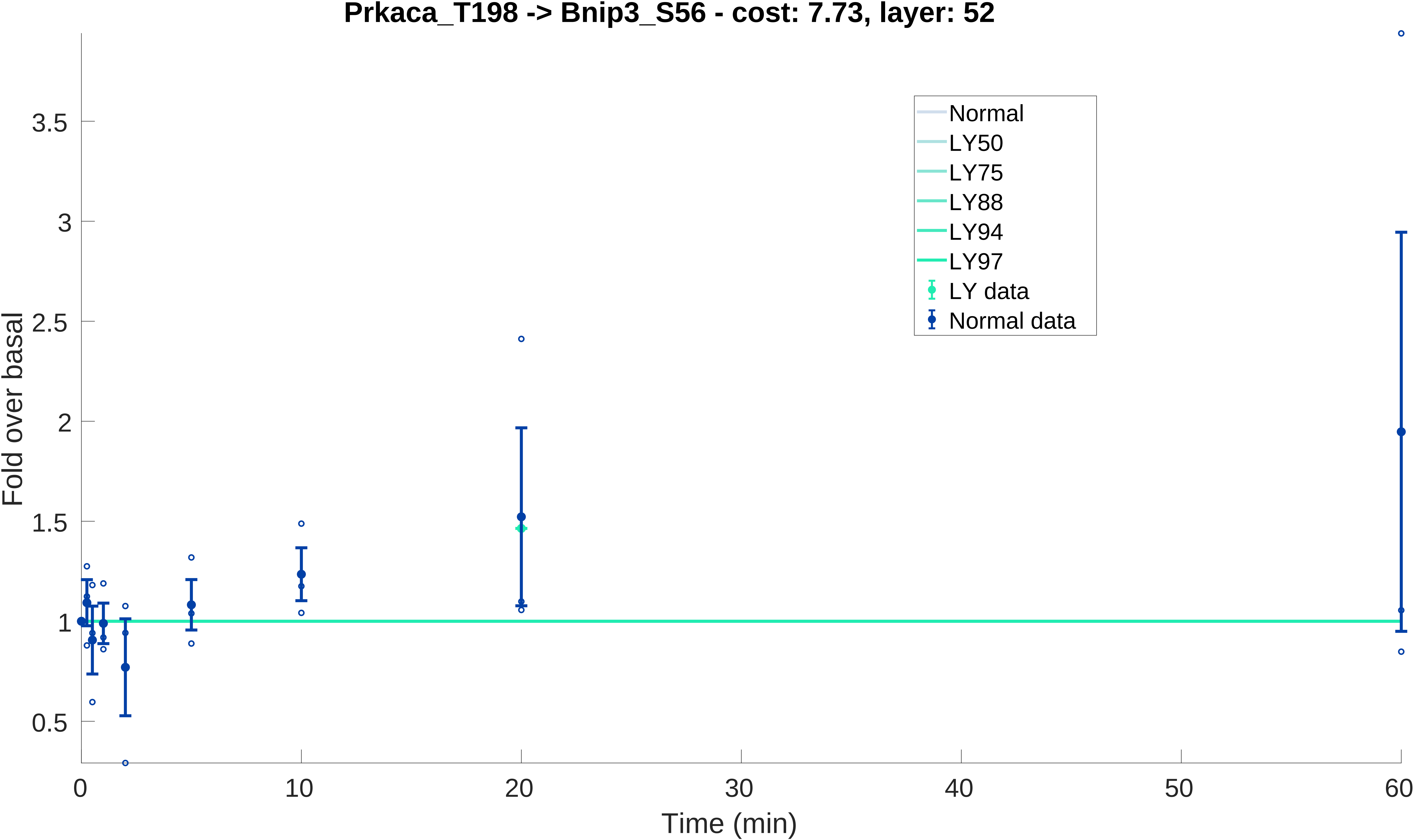

Prkaca_T198 -> Bnip3_S56 - cost: 7.73, layer: 52
Normal
LY50
LY75
LY88
LY94
LY97
LY data
Normal data
Fold over basal
3.5
3
2.5
2
1.5
1
0.5
0
10
20
30
40
50
60
Time (min)

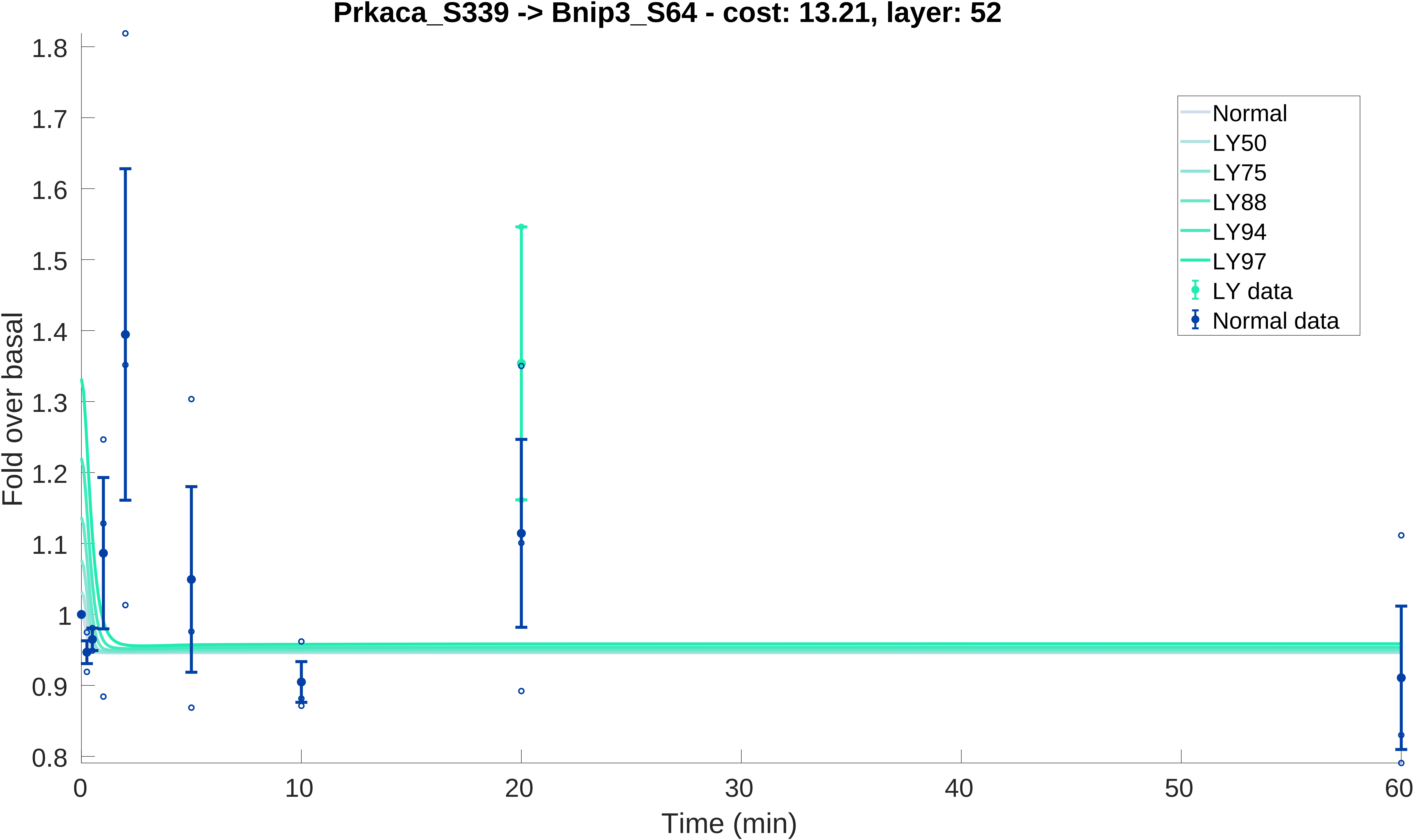

Prkaca_S339 -> Bnip3_S64 - cost: 13.21, layer: 52
Fold over basal
Time (min)
Normal
LY50
LY75
LY88
LY94
LY97
LY data
Normal data
0
10
20
30
40
50
60
0.8
0.9
1
1.1
1.2
1.3
1.4
1.5
1.6
1.7
1.8

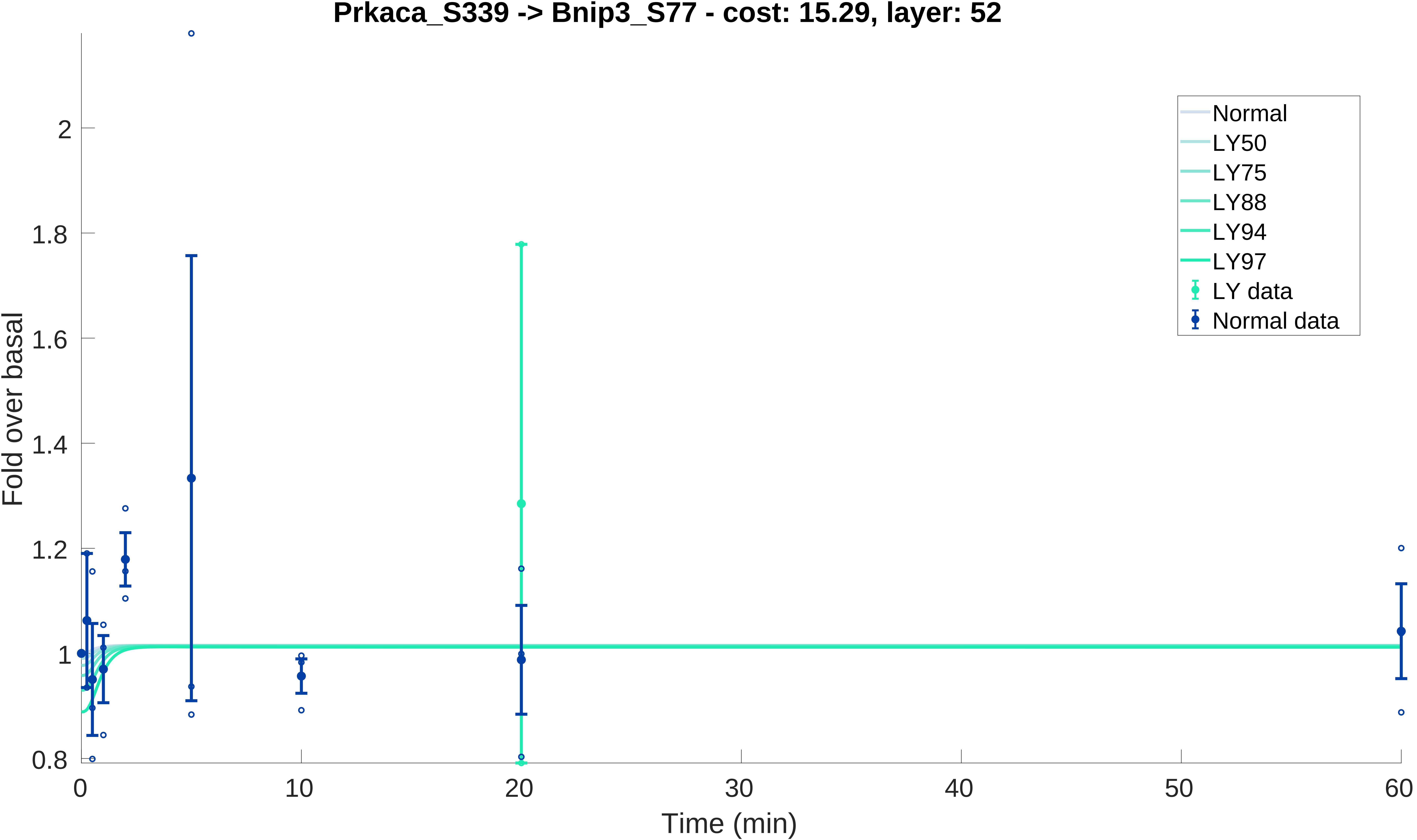
Prkaca_S339 -> Bnip3_S77 - cost: 15.29, layer: 52
Fold over basal
Time (min)
0.8
1
1.2
1.4
1.6
1.8
2
0
10
20
30
40
50
60
Normal
LY50
LY75
LY88
LY94
LY97
LY data
Normal data

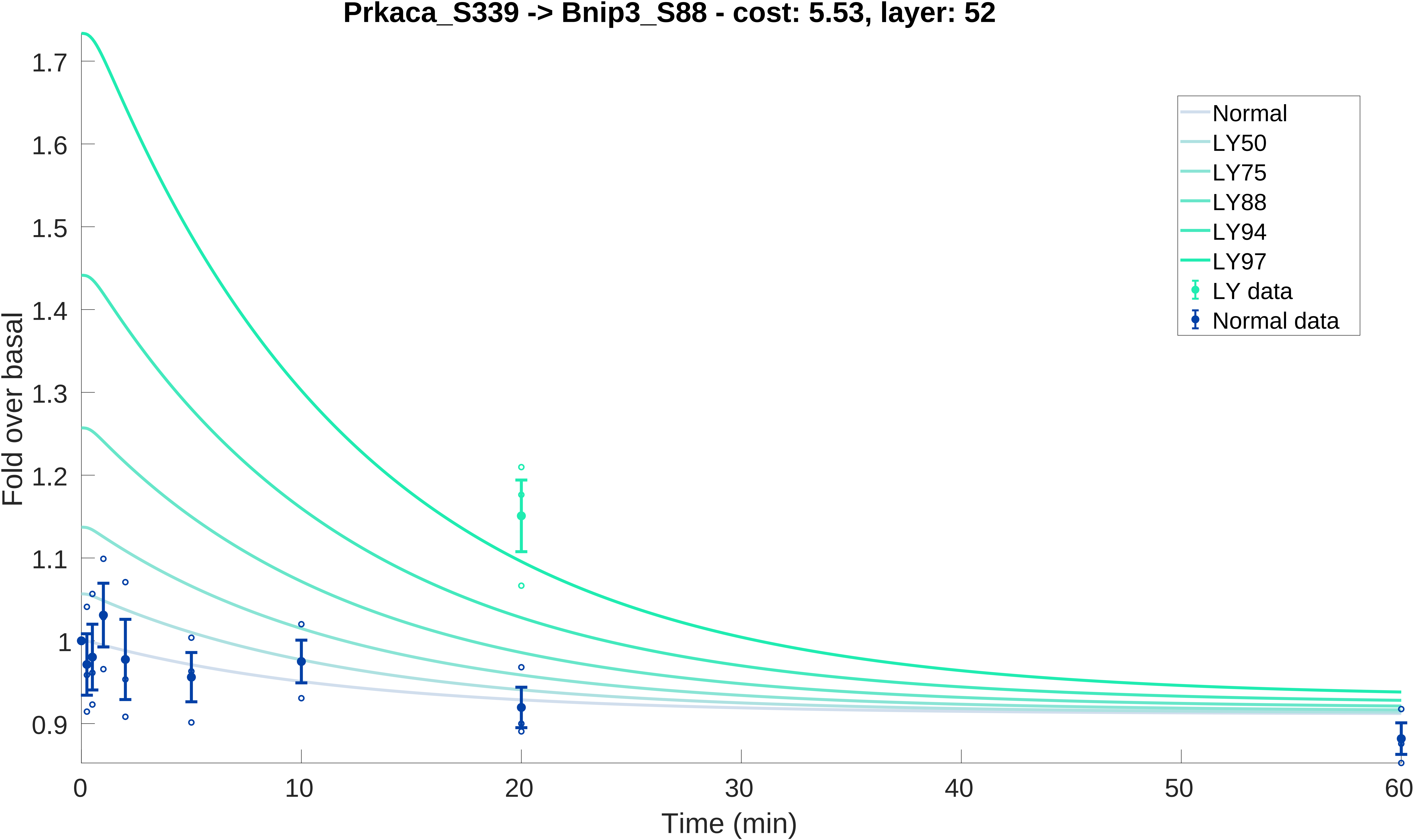
Prkaca_S339 -> Bnip3_S88 - cost: 5.53, layer: 52
Fold over basal
Time (min)
0.9
1
1.1
1.2
1.3
1.4
1.5
1.6
1.7
0
10
20
30
40
50
60
Normal
LY50
LY75
LY88
LY94
LY97
LY data
Normal data

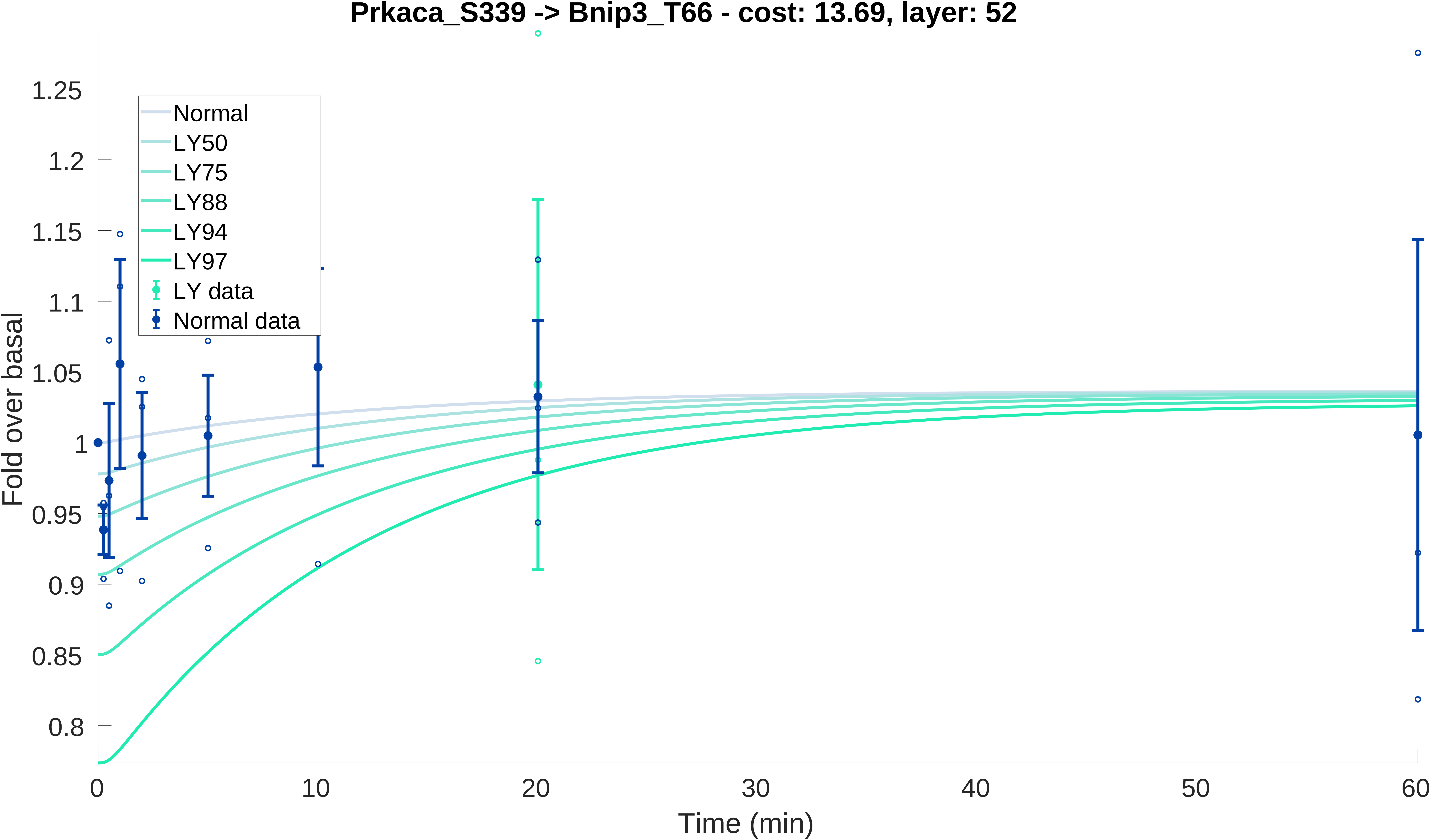

Prkaca_S339 -> Bnip3_T66 - cost: 13.69, layer: 52
Fold over basal
Time (min)
Normal
LY50
LY75
LY88
LY94
LY97
LY data
Normal data
1.25
1.2
1.15
1.1
1.05
1
0.95
0.9
0.85
0.8
0
10
20
30
40
50
60

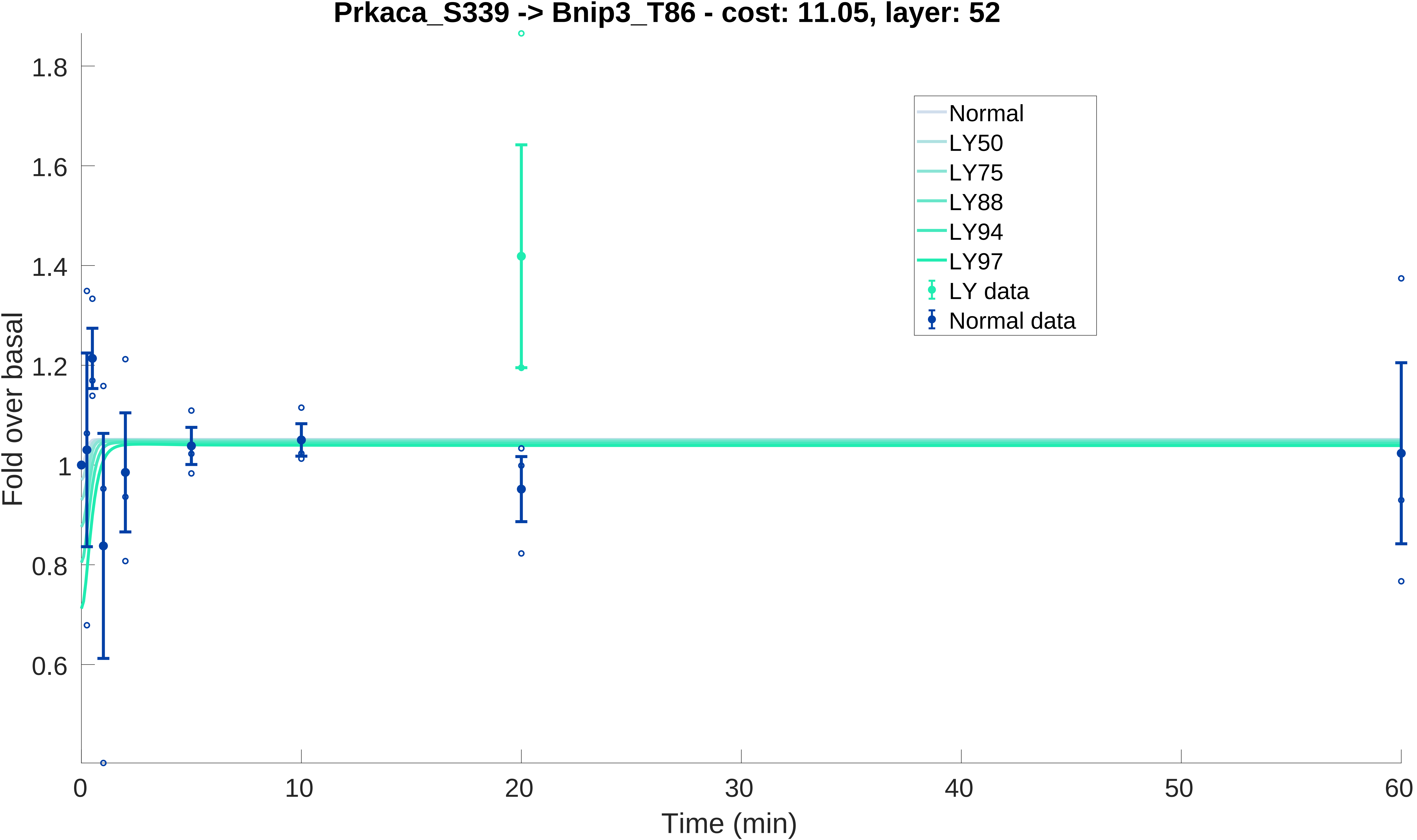

Prkaca_S339 -> Bnip3_T86 - cost: 11.05, layer: 52
Fold over basal
1.8
1.6
1.4
1.2
1
0.8
0.6
0
10
20
30
40
50
60
Time (min)
Normal
LY50
LY75
LY88
LY94
LY97
LY data
Normal data

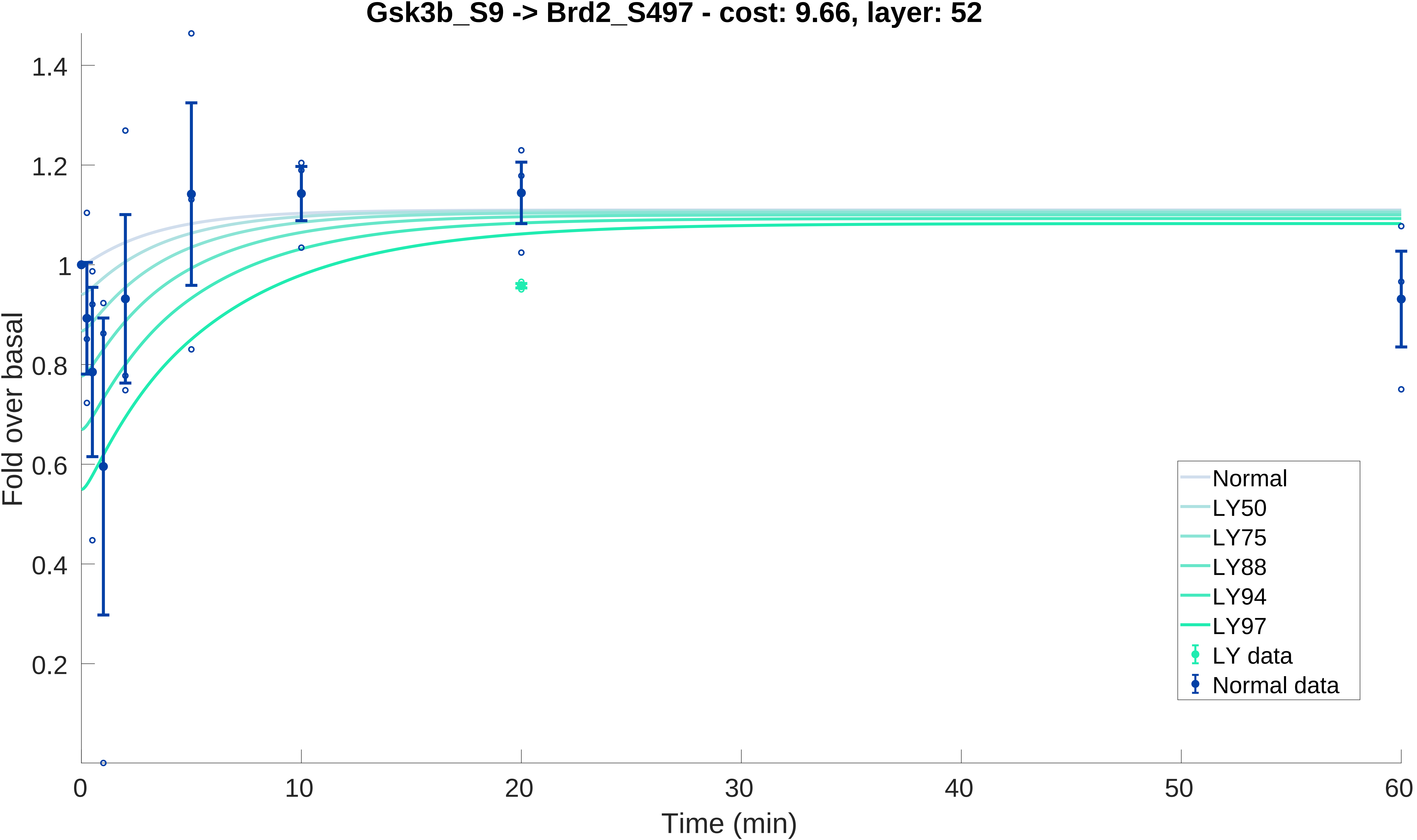

Gsk3b_S9 -> Brd2_S497 - cost: 9.66, layer: 52
Fold over basal
Time (min)
1.4
1.2
1
0.8
0.6
0.4
0.2
0
10
20
30
40
50
60
Normal
LY50
LY75
LY88
LY94
LY97
LY data
Normal data

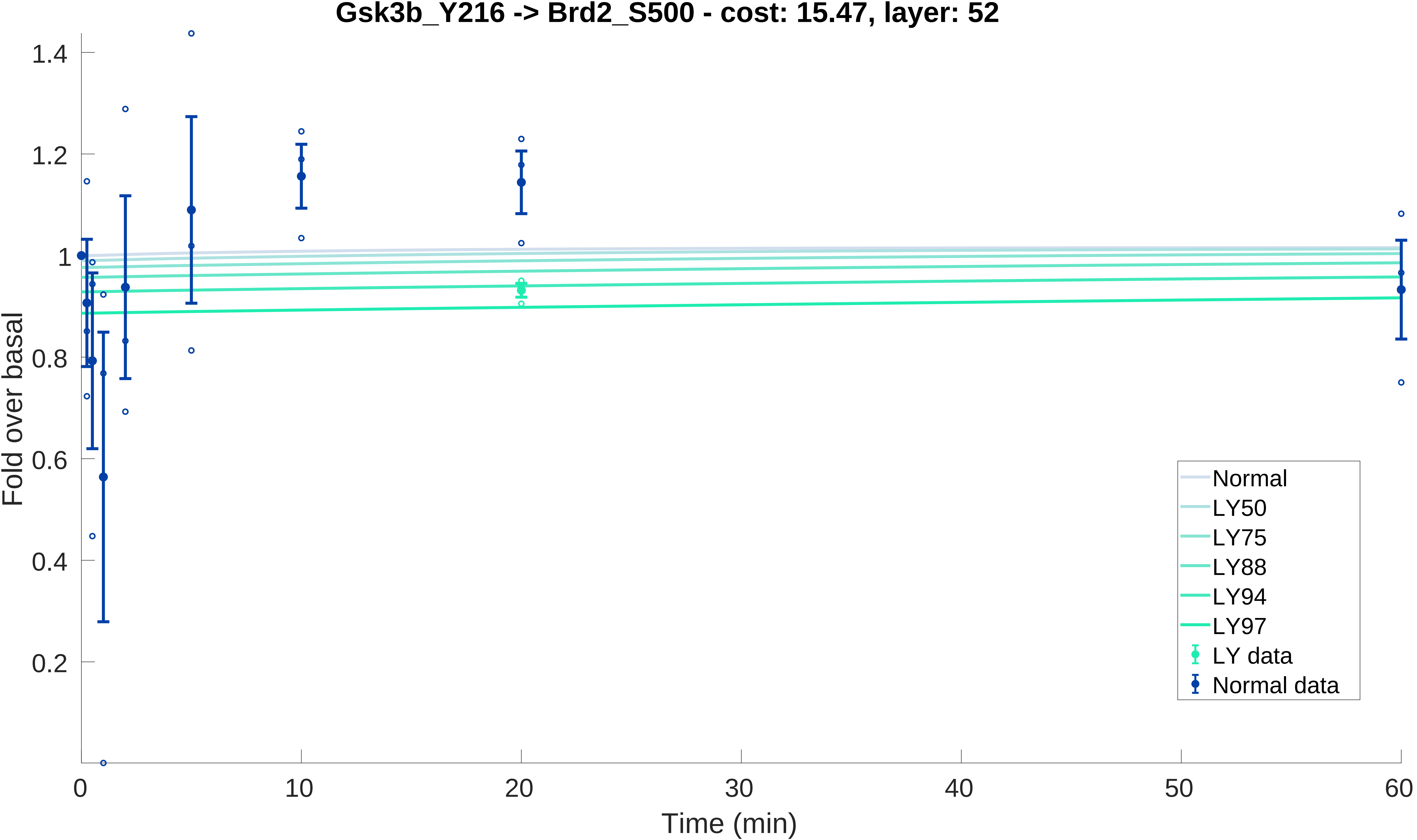

Gsk3b_Y216 -> Brd2_S500 - cost: 15.47, layer: 52
Fold over basal
Time (min)
1.4
1.2
1
0.8
0.6
0.4
0.2
0
10
20
30
40
50
60
Normal
LY50
LY75
LY88
LY94
LY97
LY data
Normal data

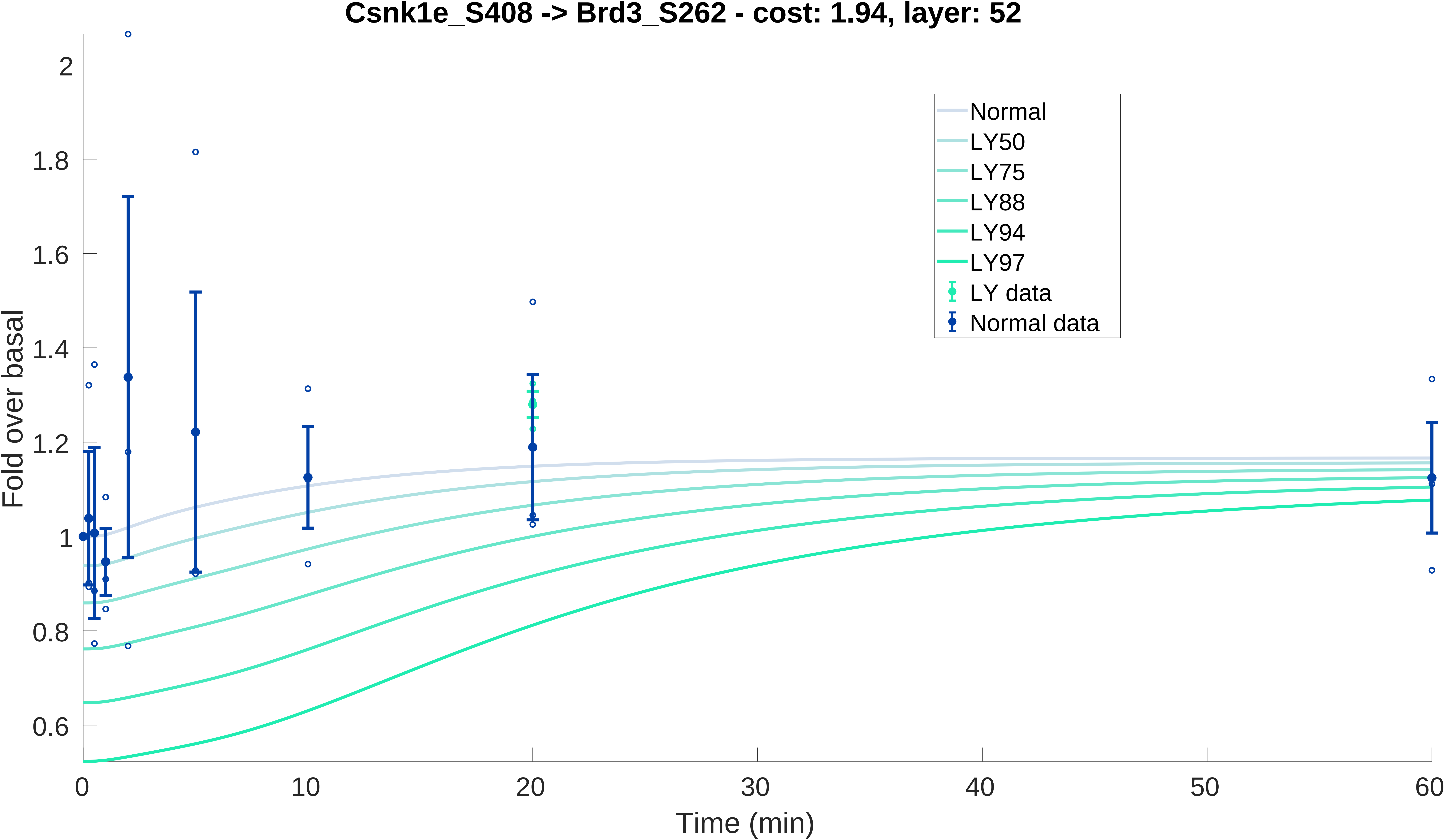

Csnk1e_S408 -> Brd3_S262 - cost: 1.94, layer: 52
Fold over basal
Time (min)
0
10
20
30
40
50
60
0.6
0.8
1
1.2
1.4
1.6
1.8
2
Normal
LY50
LY75
LY88
LY94
LY97
LY data
Normal data

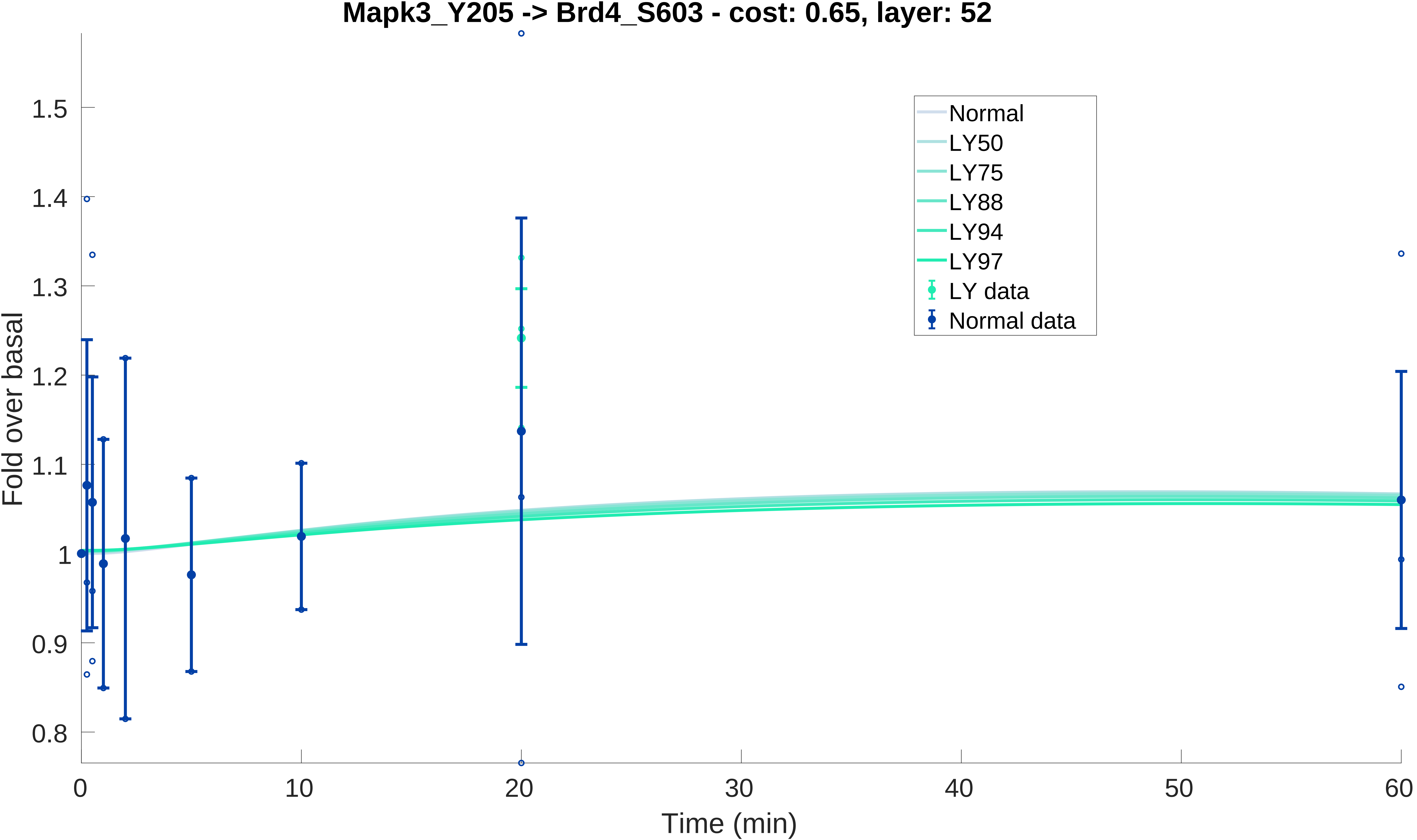

Mapk3_Y205 -> Brd4_S603 - cost: 0.65, layer: 52
Fold over basal
1.5
1.4
1.3
1.2
1.1
1
0.9
0.8
0
10
20
30
40
50
60
Time (min)
Normal
LY50
LY75
LY88
LY94
LY97
LY data
Normal data

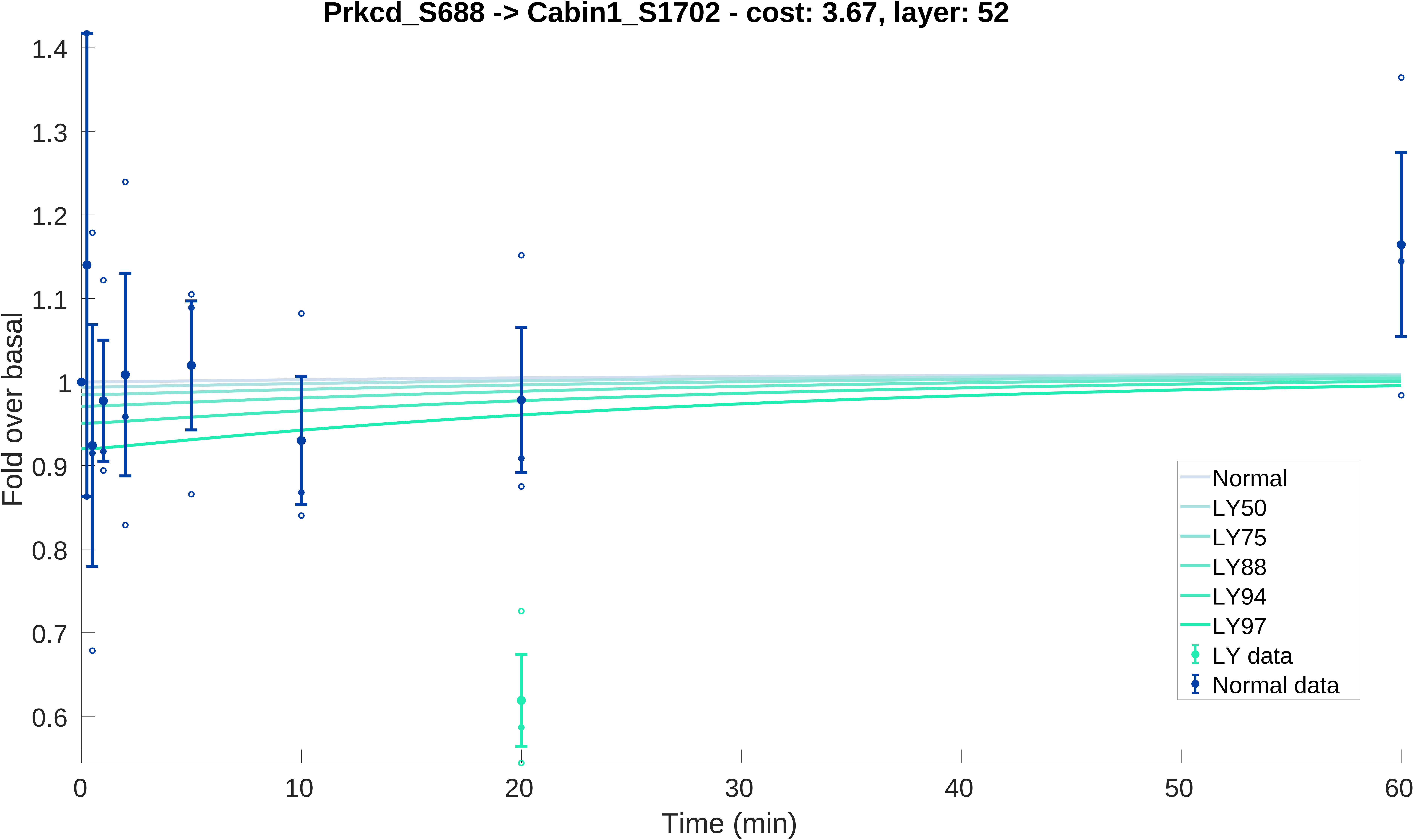

Prkcd_S688 -> Cabin1_S1702 - cost: 3.67, layer: 52
Fold over basal
1.4
1.3
1.2
1.1
1
0.9
0.8
0.7
0.6
0
10
20
30
40
50
60
Time (min)
Normal
LY50
LY75
LY88
LY94
LY97
LY data
Normal data

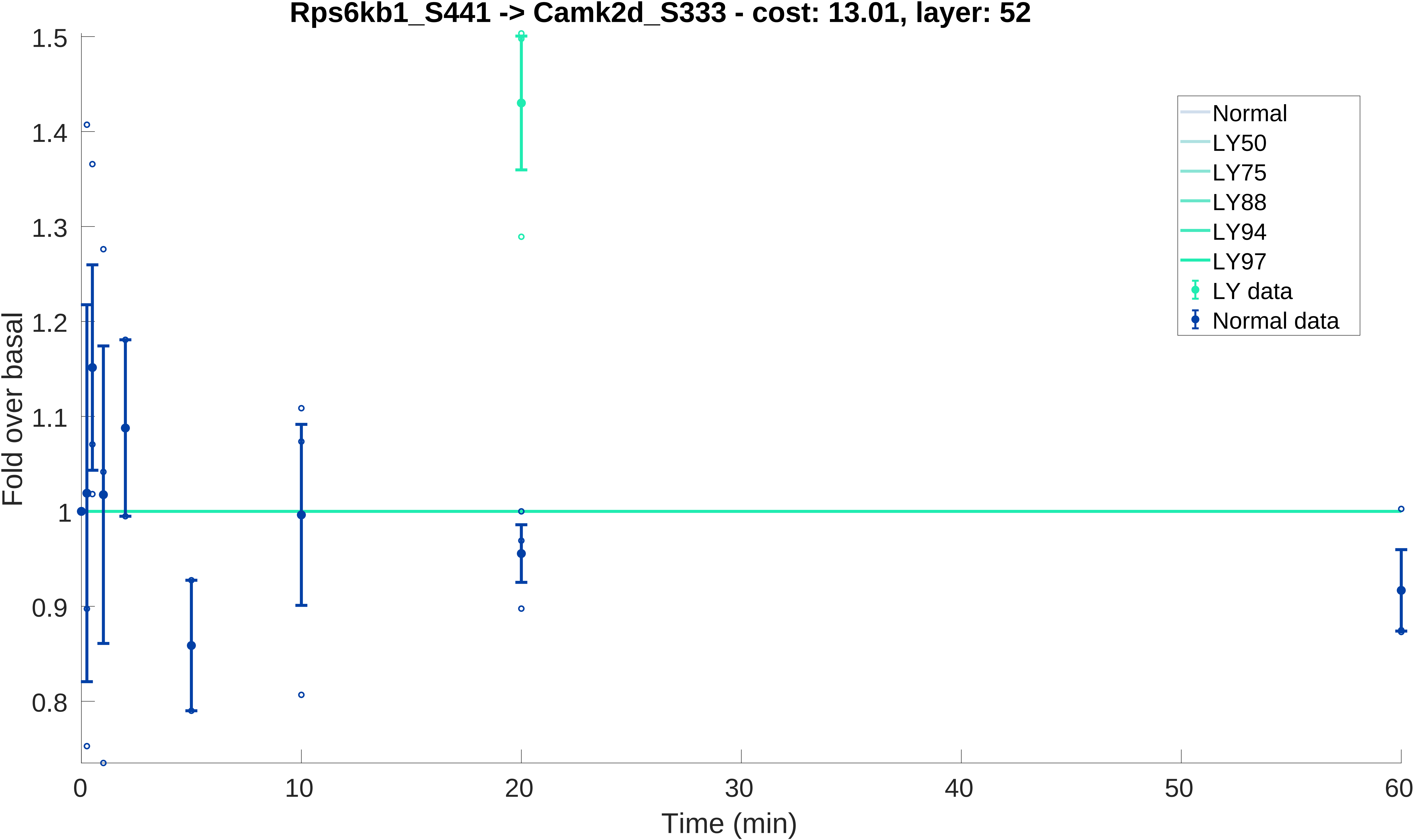

Rps6kb1_S441 -> Camk2d_S333 - cost: 13.01, layer: 52
Fold over basal
1.5
1.4
1.3
1.2
1.1
1
0.9
0.8
0
10
20
30
40
50
60
Time (min)
Normal
LY50
LY75
LY88
LY94
LY97
LY data
Normal data

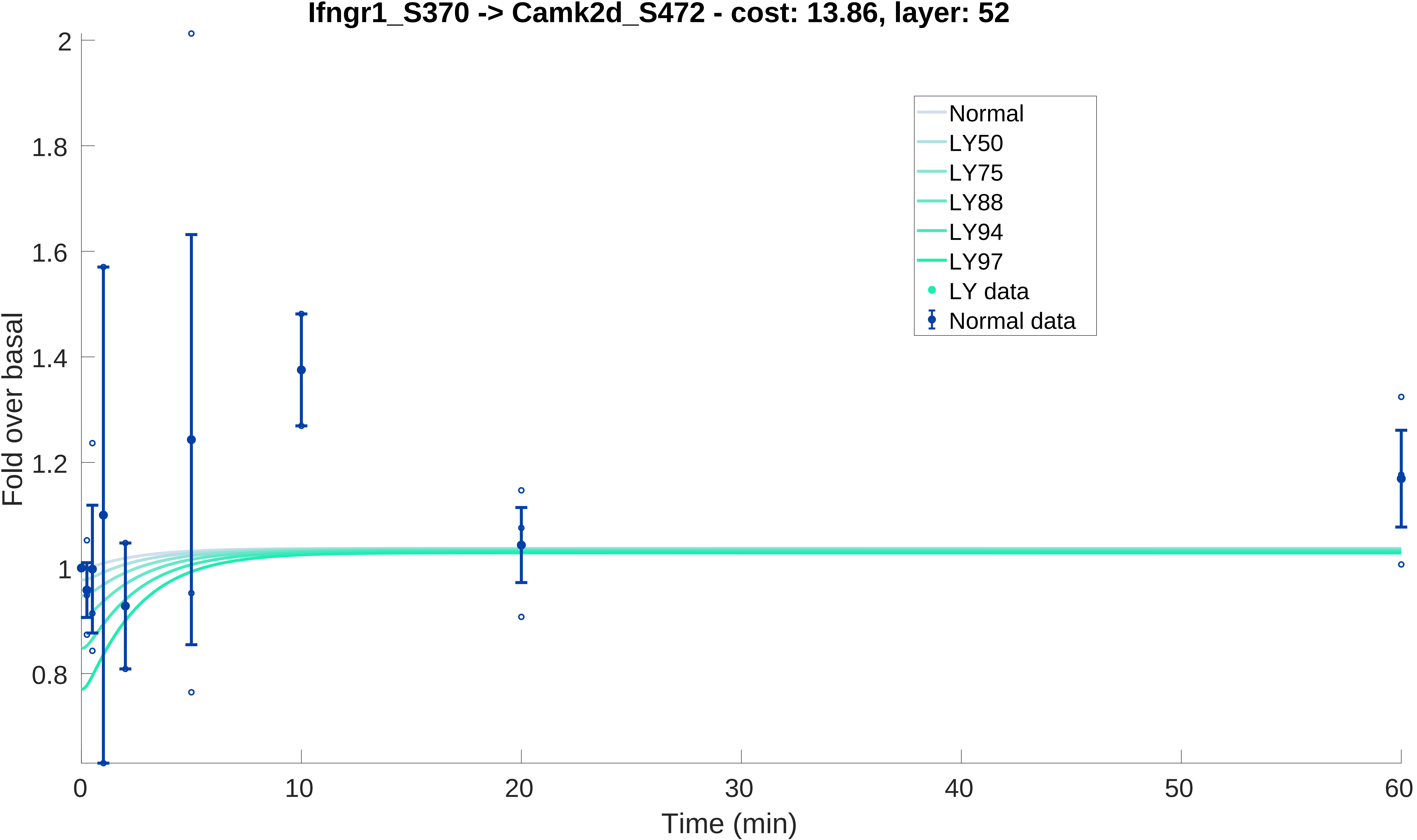

Ifngr1_S370 -> Camk2d_S472 - cost: 13.86, layer: 52
Fold over basal
Time (min)
0
10
20
30
40
50
60
0.8
1
1.2
1.4
1.6
1.8
2
Normal
LY50
LY75
LY88
LY94
LY97
LY data
Normal data

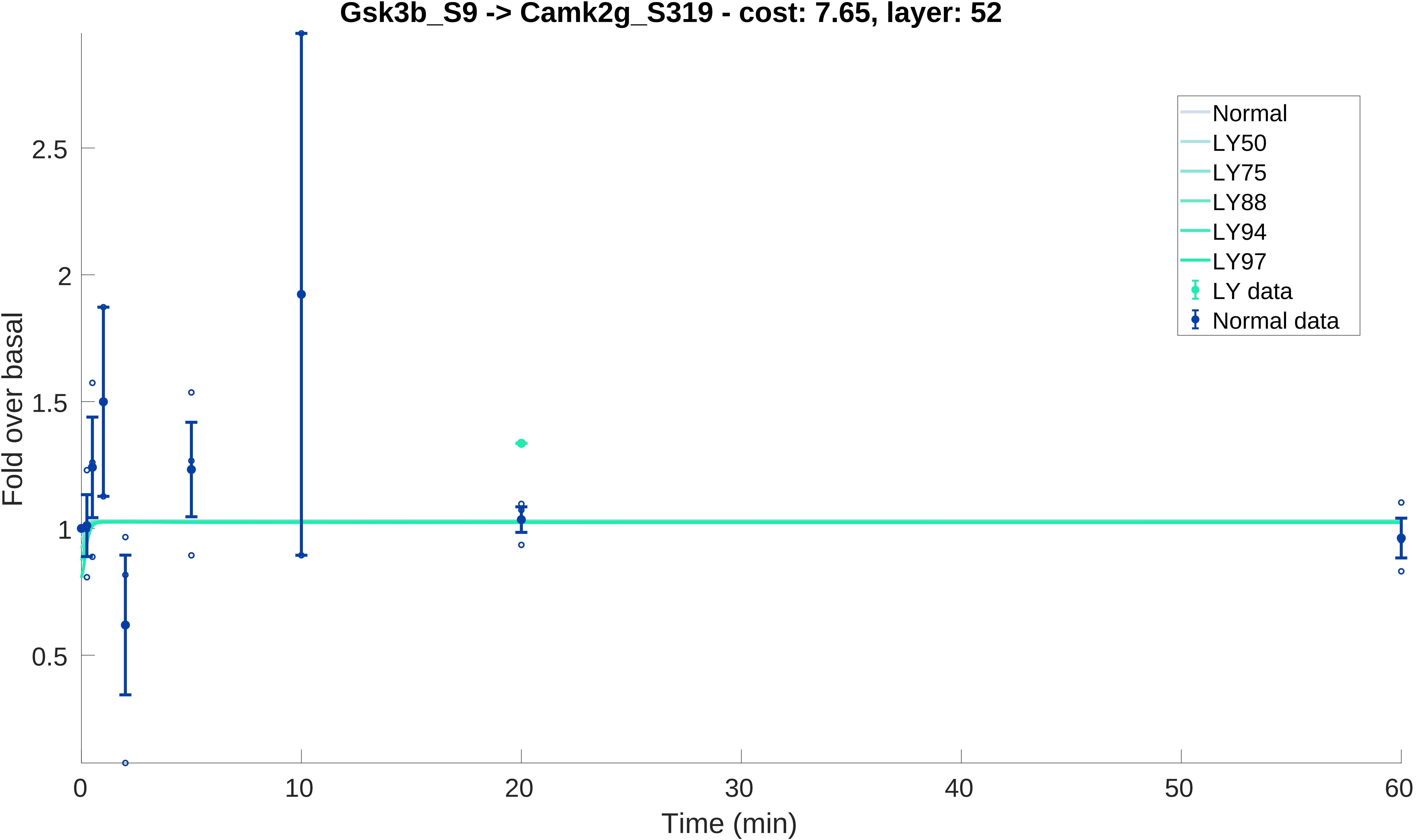

Gsk3b_S9 -> Camk2g_S319 - cost: 7.65, layer: 52
Fold over basal
Time (min)
0
10
20
30
40
50
60
0.5
1
1.5
2
2.5
Normal
LY50
LY75
LY88
LY94
LY97
LY data
Normal data

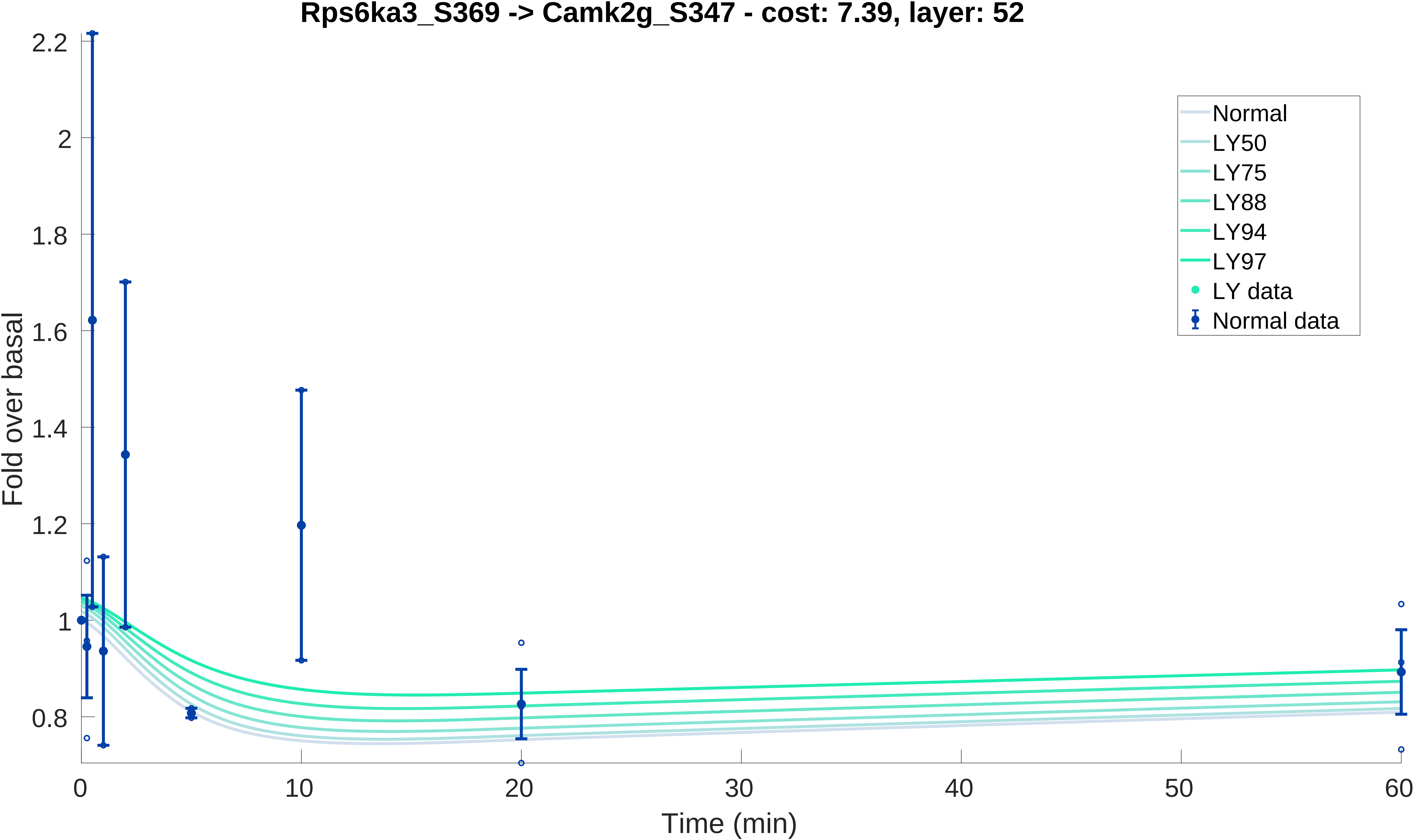
Rps6ka3_S369 -> Camk2g_S347 - cost: 7.39, layer: 52
Fold over basal
Time (min)
0
10
20
30
40
50
60
0.8
1
1.2
1.4
1.6
1.8
2
2.2
Normal
LY50
LY75
LY88
LY94
LY97
LY data
Normal data

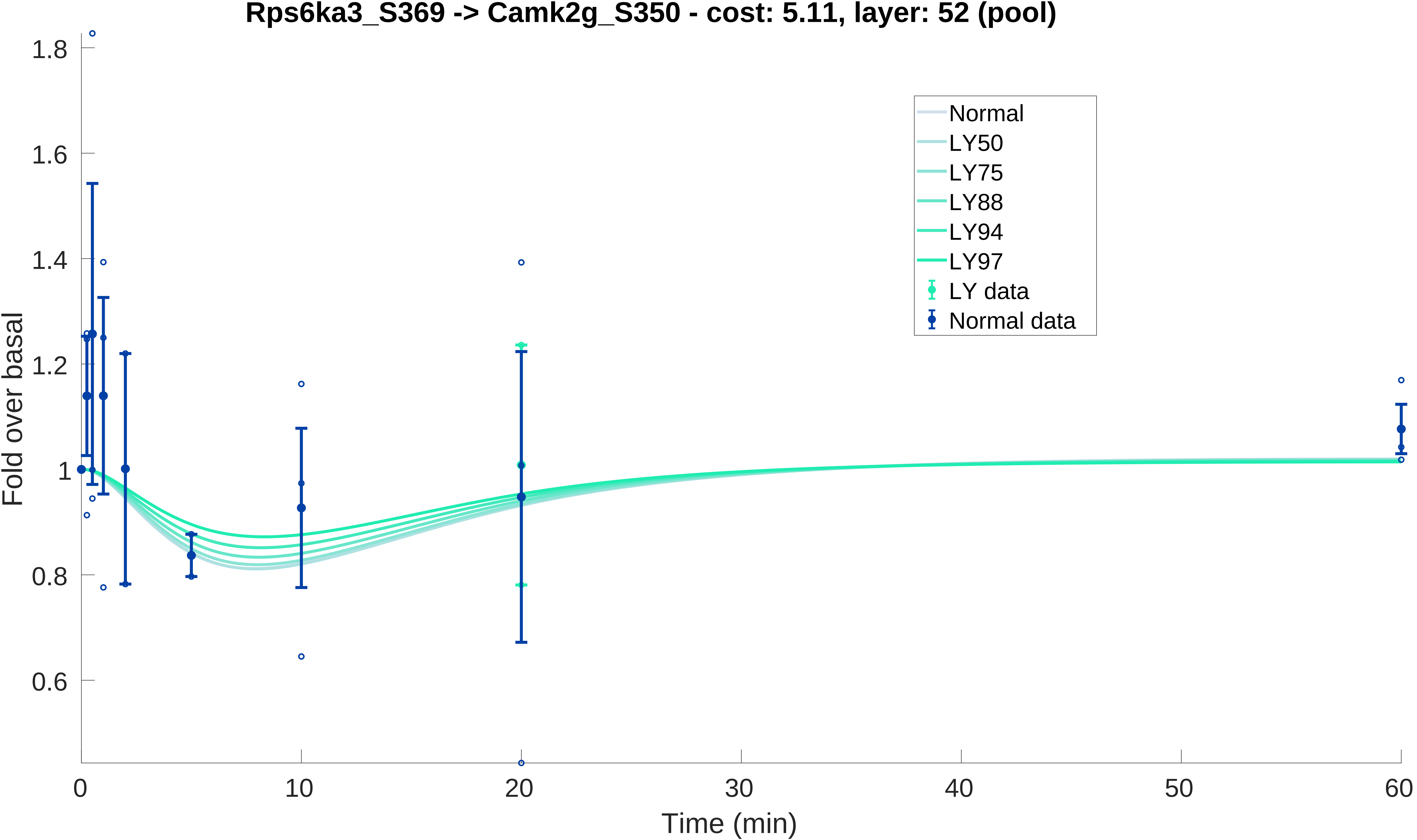

Rps6ka3_S369 -> Camk2g_S350 - cost: 5.11, layer: 52 (pool)
Fold over basal
1.8
1.6
1.4
1.2
1
0.8
0.6
0
10
20
30
40
50
60
Time (min)
Normal
LY50
LY75
LY88
LY94
LY97
LY data
Normal data

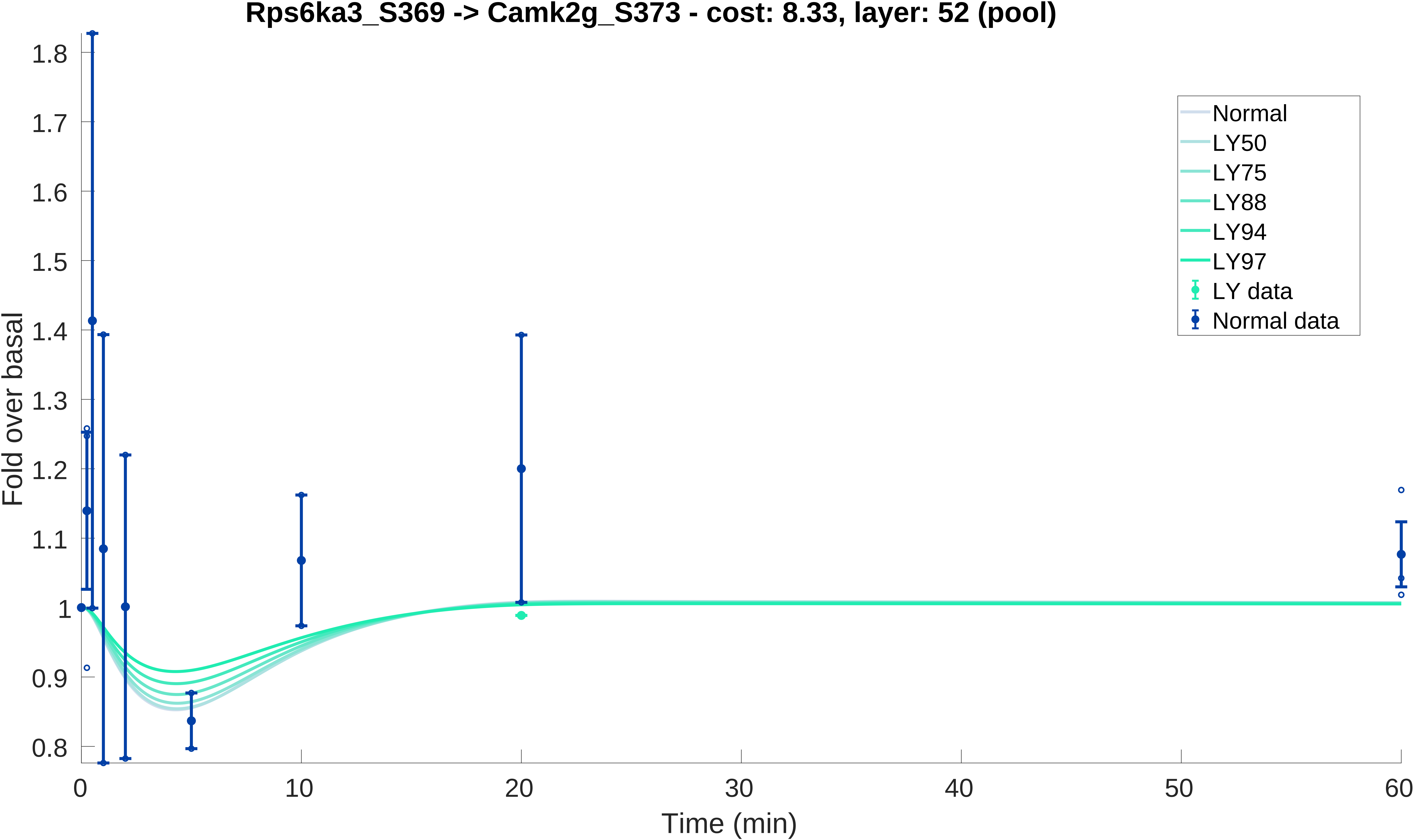
Rps6ka3_S369 -> Camk2g_S373 - cost: 8.33, layer: 52 (pool)
Fold over basal
Time (min)
Normal
LY50
LY75
LY88
LY94
LY97
LY data
Normal data
0
10
20
30
40
50
60
0.8
0.9
1
1.1
1.2
1.3
1.4
1.5
1.6
1.7
1.8

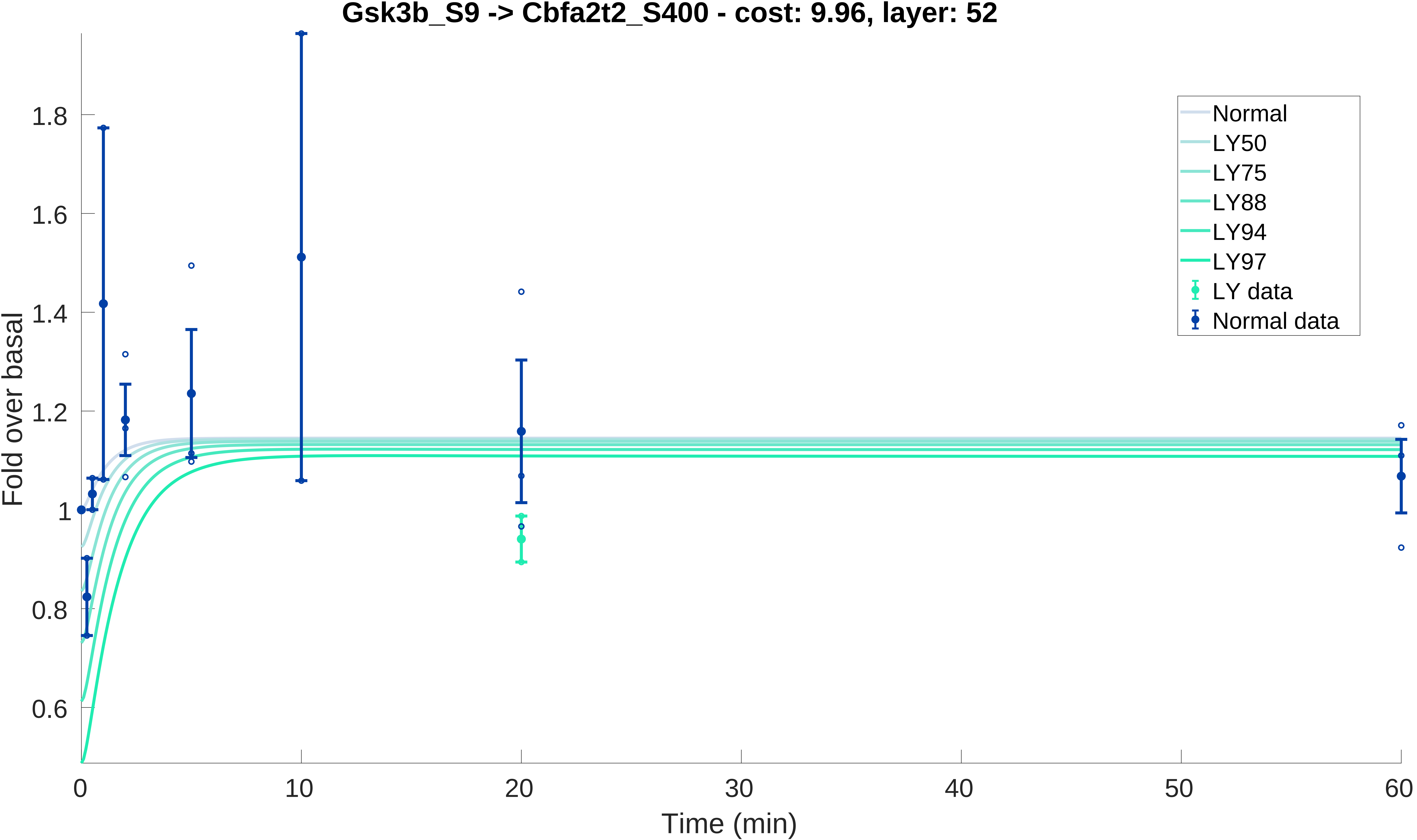
Gsk3b_S9 -> Cbfa2t2_S400 - cost: 9.96, layer: 52
Fold over basal
Time (min)
0.6
0.8
1
1.2
1.4
1.6
1.8
0
10
20
30
40
50
60
Normal
LY50
LY75
LY88
LY94
LY97
LY data
Normal data

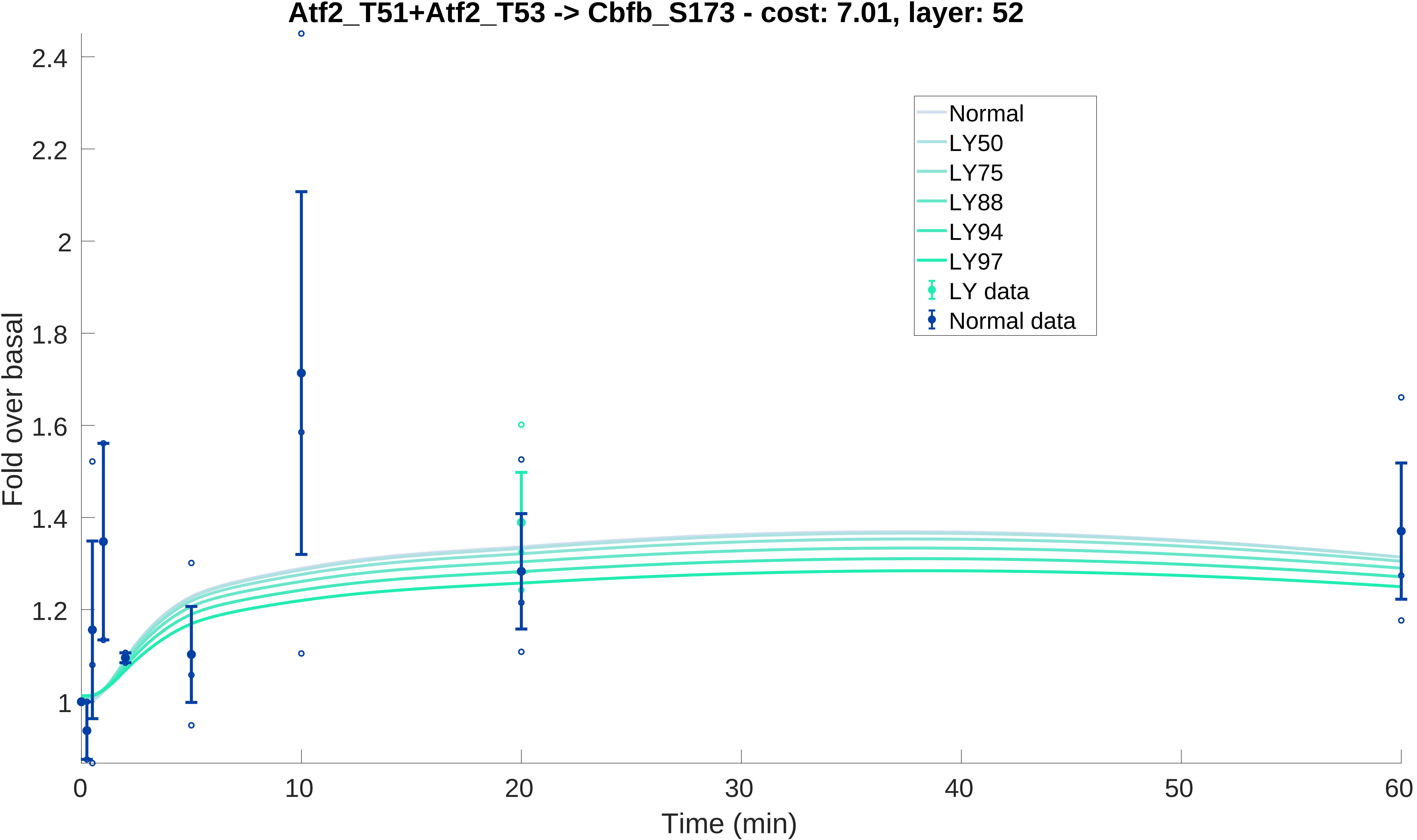

Atf2_T51+Atf2_T53 -> Cbfb_S173 - cost: 7.01, layer: 52
Fold over basal
Time (min)
Normal
LY50
LY75
LY88
LY94
LY97
LY data
Normal data
2.4
2.2
2
1.8
1.6
1.4
1.2
1
0
10
20
30
40
50
60

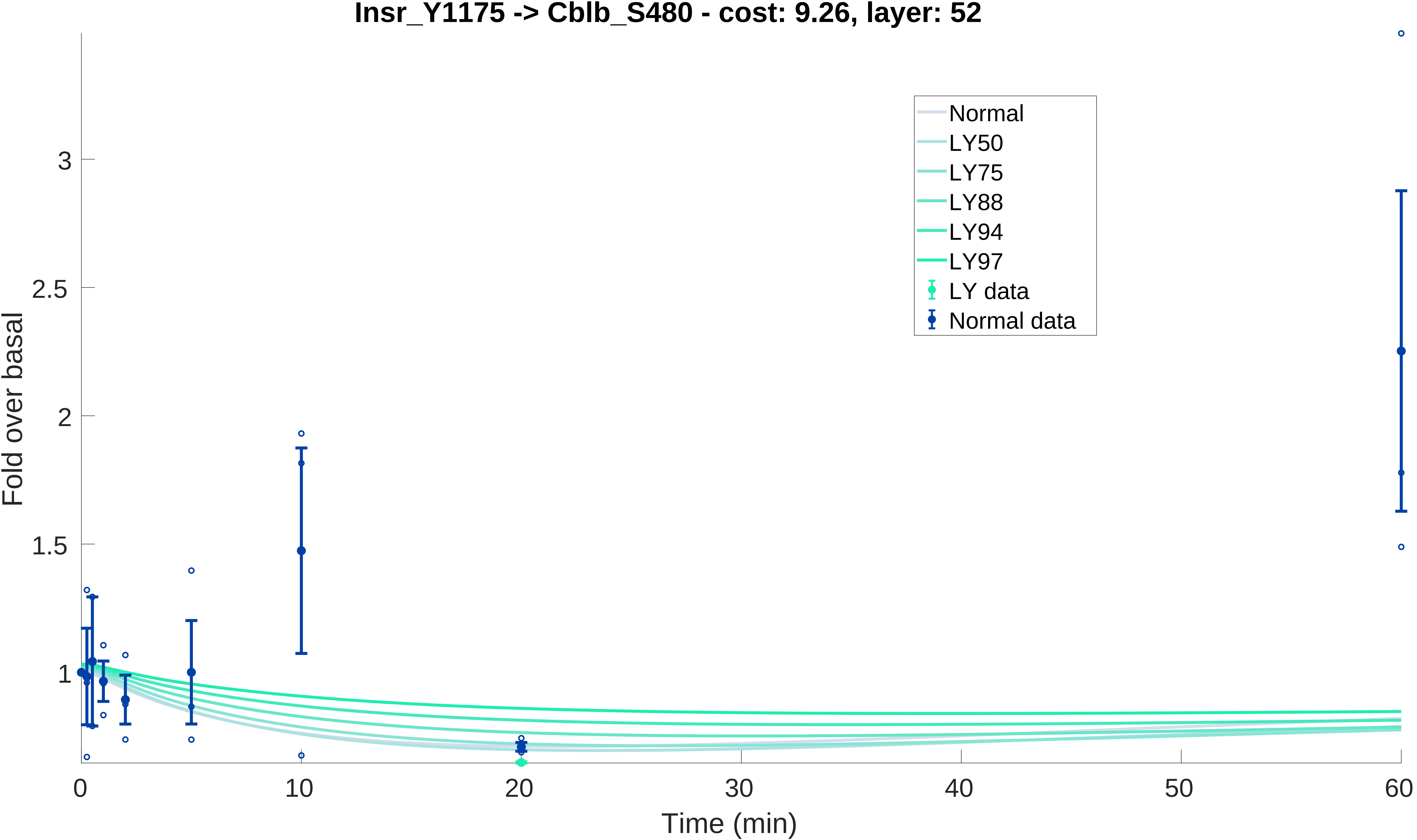

Insr_Y1175 -> Cblb_S480 - cost: 9.26, layer: 52
Fold over basal
Time (min)
Normal
LY50
LY75
LY88
LY94
LY97
LY data
Normal data
0
10
20
30
40
50
60
1
1.5
2
2.5
3

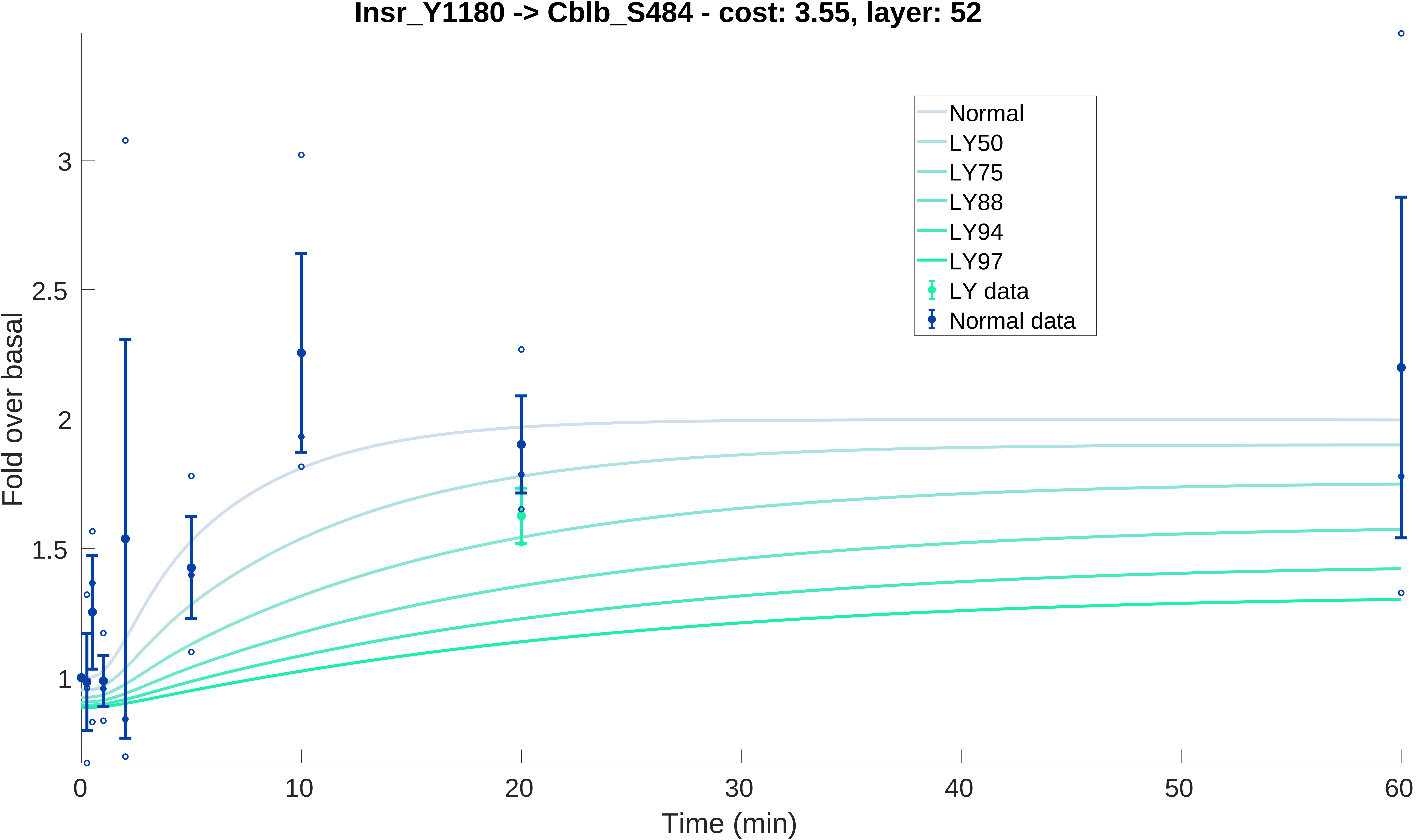

Insr_Y1180 -> Cblb_S484 - cost: 3.55, layer: 52
Fold over basal
Time (min)
0
10
20
30
40
50
60
1
1.5
2
2.5
3
Normal
LY50
LY75
LY88
LY94
LY97
LY data
Normal data

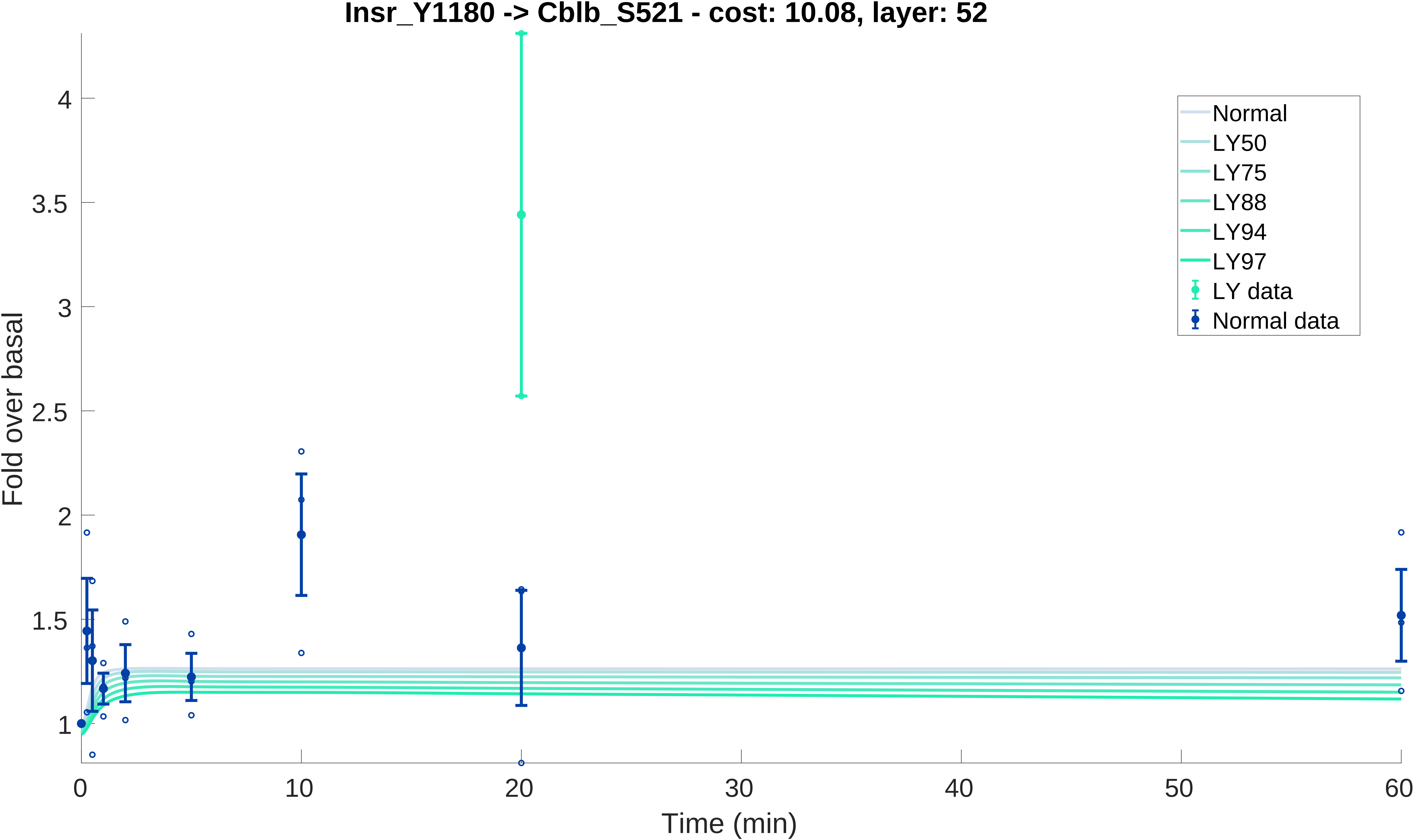

Insr_Y1180 -> Cblb_S521 - cost: 10.08, layer: 52
Fold over basal
Time (min)
0
10
20
30
40
50
60
1
1.5
2
2.5
3
3.5
4
Normal
LY50
LY75
LY88
LY94
LY97
LY data
Normal data

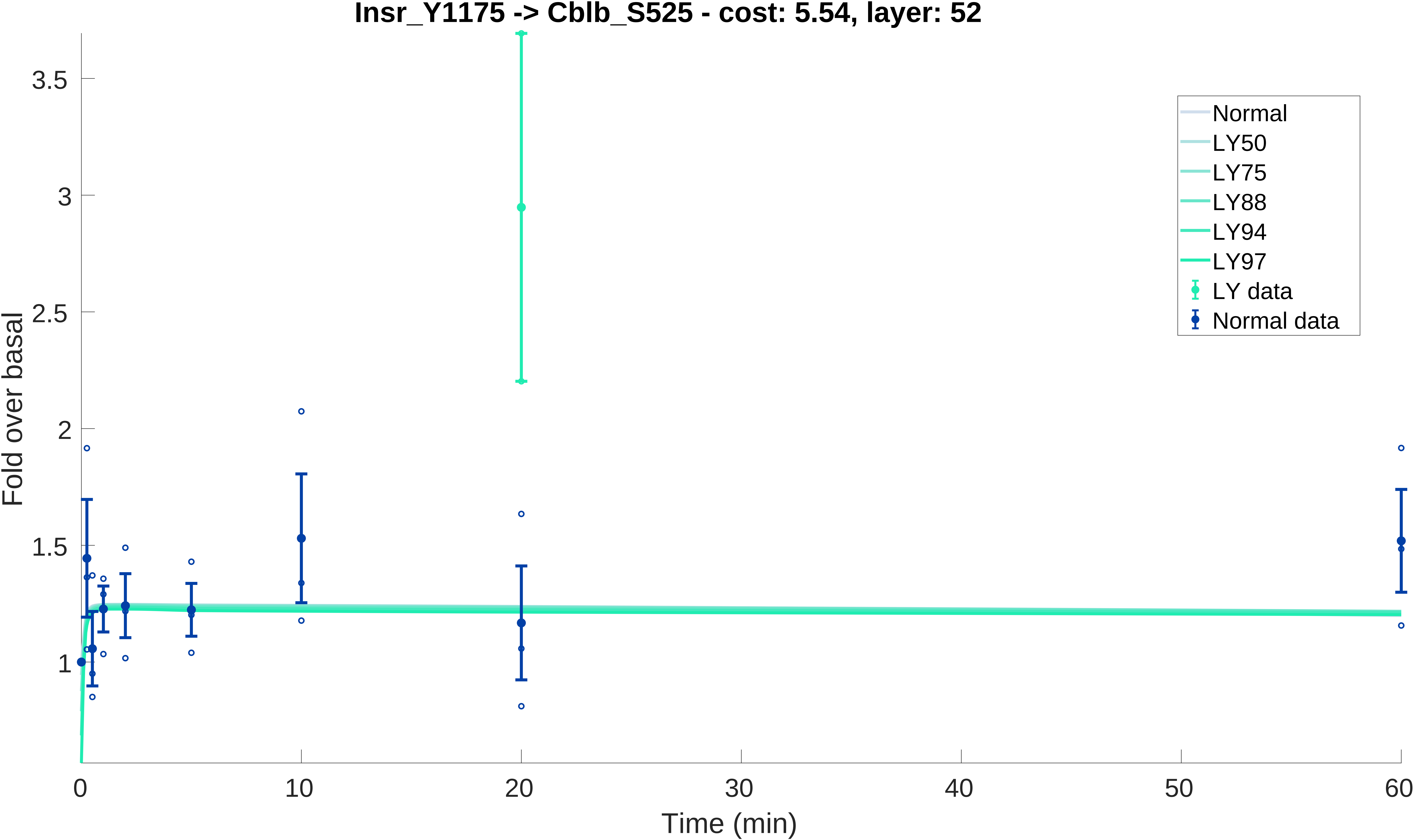
Insr_Y1175 -> Cblb_S525 - cost: 5.54, layer: 52
Fold over basal
Time (min)
Normal
LY50
LY75
LY88
LY94
LY97
LY data
Normal data
0
10
20
30
40
50
60
1
1.5
2
2.5
3
3.5

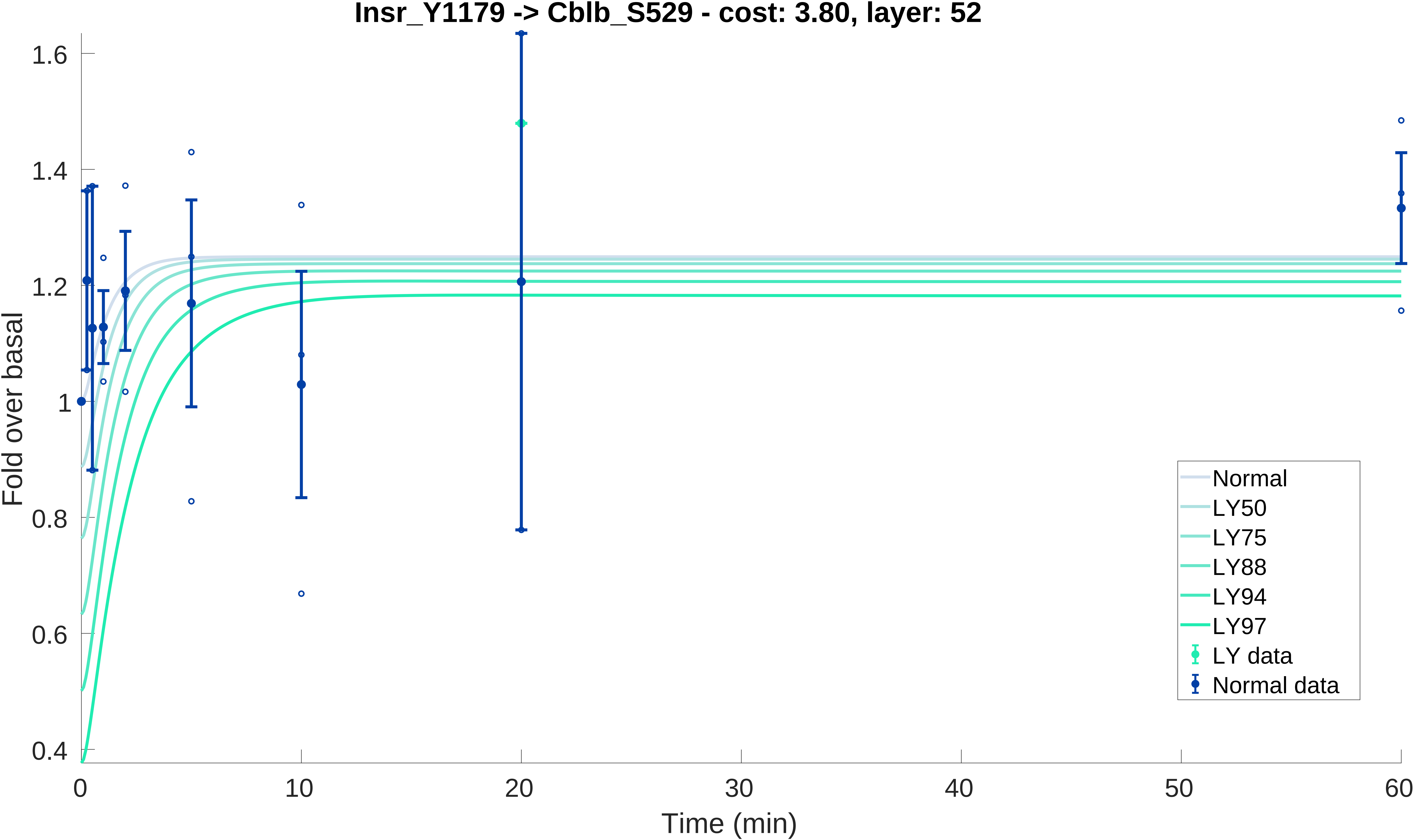

Insr_Y1179 -> Cblb_S529 - cost: 3.80, layer: 52
Fold over basal
Time (min)
1.6
1.4
1.2
1
0.8
0.6
0.4
0
10
20
30
40
50
60
Normal
LY50
LY75
LY88
LY94
LY97
LY data
Normal data

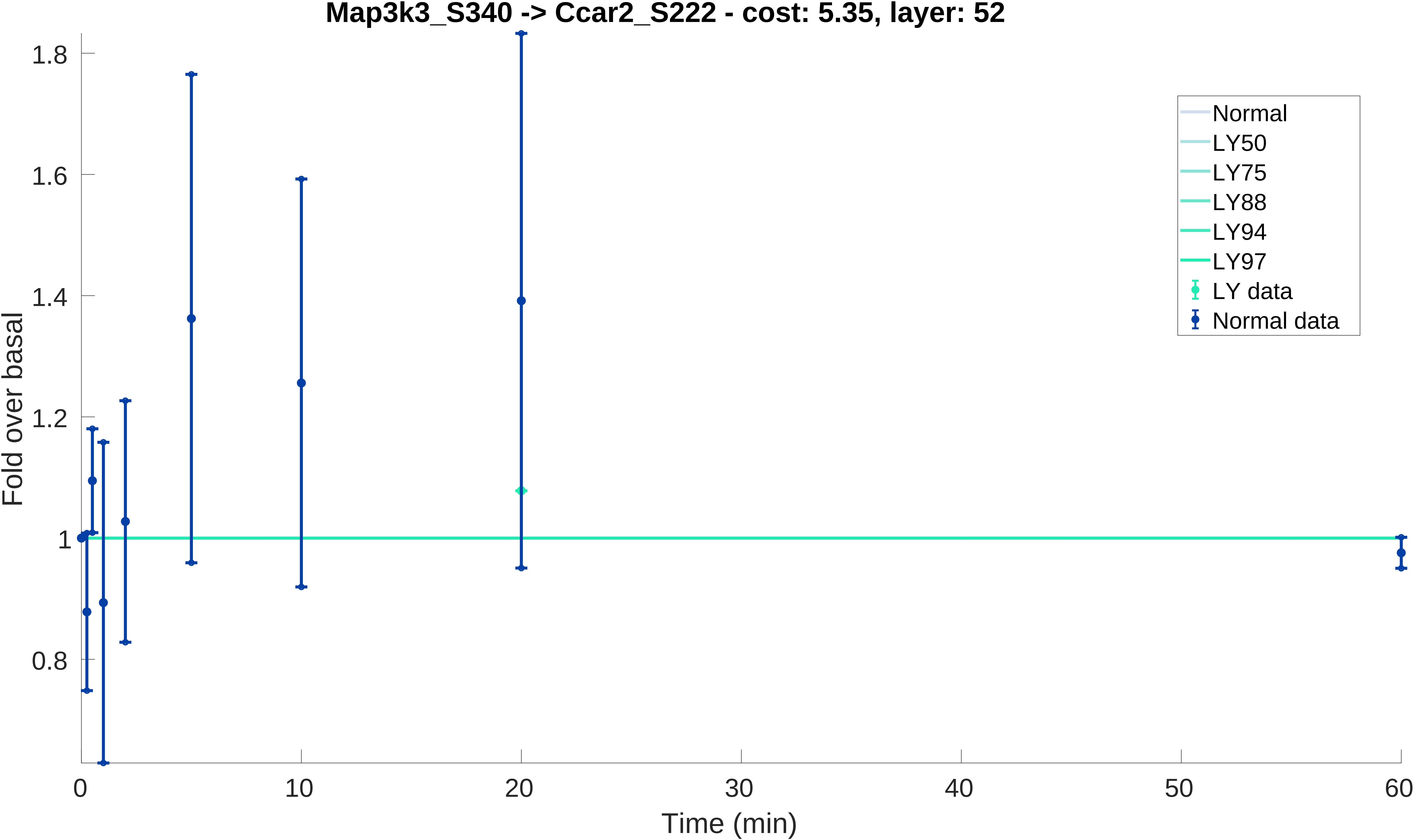

Map3k3_S340 -> Ccar2_S222 - cost: 5.35, layer: 52
Fold over basal
1.8
1.6
1.4
1.2
1
0.8
0
10
20
30
40
50
60
Time (min)
Normal
LY50
LY75
LY88
LY94
LY97
LY data
Normal data

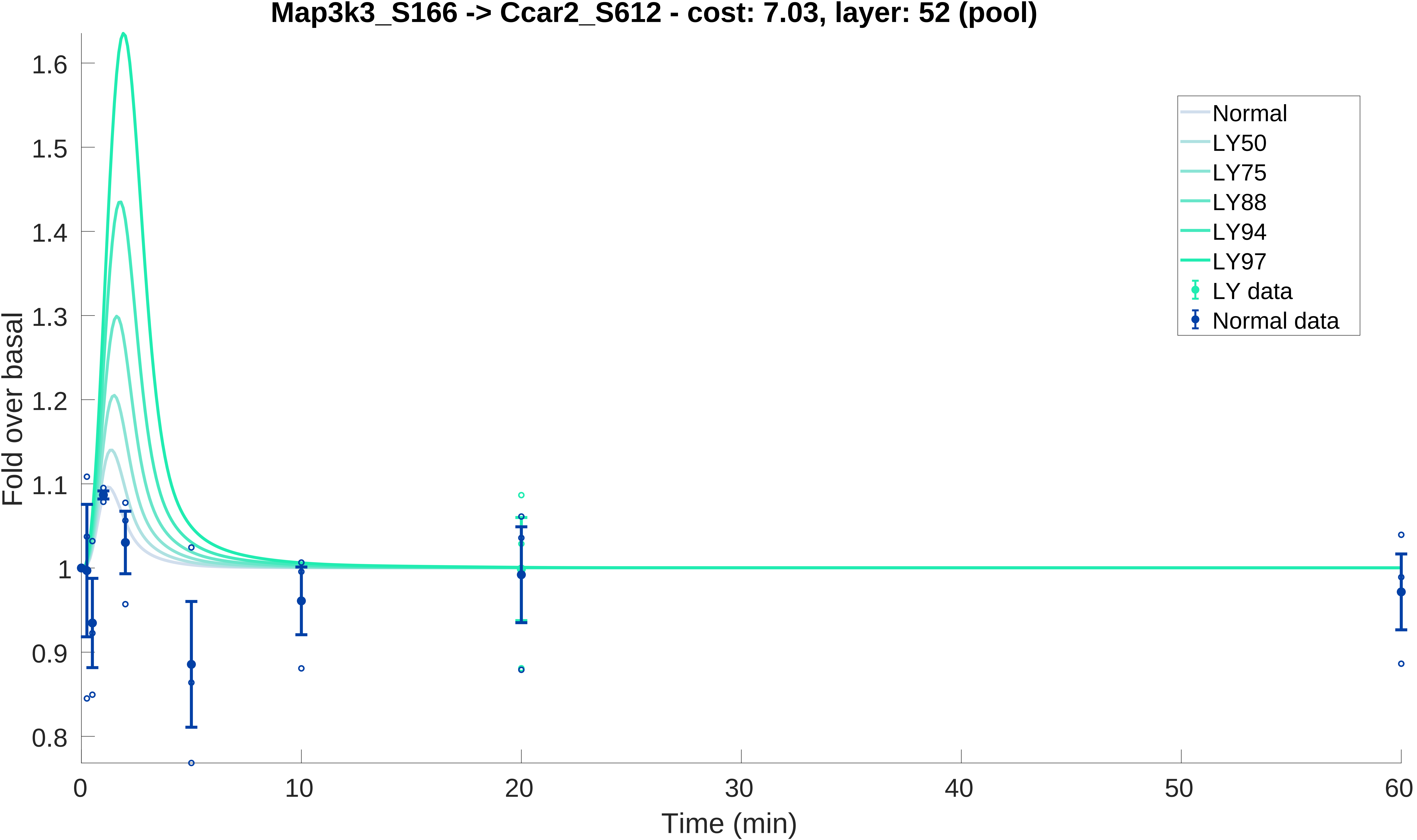

Map3k3_S166 -> Ccar2_S612 - cost: 7.03, layer: 52 (pool)
Fold over basal
1.6
1.5
1.4
1.3
1.2
1.1
1
0.9
0.8
0
10
20
30
40
50
60
Time (min)
Normal
LY50
LY75
LY88
LY94
LY97
LY data
Normal data

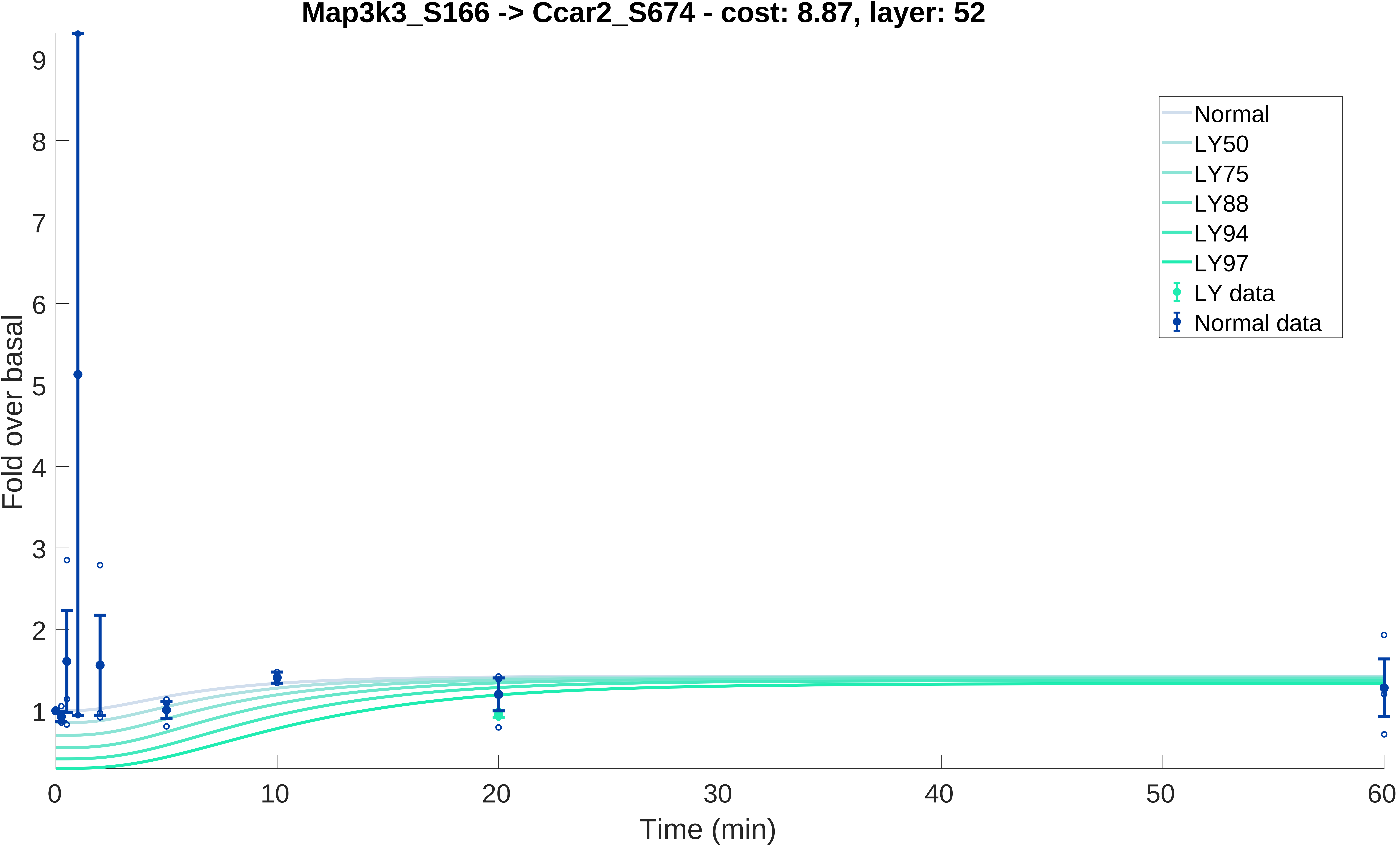

Map3k3_S166 -> Ccar2_S674 - cost: 8.87, layer: 52
Fold over basal
Time (min)
Normal
LY50
LY75
LY88
LY94
LY97
LY data
Normal data
0
10
20
30
40
50
60
1
2
3
4
5
6
7
8
9

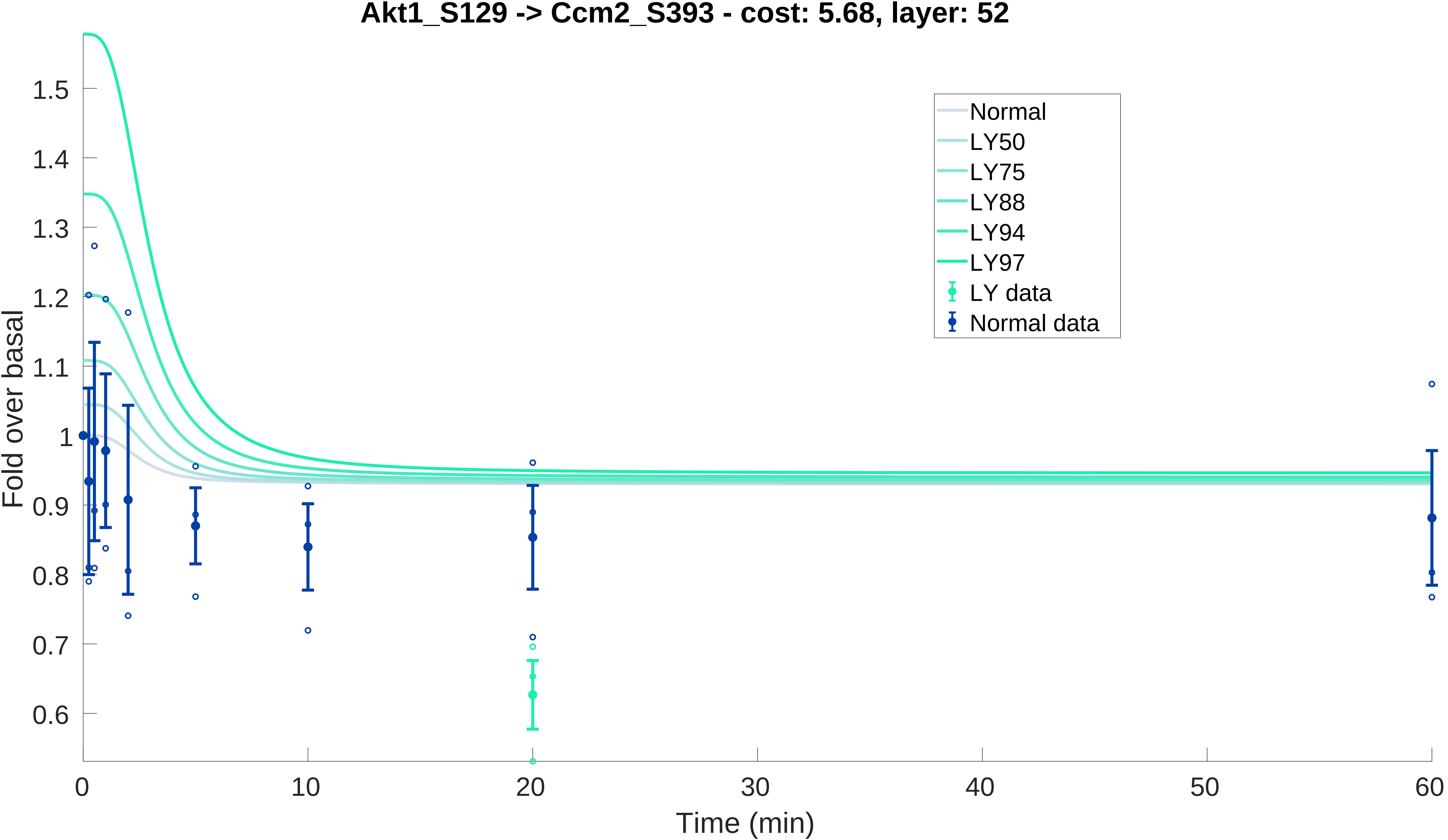

Akt1_S129 -> Ccm2_S393 - cost: 5.68, layer: 52
Fold over basal
Time (min)
1.5
1.4
1.3
1.2
1.1
1
0.9
0.8
0.7
0.6
0
10
20
30
40
50
60
Normal
LY50
LY75
LY88
LY94
LY97
LY data
Normal data

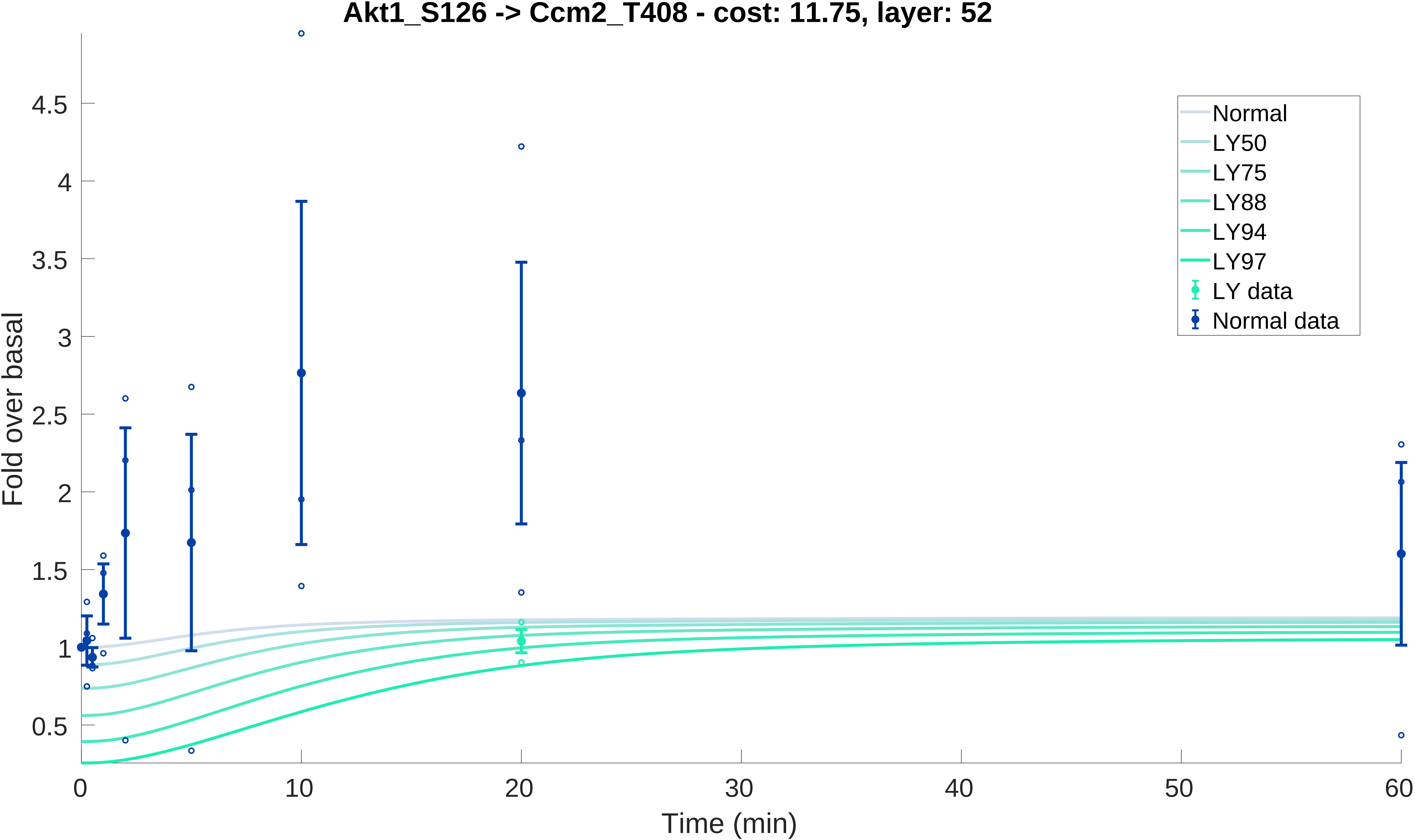

Akt1_S126 -> Ccm2_T408 - cost: 11.75, layer: 52
Fold over basal
Time (min)
0.5
1
1.5
2
2.5
3
3.5
4
4.5
0
10
20
30
40
50
60
Normal
LY50
LY75
LY88
LY94
LY97
LY data
Normal data

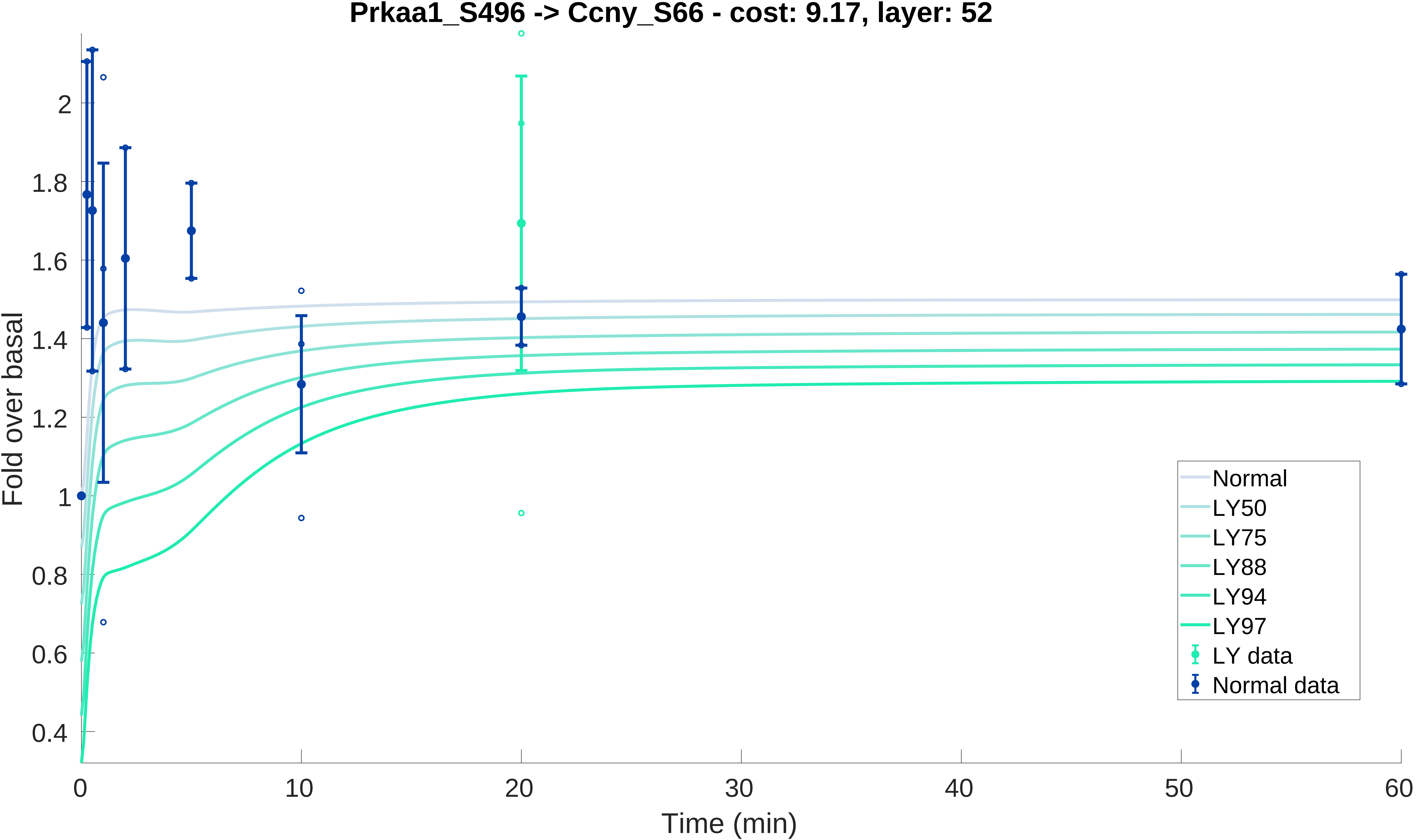

Prkaa1_S496 -> Ccny_S66 - cost: 9.17, layer: 52
Fold over basal
Time (min)
0.4
0.6
0.8
1
1.2
1.4
1.6
1.8
2
0
10
20
30
40
50
60
Normal
LY50
LY75
LY88
LY94
LY97
LY data
Normal data

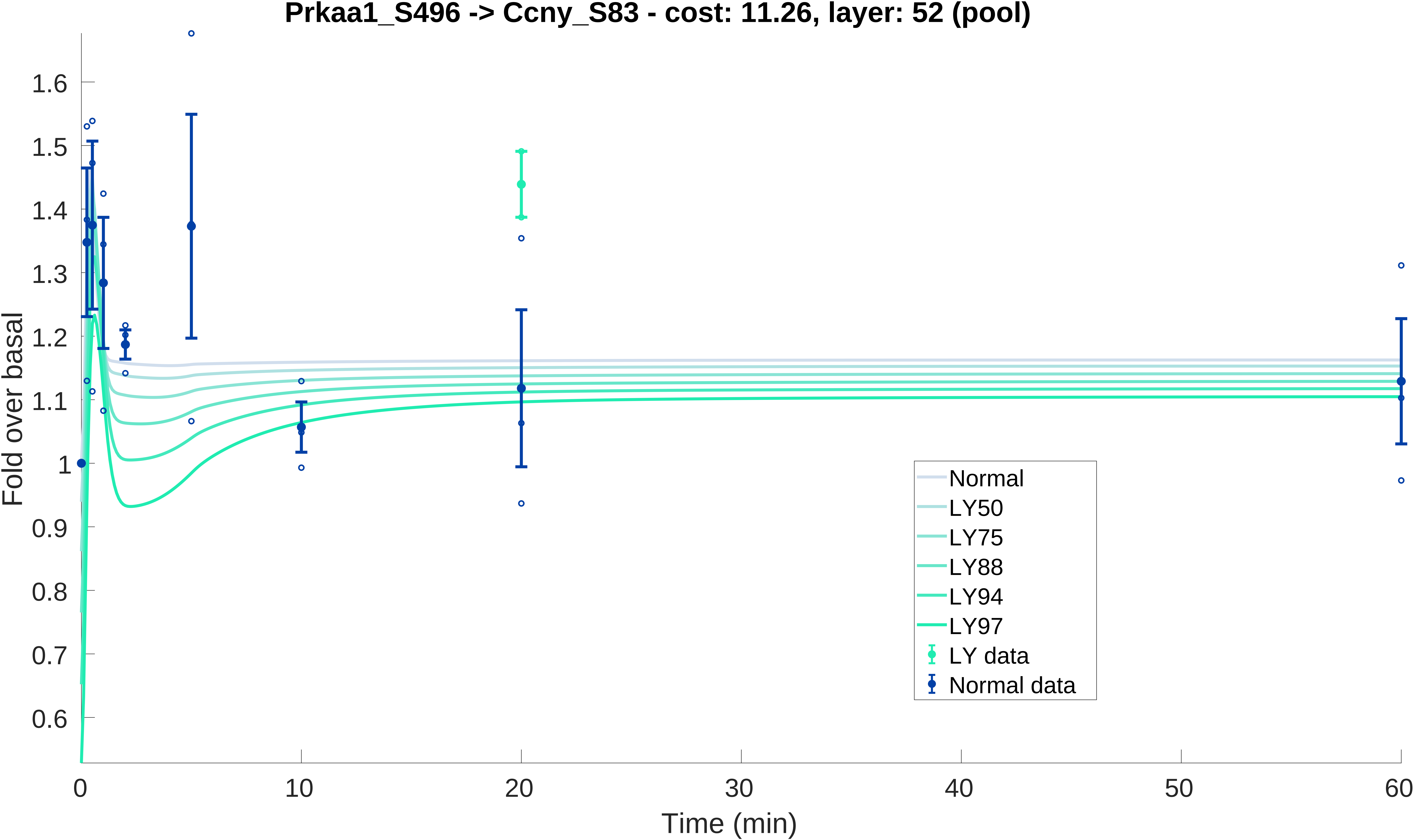

Prkaa1_S496 -> Ccny_S83 - cost: 11.26, layer: 52 (pool)
Fold over basal
1.6
1.5
1.4
1.3
1.2
1.1
1
0.9
0.8
0.7
0.6
0
10
20
30
40
50
60
Time (min)
Normal
LY50
LY75
LY88
LY94
LY97
LY data
Normal data

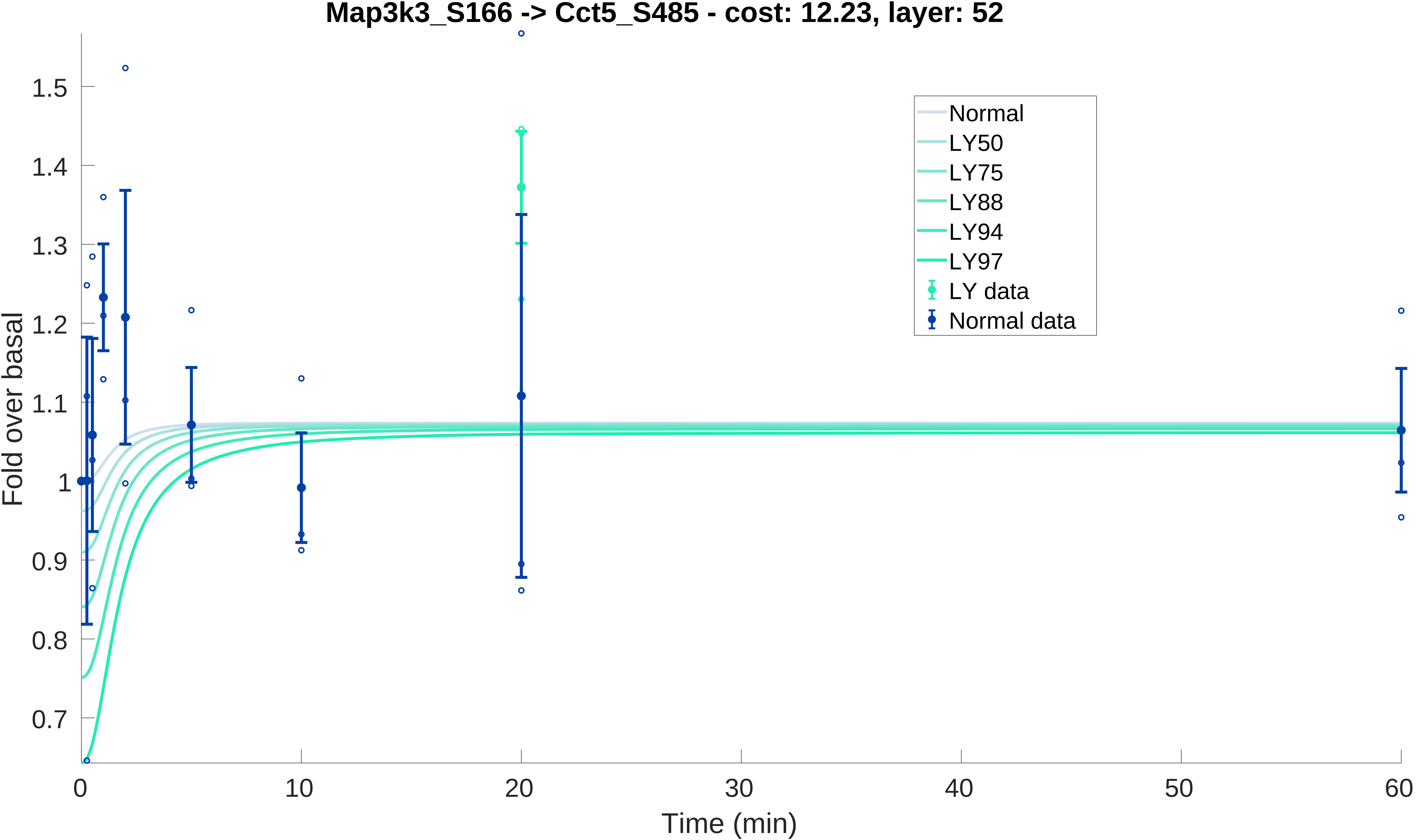

Map3k3_S166 -> Cct5_S485 - cost: 12.23, layer: 52
Fold over basal
Time (min)
0
10
20
30
40
50
60
0.7
0.8
0.9
1
1.1
1.2
1.3
1.4
1.5
Normal
LY50
LY75
LY88
LY94
LY97
LY data
Normal data

Map3k3_S166 -> Cct8_S537 - cost: 5.32, layer: 52

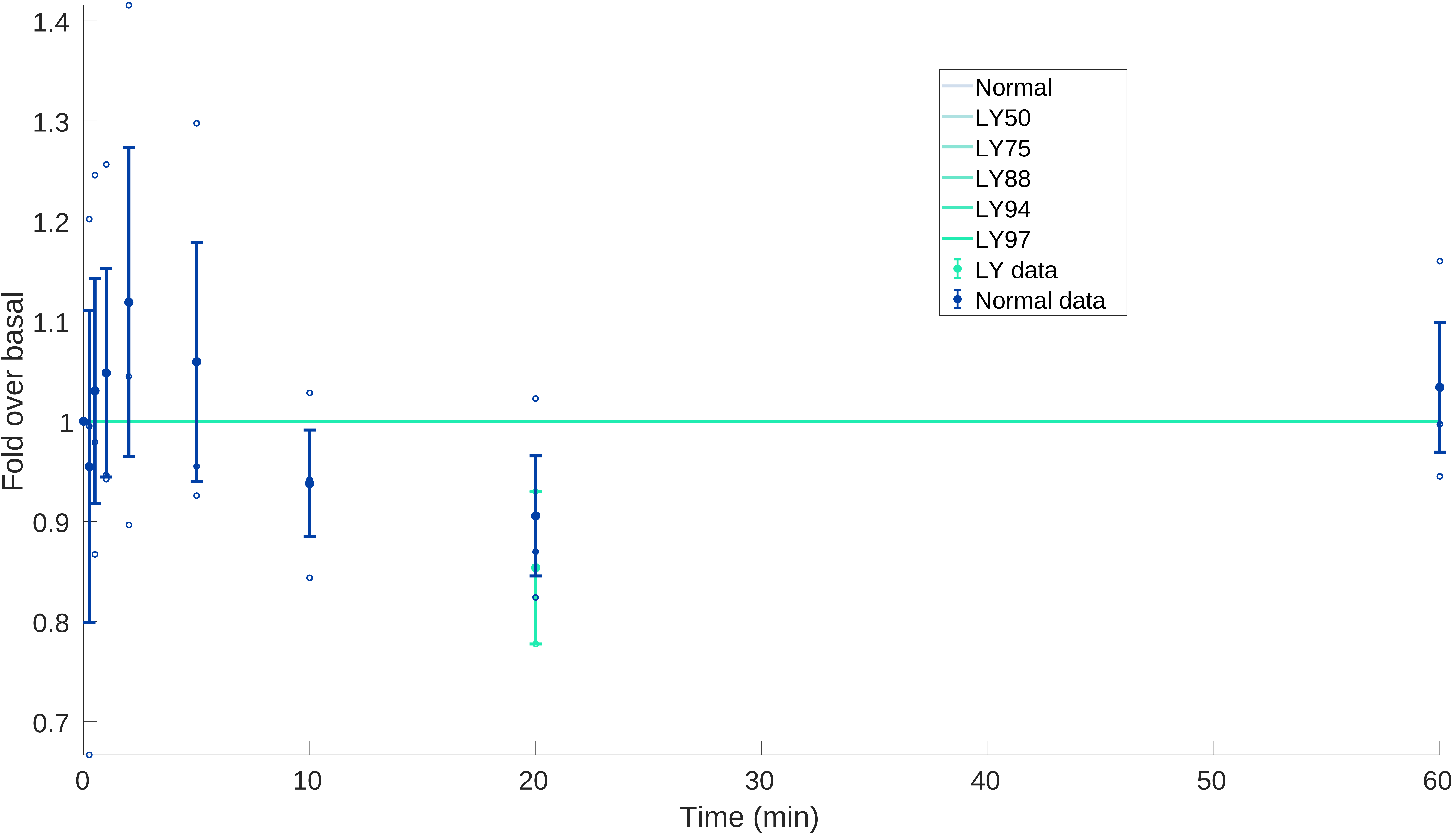

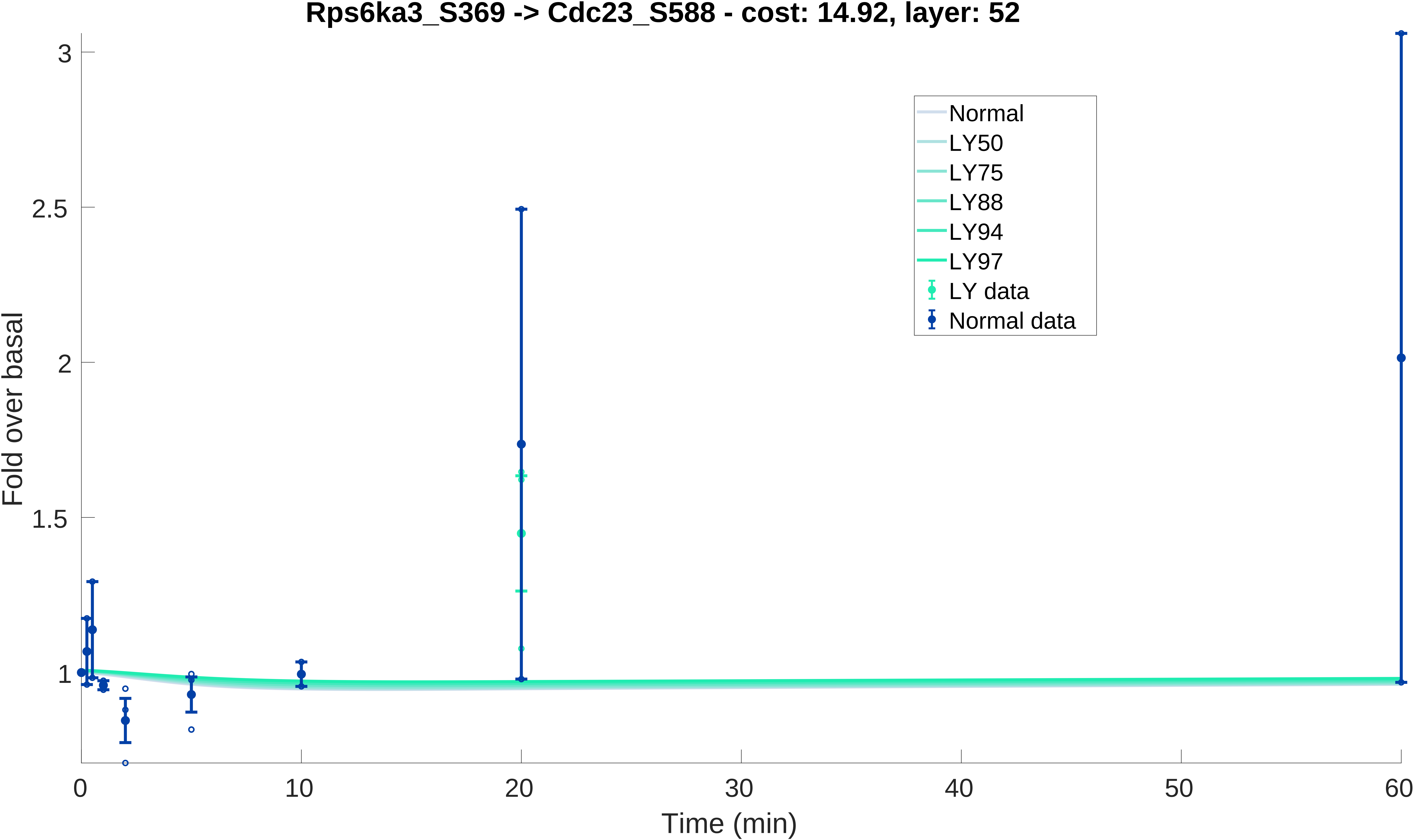

Rps6ka3_S369 -> Cdc23_S588 - cost: 14.92, layer: 52
Fold over basal
Time (min)
3
2.5
2
1.5
1
0
10
20
30
40
50
60
Normal
LY50
LY75
LY88
LY94
LY97
LY data
Normal data

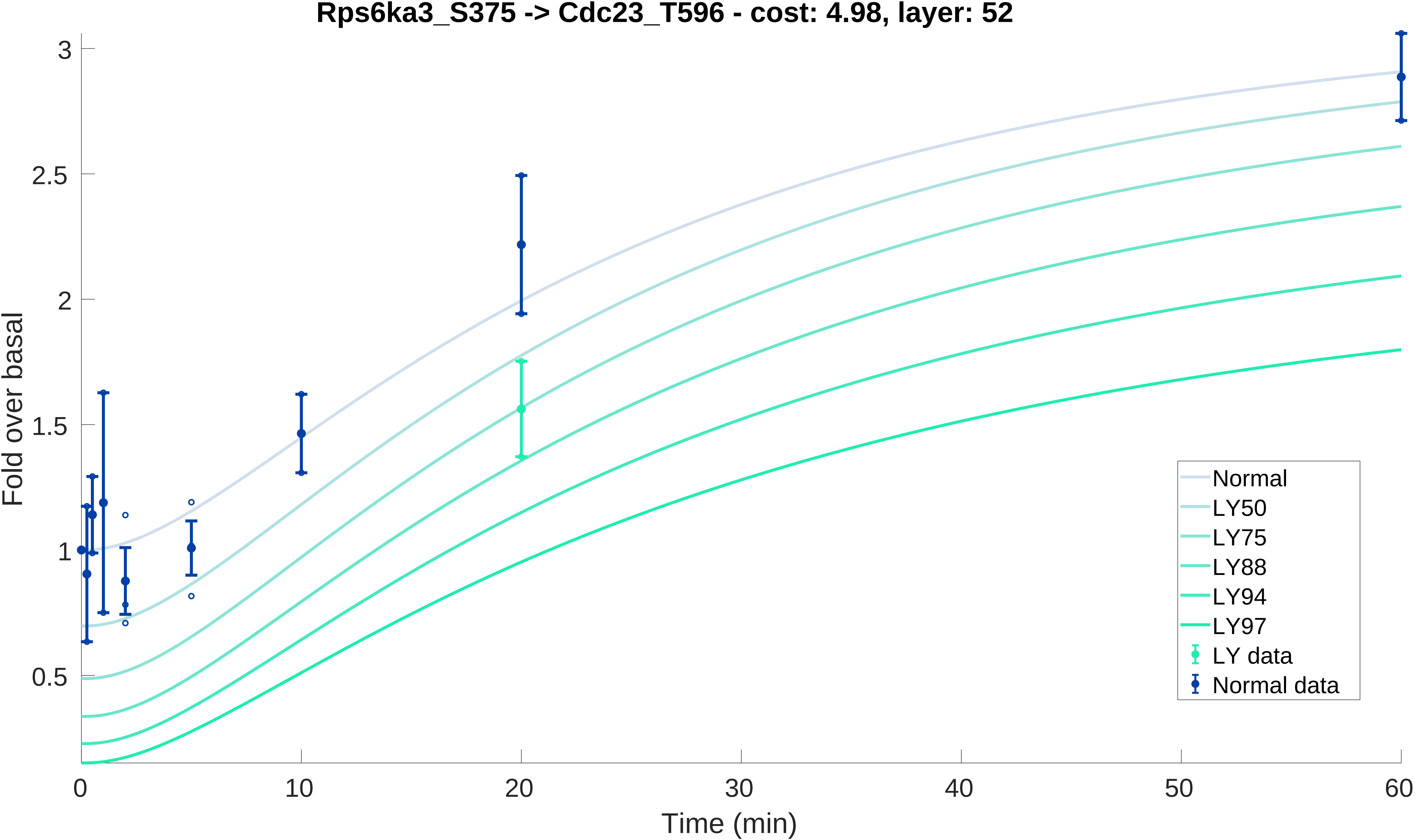

Rps6ka3_S375 -> Cdc23_T596 - cost: 4.98, layer: 52
Fold over basal
3
2.5
2
1.5
1
0.5
0
10
20
30
40
50
60
Time (min)
Normal
LY50
LY75
LY88
LY94
LY97
LY data
Normal data

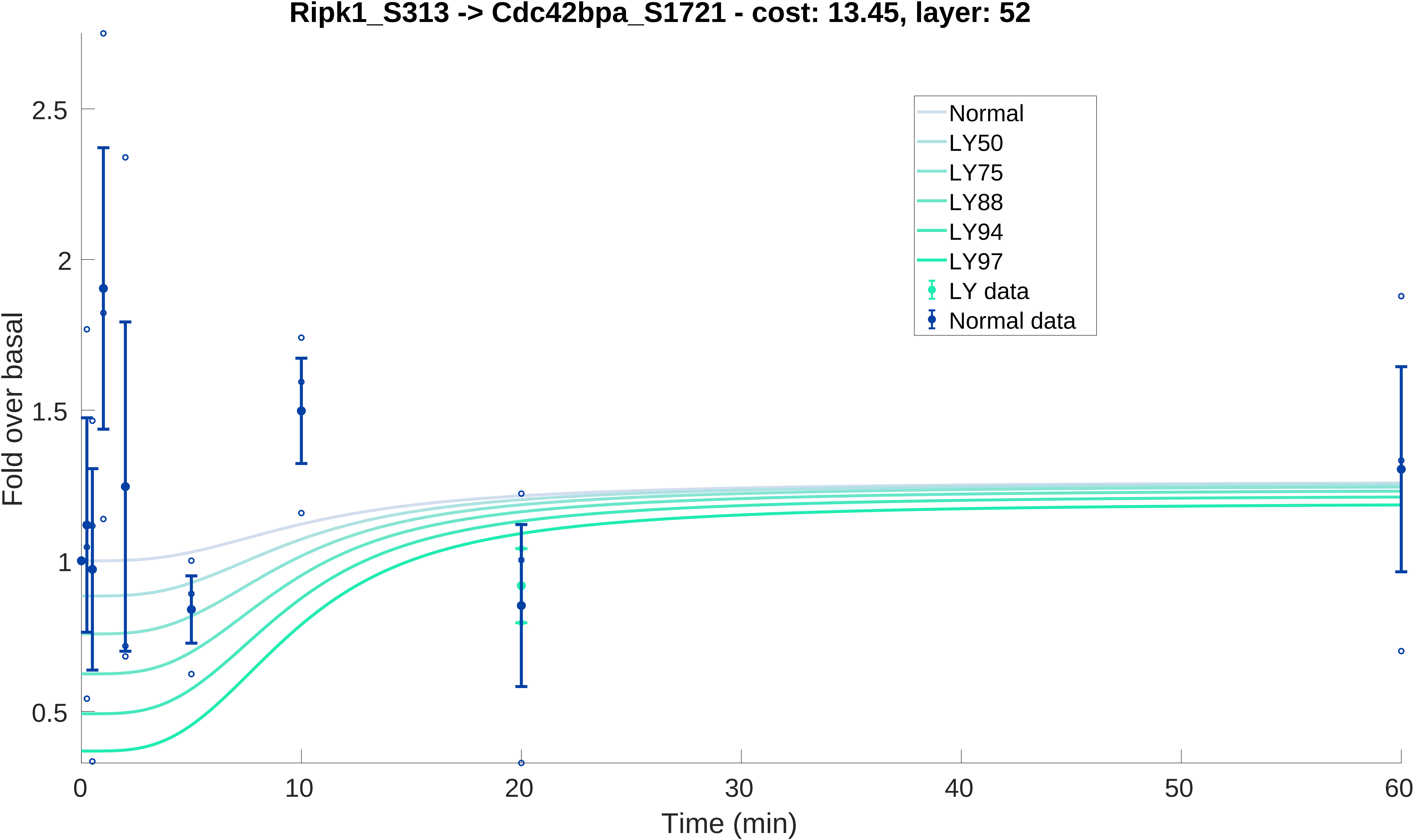

Ripk1_S313 -> Cdc42bpa_S1721 - cost: 13.45, layer: 52
Fold over basal
Time (min)
Normal
LY50
LY75
LY88
LY94
LY97
LY data
Normal data
0.5
1
1.5
2
2.5
0
10
20
30
40
50
60

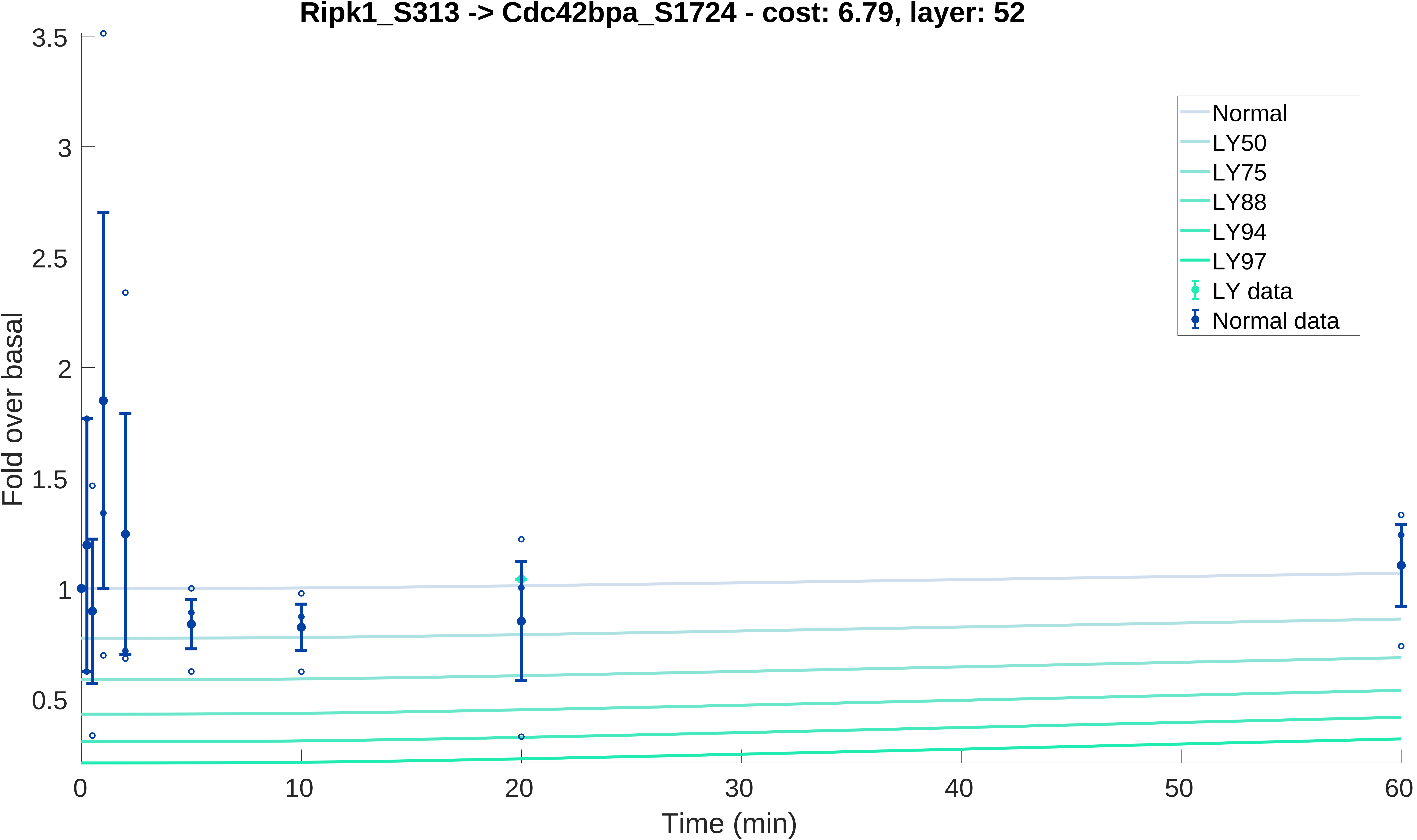

Ripk1_S313 -> Cdc42bpa_S1724 - cost: 6.79, layer: 52
Fold over basal
Time (min)
0.5
1
1.5
2
2.5
3
3.5
0
10
20
30
40
50
60
Normal
LY50
LY75
LY88
LY94
LY97
LY data
Normal data

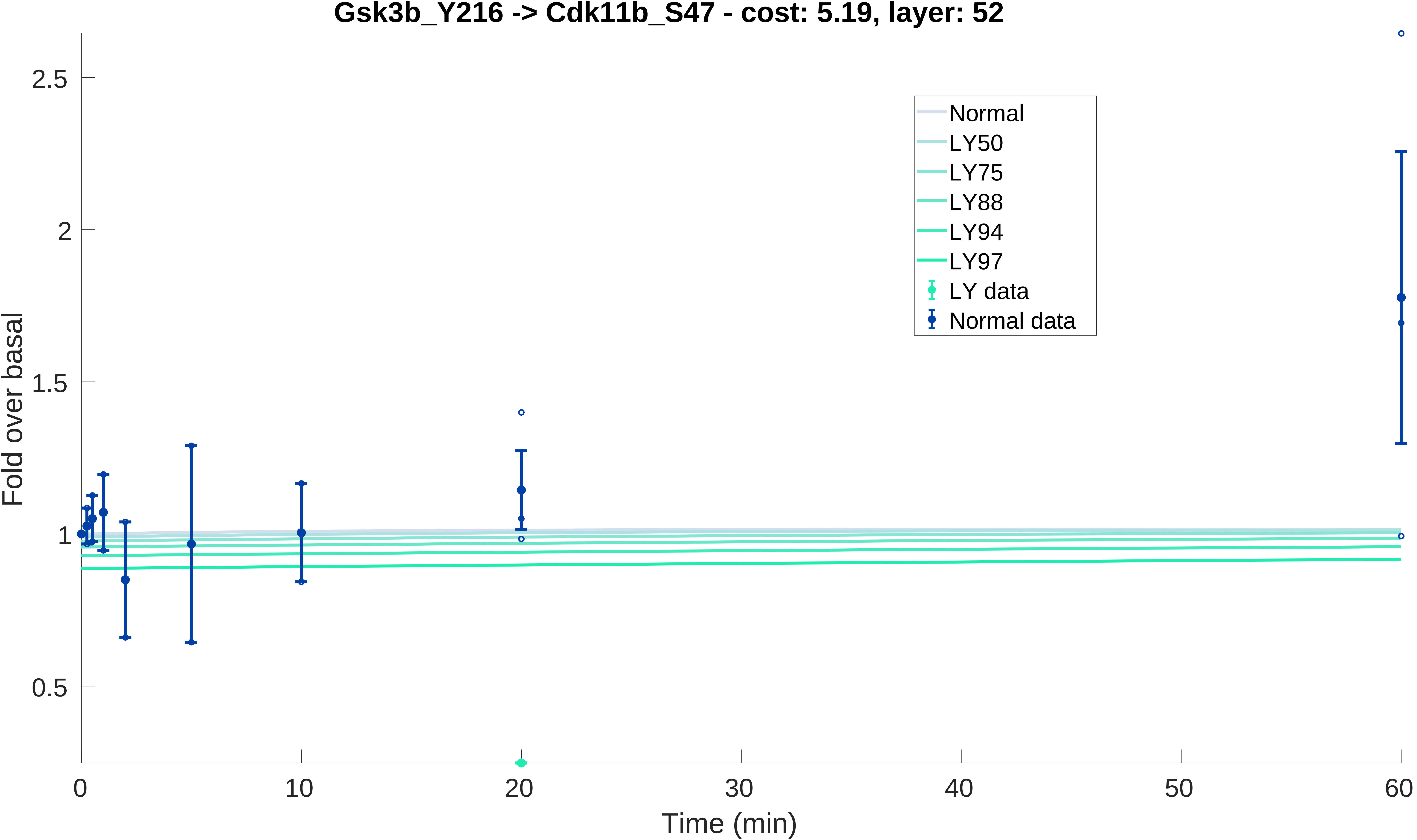

Gsk3b_Y216 -> Cdk11b_S47 - cost: 5.19, layer: 52
Normal
LY50
LY75
LY88
LY94
LY97
LY data
Normal data
Fold over basal
2.5
2
1.5
1
0.5
0
10
20
30
40
50
60
Time (min)

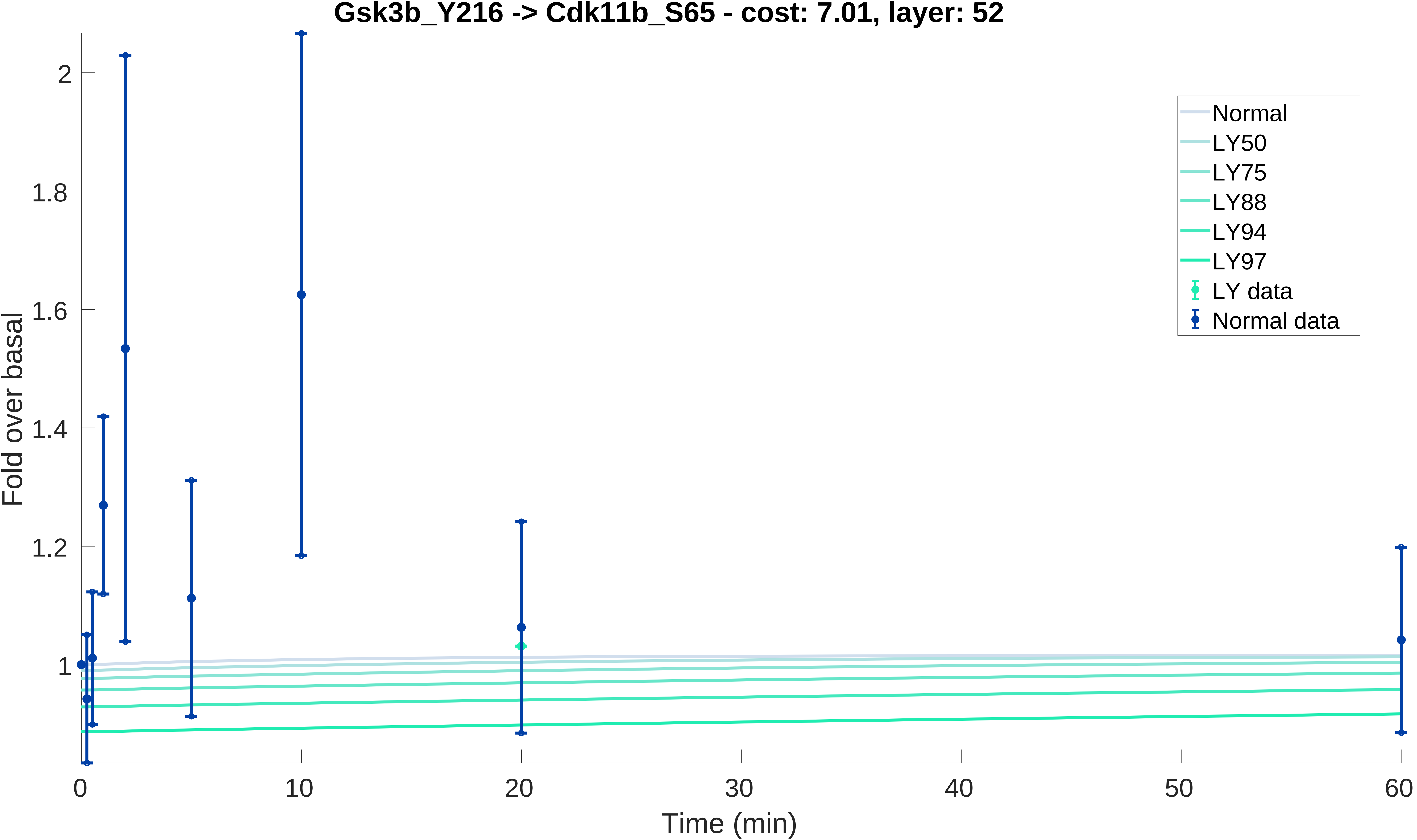

Gsk3b_Y216 -> Cdk11b_S65 - cost: 7.01, layer: 52
Fold over basal
Time (min)
2
1.8
1.6
1.4
1.2
1
0
10
20
30
40
50
60
Normal
LY50
LY75
LY88
LY94
LY97
LY data
Normal data

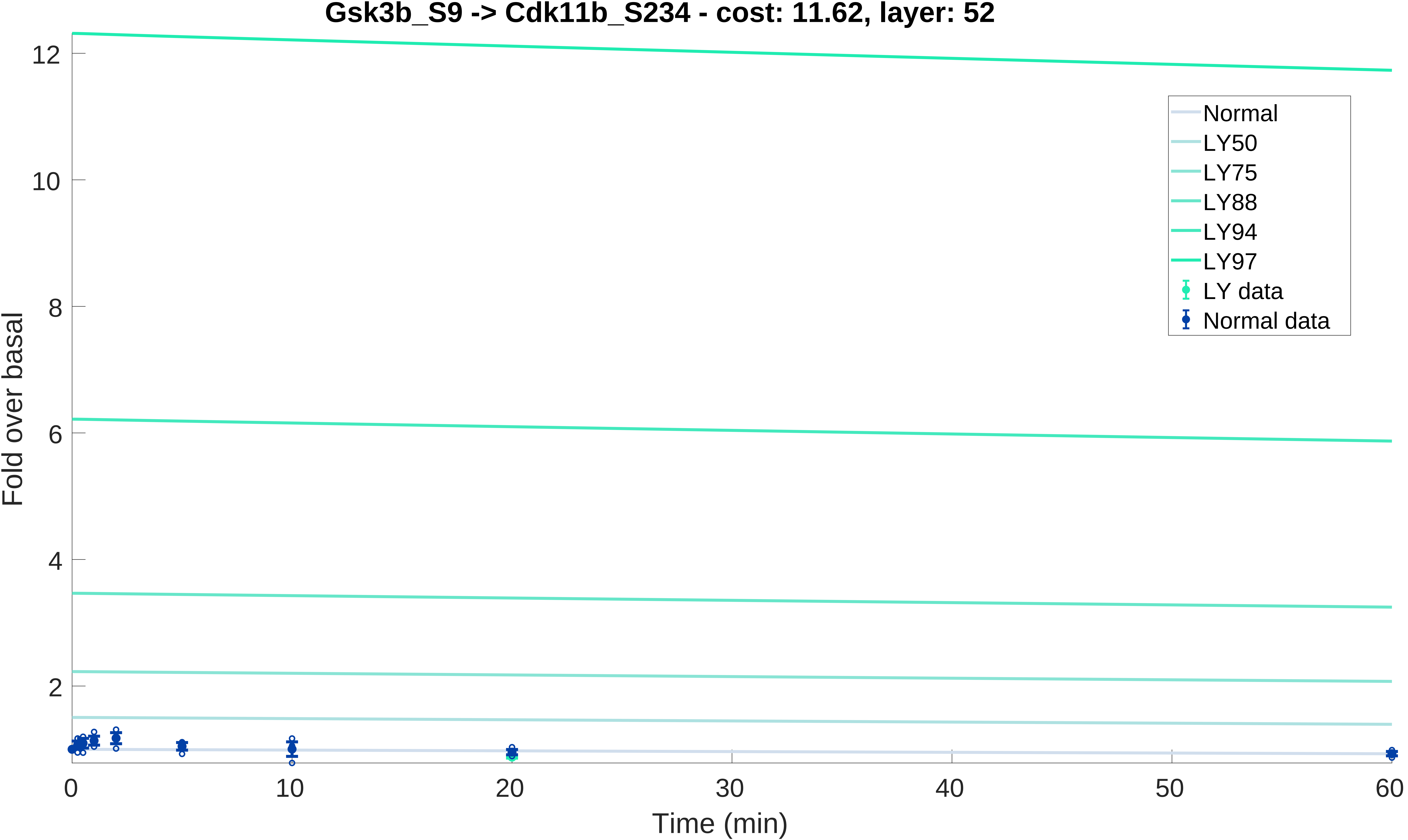

Gsk3b_S9 -> Cdk11b_S234 - cost: 11.62, layer: 52
Fold over basal
Time (min)
0
10
20
30
40
50
60
2
4
6
8
10
12
Normal
LY50
LY75
LY88
LY94
LY97
LY data
Normal data

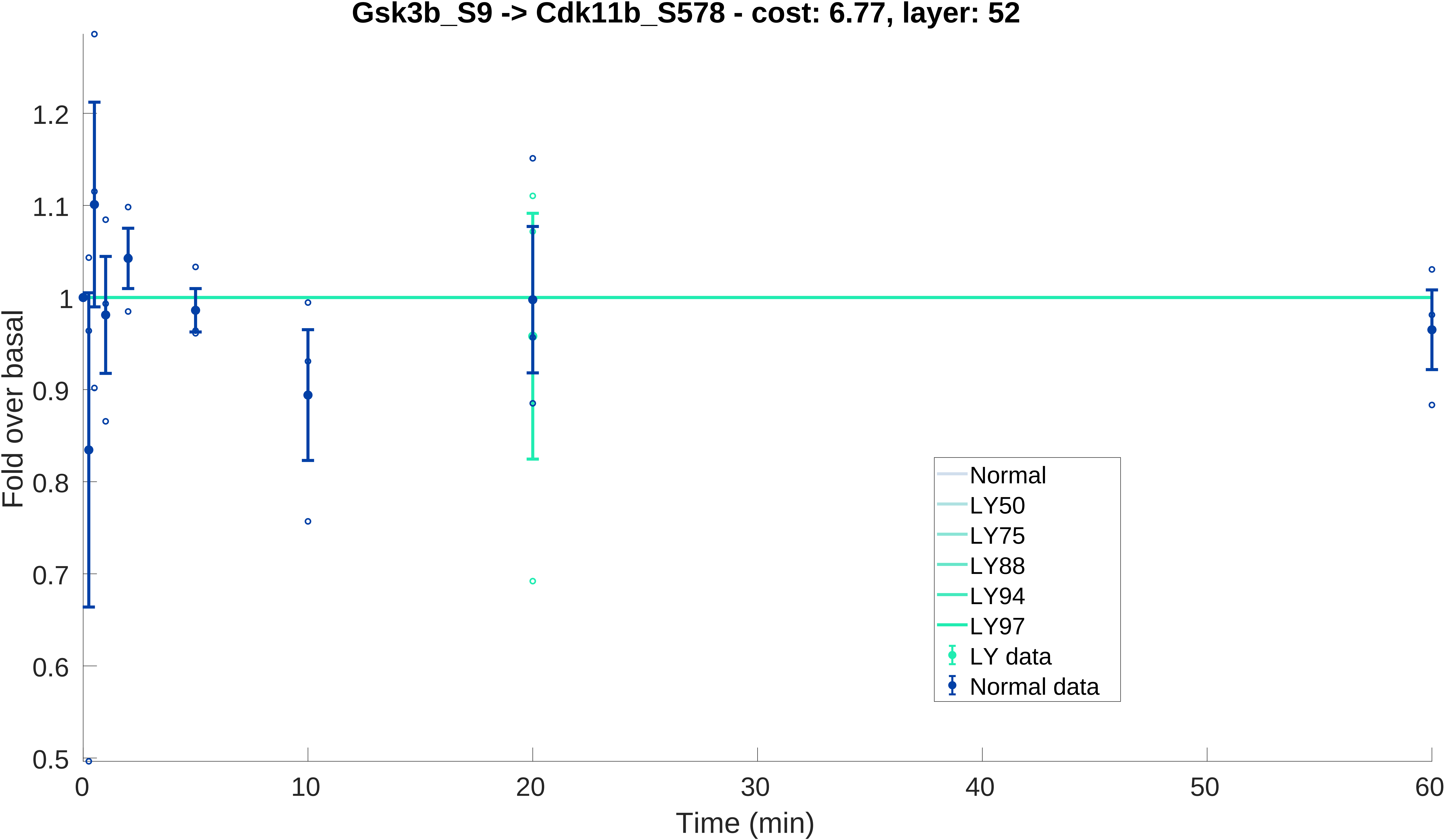

Gsk3b_S9 -> Cdk11b_S578 - cost: 6.77, layer: 52
Fold over basal
1.2
1.1
1
0.9
0.8
0.7
0.6
0.5
0
10
20
30
40
50
60
Time (min)
Normal
LY50
LY75
LY88
LY94
LY97
LY data
Normal data

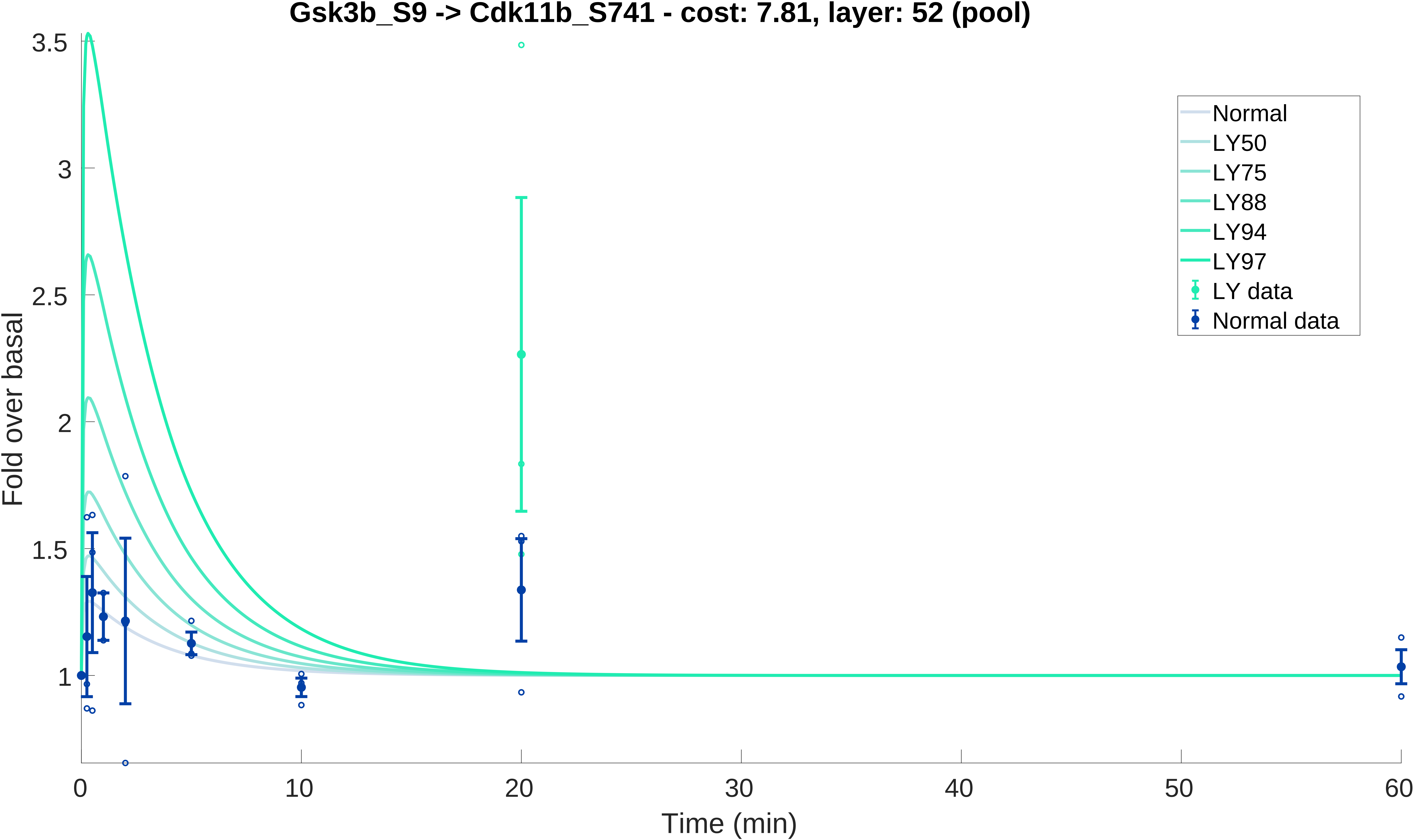
Gsk3b_S9 -> Cdk11b_S741 - cost: 7.81, layer: 52 (pool)
Fold over basal
Time (min)
Normal
LY50
LY75
LY88
LY94
LY97
LY data
Normal data
3.5
3
2.5
2
1.5
1
0
10
20
30
40
50
60

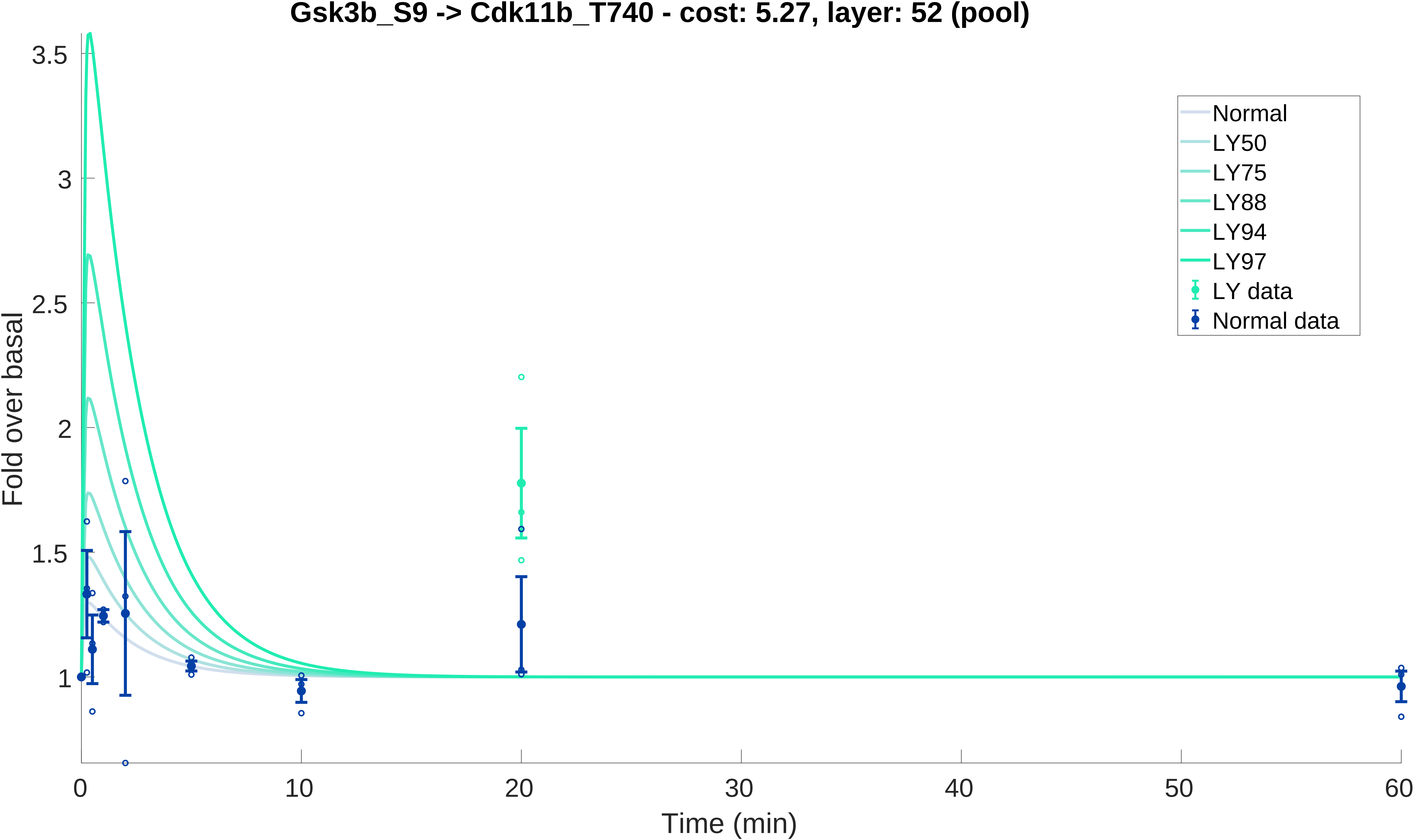
Gsk3b_S9 -> Cdk11b_T740 - cost: 5.27, layer: 52 (pool)
Fold over basal
3.5
3
2.5
2
1.5
1
Time (min)
0
10
20
30
40
50
60
Normal
LY50
LY75
LY88
LY94
LY97
LY data
Normal data

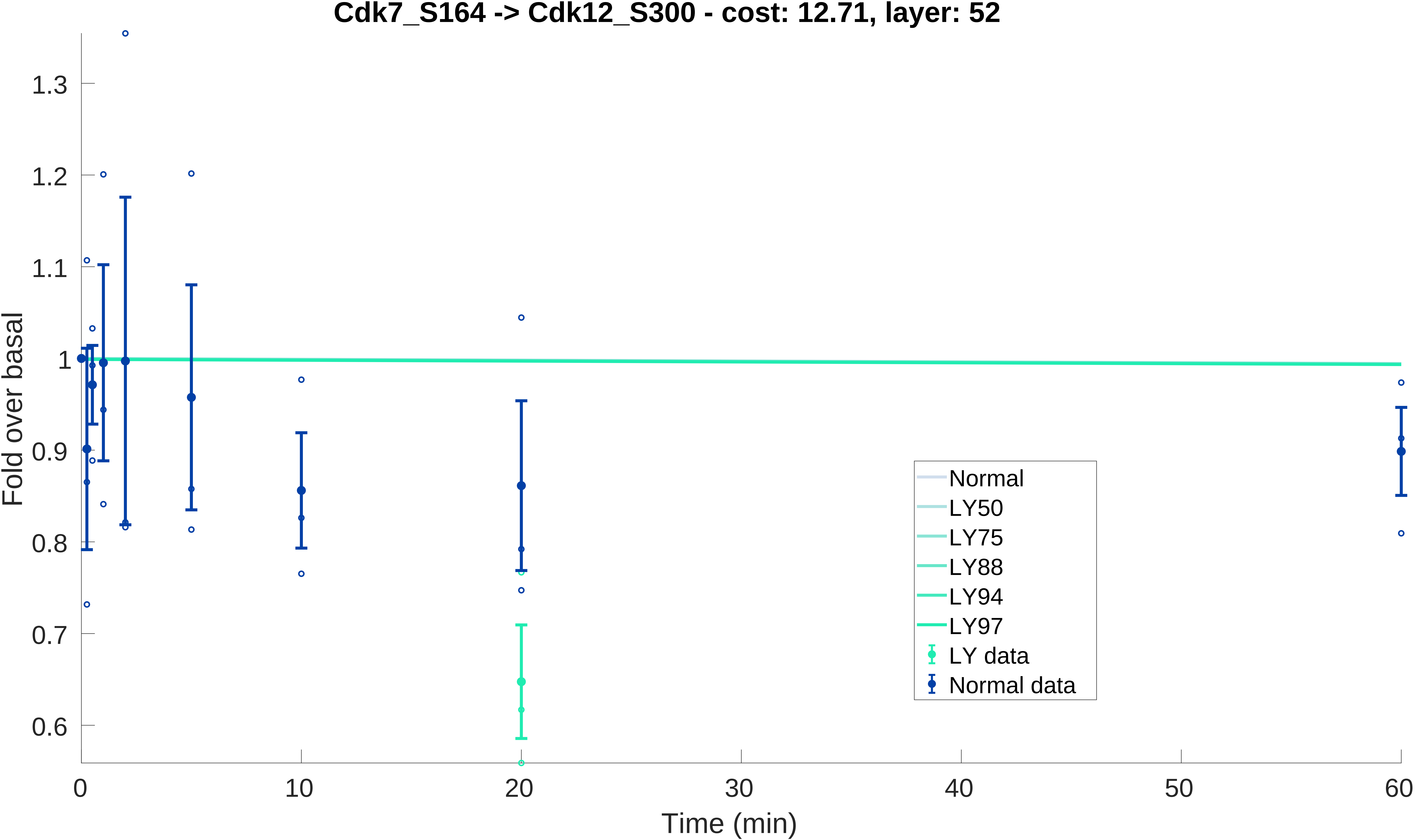

Cdk7_S164 -> Cdk12_S300 - cost: 12.71, layer: 52
Fold over basal
1.3
1.2
1.1
1
0.9
0.8
0.7
0.6
Time (min)
0
10
20
30
40
50
60
Normal
LY50
LY75
LY88
LY94
LY97
LY data
Normal data

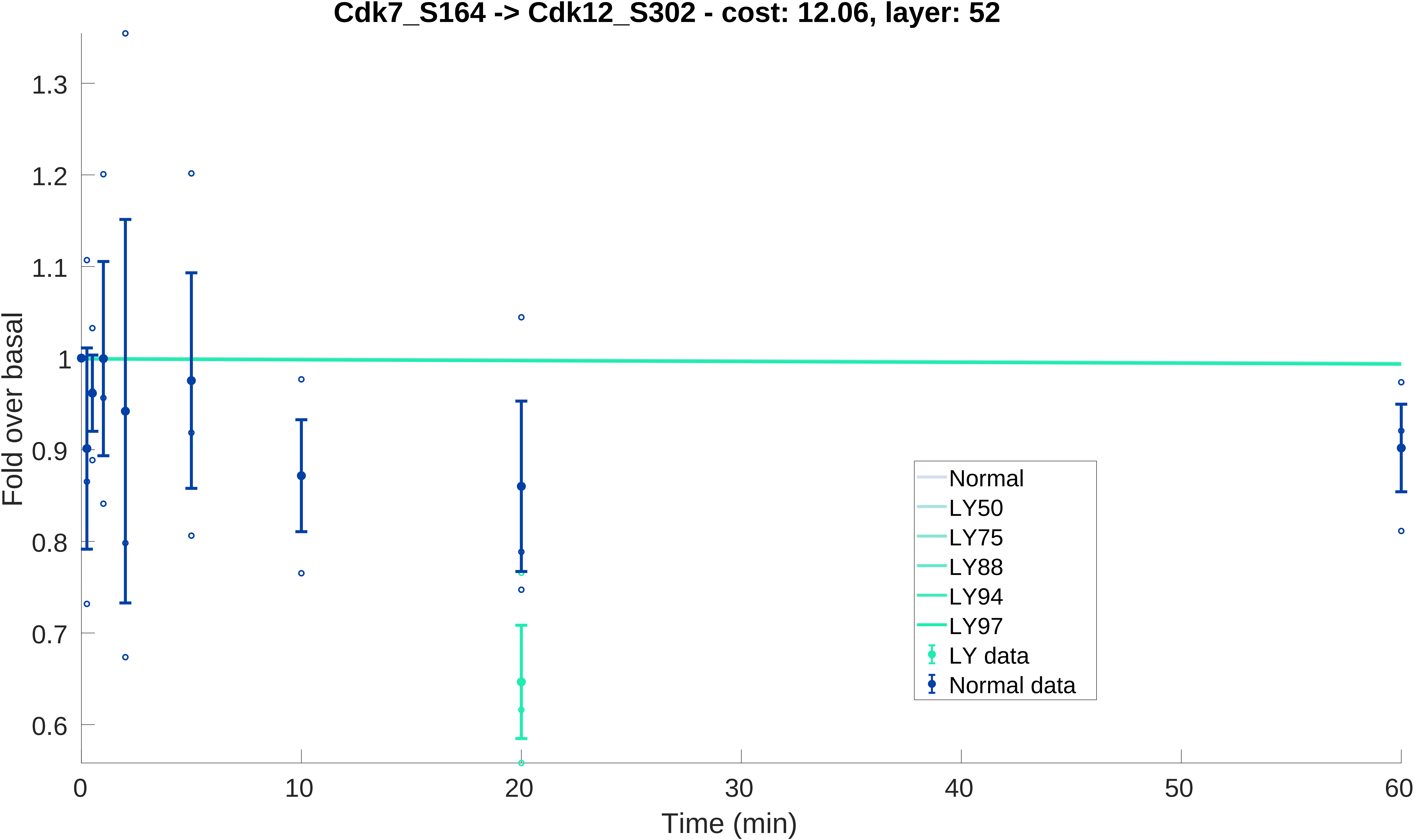

Cdk7_S164 -> Cdk12_S302 - cost: 12.06, layer: 52
Fold over basal
Time (min)
0.6
0.7
0.8
0.9
1
1.1
1.2
1.3
0
10
20
30
40
50
60
Normal
LY50
LY75
LY88
LY94
LY97
LY data
Normal data

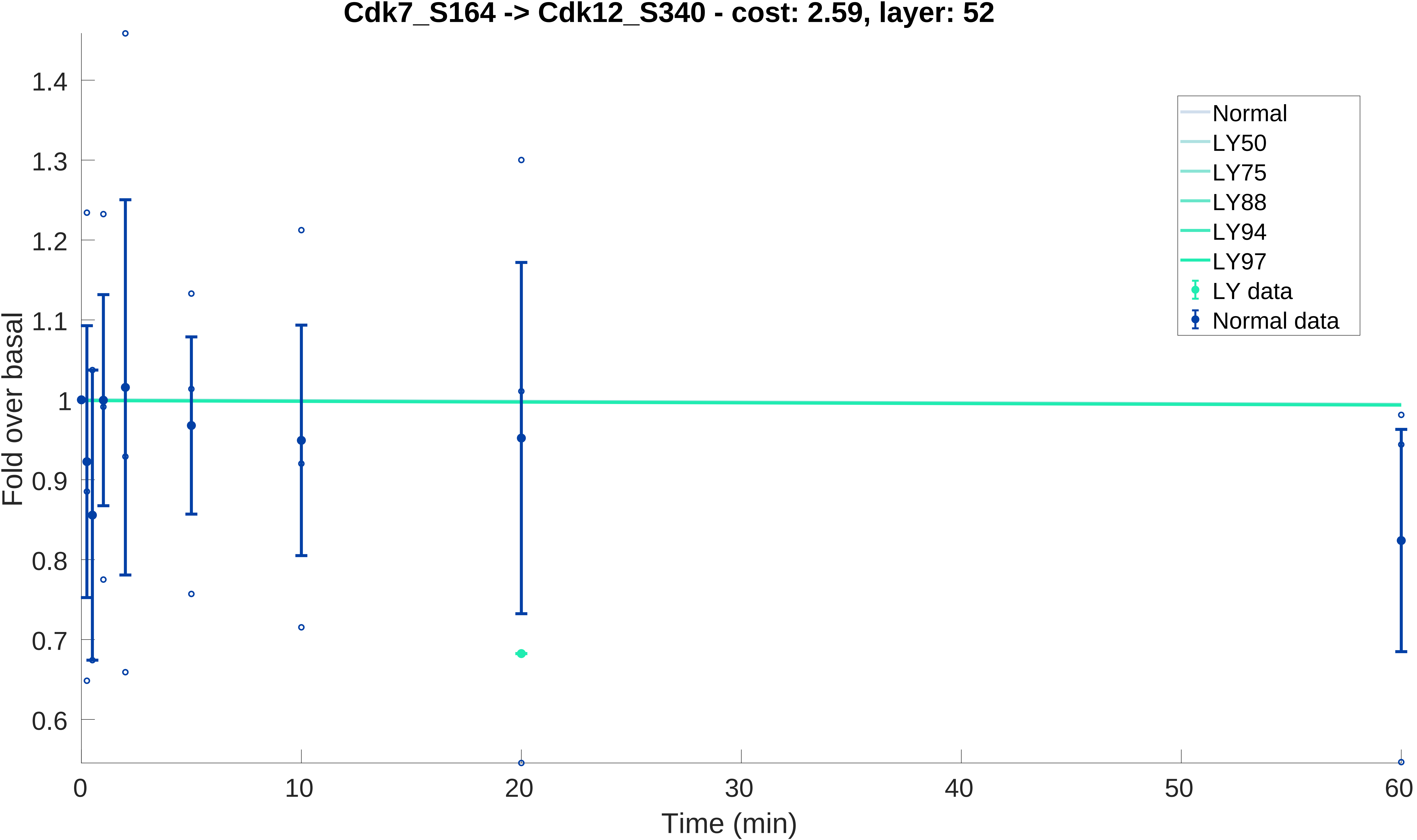

Cdk7_S164 -> Cdk12_S340 - cost: 2.59, layer: 52
Fold over basal
Time (min)
1.4
1.3
1.2
1.1
1
0.9
0.8
0.7
0.6
0
10
20
30
40
50
60
Normal
LY50
LY75
LY88
LY94
LY97
LY data
Normal data

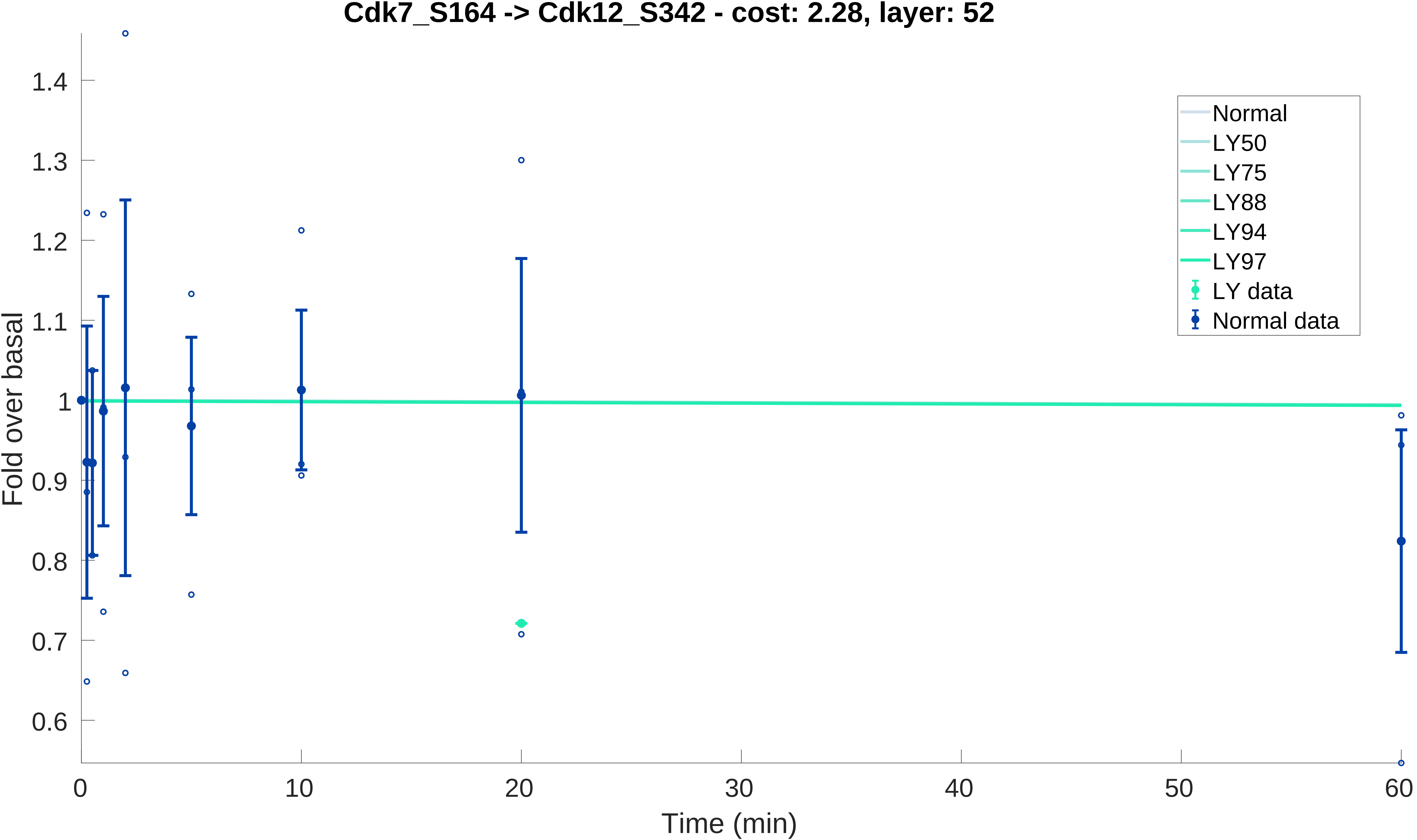

Cdk7_S164 -> Cdk12_S342 - cost: 2.28, layer: 52
Fold over basal
Time (min)
1.4
1.3
1.2
1.1
1
0.9
0.8
0.7
0.6
0
10
20
30
40
50
60
Normal
LY50
LY75
LY88
LY94
LY97
LY data
Normal data

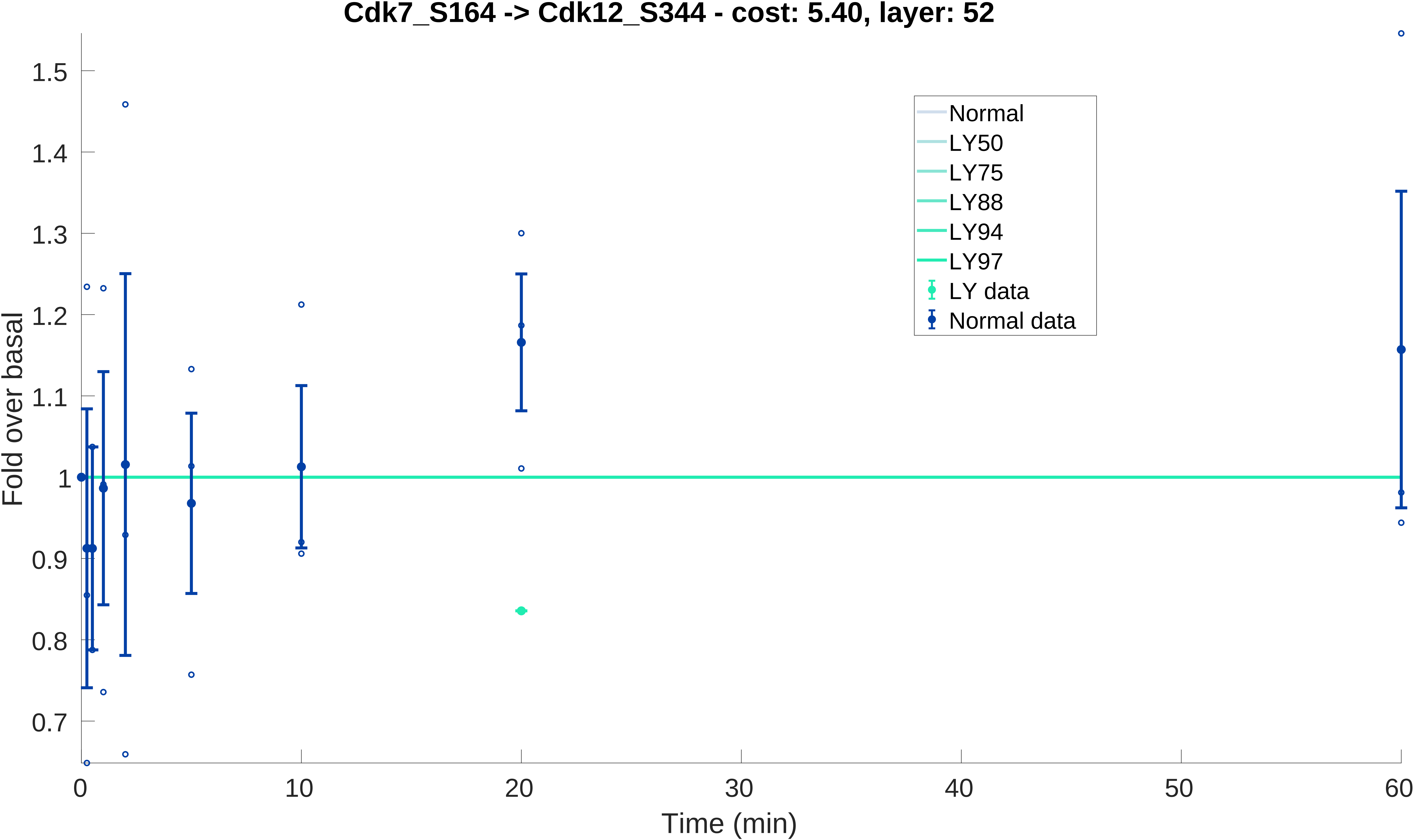
Cdk7_S164 -> Cdk12_S344 - cost: 5.40, layer: 52
Fold over basal
1.5
1.4
1.3
1.2
1.1
1
0.9
0.8
0.7
0
10
20
30
40
50
60
Time (min)
Normal
LY50
LY75
LY88
LY94
LY97
LY data
Normal data

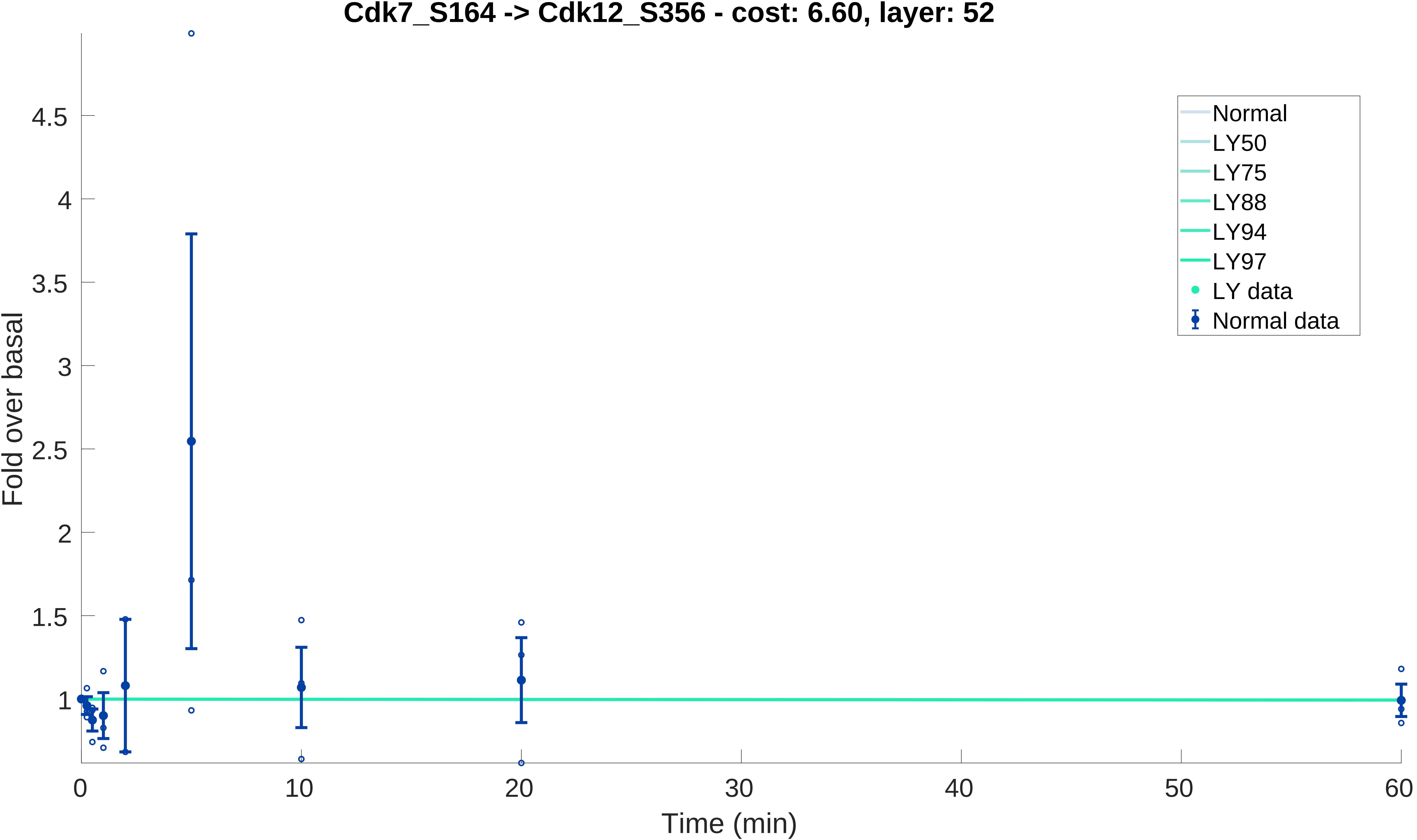
Cdk7_S164 -> Cdk12_S356 - cost: 6.60, layer: 52
Fold over basal
Time (min)
4.5
4
3.5
3
2.5
2
1.5
1
0
10
20
30
40
50
60
Normal
LY50
LY75
LY88
LY94
LY97
LY data
Normal data

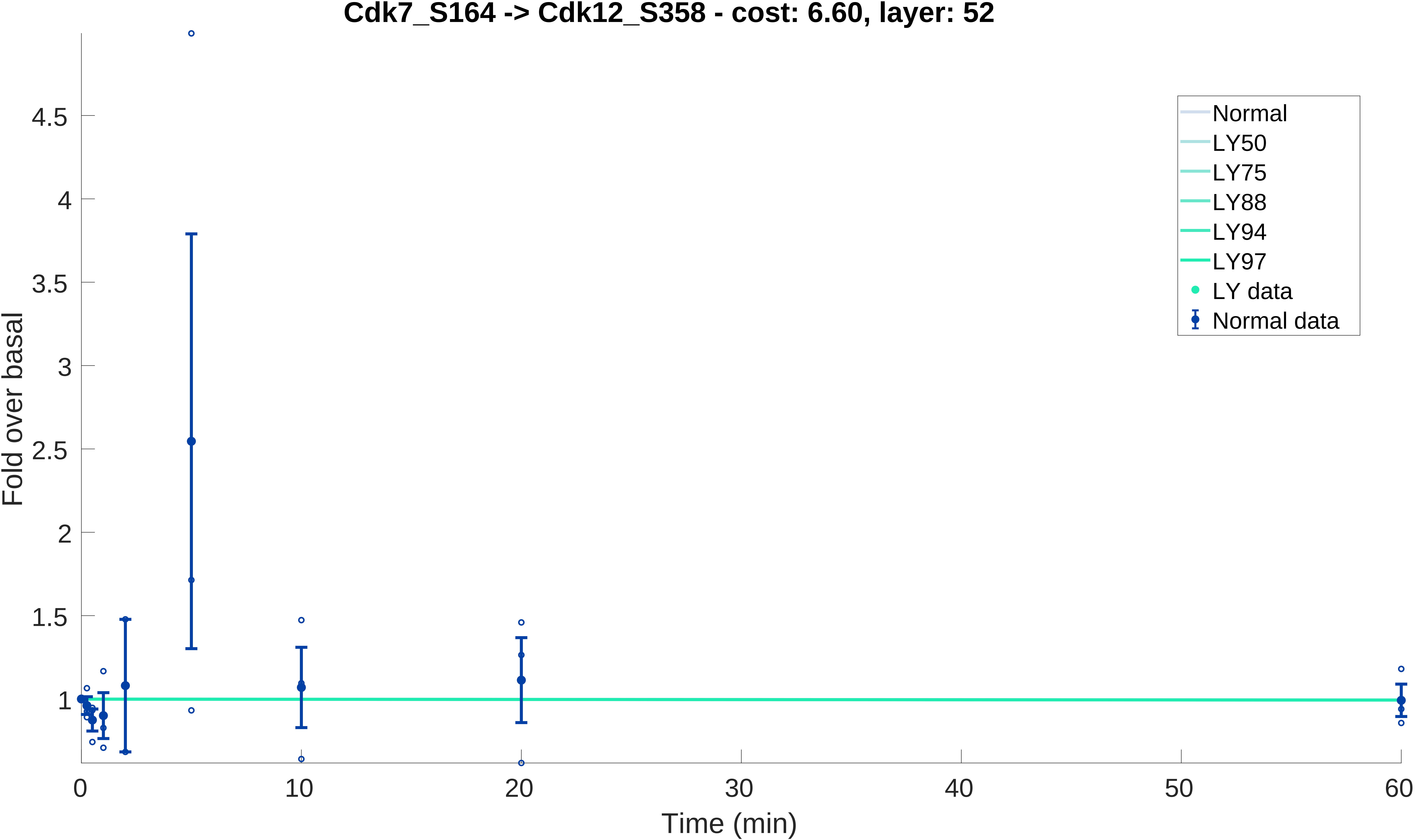

Cdk7_S164 -> Cdk12_S358 - cost: 6.60, layer: 52
Fold over basal
4.5
4
3.5
3
2.5
2
1.5
1
0
10
20
30
40
50
60
Time (min)
Normal
LY50
LY75
LY88
LY94
LY97
LY data
Normal data

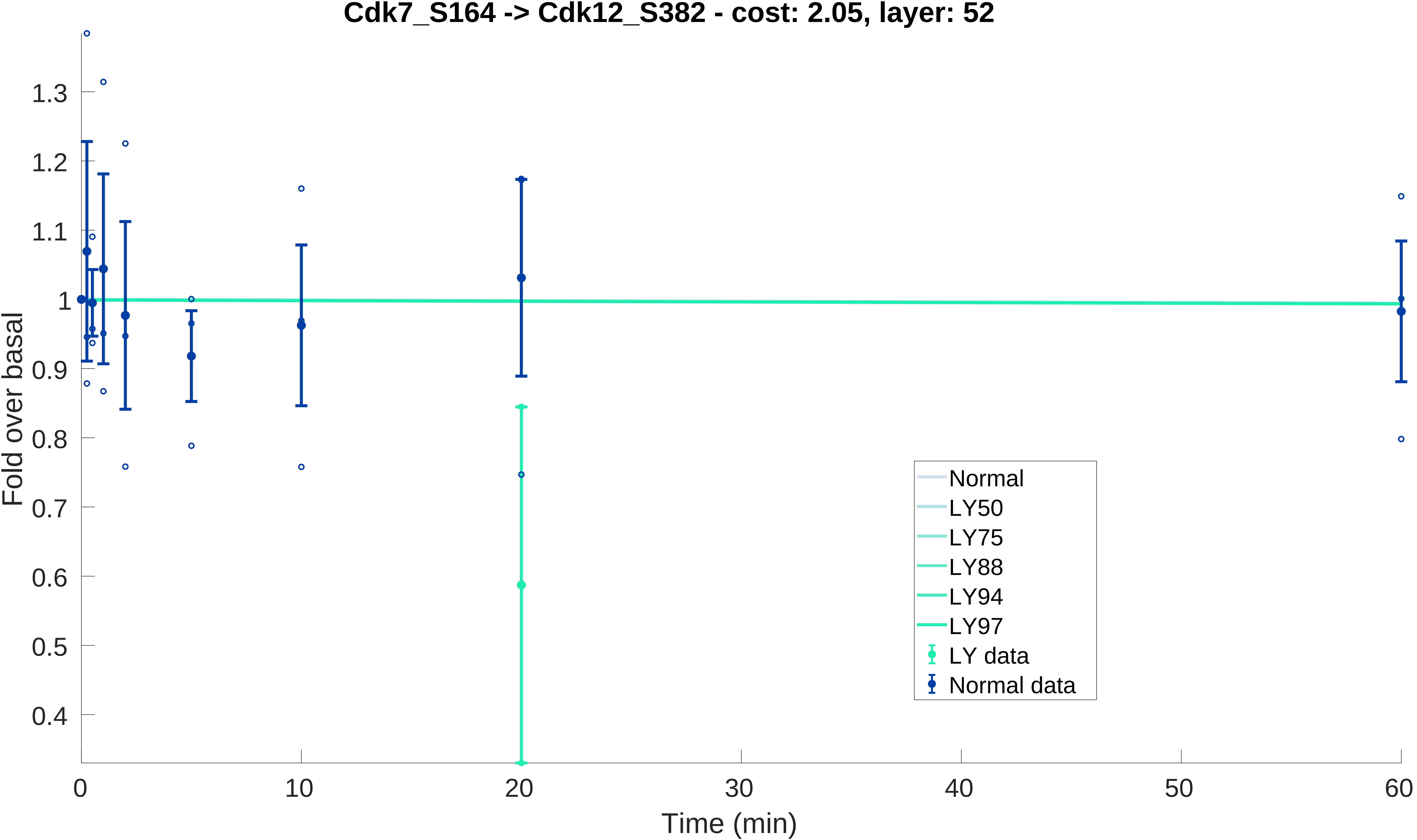

Cdk7_S164 -> Cdk12_S382 - cost: 2.05, layer: 52
Fold over basal
1.3
1.2
1.1
1
0.9
0.8
0.7
0.6
0.5
0.4
0
10
20
30
40
50
60
Time (min)
Normal
LY50
LY75
LY88
LY94
LY97
LY data
Normal data

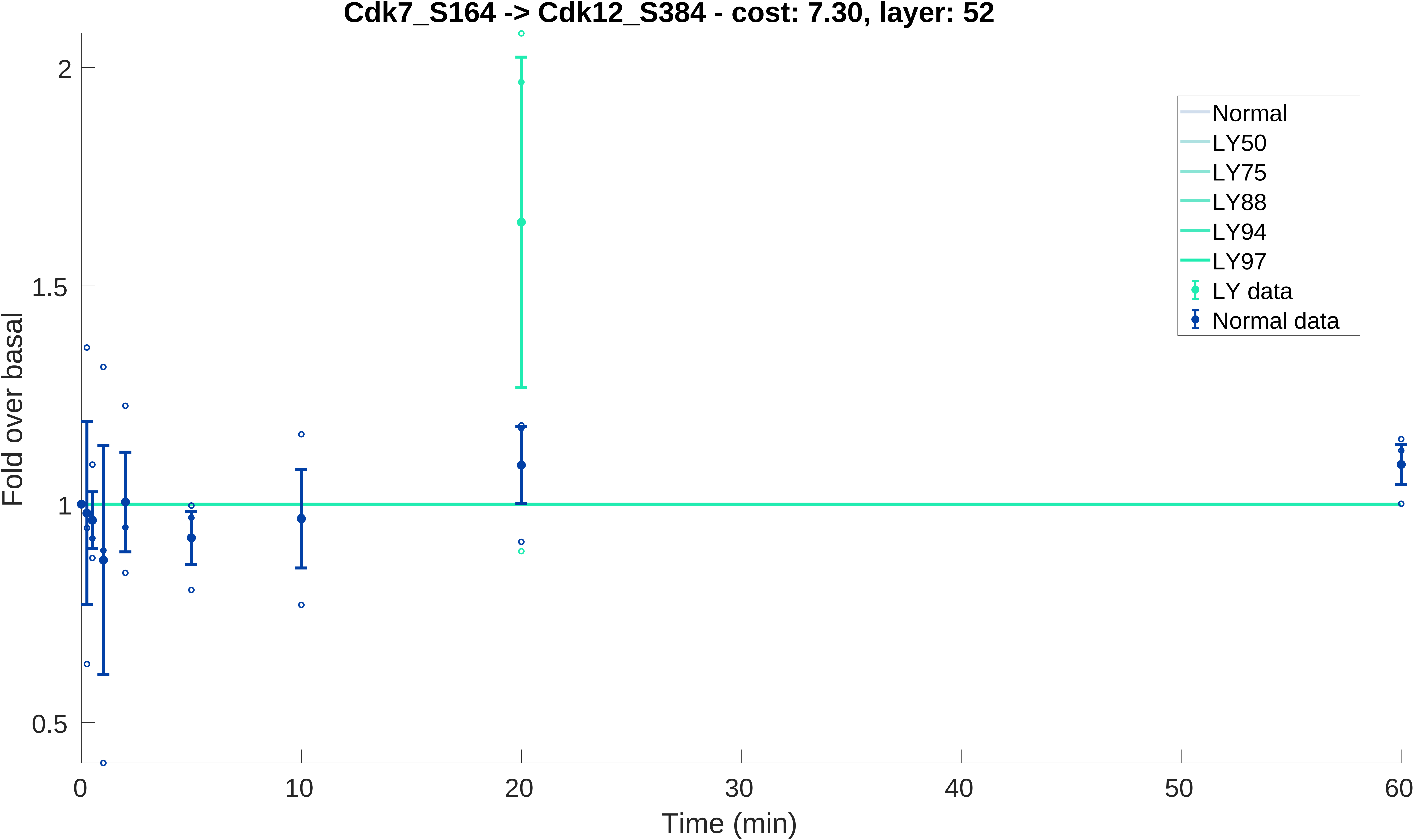

Cdk7_S164 -> Cdk12_S384 - cost: 7.30, layer: 52
Fold over basal
Time (min)
0.5
1
1.5
2
0
10
20
30
40
50
60
Normal
LY50
LY75
LY88
LY94
LY97
LY data
Normal data

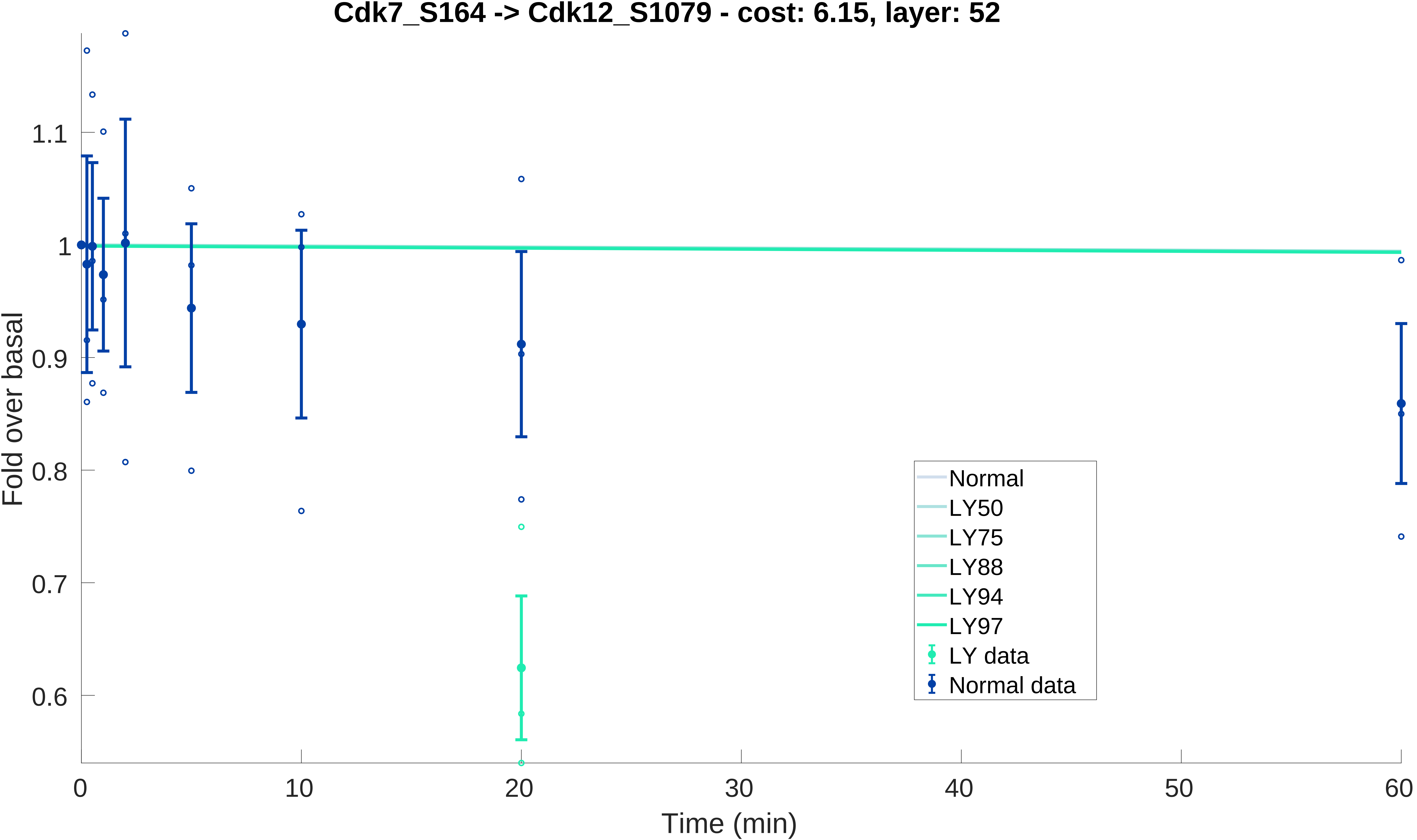

Cdk7_S164 -> Cdk12_S1079 - cost: 6.15, layer: 52
Fold over basal
Time (min)
1.1
1
0.9
0.8
0.7
0.6
0
10
20
30
40
50
60
Normal
LY50
LY75
LY88
LY94
LY97
LY data
Normal data

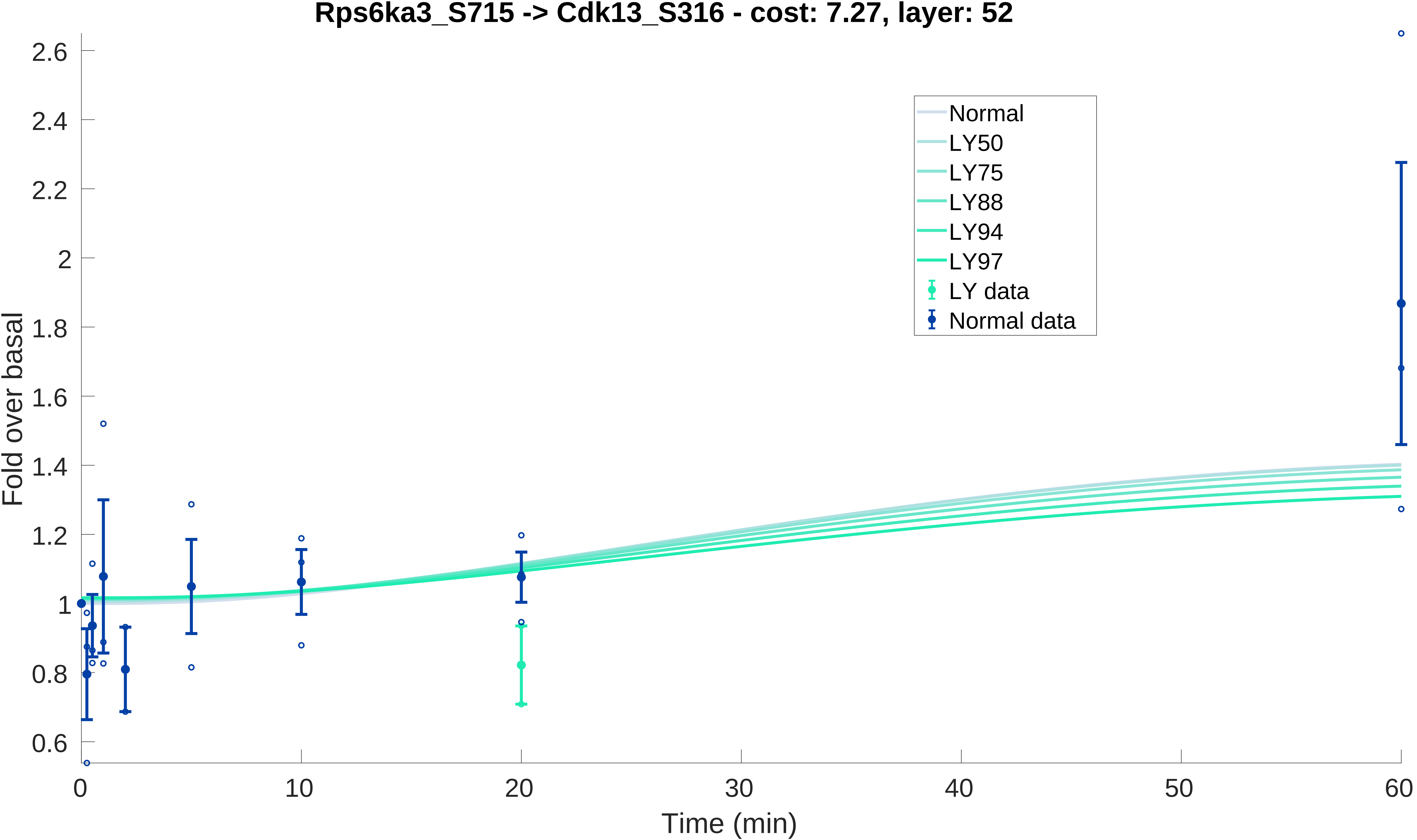

Rps6ka3_S715 -> Cdk13_S316 - cost: 7.27, layer: 52
Fold over basal
Time (min)
Normal
LY50
LY75
LY88
LY94
LY97
LY data
Normal data
2.6
2.4
2.2
2
1.8
1.6
1.4
1.2
1
0.8
0.6
0
10
20
30
40
50
60

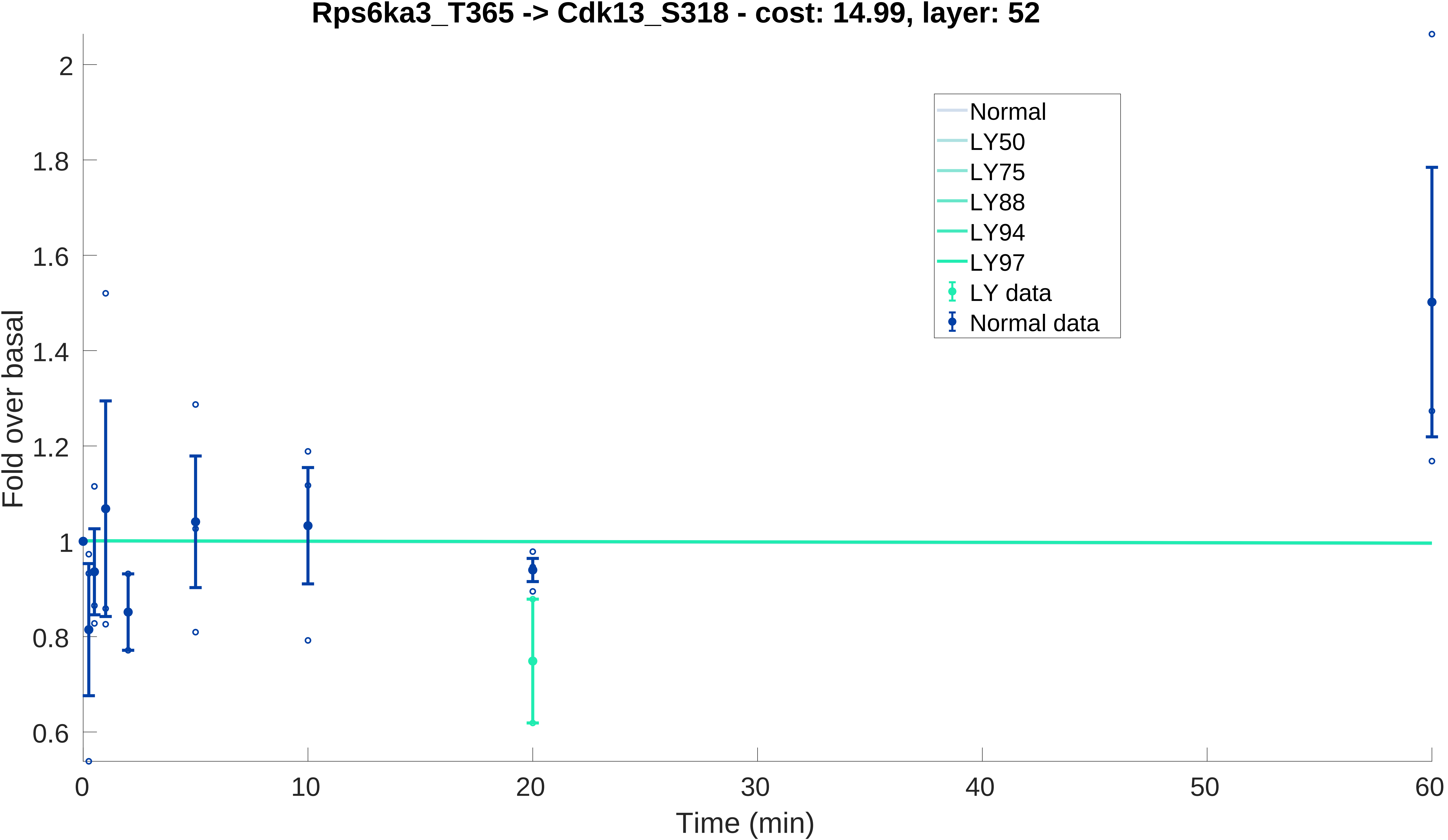

Rps6ka3_T365 -> Cdk13_S318 - cost: 14.99, layer: 52
Fold over basal
Time (min)
0
10
20
30
40
50
60
0.6
0.8
1
1.2
1.4
1.6
1.8
2
Normal
LY50
LY75
LY88
LY94
LY97
LY data
Normal data

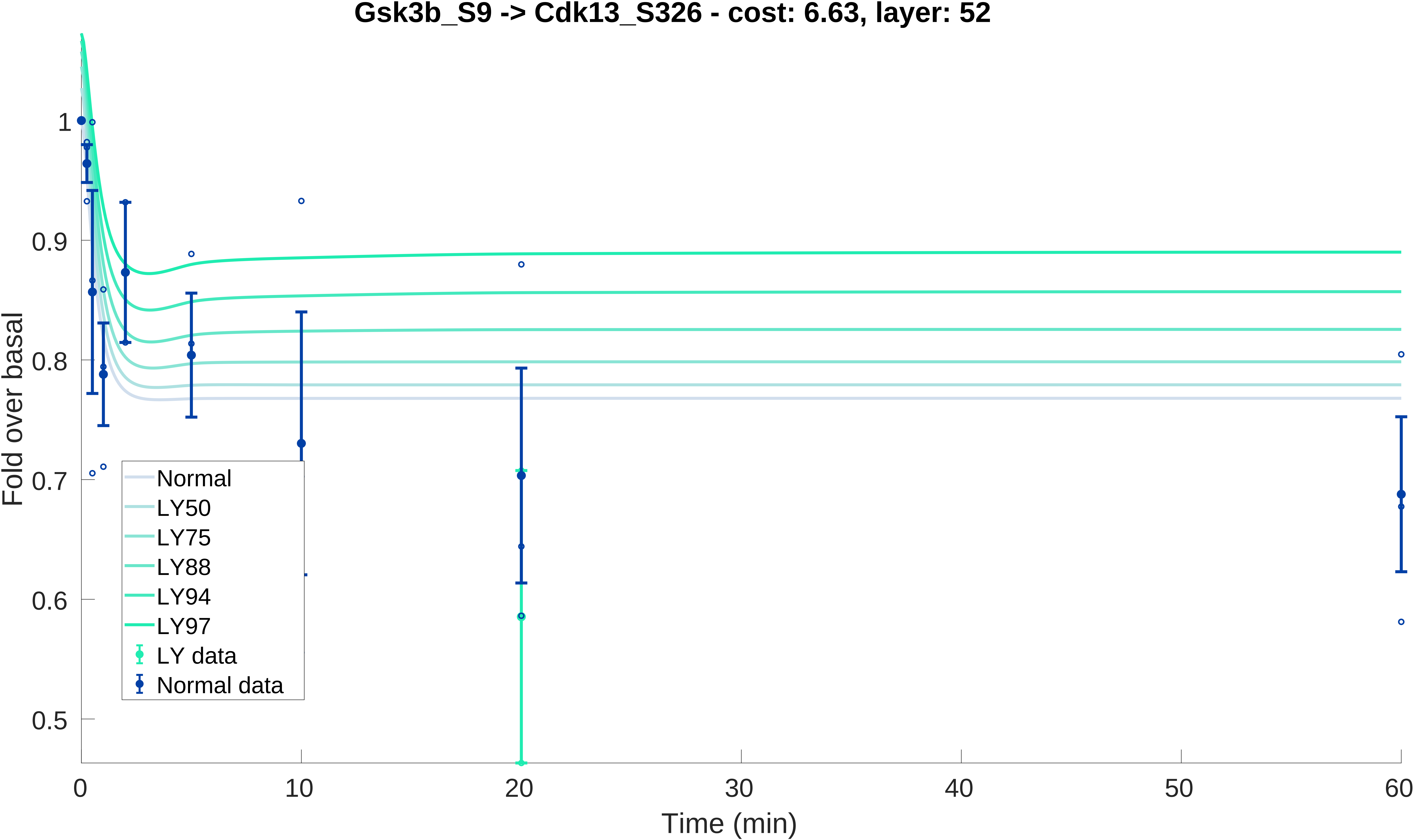

Gsk3b_S9 -> Cdk13_S326 - cost: 6.63, layer: 52
Fold over basal
Time (min)
1
0.9
0.8
0.7
0.6
0.5
0
10
20
30
40
50
60
Normal
LY50
LY75
LY88
LY94
LY97
LY data
Normal data

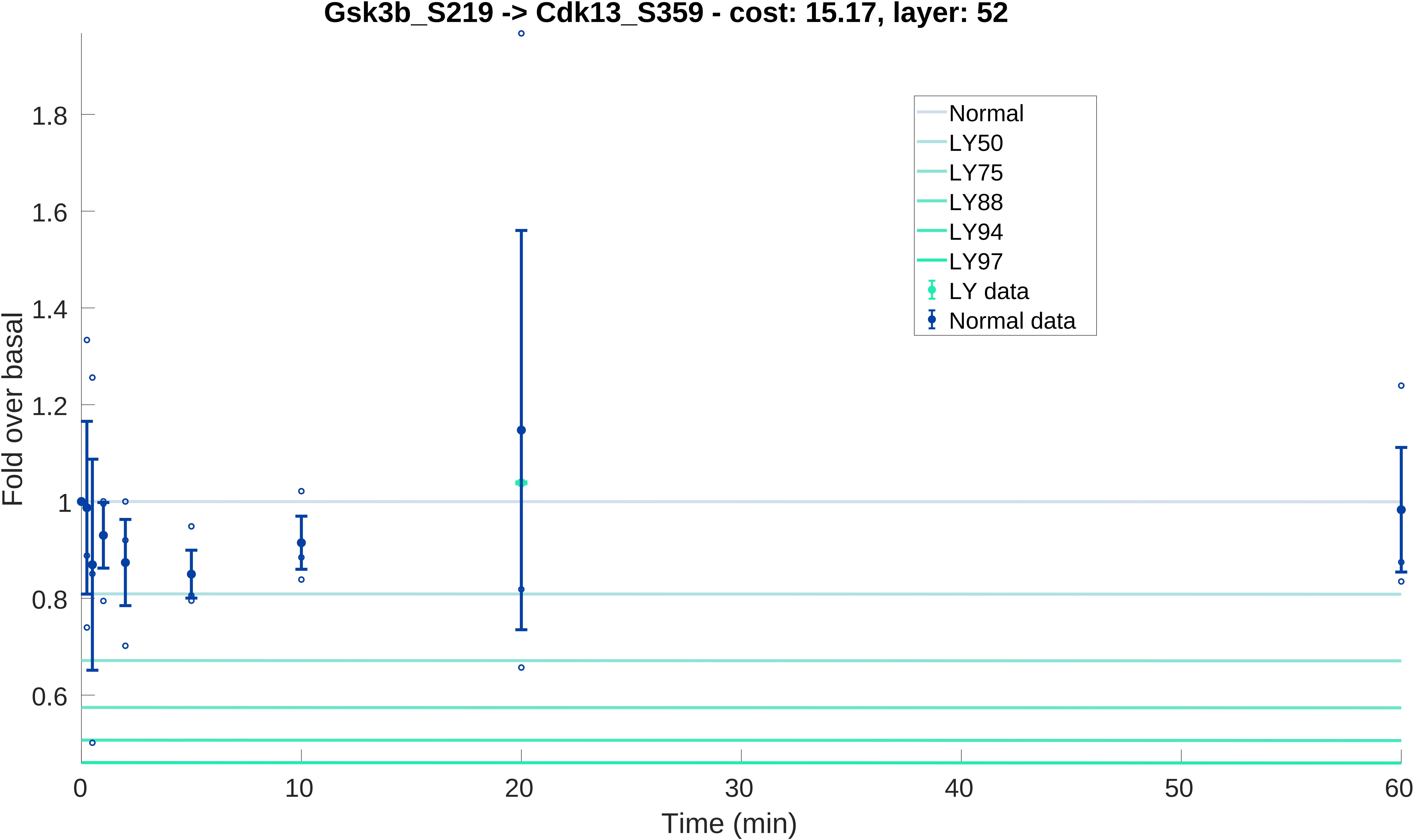

Gsk3b_S219 -> Cdk13_S359 - cost: 15.17, layer: 52
Fold over basal
1.8
1.6
1.4
1.2
1
0.8
0.6
0
10
20
30
40
50
60
Time (min)
Normal
LY50
LY75
LY88
LY94
LY97
LY data
Normal data

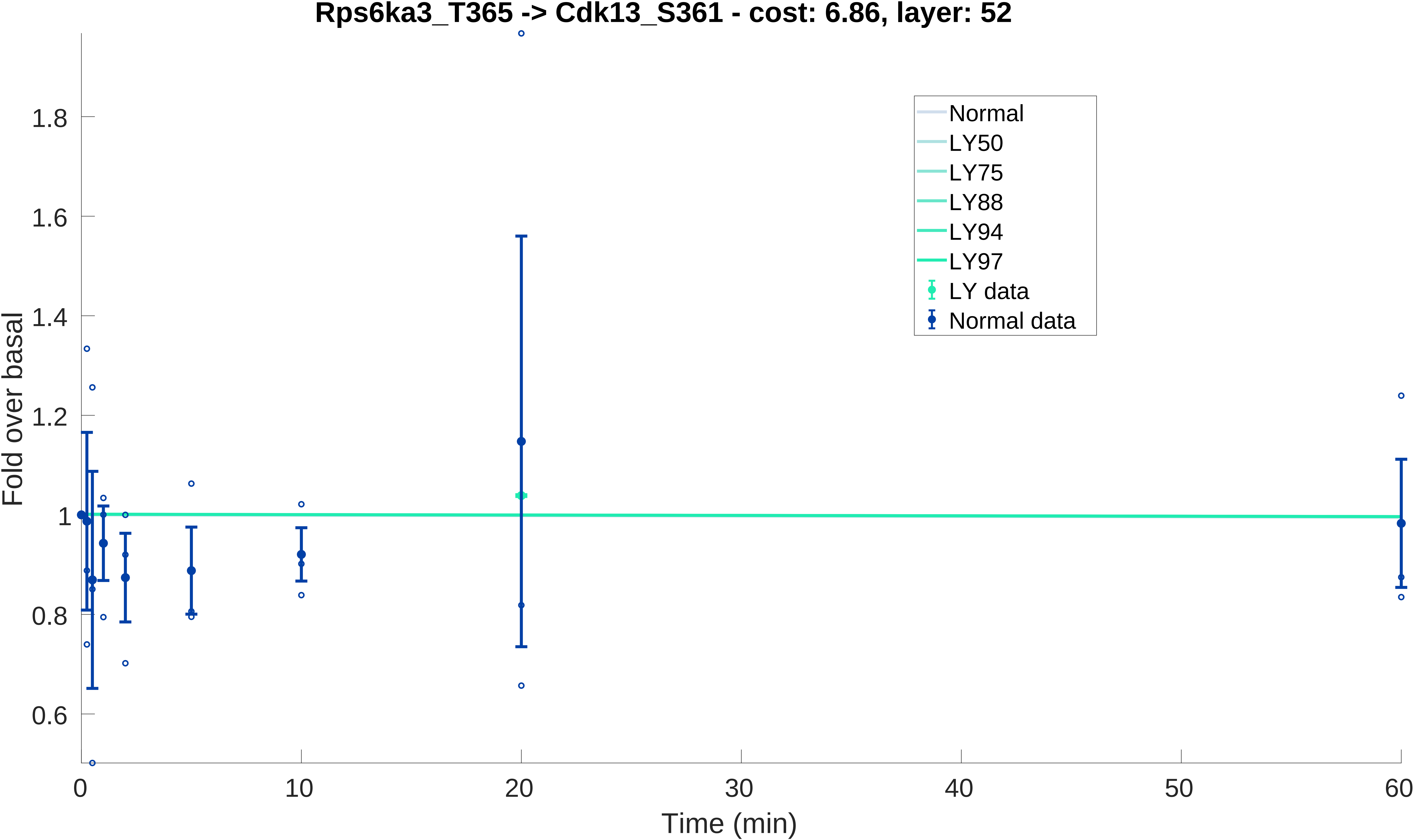

Rps6ka3_T365 -> Cdk13_S361 - cost: 6.86, layer: 52
Normal
LY50
LY75
LY88
LY94
LY97
LY data
Normal data
Fold over basal
1.8
1.6
1.4
1.2
1
0.8
0.6
0
10
20
30
40
50
60
Time (min)

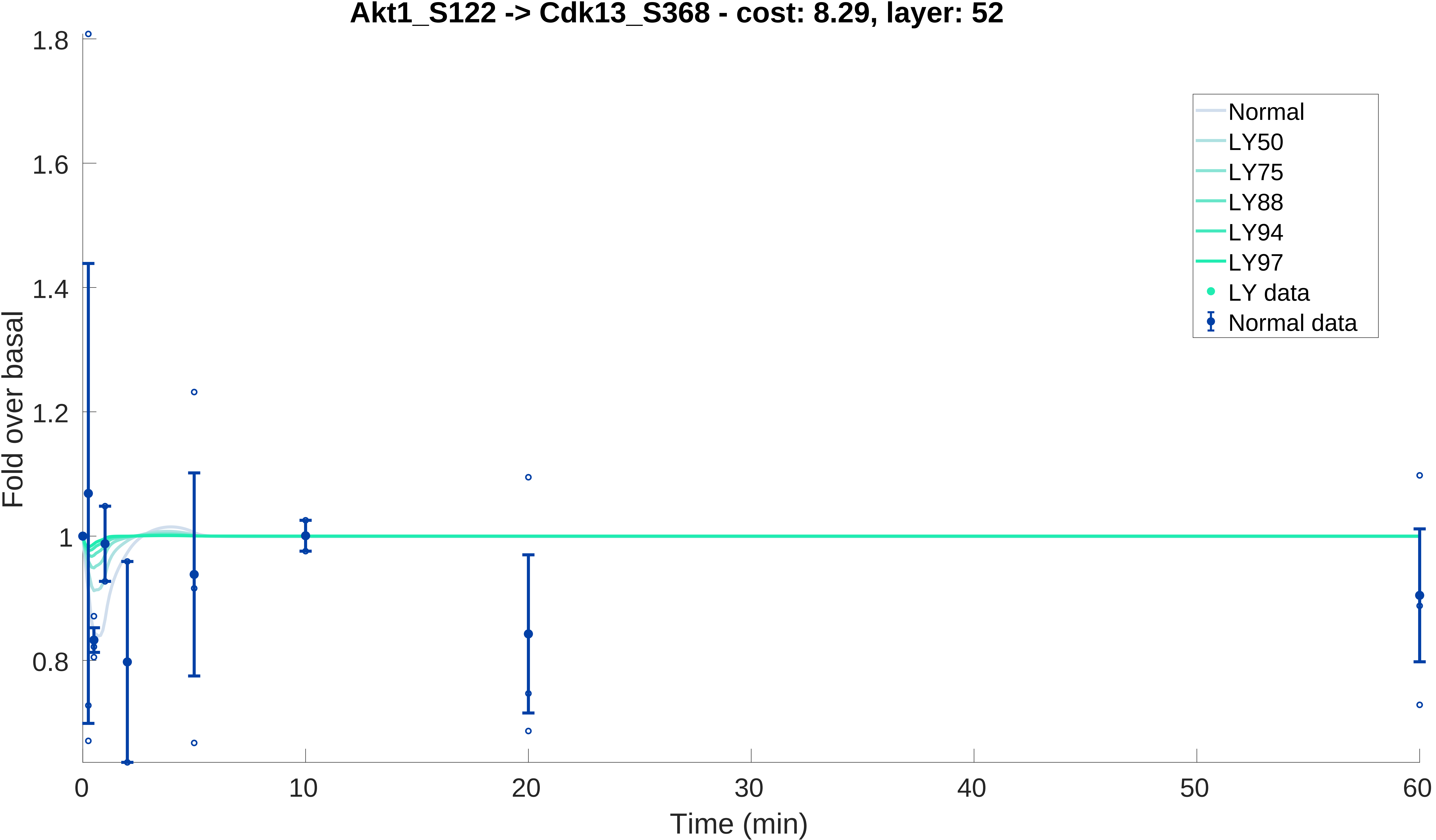

Akt1_S122 -> Cdk13_S368 - cost: 8.29, layer: 52
Fold over basal
1.8
1.6
1.4
1.2
1
0.8
0
10
20
30
40
50
60
Time (min)
Normal
LY50
LY75
LY88
LY94
LY97
LY data
Normal data

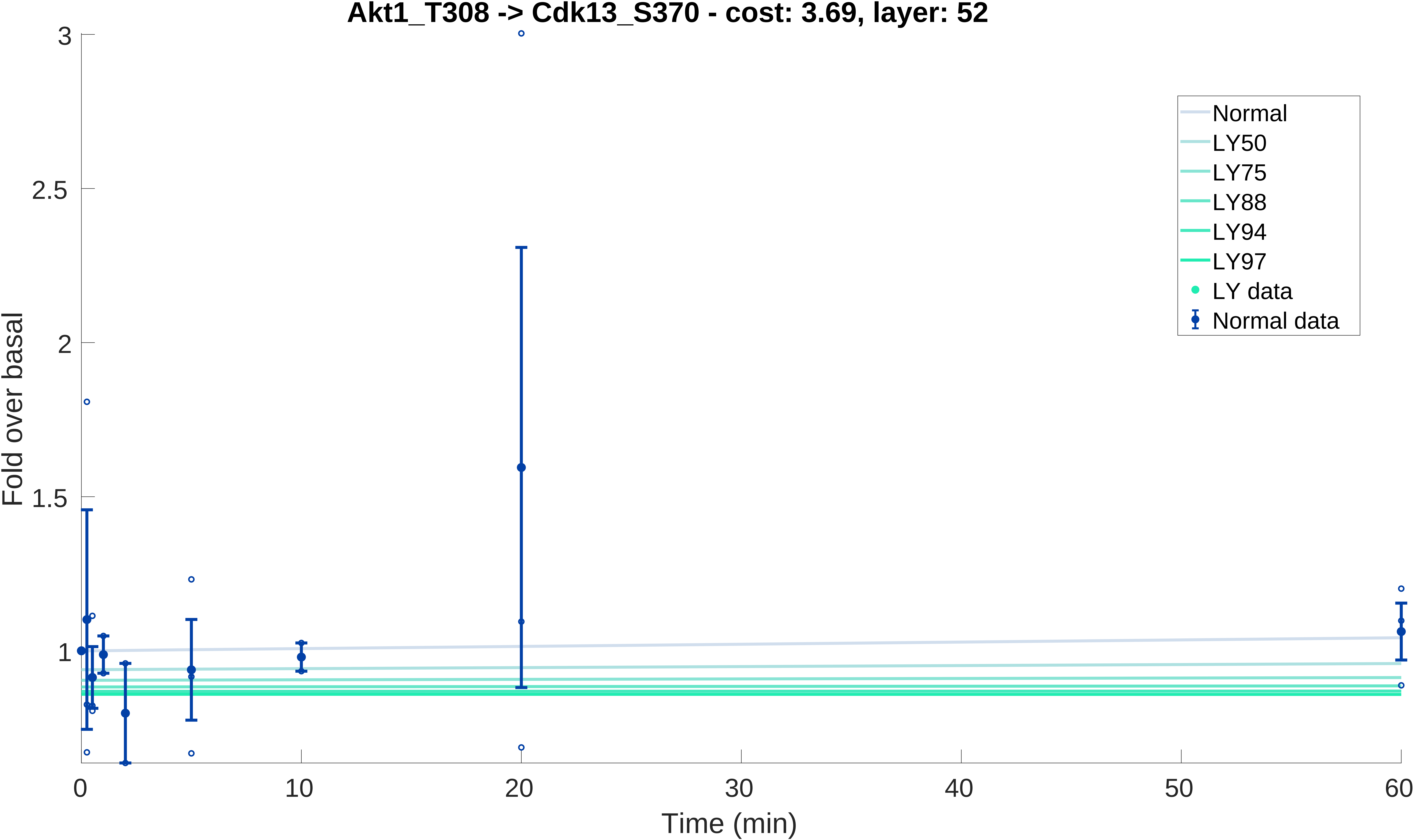

Akt1_T308 -> Cdk13_S370 - cost: 3.69, layer: 52
Fold over basal
Time (min)
3
2.5
2
1.5
1
0
10
20
30
40
50
60
Normal
LY50
LY75
LY88
LY94
LY97
LY data
Normal data

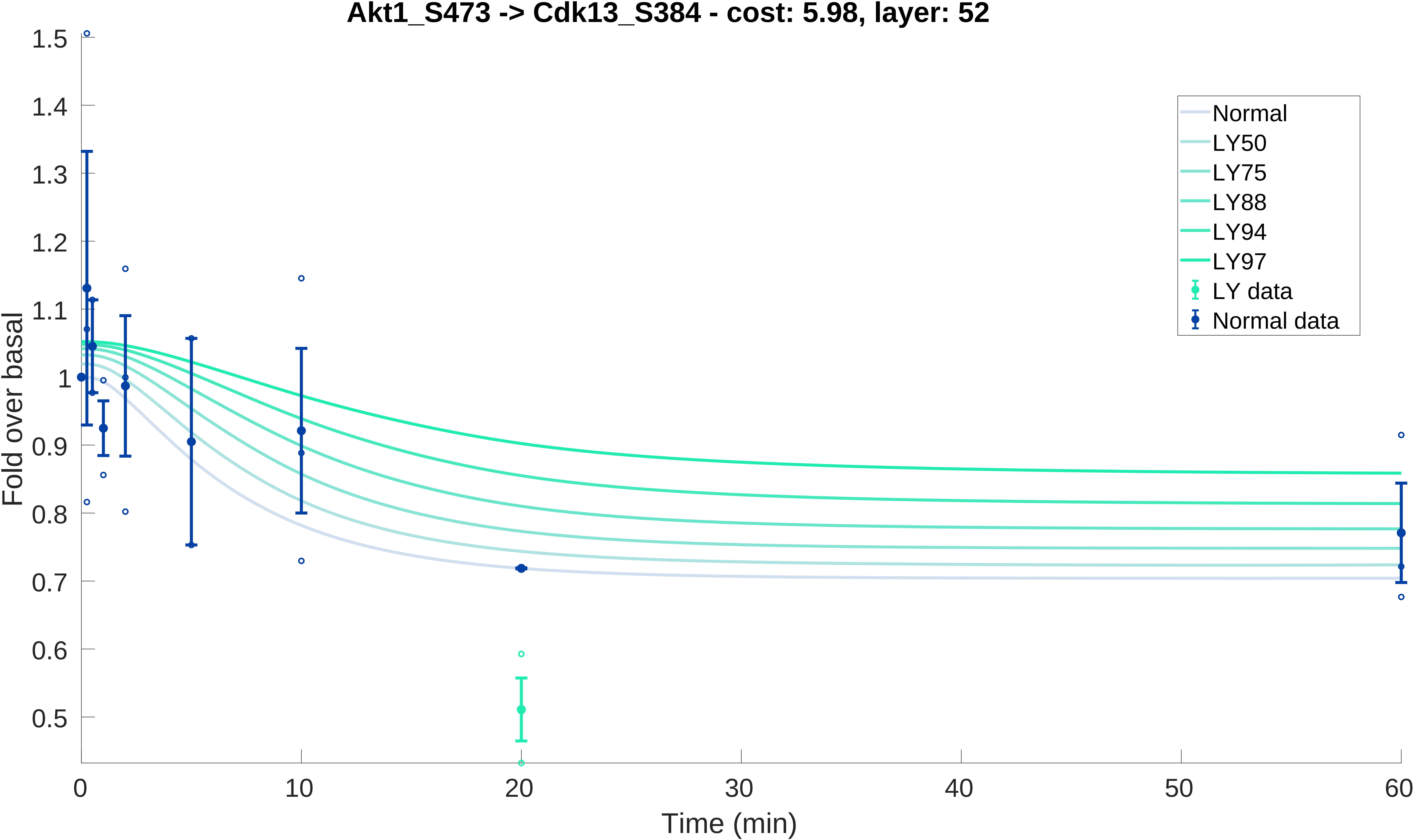
Akt1_S473 -> Cdk13_S384 - cost: 5.98, layer: 52
Fold over basal
Time (min)
1.5
1.4
1.3
1.2
1.1
1
0.9
0.8
0.7
0.6
0.5
0
10
20
30
40
50
60
Normal
LY50
LY75
LY88
LY94
LY97
LY data
Normal data

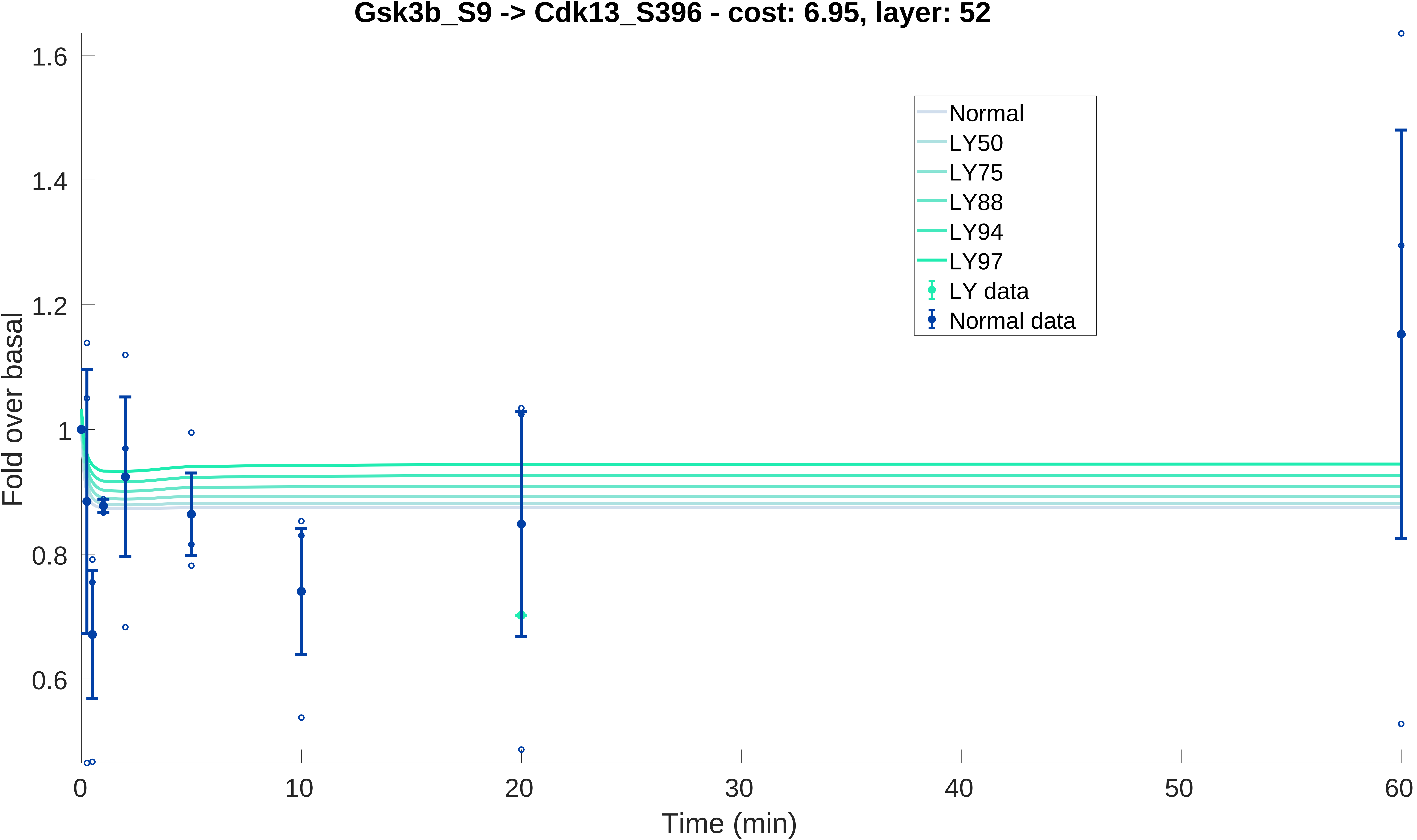

Gsk3b_S9 -> Cdk13_S396 - cost: 6.95, layer: 52
Fold over basal
1.6
1.4
1.2
1
0.8
0.6
0
10
20
30
40
50
60
Time (min)
Normal
LY50
LY75
LY88
LY94
LY97
LY data
Normal data

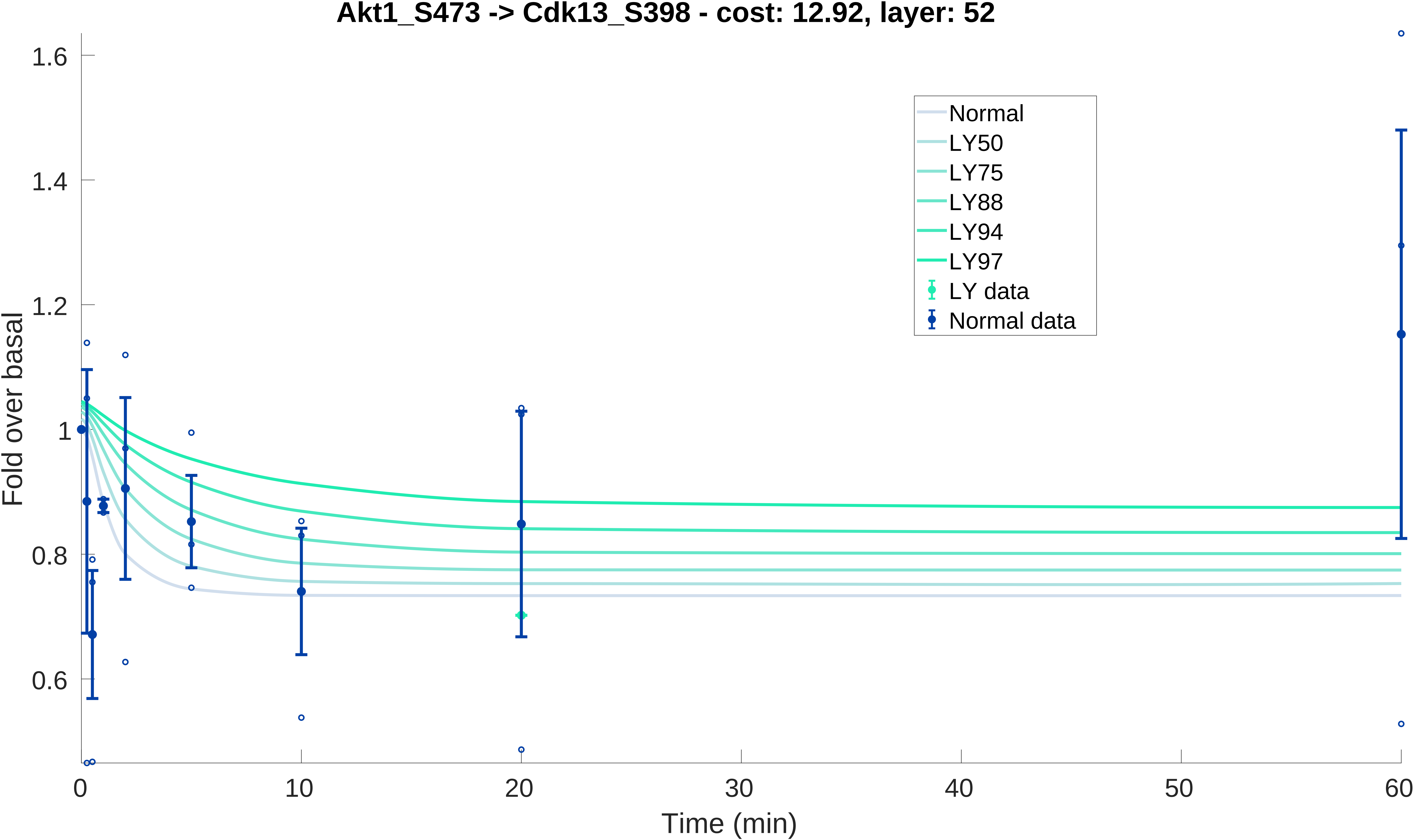

Akt1_S473 -> Cdk13_S398 - cost: 12.92, layer: 52
Fold over basal
Time (min)
1.6
1.4
1.2
1
0.8
0.6
0
10
20
30
40
50
60
Normal
LY50
LY75
LY88
LY94
LY97
LY data
Normal data

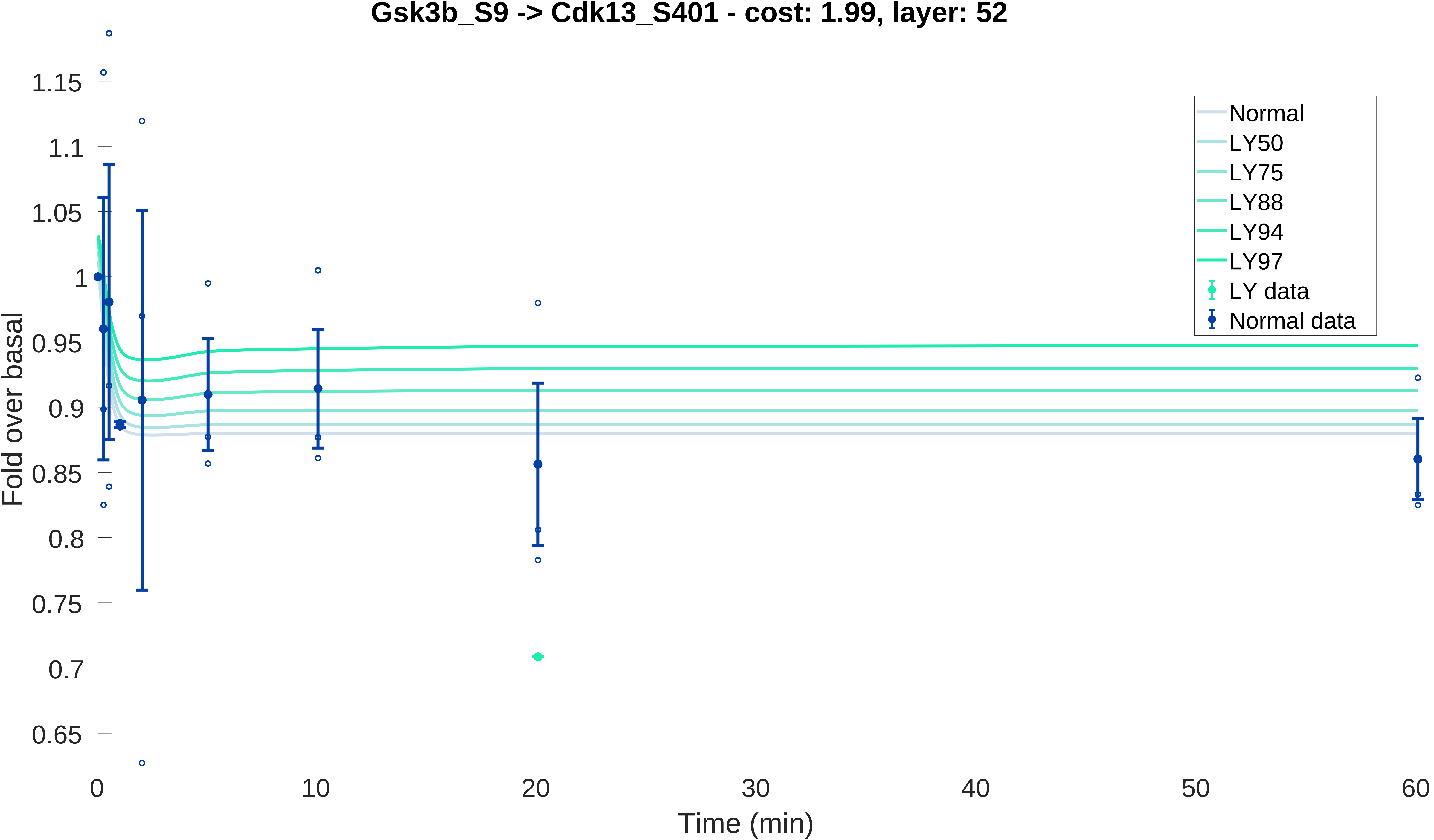

Gsk3b_S9 -> Cdk13_S401 - cost: 1.99, layer: 52
Fold over basal
Time (min)
1.15
1.1
1.05
1
0.95
0.9
0.85
0.8
0.75
0.7
0.65
0
10
20
30
40
50
60
Normal
LY50
LY75
LY88
LY94
LY97
LY data
Normal data

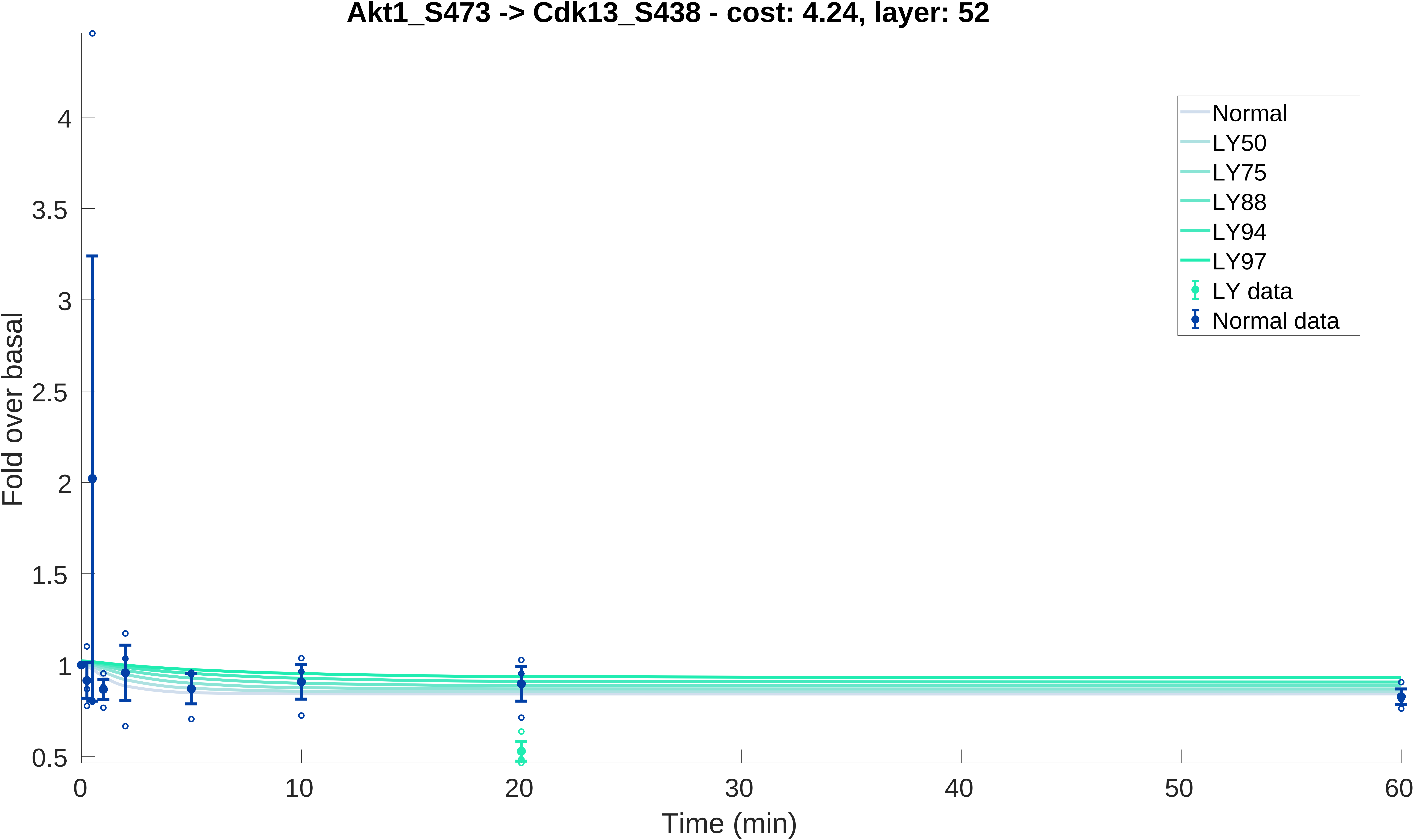

Akt1_S473 -> Cdk13_S438 - cost: 4.24, layer: 52
Fold over basal
Time (min)
0.5
1
1.5
2
2.5
3
3.5
4
0
10
20
30
40
50
60
Normal
LY50
LY75
LY88
LY94
LY97
LY data
Normal data

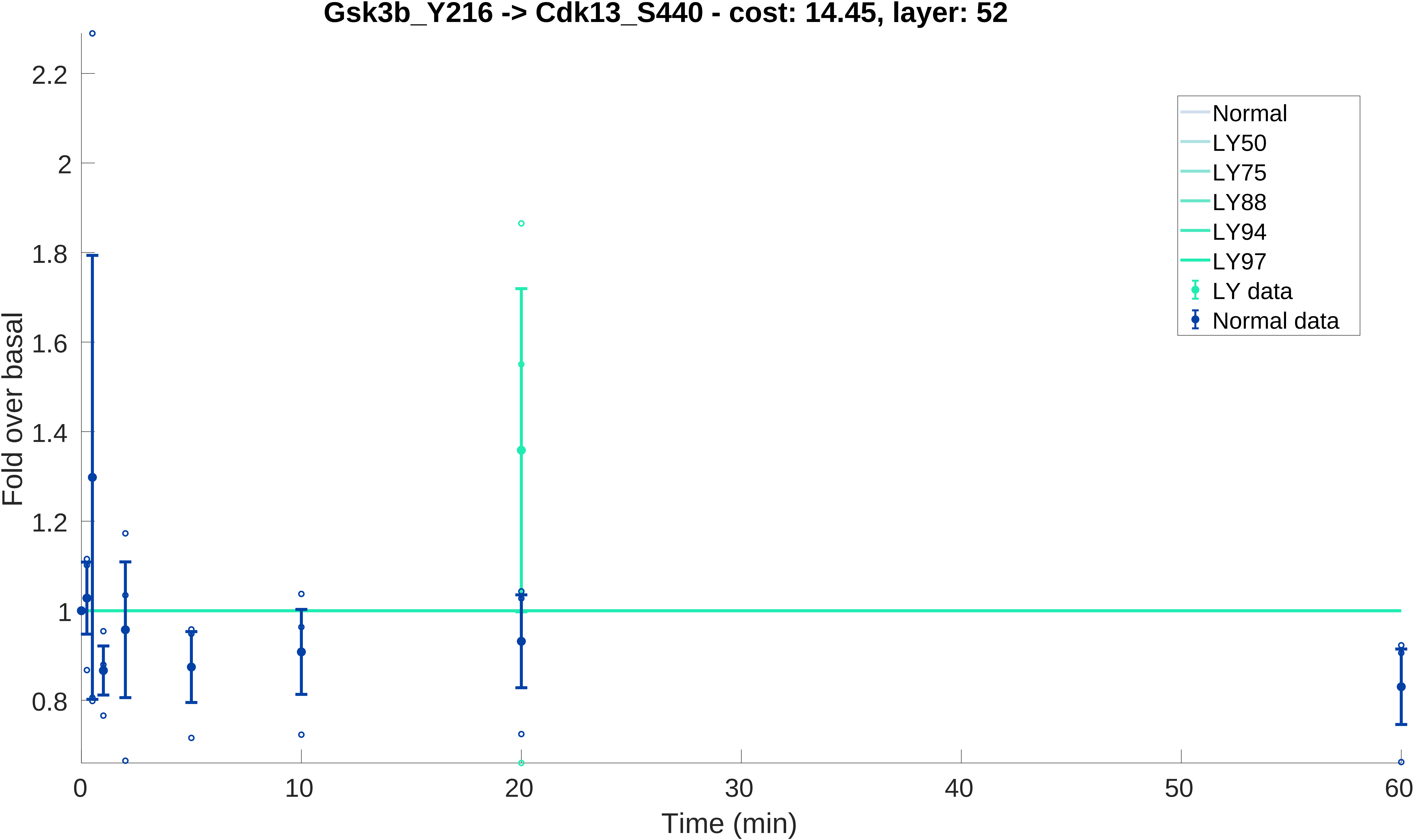
Gsk3b_Y216 -> Cdk13_S440 - cost: 14.45, layer: 52
Fold over basal
Time (min)
0
10
20
30
40
50
60
0.8
1
1.2
1.4
1.6
1.8
2
2.2
Normal
LY50
LY75
LY88
LY94
LY97
LY data
Normal data

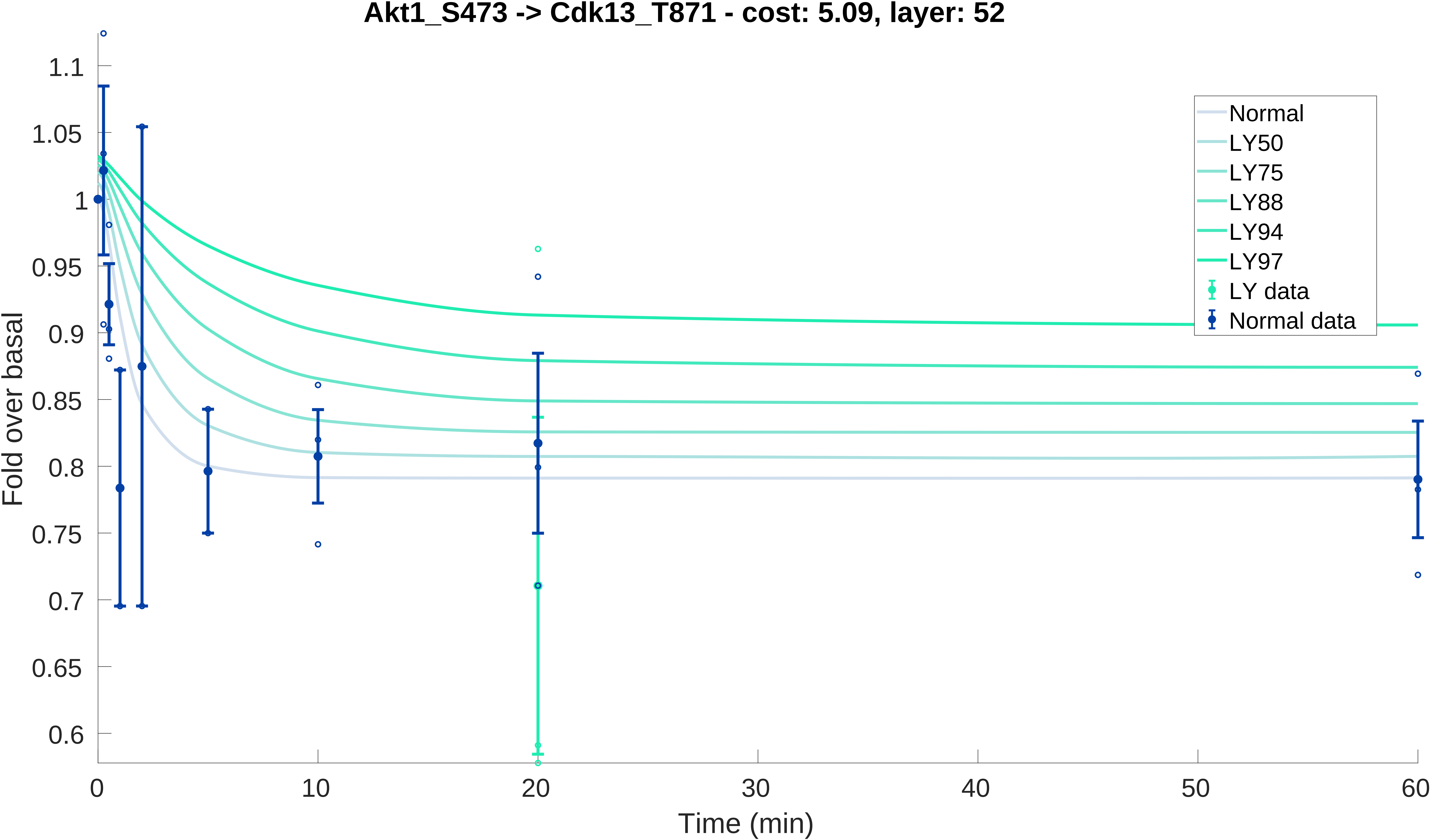

Akt1_S473 -> Cdk13_T871 - cost: 5.09, layer: 52
Fold over basal
Time (min)
1.1
1.05
1
0.95
0.9
0.85
0.8
0.75
0.7
0.65
0.6
0
10
20
30
40
50
60
Normal
LY50
LY75
LY88
LY94
LY97
LY data
Normal data

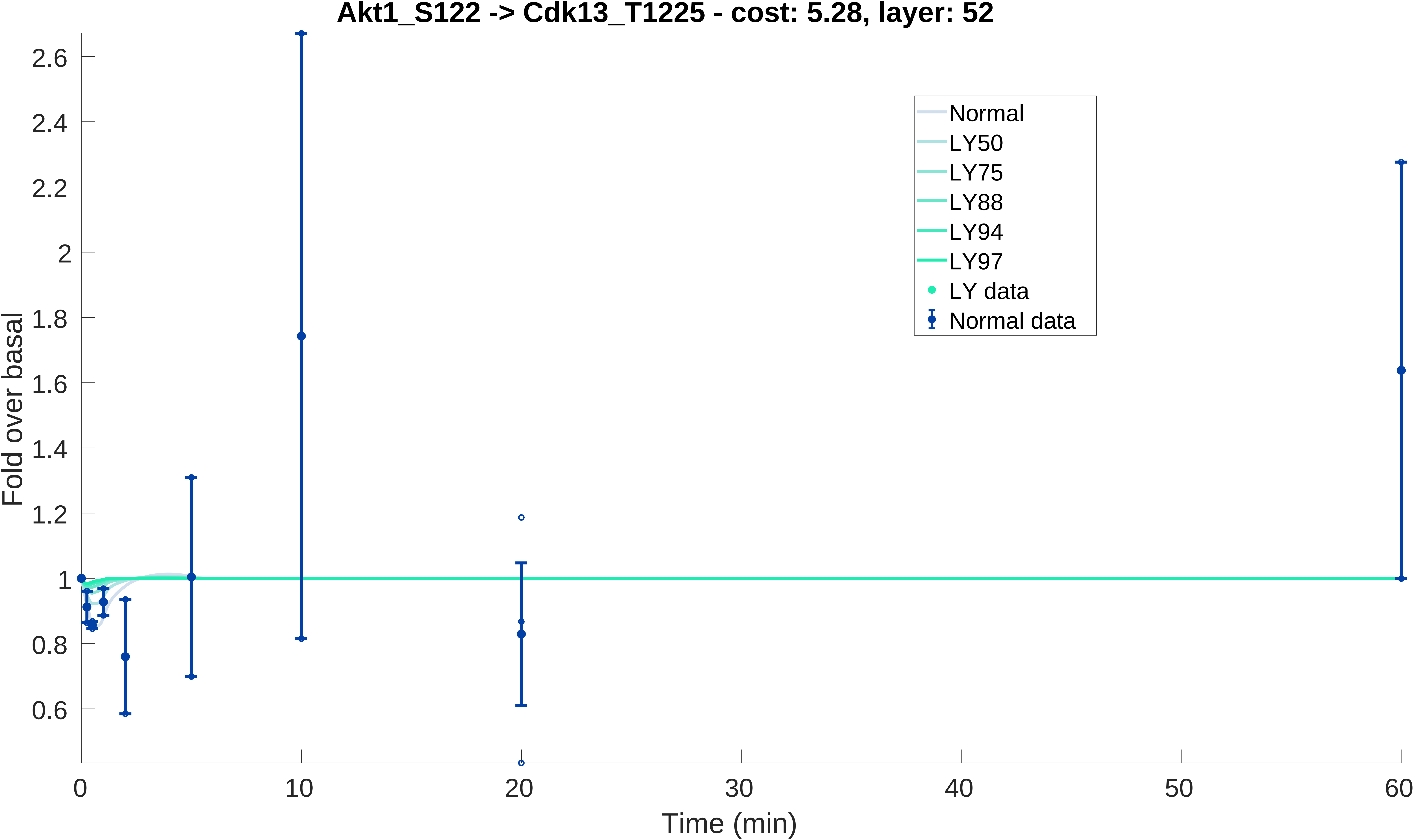

Akt1_S122 -> Cdk13_T1225 - cost: 5.28, layer: 52
Fold over basal
Time (min)
0.6
0.8
1
1.2
1.4
1.6
1.8
2
2.2
2.4
2.6
0
10
20
30
40
50
60
Normal
LY50
LY75
LY88
LY94
LY97
LY data
Normal data

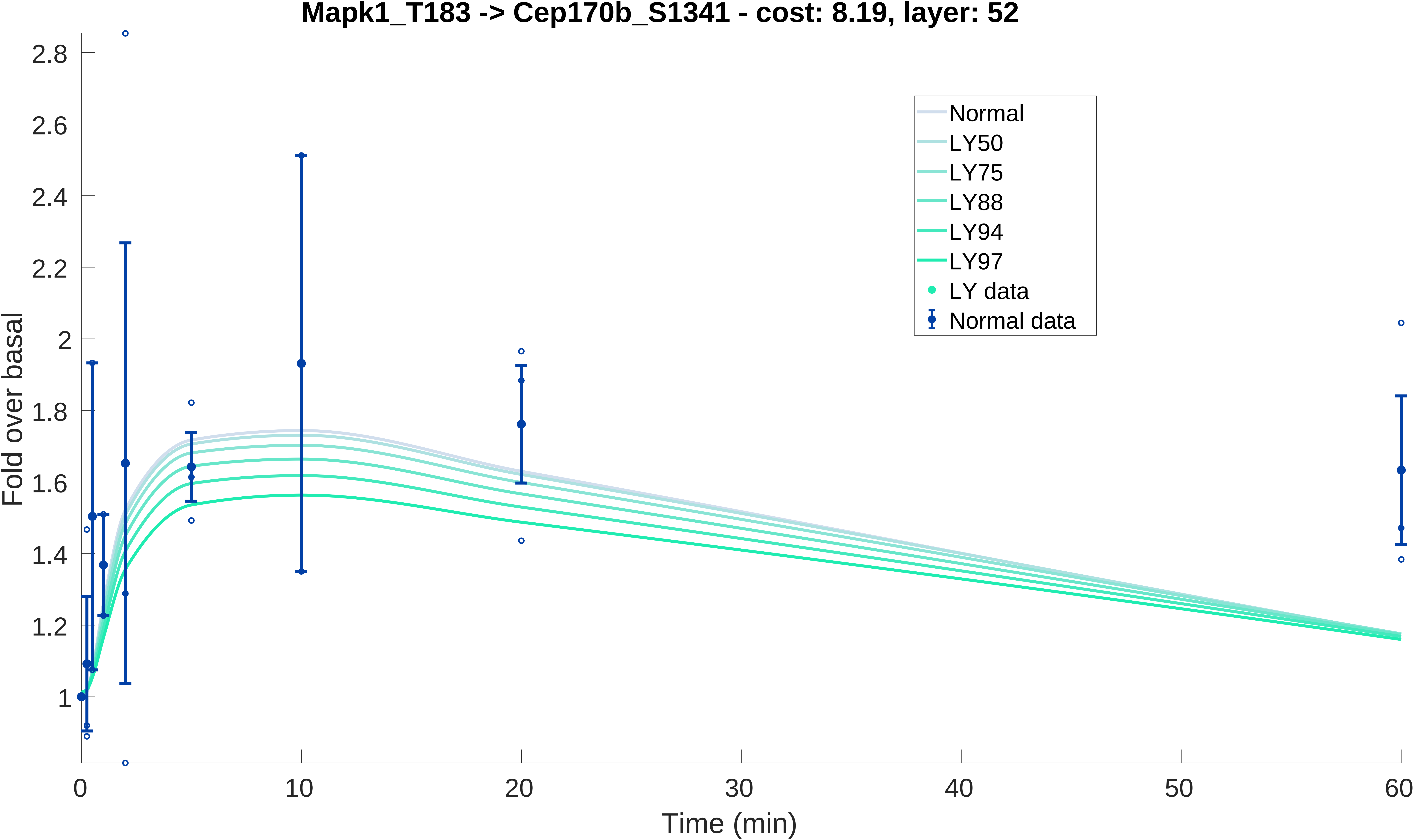

Mapk1_T183 -> Cep170b_S1341 - cost: 8.19, layer: 52
Fold over basal
Time (min)
Normal
LY50
LY75
LY88
LY94
LY97
LY data
Normal data
0
10
20
30
40
50
60
1
1.2
1.4
1.6
1.8
2
2.2
2.4
2.6
2.8

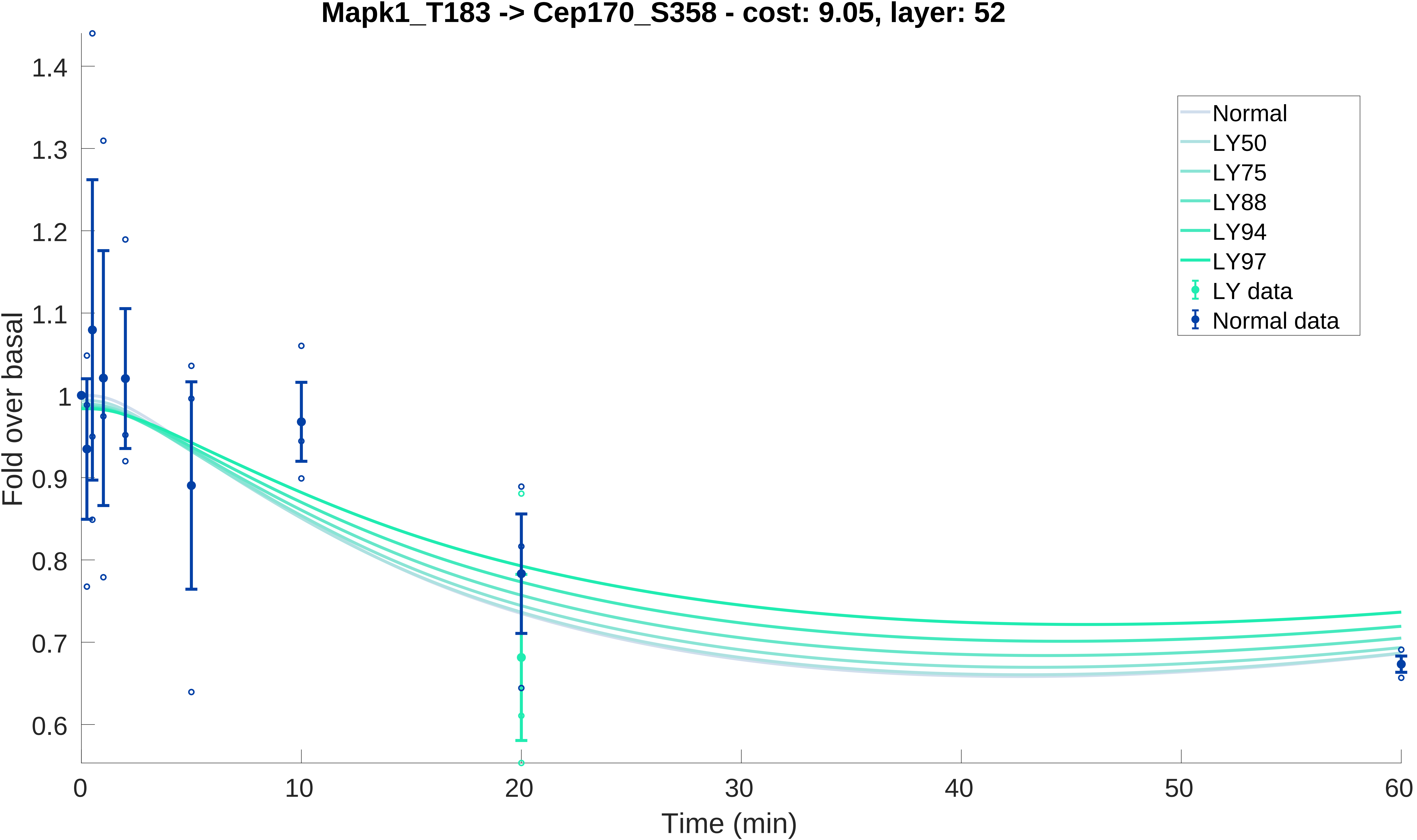

Mapk1_T183 -> Cep170_S358 - cost: 9.05, layer: 52
Fold over basal
Time (min)
Normal
LY50
LY75
LY88
LY94
LY97
LY data
Normal data
0
10
20
30
40
50
60
0.6
0.7
0.8
0.9
1
1.1
1.2
1.3
1.4

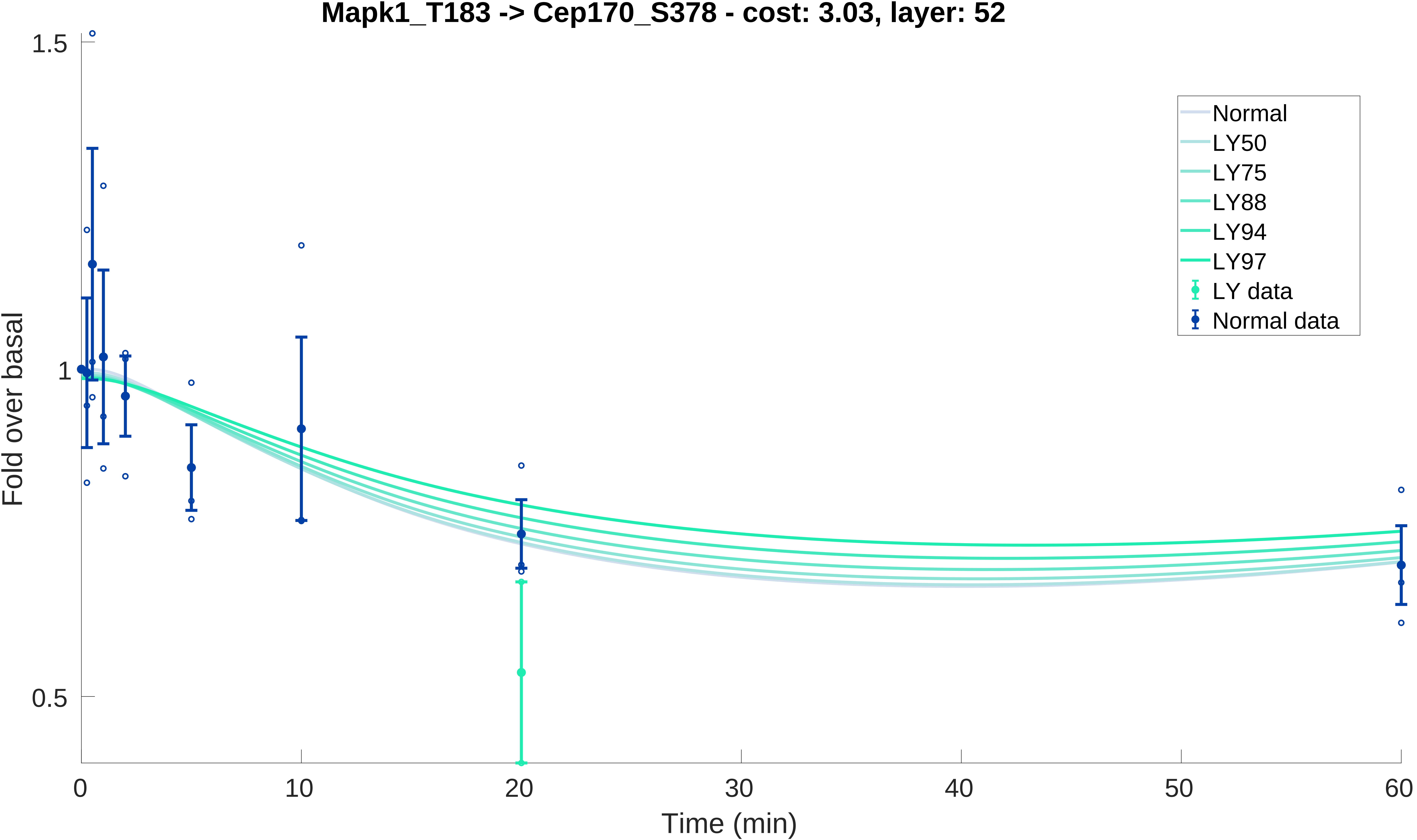

Mapk1_T183 -> Cep170_S378 - cost: 3.03, layer: 52
Fold over basal
Time (min)
1.5
1
0.5
0
10
20
30
40
50
60
Normal
LY50
LY75
LY88
LY94
LY97
LY data
Normal data

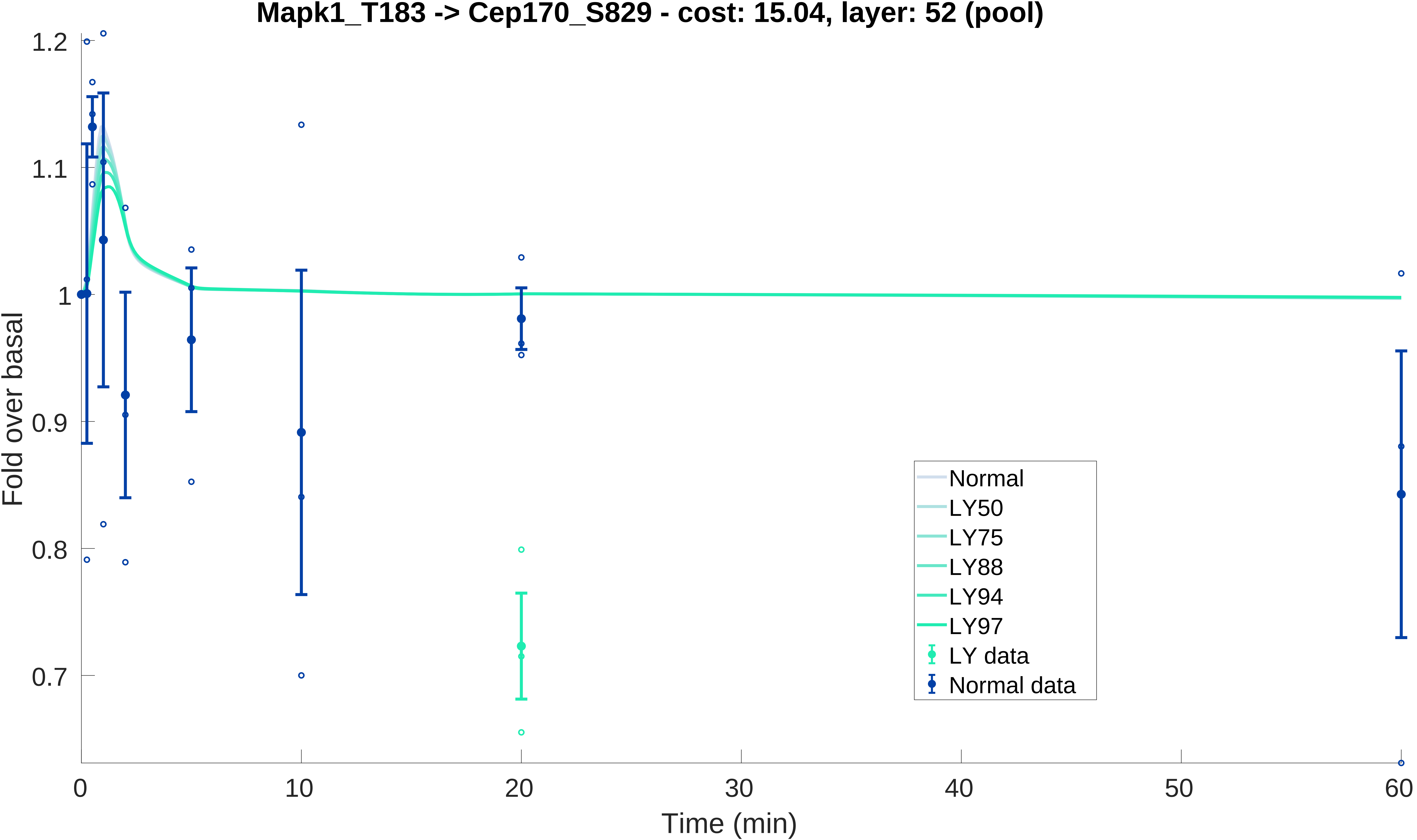

Mapk1_T183 -> Cep170_S829 - cost: 15.04, layer: 52 (pool)
Fold over basal
Time (min)
1.2
1.1
1
0.9
0.8
0.7
0
10
20
30
40
50
60
Normal
LY50
LY75
LY88
LY94
LY97
LY data
Normal data

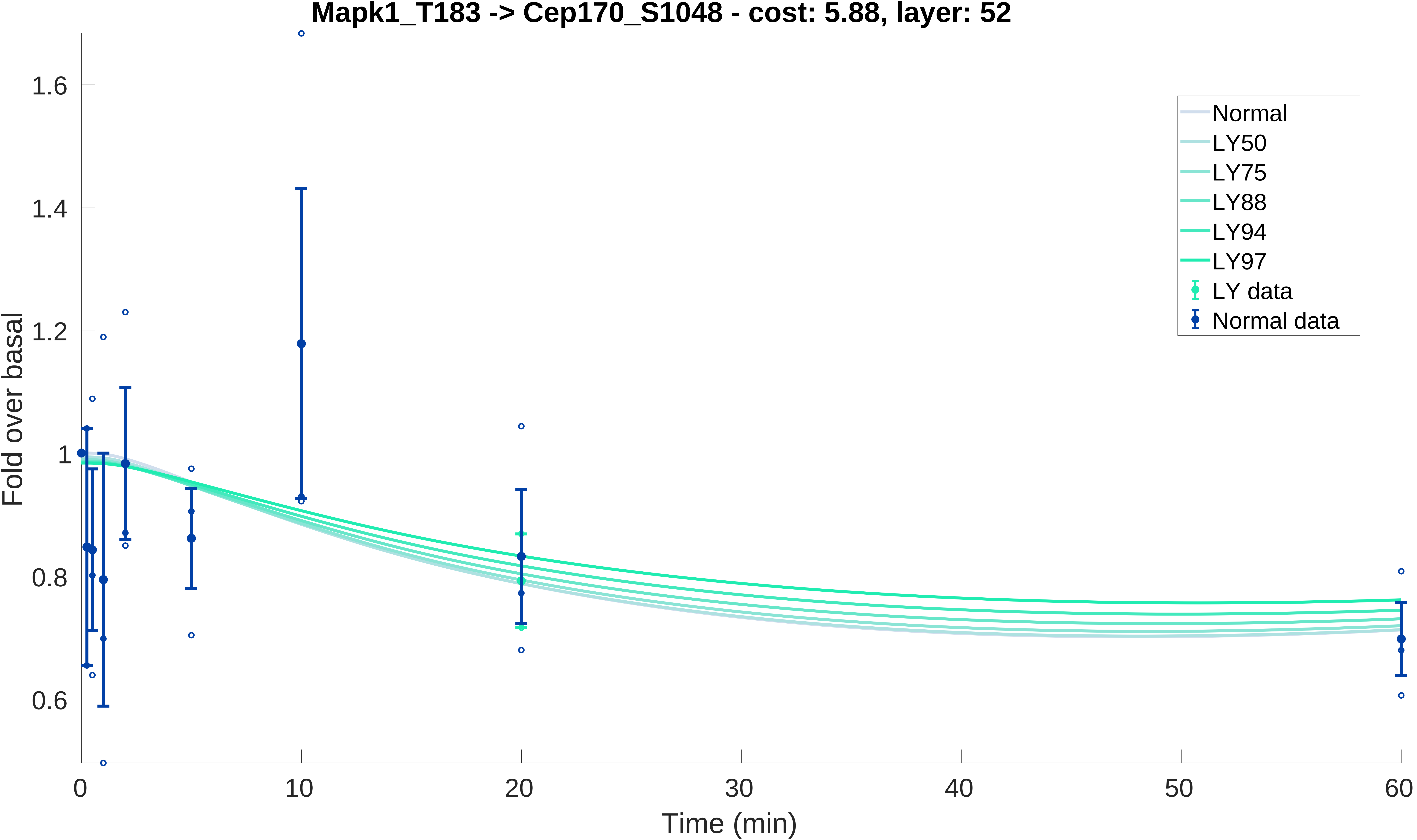
Mapk1_T183 -> Cep170_S1048 - cost: 5.88, layer: 52
Fold over basal
1.6
1.4
1.2
1
0.8
0.6
0
10
20
30
40
50
60
Time (min)
Normal
LY50
LY75
LY88
LY94
LY97
LY data
Normal data

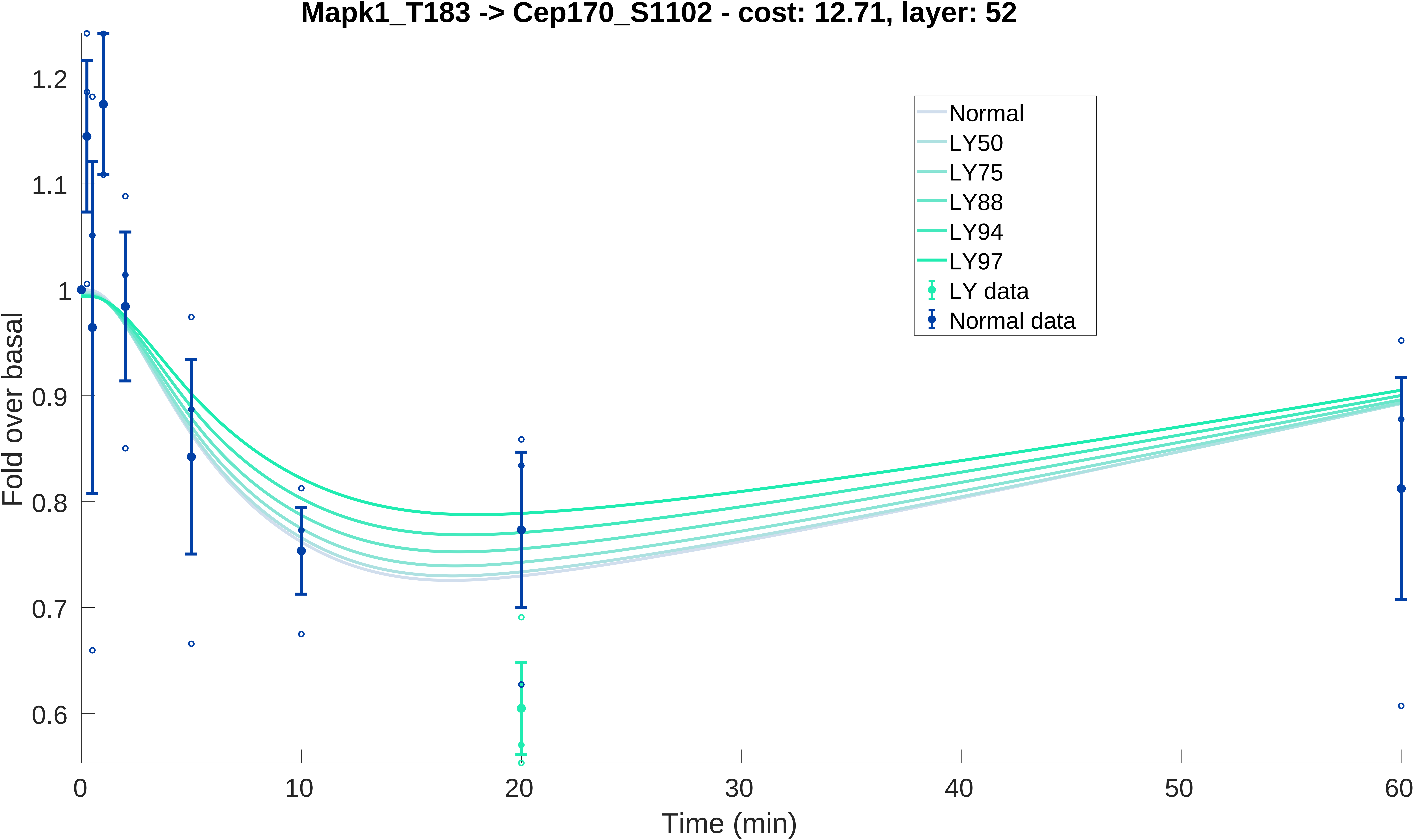

Mapk1_T183 -> Cep170_S1102 - cost: 12.71, layer: 52
Fold over basal
Time (min)
0
10
20
30
40
50
60
0.6
0.7
0.8
0.9
1
1.1
1.2
Normal
LY50
LY75
LY88
LY94
LY97
LY data
Normal data

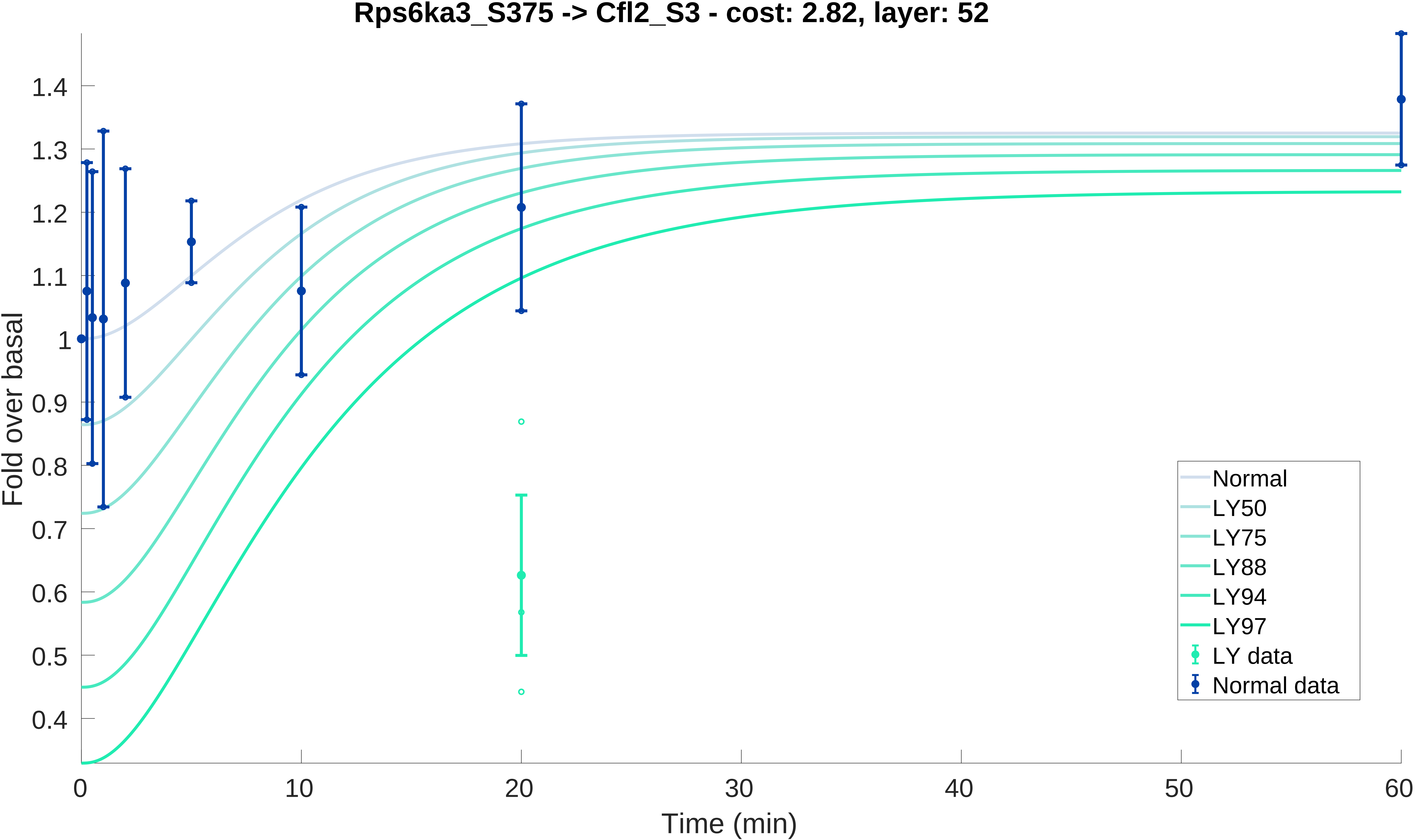
Rps6ka3_S375 -> Cfl2_S3 - cost: 2.82, layer: 52
Fold over basal
Time (min)
1.4
1.3
1.2
1.1
1
0.9
0.8
0.7
0.6
0.5
0.4
0
10
20
30
40
50
60
Normal
LY50
LY75
LY88
LY94
LY97
LY data
Normal data

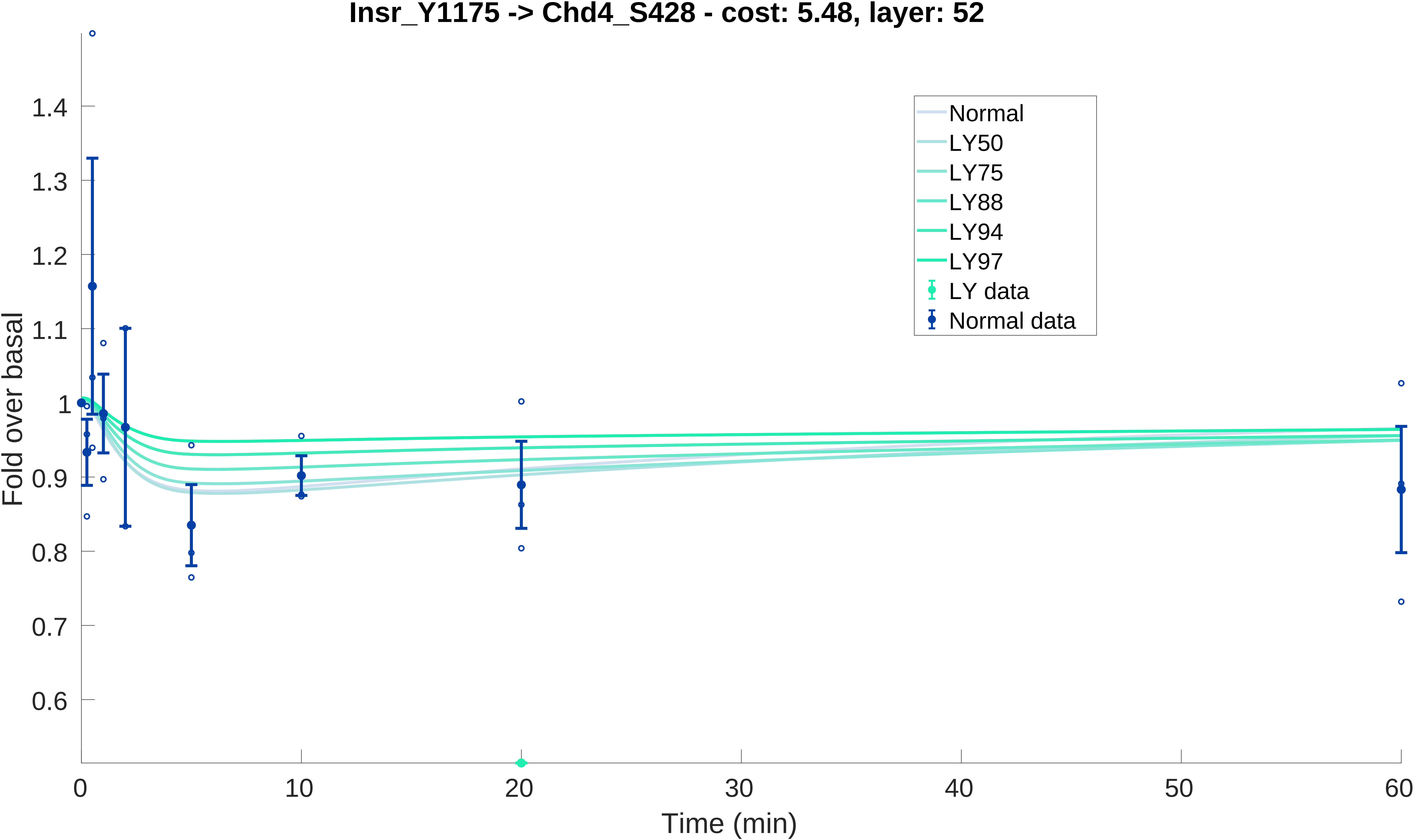
Insr_Y1175 -> Chd4_S428 - cost: 5.48, layer: 52
Fold over basal
Time (min)
1.4
1.3
1.2
1.1
1
0.9
0.8
0.7
0.6
0
10
20
30
40
50
60
Normal
LY50
LY75
LY88
LY94
LY97
LY data
Normal data

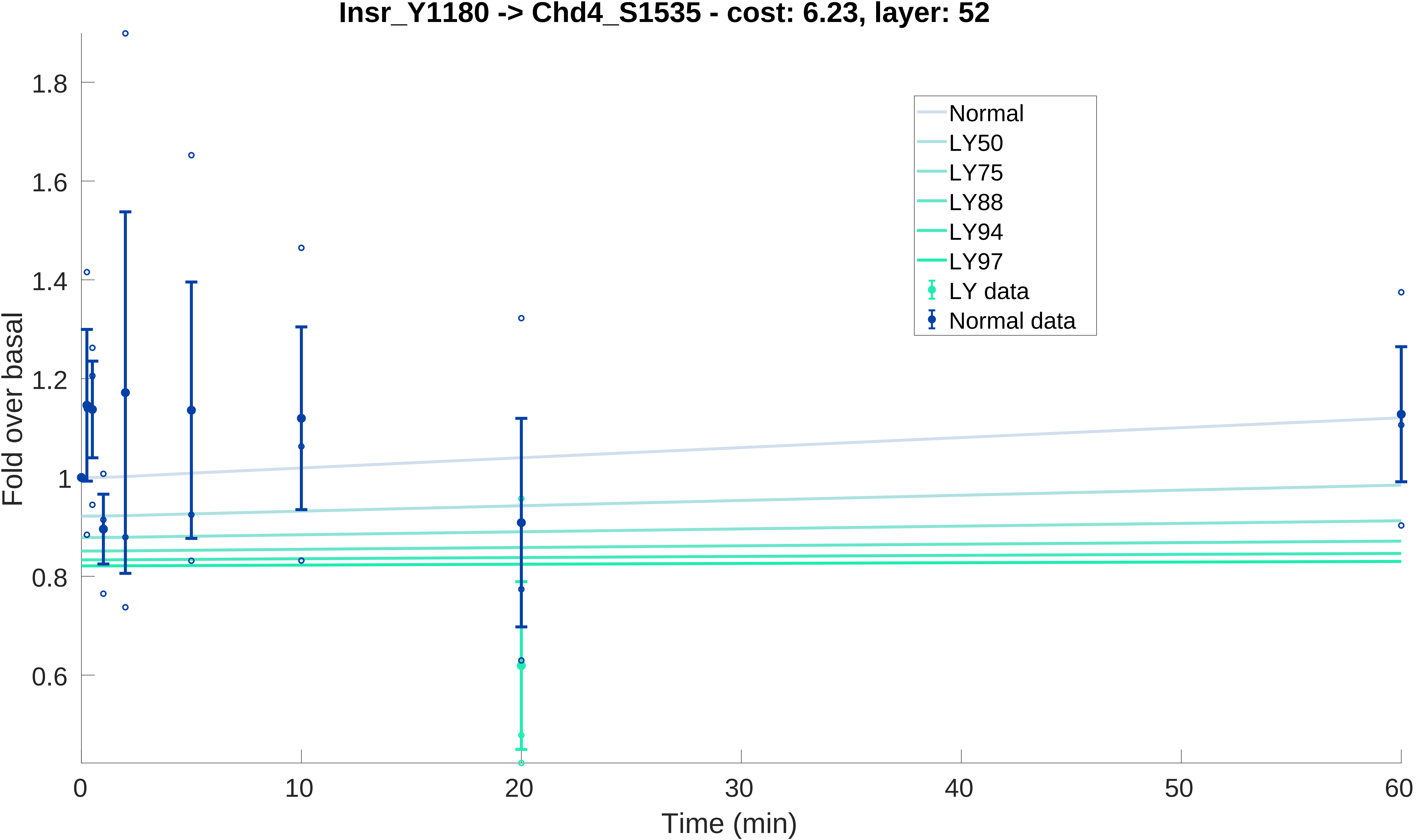
Insr_Y1180 -> Chd4_S1535 - cost: 6.23, layer: 52
Fold over basal
Time (min)
Normal
LY50
LY75
LY88
LY94
LY97
LY data
Normal data
0.6
0.8
1
1.2
1.4
1.6
1.8
0
10
20
30
40
50
60

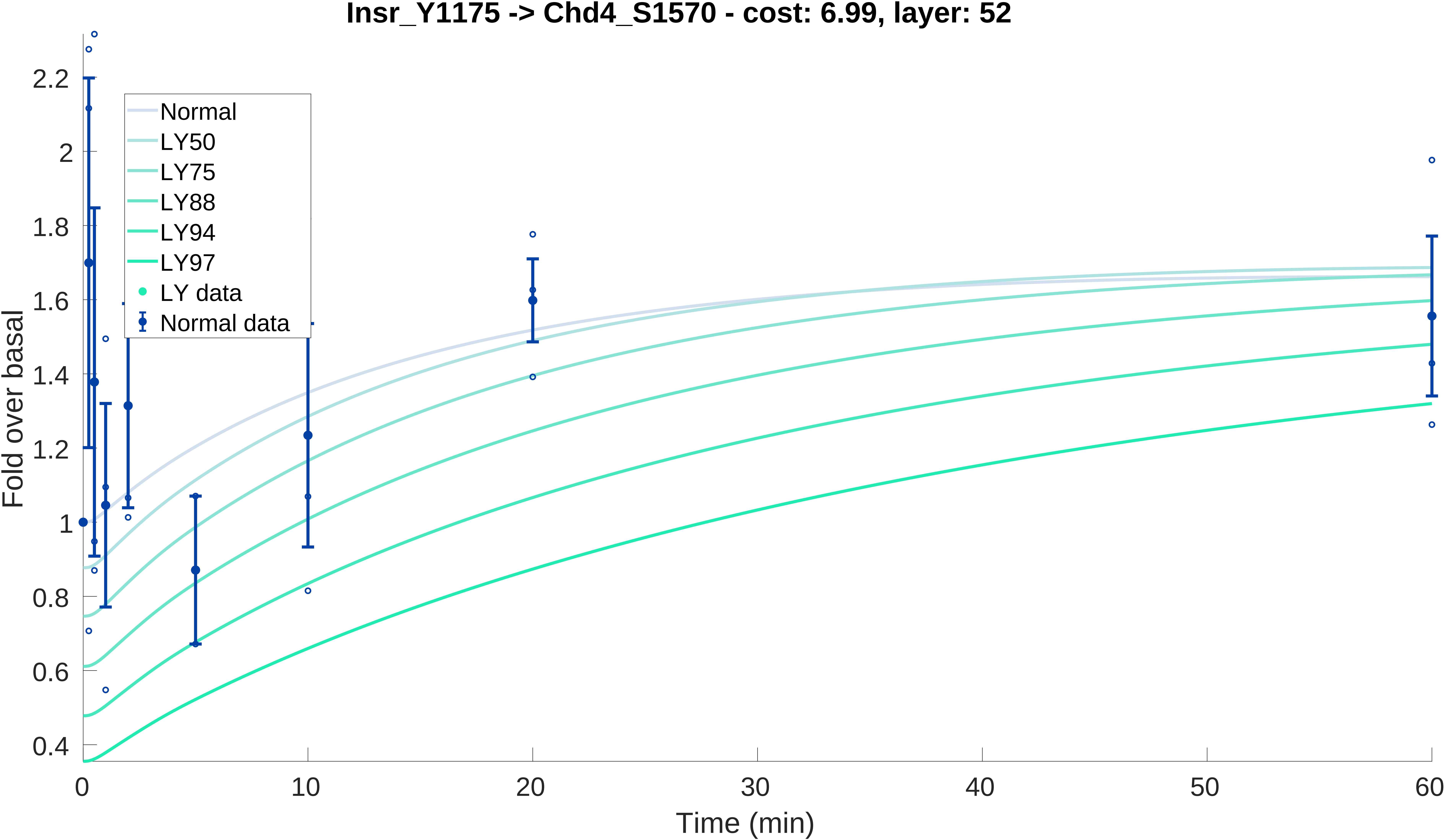

Insr_Y1175 -> Chd4_S1570 - cost: 6.99, layer: 52
Fold over basal
Time (min)
Normal
LY50
LY75
LY88
LY94
LY97
LY data
Normal data
0.4
0.6
0.8
1
1.2
1.4
1.6
1.8
2
2.2
0
10
20
30
40
50
60

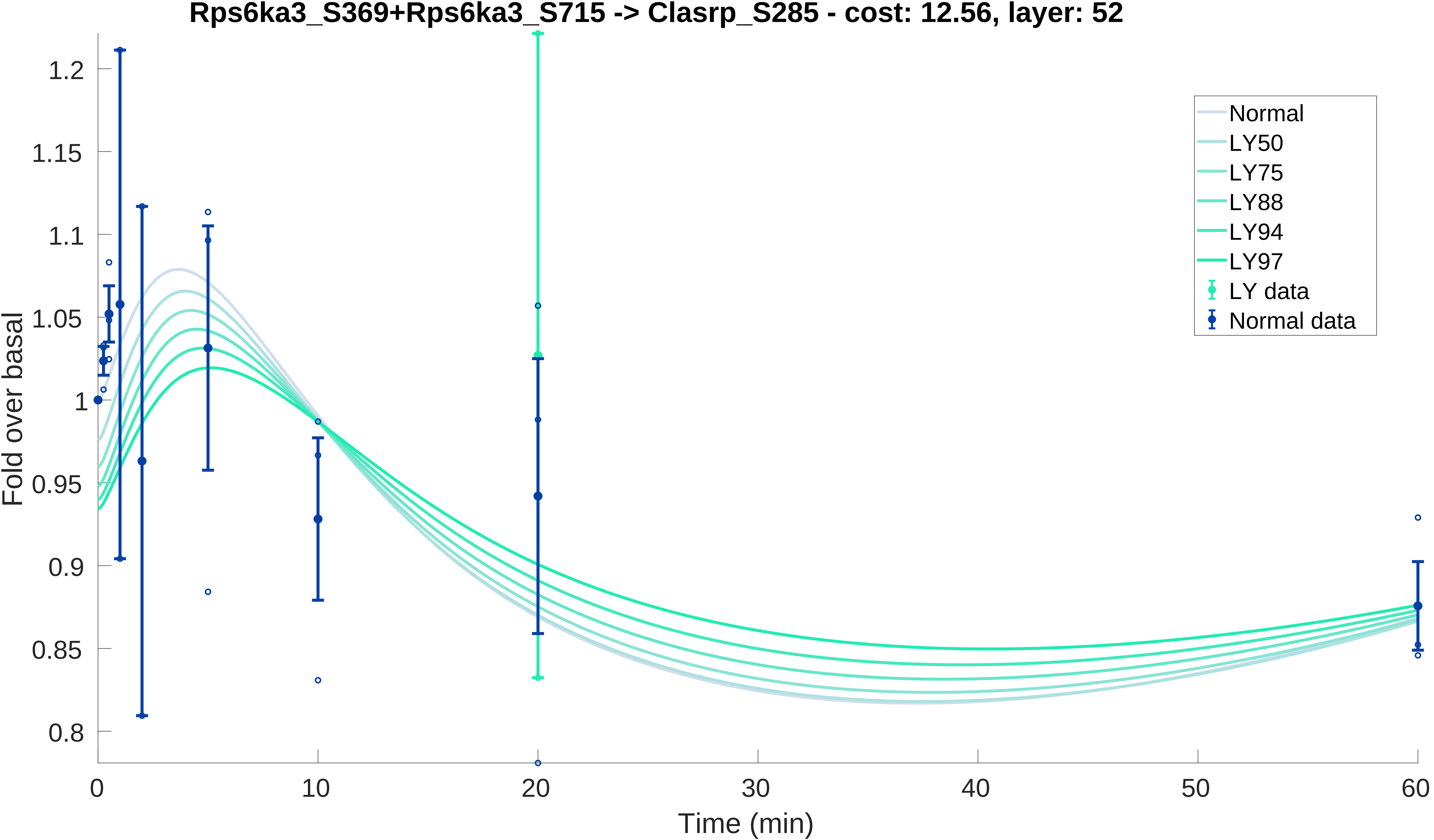

Rps6ka3_S369+Rps6ka3_S715 -> Clasrp_S285 - cost: 12.56, layer: 52
Fold over basal
1.2
1.15
1.1
1.05
1
0.95
0.9
0.85
0.8
0
10
20
30
40
50
60
Time (min)
Normal
LY50
LY75
LY88
LY94
LY97
LY data
Normal data

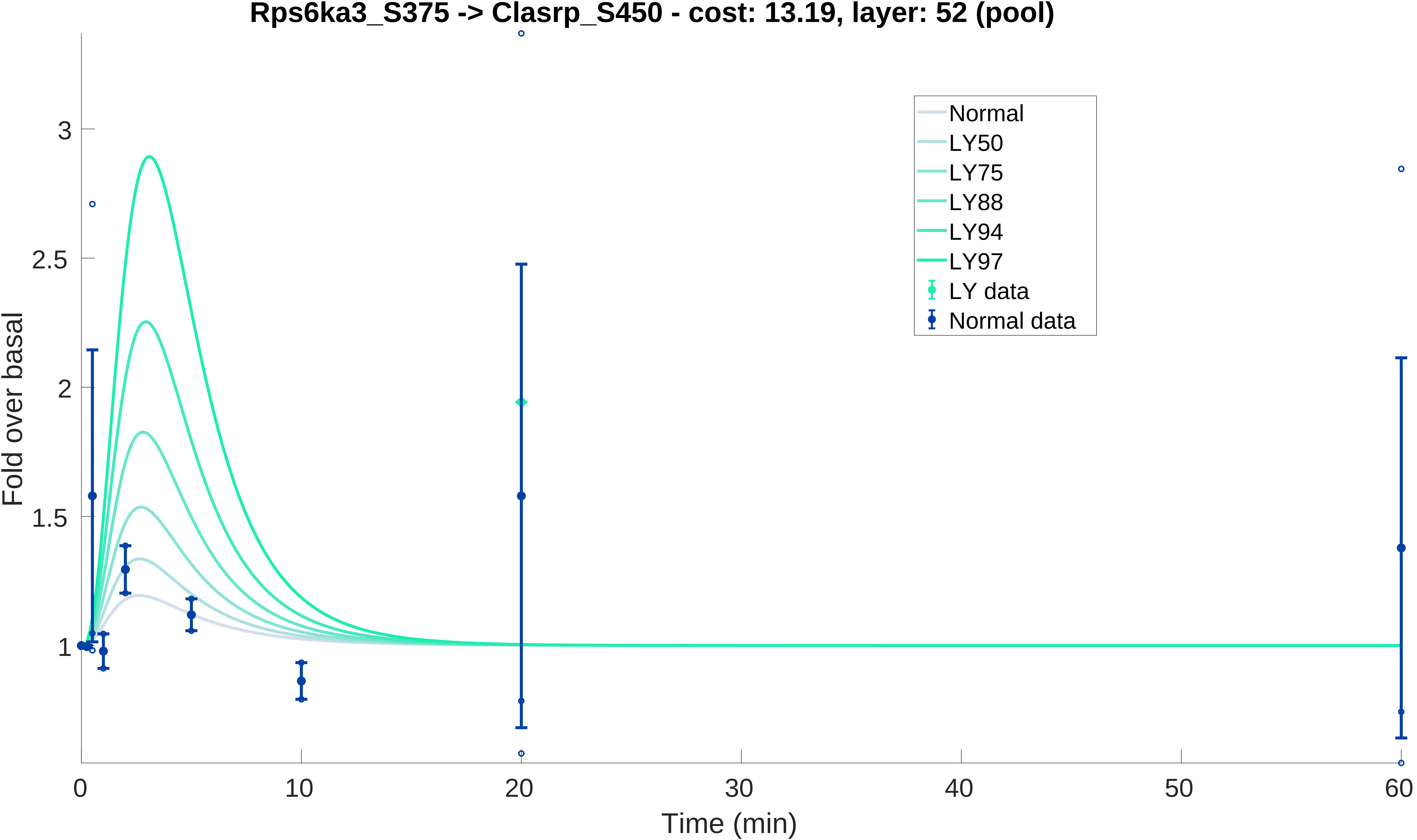

Rps6ka3_S375 -> Clasrp_S450 - cost: 13.19, layer: 52 (pool)
Fold over basal
3
2.5
2
1.5
1
0
10
20
30
40
50
60
Time (min)
Normal
LY50
LY75
LY88
LY94
LY97
LY data
Normal data

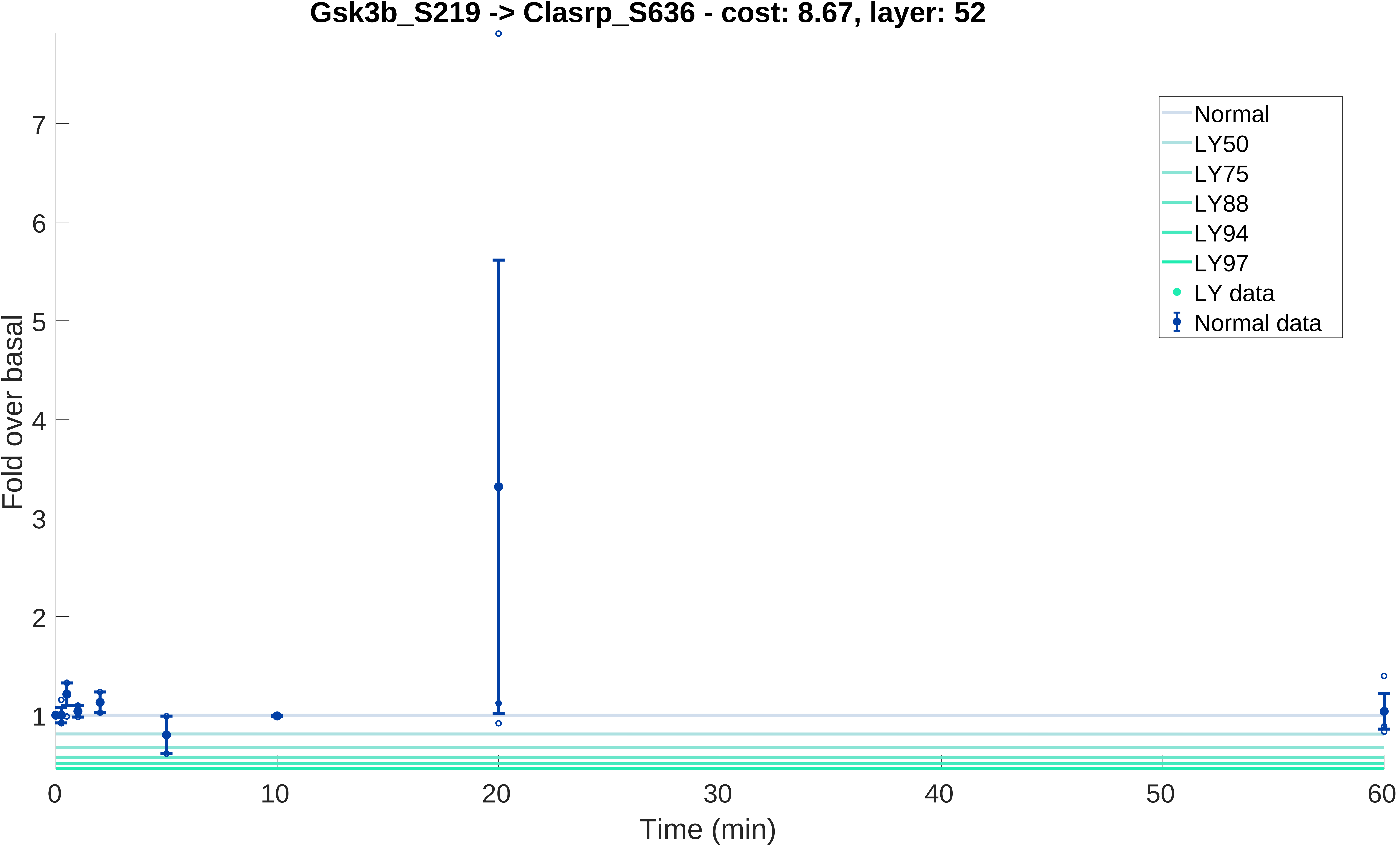
Gsk3b_S219 -> Clasrp_S636 - cost: 8.67, layer: 52
Fold over basal
Time (min)
0
10
20
30
40
50
60
1
2
3
4
5
6
7
Normal
LY50
LY75
LY88
LY94
LY97
LY data
Normal data

Akt1_S124 -> Clasrp_S639 - cost: 14.18, layer: 52

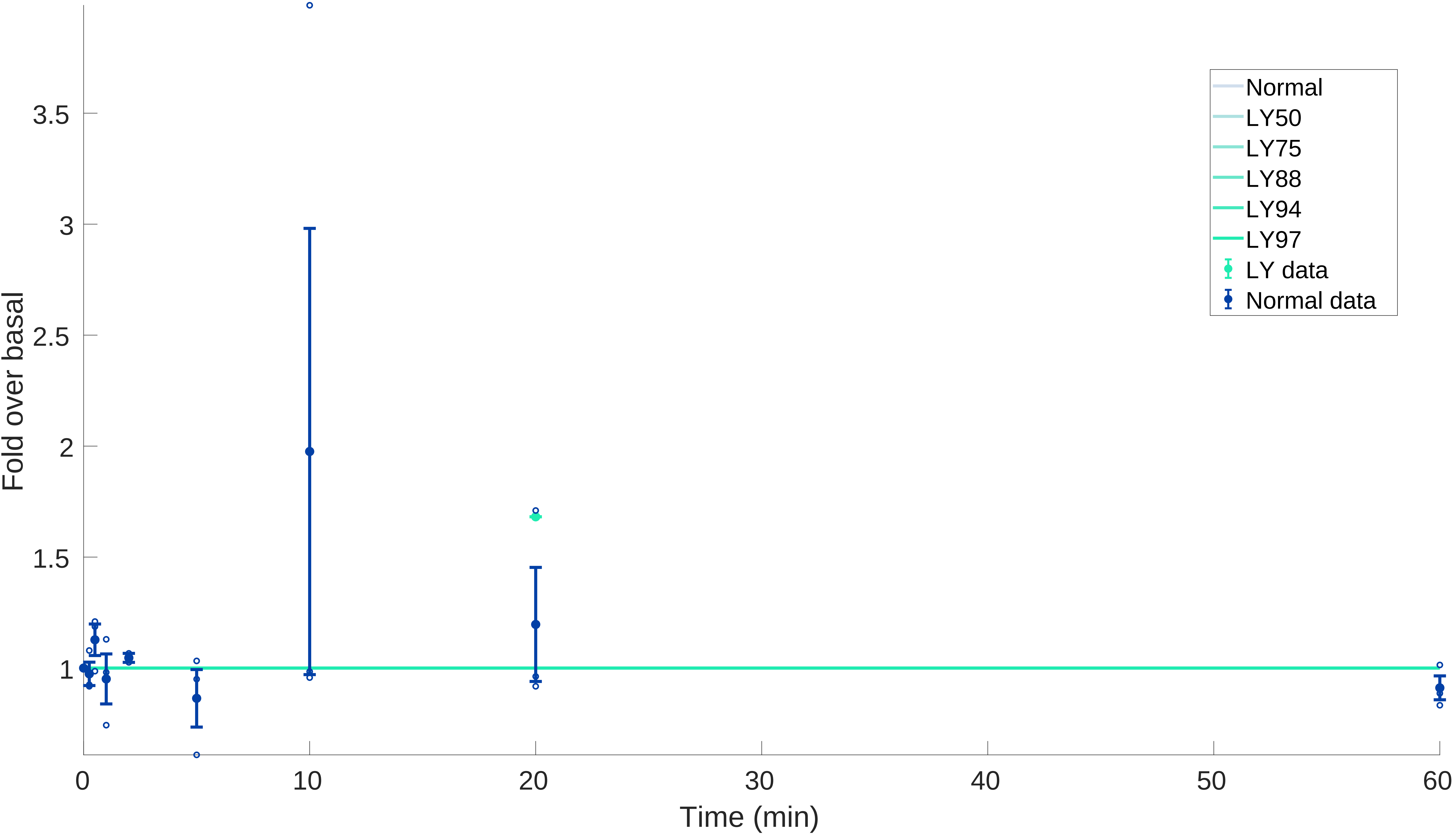

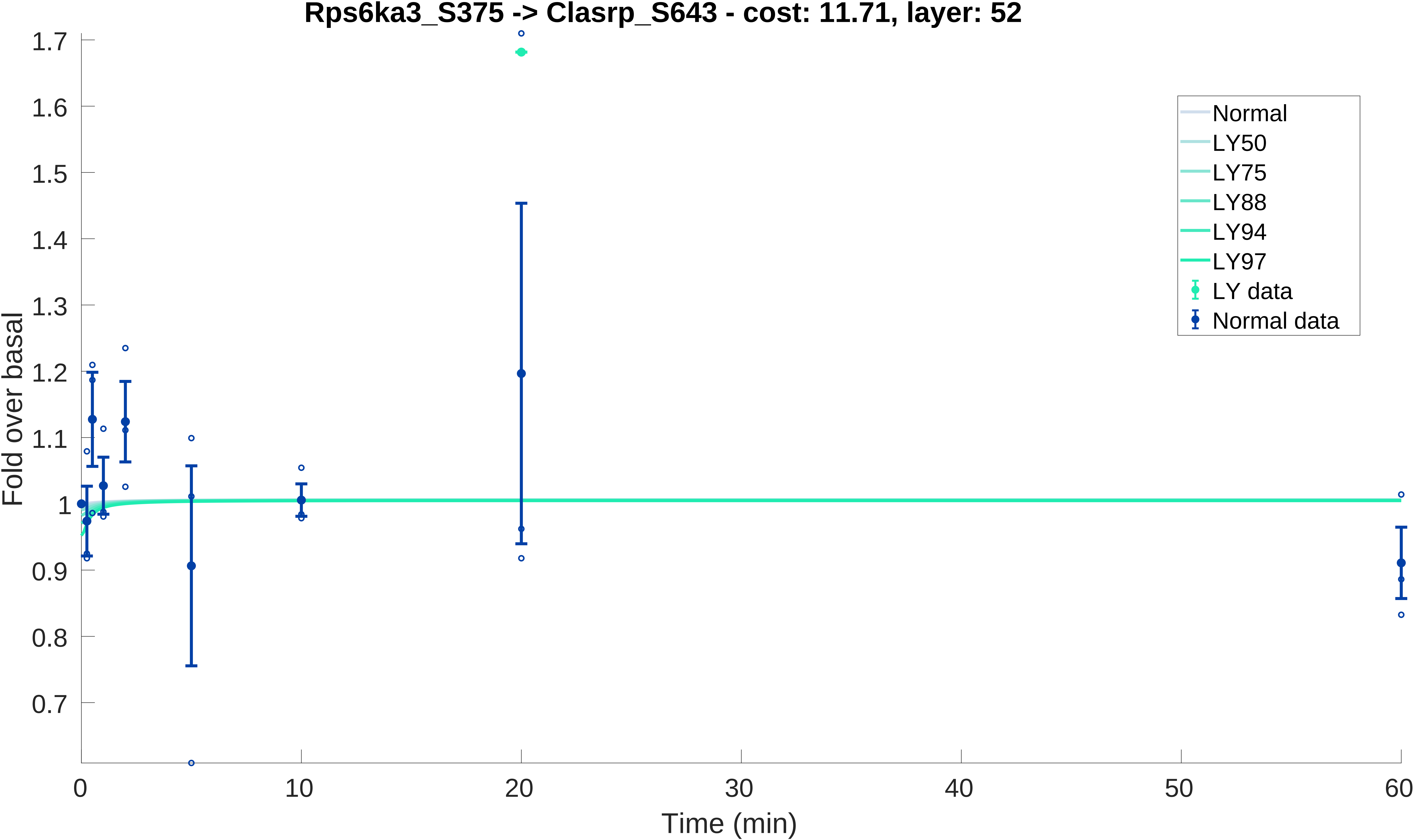
Rps6ka3_S375 -> Clasrp_S643 - cost: 11.71, layer: 52
Fold over basal
Time (min)
0.7
0.8
0.9
1
1.1
1.2
1.3
1.4
1.5
1.6
1.7
0
10
20
30
40
50
60
Normal
LY50
LY75
LY88
LY94
LY97
LY data
Normal data

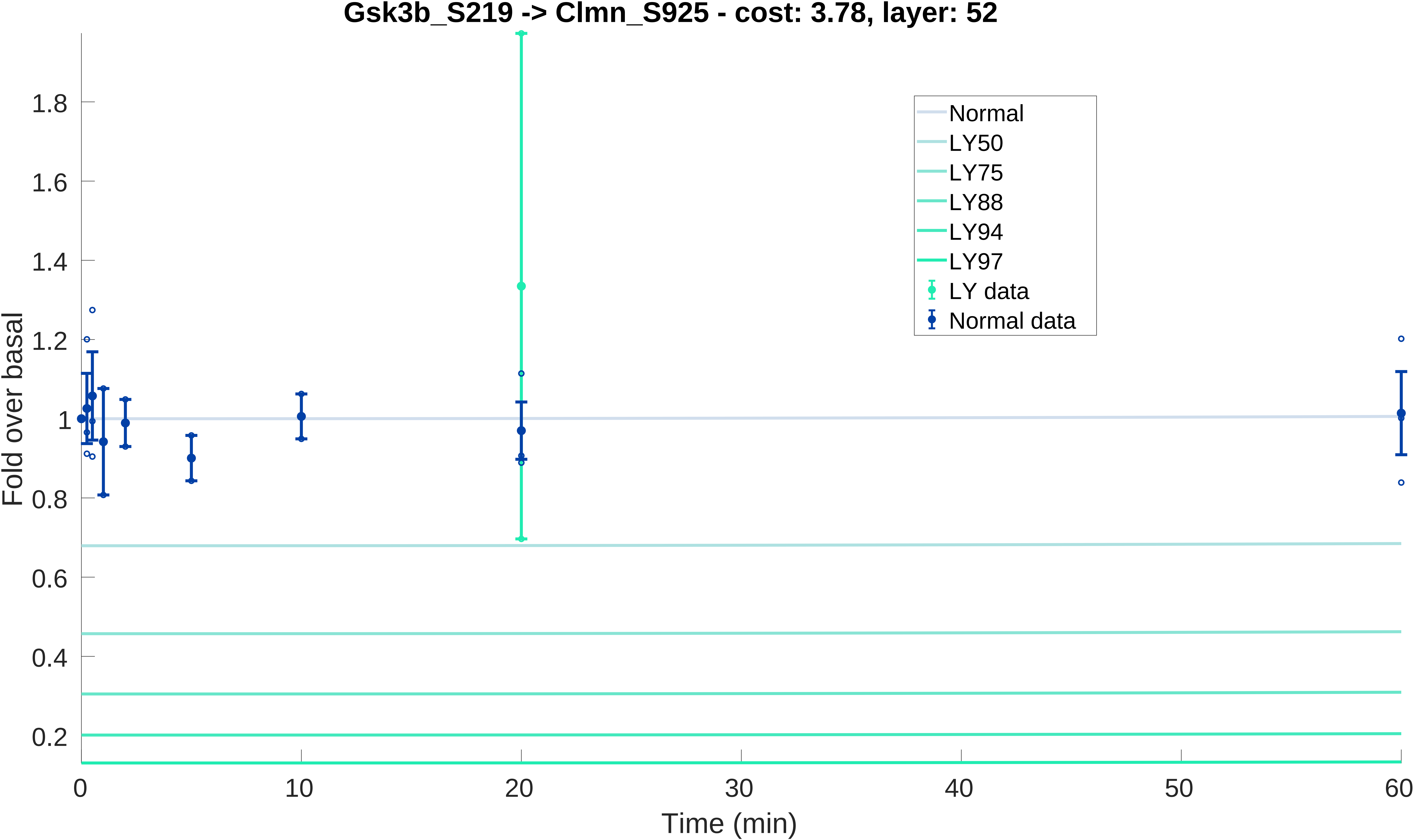
Gsk3b_S219 -> Clmn_S925 - cost: 3.78, layer: 52
Fold over basal
Time (min)
1.8
1.6
1.4
1.2
1
0.8
0.6
0.4
0.2
0
10
20
30
40
50
60
Normal
LY50
LY75
LY88
LY94
LY97
LY data
Normal data

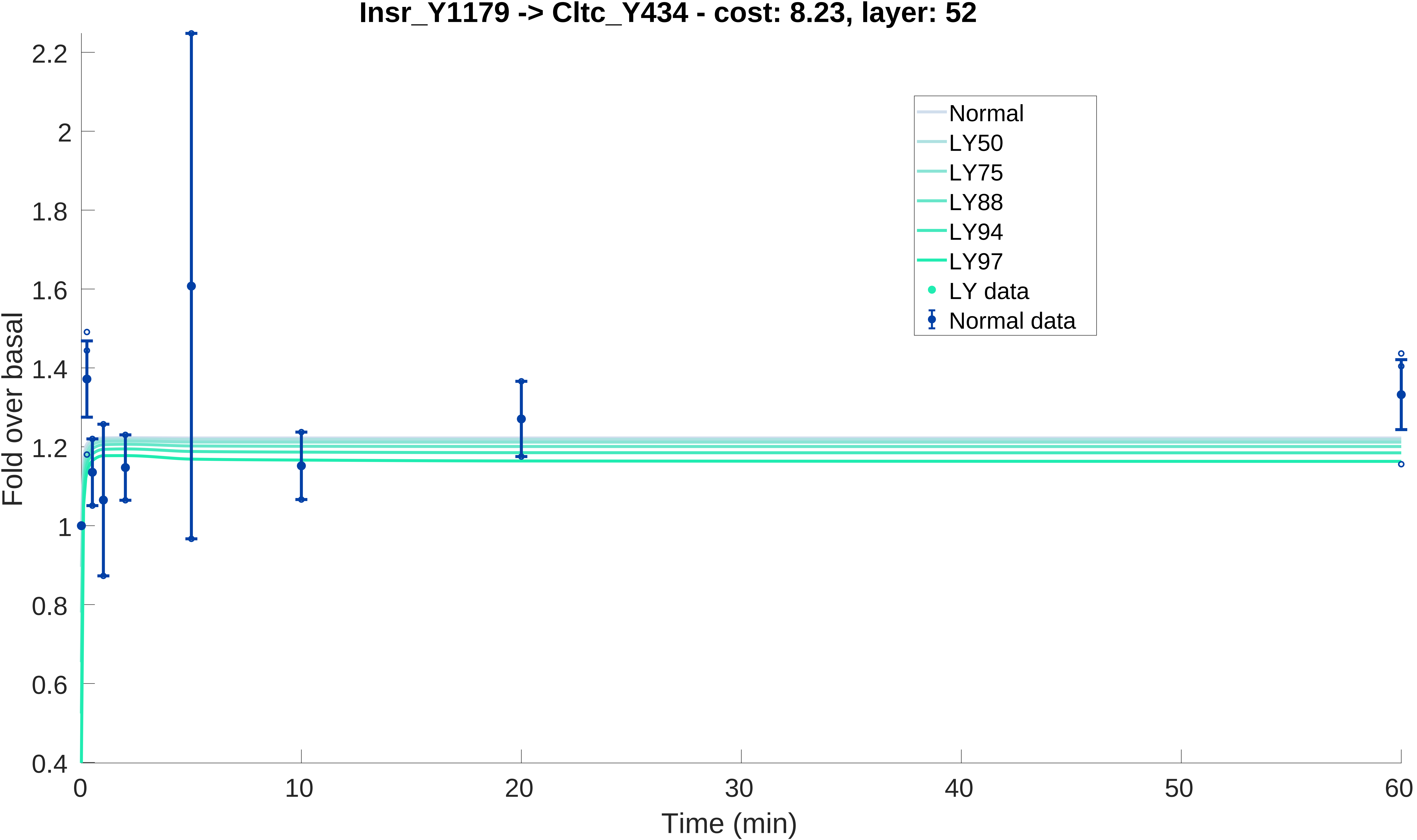
Insr_Y1179 -> Cltc_Y434 - cost: 8.23, layer: 52
Fold over basal
Time (min)
Normal
LY50
LY75
LY88
LY94
LY97
LY data
Normal data
0.4
0.6
0.8
1
1.2
1.4
1.6
1.8
2
2.2
0
10
20
30
40
50
60

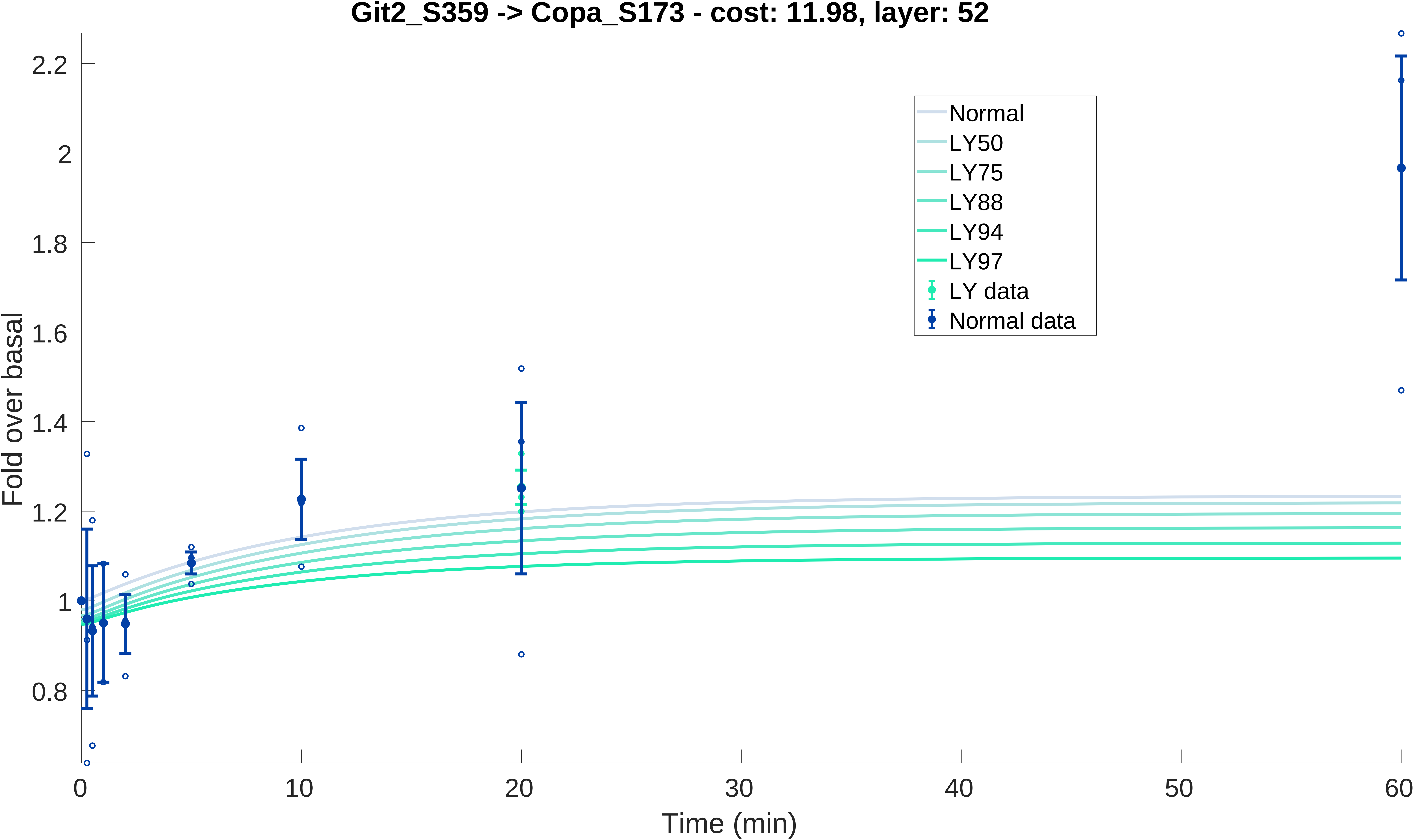
Git2_S359 -> Copa_S173 - cost: 11.98, layer: 52
Fold over basal
Time (min)
Normal
LY50
LY75
LY88
LY94
LY97
LY data
Normal data
0.8
1
1.2
1.4
1.6
1.8
2
2.2
0
10
20
30
40
50
60

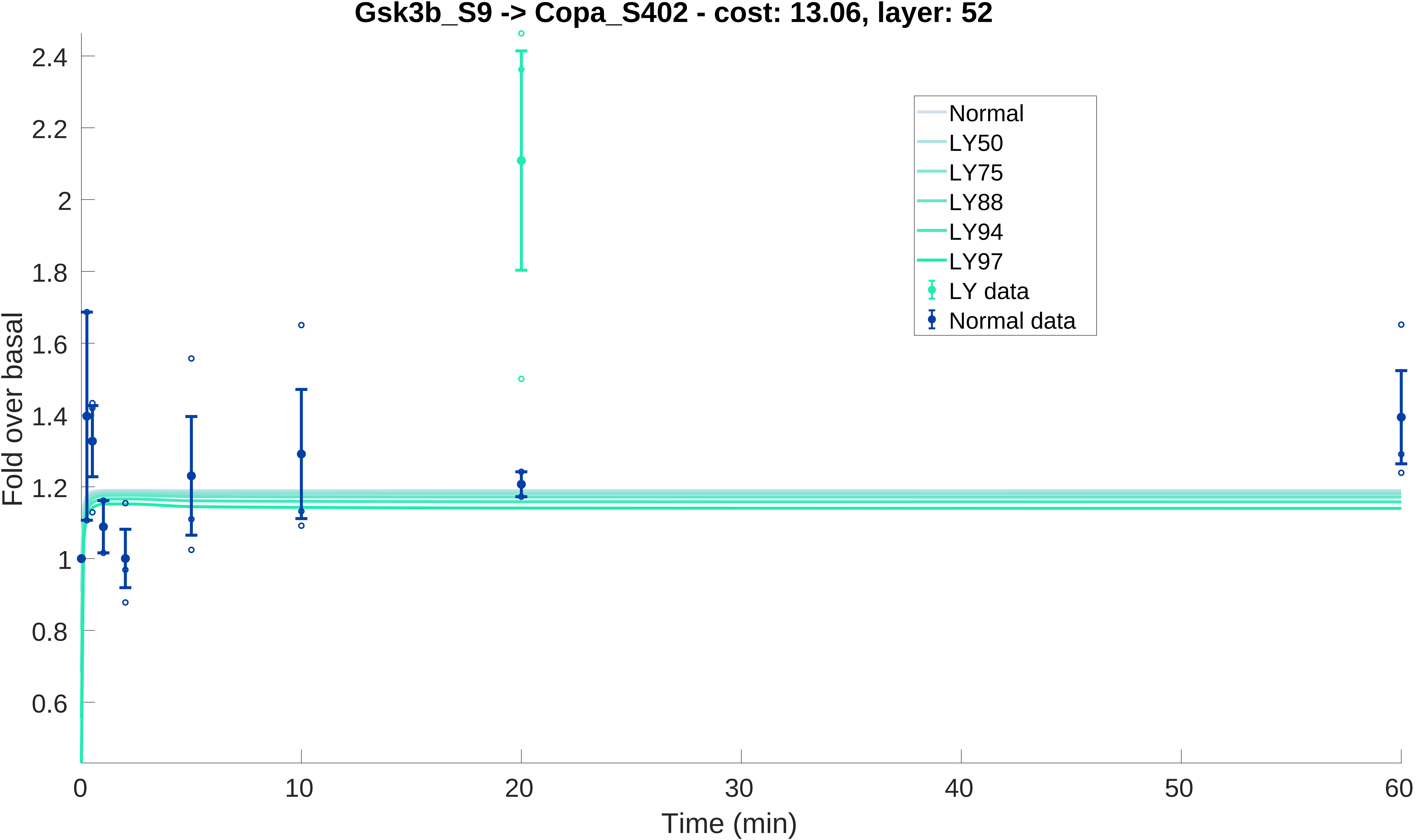
Gsk3b_S9 -> Copa_S402 - cost: 13.06, layer: 52
Fold over basal
Time (min)
Normal
LY50
LY75
LY88
LY94
LY97
LY data
Normal data
2.4
2.2
2
1.8
1.6
1.4
1.2
1
0.8
0.6
0
10
20
30
40
50
60

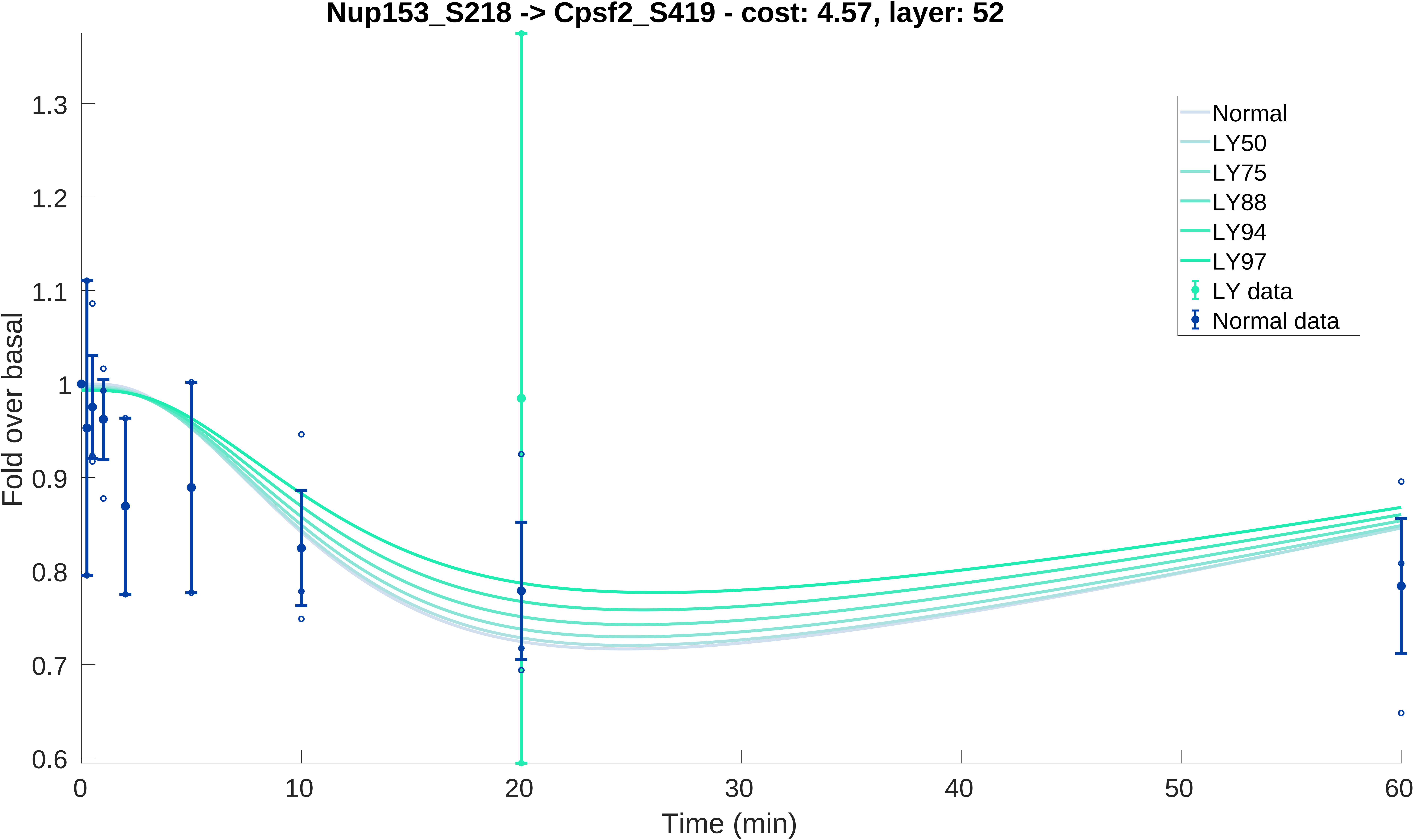

Nup153_S218 -> Cpsf2_S419 - cost: 4.57, layer: 52
Fold over basal
1.3
1.2
1.1
1
0.9
0.8
0.7
0.6
0
10
20
30
40
50
60
Time (min)
Normal
LY50
LY75
LY88
LY94
LY97
LY data
Normal data

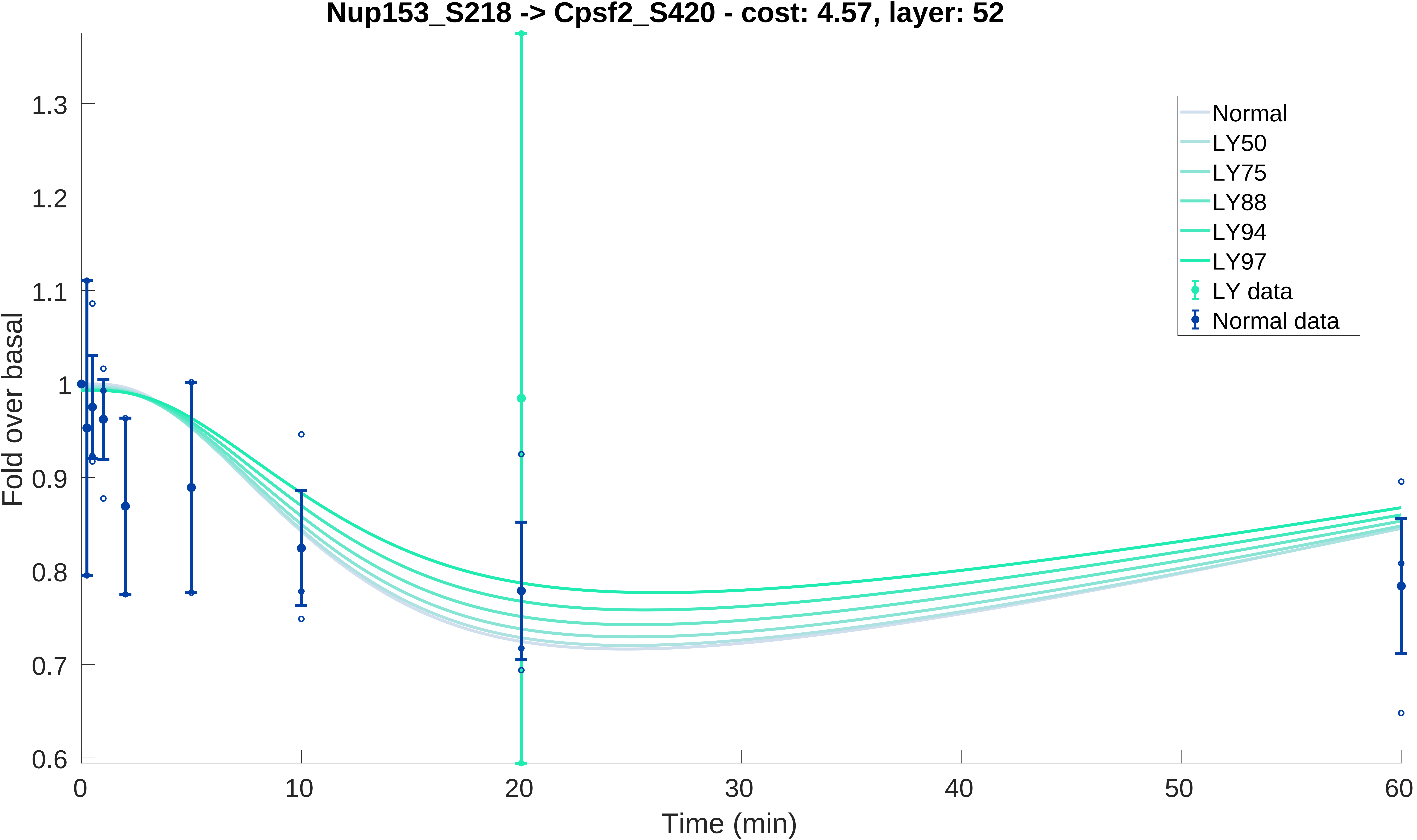

Nup153_S218 -> Cpsf2_S420 - cost: 4.57, layer: 52
Fold over basal
Time (min)
1.3
1.2
1.1
1
0.9
0.8
0.7
0.6
0
10
20
30
40
50
60
Normal
LY50
LY75
LY88
LY94
LY97
LY data
Normal data

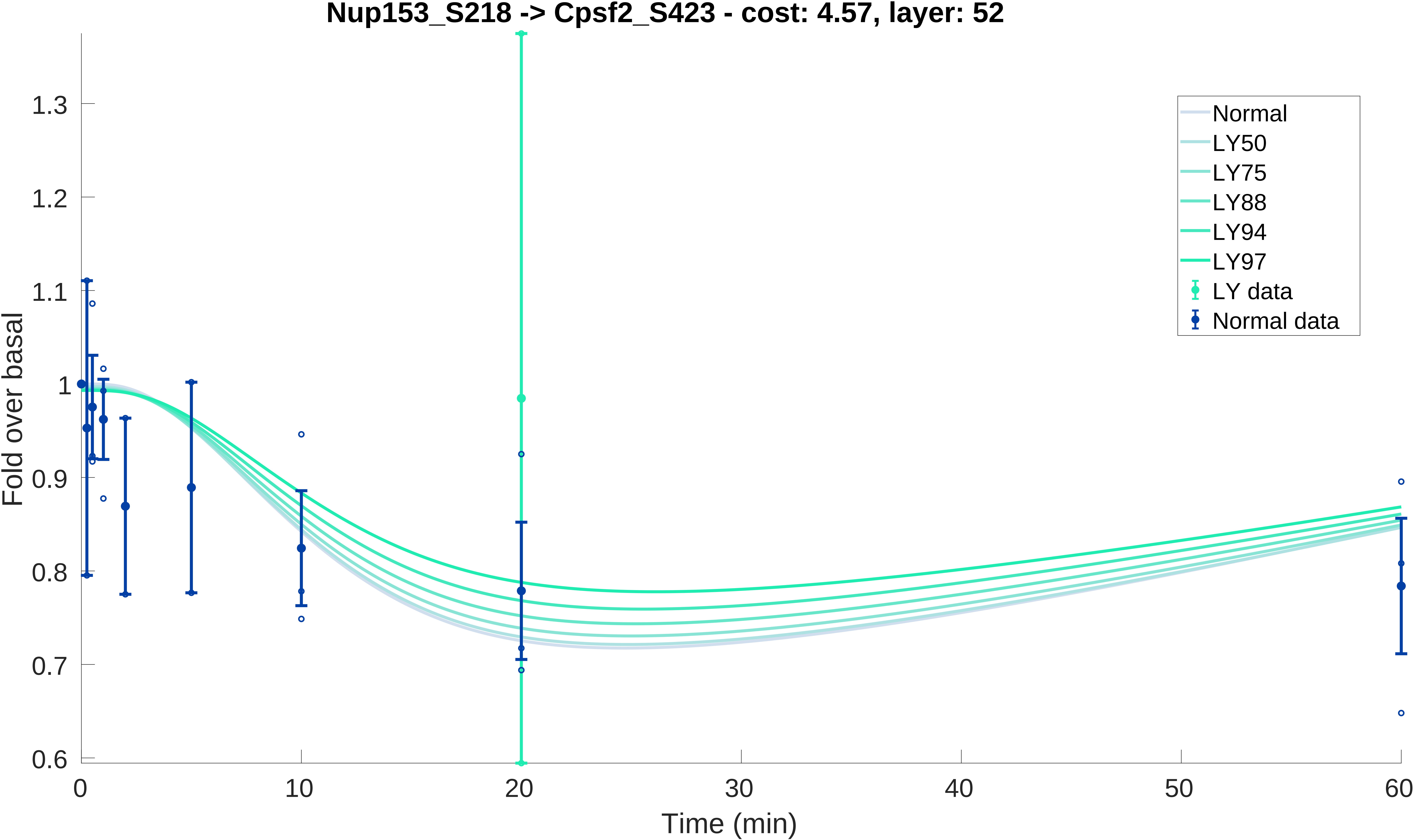
Nup153_S218 -> Cpsf2_S423 - cost: 4.57, layer: 52
Fold over basal
Time (min)
0.6
0.7
0.8
0.9
1
1.1
1.2
1.3
0
10
20
30
40
50
60
Normal
LY50
LY75
LY88
LY94
LY97
LY data
Normal data

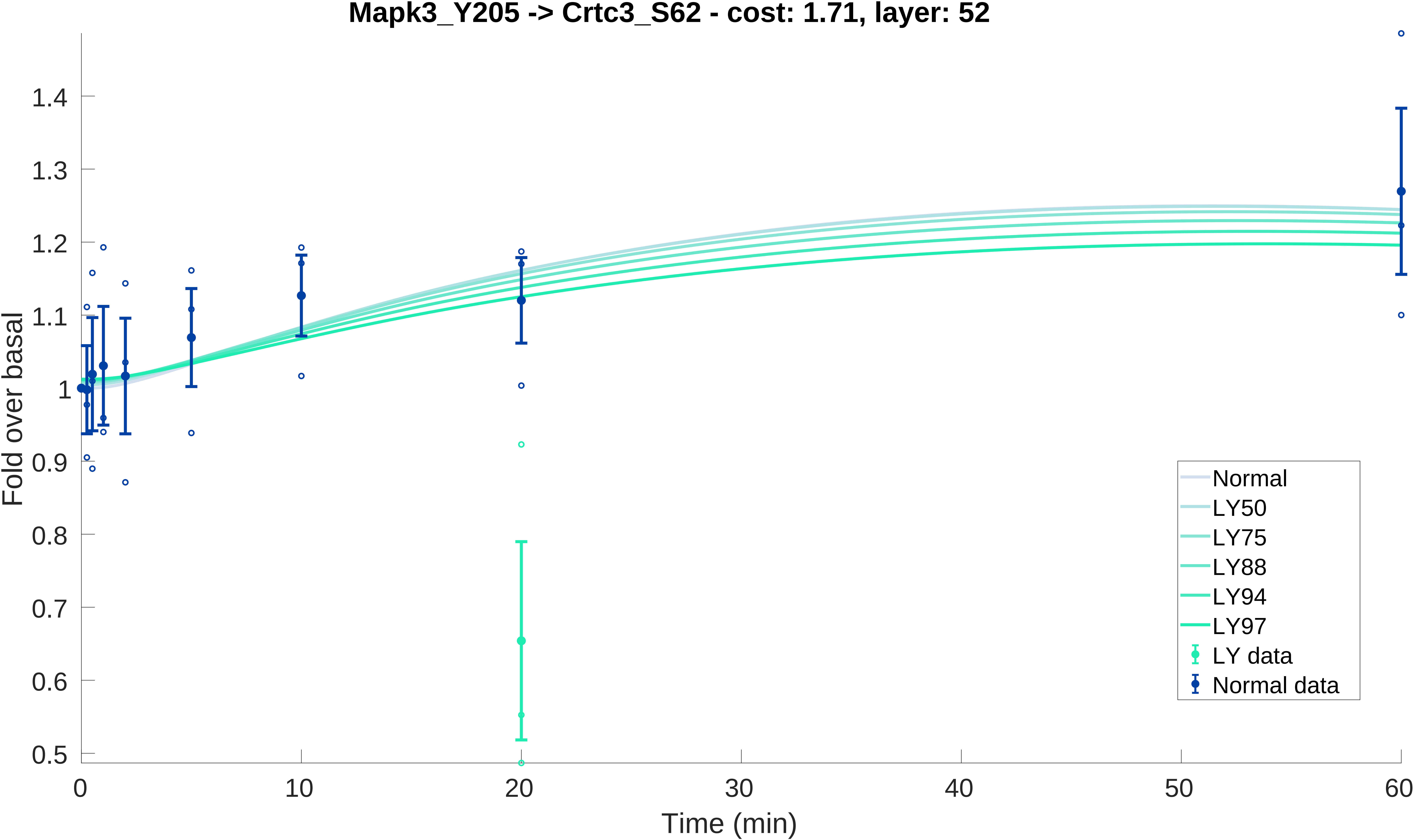

Mapk3_Y205 -> Crtc3_S62 - cost: 1.71, layer: 52
Fold over basal
1.4
1.3
1.2
1.1
1
0.9
0.8
0.7
0.6
0.5
0
10
20
30
40
50
60
Time (min)
Normal
LY50
LY75
LY88
LY94
LY97
LY data
Normal data

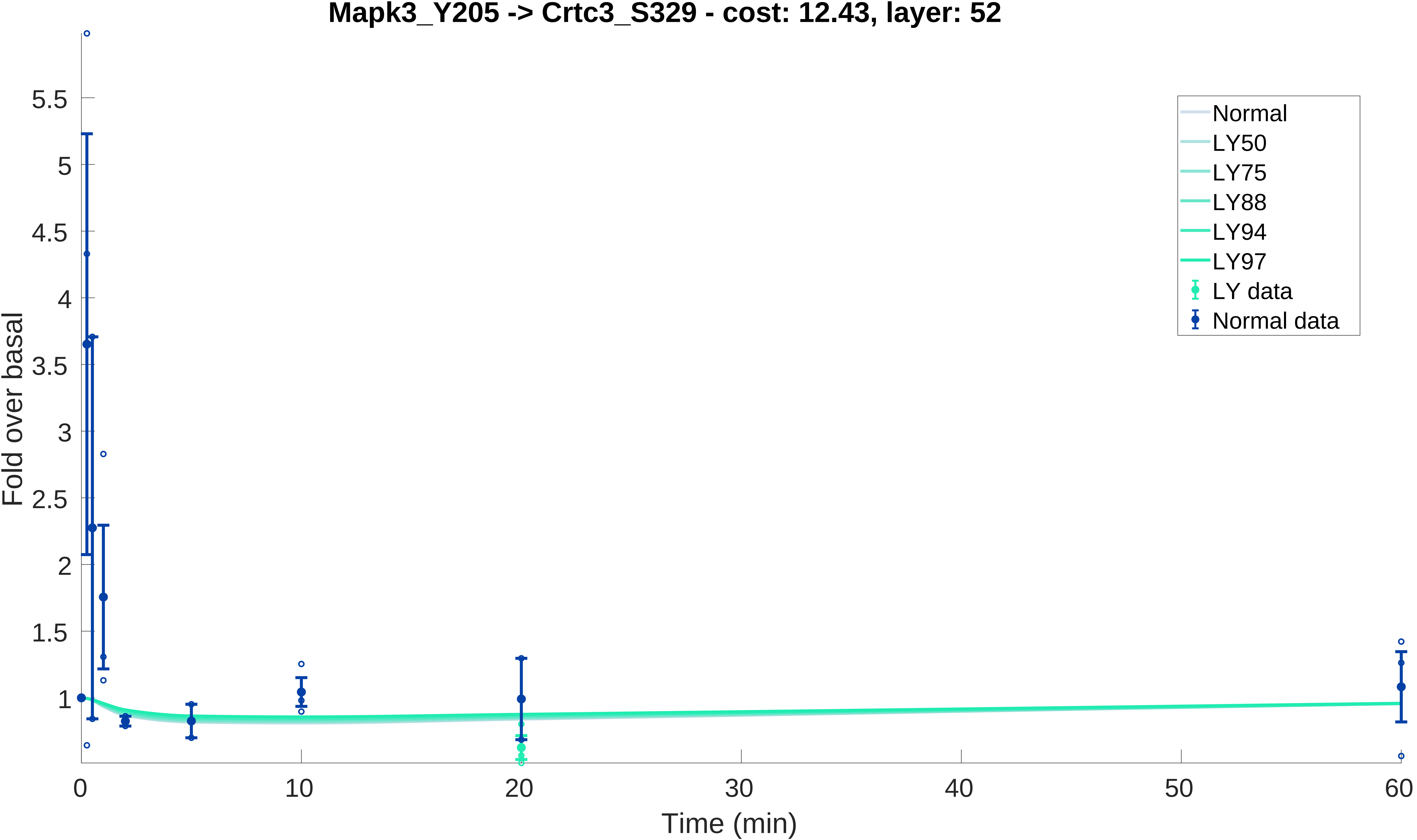
Mapk3_Y205 -> Crtc3_S329 - cost: 12.43, layer: 52
Fold over basal
5.5
5
4.5
4
3.5
3
2.5
2
1.5
1
0
10
20
30
40
50
60
Time (min)
Normal
LY50
LY75
LY88
LY94
LY97
LY data
Normal data

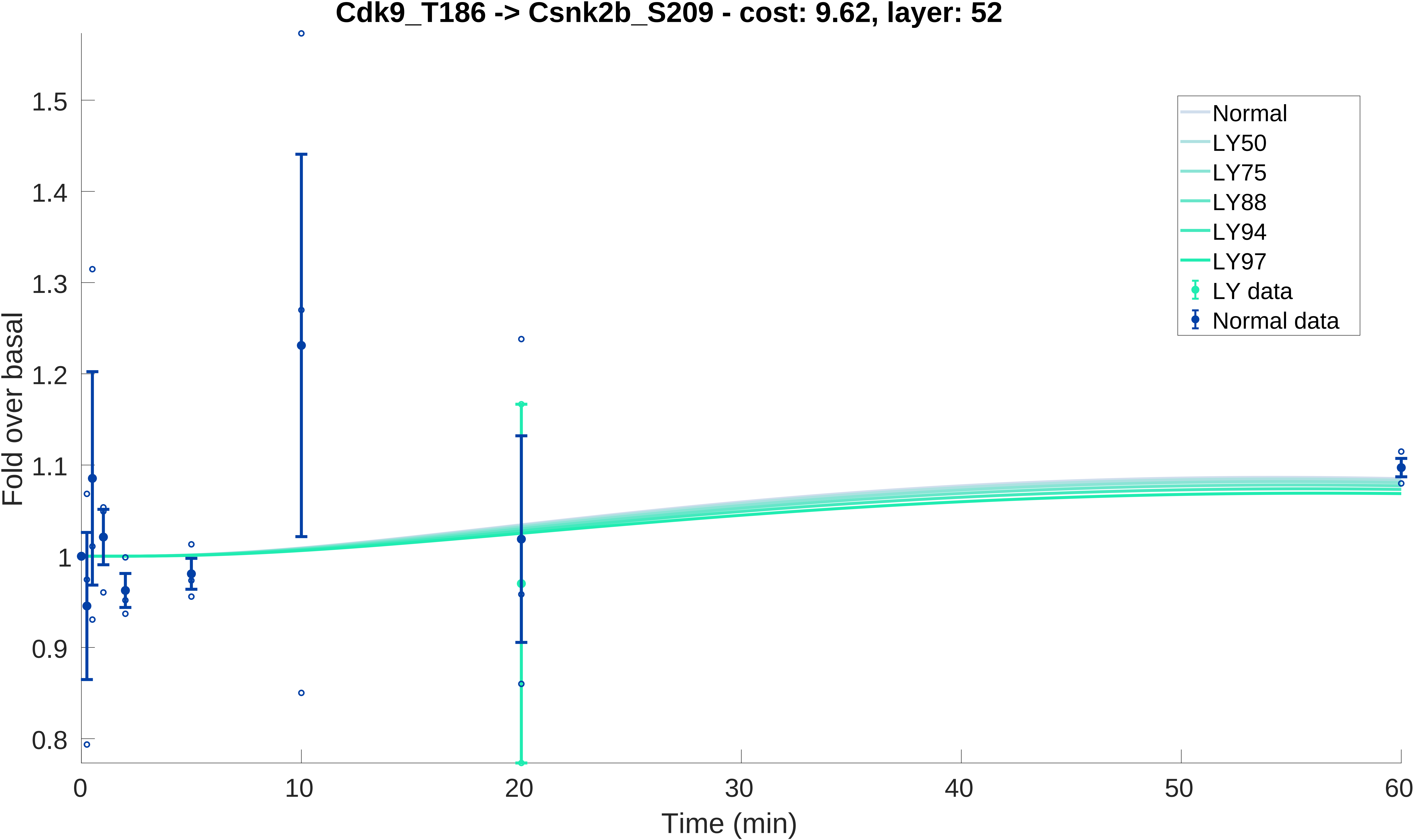

Cdk9_T186 -> Csnk2b_S209 - cost: 9.62, layer: 52
Fold over basal
Time (min)
1.5
1.4
1.3
1.2
1.1
1
0.9
0.8
0
10
20
30
40
50
60
Normal
LY50
LY75
LY88
LY94
LY97
LY data
Normal data

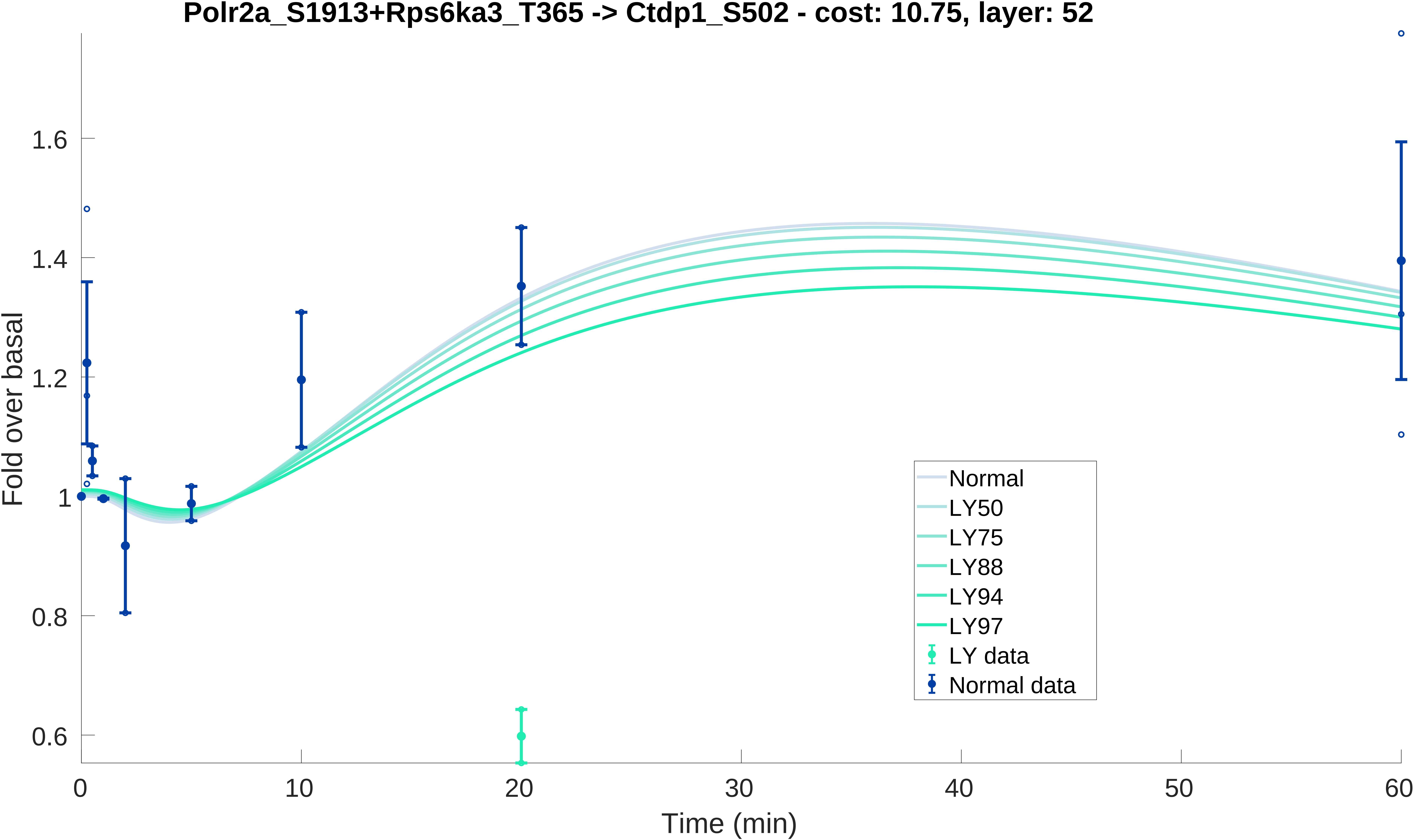
Polr2a_S1913+Rps6ka3_T365 -> Ctdp1_S502 - cost: 10.75, layer: 52
Fold over basal
1.6
1.4
1.2
1
0.8
0.6
0
10
20
30
40
50
60
Time (min)
Normal
LY50
LY75
LY88
LY94
LY97
LY data
Normal data

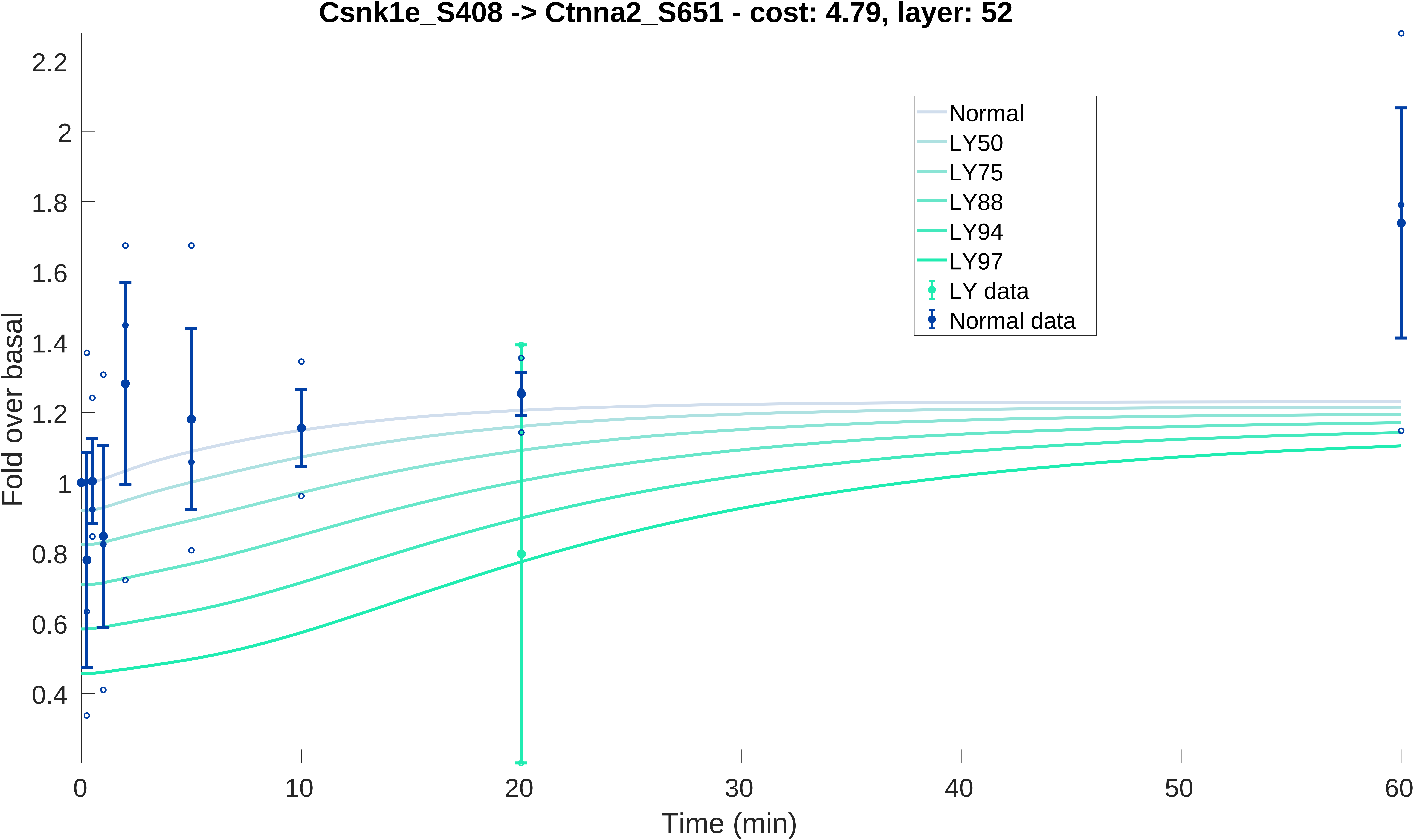

Csnk1e_S408 -> Ctnna2_S651 - cost: 4.79, layer: 52
Normal
LY50
LY75
LY88
LY94
LY97
LY data
Normal data
Fold over basal
Time (min)
2.2
2
1.8
1.6
1.4
1.2
1
0.8
0.6
0.4
0
10
20
30
40
50
60

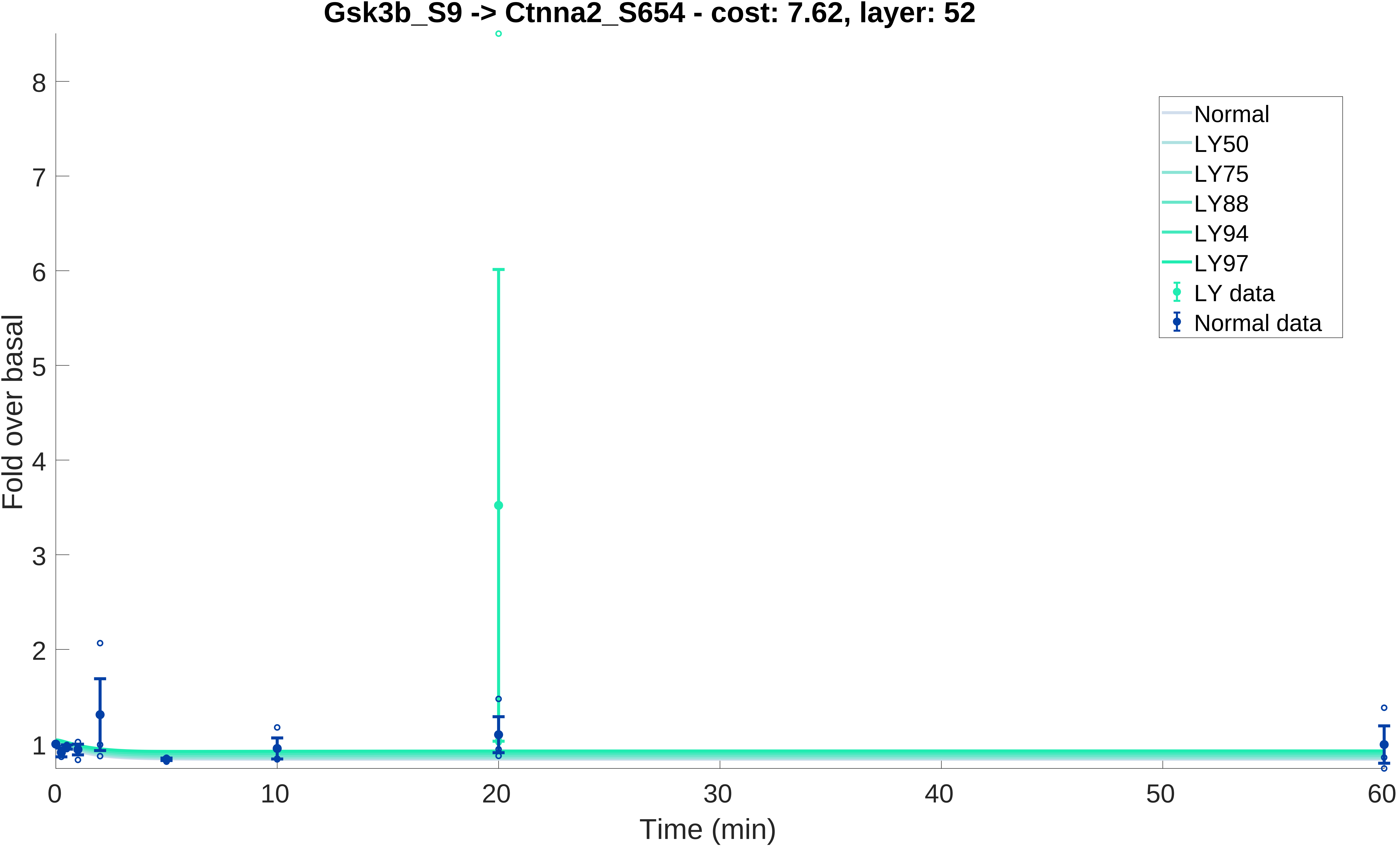
Gsk3b_S9 -> Ctnna2_S654 - cost: 7.62, layer: 52
Fold over basal
Time (min)
0
10
20
30
40
50
60
1
2
3
4
5
6
7
8
Normal
LY50
LY75
LY88
LY94
LY97
LY data
Normal data

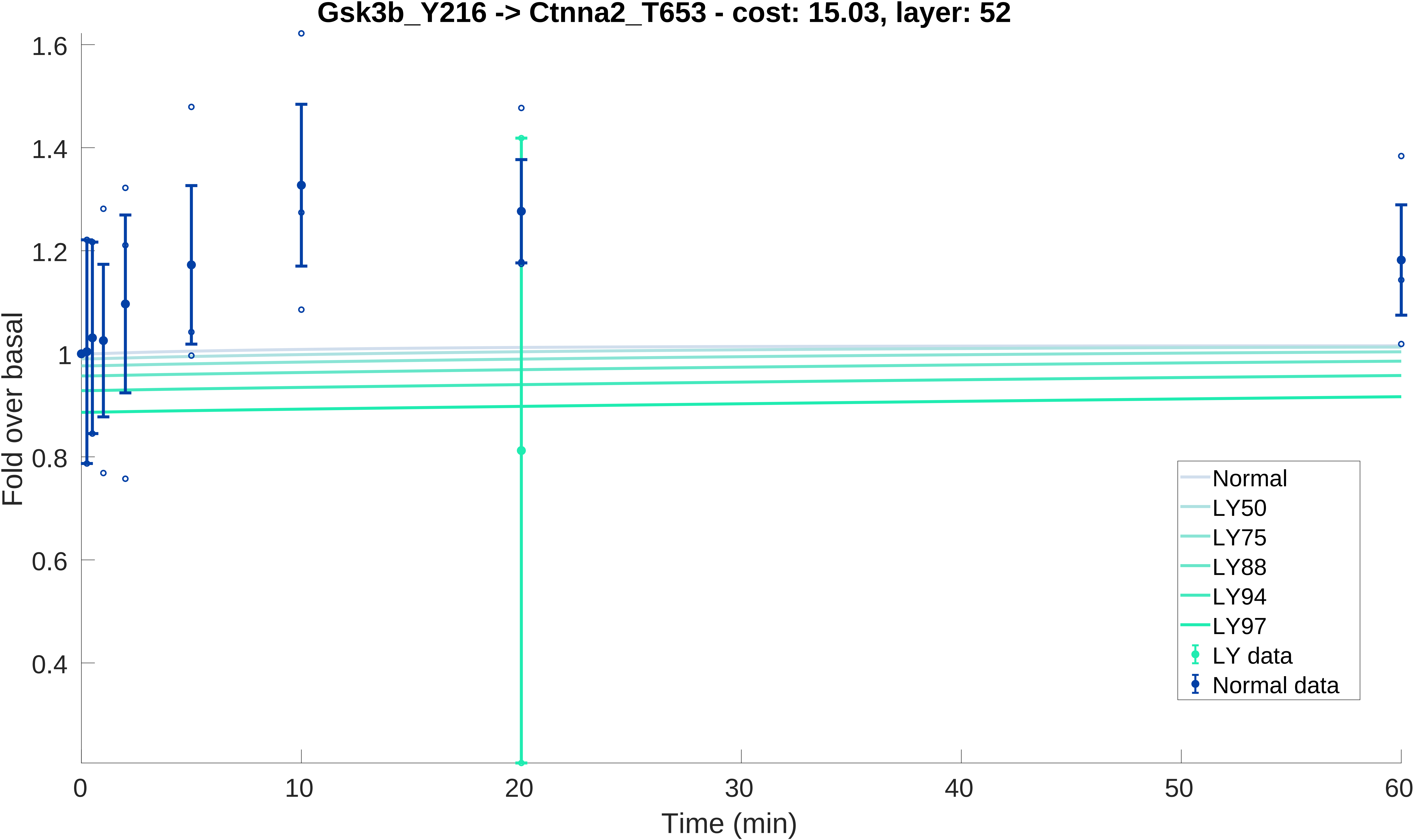

Gsk3b_Y216 -> Ctnna2_T653 - cost: 15.03, layer: 52
Fold over basal
Time (min)
0
10
20
30
40
50
60
0.4
0.6
0.8
1
1.2
1.4
1.6
Normal
LY50
LY75
LY88
LY94
LY97
LY data
Normal data

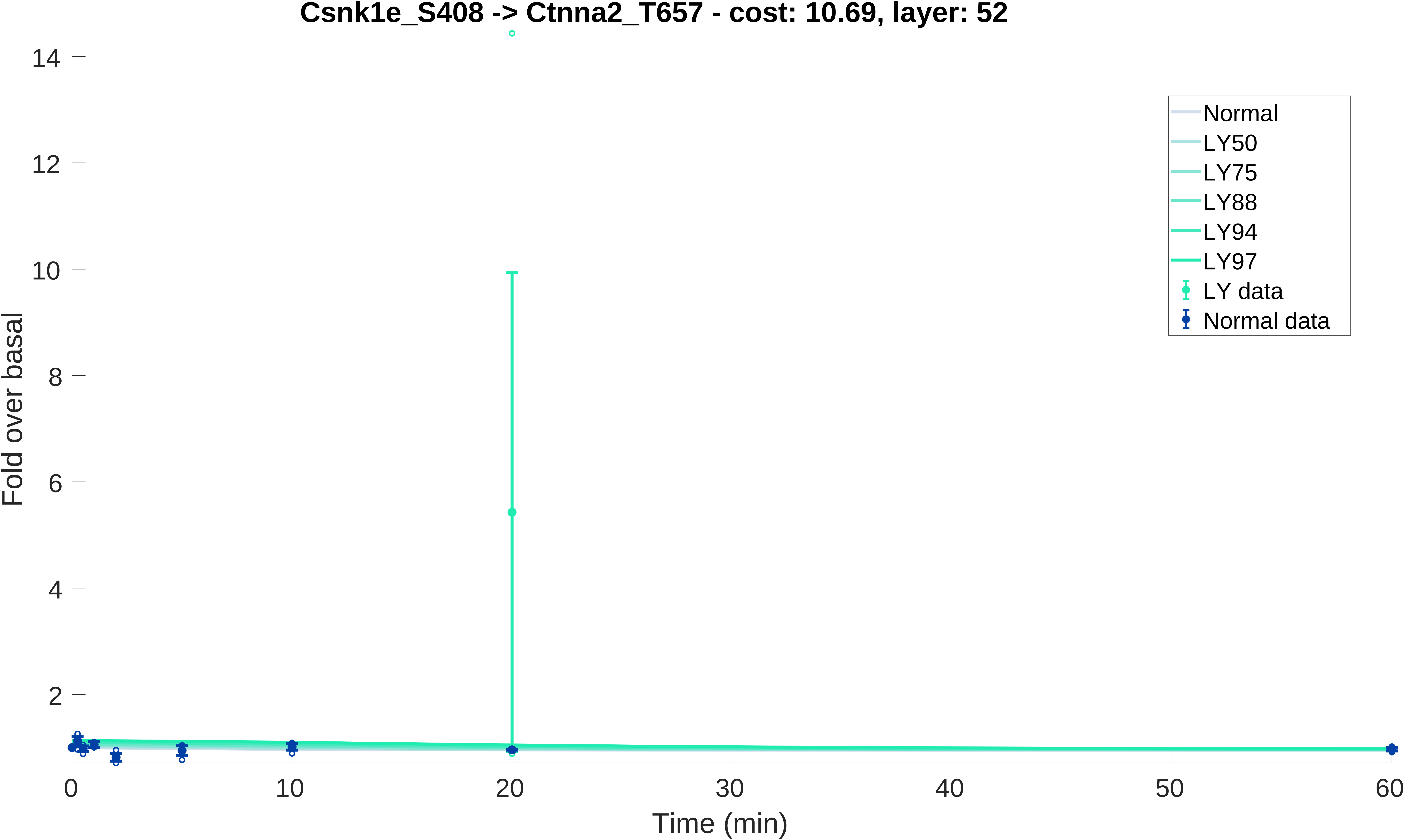

Csnk1e_S408 -> Ctnna2_T657 - cost: 10.69, layer: 52
Fold over basal
Time (min)
Normal
LY50
LY75
LY88
LY94
LY97
LY data
Normal data
0
10
20
30
40
50
60
2
4
6
8
10
12
14

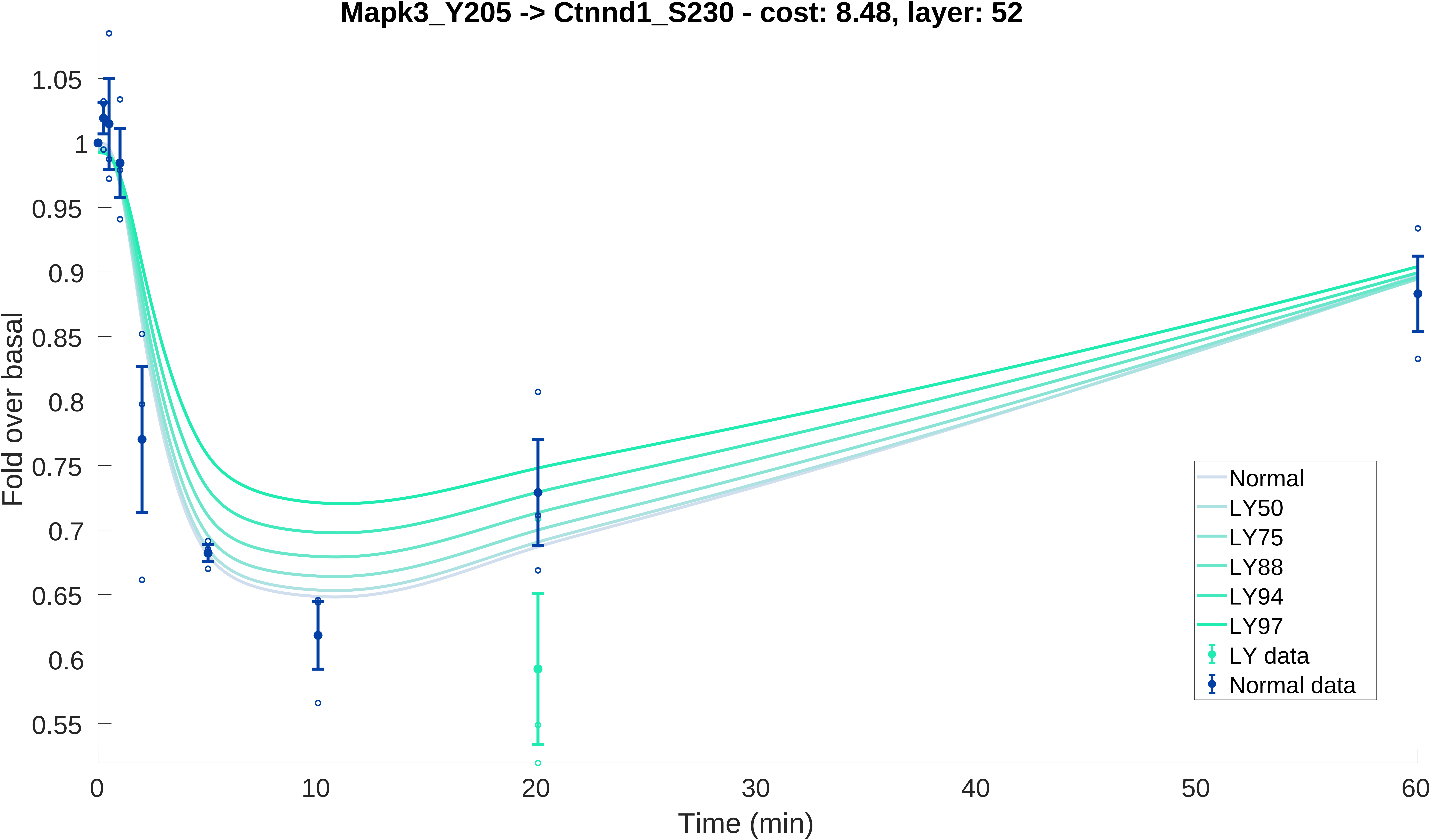
Mapk3_Y205 -> Ctnnd1_S230 - cost: 8.48, layer: 52
Fold over basal
1.05
1
0.95
0.9
0.85
0.8
0.75
0.7
0.65
0.6
0.55
0
10
20
30
40
50
60
Time (min)
Normal
LY50
LY75
LY88
LY94
LY97
LY data
Normal data

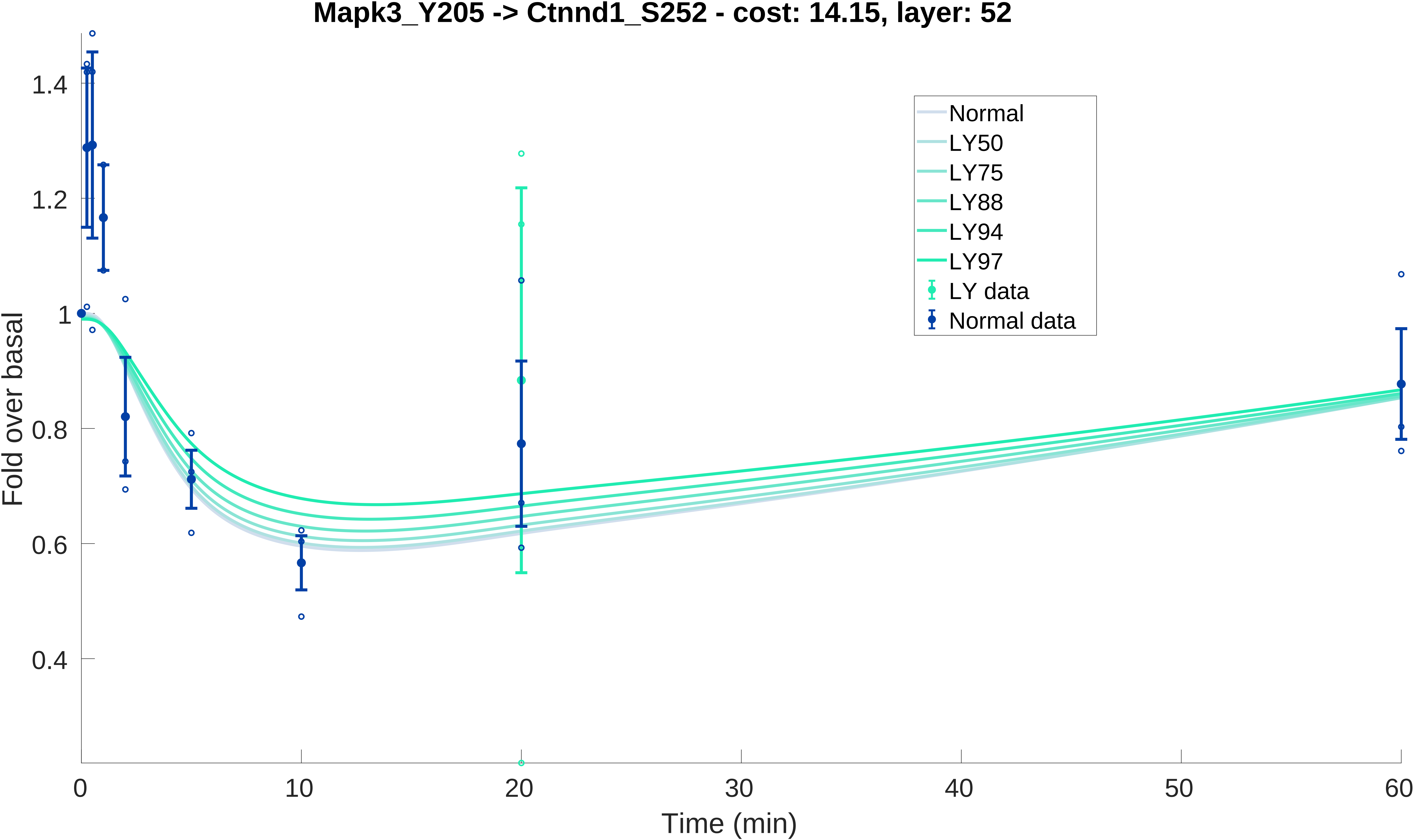

Mapk3_Y205 -> Ctnnd1_S252 - cost: 14.15, layer: 52
Fold over basal
Time (min)
1.4
1.2
1
0.8
0.6
0.4
0
10
20
30
40
50
60
Normal
LY50
LY75
LY88
LY94
LY97
LY data
Normal data

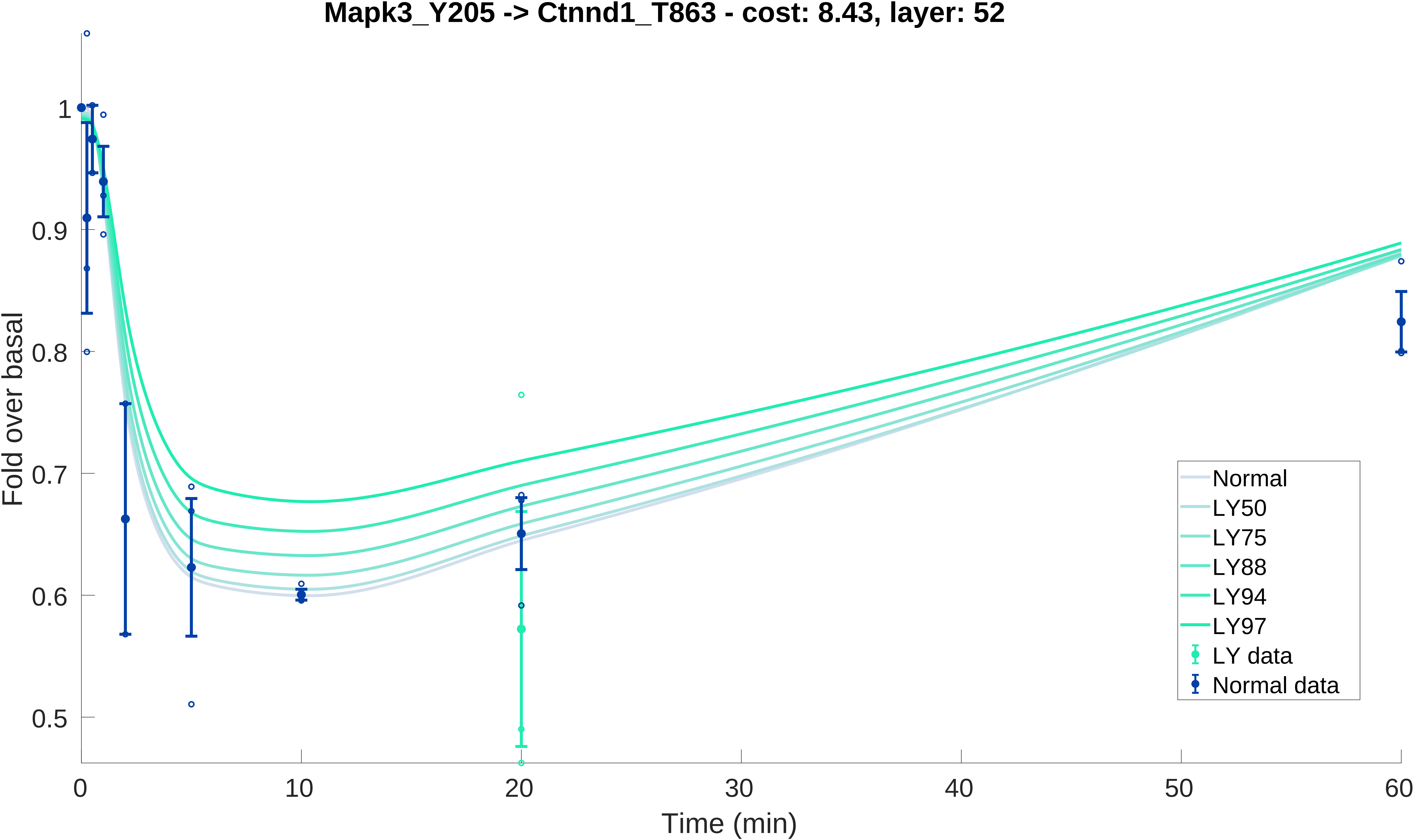

Mapk3_Y205 -> Ctnnd1_T863 - cost: 8.43, layer: 52
Fold over basal
1
0.9
0.8
0.7
0.6
0.5
0
10
20
30
40
50
60
Time (min)
Normal
LY50
LY75
LY88
LY94
LY97
LY data
Normal data

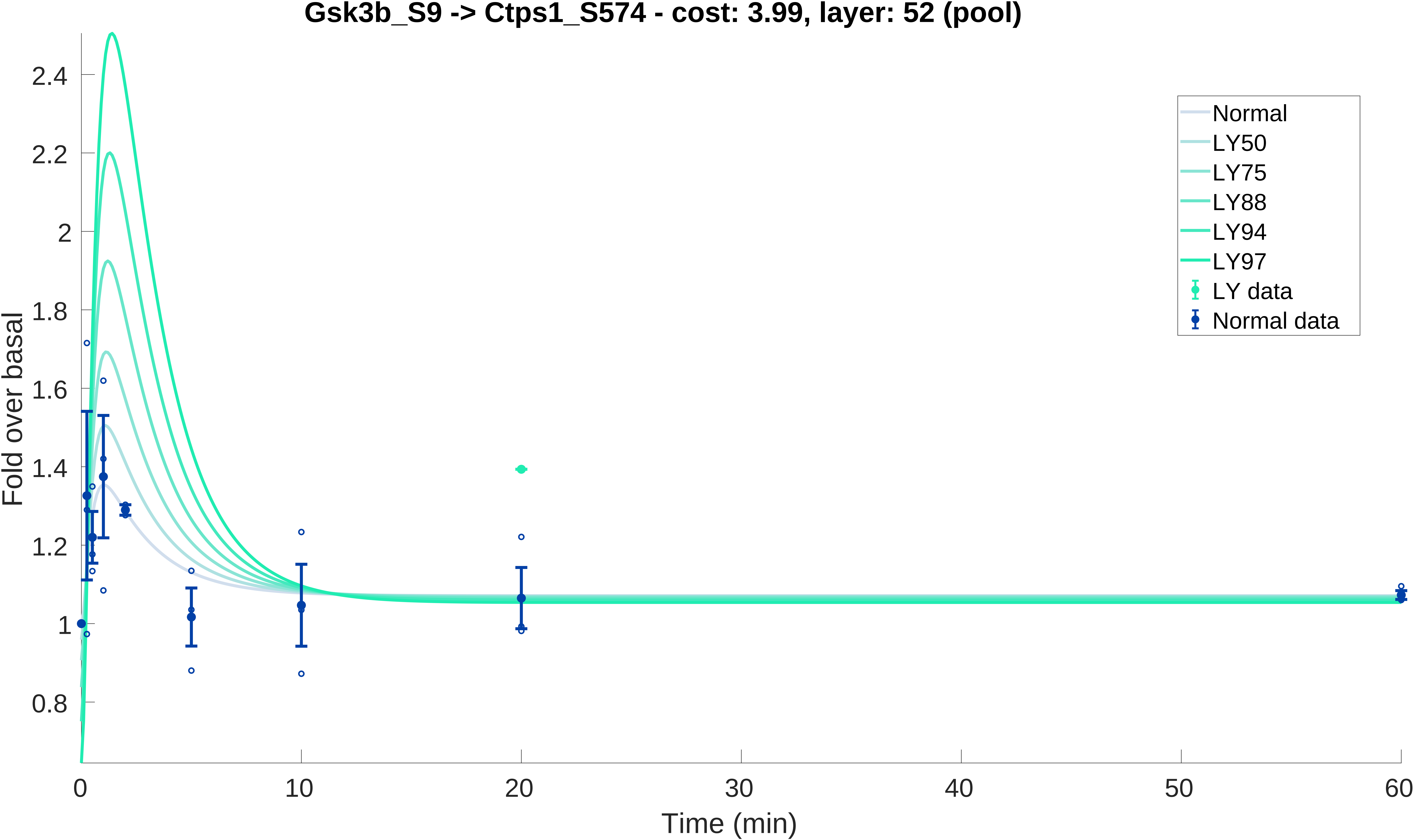

Gsk3b_S9 -> Ctps1_S574 - cost: 3.99, layer: 52 (pool)
Fold over basal
Time (min)
0.8
1
1.2
1.4
1.6
1.8
2
2.2
2.4
0
10
20
30
40
50
60
Normal
LY50
LY75
LY88
LY94
LY97
LY data
Normal data

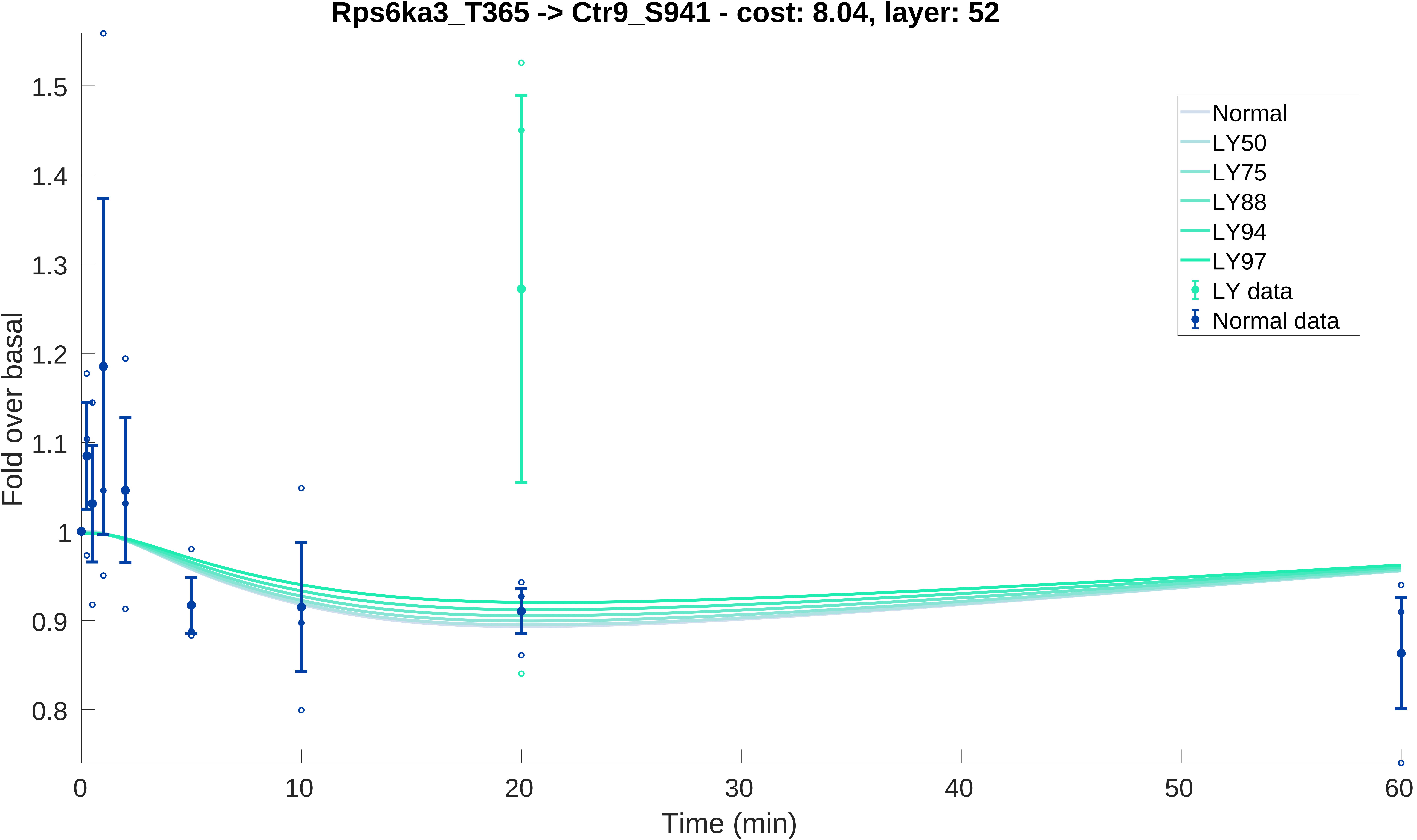

Rps6ka3_T365 -> Ctr9_S941 - cost: 8.04, layer: 52
Fold over basal
Time (min)
1.5
1.4
1.3
1.2
1.1
1
0.9
0.8
0
10
20
30
40
50
60
Normal
LY50
LY75
LY88
LY94
LY97
LY data
Normal data

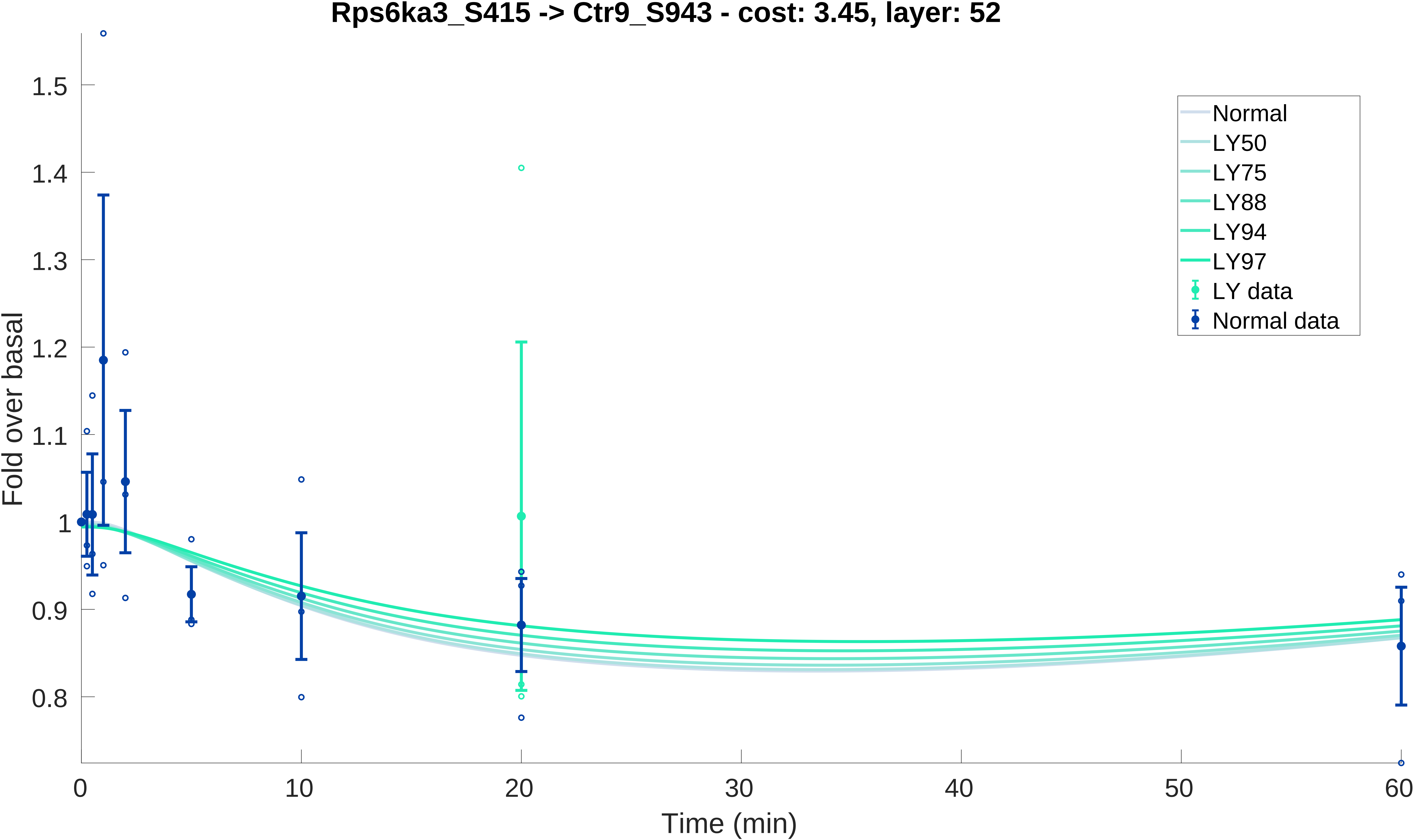

Rps6ka3_S415 -> Ctr9_S943 - cost: 3.45, layer: 52
Fold over basal
Time (min)
1.5
1.4
1.3
1.2
1.1
1
0.9
0.8
0
10
20
30
40
50
60
Normal
LY50
LY75
LY88
LY94
LY97
LY data
Normal data

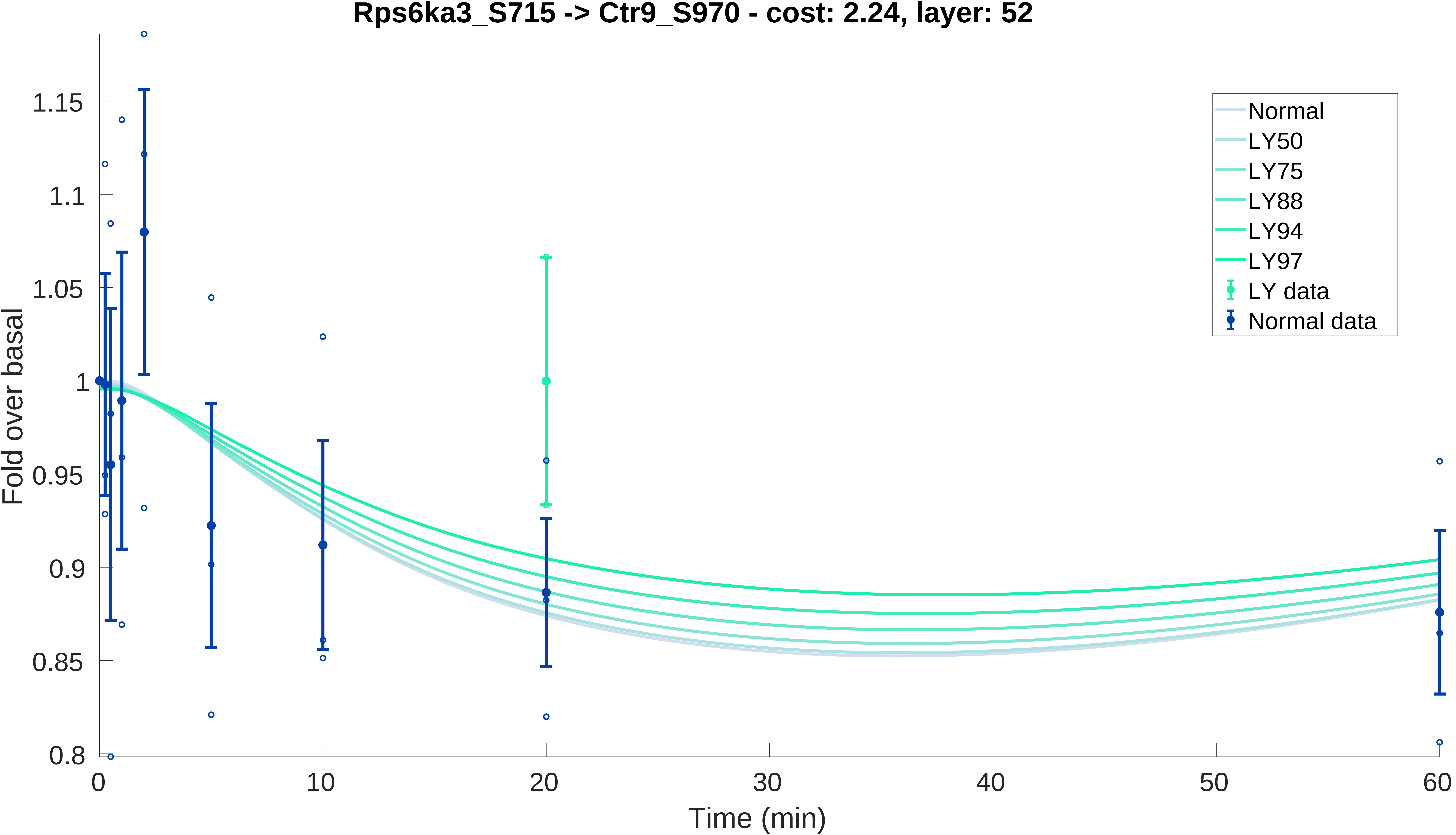
Rps6ka3_S715 -> Ctr9_S970 - cost: 2.24, layer: 52
Fold over basal
Time (min)
Normal
LY50
LY75
LY88
LY94
LY97
LY data
Normal data
0.8
0.85
0.9
0.95
1
1.05
1.1
1.15
0
10
20
30
40
50
60

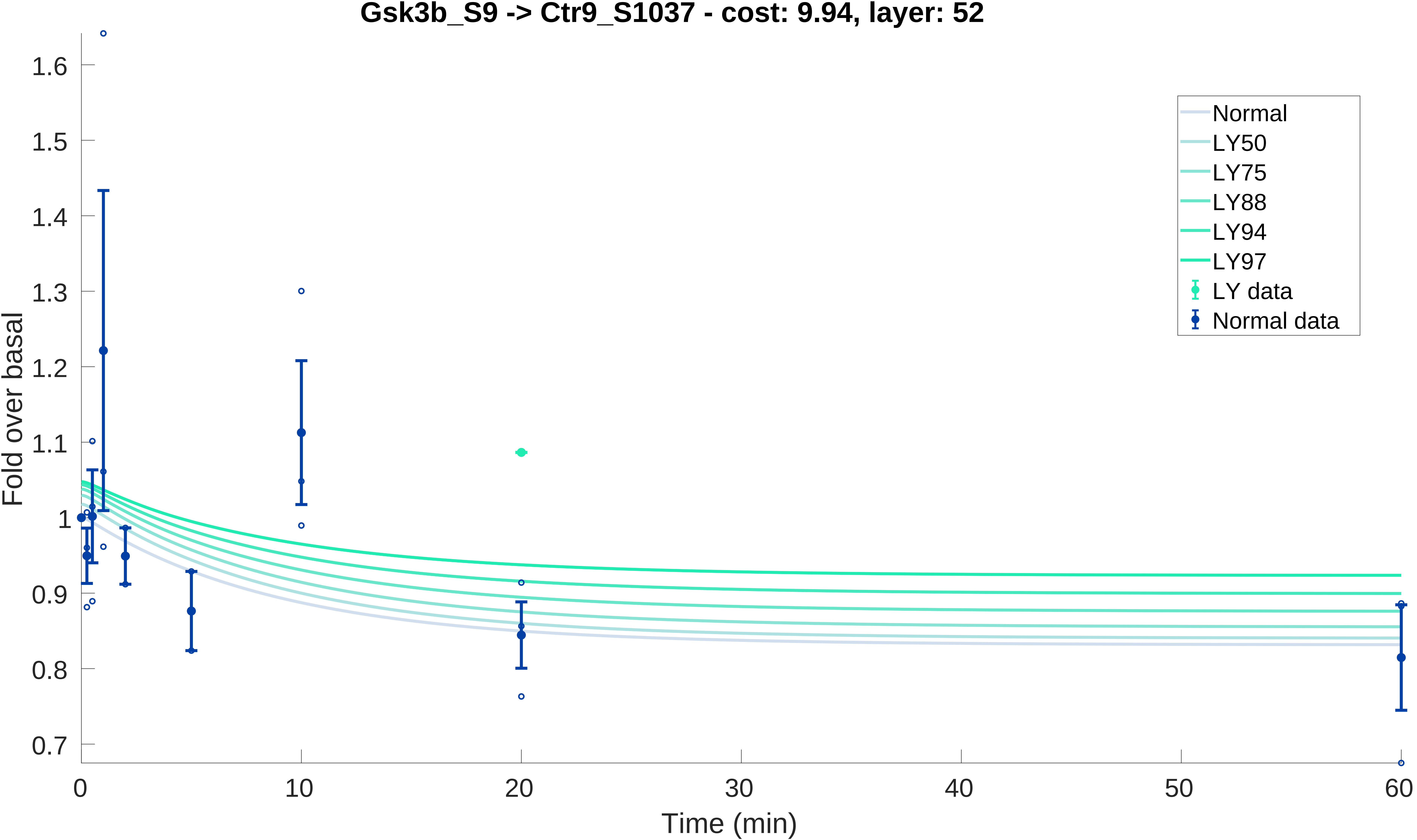
Gsk3b_S9 -> Ctr9_S1037 - cost: 9.94, layer: 52
Fold over basal
Time (min)
1.6
1.5
1.4
1.3
1.2
1.1
1
0.9
0.8
0.7
0
10
20
30
40
50
60
Normal
LY50
LY75
LY88
LY94
LY97
LY data
Normal data

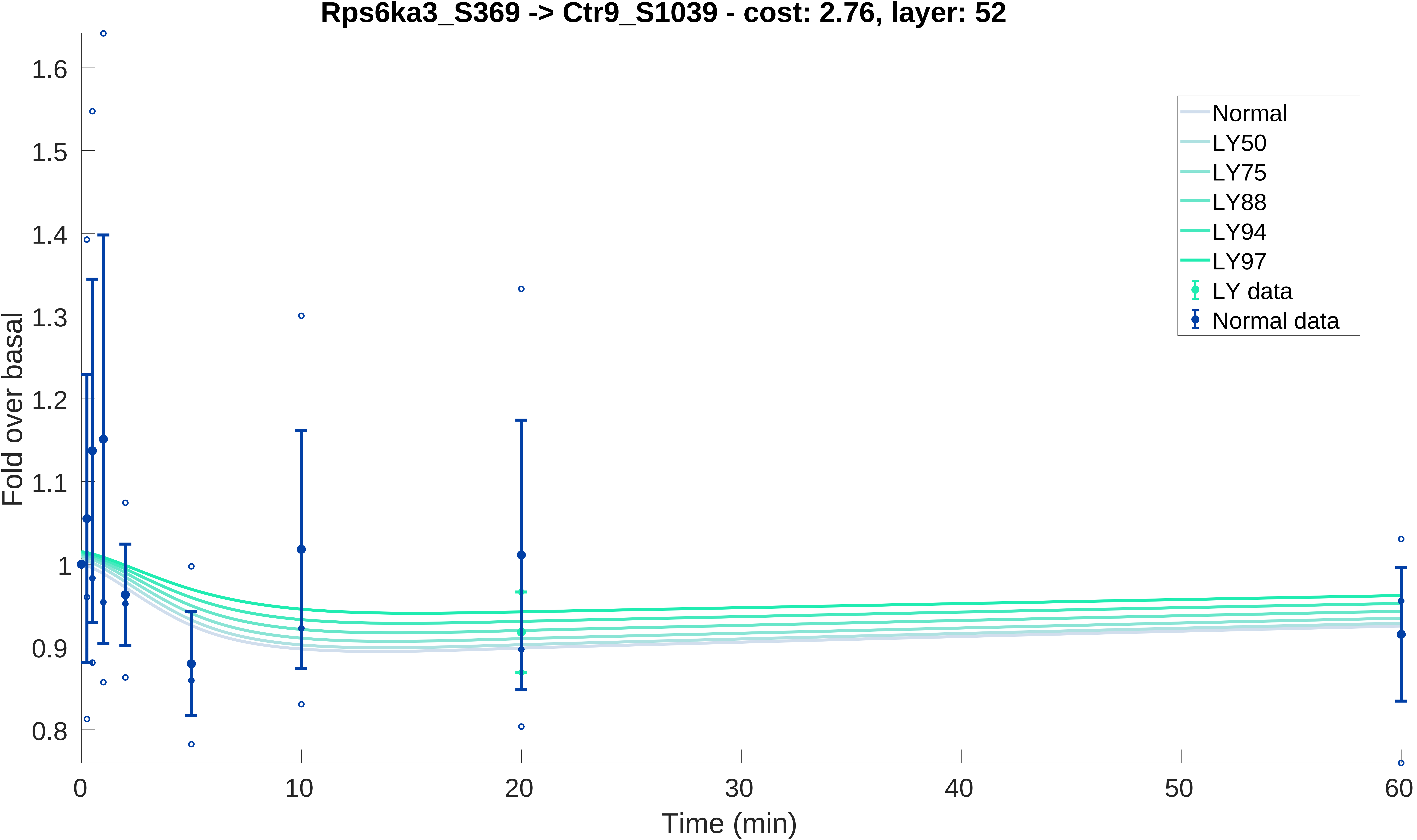

Rps6ka3_S369 -> Ctr9_S1039 - cost: 2.76, layer: 52
Fold over basal
Time (min)
0.8
0.9
1
1.1
1.2
1.3
1.4
1.5
1.6
0
10
20
30
40
50
60
Normal
LY50
LY75
LY88
LY94
LY97
LY data
Normal data

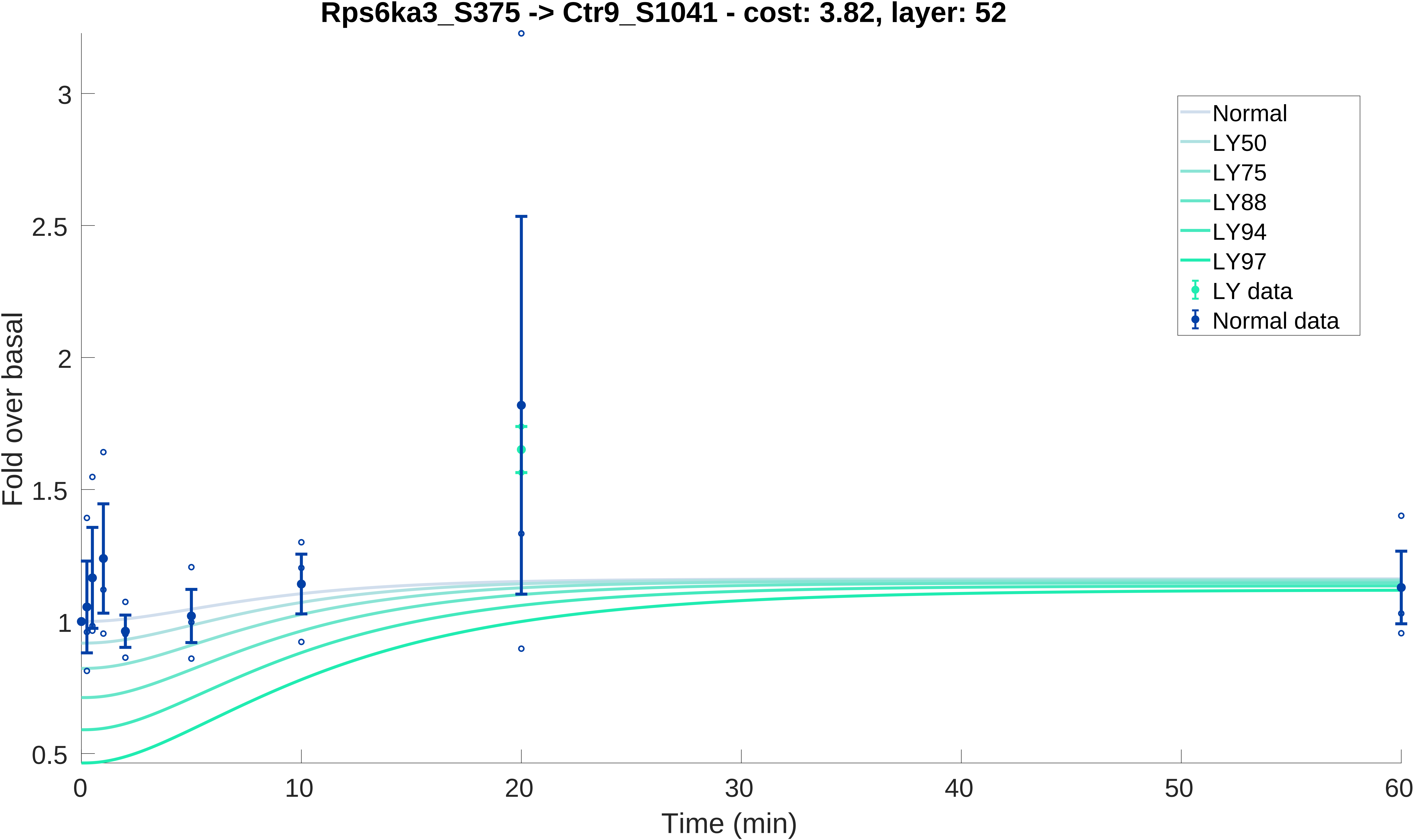

Rps6ka3_S375 -> Ctr9_S1041 - cost: 3.82, layer: 52
Fold over basal
Time (min)
0.5
1
1.5
2
2.5
3
0
10
20
30
40
50
60
Normal
LY50
LY75
LY88
LY94
LY97
LY data
Normal data

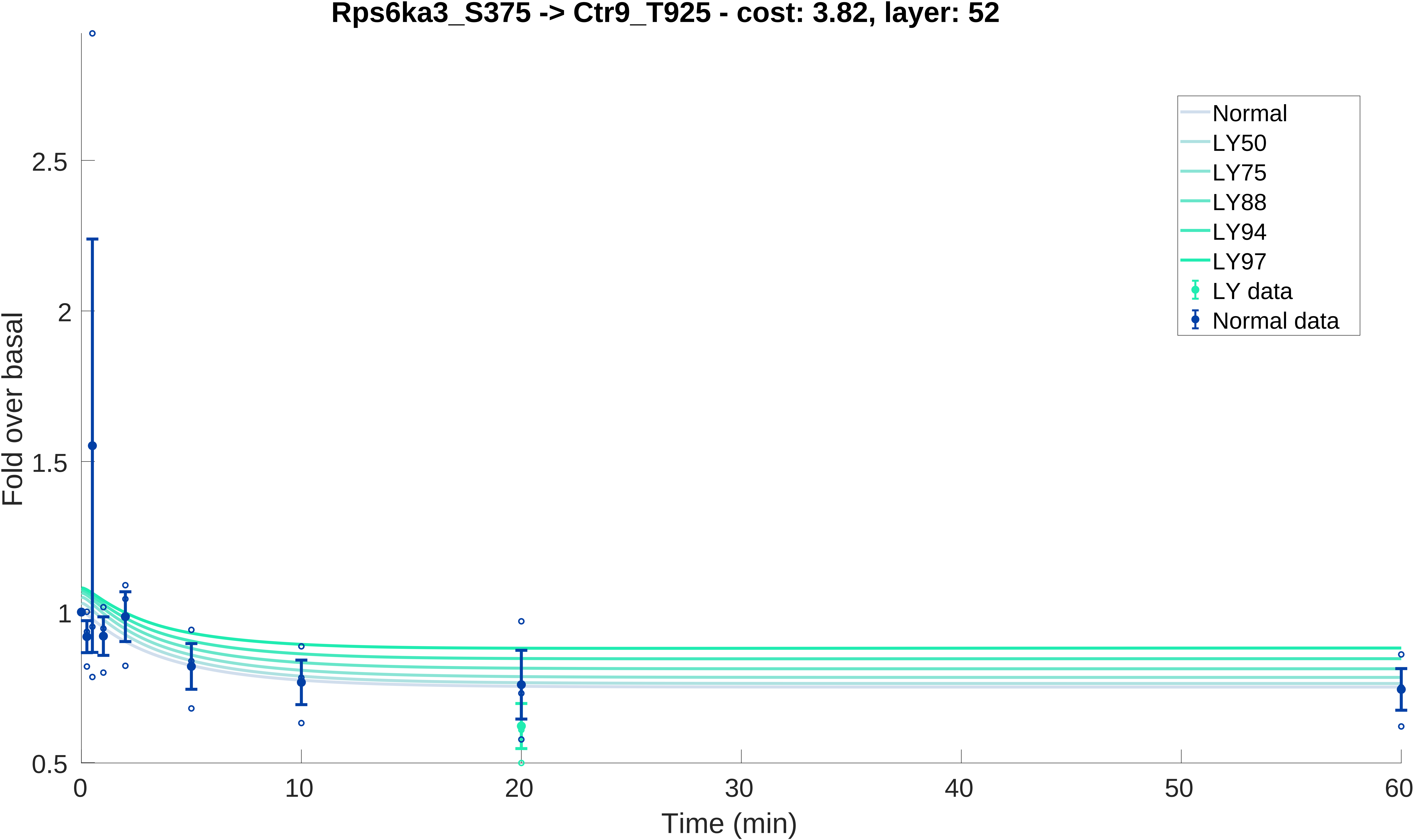
Rps6ka3_S375 -> Ctr9_T925 - cost: 3.82, layer: 52
Fold over basal
Time (min)
0.5
1
1.5
2
2.5
0
10
20
30
40
50
60
Normal
LY50
LY75
LY88
LY94
LY97
LY data
Normal data

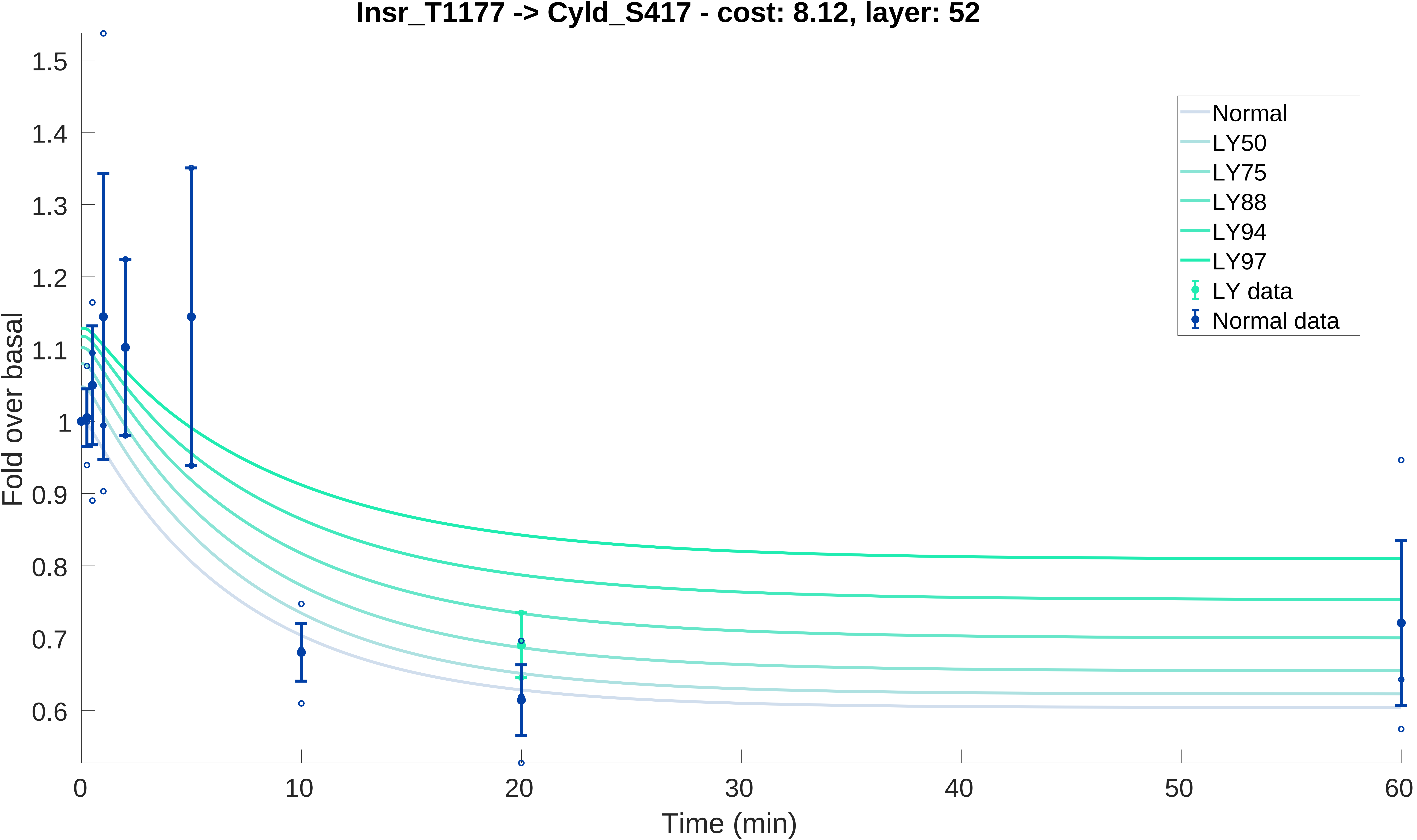

Insr_T1177 -> Cyld_S417 - cost: 8.12, layer: 52
Fold over basal
1.5
1.4
1.3
1.2
1.1
1
0.9
0.8
0.7
0.6
0
10
20
30
40
50
60
Time (min)
Normal
LY50
LY75
LY88
LY94
LY97
LY data
Normal data

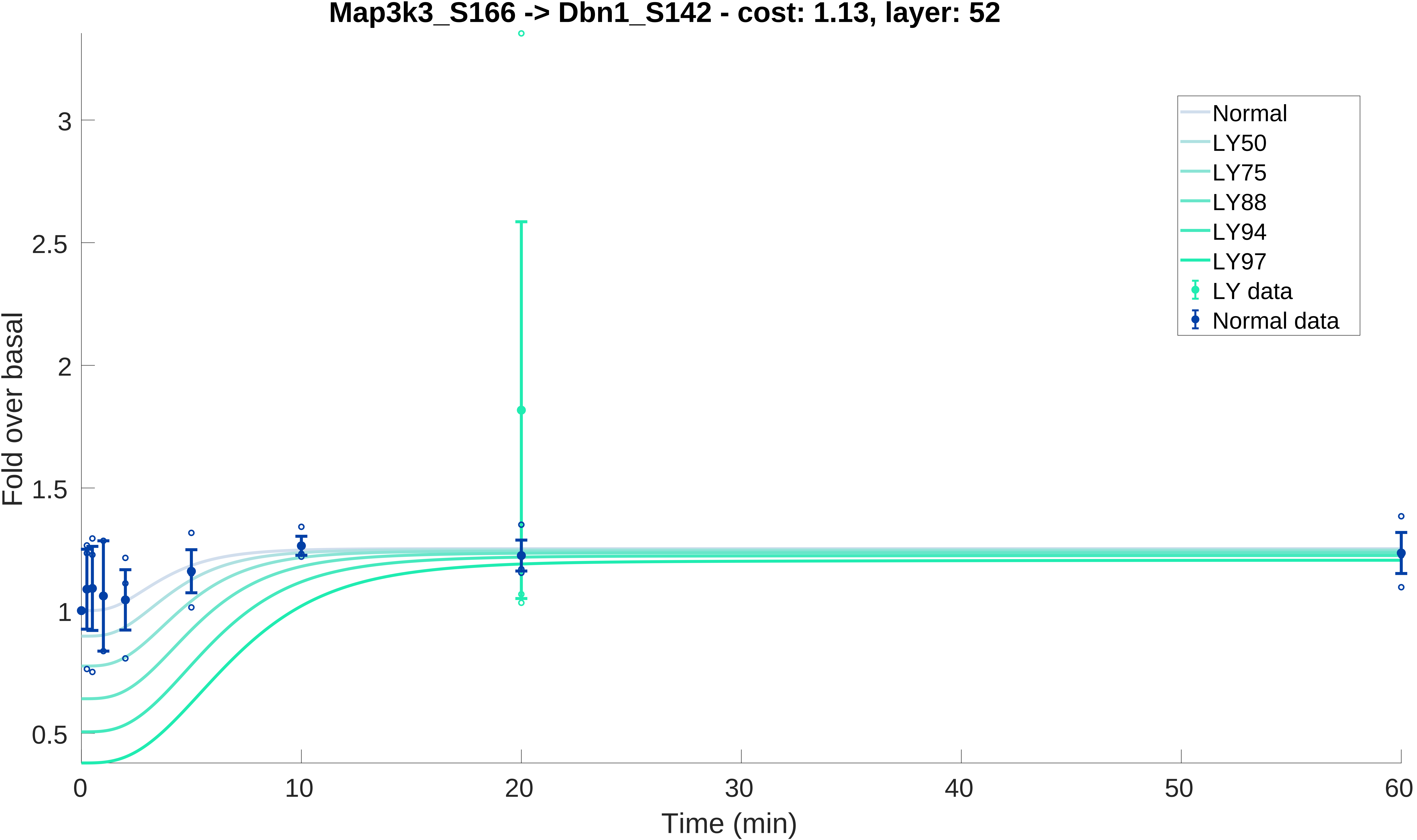

Map3k3_S166 -> Dbn1_S142 - cost: 1.13, layer: 52
Fold over basal
Time (min)
0.5
1
1.5
2
2.5
3
0
10
20
30
40
50
60
Normal
LY50
LY75
LY88
LY94
LY97
LY data
Normal data

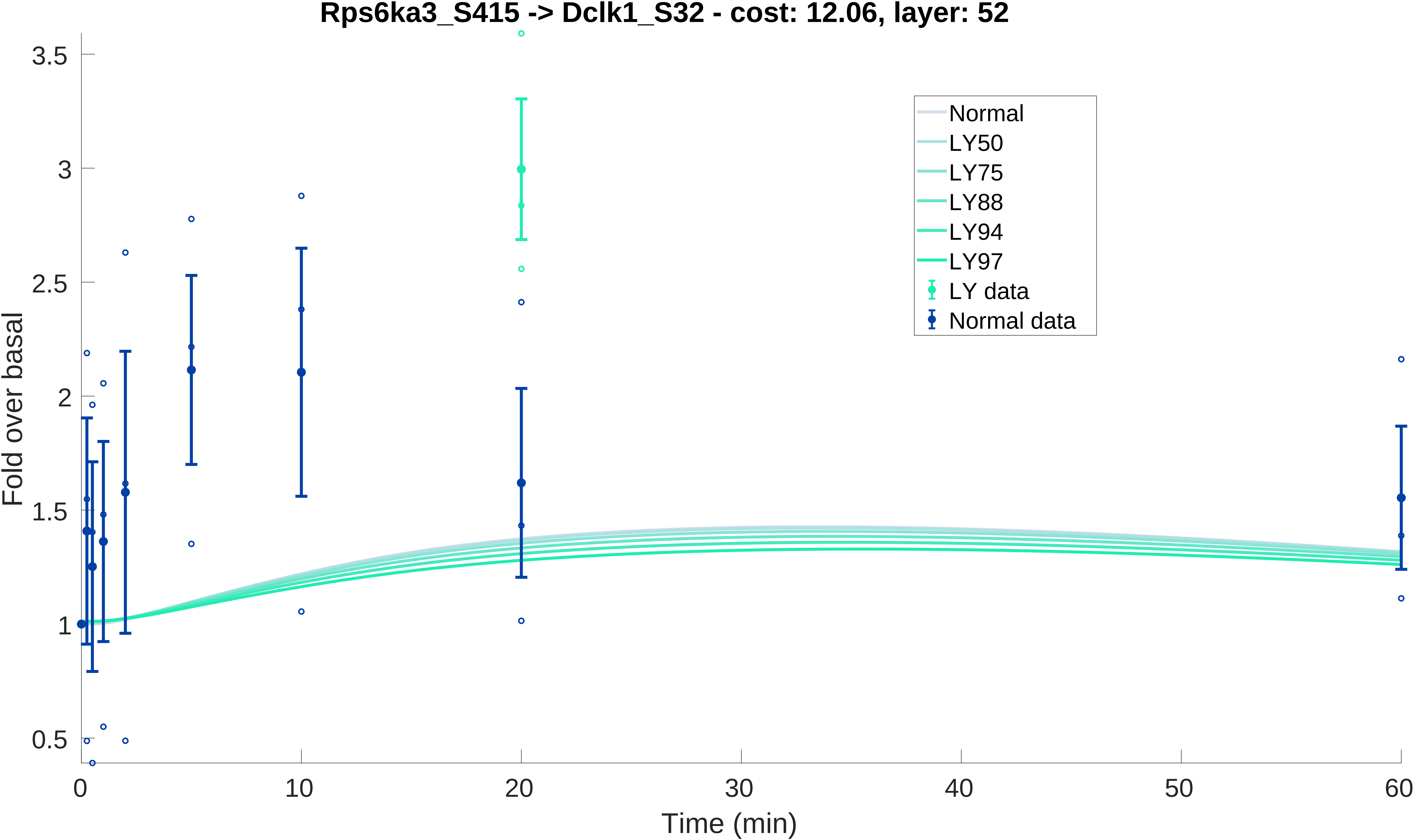

Rps6ka3_S415 -> Dclk1_S32 - cost: 12.06, layer: 52
Fold over basal
Time (min)
0.5
1
1.5
2
2.5
3
3.5
0
10
20
30
40
50
60
Normal
LY50
LY75
LY88
LY94
LY97
LY data
Normal data

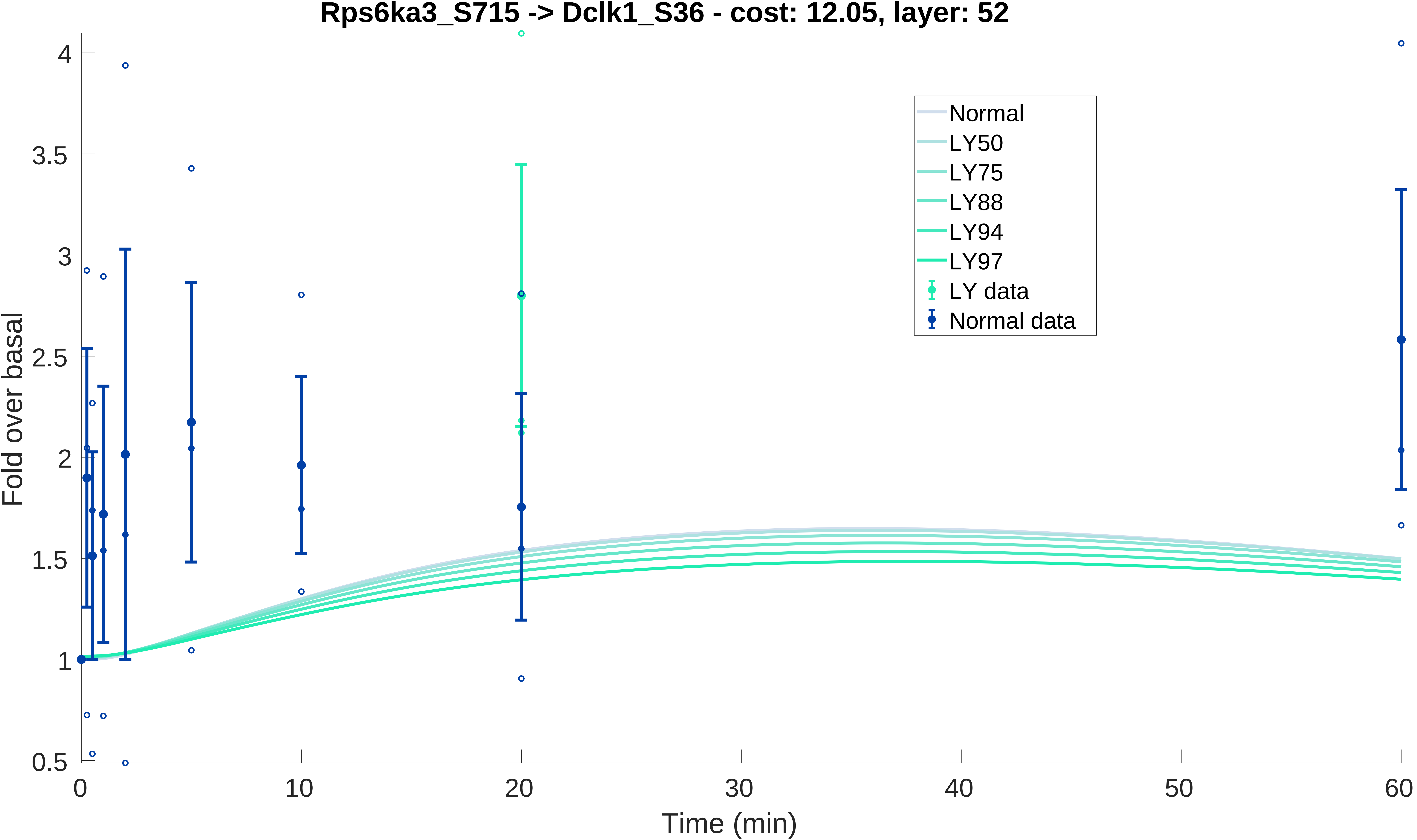

Rps6ka3_S715 -> Dclk1_S36 - cost: 12.05, layer: 52
Fold over basal
Time (min)
0.5
1
1.5
2
2.5
3
3.5
4
0
10
20
30
40
50
60
Normal
LY50
LY75
LY88
LY94
LY97
LY data
Normal data

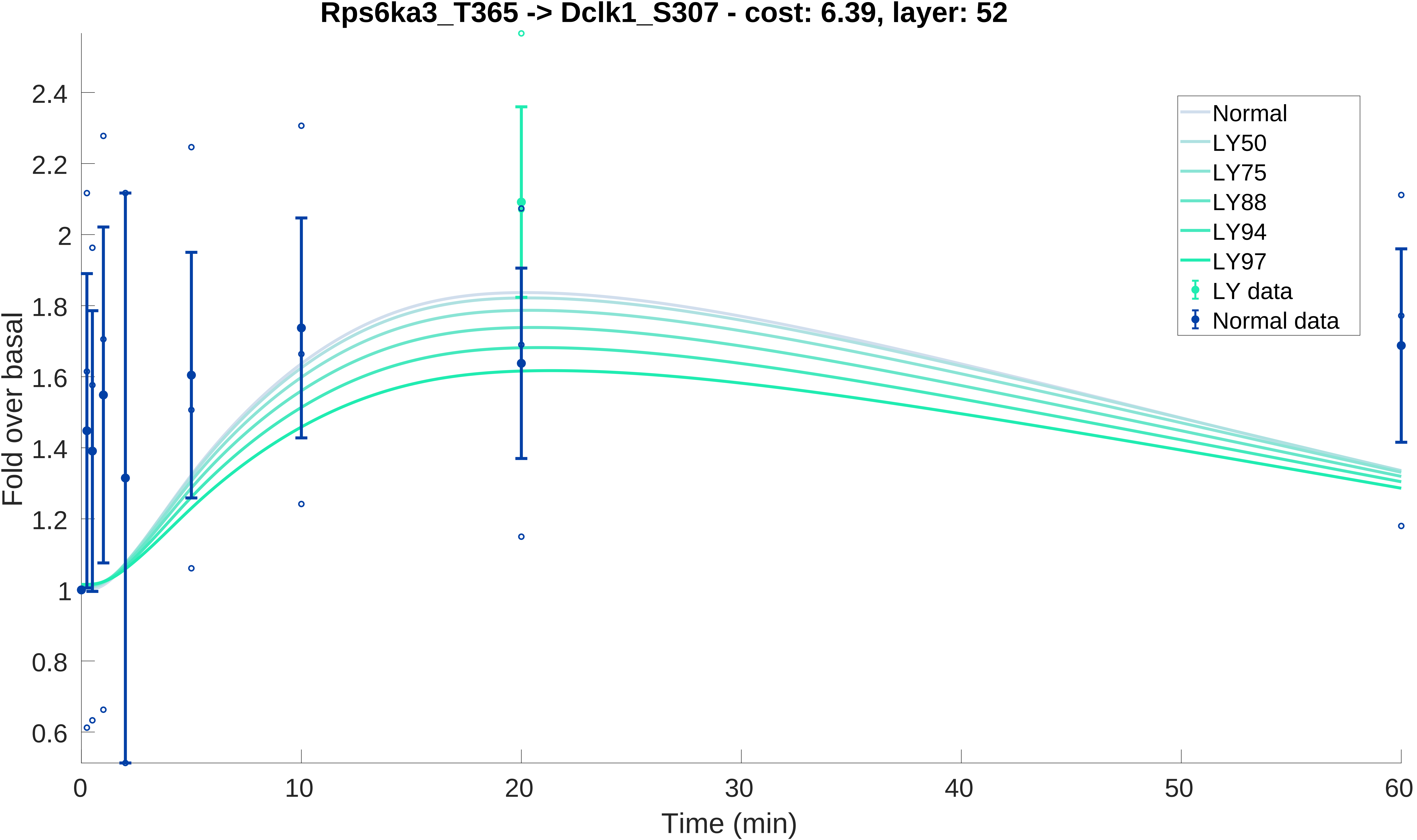
Rps6ka3_T365 -> Dclk1_S307 - cost: 6.39, layer: 52
Fold over basal
Time (min)
Normal
LY50
LY75
LY88
LY94
LY97
LY data
Normal data
0.6
0.8
1
1.2
1.4
1.6
1.8
2
2.2
2.4
0
10
20
30
40
50
60

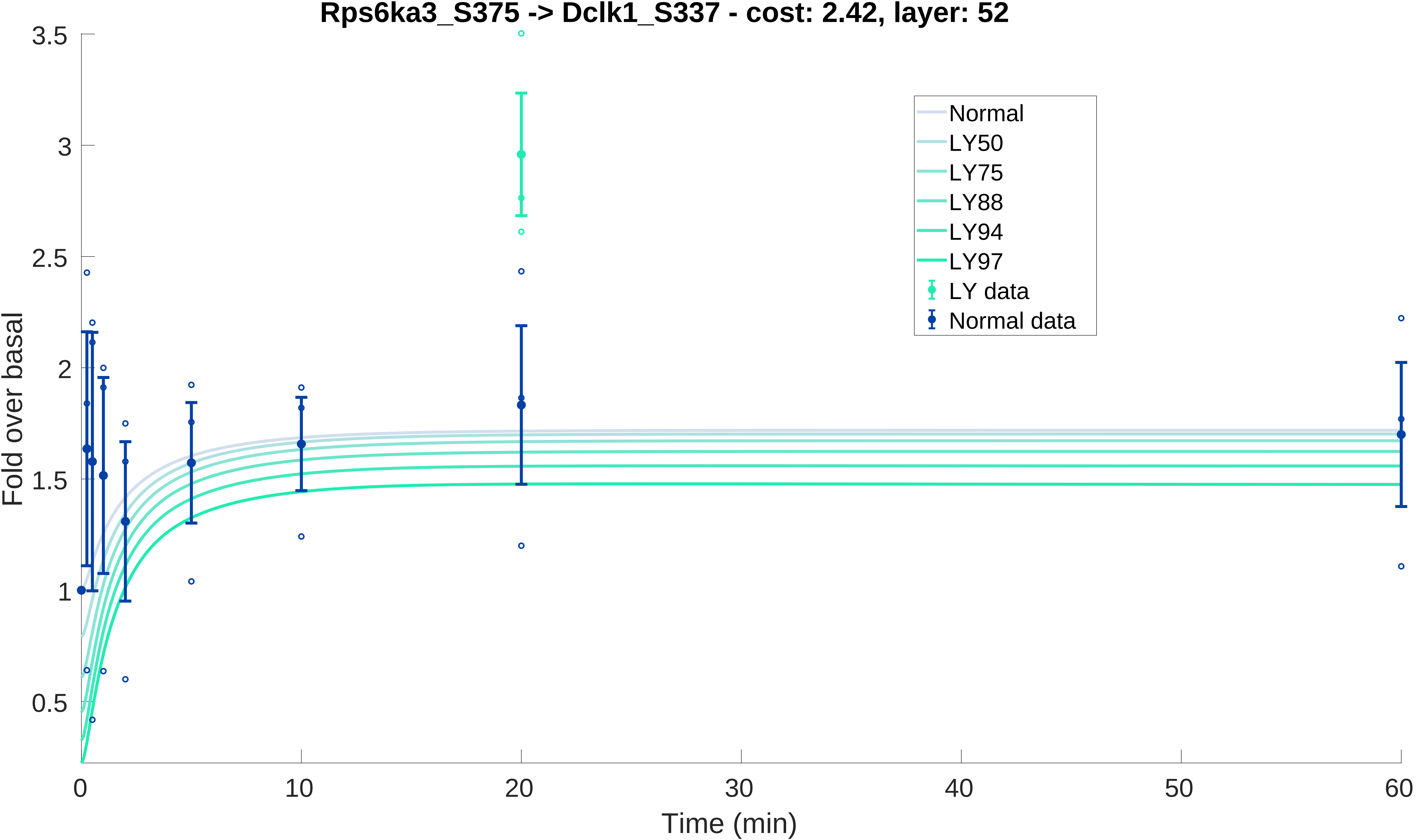

Rps6ka3_S375 -> Dclk1_S337 - cost: 2.42, layer: 52
Fold over basal
Time (min)
3.5
3
2.5
2
1.5
1
0.5
0
10
20
30
40
50
60
Normal
LY50
LY75
LY88
LY94
LY97
LY data
Normal data

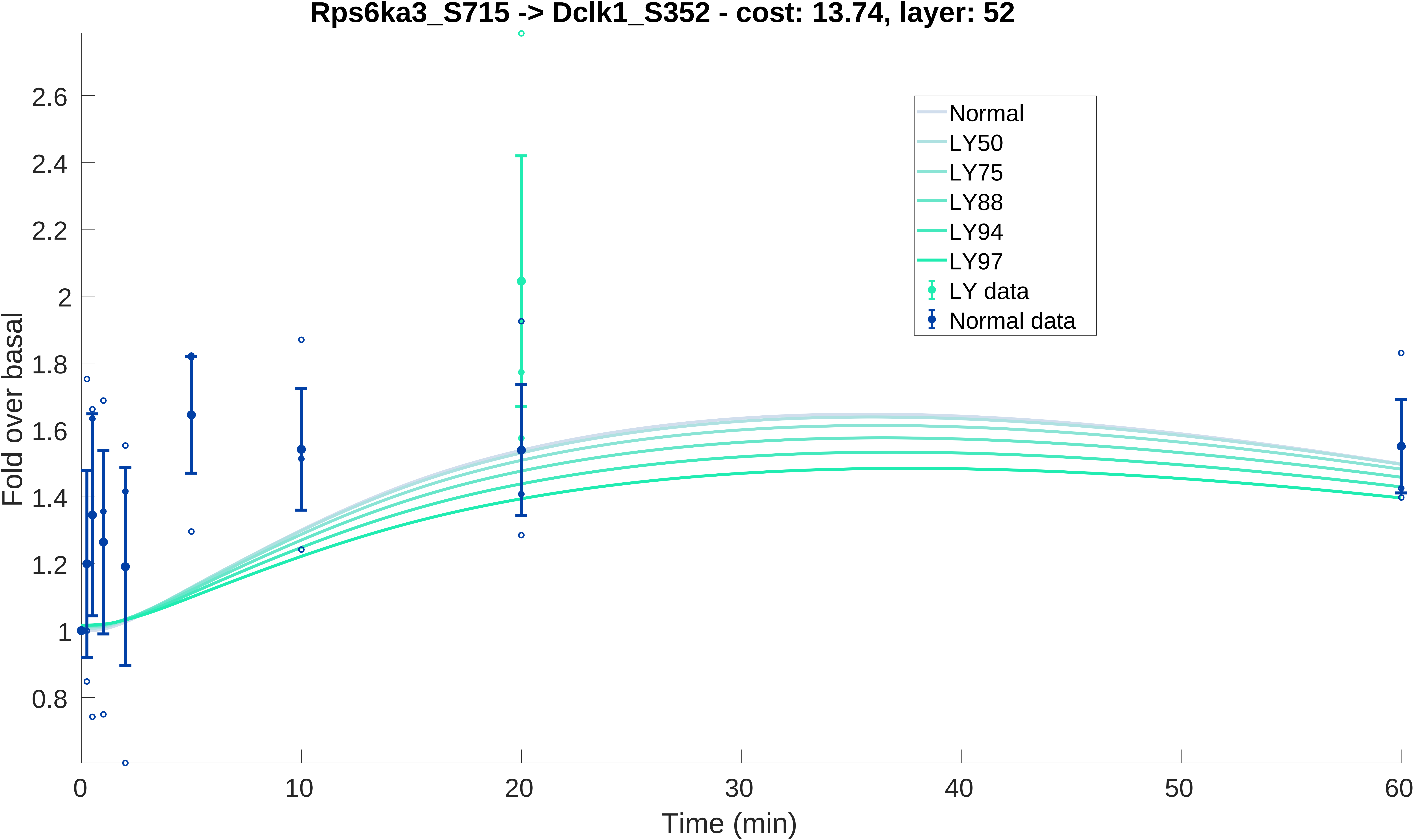

Rps6ka3_S715 -> Dclk1_S352 - cost: 13.74, layer: 52
Normal
LY50
LY75
LY88
LY94
LY97
LY data
Normal data
Fold over basal
Time (min)
0
10
20
30
40
50
60
0.8
1
1.2
1.4
1.6
1.8
2
2.2
2.4
2.6

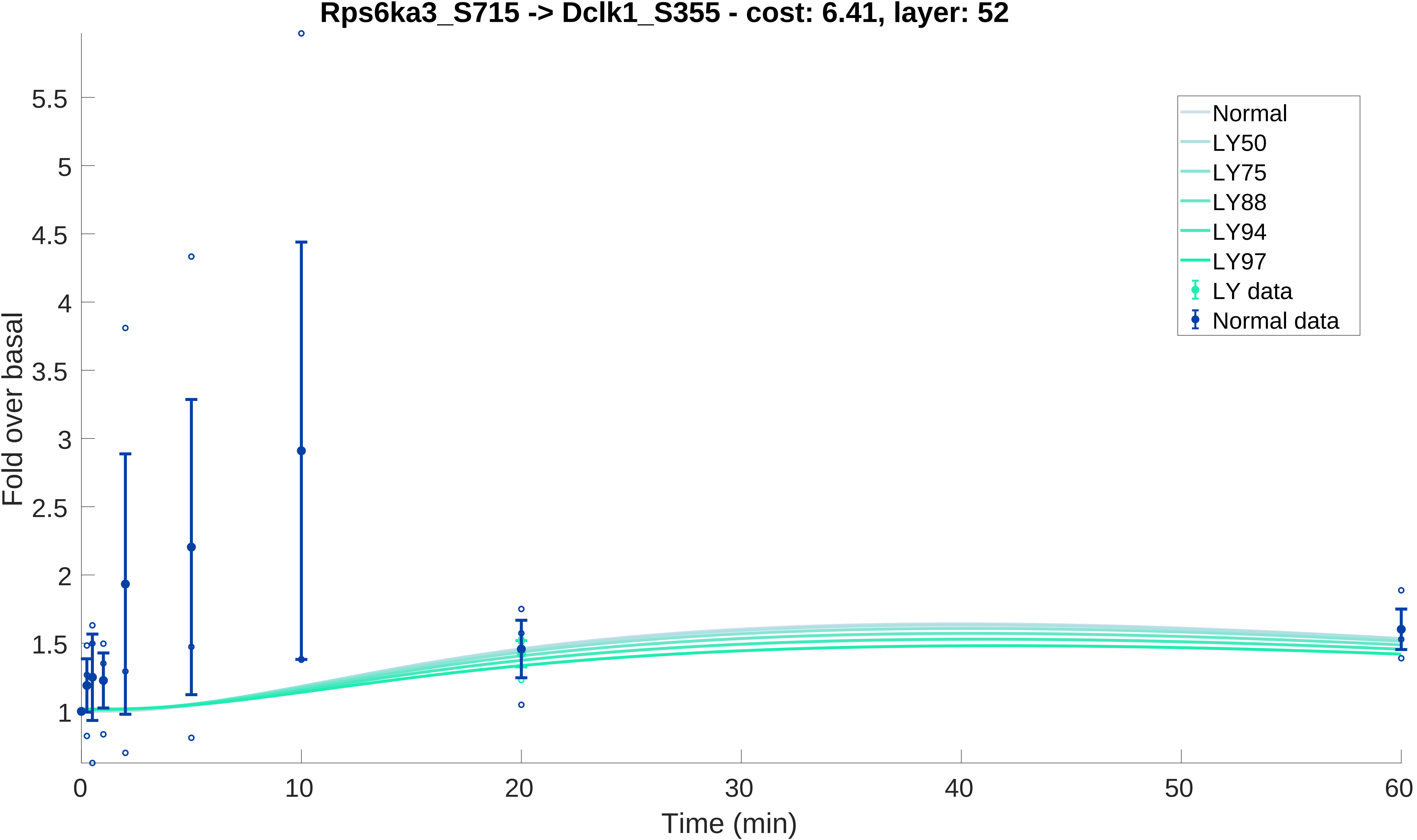

Rps6ka3_S715 -> Dclk1_S355 - cost: 6.41, layer: 52
Fold over basal
Time (min)
5.5
5
4.5
4
3.5
3
2.5
2
1.5
1
0
10
20
30
40
50
60
Normal
LY50
LY75
LY88
LY94
LY97
LY data
Normal data

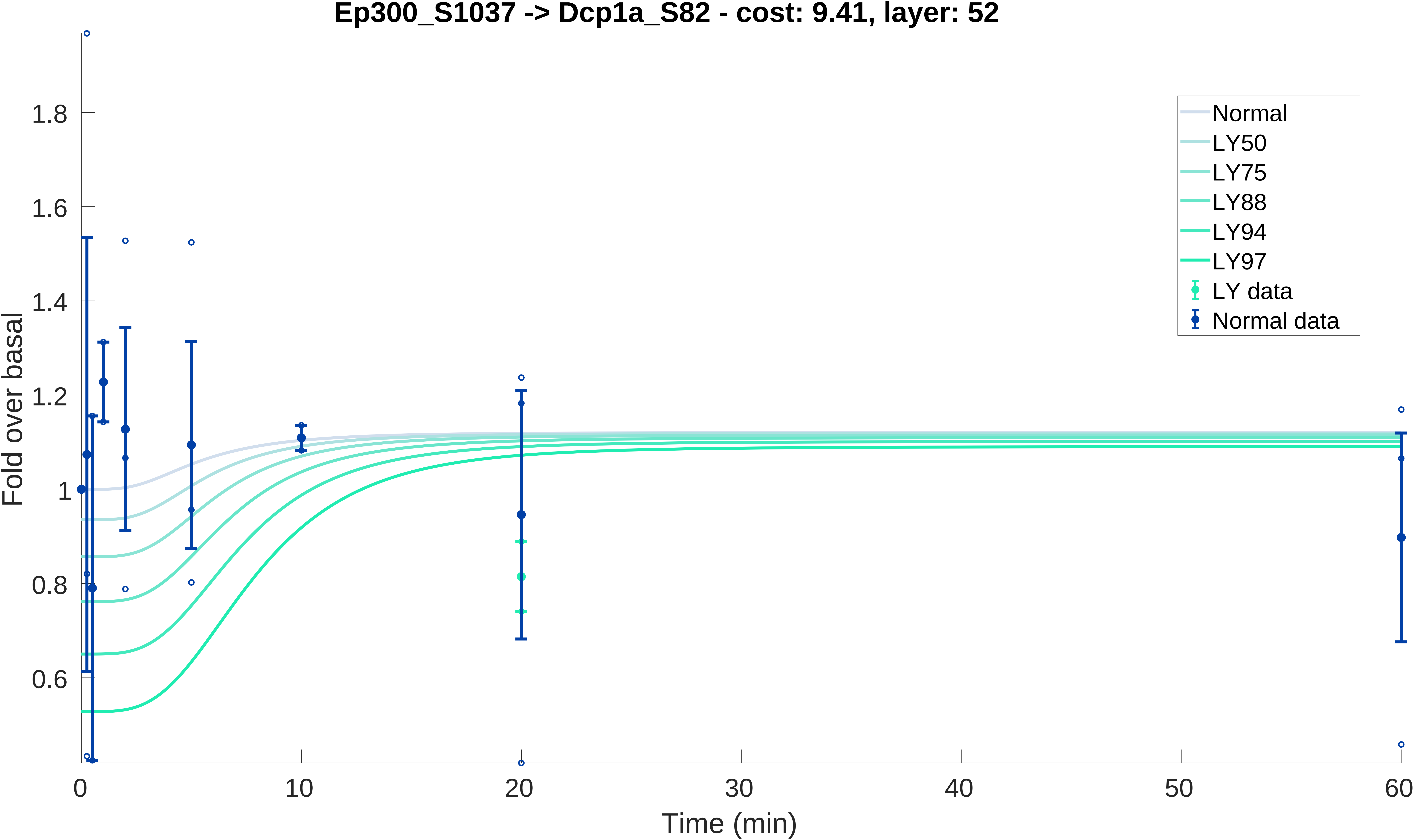
Ep300_S1037 -> Dcp1a_S82 - cost: 9.41, layer: 52
Fold over basal
Time (min)
0.6
0.8
1
1.2
1.4
1.6
1.8
0
10
20
30
40
50
60
Normal
LY50
LY75
LY88
LY94
LY97
LY data
Normal data

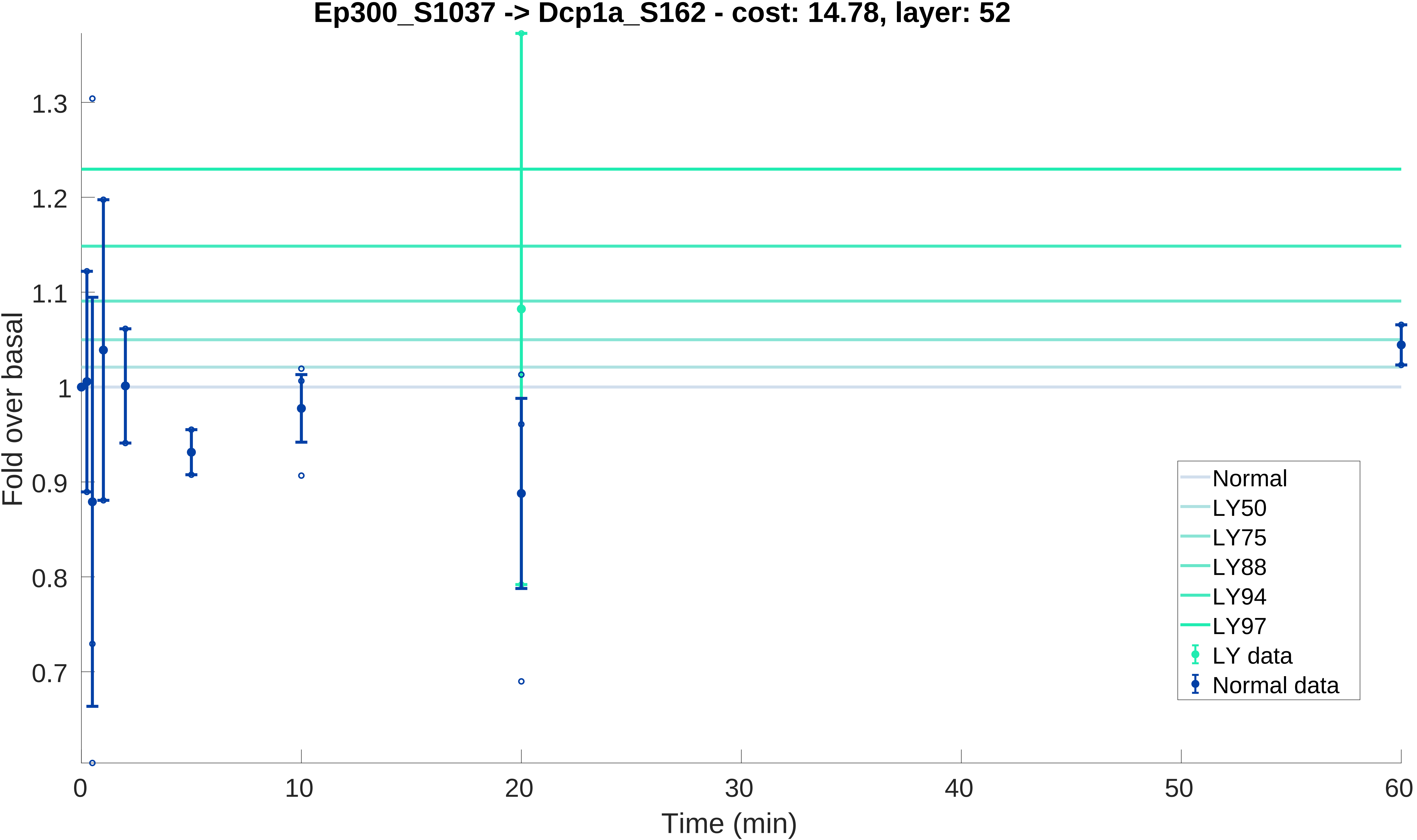

Ep300_S1037 -> Dcp1a_S162 - cost: 14.78, layer: 52
Fold over basal
1.3
1.2
1.1
1
0.9
0.8
0.7
0
10
20
30
40
50
60
Time (min)
Normal
LY50
LY75
LY88
LY94
LY97
LY data
Normal data

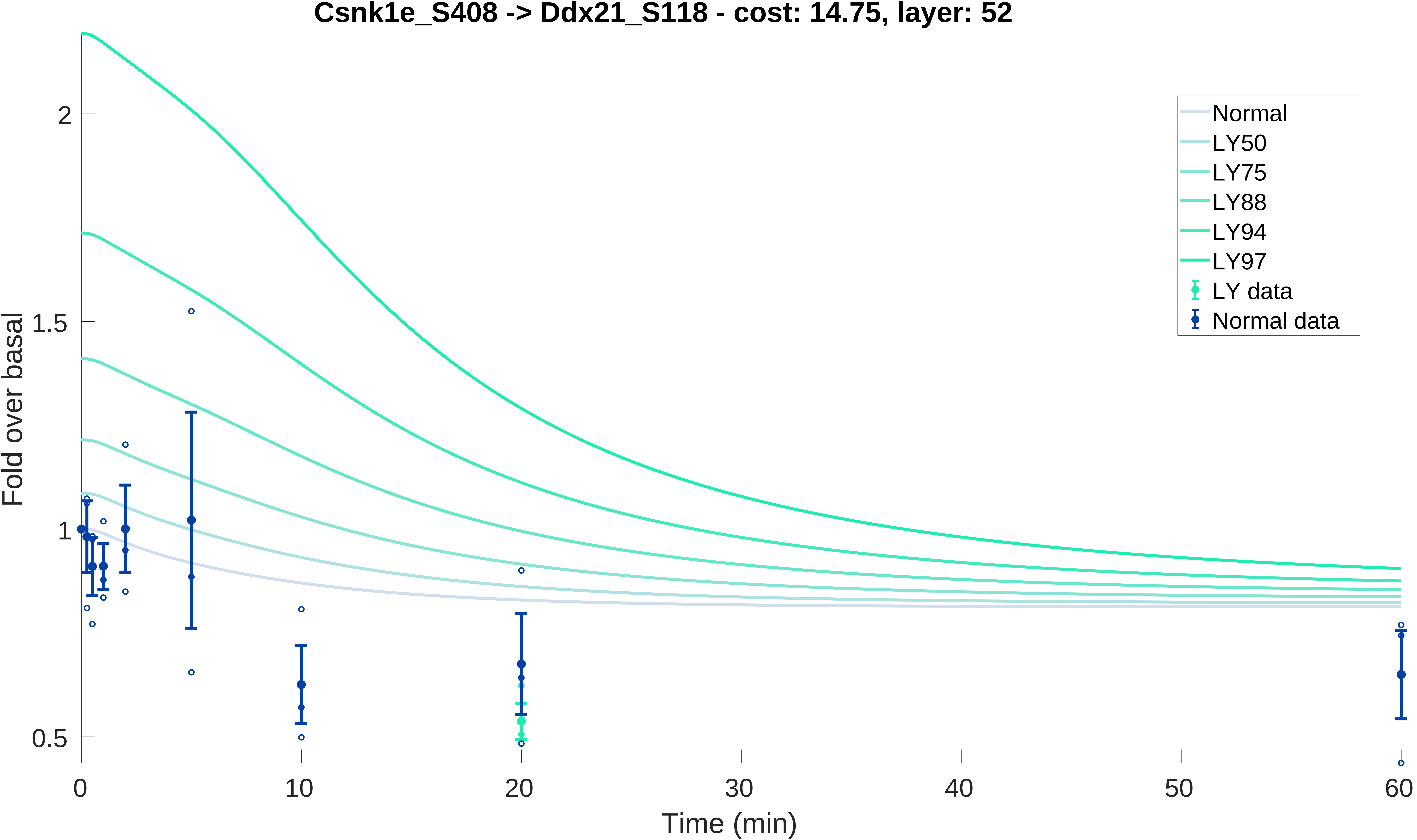

Csnk1e_S408 -> Ddx21_S118 - cost: 14.75, layer: 52
Fold over basal
Time (min)
0.5
1
1.5
2
0
10
20
30
40
50
60
Normal
LY50
LY75
LY88
LY94
LY97
LY data
Normal data

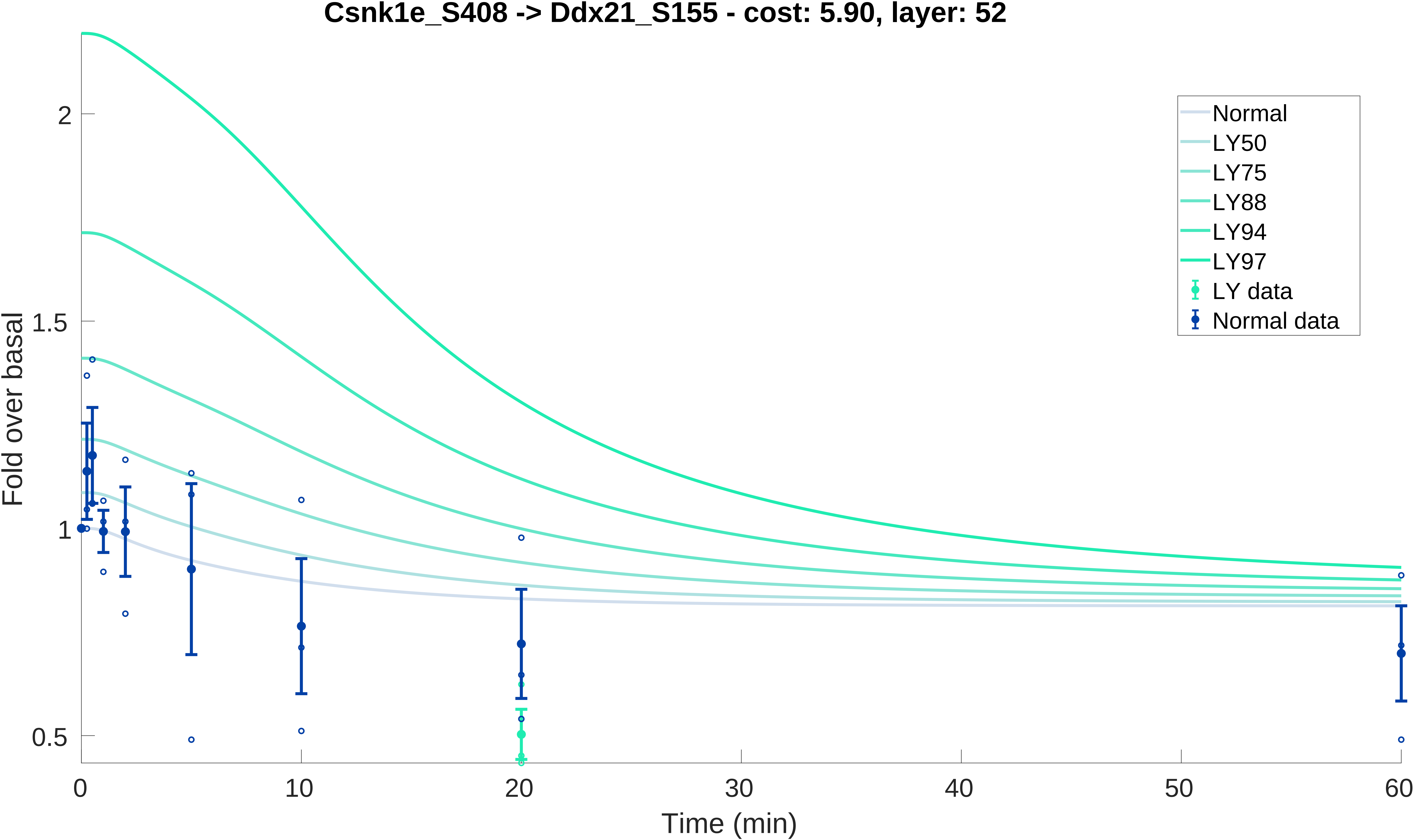

Csnk1e_S408 -> Ddx21_S155 - cost: 5.90, layer: 52
Fold over basal
2
1.5
1
0.5
0
10
20
30
40
50
60
Time (min)
Normal
LY50
LY75
LY88
LY94
LY97
LY data
Normal data

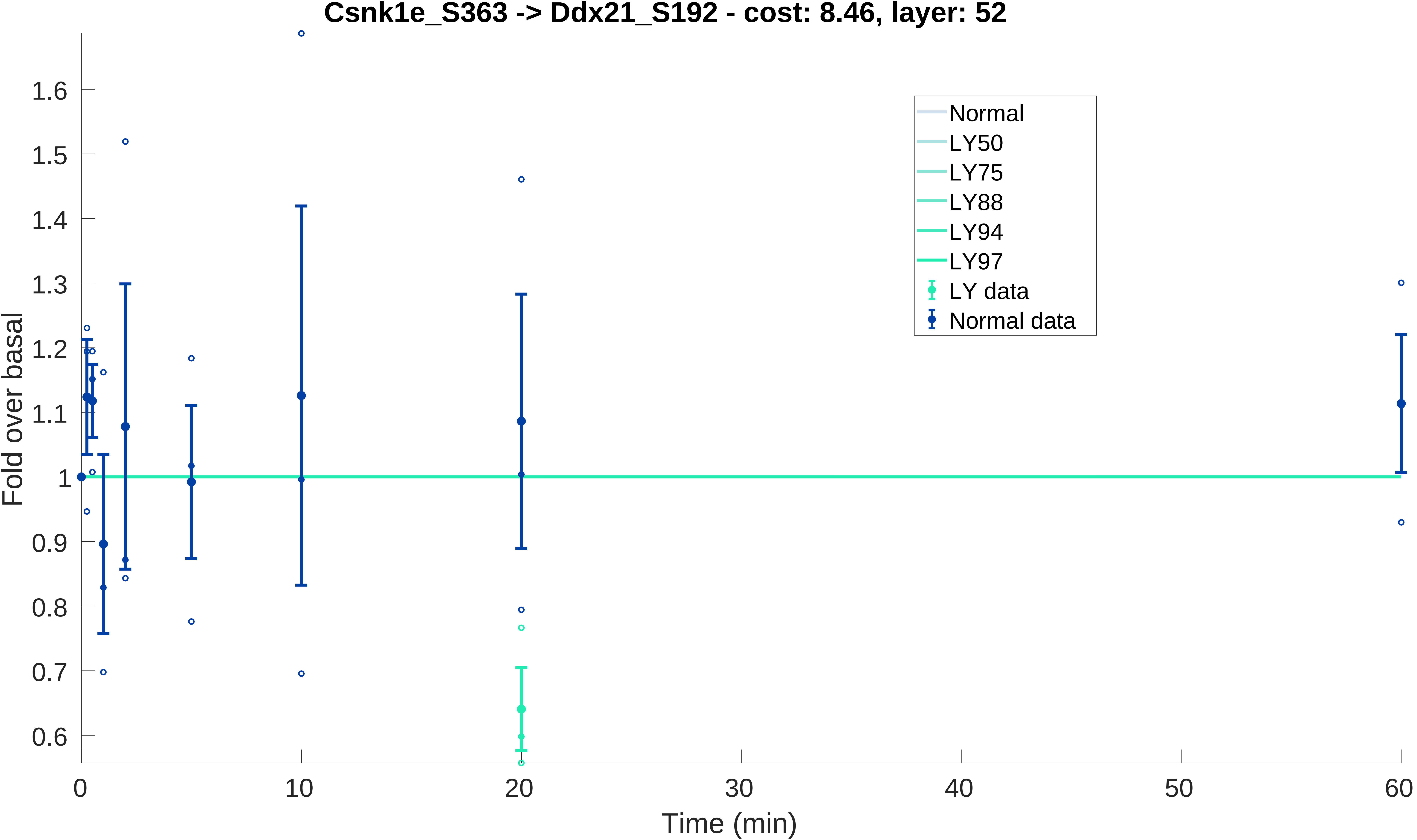

Csnk1e_S363 -> Ddx21_S192 - cost: 8.46, layer: 52
Fold over basal
Time (min)
1.6
1.5
1.4
1.3
1.2
1.1
1
0.9
0.8
0.7
0.6
0
10
20
30
40
50
60
Normal
LY50
LY75
LY88
LY94
LY97
LY data
Normal data

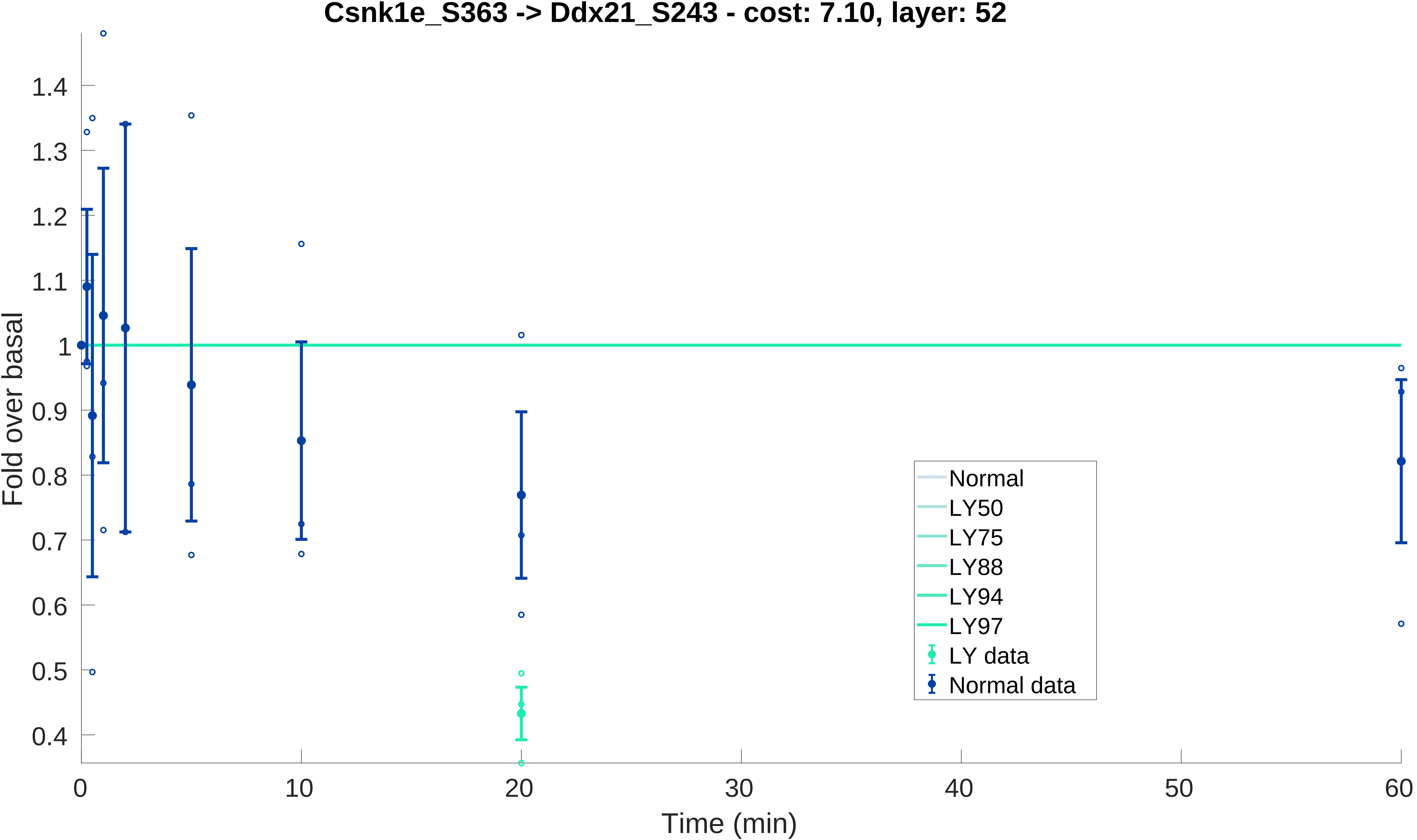

Csnk1e_S363 -> Ddx21_S243 - cost: 7.10, layer: 52
Fold over basal
1.4
1.3
1.2
1.1
1
0.9
0.8
0.7
0.6
0.5
0.4
0
10
20
30
40
50
60
Time (min)
Normal
LY50
LY75
LY88
LY94
LY97
LY data
Normal data

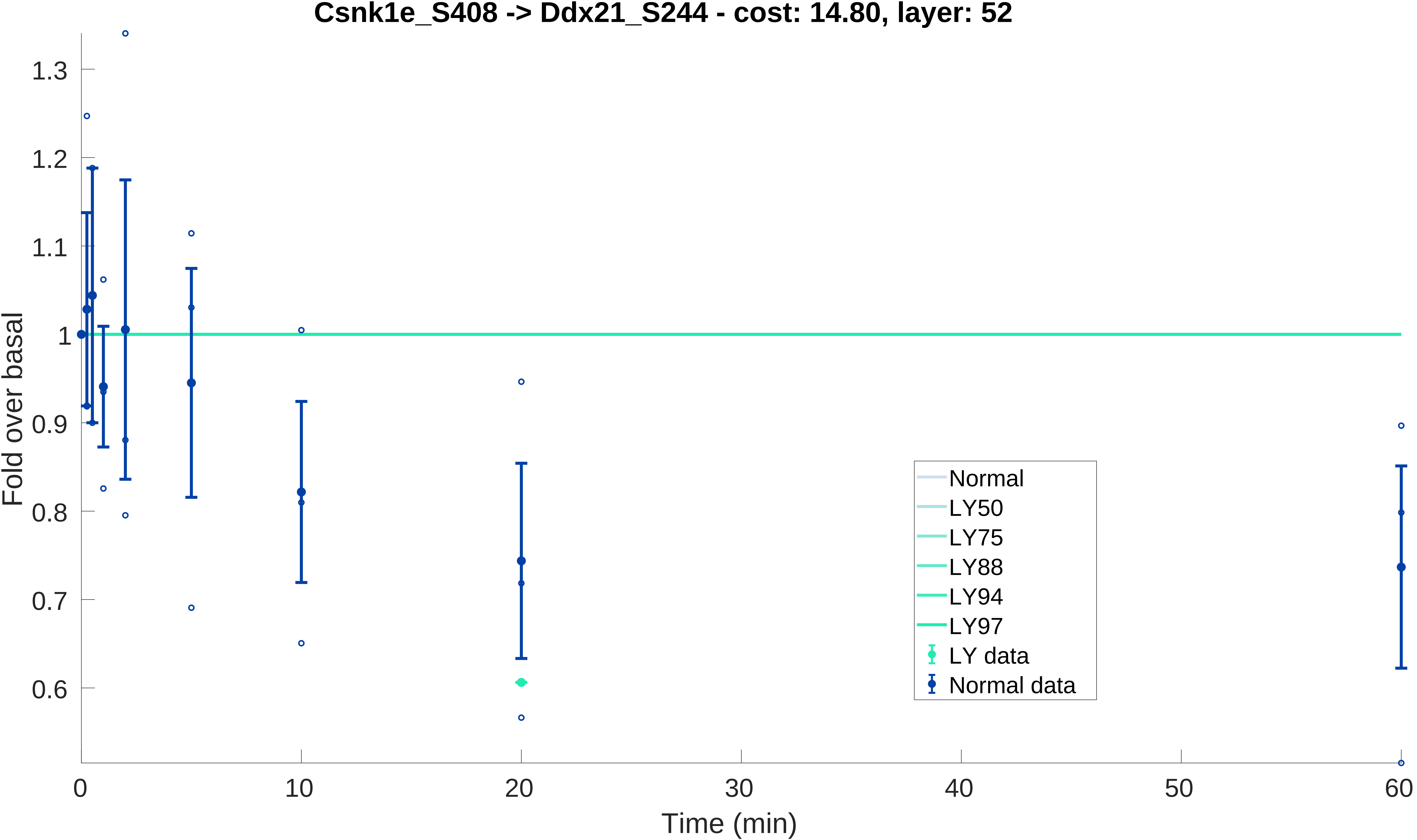

Csnk1e_S408 -> Ddx21_S244 - cost: 14.80, layer: 52
Fold over basal
1.3
1.2
1.1
1
0.9
0.8
0.7
0.6
0
10
20
30
40
50
60
Time (min)
Normal
LY50
LY75
LY88
LY94
LY97
LY data
Normal data

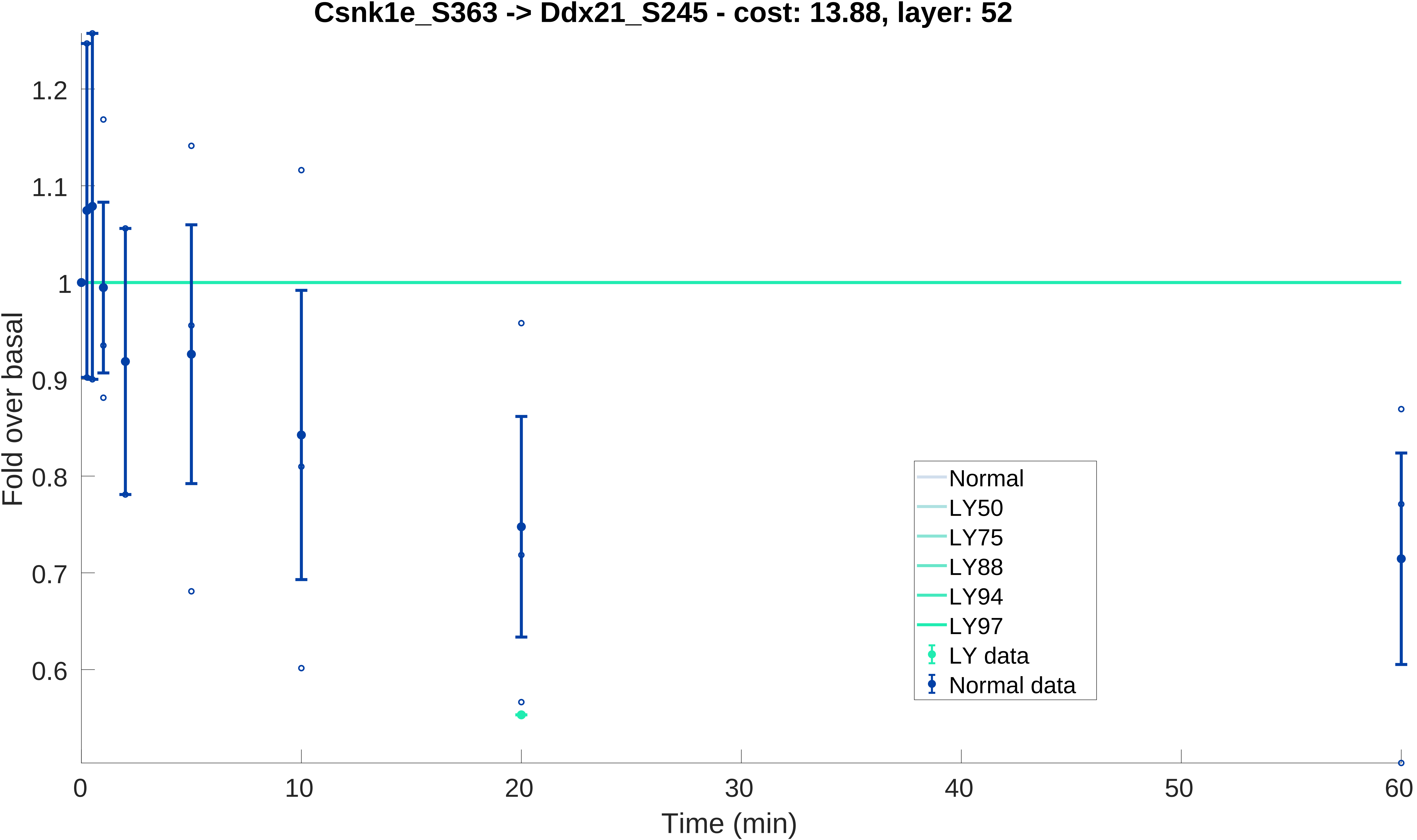

Csnk1e_S363 -> Ddx21_S245 - cost: 13.88, layer: 52
Fold over basal
1.2
1.1
1
0.9
0.8
0.7
0.6
0
10
20
30
40
50
60
Time (min)
Normal
LY50
LY75
LY88
LY94
LY97
LY data
Normal data

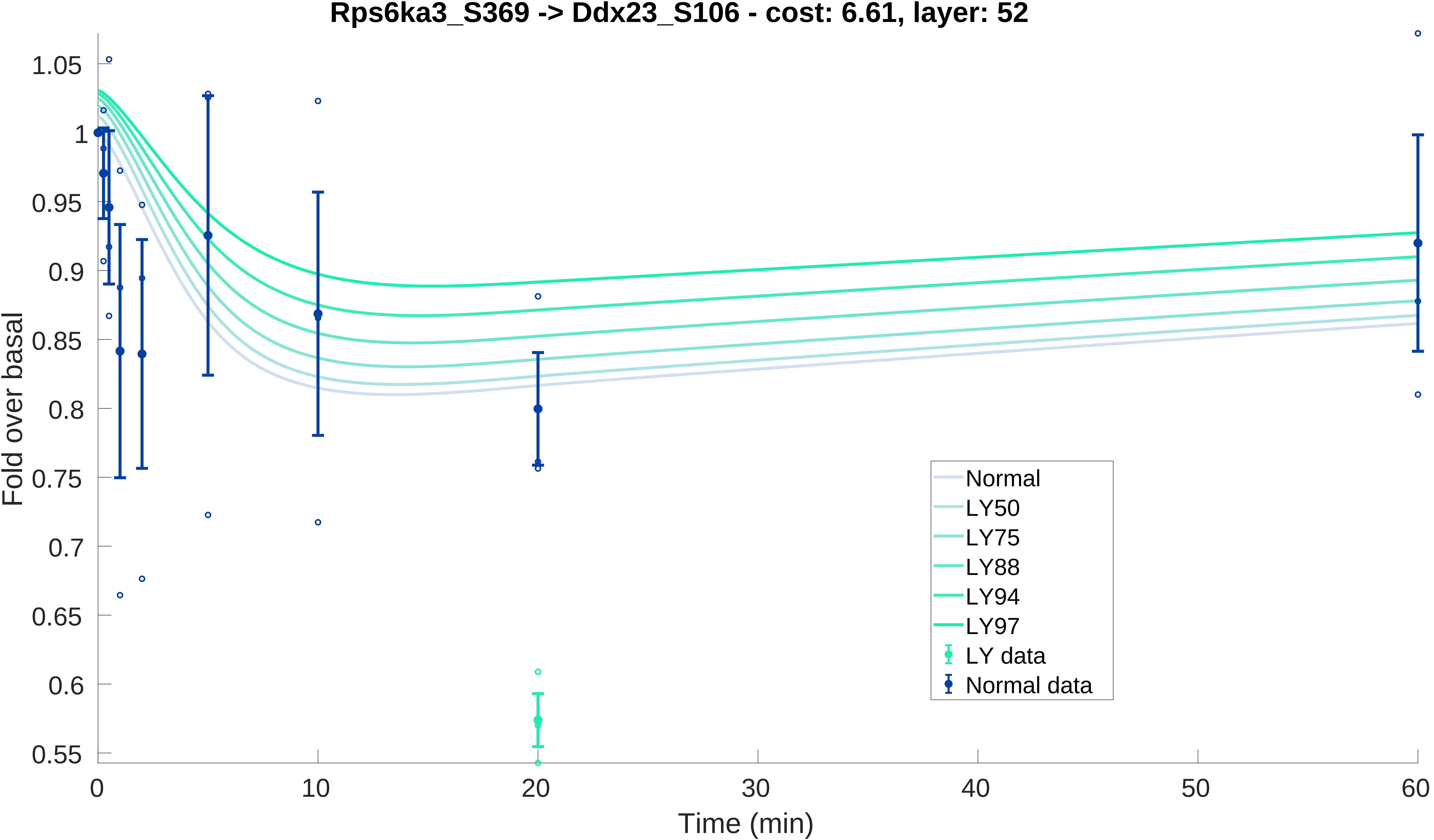

Rps6ka3_S369 -> Ddx23_S106 - cost: 6.61, layer: 52
Fold over basal
1.05
1
0.95
0.9
0.85
0.8
0.75
0.7
0.65
0.6
0.55
0
10
20
30
40
50
60
Time (min)
Normal
LY50
LY75
LY88
LY94
LY97
LY data
Normal data

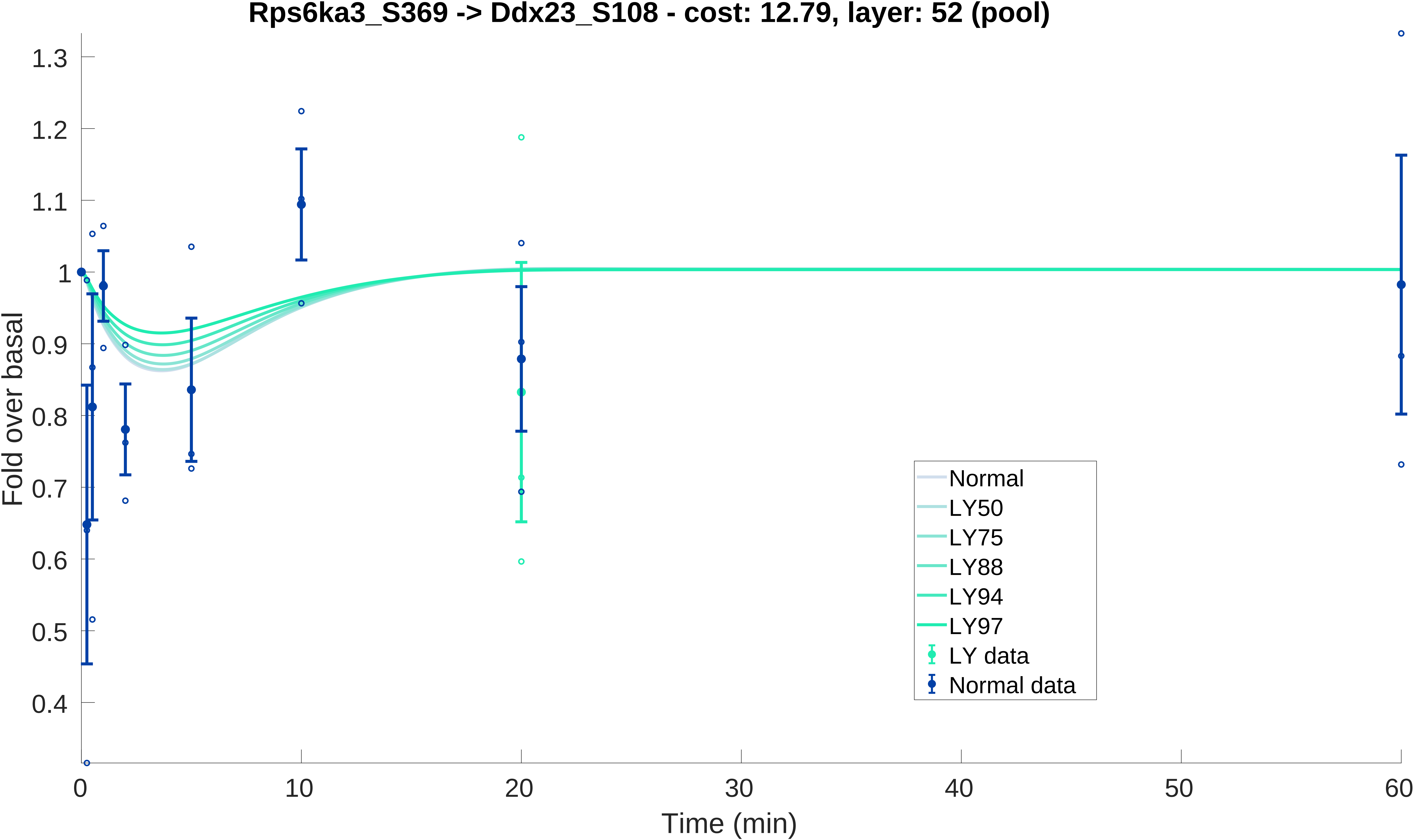
Rps6ka3_S369 -> Ddx23_S108 - cost: 12.79, layer: 52 (pool)
Fold over basal
Time (min)
1.3
1.2
1.1
1
0.9
0.8
0.7
0.6
0.5
0.4
0
10
20
30
40
50
60
Normal
LY50
LY75
LY88
LY94
LY97
LY data
Normal data

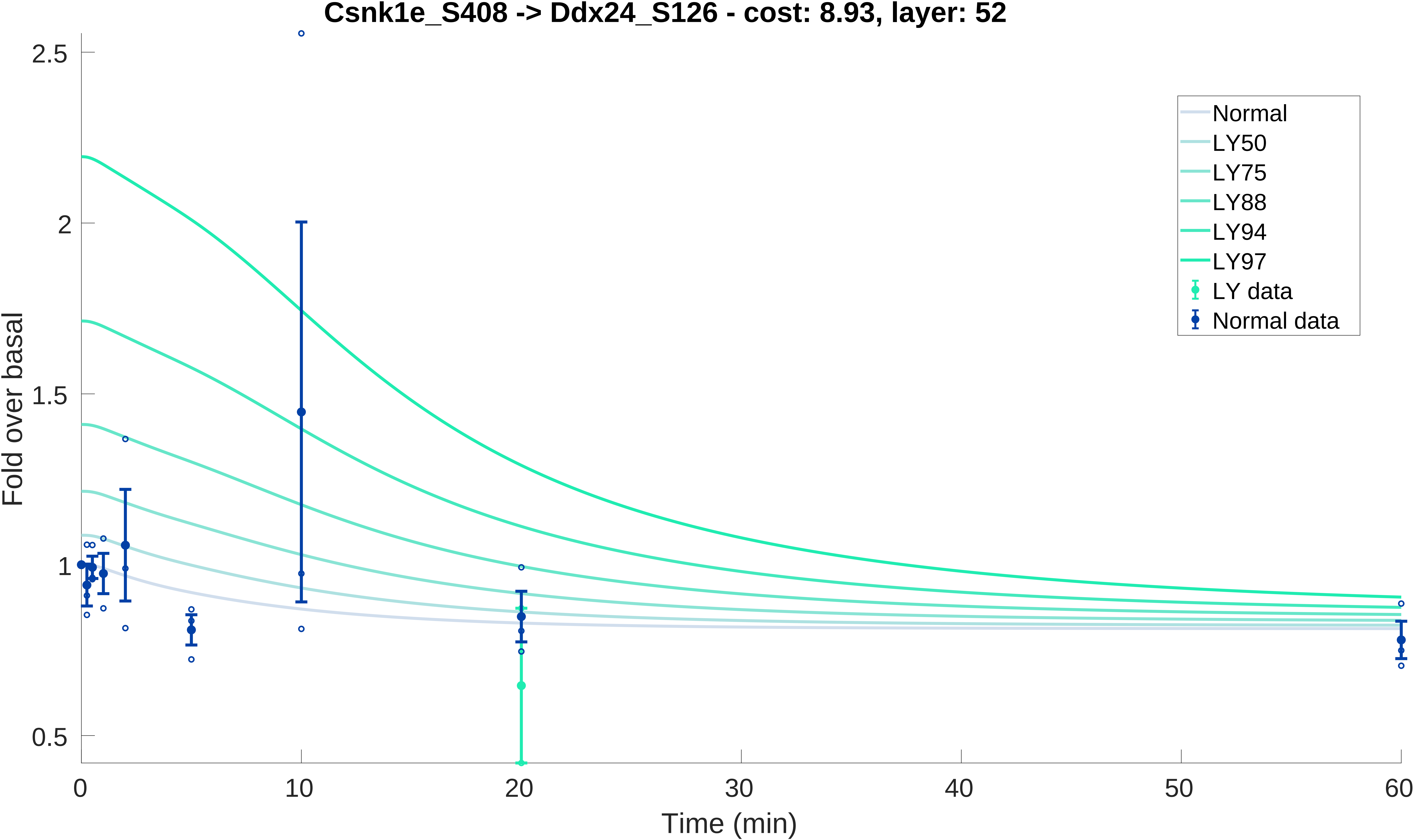

Csnk1e_S408 -> Ddx24_S126 - cost: 8.93, layer: 52
Fold over basal
Time (min)
0.5
1
1.5
2
2.5
0
10
20
30
40
50
60
Normal
LY50
LY75
LY88
LY94
LY97
LY data
Normal data

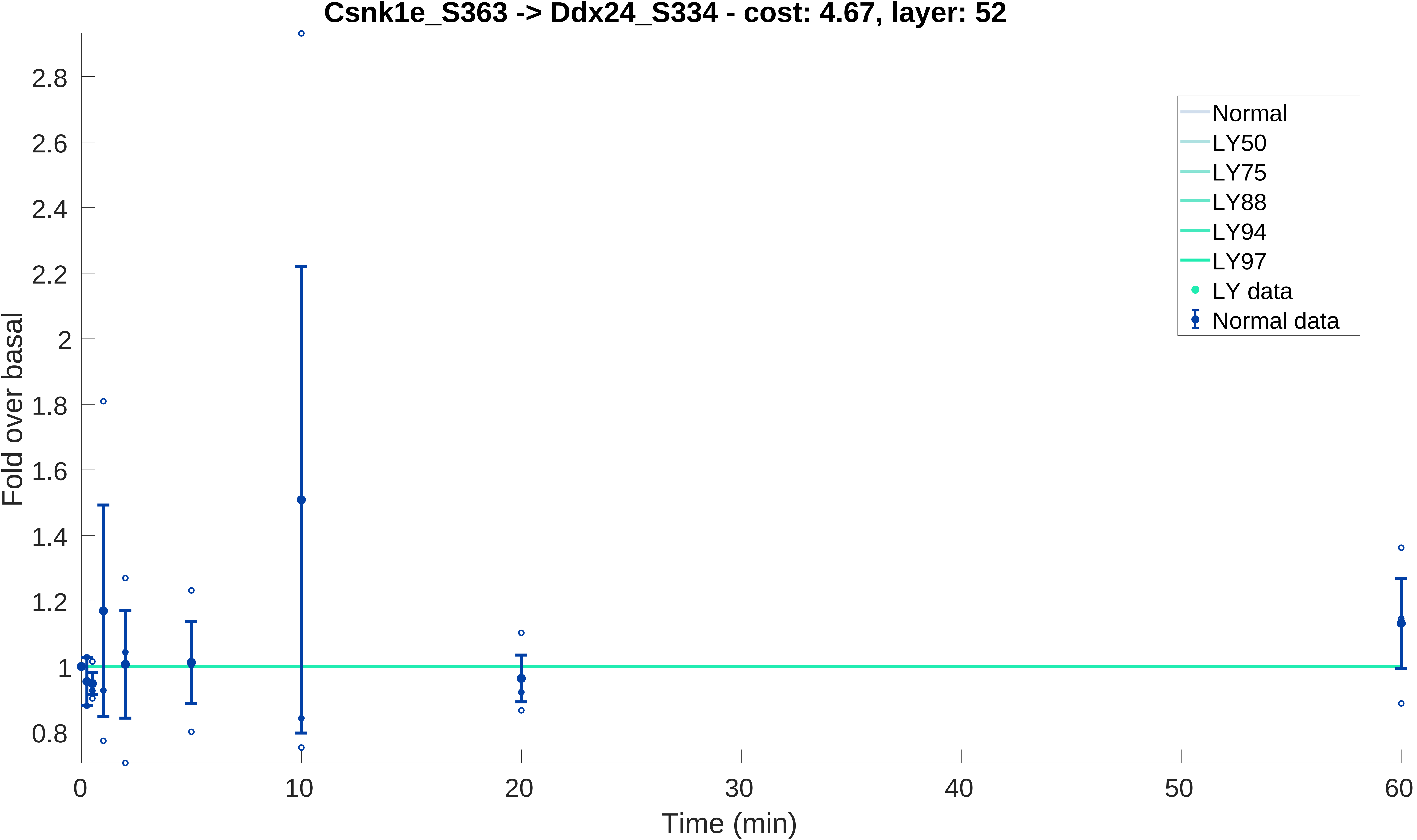

Csnk1e_S363 -> Ddx24_S334 - cost: 4.67, layer: 52
Fold over basal
Time (min)
Normal
LY50
LY75
LY88
LY94
LY97
LY data
Normal data
0.8
1
1.2
1.4
1.6
1.8
2
2.2
2.4
2.6
2.8
0
10
20
30
40
50
60

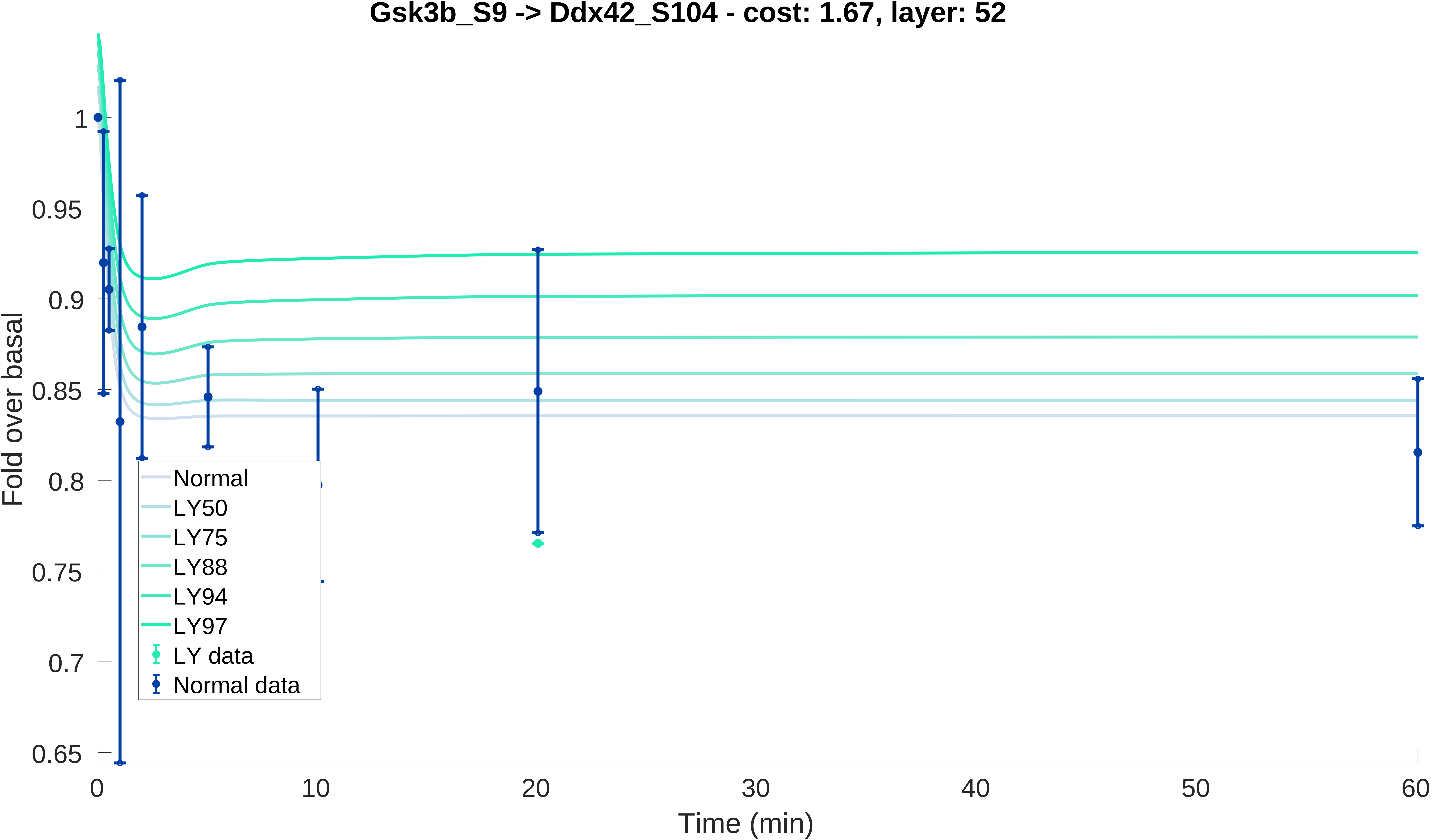
Gsk3b_S9 -> Ddx42_S104 - cost: 1.67, layer: 52
Fold over basal
Time (min)
0.65
0.7
0.75
0.8
0.85
0.9
0.95
1
0
10
20
30
40
50
60
Normal
LY50
LY75
LY88
LY94
LY97
LY data
Normal data

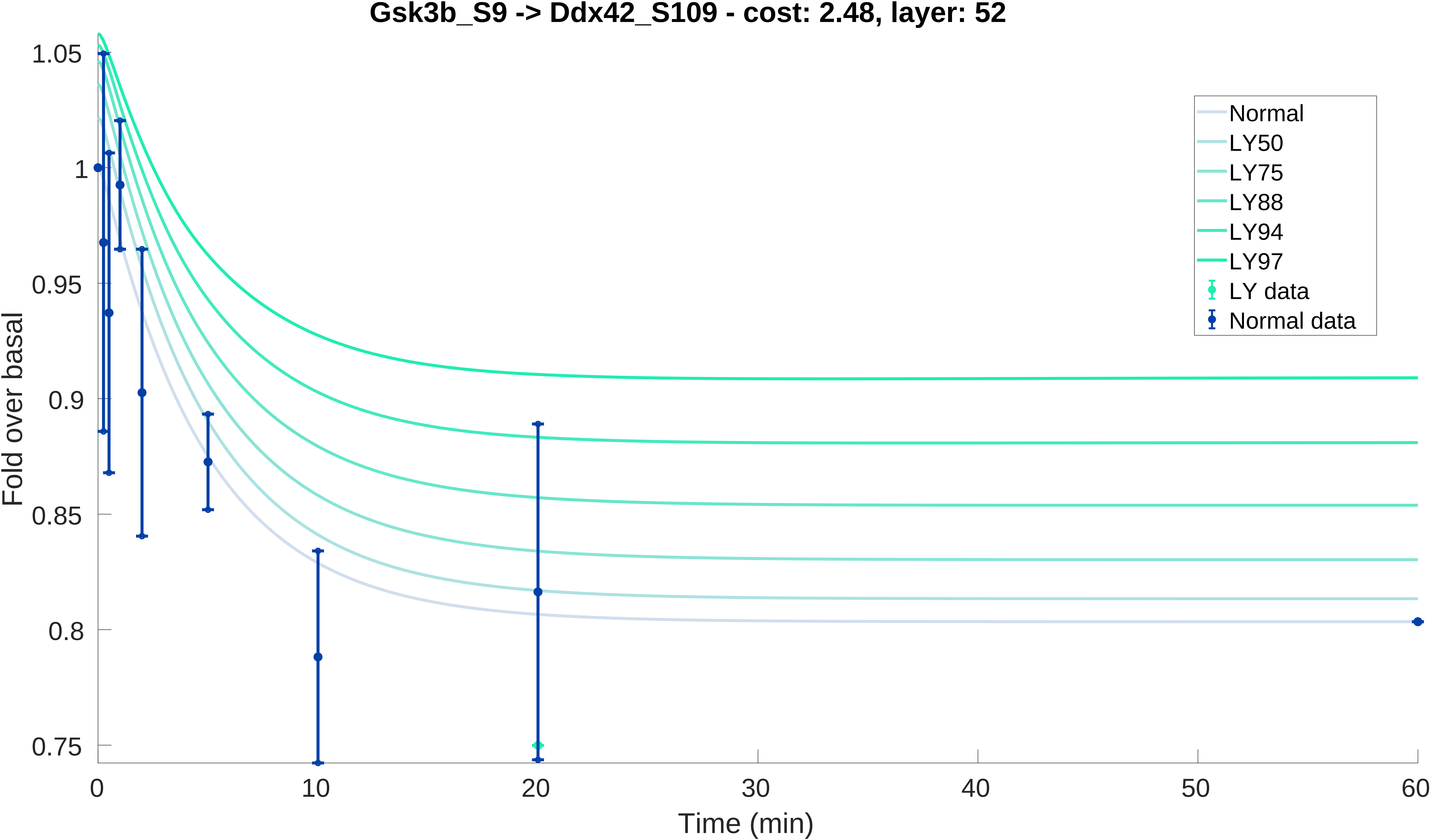

Gsk3b_S9 -> Ddx42_S109 - cost: 2.48, layer: 52
Fold over basal
1.05
1
0.95
0.9
0.85
0.8
0.75
0
10
20
30
40
50
60
Time (min)
Normal
LY50
LY75
LY88
LY94
LY97
LY data
Normal data

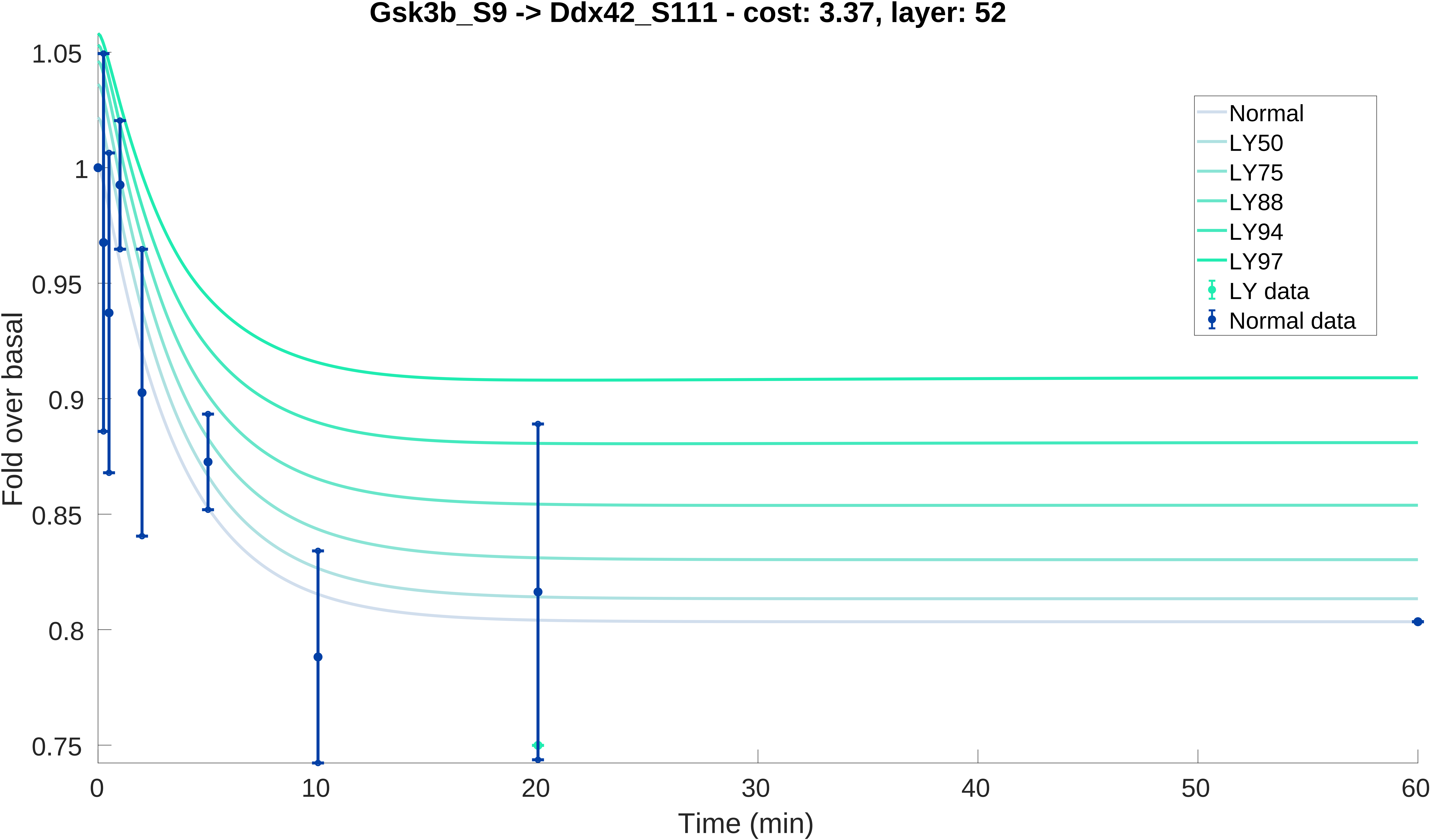

Gsk3b_S9 -> Ddx42_S111 - cost: 3.37, layer: 52
Fold over basal
Time (min)
1.05
1
0.95
0.9
0.85
0.8
0.75
0
10
20
30
40
50
60
Normal
LY50
LY75
LY88
LY94
LY97
LY data
Normal data

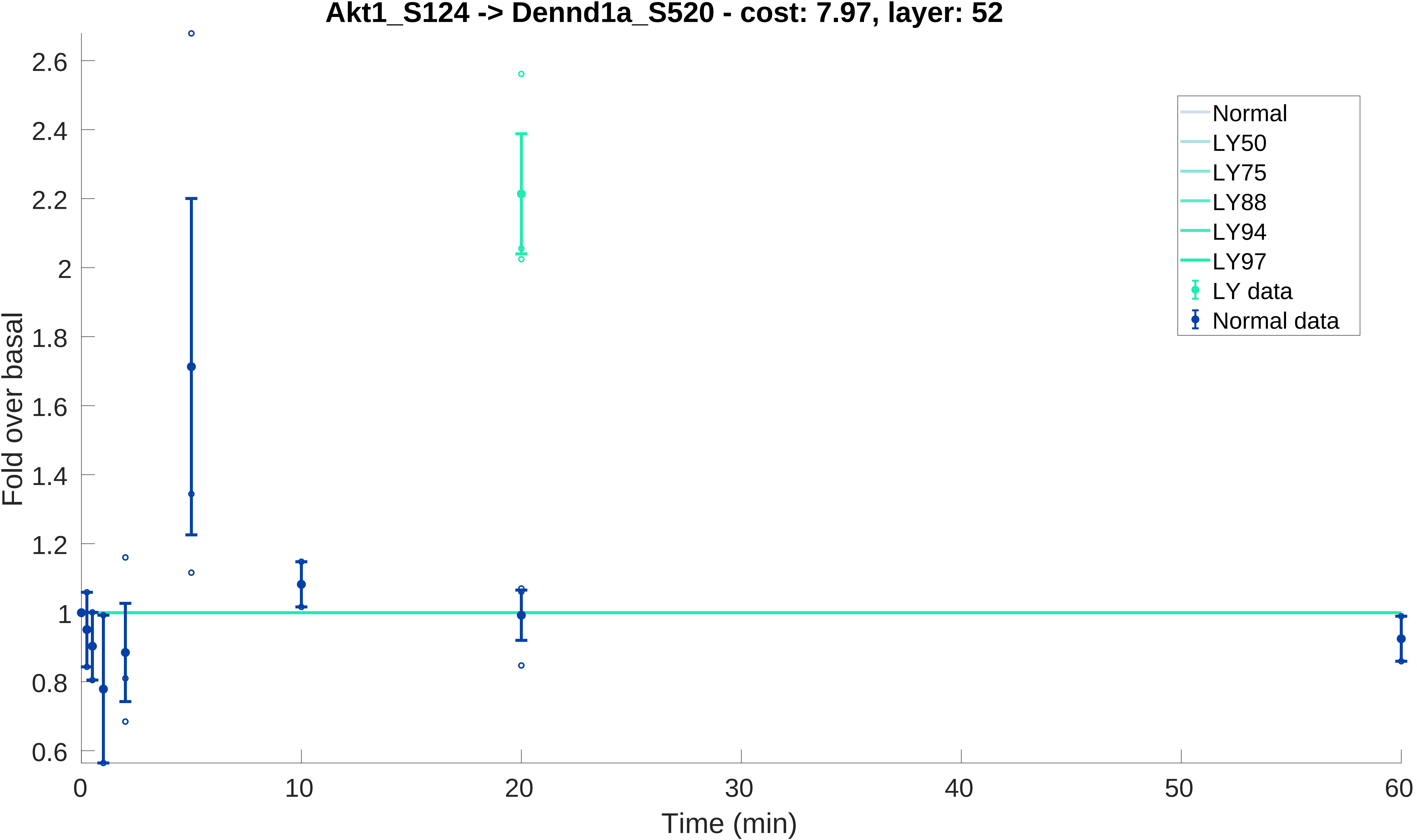

Akt1_S124 -> Dennd1a_S520 - cost: 7.97, layer: 52
Fold over basal
Time (min)
0.6
0.8
1
1.2
1.4
1.6
1.8
2
2.2
2.4
2.6
0
10
20
30
40
50
60
Normal
LY50
LY75
LY88
LY94
LY97
LY data
Normal data

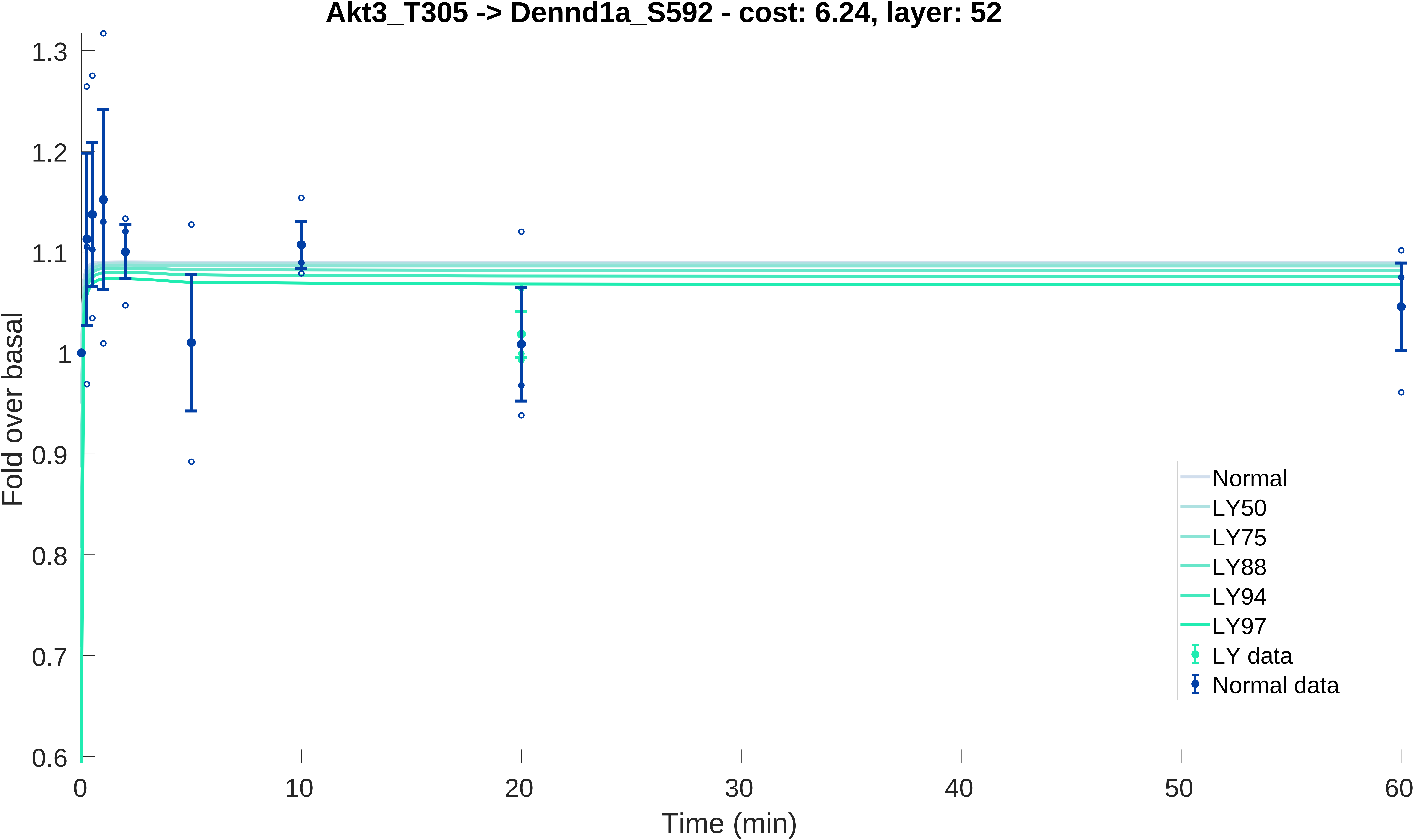

Akt3_T305 -> Dennd1a_S592 - cost: 6.24, layer: 52
Fold over basal
1.3
1.2
1.1
1
0.9
0.8
0.7
0.6
0
10
20
30
40
50
60
Time (min)
Normal
LY50
LY75
LY88
LY94
LY97
LY data
Normal data

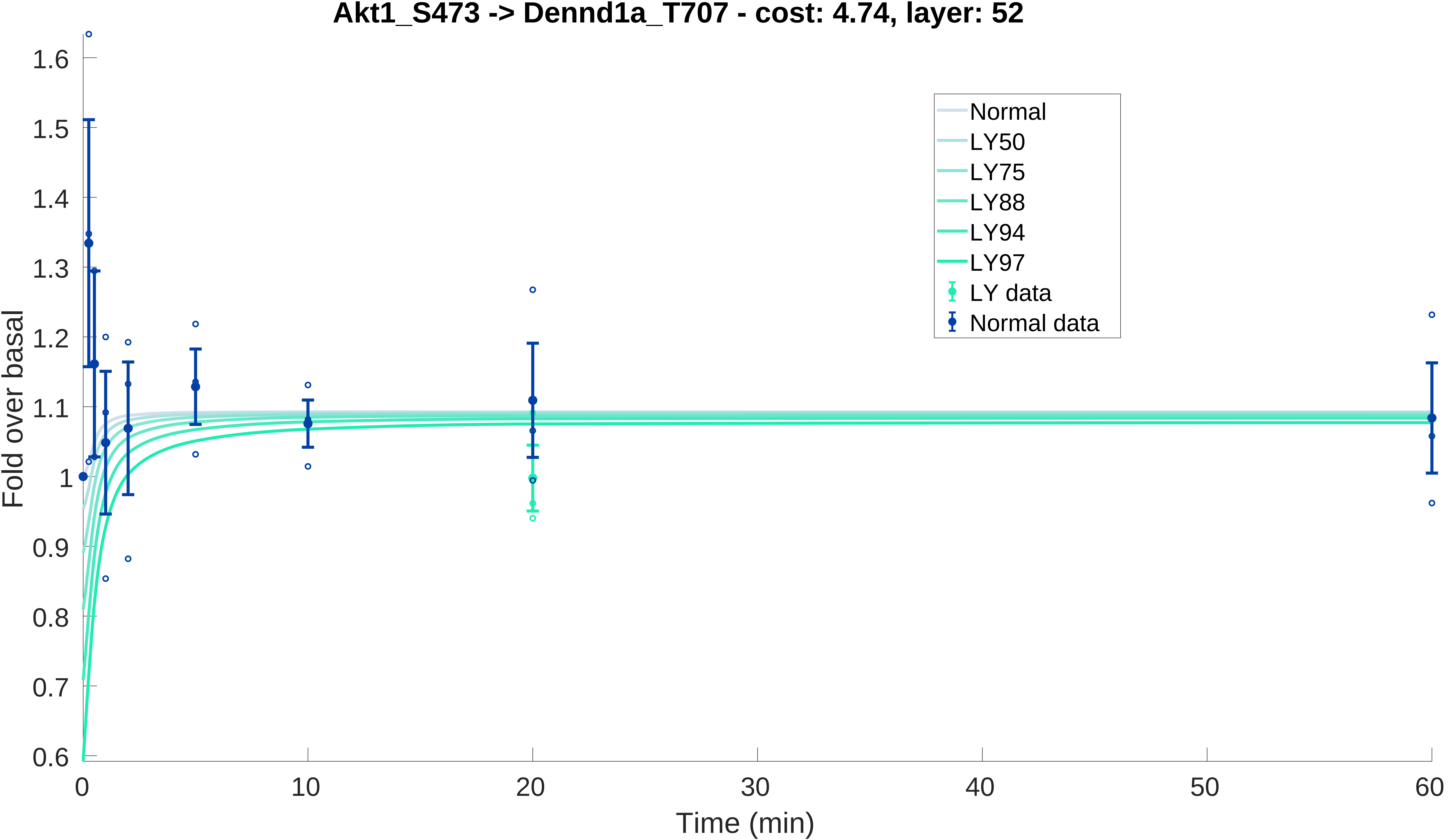
Akt1_S473 -> Dennd1a_T707 - cost: 4.74, layer: 52
Normal
LY50
LY75
LY88
LY94
LY97
LY data
Normal data
Fold over basal
Time (min)
0.6
0.7
0.8
0.9
1
1.1
1.2
1.3
1.4
1.5
1.6
0
10
20
30
40
50
60

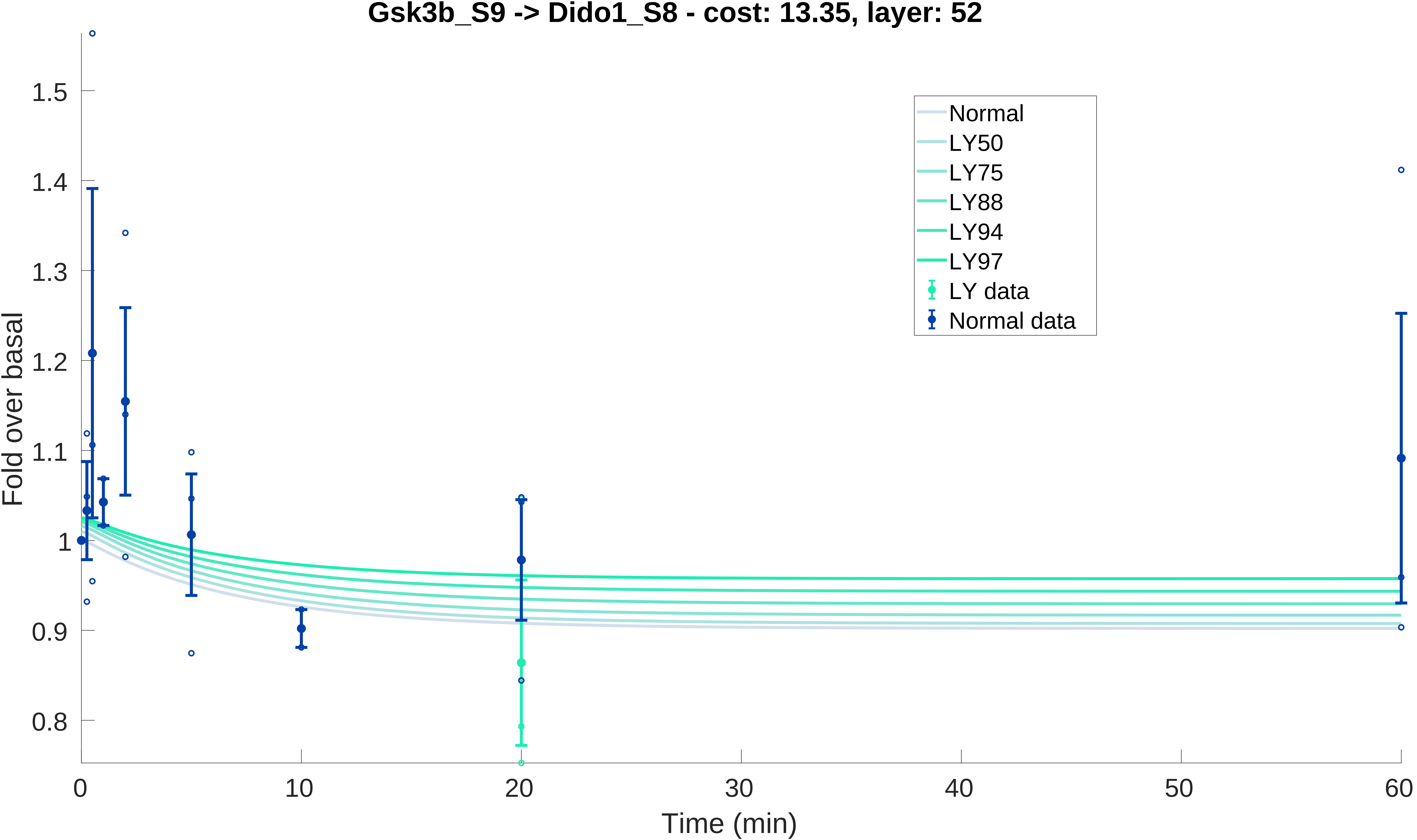
Gsk3b_S9 -> Dido1_S8 - cost: 13.35, layer: 52
Fold over basal
Time (min)
0.8
0.9
1
1.1
1.2
1.3
1.4
1.5
0
10
20
30
40
50
60
Normal
LY50
LY75
LY88
LY94
LY97
LY data
Normal data

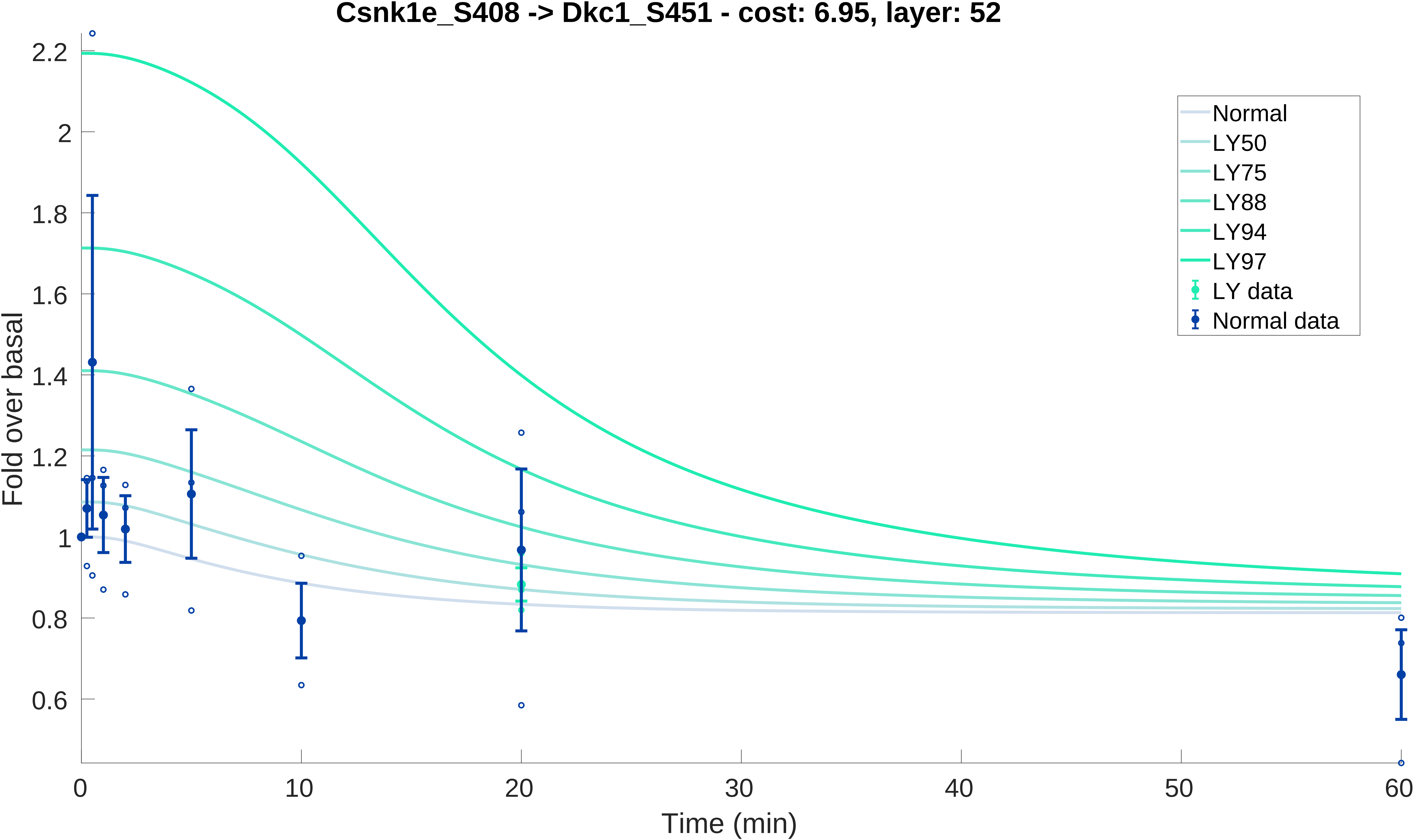
Csnk1e_S408 -> Dkc1_S451 - cost: 6.95, layer: 52
Fold over basal
Time (min)
0.6
0.8
1
1.2
1.4
1.6
1.8
2
2.2
0
10
20
30
40
50
60
Normal
LY50
LY75
LY88
LY94
LY97
LY data
Normal data

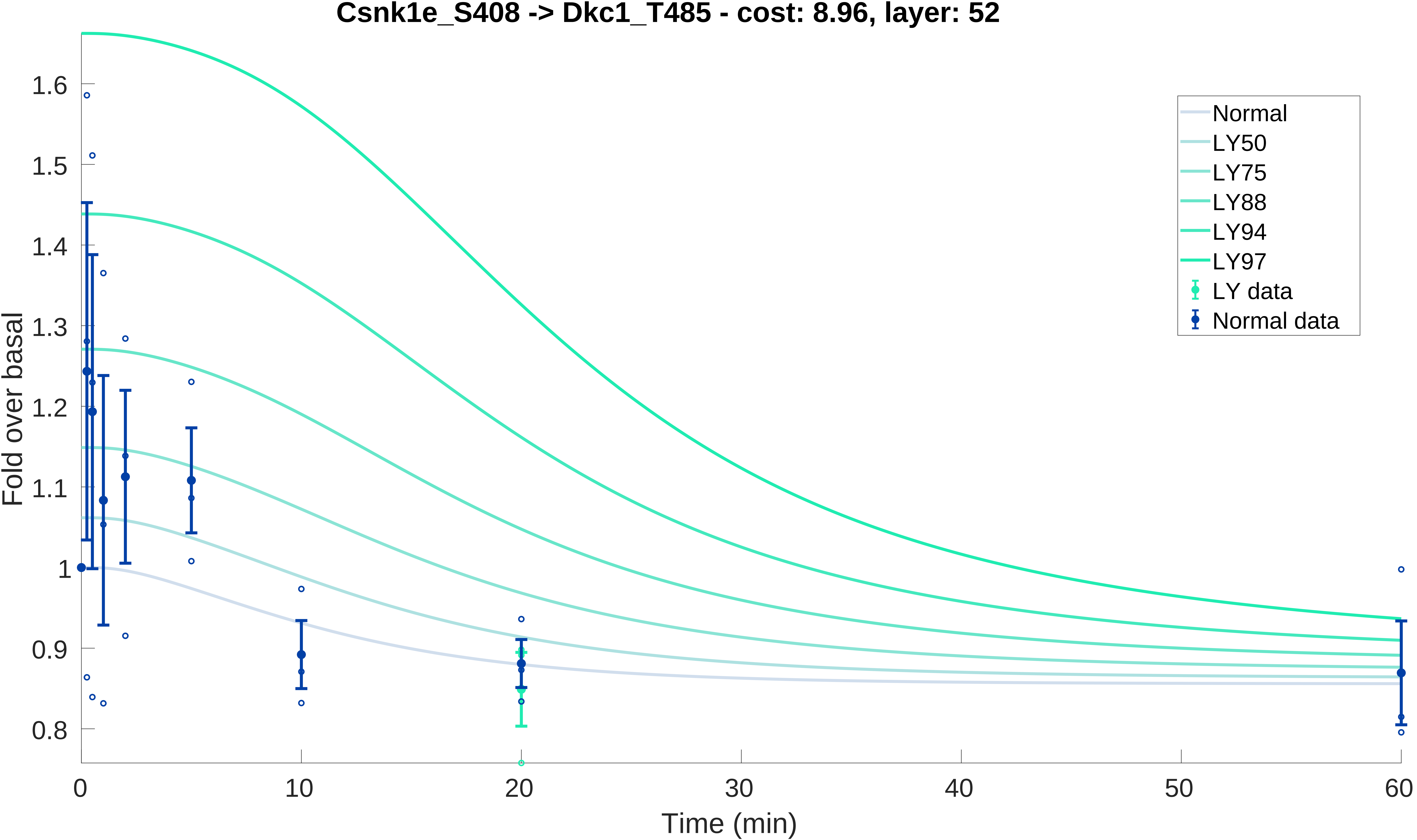
Csnk1e_S408 -> Dkc1_T485 - cost: 8.96, layer: 52
Fold over basal
Time (min)
1.6
1.5
1.4
1.3
1.2
1.1
1
0.9
0.8
0
10
20
30
40
50
60
Normal
LY50
LY75
LY88
LY94
LY97
LY data
Normal data

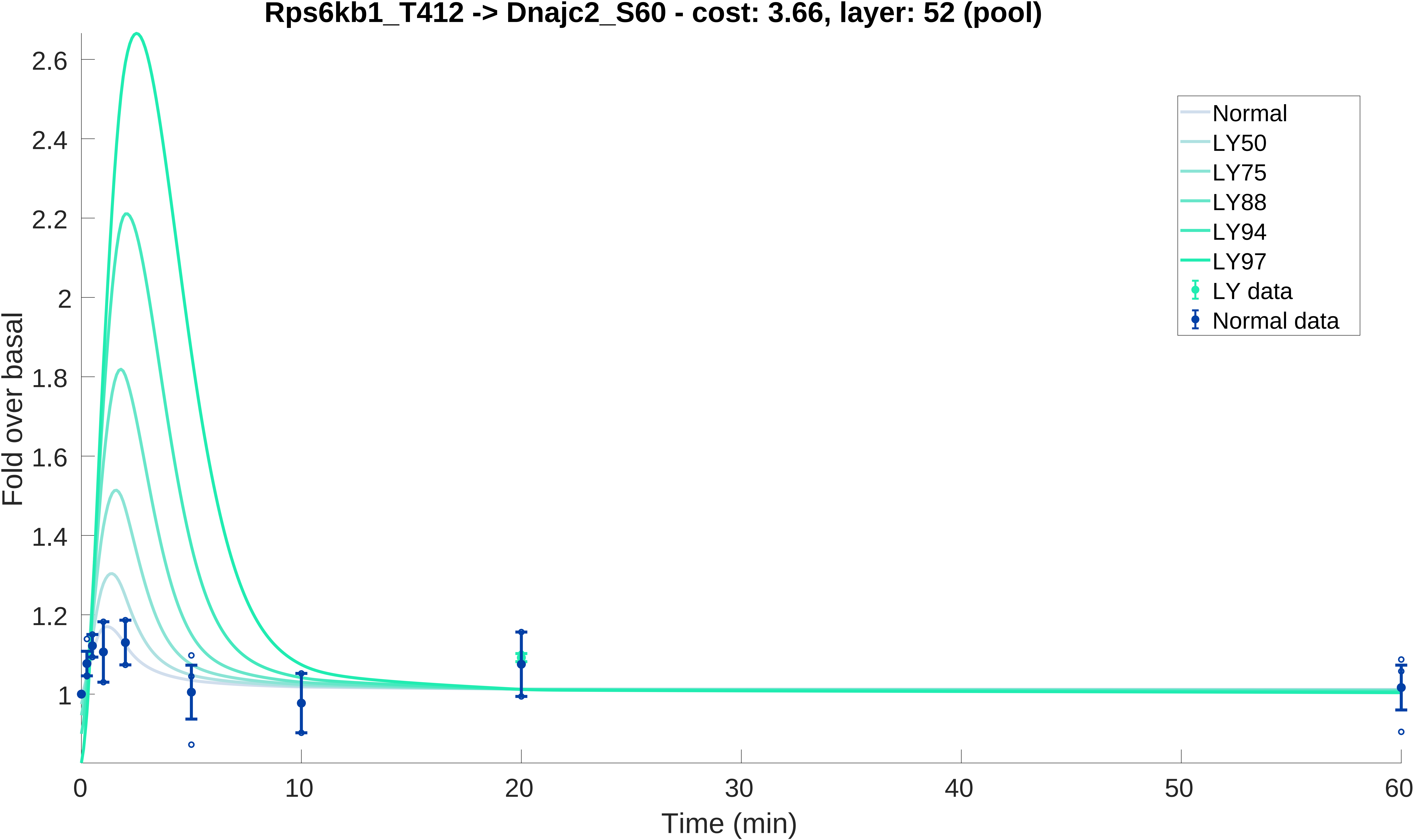

Rps6kb1_T412 -> Dnajc2_S60 - cost: 3.66, layer: 52 (pool)
Fold over basal
Time (min)
0
10
20
30
40
50
60
1
1.2
1.4
1.6
1.8
2
2.2
2.4
2.6
Normal
LY50
LY75
LY88
LY94
LY97
LY data
Normal data

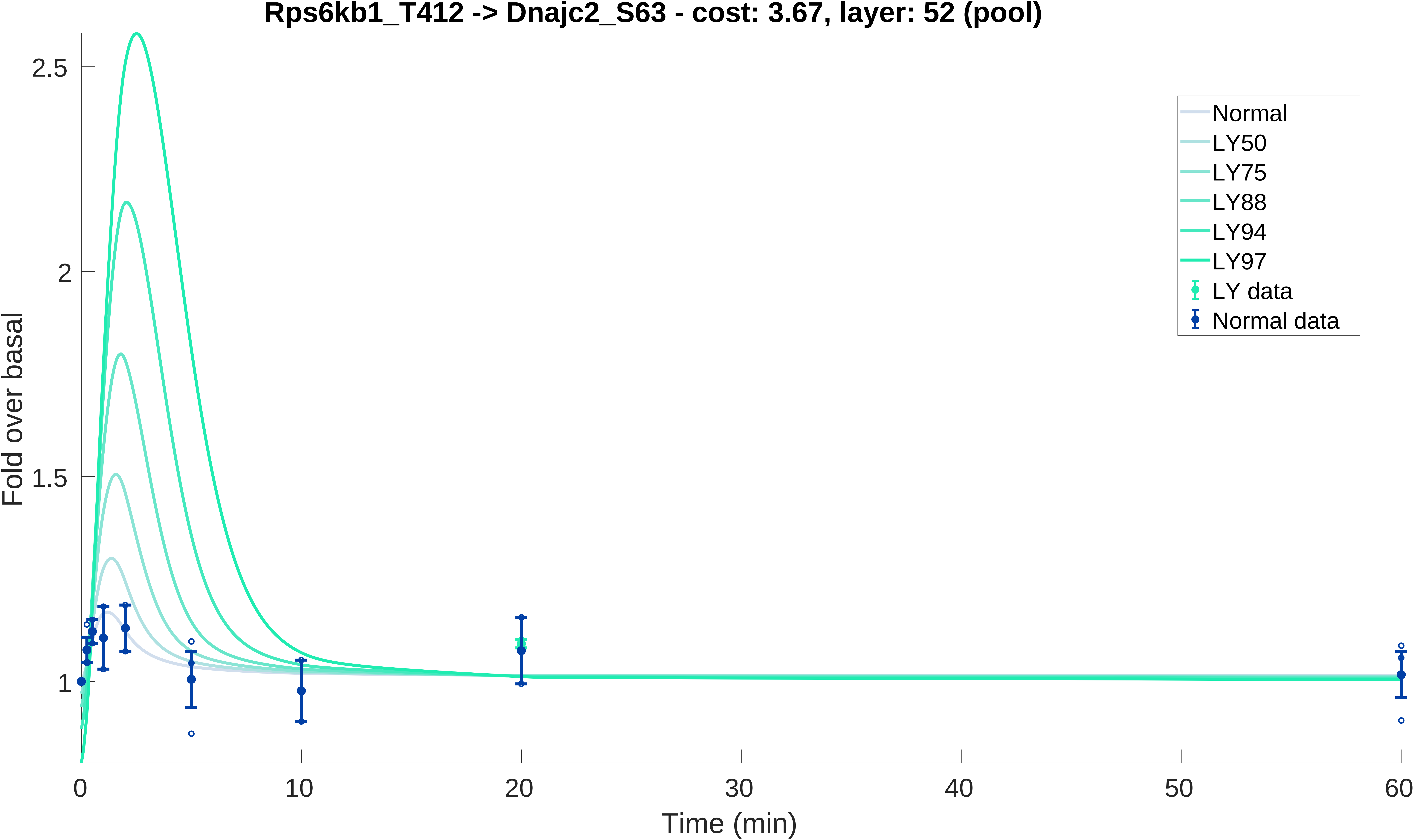

Rps6kb1_T412 -> Dnajc2_S63 - cost: 3.67, layer: 52 (pool)
Fold over basal
Time (min)
2.5
2
1.5
1
0
10
20
30
40
50
60
Normal
LY50
LY75
LY88
LY94
LY97
LY data
Normal data

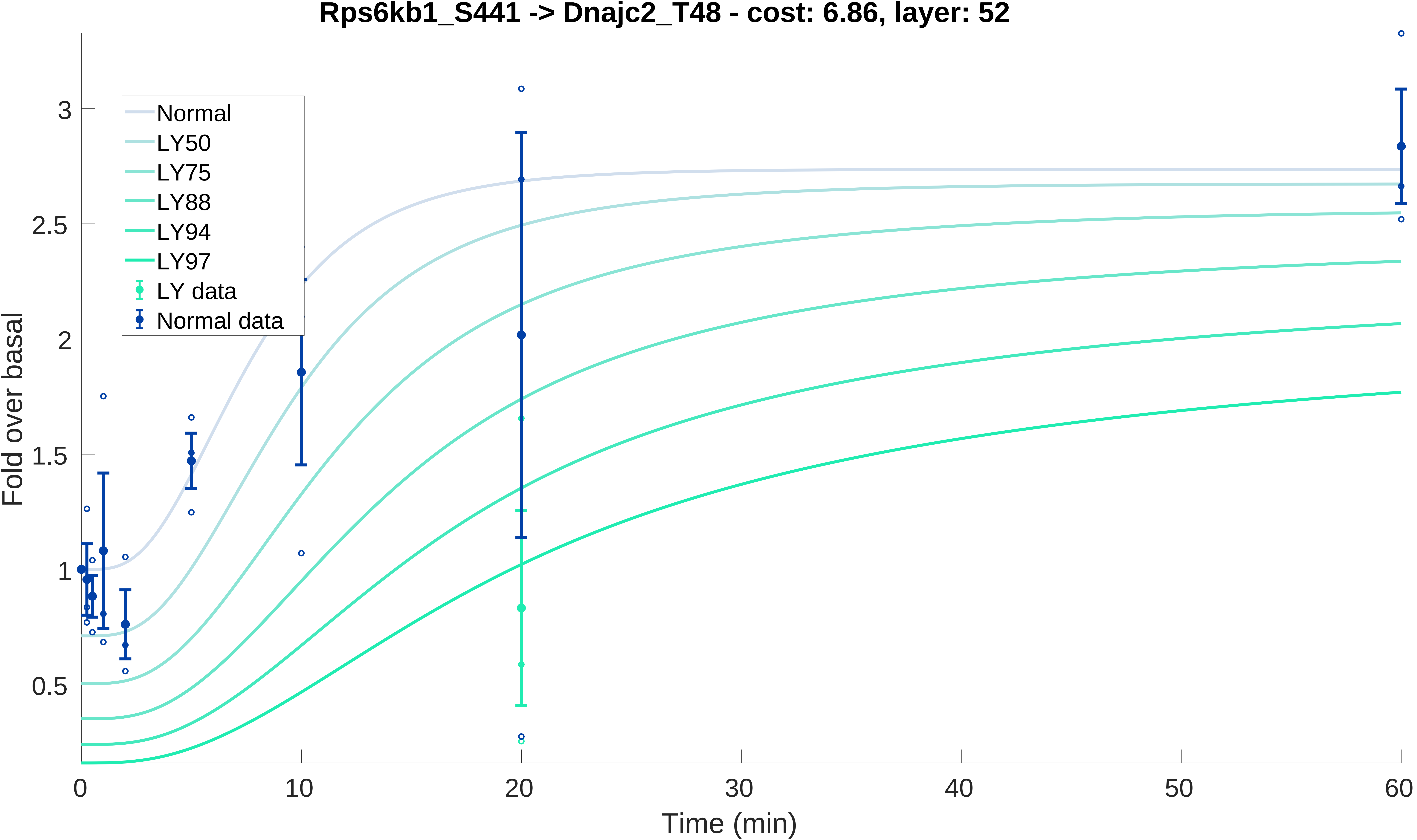

Rps6kb1_S441 -> Dnajc2_T48 - cost: 6.86, layer: 52
Normal
LY50
LY75
LY88
LY94
LY97
LY data
Normal data
Fold over basal
Time (min)
0.5
1
1.5
2
2.5
3
0
10
20
30
40
50
60

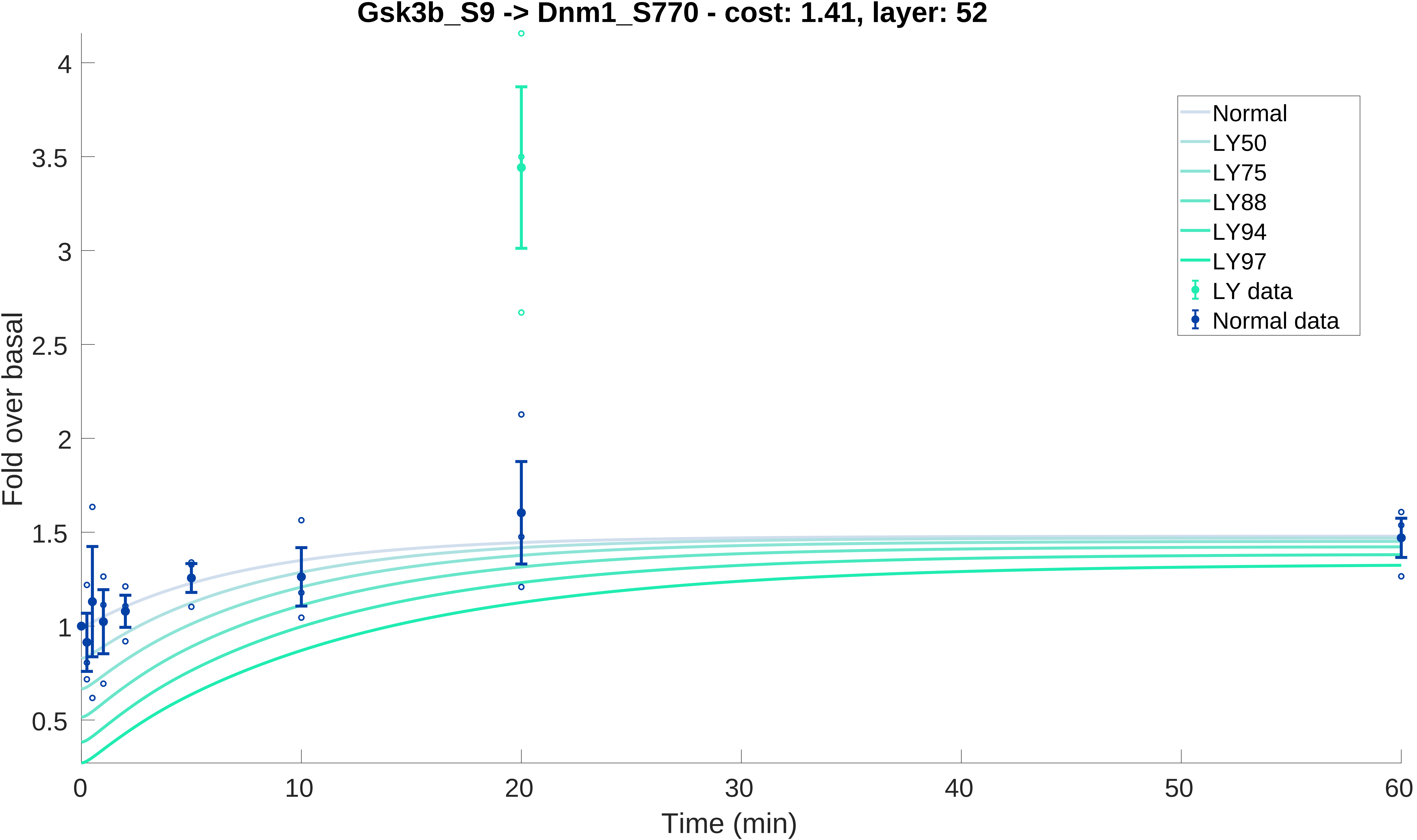

Gsk3b_S9 -> Dnm1_S770 - cost: 1.41, layer: 52
Fold over basal
Time (min)
0.5
1
1.5
2
2.5
3
3.5
4
0
10
20
30
40
50
60
Normal
LY50
LY75
LY88
LY94
LY97
LY data
Normal data

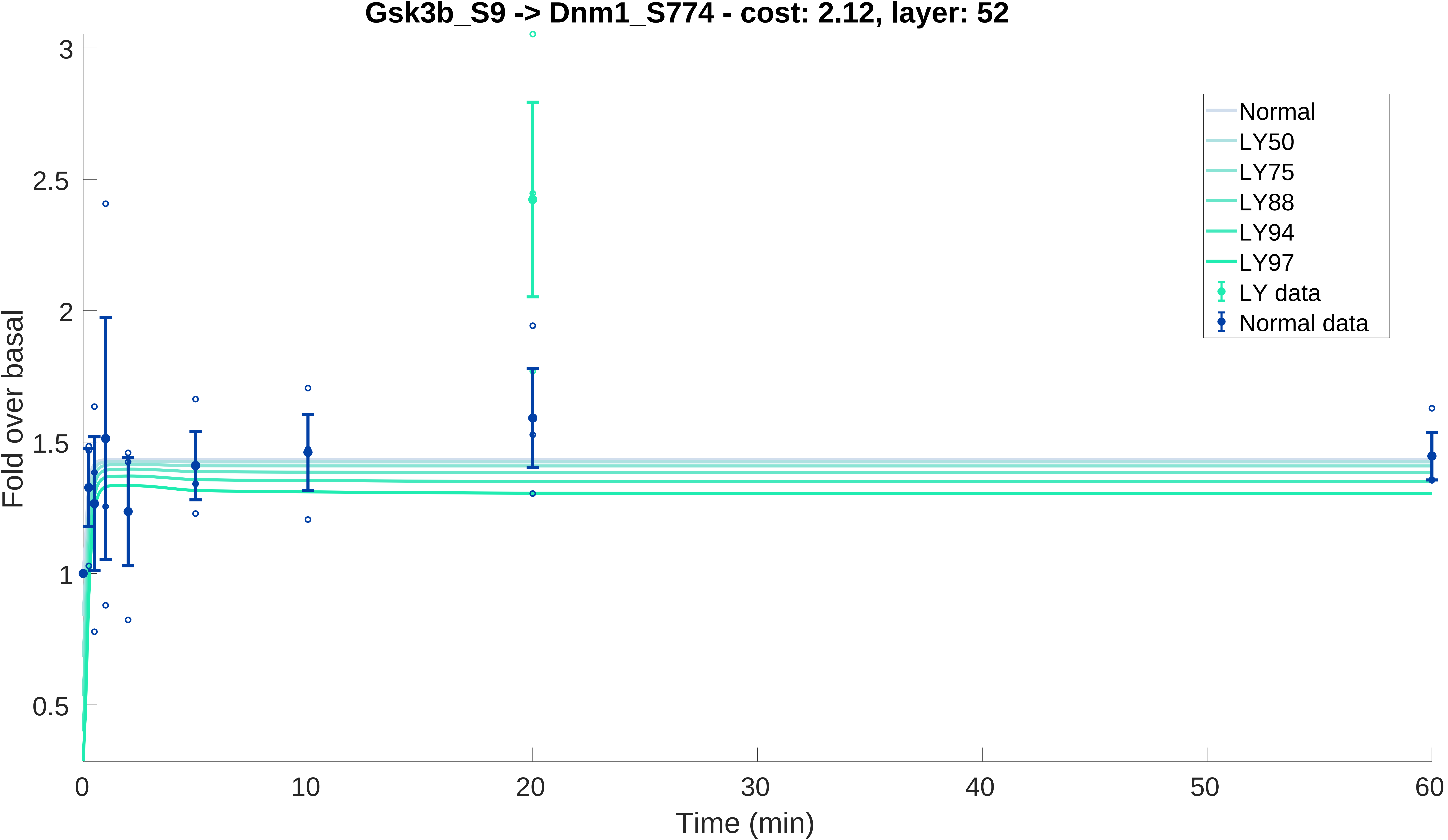
Gsk3b_S9 -> Dnm1_S774 - cost: 2.12, layer: 52
Fold over basal
Time (min)
0
0.5
1
1.5
2
2.5
3
0
10
20
30
40
50
60
Normal
LY50
LY75
LY88
LY94
LY97
LY data
Normal data

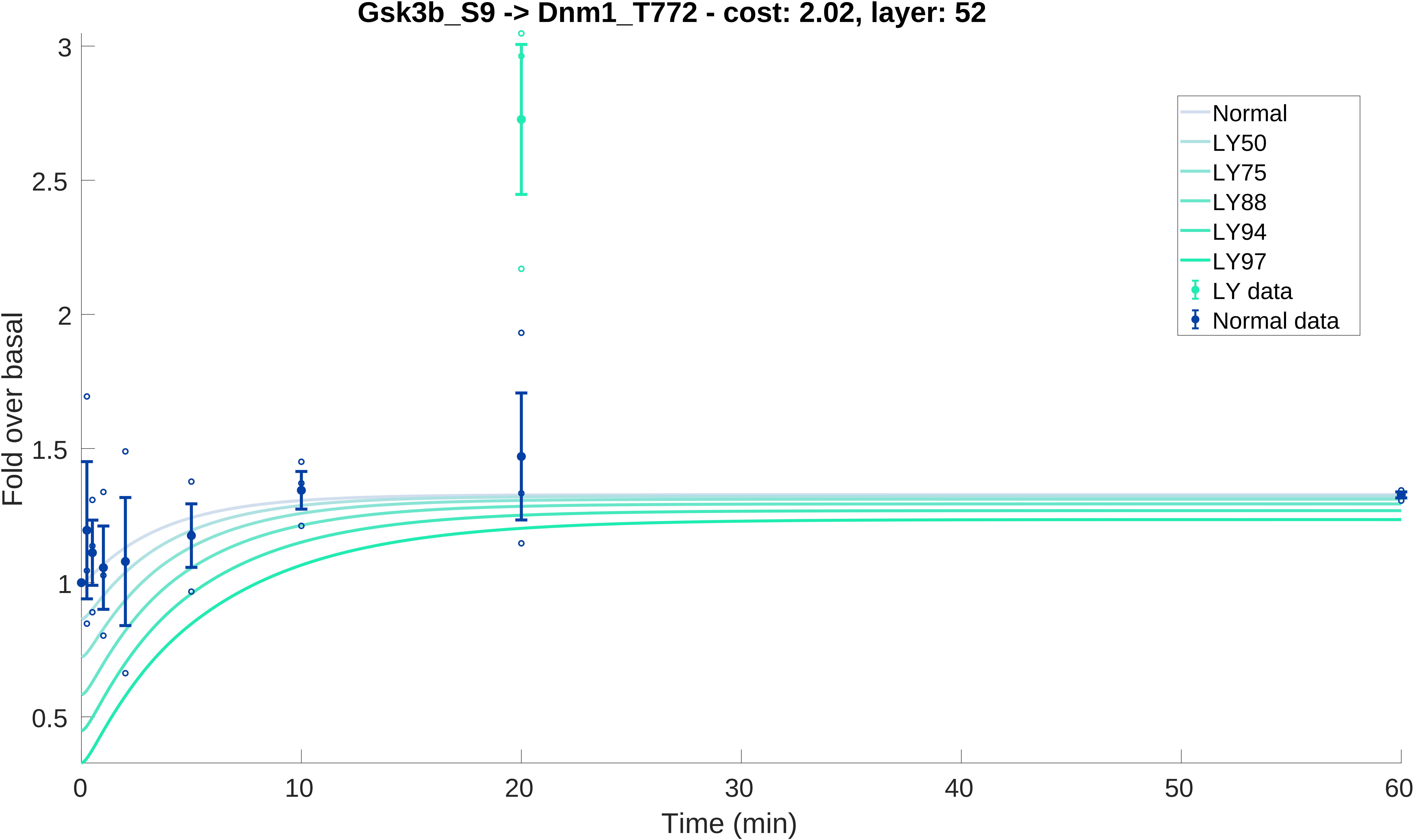
Gsk3b_S9 -> Dnm1_T772 - cost: 2.02, layer: 52
Fold over basal
Time (min)
0
10
20
30
40
50
60
0.5
1
1.5
2
2.5
3
Normal
LY50
LY75
LY88
LY94
LY97
LY data
Normal data

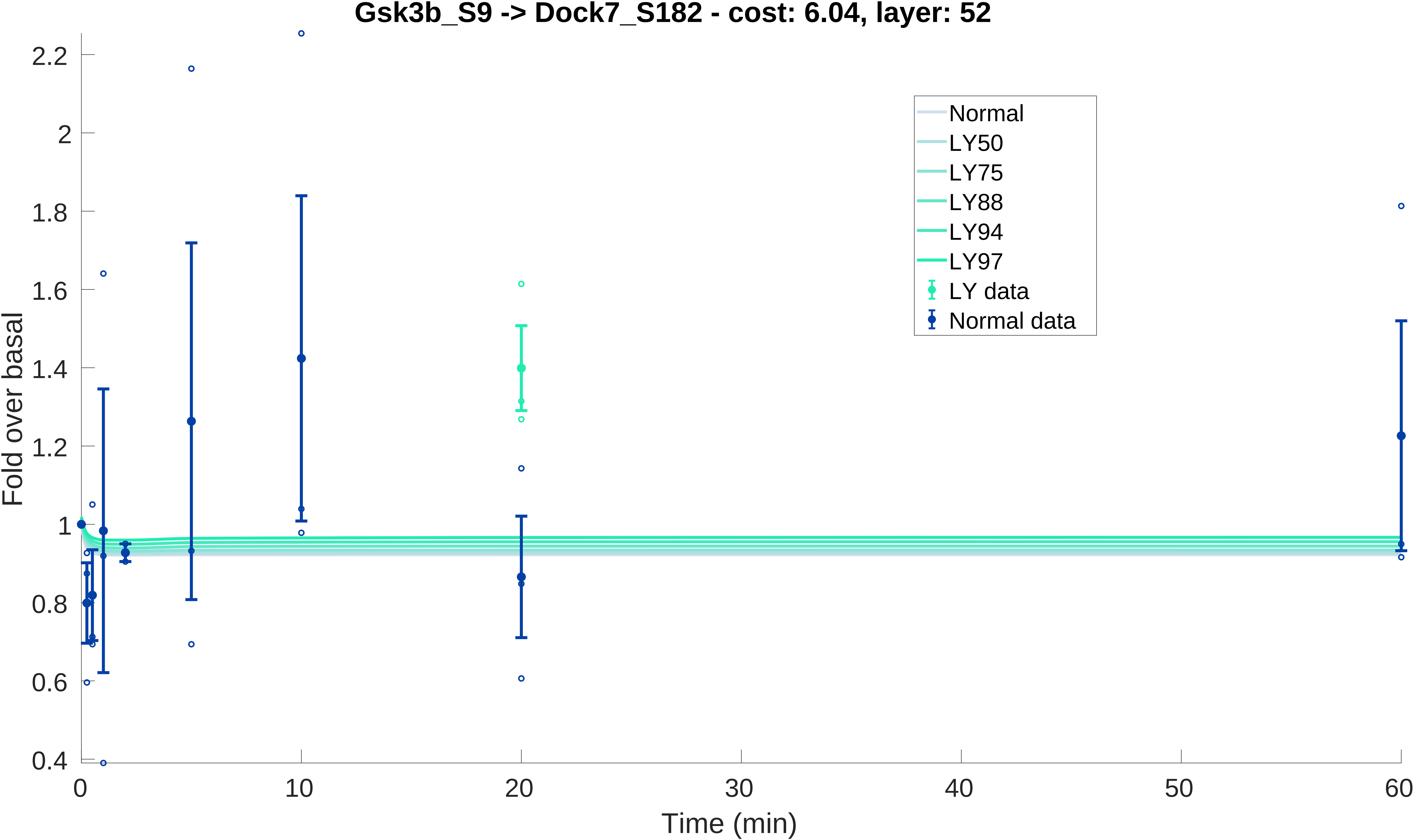

Gsk3b_S9 -> Dock7_S182 - cost: 6.04, layer: 52
Fold over basal
Time (min)
0.4
0.6
0.8
1
1.2
1.4
1.6
1.8
2
2.2
0
10
20
30
40
50
60
Normal
LY50
LY75
LY88
LY94
LY97
LY data
Normal data

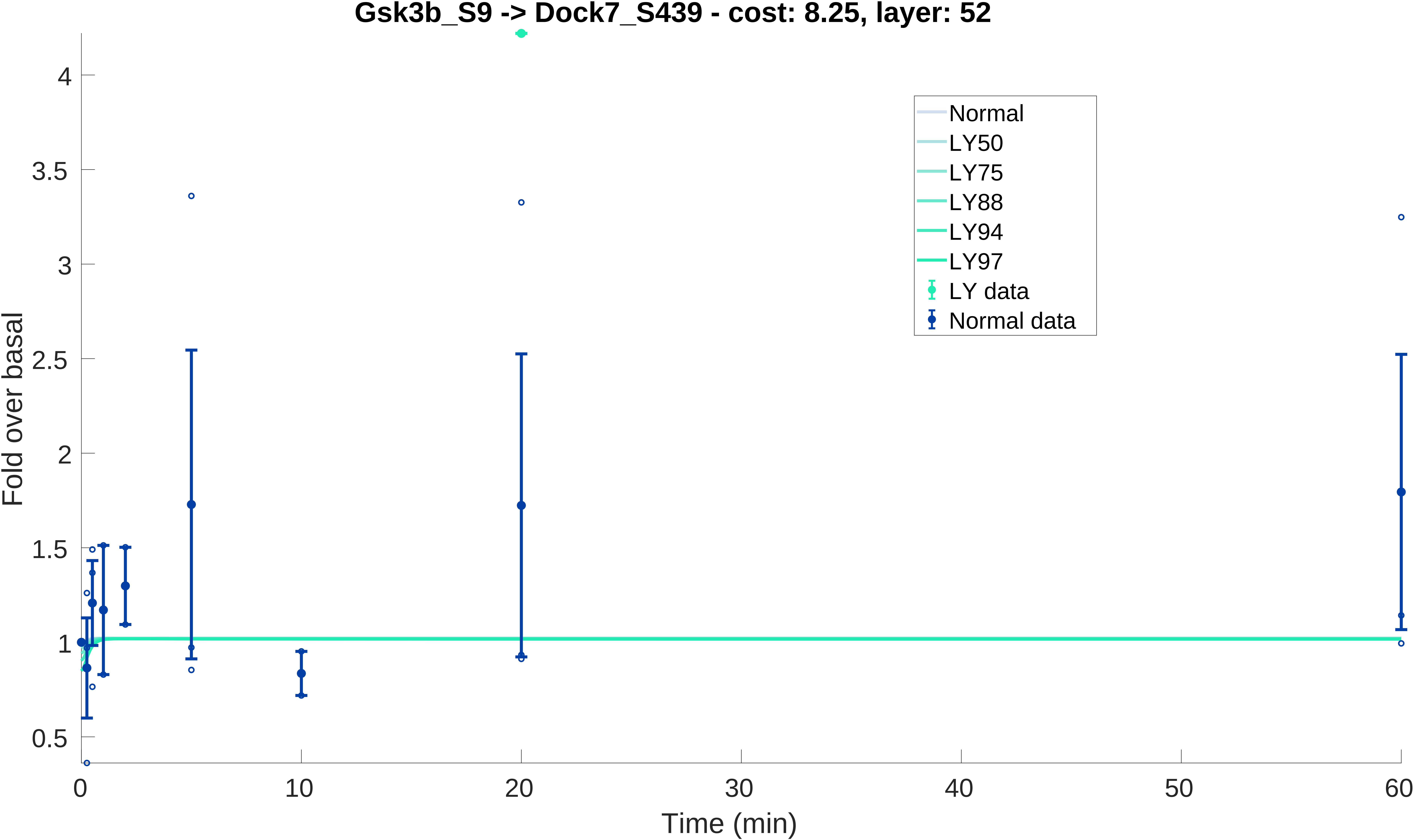
Gsk3b_S9 -> Dock7_S439 - cost: 8.25, layer: 52
Fold over basal
Time (min)
0.5
1
1.5
2
2.5
3
3.5
4
0
10
20
30
40
50
60
Normal
LY50
LY75
LY88
LY94
LY97
LY data
Normal data

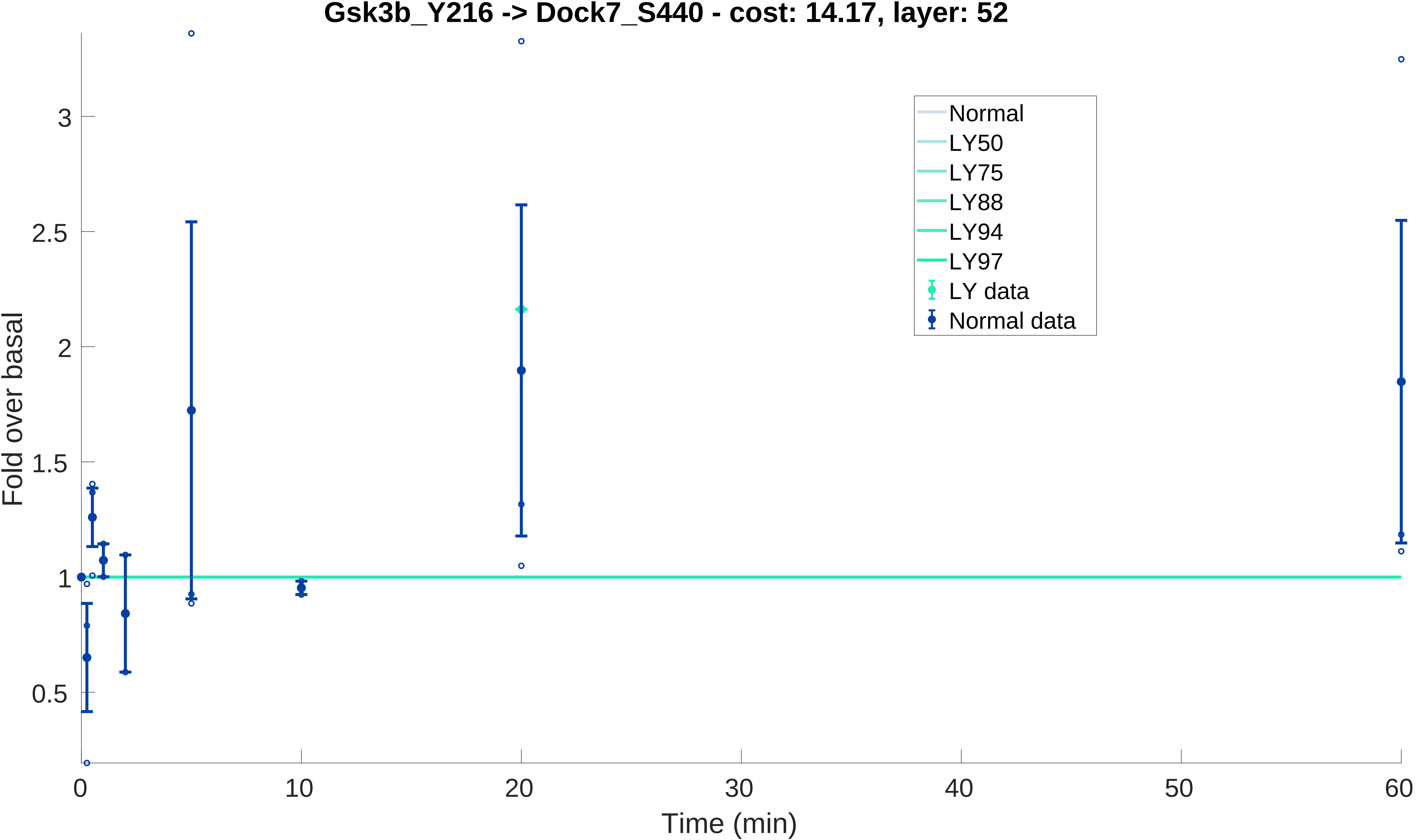

Gsk3b_Y216 -> Dock7_S440 - cost: 14.17, layer: 52
Normal
LY50
LY75
LY88
LY94
LY97
LY data
Normal data
Fold over basal
Time (min)
0.5
1
1.5
2
2.5
3
0
10
20
30
40
50
60

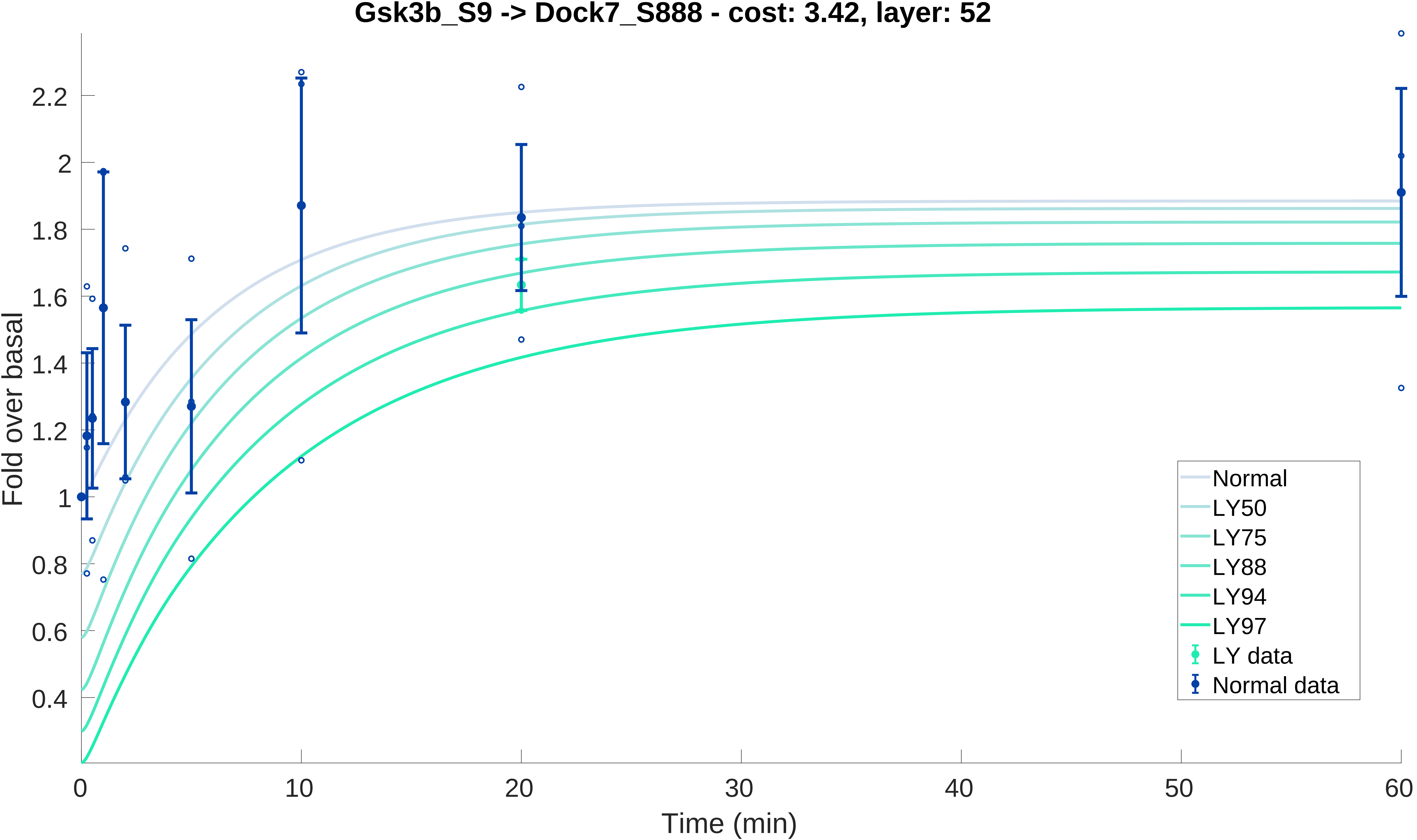

Gsk3b_S9 -> Dock7_S888 - cost: 3.42, layer: 52
Fold over basal
Time (min)
0.4
0.6
0.8
1
1.2
1.4
1.6
1.8
2
2.2
0
10
20
30
40
50
60
Normal
LY50
LY75
LY88
LY94
LY97
LY data
Normal data

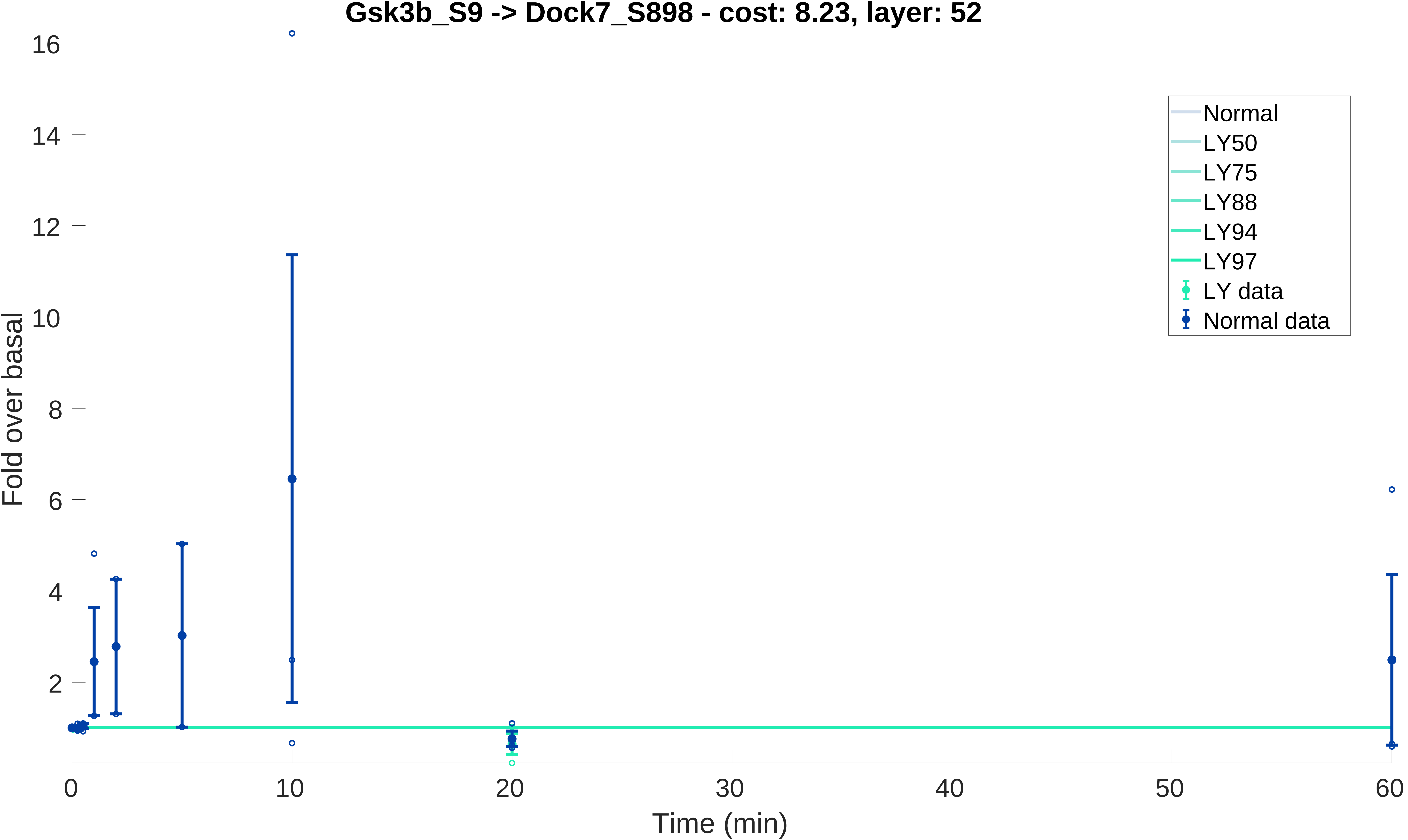

Gsk3b_S9 -> Dock7_S898 - cost: 8.23, layer: 52
Fold over basal
Time (min)
0
10
20
30
40
50
60
2
4
6
8
10
12
14
16
Normal
LY50
LY75
LY88
LY94
LY97
LY data
Normal data

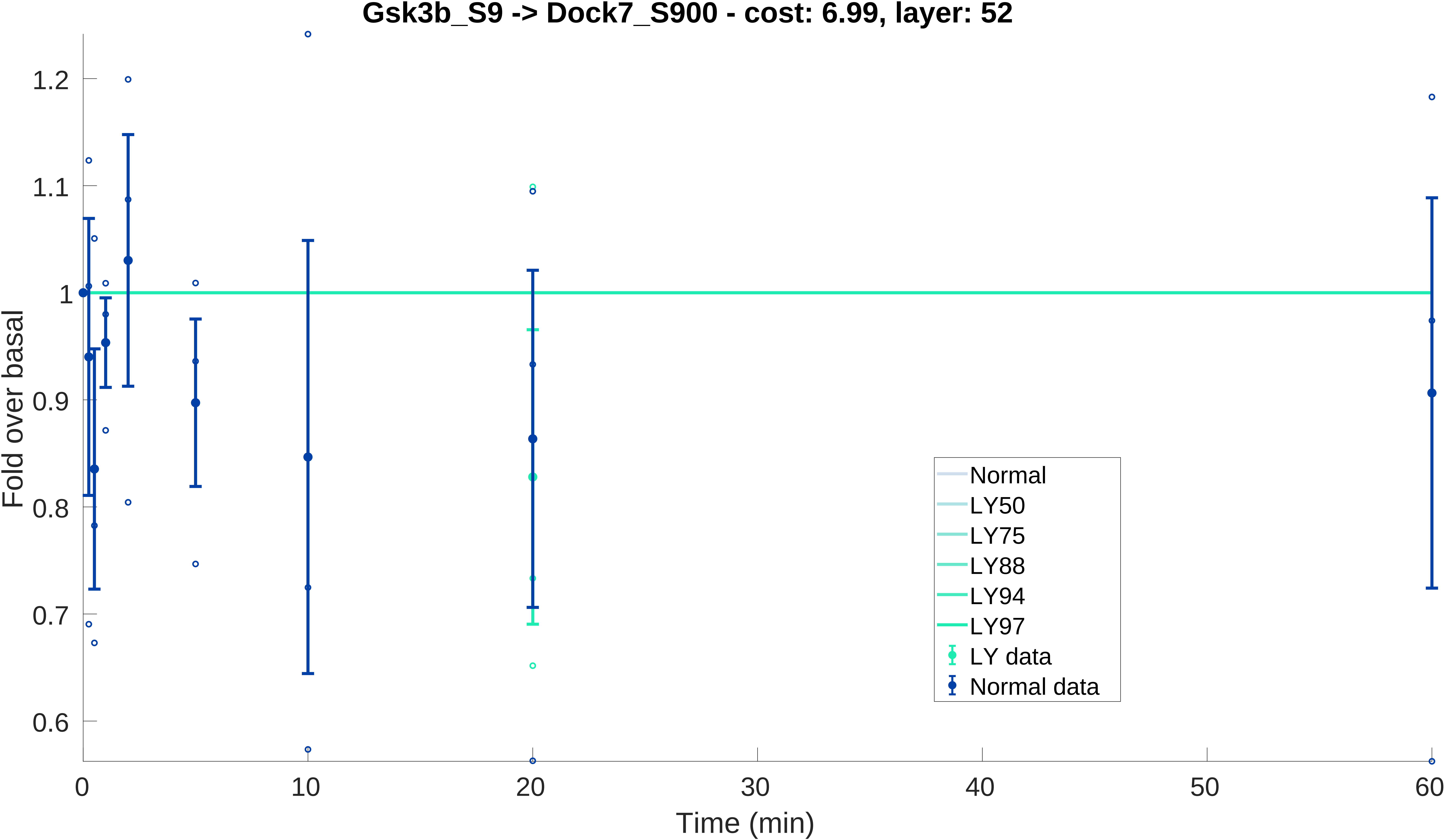
Gsk3b_S9 -> Dock7_S900 - cost: 6.99, layer: 52
Fold over basal
Time (min)
1.2
1.1
1
0.9
0.8
0.7
0.6
0
10
20
30
40
50
60
Normal
LY50
LY75
LY88
LY94
LY97
LY data
Normal data

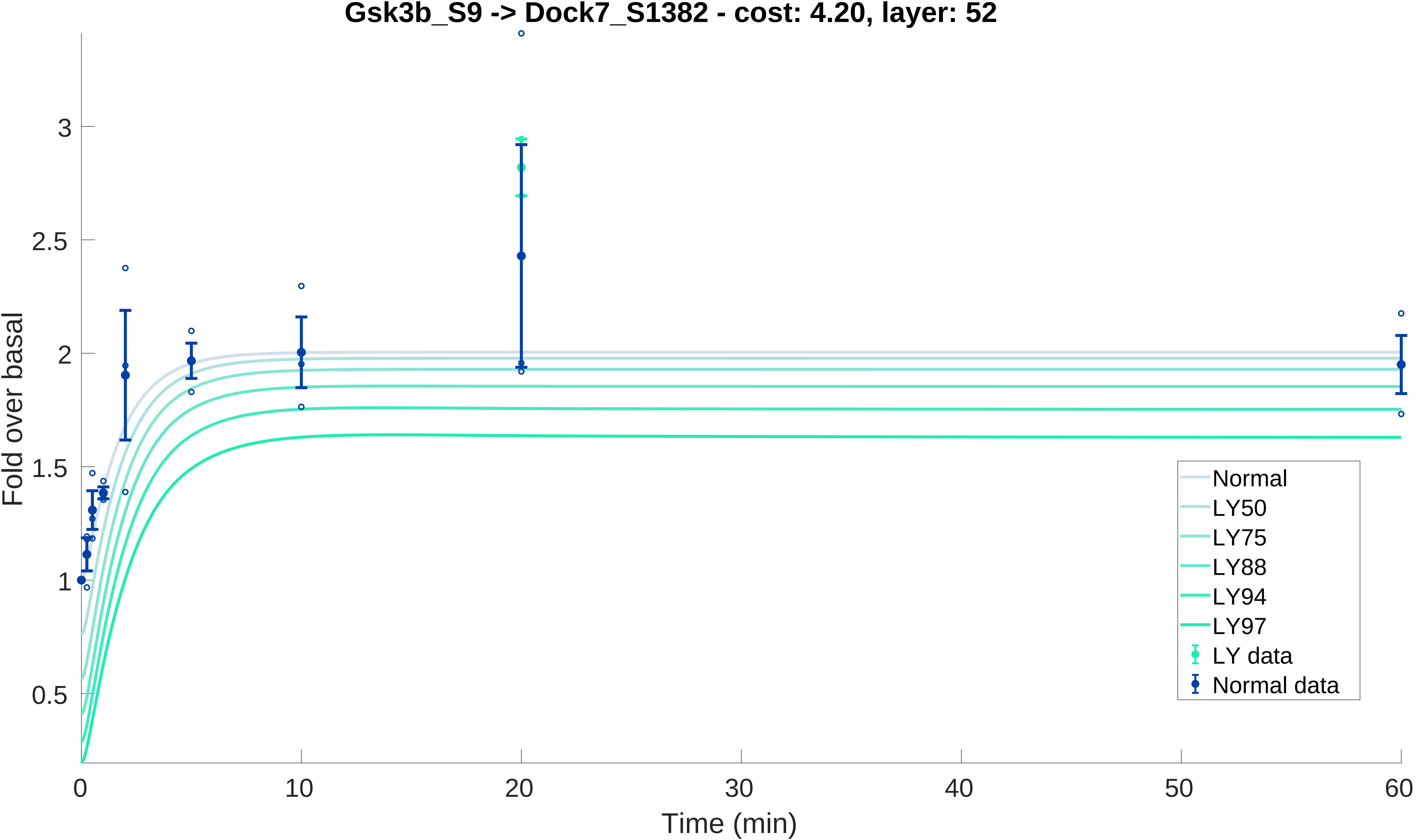
Gsk3b_S9 -> Dock7_S1382 - cost: 4.20, layer: 52
Fold over basal
Time (min)
0
0.5
1
1.5
2
2.5
3
10
20
30
40
50
60
Normal
LY50
LY75
LY88
LY94
LY97
LY data
Normal data

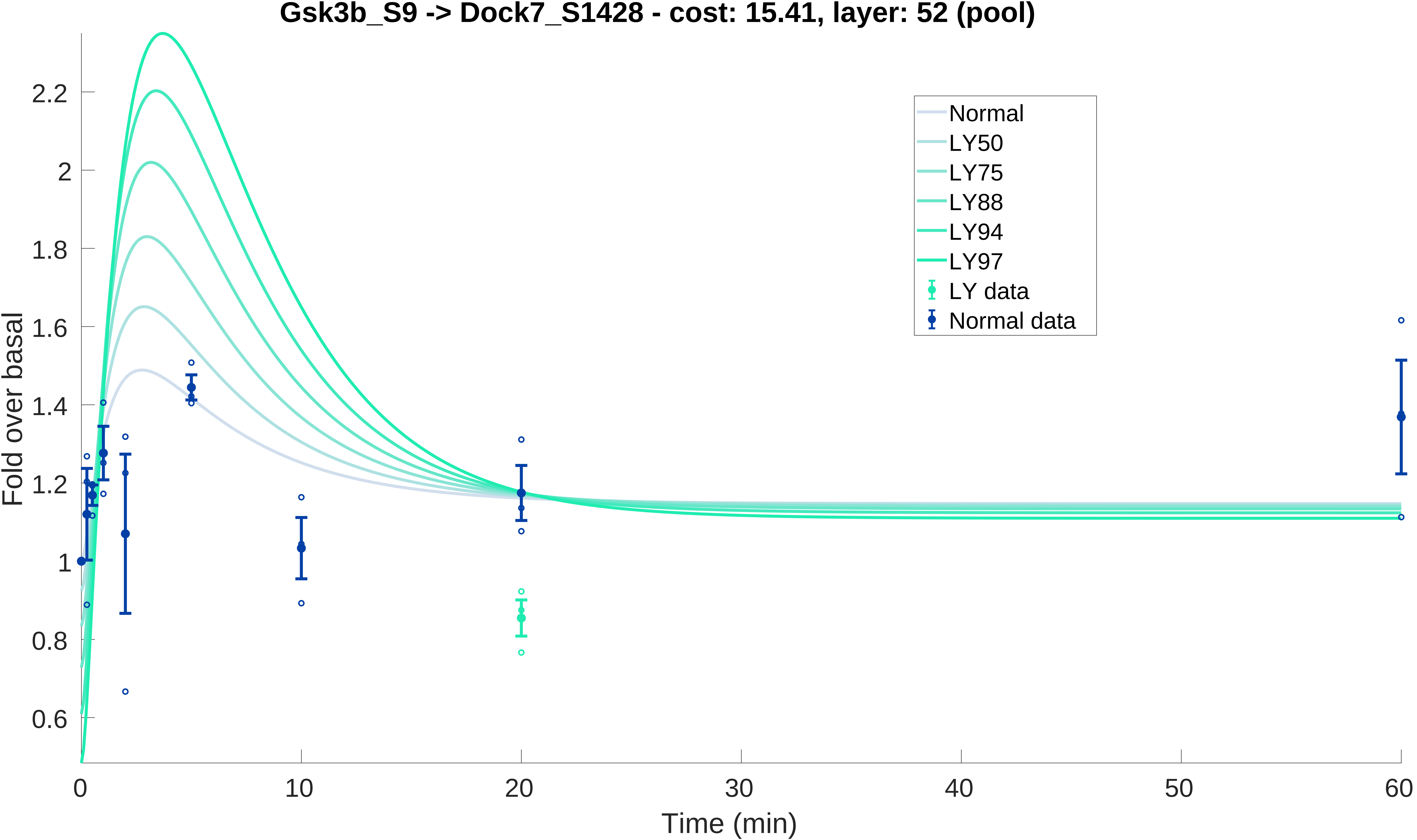

Gsk3b_S9 -> Dock7_S1428 - cost: 15.41, layer: 52 (pool)
Fold over basal
Time (min)
0.6
0.8
1
1.2
1.4
1.6
1.8
2
2.2
0
10
20
30
40
50
60
Normal
LY50
LY75
LY88
LY94
LY97
LY data
Normal data

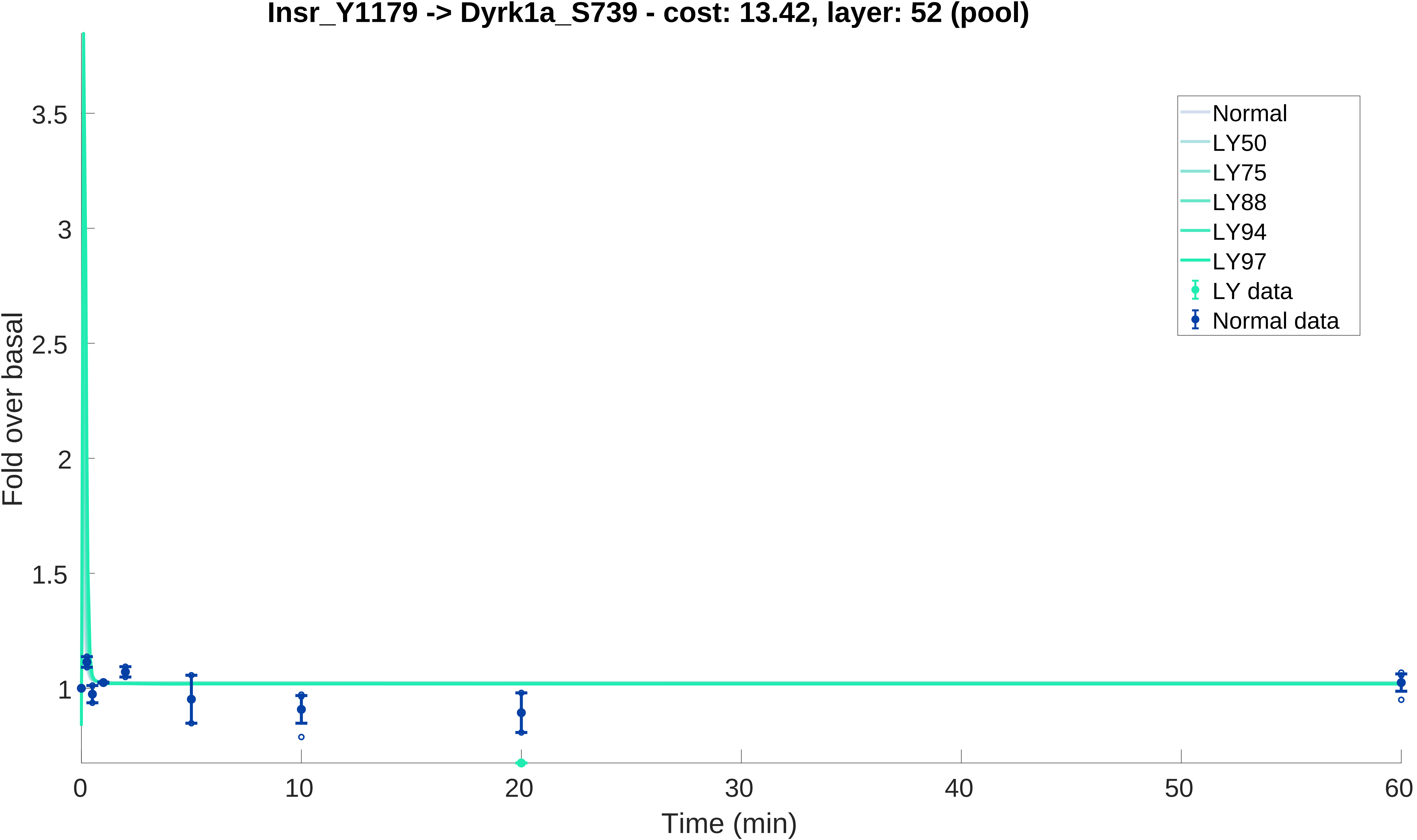

Insr_Y1179 -> Dyrk1a_S739 - cost: 13.42, layer: 52 (pool)
Fold over basal
3.5
3
2.5
2
1.5
1
0
10
20
30
40
50
60
Time (min)
Normal
LY50
LY75
LY88
LY94
LY97
LY data
Normal data

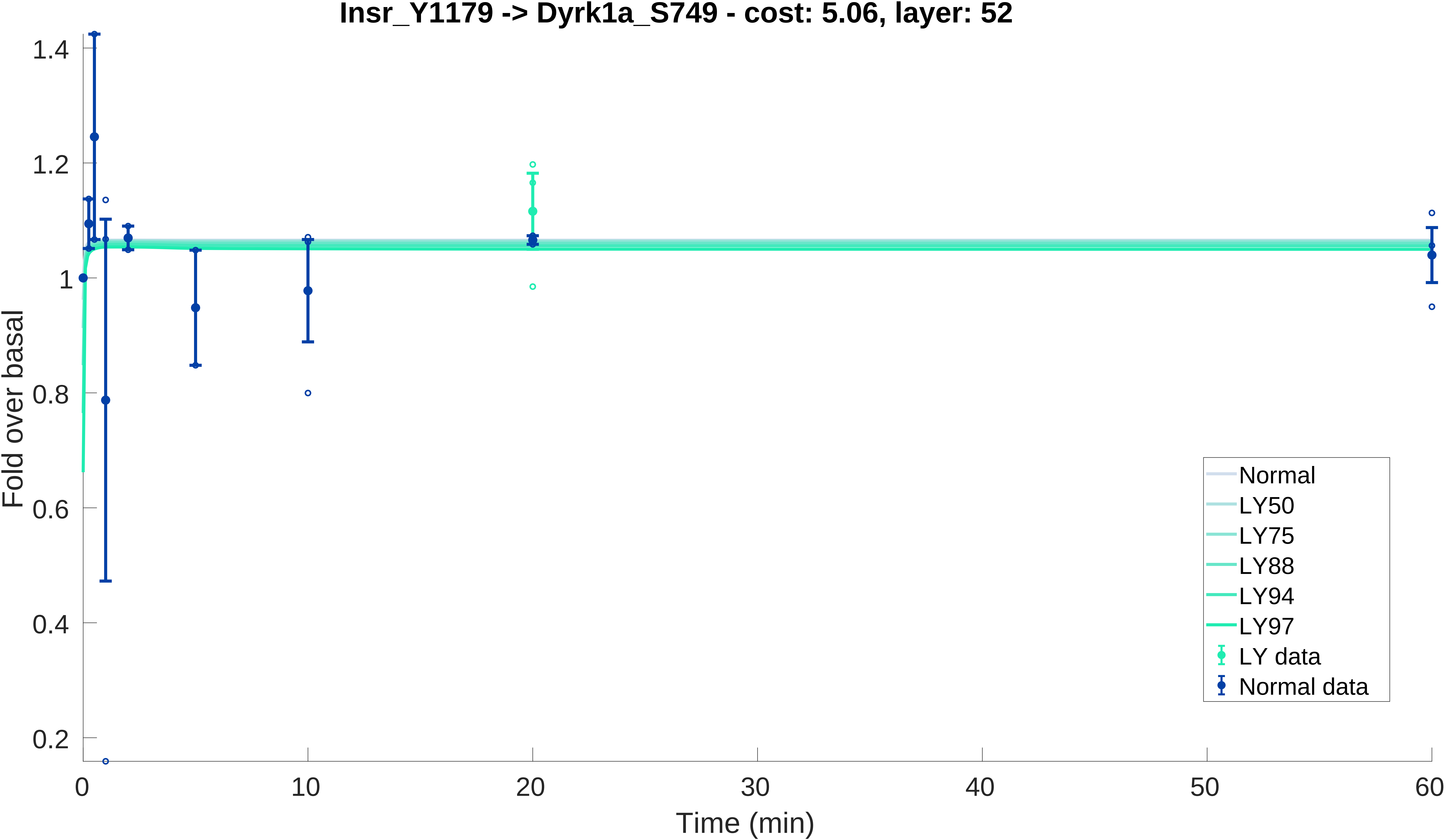

Insr_Y1179 -> Dyrk1a_S749 - cost: 5.06, layer: 52
Fold over basal
Time (min)
0
10
20
30
40
50
60
0.2
0.4
0.6
0.8
1
1.2
1.4
Normal
LY50
LY75
LY88
LY94
LY97
LY data
Normal data

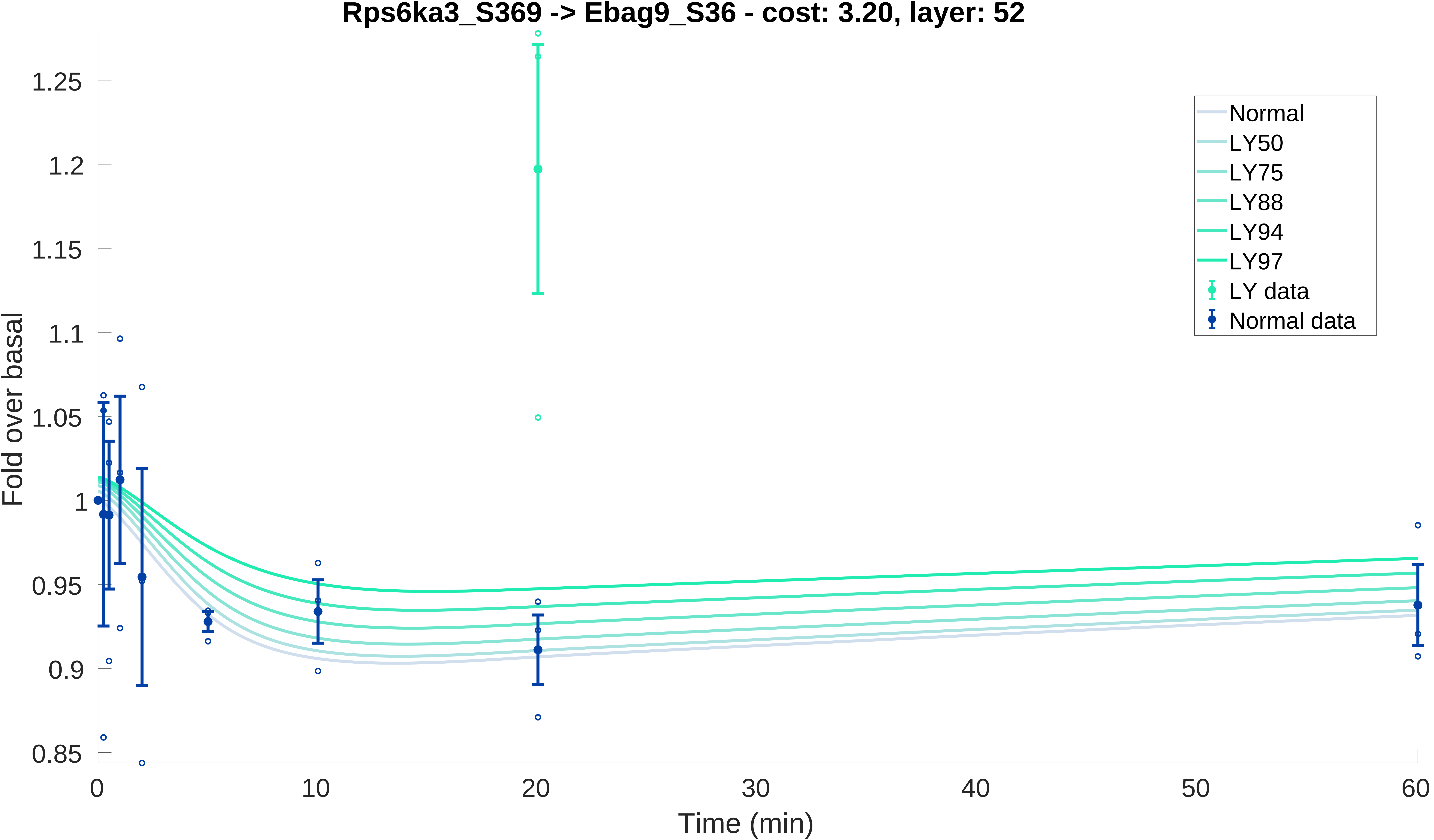

Rps6ka3_S369 -> Ebag9_S36 - cost: 3.20, layer: 52
Fold over basal
1.25
1.2
1.15
1.1
1.05
1
0.95
0.9
0.85
0
10
20
30
40
50
60
Time (min)
Normal
LY50
LY75
LY88
LY94
LY97
LY data
Normal data

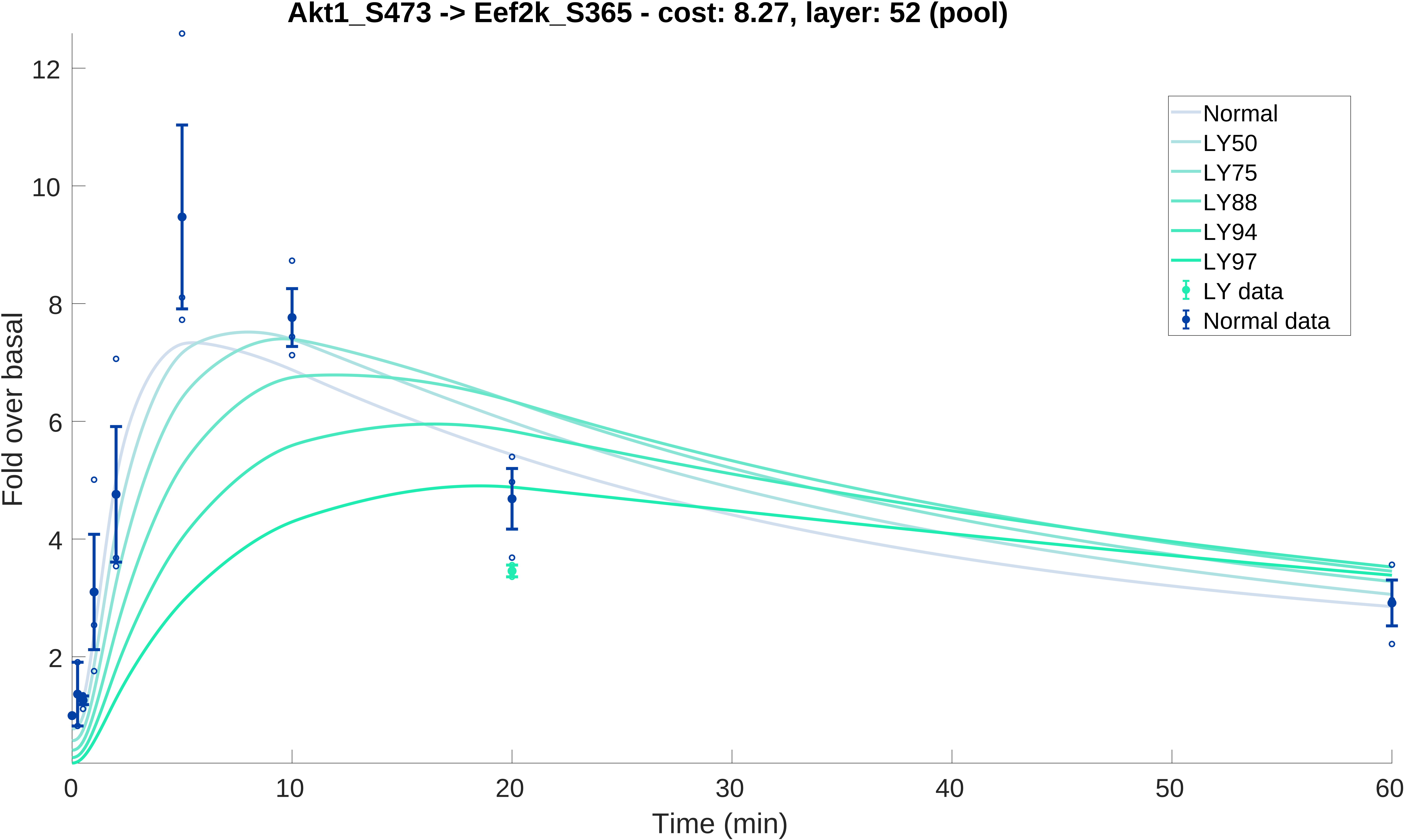

Akt1_S473 -> Eef2k_S365 - cost: 8.27, layer: 52 (pool)
Fold over basal
Time (min)
0
10
20
30
40
50
60
2
4
6
8
10
12
Normal
LY50
LY75
LY88
LY94
LY97
LY data
Normal data

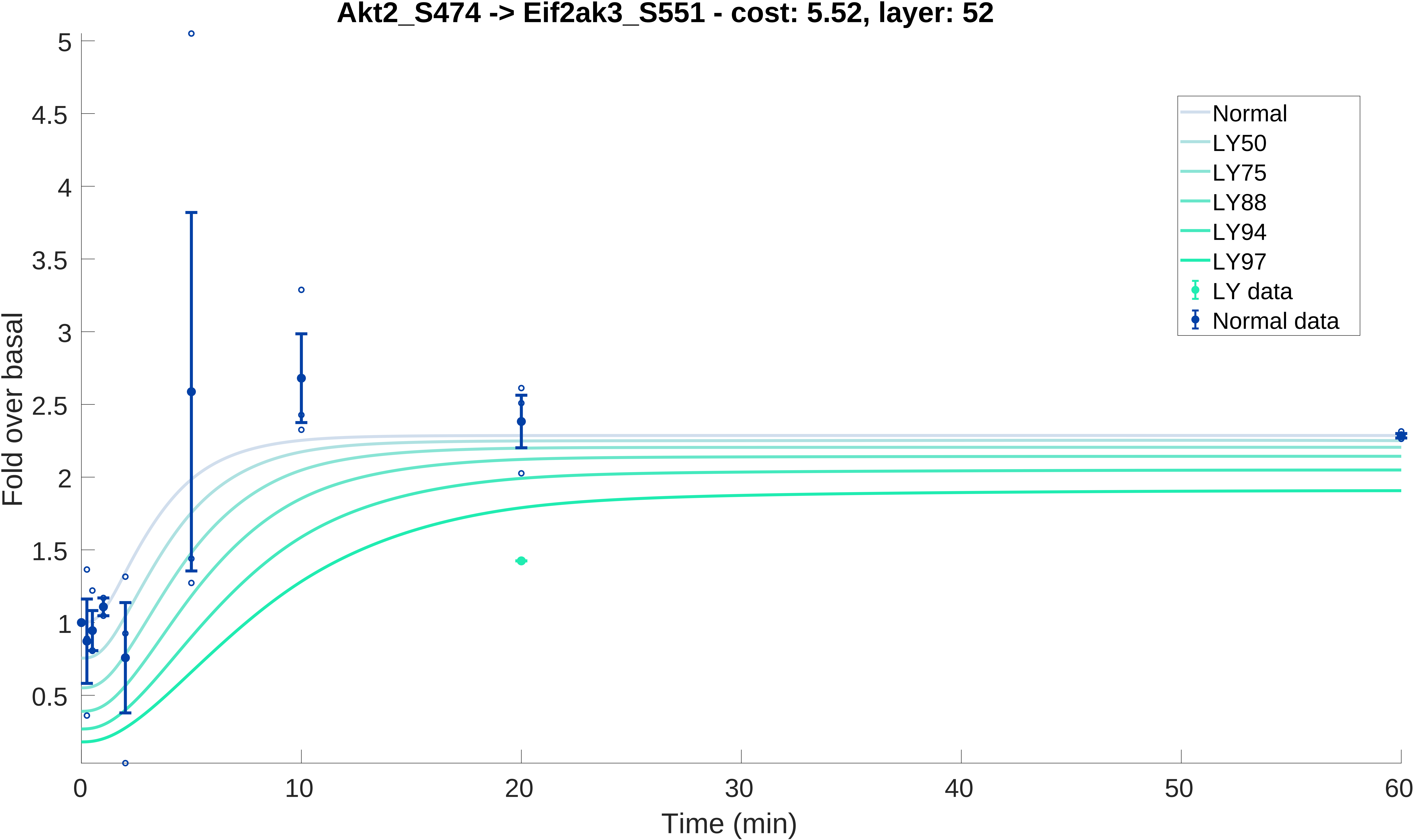

Akt2_S474 -> Eif2ak3_S551 - cost: 5.52, layer: 52
Normal
LY50
LY75
LY88
LY94
LY97
LY data
Normal data
Fold over basal
Time (min)
0
10
20
30
40
50
60
0.5
1
1.5
2
2.5
3
3.5
4
4.5
5

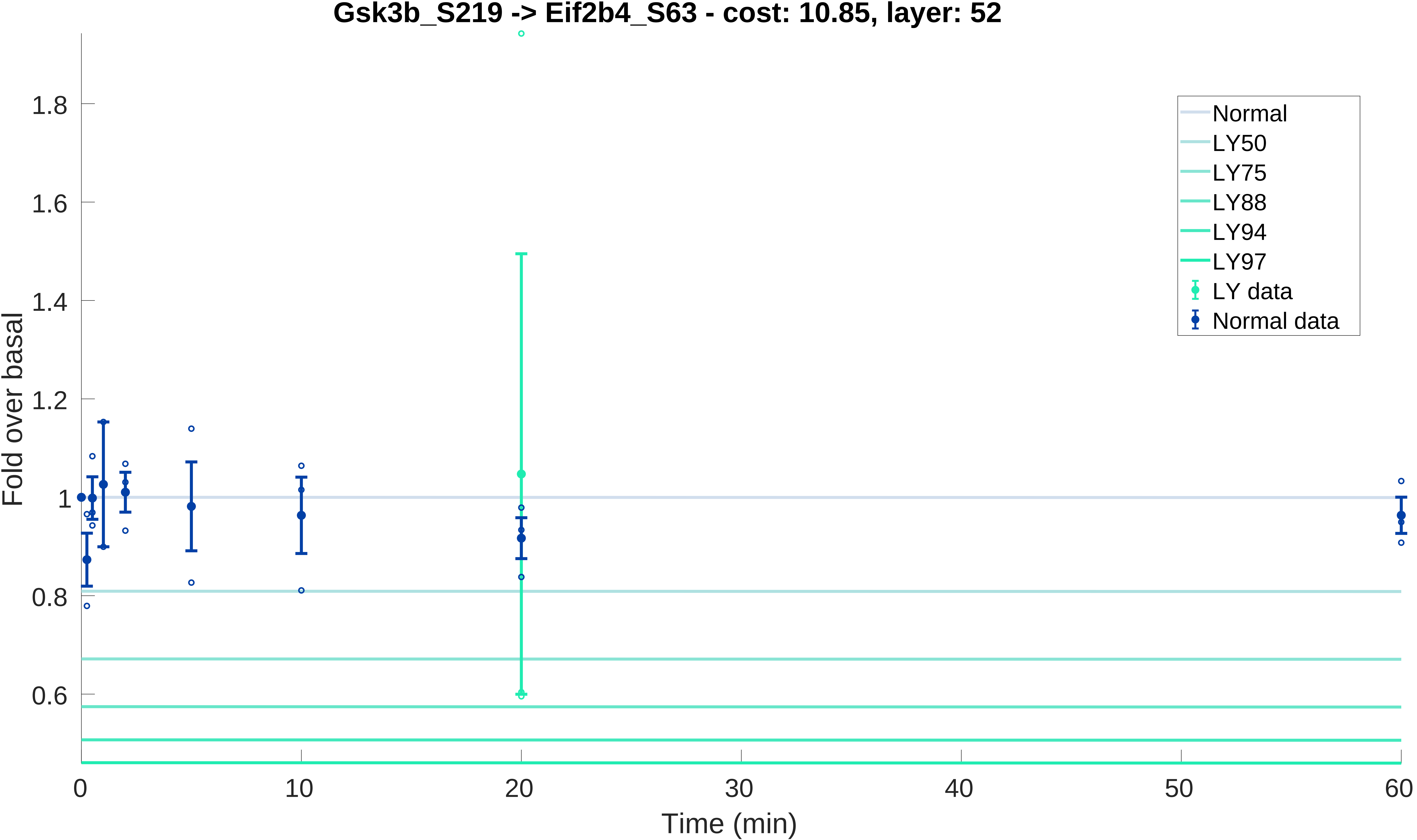

Gsk3b_S219 -> Eif2b4_S63 - cost: 10.85, layer: 52
Fold over basal
1.8
1.6
1.4
1.2
1
0.8
0.6
0
10
20
30
40
50
60
Time (min)
Normal
LY50
LY75
LY88
LY94
LY97
LY data
Normal data

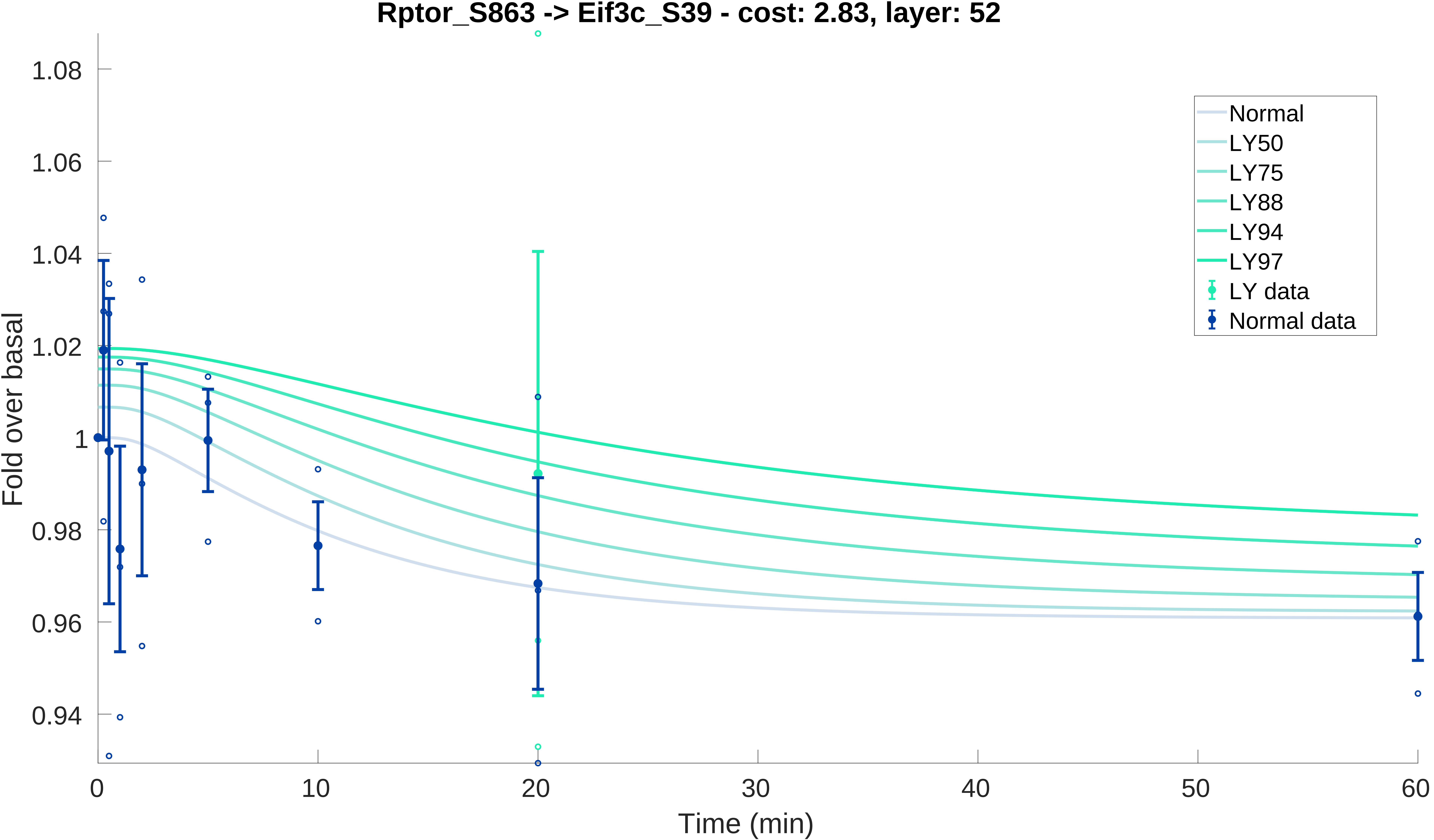
Rptor_S863 -> Eif3c_S39 - cost: 2.83, layer: 52
Fold over basal
1.08
1.06
1.04
1.02
1
0.98
0.96
0.94
0
10
20
30
40
50
60
Time (min)
Normal
LY50
LY75
LY88
LY94
LY97
LY data
Normal data

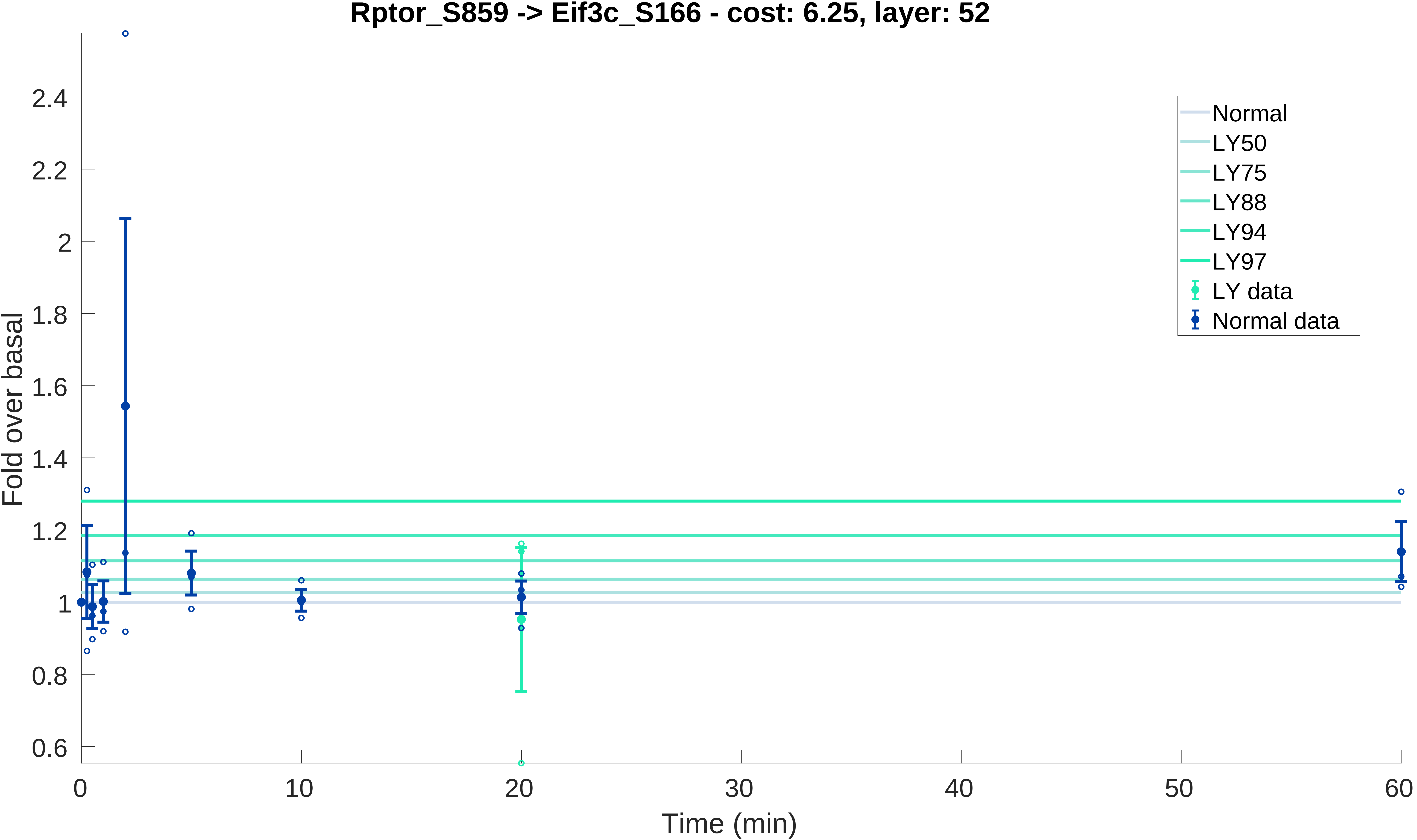

Rptor_S859 -> Eif3c_S166 - cost: 6.25, layer: 52
Normal
LY50
LY75
LY88
LY94
LY97
LY data
Normal data
Fold over basal
Time (min)
0.6
0.8
1
1.2
1.4
1.6
1.8
2
2.2
2.4
0
10
20
30
40
50
60

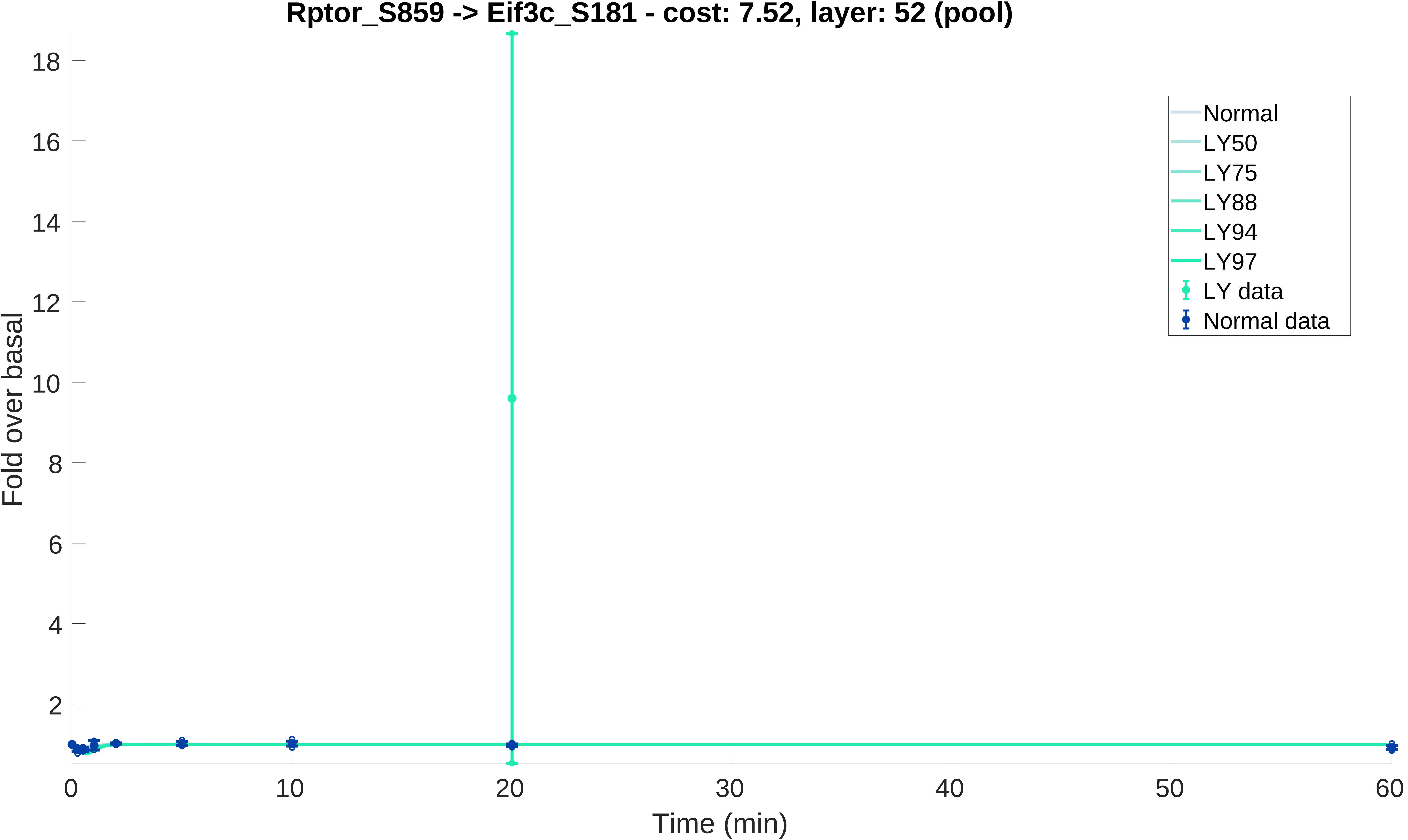
Rptor_S859 -> Eif3c_S181 - cost: 7.52, layer: 52 (pool)
Normal
LY50
LY75
LY88
LY94
LY97
LY data
Normal data
Fold over basal
Time (min)
0
10
20
30
40
50
60
2
4
6
8
10
12
14
16
18

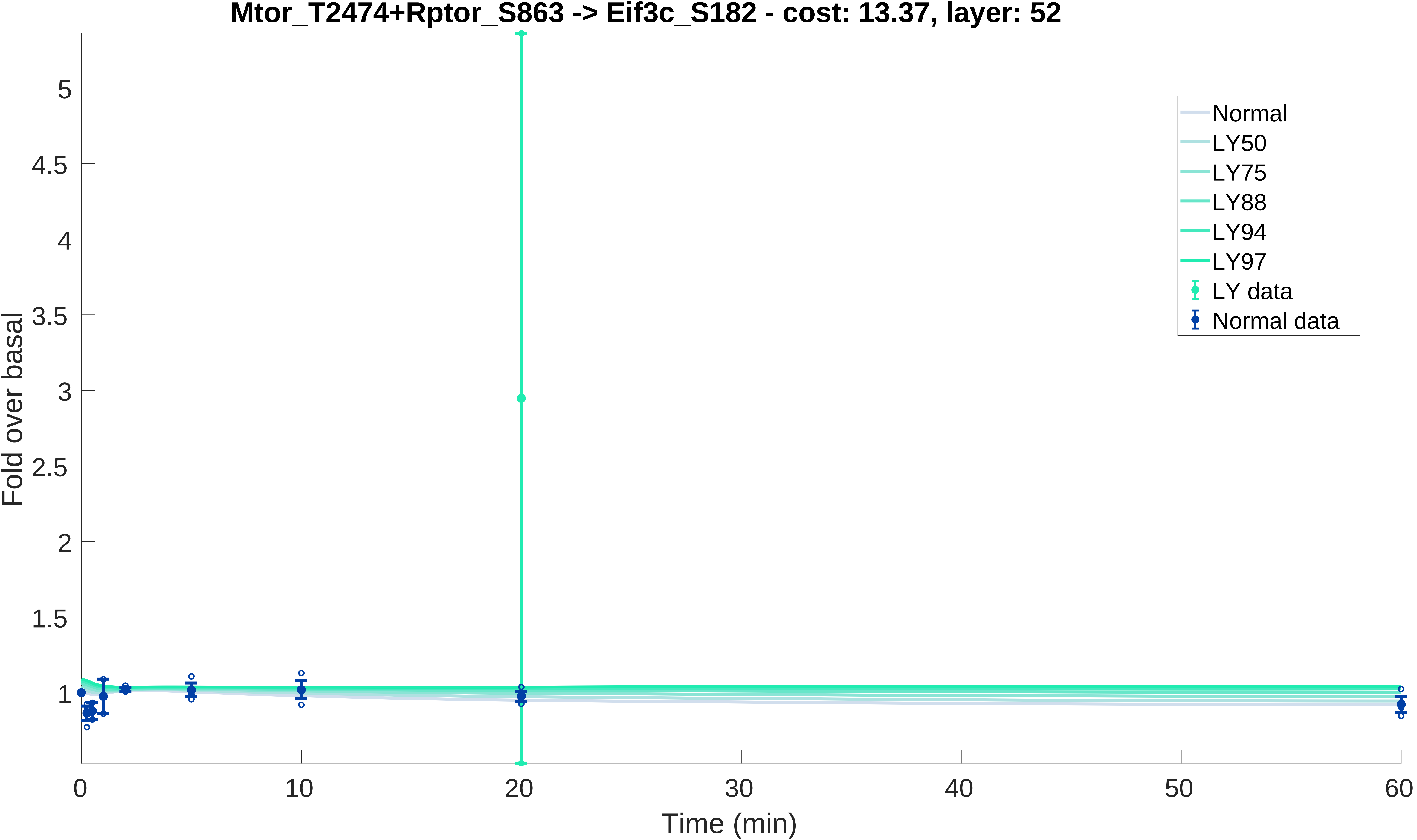

Mtor_T2474+Rptor_S863 -> Eif3c_S182 - cost: 13.37, layer: 52
Fold over basal
5
4.5
4
3.5
3
2.5
2
1.5
1
0
10
20
30
40
50
60
Time (min)
Normal
LY50
LY75
LY88
LY94
LY97
LY data
Normal data

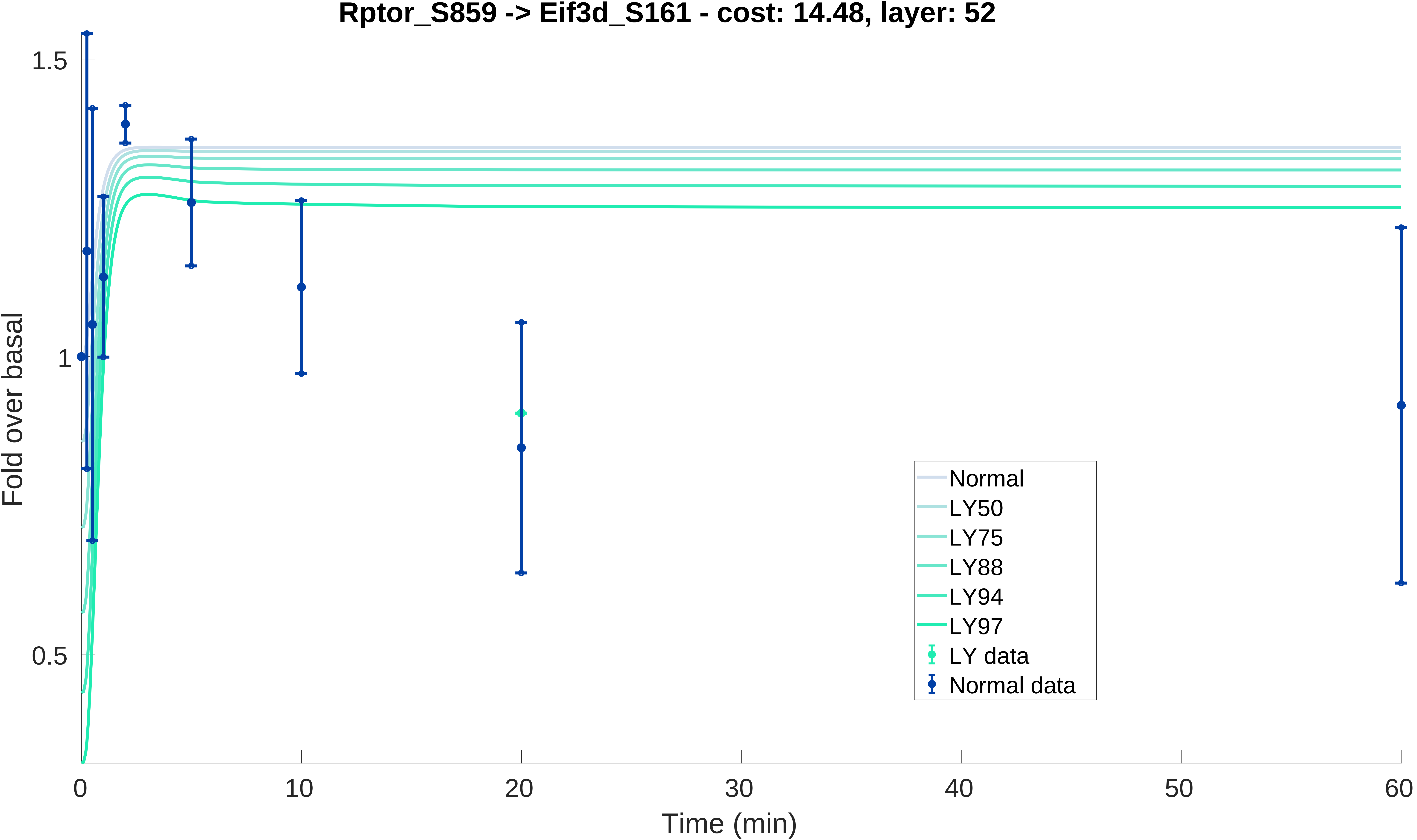
Rptor_S859 -> Eif3d_S161 - cost: 14.48, layer: 52
Fold over basal
1.5
1
0.5
0
10
20
30
40
50
60
Time (min)
Normal
LY50
LY75
LY88
LY94
LY97
LY data
Normal data

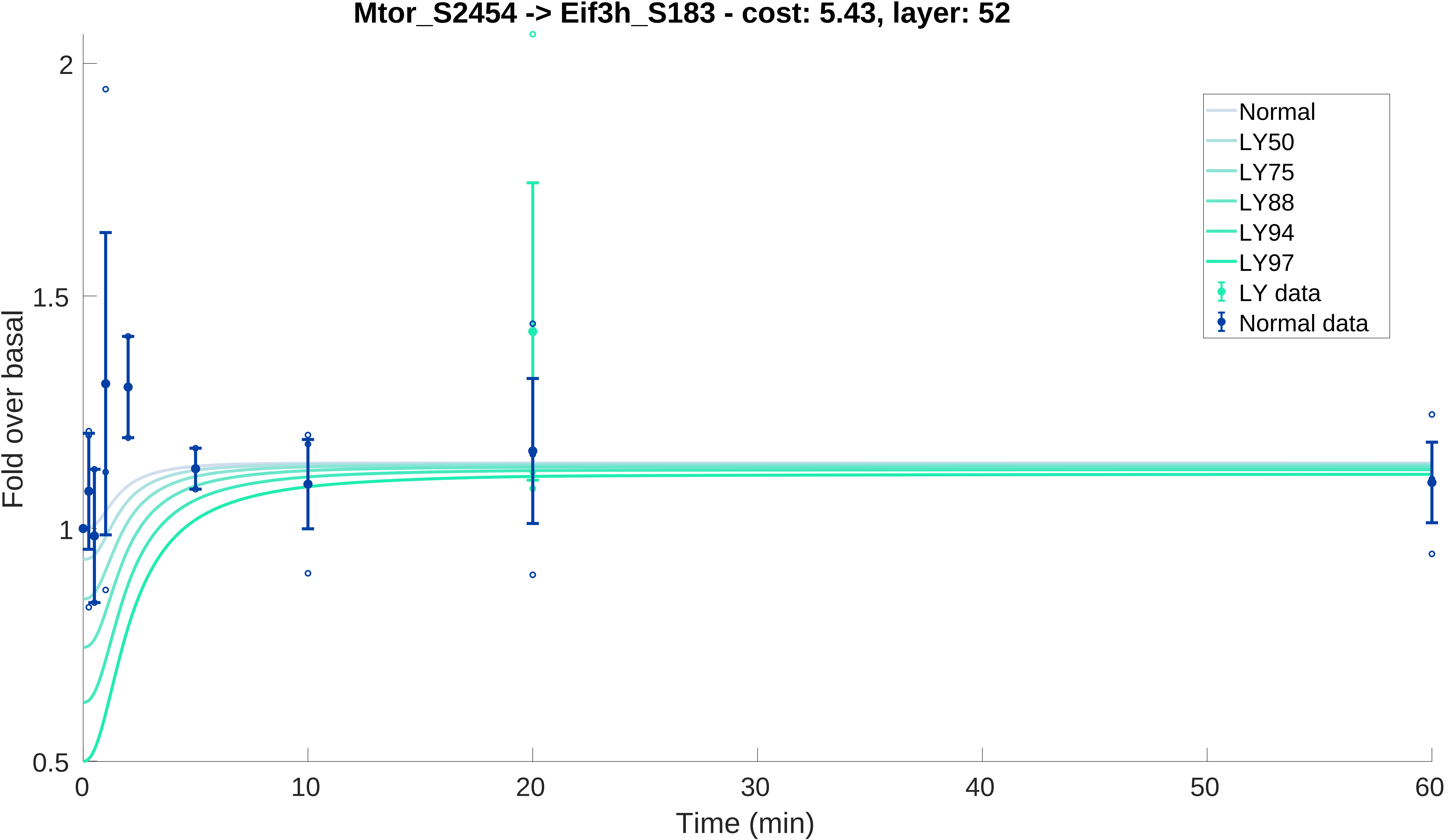

Mtor_S2454 -> Eif3h_S183 - cost: 5.43, layer: 52
Fold over basal
Time (min)
0
10
20
30
40
50
60
0.5
1
1.5
2
Normal
LY50
LY75
LY88
LY94
LY97
LY data
Normal data

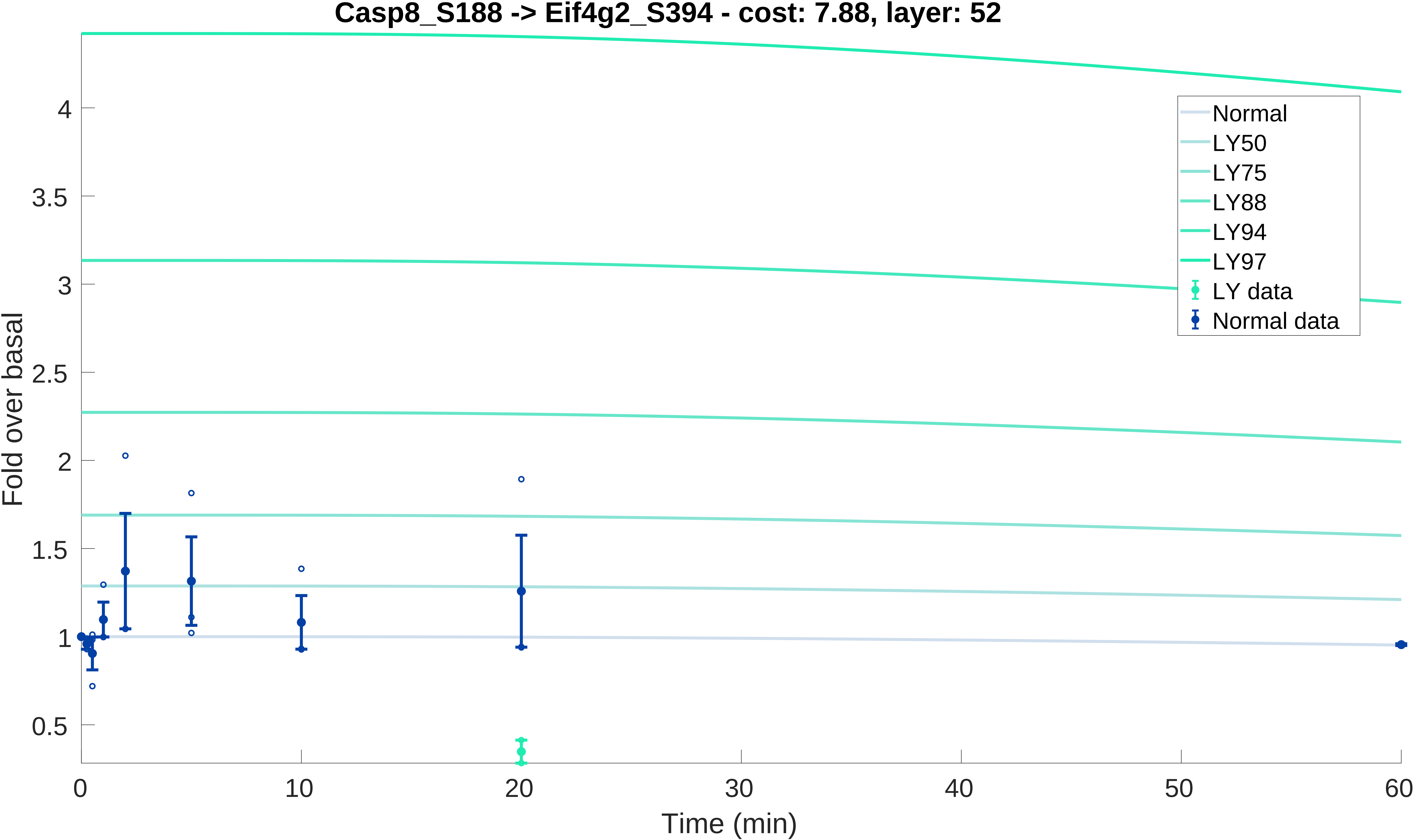

Casp8_S188 -> Eif4g2_S394 - cost: 7.88, layer: 52
Fold over basal
Time (min)
0
10
20
30
40
50
60
0.5
1
1.5
2
2.5
3
3.5
4
Normal
LY50
LY75
LY88
LY94
LY97
LY data
Normal data

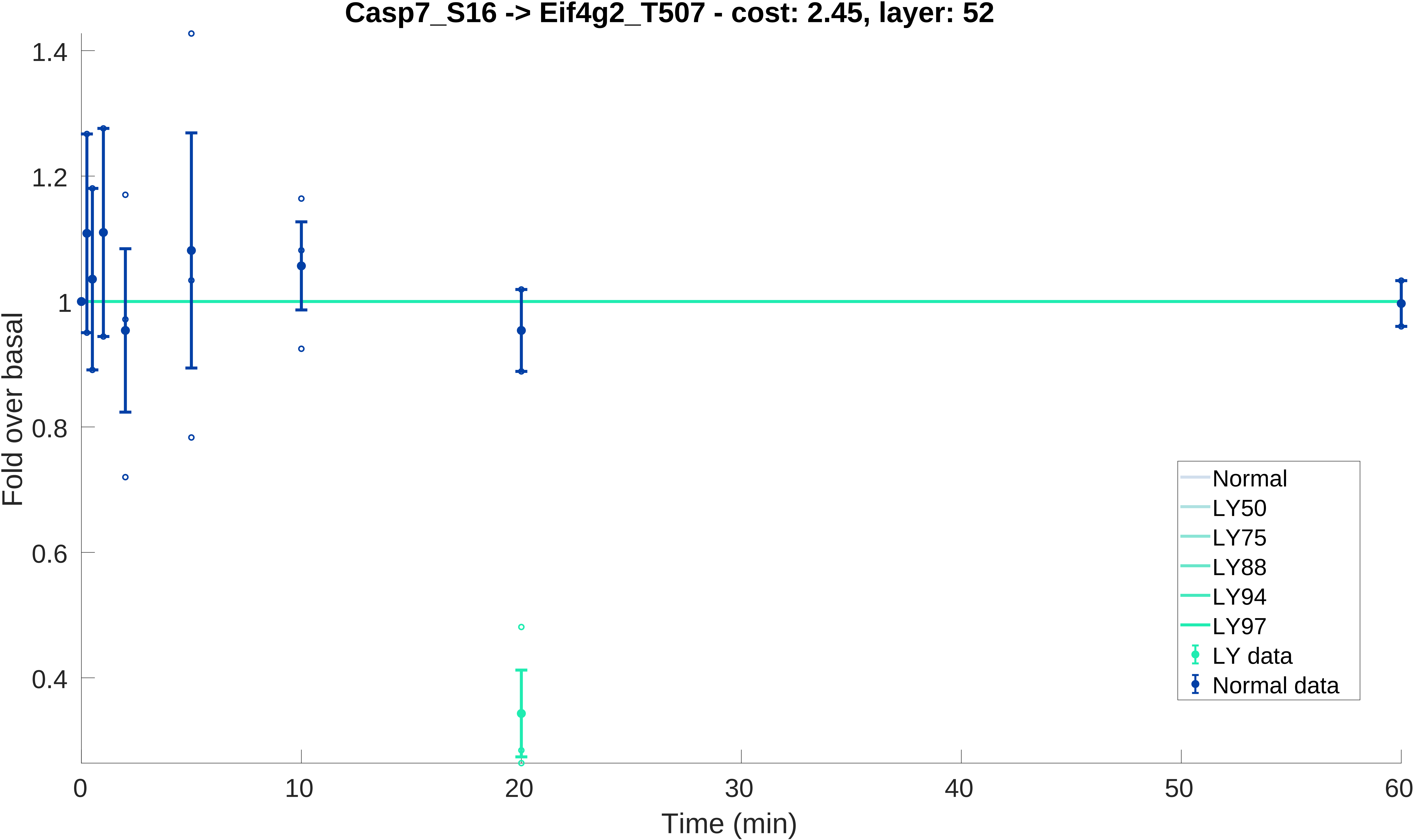
Casp7_S16 -> Eif4g2_T507 - cost: 2.45, layer: 52
Fold over basal
1.4
1.2
1
0.8
0.6
0.4
0
10
20
30
40
50
60
Time (min)
Normal
LY50
LY75
LY88
LY94
LY97
LY data
Normal data

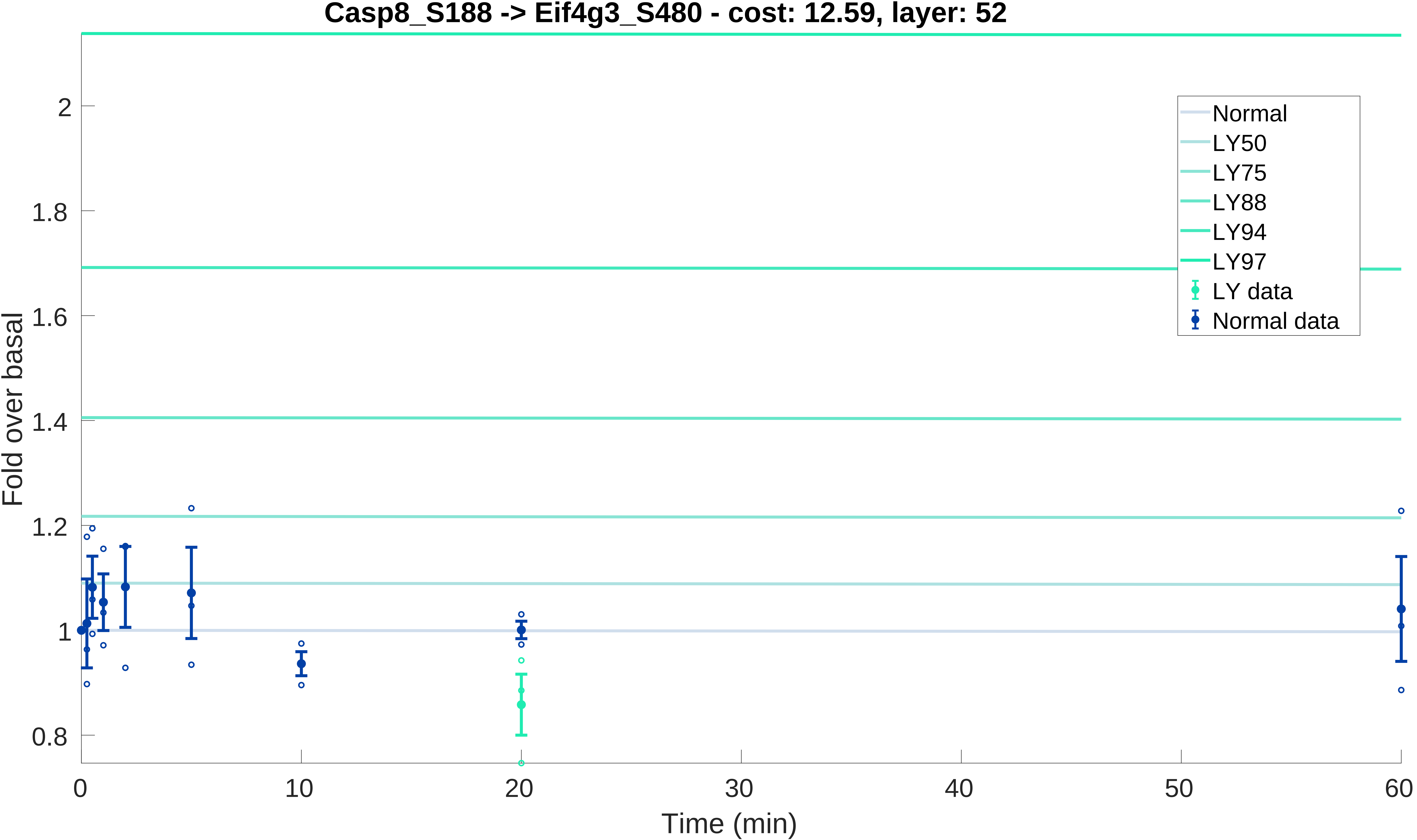

Casp8_S188 -> Eif4g3_S480 - cost: 12.59, layer: 52
Fold over basal
Time (min)
2
1.8
1.6
1.4
1.2
1
0.8
0
10
20
30
40
50
60
Normal
LY50
LY75
LY88
LY94
LY97
LY data
Normal data

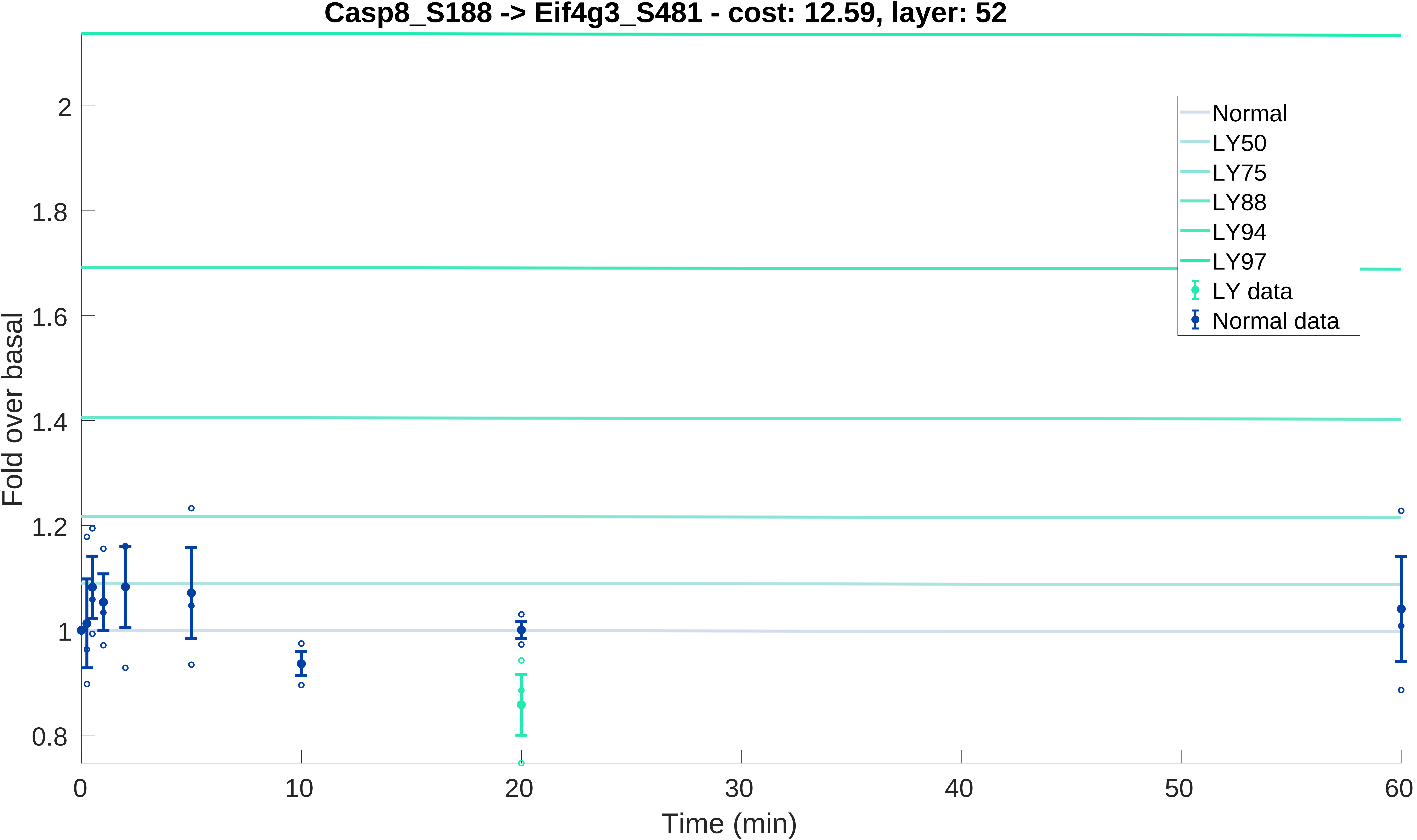
Casp8_S188 -> Eif4g3_S481 - cost: 12.59, layer: 52
Fold over basal
Time (min)
0.8
1
1.2
1.4
1.6
1.8
2
0
10
20
30
40
50
60
Normal
LY50
LY75
LY88
LY94
LY97
LY data
Normal data

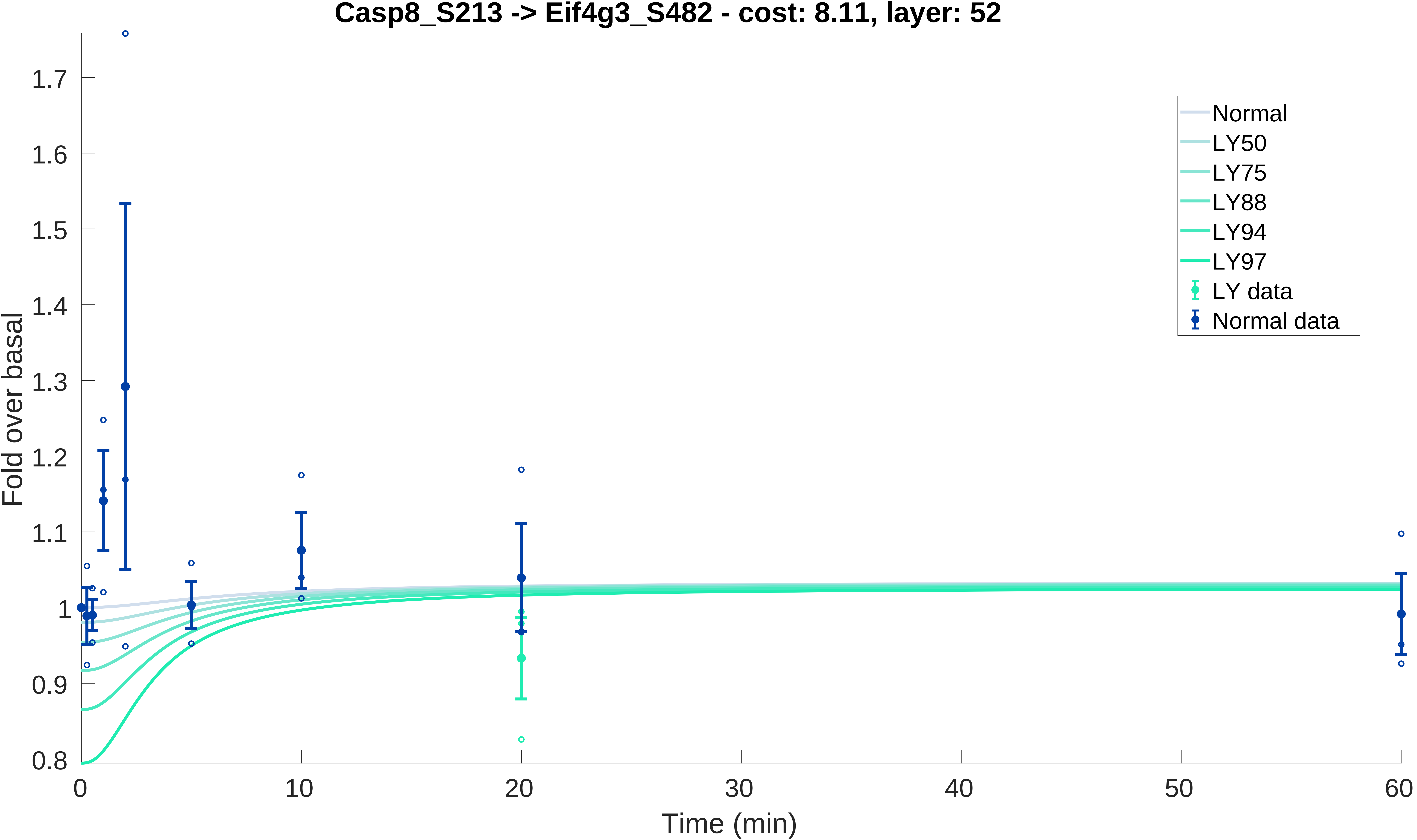

Casp8_S213 -> Eif4g3_S482 - cost: 8.11, layer: 52
Fold over basal
Time (min)
0.8
0.9
1
1.1
1.2
1.3
1.4
1.5
1.6
1.7
0
10
20
30
40
50
60
Normal
LY50
LY75
LY88
LY94
LY97
LY data
Normal data

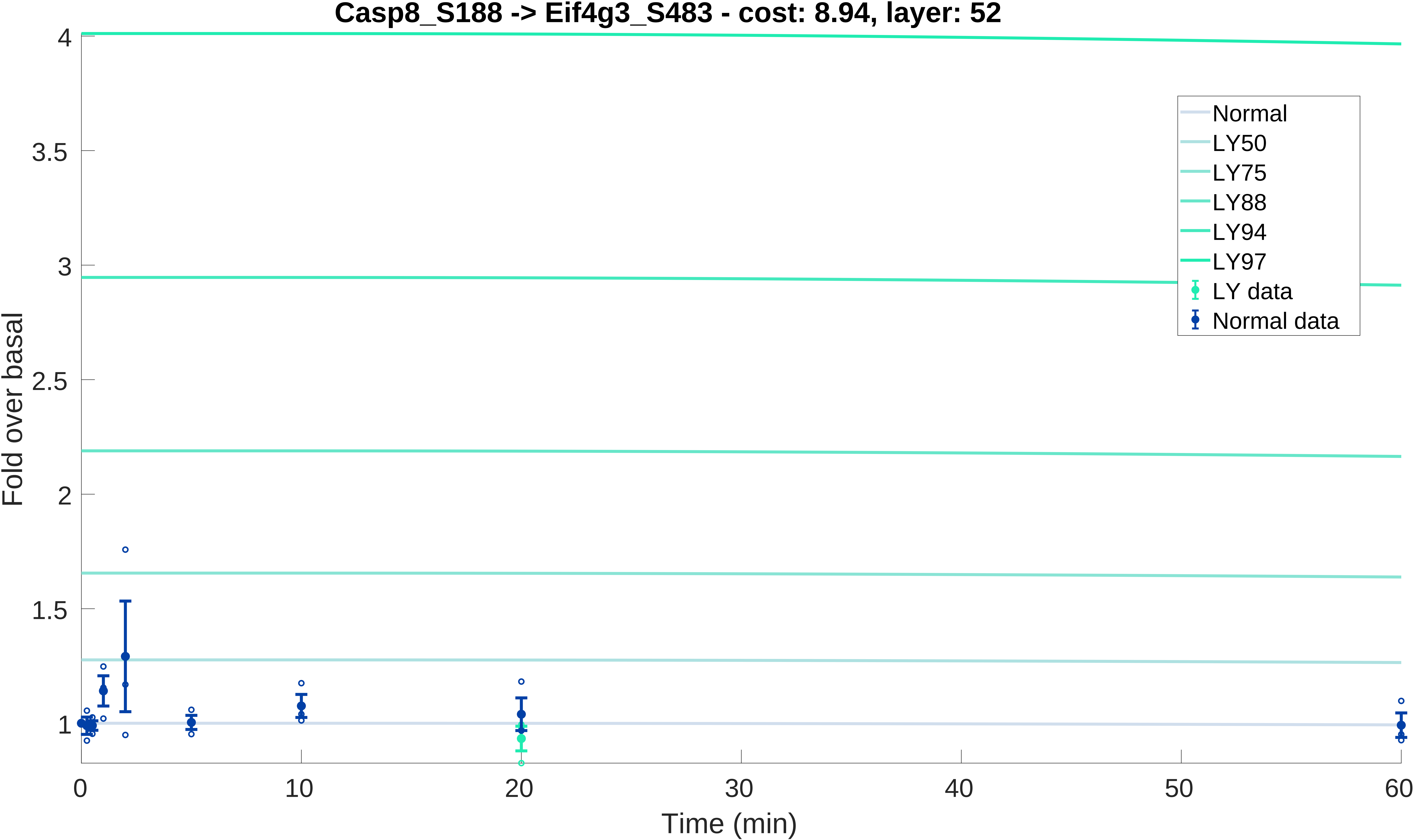

Casp8_S188 -> Eif4g3_S483 - cost: 8.94, layer: 52
Fold over basal
Time (min)
4
3.5
3
2.5
2
1.5
1
0
10
20
30
40
50
60
Normal
LY50
LY75
LY88
LY94
LY97
LY data
Normal data

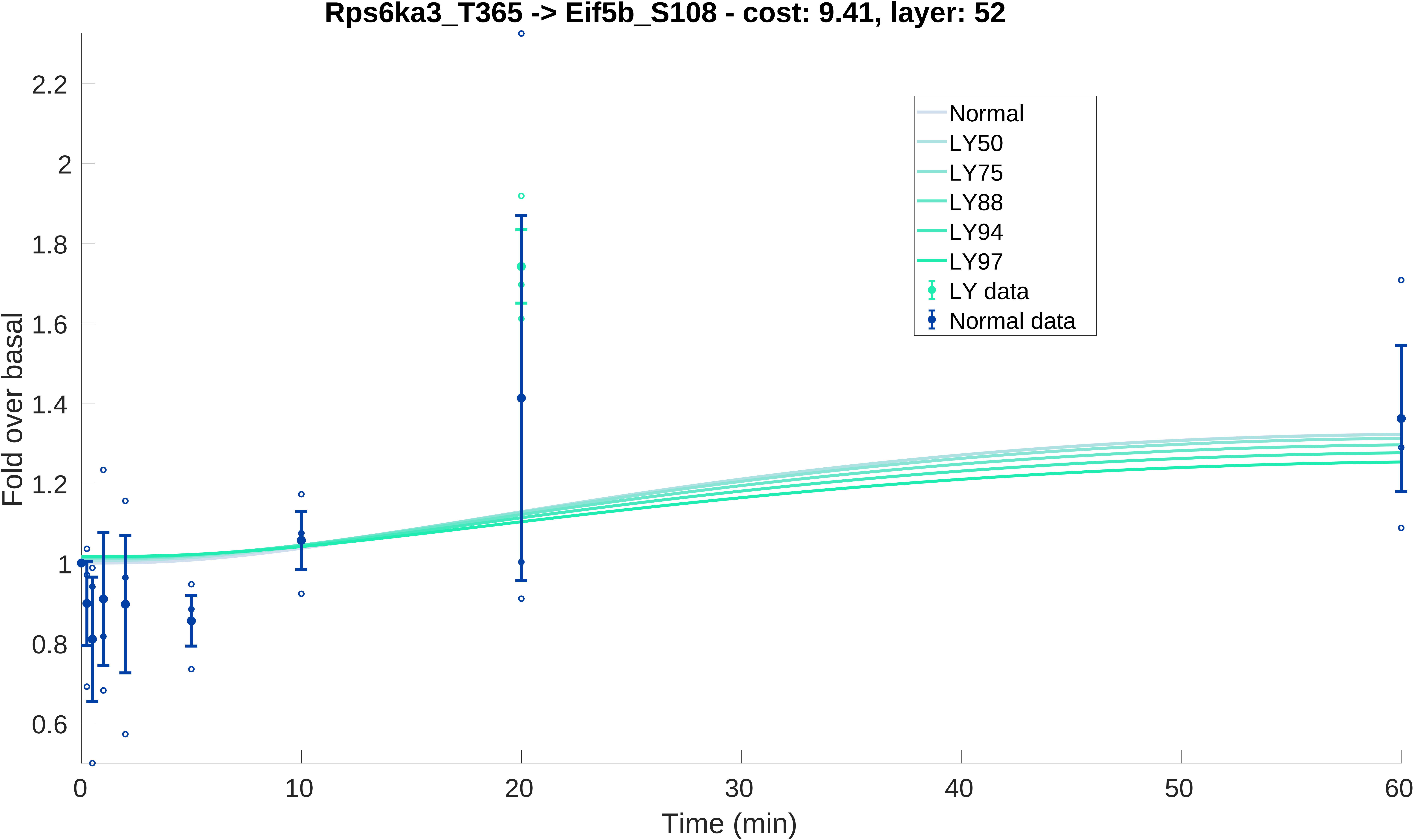
Rps6ka3_T365 -> Eif5b_S108 - cost: 9.41, layer: 52
Fold over basal
Time (min)
Normal
LY50
LY75
LY88
LY94
LY97
LY data
Normal data
0.6
0.8
1
1.2
1.4
1.6
1.8
2
2.2
0
10
20
30
40
50
60

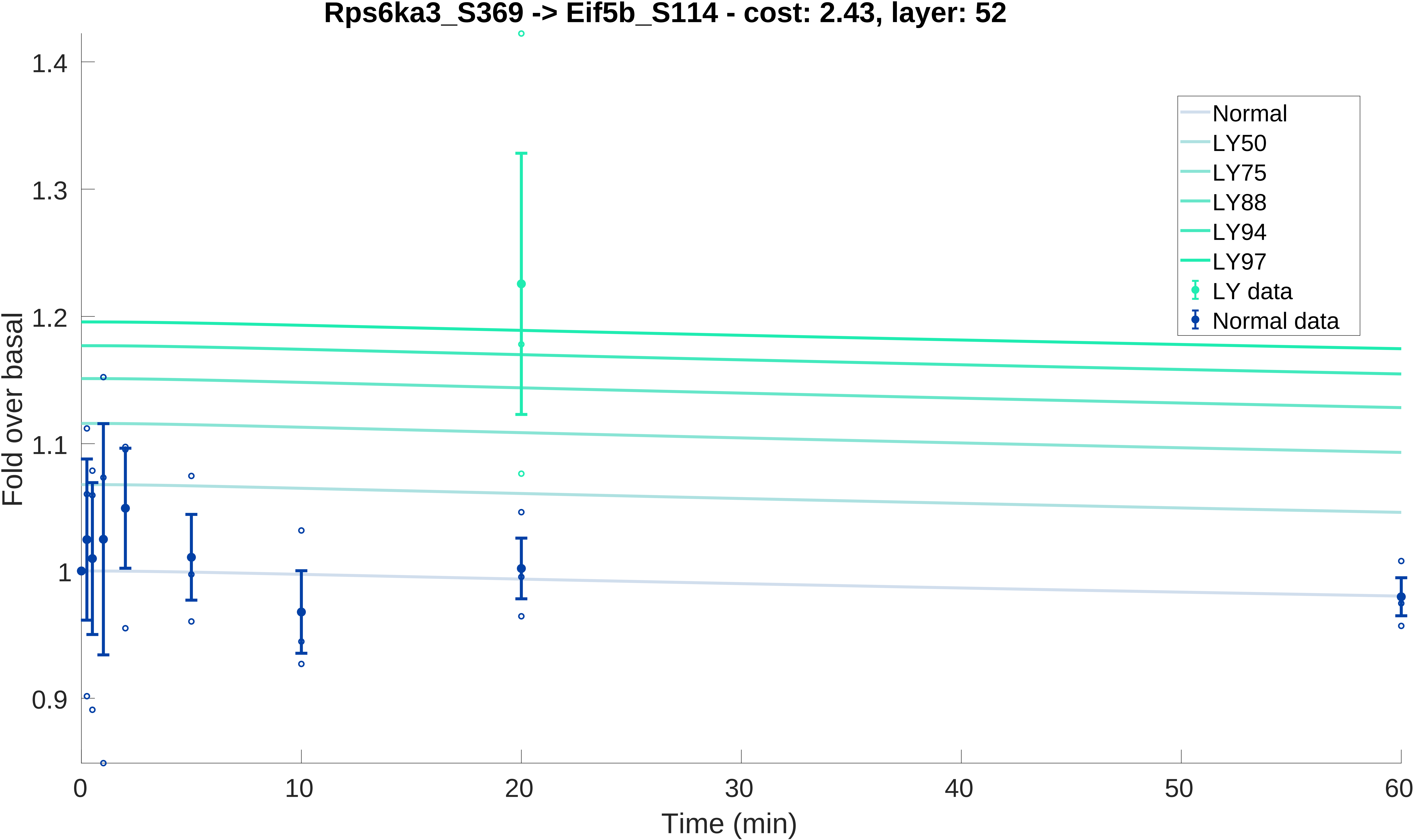

Rps6ka3_S369 -> Eif5b_S114 - cost: 2.43, layer: 52
Fold over basal
1.4
1.3
1.2
1.1
1
0.9
0
10
20
30
40
50
60
Time (min)
Normal
LY50
LY75
LY88
LY94
LY97
LY data
Normal data

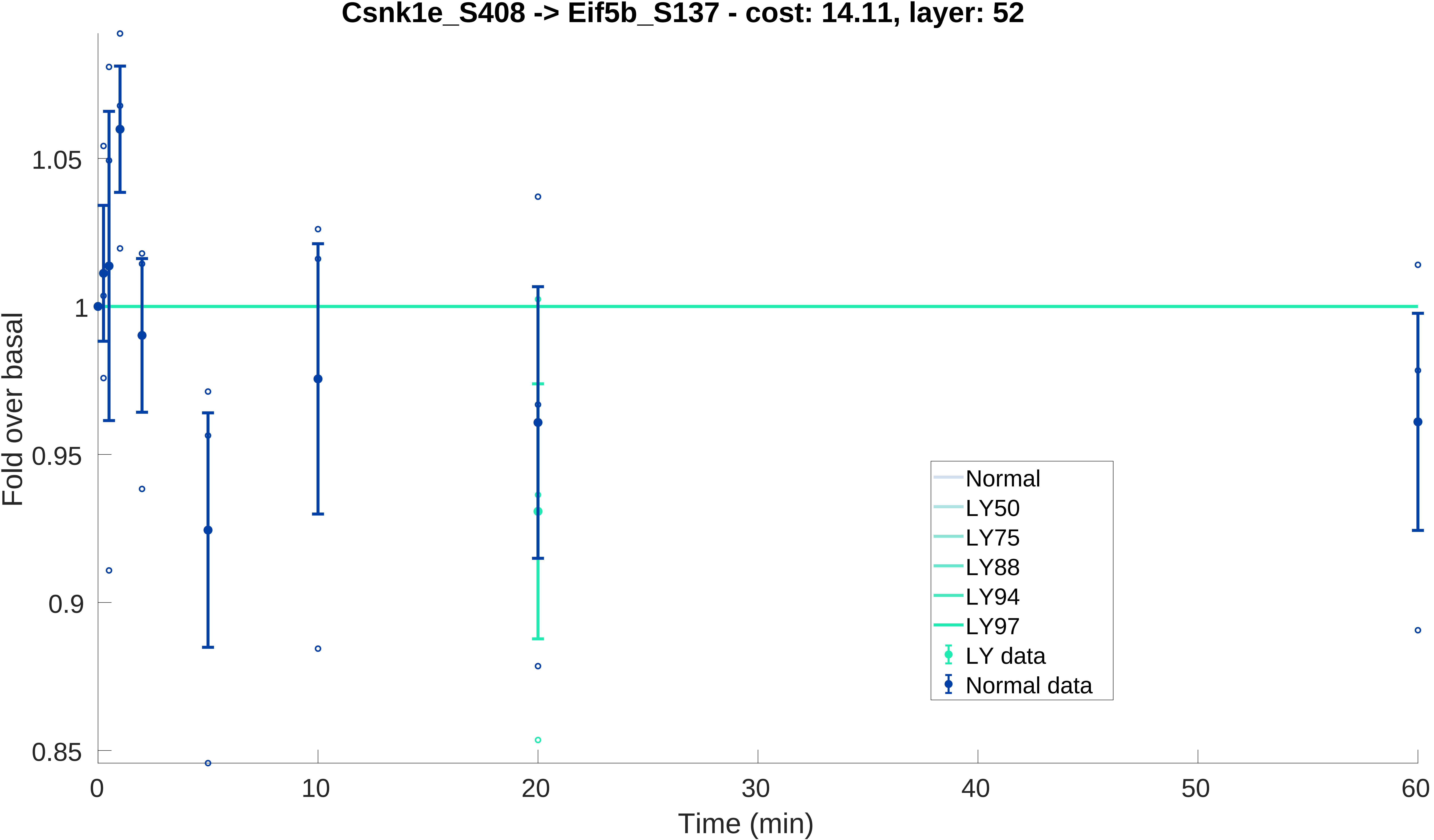

Csnk1e_S408 -> Eif5b_S137 - cost: 14.11, layer: 52
Fold over basal
1.05
1
0.95
0.9
0.85
0
10
20
30
40
50
60
Time (min)
Normal
LY50
LY75
LY88
LY94
LY97
LY data
Normal data

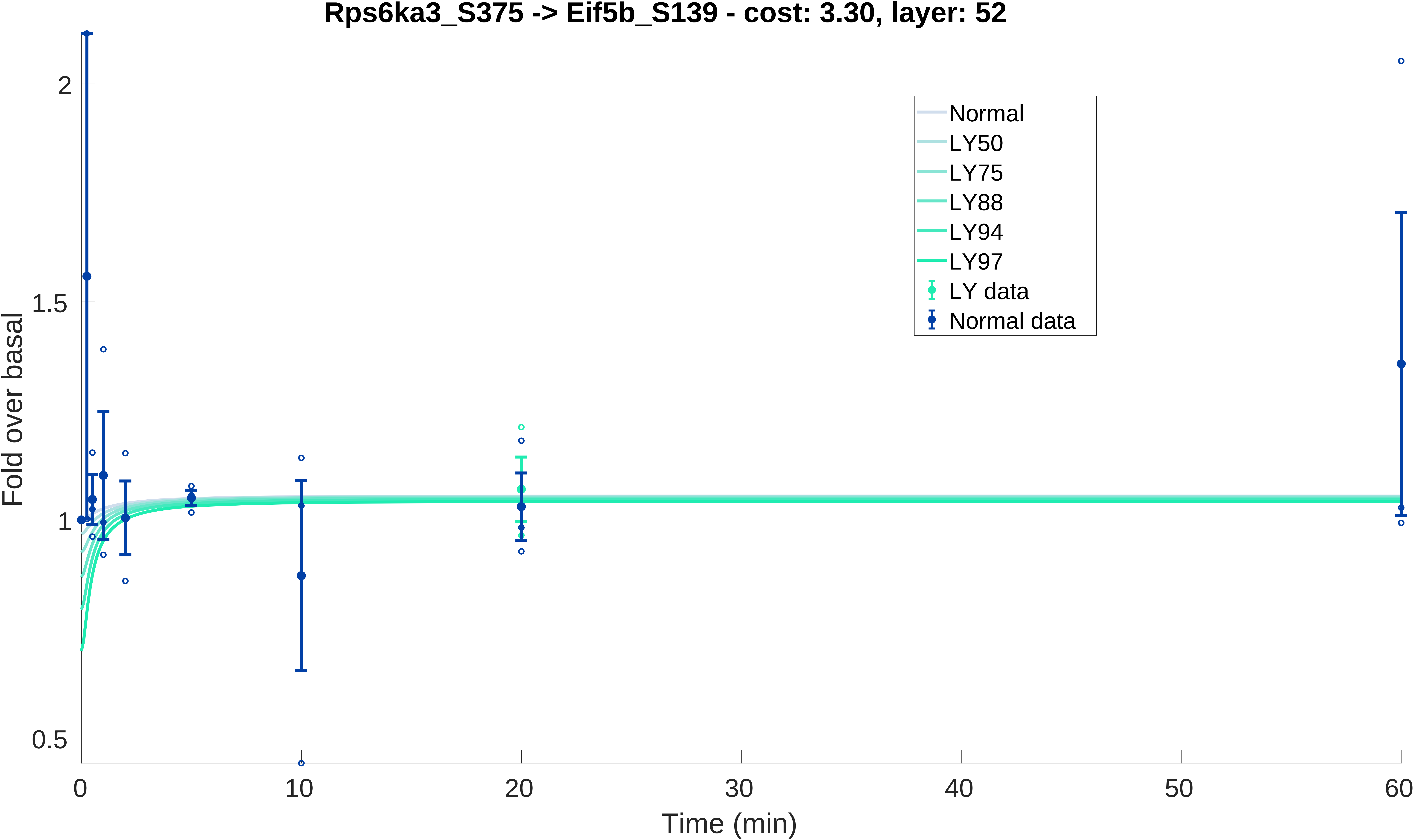

Rps6ka3_S375 -> Eif5b_S139 - cost: 3.30, layer: 52
Fold over basal
Time (min)
0.5
1
1.5
2
0
10
20
30
40
50
60
Normal
LY50
LY75
LY88
LY94
LY97
LY data
Normal data

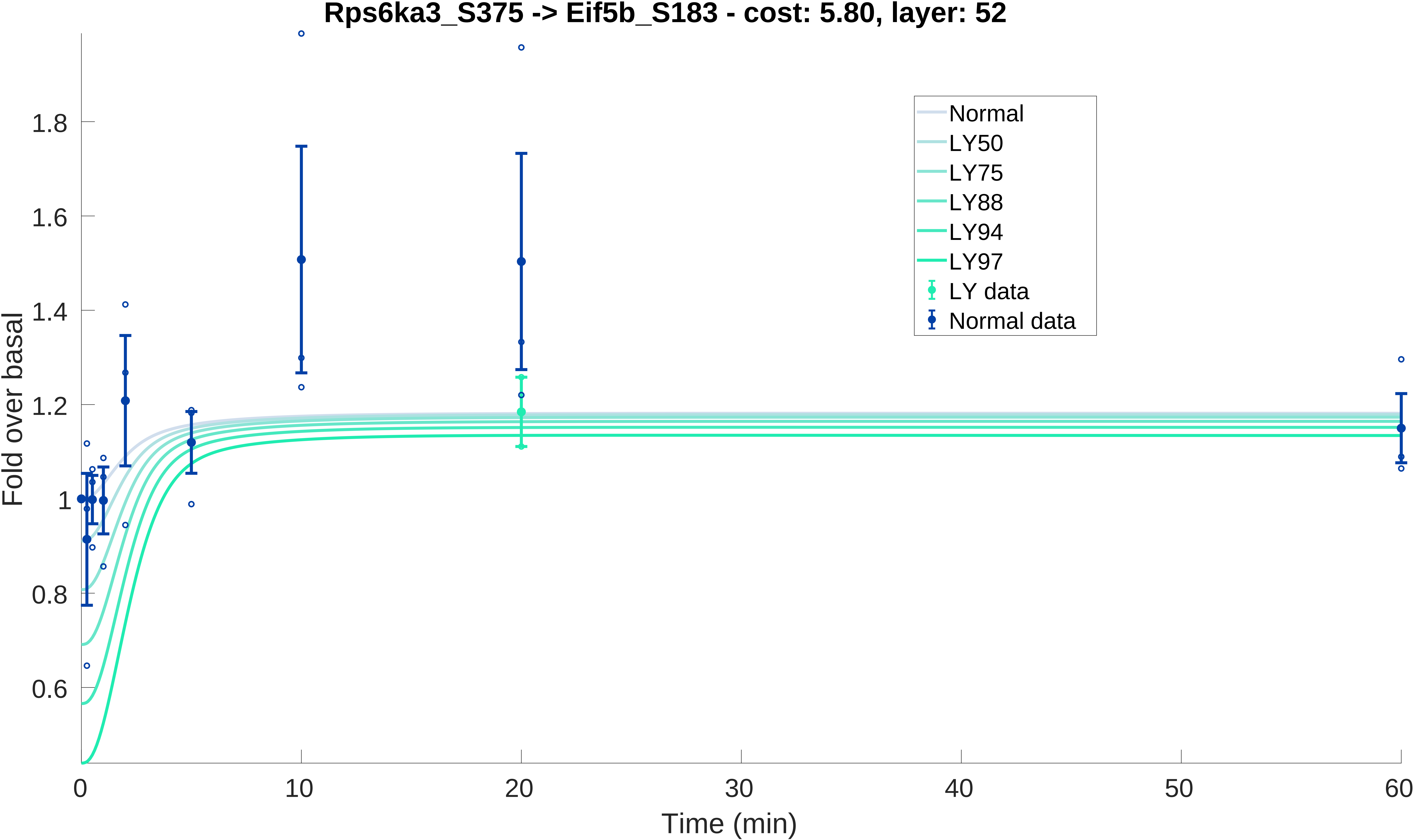

Rps6ka3_S375 -> Eif5b_S183 - cost: 5.80, layer: 52
Fold over basal
Time (min)
1.8
1.6
1.4
1.2
1
0.8
0.6
0
10
20
30
40
50
60
Normal
LY50
LY75
LY88
LY94
LY97
LY data
Normal data

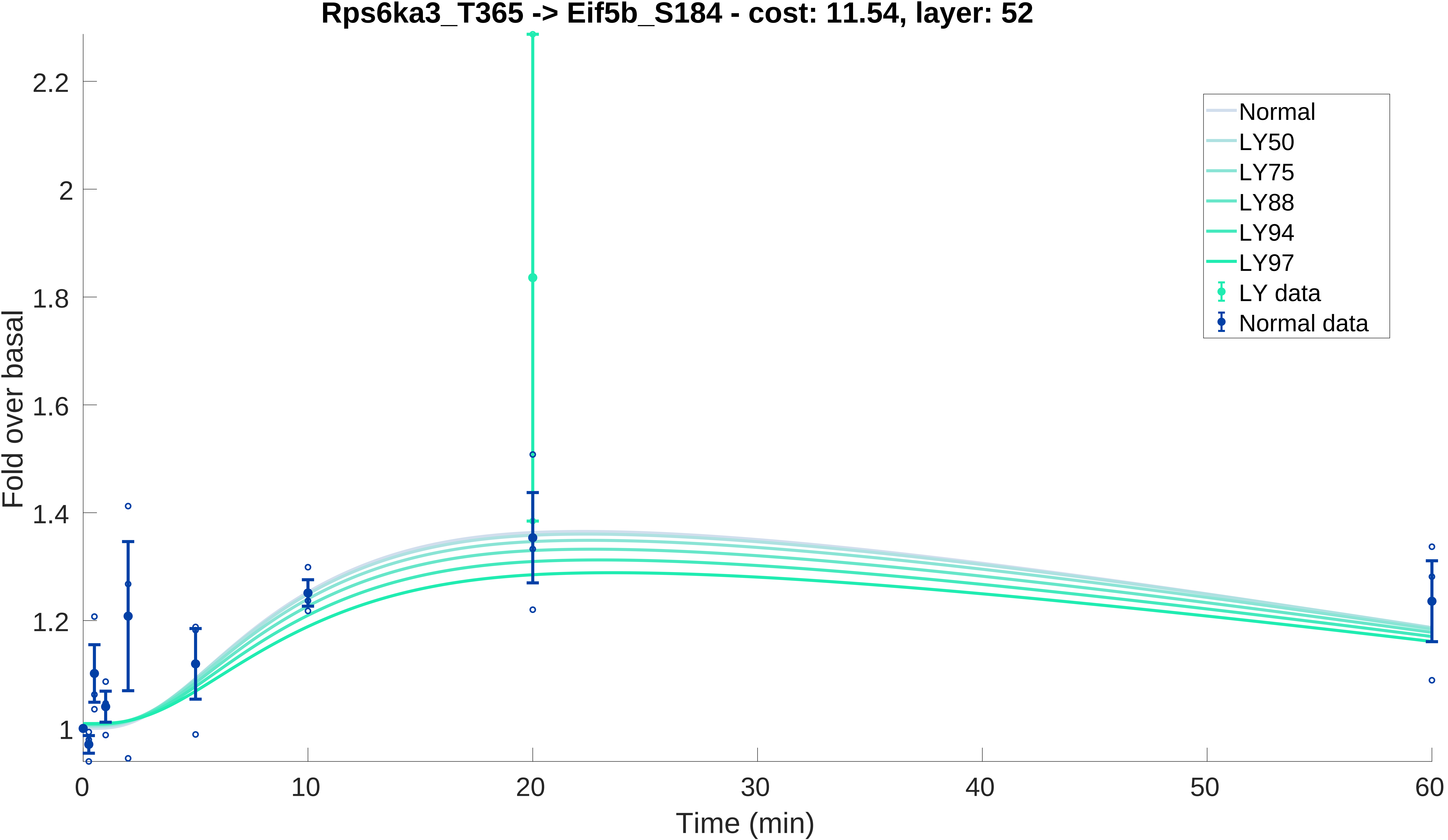

Rps6ka3_T365 -> Eif5b_S184 - cost: 11.54, layer: 52
Fold over basal
Time (min)
2.2
2
1.8
1.6
1.4
1.2
1
0
10
20
30
40
50
60
Normal
LY50
LY75
LY88
LY94
LY97
LY data
Normal data

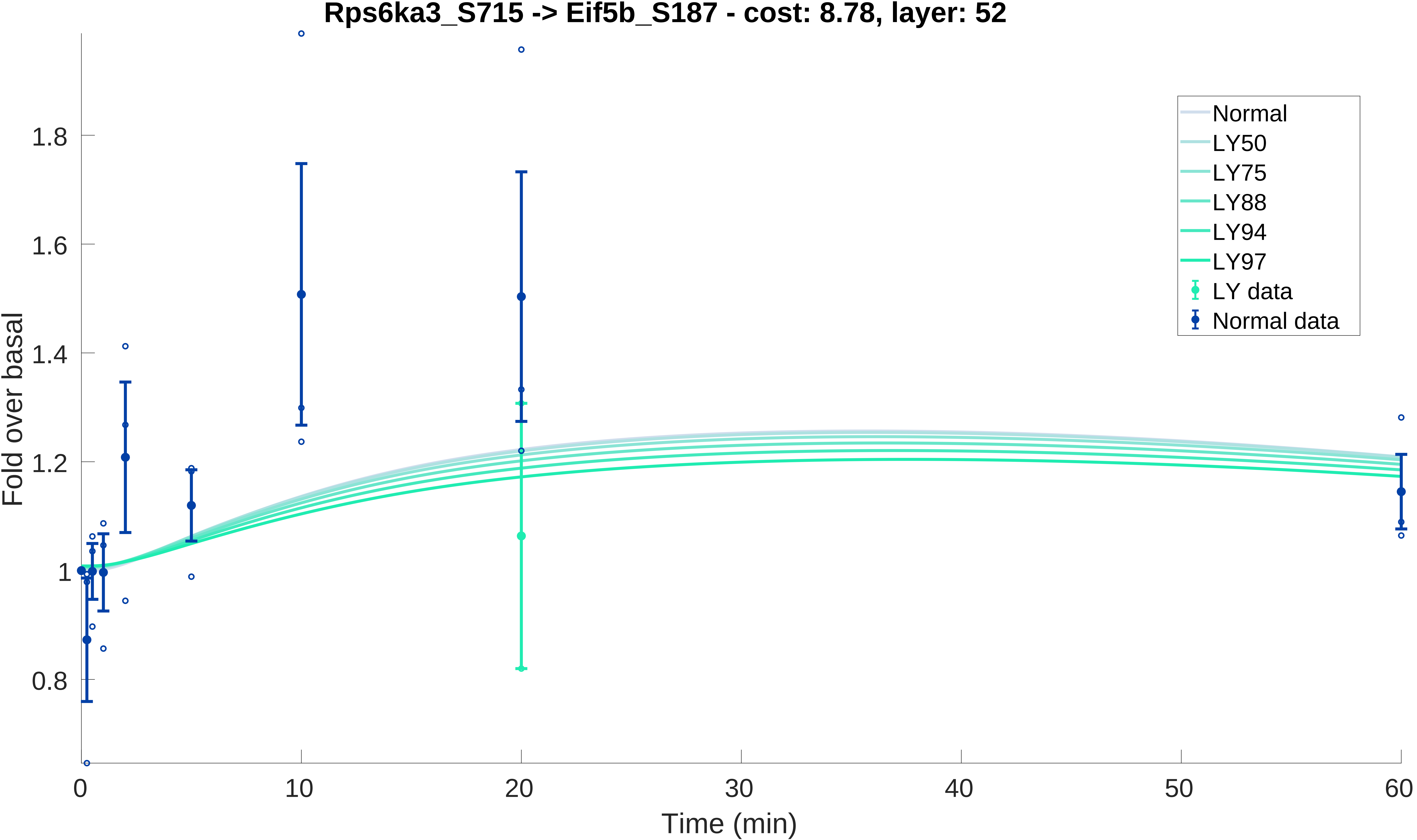

Rps6ka3_S715 -> Eif5b_S187 - cost: 8.78, layer: 52
Fold over basal
Time (min)
0
10
20
30
40
50
60
0.8
1
1.2
1.4
1.6
1.8
Normal
LY50
LY75
LY88
LY94
LY97
LY data
Normal data

Rps6ka3_S369 -> Eif5b_S215 - cost: 13.31, layer: 52

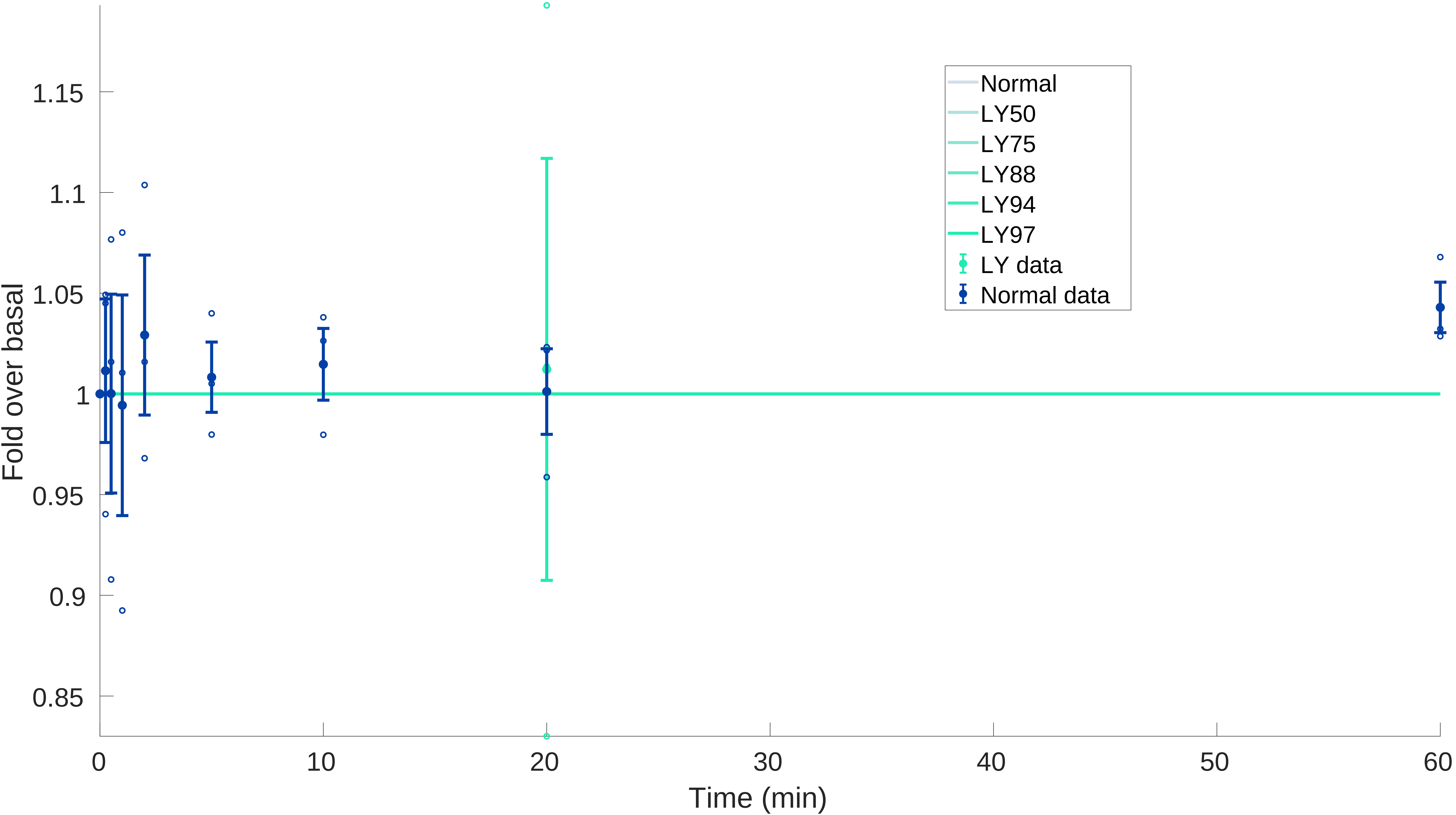

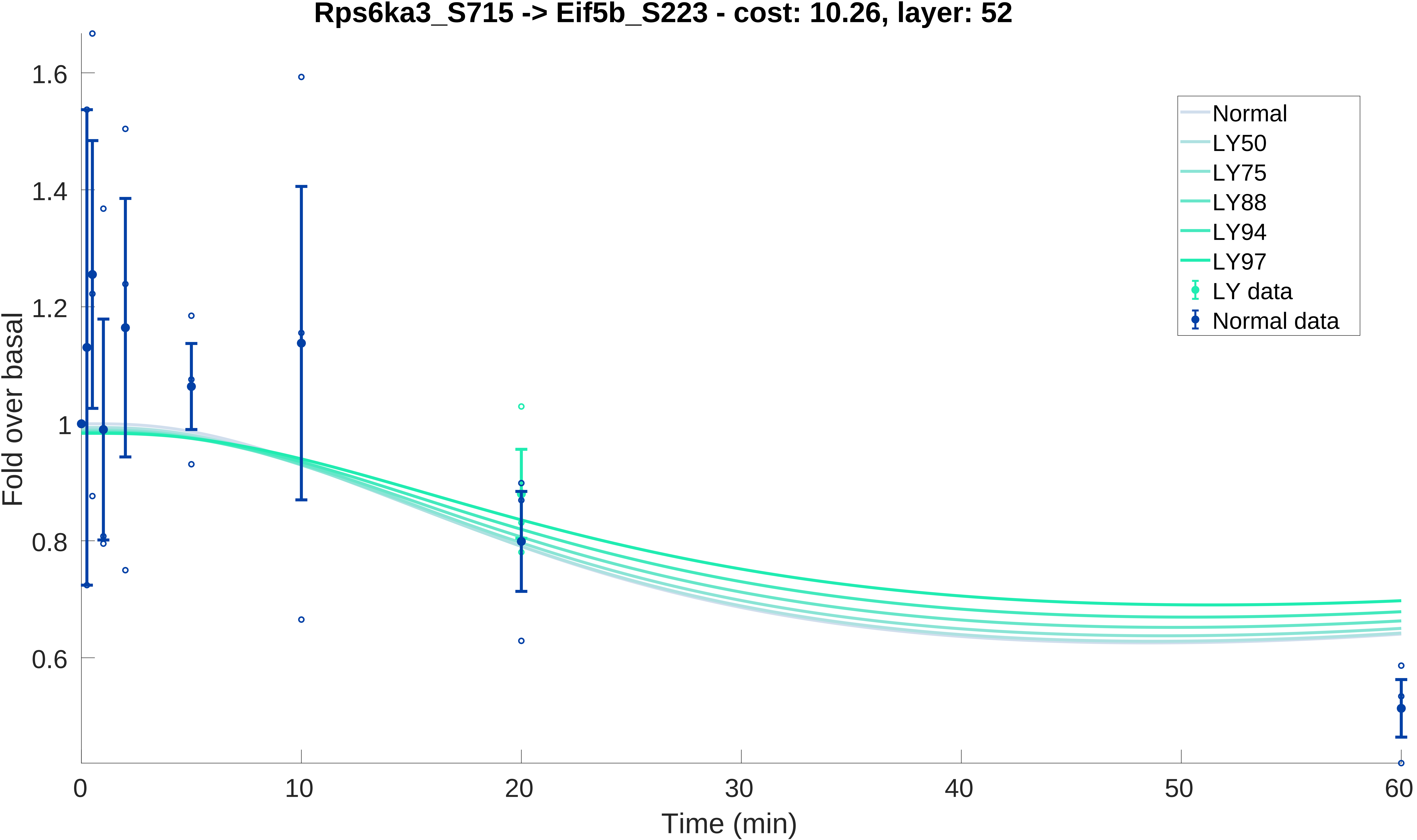

Rps6ka3_S715 -> Eif5b_S223 - cost: 10.26, layer: 52
Fold over basal
1.6
1.4
1.2
1
0.8
0.6
0
10
20
30
40
50
60
Time (min)
Normal
LY50
LY75
LY88
LY94
LY97
LY data
Normal data

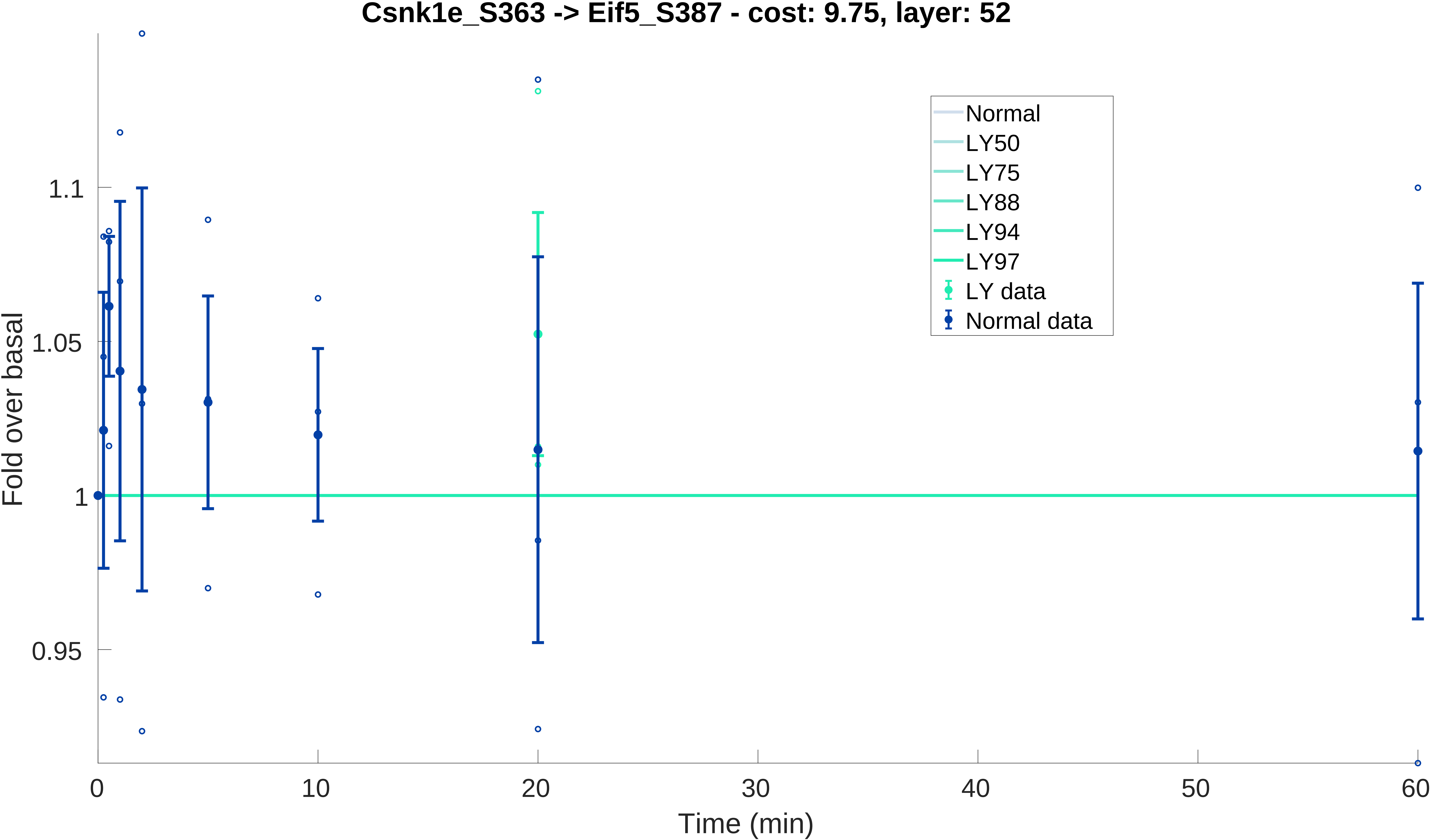

Csnk1e_S363 -> Eif5_S387 - cost: 9.75, layer: 52
Normal
LY50
LY75
LY88
LY94
LY97
LY data
Normal data
Fold over basal
1.1
1.05
1
0.95
0
10
20
30
40
50
60
Time (min)

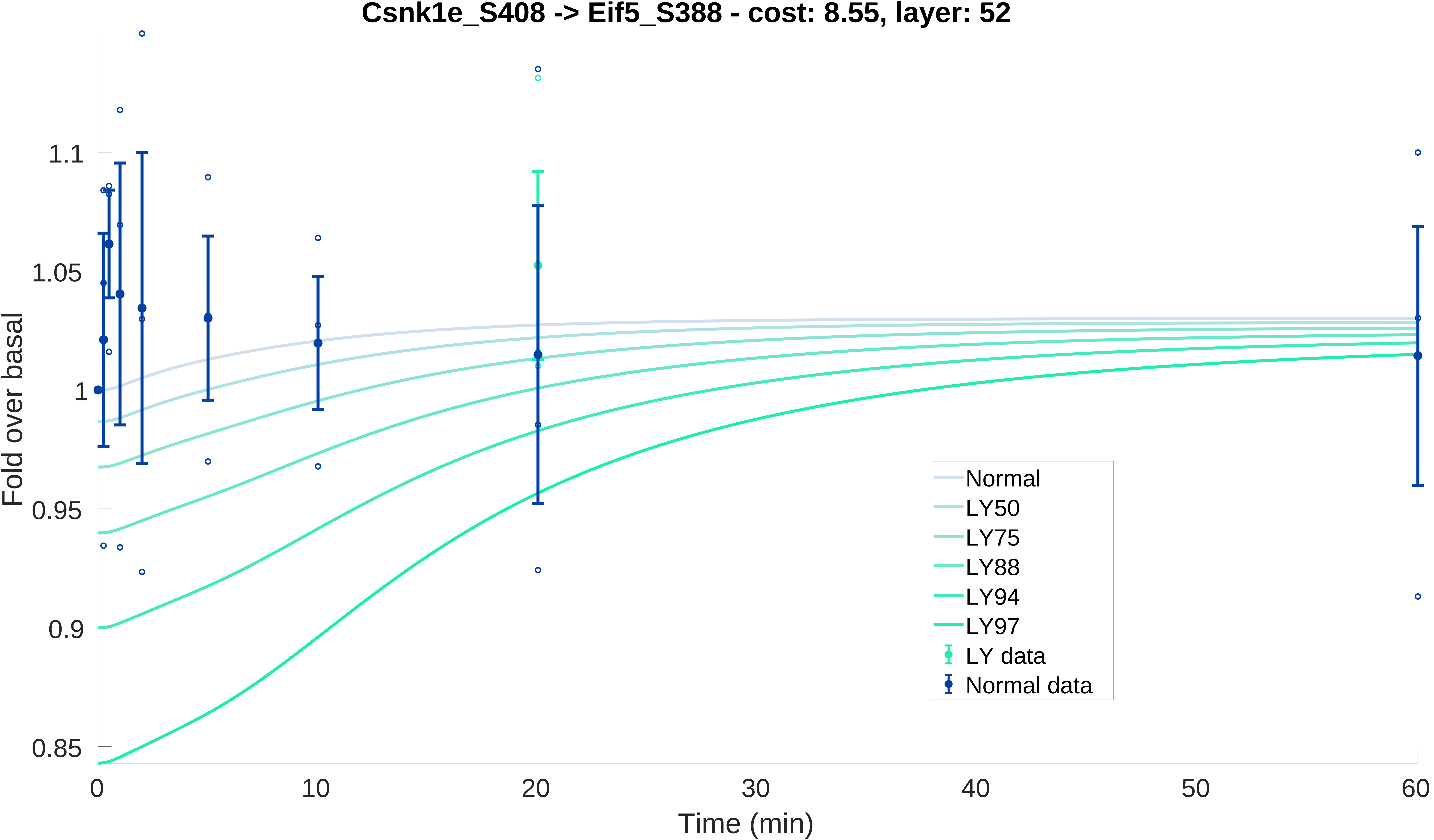
Csnk1e_S408 -> Eif5_S388 - cost: 8.55, layer: 52
Fold over basal
1.1
1.05
1
0.95
0.9
0.85
0
10
20
30
40
50
60
Time (min)
Normal
LY50
LY75
LY88
LY94
LY97
LY data
Normal data

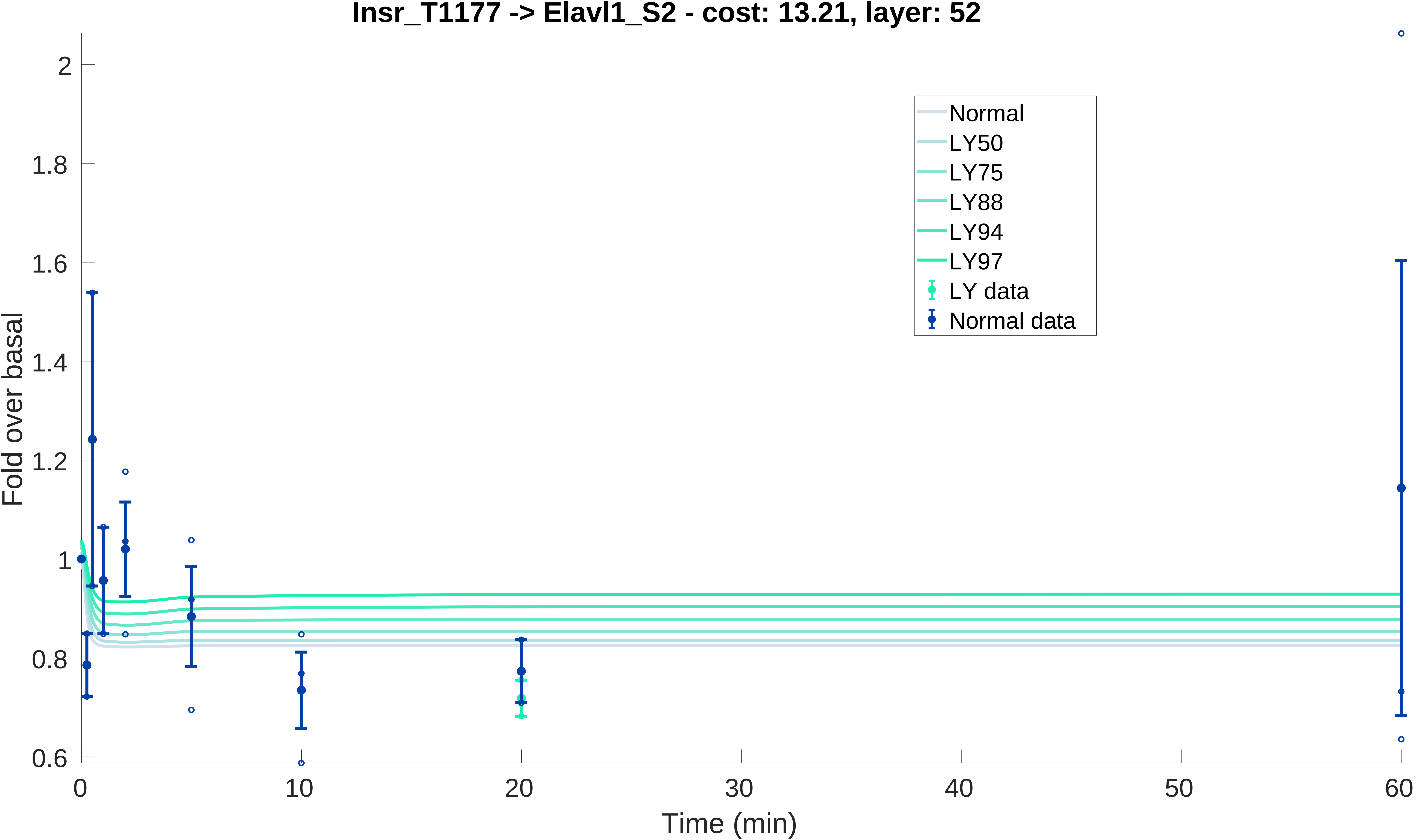
Insr_T1177 -> Elavl1_S2 - cost: 13.21, layer: 52
Fold over basal
Time (min)
Normal
LY50
LY75
LY88
LY94
LY97
LY data
Normal data
0.6
0.8
1
1.2
1.4
1.6
1.8
2
0
10
20
30
40
50
60

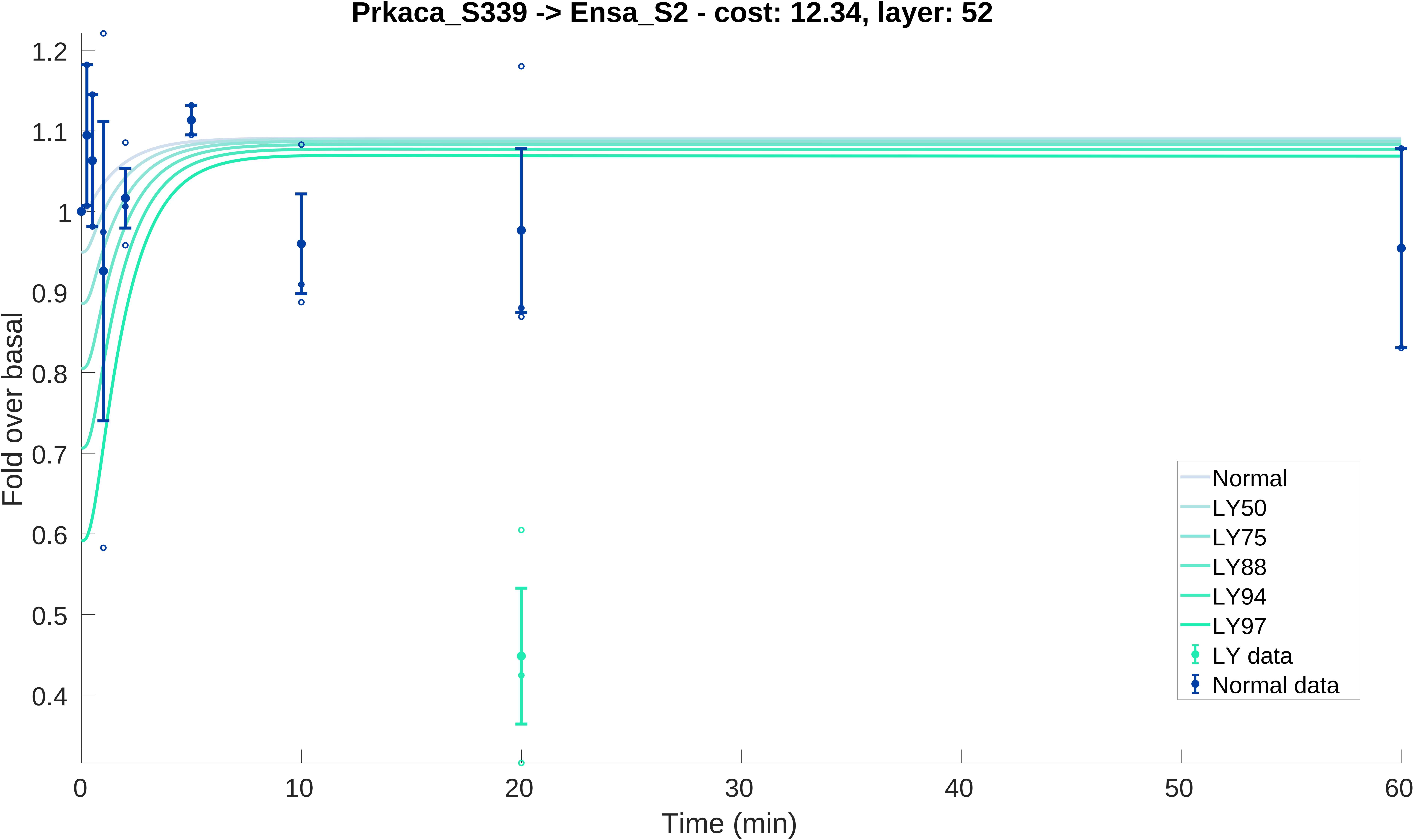
Prkaca_S339 -> Ensa_S2 - cost: 12.34, layer: 52
Fold over basal
Time (min)
0
10
20
30
40
50
60
0.4
0.5
0.6
0.7
0.8
0.9
1
1.1
1.2
Normal
LY50
LY75
LY88
LY94
LY97
LY data
Normal data

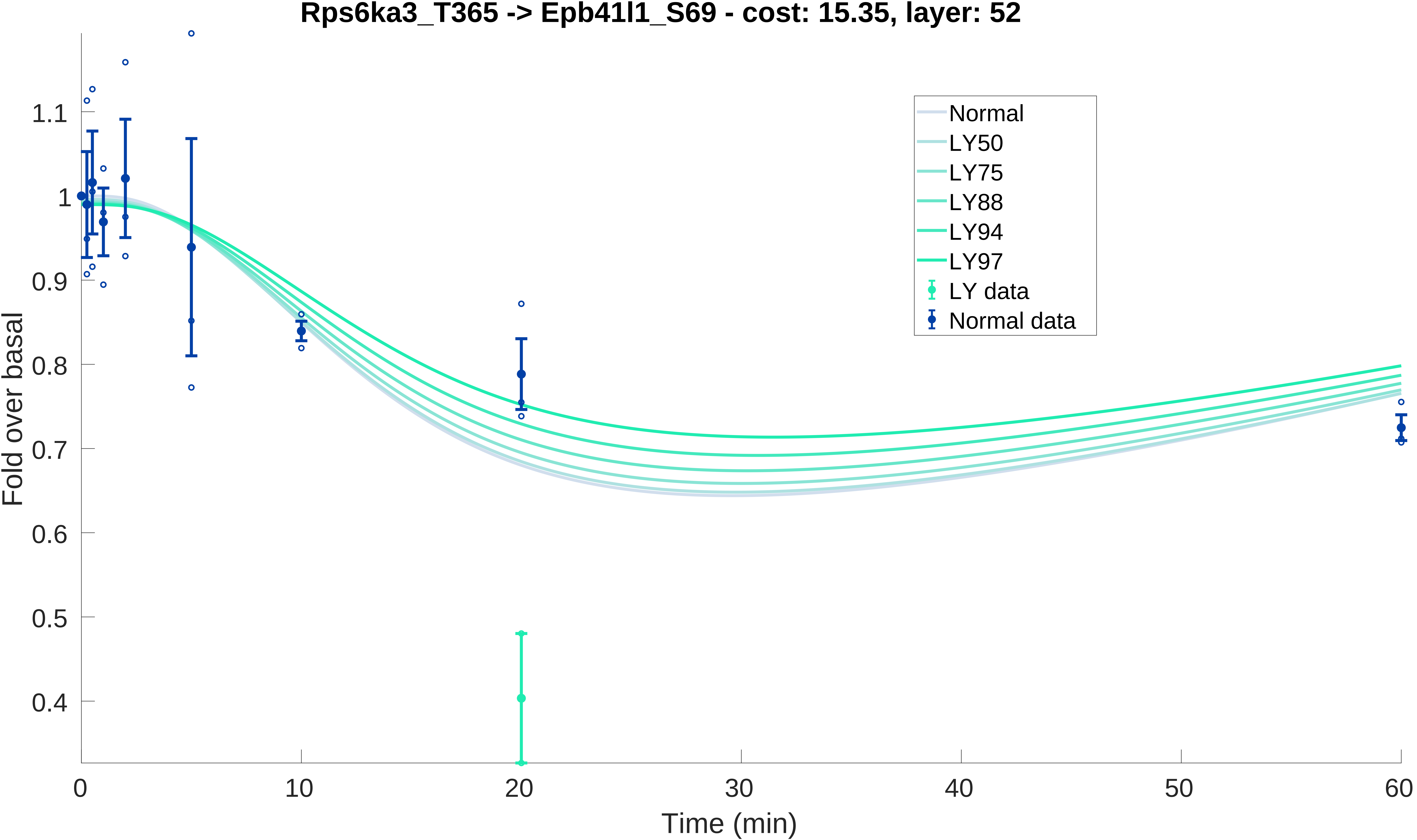
Rps6ka3_T365 -> Epb41l1_S69 - cost: 15.35, layer: 52
Fold over basal
Time (min)
0.4
0.5
0.6
0.7
0.8
0.9
1
1.1
0
10
20
30
40
50
60
Normal
LY50
LY75
LY88
LY94
LY97
LY data
Normal data

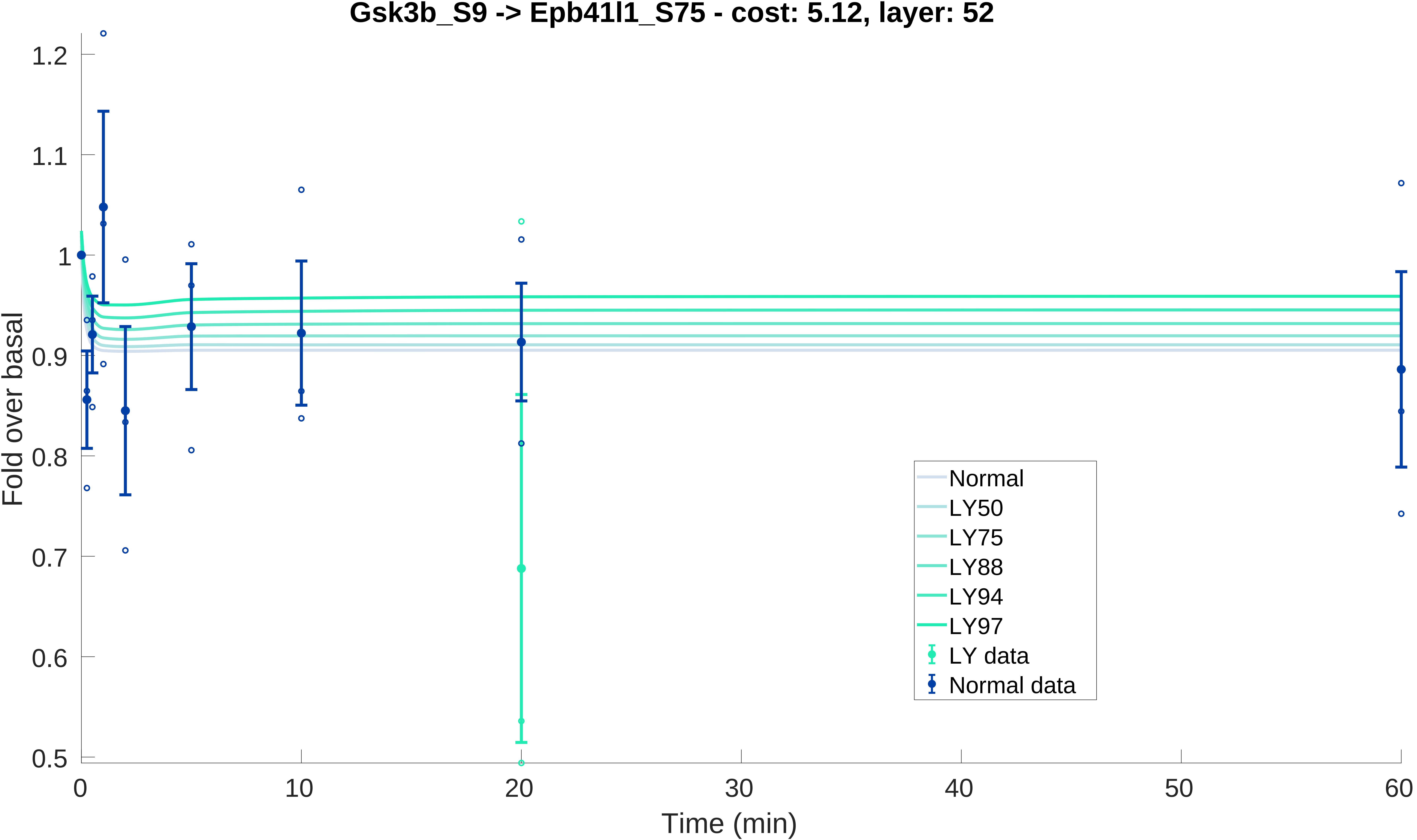

Gsk3b_S9 -> Epb41l1_S75 - cost: 5.12, layer: 52
Fold over basal
Time (min)
1.2
1.1
1
0.9
0.8
0.7
0.6
0.5
0
10
20
30
40
50
60
Normal
LY50
LY75
LY88
LY94
LY97
LY data
Normal data

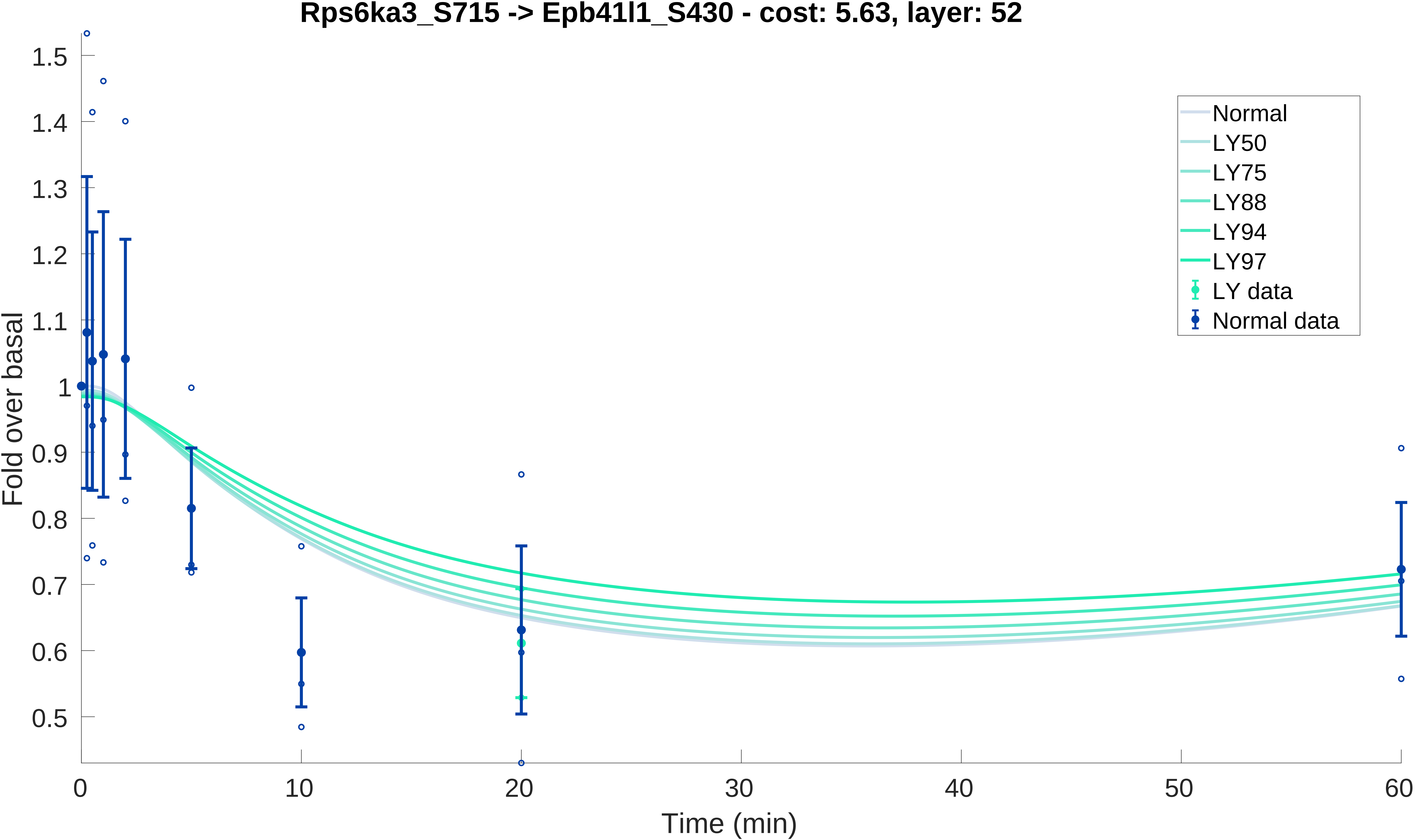
Rps6ka3_S715 -> Epb41l1_S430 - cost: 5.63, layer: 52
Fold over basal
Time (min)
Normal
LY50
LY75
LY88
LY94
LY97
LY data
Normal data
0
10
20
30
40
50
60
0.5
0.6
0.7
0.8
0.9
1
1.1
1.2
1.3
1.4
1.5

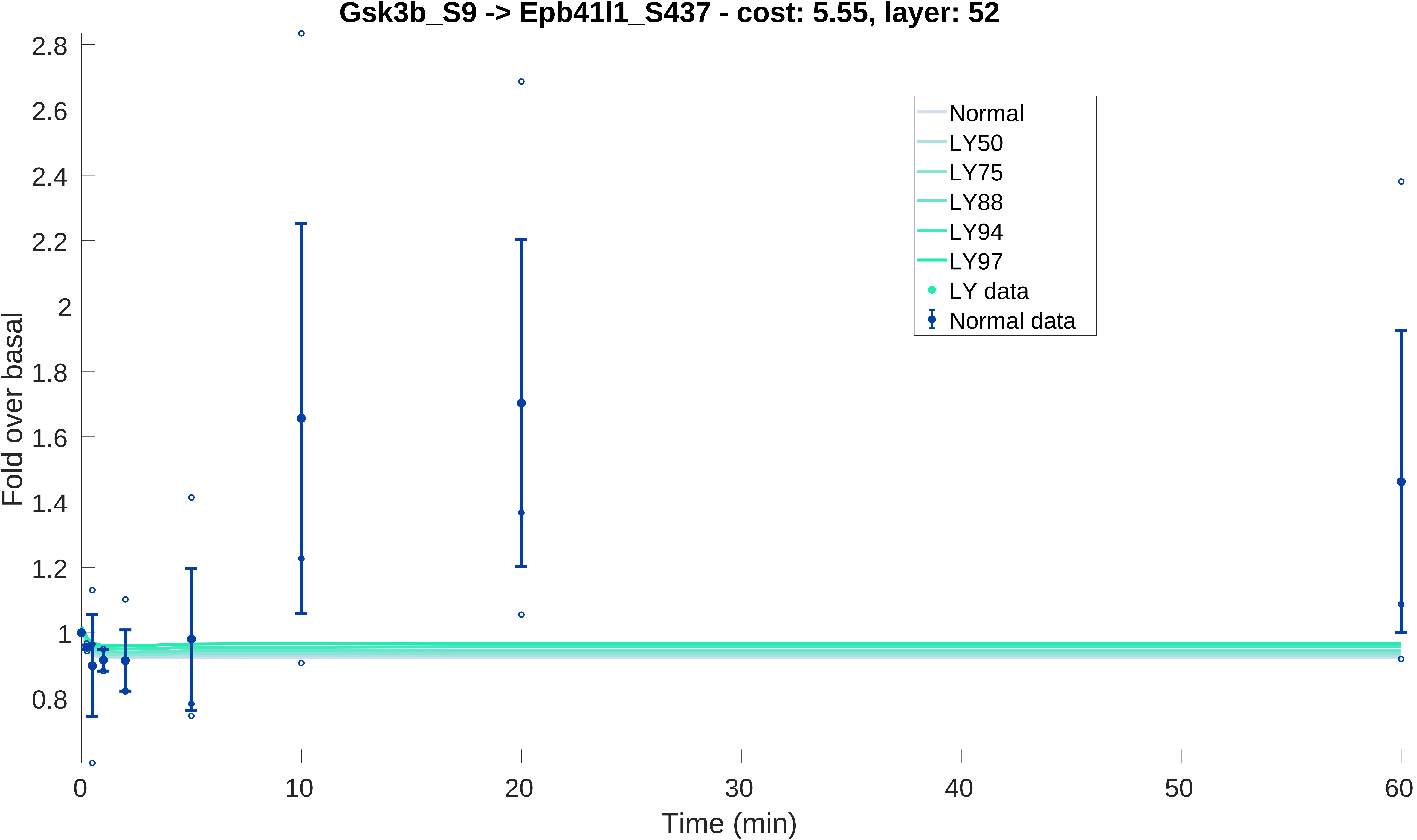

Gsk3b_S9 -> Epb41l1_S437 - cost: 5.55, layer: 52
Fold over basal
Time (min)
2.8
2.6
2.4
2.2
2
1.8
1.6
1.4
1.2
1
0.8
0
10
20
30
40
50
60
Normal
LY50
LY75
LY88
LY94
LY97
LY data
Normal data

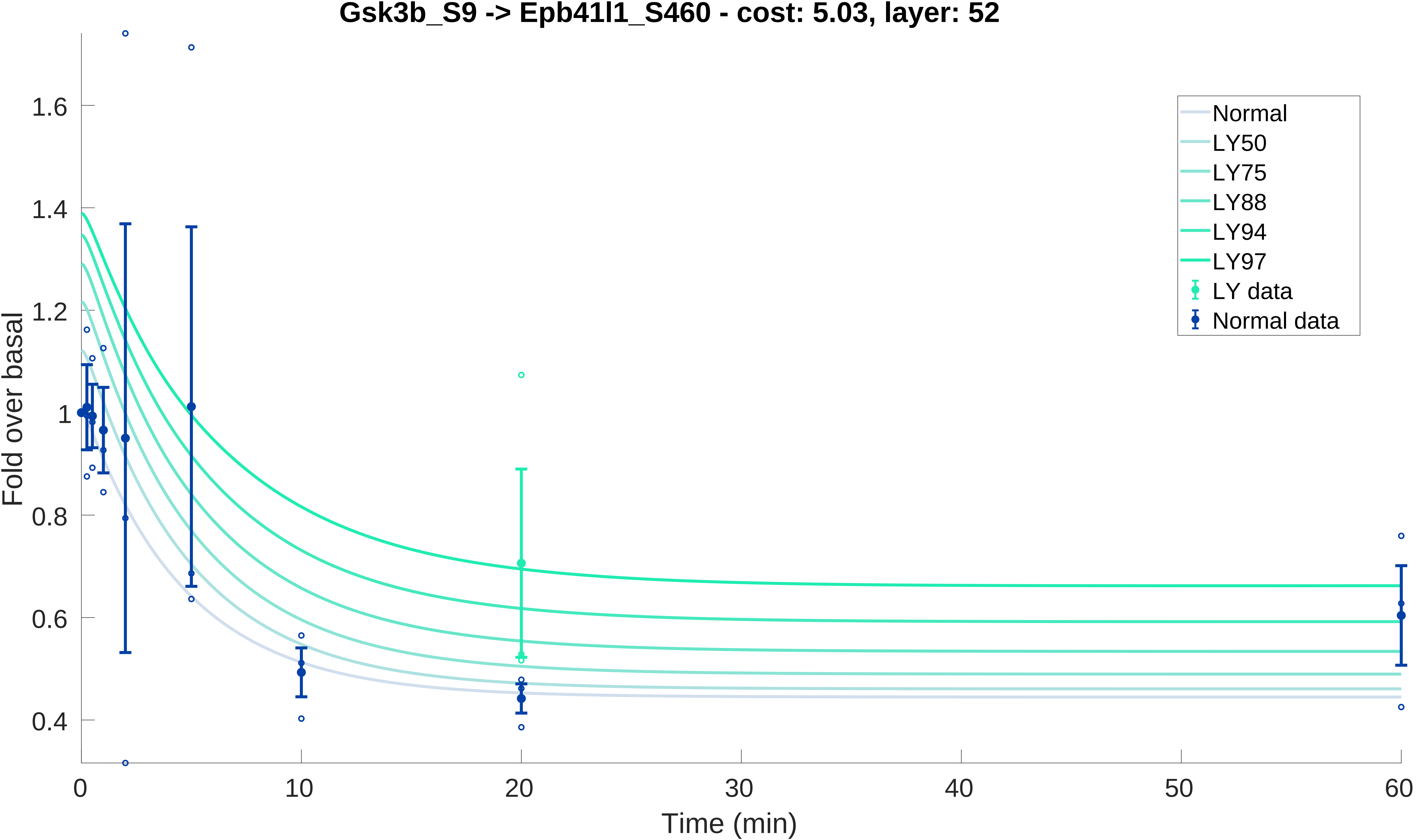

Gsk3b_S9 -> Epb41l1_S460 - cost: 5.03, layer: 52
Fold over basal
Time (min)
1.6
1.4
1.2
1
0.8
0.6
0.4
0
10
20
30
40
50
60
Normal
LY50
LY75
LY88
LY94
LY97
LY data
Normal data

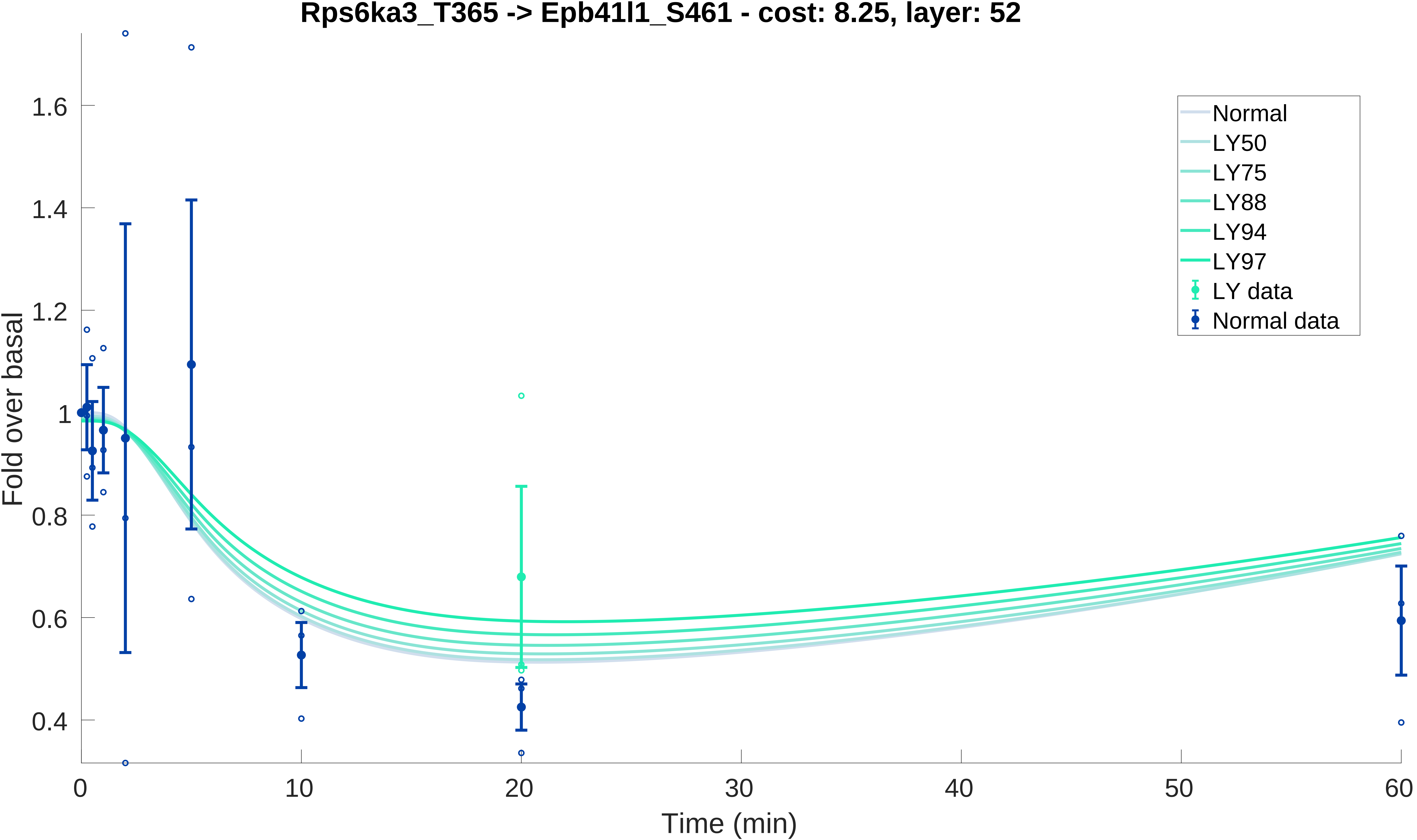

Rps6ka3_T365 -> Epb41l1_S461 - cost: 8.25, layer: 52
Fold over basal
Time (min)
0.4
0.6
0.8
1
1.2
1.4
1.6
0
10
20
30
40
50
60
Normal
LY50
LY75
LY88
LY94
LY97
LY data
Normal data

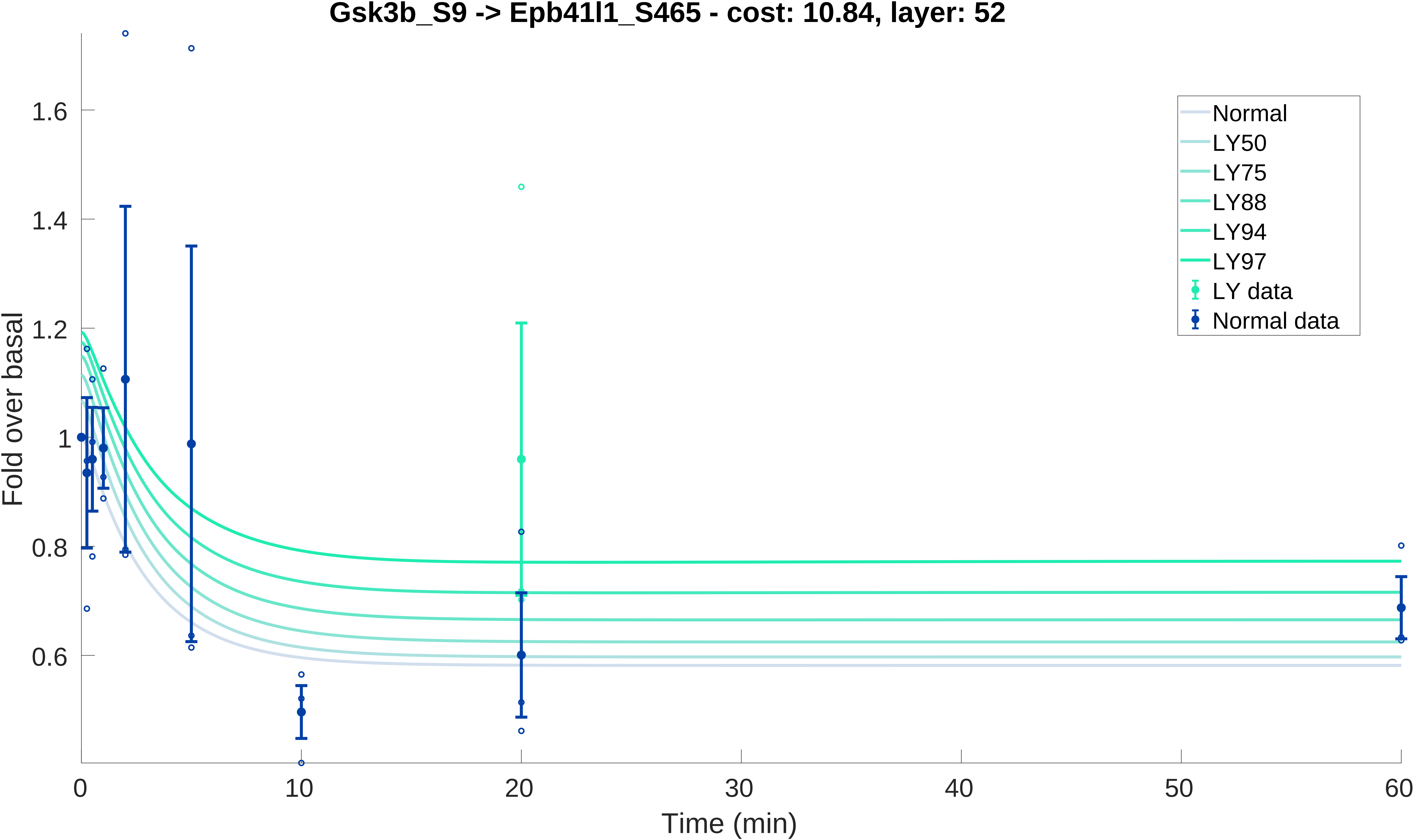

Gsk3b_S9 -> Epb41l1_S465 - cost: 10.84, layer: 52
Fold over basal
Time (min)
1.6
1.4
1.2
1
0.8
0.6
0
10
20
30
40
50
60
Normal
LY50
LY75
LY88
LY94
LY97
LY data
Normal data

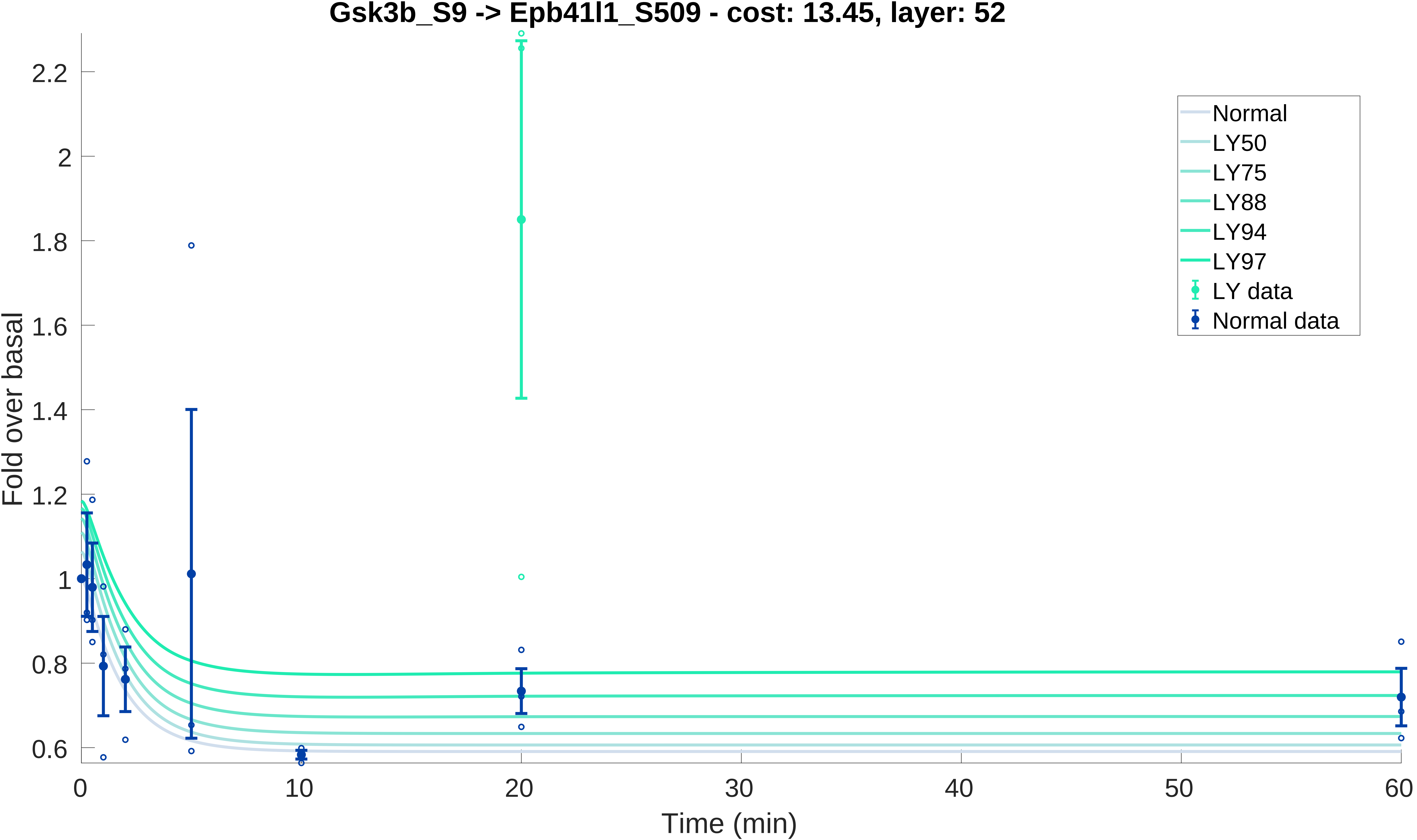

Gsk3b_S9 -> Epb41l1_S509 - cost: 13.45, layer: 52
Fold over basal
Time (min)
0
10
20
30
40
50
60
0.6
0.8
1
1.2
1.4
1.6
1.8
2
2.2
Normal
LY50
LY75
LY88
LY94
LY97
LY data
Normal data

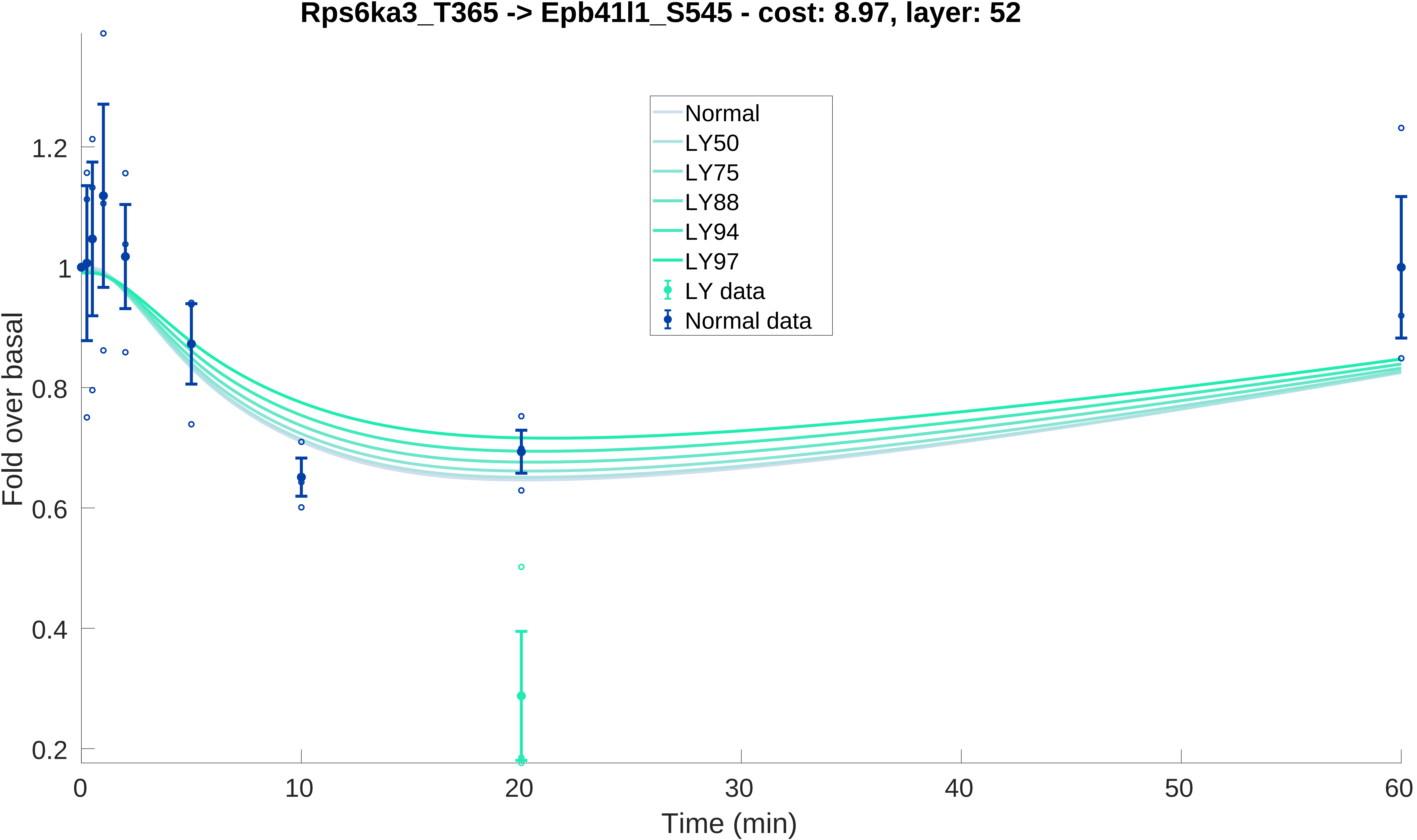

Rps6ka3_T365 -> Epb41l1_S545 - cost: 8.97, layer: 52
Fold over basal
Time (min)
Normal
LY50
LY75
LY88
LY94
LY97
LY data
Normal data
0.2
0.4
0.6
0.8
1
1.2
0
10
20
30
40
50
60

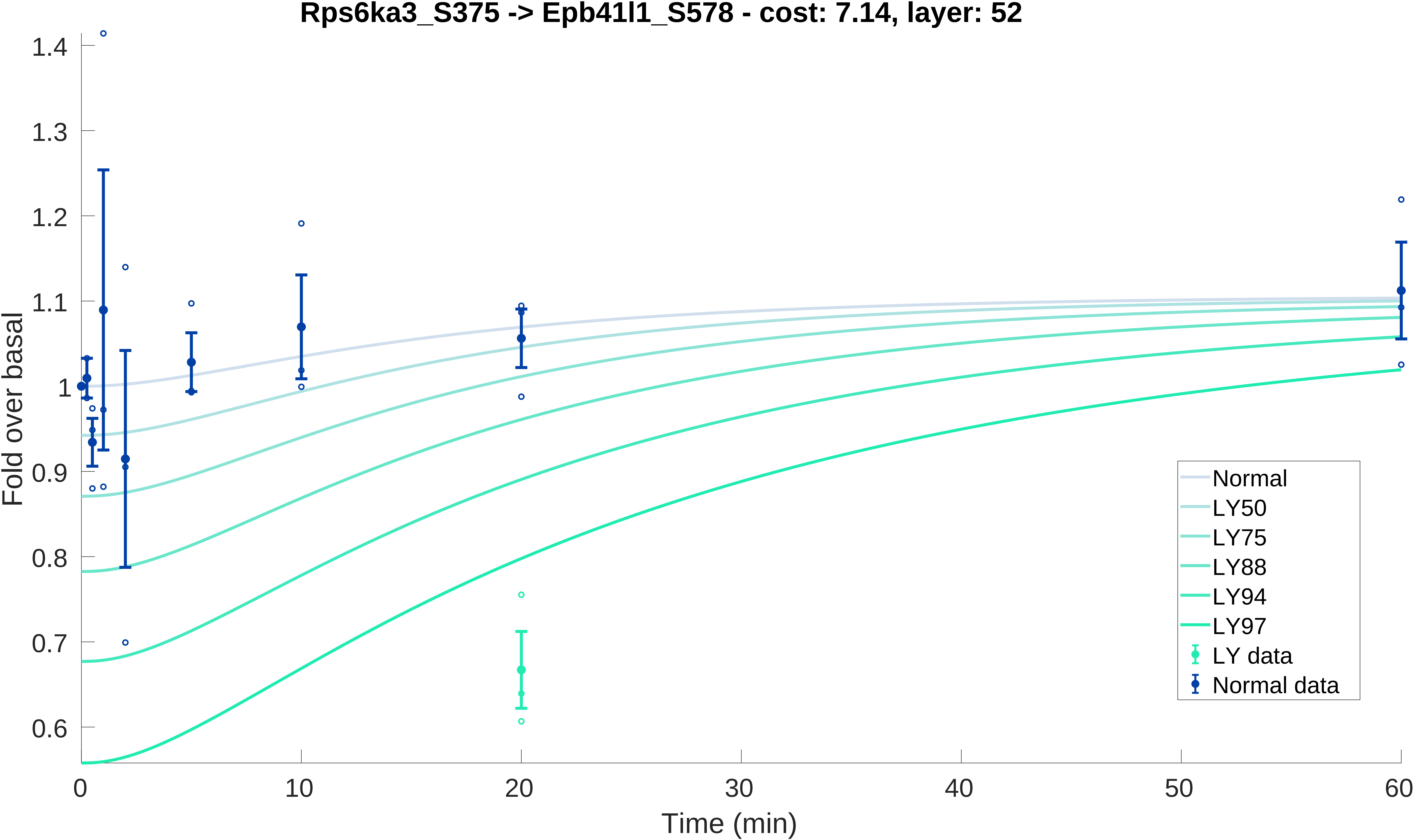
Rps6ka3_S375 -> Epb41l1_S578 - cost: 7.14, layer: 52
Fold over basal
Time (min)
1.4
1.3
1.2
1.1
1
0.9
0.8
0.7
0.6
0
10
20
30
40
50
60
Normal
LY50
LY75
LY88
LY94
LY97
LY data
Normal data

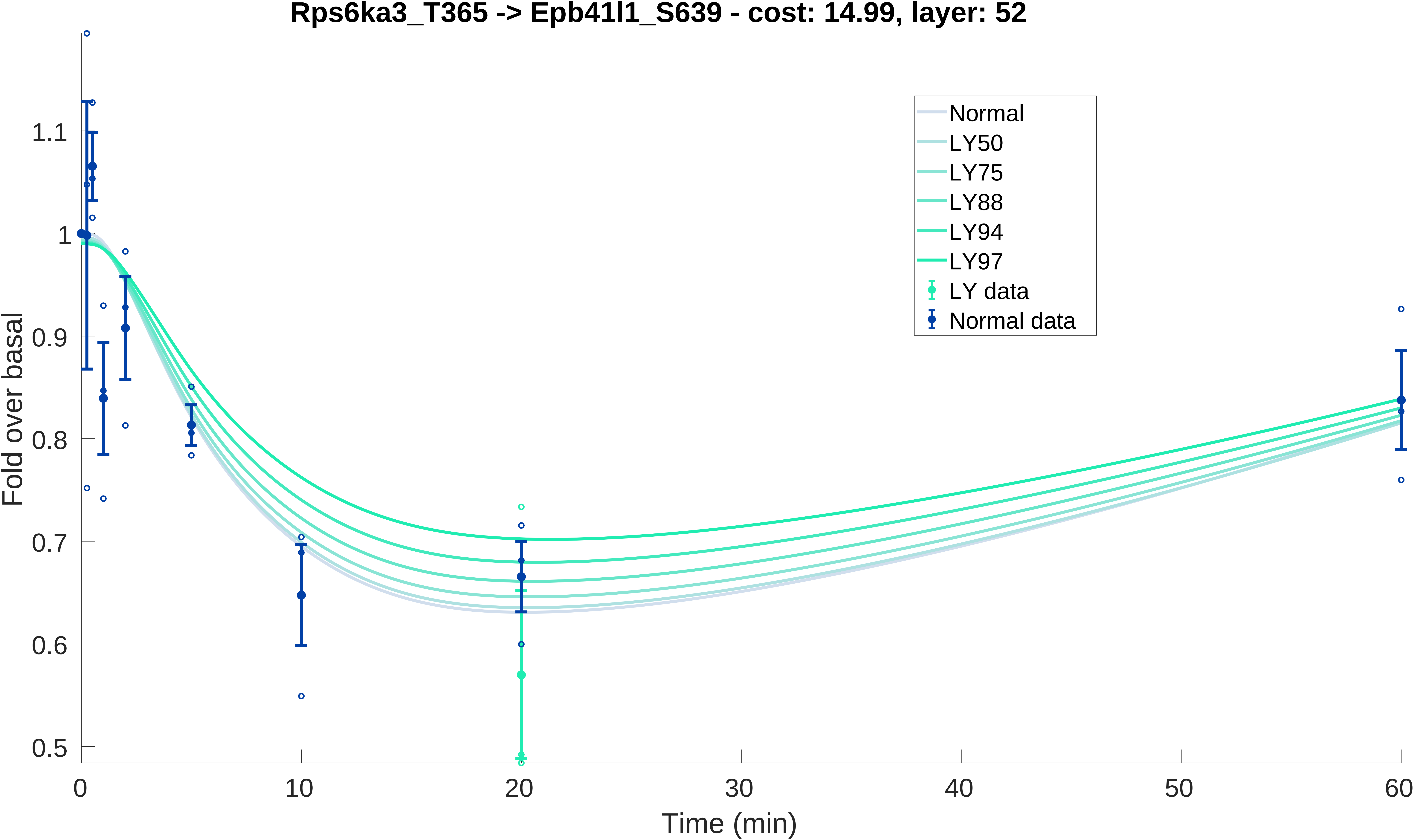

Rps6ka3_T365 -> Epb41l1_S639 - cost: 14.99, layer: 52
Fold over basal
Time (min)
0.5
0.6
0.7
0.8
0.9
1
1.1
0
10
20
30
40
50
60
Normal
LY50
LY75
LY88
LY94
LY97
LY data
Normal data

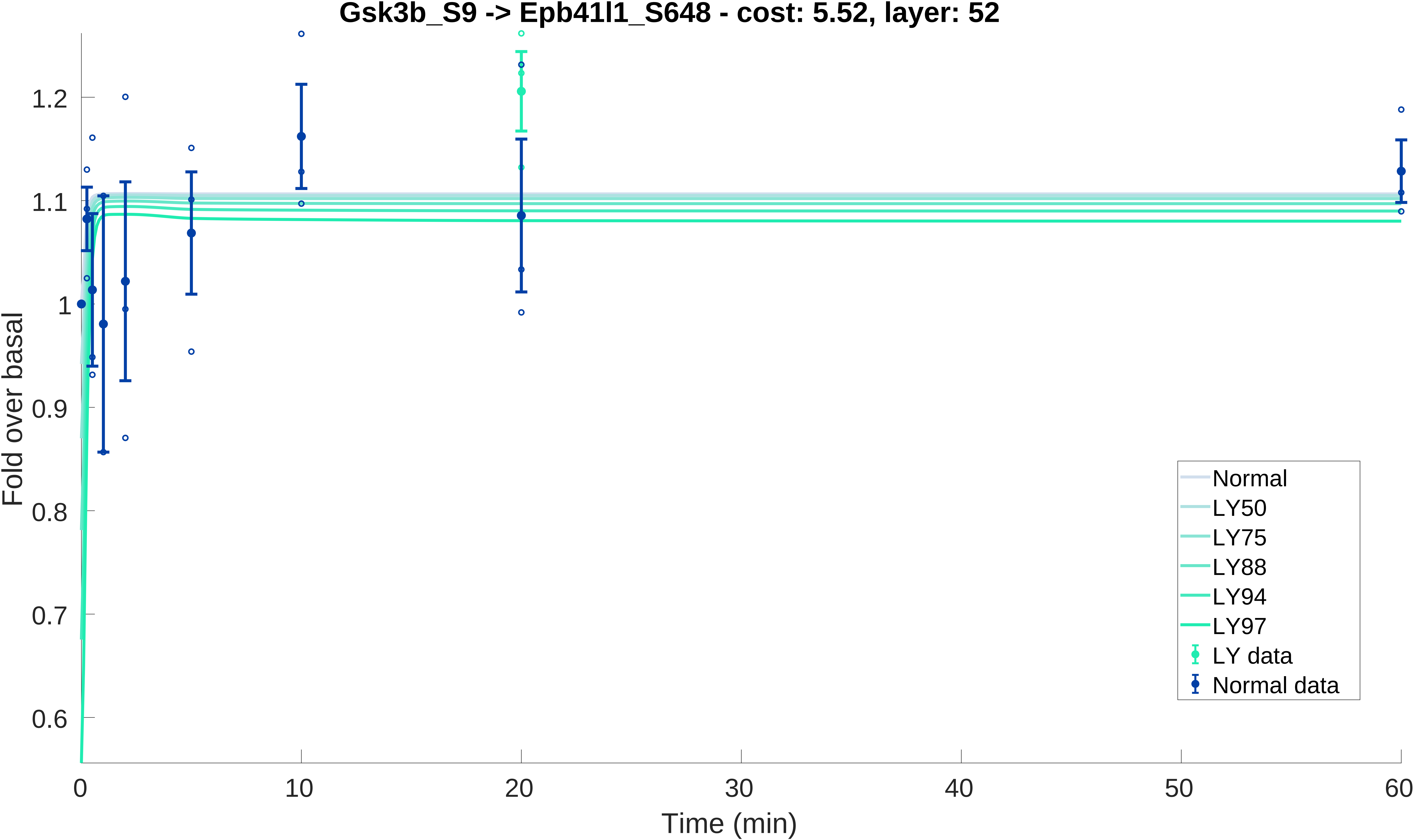

Gsk3b_S9 -> Epb41l1_S648 - cost: 5.52, layer: 52
Fold over basal
Time (min)
1.2
1.1
1
0.9
0.8
0.7
0.6
0
10
20
30
40
50
60
Normal
LY50
LY75
LY88
LY94
LY97
LY data
Normal data

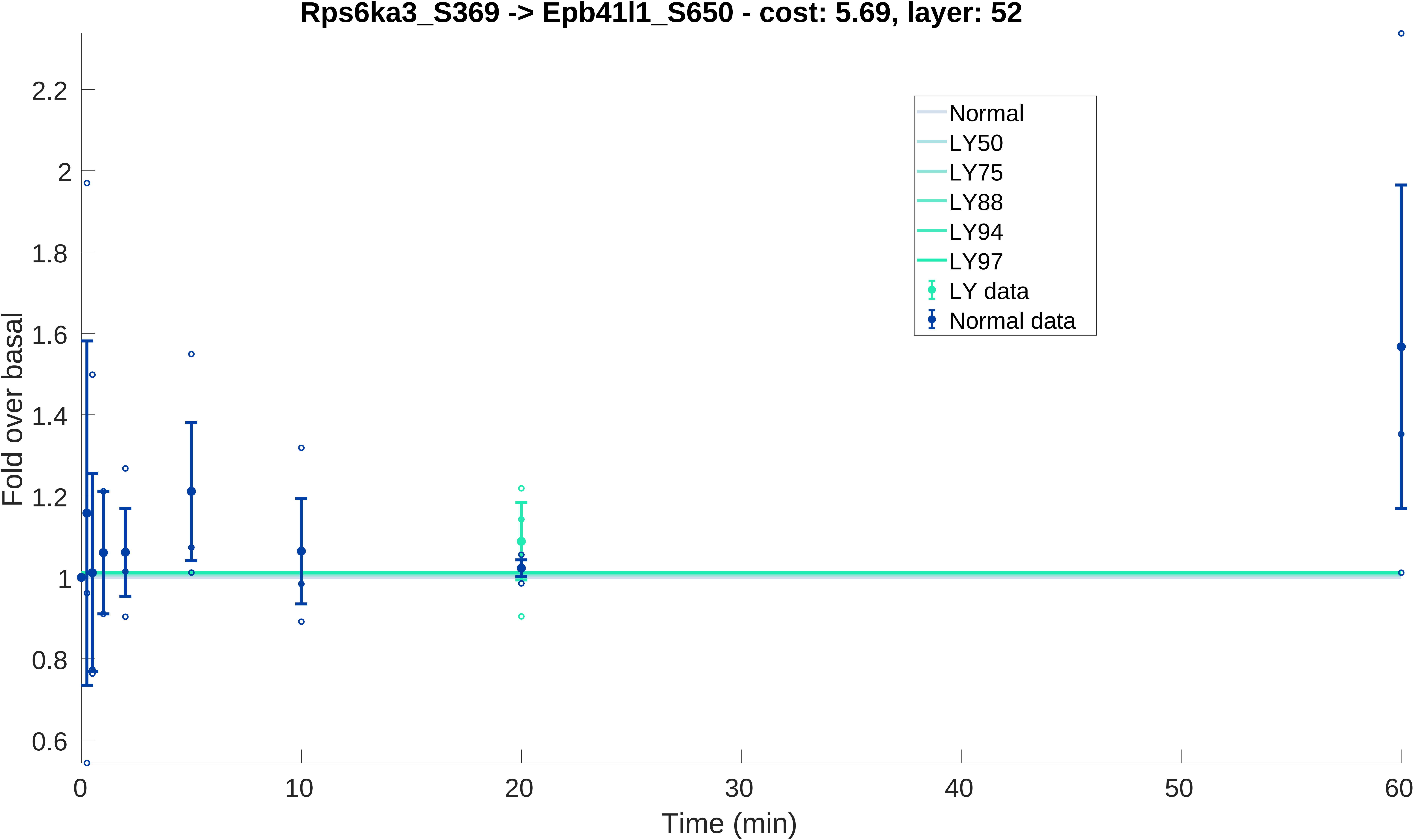

Rps6ka3_S369 -> Epb41l1_S650 - cost: 5.69, layer: 52
Fold over basal
Time (min)
Normal
LY50
LY75
LY88
LY94
LY97
LY data
Normal data
0.6
0.8
1
1.2
1.4
1.6
1.8
2
2.2
0
10
20
30
40
50
60

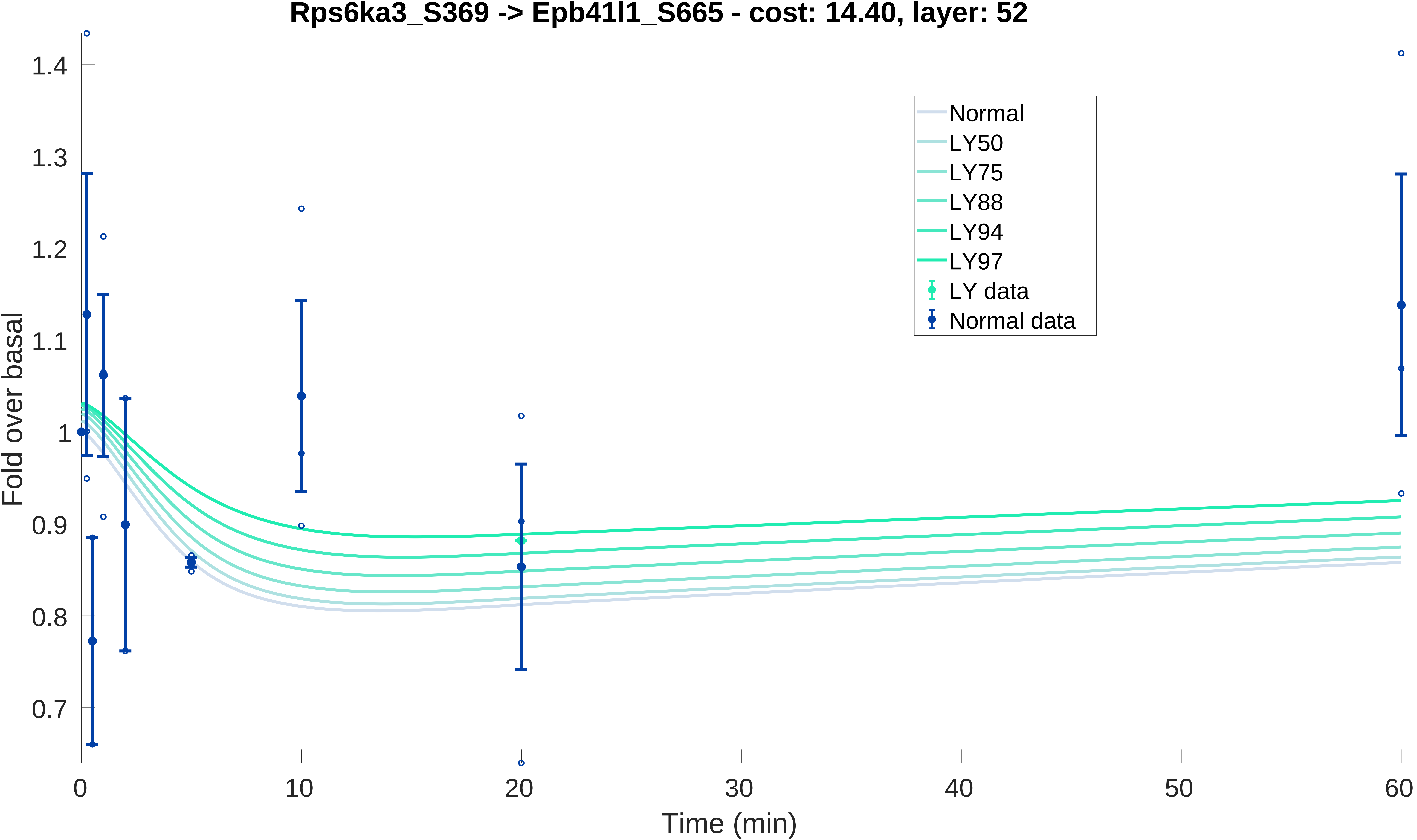
Rps6ka3_S369 -> Epb41l1_S665 - cost: 14.40, layer: 52
Fold over basal
Time (min)
Normal
LY50
LY75
LY88
LY94
LY97
LY data
Normal data
1.4
1.3
1.2
1.1
1
0.9
0.8
0.7
0
10
20
30
40
50
60

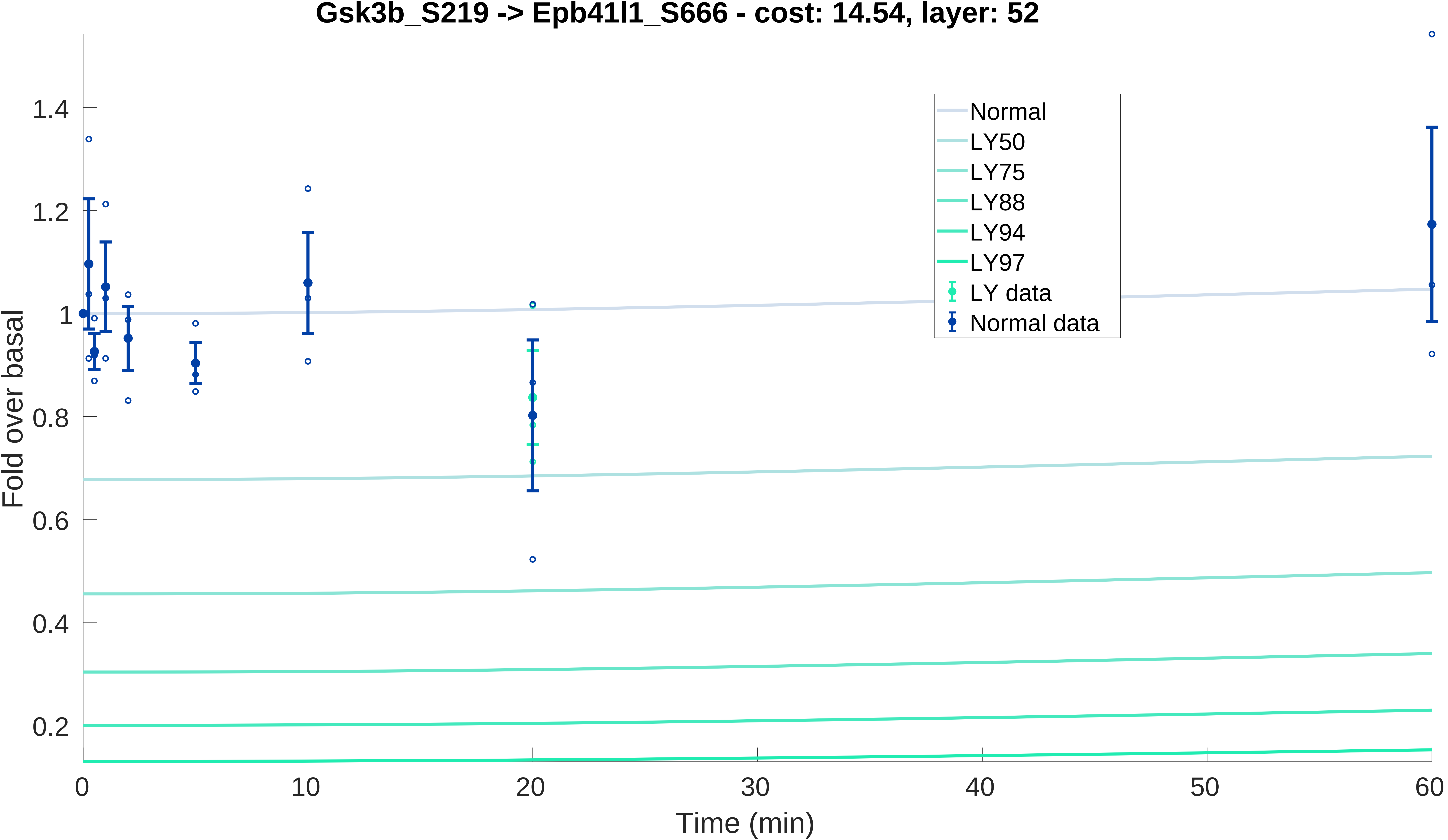
Gsk3b_S219 -> Epb41l1_S666 - cost: 14.54, layer: 52
Fold over basal
Time (min)
Normal
LY50
LY75
LY88
LY94
LY97
LY data
Normal data
0
10
20
30
40
50
60
0.2
0.4
0.6
0.8
1
1.2
1.4

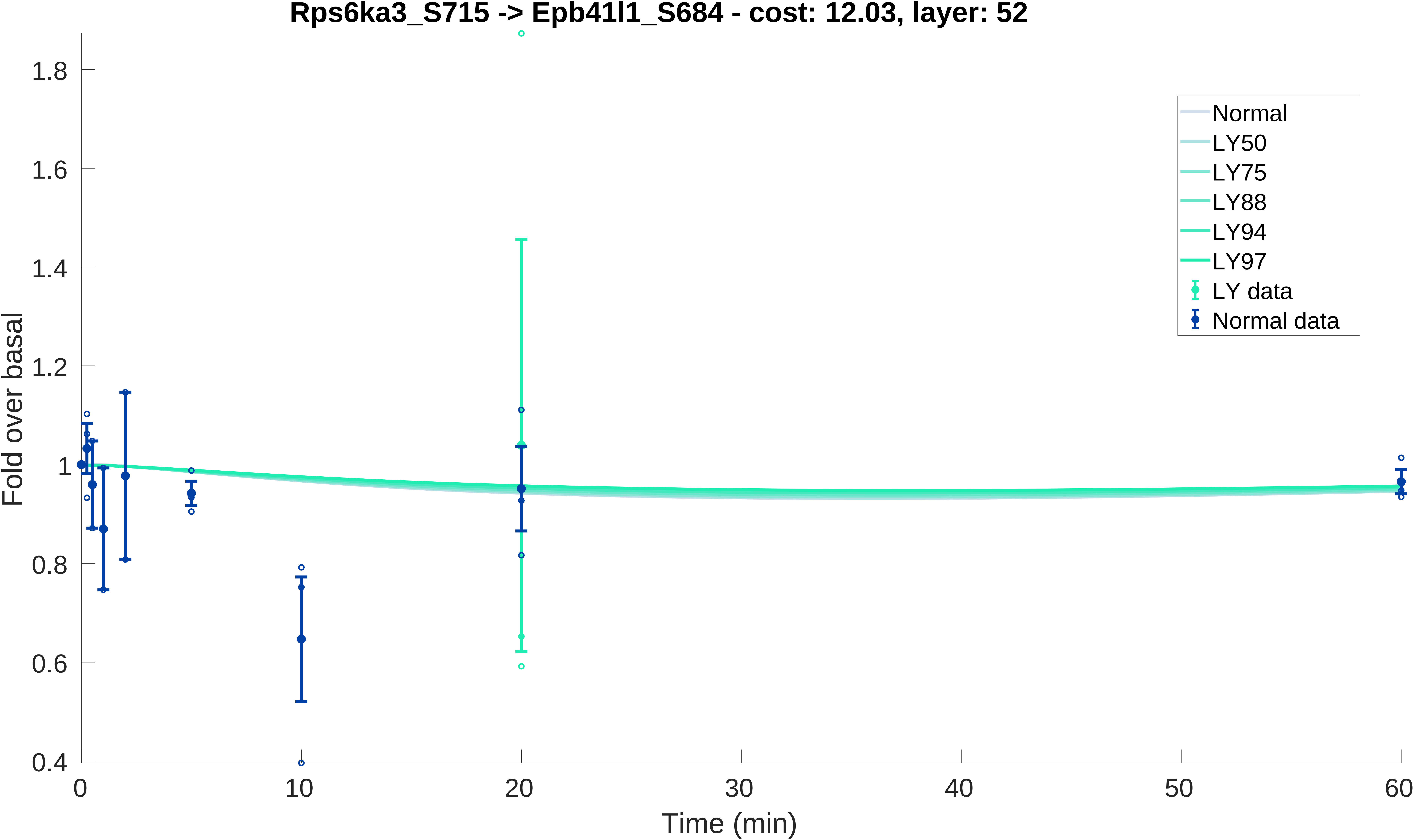

Rps6ka3_S715 -> Epb41l1_S684 - cost: 12.03, layer: 52
Fold over basal
Time (min)
1.8
1.6
1.4
1.2
1
0.8
0.6
0.4
0
10
20
30
40
50
60
Normal
LY50
LY75
LY88
LY94
LY97
LY data
Normal data

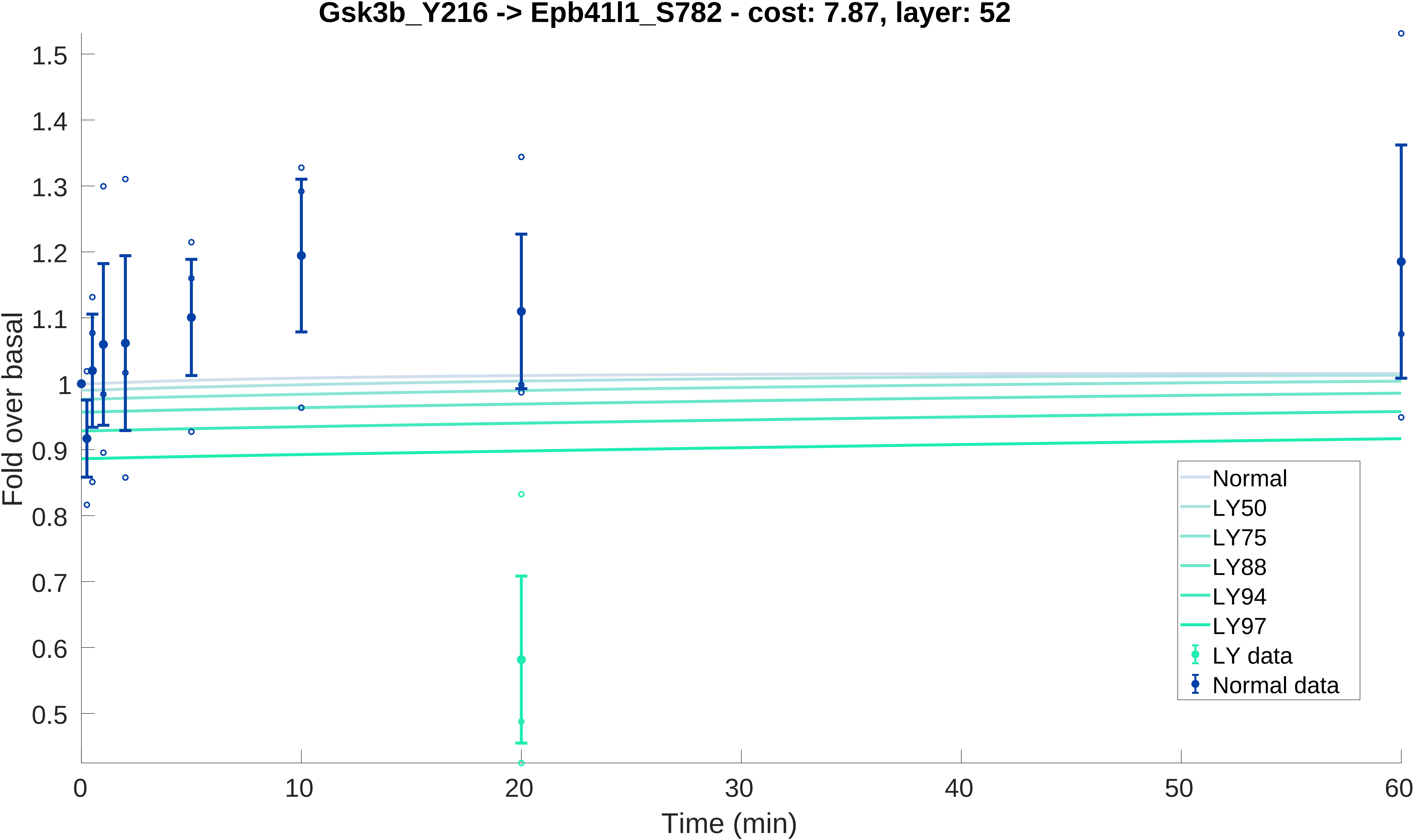
Gsk3b_Y216 -> Epb41l1_S782 - cost: 7.87, layer: 52
Fold over basal
1.5
1.4
1.3
1.2
1.1
1
0.9
0.8
0.7
0.6
0.5
0
10
20
30
40
50
60
Time (min)
Normal
LY50
LY75
LY88
LY94
LY97
LY data
Normal data

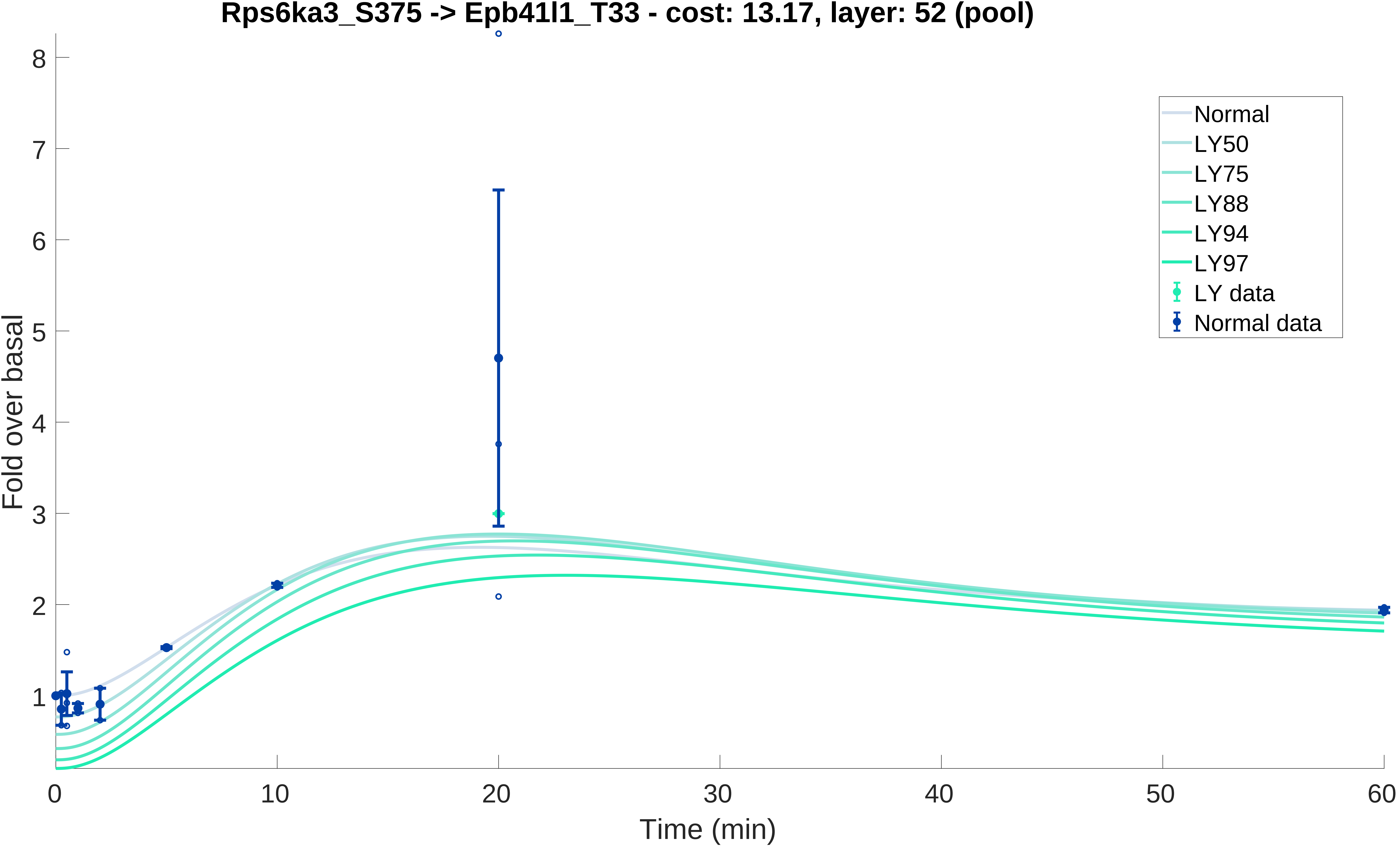
Rps6ka3_S375 -> Epb41l1_T33 - cost: 13.17, layer: 52 (pool)
Fold over basal
Time (min)
Normal
LY50
LY75
LY88
LY94
LY97
LY data
Normal data
0
10
20
30
40
50
60
1
2
3
4
5
6
7
8

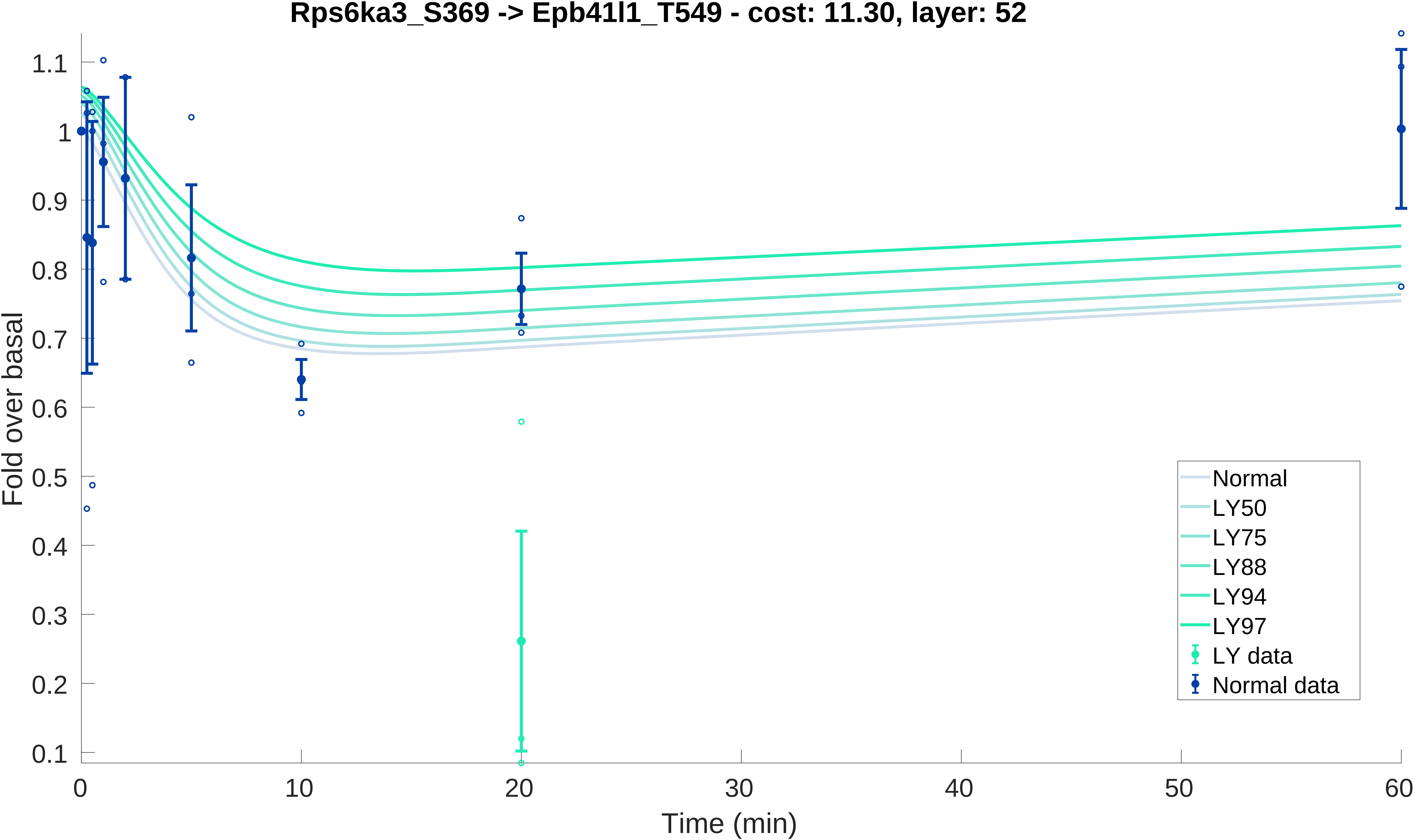

Rps6ka3_S369 -> Epb41l1_T549 - cost: 11.30, layer: 52
Fold over basal
Time (min)
1.1
1
0.9
0.8
0.7
0.6
0.5
0.4
0.3
0.2
0.1
0
10
20
30
40
50
60
Normal
LY50
LY75
LY88
LY94
LY97
LY data
Normal data

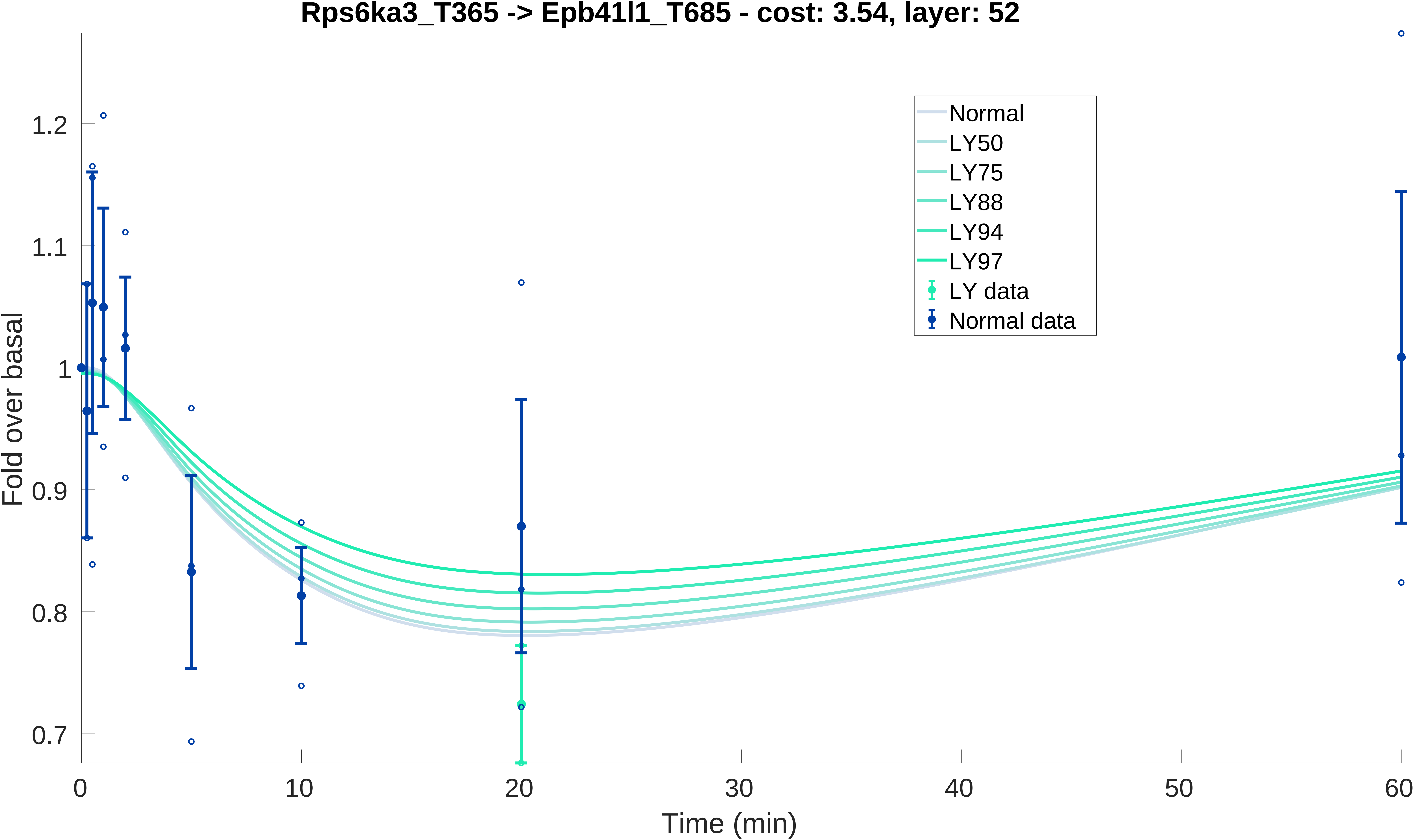

Rps6ka3_T365 -> Epb41l1_T685 - cost: 3.54, layer: 52
Fold over basal
Time (min)
1.2
1.1
1
0.9
0.8
0.7
0
10
20
30
40
50
60
Normal
LY50
LY75
LY88
LY94
LY97
LY data
Normal data

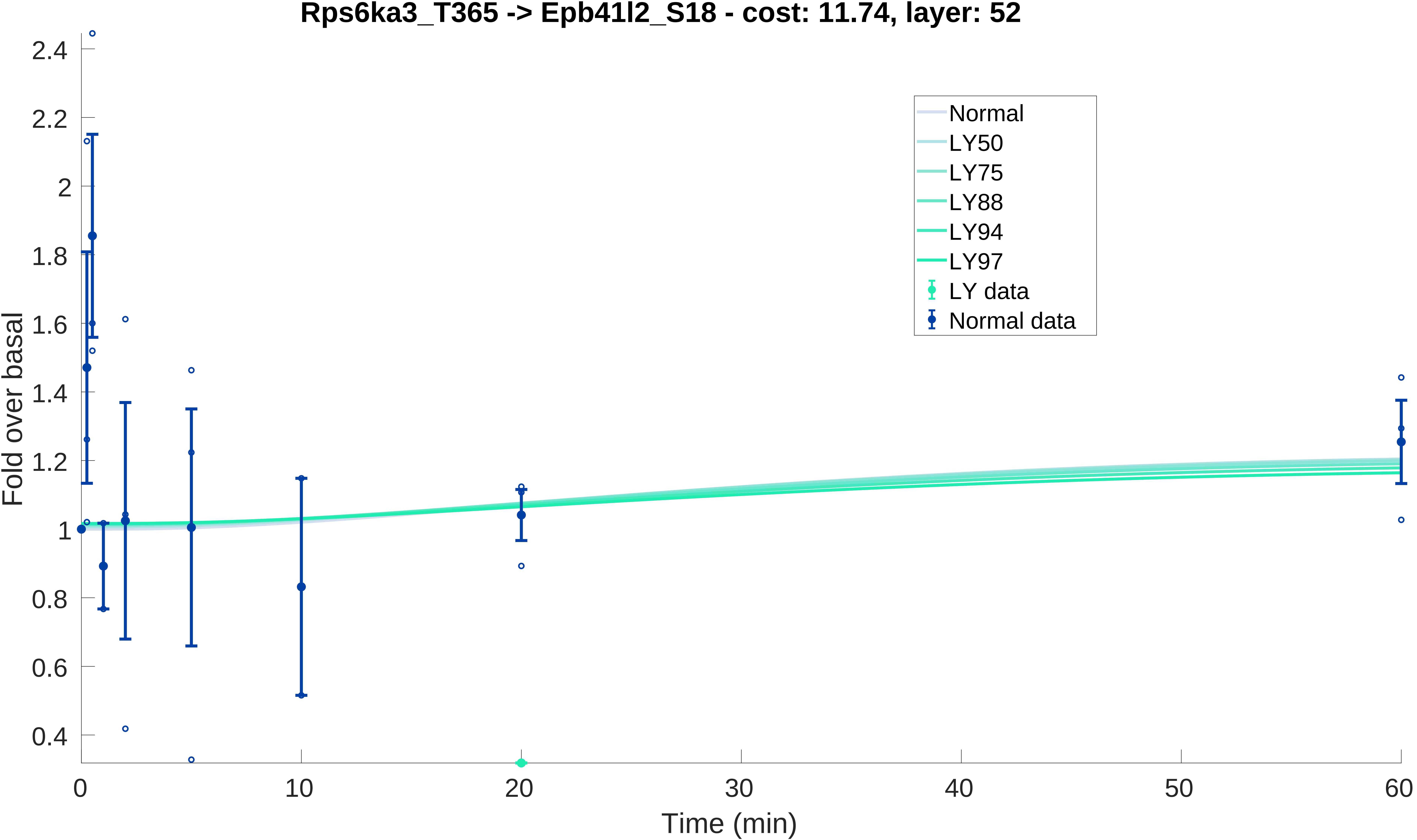

Rps6ka3_T365 -> Epb41l2_S18 - cost: 11.74, layer: 52
Fold over basal
Time (min)
2.4
2.2
2
1.8
1.6
1.4
1.2
1
0.8
0.6
0.4
0
10
20
30
40
50
60
Normal
LY50
LY75
LY88
LY94
LY97
LY data
Normal data

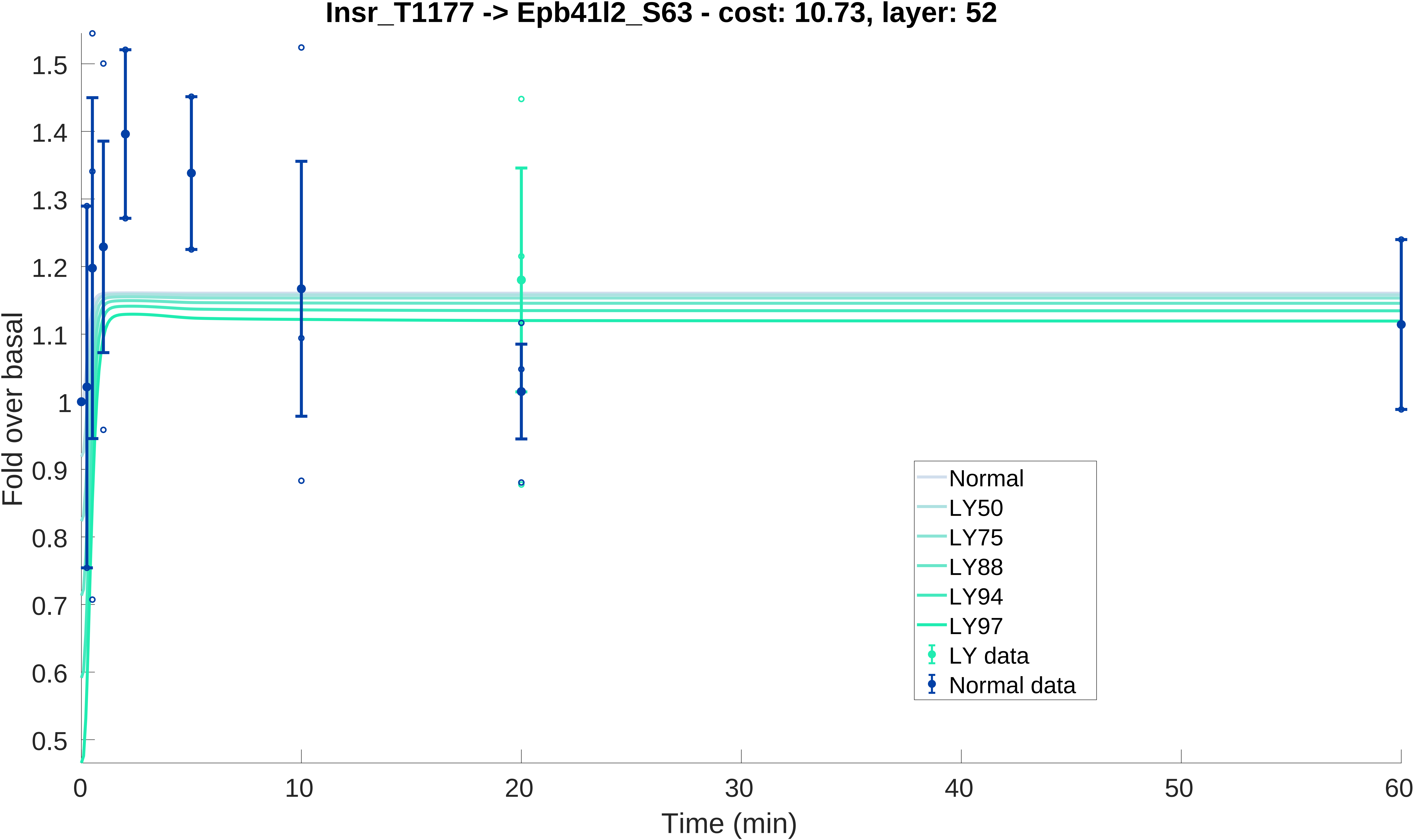
Insr_T1177 -> Epb41l2_S63 - cost: 10.73, layer: 52
Fold over basal
1.5
1.4
1.3
1.2
1.1
1
0.9
0.8
0.7
0.6
0.5
0
10
20
30
40
50
60
Time (min)
Normal
LY50
LY75
LY88
LY94
LY97
LY data
Normal data

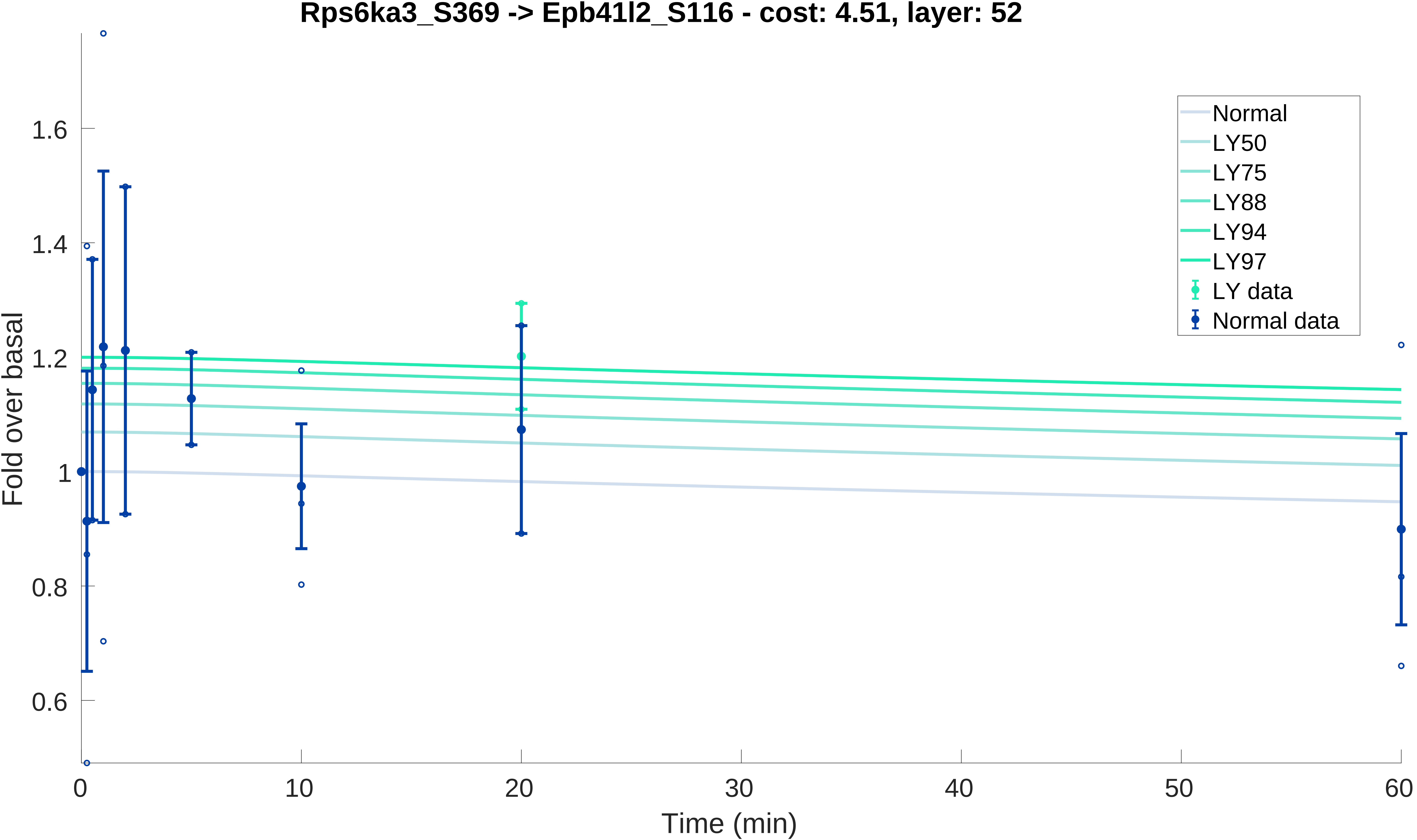

Rps6ka3_S369 -> Epb41l2_S116 - cost: 4.51, layer: 52
Fold over basal
Time (min)
1.6
1.4
1.2
1
0.8
0.6
0
10
20
30
40
50
60
Normal
LY50
LY75
LY88
LY94
LY97
LY data
Normal data

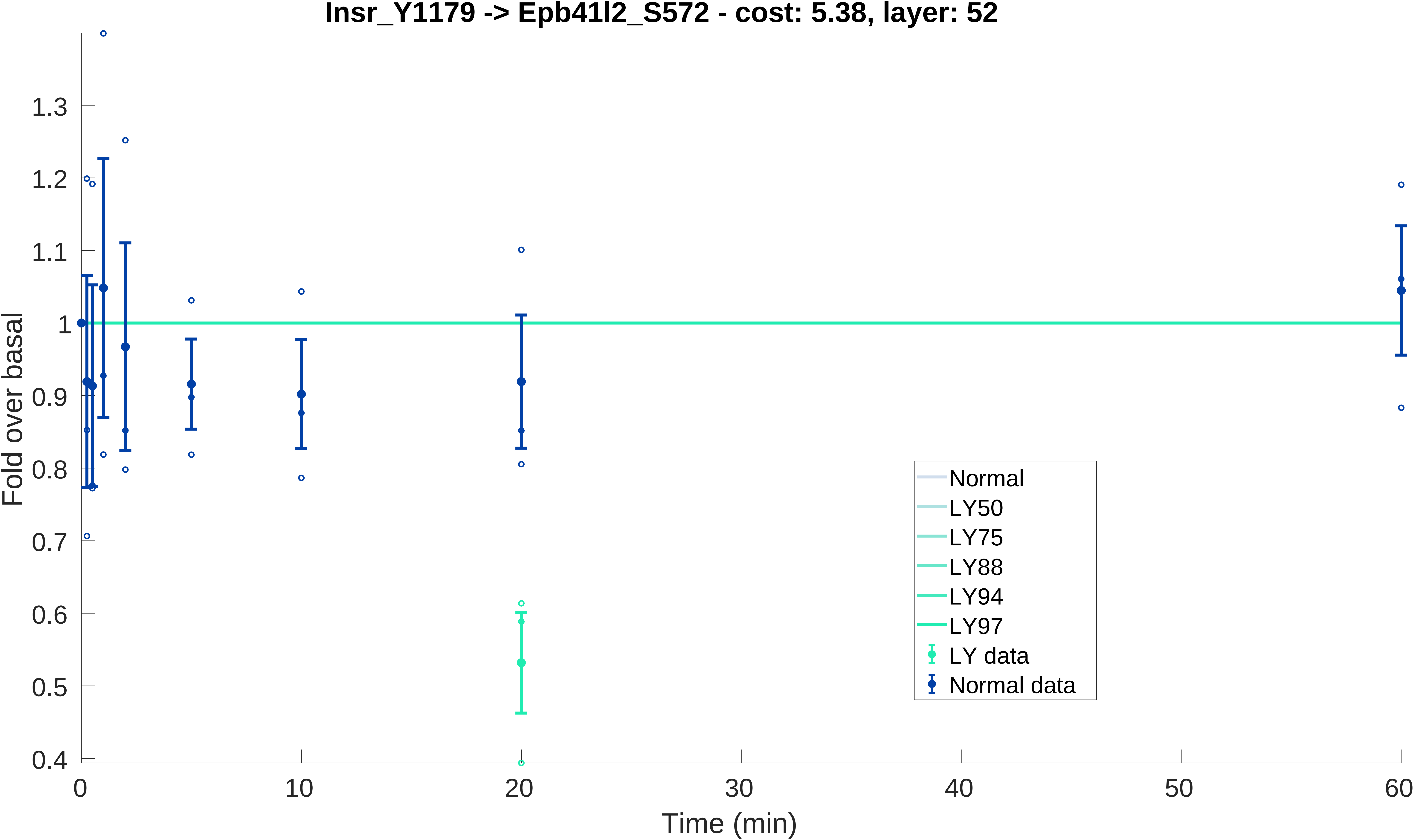
Insr_Y1179 -> Epb41l2_S572 - cost: 5.38, layer: 52
Fold over basal
Time (min)
1.3
1.2
1.1
1
0.9
0.8
0.7
0.6
0.5
0.4
0
10
20
30
40
50
60
Normal
LY50
LY75
LY88
LY94
LY97
LY data
Normal data

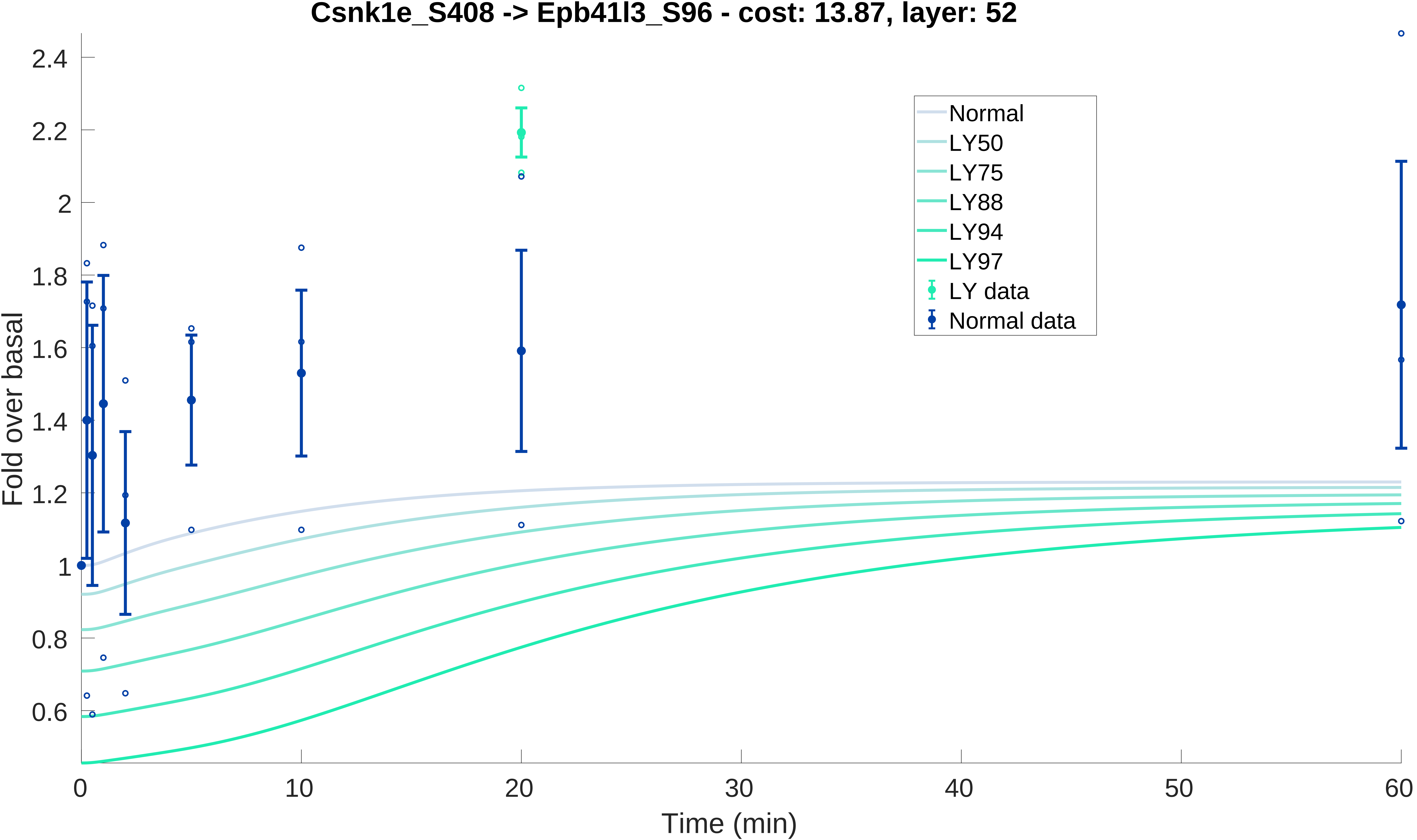

Csnk1e_S408 -> Epb41l3_S96 - cost: 13.87, layer: 52
Fold over basal
Time (min)
Normal
LY50
LY75
LY88
LY94
LY97
LY data
Normal data
2.4
2.2
2
1.8
1.6
1.4
1.2
1
0.8
0.6
0
10
20
30
40
50
60

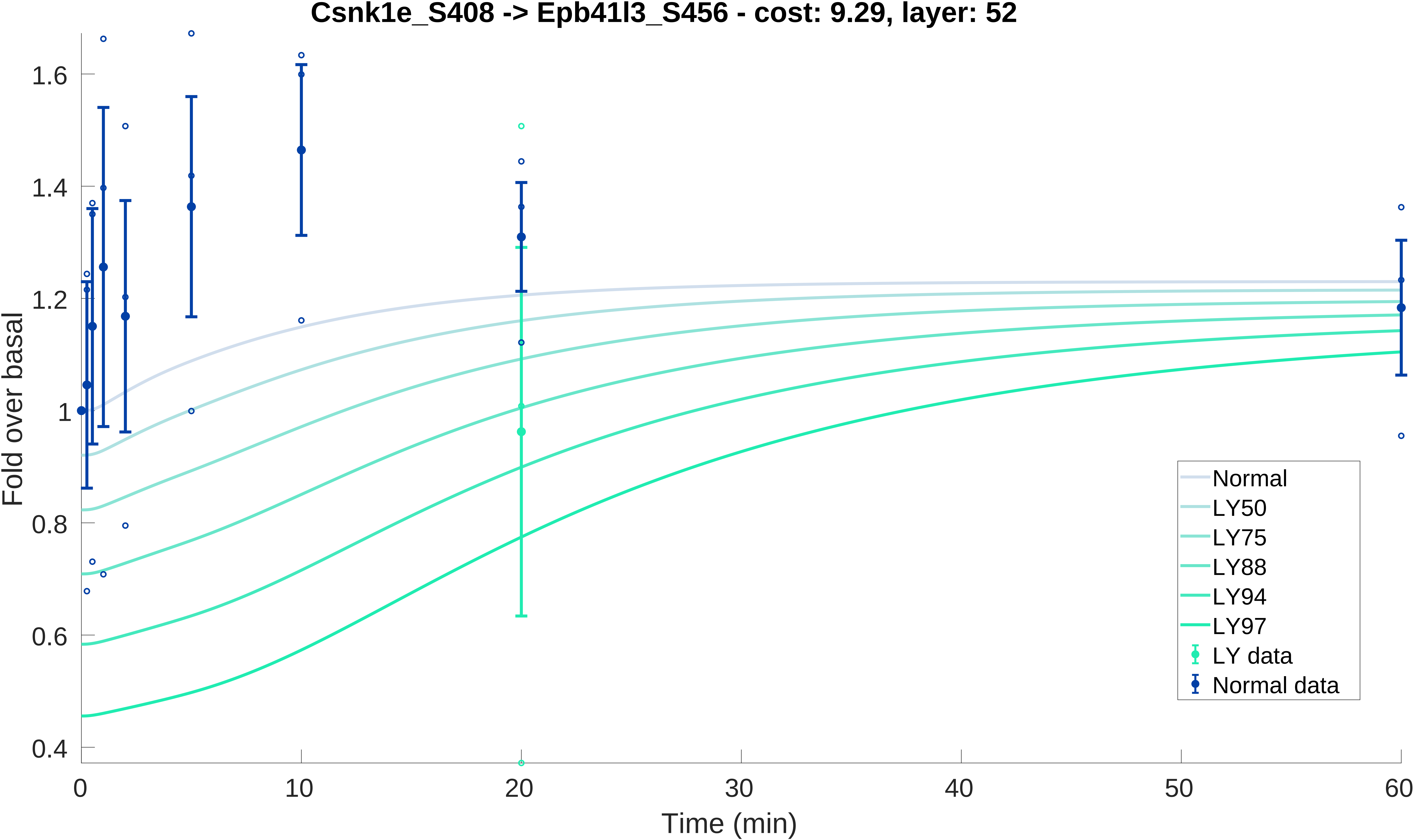

Csnk1e_S408 -> Epb41l3_S456 - cost: 9.29, layer: 52
Fold over basal
Time (min)
0.4
0.6
0.8
1
1.2
1.4
1.6
0
10
20
30
40
50
60
Normal
LY50
LY75
LY88
LY94
LY97
LY data
Normal data

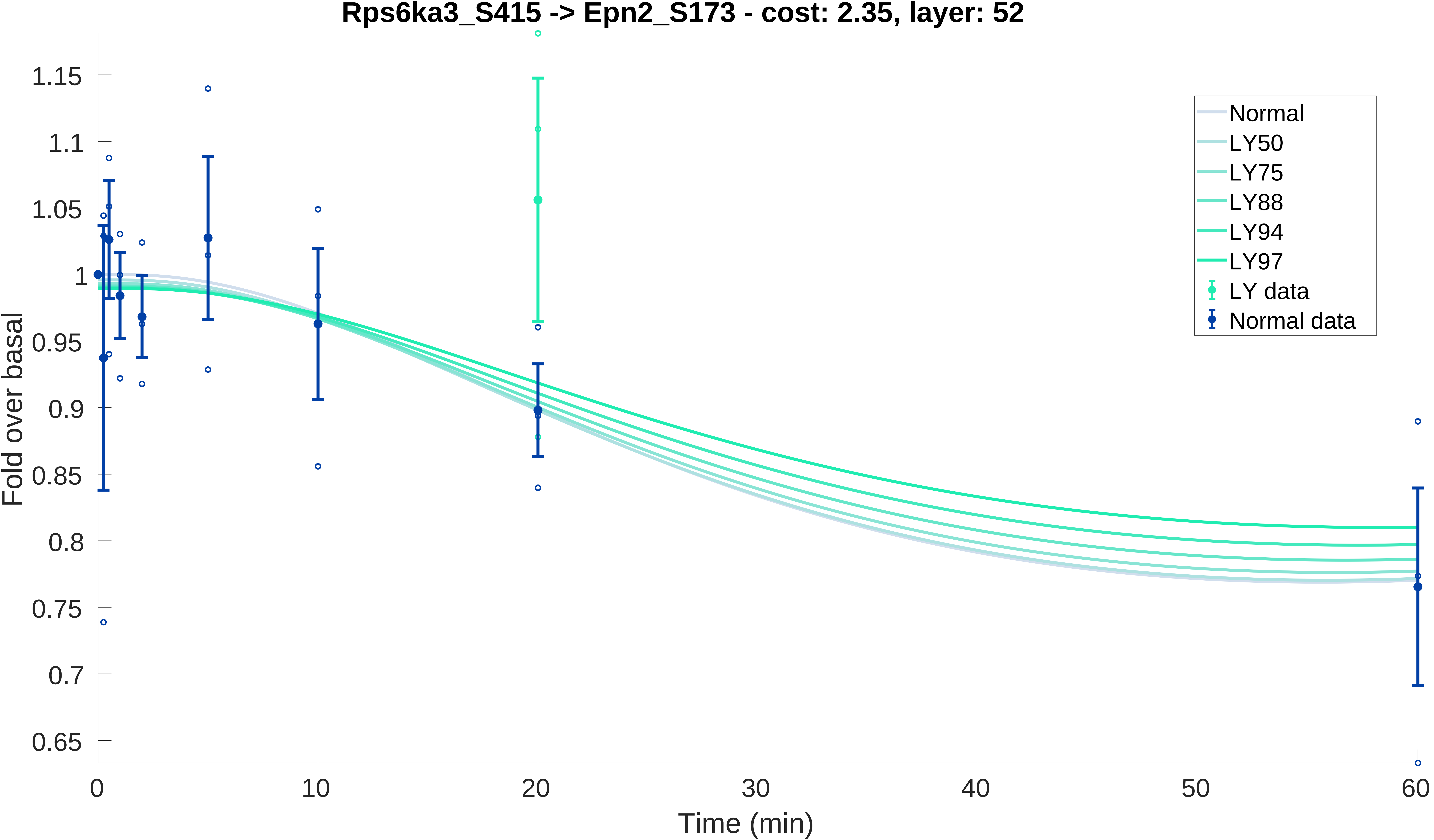

Rps6ka3_S415 -> Epn2_S173 - cost: 2.35, layer: 52
Fold over basal
Time (min)
1.15
1.1
1.05
1
0.95
0.9
0.85
0.8
0.75
0.7
0.65
0
10
20
30
40
50
60
Normal
LY50
LY75
LY88
LY94
LY97
LY data
Normal data

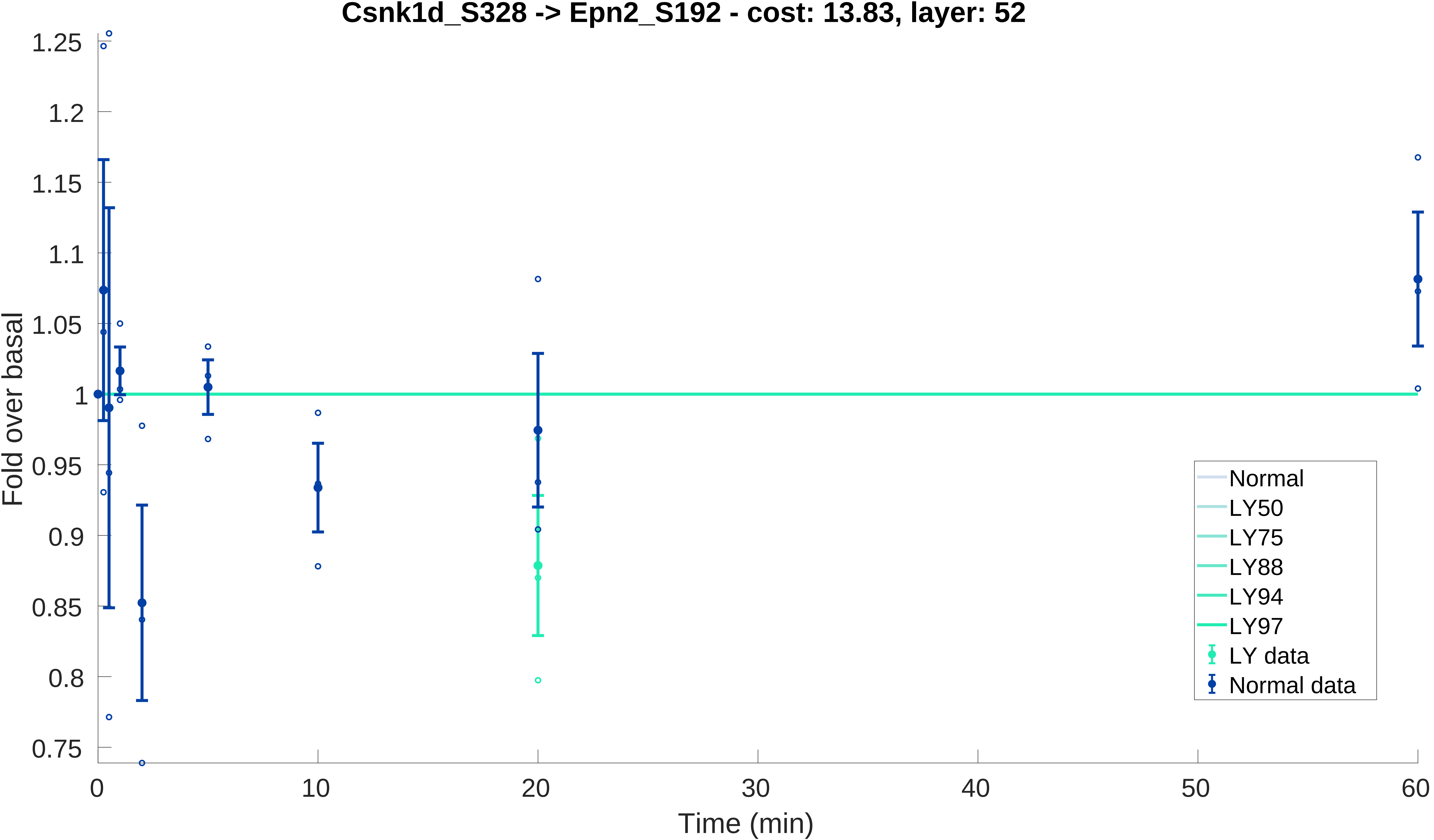

Csnk1d_S328 -> Epn2_S192 - cost: 13.83, layer: 52
Fold over basal
Time (min)
1.25
1.2
1.15
1.1
1.05
1
0.95
0.9
0.85
0.8
0.75
0
10
20
30
40
50
60
Normal
LY50
LY75
LY88
LY94
LY97
LY data
Normal data

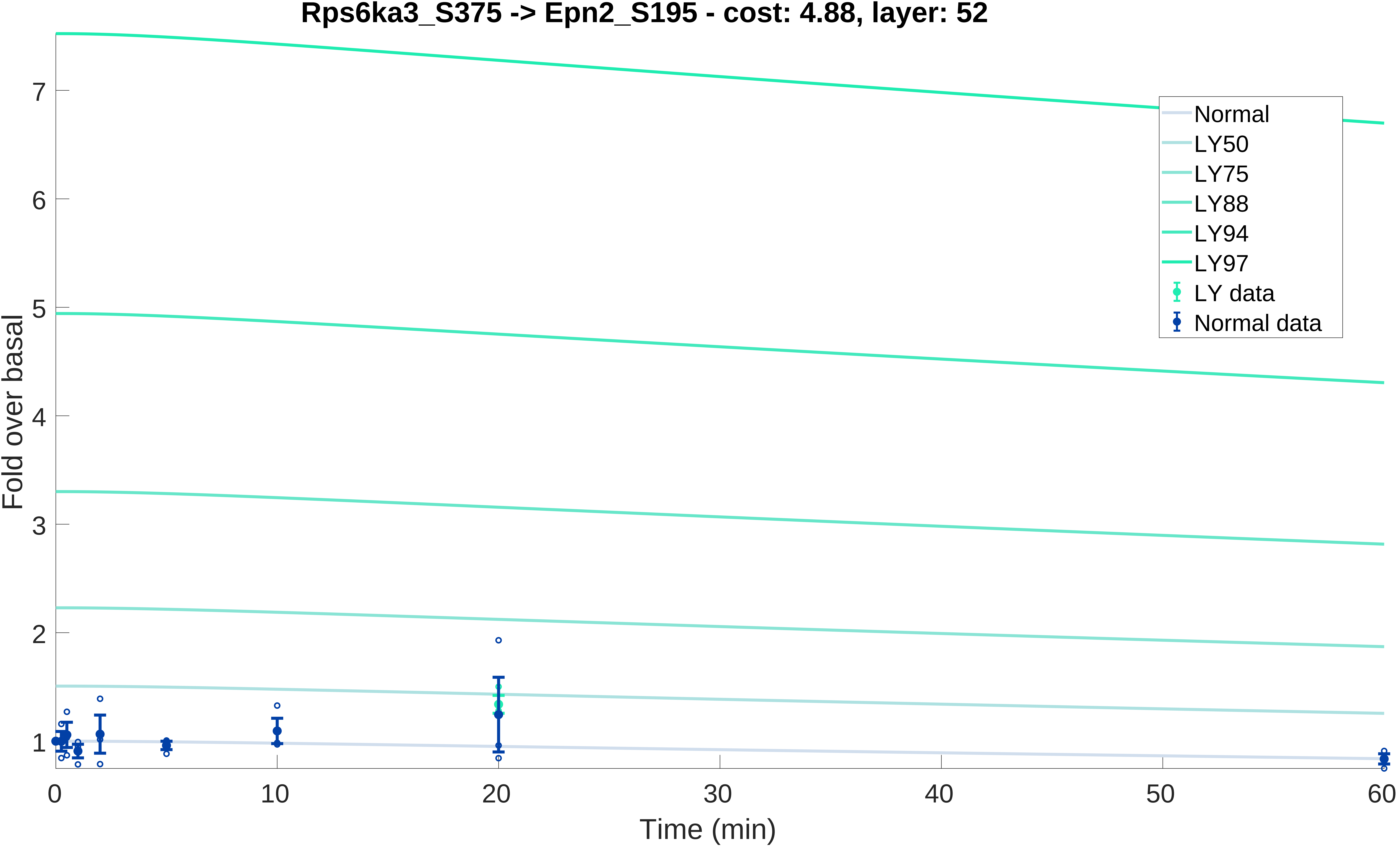

Rps6ka3_S375 -> Epn2_S195 - cost: 4.88, layer: 52
Fold over basal
Time (min)
0
10
20
30
40
50
60
1
2
3
4
5
6
7
Normal
LY50
LY75
LY88
LY94
LY97
LY data
Normal data

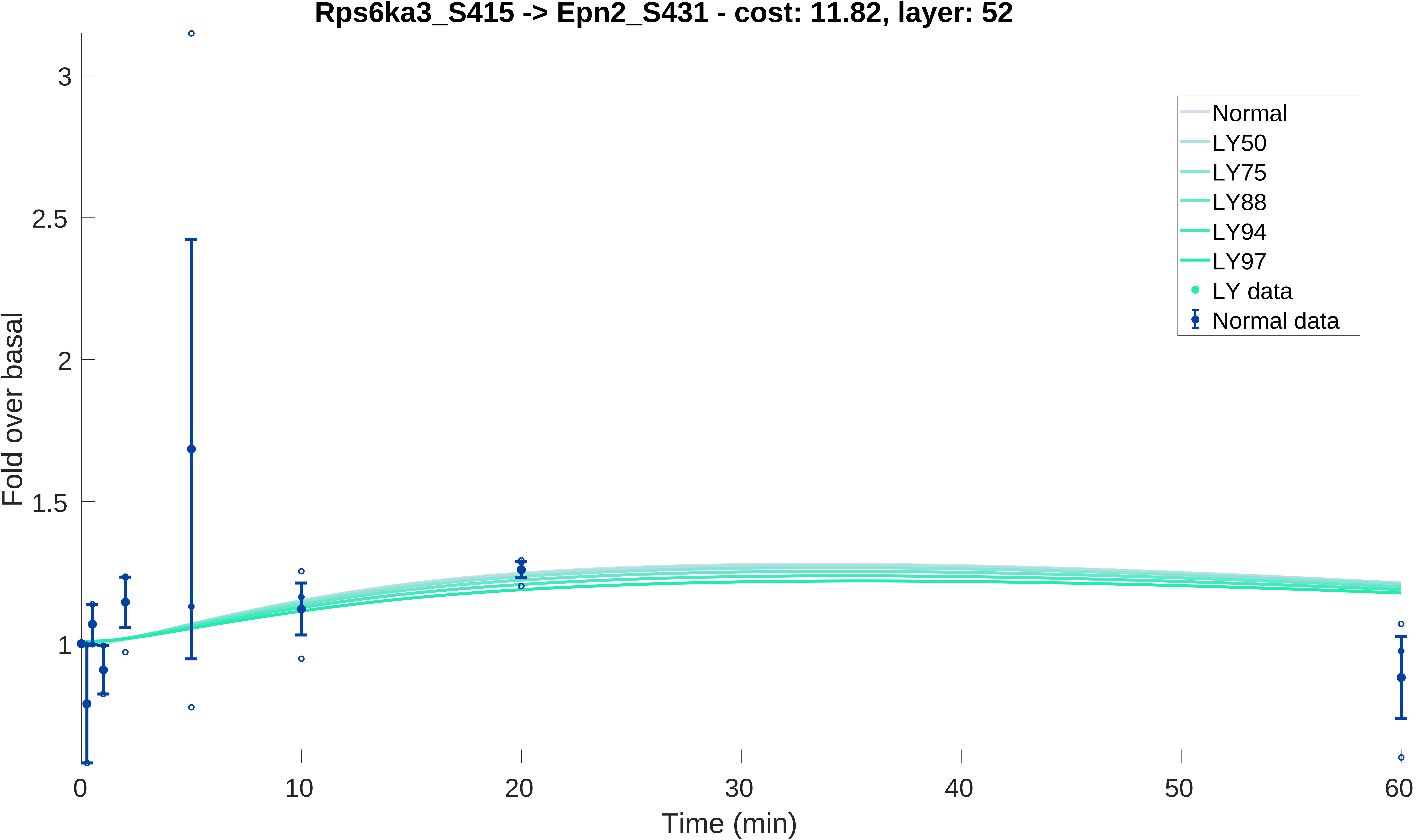

Rpsp6ka3_S415 -> Epn2_S431 - cost: 11.82, layer: 52
Fold over basal
Time (min)
0
10
20
30
40
50
60
1
1.5
2
2.5
3
Normal
LY50
LY75
LY88
LY94
LY97
LY data
Normal data

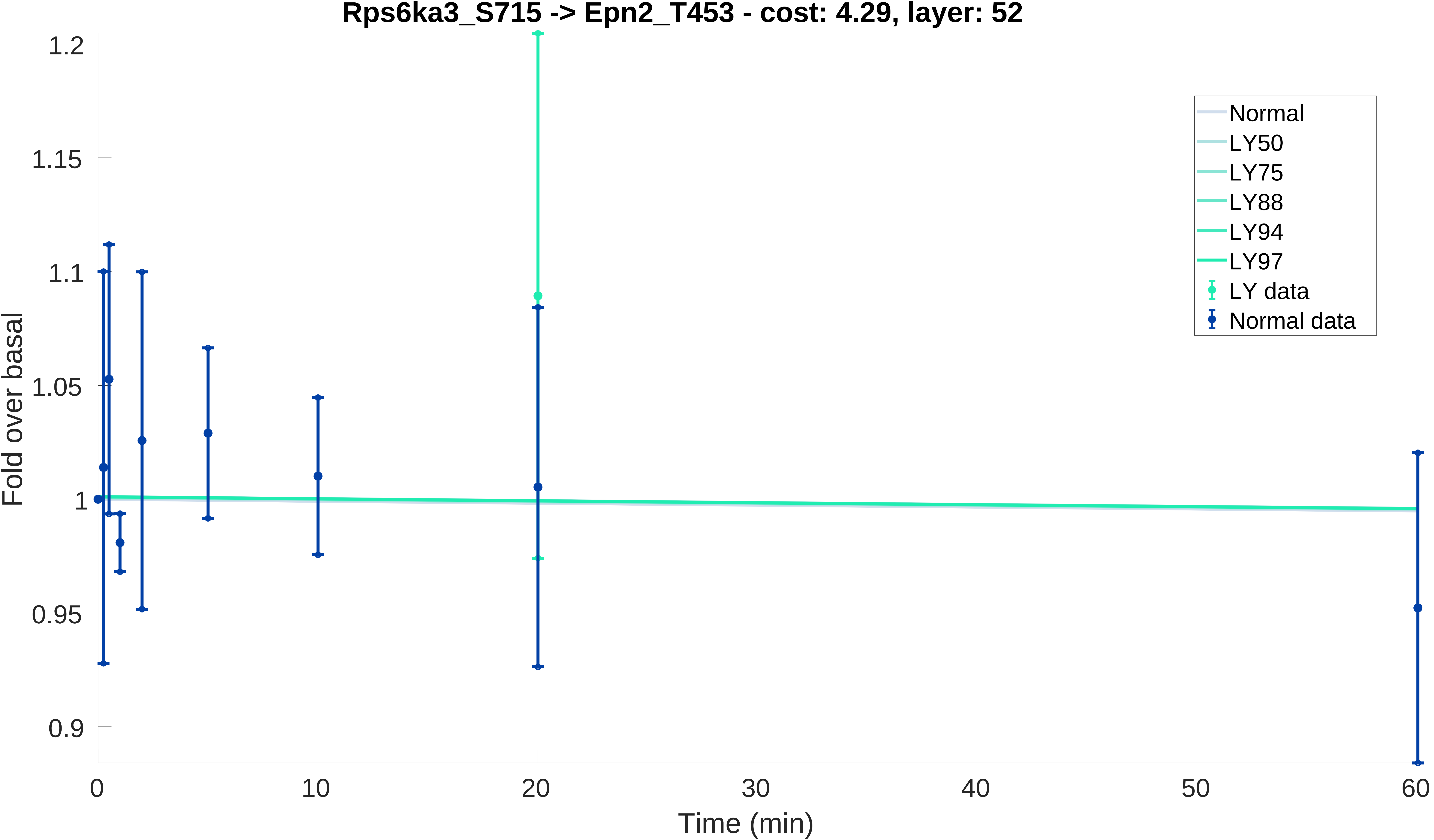

Rps6ka3_S715 -> Epn2_T453 - cost: 4.29, layer: 52
Fold over basal
Time (min)
1.2
1.15
1.1
1.05
1
0.95
0.9
0
10
20
30
40
50
60
Normal
LY50
LY75
LY88
LY94
LY97
LY data
Normal data

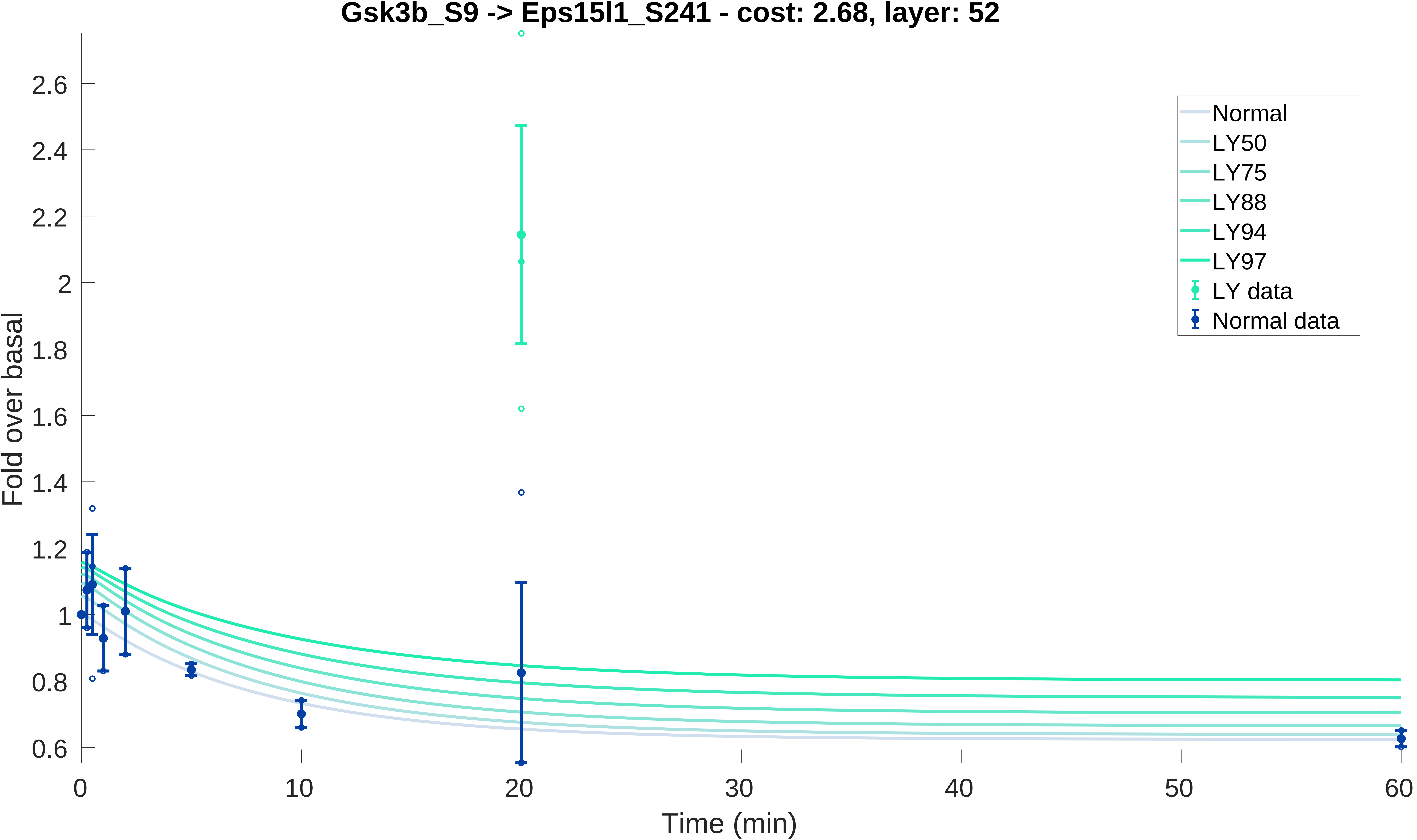

Gsk3b_S9 -> Eps15l1_S241 - cost: 2.68, layer: 52
Fold over basal
Time (min)
2.6
2.4
2.2
2
1.8
1.6
1.4
1.2
1
0.8
0.6
0
10
20
30
40
50
60
Normal
LY50
LY75
LY88
LY94
LY97
LY data
Normal data

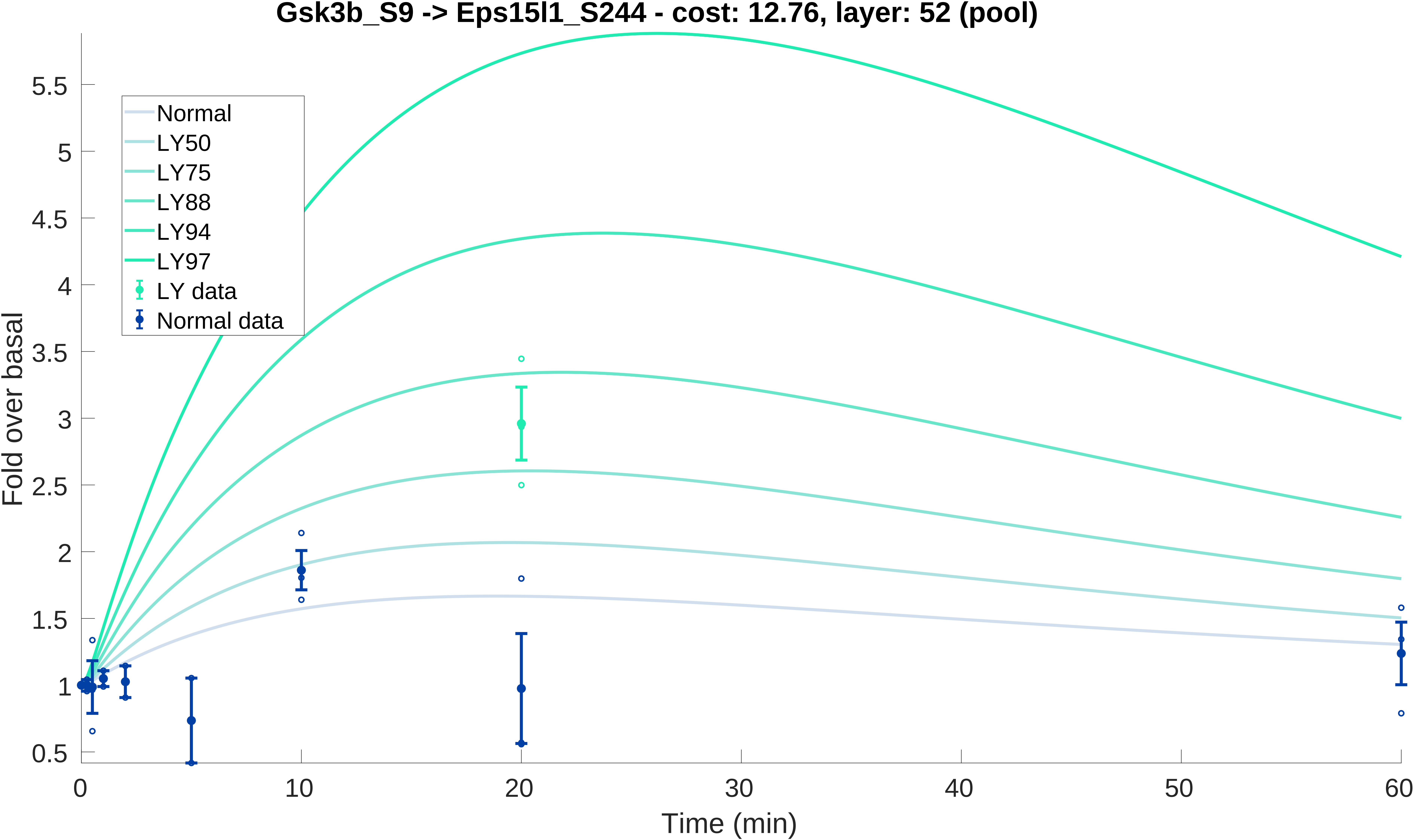

Gsk3b_S9 -> Eps15l1_S244 - cost: 12.76, layer: 52 (pool)
Normal
LY50
LY75
LY88
LY94
LY97
LY data
Normal data
Fold over basal
Time (min)
0
10
20
30
40
50
60
0.5
1
1.5
2
2.5
3
3.5
4
4.5
5
5.5

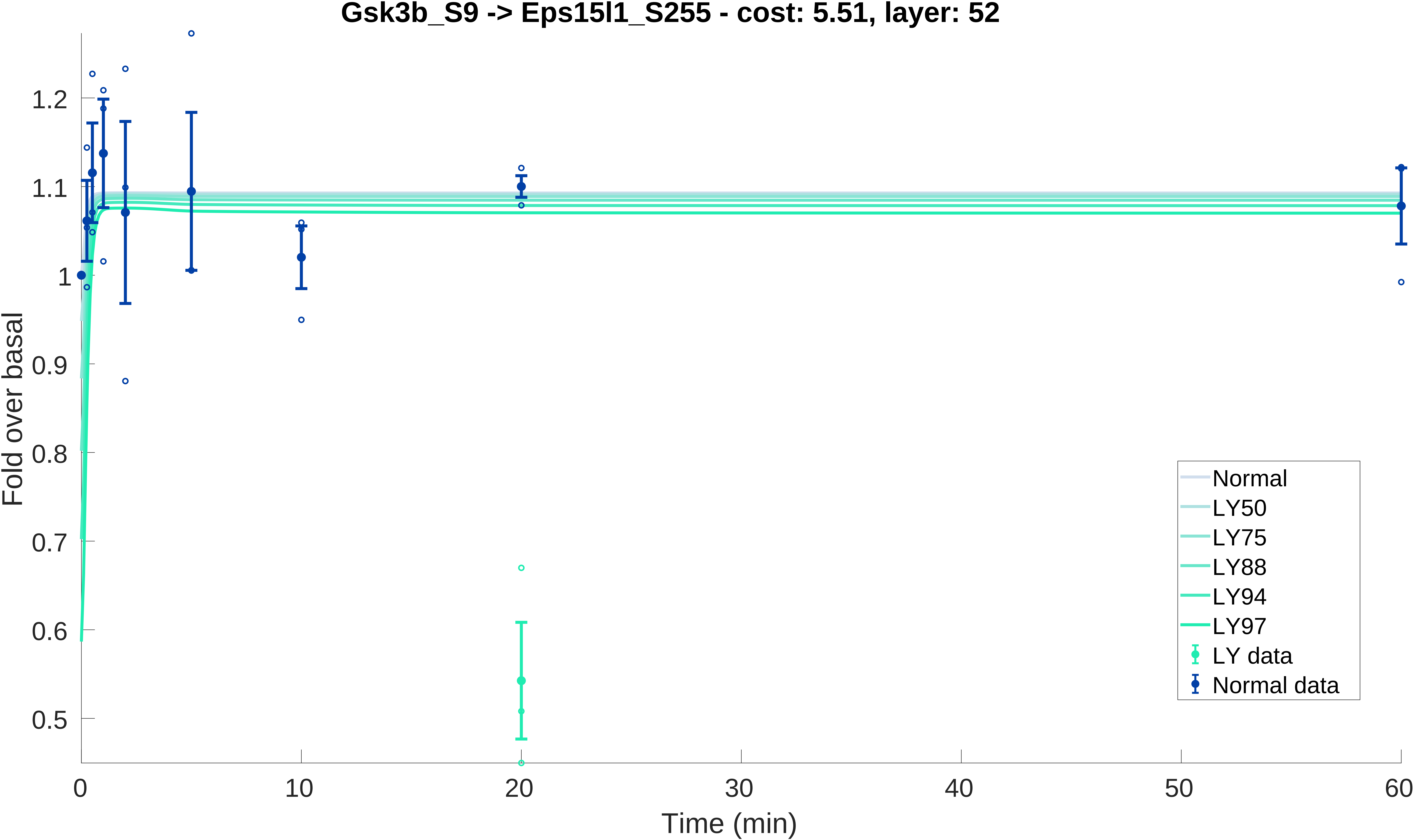

Gsk3b_S9 -> Eps15l1_S255 - cost: 5.51, layer: 52
Fold over basal
1.2
1.1
1
0.9
0.8
0.7
0.6
0.5
0
10
20
30
40
50
60
Time (min)
Normal
LY50
LY75
LY88
LY94
LY97
LY data
Normal data

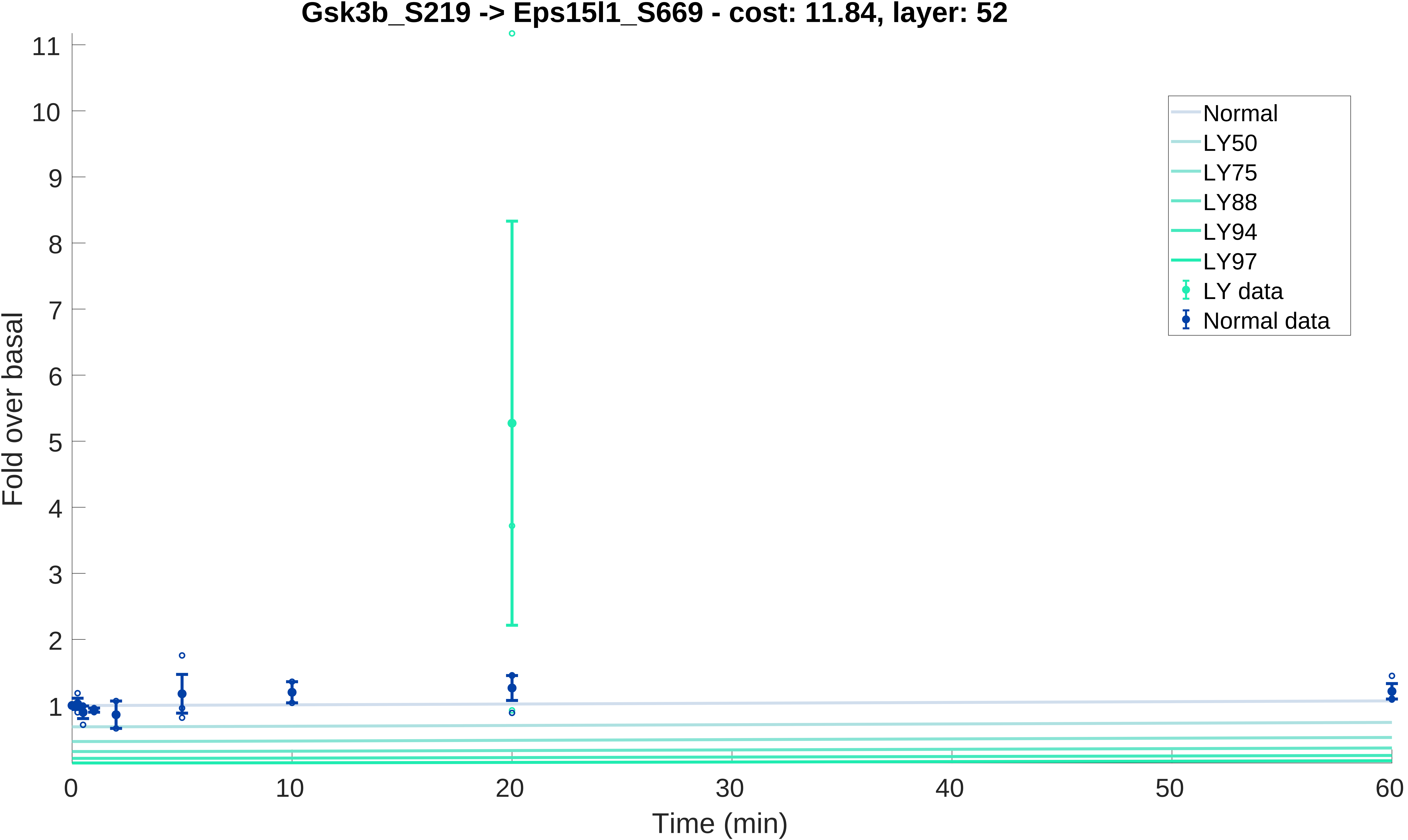

Gsk3b_S219 -> Eps15l1_S669 - cost: 11.84, layer: 52
Fold over basal
Time (min)
0
10
20
30
40
50
60
1
2
3
4
5
6
7
8
9
10
11
Normal
LY50
LY75
LY88
LY94
LY97
LY data
Normal data

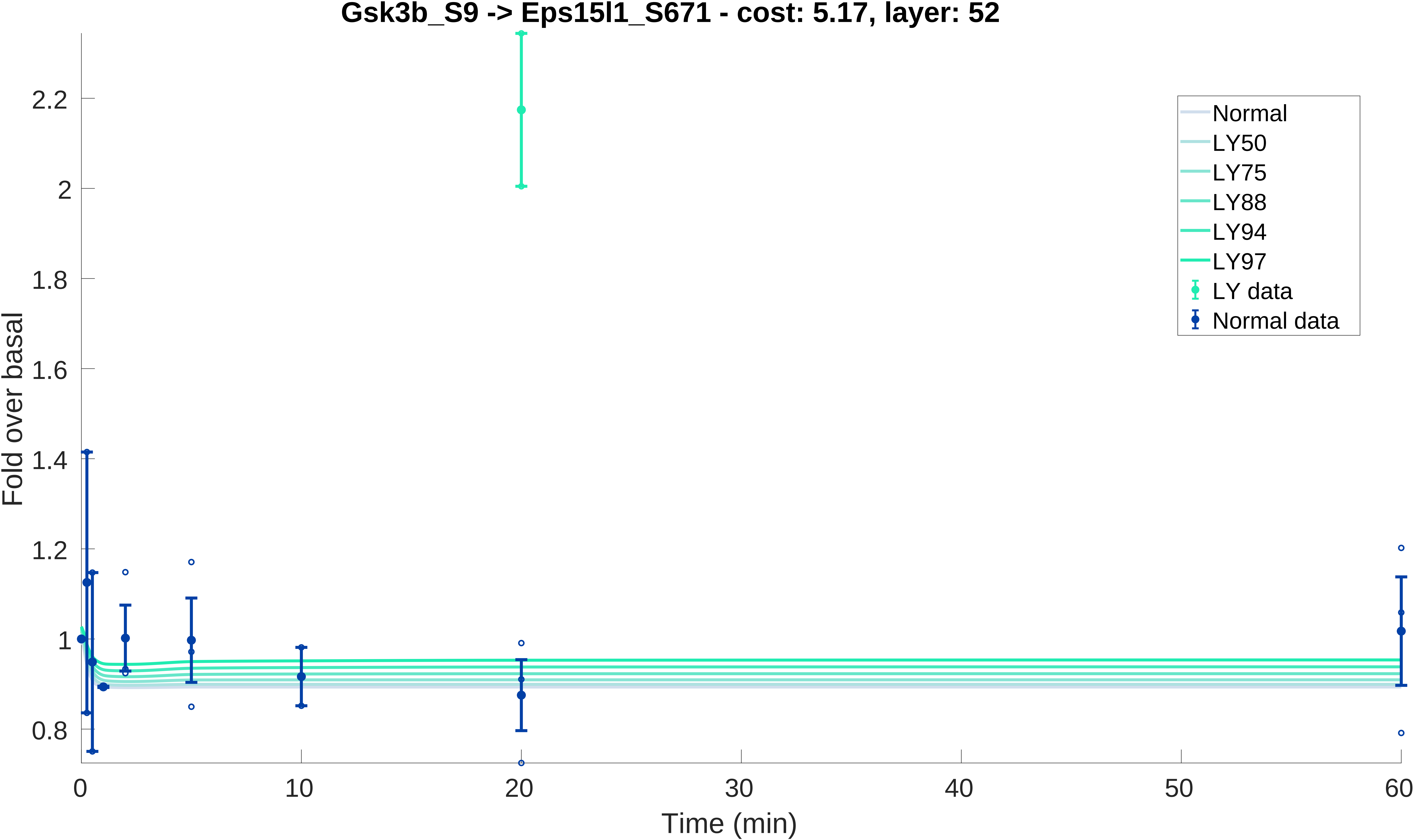

Gsk3b_S9 -> Eps15l1_S671 - cost: 5.17, layer: 52
Fold over basal
Time (min)
0.8
1
1.2
1.4
1.6
1.8
2
2.2
0
10
20
30
40
50
60
Normal
LY50
LY75
LY88
LY94
LY97
LY data
Normal data

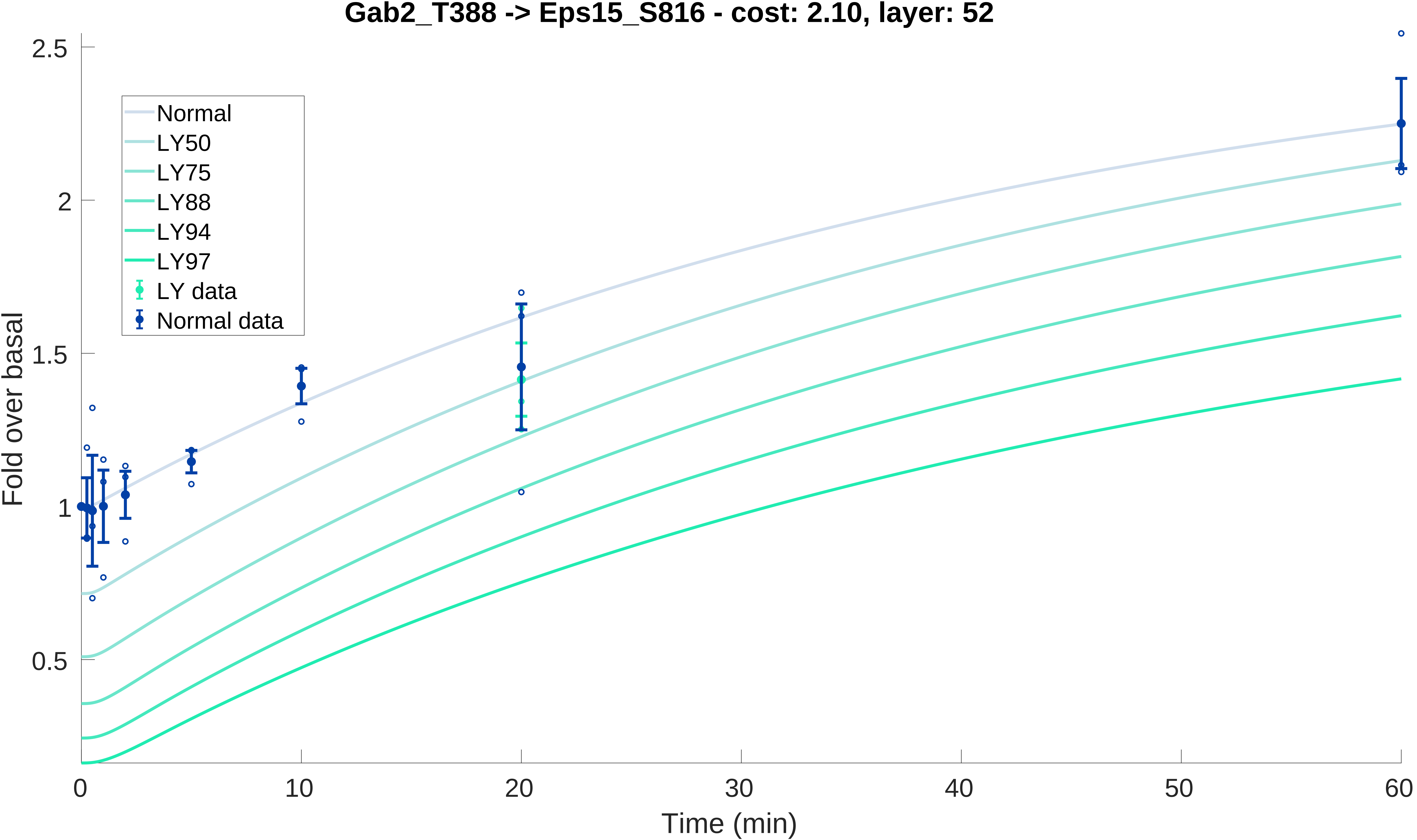

Gab2_T388 -> Eps15_S816 - cost: 2.10, layer: 52
Fold over basal
Time (min)
Normal
LY50
LY75
LY88
LY94
LY97
LY data
Normal data
2.5
2
1.5
1
0.5
0
10
20
30
40
50
60

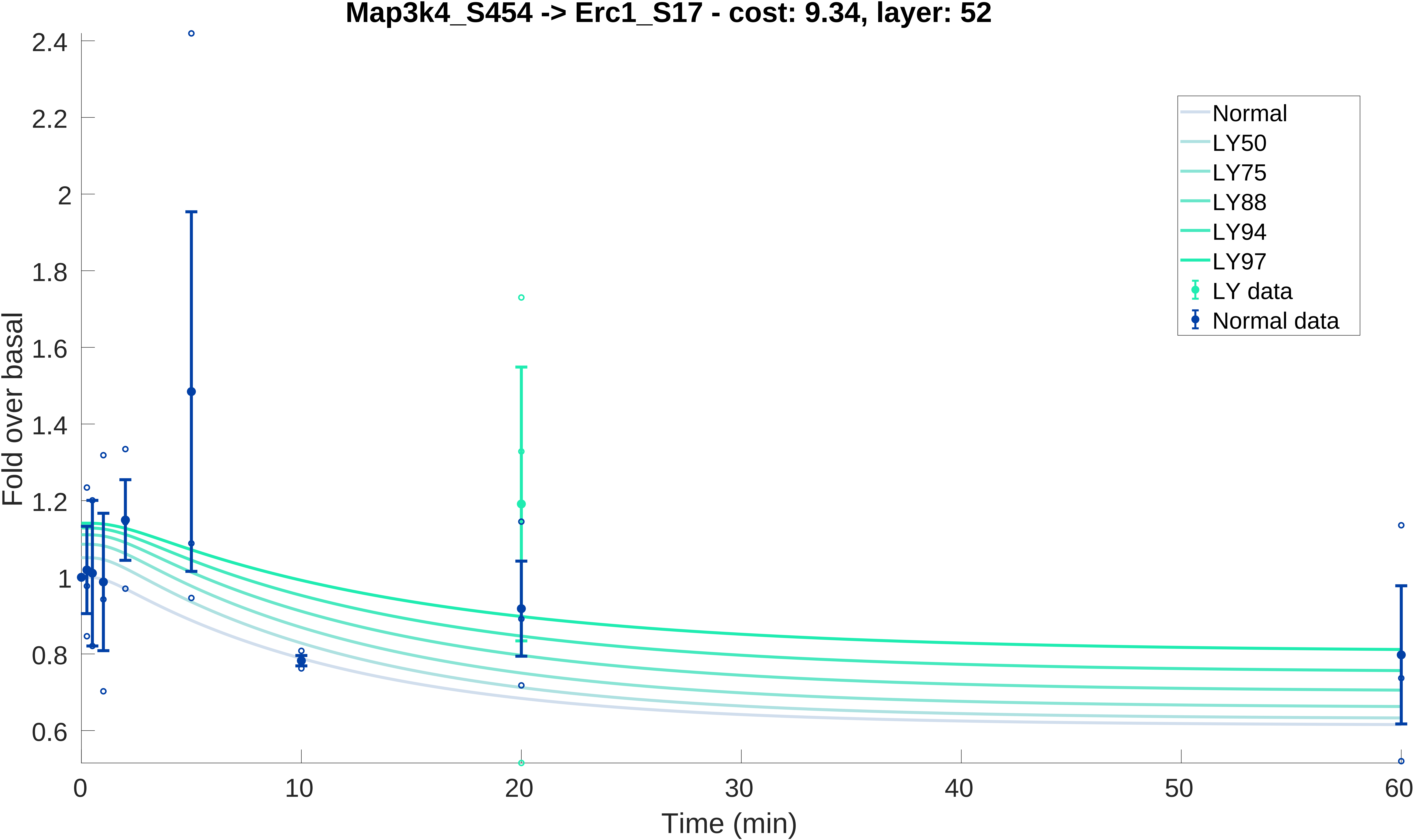

Map3k4_S454 -> Erc1_S17 - cost: 9.34, layer: 52
Fold over basal
Time (min)
Normal
LY50
LY75
LY88
LY94
LY97
LY data
Normal data
0.6
0.8
1
1.2
1.4
1.6
1.8
2
2.2
2.4
0
10
20
30
40
50
60

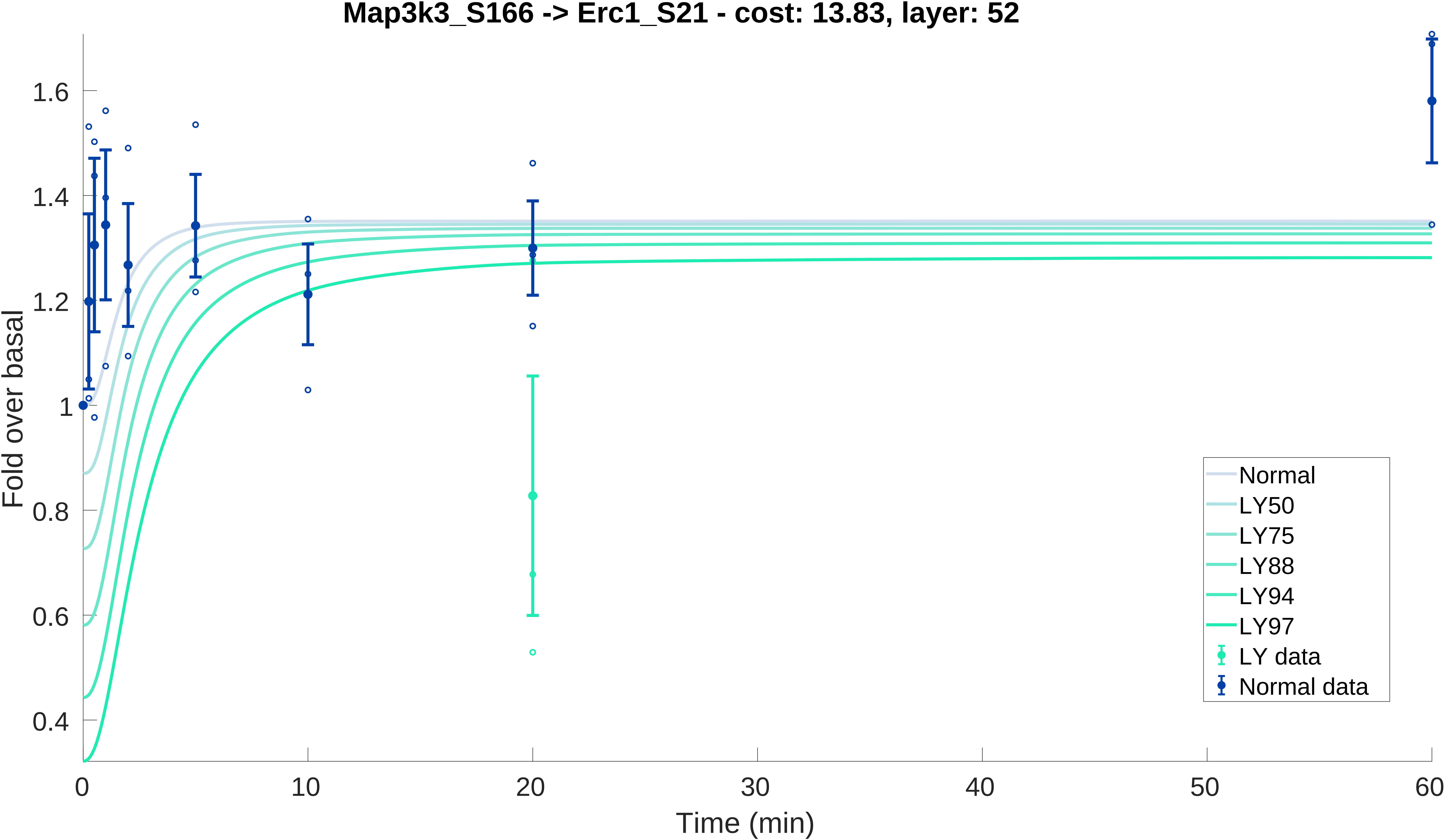

Map3k3_S166 -> Erc1_S21 - cost: 13.83, layer: 52
Fold over basal
1.6
1.4
1.2
1
0.8
0.6
0.4
0
10
20
30
40
50
60
Time (min)
Normal
LY50
LY75
LY88
LY94
LY97
LY data
Normal data

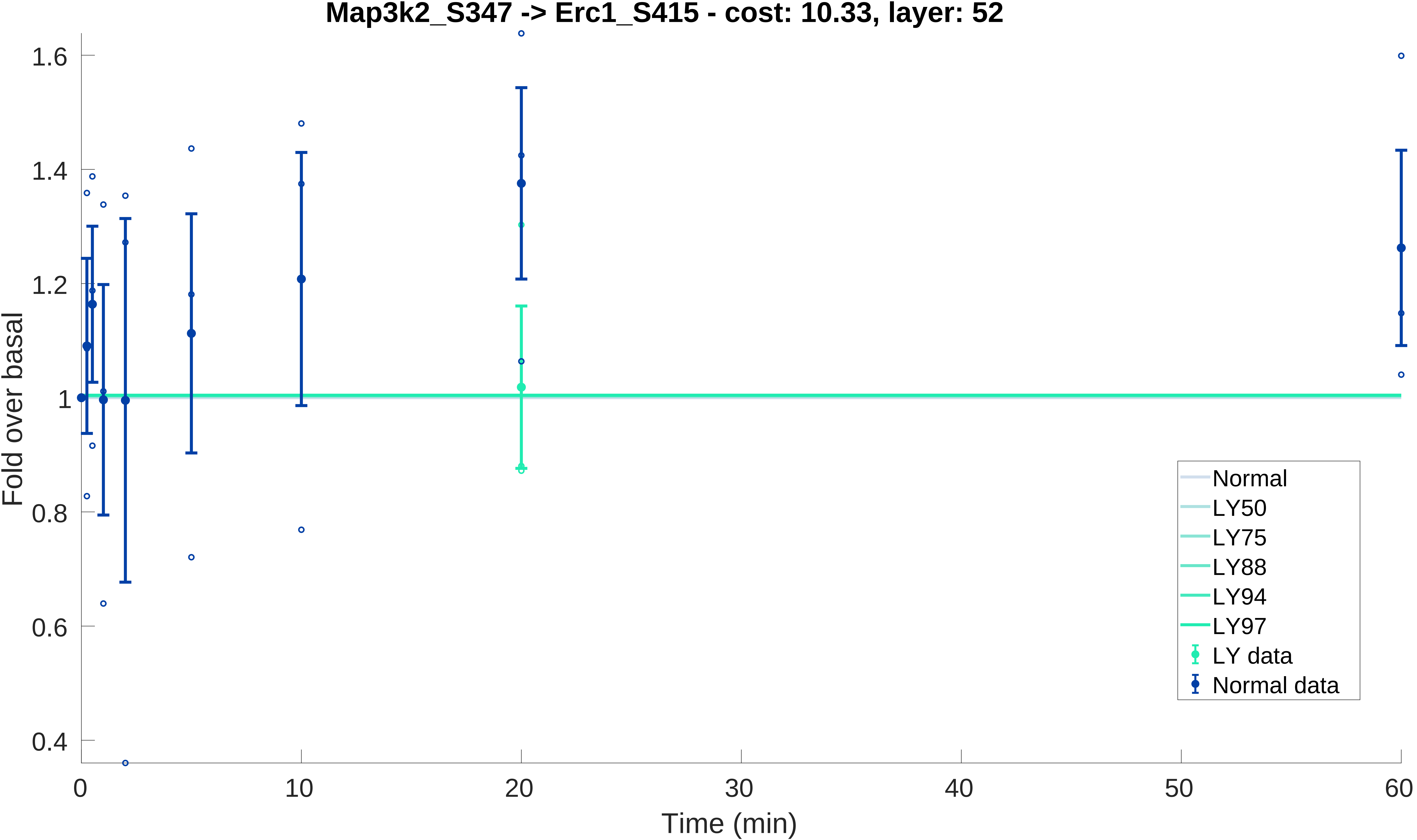

Map3k2_S347 -> Erc1_S415 - cost: 10.33, layer: 52
Fold over basal
Time (min)
0.4
0.6
0.8
1
1.2
1.4
1.6
0
10
20
30
40
50
60
Normal
LY50
LY75
LY88
LY94
LY97
LY data
Normal data

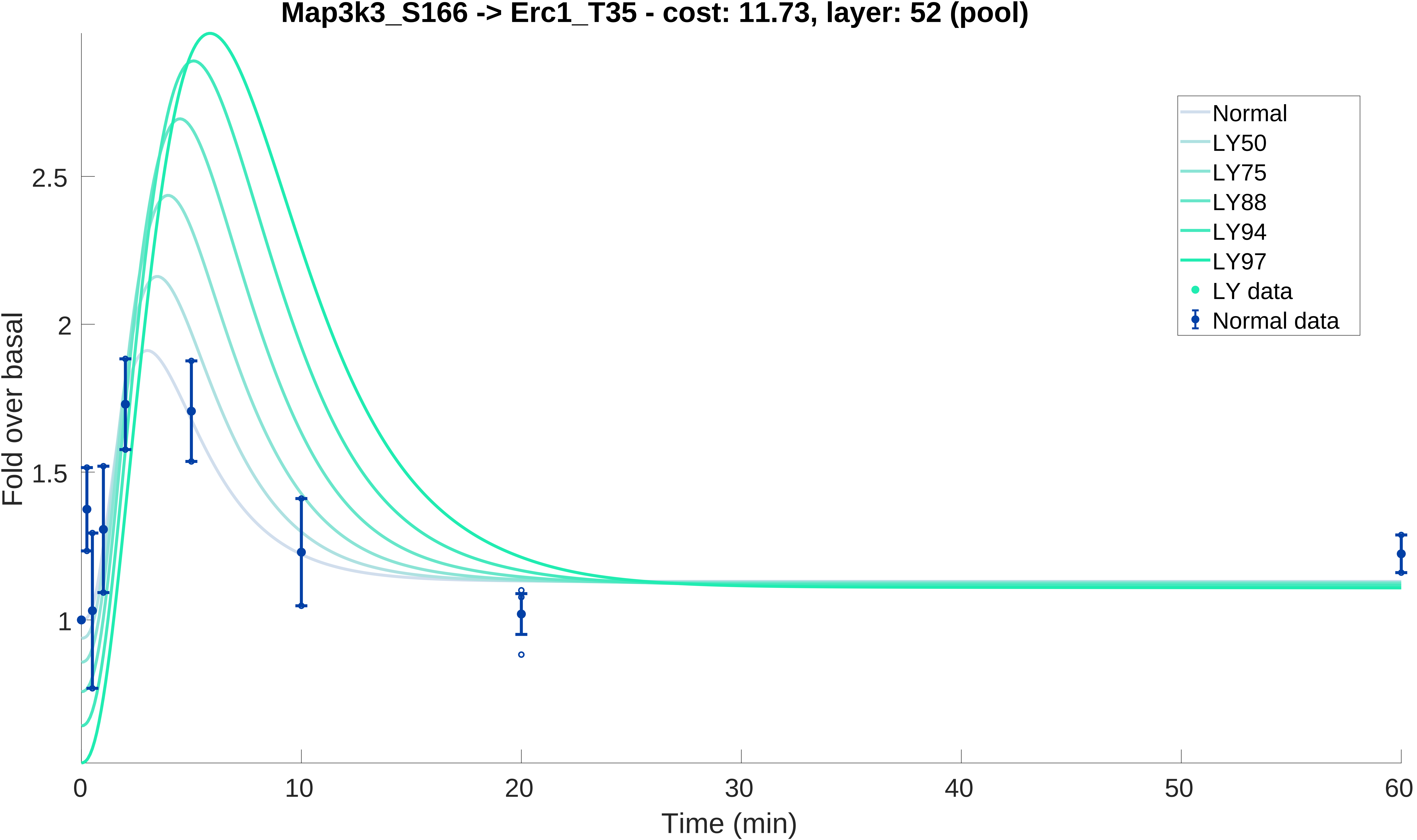
Map3k3_S166 -> Erc1_T35 - cost: 11.73, layer: 52 (pool)
Fold over basal
Time (min)
0
10
20
30
40
50
60
1
1.5
2
2.5
Normal
LY50
LY75
LY88
LY94
LY97
LY data
Normal data

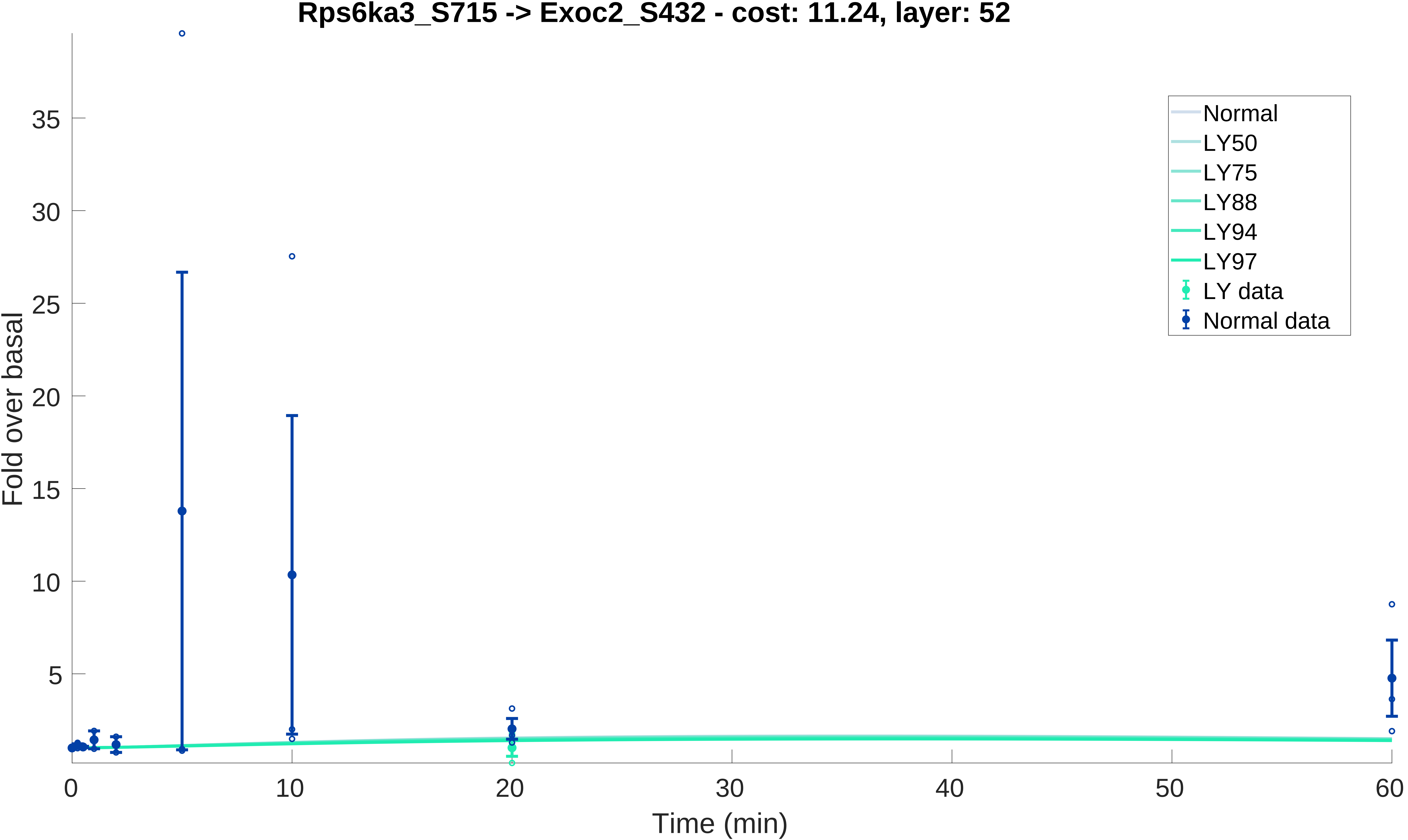

Rps6ka3_S715 -> Exoc2_S432 - cost: 11.24, layer: 52
Fold over basal
Time (min)
Normal
LY50
LY75
LY88
LY94
LY97
LY data
Normal data
0
10
20
30
40
50
60
5
10
15
20
25
30
35

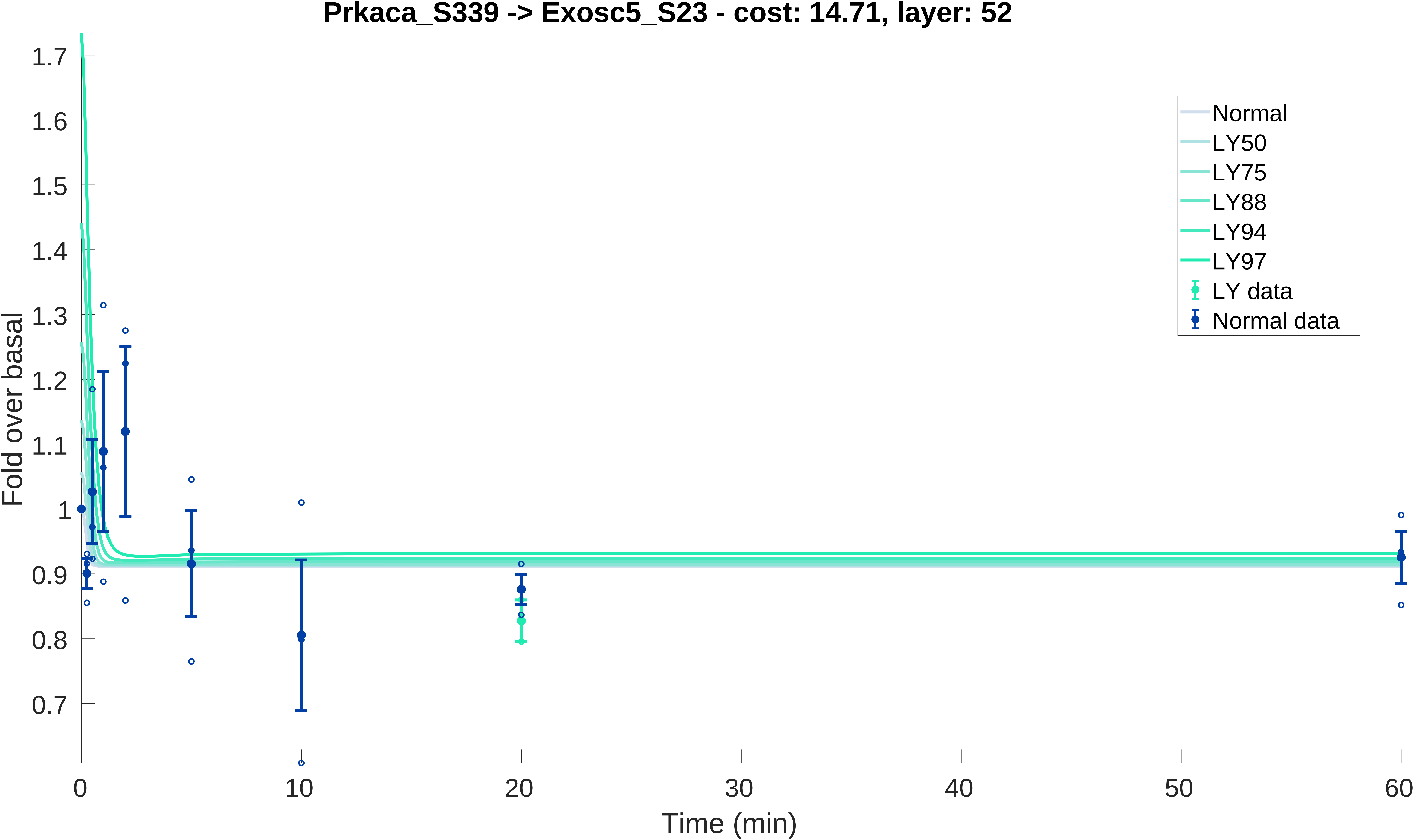

Prkaca_S339 -> Exosc5_S23 - cost: 14.71, layer: 52
Fold over basal
Time (min)
Normal
LY50
LY75
LY88
LY94
LY97
LY data
Normal data
1.7
1.6
1.5
1.4
1.3
1.2
1.1
1
0.9
0.8
0.7
0
10
20
30
40
50
60

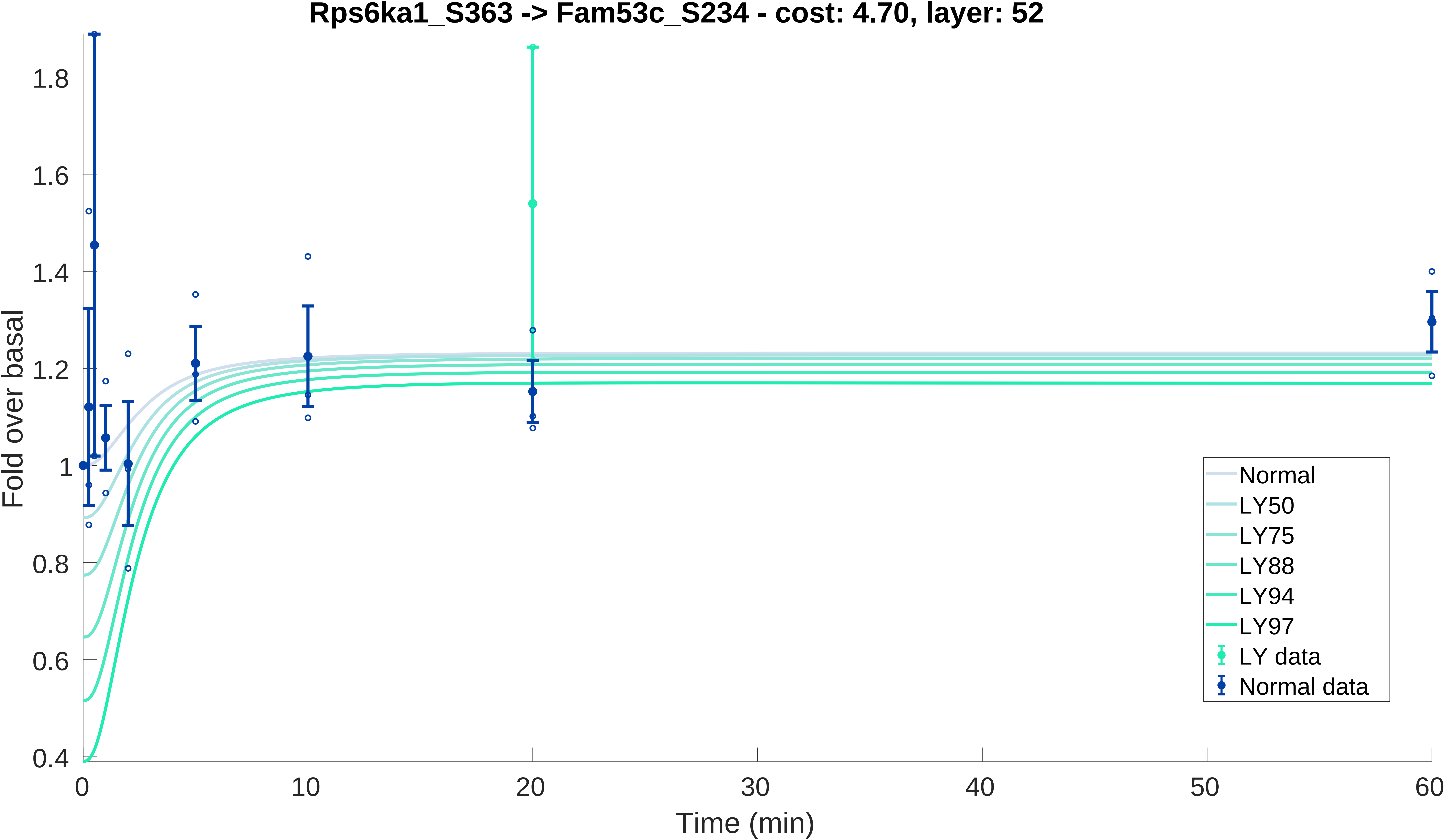

Rps6ka1_S363 -> Fam53c_S234 - cost: 4.70, layer: 52
Fold over basal
Time (min)
0.4
0.6
0.8
1
1.2
1.4
1.6
1.8
0
10
20
30
40
50
60
Normal
LY50
LY75
LY88
LY94
LY97
LY data
Normal data

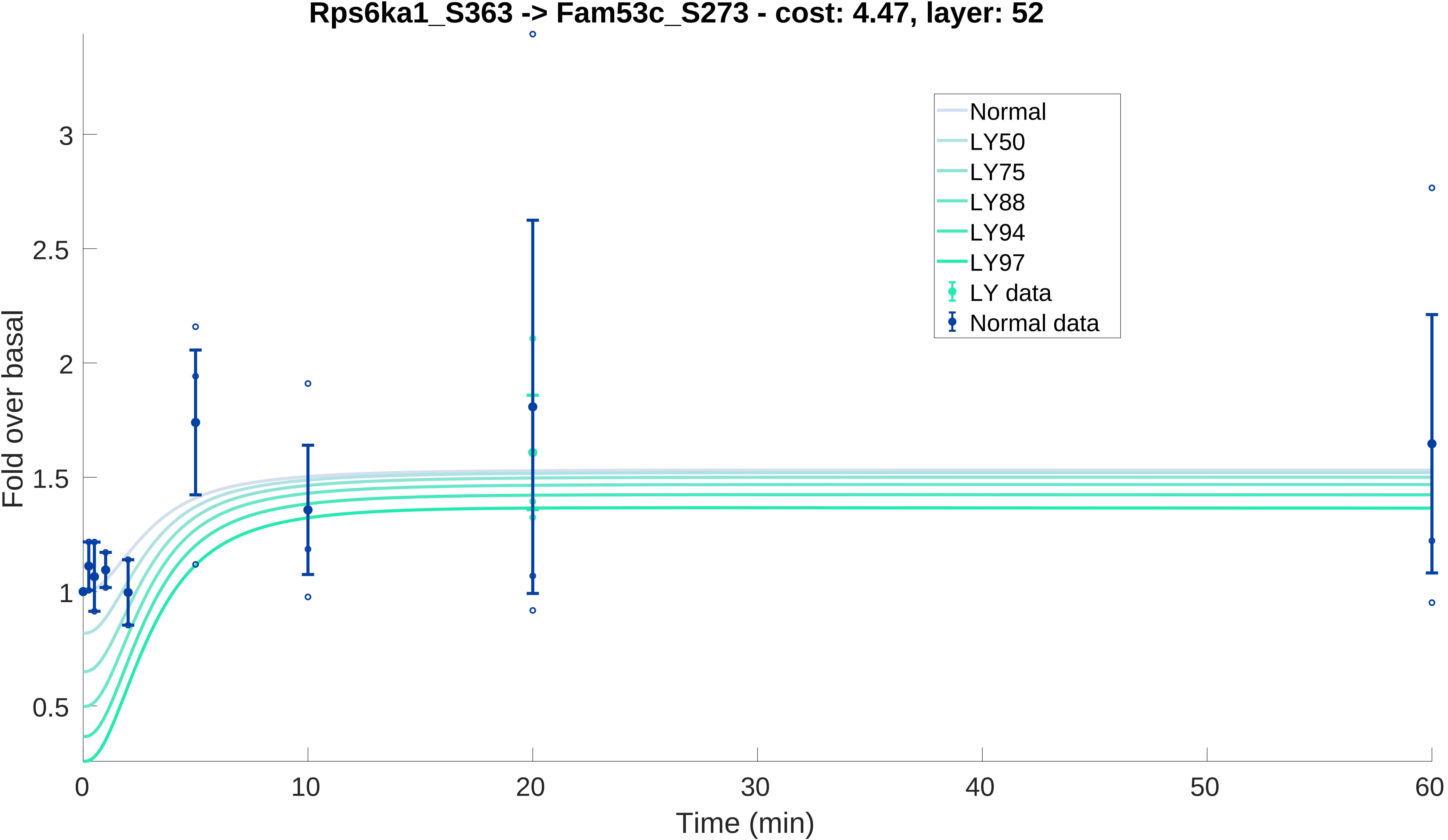

Rps6ka1_S363 -> Fam53c_S273 - cost: 4.47, layer: 52
Fold over basal
Time (min)
0.5
1
1.5
2
2.5
3
0
10
20
30
40
50
60
Normal
LY50
LY75
LY88
LY94
LY97
LY data
Normal data

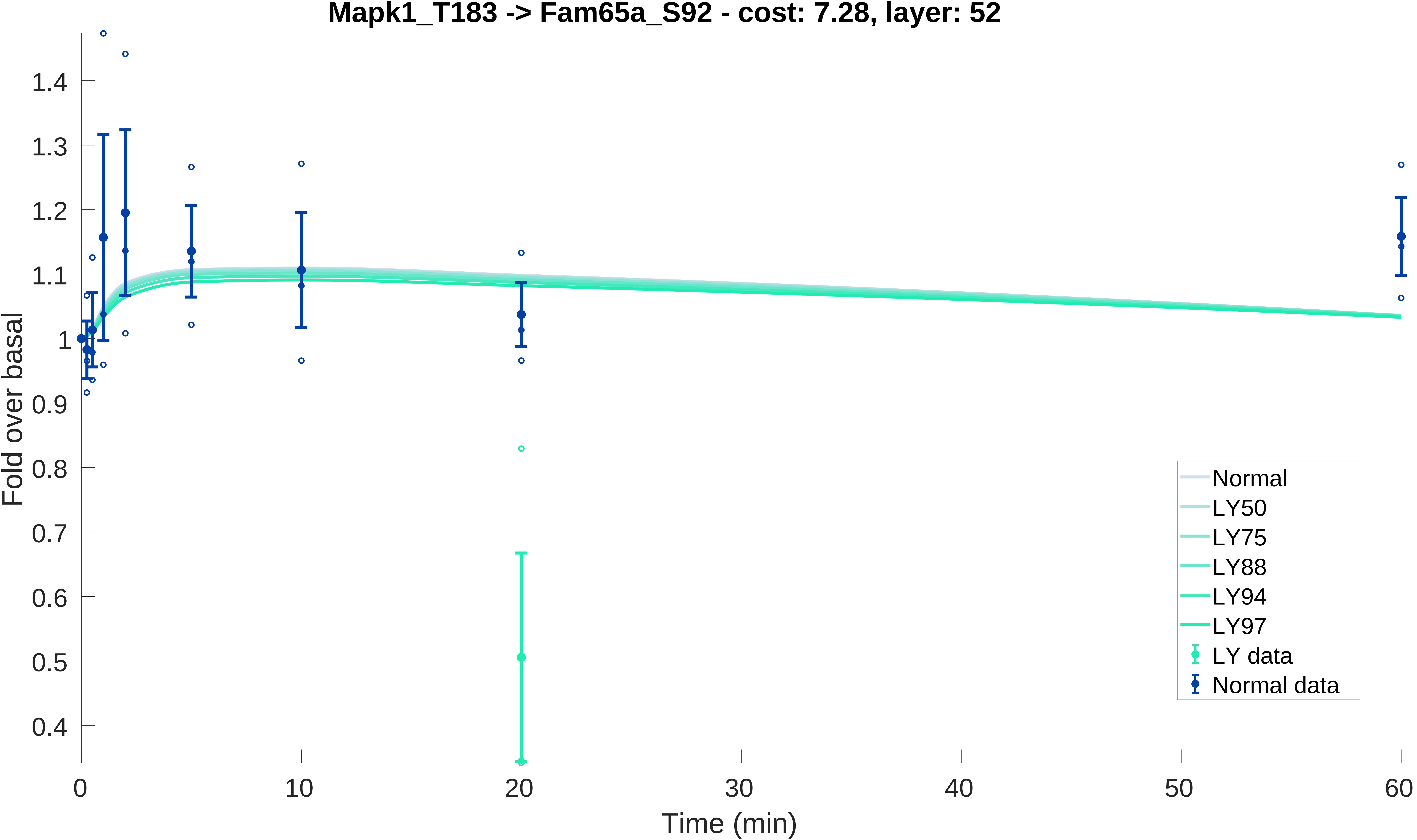
Mapk1_T183 -> Fam65a_S92 - cost: 7.28, layer: 52
Fold over basal
Time (min)
1.4
1.3
1.2
1.1
1
0.9
0.8
0.7
0.6
0.5
0.4
0
10
20
30
40
50
60
Normal
LY50
LY75
LY88
LY94
LY97
LY data
Normal data

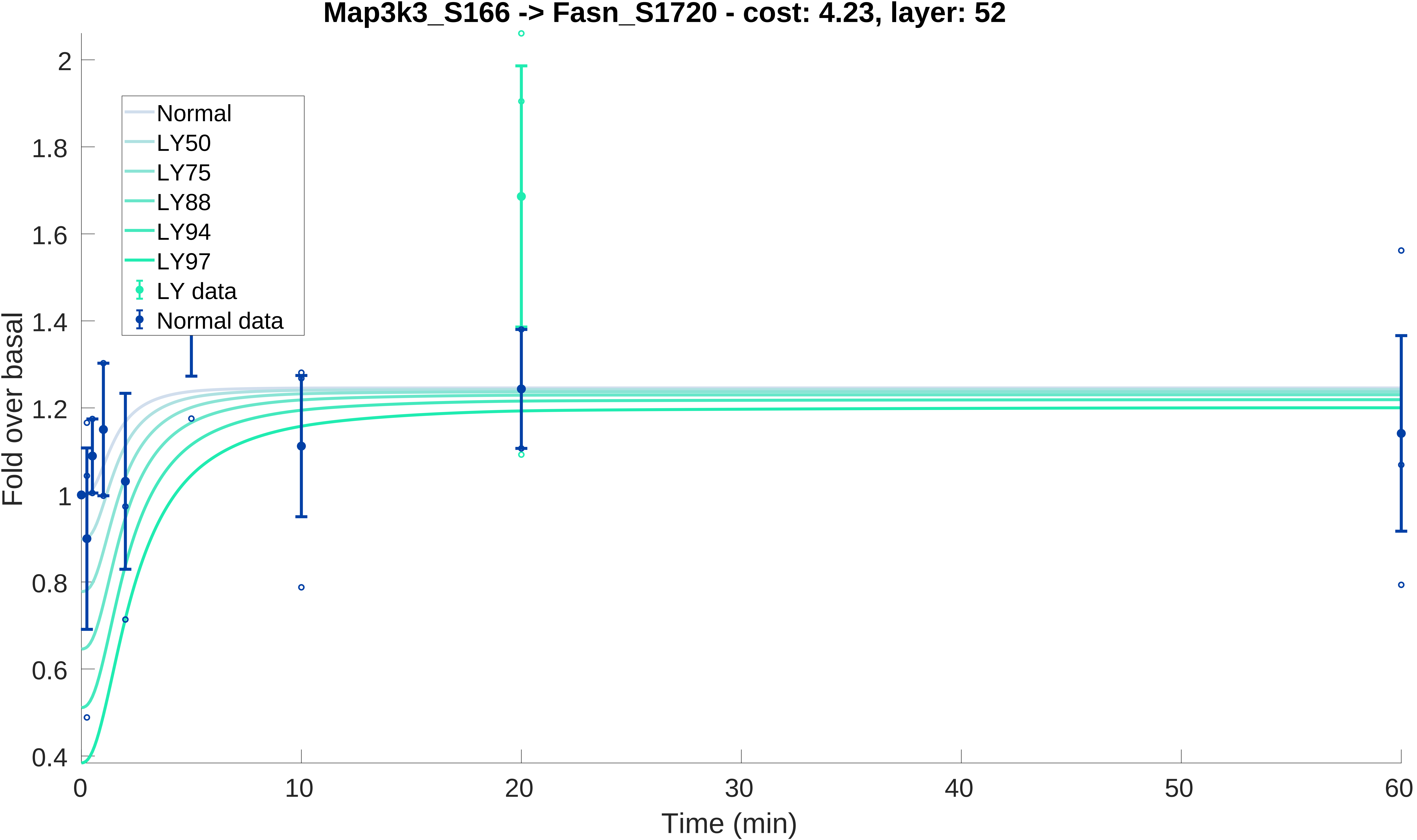

Map3k3_S166 -> Fasn_S1720 - cost: 4.23, layer: 52
Normal
LY50
LY75
LY88
LY94
LY97
LY data
Normal data
Fold over basal
Time (min)
0.4
0.6
0.8
1
1.2
1.4
1.6
1.8
2
0
10
20
30
40
50
60

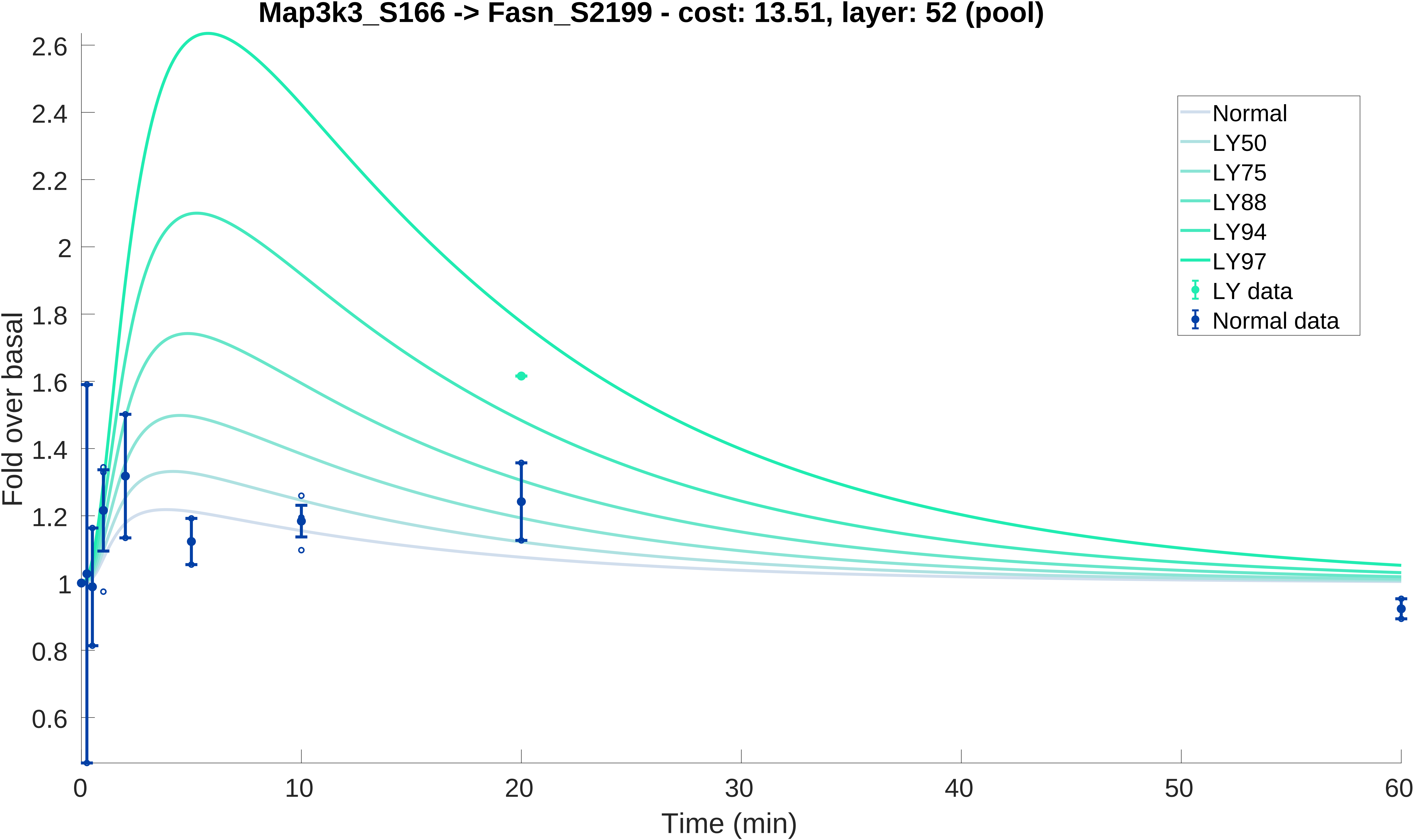
Map3k3_S166 -> Fasn_S2199 - cost: 13.51, layer: 52 (pool)
Fold over basal
Time (min)
2.6
2.4
2.2
2
1.8
1.6
1.4
1.2
1
0.8
0.6
0
10
20
30
40
50
60
Normal
LY50
LY75
LY88
LY94
LY97
LY data
Normal data

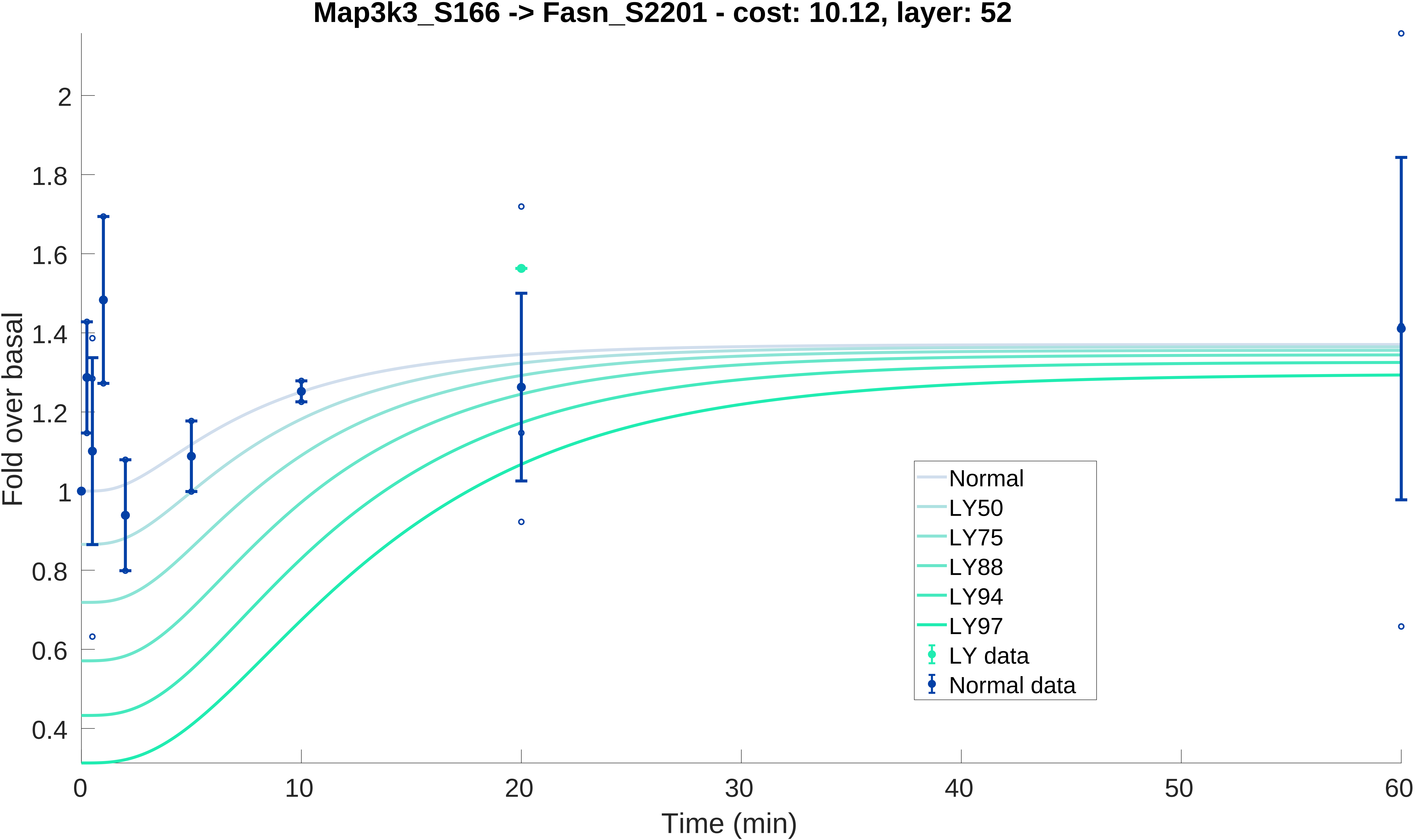

Map3k3_S166 -> Fasn_S2201 - cost: 10.12, layer: 52
Fold over basal
2
1.8
1.6
1.4
1.2
1
0.8
0.6
0.4
0
10
20
30
40
50
60
Time (min)
Normal
LY50
LY75
LY88
LY94
LY97
LY data
Normal data

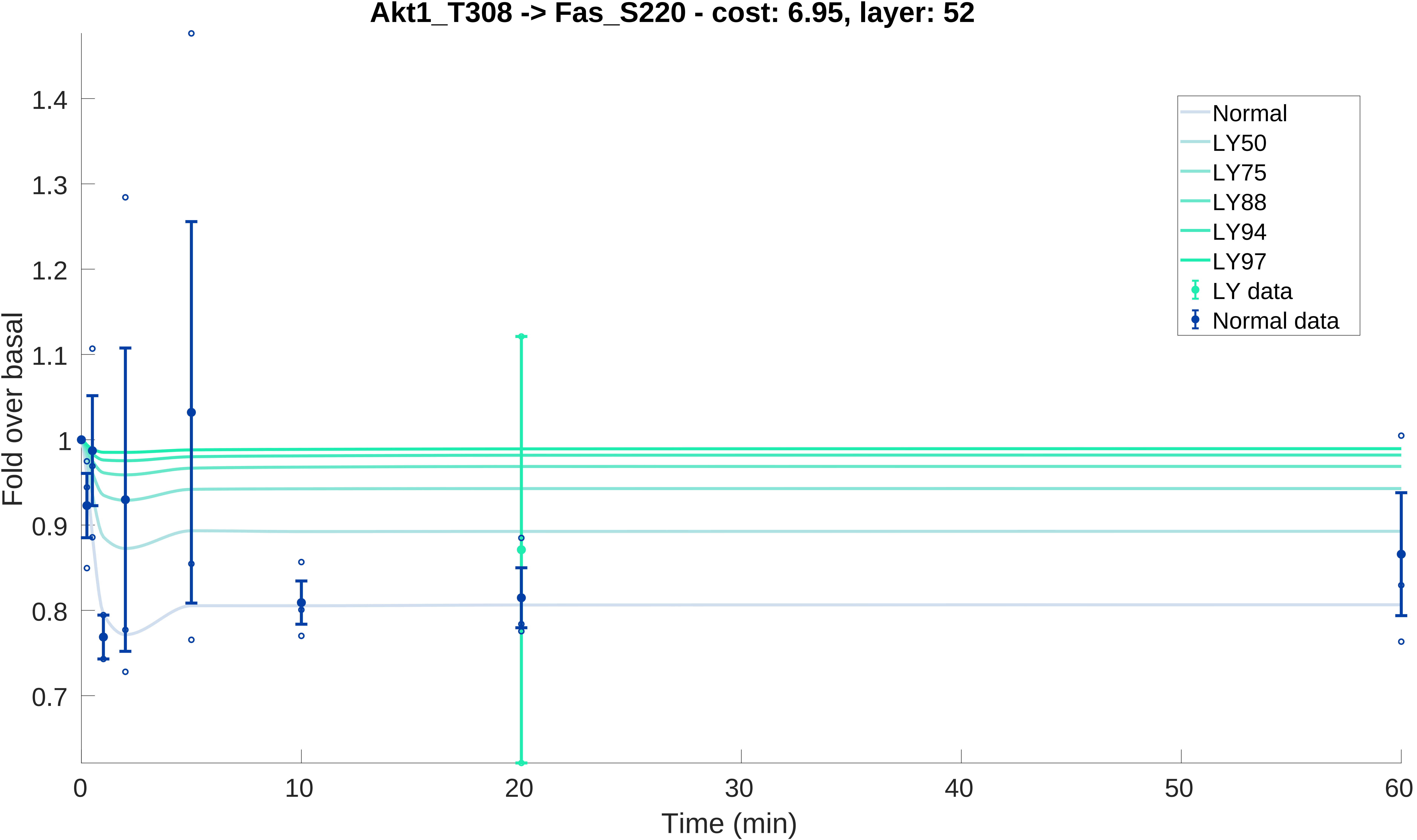

Akt1_T308 -> Fas_S220 - cost: 6.95, layer: 52
Fold over basal
Time (min)
1.4
1.3
1.2
1.1
1
0.9
0.8
0.7
0
10
20
30
40
50
60
Normal
LY50
LY75
LY88
LY94
LY97
LY data
Normal data

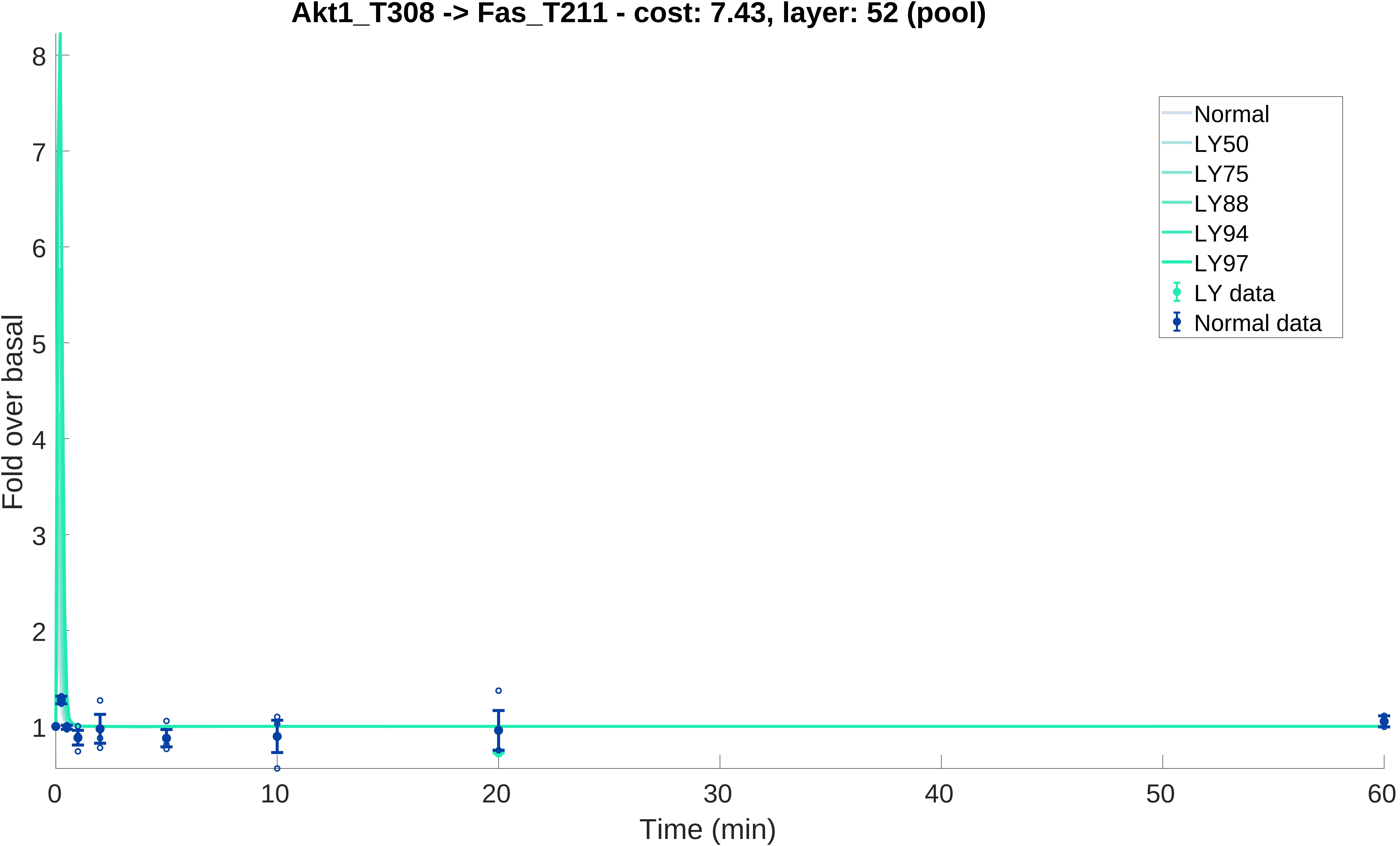

Akt1_T308 -> Fas_T211 - cost: 7.43, layer: 52 (pool)
Fold over basal
Time (min)
Normal
LY50
LY75
LY88
LY94
LY97
LY data
Normal data
0
10
20
30
40
50
60
1
2
3
4
5
6
7
8

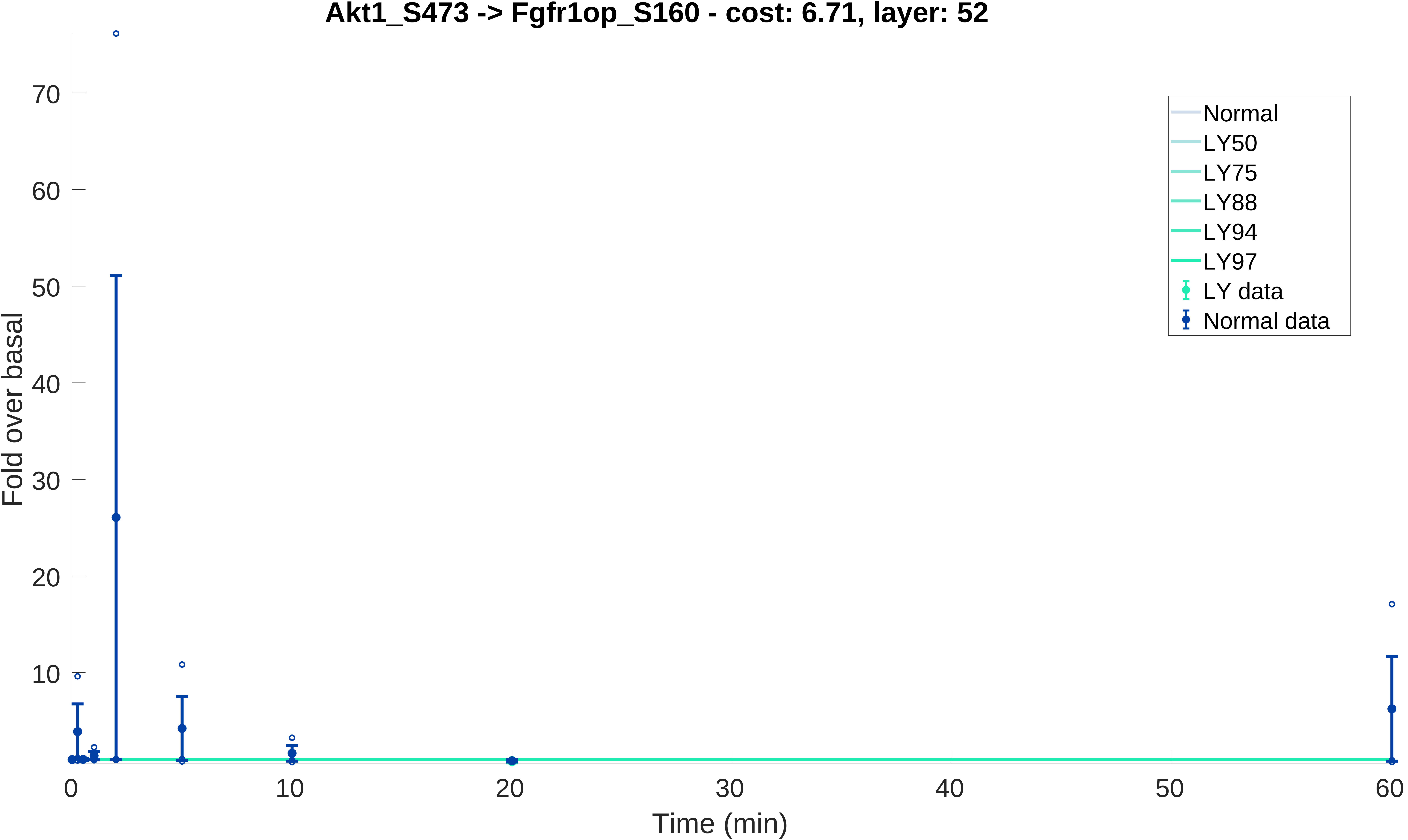

Akt1_S473 -> Fgfr1op_S160 - cost: 6.71, layer: 52
Fold over basal
Time (min)
0
10
20
30
40
50
60
70
Normal
LY50
LY75
LY88
LY94
LY97
LY data
Normal data

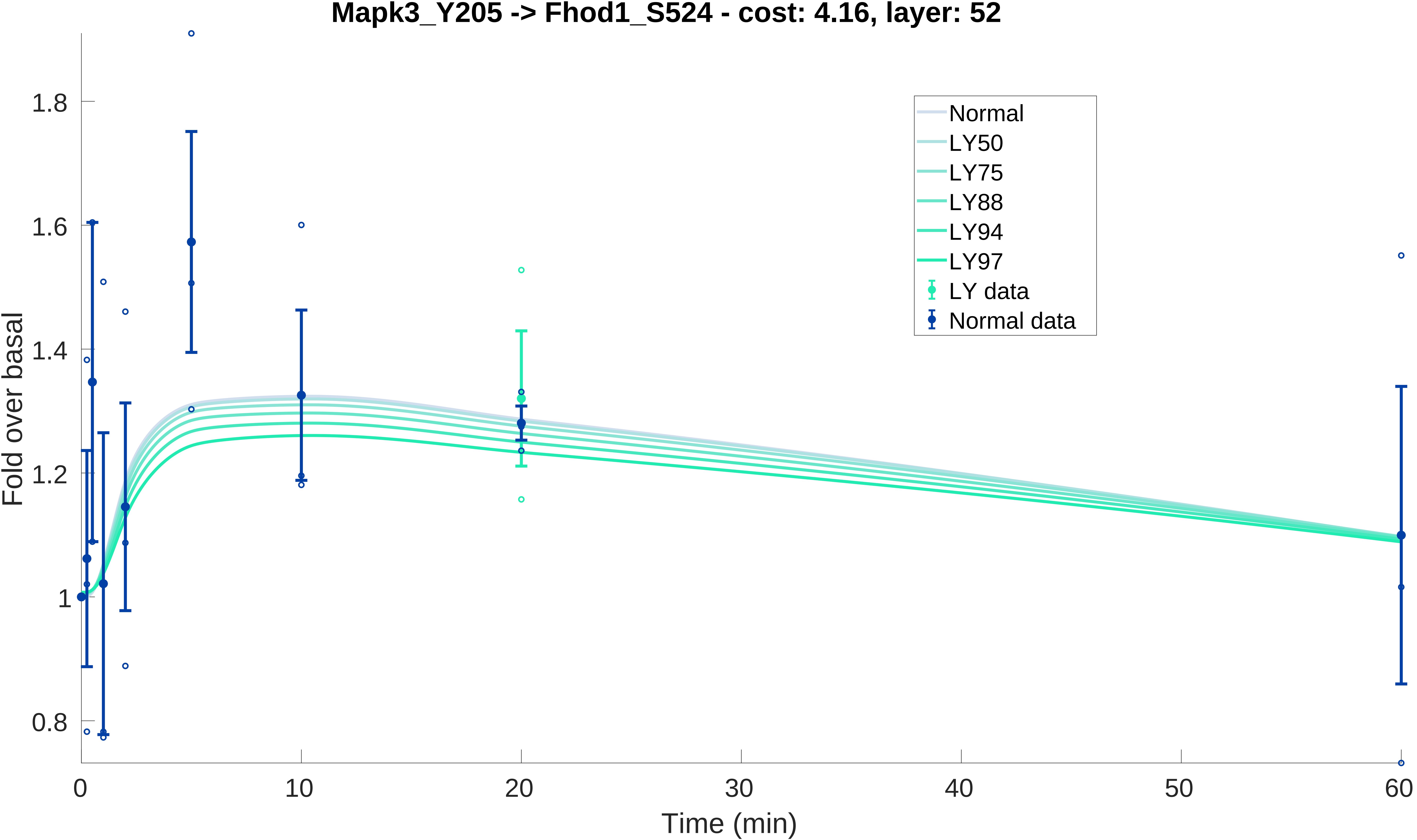

Mapk3_Y205 -> Fhod1_S524 - cost: 4.16, layer: 52
Fold over basal
Time (min)
Normal
LY50
LY75
LY88
LY94
LY97
LY data
Normal data
0.8
1
1.2
1.4
1.6
1.8
0
10
20
30
40
50
60

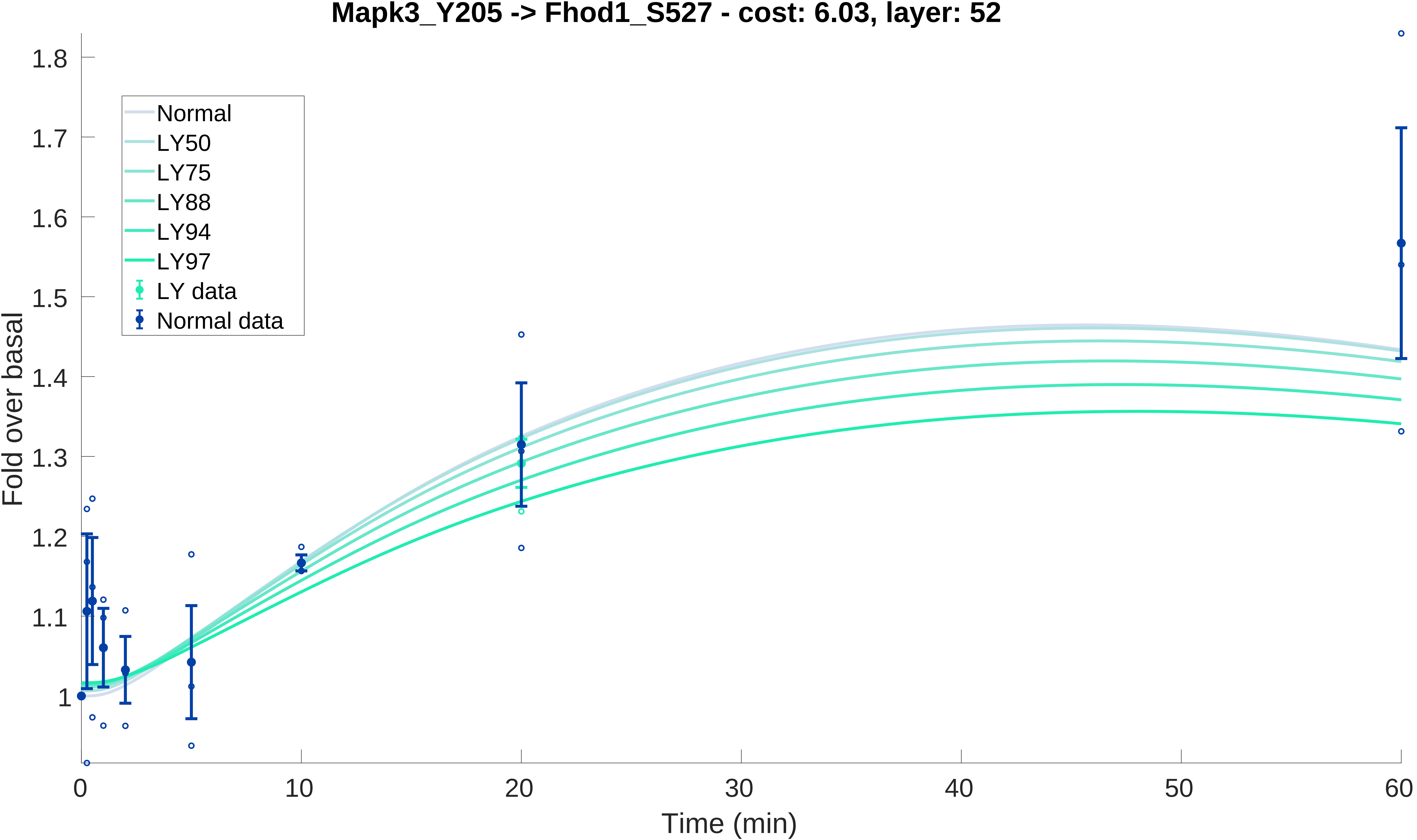

Mapk3_Y205 -> Fhod1_S527 - cost: 6.03, layer: 52
Normal
LY50
LY75
LY88
LY94
LY97
LY data
Normal data
Fold over basal
1.8
1.7
1.6
1.5
1.4
1.3
1.2
1.1
1
0
10
20
30
40
50
60
Time (min)

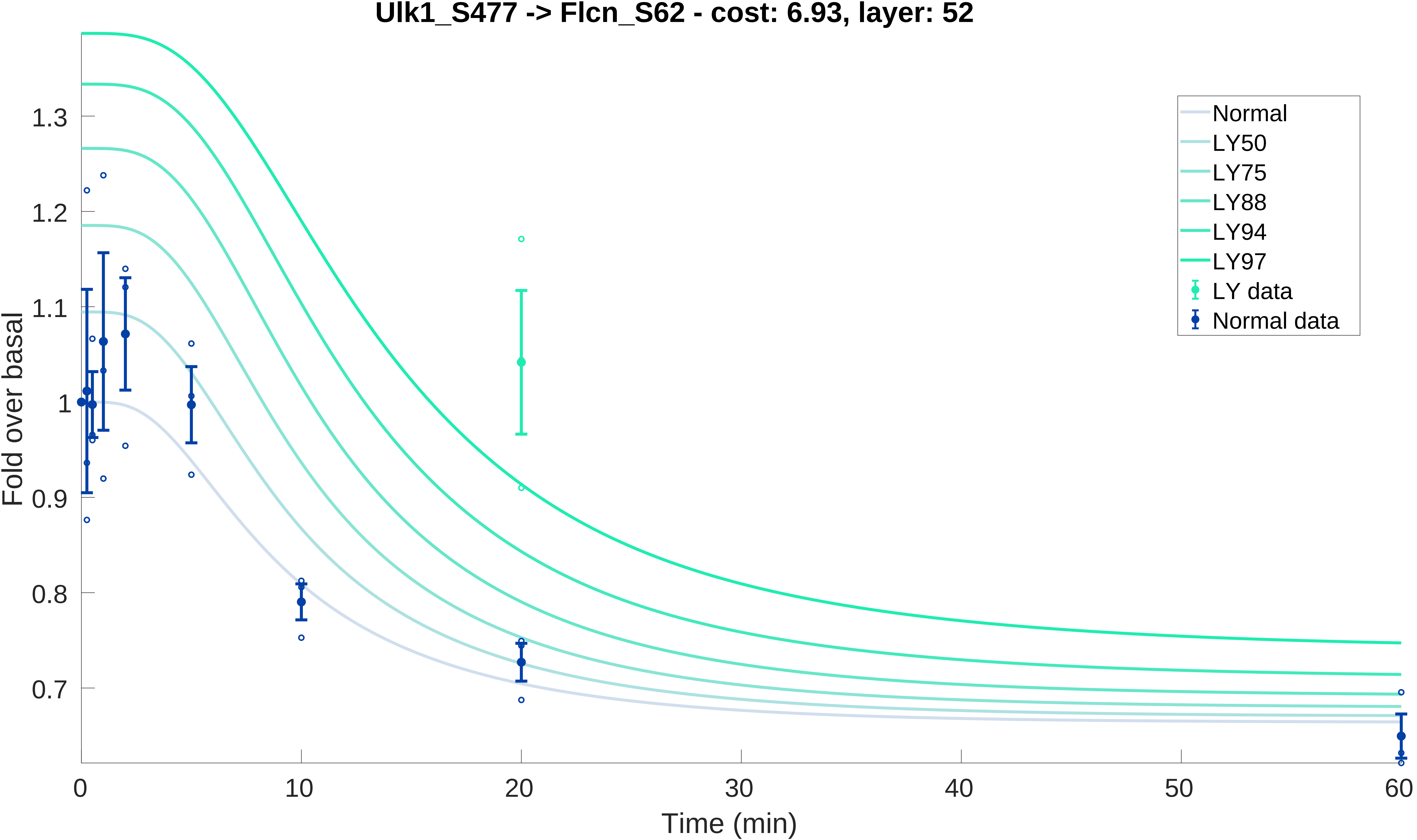

Ulk1_S477 -> Flcn_S62 - cost: 6.93, layer: 52
Fold over basal
Time (min)
Normal
LY50
LY75
LY88
LY94
LY97
LY data
Normal data
1.3
1.2
1.1
1
0.9
0.8
0.7
0
10
20
30
40
50
60

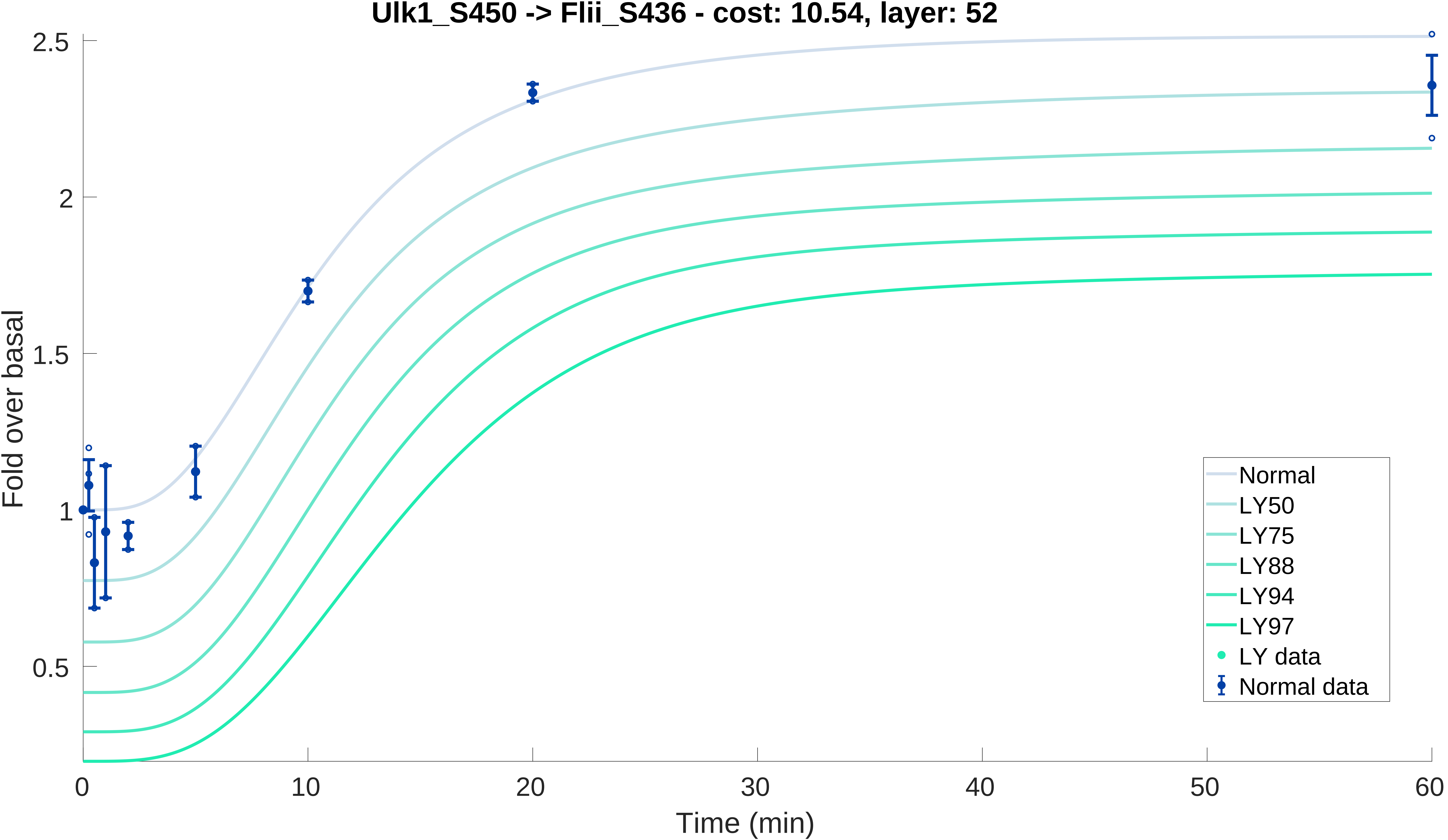

Ulk1_S450 -> Flii_S436 - cost: 10.54, layer: 52
Fold over basal
Time (min)
0
10
20
30
40
50
60
0.5
1
1.5
2
2.5
Normal
LY50
LY75
LY88
LY94
LY97
LY data
Normal data

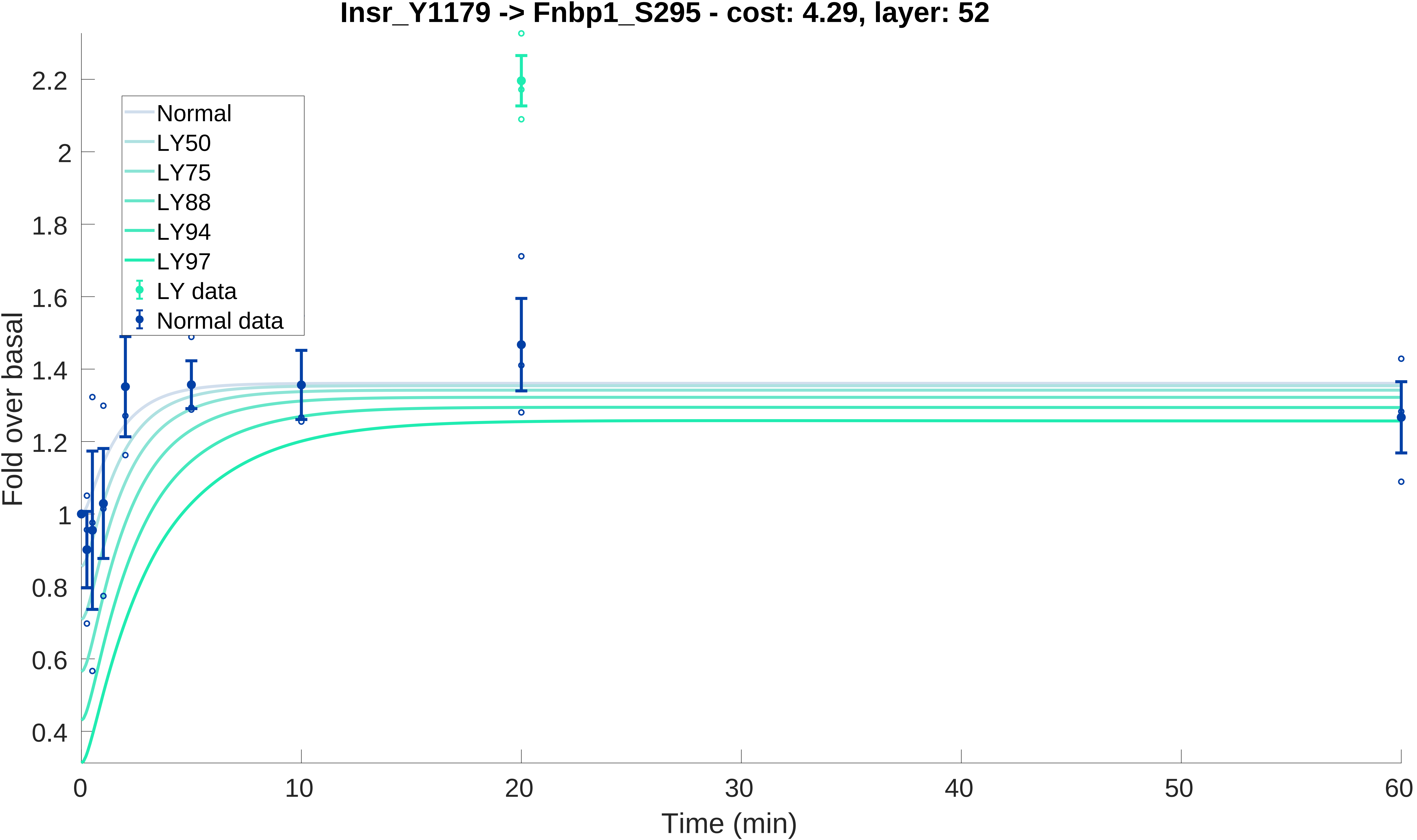

Insr_Y1179 -> Fnbp1_S295 - cost: 4.29, layer: 52
Normal
LY50
LY75
LY88
LY94
LY97
LY data
Normal data
Fold over basal
Time (min)
0
10
20
30
40
50
60
0.4
0.6
0.8
1
1.2
1.4
1.6
1.8
2
2.2

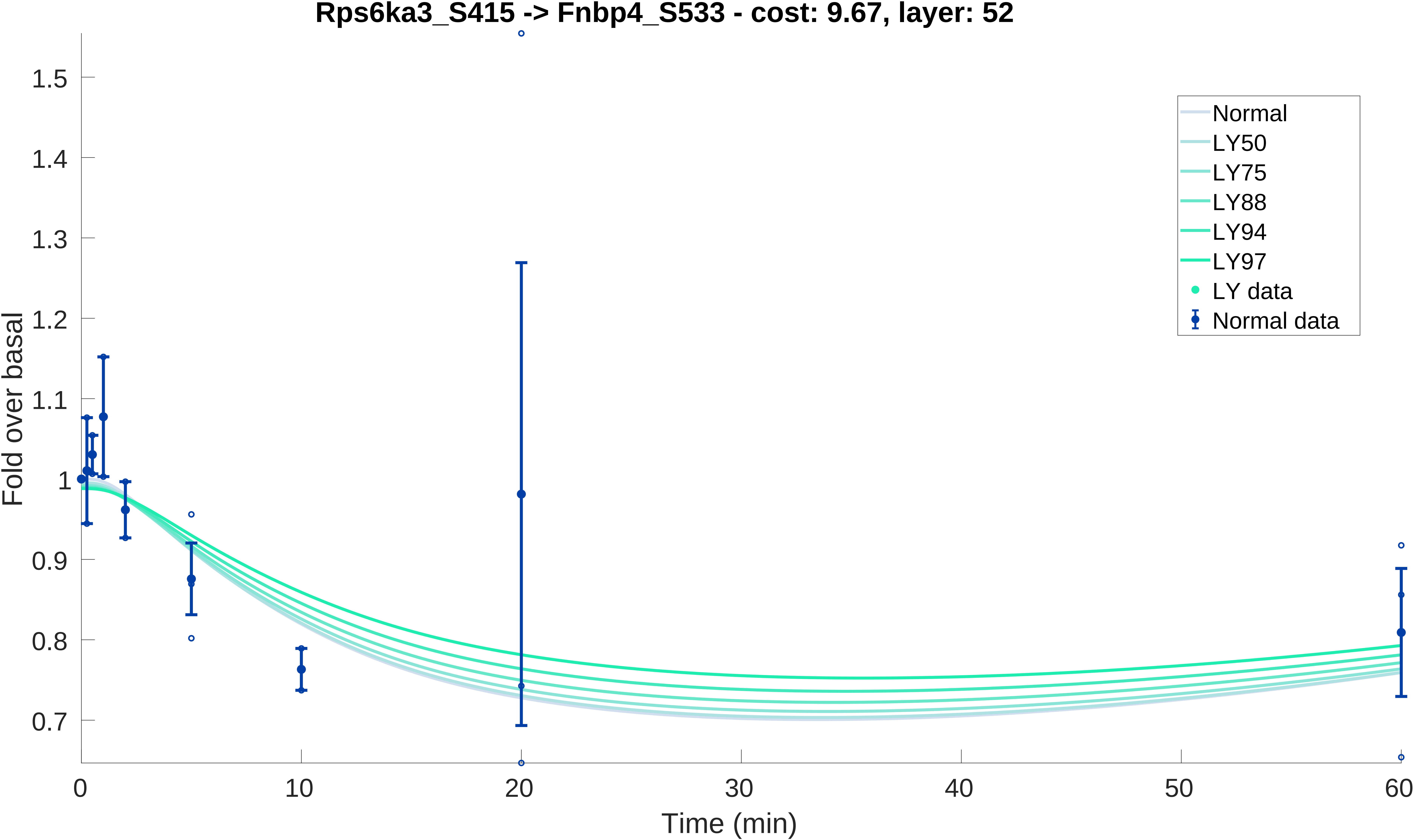

Rps6ka3_S415 -> Fnbp4_S533 - cost: 9.67, layer: 52
Fold over basal
1.5
1.4
1.3
1.2
1.1
1
0.9
0.8
0.7
0
10
20
30
40
50
60
Time (min)
Normal
LY50
LY75
LY88
LY94
LY97
LY data
Normal data

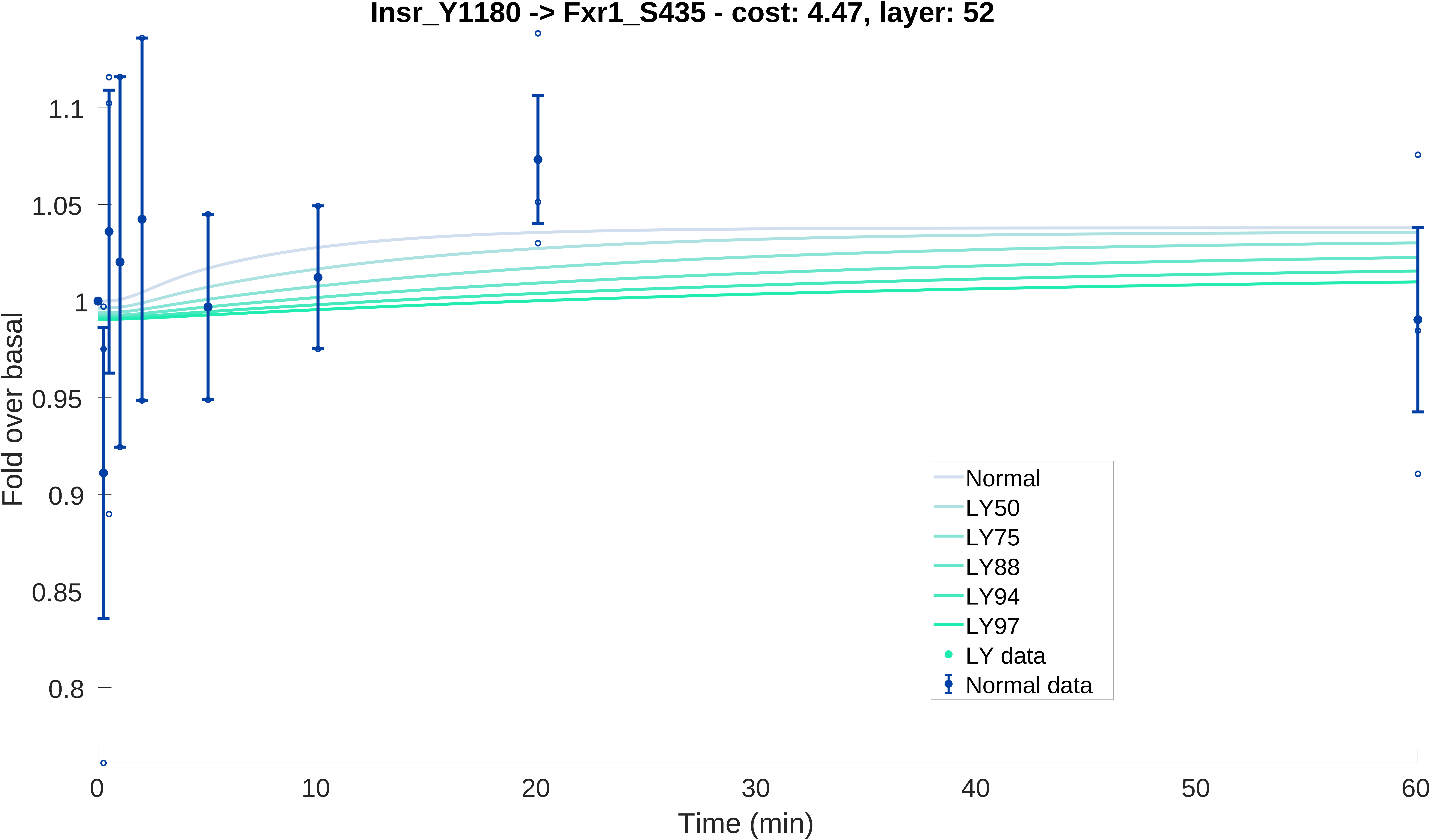
Insr_Y1180 -> Fxr1_S435 - cost: 4.47, layer: 52
Fold over basal
1.1
1.05
1
0.95
0.9
0.85
0.8
0
10
20
30
40
50
60
Time (min)
Normal
LY50
LY75
LY88
LY94
LY97
LY data
Normal data

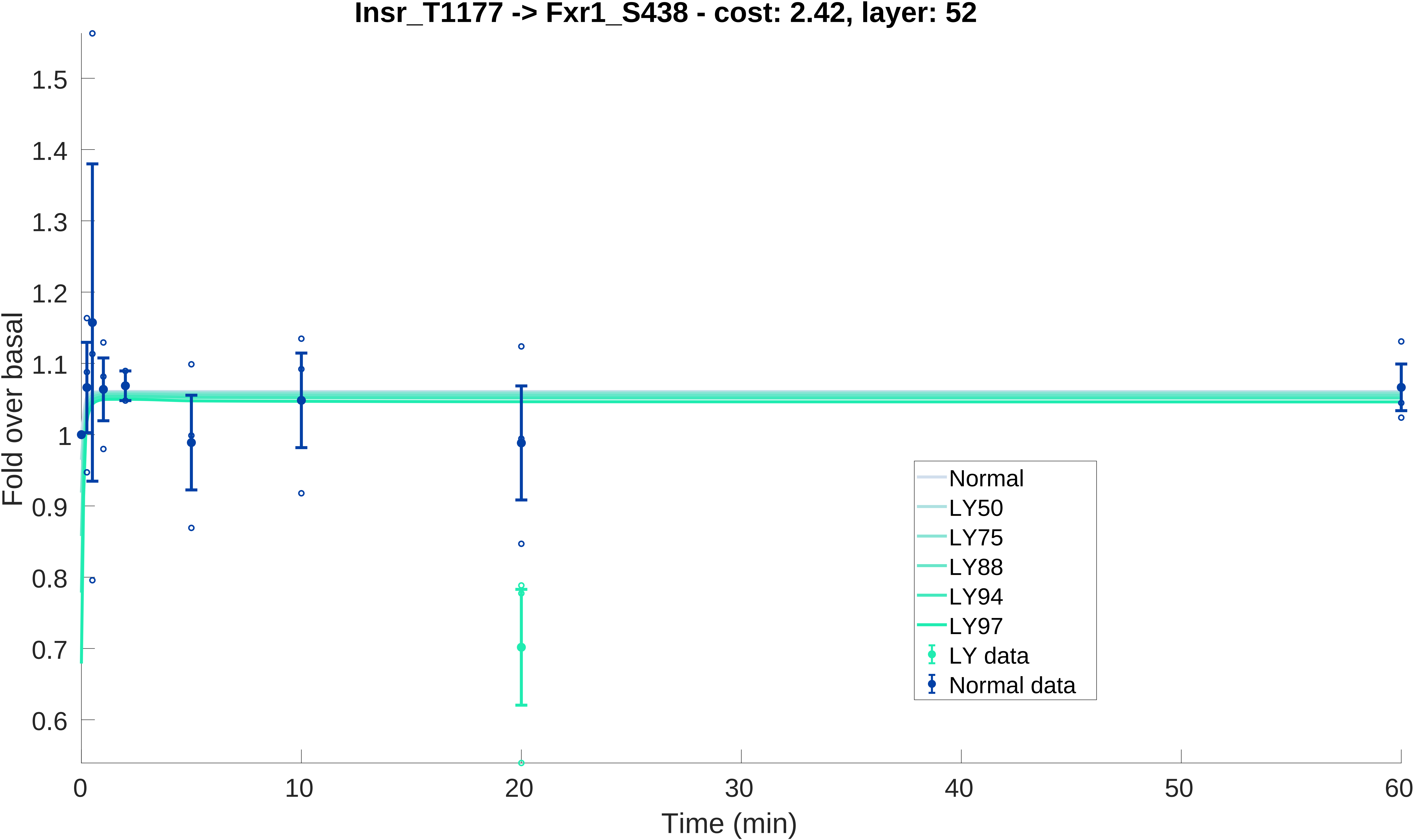

Insr_T1177 -> Fxr1_S438 - cost: 2.42, layer: 52
Fold over basal
Time (min)
1.5
1.4
1.3
1.2
1.1
1
0.9
0.8
0.7
0.6
0
10
20
30
40
50
60
Normal
LY50
LY75
LY88
LY94
LY97
LY data
Normal data

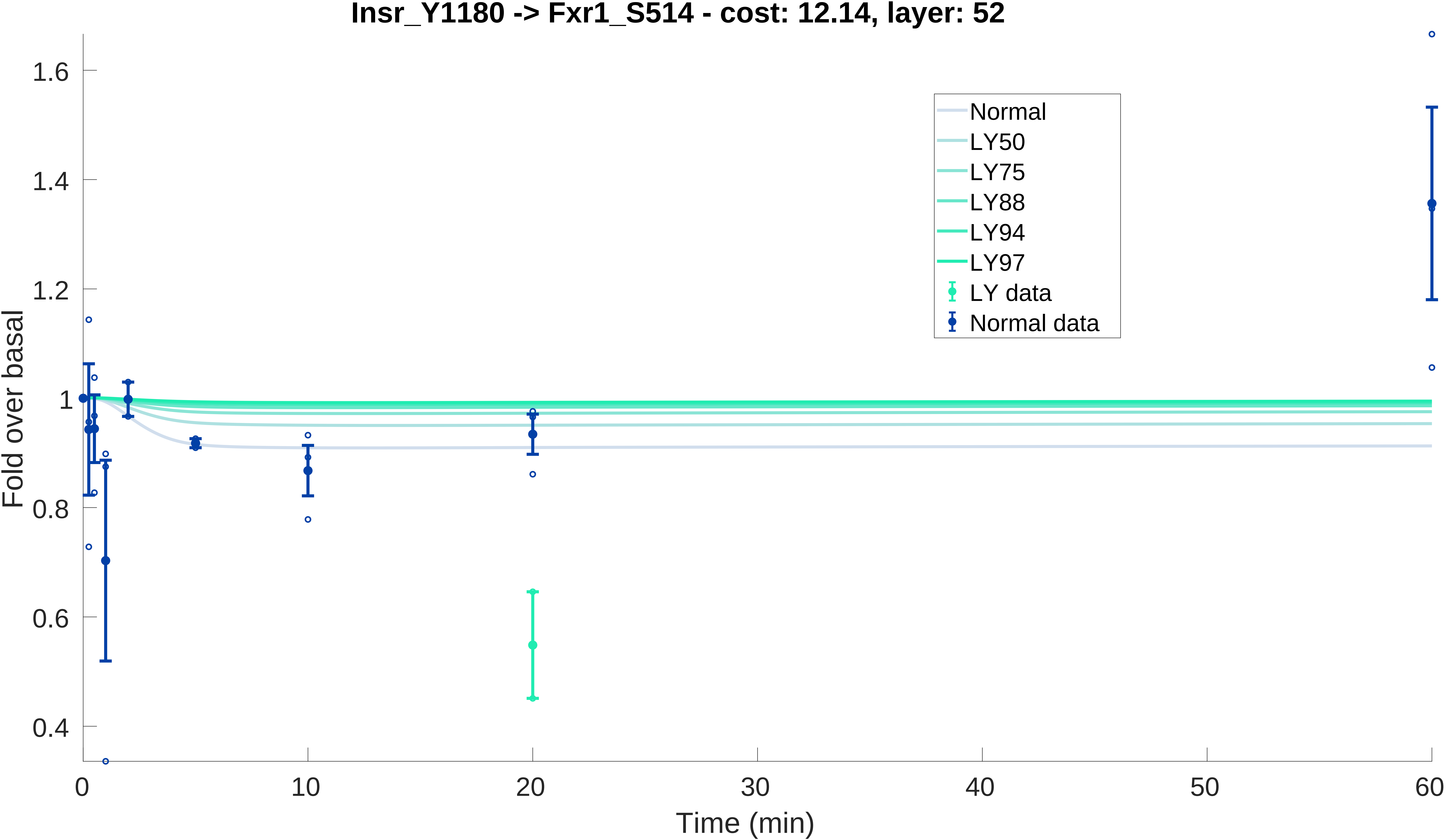

Insr_Y1180 -> Fxr1_S514 - cost: 12.14, layer: 52
Fold over basal
Time (min)
1.6
1.4
1.2
1
0.8
0.6
0.4
0
10
20
30
40
50
60
Normal
LY50
LY75
LY88
LY94
LY97
LY data
Normal data

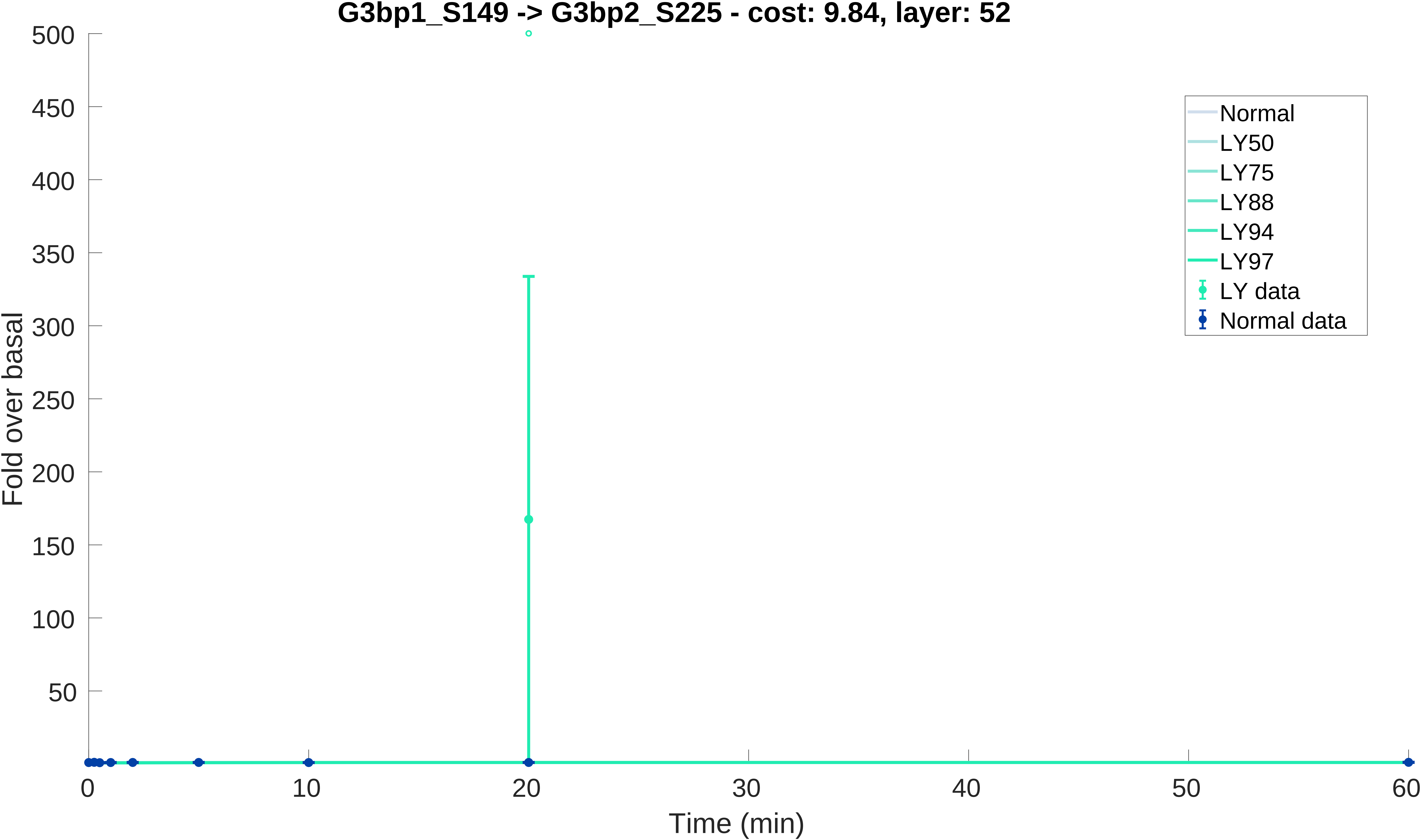

G3bp1_S149 -> G3bp2_S225 - cost: 9.84, layer: 52
Fold over basal
Time (min)
500
450
400
350
300
250
200
150
100
50
0
10
20
30
40
50
60
Normal
LY50
LY75
LY88
LY94
LY97
LY data
Normal data

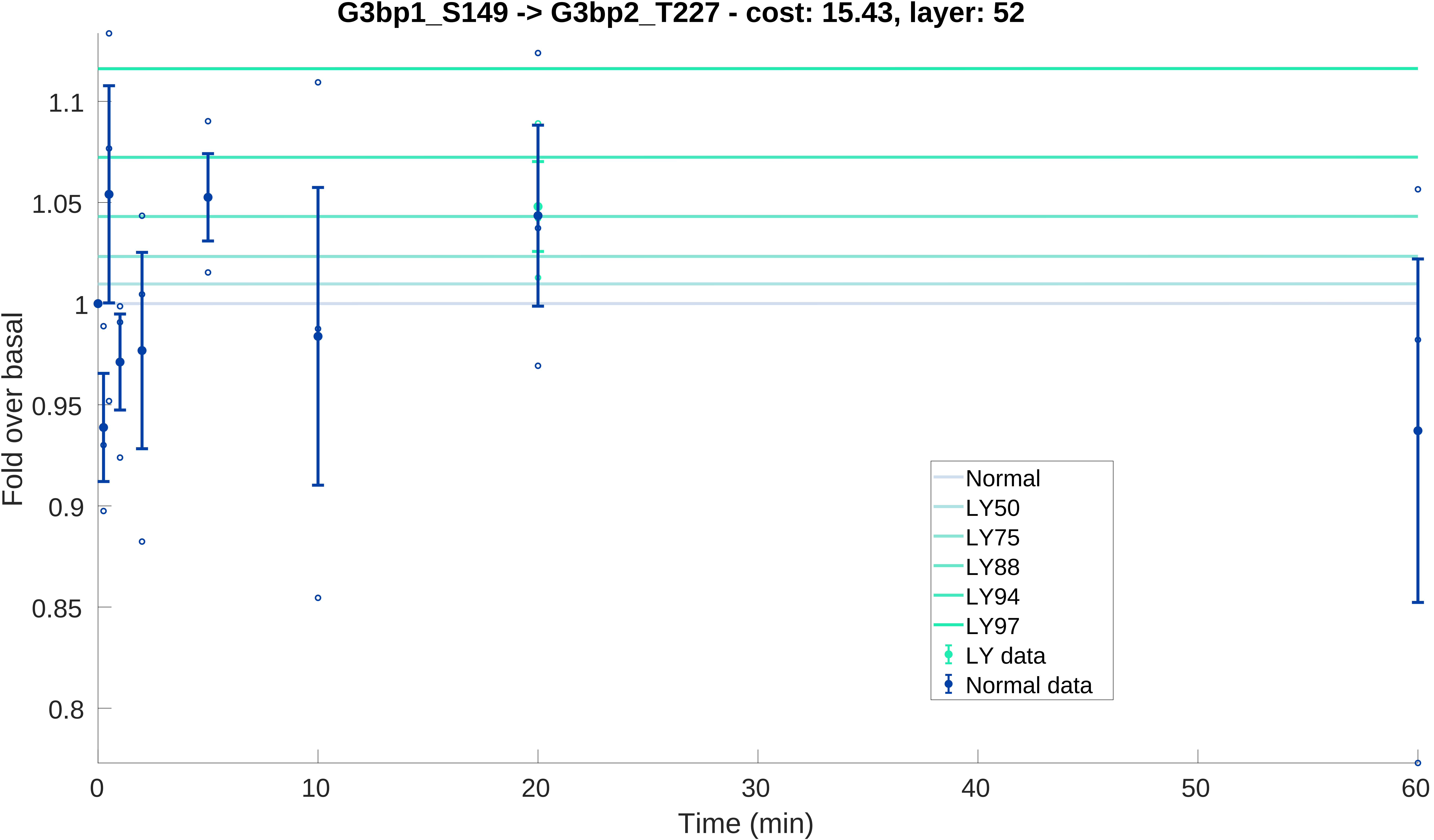

G3bp1_S149 -> G3bp2_T227 - cost: 15.43, layer: 52
Fold over basal
Time (min)
1.1
1.05
1
0.95
0.9
0.85
0.8
0
10
20
30
40
50
60
Normal
LY50
LY75
LY88
LY94
LY97
LY data
Normal data

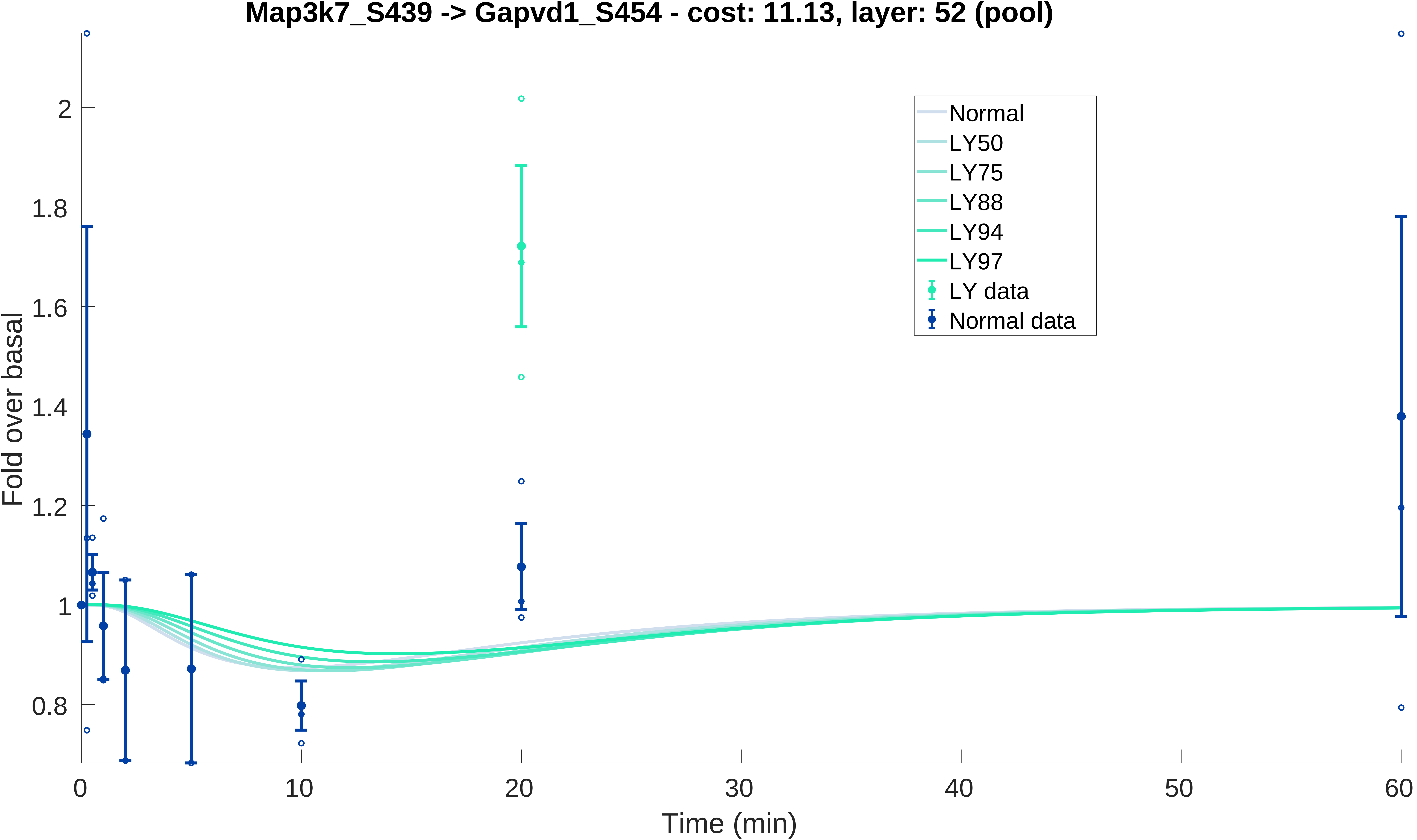

Map3k7_S439 -> Gapvd1_S454 - cost: 11.13, layer: 52 (pool)
Fold over basal
Time (min)
Normal
LY50
LY75
LY88
LY94
LY97
LY data
Normal data
0.8
1
1.2
1.4
1.6
1.8
2
0
10
20
30
40
50
60

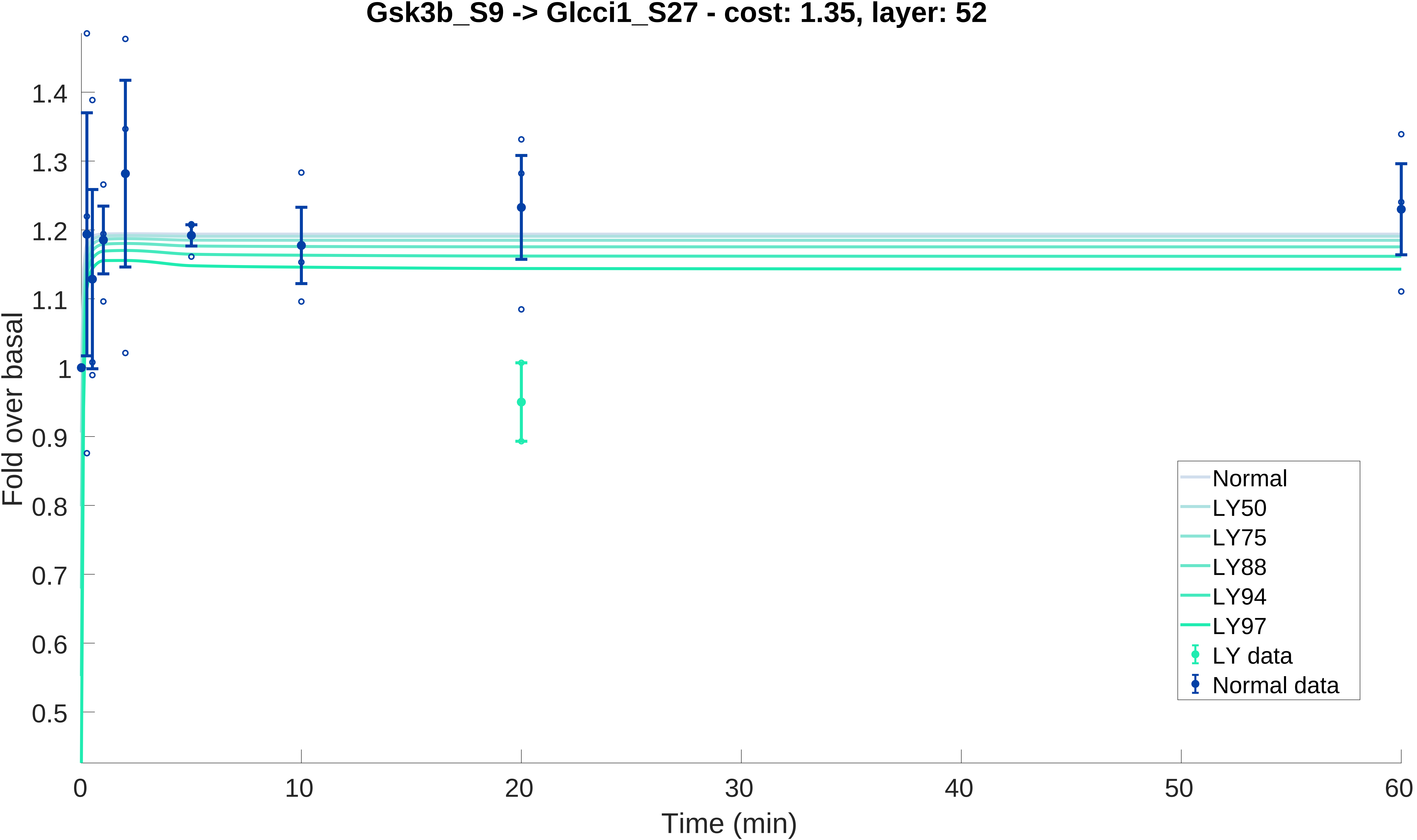

Gsk3b_S9 -> Glcci1_S27 - cost: 1.35, layer: 52
Fold over basal
Time (min)
0.5
0.6
0.7
0.8
0.9
1
1.1
1.2
1.3
1.4
0
10
20
30
40
50
60
Normal
LY50
LY75
LY88
LY94
LY97
LY data
Normal data

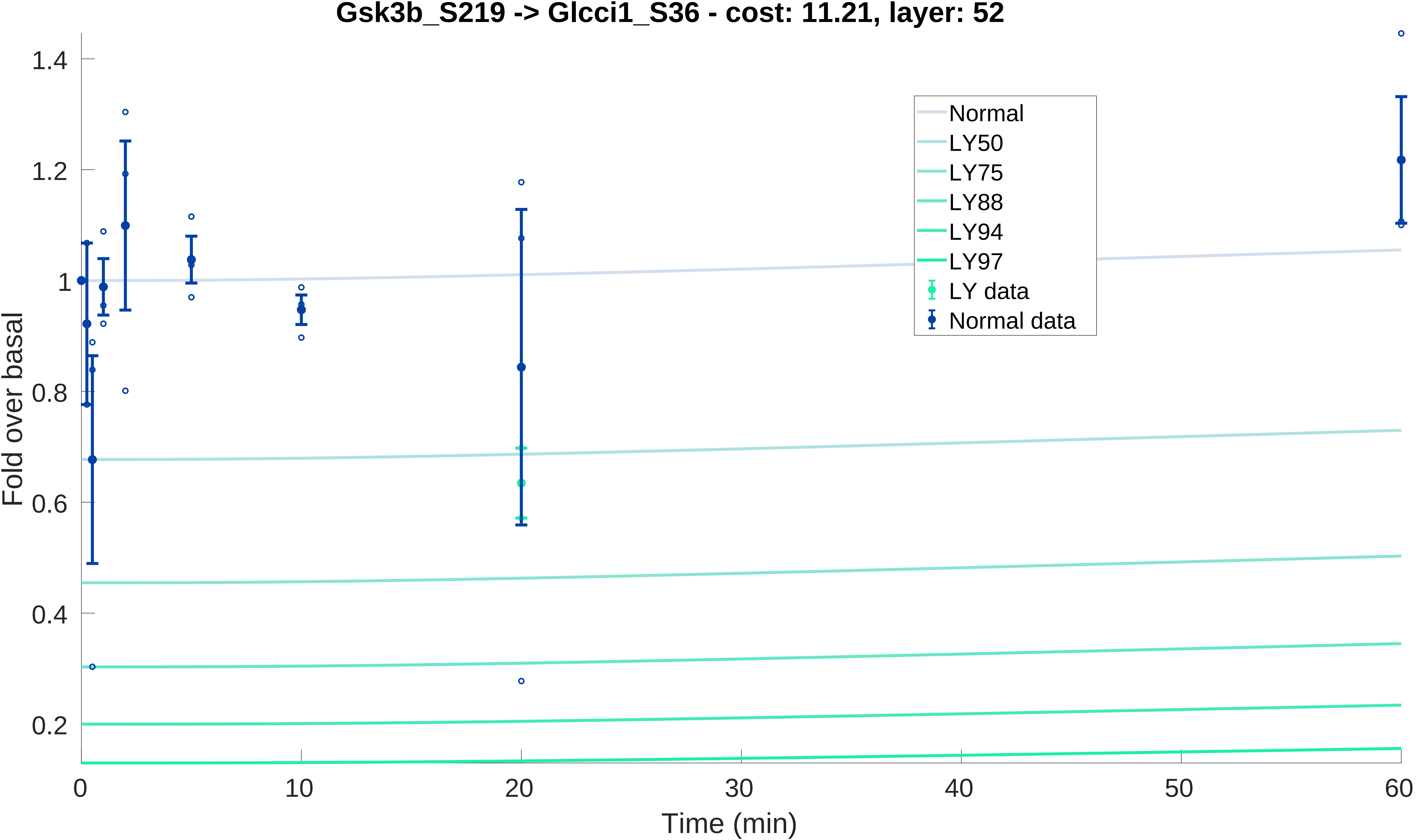

Gsk3b_S219 -> Glcci1_S36 - cost: 11.21, layer: 52
Fold over basal
Time (min)
0
10
20
30
40
50
60
0.2
0.4
0.6
0.8
1
1.2
1.4
Normal
LY50
LY75
LY88
LY94
LY97
LY data
Normal data

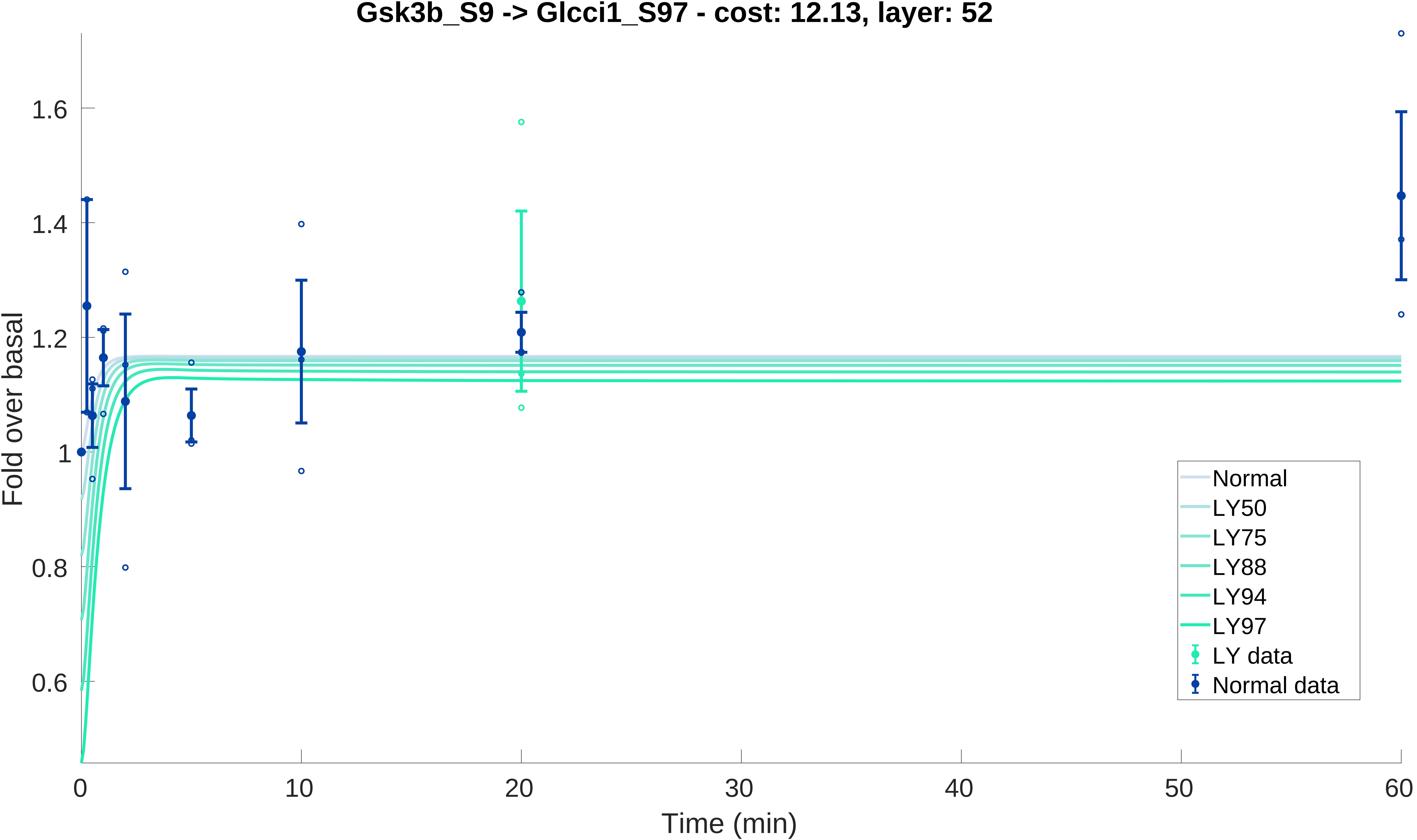

Gsk3b_S9 -> Glcci1_S97 - cost: 12.13, layer: 52
Fold over basal
1.6
1.4
1.2
1
0.8
0.6
0
10
20
30
40
50
60
Time (min)
Normal
LY50
LY75
LY88
LY94
LY97
LY data
Normal data

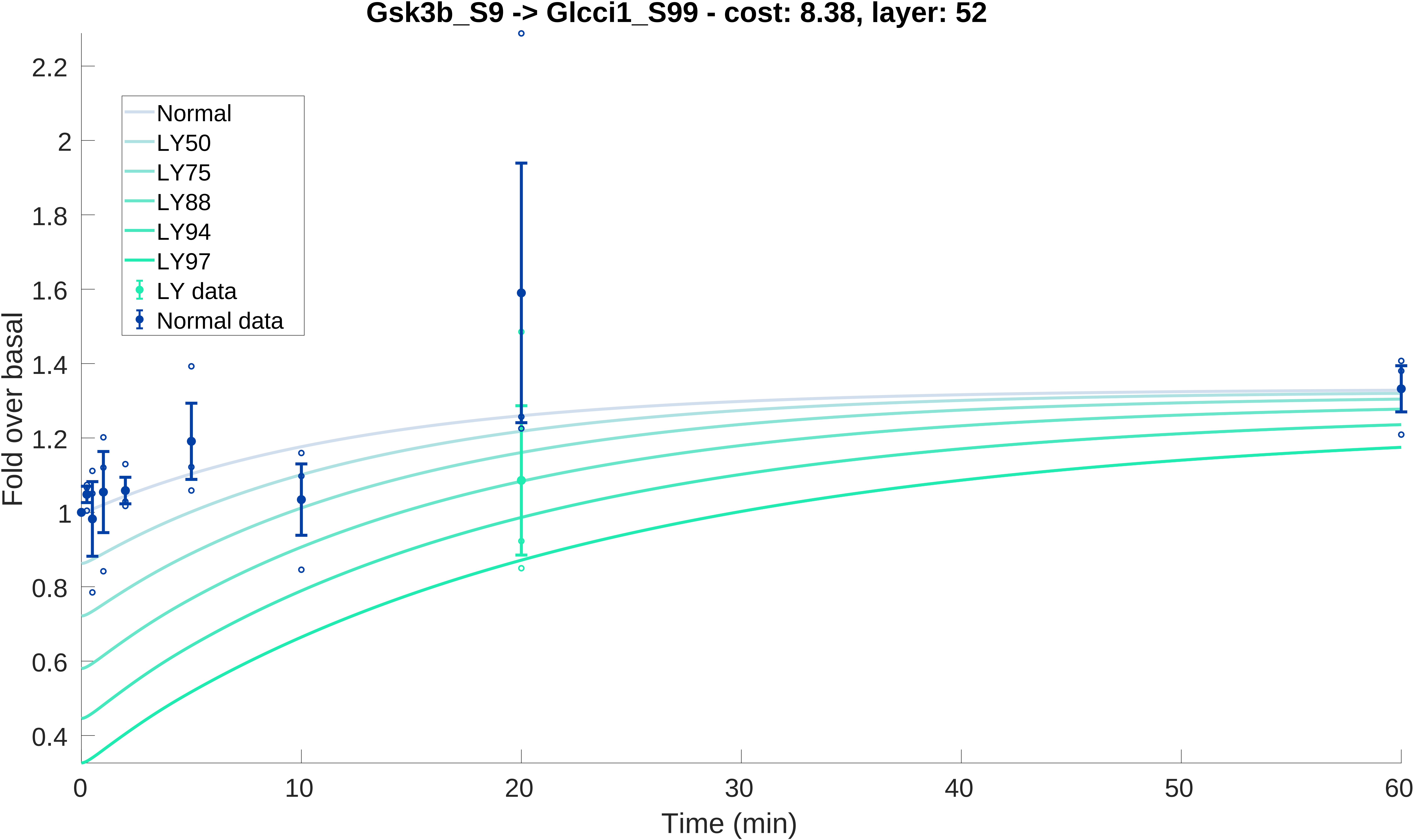
Gsk3b_S9 -> Glcci1_S99 - cost: 8.38, layer: 52
Fold over basal
Time (min)
Normal
LY50
LY75
LY88
LY94
LY97
LY data
Normal data
2.2
2
1.8
1.6
1.4
1.2
1
0.8
0.6
0.4
0
10
20
30
40
50
60

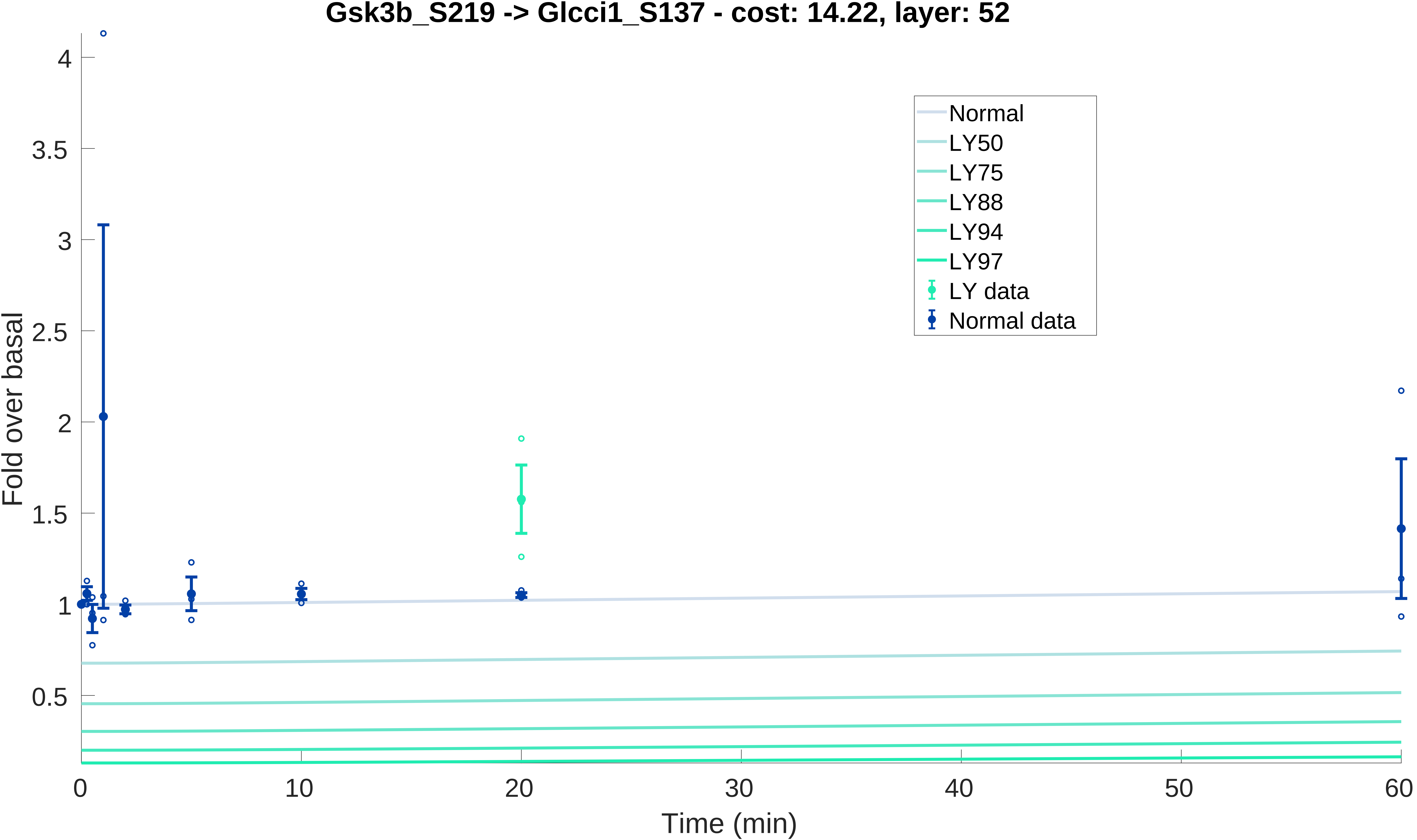

Gsk3b_S219 -> Glcci1_S137 - cost: 14.22, layer: 52
Fold over basal
Time (min)
0
10
20
30
40
50
60
0.5
1
1.5
2
2.5
3
3.5
4
Normal
LY50
LY75
LY88
LY94
LY97
LY data
Normal data

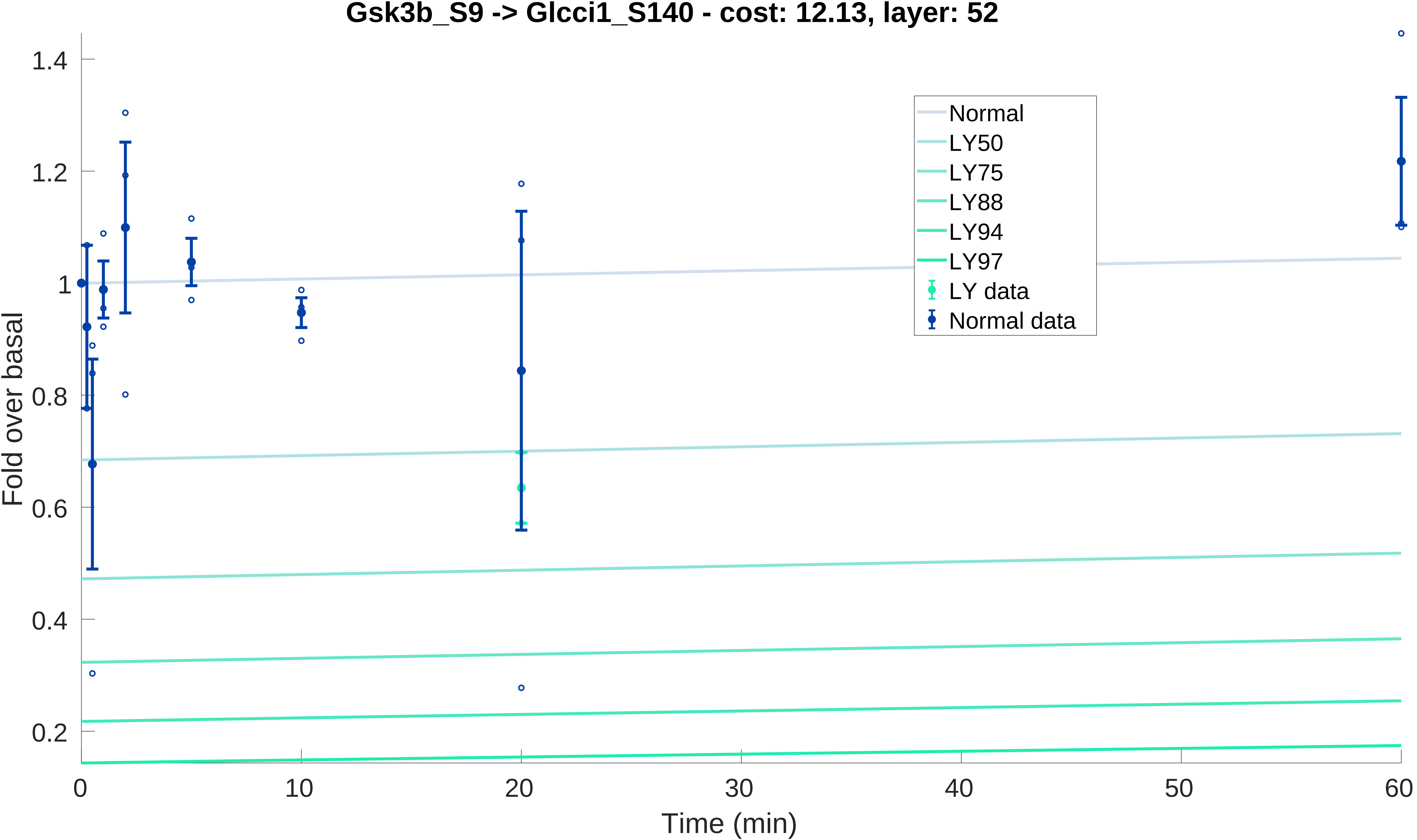
Gsk3b_S9 -> Glcci1_S140 - cost: 12.13, layer: 52
Fold over basal
Time (min)
0
10
20
30
40
50
60
0.2
0.4
0.6
0.8
1
1.2
1.4
Normal
LY50
LY75
LY88
LY94
LY97
LY data
Normal data

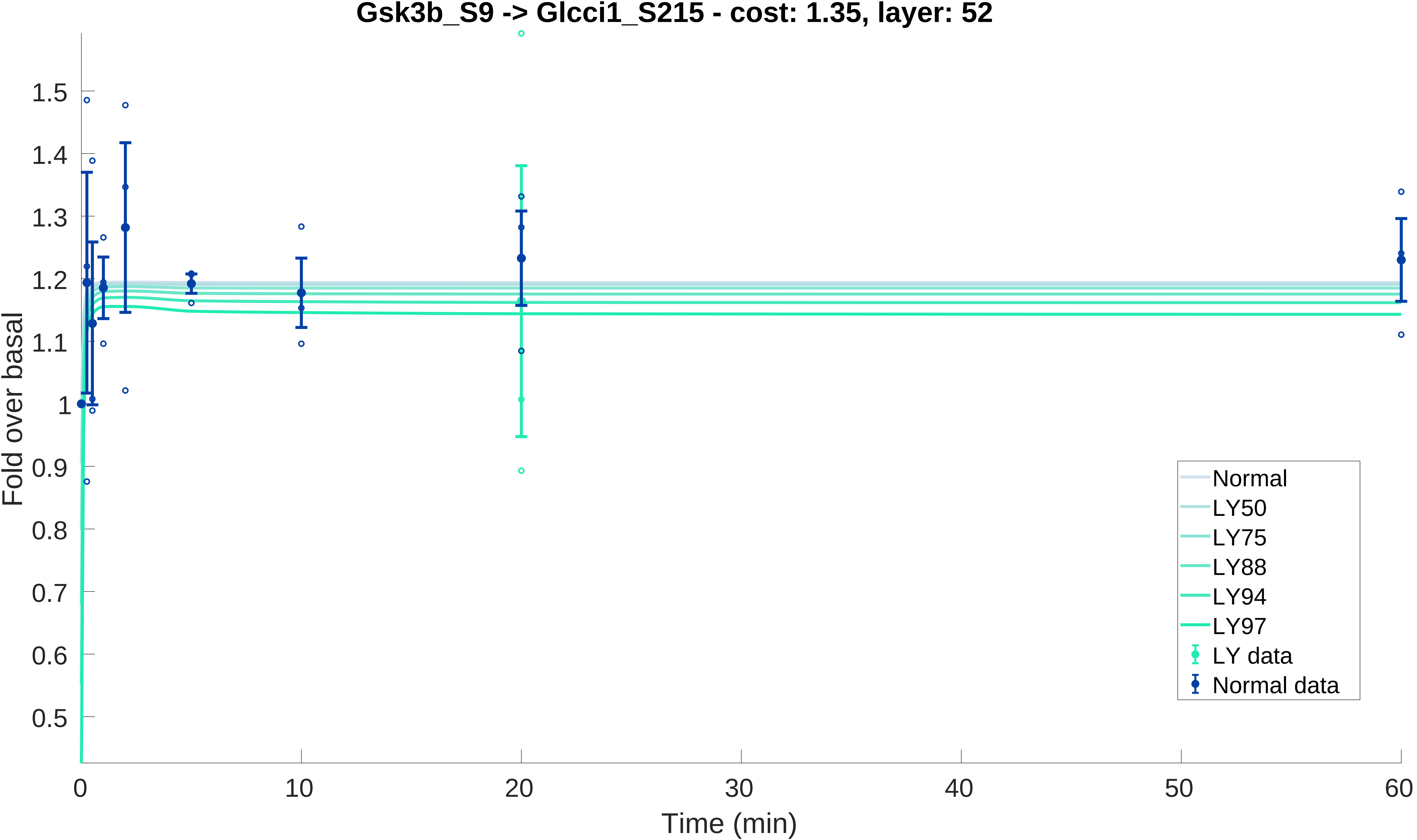

Gsk3b_S9 -> Glcci1_S215 - cost: 1.35, layer: 52
Fold over basal
Time (min)
Normal
LY50
LY75
LY88
LY94
LY97
LY data
Normal data

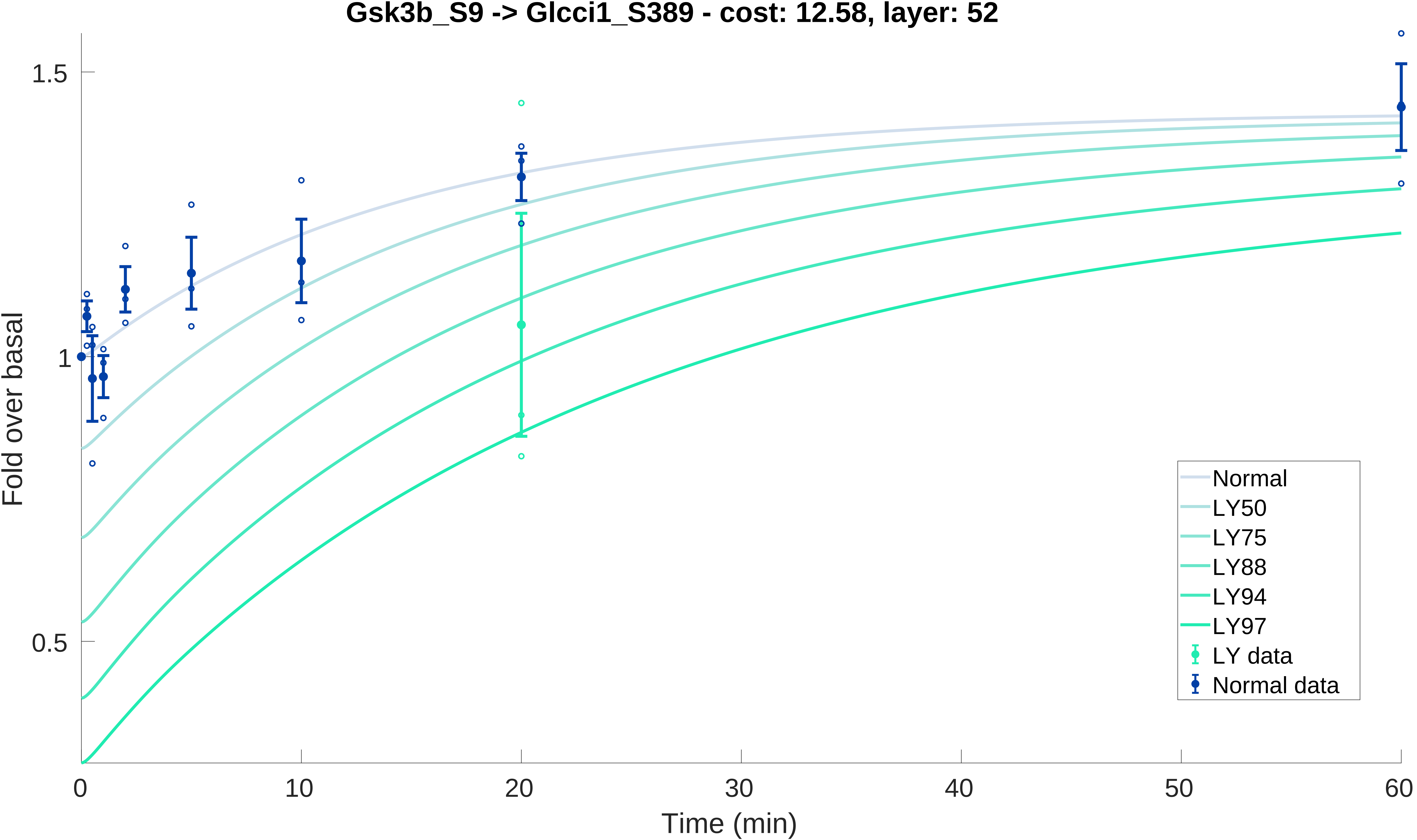

Gsk3b_S9 -> Glcci1_S389 - cost: 12.58, layer: 52
Fold over basal
1.5
1
0.5
0
10
20
30
40
50
60
Time (min)
Normal
LY50
LY75
LY88
LY94
LY97
LY data
Normal data

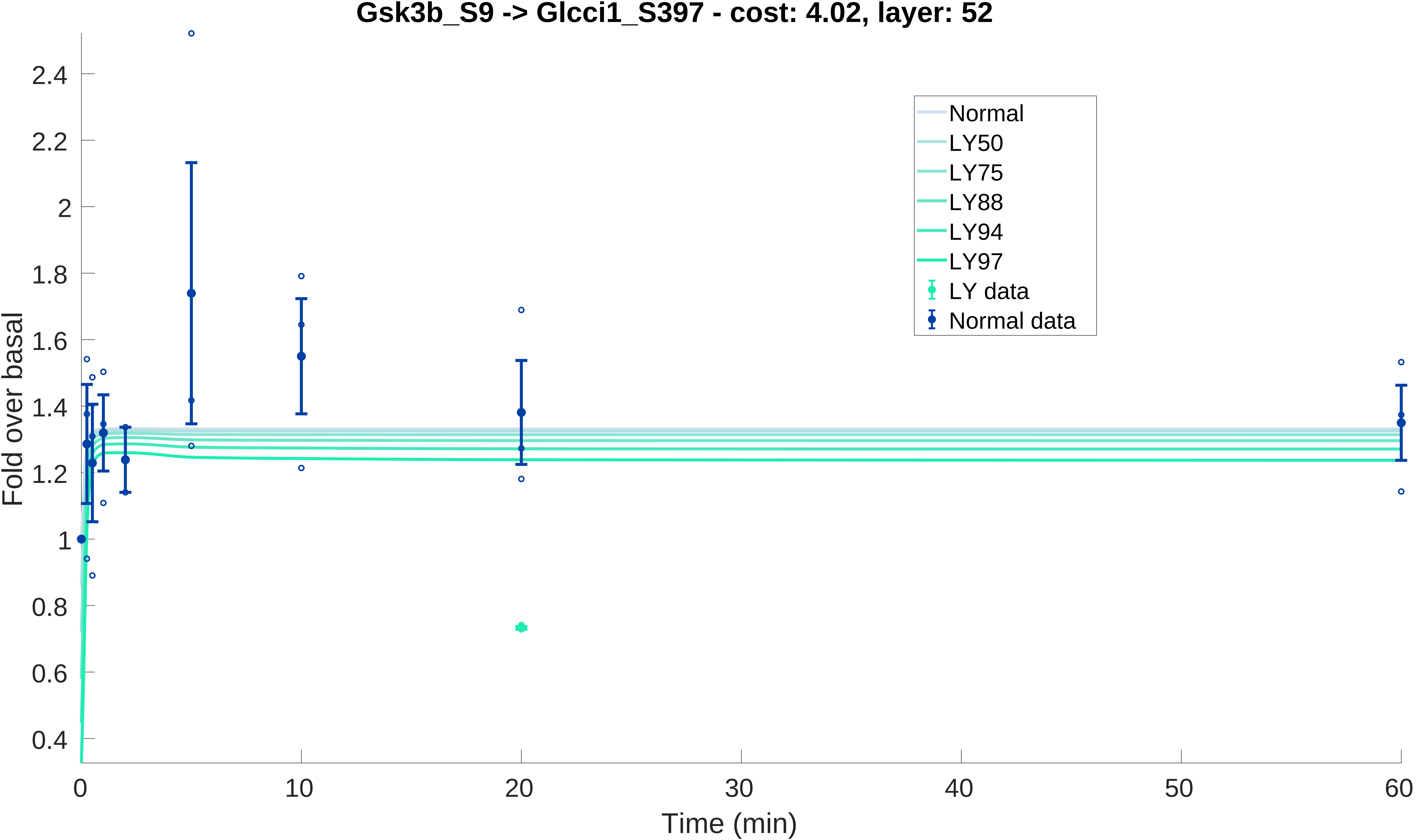

Gsk3b_S9 -> Glcci1_S397 - cost: 4.02, layer: 52
Fold over basal
Time (min)
Normal
LY50
LY75
LY88
LY94
LY97
LY data
Normal data
2.4
2.2
2
1.8
1.6
1.4
1.2
1
0.8
0.6
0.4
0
10
20
30
40
50
60

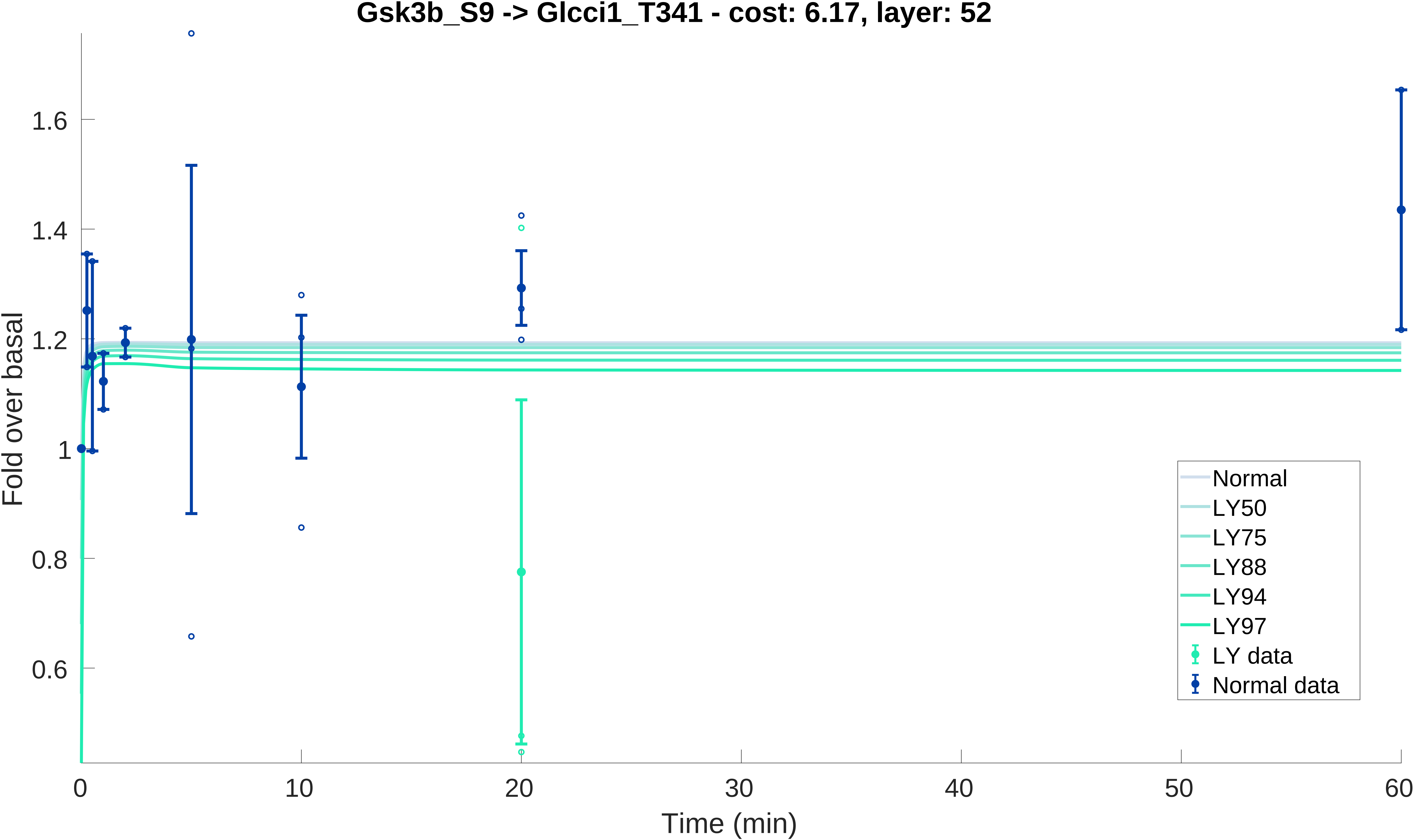

Gsk3b_S9 -> Glcci1_T341 - cost: 6.17, layer: 52
Fold over basal
1.6
1.4
1.2
1
0.8
0.6
0
10
20
30
40
50
60
Time (min)
Normal
LY50
LY75
LY88
LY94
LY97
LY data
Normal data

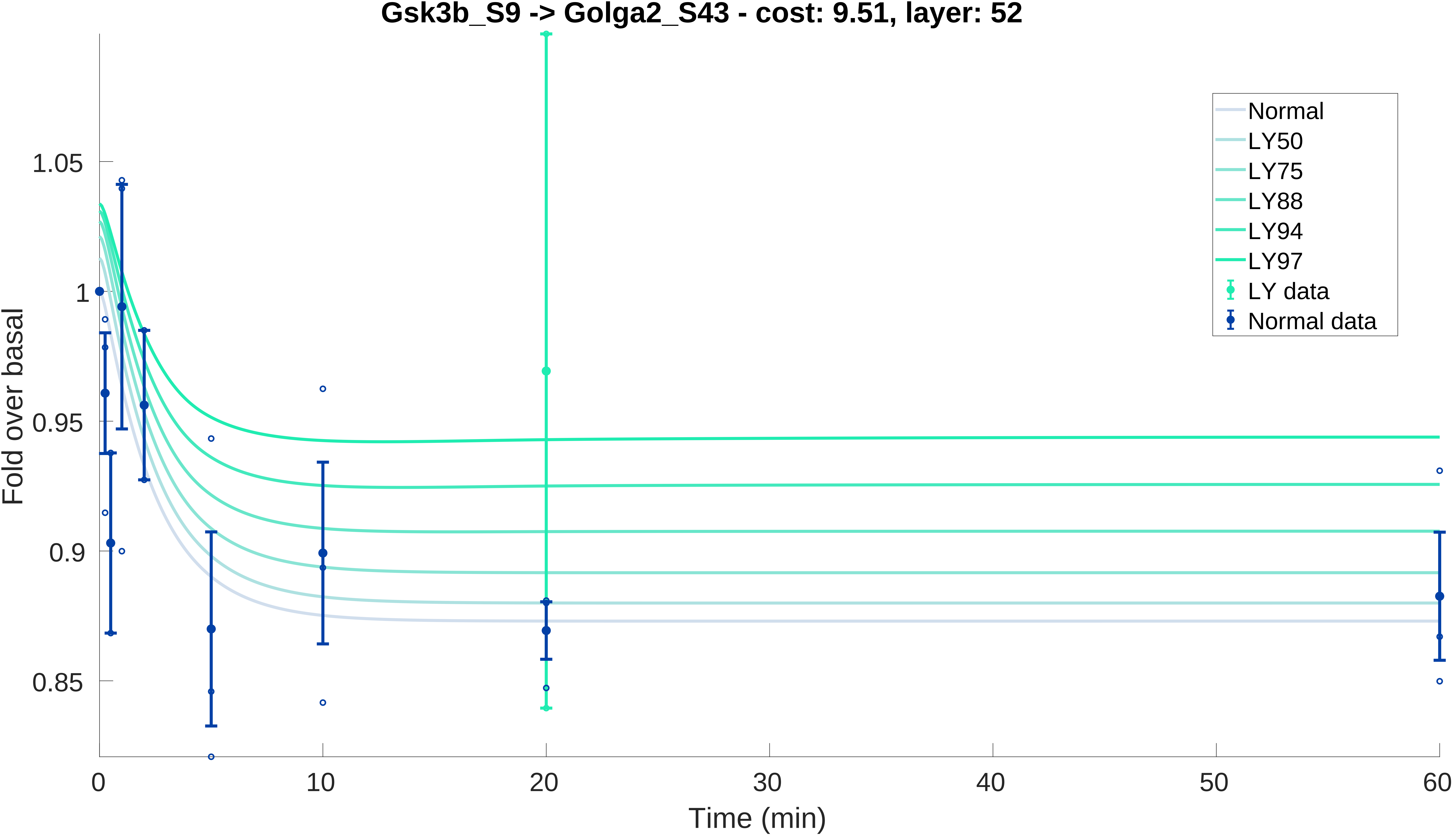

Gsk3b_S9 -> Golga2_S43 - cost: 9.51, layer: 52
Fold over basal
Time (min)
1.05
1
0.95
0.9
0.85
0
10
20
30
40
50
60
Normal
LY50
LY75
LY88
LY94
LY97
LY data
Normal data

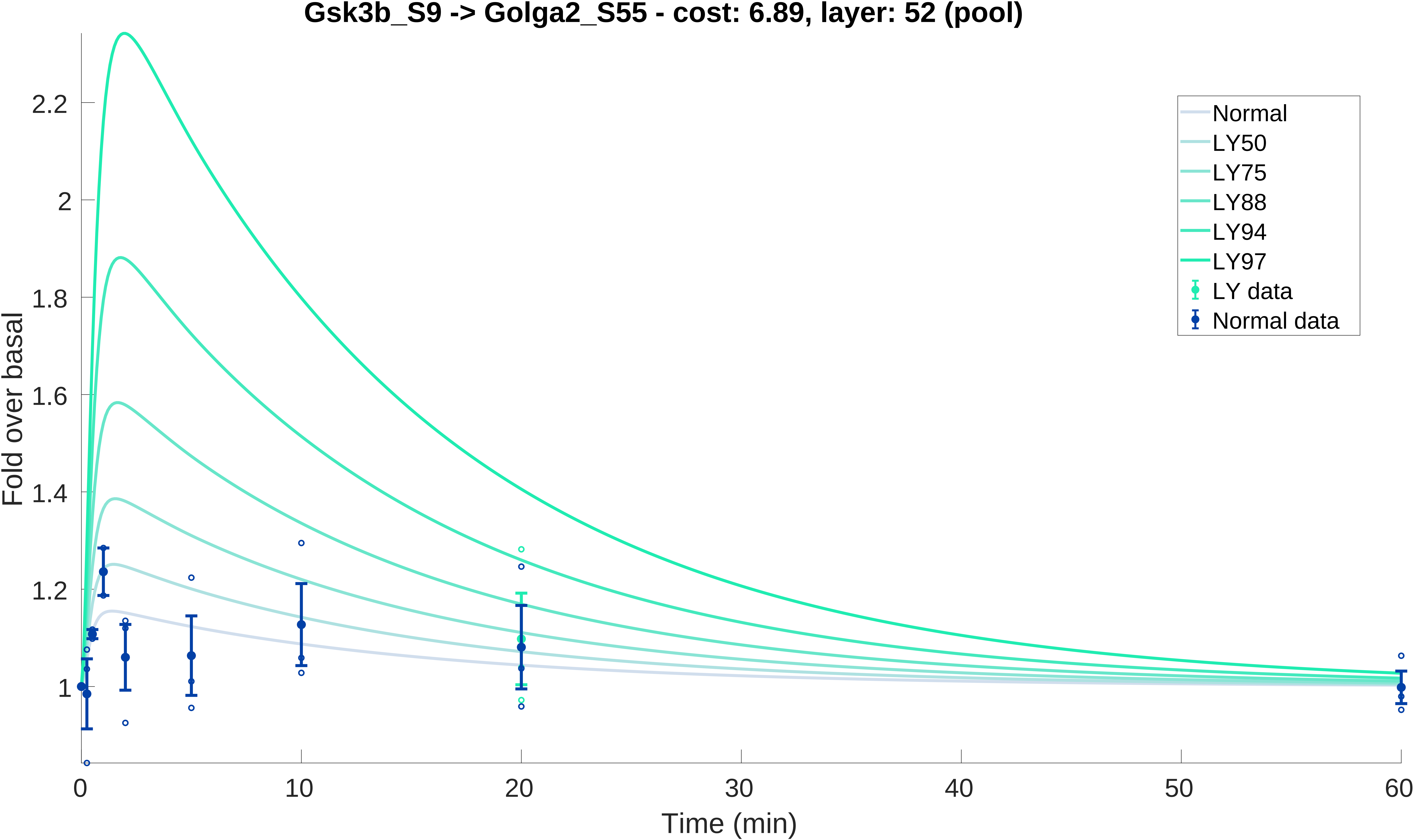
Gsk3b_S9 -> Golga2_S55 - cost: 6.89, layer: 52 (pool)
Fold over basal
Time (min)
Normal
LY50
LY75
LY88
LY94
LY97
LY data
Normal data
2.2
2
1.8
1.6
1.4
1.2
1
0
10
20
30
40
50
60

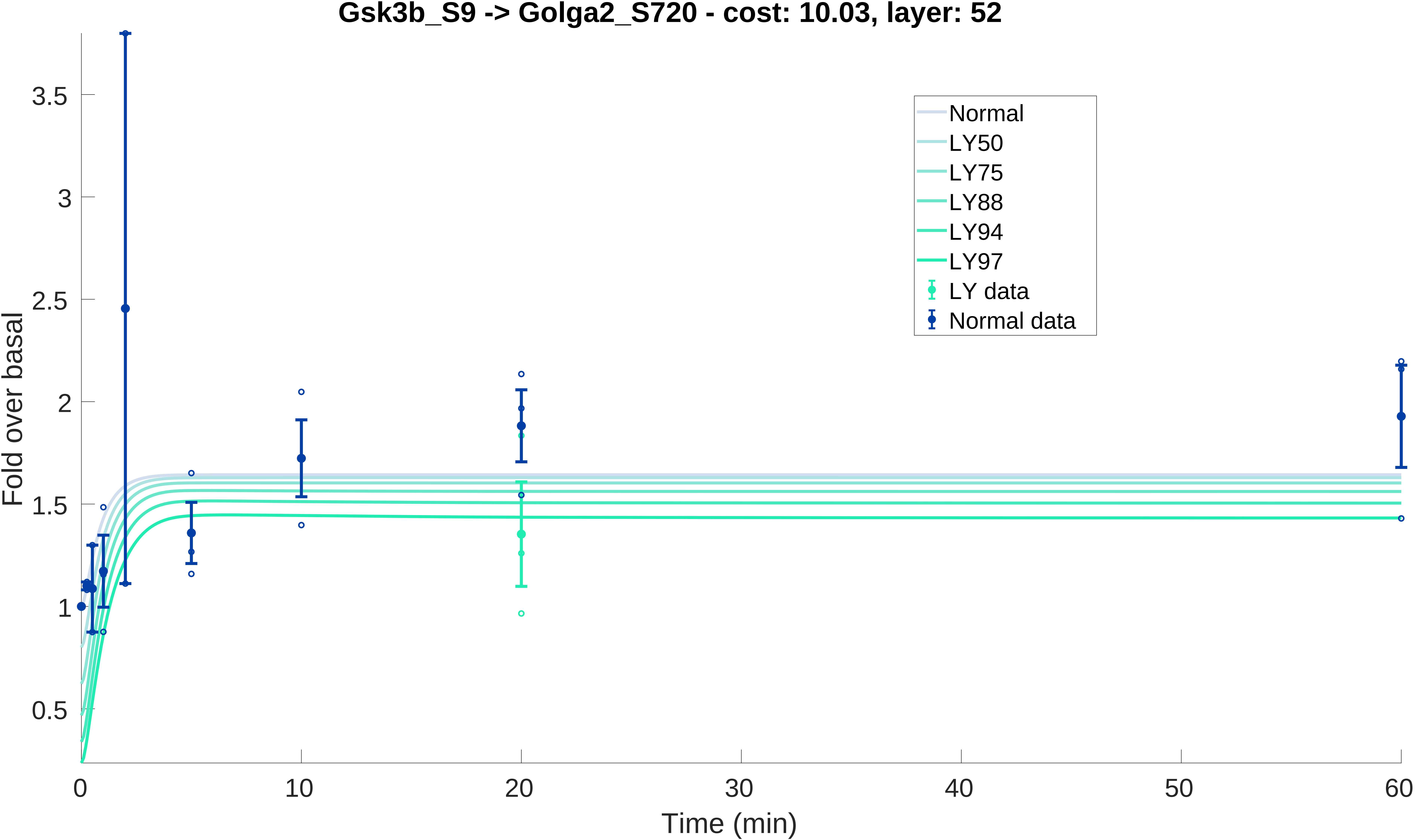
Gsk3b_S9 -> Golga2_S720 - cost: 10.03, layer: 52
Fold over basal
Time (min)
0.5
1
1.5
2
2.5
3
3.5
0
10
20
30
40
50
60
Normal
LY50
LY75
LY88
LY94
LY97
LY data
Normal data

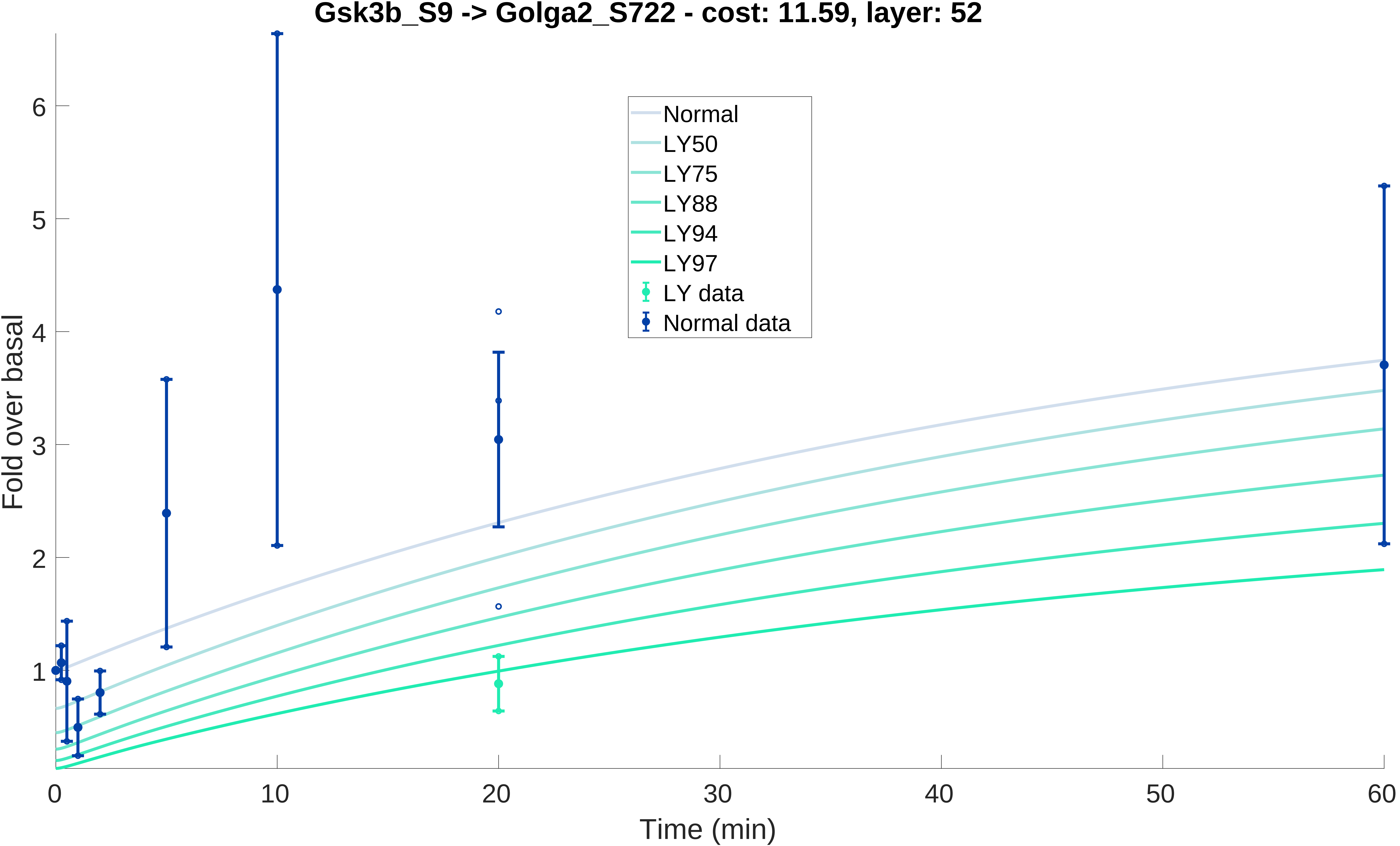
Gsk3b_S9 -> Golga2_S722 - cost: 11.59, layer: 52
Fold over basal
Time (min)
0
10
20
30
40
50
60
1
2
3
4
5
6
Normal
LY50
LY75
LY88
LY94
LY97
LY data
Normal data

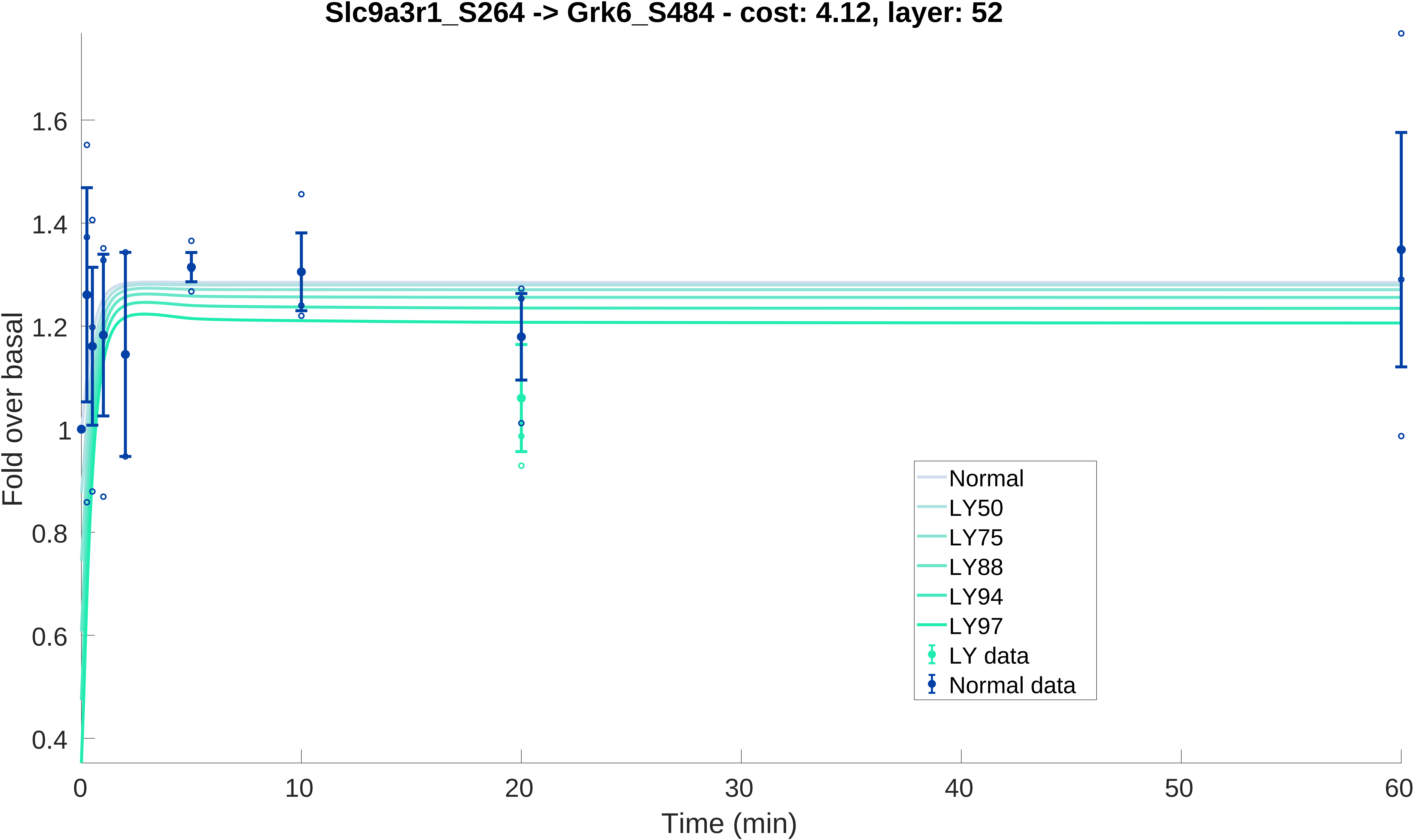
Slc9a3r1_S264 -> Grk6_S484 - cost: 4.12, layer: 52
Fold over basal
Time (min)
1.6
1.4
1.2
1
0.8
0.6
0.4
0
10
20
30
40
50
60
Normal
LY50
LY75
LY88
LY94
LY97
LY data
Normal data

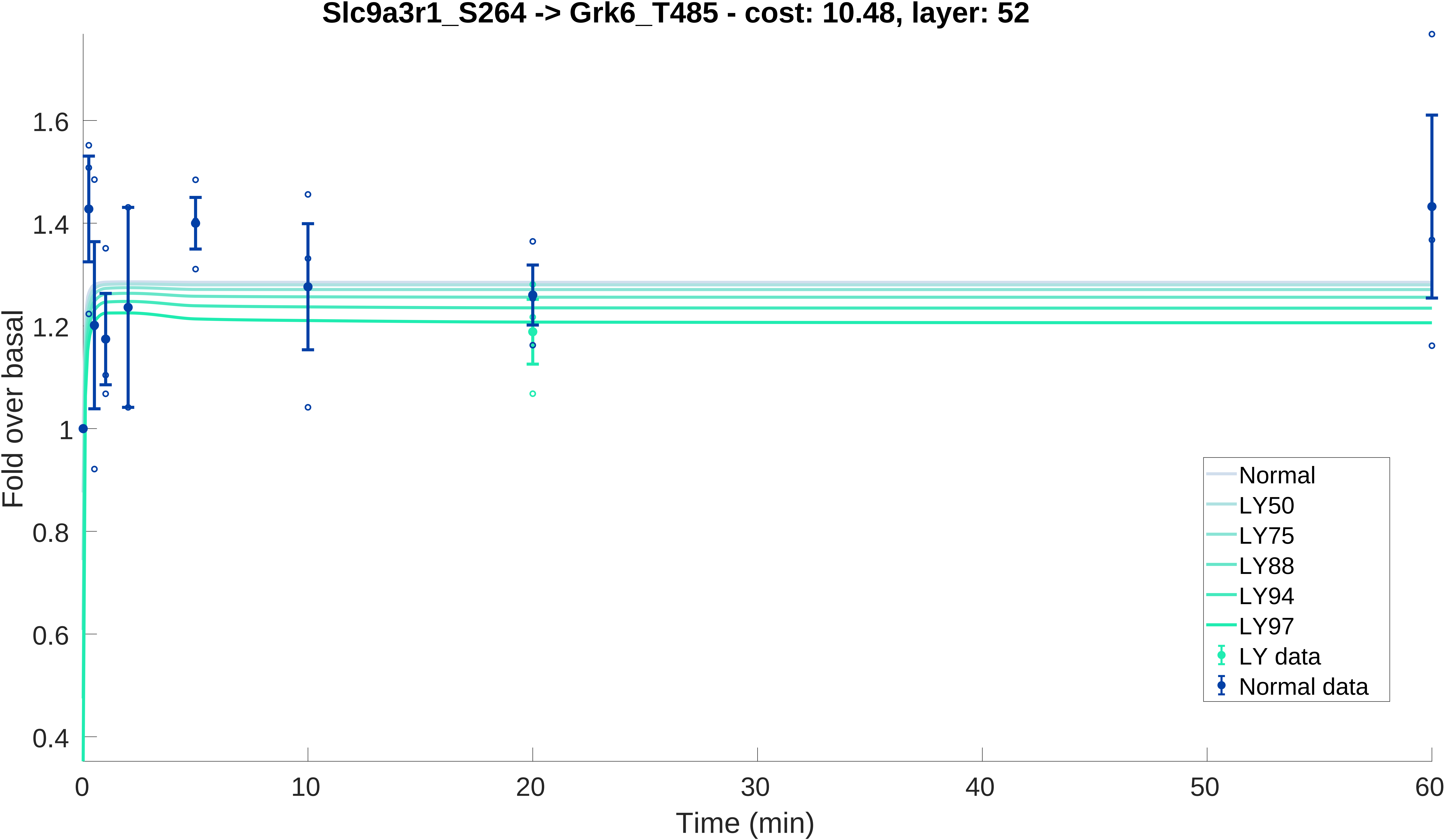

Slc9a3r1_S264 -> Grk6_T485 - cost: 10.48, layer: 52
Fold over basal
Time (min)
0.4
0.6
0.8
1
1.2
1.4
1.6
0
10
20
30
40
50
60
Normal
LY50
LY75
LY88
LY94
LY97
LY data
Normal data

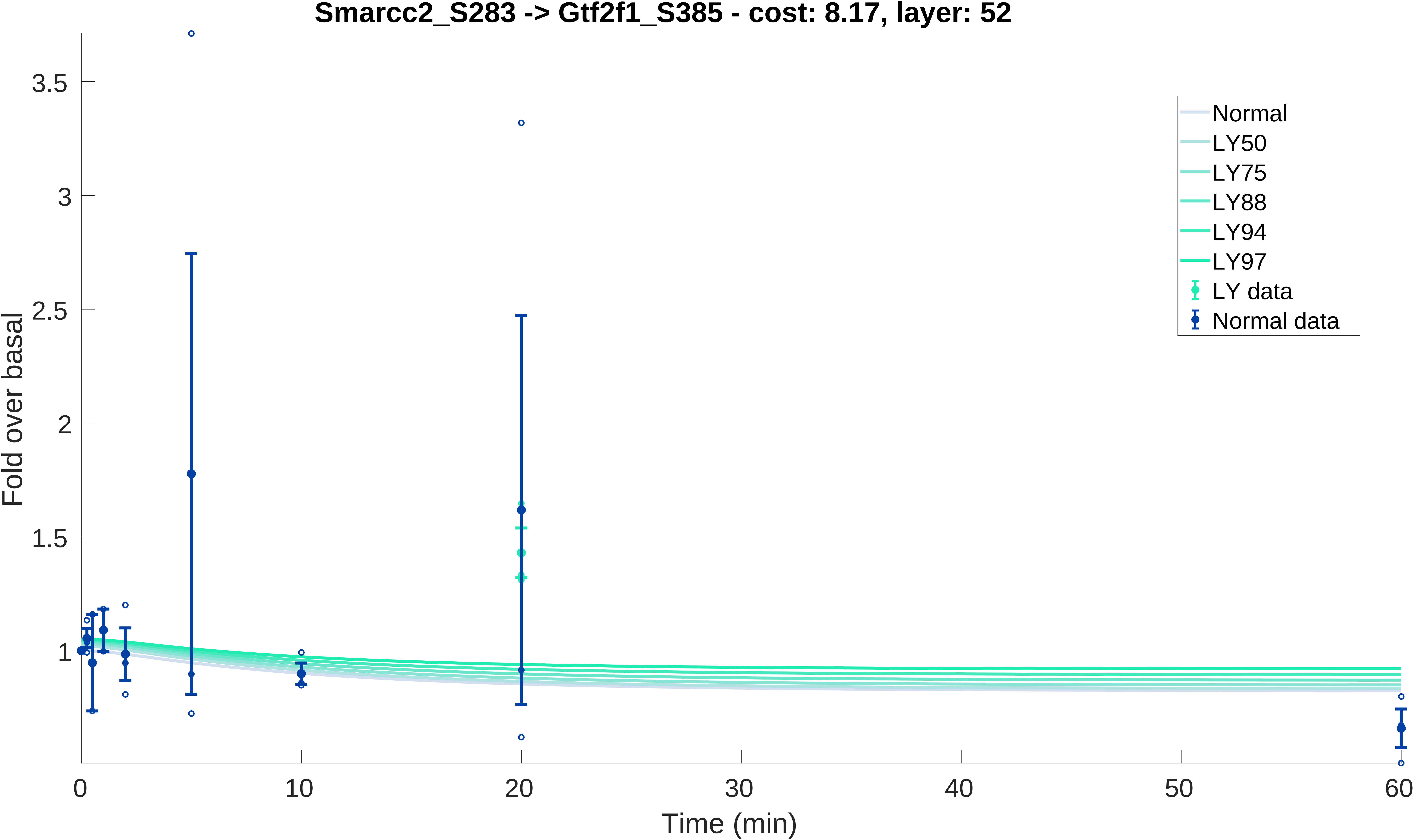
Smarcc2_S283 -> Gtf2f1_S385 - cost: 8.17, layer: 52
Fold over basal
Time (min)
3.5
3
2.5
2
1.5
1
0
10
20
30
40
50
60
Normal
LY50
LY75
LY88
LY94
LY97
LY data
Normal data

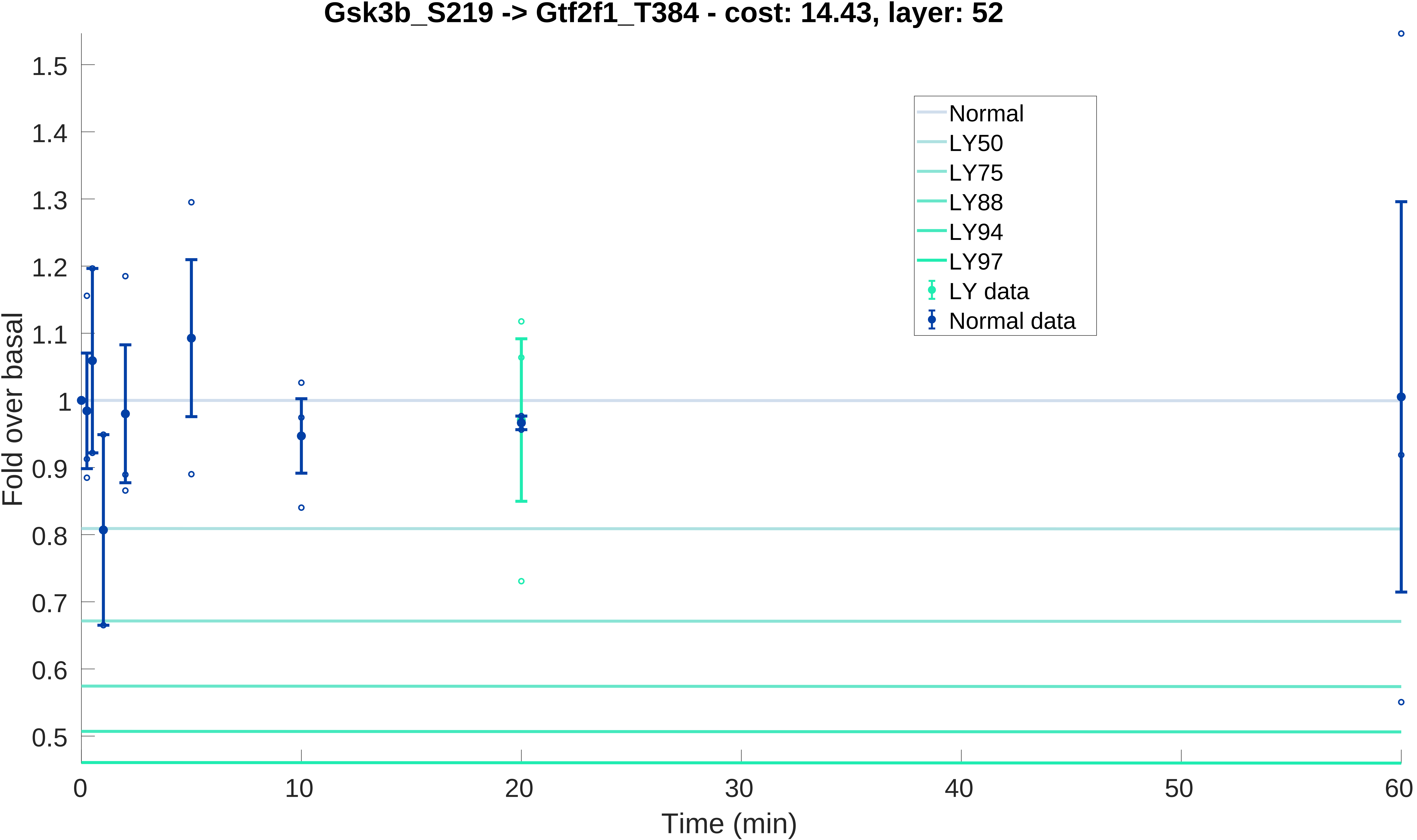

Gsk3b_S219 -> Gtf2f1_T384 - cost: 14.43, layer: 52
Fold over basal
Time (min)
Normal
LY50
LY75
LY88
LY94
LY97
LY data
Normal data
1.5
1.4
1.3
1.2
1.1
1
0.9
0.8
0.7
0.6
0.5
0
10
20
30
40
50
60

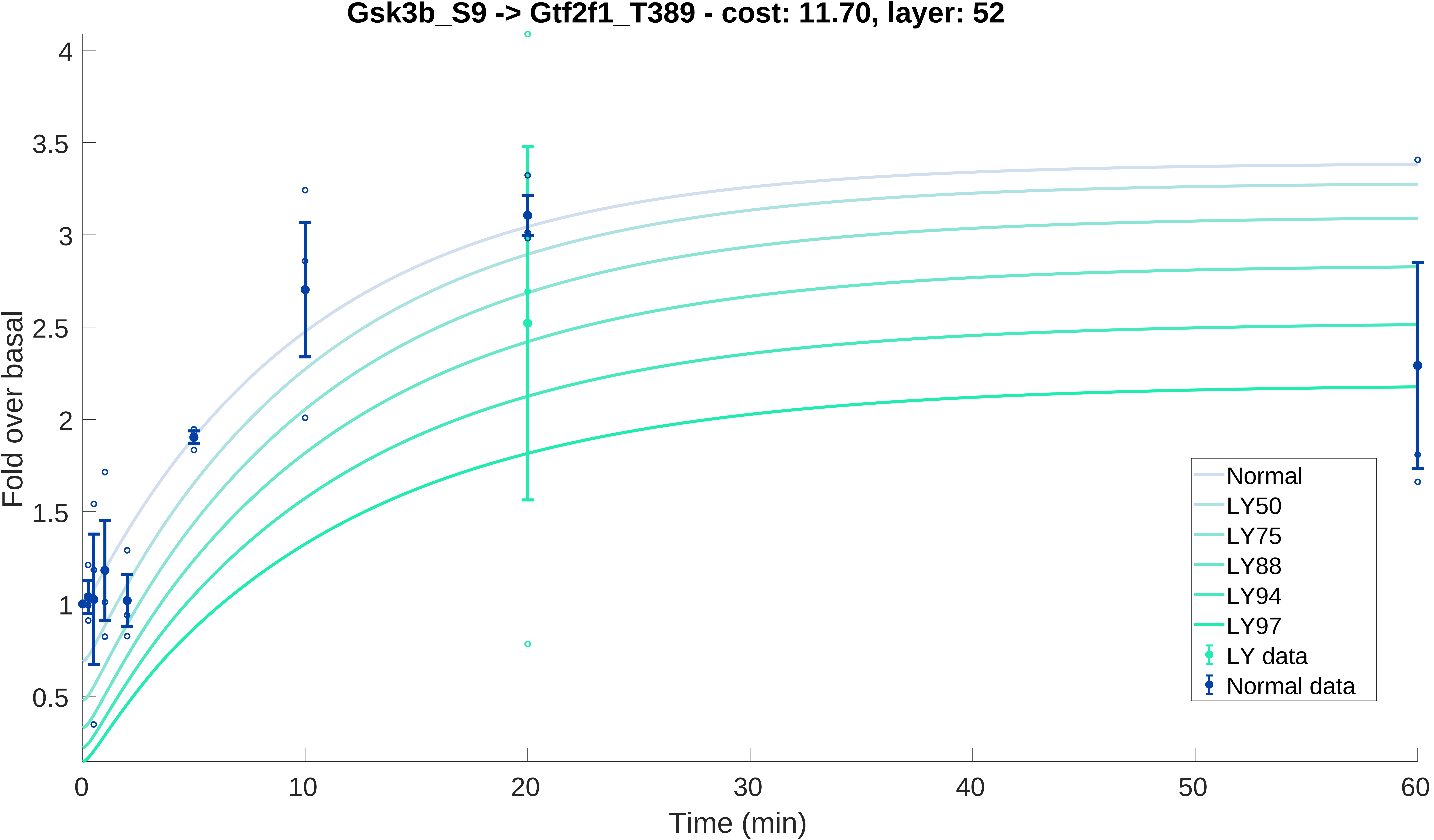

Gsk3b_S9 -> Gtf2f1_T389 - cost: 11.70, layer: 52
Fold over basal
Time (min)
0.5
1
1.5
2
2.5
3
3.5
4
0
10
20
30
40
50
60
Normal
LY50
LY75
LY88
LY94
LY97
LY data
Normal data

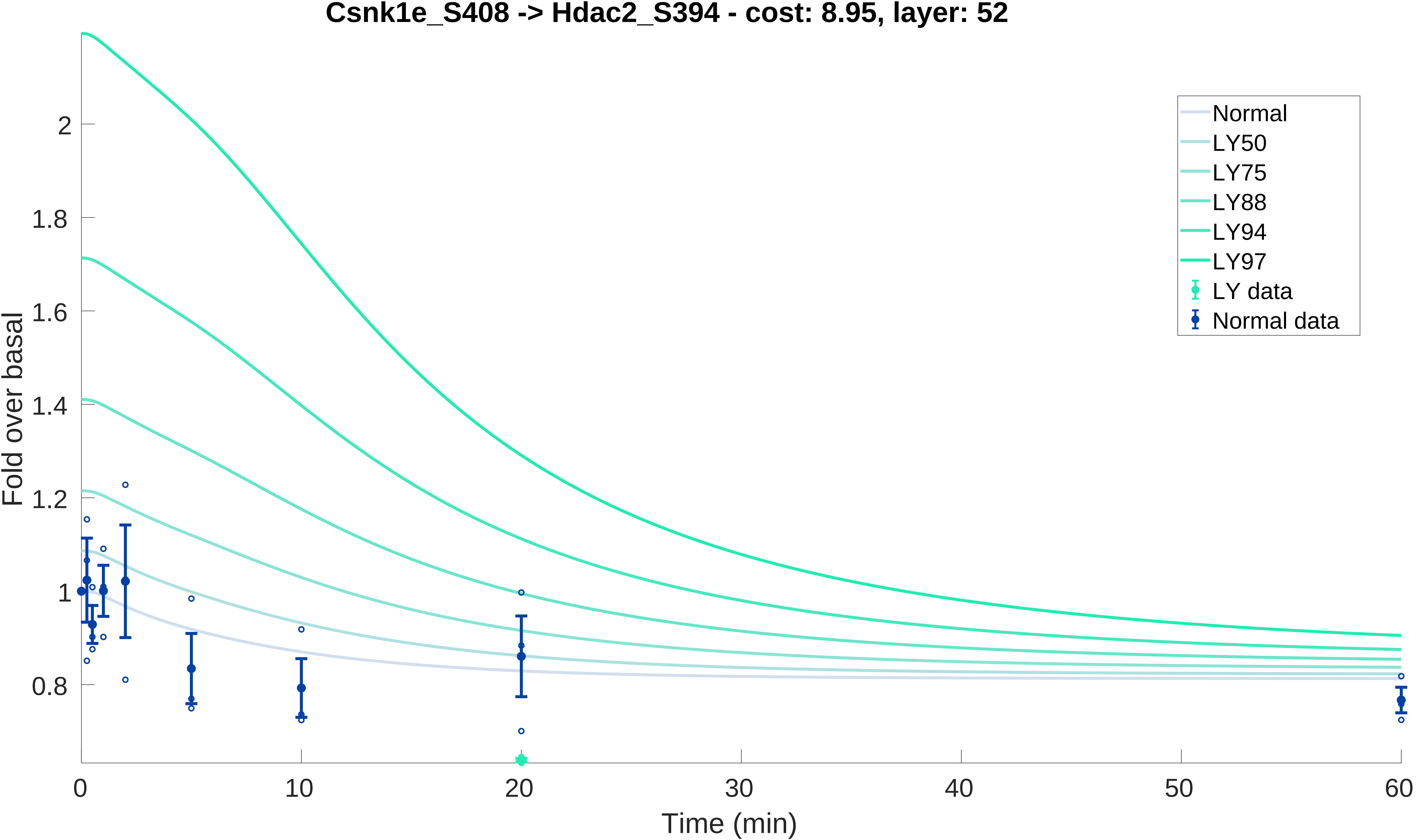
Csnk1e_S408 -> Hdac2_S394 - cost: 8.95, layer: 52
Normal
LY50
LY75
LY88
LY94
LY97
LY data
Normal data
Fold over basal
Time (min)
0.8
1
1.2
1.4
1.6
1.8
2
0
10
20
30
40
50
60

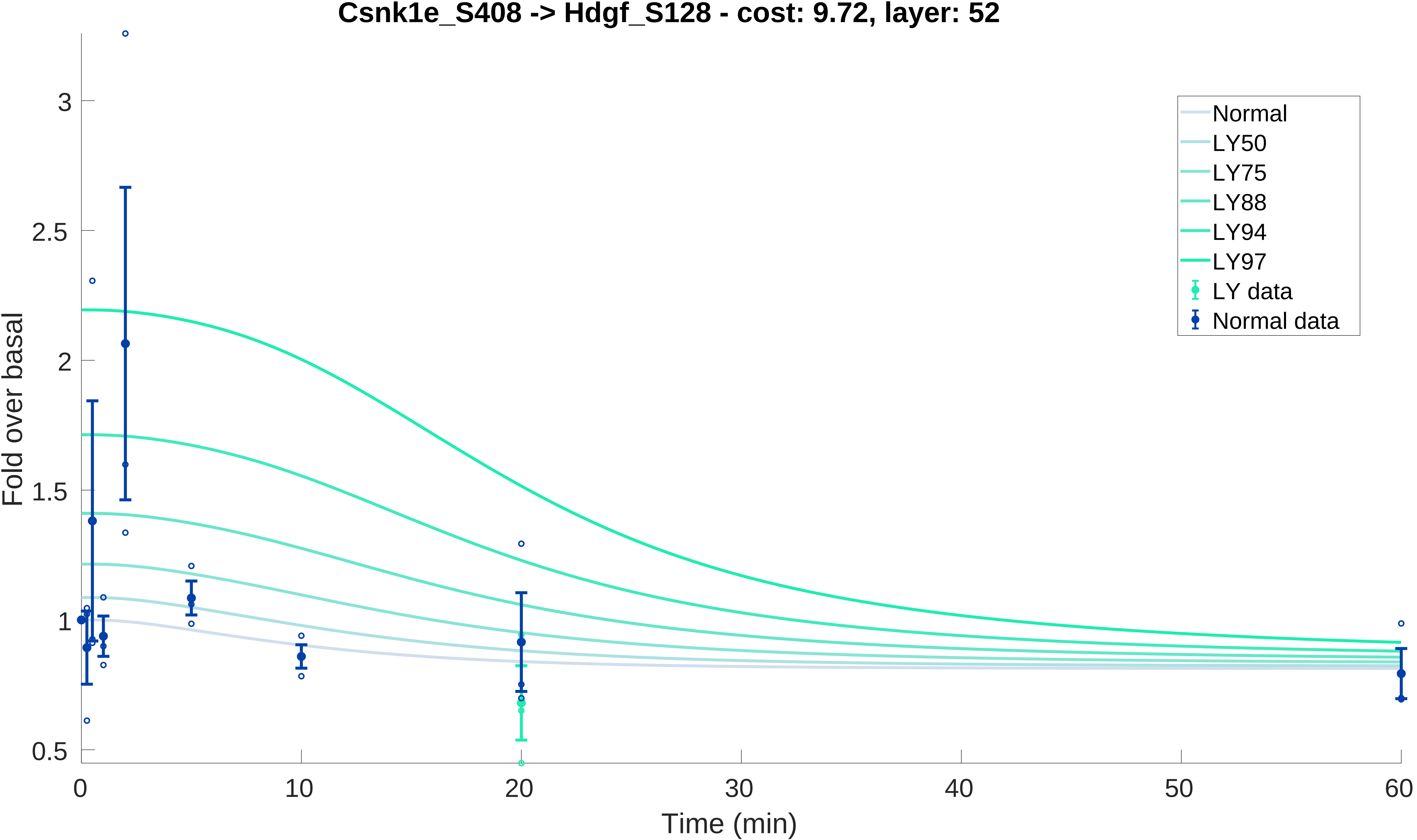
Csnk1e_S408 -> Hdgf_S128 - cost: 9.72, layer: 52
Fold over basal
Time (min)
0.5
1
1.5
2
2.5
3
0
10
20
30
40
50
60
Normal
LY50
LY75
LY88
LY94
LY97
LY data
Normal data

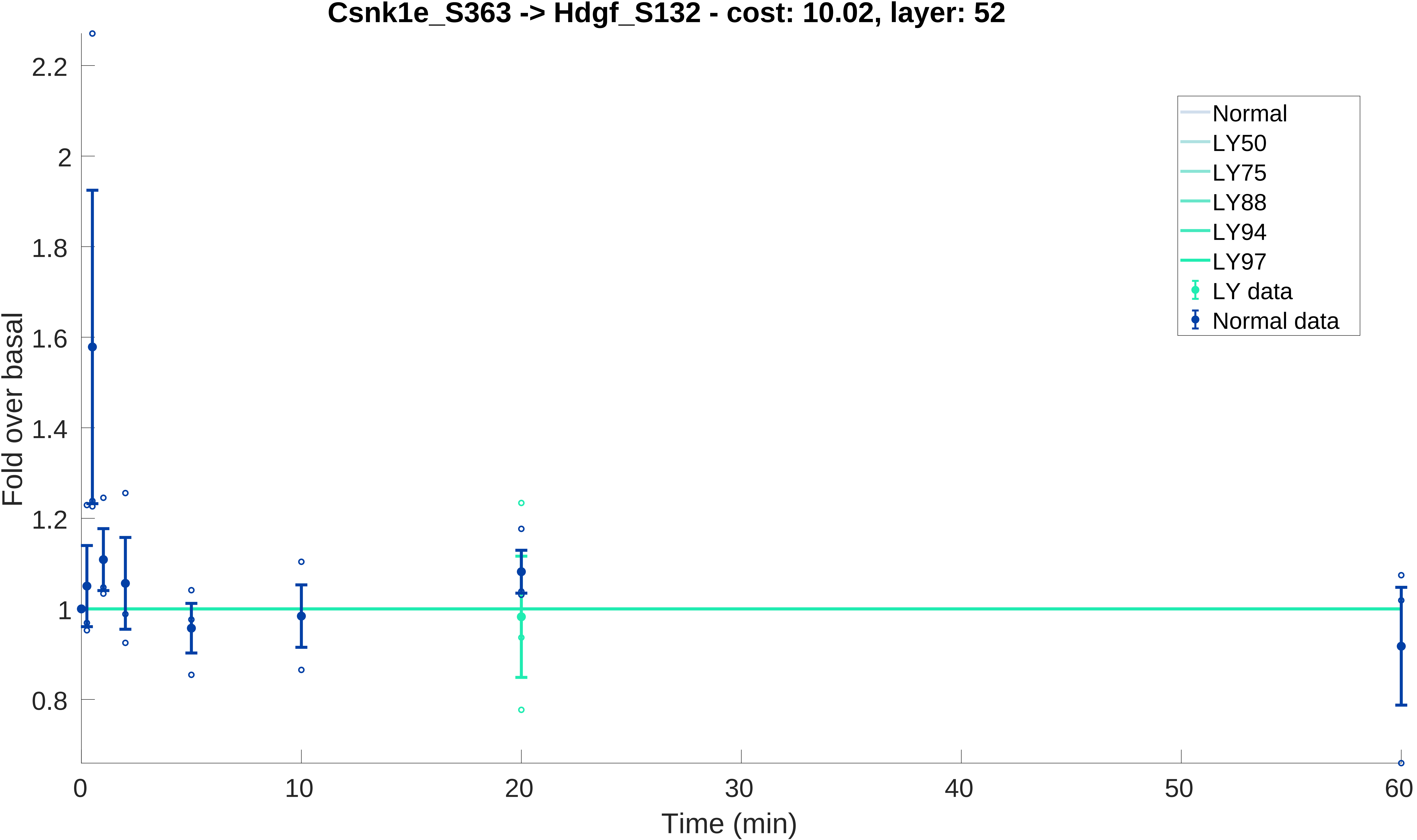
Csnk1e_S363 -> Hdgf_S132 - cost: 10.02, layer: 52
Fold over basal
Time (min)
0
10
20
30
40
50
60
0.8
1
1.2
1.4
1.6
1.8
2
2.2
Normal
LY50
LY75
LY88
LY94
LY97
LY data
Normal data

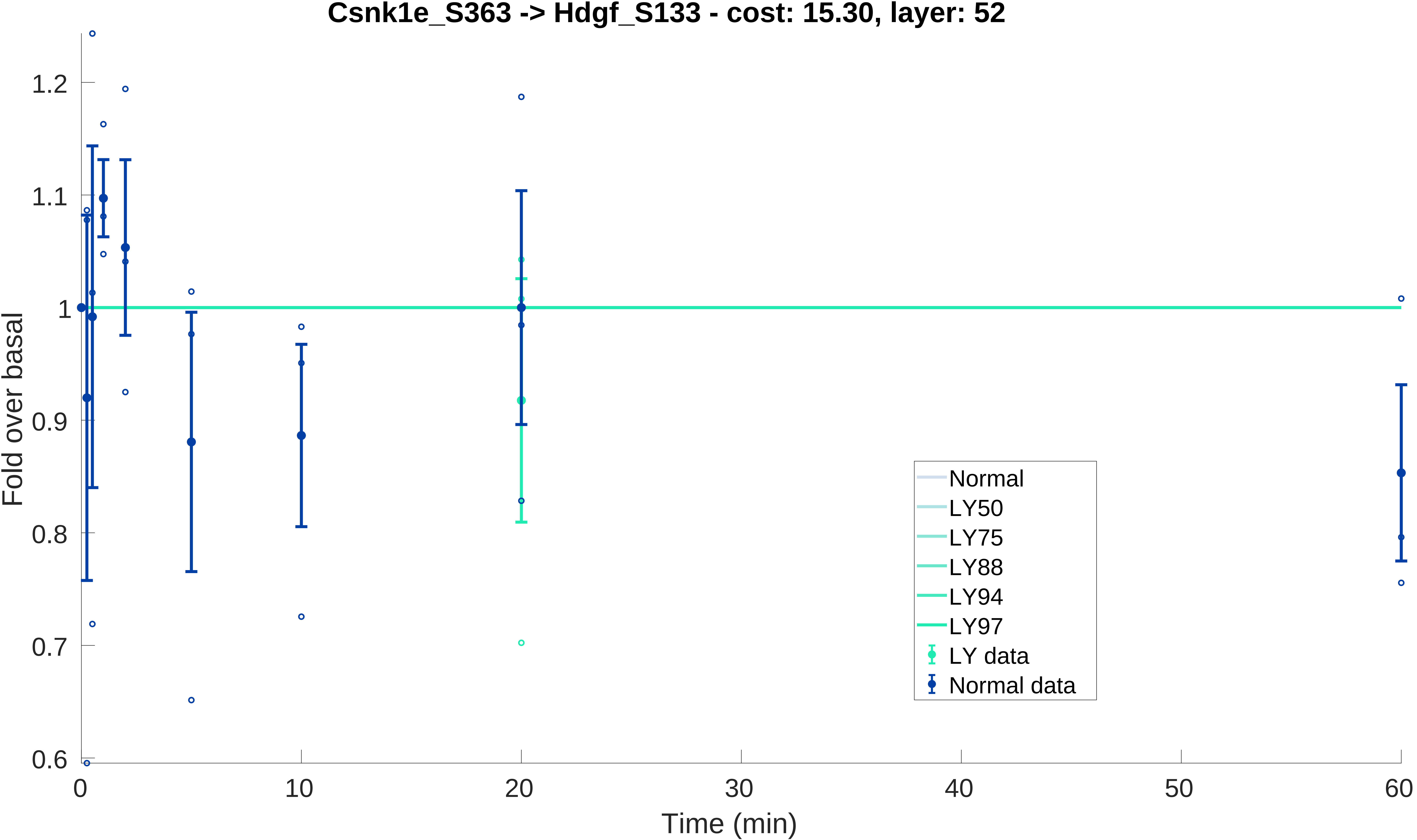
Csnk1e_S363 -> Hdgf_S133 - cost: 15.30, layer: 52
Fold over basal
Time (min)
0.6
0.7
0.8
0.9
1
1.1
1.2
0
10
20
30
40
50
60
Normal
LY50
LY75
LY88
LY94
LY97
LY data
Normal data

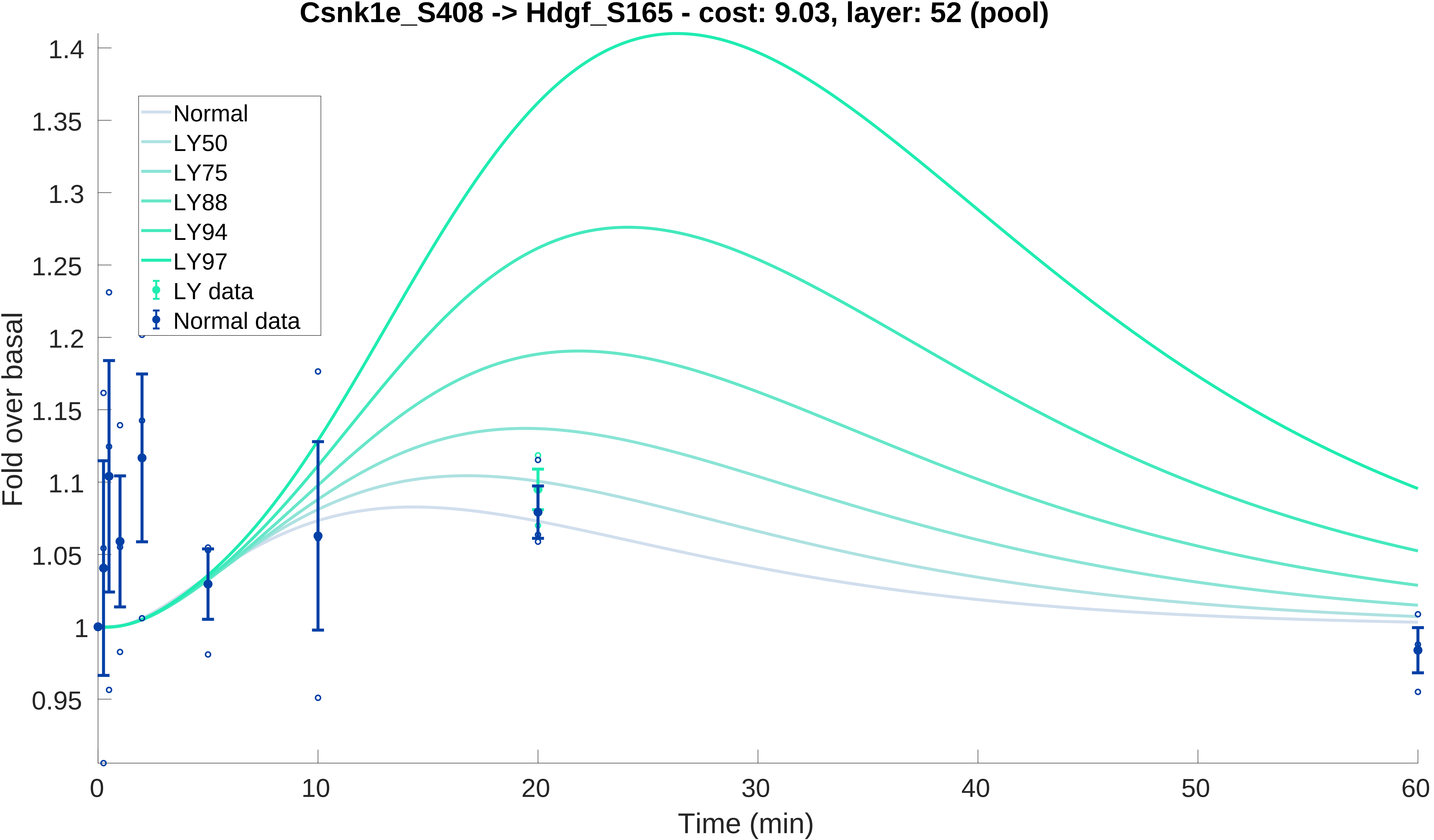

Csnk1e_S408 -> Hdgf_S165 - cost: 9.03, layer: 52 (pool)
Fold over basal
Time (min)
1.4
1.35
1.3
1.25
1.2
1.15
1.1
1.05
1
0.95
0
10
20
30
40
50
60
Normal
LY50
LY75
LY88
LY94
LY97
LY data
Normal data

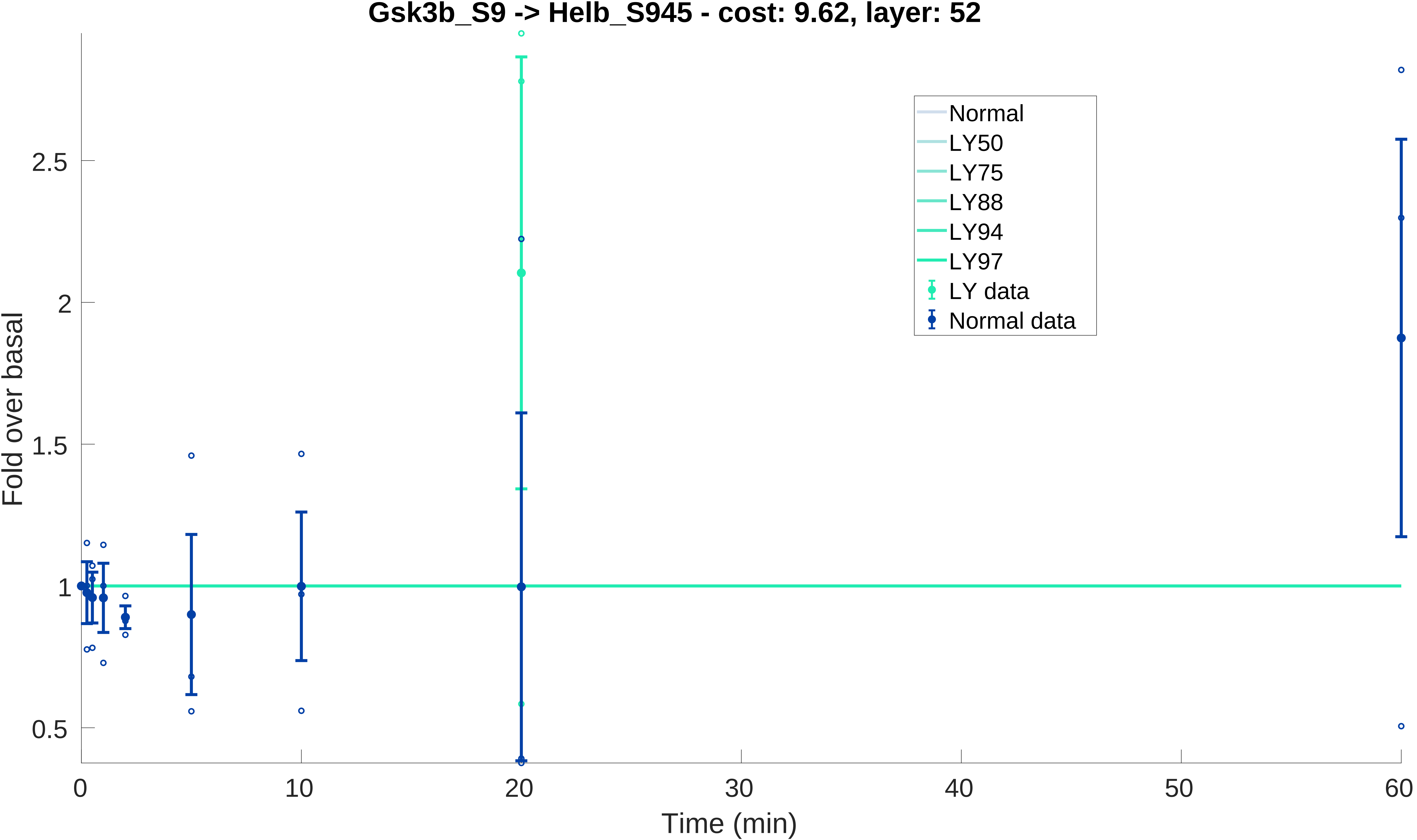
Gsk3b_S9 -> Helb_S945 - cost: 9.62, layer: 52
Fold over basal
Time (min)
0.5
1
1.5
2
2.5
0
10
20
30
40
50
60
Normal
LY50
LY75
LY88
LY94
LY97
LY data
Normal data

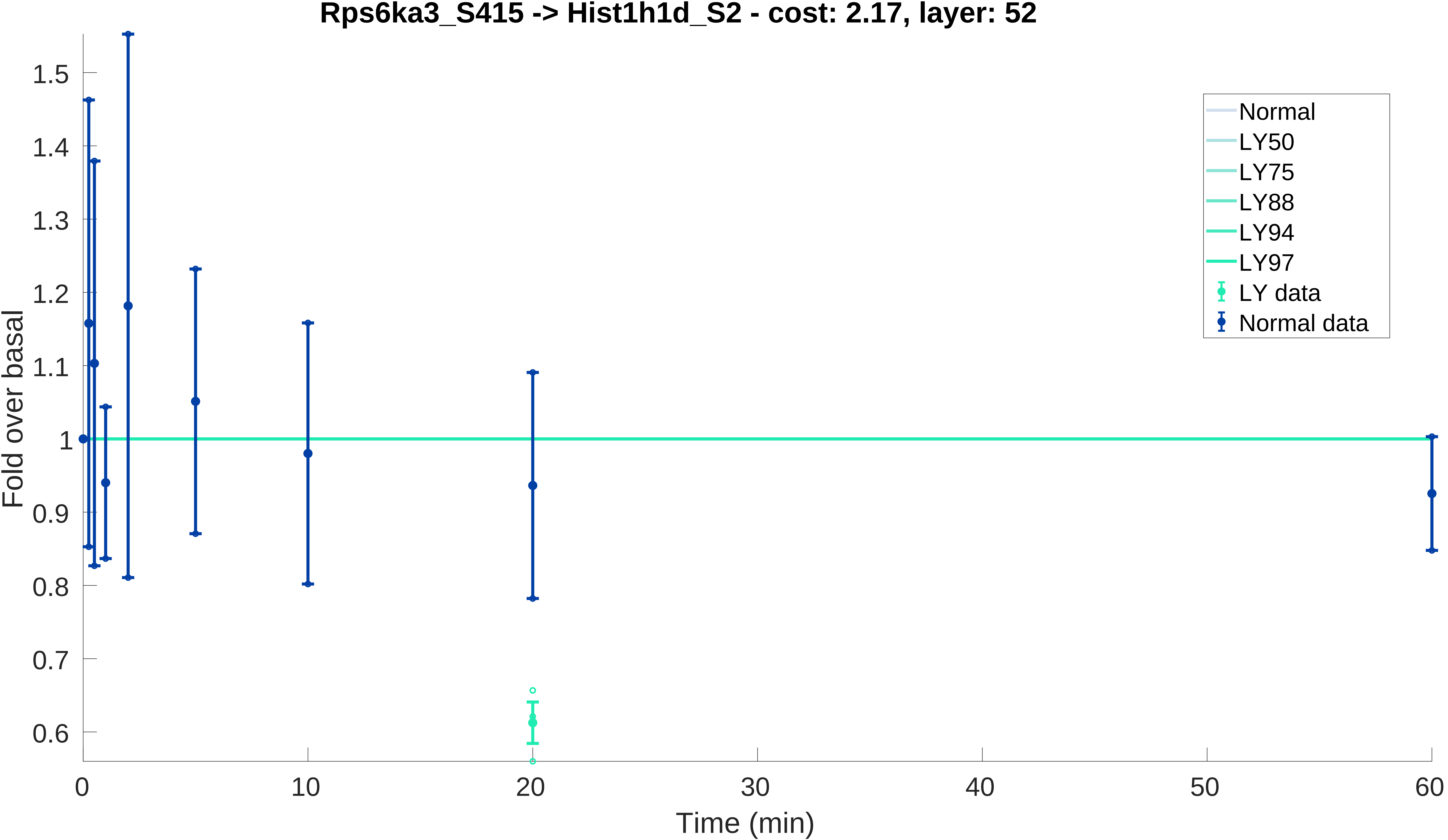
Rps6ka3_S415 -> Hist1h1d_S2 - cost: 2.17, layer: 52
Fold over basal
Time (min)
Normal
LY50
LY75
LY88
LY94
LY97
LY data
Normal data
0.6
0.7
0.8
0.9
1
1.1
1.2
1.3
1.4
1.5
0
10
20
30
40
50
60

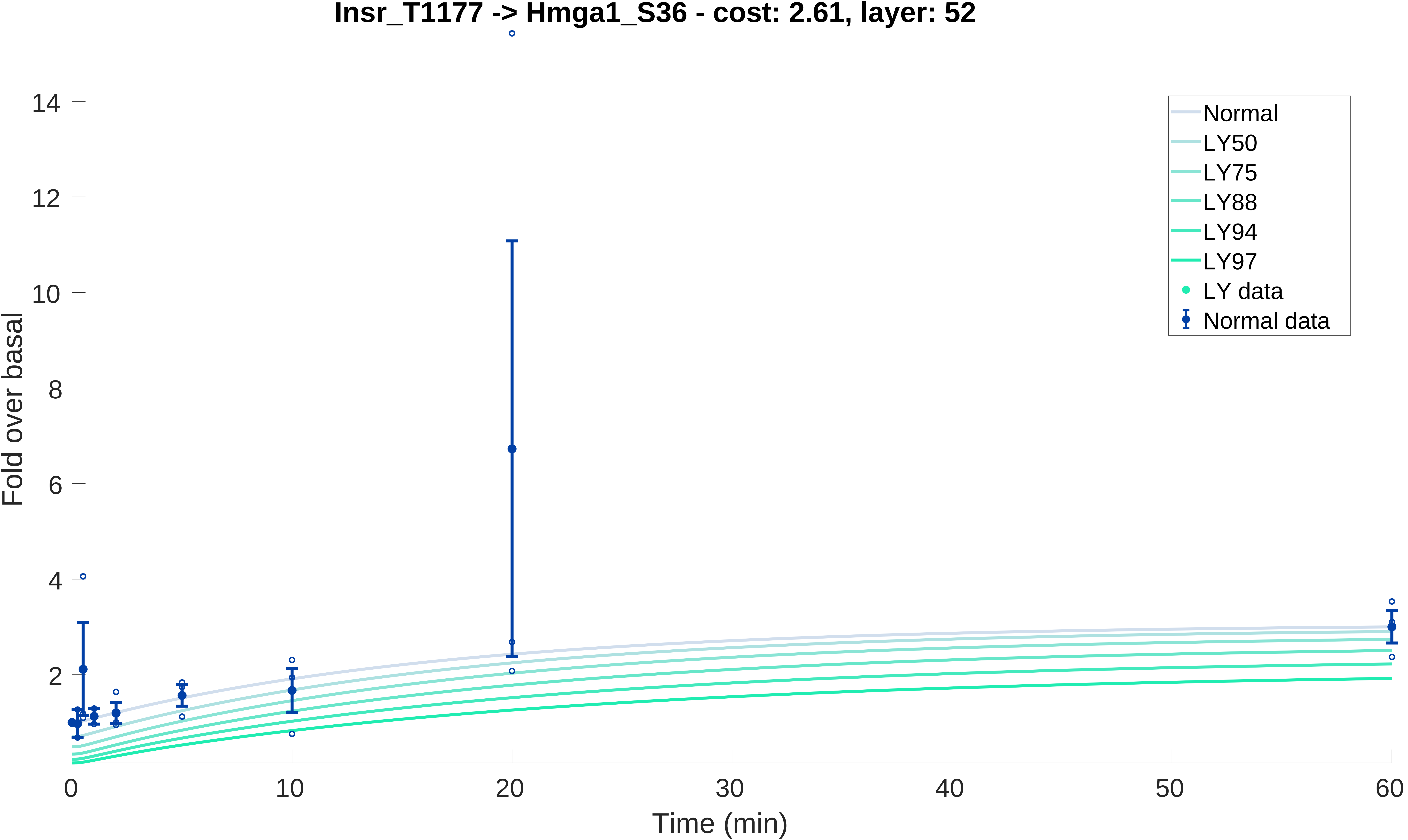

Insr_T1177 -> Hmga1_S36 - cost: 2.61, layer: 52
Fold over basal
Time (min)
0
10
20
30
40
50
60
2
4
6
8
10
12
14
Normal
LY50
LY75
LY88
LY94
LY97
LY data
Normal data

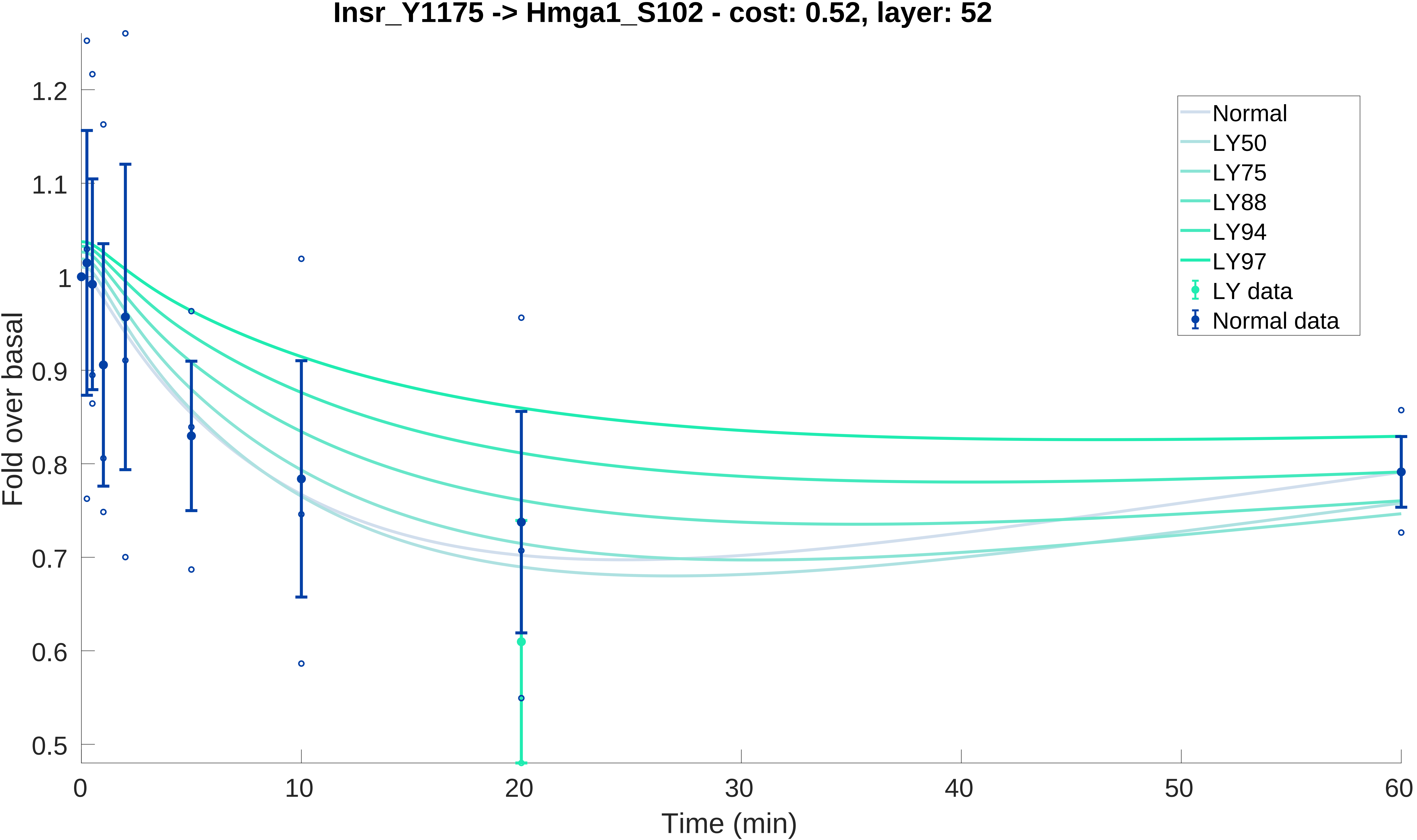

Insr_Y1175 -> Hmga1_S102 - cost: 0.52, layer: 52
Fold over basal
Time (min)
0
10
20
30
40
50
60
0.5
0.6
0.7
0.8
0.9
1
1.1
1.2
Normal
LY50
LY75
LY88
LY94
LY97
LY data
Normal data

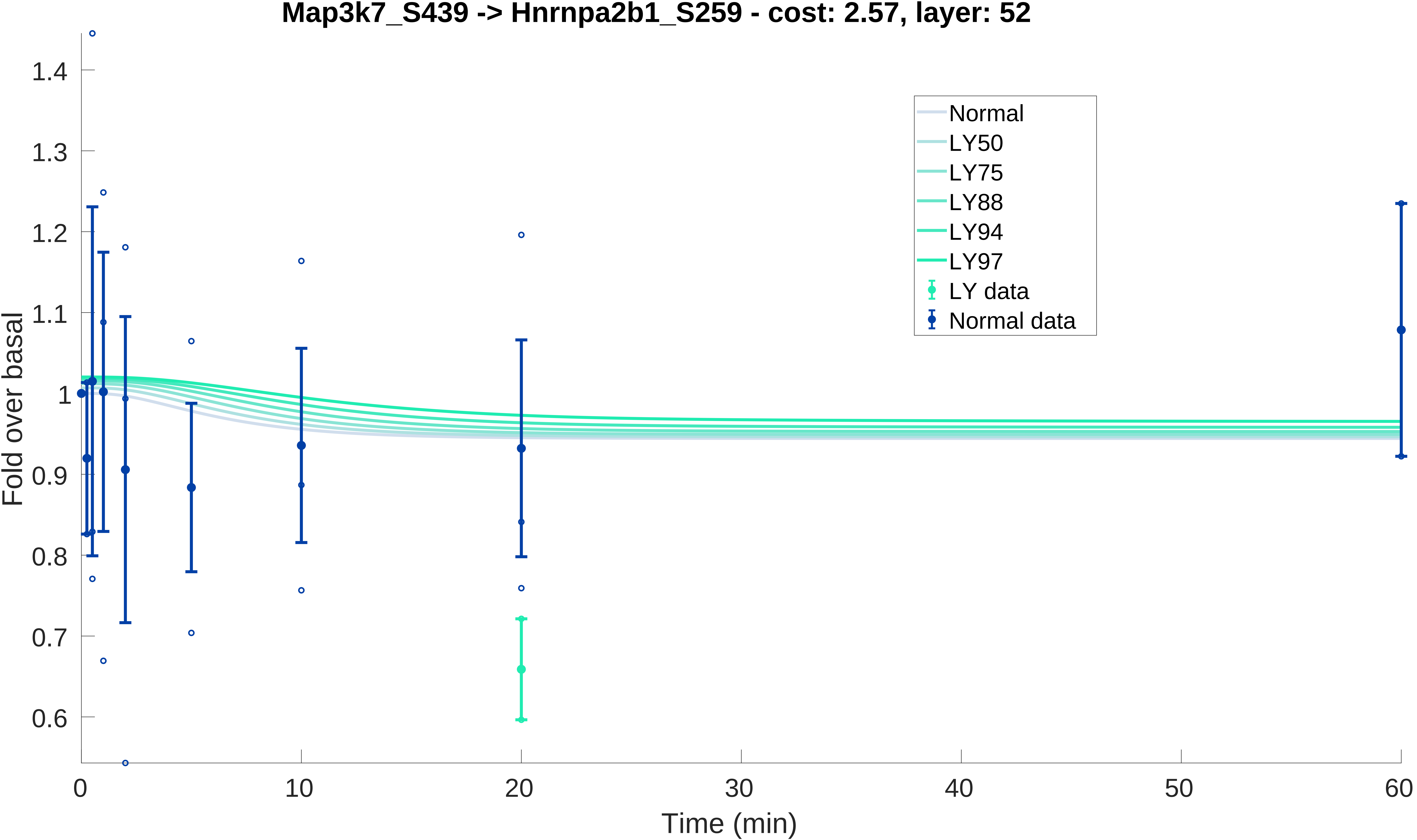

Map3k7_S439 -> Hnrnpa2b1_S259 - cost: 2.57, layer: 52
Fold over basal
Time (min)
1.4
1.3
1.2
1.1
1
0.9
0.8
0.7
0.6
0
10
20
30
40
50
60
Normal
LY50
LY75
LY88
LY94
LY97
LY data
Normal data

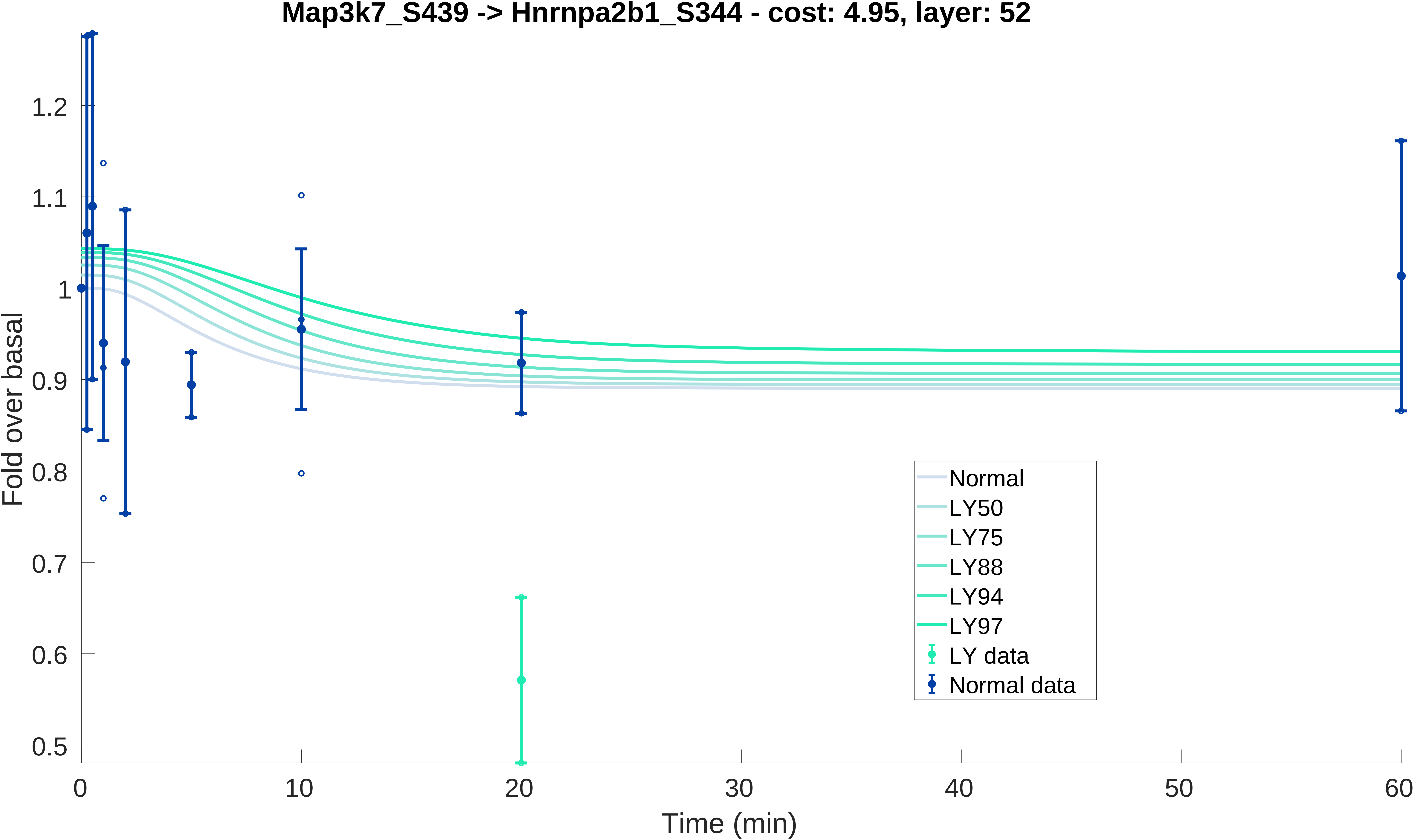

Map3k7_S439 -> Hnrnpa2b1_S344 - cost: 4.95, layer: 52
Fold over basal
Time (min)
0.5
0.6
0.7
0.8
0.9
1
1.1
1.2
0
10
20
30
40
50
60
Normal
LY50
LY75
LY88
LY94
LY97
LY data
Normal data

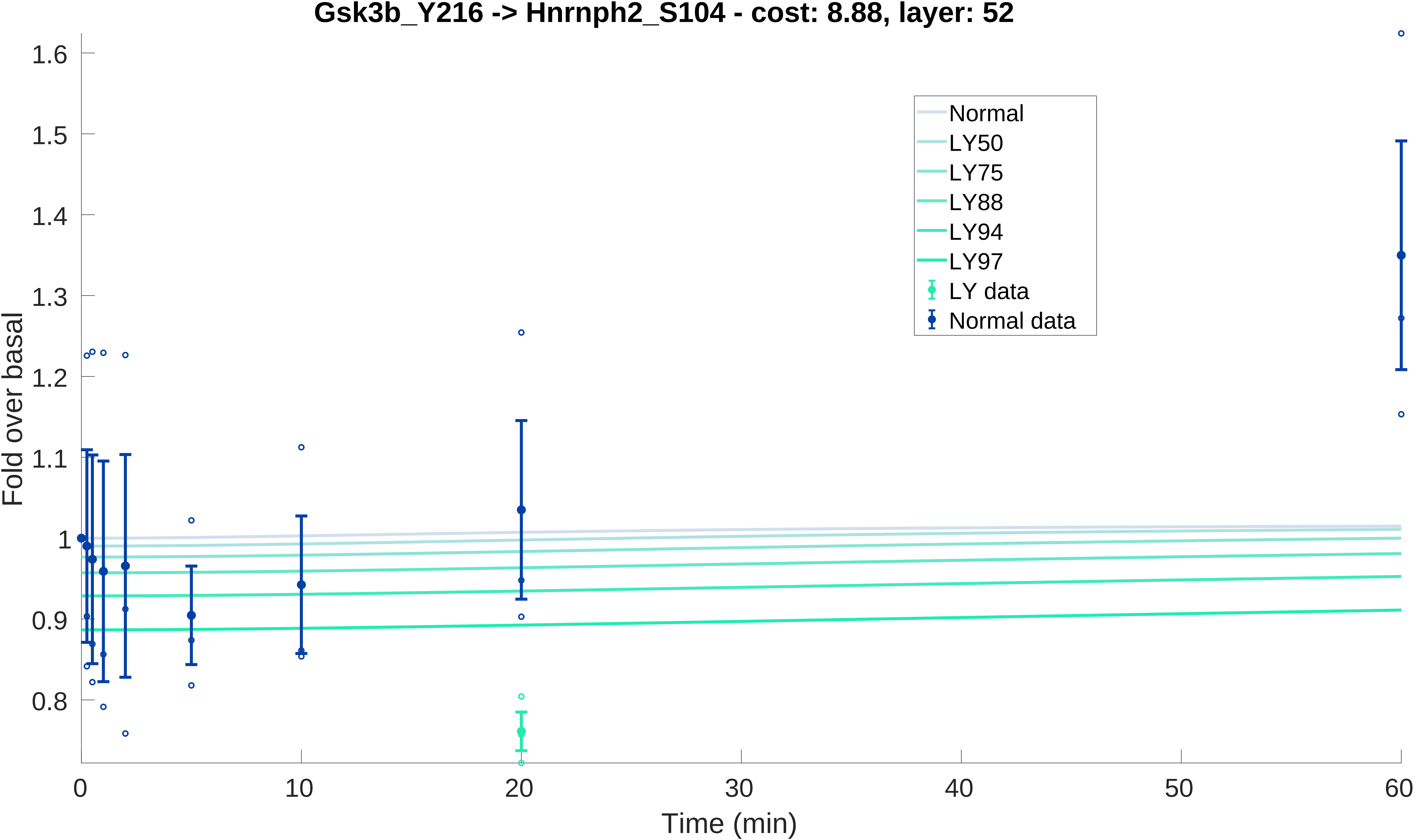
Gsk3b_Y216 -> Hnrnph2_S104 - cost: 8.88, layer: 52
Fold over basal
Time (min)
Normal
LY50
LY75
LY88
LY94
LY97
LY data
Normal data
1.6
1.5
1.4
1.3
1.2
1.1
1
0.9
0.8
0
10
20
30
40
50
60

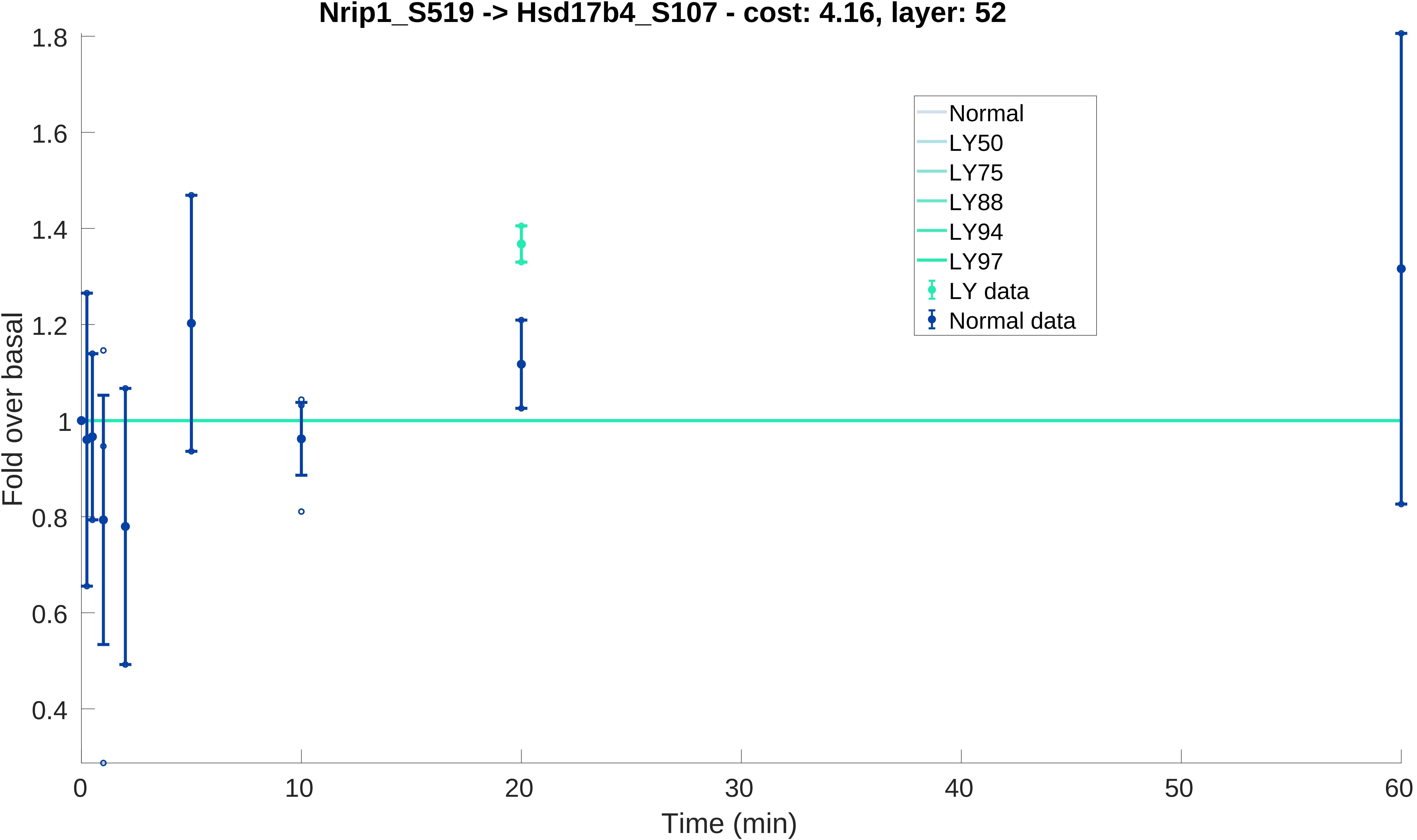
Nrip1_S519 -> Hsd17b4_S107 - cost: 4.16, layer: 52
Fold over basal
Time (min)
0
10
20
30
40
50
60
0.4
0.6
0.8
1
1.2
1.4
1.6
1.8
Normal
LY50
LY75
LY88
LY94
LY97
LY data
Normal data

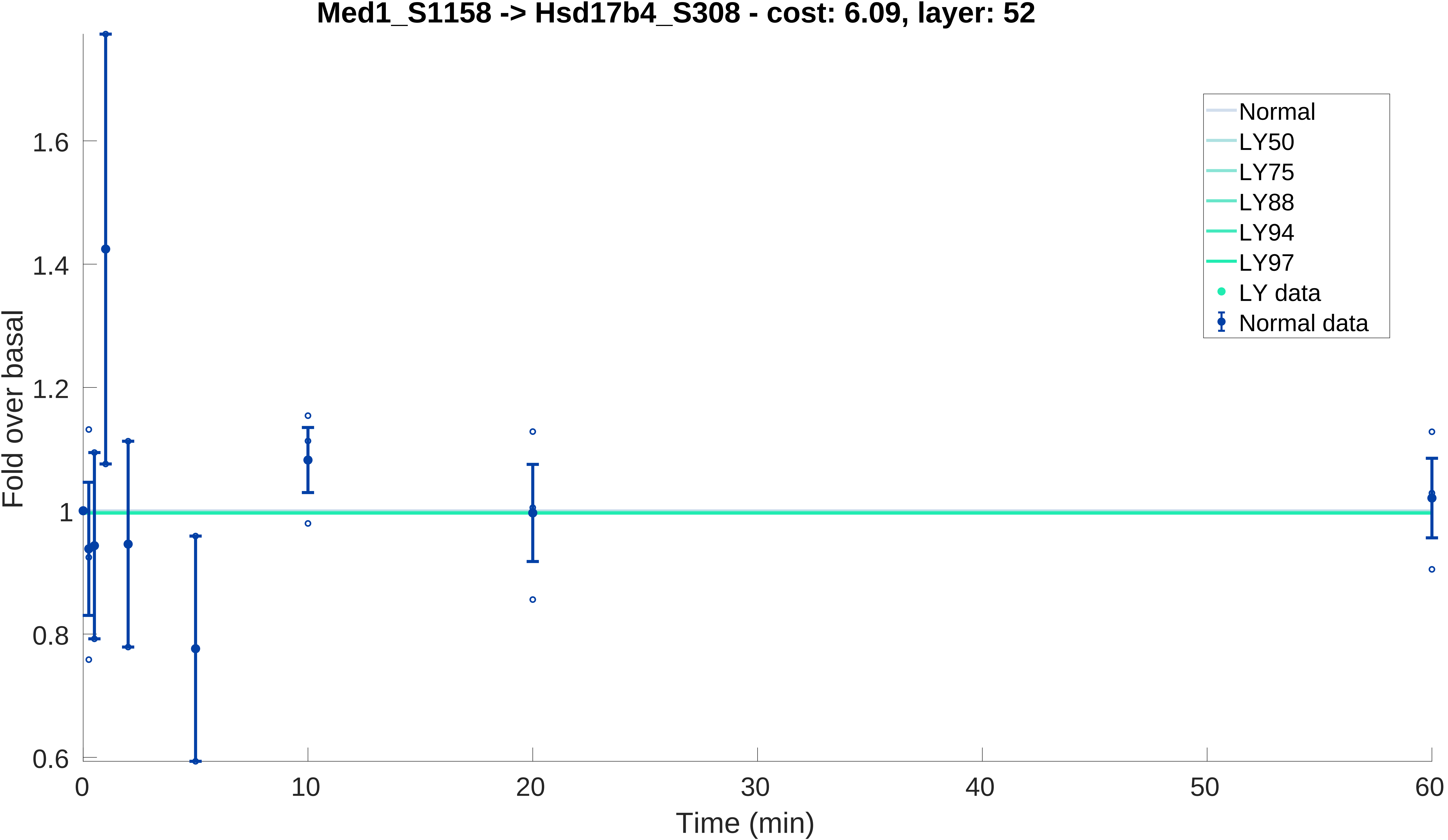

Med1_S1158 -> Hsd17b4_S308 - cost: 6.09, layer: 52
Fold over basal
Time (min)
0.6
0.8
1
1.2
1.4
1.6
0
10
20
30
40
50
60
Normal
LY50
LY75
LY88
LY94
LY97
LY data
Normal data

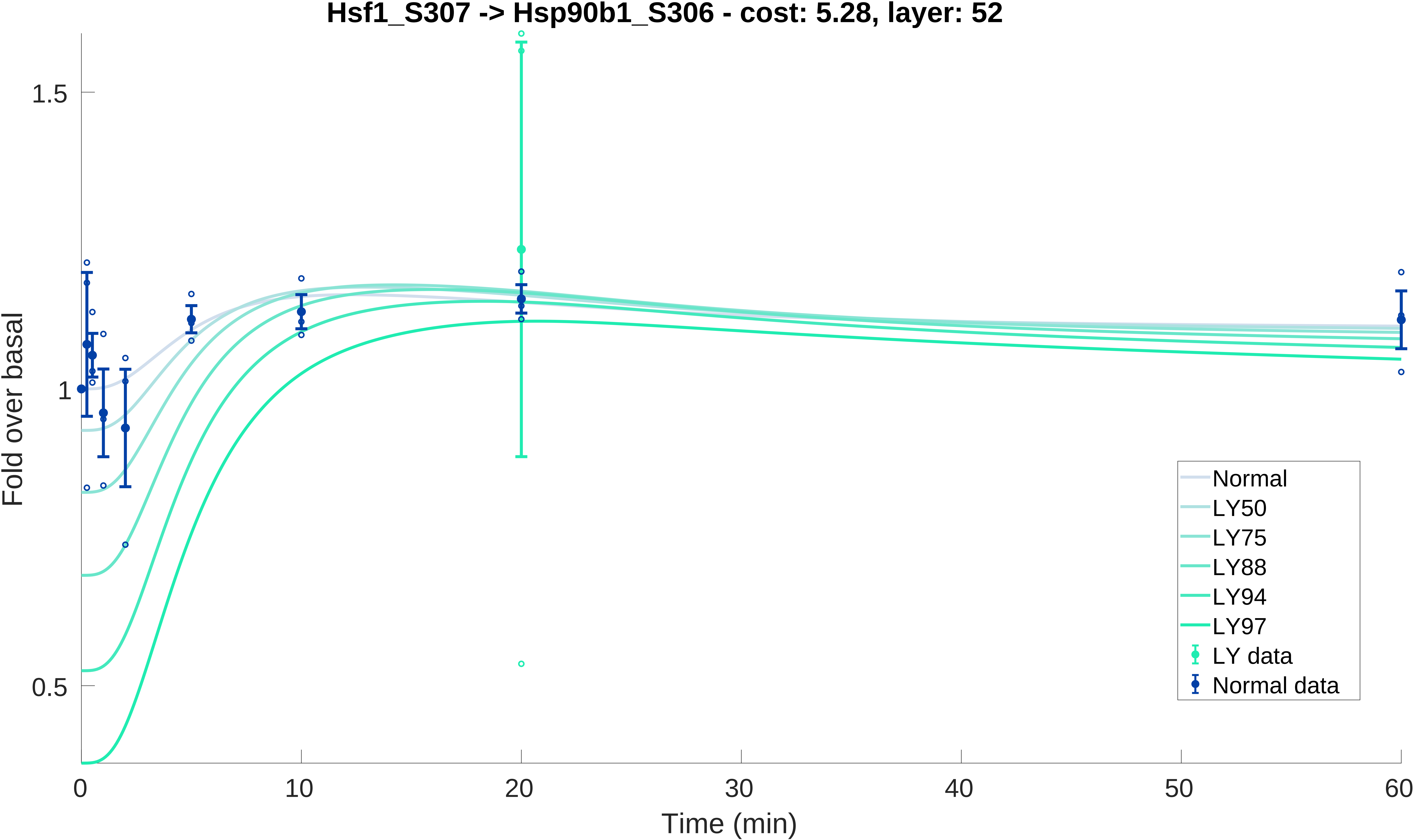

Hsf1_S307 -> Hsp90b1_S306 - cost: 5.28, layer: 52
Fold over basal
1.5
1
0.5
0
10
20
30
40
50
60
Time (min)
Normal
LY50
LY75
LY88
LY94
LY97
LY data
Normal data

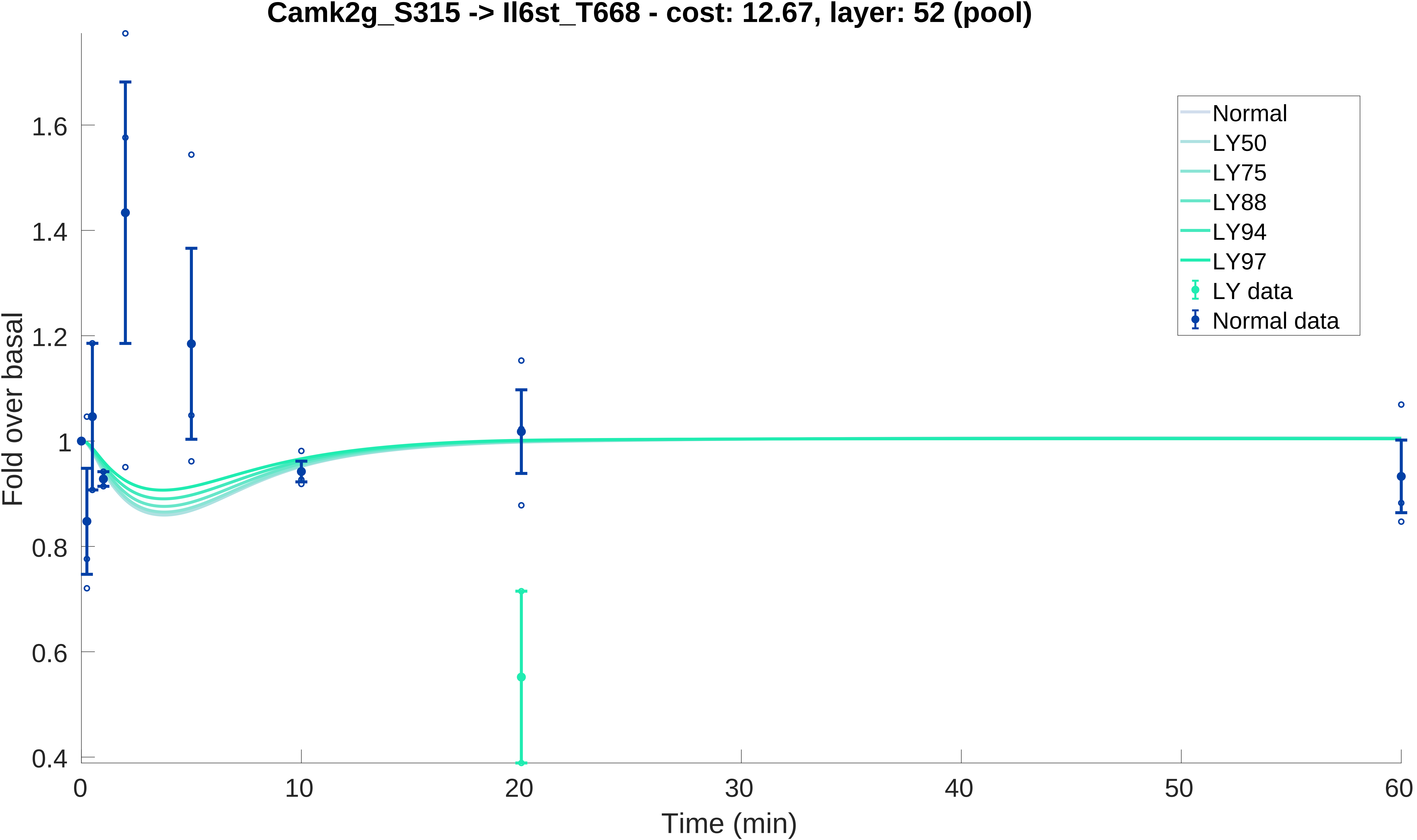

Camk2g_S315 -> Il6st_T668 - cost: 12.67, layer: 52 (pool)
Fold over basal
Time (min)
1.6
1.4
1.2
1
0.8
0.6
0.4
0
10
20
30
40
50
60
Normal
LY50
LY75
LY88
LY94
LY97
LY data
Normal data

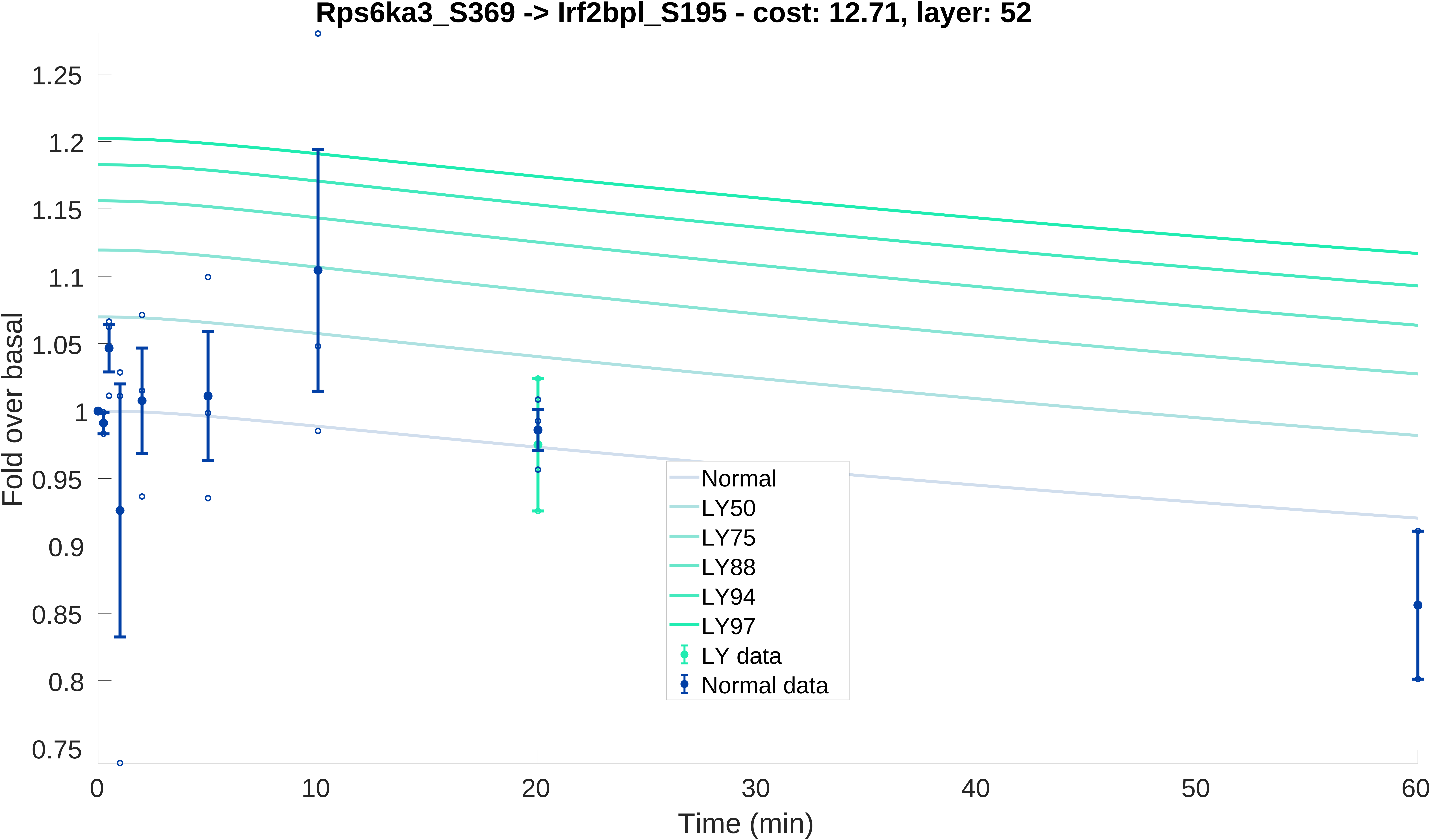

Rps6ka3_S369 -> Irf2bpl_S195 - cost: 12.71, layer: 52
Fold over basal
Time (min)
1.25
1.2
1.15
1.1
1.05
1
0.95
0.9
0.85
0.8
0.75
0
10
20
30
40
50
60
Normal
LY50
LY75
LY88
LY94
LY97
LY data
Normal data

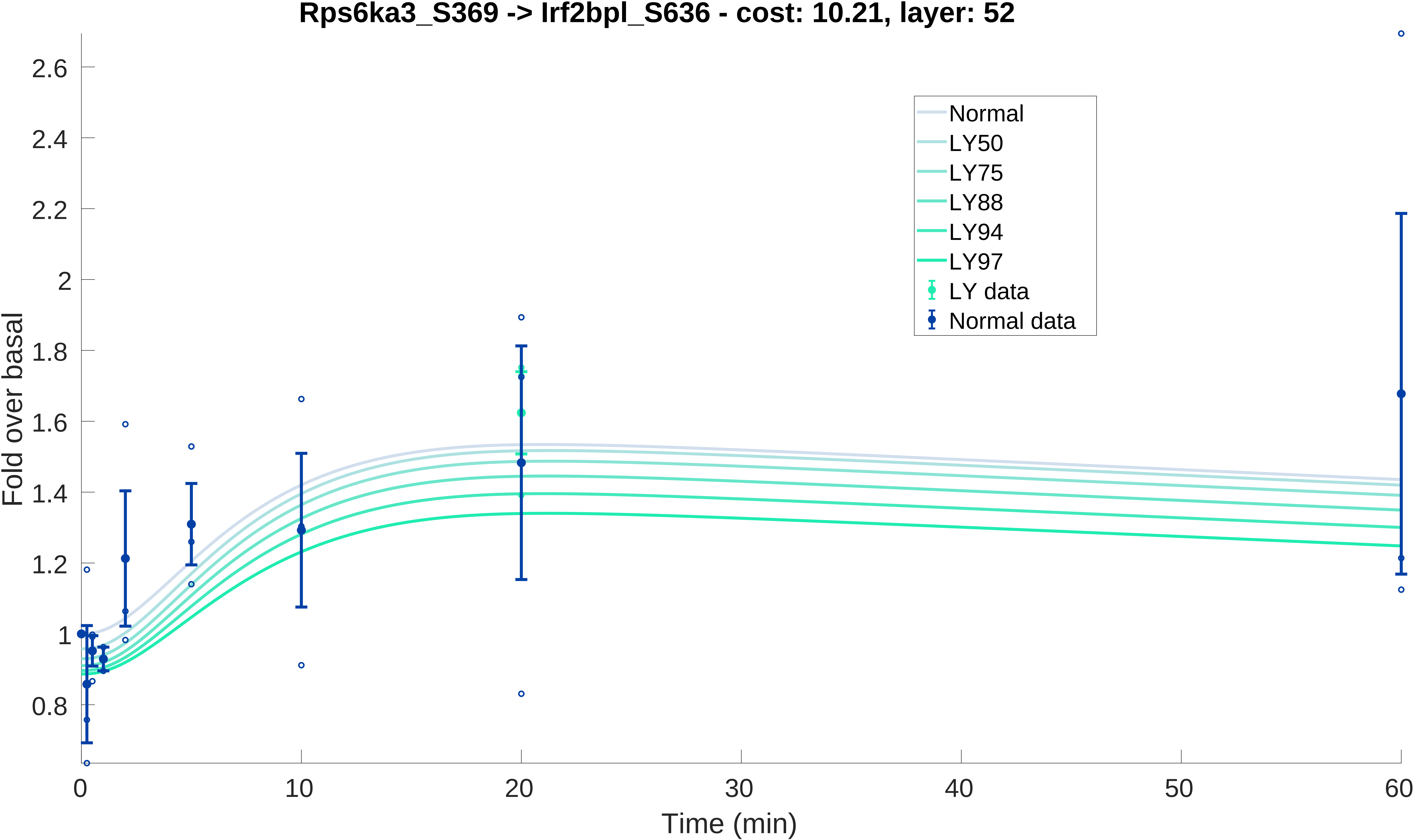

Rps6ka3_S369 -> Irf2bpl_S636 - cost: 10.21, layer: 52
Fold over basal
Time (min)
Normal
LY50
LY75
LY88
LY94
LY97
LY data
Normal data
0.8
1
1.2
1.4
1.6
1.8
2
2.2
2.4
2.6
0
10
20
30
40
50
60

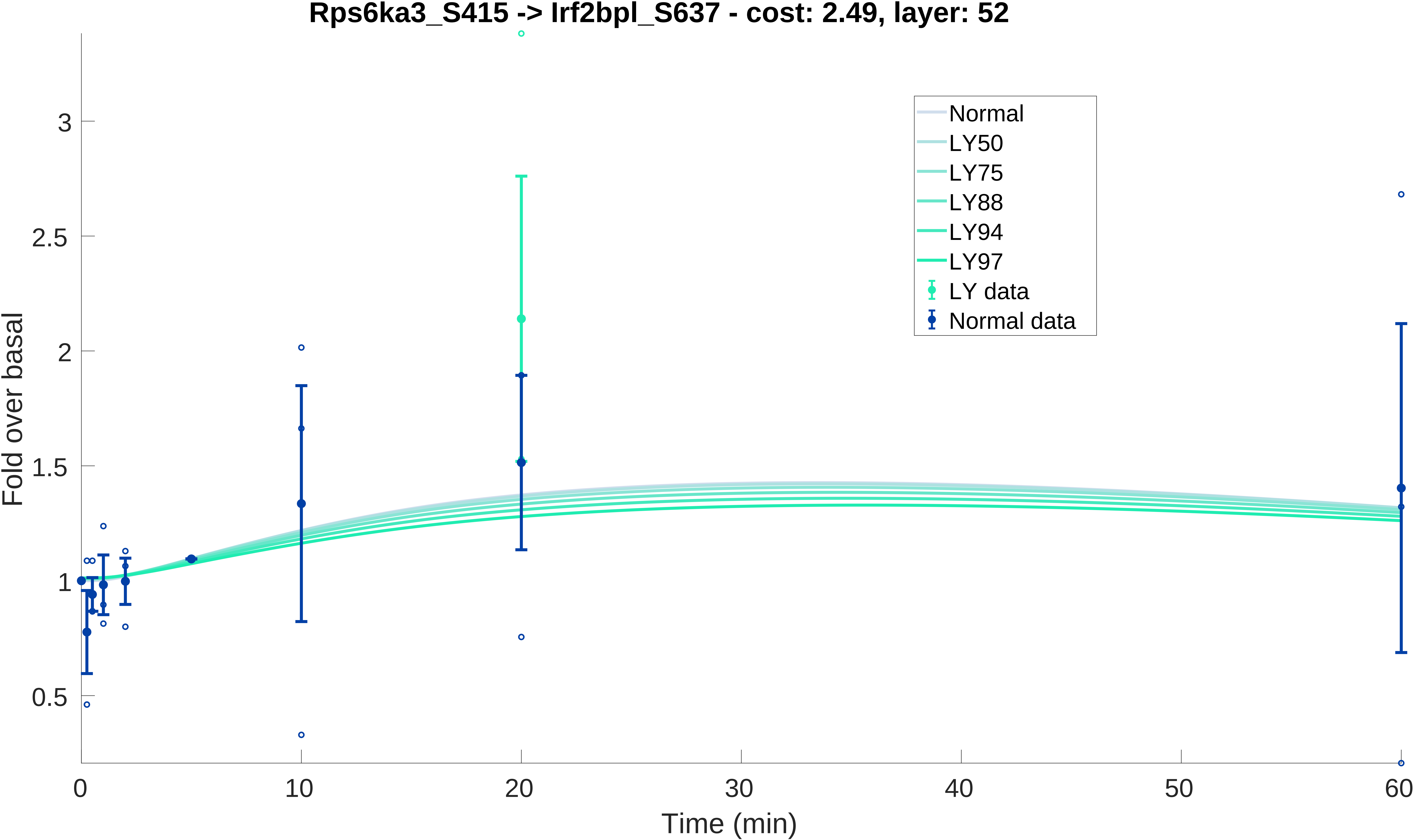

Rps6ka3_S415 -> Irf2bpl_S637 - cost: 2.49, layer: 52
Fold over basal
Time (min)
0
10
20
30
40
50
60
0.5
1
1.5
2
2.5
3
Normal
LY50
LY75
LY88
LY94
LY97
LY data
Normal data

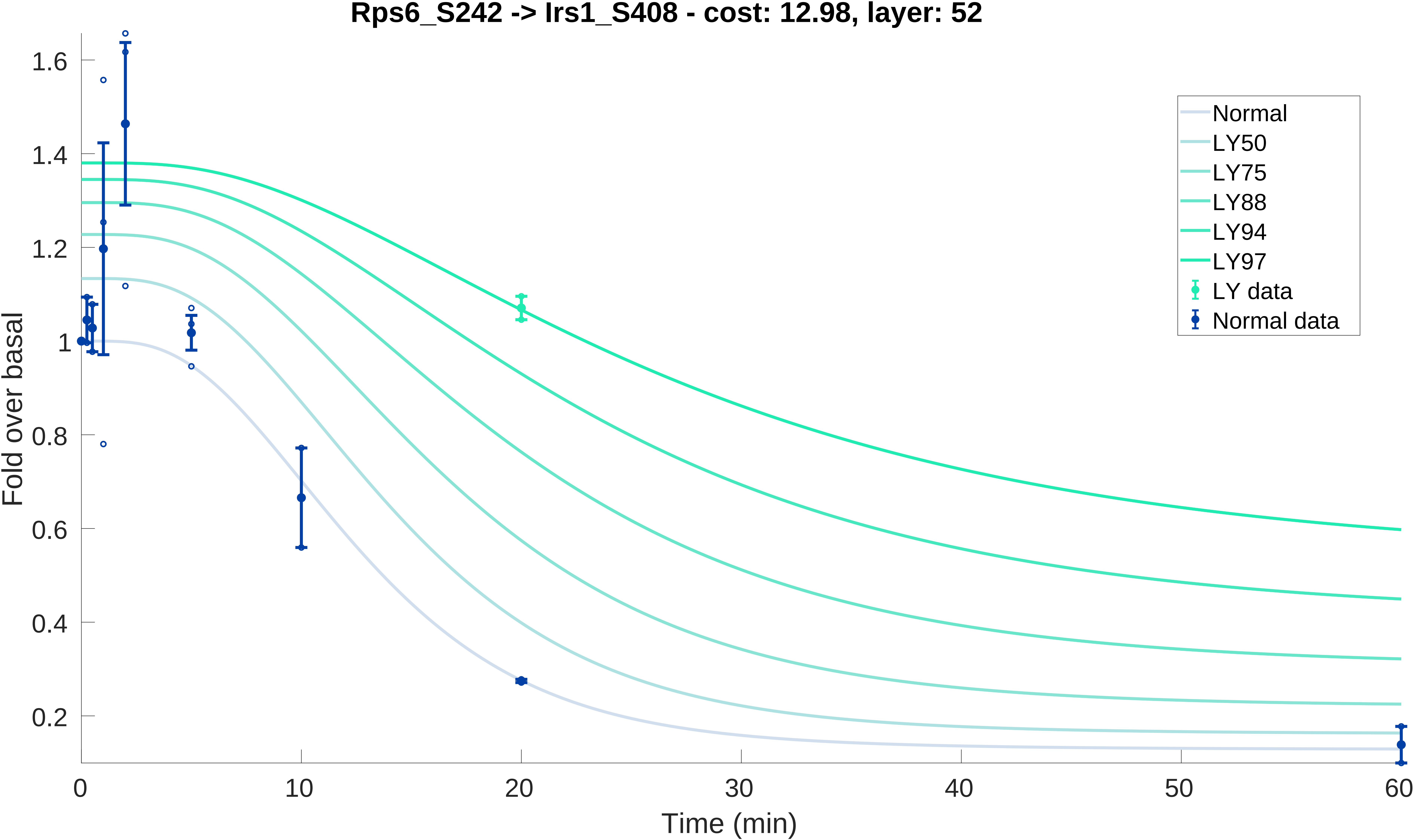
Rps6_S242 -> Irs1_S408 - cost: 12.98, layer: 52
Fold over basal
Time (min)
0
10
20
30
40
50
60
0.2
0.4
0.6
0.8
1
1.2
1.4
1.6
Normal
LY50
LY75
LY88
LY94
LY97
LY data
Normal data

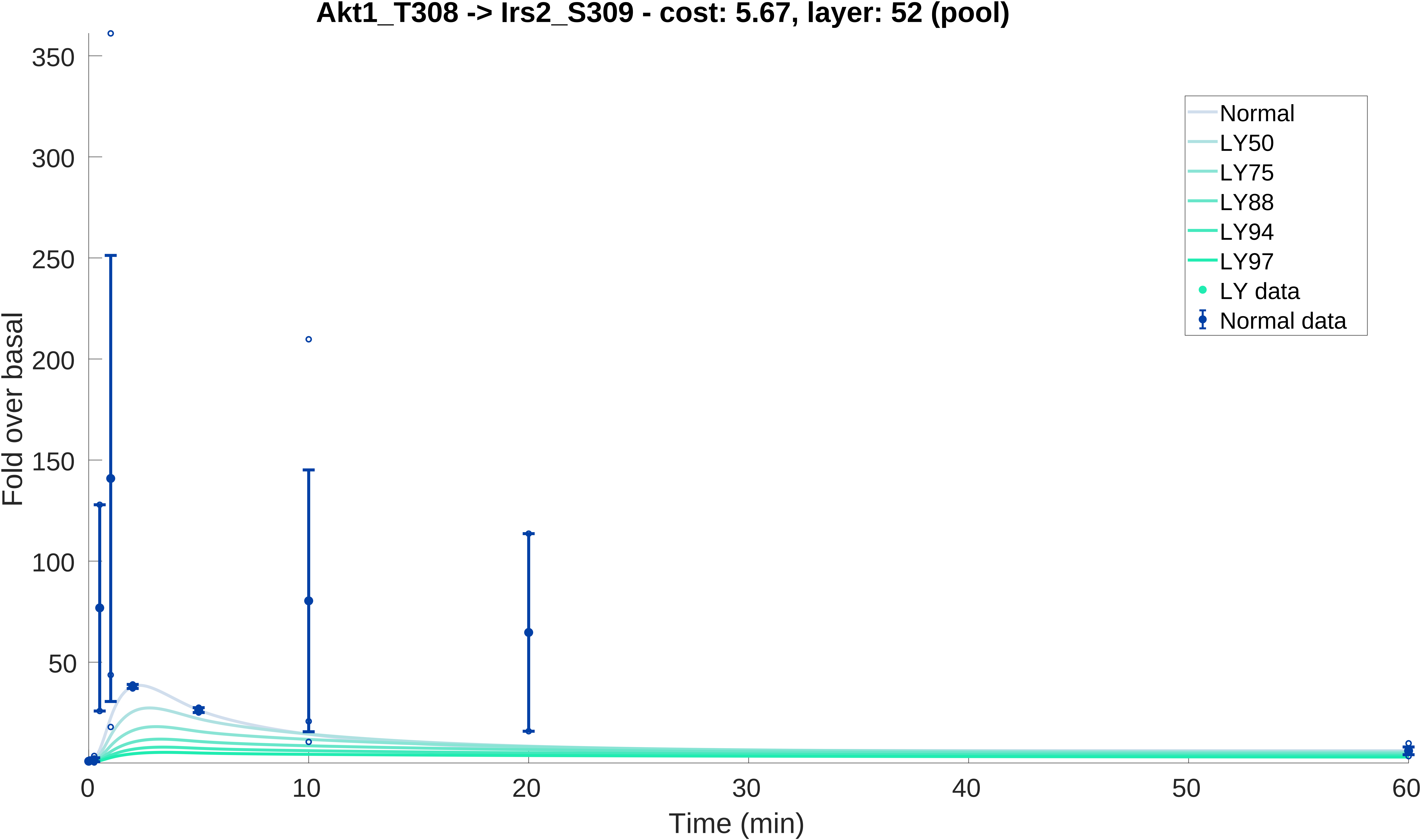

Akt1_T308 -> Irs2_S309 - cost: 5.67, layer: 52 (pool)
Fold over basal
350
300
250
200
150
100
50
0
10
20
30
40
50
60
Time (min)
Normal
LY50
LY75
LY88
LY94
LY97
LY data
Normal data

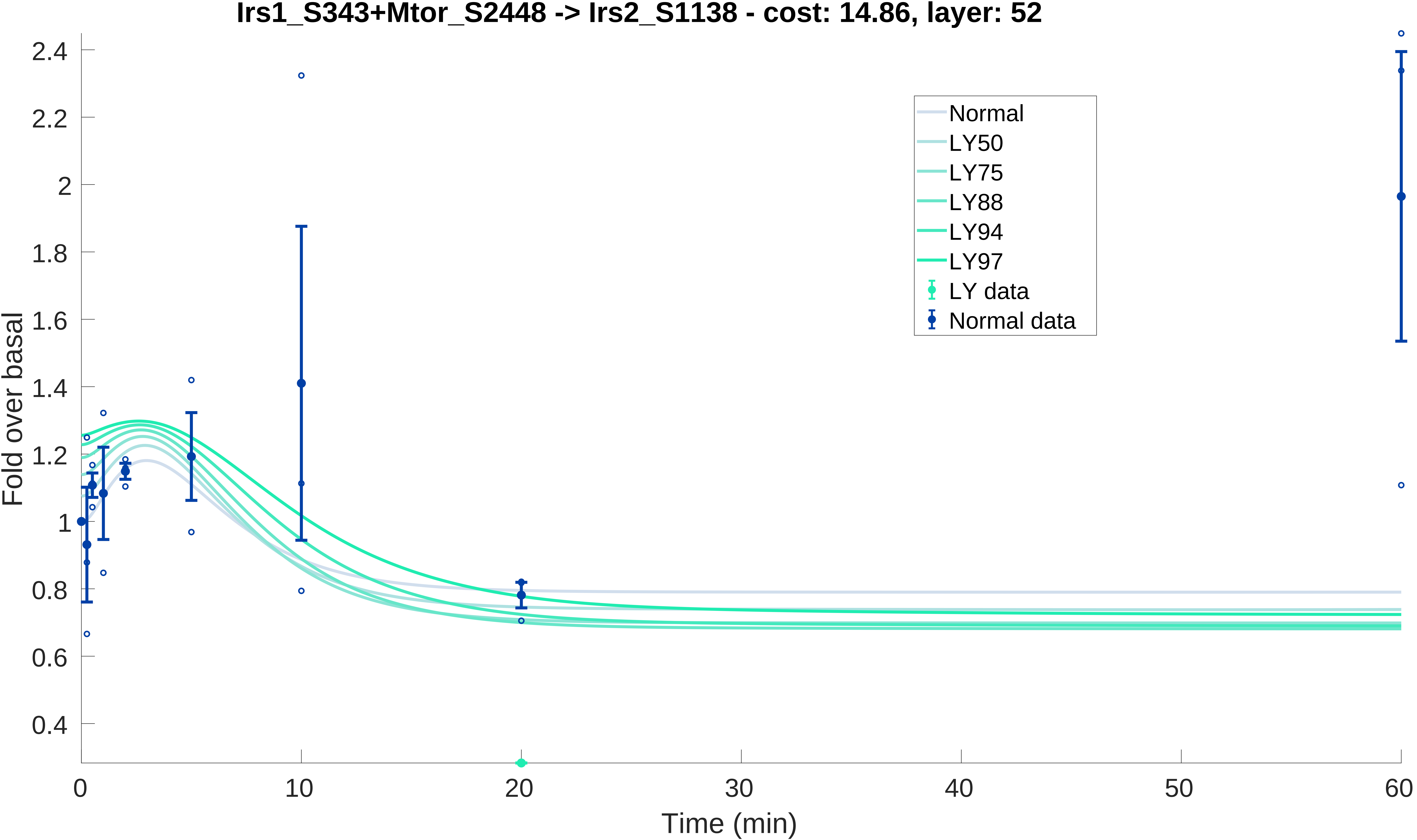
Irs1_S343+Mtor_S2448 -> Irs2_S1138 - cost: 14.86, layer: 52
Fold over basal
Time (min)
2.4
2.2
2
1.8
1.6
1.4
1.2
1
0.8
0.6
0.4
0
10
20
30
40
50
60
Normal
LY50
LY75
LY88
LY94
LY97
LY data
Normal data

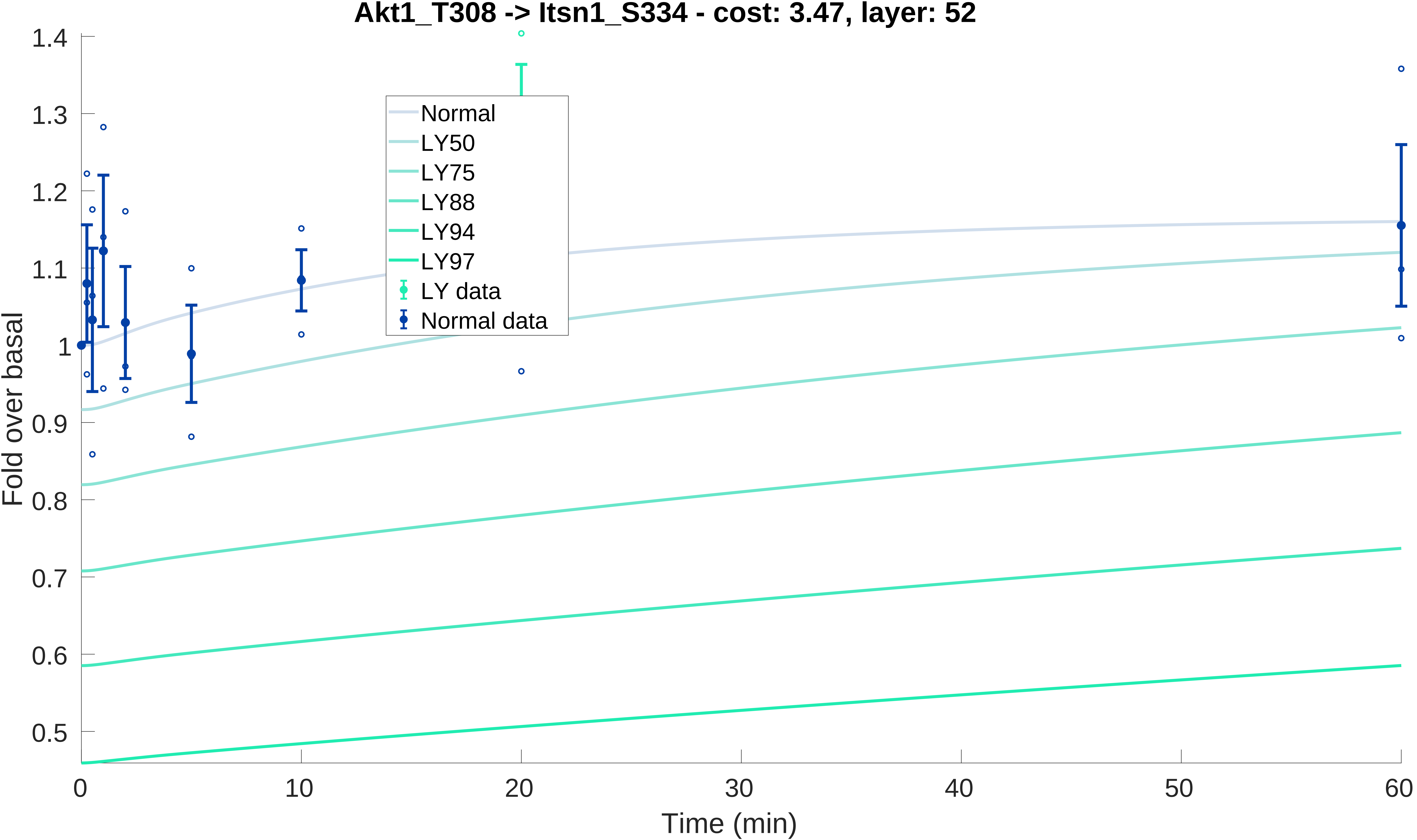

Akt1_T308 -> Itsn1_S334 - cost: 3.47, layer: 52
Normal
LY50
LY75
LY88
LY94
LY97
LY data
Normal data
Fold over basal
Time (min)
1.4
1.3
1.2
1.1
1
0.9
0.8
0.7
0.6
0.5
0
10
20
30
40
50
60

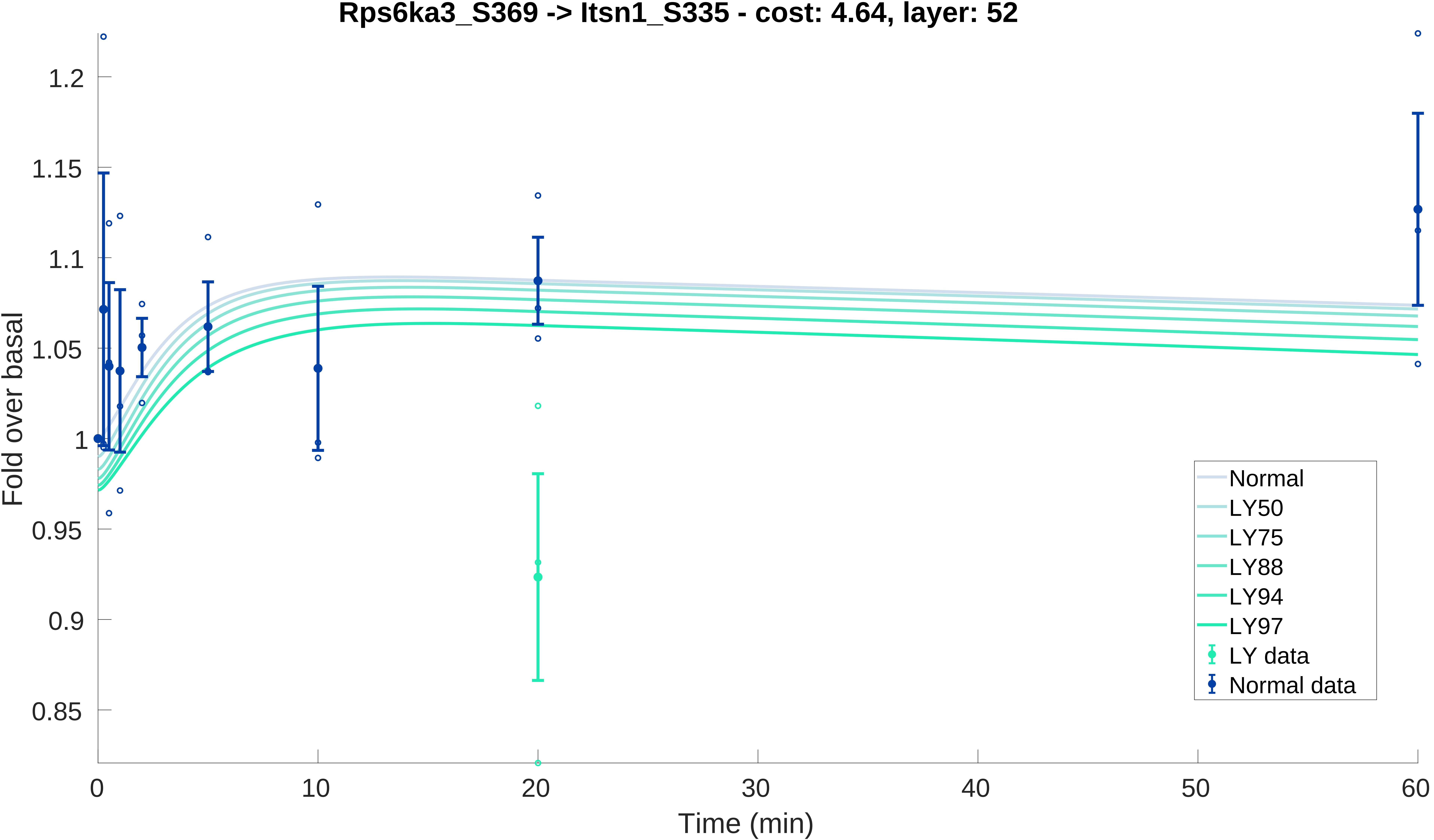
Rps6ka3_S369 -> Itsn1_S335 - cost: 4.64, layer: 52
Fold over basal
Time (min)
0
10
20
30
40
50
60
0.85
0.9
0.95
1
1.05
1.1
1.15
1.2
Normal
LY50
LY75
LY88
LY94
LY97
LY data
Normal data

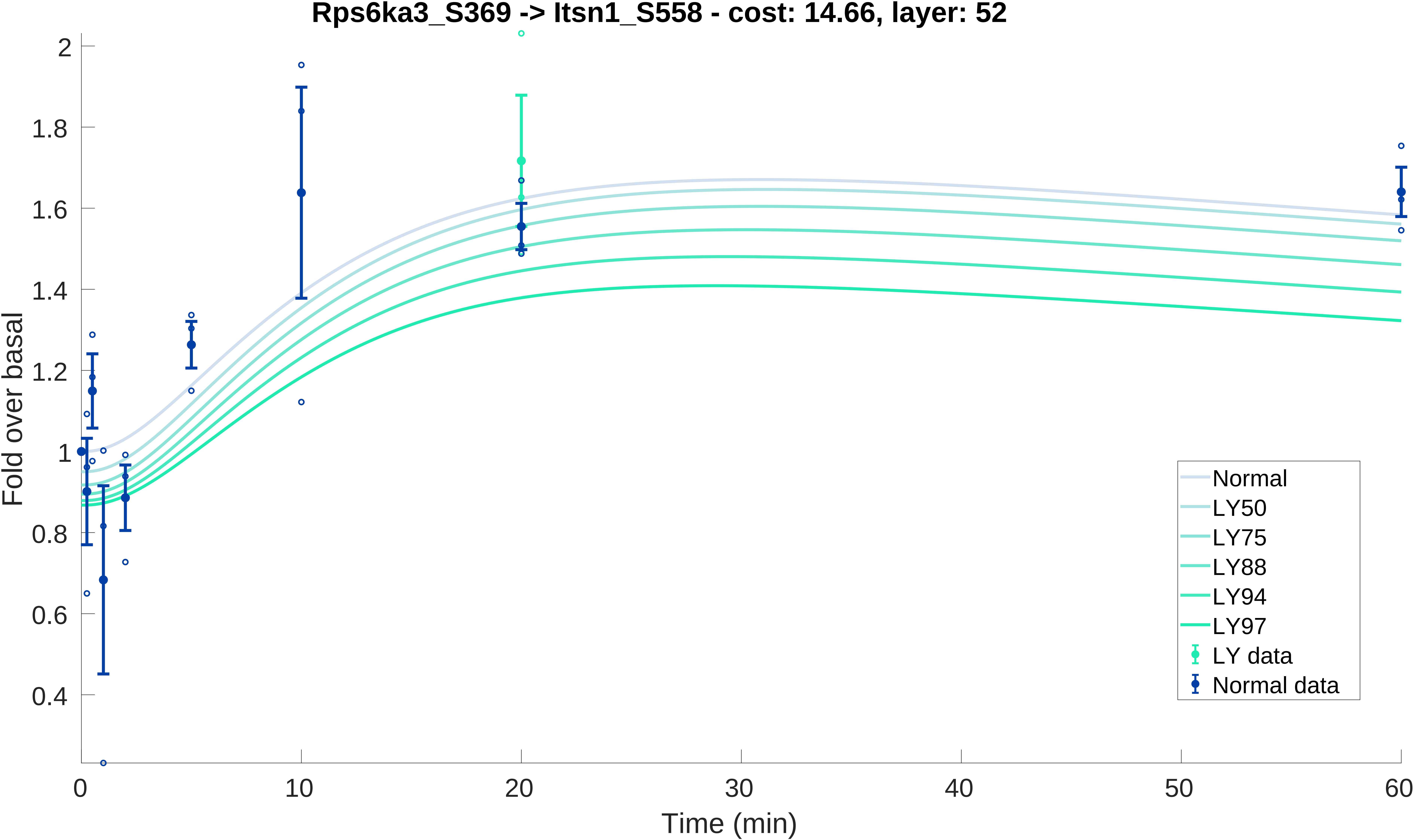

Rps6ka3_S369 -> Itsn1_S558 - cost: 14.66, layer: 52
Fold over basal
Time (min)
0.4
0.6
0.8
1
1.2
1.4
1.6
1.8
2
0
10
20
30
40
50
60
Normal
LY50
LY75
LY88
LY94
LY97
LY data
Normal data

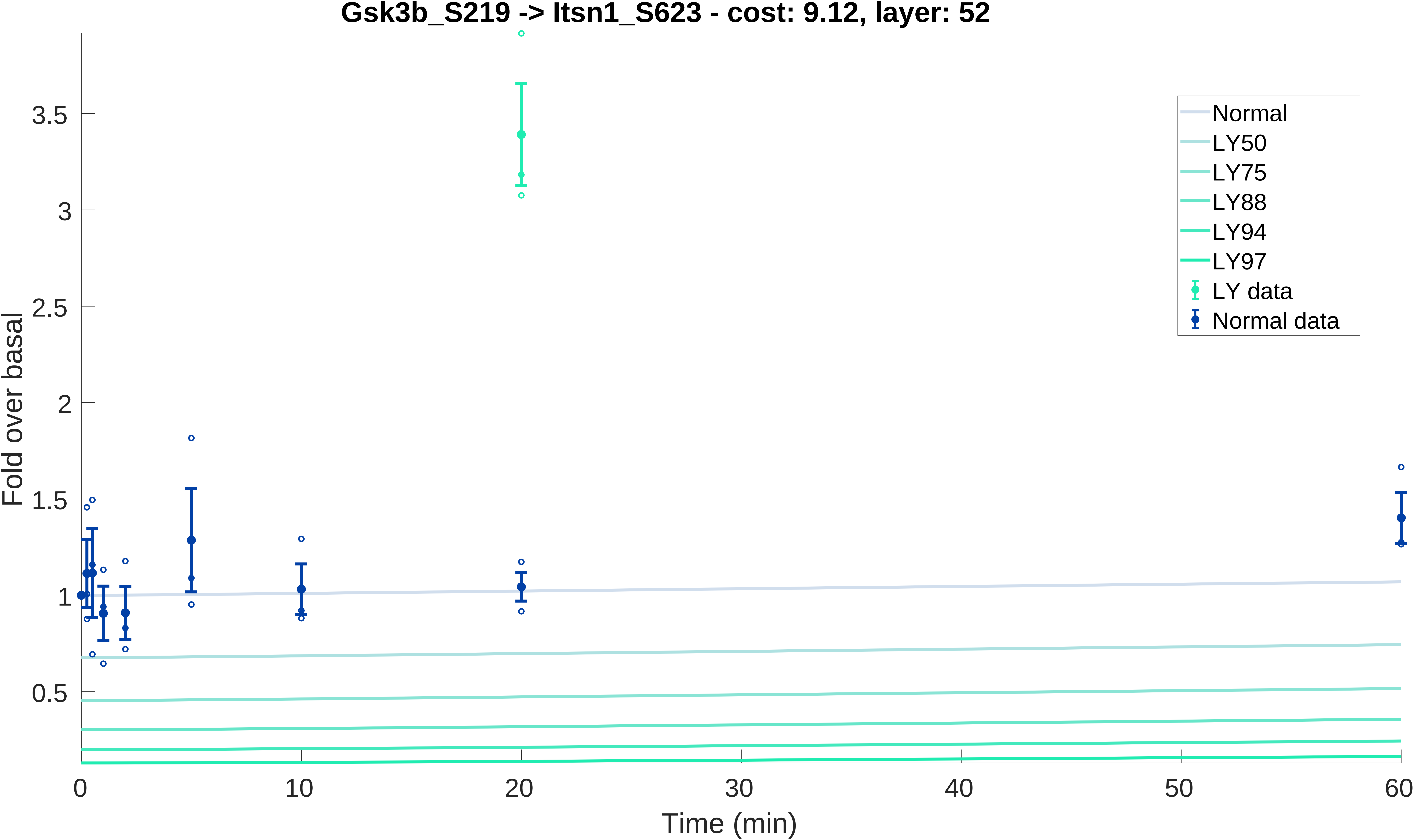
Gsk3b_S219 -> Itsn1_S623 - cost: 9.12, layer: 52
Fold over basal
Time (min)
0
10
20
30
40
50
60
0.5
1
1.5
2
2.5
3
3.5
Normal
LY50
LY75
LY88
LY94
LY97
LY data
Normal data

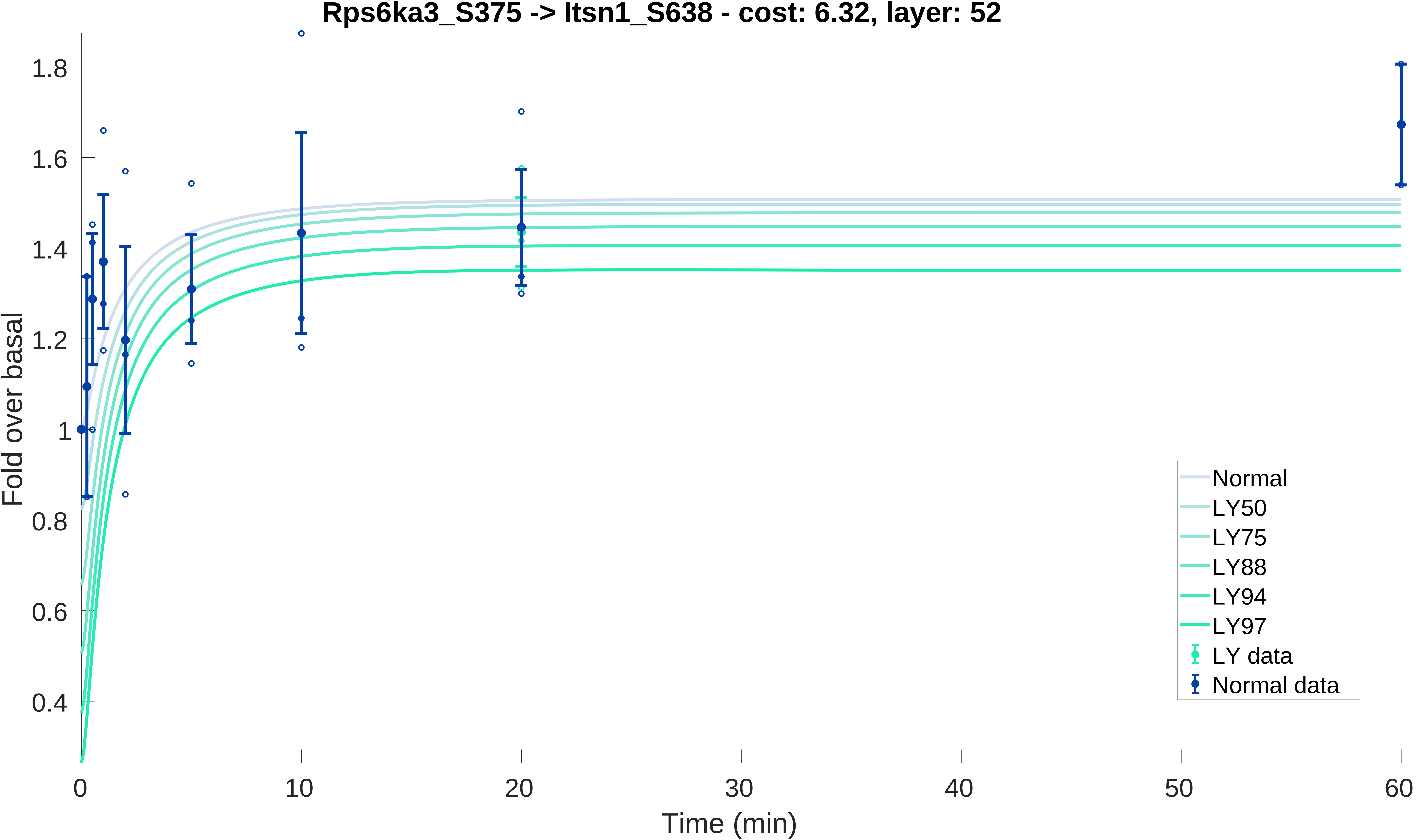

Rps6ka3_S375 -> Itsn1_S638 - cost: 6.32, layer: 52
Fold over basal
Time (min)
0.4
0.6
0.8
1
1.2
1.4
1.6
1.8
0
10
20
30
40
50
60
Normal
LY50
LY75
LY88
LY94
LY97
LY data
Normal data

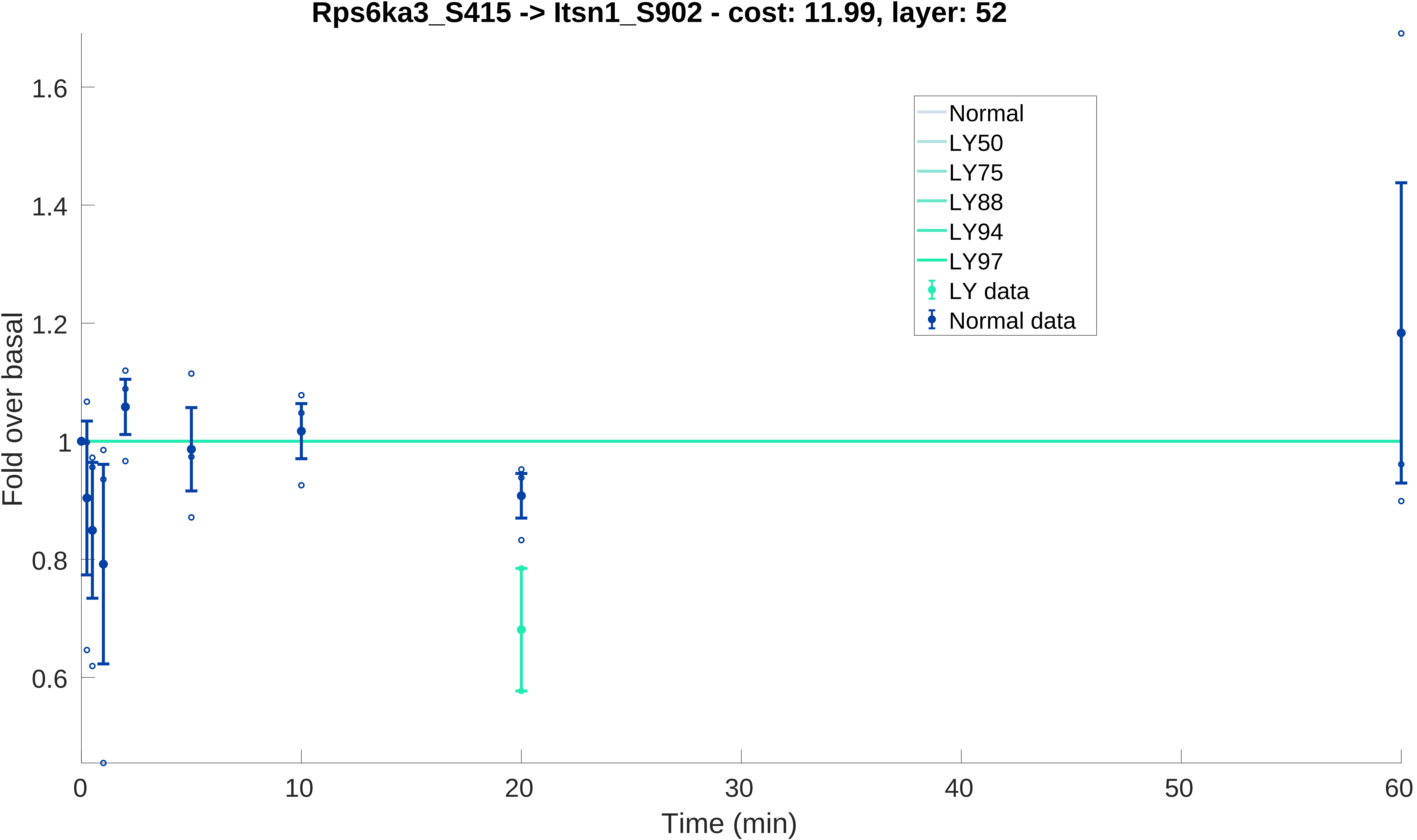

Rps6ka3_S415 -> Itsn1_S902 - cost: 11.99, layer: 52
Fold over basal
Time (min)
0.6
0.8
1
1.2
1.4
1.6
0
10
20
30
40
50
60
Normal
LY50
LY75
LY88
LY94
LY97
LY data
Normal data

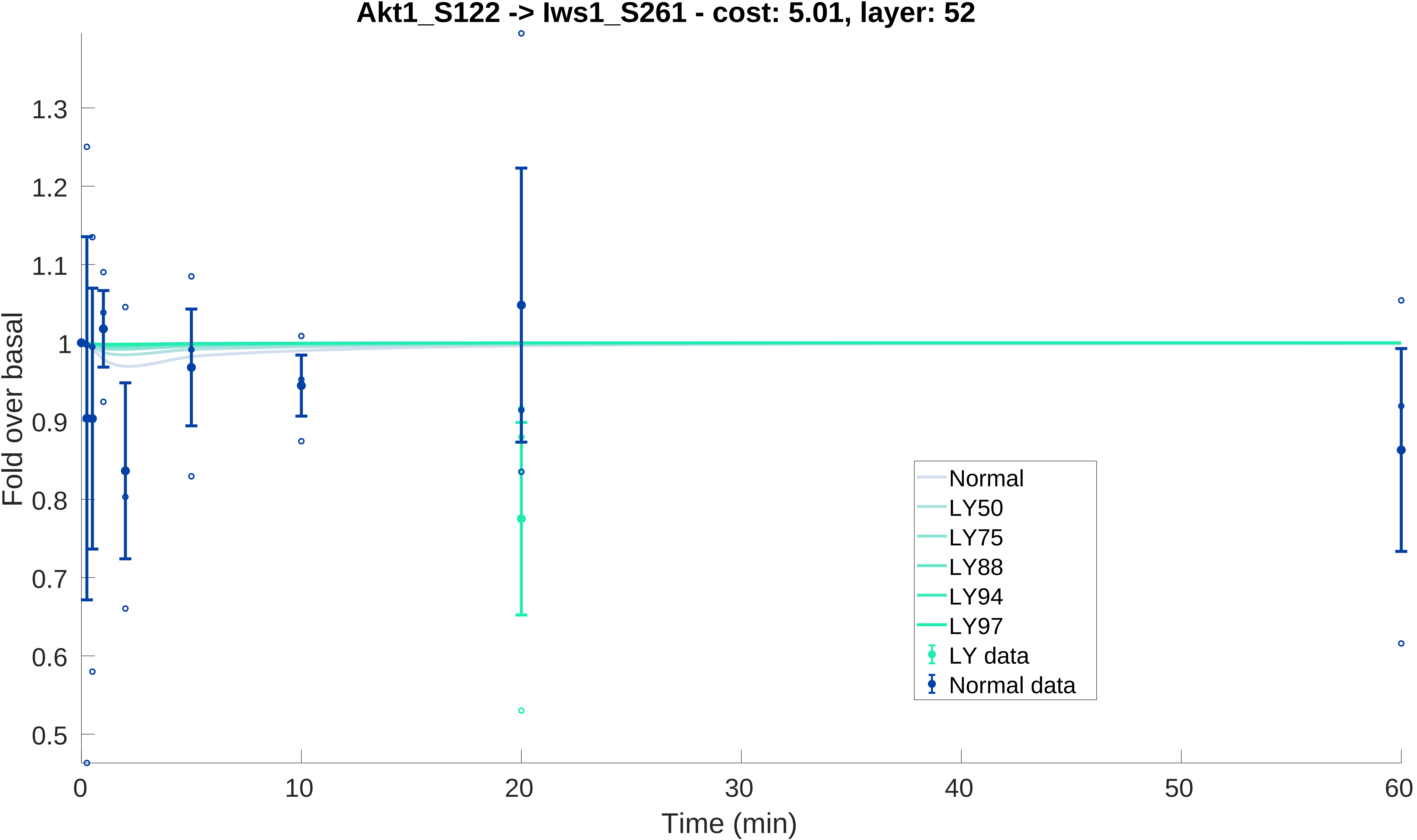
Akt1_S122 -> Iws1_S261 - cost: 5.01, layer: 52
Fold over basal
1.3
1.2
1.1
1
0.9
0.8
0.7
0.6
0.5
0
10
20
30
40
50
60
Time (min)
Normal
LY50
LY75
LY88
LY94
LY97
LY data
Normal data

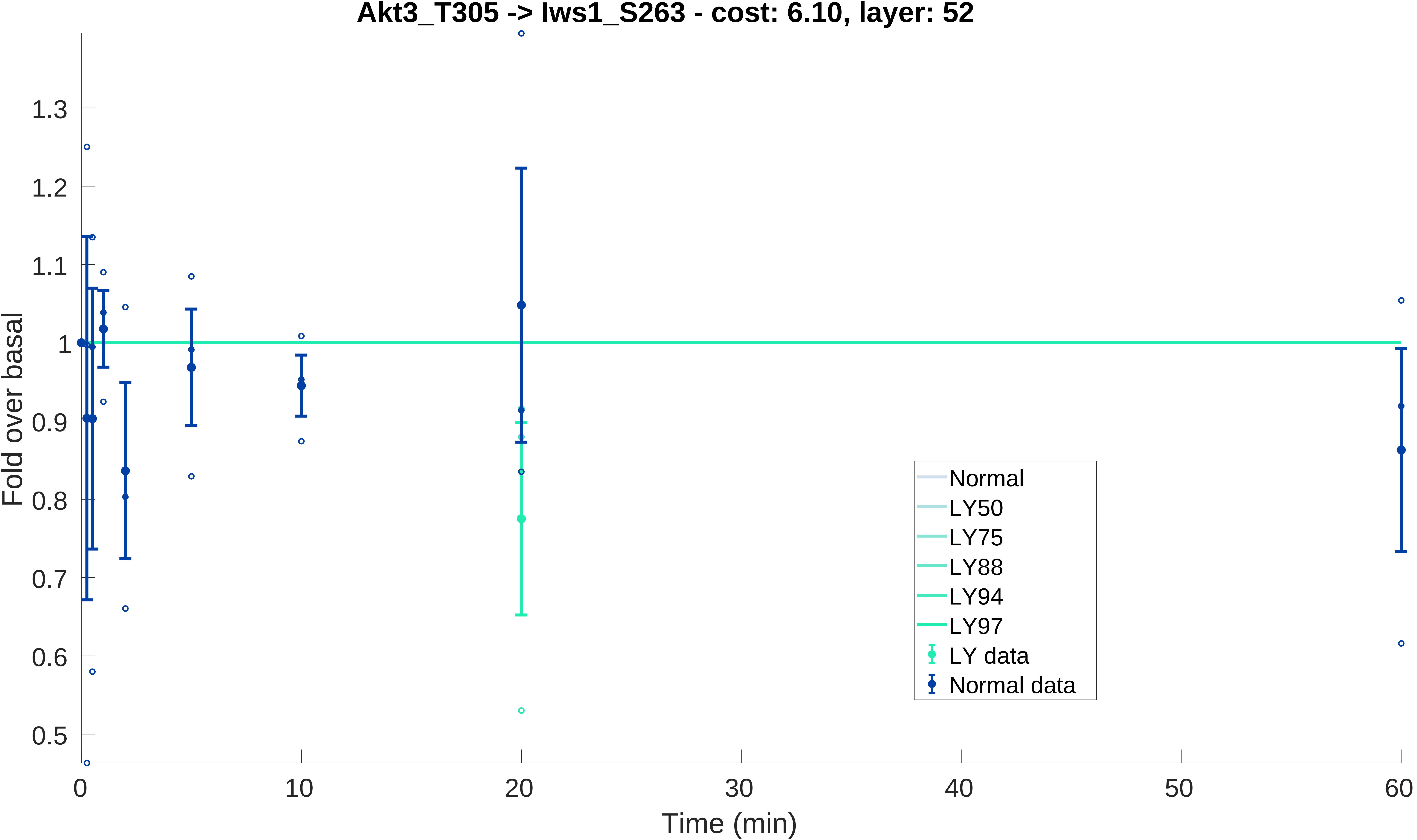

Akt3_T305 -> Iws1_S263 - cost: 6.10, layer: 52
Fold over basal
Time (min)
0.5
0.6
0.7
0.8
0.9
1
1.1
1.2
1.3
0
10
20
30
40
50
60
Normal
LY50
LY75
LY88
LY94
LY97
LY data
Normal data

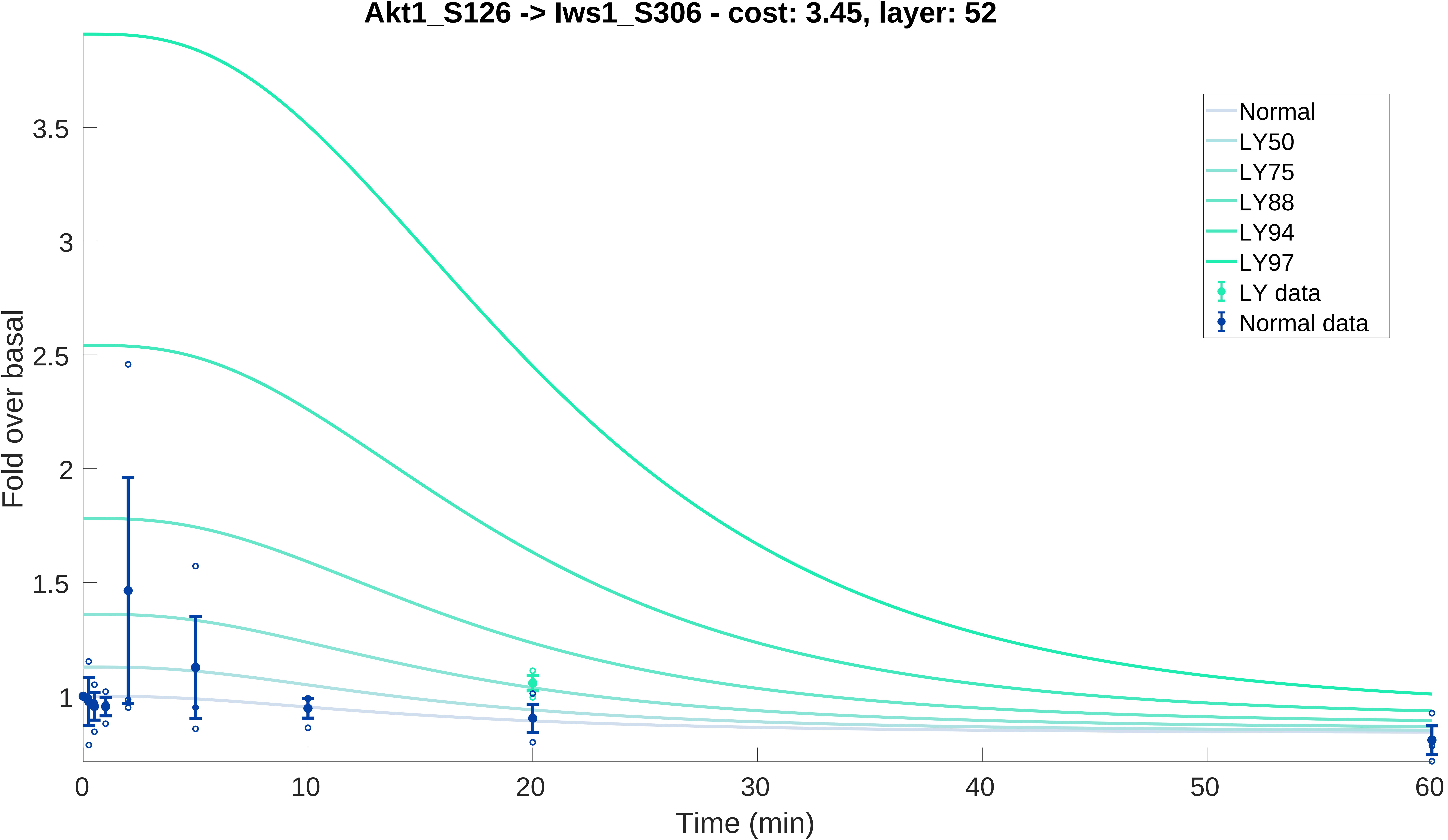

Akt1_S126 -> Iws1_S306 - cost: 3.45, layer: 52
Fold over basal
Time (min)
0
10
20
30
40
50
60
1
1.5
2
2.5
3
3.5
Normal
LY50
LY75
LY88
LY94
LY97
LY data
Normal data

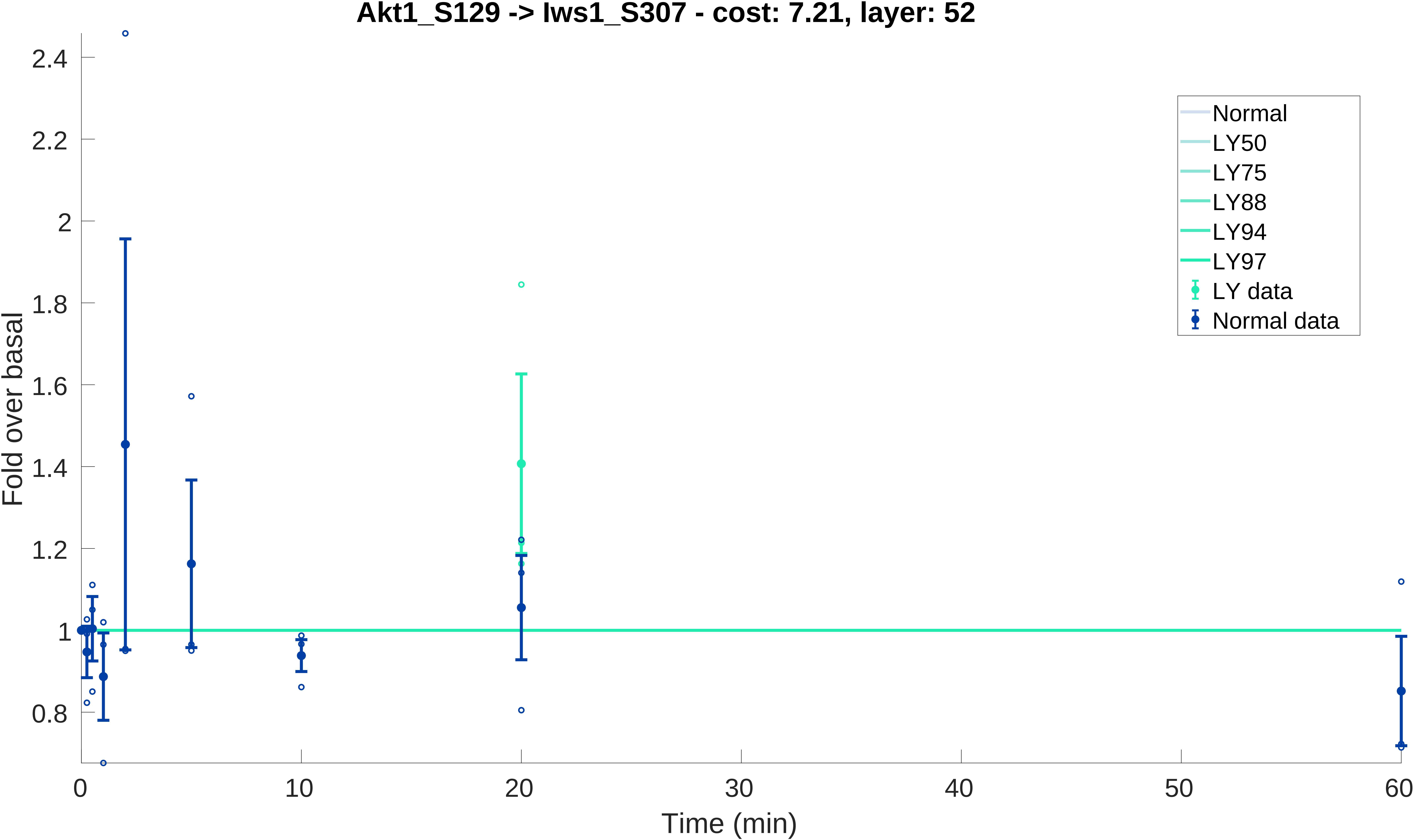
Akt1_S129 -> Iws1_S307 - cost: 7.21, layer: 52
Fold over basal
Time (min)
2.4
2.2
2
1.8
1.6
1.4
1.2
1
0.8
0
10
20
30
40
50
60
Normal
LY50
LY75
LY88
LY94
LY97
LY data
Normal data

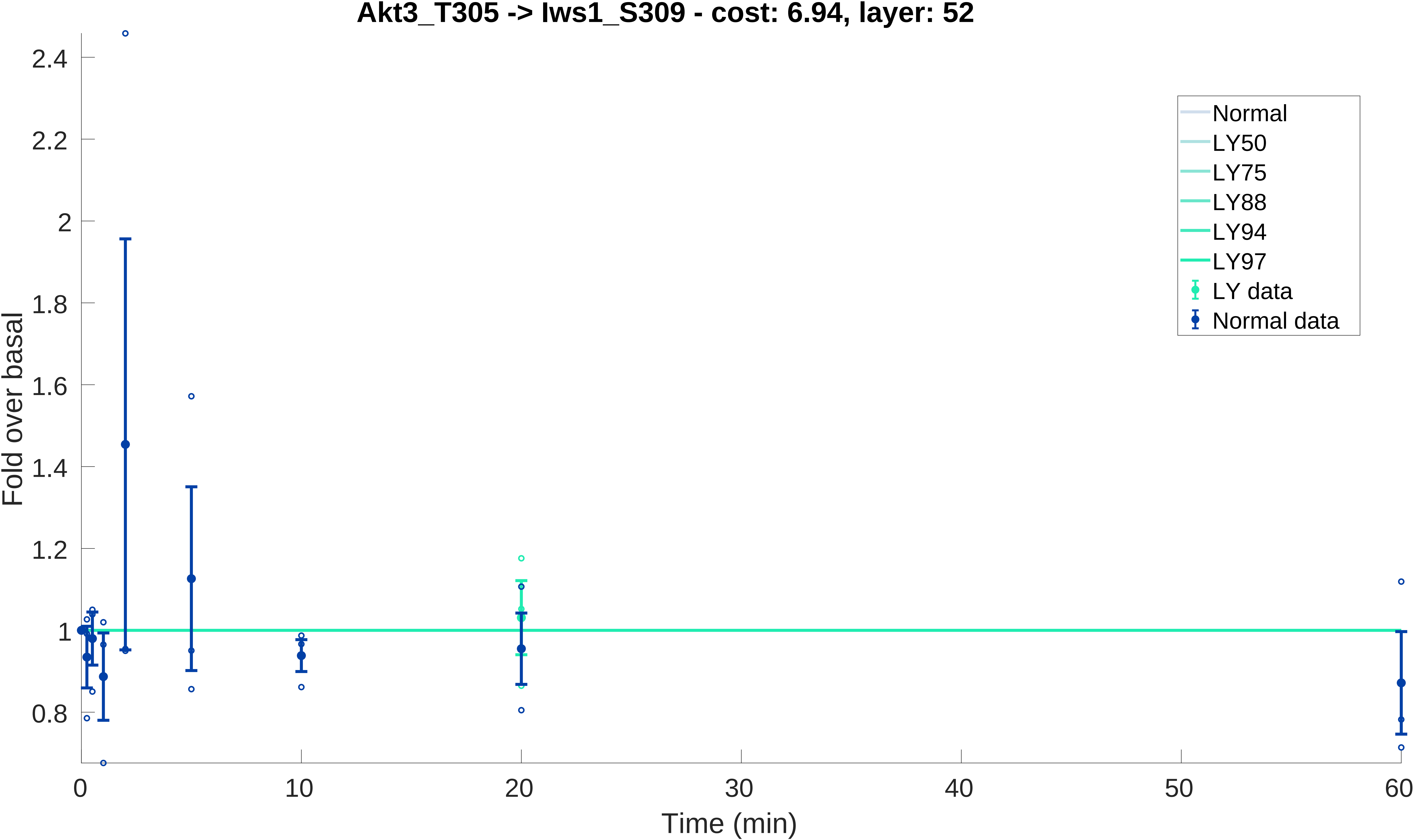

Akt3_T305 -> Iws1_S309 - cost: 6.94, layer: 52
Fold over basal
2.4
2.2
2
1.8
1.6
1.4
1.2
1
0.8
0
10
20
30
40
50
60
Time (min)
Normal
LY50
LY75
LY88
LY94
LY97
LY data
Normal data

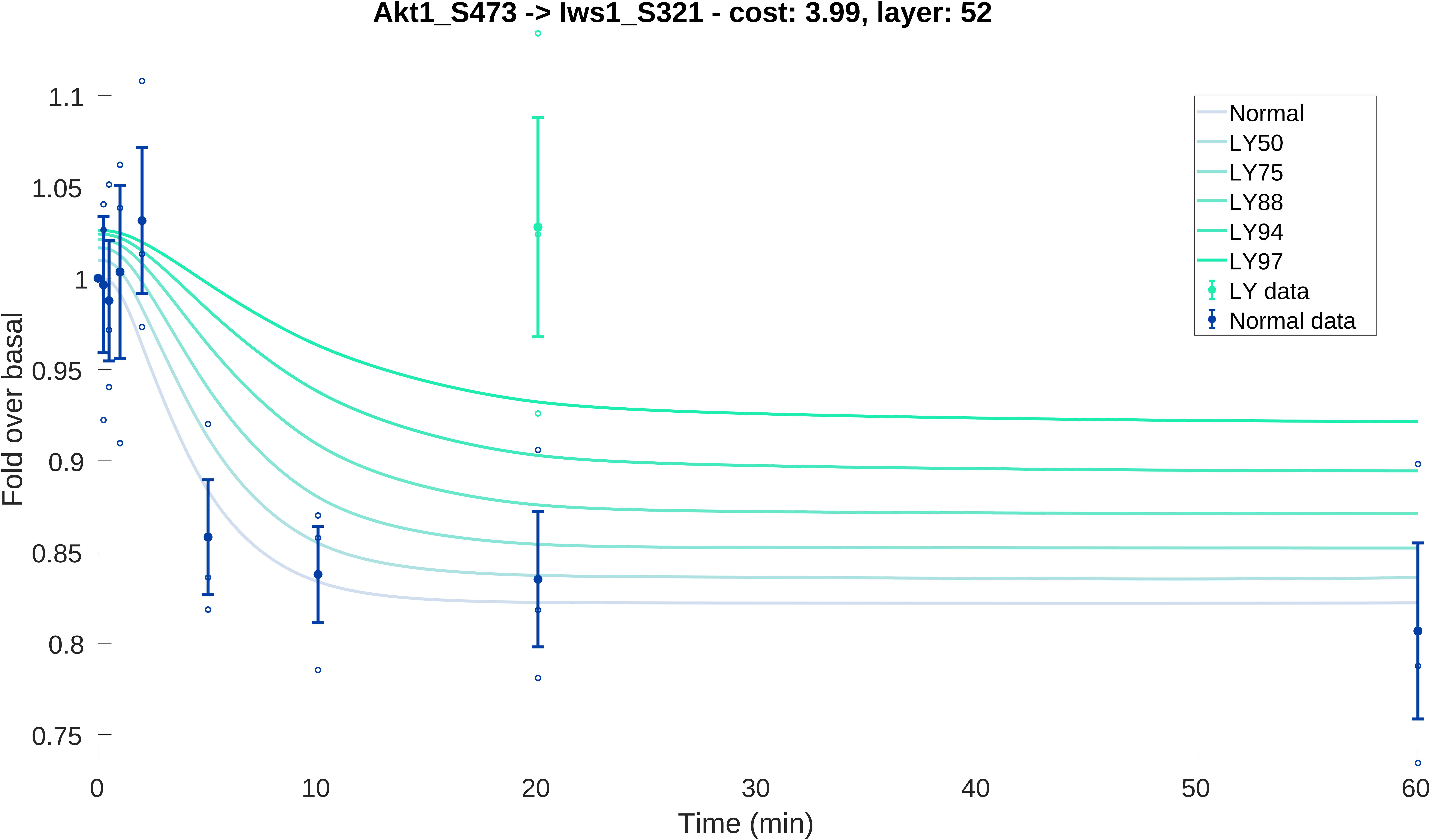
Akt1_S473 -> Iws1_S321 - cost: 3.99, layer: 52
Fold over basal
Time (min)
1.1
1.05
1
0.95
0.9
0.85
0.8
0.75
0
10
20
30
40
50
60
Normal
LY50
LY75
LY88
LY94
LY97
LY data
Normal data

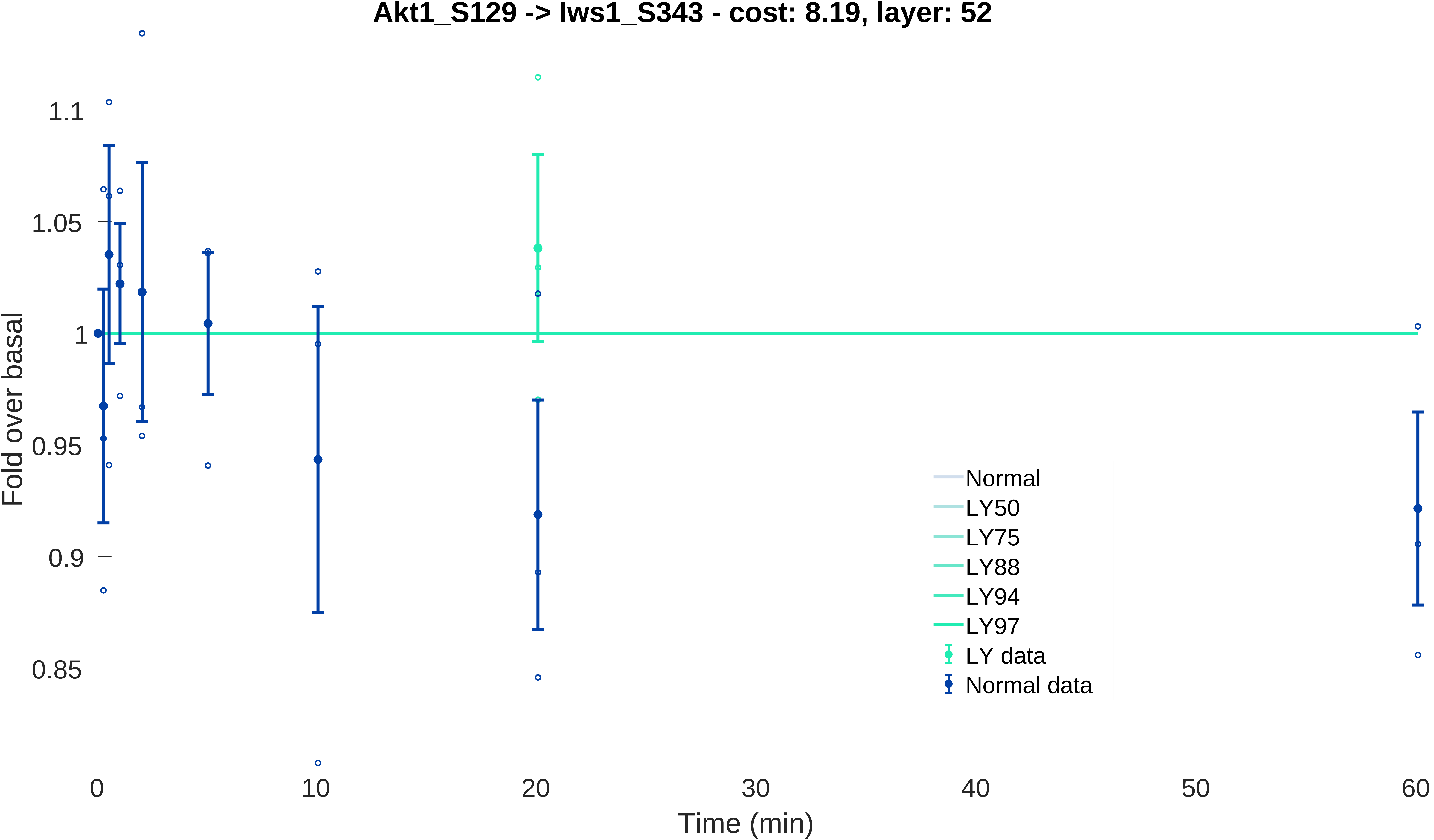

Akt1_S129 -> Iws1_S343 - cost: 8.19, layer: 52
Fold over basal
1.1
1.05
1
0.95
0.9
0.85
0
10
20
30
40
50
60
Time (min)
Normal
LY50
LY75
LY88
LY94
LY97
LY data
Normal data

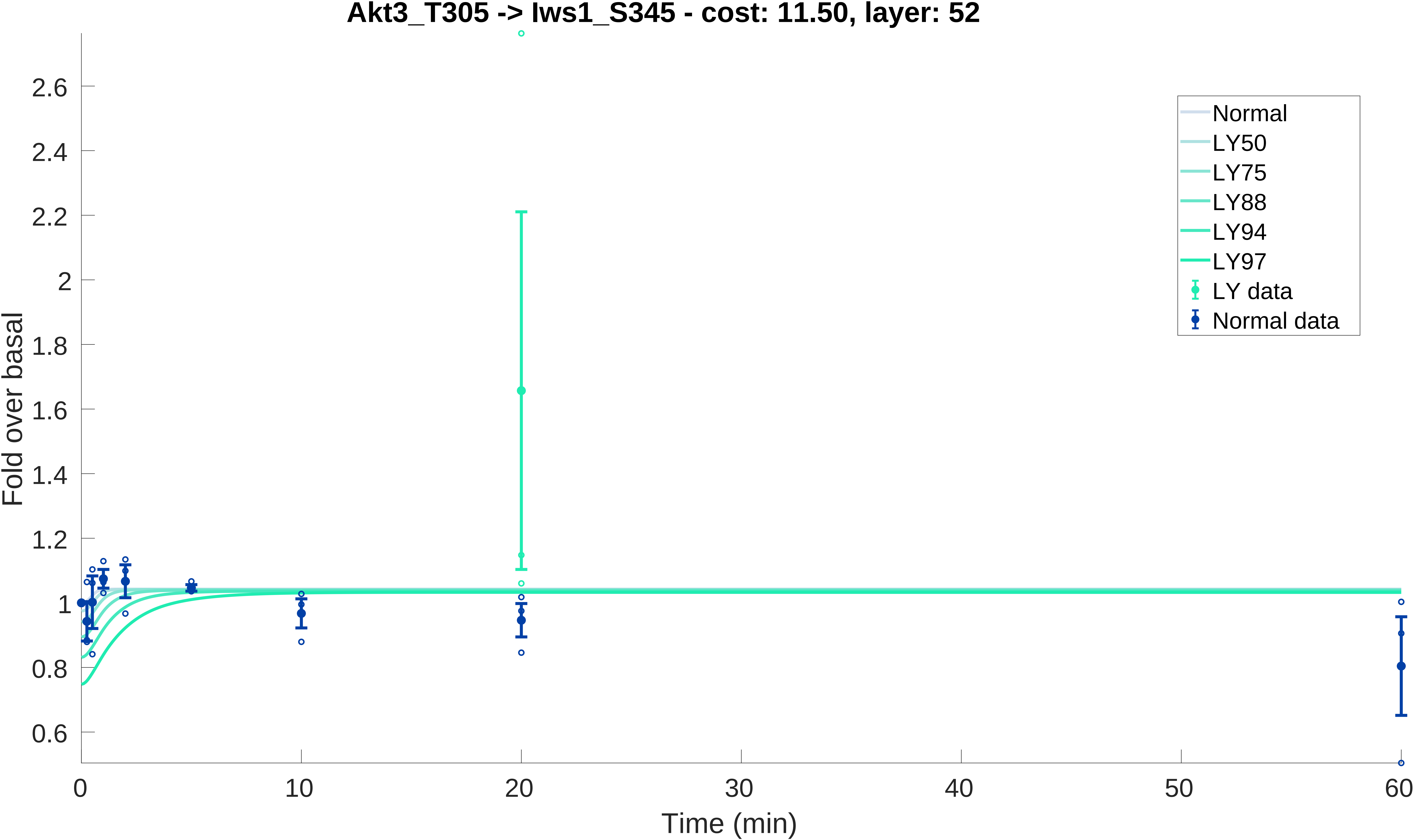

Akt3_T305 -> Iws1_S345 - cost: 11.50, layer: 52
Fold over basal
Time (min)
0
10
20
30
40
50
60
0.6
0.8
1
1.2
1.4
1.6
1.8
2
2.2
2.4
2.6
Normal
LY50
LY75
LY88
LY94
LY97
LY data
Normal data

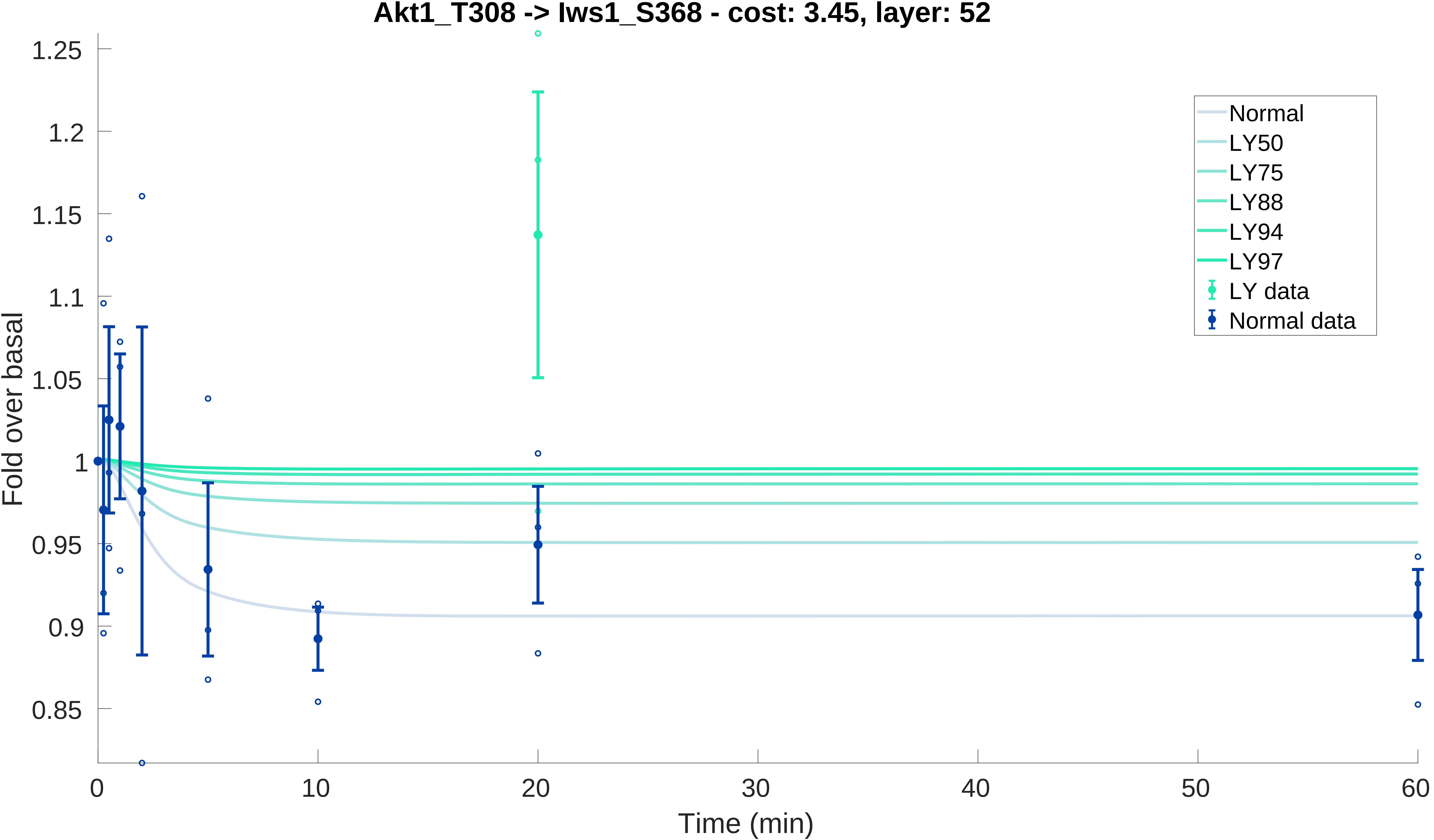
Akt1_T308 -> Iws1_S368 - cost: 3.45, layer: 52
Fold over basal
Time (min)
1.25
1.2
1.15
1.1
1.05
1
0.95
0.9
0.85
0
10
20
30
40
50
60
Normal
LY50
LY75
LY88
LY94
LY97
LY data
Normal data

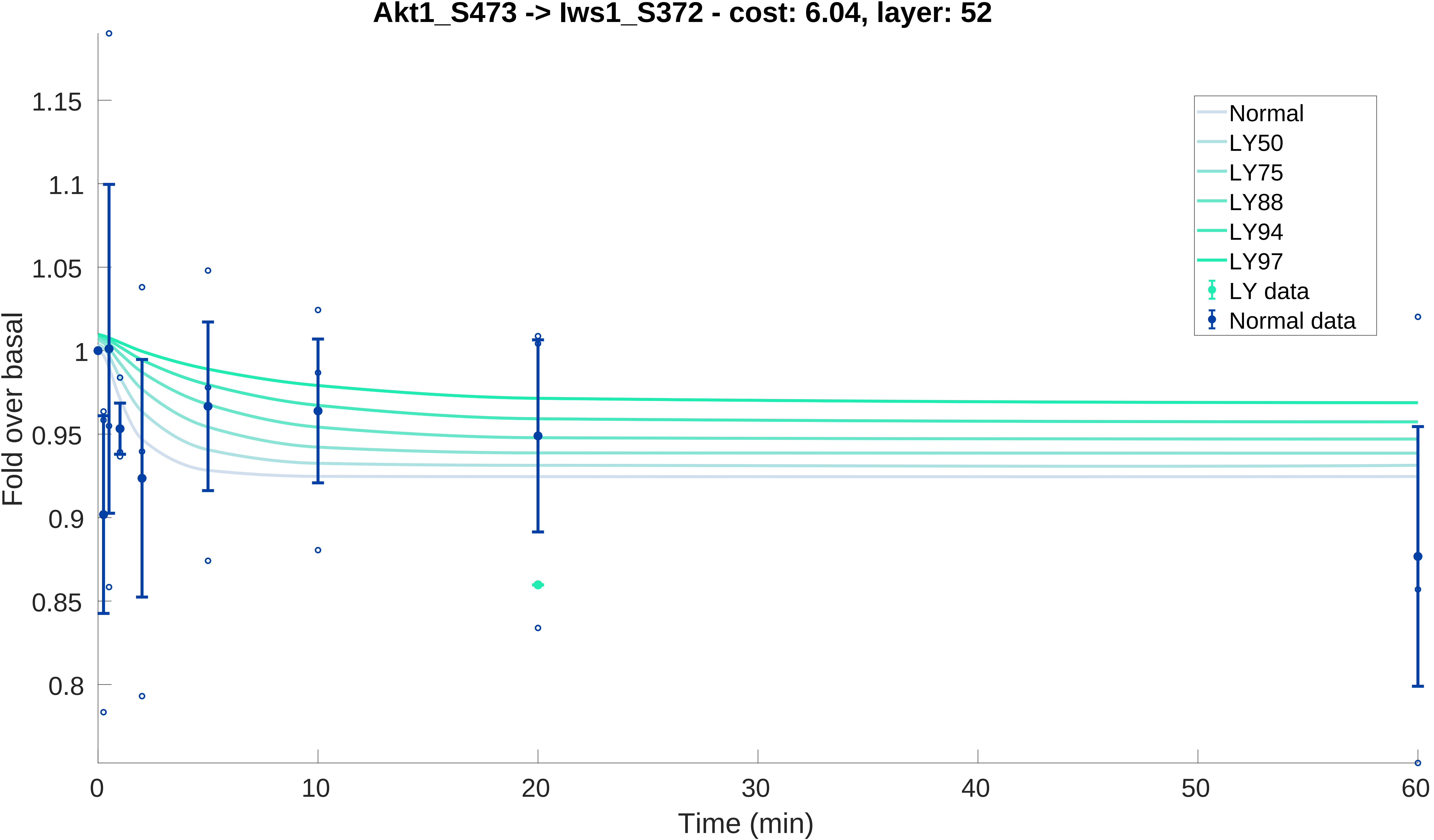

Akt1_S473 -> Iws1_S372 - cost: 6.04, layer: 52
Fold over basal
Time (min)
1.15
1.1
1.05
1
0.95
0.9
0.85
0.8
0
10
20
30
40
50
60
Normal
LY50
LY75
LY88
LY94
LY97
LY data
Normal data

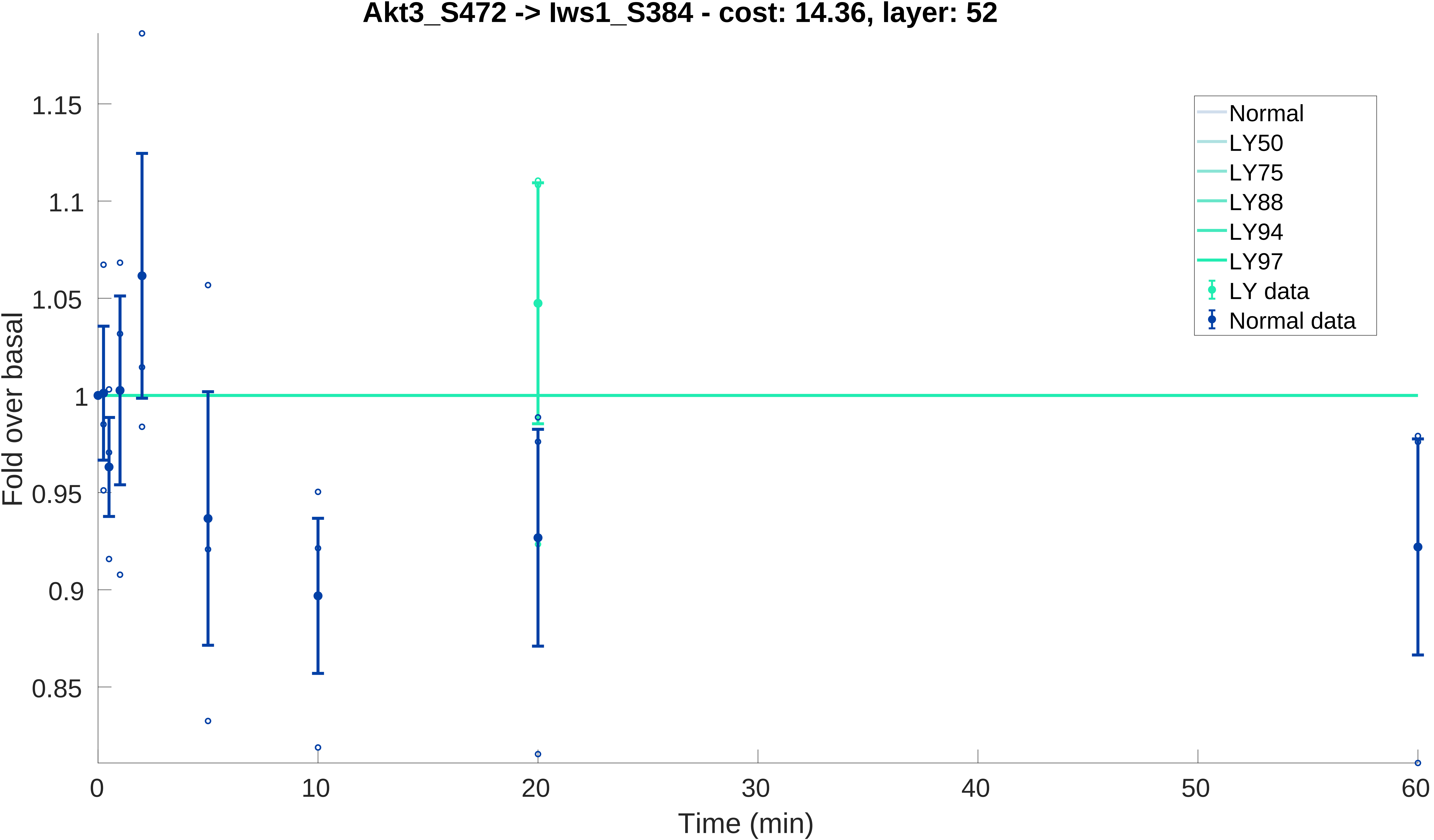
Akt3_S472 -> Iws1_S384 - cost: 14.36, layer: 52
Fold over basal
Time (min)
1.15
1.1
1.05
1
0.95
0.9
0.85
0
10
20
30
40
50
60
Normal
LY50
LY75
LY88
LY94
LY97
LY data
Normal data

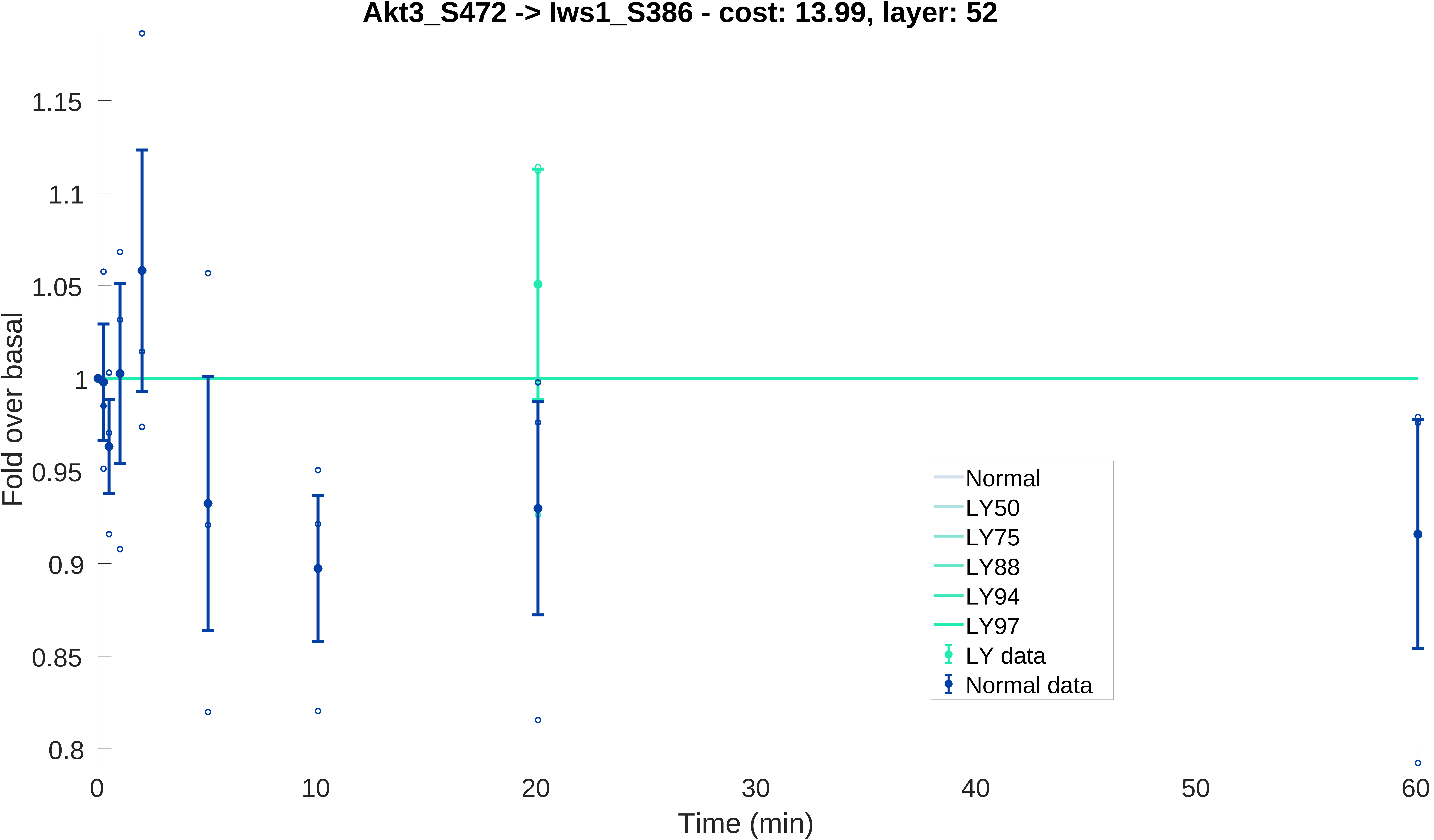

Akt3_S472 -> Iws1_S386 - cost: 13.99, layer: 52
Fold over basal
Time (min)
1.15
1.1
1.05
1
0.95
0.9
0.85
0.8
0
10
20
30
40
50
60
Normal
LY50
LY75
LY88
LY94
LY97
LY data
Normal data

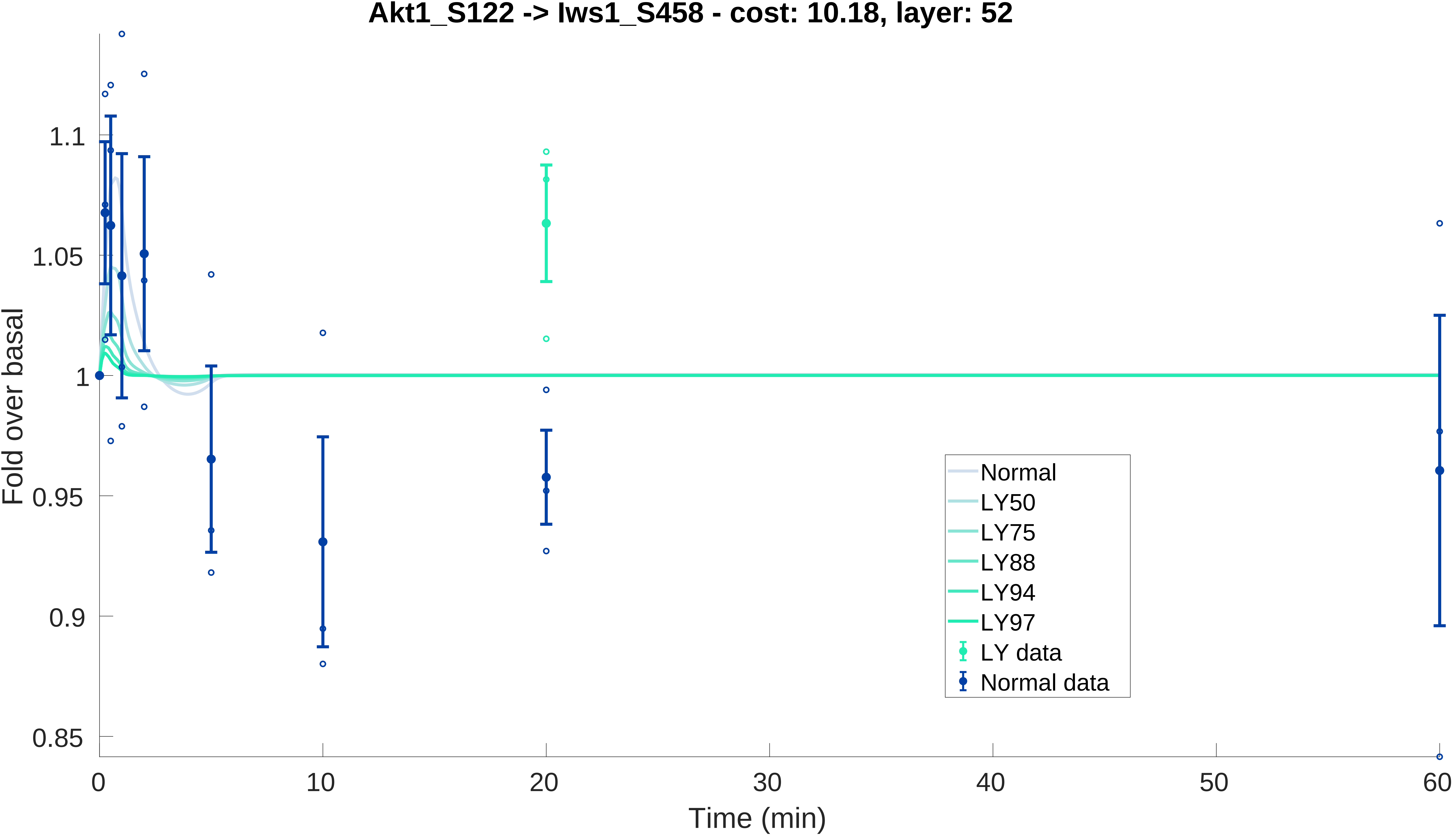

Akt1_S122 -> Iws1_S458 - cost: 10.18, layer: 52
Fold over basal
1.1
1.05
1
0.95
0.9
0.85
0
10
20
30
40
50
60
Time (min)
Normal
LY50
LY75
LY88
LY94
LY97
LY data
Normal data

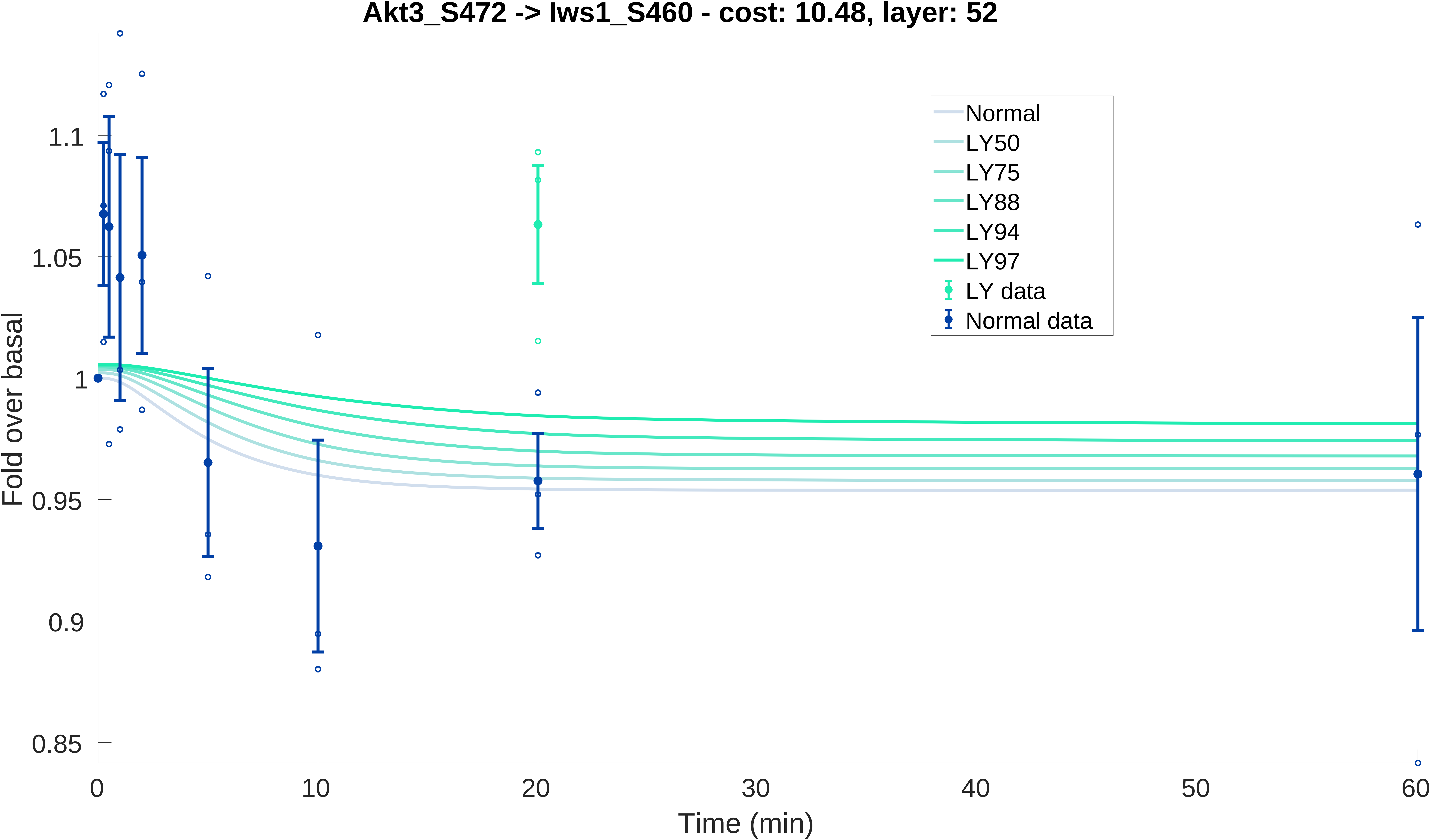

Akt3_S472 -> Iws1_S460 - cost: 10.48, layer: 52
Fold over basal
Time (min)
0.85
0.9
0.95
1
1.05
1.1
0
10
20
30
40
50
60
Normal
LY50
LY75
LY88
LY94
LY97
LY data
Normal data

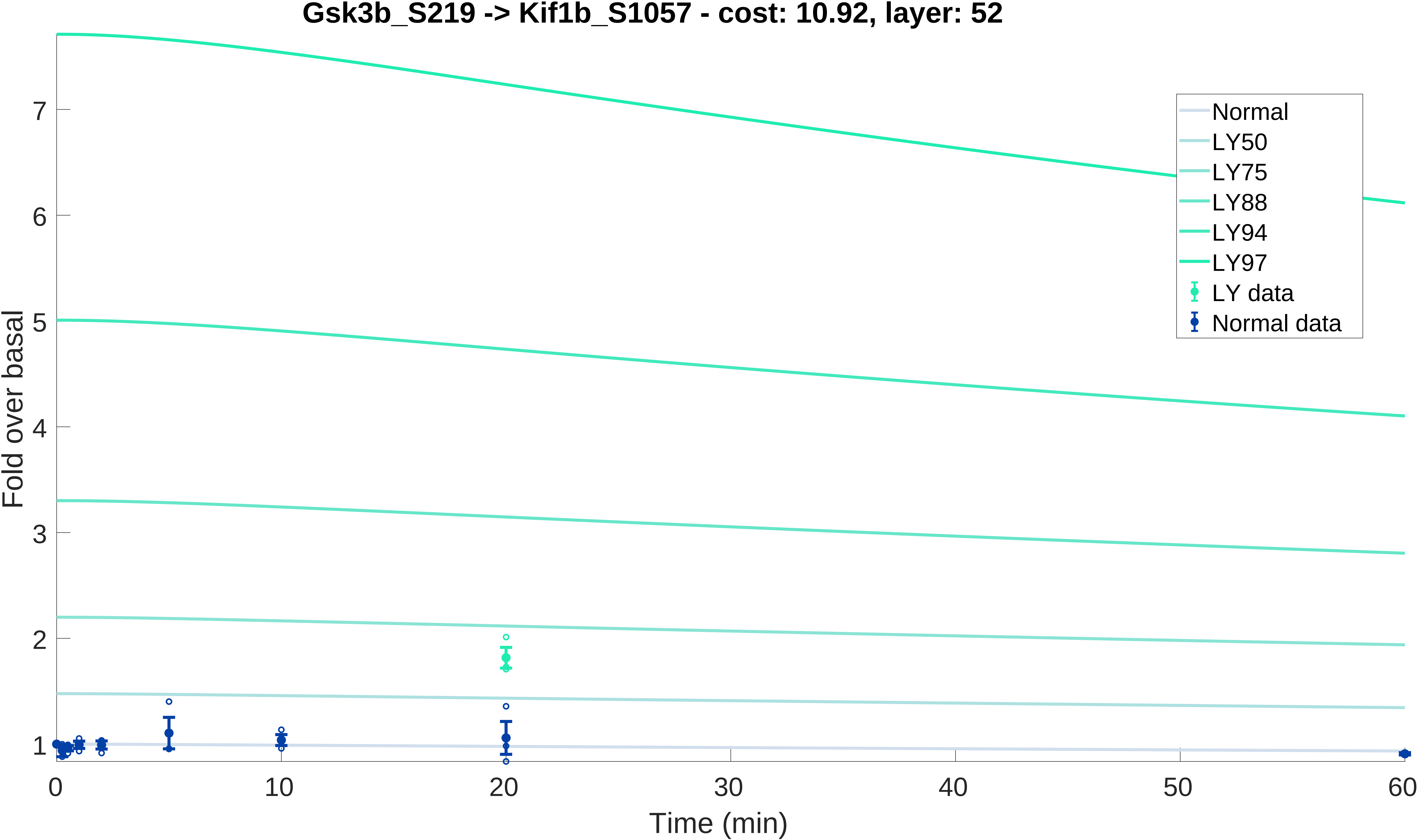
Gsk3b_S219 -> Kif1b_S1057 - cost: 10.92, layer: 52
Fold over basal
Time (min)
Normal
LY50
LY75
LY88
LY94
LY97
LY data
Normal data
0
10
20
30
40
50
60
1
2
3
4
5
6
7

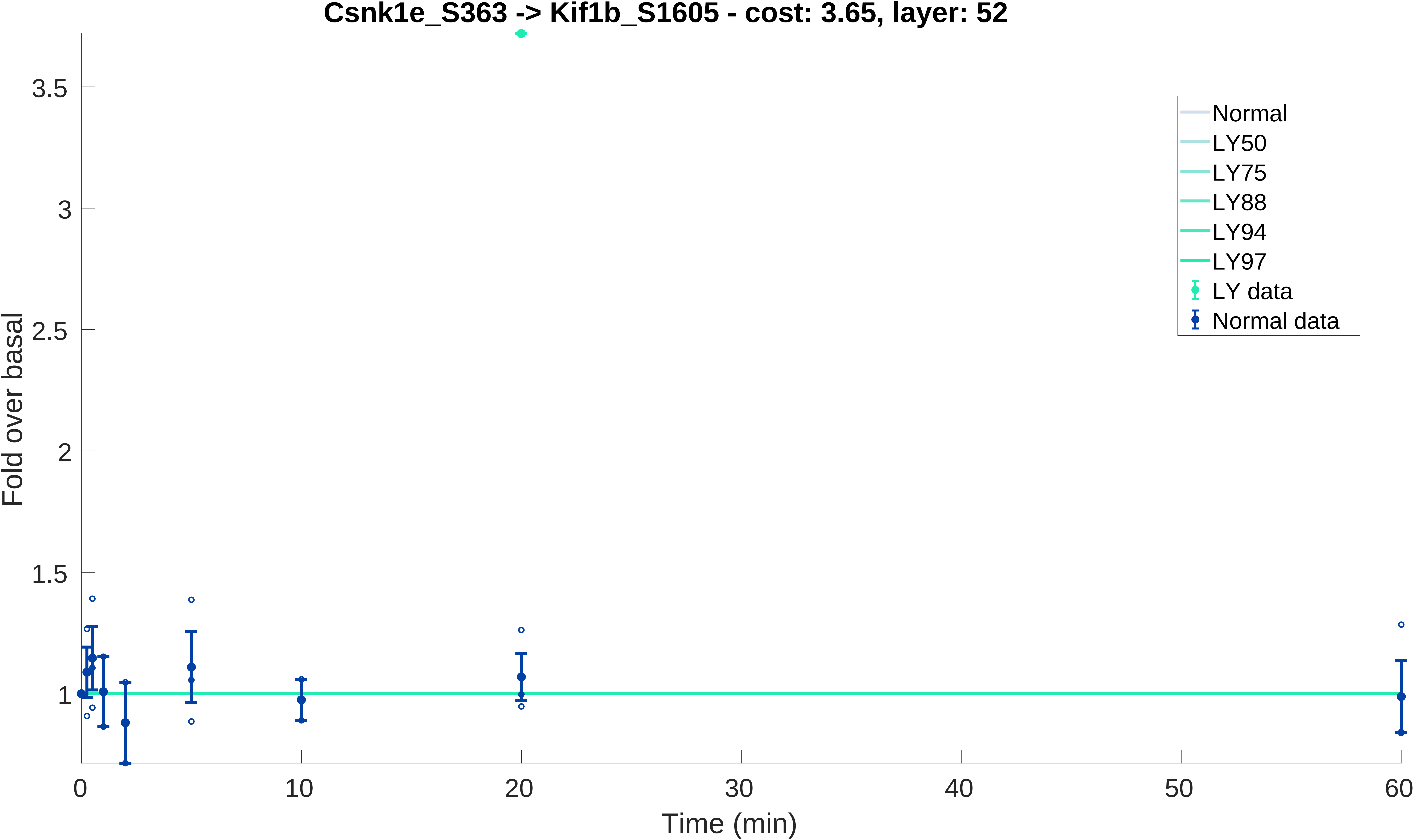
Csnk1e_S363 -> Kif1b_S1605 - cost: 3.65, layer: 52
Fold over basal
Time (min)
3.5
3
2.5
2
1.5
1
0
10
20
30
40
50
60
Normal
LY50
LY75
LY88
LY94
LY97
LY data
Normal data

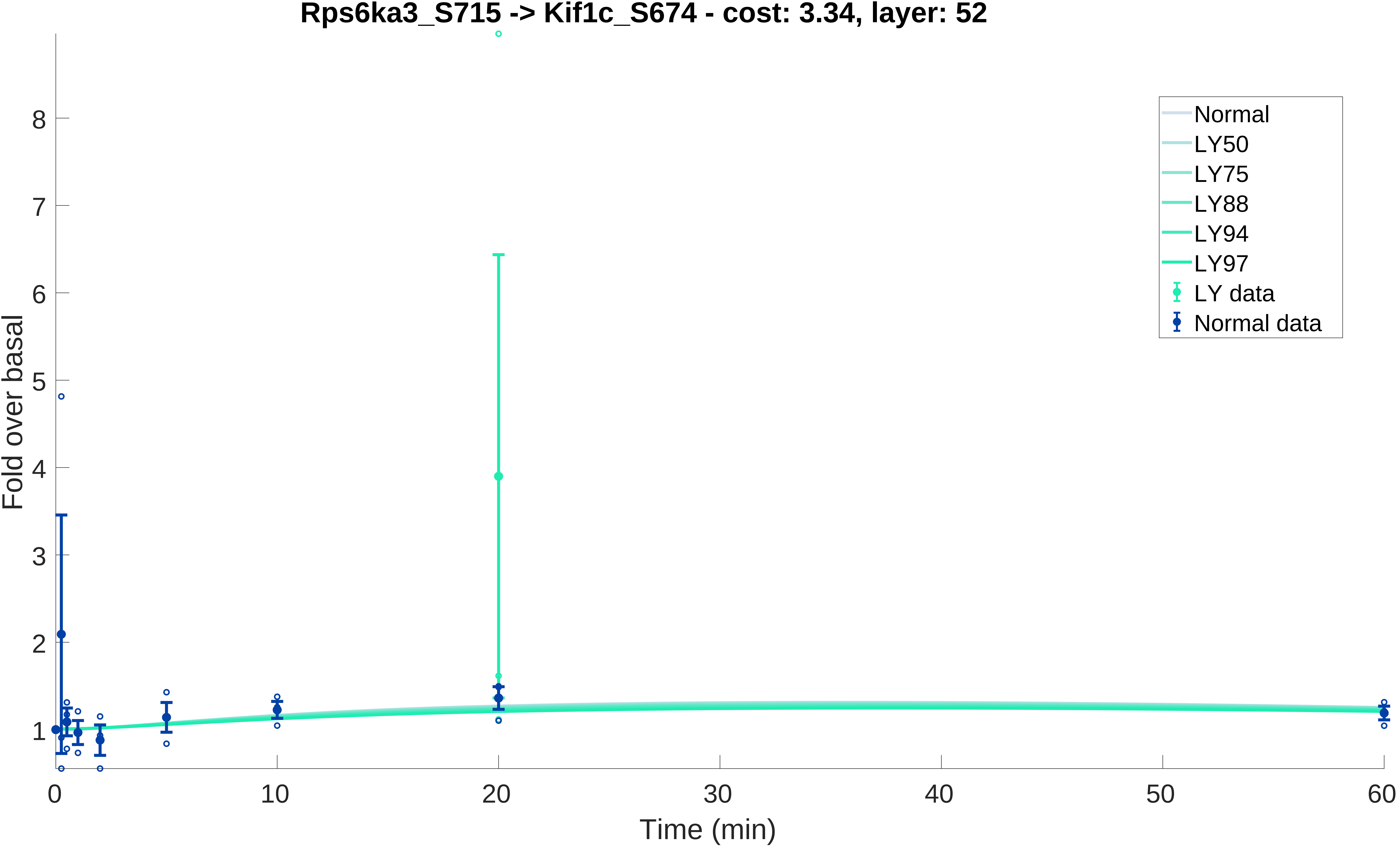
Rps6ka3_S715 -> Kif1c_S674 - cost: 3.34, layer: 52
Fold over basal
Time (min)
0
10
20
30
40
50
60
1
2
3
4
5
6
7
8
Normal
LY50
LY75
LY88
LY94
LY97
LY data
Normal data

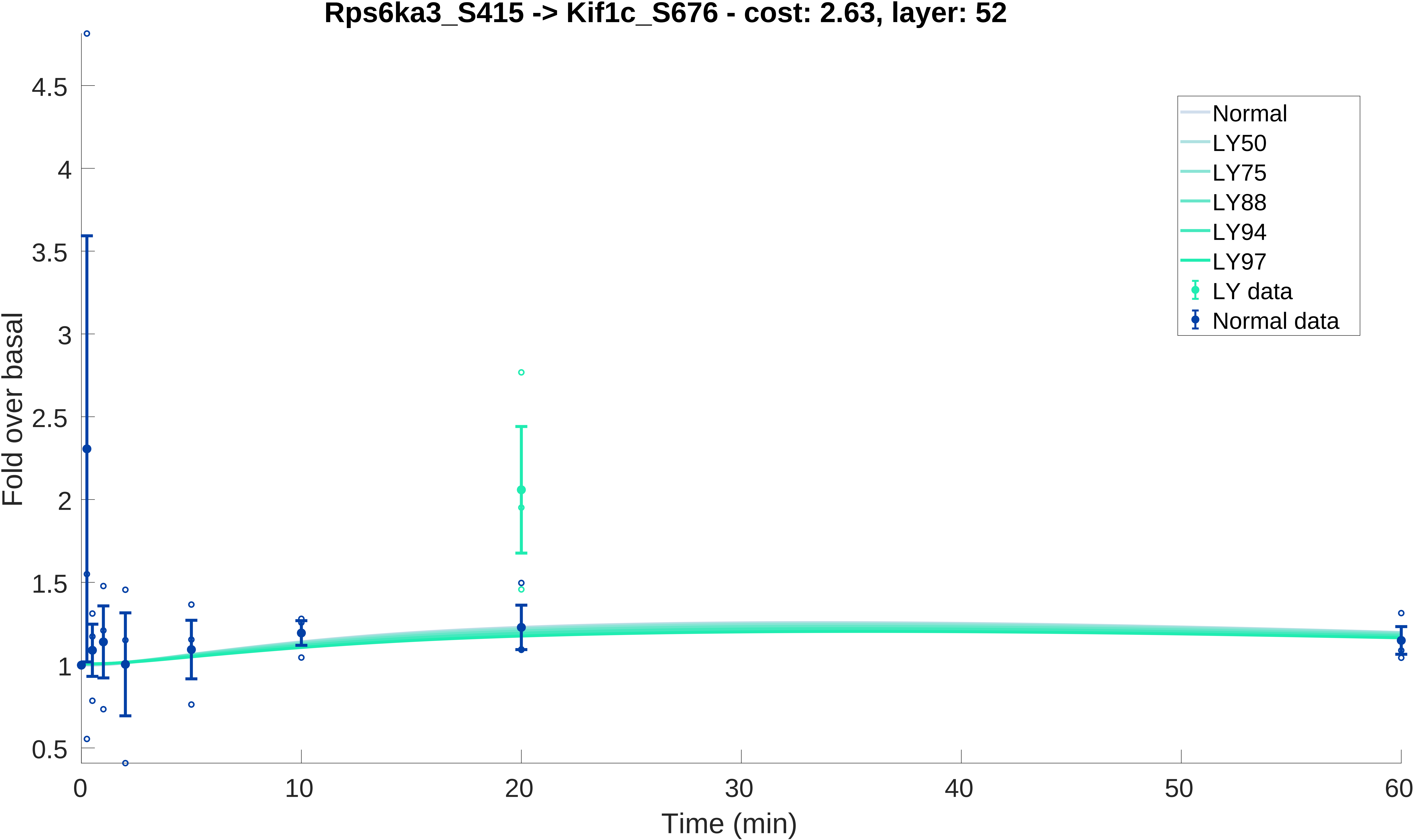

Rps6ka3_S415 -> Kif1c_S676 - cost: 2.63, layer: 52
Fold over basal
Time (min)
Normal
LY50
LY75
LY88
LY94
LY97
LY data
Normal data
0.5
1
1.5
2
2.5
3
3.5
4
4.5
0
10
20
30
40
50
60

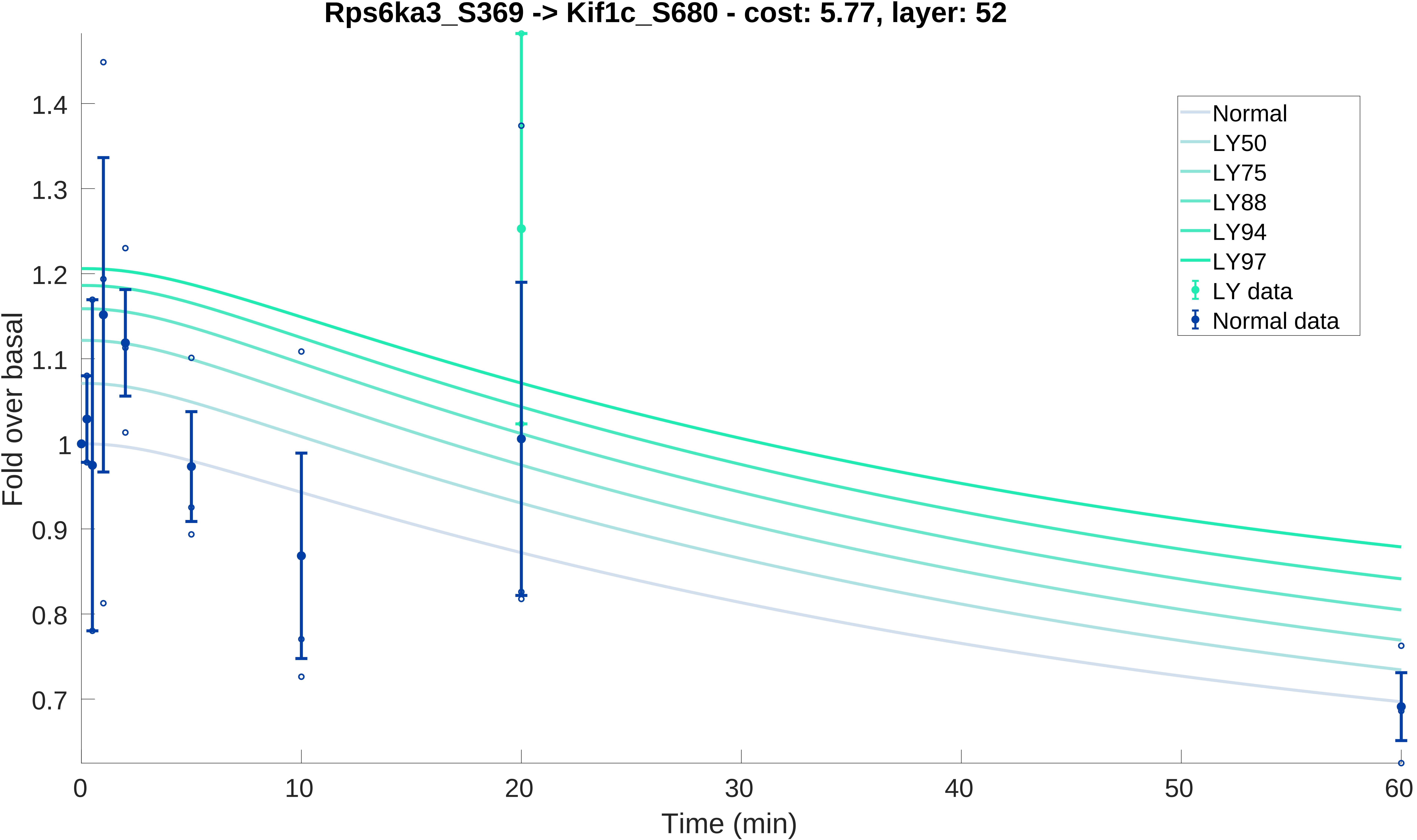
Rps6ka3_S369 -> Kif1c_S680 - cost: 5.77, layer: 52
Fold over basal
Time (min)
0.7
0.8
0.9
1
1.1
1.2
1.3
1.4
0
10
20
30
40
50
60
Normal
LY50
LY75
LY88
LY94
LY97
LY data
Normal data

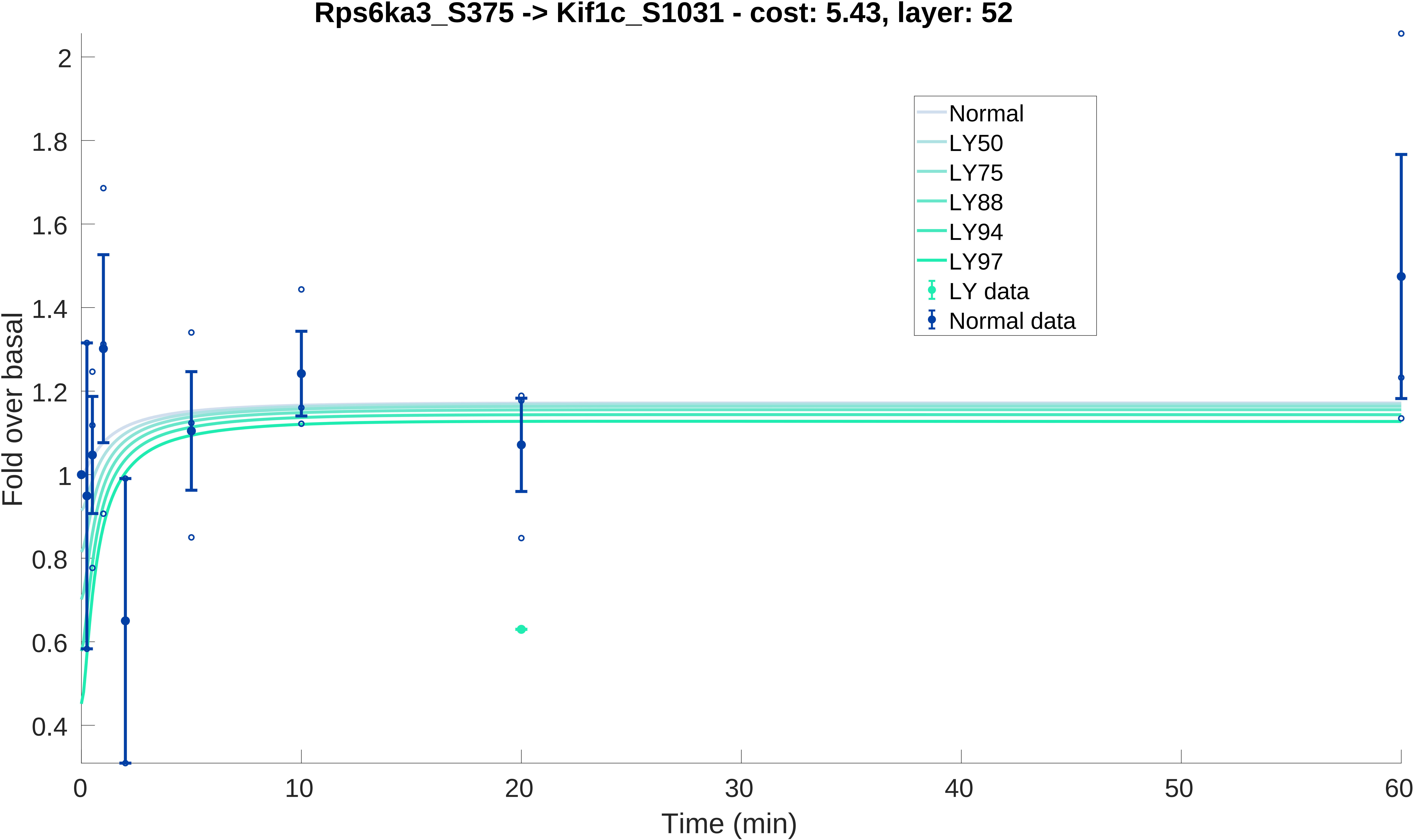

Rps6ka3_S375 -> Kif1c_S1031 - cost: 5.43, layer: 52
Fold over basal
Time (min)
Normal
LY50
LY75
LY88
LY94
LY97
LY data
Normal data
0.4
0.6
0.8
1
1.2
1.4
1.6
1.8
2
0
10
20
30
40
50
60

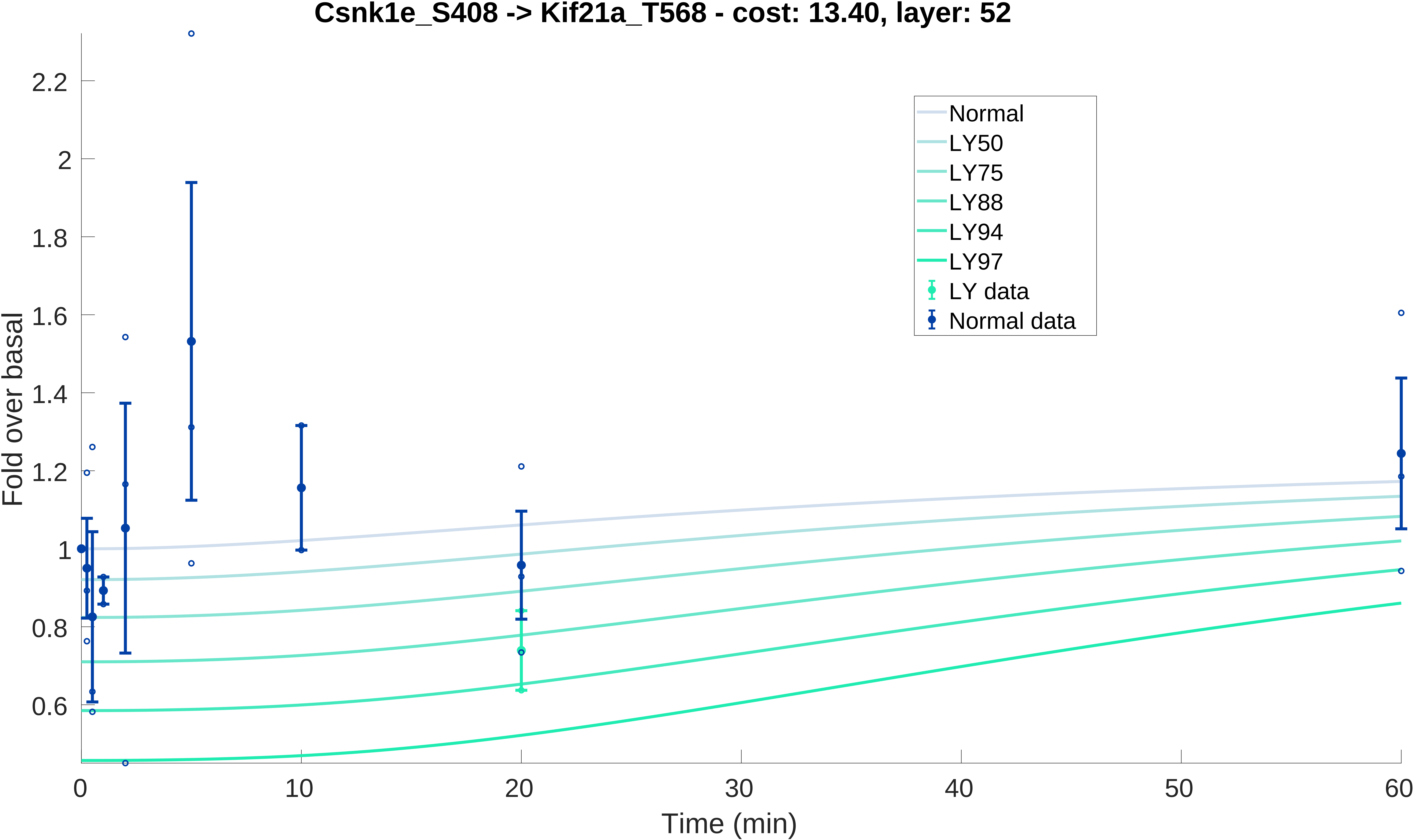

Csnk1e_S408 -> Kif21a_T568 - cost: 13.40, layer: 52
Normal
LY50
LY75
LY88
LY94
LY97
LY data
Normal data
Fold over basal
Time (min)
0
10
20
30
40
50
60
0.6
0.8
1
1.2
1.4
1.6
1.8
2
2.2

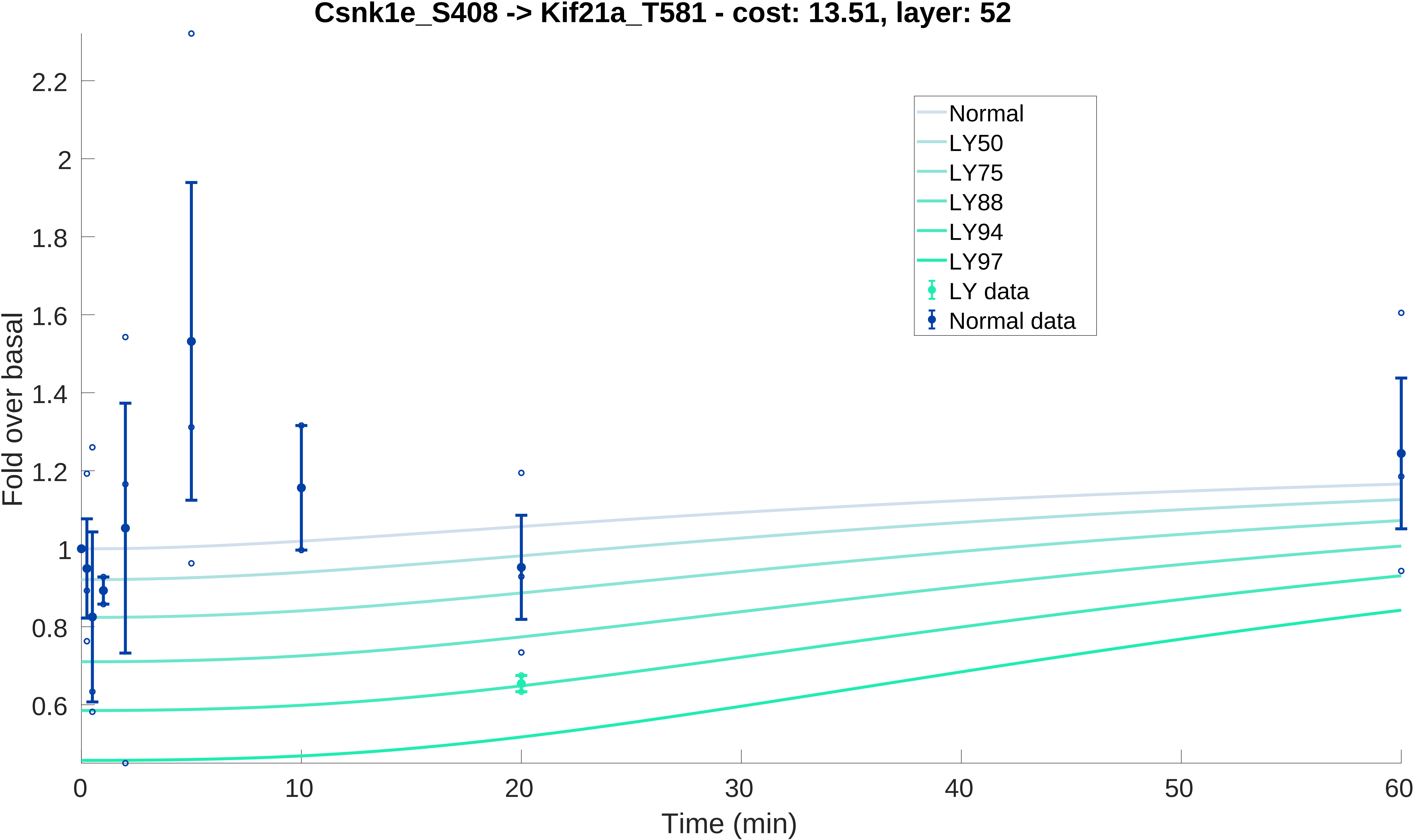

Csnk1e_S408 -> Kif21a_T581 - cost: 13.51, layer: 52
Fold over basal
Time (min)
0.6
0.8
1
1.2
1.4
1.6
1.8
2
2.2
0
10
20
30
40
50
60
Normal
LY50
LY75
LY88
LY94
LY97
LY data
Normal data

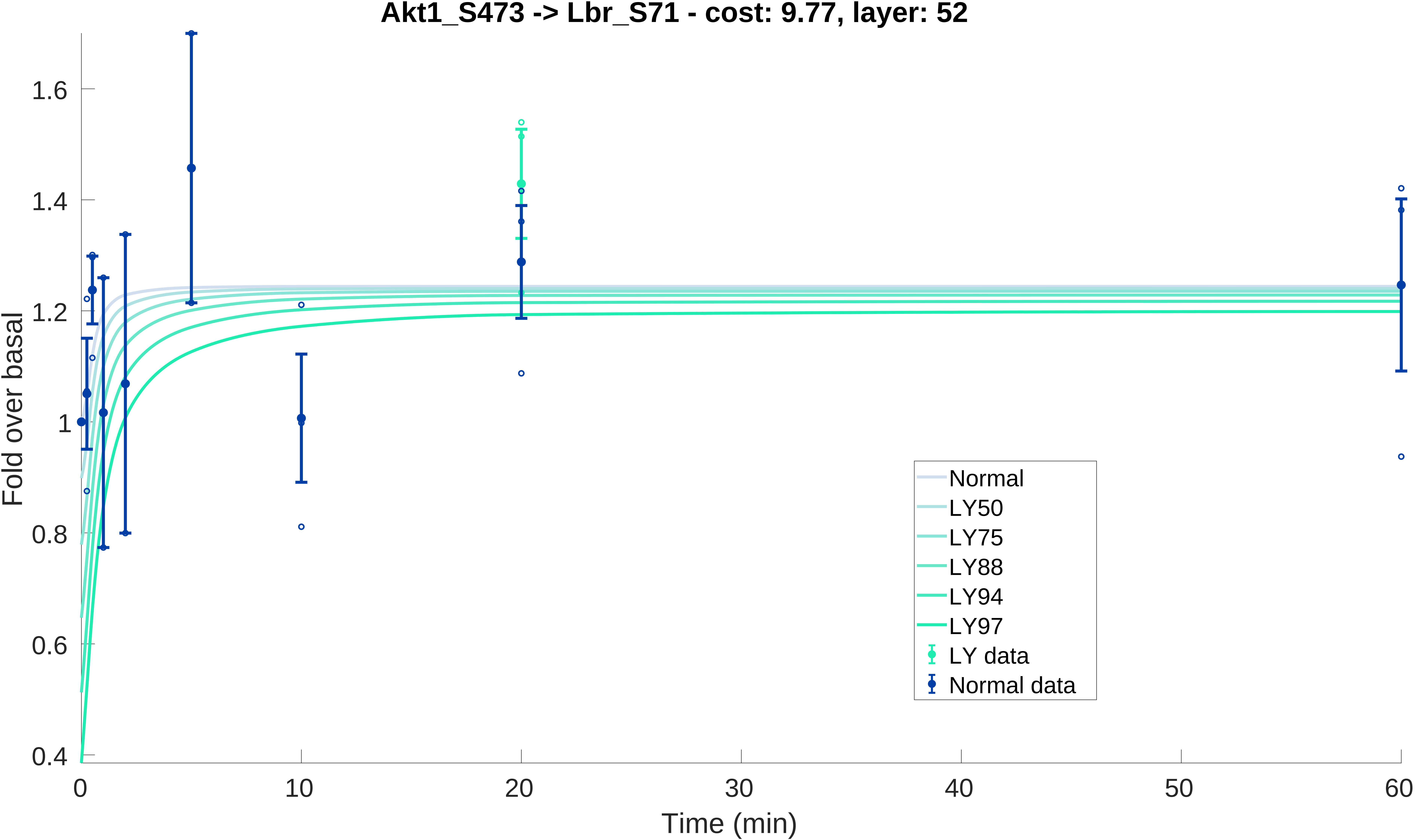

Akt1_S473 -> Lbr_S71 - cost: 9.77, layer: 52
Fold over basal
1.6
1.4
1.2
1
0.8
0.6
0.4
0
10
20
30
40
50
60
Time (min)
Normal
LY50
LY75
LY88
LY94
LY97
LY data
Normal data

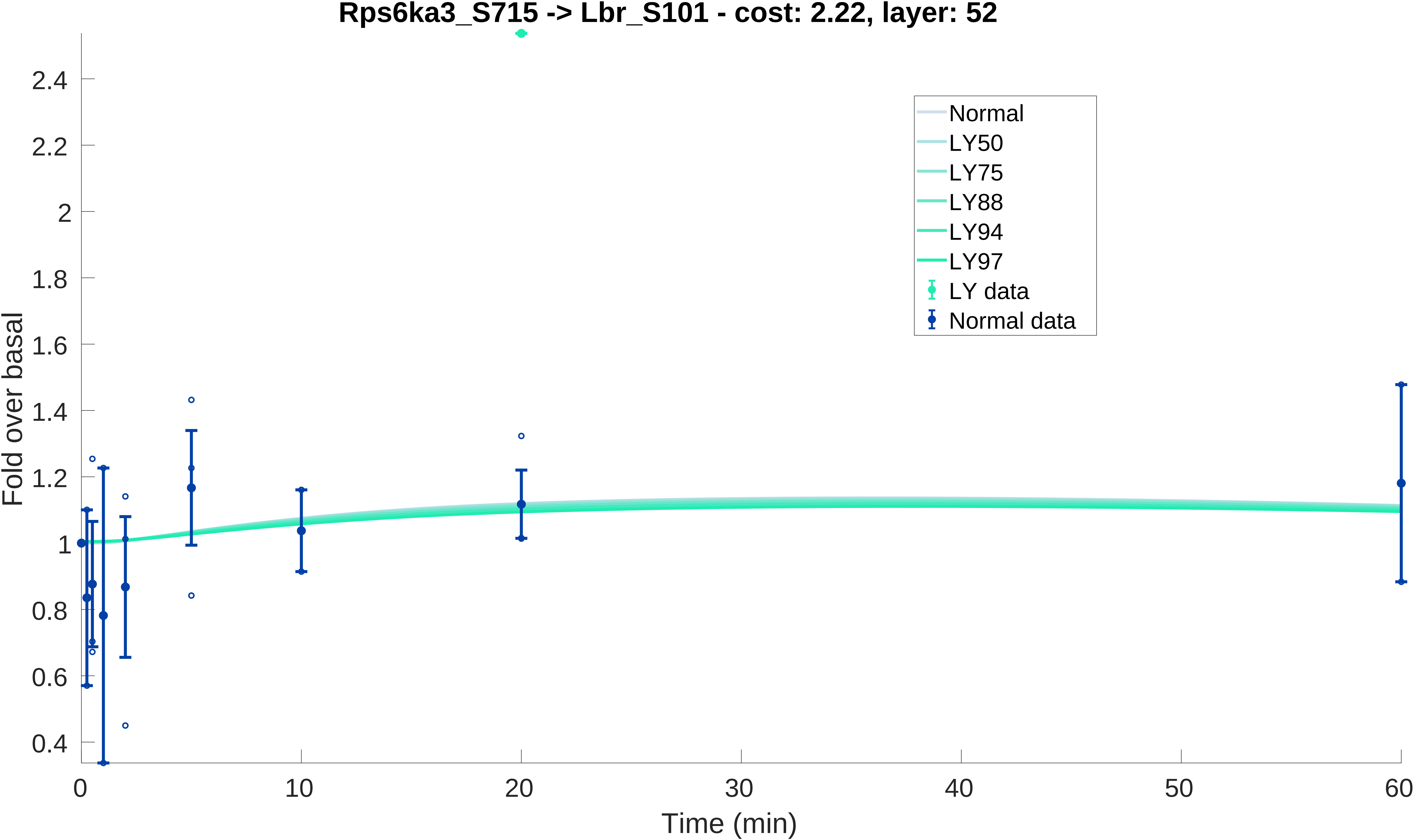

Rps6ka3_S715 -> Lbr_S101 - cost: 2.22, layer: 52
Normal
LY50
LY75
LY88
LY94
LY97
LY data
Normal data
Fold over basal
2.4
2.2
2
1.8
1.6
1.4
1.2
1
0.8
0.6
0.4
0
10
20
30
40
50
60
Time (min)

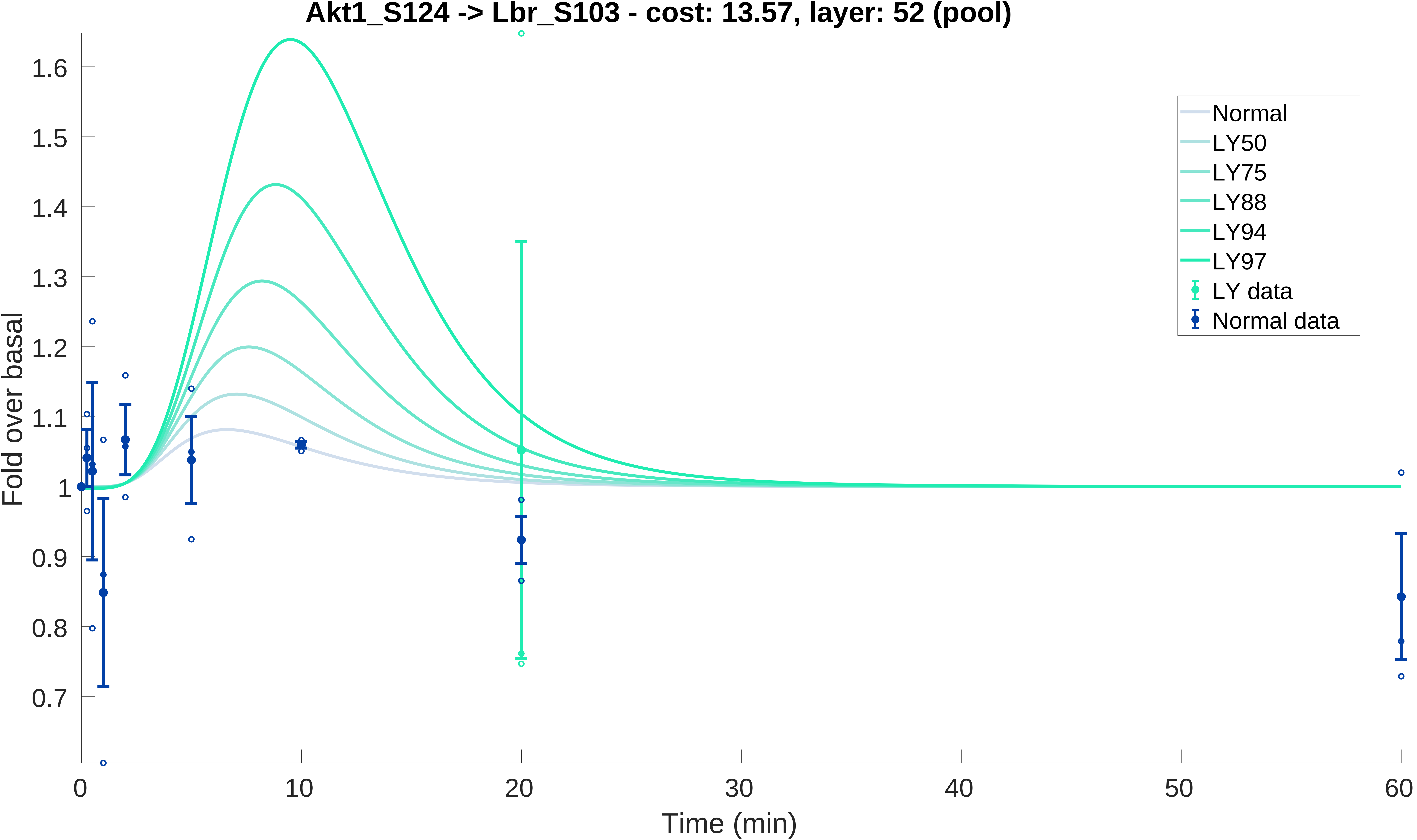

Akt1_S124 -> Lbr_S103 - cost: 13.57, layer: 52 (pool)
Fold over basal
Time (min)
1.6
1.5
1.4
1.3
1.2
1.1
1
0.9
0.8
0.7
0
10
20
30
40
50
60
Normal
LY50
LY75
LY88
LY94
LY97
LY data
Normal data

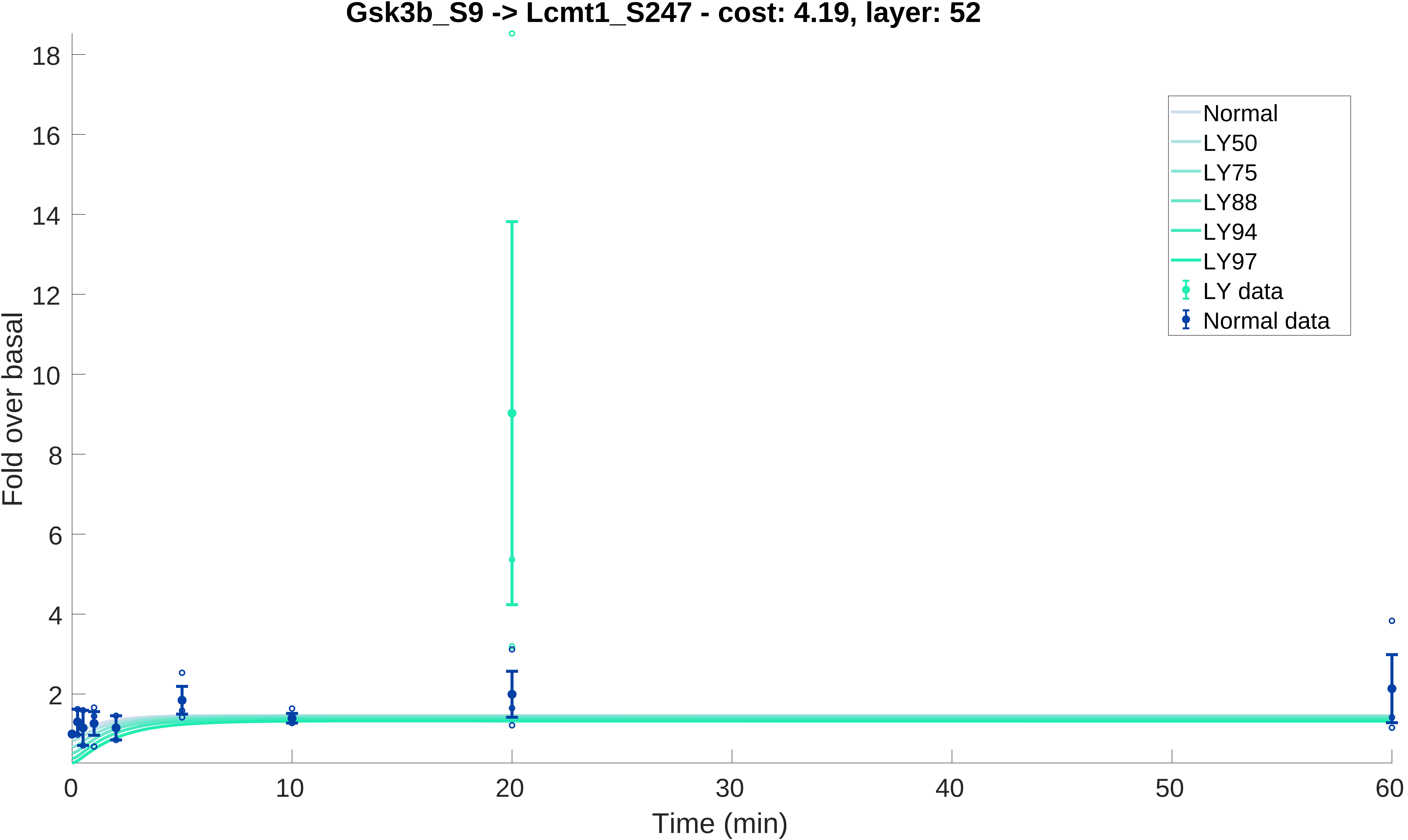

Gsk3b_S9 -> Lcmt1_S247 - cost: 4.19, layer: 52
Fold over basal
Time (min)
0
10
20
30
40
50
60
2
4
6
8
10
12
14
16
18
Normal
LY50
LY75
LY88
LY94
LY97
LY data
Normal data

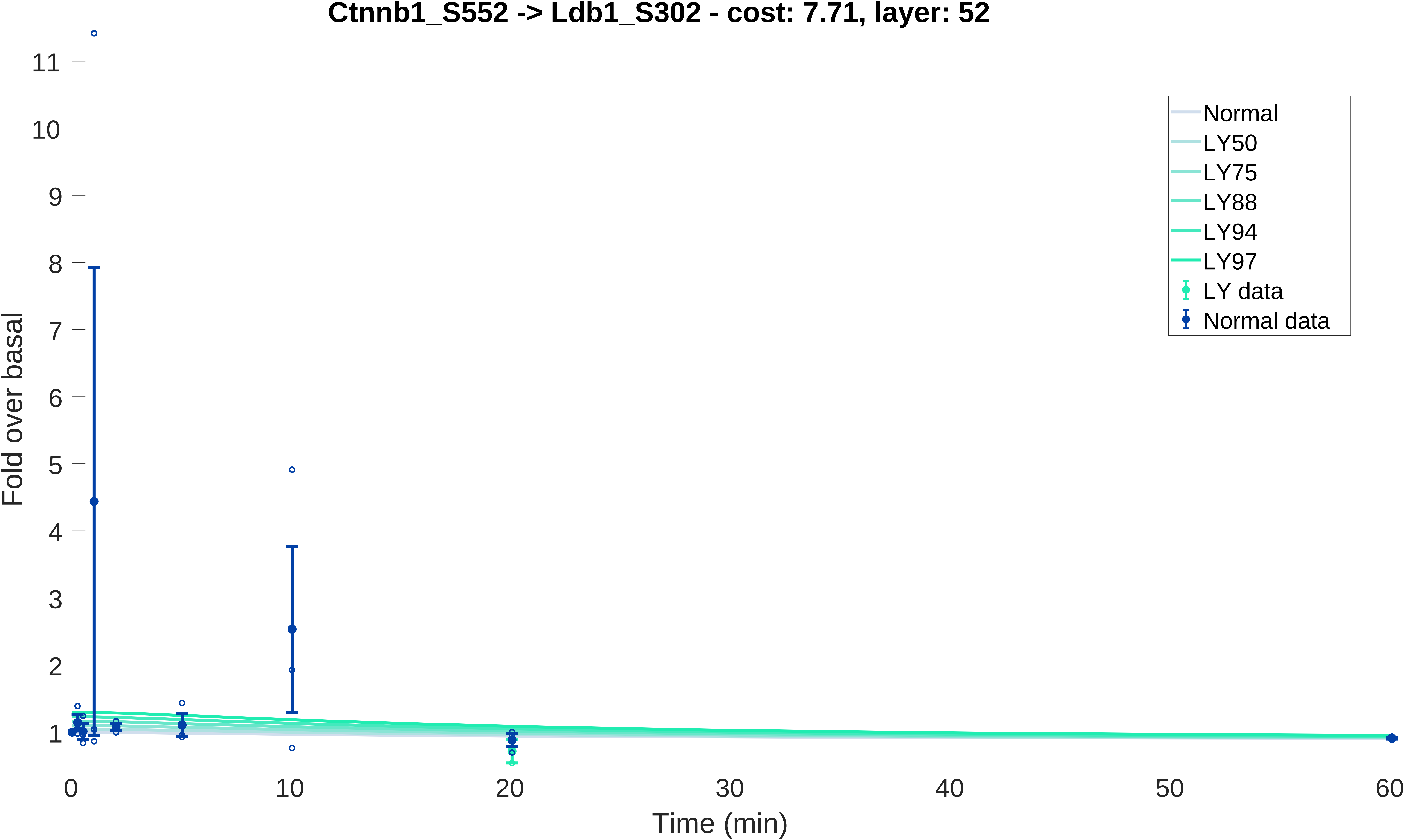
Ctnnb1_S552 -> Ldb1_S302 - cost: 7.71, layer: 52
Fold over basal
Time (min)
0
10
20
30
40
50
60
1
2
3
4
5
6
7
8
9
10
11
Normal
LY50
LY75
LY88
LY94
LY97
LY data
Normal data

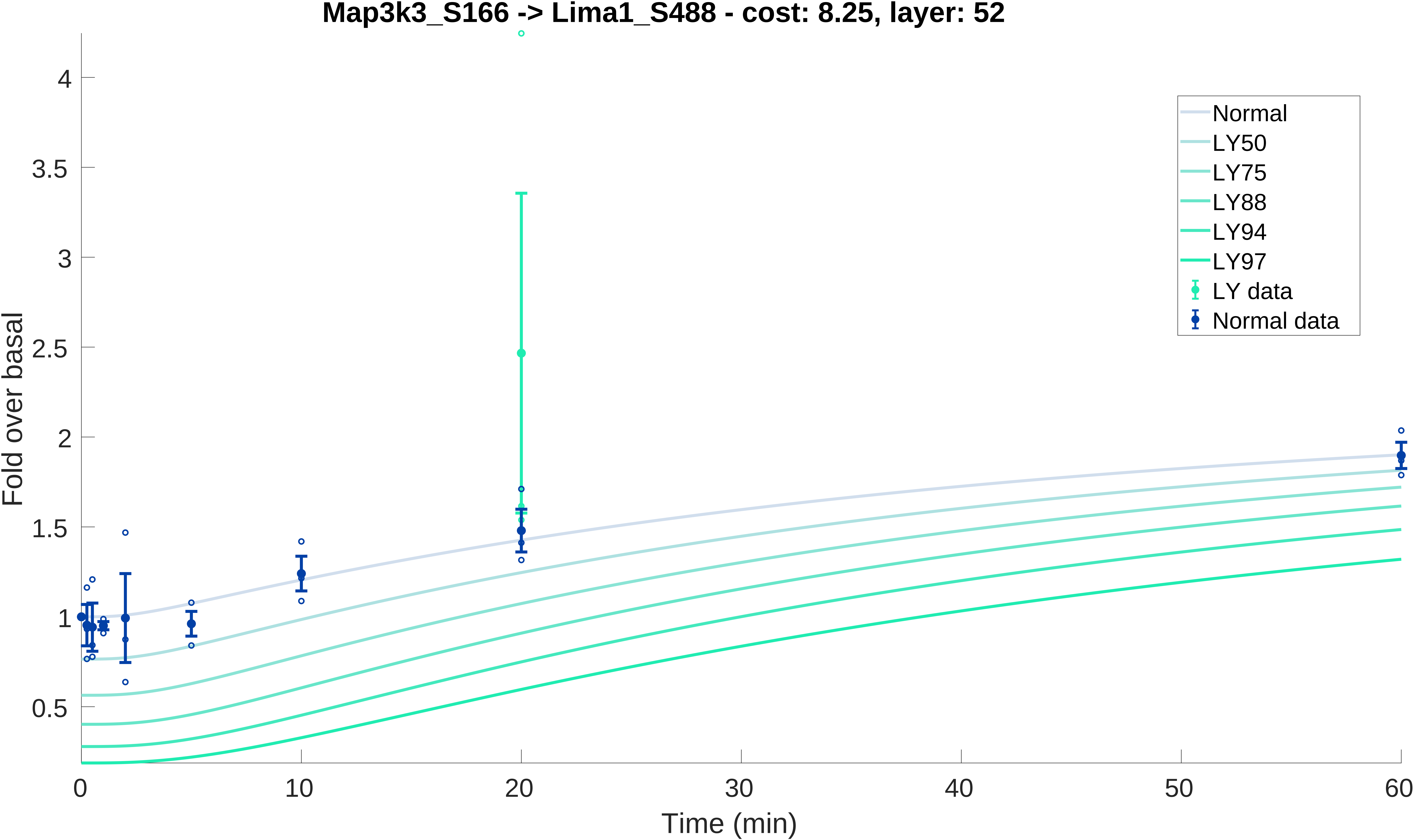
Map3k3_S166 -> Lima1_S488 - cost: 8.25, layer: 52
Fold over basal
Time (min)
Normal
LY50
LY75
LY88
LY94
LY97
LY data
Normal data
4
3.5
3
2.5
2
1.5
1
0.5
0
10
20
30
40
50
60

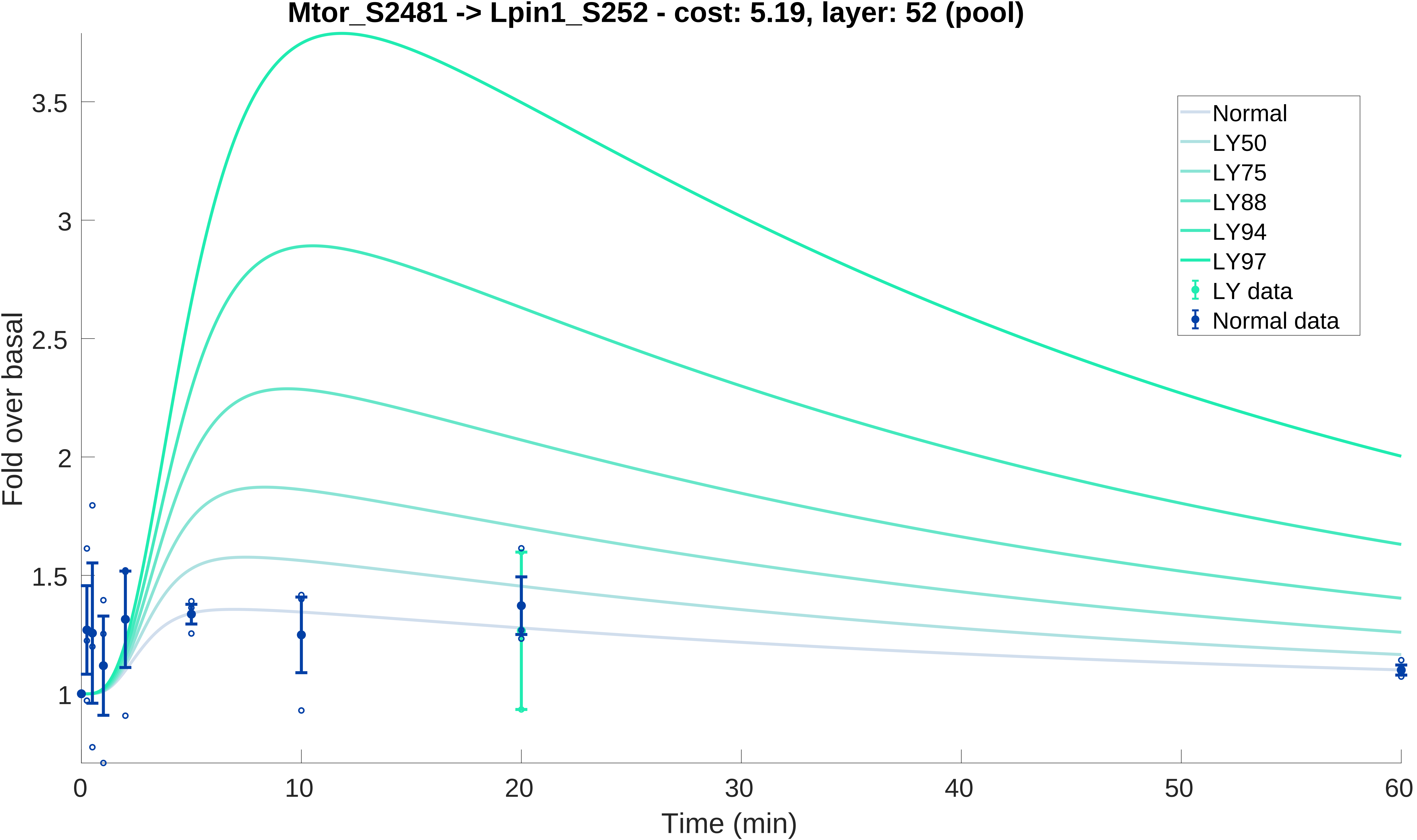
Mtor_S2481 -> Lpin1_S252 - cost: 5.19, layer: 52 (pool)
Fold over basal
Time (min)
3.5
3
2.5
2
1.5
1
0
10
20
30
40
50
60
Normal
LY50
LY75
LY88
LY94
LY97
LY data
Normal data

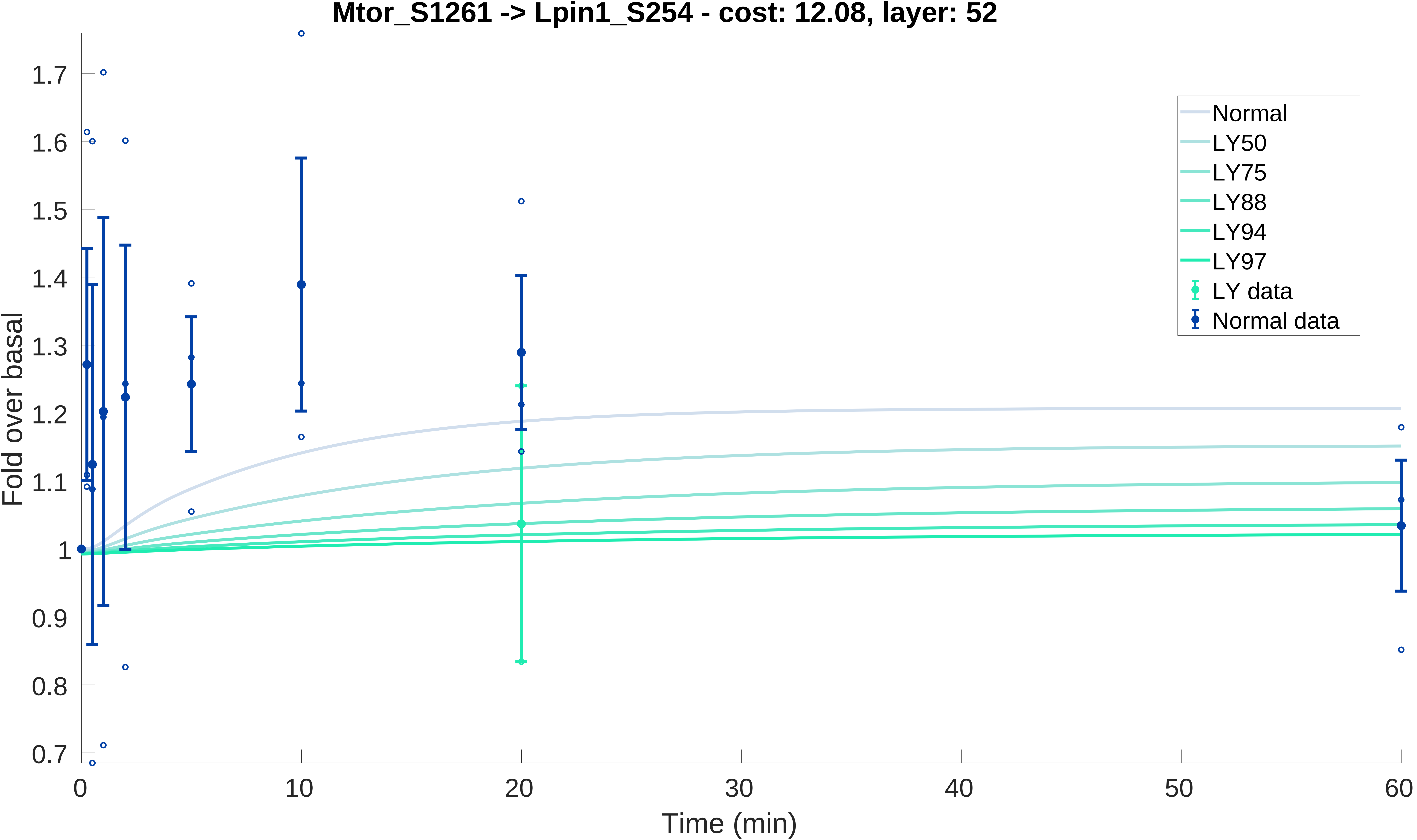
Mtor_S1261 -> Lpin1_S254 - cost: 12.08, layer: 52
Fold over basal
Time (min)
0
10
20
30
40
50
60
0.7
0.8
0.9
1
1.1
1.2
1.3
1.4
1.5
1.6
1.7
Normal
LY50
LY75
LY88
LY94
LY97
LY data
Normal data

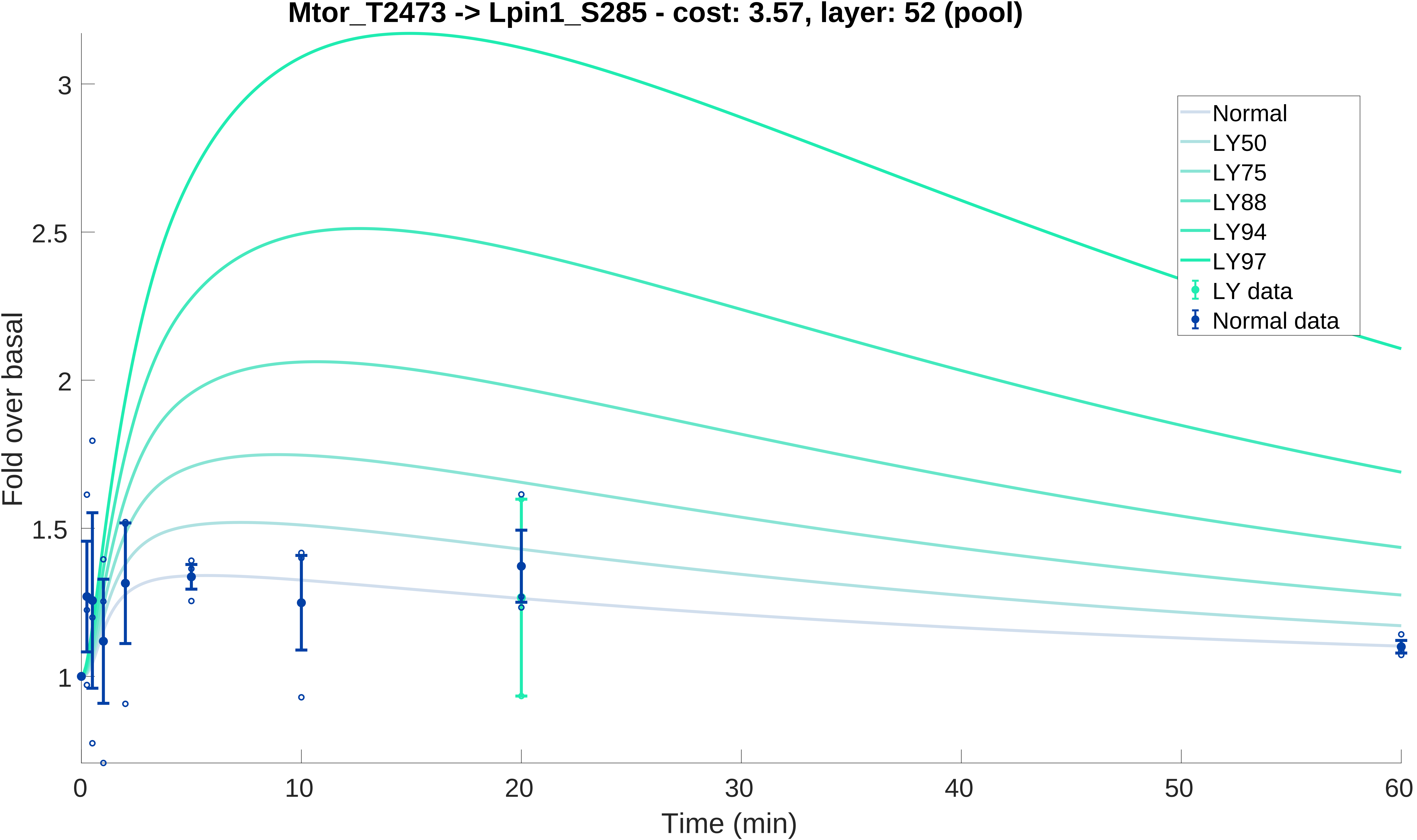

Mtor_T2473 -> Lpin1_S285 - cost: 3.57, layer: 52 (pool)
Fold over basal
Time (min)
0
10
20
30
40
50
60
1
1.5
2
2.5
3
Normal
LY50
LY75
LY88
LY94
LY97
LY data
Normal data

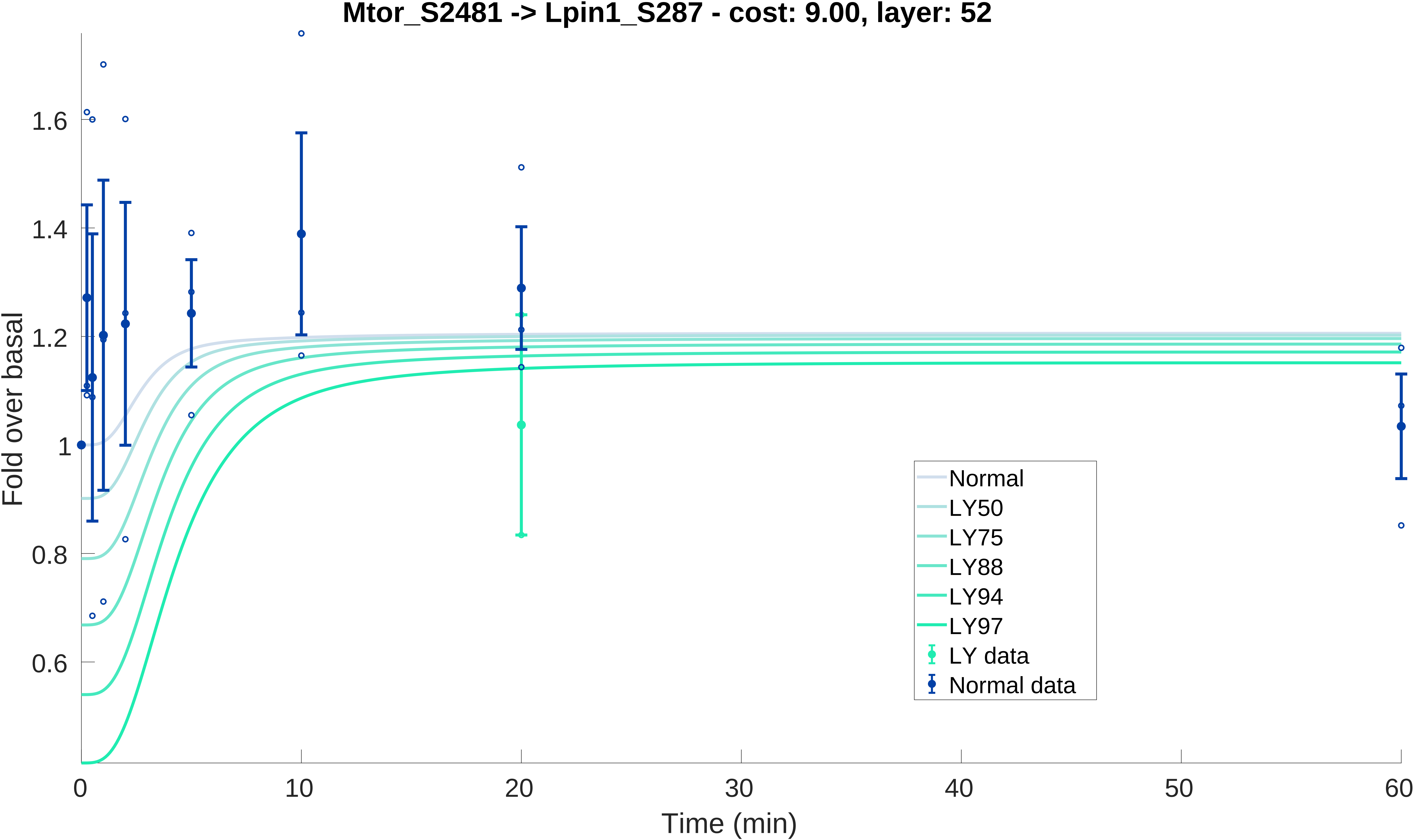
Mtor_S2481 -> Lpin1_S287 - cost: 9.00, layer: 52
Fold over basal
1.6
1.4
1.2
1
0.8
0.6
0
10
20
30
40
50
60
Time (min)
Normal
LY50
LY75
LY88
LY94
LY97
LY data
Normal data

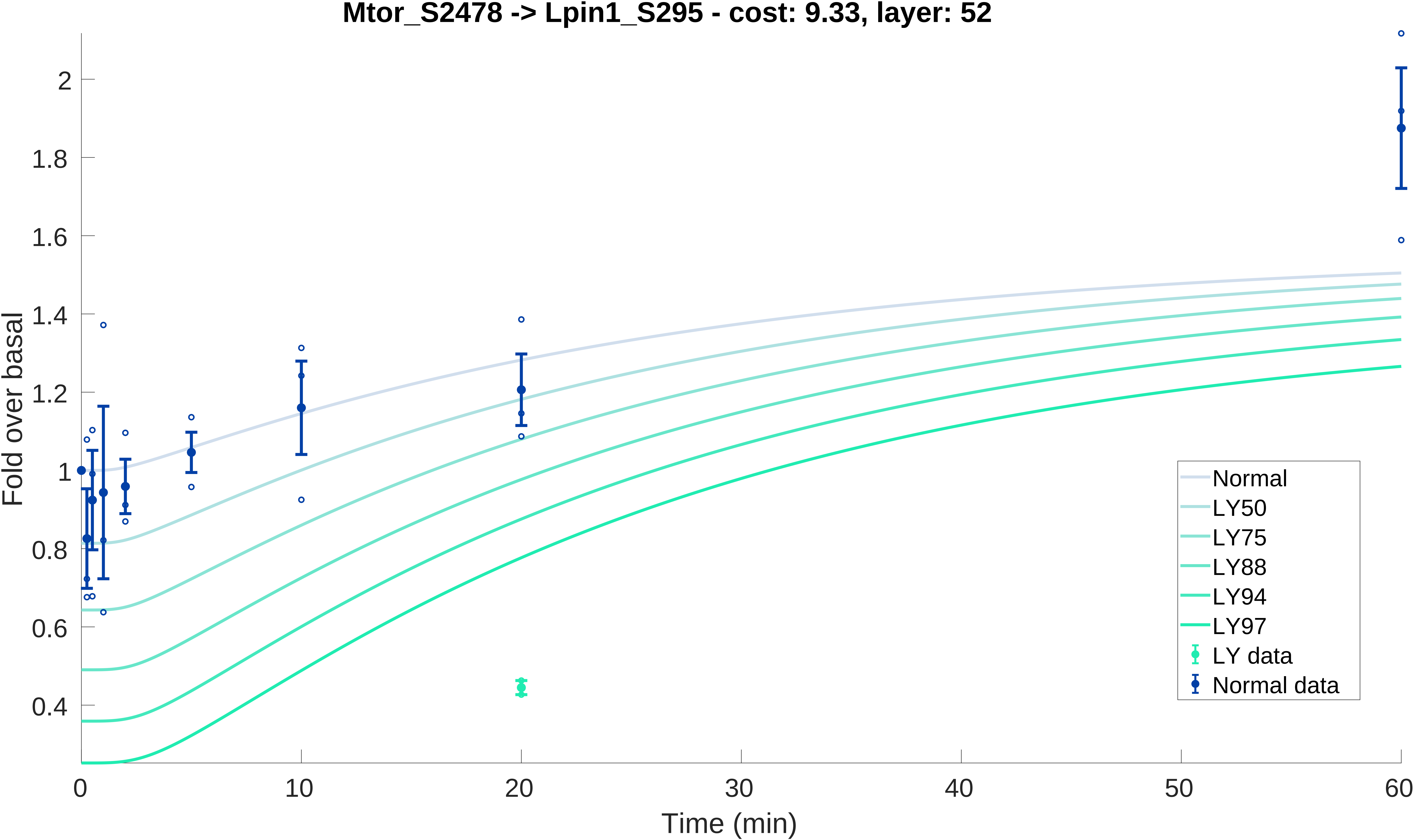

Mtor_S2478 -> Lpin1_S295 - cost: 9.33, layer: 52
Fold over basal
Time (min)
0.4
0.6
0.8
1
1.2
1.4
1.6
1.8
2
0
10
20
30
40
50
60
Normal
LY50
LY75
LY88
LY94
LY97
LY data
Normal data

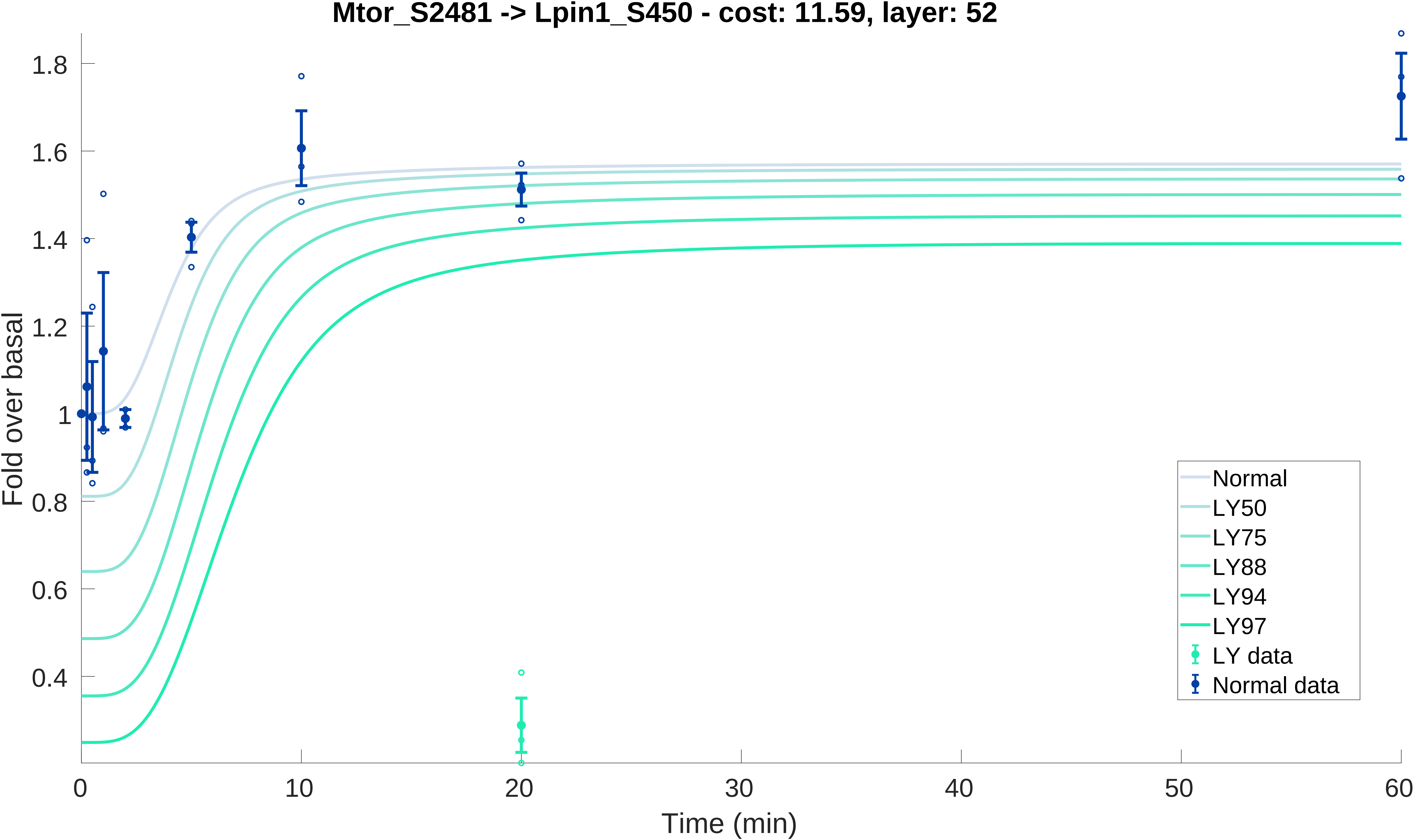

Mtor_S2481 -> Lpin1_S450 - cost: 11.59, layer: 52
Fold over basal
Time (min)
1.8
1.6
1.4
1.2
1
0.8
0.6
0.4
0
10
20
30
40
50
60
Normal
LY50
LY75
LY88
LY94
LY97
LY data
Normal data

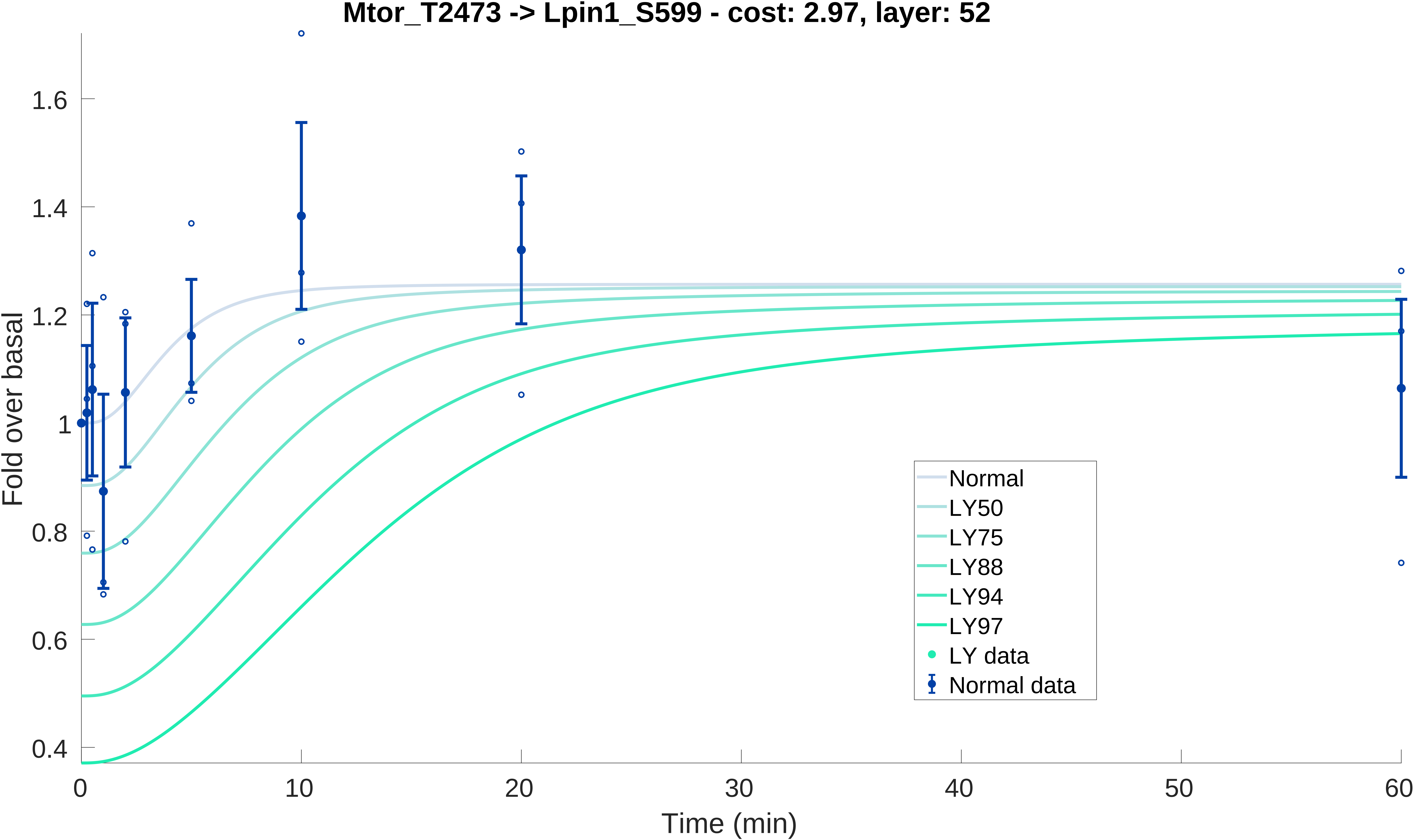
Mtor_T2473 -> Lpin1_S599 - cost: 2.97, layer: 52
Fold over basal
1.6
1.4
1.2
1
0.8
0.6
0.4
0
10
20
30
40
50
60
Time (min)
Normal
LY50
LY75
LY88
LY94
LY97
LY data
Normal data

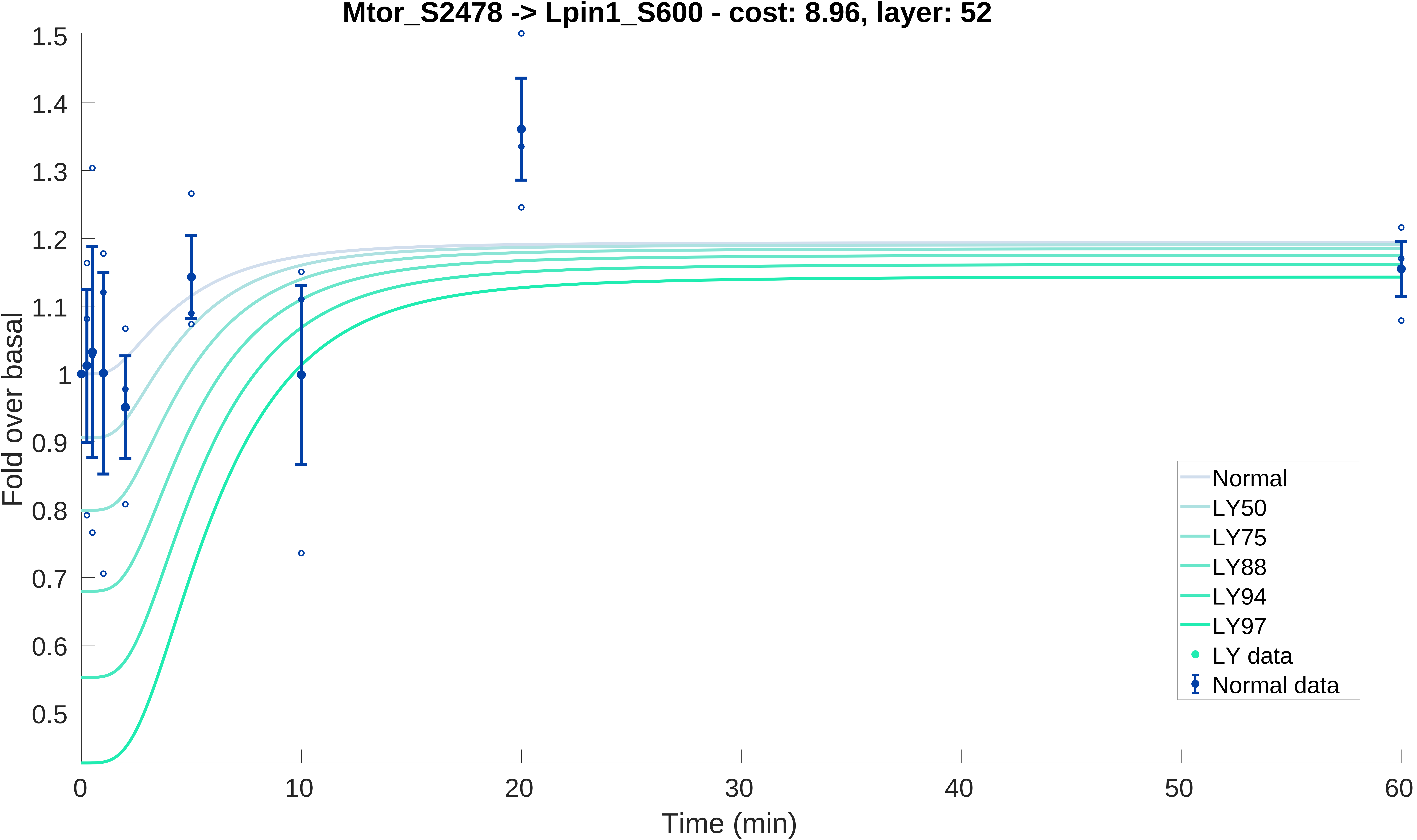

Mtor_S2478 -> Lpin1_S600 - cost: 8.96, layer: 52
Fold over basal
1.5
1.4
1.3
1.2
1.1
1
0.9
0.8
0.7
0.6
0.5
0
10
20
30
40
50
60
Time (min)
Normal
LY50
LY75
LY88
LY94
LY97
LY data
Normal data

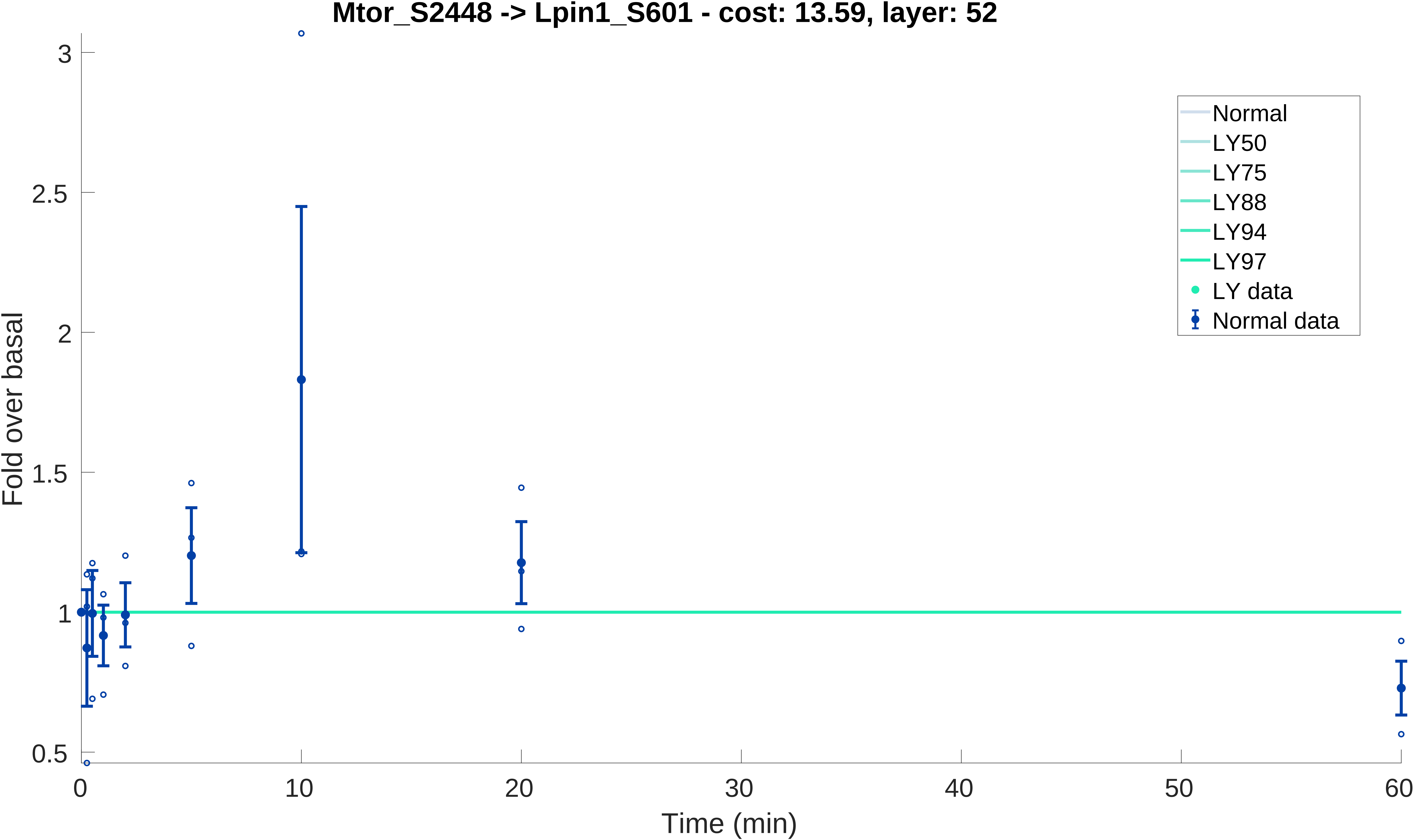
Mtor_S2448 -> Lpin1_S601 - cost: 13.59, layer: 52
Fold over basal
Time (min)
0.5
1
1.5
2
2.5
3
0
10
20
30
40
50
60
Normal
LY50
LY75
LY88
LY94
LY97
LY data
Normal data

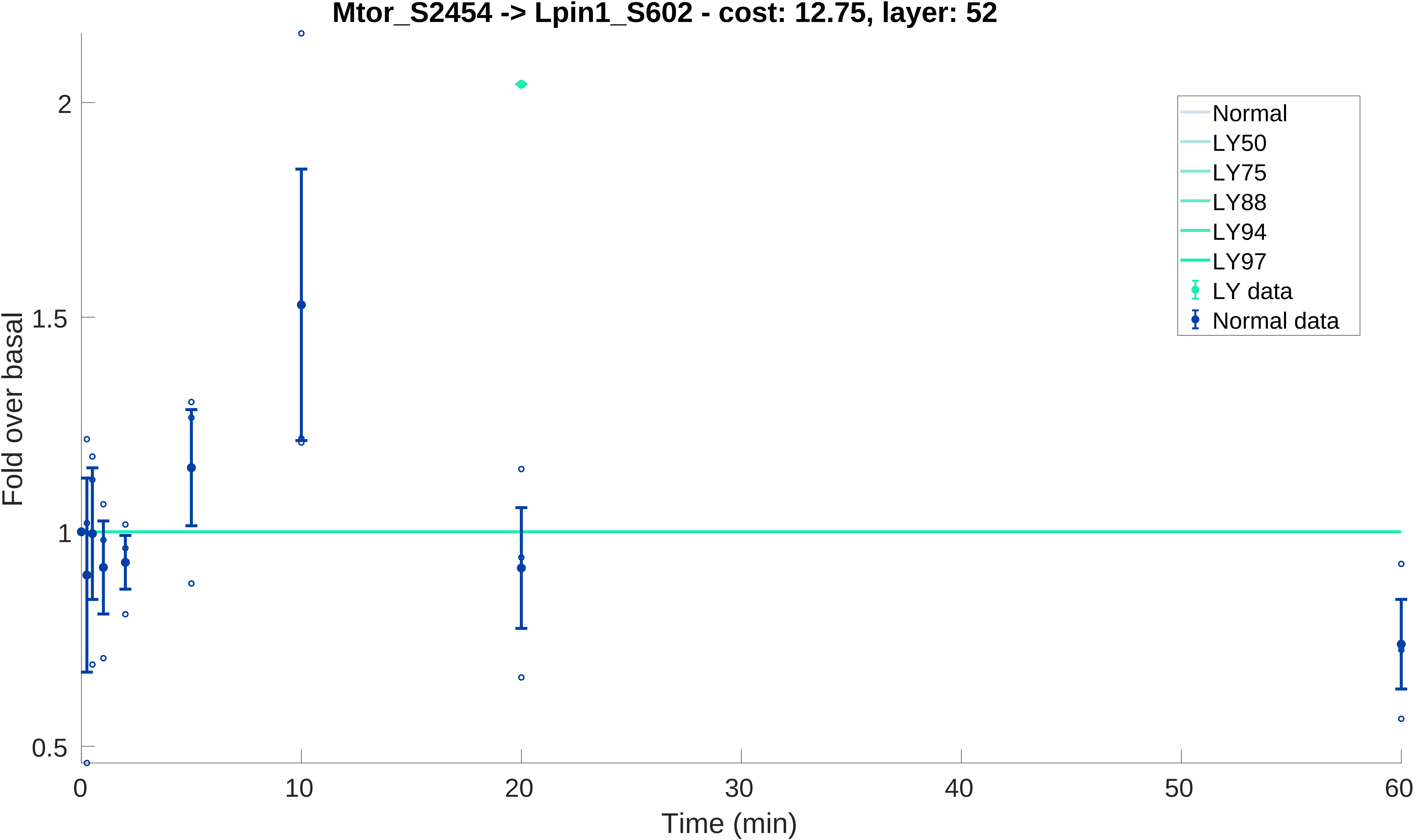

Mtor_S2454 -> Lpin1_S602 - cost: 12.75, layer: 52
Fold over basal
0.5
1
1.5
2
0
10
20
30
40
50
60
Time (min)
Normal
LY50
LY75
LY88
LY94
LY97
LY data
Normal data

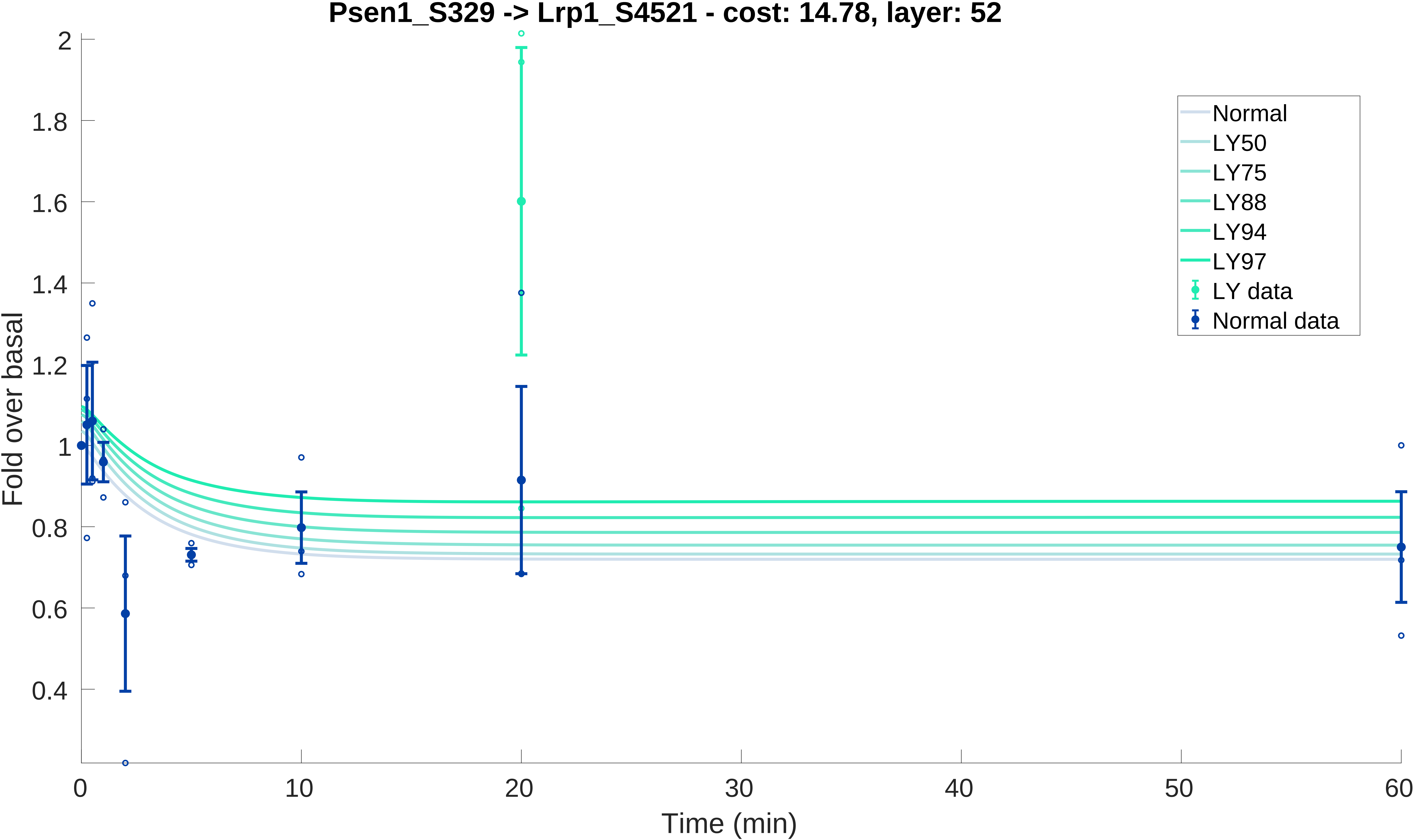

Psen1_S329 -> Lrp1_S4521 - cost: 14.78, layer: 52
Fold over basal
Time (min)
2
1.8
1.6
1.4
1.2
1
0.8
0.6
0.4
0
10
20
30
40
50
60
Normal
LY50
LY75
LY88
LY94
LY97
LY data
Normal data

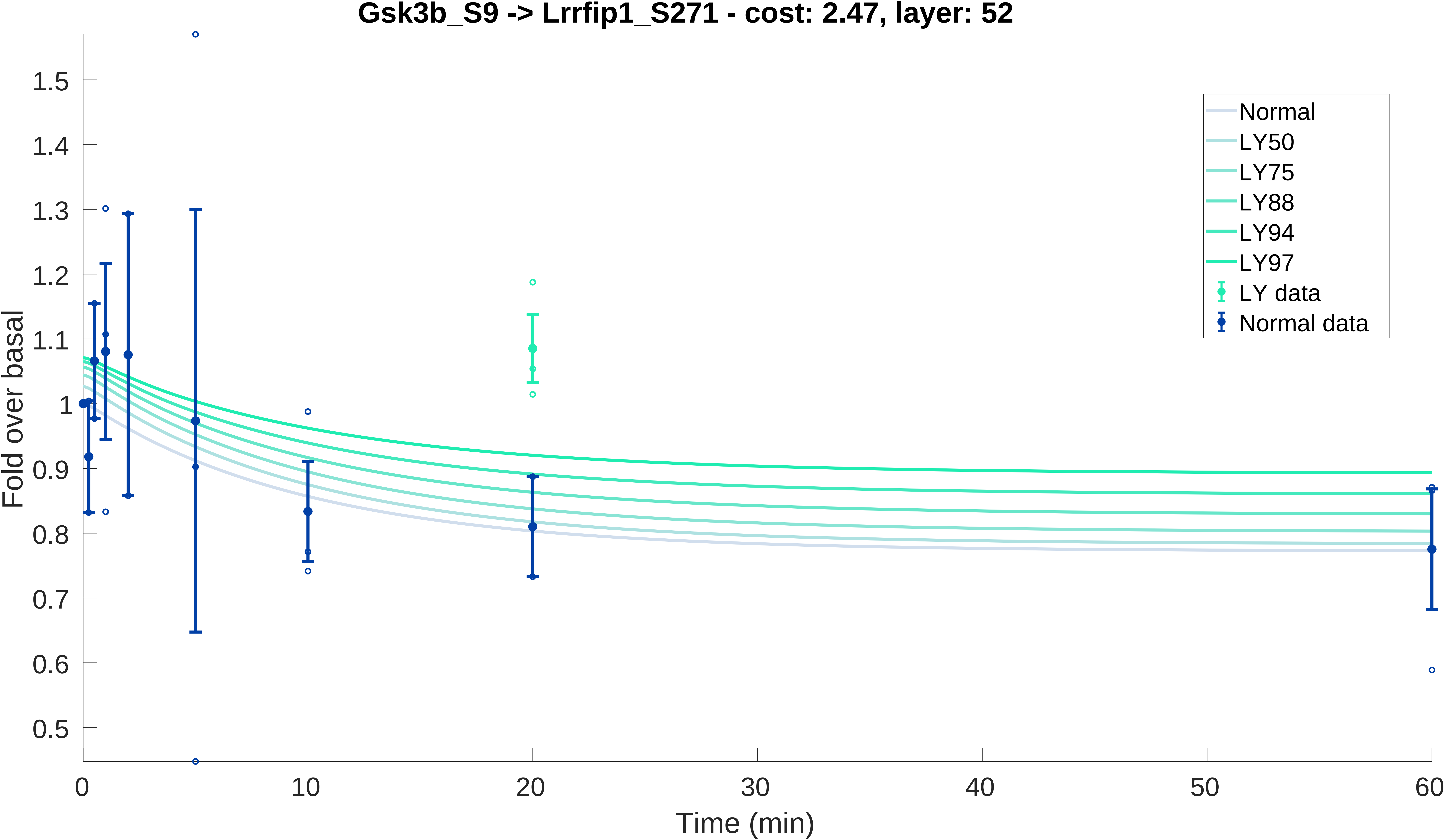
Gsk3b_S9 -> Lrrfip1_S271 - cost: 2.47, layer: 52
Fold over basal
Time (min)
1.5
1.4
1.3
1.2
1.1
1
0.9
0.8
0.7
0.6
0.5
0
10
20
30
40
50
60
Normal
LY50
LY75
LY88
LY94
LY97
LY data
Normal data

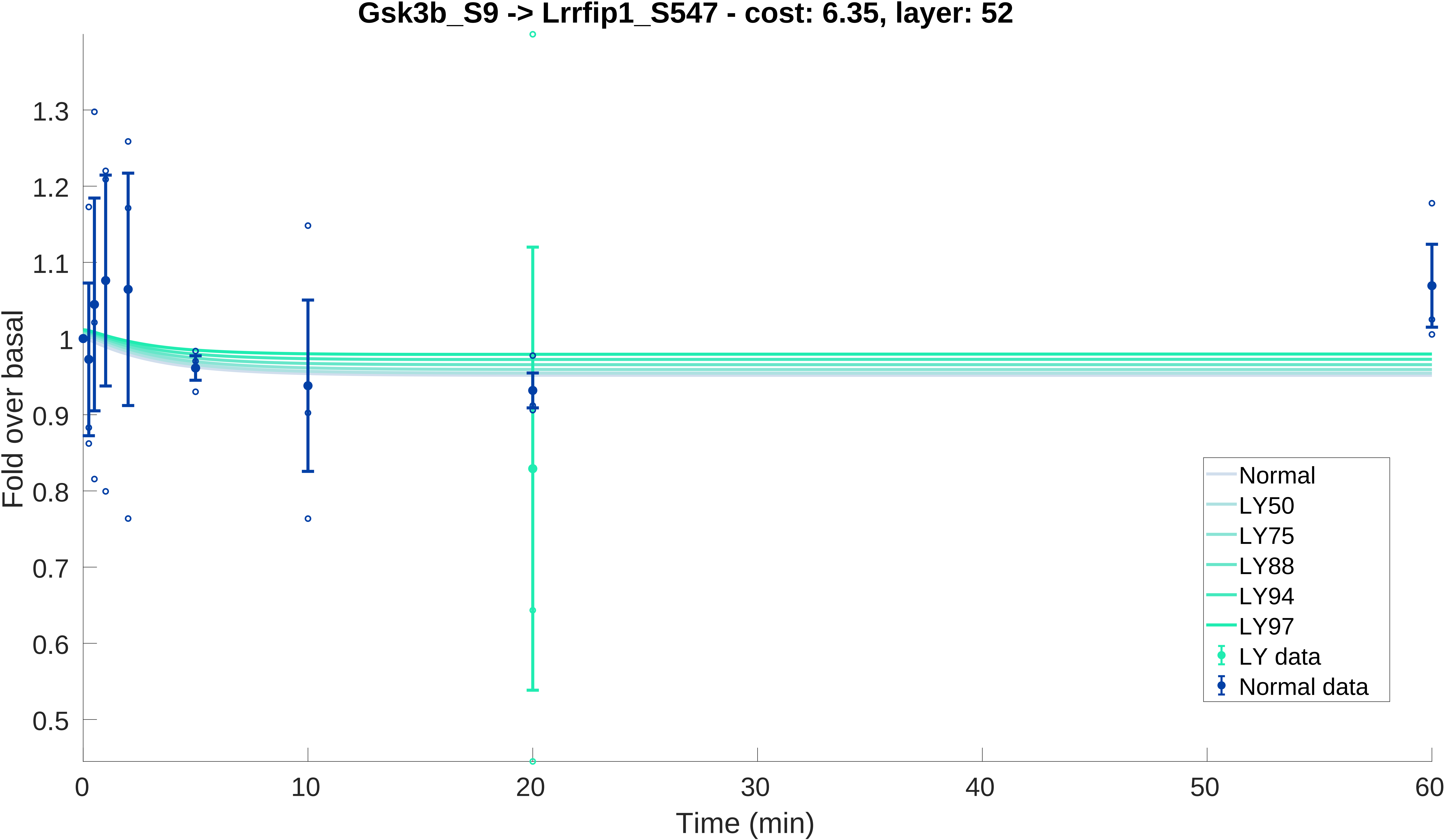

Gsk3b_S9 -> Lrrfip1_S547 - cost: 6.35, layer: 52
Fold over basal
Time (min)
0.5
0.6
0.7
0.8
0.9
1
1.1
1.2
1.3
0
10
20
30
40
50
60
Normal
LY50
LY75
LY88
LY94
LY97
LY data
Normal data

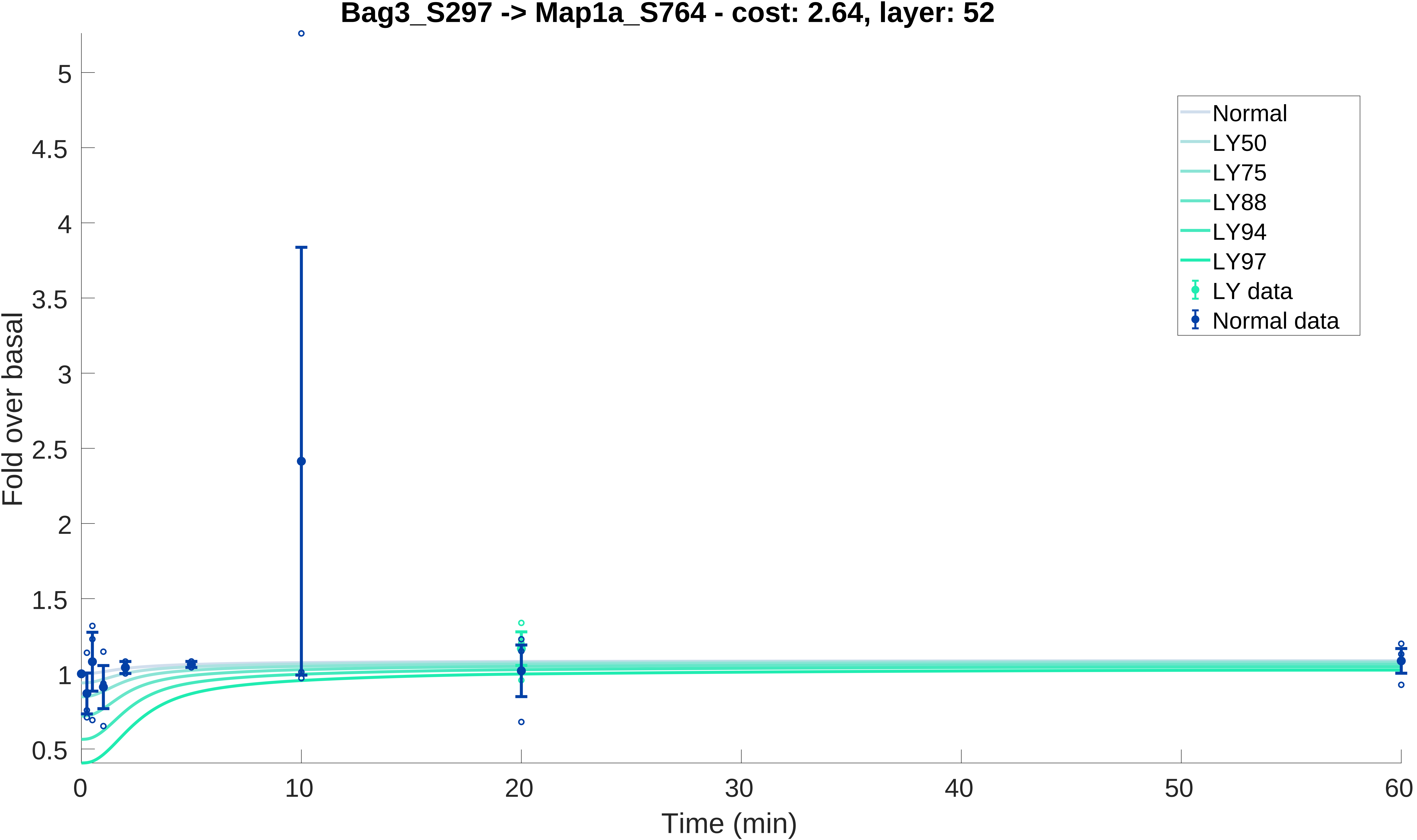

Bag3_S297 -> Map1a_S764 - cost: 2.64, layer: 52
Fold over basal
Time (min)
Normal
LY50
LY75
LY88
LY94
LY97
LY data
Normal data
0
10
20
30
40
50
60
0.5
1
1.5
2
2.5
3
3.5
4
4.5
5

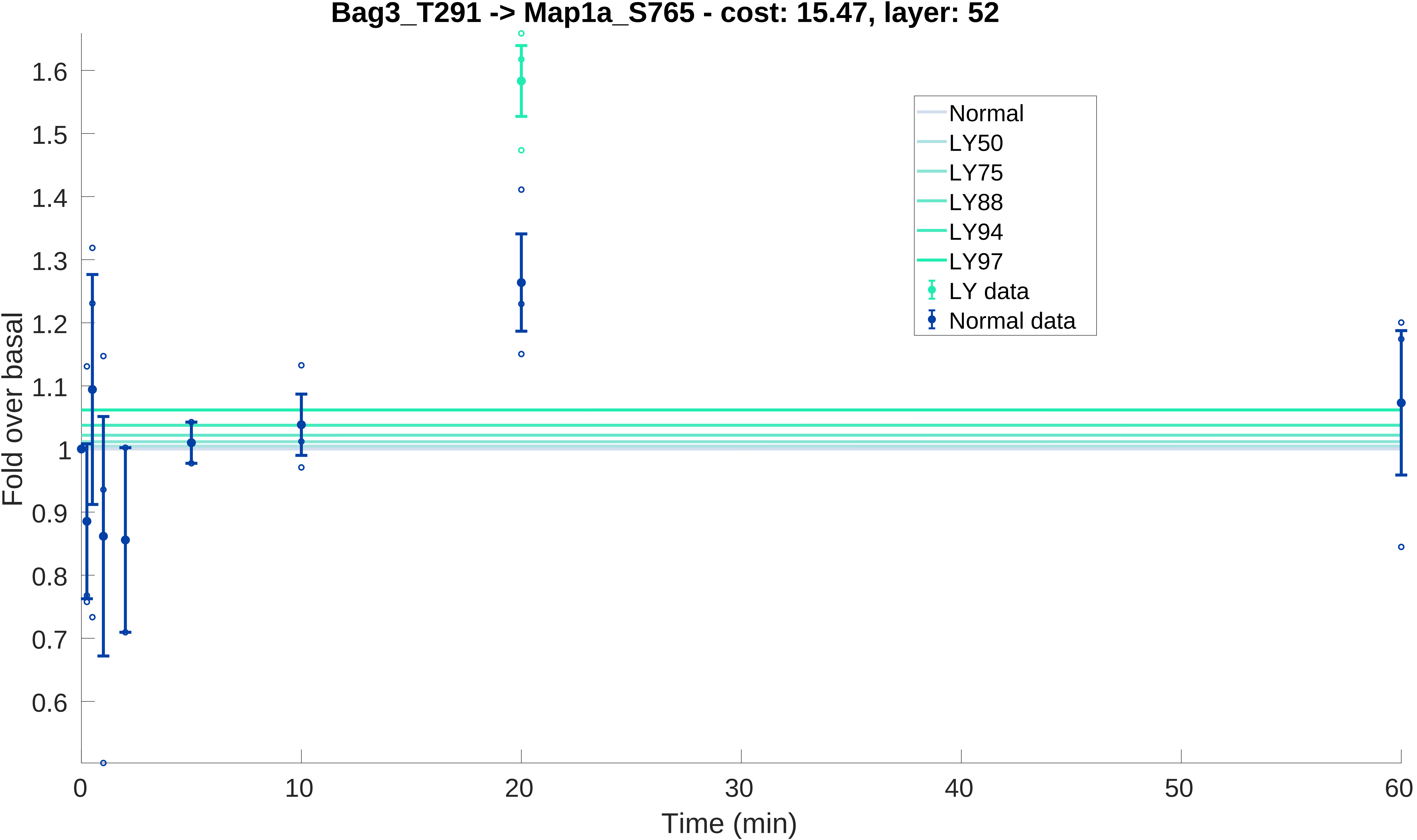

Bag3_T291 -> Map1a_S765 - cost: 15.47, layer: 52
Normal
LY50
LY75
LY88
LY94
LY97
LY data
Normal data
Fold over basal
Time (min)
1.6
1.5
1.4
1.3
1.2
1.1
1
0.9
0.8
0.7
0.6
0
10
20
30
40
50
60

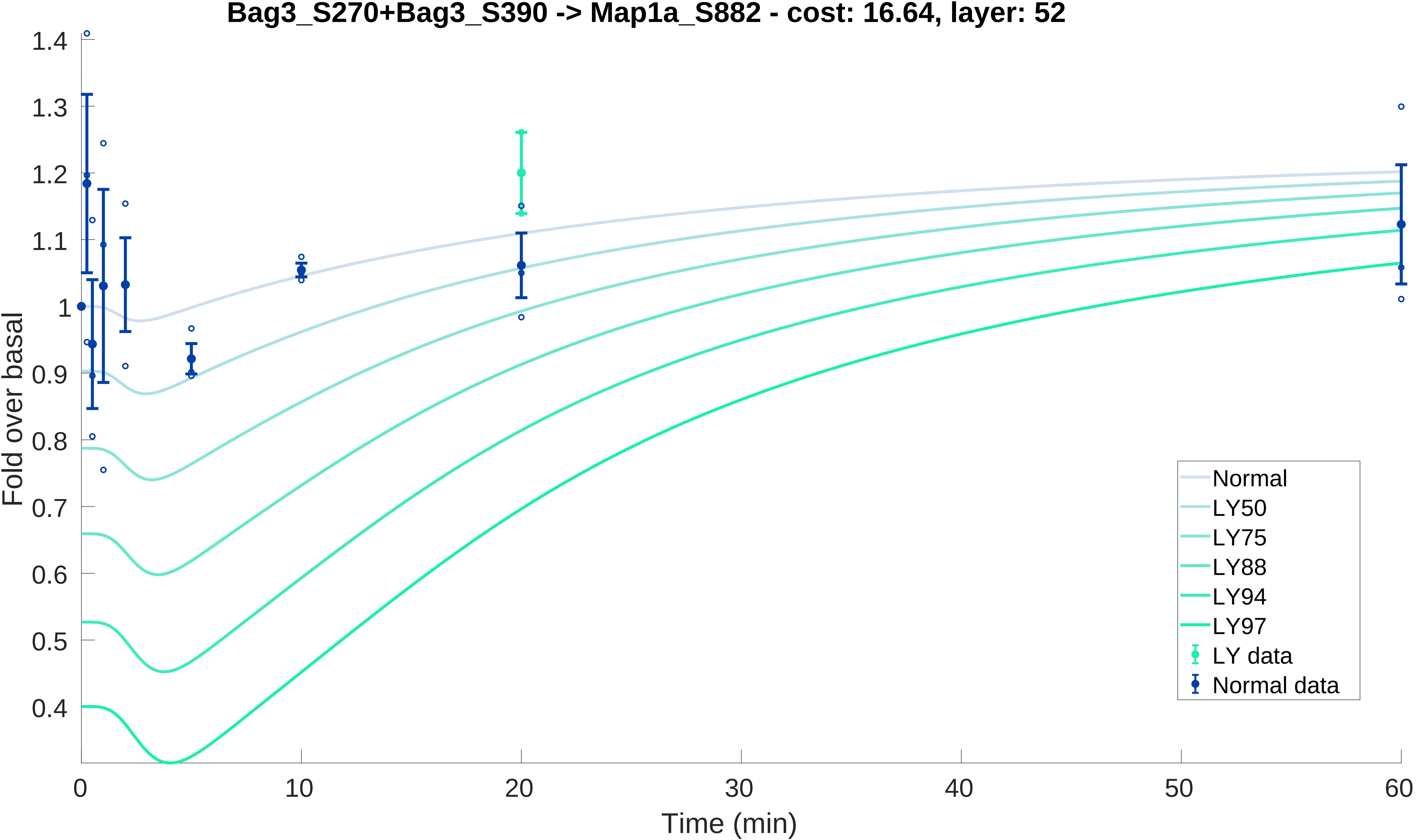

Bag3_S270+Bag3_S390 -> Map1a_S882 - cost: 16.64, layer: 52
Fold over basal
1.4
1.3
1.2
1.1
1
0.9
0.8
0.7
0.6
0.5
0.4
0
10
20
30
40
50
60
Time (min)
Normal
LY50
LY75
LY88
LY94
LY97
LY data
Normal data

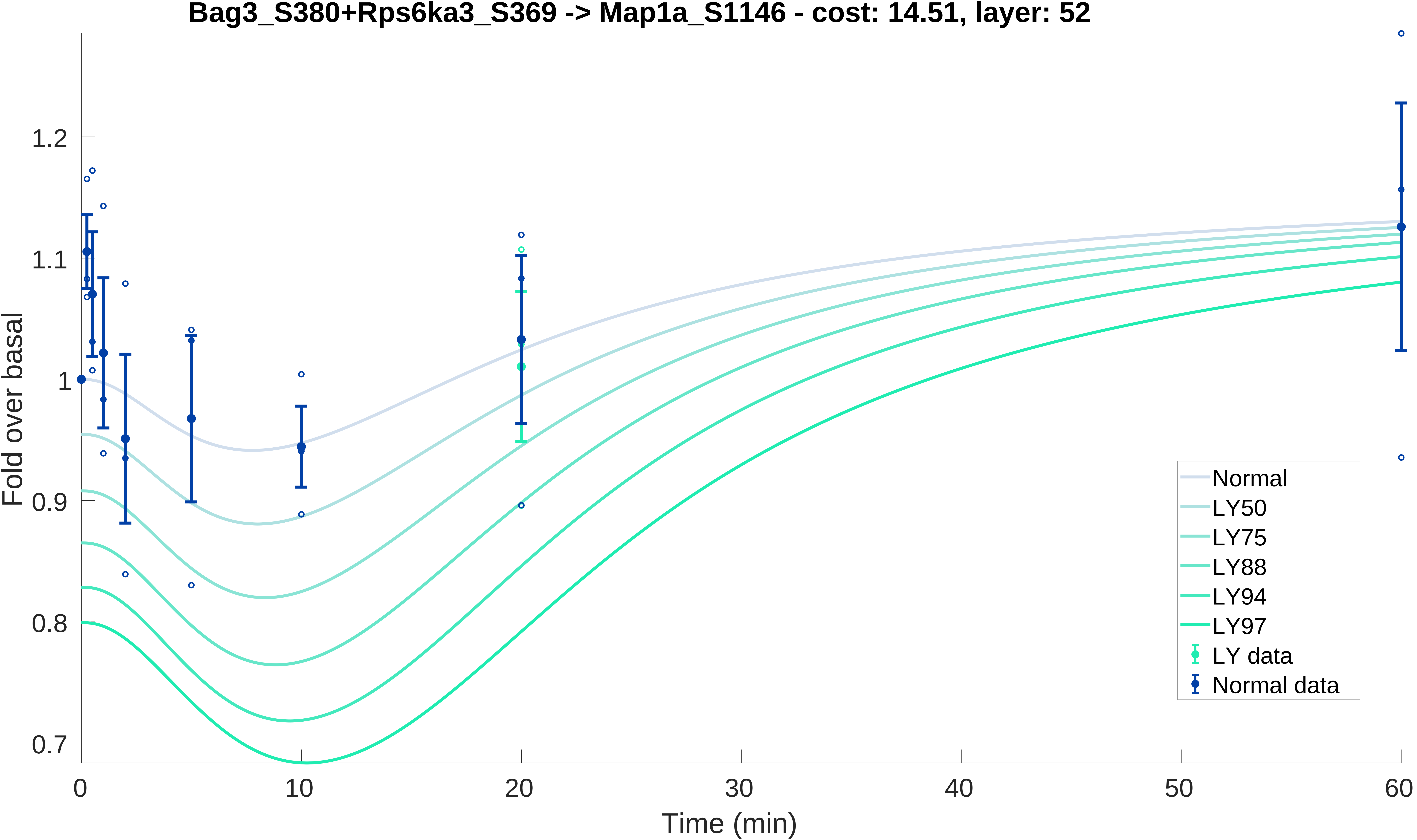
Bag3_S380+Rps6ka3_S369 -> Map1a_S1146 - cost: 14.51, layer: 52
Fold over basal
Time (min)
0.7
0.8
0.9
1
1.1
1.2
0
10
20
30
40
50
60
Normal
LY50
LY75
LY88
LY94
LY97
LY data
Normal data

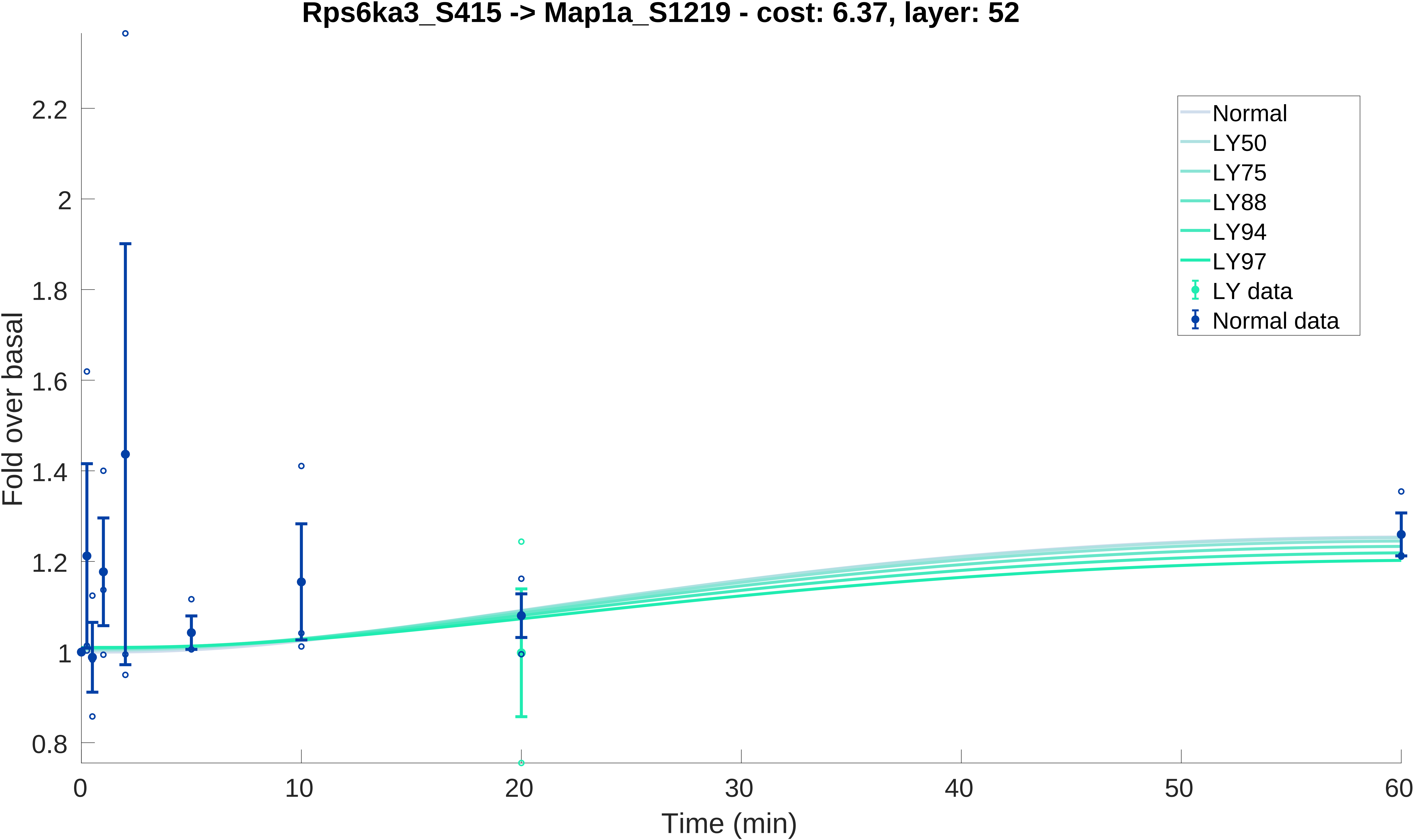

Rps6ka3_S415 -> Map1a_S1219 - cost: 6.37, layer: 52
Fold over basal
Time (min)
0.8
1
1.2
1.4
1.6
1.8
2
2.2
0
10
20
30
40
50
60
Normal
LY50
LY75
LY88
LY94
LY97
LY data
Normal data

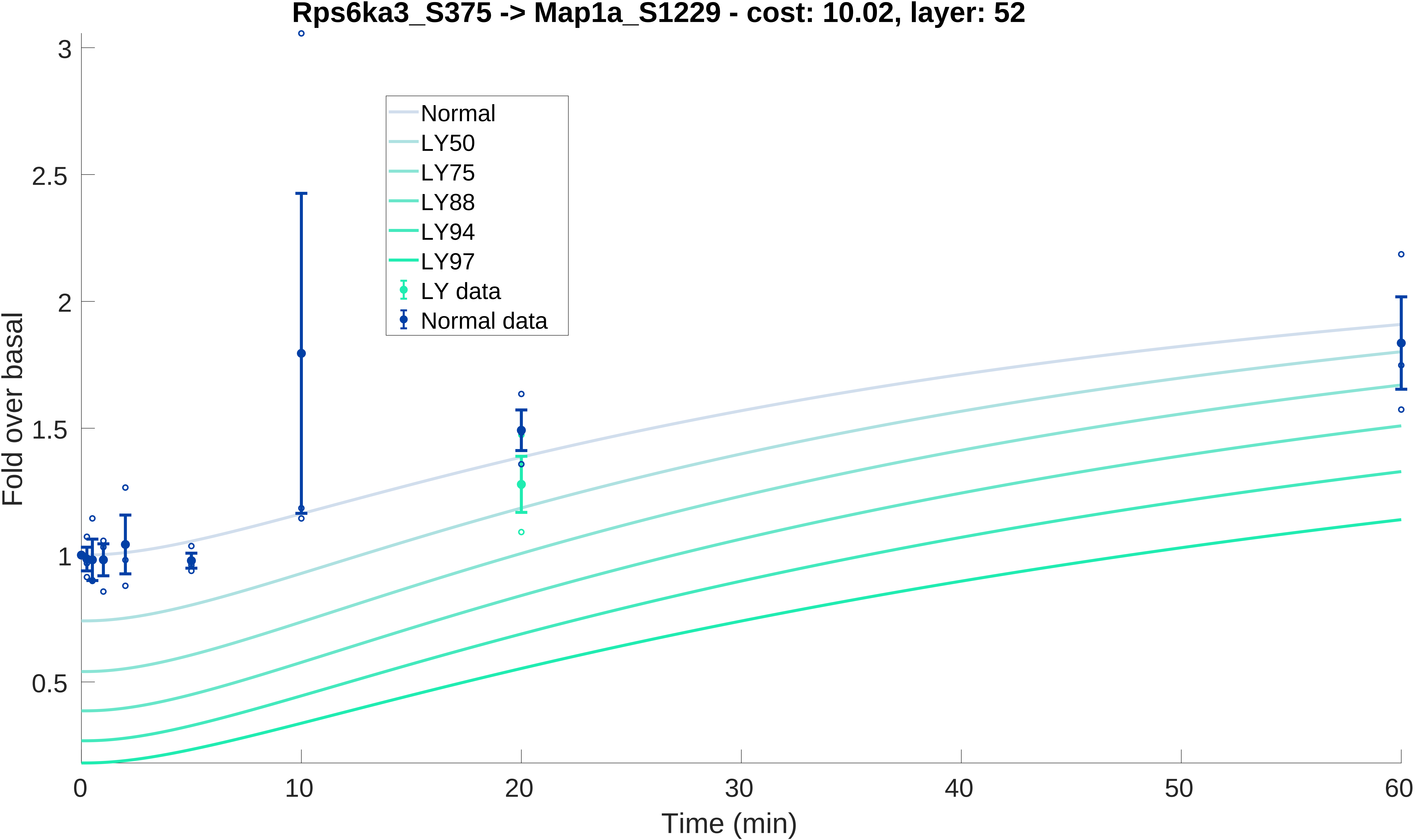

Rps6ka3_S375 -> Map1a_S1229 - cost: 10.02, layer: 52
Fold over basal
3
2.5
2
1.5
1
0.5
0
10
20
30
40
50
60
Time (min)
Normal
LY50
LY75
LY88
LY94
LY97
LY data
Normal data

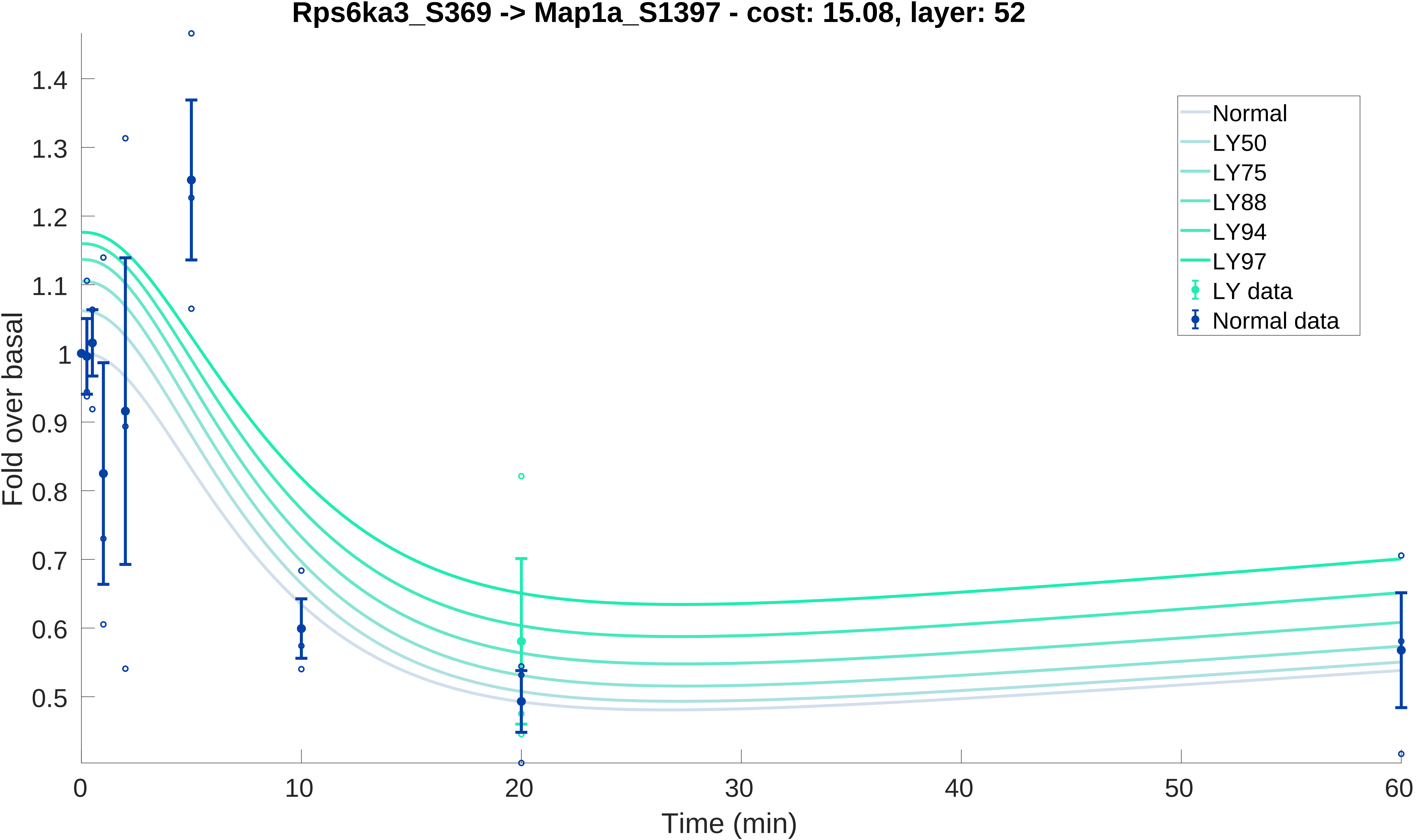

Rps6ka3_S369 -> Map1a_S1397 - cost: 15.08, layer: 52
Fold over basal
Time (min)
Normal
LY50
LY75
LY88
LY94
LY97
LY data
Normal data
1.4
1.3
1.2
1.1
1
0.9
0.8
0.7
0.6
0.5
0
10
20
30
40
50
60

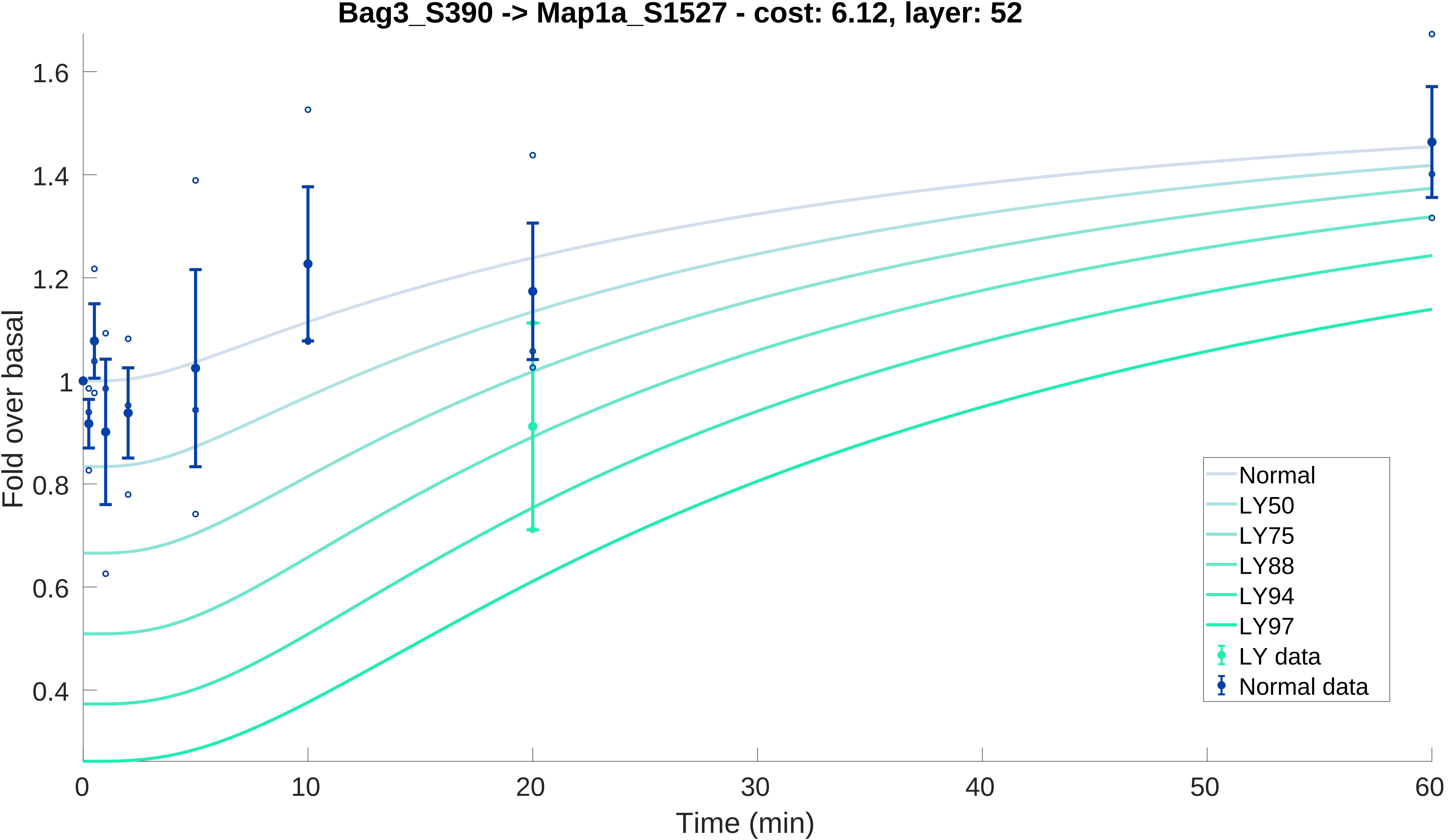
Bag3_S390 -> Map1a_S1527 - cost: 6.12, layer: 52
Fold over basal
1.6
1.4
1.2
1
0.8
0.6
0.4
0
10
20
30
40
50
60
Time (min)
Normal
LY50
LY75
LY88
LY94
LY97
LY data
Normal data

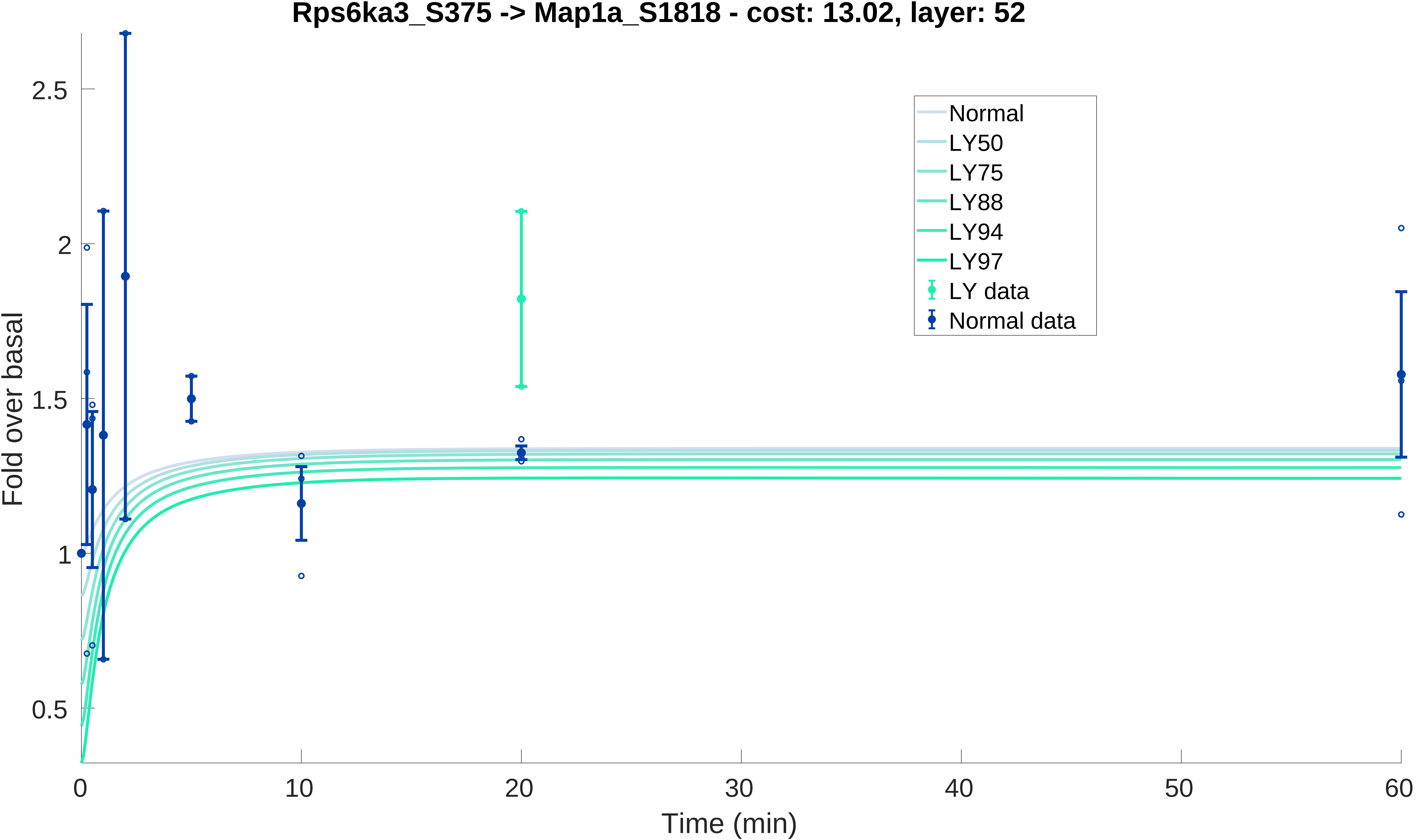
Rps6ka3_S375 -> Map1a_S1818 - cost: 13.02, layer: 52
Fold over basal
2.5
2
1.5
1
0.5
0
10
20
30
40
50
60
Time (min)
Normal
LY50
LY75
LY88
LY94
LY97
LY data
Normal data

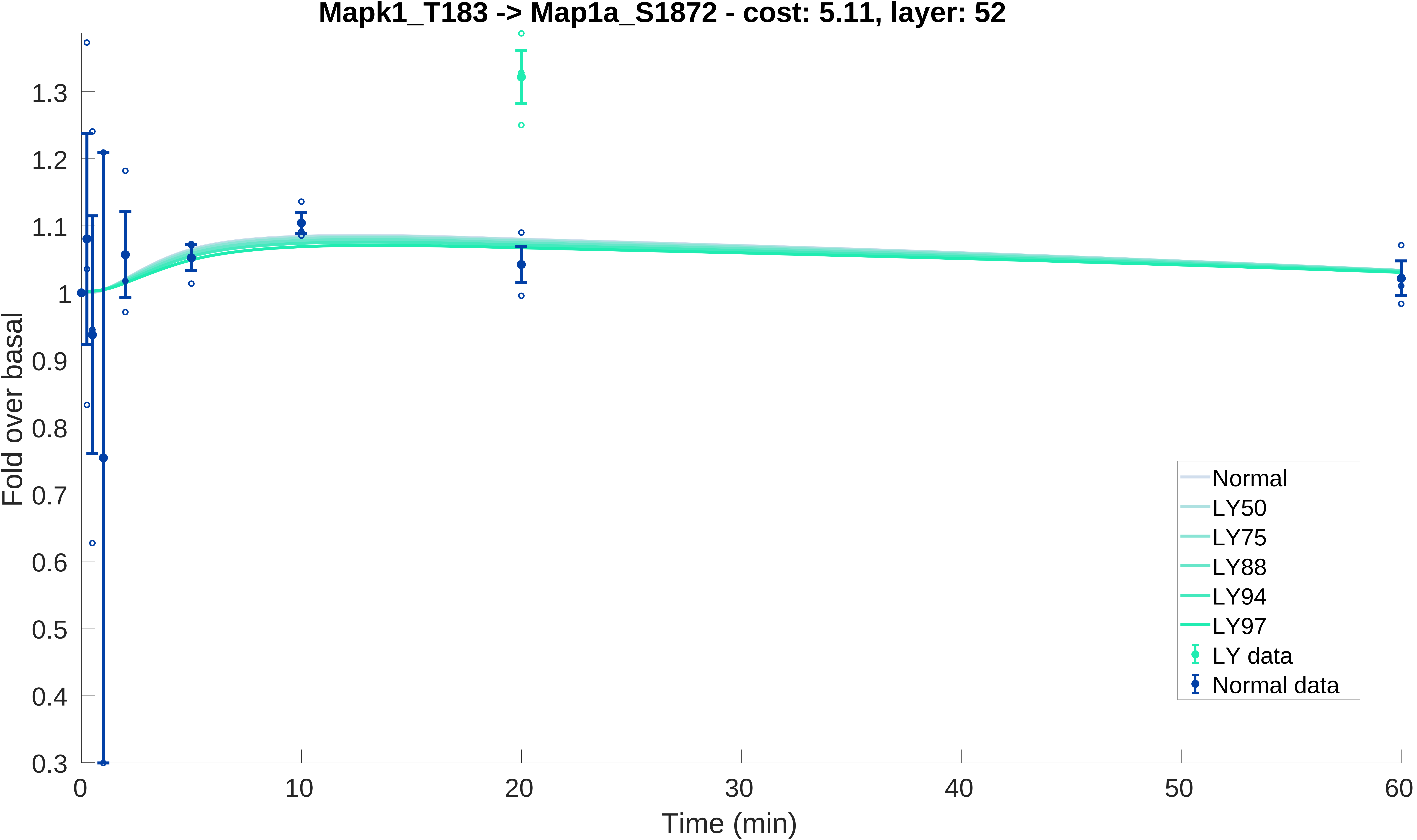

Mapk1_T183 -> Map1a_S1872 - cost: 5.11, layer: 52
Fold over basal
1.3
1.2
1.1
1
0.9
0.8
0.7
0.6
0.5
0.4
0.3
0
10
20
30
40
50
60
Time (min)
Normal
LY50
LY75
LY88
LY94
LY97
LY data
Normal data

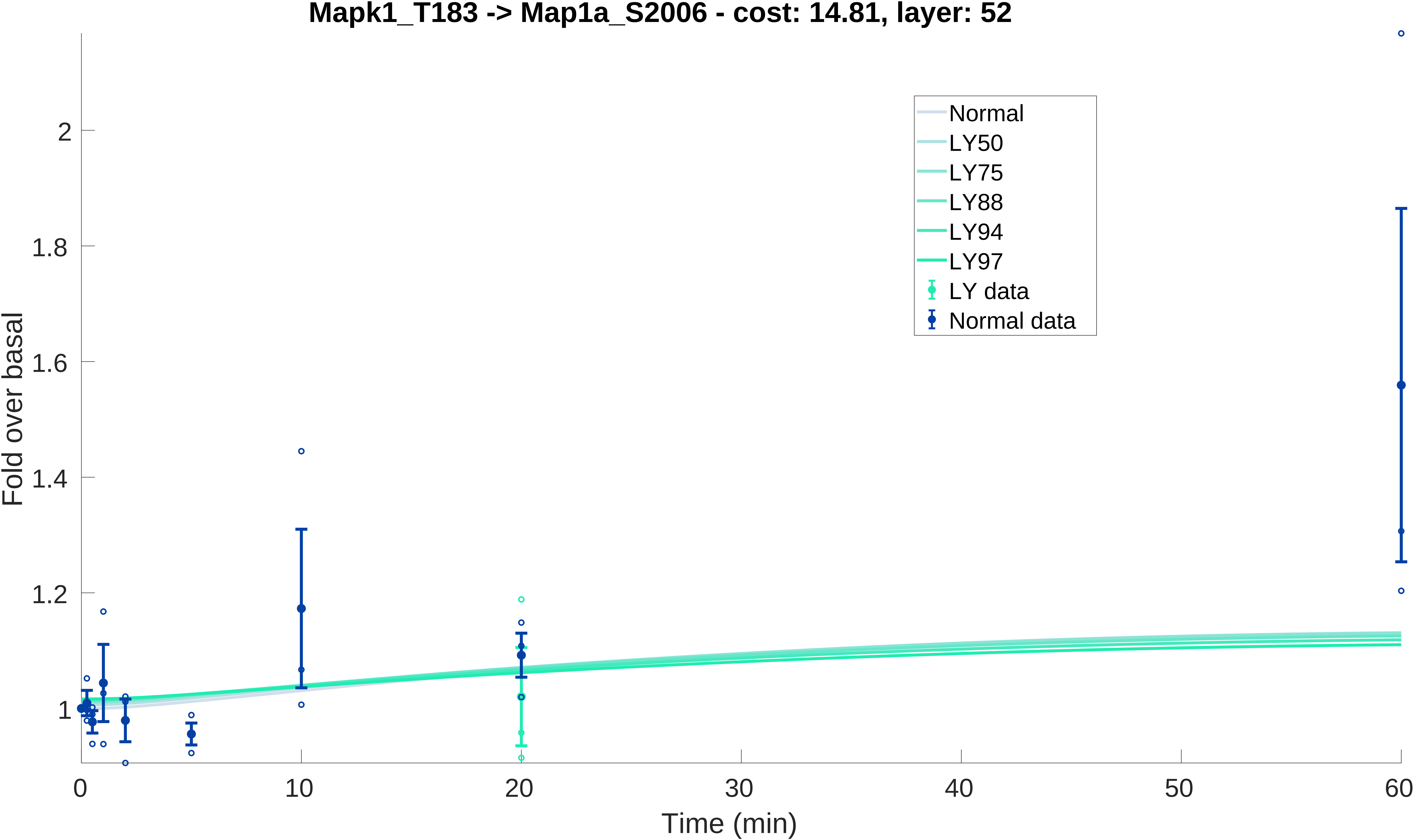
Mapk1_T183 -> Map1a_S2006 - cost: 14.81, layer: 52
Fold over basal
Time (min)
2
1.8
1.6
1.4
1.2
1
0
10
20
30
40
50
60
Normal
LY50
LY75
LY88
LY94
LY97
LY data
Normal data

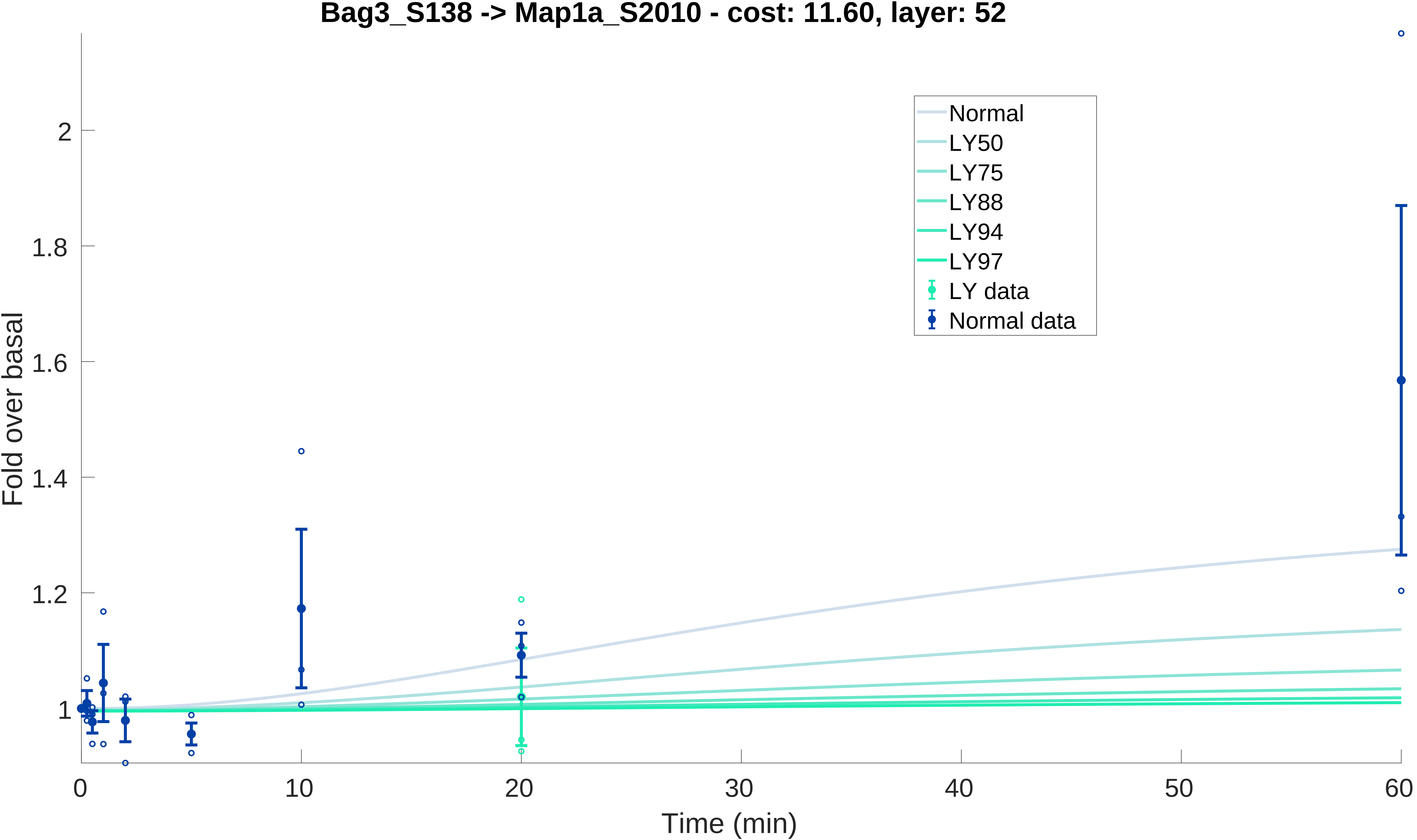

Bag3_S138 -> Map1a_S2010 - cost: 11.60, layer: 52
Fold over basal
Time (min)
2
1.8
1.6
1.4
1.2
1
0
10
20
30
40
50
60
Normal
LY50
LY75
LY88
LY94
LY97
LY data
Normal data

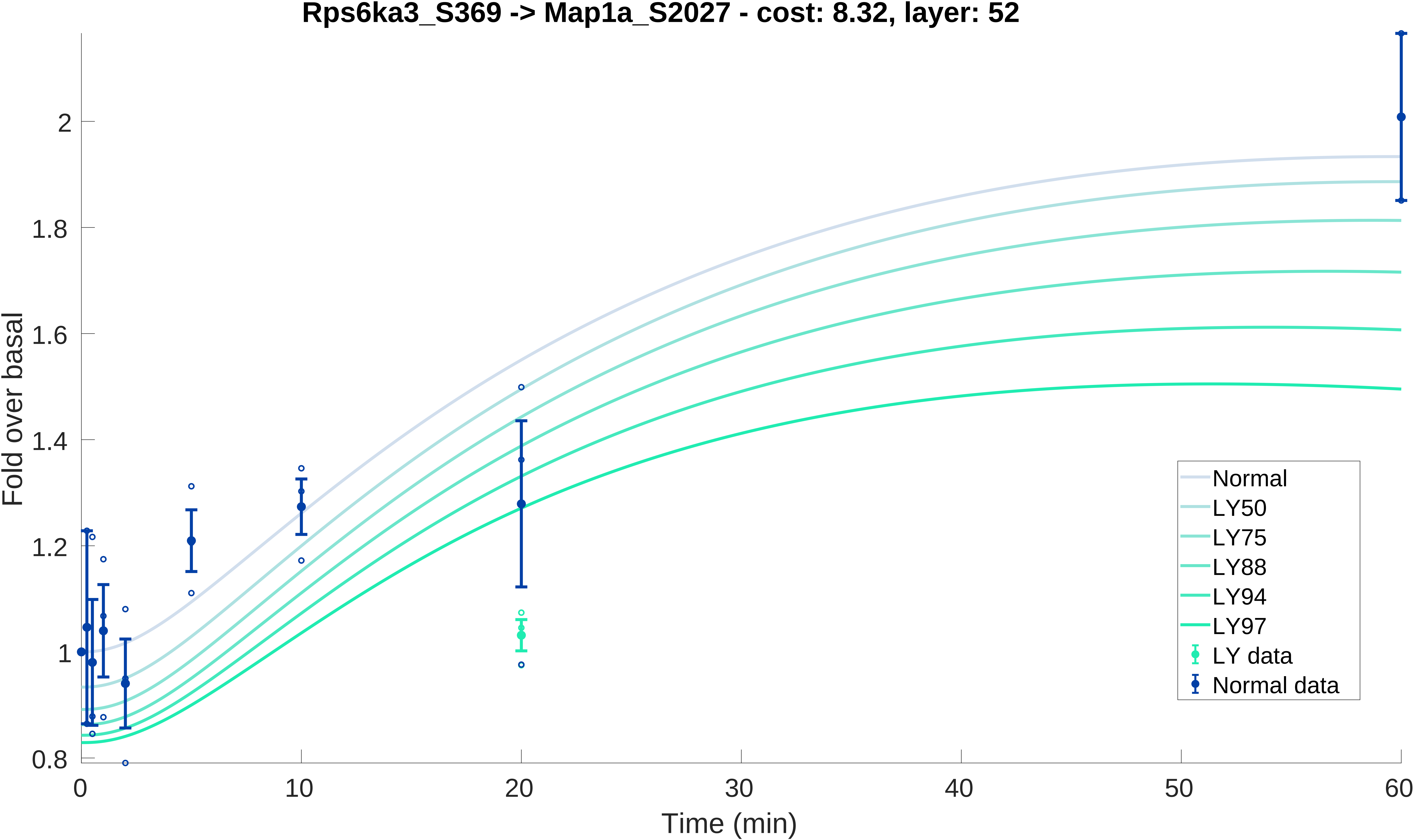

Rps6ka3_S369 -> Map1a_S2027 - cost: 8.32, layer: 52
Fold over basal
0.8
1
1.2
1.4
1.6
1.8
2
0
10
20
30
40
50
60
Time (min)
Normal
LY50
LY75
LY88
LY94
LY97
LY data
Normal data

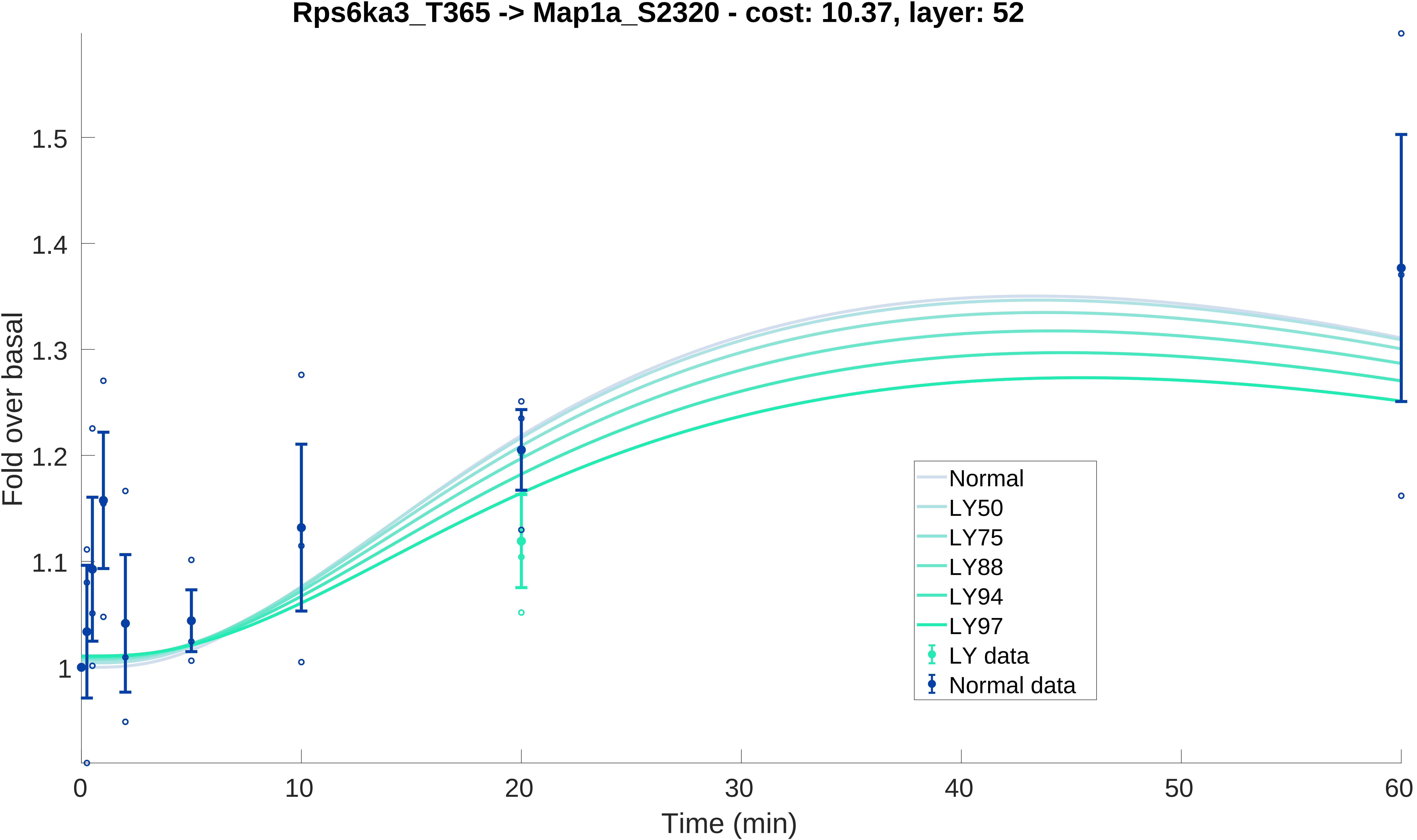

Rps6ka3_T365 -> Map1a_S2320 - cost: 10.37, layer: 52
Fold over basal
1
1.1
1.2
1.3
1.4
1.5
0
10
20
30
40
50
60
Time (min)
Normal
LY50
LY75
LY88
LY94
LY97
LY data
Normal data

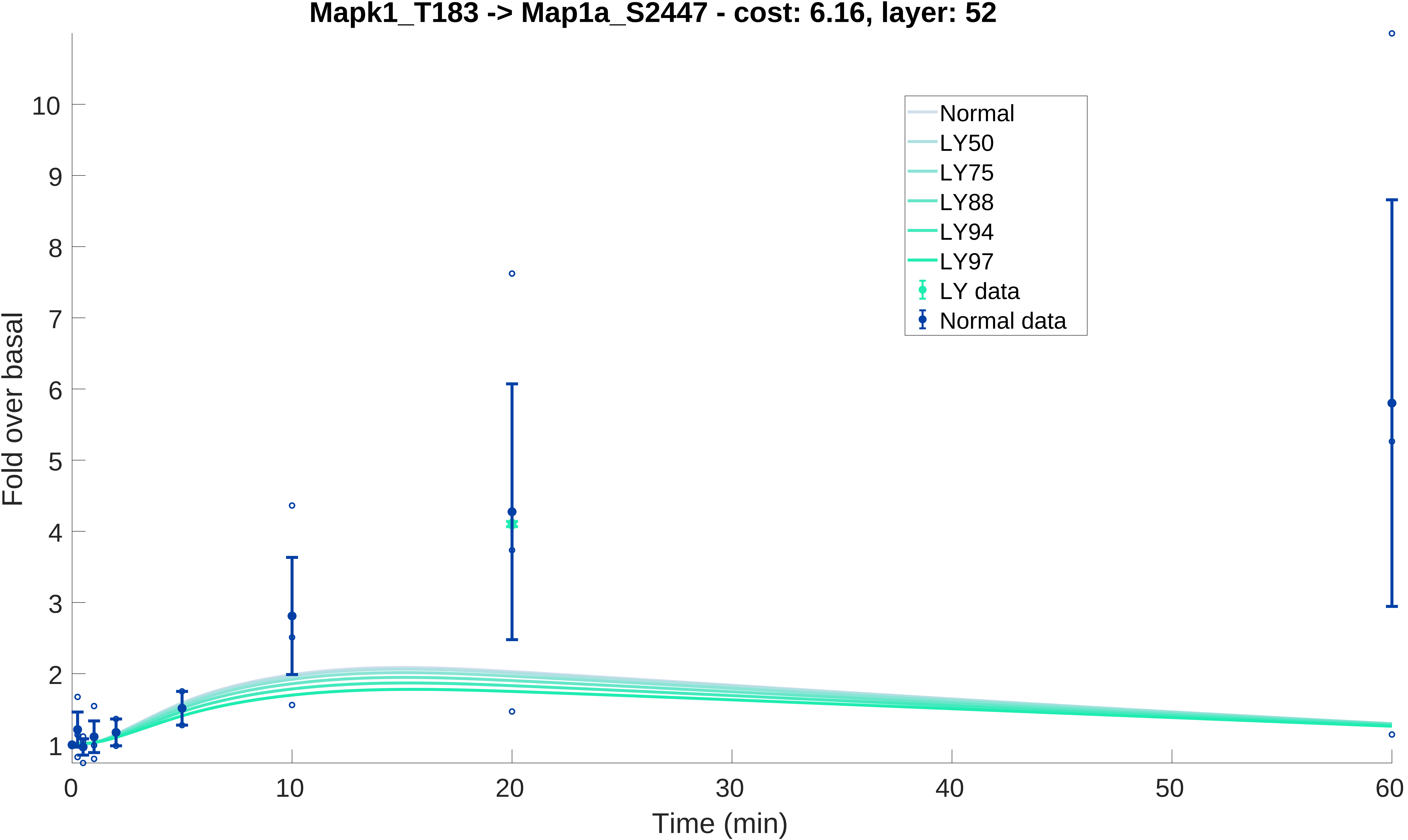

Mapk1_T183 -> Map1a_S2447 - cost: 6.16, layer: 52
Fold over basal
Time (min)
Normal
LY50
LY75
LY88
LY94
LY97
LY data
Normal data
0
10
20
30
40
50
60
1
2
3
4
5
6
7
8
9
10

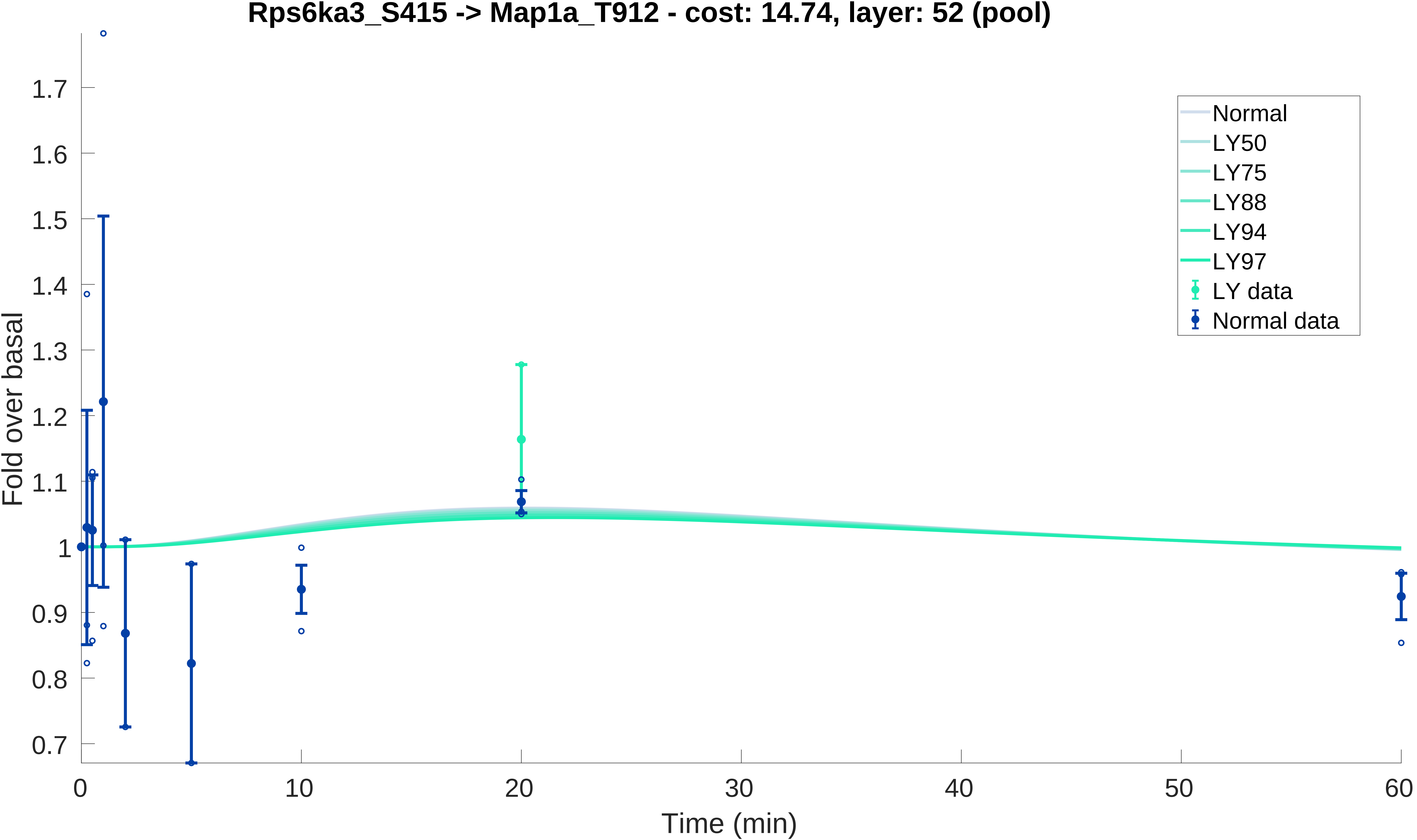

Rps6ka3_S415 -> Map1a_T912 - cost: 14.74, layer: 52 (pool)
Fold over basal
1.7
1.6
1.5
1.4
1.3
1.2
1.1
1
0.9
0.8
0.7
0
10
20
30
40
50
60
Time (min)
Normal
LY50
LY75
LY88
LY94
LY97
LY data
Normal data

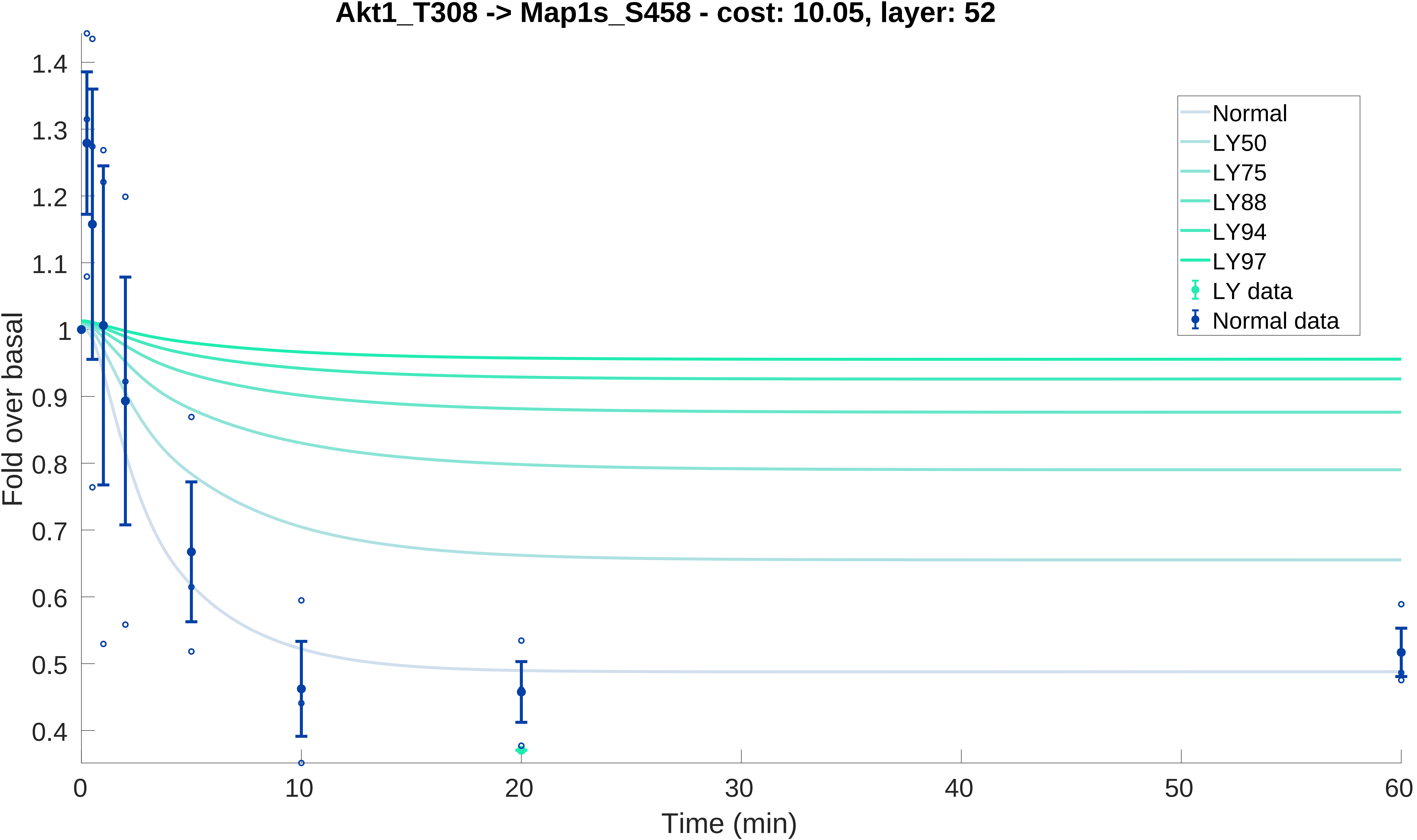

Akt1_T308 -> Map1s_S458 - cost: 10.05, layer: 52
Fold over basal
Time (min)
1.4
1.3
1.2
1.1
1
0.9
0.8
0.7
0.6
0.5
0.4
0
10
20
30
40
50
60
Normal
LY50
LY75
LY88
LY94
LY97
LY data
Normal data

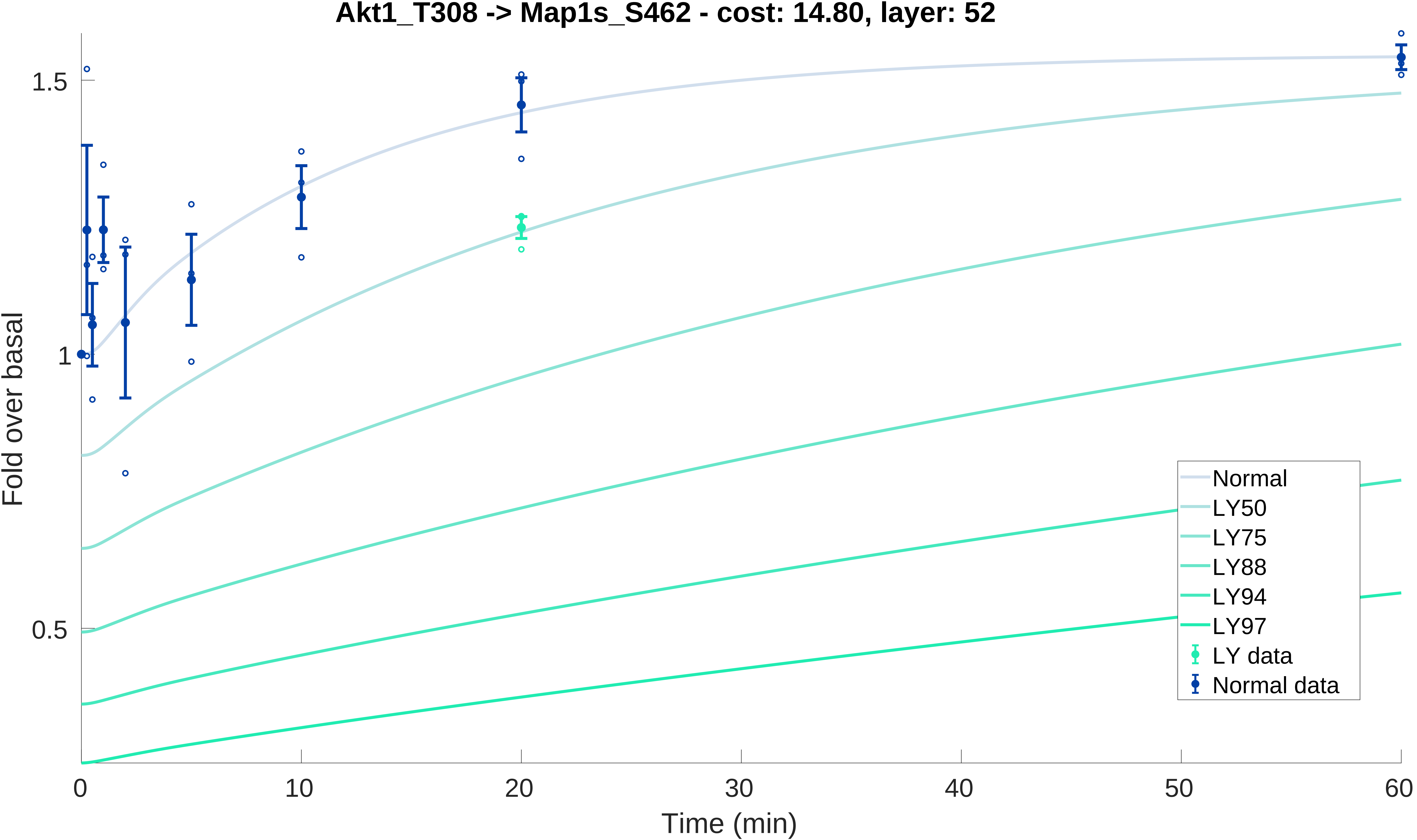

Akt1_T308 -> Map1s_S462 - cost: 14.80, layer: 52
Fold over basal
Time (min)
1.5
1
0.5
0
10
20
30
40
50
60
Normal
LY50
LY75
LY88
LY94
LY97
LY data
Normal data

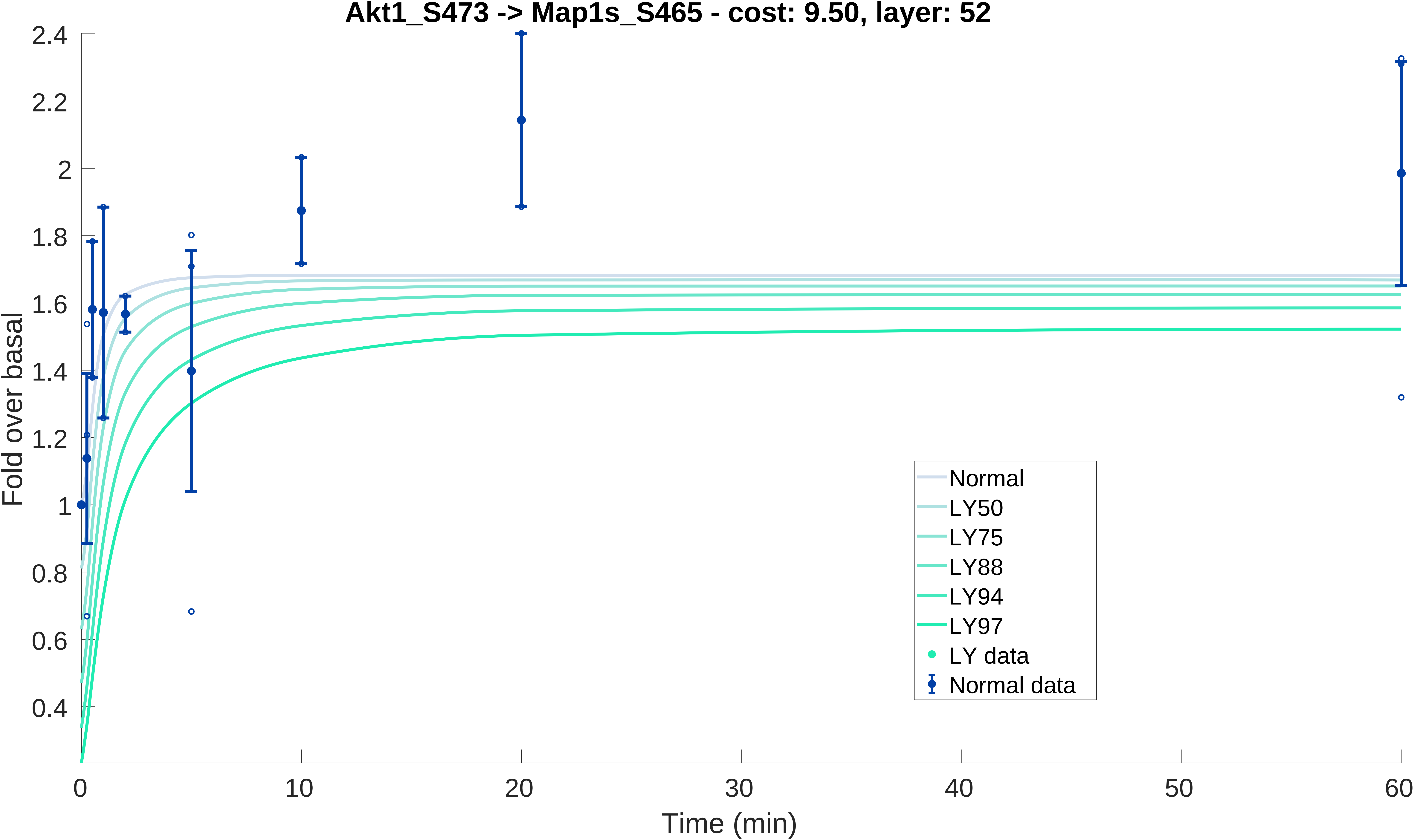

Akt1_S473 -> Map1s_S465 - cost: 9.50, layer: 52
Fold over basal
Time (min)
0.4
0.6
0.8
1
1.2
1.4
1.6
1.8
2
2.2
2.4
0
10
20
30
40
50
60
Normal
LY50
LY75
LY88
LY94
LY97
LY data
Normal data

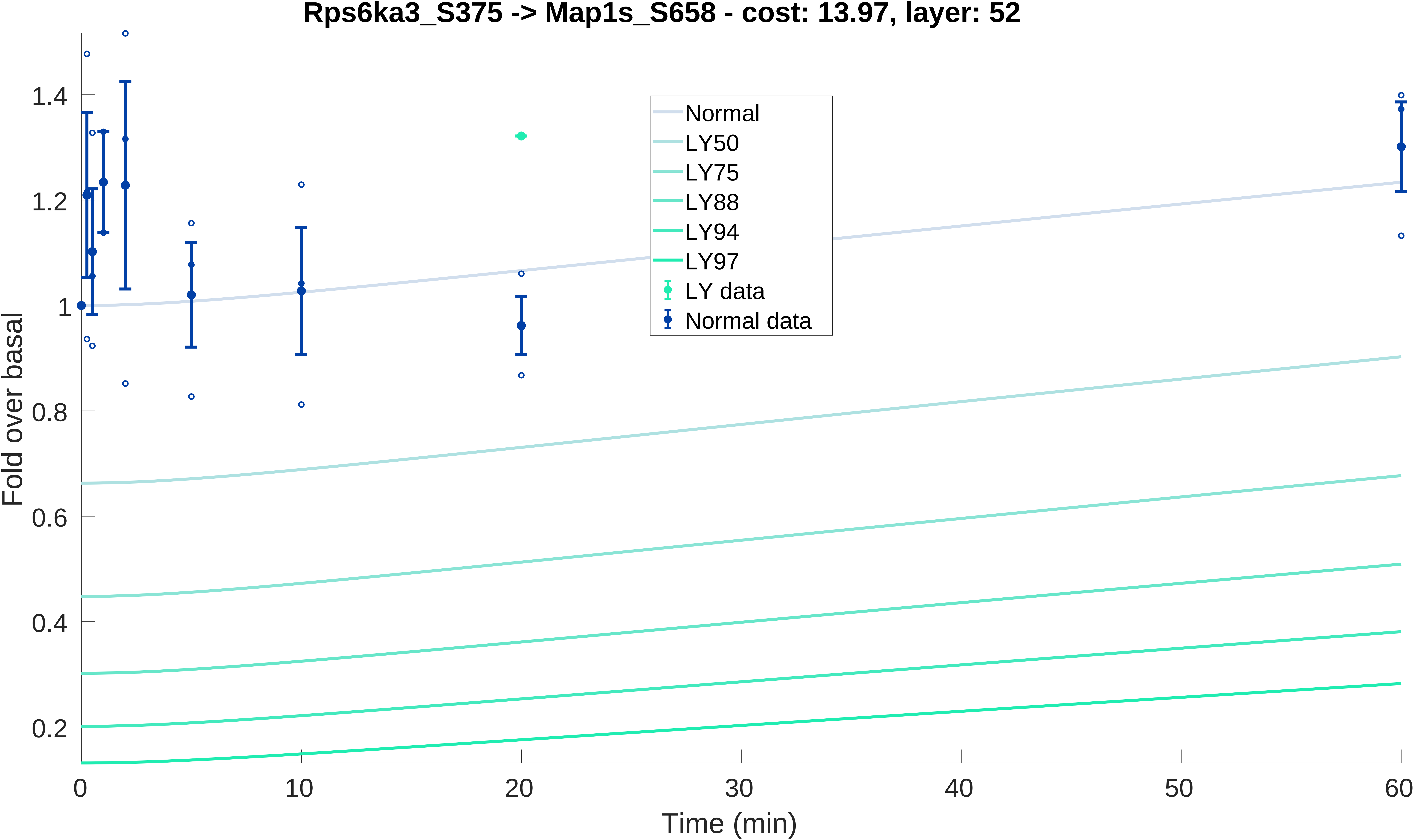

Rps6ka3_S375 -> Map1s_S658 - cost: 13.97, layer: 52
Normal
LY50
LY75
LY88
LY94
LY97
LY data
Normal data
Fold over basal
Time (min)
0.2
0.4
0.6
0.8
1
1.2
1.4
0
10
20
30
40
50
60

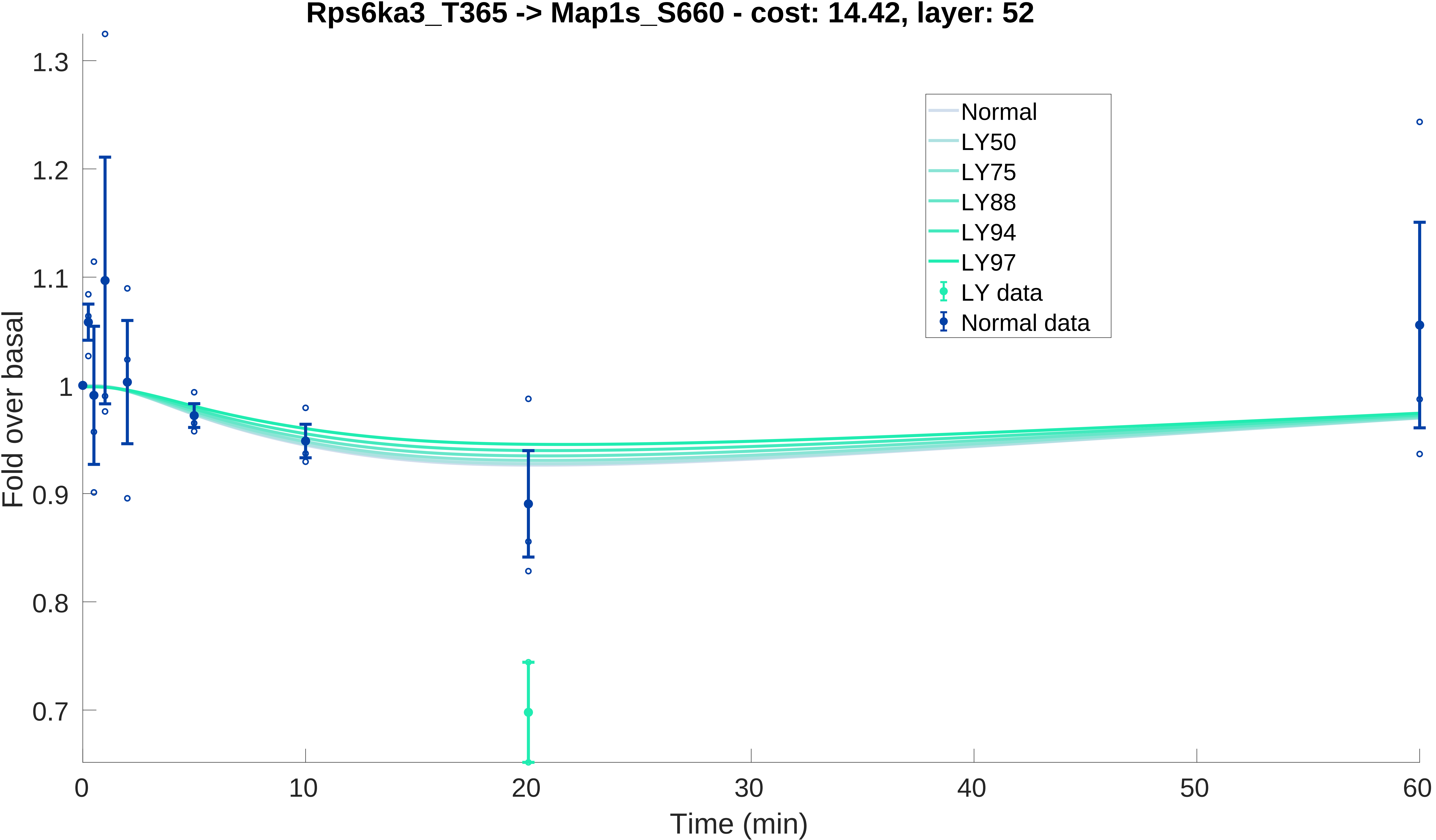

Rps6ka3_T365 -> Map1s_S660 - cost: 14.42, layer: 52
Normal
LY50
LY75
LY88
LY94
LY97
LY data
Normal data
Fold over basal
Time (min)
1.3
1.2
1.1
1
0.9
0.8
0.7
0
10
20
30
40
50
60

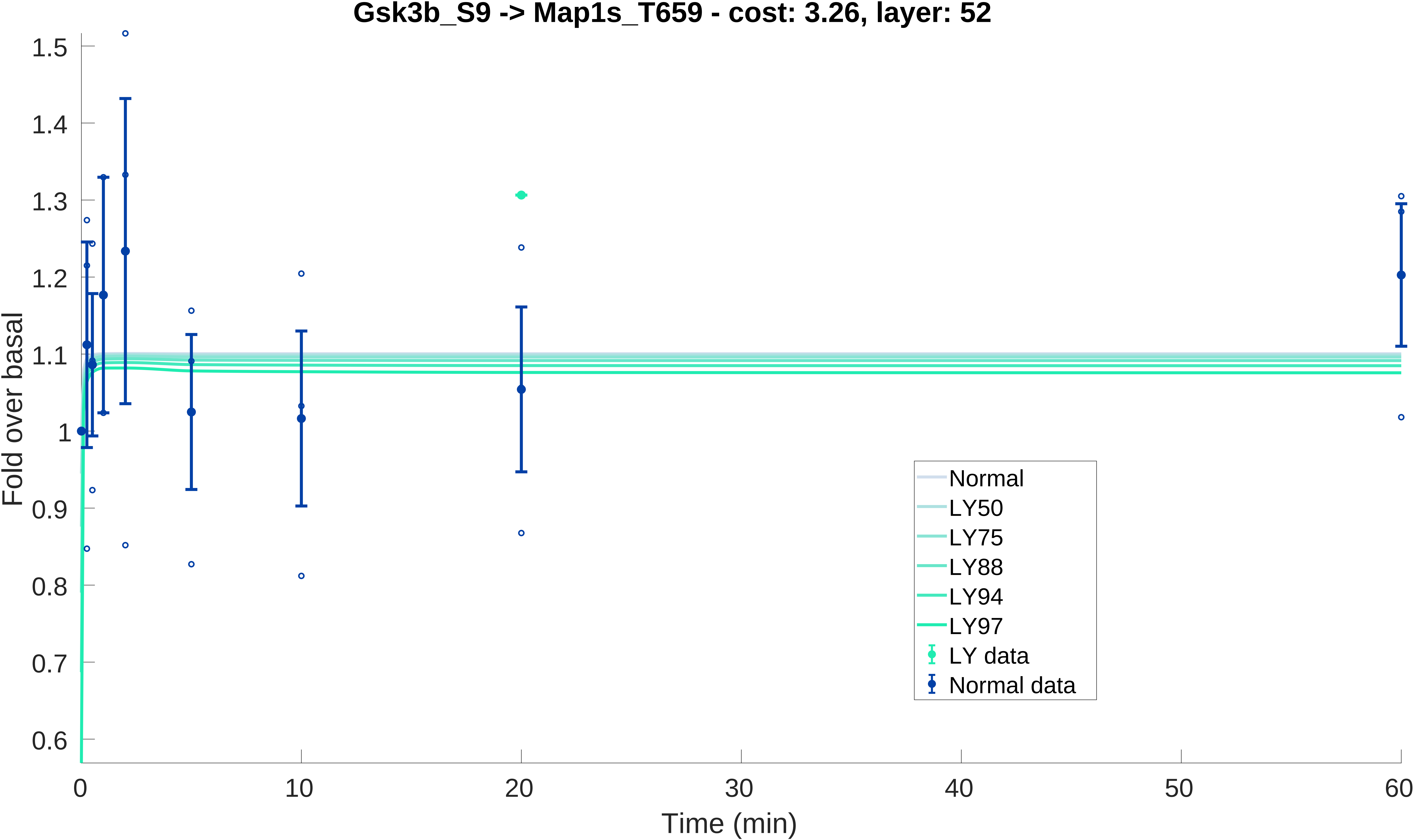
Gsk3b_S9 -> Map1s_T659 - cost: 3.26, layer: 52
Fold over basal
1.5
1.4
1.3
1.2
1.1
1
0.9
0.8
0.7
0.6
0
10
20
30
40
50
60
Time (min)
Normal
LY50
LY75
LY88
LY94
LY97
LY data
Normal data

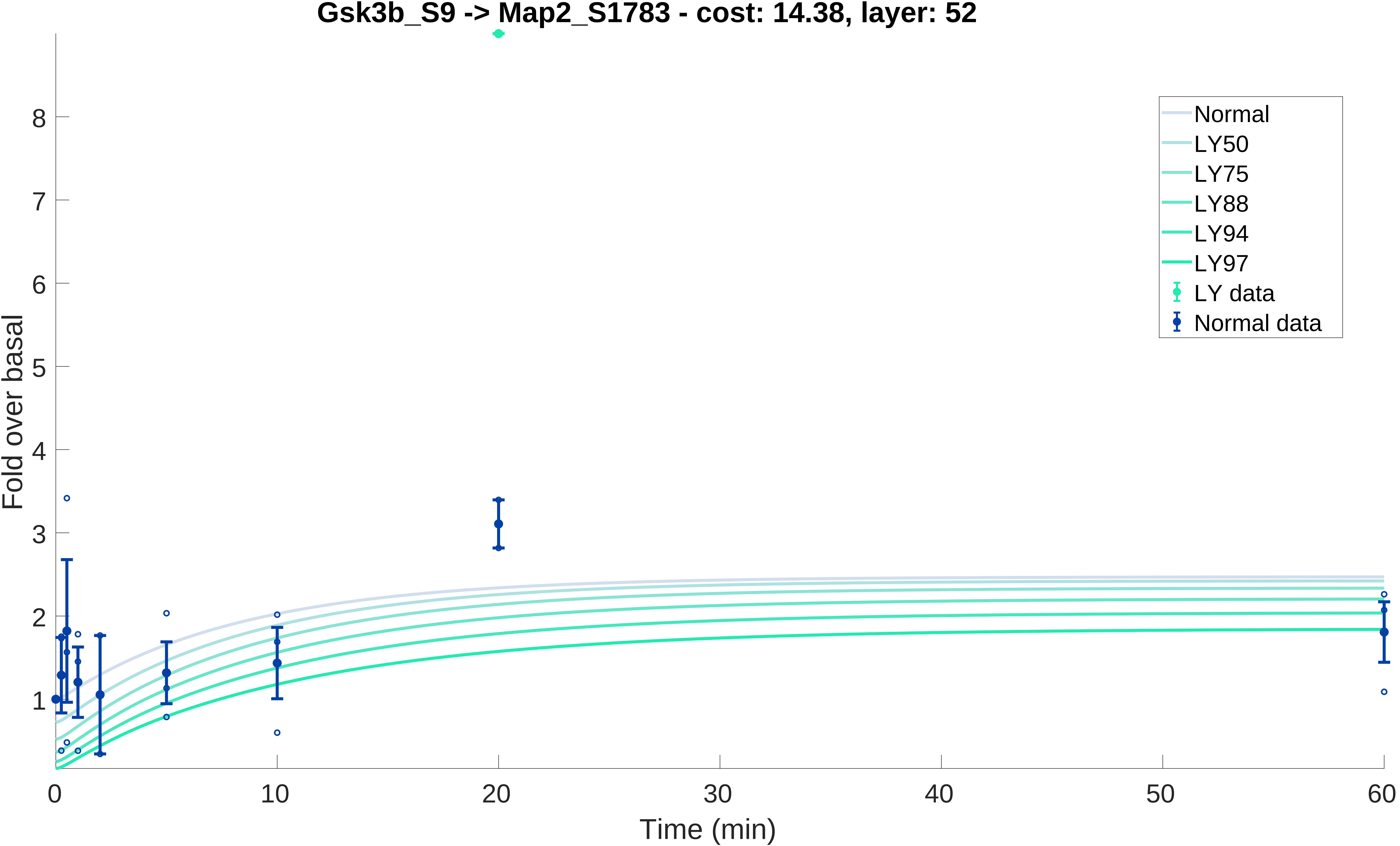

Gsk3b_S9 -> Map2_S1783 - cost: 14.38, layer: 52
Fold over basal
Time (min)
0
10
20
30
40
50
60
1
2
3
4
5
6
7
8
Normal
LY50
LY75
LY88
LY94
LY97
LY data
Normal data

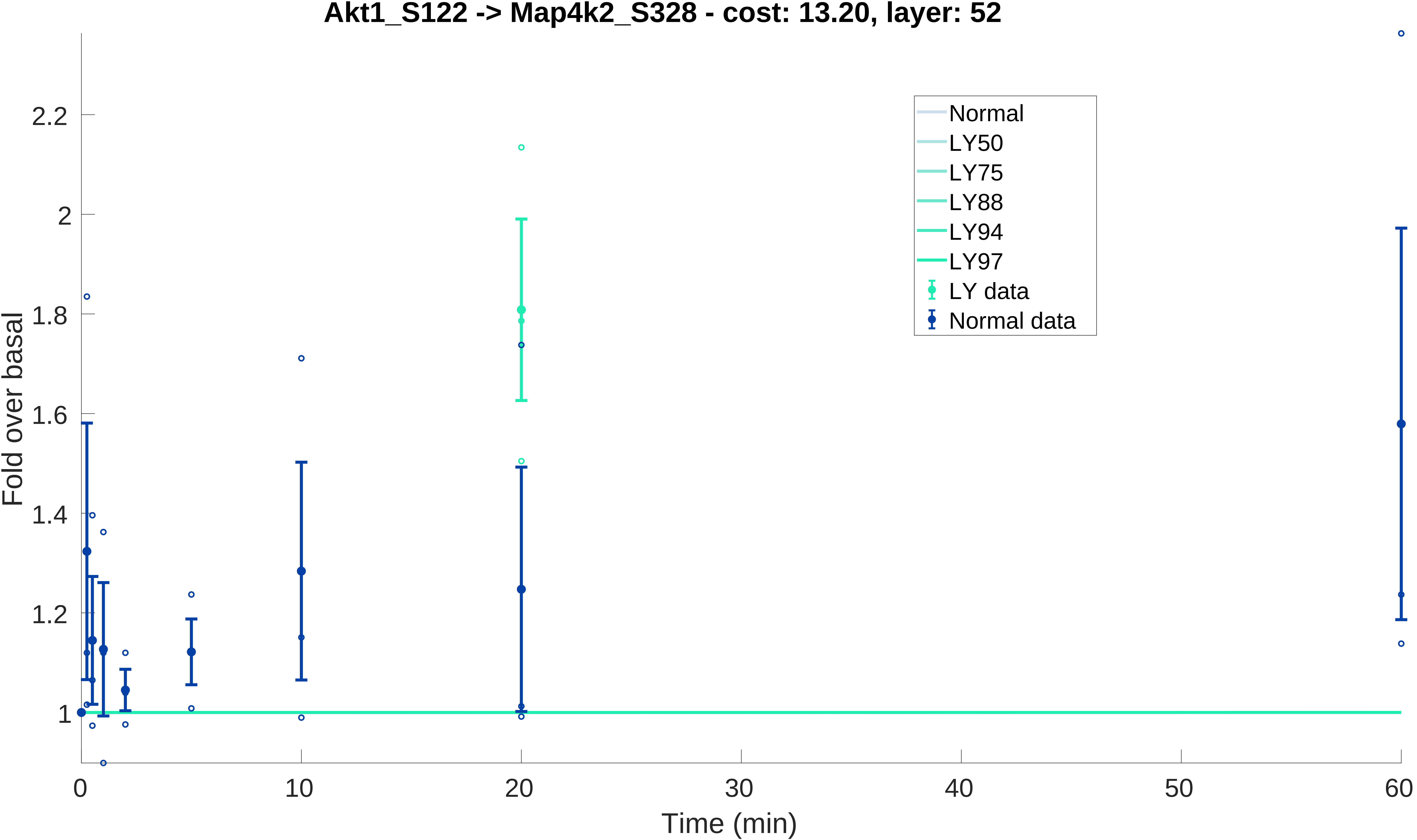

Akt1_S122 -> Map4k2_S328 - cost: 13.20, layer: 52
Fold over basal
Time (min)
0
10
20
30
40
50
60
1
1.2
1.4
1.6
1.8
2
2.2
Normal
LY50
LY75
LY88
LY94
LY97
LY data
Normal data

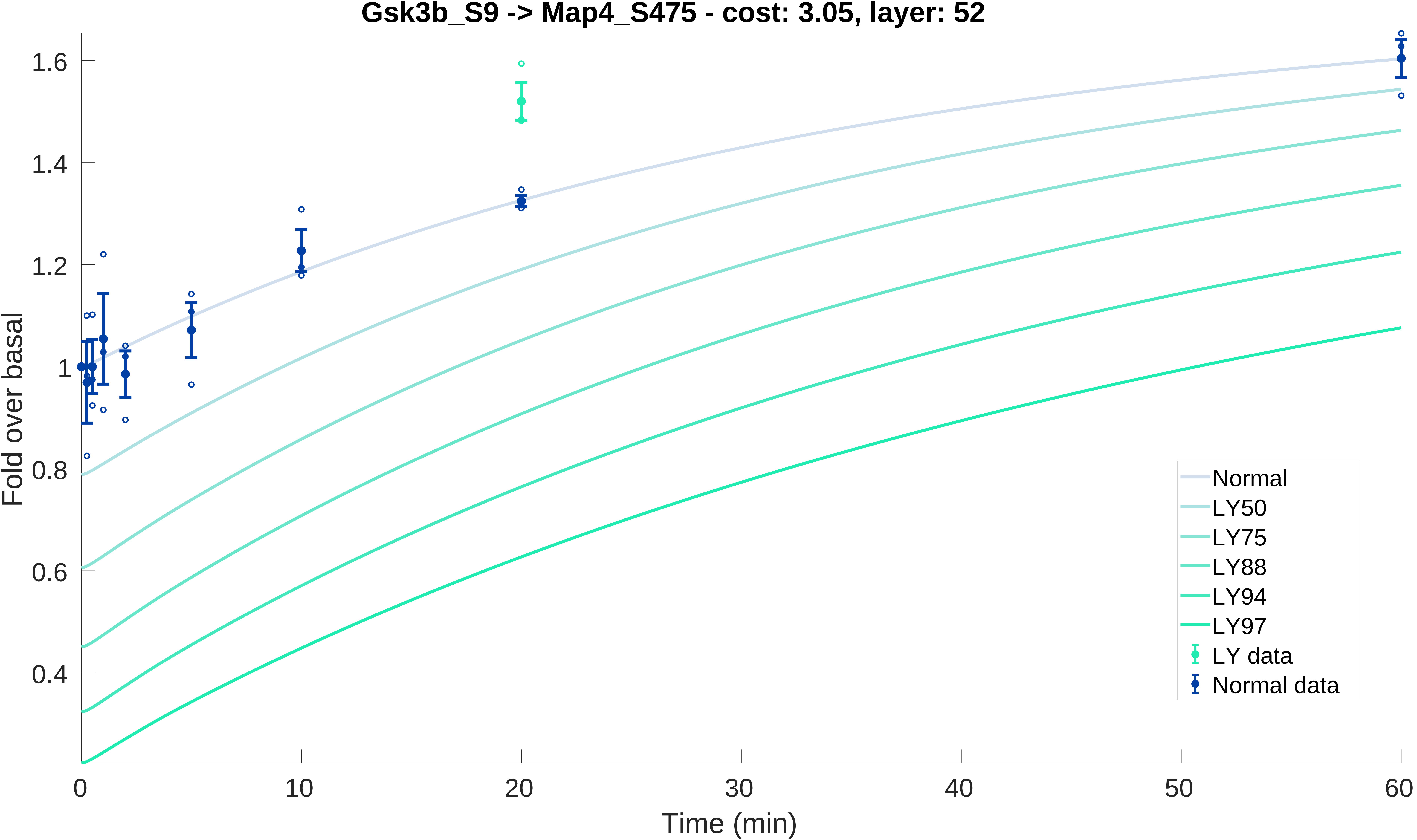
Gsk3b_S9 -> Map4_S475 - cost: 3.05, layer: 52
Fold over basal
Time (min)
0
10
20
30
40
50
60
0.4
0.6
0.8
1
1.2
1.4
1.6
Normal
LY50
LY75
LY88
LY94
LY97
LY data
Normal data

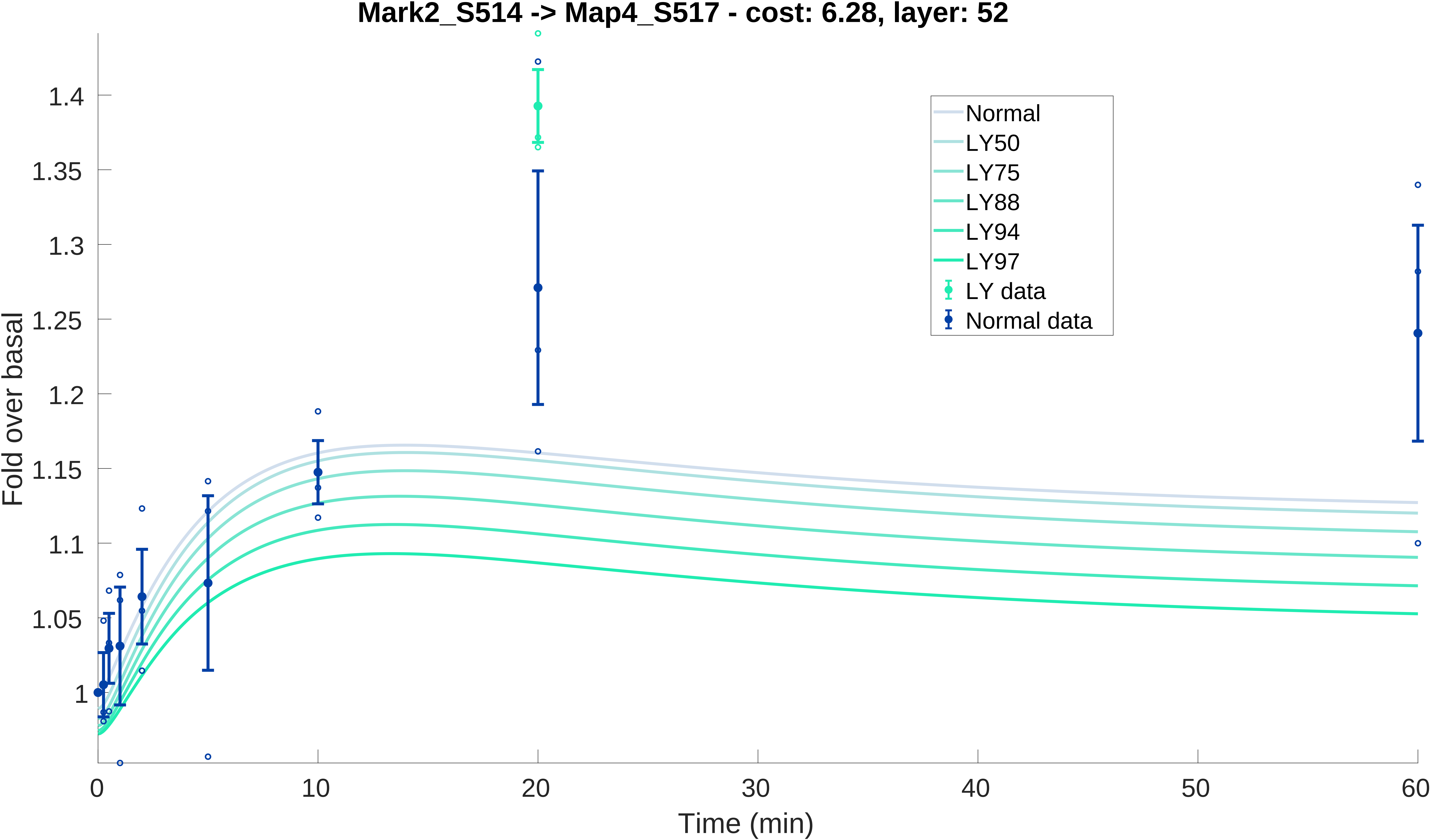
Mark2_S514 -> Map4_S517 - cost: 6.28, layer: 52
Fold over basal
1.4
1.35
1.3
1.25
1.2
1.15
1.1
1.05
1
0
10
20
30
40
50
60
Time (min)
Normal
LY50
LY75
LY88
LY94
LY97
LY data
Normal data

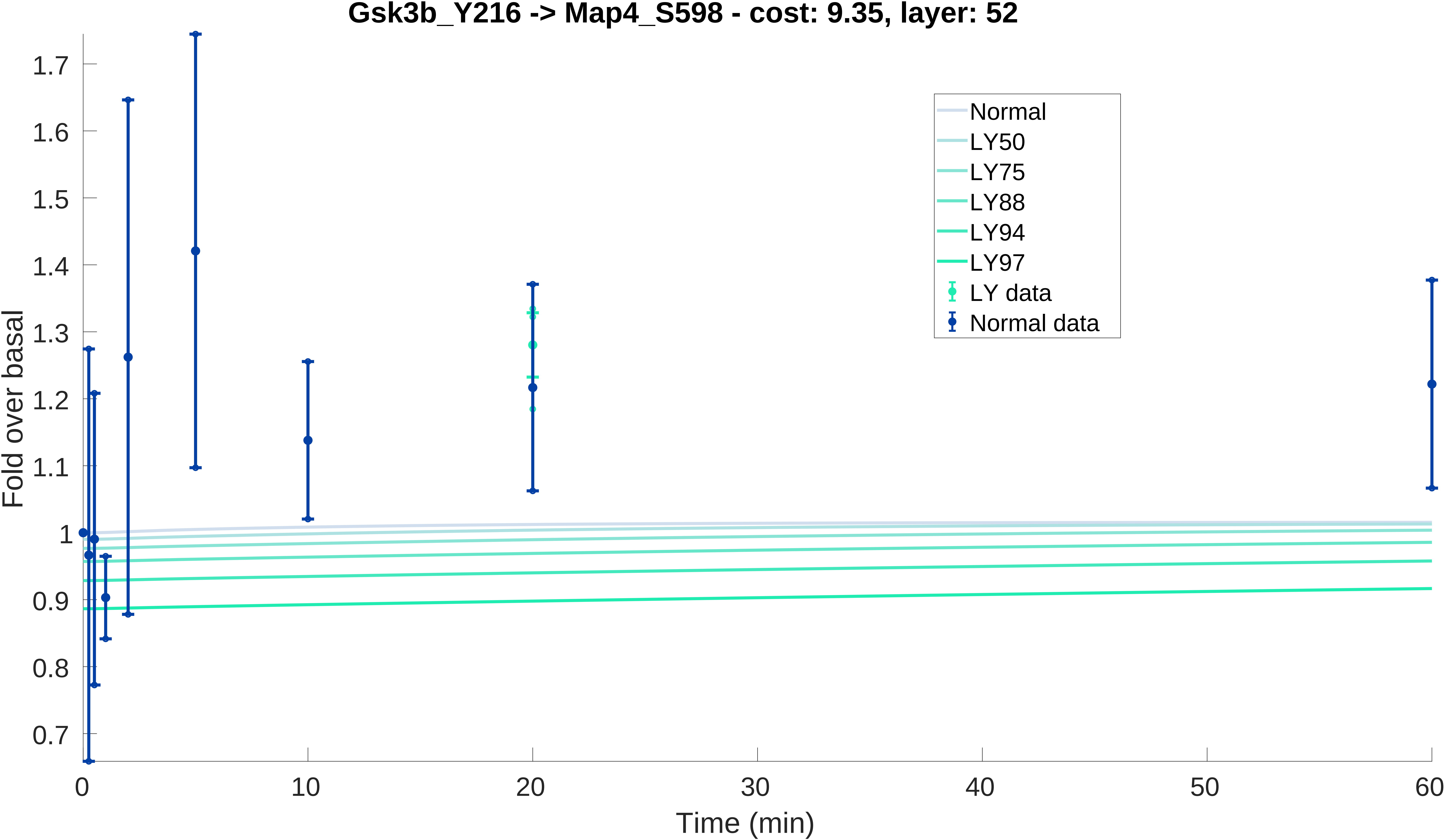

Gsk3b_Y216 -> Map4_S598 - cost: 9.35, layer: 52
Fold over basal
Time (min)
0.7
0.8
0.9
1
1.1
1.2
1.3
1.4
1.5
1.6
1.7
0
10
20
30
40
50
60
Normal
LY50
LY75
LY88
LY94
LY97
LY data
Normal data

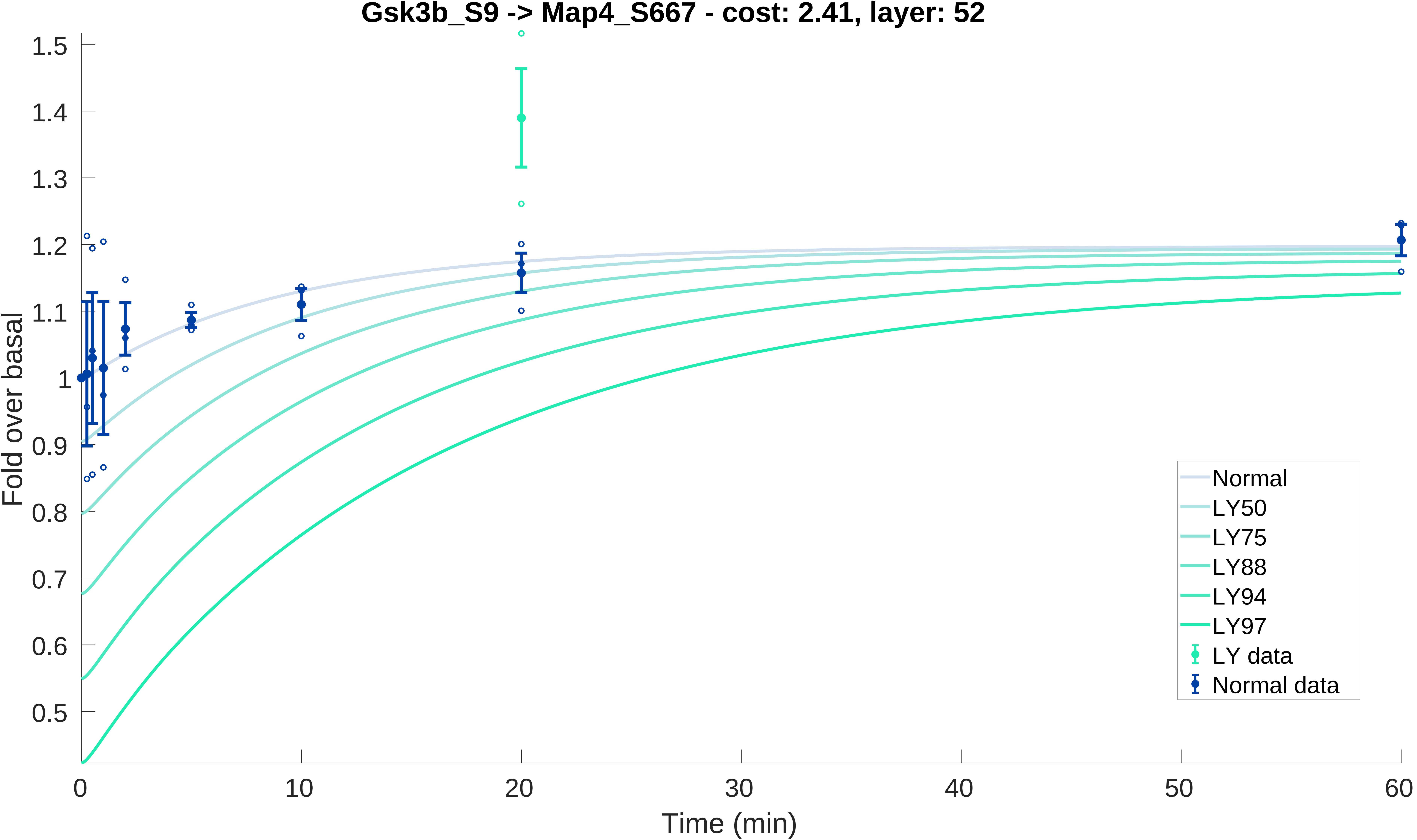
Gsk3b_S9 -> Map4_S667 - cost: 2.41, layer: 52
Fold over basal
Time (min)
1.5
1.4
1.3
1.2
1.1
1
0.9
0.8
0.7
0.6
0.5
0
10
20
30
40
50
60
Normal
LY50
LY75
LY88
LY94
LY97
LY data
Normal data

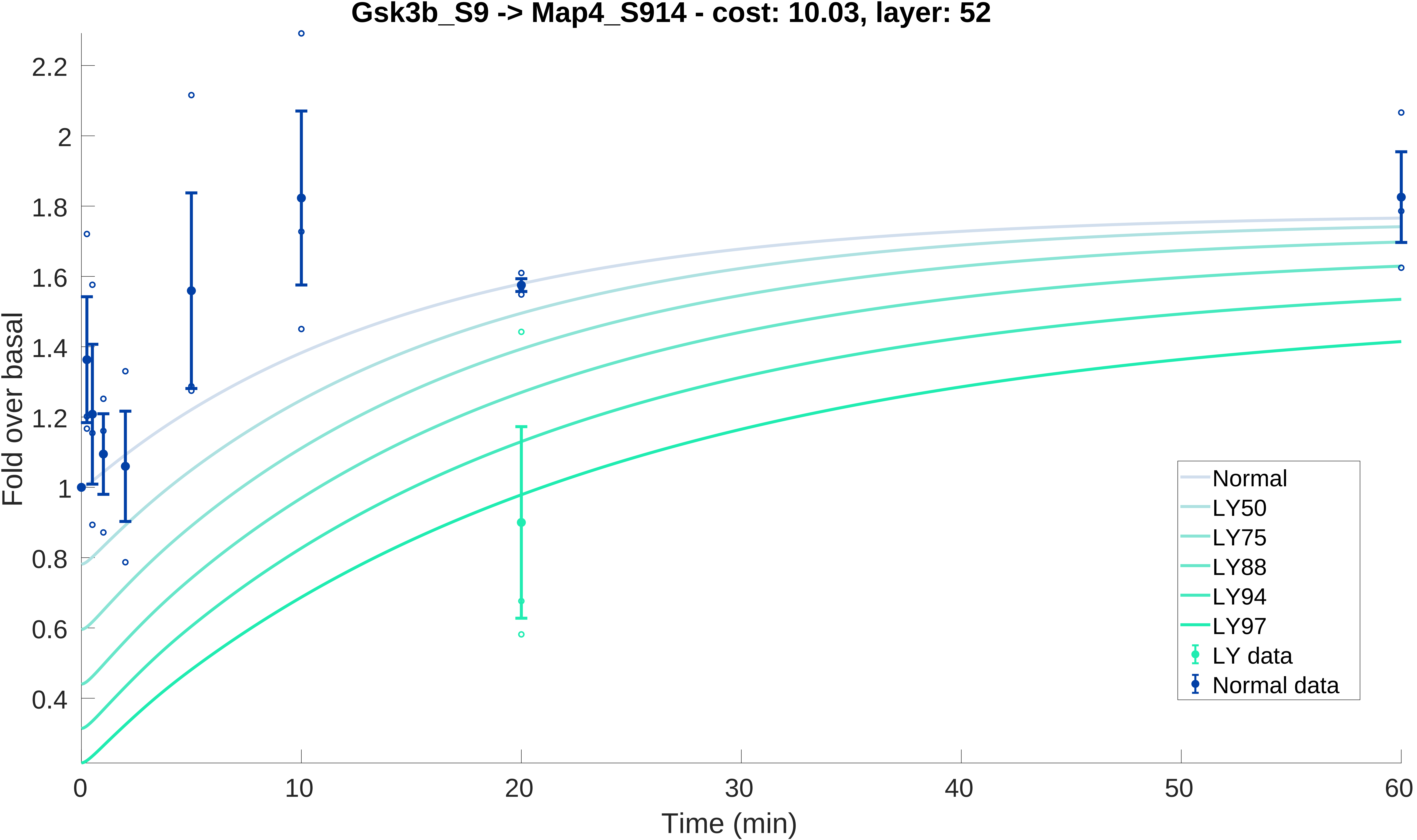

Gsk3b_S9 -> Map4_S914 - cost: 10.03, layer: 52
Fold over basal
Time (min)
2.2
2
1.8
1.6
1.4
1.2
1
0.8
0.6
0.4
0
10
20
30
40
50
60
Normal
LY50
LY75
LY88
LY94
LY97
LY data
Normal data

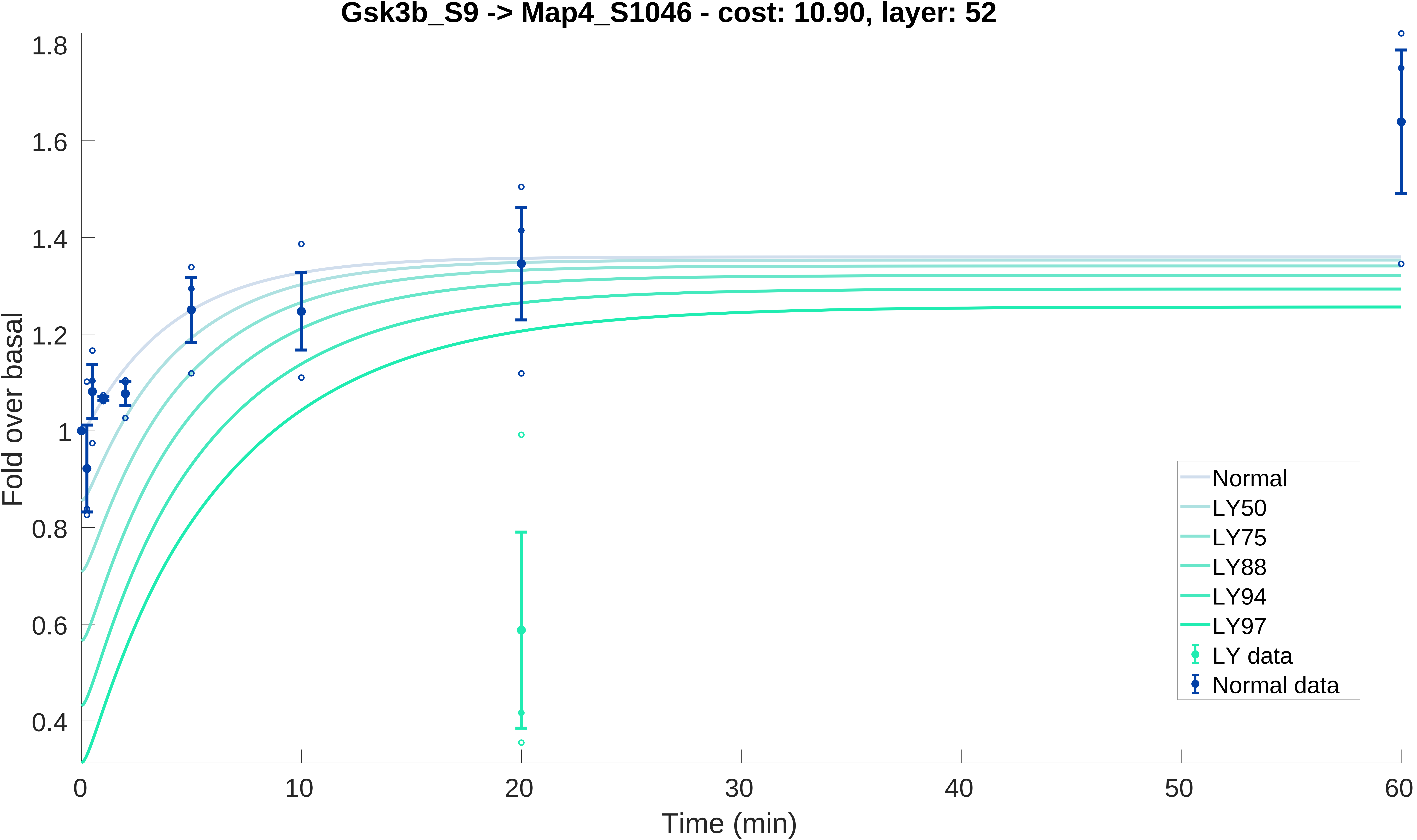

Gsk3b_S9 -> Map4_S1046 - cost: 10.90, layer: 52
Fold over basal
Time (min)
1.8
1.6
1.4
1.2
1
0.8
0.6
0.4
0
10
20
30
40
50
60
Normal
LY50
LY75
LY88
LY94
LY97
LY data
Normal data

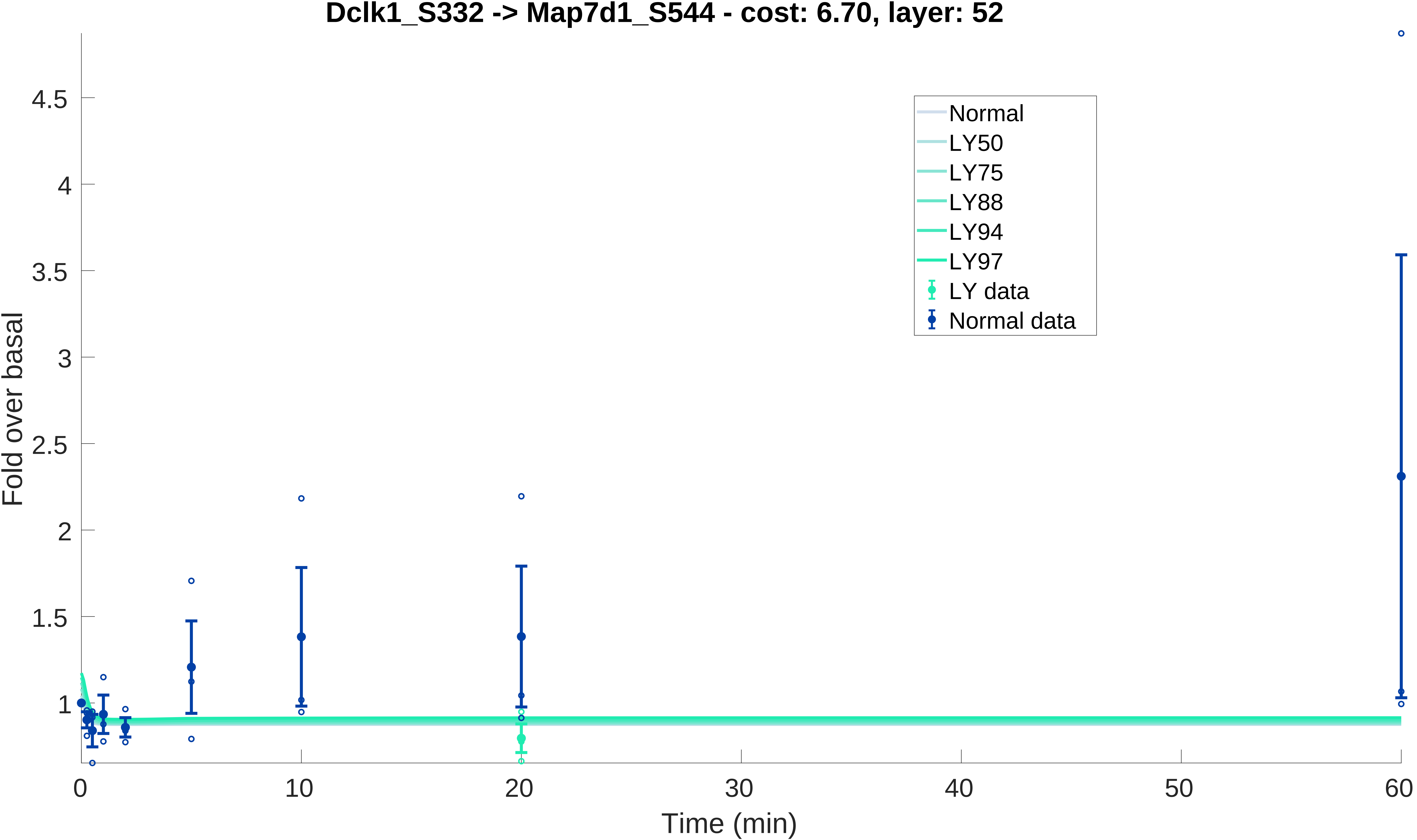

Dclk1_S332 -> Map7d1_S544 - cost: 6.70, layer: 52
Fold over basal
Time (min)
Normal
LY50
LY75
LY88
LY94
LY97
LY data
Normal data
4.5
4
3.5
3
2.5
2
1.5
1
0
10
20
30
40
50
60

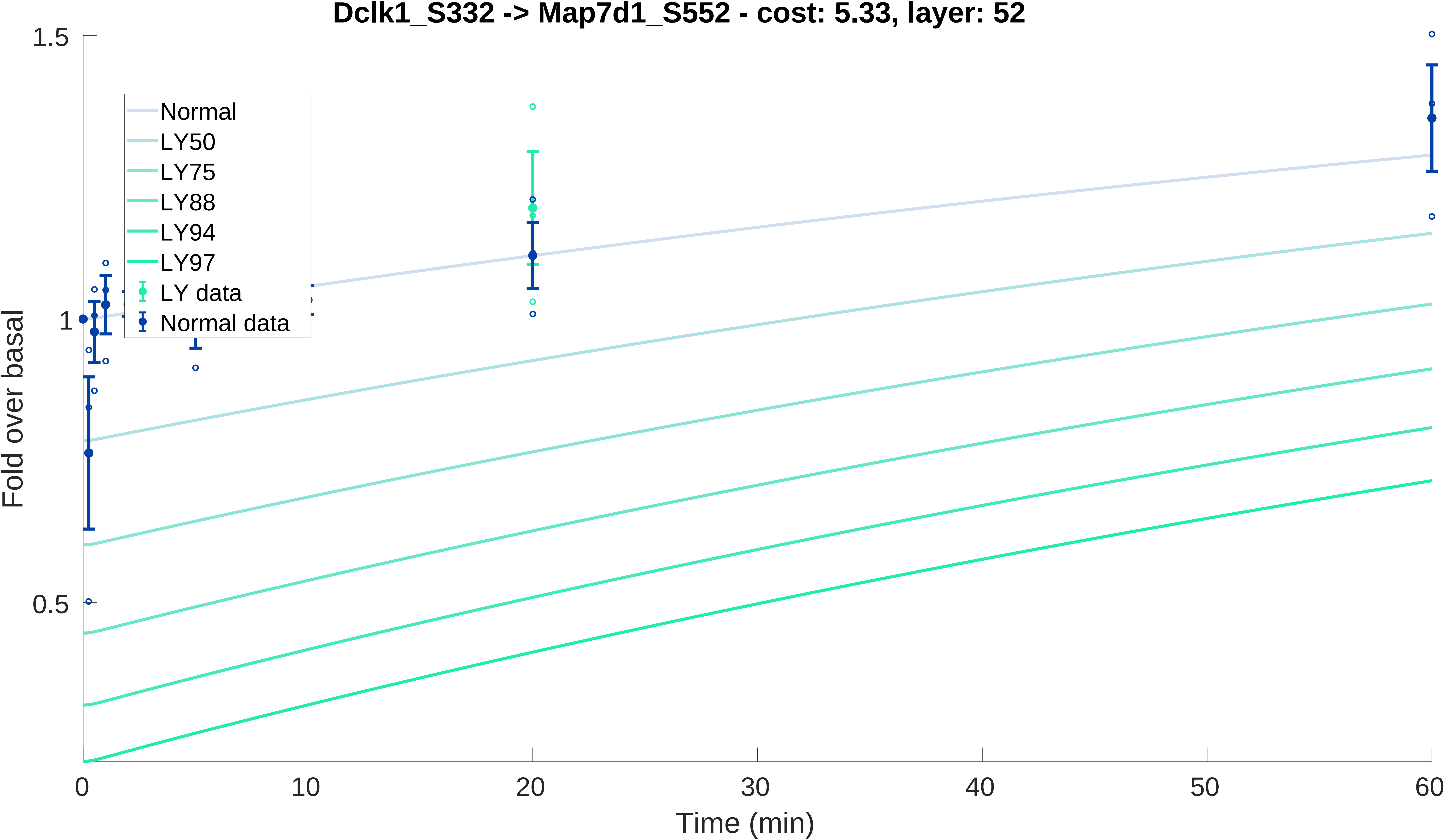

Dclk1_S332 -> Map7d1_S552 - cost: 5.33, layer: 52
Fold over basal
Time (min)
1.5
1
0.5
0
10
20
30
40
50
60
Normal
LY50
LY75
LY88
LY94
LY97
LY data
Normal data

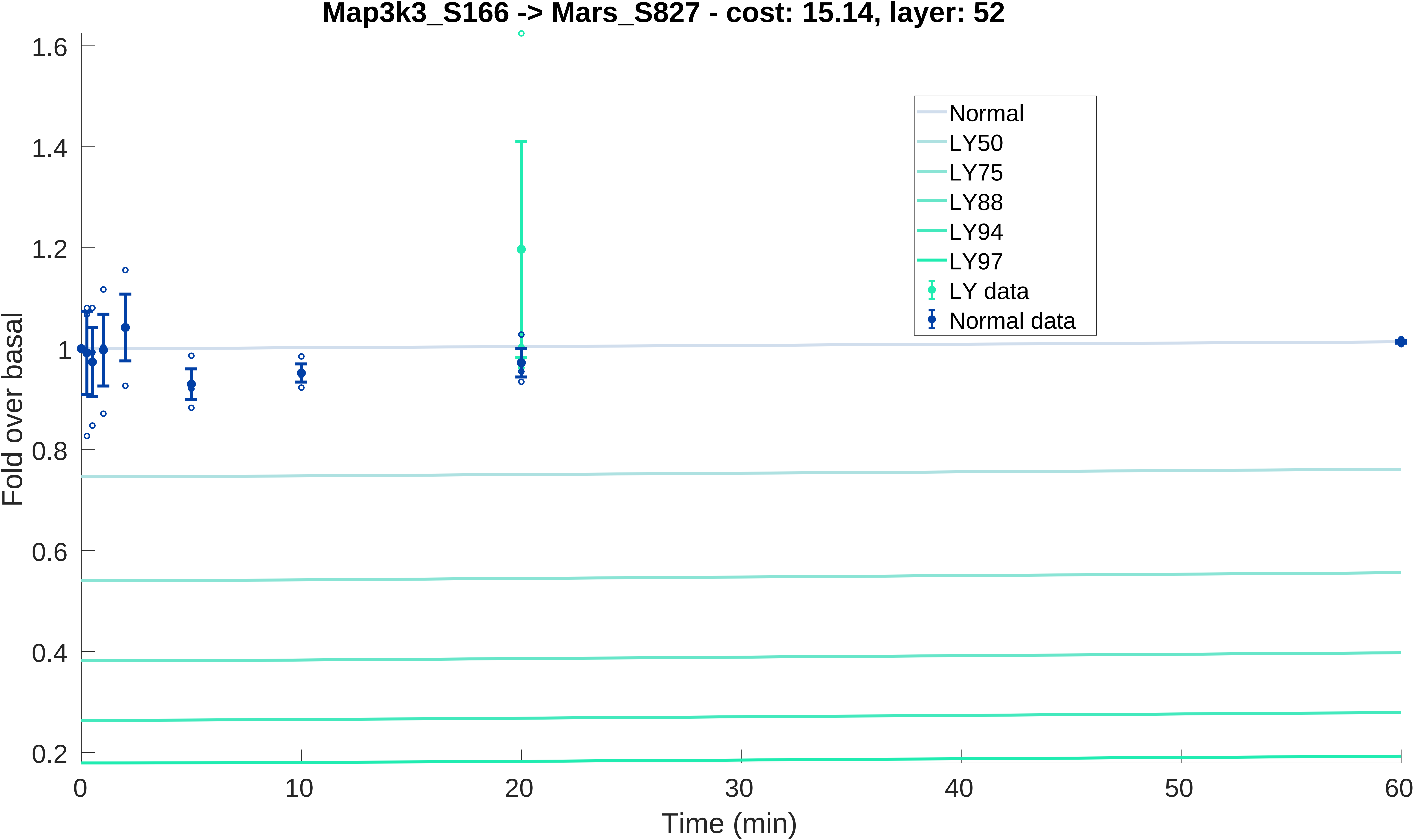

Map3k3_S166 -> Mars_S827 - cost: 15.14, layer: 52
Fold over basal
Time (min)
1.6
1.4
1.2
1
0.8
0.6
0.4
0.2
0
10
20
30
40
50
60
Normal
LY50
LY75
LY88
LY94
LY97
LY data
Normal data

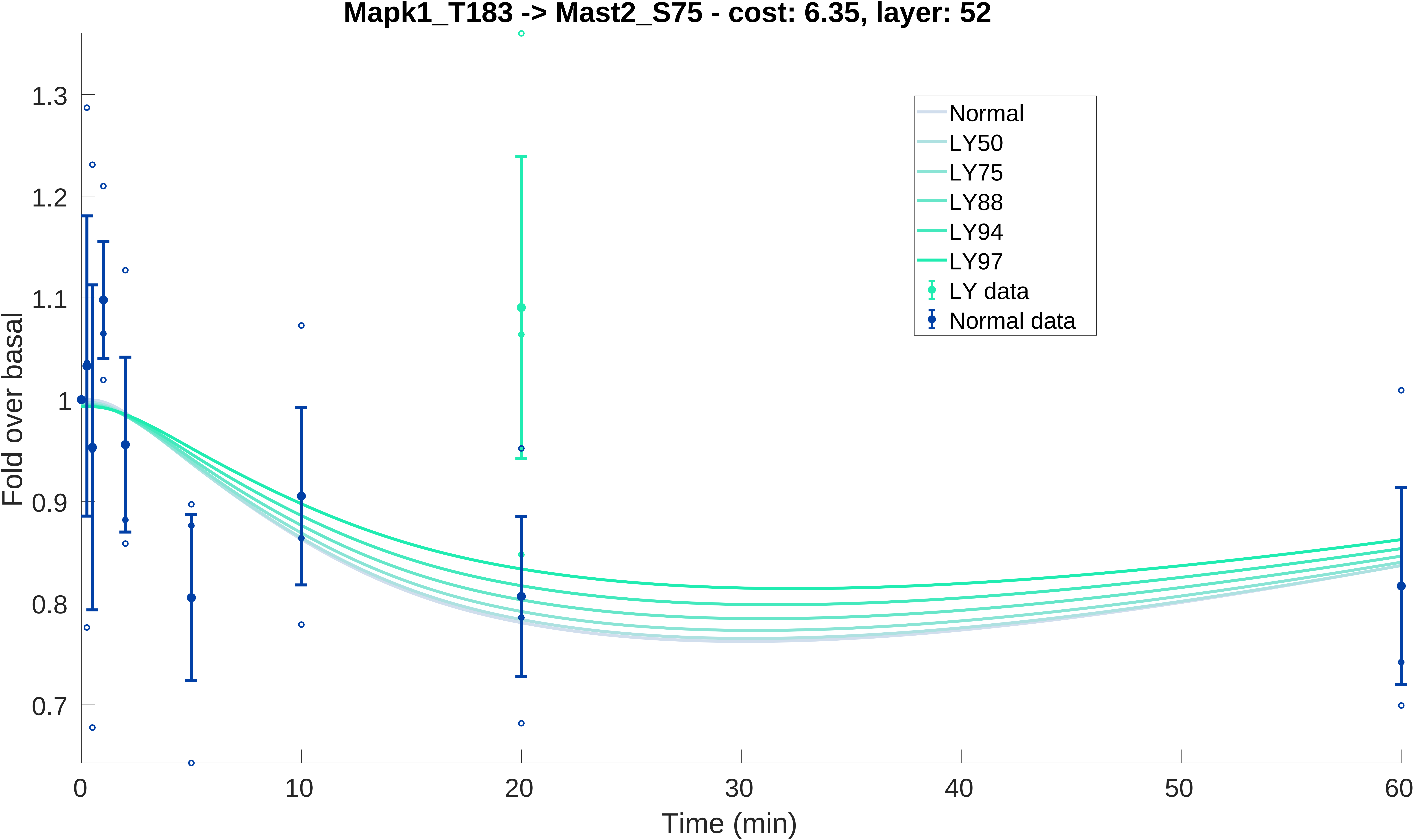
Mapk1_T183 -> Mast2_S75 - cost: 6.35, layer: 52
Fold over basal
Time (min)
1.3
1.2
1.1
1
0.9
0.8
0.7
0
10
20
30
40
50
60
Normal
LY50
LY75
LY88
LY94
LY97
LY data
Normal data

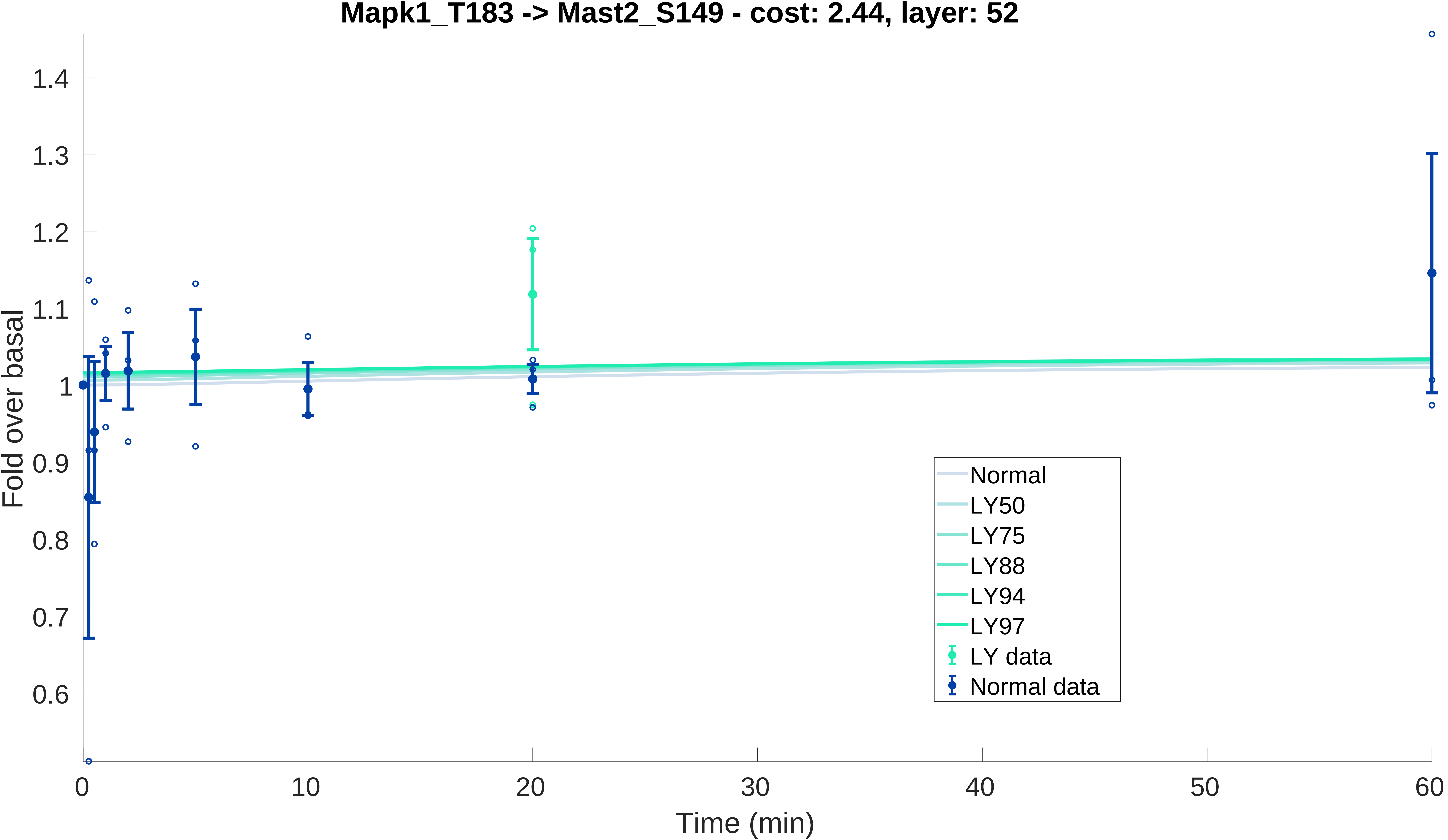
Mapk1_T183 -> Mast2_S149 - cost: 2.44, layer: 52
Fold over basal
1.4
1.3
1.2
1.1
1
0.9
0.8
0.7
0.6
0
10
20
30
40
50
60
Time (min)
Normal
LY50
LY75
LY88
LY94
LY97
LY data
Normal data

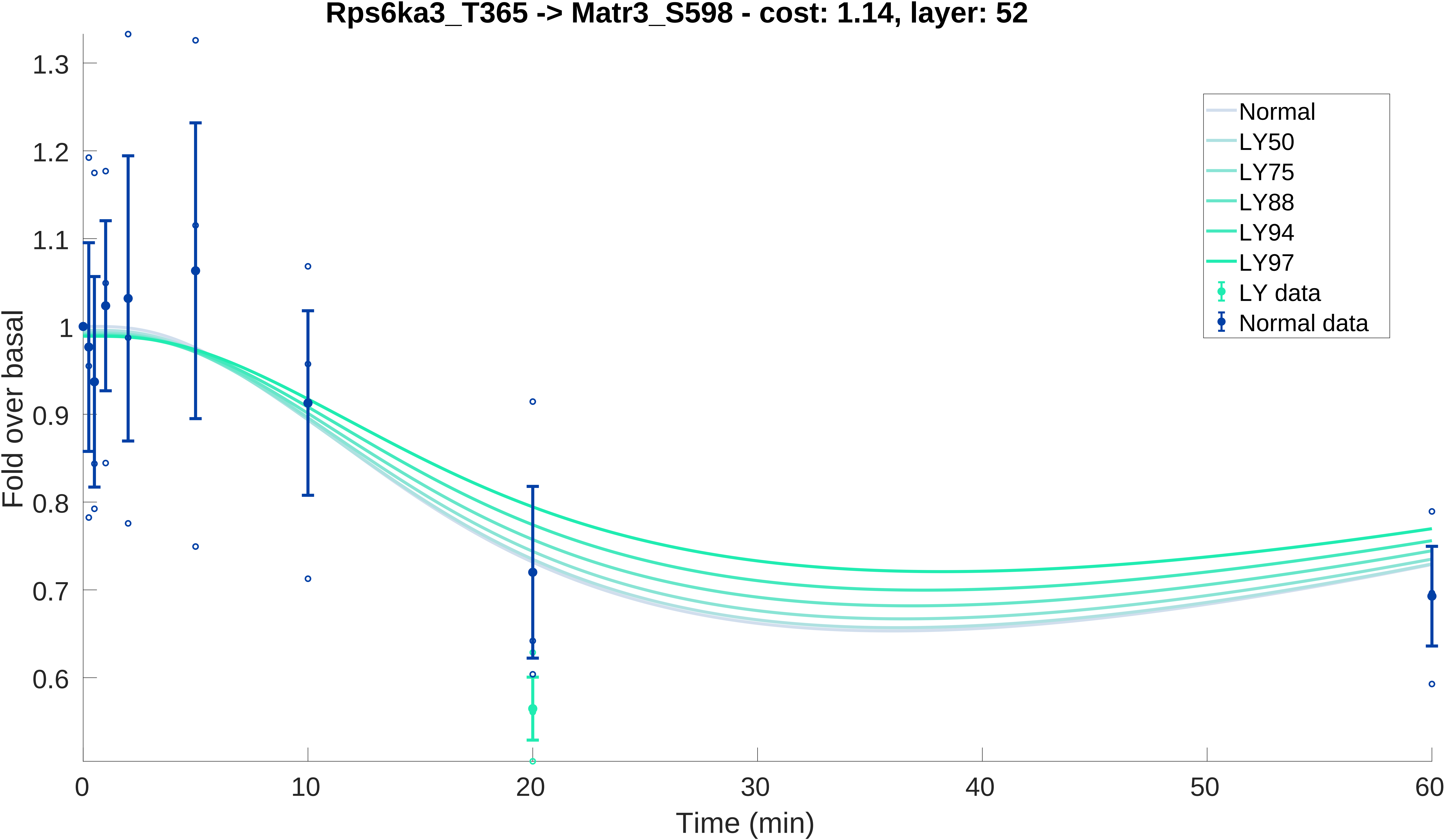

Rps6ka3_T365 -> Matr3_S598 - cost: 1.14, layer: 52
Fold over basal
Time (min)
1.3
1.2
1.1
1
0.9
0.8
0.7
0.6
0
10
20
30
40
50
60
Normal
LY50
LY75
LY88
LY94
LY97
LY data
Normal data

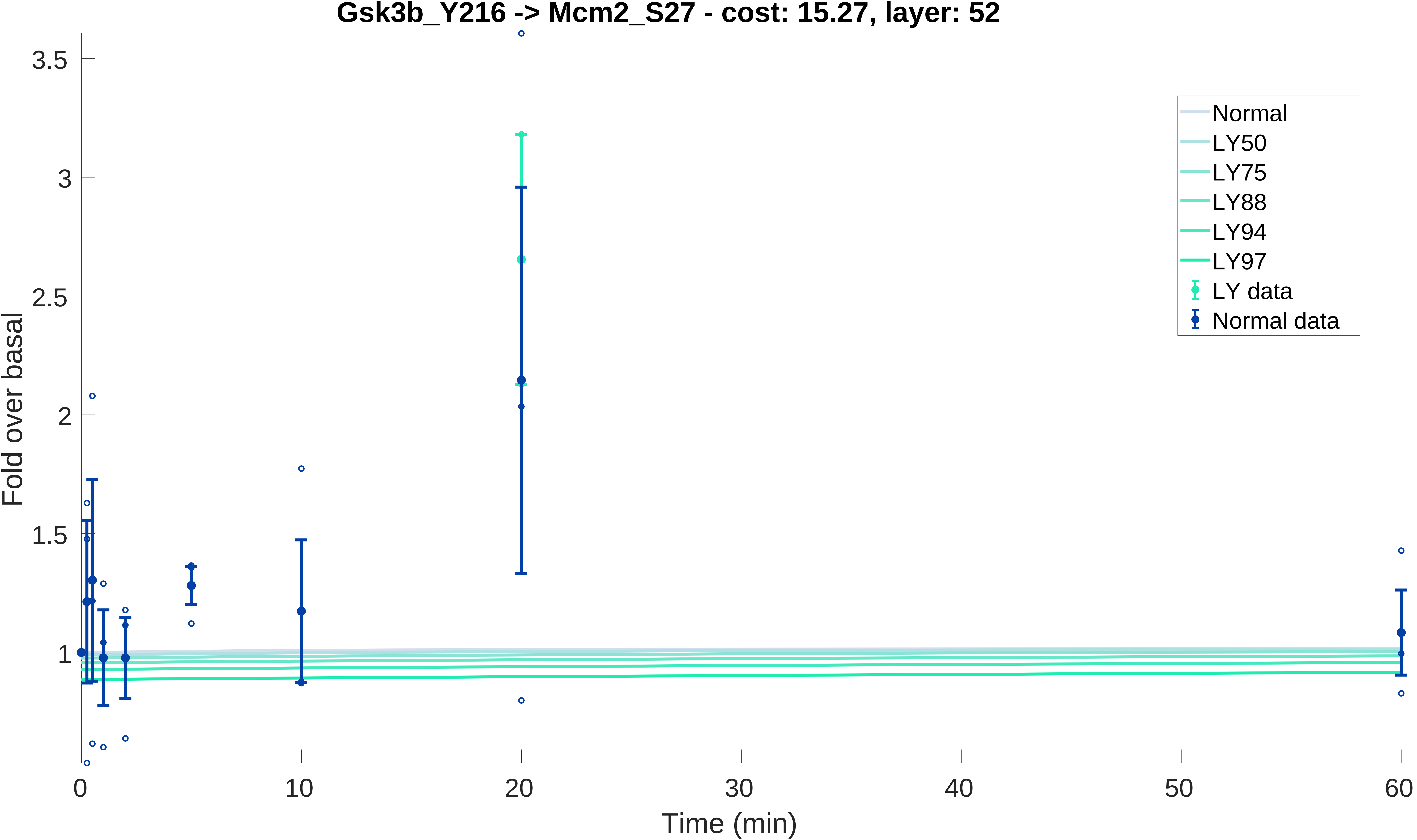

Gsk3b_Y216 -> Mcm2_S27 - cost: 15.27, layer: 52
Fold over basal
Time (min)
3.5
3
2.5
2
1.5
1
0
10
20
30
40
50
60
Normal
LY50
LY75
LY88
LY94
LY97
LY data
Normal data

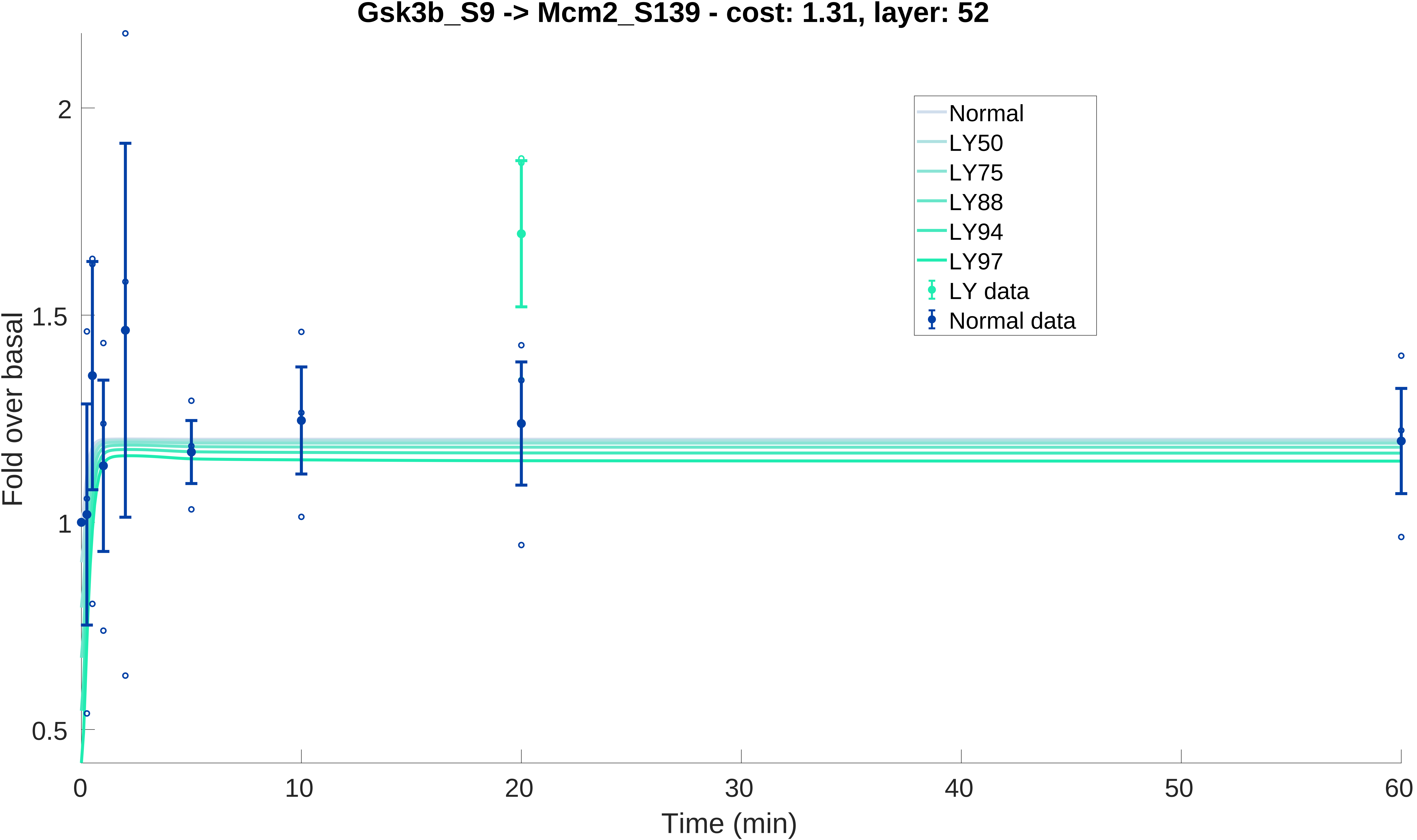

Gsk3b_S9 -> Mcm2_S139 - cost: 1.31, layer: 52
Fold over basal
Time (min)
Normal
LY50
LY75
LY88
LY94
LY97
LY data
Normal data
0.5
1
1.5
2
0
10
20
30
40
50
60

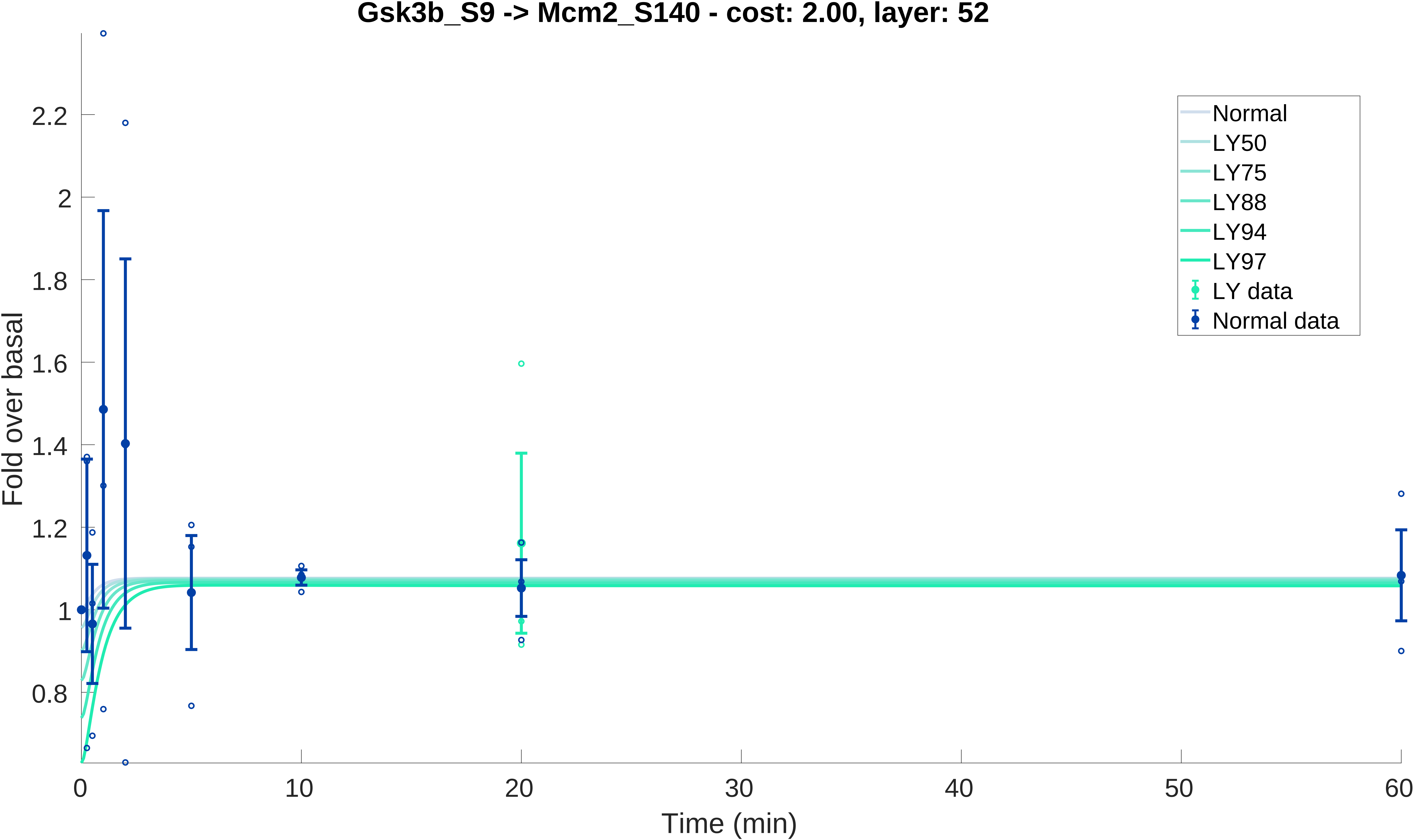

Gsk3b_S9 -> Mcm2_S140 - cost: 2.00, layer: 52
Fold over basal
Time (min)
Normal
LY50
LY75
LY88
LY94
LY97
LY data
Normal data
0.8
1
1.2
1.4
1.6
1.8
2
2.2
0
10
20
30
40
50
60

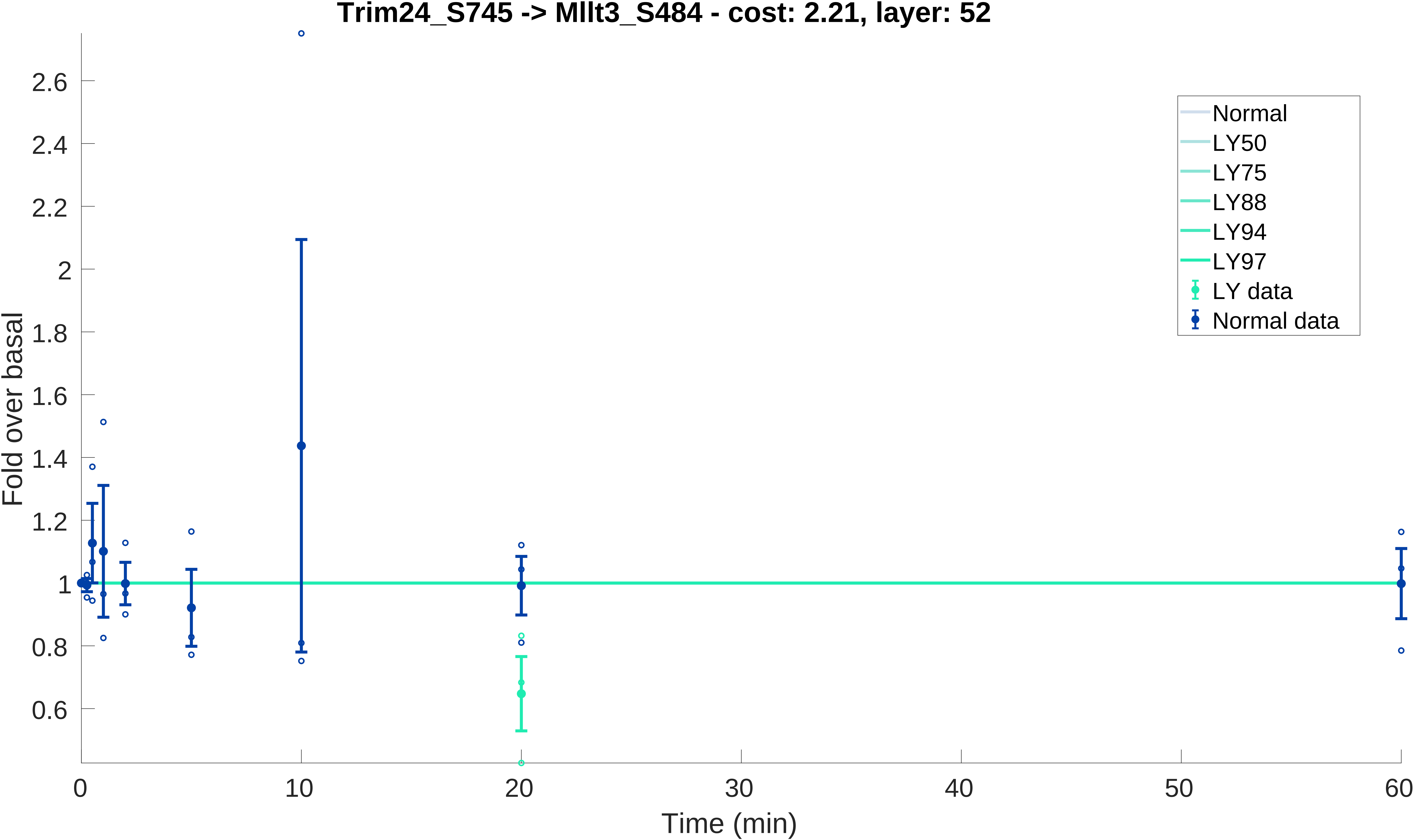
Trim24_S745 -> Mllt3_S484 - cost: 2.21, layer: 52
Fold over basal
Time (min)
0
10
20
30
40
50
60
0.6
0.8
1
1.2
1.4
1.6
1.8
2
2.2
2.4
2.6
Normal
LY50
LY75
LY88
LY94
LY97
LY data
Normal data

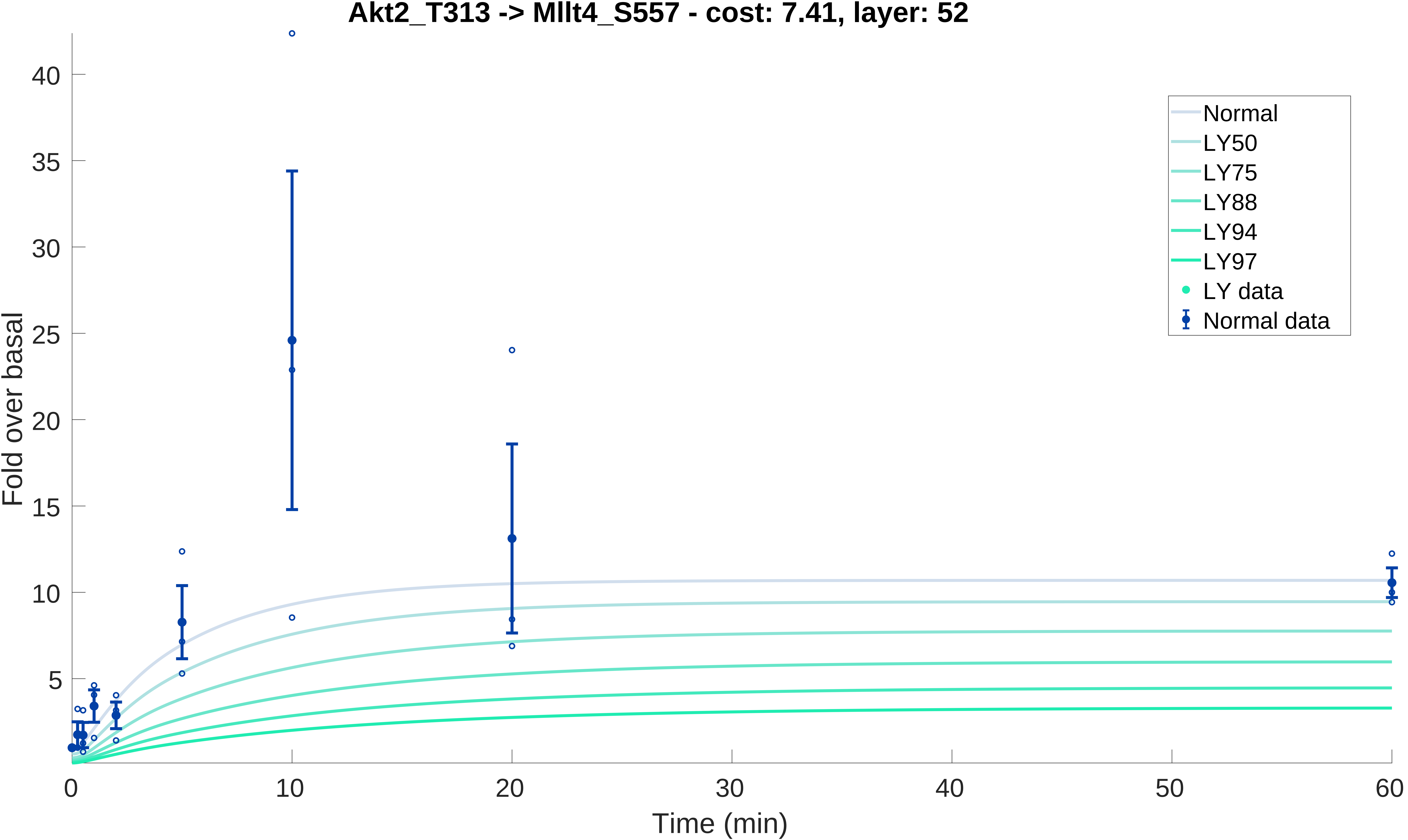

Akt2_T313 -> Mllt4_S557 - cost: 7.41, layer: 52
Fold over basal
Time (min)
0
10
20
30
40
50
60
5
10
15
20
25
30
35
40
Normal
LY50
LY75
LY88
LY94
LY97
LY data
Normal data

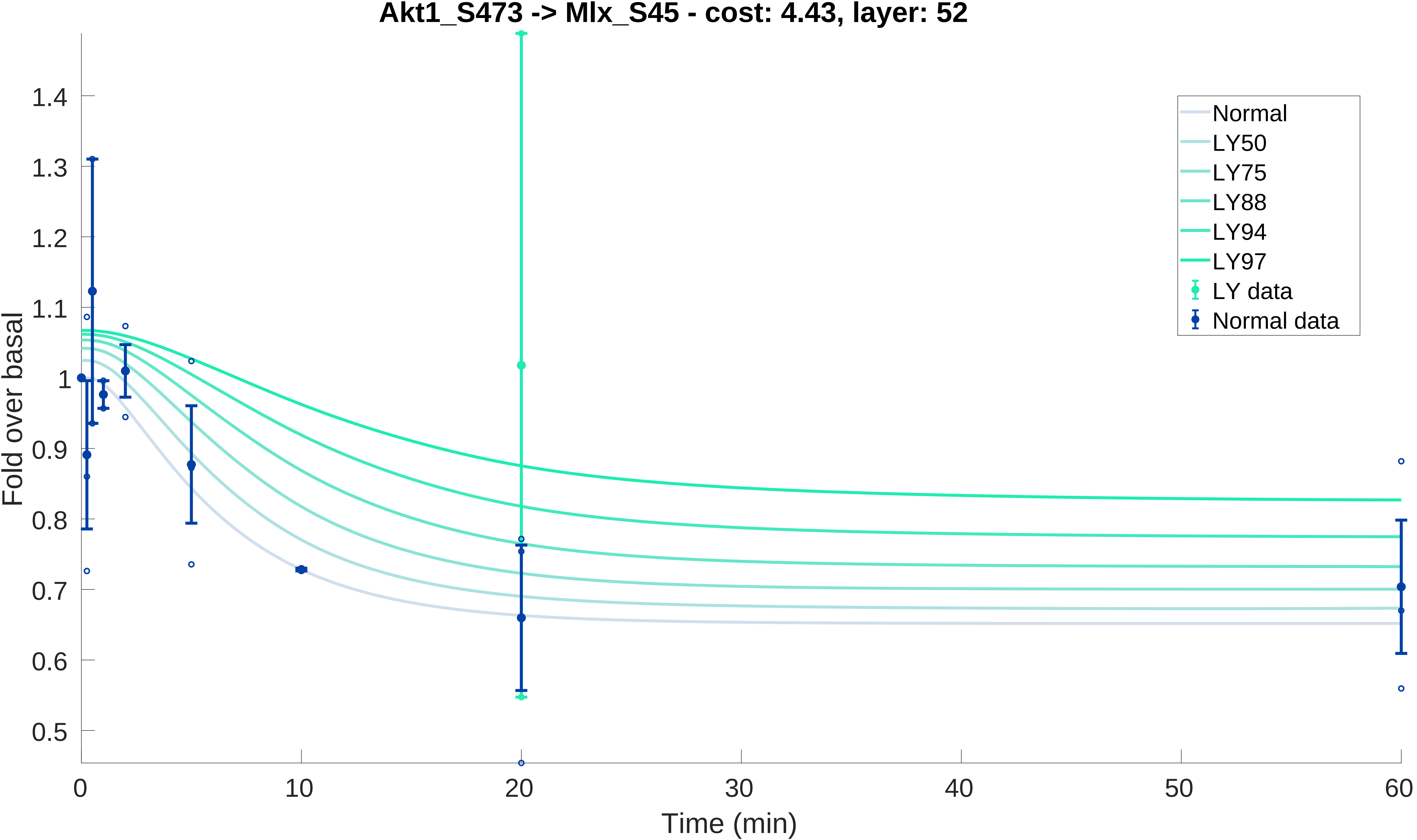
Akt1_S473 -> Mlx_S45 - cost: 4.43, layer: 52
Fold over basal
1.4
1.3
1.2
1.1
1
0.9
0.8
0.7
0.6
0.5
0
10
20
30
40
50
60
Time (min)
Normal
LY50
LY75
LY88
LY94
LY97
LY data
Normal data

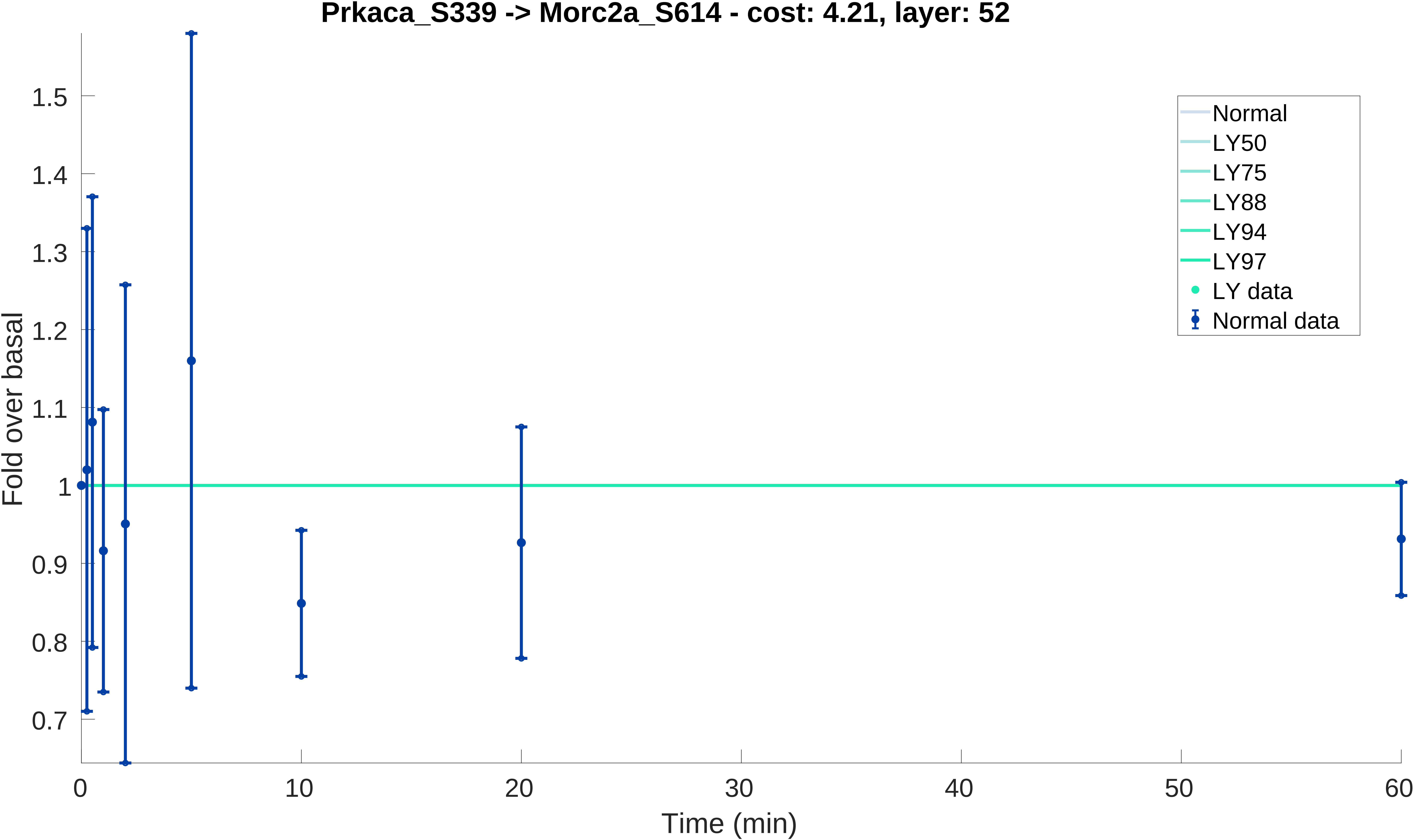

Prkaca_S339 -> Morc2a_S614 - cost: 4.21, layer: 52
Fold over basal
1.5
1.4
1.3
1.2
1.1
1
0.9
0.8
0.7
0
10
20
30
40
50
60
Time (min)
Normal
LY50
LY75
LY88
LY94
LY97
LY data
Normal data

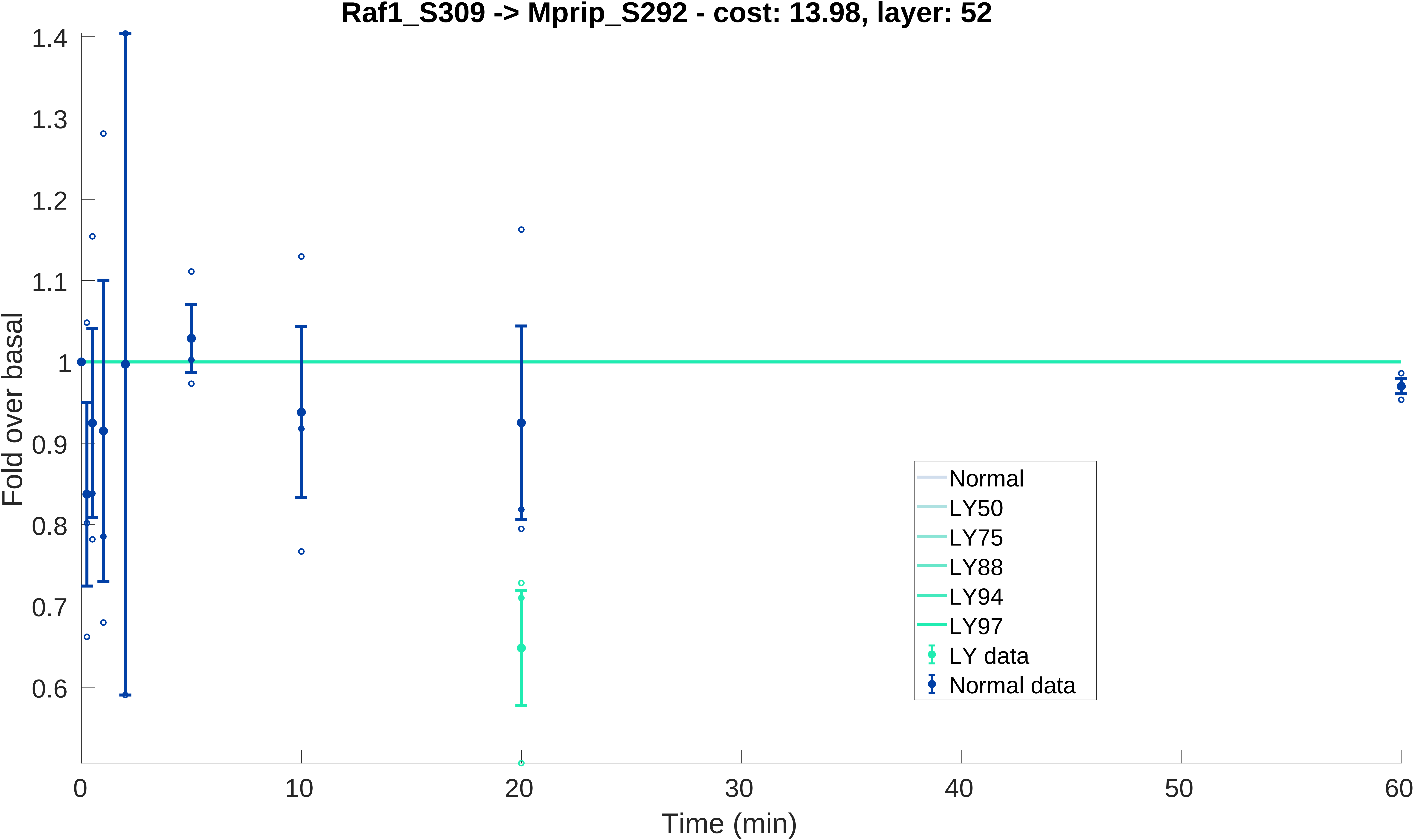

Raf1_S309 -> Mprip_S292 - cost: 13.98, layer: 52
Fold over basal
1.4
1.3
1.2
1.1
1
0.9
0.8
0.7
0.6
Time (min)
0
10
20
30
40
50
60
Normal
LY50
LY75
LY88
LY94
LY97
LY data
Normal data

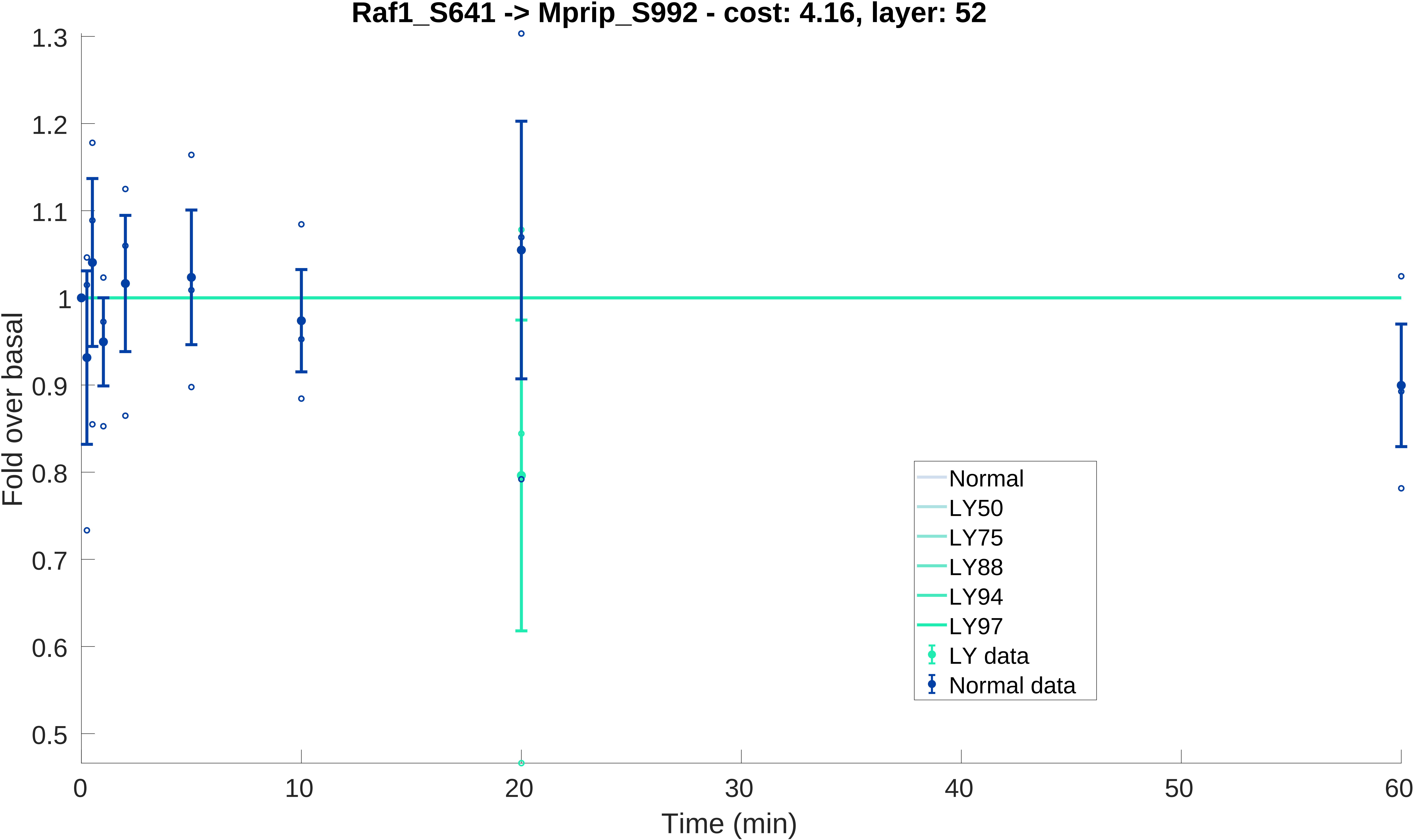

Raf1_S641 -> Mprip_S992 - cost: 4.16, layer: 52
Fold over basal
1.3
1.2
1.1
1
0.9
0.8
0.7
0.6
0.5
0
10
20
30
40
50
60
Time (min)
Normal
LY50
LY75
LY88
LY94
LY97
LY data
Normal data

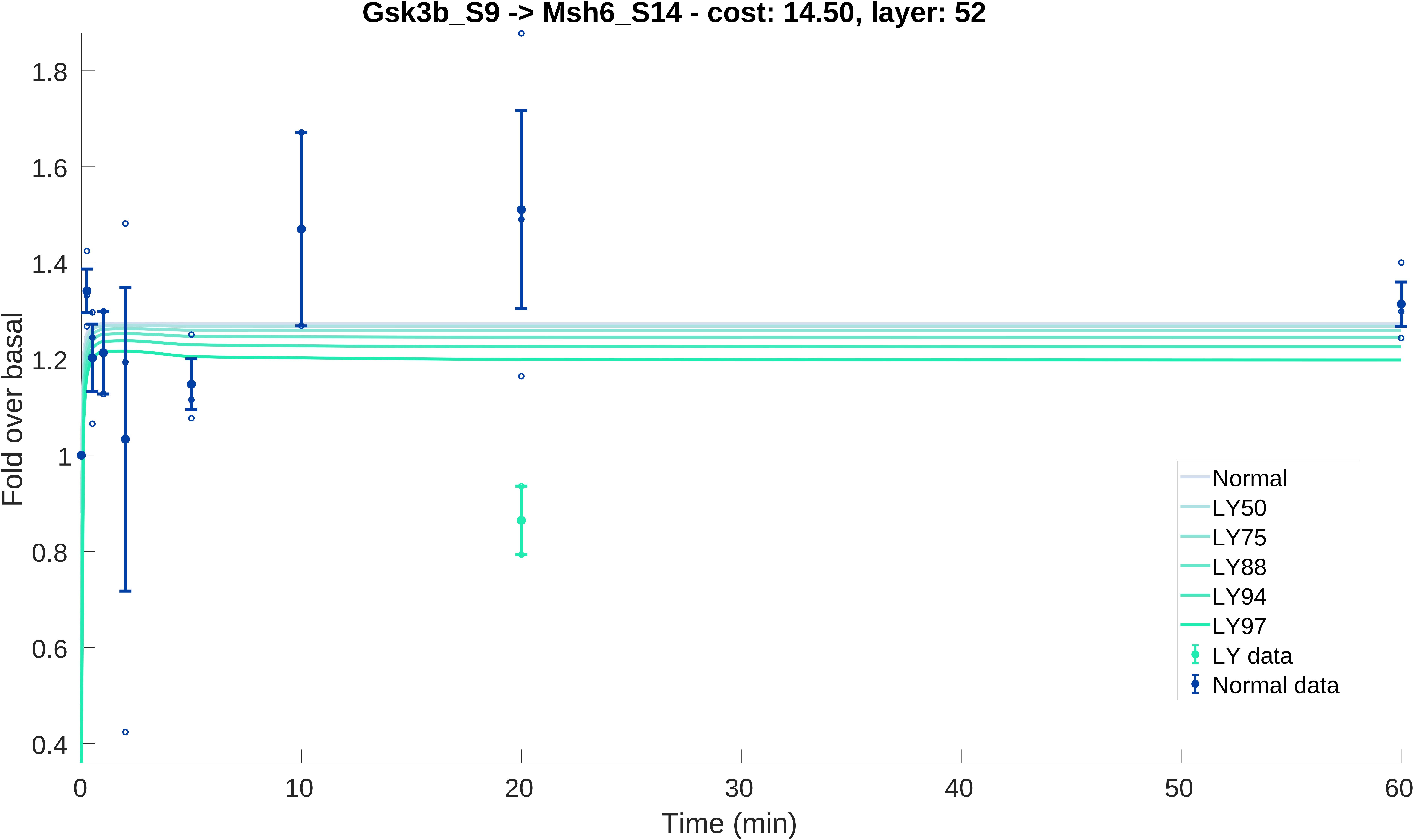

Gsk3b_S9 -> Msh6_S14 - cost: 14.50, layer: 52
Fold over basal
Time (min)
0.4
0.6
0.8
1
1.2
1.4
1.6
1.8
0
10
20
30
40
50
60
Normal
LY50
LY75
LY88
LY94
LY97
LY data
Normal data

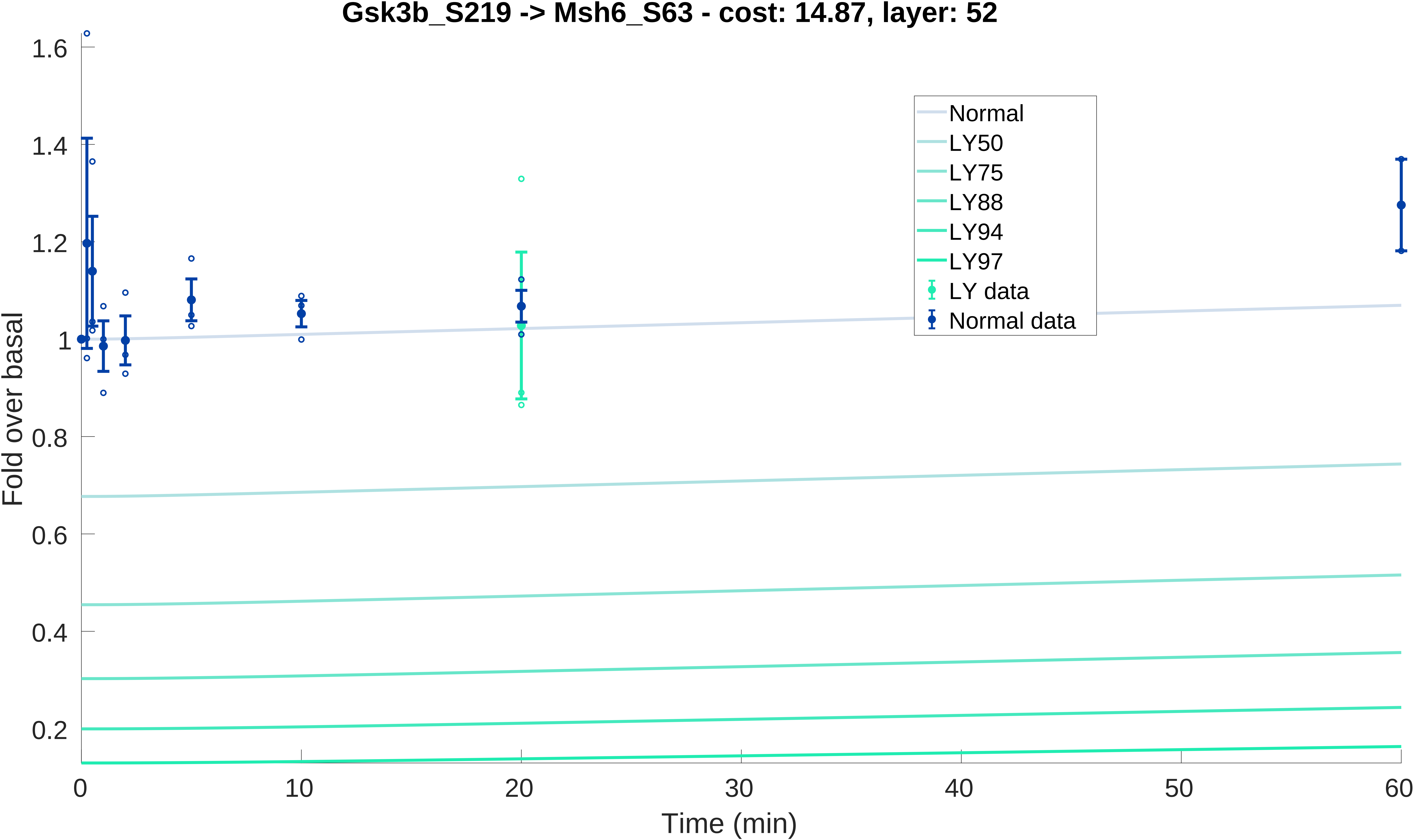

Gsk3b_S219 -> Msh6_S63 - cost: 14.87, layer: 52
Fold over basal
Time (min)
0
10
20
30
40
50
60
0.2
0.4
0.6
0.8
1
1.2
1.4
1.6
Normal
LY50
LY75
LY88
LY94
LY97
LY data
Normal data

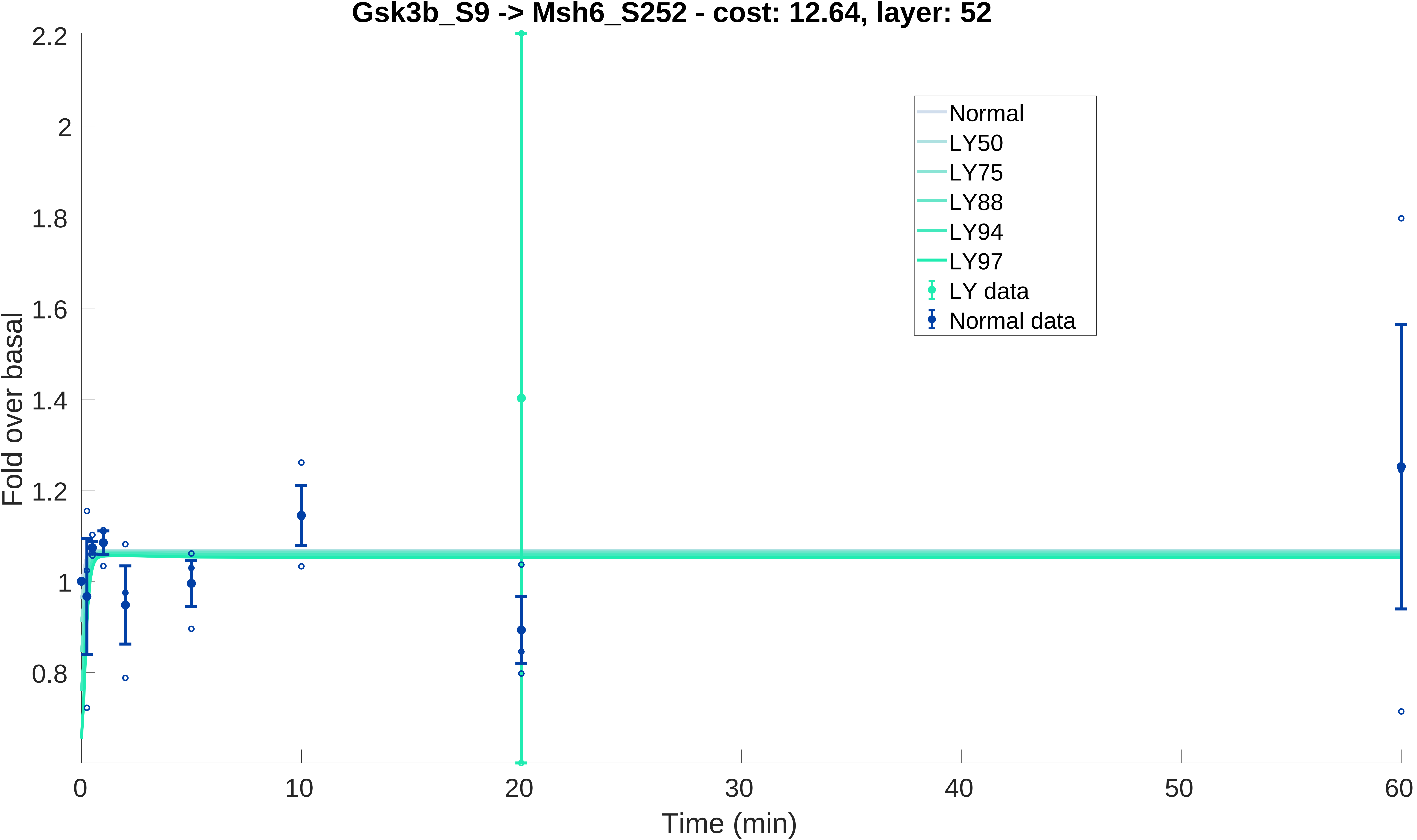

Gsk3b_S9 -> Msh6_S252 - cost: 12.64, layer: 52
Fold over basal
Time (min)
Normal
LY50
LY75
LY88
LY94
LY97
LY data
Normal data
2.2
2
1.8
1.6
1.4
1.2
1
0.8
0
10
20
30
40
50
60

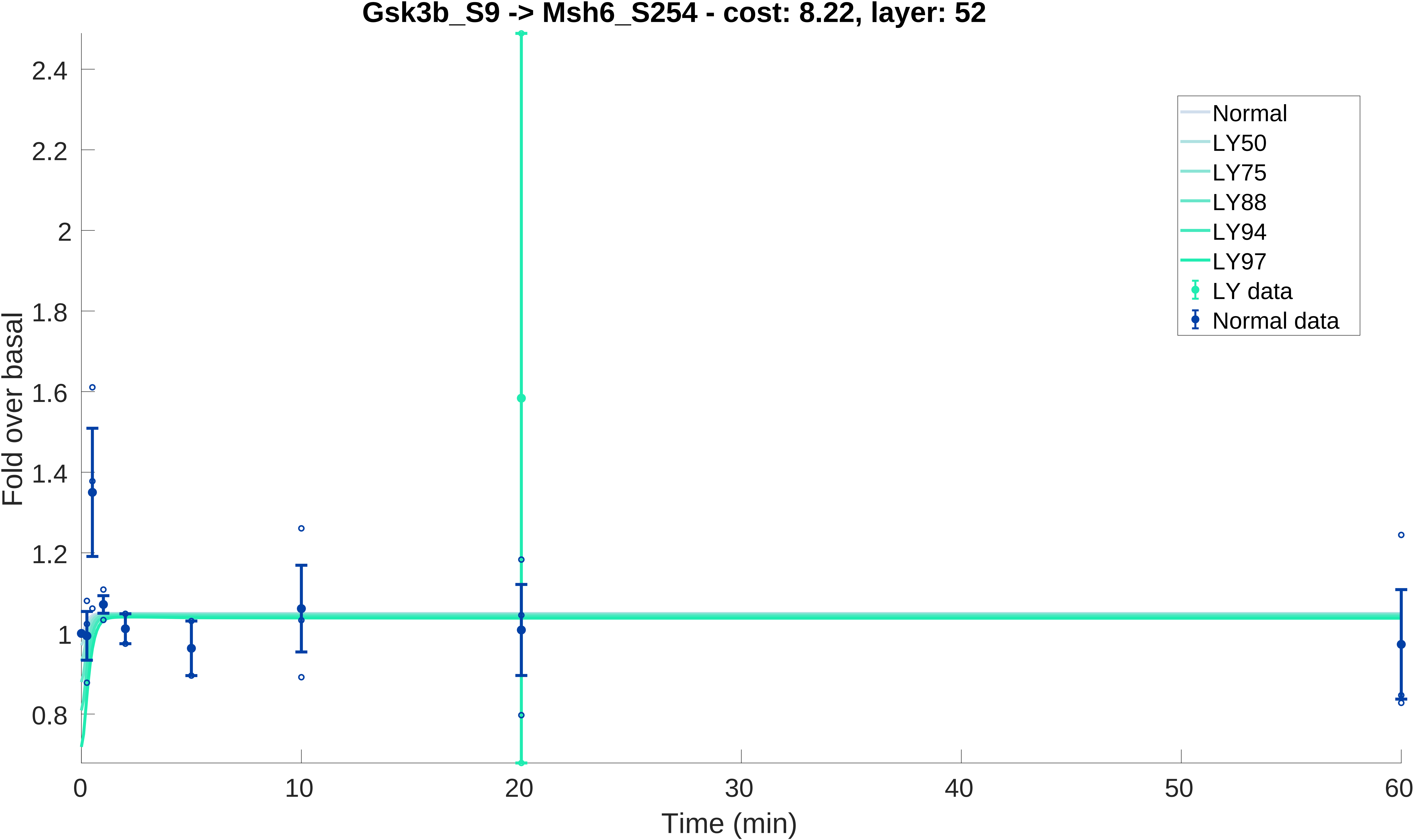

Gsk3b_S9 -> Msh6_S254 - cost: 8.22, layer: 52
Fold over basal
Time (min)
0.8
1
1.2
1.4
1.6
1.8
2
2.2
2.4
0
10
20
30
40
50
60
Normal
LY50
LY75
LY88
LY94
LY97
LY data
Normal data

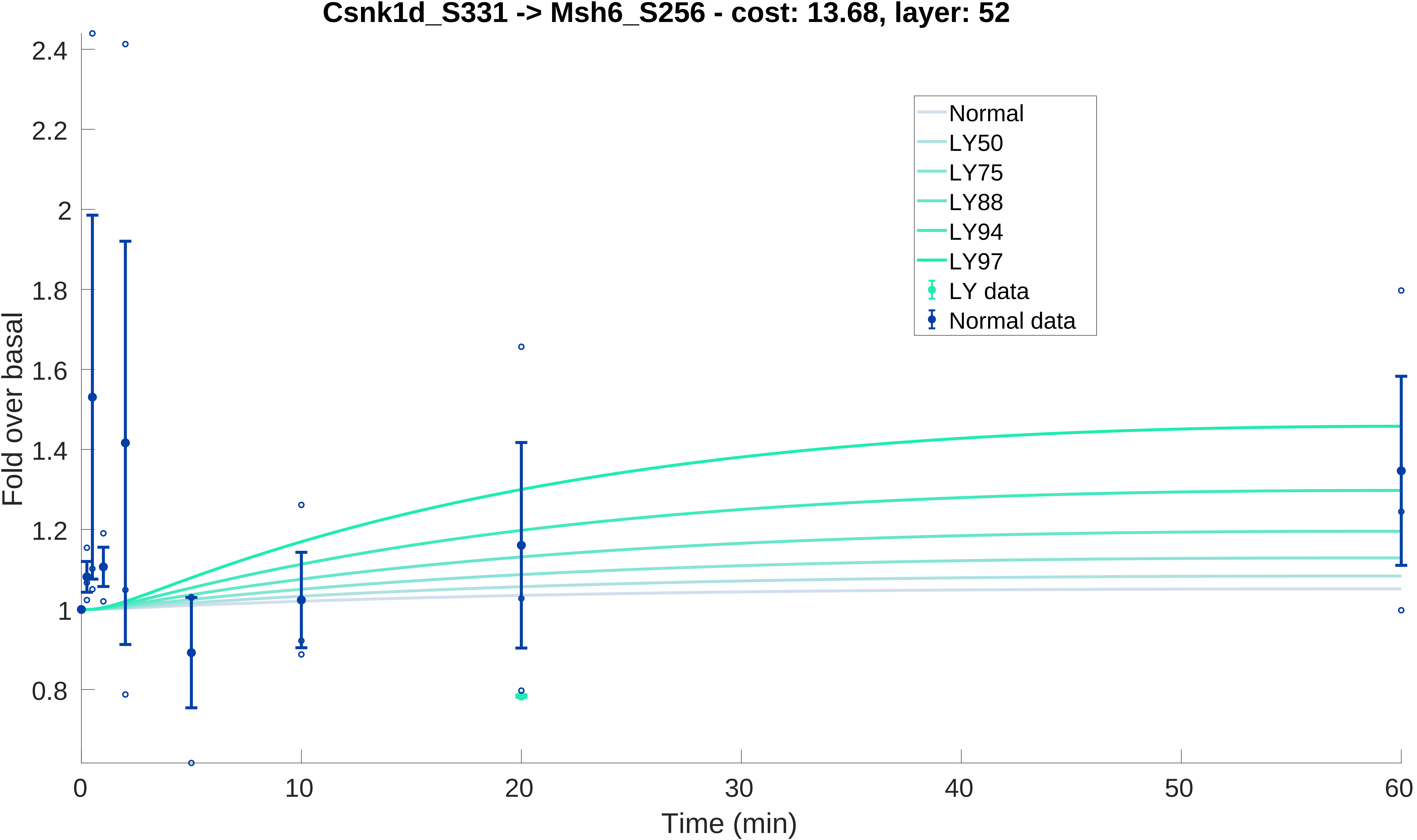
Csnk1d_S331 -> Msh6_S256 - cost: 13.68, layer: 52
Fold over basal
Time (min)
2.4
2.2
2
1.8
1.6
1.4
1.2
1
0.8
0
10
20
30
40
50
60
Normal
LY50
LY75
LY88
LY94
LY97
LY data
Normal data

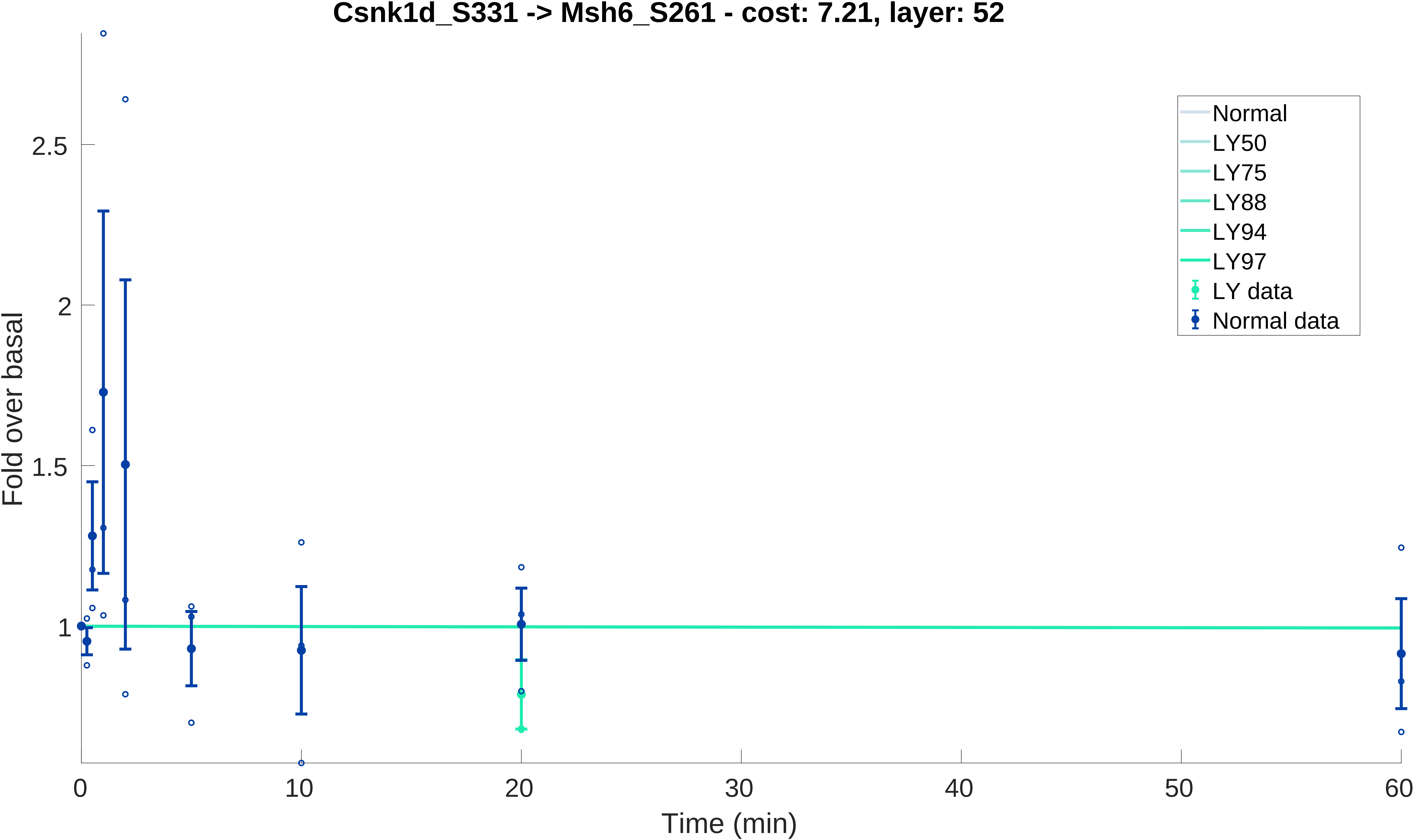

Csnk1d_S331 -> Msh6_S261 - cost: 7.21, layer: 52
Fold over basal
Time (min)
2.5
2
1.5
1
0
10
20
30
40
50
60
Normal
LY50
LY75
LY88
LY94
LY97
LY data
Normal data

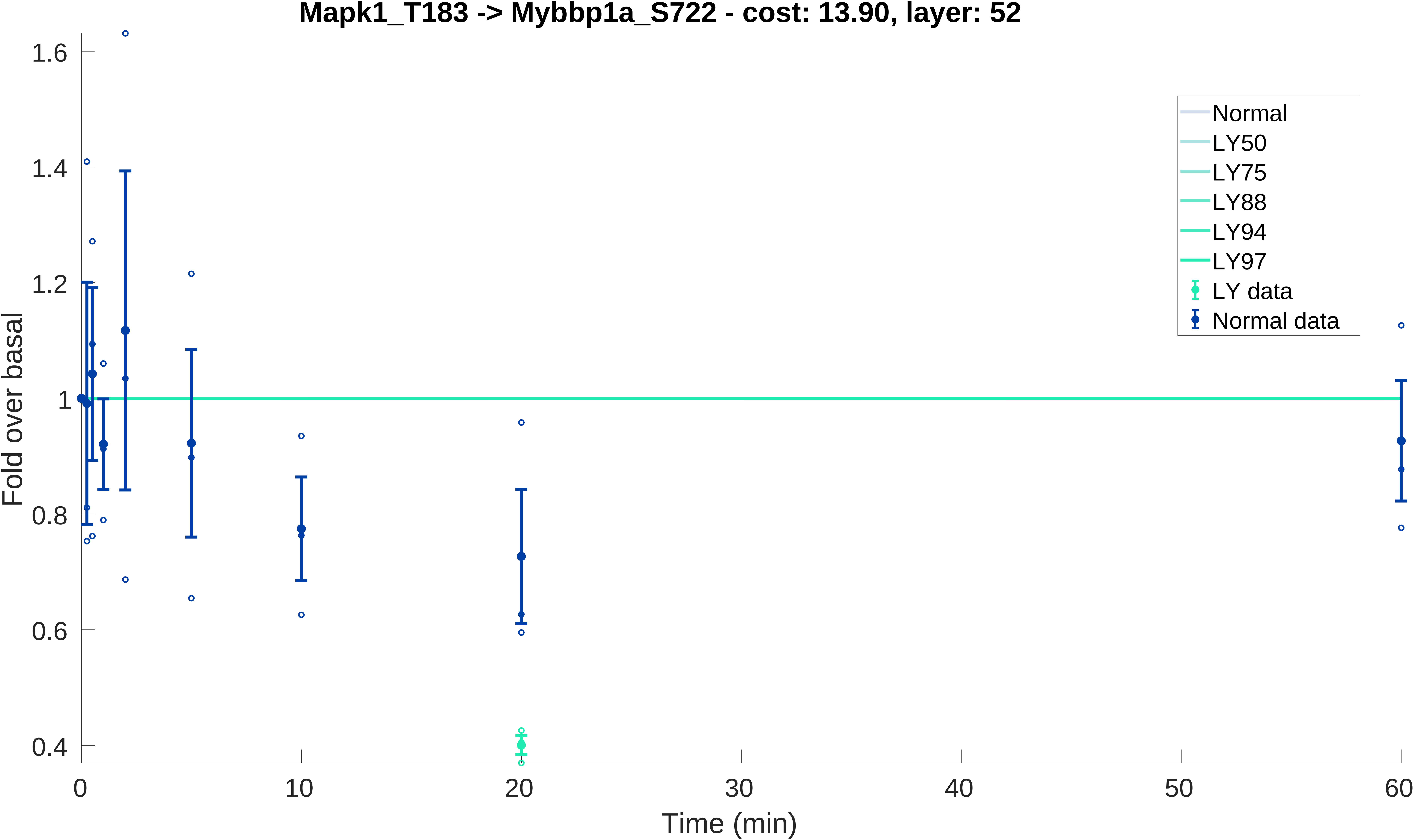
Mapk1_T183 -> Mybbp1a_S722 - cost: 13.90, layer: 52
Fold over basal
Time (min)
0
10
20
30
40
50
60
0.4
0.6
0.8
1
1.2
1.4
1.6
Normal
LY50
LY75
LY88
LY94
LY97
LY data
Normal data

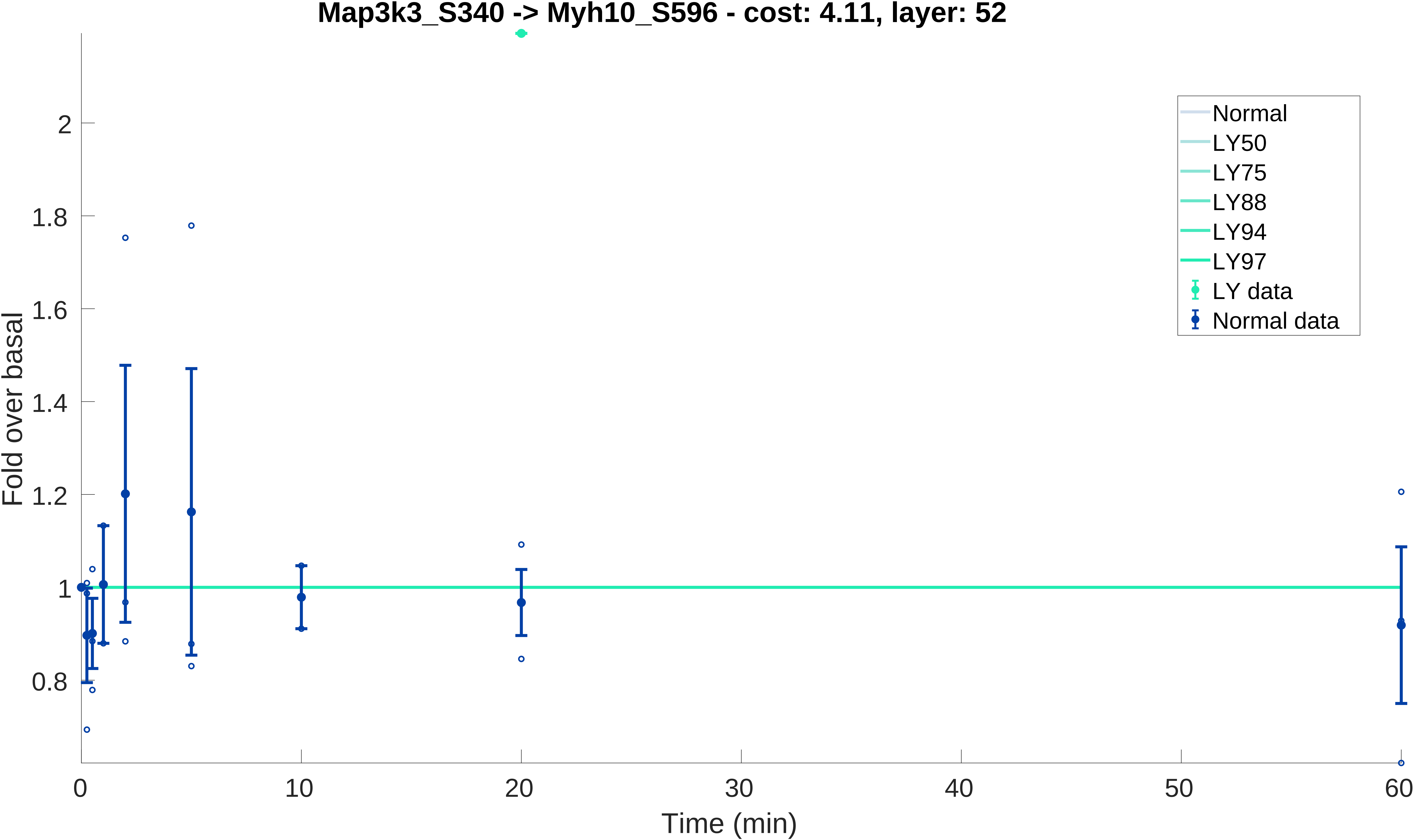

Map3k3_S340 -> Myh10_S596 - cost: 4.11, layer: 52
Fold over basal
Time (min)
0
10
20
30
40
50
60
0.8
1
1.2
1.4
1.6
1.8
2
Normal
LY50
LY75
LY88
LY94
LY97
LY data
Normal data

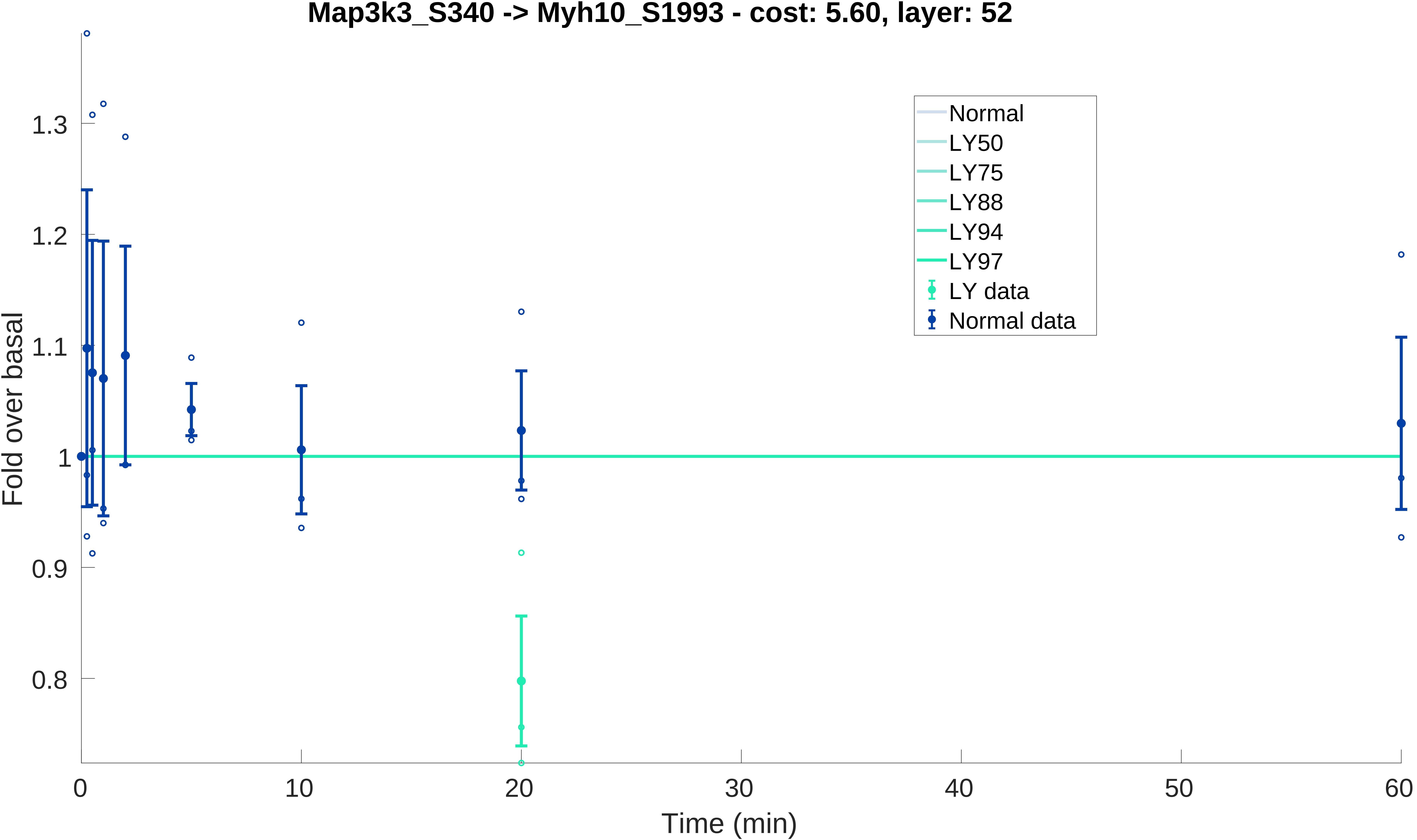

Map3k3_S340 -> Myh10_S1993 - cost: 5.60, layer: 52
Fold over basal
Time (min)
0.8
0.9
1
1.1
1.2
1.3
0
10
20
30
40
50
60
Normal
LY50
LY75
LY88
LY94
LY97
LY data
Normal data

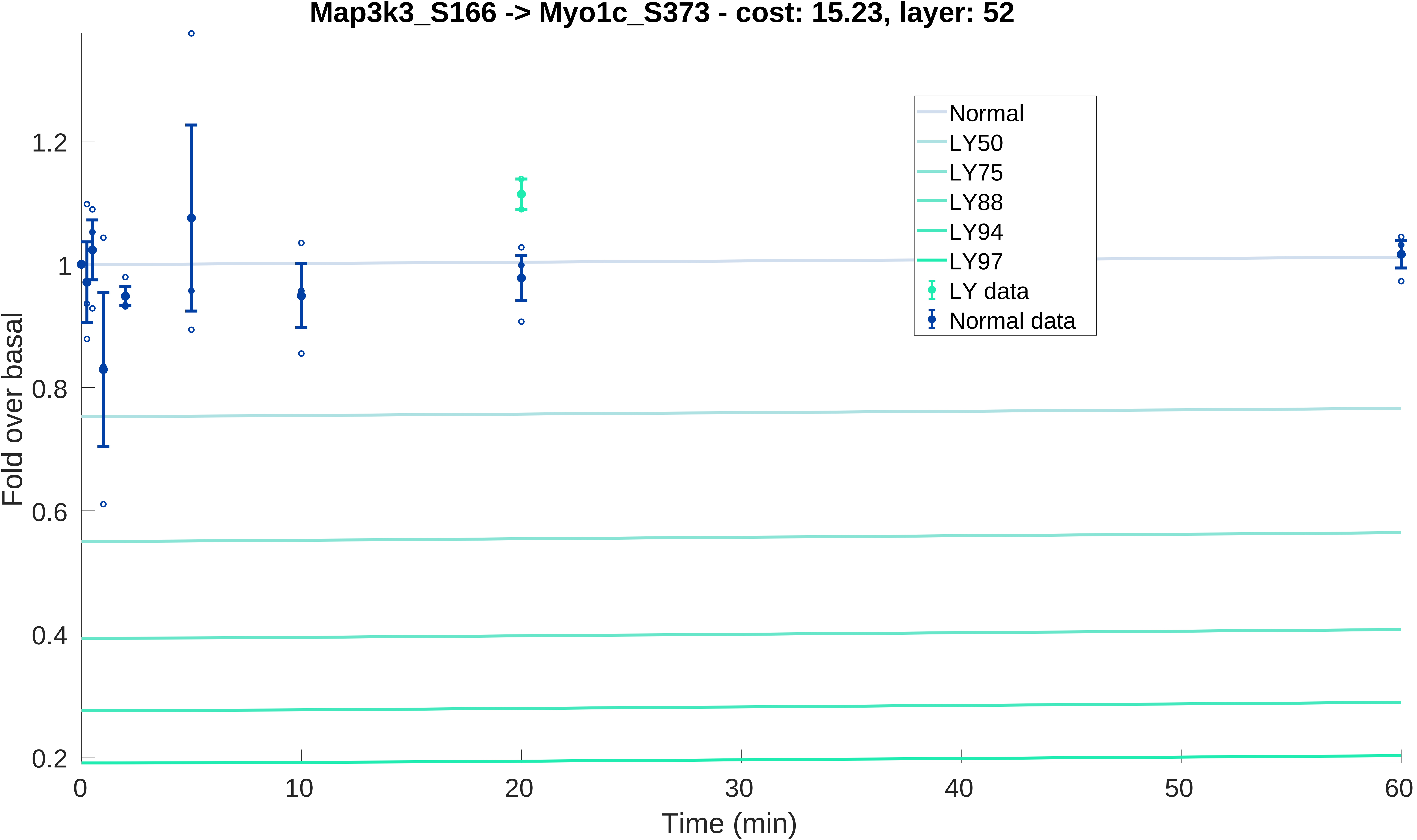

Map3k3_S166 -> Myo1c_S373 - cost: 15.23, layer: 52
Normal
LY50
LY75
LY88
LY94
LY97
LY data
Normal data
Fold over basal
1.2
1
0.8
0.6
0.4
0.2
0
10
20
30
40
50
60
Time (min)

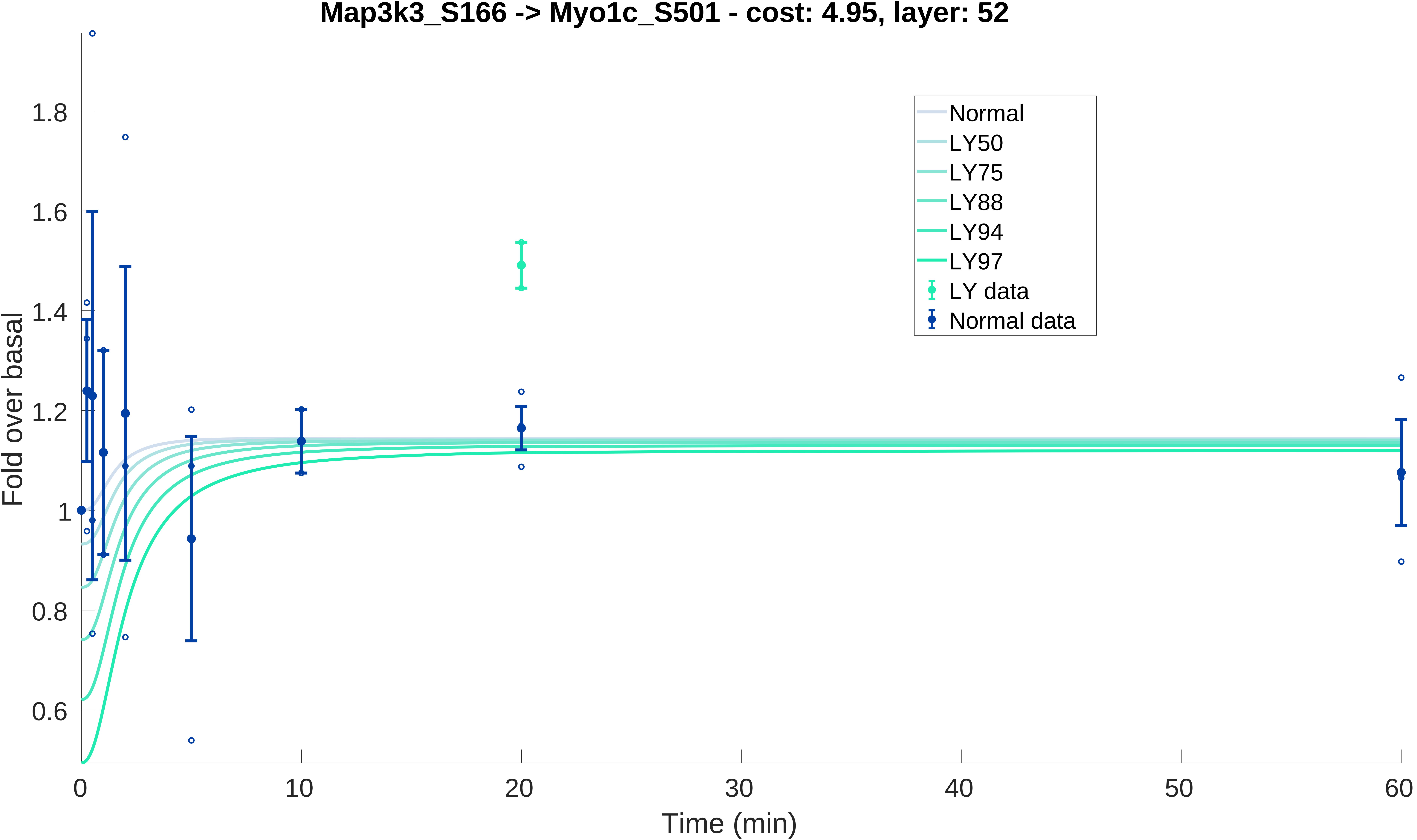

Map3k3_S166 -> Myo1c_S501 - cost: 4.95, layer: 52
Fold over basal
Time (min)
Normal
LY50
LY75
LY88
LY94
LY97
LY data
Normal data
0.6
0.8
1
1.2
1.4
1.6
1.8
0
10
20
30
40
50
60

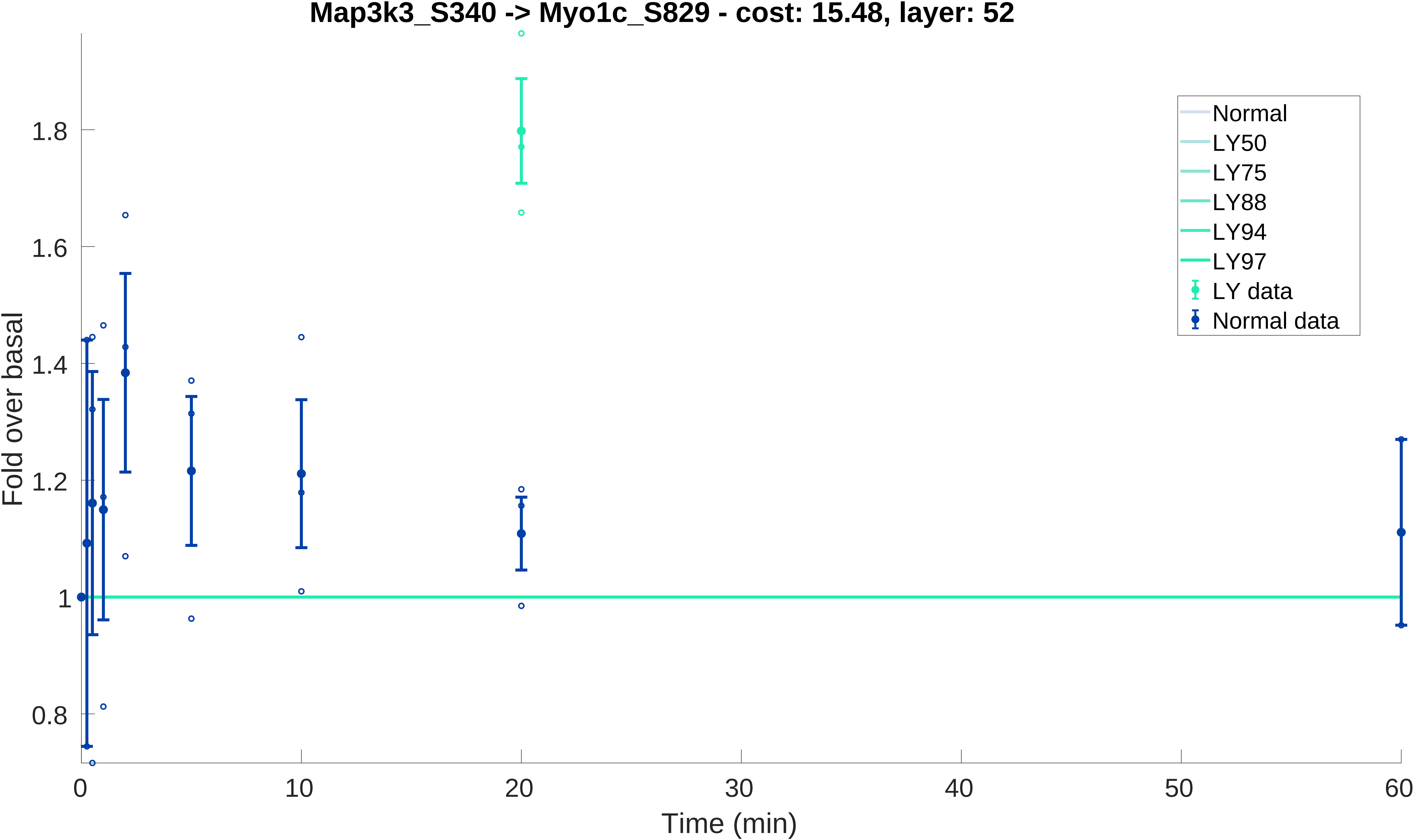
Map3k3_S340 -> Myo1c_S829 - cost: 15.48, layer: 52
Fold over basal
0.8
1
1.2
1.4
1.6
1.8
Time (min)
0
10
20
30
40
50
60
Normal
LY50
LY75
LY88
LY94
LY97
LY data
Normal data

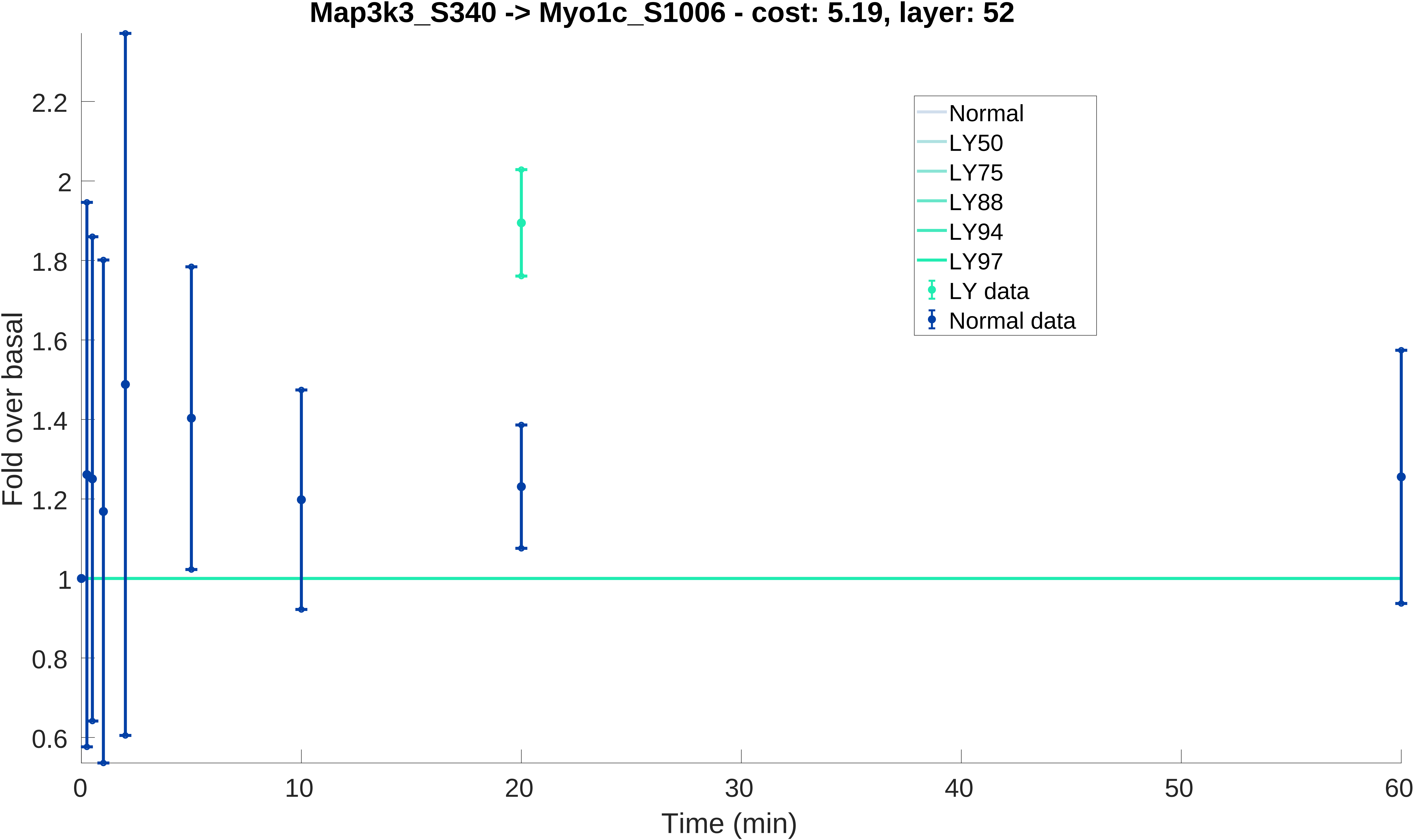

Map3k3_S340 -> Myo1c_S1006 - cost: 5.19, layer: 52
Fold over basal
Time (min)
0.6
0.8
1
1.2
1.4
1.6
1.8
2
2.2
0
10
20
30
40
50
60
Normal
LY50
LY75
LY88
LY94
LY97
LY data
Normal data

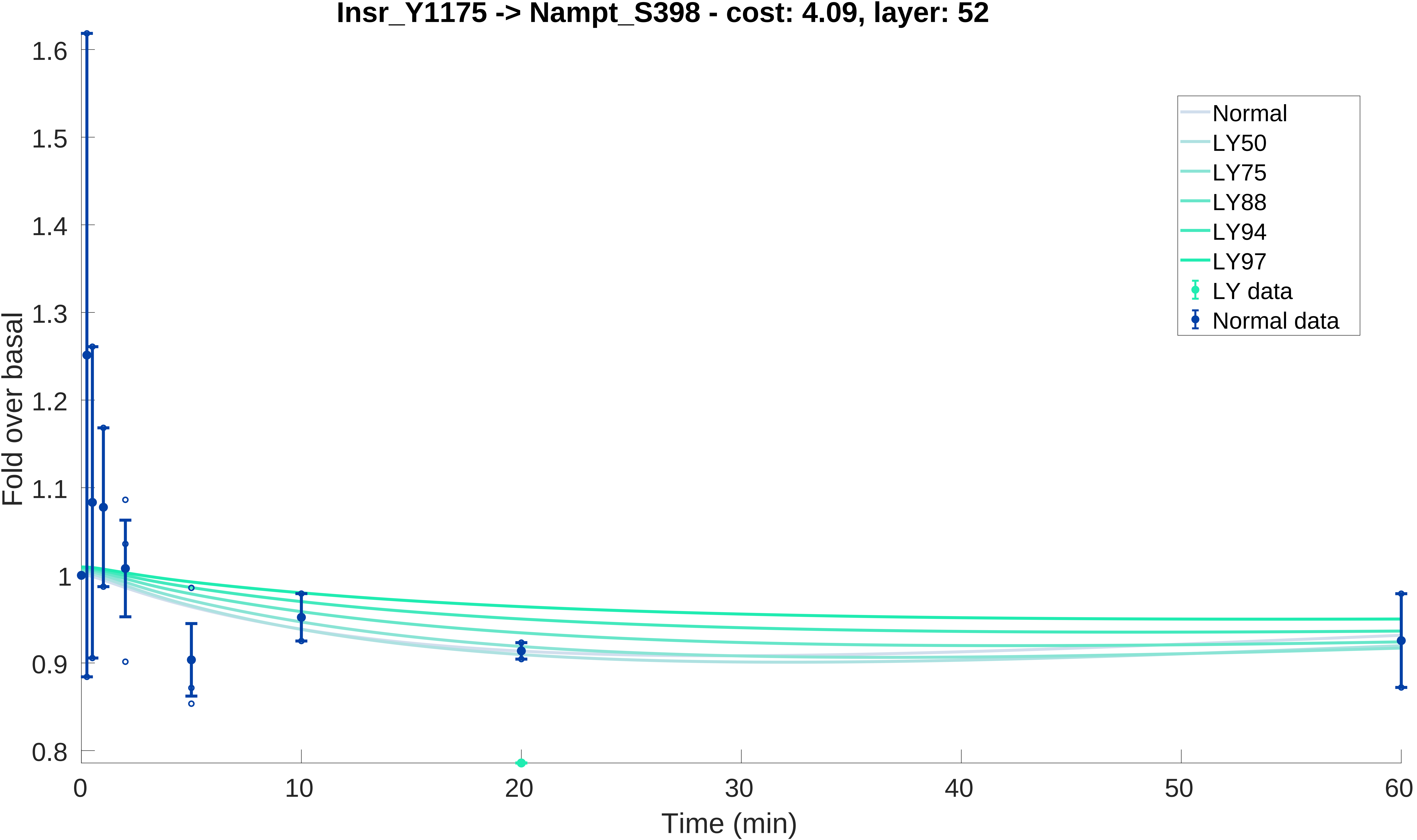

Insr_Y1175 -> Nampt_S398 - cost: 4.09, layer: 52
Fold over basal
Time (min)
0.8
0.9
1
1.1
1.2
1.3
1.4
1.5
1.6
0
10
20
30
40
50
60
Normal
LY50
LY75
LY88
LY94
LY97
LY data
Normal data

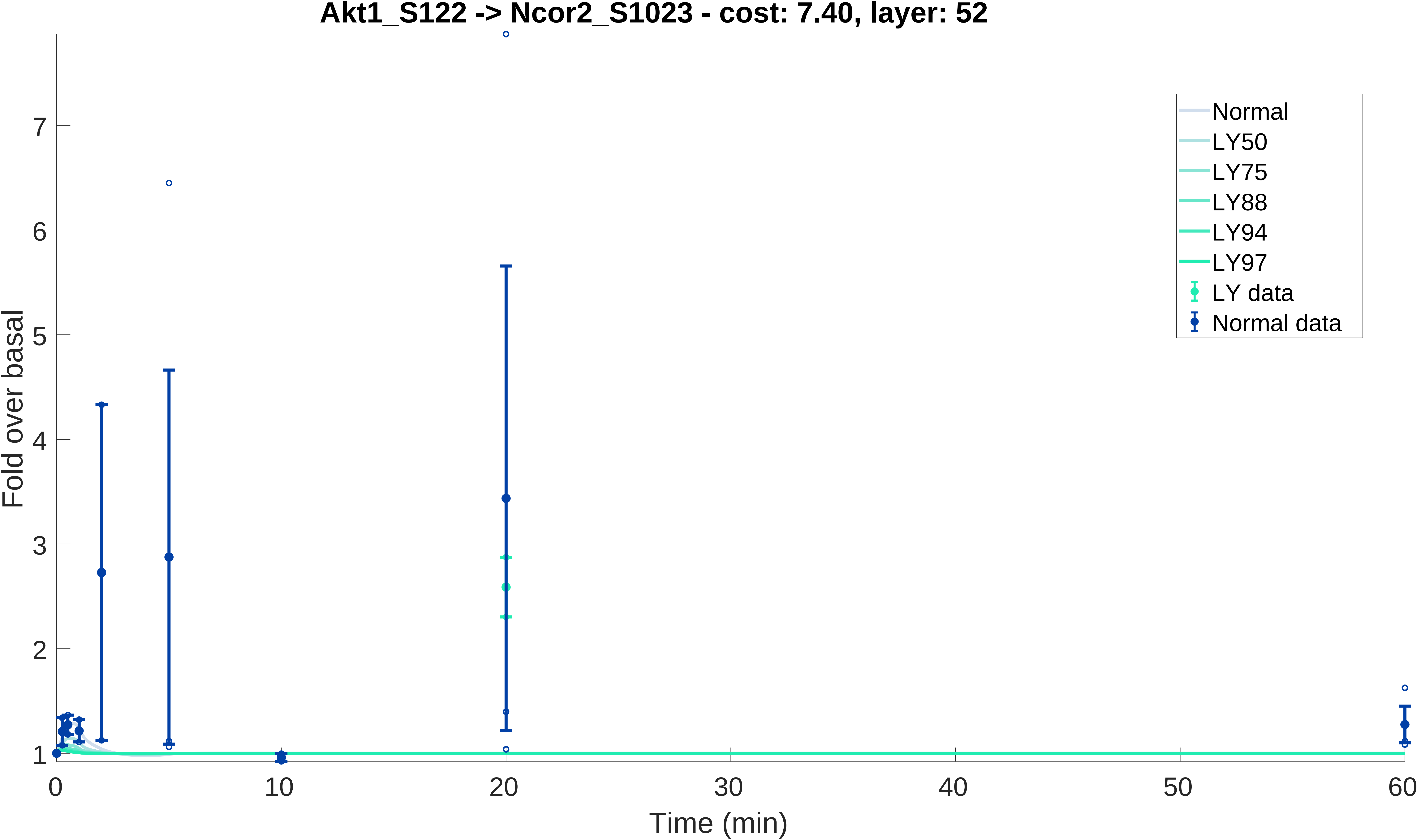
Akt1_S122 -> Ncor2_S1023 - cost: 7.40, layer: 52
Fold over basal
Time (min)
1
2
3
4
5
6
7
0
10
20
30
40
50
60
Normal
LY50
LY75
LY88
LY94
LY97
LY data
Normal data

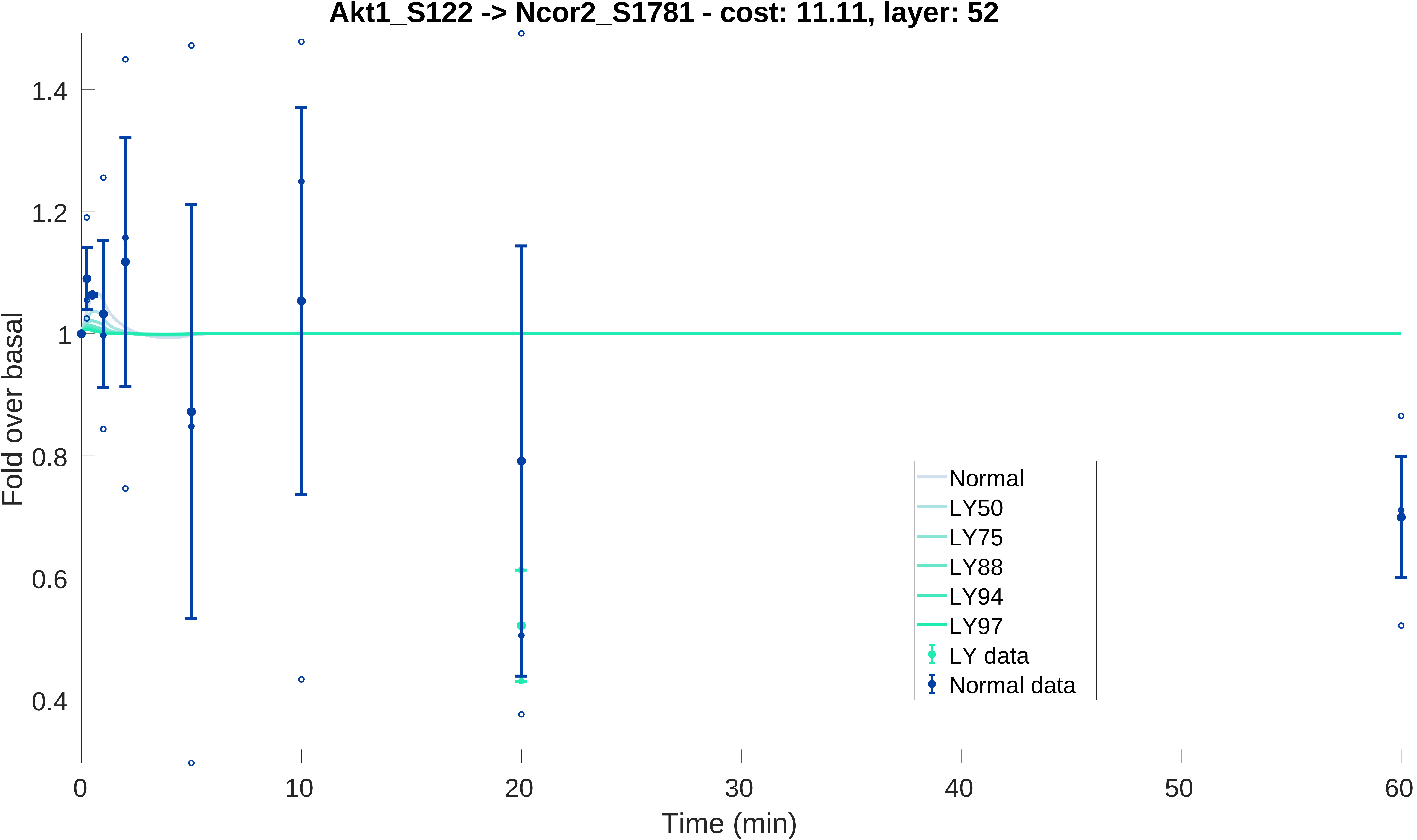

Akt1_S122 -> Ncor2_S1781 - cost: 11.11, layer: 52
Fold over basal
1.4
1.2
1
0.8
0.6
0.4
0
10
20
30
40
50
60
Time (min)
Normal
LY50
LY75
LY88
LY94
LY97
LY data
Normal data

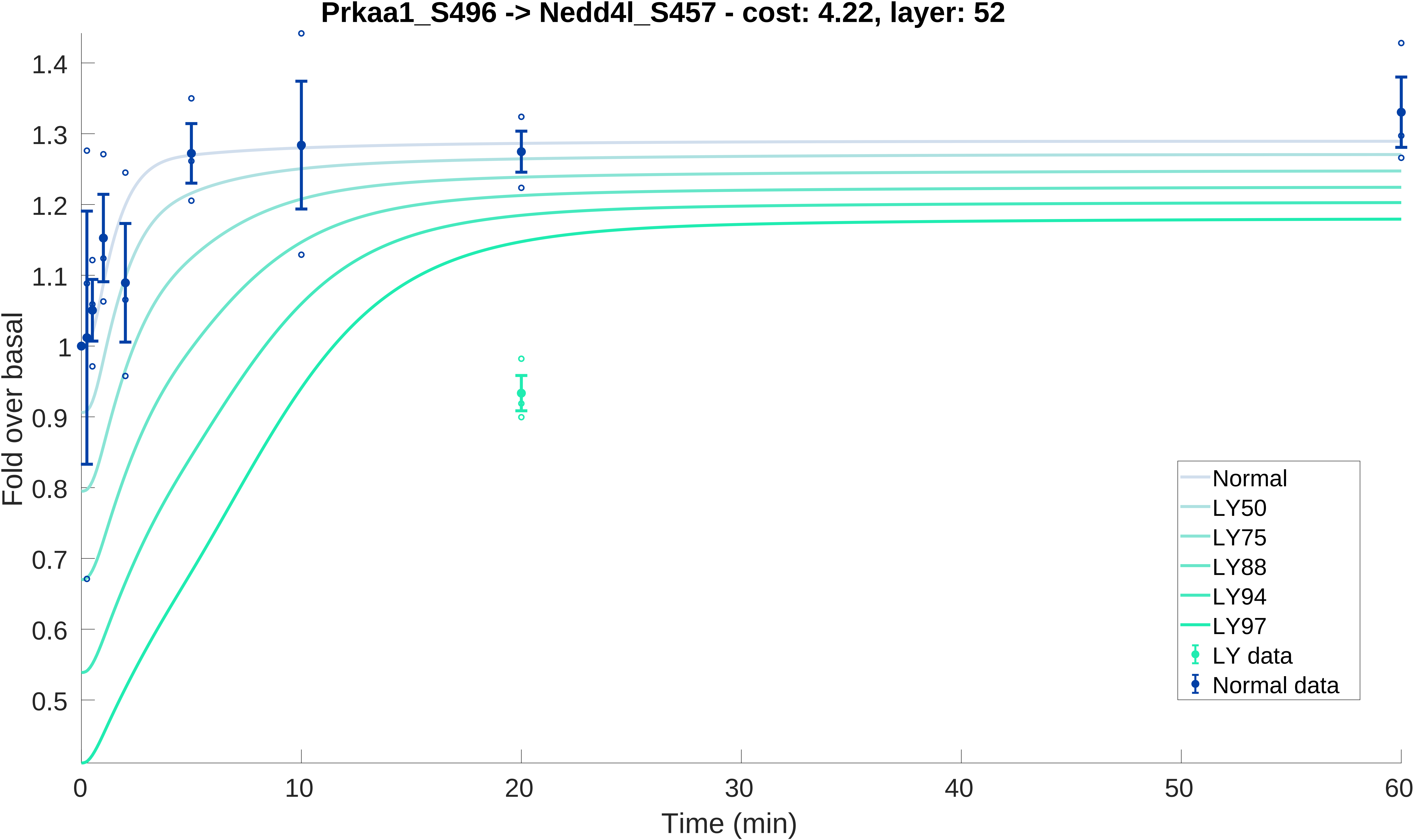

Prkaa1_S496 -> Nedd4l_S457 - cost: 4.22, layer: 52
Fold over basal
Time (min)
1.4
1.3
1.2
1.1
1
0.9
0.8
0.7
0.6
0.5
0
10
20
30
40
50
60
Normal
LY50
LY75
LY88
LY94
LY97
LY data
Normal data

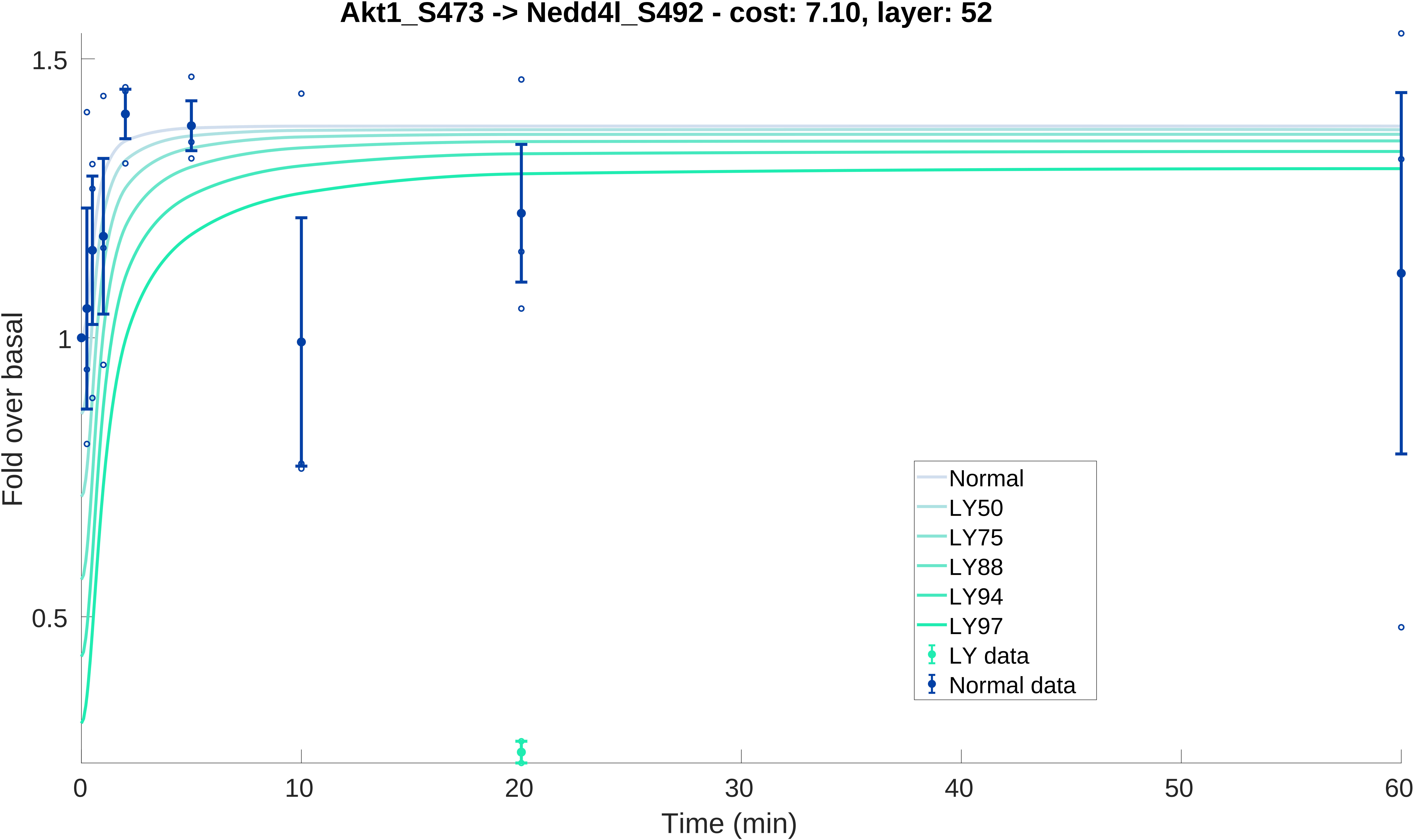

Akt1_S473 -> Nedd4l_S492 - cost: 7.10, layer: 52
Fold over basal
Time (min)
1.5
1
0.5
0
10
20
30
40
50
60
Normal
LY50
LY75
LY88
LY94
LY97
LY data
Normal data

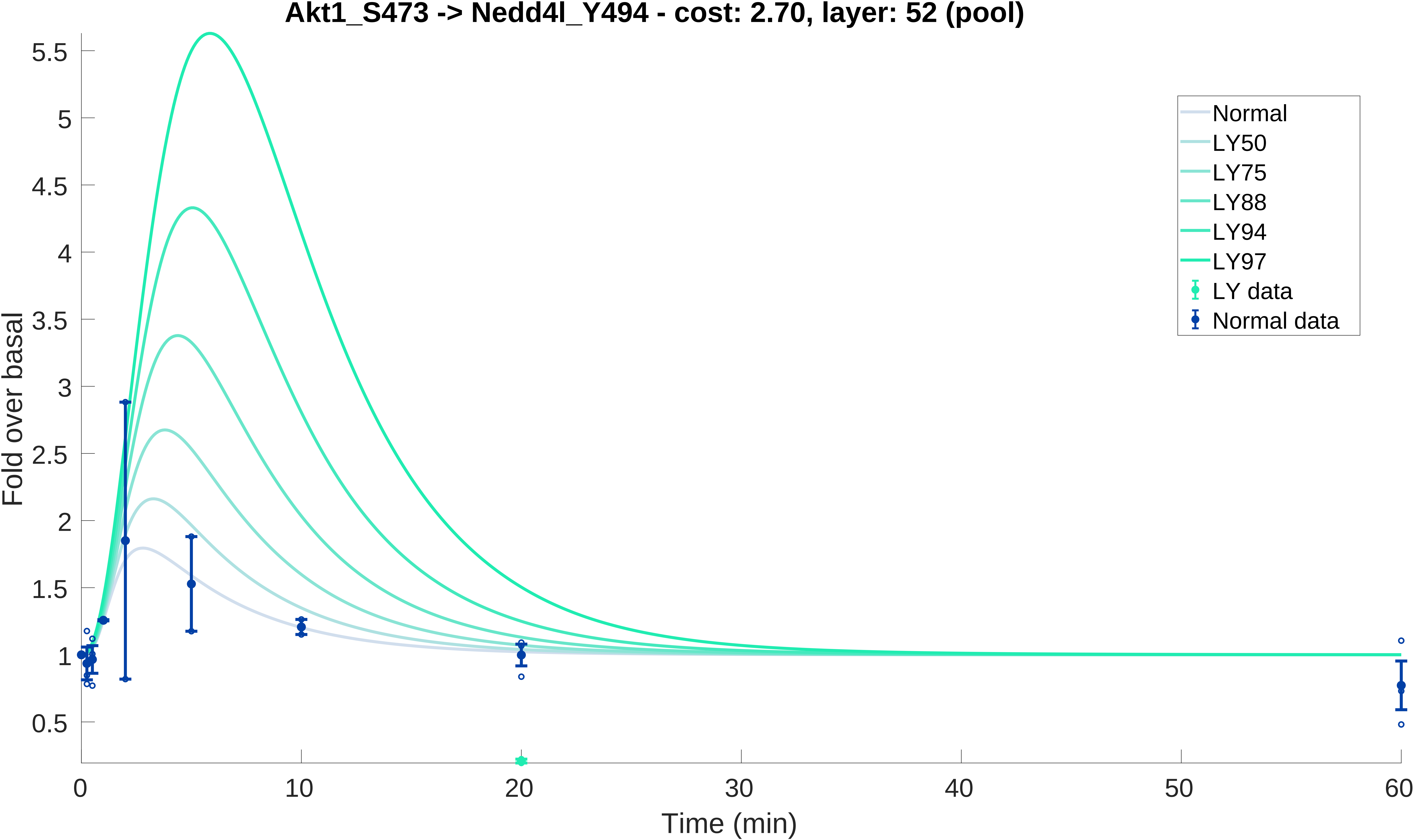
Akt1_S473 -> Nedd4l_Y494 - cost: 2.70, layer: 52 (pool)
Fold over basal
5.5
5
4.5
4
3.5
3
2.5
2
1.5
1
0.5
0
10
20
30
40
50
60
Time (min)
Normal
LY50
LY75
LY88
LY94
LY97
LY data
Normal data

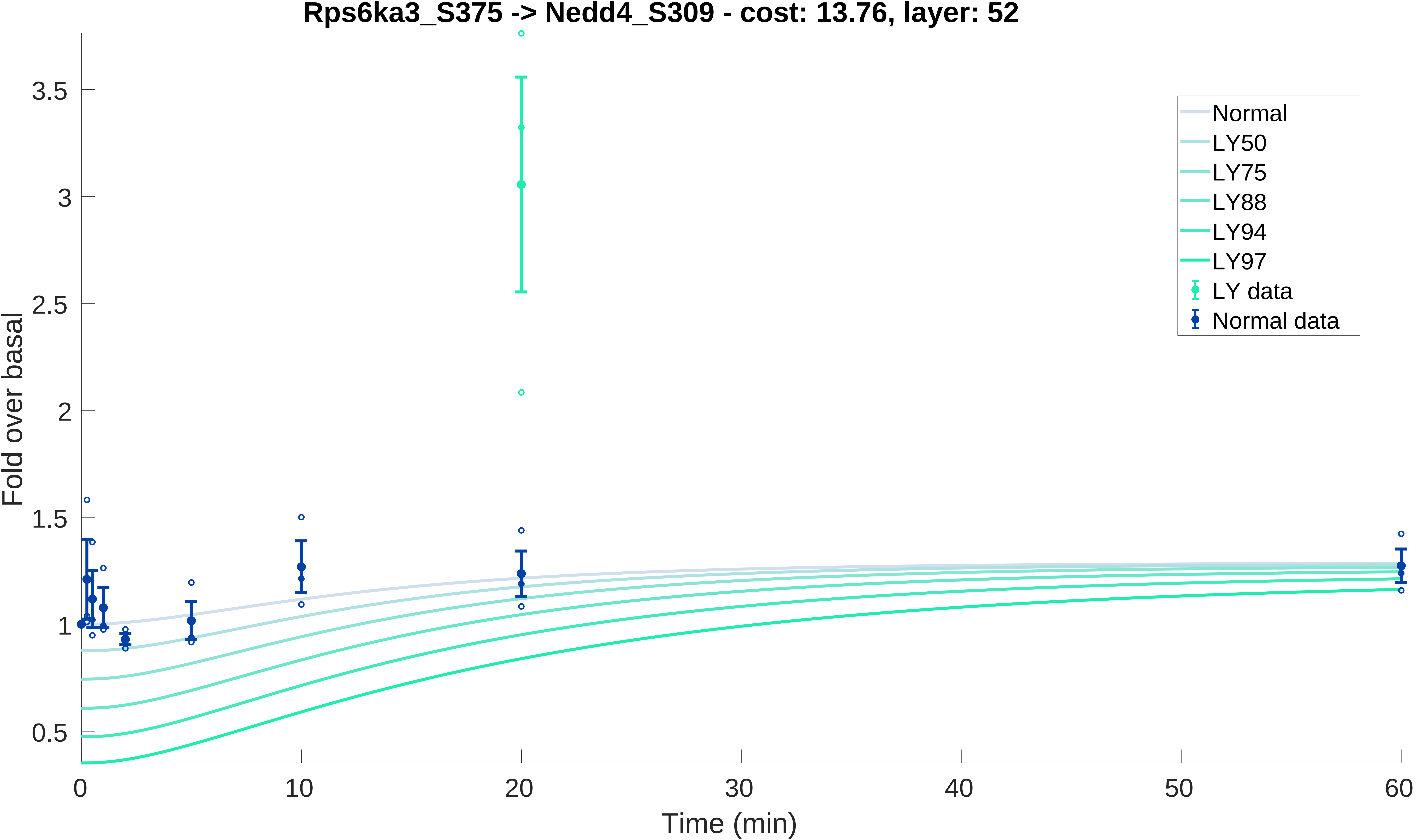

Rps6ka3_S375 -> Nedd4_S309 - cost: 13.76, layer: 52
Fold over basal
Time (min)
0.5
1
1.5
2
2.5
3
3.5
0
10
20
30
40
50
60
Normal
LY50
LY75
LY88
LY94
LY97
LY data
Normal data

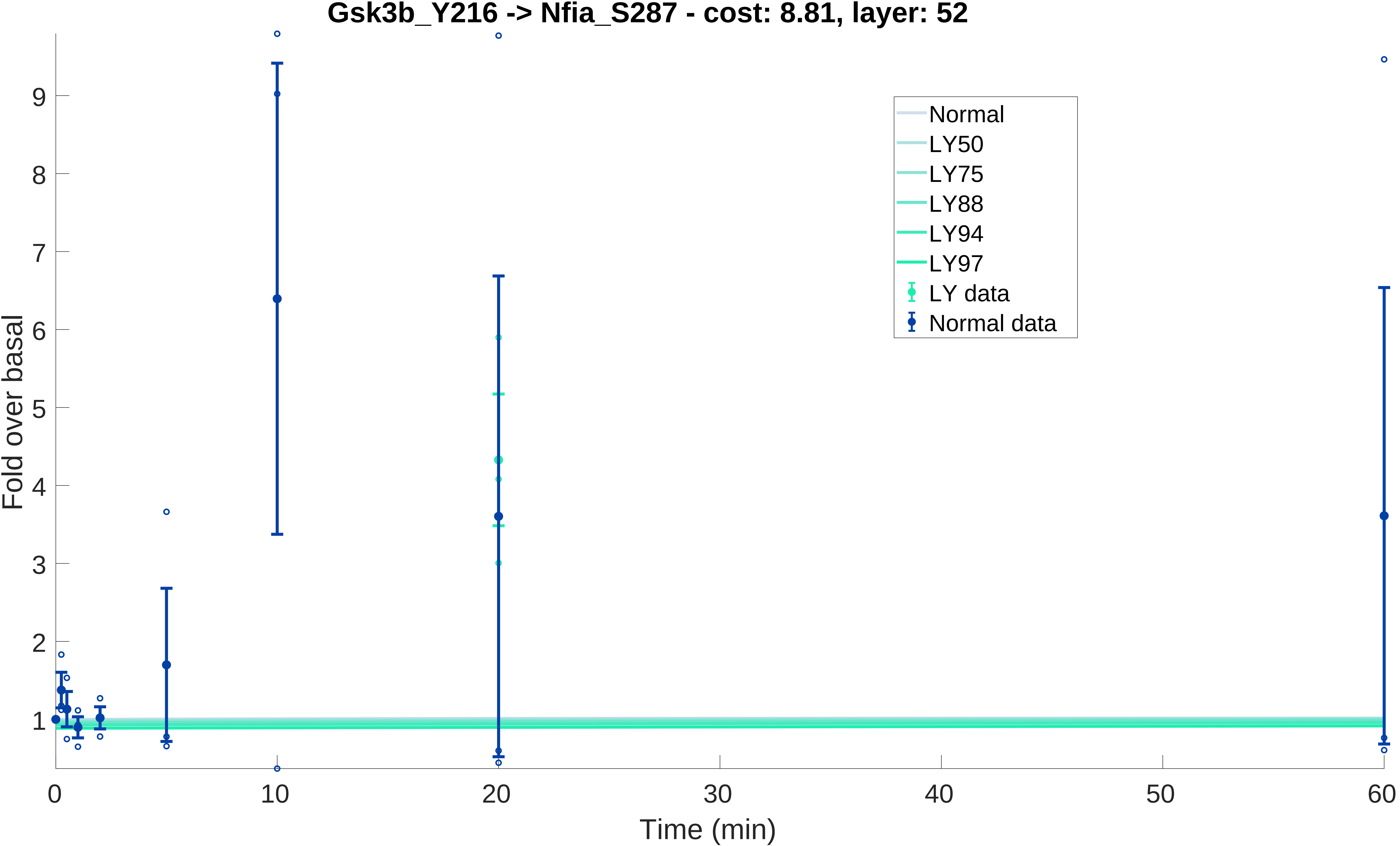
Gsk3b_Y216 -> Nfia_S287 - cost: 8.81, layer: 52
Fold over basal
Time (min)
Normal
LY50
LY75
LY88
LY94
LY97
LY data
Normal data
0
10
20
30
40
50
60
1
2
3
4
5
6
7
8
9

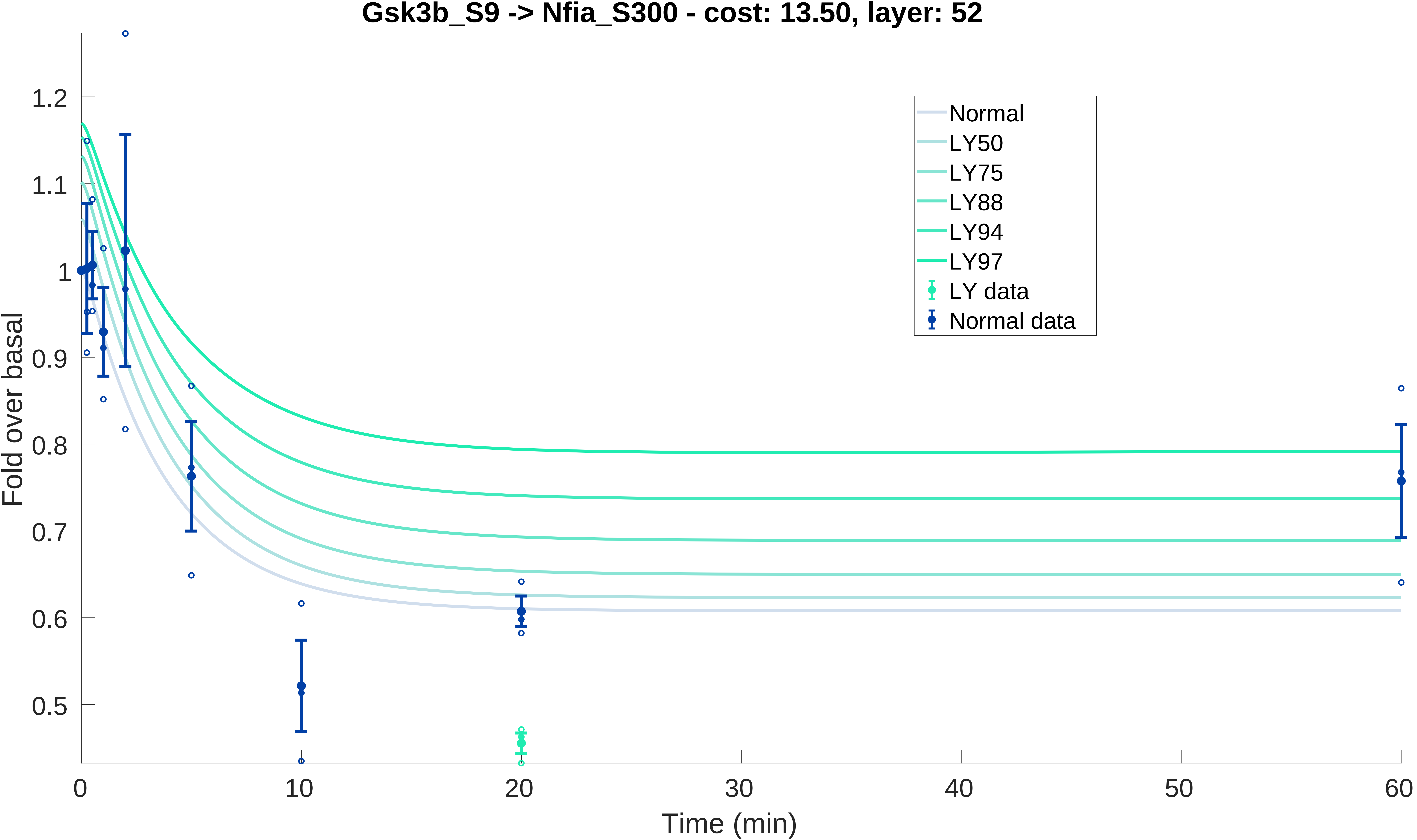
Gsk3b_S9 -> Nfia_S300 - cost: 13.50, layer: 52
Normal
LY50
LY75
LY88
LY94
LY97
LY data
Normal data
Fold over basal
Time (min)
0
10
20
30
40
50
60
0.5
0.6
0.7
0.8
0.9
1
1.1
1.2

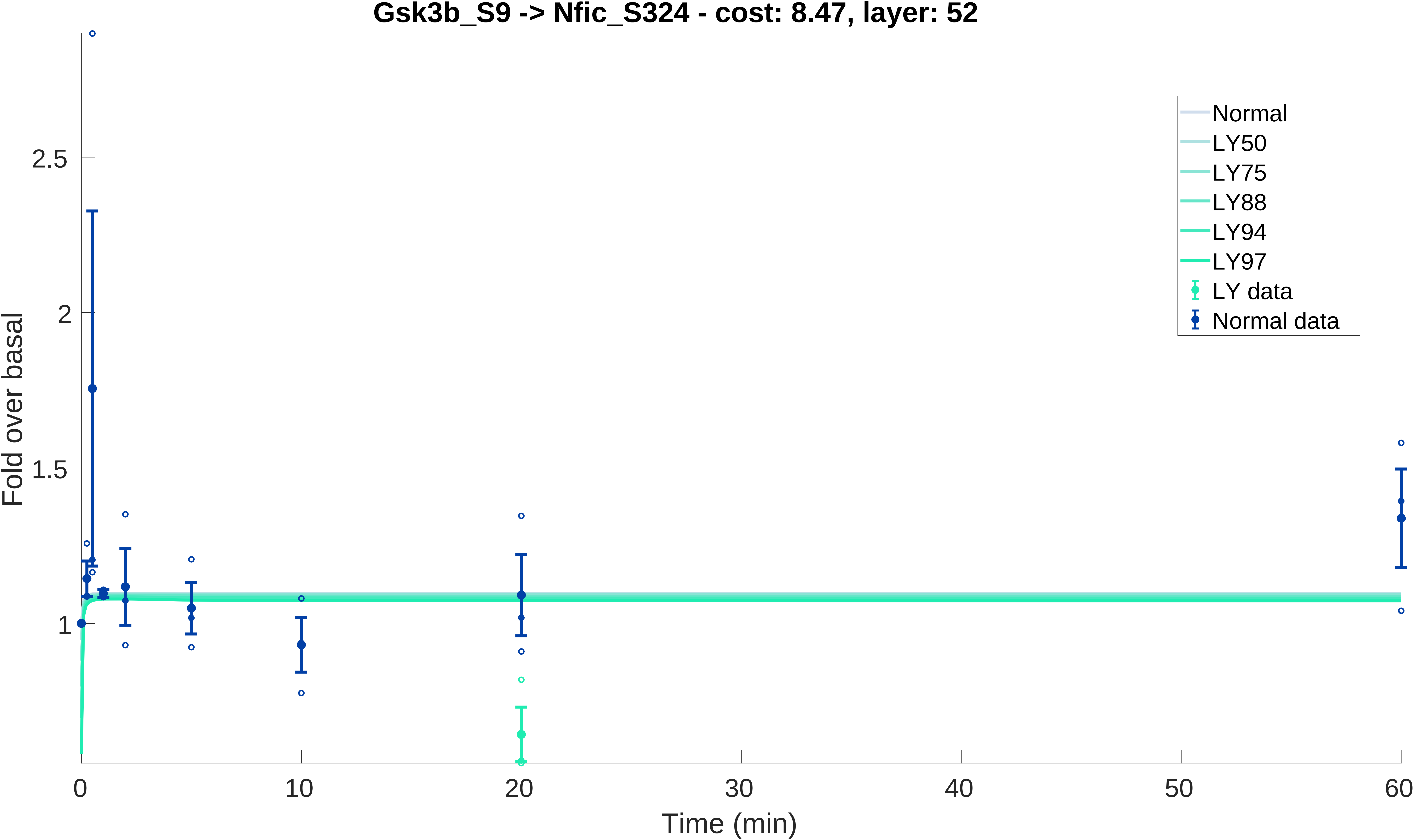
Gsk3b_S9 -> Nfic_S324 - cost: 8.47, layer: 52
Fold over basal
Time (min)
2.5
2
1.5
1
0
10
20
30
40
50
60
Normal
LY50
LY75
LY88
LY94
LY97
LY data
Normal data

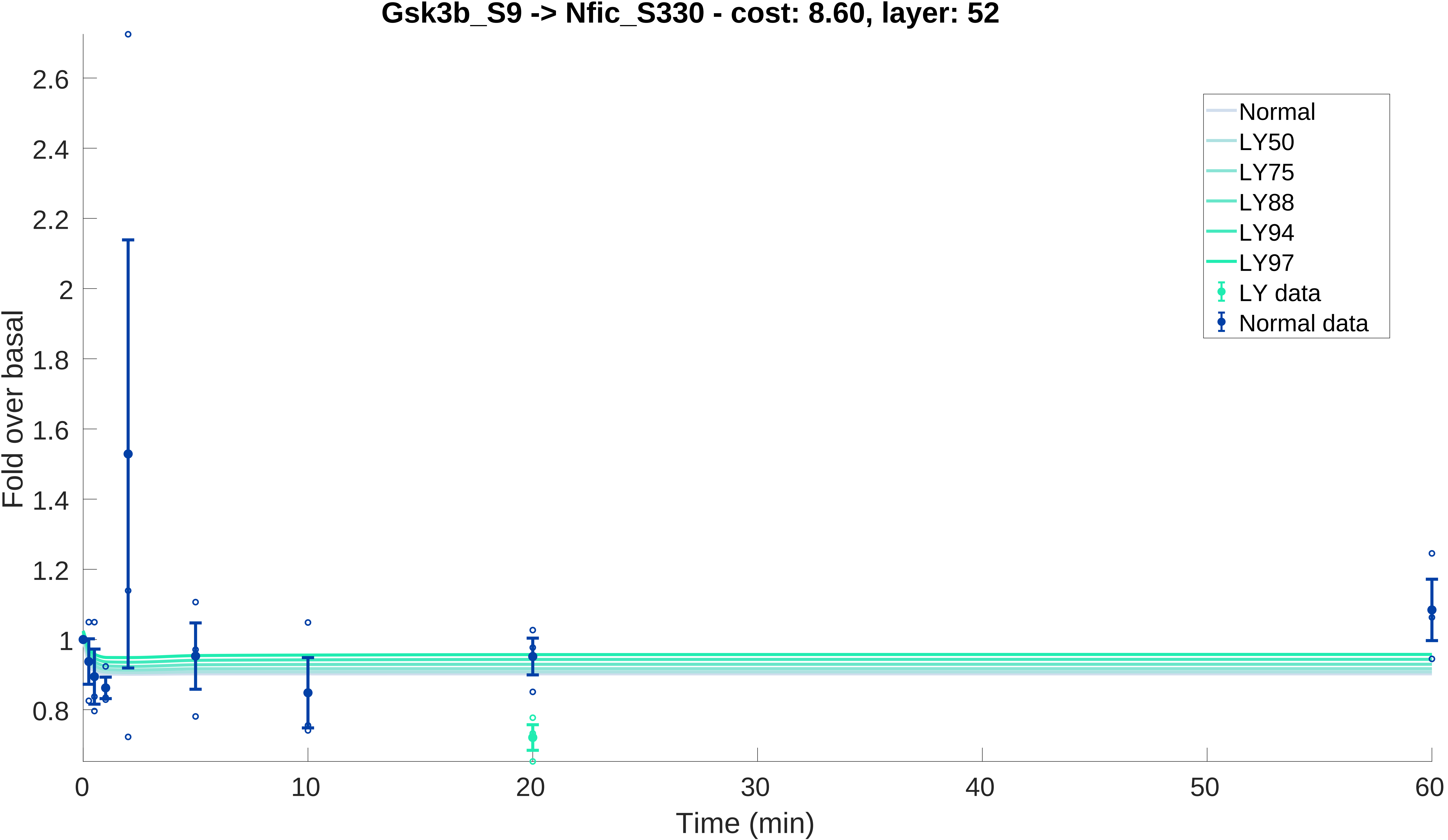

Gsk3b_S9 -> Nfic_S330 - cost: 8.60, layer: 52
Fold over basal
Time (min)
0.8
1
1.2
1.4
1.6
1.8
2
2.2
2.4
2.6
0
10
20
30
40
50
60
Normal
LY50
LY75
LY88
LY94
LY97
LY data
Normal data

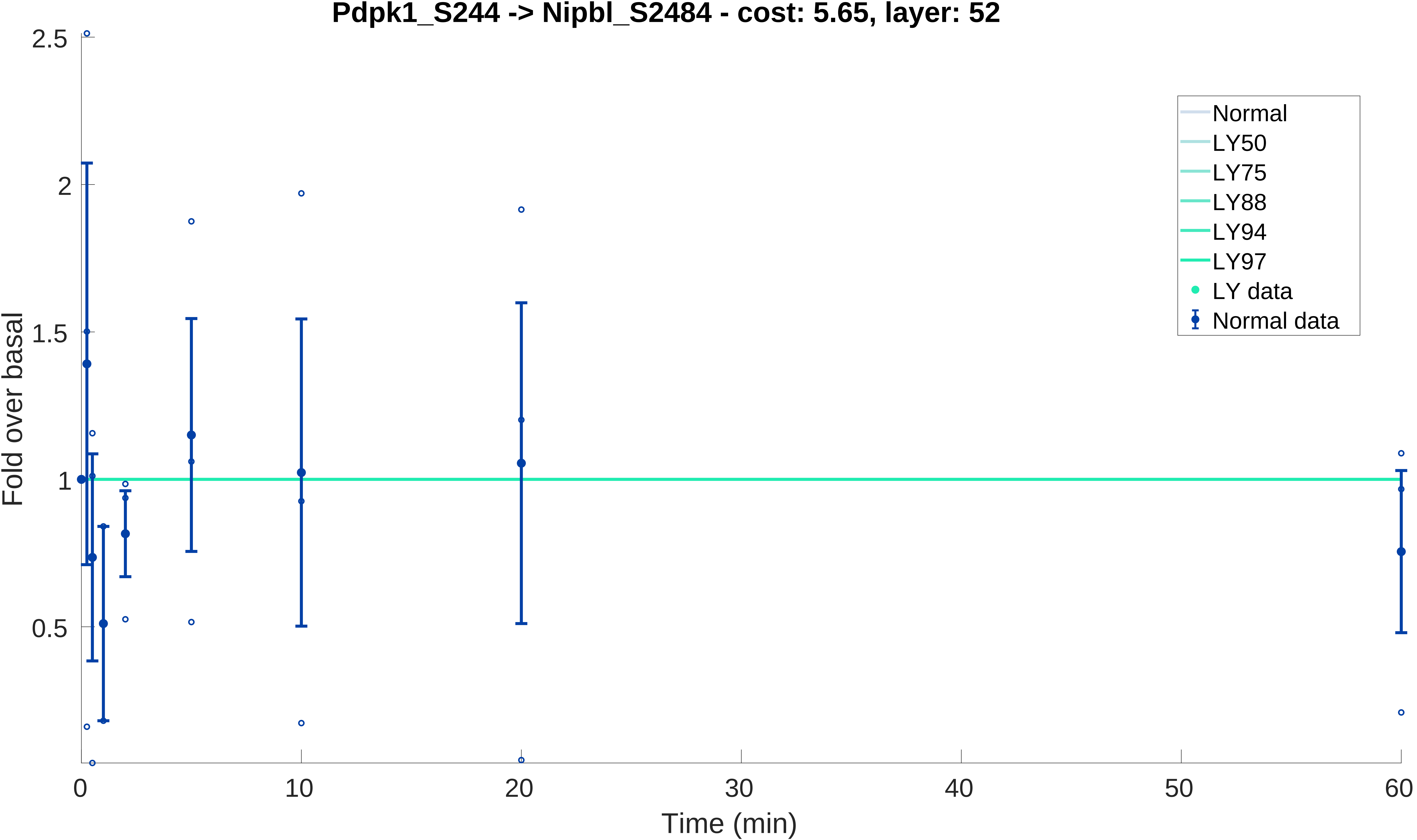

Pdpk1_S244 -> Nipbl_S2484 - cost: 5.65, layer: 52
Fold over basal
Time (min)
0
10
20
30
40
50
60
0.5
1
1.5
2
2.5
Normal
LY50
LY75
LY88
LY94
LY97
LY data
Normal data

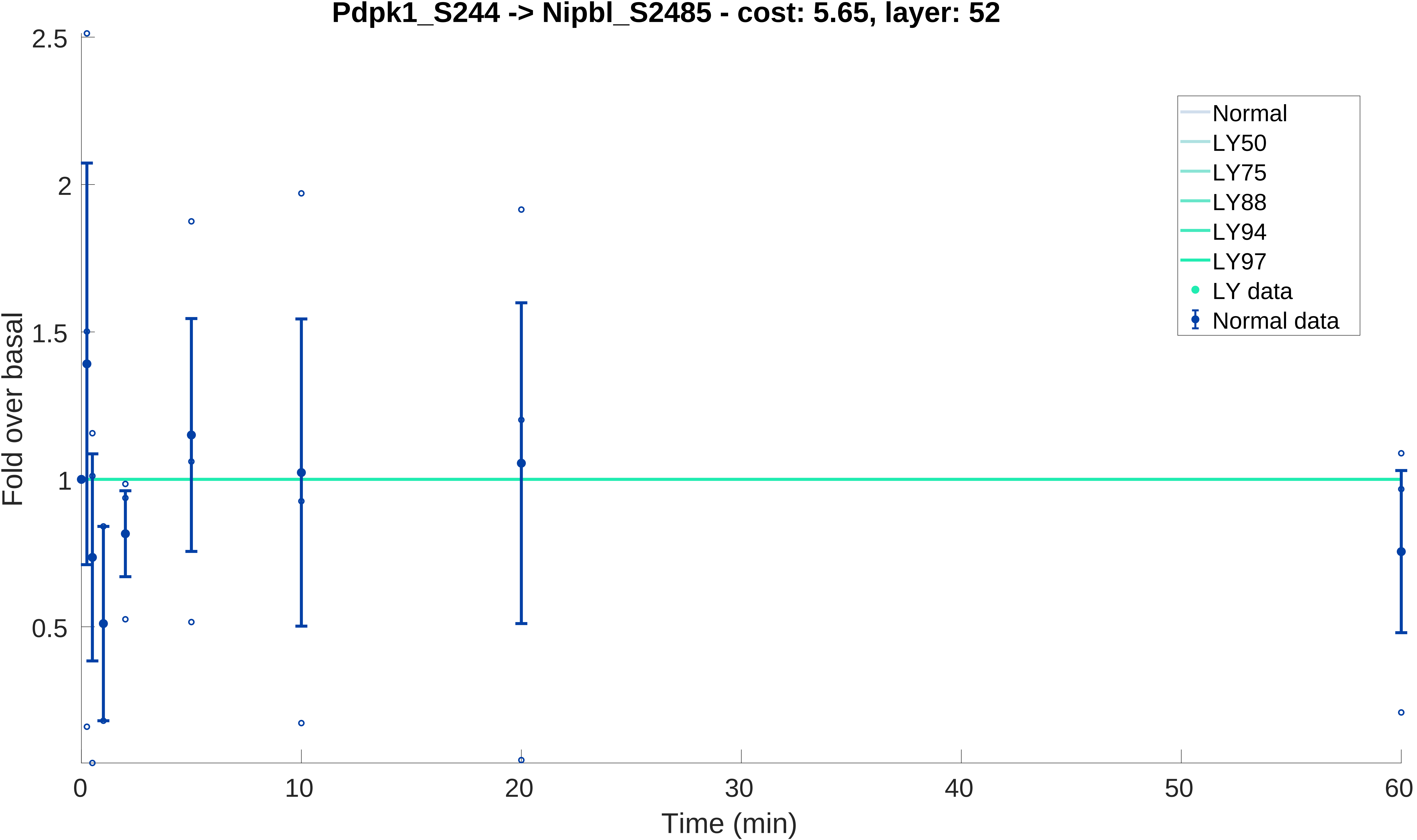

Pdpk1_S244 -> Nipbl_S2485 - cost: 5.65, layer: 52
Fold over basal
Time (min)
2.5
2
1.5
1
0.5
0
10
20
30
40
50
60
Normal
LY50
LY75
LY88
LY94
LY97
LY data
Normal data

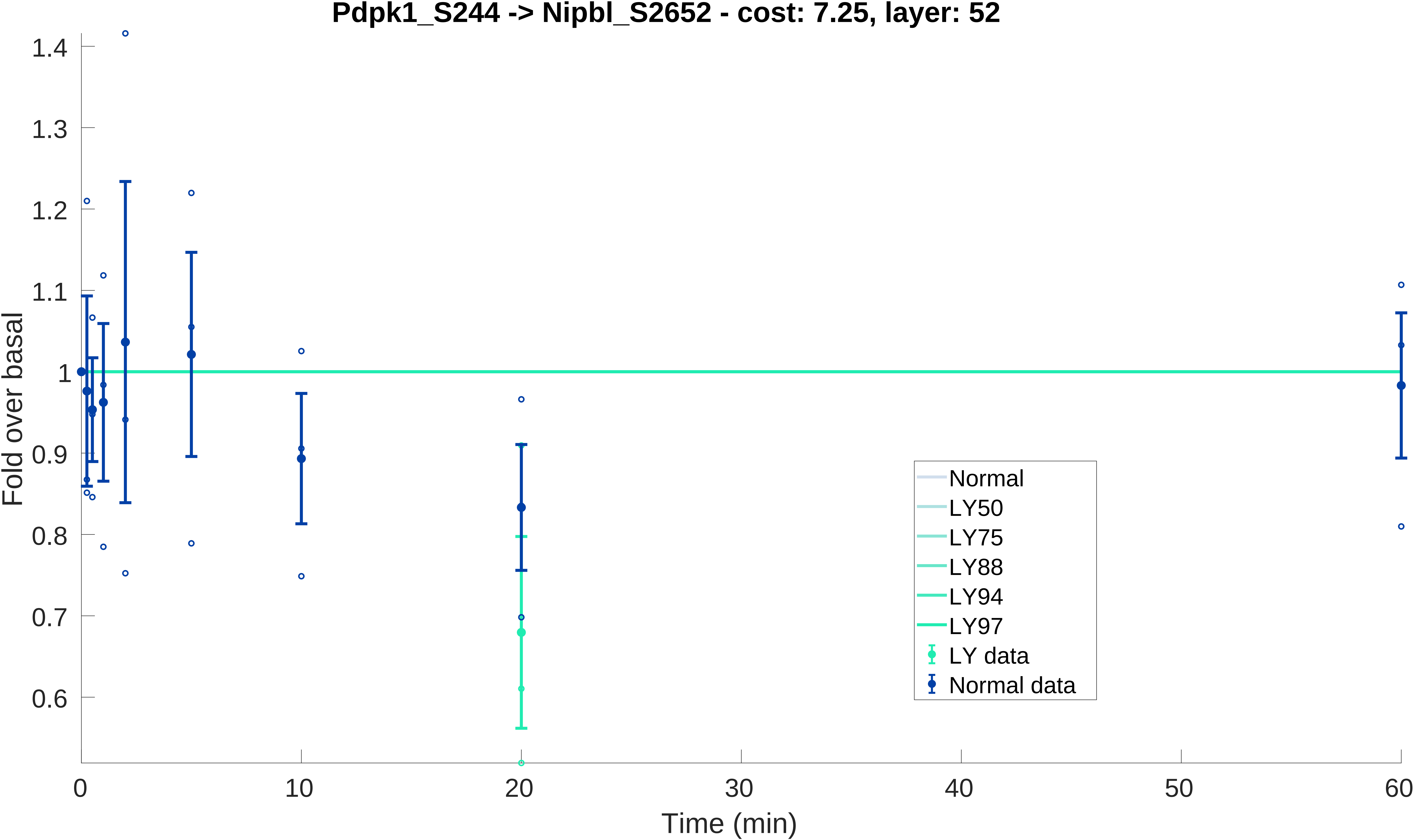

Pdpk1_S244 -> Nipbl_S2652 - cost: 7.25, layer: 52
Fold over basal
1.4
1.3
1.2
1.1
1
0.9
0.8
0.7
0.6
0
10
20
30
40
50
60
Time (min)
Normal
LY50
LY75
LY88
LY94
LY97
LY data
Normal data

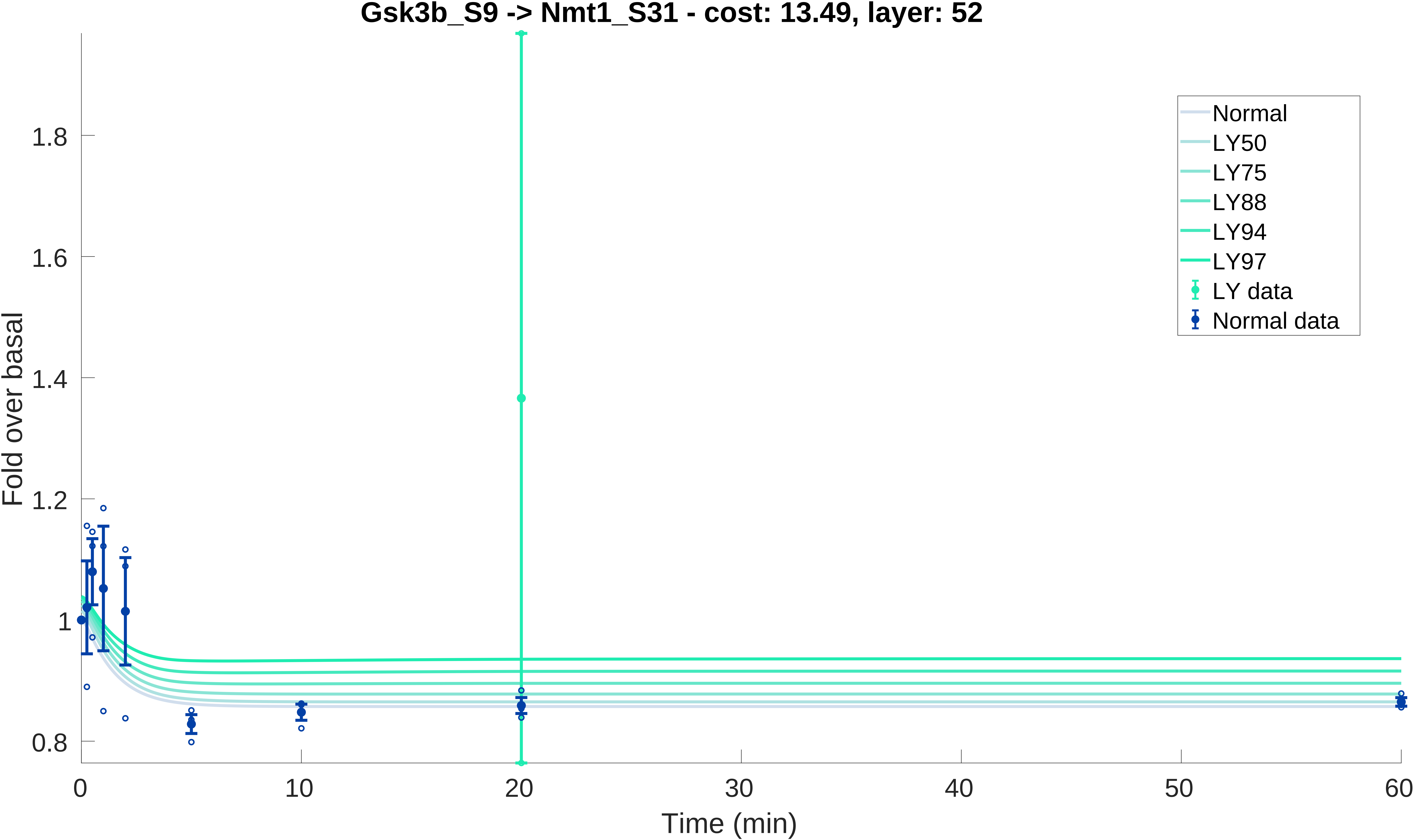

Gsk3b_S9 -> Nmt1_S31 - cost: 13.49, layer: 52
Fold over basal
Time (min)
0.8
1
1.2
1.4
1.6
1.8
0
10
20
30
40
50
60
Normal
LY50
LY75
LY88
LY94
LY97
LY data
Normal data

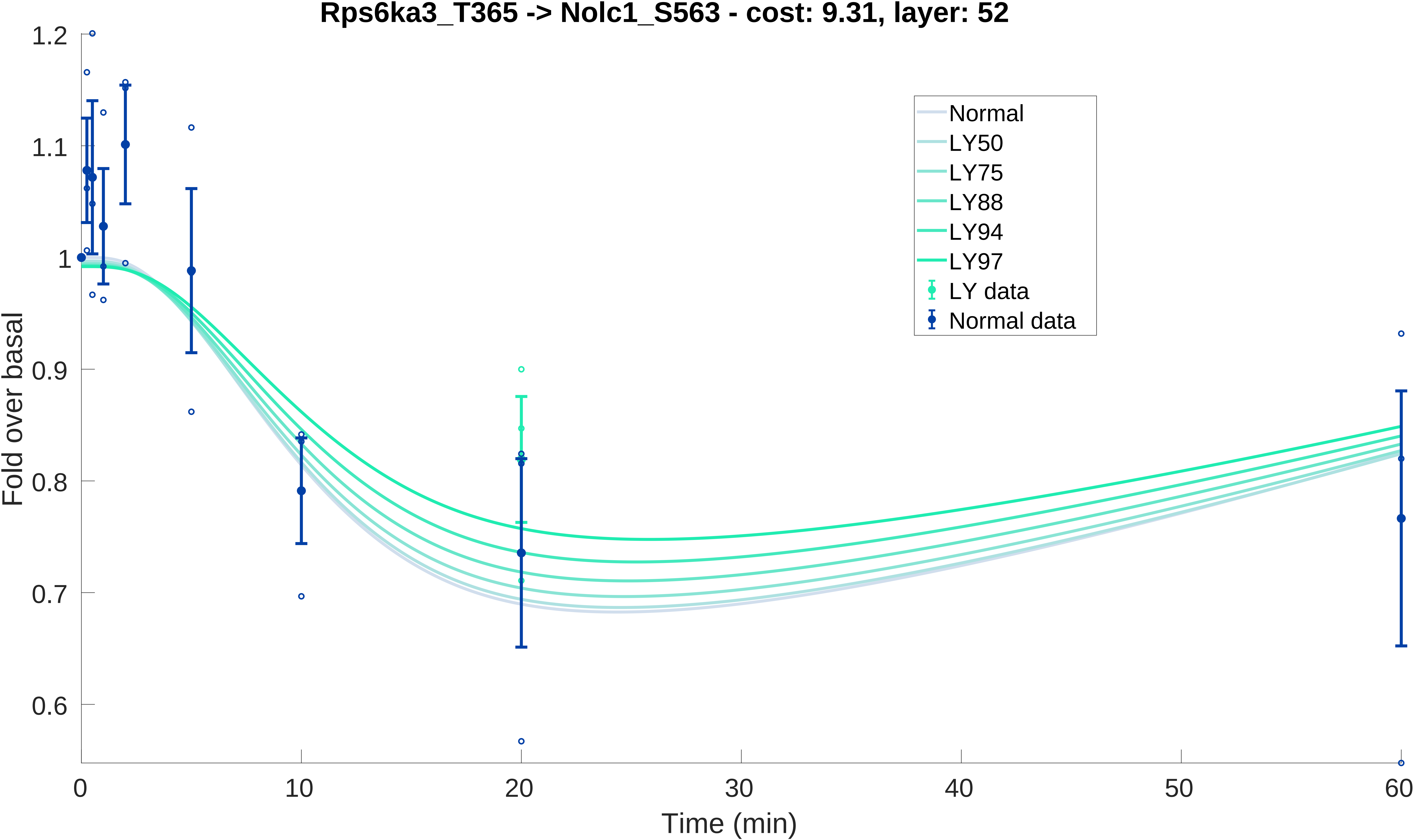

Rps6ka3_T365 -> Nolc1_S563 - cost: 9.31, layer: 52
Fold over basal
Time (min)
1.2
1.1
1
0.9
0.8
0.7
0.6
0
10
20
30
40
50
60
Normal
LY50
LY75
LY88
LY94
LY97
LY data
Normal data

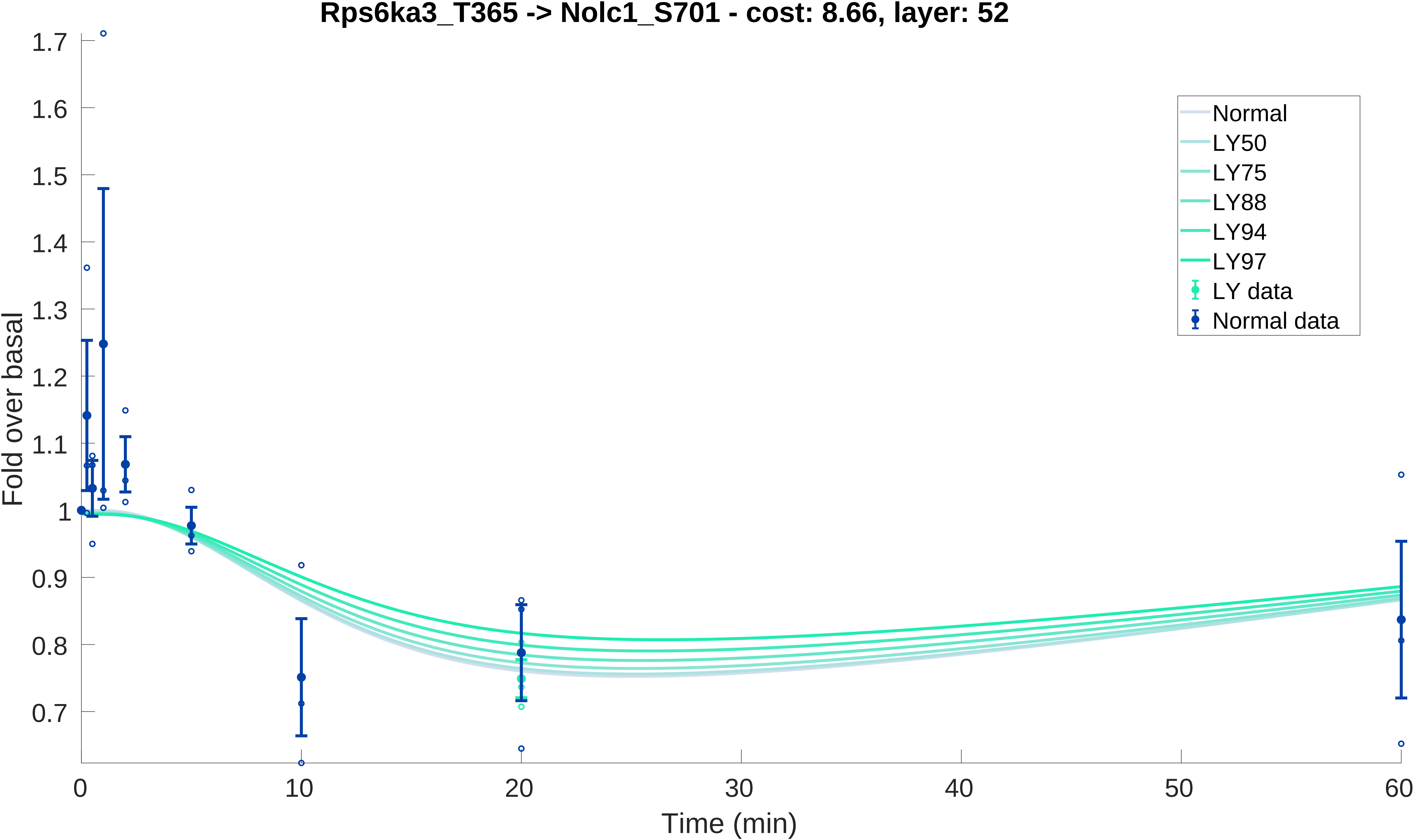

Rps6ka3_T365 -> Nolc1_S701 - cost: 8.66, layer: 52
Fold over basal
Time (min)
1.7
1.6
1.5
1.4
1.3
1.2
1.1
1
0.9
0.8
0.7
0
10
20
30
40
50
60
Normal
LY50
LY75
LY88
LY94
LY97
LY data
Normal data

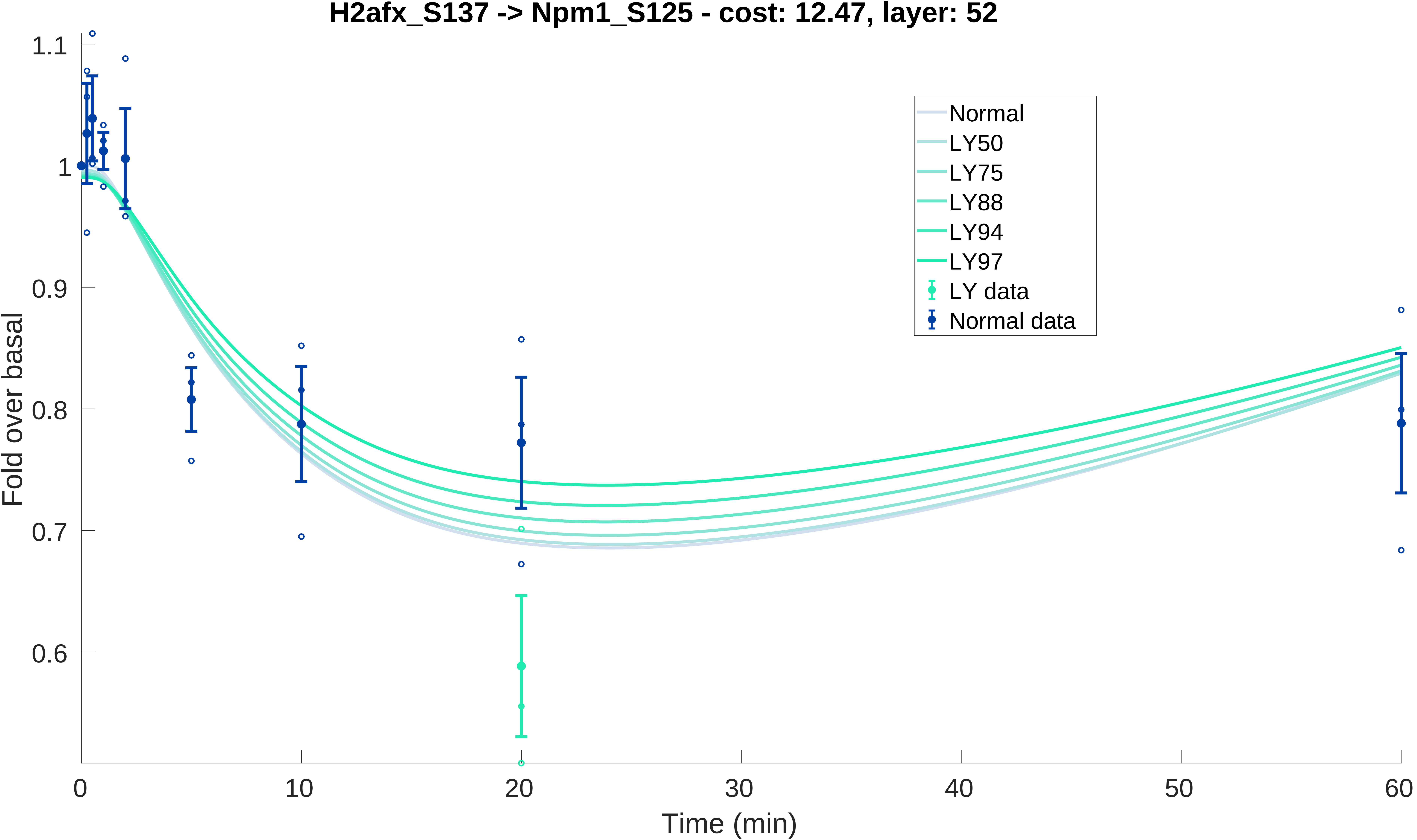

H2afx_S137 -> Npm1_S125 - cost: 12.47, layer: 52
Fold over basal
Time (min)
1.1
1
0.9
0.8
0.7
0.6
0
10
20
30
40
50
60
Normal
LY50
LY75
LY88
LY94
LY97
LY data
Normal data

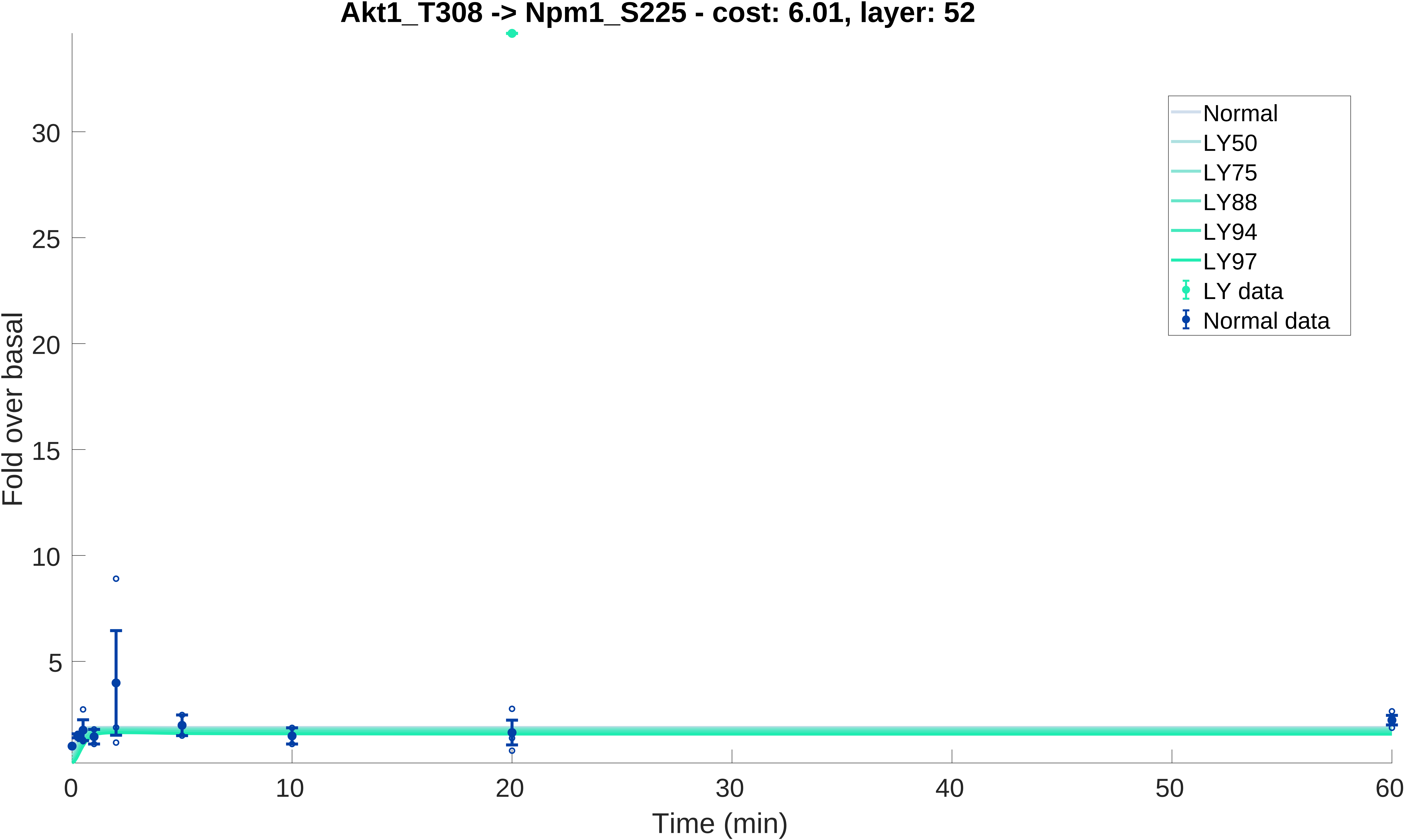
Akt1_T308 -> Npm1_S225 - cost: 6.01, layer: 52
Fold over basal
Time (min)
0
10
20
30
40
50
60
5
10
15
20
25
30
Normal
LY50
LY75
LY88
LY94
LY97
LY data
Normal data

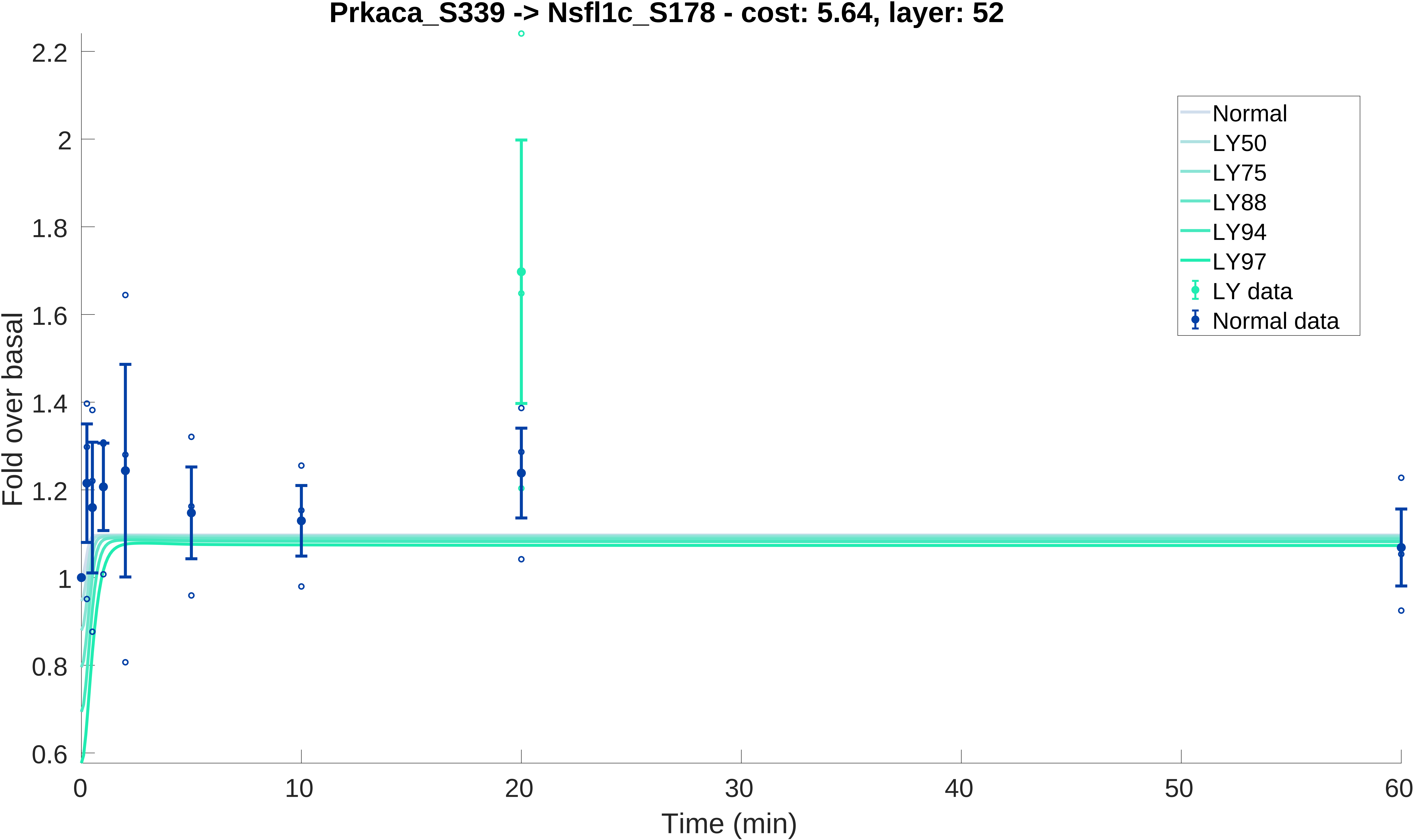
Prkaca_S339 -> Nsfl1c_S178 - cost: 5.64, layer: 52
Fold over basal
Time (min)
Normal
LY50
LY75
LY88
LY94
LY97
LY data
Normal data
0.6
0.8
1
1.2
1.4
1.6
1.8
2
2.2
0
10
20
30
40
50
60

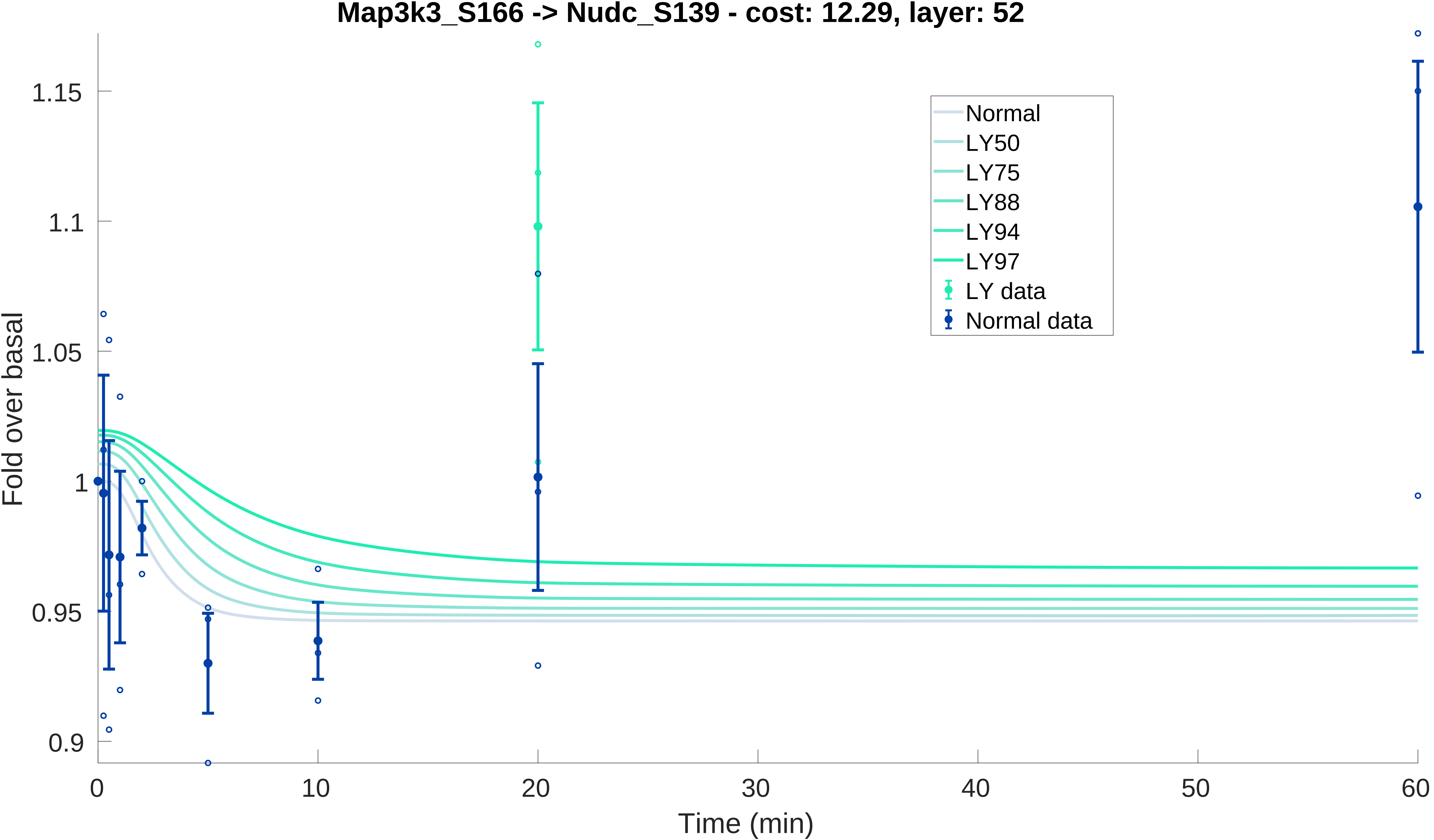

Map3k3_S166 -> Nudc_S139 - cost: 12.29, layer: 52
Fold over basal
Time (min)
0.9
0.95
1
1.05
1.1
1.15
0
10
20
30
40
50
60
Normal
LY50
LY75
LY88
LY94
LY97
LY data
Normal data

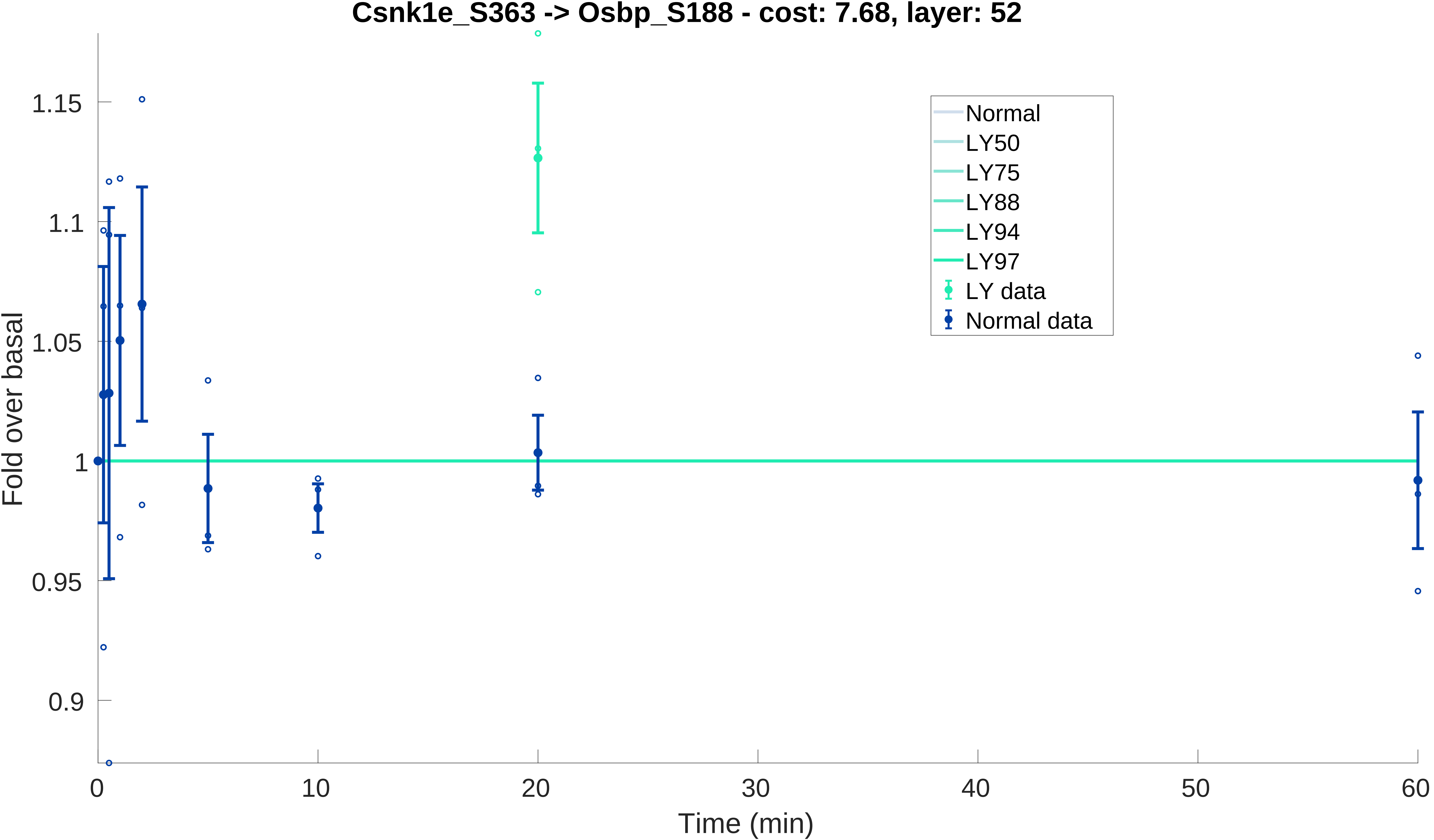

Csnk1e_S363 -> Osbp_S188 - cost: 7.68, layer: 52
Fold over basal
1.15
1.1
1.05
1
0.95
0.9
0
10
20
30
40
50
60
Time (min)
Normal
LY50
LY75
LY88
LY94
LY97
LY data
Normal data

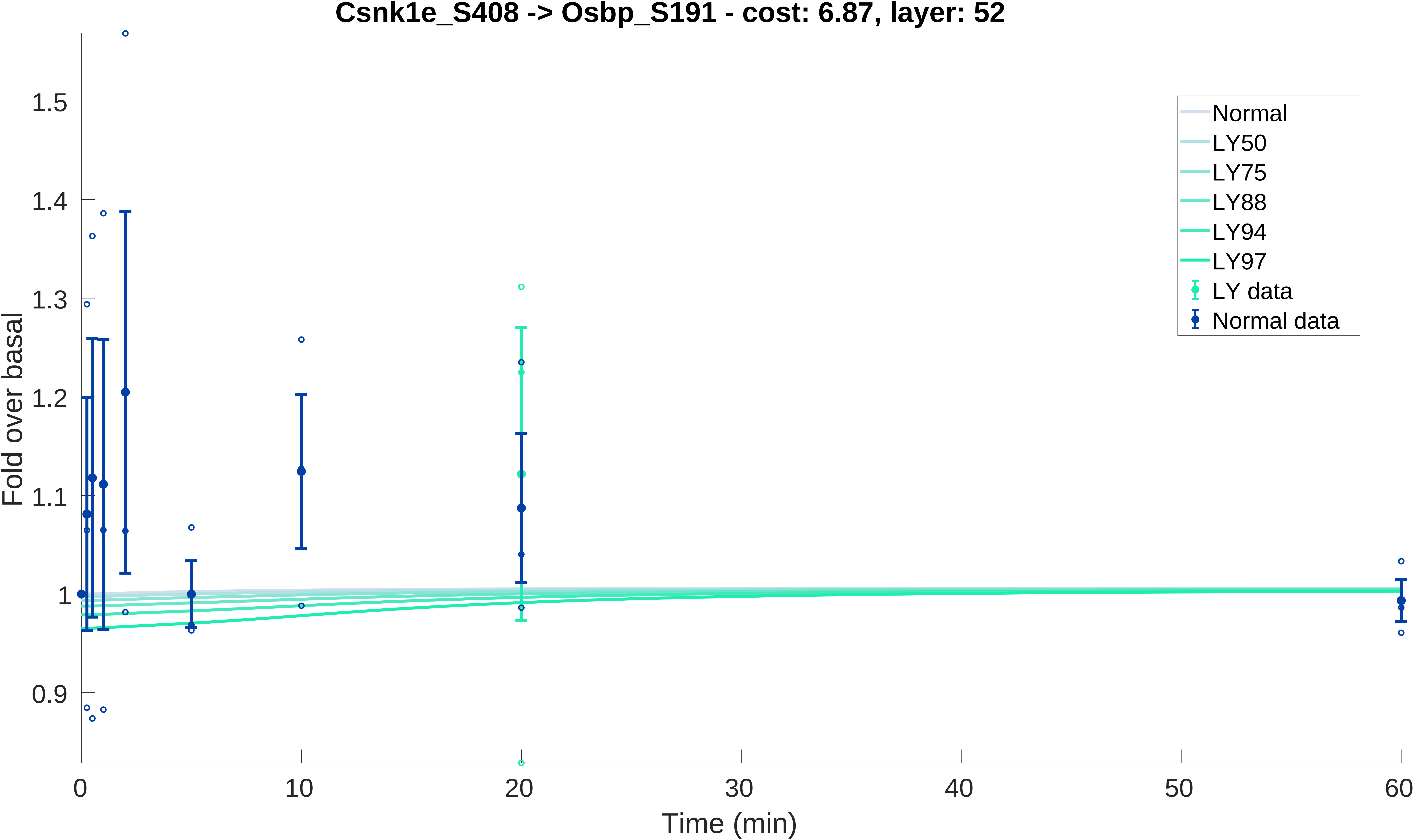

Csnk1e_S408 -> Osbp_S191 - cost: 6.87, layer: 52
Fold over basal
Time (min)
Normal
LY50
LY75
LY88
LY94
LY97
LY data
Normal data
0.9
1
1.1
1.2
1.3
1.4
1.5
0
10
20
30
40
50
60

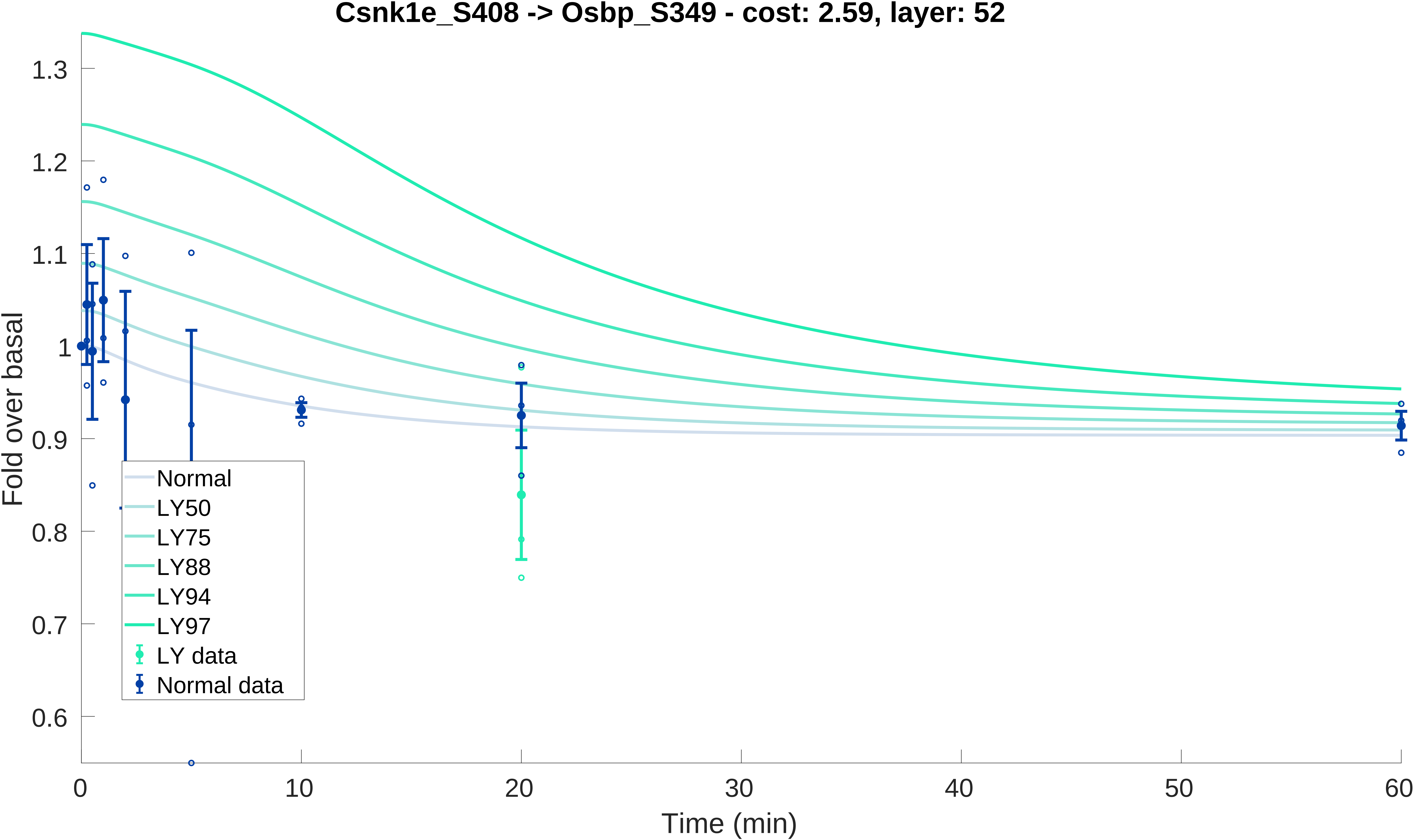
Csnk1e_S408 -> Osbp_S349 - cost: 2.59, layer: 52
Fold over basal
Time (min)
1.3
1.2
1.1
1
0.9
0.8
0.7
0.6
0
10
20
30
40
50
60
Normal
LY50
LY75
LY88
LY94
LY97
LY data
Normal data

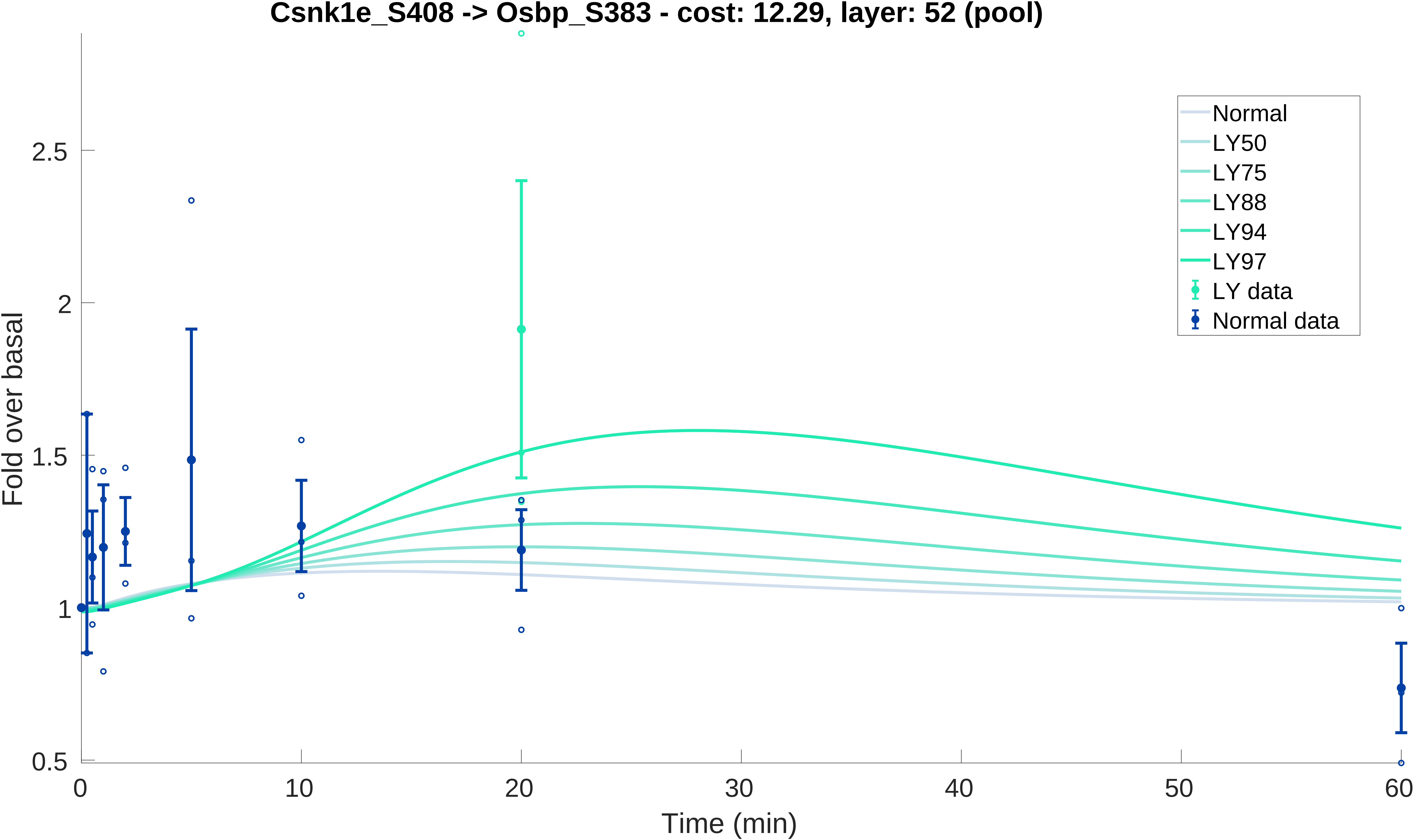

Csnk1e_S408 -> Osbp_S383 - cost: 12.29, layer: 52 (pool)
Fold over basal
Time (min)
Normal
LY50
LY75
LY88
LY94
LY97
LY data
Normal data
0.5
1
1.5
2
2.5
0
10
20
30
40
50
60

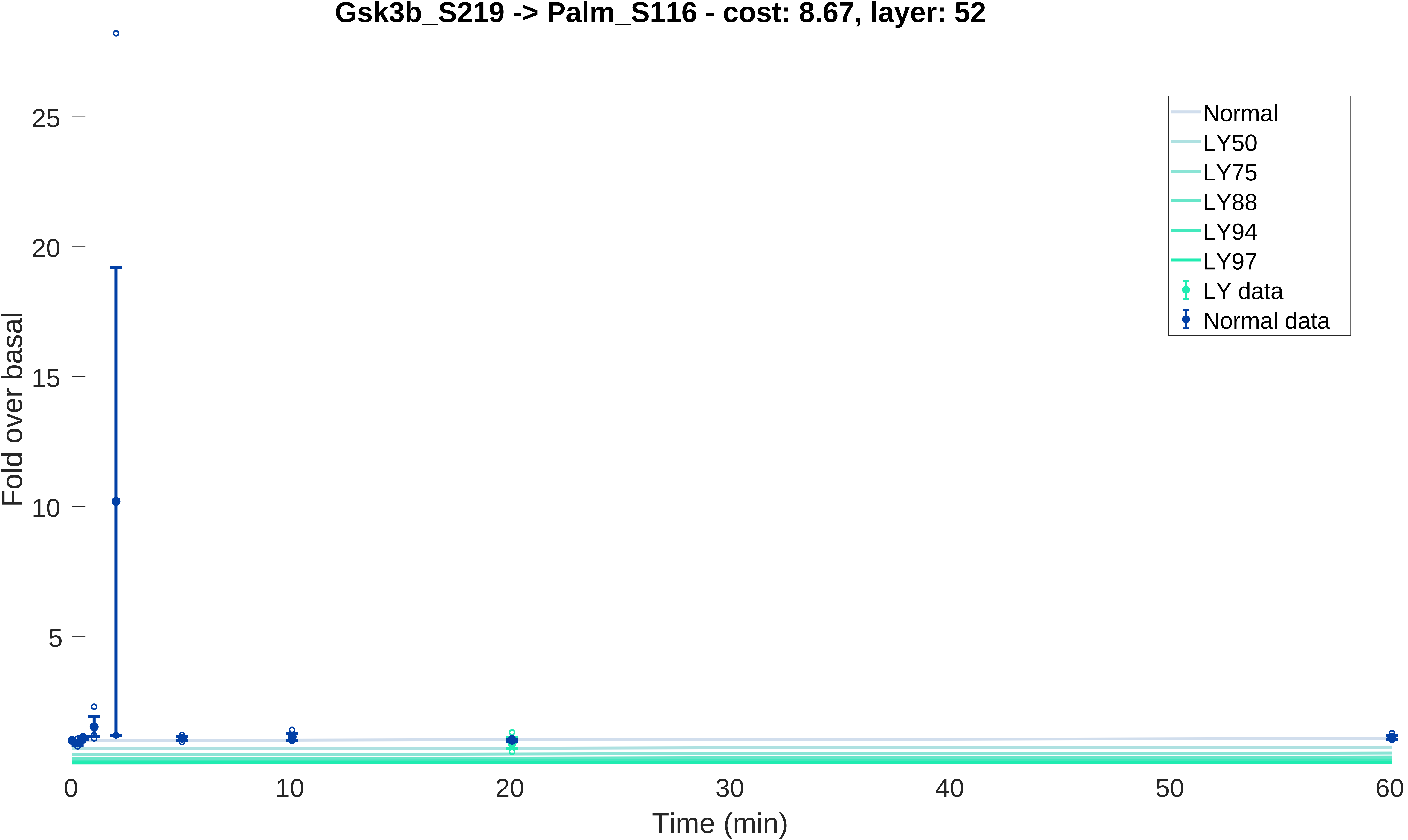
Gsk3b_S219 -> Palm_S116 - cost: 8.67, layer: 52
Fold over basal
Time (min)
0
5
10
15
20
25
30
40
50
60
Normal
LY50
LY75
LY88
LY94
LY97
LY data
Normal data

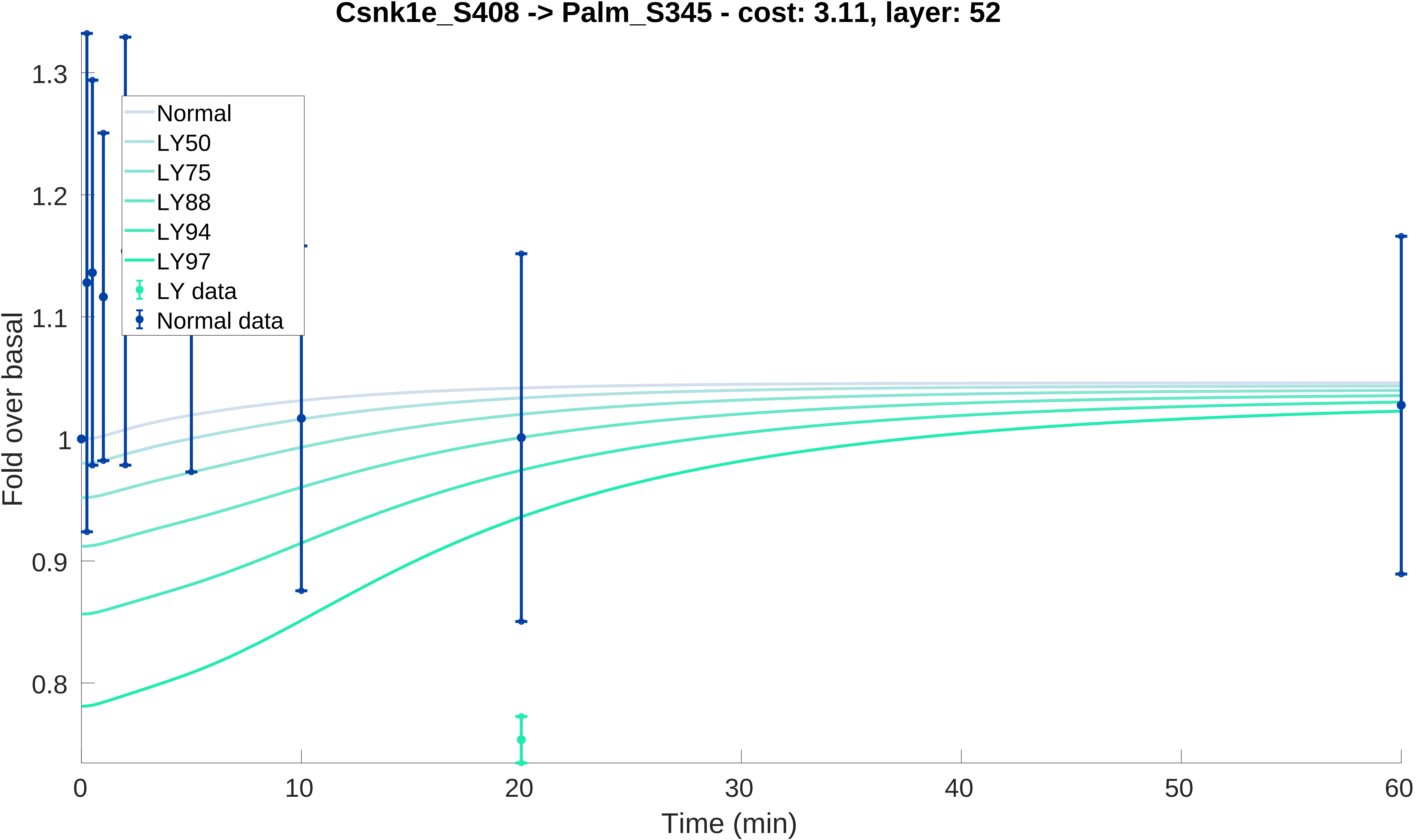
Csnk1e_S408 -> Palm_S345 - cost: 3.11, layer: 52
Normal
LY50
LY75
LY88
LY94
LY97
LY data
Normal data
Fold over basal
Time (min)
1.3
1.2
1.1
1
0.9
0.8
0
10
20
30
40
50
60

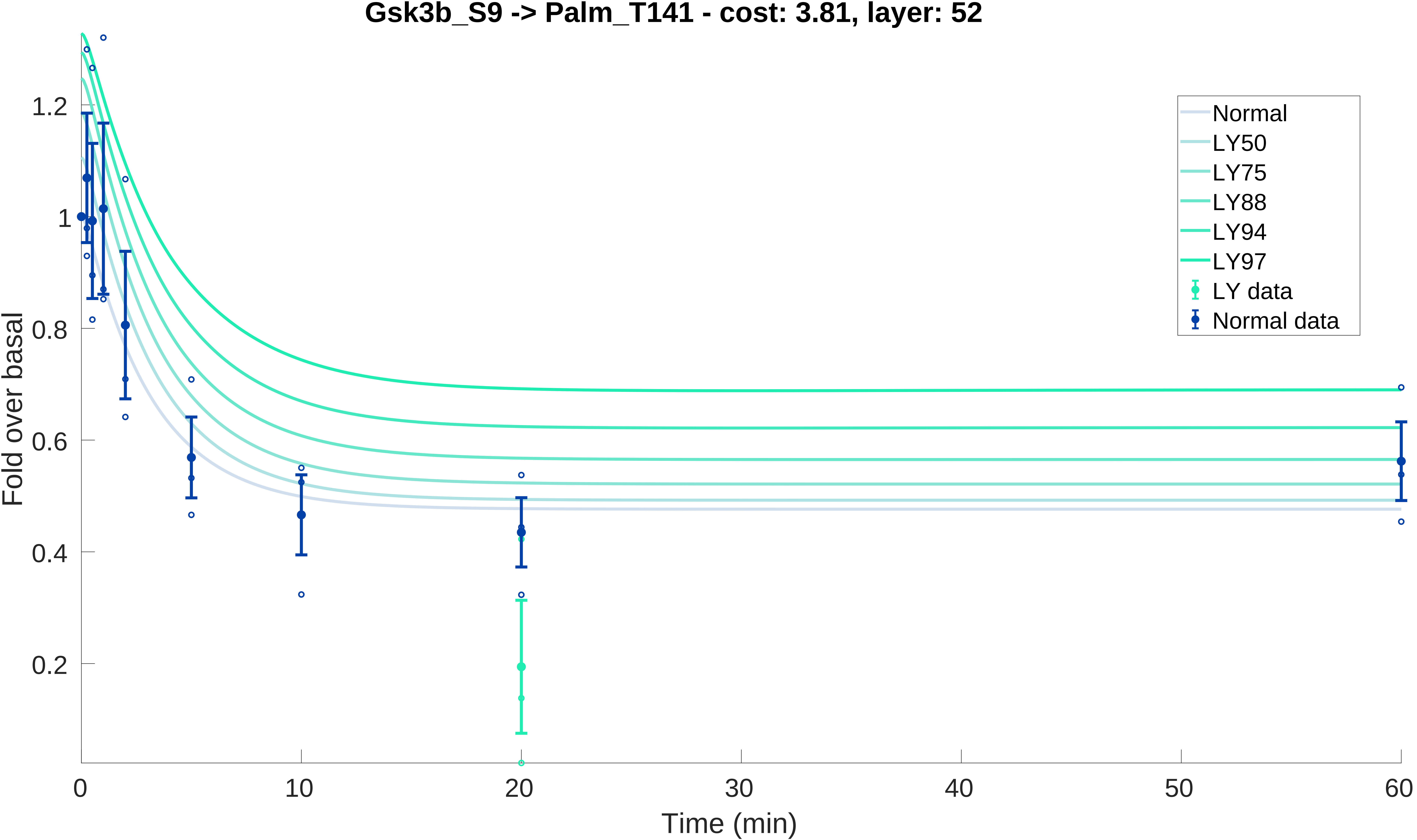

Gsk3b_S9 -> Palm_T141 - cost: 3.81, layer: 52
Fold over basal
Time (min)
Normal
LY50
LY75
LY88
LY94
LY97
LY data
Normal data
0.2
0.4
0.6
0.8
1
1.2
0
10
20
30
40
50
60

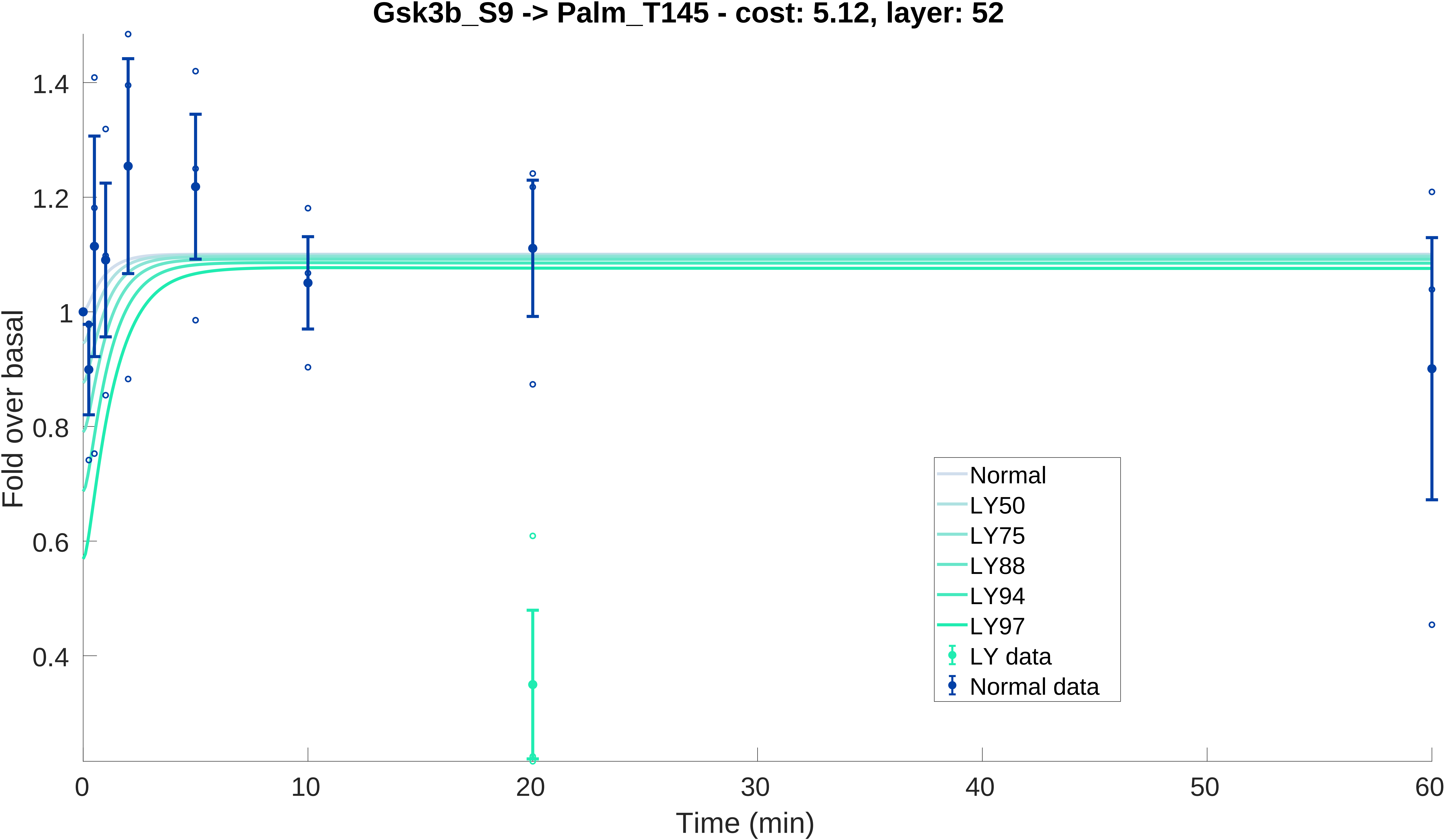

Gsk3b_S9 -> Palm_T145 - cost: 5.12, layer: 52
Fold over basal
1.4
1.2
1
0.8
0.6
0.4
0
10
20
30
40
50
60
Time (min)
Normal
LY50
LY75
LY88
LY94
LY97
LY data
Normal data

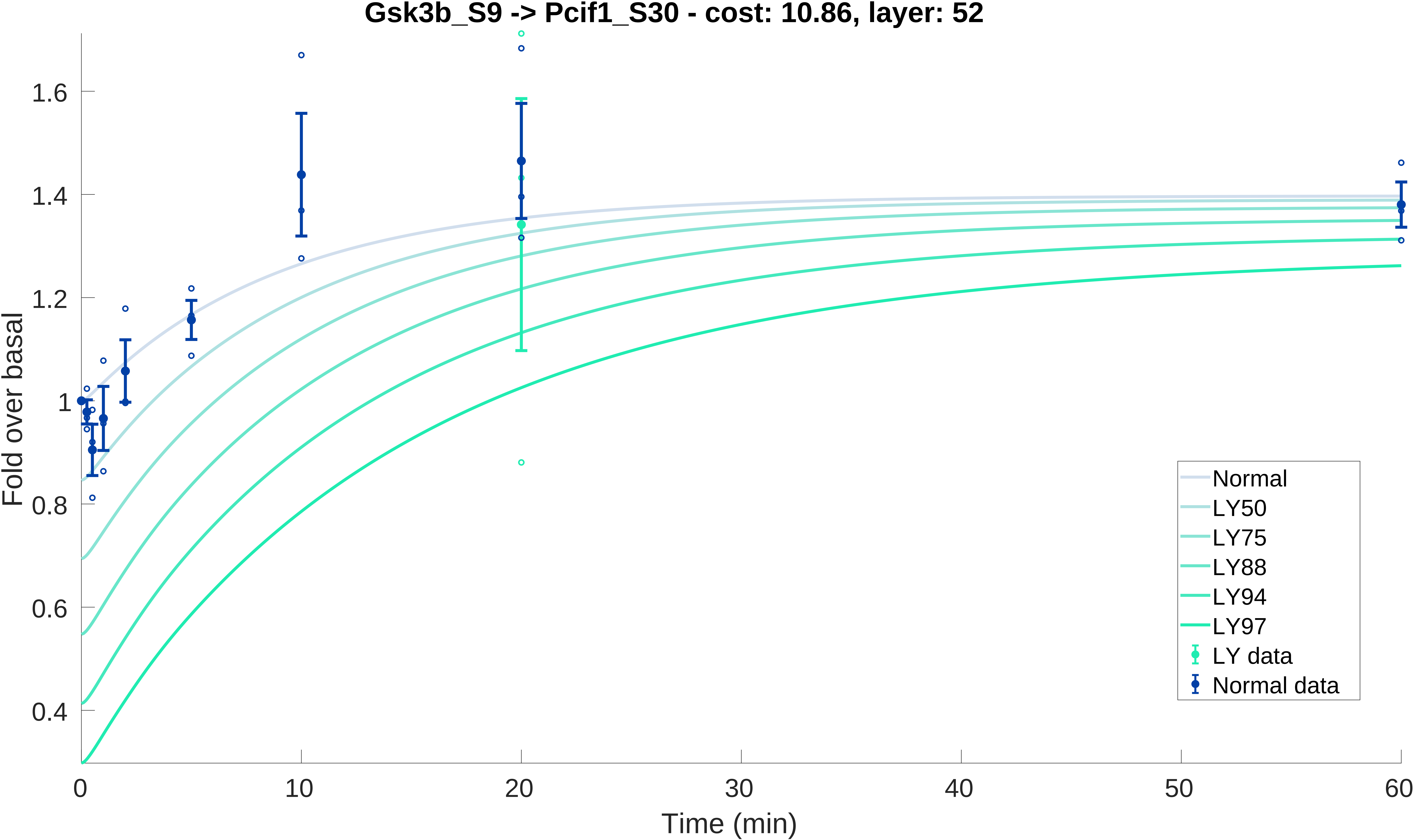

Gsk3b_S9 -> Pcif1_S30 - cost: 10.86, layer: 52
Fold over basal
Time (min)
0
10
20
30
40
50
60
0.4
0.6
0.8
1
1.2
1.4
1.6
Normal
LY50
LY75
LY88
LY94
LY97
LY data
Normal data

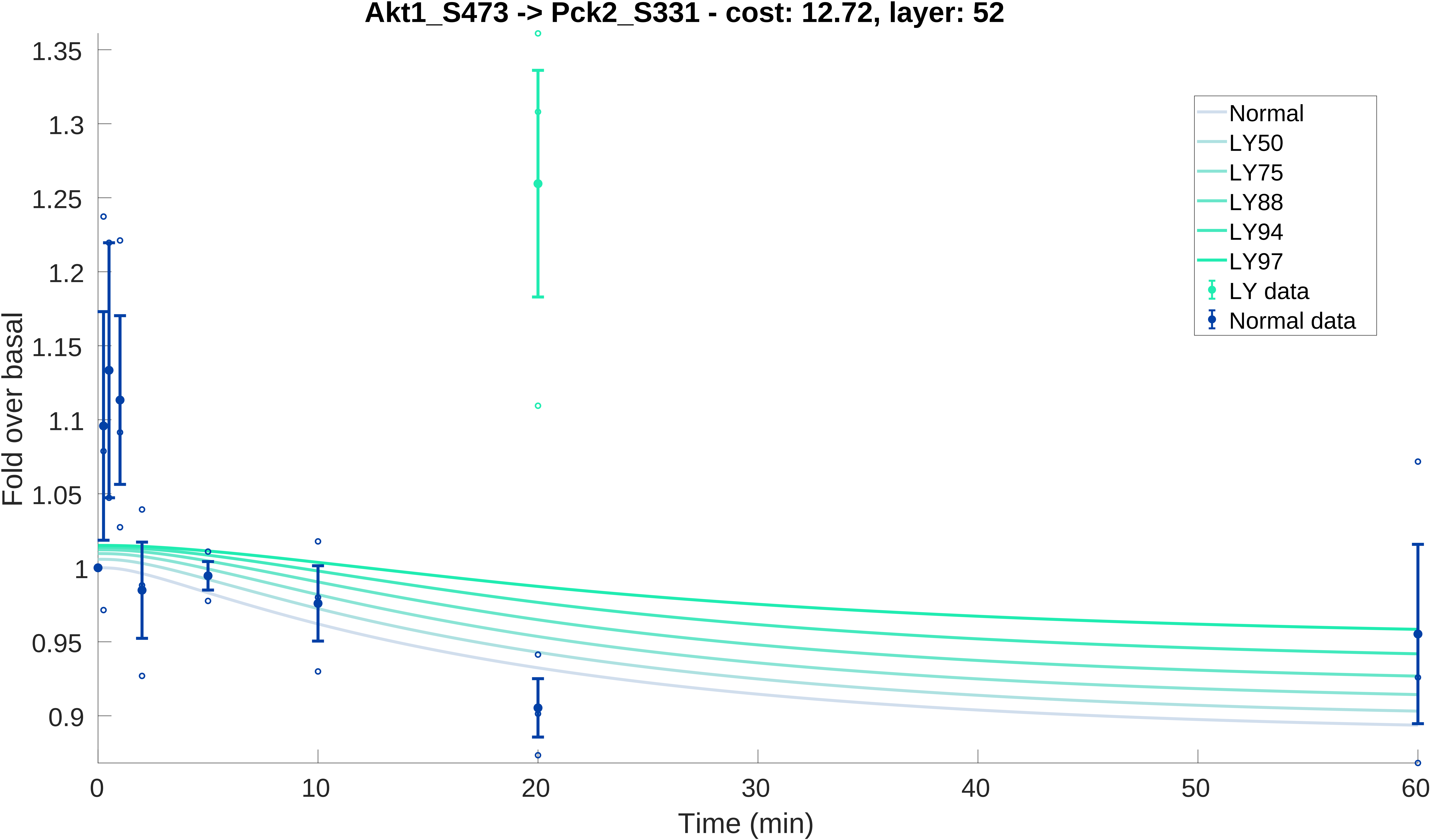

Akt1_S473 -> Pck2_S331 - cost: 12.72, layer: 52
Fold over basal
1.35
1.3
1.25
1.2
1.15
1.1
1.05
1
0.95
0.9
0
10
20
30
40
50
60
Time (min)
Normal
LY50
LY75
LY88
LY94
LY97
LY data
Normal data

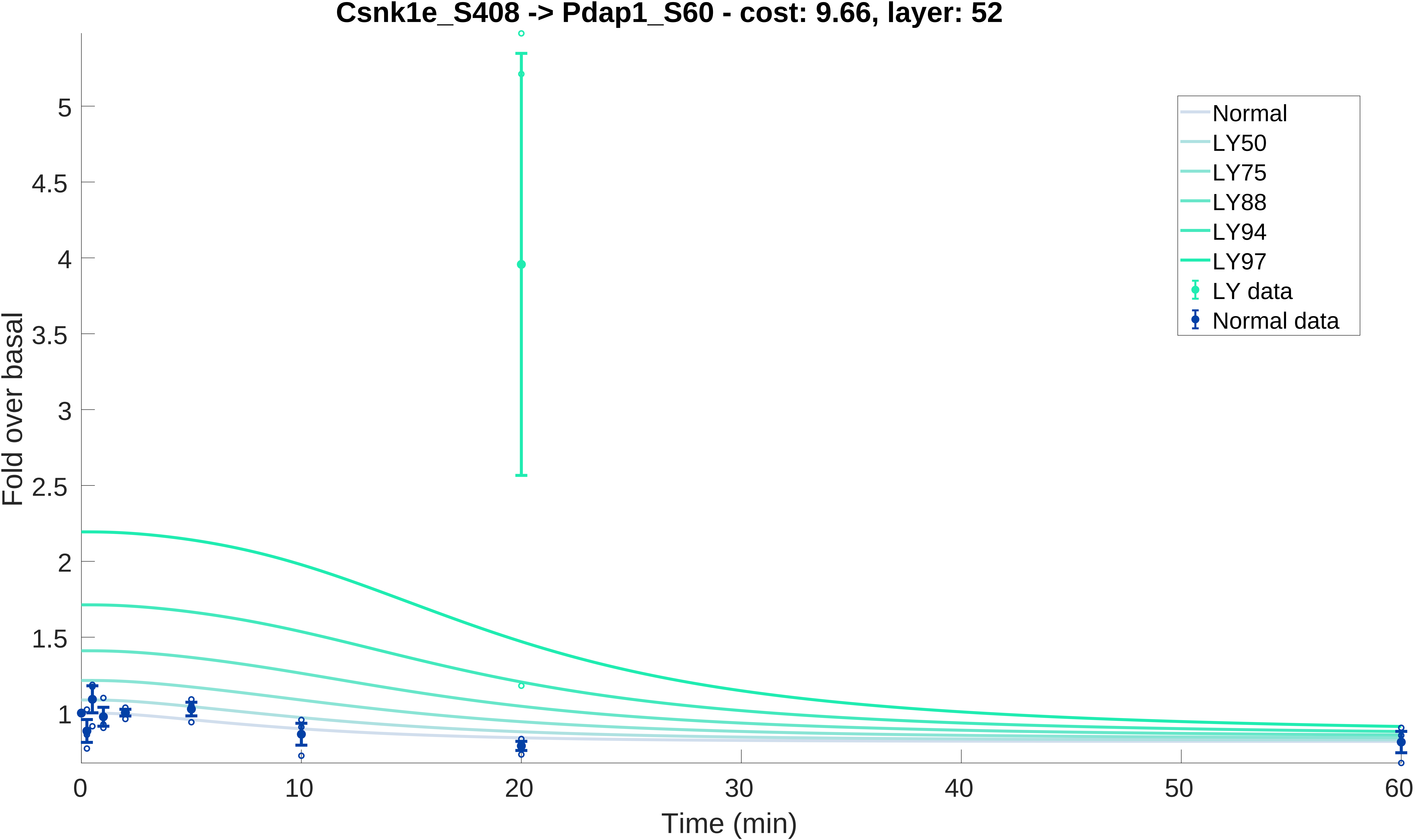
Csnk1e_S408 -> Pdap1_S60 - cost: 9.66, layer: 52
Fold over basal
Time (min)
0
10
20
30
40
50
60
1
1.5
2
2.5
3
3.5
4
4.5
5
Normal
LY50
LY75
LY88
LY94
LY97
LY data
Normal data

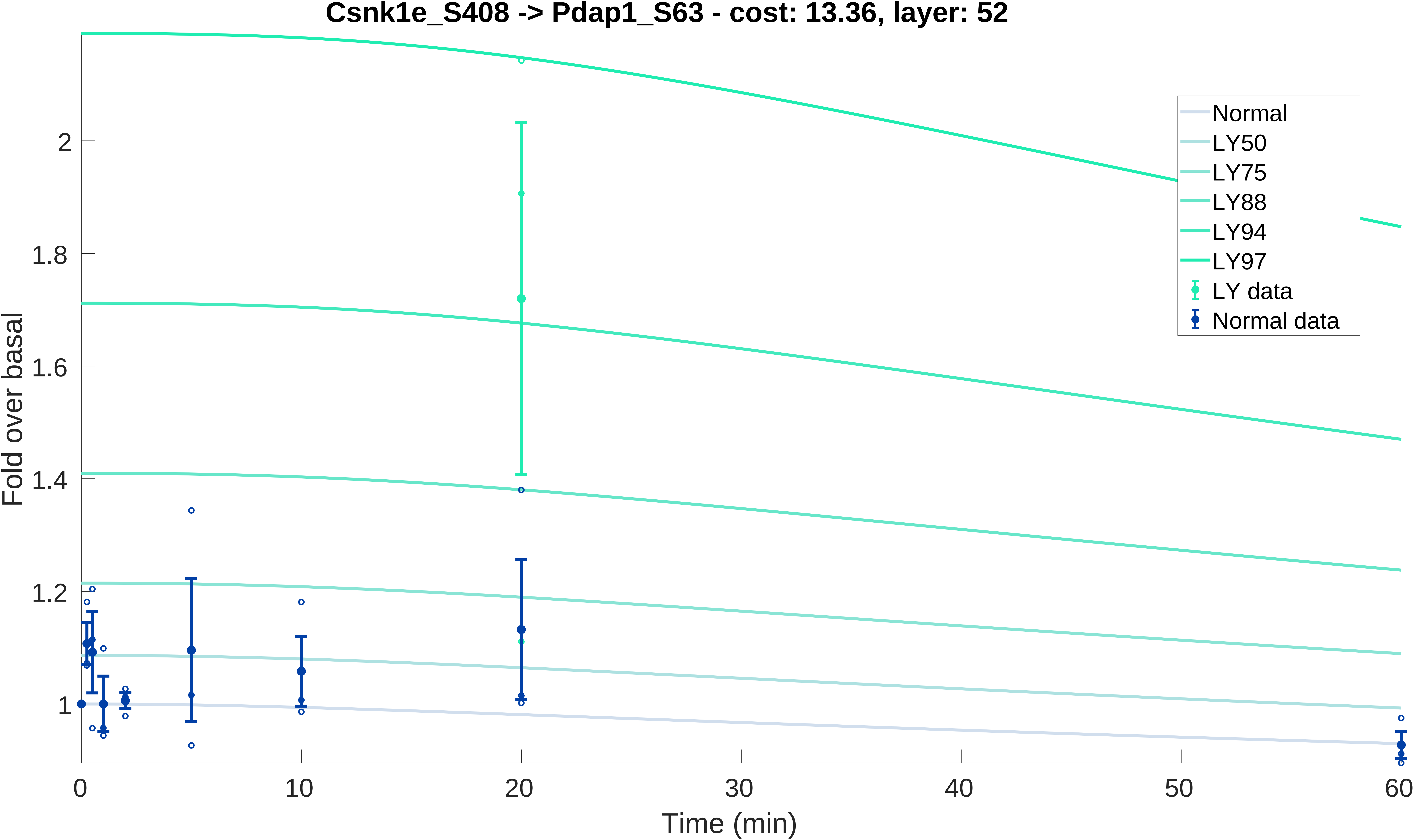
Csnk1e_S408 -> Pdap1_S63 - cost: 13.36, layer: 52
Fold over basal
2
1.8
1.6
1.4
1.2
1
0
10
20
30
40
50
60
Time (min)
Normal
LY50
LY75
LY88
LY94
LY97
LY data
Normal data

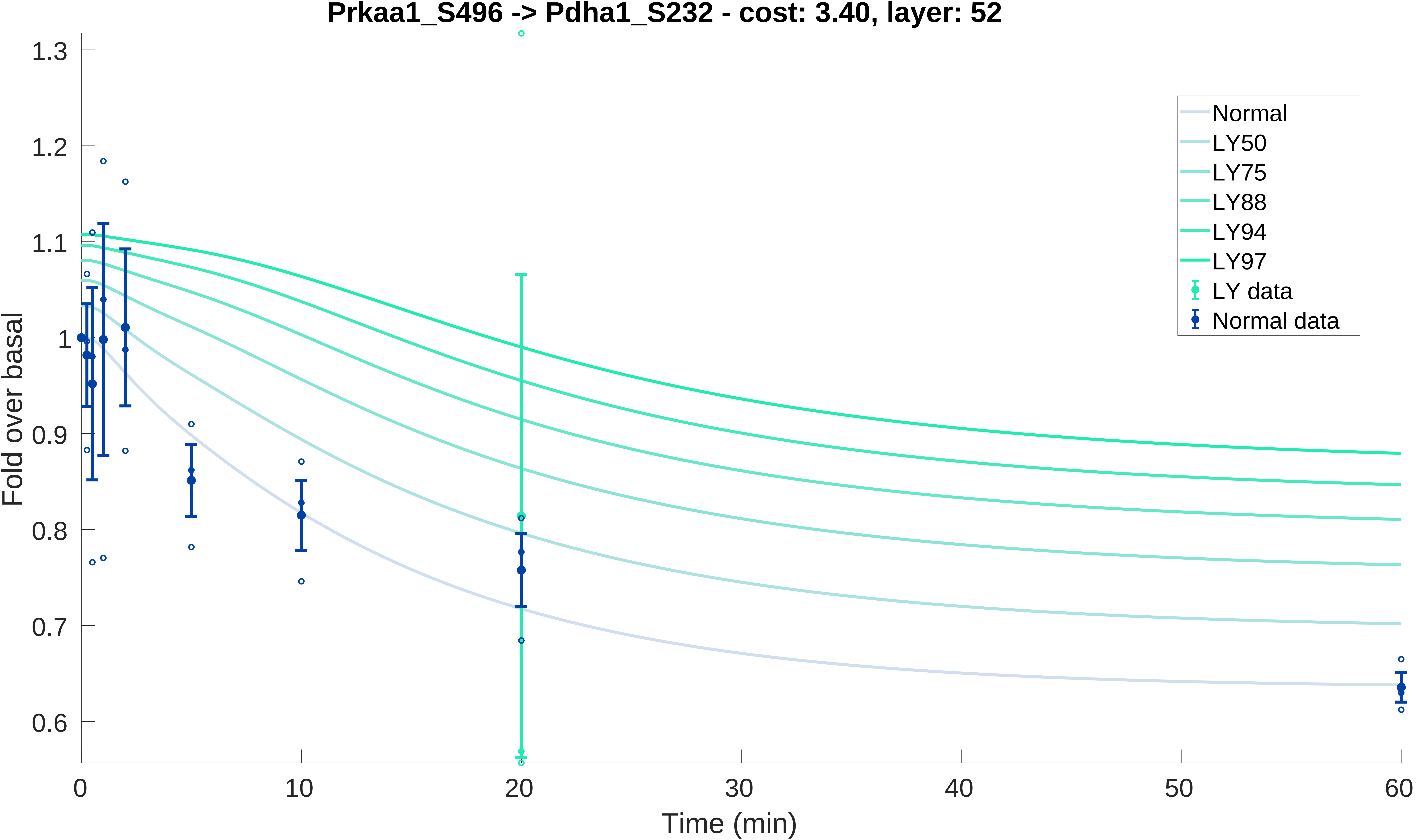

Prkaa1_S496 -> Pdha1_S232 - cost: 3.40, layer: 52
Fold over basal
1.3
1.2
1.1
1
0.9
0.8
0.7
0.6
0
10
20
30
40
50
60
Time (min)
Normal
LY50
LY75
LY88
LY94
LY97
LY data
Normal data

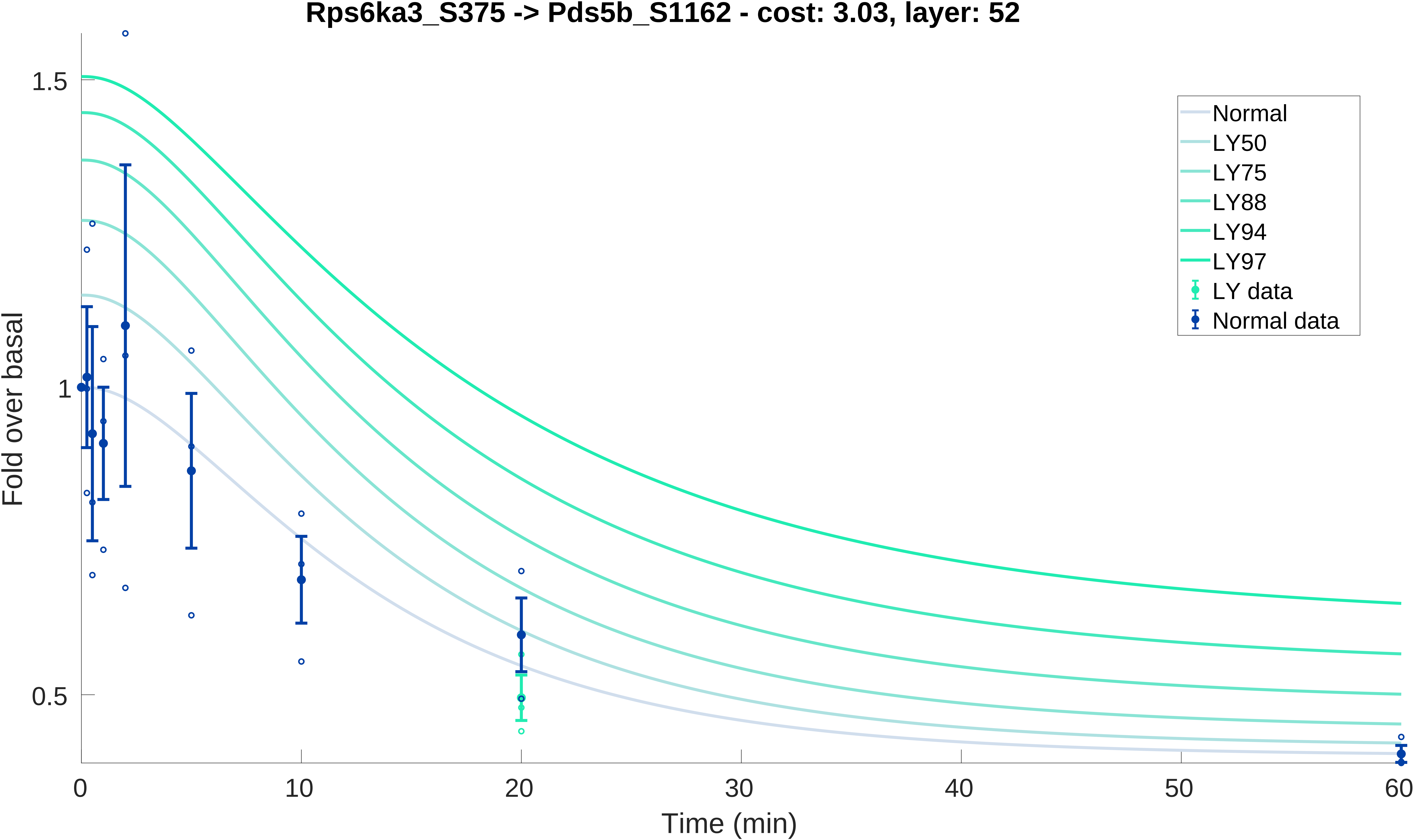

Rps6ka3_S375 -> Pds5b_S1162 - cost: 3.03, layer: 52
Fold over basal
Time (min)
1.5
1
0.5
0
10
20
30
40
50
60
Normal
LY50
LY75
LY88
LY94
LY97
LY data
Normal data

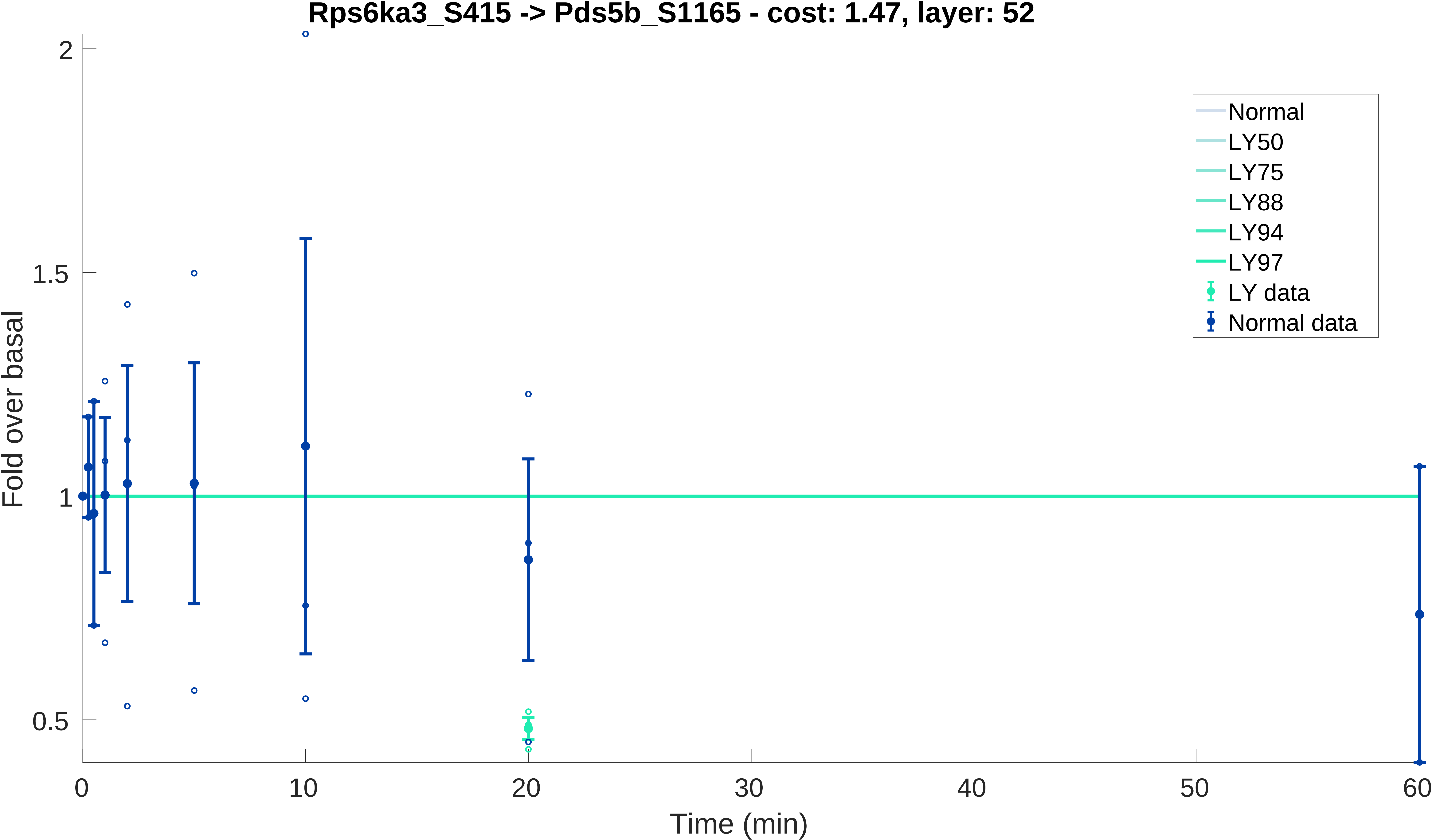

Rps6ka3_S415 -> Pds5b_S1165 - cost: 1.47, layer: 52
Fold over basal
Time (min)
2
1.5
1
0.5
0
10
20
30
40
50
60
Normal
LY50
LY75
LY88
LY94
LY97
LY data
Normal data

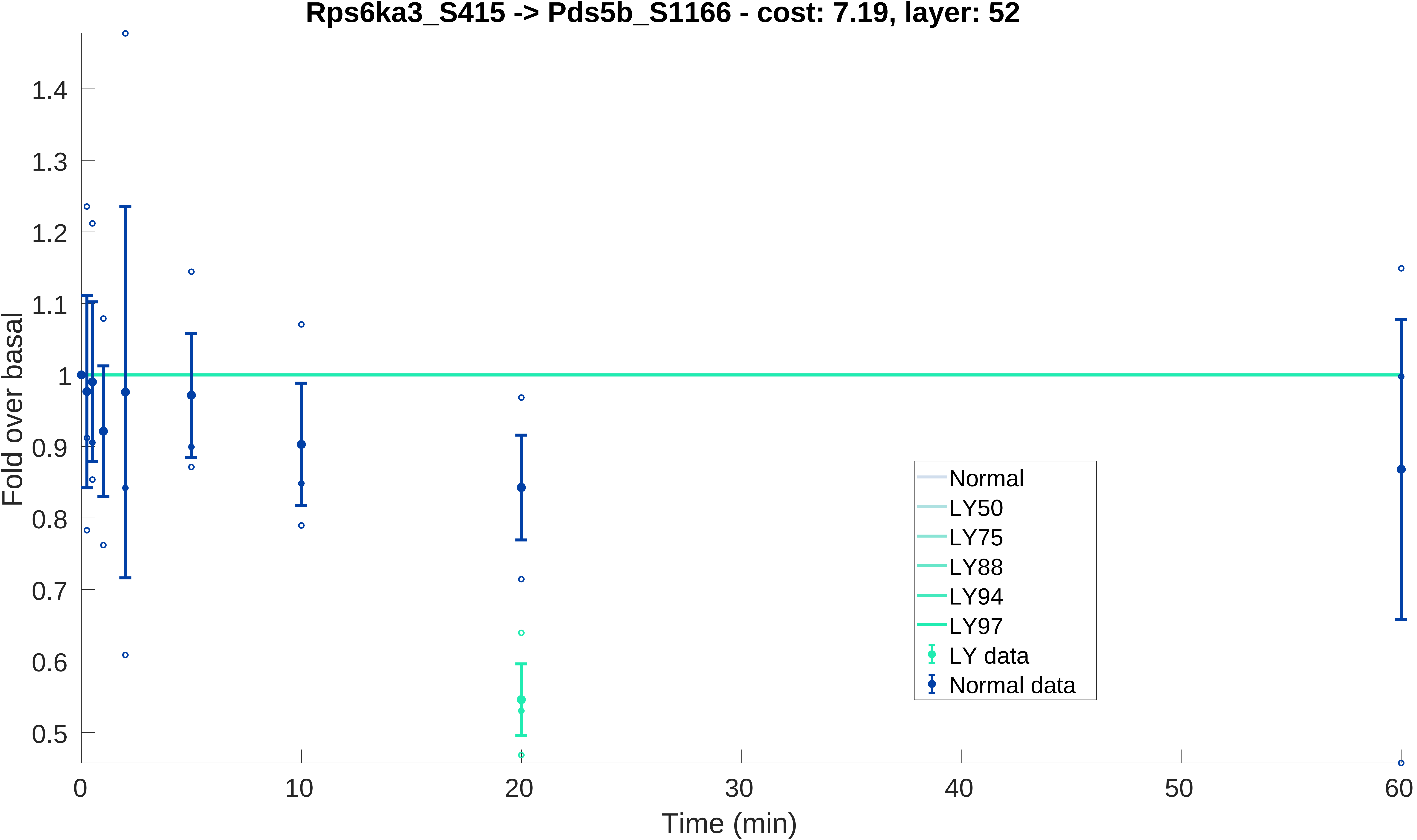

Rps6ka3_S415 -> Pds5b_S1166 - cost: 7.19, layer: 52
Fold over basal
Time (min)
1.4
1.3
1.2
1.1
1
0.9
0.8
0.7
0.6
0.5
0
10
20
30
40
50
60
Normal
LY50
LY75
LY88
LY94
LY97
LY data
Normal data

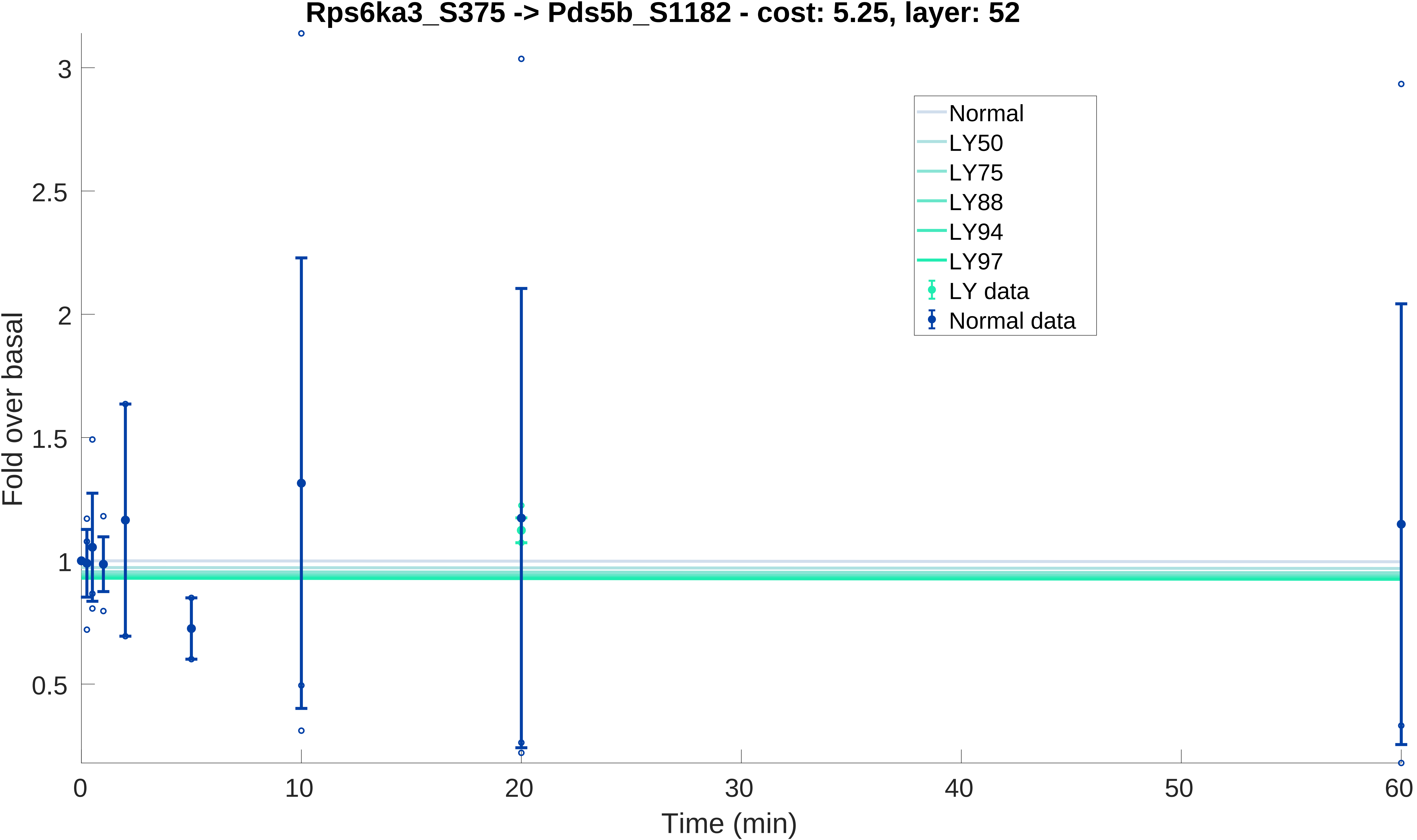

Rps6ka3_S375 -> Pds5b_S1182 - cost: 5.25, layer: 52
Fold over basal
Time (min)
0.5
1
1.5
2
2.5
3
0
10
20
30
40
50
60
Normal
LY50
LY75
LY88
LY94
LY97
LY data
Normal data

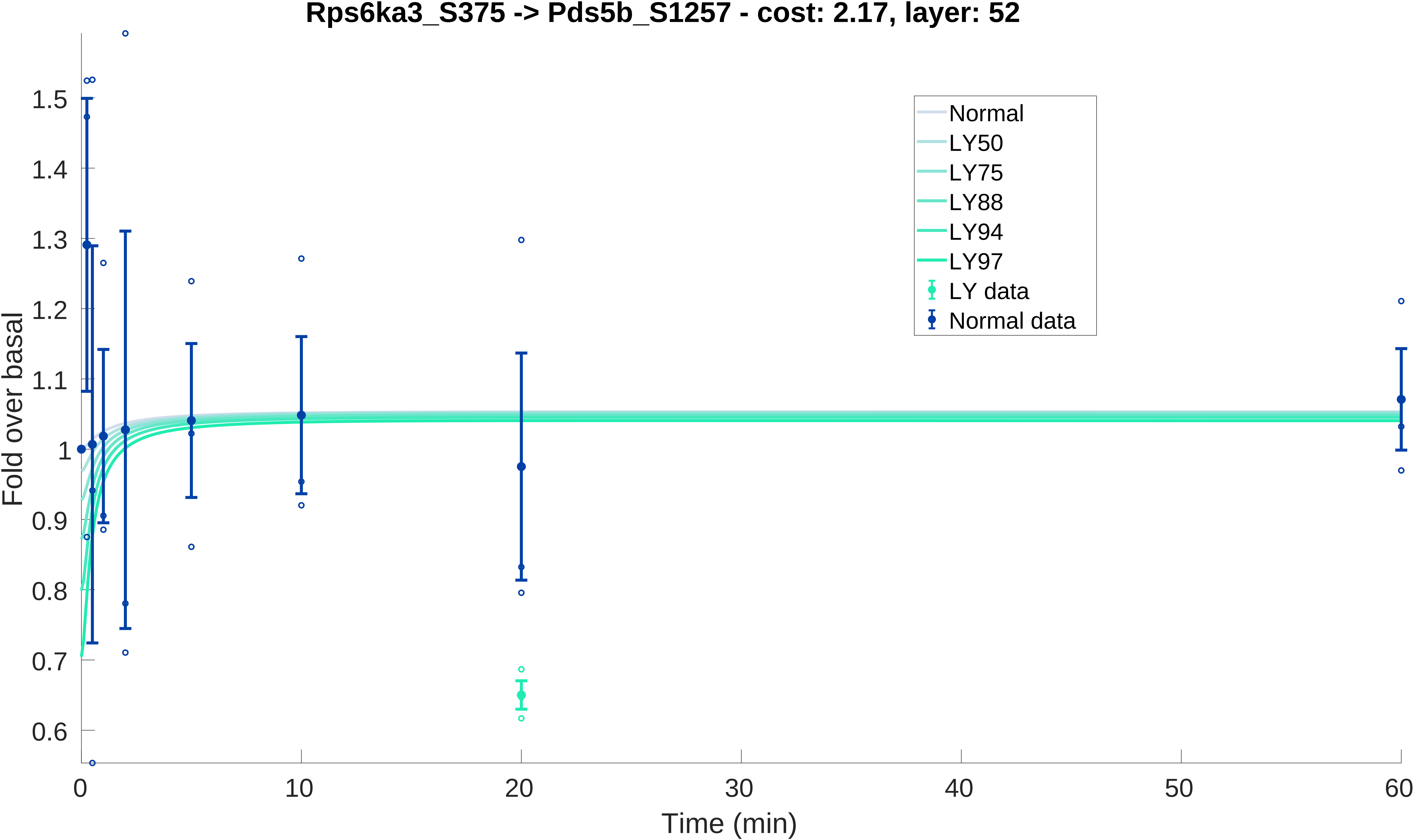

Rps6ka3_S375 -> Pds5b_S1257 - cost: 2.17, layer: 52
Normal
LY50
LY75
LY88
LY94
LY97
LY data
Normal data
Fold over basal
Time (min)
0.6
0.7
0.8
0.9
1
1.1
1.2
1.3
1.4
1.5
0
10
20
30
40
50
60

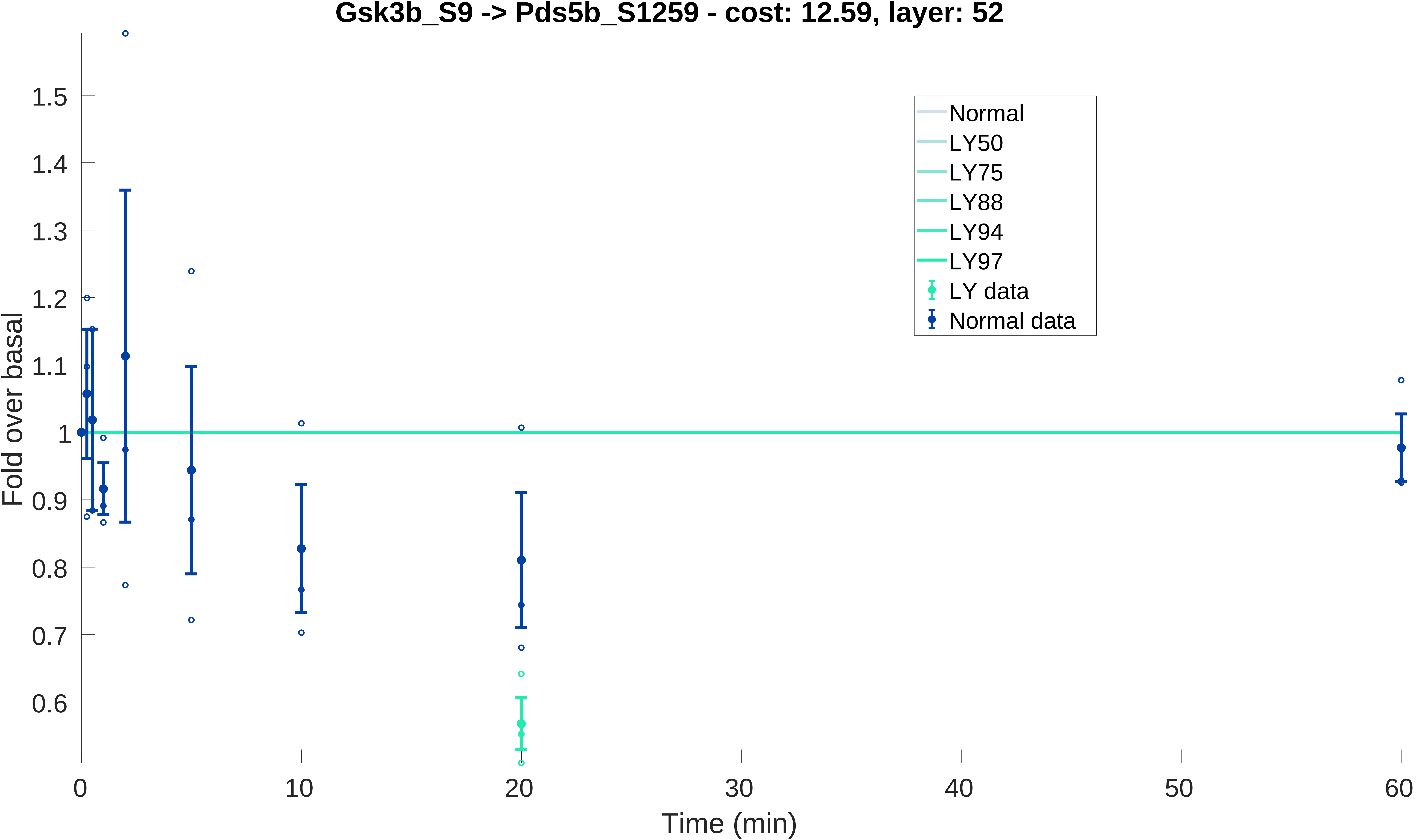

Gsk3b_S9 -> Pds5b_S1259 - cost: 12.59, layer: 52
Fold over basal
Time (min)
Normal
LY50
LY75
LY88
LY94
LY97
LY data
Normal data
0
10
20
30
40
50
60
0.6
0.7
0.8
0.9
1
1.1
1.2
1.3
1.4
1.5

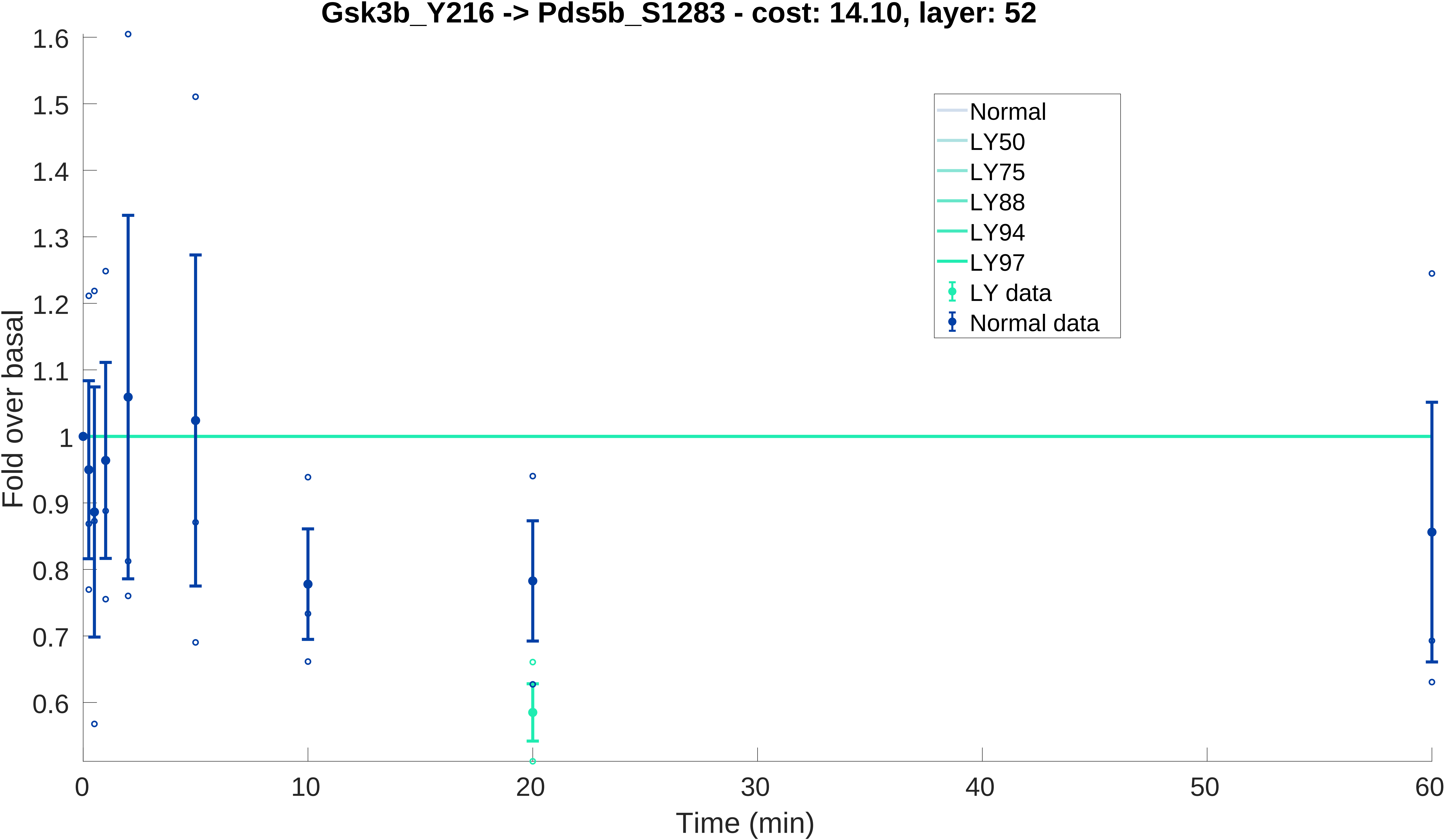

Gsk3b_Y216 -> Pds5b_S1283 - cost: 14.10, layer: 52
Fold over basal
Time (min)
1.6
1.5
1.4
1.3
1.2
1.1
1
0.9
0.8
0.7
0.6
0
10
20
30
40
50
60
Normal
LY50
LY75
LY88
LY94
LY97
LY data
Normal data

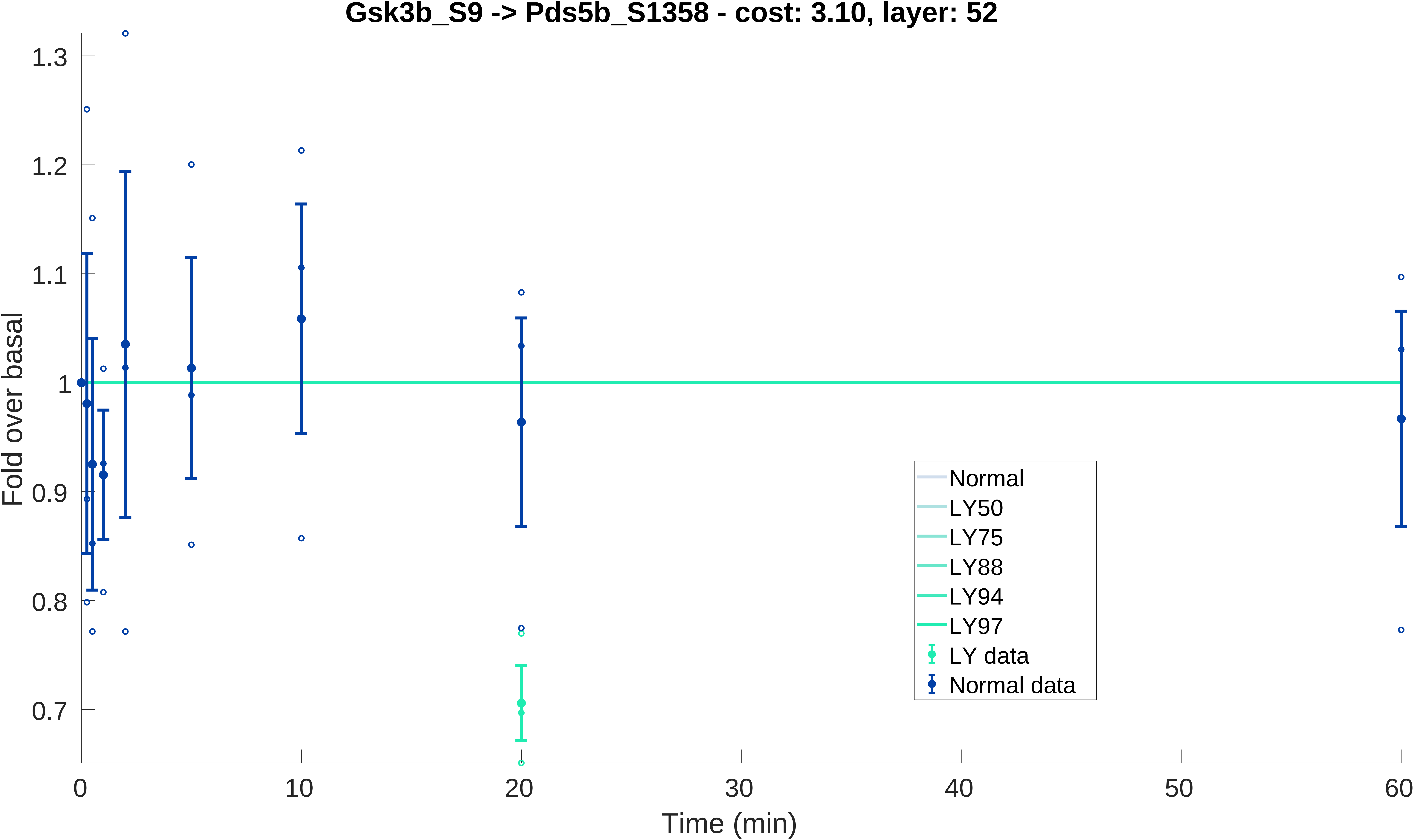

Gsk3b_S9 -> Pds5b_S1358 - cost: 3.10, layer: 52
Fold over basal
Time (min)
1.3
1.2
1.1
1
0.9
0.8
0.7
0
10
20
30
40
50
60
Normal
LY50
LY75
LY88
LY94
LY97
LY data
Normal data

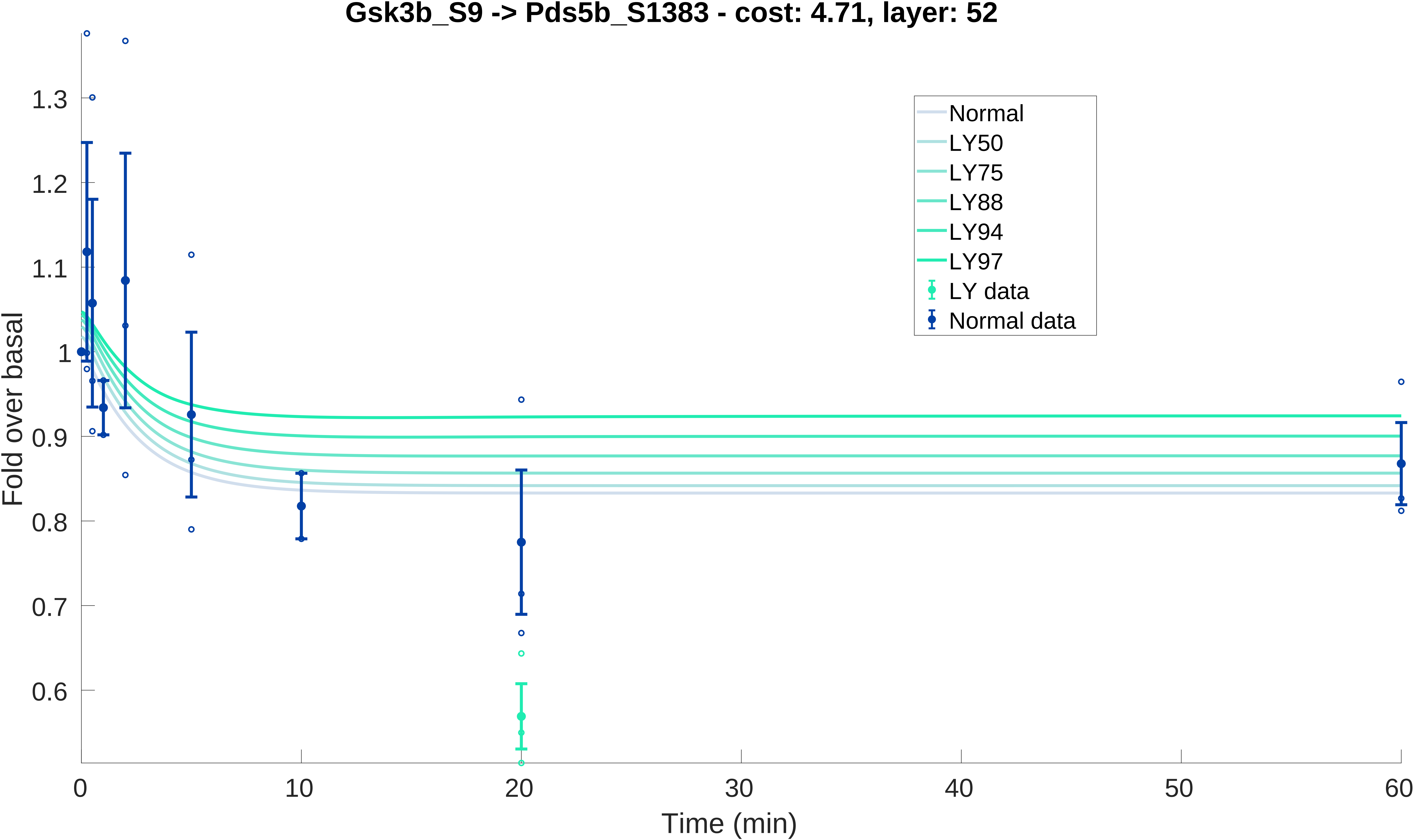

Gsk3b_S9 -> Pds5b_S1383 - cost: 4.71, layer: 52
Fold over basal
Time (min)
Normal
LY50
LY75
LY88
LY94
LY97
LY data
Normal data
1.3
1.2
1.1
1
0.9
0.8
0.7
0.6
0
10
20
30
40
50
60

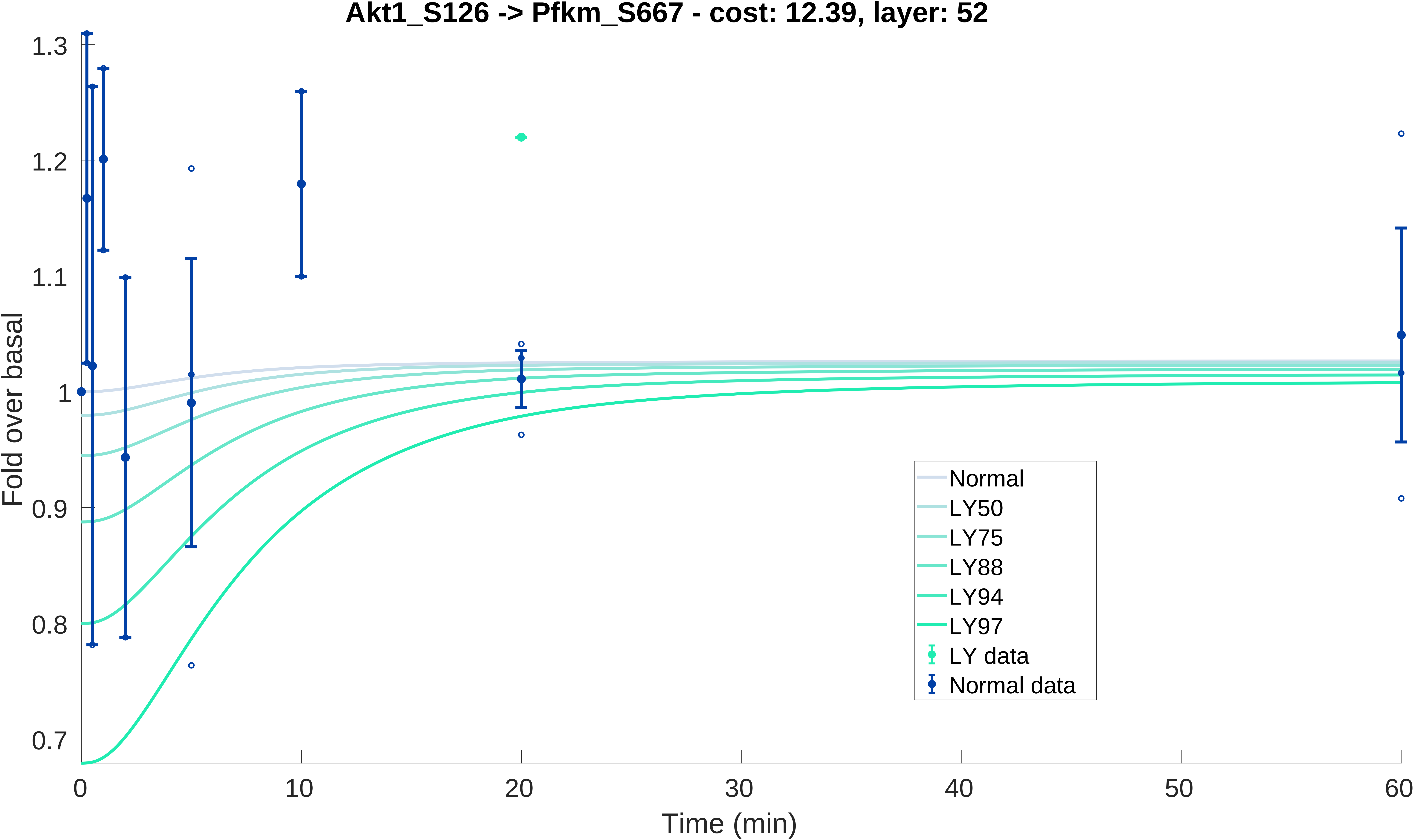

Akt1_S126 -> Pfkm_S667 - cost: 12.39, layer: 52
Fold over basal
1.3
1.2
1.1
1
0.9
0.8
0.7
0
10
20
30
40
50
60
Time (min)
Normal
LY50
LY75
LY88
LY94
LY97
LY data
Normal data

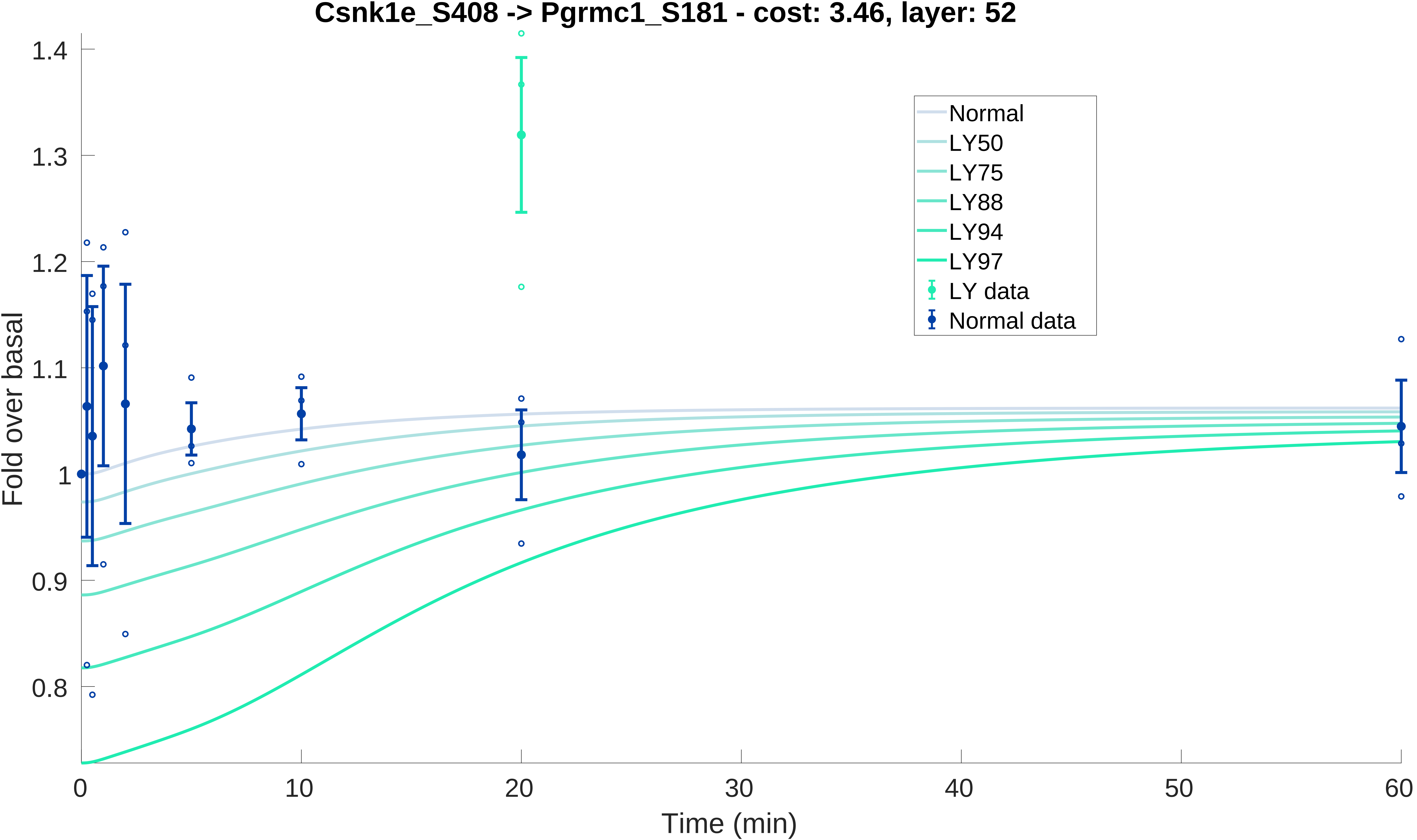

Csnk1e_S408 -> Pgrmc1_S181 - cost: 3.46, layer: 52
Fold over basal
1.4
1.3
1.2
1.1
1
0.9
0.8
0
10
20
30
40
50
60
Time (min)
Normal
LY50
LY75
LY88
LY94
LY97
LY data
Normal data

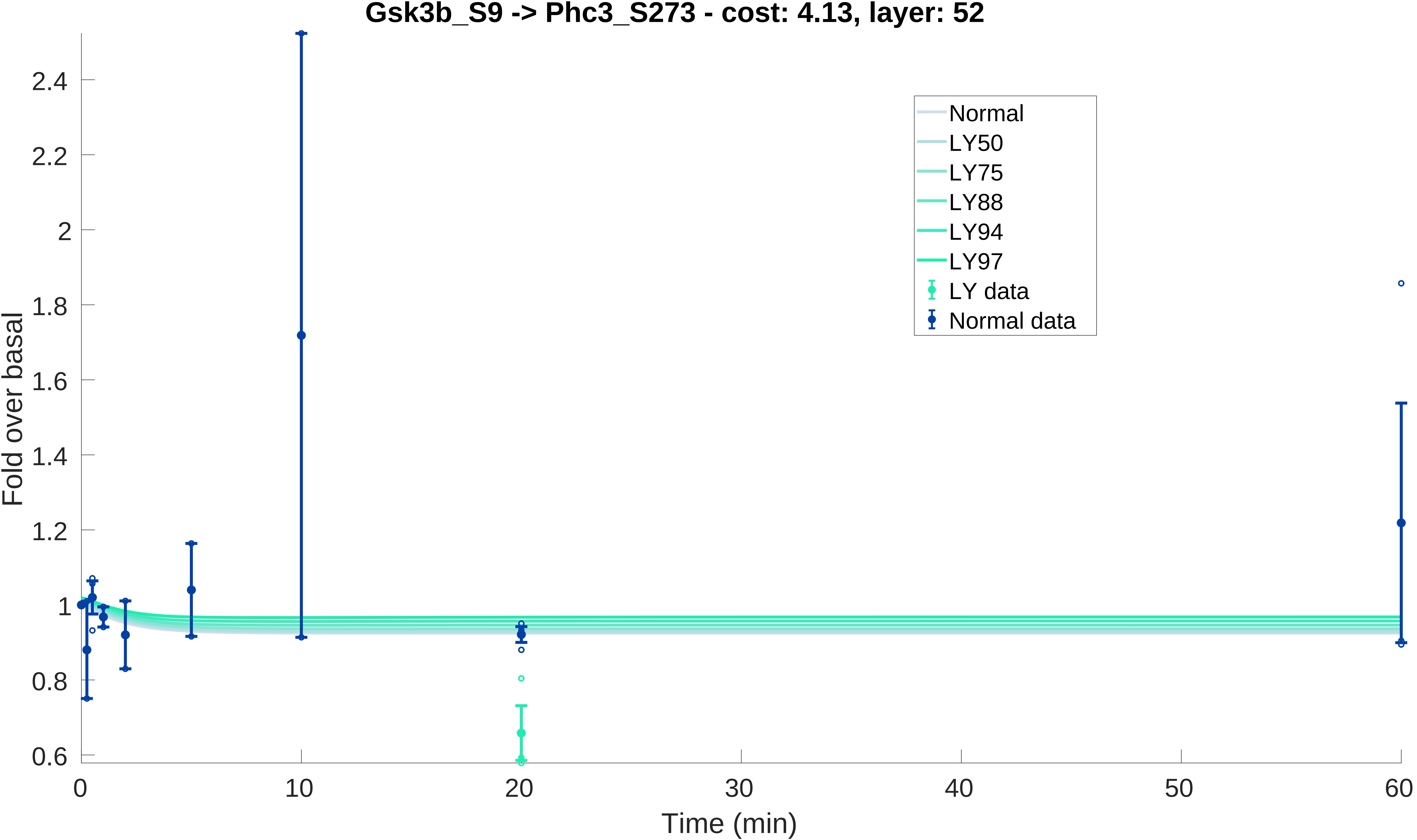

Gsk3b_S9 -> Phc3_S273 - cost: 4.13, layer: 52
Fold over basal
Time (min)
Normal
LY50
LY75
LY88
LY94
LY97
LY data
Normal data
0.6
0.8
1
1.2
1.4
1.6
1.8
2
2.2
2.4
0
10
20
30
40
50
60

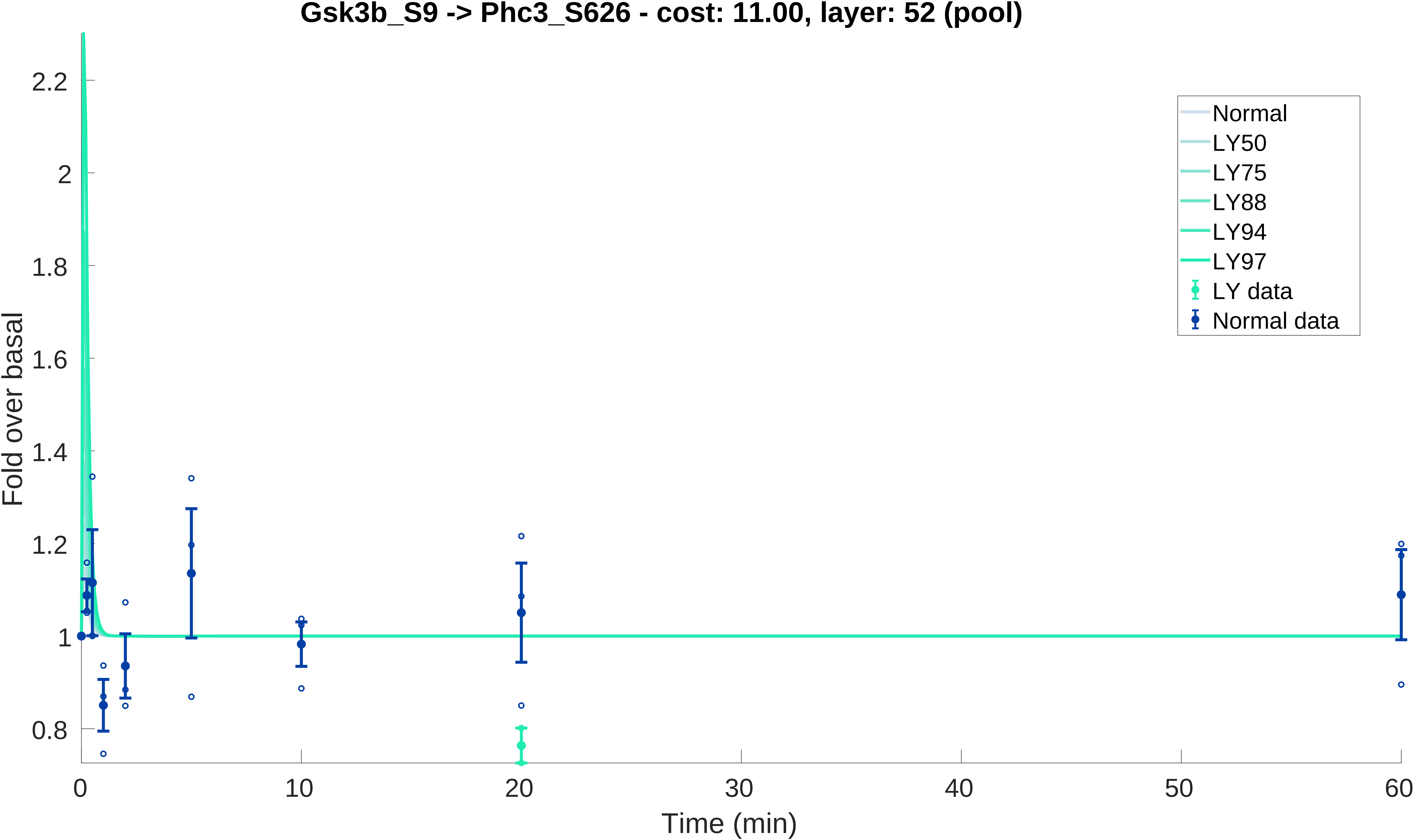

Gsk3b_S9 -> Phc3_S626 - cost: 11.00, layer: 52 (pool)
Fold over basal
Time (min)
Normal
LY50
LY75
LY88
LY94
LY97
LY data
Normal data
2.2
2
1.8
1.6
1.4
1.2
1
0.8
0
10
20
30
40
50
60

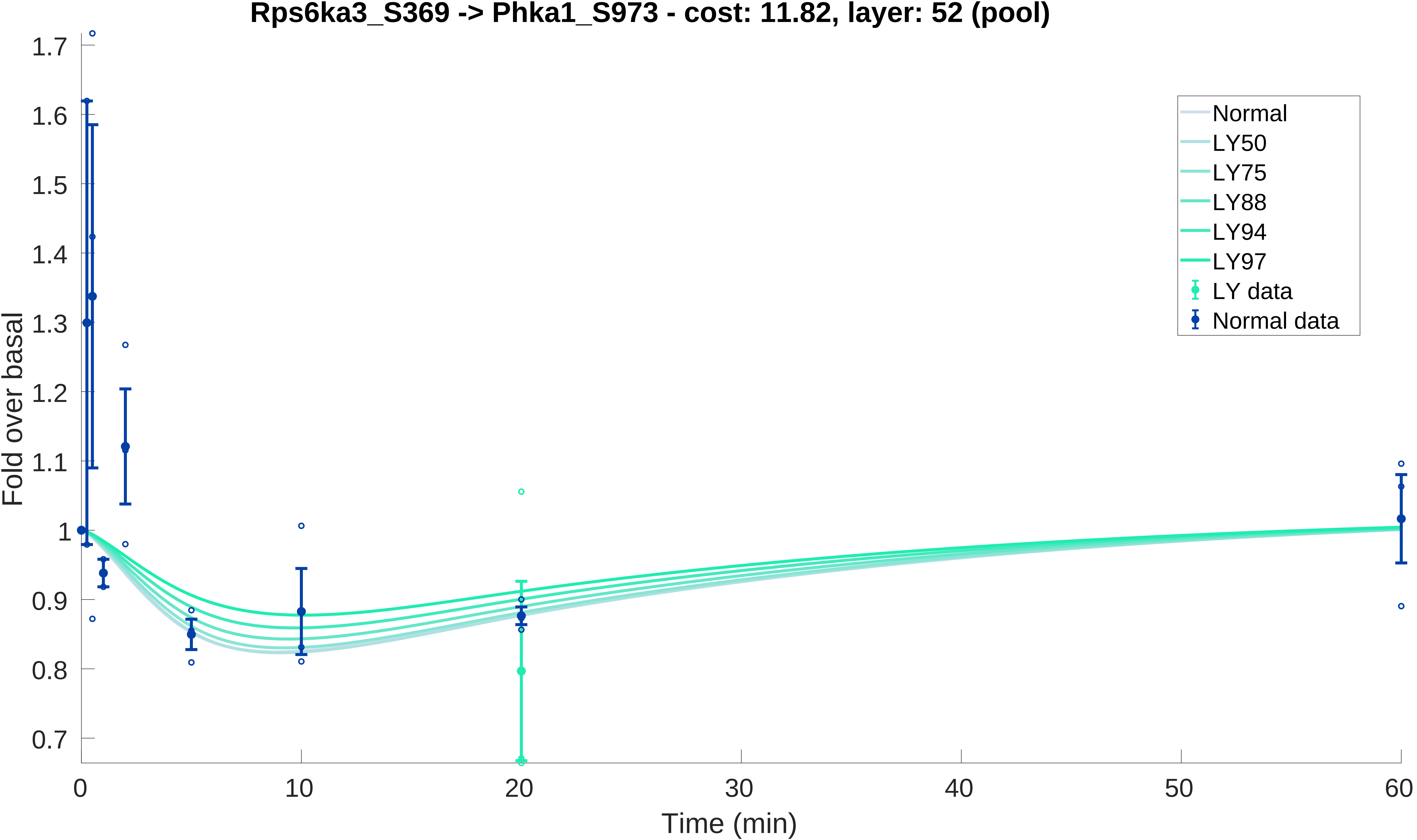

Rps6ka3_S369 -> Phka1_S973 - cost: 11.82, layer: 52 (pool)
Fold over basal
Time (min)
1.7
1.6
1.5
1.4
1.3
1.2
1.1
1
0.9
0.8
0.7
0
10
20
30
40
50
60
Normal
LY50
LY75
LY88
LY94
LY97
LY data
Normal data

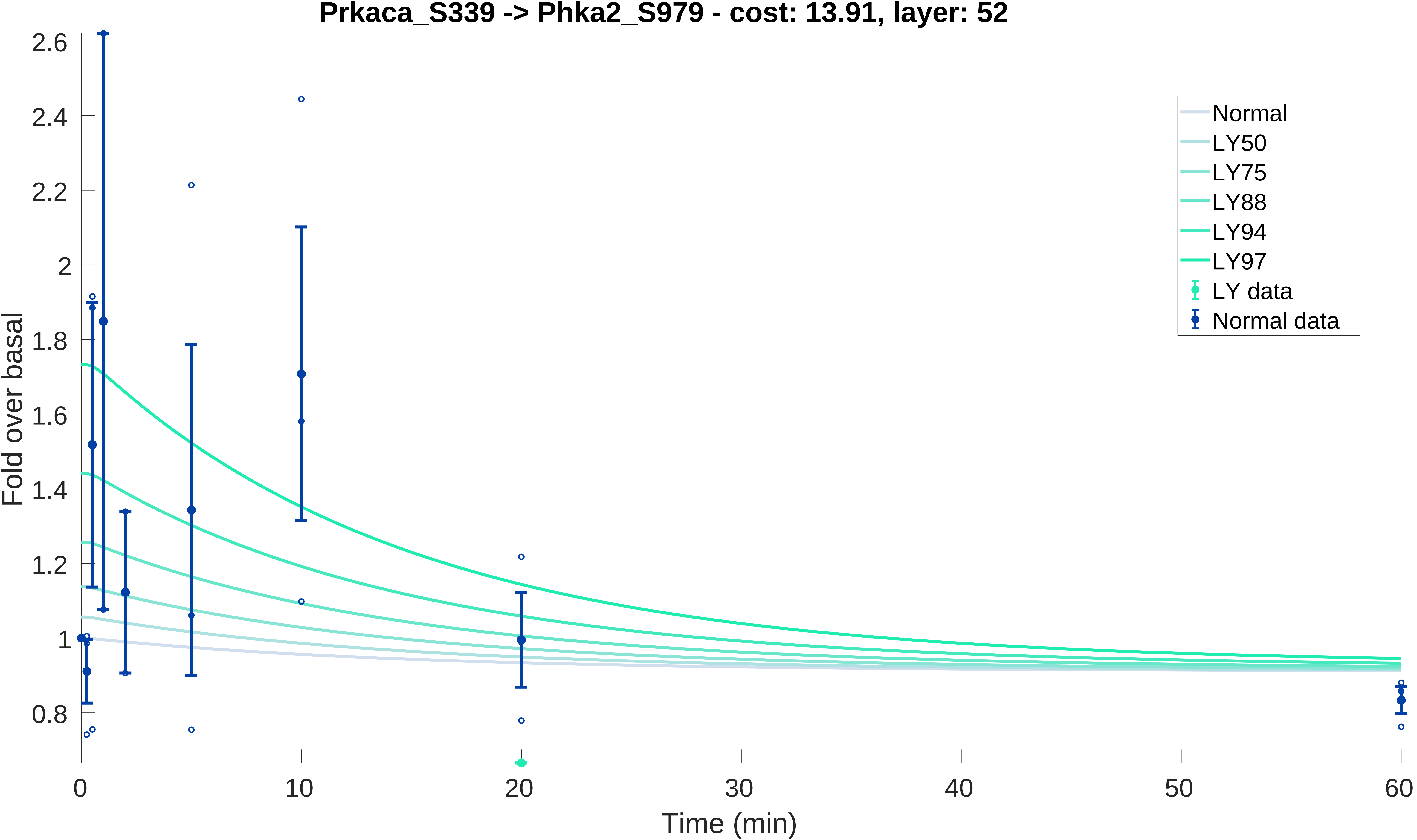

Prkaca_S339 -> Phka2_S979 - cost: 13.91, layer: 52
Fold over basal
Time (min)
2.6
2.4
2.2
2
1.8
1.6
1.4
1.2
1
0.8
0
10
20
30
40
50
60
Normal
LY50
LY75
LY88
LY94
LY97
LY data
Normal data

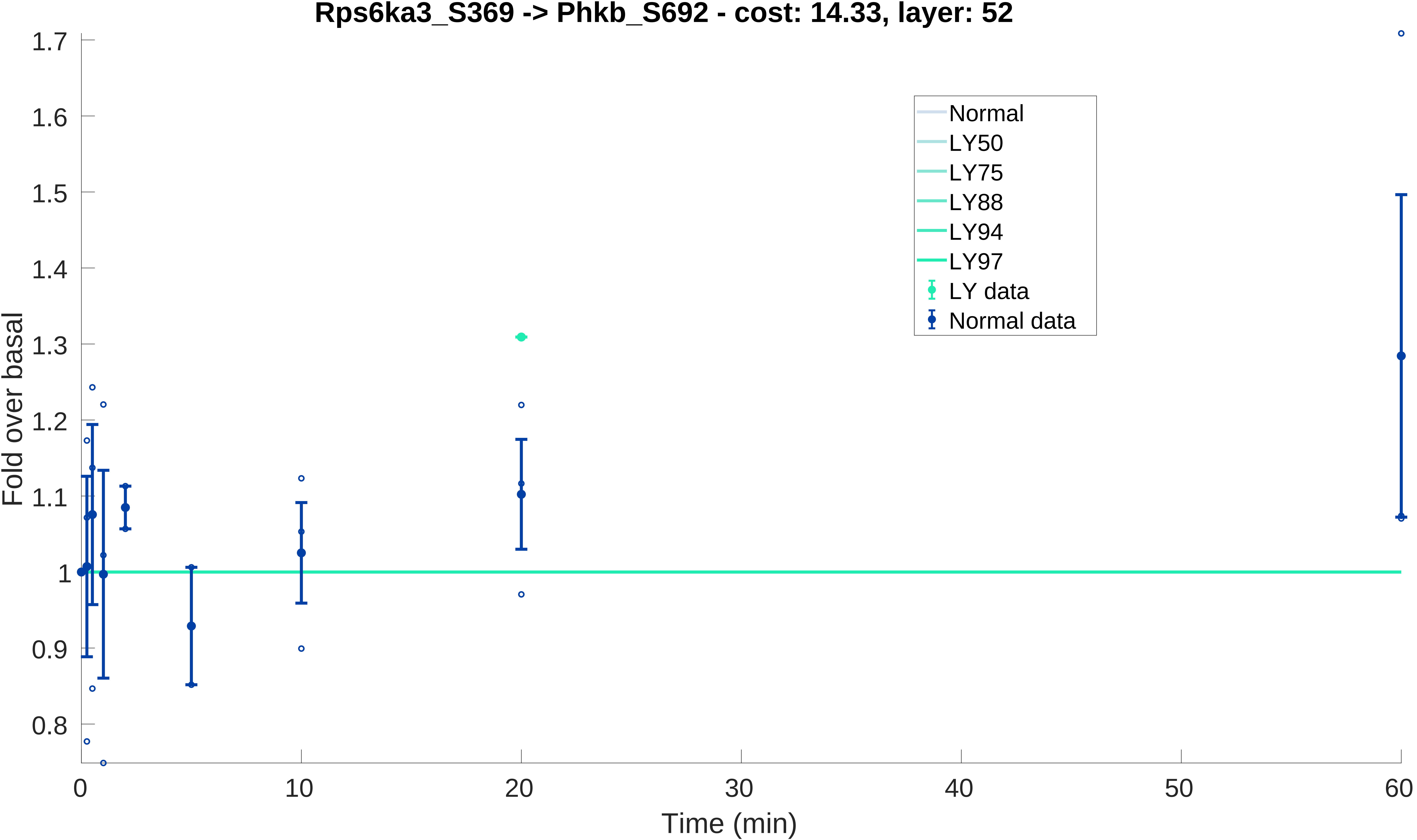

Rps6ka3_S369 -> Phkb_S692 - cost: 14.33, layer: 52
Fold over basal
1.7
1.6
1.5
1.4
1.3
1.2
1.1
1
0.9
0.8
0
10
20
30
40
50
60
Time (min)
Normal
LY50
LY75
LY88
LY94
LY97
LY data
Normal data

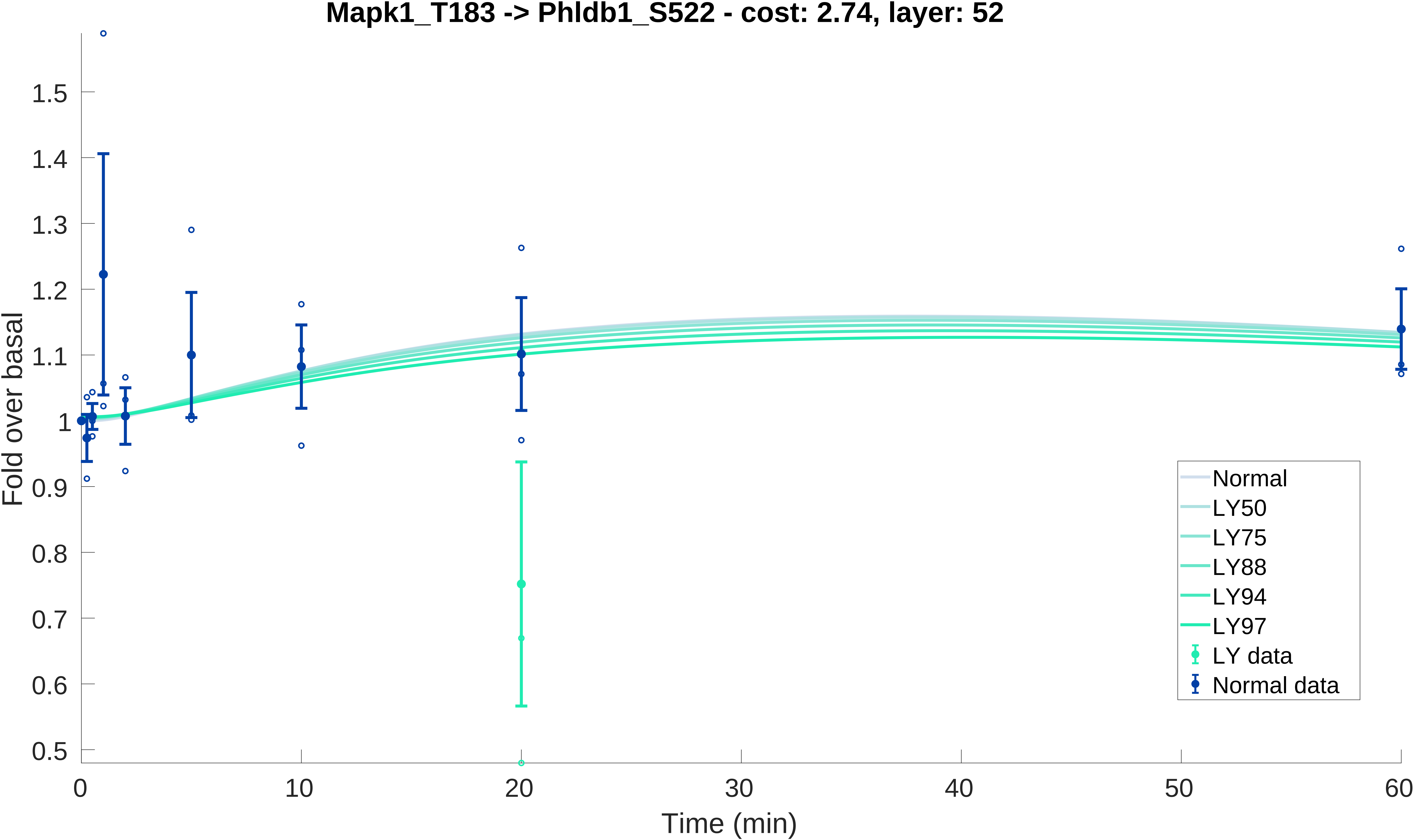

Mapk1_T183 -> Phldb1_S522 - cost: 2.74, layer: 52
Fold over basal
1.5
1.4
1.3
1.2
1.1
1
0.9
0.8
0.7
0.6
0.5
0
10
20
30
40
50
60
Time (min)
Normal
LY50
LY75
LY88
LY94
LY97
LY data
Normal data

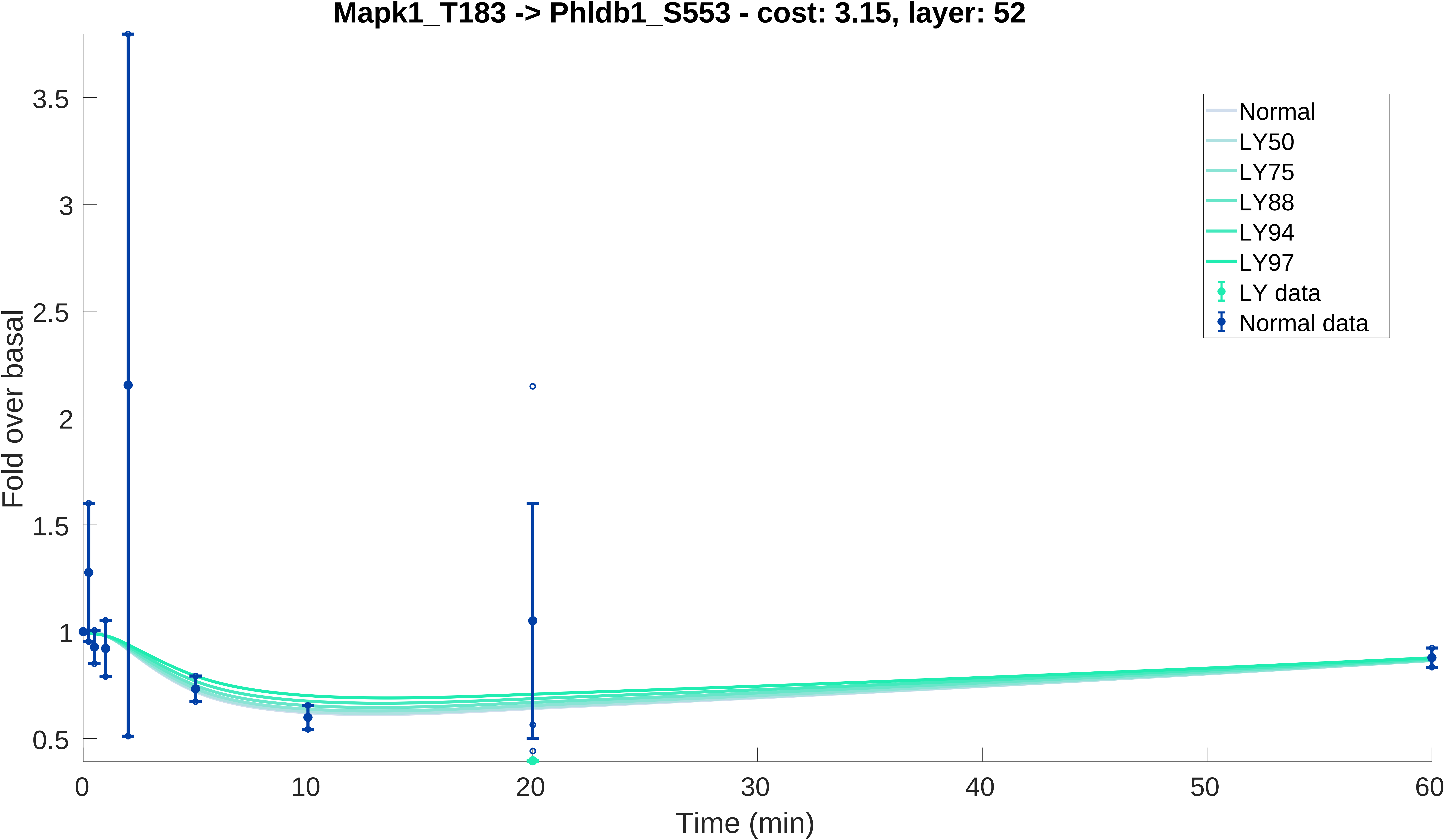

Mapk1_T183 -> Phldb1_S553 - cost: 3.15, layer: 52
Fold over basal
Time (min)
0.5
1
1.5
2
2.5
3
3.5
0
10
20
30
40
50
60
Normal
LY50
LY75
LY88
LY94
LY97
LY data
Normal data

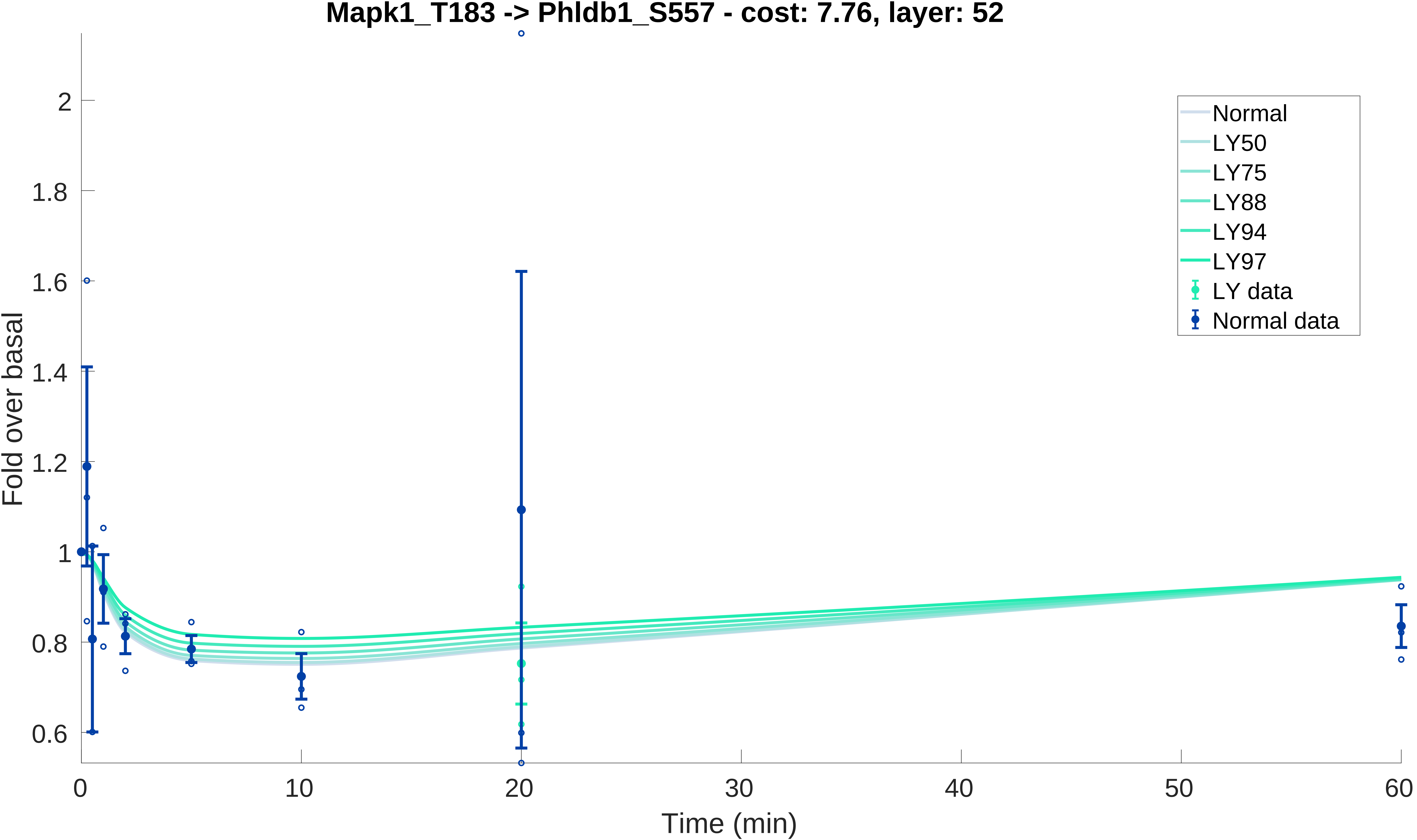

Mapk1_T183 -> Phldb1_S557 - cost: 7.76, layer: 52
Fold over basal
Time (min)
0.6
0.8
1
1.2
1.4
1.6
1.8
2
0
10
20
30
40
50
60
Normal
LY50
LY75
LY88
LY94
LY97
LY data
Normal data

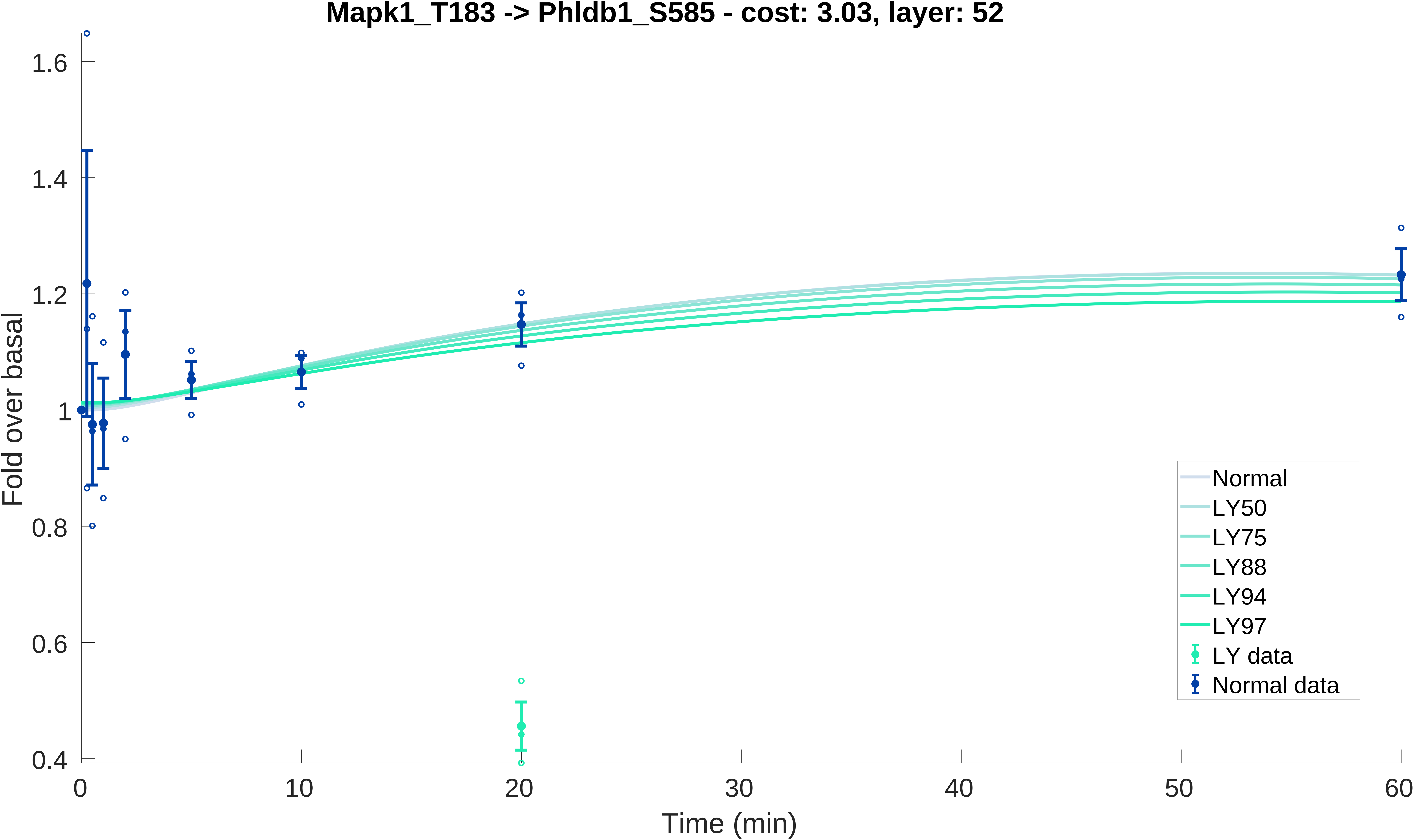

Mapk1_T183 -> Phldb1_S585 - cost: 3.03, layer: 52
Fold over basal
Time (min)
0.4
0.6
0.8
1
1.2
1.4
1.6
0
10
20
30
40
50
60
Normal
LY50
LY75
LY88
LY94
LY97
LY data
Normal data

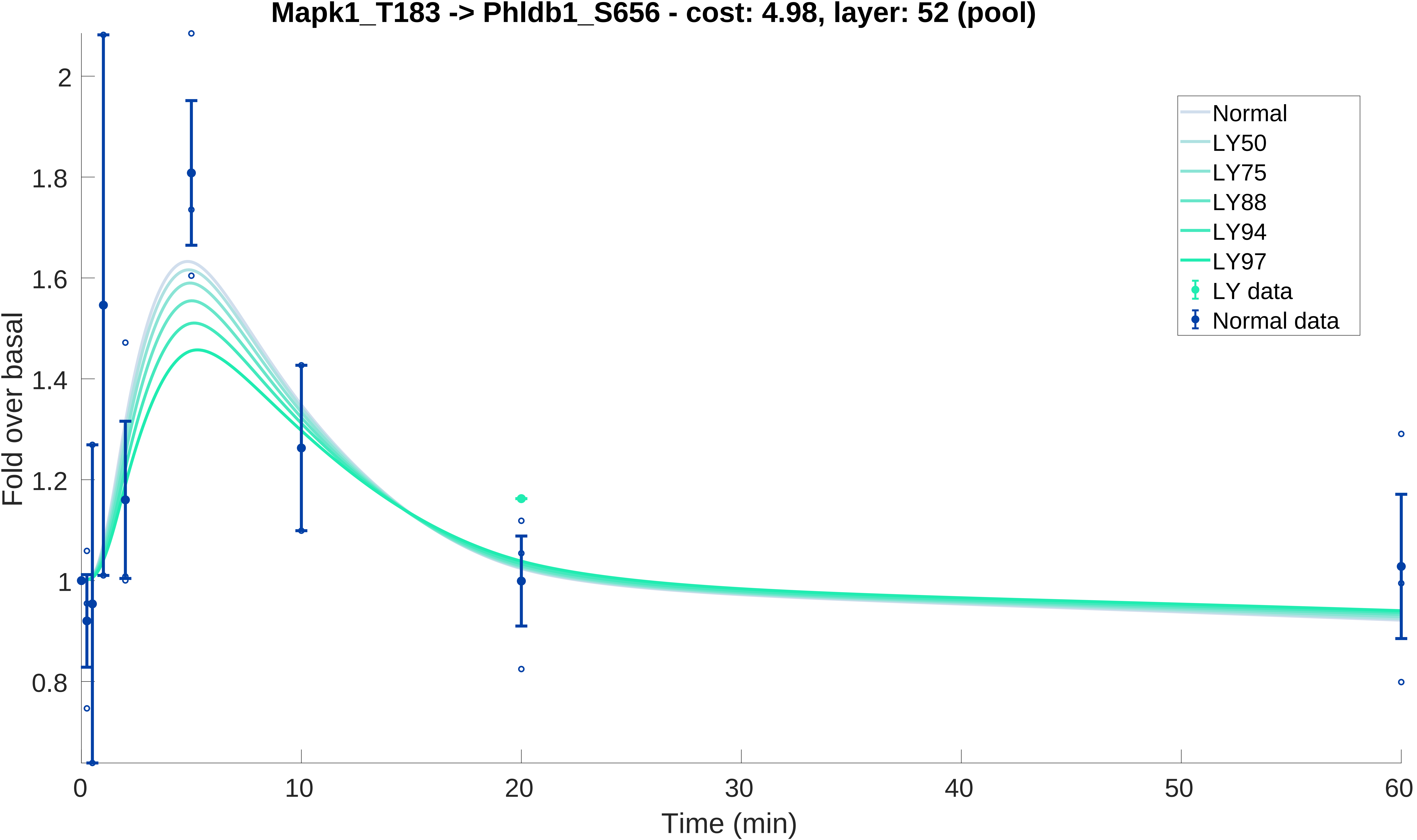

Mapk1_T183 -> Phldb1_S656 - cost: 4.98, layer: 52 (pool)
Fold over basal
Time (min)
0.8
1
1.2
1.4
1.6
1.8
2
0
10
20
30
40
50
60
Normal
LY50
LY75
LY88
LY94
LY97
LY data
Normal data

Mapk1_T183 -> Phldb1_S687 - cost: 3.38, layer: 52 (pool)

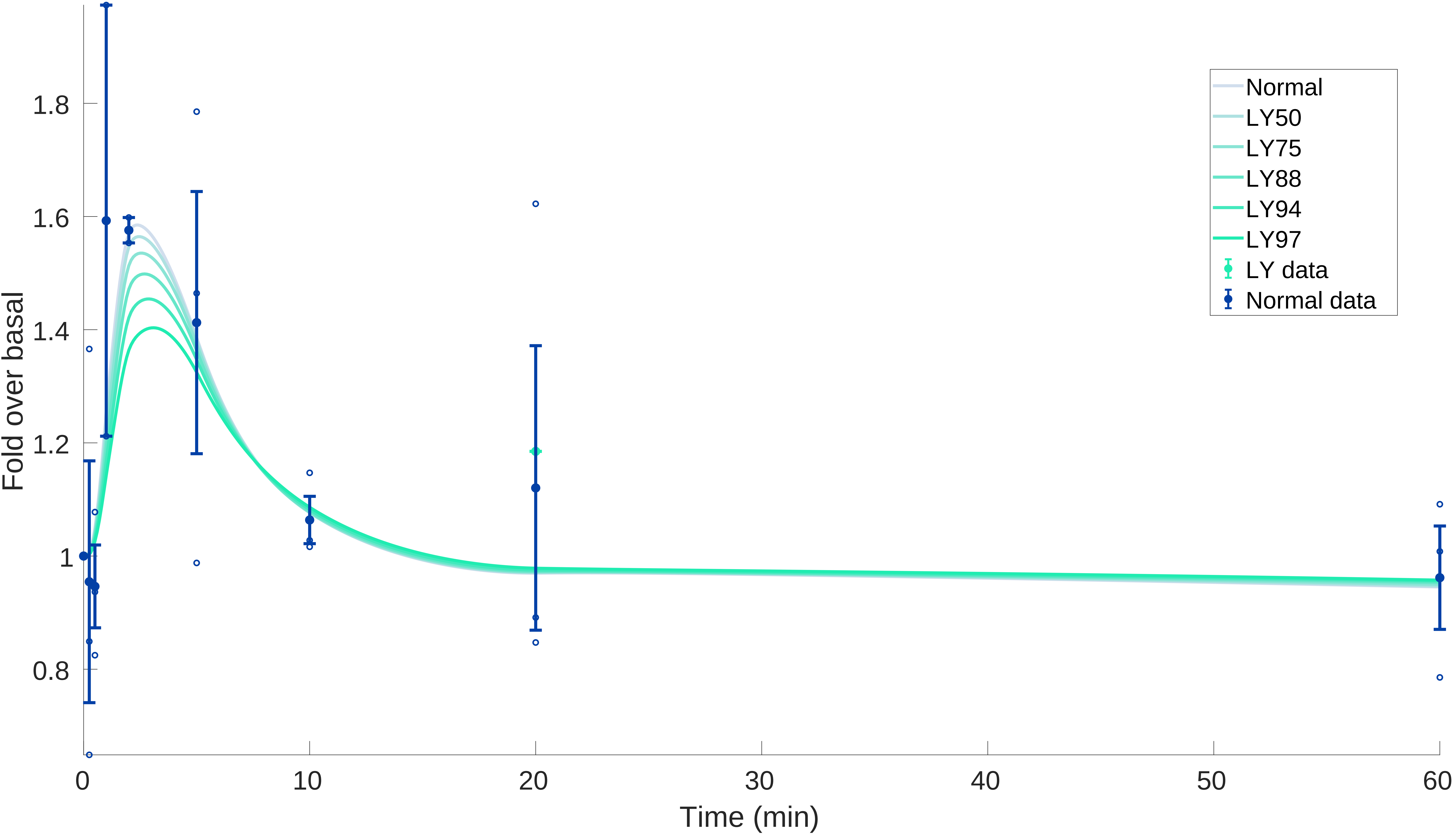

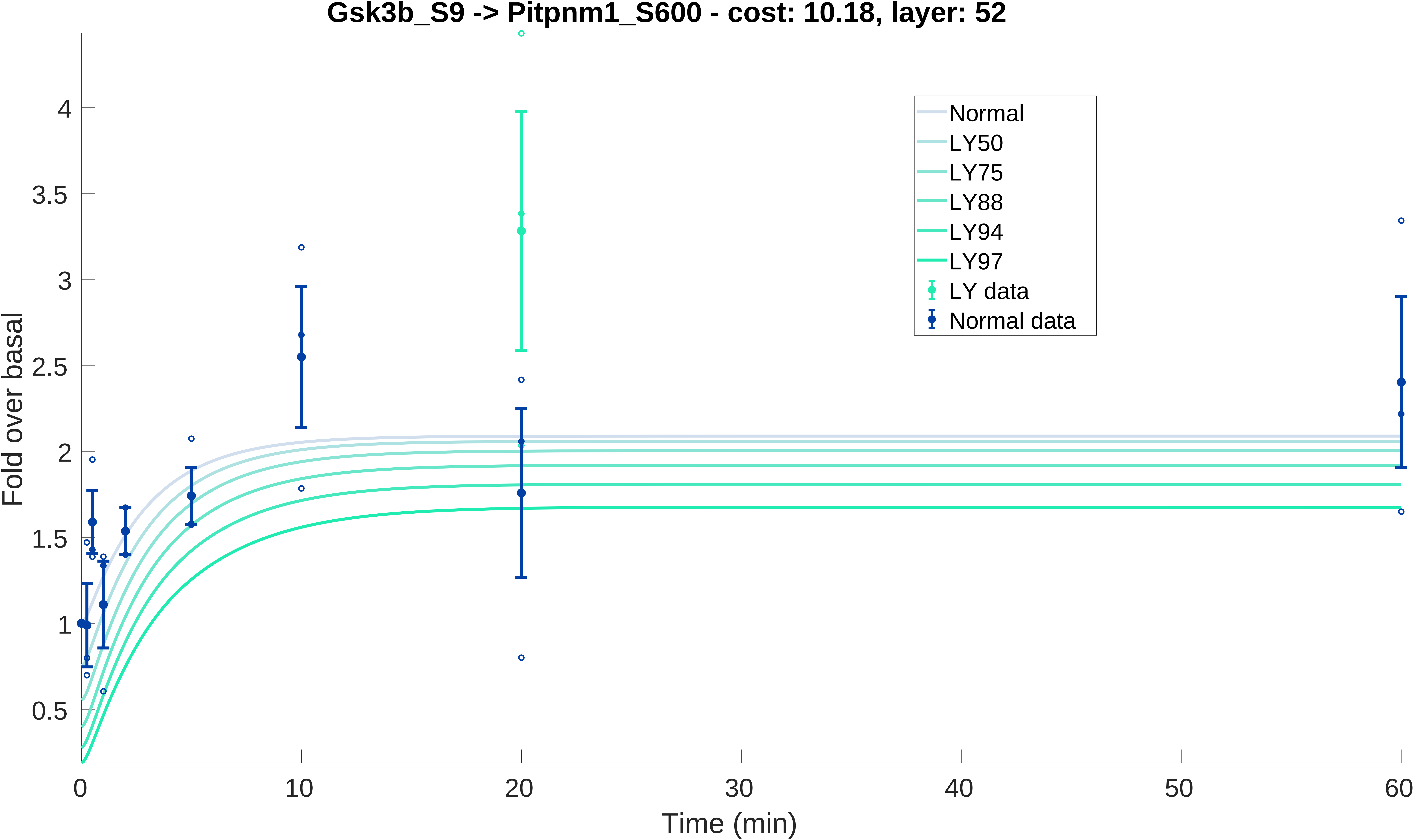

Gsk3b_S9 -> Pitpnm1_S600 - cost: 10.18, layer: 52
Fold over basal
Time (min)
0.5
1
1.5
2
2.5
3
3.5
4
0
10
20
30
40
50
60
Normal
LY50
LY75
LY88
LY94
LY97
LY data
Normal data

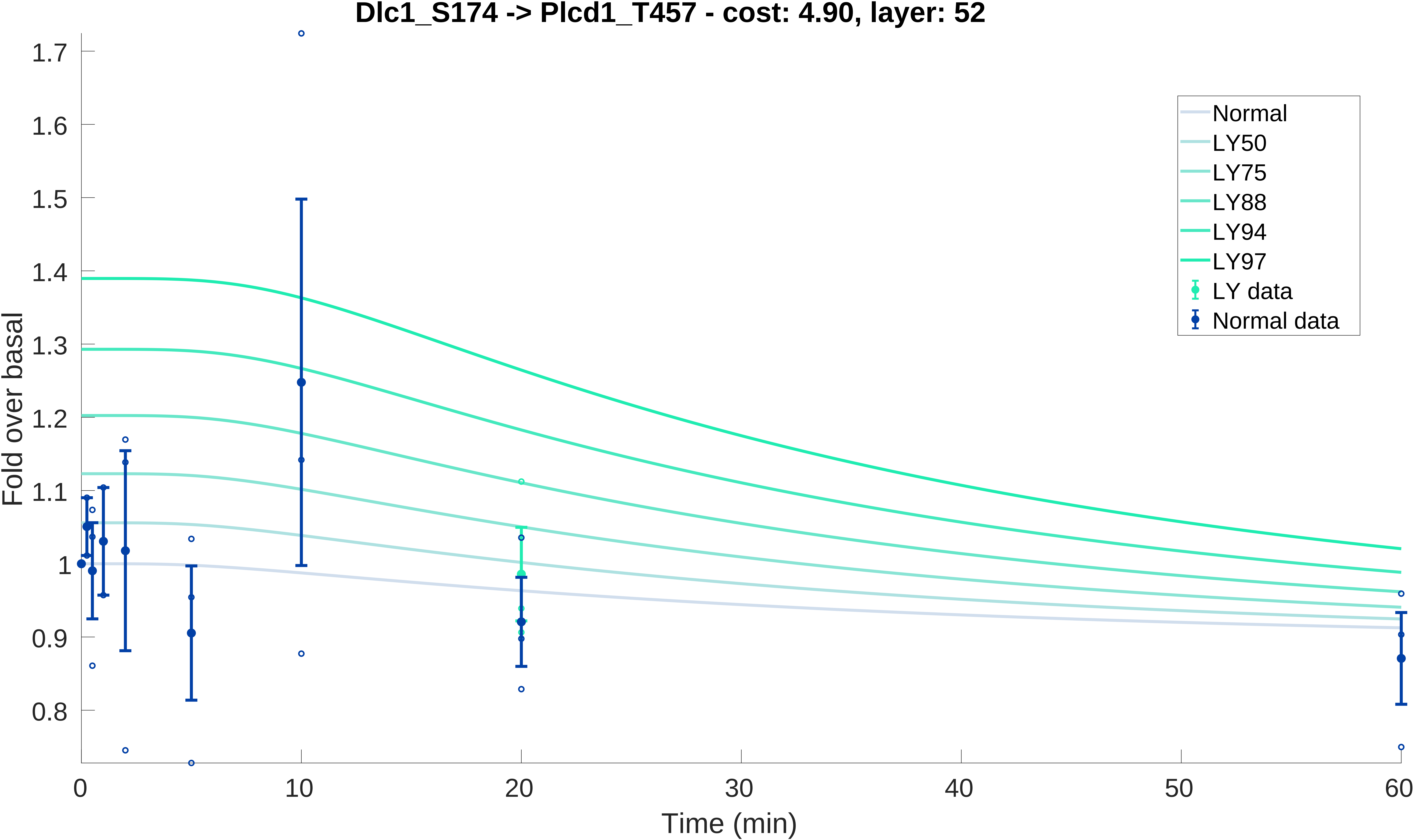

Dlc1_S174 -> Plcd1_T457 - cost: 4.90, layer: 52
Fold over basal
Time (min)
Normal
LY50
LY75
LY88
LY94
LY97
LY data
Normal data
1.7
1.6
1.5
1.4
1.3
1.2
1.1
1
0.9
0.8
0
10
20
30
40
50
60

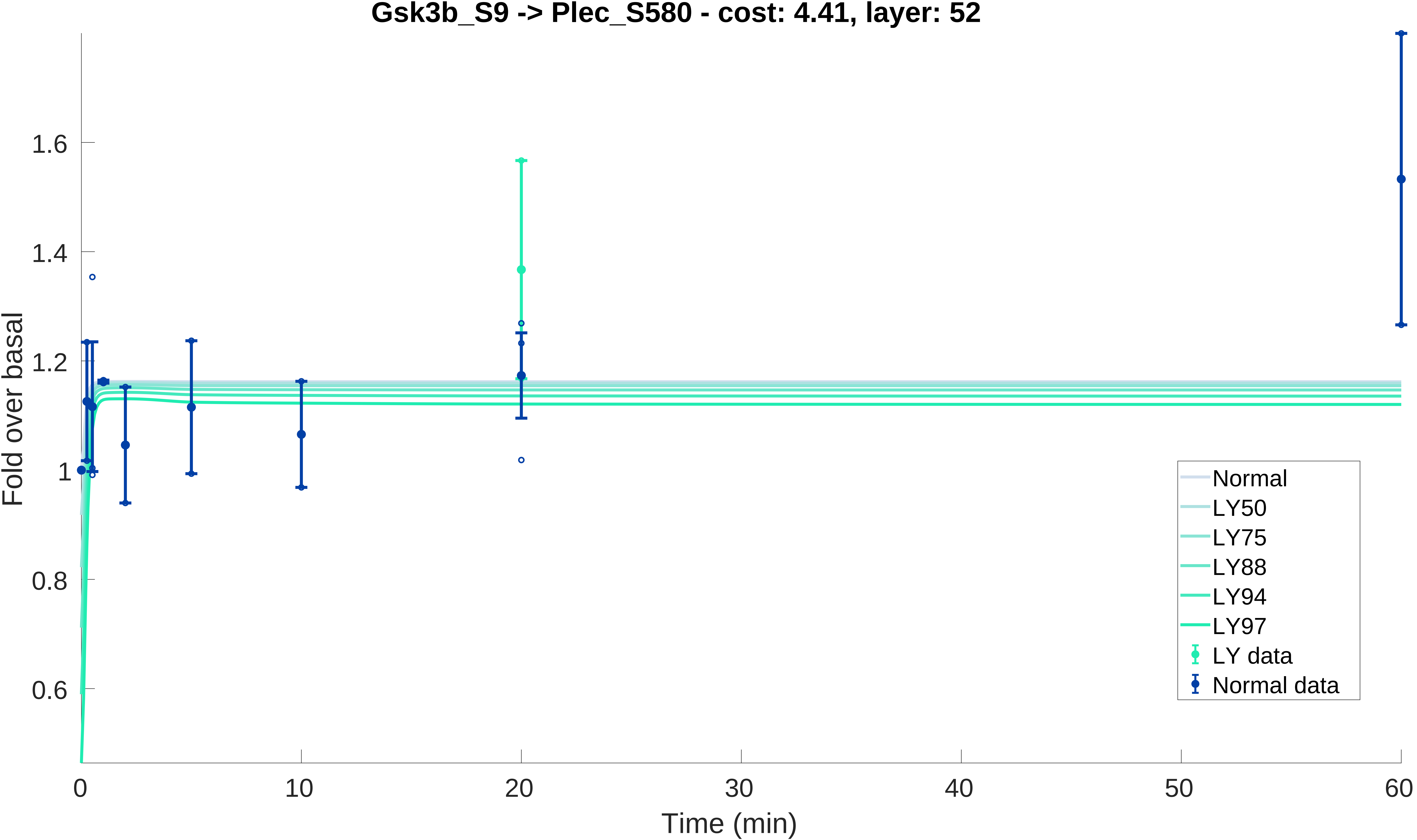
Gsk3b_S9 -> Plec_S580 - cost: 4.41, layer: 52
Fold over basal
Time (min)
0.6
0.8
1
1.2
1.4
1.6
0
10
20
30
40
50
60
Normal
LY50
LY75
LY88
LY94
LY97
LY data
Normal data

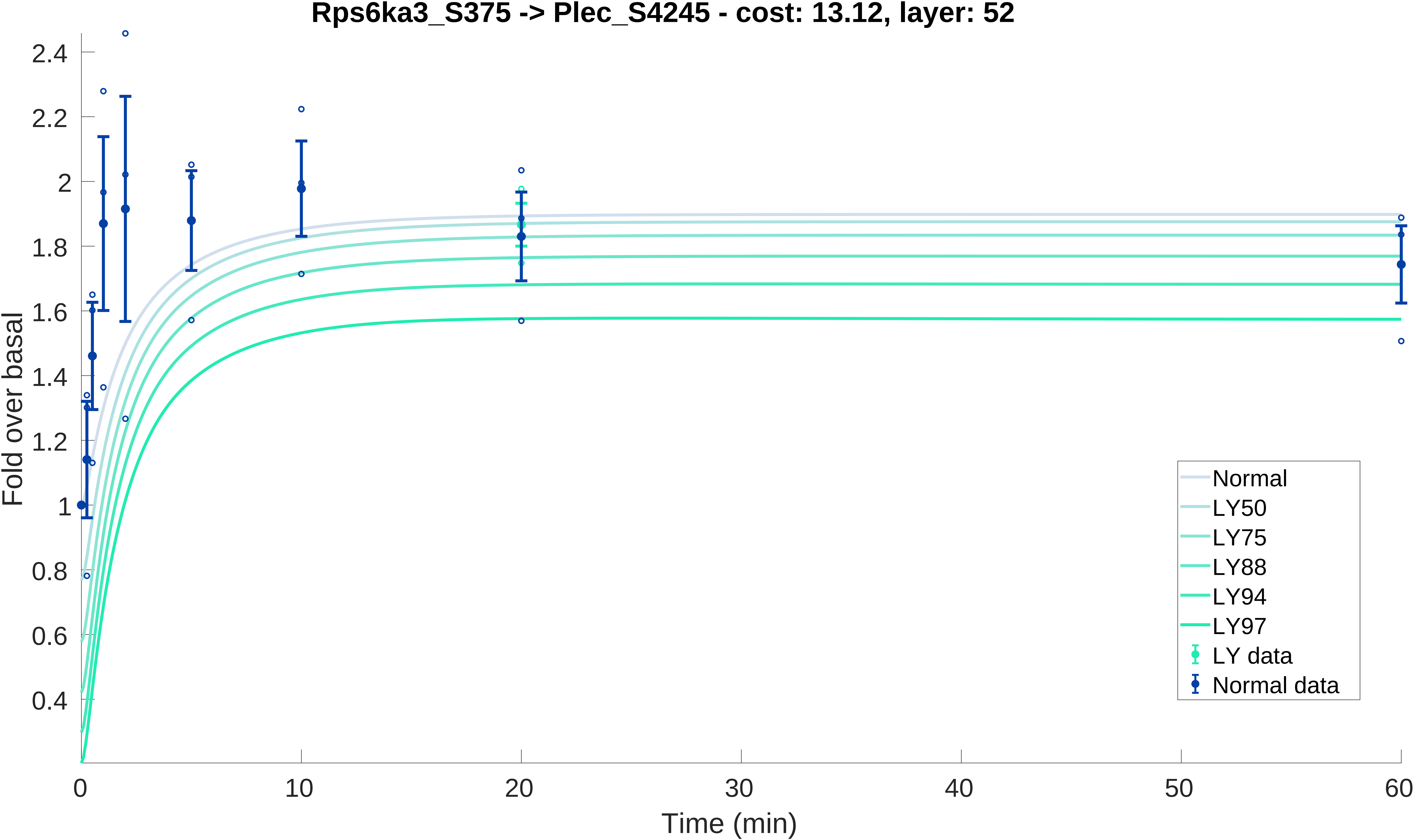
Rps6ka3_S375 -> Plec_S4245 - cost: 13.12, layer: 52
Fold over basal
2.4
2.2
2
1.8
1.6
1.4
1.2
1
0.8
0.6
0.4
0
10
20
30
40
50
60
Time (min)
Normal
LY50
LY75
LY88
LY94
LY97
LY data
Normal data

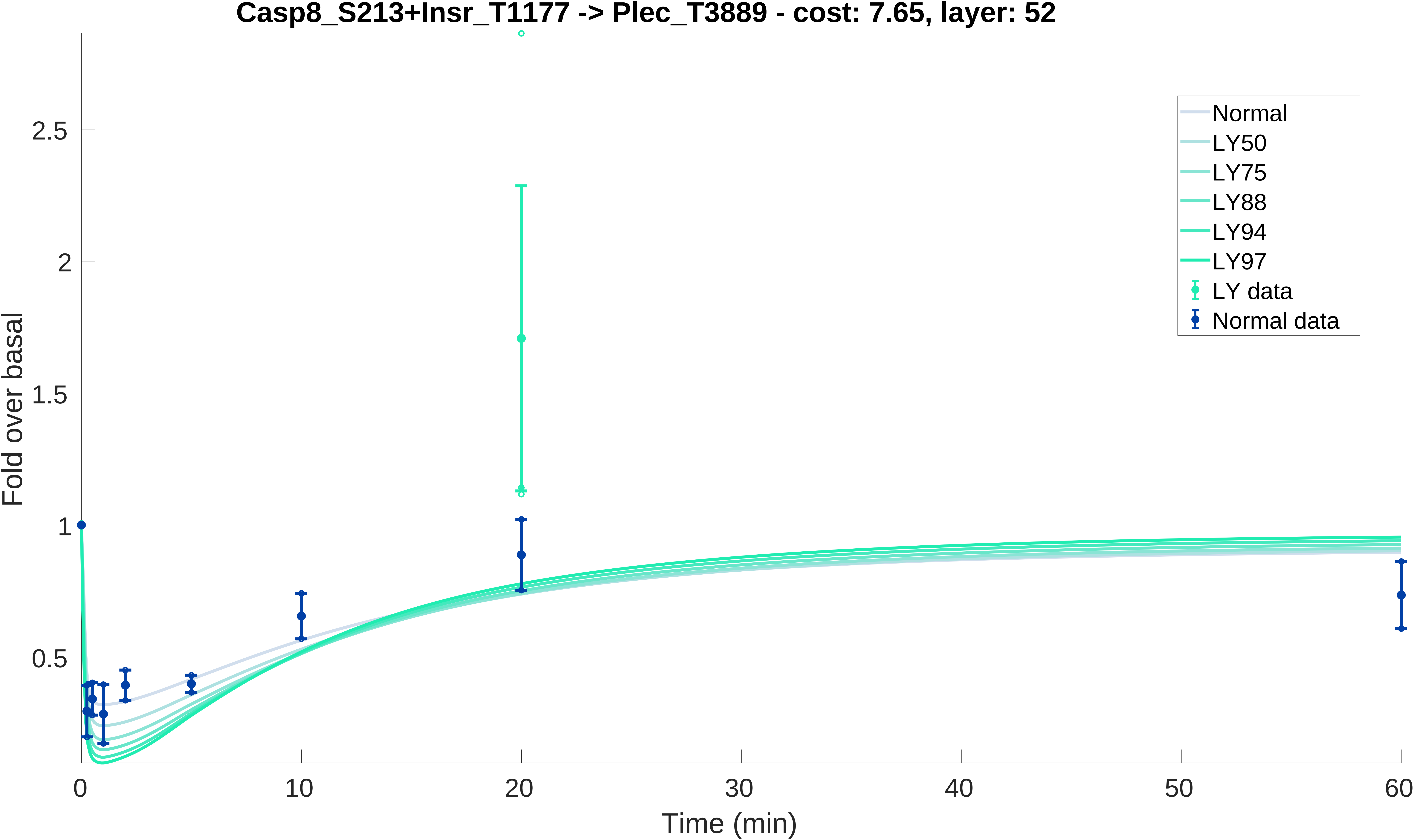

Casp8_S213+Insr_T1177 -> Plec_T3889 - cost: 7.65, layer: 52
Fold over basal
2.5
2
1.5
1
0.5
0
10
20
30
40
50
60
Time (min)
Normal
LY50
LY75
LY88
LY94
LY97
LY data
Normal data

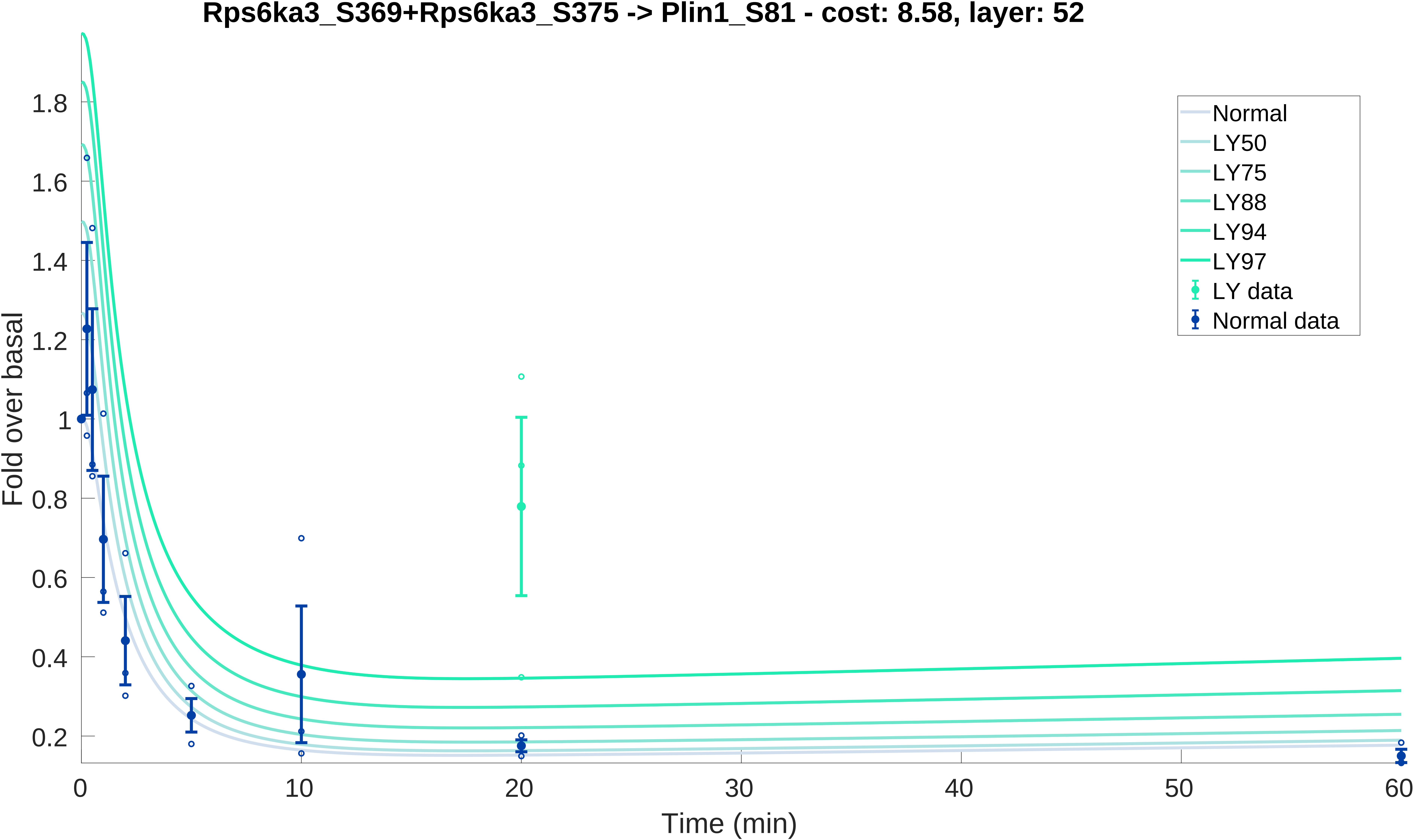

Rps6ka3_S369+Rps6ka3_S375 -> Plin1_S81 - cost: 8.58, layer: 52
Fold over basal
Time (min)
1.8
1.6
1.4
1.2
1
0.8
0.6
0.4
0.2
0
10
20
30
40
50
60
Normal
LY50
LY75
LY88
LY94
LY97
LY data
Normal data

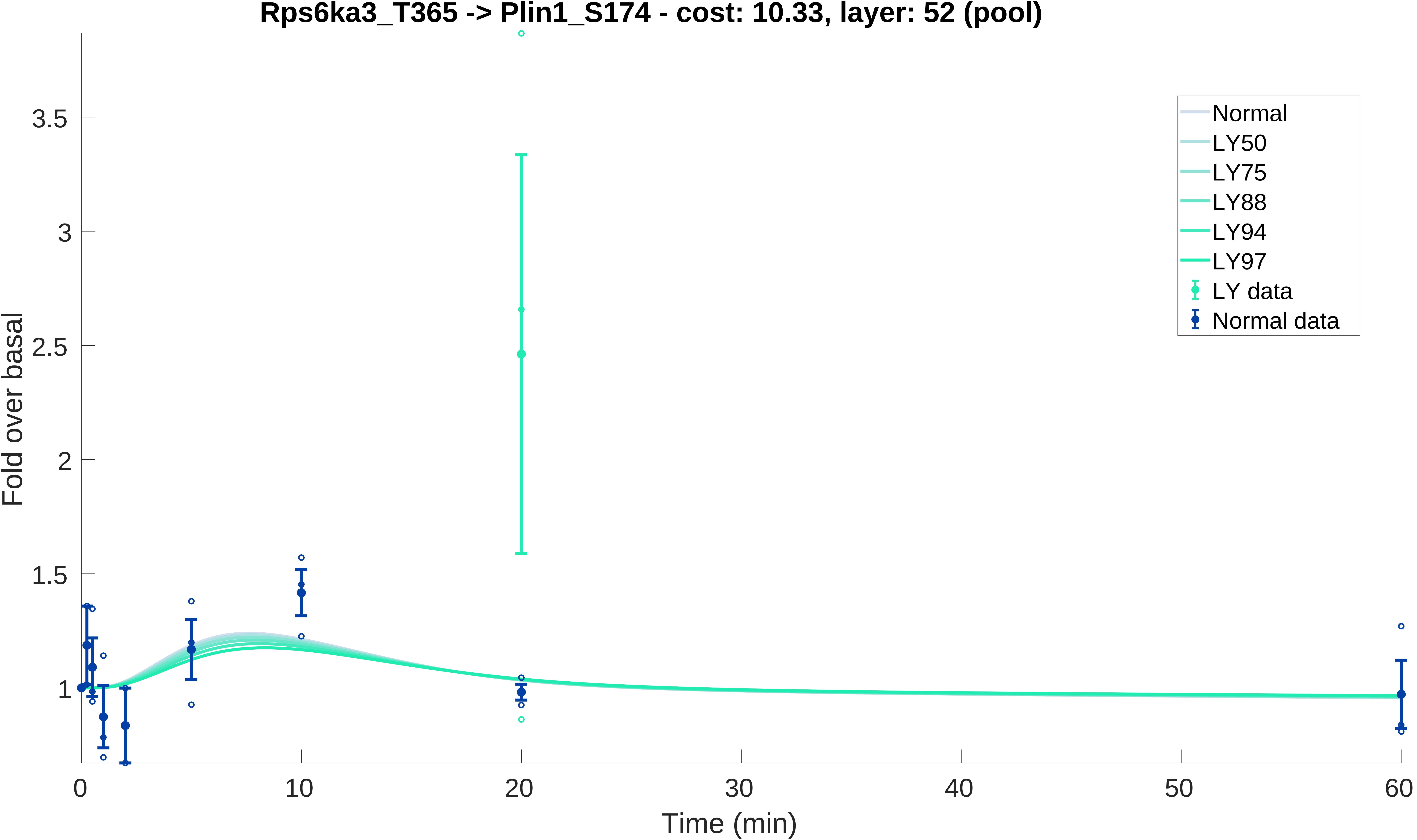
Rps6ka3_T365 -> Plin1_S174 - cost: 10.33, layer: 52 (pool)
Fold over basal
3.5
3
2.5
2
1.5
1
0
10
20
30
40
50
60
Time (min)
Normal
LY50
LY75
LY88
LY94
LY97
LY data
Normal data

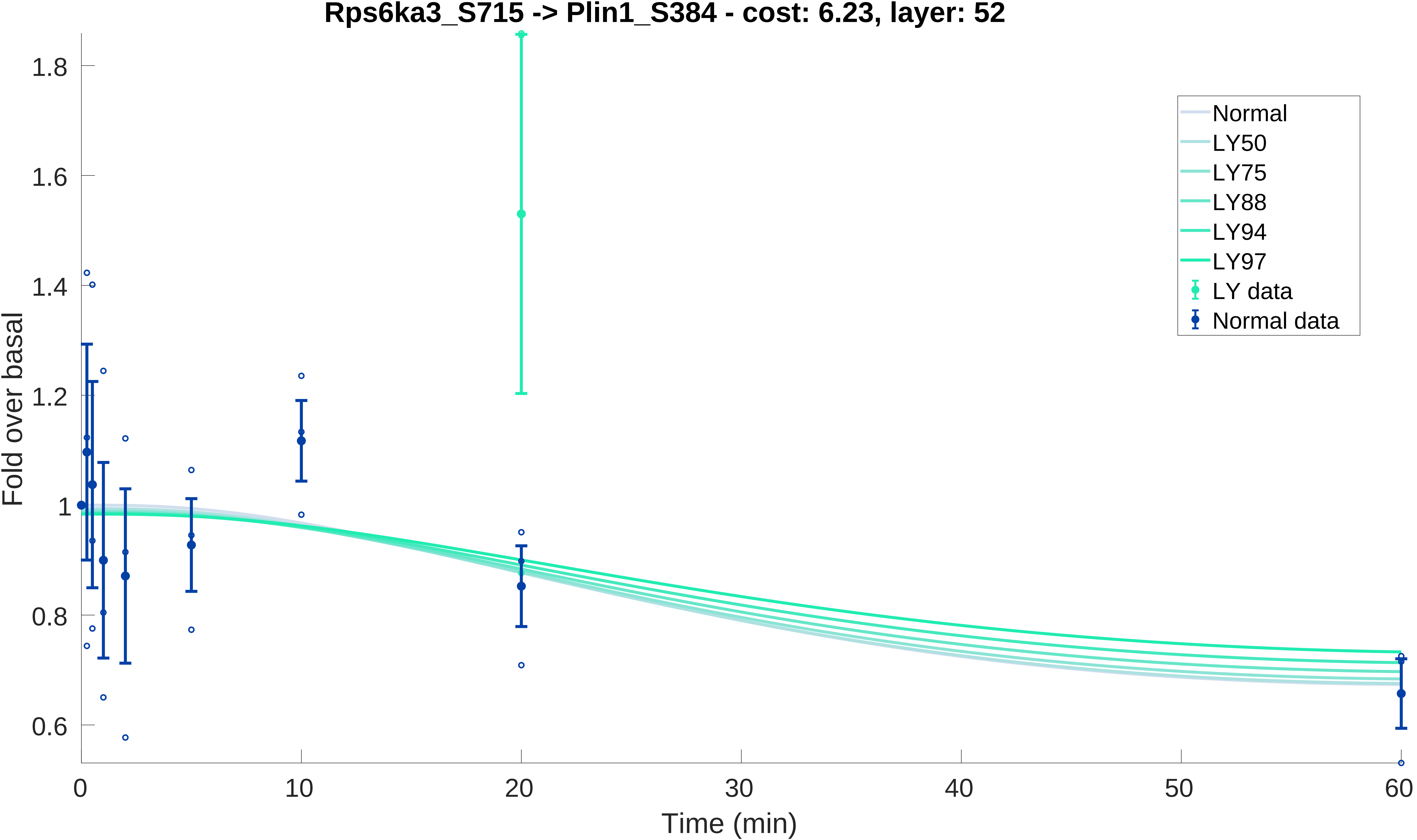

Rps6ka3_S715 -> Plin1_S384 - cost: 6.23, layer: 52
Fold over basal
Time (min)
1.8
1.6
1.4
1.2
1
0.8
0.6
0
10
20
30
40
50
60
Normal
LY50
LY75
LY88
LY94
LY97
LY data
Normal data

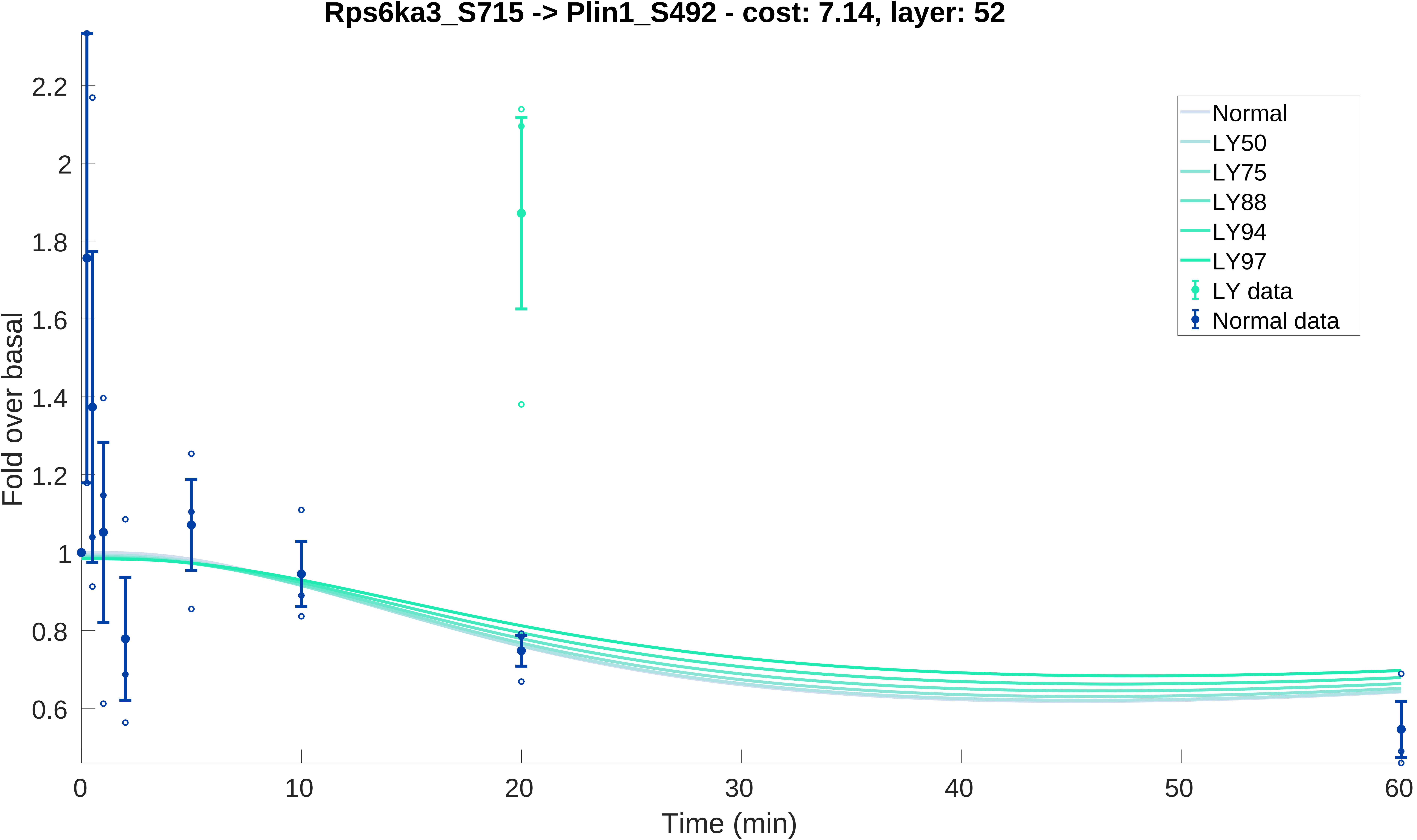

Rps6ka3_S715 -> Plin1_S492 - cost: 7.14, layer: 52
Fold over basal
Time (min)
0.6
0.8
1
1.2
1.4
1.6
1.8
2
2.2
0
10
20
30
40
50
60
Normal
LY50
LY75
LY88
LY94
LY97
LY data
Normal data

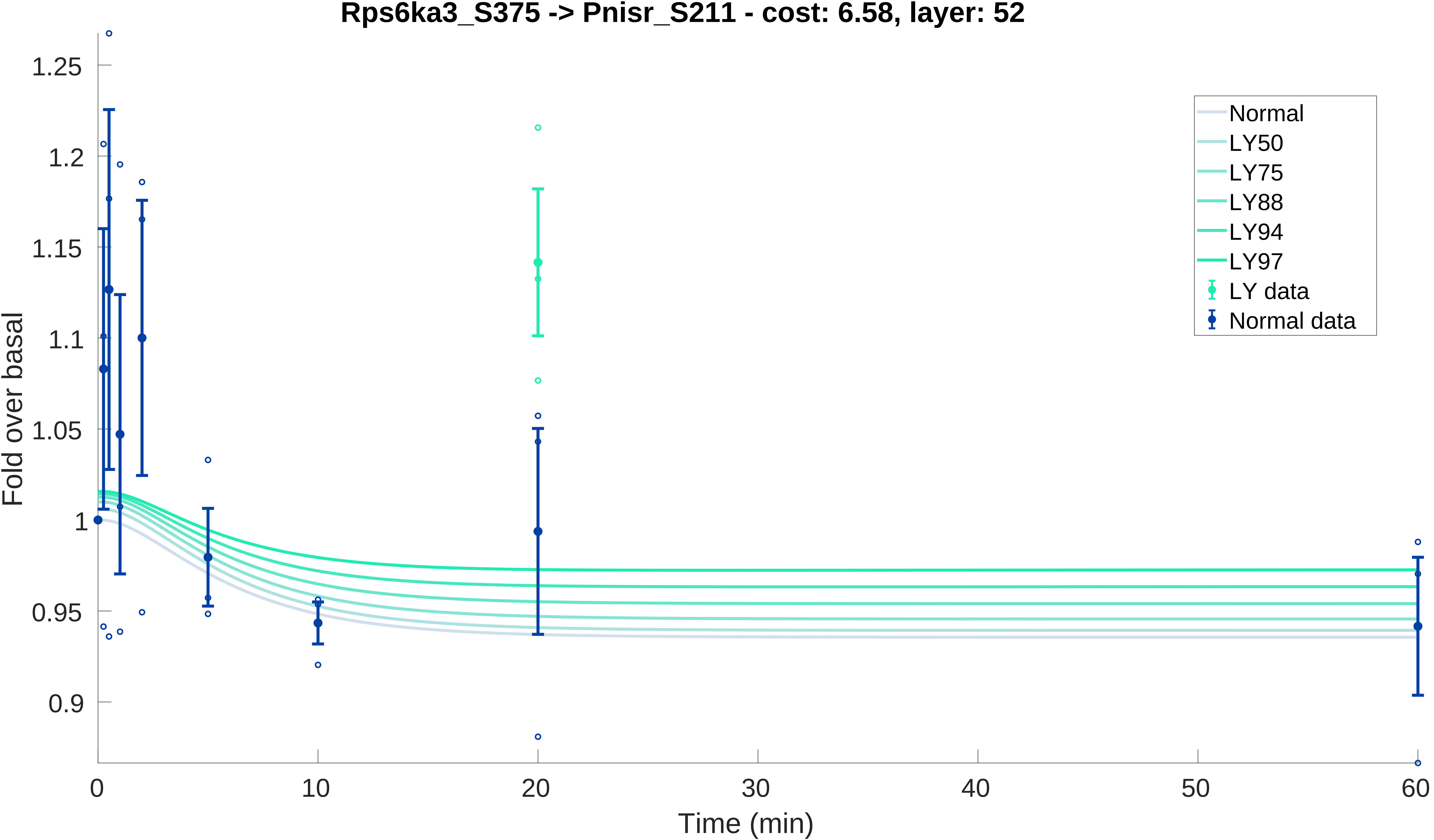
Rps6ka3_S375 -> Pnisr_S211 - cost: 6.58, layer: 52
Fold over basal
1.25
1.2
1.15
1.1
1.05
1
0.95
0.9
0
10
20
30
40
50
60
Time (min)
Normal
LY50
LY75
LY88
LY94
LY97
LY data
Normal data

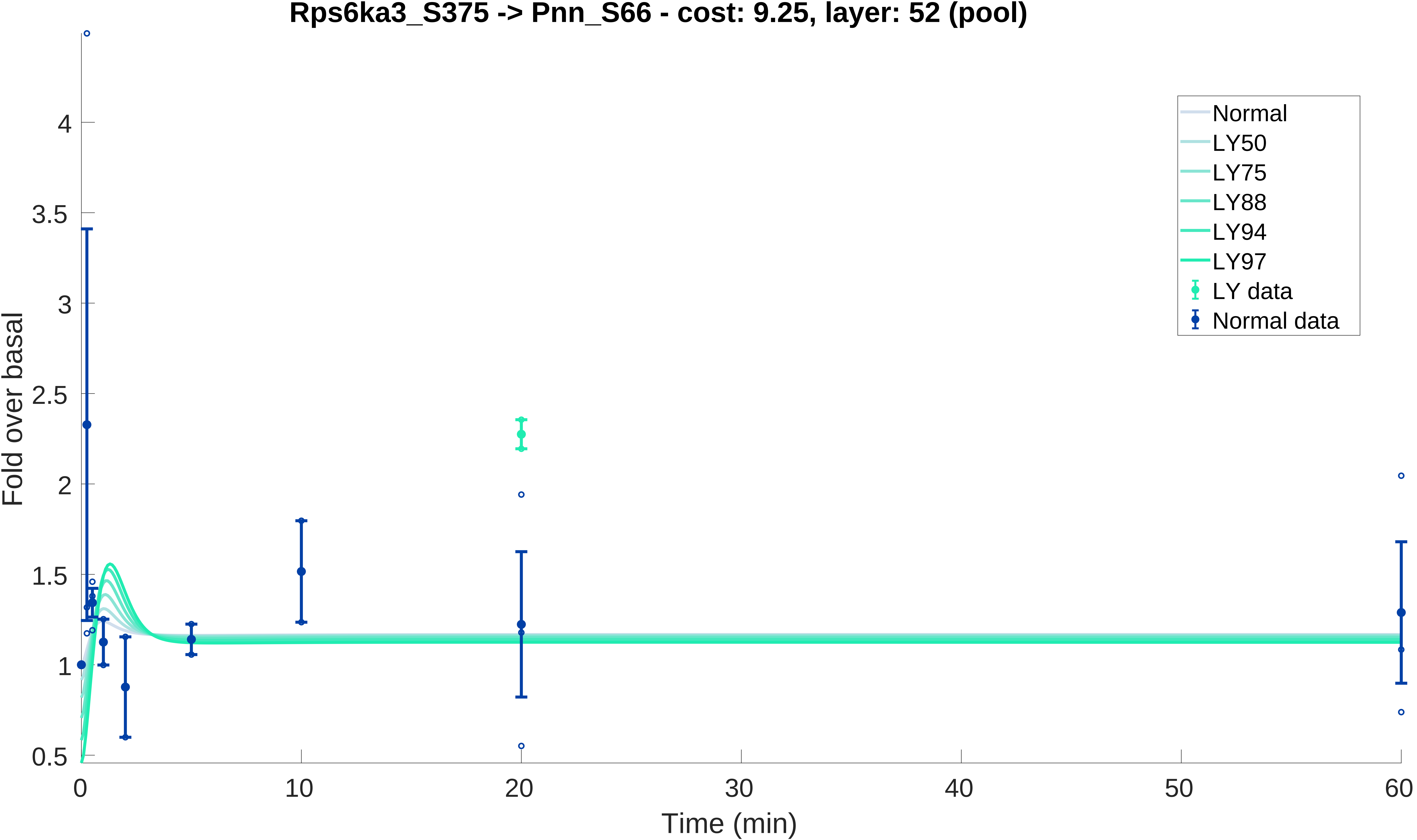
Rps6ka3_S375 -> Pnn_S66 - cost: 9.25, layer: 52 (pool)
Fold over basal
Time (min)
0.5
1
1.5
2
2.5
3
3.5
4
0
10
20
30
40
50
60
Normal
LY50
LY75
LY88
LY94
LY97
LY data
Normal data

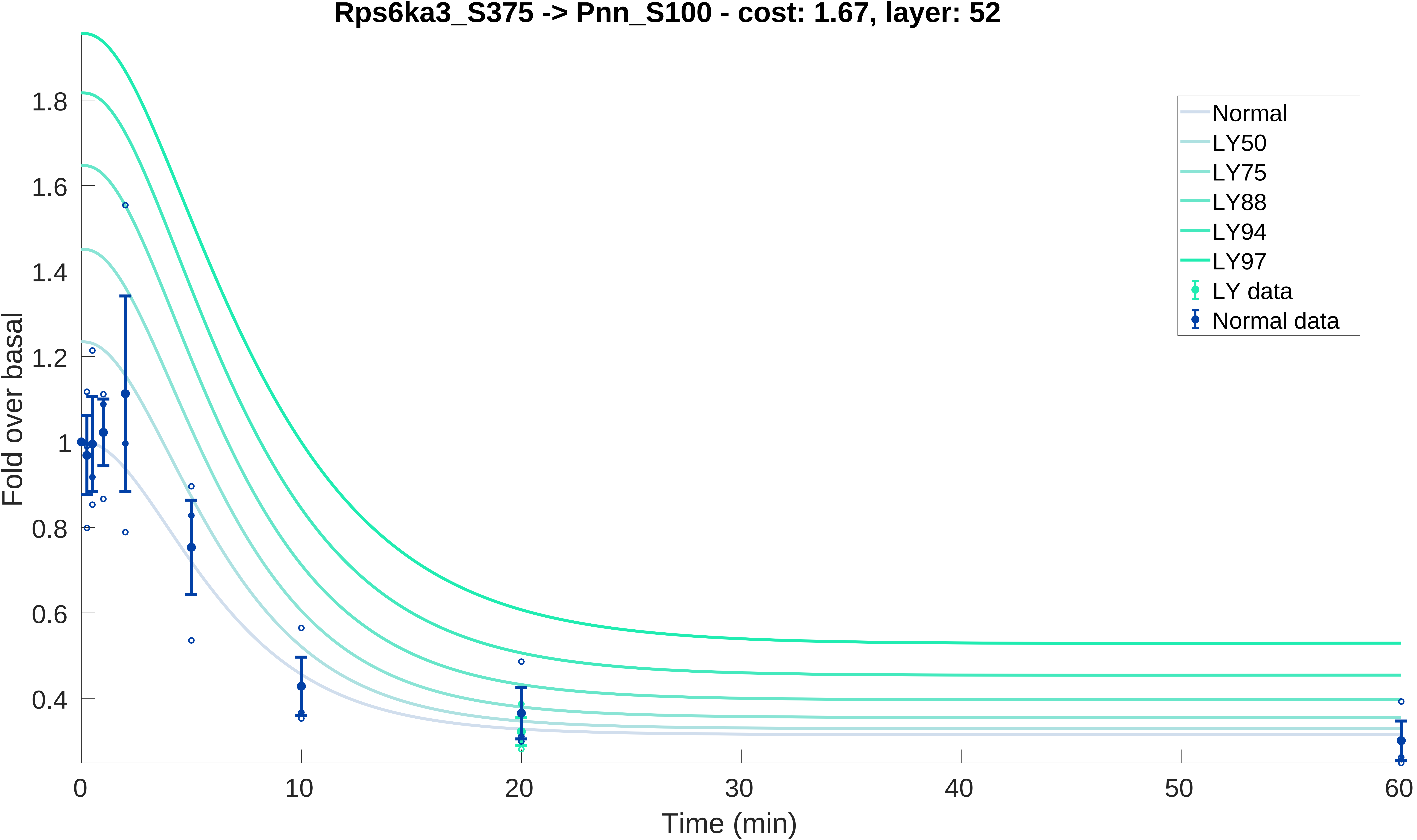
Rps6ka3_S375 -> Pnn_S100 - cost: 1.67, layer: 52
Fold over basal
1.8
1.6
1.4
1.2
1
0.8
0.6
0.4
0
10
20
30
40
50
60
Time (min)
Normal
LY50
LY75
LY88
LY94
LY97
LY data
Normal data

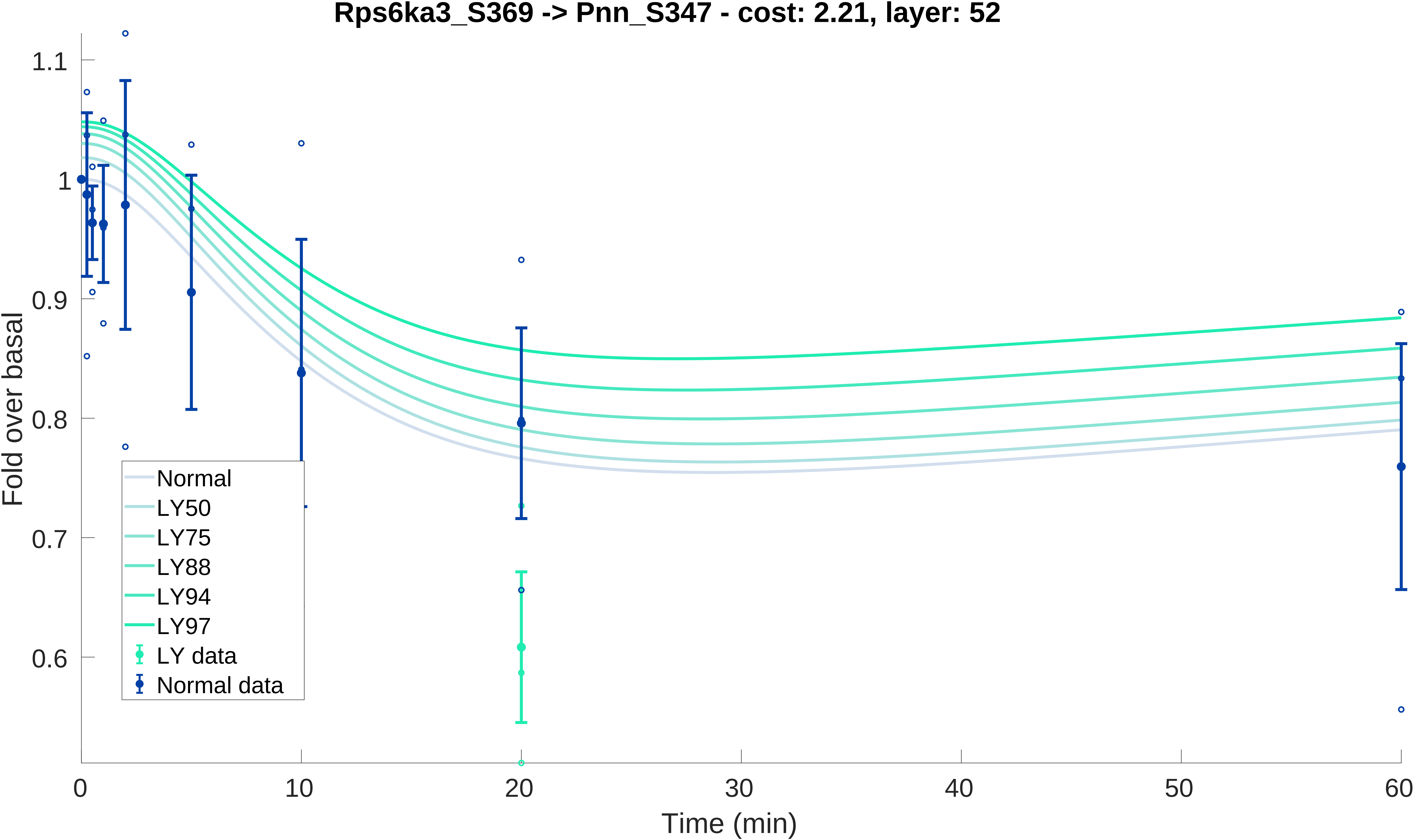

Rps6ka3_S369 -> Pnn_S347 - cost: 2.21, layer: 52
Fold over basal
1.1
1
0.9
0.8
0.7
0.6
0
10
20
30
40
50
60
Time (min)
Normal
LY50
LY75
LY88
LY94
LY97
LY data
Normal data

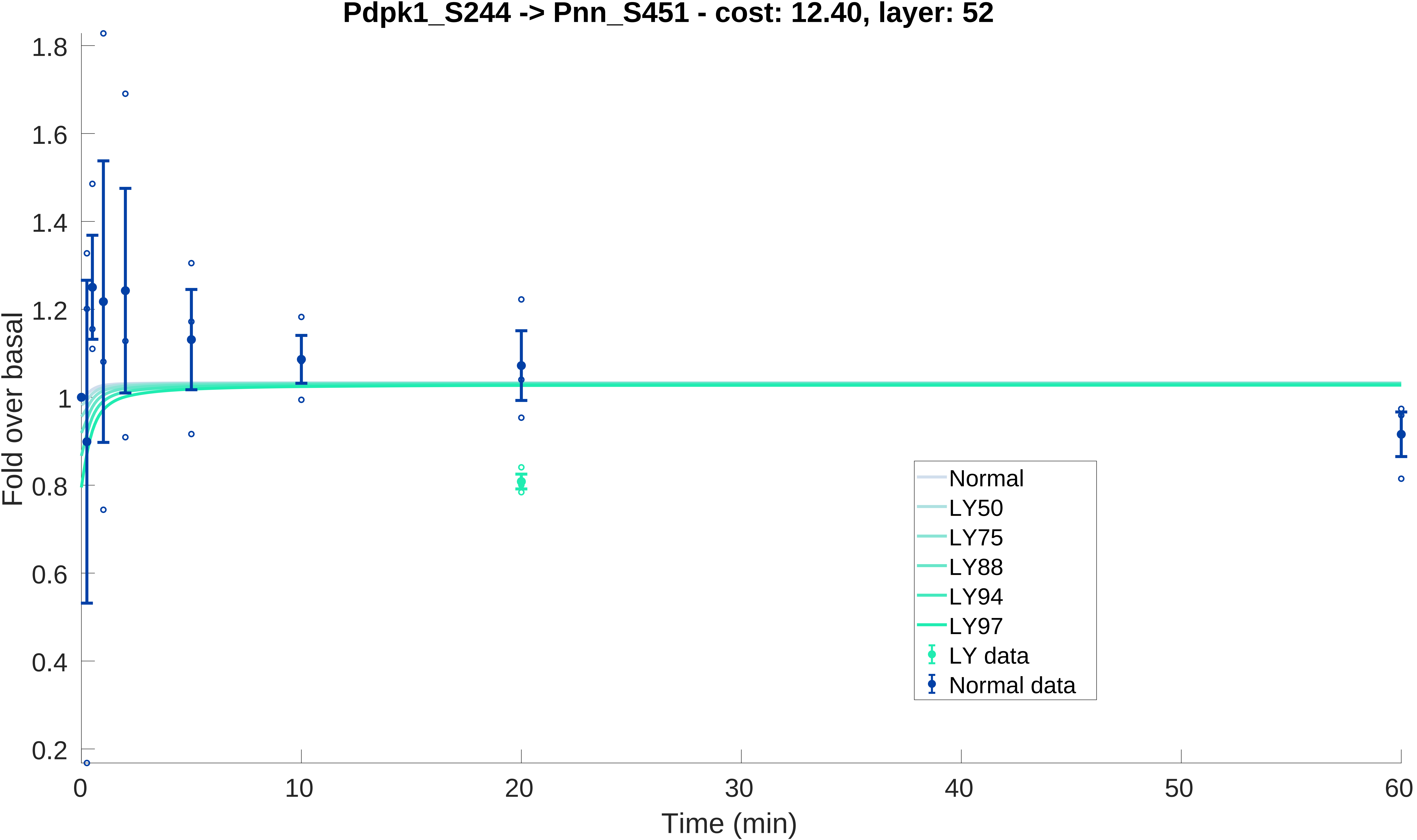

Pdpk1_S244 -> Pnn_S451 - cost: 12.40, layer: 52
1.8
1.6
1.4
1.2
1
0.8
0.6
0.4
0.2
Fold over basal
0
10
20
30
40
50
60
Time (min)
Normal
LY50
LY75
LY88
LY94
LY97
LY data
Normal data

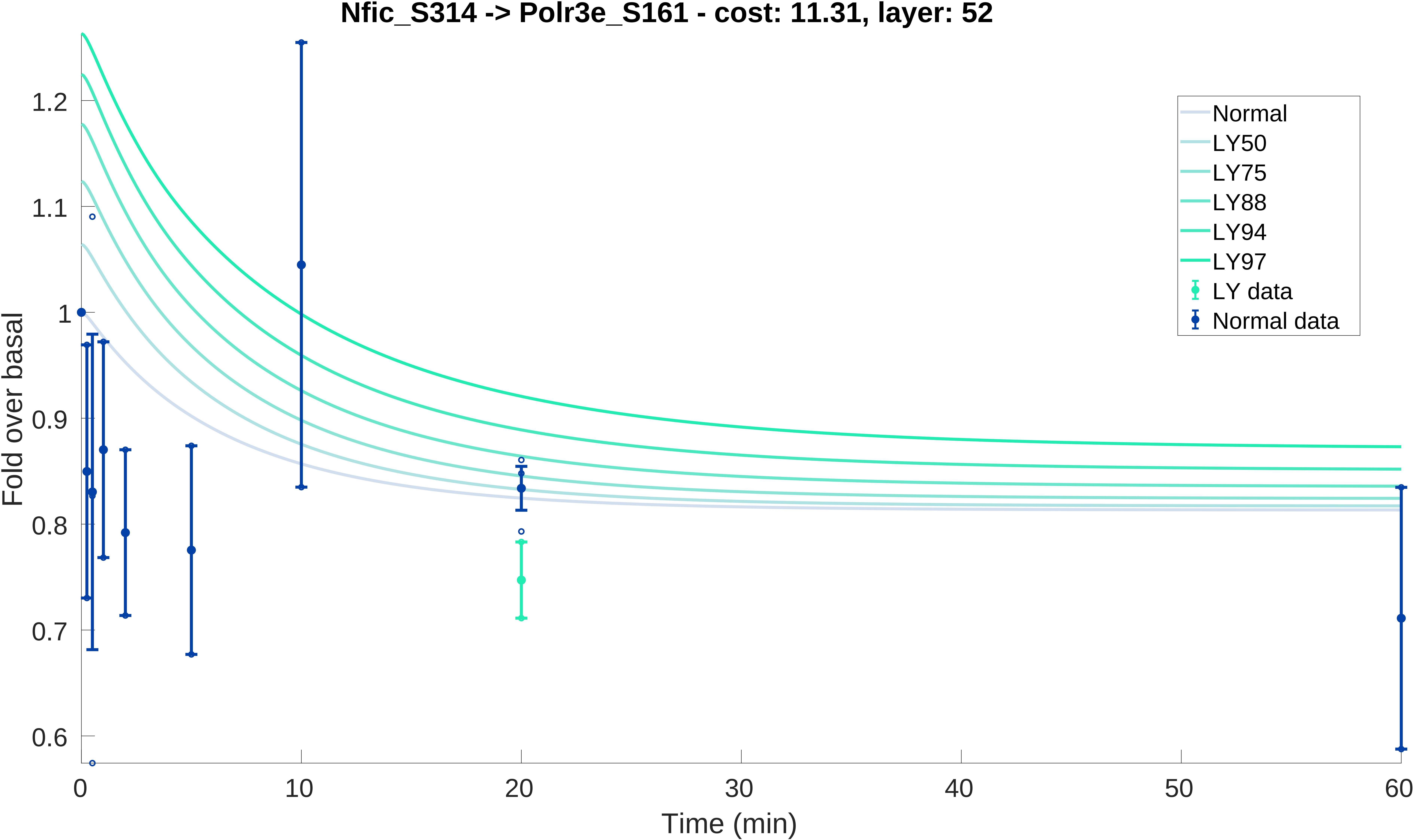

Nfic_S314 -> Polr3e_S161 - cost: 11.31, layer: 52
Fold over basal
Time (min)
0.6
0.7
0.8
0.9
1
1.1
1.2
0
10
20
30
40
50
60
Normal
LY50
LY75
LY88
LY94
LY97
LY data
Normal data

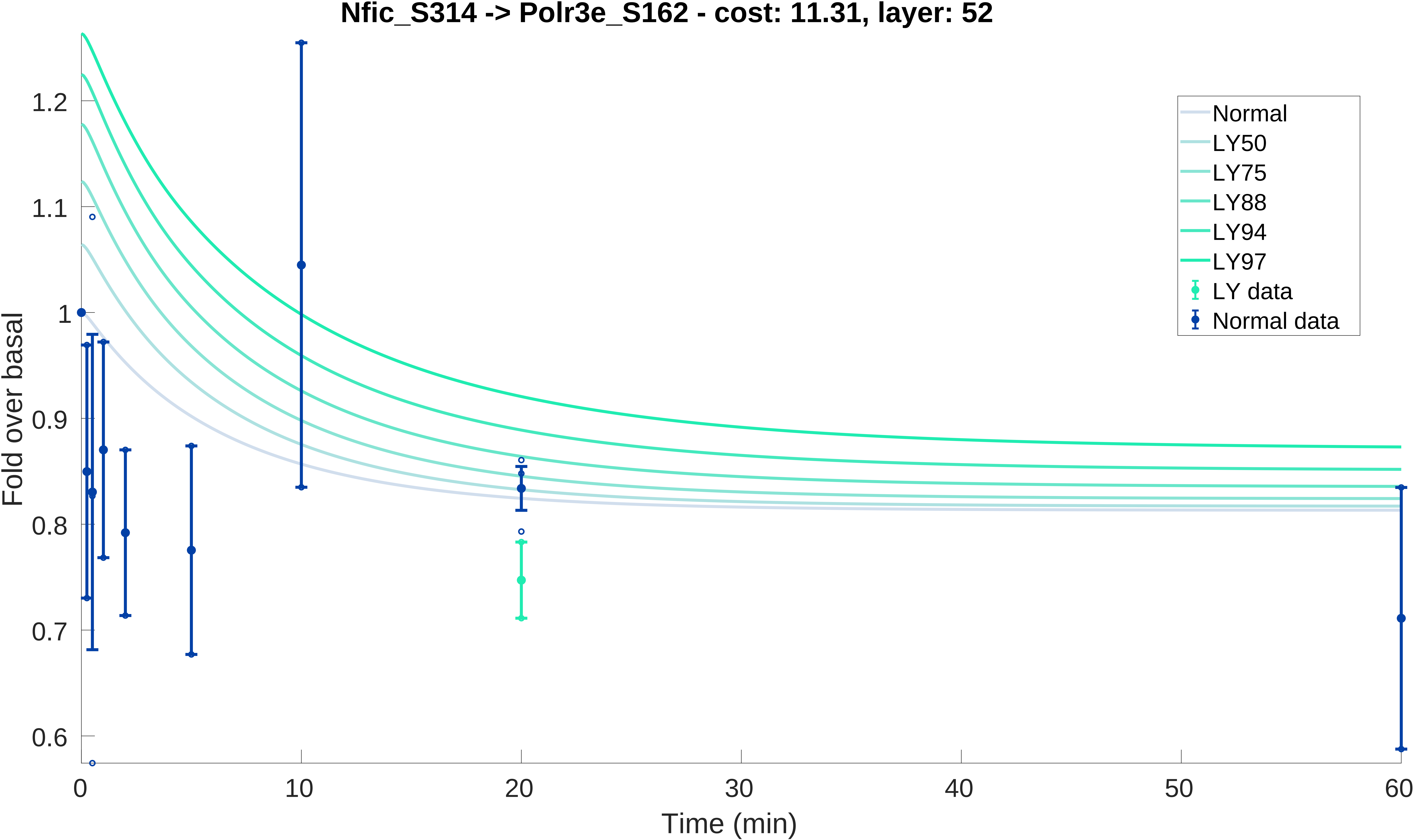

Nfic_S314 -> Polr3e_S162 - cost: 11.31, layer: 52
Fold over basal
Time (min)
1.2
1.1
1
0.9
0.8
0.7
0.6
0
10
20
30
40
50
60
Normal
LY50
LY75
LY88
LY94
LY97
LY data
Normal data

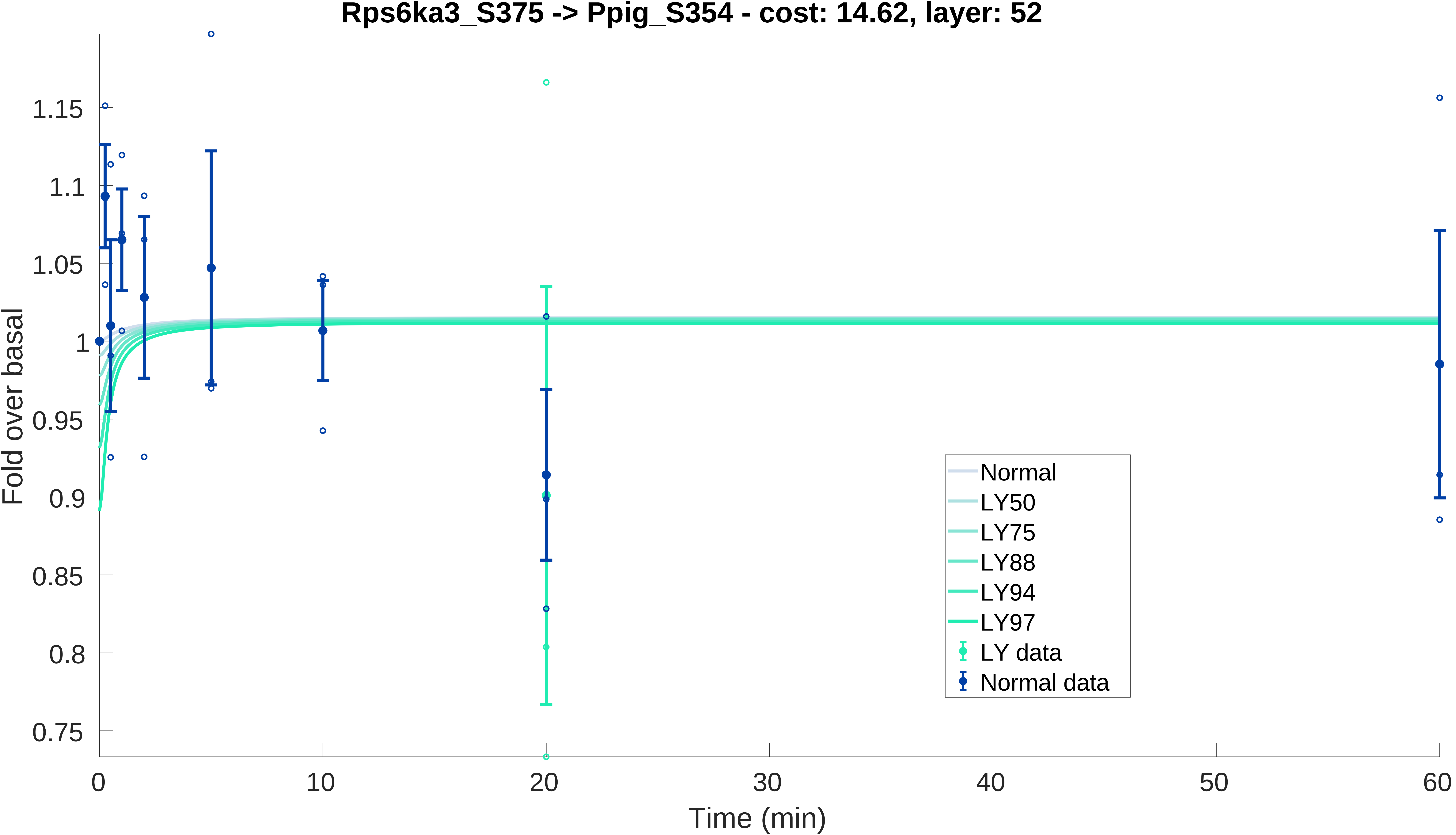

Rps6ka3_S375 -> Ppig_S354 - cost: 14.62, layer: 52
Fold over basal
Time (min)
1.15
1.1
1.05
1
0.95
0.9
0.85
0.8
0.75
0
10
20
30
40
50
60
Normal
LY50
LY75
LY88
LY94
LY97
LY data
Normal data

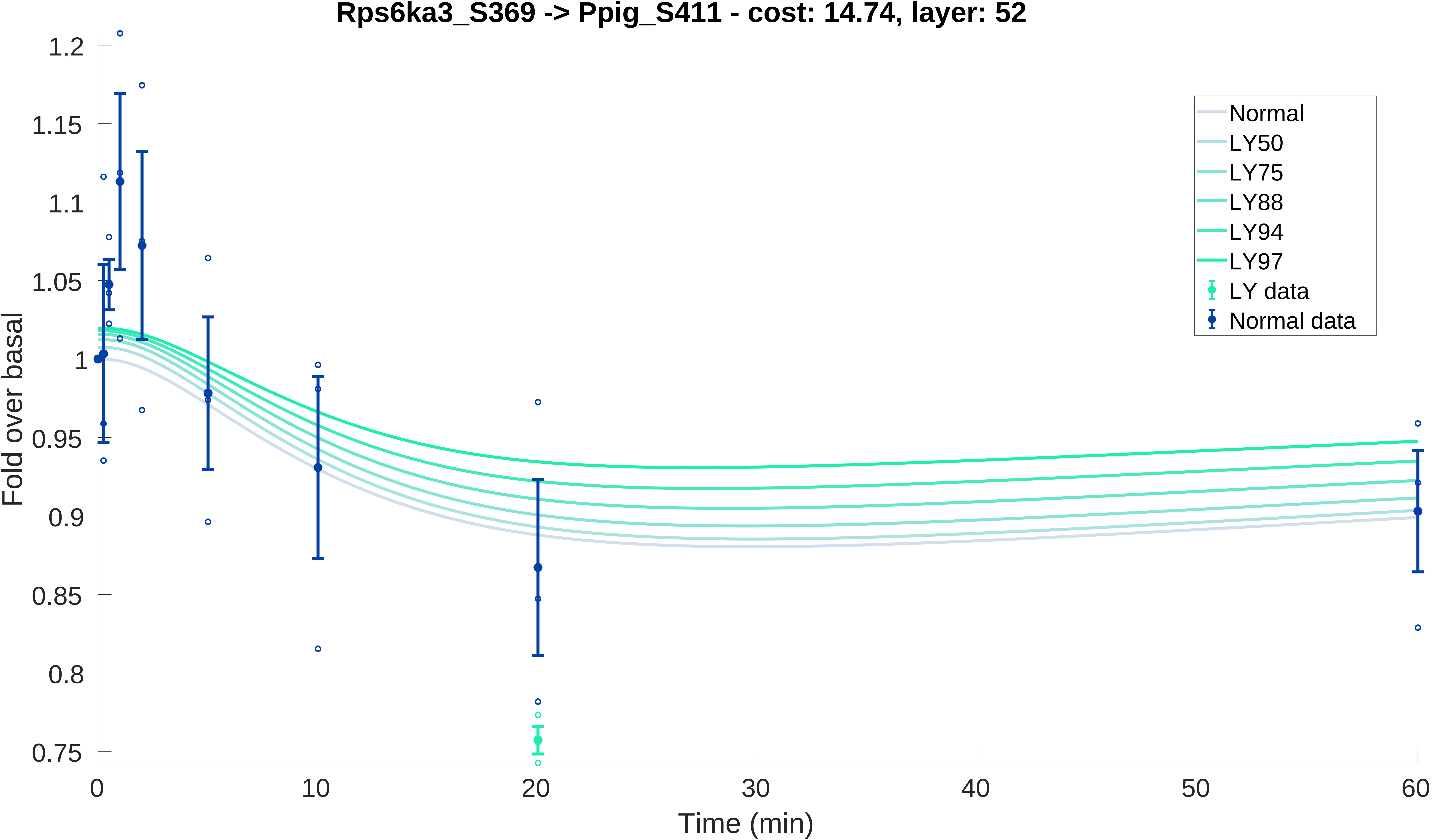

Rps6ka3_S369 -> Ppig_S411 - cost: 14.74, layer: 52
Fold over basal
Time (min)
1.2
1.15
1.1
1.05
1
0.95
0.9
0.85
0.8
0.75
0
10
20
30
40
50
60
Normal
LY50
LY75
LY88
LY94
LY97
LY data
Normal data

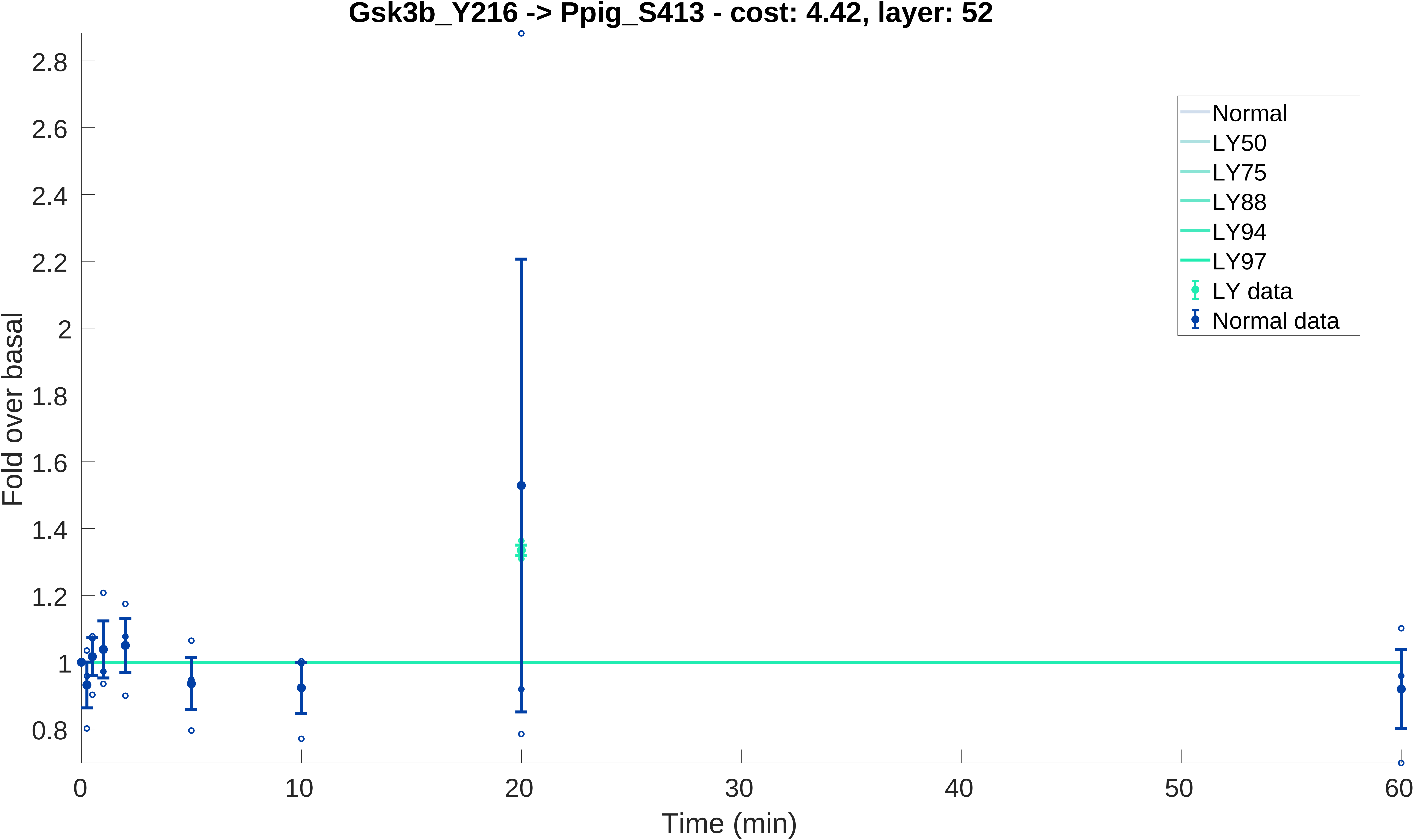
Gsk3b_Y216 -> Ppig_S413 - cost: 4.42, layer: 52
Fold over basal
Time (min)
2.8
2.6
2.4
2.2
2
1.8
1.6
1.4
1.2
1
0.8
0
10
20
30
40
50
60
Normal
LY50
LY75
LY88
LY94
LY97
LY data
Normal data

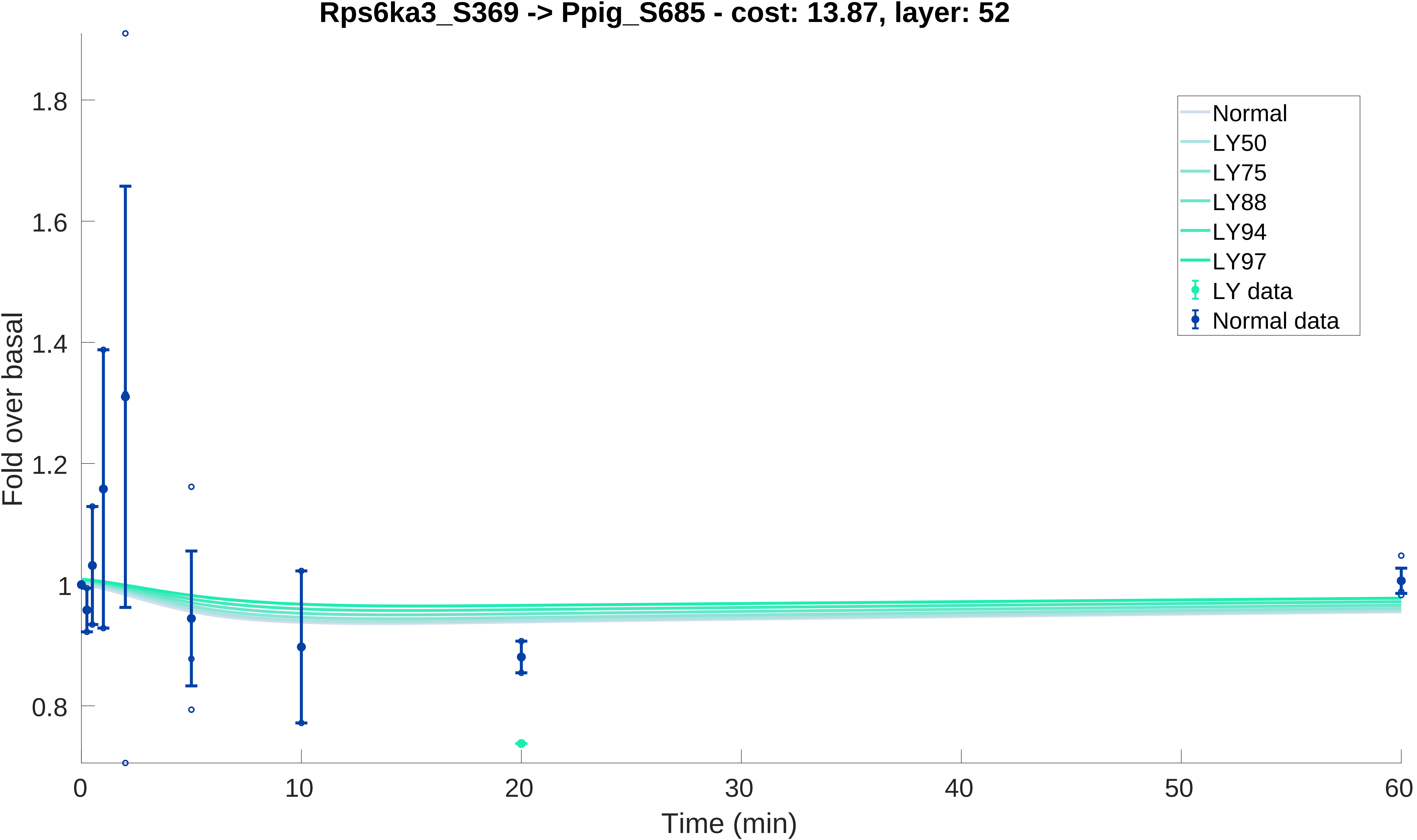

Rps6ka3_S369 -> Ppig_S685 - cost: 13.87, layer: 52
Fold over basal
Time (min)
0.8
1
1.2
1.4
1.6
1.8
0
10
20
30
40
50
60
Normal
LY50
LY75
LY88
LY94
LY97
LY data
Normal data

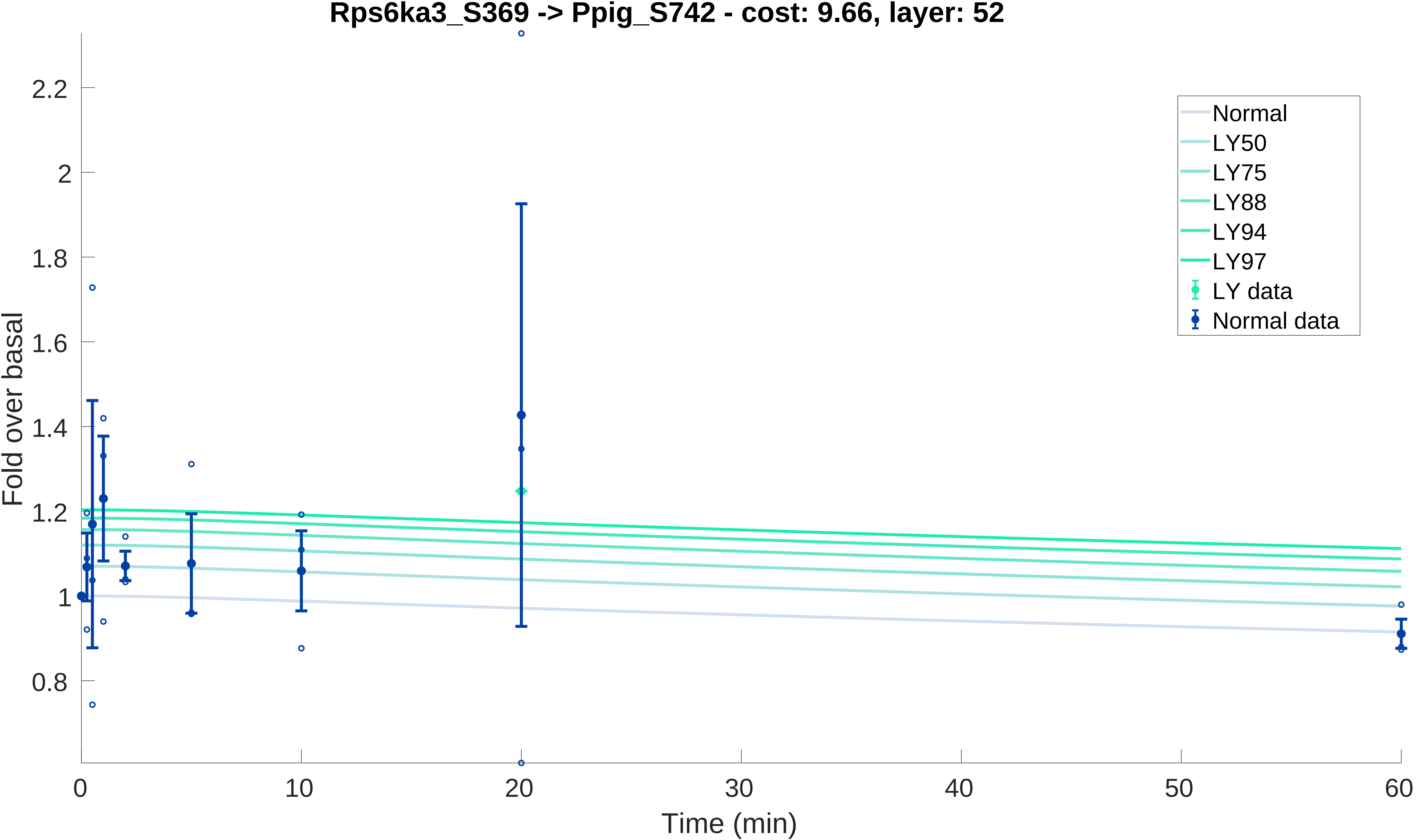

Rps6ka3_S369 -> Ppig_S742 - cost: 9.66, layer: 52
Fold over basal
Time (min)
0.8
1
1.2
1.4
1.6
1.8
2
2.2
0
10
20
30
40
50
60
Normal
LY50
LY75
LY88
LY94
LY97
LY data
Normal data

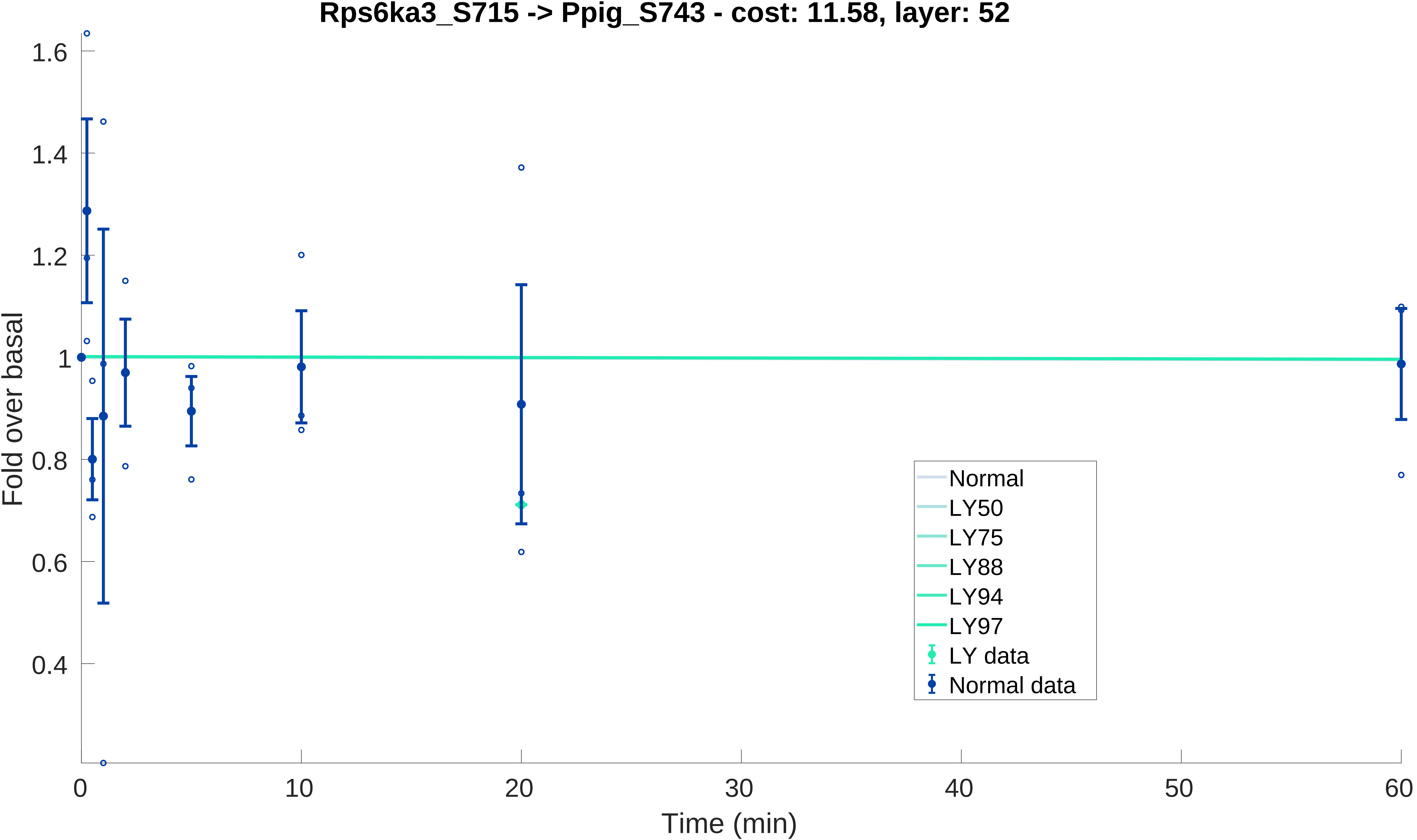
Rps6ka3_S715 -> Ppig_S743 - cost: 11.58, layer: 52
Fold over basal
1.6
1.4
1.2
1
0.8
0.6
0.4
0
10
20
30
40
50
60
Time (min)
Normal
LY50
LY75
LY88
LY94
LY97
LY data
Normal data

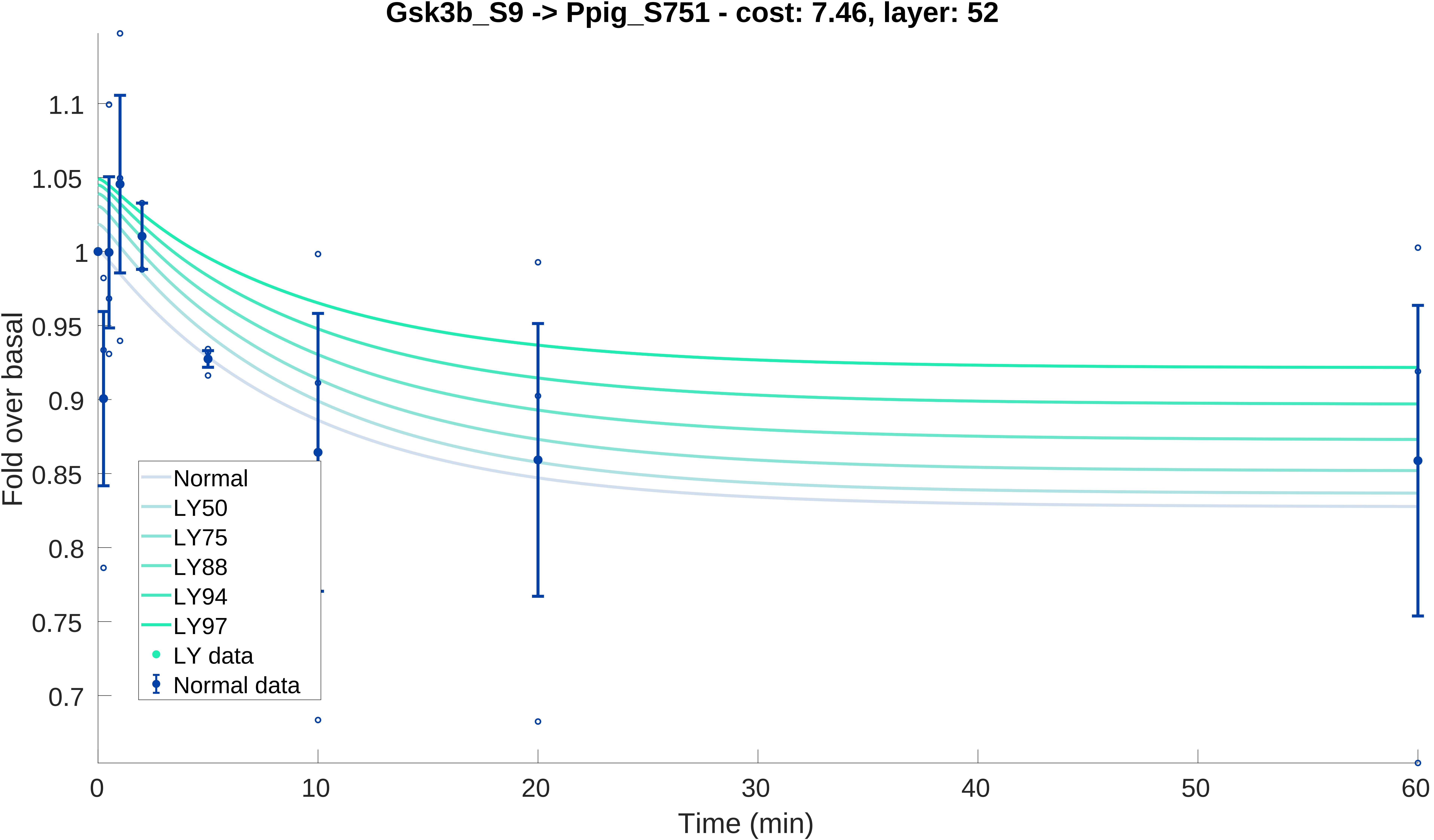

Gsk3b_S9 -> Ppig_S751 - cost: 7.46, layer: 52
Fold over basal
1.1
1.05
1
0.95
0.9
0.85
0.8
0.75
0.7
Time (min)
0
10
20
30
40
50
60
Normal
LY50
LY75
LY88
LY94
LY97
LY data
Normal data

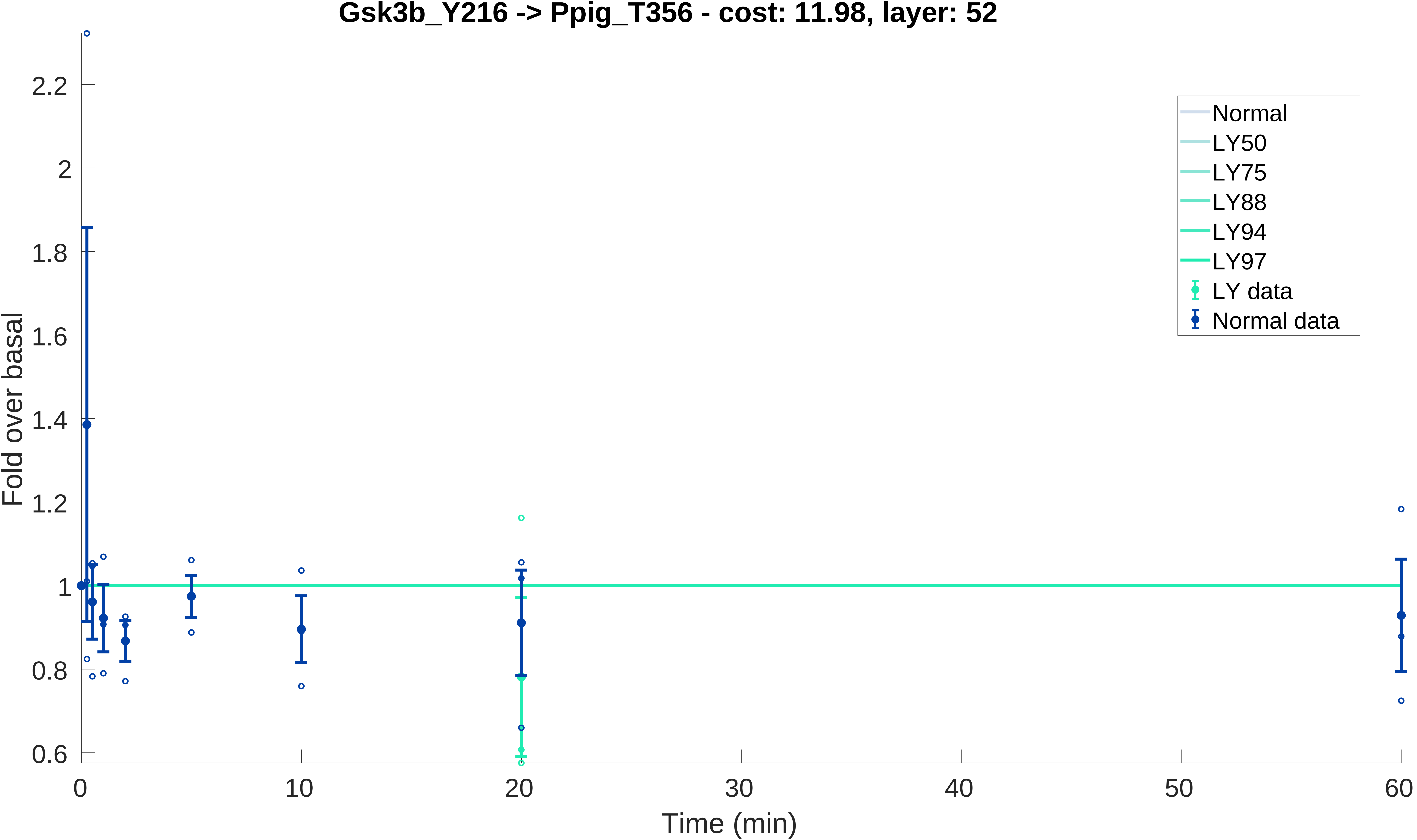

Gsk3b_Y216 -> Ppig_T356 - cost: 11.98, layer: 52
Fold over basal
Time (min)
Normal
LY50
LY75
LY88
LY94
LY97
LY data
Normal data
0.6
0.8
1
1.2
1.4
1.6
1.8
2
2.2
0
10
20
30
40
50
60

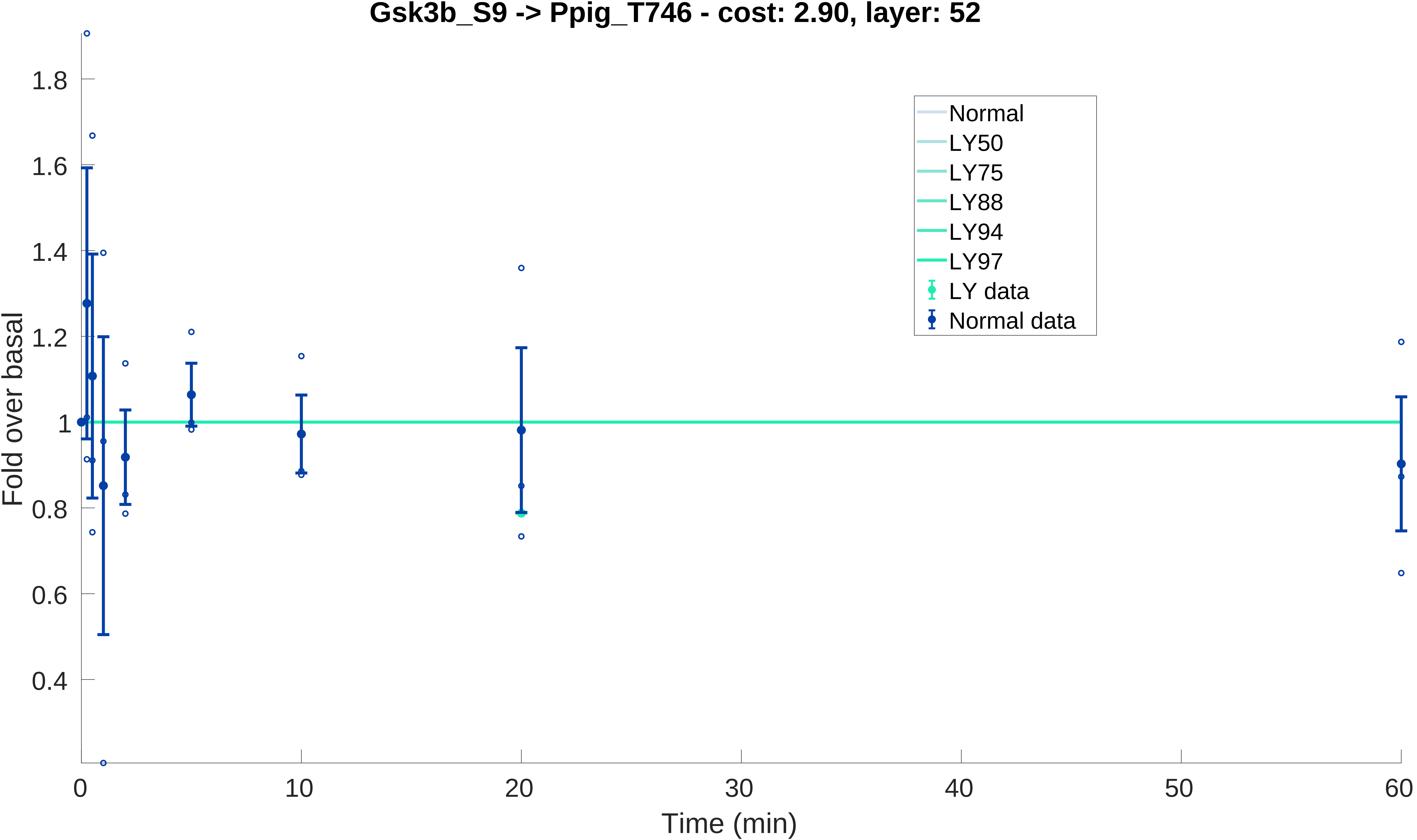

Gsk3b_S9 -> Ppig_T746 - cost: 2.90, layer: 52
Fold over basal
1.8
1.6
1.4
1.2
1
0.8
0.6
0.4
0
10
20
30
40
50
60
Time (min)
Normal
LY50
LY75
LY88
LY94
LY97
LY data
Normal data

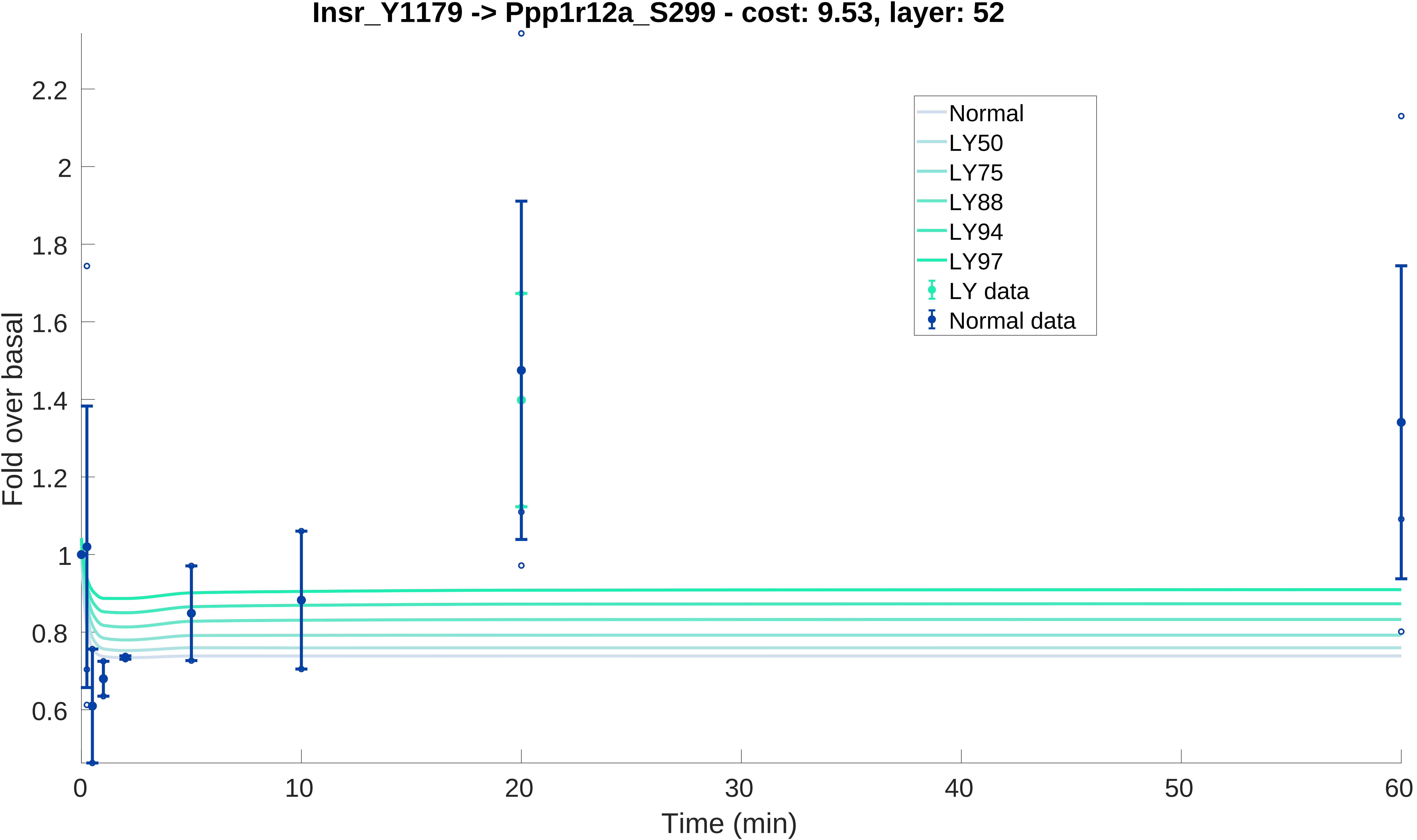

Insr_Y1179 -> Ppp1r12a_S299 - cost: 9.53, layer: 52
Normal
LY50
LY75
LY88
LY94
LY97
LY data
Normal data
Fold over basal
Time (min)
2.2
2
1.8
1.6
1.4
1.2
1
0.8
0.6
0
10
20
30
40
50
60

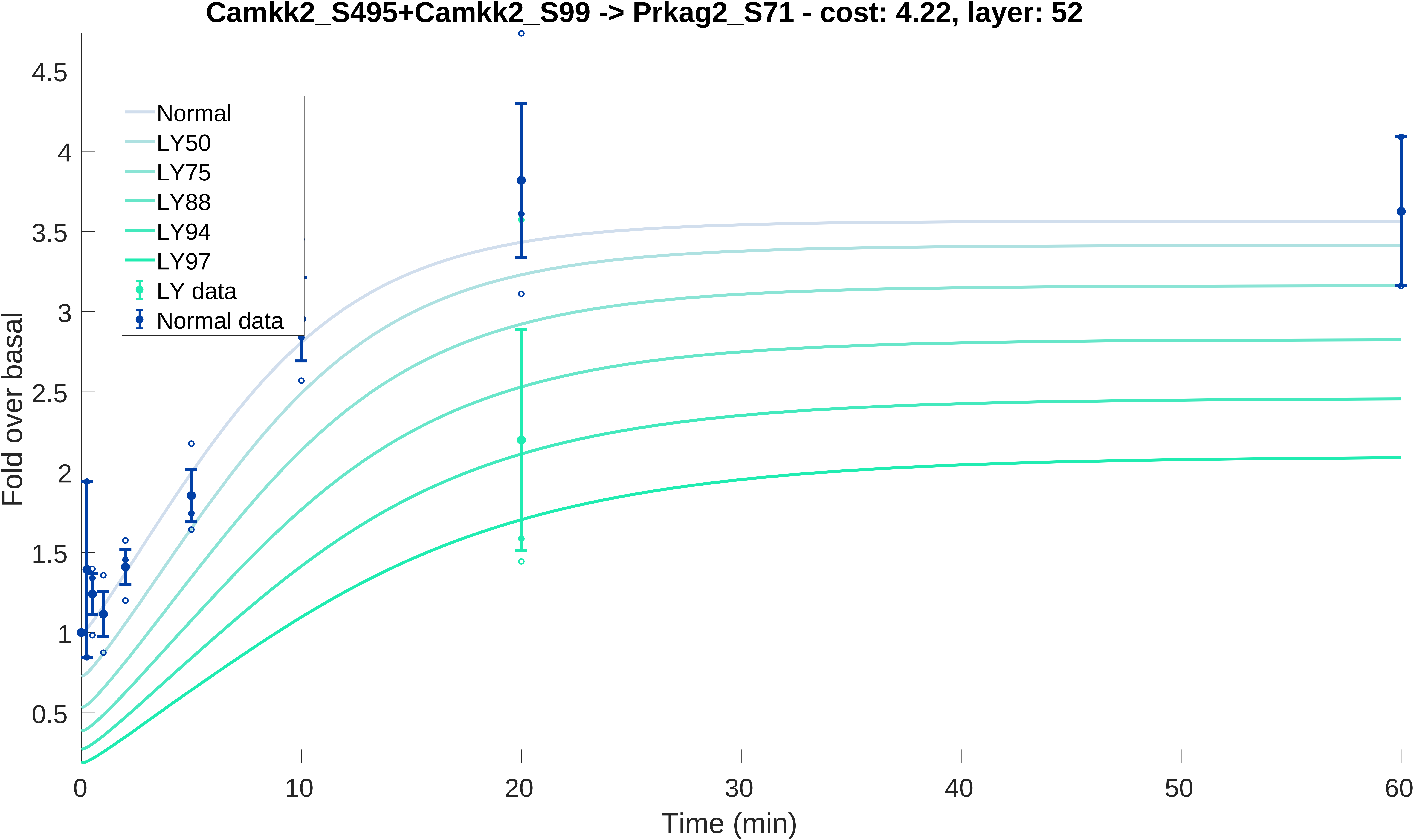
Camkk2_S495+Camkk2_S99 -> Prkag2_S71 - cost: 4.22, layer: 52
Normal
LY50
LY75
LY88
LY94
LY97
LY data
Normal data
Fold over basal
Time (min)
0.5
1
1.5
2
2.5
3
3.5
4
4.5
0
10
20
30
40
50
60

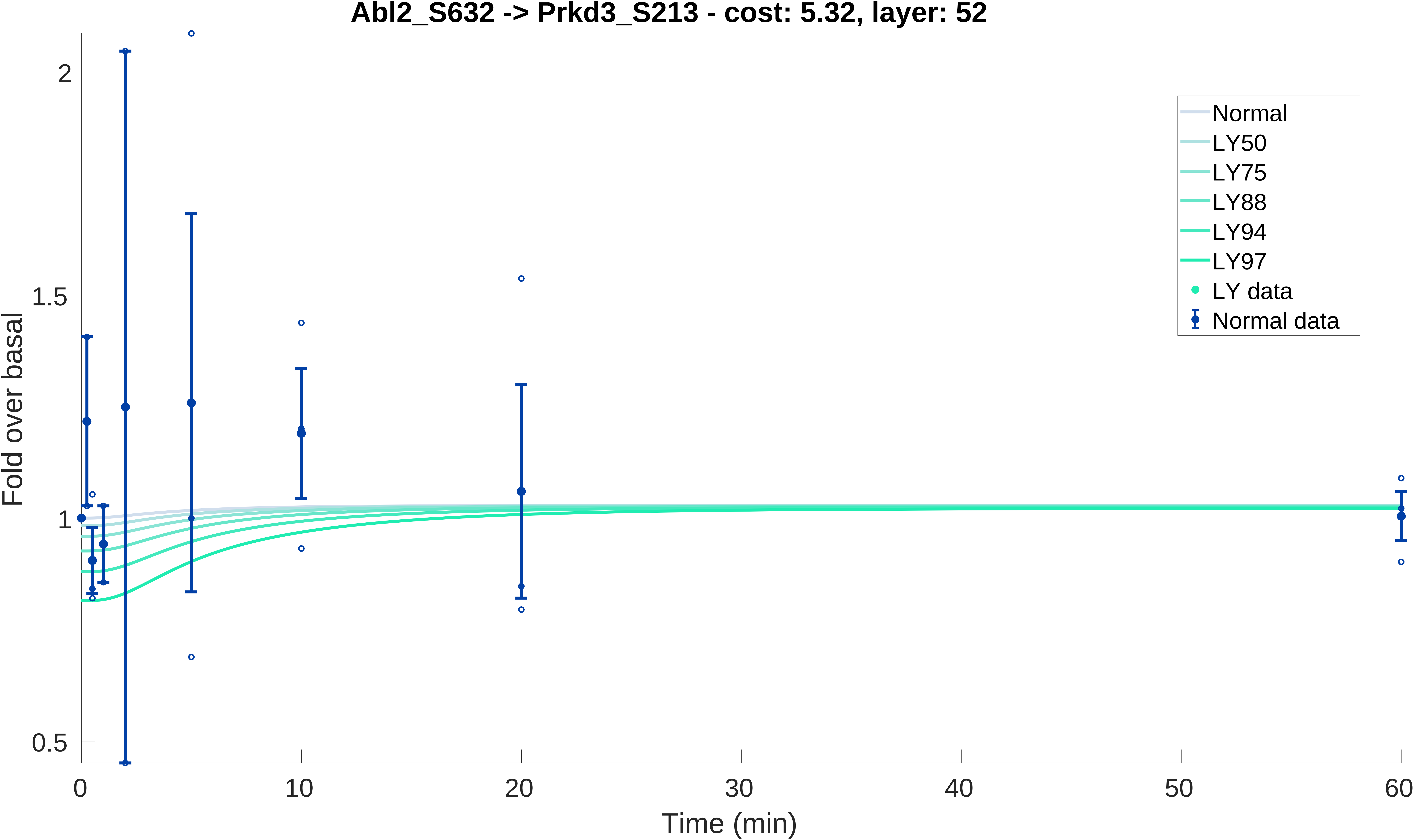
Abl2_S632 -> Prkd3_S213 - cost: 5.32, layer: 52
Fold over basal
Time (min)
0
10
20
30
40
50
60
0.5
1
1.5
2
Normal
LY50
LY75
LY88
LY94
LY97
LY data
Normal data

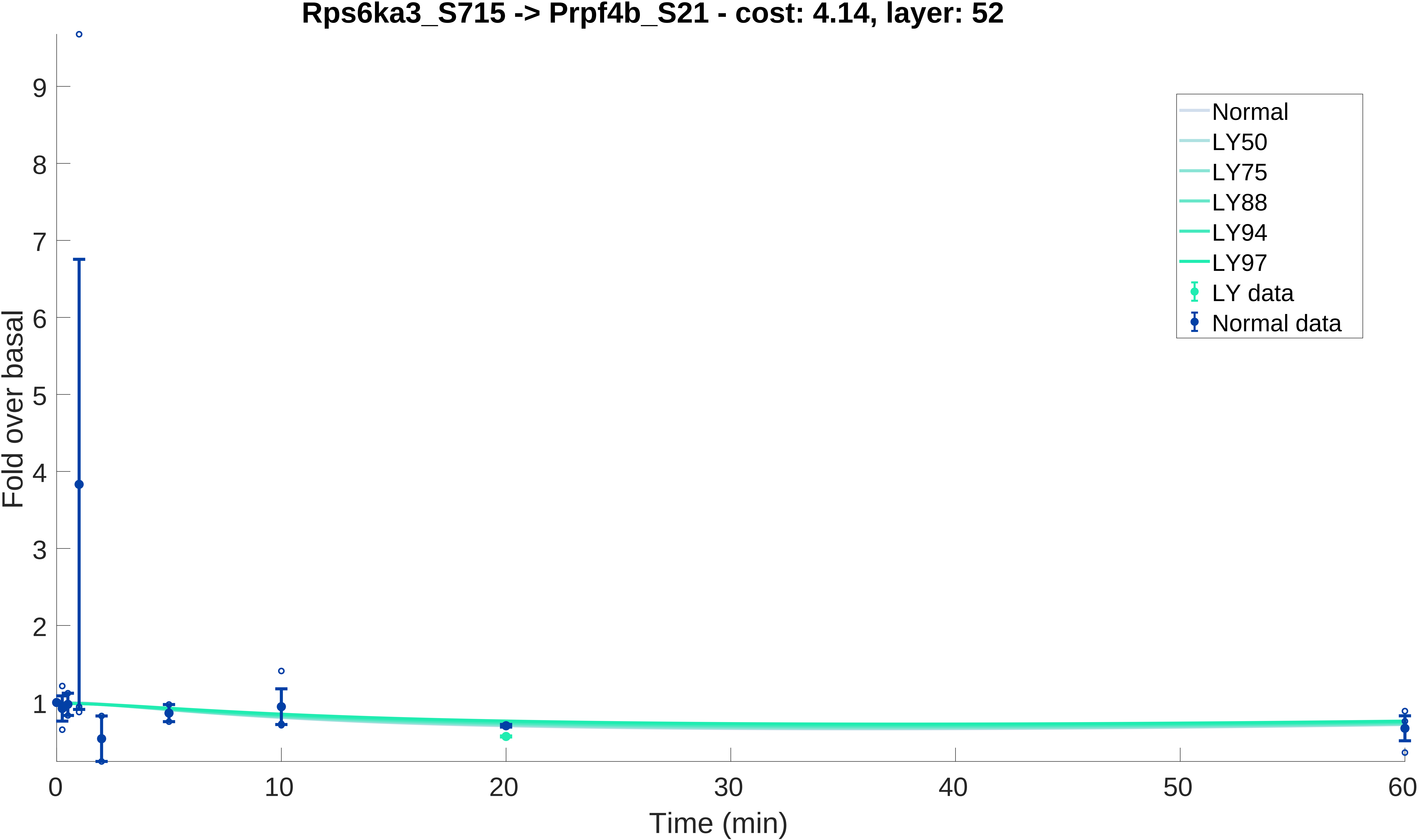
Rps6ka3_S715 -> Prpf4b_S21 - cost: 4.14, layer: 52
Fold over basal
Time (min)
0
10
20
30
40
50
60
1
2
3
4
5
6
7
8
9
Normal
LY50
LY75
LY88
LY94
LY97
LY data
Normal data

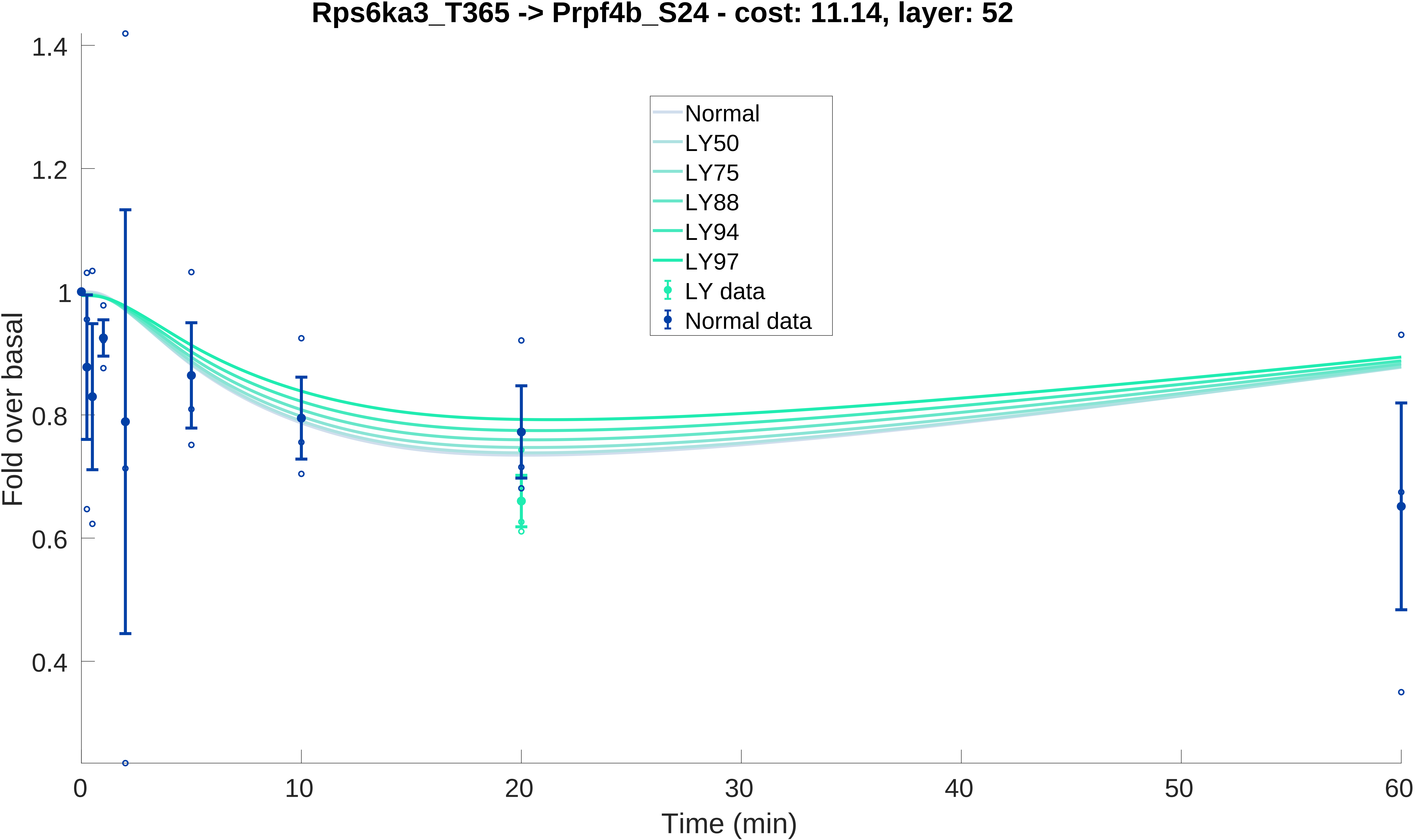

Rps6ka3_T365 -> Prpf4b_S24 - cost: 11.14, layer: 52
Fold over basal
Time (min)
1.4
1.2
1
0.8
0.6
0.4
0
10
20
30
40
50
60
Normal
LY50
LY75
LY88
LY94
LY97
LY data
Normal data

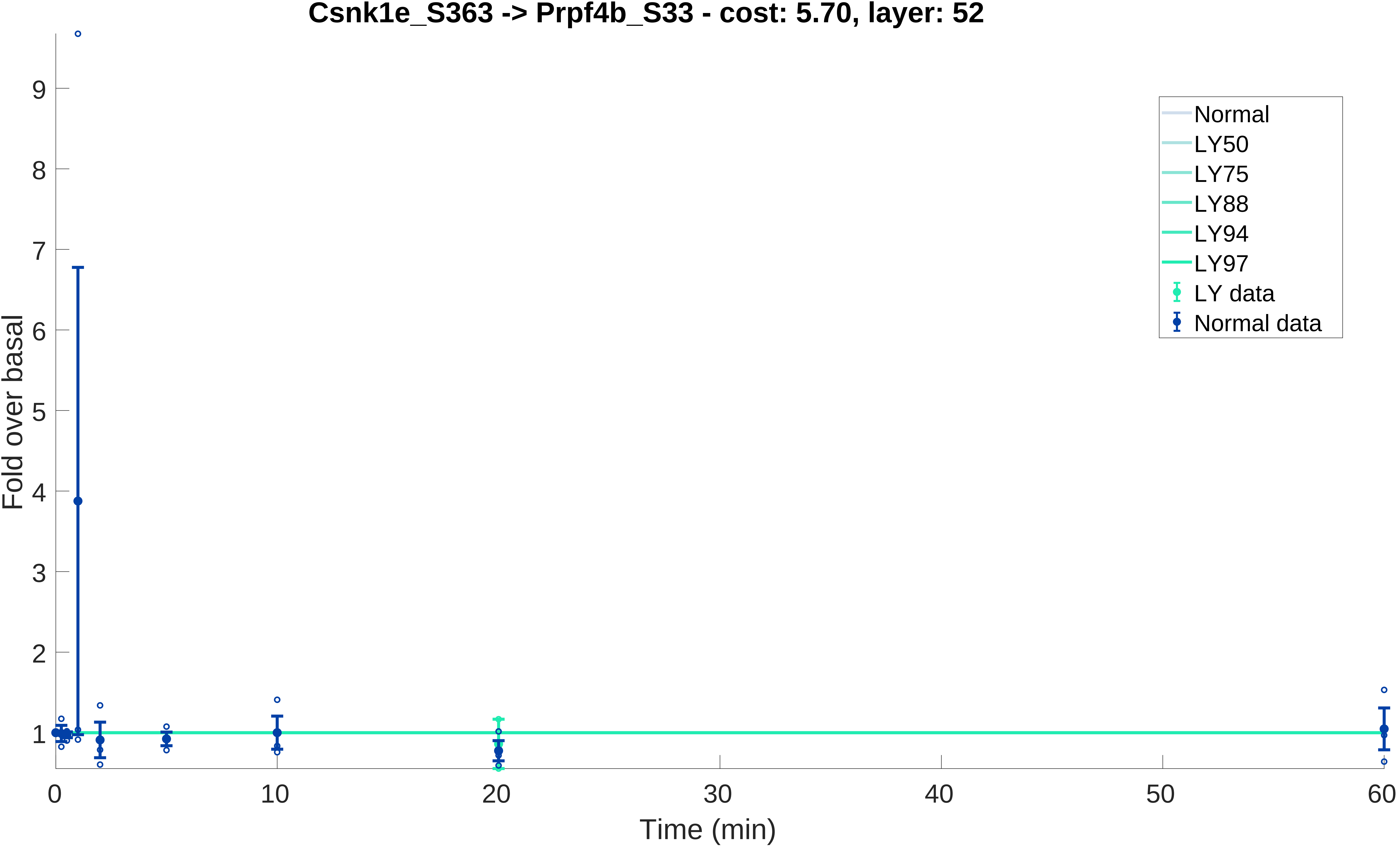

Csnk1e_S363 -> Prpf4b_S33 - cost: 5.70, layer: 52
Fold over basal
Time (min)
0
10
20
30
40
50
60
1
2
3
4
5
6
7
8
9
Normal
LY50
LY75
LY88
LY94
LY97
LY data
Normal data

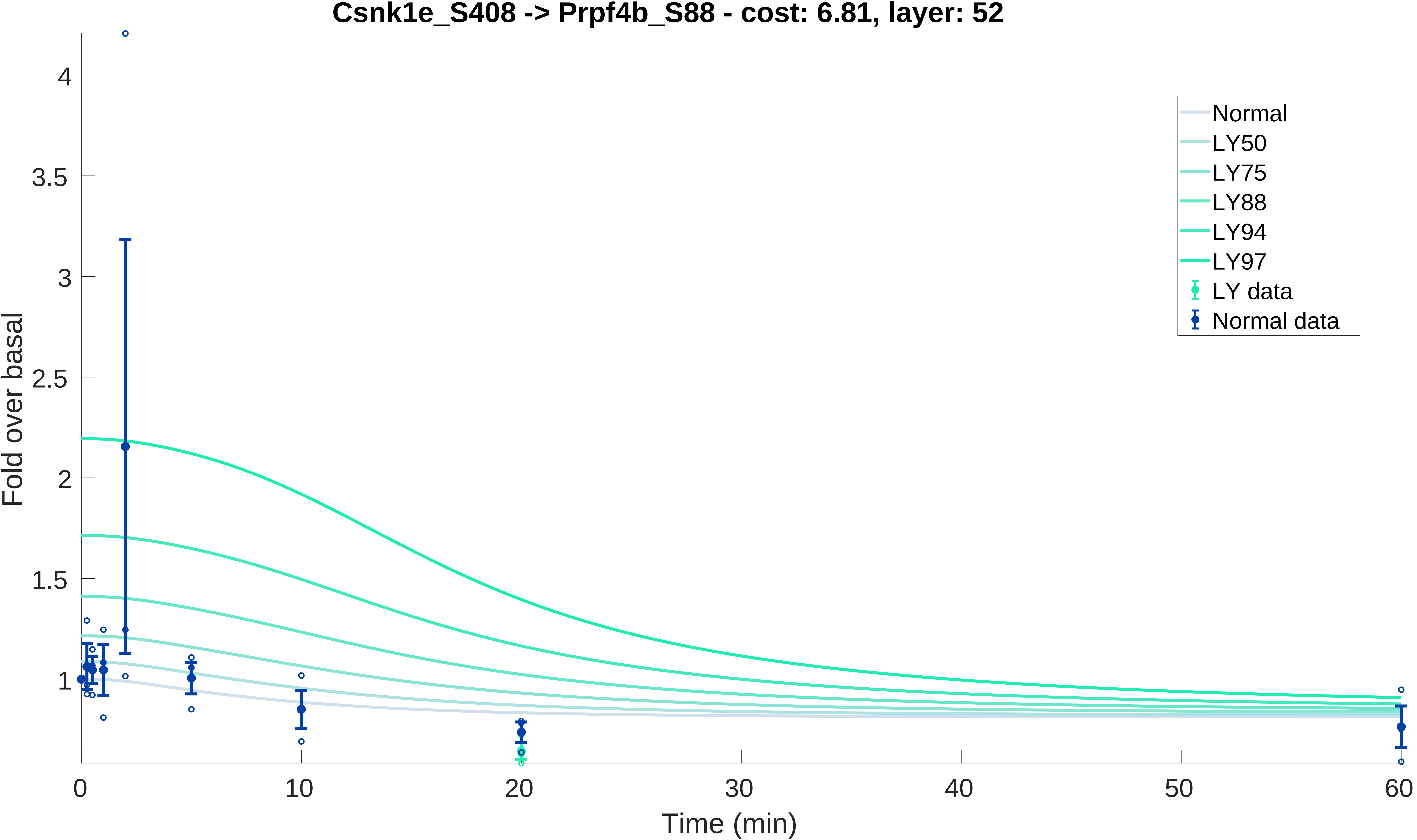

Csnk1e_S408 -> Prpf4b_S88 - cost: 6.81, layer: 52
Fold over basal
Time (min)
Normal
LY50
LY75
LY88
LY94
LY97
LY data
Normal data
0
10
20
30
40
50
60
1
1.5
2
2.5
3
3.5
4

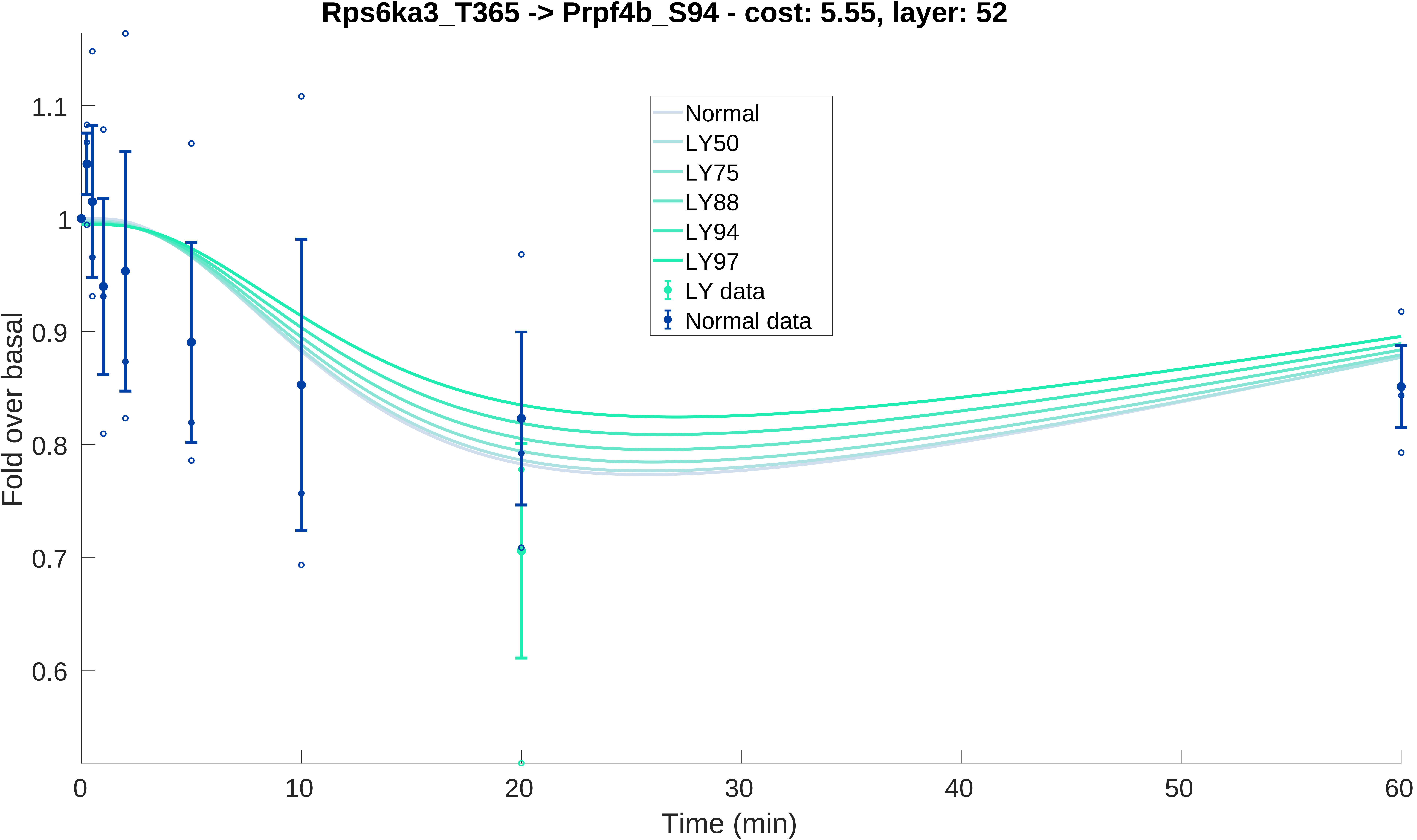

Rps6ka3_T365 -> Prpf4b_S94 - cost: 5.55, layer: 52
Fold over basal
Time (min)
0.6
0.7
0.8
0.9
1
1.1
0
10
20
30
40
50
60
Normal
LY50
LY75
LY88
LY94
LY97
LY data
Normal data

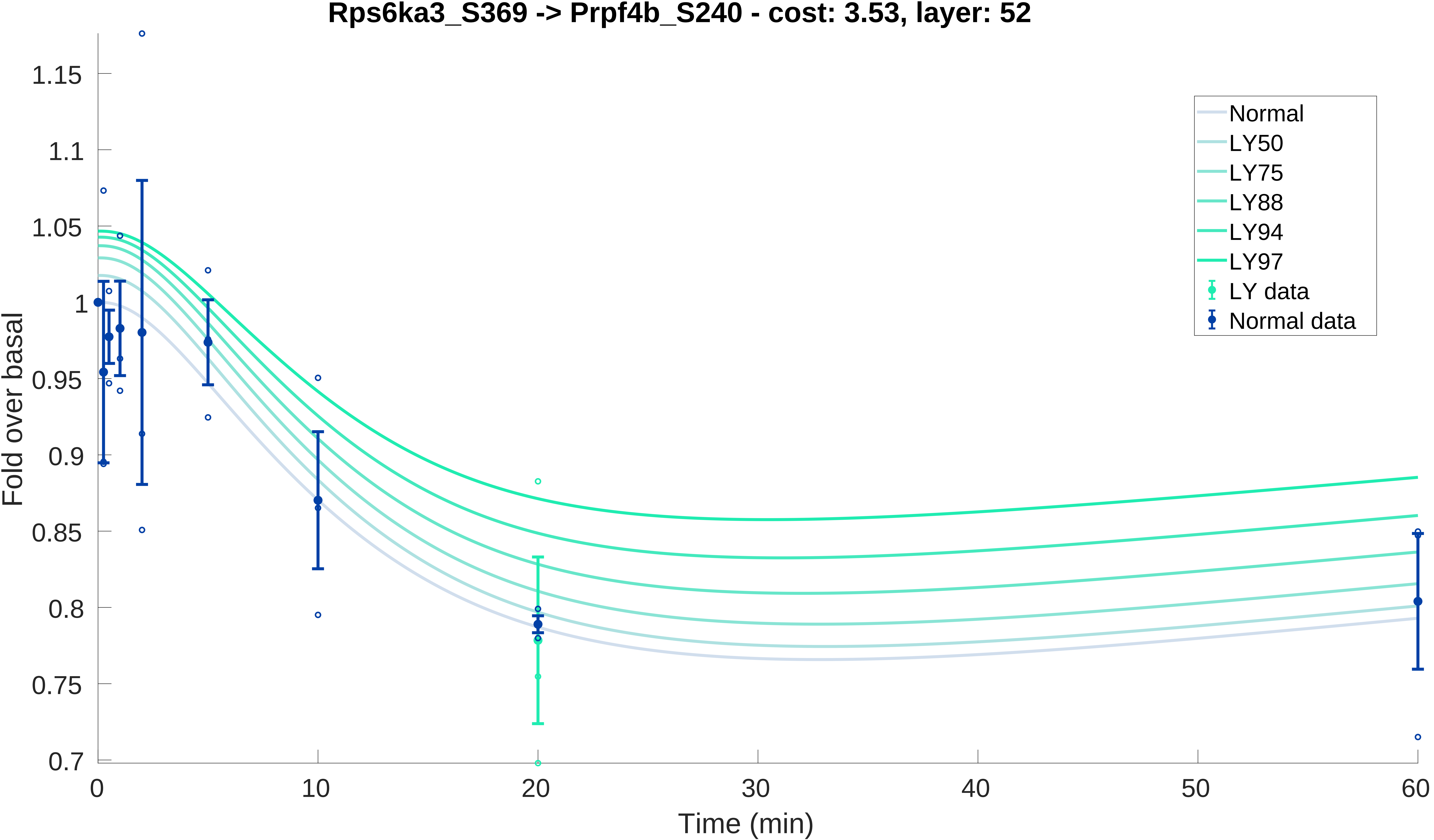

Rps6ka3_S369 -> Prpf4b_S240 - cost: 3.53, layer: 52
Fold over basal
Time (min)
Normal
LY50
LY75
LY88
LY94
LY97
LY data
Normal data
0.7
0.75
0.8
0.85
0.9
0.95
1
1.05
1.1
1.15
0
10
20
30
40
50
60

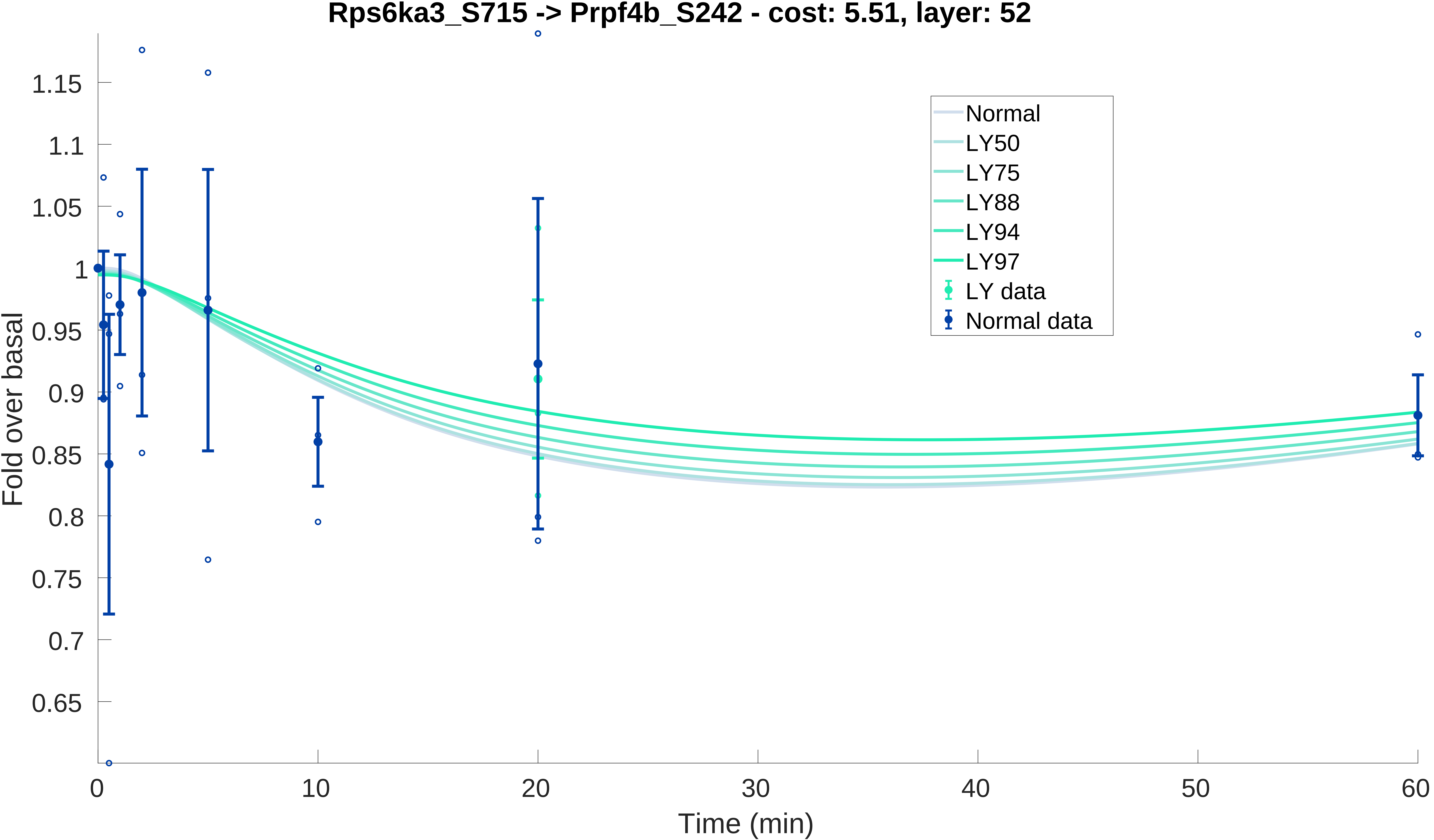

Rps6ka3_S715 -> Prpf4b_S242 - cost: 5.51, layer: 52
Fold over basal
1.15
1.1
1.05
1
0.95
0.9
0.85
0.8
0.75
0.7
0.65
0
10
20
30
40
50
60
Time (min)
Normal
LY50
LY75
LY88
LY94
LY97
LY data
Normal data

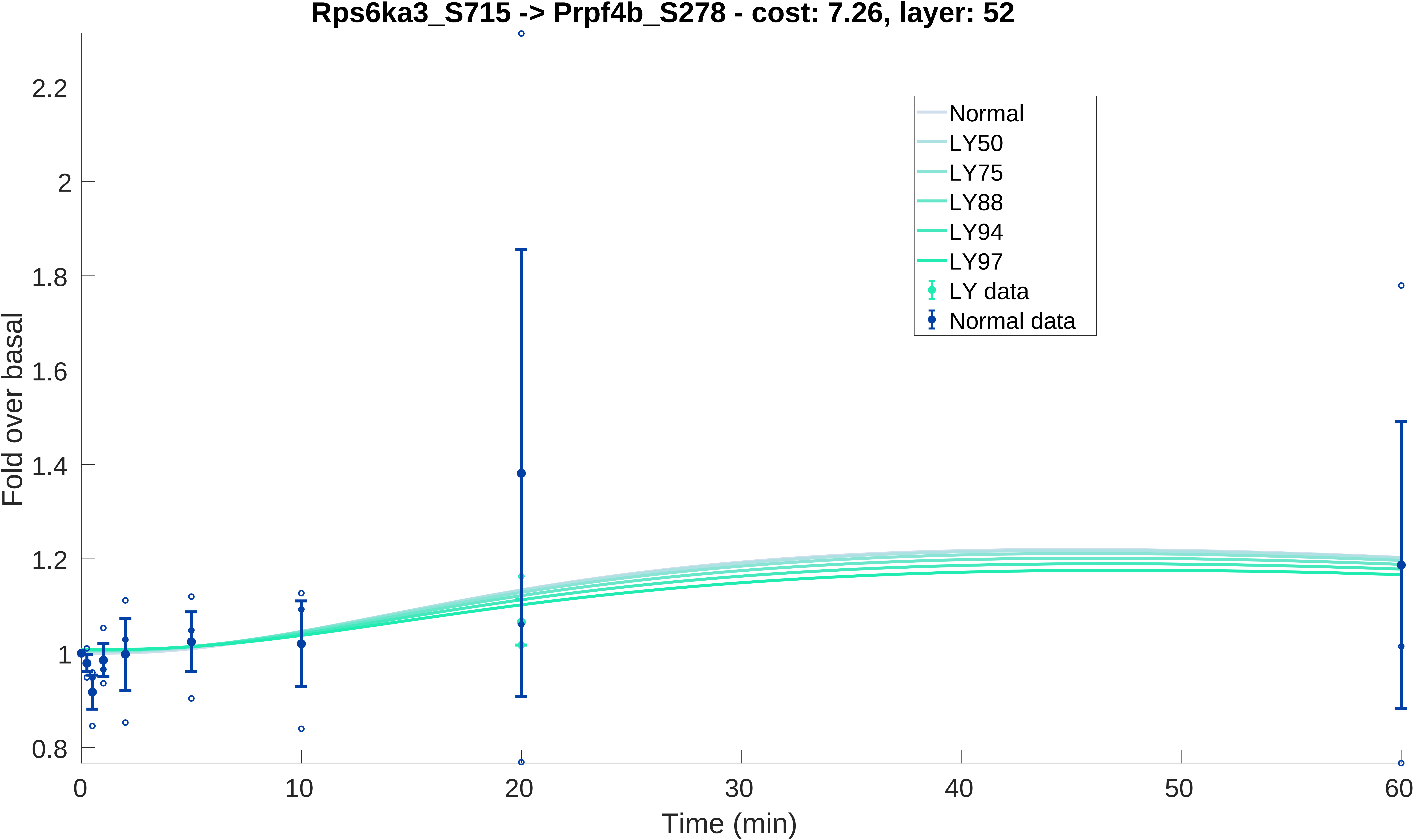
Rps6ka3_S715 -> Prpf4b_S278 - cost: 7.26, layer: 52
Fold over basal
Time (min)
0.8
1
1.2
1.4
1.6
1.8
2
2.2
0
10
20
30
40
50
60
Normal
LY50
LY75
LY88
LY94
LY97
LY data
Normal data

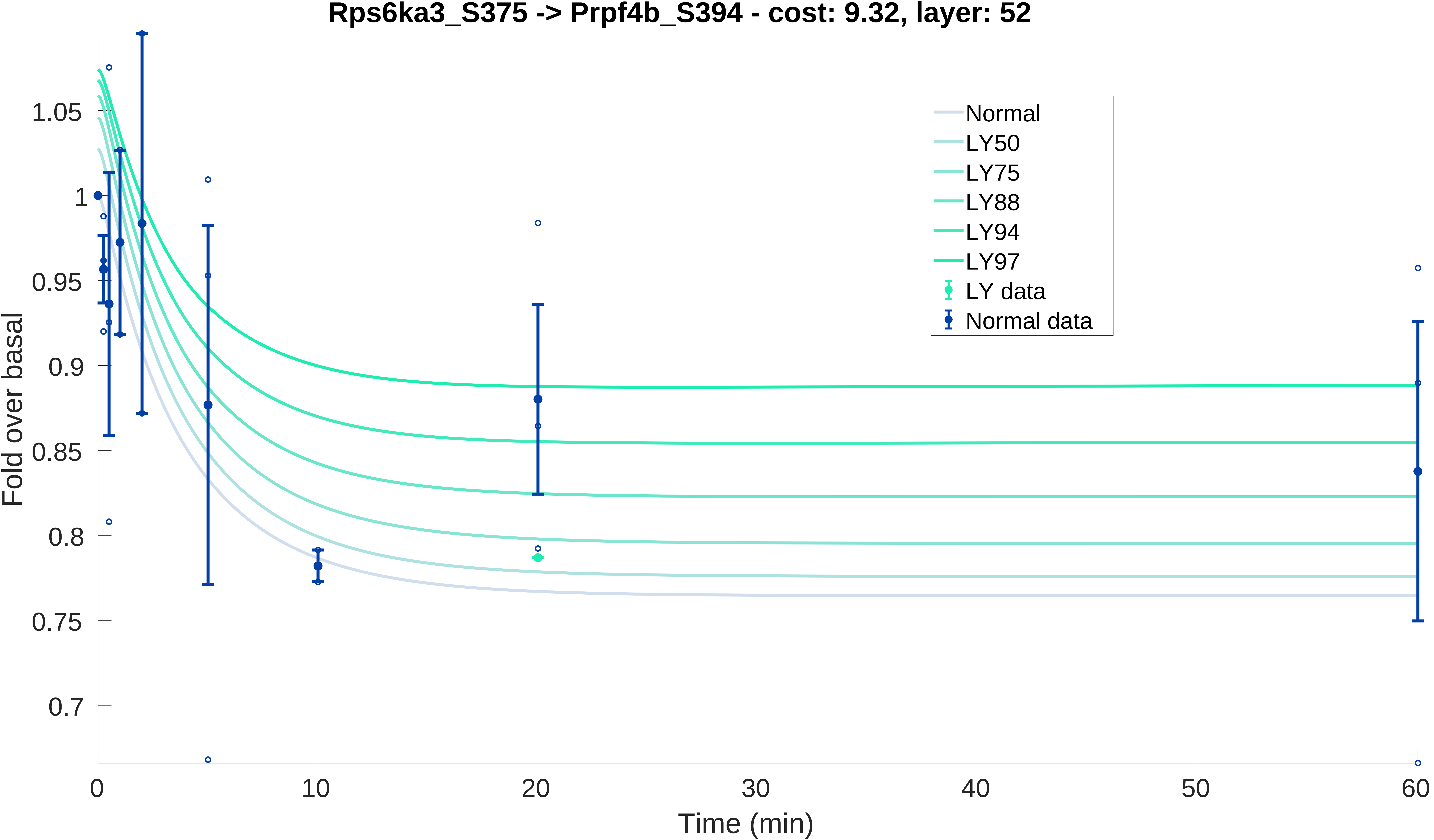

Rps6ka3_S375 -> Prpf4b_S394 - cost: 9.32, layer: 52
Fold over basal
Time (min)
Normal
LY50
LY75
LY88
LY94
LY97
LY data
Normal data
1.05
1
0.95
0.9
0.85
0.8
0.75
0.7
0
10
20
30
40
50
60

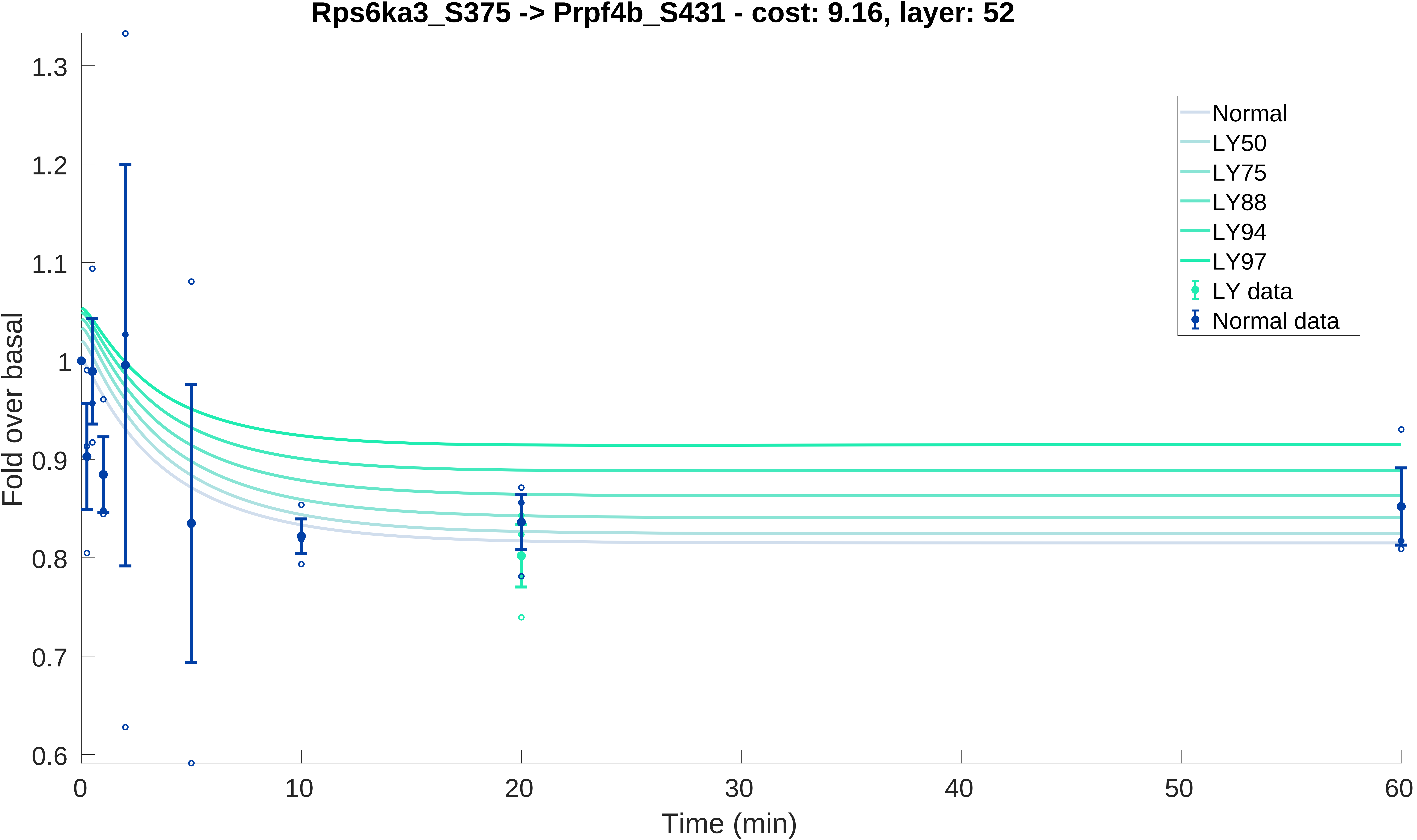

Rps6ka3_S375 -> Prpf4b_S431 - cost: 9.16, layer: 52
Fold over basal
Time (min)
Normal
LY50
LY75
LY88
LY94
LY97
LY data
Normal data
1.3
1.2
1.1
1
0.9
0.8
0.7
0.6
0
10
20
30
40
50
60

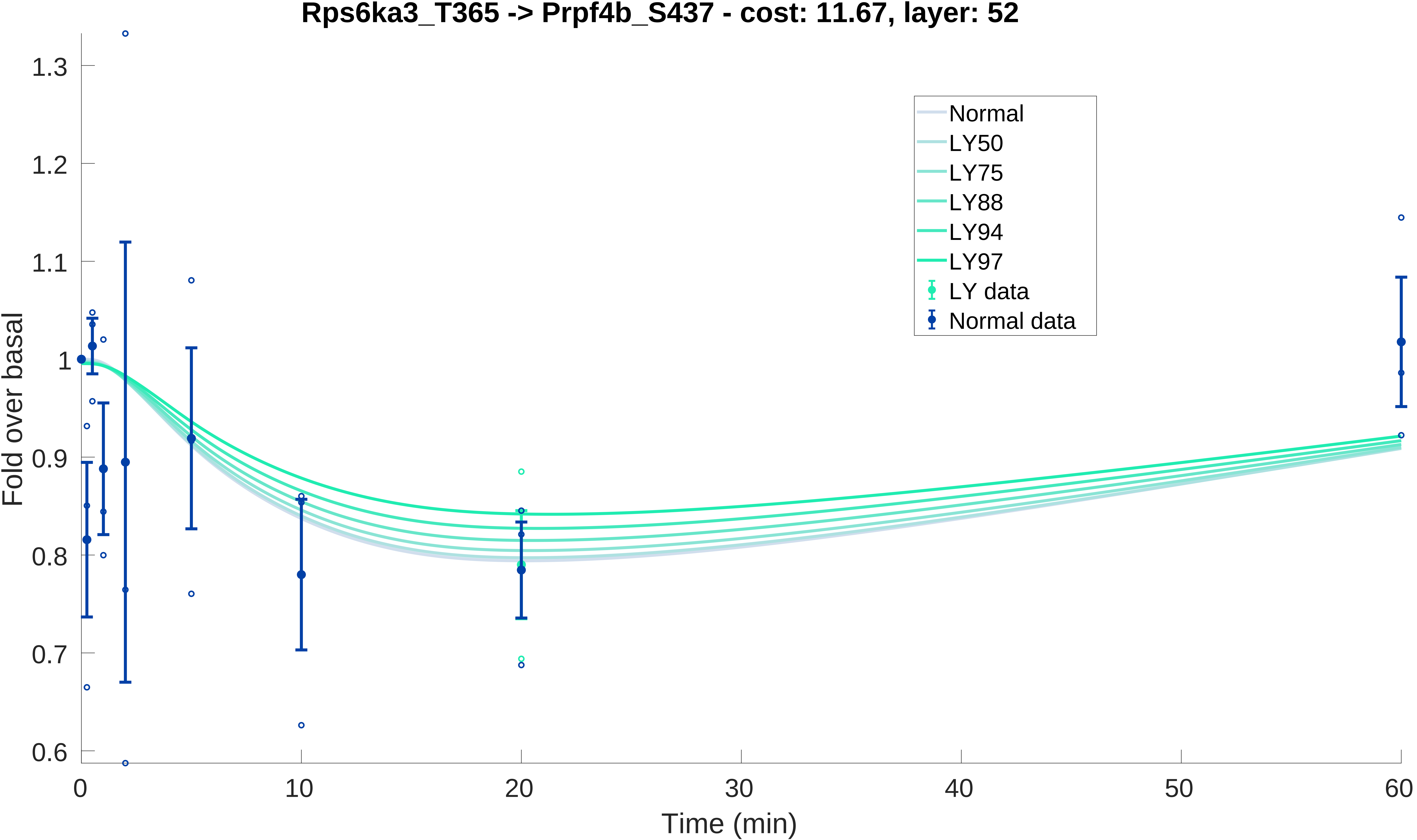

Rps6ka3_T365 -> Prpf4b_S437 - cost: 11.67, layer: 52
Fold over basal
Time (min)
1.3
1.2
1.1
1
0.9
0.8
0.7
0.6
0
10
20
30
40
50
60
Normal
LY50
LY75
LY88
LY94
LY97
LY data
Normal data

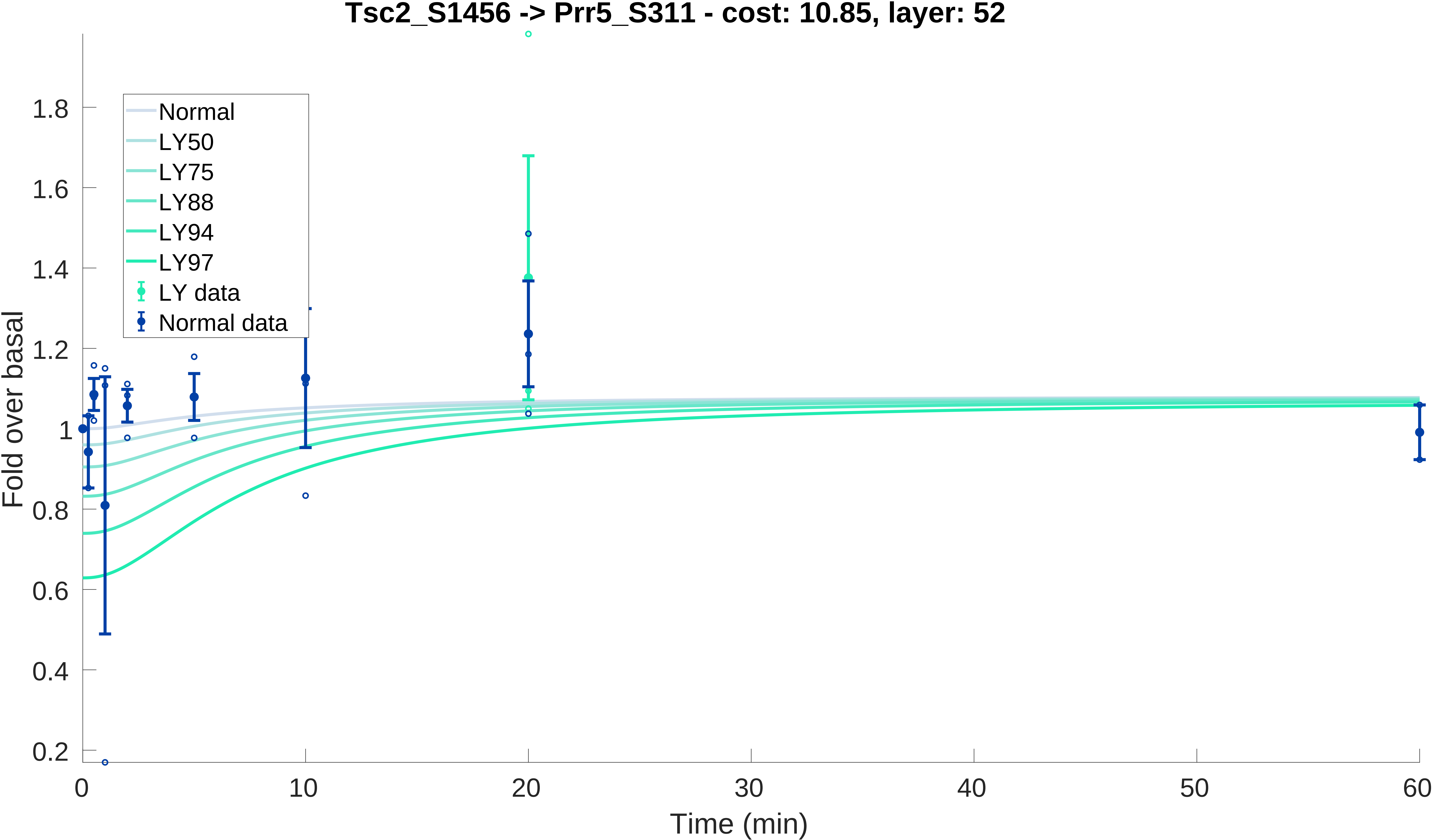

Tsc2_S1456 -> Prr5_S311 - cost: 10.85, layer: 52
Normal
LY50
LY75
LY88
LY94
LY97
LY data
Normal data
Fold over basal
Time (min)
0.2
0.4
0.6
0.8
1
1.2
1.4
1.6
1.8
0
10
20
30
40
50
60

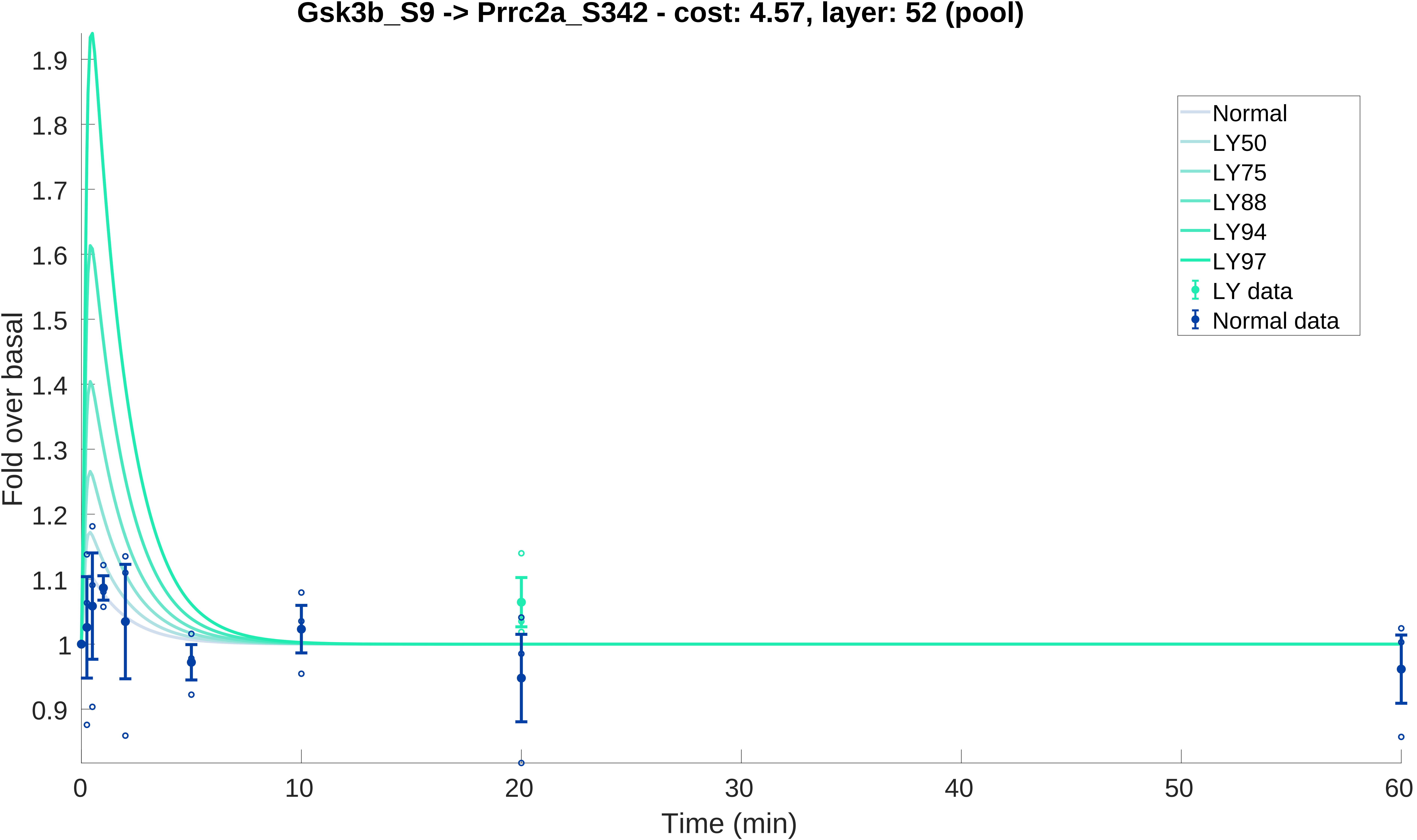

Gsk3b_S9 -> Prrc2a_S342 - cost: 4.57, layer: 52 (pool)
Fold over basal
Time (min)
0
10
20
30
40
50
60
0.9
1
1.1
1.2
1.3
1.4
1.5
1.6
1.7
1.8
1.9
Normal
LY50
LY75
LY88
LY94
LY97
LY data
Normal data

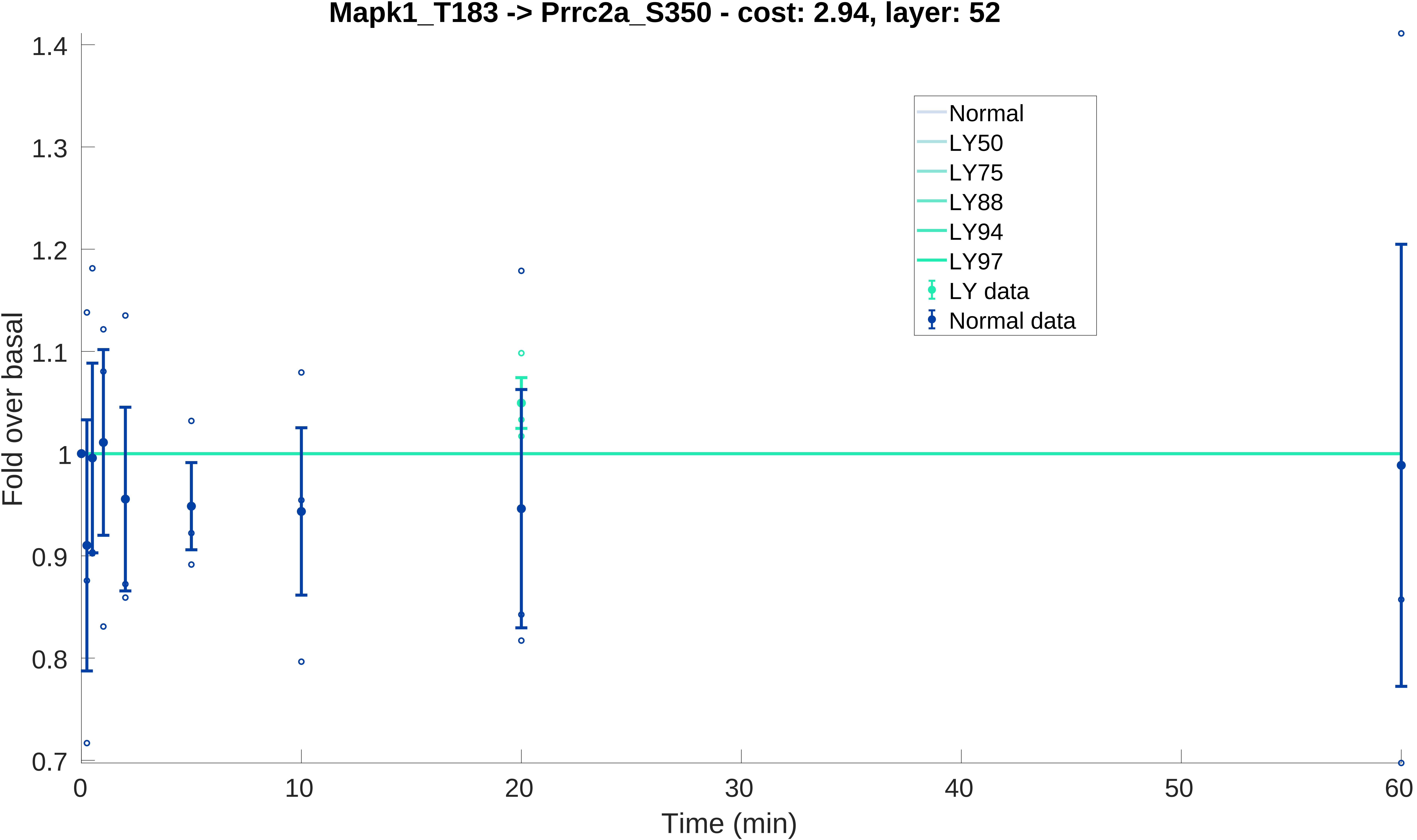

Mapk1_T183 -> Prrc2a_S350 - cost: 2.94, layer: 52
Fold over basal
Time (min)
1.4
1.3
1.2
1.1
1
0.9
0.8
0.7
0
10
20
30
40
50
60
Normal
LY50
LY75
LY88
LY94
LY97
LY data
Normal data

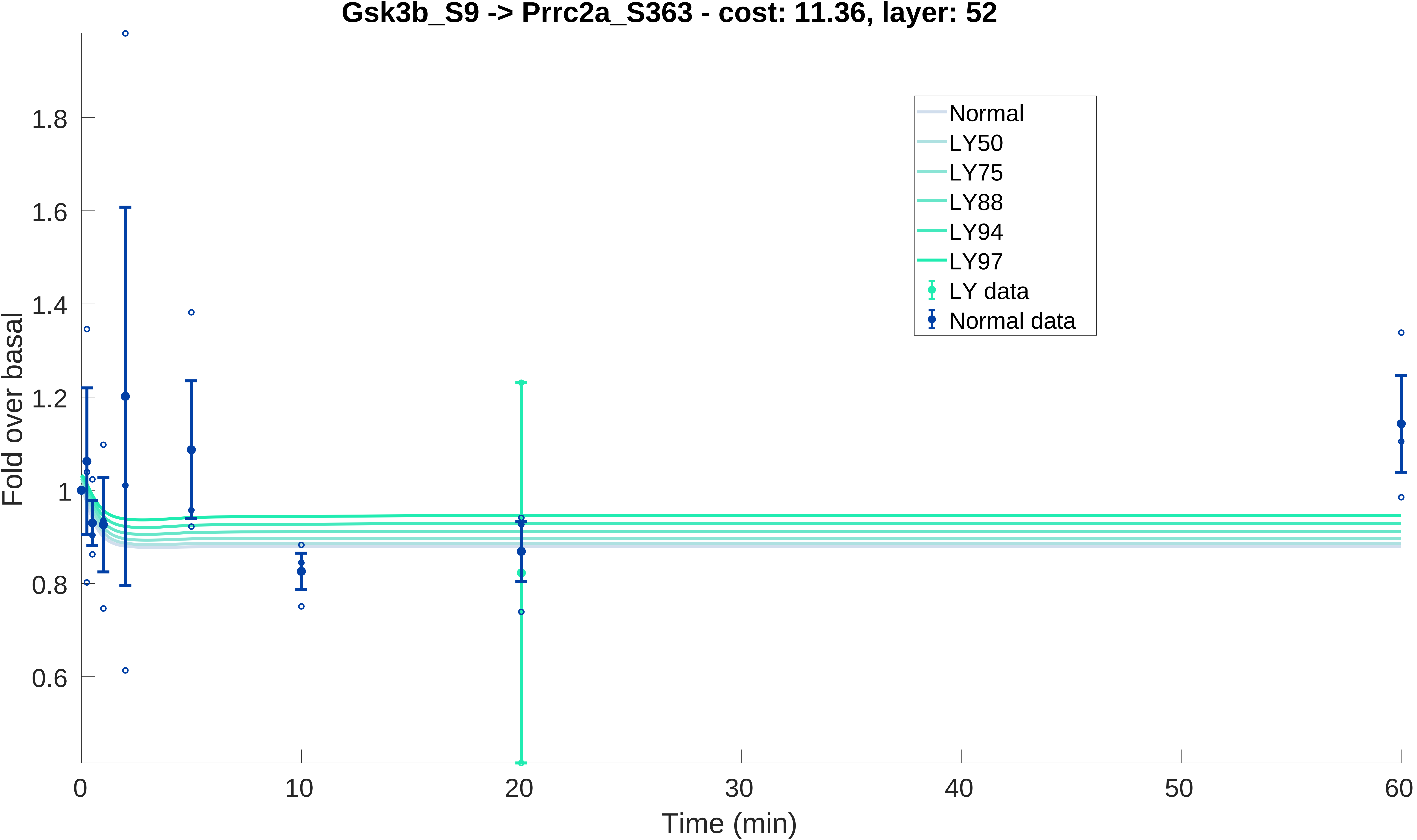

Gsk3b_S9 -> Prrc2a_S363 - cost: 11.36, layer: 52
Normal
LY50
LY75
LY88
LY94
LY97
LY data
Normal data
Fold over basal
Time (min)
1.8
1.6
1.4
1.2
1
0.8
0.6
0
10
20
30
40
50
60

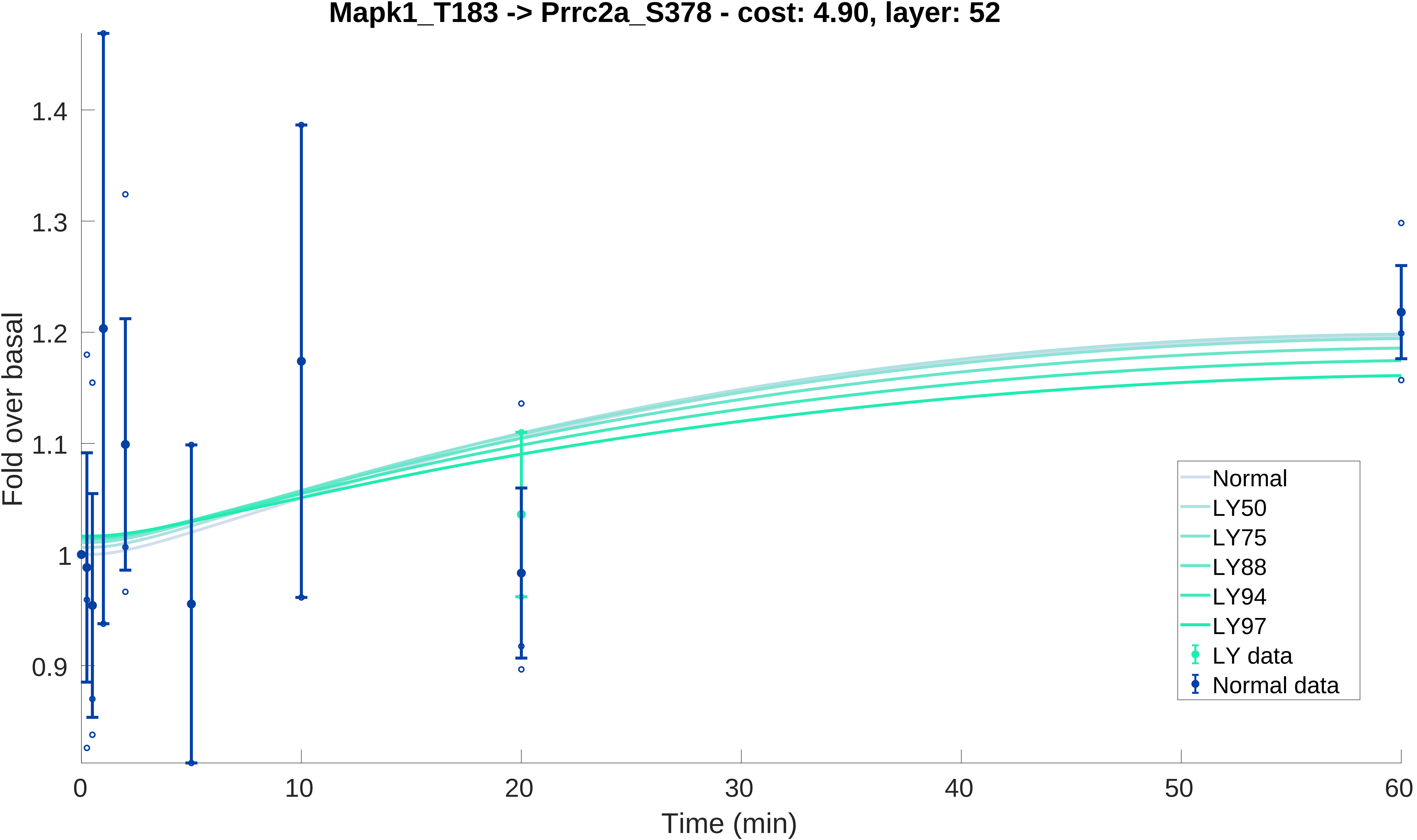
Mapk1_T183 -> Prrc2a_S378 - cost: 4.90, layer: 52
Fold over basal
Time (min)
1.4
1.3
1.2
1.1
1
0.9
0
10
20
30
40
50
60
Normal
LY50
LY75
LY88
LY94
LY97
LY data
Normal data

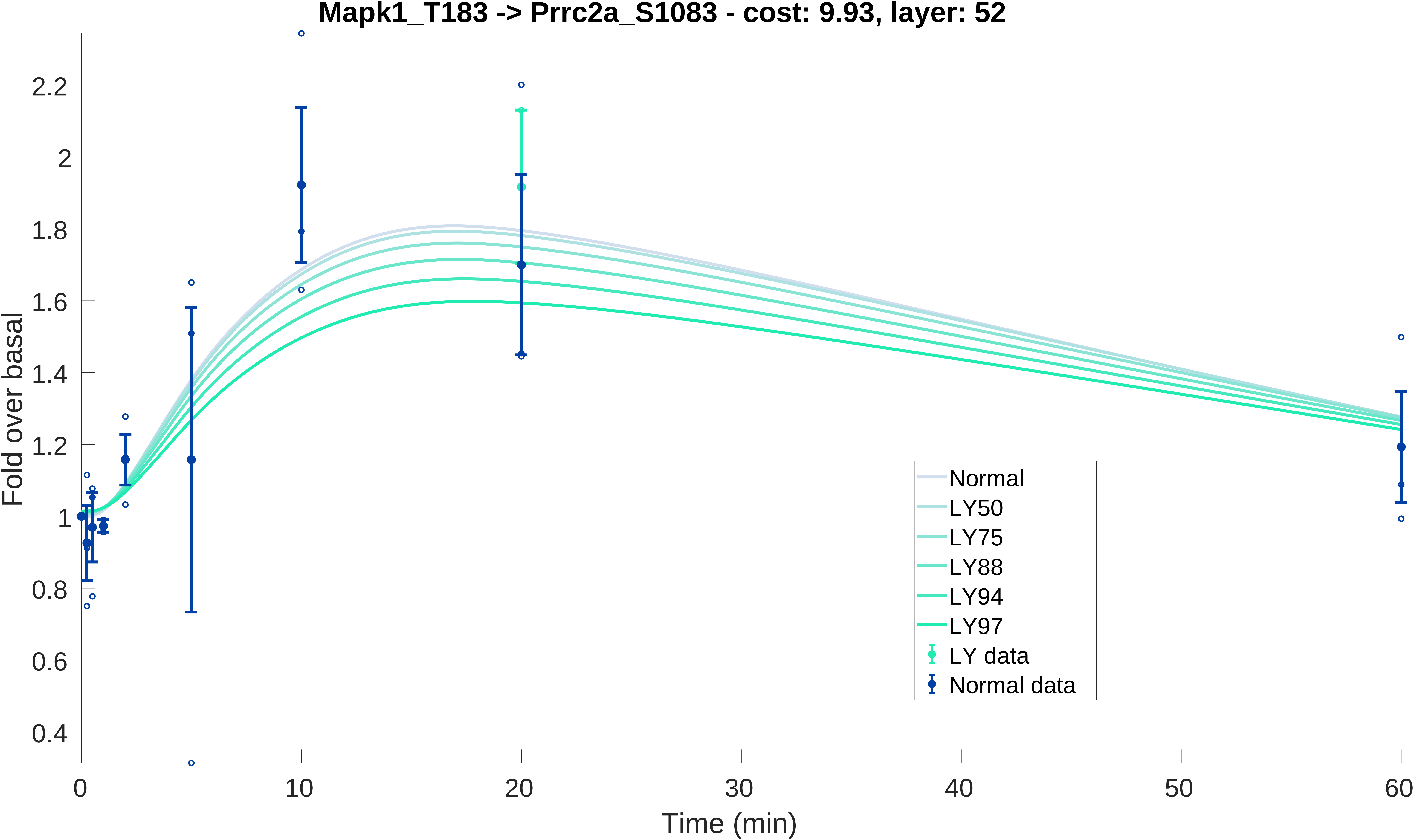

Mapk1_T183 -> Prrc2a_S1083 - cost: 9.93, layer: 52
Fold over basal
Time (min)
0
10
20
30
40
50
60
0.4
0.6
0.8
1
1.2
1.4
1.6
1.8
2
2.2
Normal
LY50
LY75
LY88
LY94
LY97
LY data
Normal data

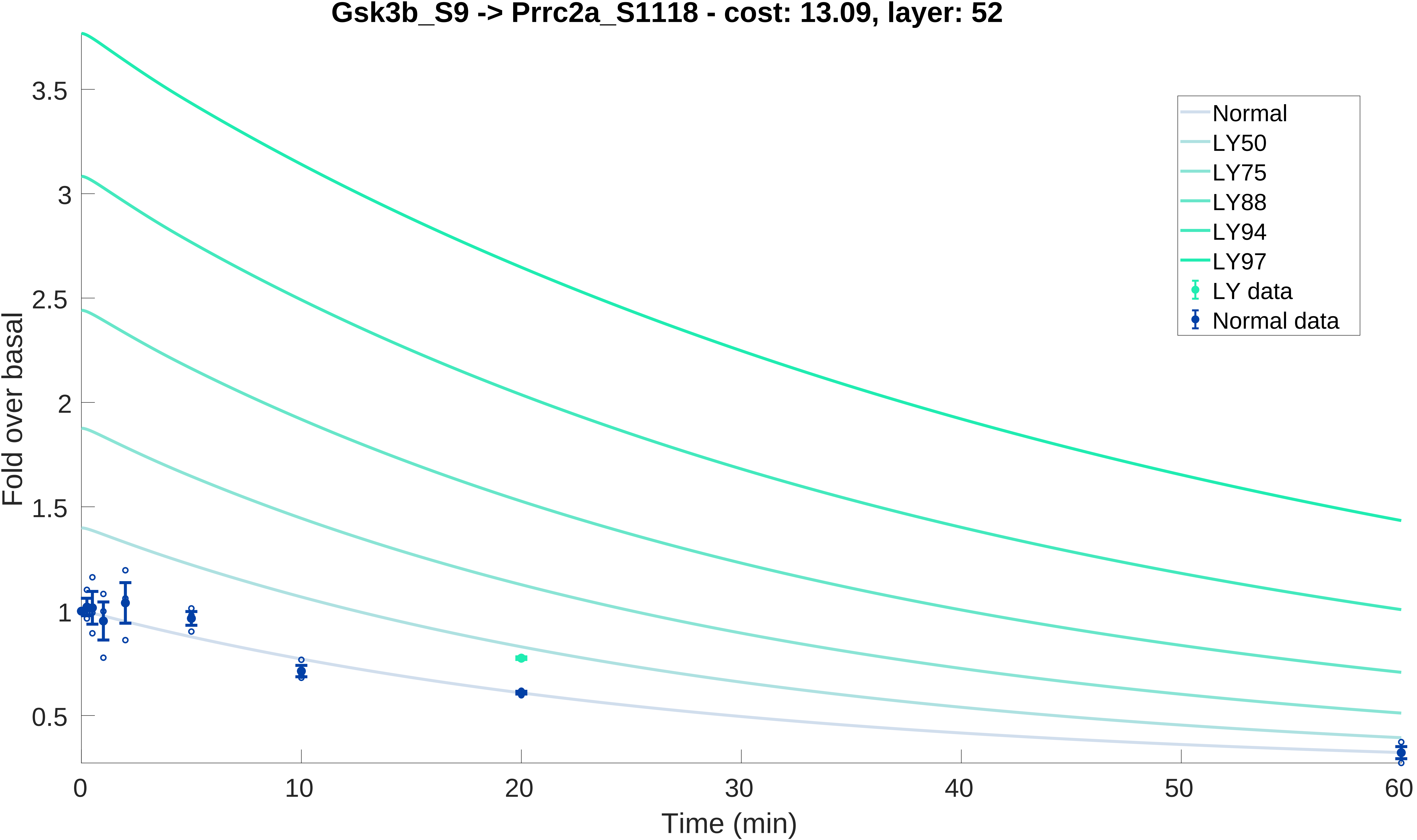

Gsk3b_S9 -> Prrc2a_S1118 - cost: 13.09, layer: 52
Fold over basal
Time (min)
3.5
3
2.5
2
1.5
1
0.5
0
10
20
30
40
50
60
Normal
LY50
LY75
LY88
LY94
LY97
LY data
Normal data

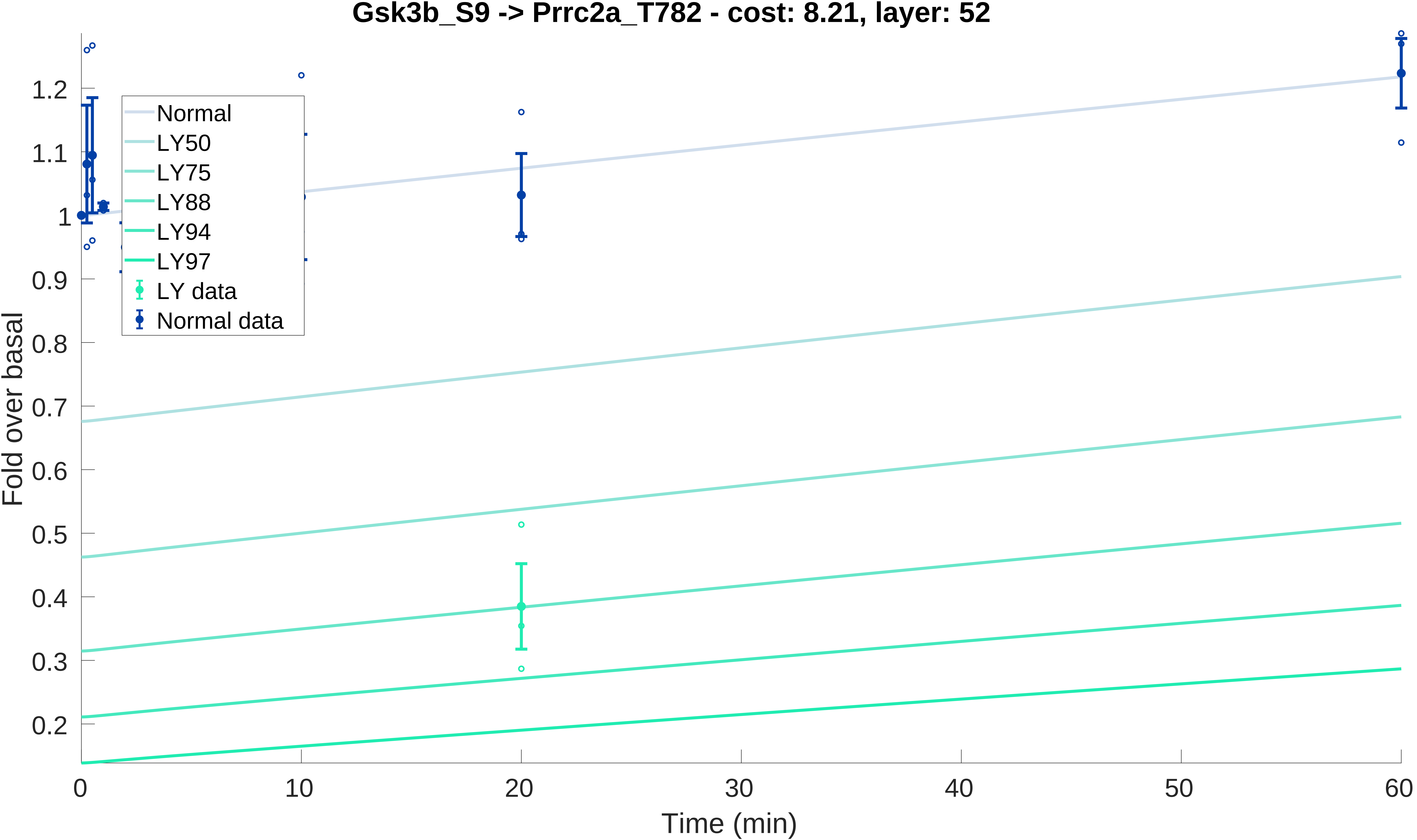

Gsk3b_S9 -> Prrc2a_T782 - cost: 8.21, layer: 52
Normal
LY50
LY75
LY88
LY94
LY97
LY data
Normal data
Fold over basal
Time (min)
0
10
20
30
40
50
60
0.2
0.3
0.4
0.5
0.6
0.7
0.8
0.9
1
1.1
1.2

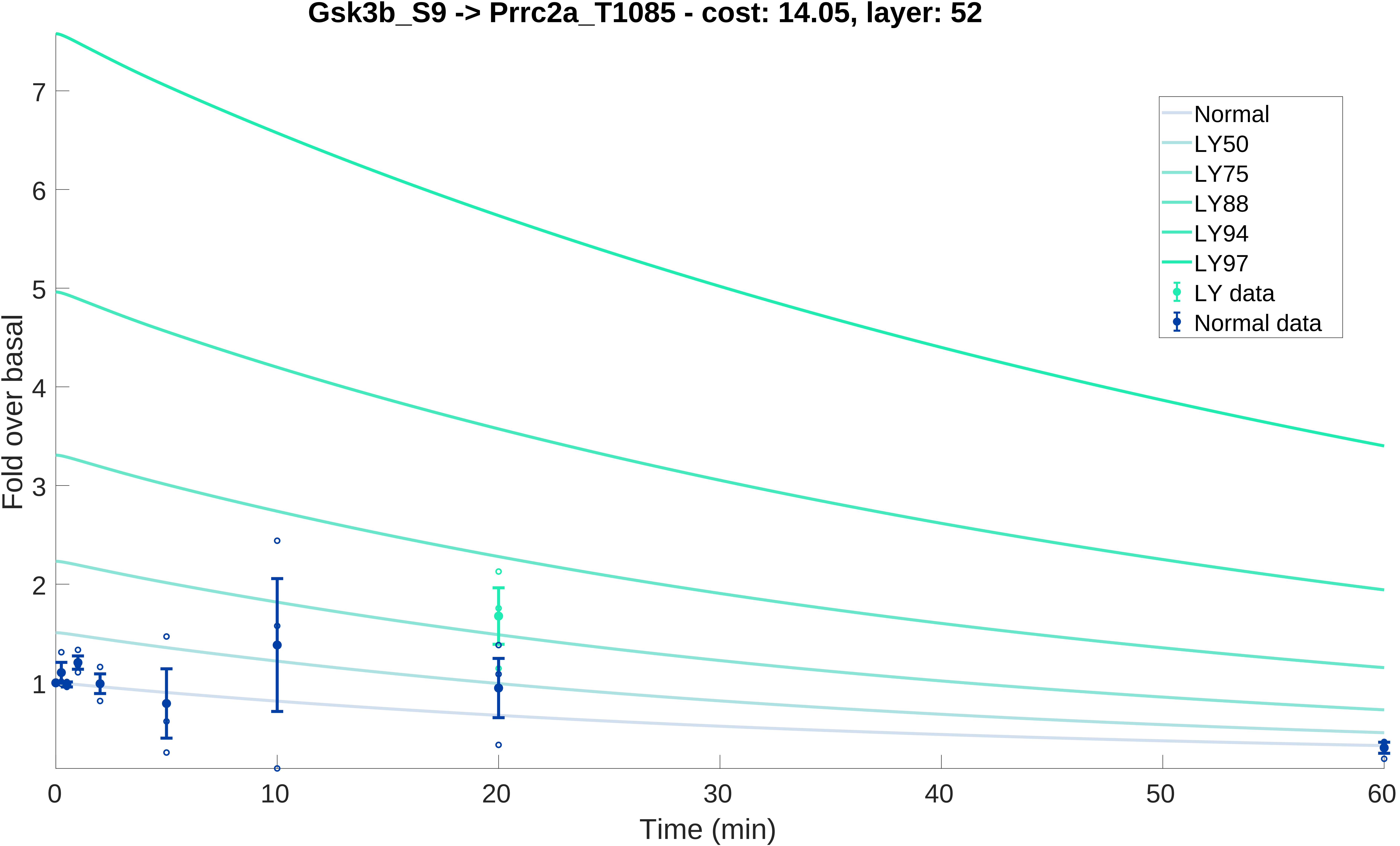

Gsk3b_S9 -> Prrc2a_T1085 - cost: 14.05, layer: 52
Fold over basal
Time (min)
Normal
LY50
LY75
LY88
LY94
LY97
LY data
Normal data
0
10
20
30
40
50
60
1
2
3
4
5
6
7

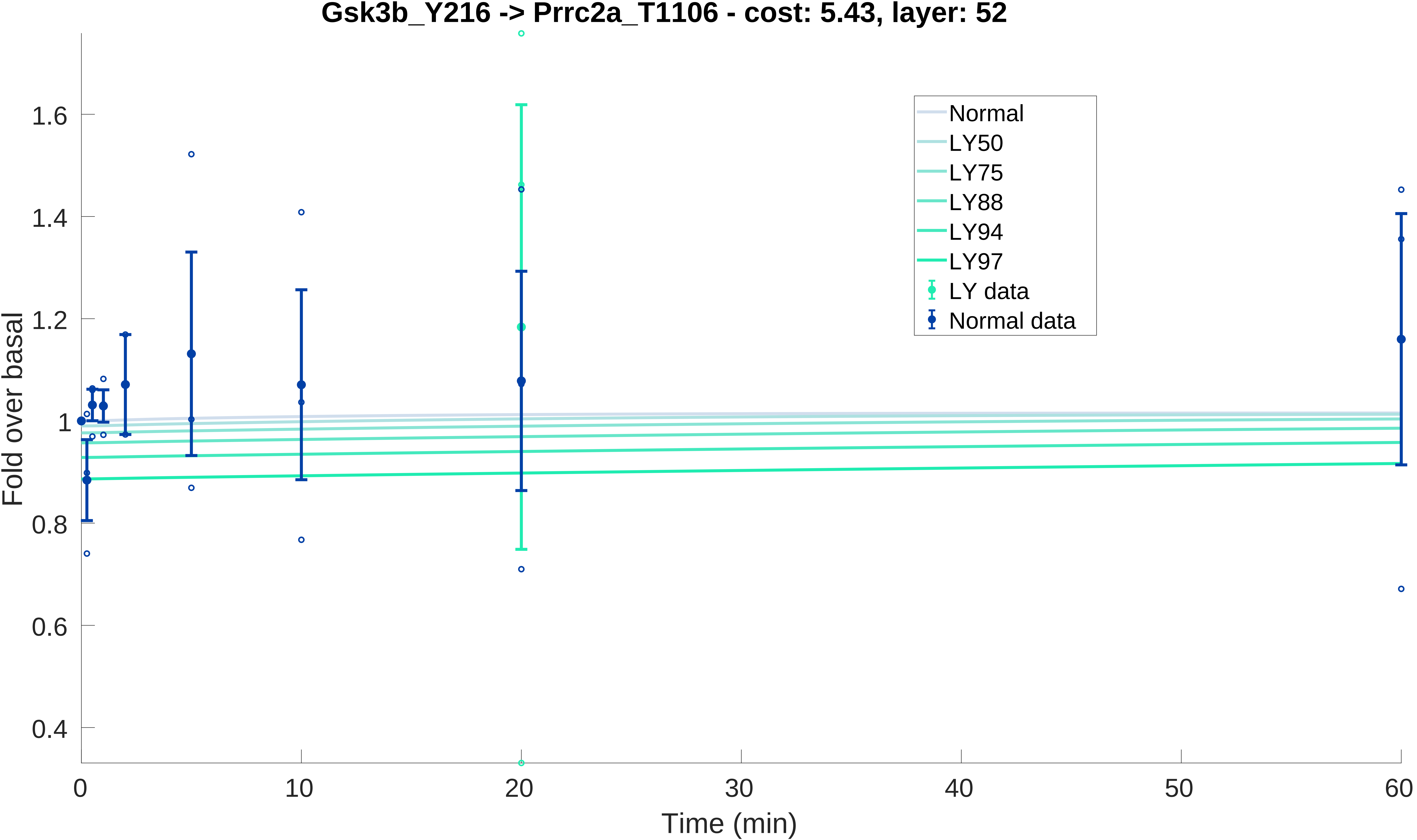

Gsk3b_Y216 -> Prrc2a_T1106 - cost: 5.43, layer: 52
Fold over basal
1.6
1.4
1.2
1
0.8
0.6
0.4
0
10
20
30
40
50
60
Time (min)
Normal
LY50
LY75
LY88
LY94
LY97
LY data
Normal data

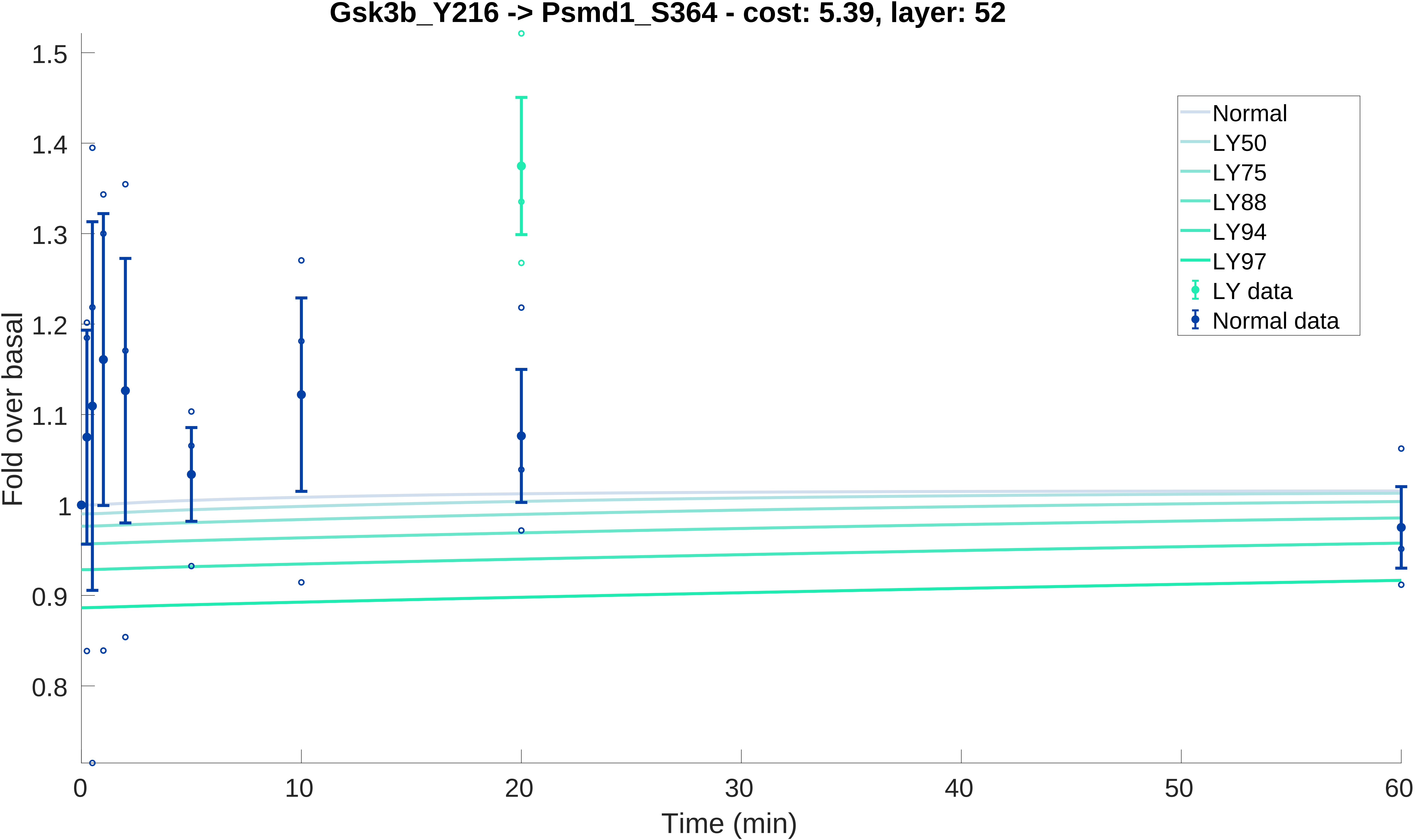

Gsk3b_Y216 -> Psmd1_S364 - cost: 5.39, layer: 52
Fold over basal
1.5
1.4
1.3
1.2
1.1
1
0.9
0.8
0
10
20
30
40
50
60
Time (min)
Normal
LY50
LY75
LY88
LY94
LY97
LY data
Normal data

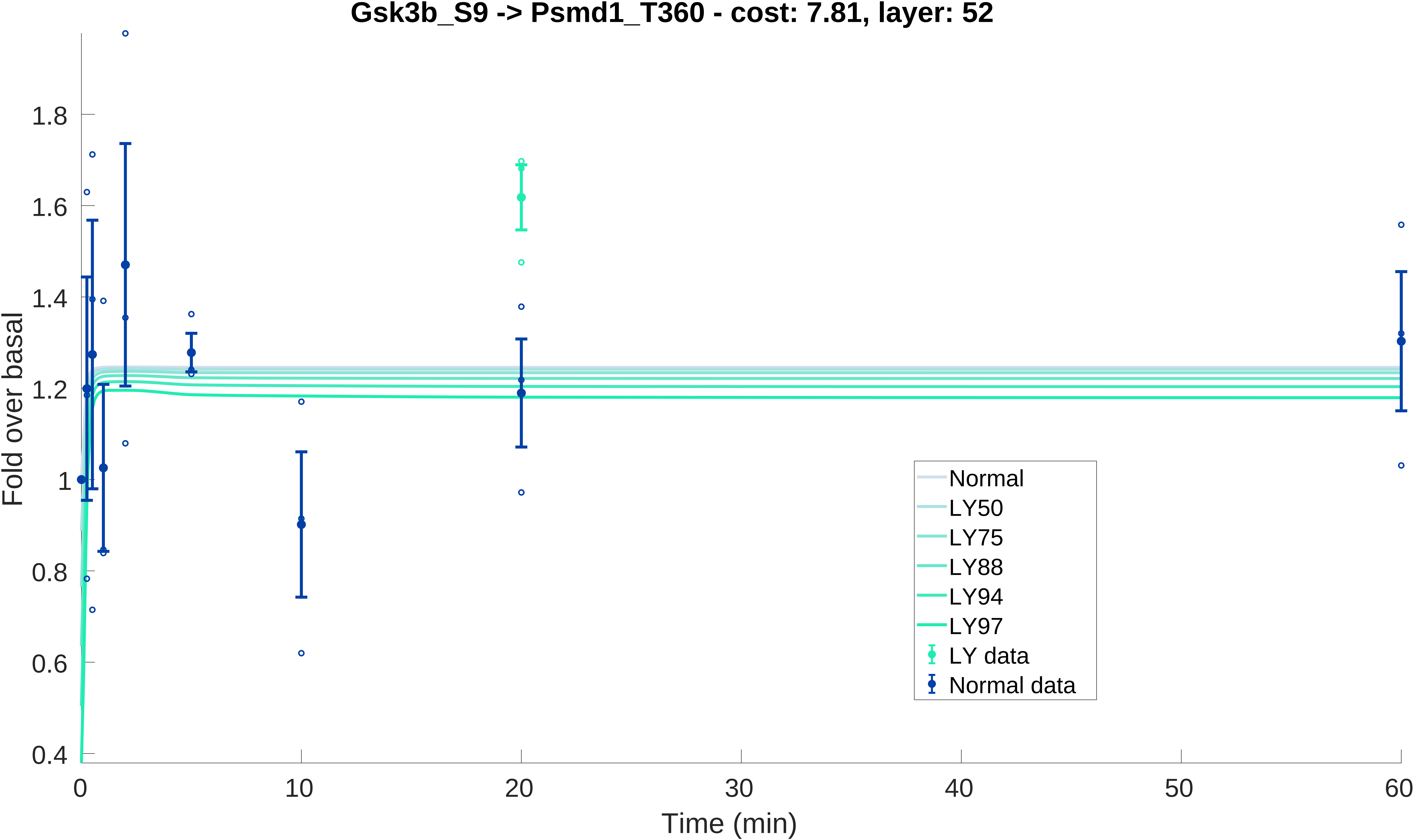
Gsk3b_S9 -> Psmd1_T360 - cost: 7.81, layer: 52
Fold over basal
1.8
1.6
1.4
1.2
1
0.8
0.6
0.4
0
10
20
30
40
50
60
Time (min)
Normal
LY50
LY75
LY88
LY94
LY97
LY data
Normal data

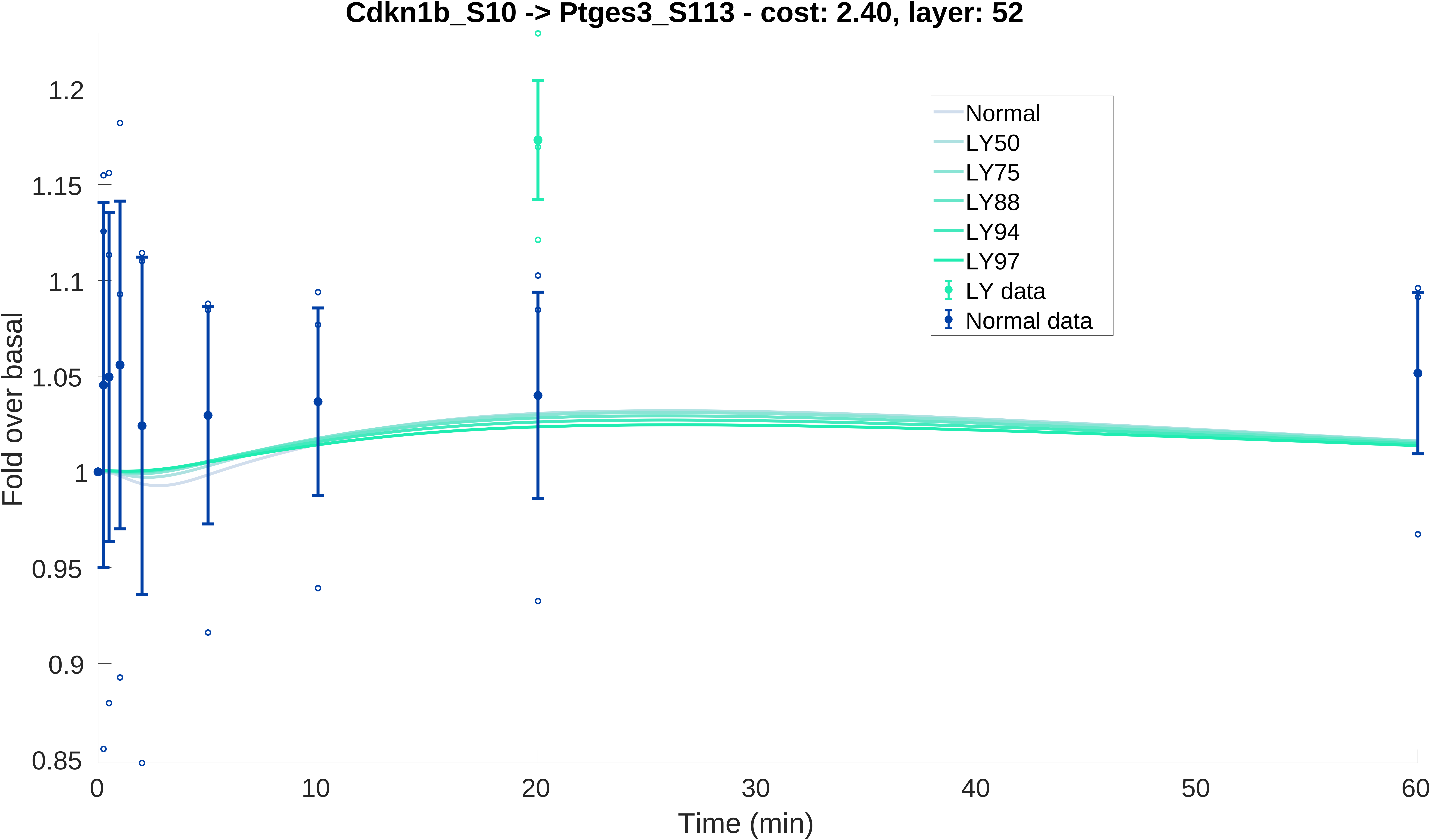

Cdkn1b_S10 -> Ptges3_S113 - cost: 2.40, layer: 52
Fold over basal
Time (min)
1.2
1.15
1.1
1.05
1
0.95
0.9
0.85
0
10
20
30
40
50
60
Normal
LY50
LY75
LY88
LY94
LY97
LY data
Normal data

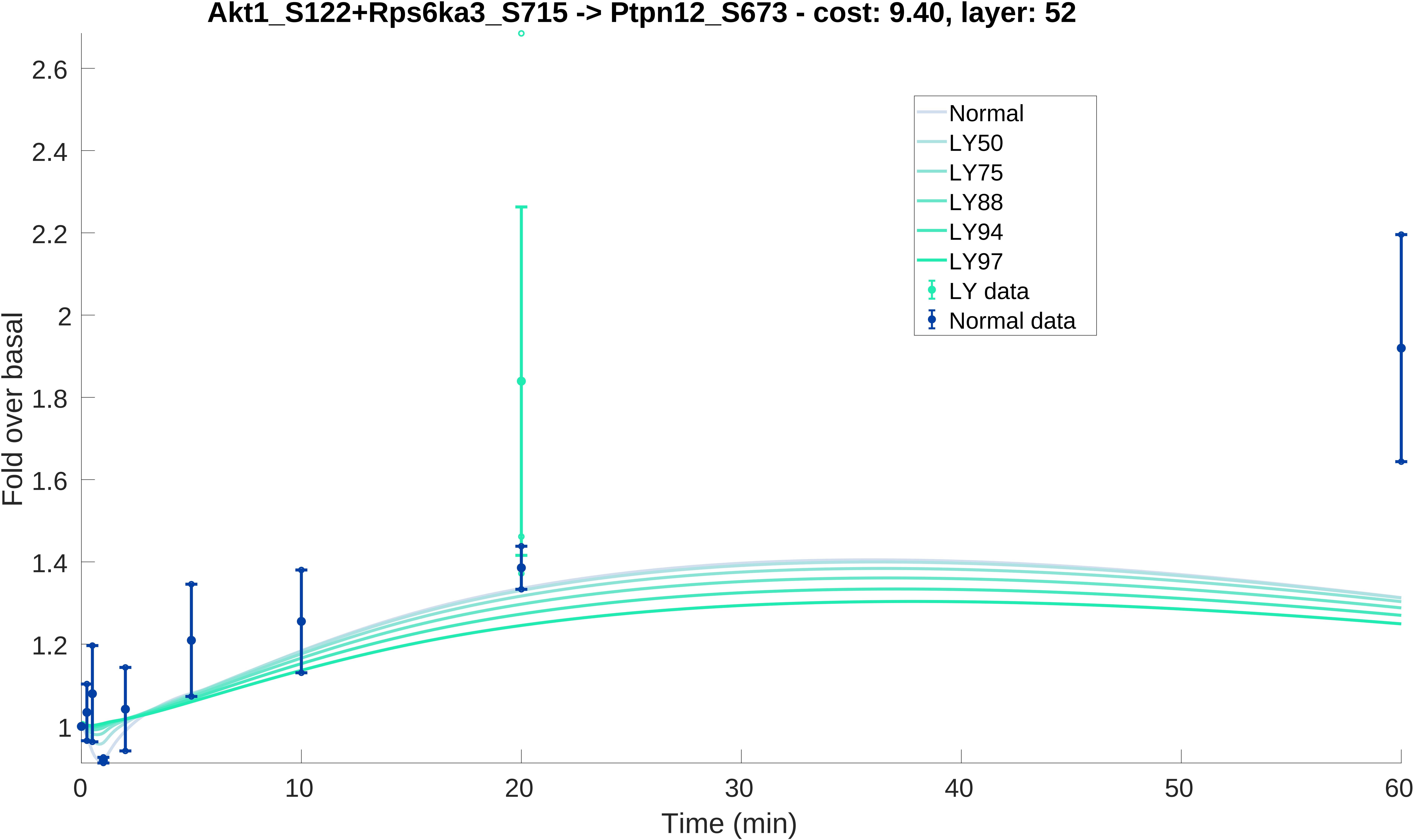

Akt1_S122+Rps6ka3_S715 -> Ptpn12_S673 - cost: 9.40, layer: 52
Fold over basal
Time (min)
2.6
2.4
2.2
2
1.8
1.6
1.4
1.2
1
0
10
20
30
40
50
60
Normal
LY50
LY75
LY88
LY94
LY97
LY data
Normal data

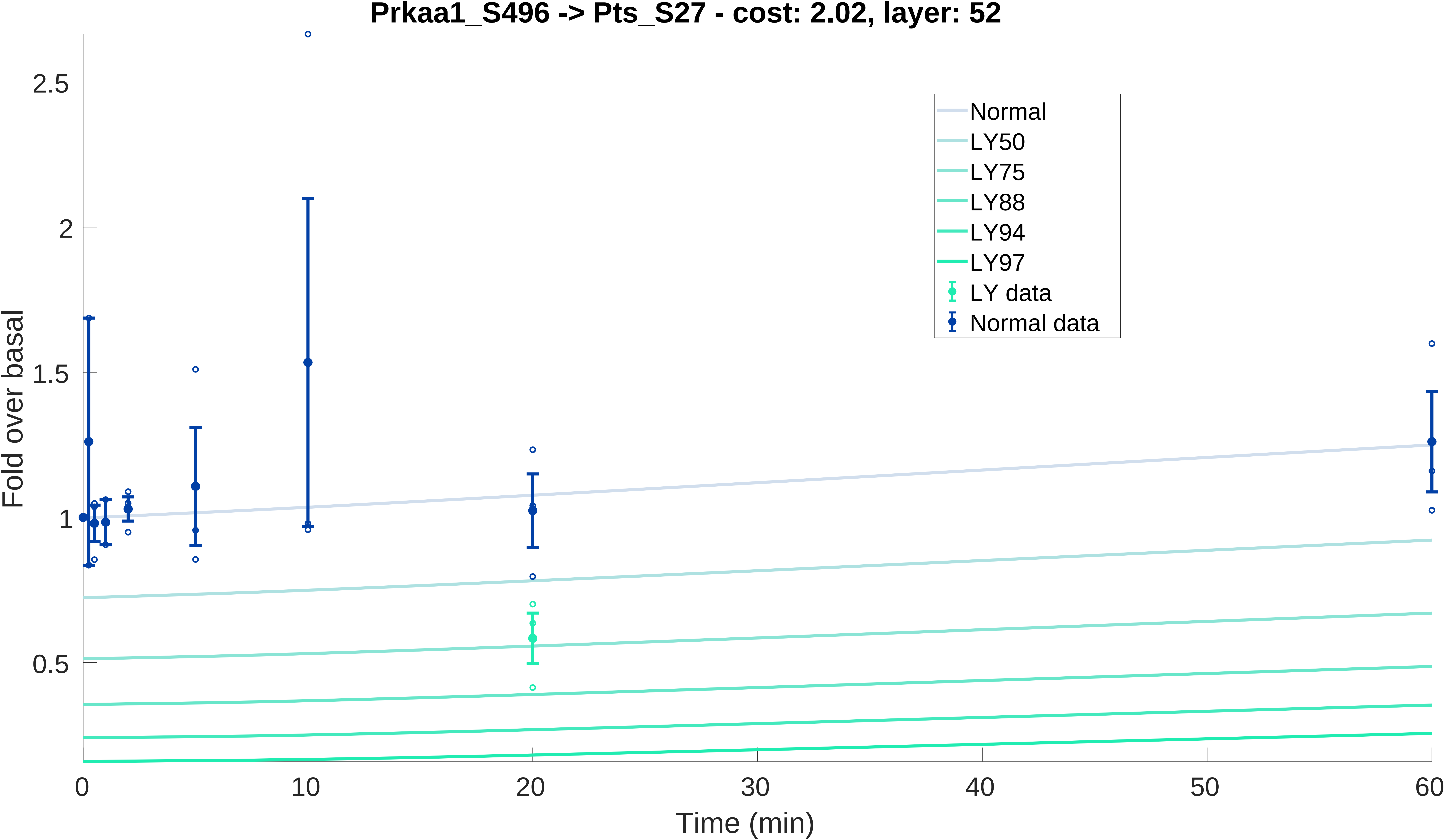

Prkaa1_S496 -> Pts_S27 - cost: 2.02, layer: 52
Fold over basal
Time (min)
0
10
20
30
40
50
60
0.5
1
1.5
2
2.5
Normal
LY50
LY75
LY88
LY94
LY97
LY data
Normal data

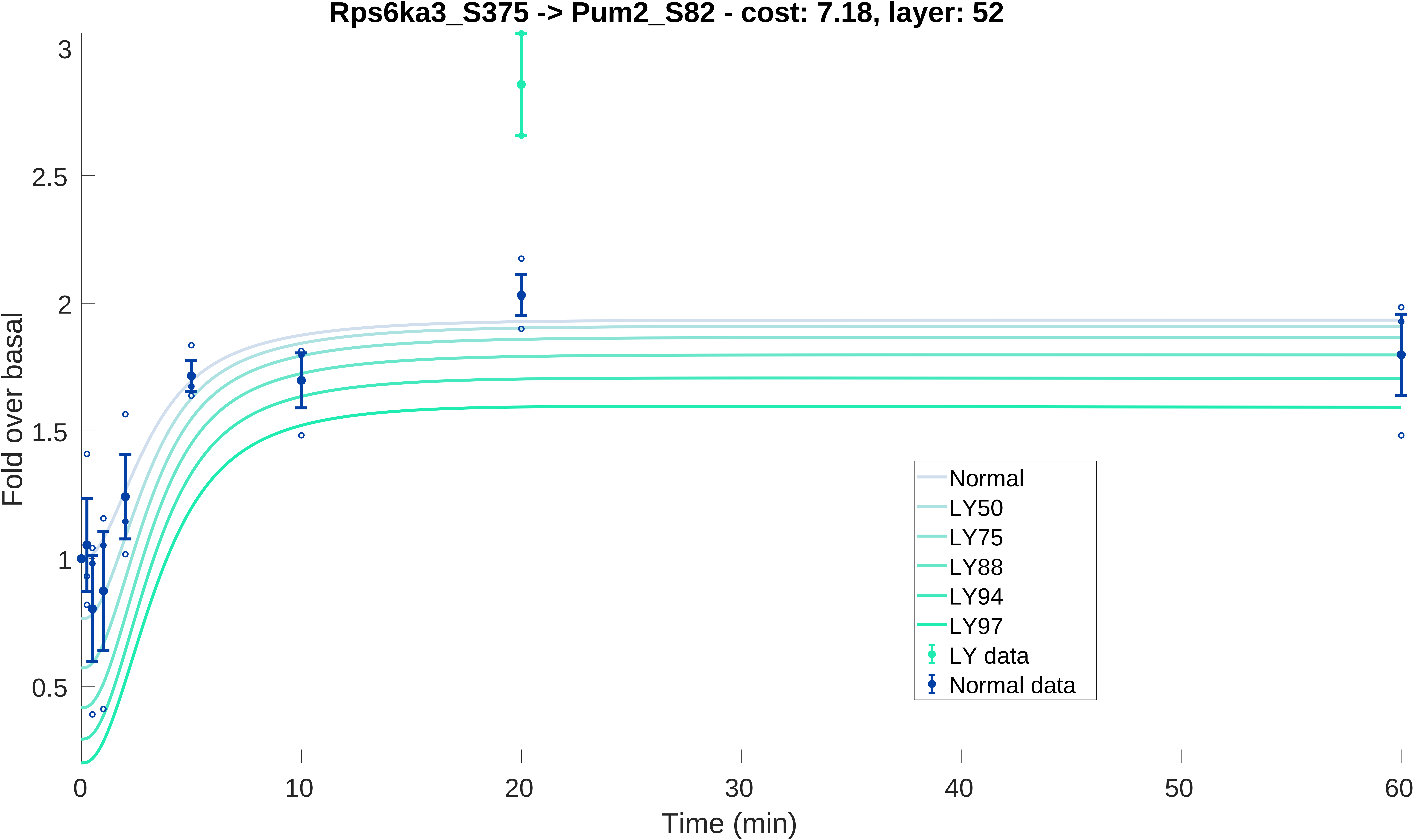

Rps6ka3_S375 -> Pum2_S82 - cost: 7.18, layer: 52
Fold over basal
Time (min)
0.5
1
1.5
2
2.5
3
0
10
20
30
40
50
60
Normal
LY50
LY75
LY88
LY94
LY97
LY data
Normal data

Rps6ka3_S375 -> Pum2_S136 - cost: 1.09, layer: 52 (pool)

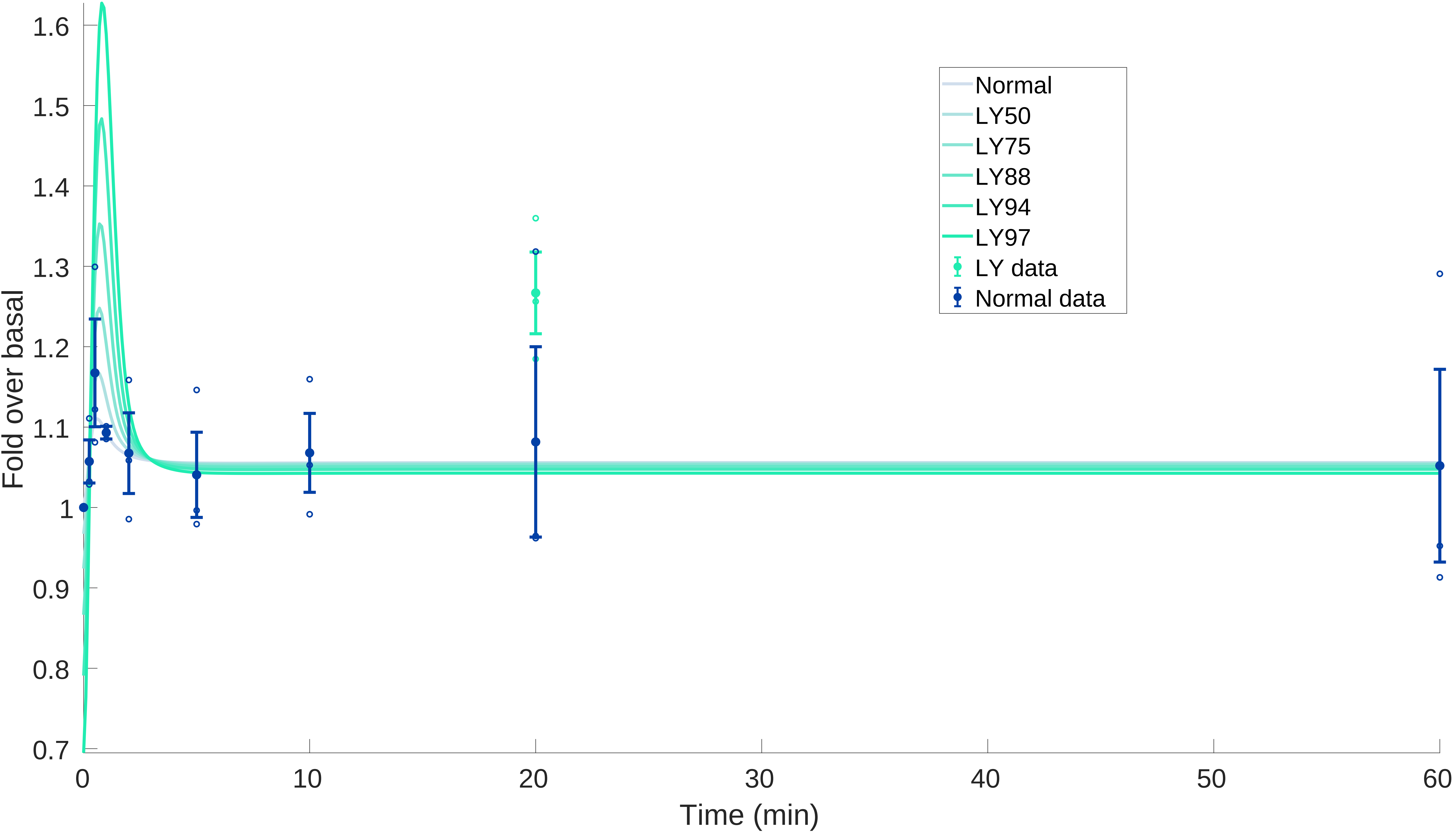

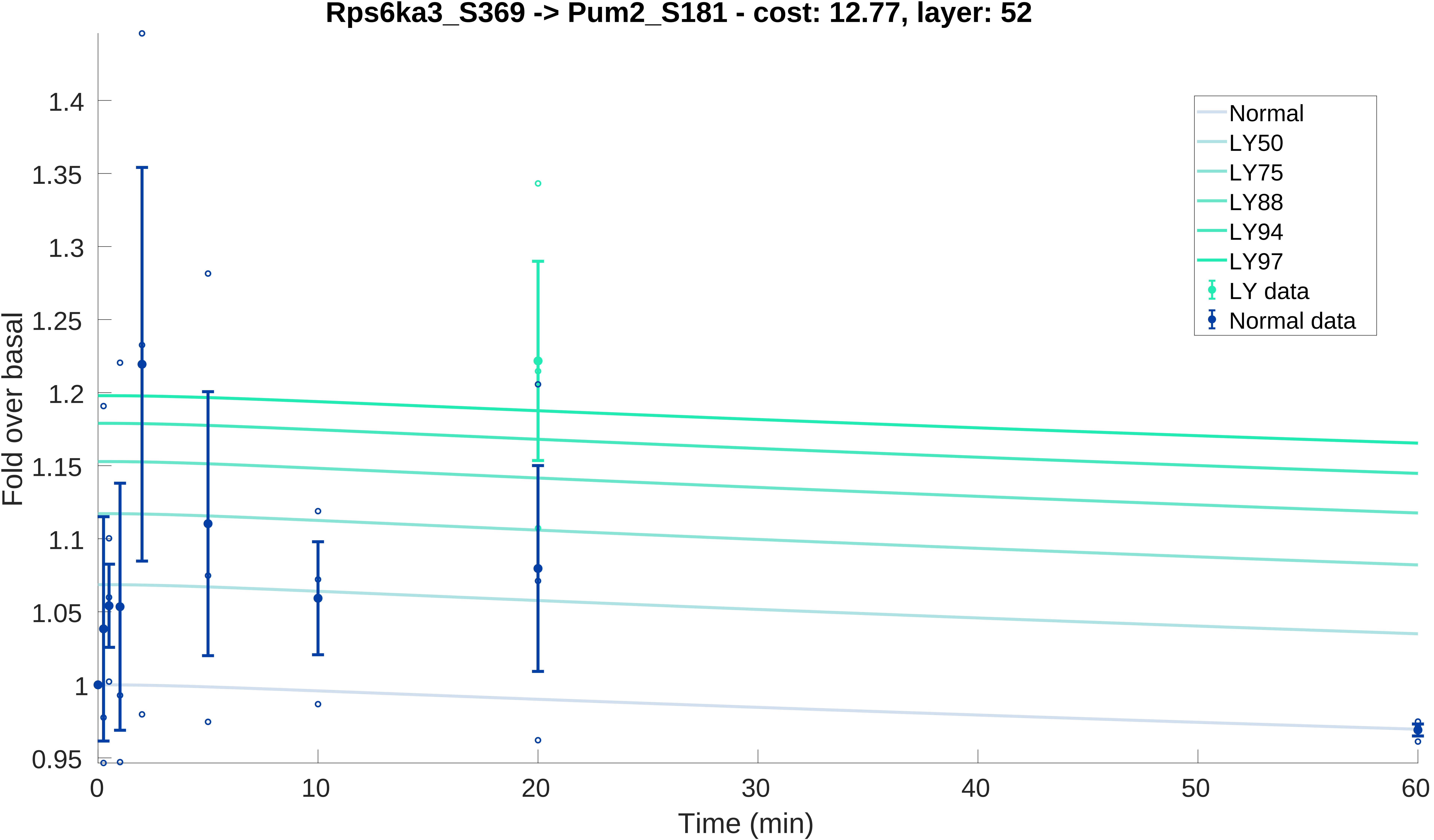

Rps6ka3_S369 -> Pum2_S181 - cost: 12.77, layer: 52
Fold over basal
Time (min)
0.95
1
1.05
1.1
1.15
1.2
1.25
1.3
1.35
1.4
0
10
20
30
40
50
60
Normal
LY50
LY75
LY88
LY94
LY97
LY data
Normal data

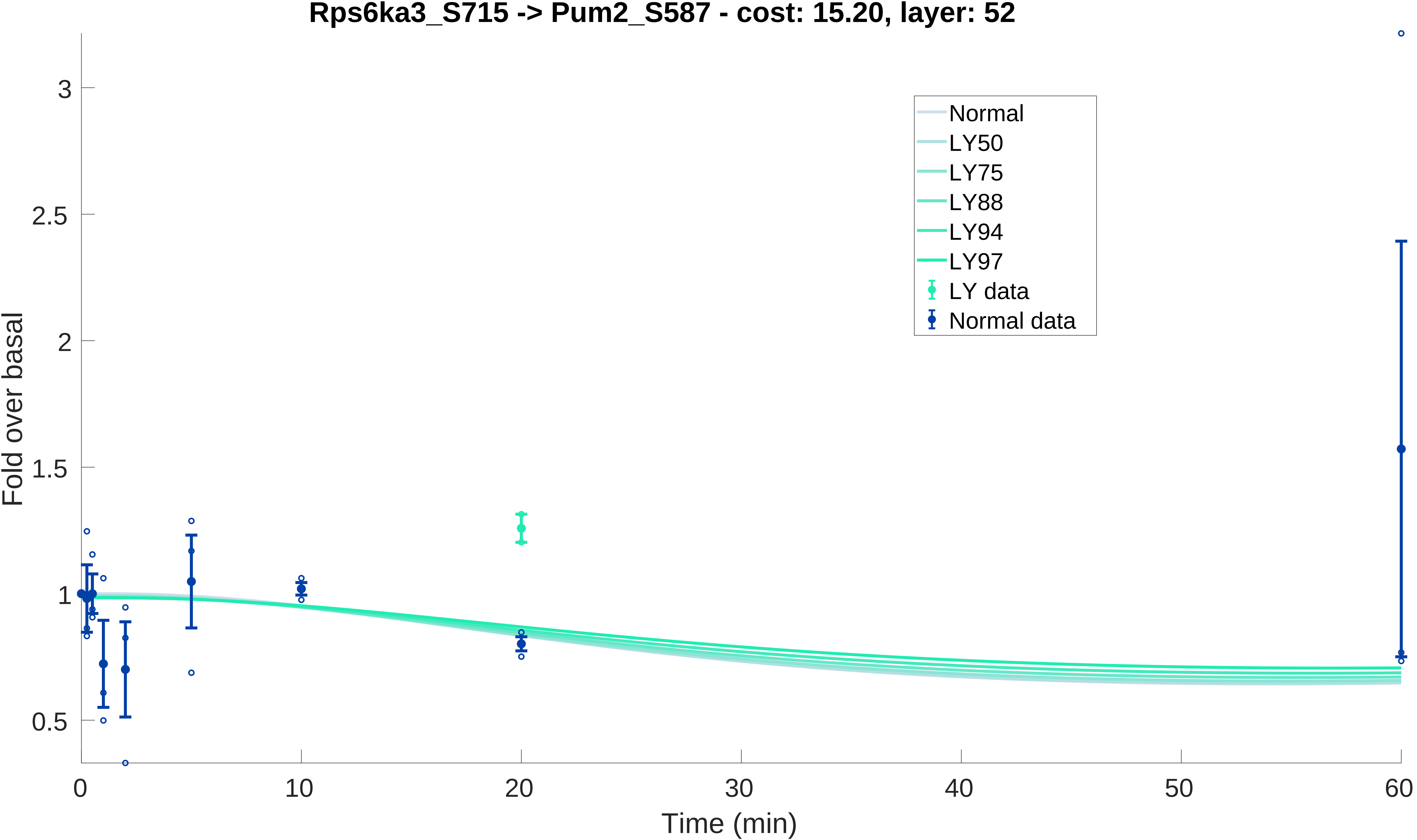

Rps6ka3_S715 -> Pum2_S587 - cost: 15.20, layer: 52
Fold over basal
Time (min)
Normal
LY50
LY75
LY88
LY94
LY97
LY data
Normal data

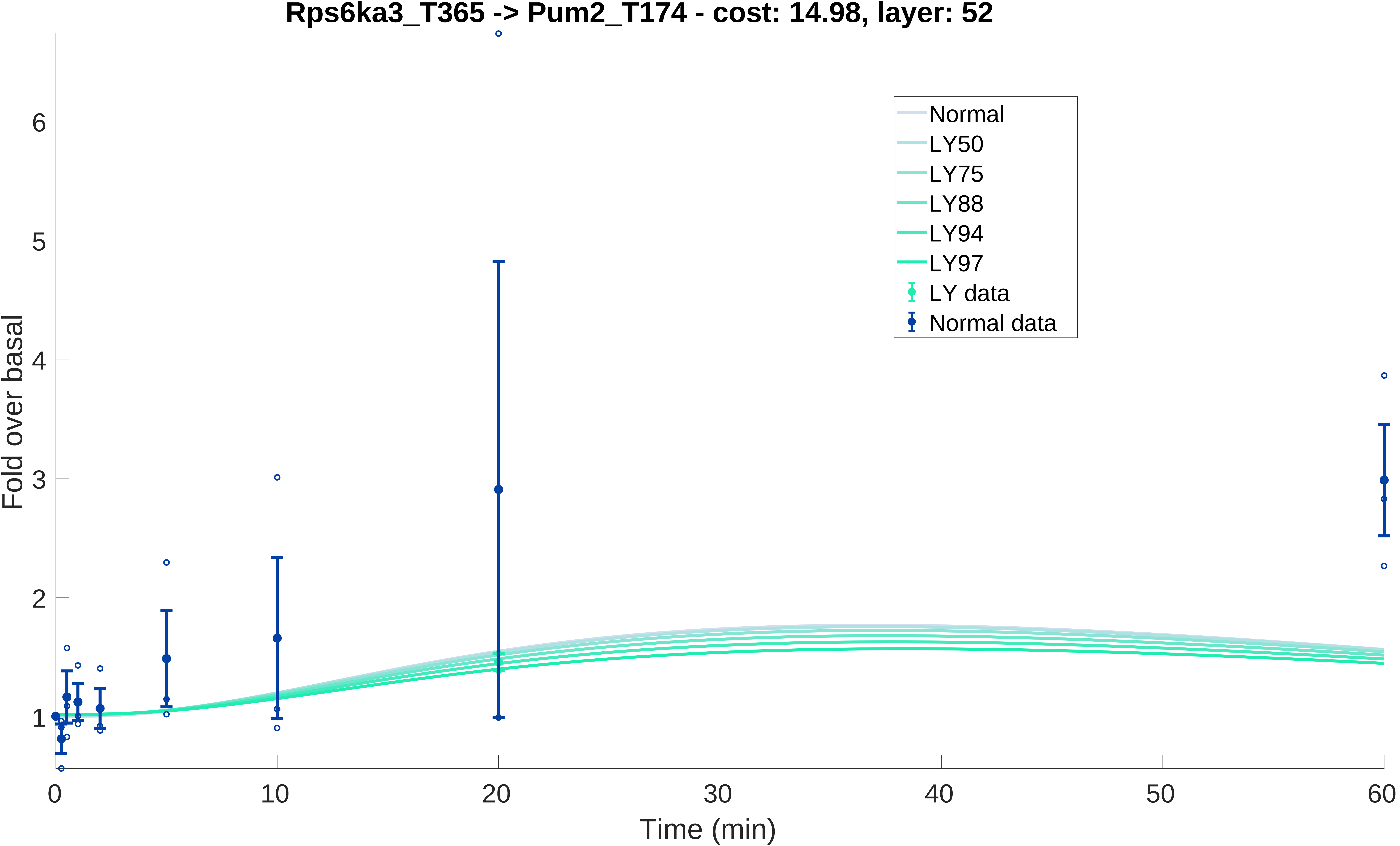

Rps6ka3_T365 -> Pum2_T174 - cost: 14.98, layer: 52
Fold over basal
Time (min)
Normal
LY50
LY75
LY88
LY94
LY97
LY data
Normal data
1
2
3
4
5
6
0
10
20
30
40
50
60

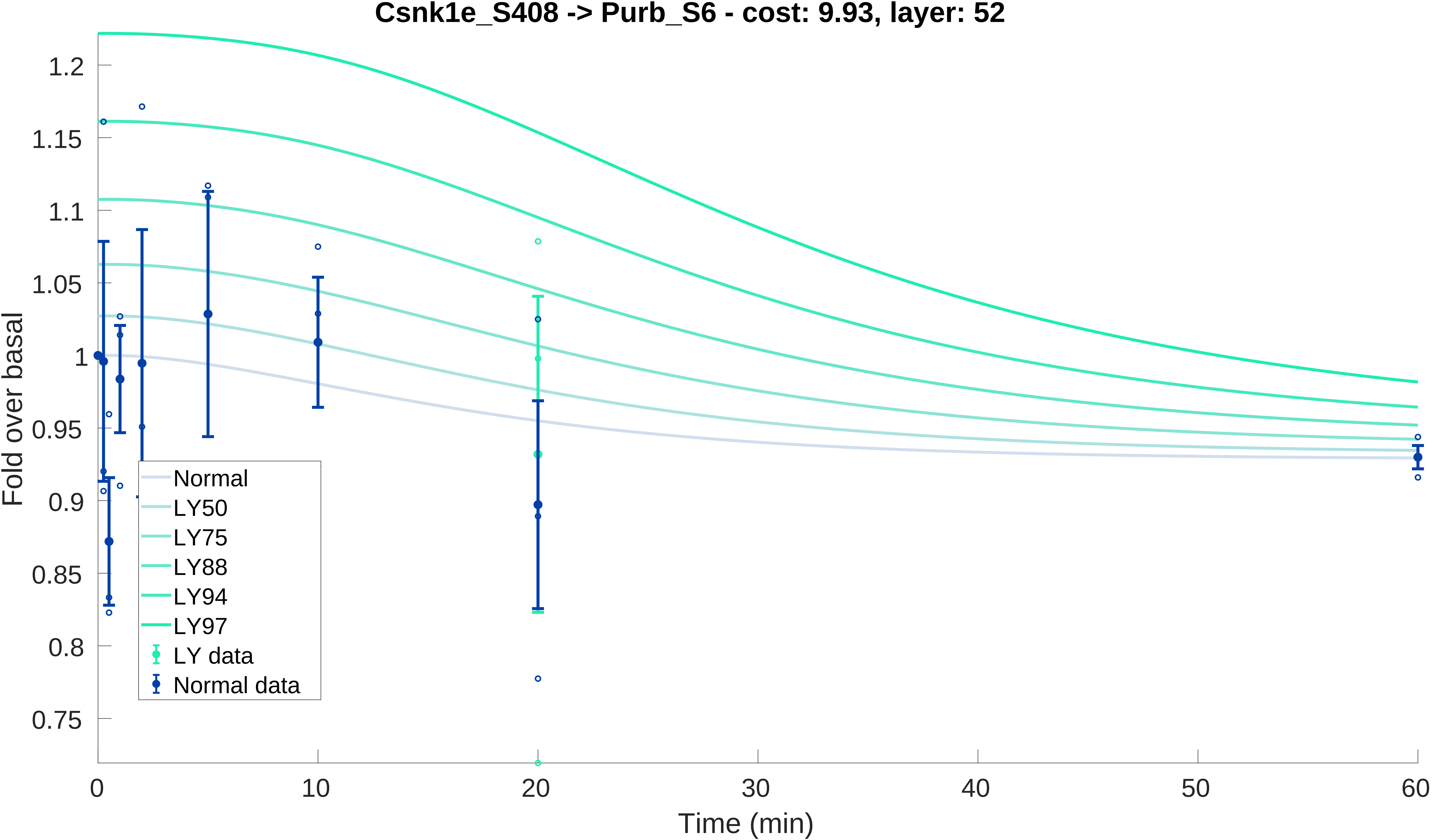

Csnk1e_S408 -> Purb_S6 - cost: 9.93, layer: 52
Fold over basal
1.2
1.15
1.1
1.05
1
0.95
0.9
0.85
0.8
0.75
0
10
20
30
40
50
60
Time (min)
Normal
LY50
LY75
LY88
LY94
LY97
LY data
Normal data

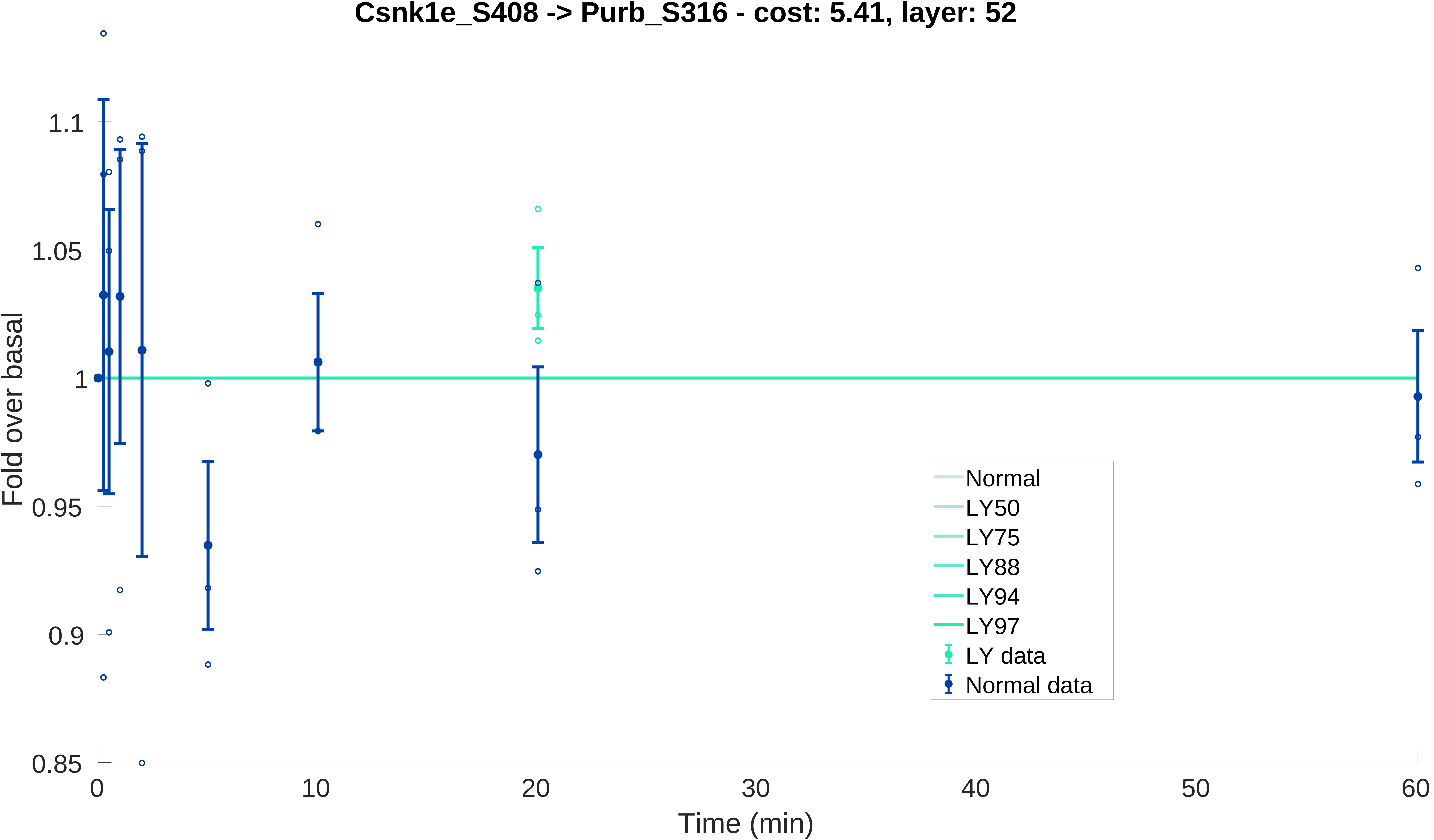
Csnk1e_S408 -> Purb_S316 - cost: 5.41, layer: 52
Fold over basal
Time (min)
0.85
0.9
0.95
1
1.05
1.1
0
10
20
30
40
50
60
Normal
LY50
LY75
LY88
LY94
LY97
LY data
Normal data

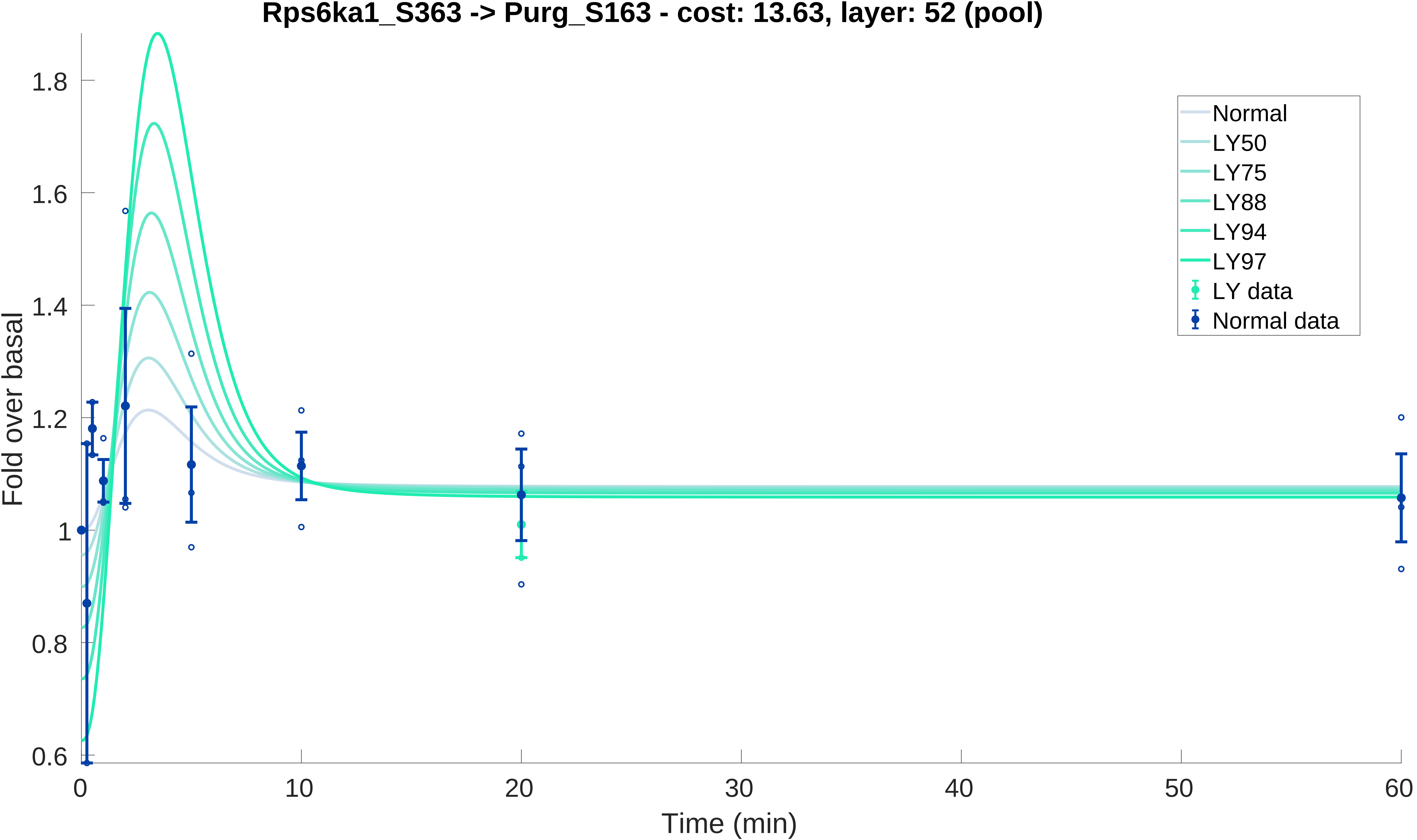

Rps6ka1_S363 -> Purg_S163 - cost: 13.63, layer: 52 (pool)
Fold over basal
Time (min)
0.6
0.8
1
1.2
1.4
1.6
1.8
0
10
20
30
40
50
60
Normal
LY50
LY75
LY88
LY94
LY97
LY data
Normal data

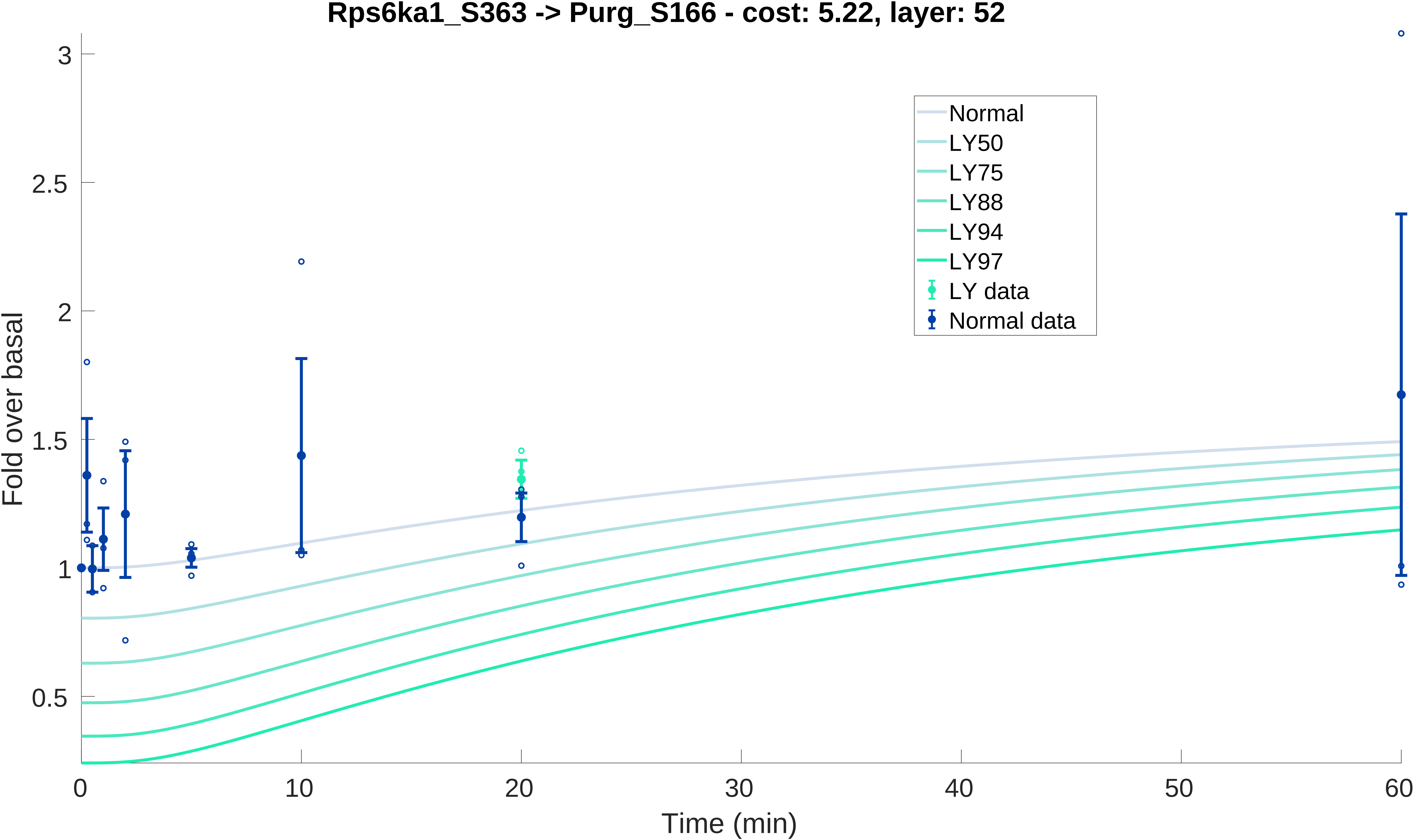

Rps6ka1_S363 -> Purg_S166 - cost: 5.22, layer: 52
Fold over basal
Time (min)
0.5
1
1.5
2
2.5
3
0
10
20
30
40
50
60
Normal
LY50
LY75
LY88
LY94
LY97
LY data
Normal data

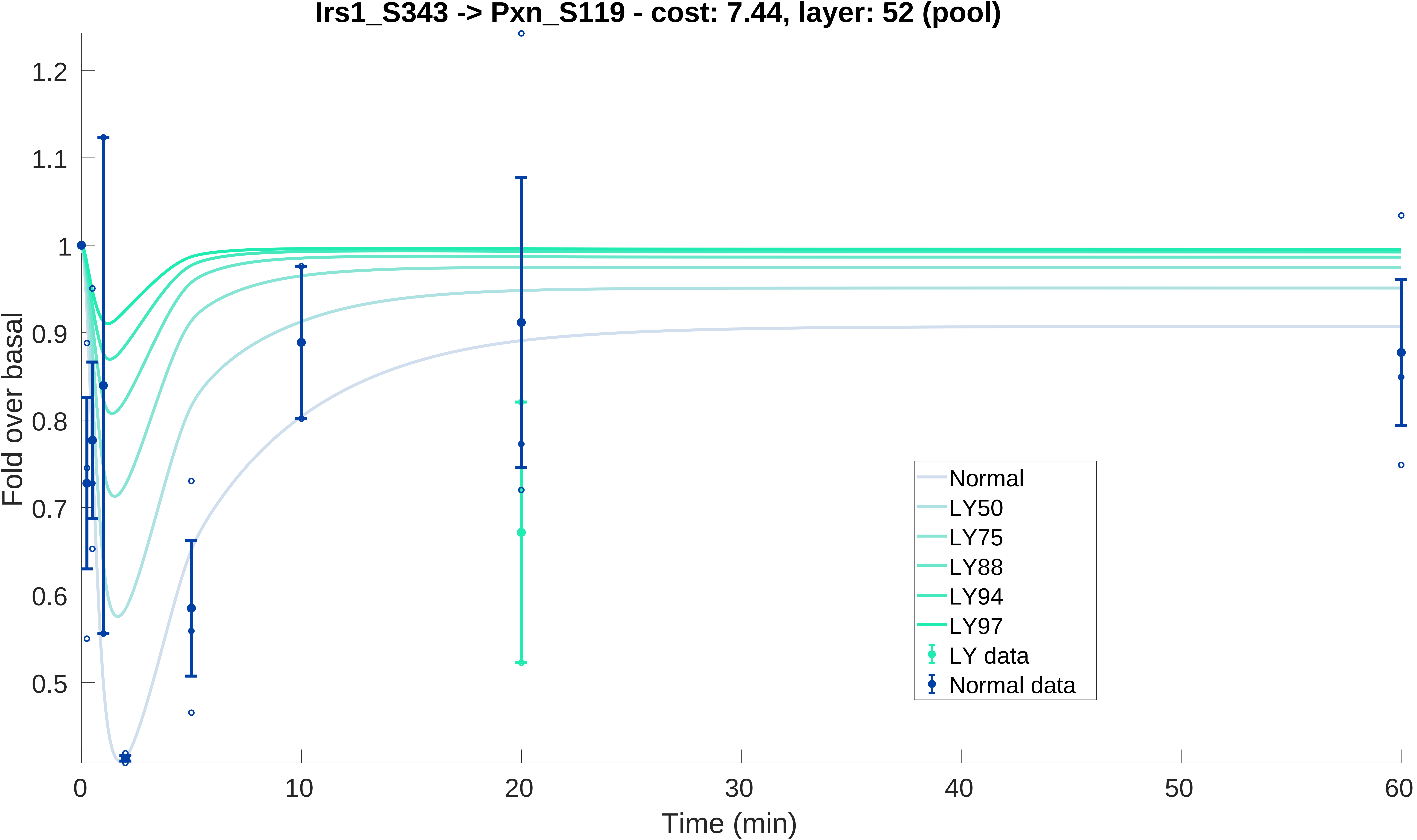

Irs1_S343 -> Pxn_S119 - cost: 7.44, layer: 52 (pool)
Fold over basal
1.2
1.1
1
0.9
0.8
0.7
0.6
0.5
0
10
20
30
40
50
60
Time (min)
Normal
LY50
LY75
LY88
LY94
LY97
LY data
Normal data

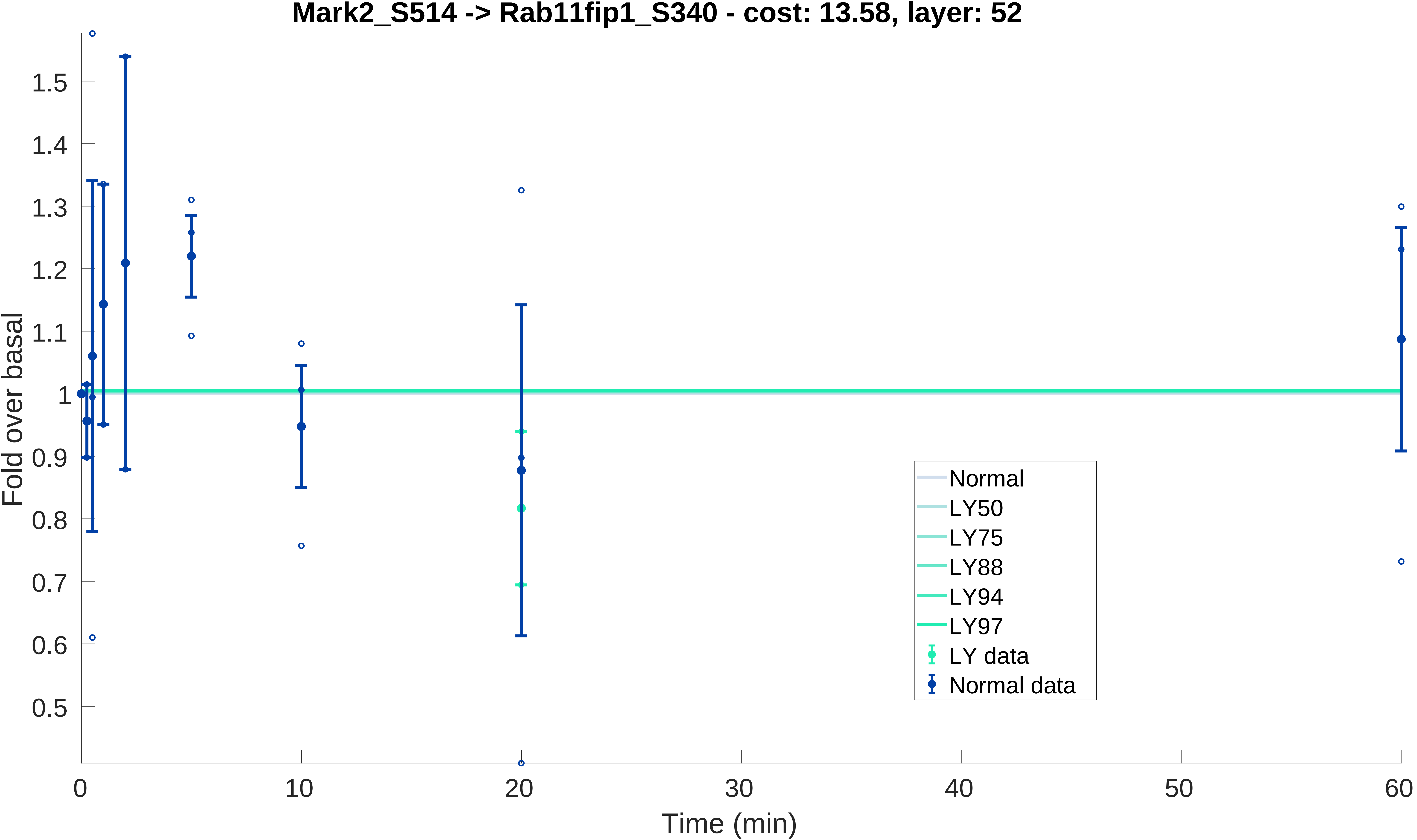

Mark2_S514 -> Rab11fip1_S340 - cost: 13.58, layer: 52
Fold over basal
Time (min)
0.5
0.6
0.7
0.8
0.9
1
1.1
1.2
1.3
1.4
1.5
0
10
20
30
40
50
60
Normal
LY50
LY75
LY88
LY94
LY97
LY data
Normal data

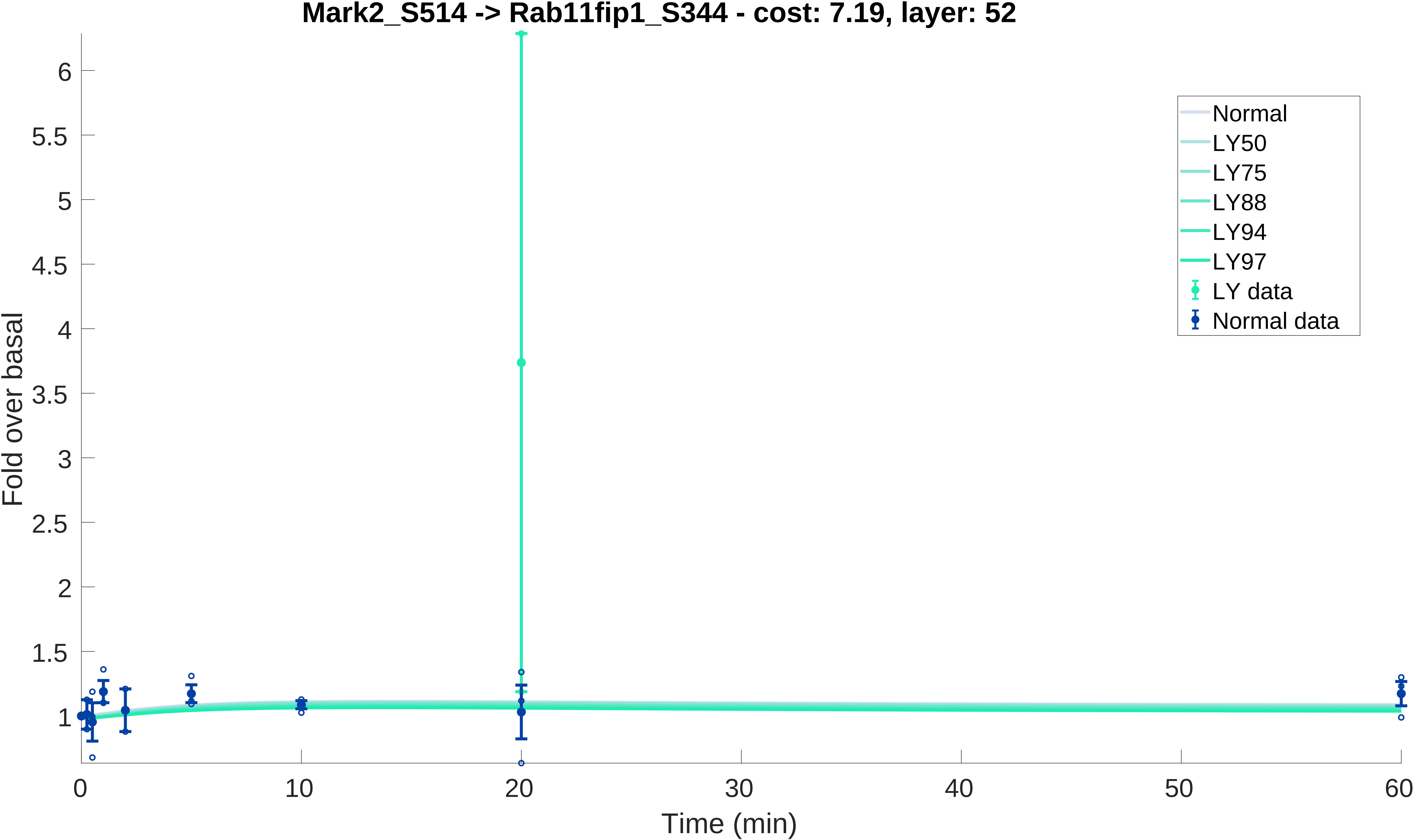

Mark2_S514 -> Rab11fip1_S344 - cost: 7.19, layer: 52
Fold over basal
Time (min)
6
5.5
5
4.5
4
3.5
3
2.5
2
1.5
1
0
10
20
30
40
50
60
Normal
LY50
LY75
LY88
LY94
LY97
LY data
Normal data

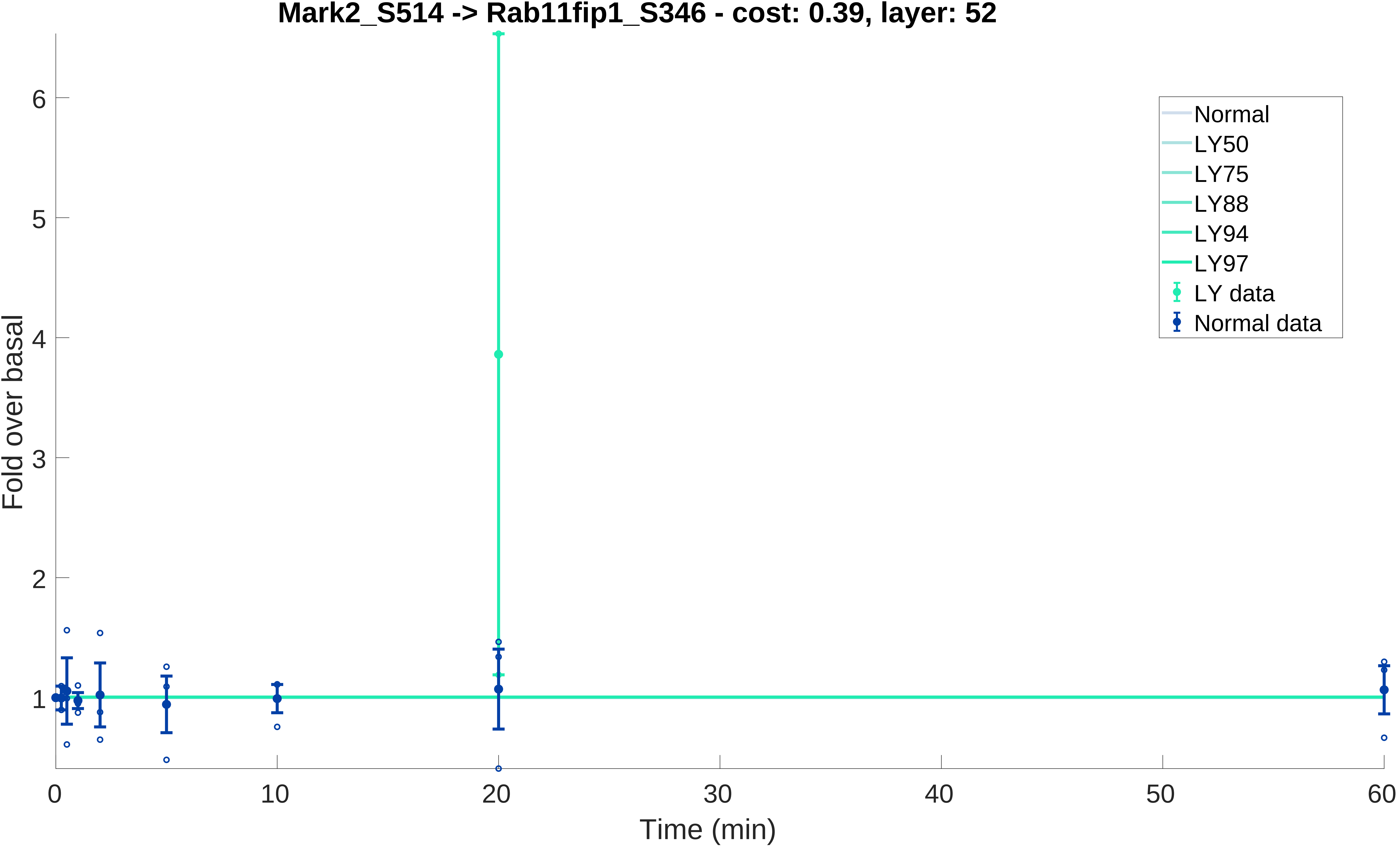

Mark2_S514 -> Rab11fip1_S346 - cost: 0.39, layer: 52
Fold over basal
Time (min)
0
10
20
30
40
50
60
1
2
3
4
5
6
Normal
LY50
LY75
LY88
LY94
LY97
LY data
Normal data

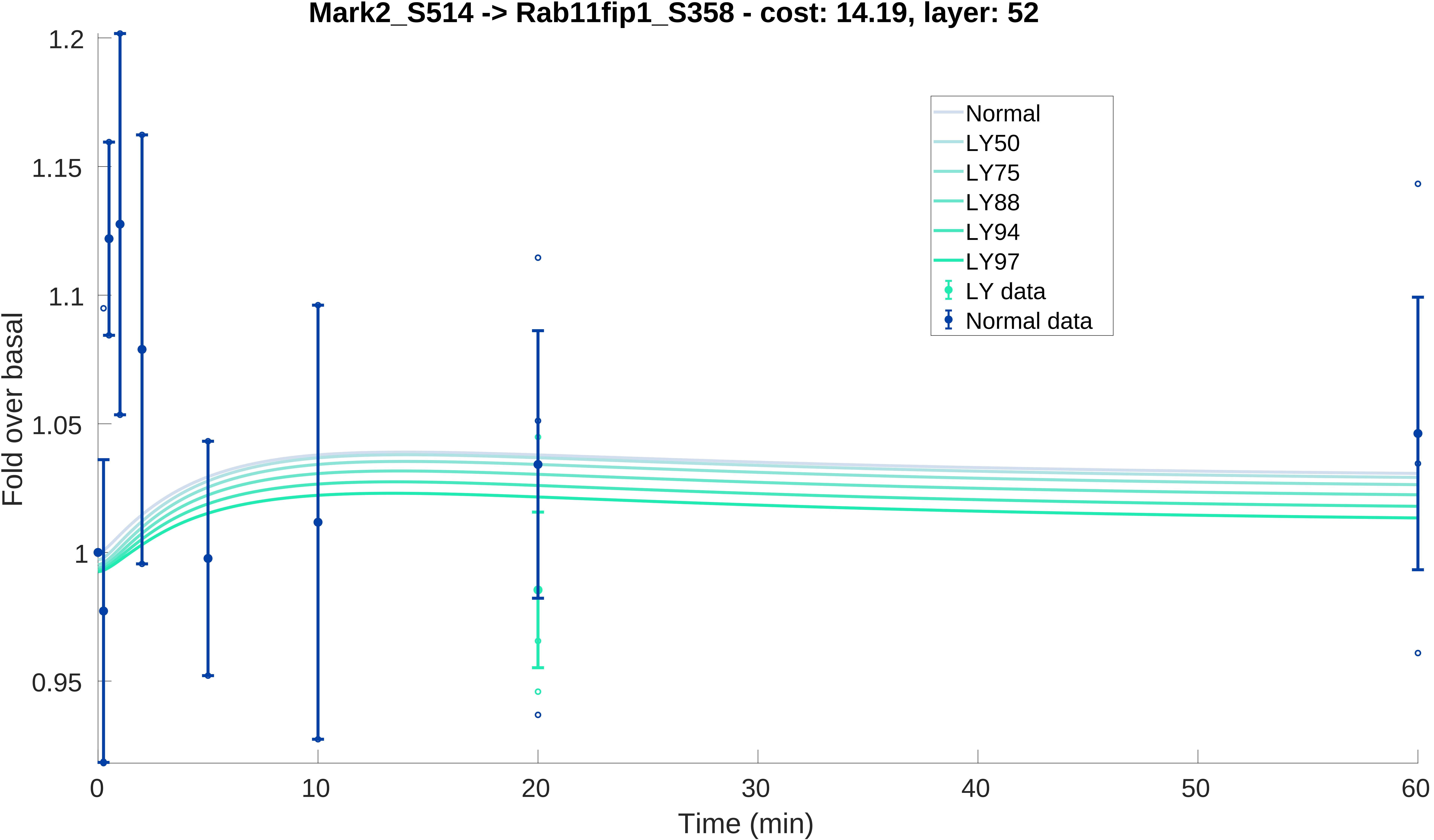

Mark2_S514 -> Rab11fip1_S358 - cost: 14.19, layer: 52
Fold over basal
Time (min)
1.2
1.15
1.1
1.05
1
0.95
0
10
20
30
40
50
60
Normal
LY50
LY75
LY88
LY94
LY97
LY data
Normal data

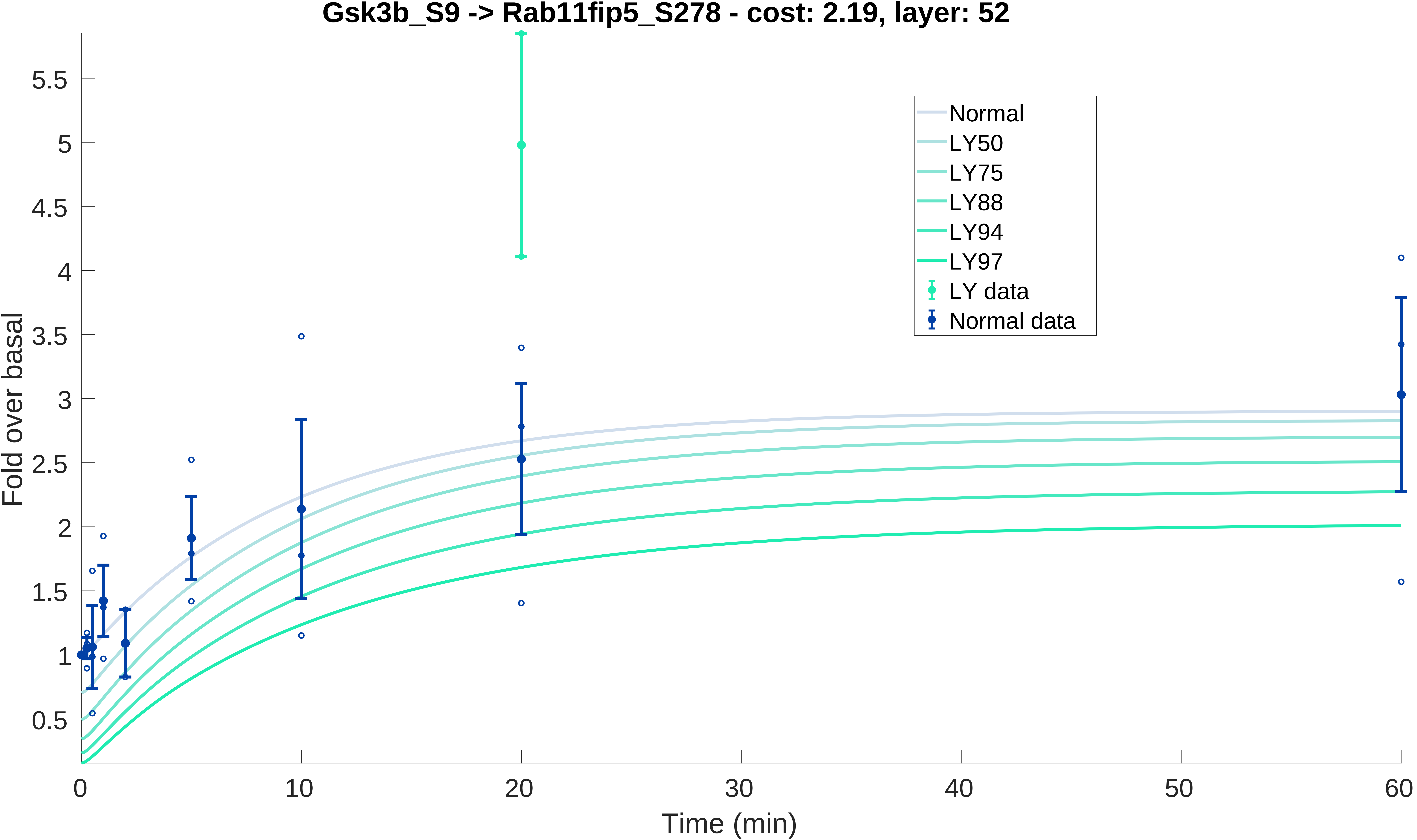
Gsk3b_S9 -> Rab11fip5_S278 - cost: 2.19, layer: 52
Fold over basal
Time (min)
0
10
20
30
40
50
60
0.5
1
1.5
2
2.5
3
3.5
4
4.5
5
5.5
Normal
LY50
LY75
LY88
LY94
LY97
LY data
Normal data

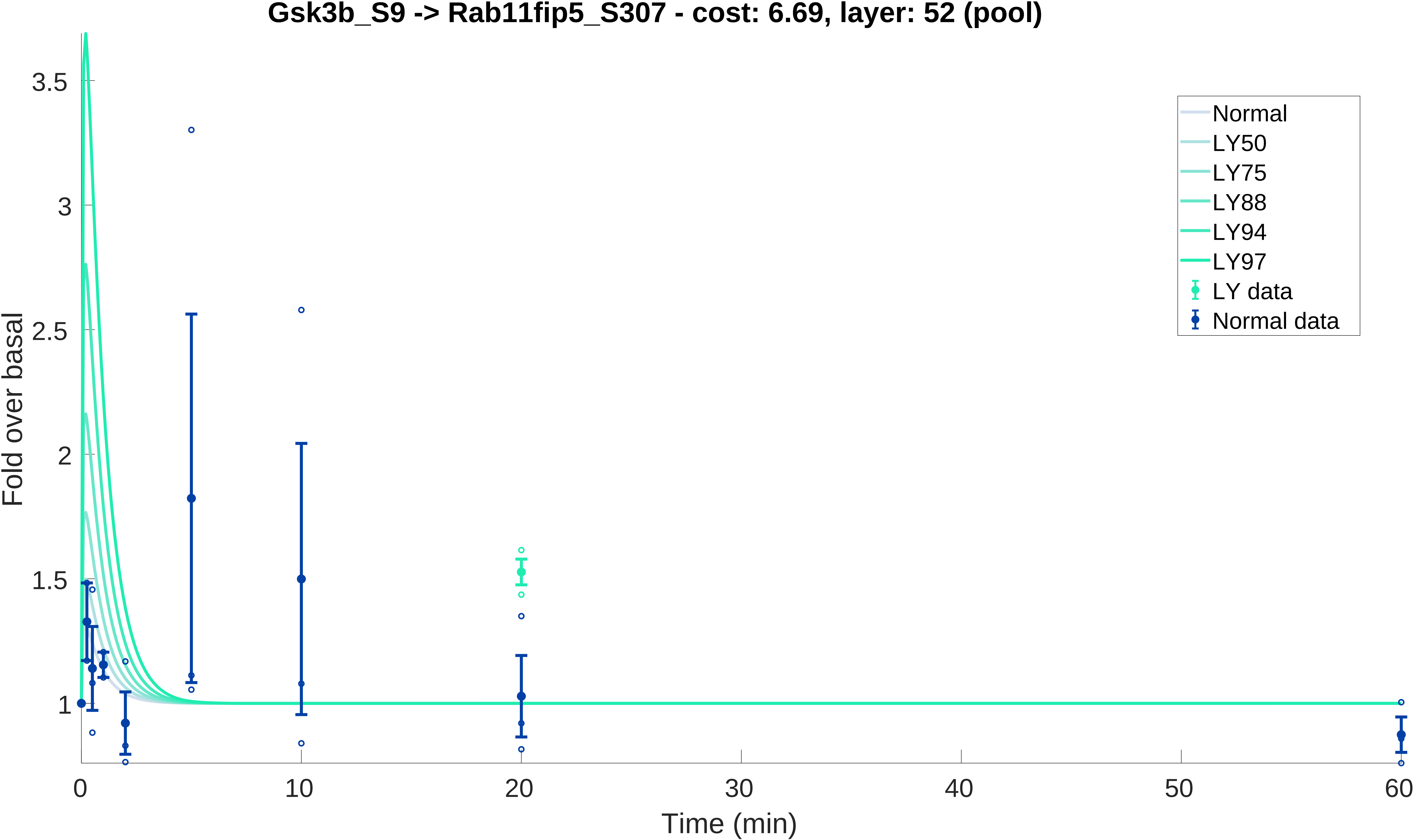

Gsk3b_S9 -> Rab11fip5_S307 - cost: 6.69, layer: 52 (pool)
Fold over basal
3.5
3
2.5
2
1.5
1
0
10
20
30
40
50
60
Time (min)
Normal
LY50
LY75
LY88
LY94
LY97
LY data
Normal data

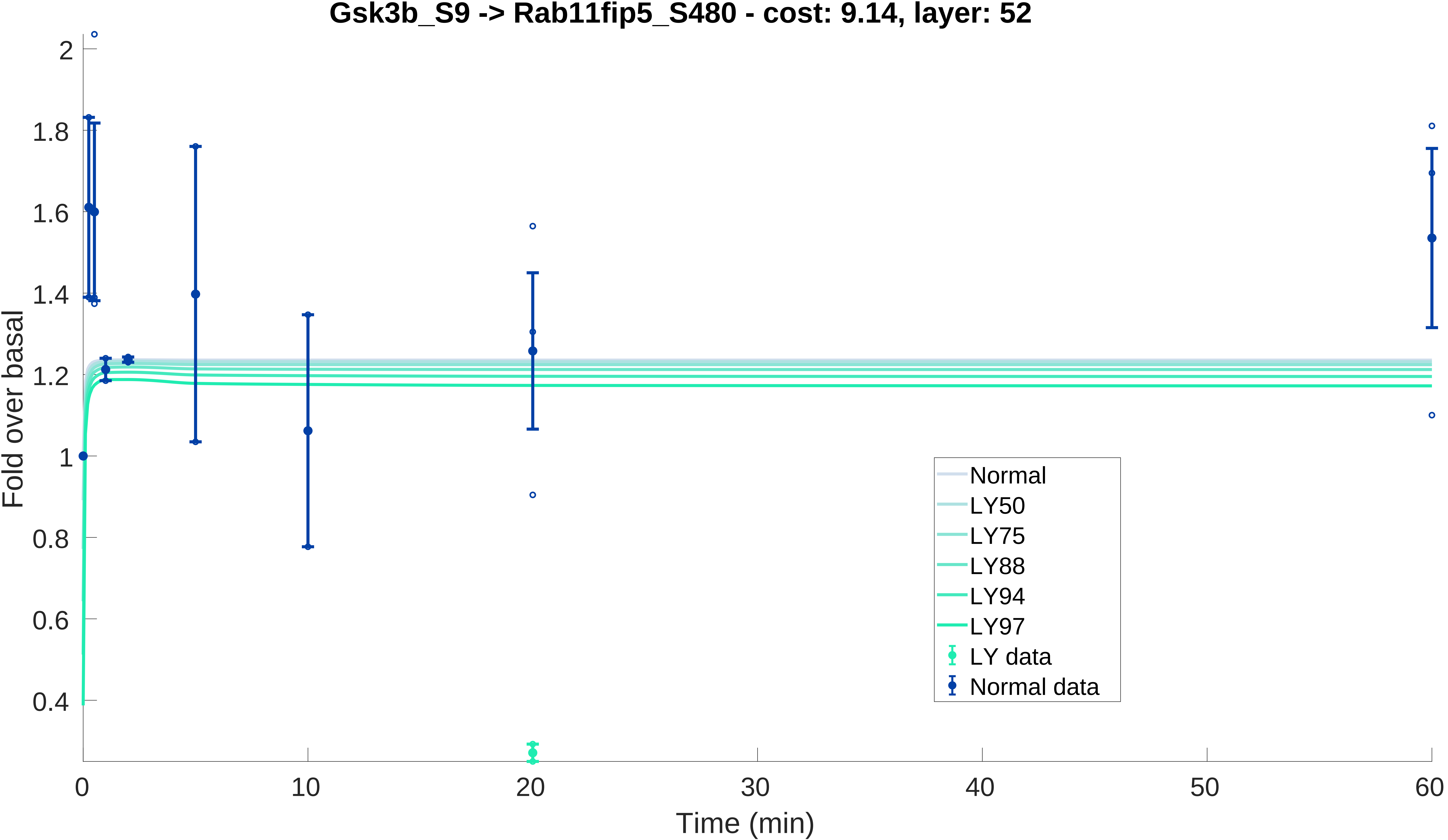

Gsk3b_S9 -> Rab11fip5_S480 - cost: 9.14, layer: 52
Fold over basal
Time (min)
0
10
20
30
40
50
60
0.4
0.6
0.8
1
1.2
1.4
1.6
1.8
2
Normal
LY50
LY75
LY88
LY94
LY97
LY data
Normal data

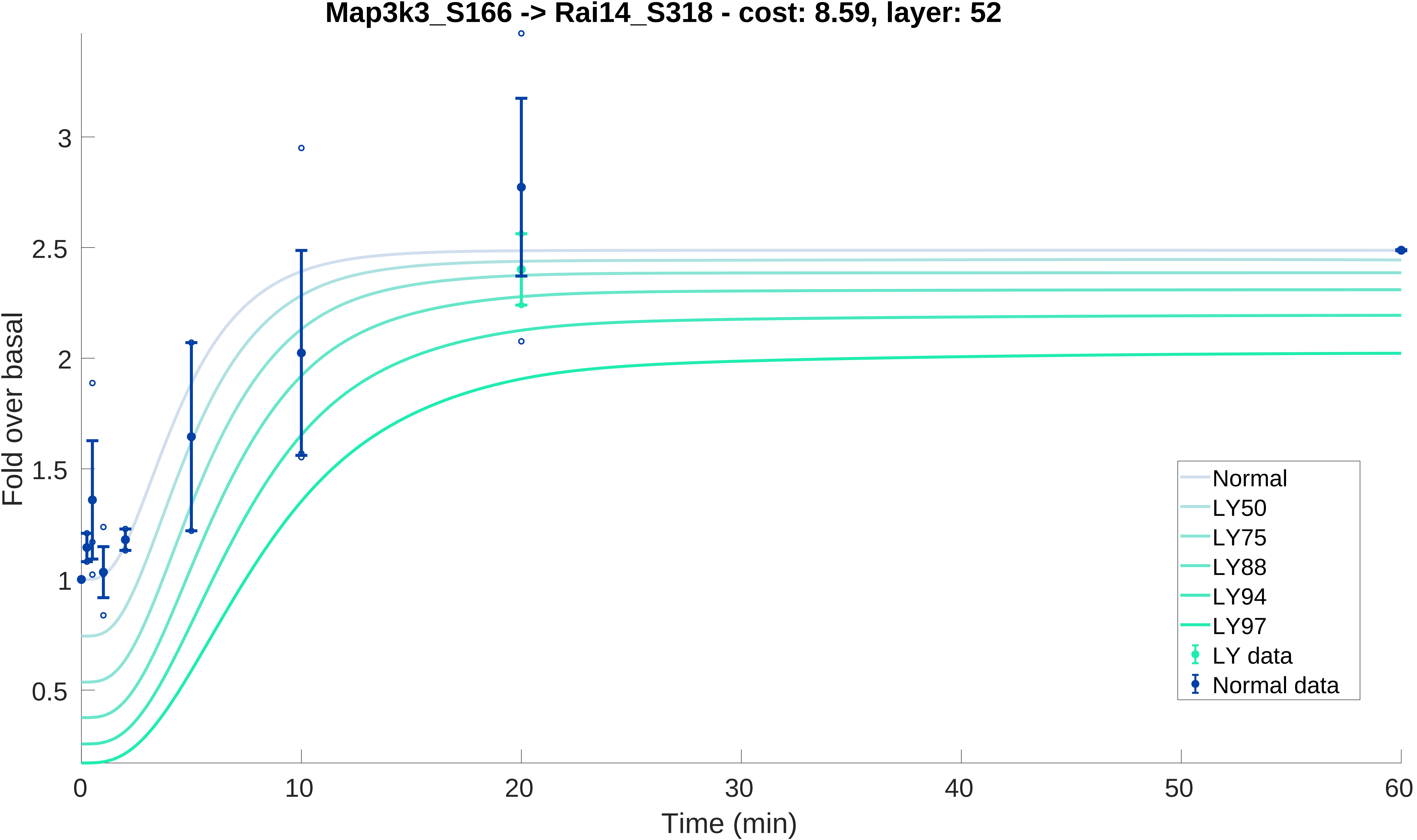
Map3k3_S166 -> Rai14_S318 - cost: 8.59, layer: 52
Fold over basal
3
2.5
2
1.5
1
0.5
0
10
20
30
40
50
60
Time (min)
Normal
LY50
LY75
LY88
LY94
LY97
LY data
Normal data

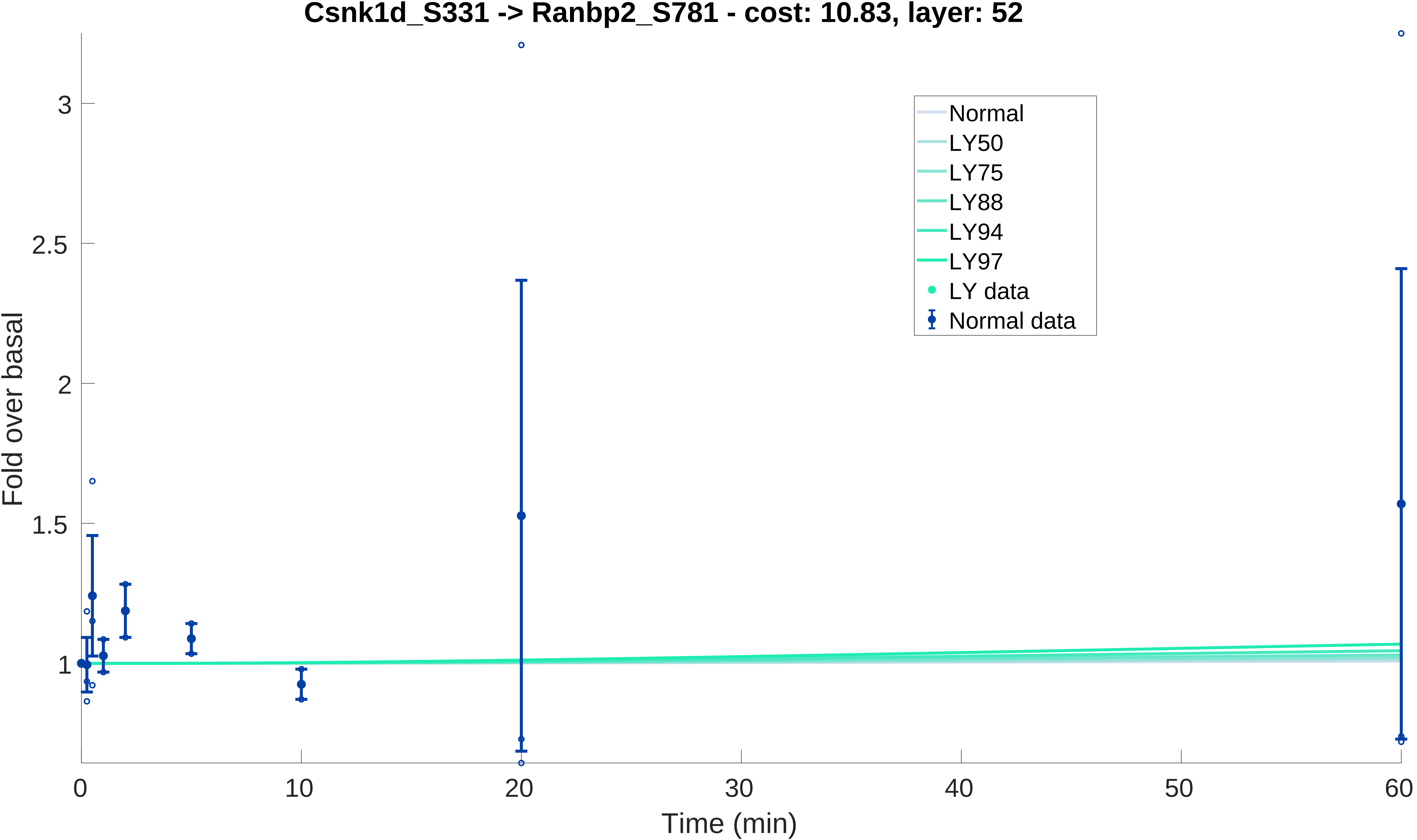

Csnk1d_S331 -> Ranbp2_S781 - cost: 10.83, layer: 52
Normal
LY50
LY75
LY88
LY94
LY97
LY data
Normal data
Fold over basal
3
2.5
2
1.5
1
0
10
20
30
40
50
60
Time (min)

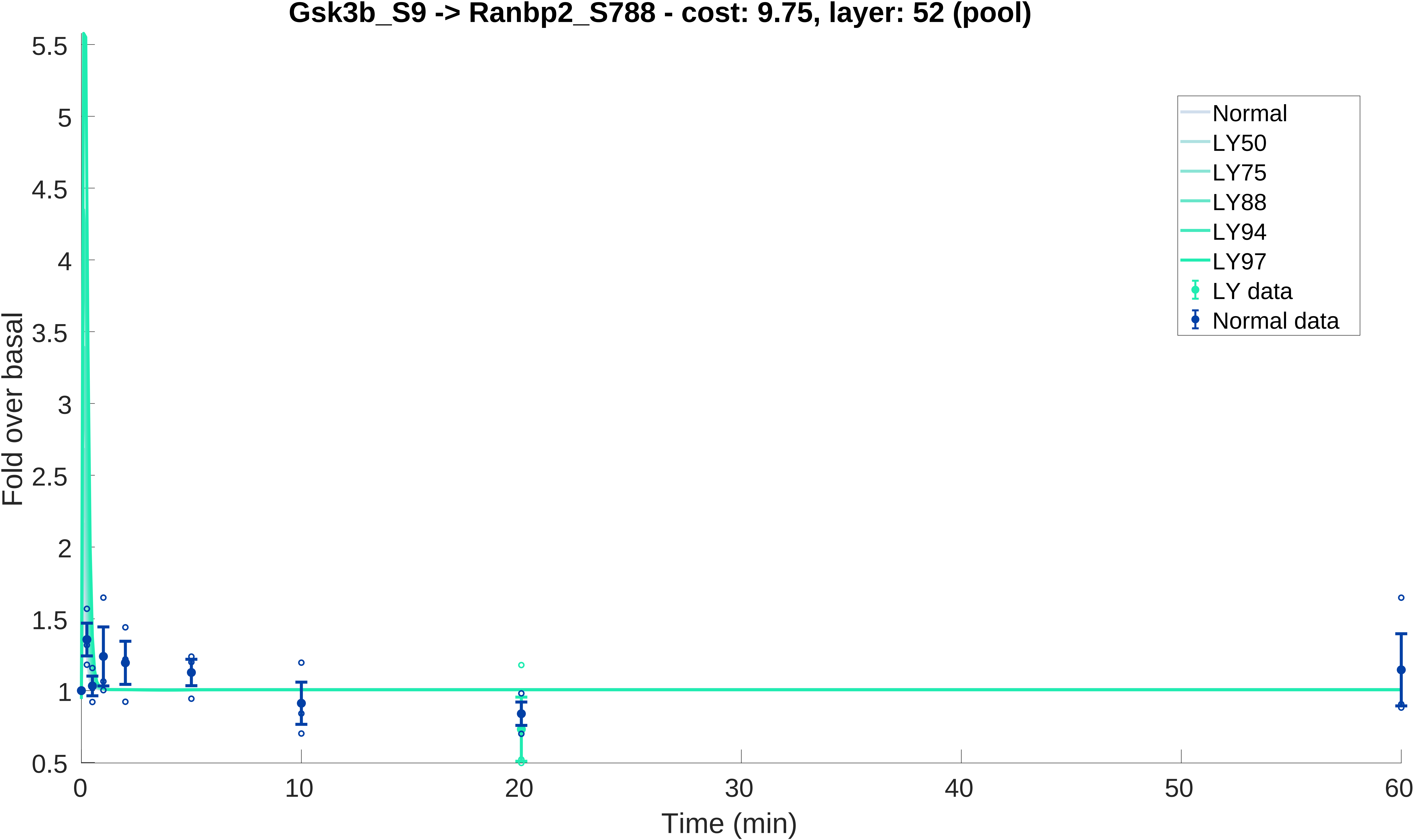

Gsk3b_S9 -> Ranbp2_S788 - cost: 9.75, layer: 52 (pool)
Fold over basal
Time (min)
0.5
1
1.5
2
2.5
3
3.5
4
4.5
5
5.5
0
10
20
30
40
50
60
Normal
LY50
LY75
LY88
LY94
LY97
LY data
Normal data

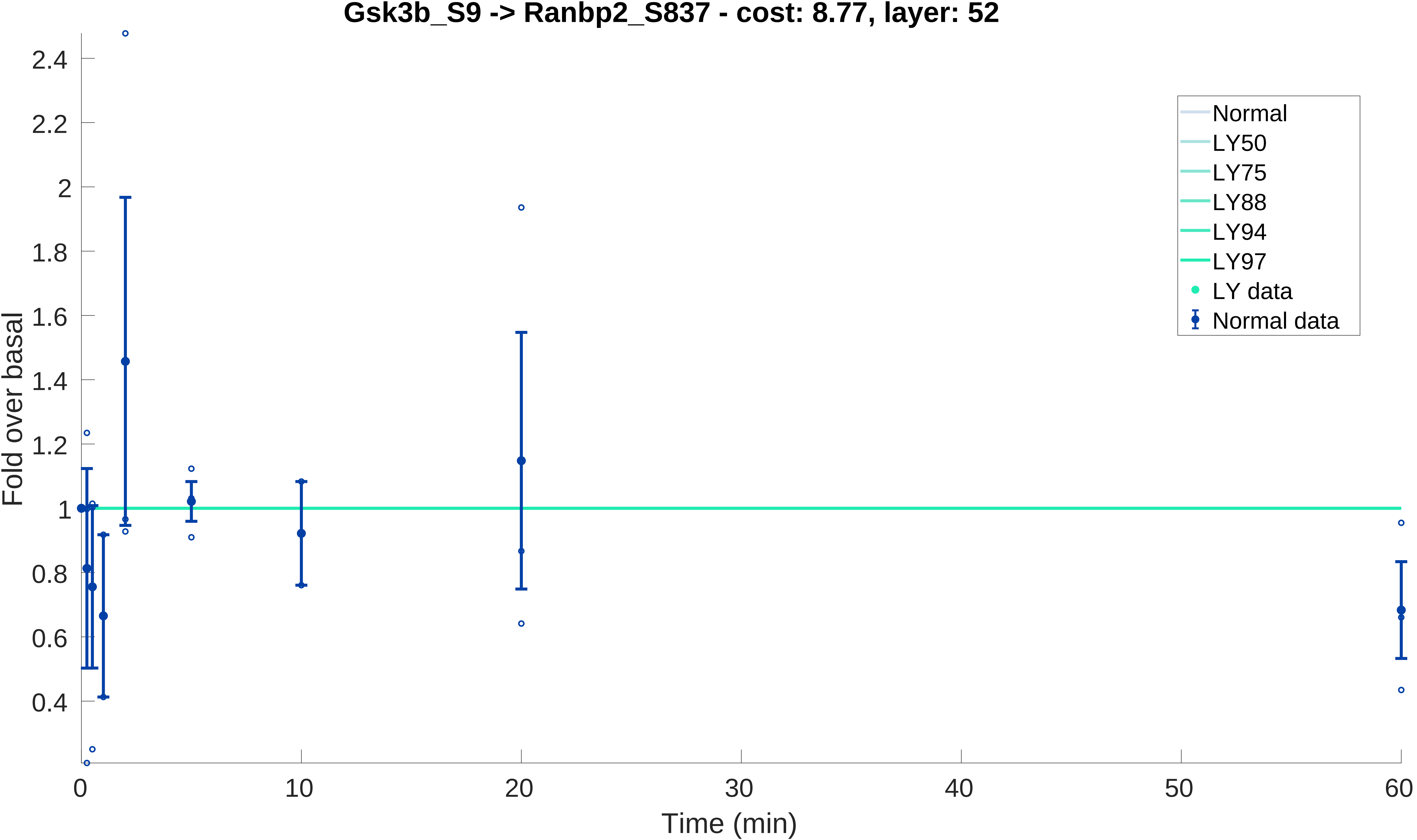

Gsk3b_S9 -> Ranbp2_S837 - cost: 8.77, layer: 52
Fold over basal
Time (min)
0.4
0.6
0.8
1
1.2
1.4
1.6
1.8
2
2.2
2.4
0
10
20
30
40
50
60
Normal
LY50
LY75
LY88
LY94
LY97
LY data
Normal data

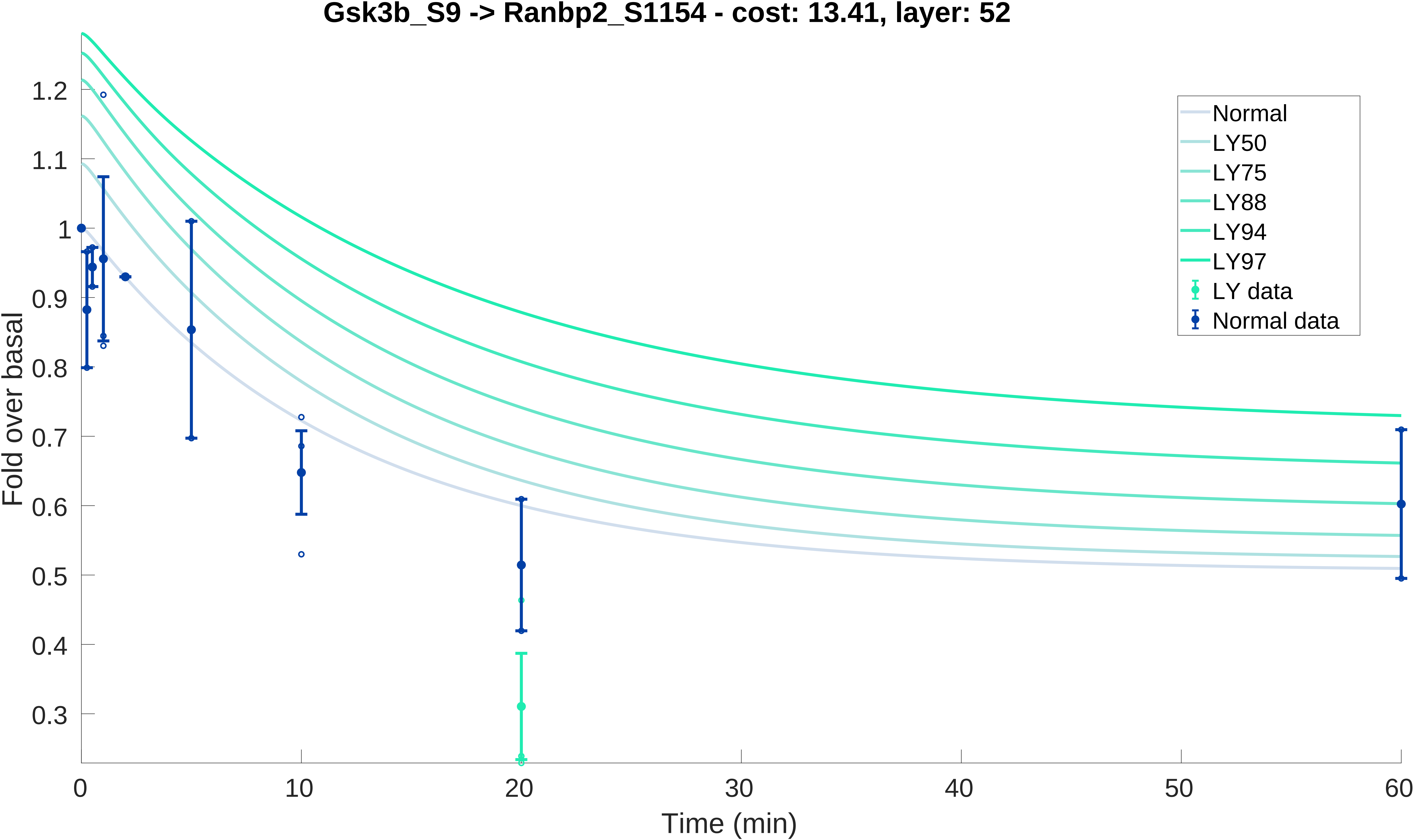

Gsk3b_S9 -> Ranbp2_S1154 - cost: 13.41, layer: 52
Fold over basal
Time (min)
0
10
20
30
40
50
60
0.3
0.4
0.5
0.6
0.7
0.8
0.9
1
1.1
1.2
Normal
LY50
LY75
LY88
LY94
LY97
LY data
Normal data

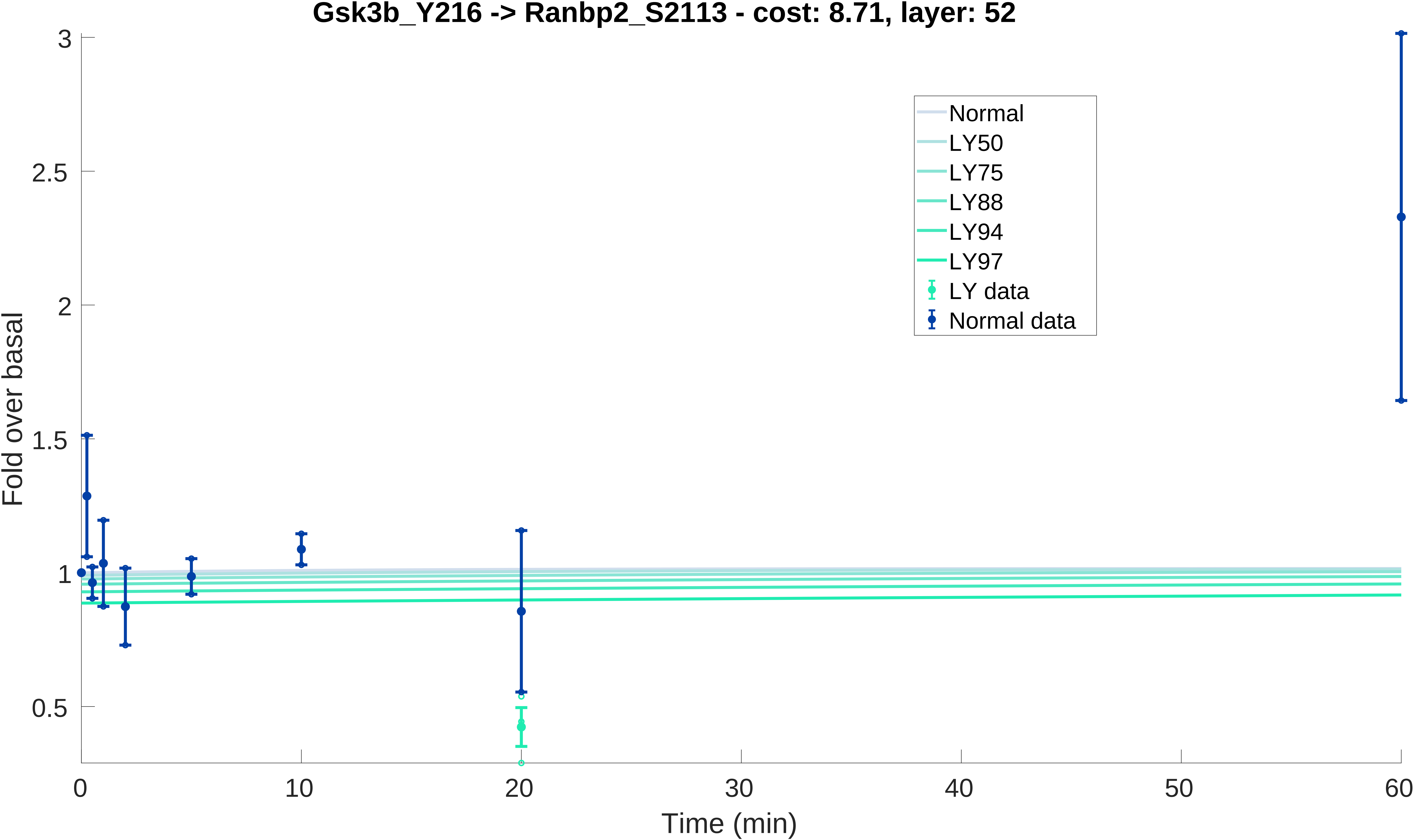

Gsk3b_Y216 -> Ranbp2_S2113 - cost: 8.71, layer: 52
Fold over basal
Time (min)
Normal
LY50
LY75
LY88
LY94
LY97
LY data
Normal data
0
10
20
30
40
50
60
0.5
1
1.5
2
2.5
3

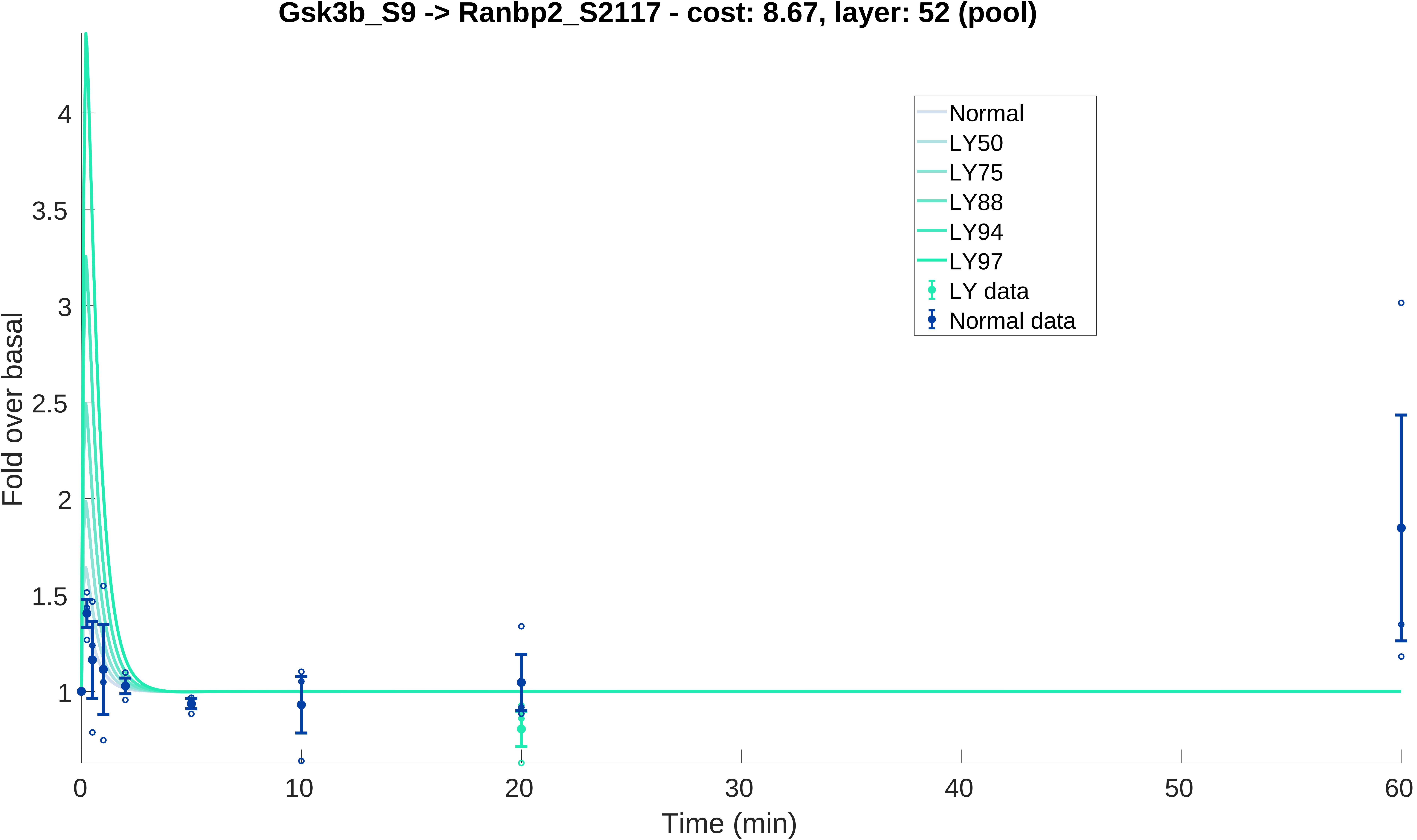
Gsk3b_S9 -> Ranbp2_S2117 - cost: 8.67, layer: 52 (pool)
Fold over basal
Time (min)
Normal
LY50
LY75
LY88
LY94
LY97
LY data
Normal data
4
3.5
3
2.5
2
1.5
1
0
10
20
30
40
50
60

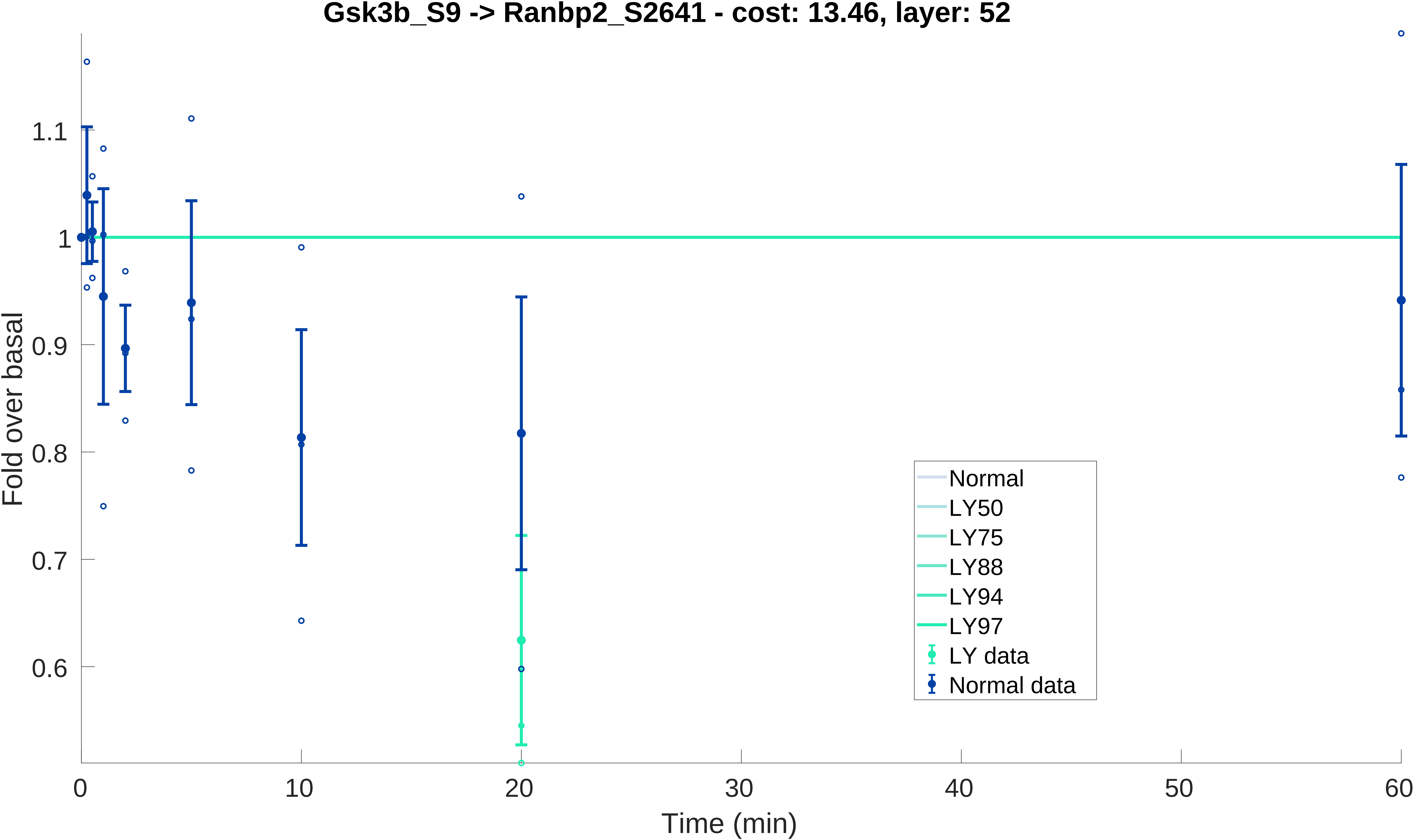

Gsk3b_S9 -> Ranbp2_S2641 - cost: 13.46, layer: 52
Fold over basal
1.1
1
0.9
0.8
0.7
0.6
0
10
20
30
40
50
60
Time (min)
Normal
LY50
LY75
LY88
LY94
LY97
LY data
Normal data

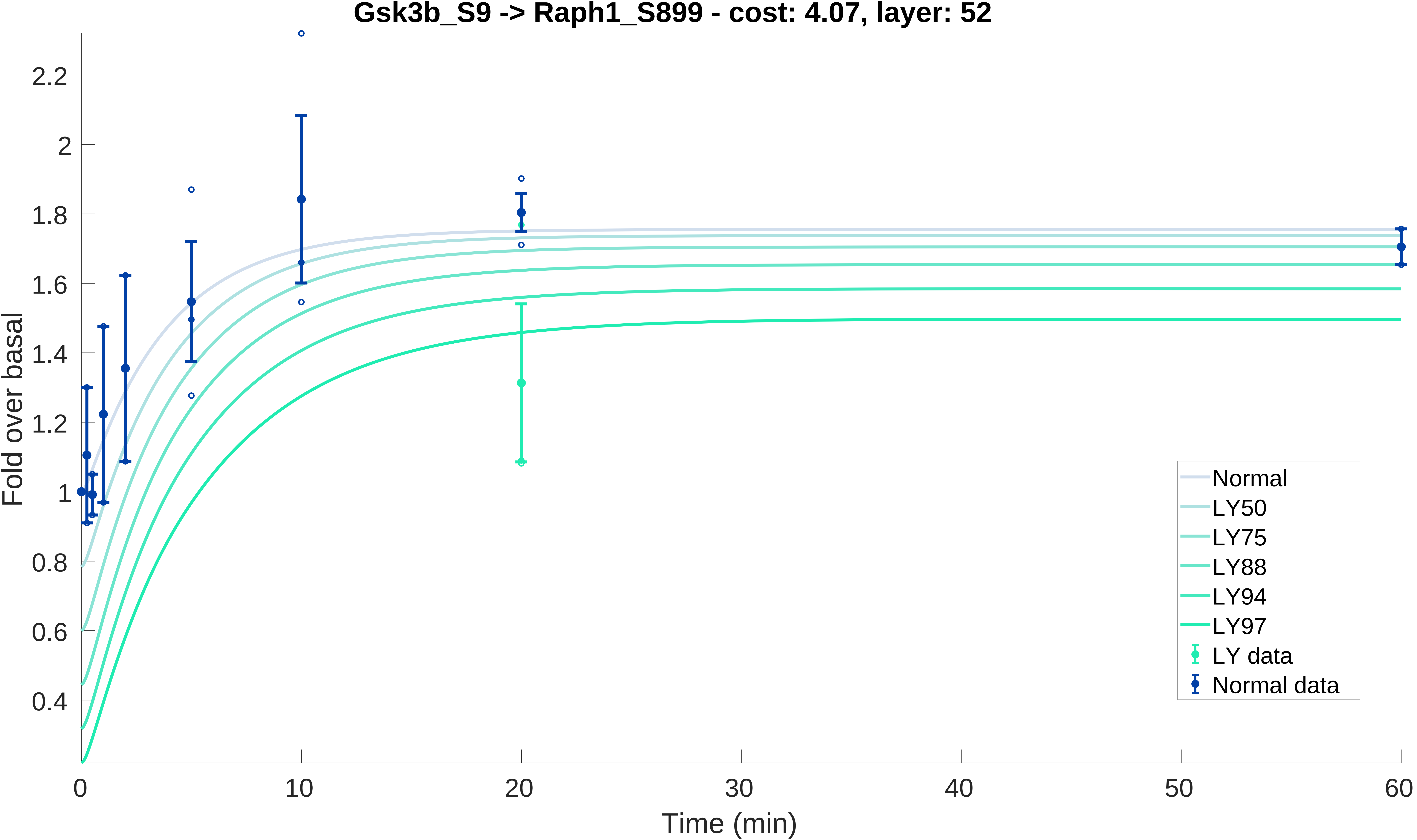
Gsk3b_S9 -> Raph1_S899 - cost: 4.07, layer: 52
Fold over basal
Time (min)
0
10
20
30
40
50
60
0.4
0.6
0.8
1
1.2
1.4
1.6
1.8
2
2.2
Normal
LY50
LY75
LY88
LY94
LY97
LY data
Normal data

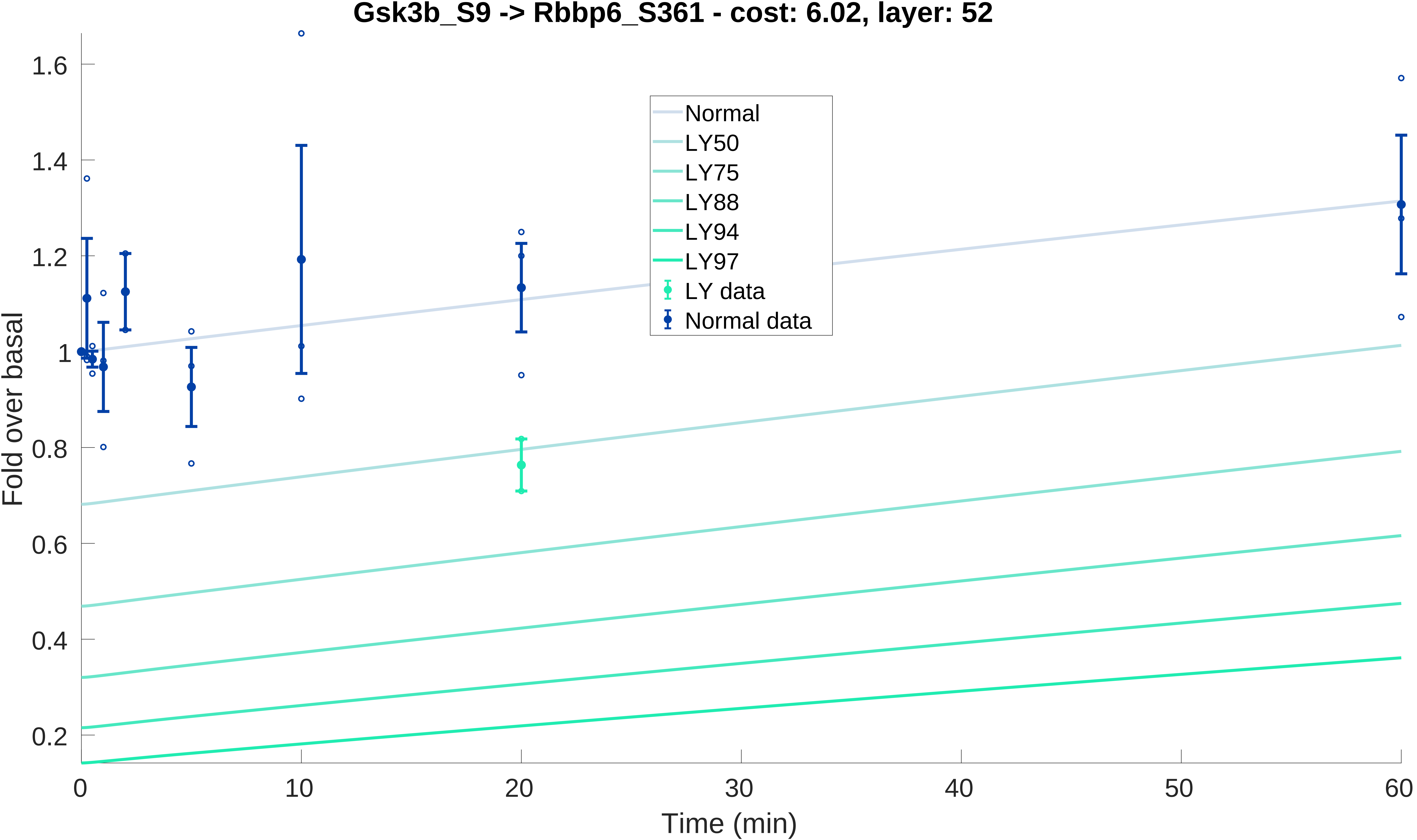

Gsk3b_S9 -> Rbbp6_S361 - cost: 6.02, layer: 52
Normal
LY50
LY75
LY88
LY94
LY97
LY data
Normal data
Fold over basal
Time (min)
0
10
20
30
40
50
60
0.2
0.4
0.6
0.8
1
1.2
1.4
1.6

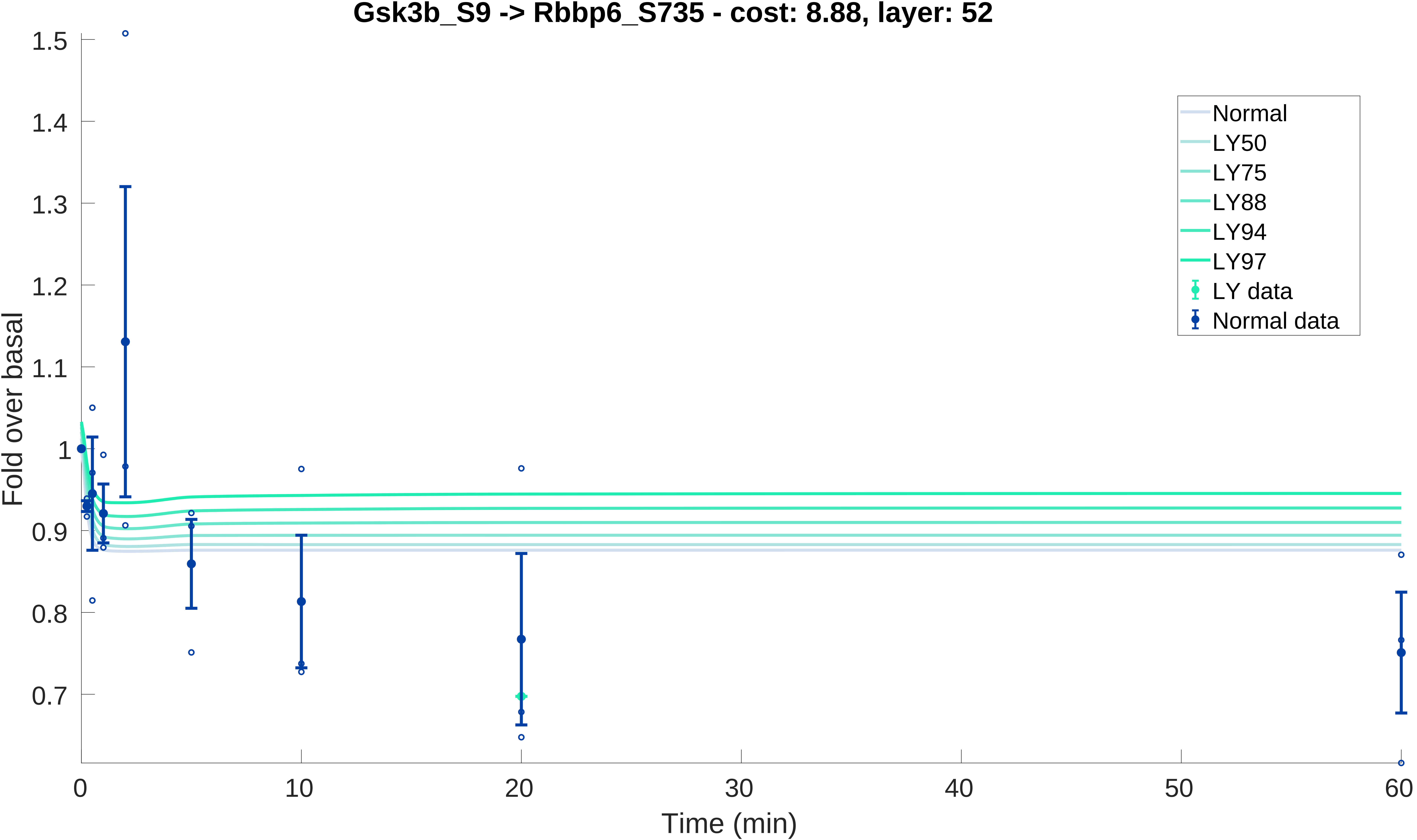
Gsk3b_S9 -> Rbbp6_S735 - cost: 8.88, layer: 52
Fold over basal
Time (min)
1.5
1.4
1.3
1.2
1.1
1
0.9
0.8
0.7
0
10
20
30
40
50
60
Normal
LY50
LY75
LY88
LY94
LY97
LY data
Normal data

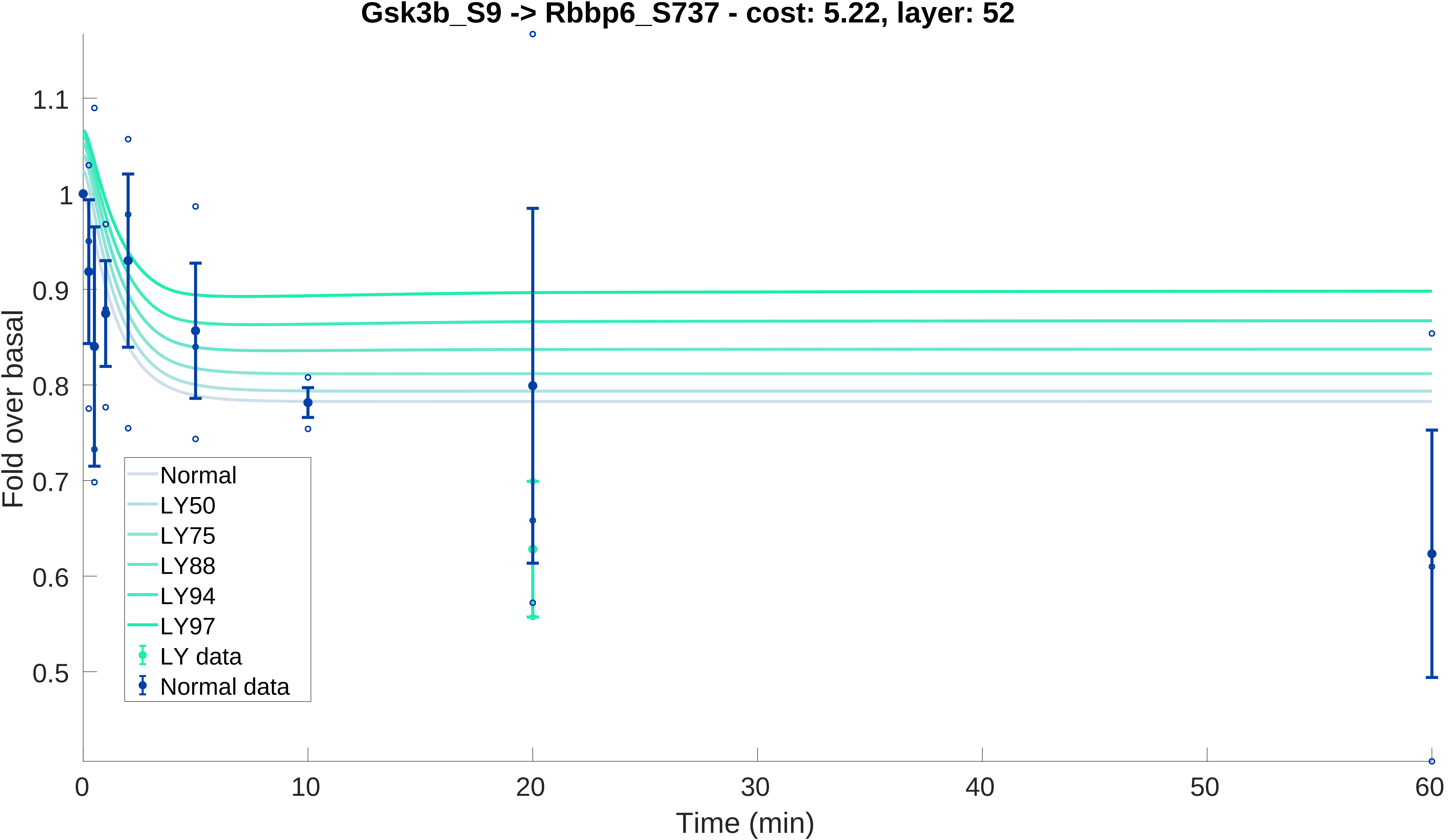

Gsk3b_S9 -> Rbbp6_S737 - cost: 5.22, layer: 52
Fold over basal
Time (min)
Normal
LY50
LY75
LY88
LY94
LY97
LY data
Normal data
1.1
1
0.9
0.8
0.7
0.6
0.5
0
10
20
30
40
50
60

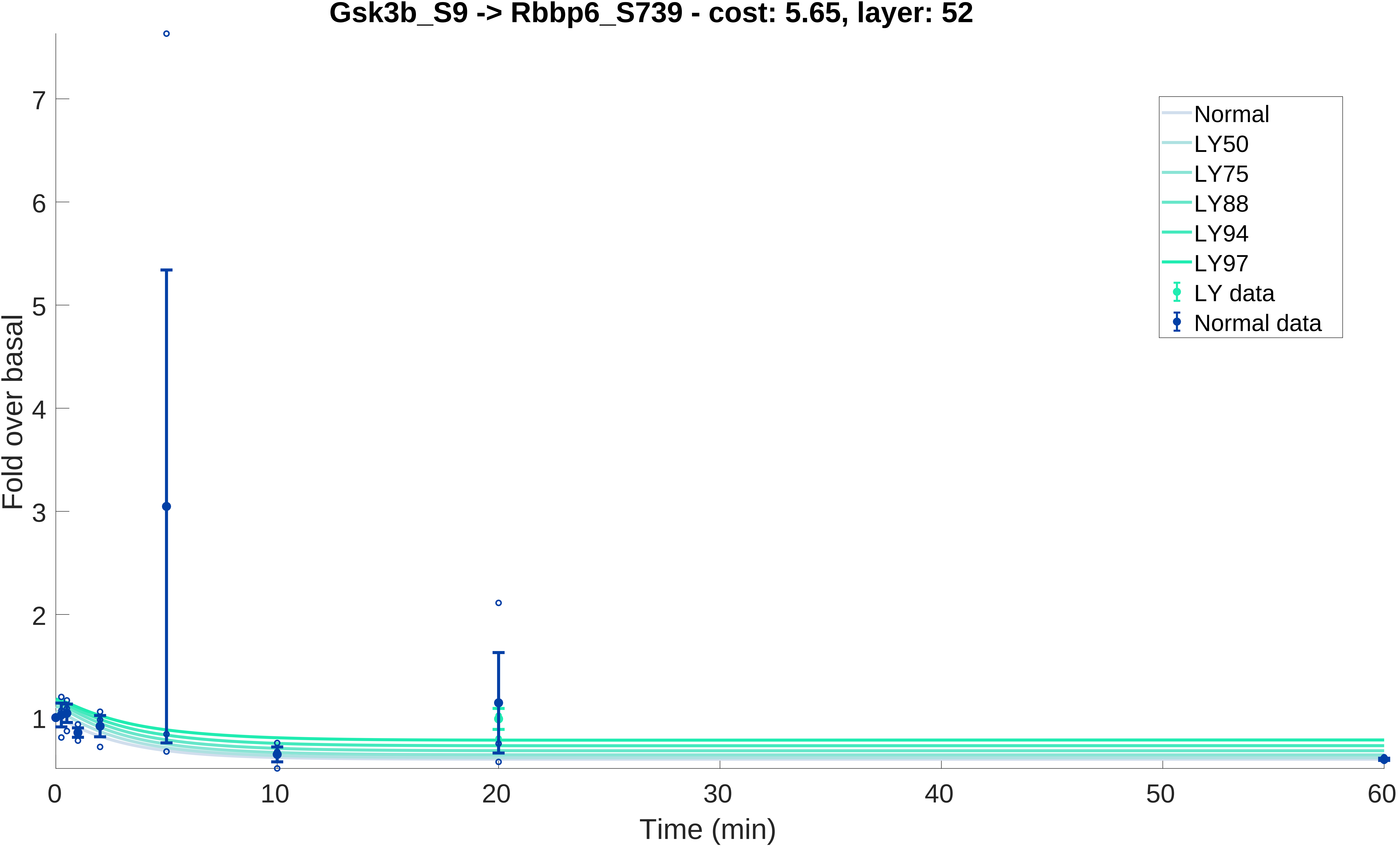

Gsk3b_S9 -> Rbbp6_S739 - cost: 5.65, layer: 52
Fold over basal
Time (min)
Normal
LY50
LY75
LY88
LY94
LY97
LY data
Normal data
0
10
20
30
40
50
60
1
2
3
4
5
6
7

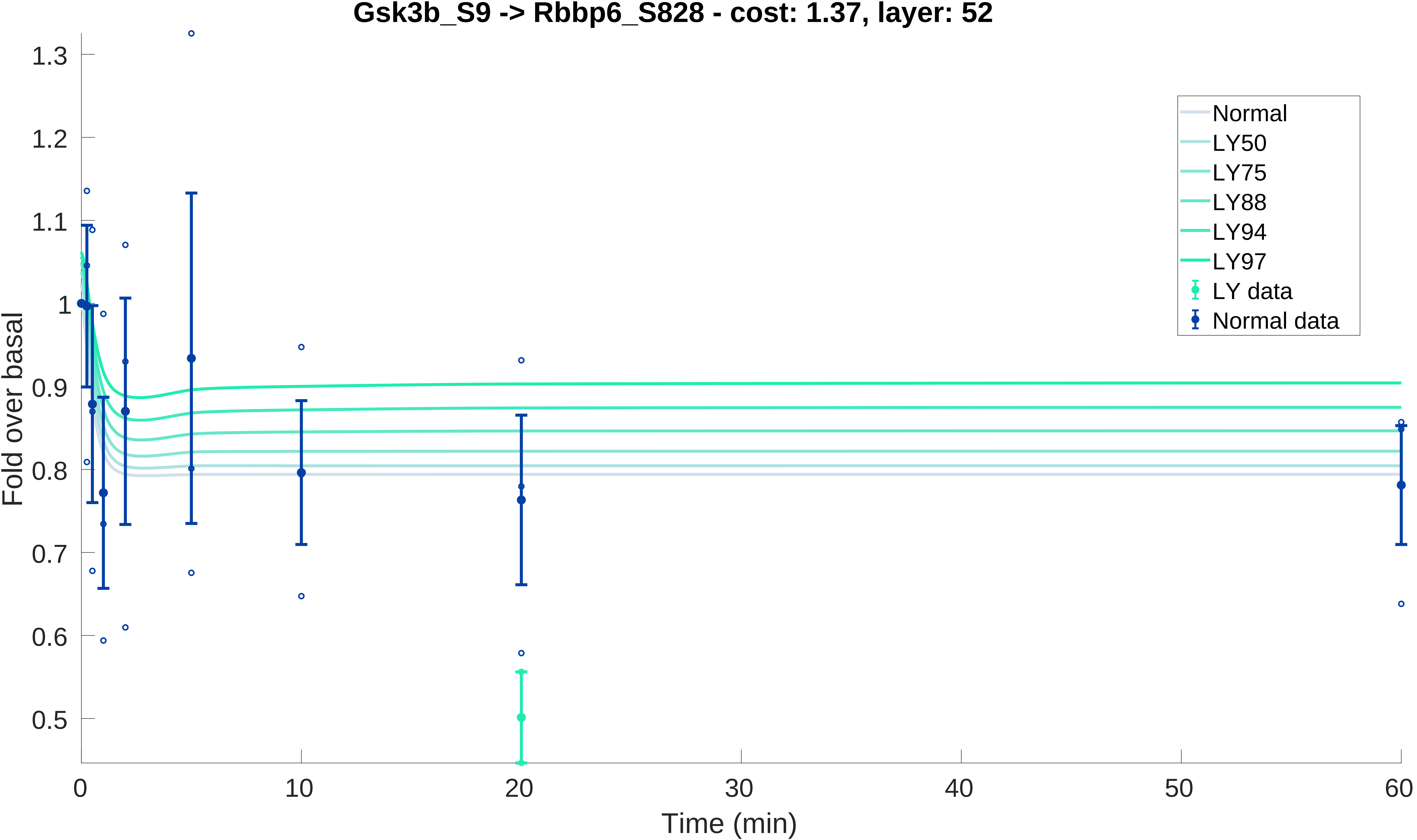
Gsk3b_S9 -> Rbbp6_S828 - cost: 1.37, layer: 52
Fold over basal
Time (min)
Normal
LY50
LY75
LY88
LY94
LY97
LY data
Normal data
1.3
1.2
1.1
1
0.9
0.8
0.7
0.6
0.5
0
10
20
30
40
50
60

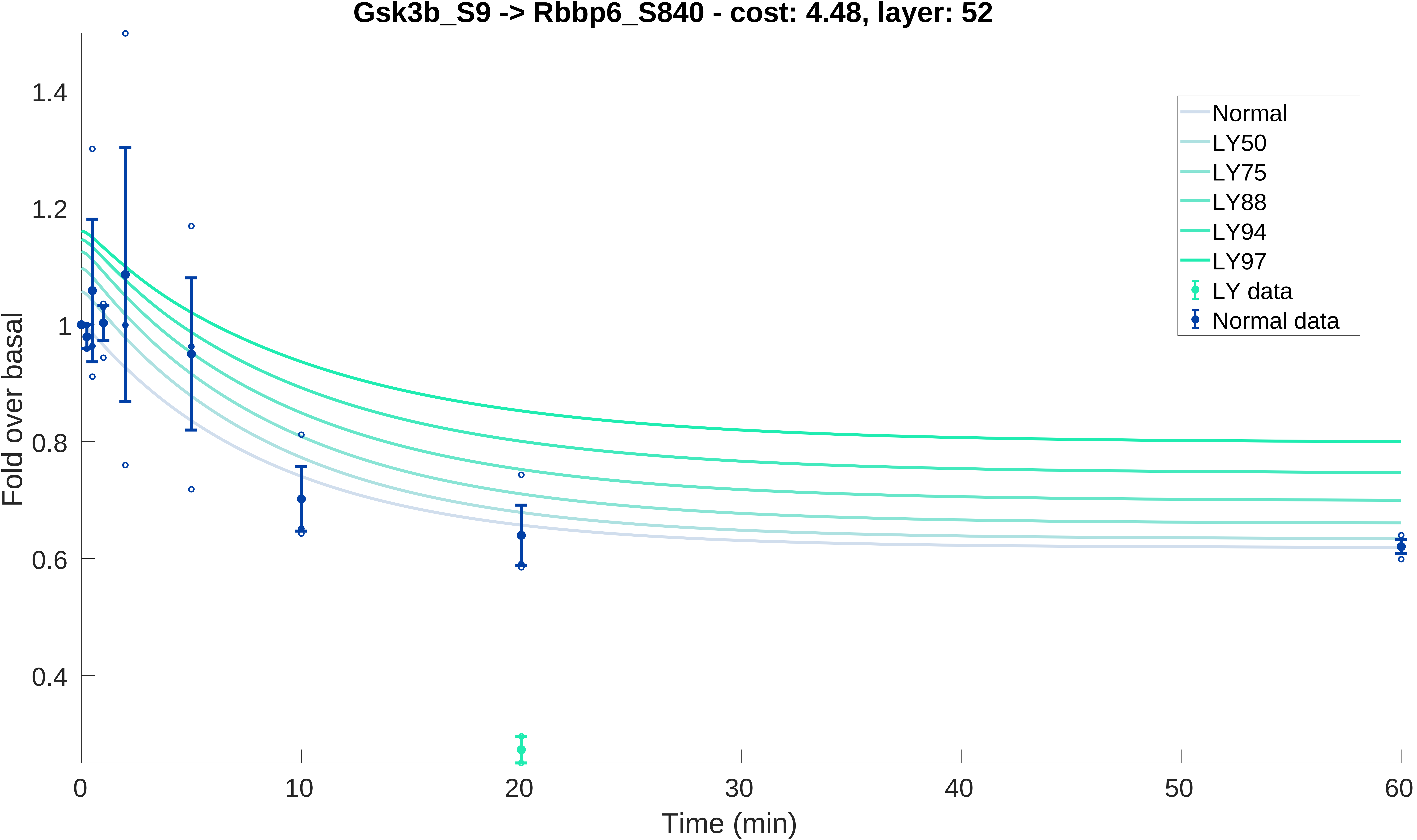
Gsk3b_S9 -> Rbbp6_S840 - cost: 4.48, layer: 52
Fold over basal
1.4
1.2
1
0.8
0.6
0.4
0
10
20
30
40
50
60
Time (min)
Normal
LY50
LY75
LY88
LY94
LY97
LY data
Normal data

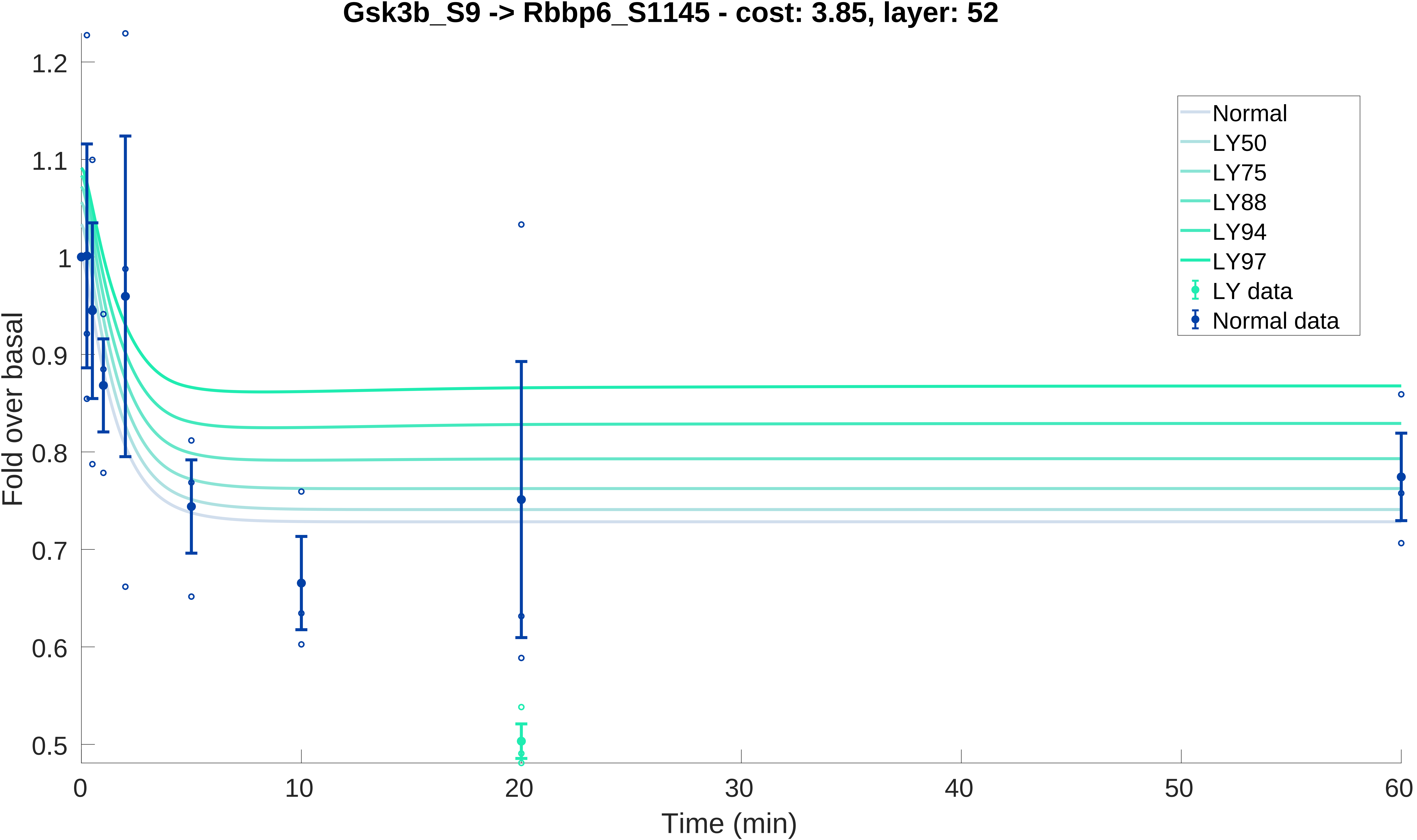
Gsk3b_S9 -> Rbbp6_S1145 - cost: 3.85, layer: 52
Fold over basal
1.2
1.1
1
0.9
0.8
0.7
0.6
0.5
0
10
20
30
40
50
60
Time (min)
Normal
LY50
LY75
LY88
LY94
LY97
LY data
Normal data

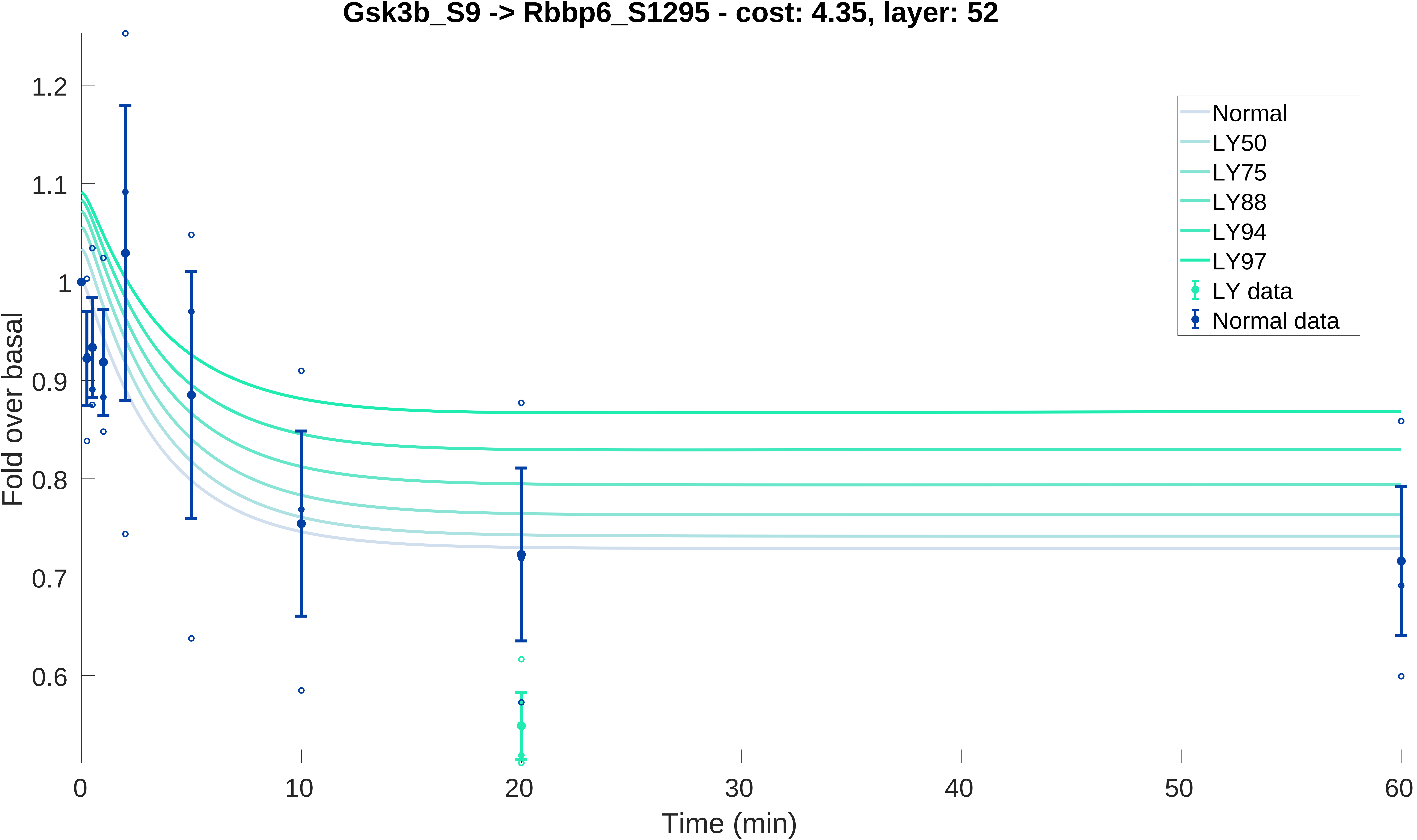
Gsk3b_S9 -> Rbbp6_S1295 - cost: 4.35, layer: 52
Fold over basal
Time (min)
1.2
1.1
1
0.9
0.8
0.7
0.6
0
10
20
30
40
50
60
Normal
LY50
LY75
LY88
LY94
LY97
LY data
Normal data

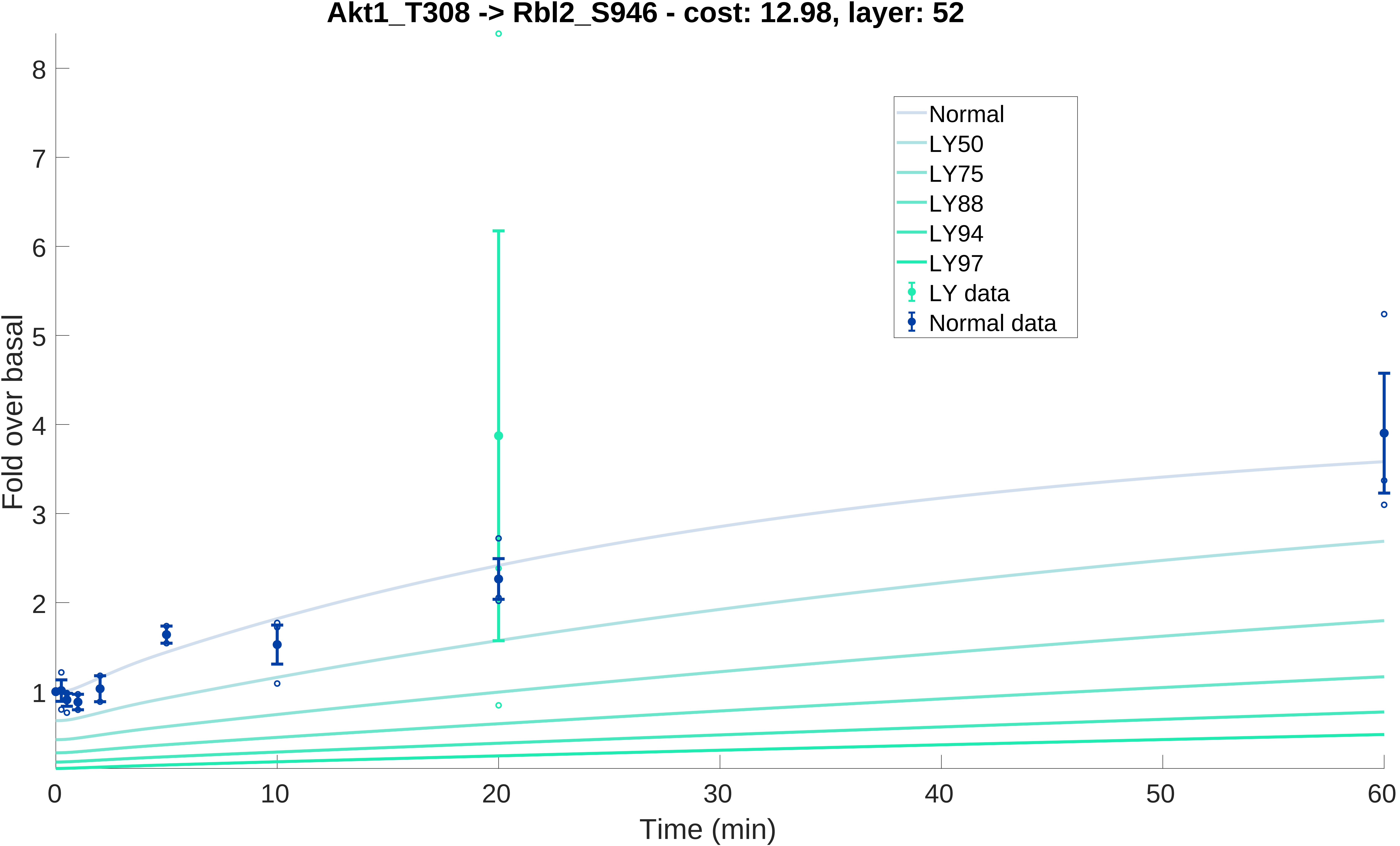

Akt1_T308 -> Rbl2_S946 - cost: 12.98, layer: 52
Fold over basal
Time (min)
0
10
20
30
40
50
60
1
2
3
4
5
6
7
8
Normal
LY50
LY75
LY88
LY94
LY97
LY data
Normal data

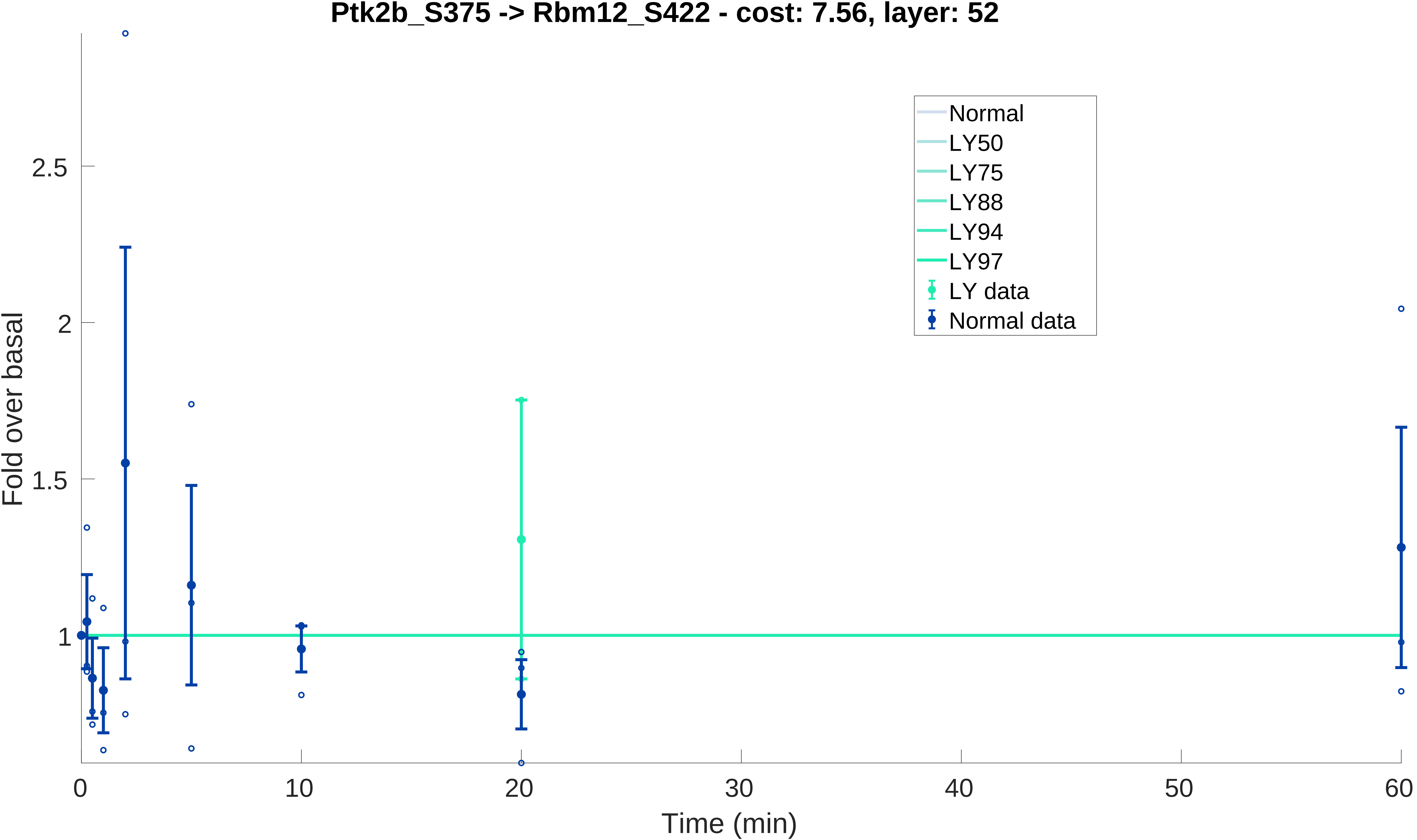
Ptk2b_S375 -> Rbm12_S422 - cost: 7.56, layer: 52
Fold over basal
Time (min)
Normal
LY50
LY75
LY88
LY94
LY97
LY data
Normal data
2.5
2
1.5
1
0
10
20
30
40
50
60

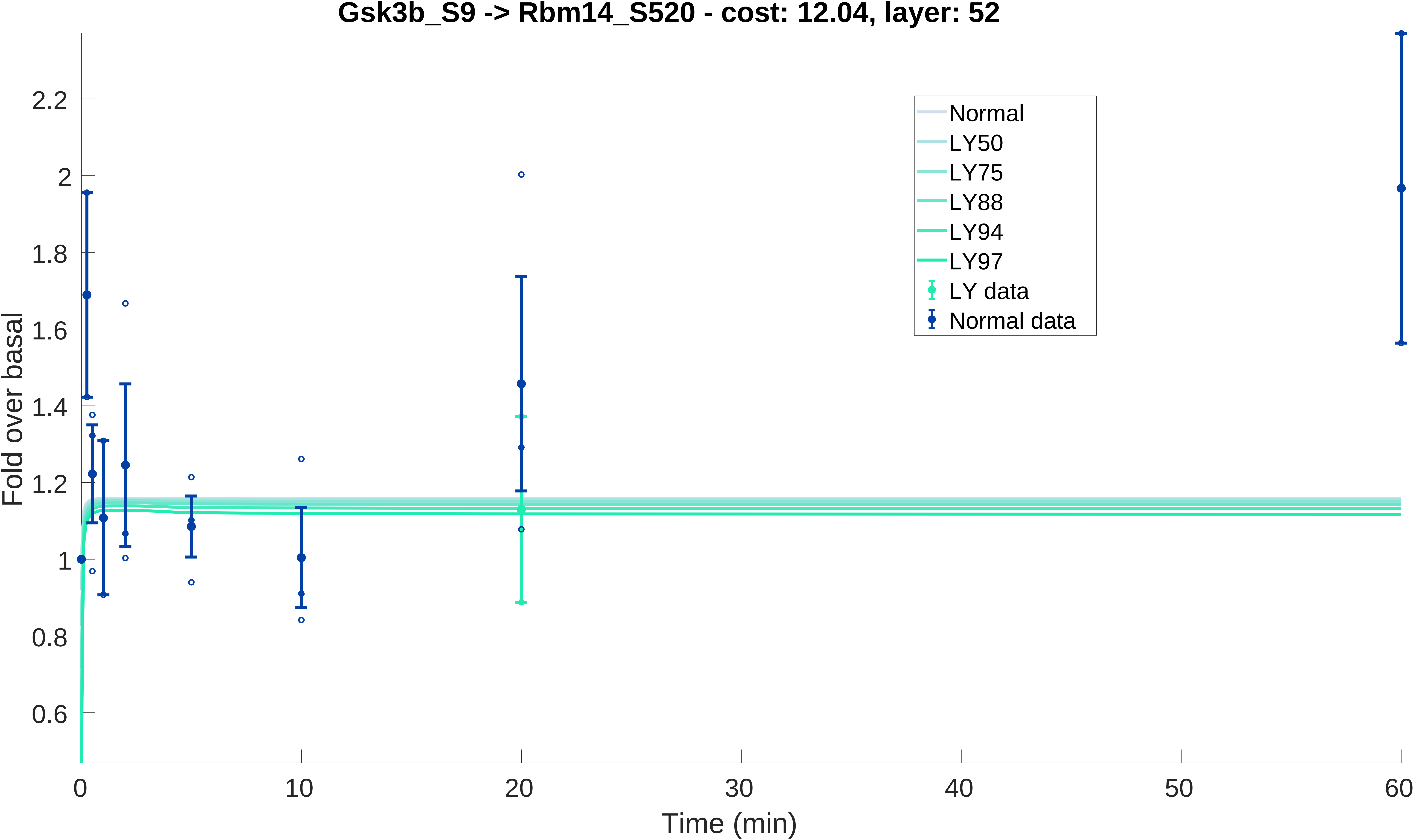

Gsk3b_S9 -> Rbm14_S520 - cost: 12.04, layer: 52
Normal
LY50
LY75
LY88
LY94
LY97
LY data
Normal data
Fold over basal
Time (min)
0.6
0.8
1
1.2
1.4
1.6
1.8
2
2.2
0
10
20
30
40
50
60

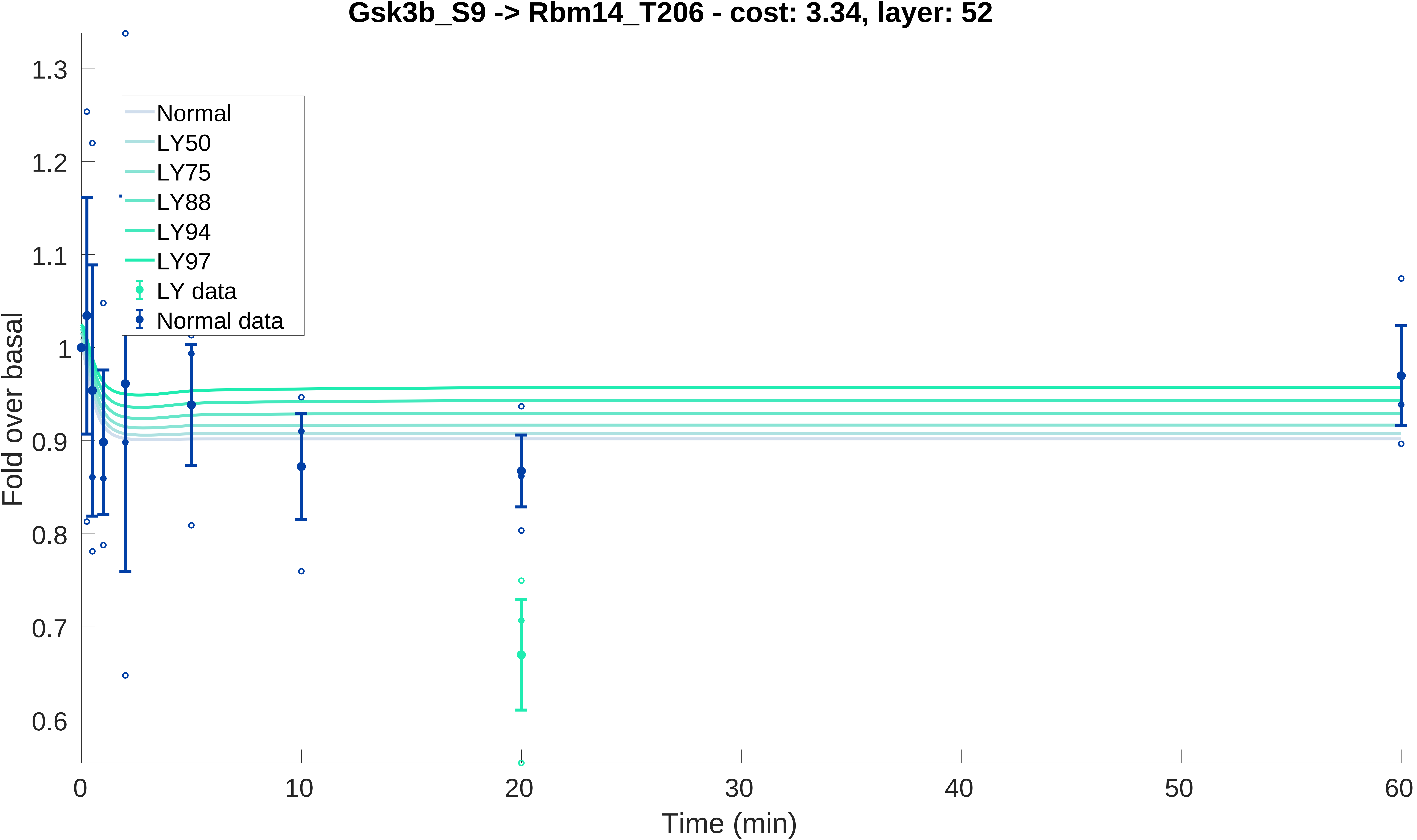

Gsk3b_S9 -> Rbm14_T206 - cost: 3.34, layer: 52
Normal
LY50
LY75
LY88
LY94
LY97
LY data
Normal data
Fold over basal
Time (min)
1.3
1.2
1.1
1
0.9
0.8
0.7
0.6
0
10
20
30
40
50
60

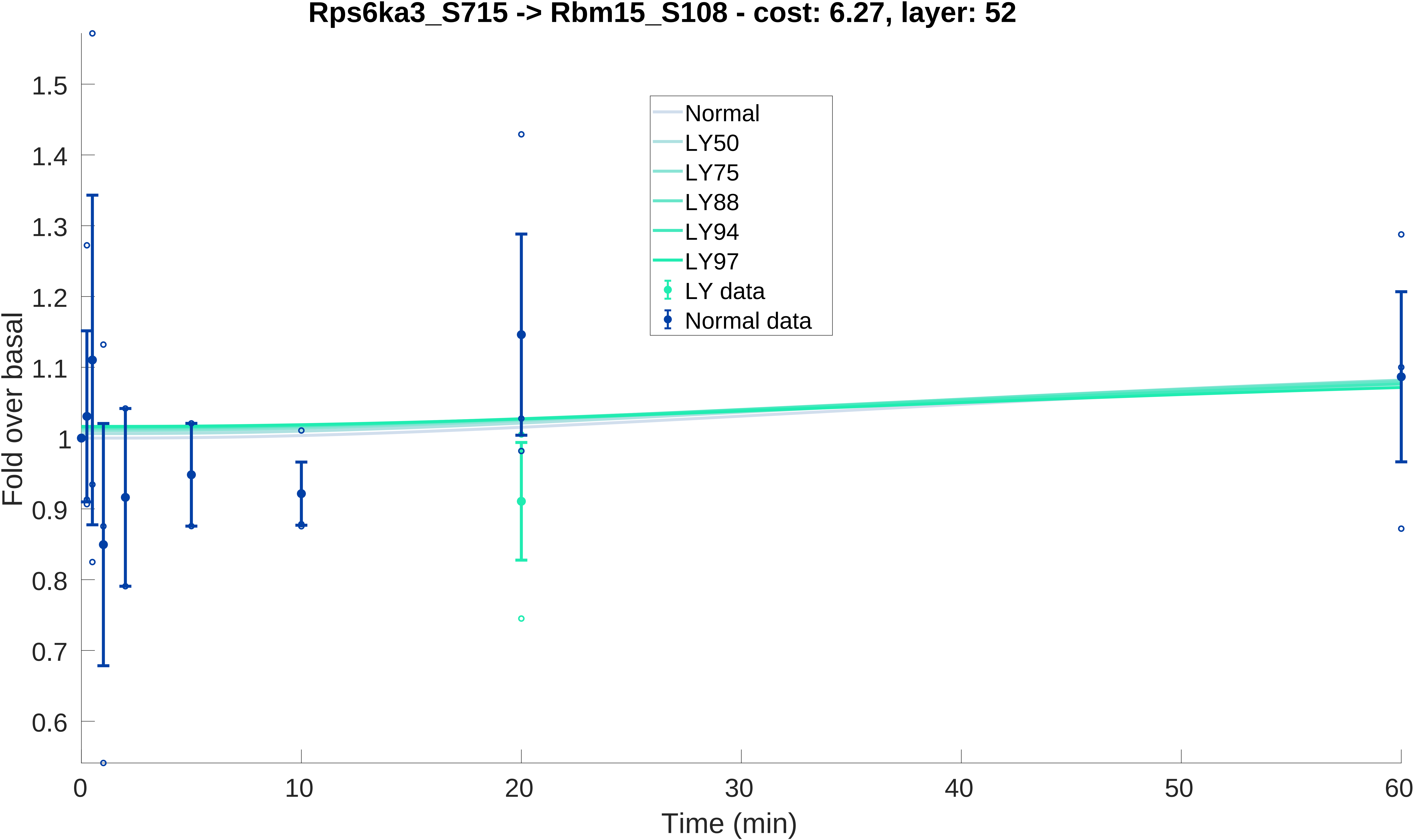

Rps6ka3_S715 -> Rbm15_S108 - cost: 6.27, layer: 52
Fold over basal
Time (min)
Normal
LY50
LY75
LY88
LY94
LY97
LY data
Normal data
1.5
1.4
1.3
1.2
1.1
1
0.9
0.8
0.7
0.6
0
10
20
30
40
50
60

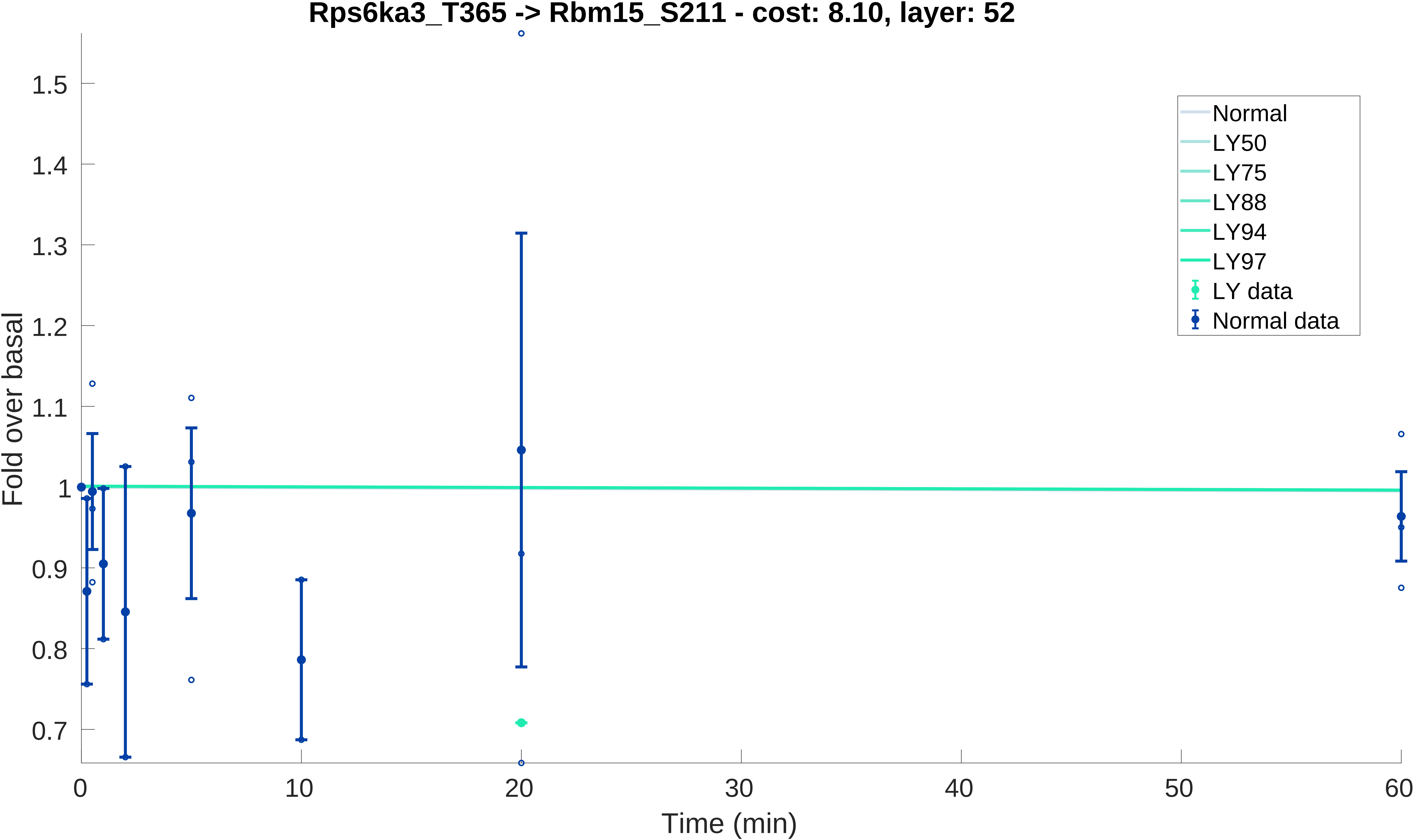

Rps6ka3_T365 -> Rbm15_S211 - cost: 8.10, layer: 52
Fold over basal
1.5
1.4
1.3
1.2
1.1
1
0.9
0.8
0.7
0
10
20
30
40
50
60
Time (min)
Normal
LY50
LY75
LY88
LY94
LY97
LY data
Normal data

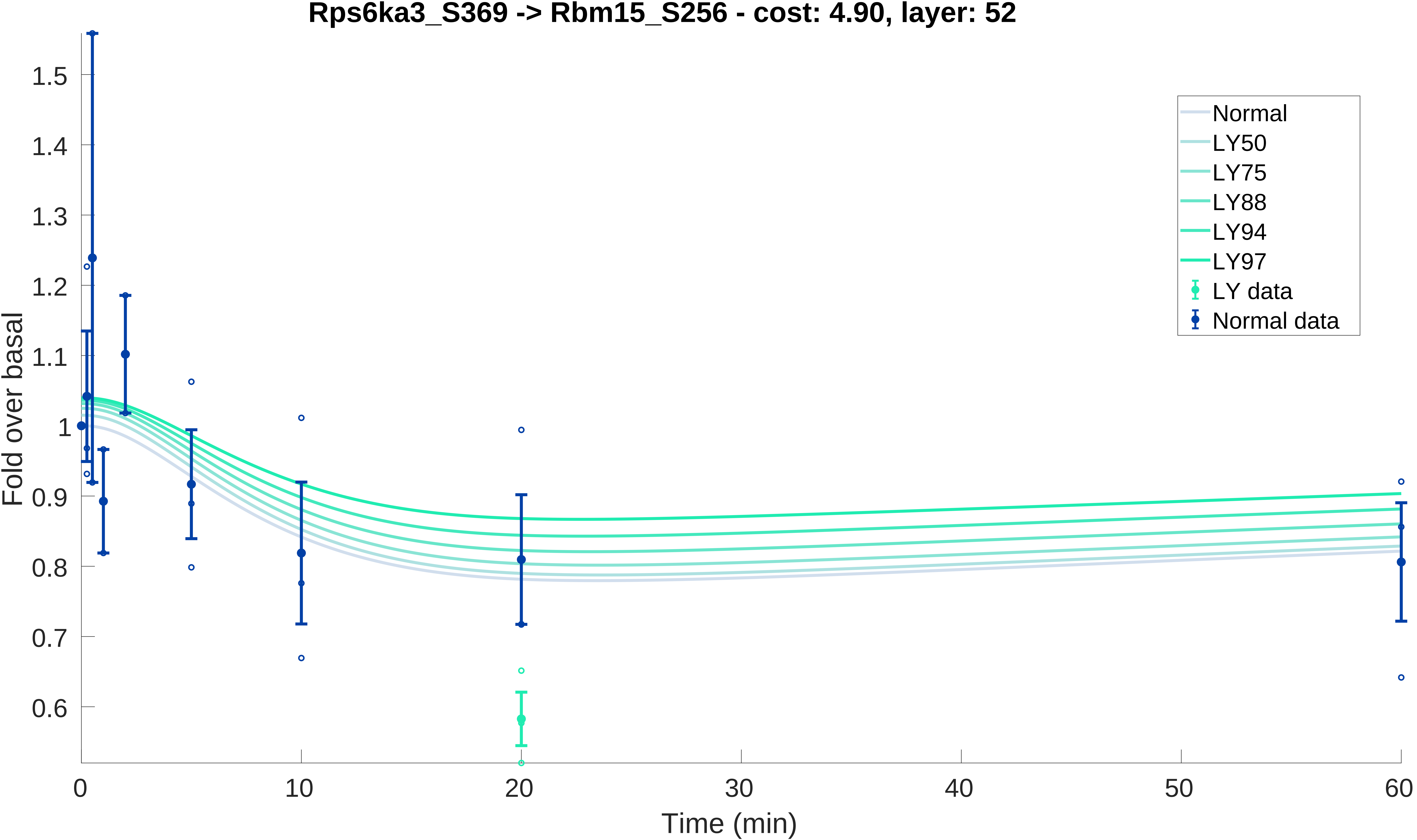

Rps6ka3_S369 -> Rbm15_S256 - cost: 4.90, layer: 52
Fold over basal
Time (min)
0.6
0.7
0.8
0.9
1
1.1
1.2
1.3
1.4
1.5
0
10
20
30
40
50
60
Normal
LY50
LY75
LY88
LY94
LY97
LY data
Normal data

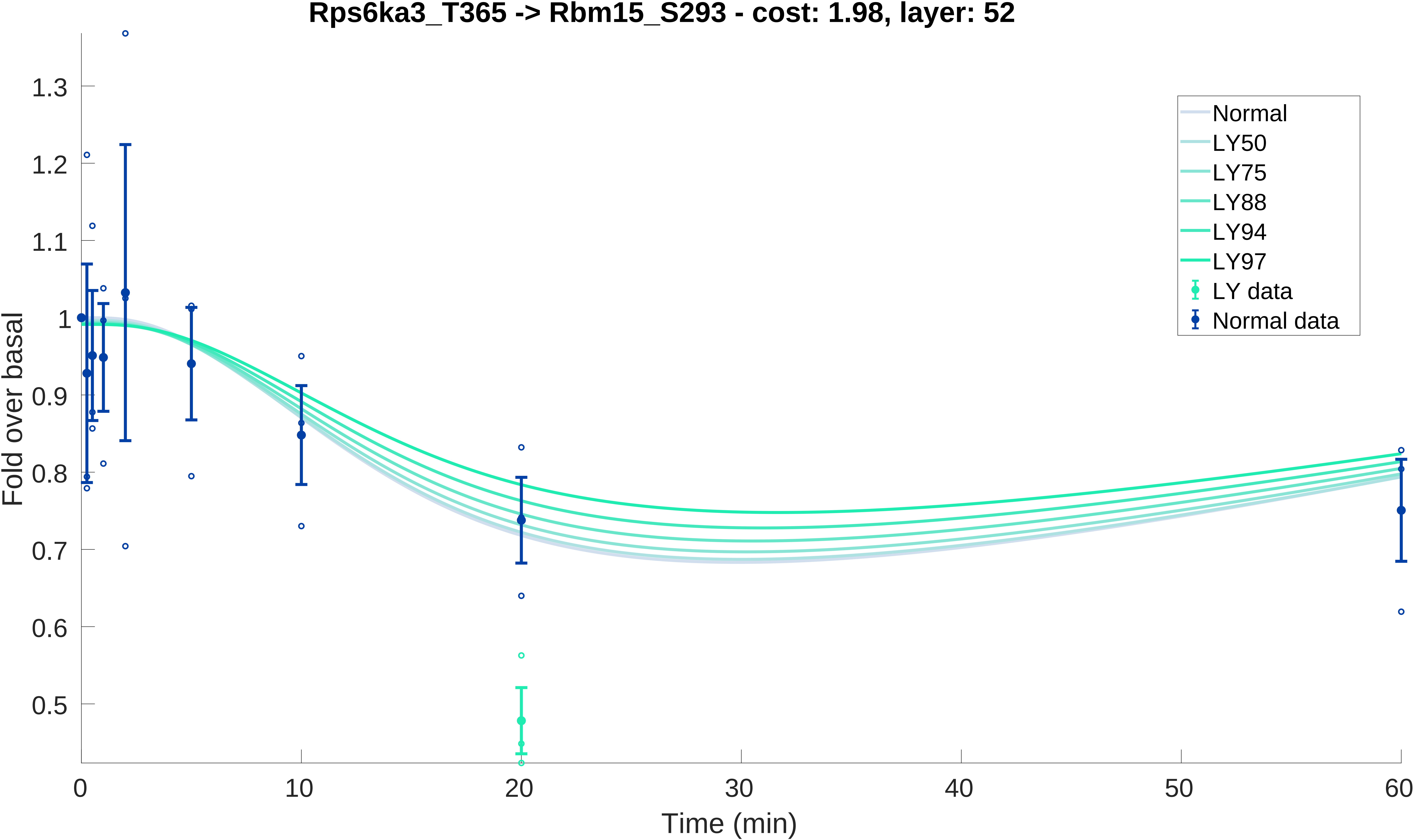
Rps6ka3_T365 -> Rbm15_S293 - cost: 1.98, layer: 52
Fold over basal
Time (min)
1.3
1.2
1.1
1
0.9
0.8
0.7
0.6
0.5
0
10
20
30
40
50
60
Normal
LY50
LY75
LY88
LY94
LY97
LY data
Normal data

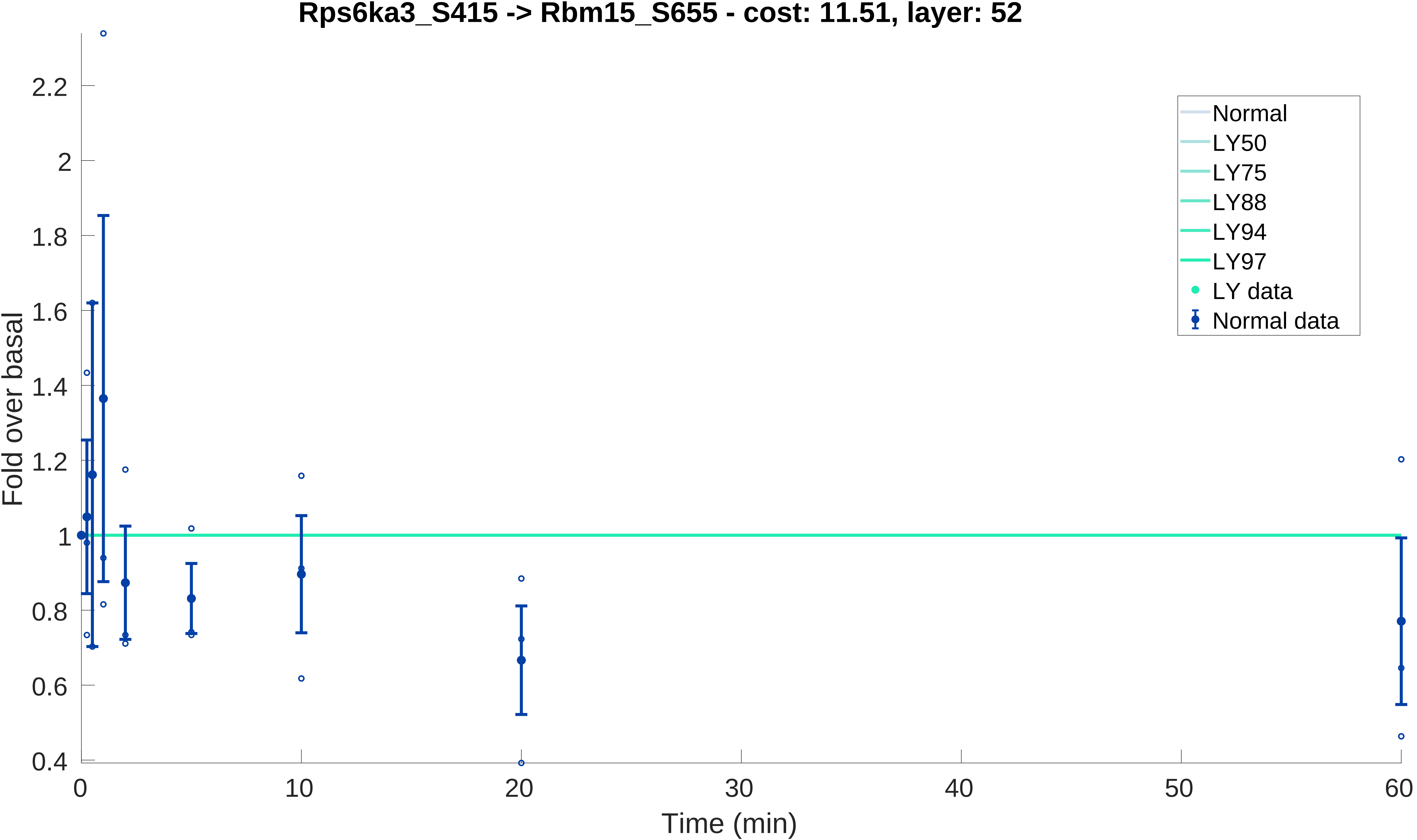
Rps6ka3_S415 -> Rbm15_S655 - cost: 11.51, layer: 52
Fold over basal
Time (min)
0.4
0.6
0.8
1
1.2
1.4
1.6
1.8
2
2.2
0
10
20
30
40
50
60
Normal
LY50
LY75
LY88
LY94
LY97
LY data
Normal data

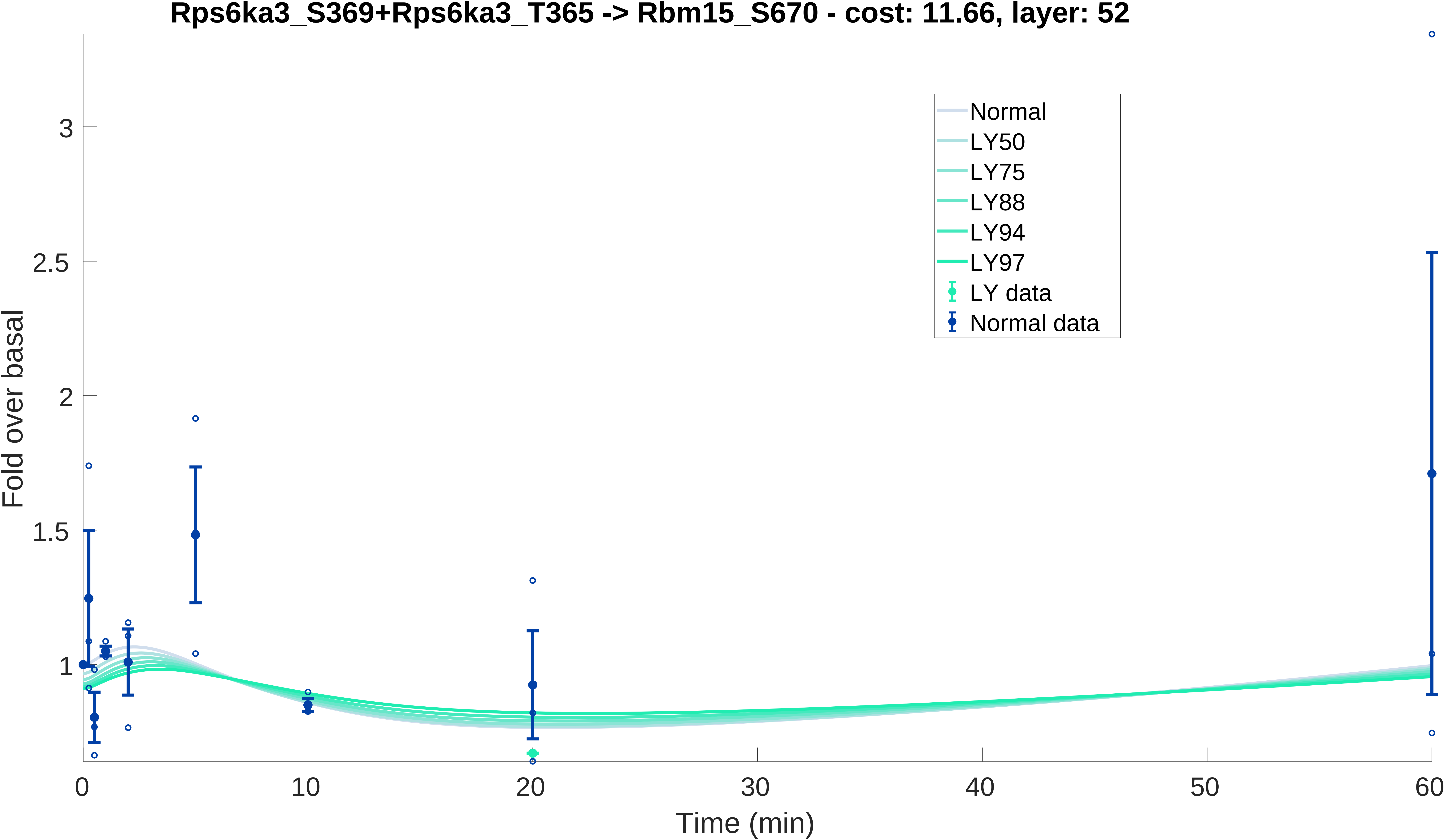

Rps6ka3_S369+Rps6ka3_T365 -> Rbm15_S670 - cost: 11.66, layer: 52
Fold over basal
Time (min)
Normal
LY50
LY75
LY88
LY94
LY97
LY data
Normal data
3
2.5
2
1.5
1
0
10
20
30
40
50
60

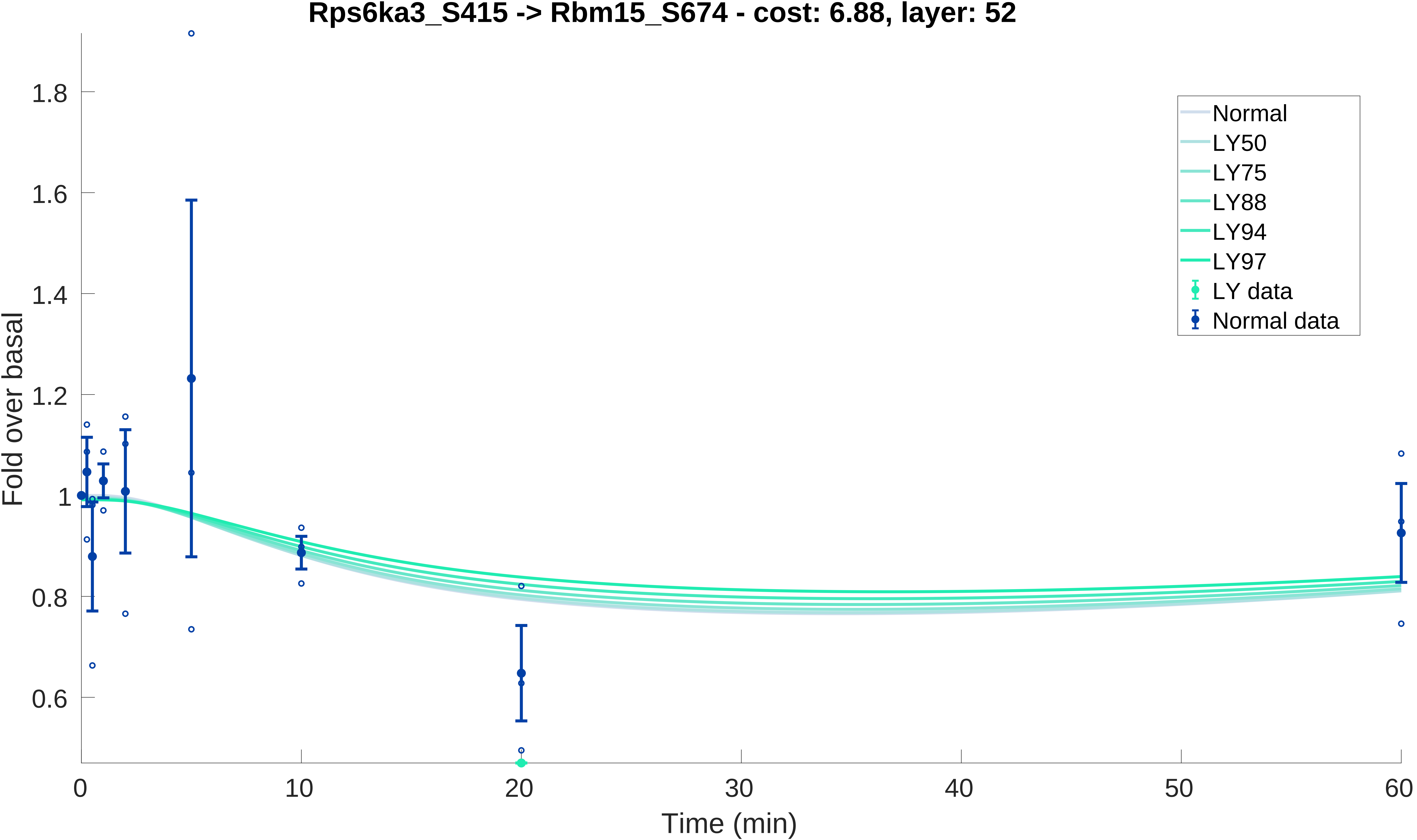

Rps6ka3_S415 -> Rbm15_S674 - cost: 6.88, layer: 52
Fold over basal
Time (min)
0.6
0.8
1
1.2
1.4
1.6
1.8
0
10
20
30
40
50
60
Normal
LY50
LY75
LY88
LY94
LY97
LY data
Normal data

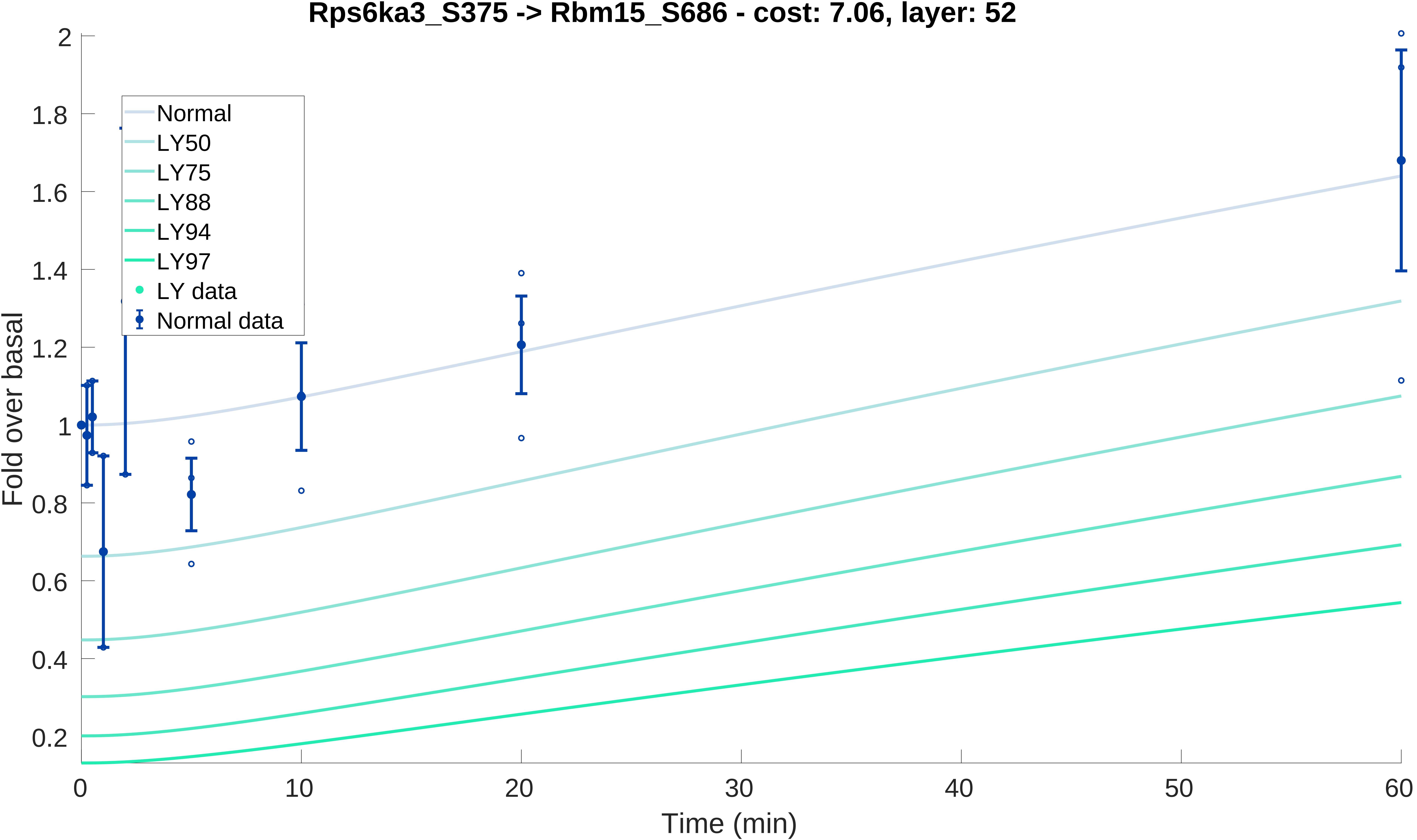
Rps6ka3_S375 -> Rbm15_S686 - cost: 7.06, layer: 52
Normal
LY50
LY75
LY88
LY94
LY97
LY data
Normal data
Fold over basal
Time (min)
2
1.8
1.6
1.4
1.2
1
0.8
0.6
0.4
0.2
0
10
20
30
40
50
60

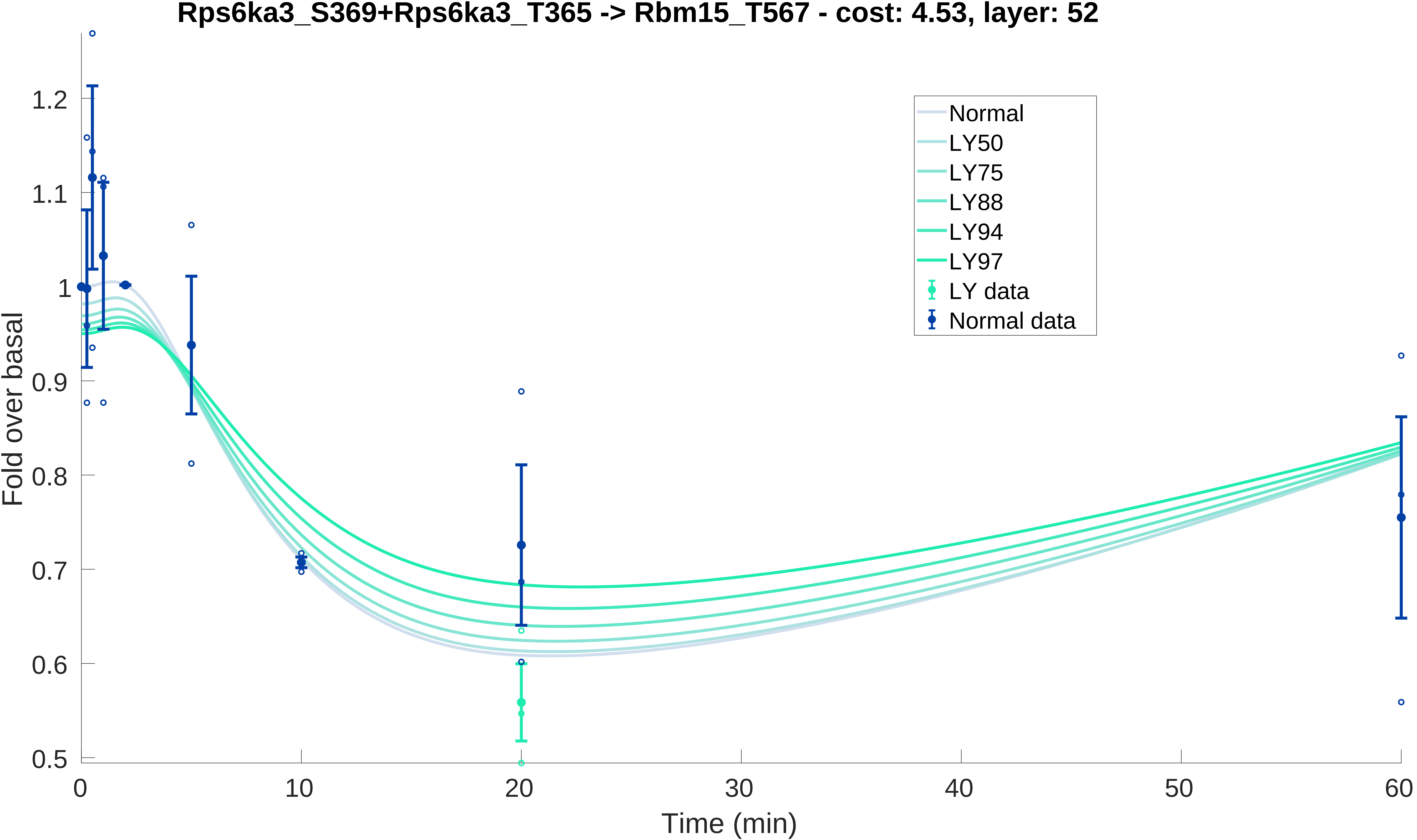
Rps6ka3_S369+Rps6ka3_T365 -> Rbm15_T567 - cost: 4.53, layer: 52
Fold over basal
Time (min)
1.2
1.1
1
0.9
0.8
0.7
0.6
0.5
0
10
20
30
40
50
60
Normal
LY50
LY75
LY88
LY94
LY97
LY data
Normal data

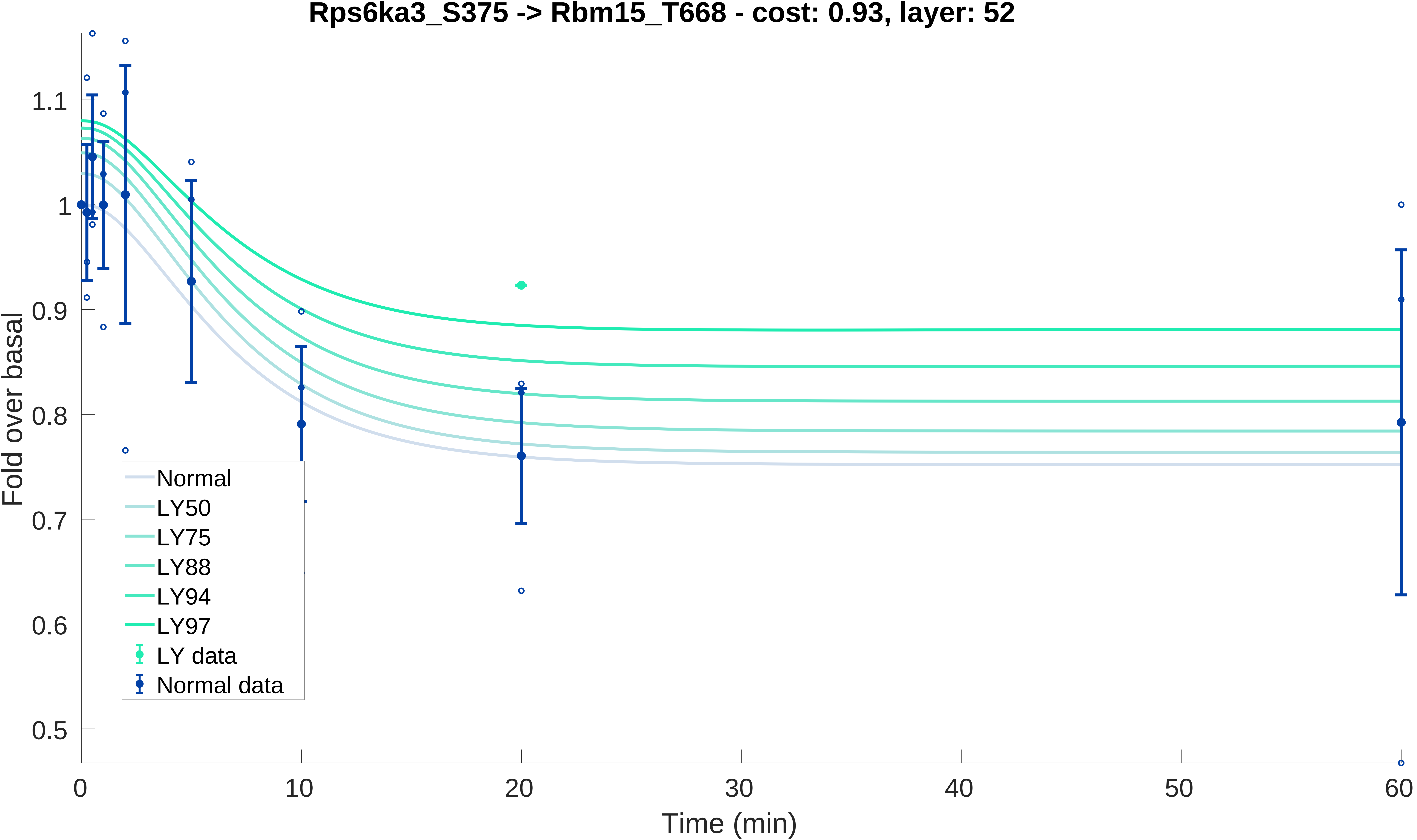

Rps6ka3_S375 -> Rbm15_T668 - cost: 0.93, layer: 52
Fold over basal
Time (min)
1.1
1
0.9
0.8
0.7
0.6
0.5
0
10
20
30
40
50
60
Normal
LY50
LY75
LY88
LY94
LY97
LY data
Normal data

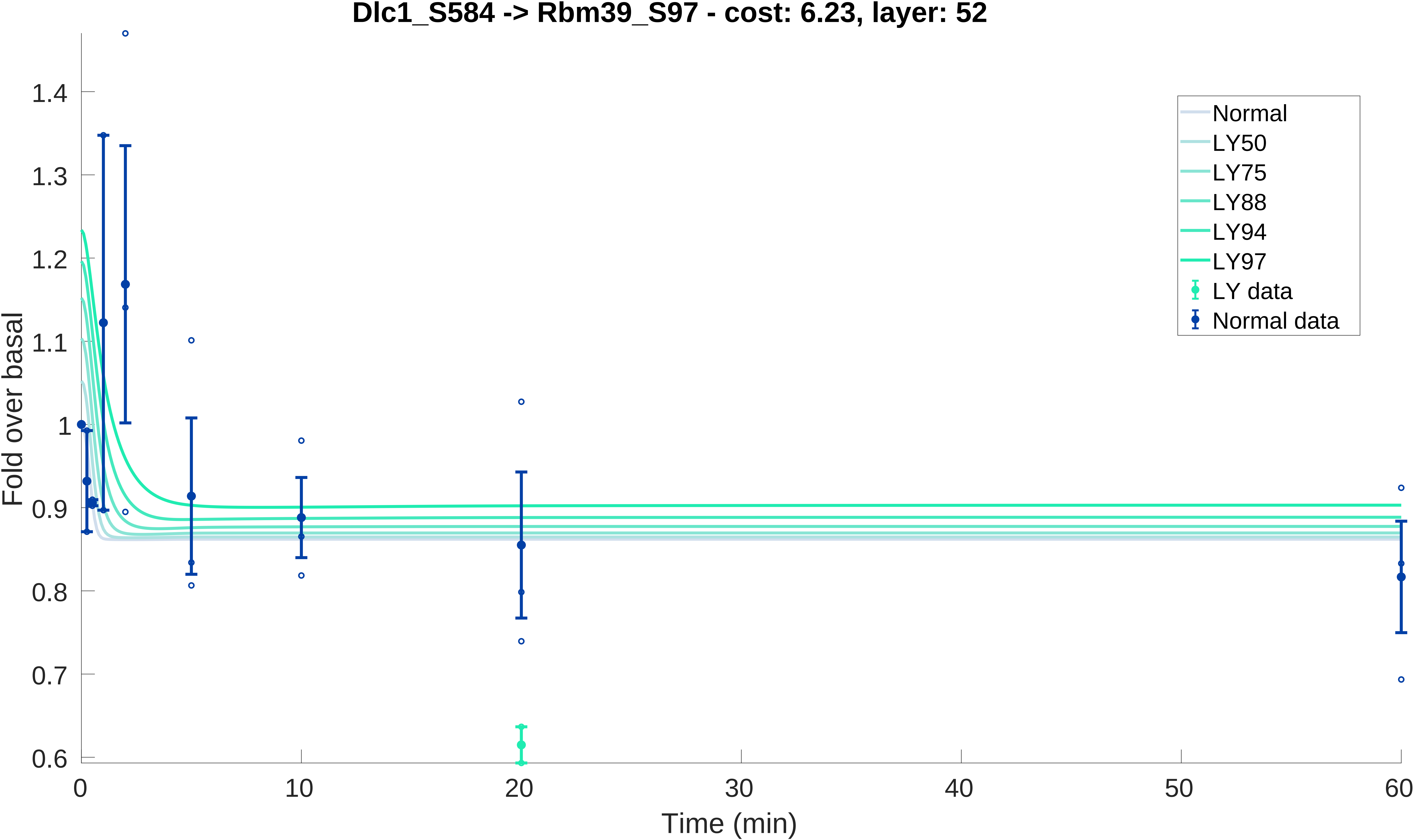
Dlc1_S584 -> Rbm39_S97 - cost: 6.23, layer: 52
Fold over basal
Time (min)
1.4
1.3
1.2
1.1
1
0.9
0.8
0.7
0.6
0
10
20
30
40
50
60
Normal
LY50
LY75
LY88
LY94
LY97
LY data
Normal data

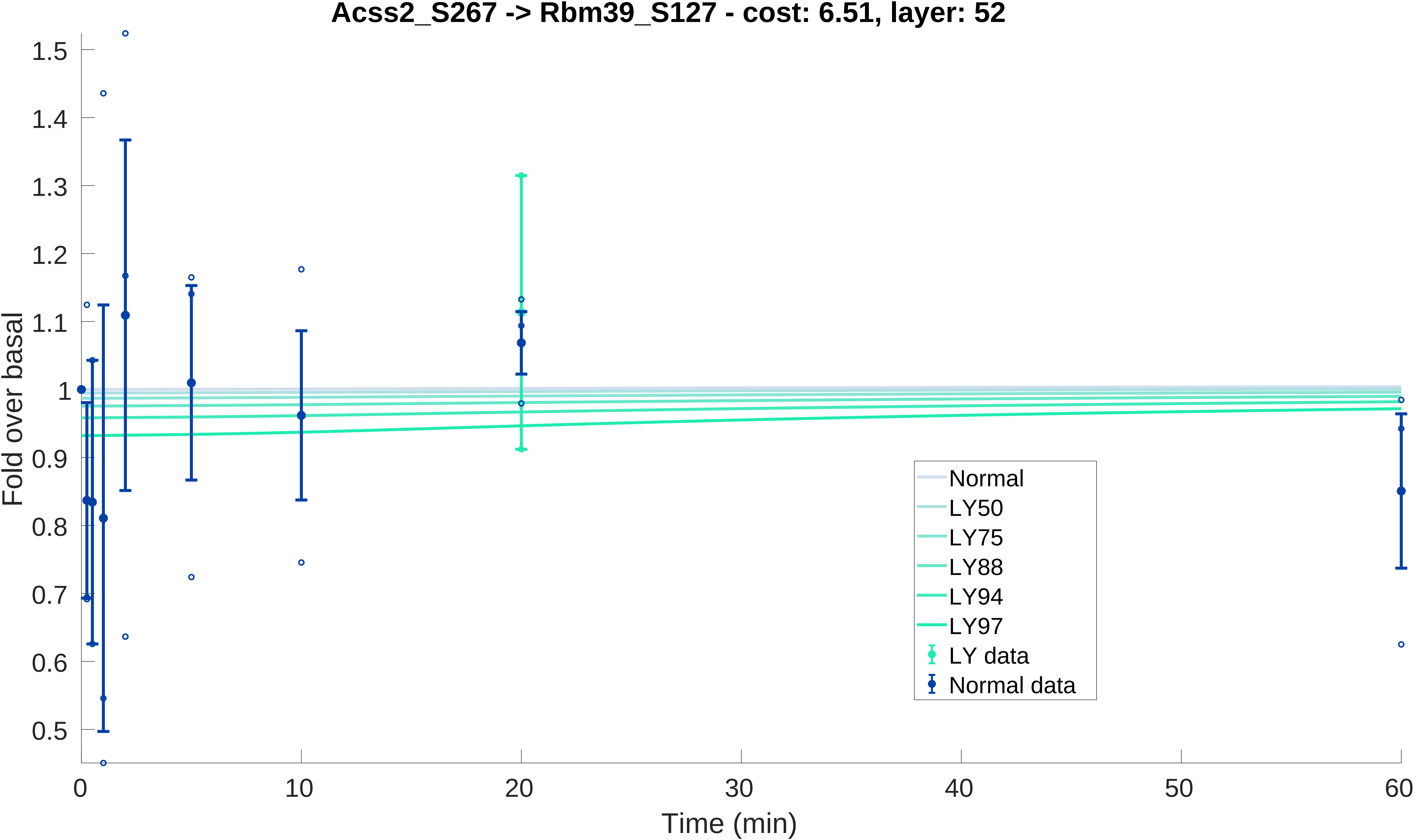

Acss2_S267 -> Rbm39_S127 - cost: 6.51, layer: 52
1.5
1.4
1.3
1.2
1.1
1
0.9
0.8
0.7
0.6
0.5
Fold over basal
0
10
20
30
40
50
60
Time (min)
Normal
LY50
LY75
LY88
LY94
LY97
LY data
Normal data

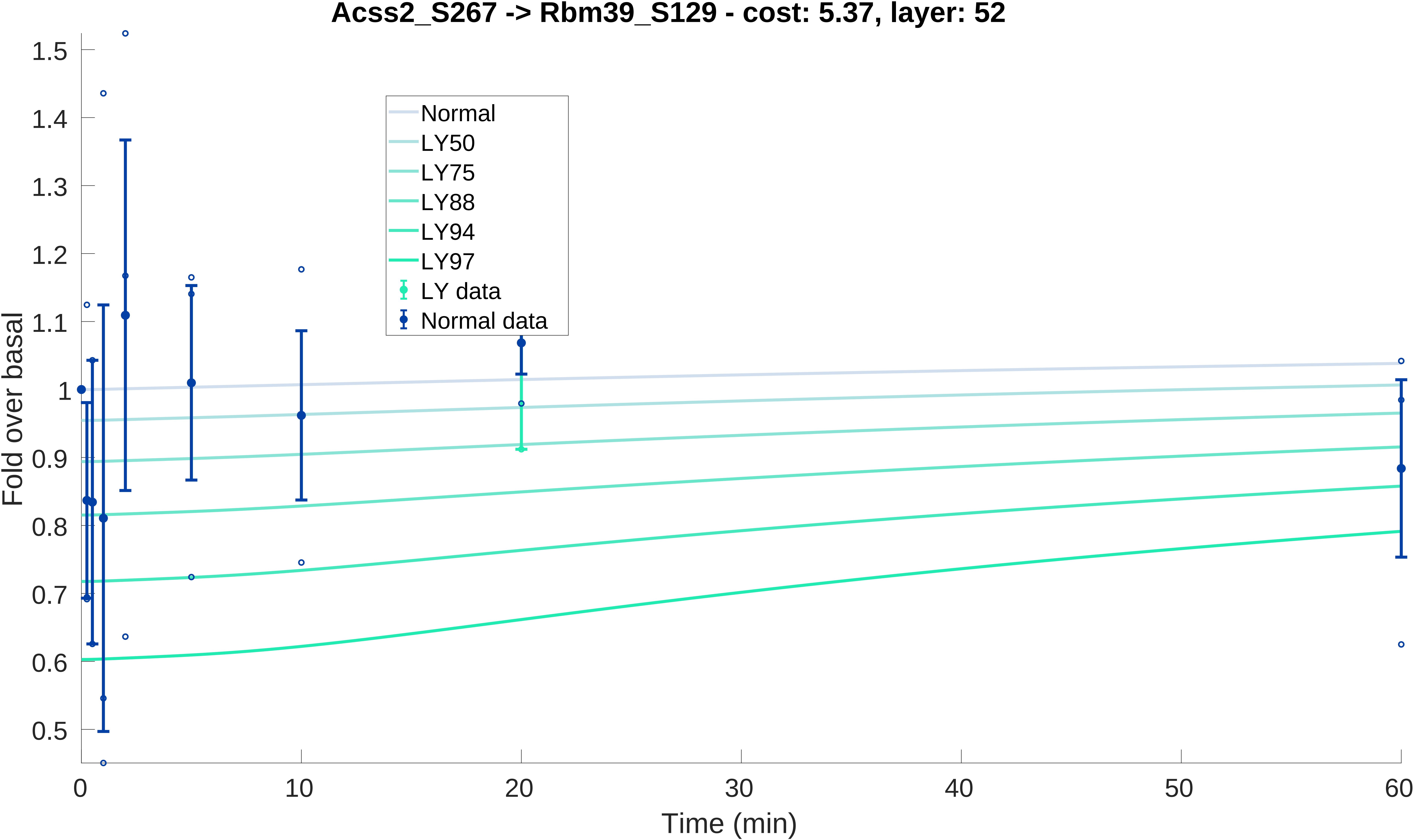

Acss2_S267 -> Rbm39_S129 - cost: 5.37, layer: 52
Fold over basal
Time (min)
Normal
LY50
LY75
LY88
LY94
LY97
LY data
Normal data
1.5
1.4
1.3
1.2
1.1
1
0.9
0.8
0.7
0.6
0.5
0
10
20
30
40
50
60

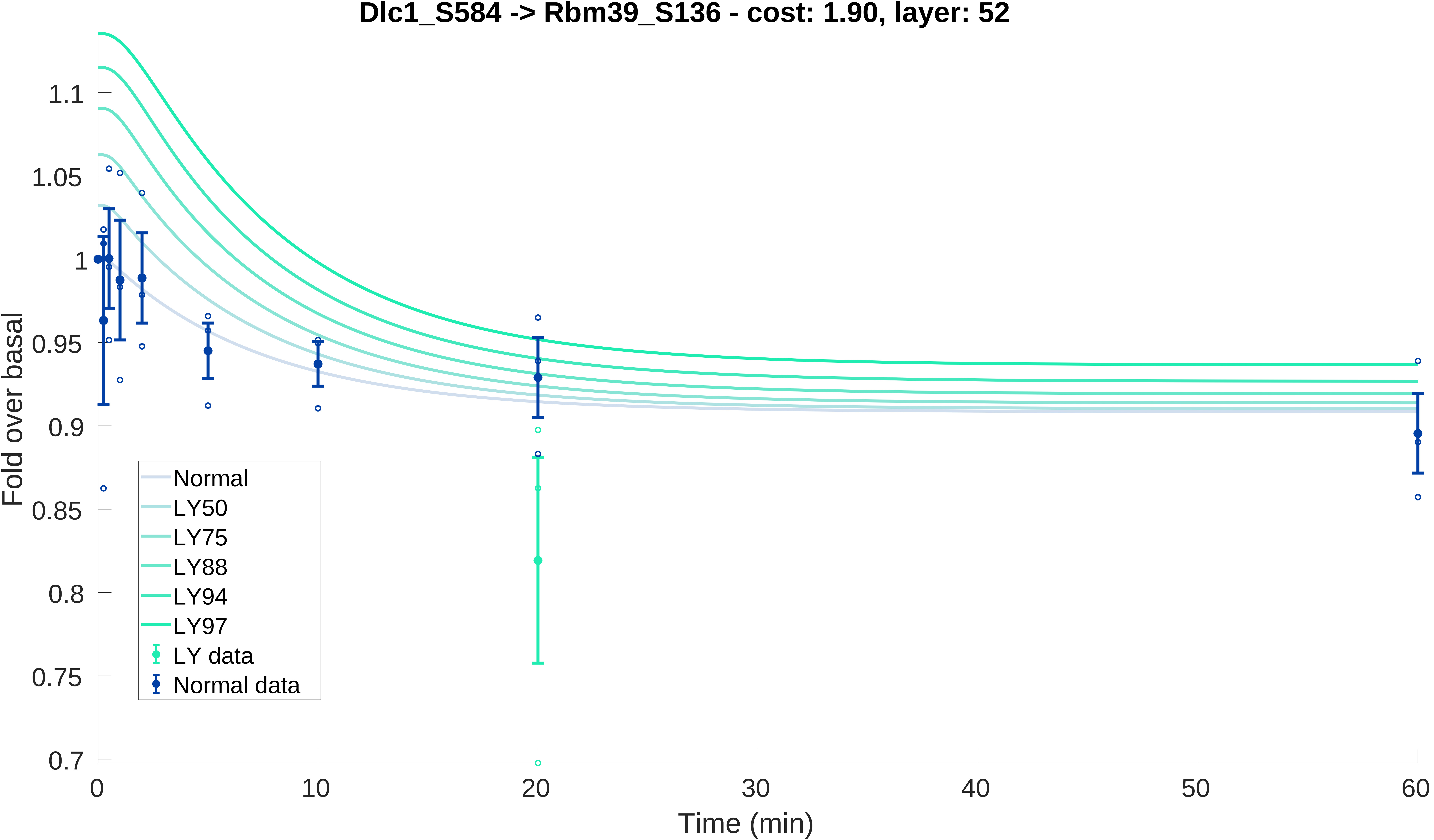
Dlc1_S584 -> Rbm39_S136 - cost: 1.90, layer: 52
Fold over basal
Time (min)
1.1
1.05
1
0.95
0.9
0.85
0.8
0.75
0.7
0
10
20
30
40
50
60
Normal
LY50
LY75
LY88
LY94
LY97
LY data
Normal data

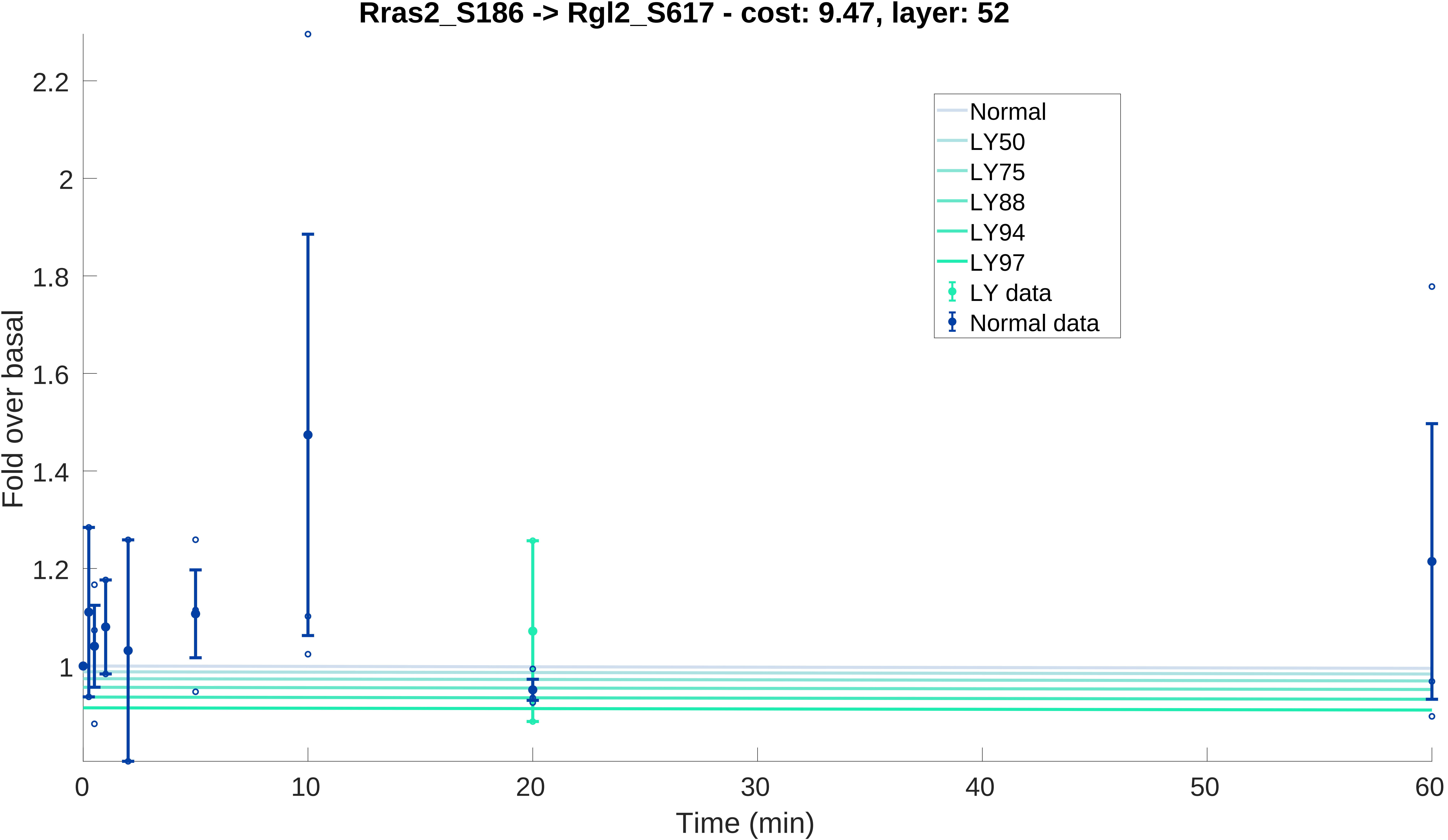

Rras2_S186 -> Rgl2_S617 - cost: 9.47, layer: 52
Fold over basal
2.2
2
1.8
1.6
1.4
1.2
1
0
10
20
30
40
50
60
Time (min)
Normal
LY50
LY75
LY88
LY94
LY97
LY data
Normal data

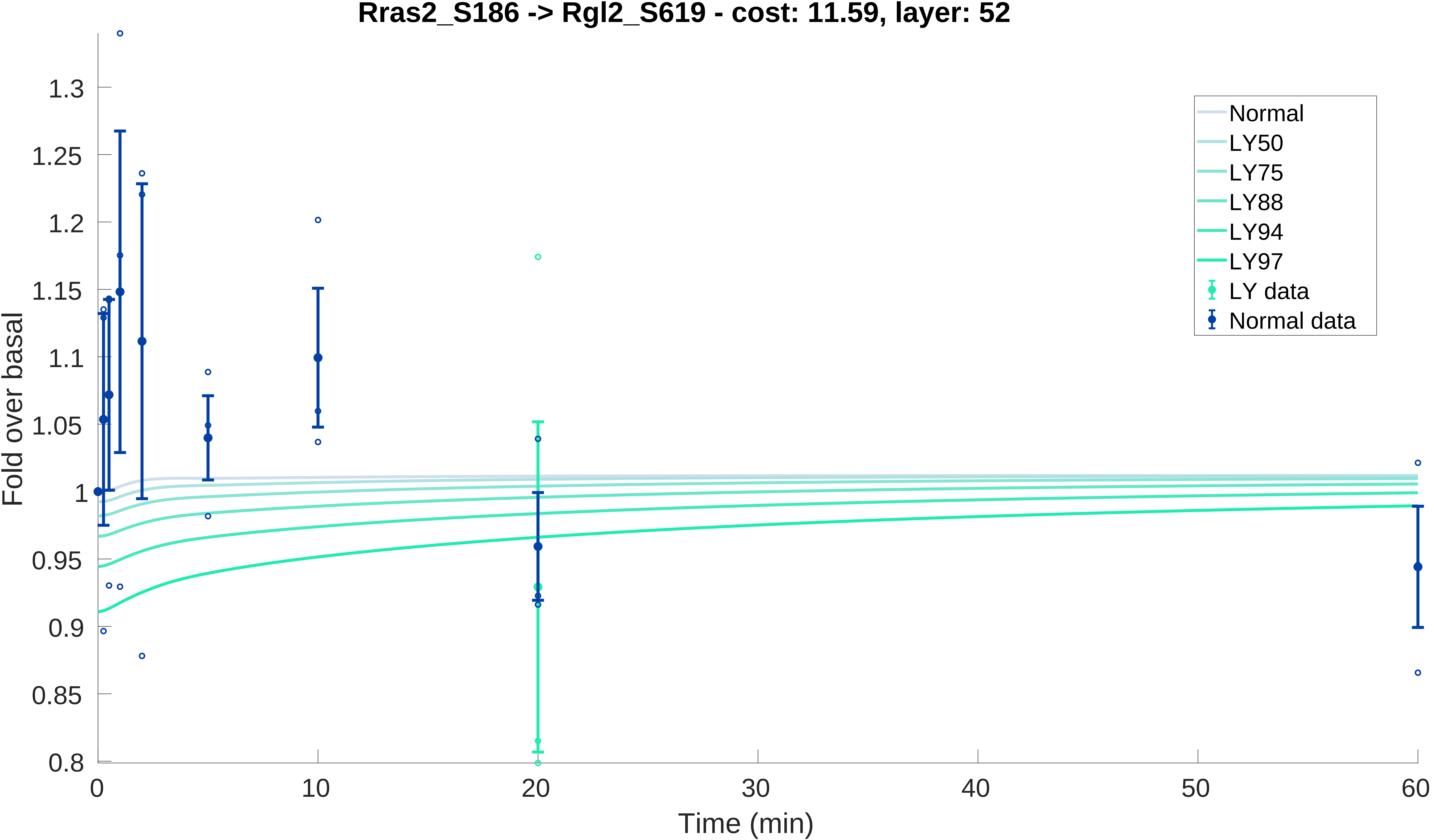
Rras2_S186 -> Rgl2_S619 - cost: 11.59, layer: 52
Fold over basal
Time (min)
1.3
1.25
1.2
1.15
1.1
1.05
1
0.95
0.9
0.85
0.8
0
10
20
30
40
50
60
Normal
LY50
LY75
LY88
LY94
LY97
LY data
Normal data

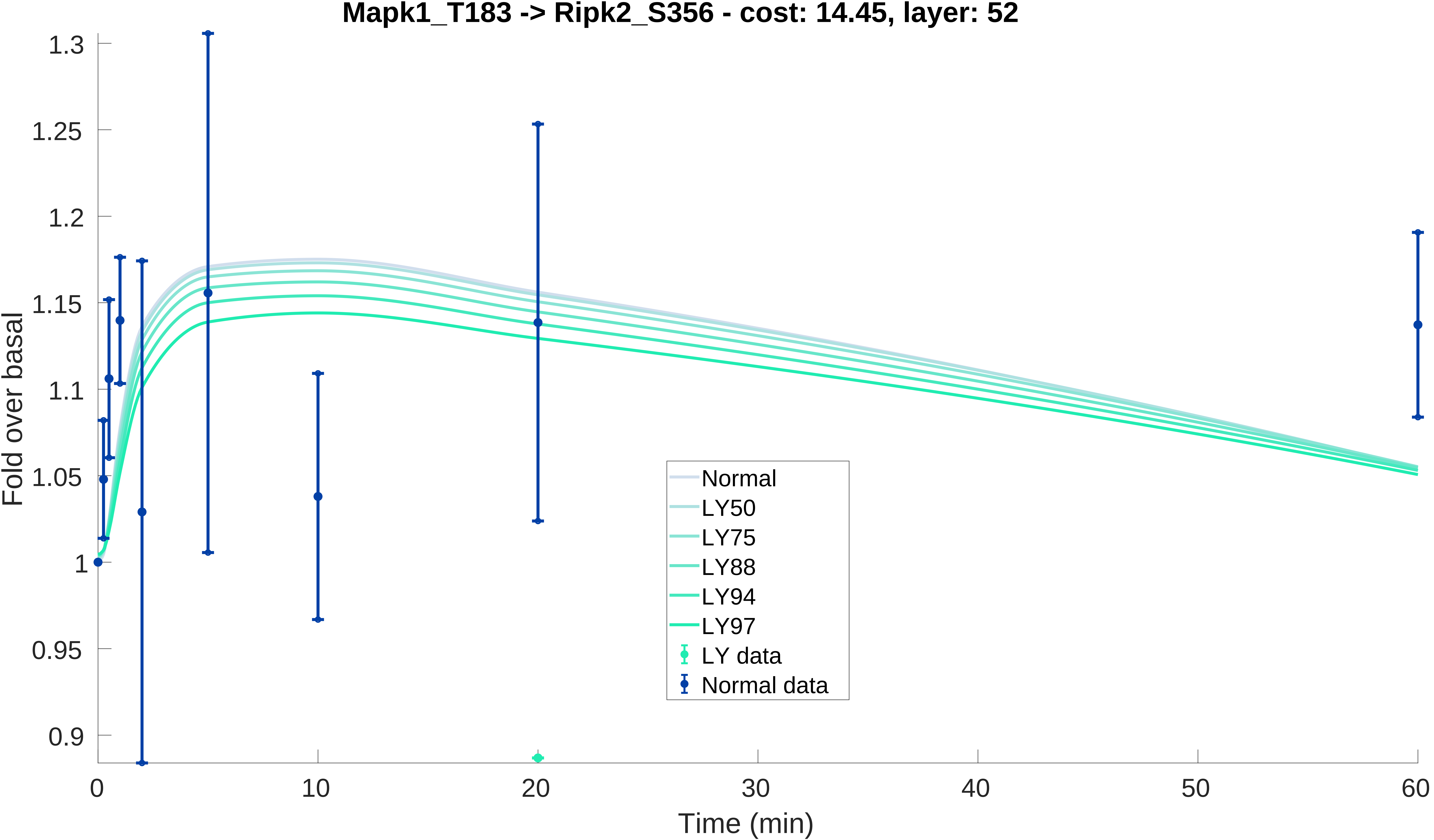

Mapk1_T183 -> Ripk2_S356 - cost: 14.45, layer: 52
Fold over basal
Time (min)
0.9
0.95
1
1.05
1.1
1.15
1.2
1.25
1.3
0
10
20
30
40
50
60
Normal
LY50
LY75
LY88
LY94
LY97
LY data
Normal data

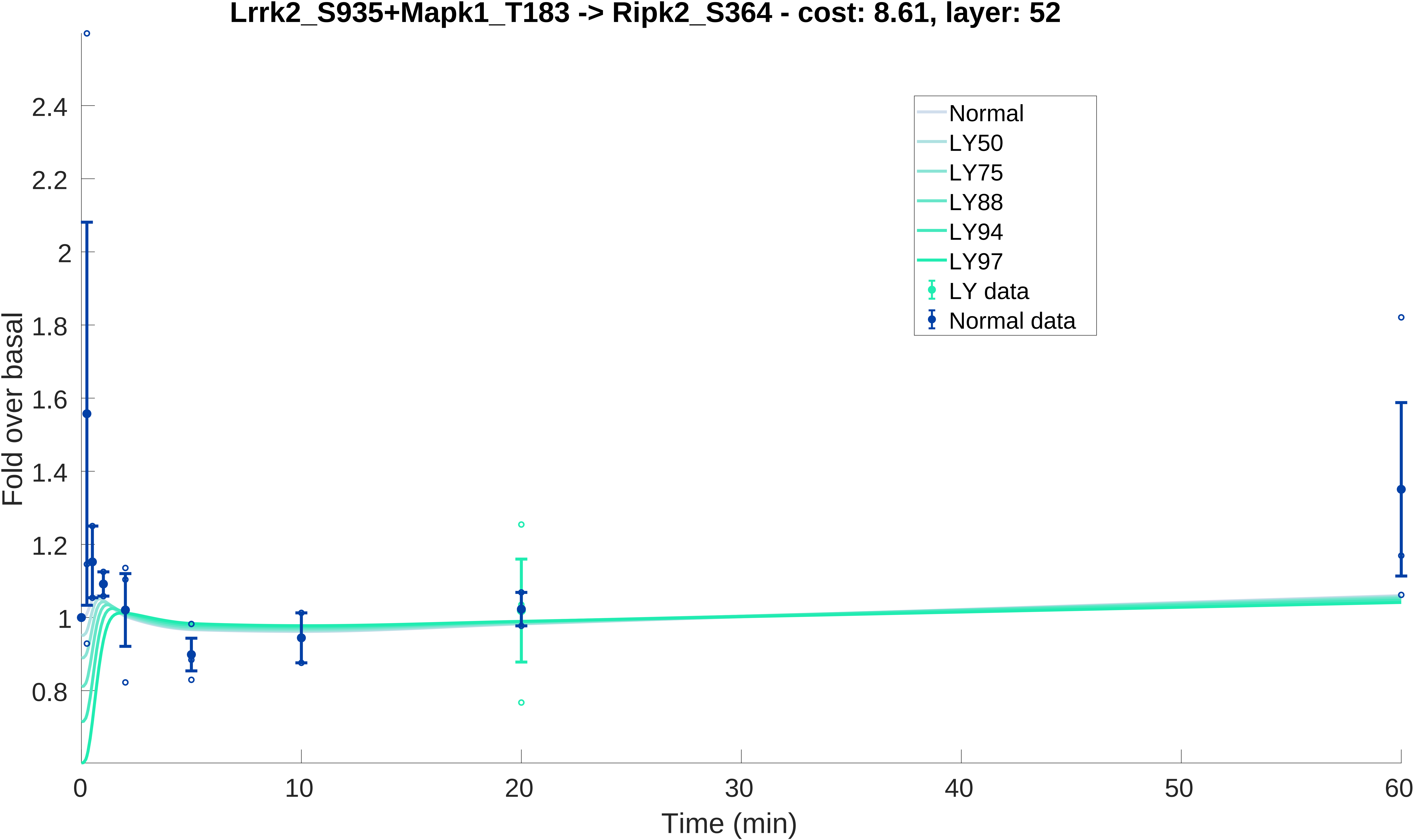

Lrrk2_S935+Mapk1_T183 -> Ripk2_S364 - cost: 8.61, layer: 52
Fold over basal
Time (min)
0.8
1
1.2
1.4
1.6
1.8
2
2.2
2.4
0
10
20
30
40
50
60
Normal
LY50
LY75
LY88
LY94
LY97
LY data
Normal data

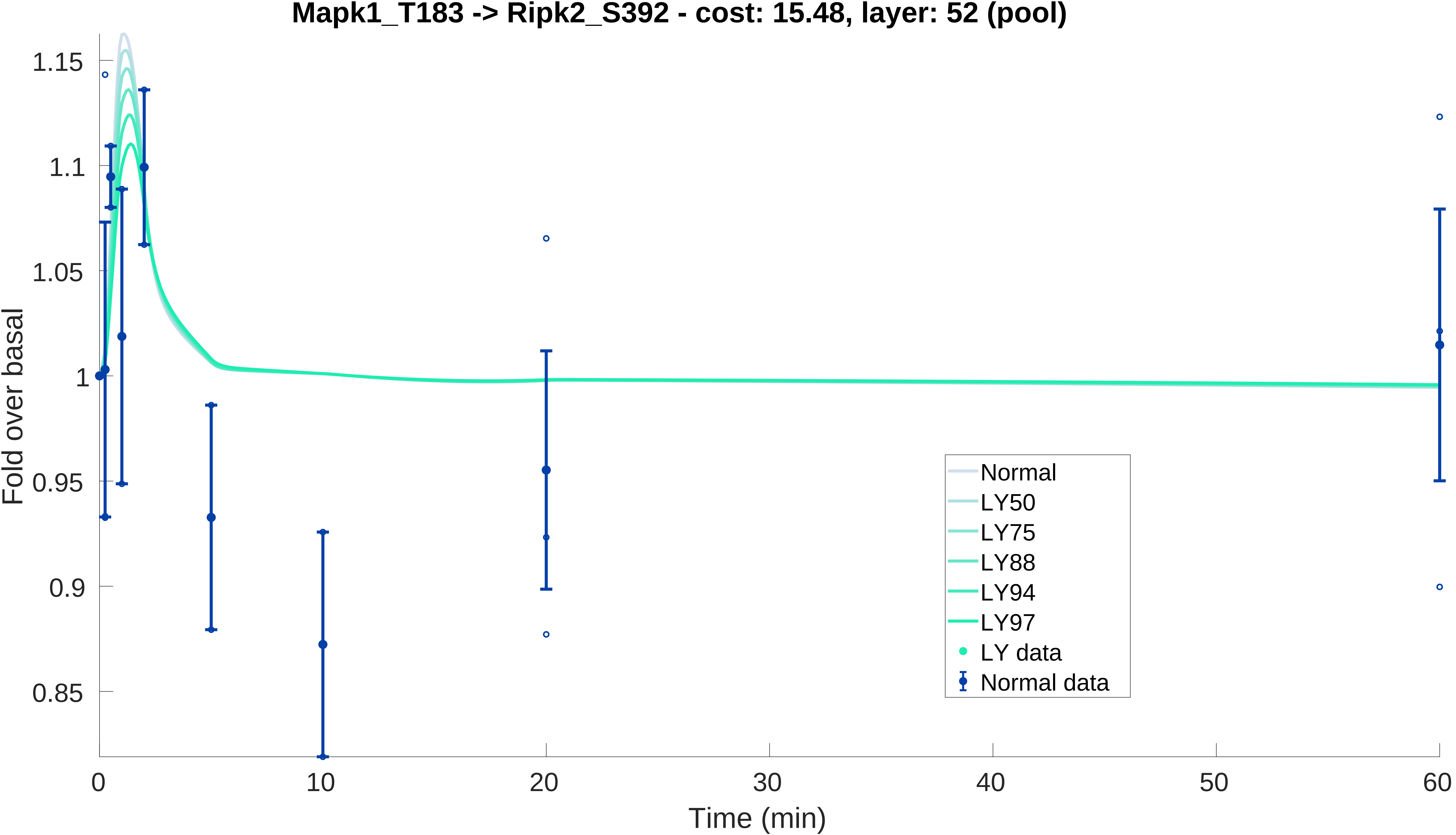

Mapk1_T183 -> Ripk2_S392 - cost: 15.48, layer: 52 (pool)
Fold over basal
Time (min)
1.15
1.1
1.05
1
0.95
0.9
0.85
0
10
20
30
40
50
60
Normal
LY50
LY75
LY88
LY94
LY97
LY data
Normal data

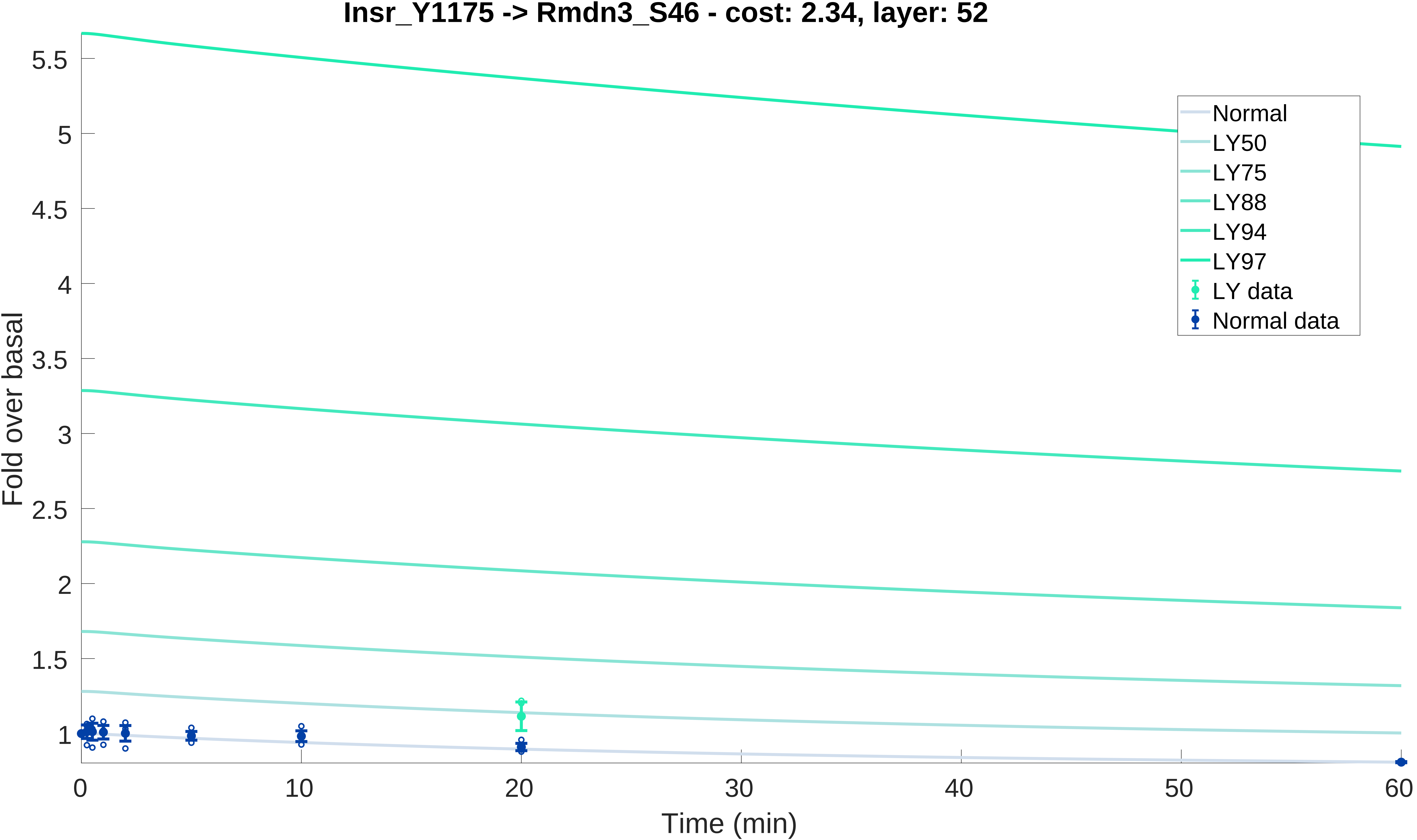

Insr_Y1175 -> Rmdn3_S46 - cost: 2.34, layer: 52
Fold over basal
5.5
5
4.5
4
3.5
3
2.5
2
1.5
1
0
10
20
30
40
50
60
Time (min)
Normal
LY50
LY75
LY88
LY94
LY97
LY data
Normal data

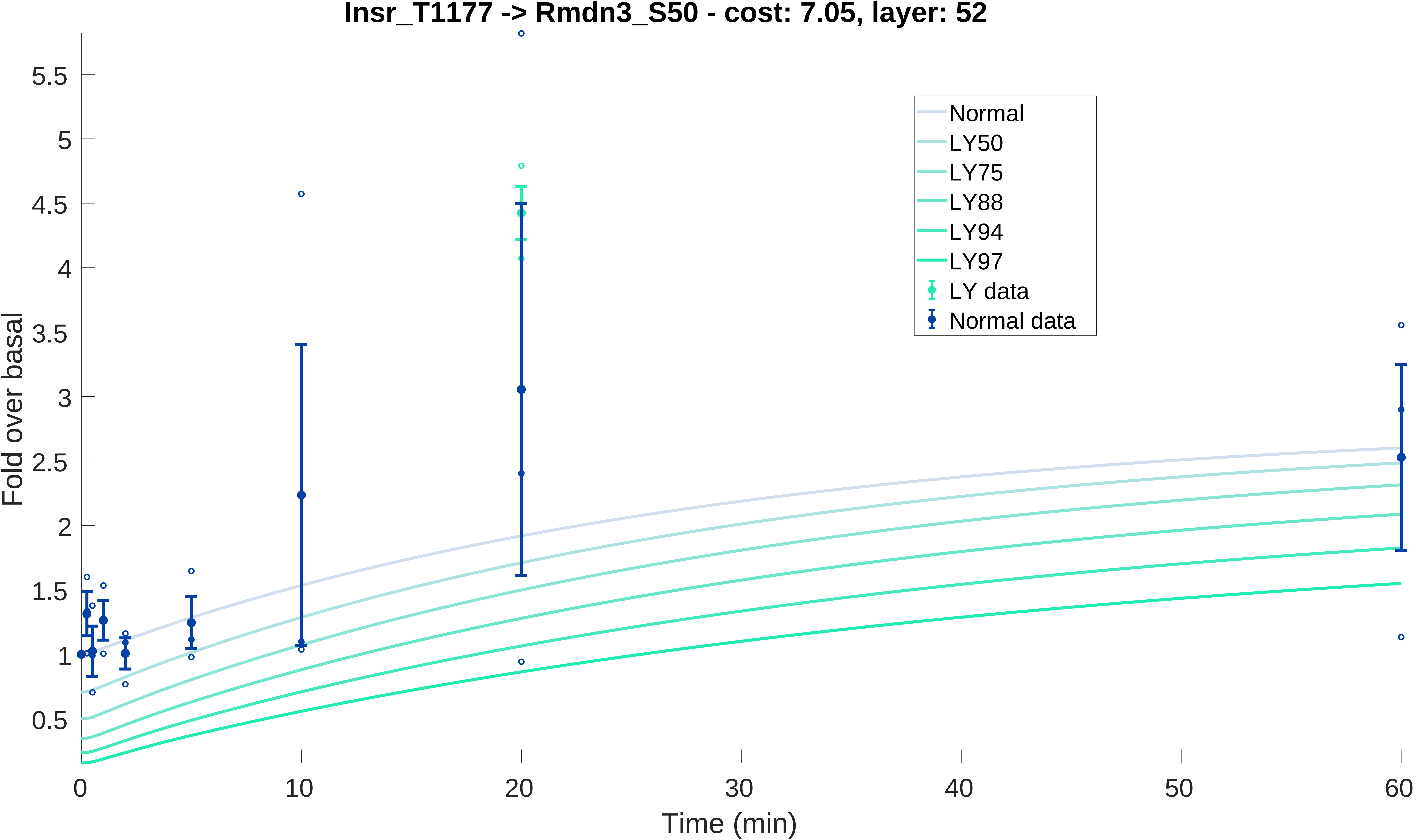

Insr_T1177 -> Rmdn3_S50 - cost: 7.05, layer: 52
Fold over basal
Time (min)
0.5
1
1.5
2
2.5
3
3.5
4
4.5
5
5.5
0
10
20
30
40
50
60
Normal
LY50
LY75
LY88
LY94
LY97
LY data
Normal data

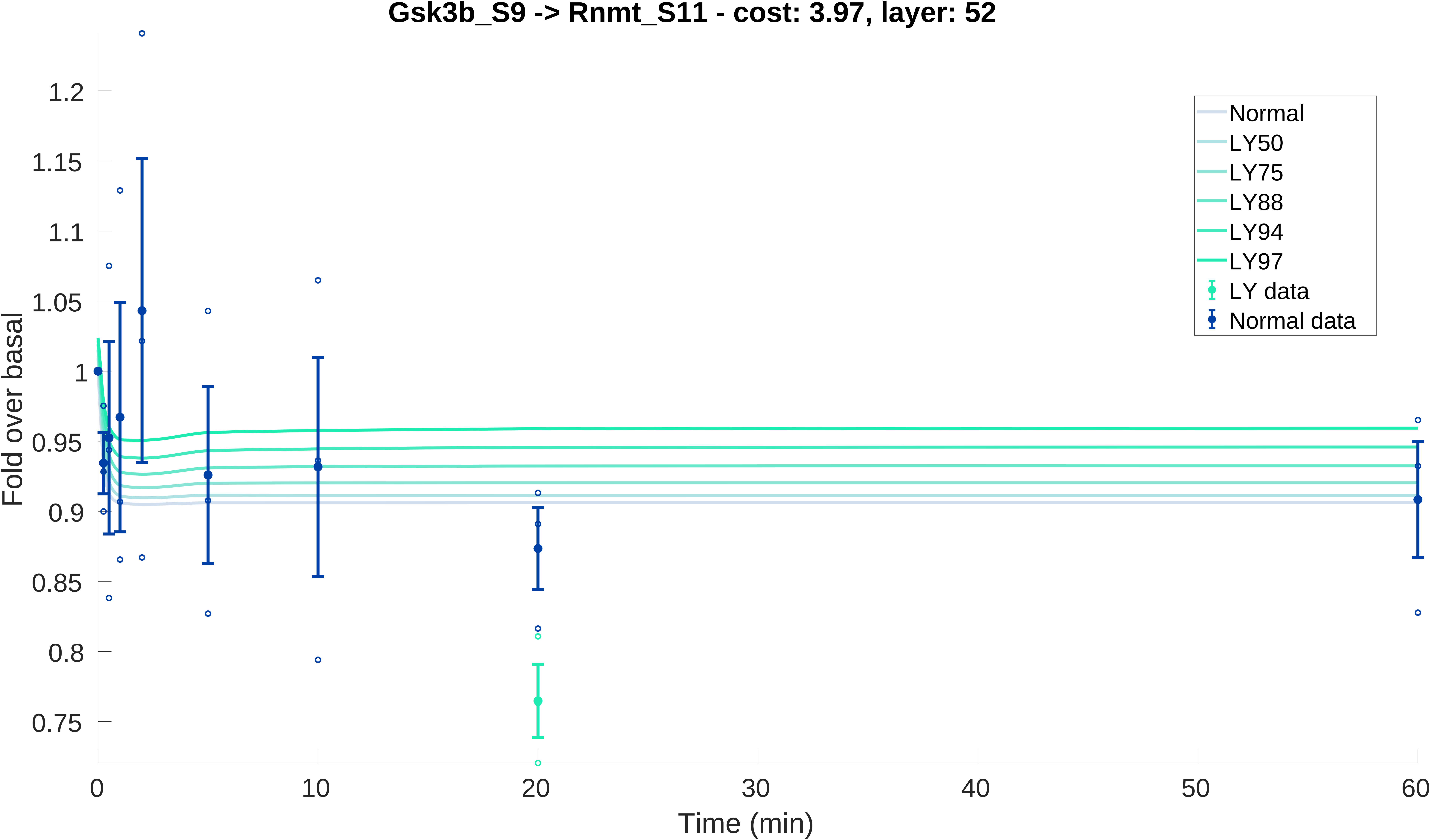
Gsk3b_S9 -> Rnmt_S11 - cost: 3.97, layer: 52
Fold over basal
Time (min)
Normal
LY50
LY75
LY88
LY94
LY97
LY data
Normal data
1.2
1.15
1.1
1.05
1
0.95
0.9
0.85
0.8
0.75
0
10
20
30
40
50
60

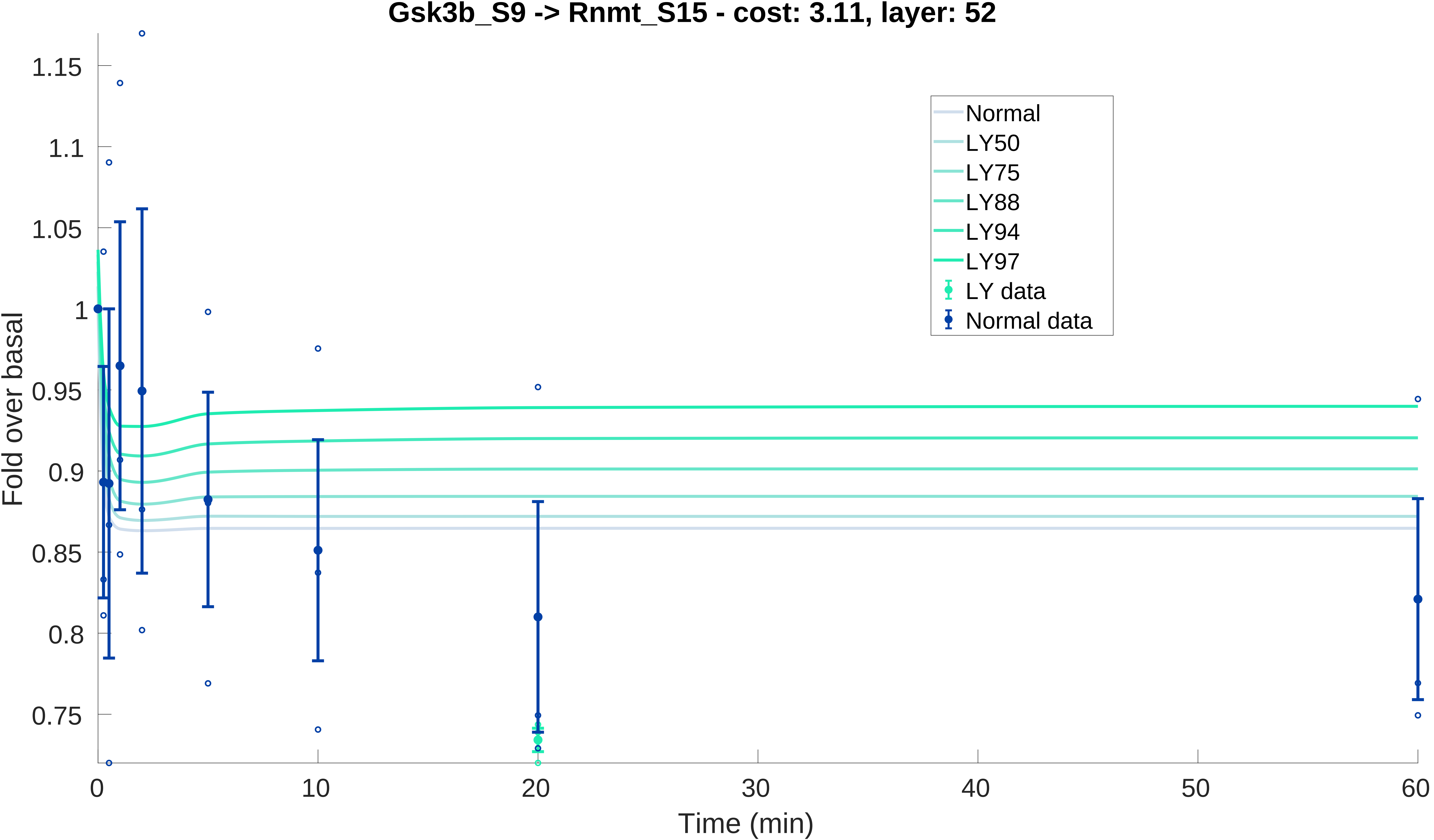
Gsk3b_S9 -> Rnmt_S15 - cost: 3.11, layer: 52
Fold over basal
1.15
1.1
1.05
1
0.95
0.9
0.85
0.8
0.75
0
10
20
30
40
50
60
Time (min)
Normal
LY50
LY75
LY88
LY94
LY97
LY data
Normal data

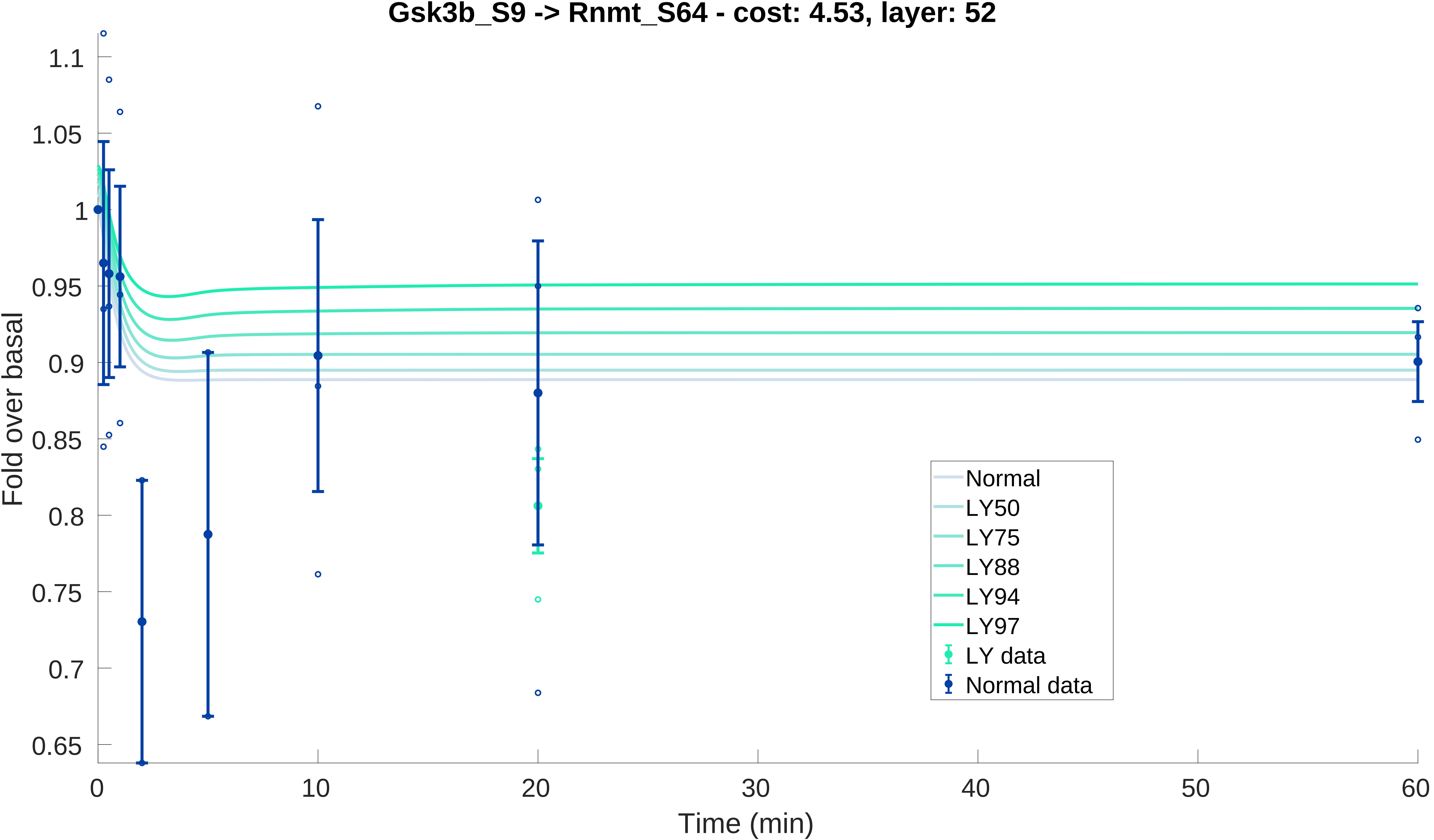

Gsk3b_S9 -> Rnmt_S64 - cost: 4.53, layer: 52
Fold over basal
1.1
1.05
1
0.95
0.9
0.85
0.8
0.75
0.7
0.65
0
10
20
30
40
50
60
Time (min)
Normal
LY50
LY75
LY88
LY94
LY97
LY data
Normal data

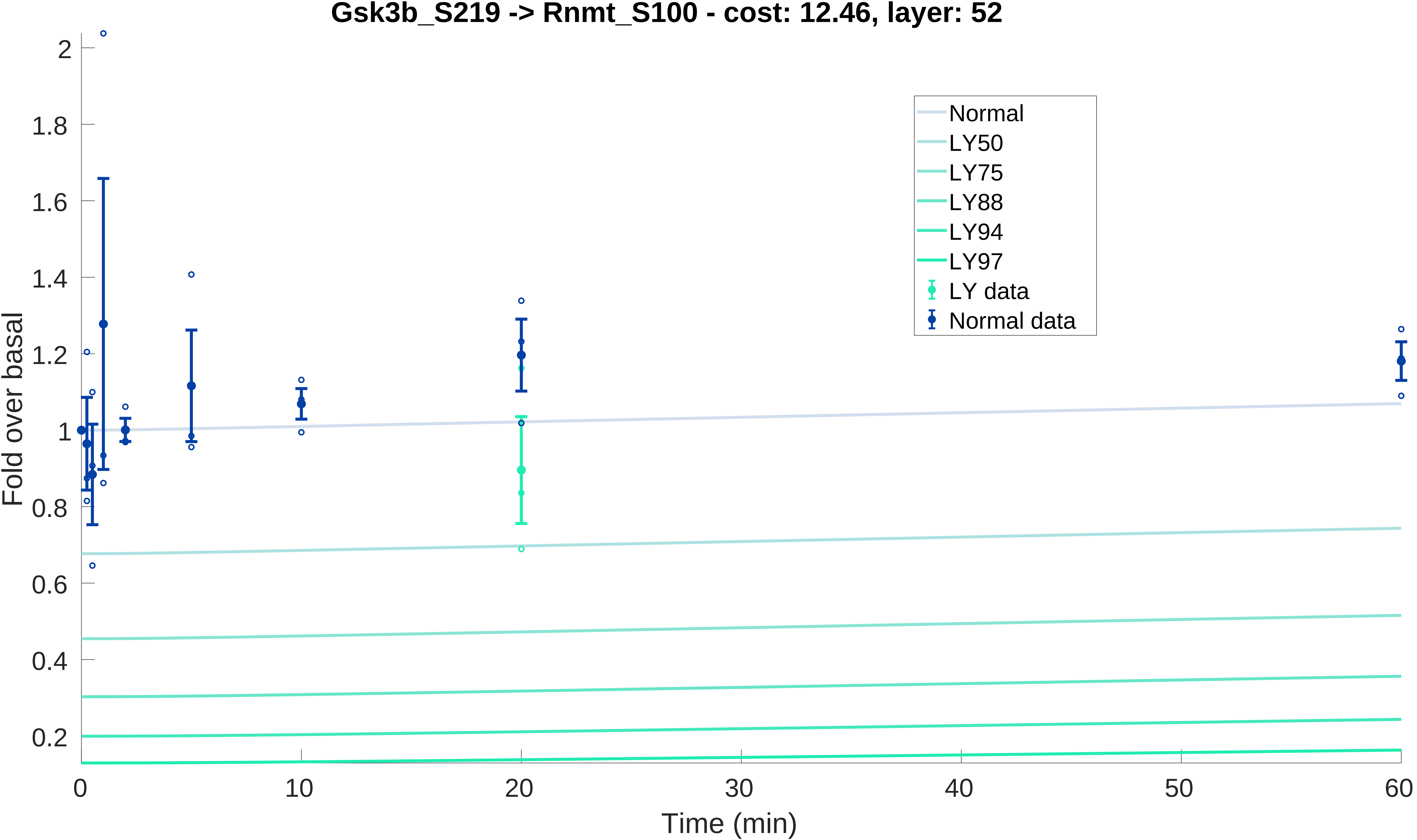

Gsk3b_S219 -> Rnmt_S100 - cost: 12.46, layer: 52
Fold over basal
Time (min)
Normal
LY50
LY75
LY88
LY94
LY97
LY data
Normal data
0.2
0.4
0.6
0.8
1
1.2
1.4
1.6
1.8
2
0
10
20
30
40
50
60

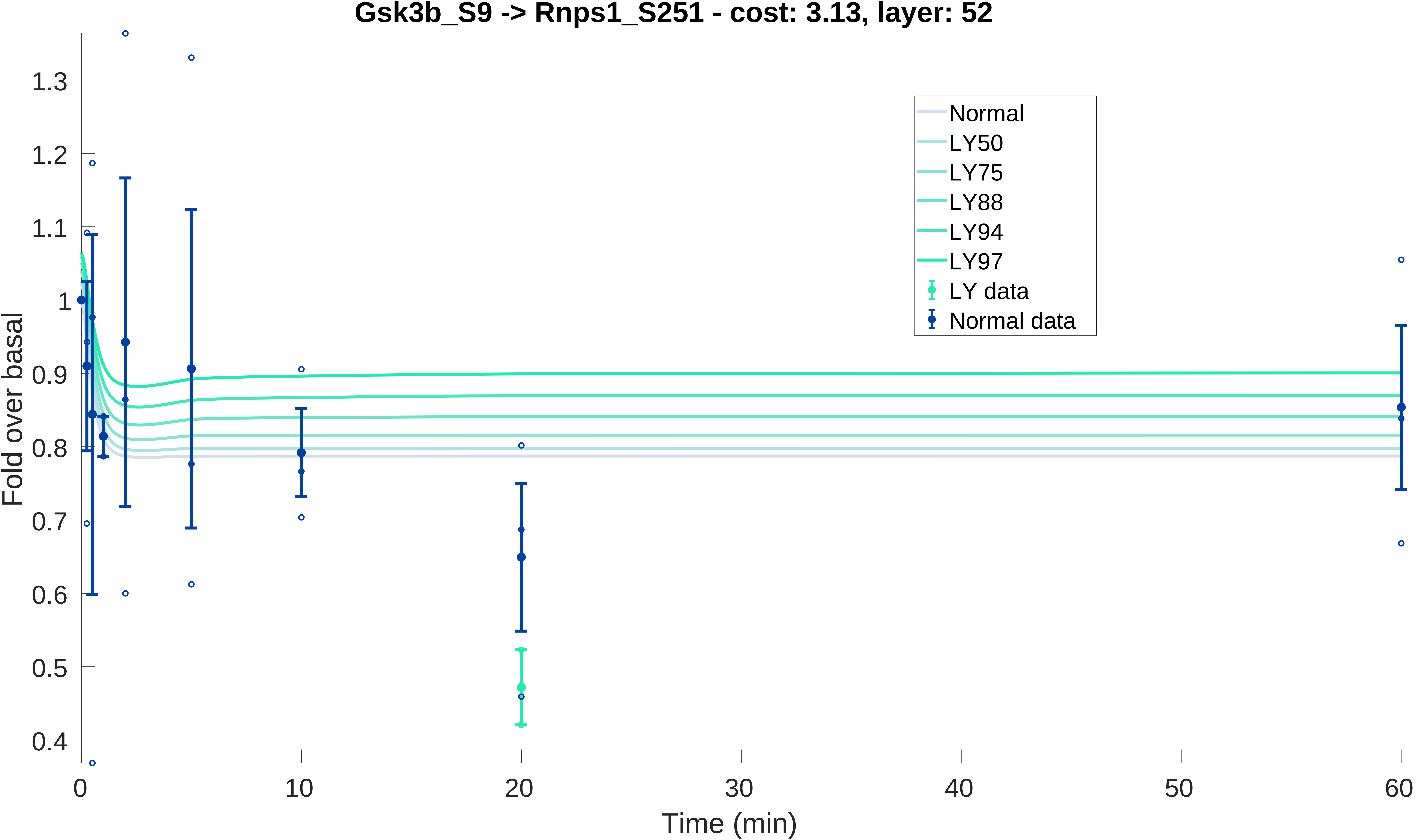

Gsk3b_S9 -> Rnps1_S251 - cost: 3.13, layer: 52
Fold over basal
Time (min)
Normal
LY50
LY75
LY88
LY94
LY97
LY data
Normal data
1.3
1.2
1.1
1
0.9
0.8
0.7
0.6
0.5
0.4
0
10
20
30
40
50
60

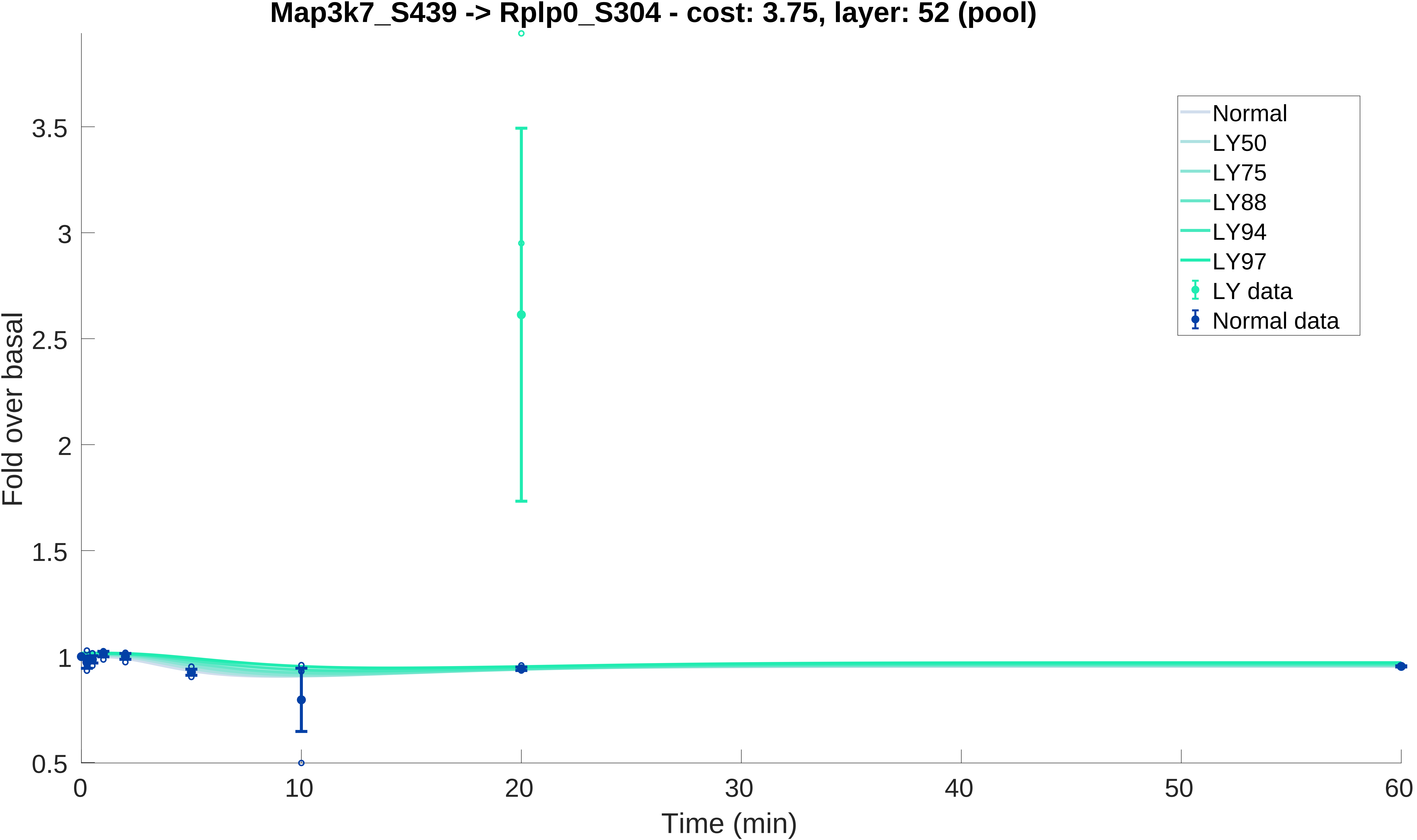

Map3k7_S439 -> Rplp0_S304 - cost: 3.75, layer: 52 (pool)
Fold over basal
3.5
3
2.5
2
1.5
1
0.5
0
10
20
30
40
50
60
Time (min)
Normal
LY50
LY75
LY88
LY94
LY97
LY data
Normal data

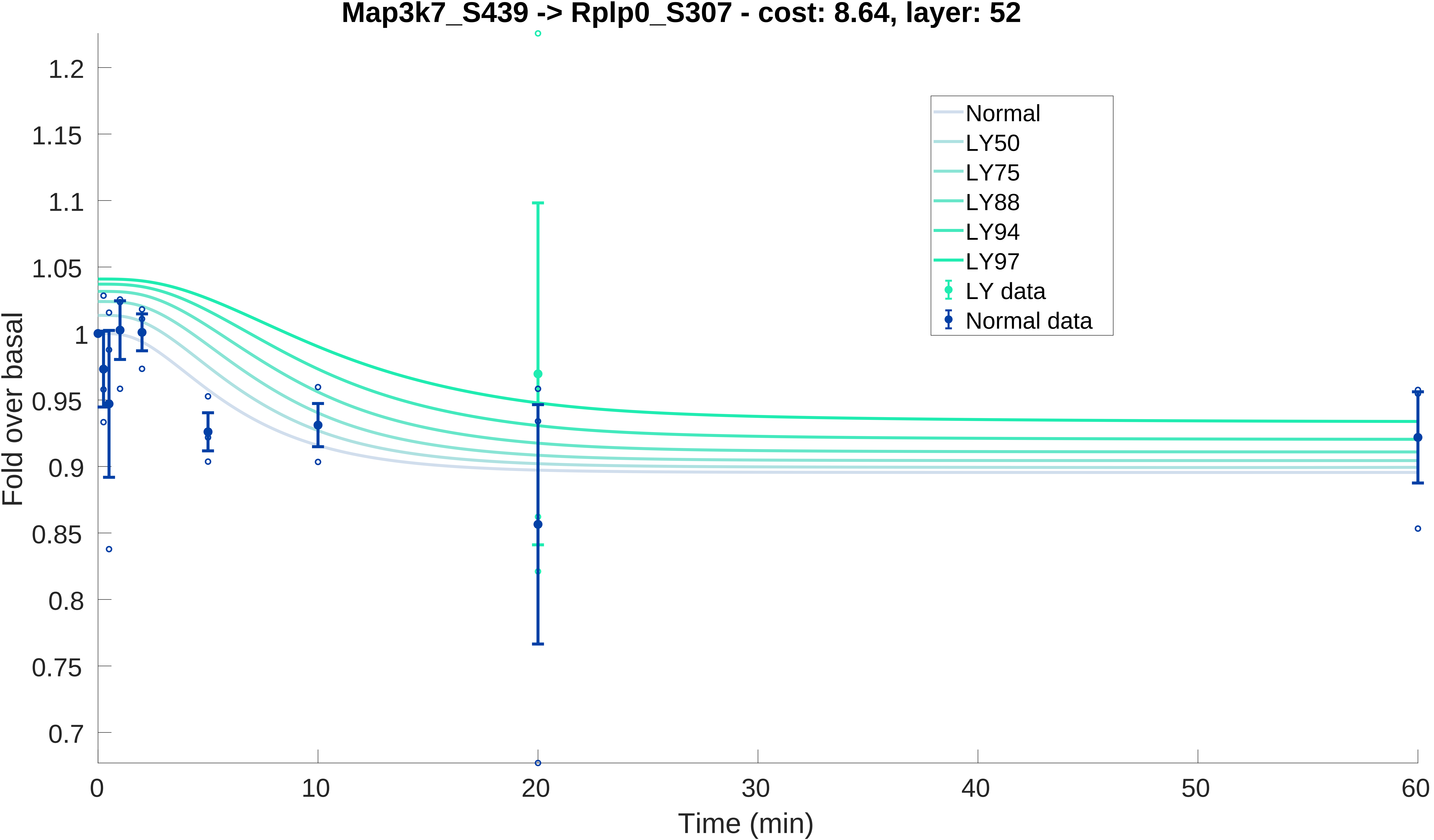

Map3k7_S439 -> Rplp0_S307 - cost: 8.64, layer: 52
Fold over basal
Time (min)
1.2
1.15
1.1
1.05
1
0.95
0.9
0.85
0.8
0.75
0.7
0
10
20
30
40
50
60
Normal
LY50
LY75
LY88
LY94
LY97
LY data
Normal data

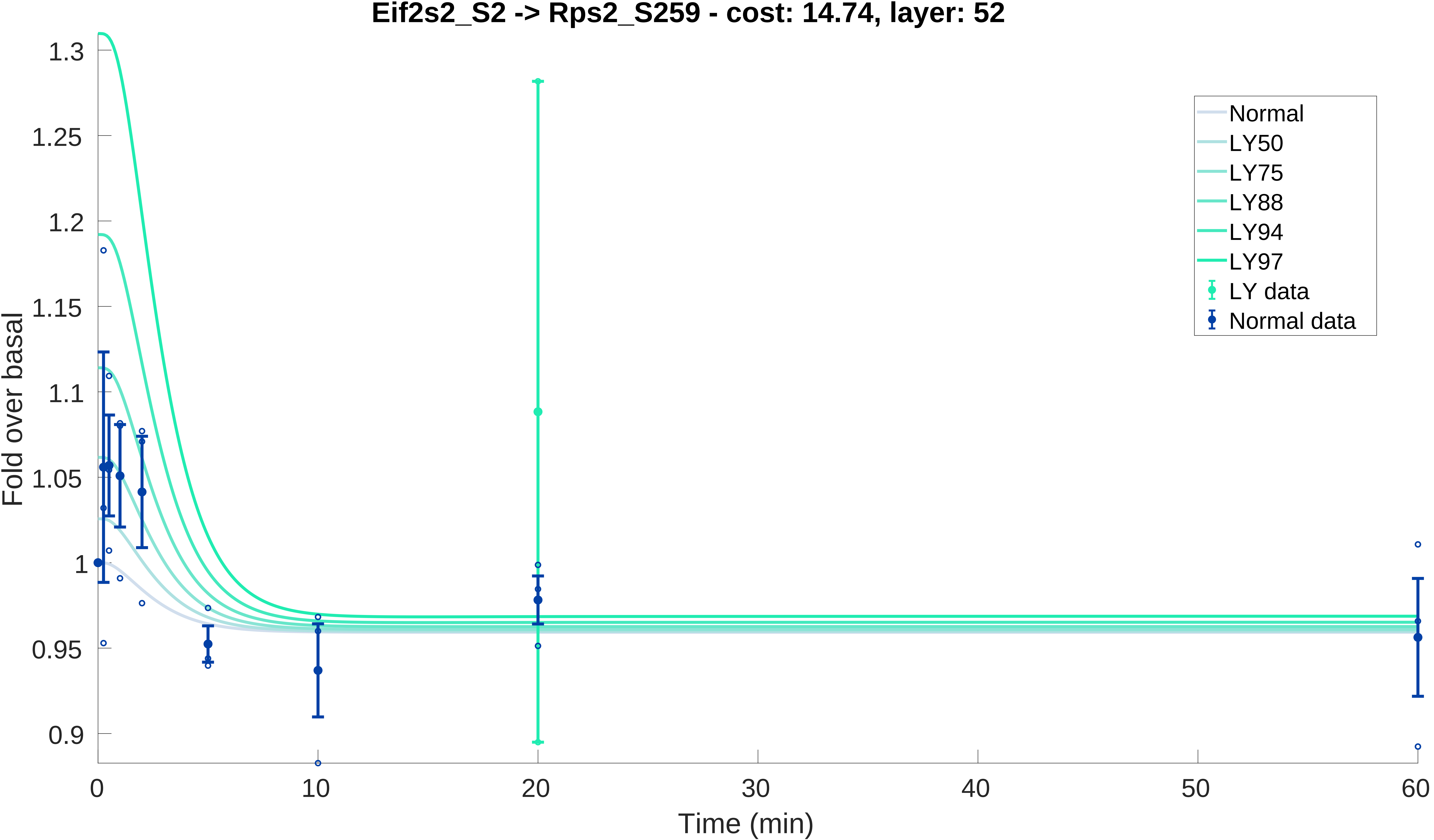

Eif2s2_S2 -> Rps2_S259 - cost: 14.74, layer: 52
Fold over basal
1.3
1.25
1.2
1.15
1.1
1.05
1
0.95
0.9
0
10
20
30
40
50
60
Time (min)
Normal
LY50
LY75
LY88
LY94
LY97
LY data
Normal data

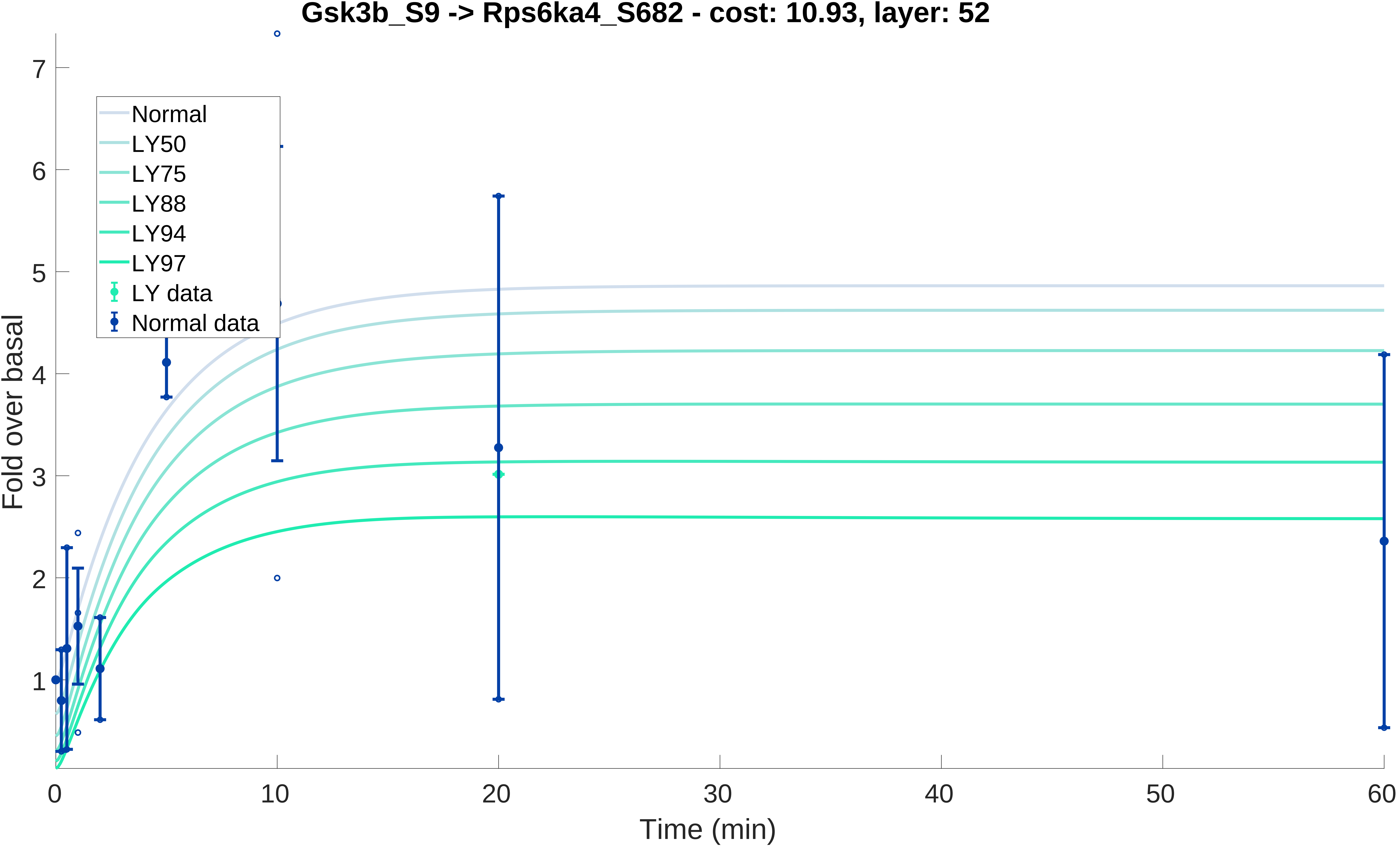

Gsk3b_S9 -> Rps6ka4_S682 - cost: 10.93, layer: 52
Fold over basal
Time (min)
Normal
LY50
LY75
LY88
LY94
LY97
LY data
Normal data
0
10
20
30
40
50
60
1
2
3
4
5
6
7

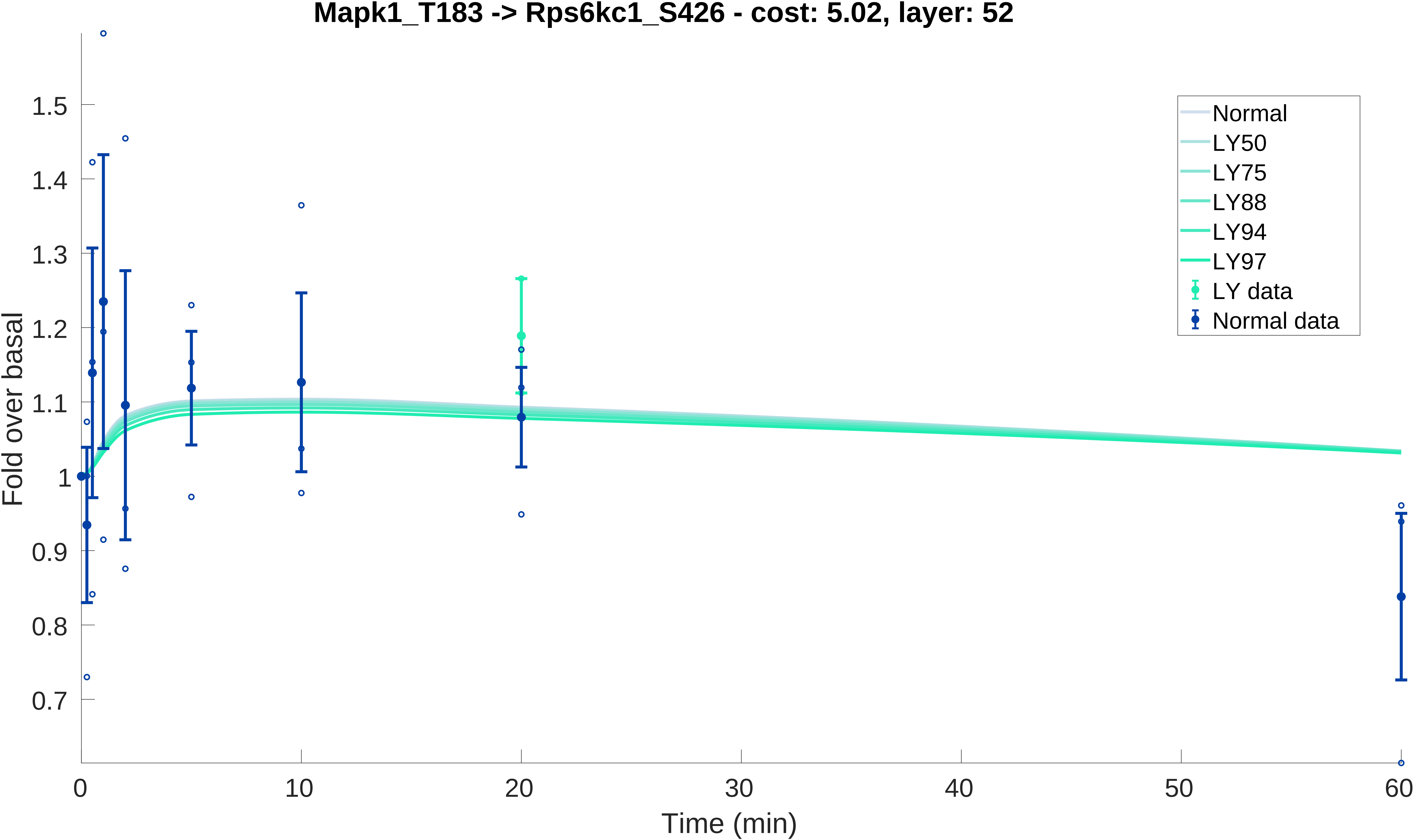

Mapk1_T183 -> Rps6kc1_S426 - cost: 5.02, layer: 52
Fold over basal
Time (min)
0
10
20
30
40
50
60
0.7
0.8
0.9
1
1.1
1.2
1.3
1.4
1.5
Normal
LY50
LY75
LY88
LY94
LY97
LY data
Normal data

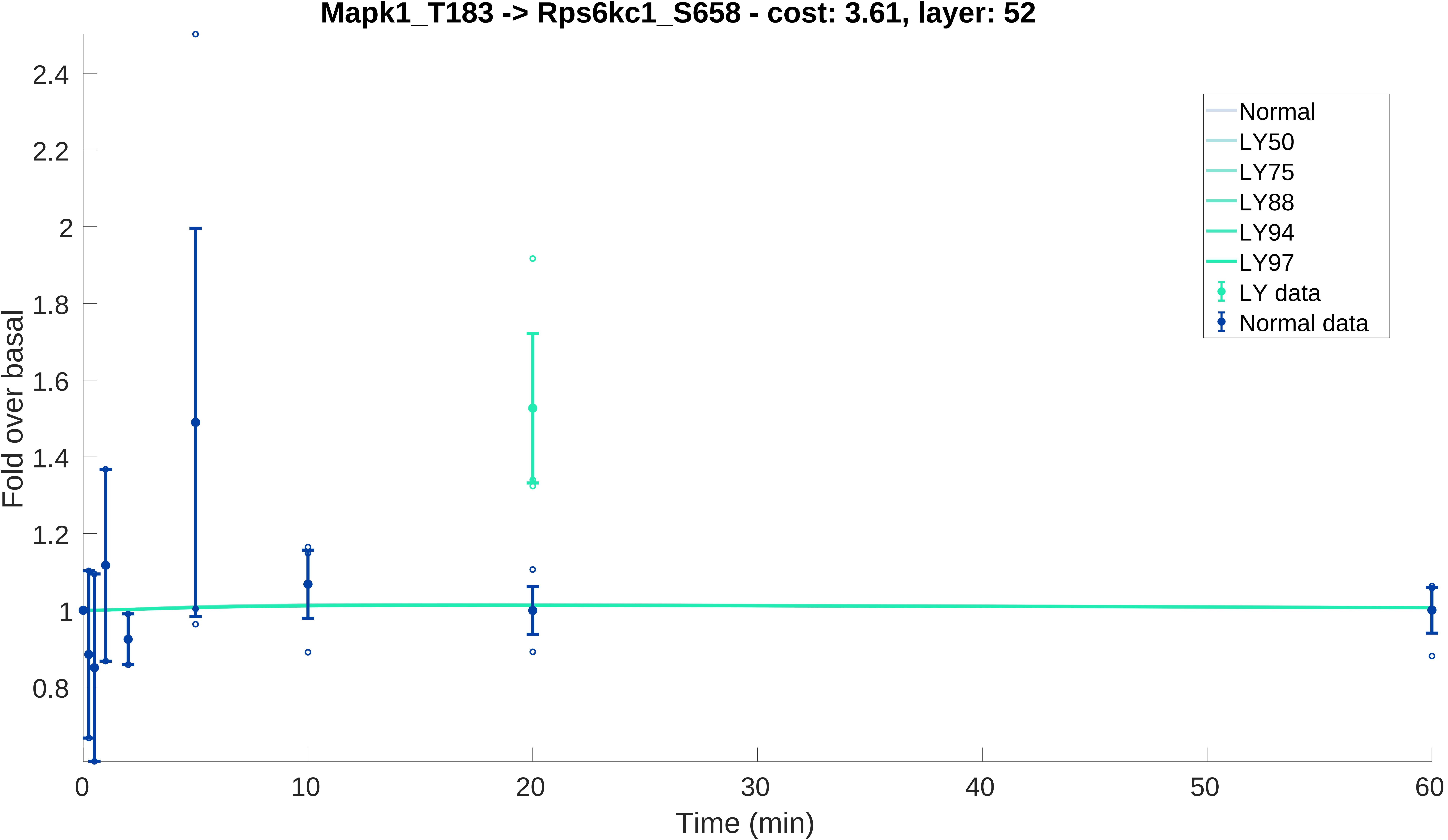

Mapk1_T183 -> Rps6kc1_S658 - cost: 3.61, layer: 52
Fold over basal
Time (min)
0.8
1
1.2
1.4
1.6
1.8
2
2.2
2.4
0
10
20
30
40
50
60
Normal
LY50
LY75
LY88
LY94
LY97
LY data
Normal data

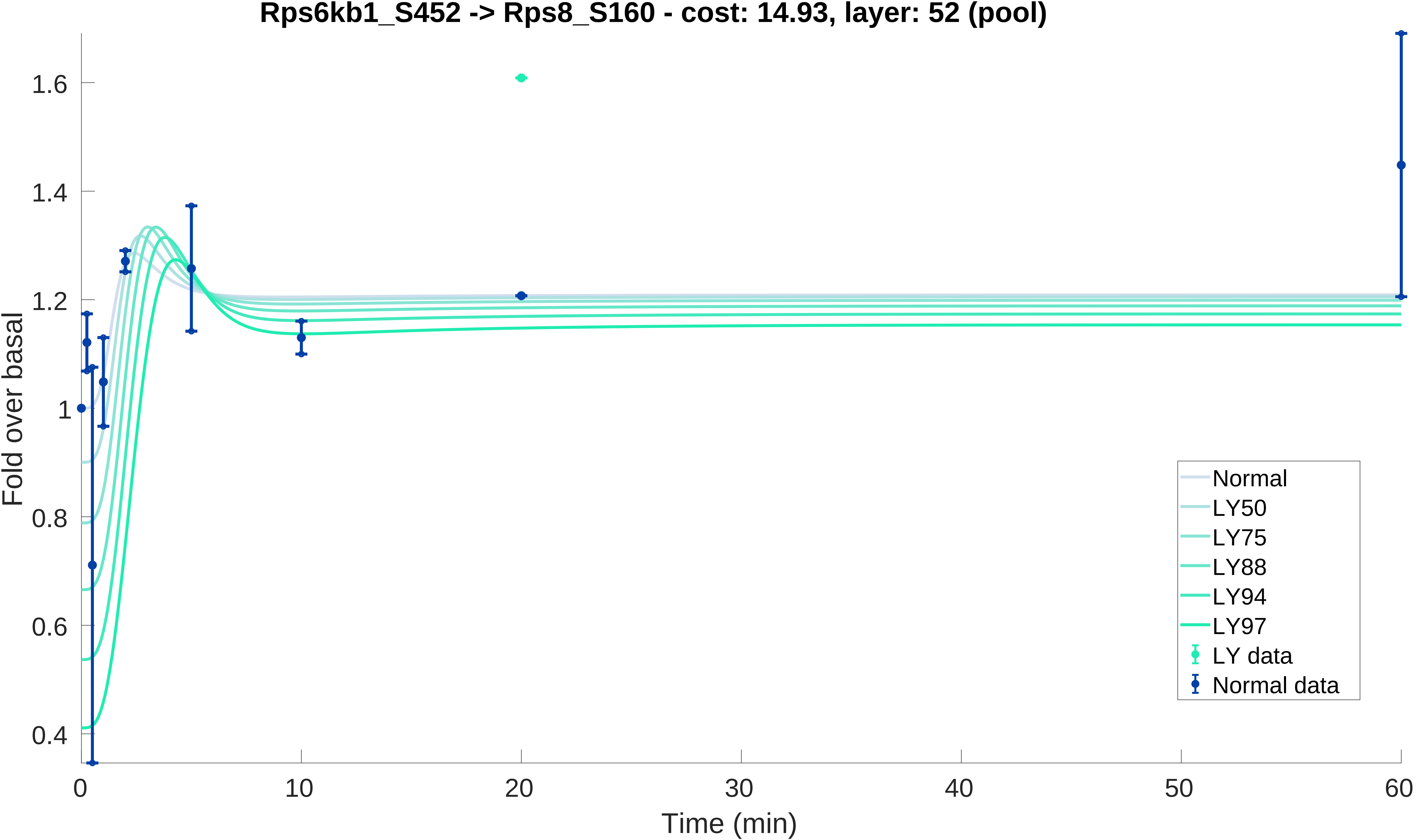

Rps6kb1_S452 -> Rps8_S160 - cost: 14.93, layer: 52 (pool)
Fold over basal
Time (min)
0.4
0.6
0.8
1
1.2
1.4
1.6
0
10
20
30
40
50
60
Normal
LY50
LY75
LY88
LY94
LY97
LY data
Normal data

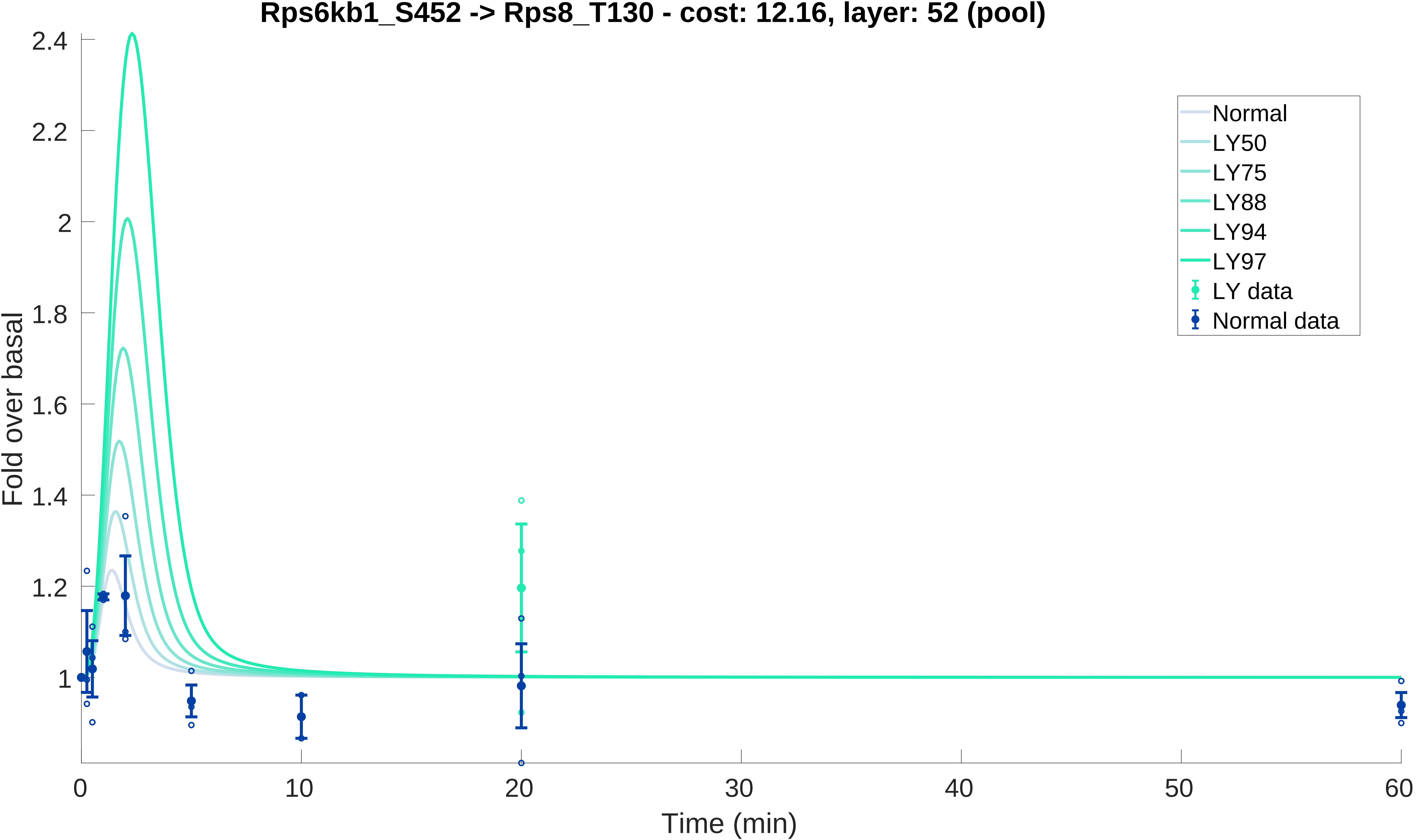

Rps6kb1_S452 -> Rps8_T130 - cost: 12.16, layer: 52 (pool)
Fold over basal
Time (min)
2.4
2.2
2
1.8
1.6
1.4
1.2
1
0
10
20
30
40
50
60
Normal
LY50
LY75
LY88
LY94
LY97
LY data
Normal data

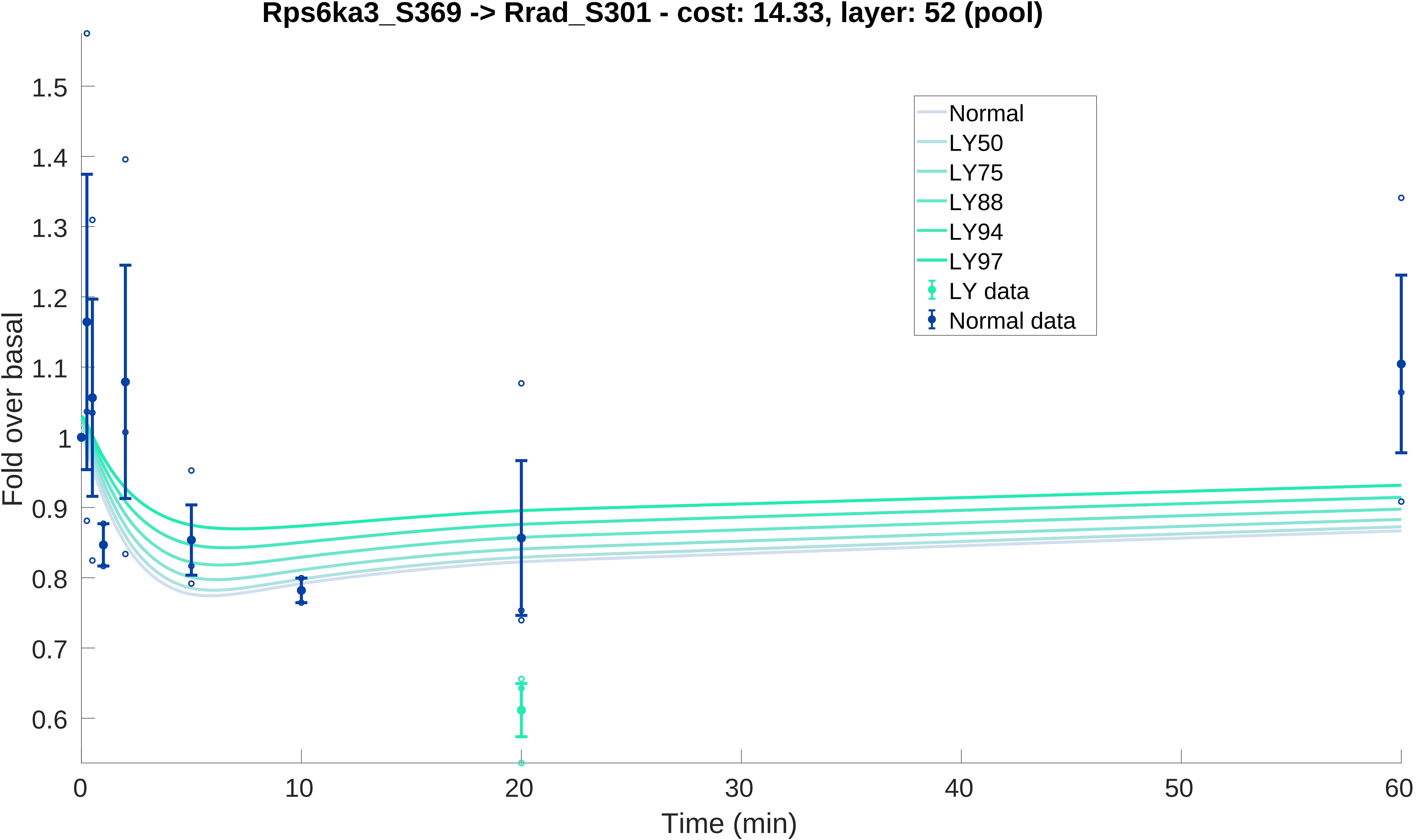

Rps6ka3_S369 -> Rrad_S301 - cost: 14.33, layer: 52 (pool)
Fold over basal
Time (min)
Normal
LY50
LY75
LY88
LY94
LY97
LY data
Normal data
1.5
1.4
1.3
1.2
1.1
1
0.9
0.8
0.7
0.6
0
10
20
30
40
50
60

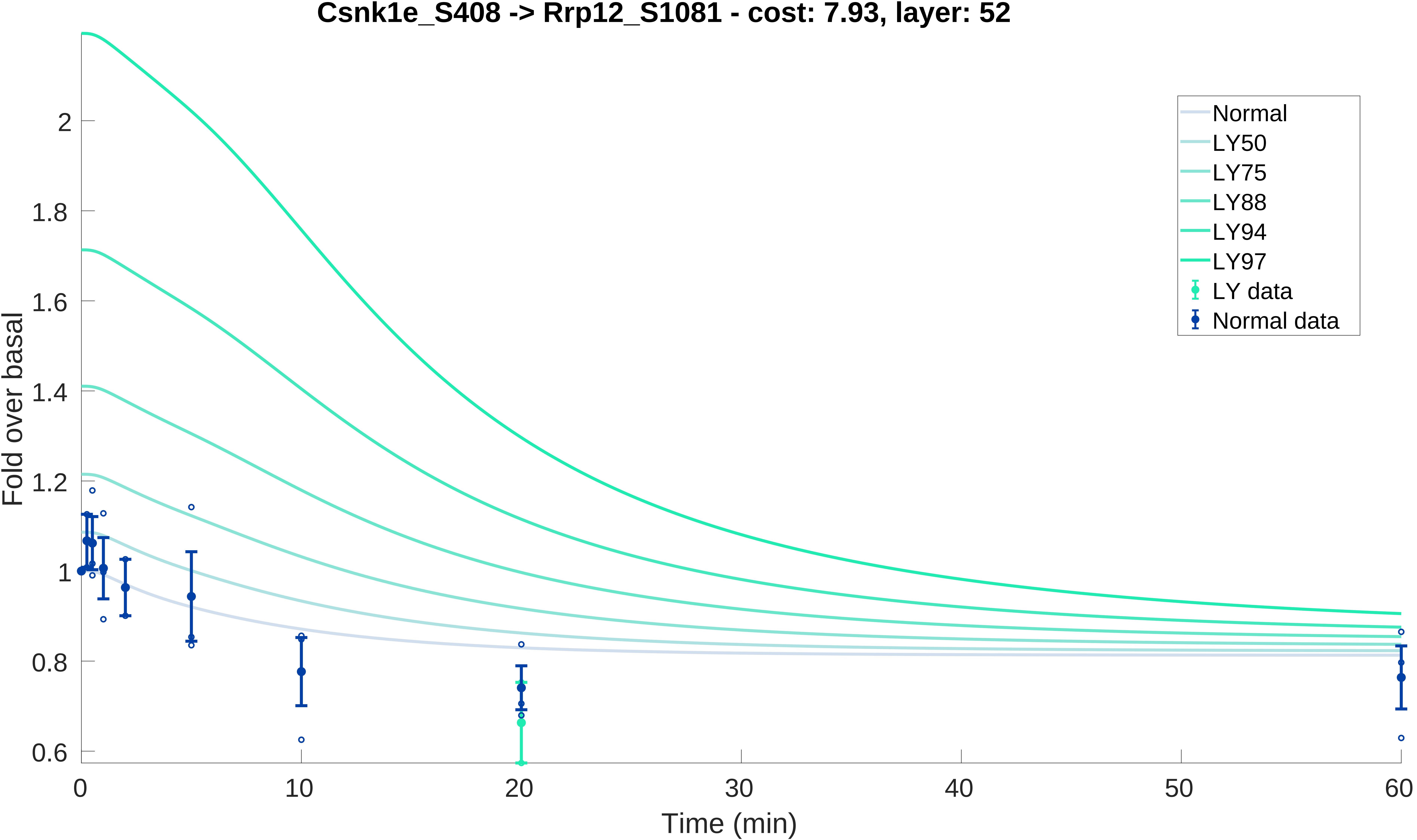
Csnk1e_S408 -> Rrp12_S1081 - cost: 7.93, layer: 52
Fold over basal
Time (min)
Normal
LY50
LY75
LY88
LY94
LY97
LY data
Normal data
0.6
0.8
1
1.2
1.4
1.6
1.8
2
0
10
20
30
40
50
60

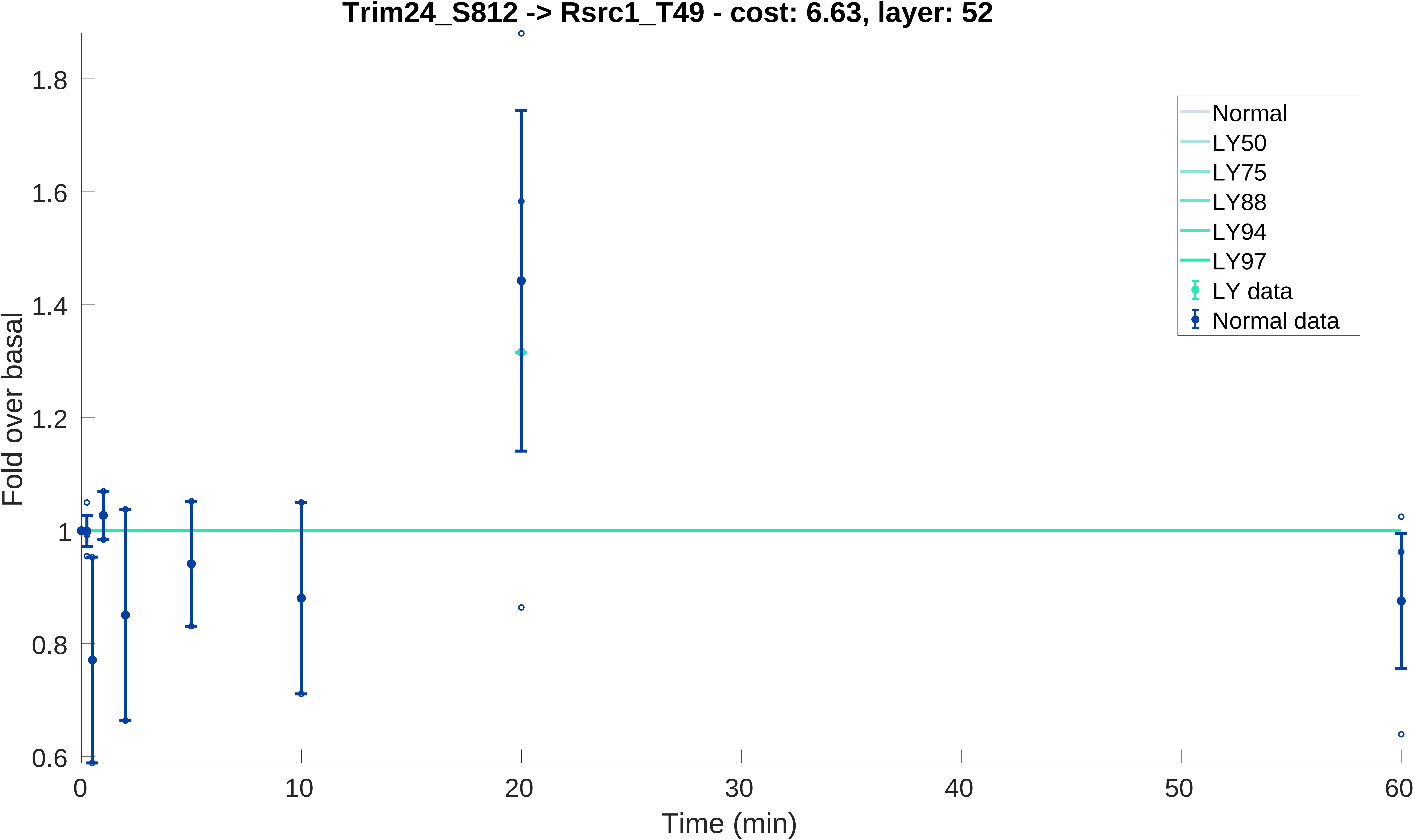
Trim24_S812 -> Rsrc1_T49 - cost: 6.63, layer: 52
Fold over basal
Time (min)
Normal
LY50
LY75
LY88
LY94
LY97
LY data
Normal data
1.8
1.6
1.4
1.2
1
0.8
0.6
0
10
20
30
40
50
60

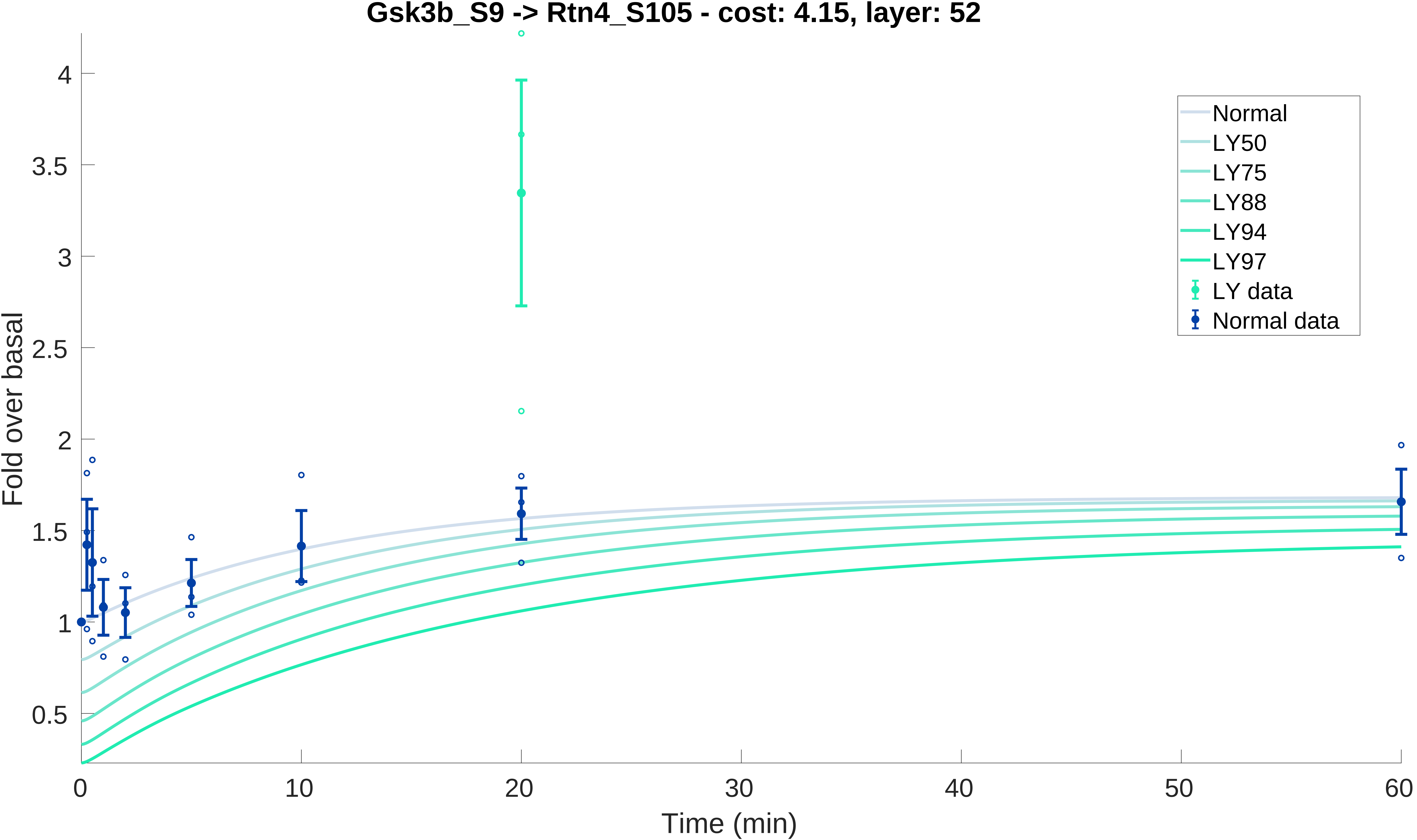

Gsk3b_S9 -> Rtn4_S105 - cost: 4.15, layer: 52
Fold over basal
Time (min)
0.5
1
1.5
2
2.5
3
3.5
4
0
10
20
30
40
50
60
Normal
LY50
LY75
LY88
LY94
LY97
LY data
Normal data

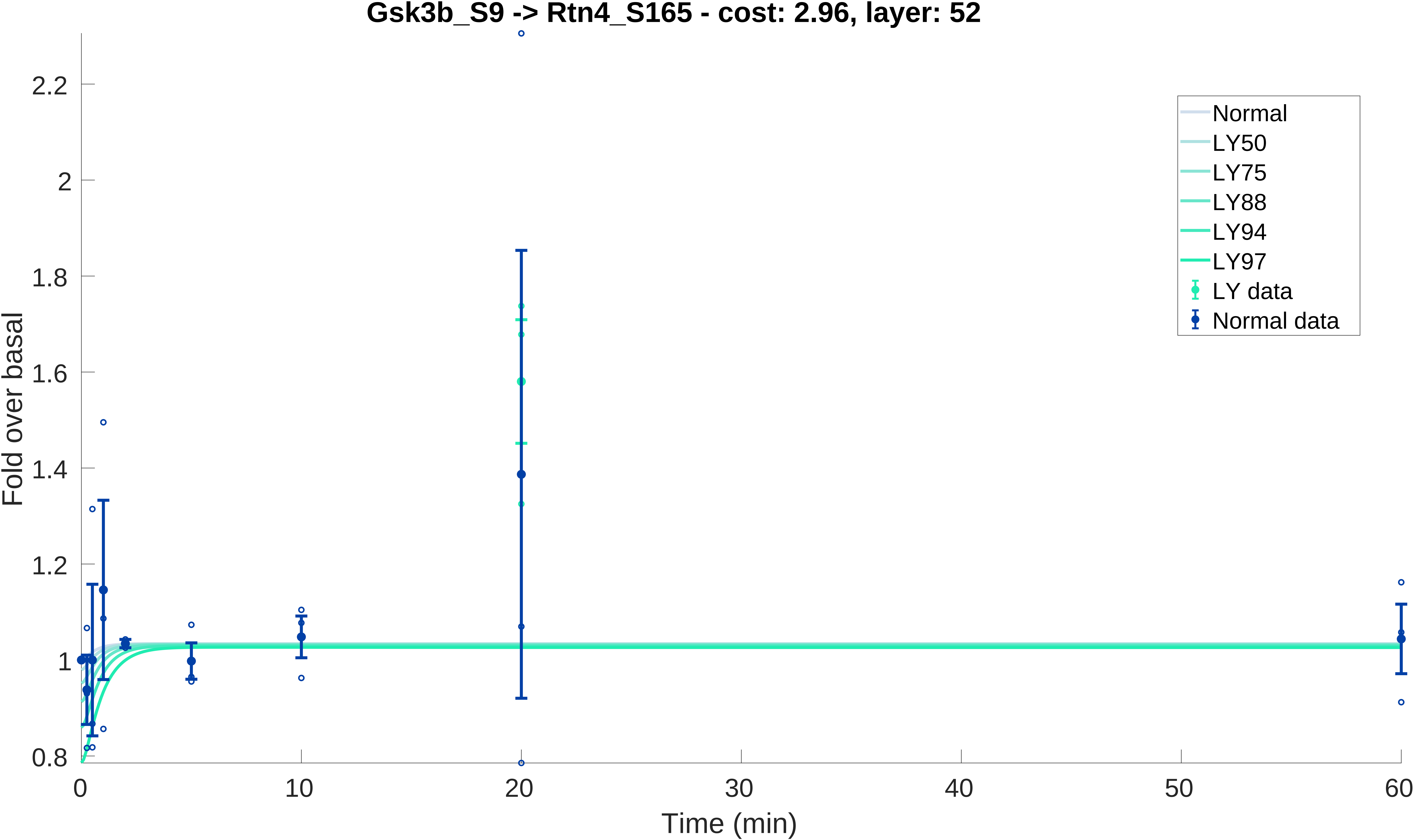
Gsk3b_S9 -> Rtn4_S165 - cost: 2.96, layer: 52
Fold over basal
Time (min)
0.8
1
1.2
1.4
1.6
1.8
2
2.2
0
10
20
30
40
50
60
Normal
LY50
LY75
LY88
LY94
LY97
LY data
Normal data

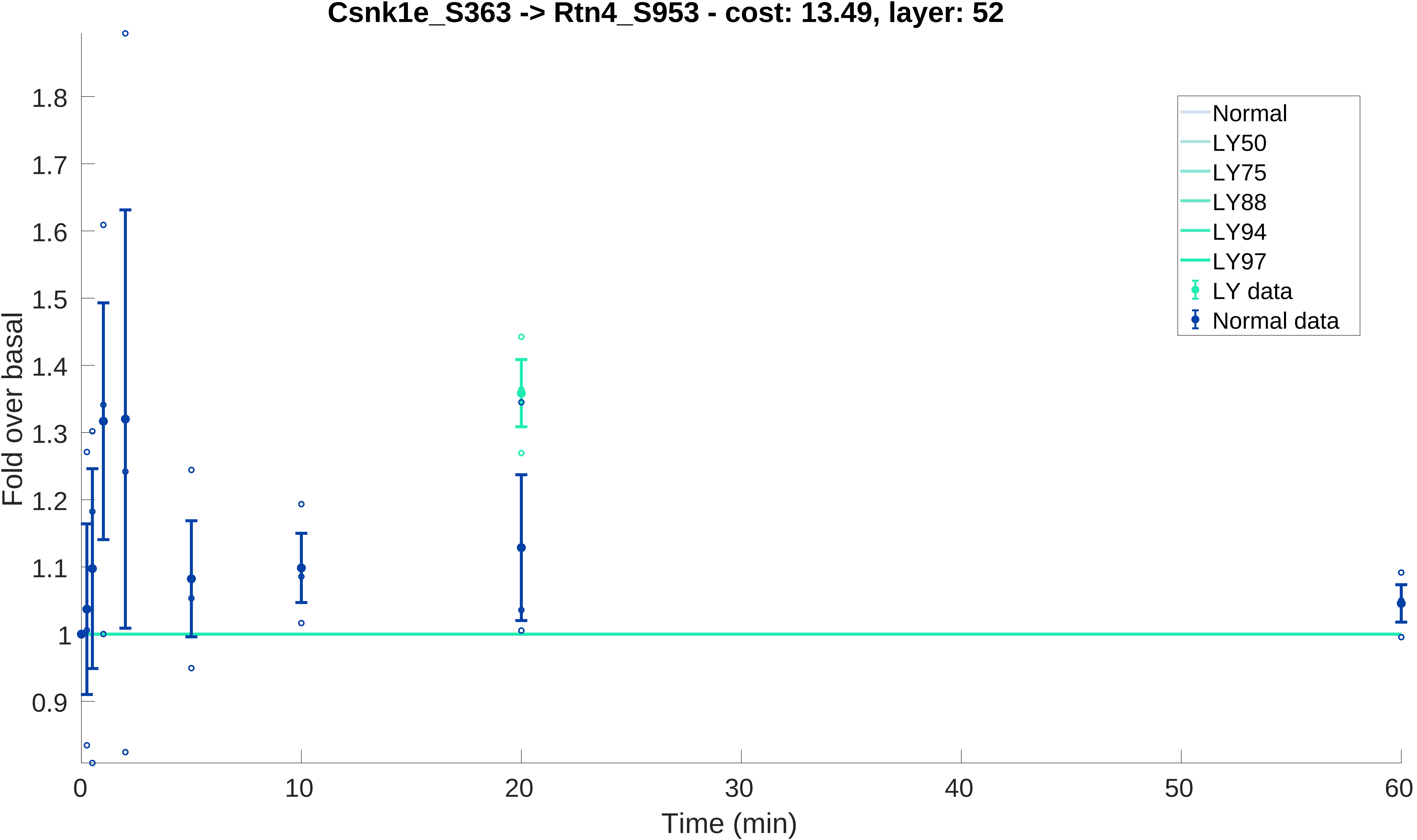
Csnk1e_S363 -> Rtn4_S953 - cost: 13.49, layer: 52
Fold over basal
Time (min)
0.9
1
1.1
1.2
1.3
1.4
1.5
1.6
1.7
1.8
0
10
20
30
40
50
60
Normal
LY50
LY75
LY88
LY94
LY97
LY data
Normal data

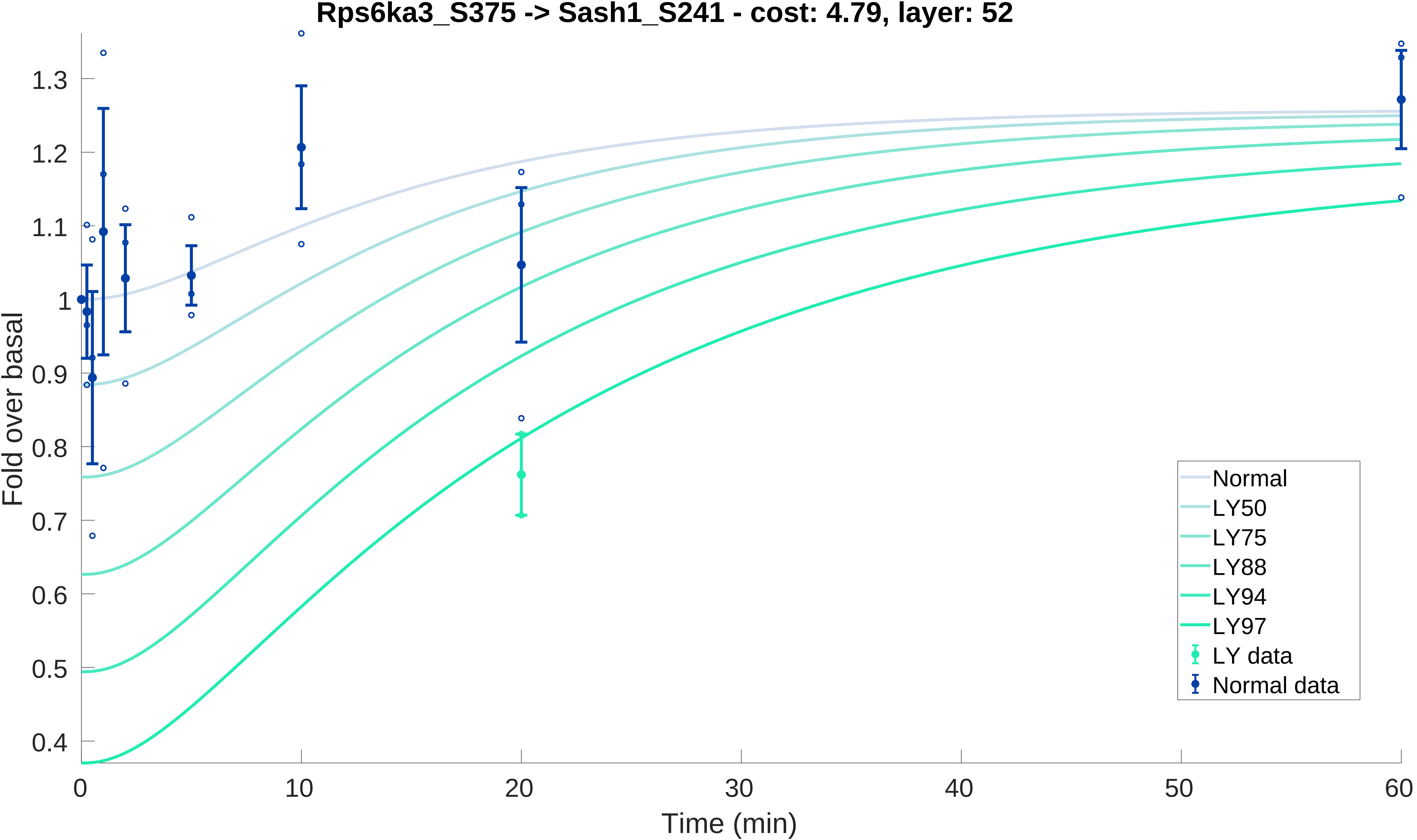
Rps6ka3_S375 -> Sash1_S241 - cost: 4.79, layer: 52
Fold over basal
1.3
1.2
1.1
1
0.9
0.8
0.7
0.6
0.5
0.4
0
10
20
30
40
50
60
Time (min)
Normal
LY50
LY75
LY88
LY94
LY97
LY data
Normal data

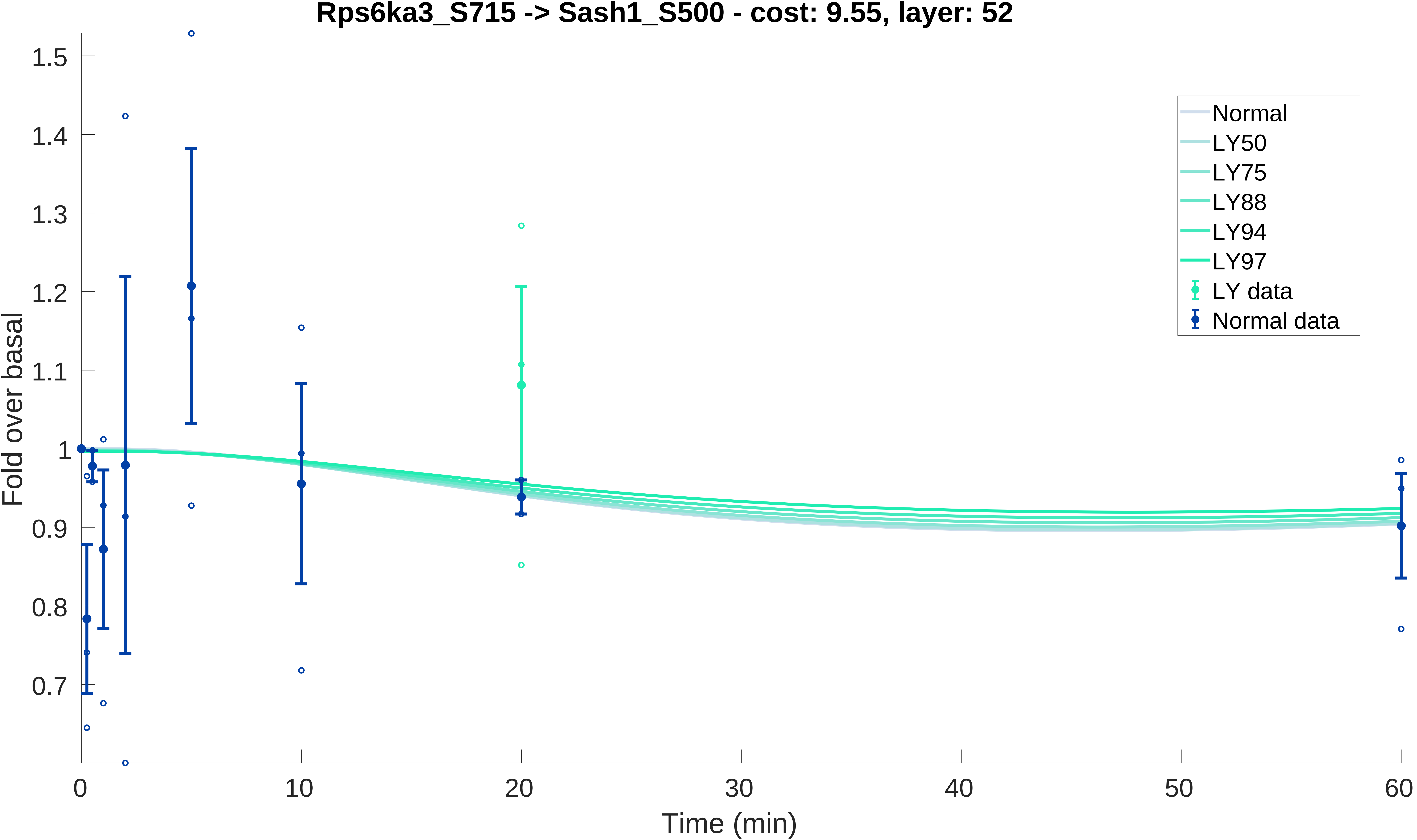

Rps6ka3_S715 -> Sash1_S500 - cost: 9.55, layer: 52
Fold over basal
Time (min)
0
10
20
30
40
50
60
0.7
0.8
0.9
1
1.1
1.2
1.3
1.4
1.5
Normal
LY50
LY75
LY88
LY94
LY97
LY data
Normal data

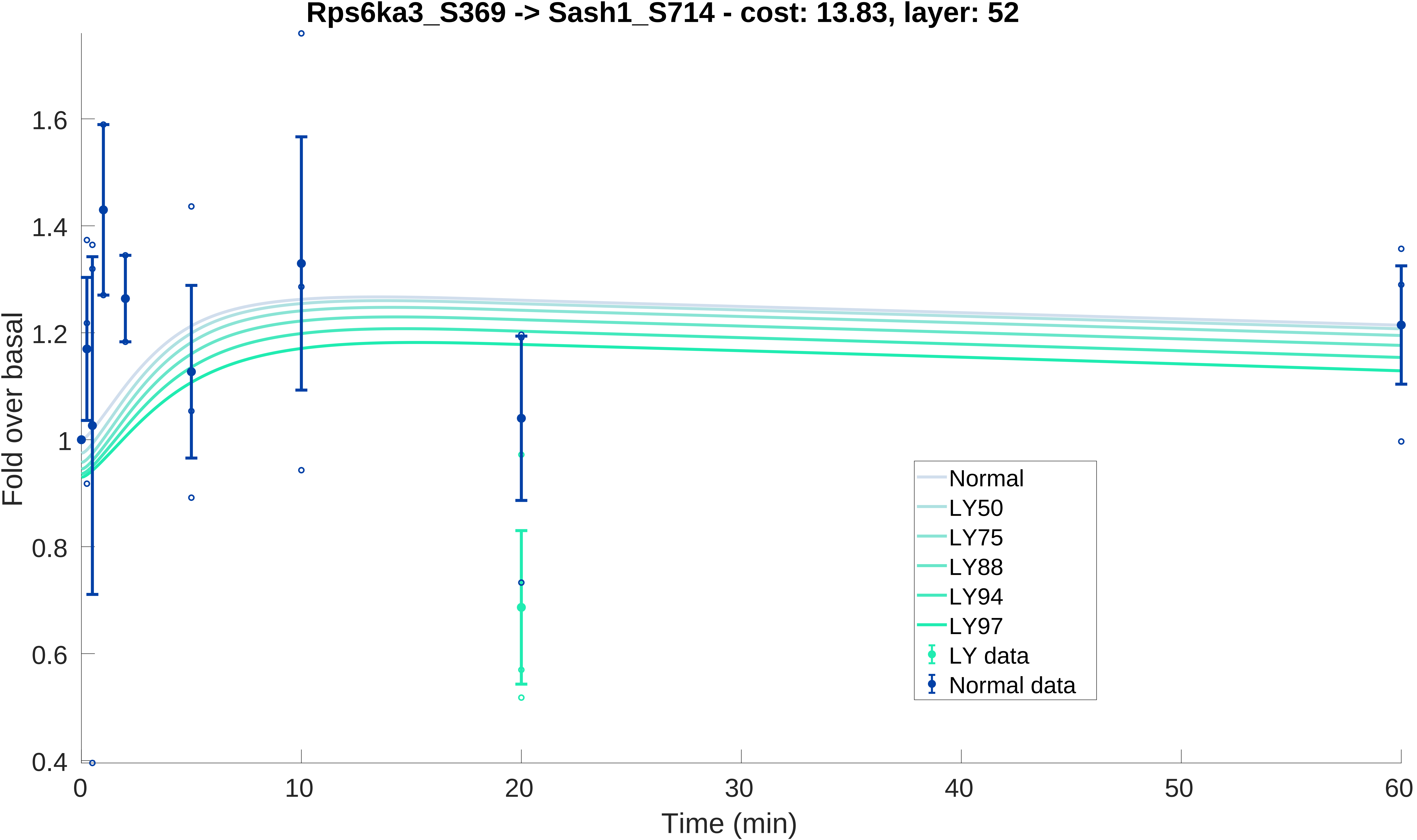
Rps6ka3_S369 -> Sash1_S714 - cost: 13.83, layer: 52
Fold over basal
Time (min)
0.4
0.6
0.8
1
1.2
1.4
1.6
0
10
20
30
40
50
60
Normal
LY50
LY75
LY88
LY94
LY97
LY data
Normal data

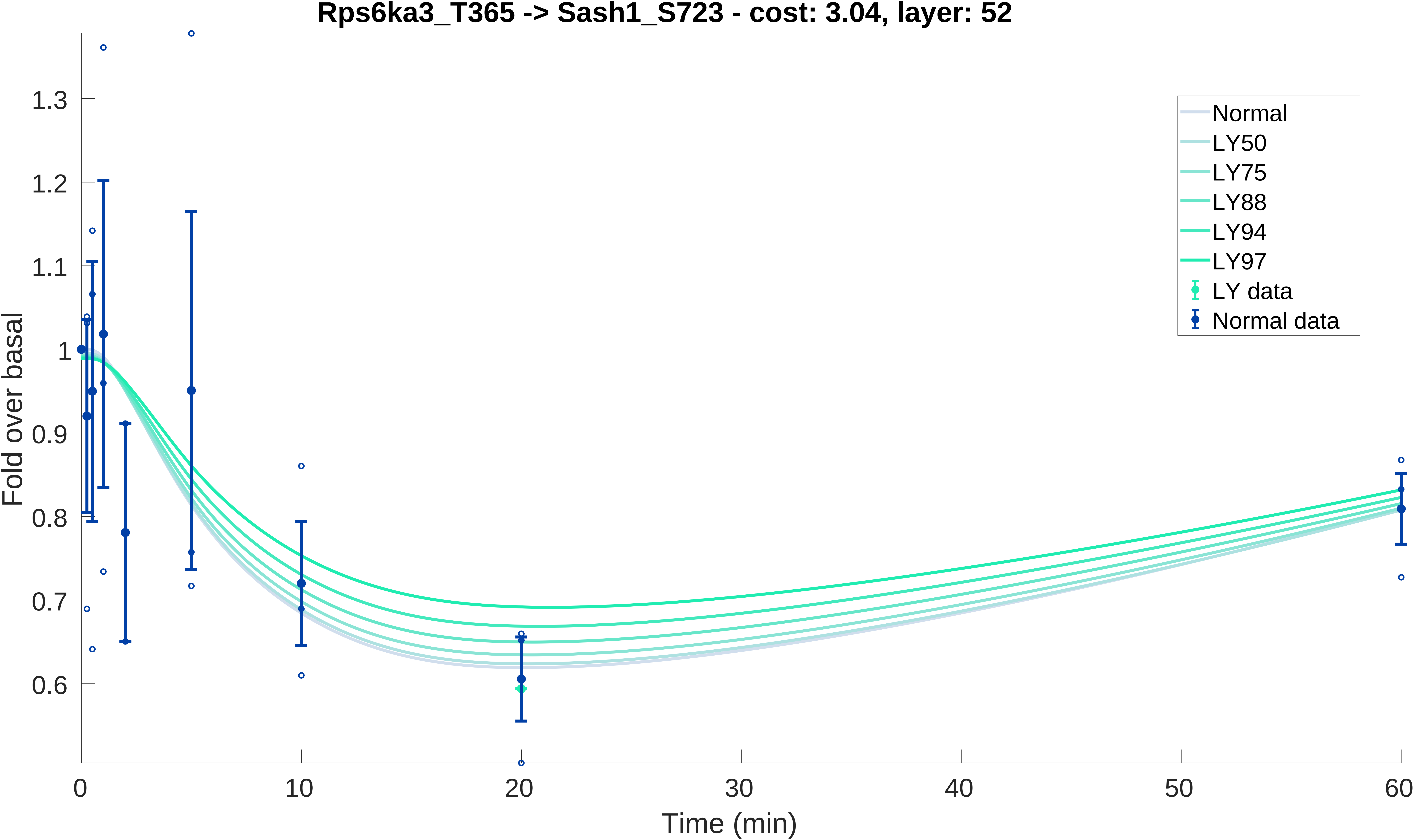

Rps6ka3_T365 -> Sash1_S723 - cost: 3.04, layer: 52
Fold over basal
1.3
1.2
1.1
1
0.9
0.8
0.7
0.6
0
10
20
30
40
50
60
Time (min)
Normal
LY50
LY75
LY88
LY94
LY97
LY data
Normal data

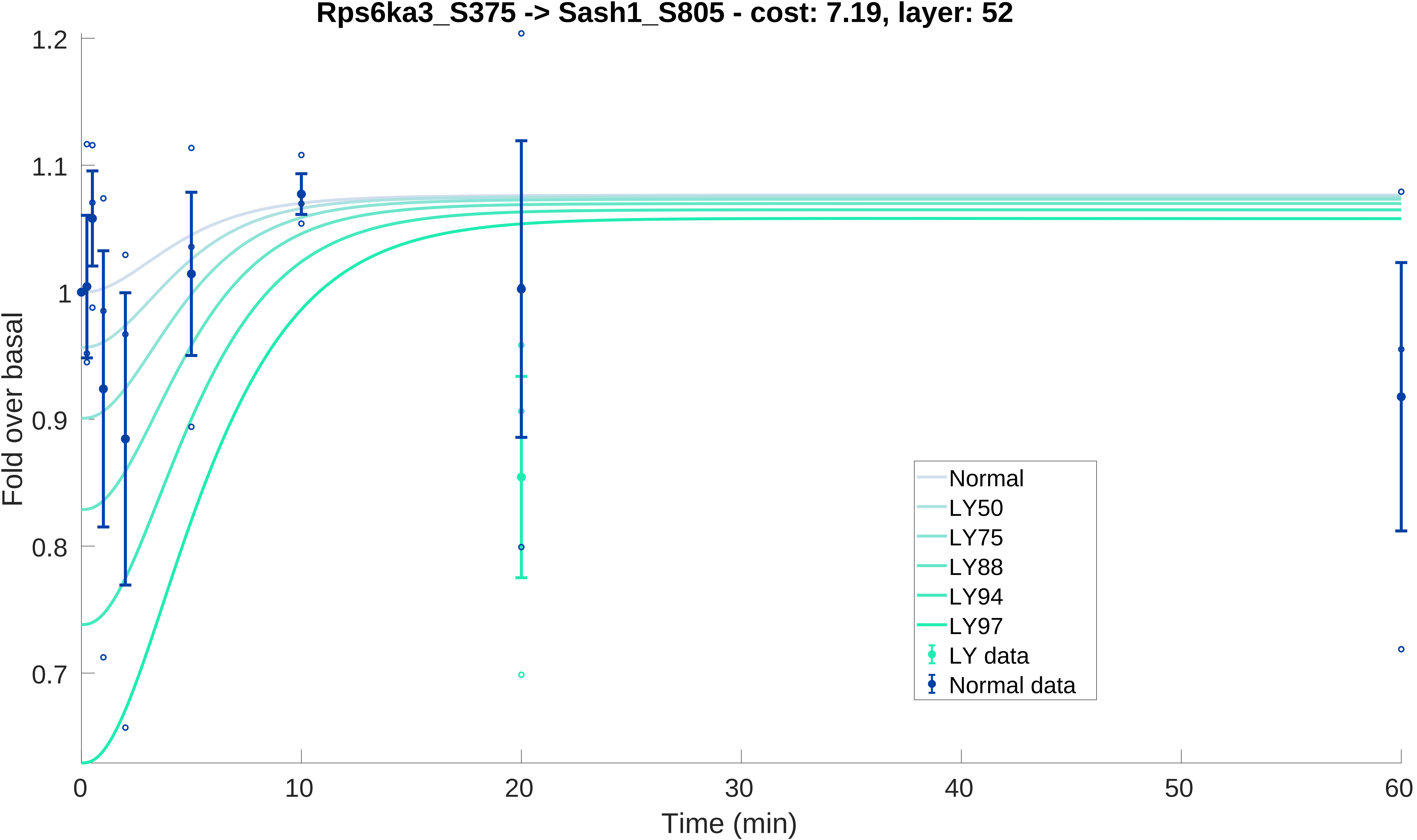

Rps6ka3_S375 -> Sash1_S805 - cost: 7.19, layer: 52
Fold over basal
Time (min)
1.2
1.1
1
0.9
0.8
0.7
0
10
20
30
40
50
60
Normal
LY50
LY75
LY88
LY94
LY97
LY data
Normal data

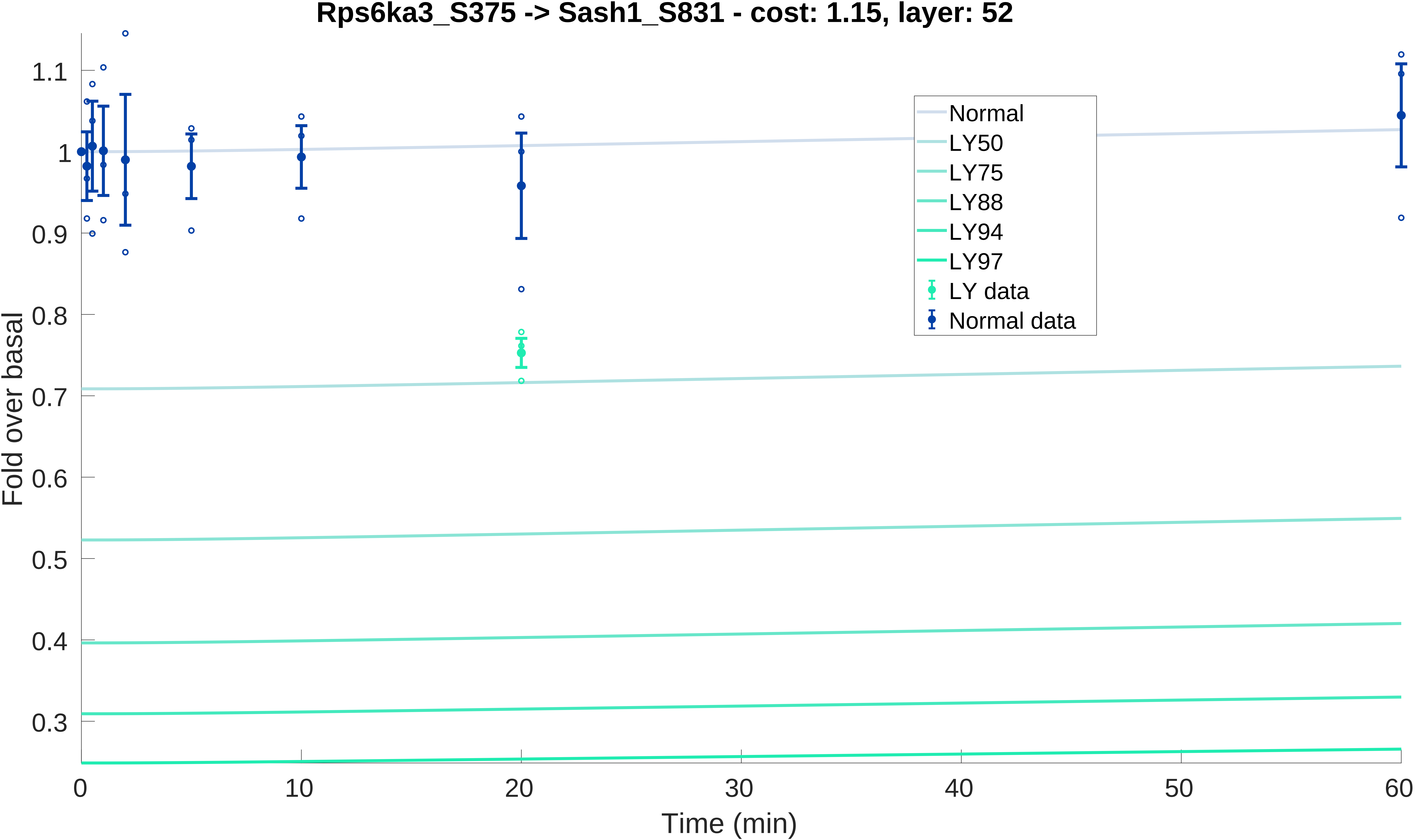
Rps6ka3_S375 -> Sash1_S831 - cost: 1.15, layer: 52
Fold over basal
Time (min)
Normal
LY50
LY75
LY88
LY94
LY97
LY data
Normal data
1.1
1
0.9
0.8
0.7
0.6
0.5
0.4
0.3
0
10
20
30
40
50
60

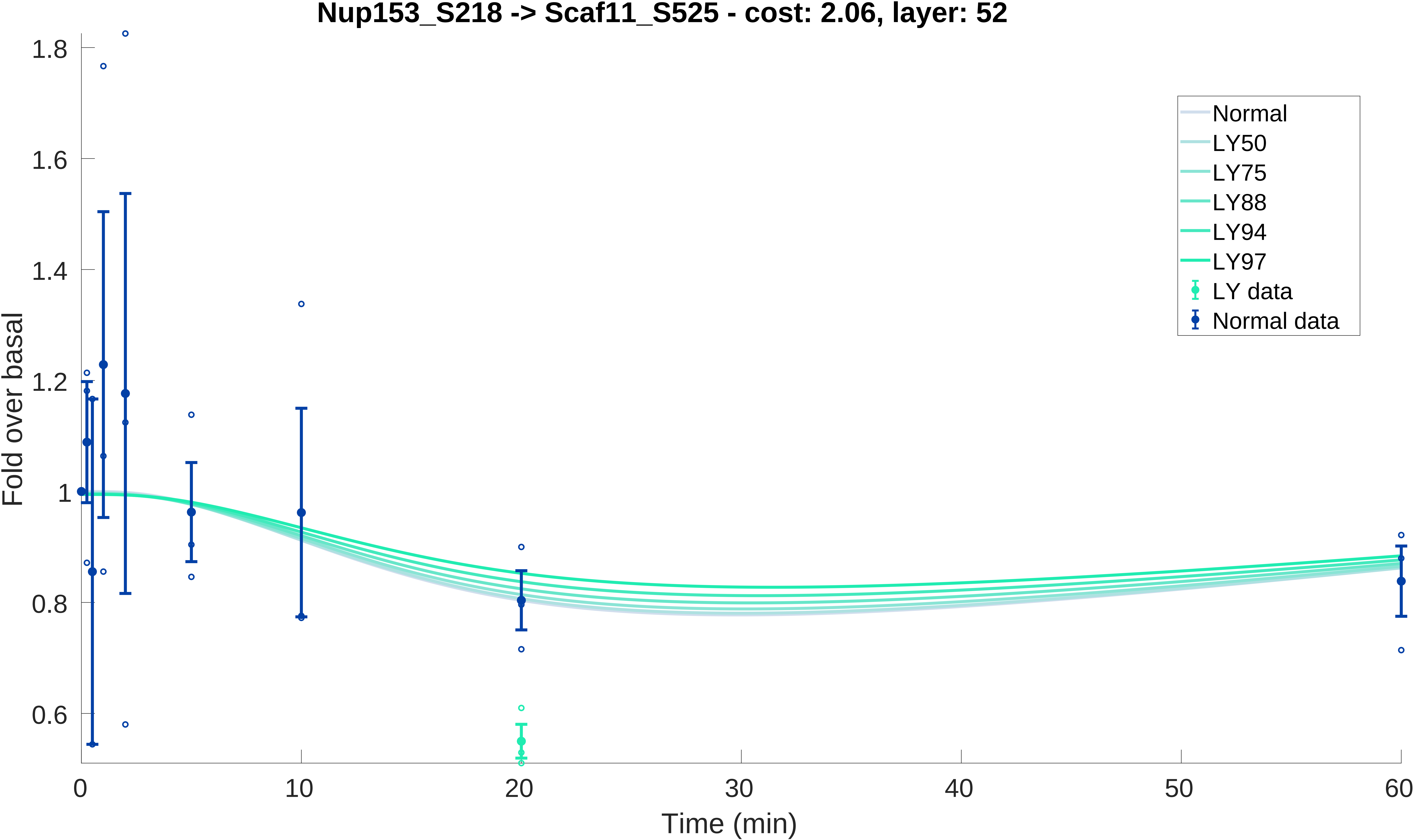
Nup153_S218 -> Scaf11_S525 - cost: 2.06, layer: 52
Fold over basal
Time (min)
0
10
20
30
40
50
60
0.6
0.8
1
1.2
1.4
1.6
1.8
Normal
LY50
LY75
LY88
LY94
LY97
LY data
Normal data

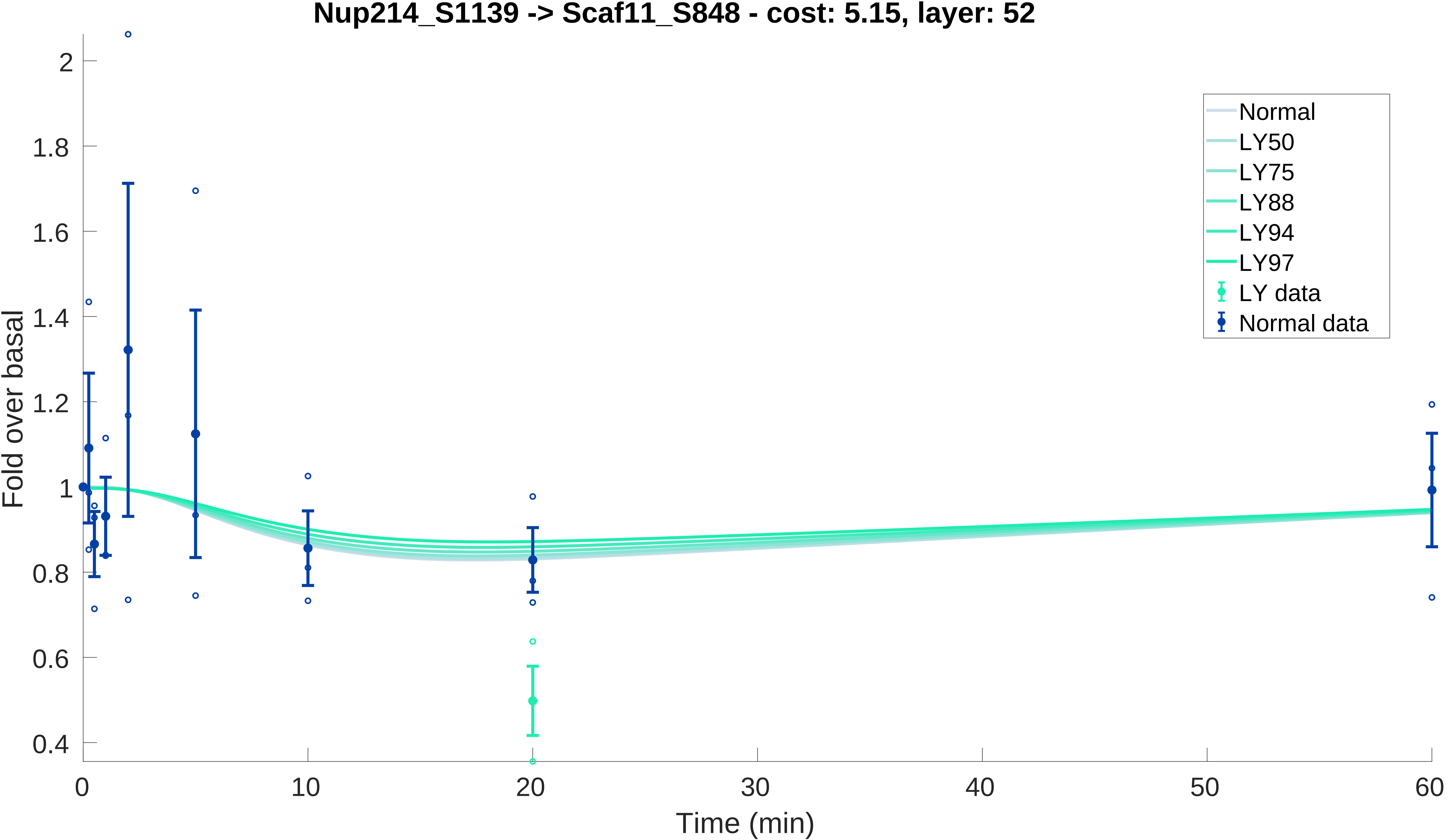

Nup214_S1139 -> Scaf11_S848 - cost: 5.15, layer: 52
Fold over basal
Time (min)
0.4
0.6
0.8
1
1.2
1.4
1.6
1.8
2
0
10
20
30
40
50
60
Normal
LY50
LY75
LY88
LY94
LY97
LY data
Normal data

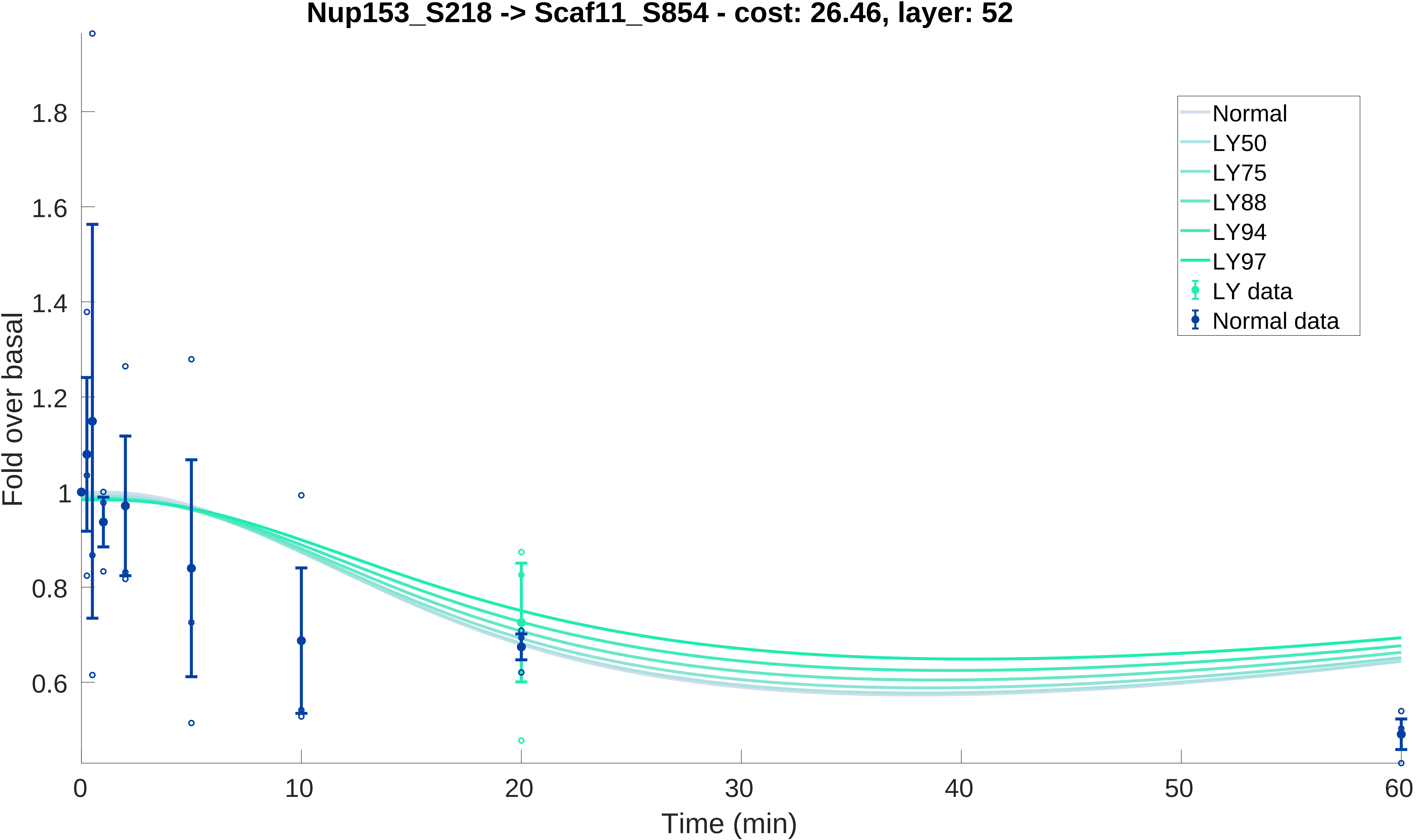

Nup153_S218 -> Scaf11_S854 - cost: 26.46, layer: 52
Fold over basal
Time (min)
1.8
1.6
1.4
1.2
1
0.8
0.6
0
10
20
30
40
50
60
Normal
LY50
LY75
LY88
LY94
LY97
LY data
Normal data

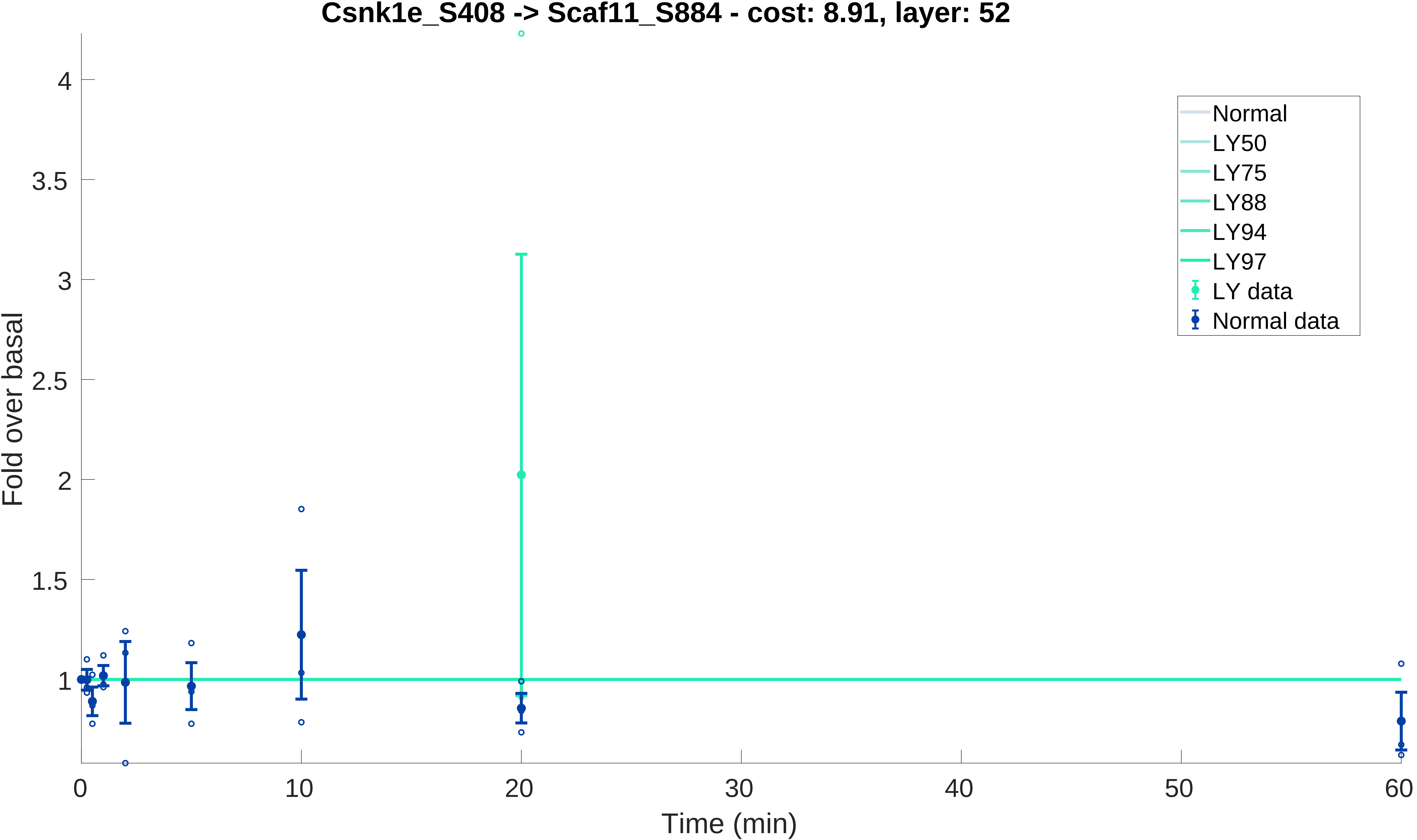

Csnk1e_S408 -> Scaf11_S884 - cost: 8.91, layer: 52
Fold over basal
Time (min)
Normal
LY50
LY75
LY88
LY94
LY97
LY data
Normal data
0
10
20
30
40
50
60
1
1.5
2
2.5
3
3.5
4

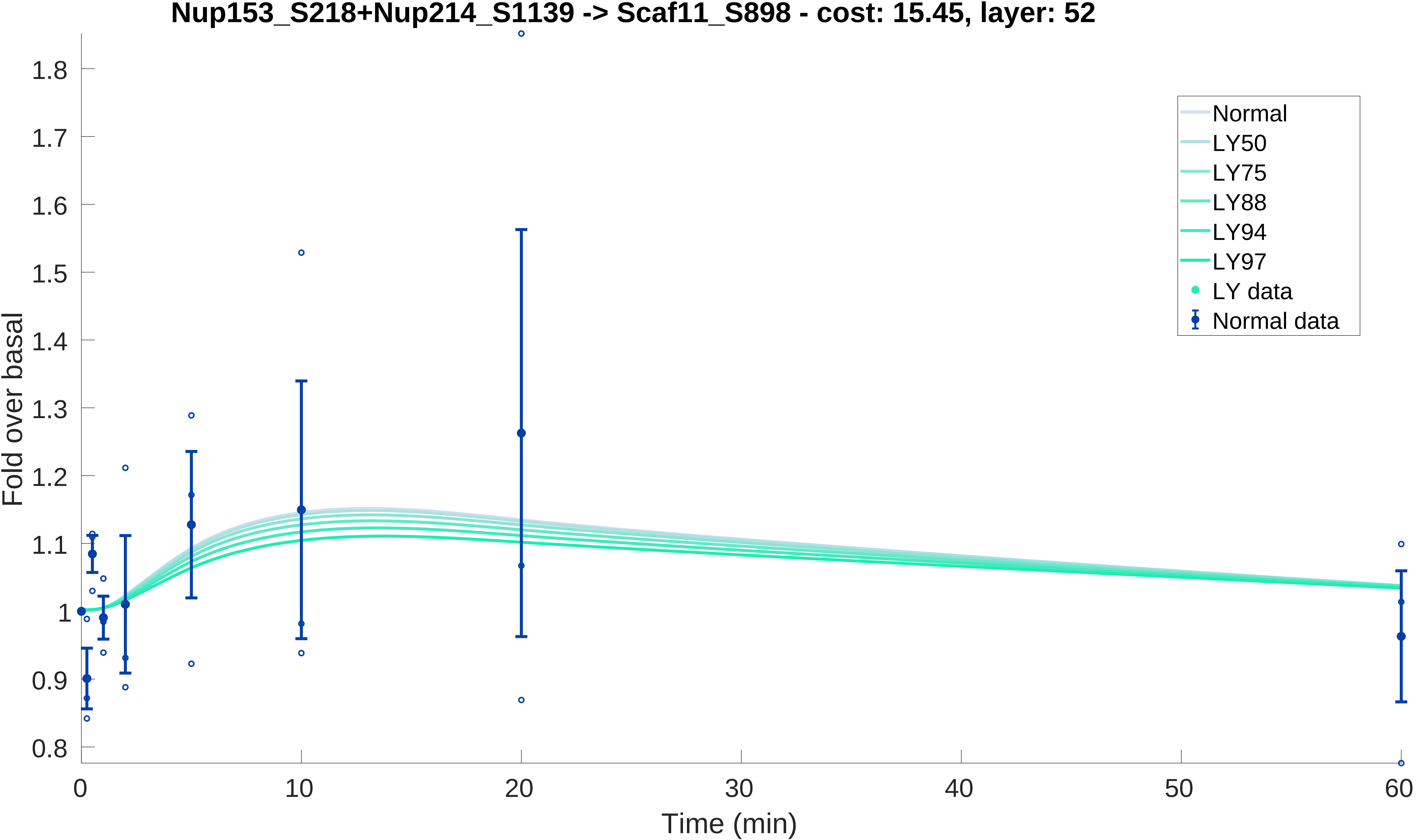

Nup153_S218+Nup214_S1139 -> Scaf11_S898 - cost: 15.45, layer: 52
Fold over basal
Time (min)
0.8
0.9
1
1.1
1.2
1.3
1.4
1.5
1.6
1.7
1.8
0
10
20
30
40
50
60
Normal
LY50
LY75
LY88
LY94
LY97
LY data
Normal data

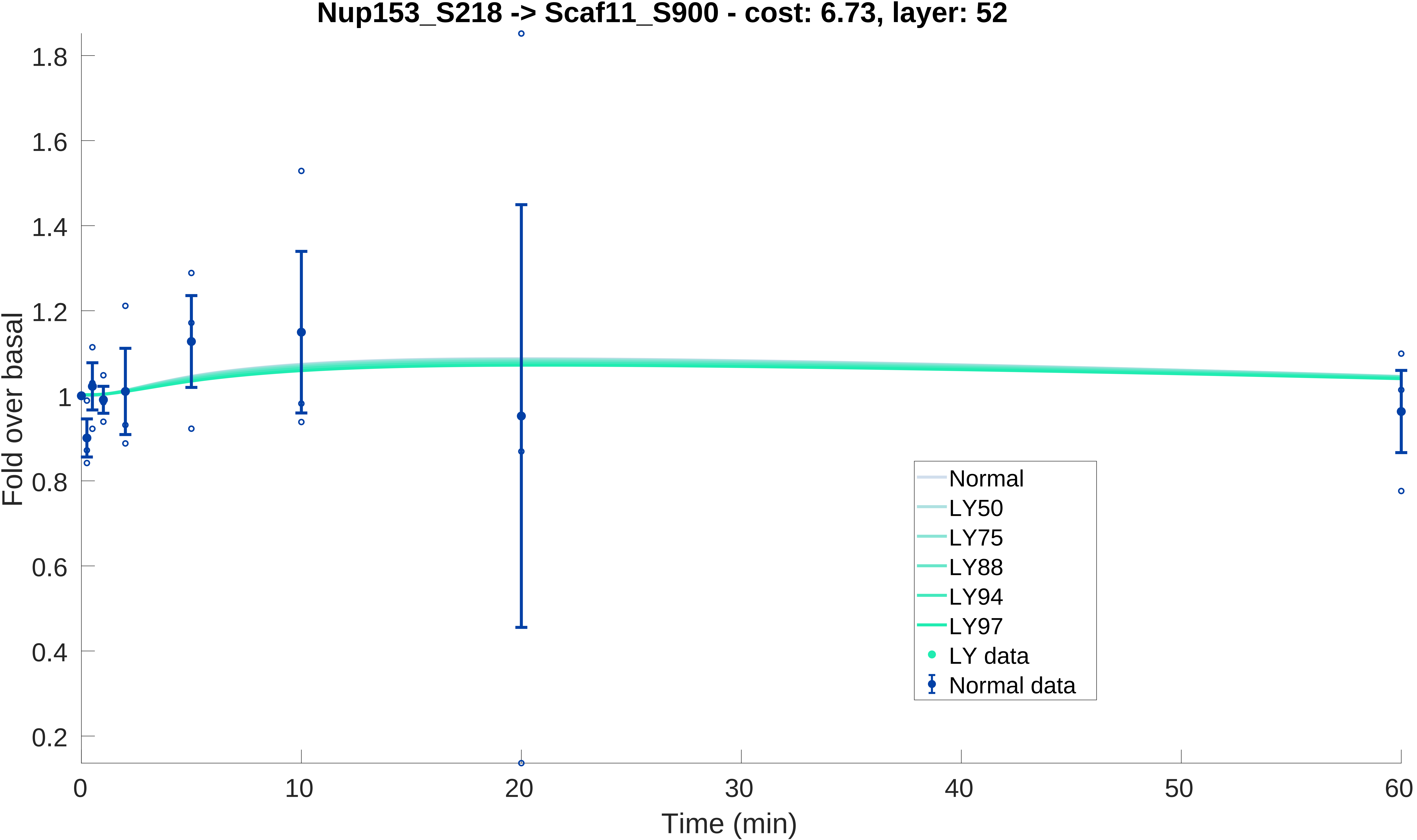

Nup153_S218 -> Scaf11_S900 - cost: 6.73, layer: 52
Fold over basal
Time (min)
1.8
1.6
1.4
1.2
1
0.8
0.6
0.4
0.2
0
10
20
30
40
50
60
Normal
LY50
LY75
LY88
LY94
LY97
LY data
Normal data

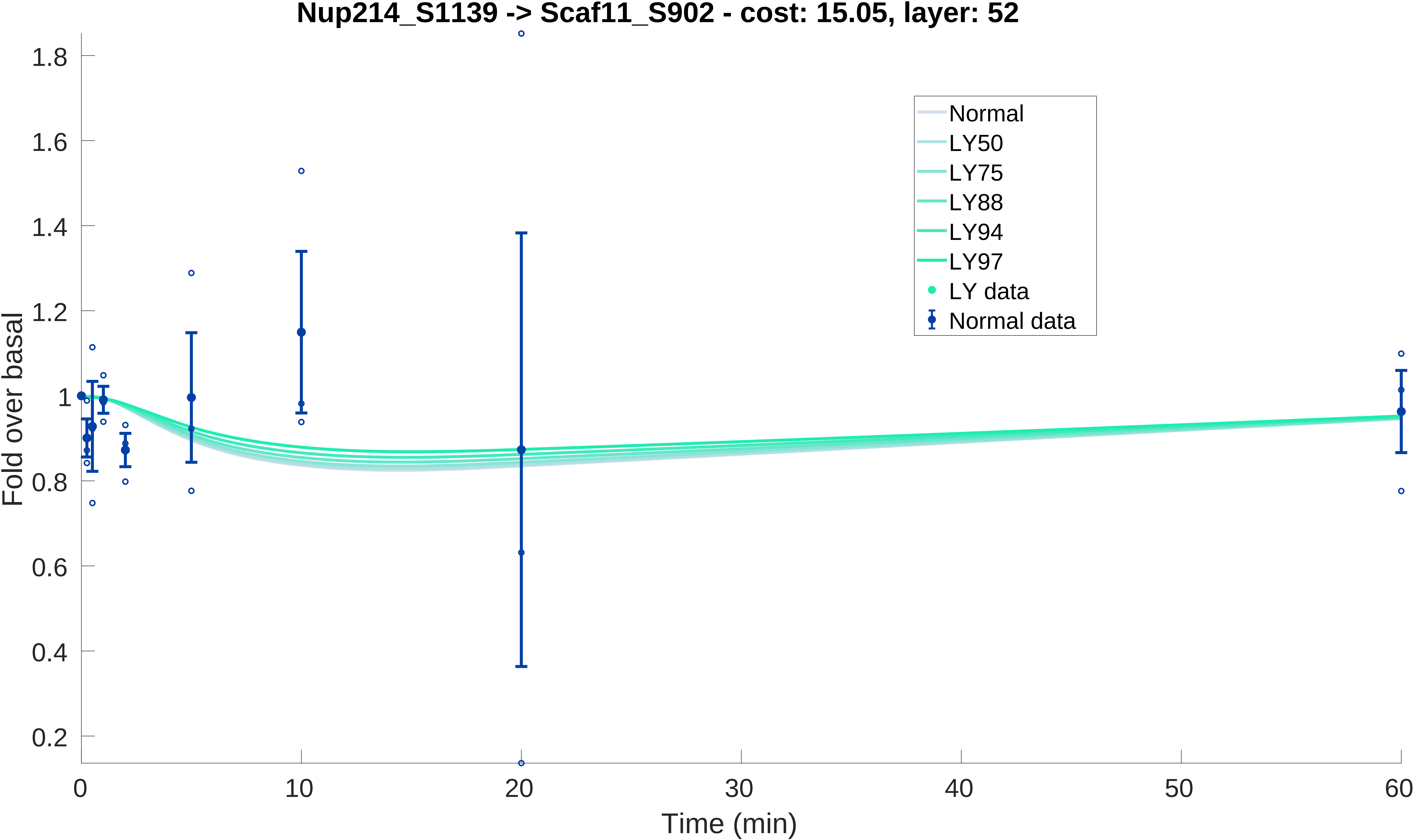

Nup214_S1139 -> Scaf11_S902 - cost: 15.05, layer: 52
Fold over basal
Time (min)
1.8
1.6
1.4
1.2
1
0.8
0.6
0.4
0.2
0
10
20
30
40
50
60
Normal
LY50
LY75
LY88
LY94
LY97
LY data
Normal data

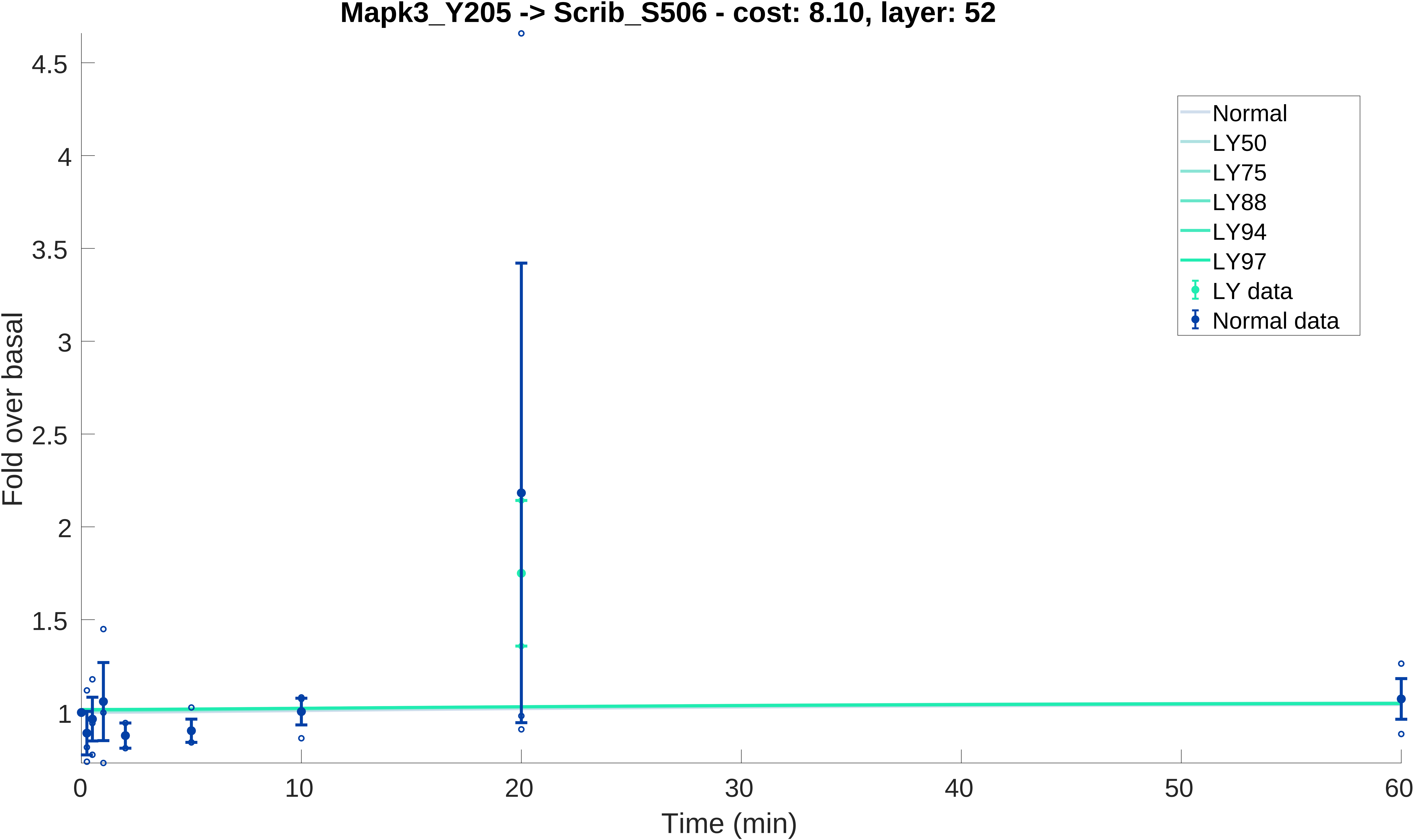
Mapk3_Y205 -> Scrib_S506 - cost: 8.10, layer: 52
Fold over basal
4.5
4
3.5
3
2.5
2
1.5
1
0
10
20
30
40
50
60
Time (min)
Normal
LY50
LY75
LY88
LY94
LY97
LY data
Normal data

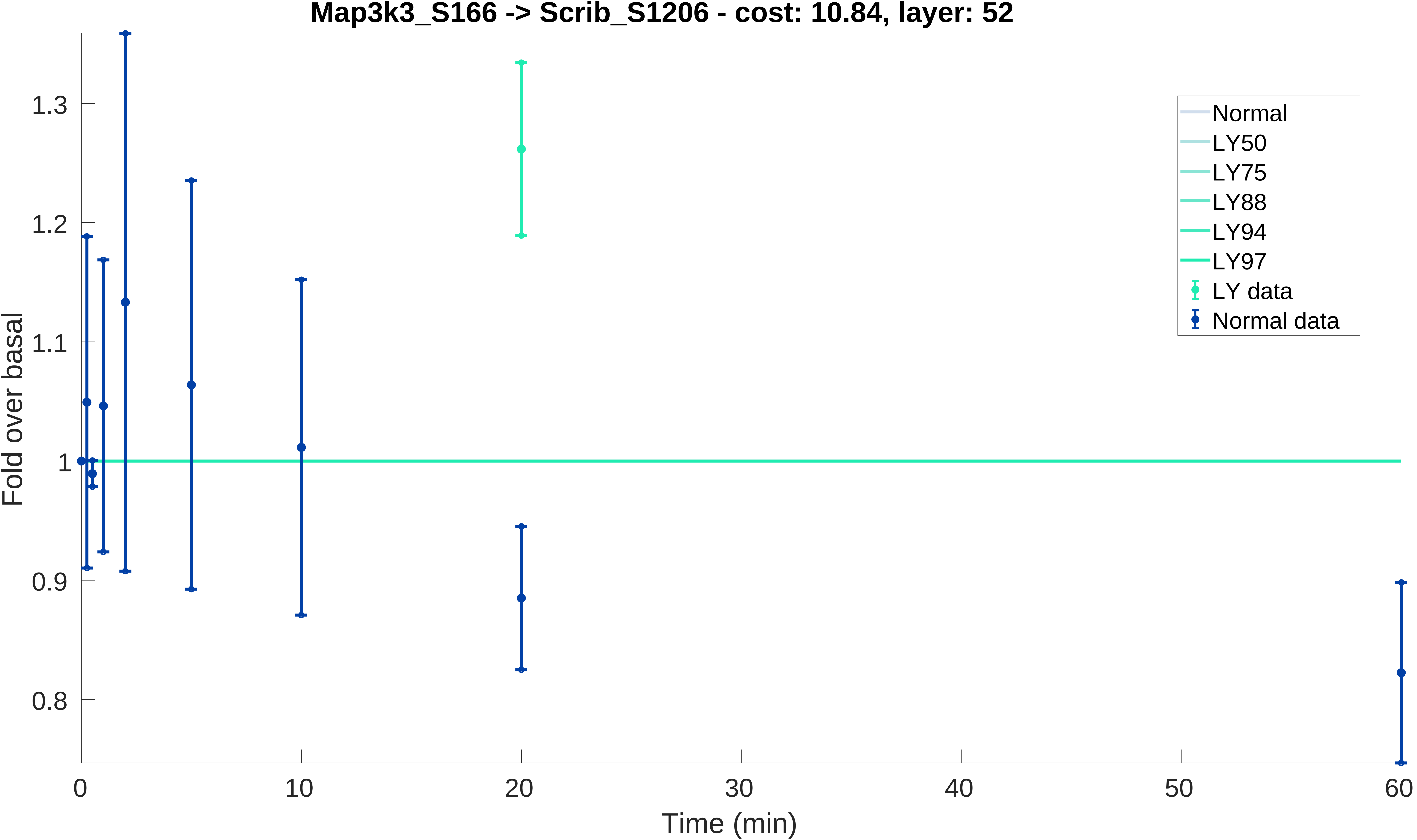

Map3k3_S166 -> Scrib_S1206 - cost: 10.84, layer: 52
Fold over basal
Time (min)
0.8
0.9
1
1.1
1.2
1.3
0
10
20
30
40
50
60
Normal
LY50
LY75
LY88
LY94
LY97
LY data
Normal data

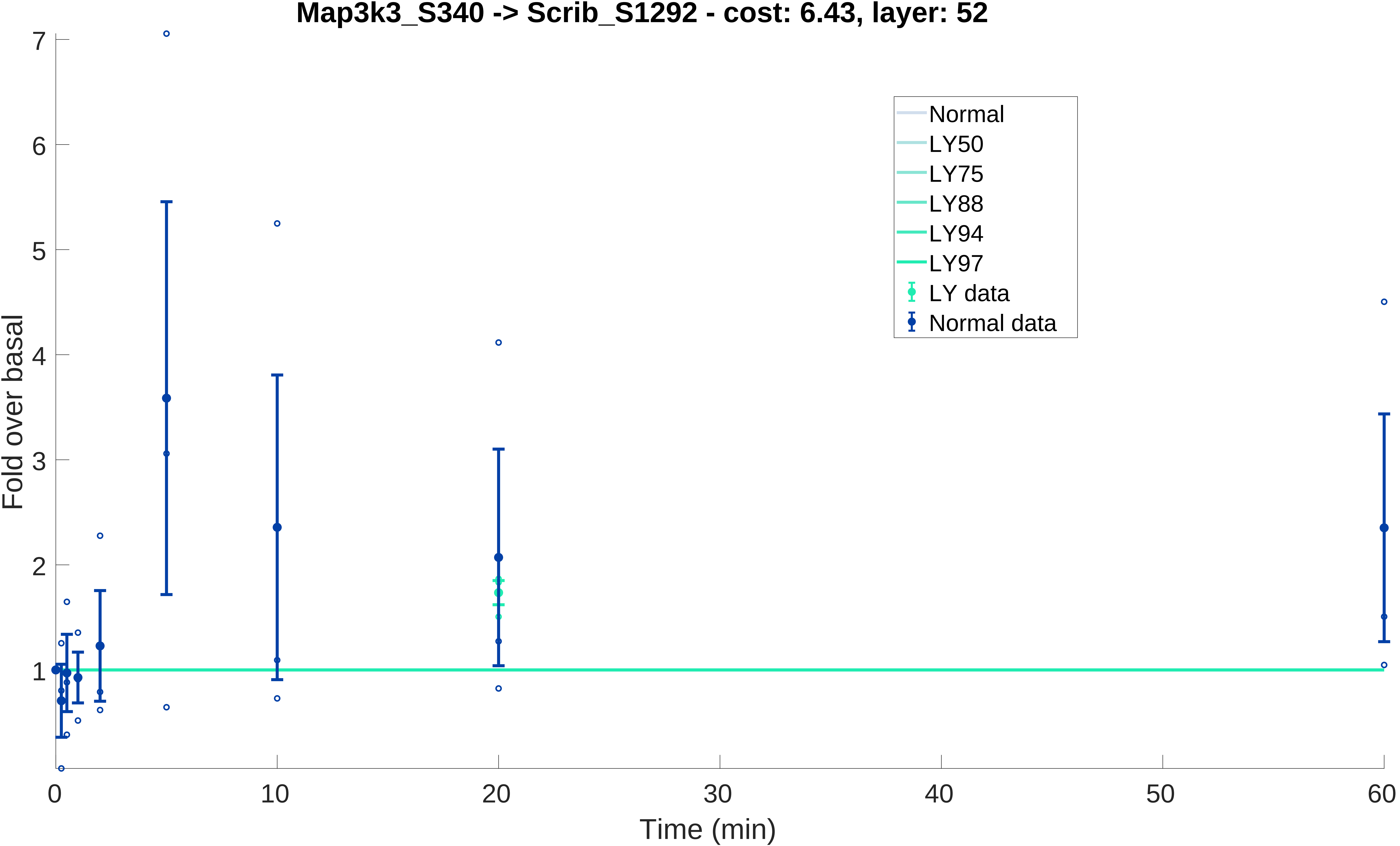

Map3k3_S340 -> Scrib_S1292 - cost: 6.43, layer: 52
Fold over basal
Time (min)
Normal
LY50
LY75
LY88
LY94
LY97
LY data
Normal data
1
2
3
4
5
6
7
0
10
20
30
40
50
60

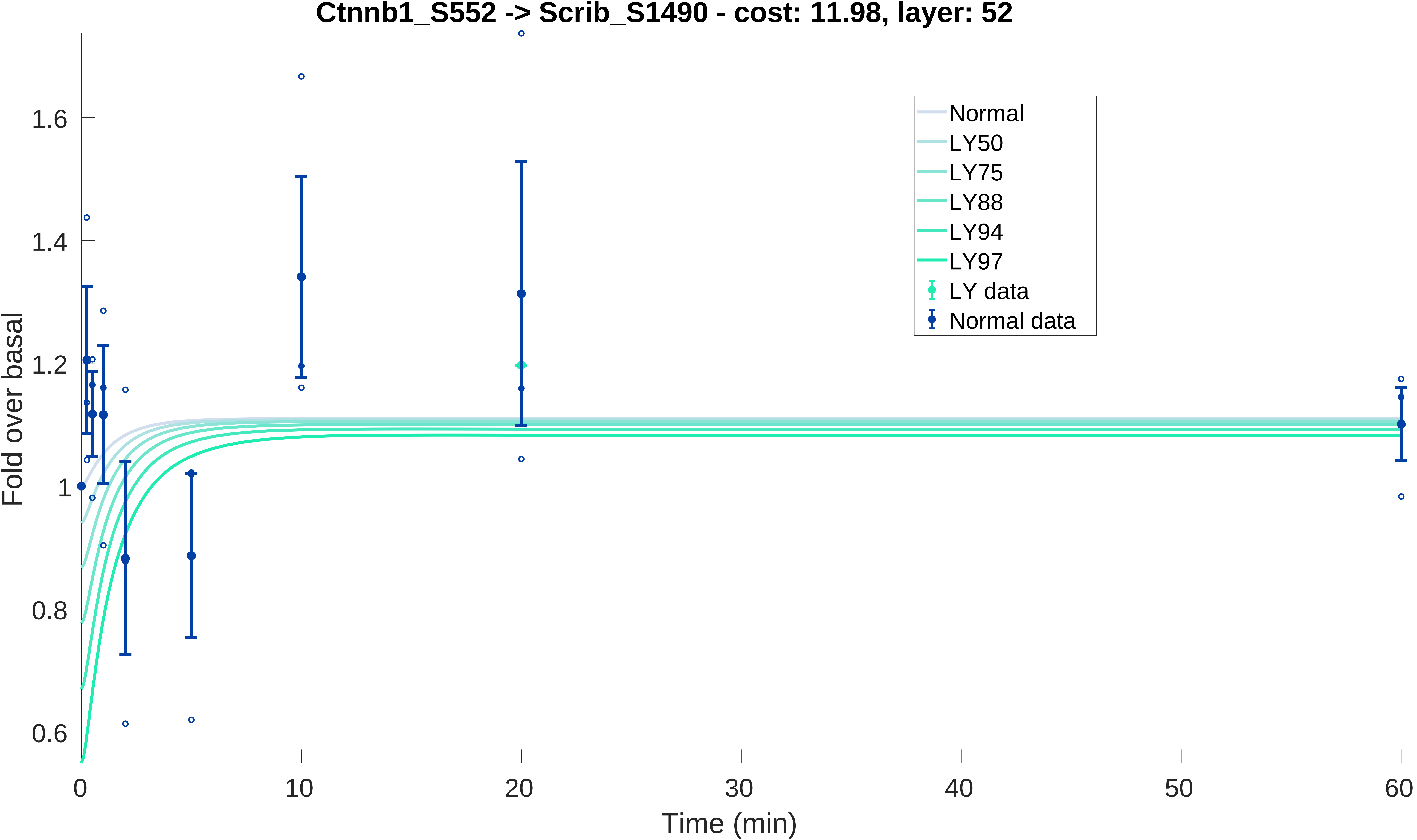

Ctnnb1_S552 -> Scrib_S1490 - cost: 11.98, layer: 52
Normal
LY50
LY75
LY88
LY94
LY97
LY data
Normal data
Fold over basal
Time (min)
0.6
0.8
1
1.2
1.4
1.6
0
10
20
30
40
50
60

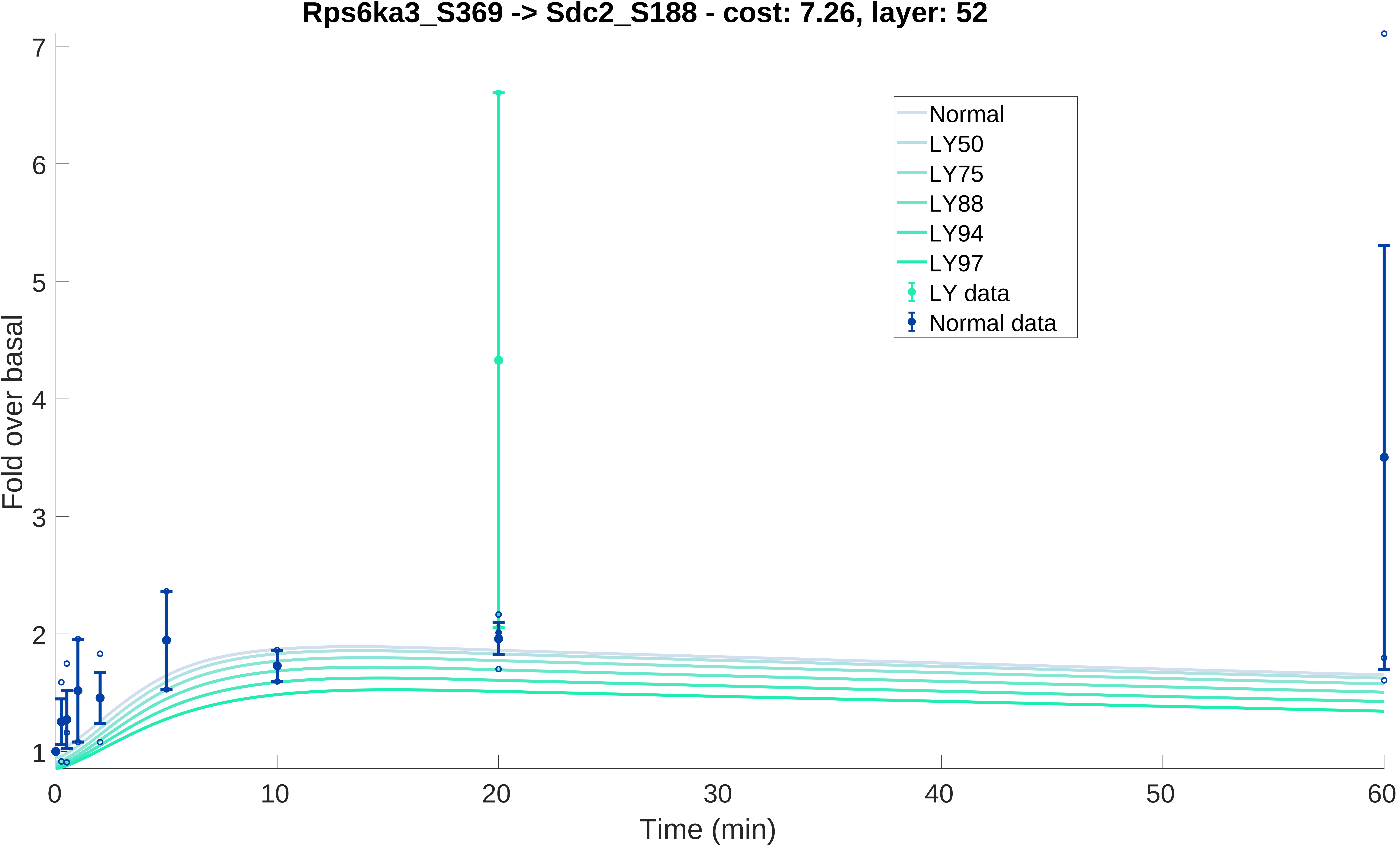
Rps6ka3_S369 -> Sdc2_S188 - cost: 7.26, layer: 52
Fold over basal
Time (min)
0
10
20
30
40
50
60
1
2
3
4
5
6
7
Normal
LY50
LY75
LY88
LY94
LY97
LY data
Normal data

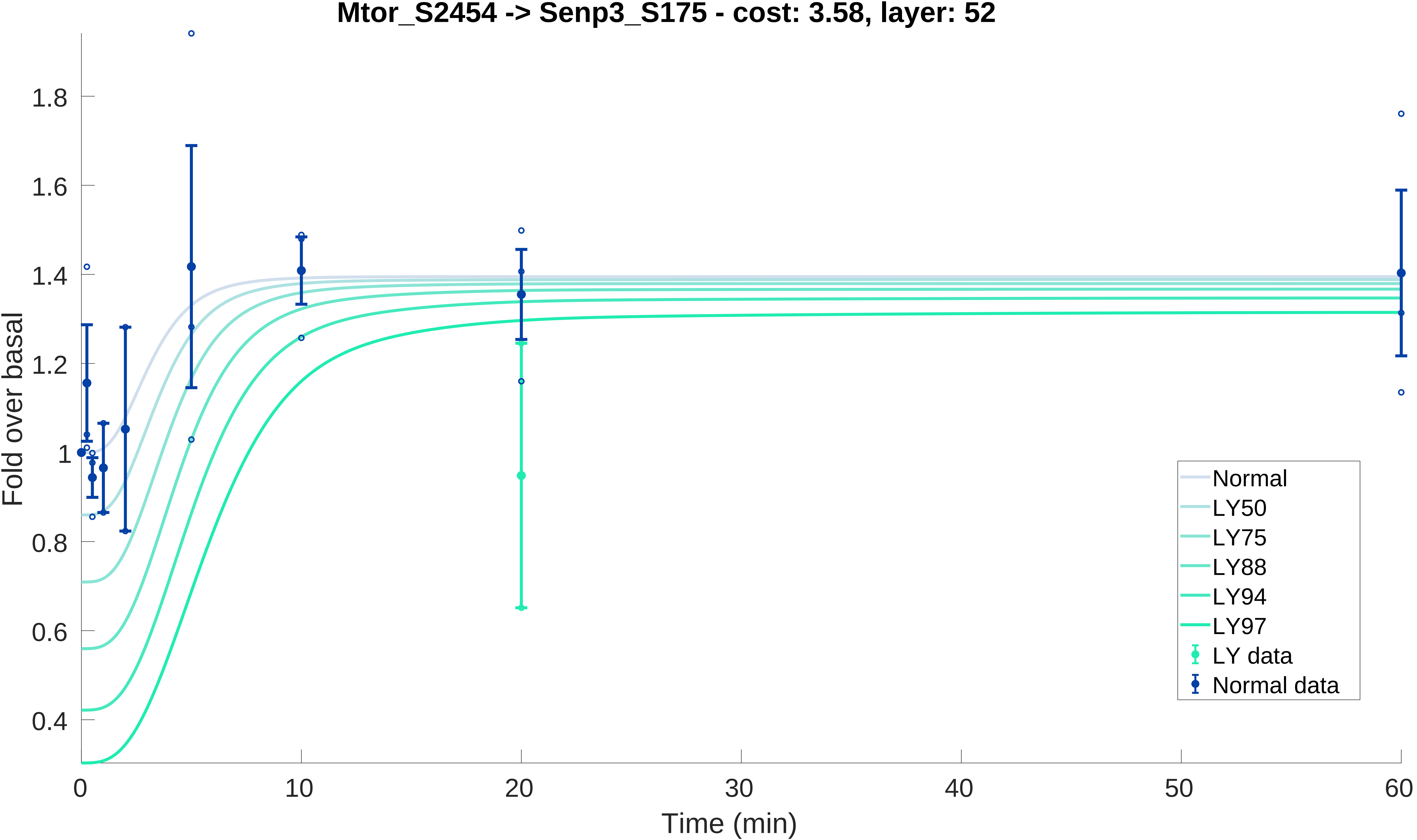

Mtor_S2454 -> Senp3_S175 - cost: 3.58, layer: 52
Fold over basal
1.8
1.6
1.4
1.2
1
0.8
0.6
0.4
0
10
20
30
40
50
60
Time (min)
Normal
LY50
LY75
LY88
LY94
LY97
LY data
Normal data

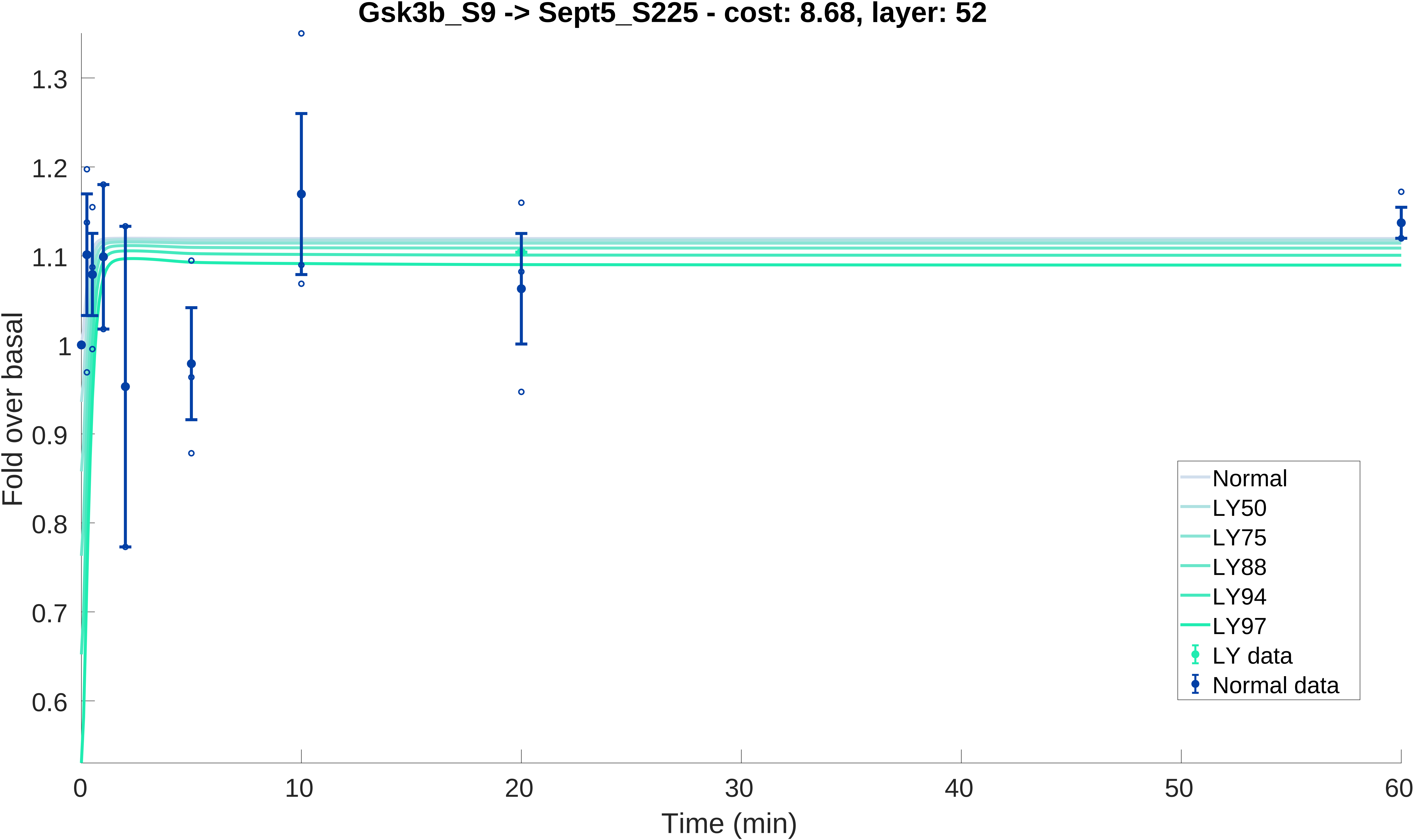

Gsk3b_S9 -> Sept5_S225 - cost: 8.68, layer: 52
Fold over basal
1.3
1.2
1.1
1
0.9
0.8
0.7
0.6
Time (min)
0
10
20
30
40
50
60
Normal
LY50
LY75
LY88
LY94
LY97
LY data
Normal data

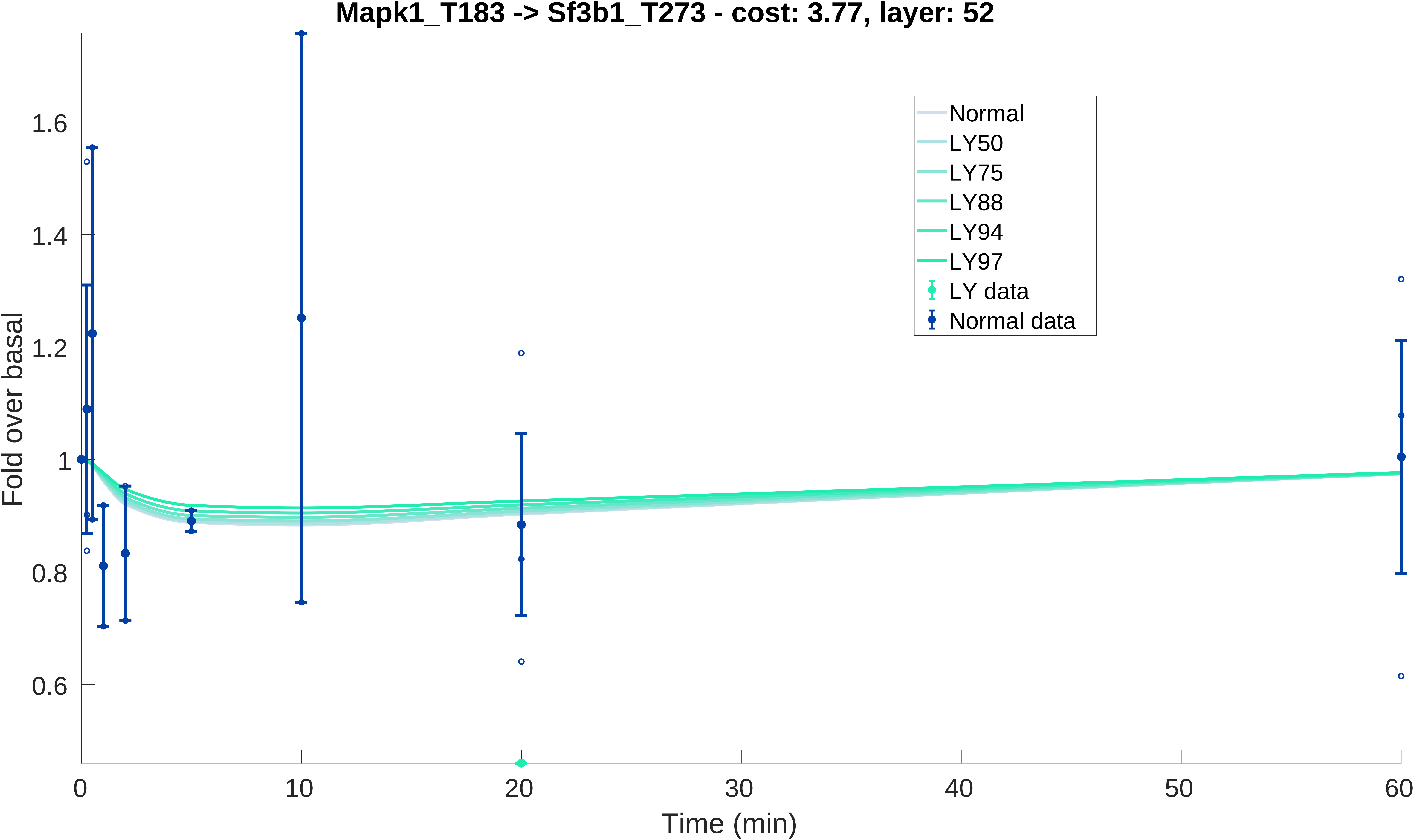

Mapk1_T183 -> Sf3b1_T273 - cost: 3.77, layer: 52
Fold over basal
Time (min)
Normal
LY50
LY75
LY88
LY94
LY97
LY data
Normal data
0.6
0.8
1
1.2
1.4
1.6
0
10
20
30
40
50
60

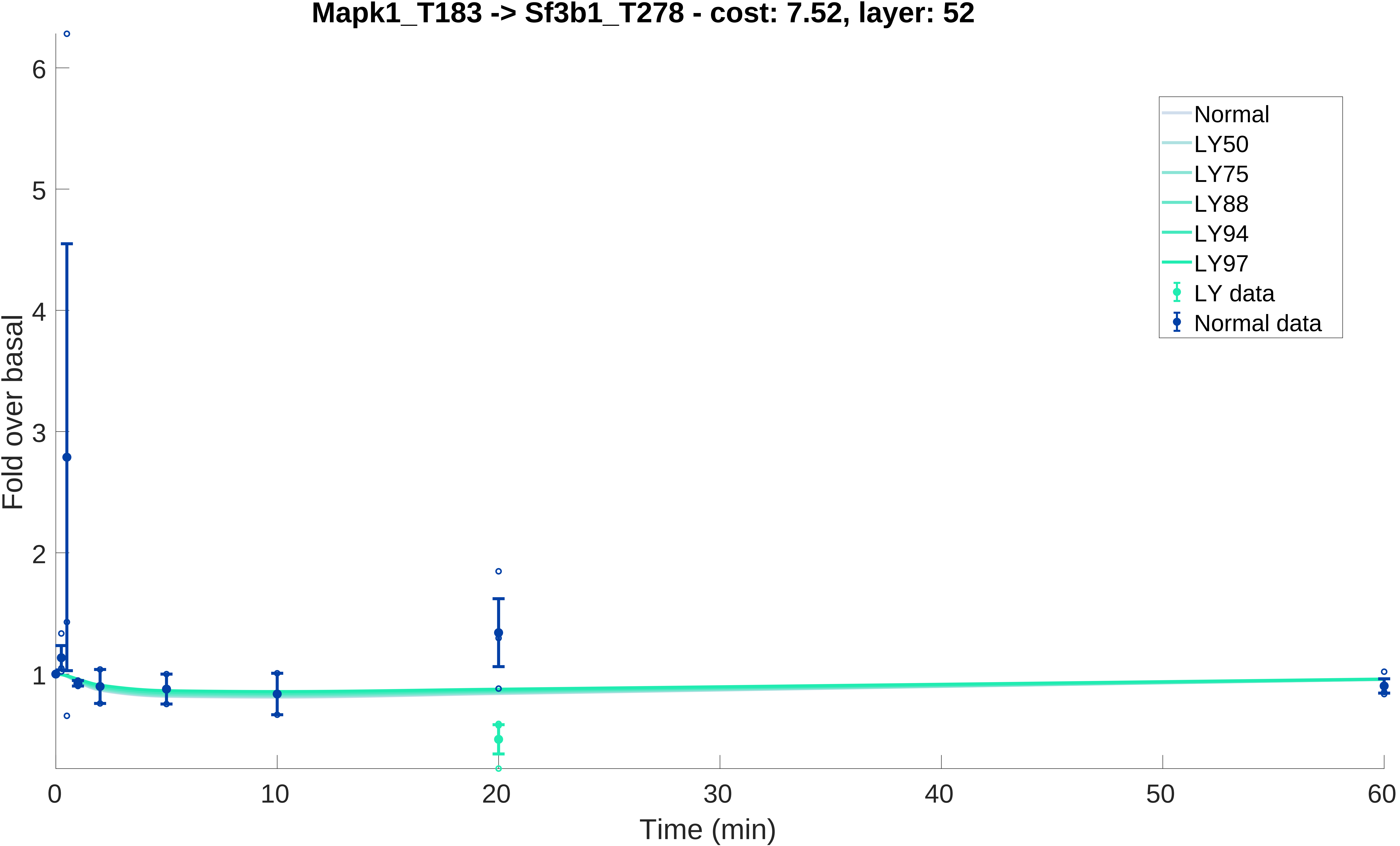
Mapk1_T183 -> Sf3b1_T278 - cost: 7.52, layer: 52
Fold over basal
Time (min)
0
10
20
30
40
50
60
1
2
3
4
5
6
Normal
LY50
LY75
LY88
LY94
LY97
LY data
Normal data

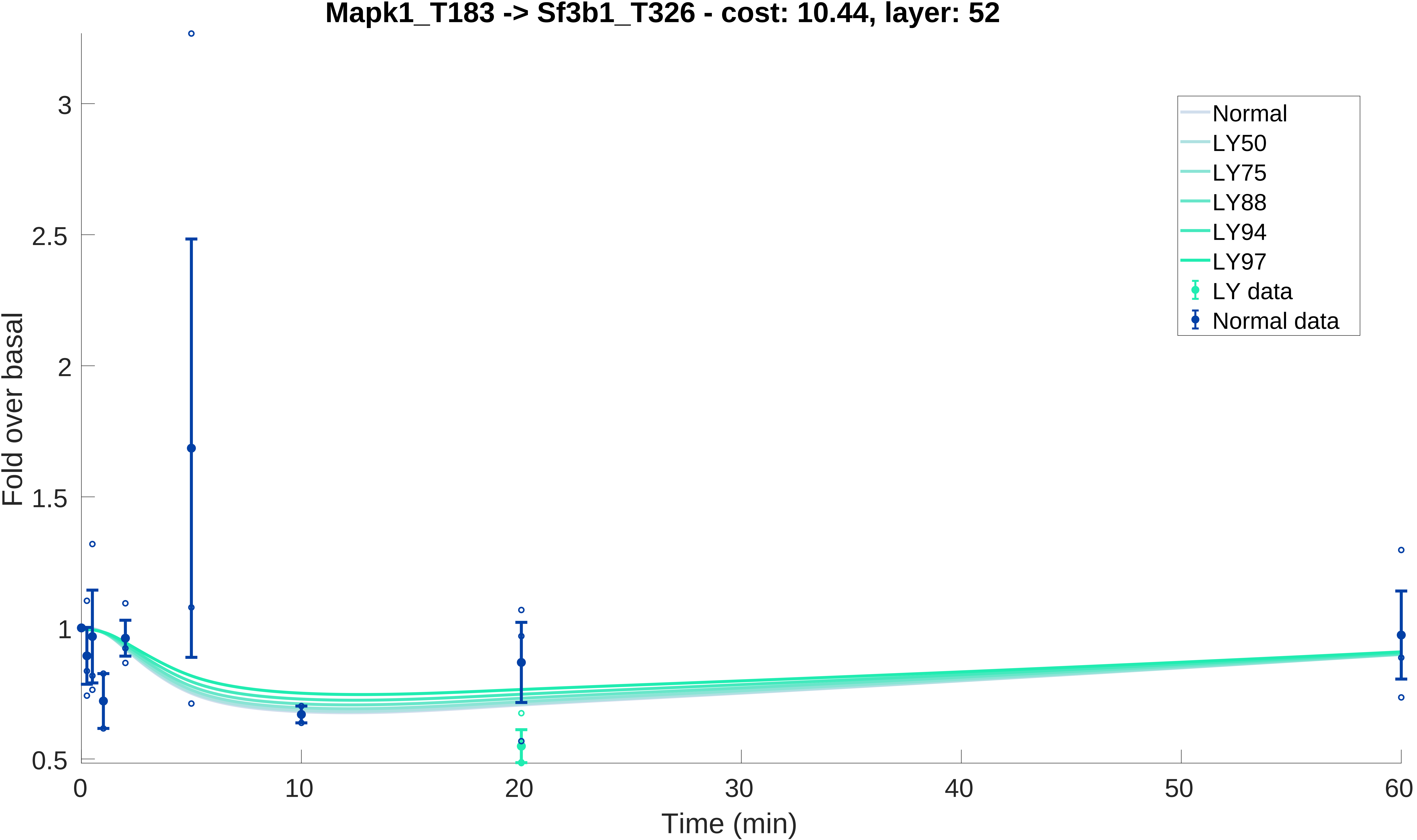

Mapk1_T183 -> Sf3b1_T326 - cost: 10.44, layer: 52
Fold over basal
Time (min)
0.5
1
1.5
2
2.5
3
0
10
20
30
40
50
60
Normal
LY50
LY75
LY88
LY94
LY97
LY data
Normal data

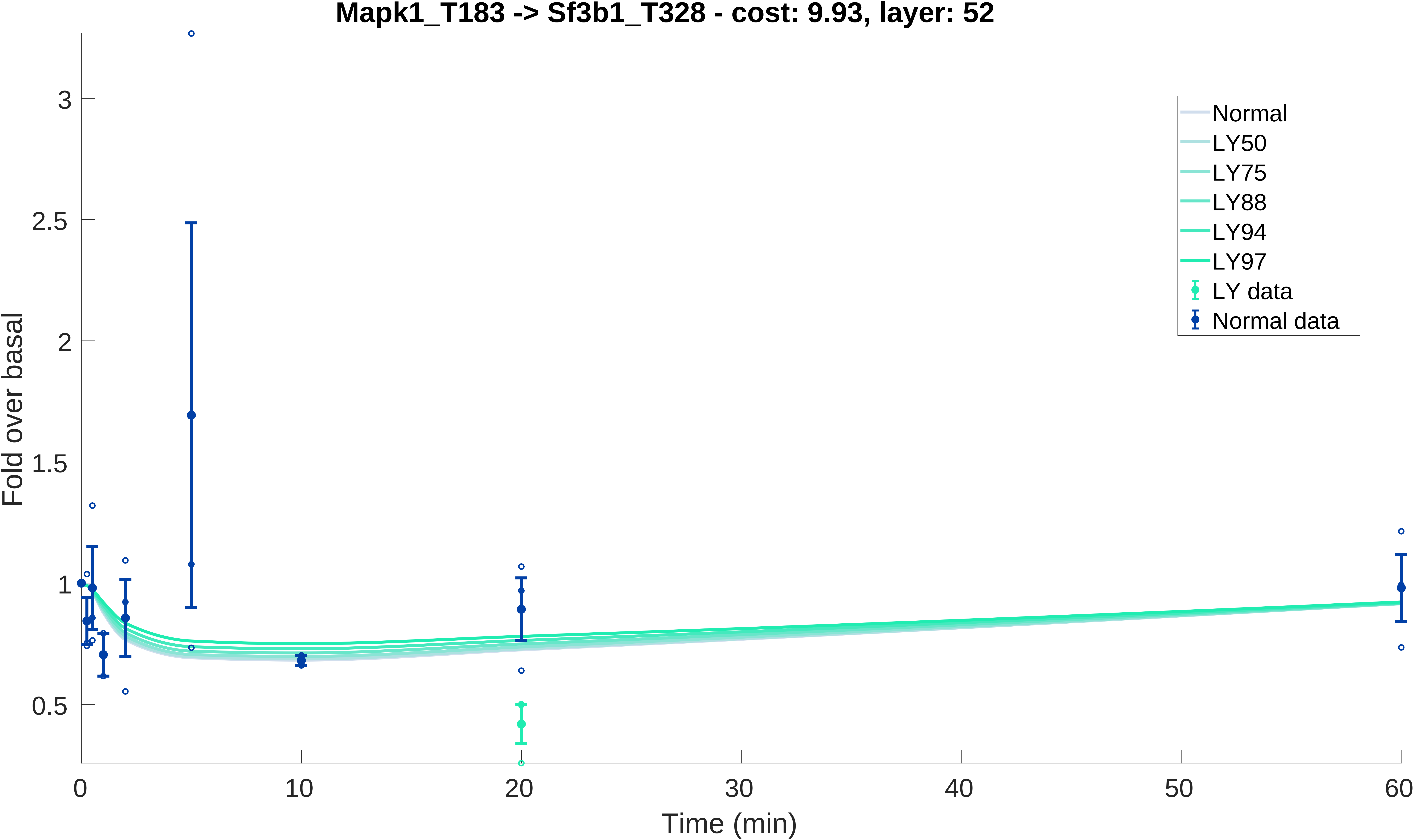

Mapk1_T183 -> Sf3b1_T328 - cost: 9.93, layer: 52
Fold over basal
Time (min)
0.5
1
1.5
2
2.5
3
0
10
20
30
40
50
60
Normal
LY50
LY75
LY88
LY94
LY97
LY data
Normal data

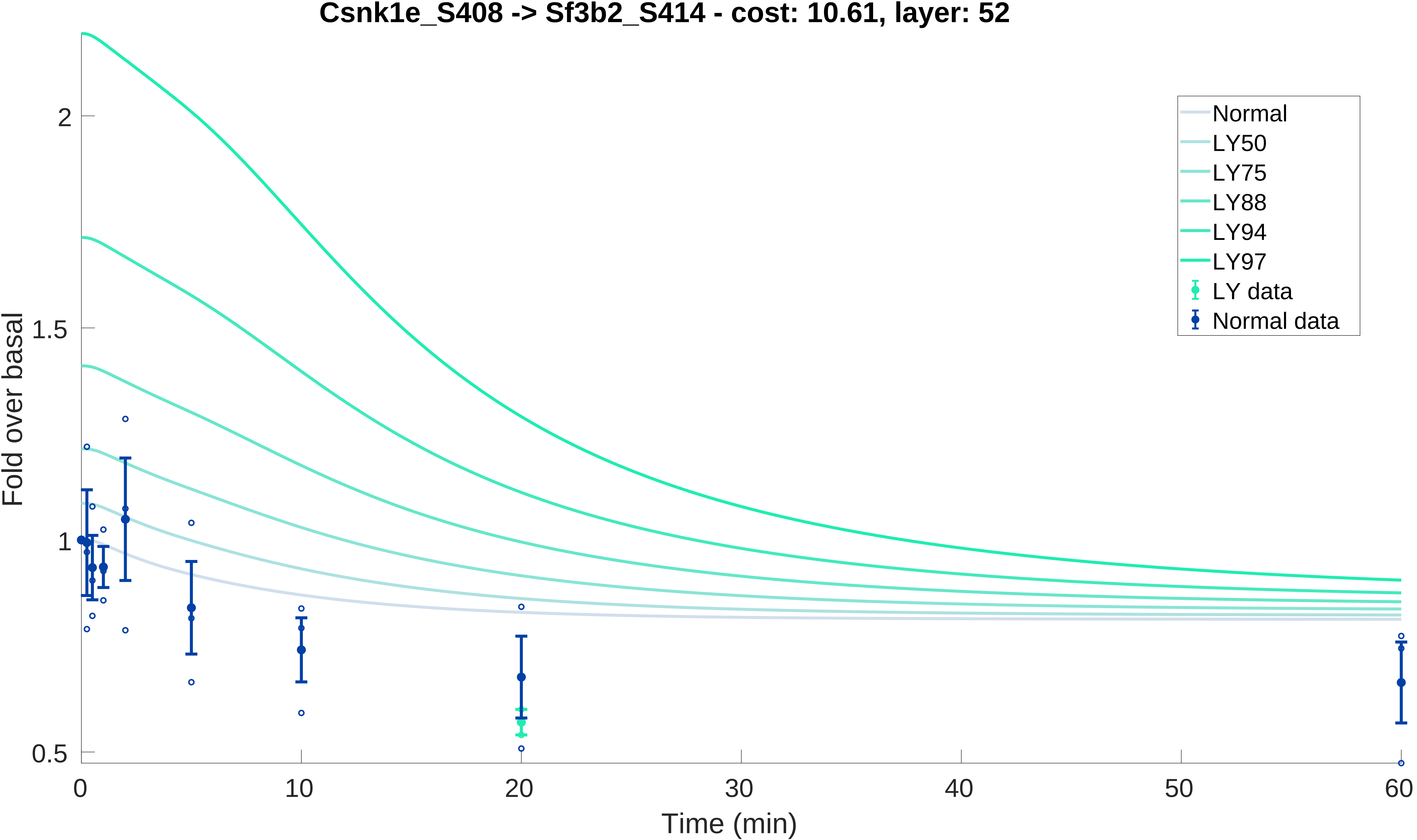
Csnk1e_S408 -> Sf3b2_S414 - cost: 10.61, layer: 52
Fold over basal
Time (min)
0.5
1
1.5
2
0
10
20
30
40
50
60
Normal
LY50
LY75
LY88
LY94
LY97
LY data
Normal data

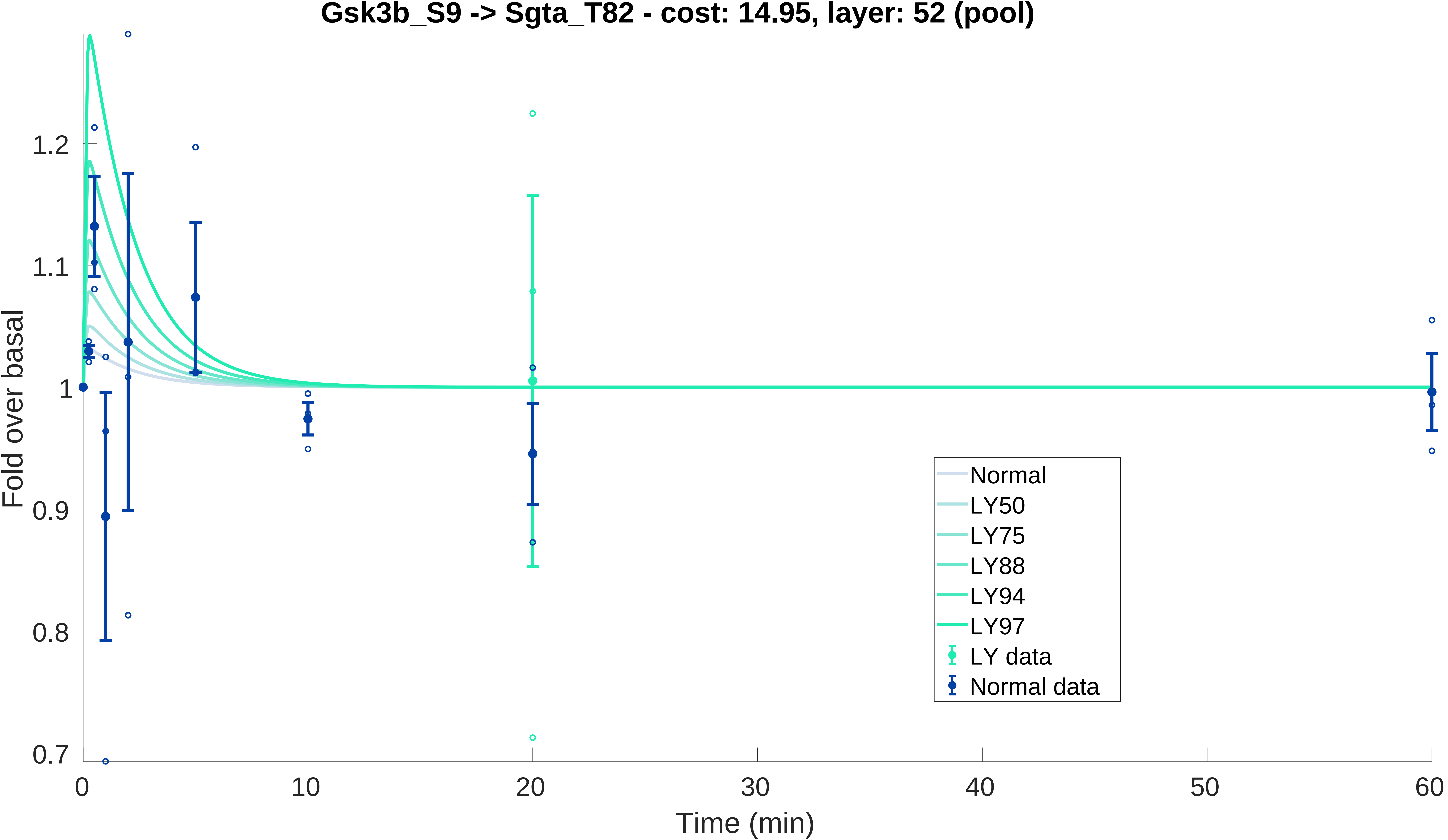
Gsk3b_S9 -> Sgta_T82 - cost: 14.95, layer: 52 (pool)
Fold over basal
Time (min)
0.7
0.8
0.9
1
1.1
1.2
0
10
20
30
40
50
60
Normal
LY50
LY75
LY88
LY94
LY97
LY data
Normal data

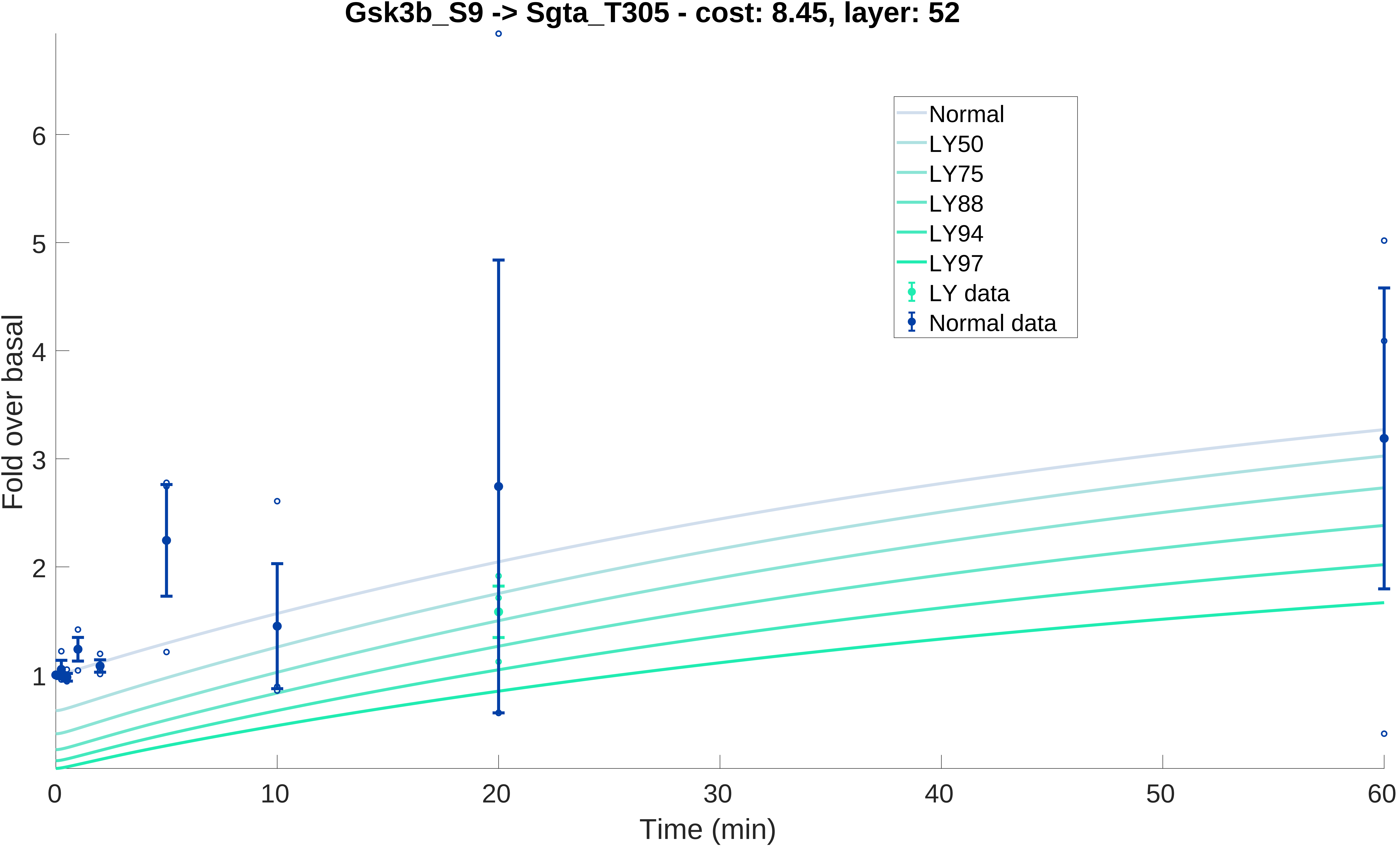

Gsk3b_S9 -> Sgta_T305 - cost: 8.45, layer: 52
Fold over basal
Time (min)
0
10
20
30
40
50
60
1
2
3
4
5
6
Normal
LY50
LY75
LY88
LY94
LY97
LY data
Normal data

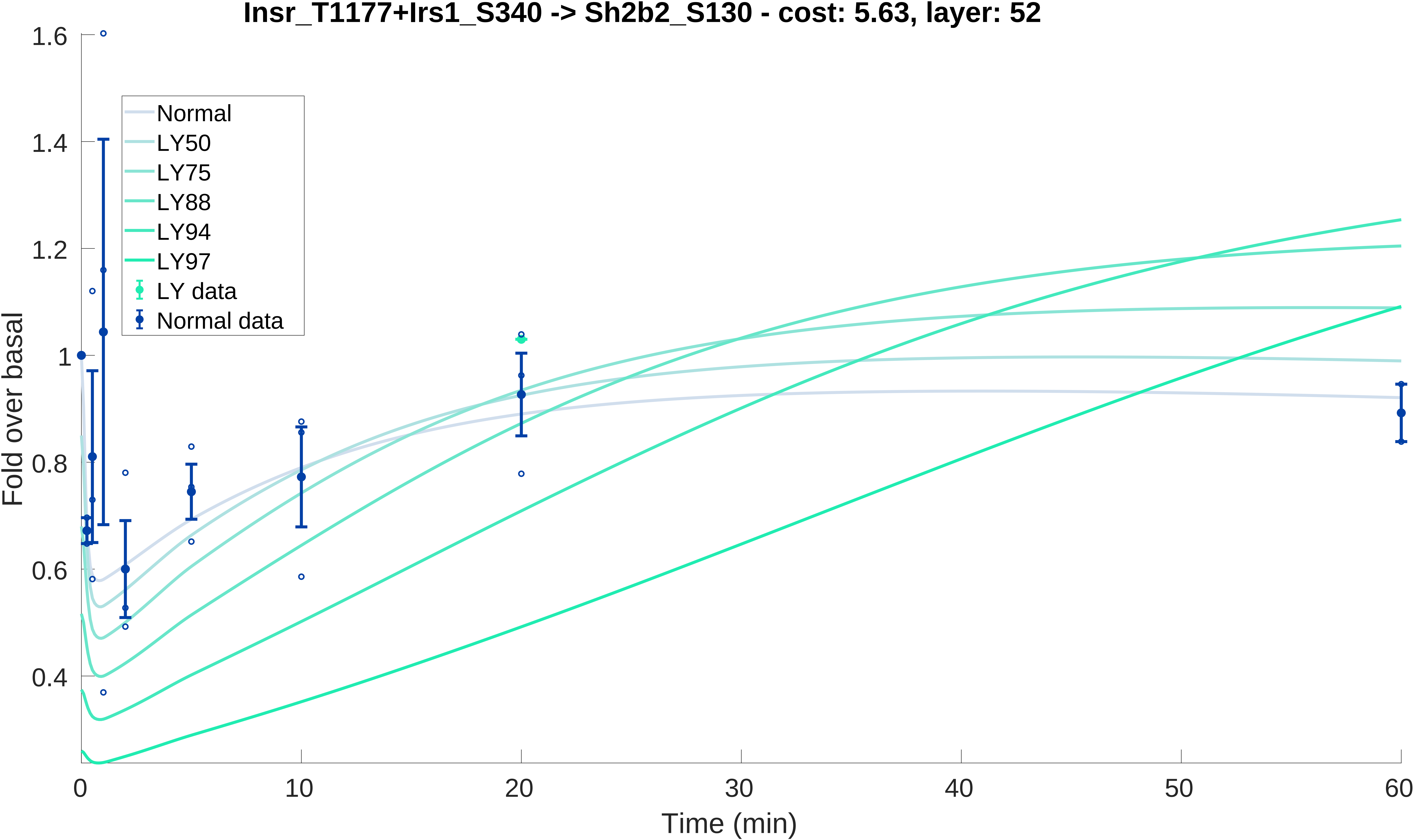

Insr_T1177+Irs1_S340 -> Sh2b2_S130 - cost: 5.63, layer: 52
Fold over basal
Time (min)
Normal
LY50
LY75
LY88
LY94
LY97
LY data
Normal data
0
10
20
30
40
50
60
0.4
0.6
0.8
1
1.2
1.4
1.6

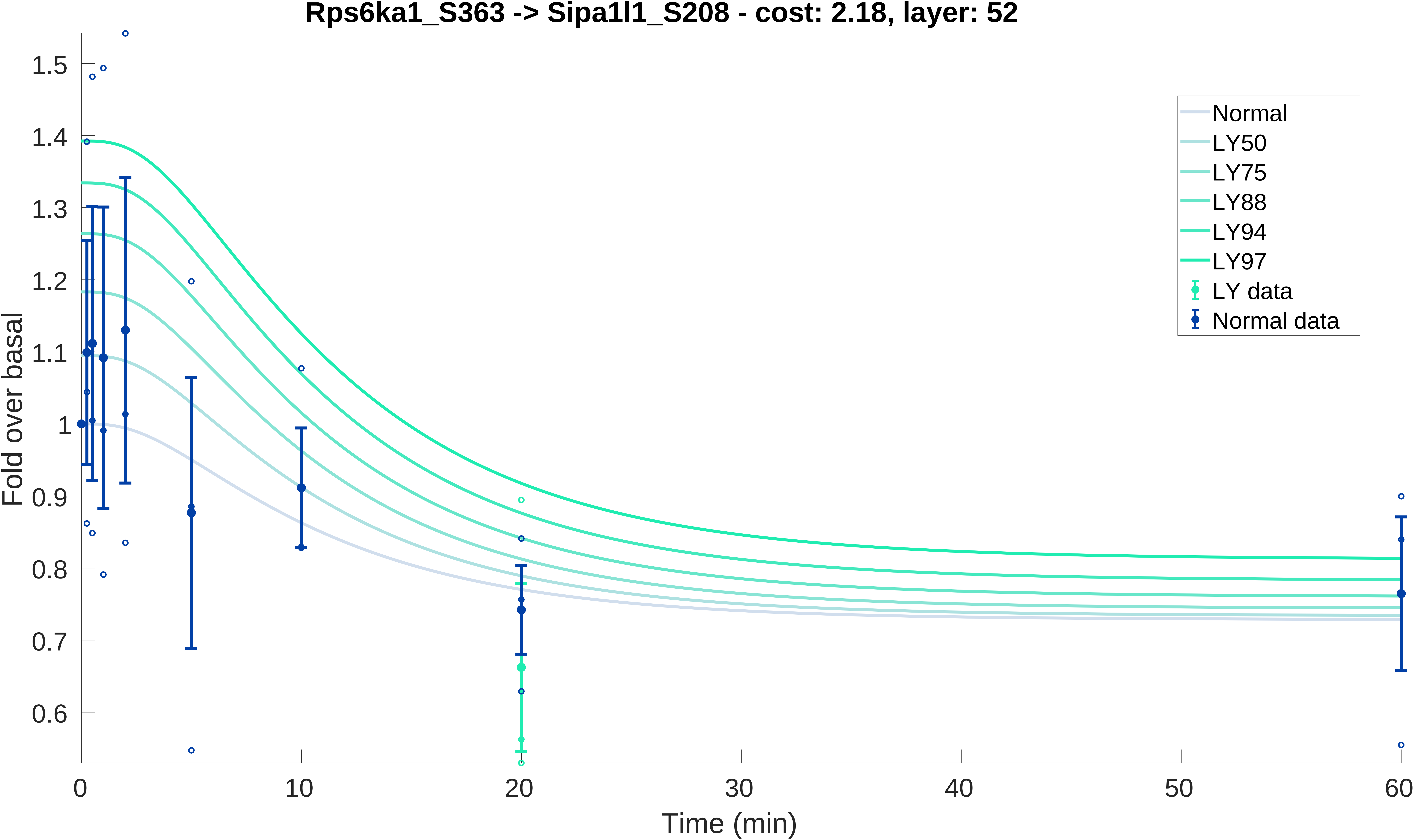

Rps6ka1_S363 -> Sipa1l1_S208 - cost: 2.18, layer: 52
Fold over basal
Time (min)
1.5
1.4
1.3
1.2
1.1
1
0.9
0.8
0.7
0.6
0
10
20
30
40
50
60
Normal
LY50
LY75
LY88
LY94
LY97
LY data
Normal data

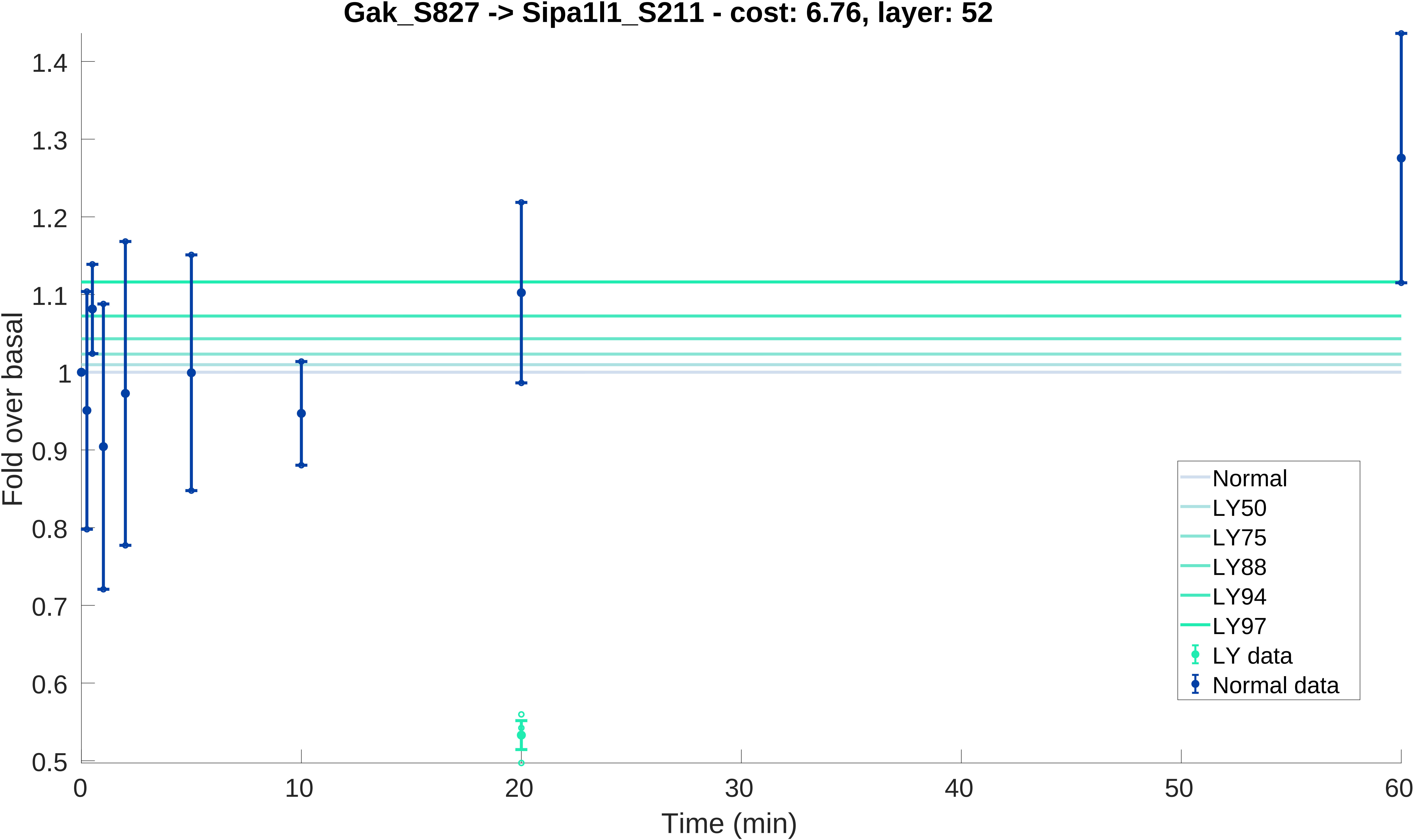

Gak_S827 -> Sipa1l1_S211 - cost: 6.76, layer: 52
Fold over basal
Time (min)
1.4
1.3
1.2
1.1
1
0.9
0.8
0.7
0.6
0.5
0
10
20
30
40
50
60
Normal
LY50
LY75
LY88
LY94
LY97
LY data
Normal data

Gak_S827 -> Sipa1l1_S1181 - cost: 2.37, layer: 52

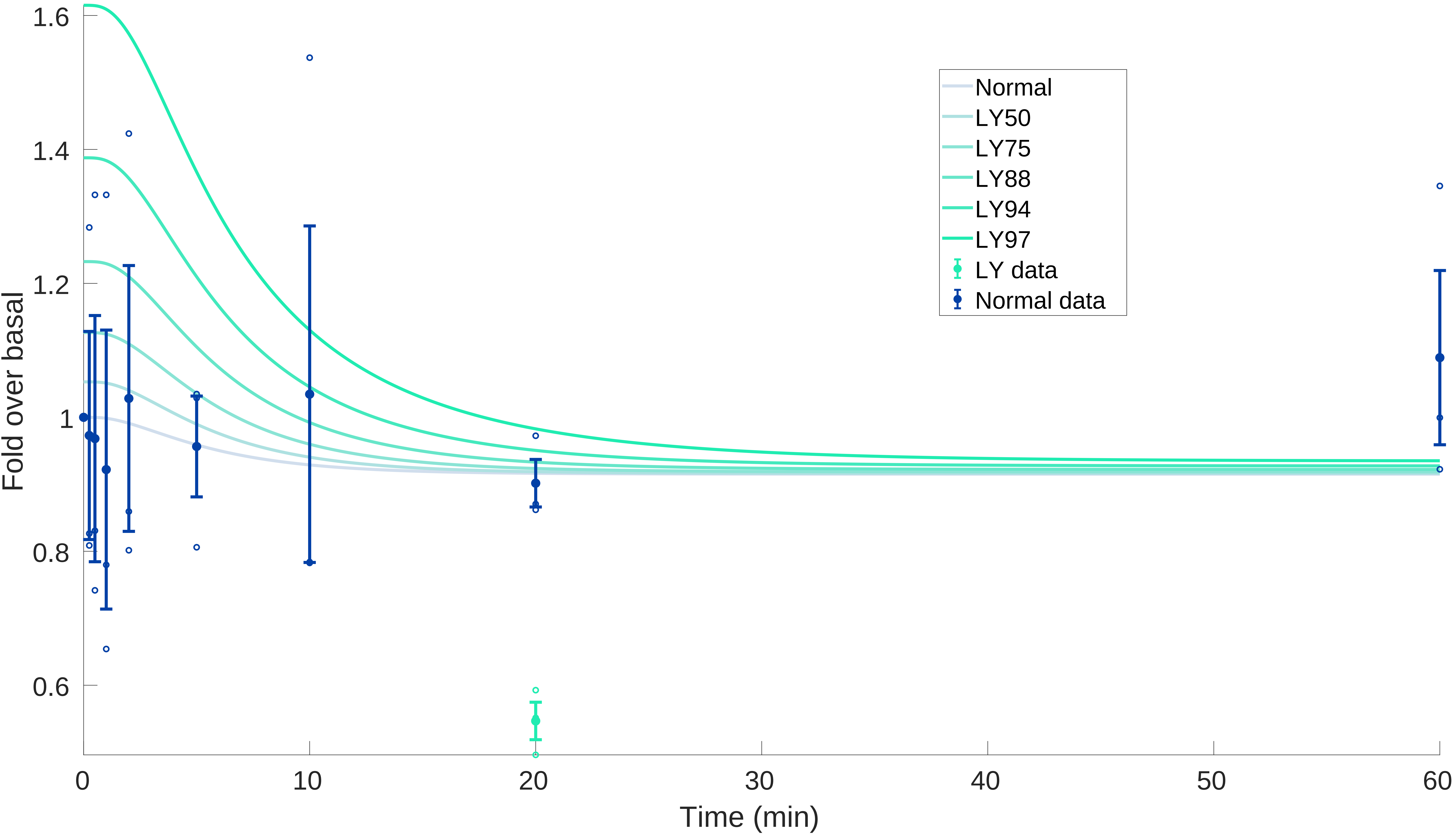

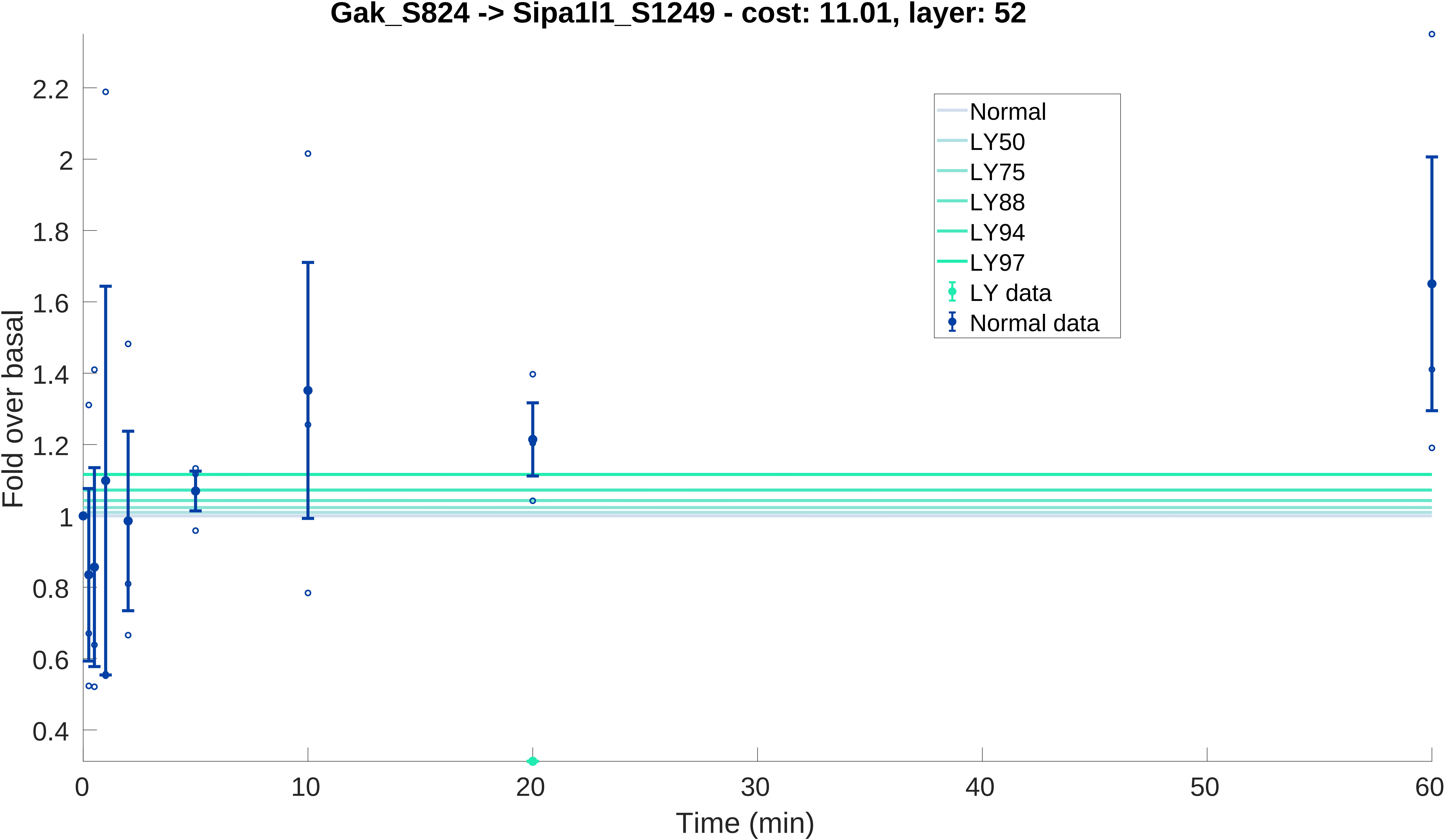
Gak_S824 -> Sipa1l1_S1249 - cost: 11.01, layer: 52
Fold over basal
Time (min)
0
10
20
30
40
50
60
0.4
0.6
0.8
1
1.2
1.4
1.6
1.8
2
2.2
Normal
LY50
LY75
LY88
LY94
LY97
LY data
Normal data

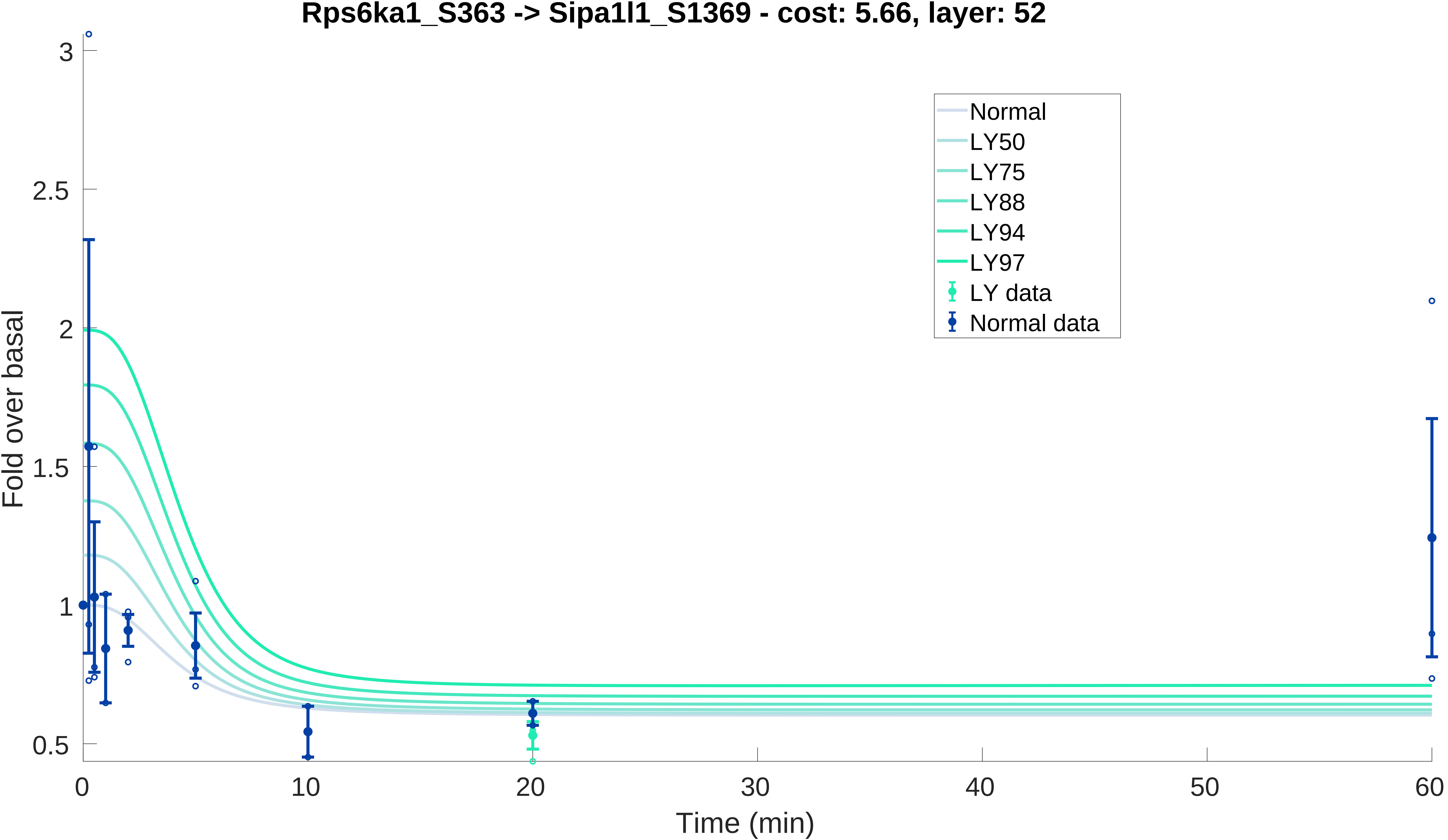

Rps6ka1_S363 -> Sipa1l1_S1369 - cost: 5.66, layer: 52
Fold over basal
Time (min)
Normal
LY50
LY75
LY88
LY94
LY97
LY data
Normal data
0.5
1
1.5
2
2.5
3
0
10
20
30
40
50
60

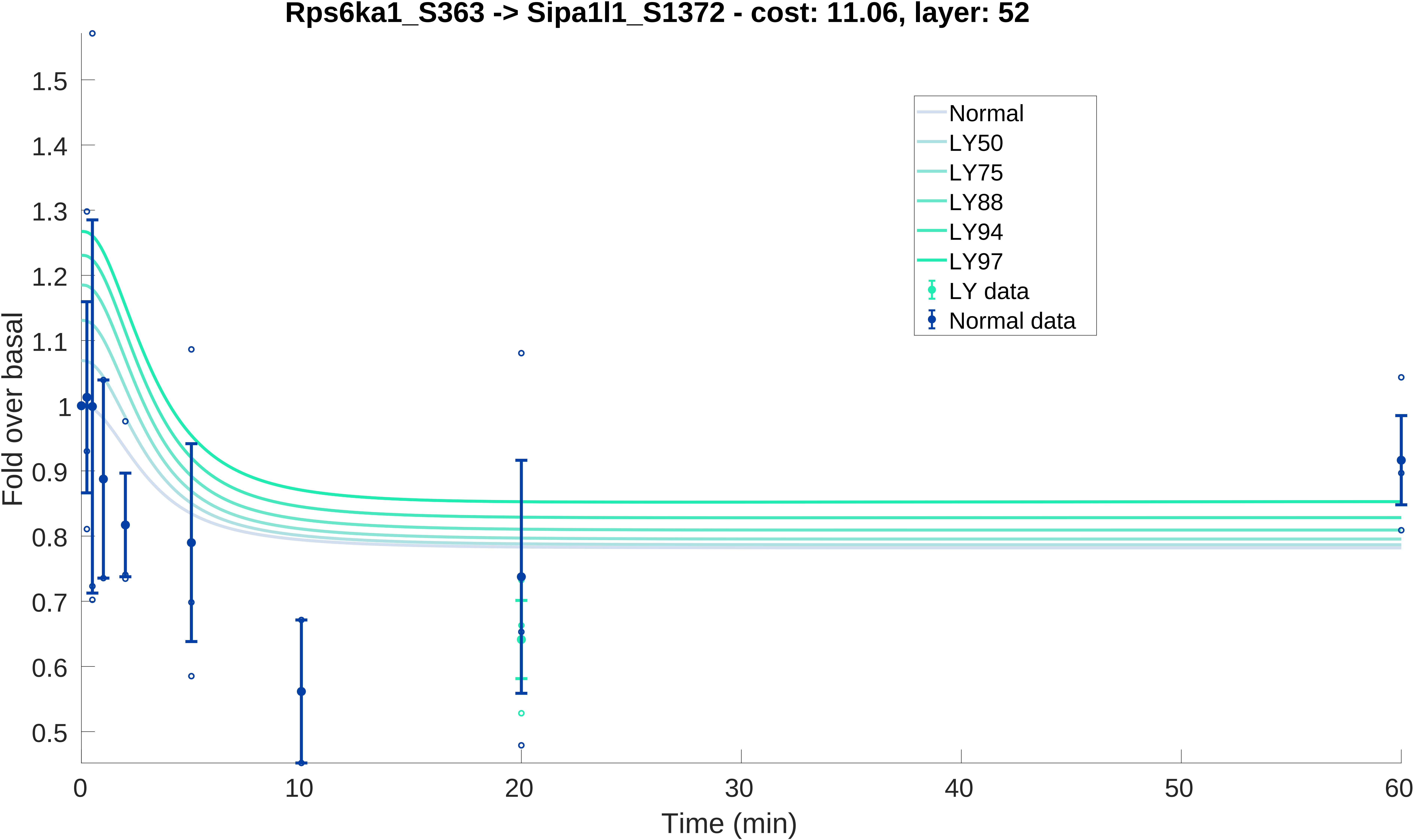

Rps6ka1_S363 -> Sipa1l1_S1372 - cost: 11.06, layer: 52
Fold over basal
Time (min)
Normal
LY50
LY75
LY88
LY94
LY97
LY data
Normal data
1.5
1.4
1.3
1.2
1.1
1
0.9
0.8
0.7
0.6
0.5
0
10
20
30
40
50
60

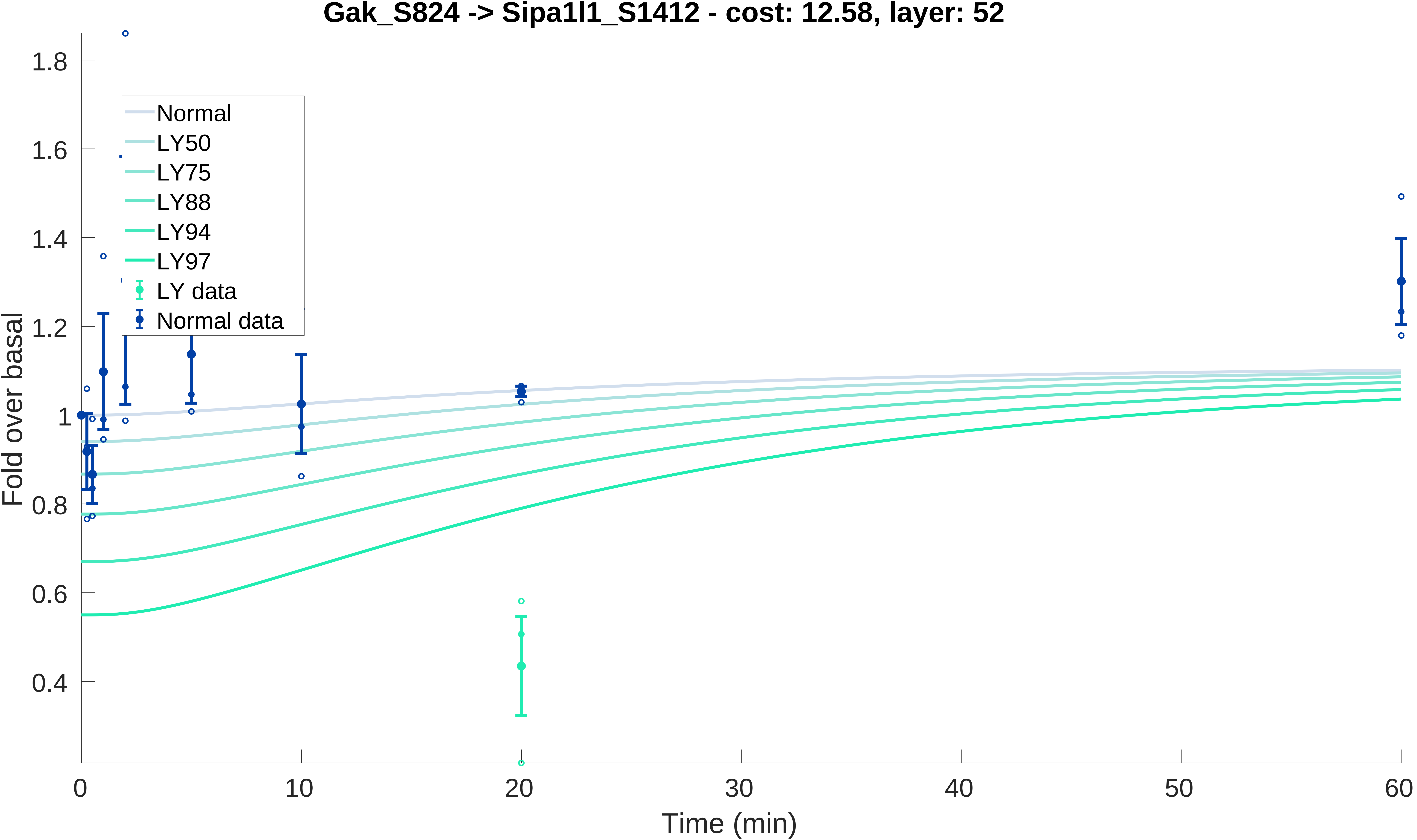

Gak_S824 -> Sipa1l1_S1412 - cost: 12.58, layer: 52
Normal
LY50
LY75
LY88
LY94
LY97
LY data
Normal data
Fold over basal
Time (min)
0.4
0.6
0.8
1
1.2
1.4
1.6
1.8
0
10
20
30
40
50
60

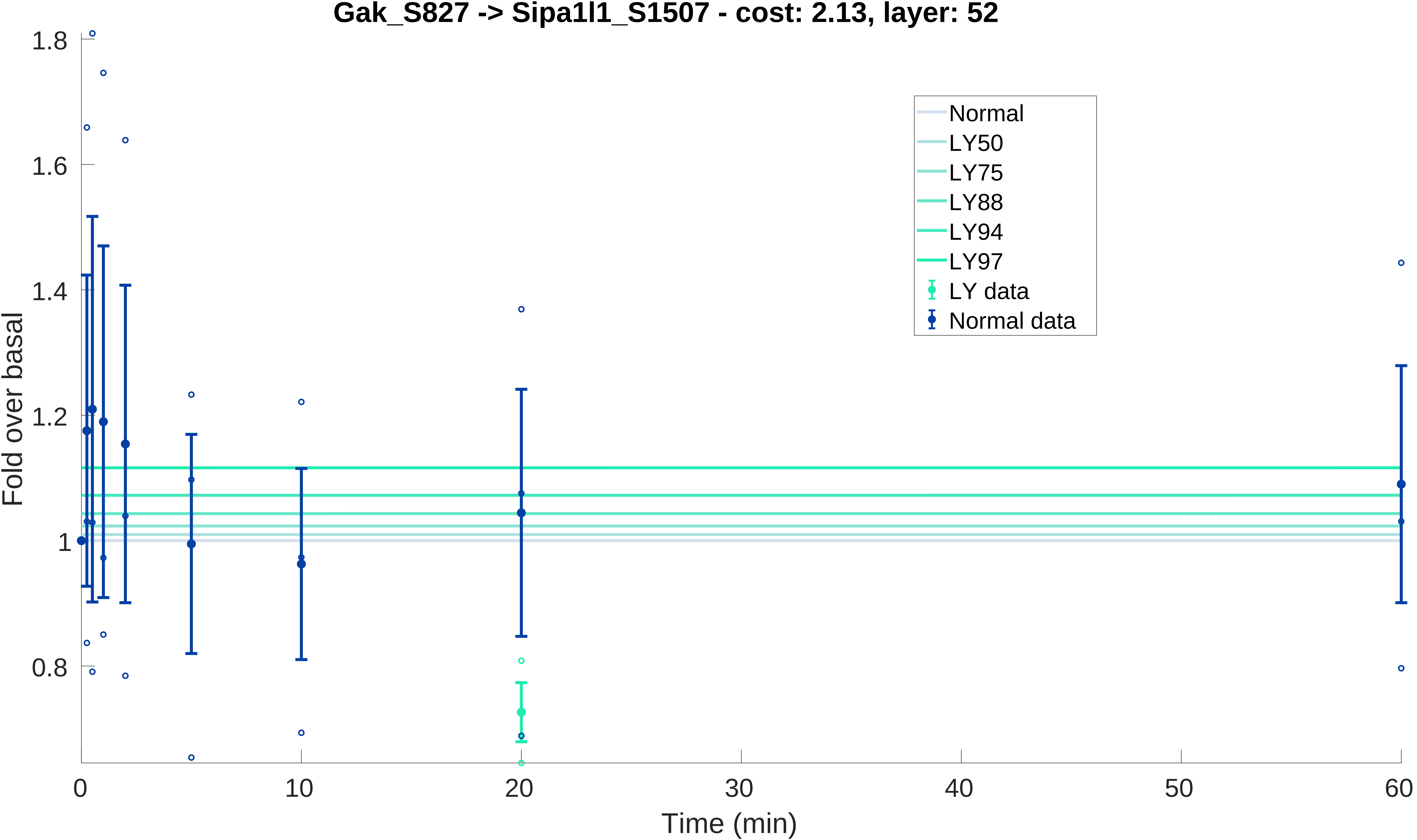

Gak_S827 -> Sipa1l1_S1507 - cost: 2.13, layer: 52
Fold over basal
Time (min)
Normal
LY50
LY75
LY88
LY94
LY97
LY data
Normal data
1.8
1.6
1.4
1.2
1
0.8
0
10
20
30
40
50
60

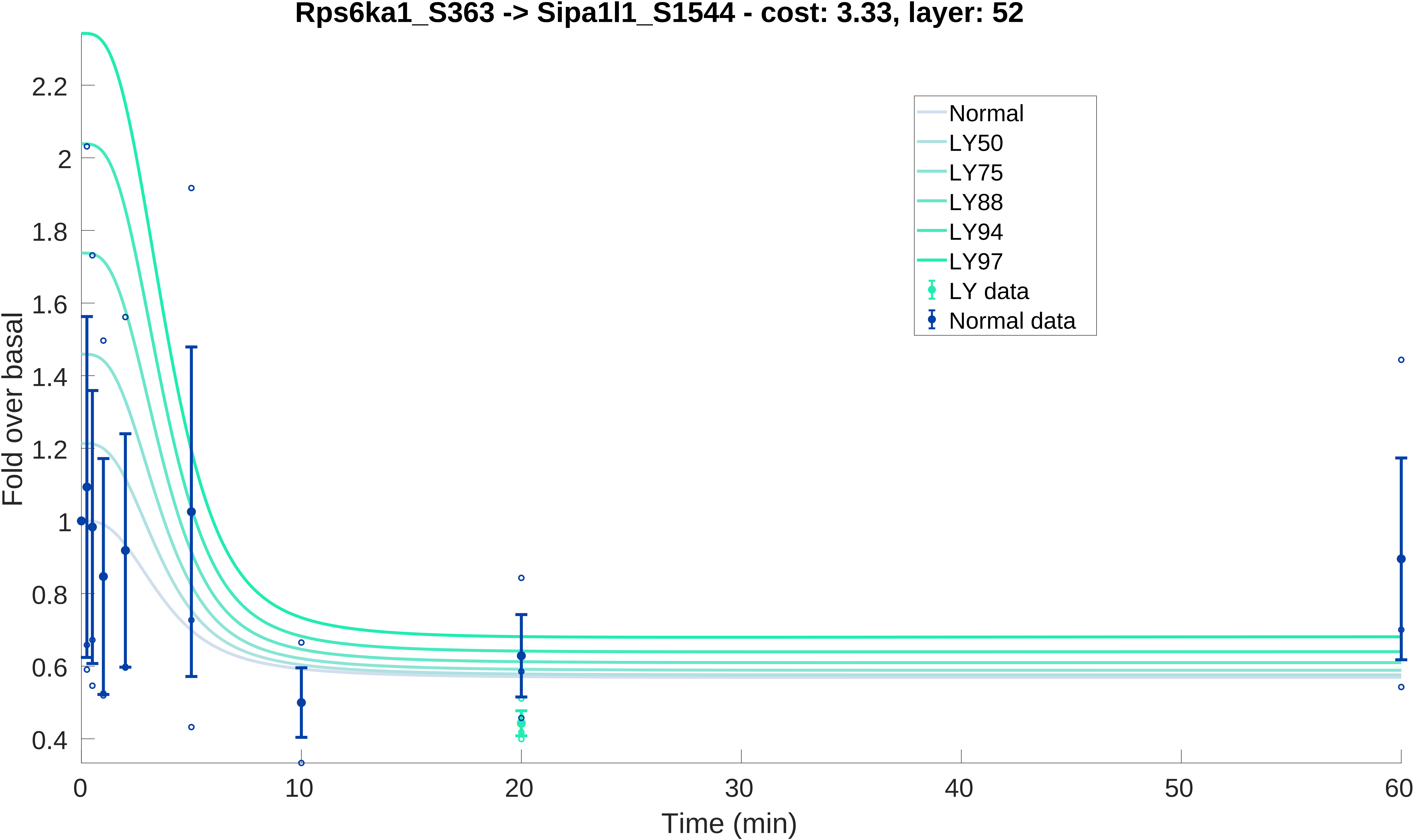
Rps6ka1_S363 -> Sipa1l1_S1544 - cost: 3.33, layer: 52
Fold over basal
Time (min)
Normal
LY50
LY75
LY88
LY94
LY97
LY data
Normal data
0.4
0.6
0.8
1
1.2
1.4
1.6
1.8
2
2.2
0
10
20
30
40
50
60

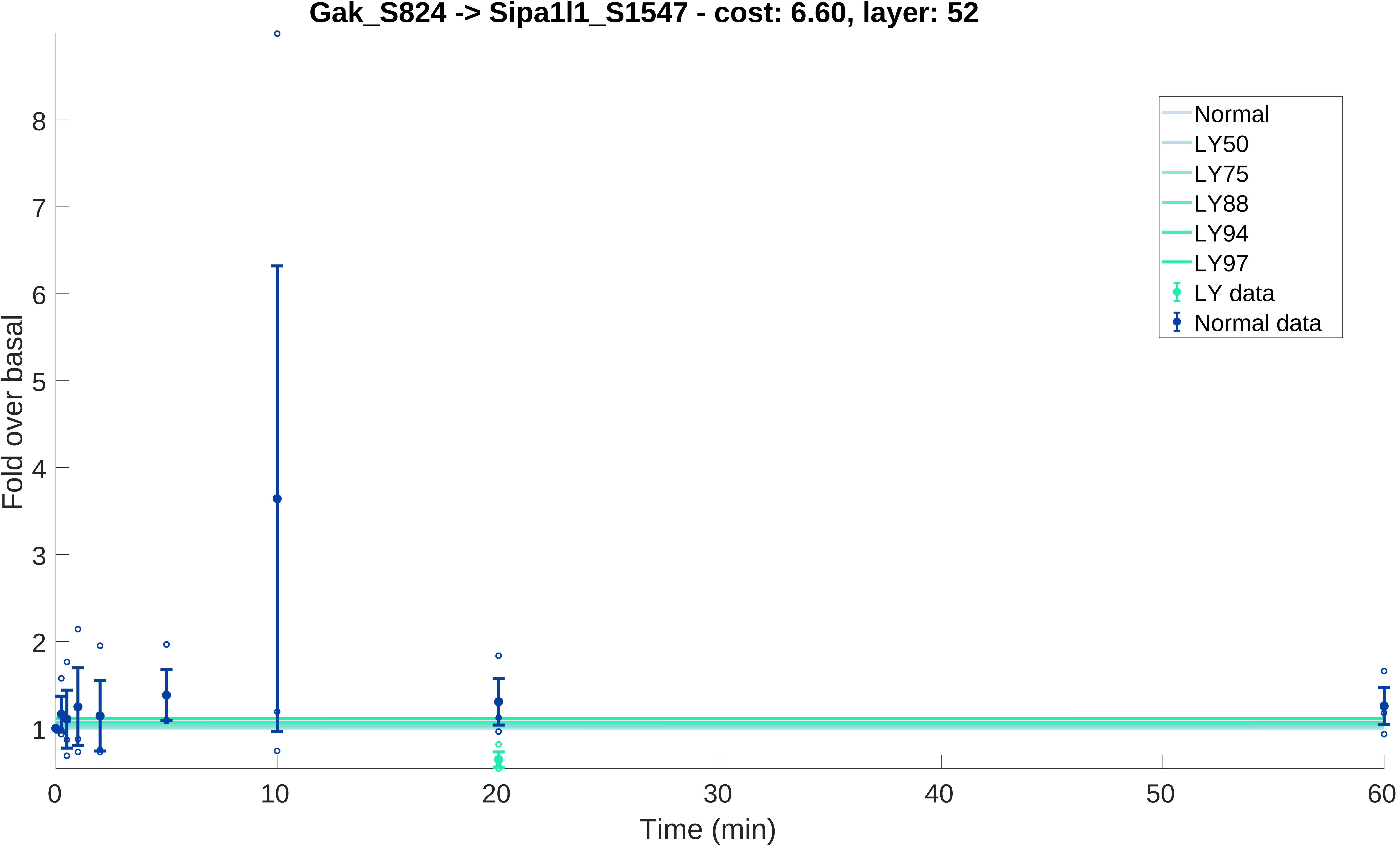
Gak_S824 -> Sipa1l1_S1547 - cost: 6.60, layer: 52
Fold over basal
Time (min)
Normal
LY50
LY75
LY88
LY94
LY97
LY data
Normal data
0
10
20
30
40
50
60
1
2
3
4
5
6
7
8

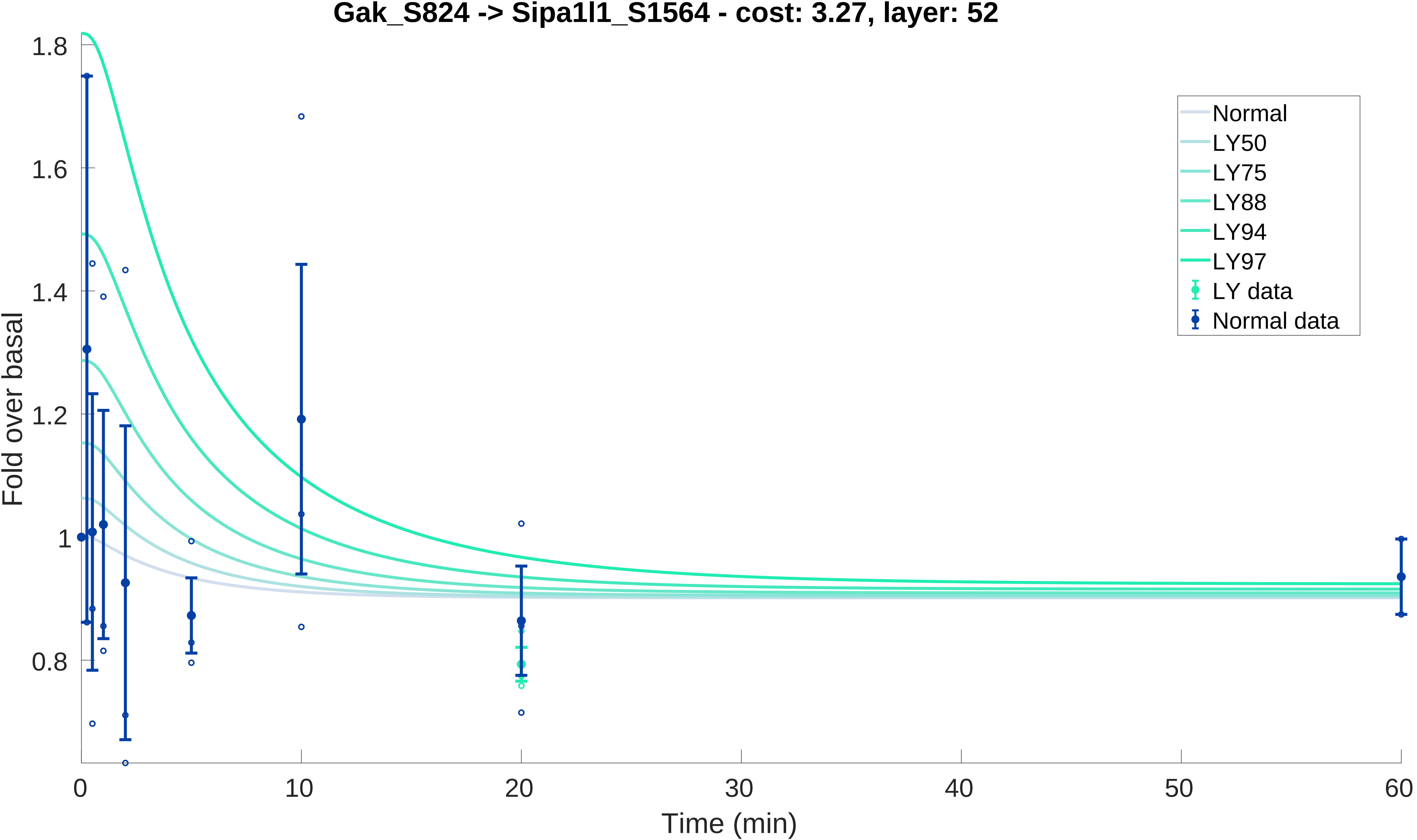

Gak_S824 -> Sipa1l1_S1564 - cost: 3.27, layer: 52
Fold over basal
Time (min)
0
10
20
30
40
50
60
0.8
1
1.2
1.4
1.6
1.8
Normal
LY50
LY75
LY88
LY94
LY97
LY data
Normal data

Rps6ka1_S363 -> Sipa1l1_S1611 - cost: 7.85, layer: 52

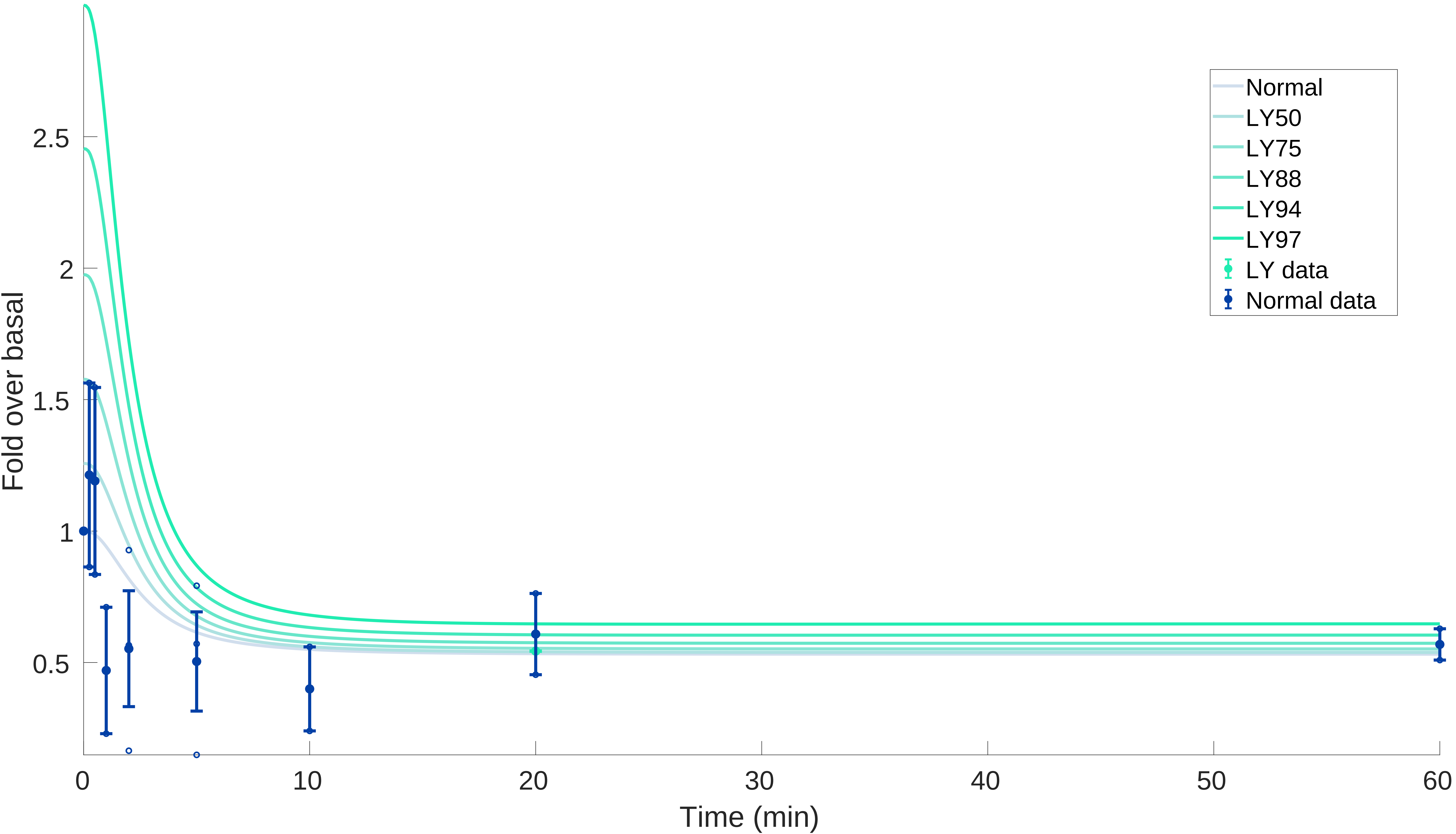

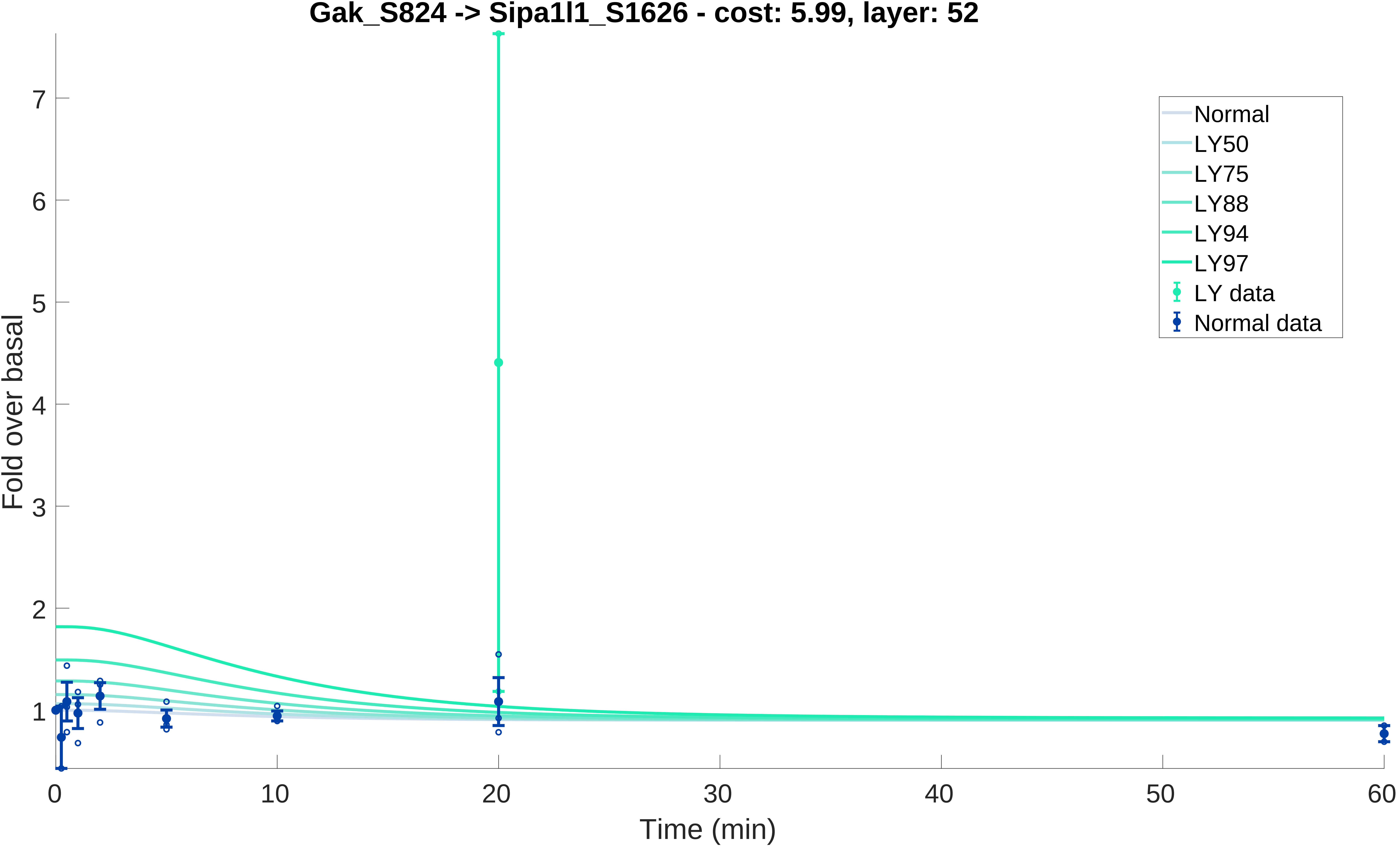

Gak_S824 -> Sipa1l1_S1626 - cost: 5.99, layer: 52
Fold over basal
Time (min)
Normal
LY50
LY75
LY88
LY94
LY97
LY data
Normal data
0
10
20
30
40
50
60
1
2
3
4
5
6
7

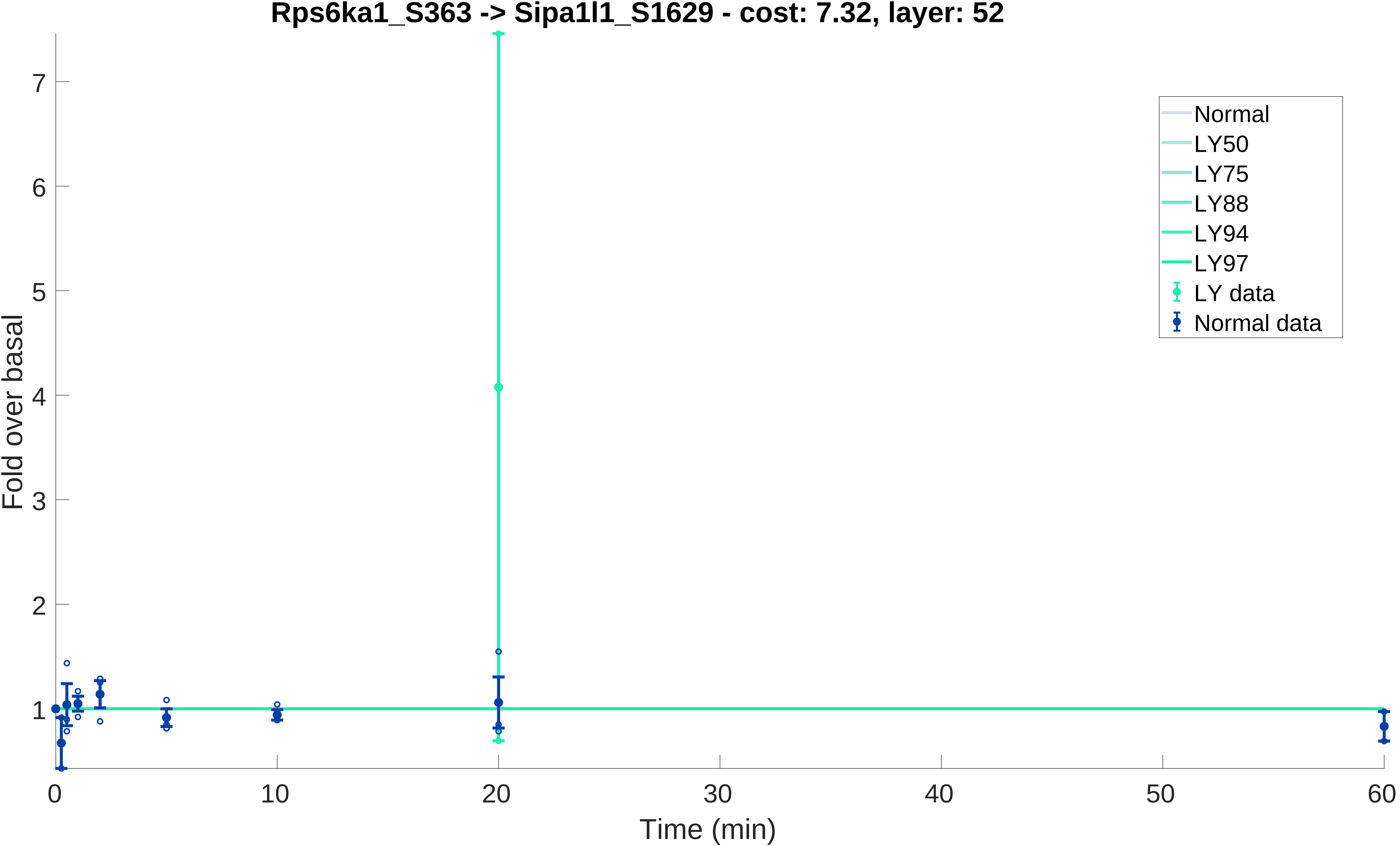

Rps6ka1_S363 -> Sipa1l1_S1629 - cost: 7.32, layer: 52
Fold over basal
Time (min)
0
10
20
30
40
50
60
1
2
3
4
5
6
7
Normal
LY50
LY75
LY88
LY94
LY97
LY data
Normal data

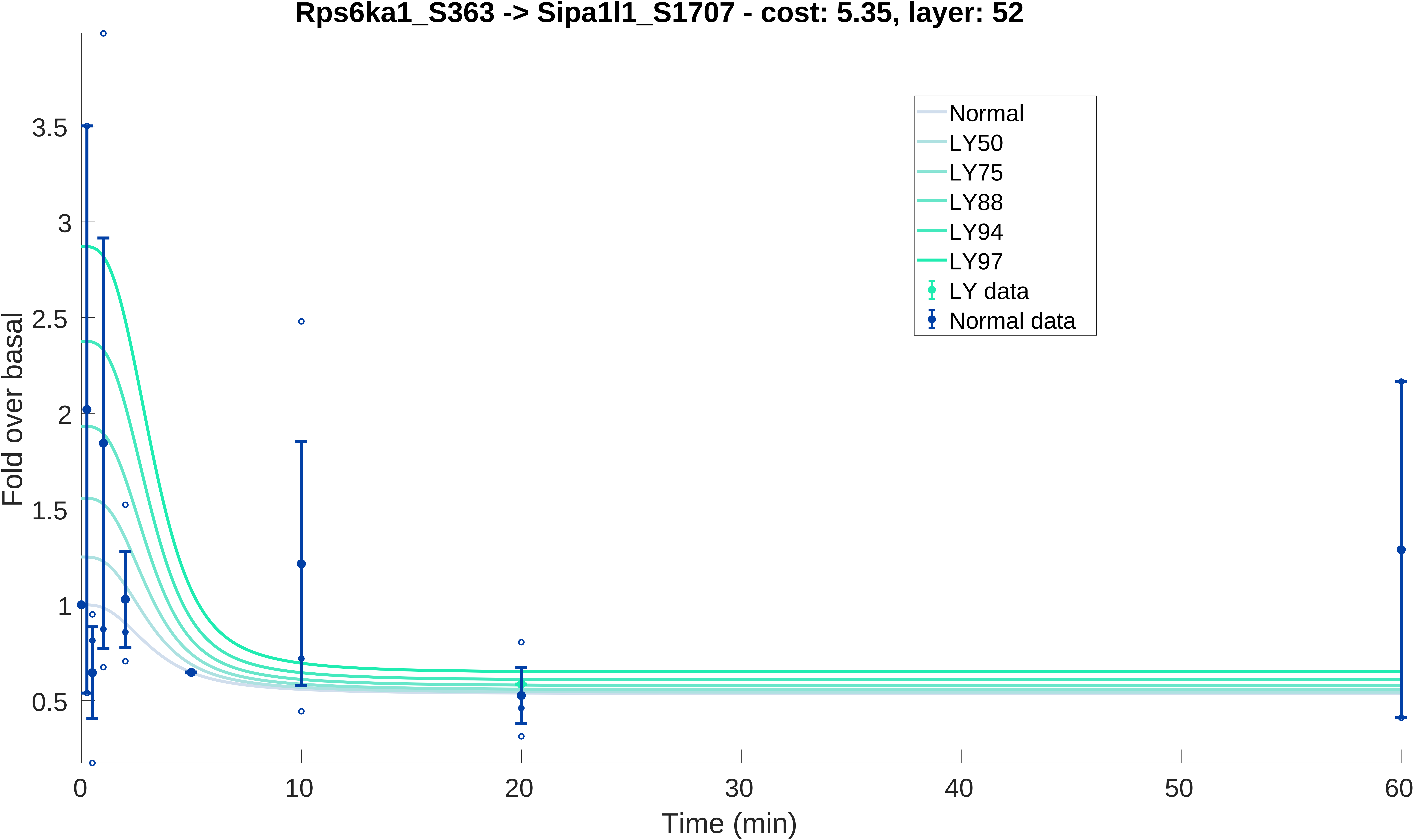

Rps6ka1_S363 -> Sipa1l1_S1707 - cost: 5.35, layer: 52
Fold over basal
3.5
3
2.5
2
1.5
1
0.5
0
10
20
30
40
50
60
Time (min)
Normal
LY50
LY75
LY88
LY94
LY97
LY data
Normal data

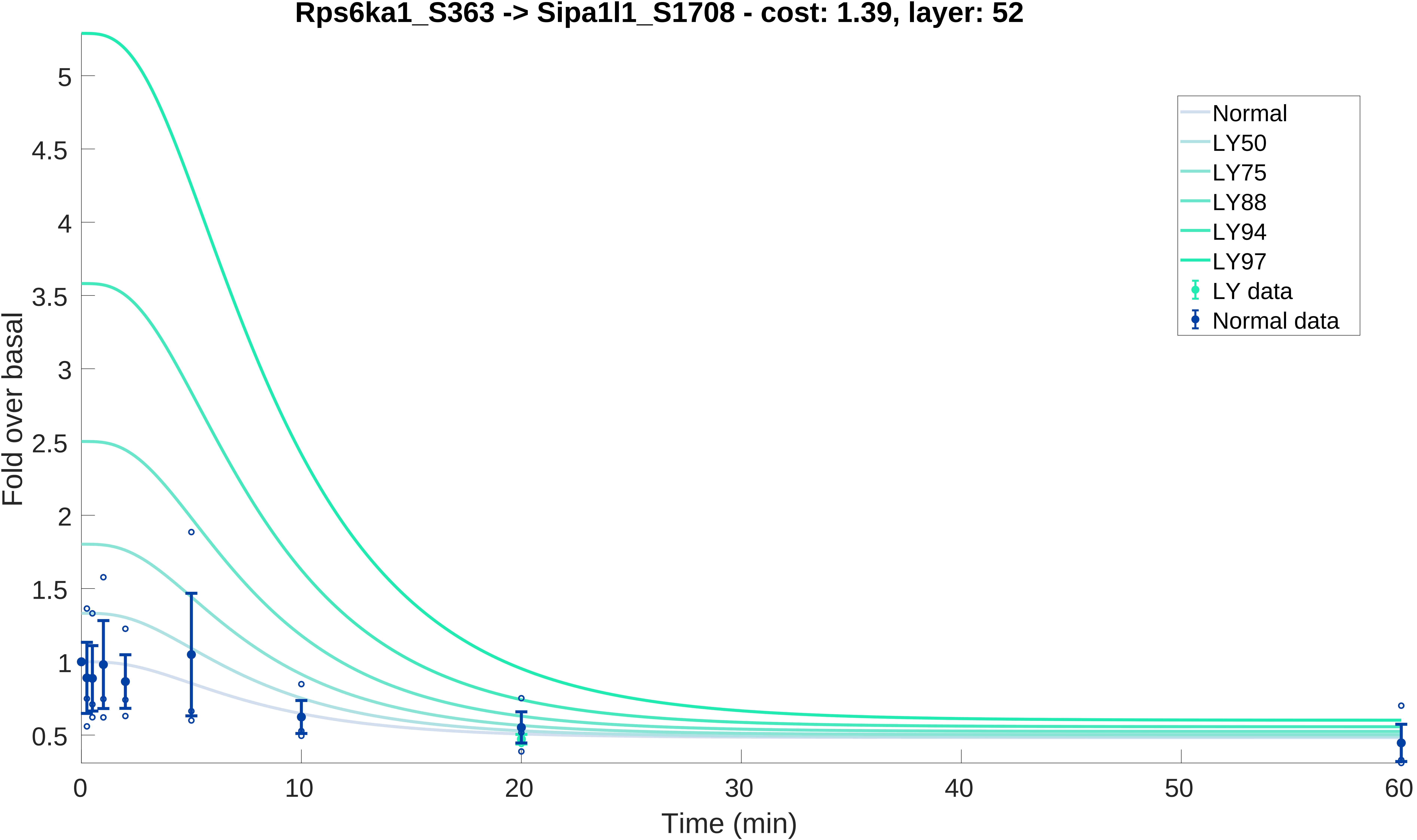
Rps6ka1_S363 -> Sipa1l1_S1708 - cost: 1.39, layer: 52
Fold over basal
Time (min)
0
10
20
30
40
50
60
0.5
1
1.5
2
2.5
3
3.5
4
4.5
5
Normal
LY50
LY75
LY88
LY94
LY97
LY data
Normal data

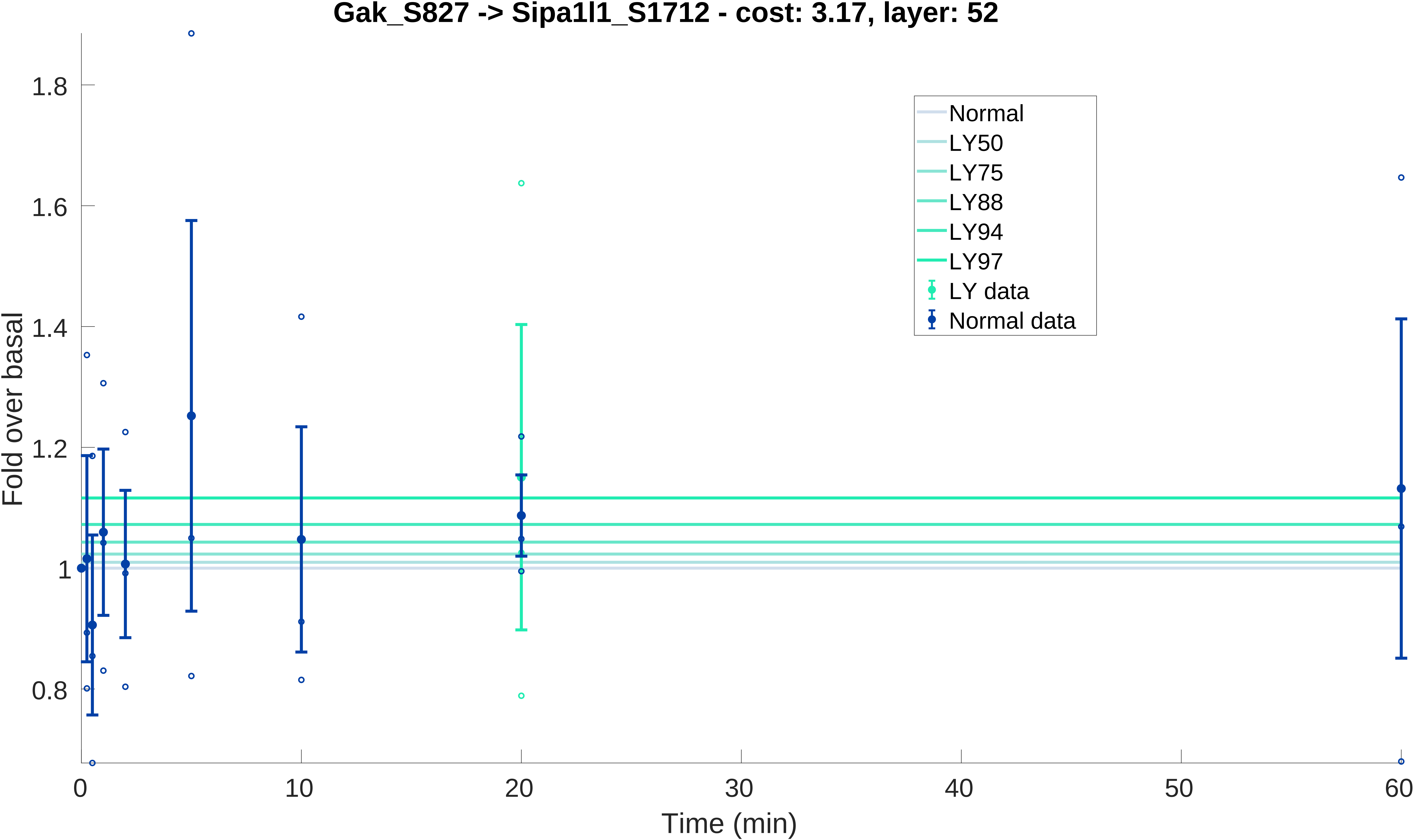

Gak_S827 -> Sipa1l1_S1712 - cost: 3.17, layer: 52
Fold over basal
1.8
1.6
1.4
1.2
1
0.8
0
10
20
30
40
50
60
Time (min)
Normal
LY50
LY75
LY88
LY94
LY97
LY data
Normal data

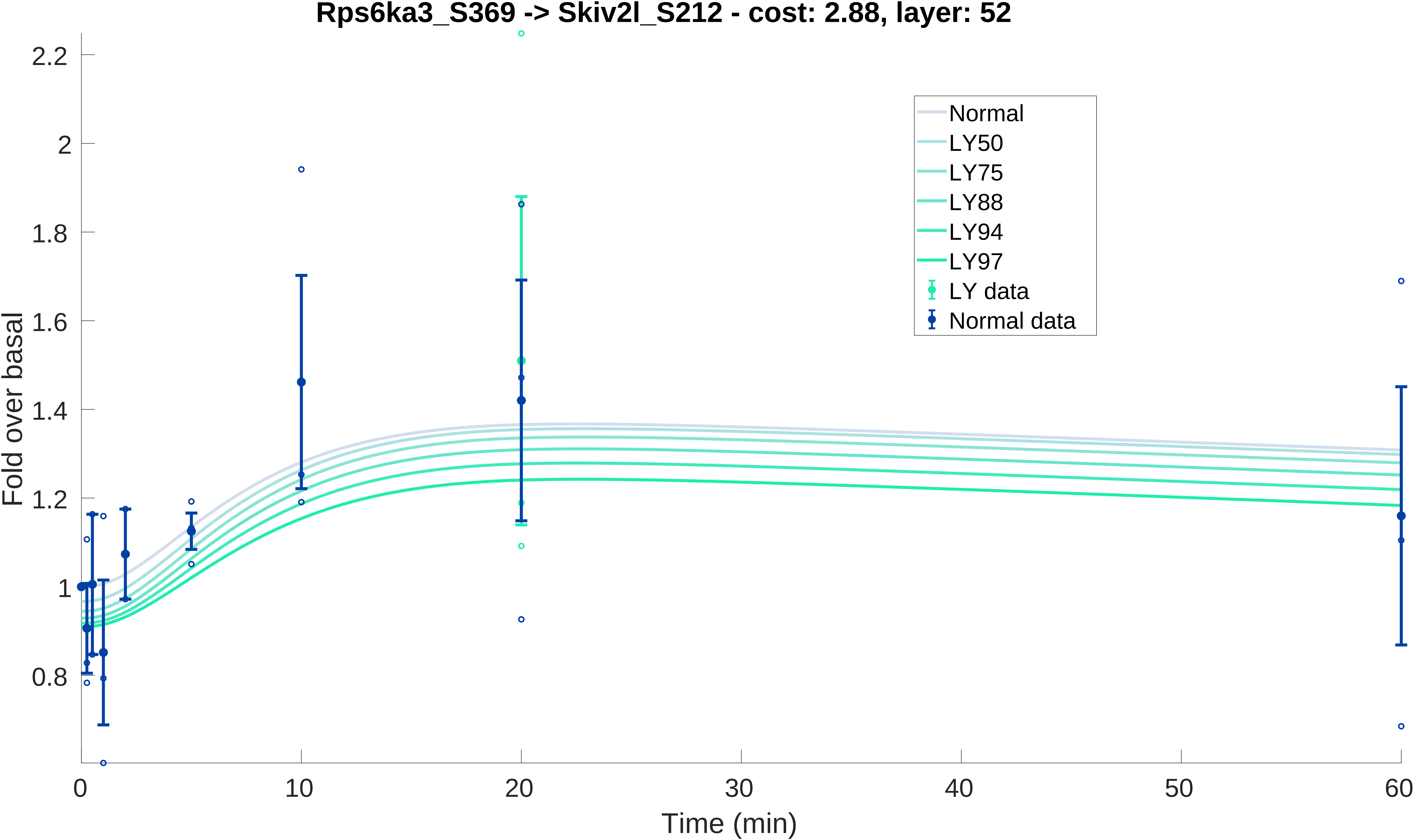

Rps6ka3_S369 -> Skiv2l_S212 - cost: 2.88, layer: 52
Normal
LY50
LY75
LY88
LY94
LY97
LY data
Normal data
Fold over basal
Time (min)
2.2
2
1.8
1.6
1.4
1.2
1
0.8
0
10
20
30
40
50
60

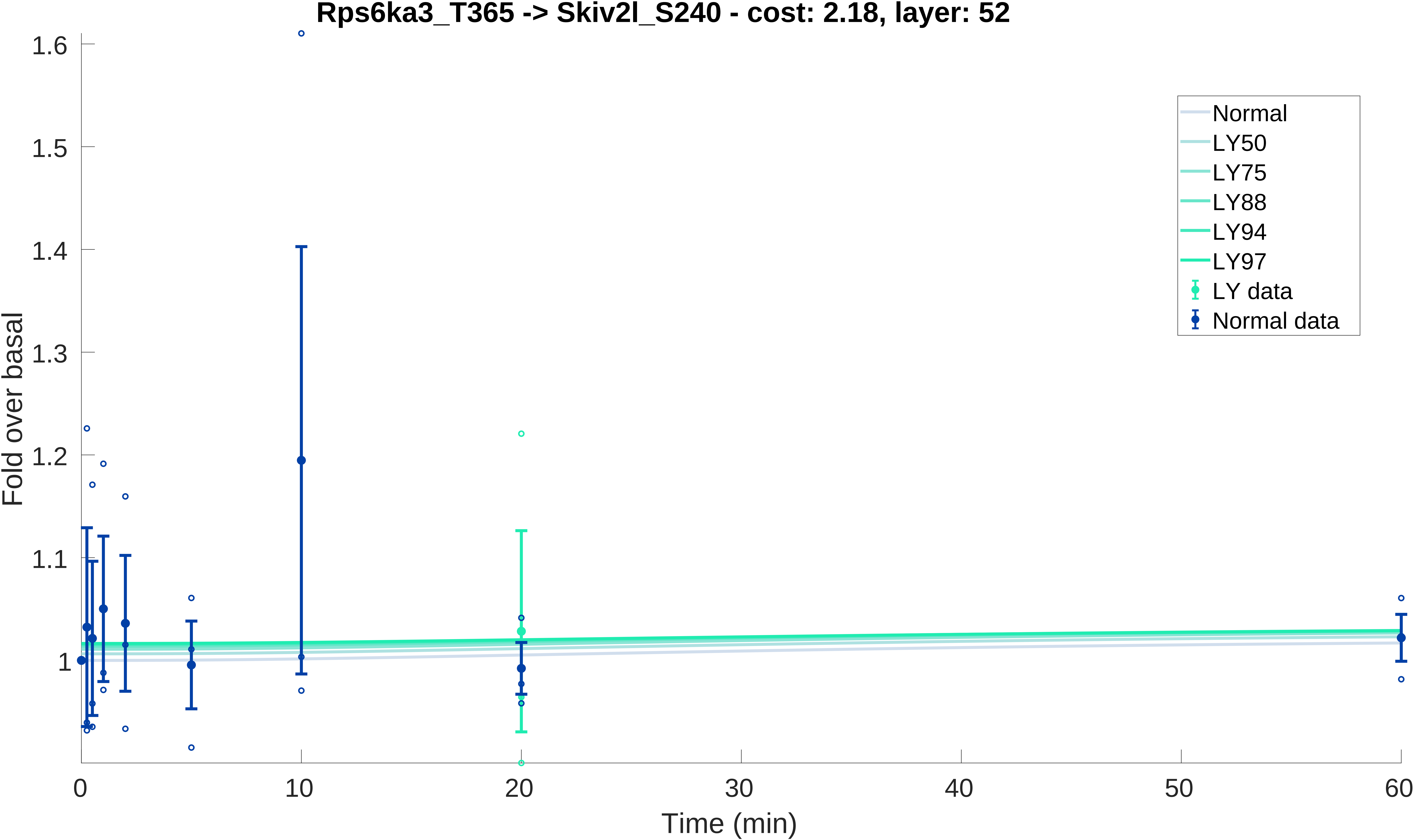
Rps6ka3_T365 -> Skiv2l_S240 - cost: 2.18, layer: 52
Fold over basal
1.6
1.5
1.4
1.3
1.2
1.1
1
0
10
20
30
40
50
60
Time (min)
Normal
LY50
LY75
LY88
LY94
LY97
LY data
Normal data

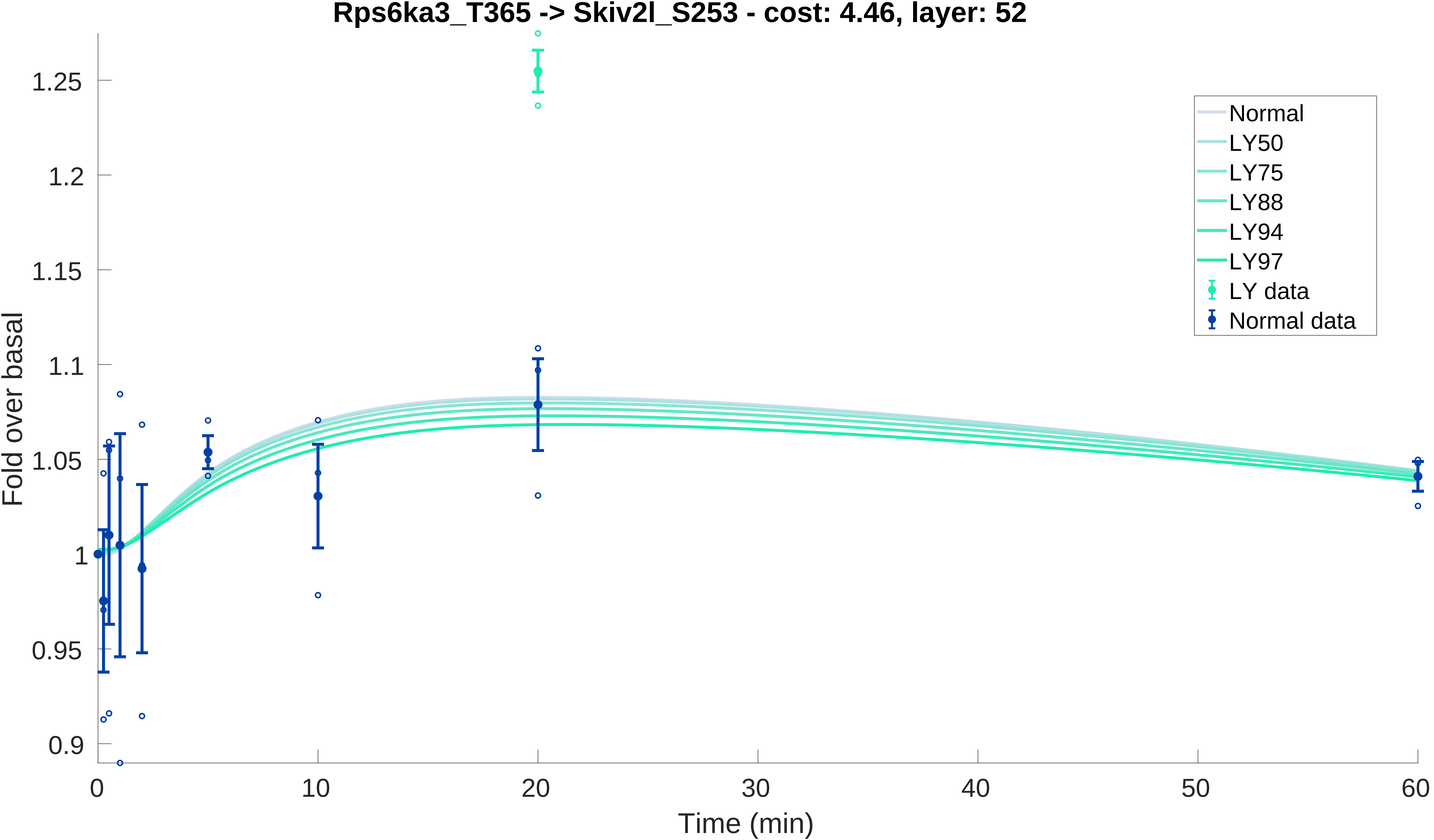

Rps6ka3_T365 -> Skiv2l_S253 - cost: 4.46, layer: 52
Fold over basal
1.25
1.2
1.15
1.1
1.05
1
0.95
0.9
Time (min)
0
10
20
30
40
50
60
Normal
LY50
LY75
LY88
LY94
LY97
LY data
Normal data

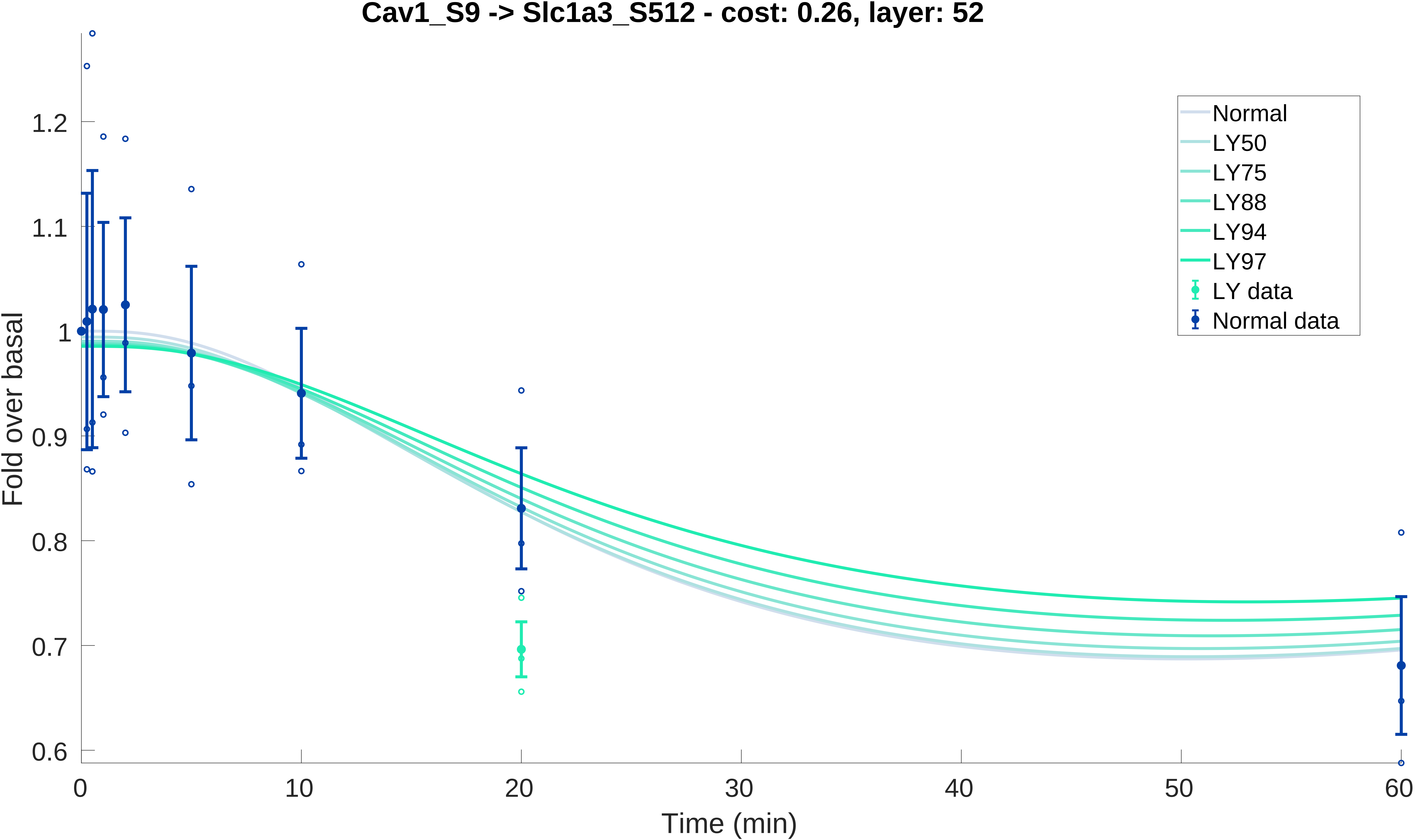

Cav1_S9 -> Slc1a3_S512 - cost: 0.26, layer: 52
Fold over basal
Time (min)
0.6
0.7
0.8
0.9
1
1.1
1.2
0
10
20
30
40
50
60
Normal
LY50
LY75
LY88
LY94
LY97
LY data
Normal data

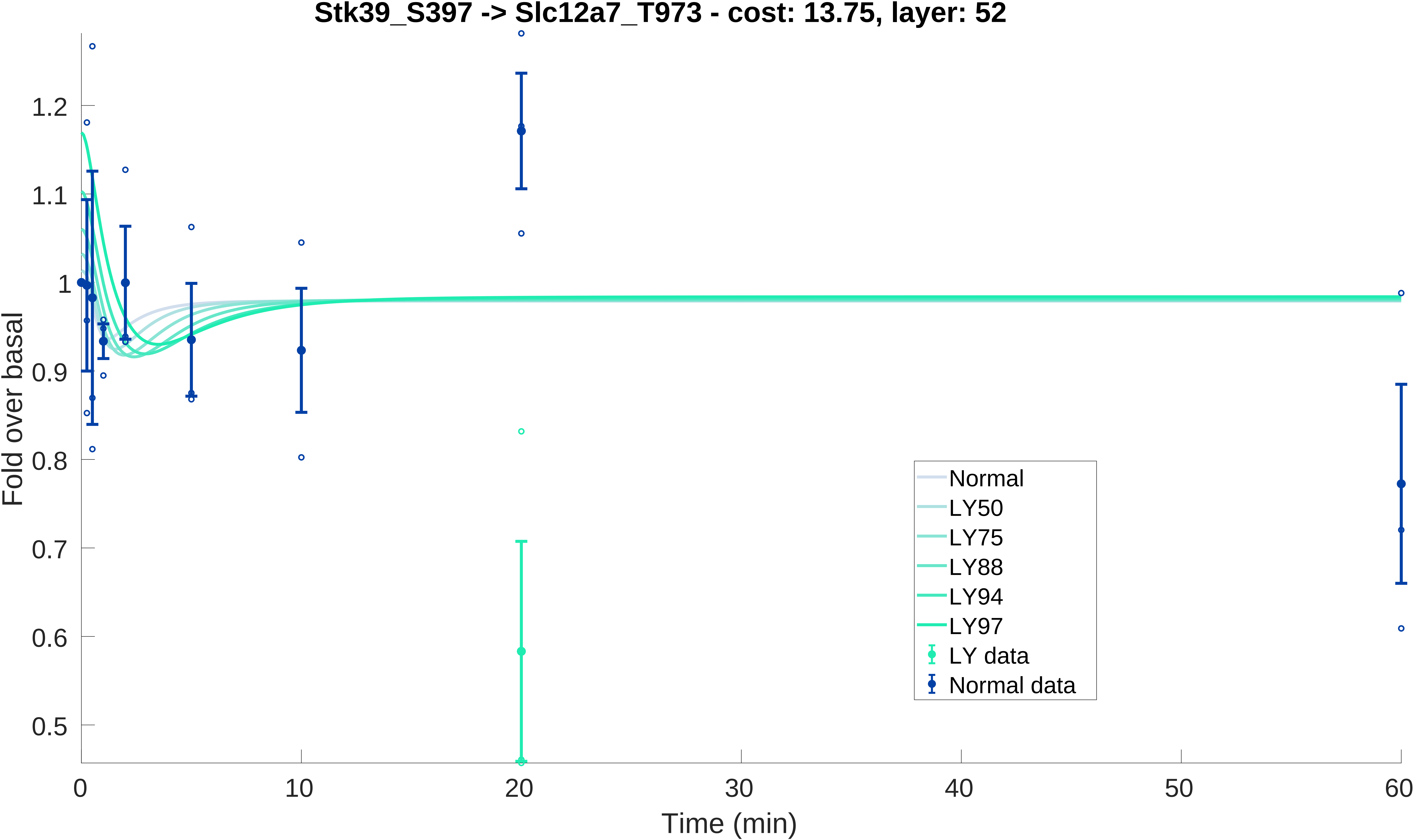

Stk39_S397 -> Slc12a7_T973 - cost: 13.75, layer: 52
Fold over basal
Time (min)
1.2
1.1
1
0.9
0.8
0.7
0.6
0.5
0
10
20
30
40
50
60
Normal
LY50
LY75
LY88
LY94
LY97
LY data
Normal data

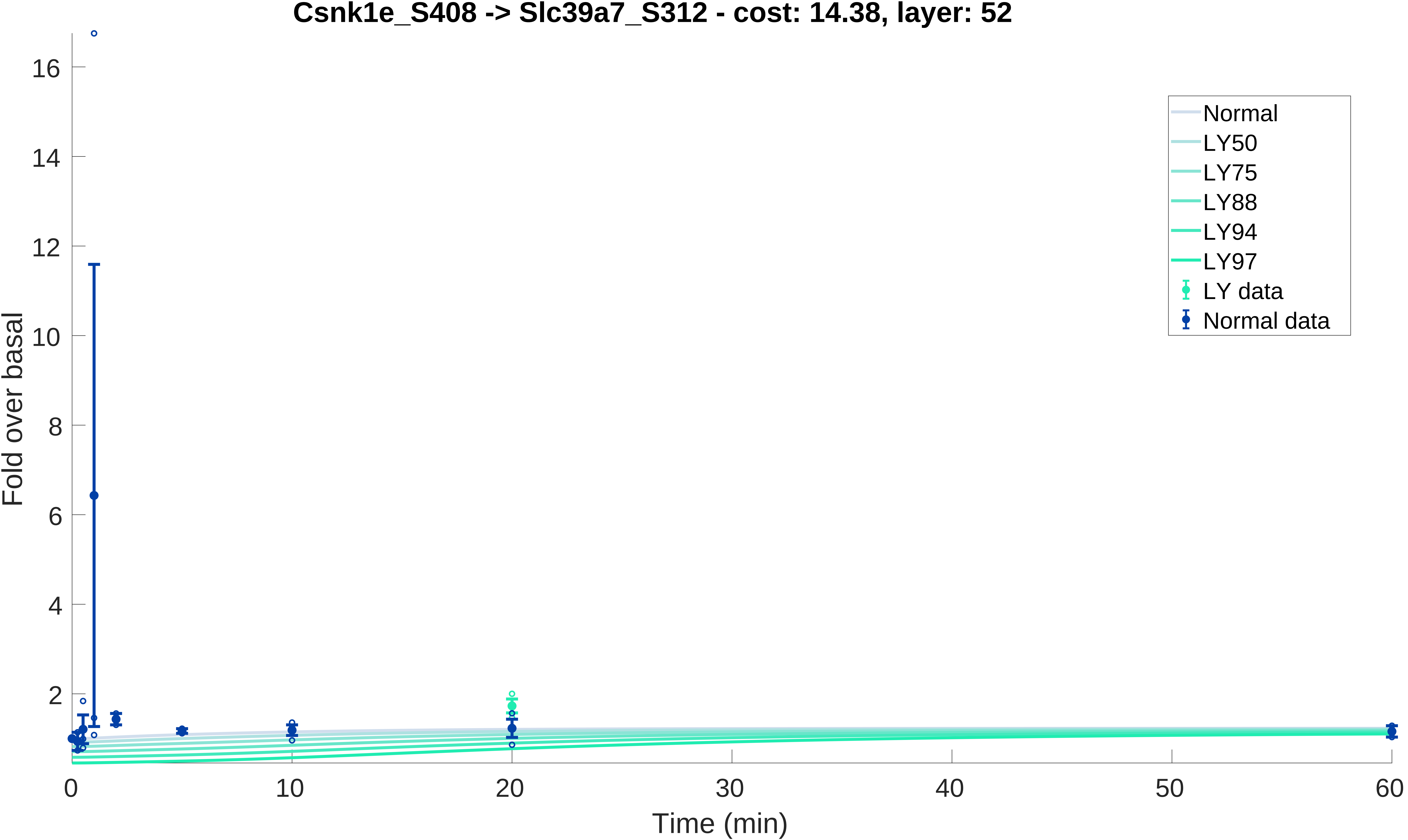

Csnk1e_S408 -> Slc39a7_S312 - cost: 14.38, layer: 52
Fold over basal
16
14
12
10
8
6
4
2
0
10
20
30
40
50
60
Time (min)
Normal
LY50
LY75
LY88
LY94
LY97
LY data
Normal data

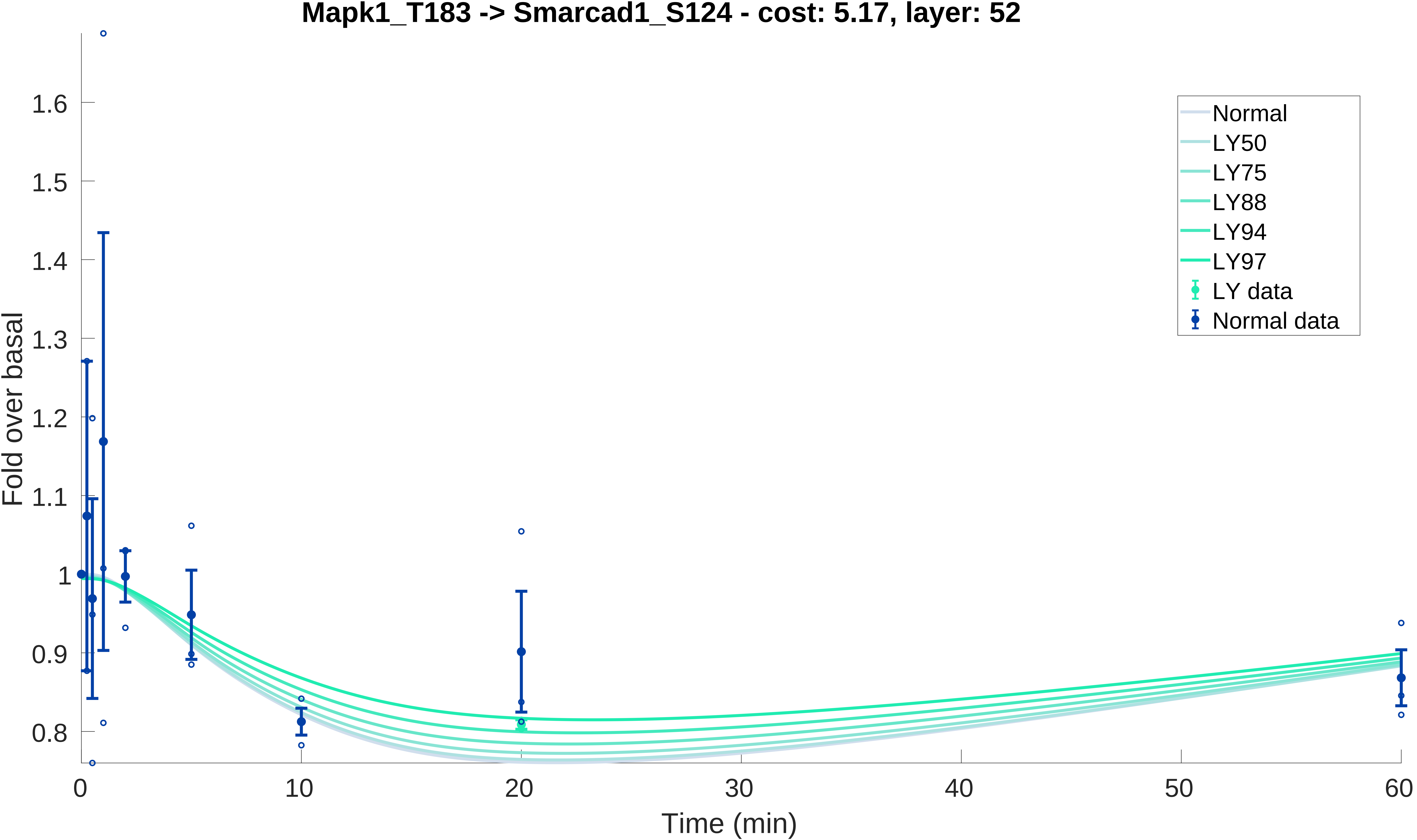

Mapk1_T183 -> Smarcad1_S124 - cost: 5.17, layer: 52
Fold over basal
1.6
1.5
1.4
1.3
1.2
1.1
1
0.9
0.8
0
10
20
30
40
50
60
Time (min)
Normal
LY50
LY75
LY88
LY94
LY97
LY data
Normal data

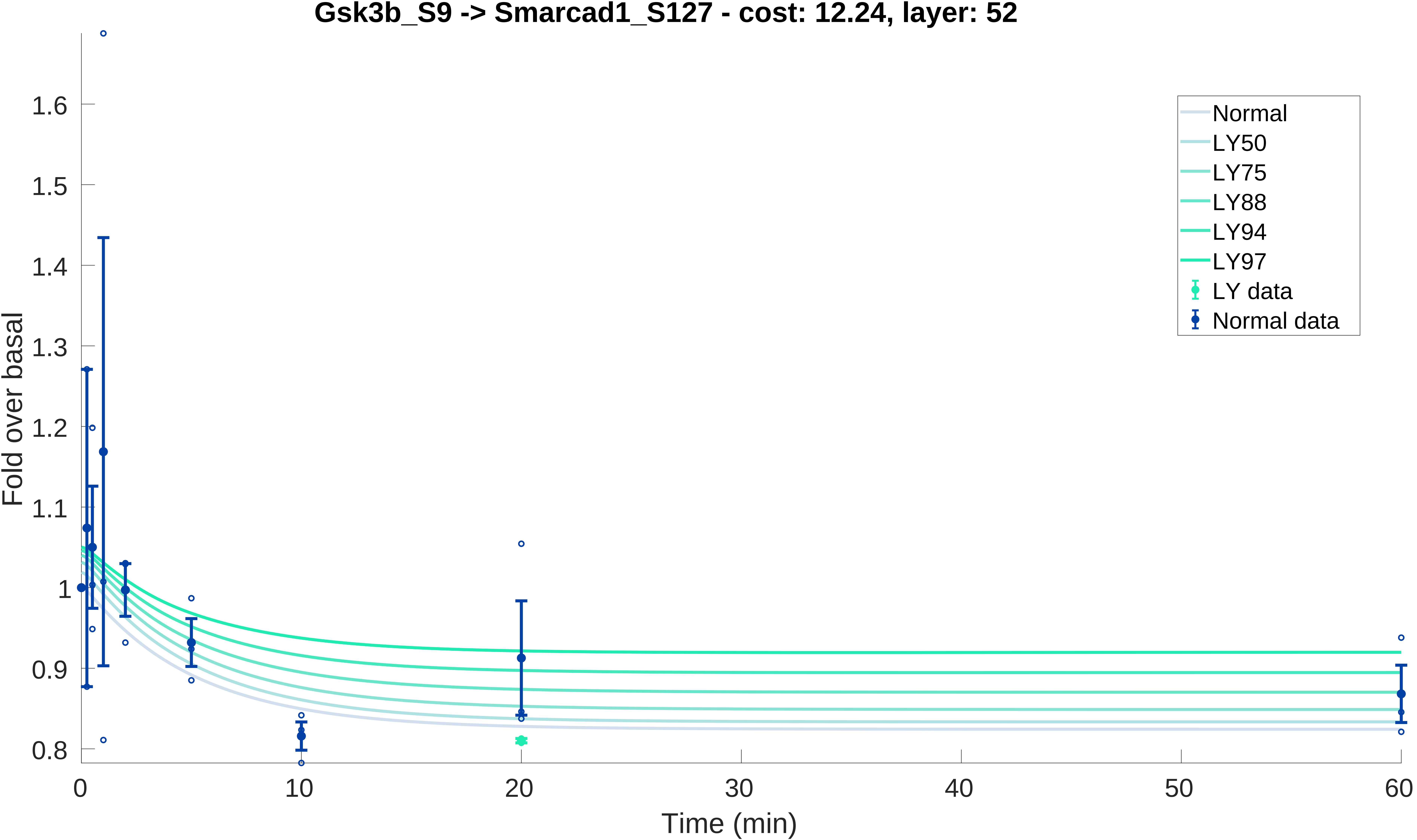

Gsk3b_S9 -> Smarcad1_S127 - cost: 12.24, layer: 52
Fold over basal
Time (min)
1.6
1.5
1.4
1.3
1.2
1.1
1
0.9
0.8
0
10
20
30
40
50
60
Normal
LY50
LY75
LY88
LY94
LY97
LY data
Normal data

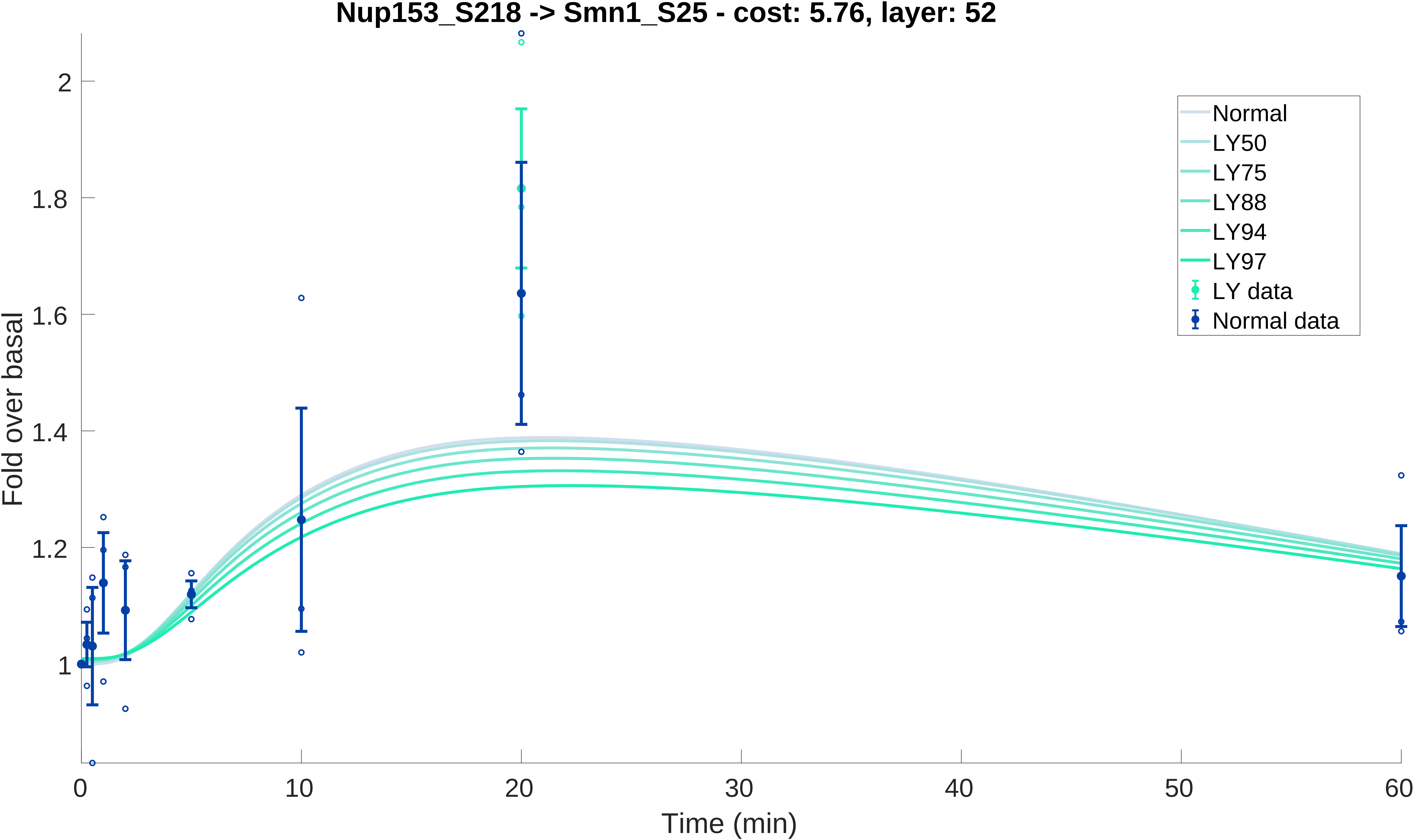
Nup153_S218 -> Smn1_S25 - cost: 5.76, layer: 52
Fold over basal
Time (min)
2
1.8
1.6
1.4
1.2
1
0
10
20
30
40
50
60
Normal
LY50
LY75
LY88
LY94
LY97
LY data
Normal data

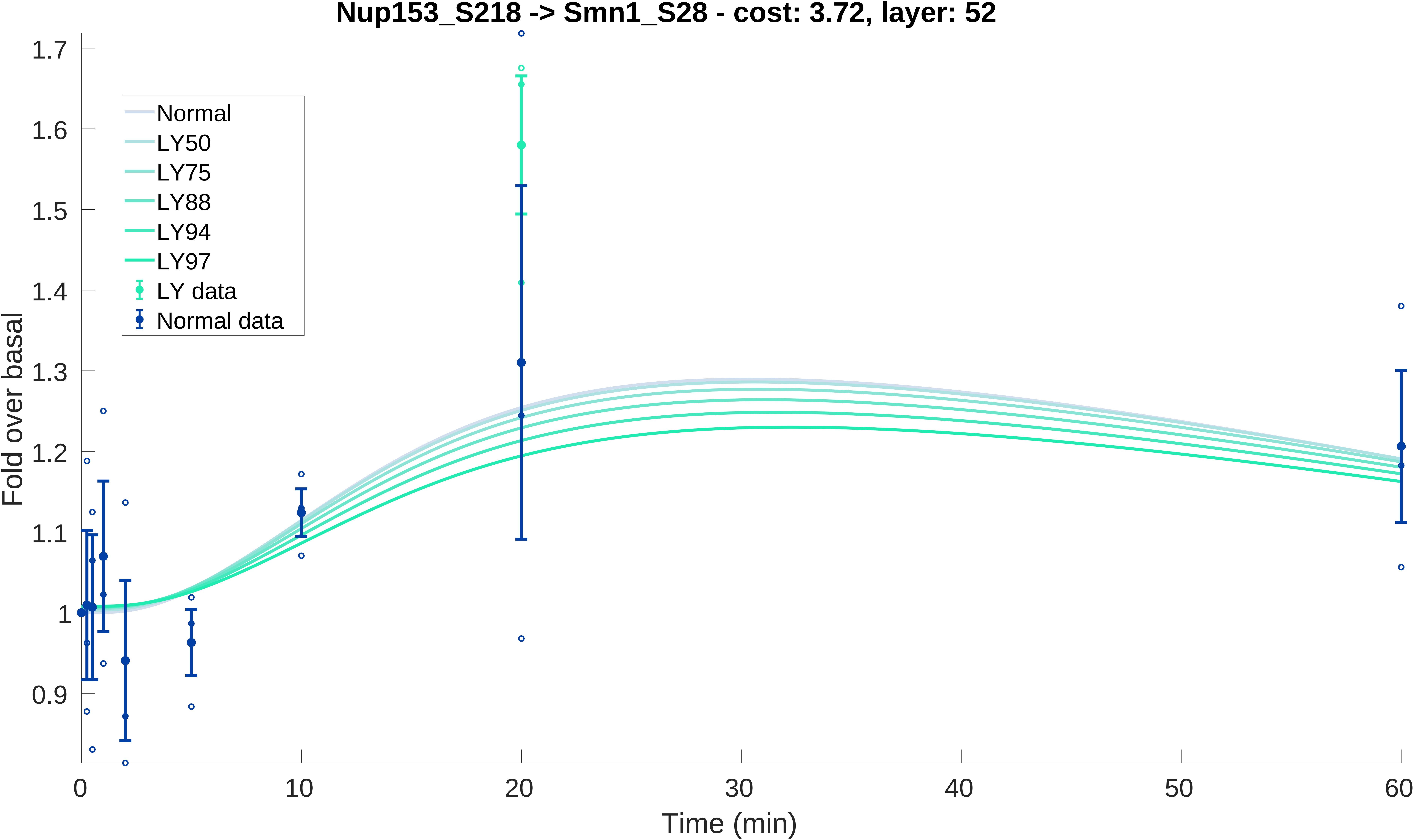
Nup153_S218 -> Smn1_S28 - cost: 3.72, layer: 52
Normal
LY50
LY75
LY88
LY94
LY97
LY data
Normal data
Fold over basal
Time (min)
0
10
20
30
40
50
60
0.9
1
1.1
1.2
1.3
1.4
1.5
1.6
1.7

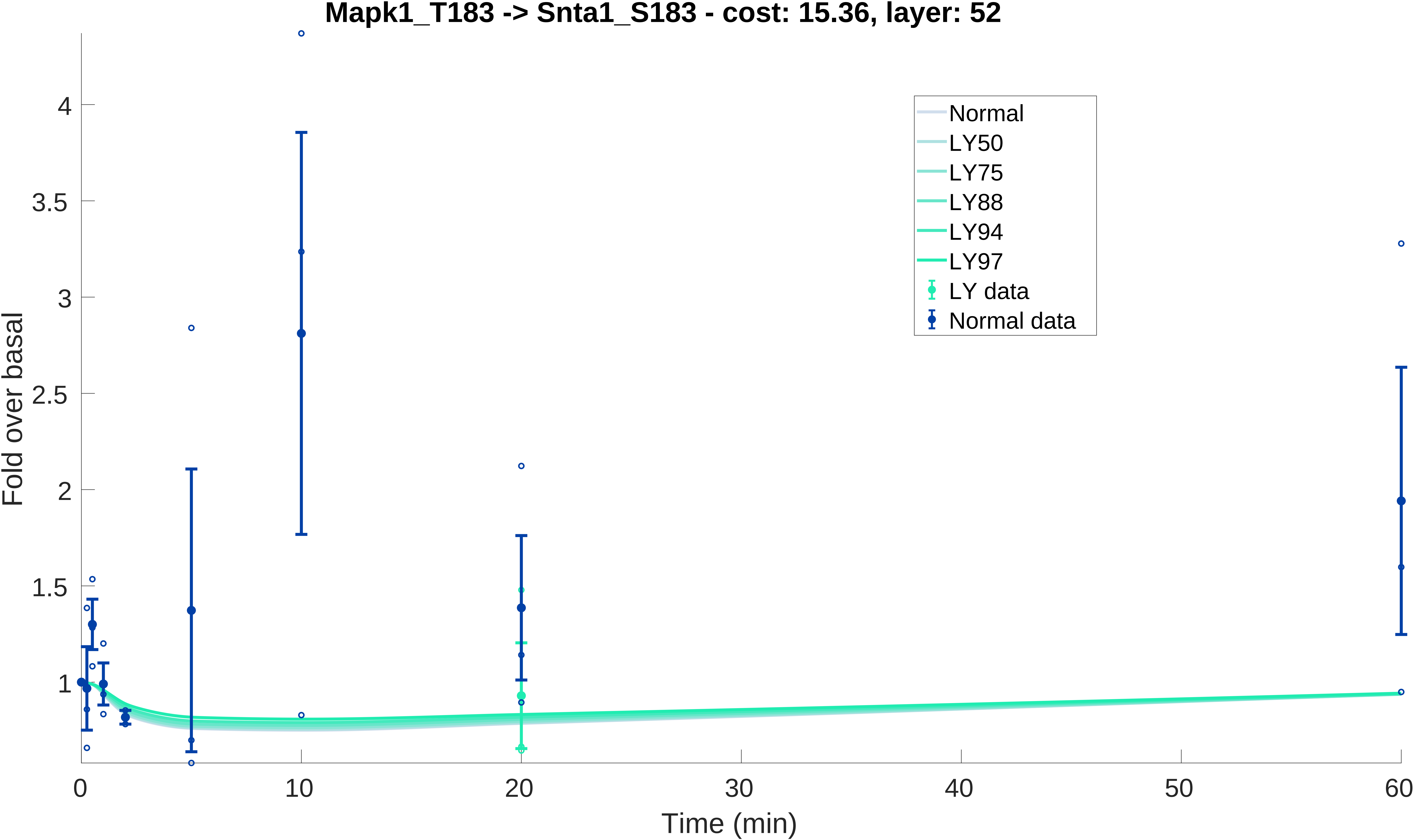

Mapk1_T183 -> Snta1_S183 - cost: 15.36, layer: 52
Fold over basal
Time (min)
0
10
20
30
40
50
60
1
1.5
2
2.5
3
3.5
4
Normal
LY50
LY75
LY88
LY94
LY97
LY data
Normal data

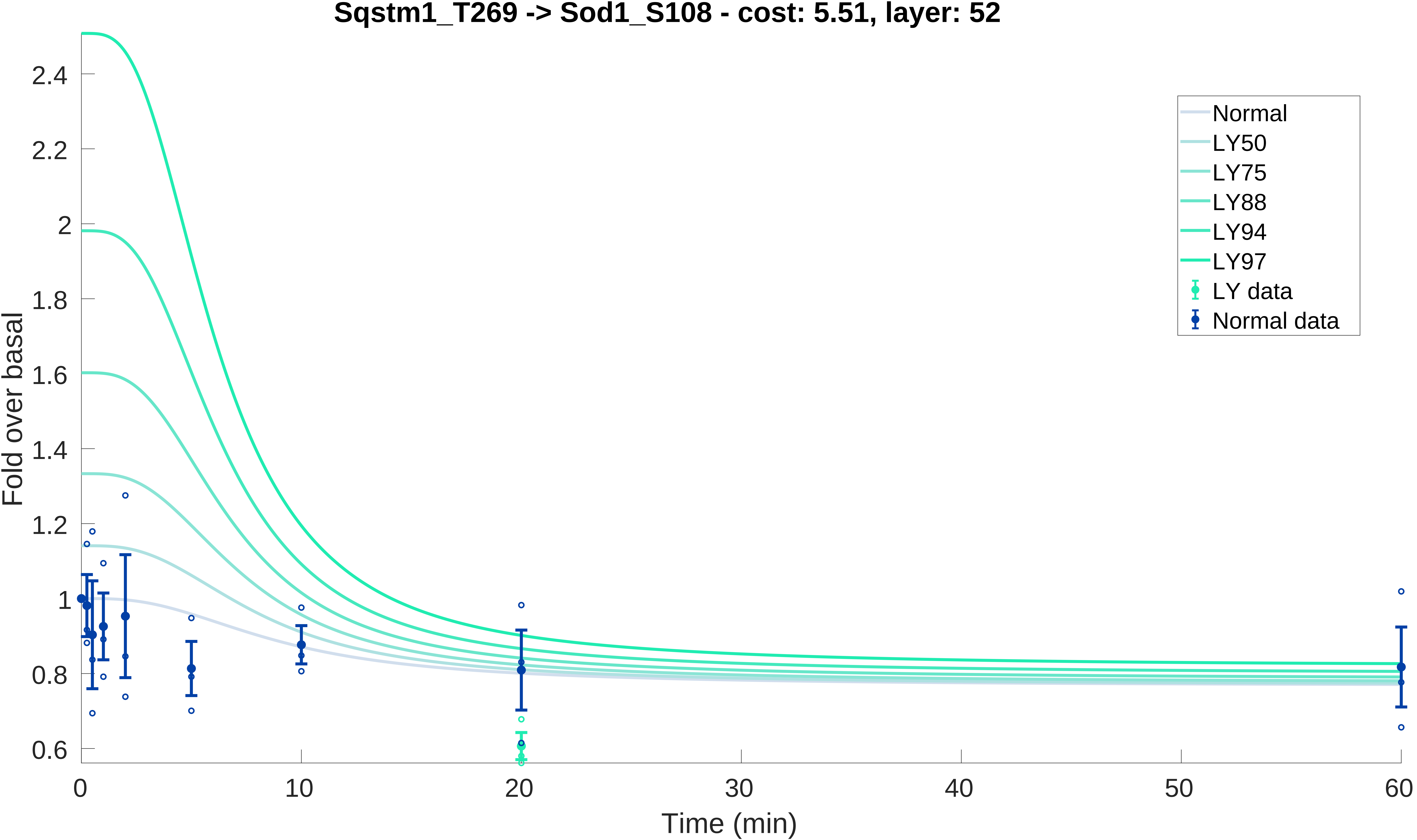

Sqstm1_T269 -> Sod1_S108 - cost: 5.51, layer: 52
Fold over basal
Time (min)
0.6
0.8
1
1.2
1.4
1.6
1.8
2
2.2
2.4
0
10
20
30
40
50
60
Normal
LY50
LY75
LY88
LY94
LY97
LY data
Normal data

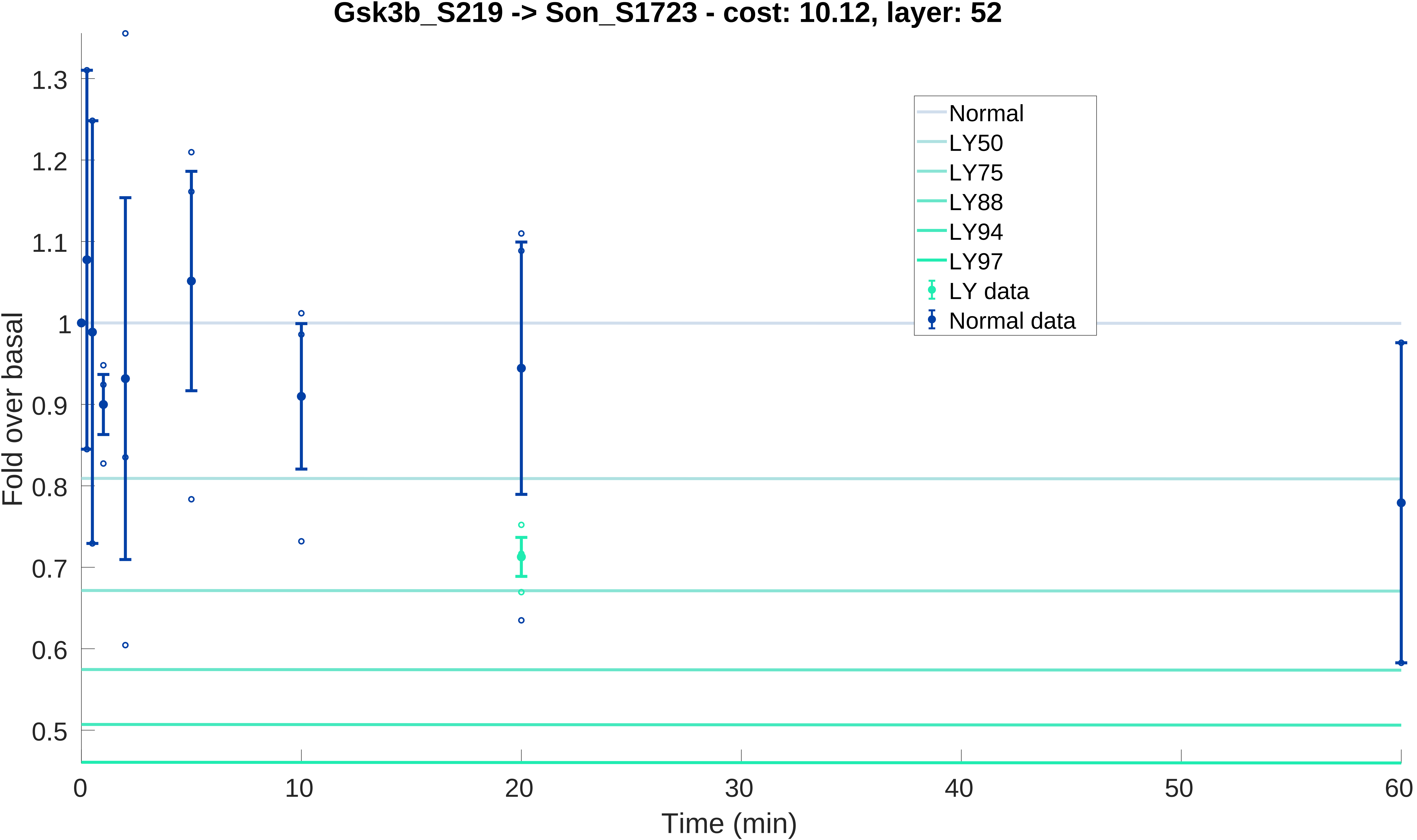

Gsk3b_S219 -> Son_S1723 - cost: 10.12, layer: 52
Fold over basal
Time (min)
Normal
LY50
LY75
LY88
LY94
LY97
LY data
Normal data
0.5
0.6
0.7
0.8
0.9
1
1.1
1.2
1.3
0
10
20
30
40
50
60

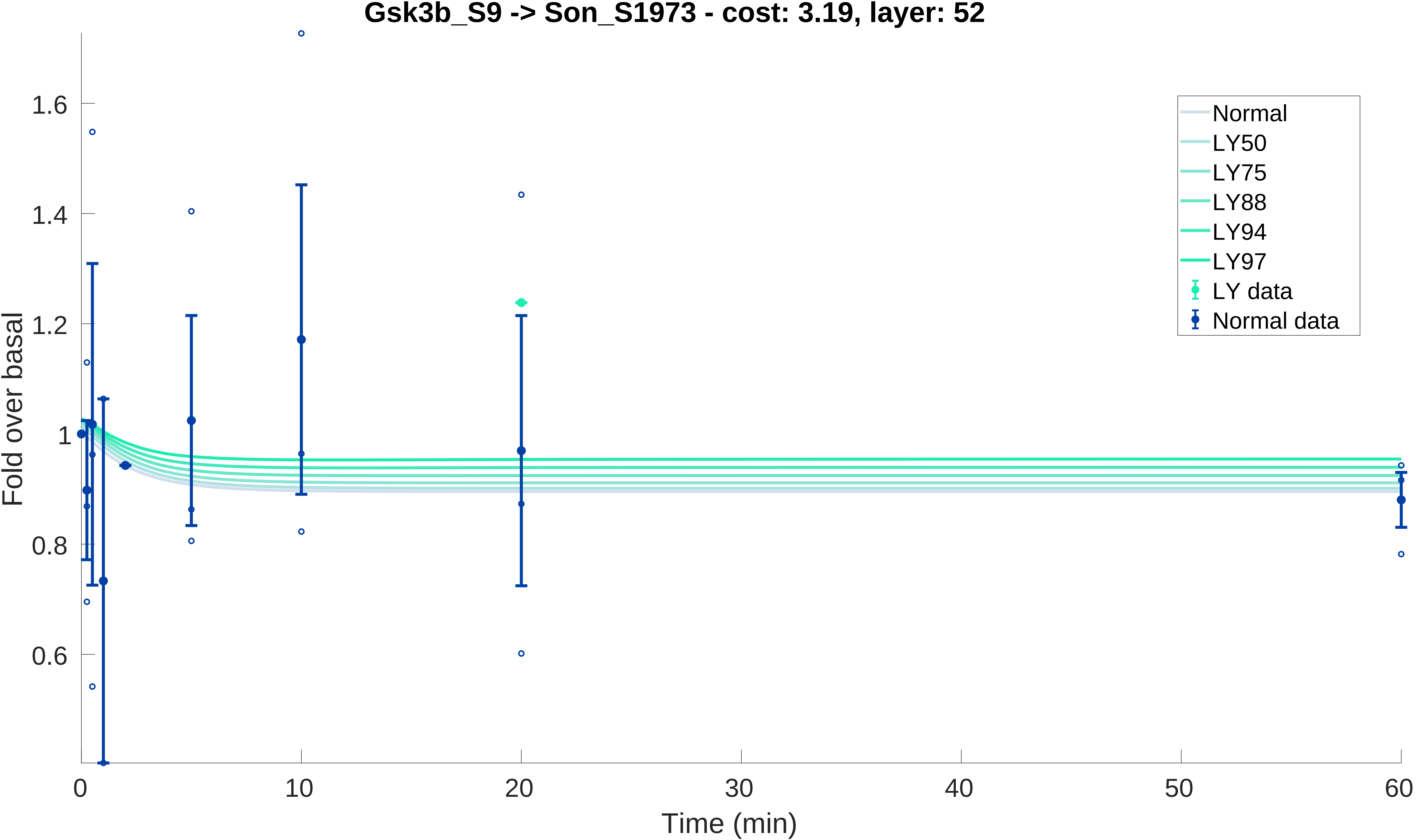
Gsk3b_S9 -> Son_S1973 - cost: 3.19, layer: 52
Fold over basal
Time (min)
0.6
0.8
1
1.2
1.4
1.6
0
10
20
30
40
50
60
Normal
LY50
LY75
LY88
LY94
LY97
LY data
Normal data

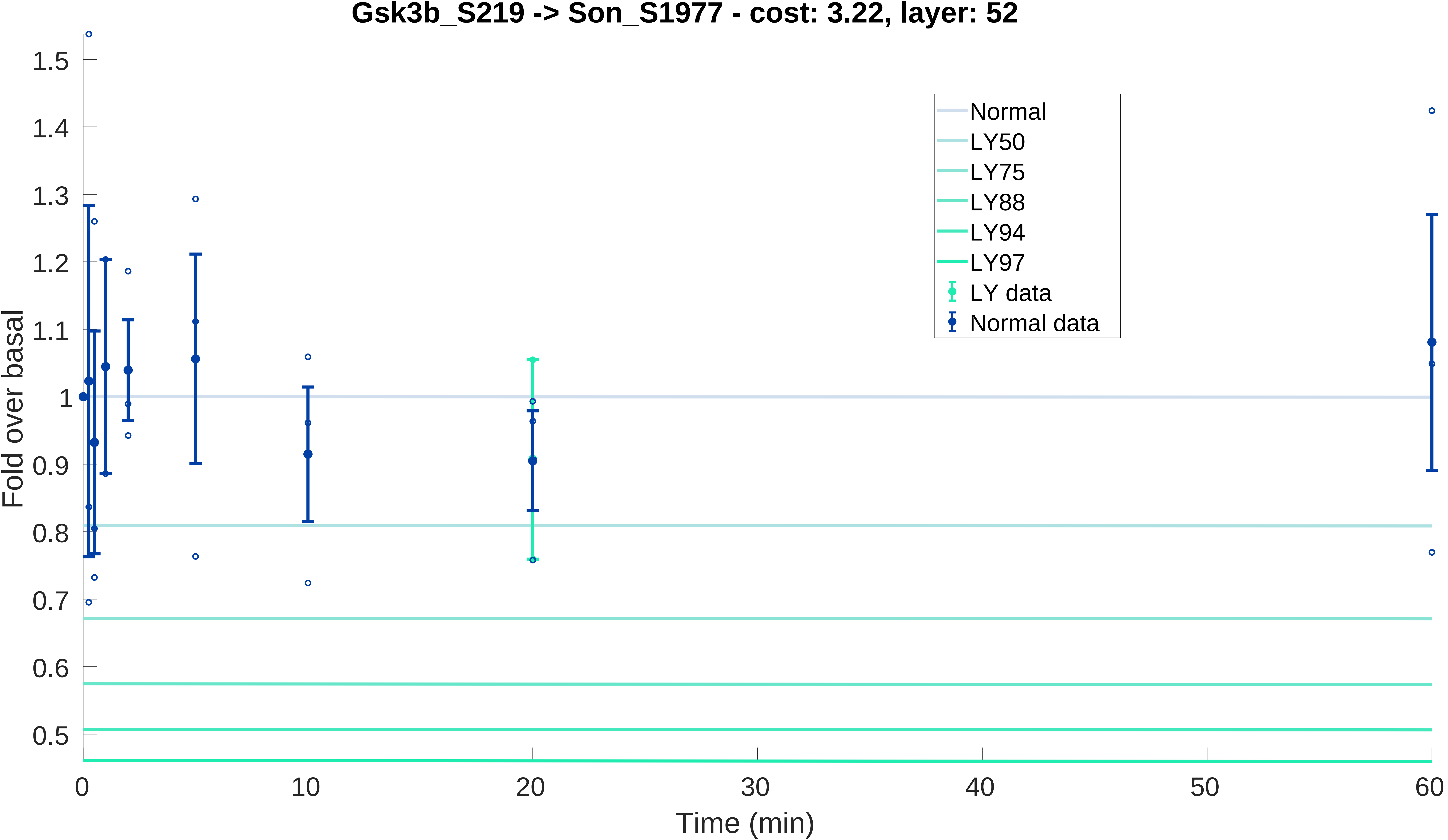
Gsk3b_S219 -> Son_S1977 - cost: 3.22, layer: 52
Fold over basal
Time (min)
0
10
20
30
40
50
60
1.5
1.4
1.3
1.2
1.1
1
0.9
0.8
0.7
0.6
0.5
Normal
LY50
LY75
LY88
LY94
LY97
LY data
Normal data

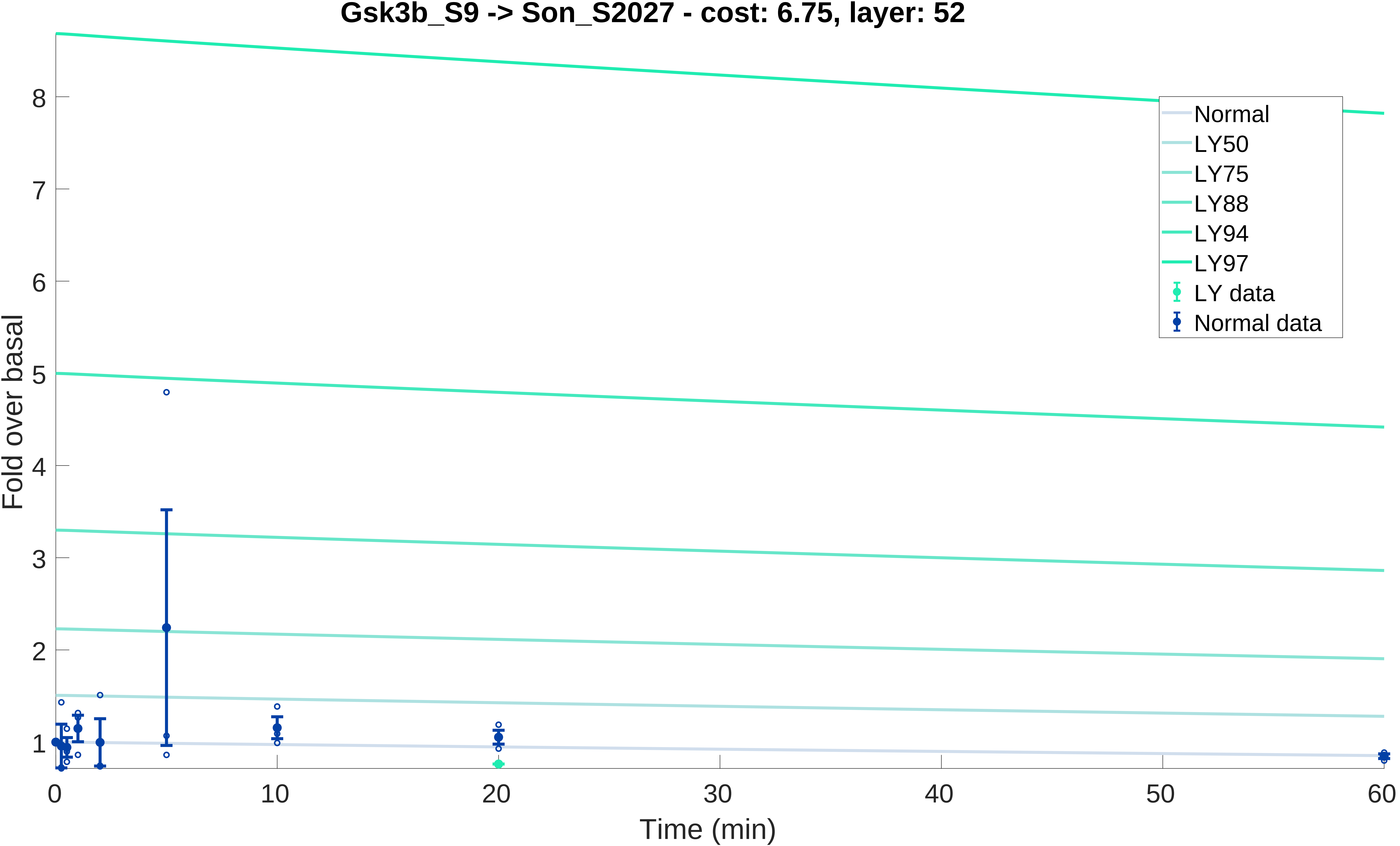
Gsk3b_S9 -> Son_S2027 - cost: 6.75, layer: 52
Fold over basal
Time (min)
0
10
20
30
40
50
60
1
2
3
4
5
6
7
8
Normal
LY50
LY75
LY88
LY94
LY97
LY data
Normal data

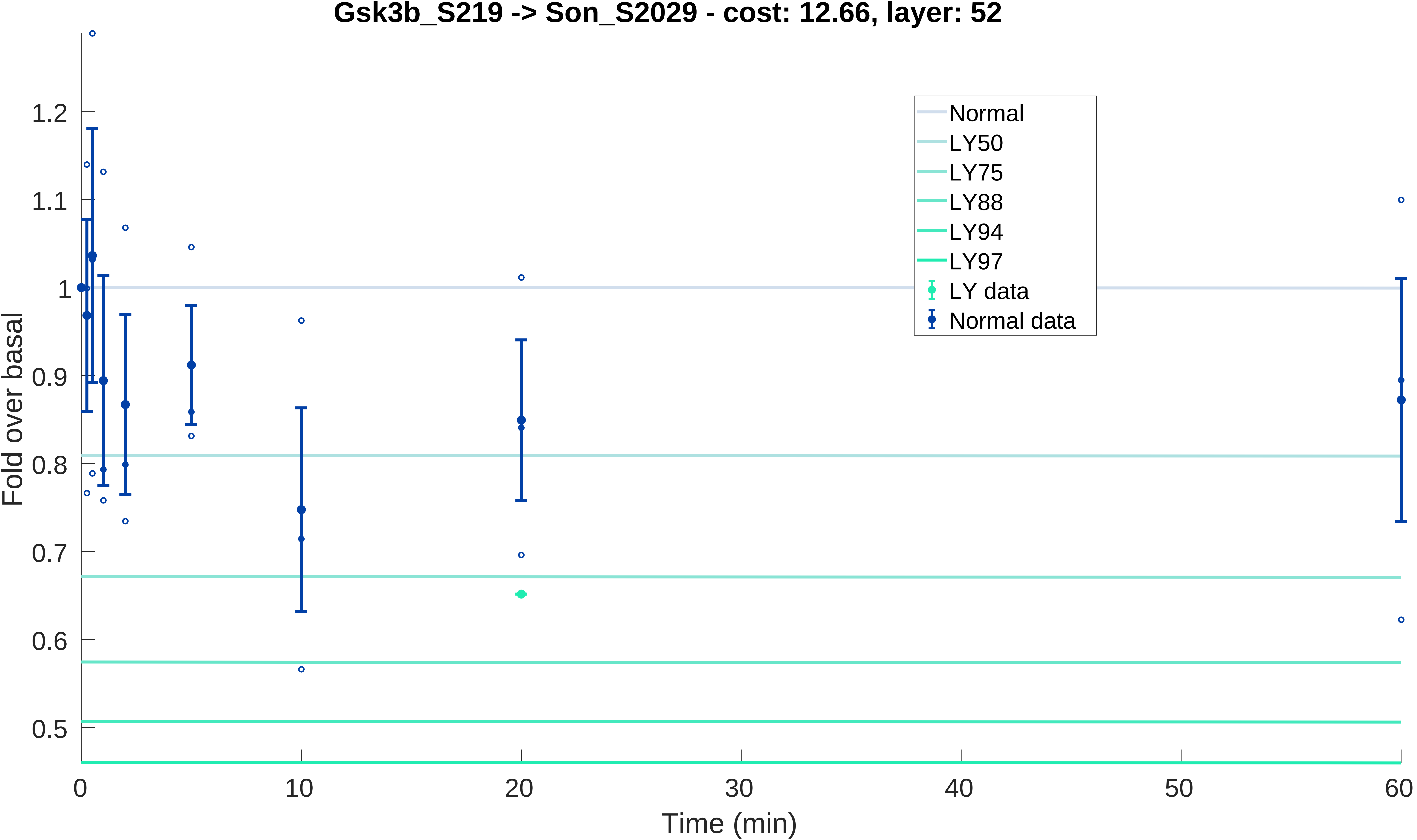
Gsk3b_S219 -> Son_S2029 - cost: 12.66, layer: 52
Fold over basal
Time (min)
1.2
1.1
1
0.9
0.8
0.7
0.6
0.5
0
10
20
30
40
50
60
Normal
LY50
LY75
LY88
LY94
LY97
LY data
Normal data

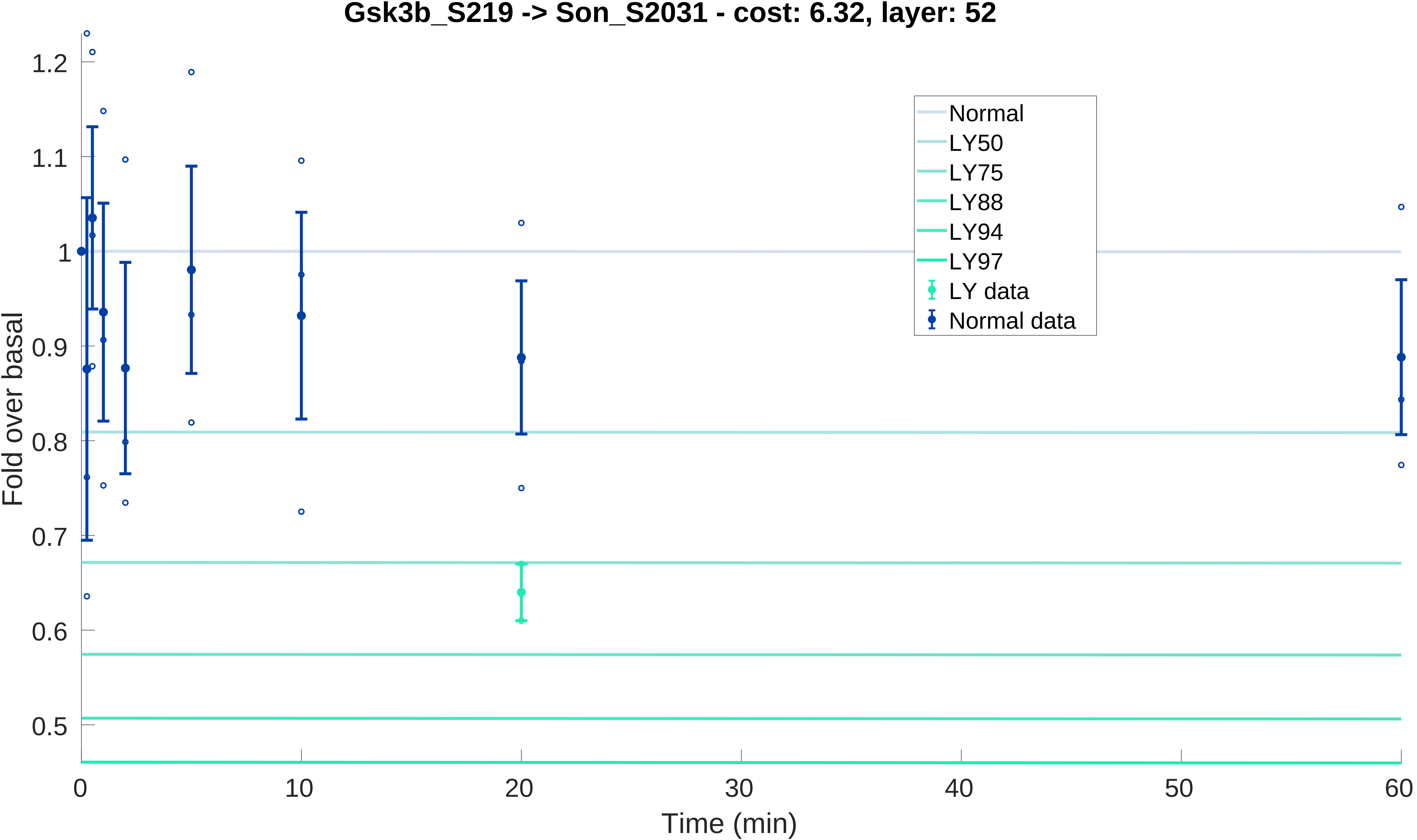

Gsk3b_S219 -> Son_S2031 - cost: 6.32, layer: 52
Fold over basal
Time (min)
1.2
1.1
1
0.9
0.8
0.7
0.6
0.5
0
10
20
30
40
50
60
Normal
LY50
LY75
LY88
LY94
LY97
LY data
Normal data

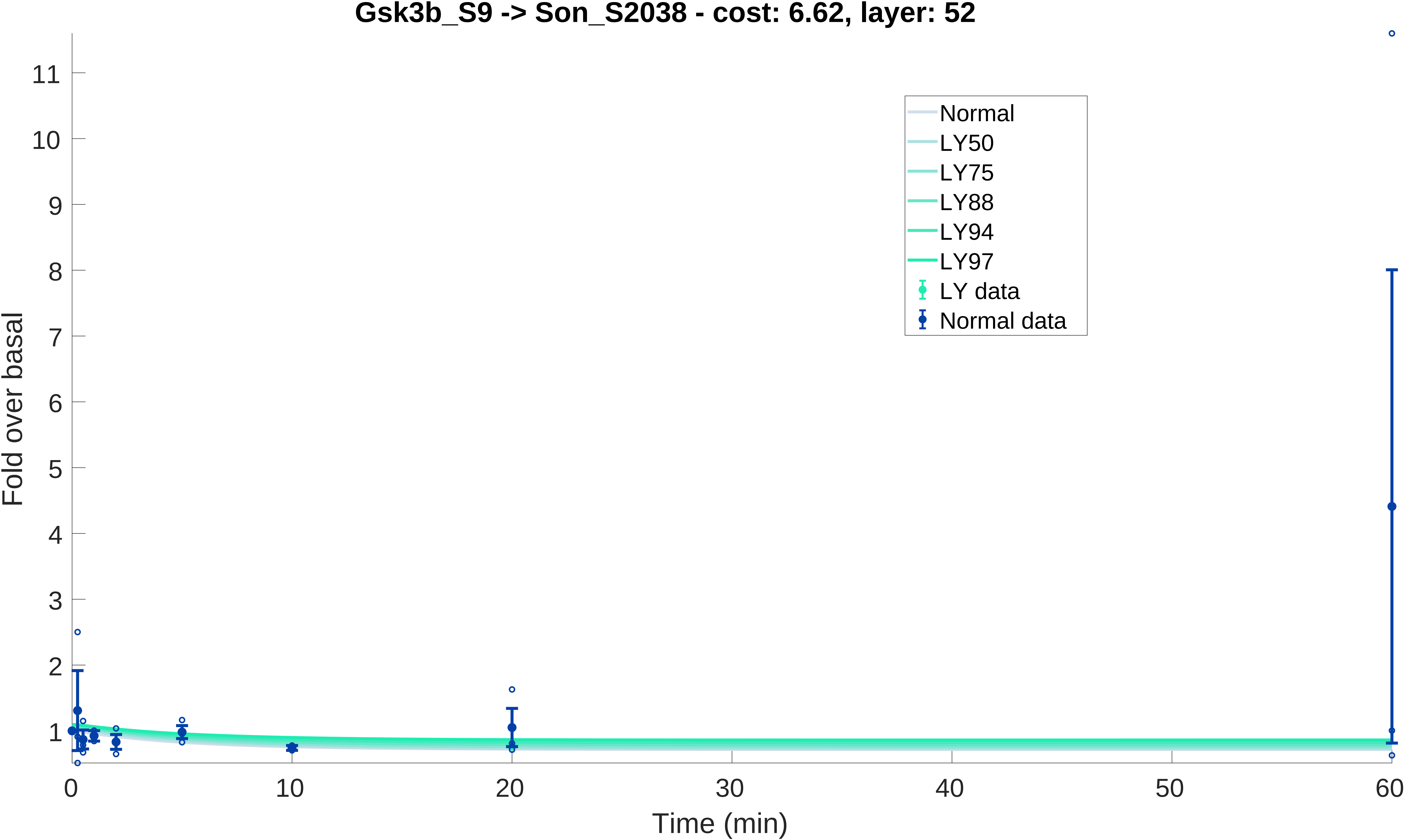

Gsk3b_S9 -> Son_S2038 - cost: 6.62, layer: 52
Fold over basal
Time (min)
Normal
LY50
LY75
LY88
LY94
LY97
LY data
Normal data
0
10
20
30
40
50
60
1
2
3
4
5
6
7
8
9
10
11

Gsk3b_Y216 -> Son_S2047 - cost: 11.25, layer: 52

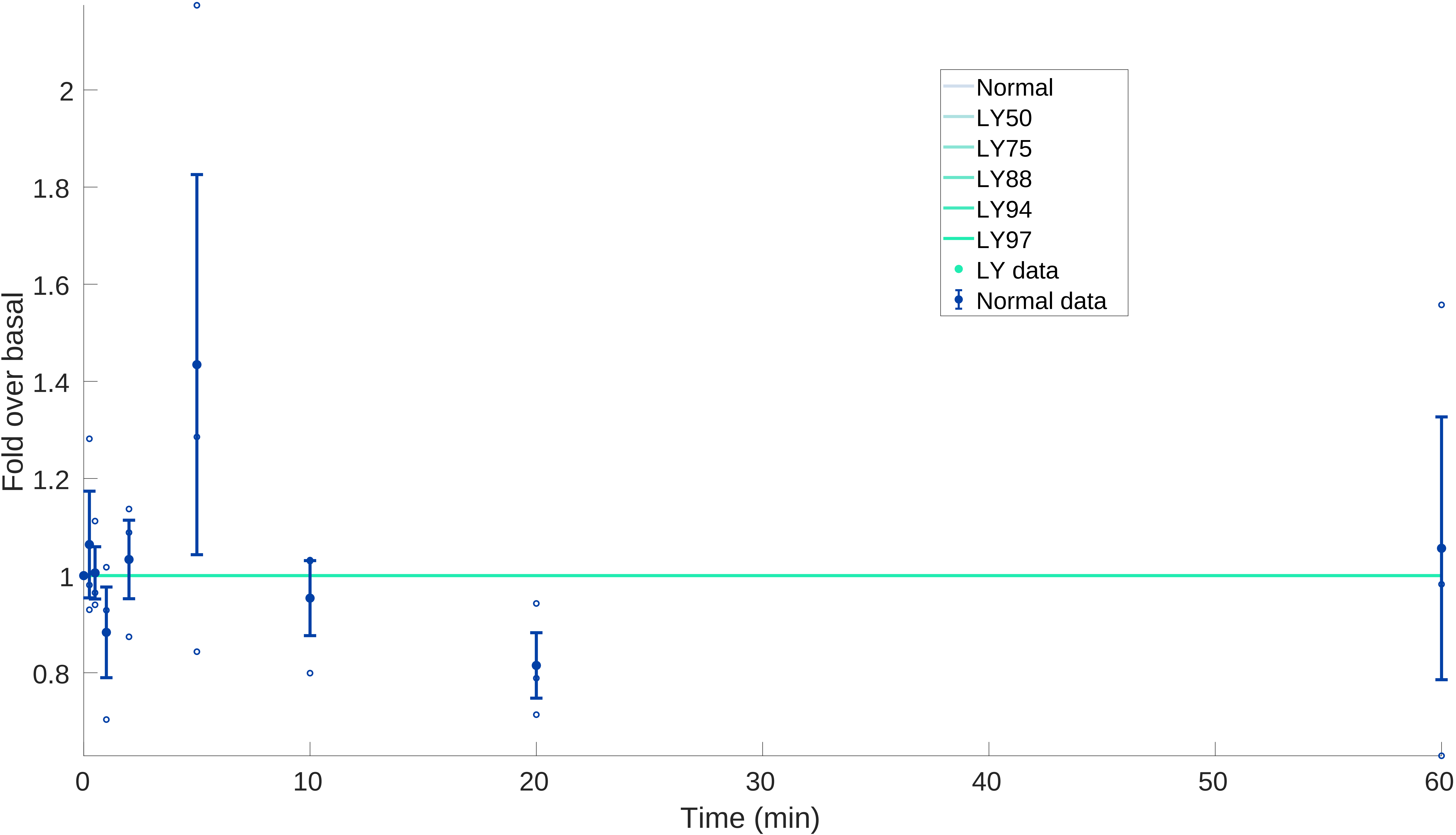

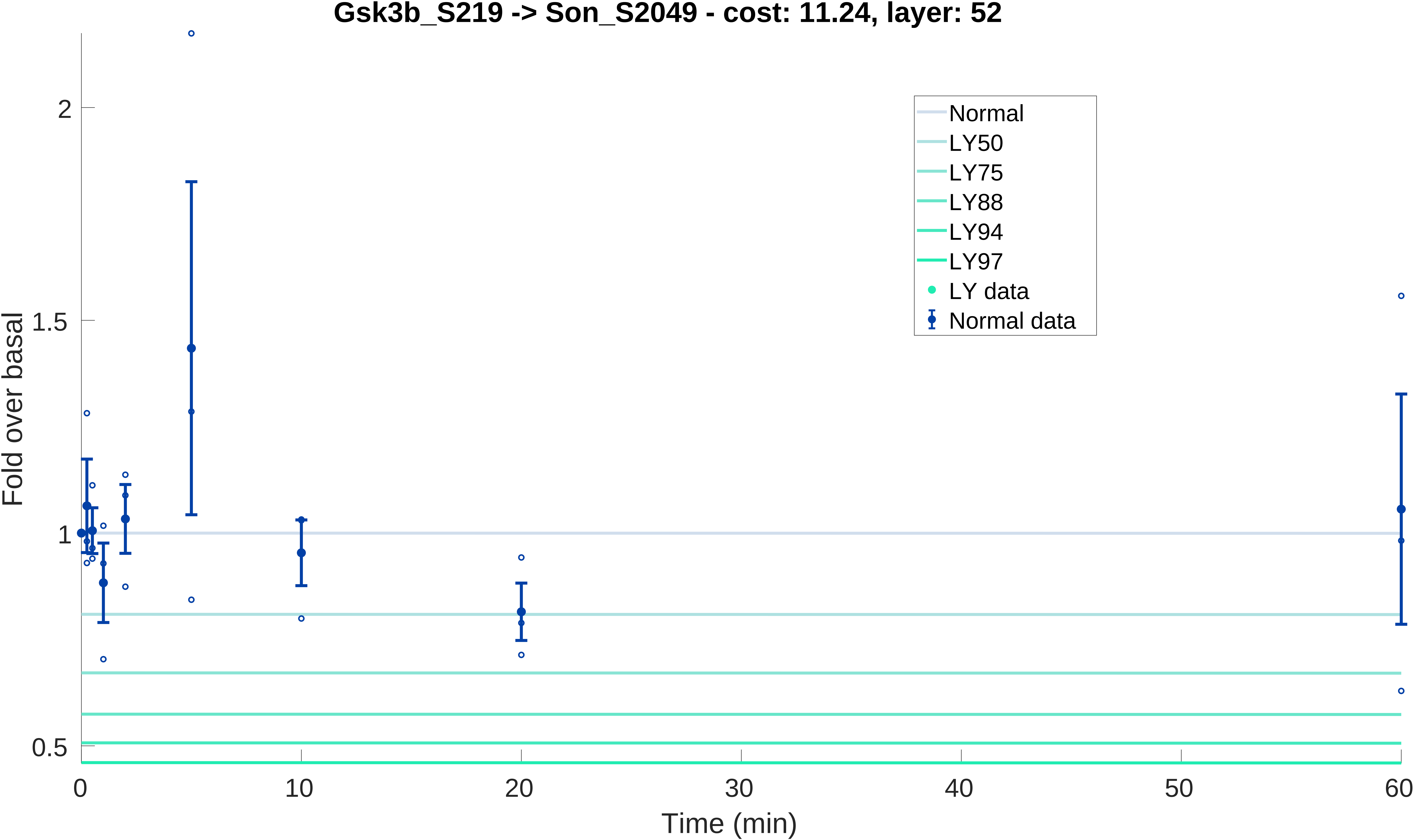

Gsk3b_S219 -> Son_S2049 - cost: 11.24, layer: 52
Normal
LY50
LY75
LY88
LY94
LY97
LY data
Normal data
Fold over basal
2
1.5
1
0.5
0
10
20
30
40
50
60
Time (min)

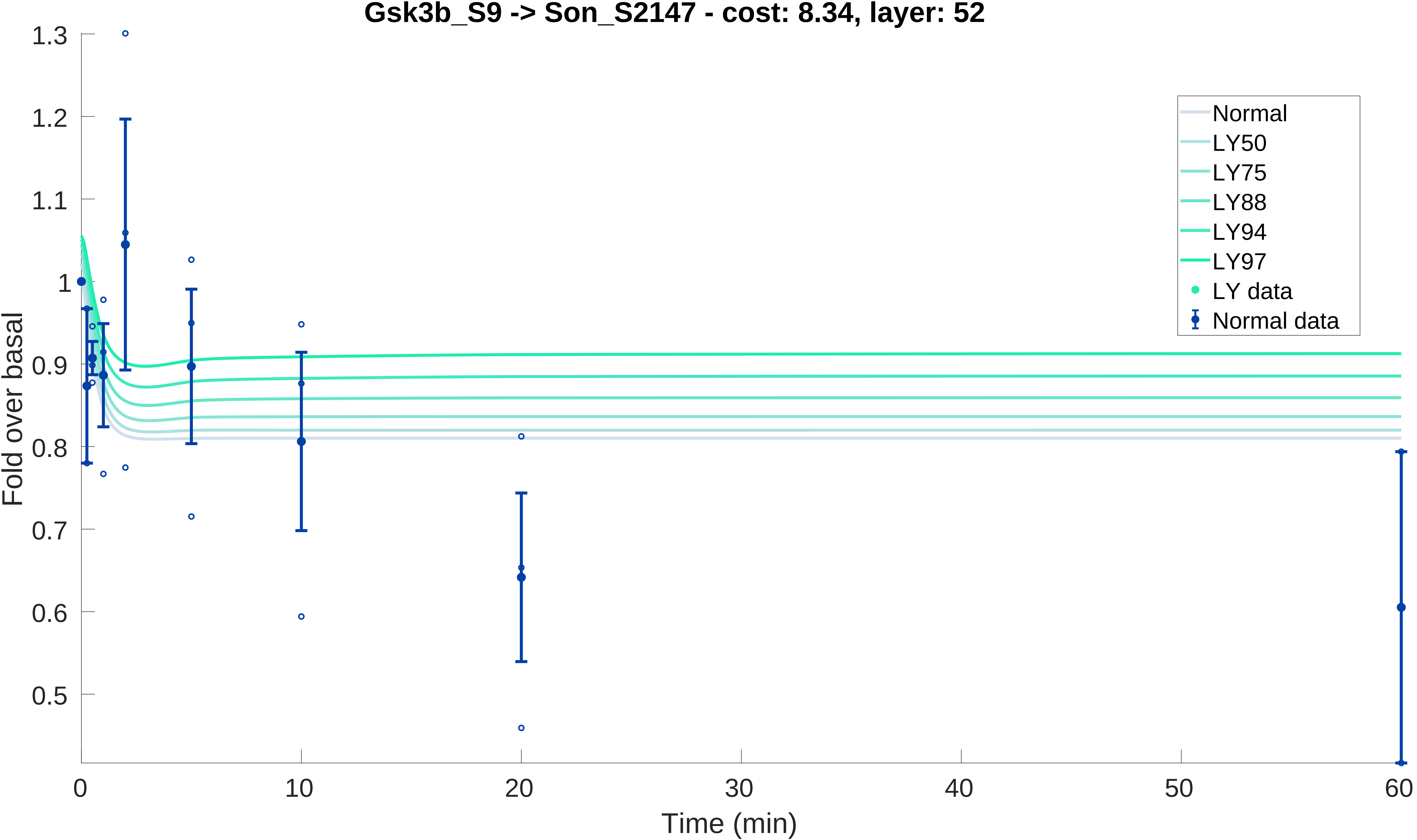

Gsk3b_S9 -> Son_S2147 - cost: 8.34, layer: 52
Fold over basal
Time (min)
1.3
1.2
1.1
1
0.9
0.8
0.7
0.6
0.5
0
10
20
30
40
50
60
Normal
LY50
LY75
LY88
LY94
LY97
LY data
Normal data

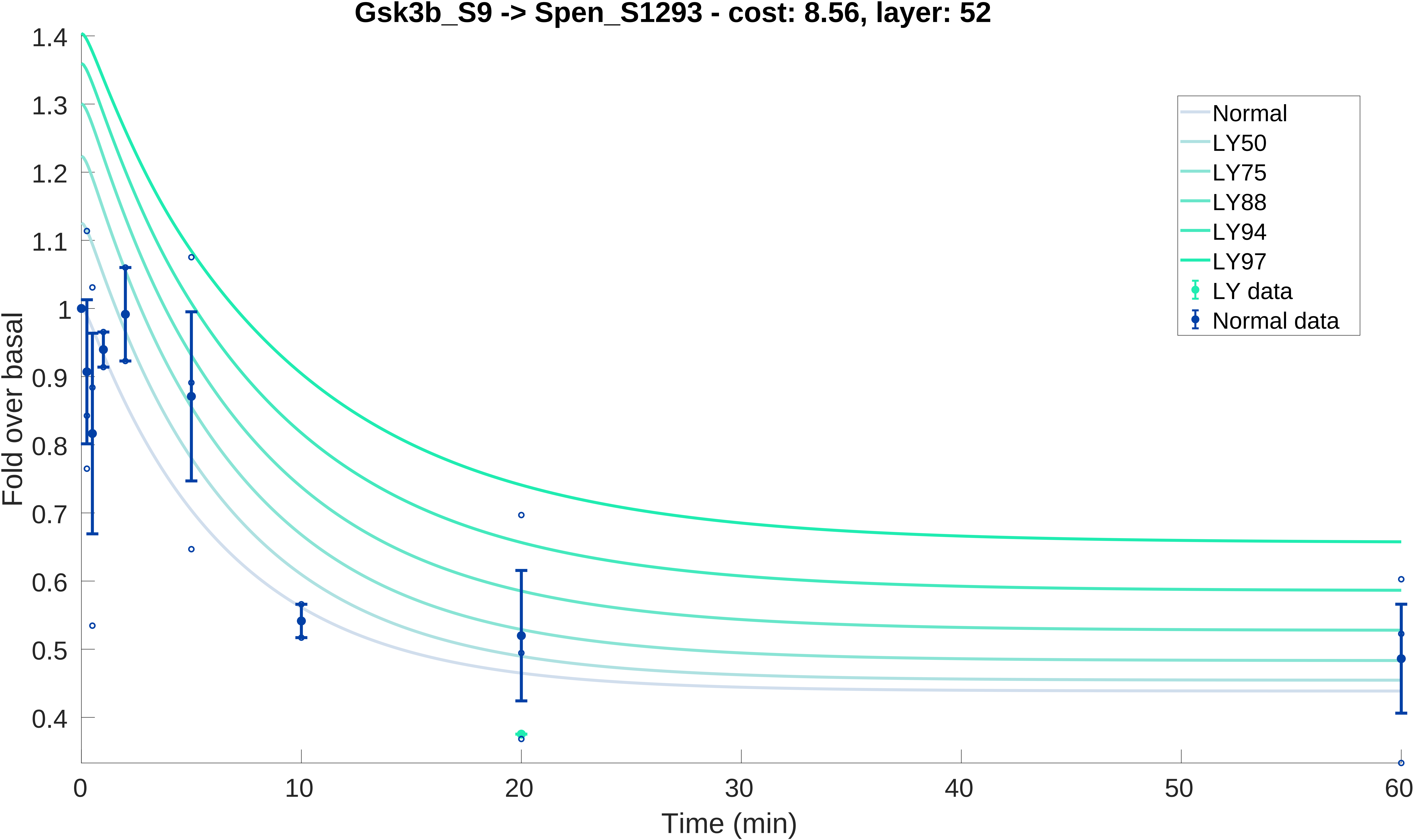
Gsk3b_S9 -> Spen_S1293 - cost: 8.56, layer: 52
Fold over basal
Time (min)
1.4
1.3
1.2
1.1
1
0.9
0.8
0.7
0.6
0.5
0.4
0
10
20
30
40
50
60
Normal
LY50
LY75
LY88
LY94
LY97
LY data
Normal data

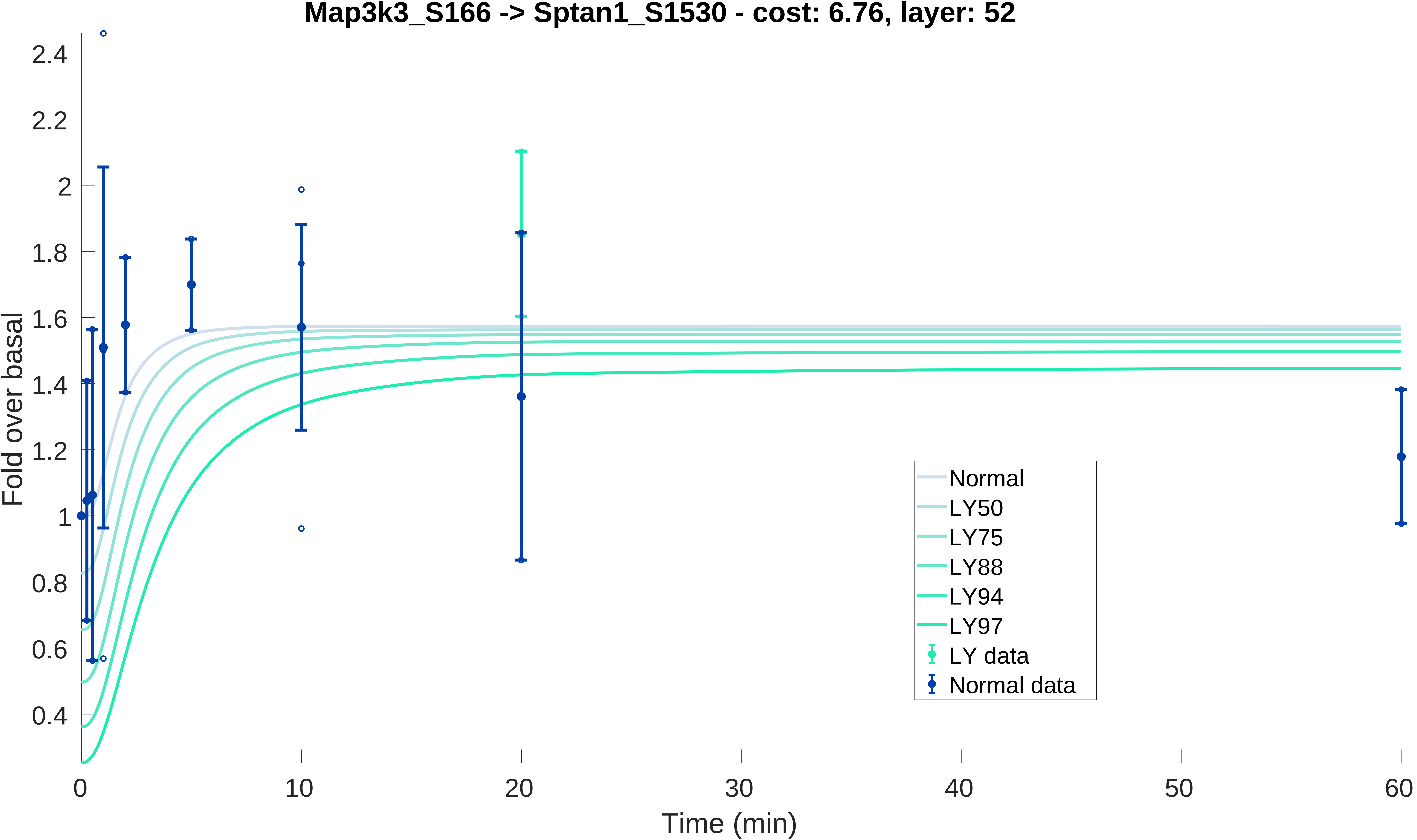
Map3k3_S166 -> Sptan1_S1530 - cost: 6.76, layer: 52
Fold over basal
Time (min)
2.4
2.2
2
1.8
1.6
1.4
1.2
1
0.8
0.6
0.4
0
10
20
30
40
50
60
Normal
LY50
LY75
LY88
LY94
LY97
LY data
Normal data

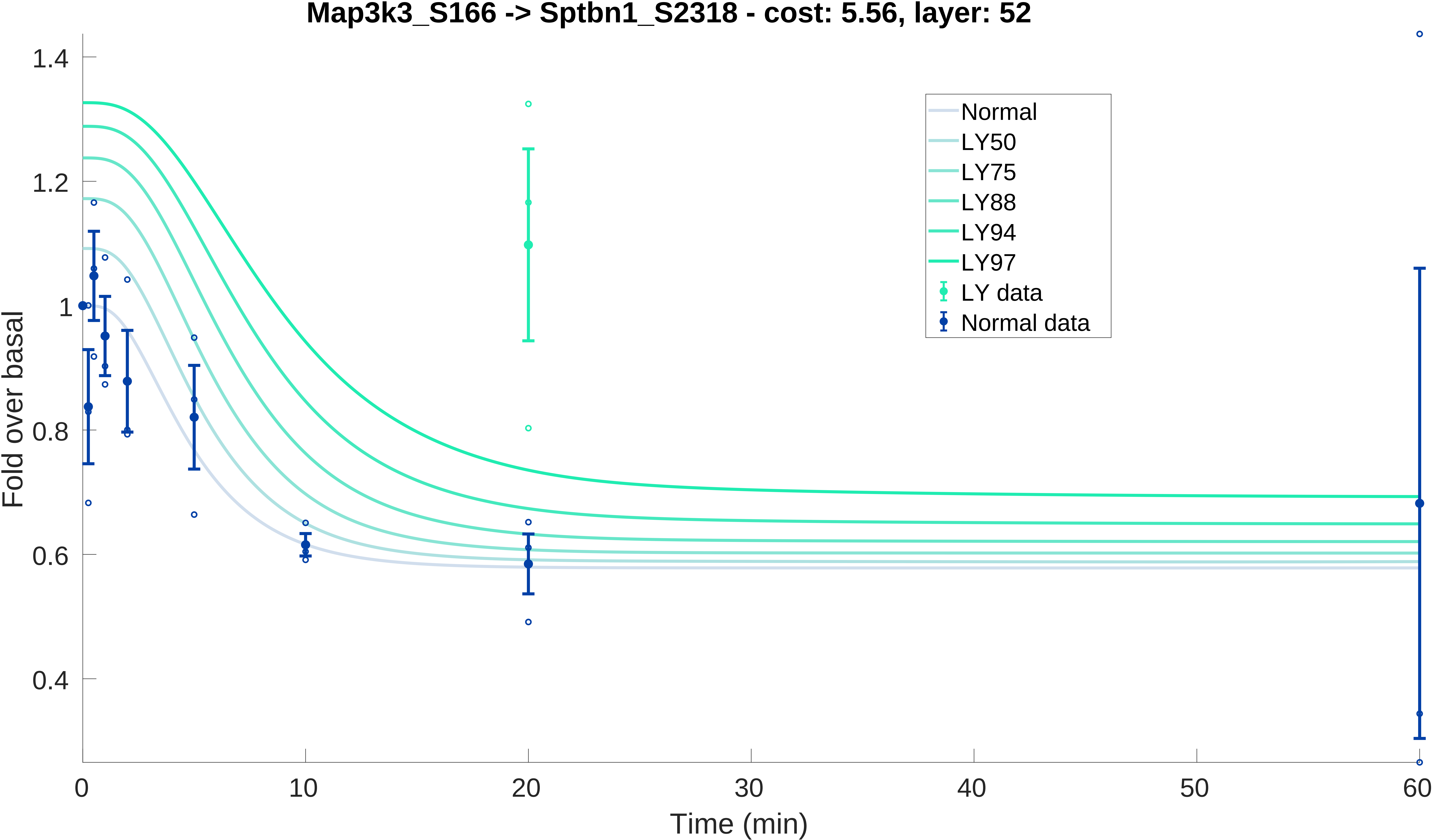
Map3k3_S166 -> Sptbn1_S2318 - cost: 5.56, layer: 52
Fold over basal
Time (min)
0.4
0.6
0.8
1
1.2
1.4
0
10
20
30
40
50
60
Normal
LY50
LY75
LY88
LY94
LY97
LY data
Normal data

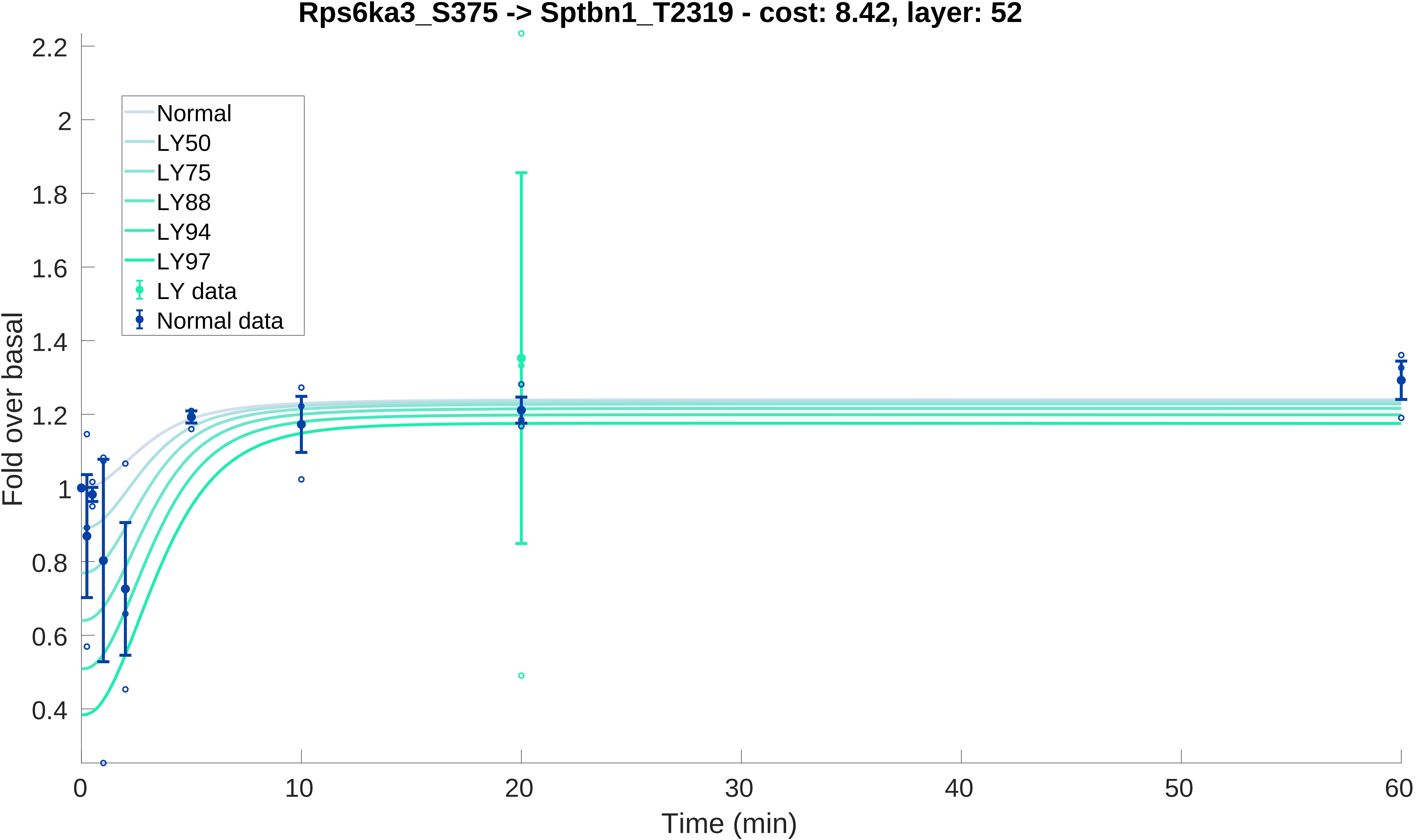

Rps6ka3_S375 -> Sptbn1_T2319 - cost: 8.42, layer: 52
Fold over basal
Time (min)
Normal
LY50
LY75
LY88
LY94
LY97
LY data
Normal data
2.2
2
1.8
1.6
1.4
1.2
1
0.8
0.6
0.4
0
10
20
30
40
50
60

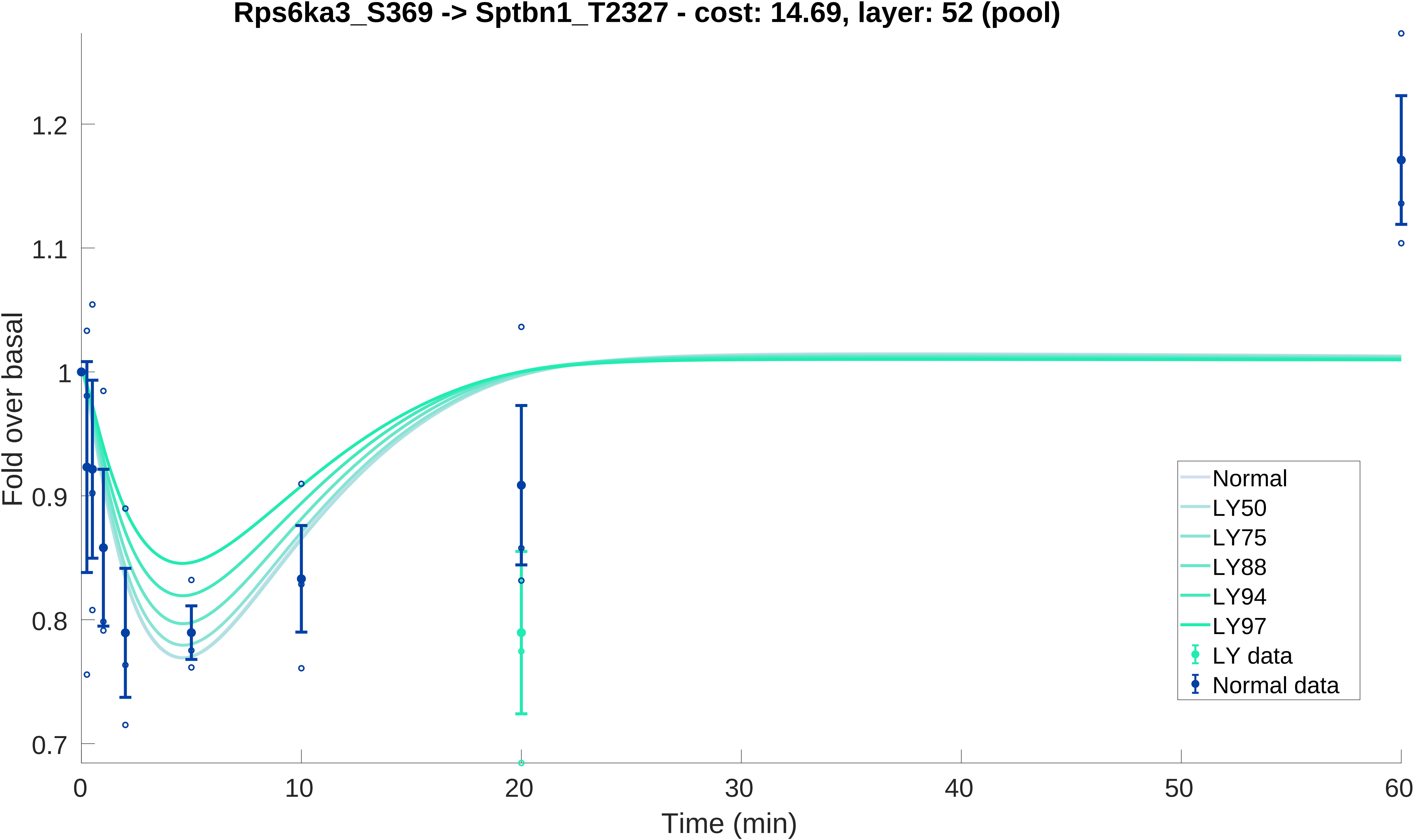

Rps6ka3_S369 -> Sptbn1_T2327 - cost: 14.69, layer: 52 (pool)
Fold over basal
1.2
1.1
1
0.9
0.8
0.7
0
10
20
30
40
50
60
Time (min)
Normal
LY50
LY75
LY88
LY94
LY97
LY data
Normal data

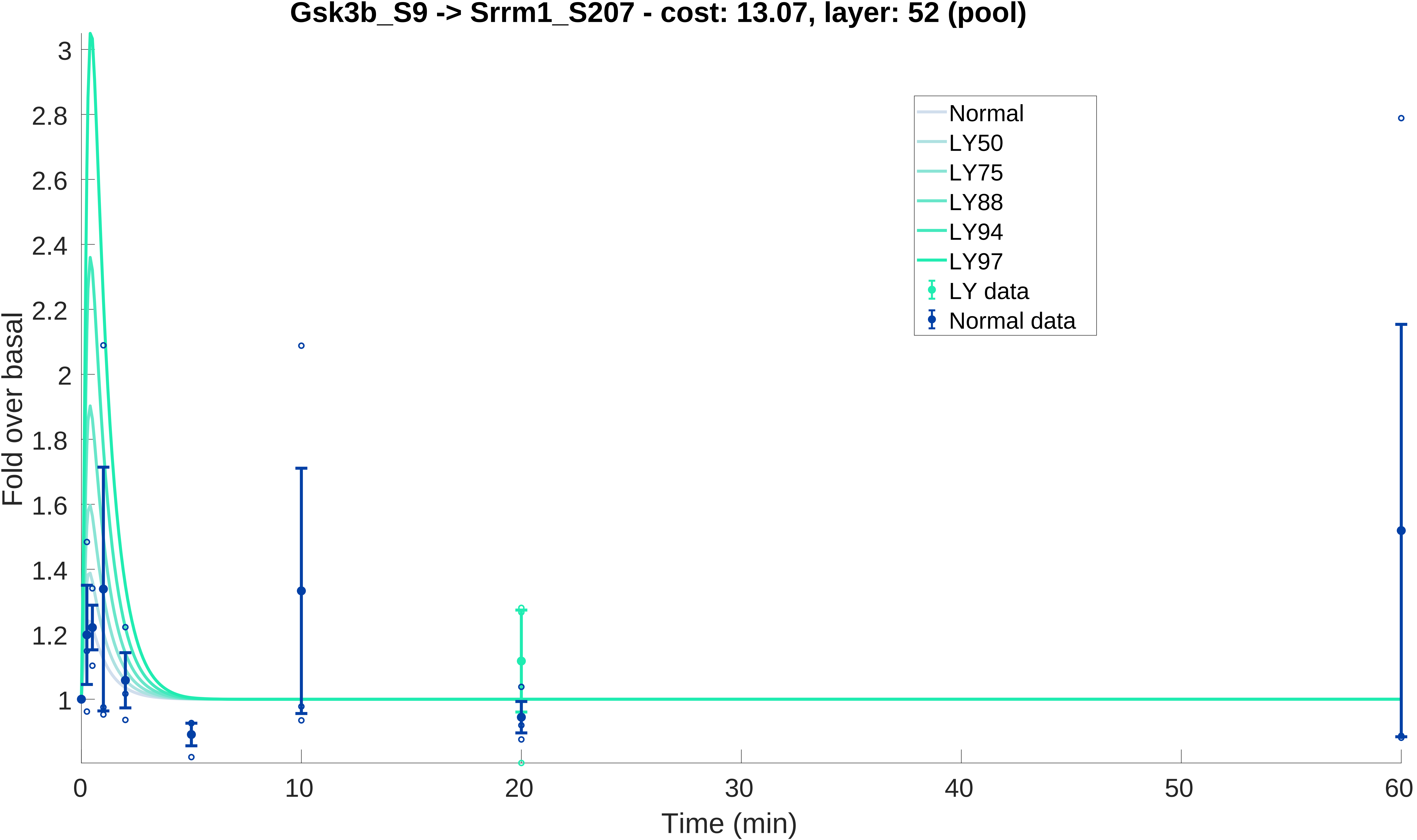
Gsk3b_S9 -> Srrm1_S207 - cost: 13.07, layer: 52 (pool)
Fold over basal
Time (min)
3
2.8
2.6
2.4
2.2
2
1.8
1.6
1.4
1.2
1
0
10
20
30
40
50
60
Normal
LY50
LY75
LY88
LY94
LY97
LY data
Normal data

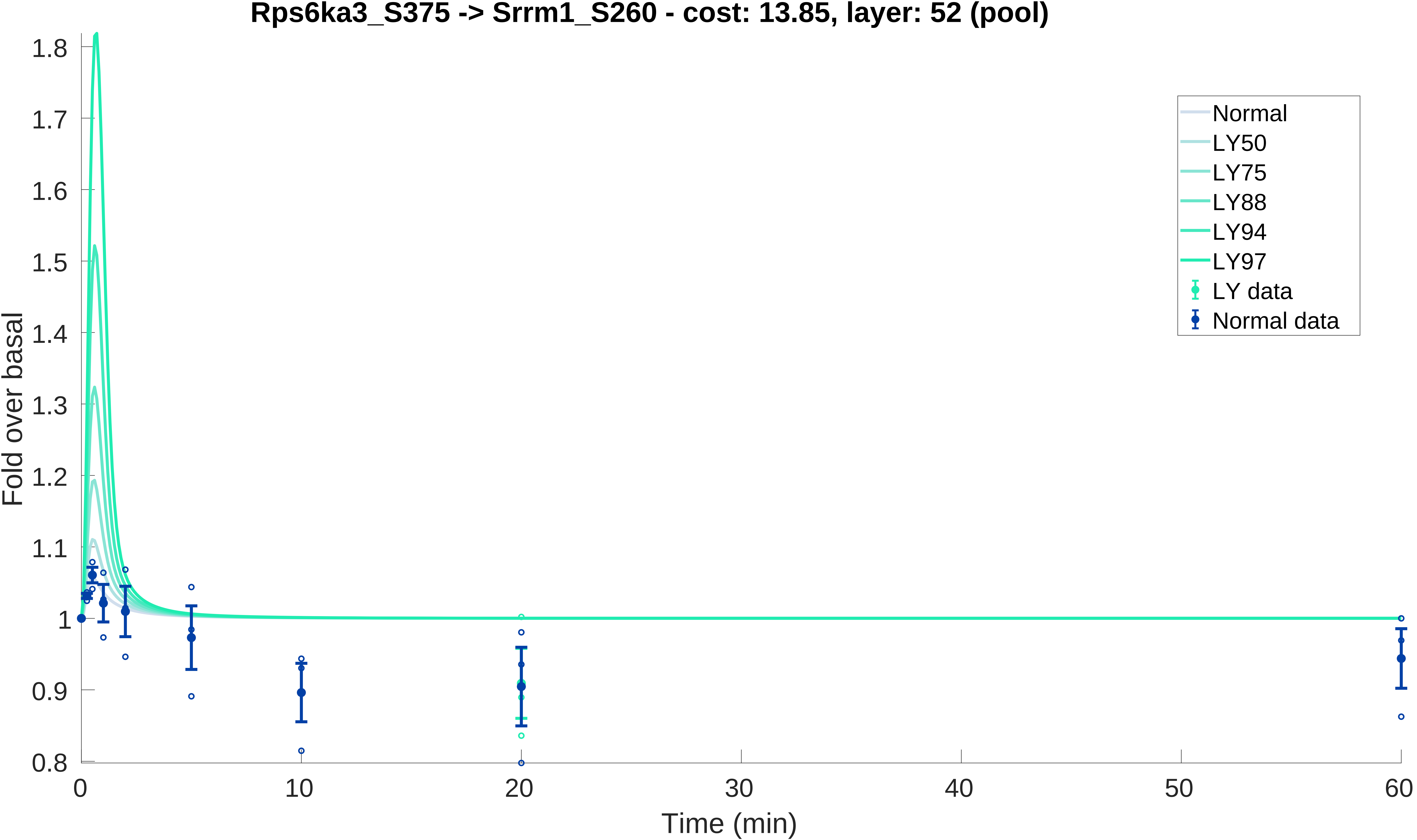
Rps6ka3_S375 -> Srrm1_S260 - cost: 13.85, layer: 52 (pool)
Fold over basal
1.8
1.7
1.6
1.5
1.4
1.3
1.2
1.1
1
0.9
0.8
0
10
20
30
40
50
60
Time (min)
Normal
LY50
LY75
LY88
LY94
LY97
LY data
Normal data

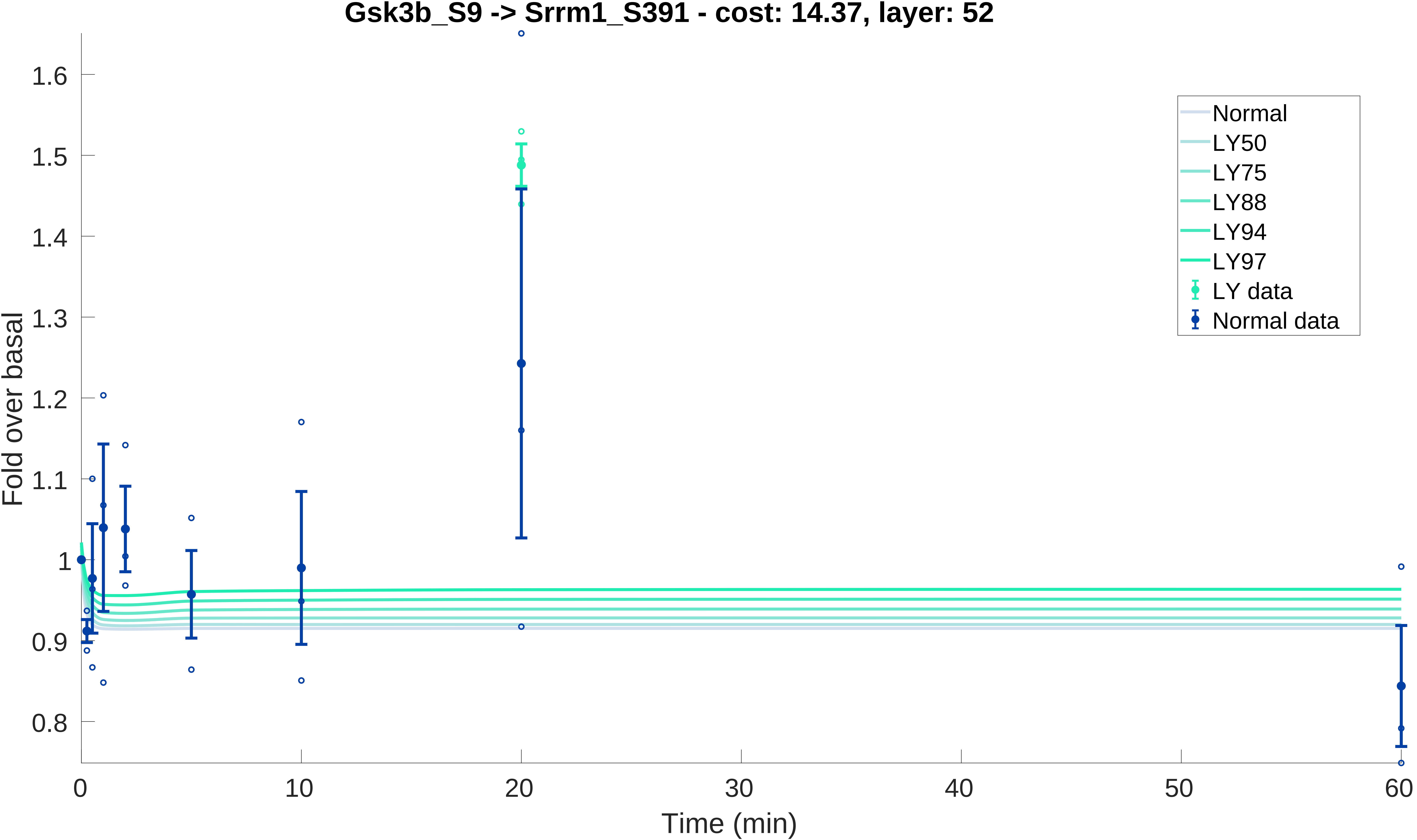

Gsk3b_S9 -> Srrm1_S391 - cost: 14.37, layer: 52
Fold over basal
Time (min)
0.8
0.9
1
1.1
1.2
1.3
1.4
1.5
1.6
0
10
20
30
40
50
60
Normal
LY50
LY75
LY88
LY94
LY97
LY data
Normal data

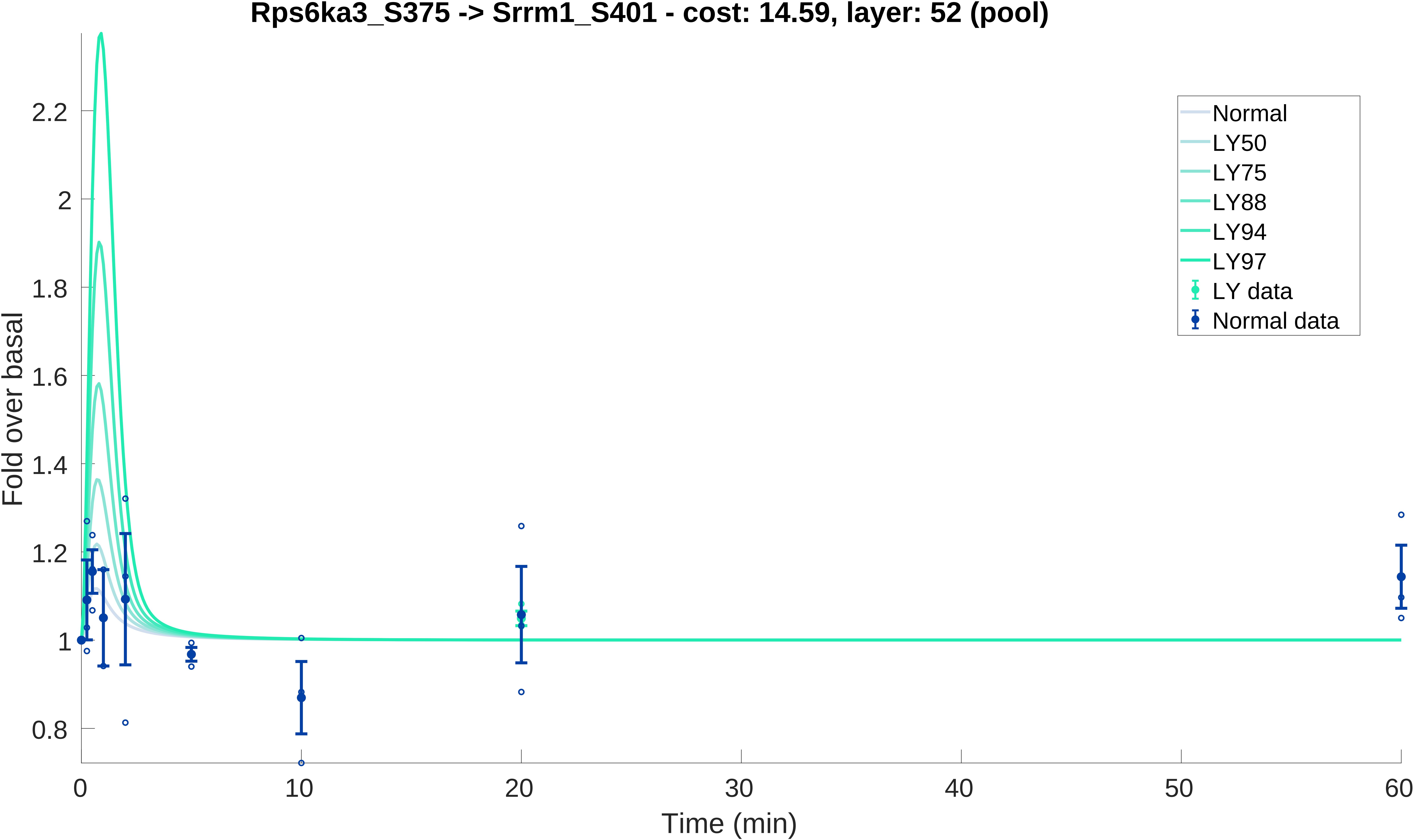
Rps6ka3_S375 -> Srrm1_S401 - cost: 14.59, layer: 52 (pool)
Fold over basal
2.2
2
1.8
1.6
1.4
1.2
1
0.8
0
10
20
30
40
50
60
Time (min)
Normal
LY50
LY75
LY88
LY94
LY97
LY data
Normal data

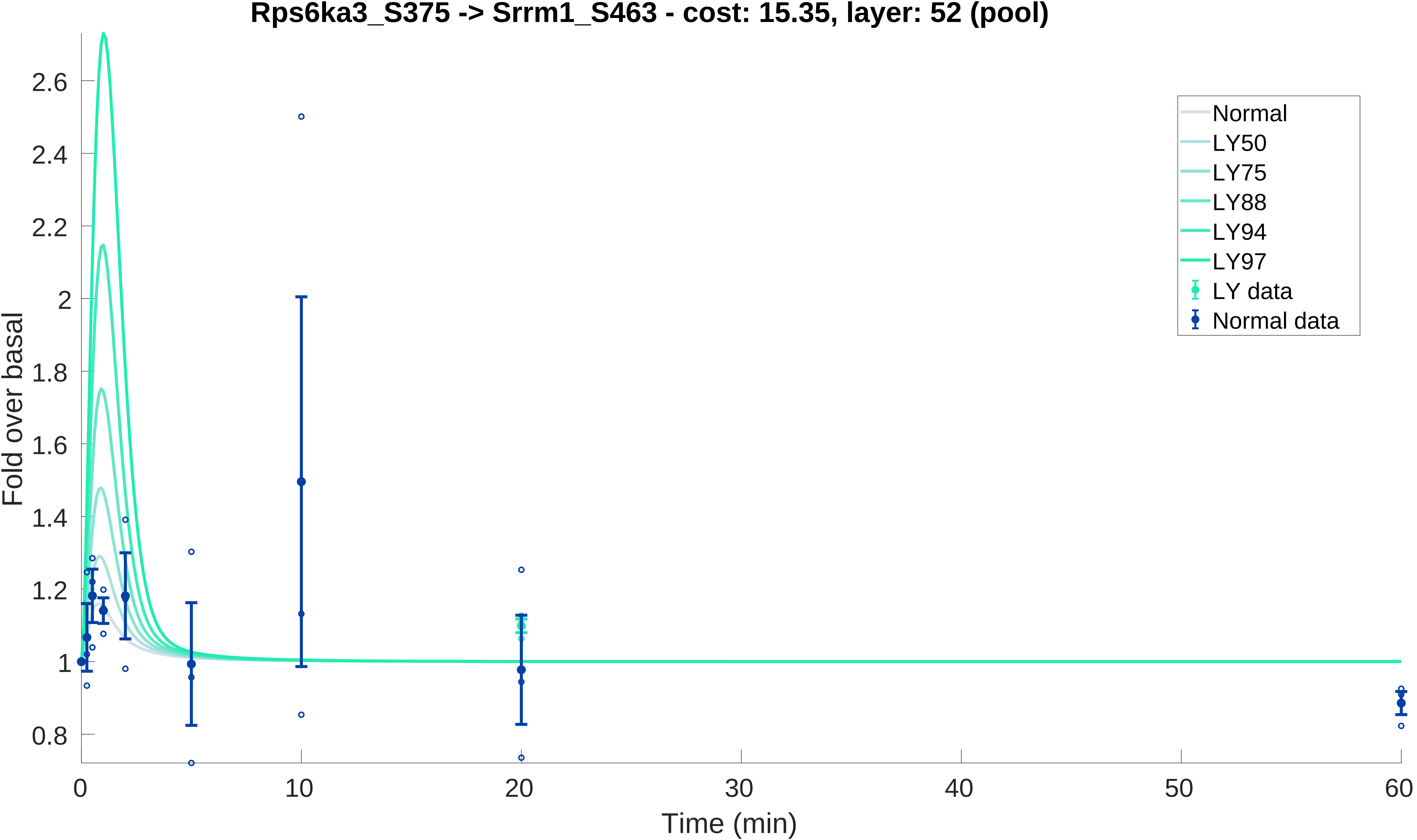

Rps6ka3_S375 -> Srrm1_S463 - cost: 15.35, layer: 52 (pool)
Fold over basal
Time (min)
0.8
1
1.2
1.4
1.6
1.8
2
2.2
2.4
2.6
0
10
20
30
40
50
60
Normal
LY50
LY75
LY88
LY94
LY97
LY data
Normal data

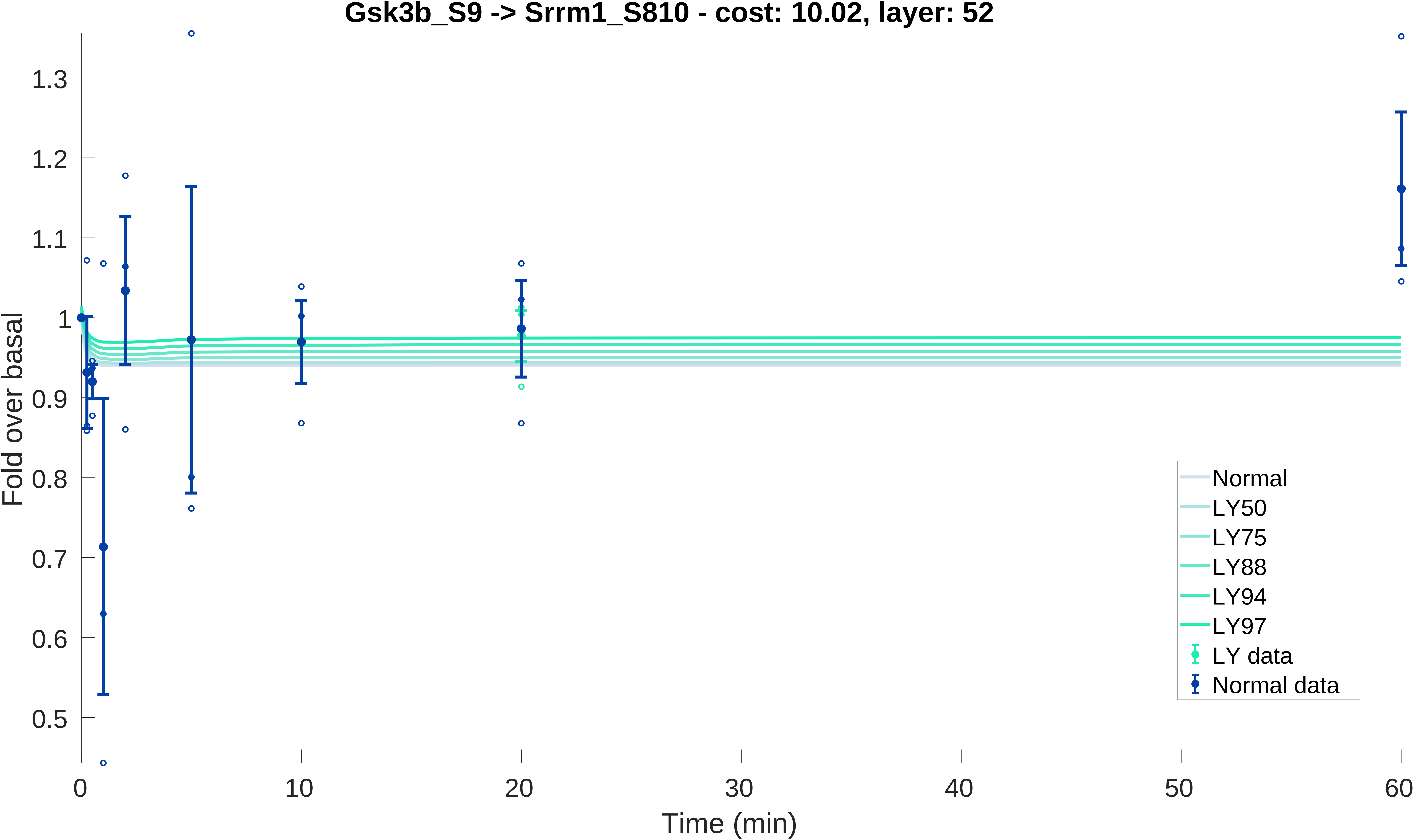

Gsk3b_S9 -> Srrm1_S810 - cost: 10.02, layer: 52
Fold over basal
Time (min)
Normal
LY50
LY75
LY88
LY94
LY97
LY data
Normal data

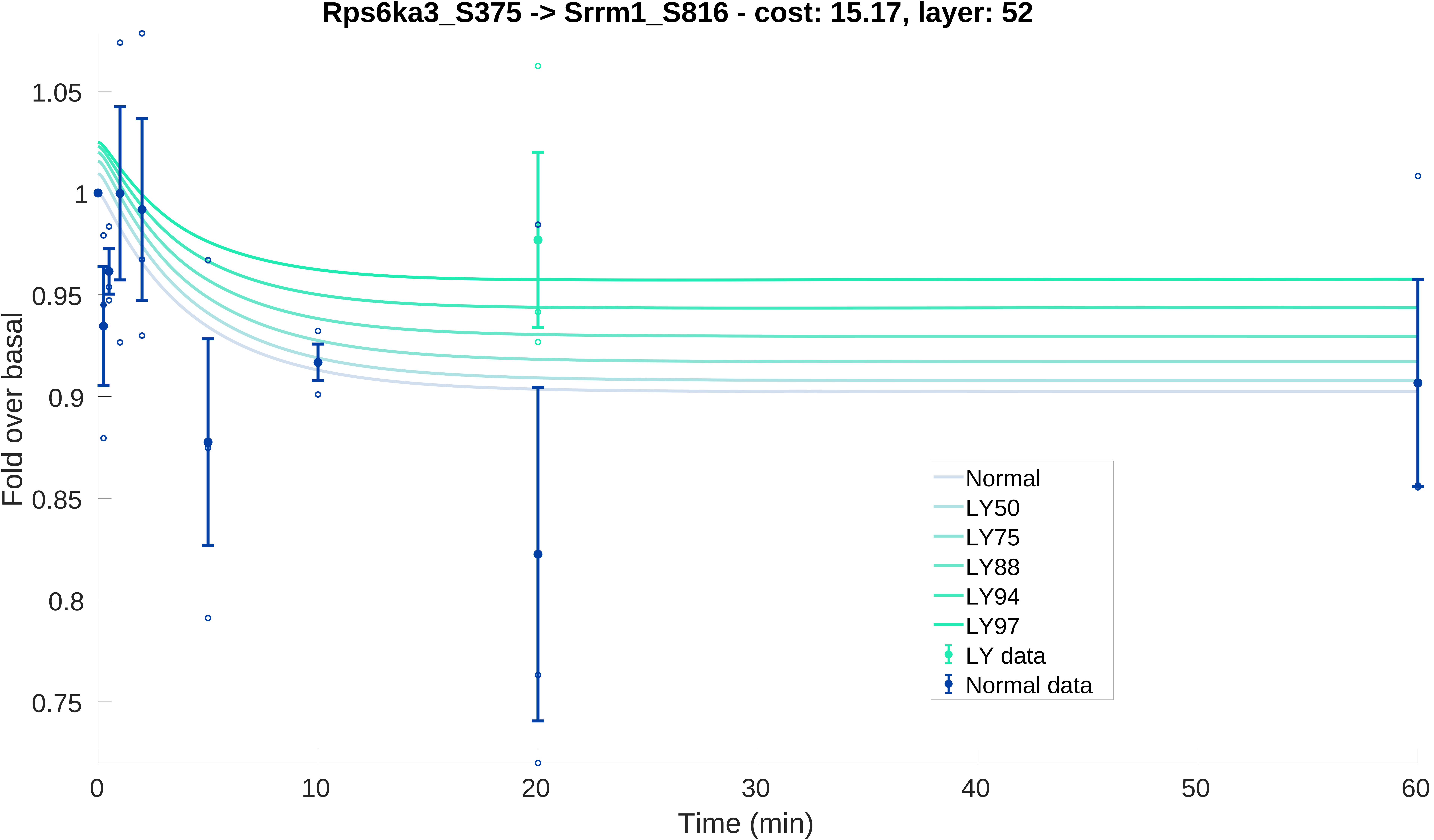
Rps6ka3_S375 -> Srrm1_S816 - cost: 15.17, layer: 52
Fold over basal
1.05
1
0.95
0.9
0.85
0.8
0.75
0
10
20
30
40
50
60
Time (min)
Normal
LY50
LY75
LY88
LY94
LY97
LY data
Normal data

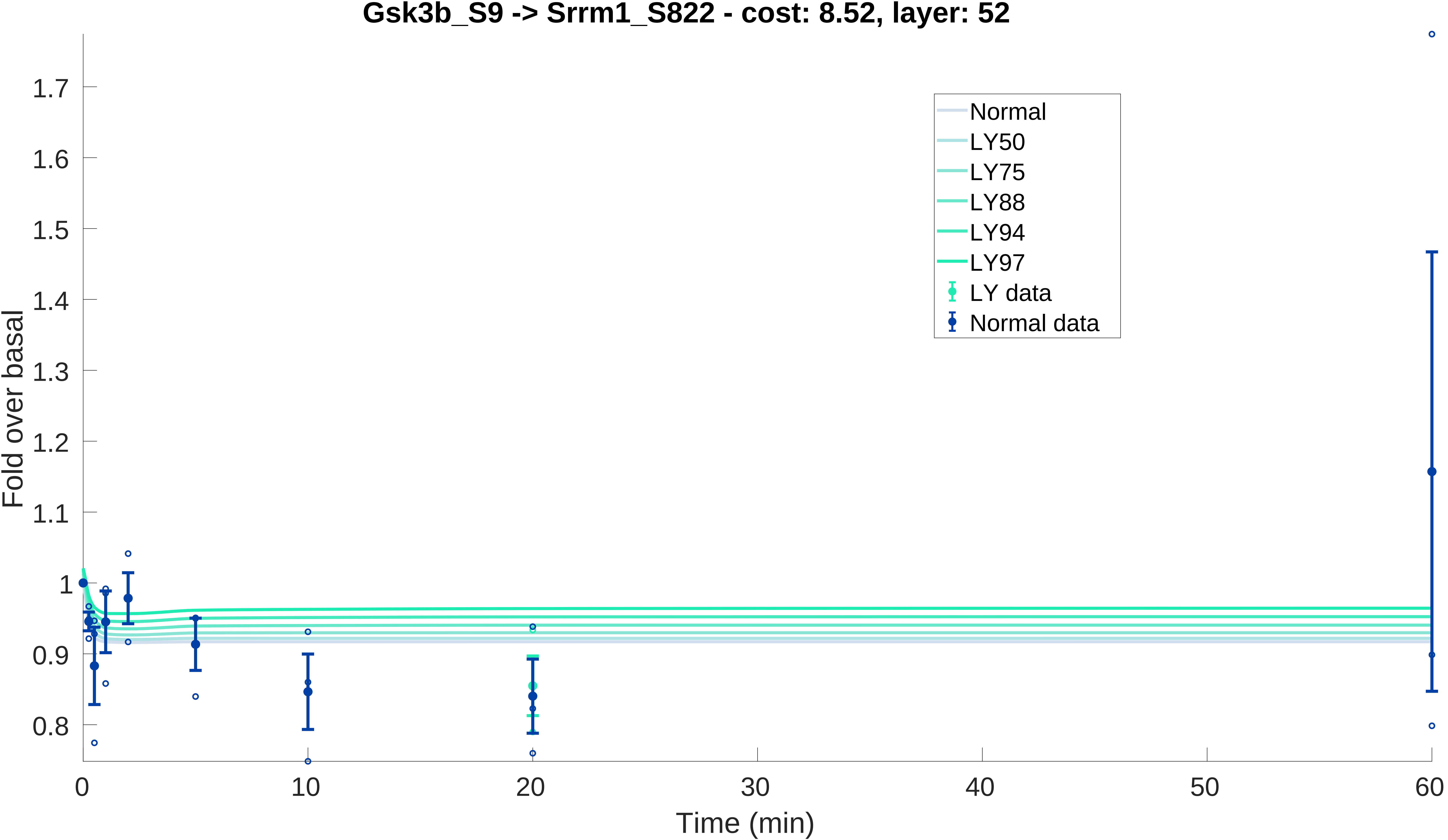

Gsk3b_S9 -> Srrm1_S822 - cost: 8.52, layer: 52
Fold over basal
Time (min)
0.8
0.9
1
1.1
1.2
1.3
1.4
1.5
1.6
1.7
0
10
20
30
40
50
60
Normal
LY50
LY75
LY88
LY94
LY97
LY data
Normal data

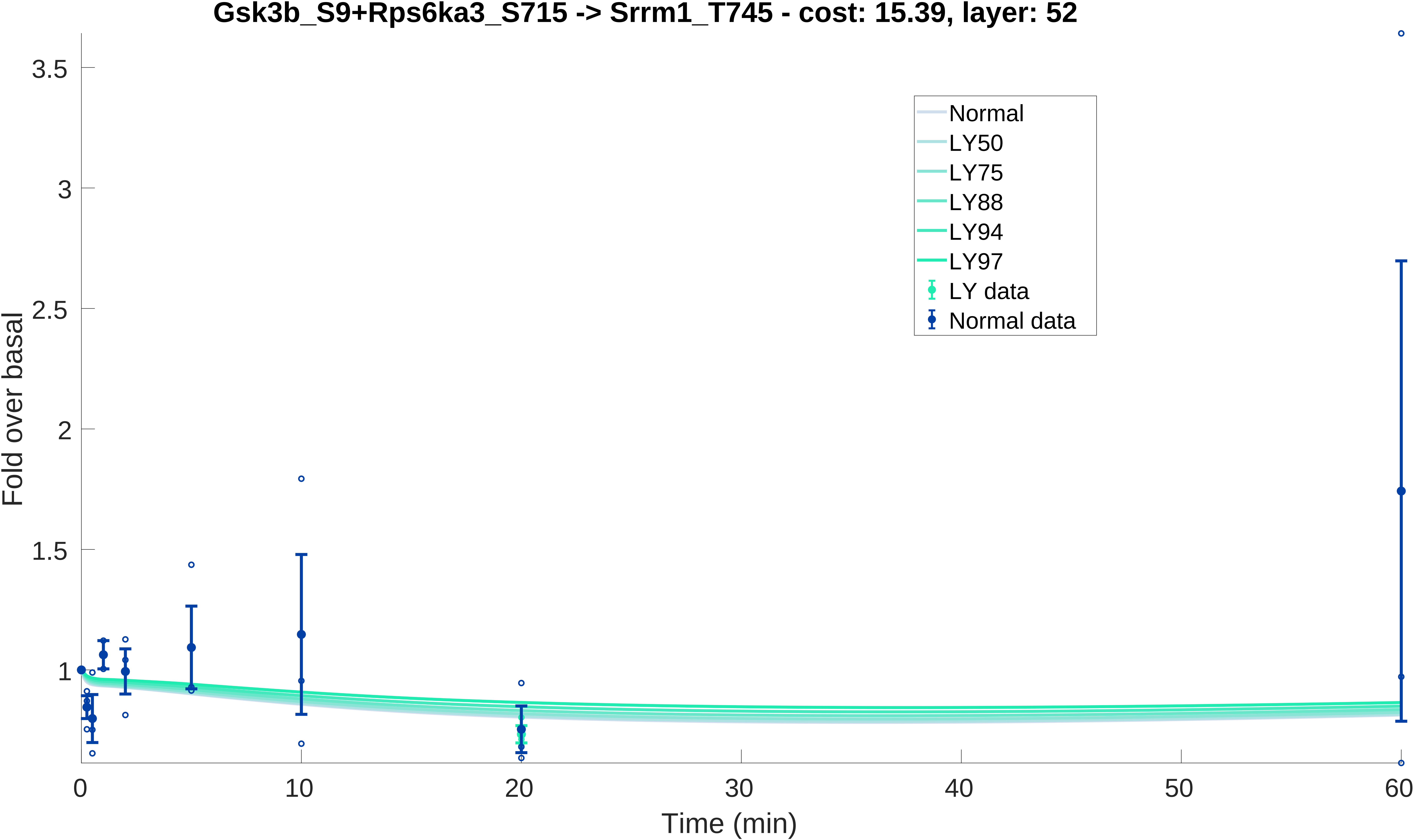
Gsk3b_S9+Rps6ka3_S715 -> Srrm1_T745 - cost: 15.39, layer: 52
Fold over basal
3.5
3
2.5
2
1.5
1
0
10
20
30
40
50
60
Time (min)
Normal
LY50
LY75
LY88
LY94
LY97
LY data
Normal data

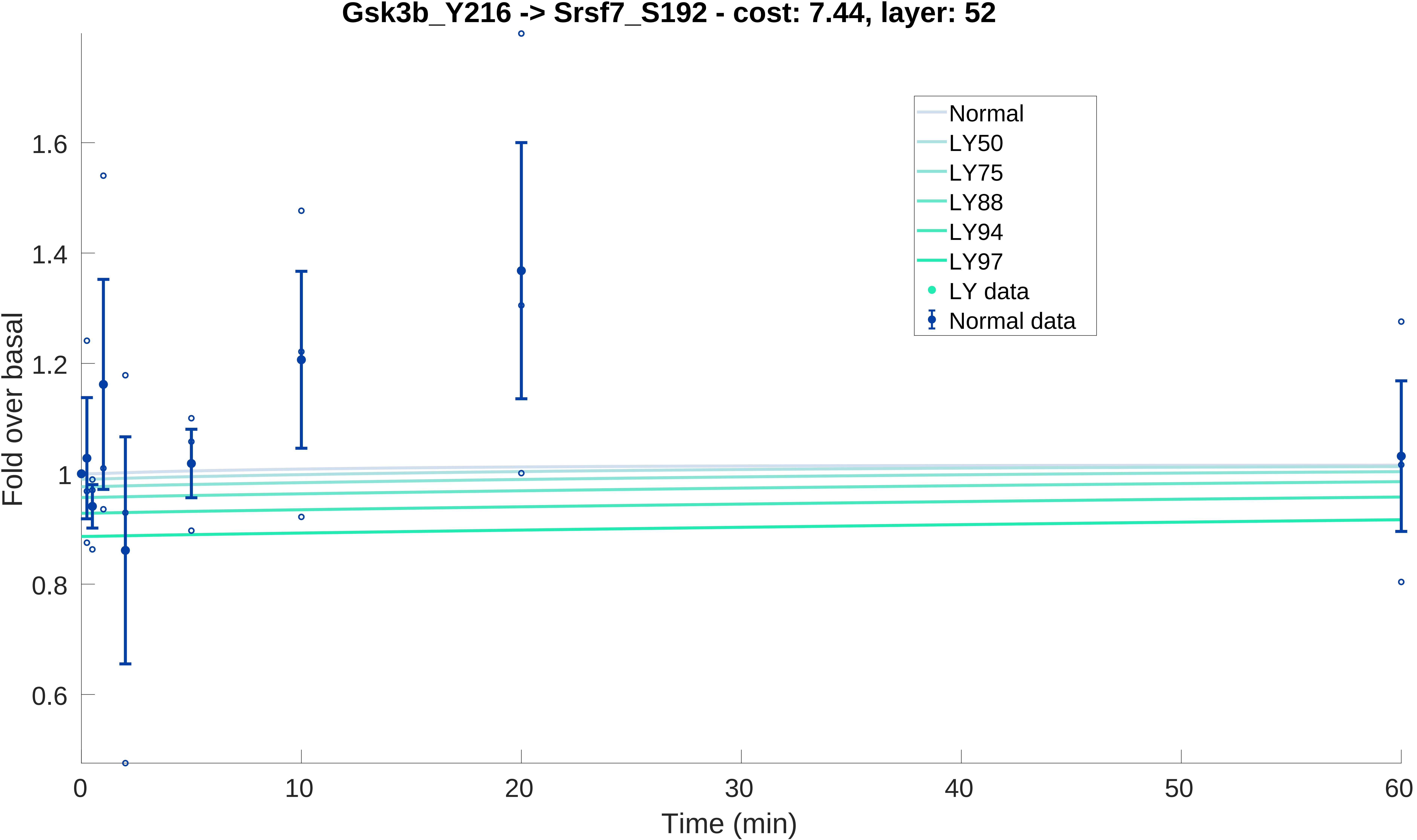

Gsk3b_Y216 -> Srsf7_S192 - cost: 7.44, layer: 52
Fold over basal
Time (min)
0.6
0.8
1
1.2
1.4
1.6
0
10
20
30
40
50
60
Normal
LY50
LY75
LY88
LY94
LY97
LY data
Normal data

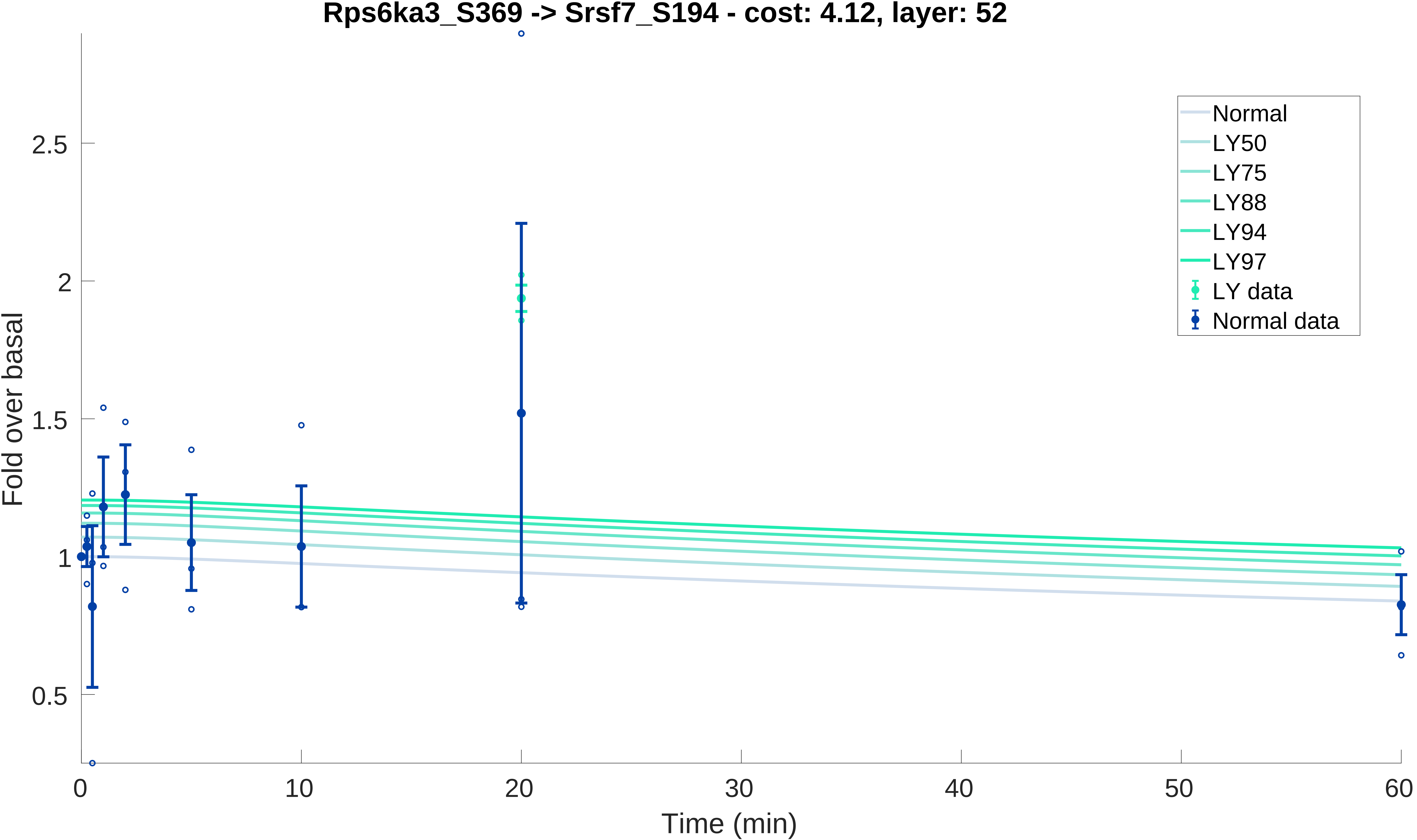

Rps6ka3_S369 -> Srsf7_S194 - cost: 4.12, layer: 52
Fold over basal
Time (min)
2.5
2
1.5
1
0.5
0
10
20
30
40
50
60
Normal
LY50
LY75
LY88
LY94
LY97
LY data
Normal data

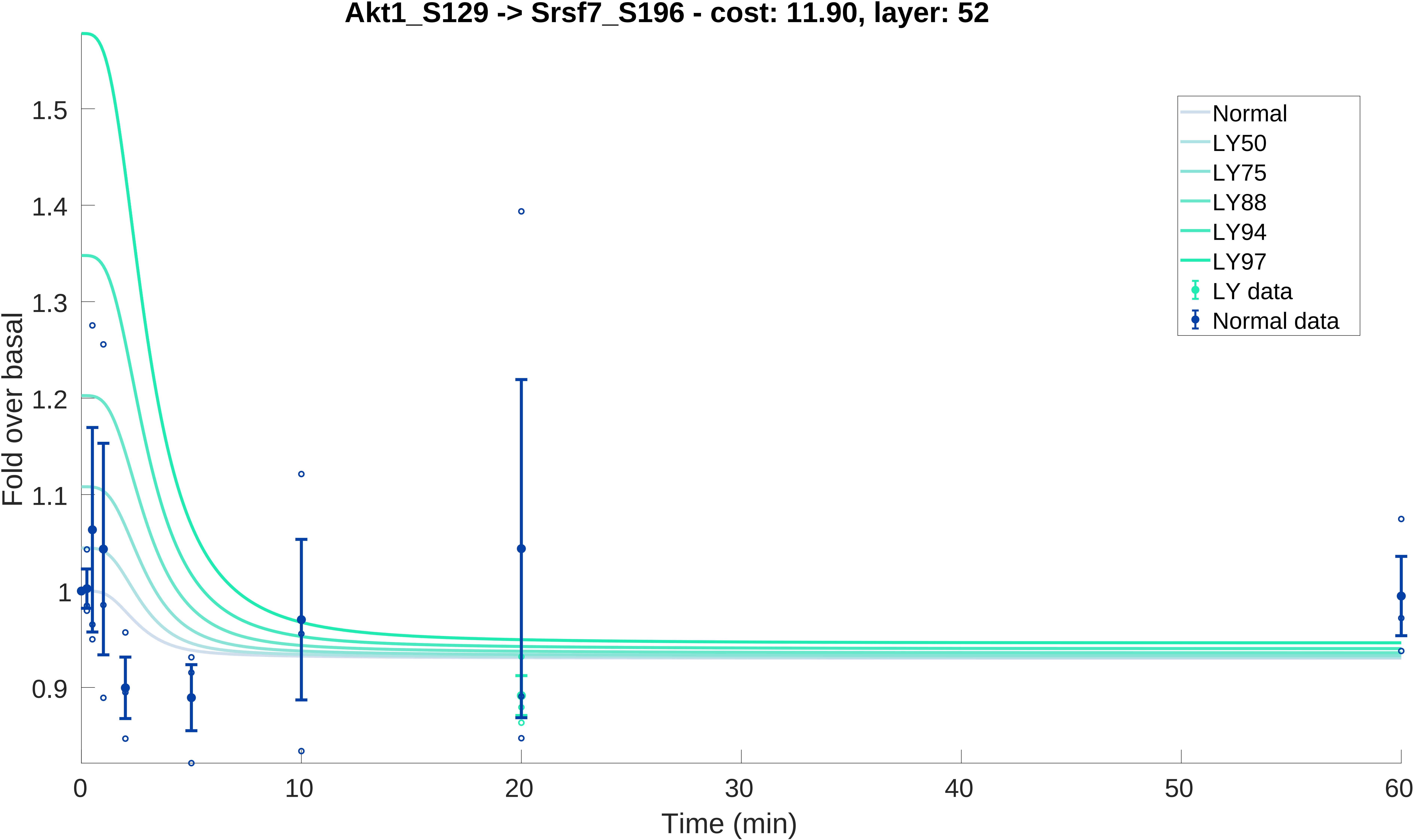

Akt1_S129 -> Srsf7_S196 - cost: 11.90, layer: 52
Fold over basal
1.5
1.4
1.3
1.2
1.1
1
0.9
0
10
20
30
40
50
60
Time (min)
Normal
LY50
LY75
LY88
LY94
LY97
LY data
Normal data

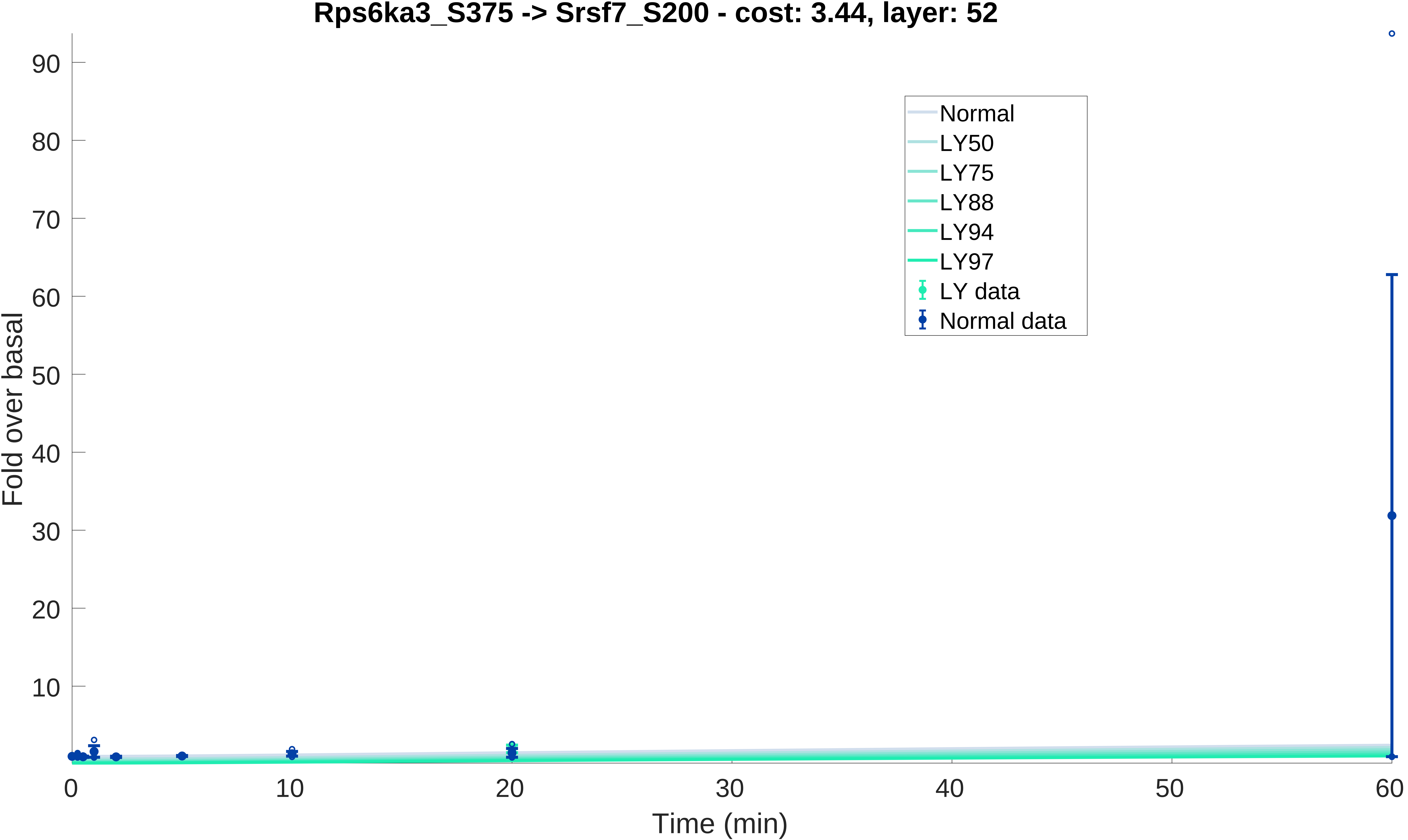

Rps6ka3_S375 -> Srsf7_S200 - cost: 3.44, layer: 52
Fold over basal
Time (min)
Normal
LY50
LY75
LY88
LY94
LY97
LY data
Normal data
0
10
20
30
40
50
60
70
80
90

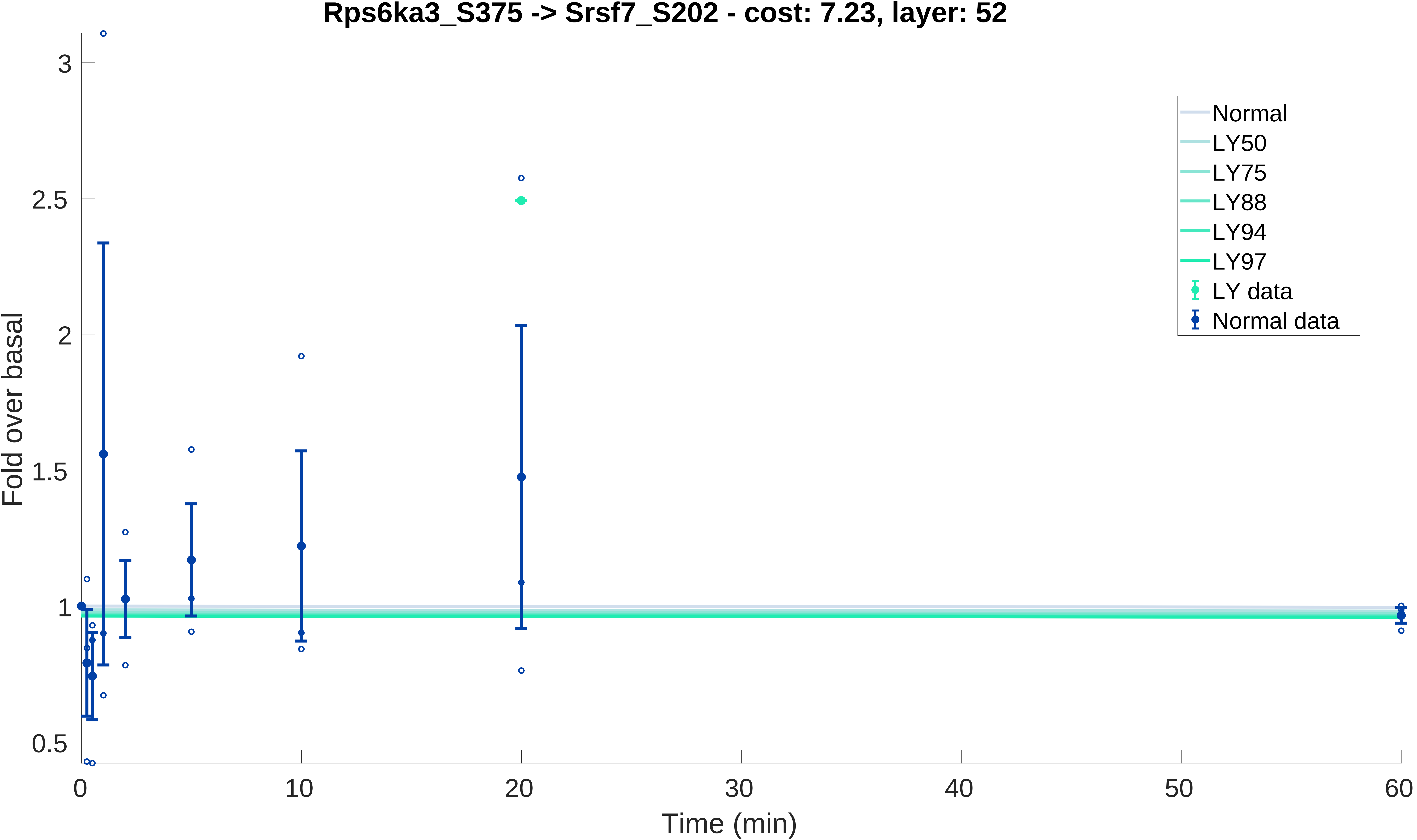
Rps6ka3_S375 -> Srsf7_S202 - cost: 7.23, layer: 52
Fold over basal
Time (min)
0.5
1
1.5
2
2.5
3
0
10
20
30
40
50
60
Normal
LY50
LY75
LY88
LY94
LY97
LY data
Normal data

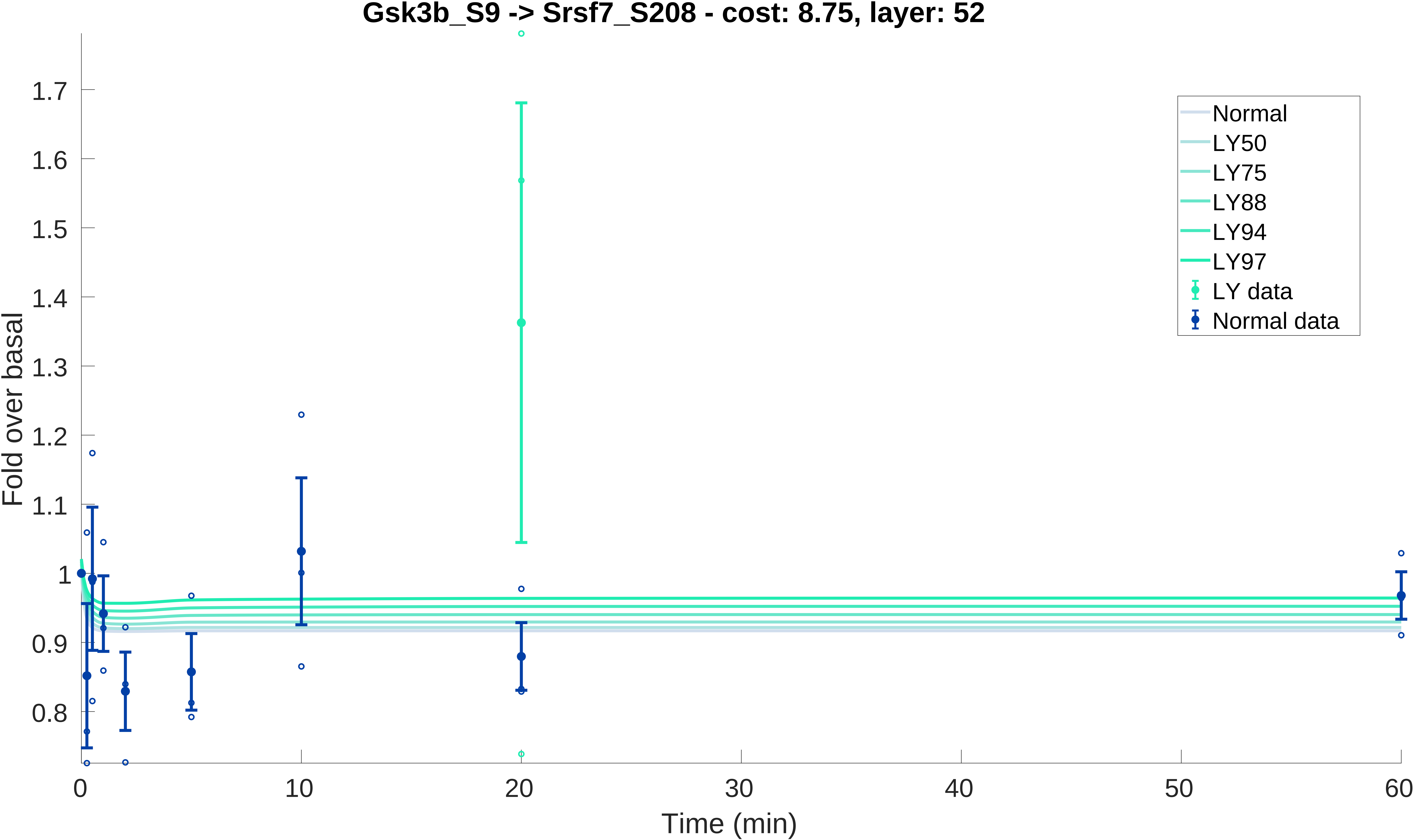

Gsk3b_S9 -> Srsf7_S208 - cost: 8.75, layer: 52
Fold over basal
Time (min)
0
10
20
30
40
50
60
0.8
0.9
1
1.1
1.2
1.3
1.4
1.5
1.6
1.7
Normal
LY50
LY75
LY88
LY94
LY97
LY data
Normal data

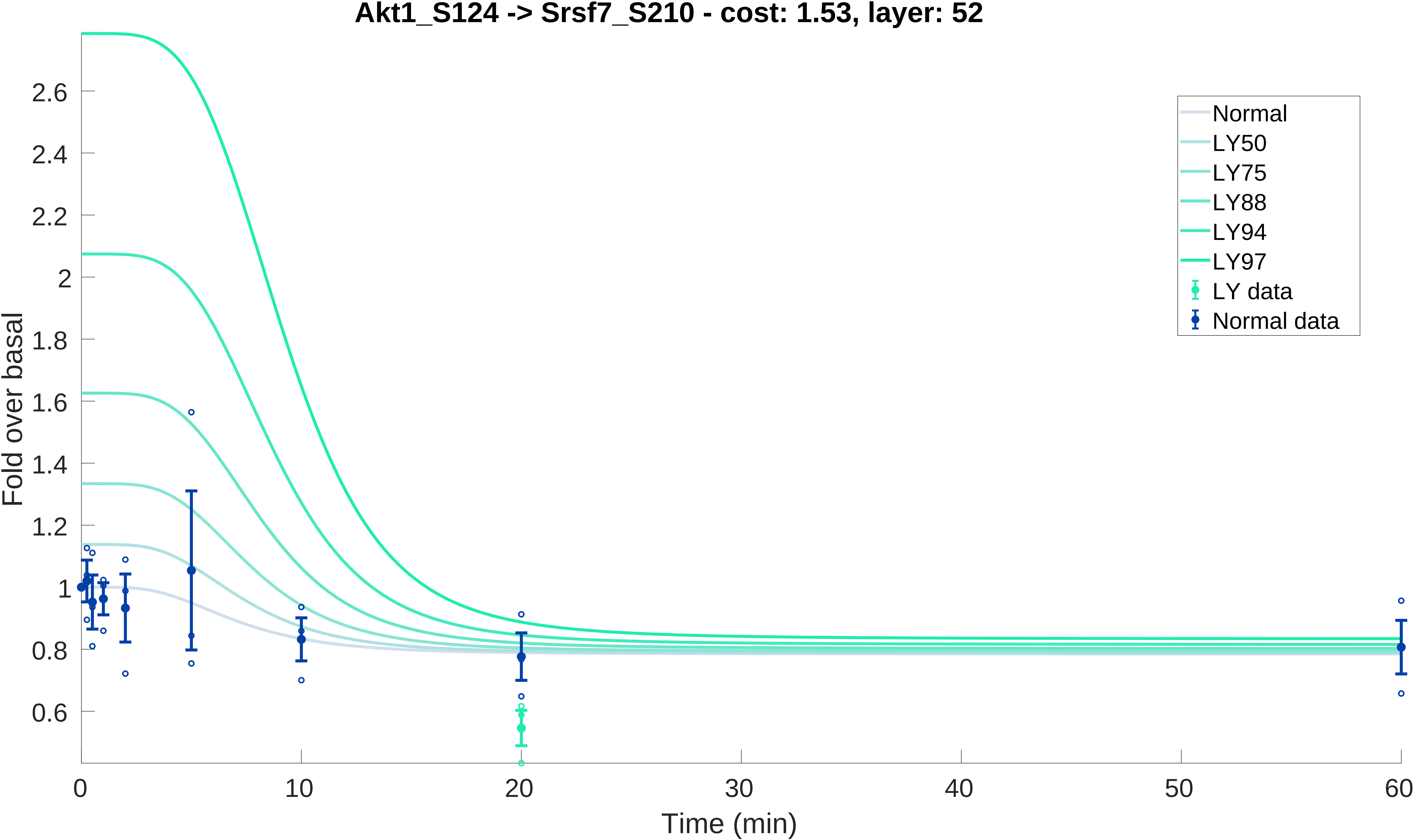
Akt1_S124 -> Srsf7_S210 - cost: 1.53, layer: 52
Fold over basal
2.6
2.4
2.2
2
1.8
1.6
1.4
1.2
1
0.8
0.6
0
10
20
30
40
50
60
Time (min)
Normal
LY50
LY75
LY88
LY94
LY97
LY data
Normal data

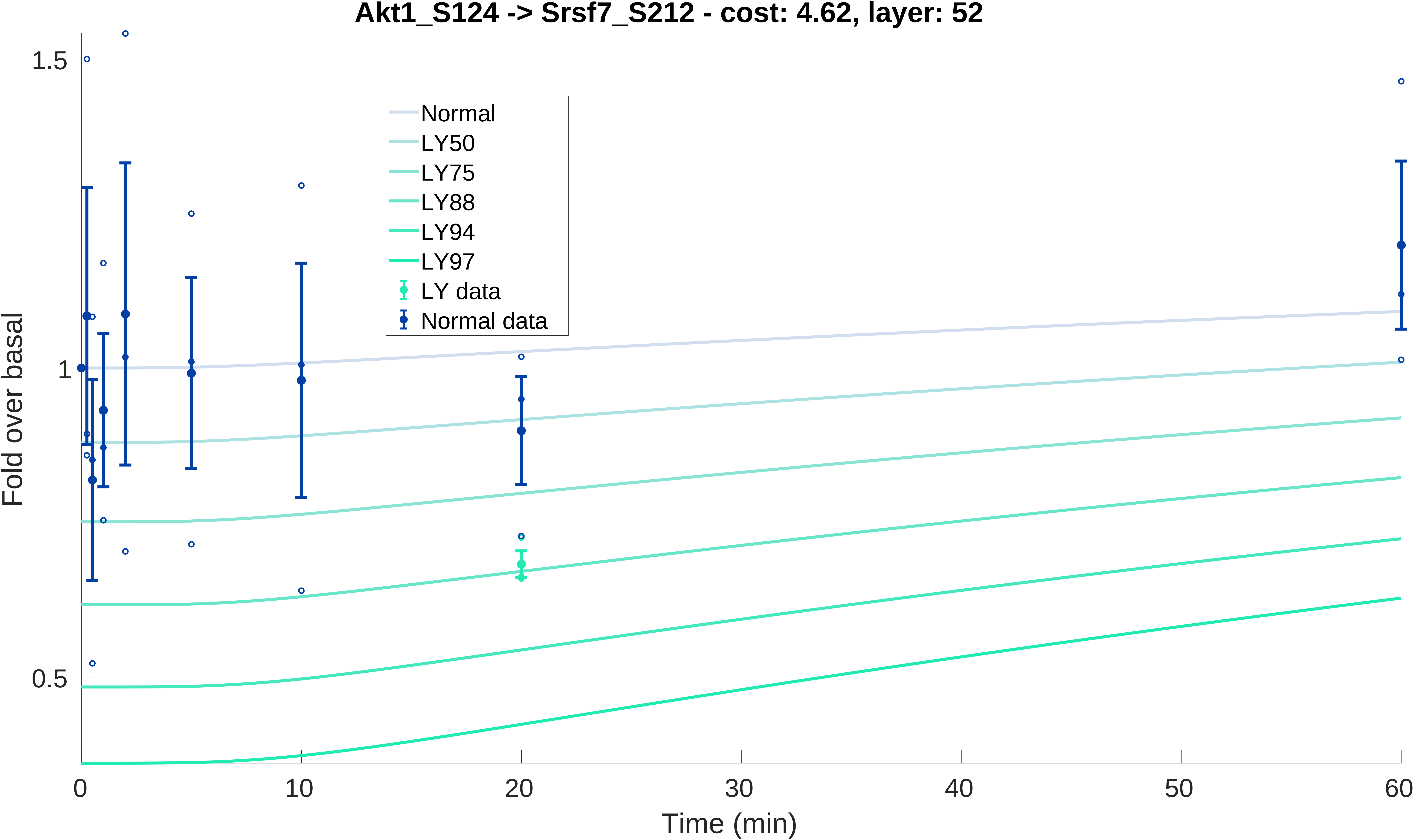

Akt1_S124 -> Srsf7_S212 - cost: 4.62, layer: 52
Fold over basal
Time (min)
1.5
1
0.5
0
10
20
30
40
50
60
Normal
LY50
LY75
LY88
LY94
LY97
LY data
Normal data

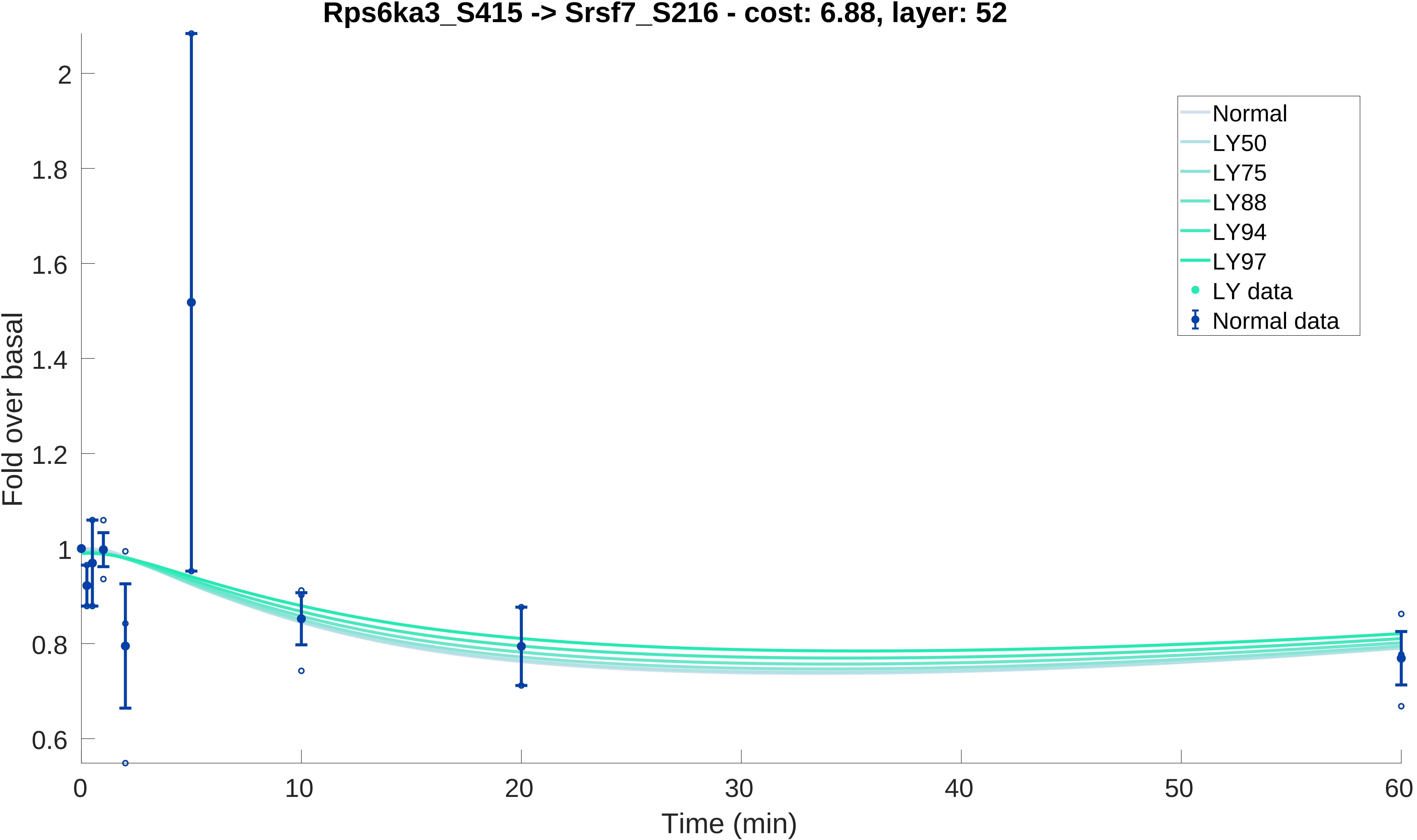

Rps6ka3_S415 -> Srsf7_S216 - cost: 6.88, layer: 52
Fold over basal
Time (min)
0.6
0.8
1
1.2
1.4
1.6
1.8
2
0
10
20
30
40
50
60
Normal
LY50
LY75
LY88
LY94
LY97
LY data
Normal data

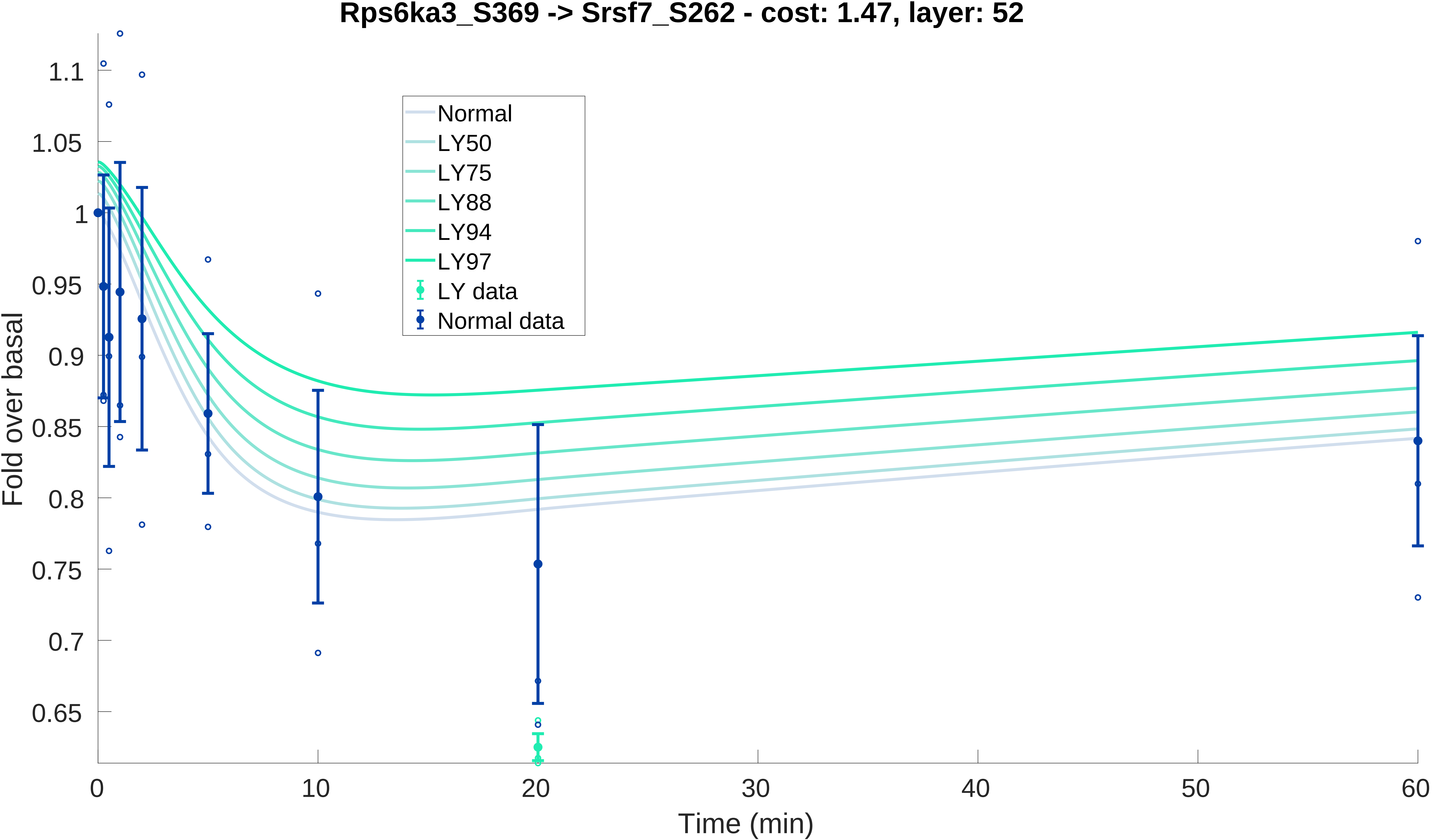

Rps6ka3_S369 -> Srsf7_S262 - cost: 1.47, layer: 52
Fold over basal
1.1
1.05
1
0.95
0.9
0.85
0.8
0.75
0.7
0.65
0
10
20
30
40
50
60
Time (min)
Normal
LY50
LY75
LY88
LY94
LY97
LY data
Normal data

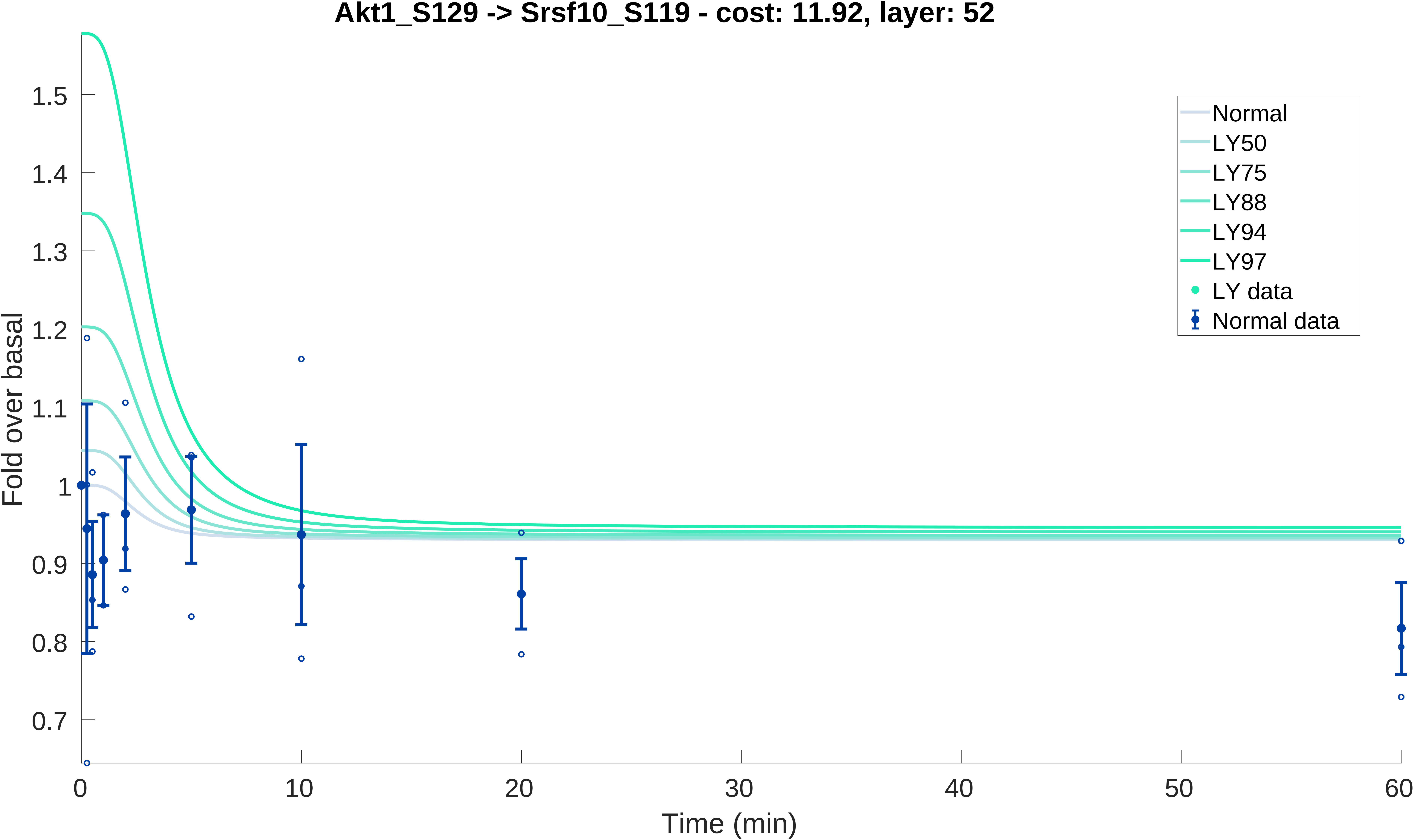

Akt1_S129 -> Srsf10_S119 - cost: 11.92, layer: 52
Fold over basal
Time (min)
1.5
1.4
1.3
1.2
1.1
1
0.9
0.8
0.7
0
10
20
30
40
50
60
Normal
LY50
LY75
LY88
LY94
LY97
LY data
Normal data

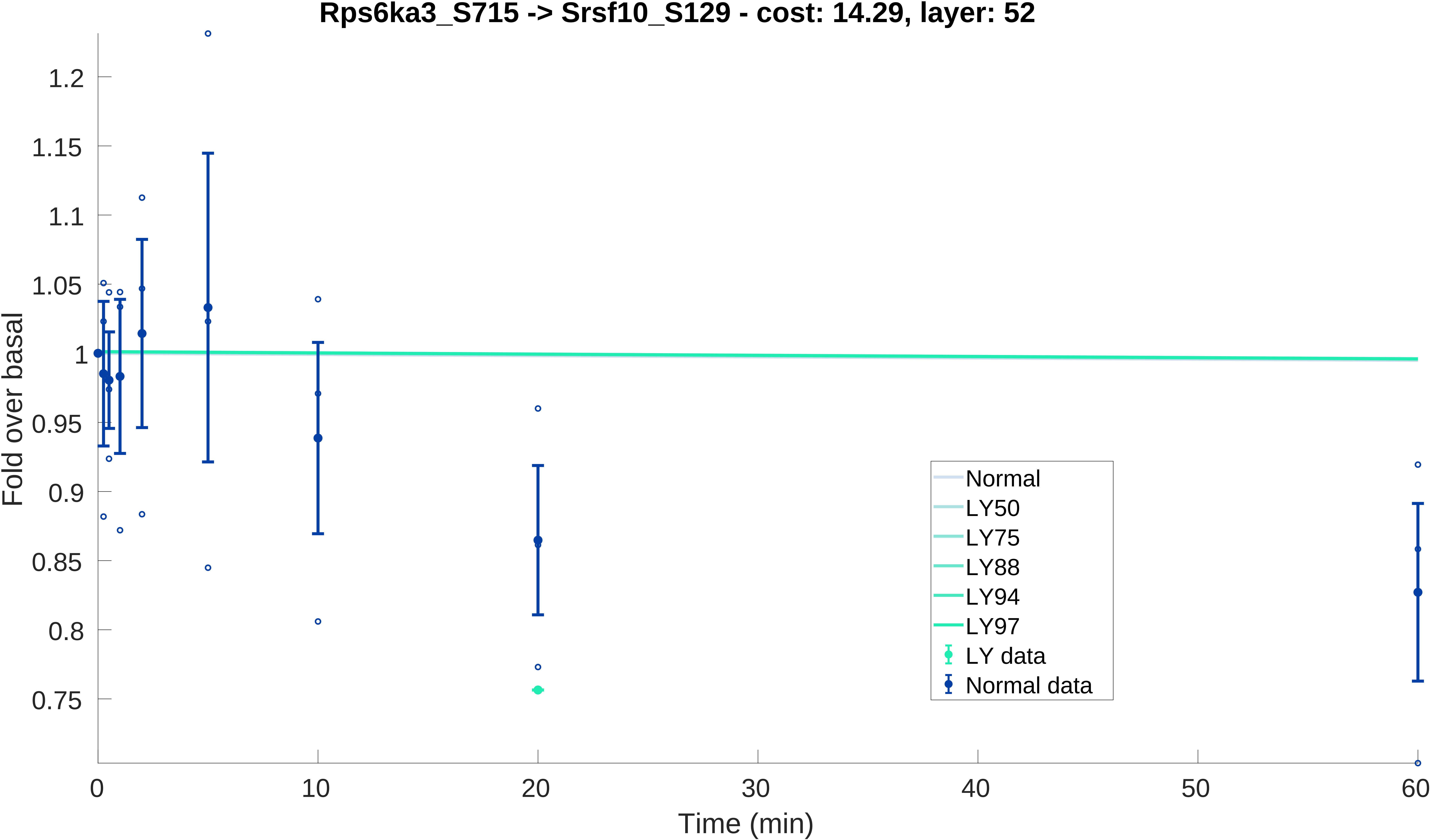

Rps6ka3_S715 -> Srsf10_S129 - cost: 14.29, layer: 52
Fold over basal
1.2
1.15
1.1
1.05
1
0.95
0.9
0.85
0.8
0.75
0
10
20
30
40
50
60
Time (min)
Normal
LY50
LY75
LY88
LY94
LY97
LY data
Normal data

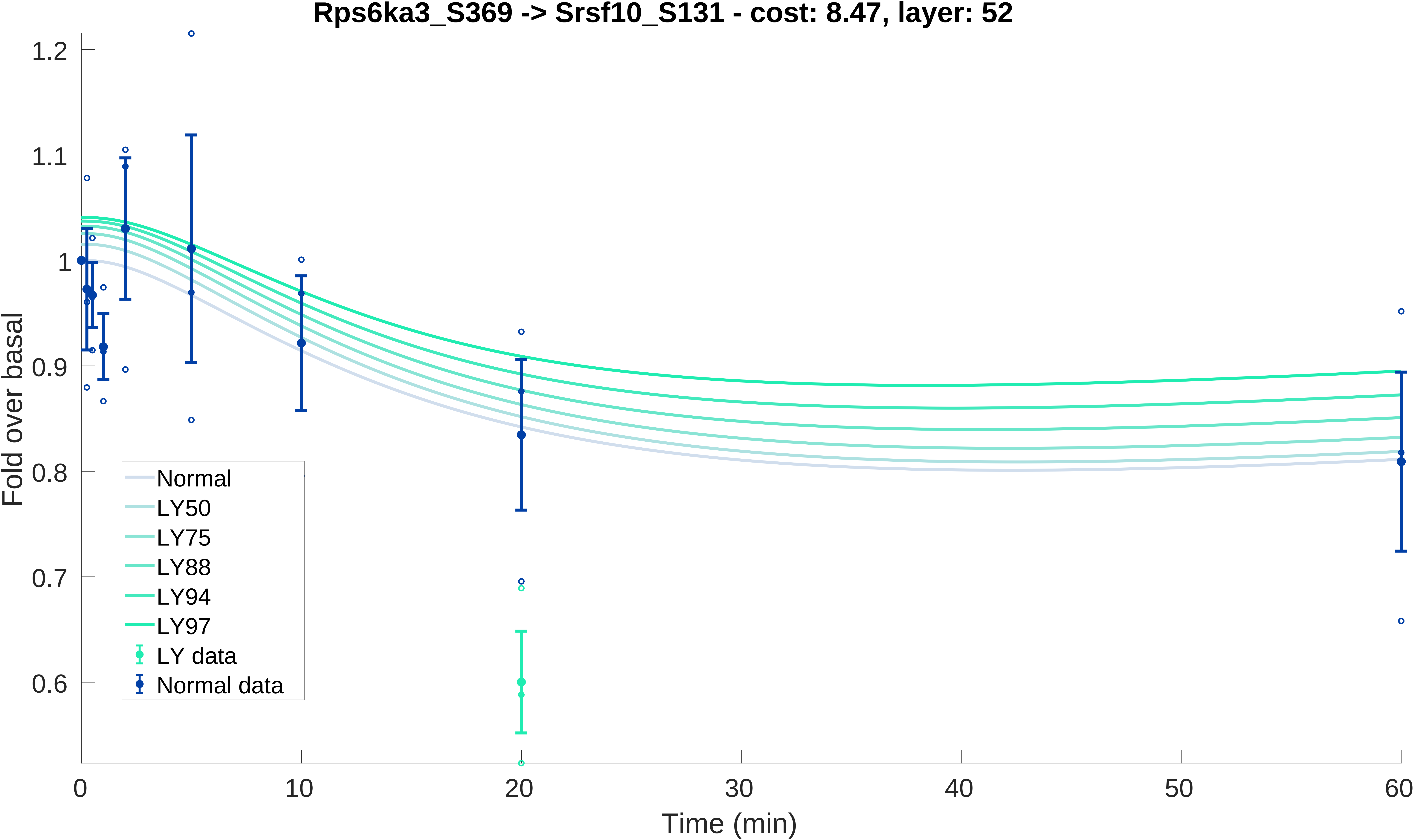
Rps6ka3_S369 -> Srsf10_S131 - cost: 8.47, layer: 52
Fold over basal
1.2
1.1
1
0.9
0.8
0.7
0.6
Time (min)
0
10
20
30
40
50
60
Normal
LY50
LY75
LY88
LY94
LY97
LY data
Normal data

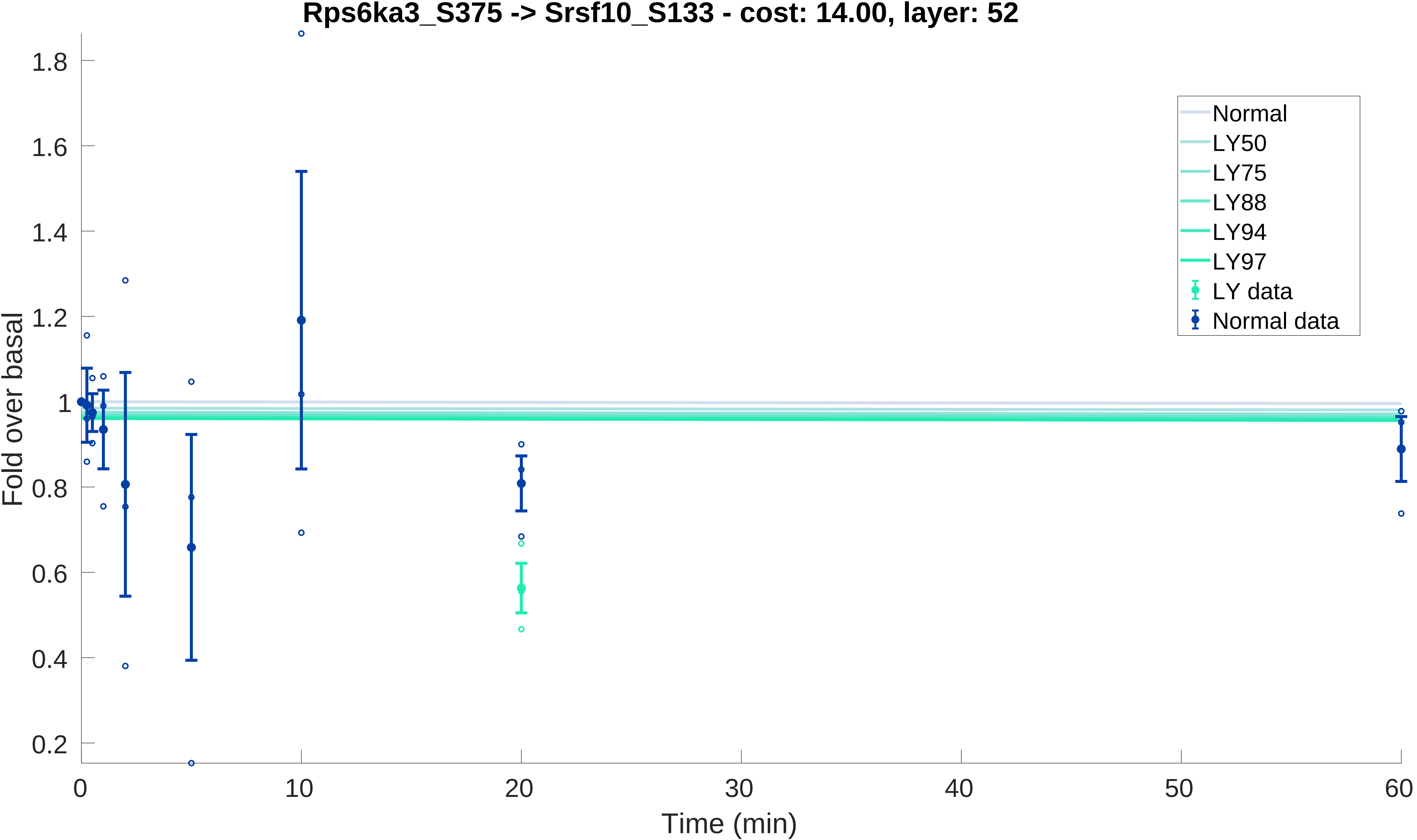
Rps6ka3_S375 -> Srsf10_S133 - cost: 14.00, layer: 52
Fold over basal
Time (min)
Normal
LY50
LY75
LY88
LY94
LY97
LY data
Normal data
1.8
1.6
1.4
1.2
1
0.8
0.6
0.4
0.2
0
10
20
30
40
50
60

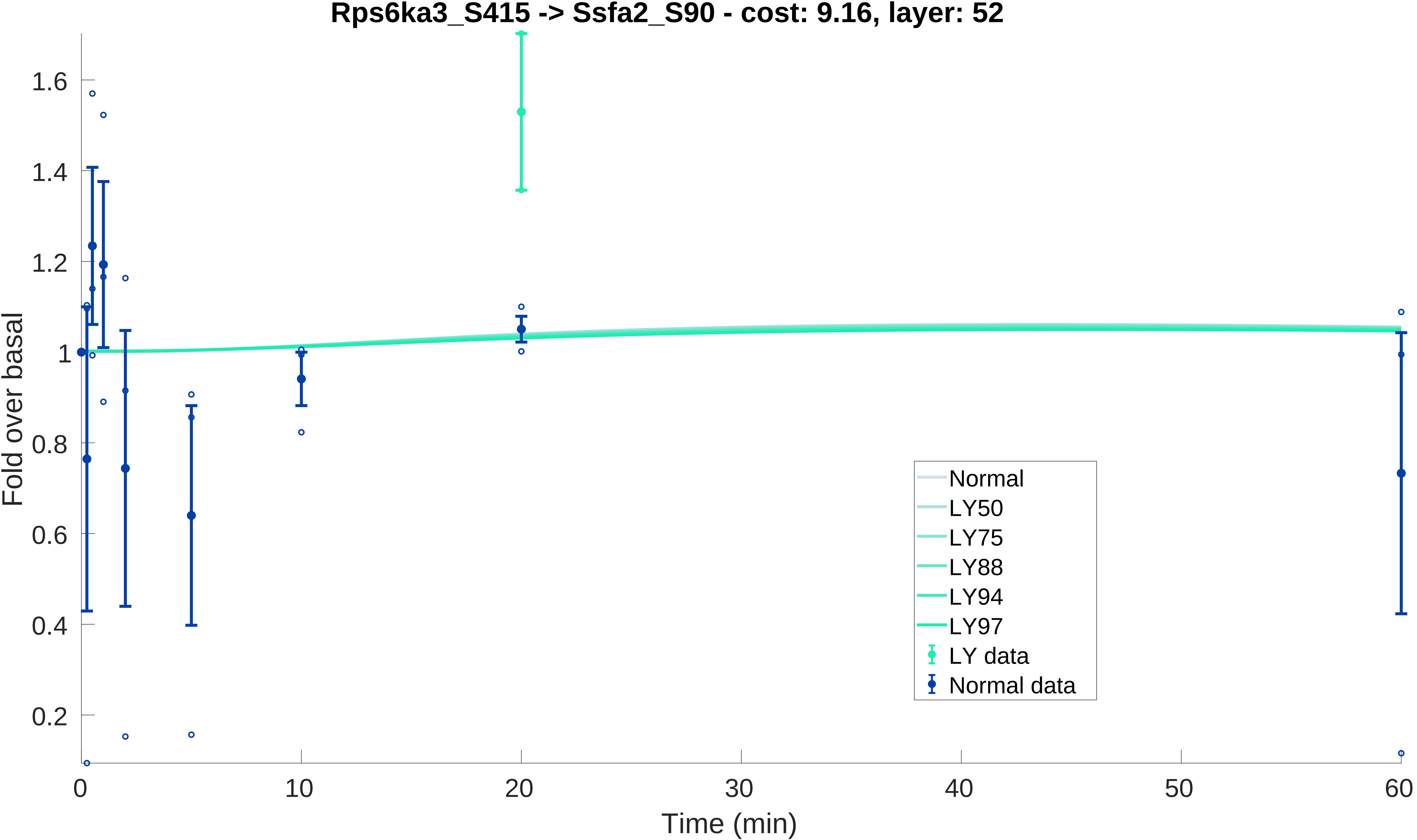

Rps6ka3_S415 -> Ssfa2_S90 - cost: 9.16, layer: 52
Fold over basal
1.6
1.4
1.2
1
0.8
0.6
0.4
0.2
0
10
20
30
40
50
60
Time (min)
Normal
LY50
LY75
LY88
LY94
LY97
LY data
Normal data

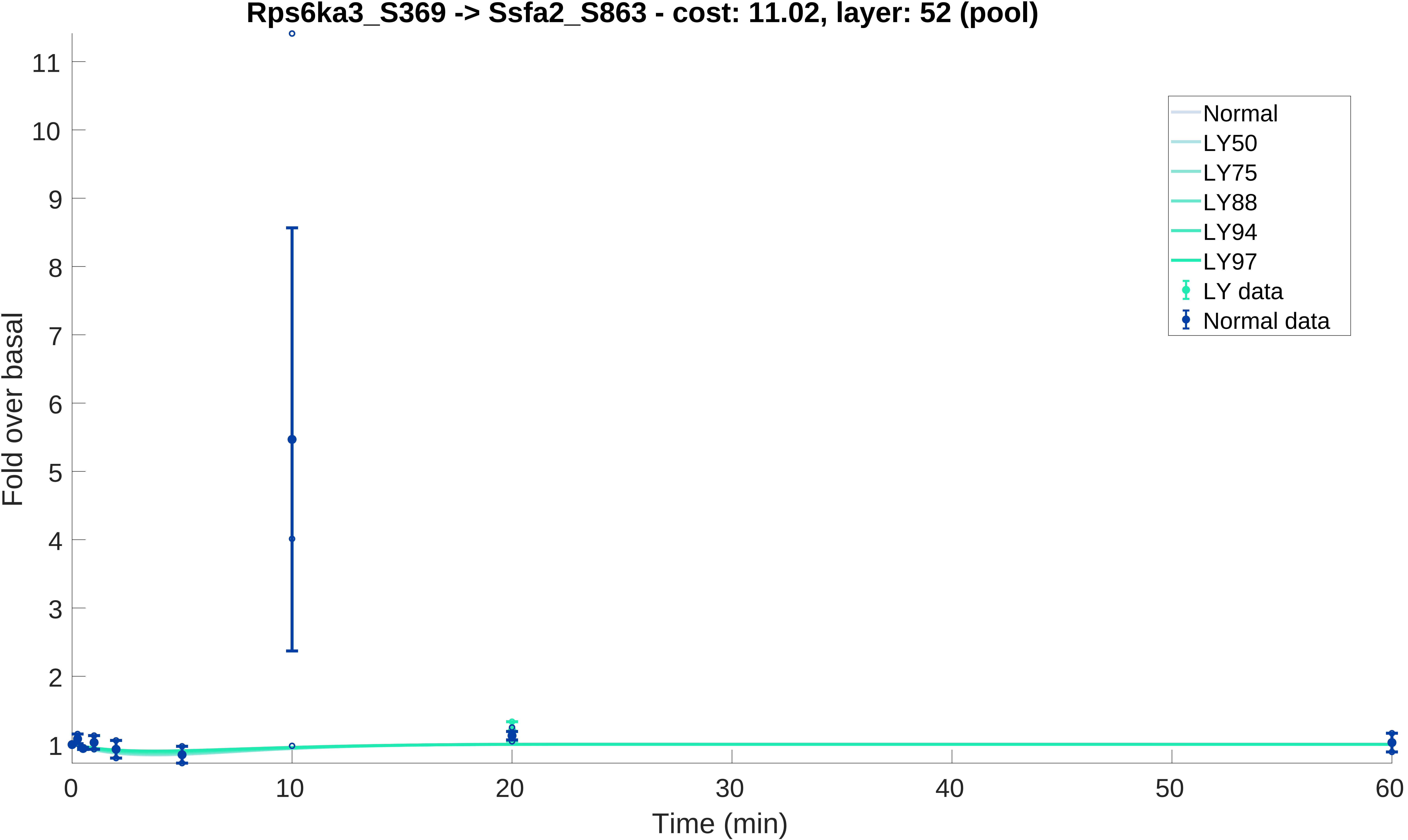

Rps6ka3_S369 -> Ssfa2_S863 - cost: 11.02, layer: 52 (pool)
Fold over basal
Time (min)
Normal
LY50
LY75
LY88
LY94
LY97
LY data
Normal data
0
10
20
30
40
50
60
1
2
3
4
5
6
7
8
9
10
11

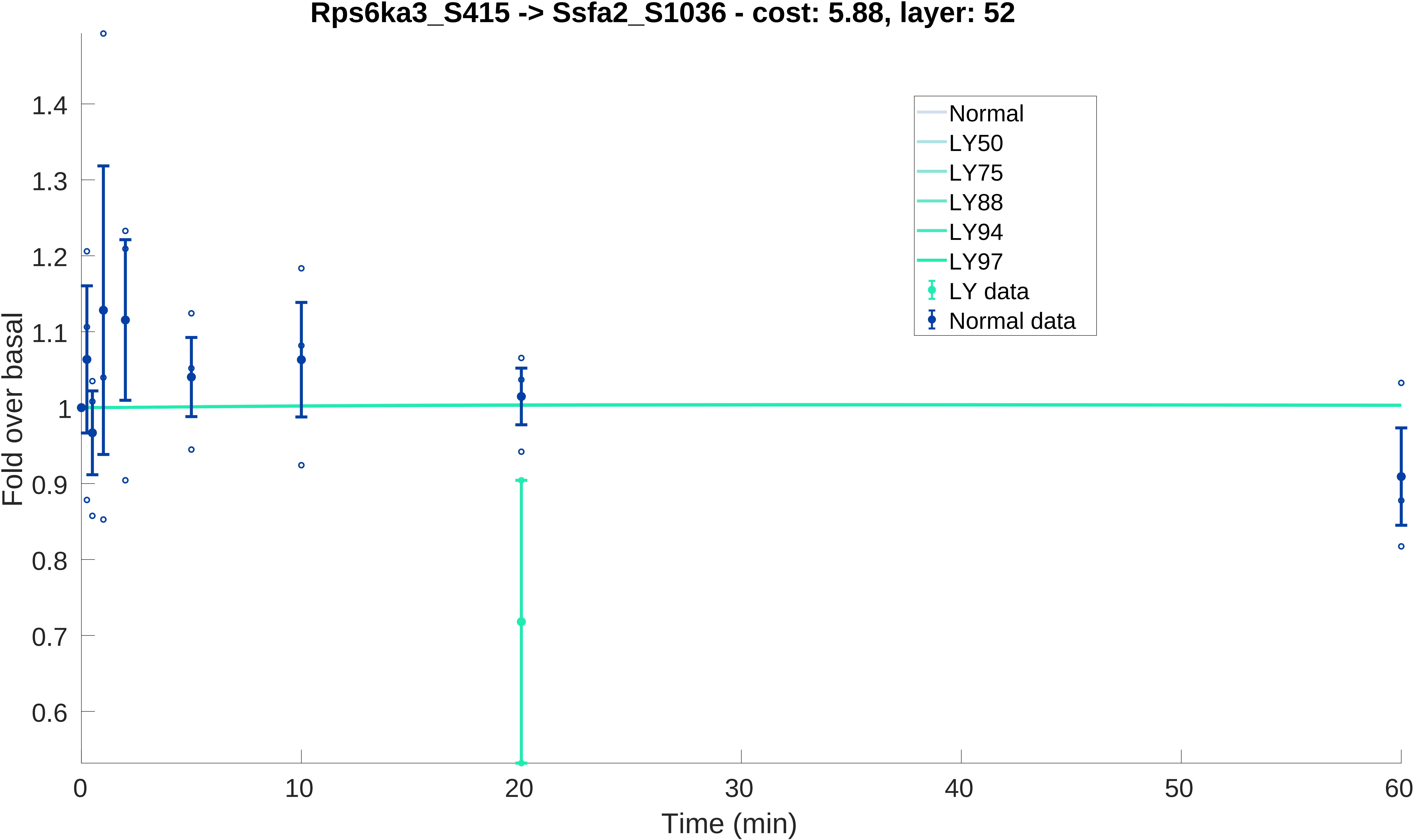

Rps6ka3_S415 -> Ssfa2_S1036 - cost: 5.88, layer: 52
Fold over basal
Time (min)
1.4
1.3
1.2
1.1
1
0.9
0.8
0.7
0.6
0
10
20
30
40
50
60
Normal
LY50
LY75
LY88
LY94
LY97
LY data
Normal data

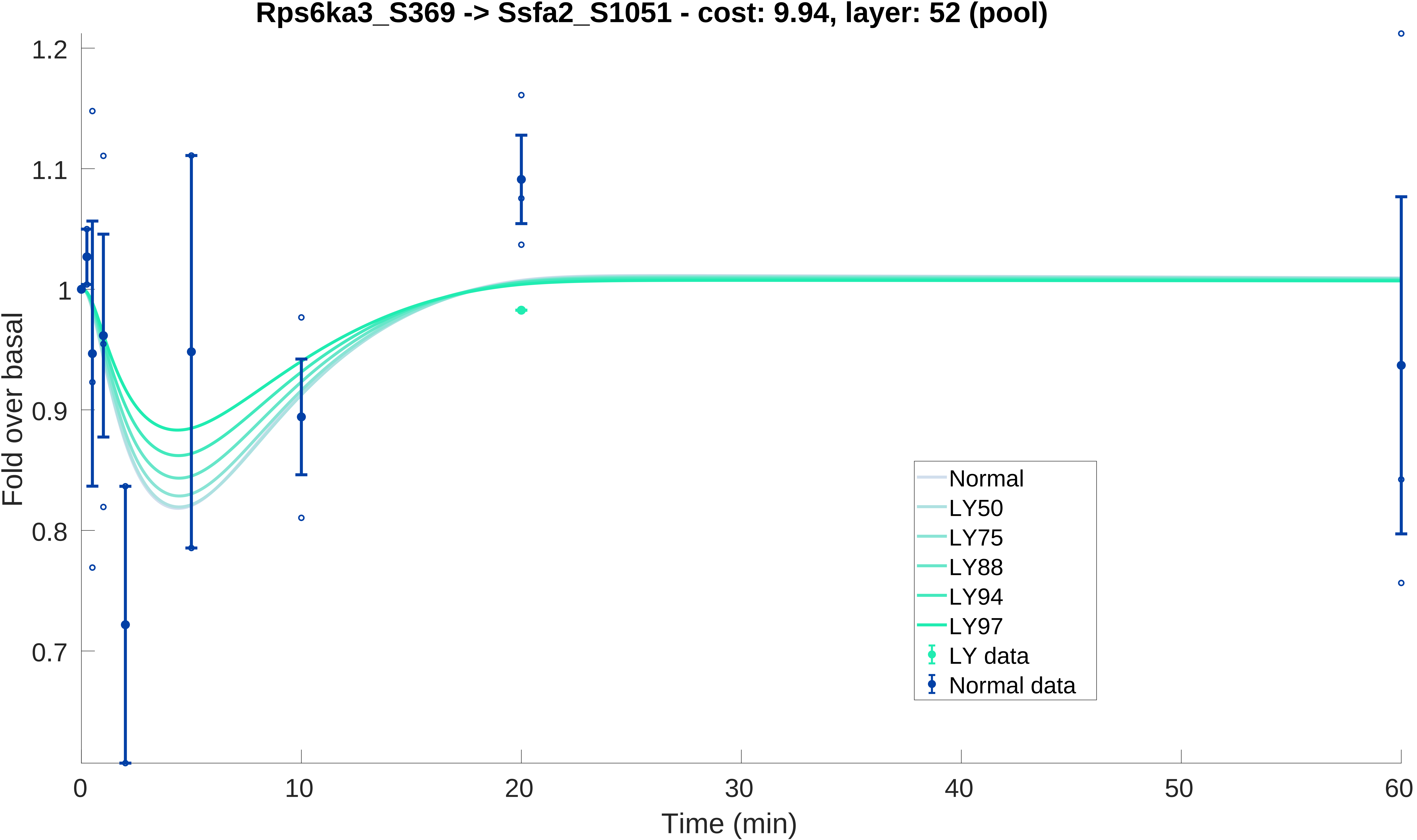

Rps6ka3_S369 -> Ssfa2_S1051 - cost: 9.94, layer: 52 (pool)
1.2
1.1
1
0.9
0.8
0.7
Fold over basal
0
10
20
30
40
50
60
Time (min)
Normal
LY50
LY75
LY88
LY94
LY97
LY data
Normal data

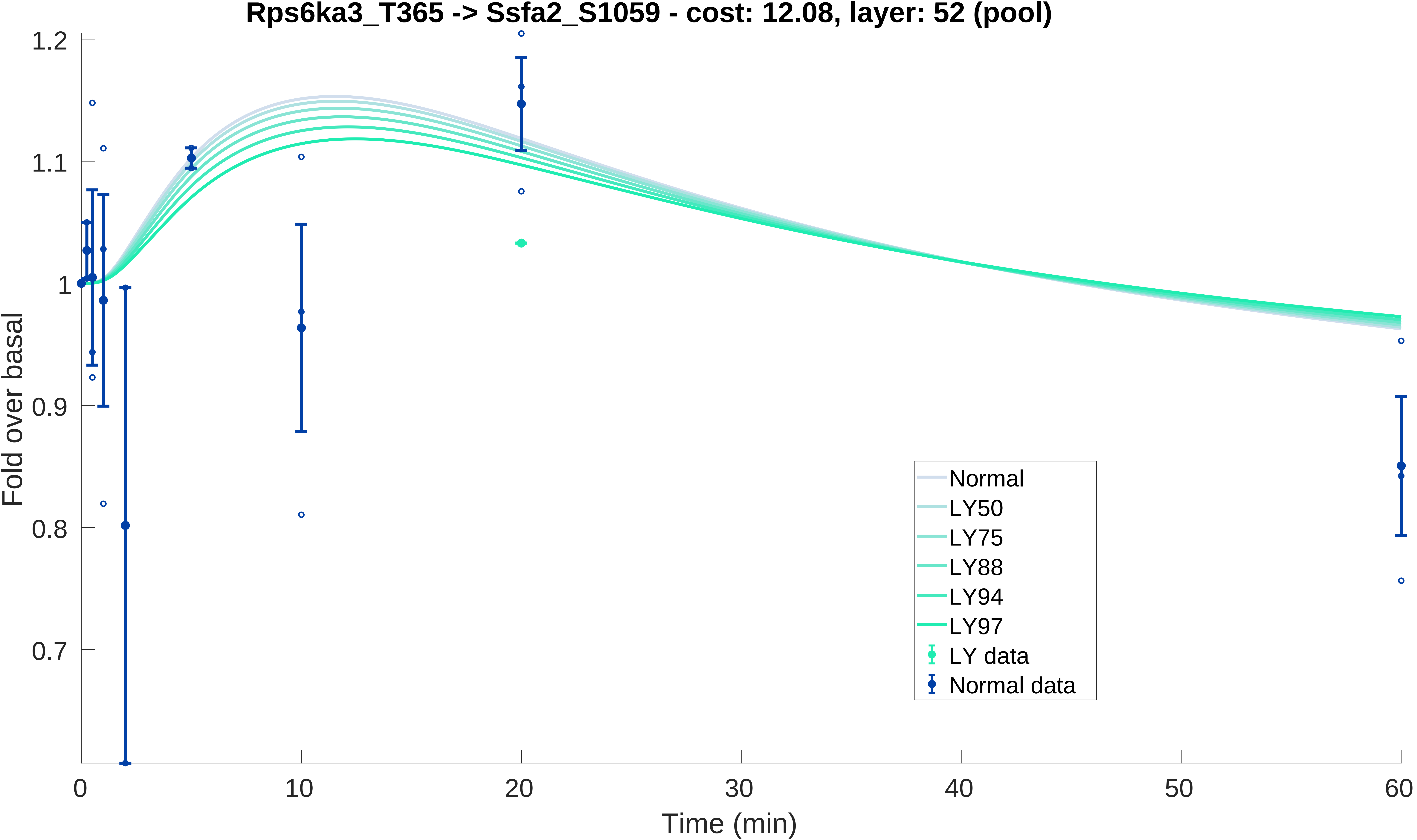

Rps6ka3_T365 -> Ssfa2_S1059 - cost: 12.08, layer: 52 (pool)
Fold over basal
Time (min)
0
10
20
30
40
50
60
0.7
0.8
0.9
1
1.1
1.2
Normal
LY50
LY75
LY88
LY94
LY97
LY data
Normal data

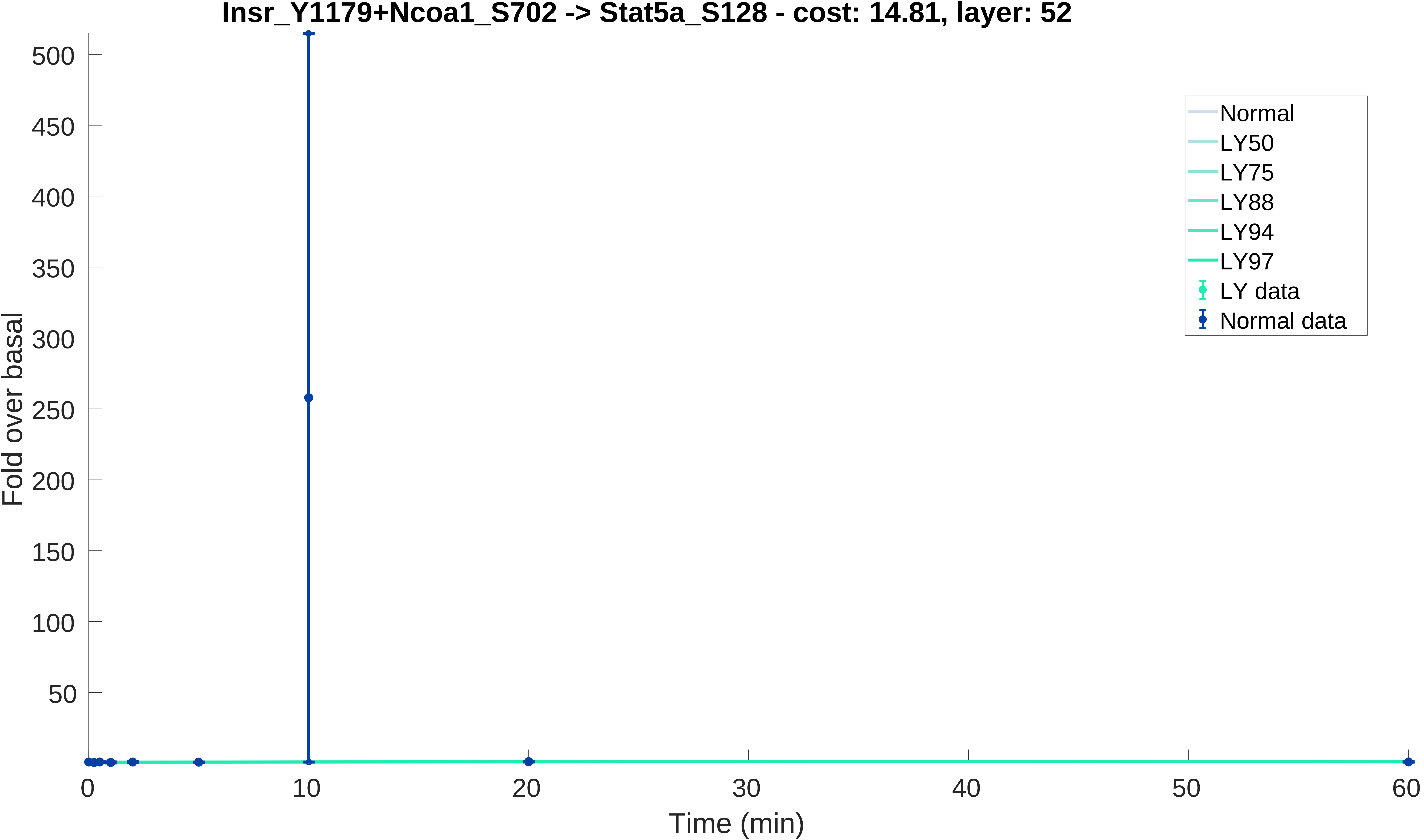
Insr_Y1179+Ncoa1_S702 -> Stat5a_S128 - cost: 14.81, layer: 52
Fold over basal
Time (min)
Normal
LY50
LY75
LY88
LY94
LY97
LY data
Normal data
500
450
400
350
300
250
200
150
100
50
0
10
20
30
40
50
60

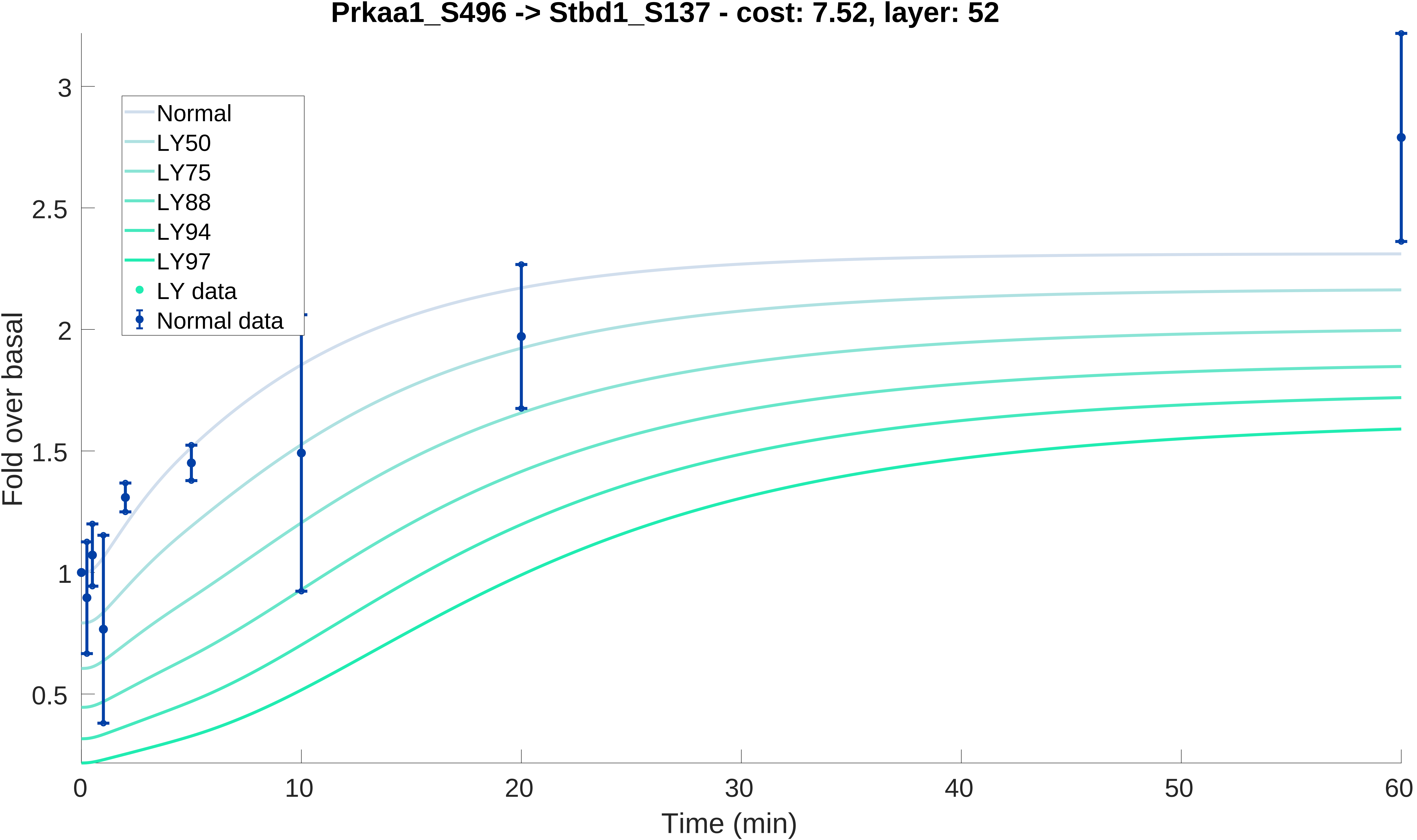
Prkaa1_S496 -> Stbd1_S137 - cost: 7.52, layer: 52
Normal
LY50
LY75
LY88
LY94
LY97
LY data
Normal data
Fold over basal
Time (min)
0
10
20
30
40
50
60
0.5
1
1.5
2
2.5
3

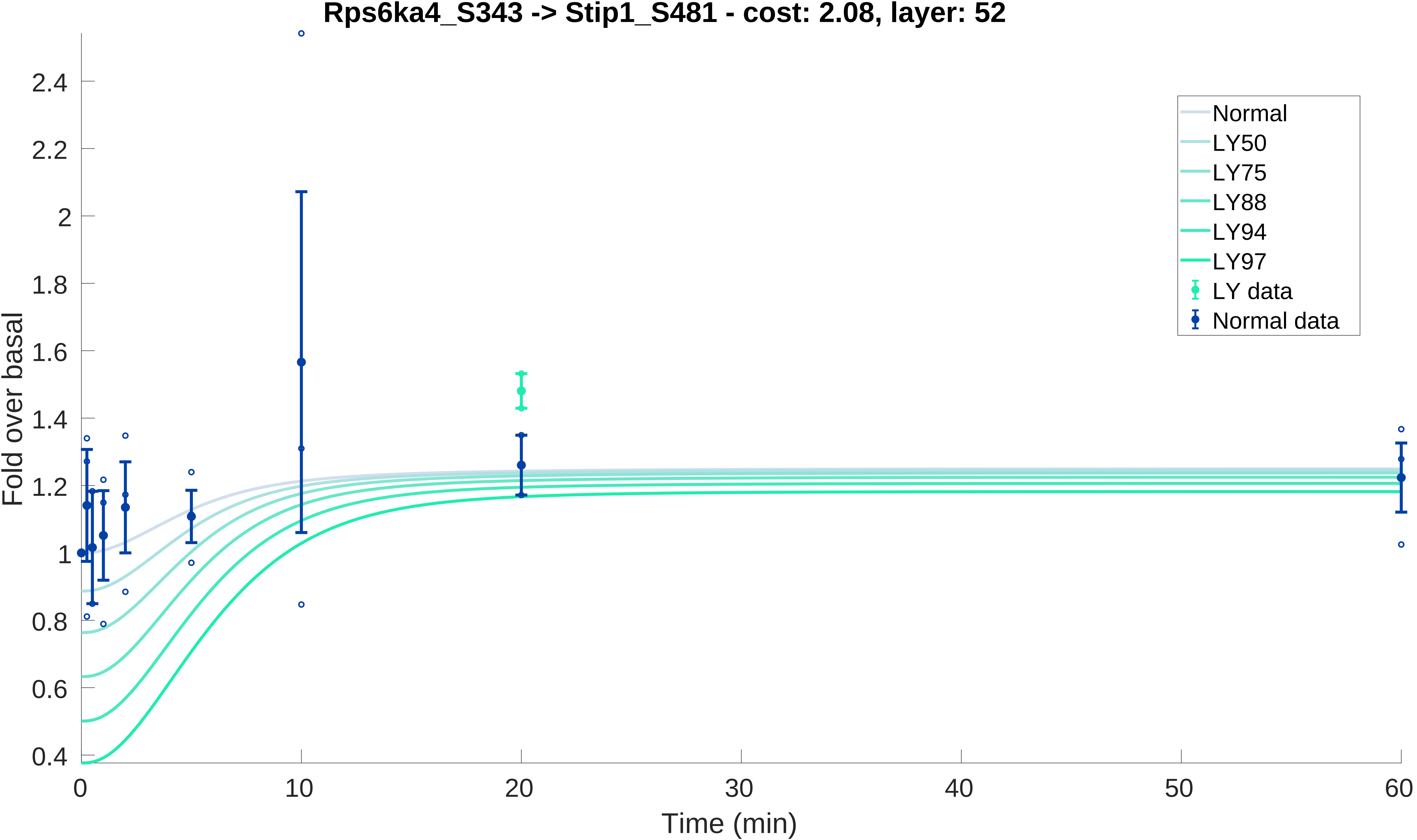
Rps6ka4_S343 -> Stip1_S481 - cost: 2.08, layer: 52
Fold over basal
Time (min)
0.4
0.6
0.8
1
1.2
1.4
1.6
1.8
2
2.2
2.4
0
10
20
30
40
50
60
Normal
LY50
LY75
LY88
LY94
LY97
LY data
Normal data

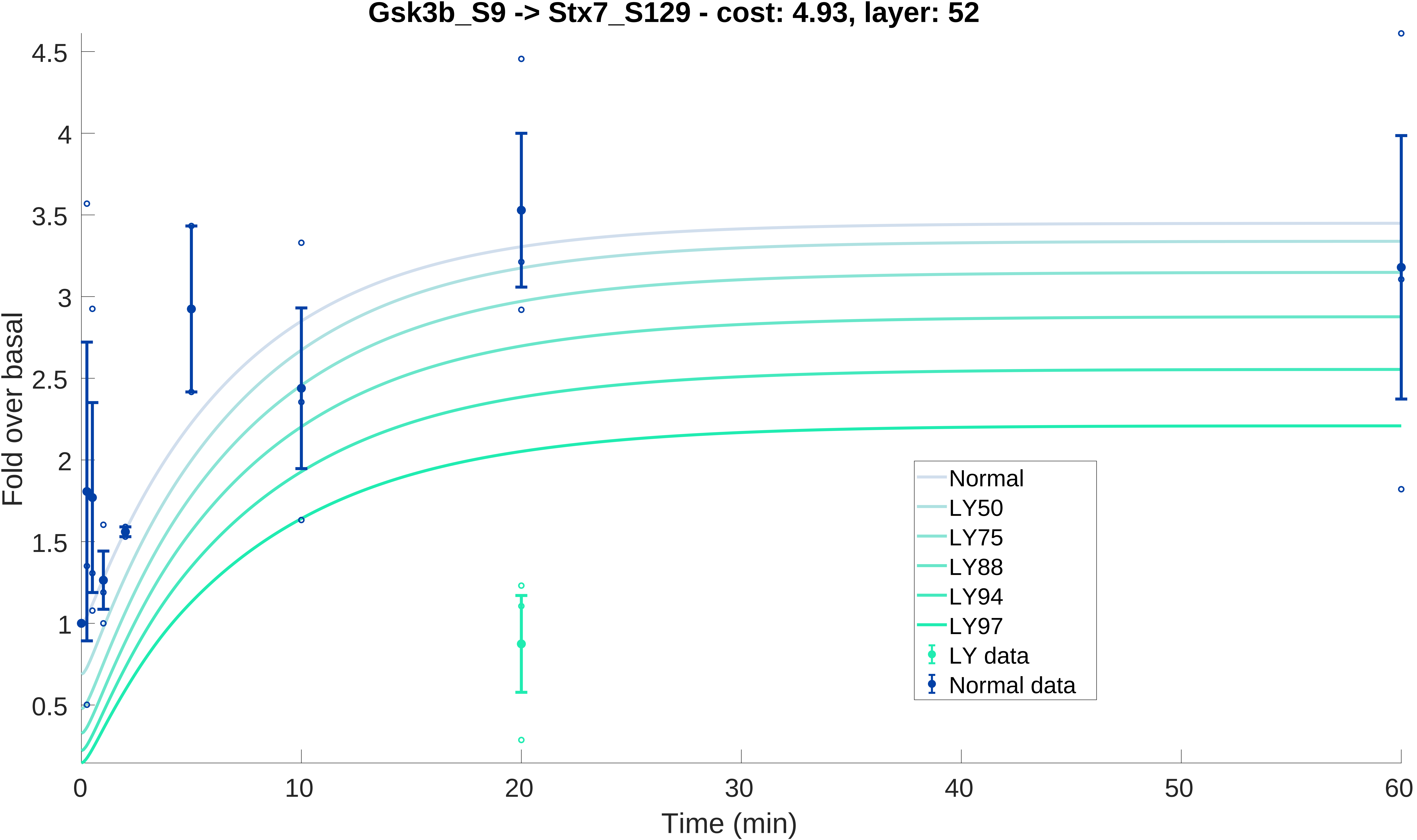
Gsk3b_S9 -> Stx7_S129 - cost: 4.93, layer: 52
Fold over basal
Time (min)
0.5
1
1.5
2
2.5
3
3.5
4
4.5
0
10
20
30
40
50
60
Normal
LY50
LY75
LY88
LY94
LY97
LY data
Normal data

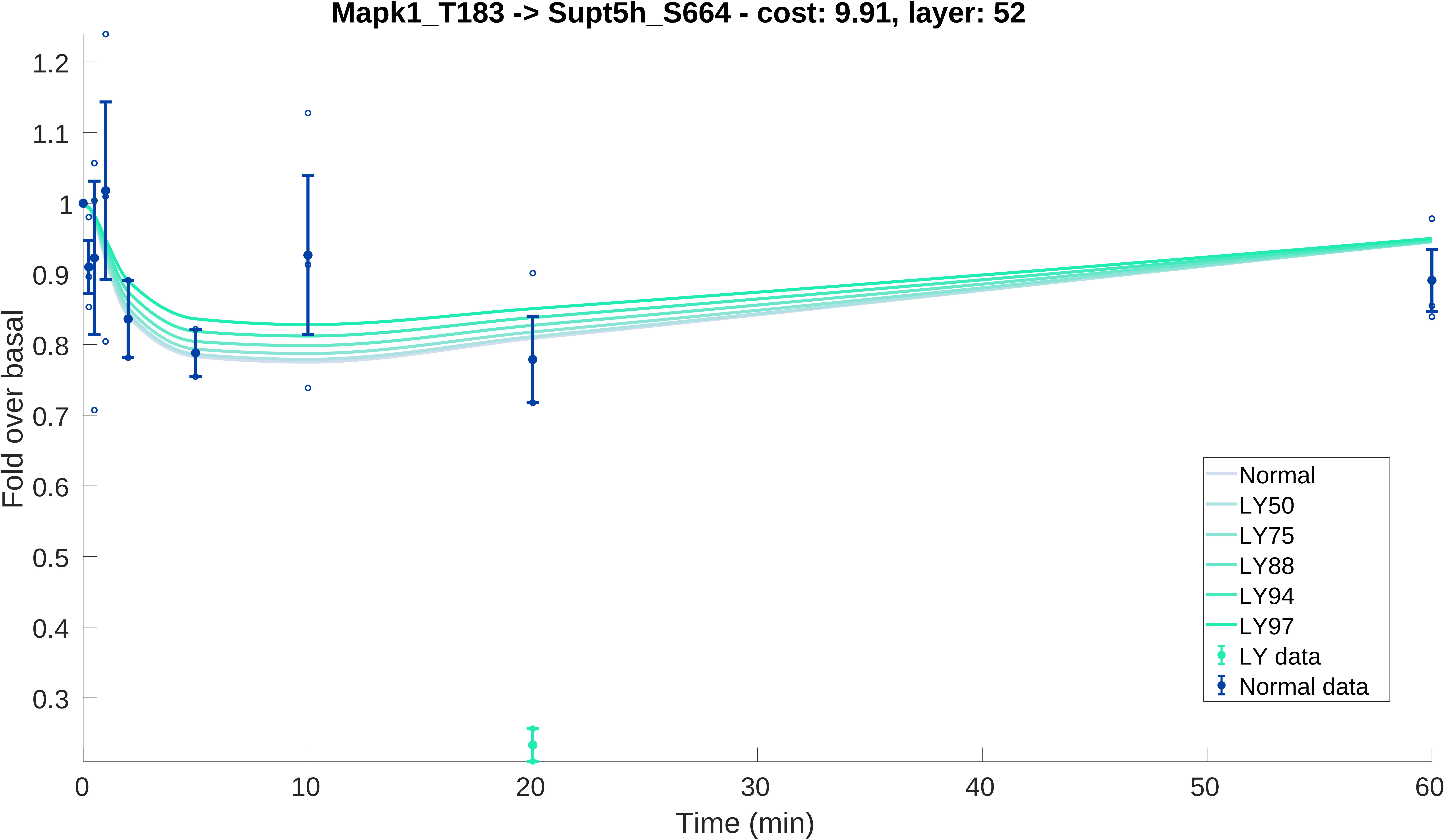

Mapk1_T183 -> Supt5h_S664 - cost: 9.91, layer: 52
Fold over basal
1.2
1.1
1
0.9
0.8
0.7
0.6
0.5
0.4
0.3
0
10
20
30
40
50
60
Time (min)
Normal
LY50
LY75
LY88
LY94
LY97
LY data
Normal data

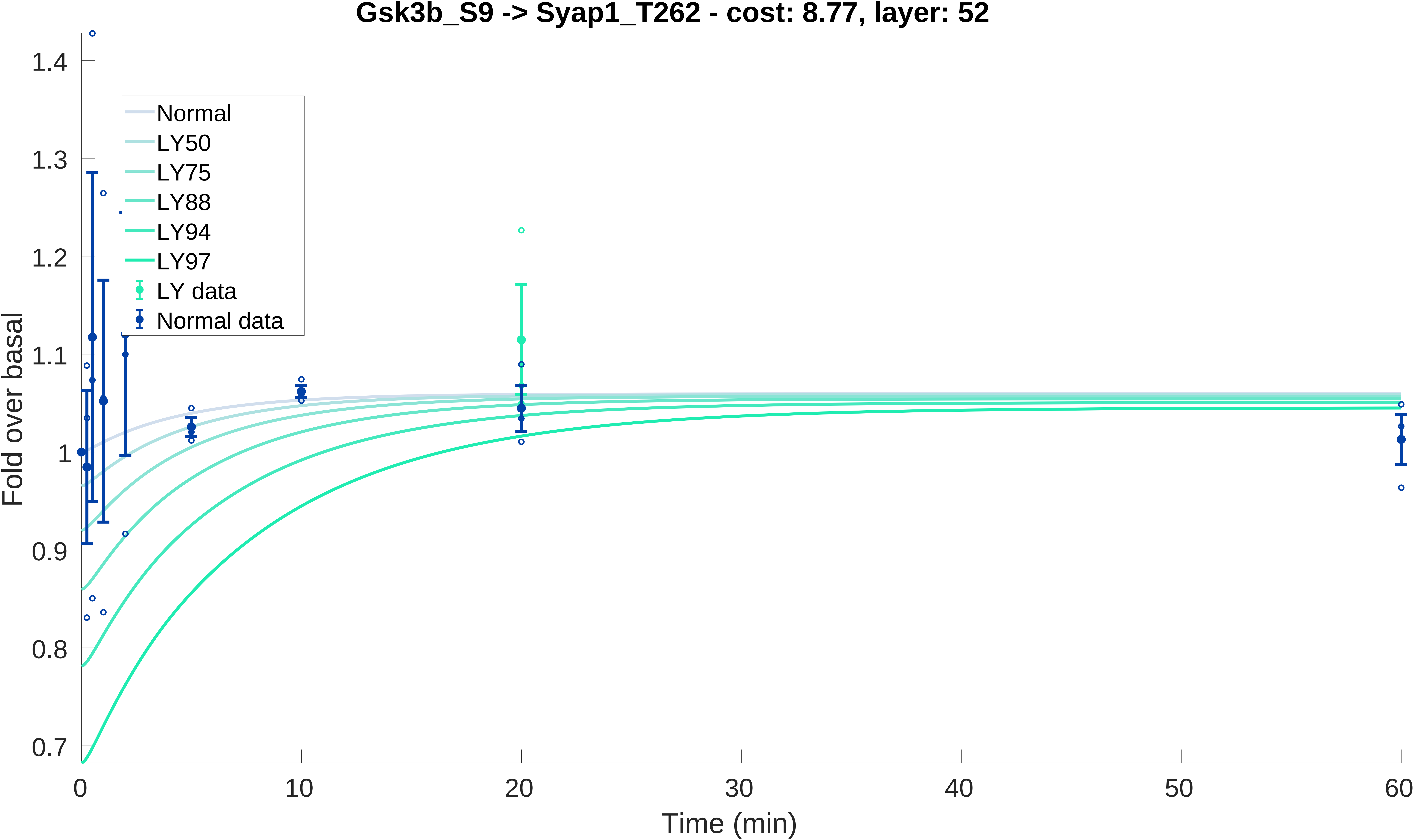
Gsk3b_S9 -> Syap1_T262 - cost: 8.77, layer: 52
Normal
LY50
LY75
LY88
LY94
LY97
LY data
Normal data
Fold over basal
Time (min)
0
10
20
30
40
50
60
0.7
0.8
0.9
1
1.1
1.2
1.3
1.4

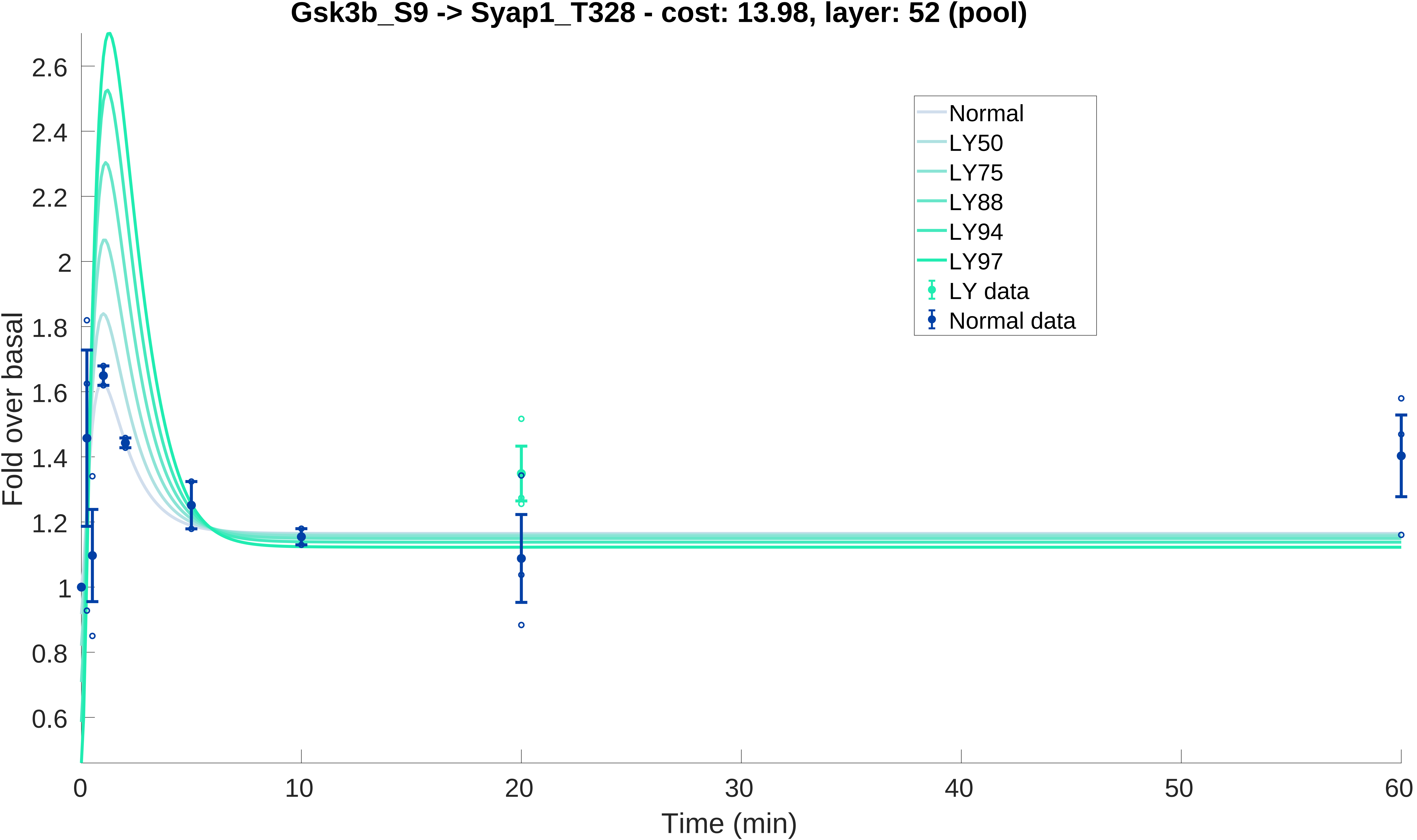

Gsk3b_S9 -> Syap1_T328 - cost: 13.98, layer: 52 (pool)
Fold over basal
Time (min)
0.6
0.8
1
1.2
1.4
1.6
1.8
2
2.2
2.4
2.6
0
10
20
30
40
50
60
Normal
LY50
LY75
LY88
LY94
LY97
LY data
Normal data

Gsk3b_S9 -> Synpo_S672 - cost: 0.31, layer: 52

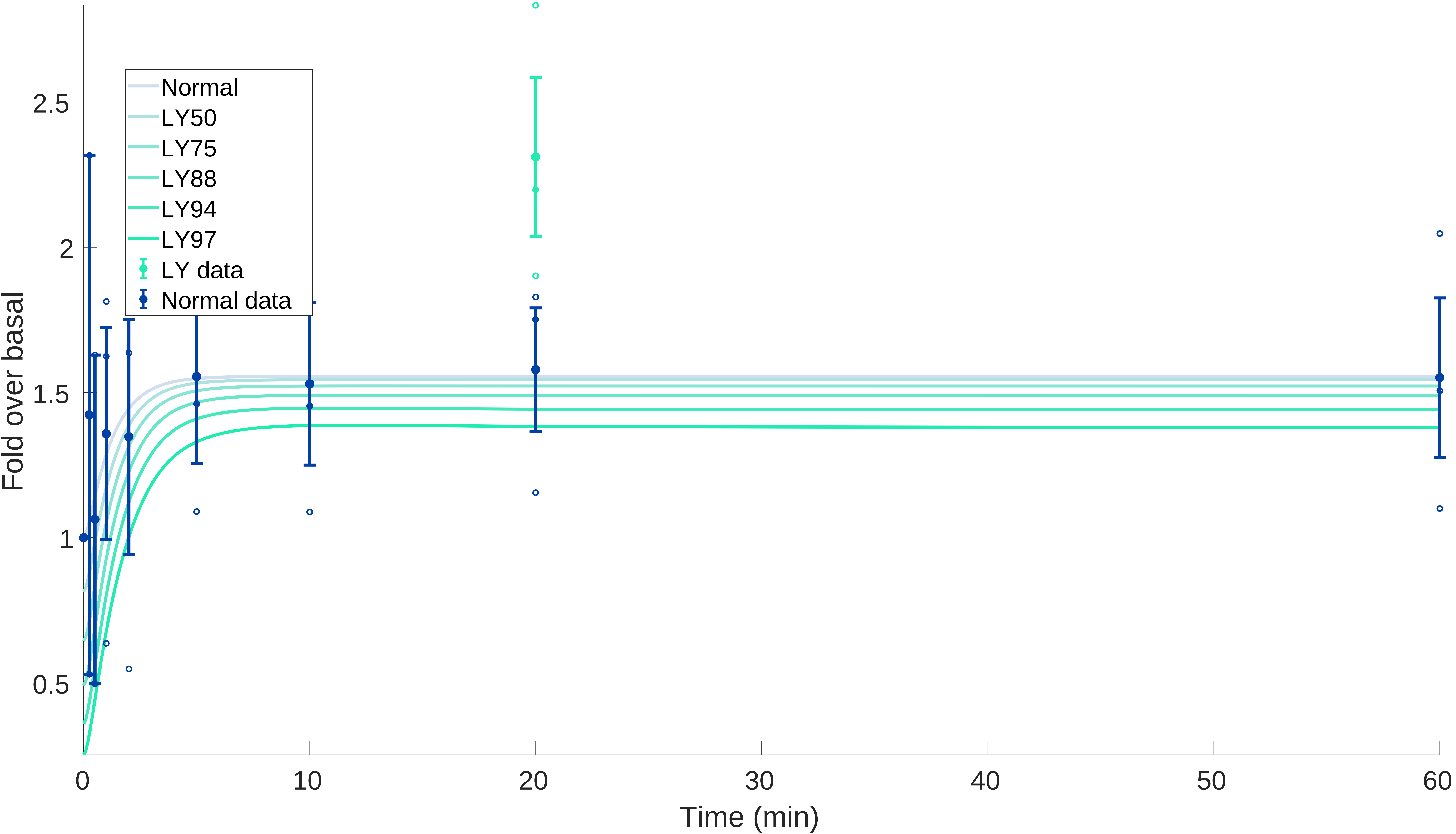

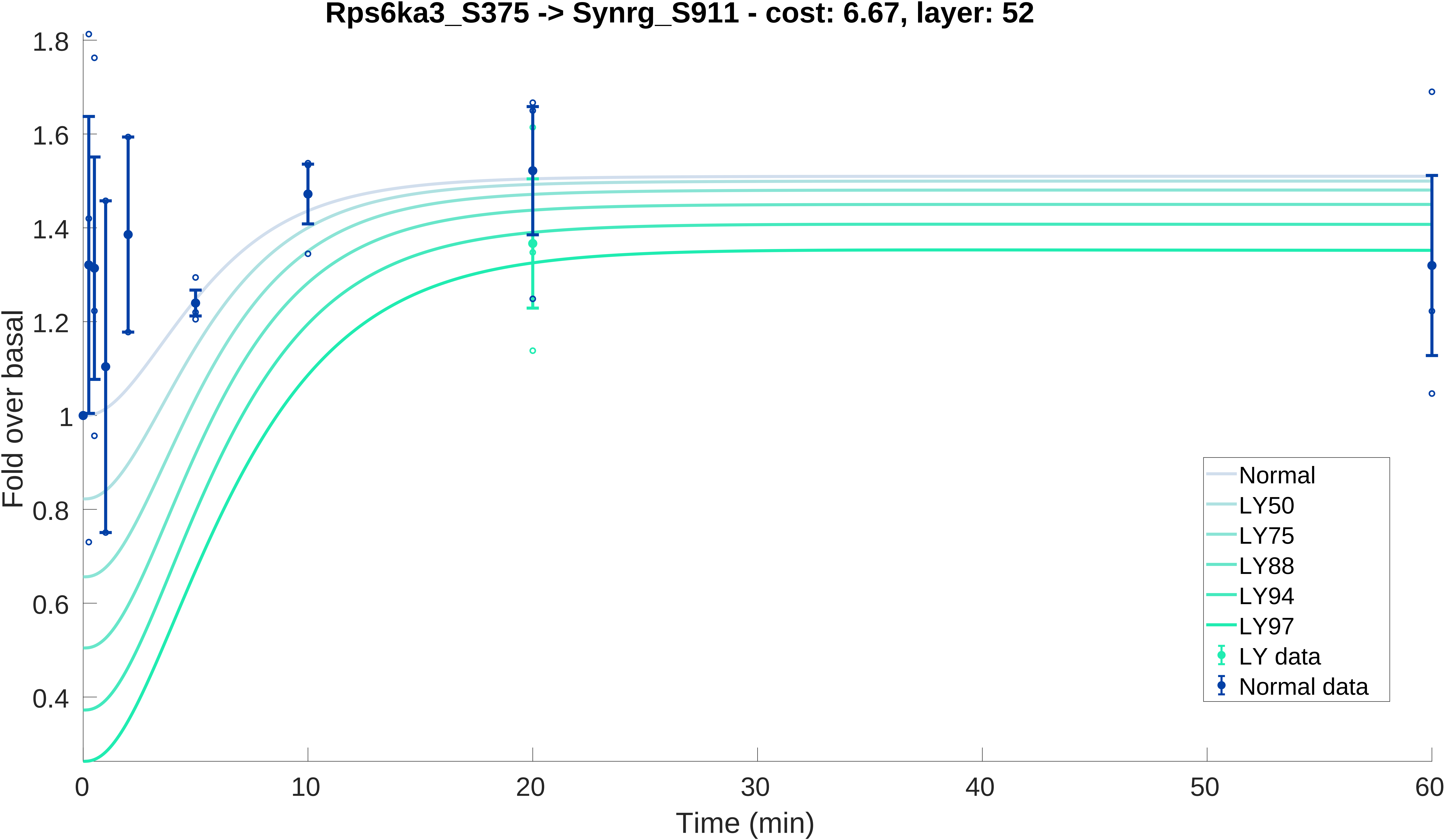
Rps6ka3_S375 -> Synrg_S911 - cost: 6.67, layer: 52
Fold over basal
Time (min)
0
10
20
30
40
50
60
0.4
0.6
0.8
1
1.2
1.4
1.6
1.8
Normal
LY50
LY75
LY88
LY94
LY97
LY data
Normal data

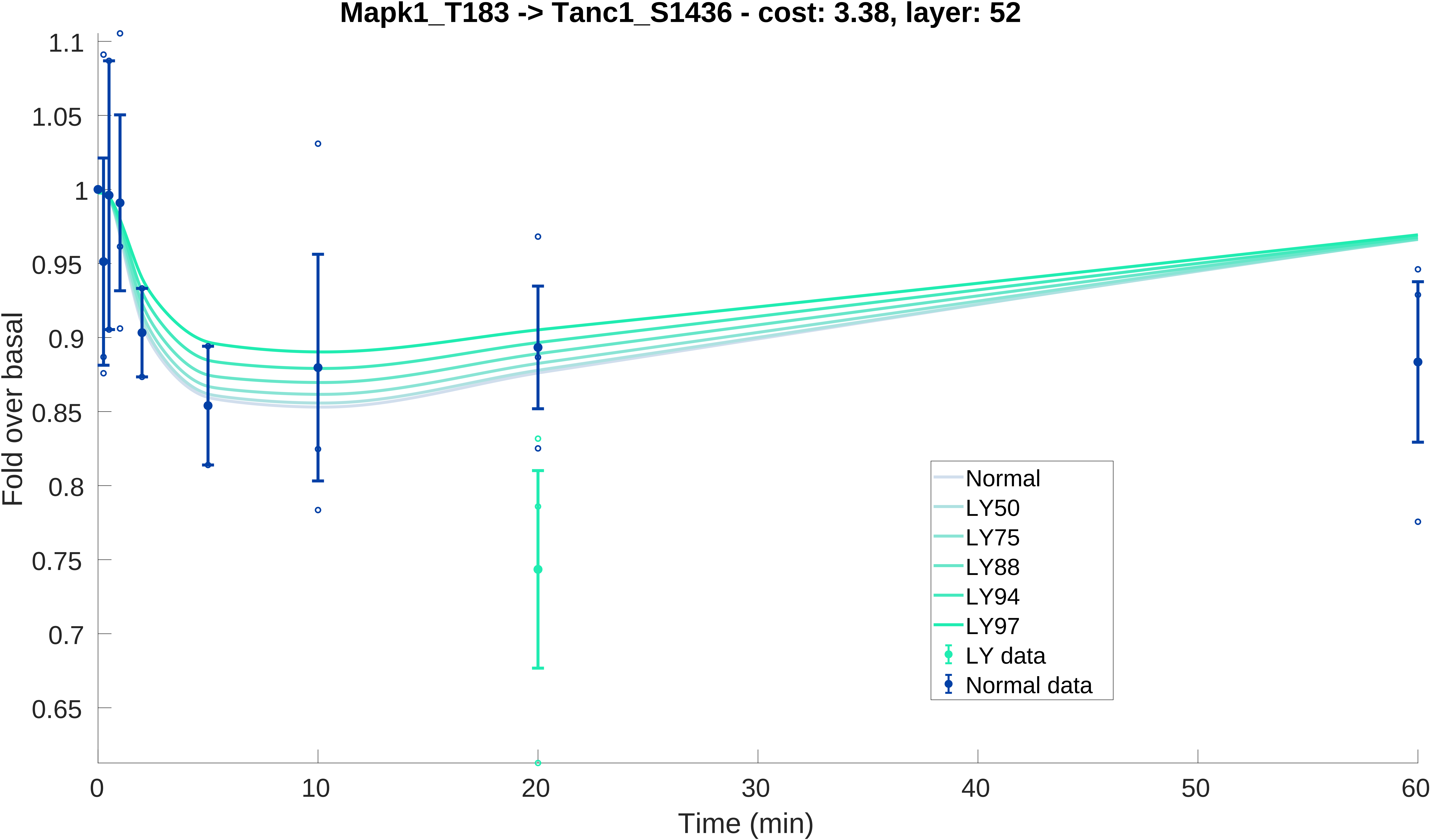

Mapk1_T183 -> Tanc1_S1436 - cost: 3.38, layer: 52
Fold over basal
Time (min)
1.1
1.05
1
0.95
0.9
0.85
0.8
0.75
0.7
0.65
0
10
20
30
40
50
60
Normal
LY50
LY75
LY88
LY94
LY97
LY data
Normal data

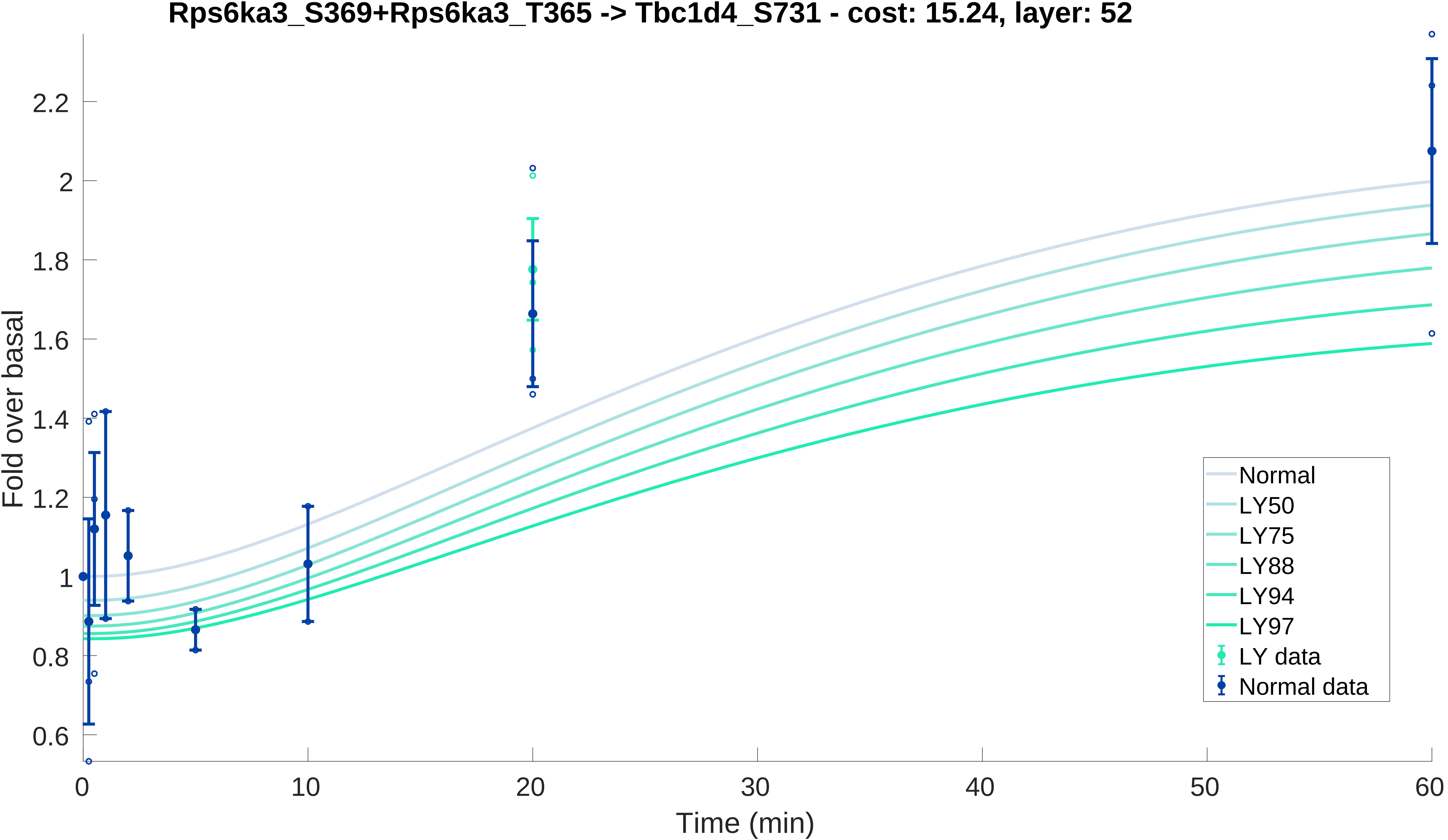
Rps6ka3_S369+Rps6ka3_T365 -> Tbc1d4_S731 - cost: 15.24, layer: 52
Fold over basal
Time (min)
0.6
0.8
1
1.2
1.4
1.6
1.8
2
2.2
0
10
20
30
40
50
60
Normal
LY50
LY75
LY88
LY94
LY97
LY data
Normal data

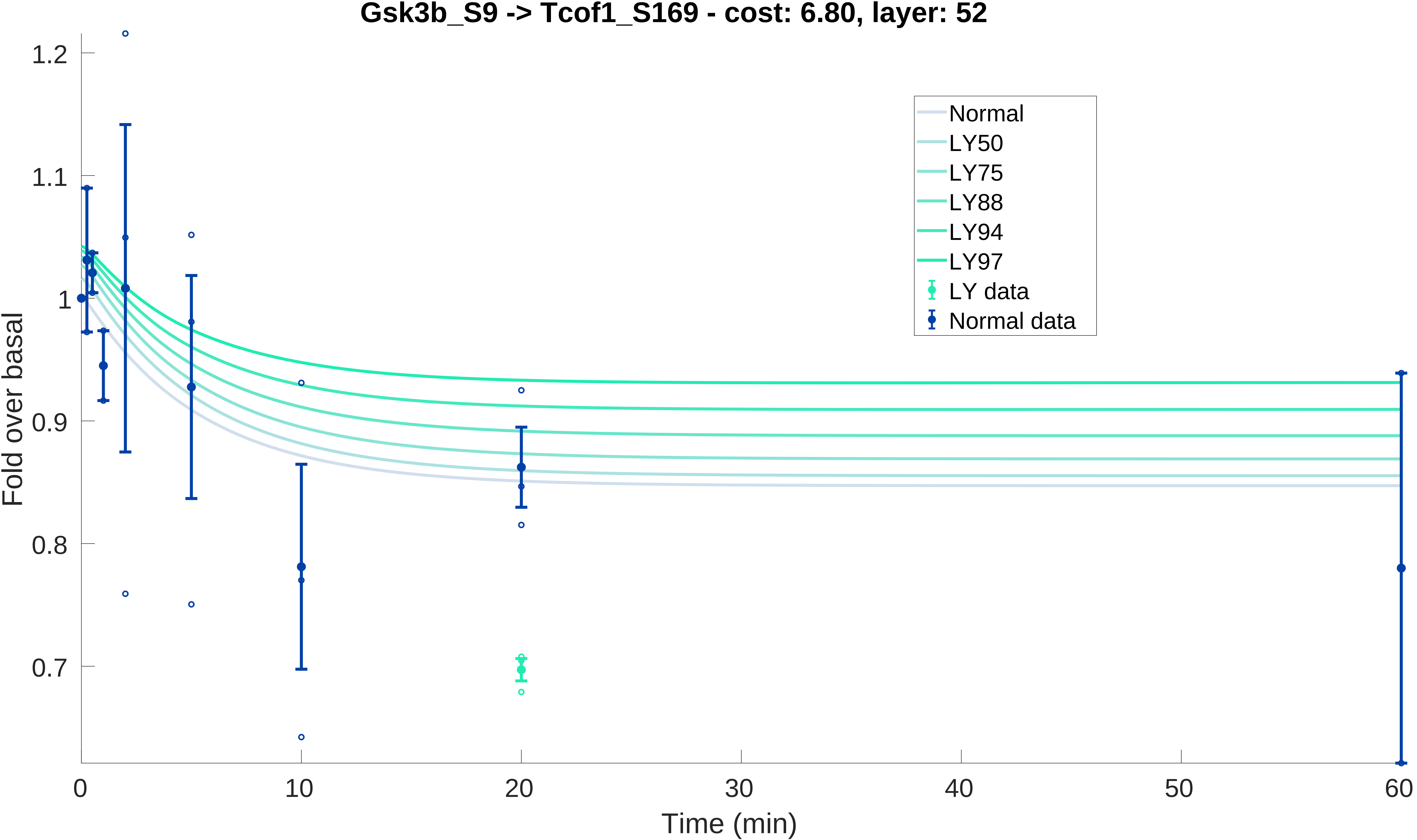

Gsk3b_S9 -> Tcof1_S169 - cost: 6.80, layer: 52
Fold over basal
Time (min)
1.2
1.1
1
0.9
0.8
0.7
0
10
20
30
40
50
60
Normal
LY50
LY75
LY88
LY94
LY97
LY data
Normal data

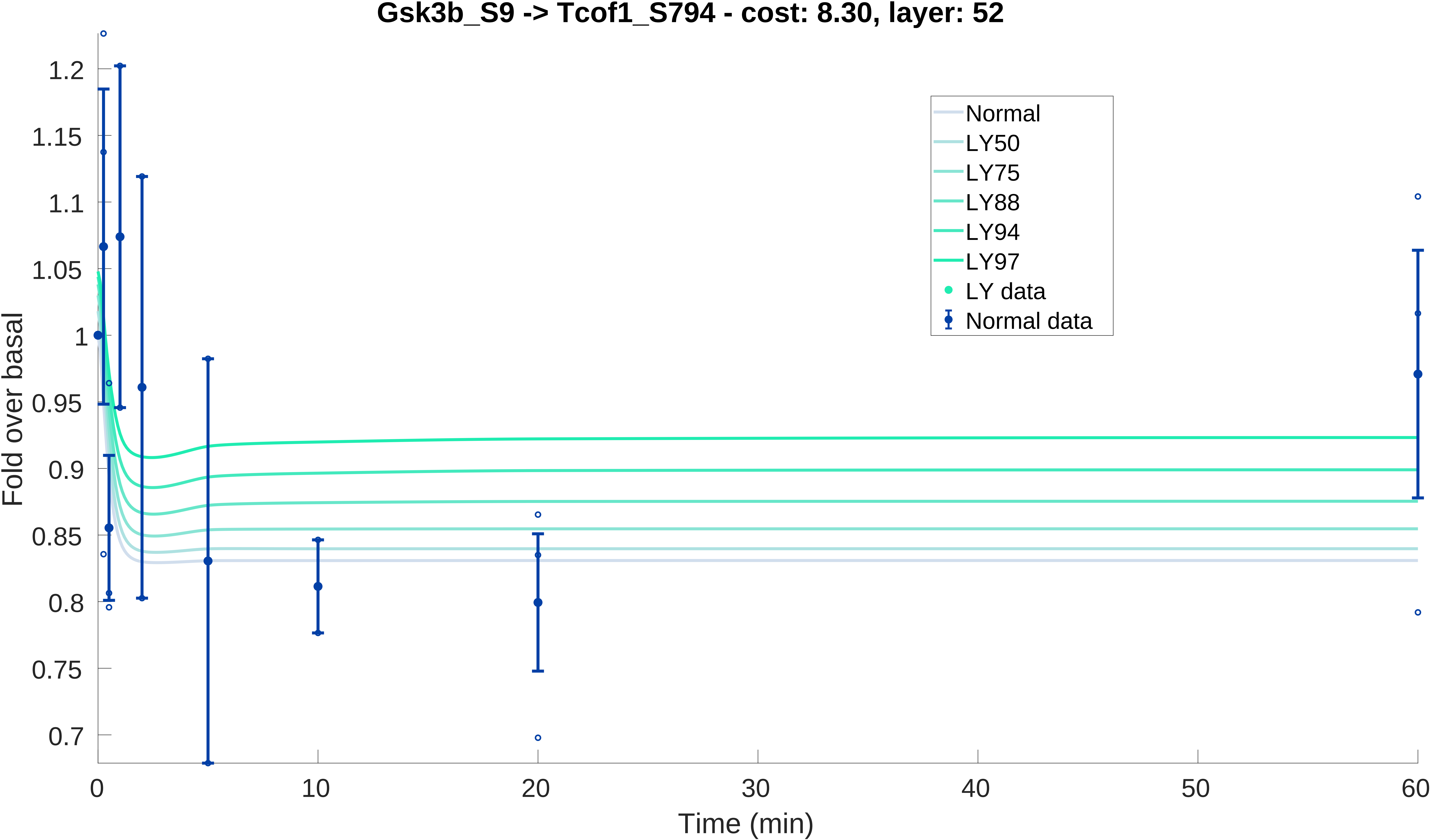
Gsk3b_S9 -> Tcof1_S794 - cost: 8.30, layer: 52
Fold over basal
Time (min)
1.2
1.15
1.1
1.05
1
0.95
0.9
0.85
0.8
0.75
0.7
0
10
20
30
40
50
60
Normal
LY50
LY75
LY88
LY94
LY97
LY data
Normal data

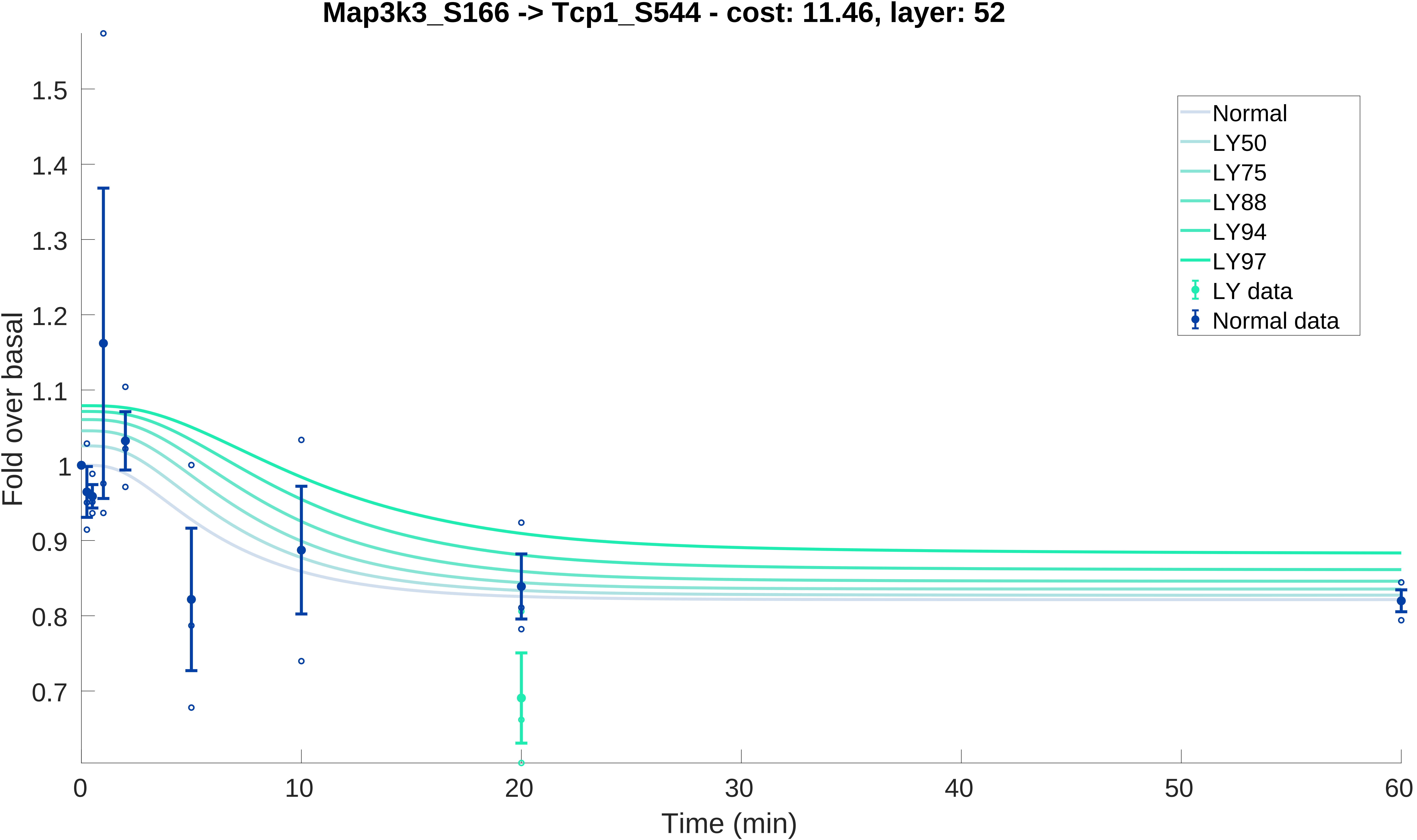
Map3k3_S166 -> Tcp1_S544 - cost: 11.46, layer: 52
Fold over basal
Time (min)
Normal
LY50
LY75
LY88
LY94
LY97
LY data
Normal data
1.5
1.4
1.3
1.2
1.1
1
0.9
0.8
0.7
0
10
20
30
40
50
60

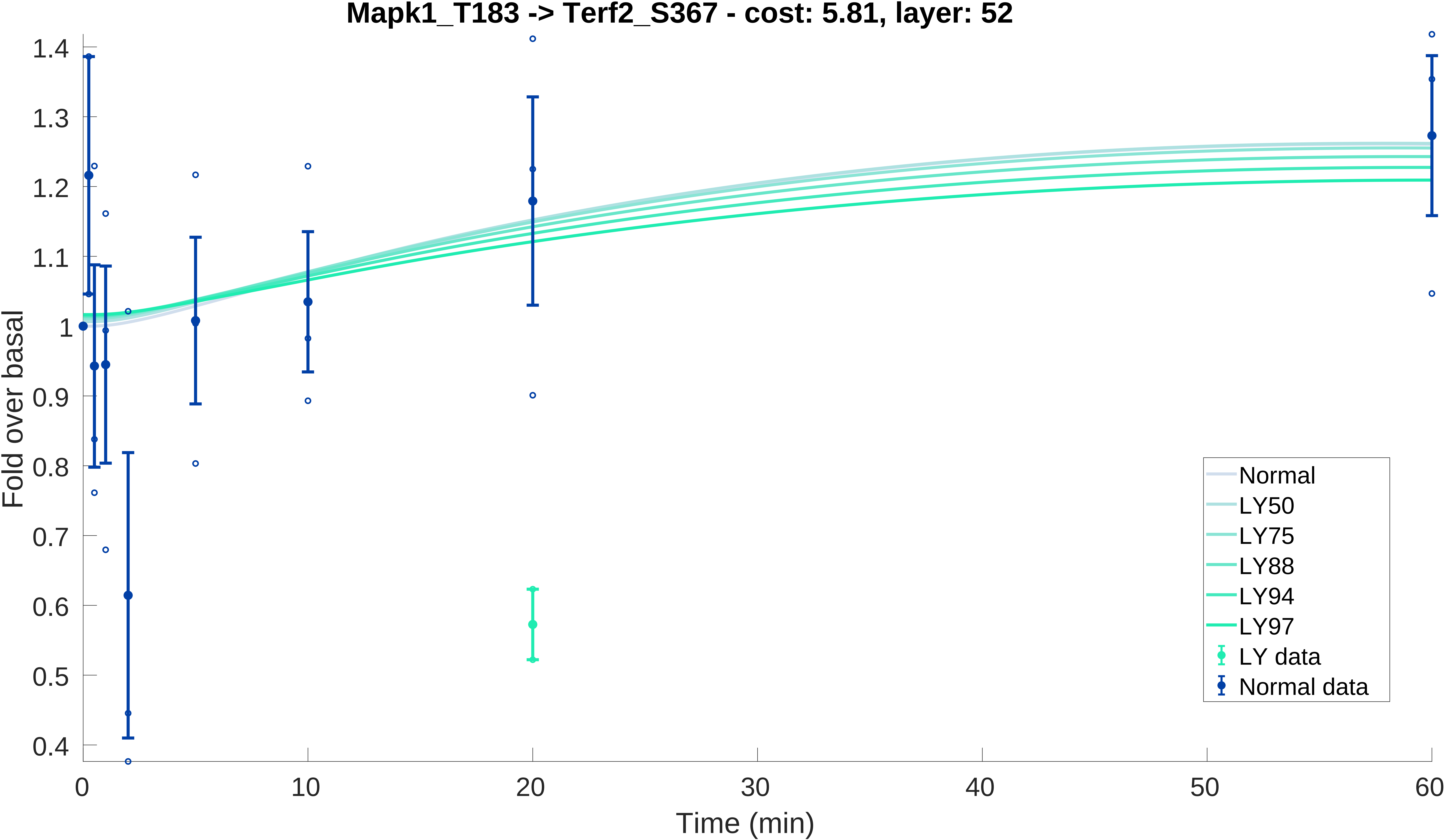
Mapk1_T183 -> Terf2_S367 - cost: 5.81, layer: 52
Fold over basal
Time (min)
0.4
0.5
0.6
0.7
0.8
0.9
1
1.1
1.2
1.3
1.4
0
10
20
30
40
50
60
Normal
LY50
LY75
LY88
LY94
LY97
LY data
Normal data

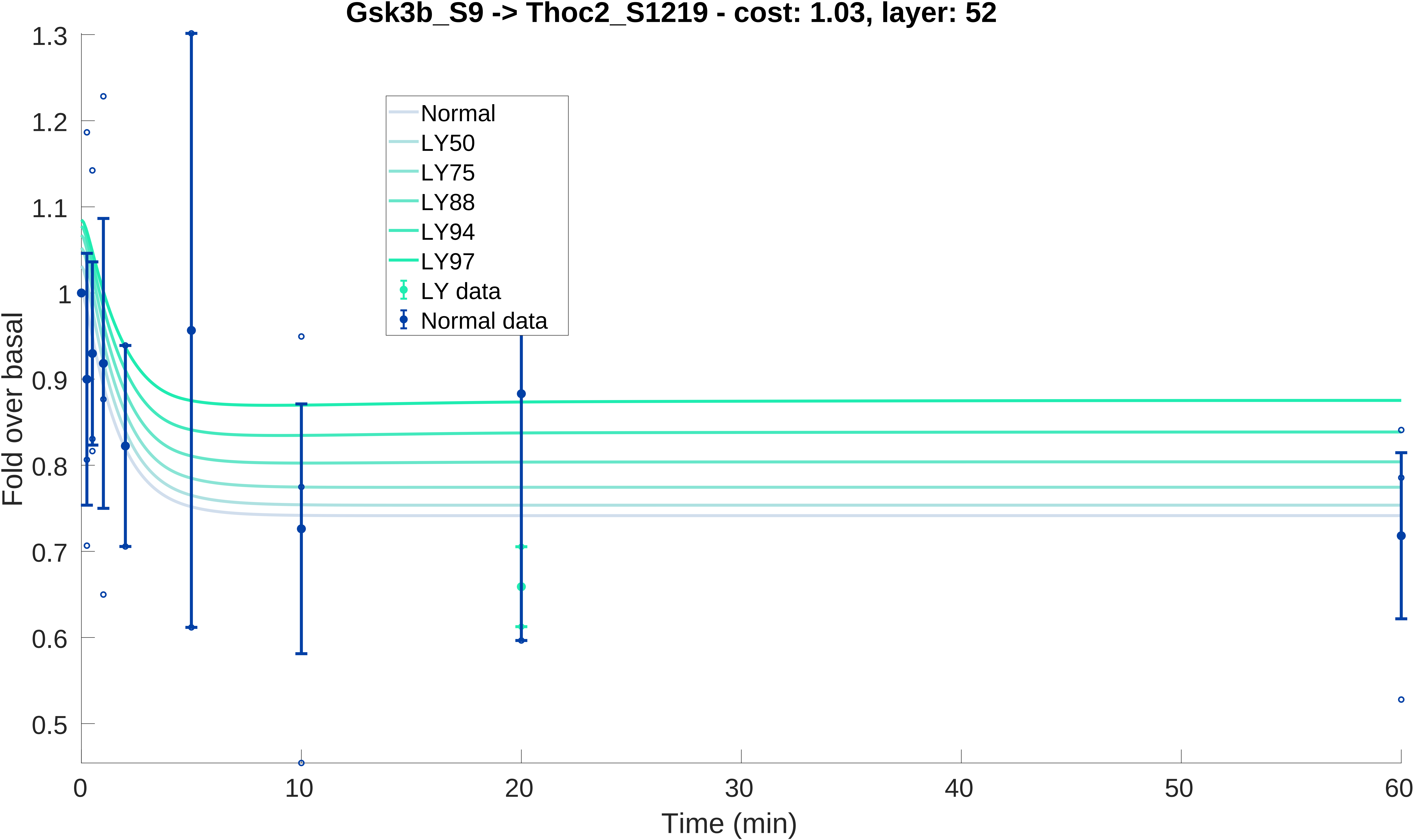

Gsk3b_S9 -> Thoc2_S1219 - cost: 1.03, layer: 52
Fold over basal
Time (min)
1.3
1.2
1.1
1
0.9
0.8
0.7
0.6
0.5
0
10
20
30
40
50
60
Normal
LY50
LY75
LY88
LY94
LY97
LY data
Normal data

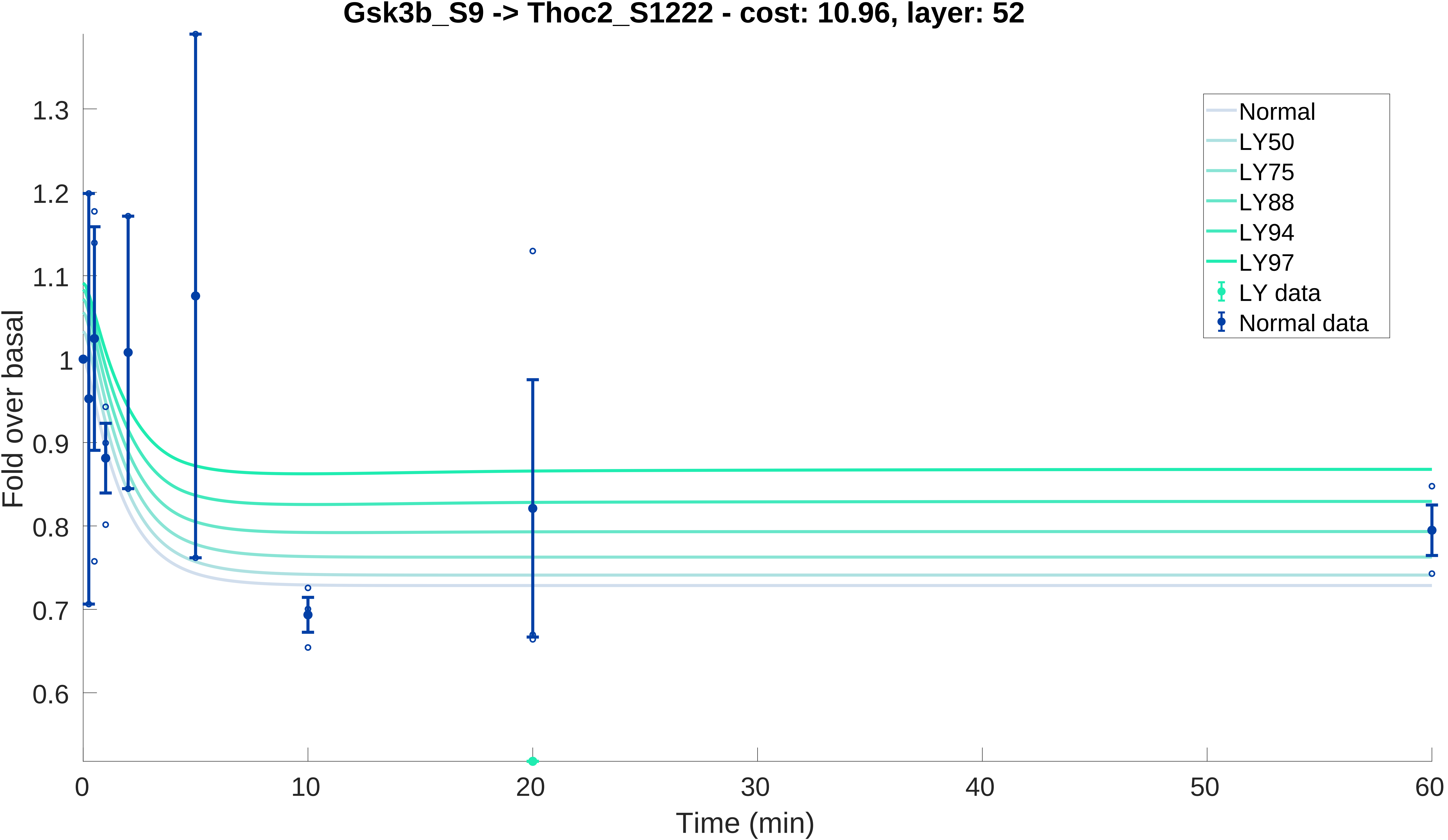

Gsk3b_S9 -> Thoc2_S1222 - cost: 10.96, layer: 52
Fold over basal
Time (min)
0.6
0.7
0.8
0.9
1
1.1
1.2
1.3
0
10
20
30
40
50
60
Normal
LY50
LY75
LY88
LY94
LY97
LY data
Normal data

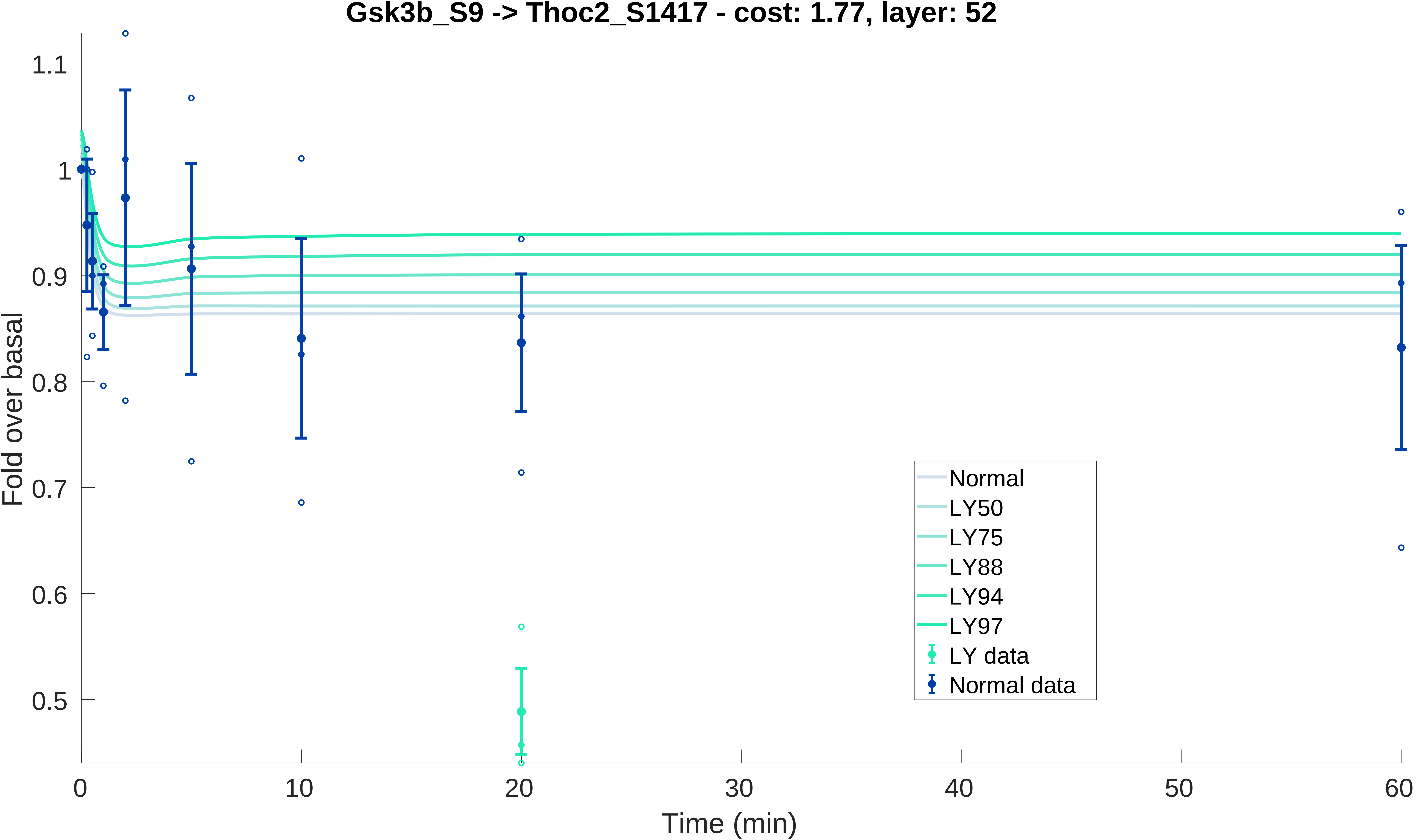

Gsk3b_S9 -> Thoc2_S1417 - cost: 1.77, layer: 52
Fold over basal
1.1
1
0.9
0.8
0.7
0.6
0.5
0
10
20
30
40
50
60
Time (min)
Normal
LY50
LY75
LY88
LY94
LY97
LY data
Normal data

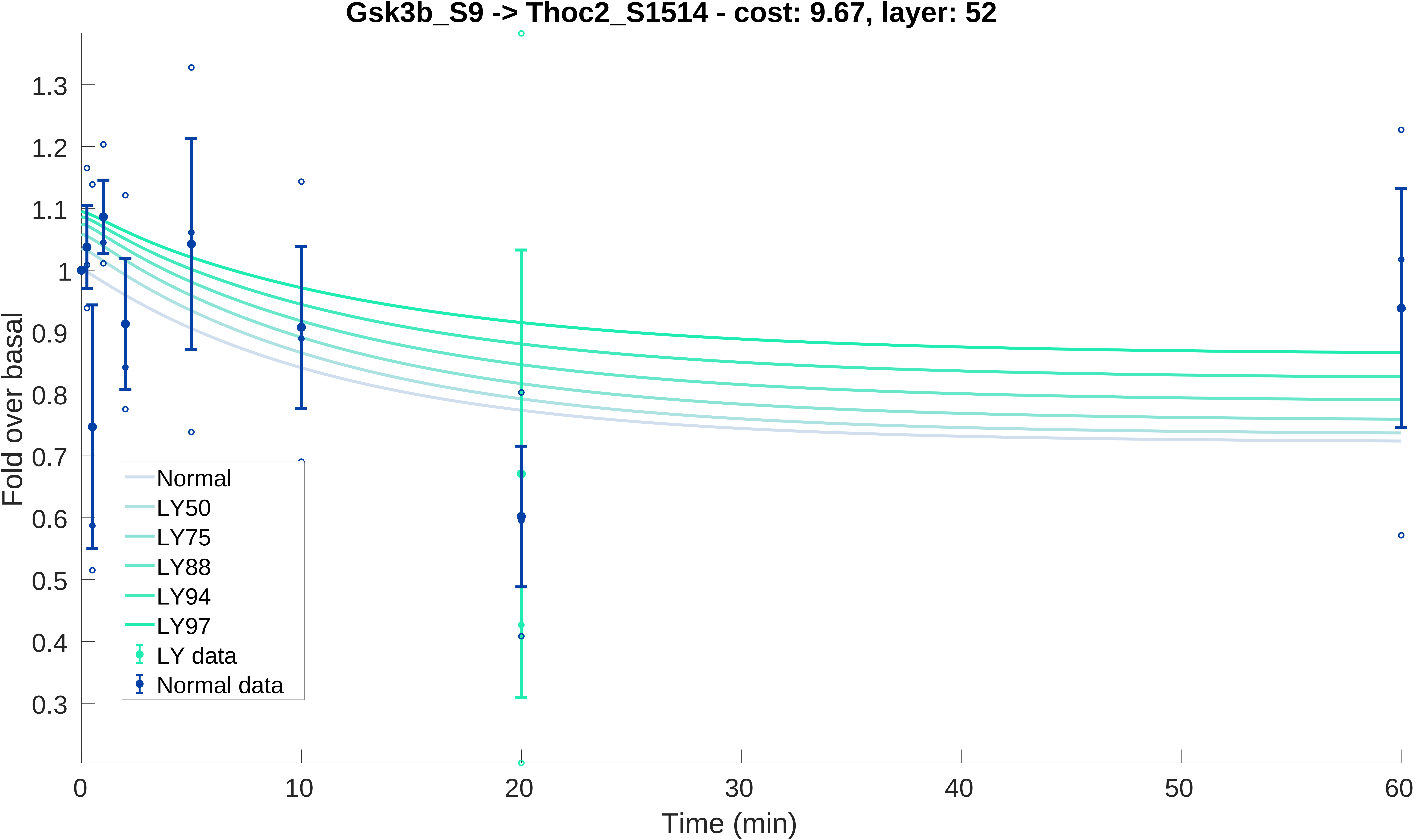
Gsk3b_S9 -> Thoc2_S1514 - cost: 9.67, layer: 52
Fold over basal
1.3
1.2
1.1
1
0.9
0.8
0.7
0.6
0.5
0.4
0.3
0
10
20
30
40
50
60
Time (min)
Normal
LY50
LY75
LY88
LY94
LY97
LY data
Normal data

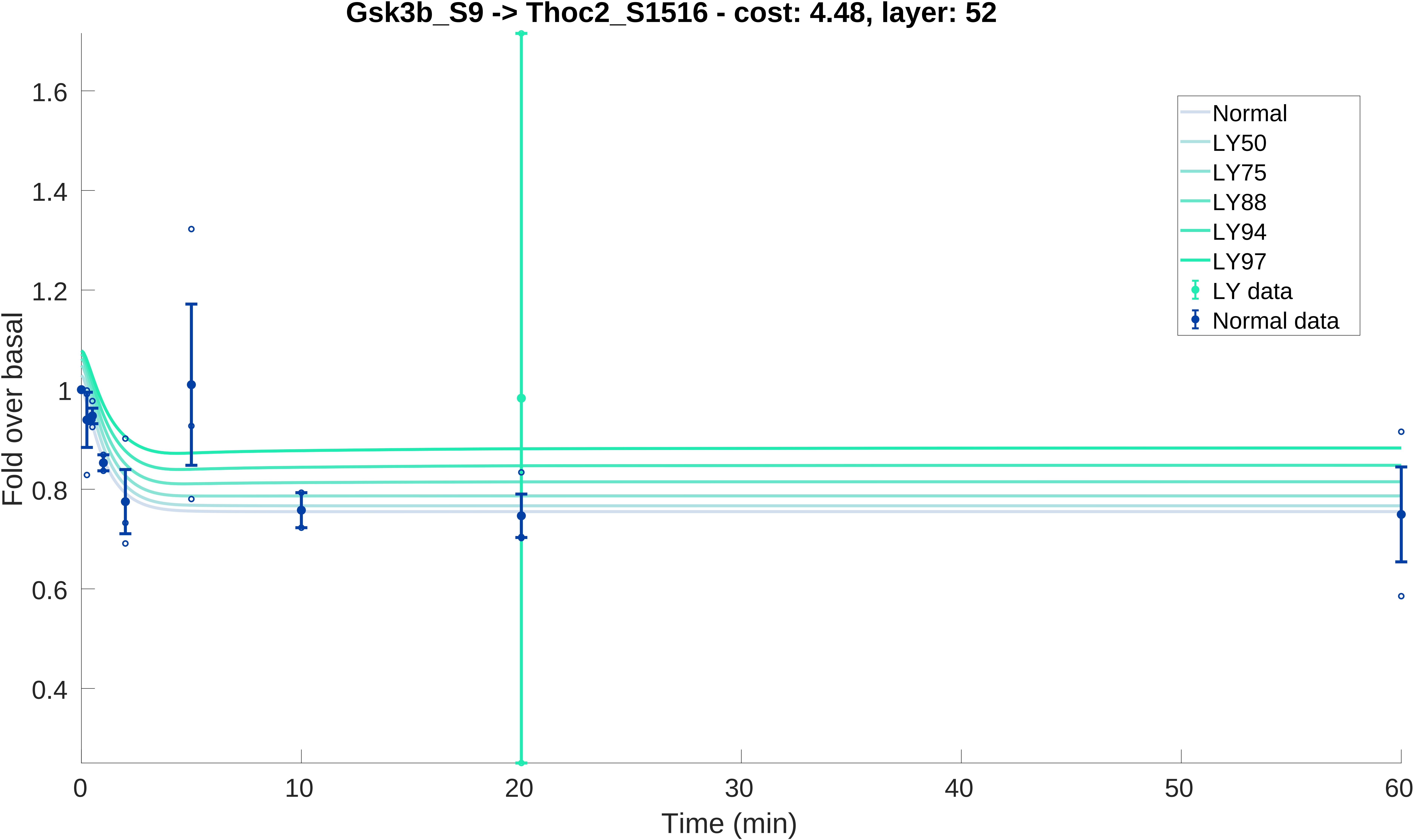

Gsk3b_S9 -> Thoc2_S1516 - cost: 4.48, layer: 52
Fold over basal
Time (min)
1.6
1.4
1.2
1
0.8
0.6
0.4
0
10
20
30
40
50
60
Normal
LY50
LY75
LY88
LY94
LY97
LY data
Normal data

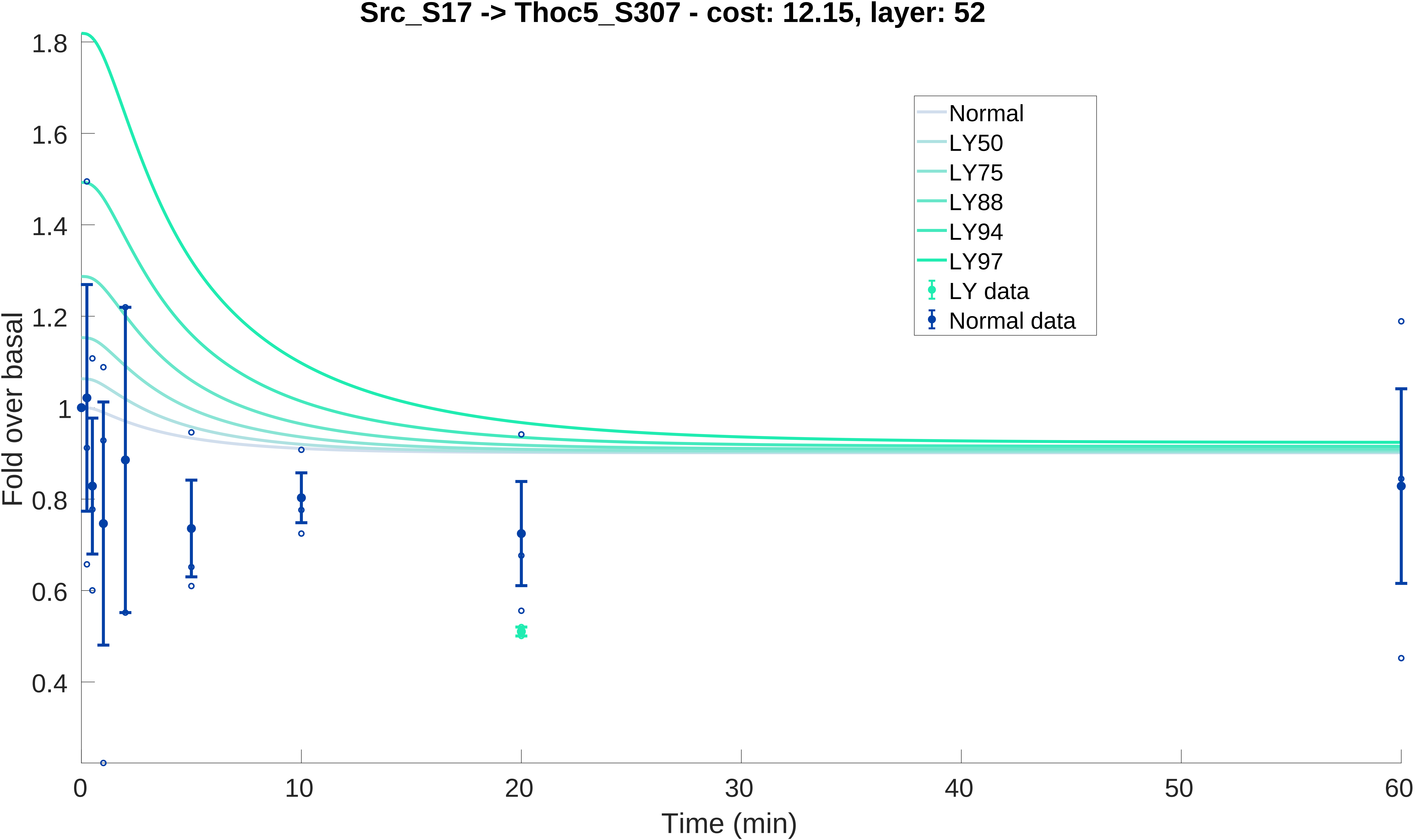

Src_S17 -> Thoc5_S307 - cost: 12.15, layer: 52
Normal
LY50
LY75
LY88
LY94
LY97
LY data
Normal data
Fold over basal
Time (min)
1.8
1.6
1.4
1.2
1
0.8
0.6
0.4
0
10
20
30
40
50
60

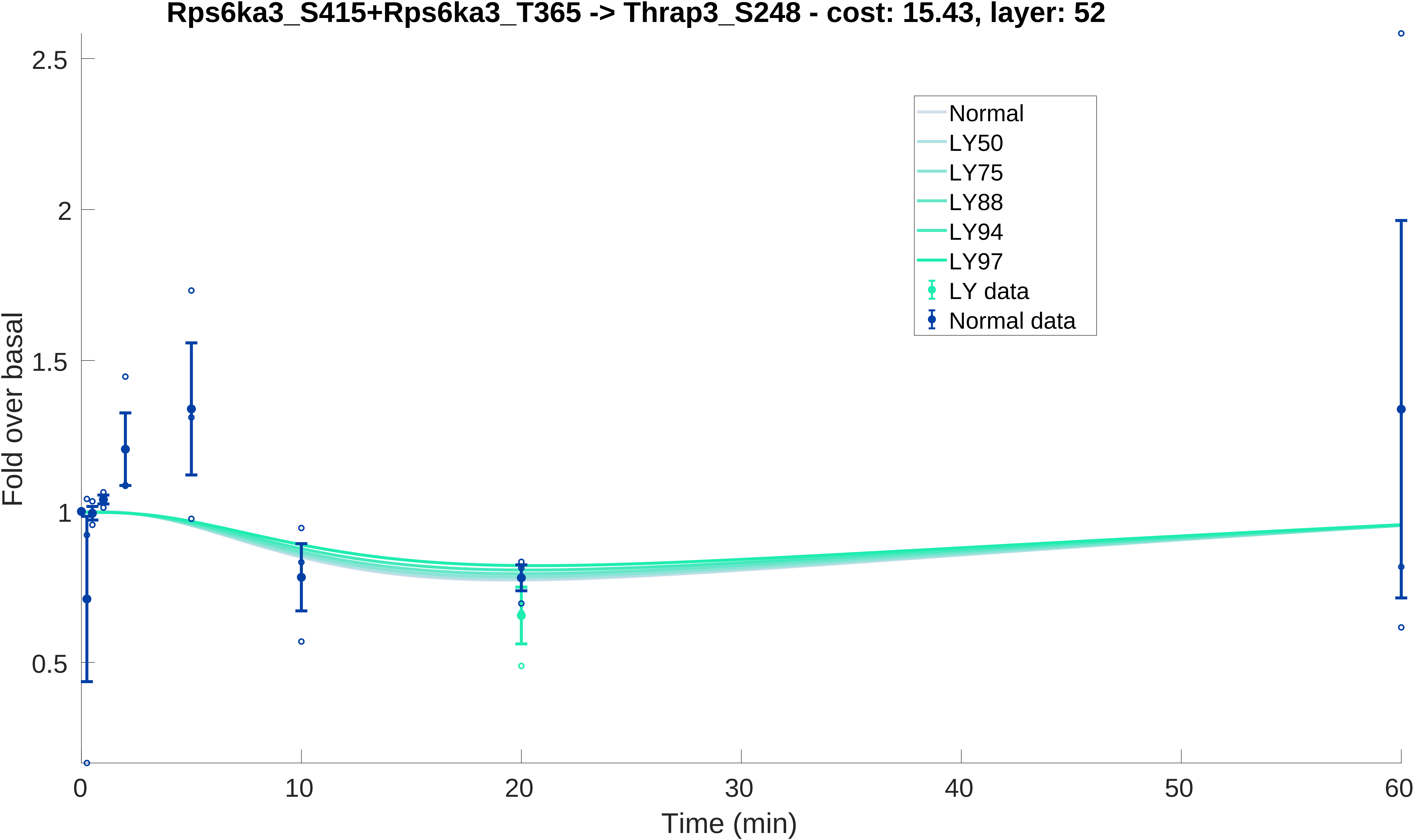

Rps6ka3_S415+Rps6ka3_T365 -> Thrap3_S248 - cost: 15.43, layer: 52
Fold over basal
Time (min)
0.5
1
1.5
2
2.5
0
10
20
30
40
50
60
Normal
LY50
LY75
LY88
LY94
LY97
LY data
Normal data

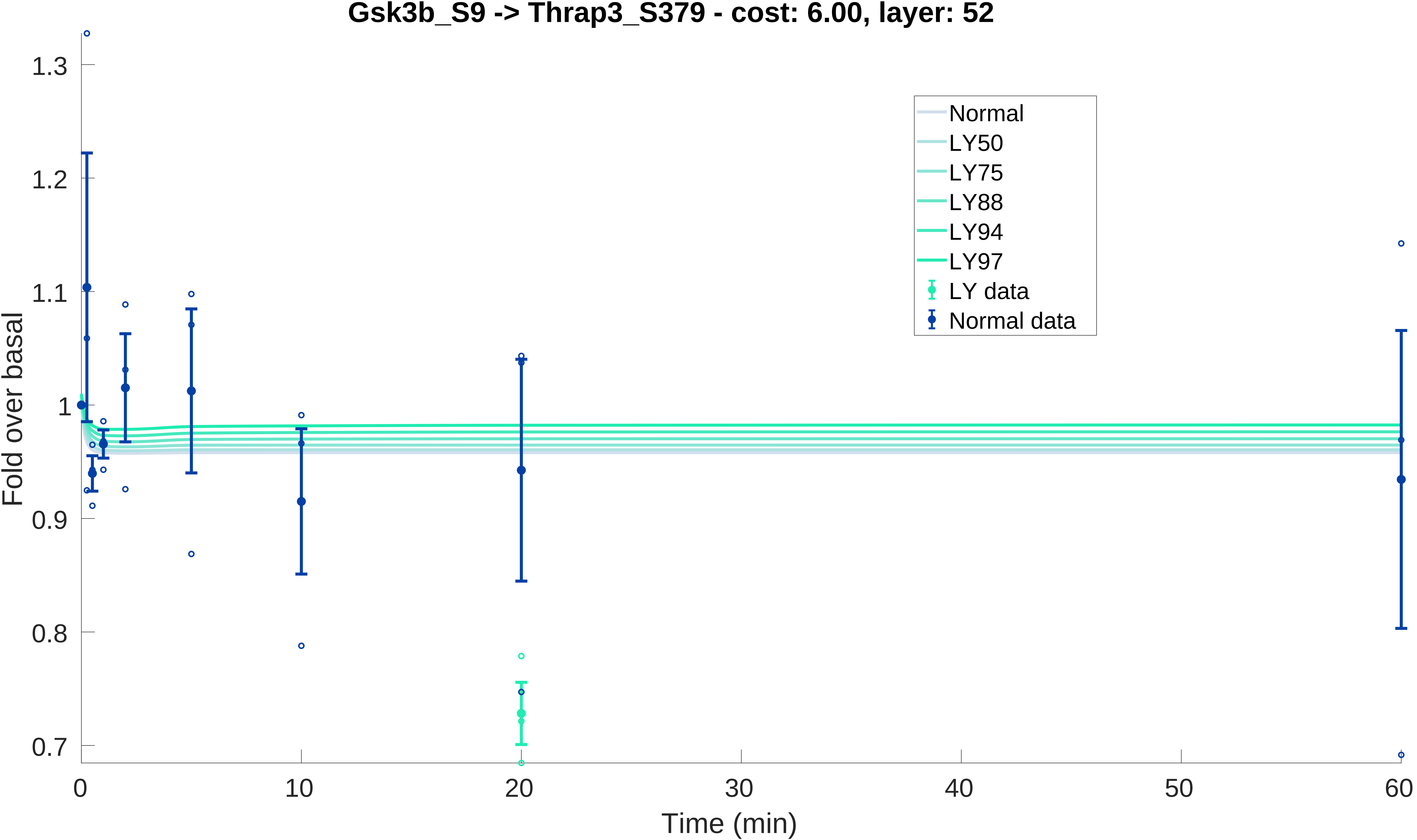
Gsk3b_S9 -> Thrap3_S379 - cost: 6.00, layer: 52
Fold over basal
Time (min)
1.3
1.2
1.1
1
0.9
0.8
0.7
0
10
20
30
40
50
60
Normal
LY50
LY75
LY88
LY94
LY97
LY data
Normal data

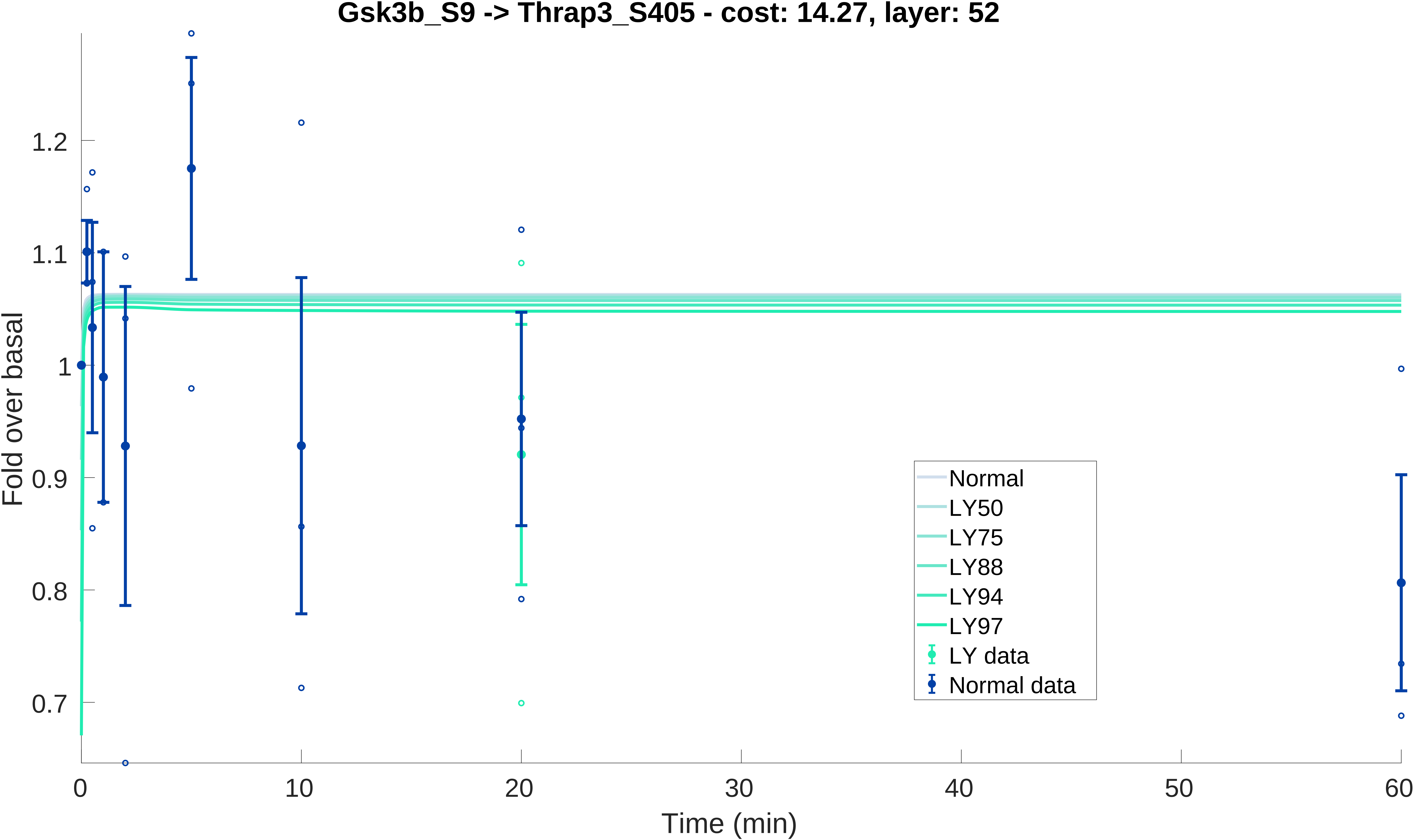

Gsk3b_S9 -> Thrap3_S405 - cost: 14.27, layer: 52
Fold over basal
1.2
1.1
1
0.9
0.8
0.7
0
10
20
30
40
50
60
Time (min)
Normal
LY50
LY75
LY88
LY94
LY97
LY data
Normal data

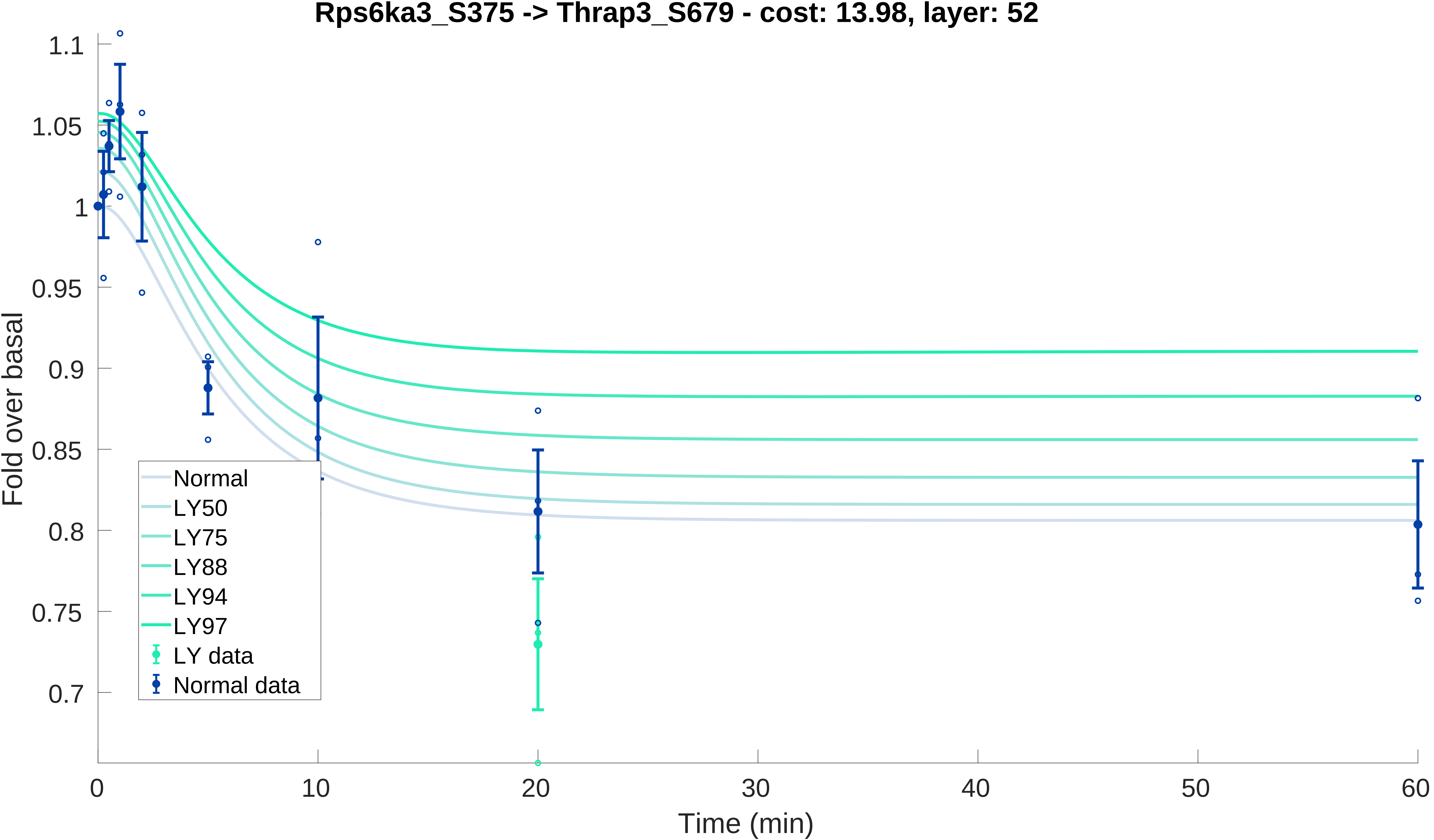

Rps6ka3_S375 -> Thrap3_S679 - cost: 13.98, layer: 52
Fold over basal
Time (min)
1.1
1.05
1
0.95
0.9
0.85
0.8
0.75
0.7
0
10
20
30
40
50
60
Normal
LY50
LY75
LY88
LY94
LY97
LY data
Normal data

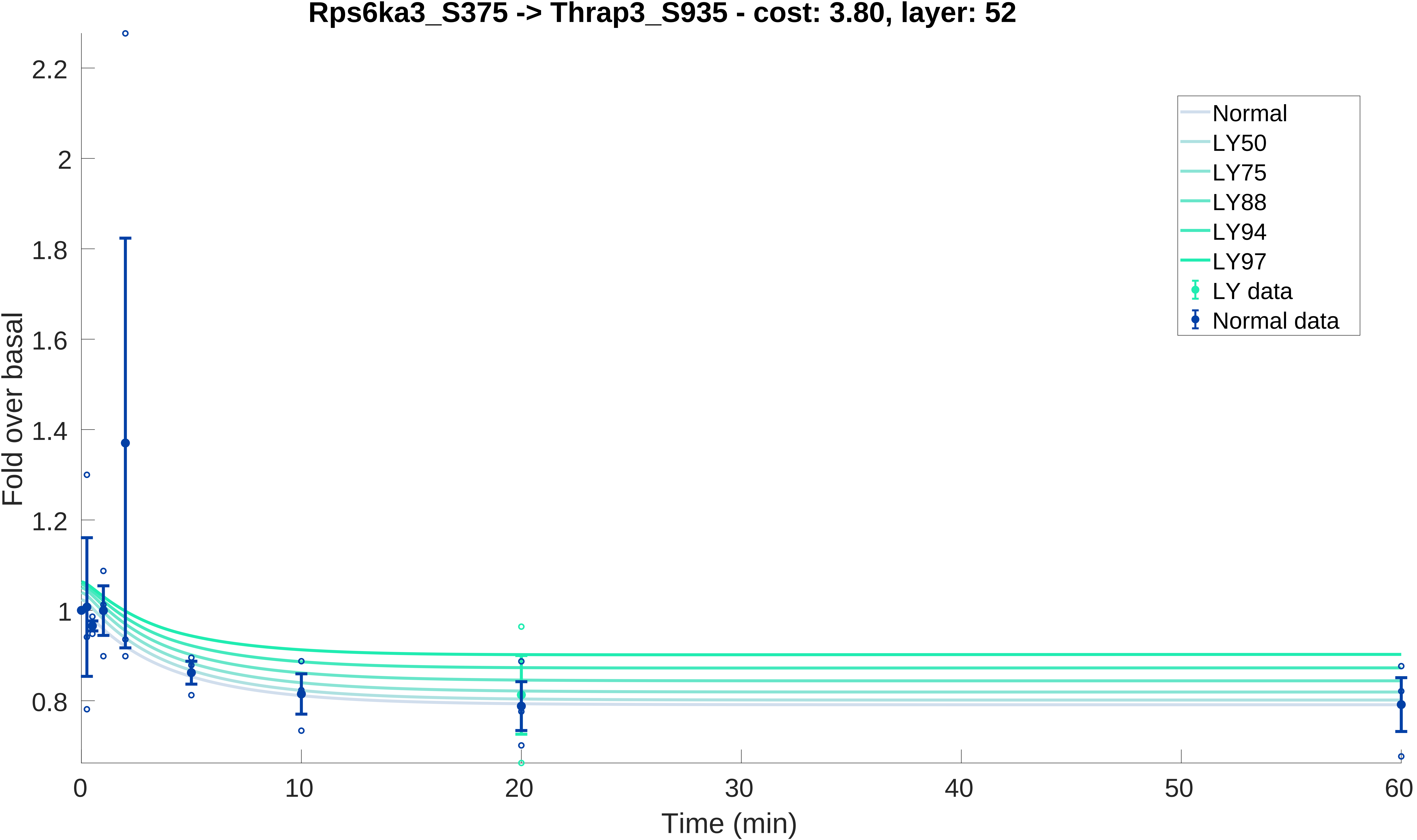

Rps6ka3_S375 -> Thrap3_S935 - cost: 3.80, layer: 52
Fold over basal
Time (min)
0.8
1
1.2
1.4
1.6
1.8
2
2.2
0
10
20
30
40
50
60
Normal
LY50
LY75
LY88
LY94
LY97
LY data
Normal data

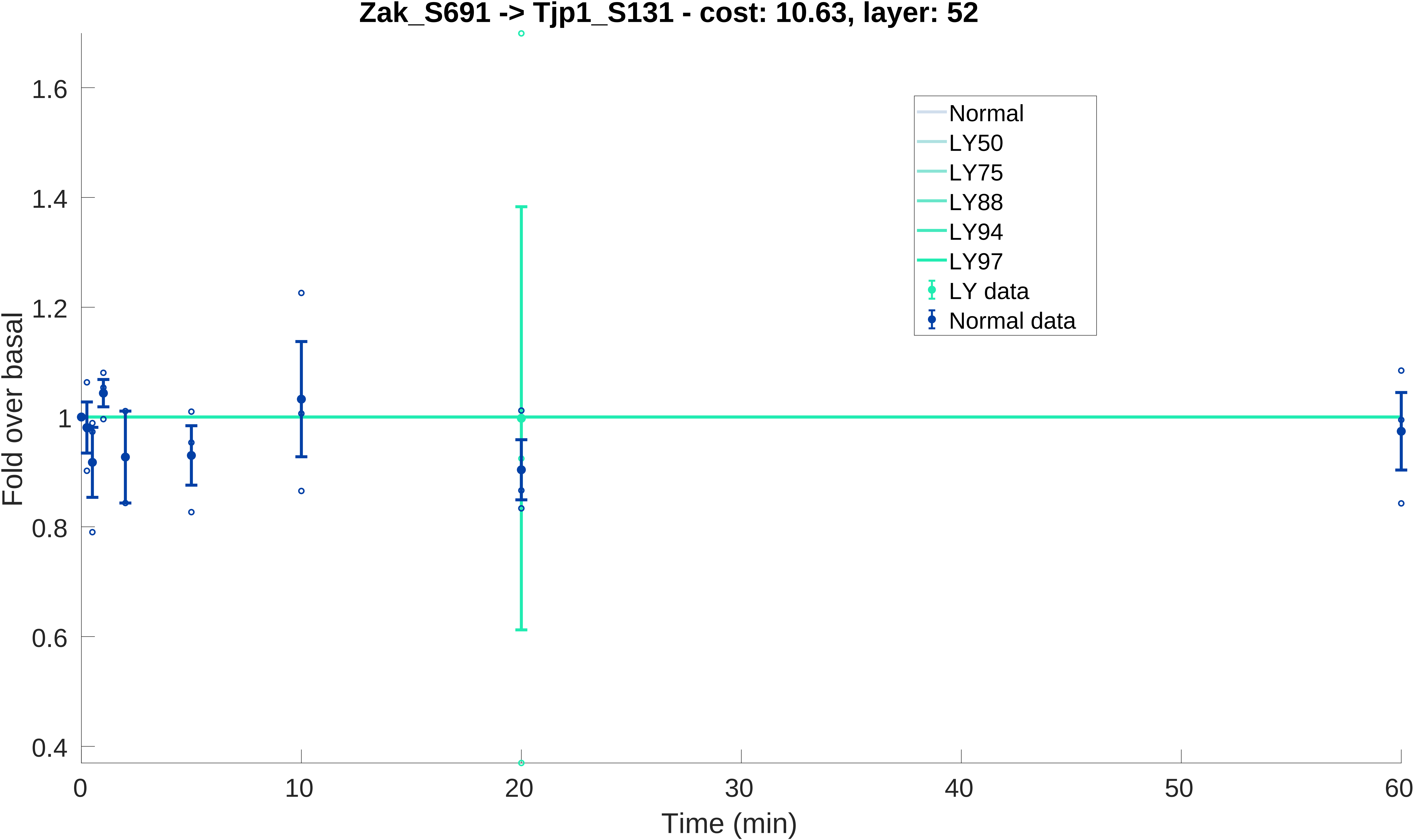

Zak_S691 -> Tjp1_S131 - cost: 10.63, layer: 52
Fold over basal
1.6
1.4
1.2
1
0.8
0.6
0.4
0
10
20
30
40
50
60
Time (min)
Normal
LY50
LY75
LY88
LY94
LY97
LY data
Normal data

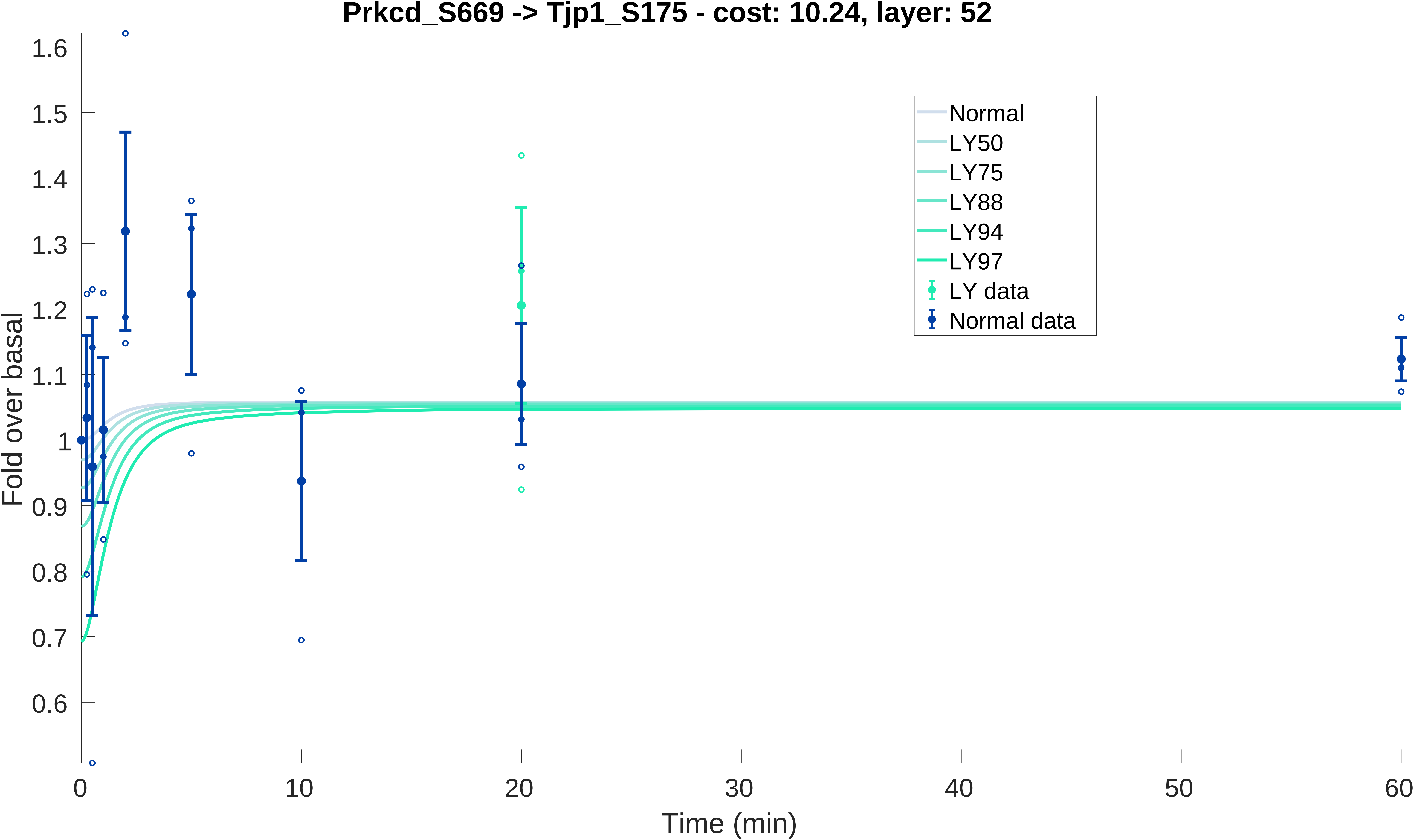

Prkcd_S669 -> Tjp1_S175 - cost: 10.24, layer: 52
Fold over basal
Time (min)
1.6
1.5
1.4
1.3
1.2
1.1
1
0.9
0.8
0.7
0.6
0
10
20
30
40
50
60
Normal
LY50
LY75
LY88
LY94
LY97
LY data
Normal data

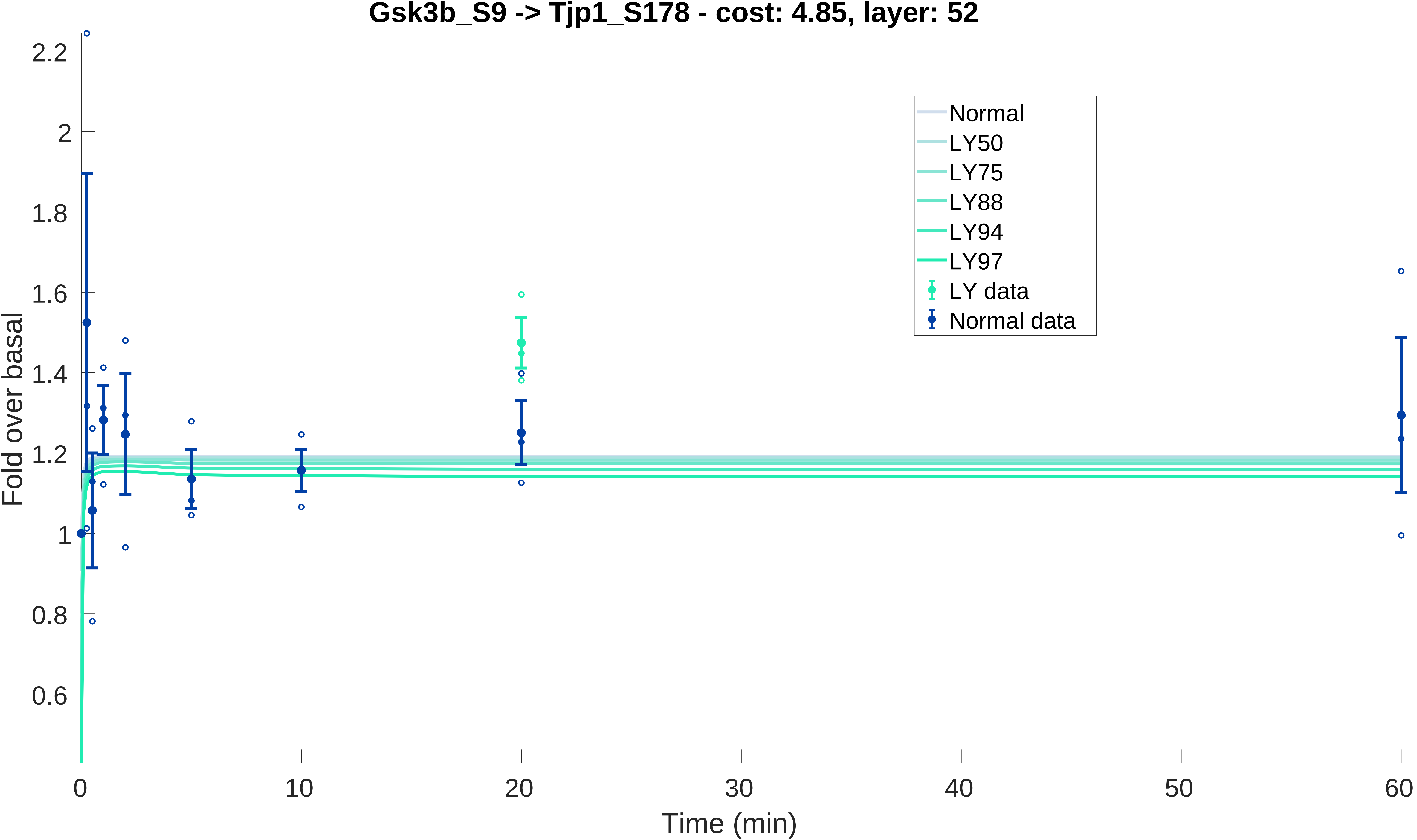

Gsk3b_S9 -> Tjp1_S178 - cost: 4.85, layer: 52
Fold over basal
Time (min)
Normal
LY50
LY75
LY88
LY94
LY97
LY data
Normal data
0.6
0.8
1
1.2
1.4
1.6
1.8
2
2.2
0
10
20
30
40
50
60

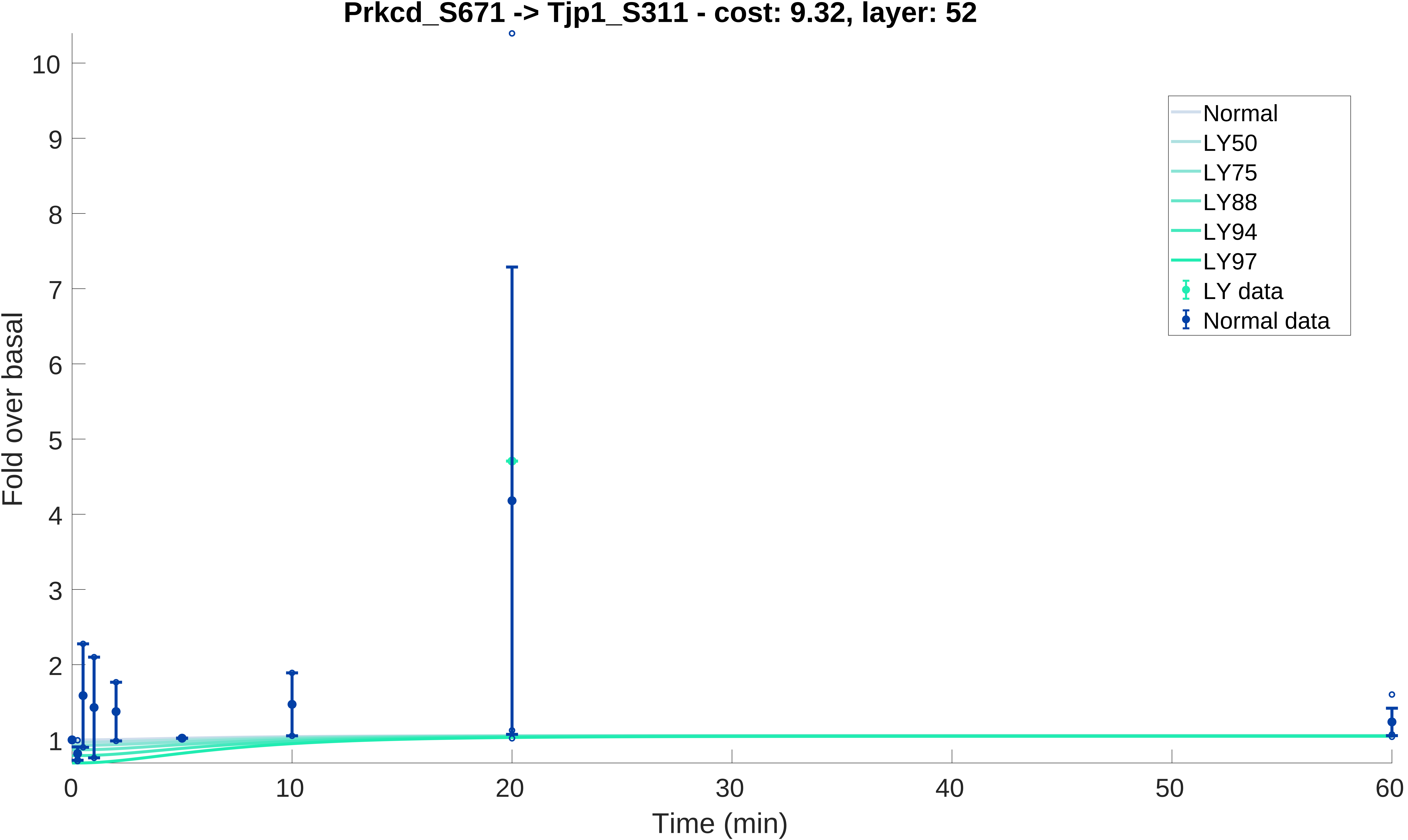

Prkcd_S671 -> Tjp1_S311 - cost: 9.32, layer: 52
Fold over basal
Time (min)
10
9
8
7
6
5
4
3
2
1
0
10
20
30
40
50
60
Normal
LY50
LY75
LY88
LY94
LY97
LY data
Normal data

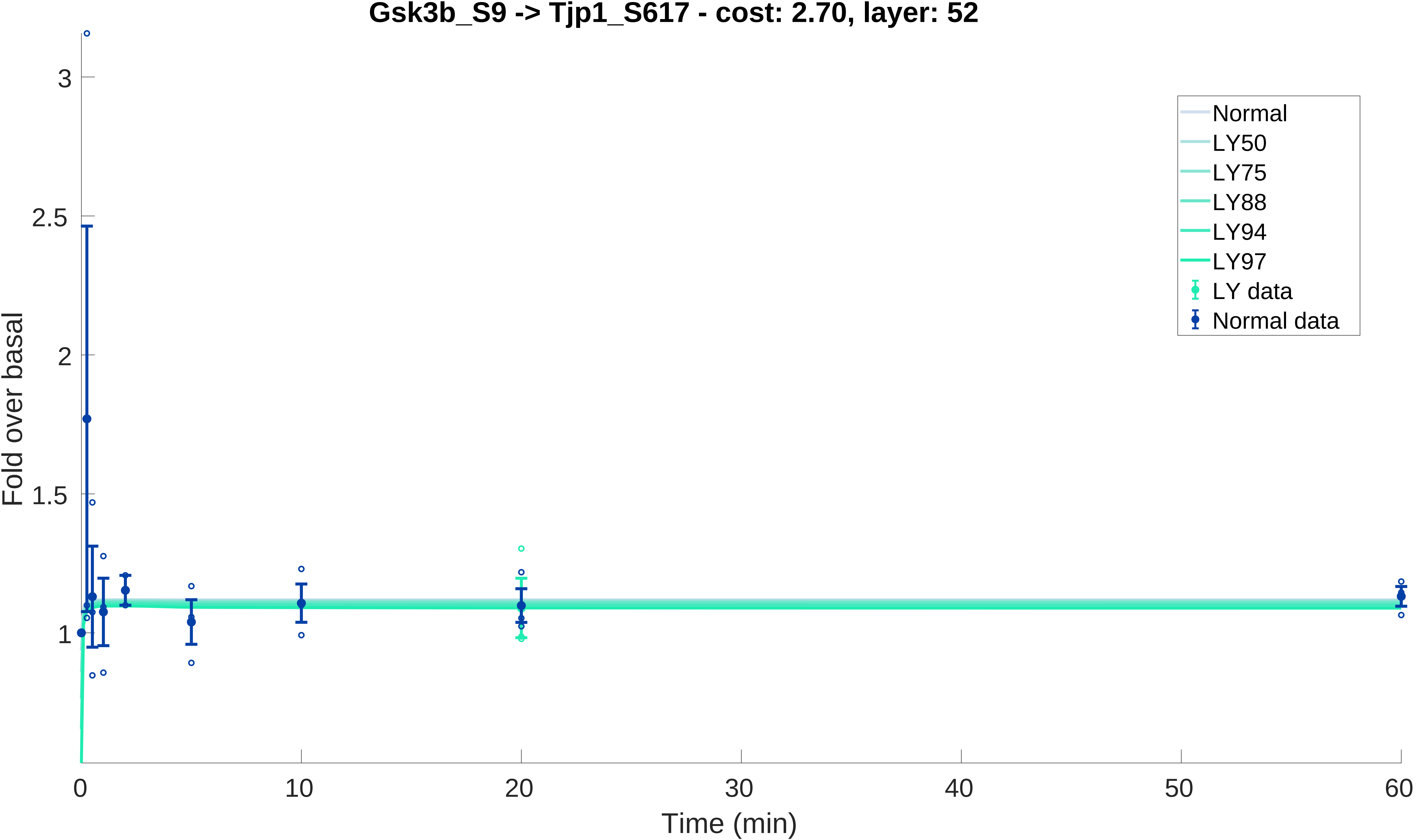

Gsk3b_S9 -> Tjp1_S617 - cost: 2.70, layer: 52
Fold over basal
Time (min)
3
2.5
2
1.5
1
0
10
20
30
40
50
60
Normal
LY50
LY75
LY88
LY94
LY97
LY data
Normal data

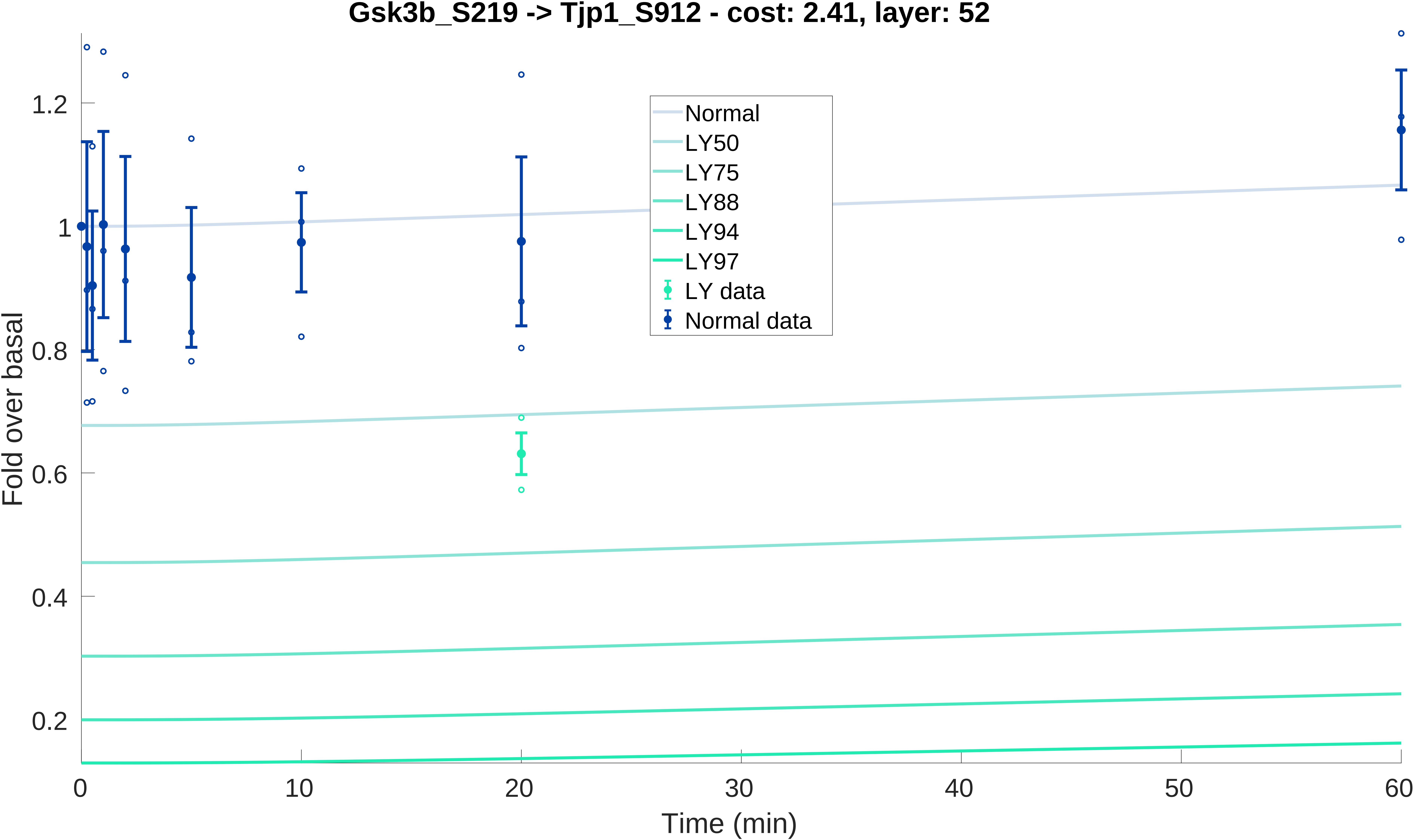

Gsk3b_S219 -> Tjp1_S912 - cost: 2.41, layer: 52
Fold over basal
Time (min)
Normal
LY50
LY75
LY88
LY94
LY97
LY data
Normal data
1.2
1
0.8
0.6
0.4
0.2
0
10
20
30
40
50
60

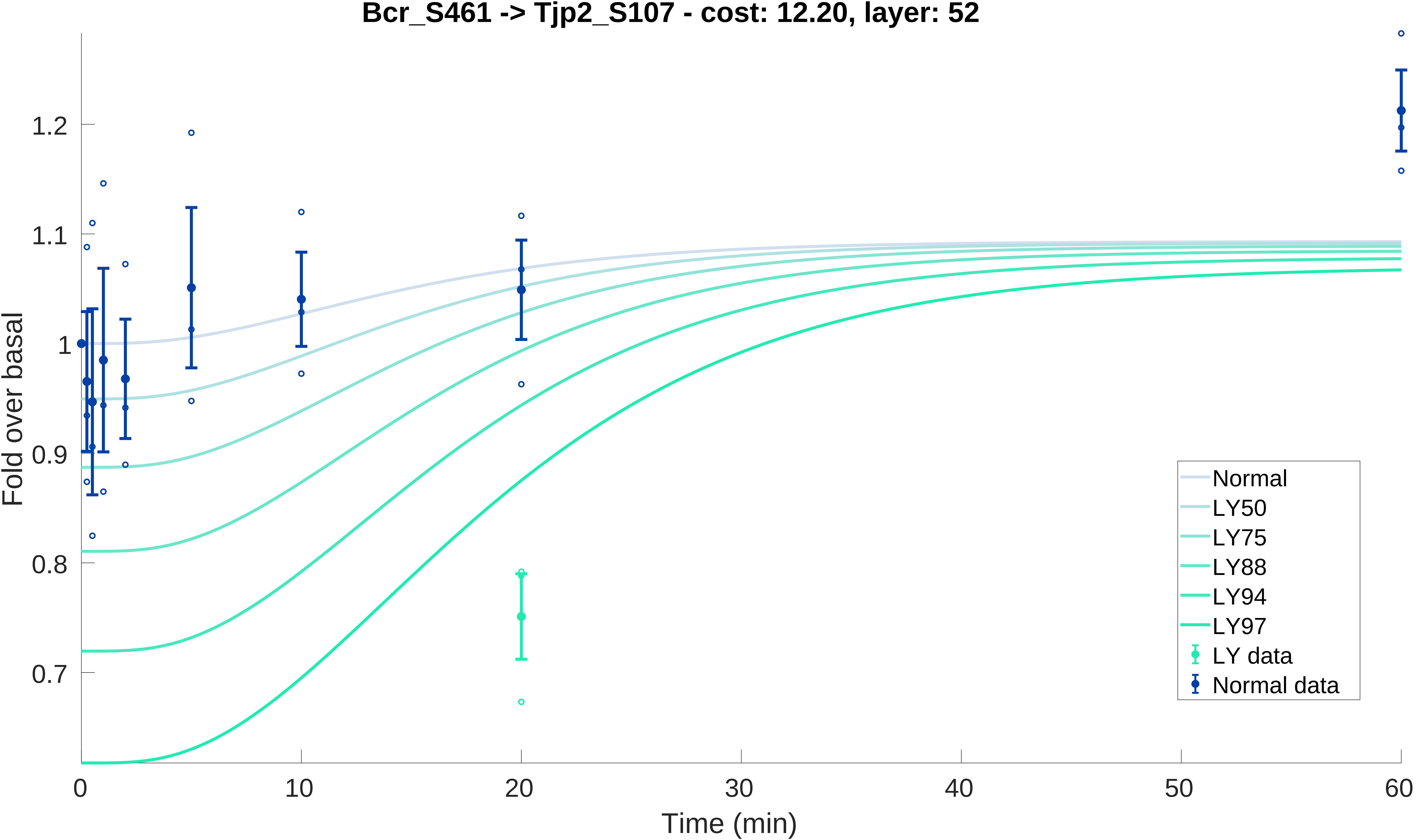

Bcr_S461 -> Tjp2_S107 - cost: 12.20, layer: 52
Fold over basal
1.2
1.1
1
0.9
0.8
0.7
0
10
20
30
40
50
60
Time (min)
Normal
LY50
LY75
LY88
LY94
LY97
LY data
Normal data

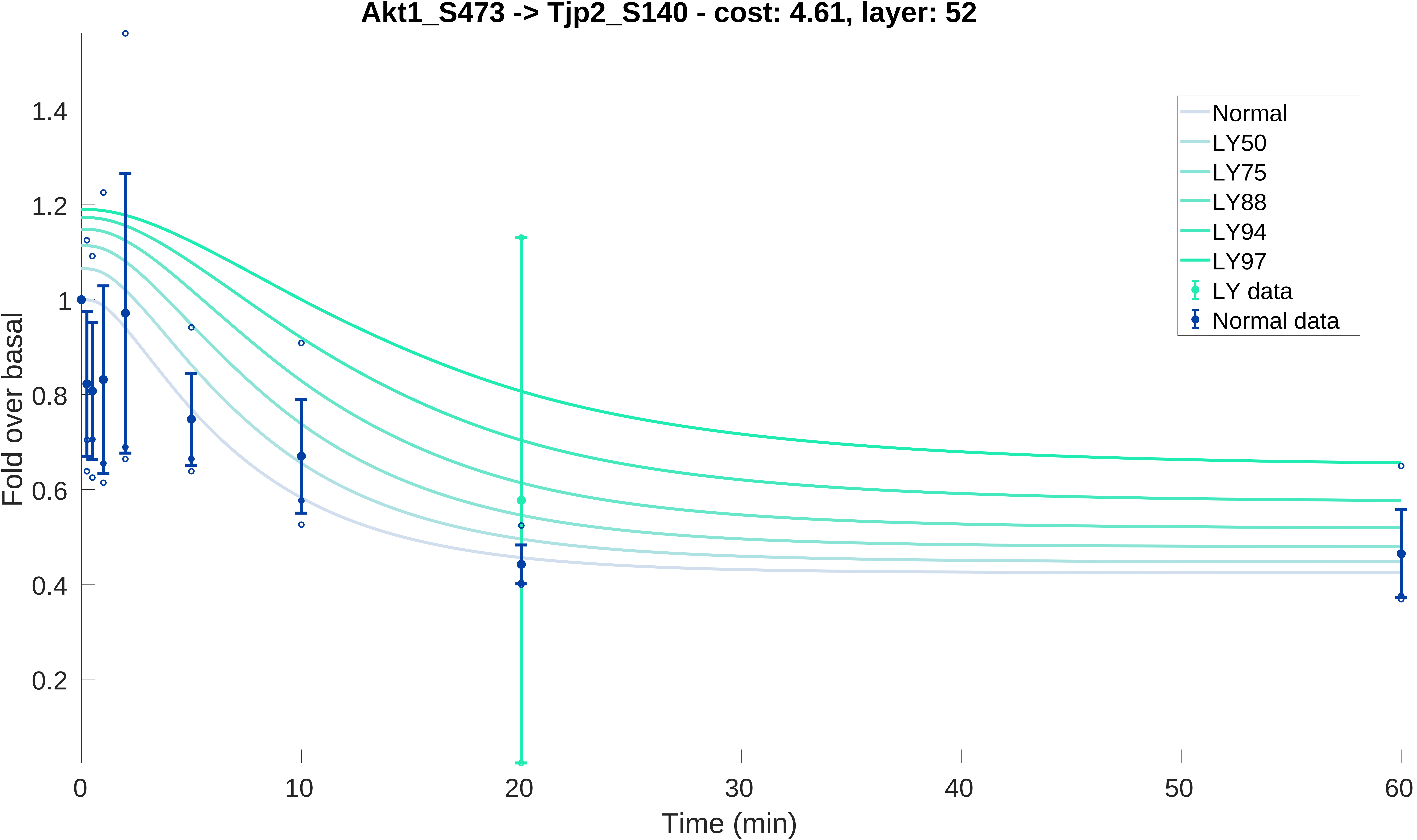

Akt1_S473 -> Tjp2_S140 - cost: 4.61, layer: 52
Fold over basal
Time (min)
Normal
LY50
LY75
LY88
LY94
LY97
LY data
Normal data

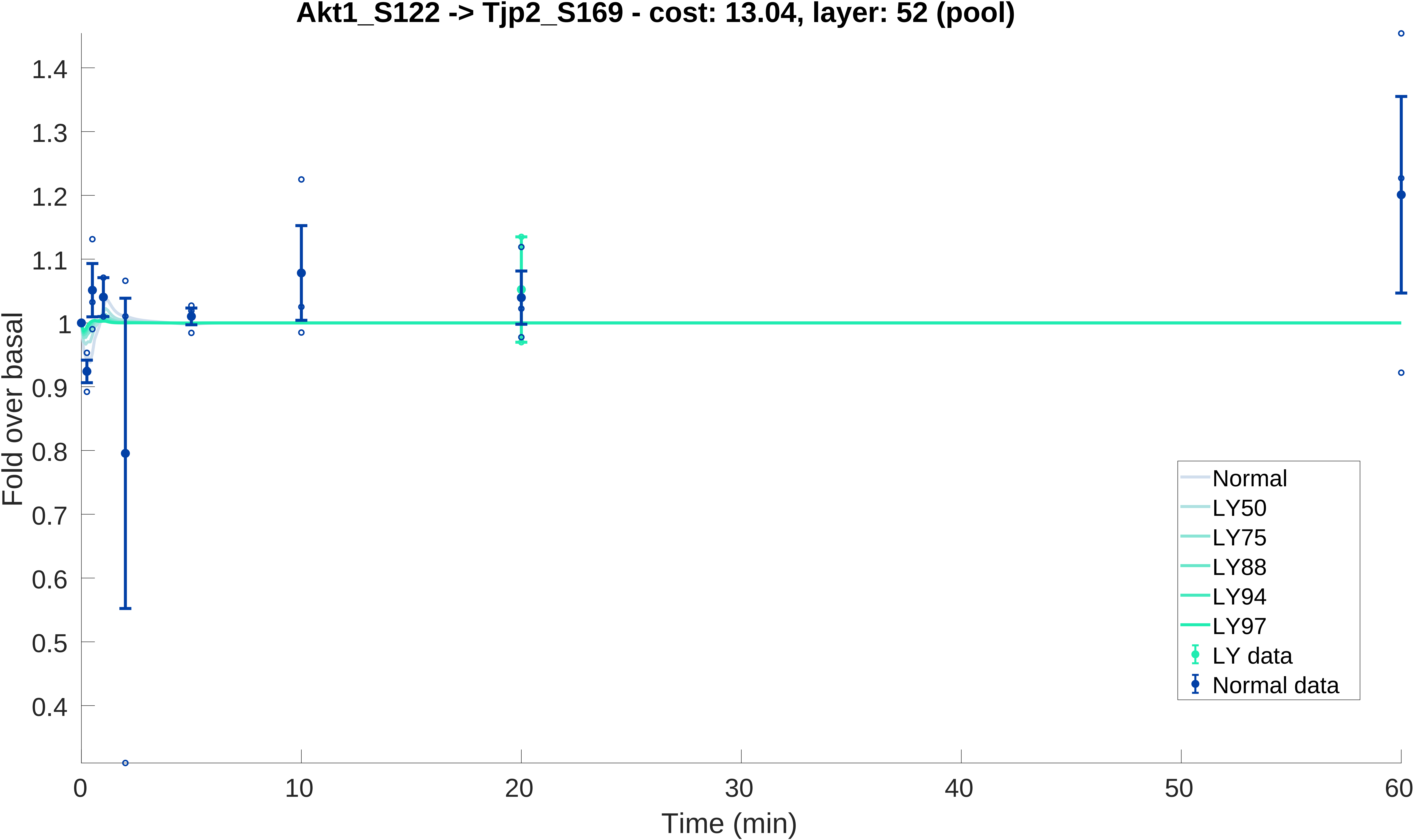

Akt1_S122 -> Tjp2_S169 - cost: 13.04, layer: 52 (pool)
Fold over basal
1.4
1.3
1.2
1.1
1
0.9
0.8
0.7
0.6
0.5
0.4
0
10
20
30
40
50
60
Time (min)
Normal
LY50
LY75
LY88
LY94
LY97
LY data
Normal data

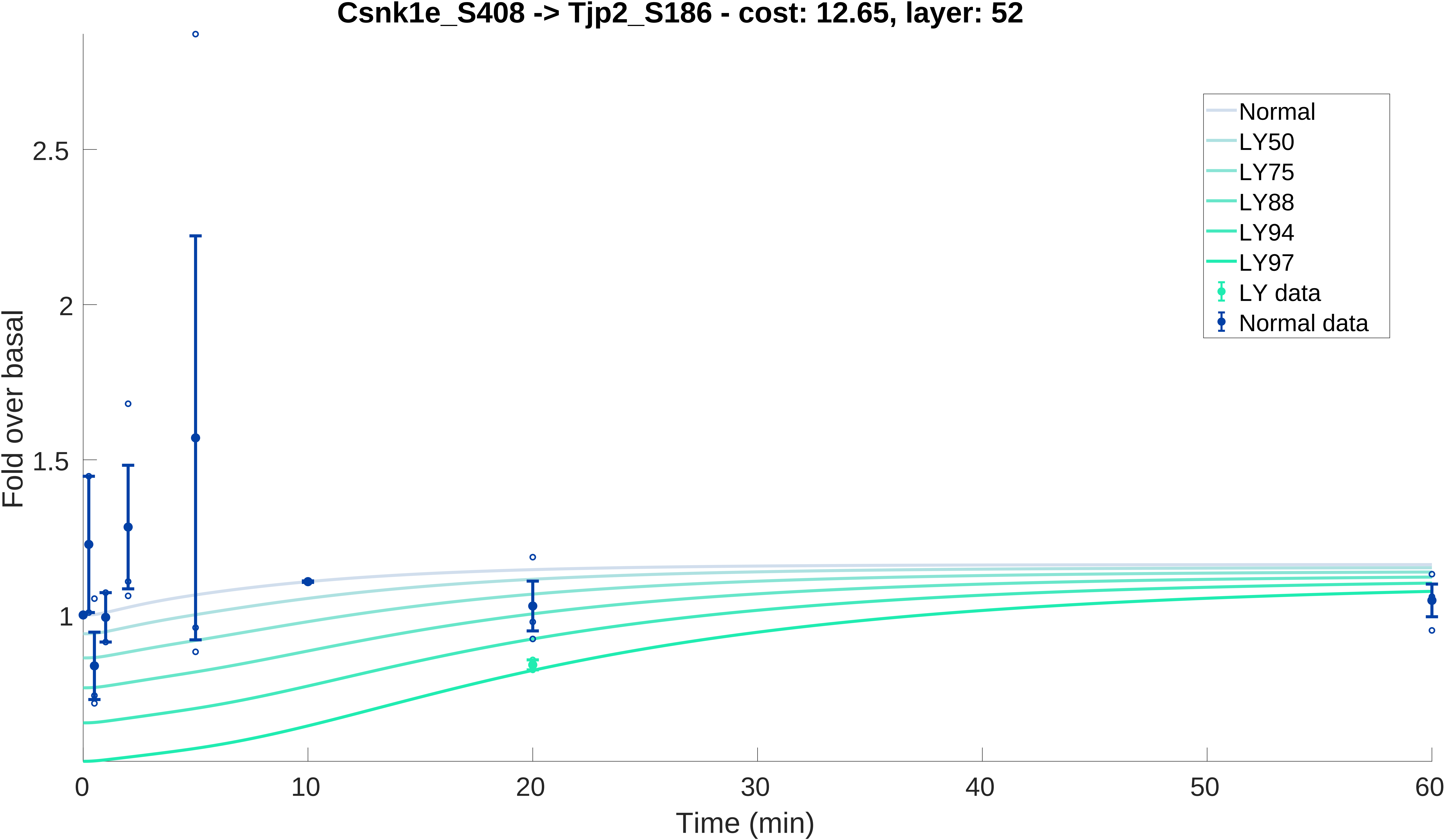

Csnk1e_S408 -> Tjp2_S186 - cost: 12.65, layer: 52
Fold over basal
2.5
2
1.5
1
Time (min)
0
10
20
30
40
50
60
Normal
LY50
LY75
LY88
LY94
LY97
LY data
Normal data

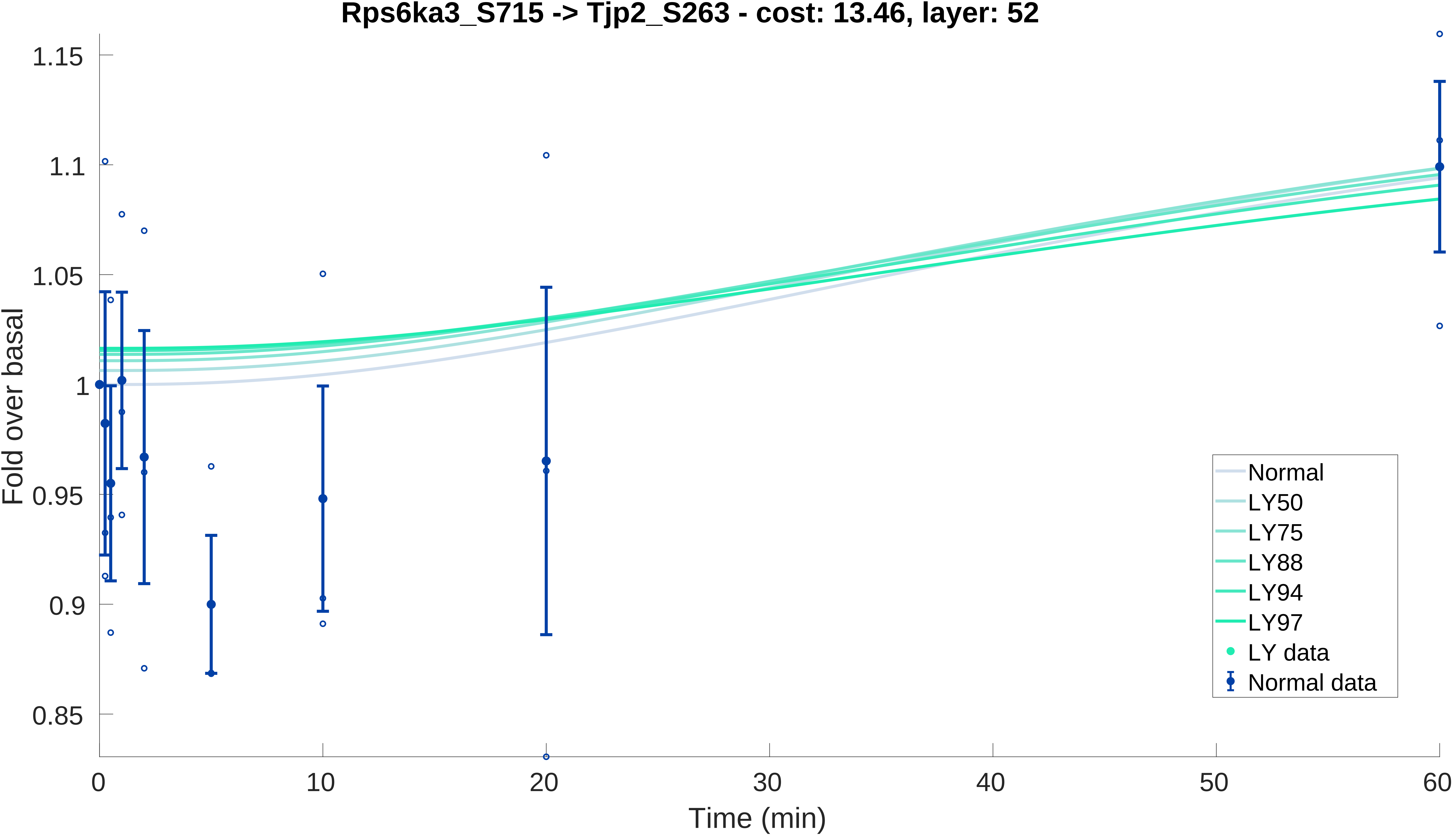

Rps6ka3_S715 -> Tjp2_S263 - cost: 13.46, layer: 52
Fold over basal
1.15
1.1
1.05
1
0.95
0.9
0.85
0
10
20
30
40
50
60
Time (min)
Normal
LY50
LY75
LY88
LY94
LY97
LY data
Normal data

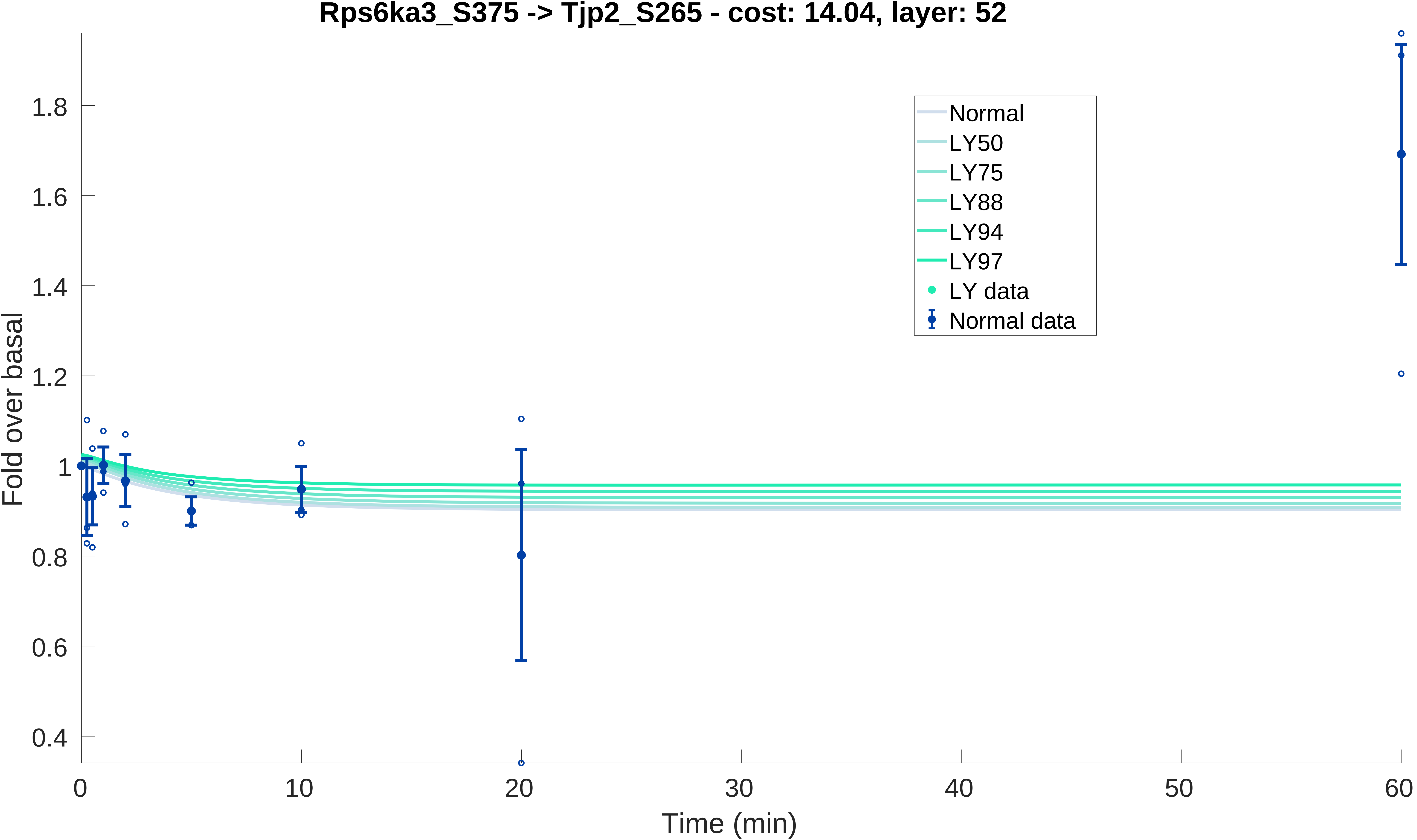

Rps6ka3_S375 -> Tjp2_S265 - cost: 14.04, layer: 52
Normal
LY50
LY75
LY88
LY94
LY97
LY data
Normal data
Fold over basal
1.8
1.6
1.4
1.2
1
0.8
0.6
0.4
0
10
20
30
40
50
60
Time (min)

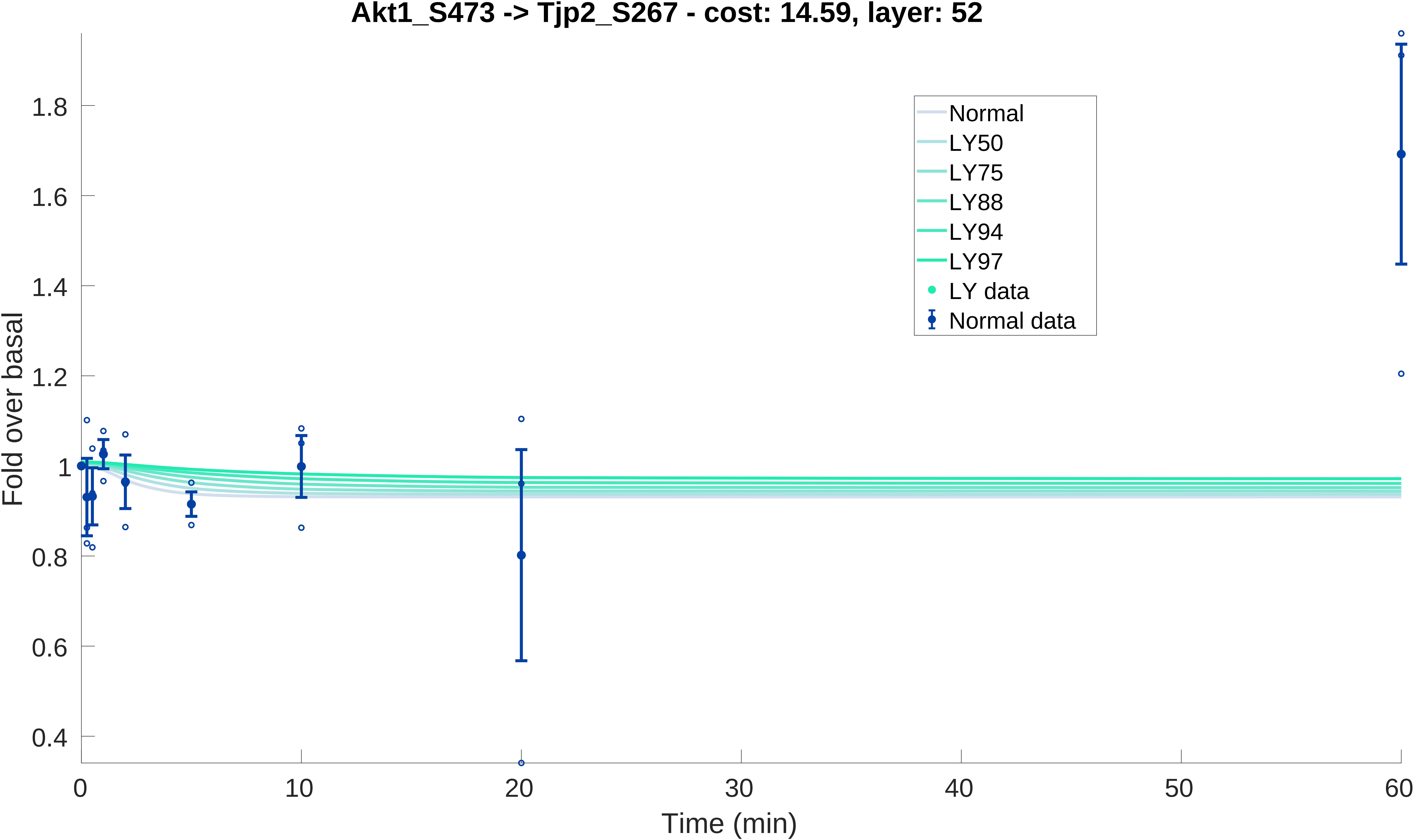
Akt1_S473 -> Tjp2_S267 - cost: 14.59, layer: 52
Fold over basal
Time (min)
Normal
LY50
LY75
LY88
LY94
LY97
LY data
Normal data
0.4
0.6
0.8
1
1.2
1.4
1.6
1.8
0
10
20
30
40
50
60

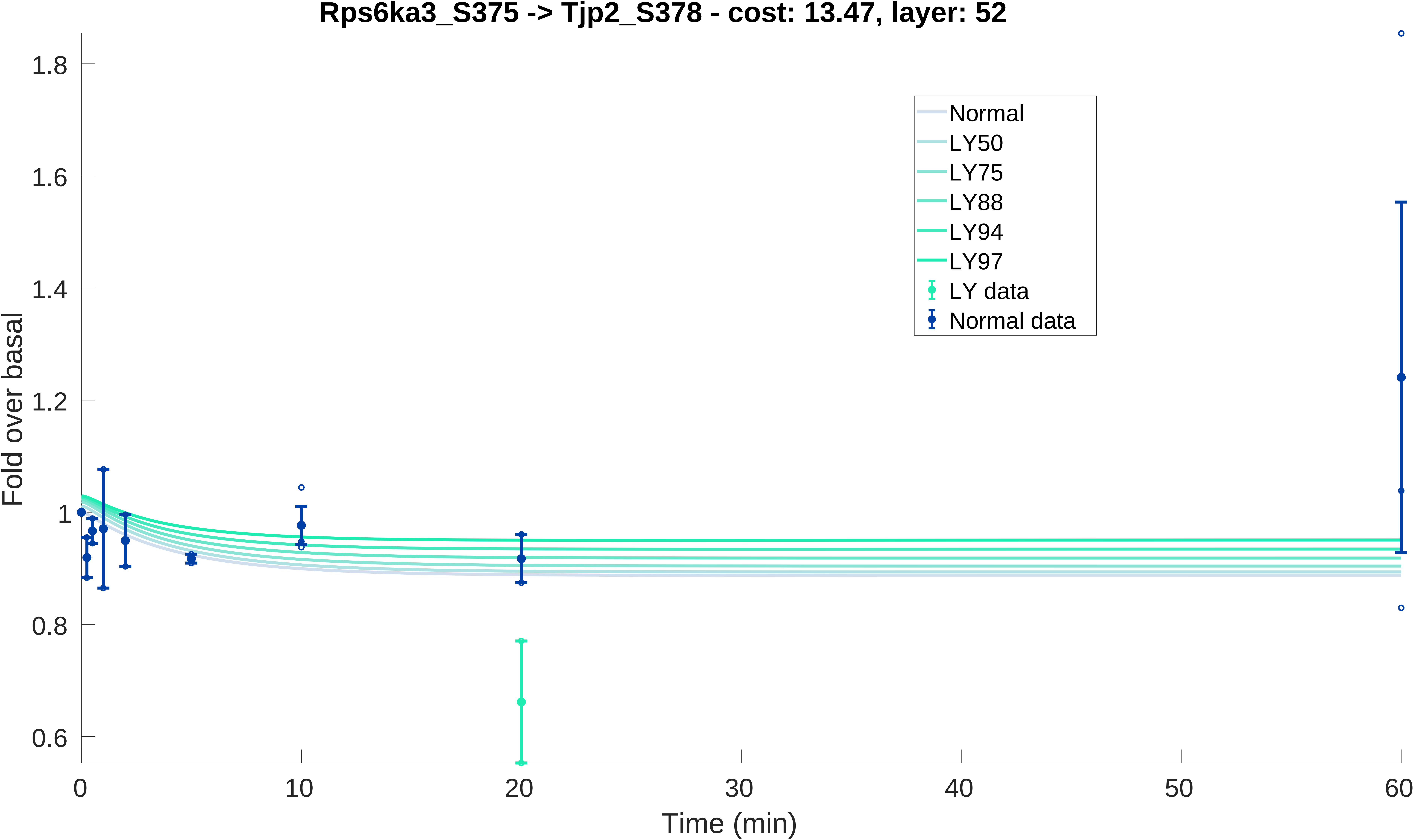

Rps6ka3_S375 -> Tjp2_S378 - cost: 13.47, layer: 52
Normal
LY50
LY75
LY88
LY94
LY97
LY data
Normal data
Fold over basal
Time (min)
0
10
20
30
40
50
60
0.6
0.8
1
1.2
1.4
1.6
1.8

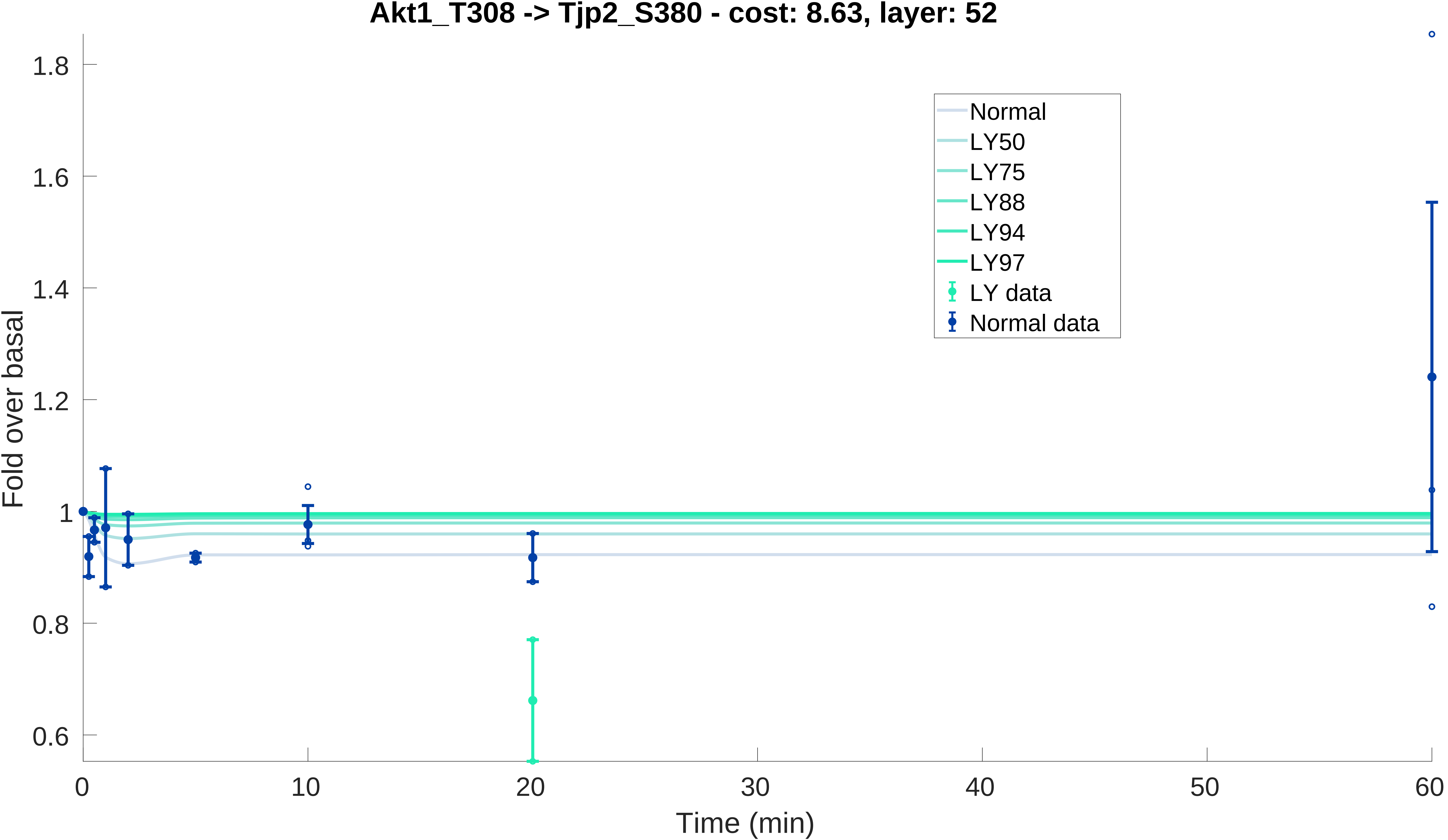
Akt1_T308 -> Tjp2_S380 - cost: 8.63, layer: 52
Fold over basal
1.8
1.6
1.4
1.2
1
0.8
0.6
Normal
LY50
LY75
LY88
LY94
LY97
LY data
Normal data
0
10
20
30
40
50
60
Time (min)

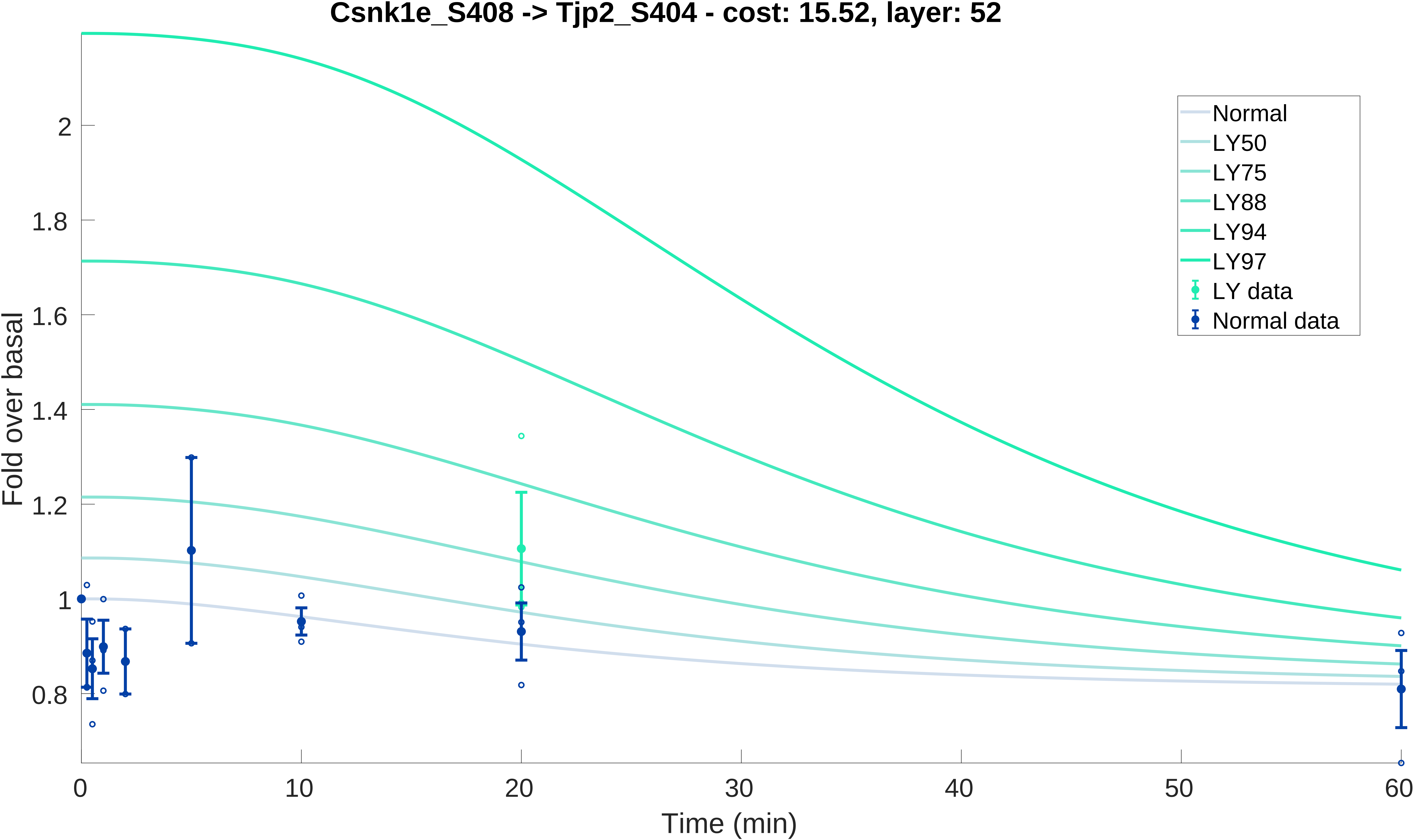

Csnk1e_S408 -> Tjp2_S404 - cost: 15.52, layer: 52
Normal
LY50
LY75
LY88
LY94
LY97
LY data
Normal data
Fold over basal
Time (min)
0
10
20
30
40
50
60
0.8
1
1.2
1.4
1.6
1.8
2

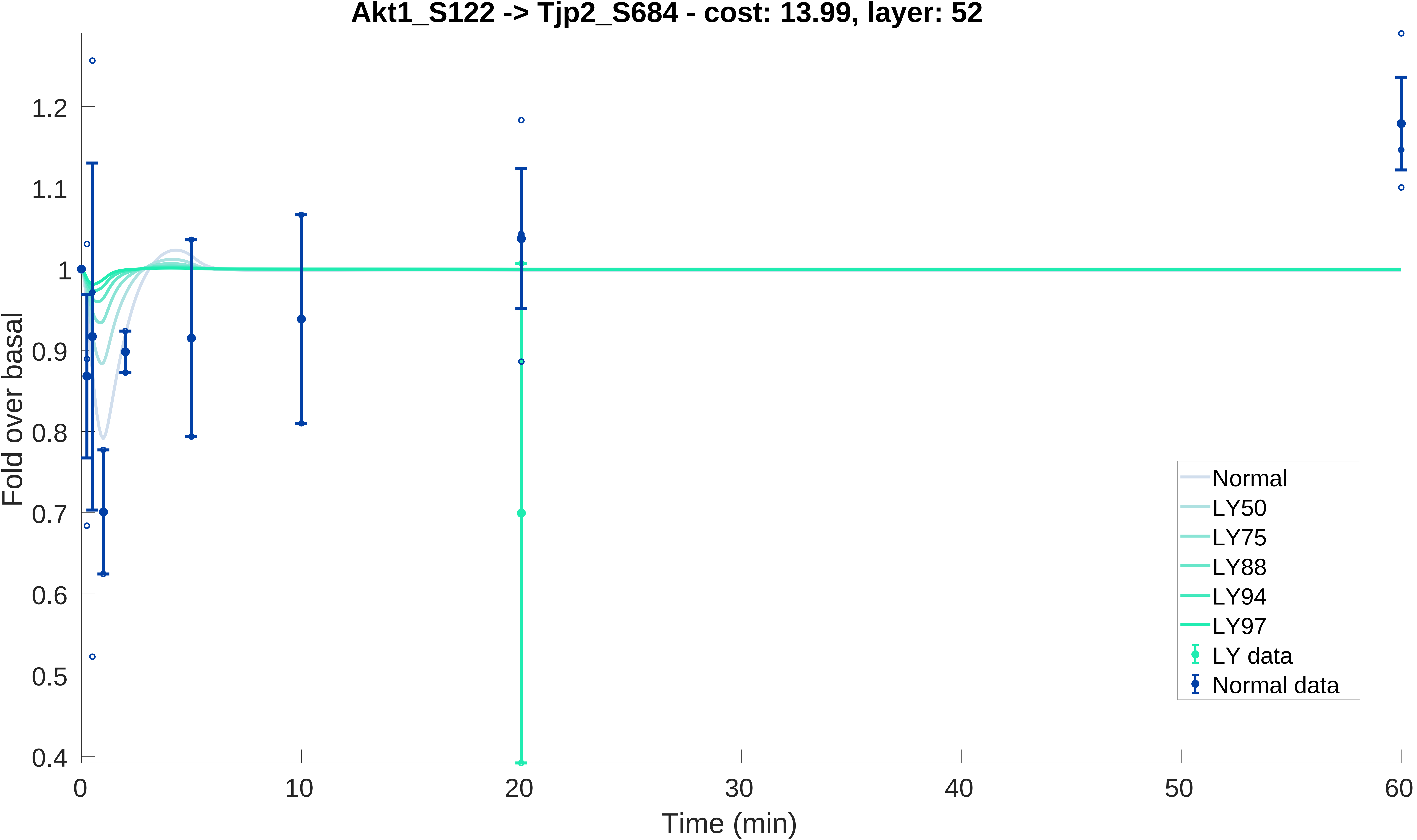

Akt1_S122 -> Tjp2_S684 - cost: 13.99, layer: 52
Fold over basal
1.2
1.1
1
0.9
0.8
0.7
0.6
0.5
0.4
0
10
20
30
40
50
60
Time (min)
Normal
LY50
LY75
LY88
LY94
LY97
LY data
Normal data

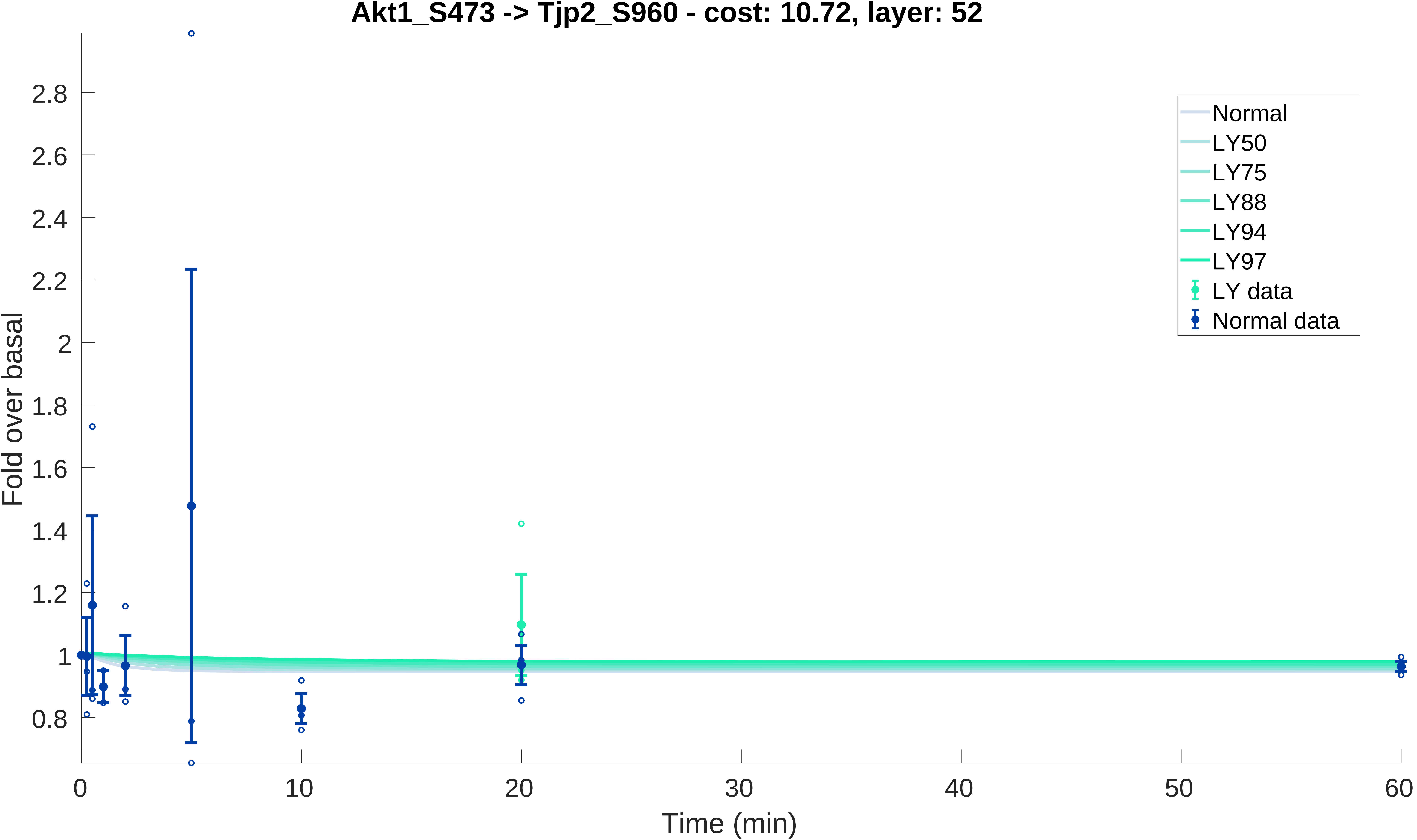
Akt1_S473 -> Tjp2_S960 - cost: 10.72, layer: 52
Fold over basal
Time (min)
0
10
20
30
40
50
60
0.8
1
1.2
1.4
1.6
1.8
2
2.2
2.4
2.6
2.8
Normal
LY50
LY75
LY88
LY94
LY97
LY data
Normal data

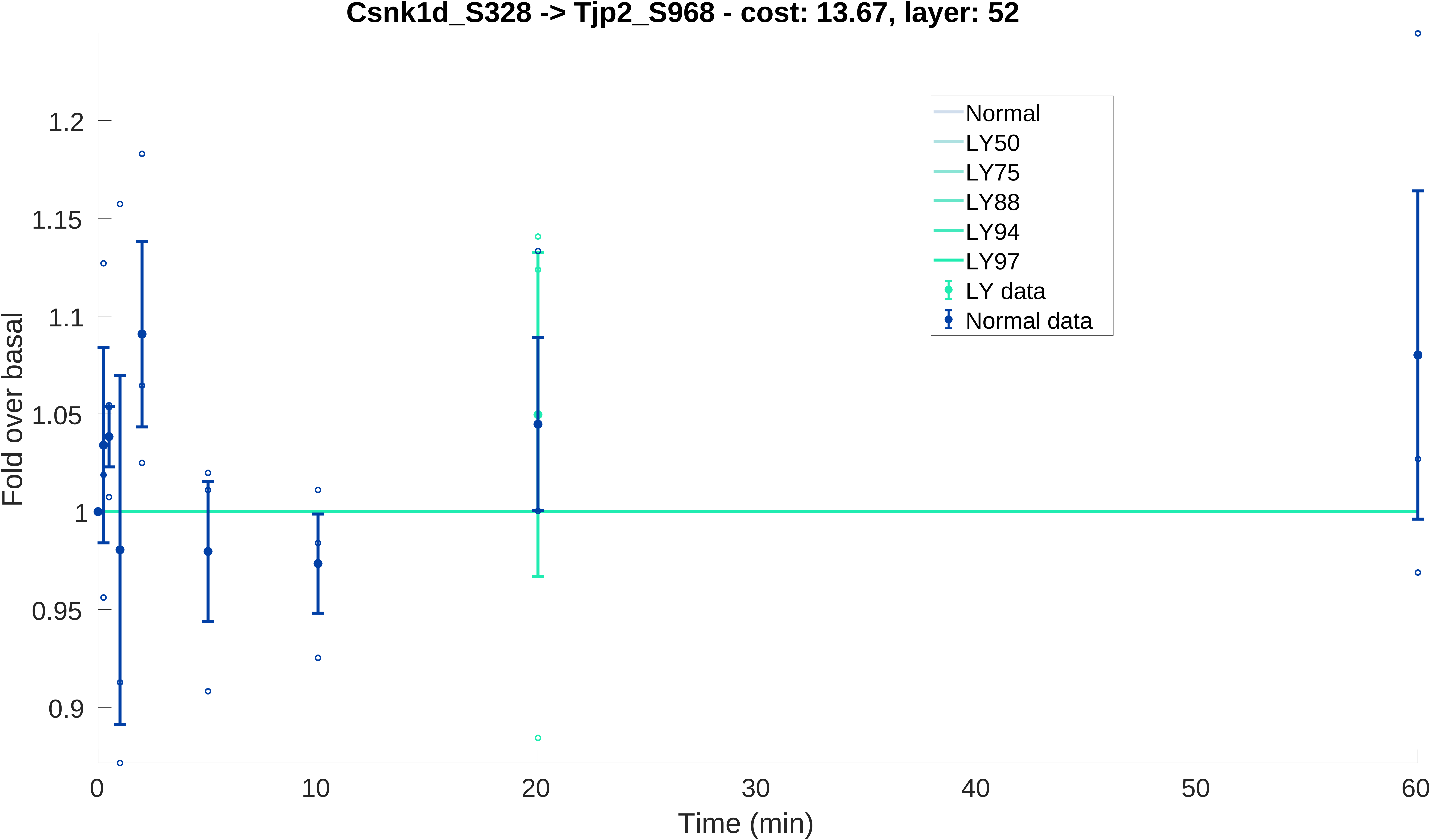

Csnk1d_S328 -> Tjp2_S968 - cost: 13.67, layer: 52
Fold over basal
Time (min)
1.2
1.15
1.1
1.05
1
0.95
0.9
0
10
20
30
40
50
60
Normal
LY50
LY75
LY88
LY94
LY97
LY data
Normal data

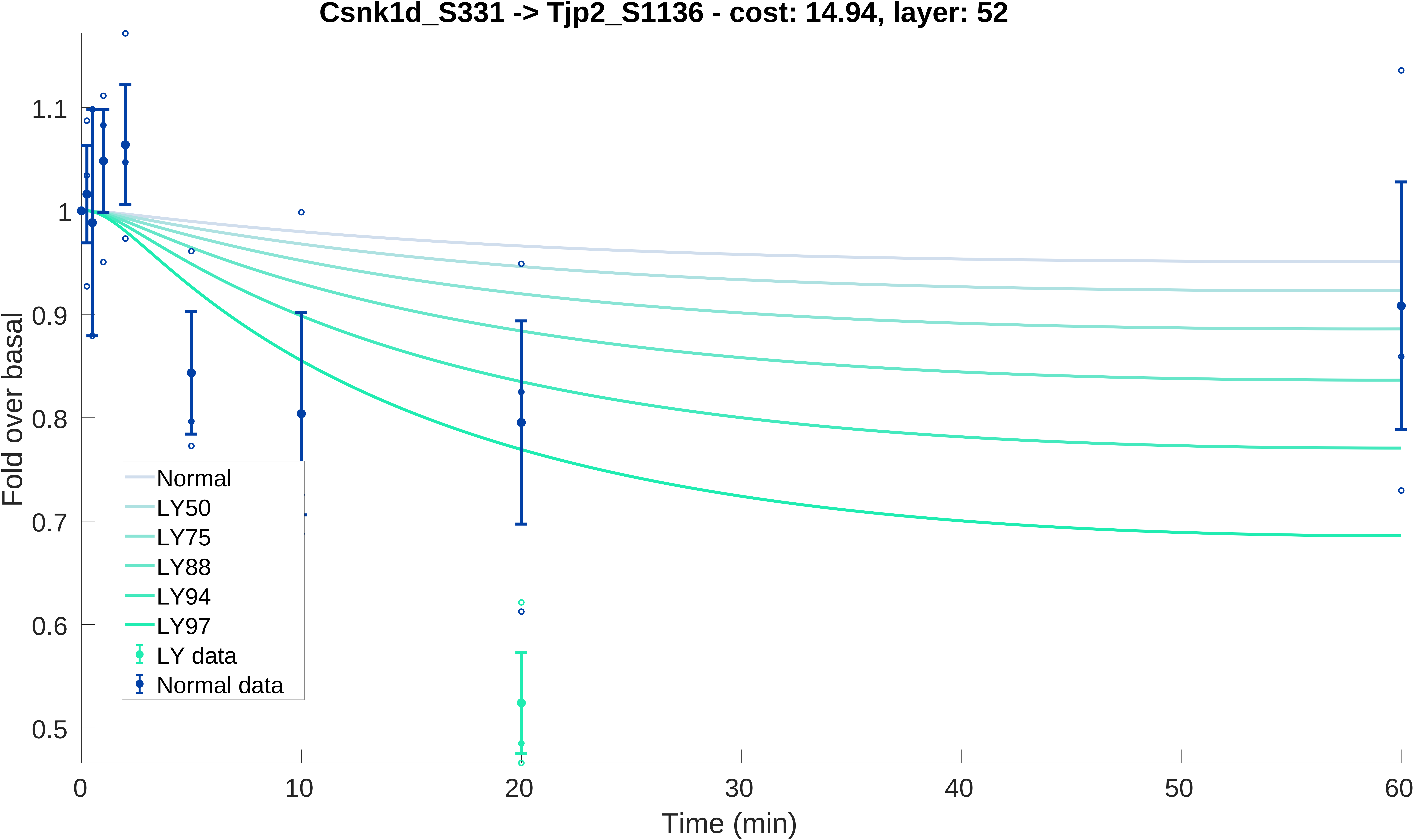

Csnk1d_S331 -> Tjp2_S1136 - cost: 14.94, layer: 52
Fold over basal
Time (min)
1.1
1
0.9
0.8
0.7
0.6
0.5
0
10
20
30
40
50
60
Normal
LY50
LY75
LY88
LY94
LY97
LY data
Normal data

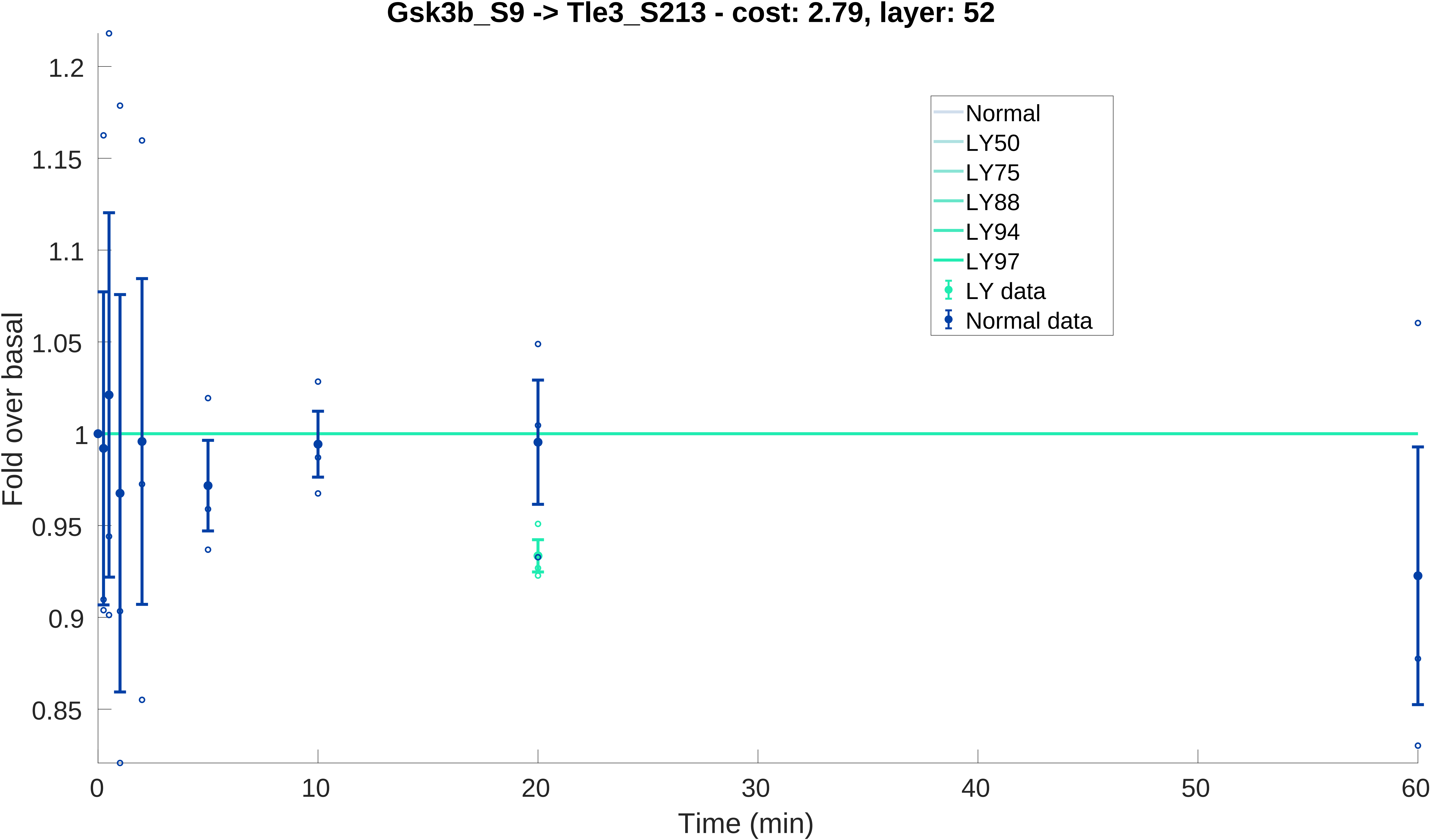

Gsk3b_S9 -> Tle3_S213 - cost: 2.79, layer: 52
Fold over basal
1.2
1.15
1.1
1.05
1
0.95
0.9
0.85
Time (min)
0
10
20
30
40
50
60
Normal
LY50
LY75
LY88
LY94
LY97
LY data
Normal data

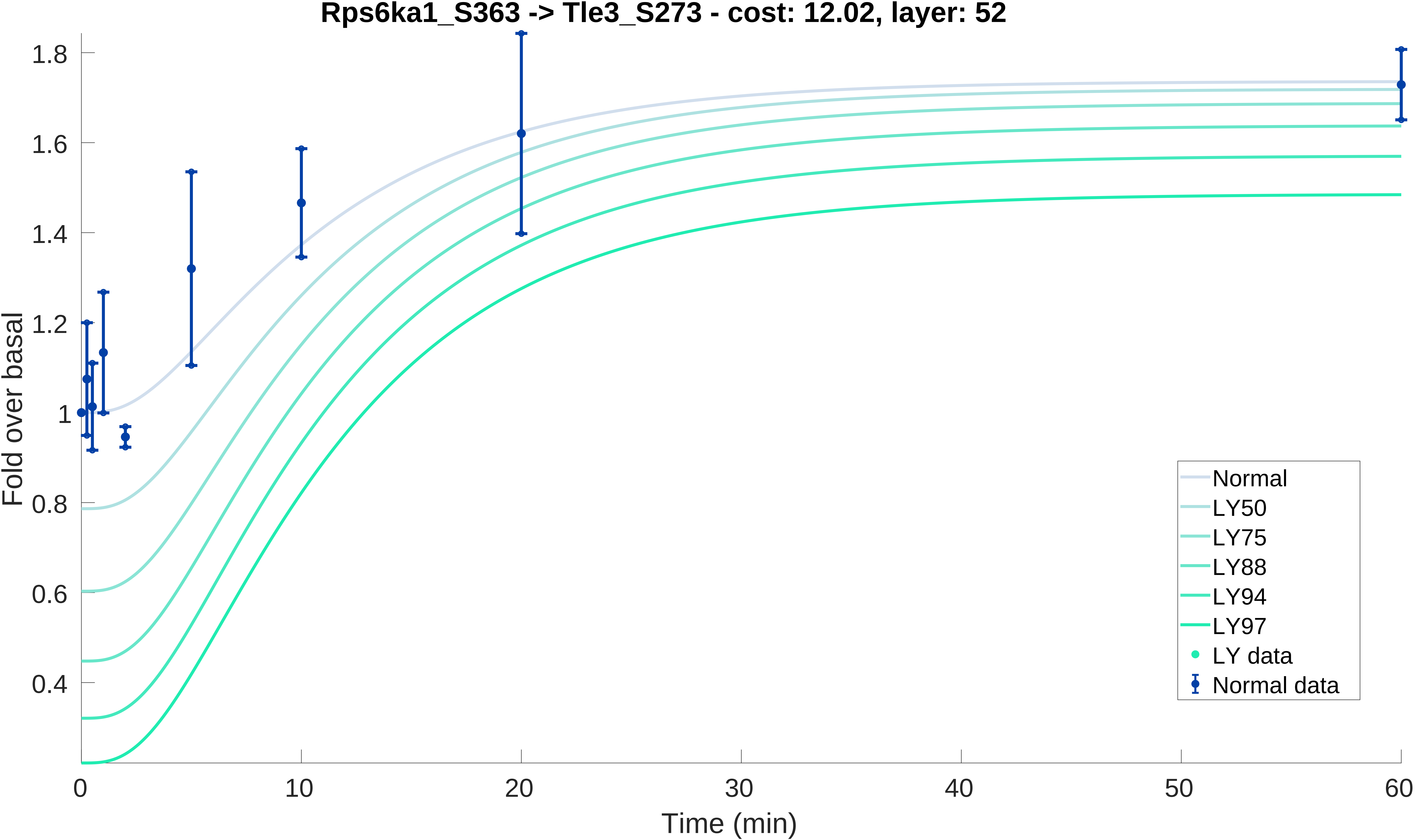

Rps6ka1_S363 -> Tle3_S273 - cost: 12.02, layer: 52
Fold over basal
1.8
1.6
1.4
1.2
1
0.8
0.6
0.4
0
10
20
30
40
50
60
Time (min)
Normal
LY50
LY75
LY88
LY94
LY97
LY data
Normal data

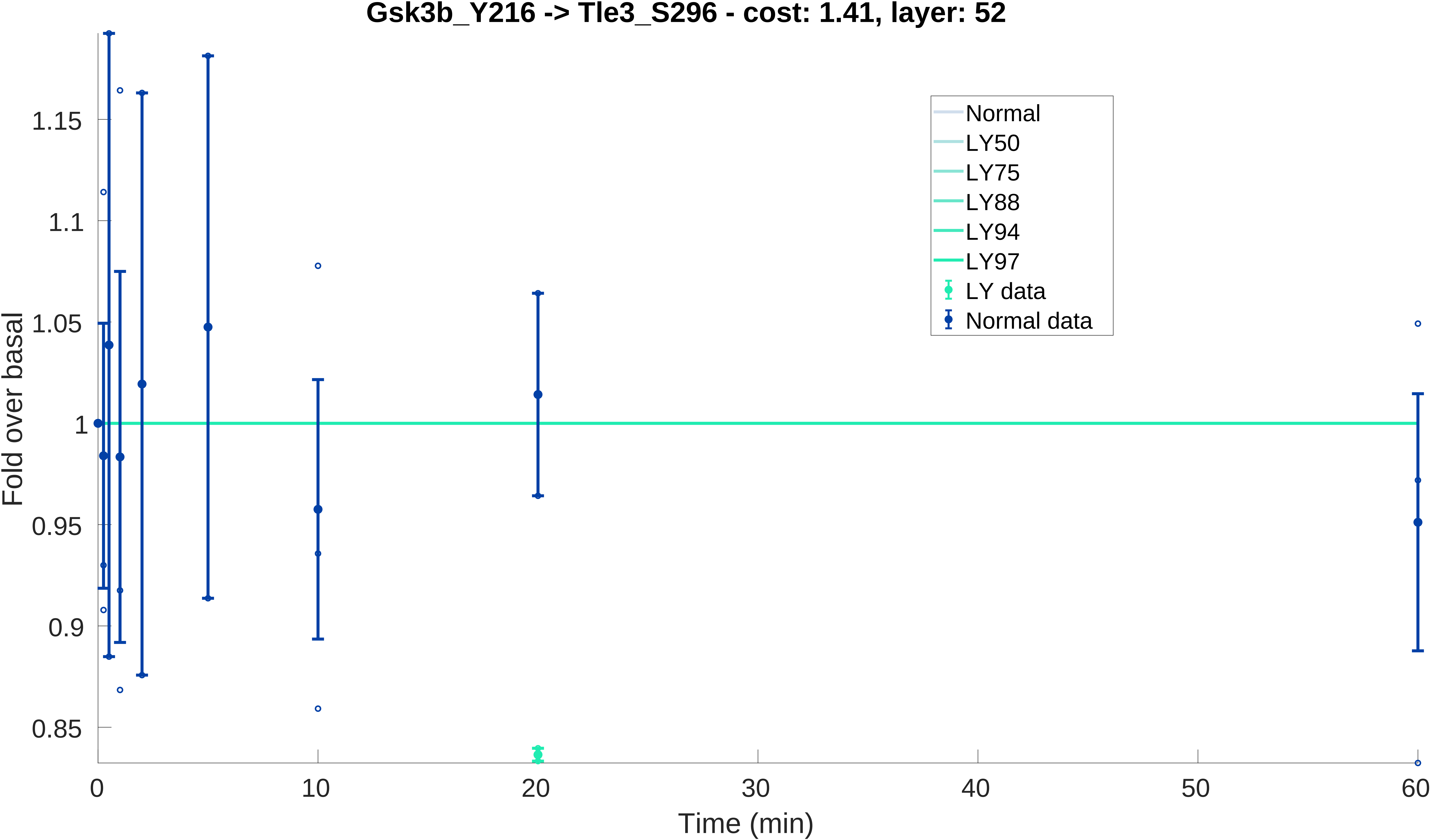

Gsk3b_Y216 -> Tle3_S296 - cost: 1.41, layer: 52
Normal
LY50
LY75
LY88
LY94
LY97
LY data
Normal data
Fold over basal
1.15
1.1
1.05
1
0.95
0.9
0.85
0
10
20
30
40
50
60
Time (min)

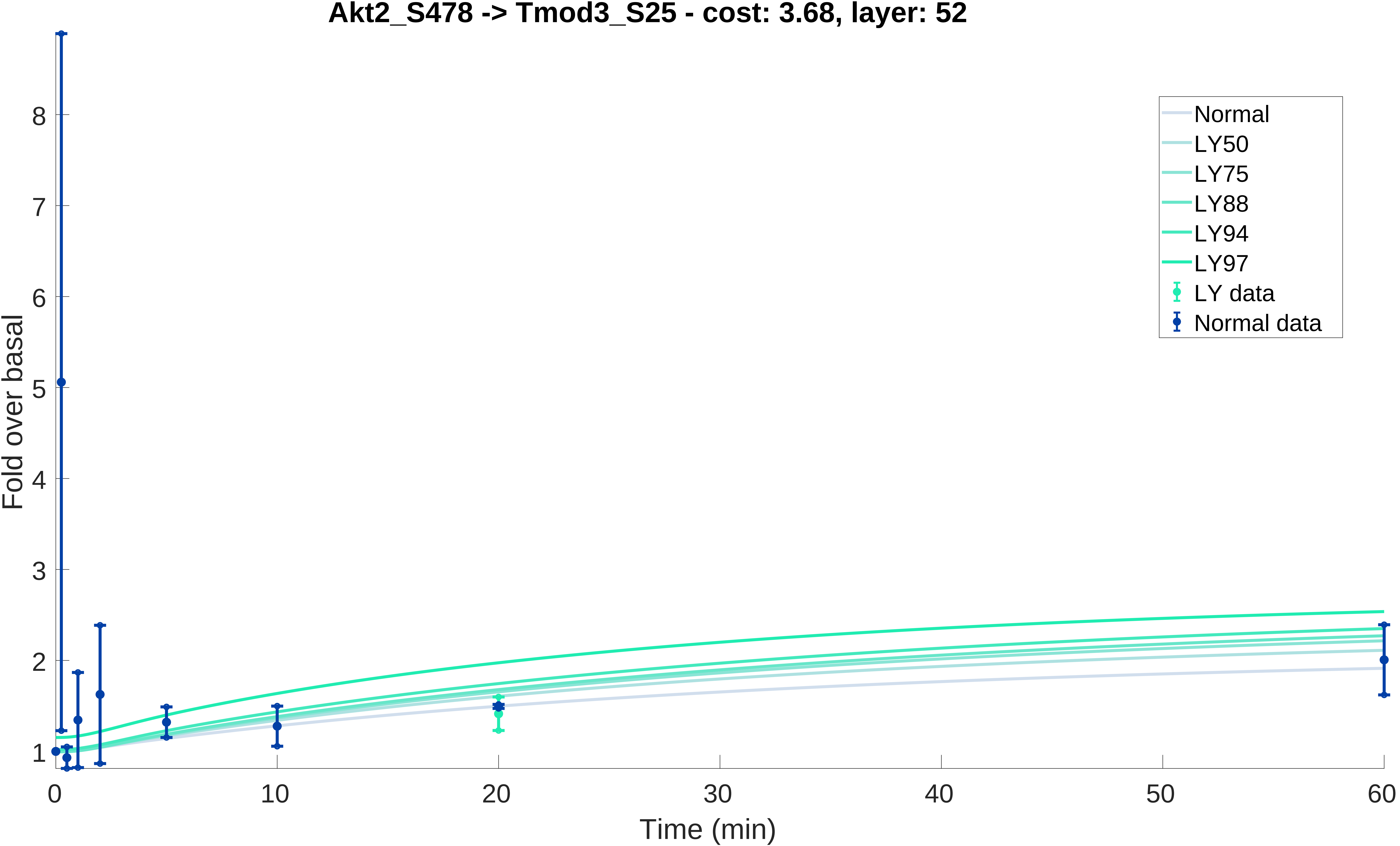

Akt2_S478 -> Tmod3_S25 - cost: 3.68, layer: 52
Fold over basal
Time (min)
Normal
LY50
LY75
LY88
LY94
LY97
LY data
Normal data
0
10
20
30
40
50
60
1
2
3
4
5
6
7
8

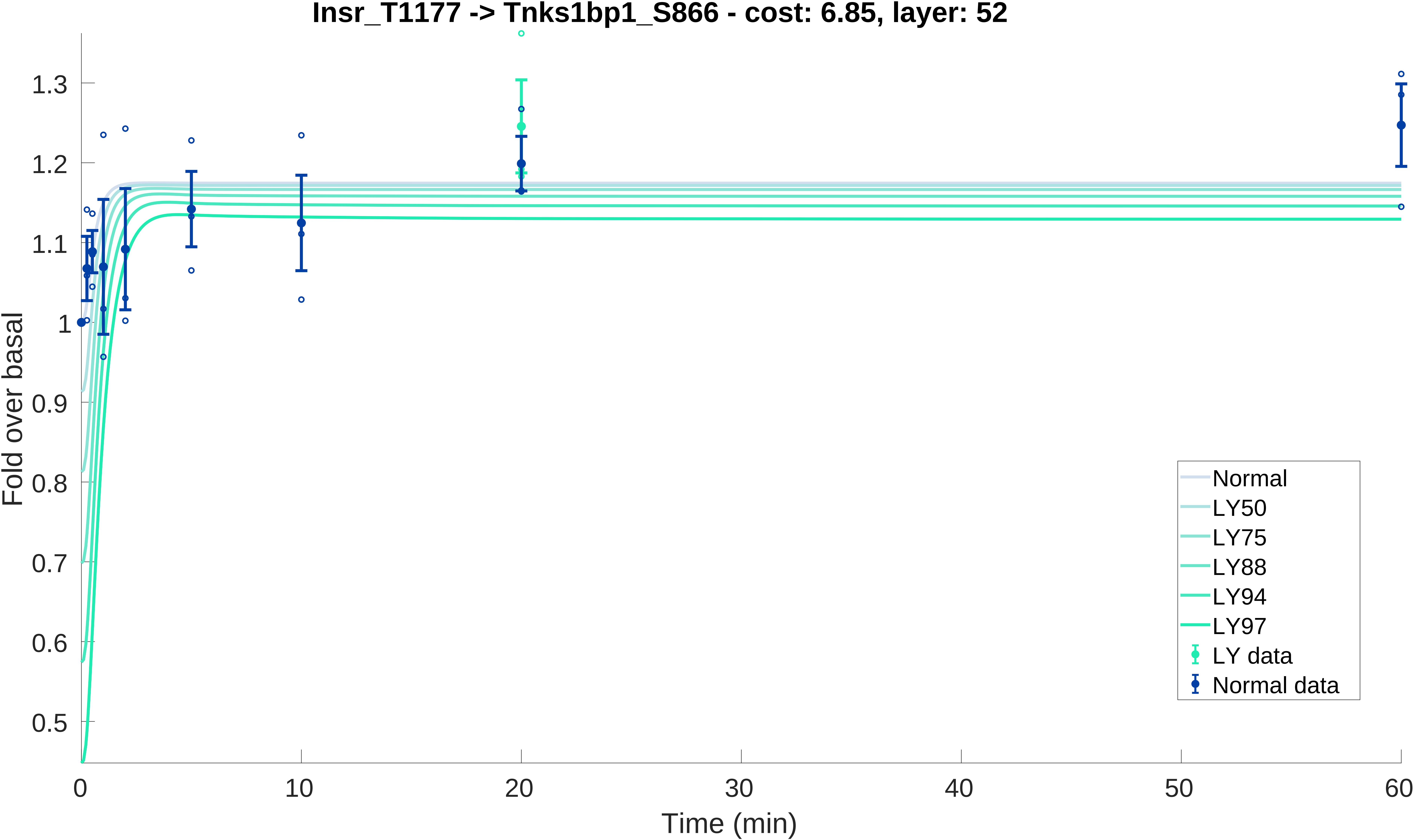

Insr_T1177 -> Tnks1bp1_S866 - cost: 6.85, layer: 52
Fold over basal
Time (min)
0.5
0.6
0.7
0.8
0.9
1
1.1
1.2
1.3
0
10
20
30
40
50
60
Normal
LY50
LY75
LY88
LY94
LY97
LY data
Normal data

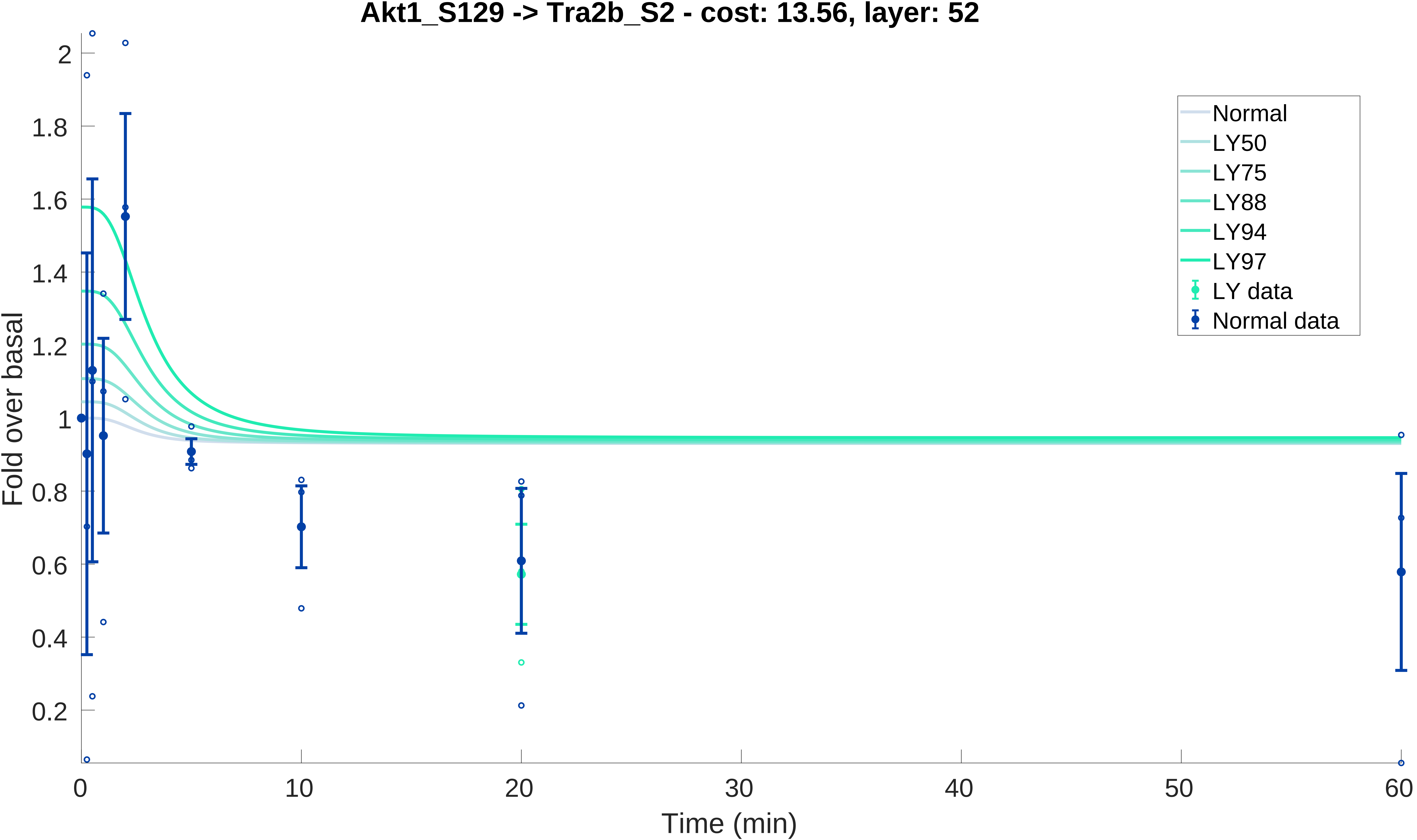

Akt1_S129 -> Tra2b_S2 - cost: 13.56, layer: 52
Fold over basal
Time (min)
0
10
20
30
40
50
60
0.2
0.4
0.6
0.8
1
1.2
1.4
1.6
1.8
2
Normal
LY50
LY75
LY88
LY94
LY97
LY data
Normal data

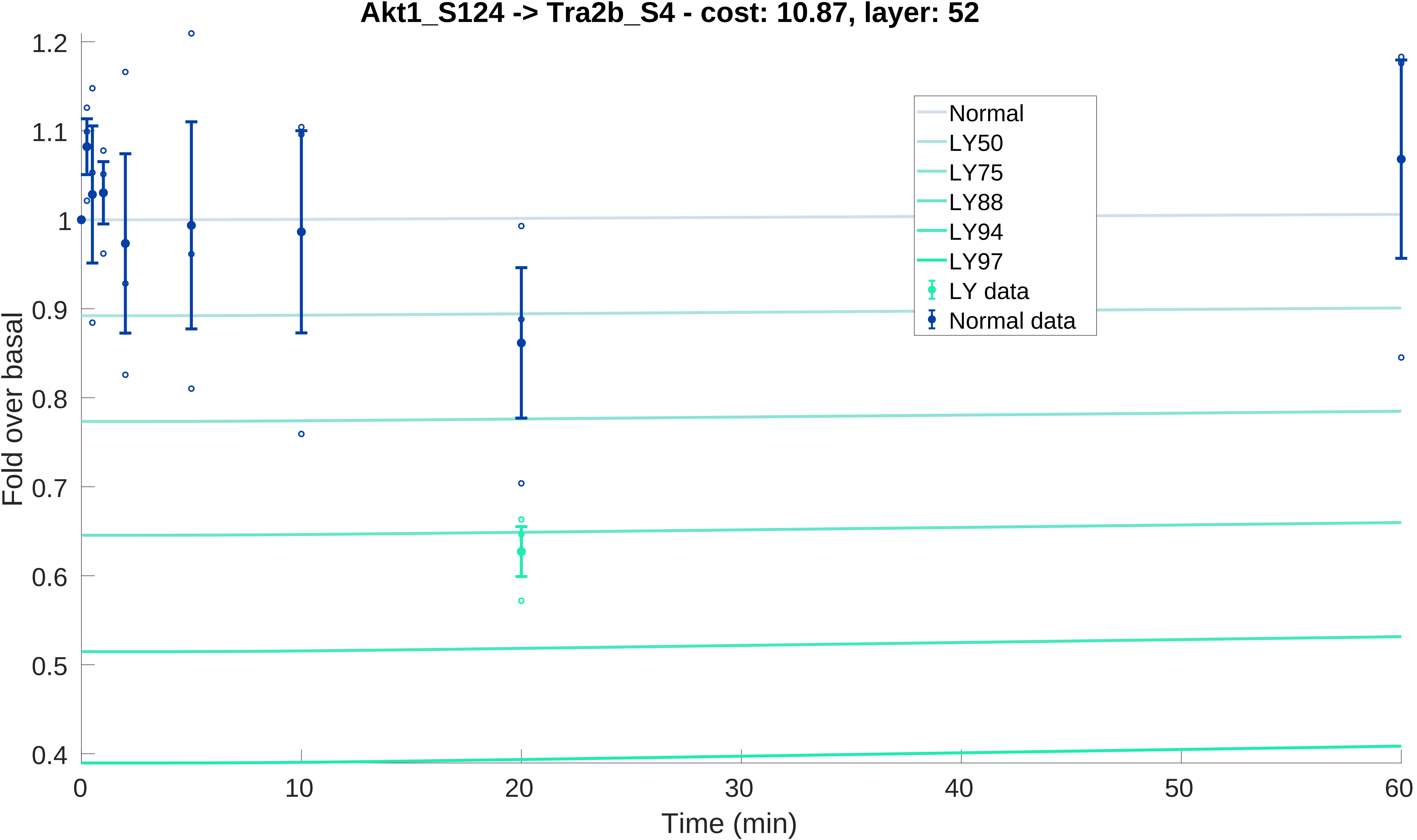
Akt1_S124 -> Tra2b_S4 - cost: 10.87, layer: 52
Fold over basal
1.2
1.1
1
0.9
0.8
0.7
0.6
0.5
0.4
0
10
20
30
40
50
60
Time (min)
Normal
LY50
LY75
LY88
LY94
LY97
LY data
Normal data

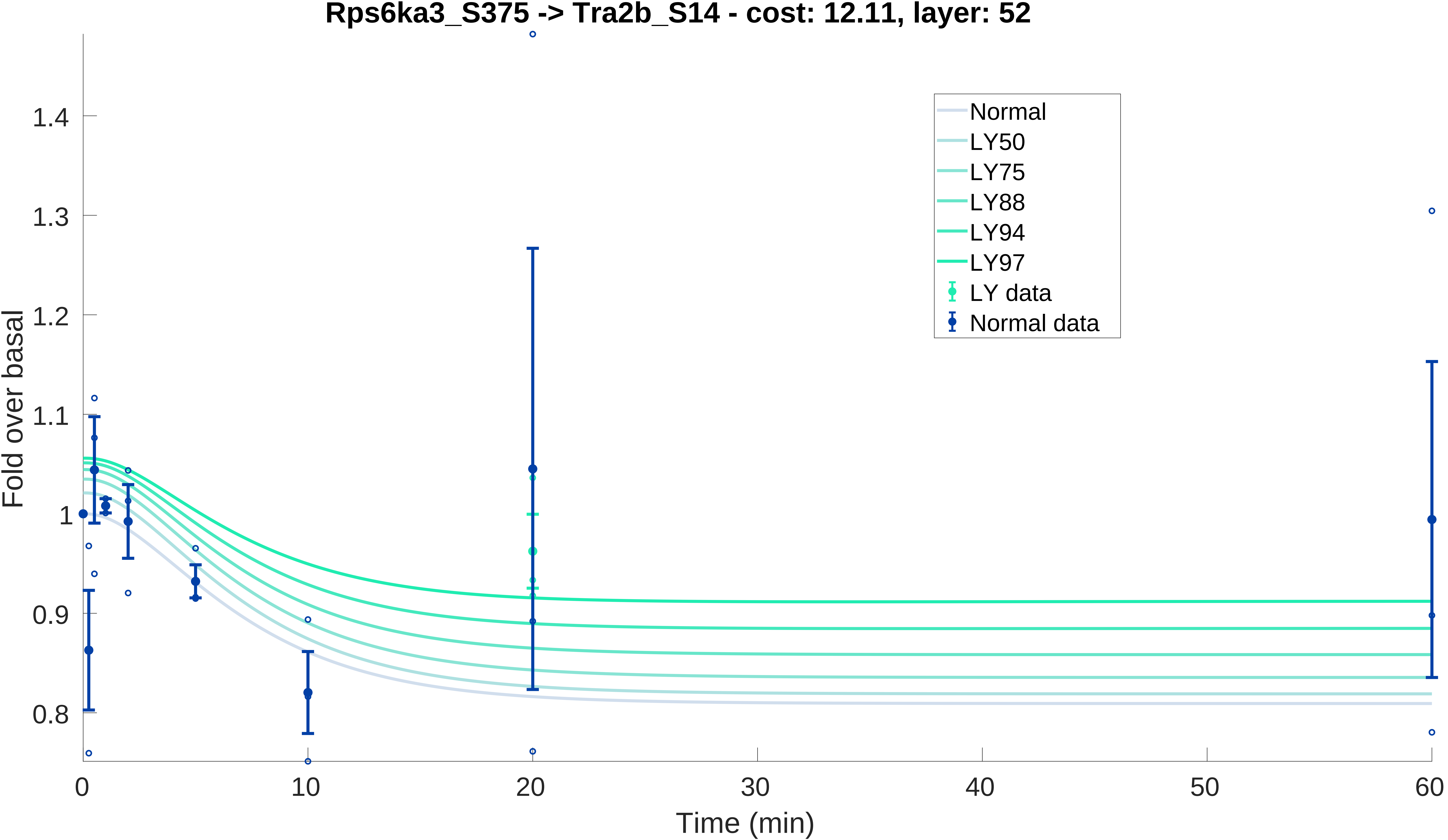
Rps6ka3_S375 -> Tra2b_S14 - cost: 12.11, layer: 52
Fold over basal
Time (min)
0.8
0.9
1
1.1
1.2
1.3
1.4
0
10
20
30
40
50
60
Normal
LY50
LY75
LY88
LY94
LY97
LY data
Normal data

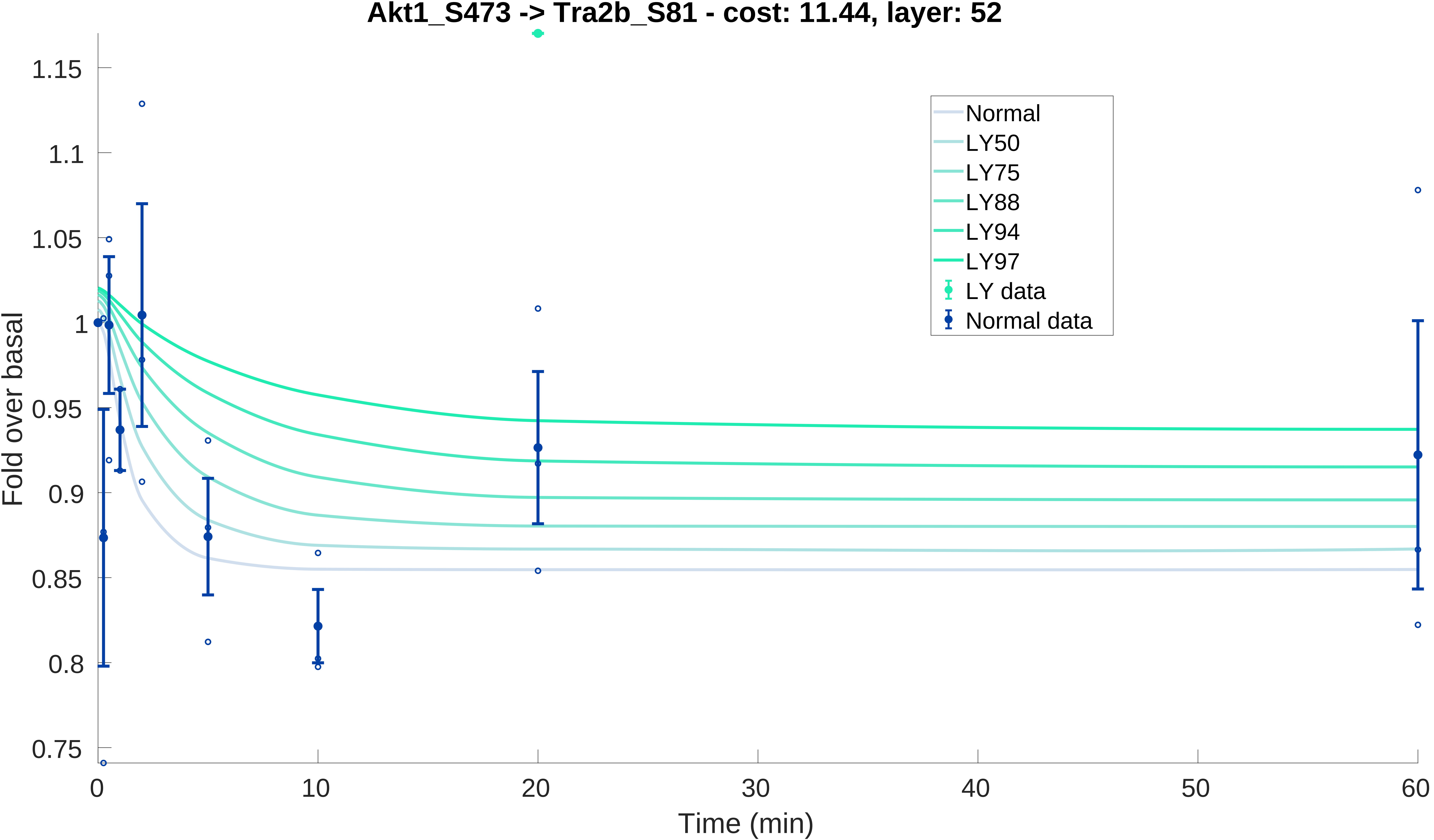

Akt1_S473 -> Tra2b_S81 - cost: 11.44, layer: 52
Fold over basal
Time (min)
1.15
1.1
1.05
1
0.95
0.9
0.85
0.8
0.75
0
10
20
30
40
50
60
Normal
LY50
LY75
LY88
LY94
LY97
LY data
Normal data

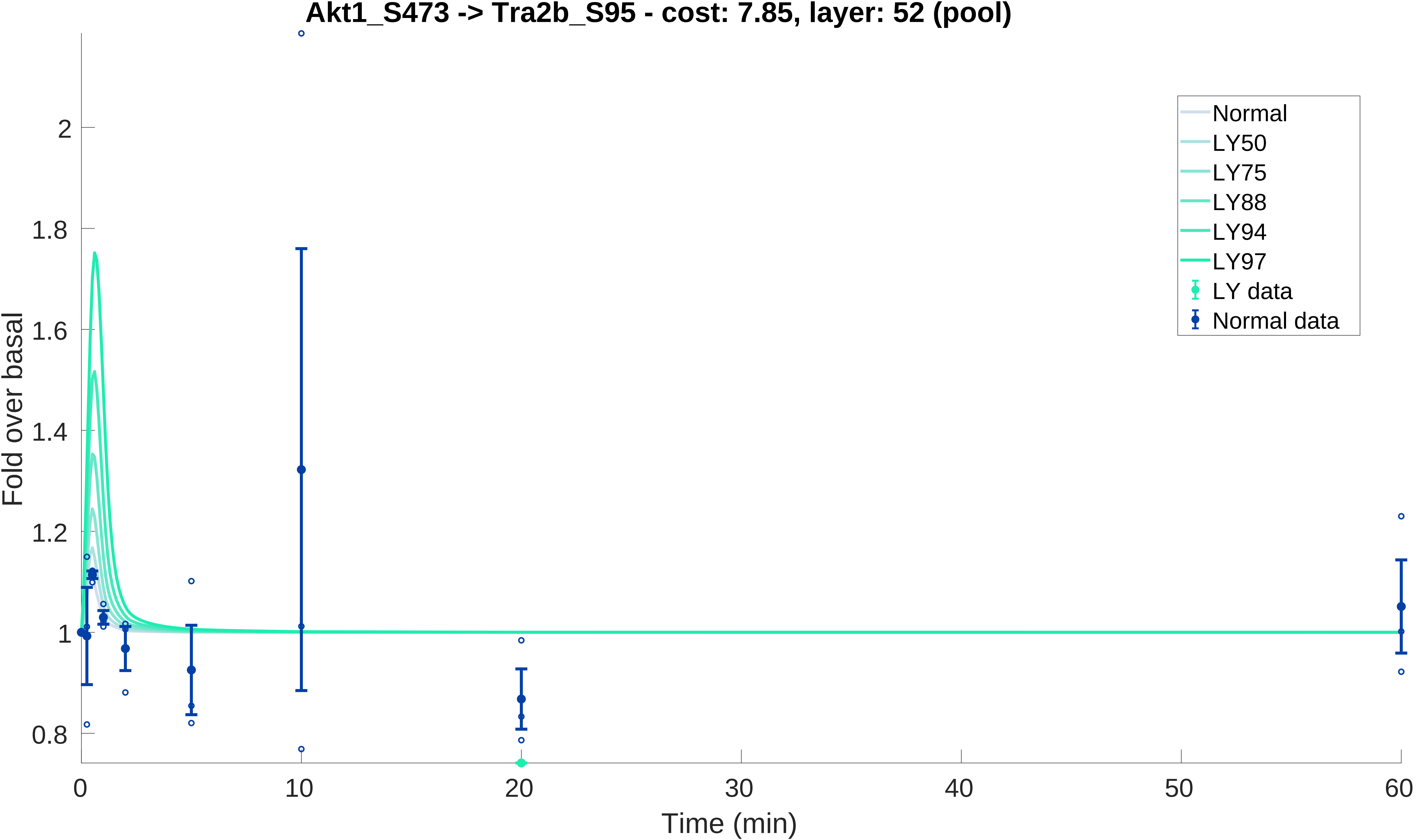

Akt1_S473 -> Tra2b_S95 - cost: 7.85, layer: 52 (pool)
Fold over basal
Time (min)
0.8
1
1.2
1.4
1.6
1.8
2
0
10
20
30
40
50
60
Normal
LY50
LY75
LY88
LY94
LY97
LY data
Normal data

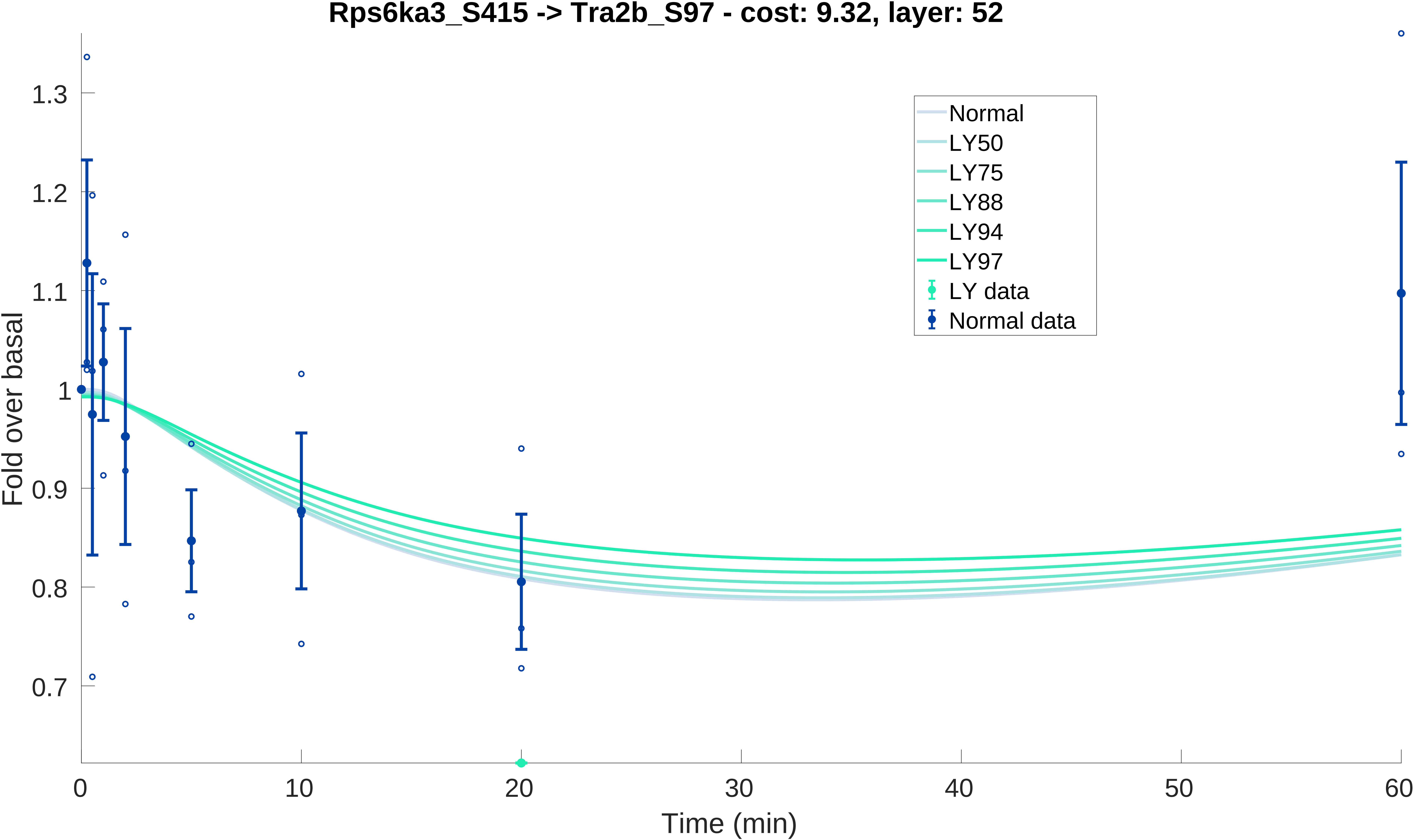

Rps6ka3_S415 -> Tra2b_S97 - cost: 9.32, layer: 52
Fold over basal
Time (min)
1.3
1.2
1.1
1
0.9
0.8
0.7
0
10
20
30
40
50
60
Normal
LY50
LY75
LY88
LY94
LY97
LY data
Normal data

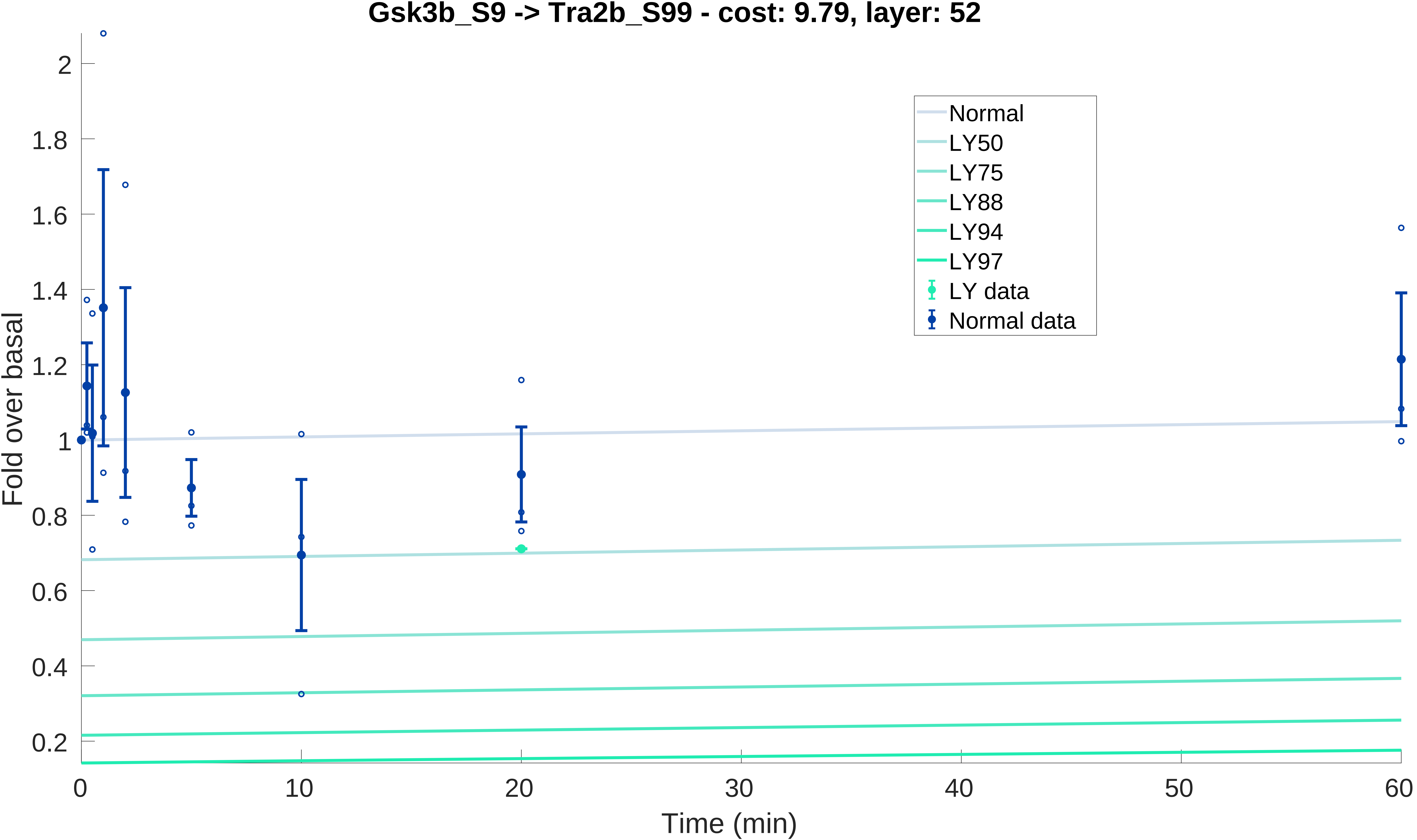

Gsk3b_S9 -> Tra2b_S99 - cost: 9.79, layer: 52
Fold over basal
Time (min)
Normal
LY50
LY75
LY88
LY94
LY97
LY data
Normal data
0
10
20
30
40
50
60
0.2
0.4
0.6
0.8
1
1.2
1.4
1.6
1.8
2

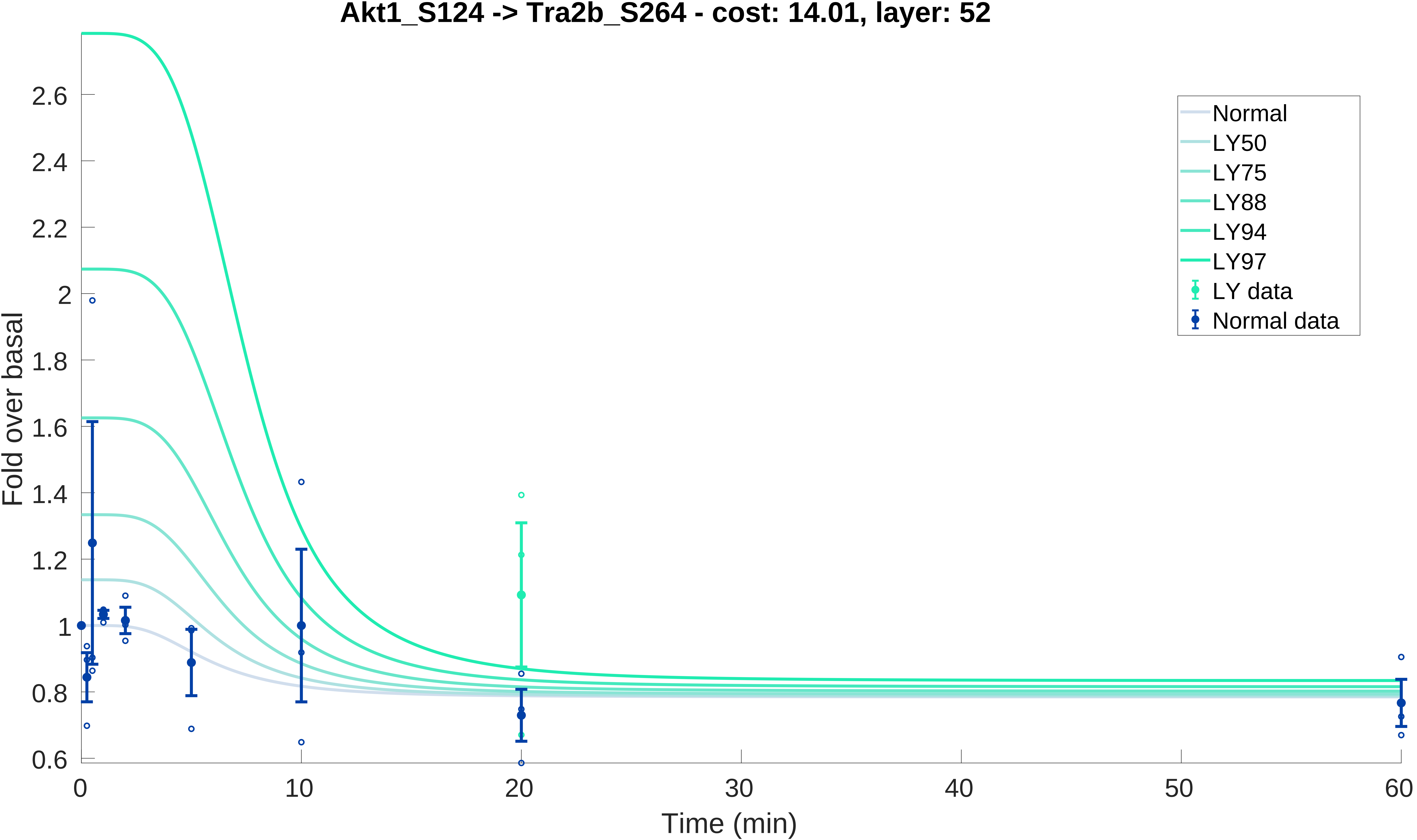

Akt1_S124 -> Tra2b_S264 - cost: 14.01, layer: 52
Fold over basal
2.6
2.4
2.2
2
1.8
1.6
1.4
1.2
1
0.8
0.6
0
10
20
30
40
50
60
Time (min)
Normal
LY50
LY75
LY88
LY94
LY97
LY data
Normal data

Akt1_S122 -> Tra2b_S266 - cost: 8.43, layer: 52

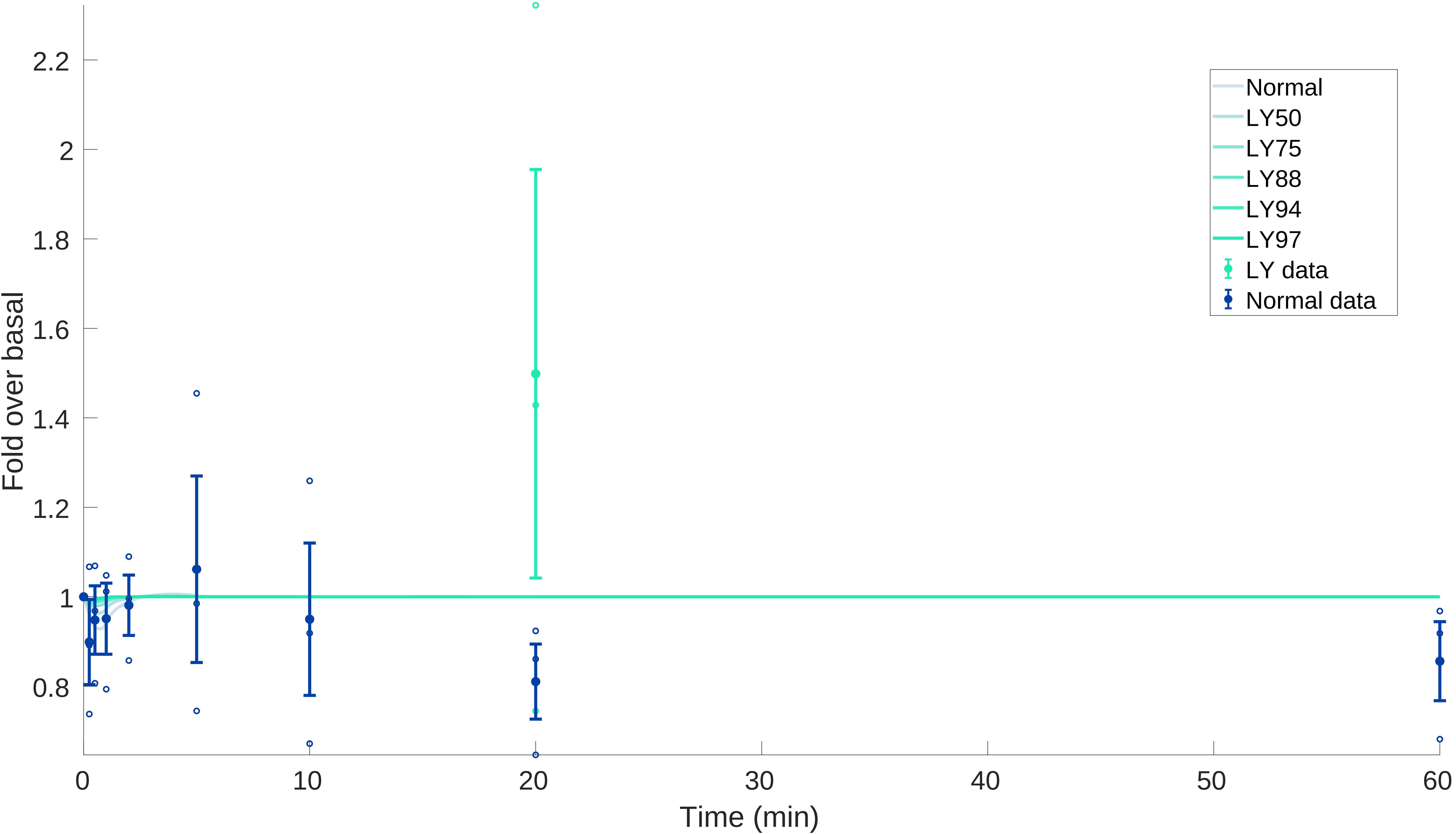

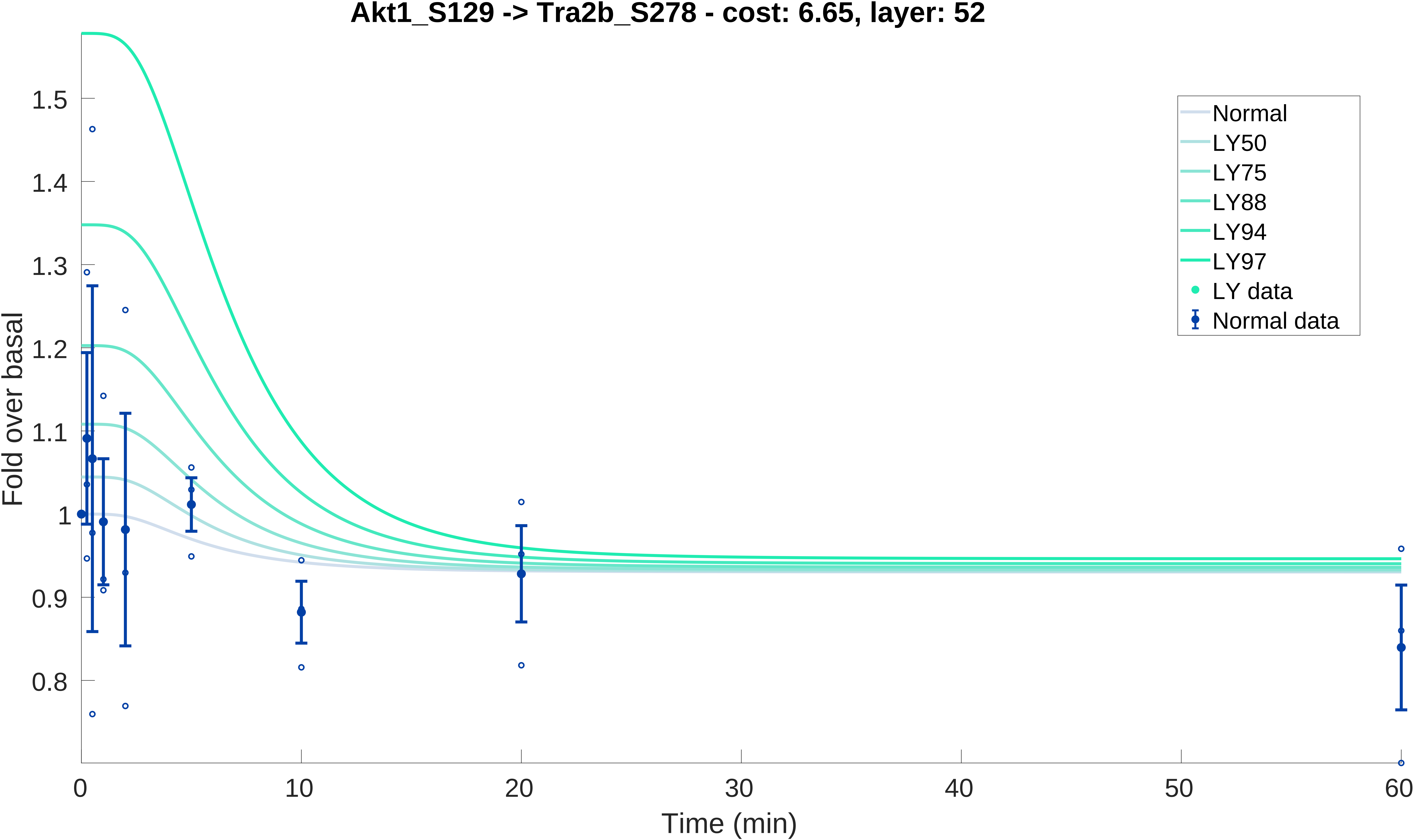

Akt1_S129 -> Tra2b_S278 - cost: 6.65, layer: 52
Fold over basal
Time (min)
Normal
LY50
LY75
LY88
LY94
LY97
LY data
Normal data
1.5
1.4
1.3
1.2
1.1
1
0.9
0.8
0
10
20
30
40
50
60

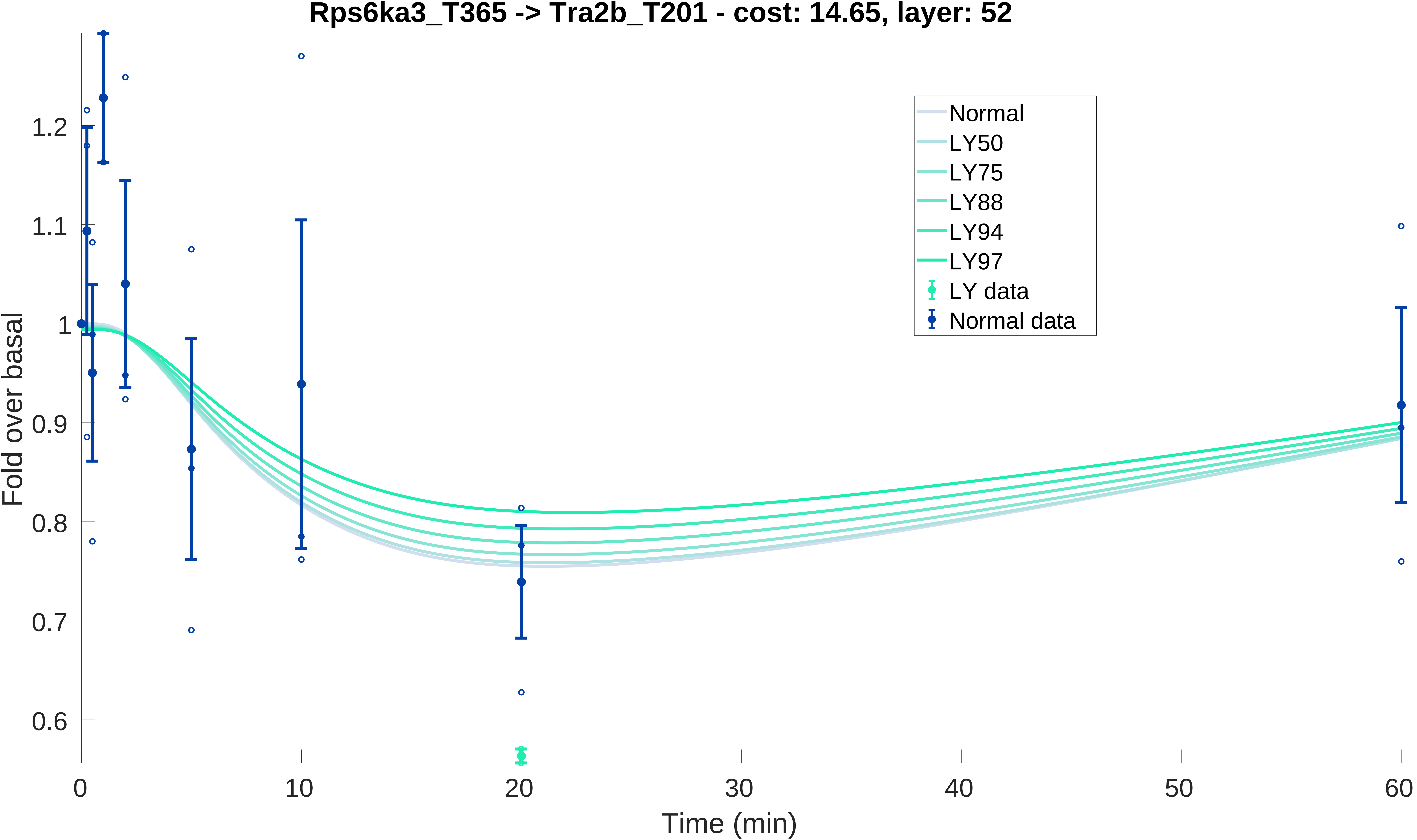

Rps6ka3_T365 -> Tra2b_T201 - cost: 14.65, layer: 52
Normal
LY50
LY75
LY88
LY94
LY97
LY data
Normal data
Fold over basal
Time (min)
0.6
0.7
0.8
0.9
1
1.1
1.2
0
10
20
30
40
50
60

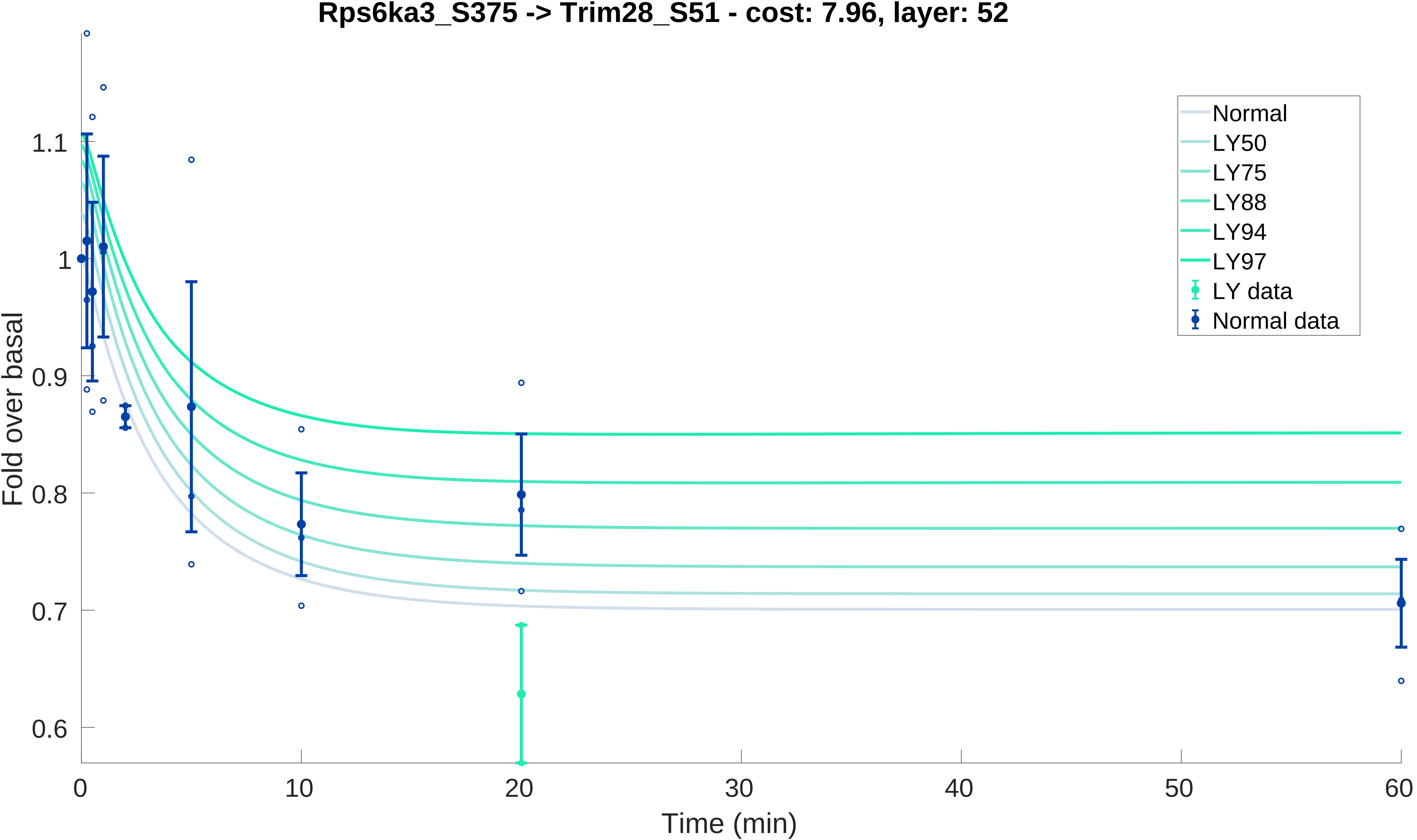

Rps6ka3_S375 -> Trim28_S51 - cost: 7.96, layer: 52
Fold over basal
Time (min)
0
10
20
30
40
50
60
0.6
0.7
0.8
0.9
1
1.1
Normal
LY50
LY75
LY88
LY94
LY97
LY data
Normal data

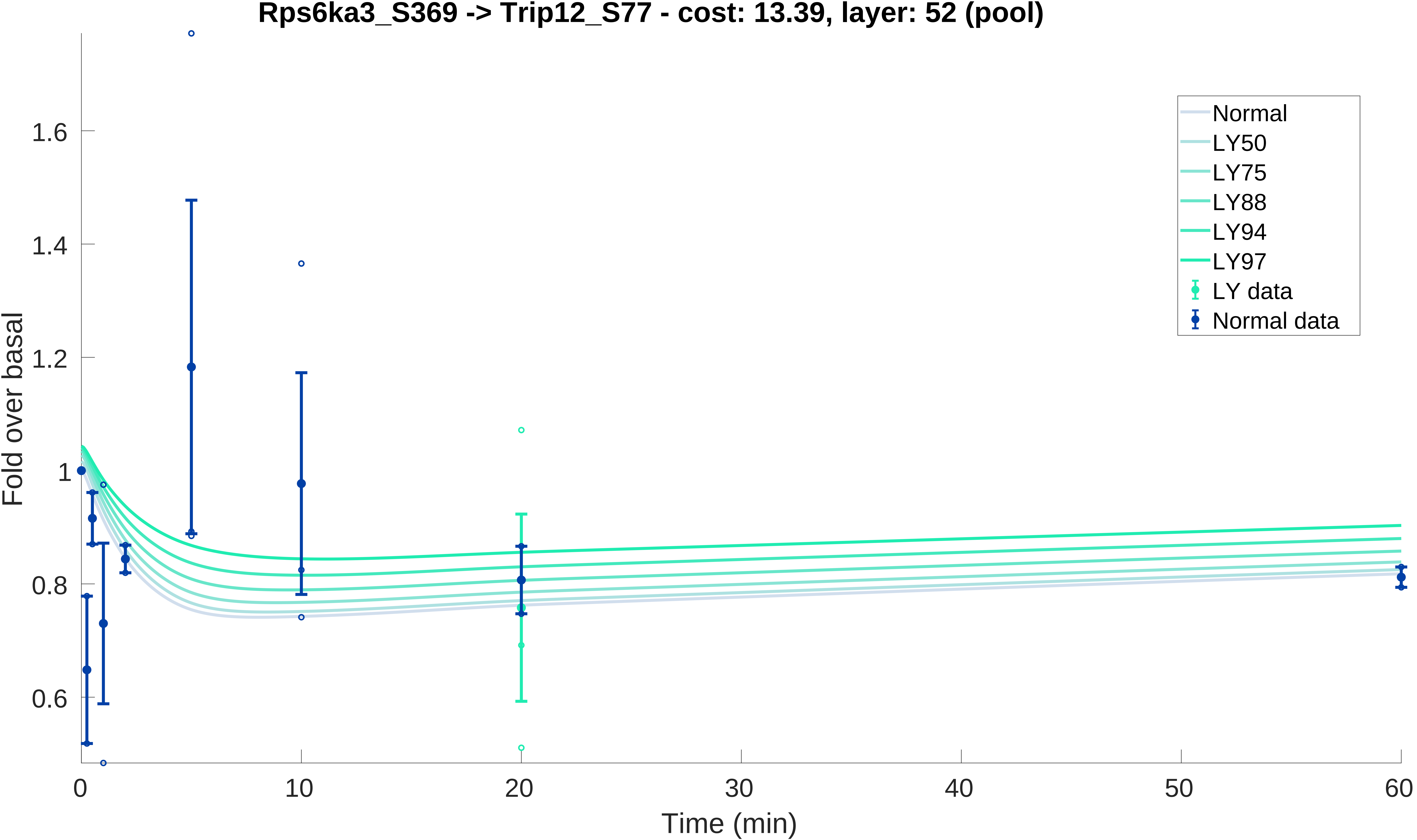
Rps6ka3_S369 -> Trip12_S77 - cost: 13.39, layer: 52 (pool)
Fold over basal
Time (min)
0.6
0.8
1
1.2
1.4
1.6
0
10
20
30
40
50
60
Normal
LY50
LY75
LY88
LY94
LY97
LY data
Normal data

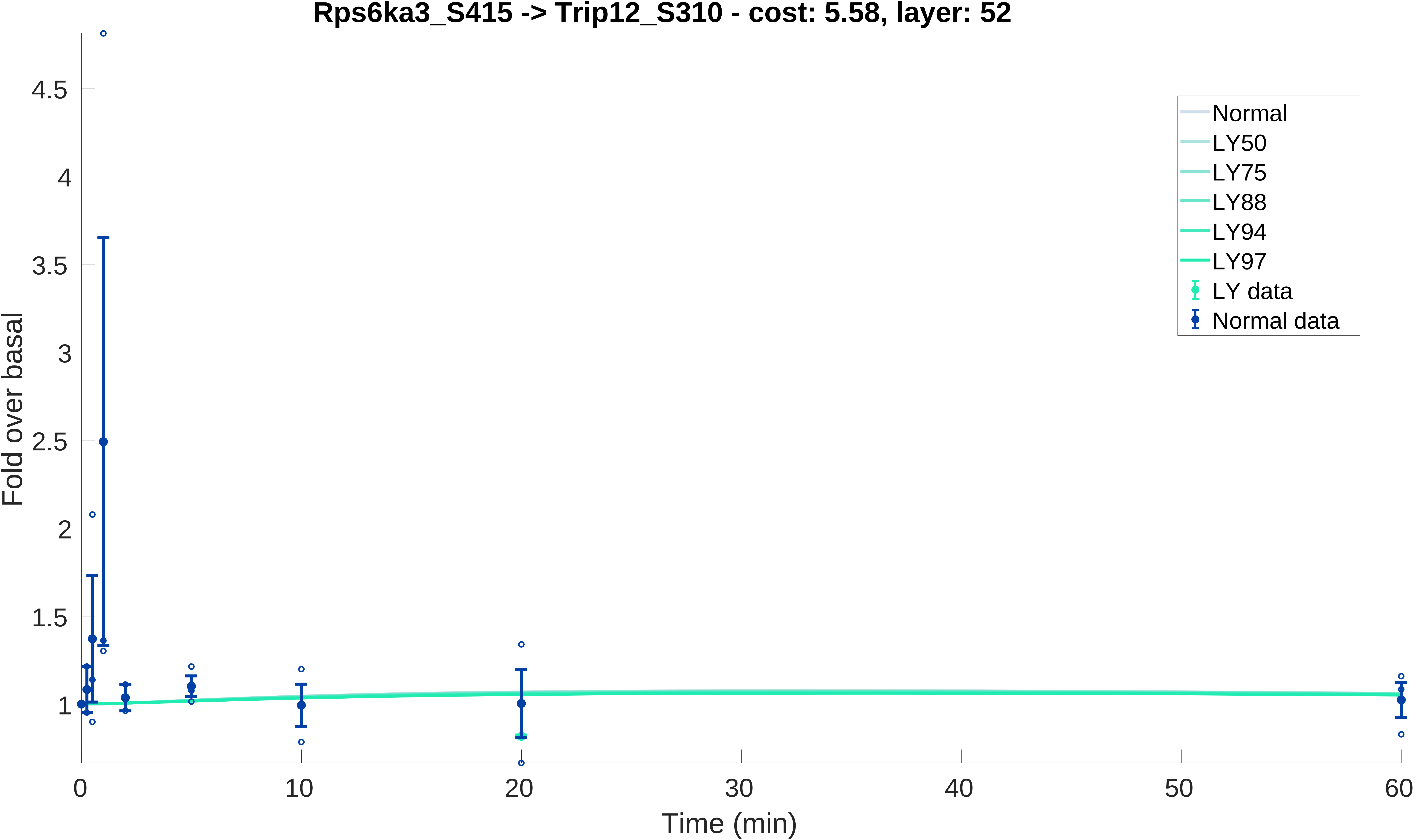

Rps6ka3_S415 -> Trip12_S310 - cost: 5.58, layer: 52
Fold over basal
Time (min)
4.5
4
3.5
3
2.5
2
1.5
1
0
10
20
30
40
50
60
Normal
LY50
LY75
LY88
LY94
LY97
LY data
Normal data

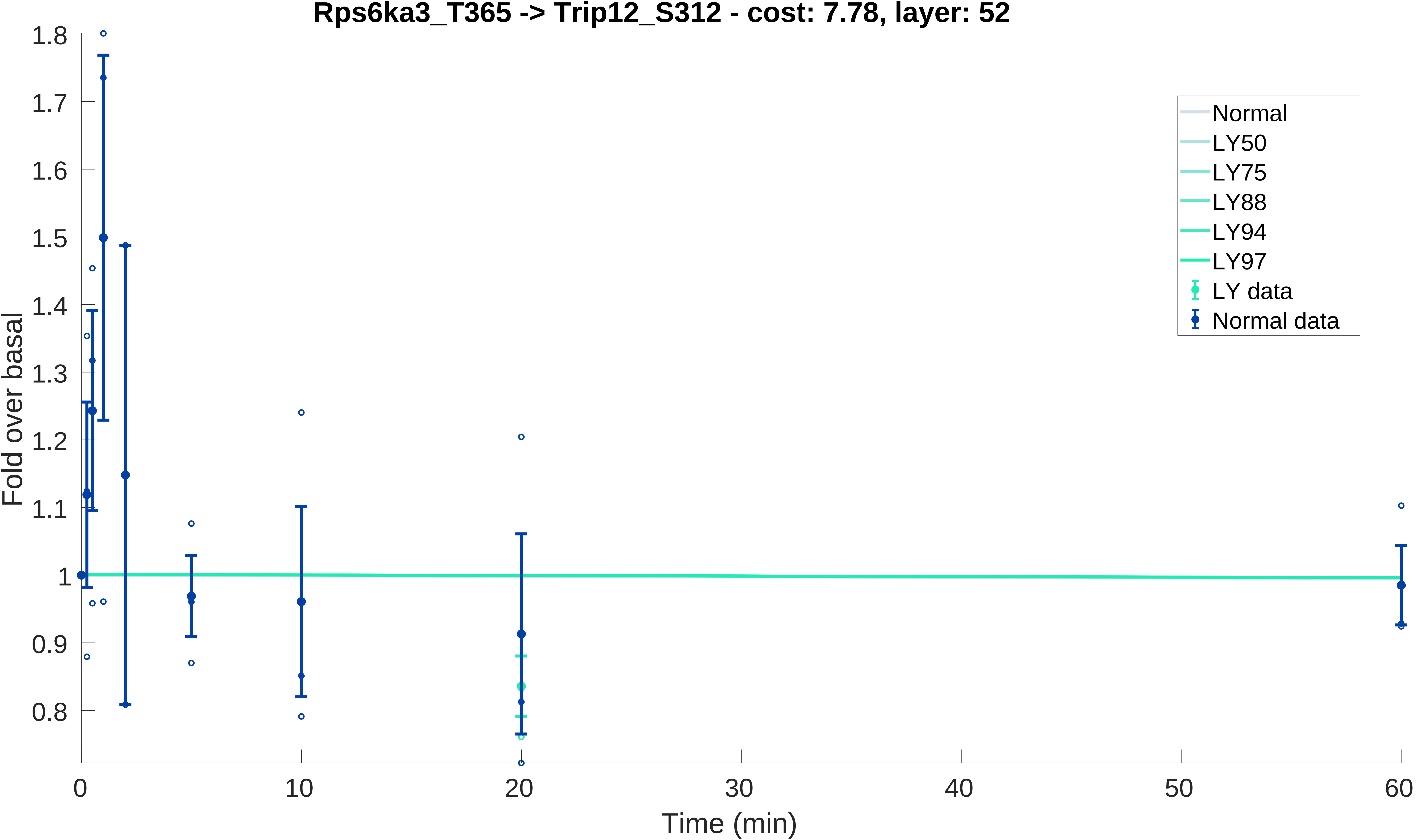

Rps6ka3_T365 -> Trip12_S312 - cost: 7.78, layer: 52
Fold over basal
Time (min)
0
10
20
30
40
50
60
0.8
0.9
1
1.1
1.2
1.3
1.4
1.5
1.6
1.7
1.8
Normal
LY50
LY75
LY88
LY94
LY97
LY data
Normal data

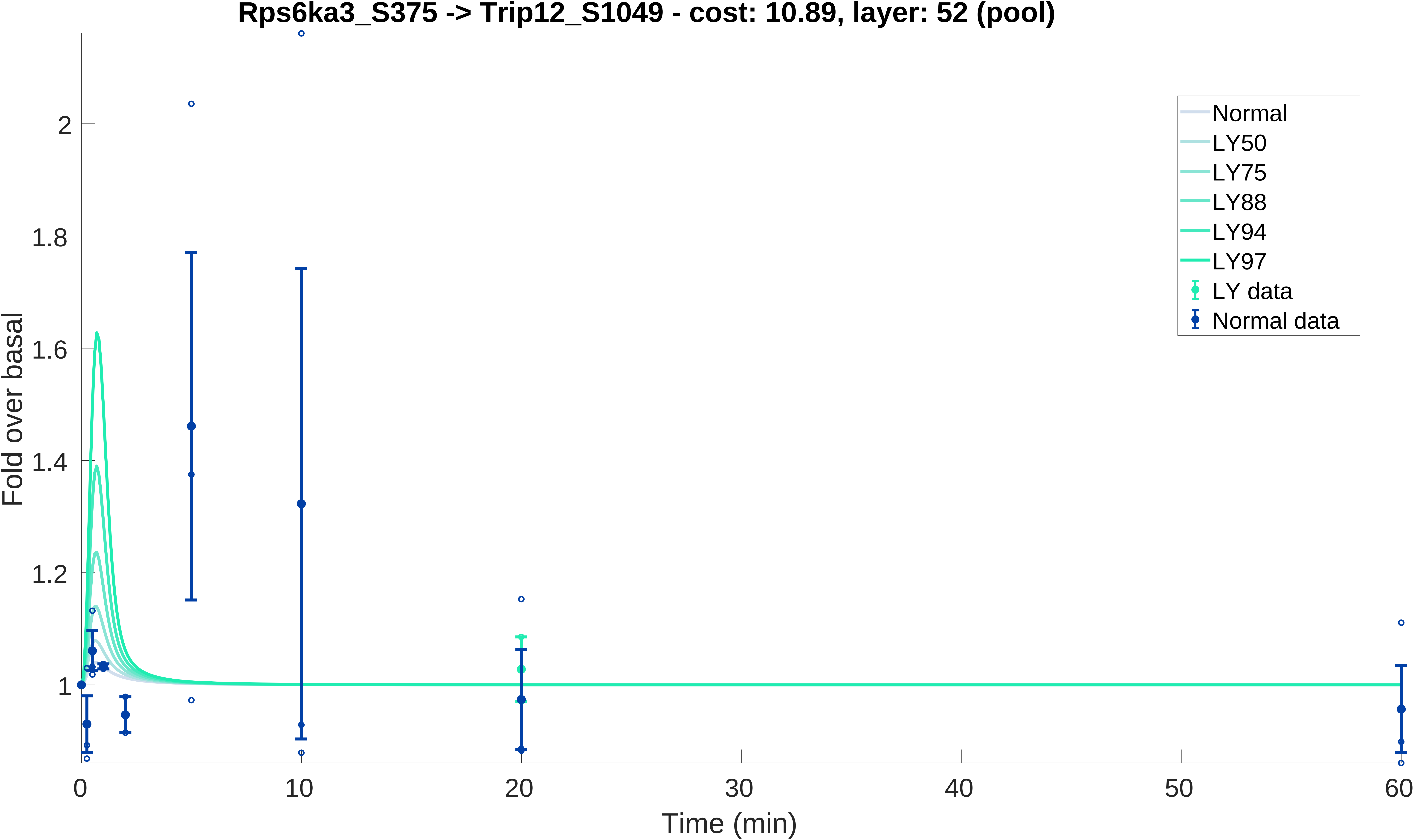
Rps6ka3_S375 -> Trip12_S1049 - cost: 10.89, layer: 52 (pool)
Fold over basal
Time (min)
Normal
LY50
LY75
LY88
LY94
LY97
LY data
Normal data
2
1.8
1.6
1.4
1.2
1
0
10
20
30
40
50
60

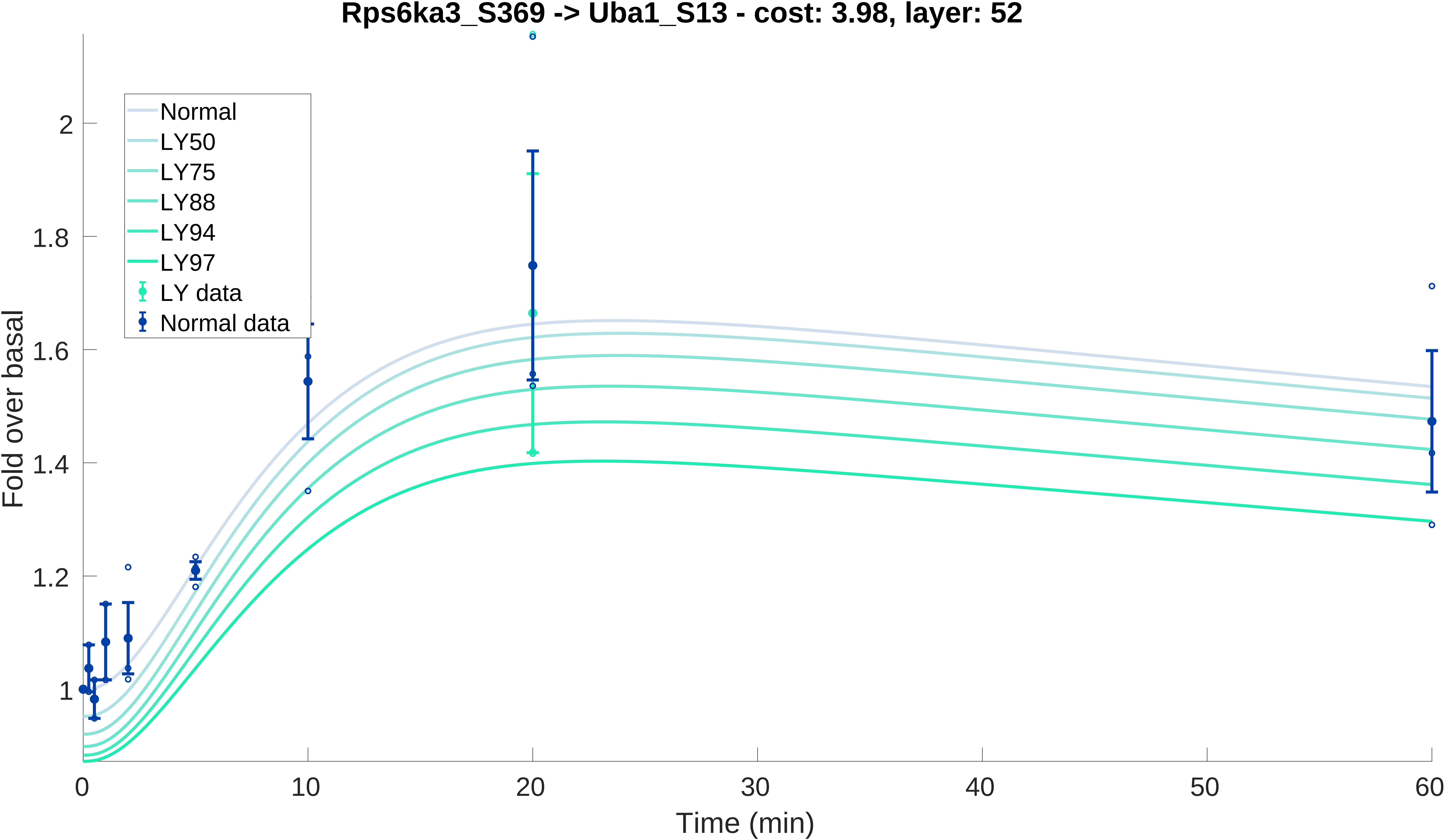

Rps6ka3_S369 -> Uba1_S13 - cost: 3.98, layer: 52
Normal
LY50
LY75
LY88
LY94
LY97
LY data
Normal data
Fold over basal
2
1.8
1.6
1.4
1.2
1
0
10
20
30
40
50
60
Time (min)

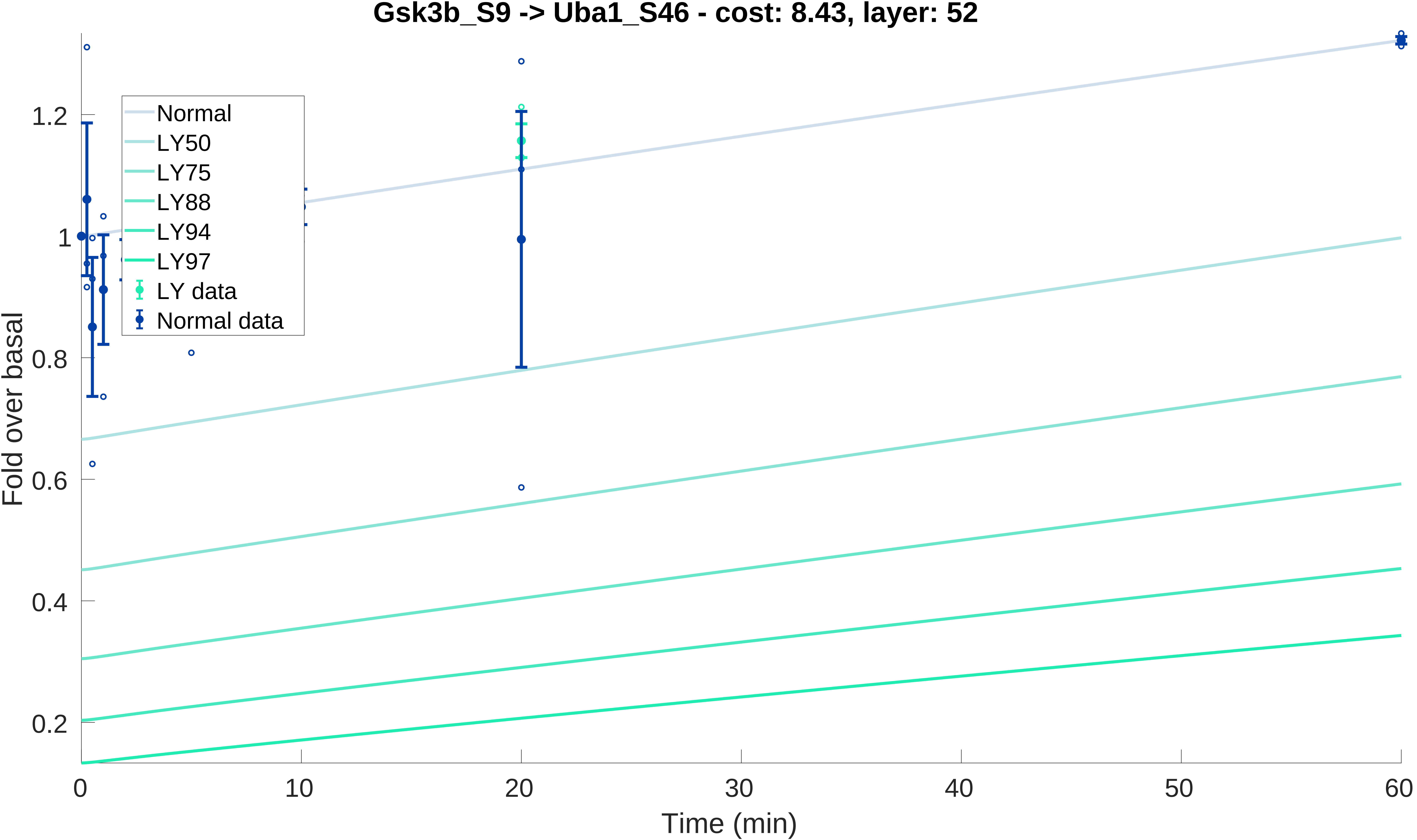

Gsk3b_S9 -> Uba1_S46 - cost: 8.43, layer: 52
Normal
LY50
LY75
LY88
LY94
LY97
LY data
Normal data
Fold over basal
Time (min)
0.2
0.4
0.6
0.8
1
1.2
0
10
20
30
40
50
60

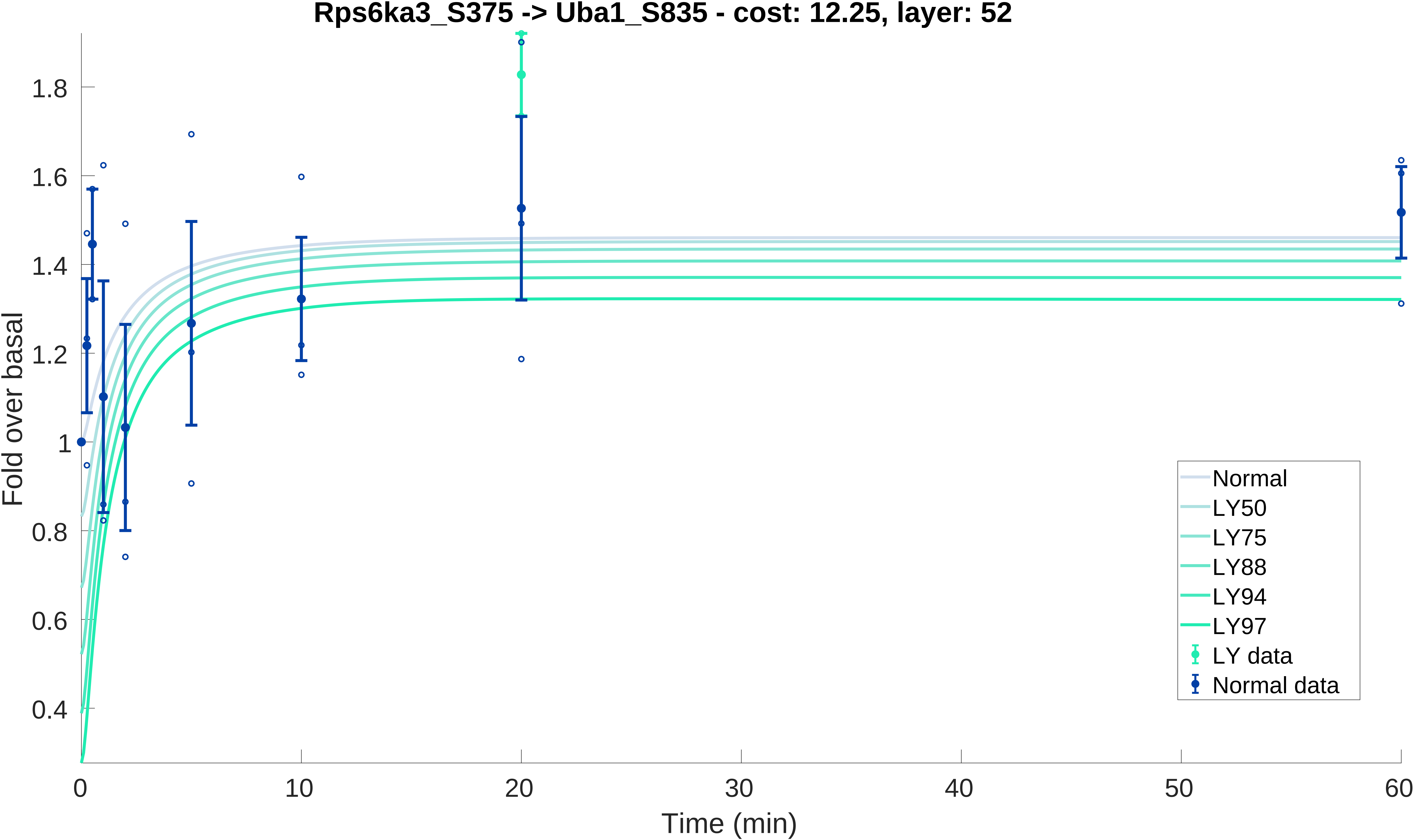

Rps6ka3_S375 -> Uba1_S835 - cost: 12.25, layer: 52
Fold over basal
1.8
1.6
1.4
1.2
1
0.8
0.6
0.4
0
10
20
30
40
50
60
Time (min)
Normal
LY50
LY75
LY88
LY94
LY97
LY data
Normal data

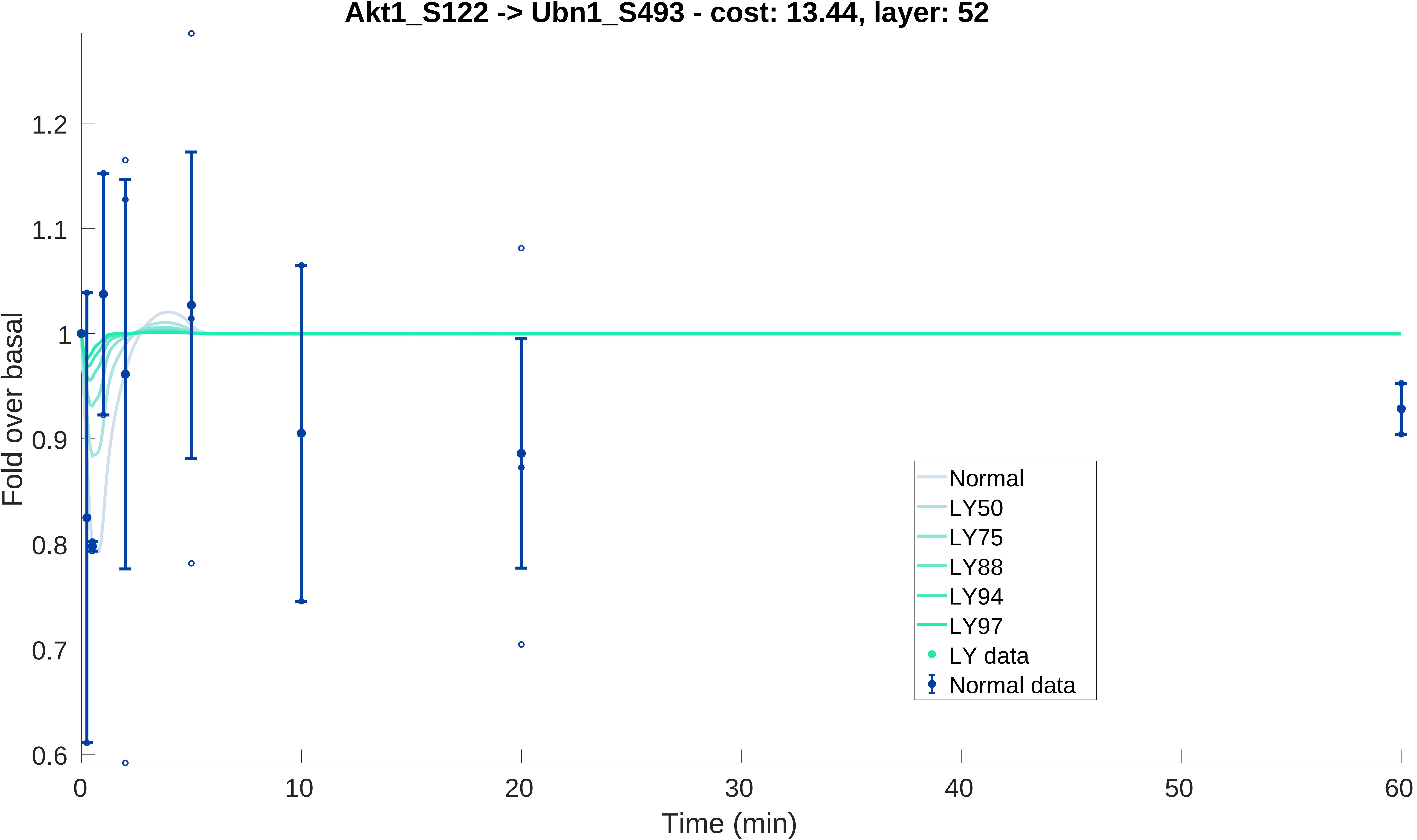
Akt1_S122 -> Ubn1_S493 - cost: 13.44, layer: 52
Fold over basal
Time (min)
0
10
20
30
40
50
60
0.6
0.7
0.8
0.9
1
1.1
1.2
Normal
LY50
LY75
LY88
LY94
LY97
LY data
Normal data

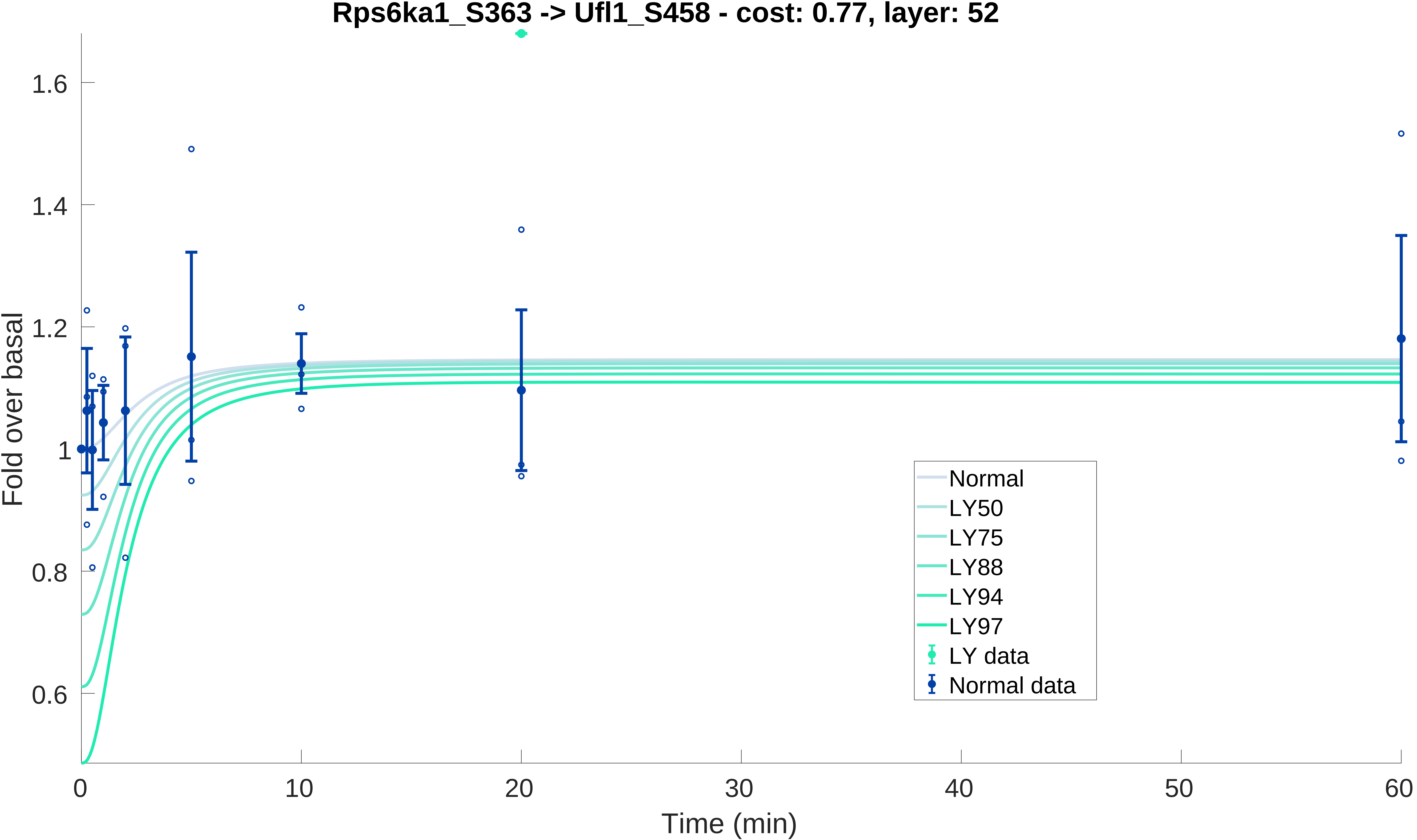
Rps6ka1_S363 -> Ufl1_S458 - cost: 0.77, layer: 52
Fold over basal
1.6
1.4
1.2
1
0.8
0.6
0
10
20
30
40
50
60
Time (min)
Normal
LY50
LY75
LY88
LY94
LY97
LY data
Normal data

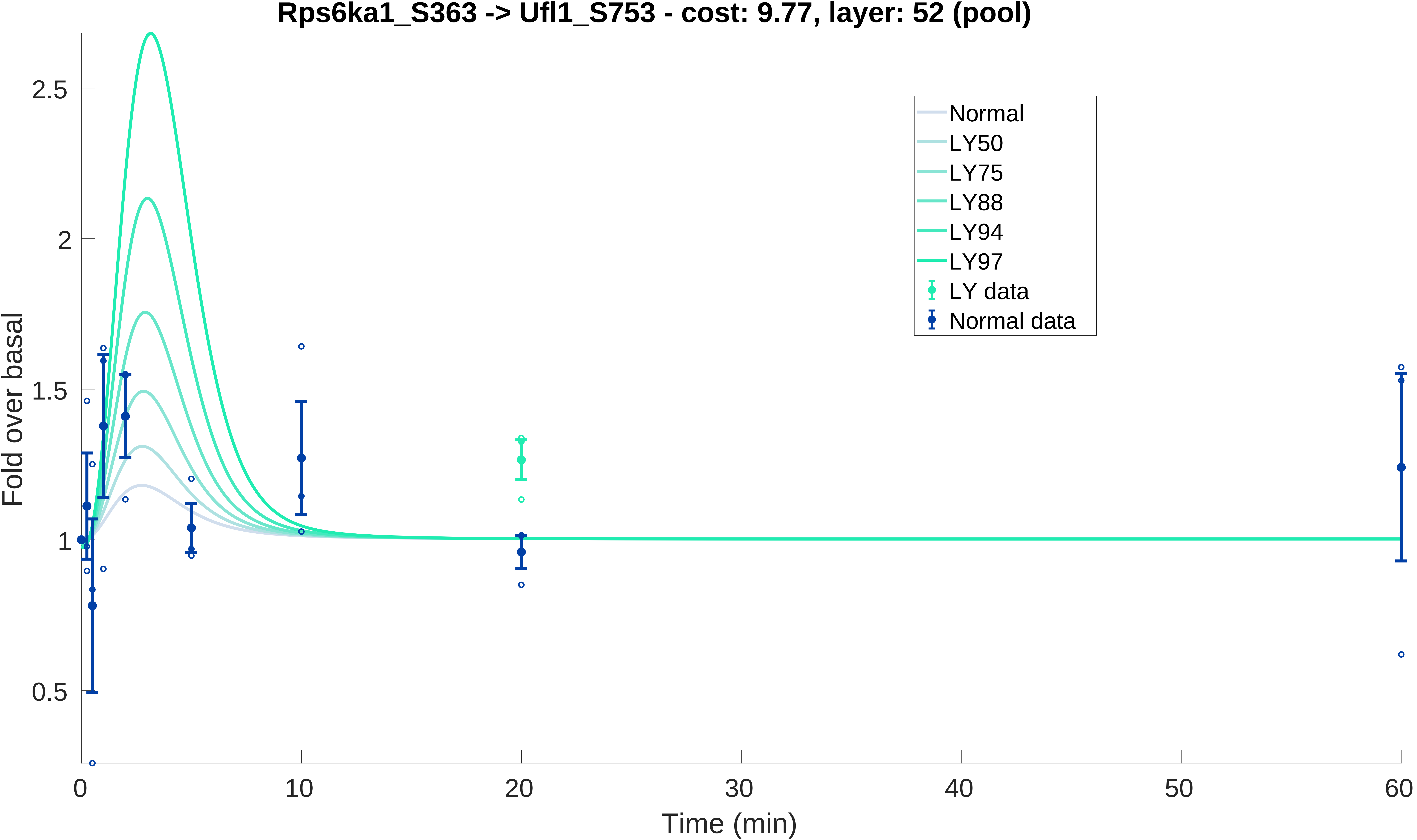

Rps6ka1_S363 -> Ufl1_S753 - cost: 9.77, layer: 52 (pool)
Fold over basal
Time (min)
0.5
1
1.5
2
2.5
0
10
20
30
40
50
60
Normal
LY50
LY75
LY88
LY94
LY97
LY data
Normal data

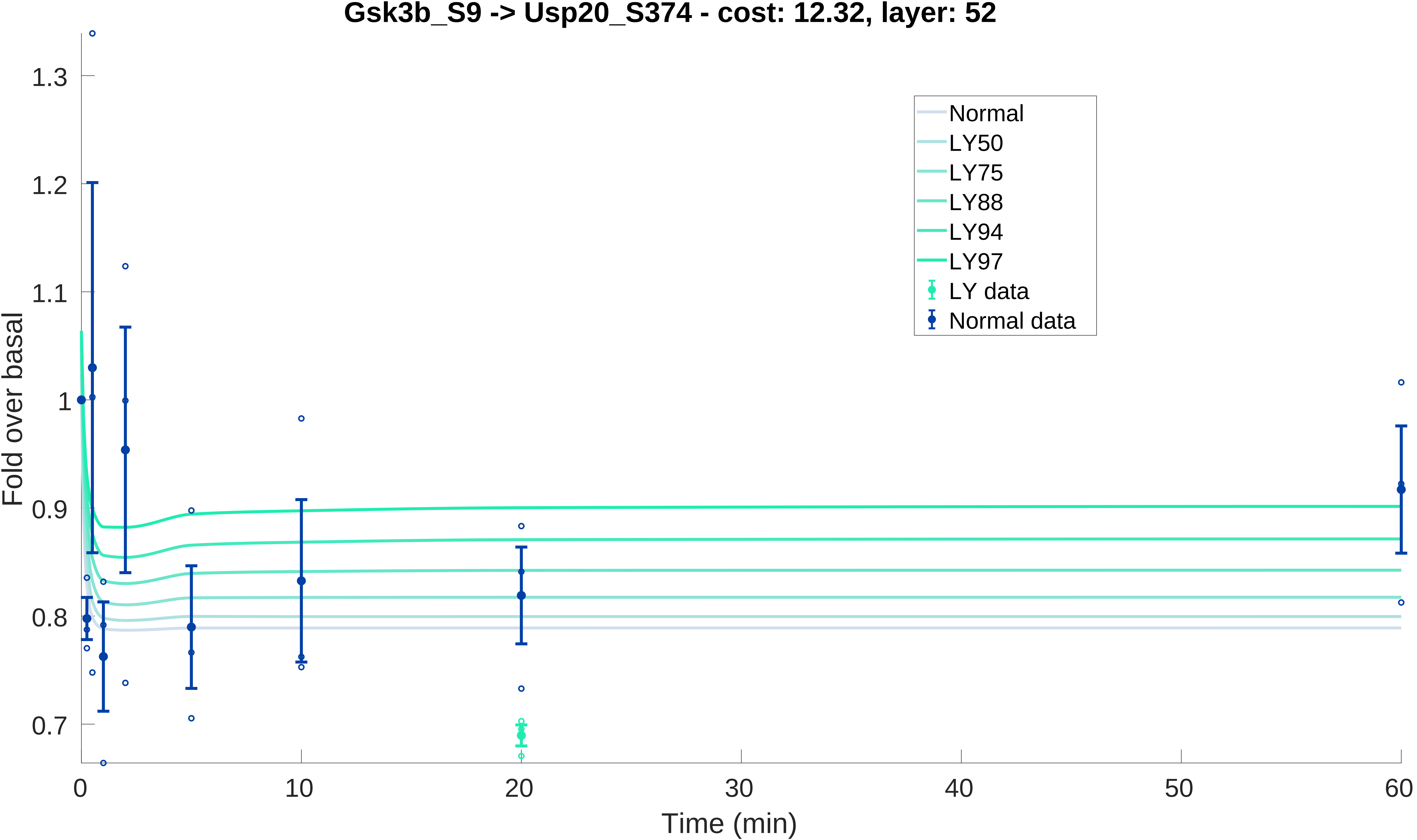

Gsk3b_S9 -> Usp20_S374 - cost: 12.32, layer: 52
Fold over basal
1.3
1.2
1.1
1
0.9
0.8
0.7
0
10
20
30
40
50
60
Time (min)
Normal
LY50
LY75
LY88
LY94
LY97
LY data
Normal data

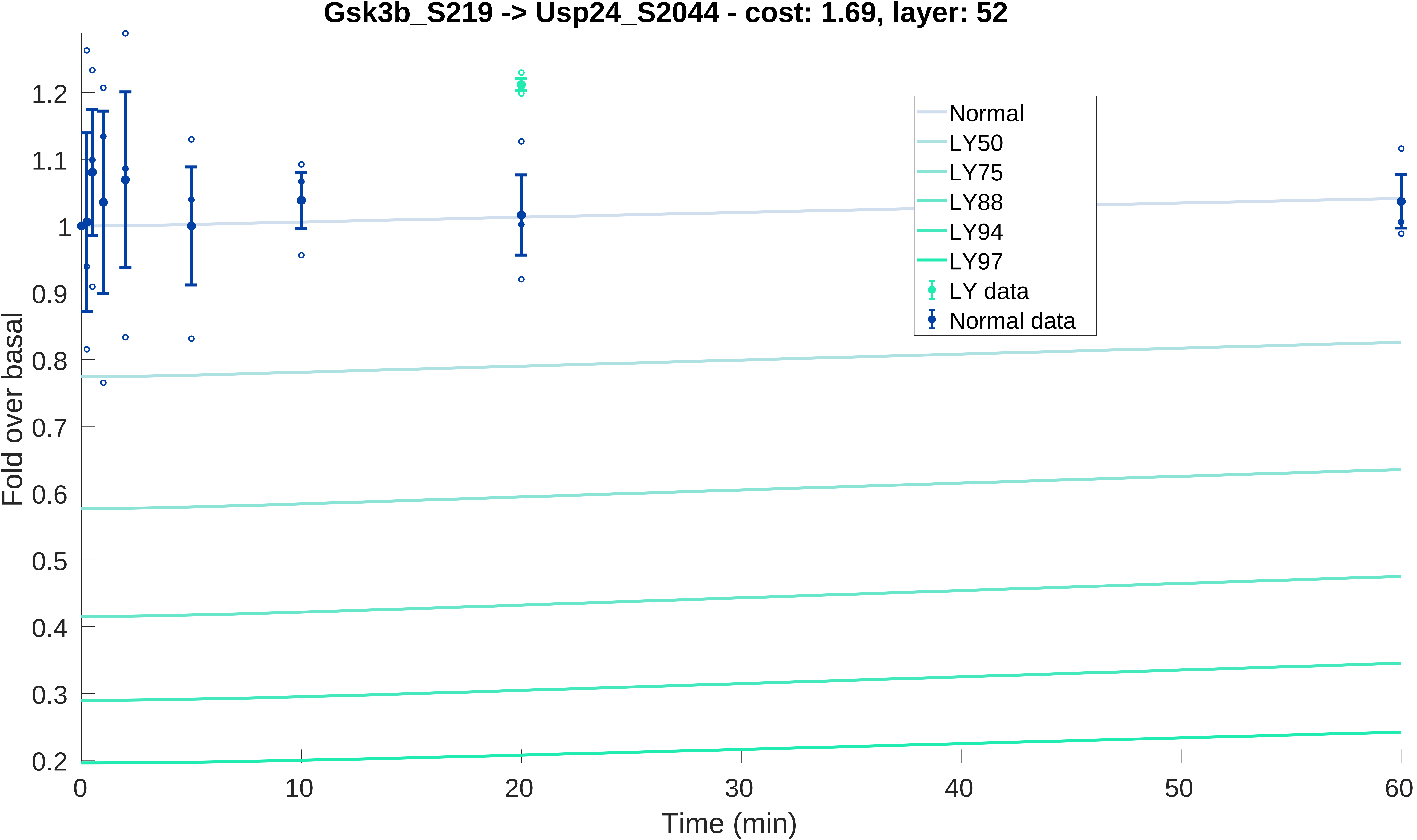

Gsk3b_S219 -> Usp24_S2044 - cost: 1.69, layer: 52
Fold over basal
1.2
1.1
1
0.9
0.8
0.7
0.6
0.5
0.4
0.3
0.2
0
10
20
30
40
50
60
Time (min)
Normal
LY50
LY75
LY88
LY94
LY97
LY data
Normal data

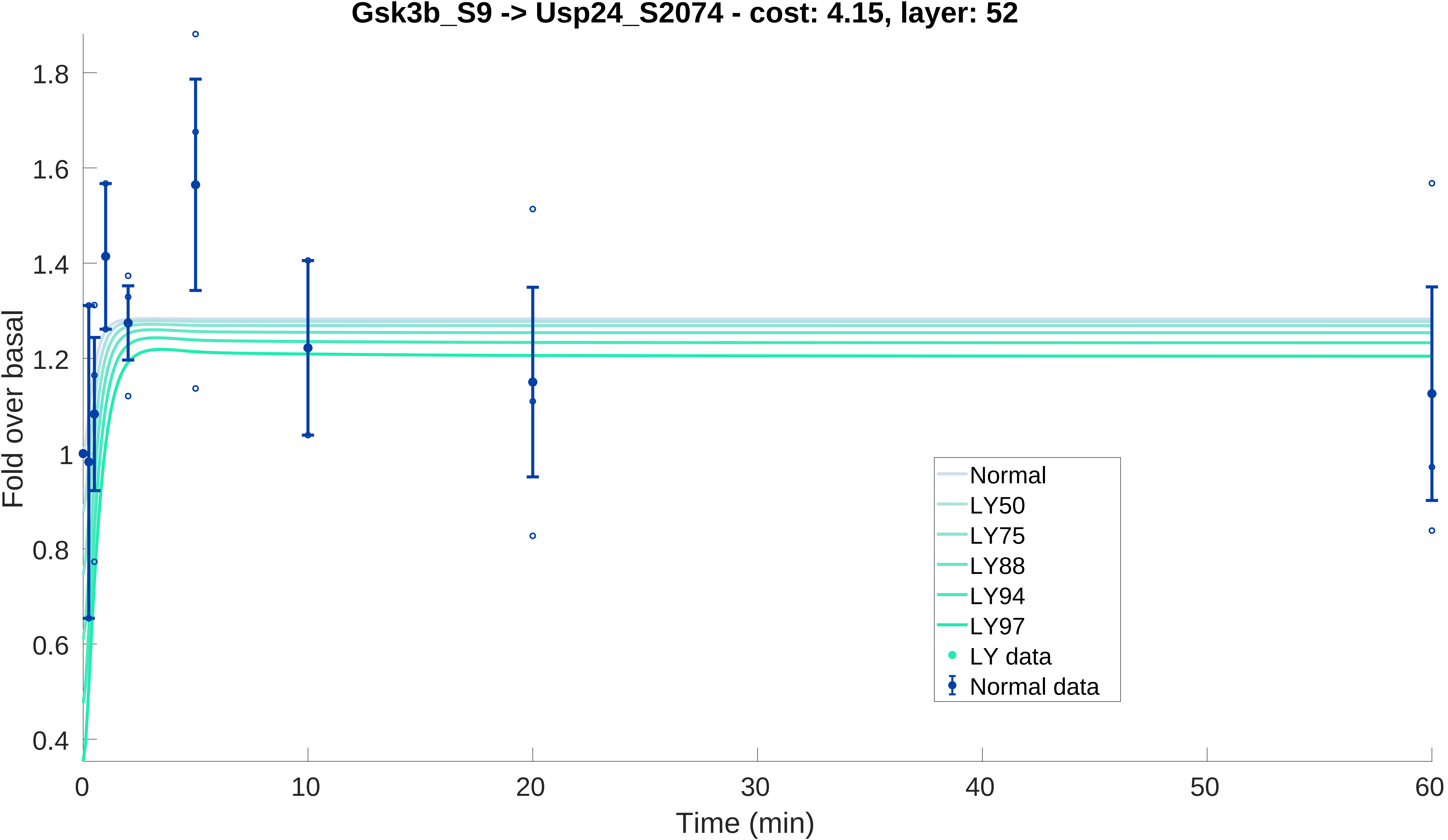

Gsk3b_S9 -> Usp24_S2074 - cost: 4.15, layer: 52
Fold over basal
Time (min)
0.4
0.6
0.8
1
1.2
1.4
1.6
1.8
0
10
20
30
40
50
60
Normal
LY50
LY75
LY88
LY94
LY97
LY data
Normal data

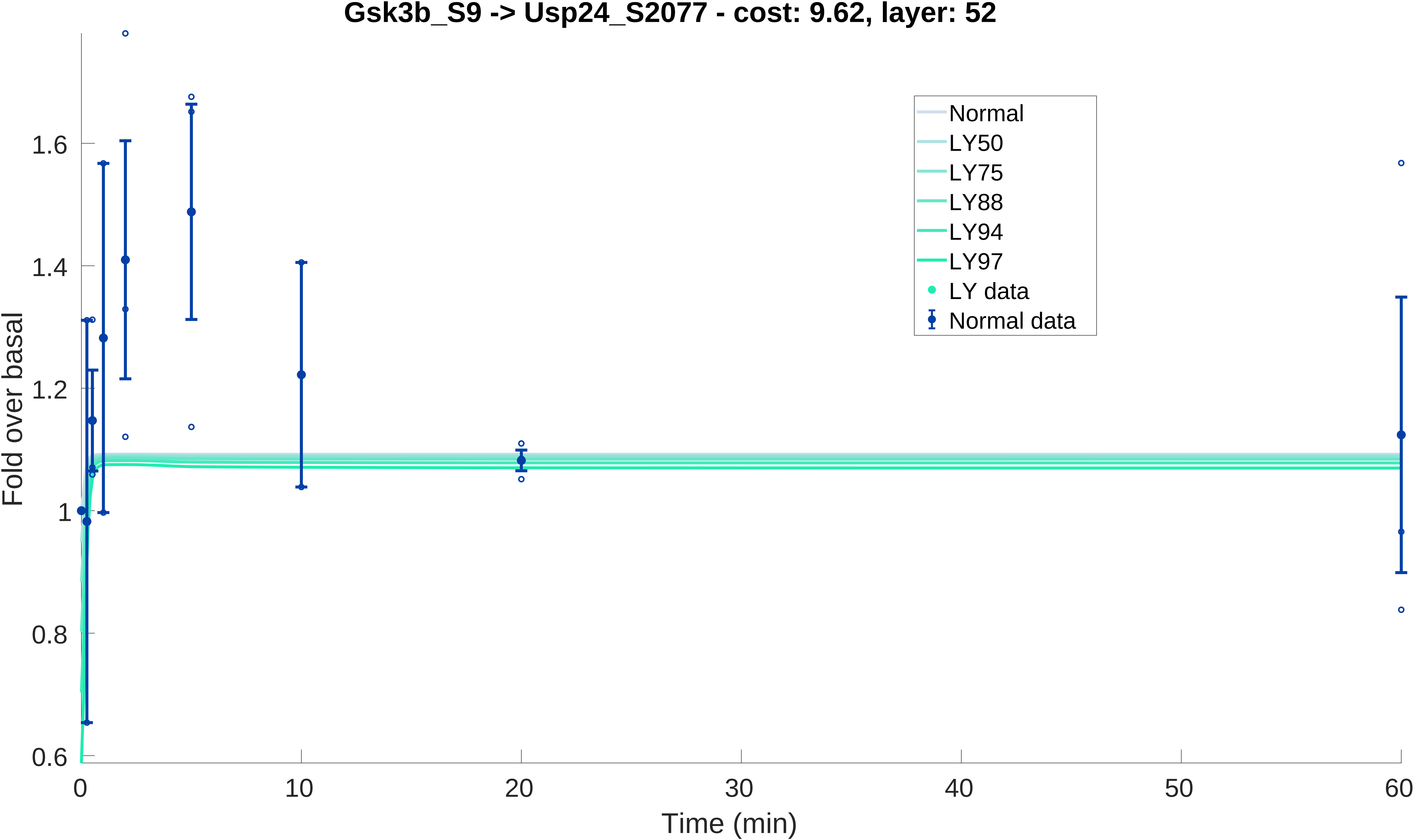
Gsk3b_S9 -> Usp24_S2077 - cost: 9.62, layer: 52
Fold over basal
Time (min)
0.6
0.8
1
1.2
1.4
1.6
0
10
20
30
40
50
60
Normal
LY50
LY75
LY88
LY94
LY97
LY data
Normal data

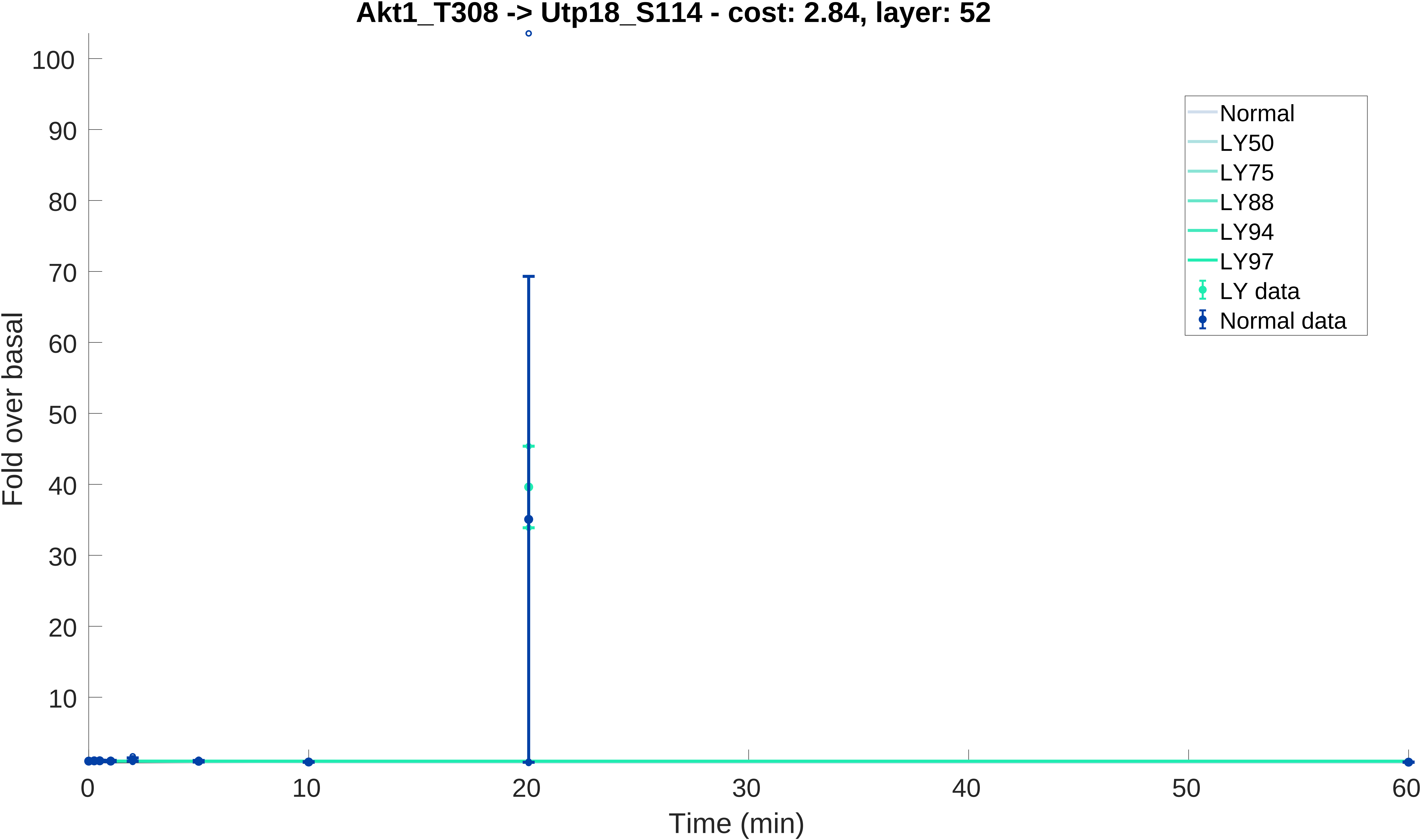

Akt1_T308 -> Utp18_S114 - cost: 2.84, layer: 52
Fold over basal
Time (min)
0
10
20
30
40
50
60
70
80
90
100
Normal
LY50
LY75
LY88
LY94
LY97
LY data
Normal data

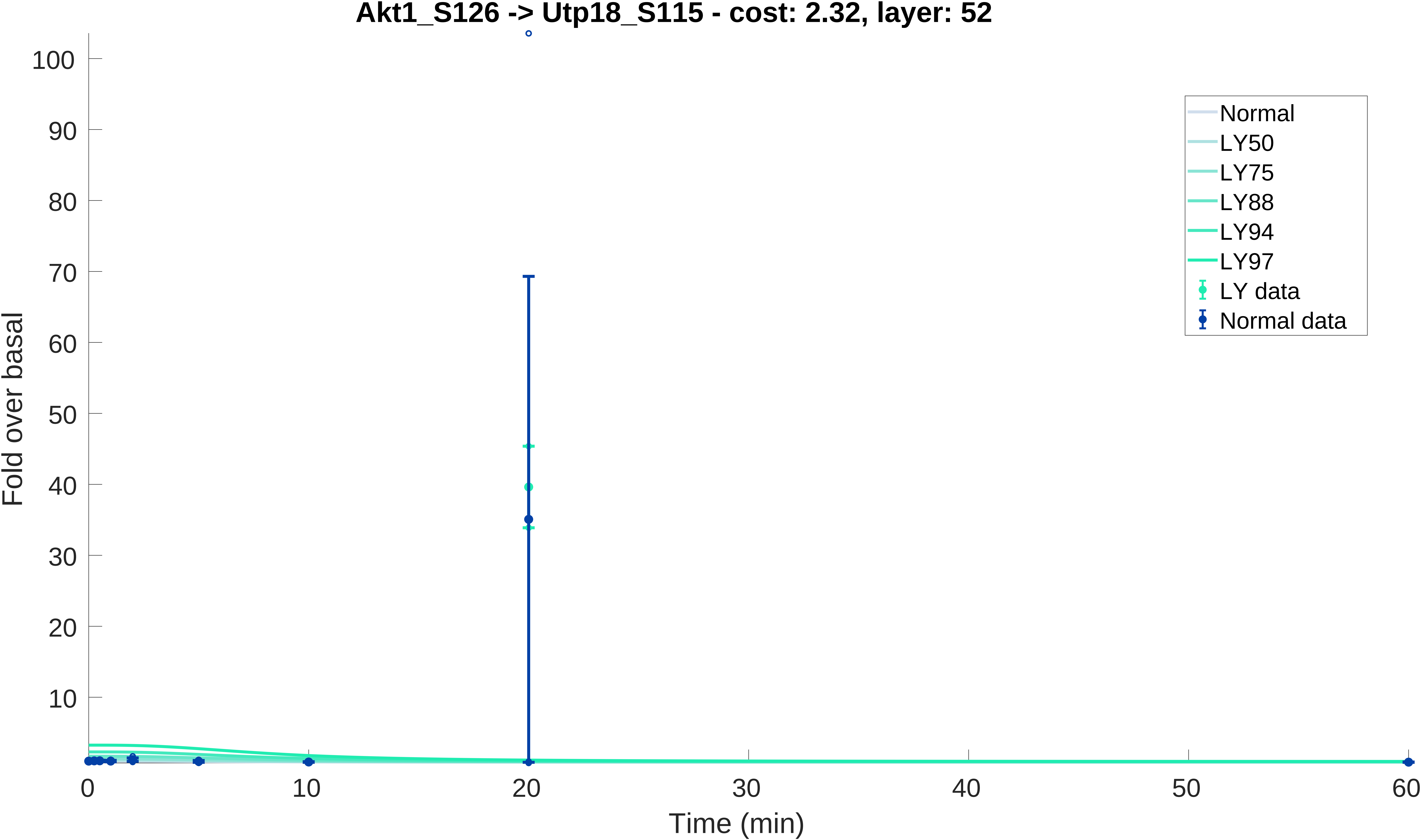

Akt1_S126 -> Utp18_S115 - cost: 2.32, layer: 52
Fold over basal
Time (min)
100
90
80
70
60
50
40
30
20
10
0
10
20
30
40
50
60
Normal
LY50
LY75
LY88
LY94
LY97
LY data
Normal data

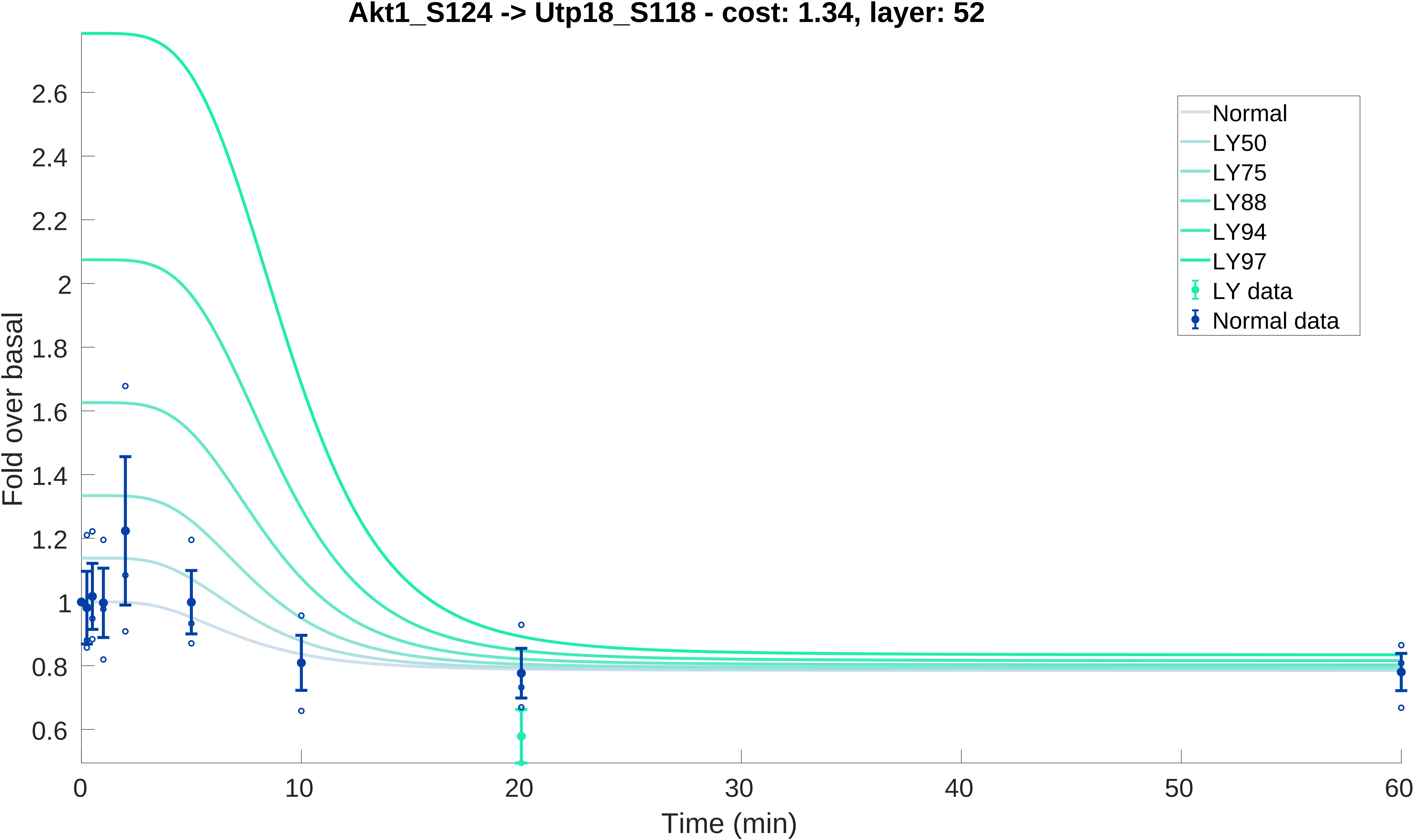

Akt1_S124 -> Utp18_S118 - cost: 1.34, layer: 52
Fold over basal
Time (min)
0.6
0.8
1
1.2
1.4
1.6
1.8
2
2.2
2.4
2.6
0
10
20
30
40
50
60
Normal
LY50
LY75
LY88
LY94
LY97
LY data
Normal data

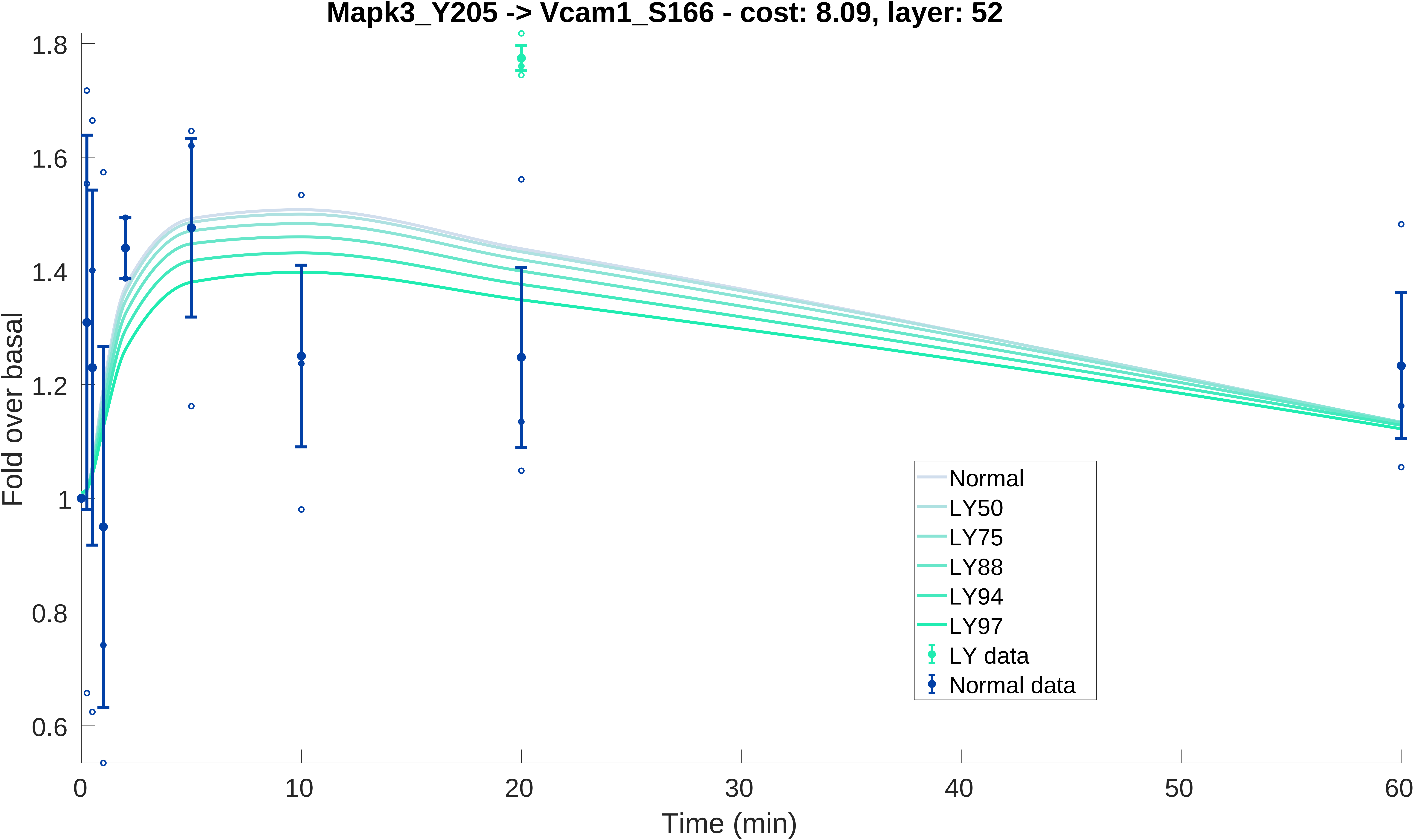

Mapk3_Y205 -> Vcam1_S166 - cost: 8.09, layer: 52
Fold over basal
1.8
1.6
1.4
1.2
1
0.8
0.6
0
10
20
30
40
50
60
Time (min)
Normal
LY50
LY75
LY88
LY94
LY97
LY data
Normal data

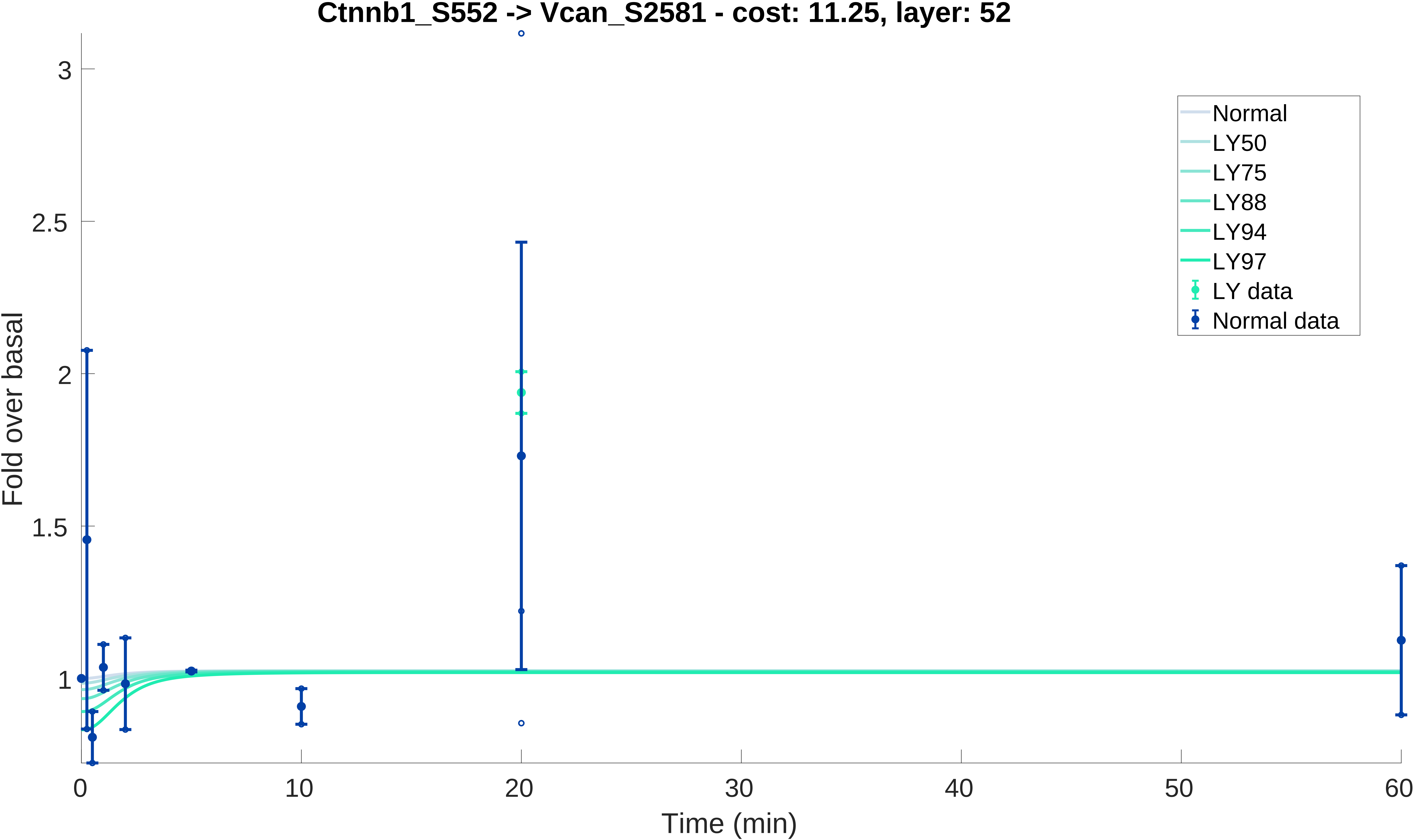

Ctnnb1_S552 -> Vcan_S2581 - cost: 11.25, layer: 52
Fold over basal
Time (min)
0
10
20
30
40
50
60
1
1.5
2
2.5
3
Normal
LY50
LY75
LY88
LY94
LY97
LY data
Normal data

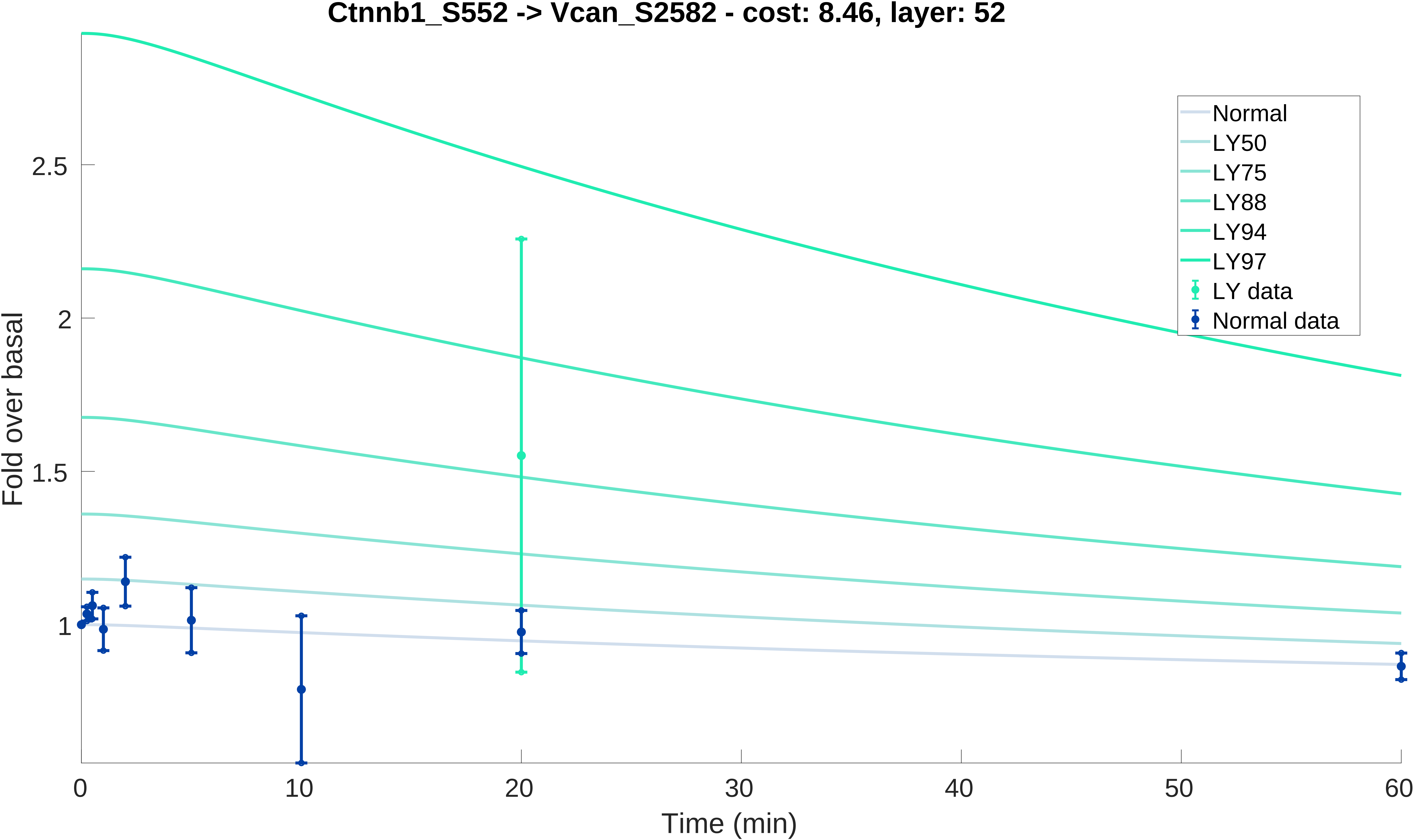

Ctnnb1_S552 -> Vcan_S2582 - cost: 8.46, layer: 52
Fold over basal
Time (min)
2.5
2
1.5
1
0
10
20
30
40
50
60
Normal
LY50
LY75
LY88
LY94
LY97
LY data
Normal data

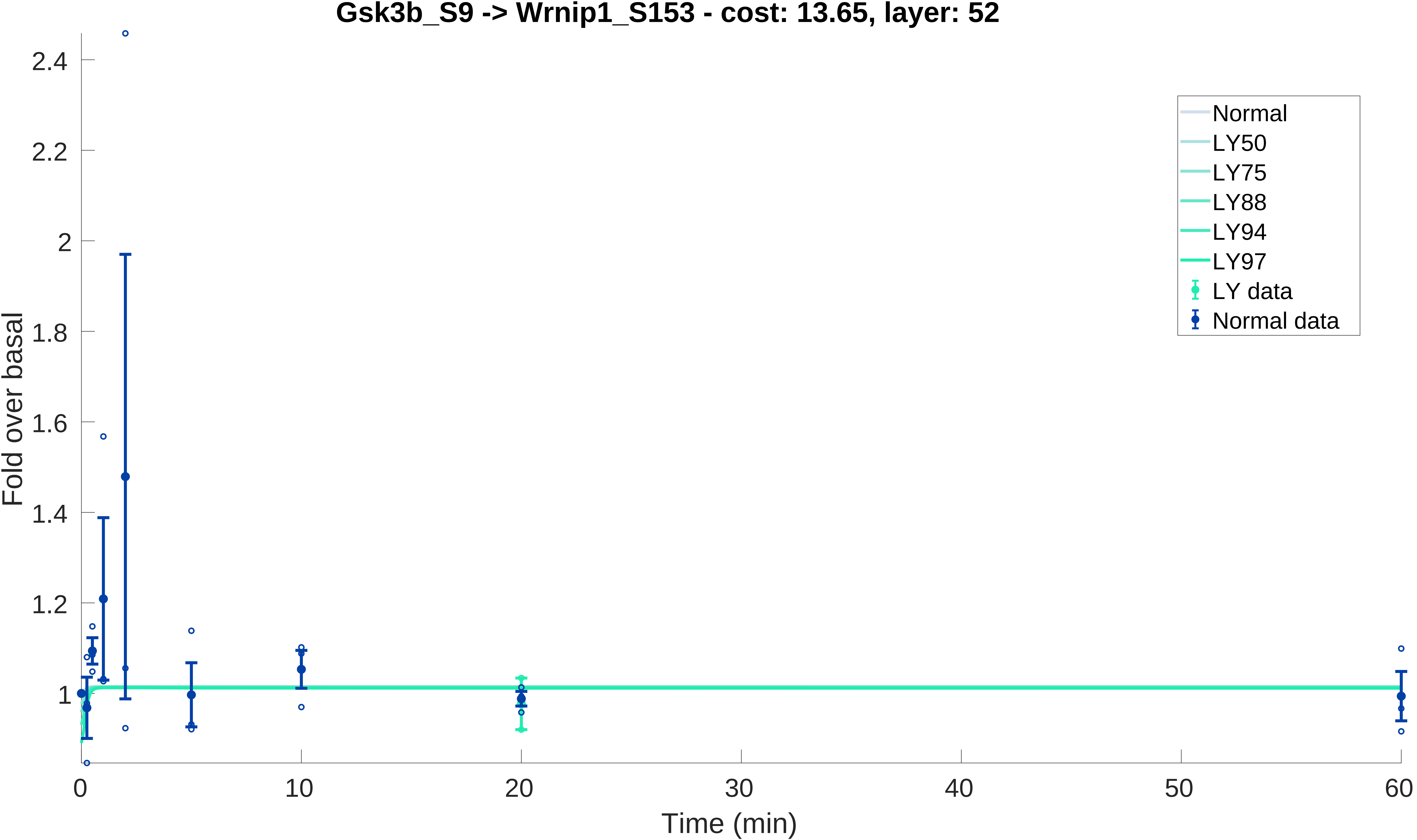
Gsk3b_S9 -> Wrnip1_S153 - cost: 13.65, layer: 52
Fold over basal
Time (min)
2.4
2.2
2
1.8
1.6
1.4
1.2
1
0
10
20
30
40
50
60
Normal
LY50
LY75
LY88
LY94
LY97
LY data
Normal data

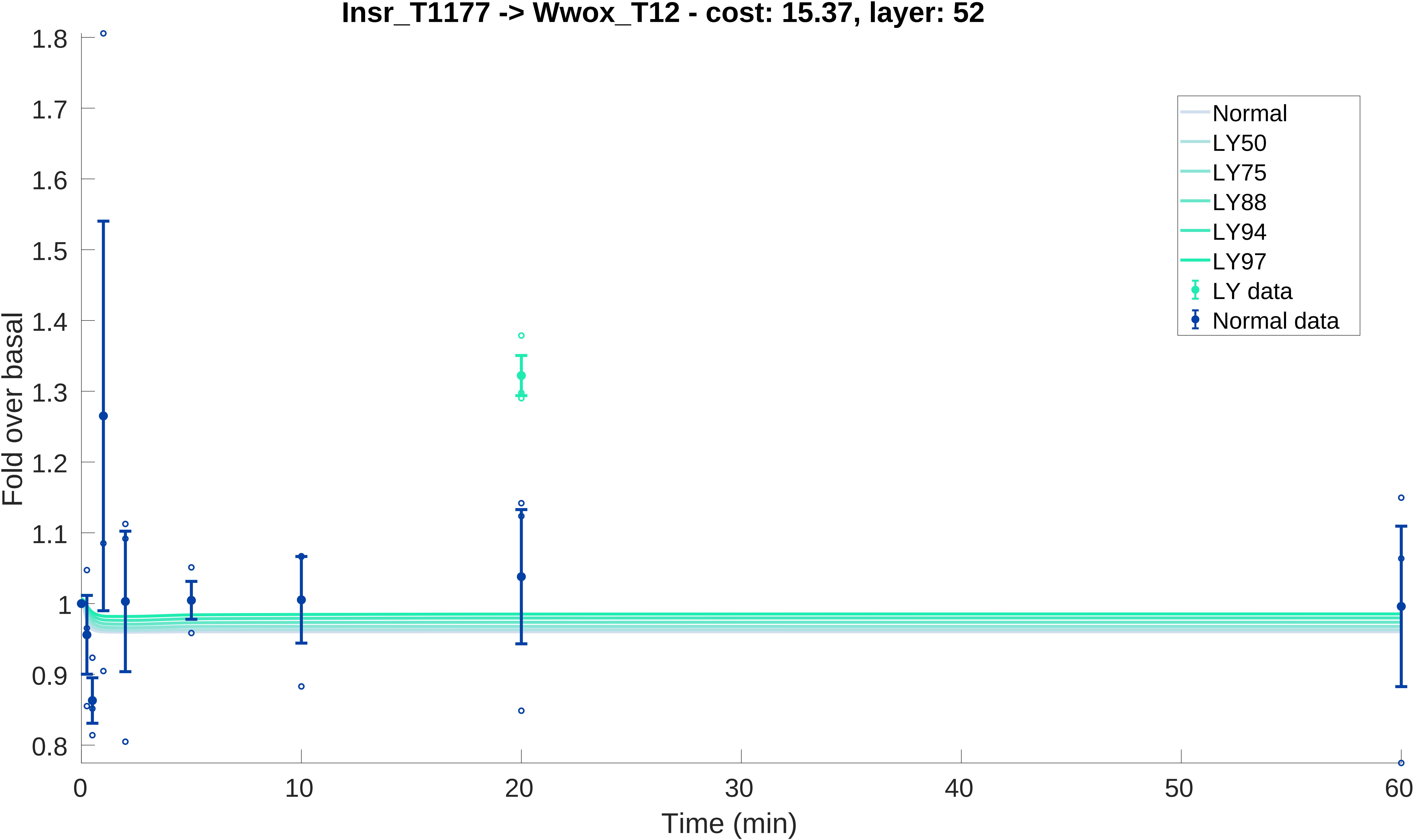
Insr_T1177 -> Wwox_T12 - cost: 15.37, layer: 52
Fold over basal
1.8
1.7
1.6
1.5
1.4
1.3
1.2
1.1
1
0.9
0.8
0
10
20
30
40
50
60
Time (min)
Normal
LY50
LY75
LY88
LY94
LY97
LY data
Normal data

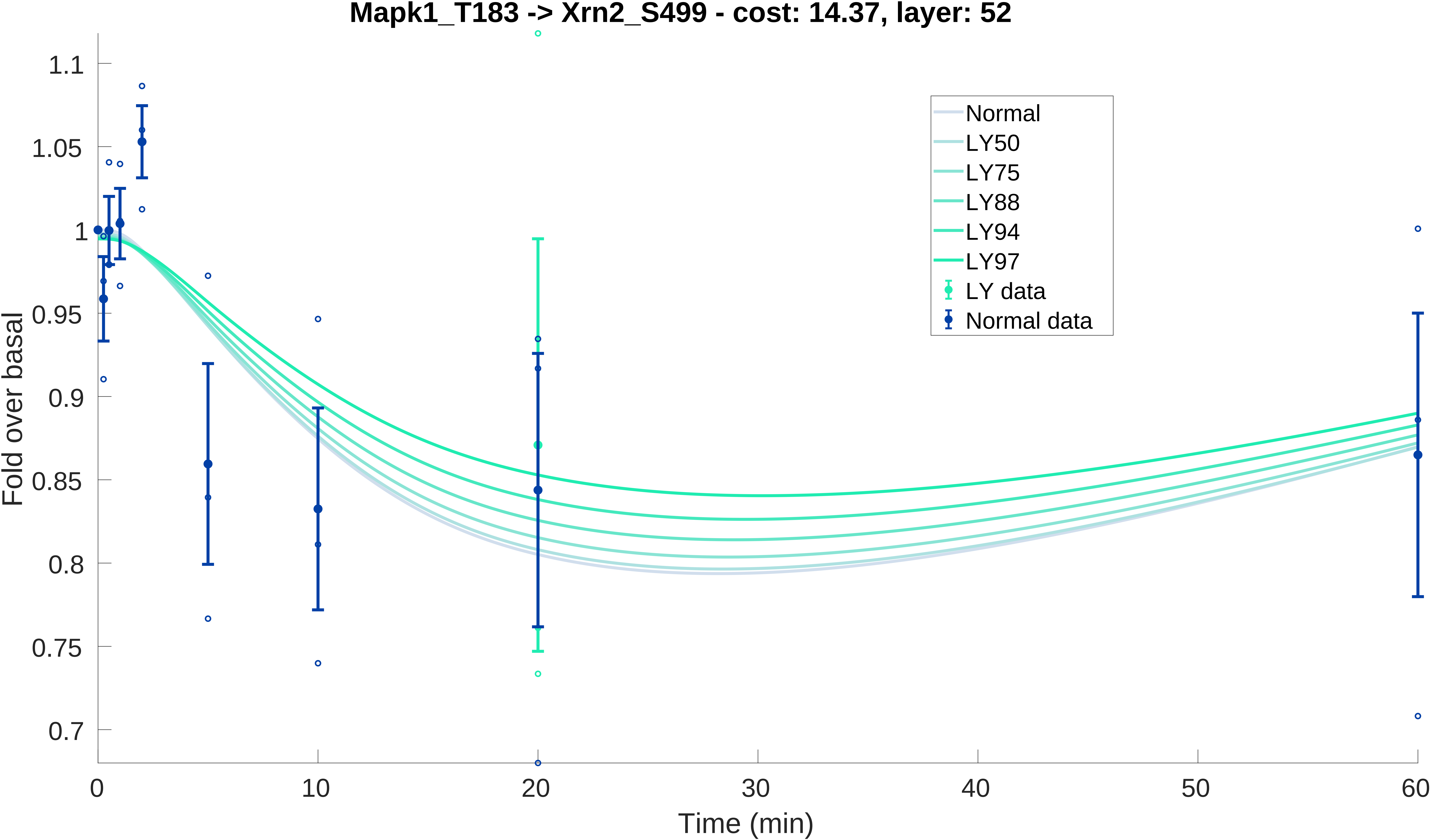
Mapk1_T183 -> Xrn2_S499 - cost: 14.37, layer: 52
Fold over basal
Time (min)
1.1
1.05
1
0.95
0.9
0.85
0.8
0.75
0.7
0
10
20
30
40
50
60
Normal
LY50
LY75
LY88
LY94
LY97
LY data
Normal data

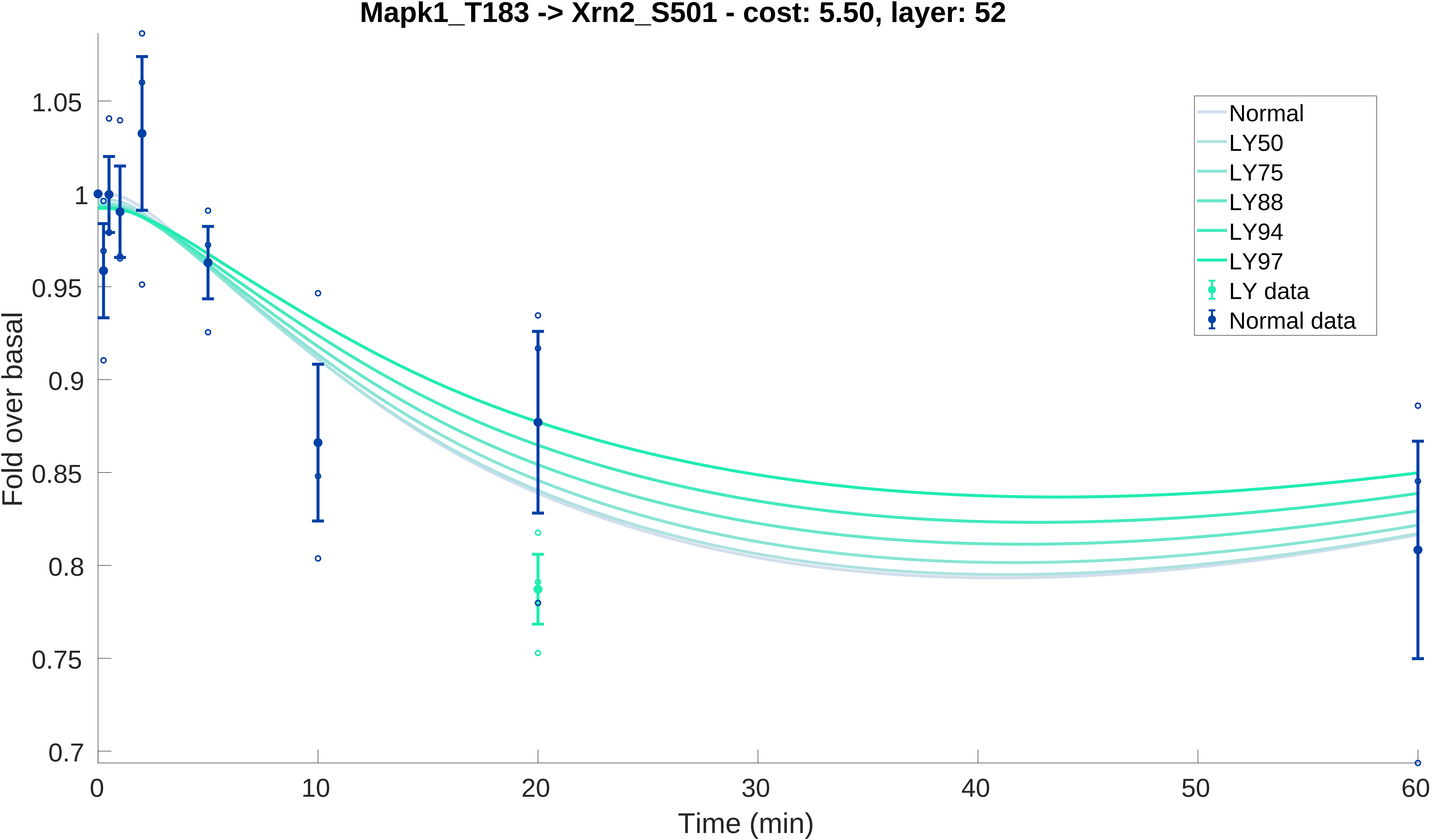
Mapk1_T183 -> Xrn2_S501 - cost: 5.50, layer: 52
Fold over basal
Time (min)
1.05
1
0.95
0.9
0.85
0.8
0.75
0.7
0
10
20
30
40
50
60
Normal
LY50
LY75
LY88
LY94
LY97
LY data
Normal data

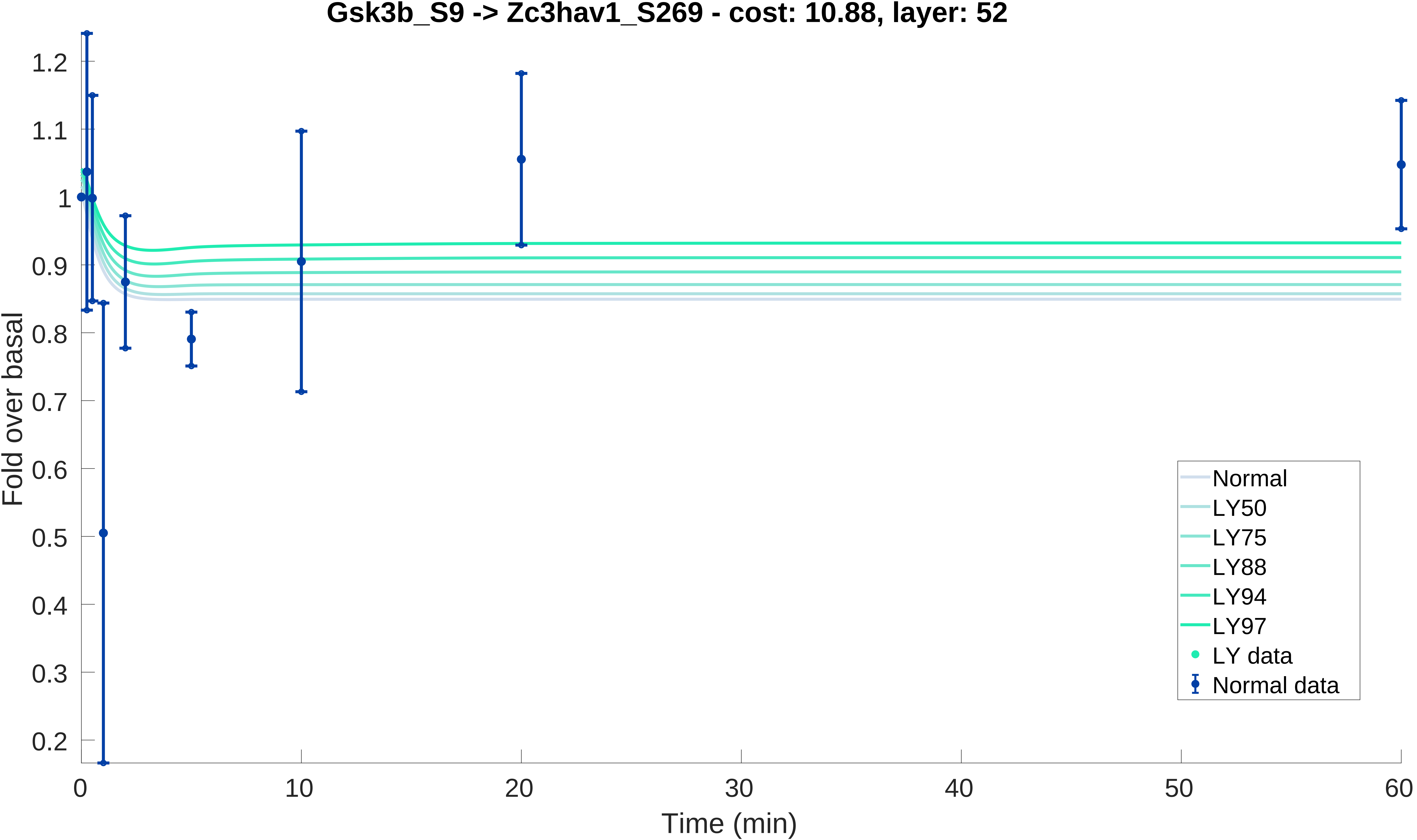

Gsk3b_S9 -> Zc3hav1_S269 - cost: 10.88, layer: 52
Fold over basal
Time (min)
0.2
0.3
0.4
0.5
0.6
0.7
0.8
0.9
1
1.1
1.2
0
10
20
30
40
50
60
Normal
LY50
LY75
LY88
LY94
LY97
LY data
Normal data

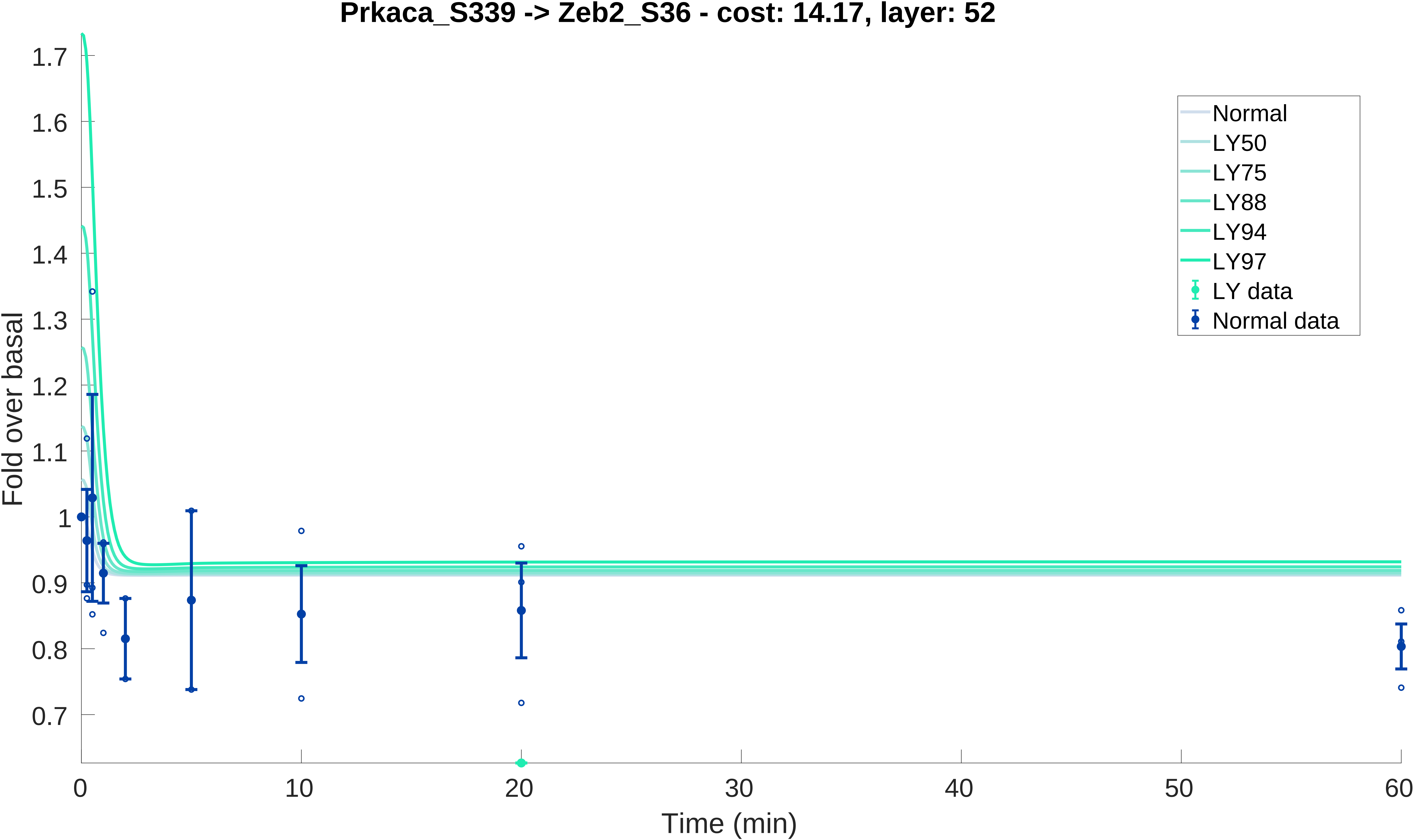

Prkaca_S339 -> Zeb2_S36 - cost: 14.17, layer: 52
Fold over basal
Time (min)
0
10
20
30
40
50
60
0.7
0.8
0.9
1
1.1
1.2
1.3
1.4
1.5
1.6
1.7
Normal
LY50
LY75
LY88
LY94
LY97
LY data
Normal data

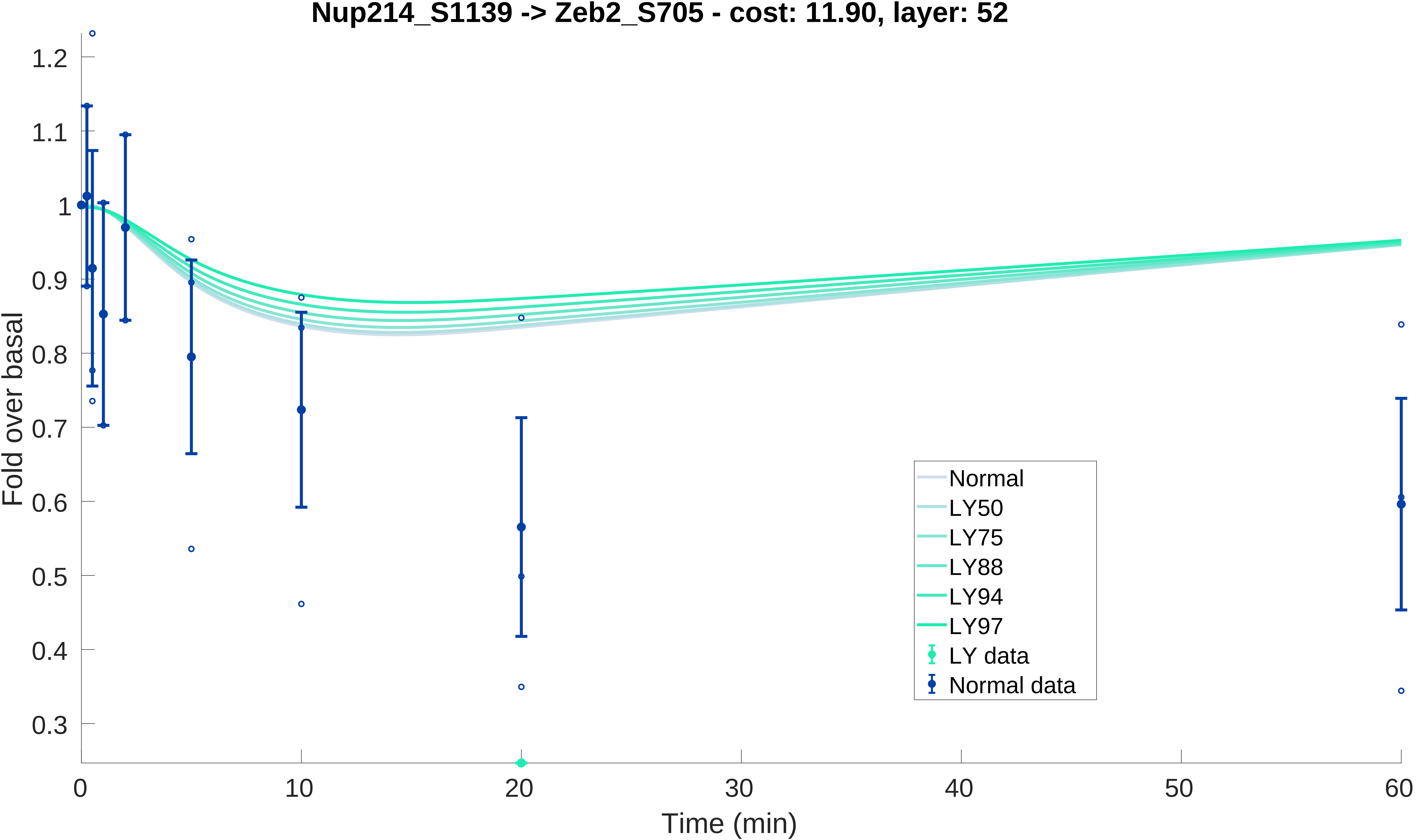
Nup214_S1139 -> Zeb2_S705 - cost: 11.90, layer: 52
Fold over basal
Time (min)
1.2
1.1
1
0.9
0.8
0.7
0.6
0.5
0.4
0.3
0
10
20
30
40
50
60
Normal
LY50
LY75
LY88
LY94
LY97
LY data
Normal data

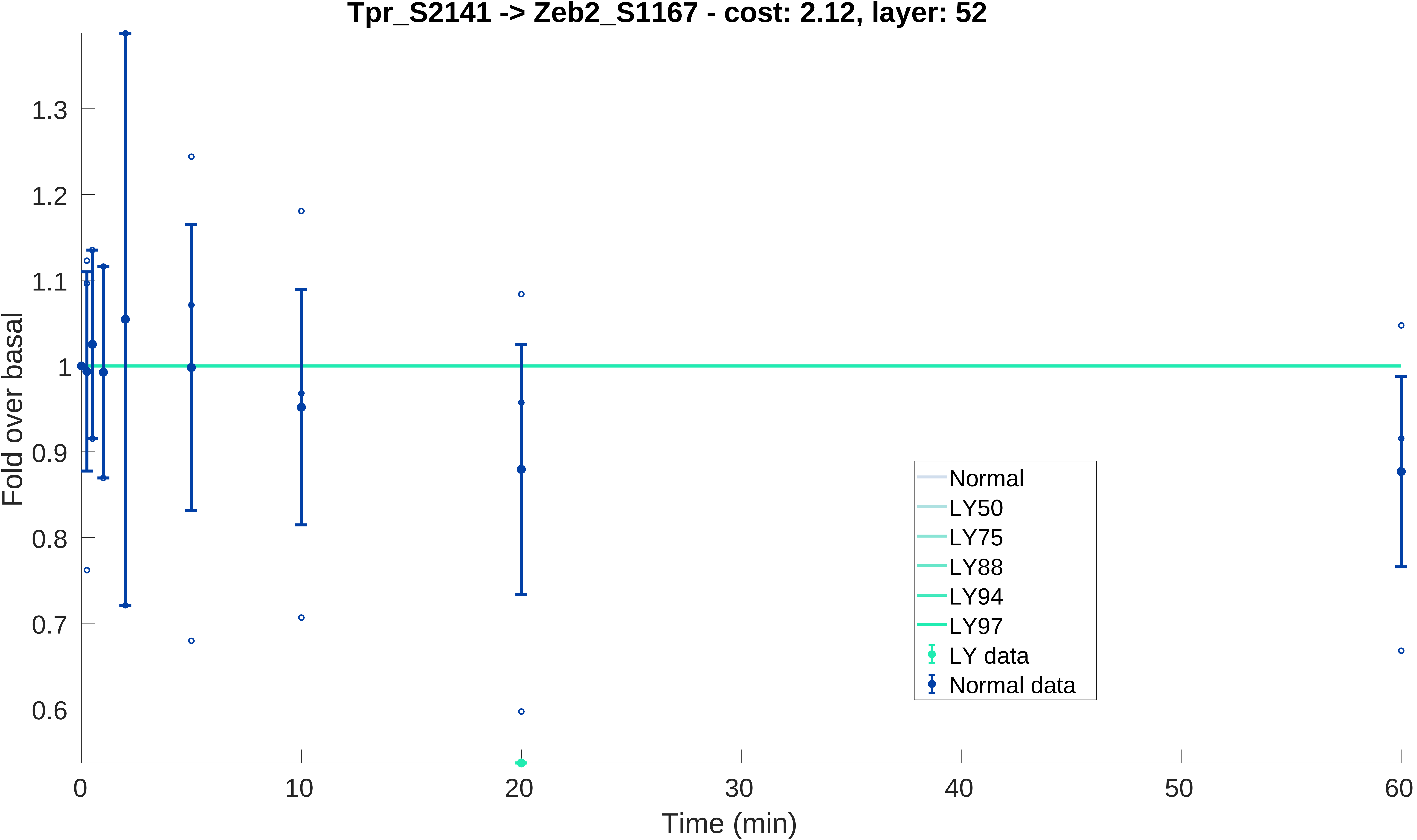

Tpr_S2141 -> Zeb2_S1167 - cost: 2.12, layer: 52
Fold over basal
Time (min)
0
10
20
30
40
50
60
0.6
0.7
0.8
0.9
1
1.1
1.2
1.3
Normal
LY50
LY75
LY88
LY94
LY97
LY data
Normal data

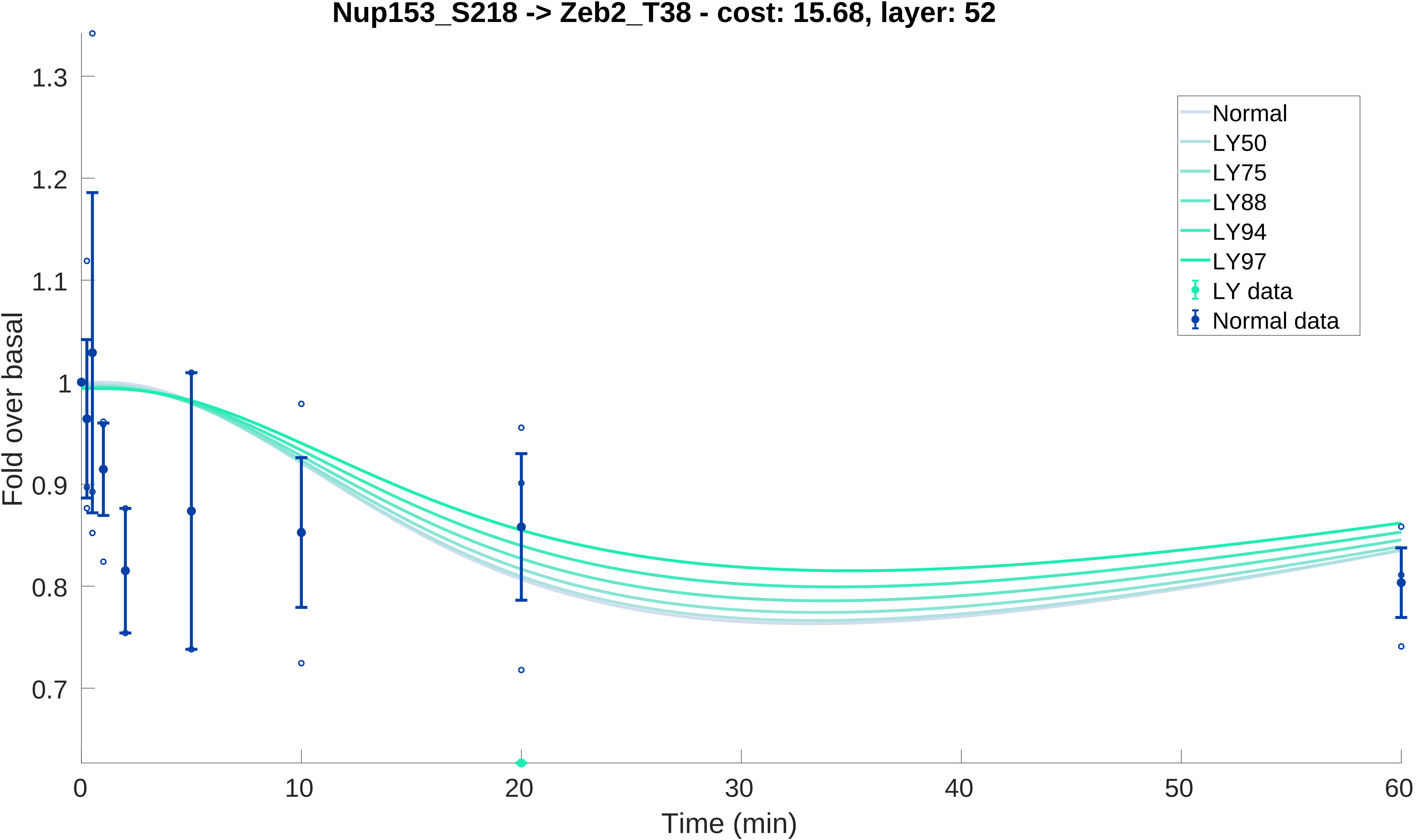

Nup153_S218 -> Zeb2_T38 - cost: 15.68, layer: 52
Fold over basal
Time (min)
1.3
1.2
1.1
1
0.9
0.8
0.7
0
10
20
30
40
50
60
Normal
LY50
LY75
LY88
LY94
LY97
LY data
Normal data

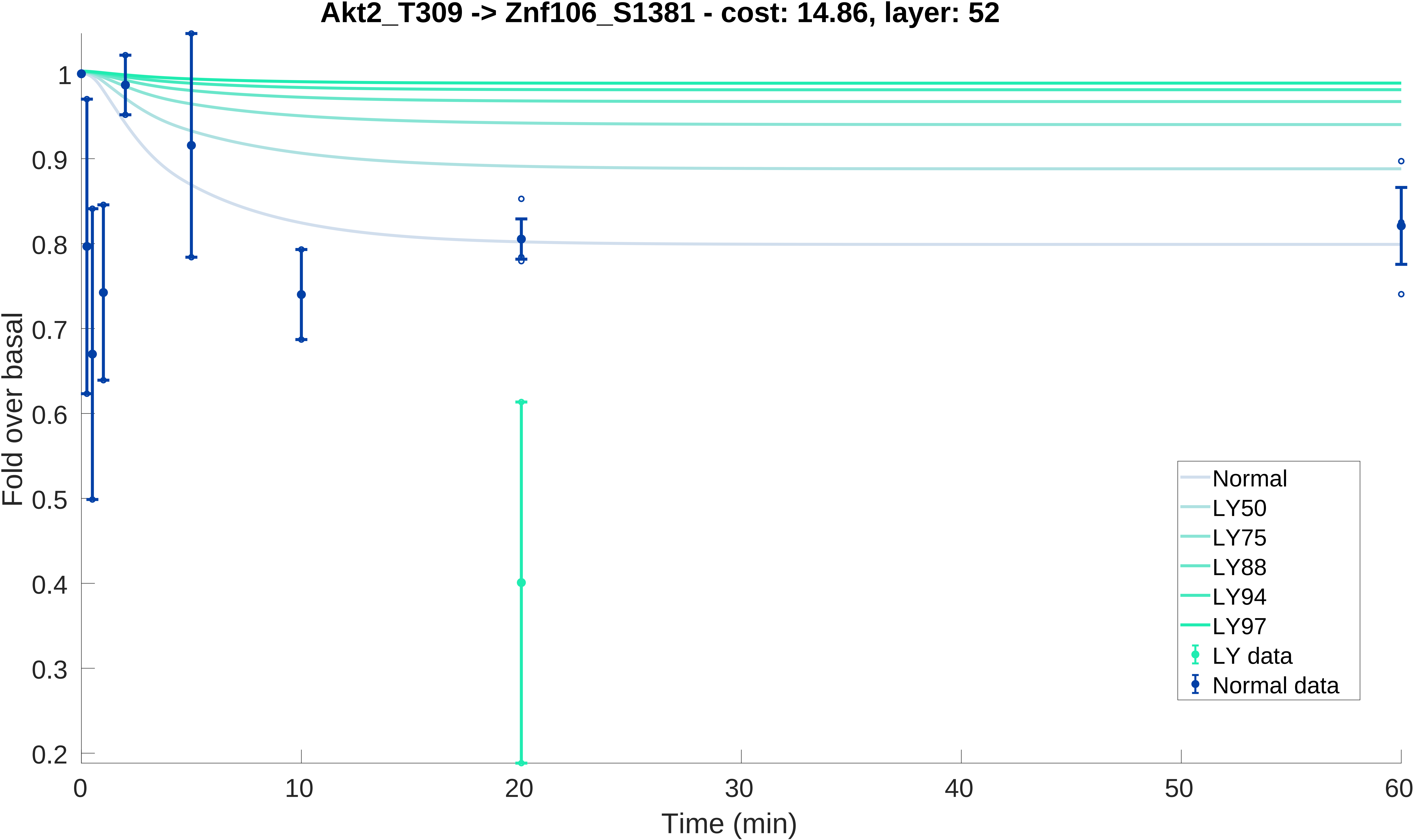

Akt2_T309 -> Znf106_S1381 - cost: 14.86, layer: 52
Fold over basal
Time (min)
0.2
0.3
0.4
0.5
0.6
0.7
0.8
0.9
1
0
10
20
30
40
50
60
Normal
LY50
LY75
LY88
LY94
LY97
LY data
Normal data

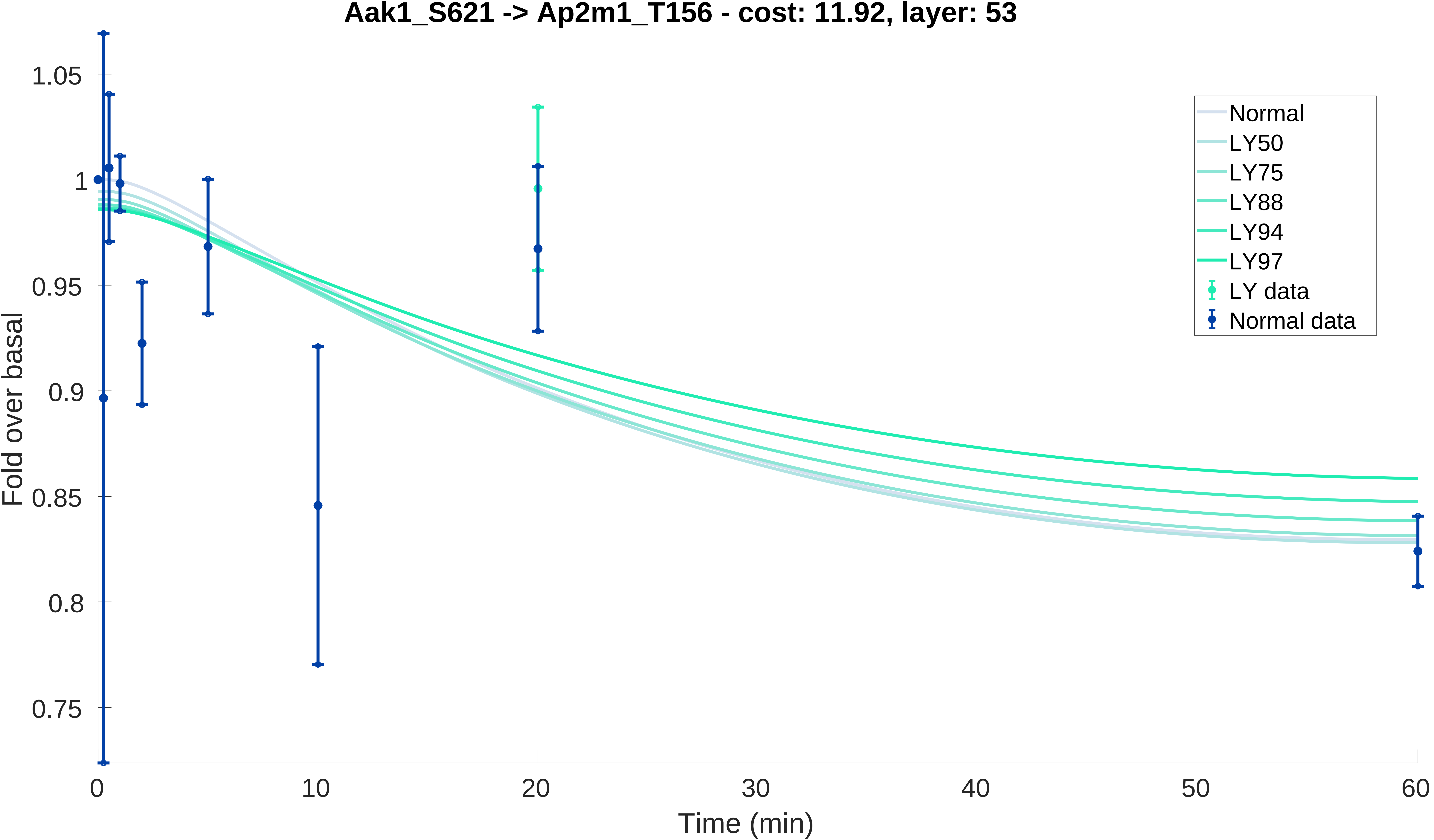
Aak1_S621 -> Ap2m1_T156 - cost: 11.92, layer: 53
Fold over basal
1.05
1
0.95
0.9
0.85
0.8
0.75
0
10
20
30
40
50
60
Time (min)
Normal
LY50
LY75
LY88
LY94
LY97
LY data
Normal data

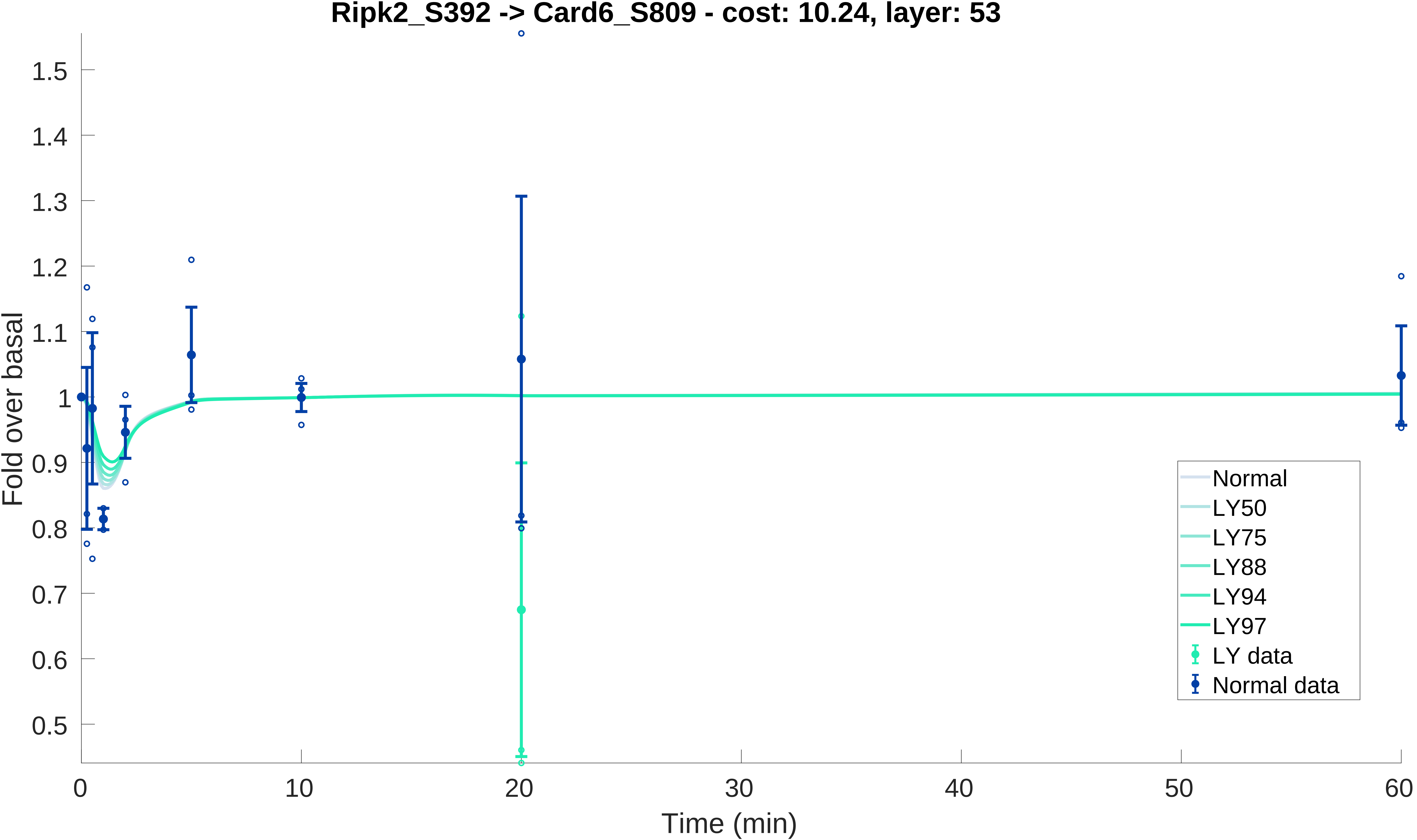

Ripk2_S392 -> Card6_S809 - cost: 10.24, layer: 53
Fold over basal
1.5
1.4
1.3
1.2
1.1
1
0.9
0.8
0.7
0.6
0.5
0
10
20
30
40
50
60
Time (min)
Normal
LY50
LY75
LY88
LY94
LY97
LY data
Normal data

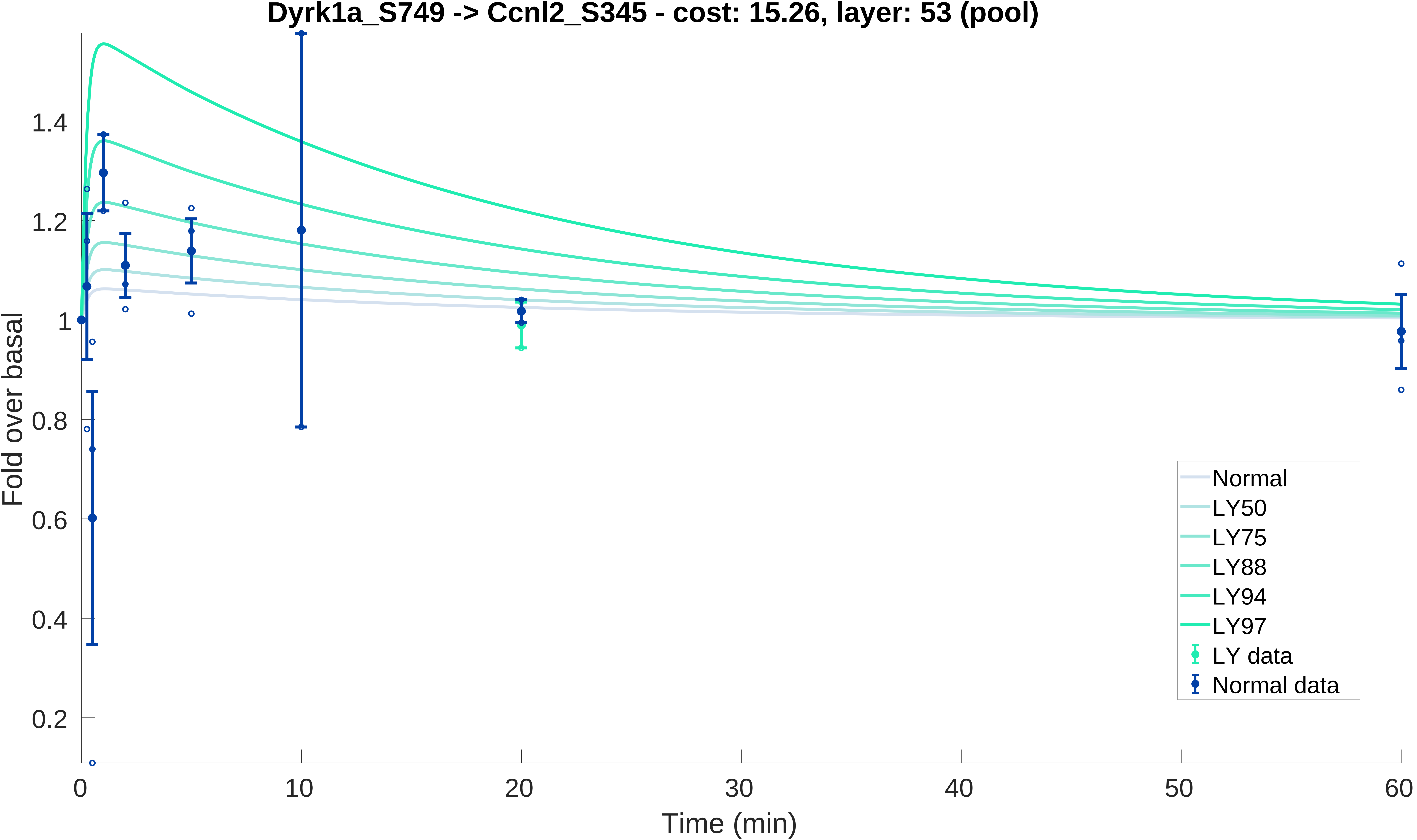
Dyrk1a_S749 -> Ccnl2_S345 - cost: 15.26, layer: 53 (pool)
Fold over basal
1.4
1.2
1
0.8
0.6
0.4
0.2
0
10
20
30
40
50
60
Time (min)
Normal
LY50
LY75
LY88
LY94
LY97
LY data
Normal data

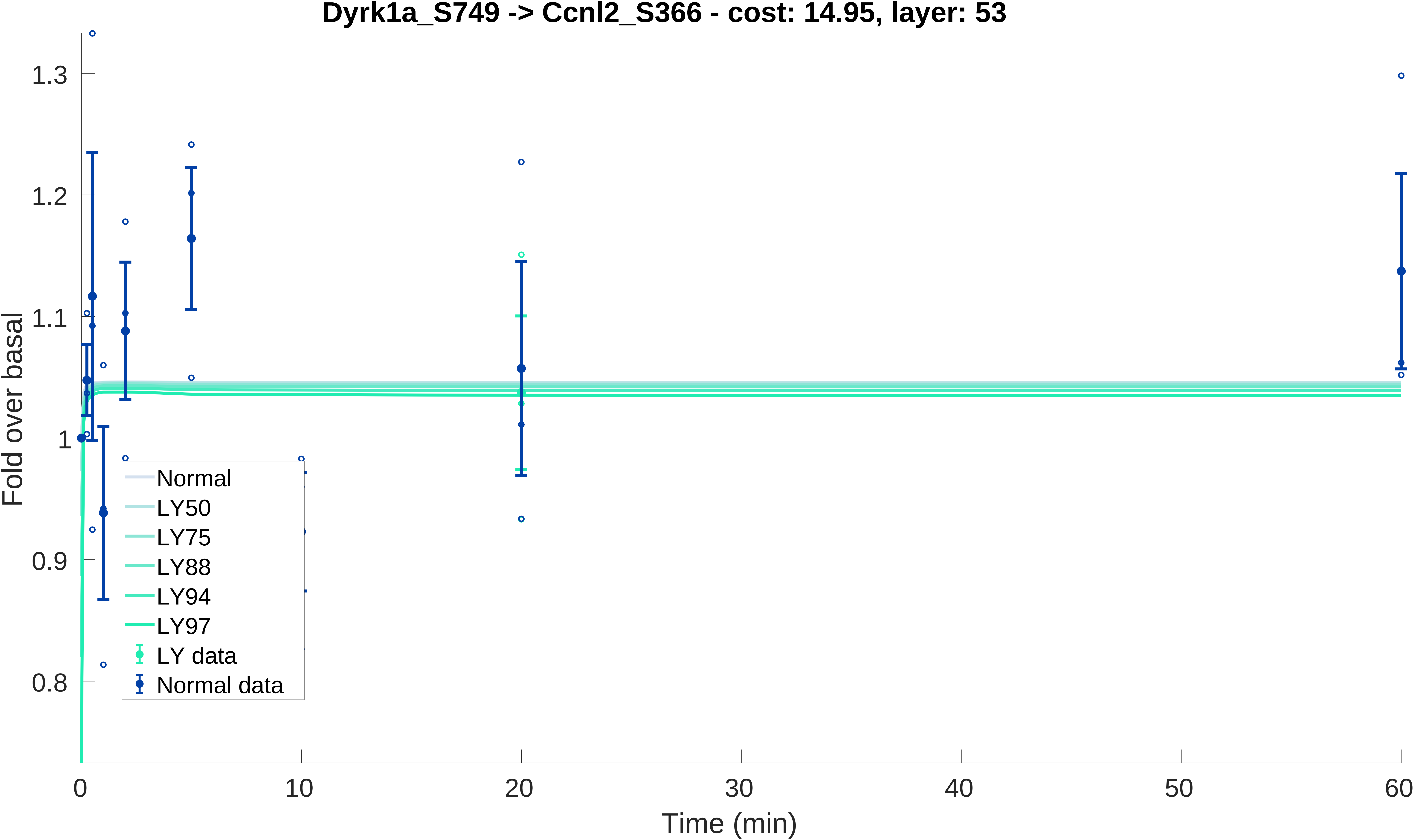
Dyrk1a_S749 -> Ccnl2_S366 - cost: 14.95, layer: 53
Fold over basal
Time (min)
1.3
1.2
1.1
1
0.9
0.8
0
10
20
30
40
50
60
Normal
LY50
LY75
LY88
LY94
LY97
LY data
Normal data

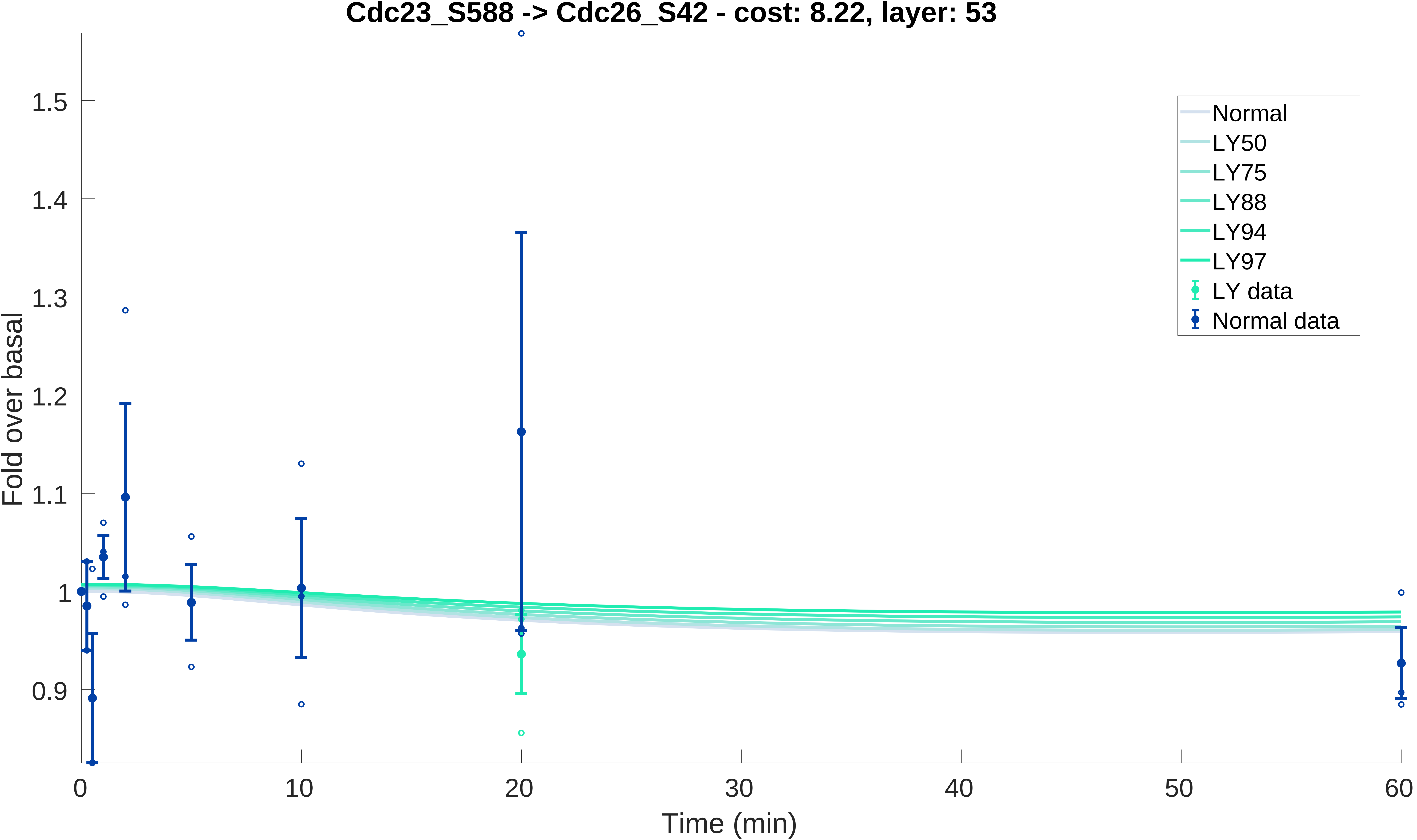

Cdc23_S588 -> Cdc26_S42 - cost: 8.22, layer: 53
Fold over basal
1.5
1.4
1.3
1.2
1.1
1
0.9
Time (min)
0
10
20
30
40
50
60
Normal
LY50
LY75
LY88
LY94
LY97
LY data
Normal data

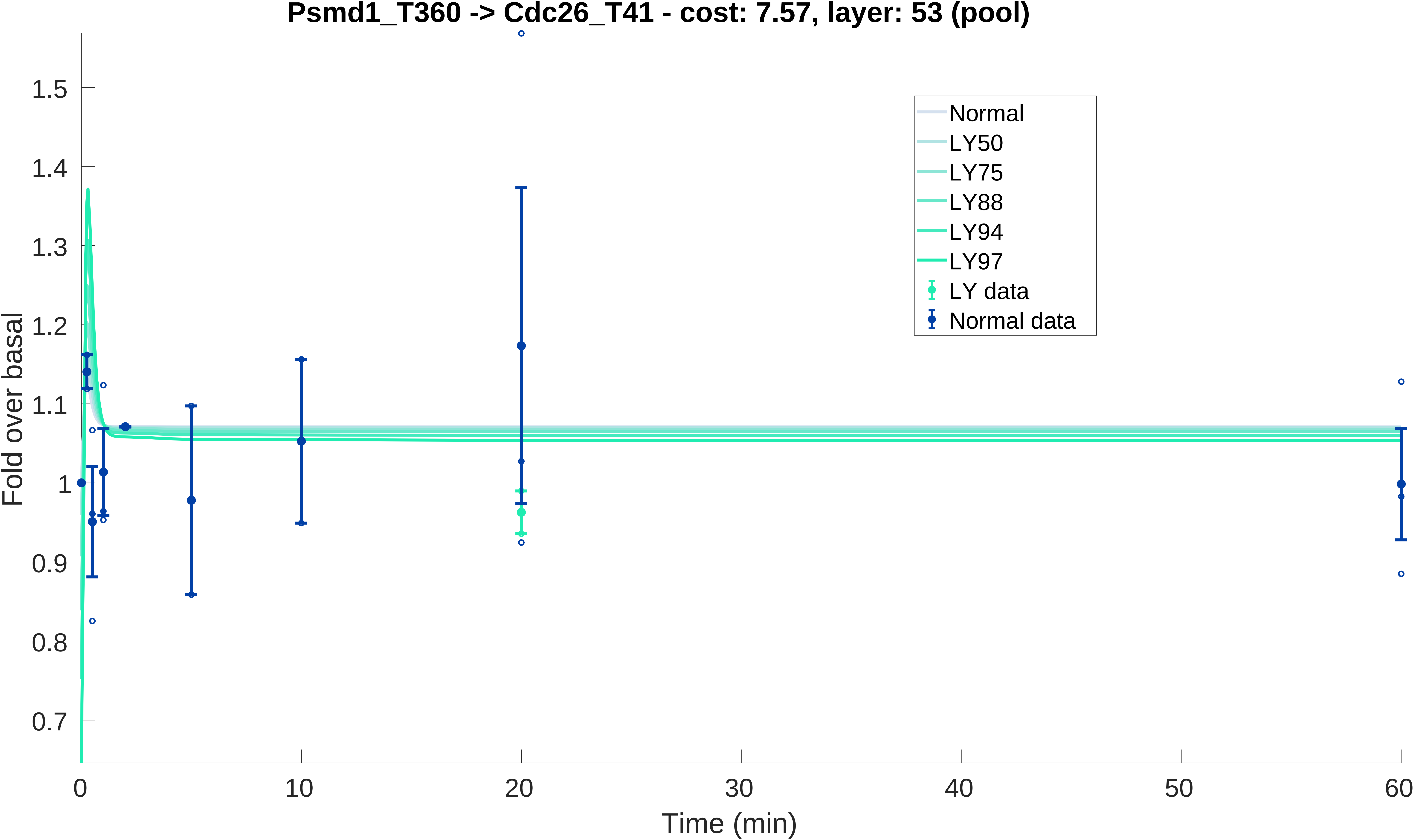
Psmd1_T360 -> Cdc26_T41 - cost: 7.57, layer: 53 (pool)
Fold over basal
Time (min)
Normal
LY50
LY75
LY88
LY94
LY97
LY data
Normal data
0.7
0.8
0.9
1
1.1
1.2
1.3
1.4
1.5
0
10
20
30
40
50
60

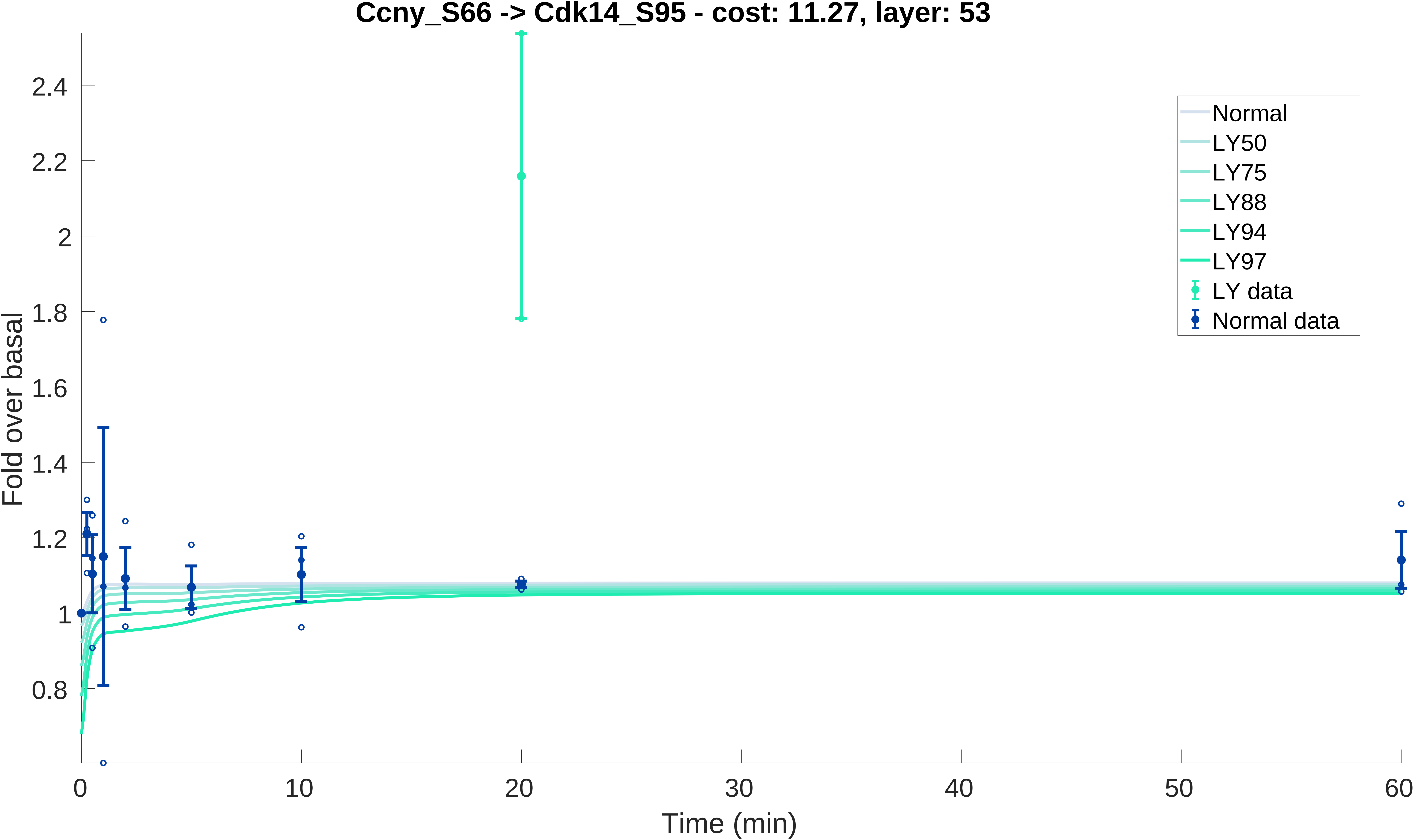

Ccny_S66 -> Cdk14_S95 - cost: 11.27, layer: 53
Fold over basal
Time (min)
2.4
2.2
2
1.8
1.6
1.4
1.2
1
0.8
0
10
20
30
40
50
60
Normal
LY50
LY75
LY88
LY94
LY97
LY data
Normal data

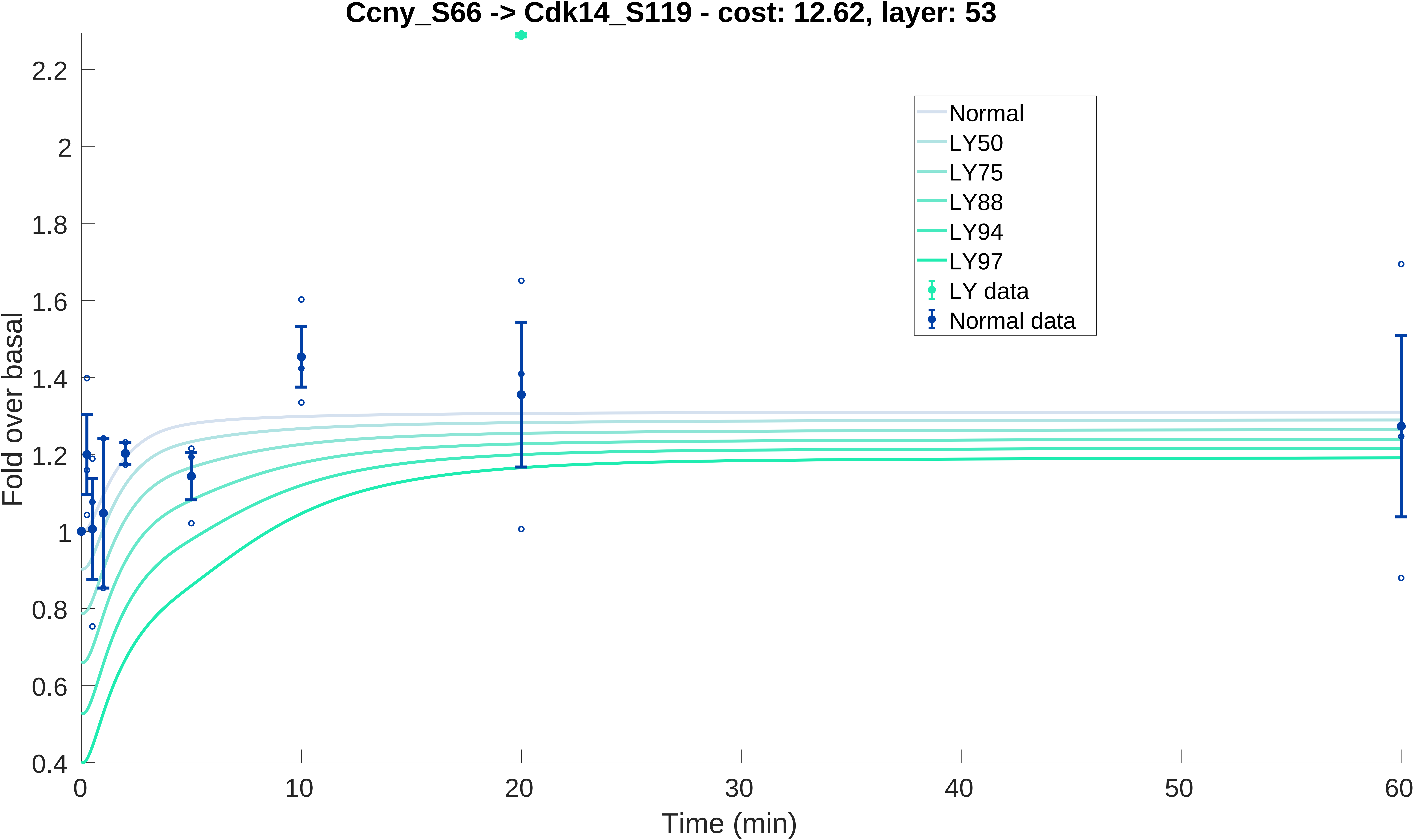

Ccny_S66 -> Cdk14_S119 - cost: 12.62, layer: 53
Fold over basal
Time (min)
0
10
20
30
40
50
60
0.4
0.6
0.8
1
1.2
1.4
1.6
1.8
2
2.2
Normal
LY50
LY75
LY88
LY94
LY97
LY data
Normal data

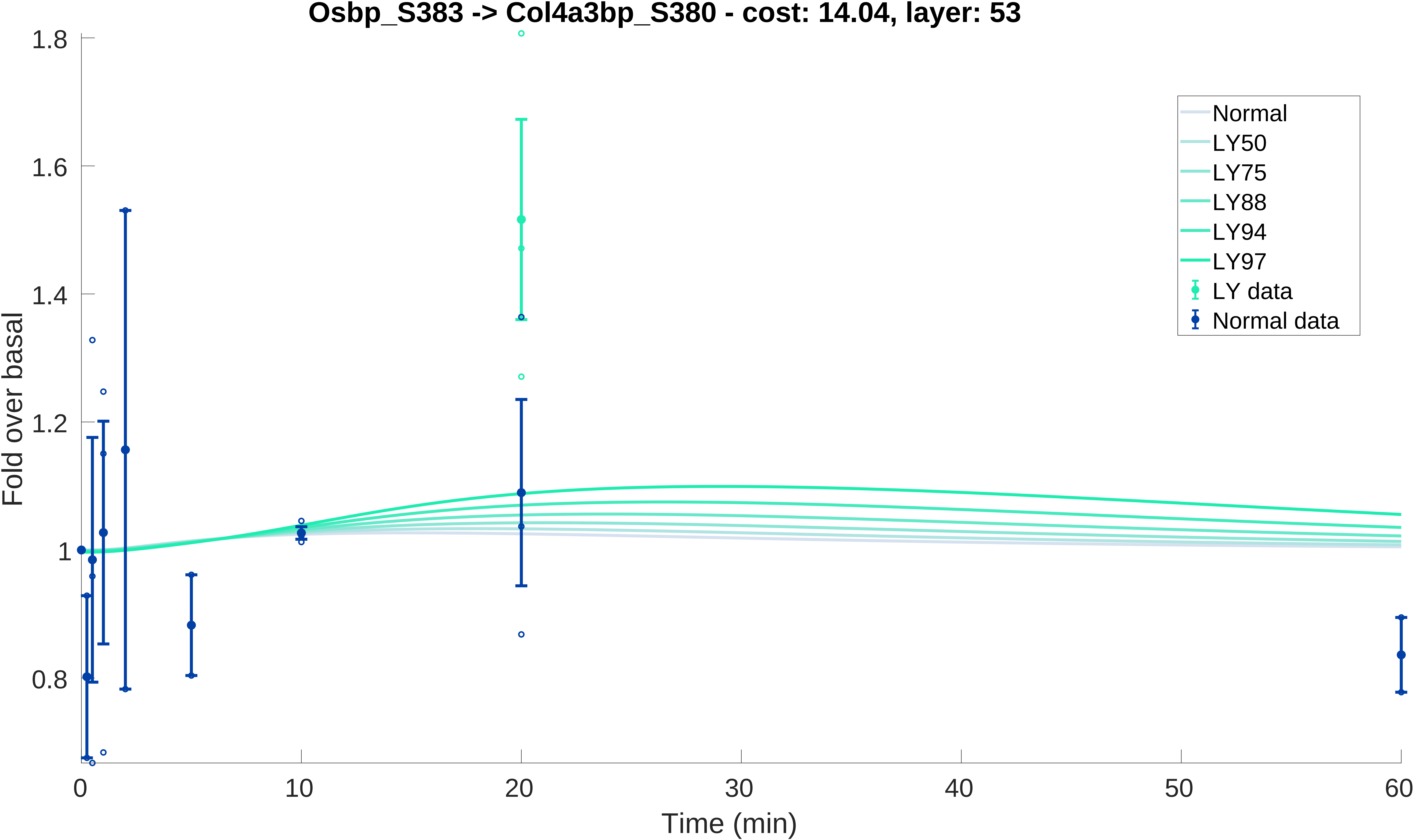
Osbp_S383 -> Col4a3bp_S380 - cost: 14.04, layer: 53
Fold over basal
Time (min)
1.8
1.6
1.4
1.2
1
0.8
0
10
20
30
40
50
60
Normal
LY50
LY75
LY88
LY94
LY97
LY data
Normal data

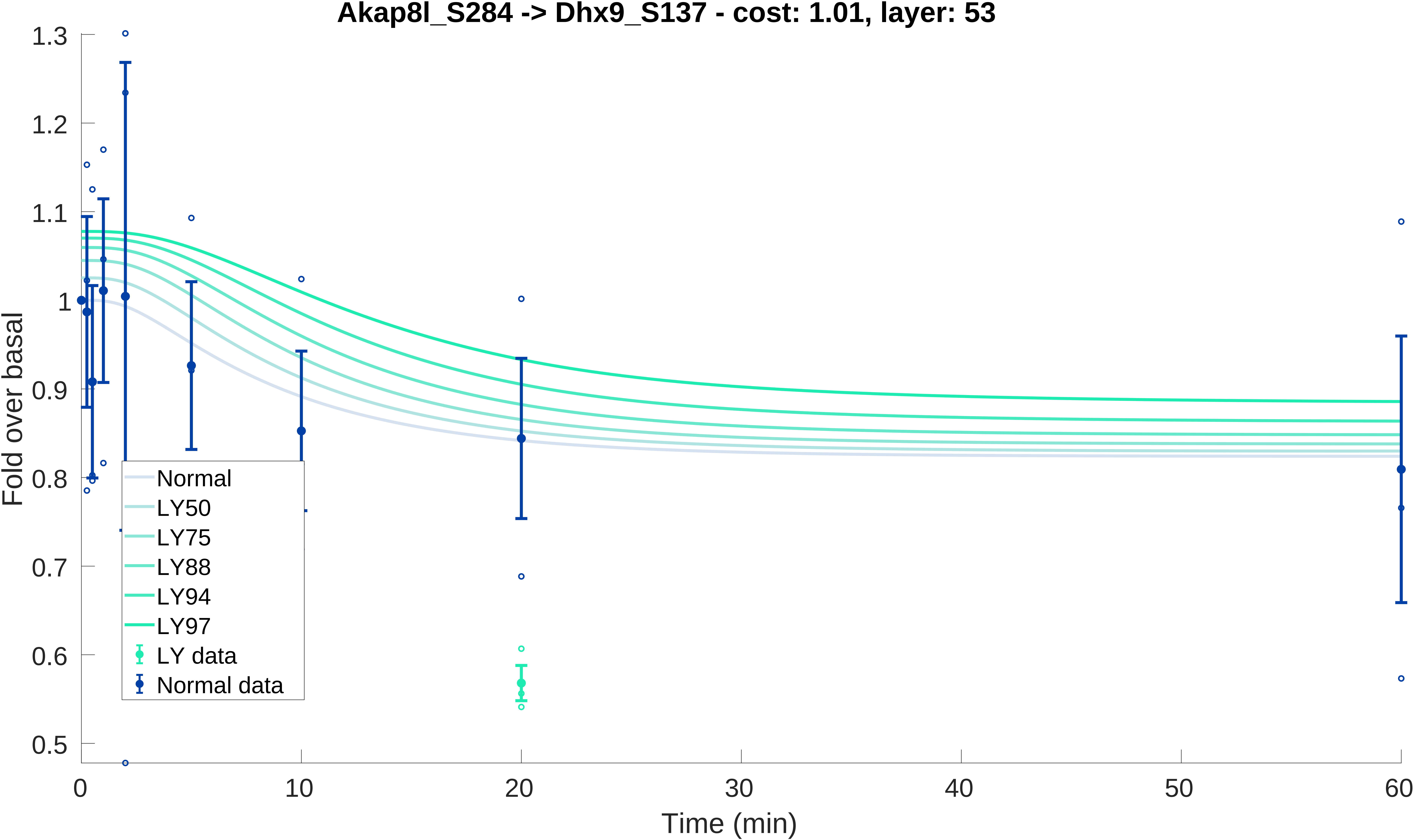

Akap8l_S284 -> Dhx9_S137 - cost: 1.01, layer: 53
Fold over basal
Time (min)
1.3
1.2
1.1
1
0.9
0.8
0.7
0.6
0.5
0
10
20
30
40
50
60
Normal
LY50
LY75
LY88
LY94
LY97
LY data
Normal data

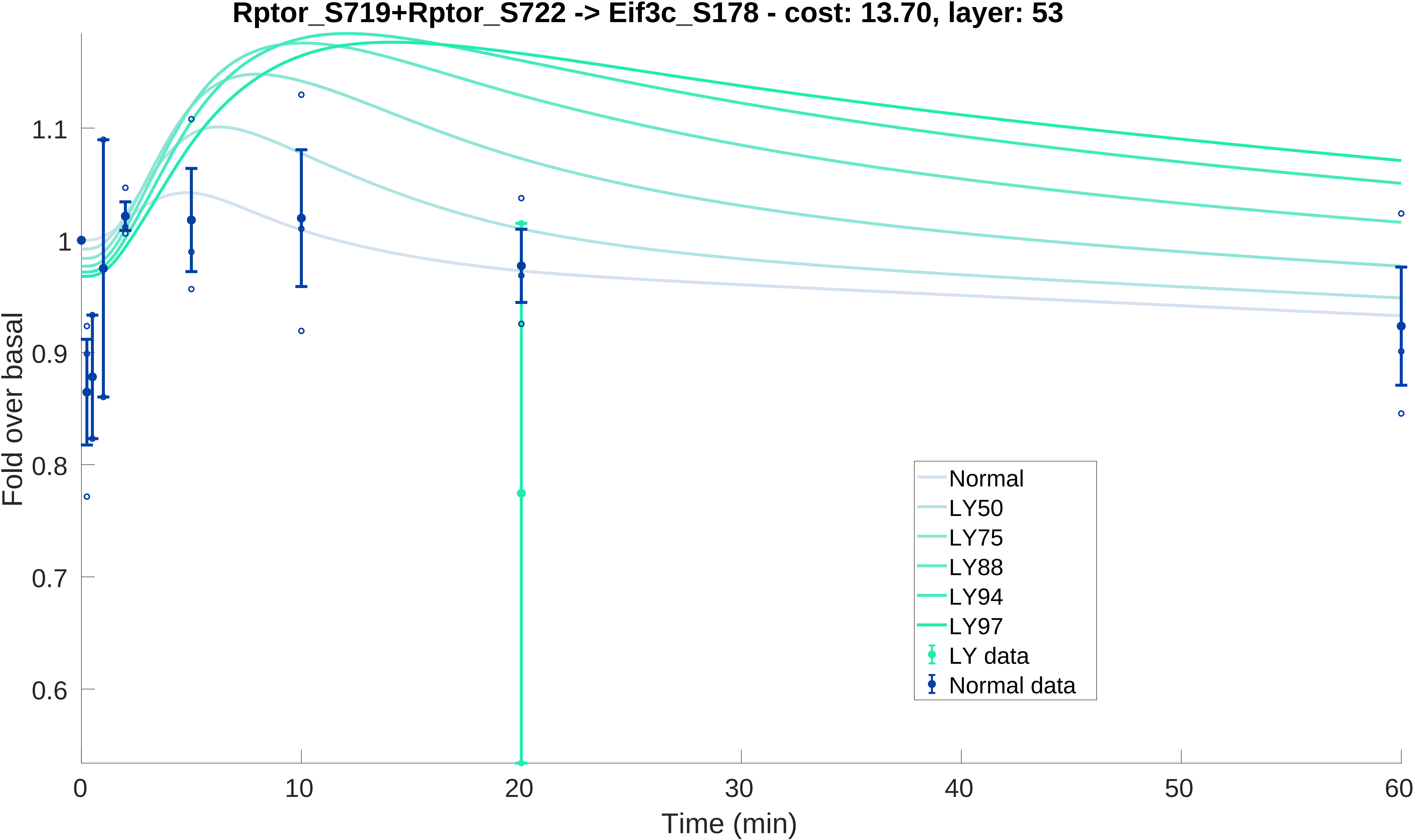
Rptor_S719+Rptor_S722 -> Eif3c_S178 - cost: 13.70, layer: 53
Fold over basal
Time (min)
0.6
0.7
0.8
0.9
1
1.1
0
10
20
30
40
50
60
Normal
LY50
LY75
LY88
LY94
LY97
LY data
Normal data

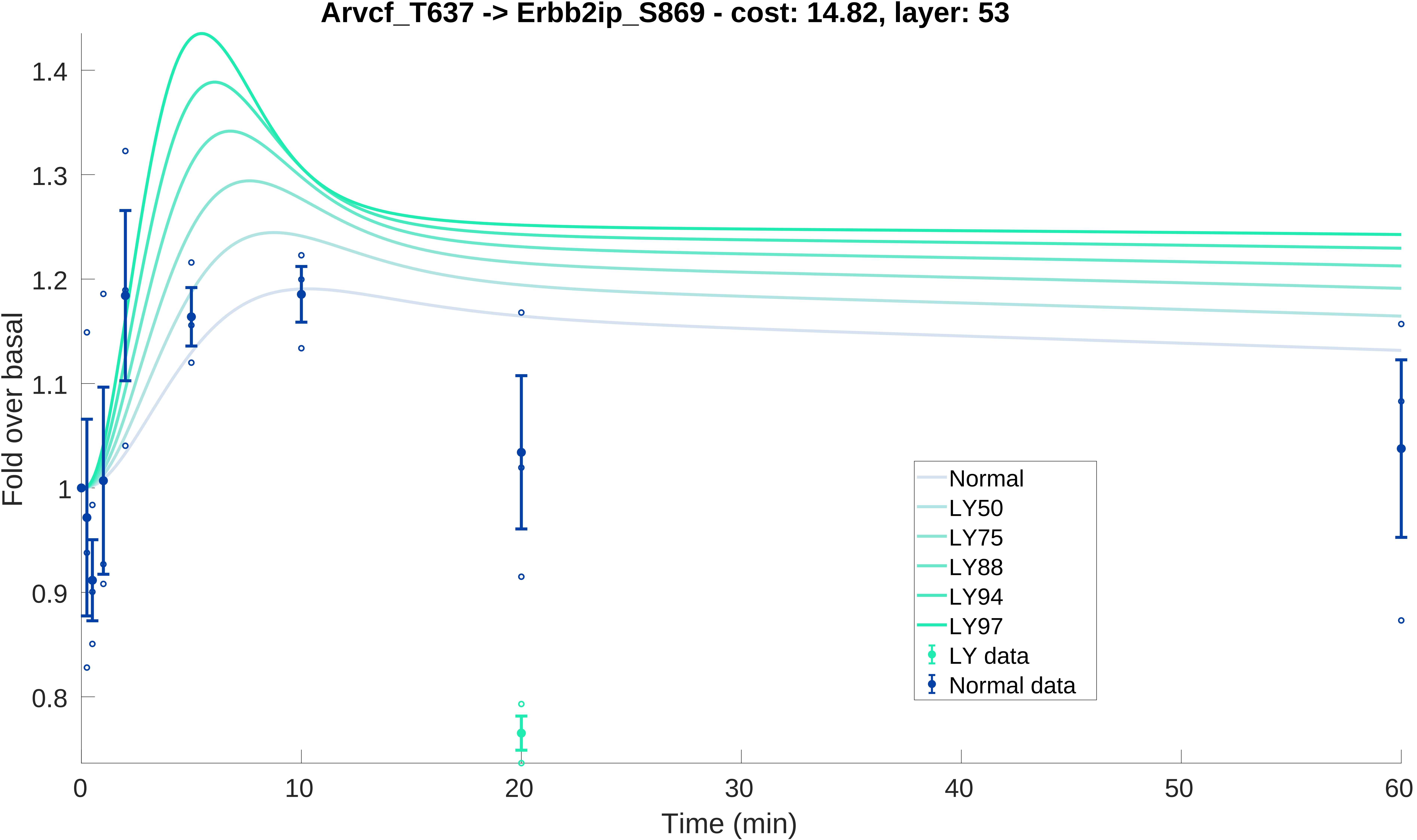

Arvcf_T637 -> Erbb2ip_S869 - cost: 14.82, layer: 53
Fold over basal
1.4
1.3
1.2
1.1
1
0.9
0.8
0
10
20
30
40
50
60
Time (min)
Normal
LY50
LY75
LY88
LY94
LY97
LY data
Normal data

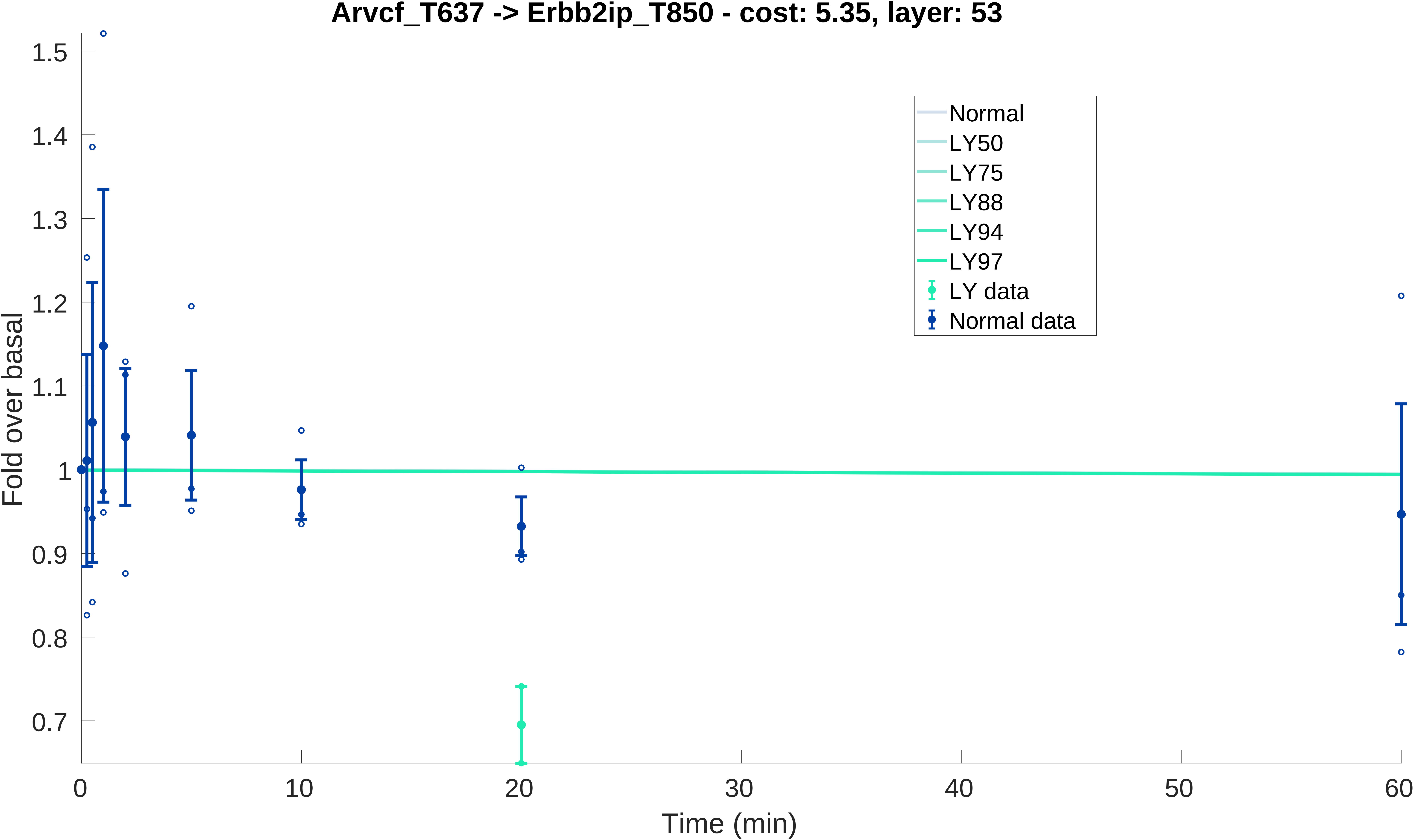

Arvcf_T637 -> Erbb2ip_T850 - cost: 5.35, layer: 53
Fold over basal
Time (min)
0.7
0.8
0.9
1
1.1
1.2
1.3
1.4
1.5
0
10
20
30
40
50
60
Normal
LY50
LY75
LY88
LY94
LY97
LY data
Normal data

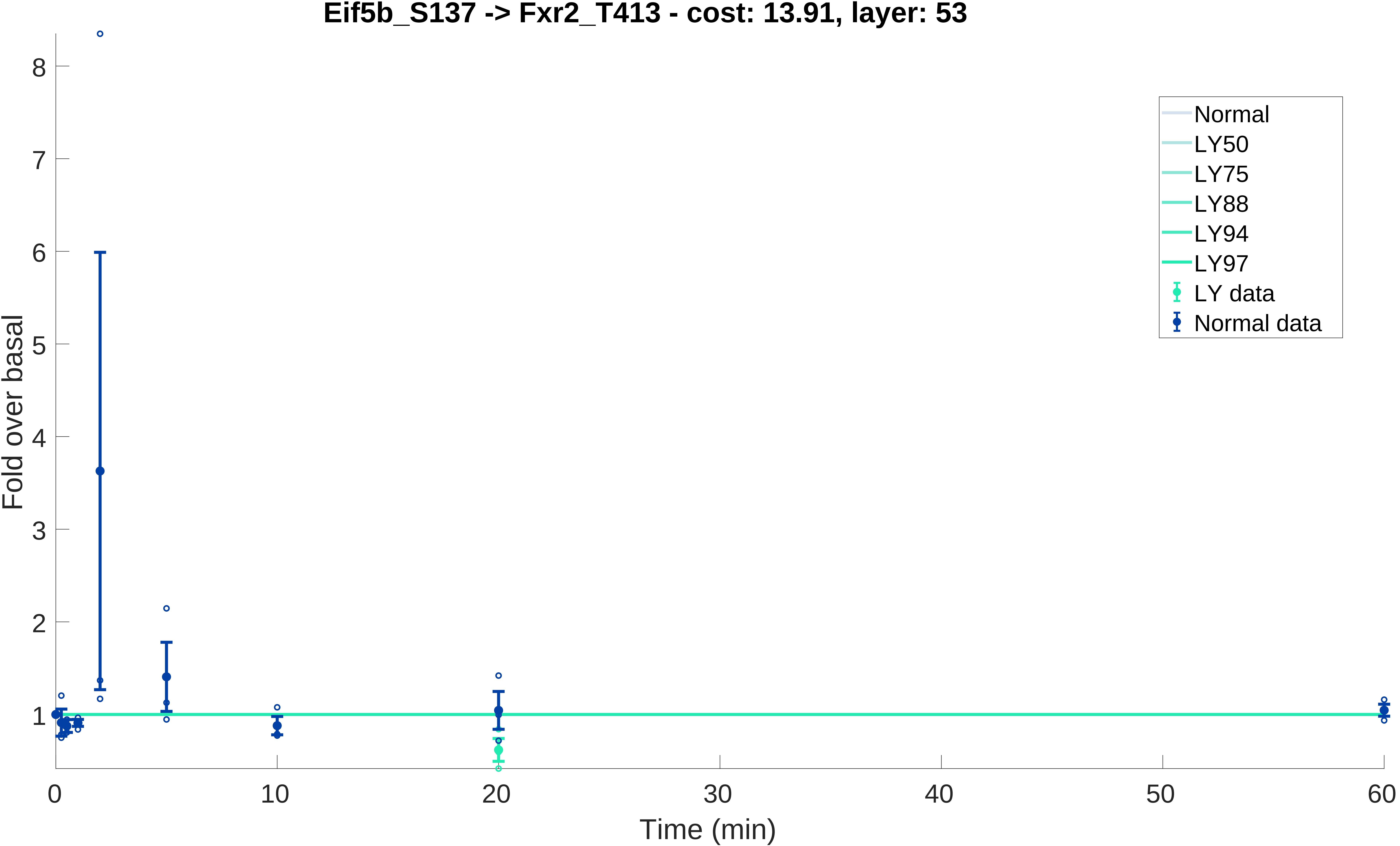

Eif5b_S137 -> Fxr2_T413 - cost: 13.91, layer: 53
Fold over basal
Time (min)
0
10
20
30
40
50
60
1
2
3
4
5
6
7
8
Normal
LY50
LY75
LY88
LY94
LY97
LY data
Normal data

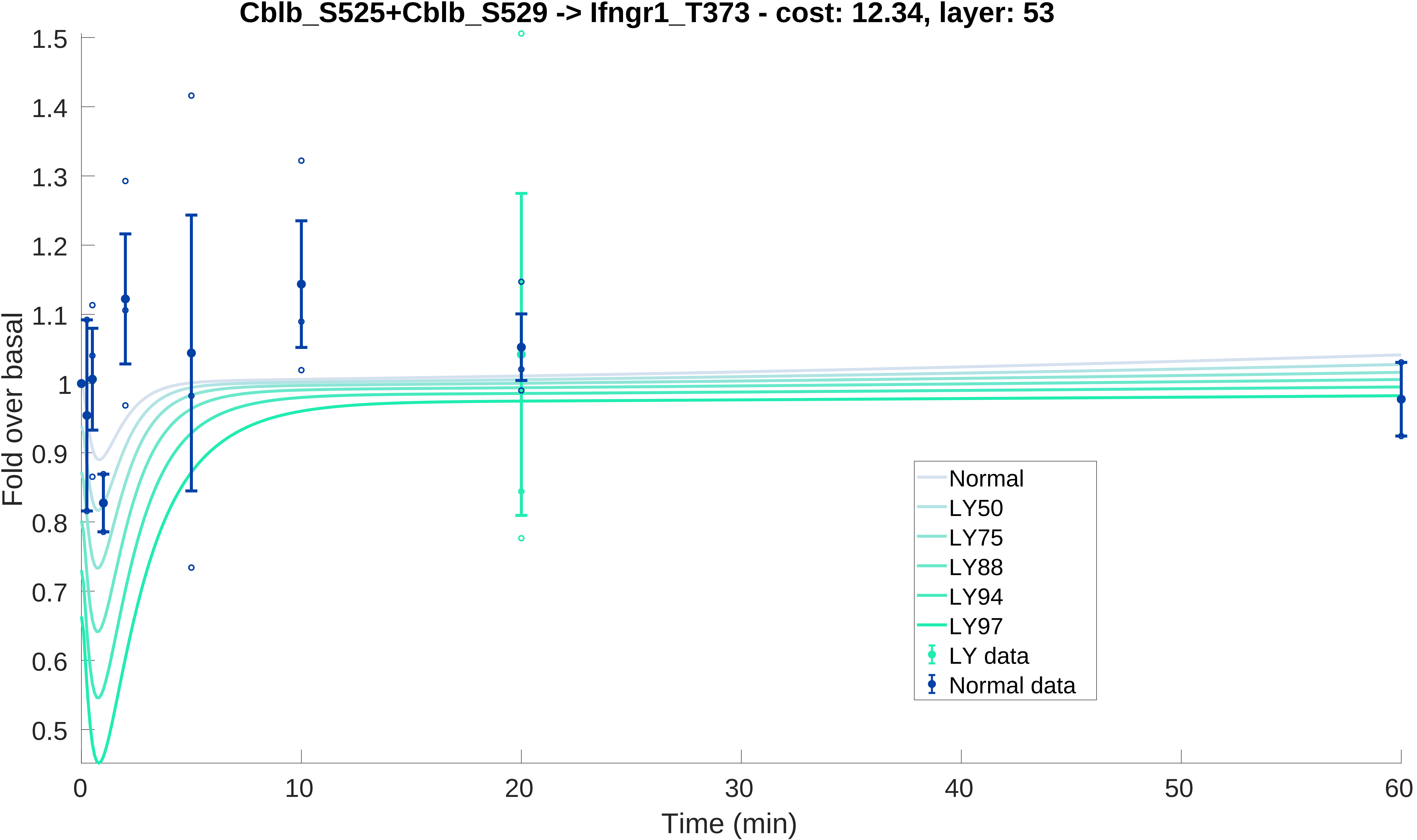

Cblb_S525+Cblb_S529 -> Ifngr1_T373 - cost: 12.34, layer: 53
Fold over basal
Time (min)
1.5
1.4
1.3
1.2
1.1
1
0.9
0.8
0.7
0.6
0.5
0
10
20
30
40
50
60
Normal
LY50
LY75
LY88
LY94
LY97
LY data
Normal data

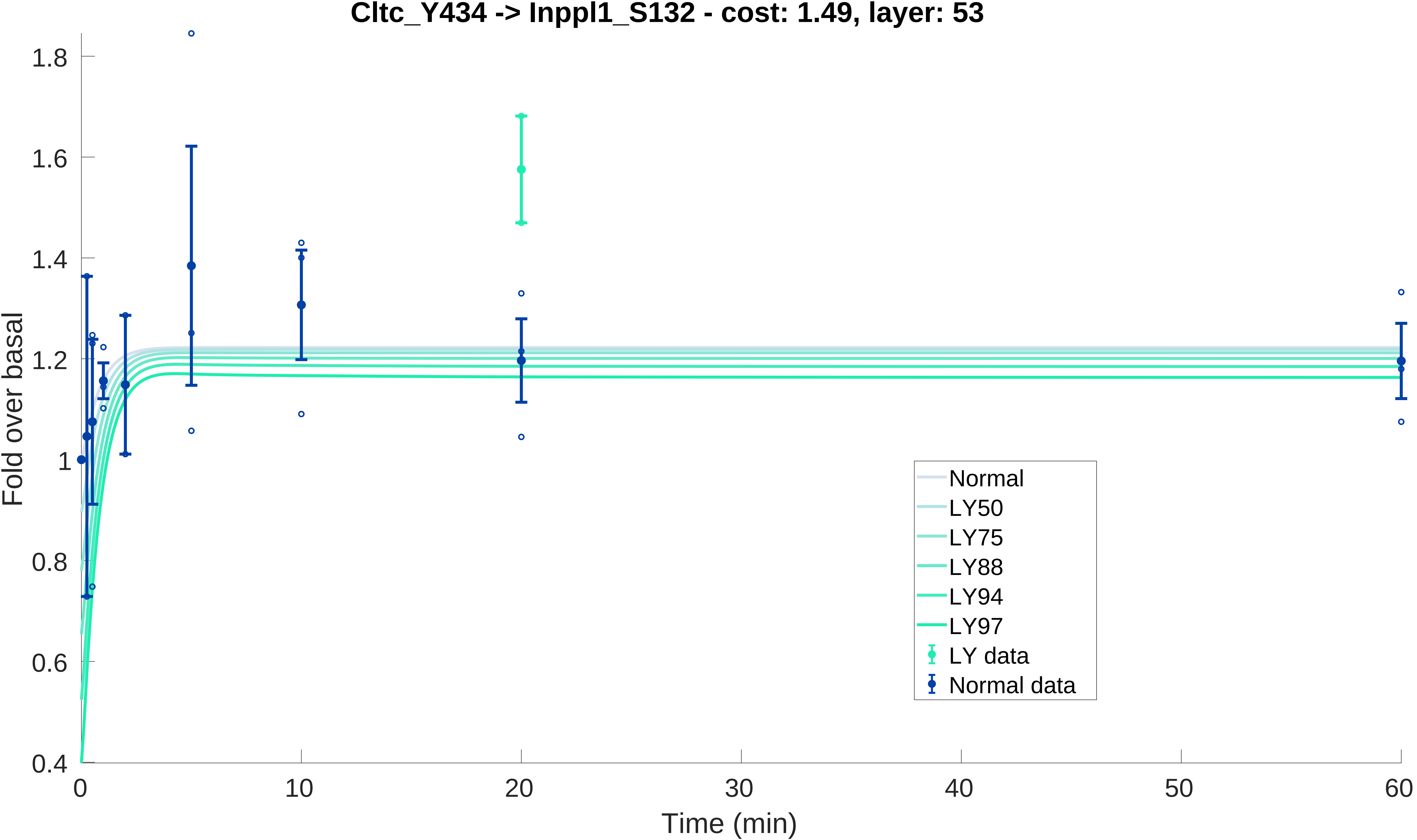

Cltc_Y434 -> Inppl1_S132 - cost: 1.49, layer: 53
Fold over basal
Time (min)
0.4
0.6
0.8
1
1.2
1.4
1.6
1.8
0
10
20
30
40
50
60
Normal
LY50
LY75
LY88
LY94
LY97
LY data
Normal data

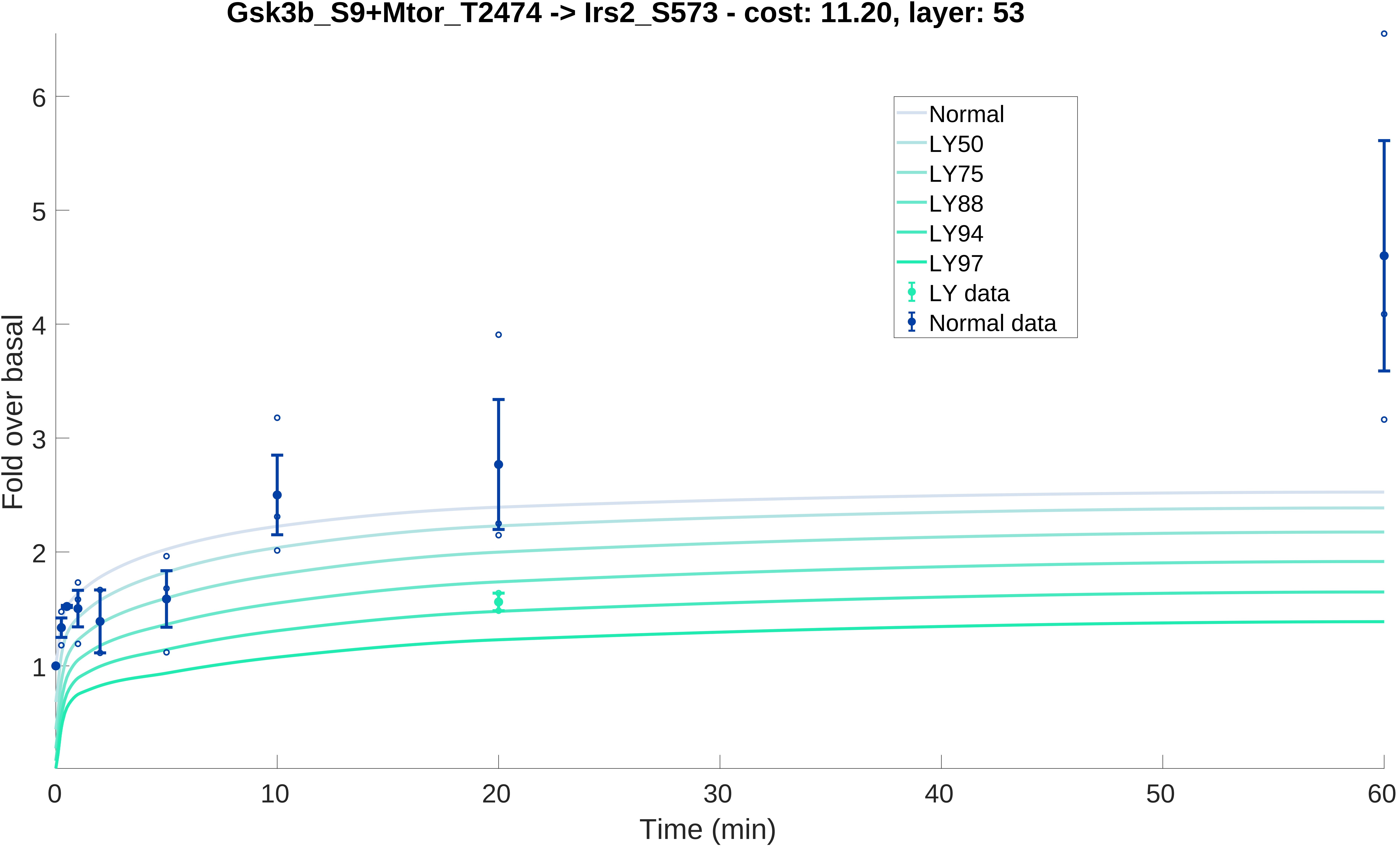

Gsk3b_S9+Mtor_T2474 -> Irs2_S573 - cost: 11.20, layer: 53
Normal
LY50
LY75
LY88
LY94
LY97
LY data
Normal data
Fold over basal
Time (min)
0
10
20
30
40
50
60
1
2
3
4
5
6

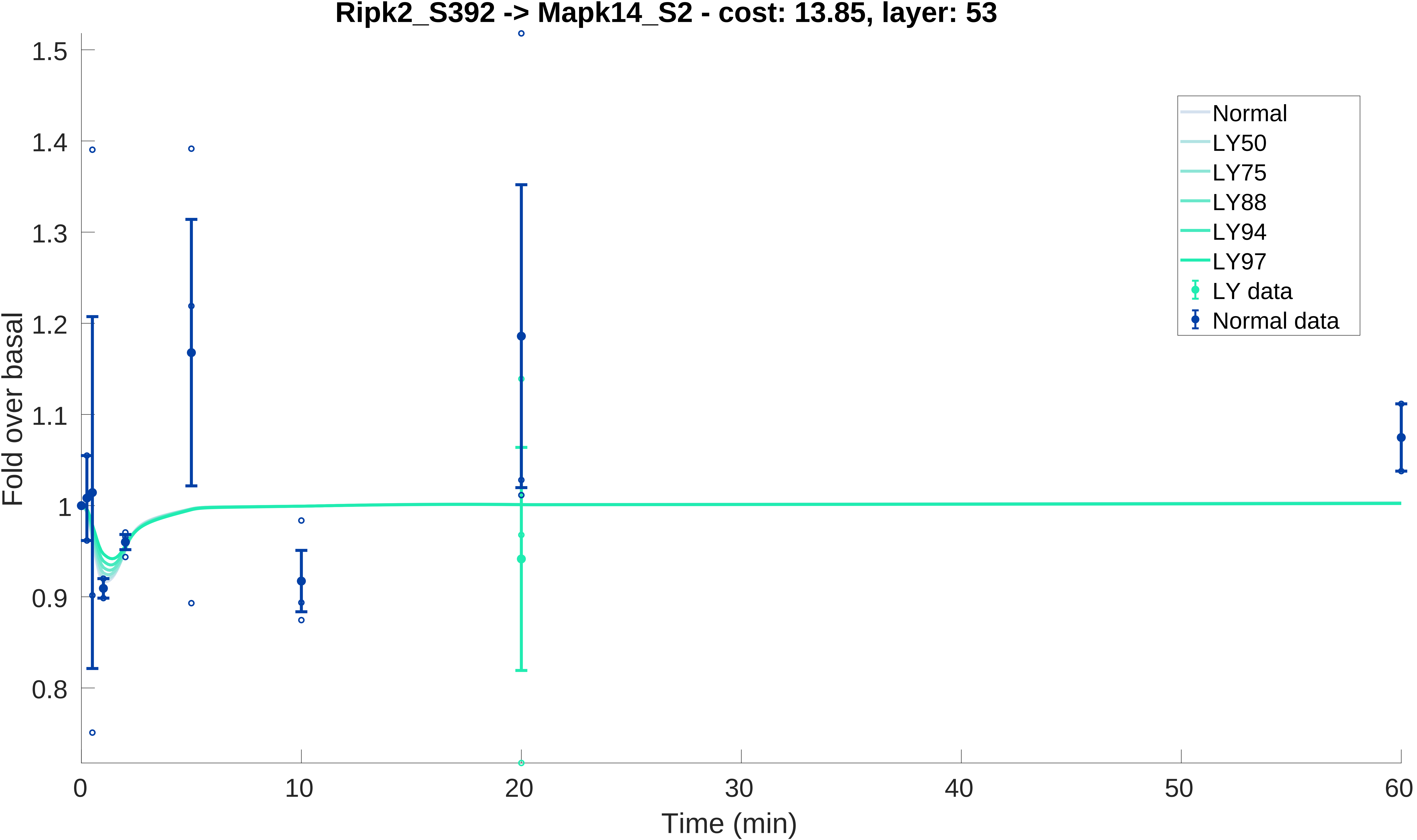

Ripk2_S392 -> Mapk14_S2 - cost: 13.85, layer: 53
Fold over basal
1.5
1.4
1.3
1.2
1.1
1
0.9
0.8
0
10
20
30
40
50
60
Time (min)
Normal
LY50
LY75
LY88
LY94
LY97
LY data
Normal data

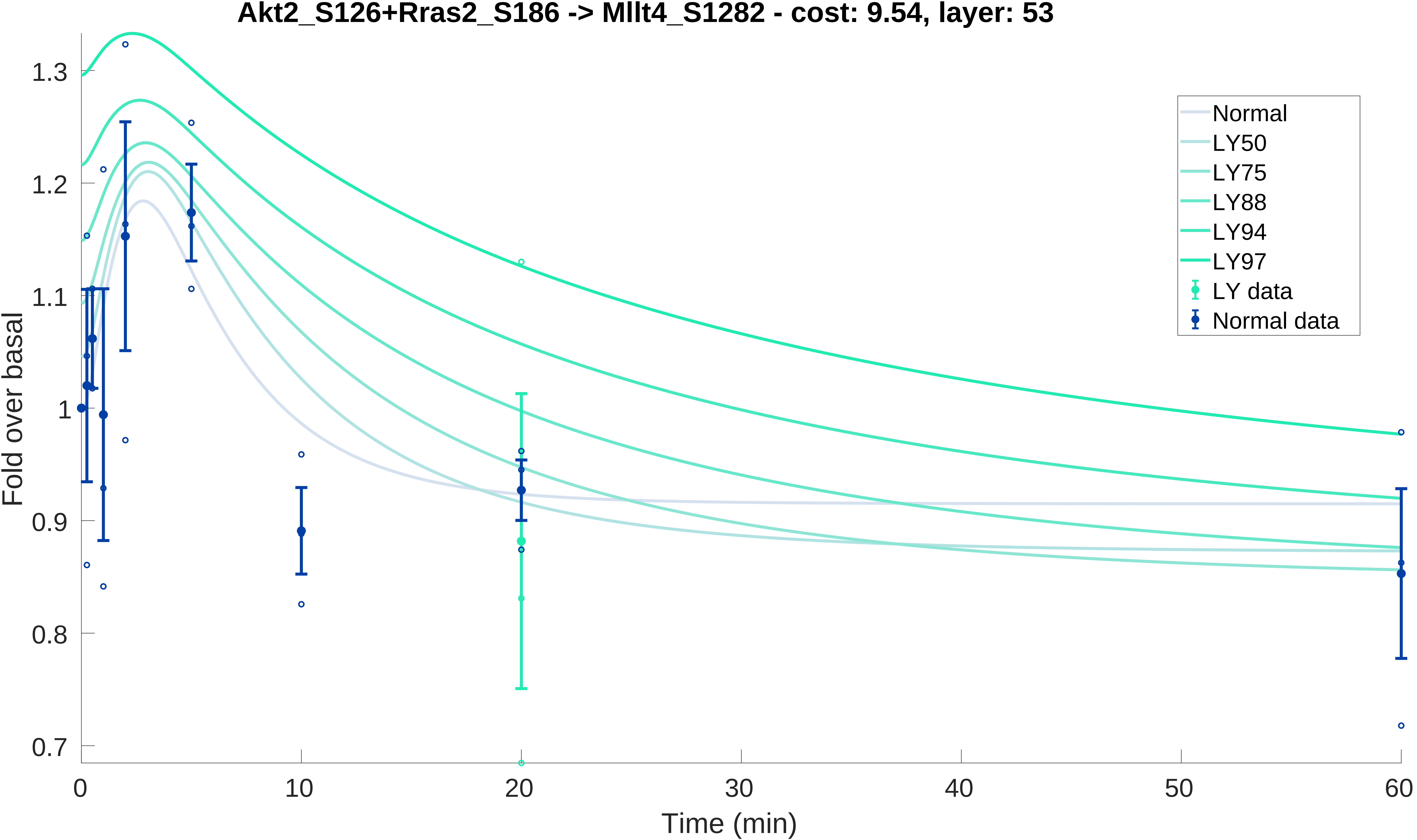

Akt2_S126+Rras2_S186 -> Mllt4_S1282 - cost: 9.54, layer: 53
Fold over basal
Time (min)
1.3
1.2
1.1
1
0.9
0.8
0.7
0
10
20
30
40
50
60
Normal
LY50
LY75
LY88
LY94
LY97
LY data
Normal data

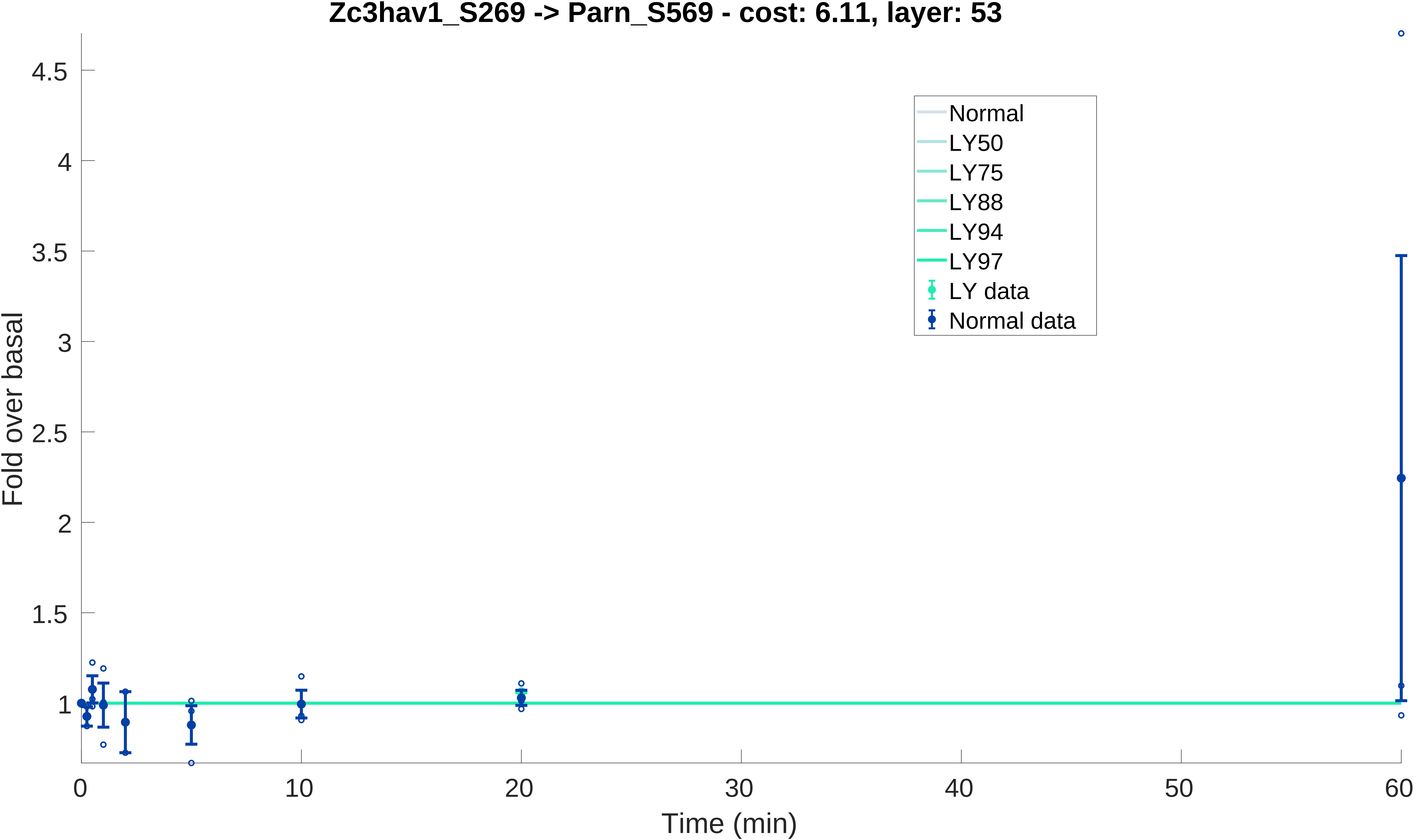

Zc3hav1_S269 -> Parn_S569 - cost: 6.11, layer: 53
Fold over basal
Time (min)
4.5
4
3.5
3
2.5
2
1.5
1
0
10
20
30
40
50
60
Normal
LY50
LY75
LY88
LY94
LY97
LY data
Normal data

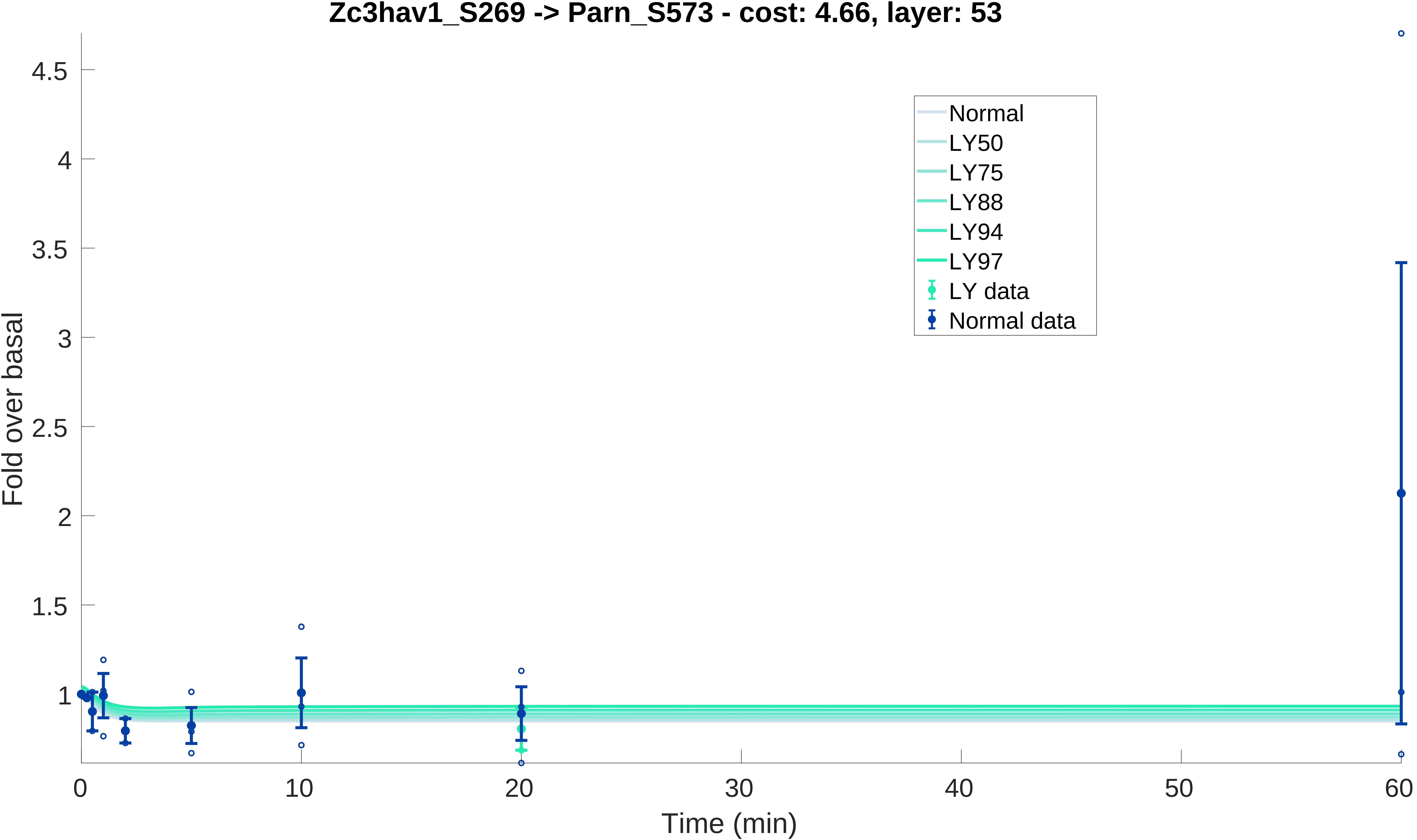

Zc3hav1_S269 -> Parn_S573 - cost: 4.66, layer: 53
Fold over basal
Time (min)
Normal
LY50
LY75
LY88
LY94
LY97
LY data
Normal data

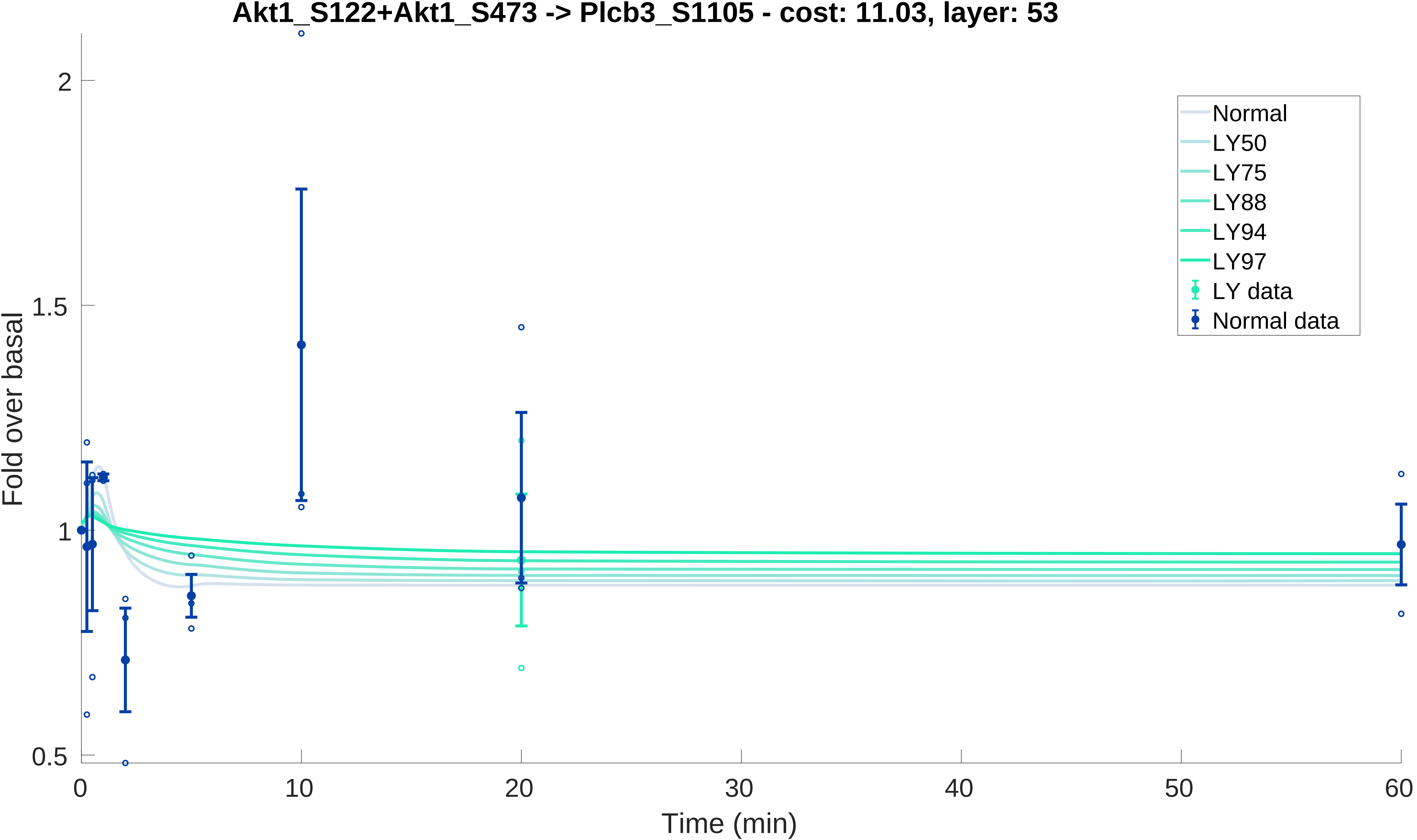

Akt1_S122+Akt1_S473 -> Plcb3_S1105 - cost: 11.03, layer: 53
Fold over basal
Time (min)
0.5
1
1.5
2
0
10
20
30
40
50
60
Normal
LY50
LY75
LY88
LY94
LY97
LY data
Normal data

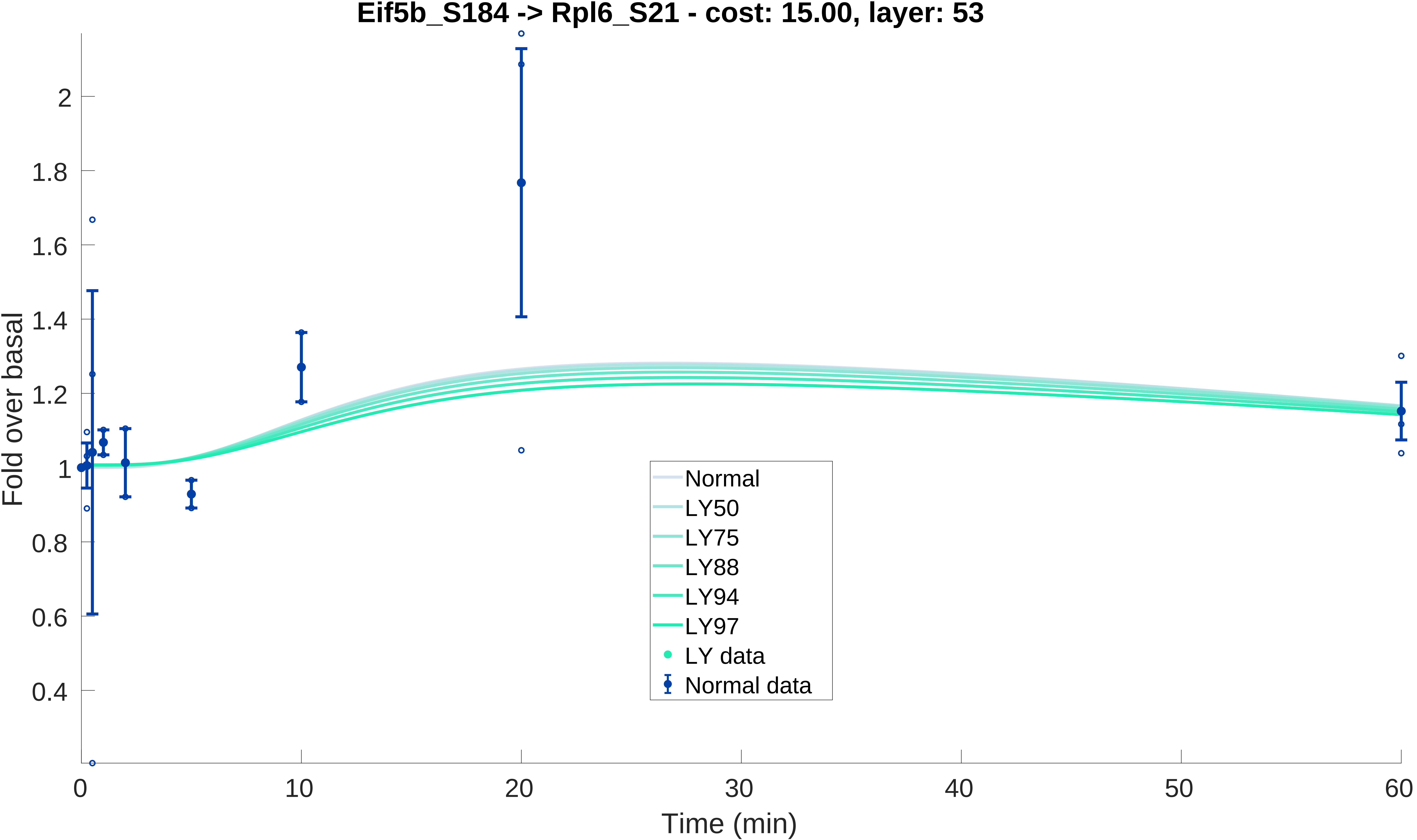

Eif5b_S184 -> Rpl6_S21 - cost: 15.00, layer: 53
Fold over basal
2
1.8
1.6
1.4
1.2
1
0.8
0.6
0.4
0
10
20
30
40
50
60
Time (min)
Normal
LY50
LY75
LY88
LY94
LY97
LY data
Normal data

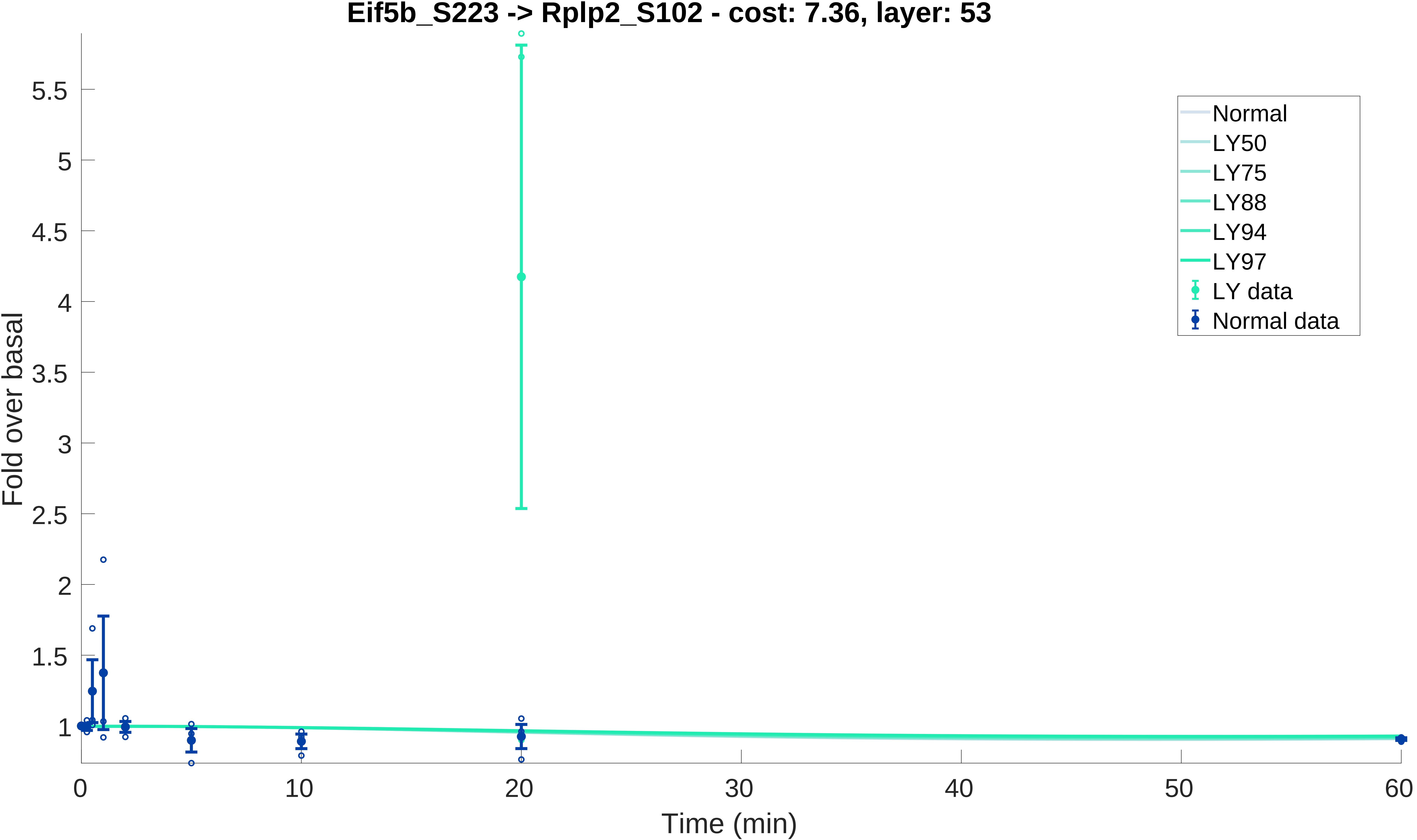

Eif5b_S223 -> Rplp2_S102 - cost: 7.36, layer: 53
Fold over basal
Time (min)
0
10
20
30
40
50
60
1
1.5
2
2.5
3
3.5
4
4.5
5
5.5
Normal
LY50
LY75
LY88
LY94
LY97
LY data
Normal data

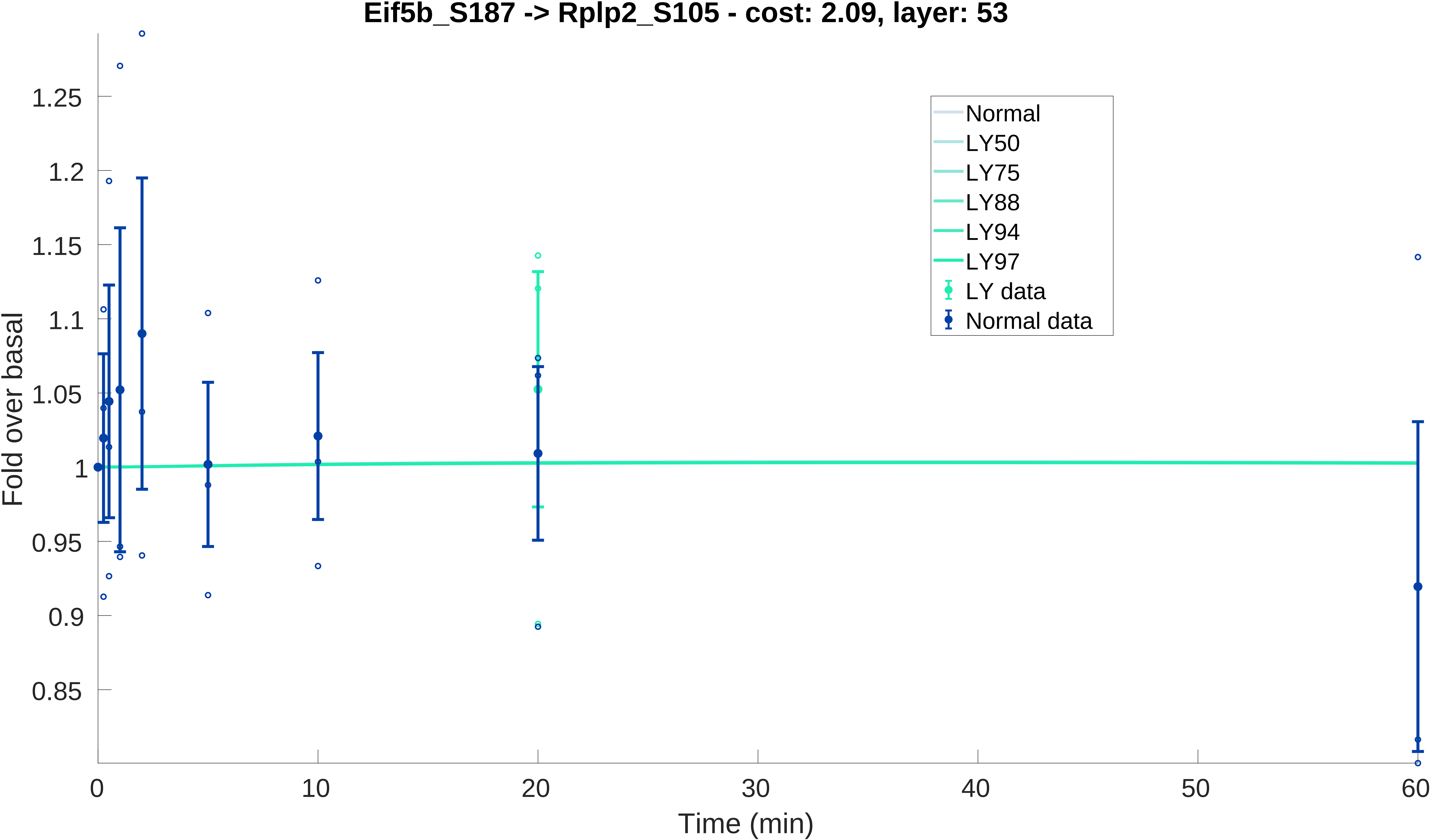

Eif5b_S187 -> Rplp2_S105 - cost: 2.09, layer: 53
Fold over basal
1.25
1.2
1.15
1.1
1.05
1
0.95
0.9
0.85
0
10
20
30
40
50
60
Time (min)
Normal
LY50
LY75
LY88
LY94
LY97
LY data
Normal data

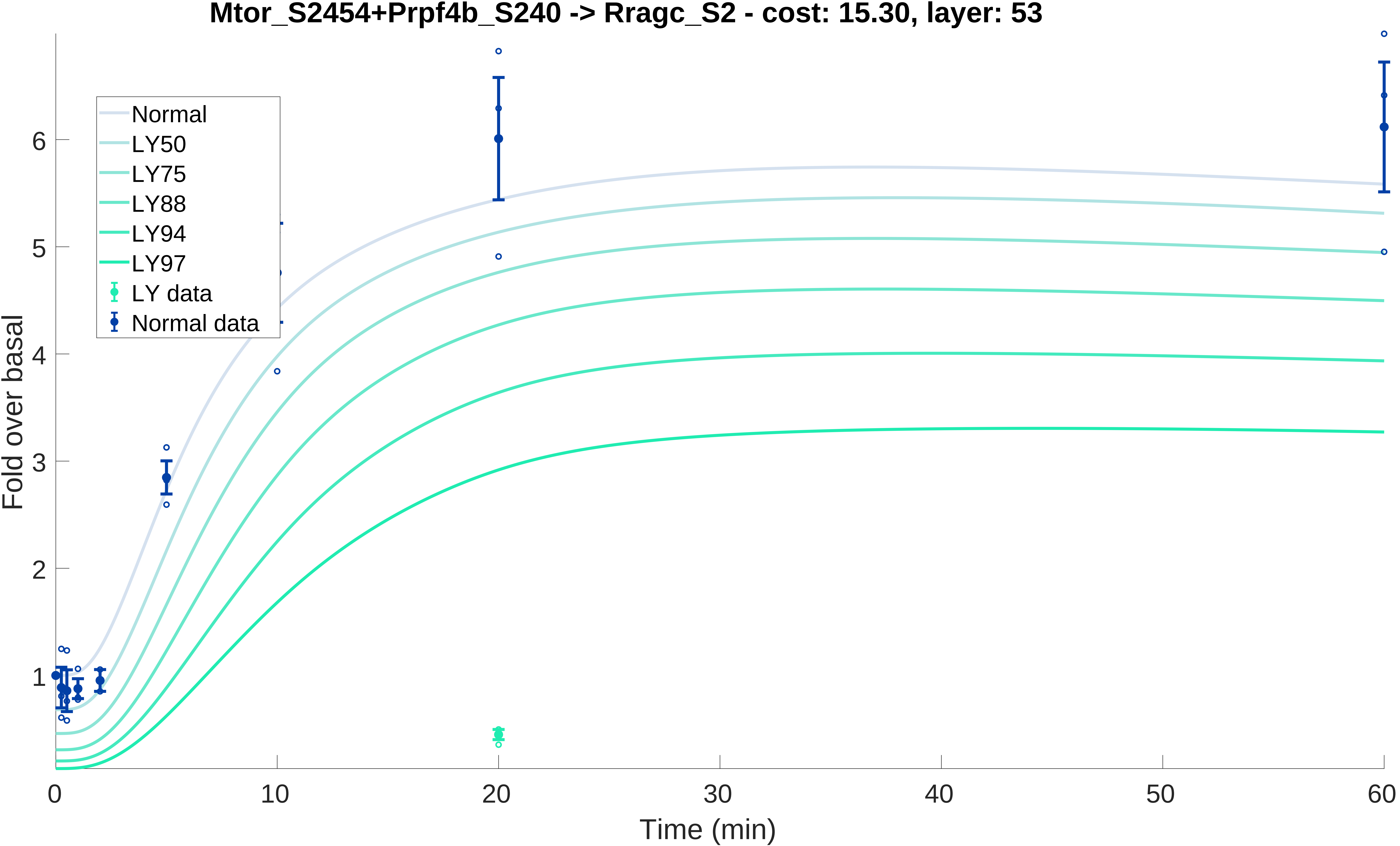

Mtor_S2454+Prpf4b_S240 -> Rragc_S2 - cost: 15.30, layer: 53
Normal
LY50
LY75
LY88
LY94
LY97
LY data
Normal data
Fold over basal
1
2
3
4
5
6
0
10
20
30
40
50
60
Time (min)

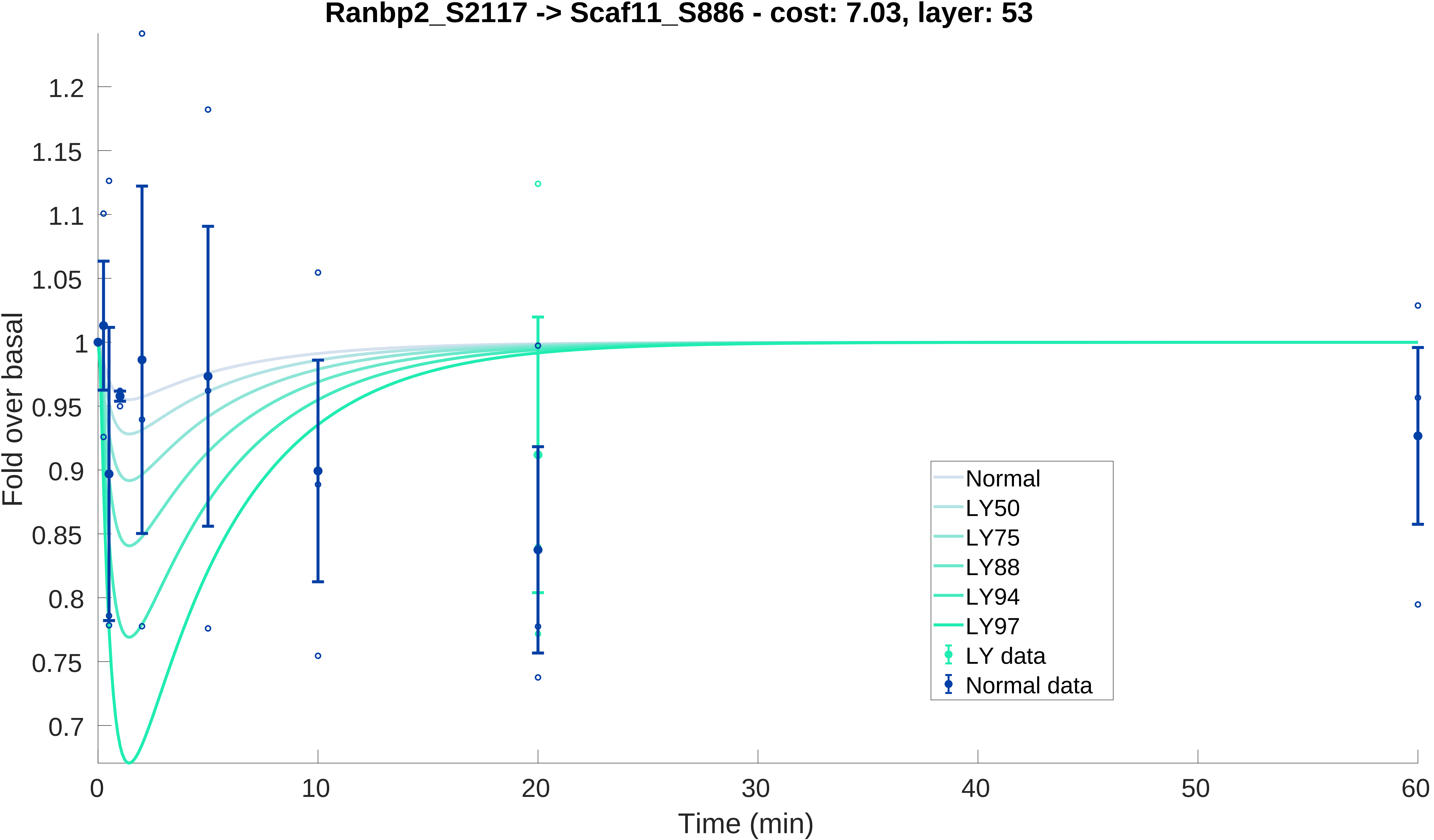
Ranbp2_S2117 -> Scaf11_S886 - cost: 7.03, layer: 53
Fold over basal
1.2
1.15
1.1
1.05
1
0.95
0.9
0.85
0.8
0.75
0.7
0
10
20
30
40
50
60
Time (min)
Normal
LY50
LY75
LY88
LY94
LY97
LY data
Normal data

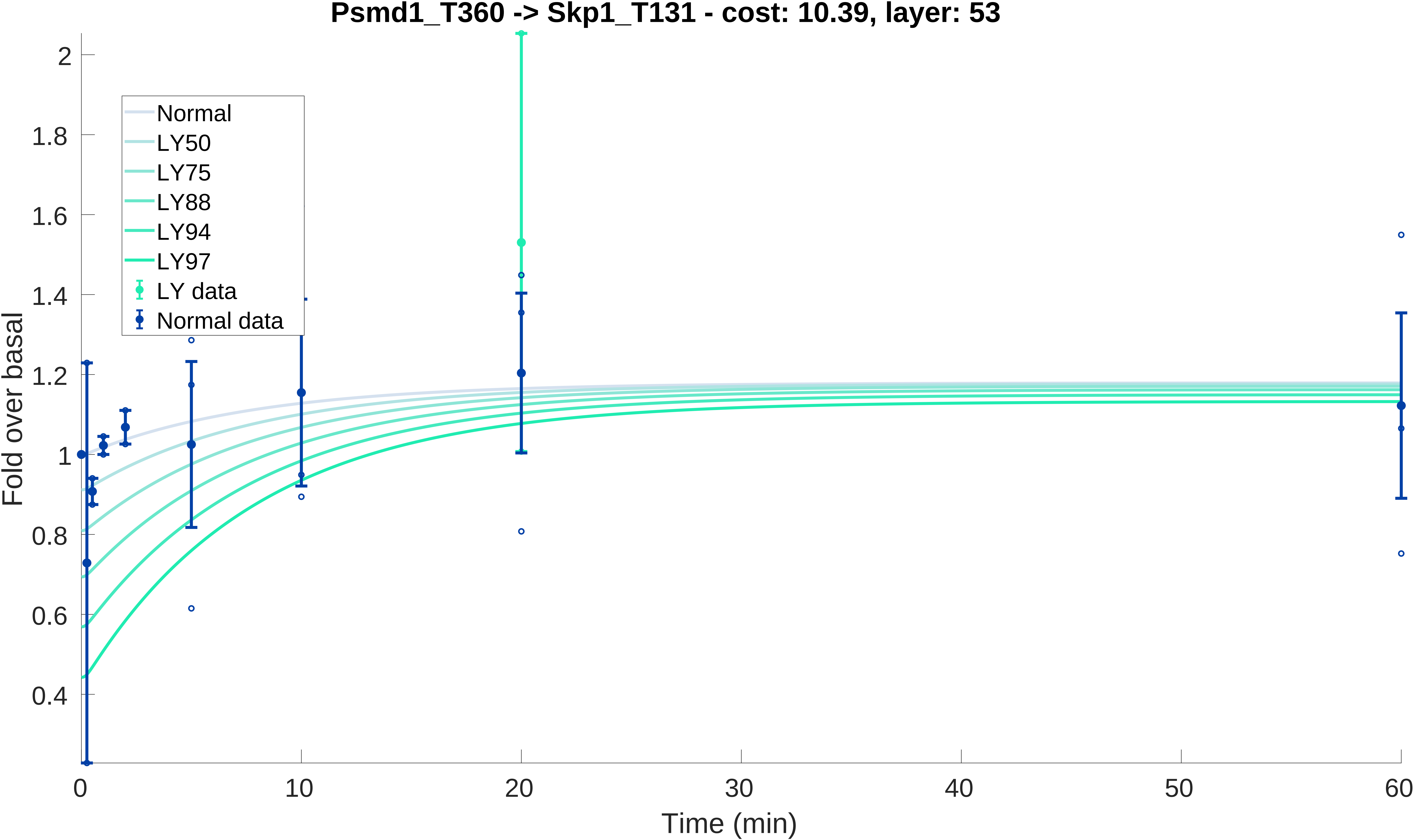

Psmd1_T360 -> Skp1_T131 - cost: 10.39, layer: 53
Normal
LY50
LY75
LY88
LY94
LY97
LY data
Normal data
Fold over basal
Time (min)
0
10
20
30
40
50
60
0.4
0.6
0.8
1
1.2
1.4
1.6
1.8
2

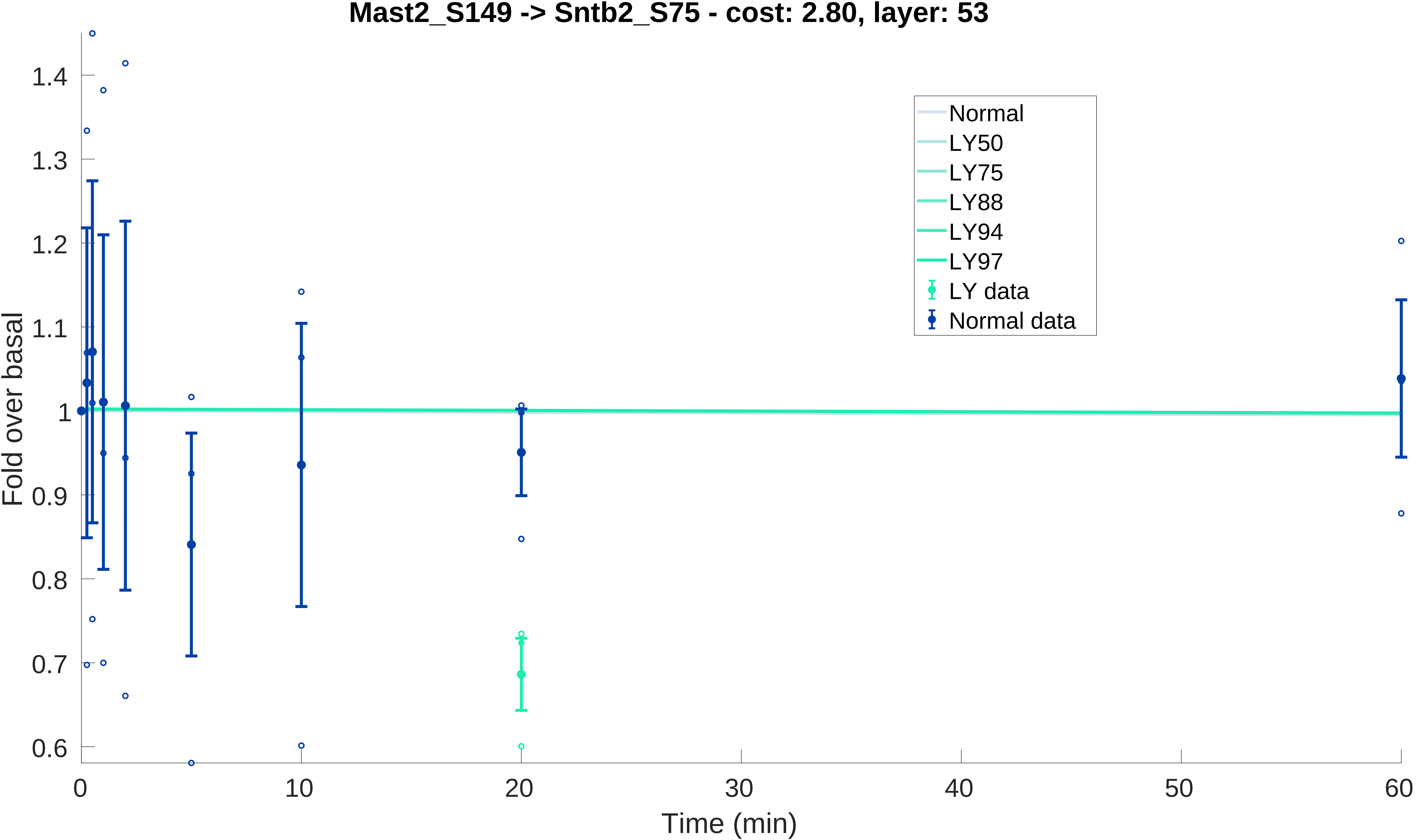
Mast2_S149 -> Sntb2_S75 - cost: 2.80, layer: 53
Normal
LY50
LY75
LY88
LY94
LY97
LY data
Normal data
Fold over basal
Time (min)
1.4
1.3
1.2
1.1
1
0.9
0.8
0.7
0.6
0
10
20
30
40
50
60

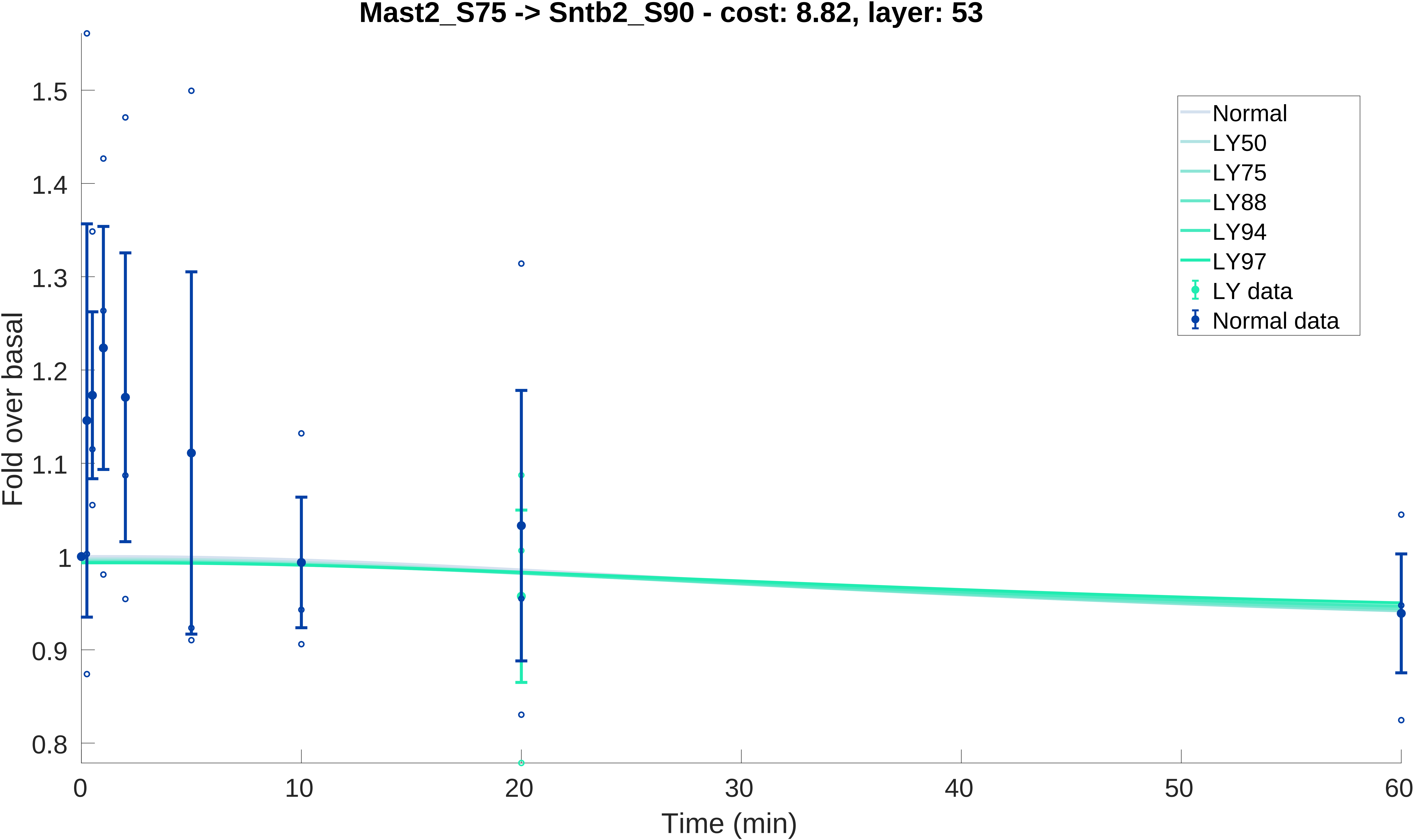
Mast2_S75 -> Sntb2_S90 - cost: 8.82, layer: 53
Fold over basal
Time (min)
Normal
LY50
LY75
LY88
LY94
LY97
LY data
Normal data
1.5
1.4
1.3
1.2
1.1
1
0.9
0.8
0
10
20
30
40
50
60

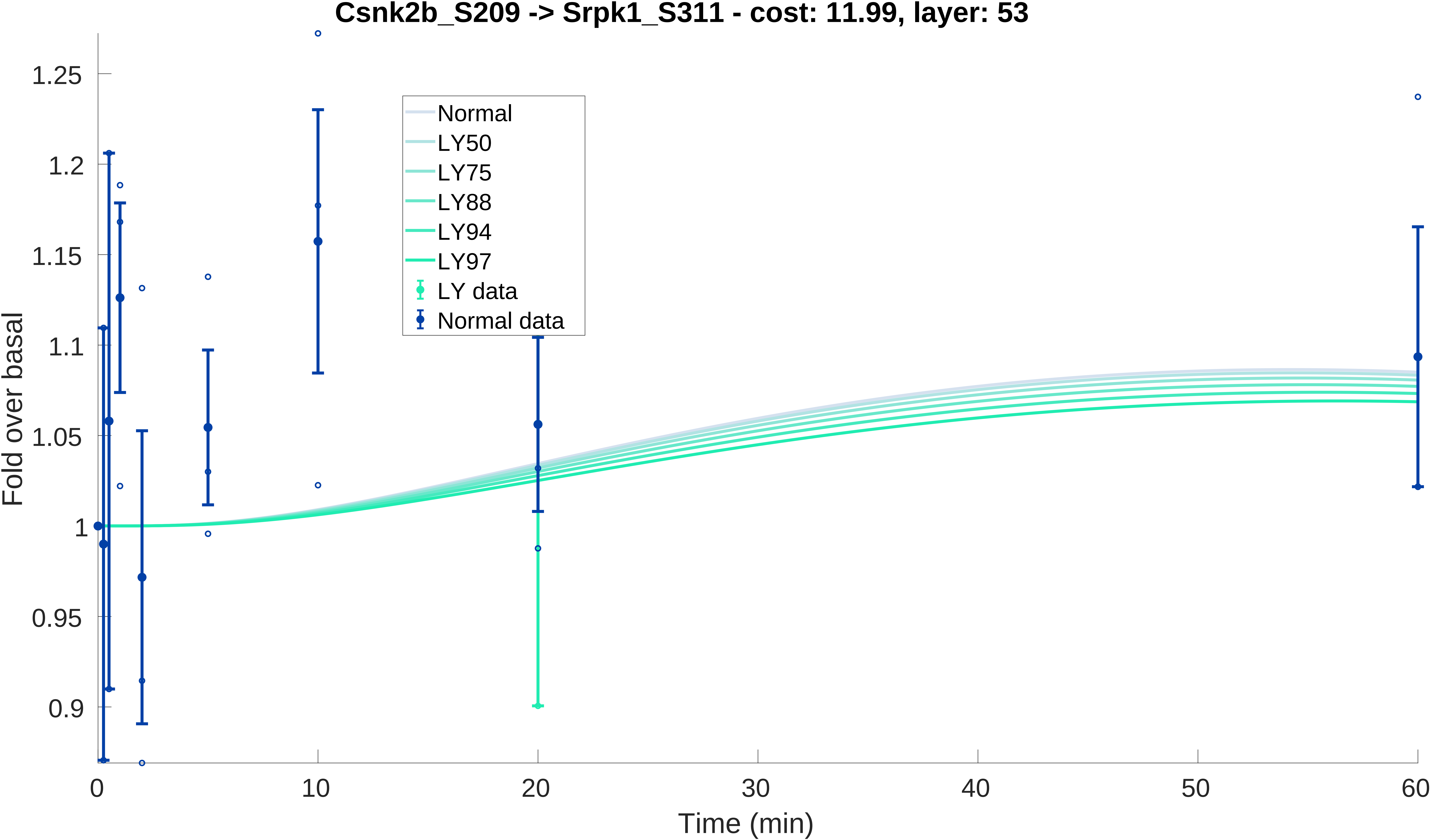

Csnk2b_S209 -> Srpk1_S311 - cost: 11.99, layer: 53
Fold over basal
Time (min)
1.25
1.2
1.15
1.1
1.05
1
0.95
0.9
0
10
20
30
40
50
60
Normal
LY50
LY75
LY88
LY94
LY97
LY data
Normal data

Csnk2b_S209 -> Srpk1_S450 - cost: 13.92, layer: 53

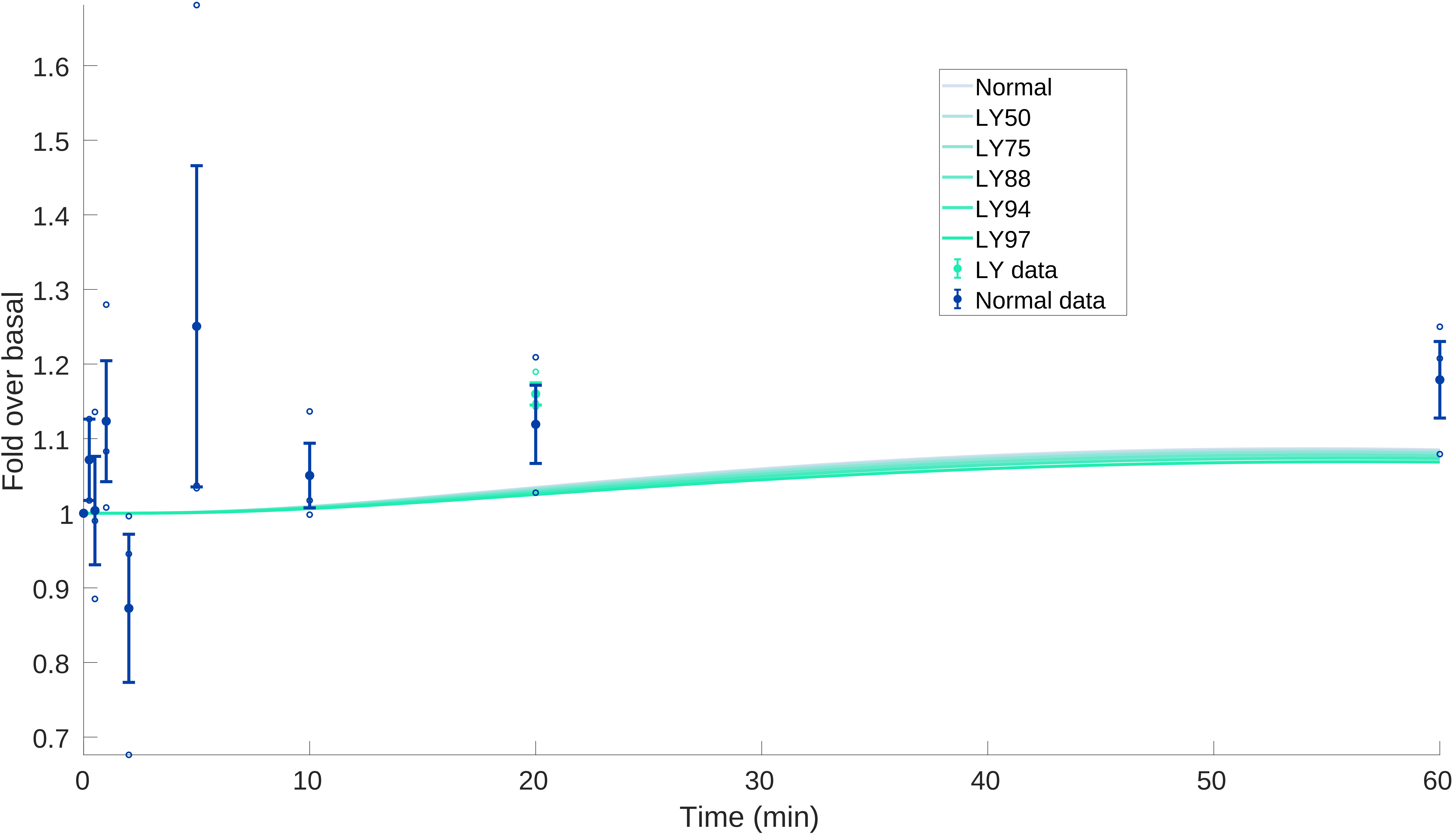

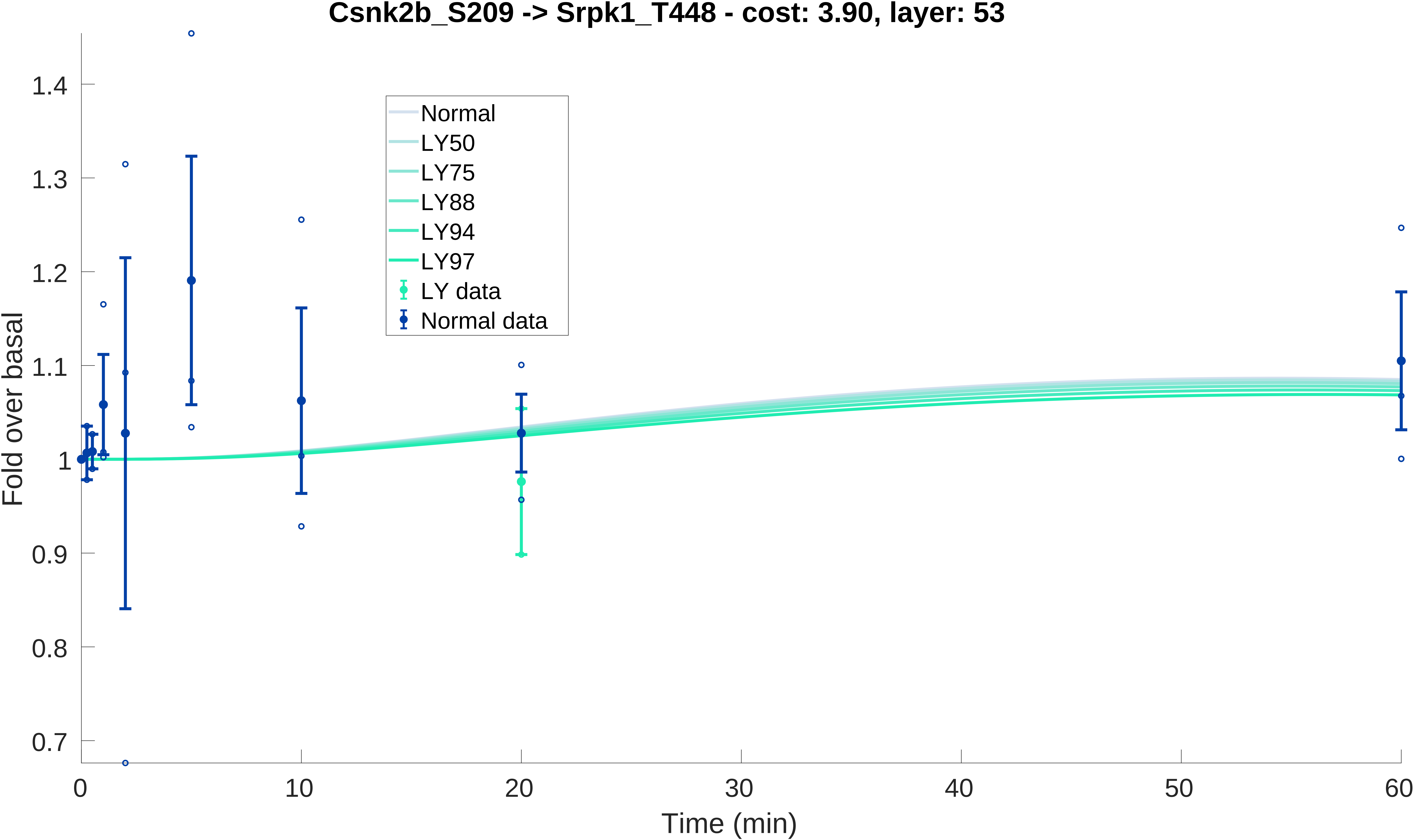

Csnk2b_S209 -> Srpk1_T448 - cost: 3.90, layer: 53
Normal
LY50
LY75
LY88
LY94
LY97
LY data
Normal data
Fold over basal
Time (min)
1.4
1.3
1.2
1.1
1
0.9
0.8
0.7
0
10
20
30
40
50
60

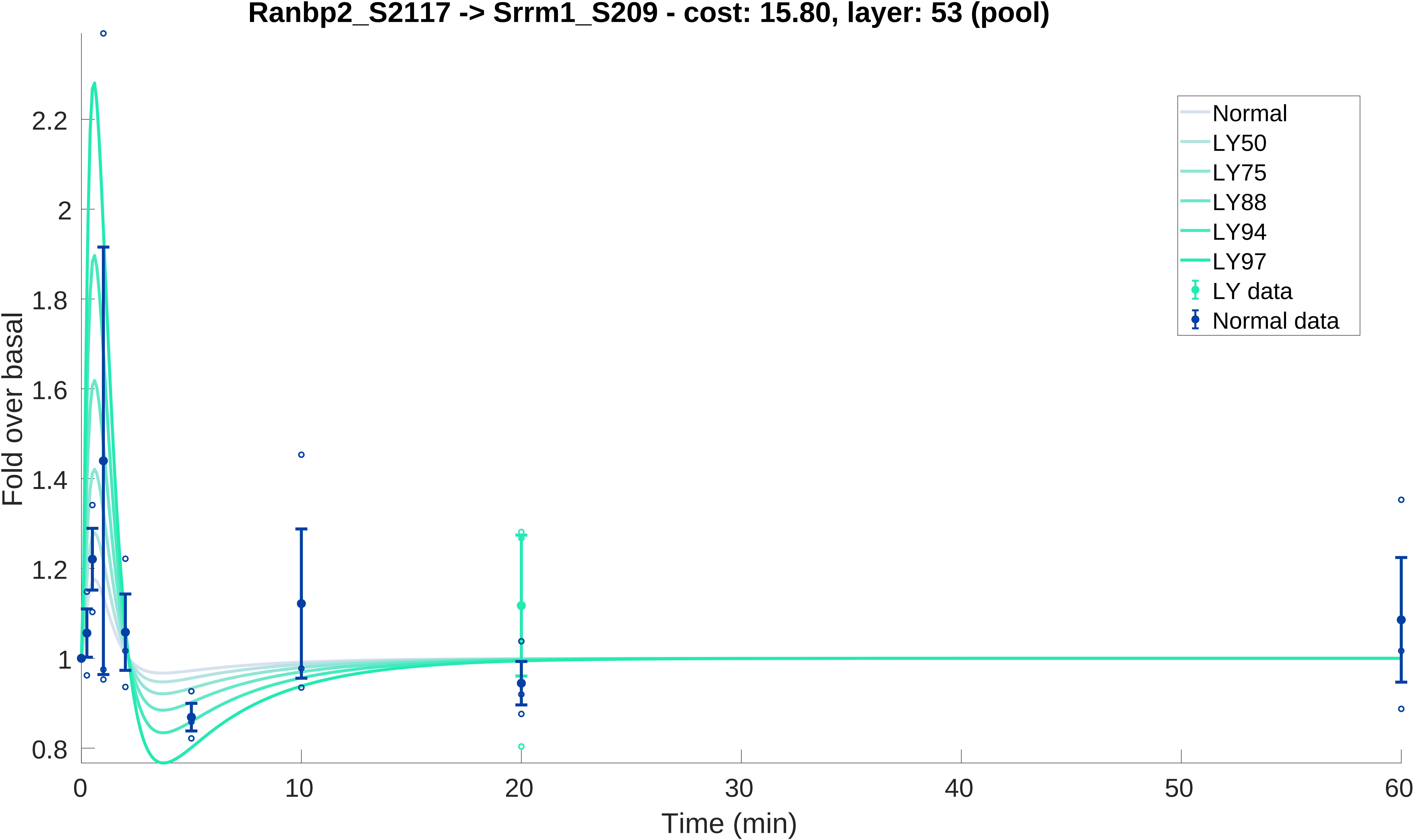

Ranbp2_S2117 -> Srrm1_S209 - cost: 15.80, layer: 53 (pool)
Fold over basal
0.8
1
1.2
1.4
1.6
1.8
2
2.2
0
10
20
30
40
50
60
Time (min)
Normal
LY50
LY75
LY88
LY94
LY97
LY data
Normal data

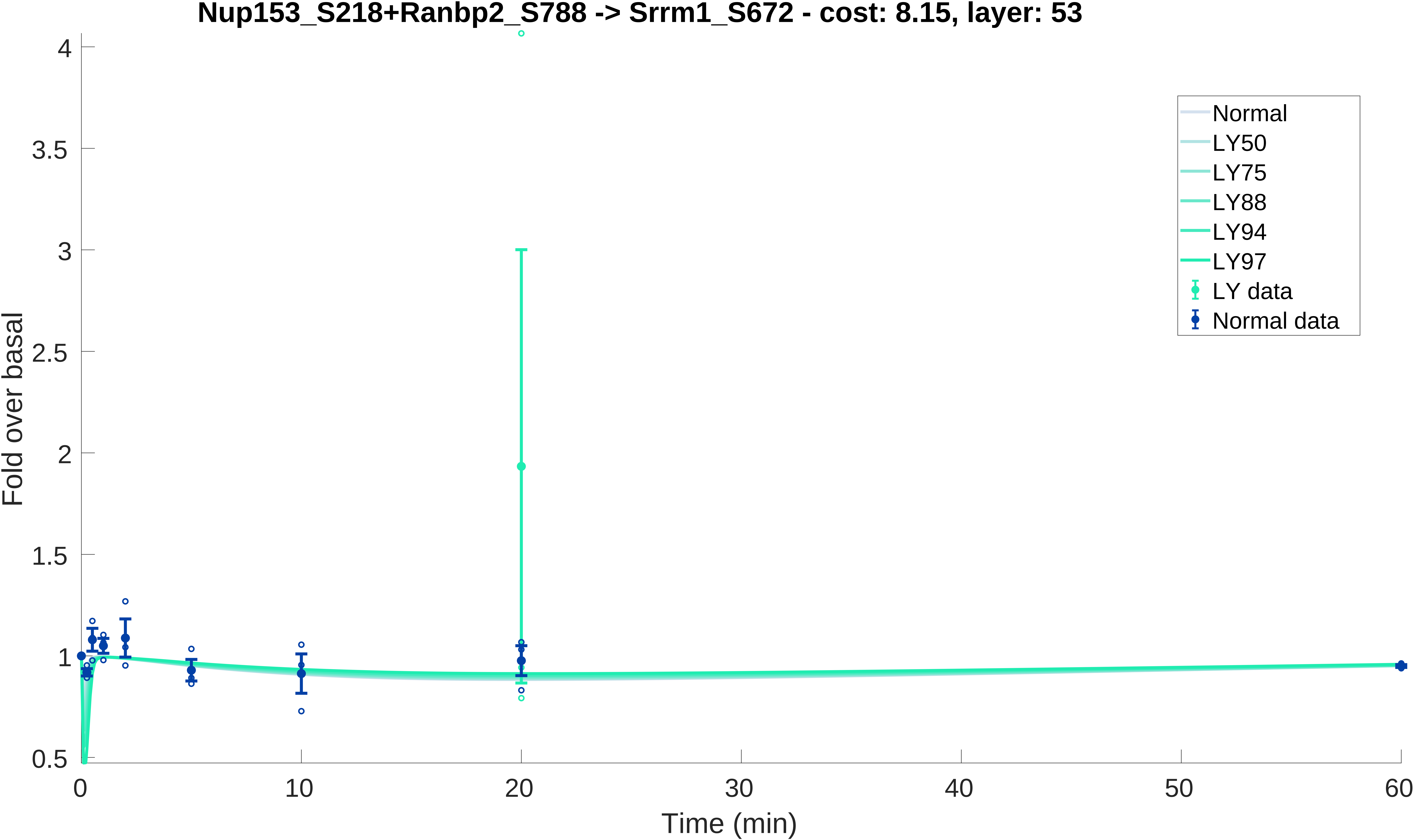

Nup153_S218+Ranbp2_S788 -> Srrm1_S672 - cost: 8.15, layer: 53
Fold over basal
Time (min)
0.5
1
1.5
2
2.5
3
3.5
4
0
10
20
30
40
50
60
Normal
LY50
LY75
LY88
LY94
LY97
LY data
Normal data

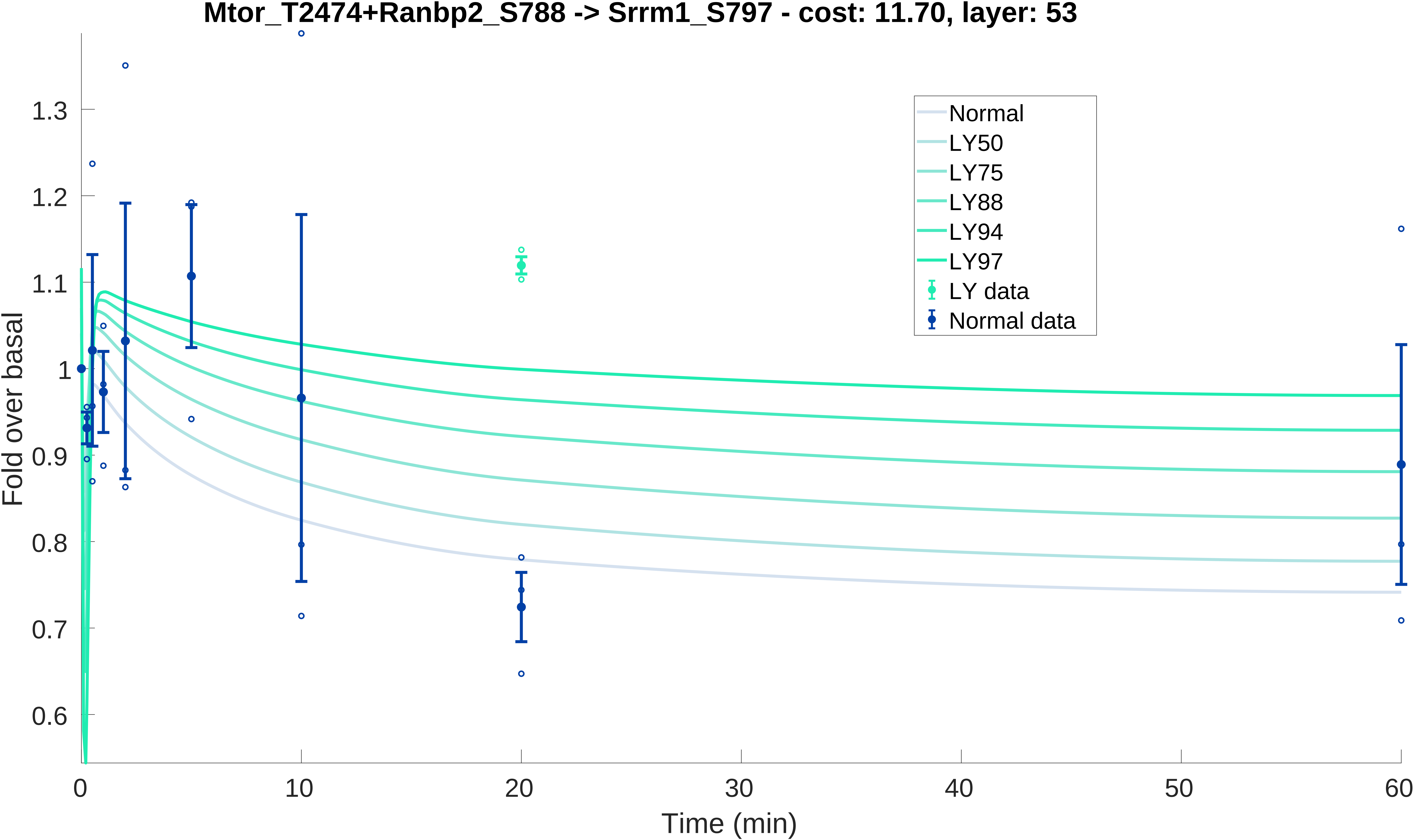
Mtor_T2474+Ranbp2_S788 -> Srrm1_S797 - cost: 11.70, layer: 53
Fold over basal
Time (min)
0.6
0.7
0.8
0.9
1
1.1
1.2
1.3
0
10
20
30
40
50
60
Normal
LY50
LY75
LY88
LY94
LY97
LY data
Normal data

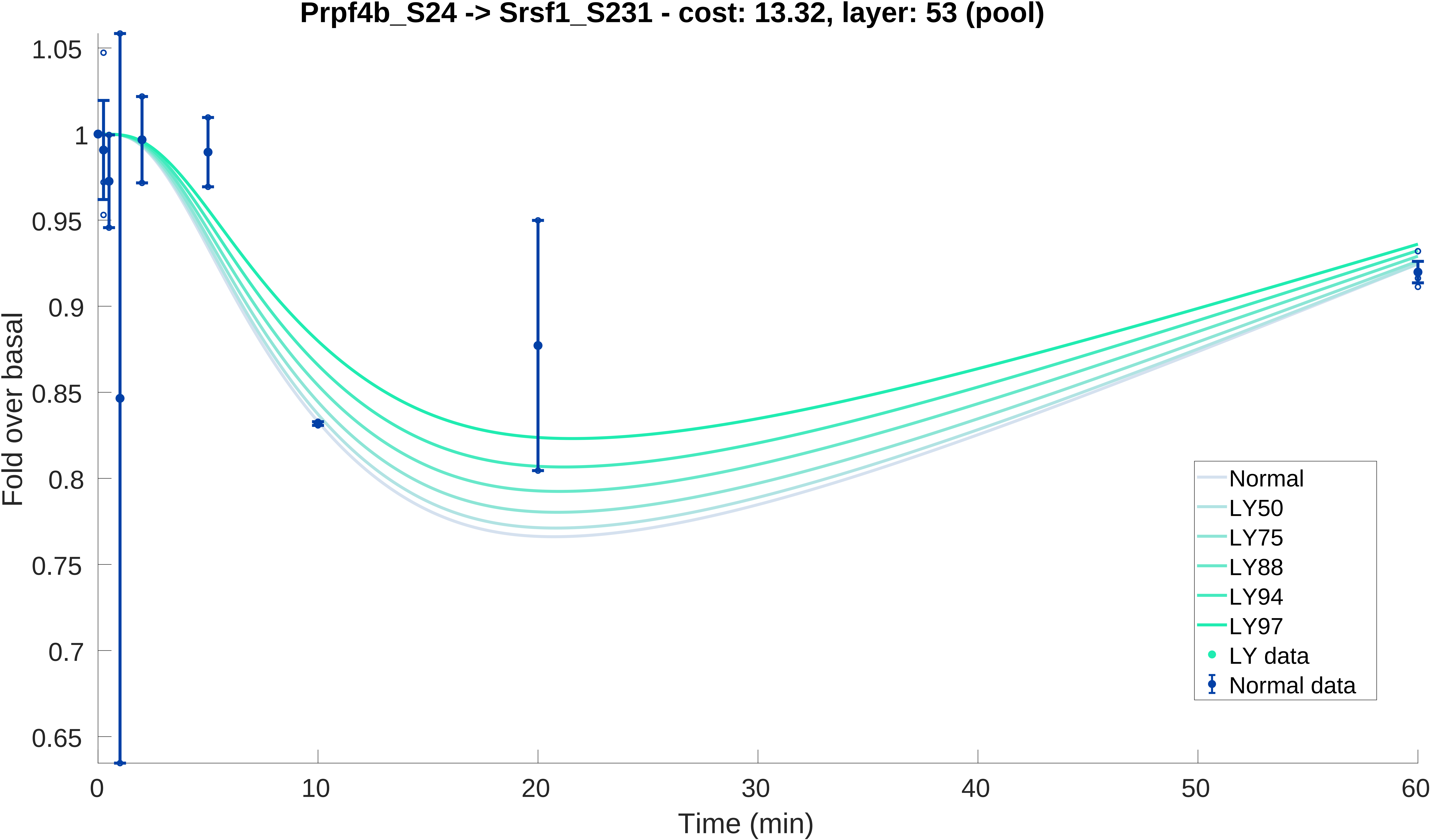

Prpf4b_S24 -> Srsf1_S231 - cost: 13.32, layer: 53 (pool)
1.05
1
0.95
0.9
0.85
0.8
0.75
0.7
0.65
Fold over basal
0
10
20
30
40
50
60
Time (min)
Normal
LY50
LY75
LY88
LY94
LY97
LY data
Normal data

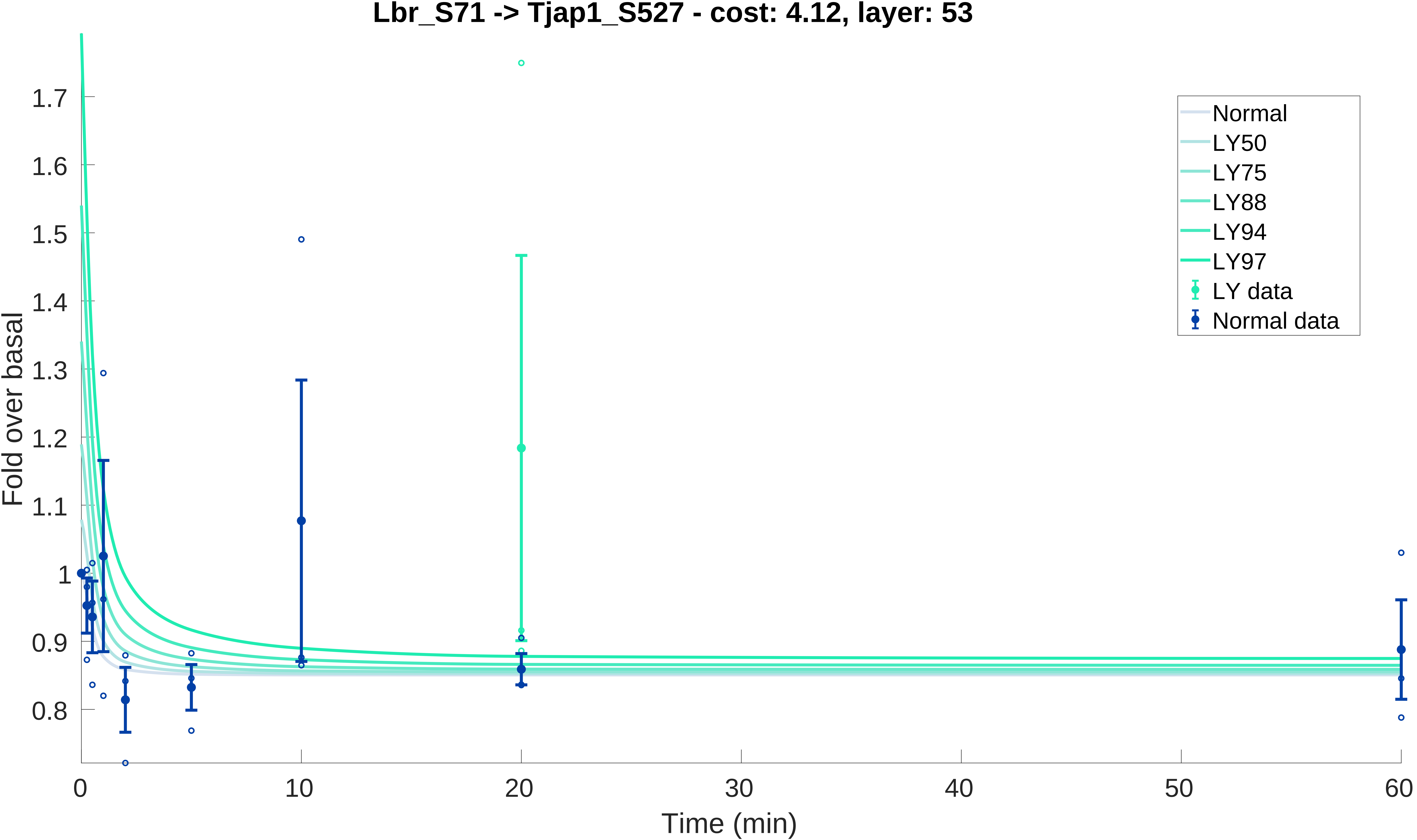
Lbr_S71 -> Tjap1_S527 - cost: 4.12, layer: 53
Fold over basal
Time (min)
0
10
20
30
40
50
60
0.8
0.9
1
1.1
1.2
1.3
1.4
1.5
1.6
1.7
Normal
LY50
LY75
LY88
LY94
LY97
LY data
Normal data

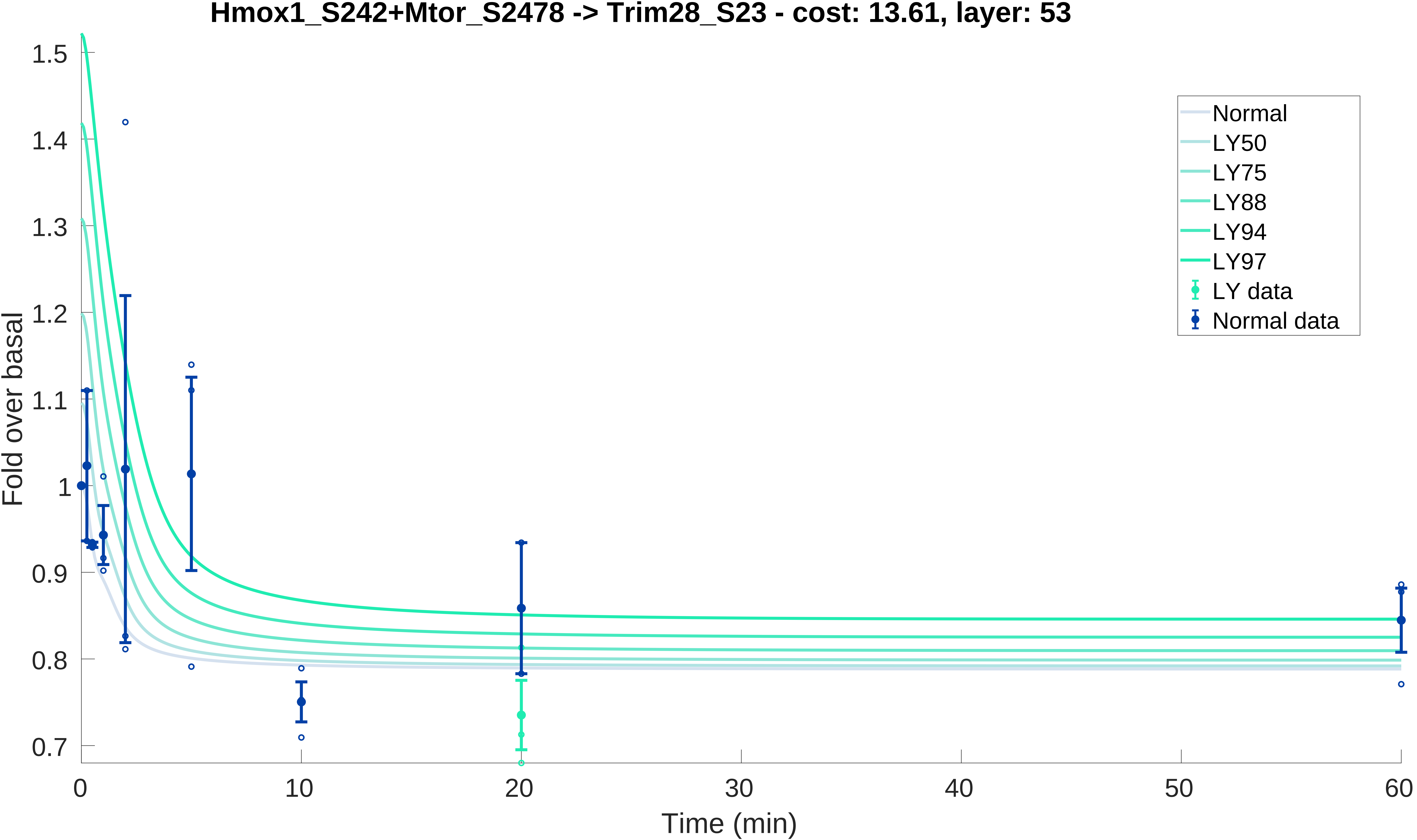

Hmox1_S242+Mtor_S2478 -> Trim28_S23 - cost: 13.61, layer: 53
Fold over basal
Time (min)
0.7
0.8
0.9
1
1.1
1.2
1.3
1.4
1.5
0
10
20
30
40
50
60
Normal
LY50
LY75
LY88
LY94
LY97
LY data
Normal data

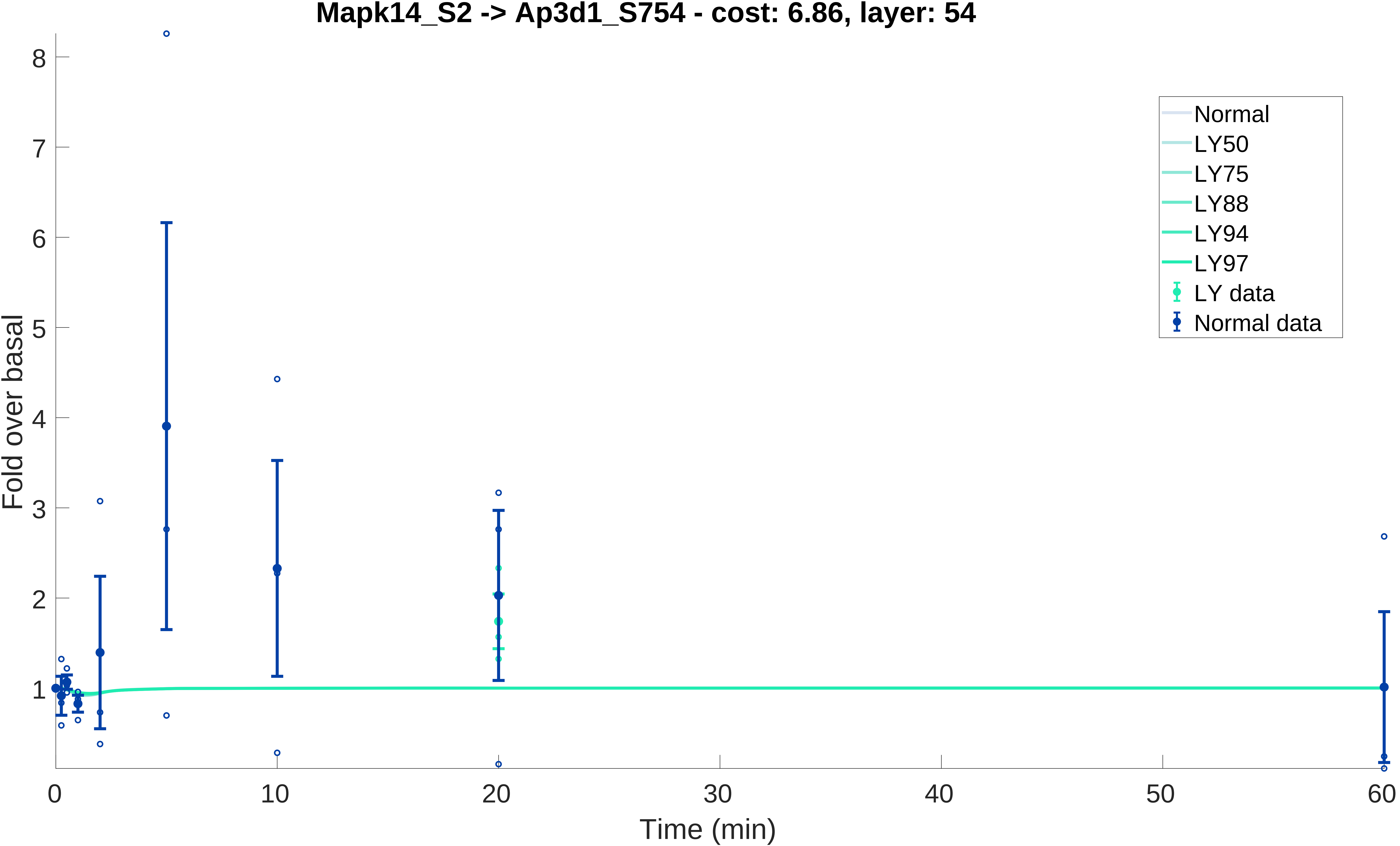

Mapk14_S2 -> Ap3d1_S754 - cost: 6.86, layer: 54
Fold over basal
Time (min)
Normal
LY50
LY75
LY88
LY94
LY97
LY data
Normal data
0
10
20
30
40
50
60
1
2
3
4
5
6
7
8

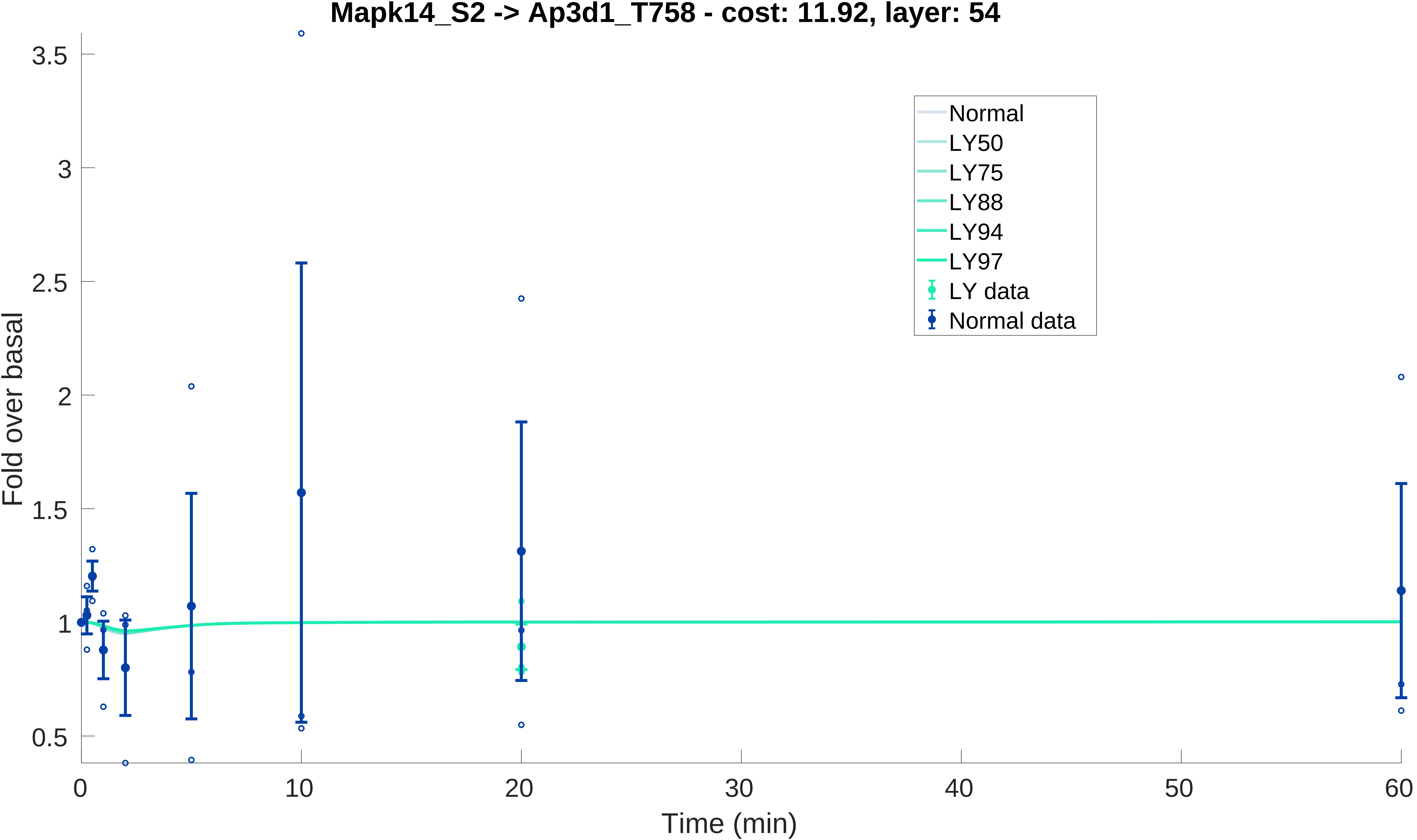

Mapk14_S2 -> Ap3d1_T758 - cost: 11.92, layer: 54
Fold over basal
3.5
3
2.5
2
1.5
1
0.5
0
10
20
30
40
50
60
Time (min)
Normal
LY50
LY75
LY88
LY94
LY97
LY data
Normal data

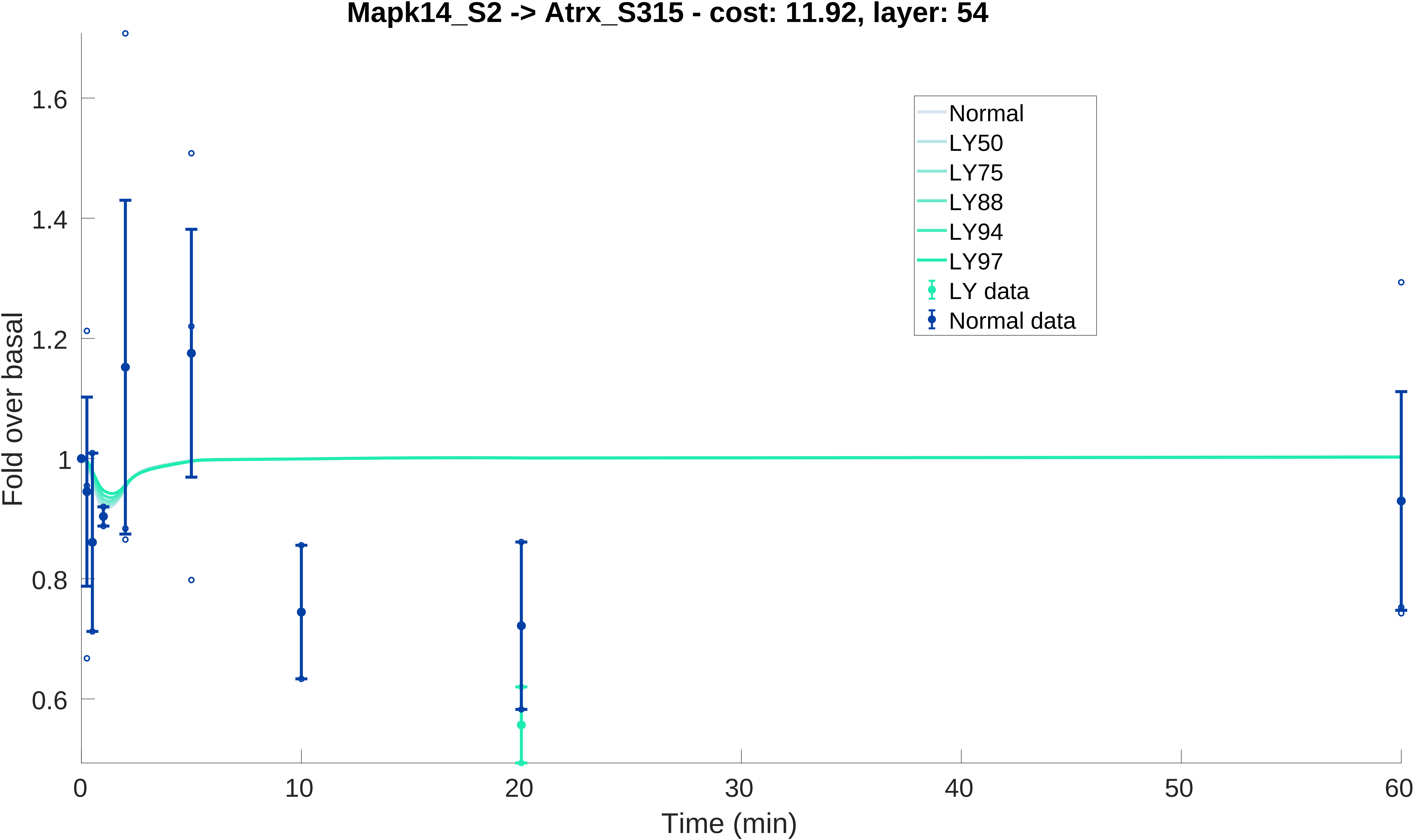
Mapk14_S2 -> Atrx_S315 - cost: 11.92, layer: 54
Fold over basal
Time (min)
1.6
1.4
1.2
1
0.8
0.6
0
10
20
30
40
50
60
Normal
LY50
LY75
LY88
LY94
LY97
LY data
Normal data

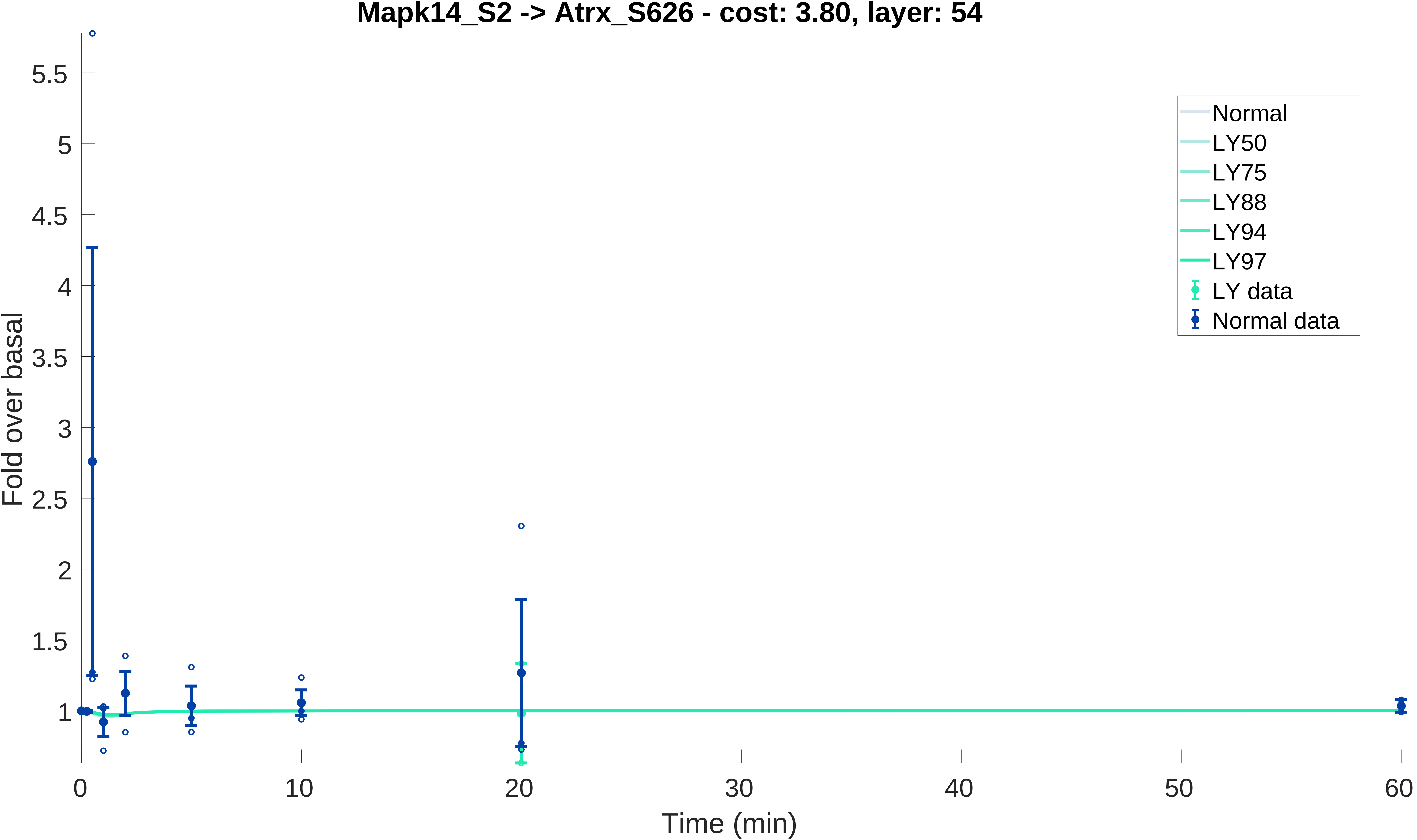
Mapk14_S2 -> Atrx_S626 - cost: 3.80, layer: 54
Fold over basal
5.5
5
4.5
4
3.5
3
2.5
2
1.5
1
0
10
20
30
40
50
60
Time (min)
Normal
LY50
LY75
LY88
LY94
LY97
LY data
Normal data

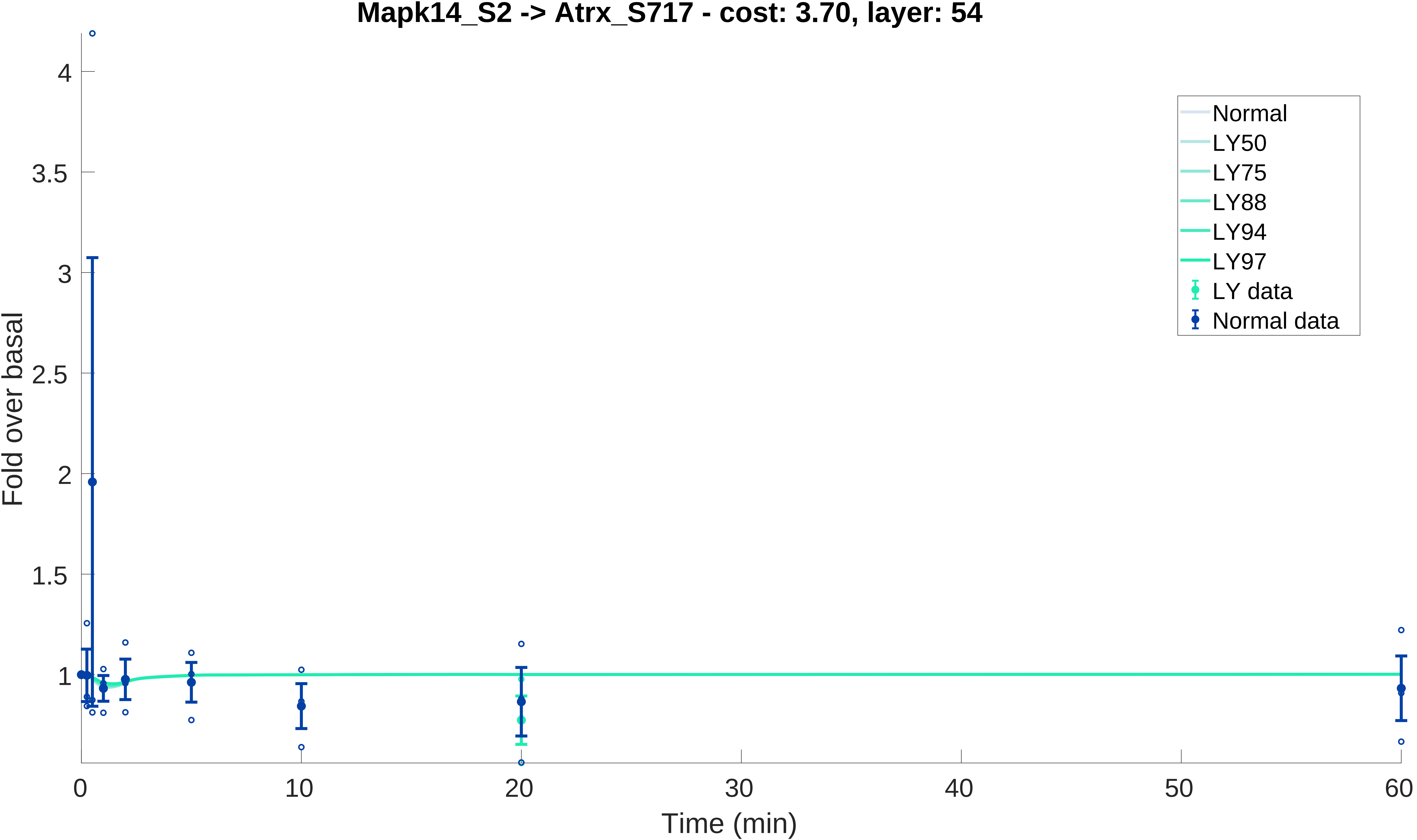

Mapk14_S2 -> Atrx_S717 - cost: 3.70, layer: 54
Fold over basal
Time (min)
Normal
LY50
LY75
LY88
LY94
LY97
LY data
Normal data
4
3.5
3
2.5
2
1.5
1
0
10
20
30
40
50
60

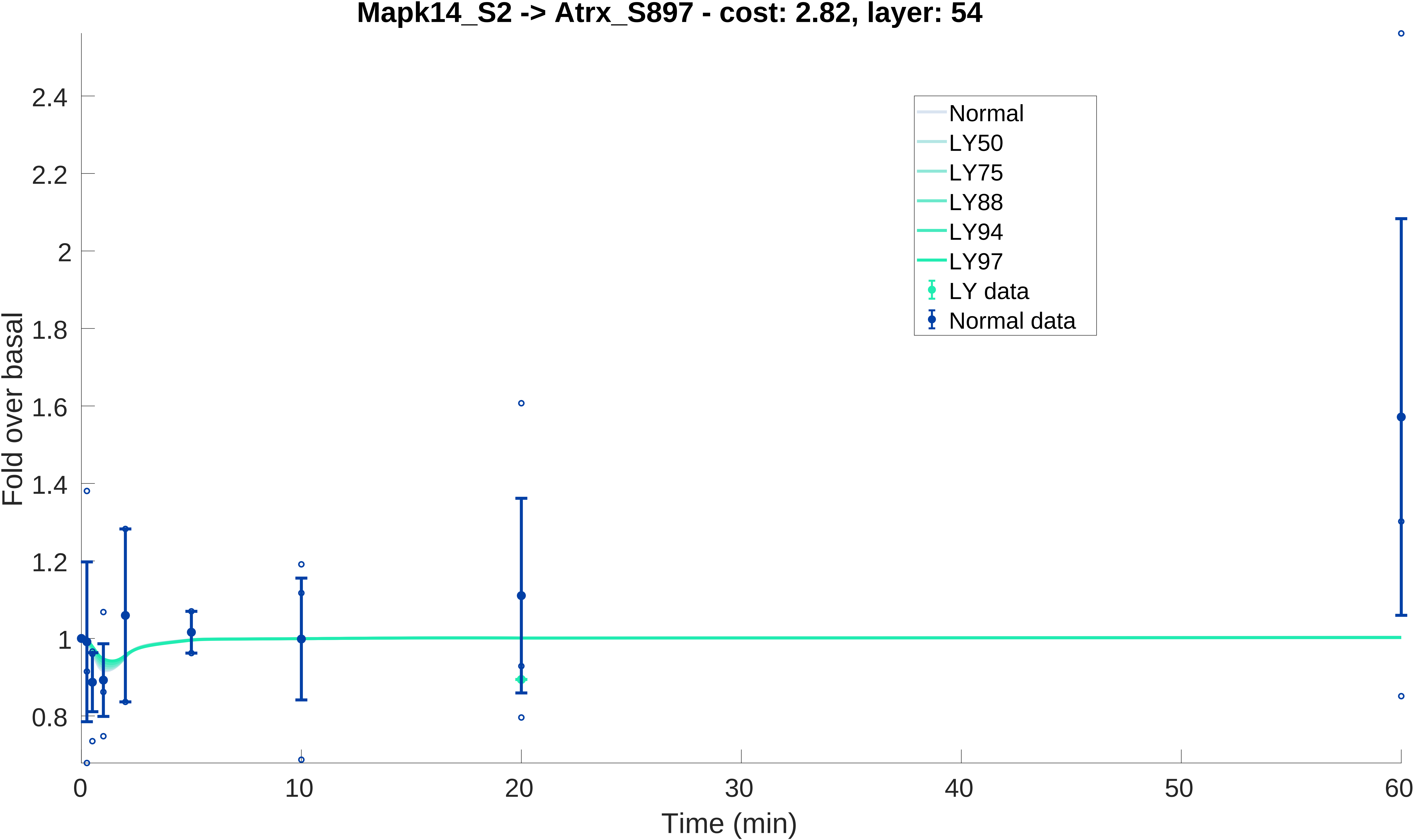
Mapk14_S2 -> Atrx_S897 - cost: 2.82, layer: 54
Fold over basal
Time (min)
0.8
1
1.2
1.4
1.6
1.8
2
2.2
2.4
0
10
20
30
40
50
60
Normal
LY50
LY75
LY88
LY94
LY97
LY data
Normal data

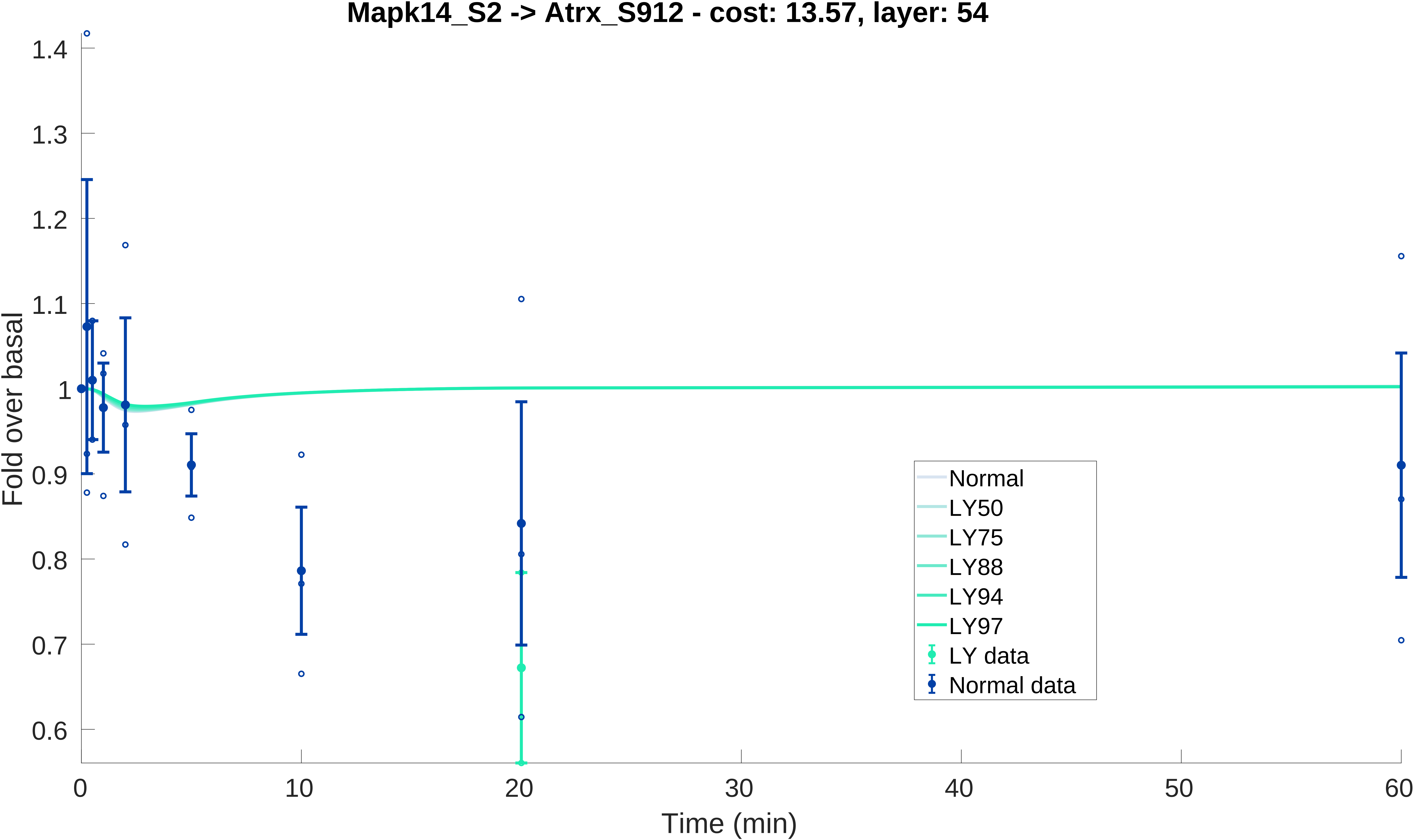

Mapk14_S2 -> Atrx_S912 - cost: 13.57, layer: 54
Fold over basal
1.4
1.3
1.2
1.1
1
0.9
0.8
0.7
0.6
0
10
20
30
40
50
60
Time (min)
Normal
LY50
LY75
LY88
LY94
LY97
LY data
Normal data

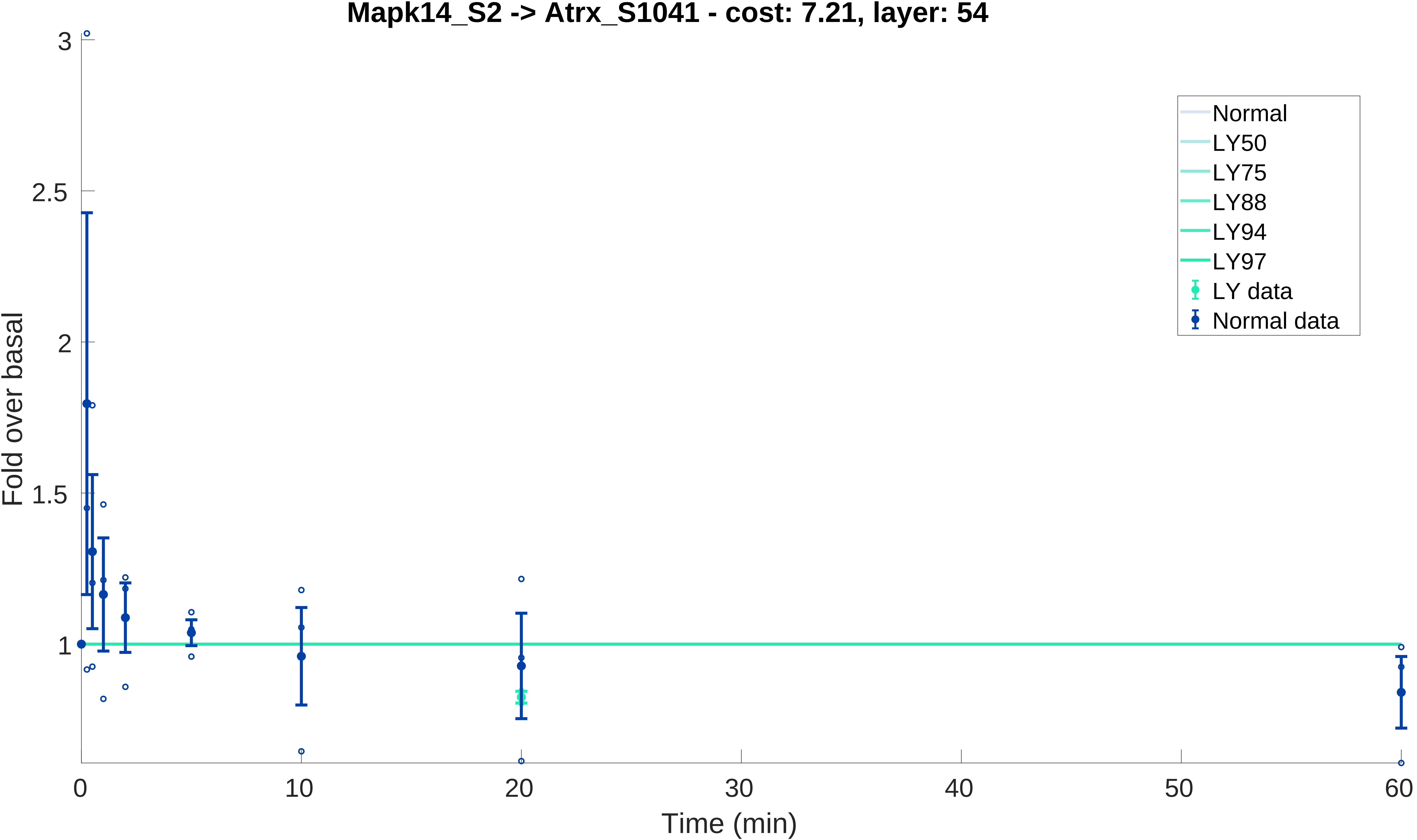

Mapk14_S2 -> Atrx_S1041 - cost: 7.21, layer: 54
Fold over basal
Time (min)
0
10
20
30
40
50
60
1
1.5
2
2.5
3
Normal
LY50
LY75
LY88
LY94
LY97
LY data
Normal data

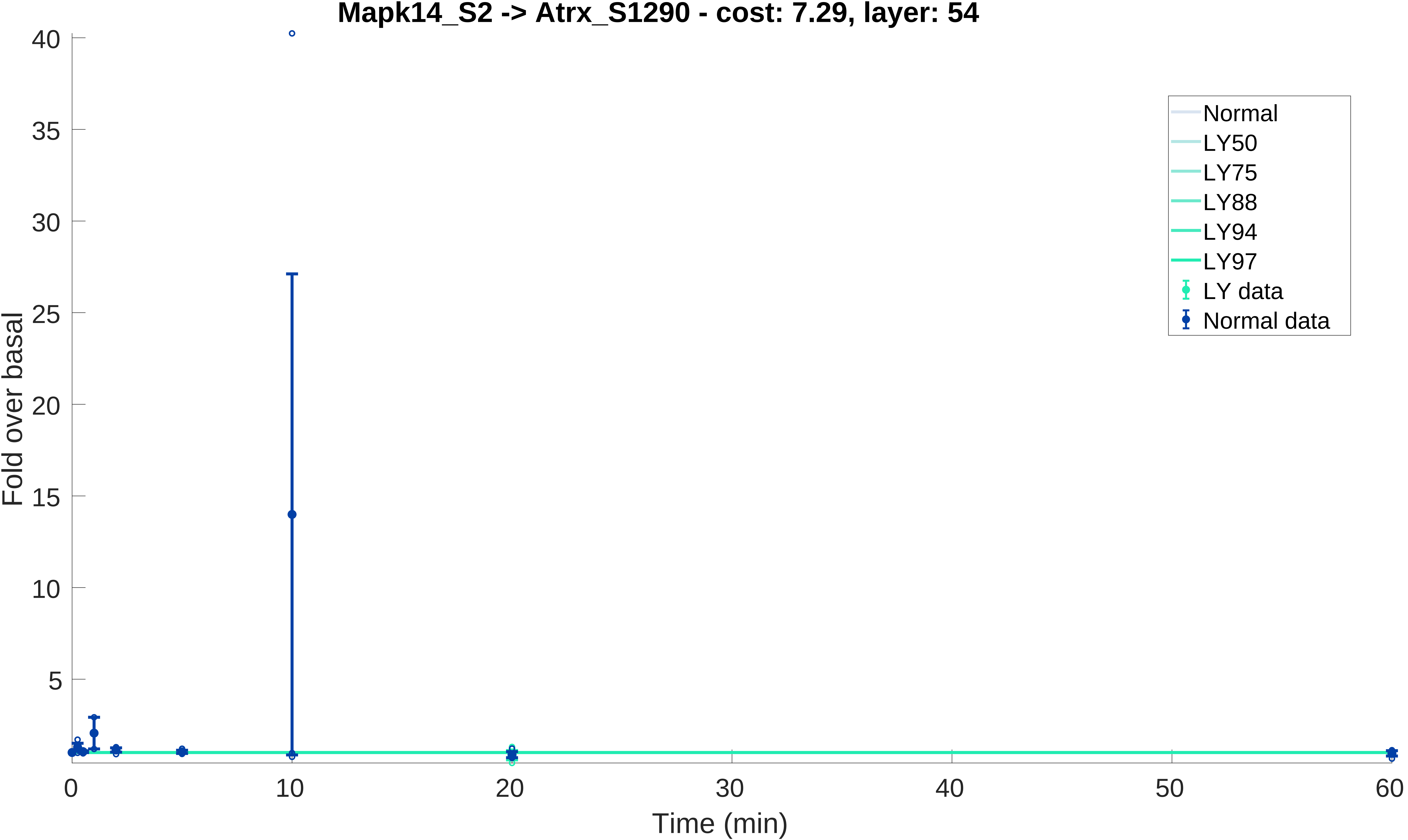

Mapk14_S2 -> Atrx_S1290 - cost: 7.29, layer: 54
Fold over basal
40
35
30
25
20
15
10
5
0
10
20
30
40
50
60
Time (min)
Normal
LY50
LY75
LY88
LY94
LY97
LY data
Normal data

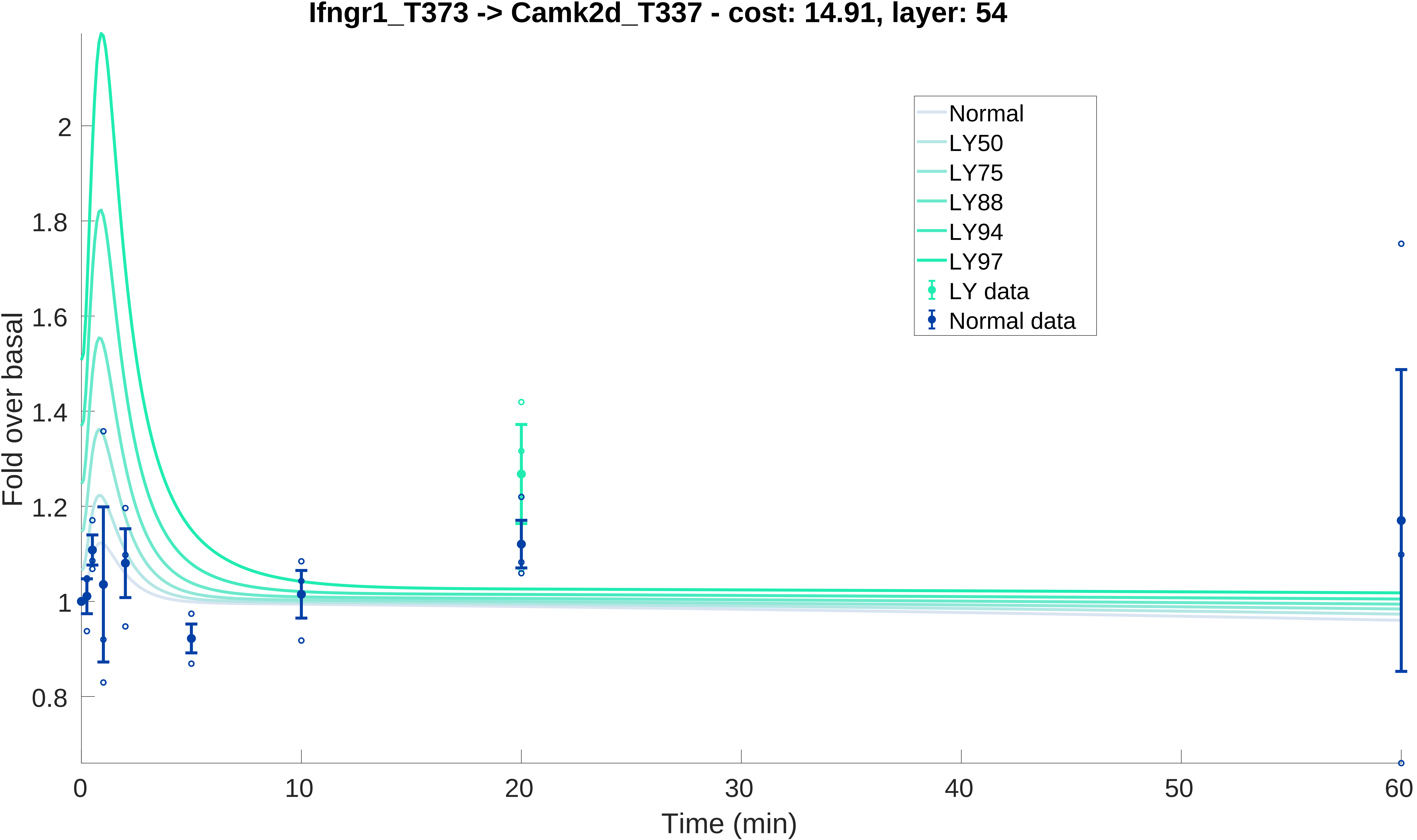

Ifngr1_T373 -> Camk2d_T337 - cost: 14.91, layer: 54
Fold over basal
Time (min)
0.8
1
1.2
1.4
1.6
1.8
2
0
10
20
30
40
50
60
Normal
LY50
LY75
LY88
LY94
LY97
LY data
Normal data

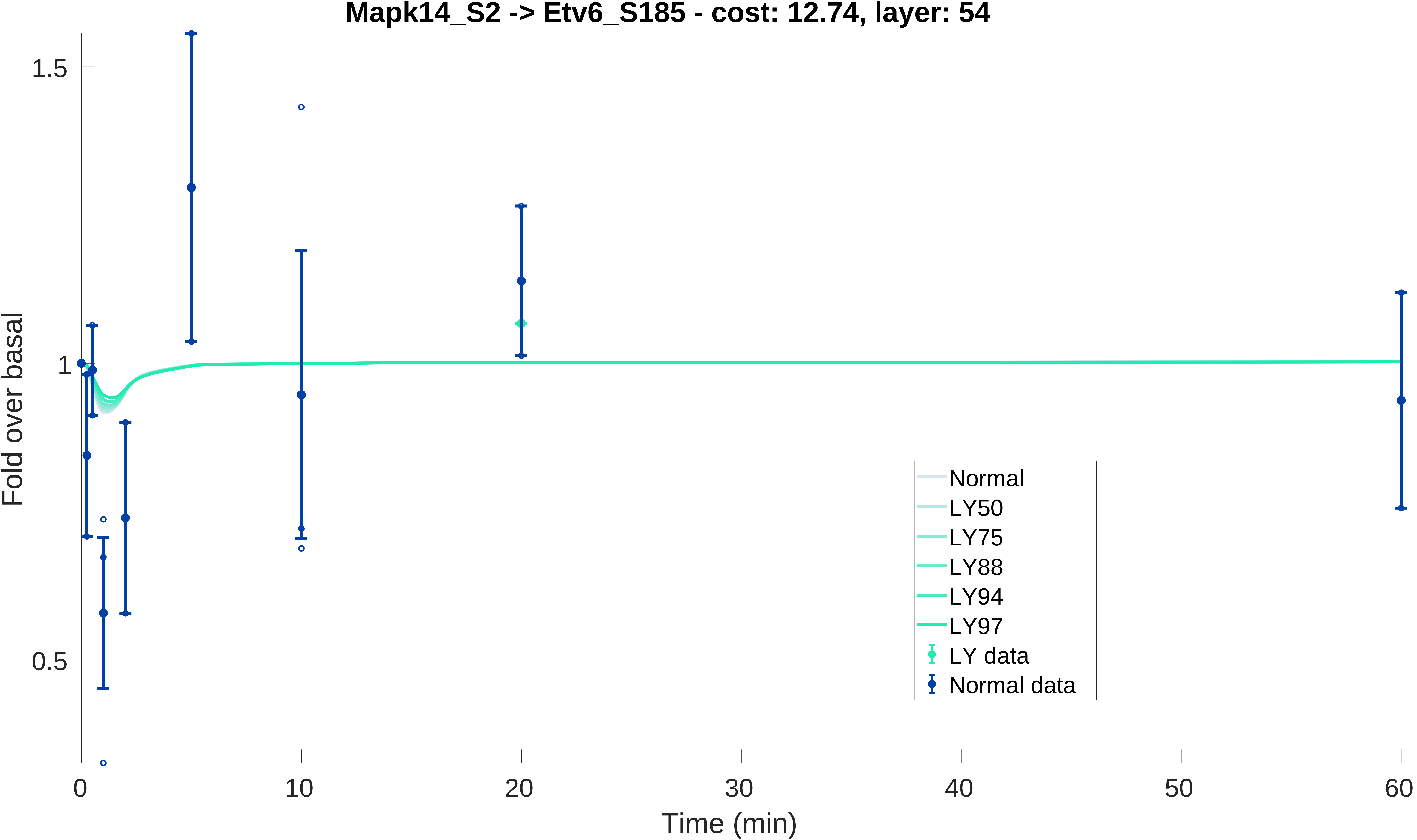

Mapk14_S2 -> Etv6_S185 - cost: 12.74, layer: 54
Fold over basal
1.5
1
0.5
0
10
20
30
40
50
60
Time (min)
Normal
LY50
LY75
LY88
LY94
LY97
LY data
Normal data

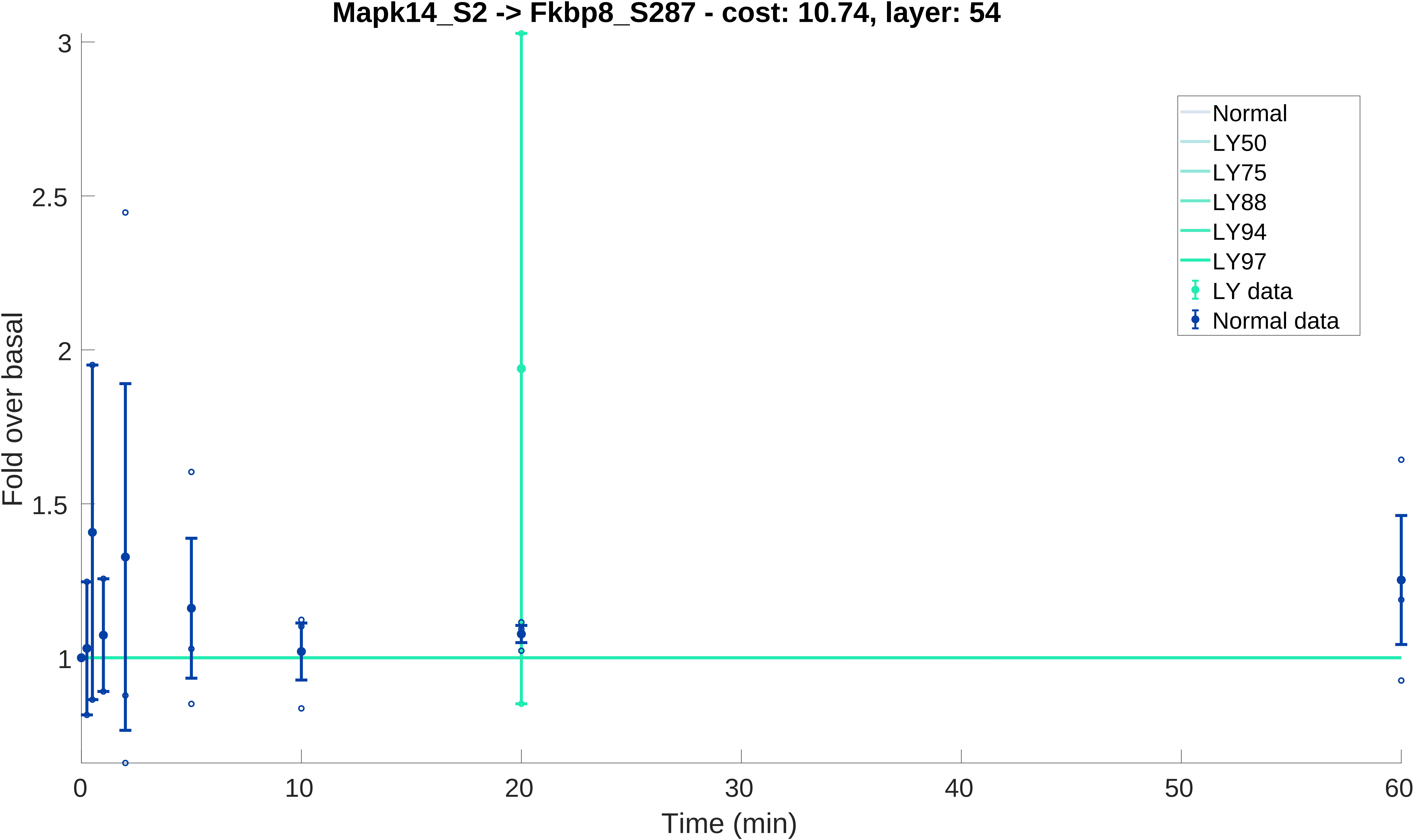
Mapk14_S2 -> Fkbp8_S287 - cost: 10.74, layer: 54
Fold over basal
Time (min)
0
10
20
30
40
50
60
1
1.5
2
2.5
3
Normal
LY50
LY75
LY88
LY94
LY97
LY data
Normal data

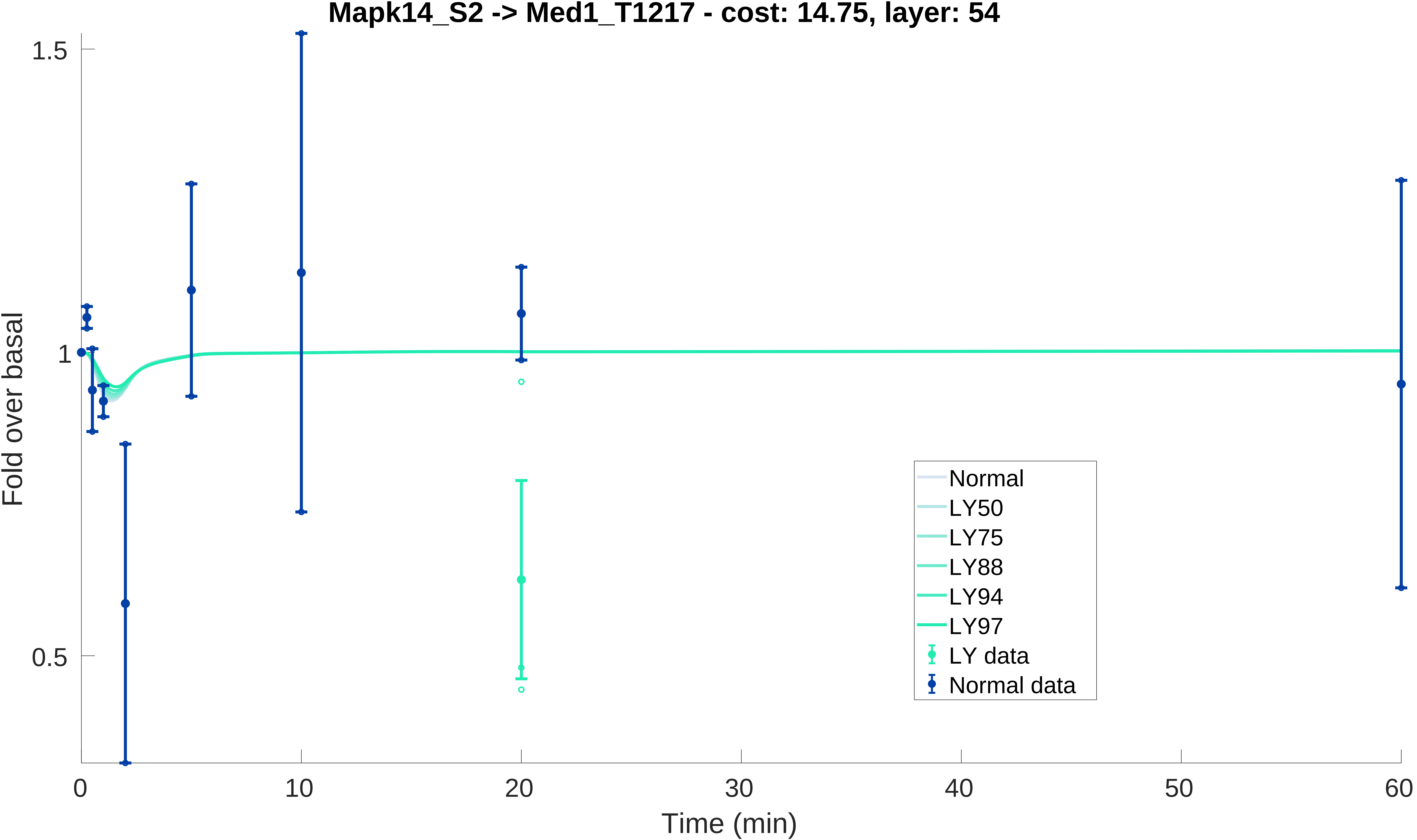

Mapk14_S2 -> Med1_T1217 - cost: 14.75, layer: 54
Fold over basal
1.5
1
0.5
0
10
20
30
40
50
60
Time (min)
Normal
LY50
LY75
LY88
LY94
LY97
LY data
Normal data

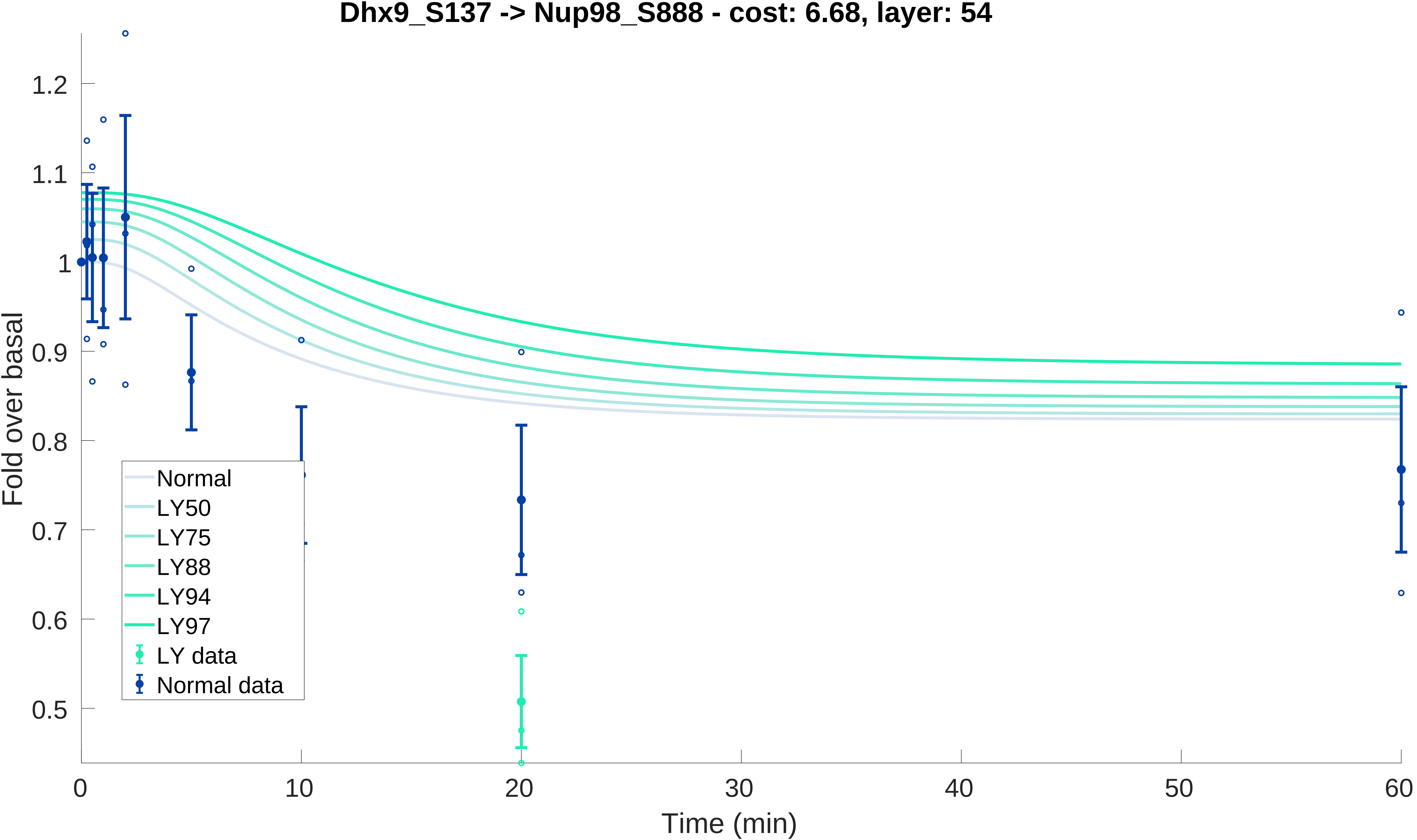

Dhx9_S137 -> Nup98_S888 - cost: 6.68, layer: 54
Fold over basal
Time (min)
1.2
1.1
1
0.9
0.8
0.7
0.6
0.5
0
10
20
30
40
50
60
Normal
LY50
LY75
LY88
LY94
LY97
LY data
Normal data

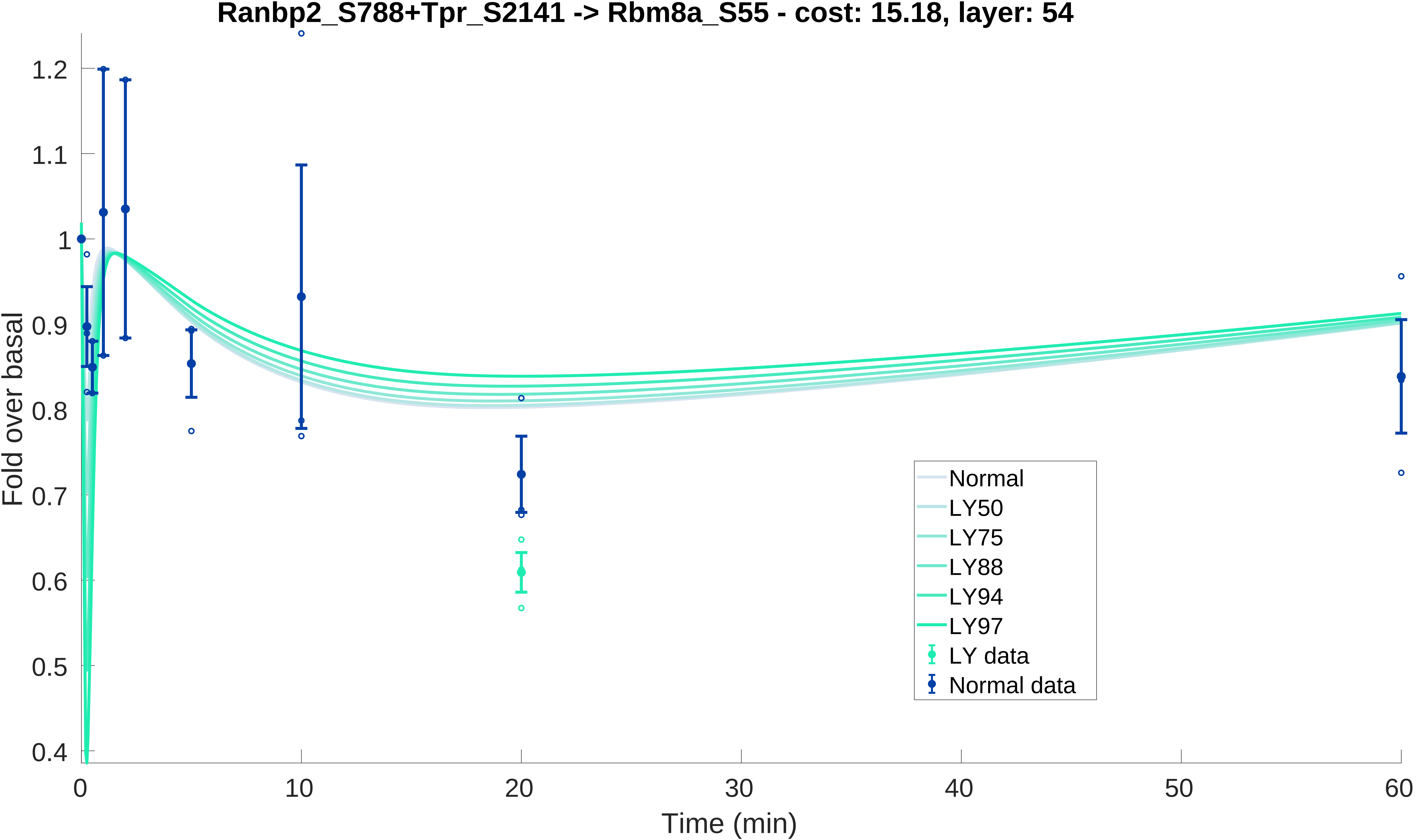

Ranbp2_S788+Tpr_S2141 -> Rbm8a_S55 - cost: 15.18, layer: 54
Fold over basal
Time (min)
0.4
0.5
0.6
0.7
0.8
0.9
1
1.1
1.2
0
10
20
30
40
50
60
Normal
LY50
LY75
LY88
LY94
LY97
LY data
Normal data

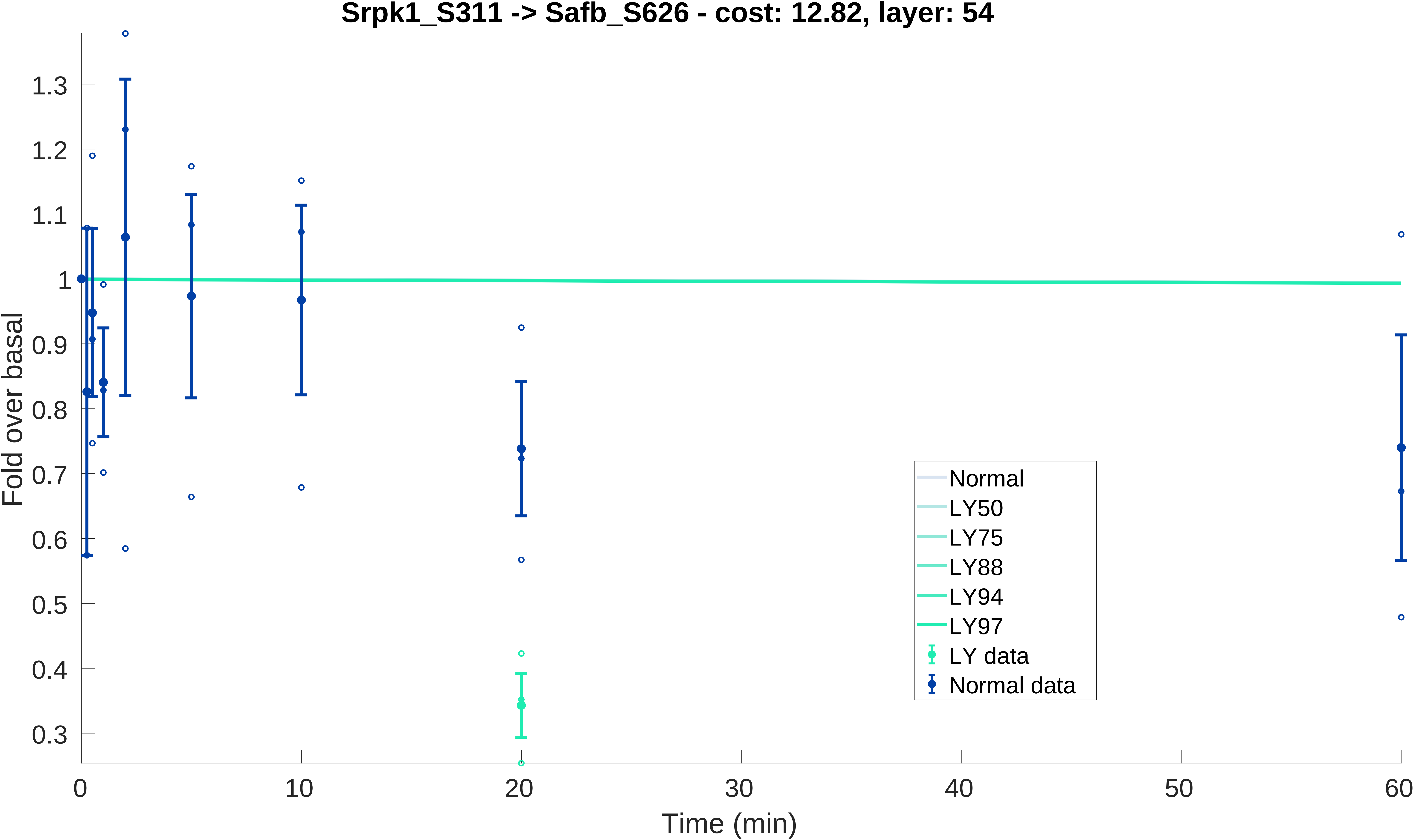

Srpk1_S311 -> Safb_S626 - cost: 12.82, layer: 54
Fold over basal
Time (min)
0
10
20
30
40
50
60
0.3
0.4
0.5
0.6
0.7
0.8
0.9
1
1.1
1.2
1.3
Normal
LY50
LY75
LY88
LY94
LY97
LY data
Normal data

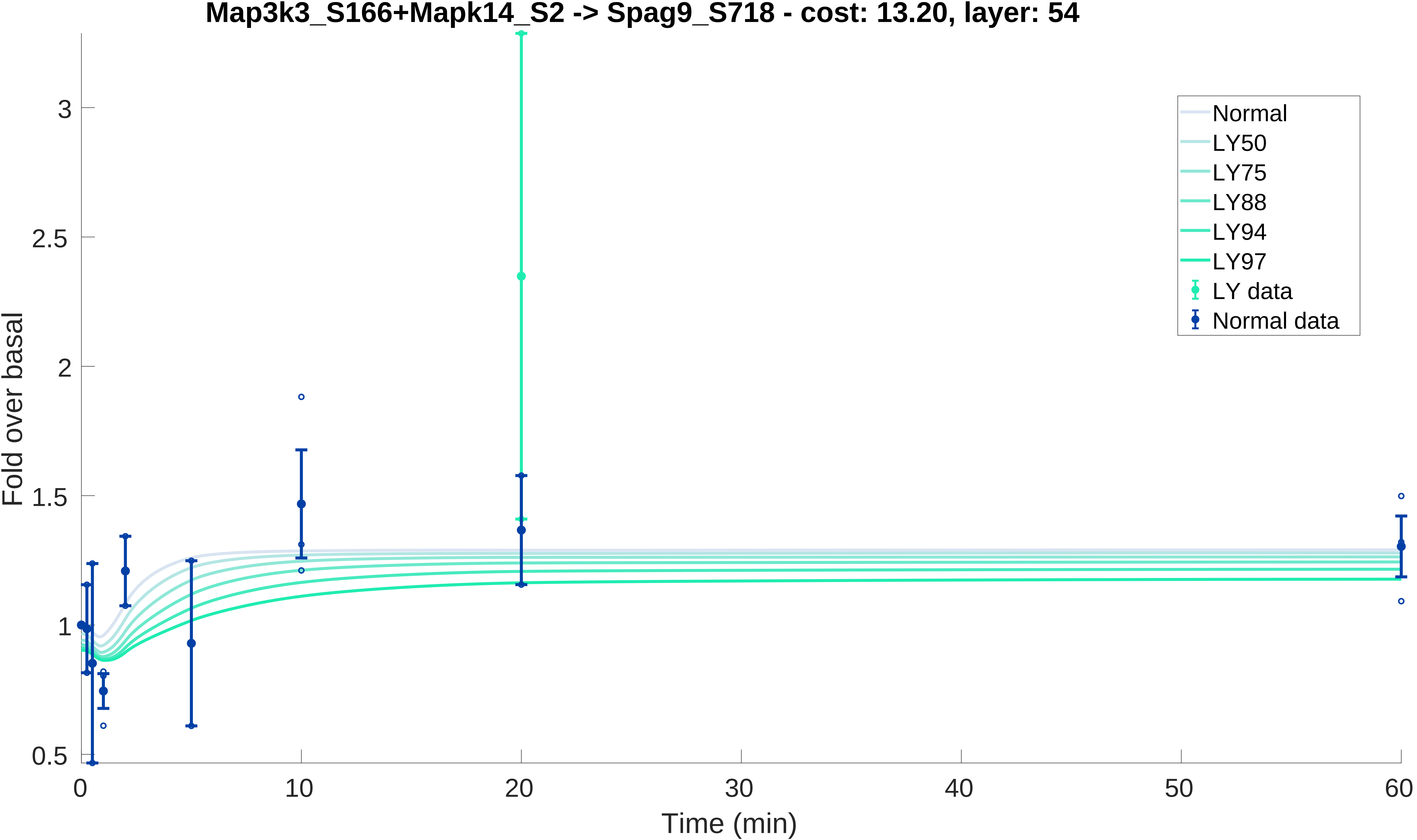
Map3k3_S166+Mapk14_S2 -> Spag9_S718 - cost: 13.20, layer: 54
Fold over basal
Time (min)
0.5
1
1.5
2
2.5
3
0
10
20
30
40
50
60
Normal
LY50
LY75
LY88
LY94
LY97
LY data
Normal data

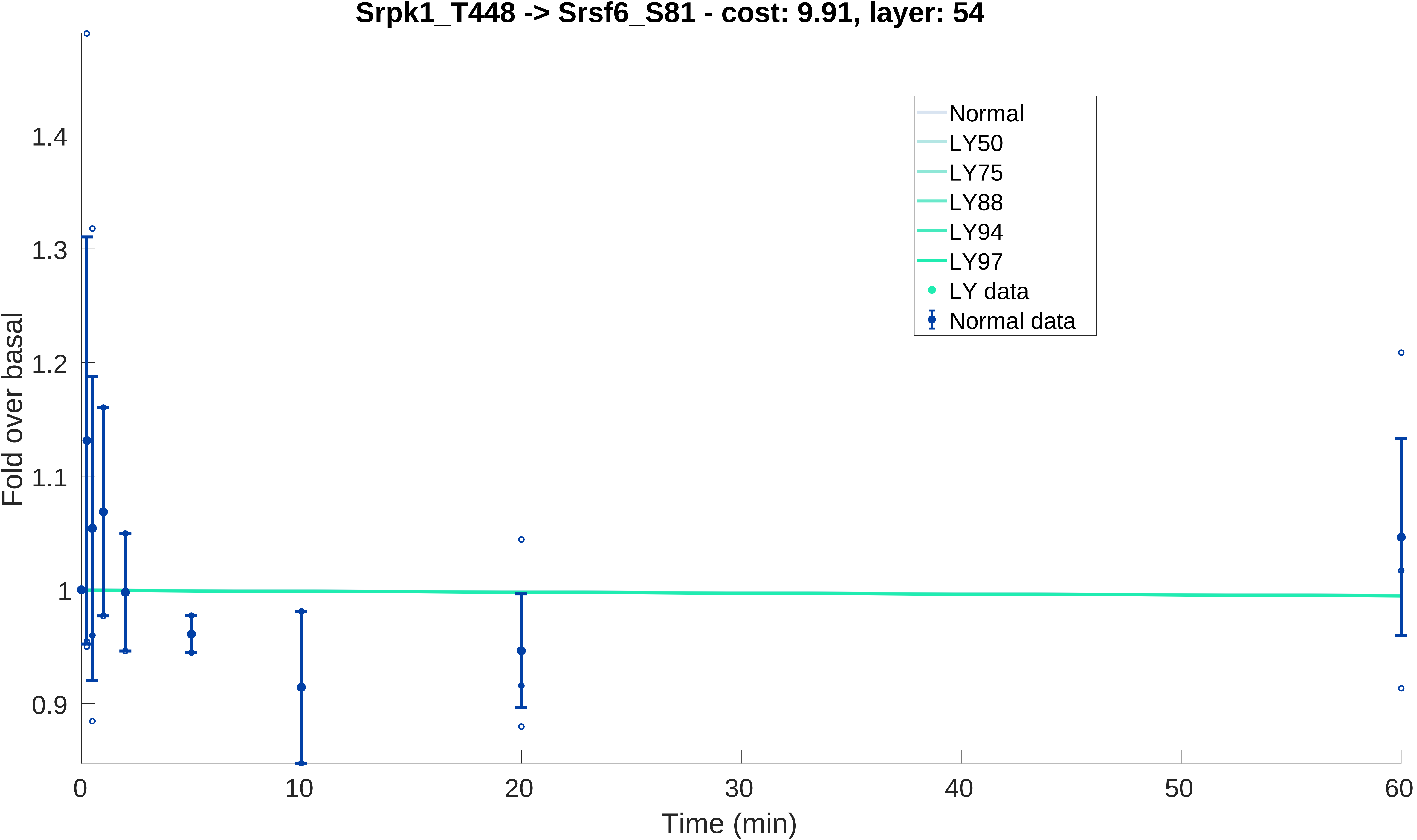

Srpk1_T448 -> Srsf6_S81 - cost: 9.91, layer: 54
Fold over basal
1.4
1.3
1.2
1.1
1
0.9
0
10
20
30
40
50
60
Time (min)
Normal
LY50
LY75
LY88
LY94
LY97
LY data
Normal data

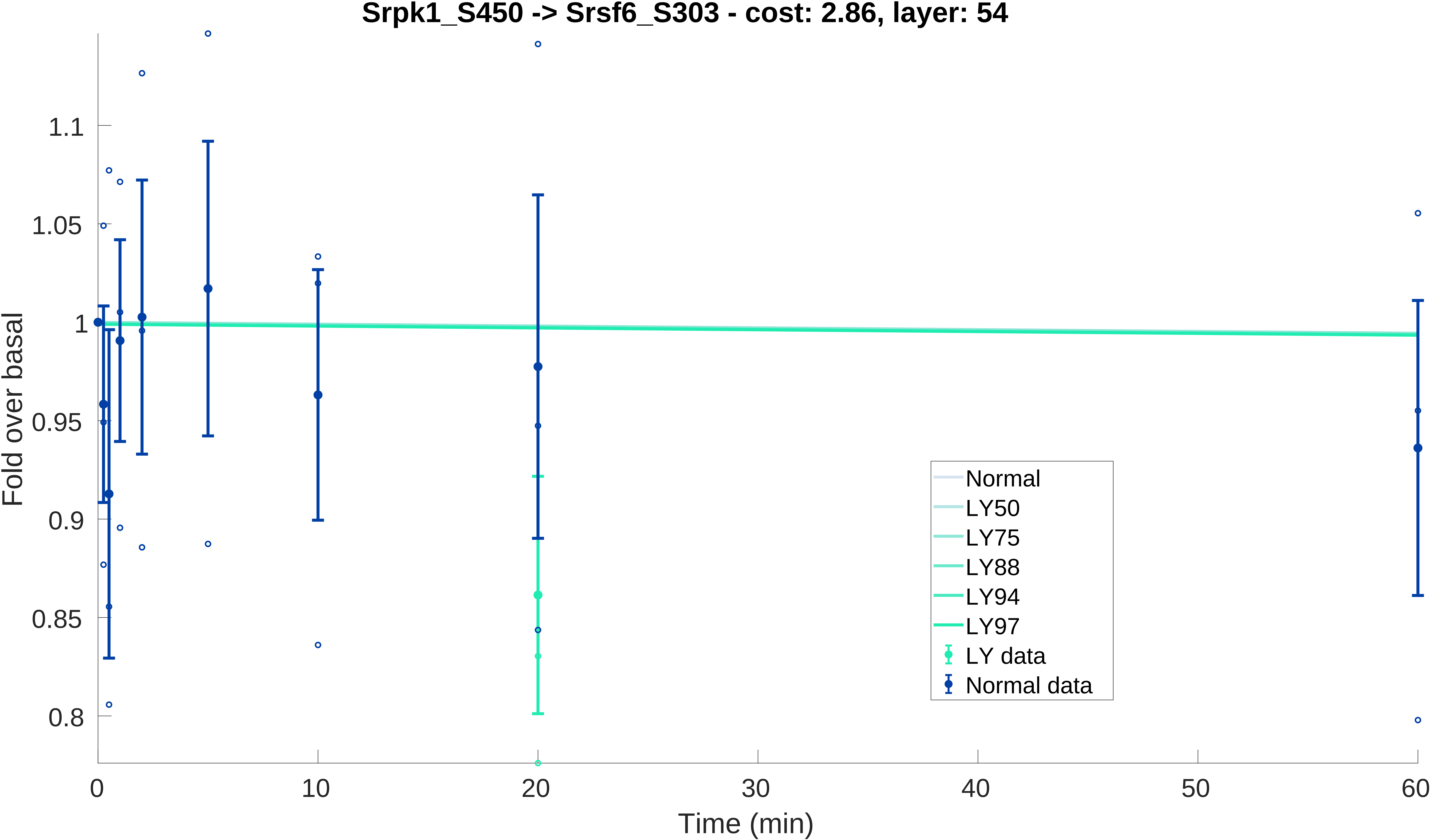
Srpk1_S450 -> Srsf6_S303 - cost: 2.86, layer: 54
Fold over basal
Time (min)
1.1
1.05
1
0.95
0.9
0.85
0.8
0
10
20
30
40
50
60
Normal
LY50
LY75
LY88
LY94
LY97
LY data
Normal data

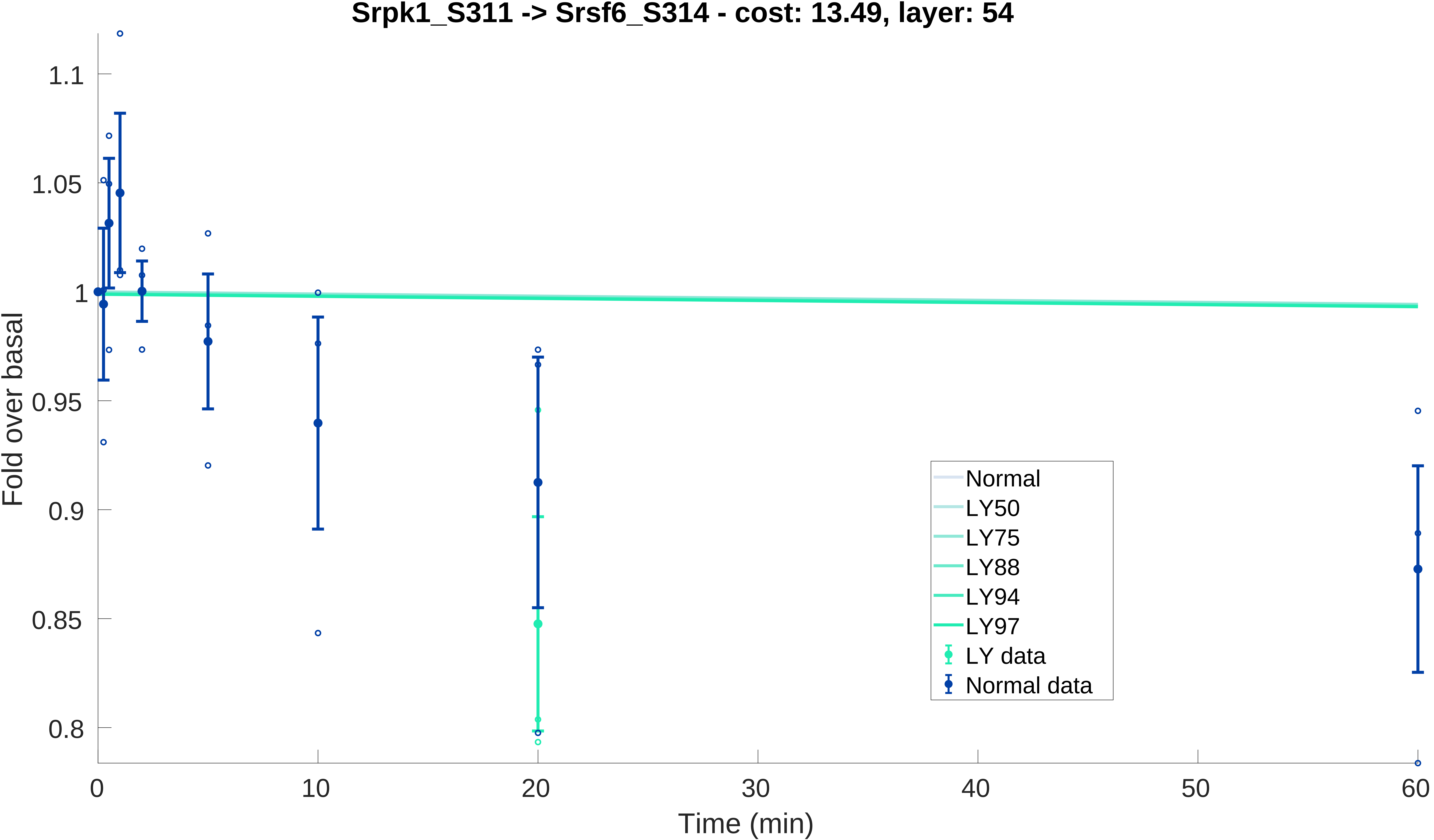

Srpk1_S311 -> Srsf6_S314 - cost: 13.49, layer: 54
Fold over basal
1.1
1.05
1
0.95
0.9
0.85
0.8
0
10
20
30
40
50
60
Time (min)
Normal
LY50
LY75
LY88
LY94
LY97
LY data
Normal data

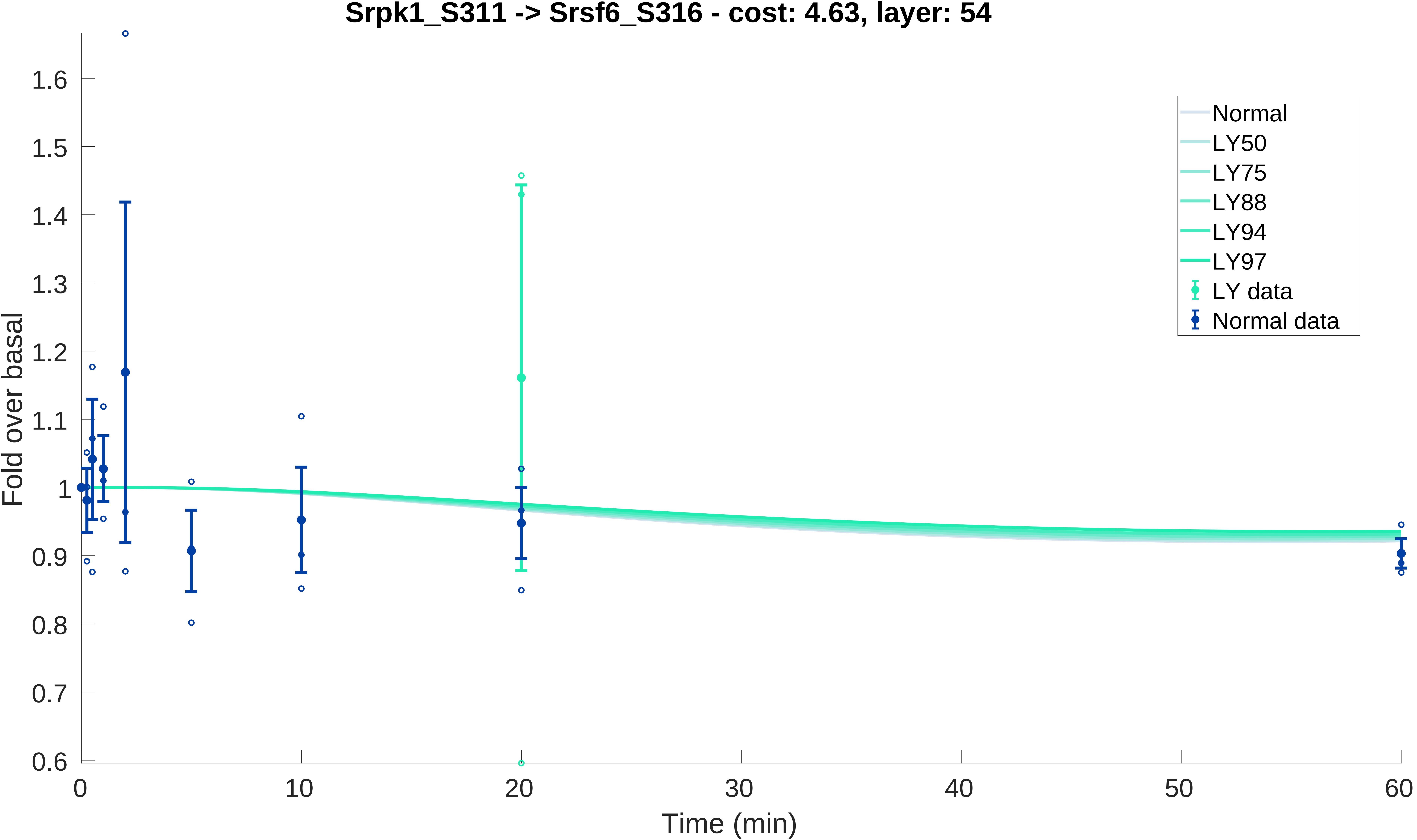

Srpk1_S311 -> Srsf6_S316 - cost: 4.63, layer: 54
Fold over basal
1.6
1.5
1.4
1.3
1.2
1.1
1
0.9
0.8
0.7
0.6
0
10
20
30
40
50
60
Time (min)
Normal
LY50
LY75
LY88
LY94
LY97
LY data
Normal data

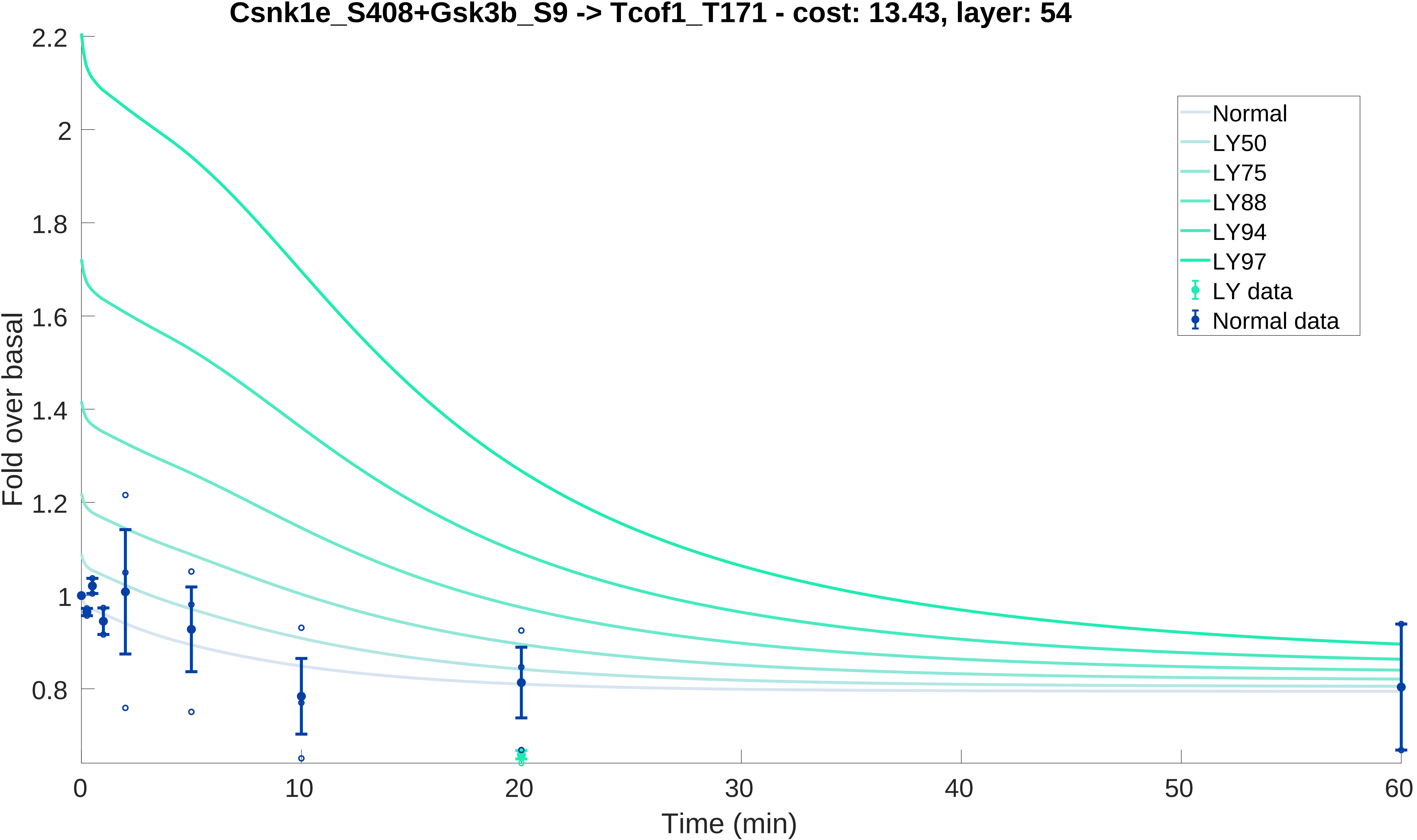

Csnk1e_S408+Gsk3b_S9 -> Tcof1_T171 - cost: 13.43, layer: 54
Fold over basal
Time (min)
2.2
2
1.8
1.6
1.4
1.2
1
0.8
0
10
20
30
40
50
60
Normal
LY50
LY75
LY88
LY94
LY97
LY data
Normal data

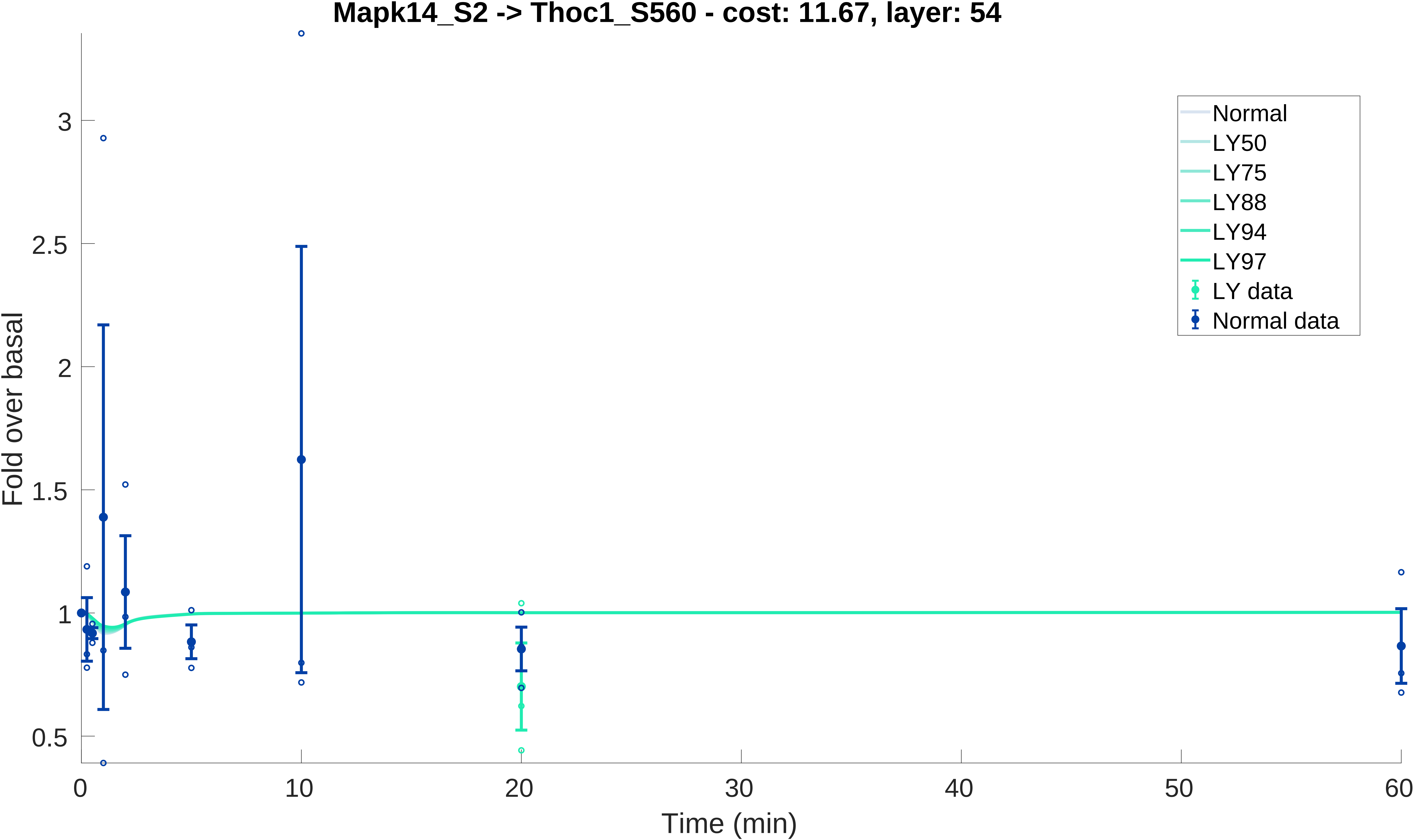

Mapk14_S2 -> Thoc1_S560 - cost: 11.67, layer: 54
Normal
LY50
LY75
LY88
LY94
LY97
LY data
Normal data
Fold over basal
Time (min)
0.5
1
1.5
2
2.5
3
0
10
20
30
40
50
60

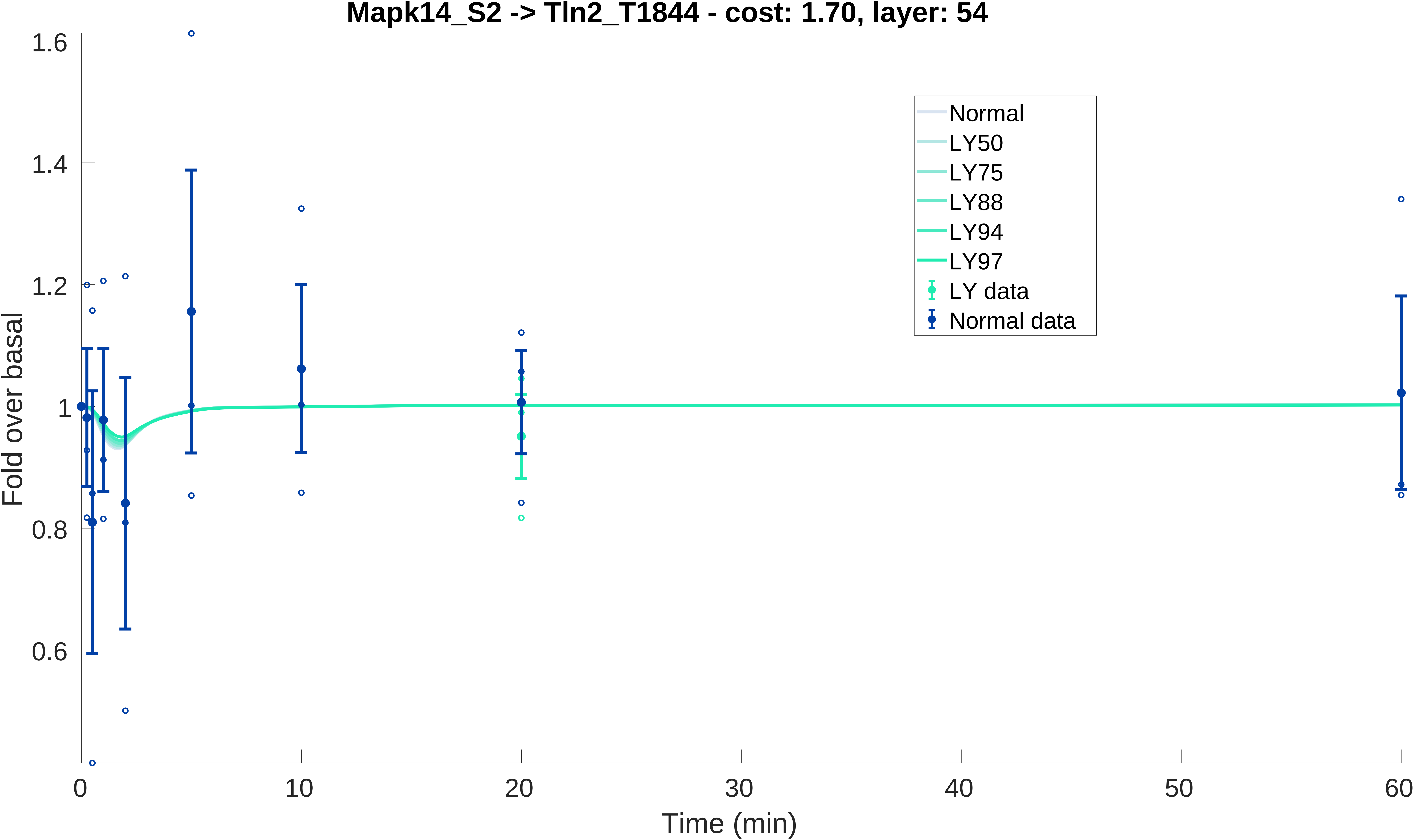

Mapk14_S2 -> Tln2_T1844 - cost: 1.70, layer: 54
Fold over basal
Time (min)
1.6
1.4
1.2
1
0.8
0.6
0
10
20
30
40
50
60
Normal
LY50
LY75
LY88
LY94
LY97
LY data
Normal data

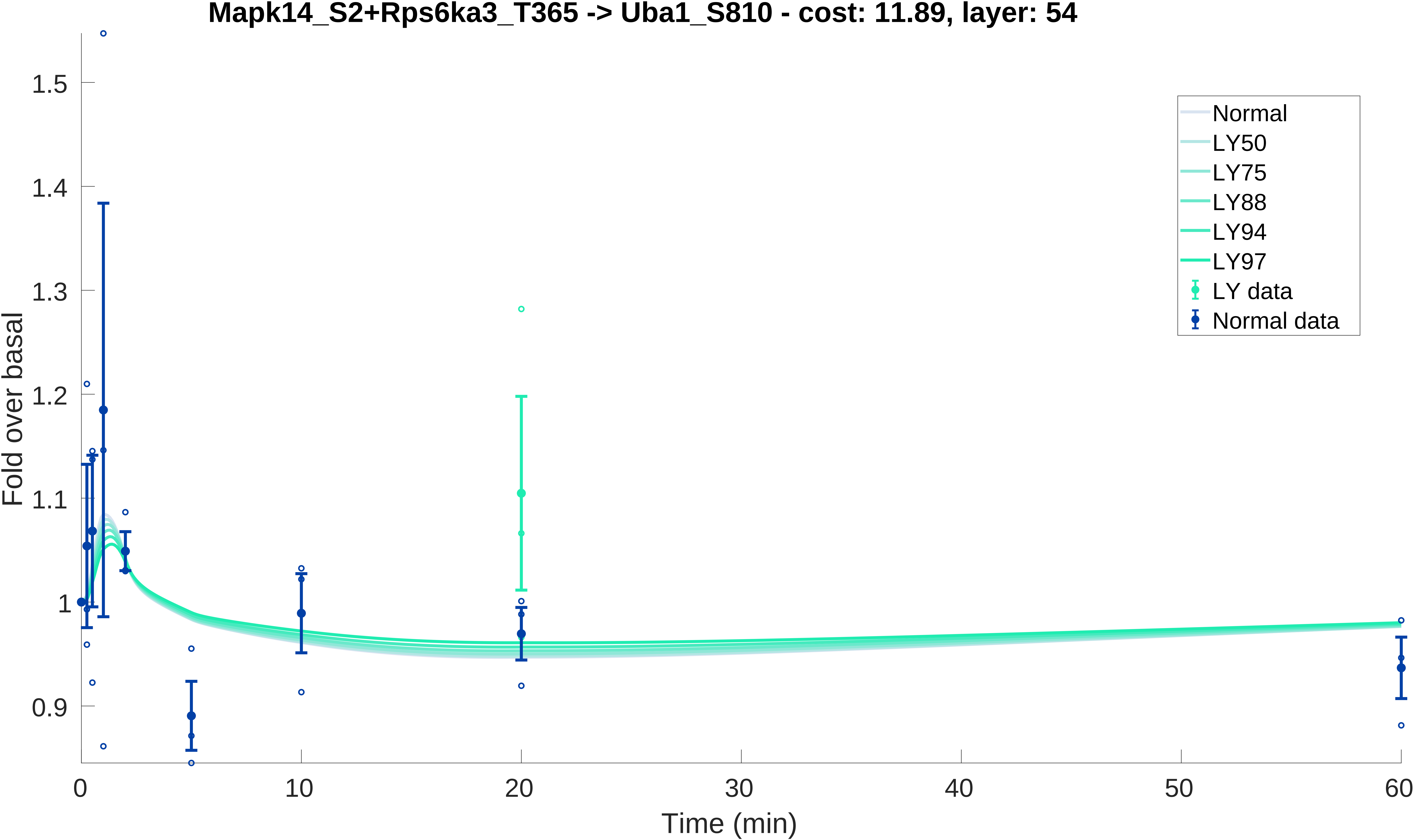

Mapk14_S2+Rps6ka3_T365 -> Uba1_S810 - cost: 11.89, layer: 54
Fold over basal
Time (min)
Normal
LY50
LY75
LY88
LY94
LY97
LY data
Normal data
0.9
1
1.1
1.2
1.3
1.4
1.5
0
10
20
30
40
50
60

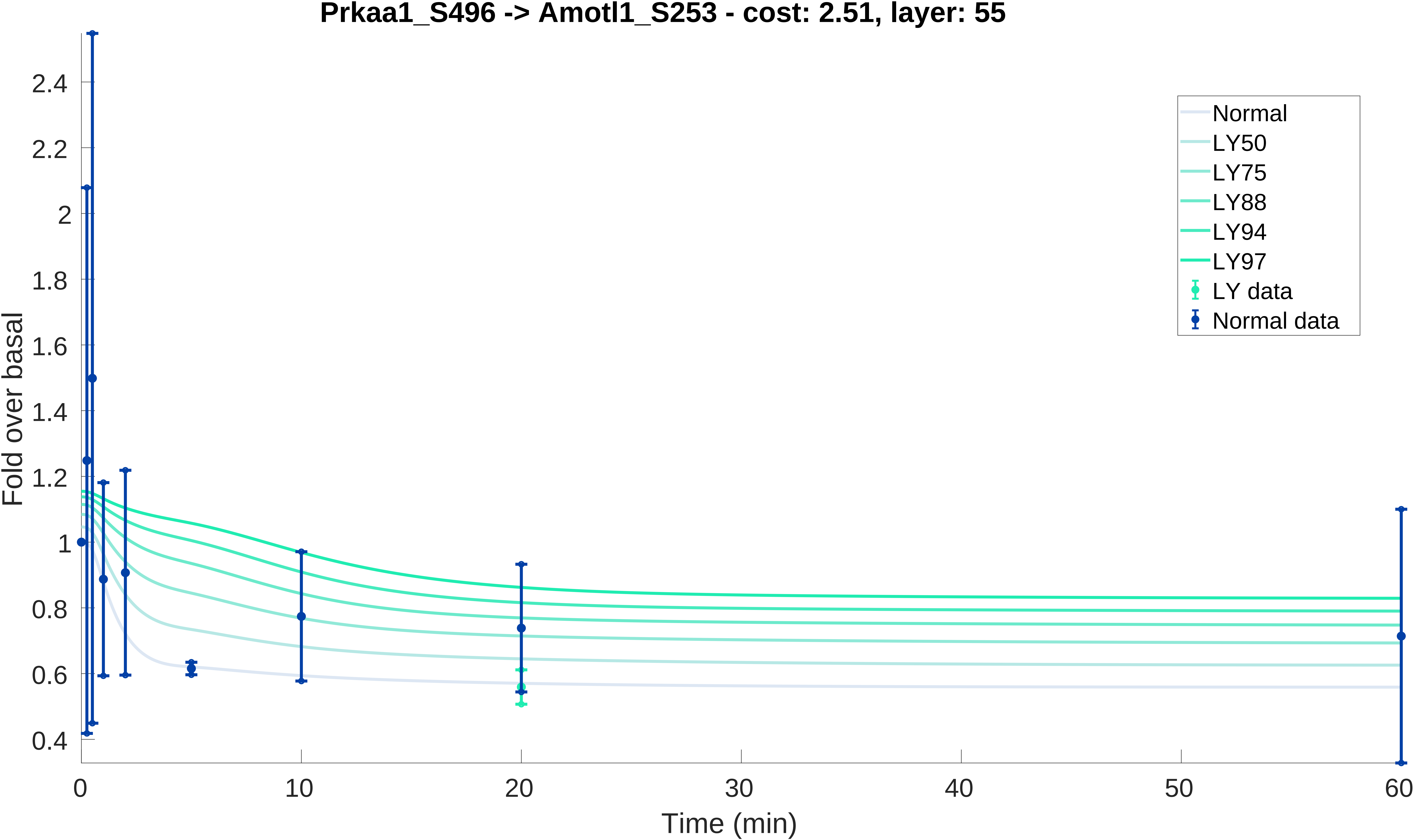

Prkaa1_S496 -> Amotl1_S253 - cost: 2.51, layer: 55
Fold over basal
2.4
2.2
2
1.8
1.6
1.4
1.2
1
0.8
0.6
0.4
0
10
20
30
40
50
60
Time (min)
Normal
LY50
LY75
LY88
LY94
LY97
LY data
Normal data

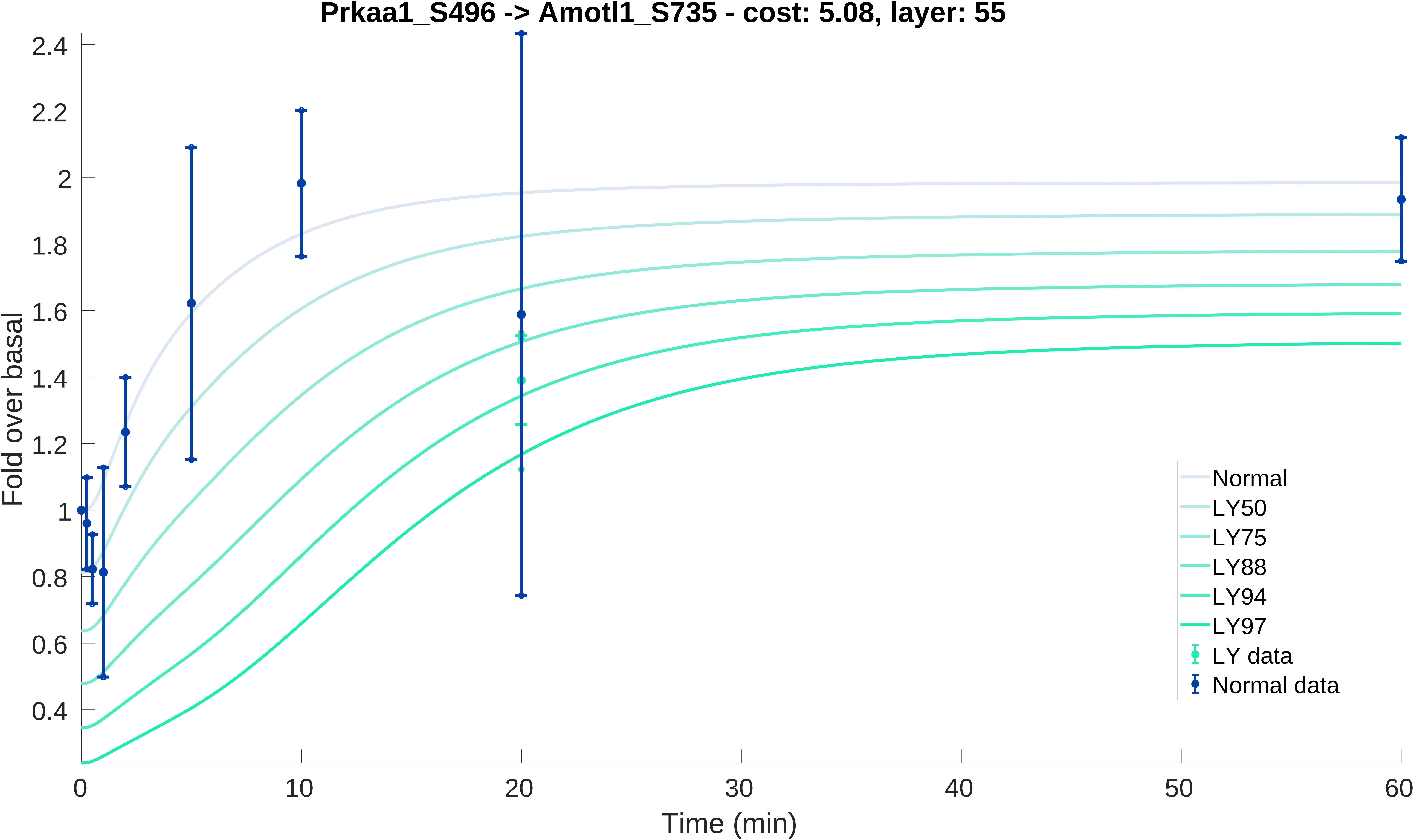

Prkaa1_S496 -> Amotl1_S735 - cost: 5.08, layer: 55
Fold over basal
2.4
2.2
2
1.8
1.6
1.4
1.2
1
0.8
0.6
0.4
0
10
20
30
40
50
60
Time (min)
Normal
LY50
LY75
LY88
LY94
LY97
LY data
Normal data

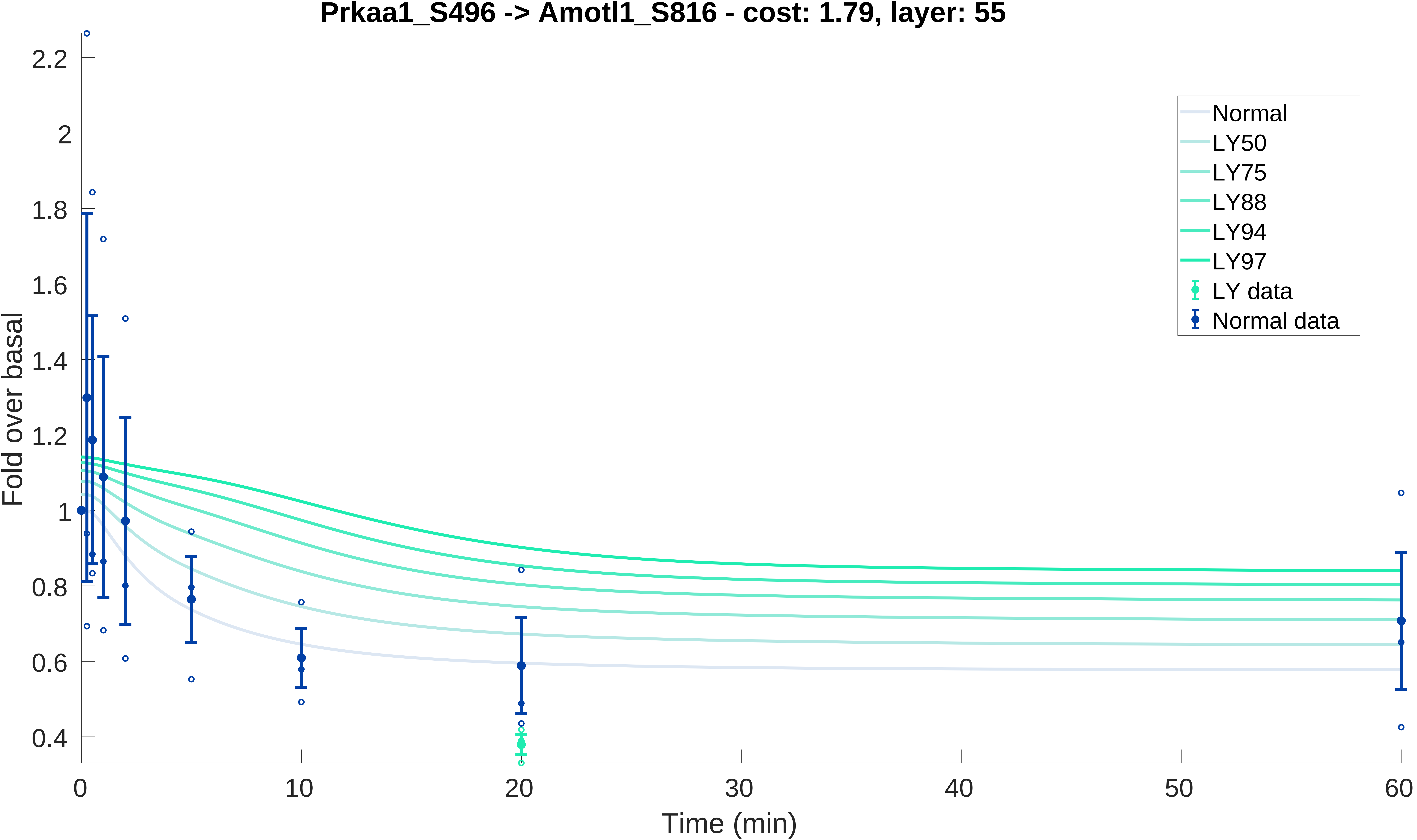

Prkaa1_S496 -> Amotl1_S816 - cost: 1.79, layer: 55
Fold over basal
Time (min)
Normal
LY50
LY75
LY88
LY94
LY97
LY data
Normal data

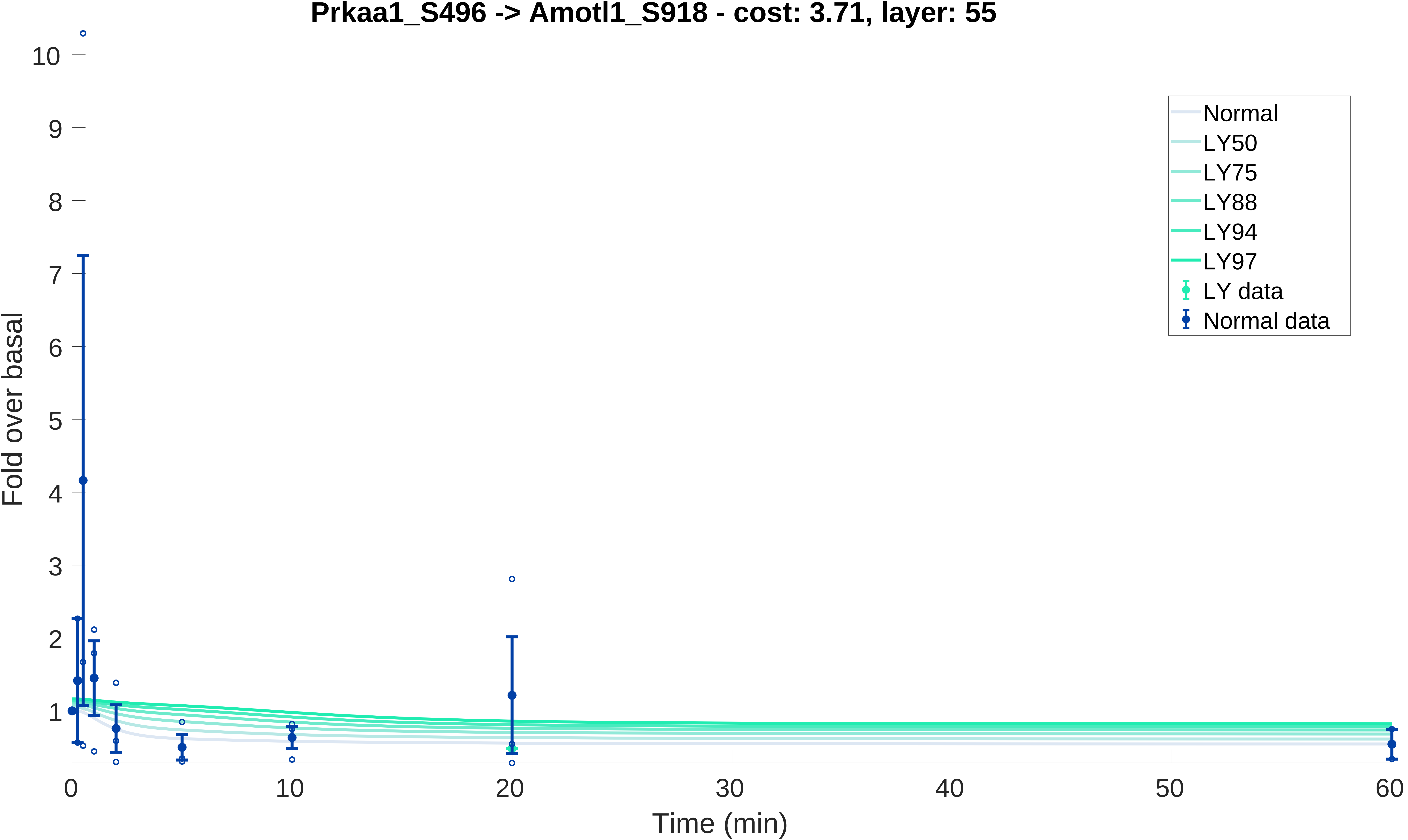

Prrkaa1_S496 -> Amotl1_S918 - cost: 3.71, layer: 55
Fold over basal
Time (min)
0
10
20
30
40
50
60
1
2
3
4
5
6
7
8
9
10
Normal
LY50
LY75
LY88
LY94
LY97
LY data
Normal data

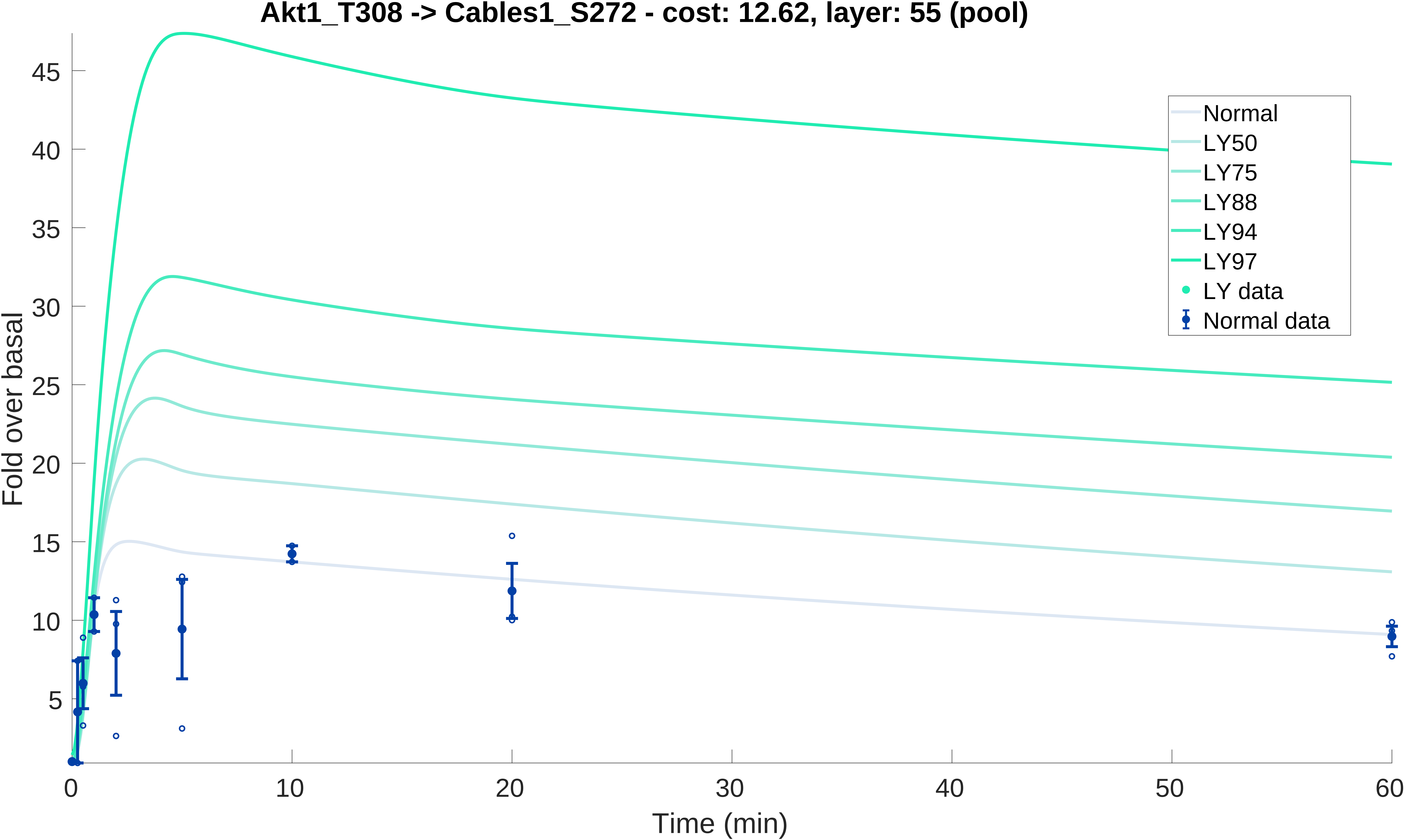

Akt1_T308 -> Cables1_S272 - cost: 12.62, layer: 55 (pool)
Fold over basal
Time (min)
45
40
35
30
25
20
15
10
5
0
10
20
30
40
50
60
Normal
LY50
LY75
LY88
LY94
LY97
LY data
Normal data

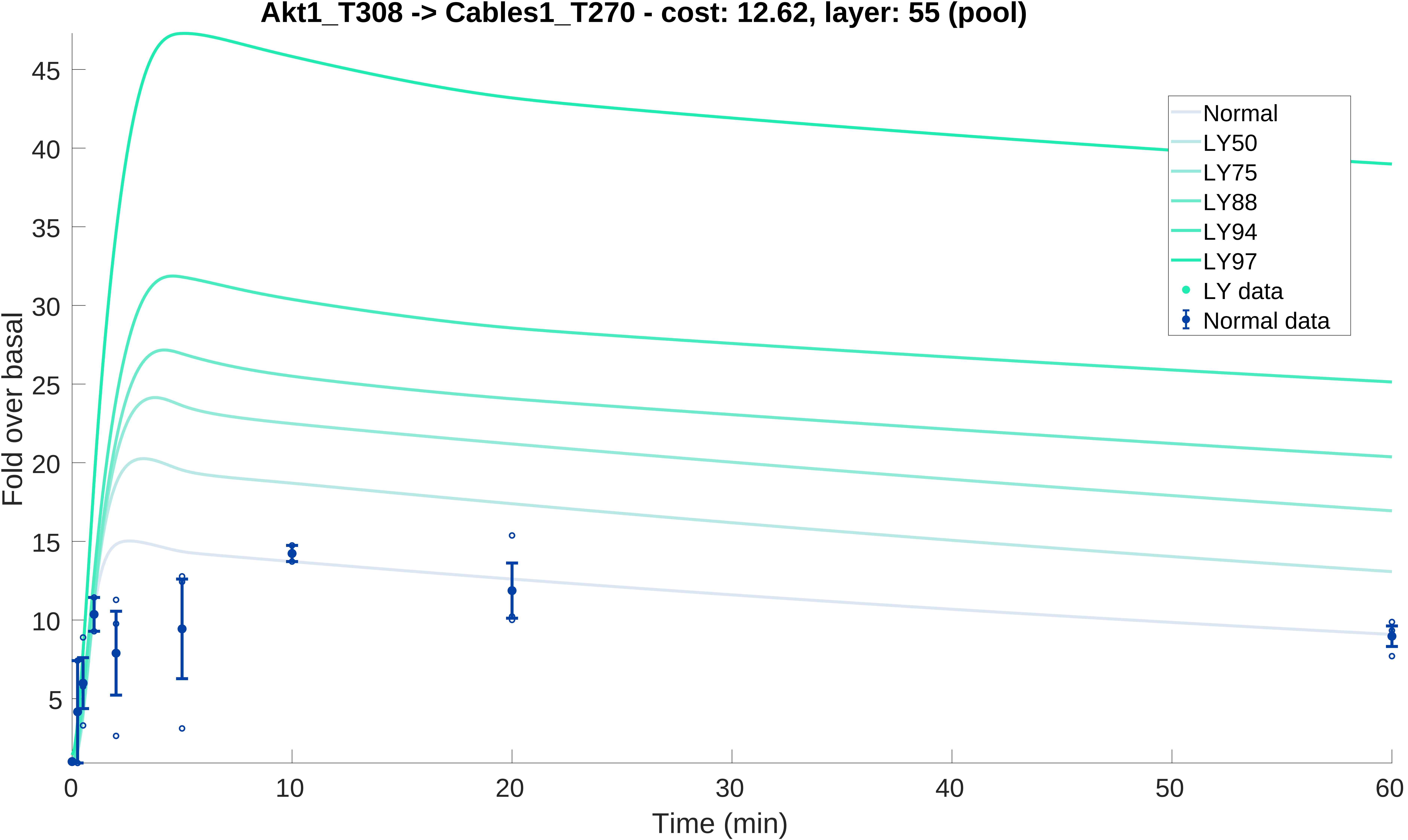

Akt1_T308 -> Cables1_T270 - cost: 12.62, layer: 55 (pool)
Fold over basal
Time (min)
Normal
LY50
LY75
LY88
LY94
LY97
LY data
Normal data
45
40
35
30
25
20
15
10
5
0
10
20
30
40
50
60

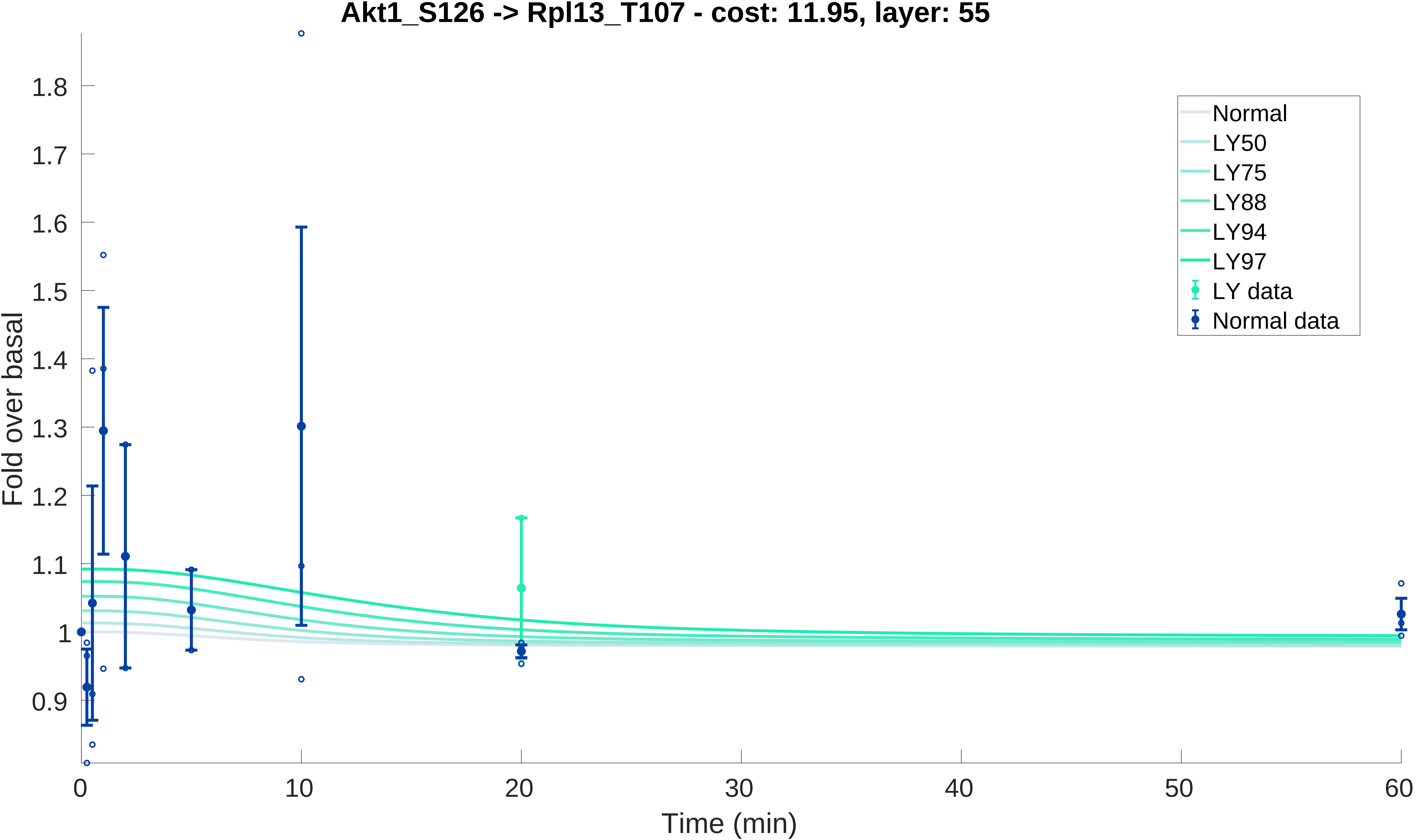

Akt1_S126 -> Rpl13_T107 - cost: 11.95, layer: 55
Fold over basal
Time (min)
0.9
1
1.1
1.2
1.3
1.4
1.5
1.6
1.7
1.8
0
10
20
30
40
50
60
Normal
LY50
LY75
LY88
LY94
LY97
LY data
Normal data

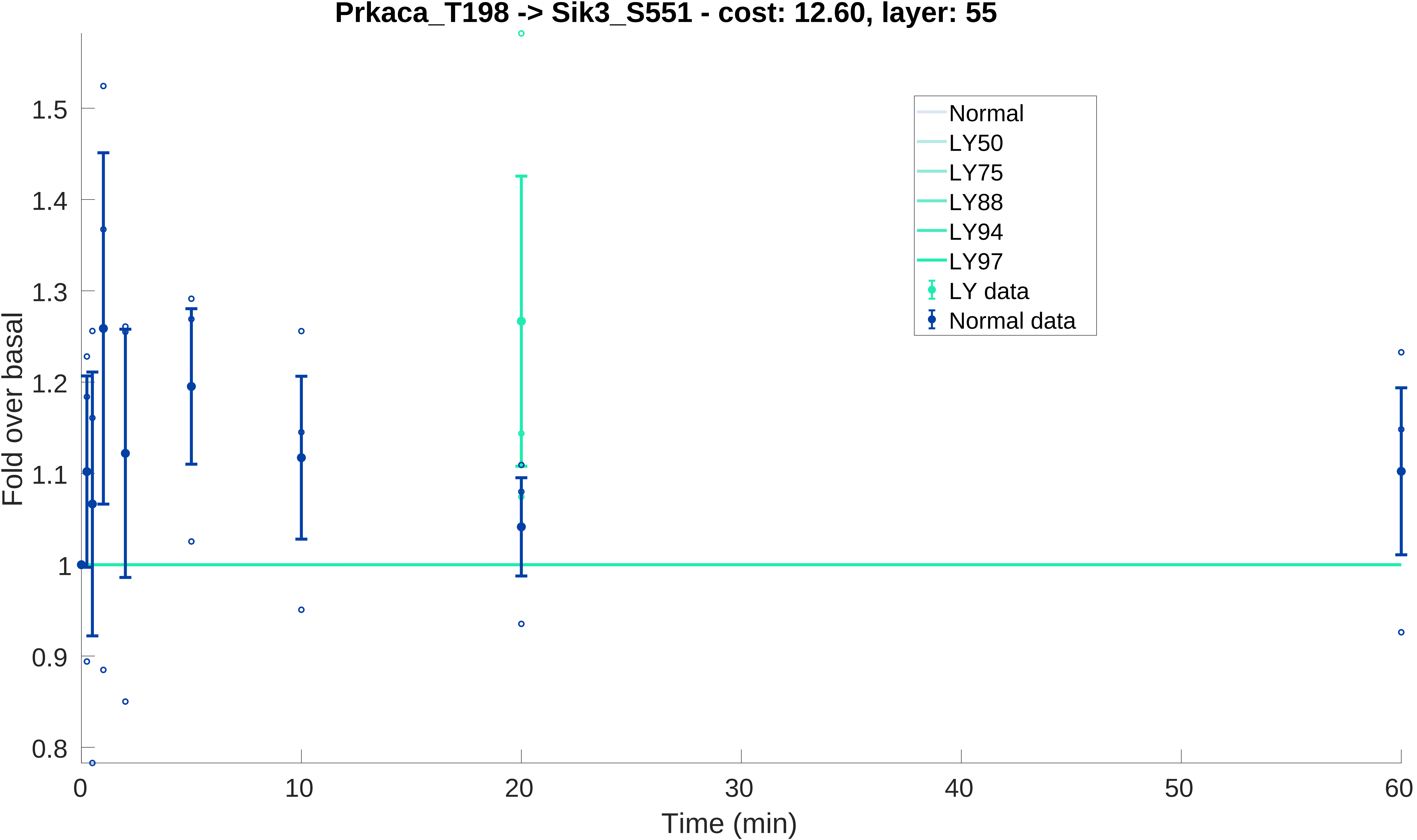

Prkaca_T198 -> Sik3_S551 - cost: 12.60, layer: 55
Fold over basal
Time (min)
0.8
0.9
1
1.1
1.2
1.3
1.4
1.5
0
10
20
30
40
50
60
Normal
LY50
LY75
LY88
LY94
LY97
LY data
Normal data

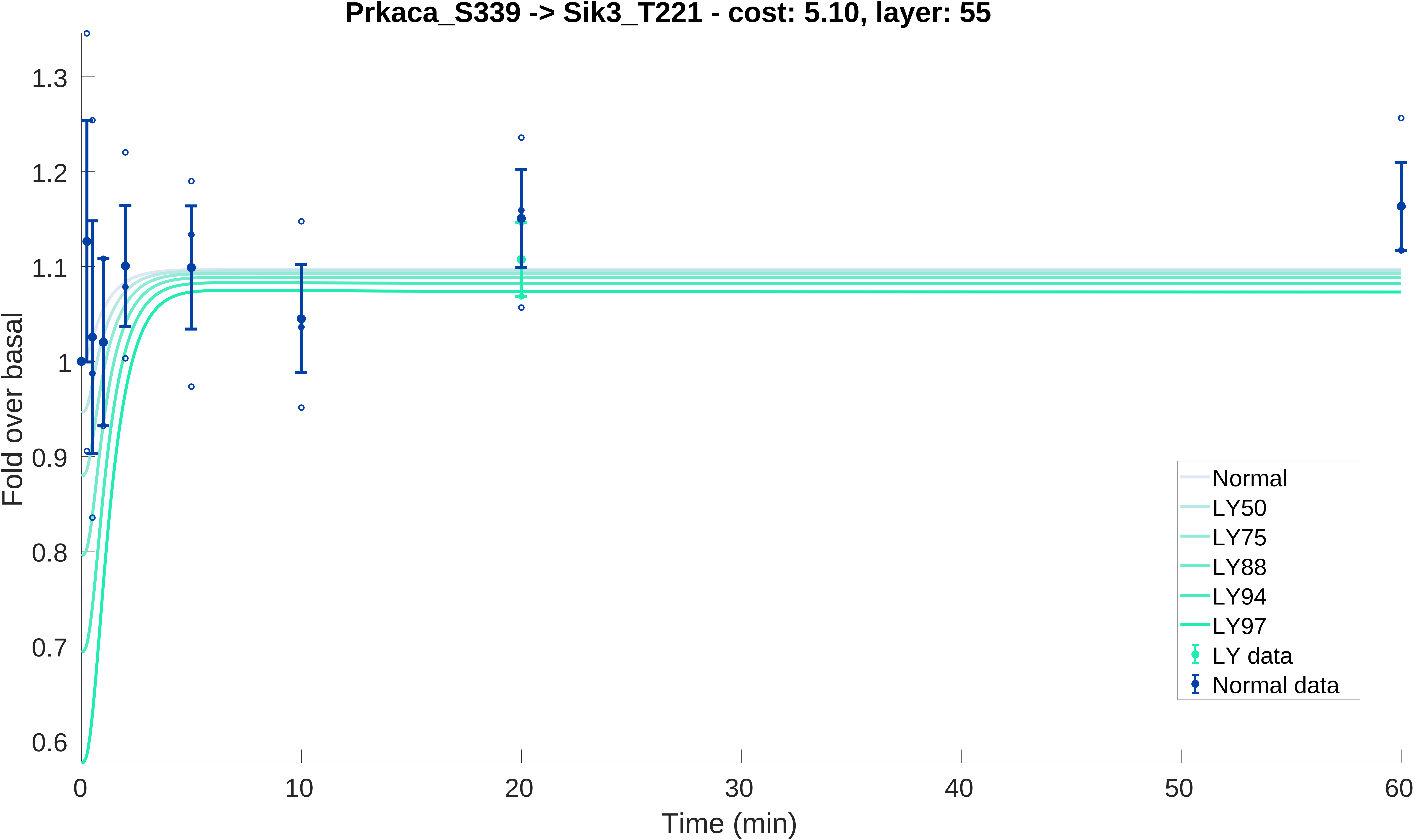
Prrkaca_S339 -> Sik3_T221 - cost: 5.10, layer: 55
Fold over basal
Time (min)
1.3
1.2
1.1
1
0.9
0.8
0.7
0.6
0
10
20
30
40
50
60
Normal
LY50
LY75
LY88
LY94
LY97
LY data
Normal data

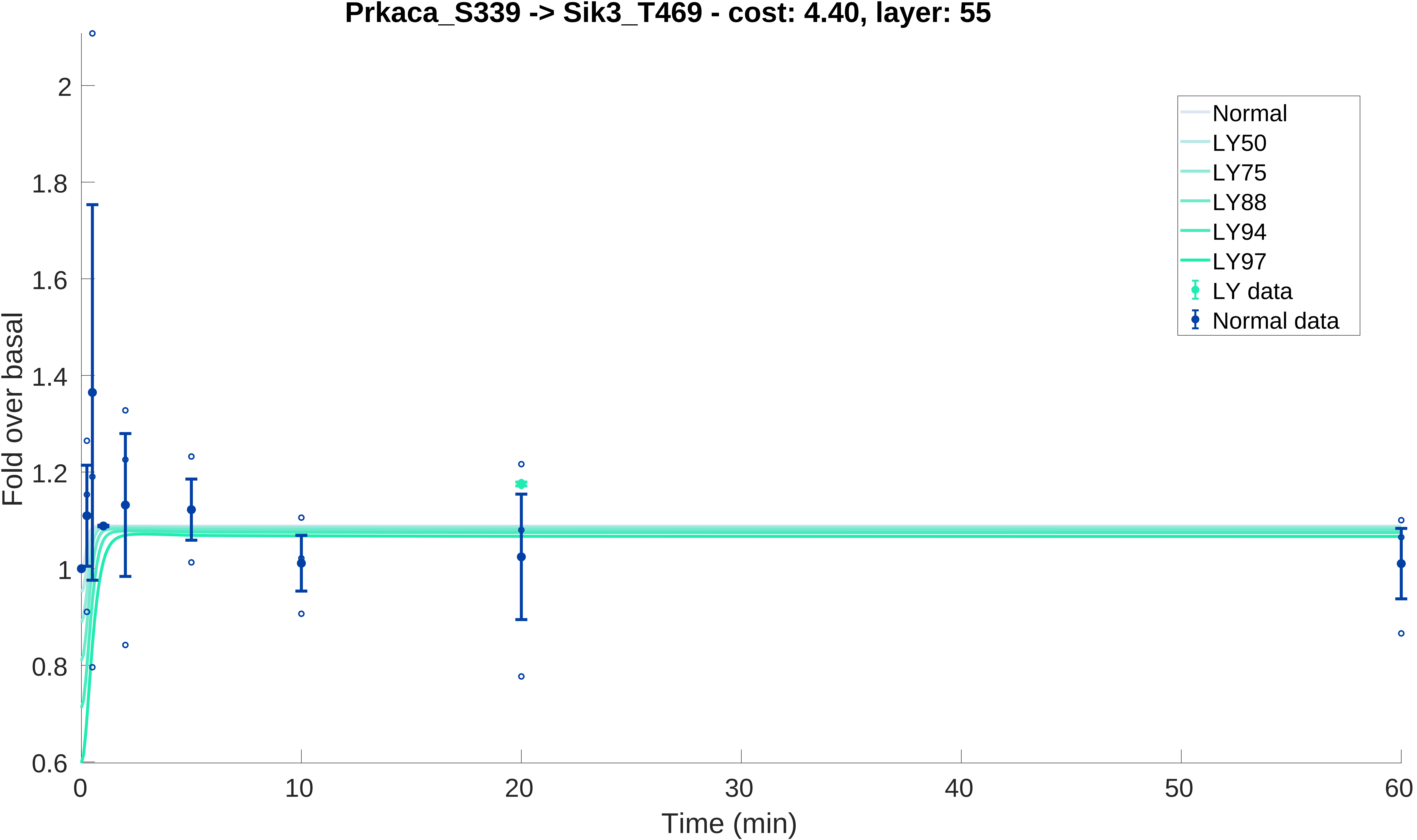
Prkaca_S339 -> Sik3_T469 - cost: 4.40, layer: 55
Fold over basal
Time (min)
0.6
0.8
1
1.2
1.4
1.6
1.8
2
0
10
20
30
40
50
60
Normal
LY50
LY75
LY88
LY94
LY97
LY data
Normal data

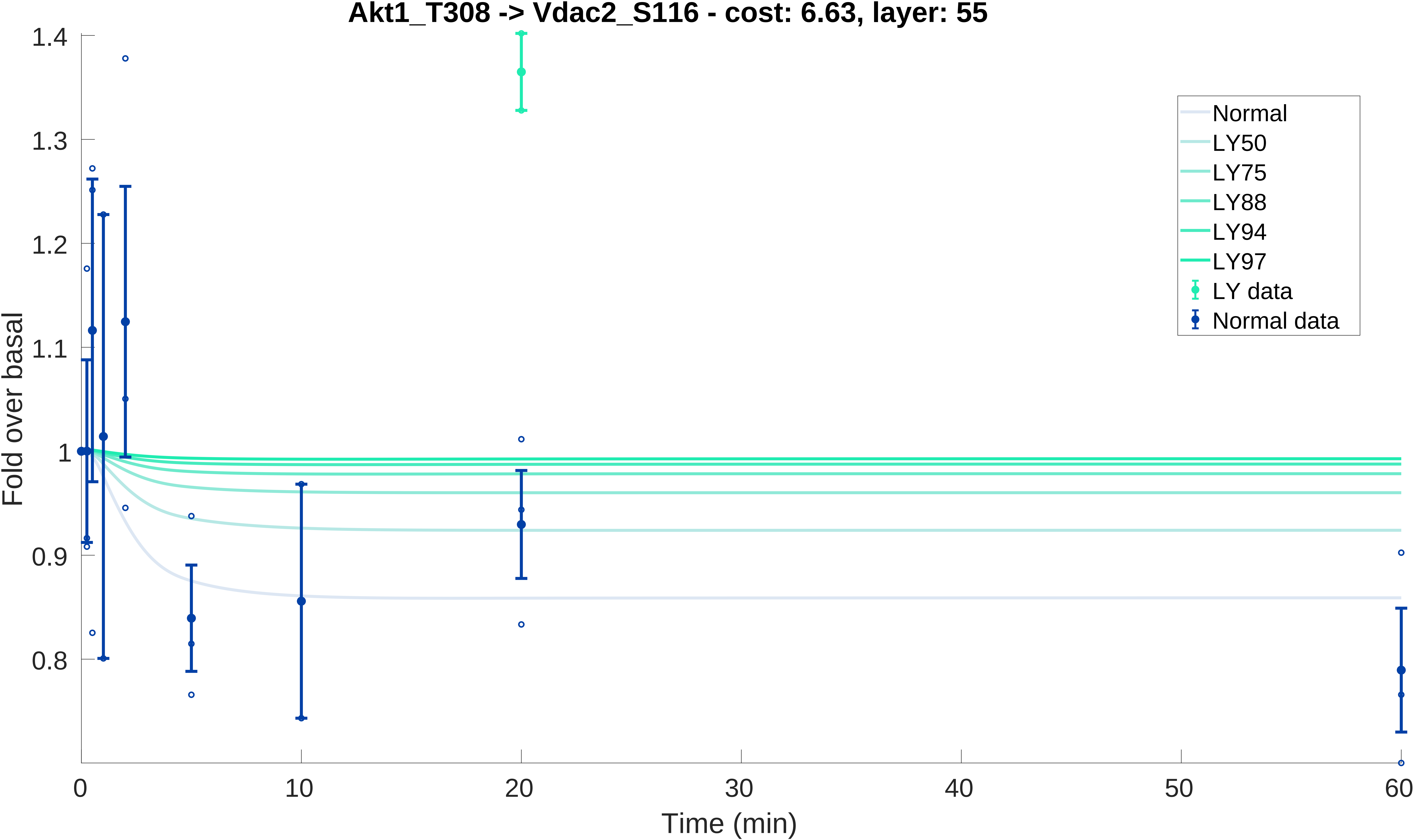
Akt1_T308 -> Vdac2_S116 - cost: 6.63, layer: 55
Fold over basal
Time (min)
1.4
1.3
1.2
1.1
1
0.9
0.8
0
10
20
30
40
50
60
Normal
LY50
LY75
LY88
LY94
LY97
LY data
Normal data

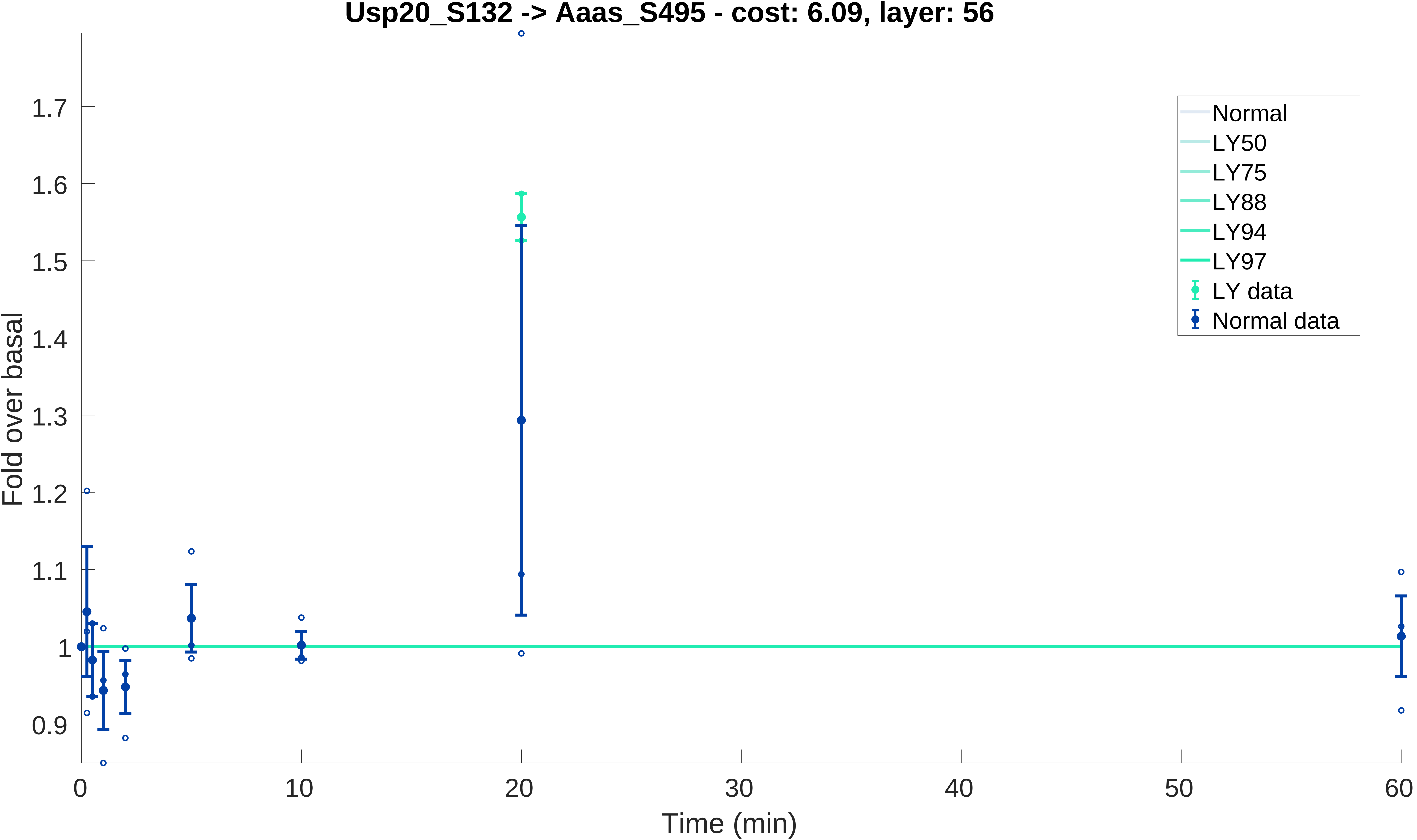
Usp20_S132 -> Aaas_S495 - cost: 6.09, layer: 56
Fold over basal
Time (min)
0
10
20
30
40
50
60
0.9
1
1.1
1.2
1.3
1.4
1.5
1.6
1.7
Normal
LY50
LY75
LY88
LY94
LY97
LY data
Normal data

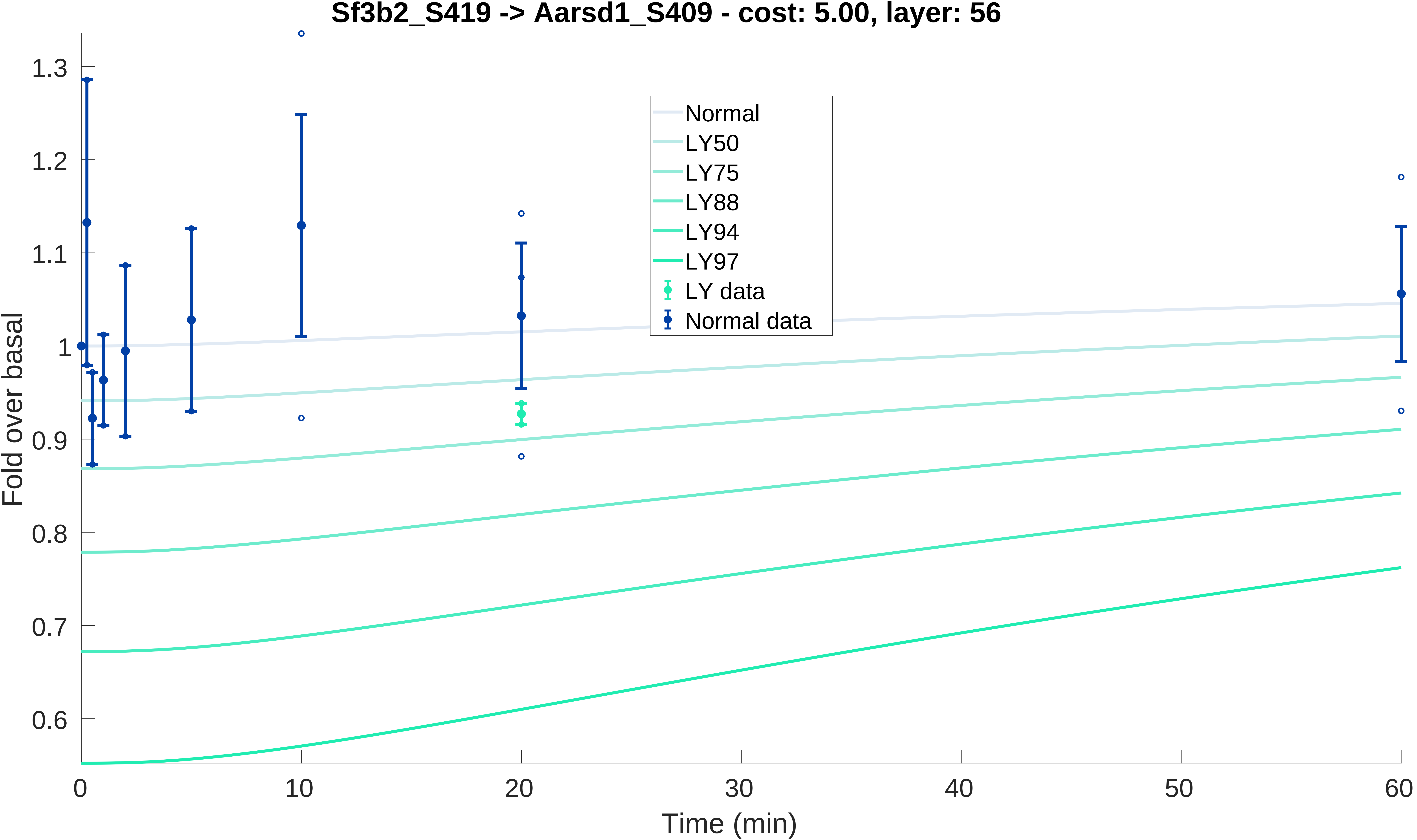

Sf3b2_S419 -> Aarsd1_S409 - cost: 5.00, layer: 56
Fold over basal
Time (min)
0.6
0.7
0.8
0.9
1
1.1
1.2
1.3
0
10
20
30
40
50
60
Normal
LY50
LY75
LY88
LY94
LY97
LY data
Normal data

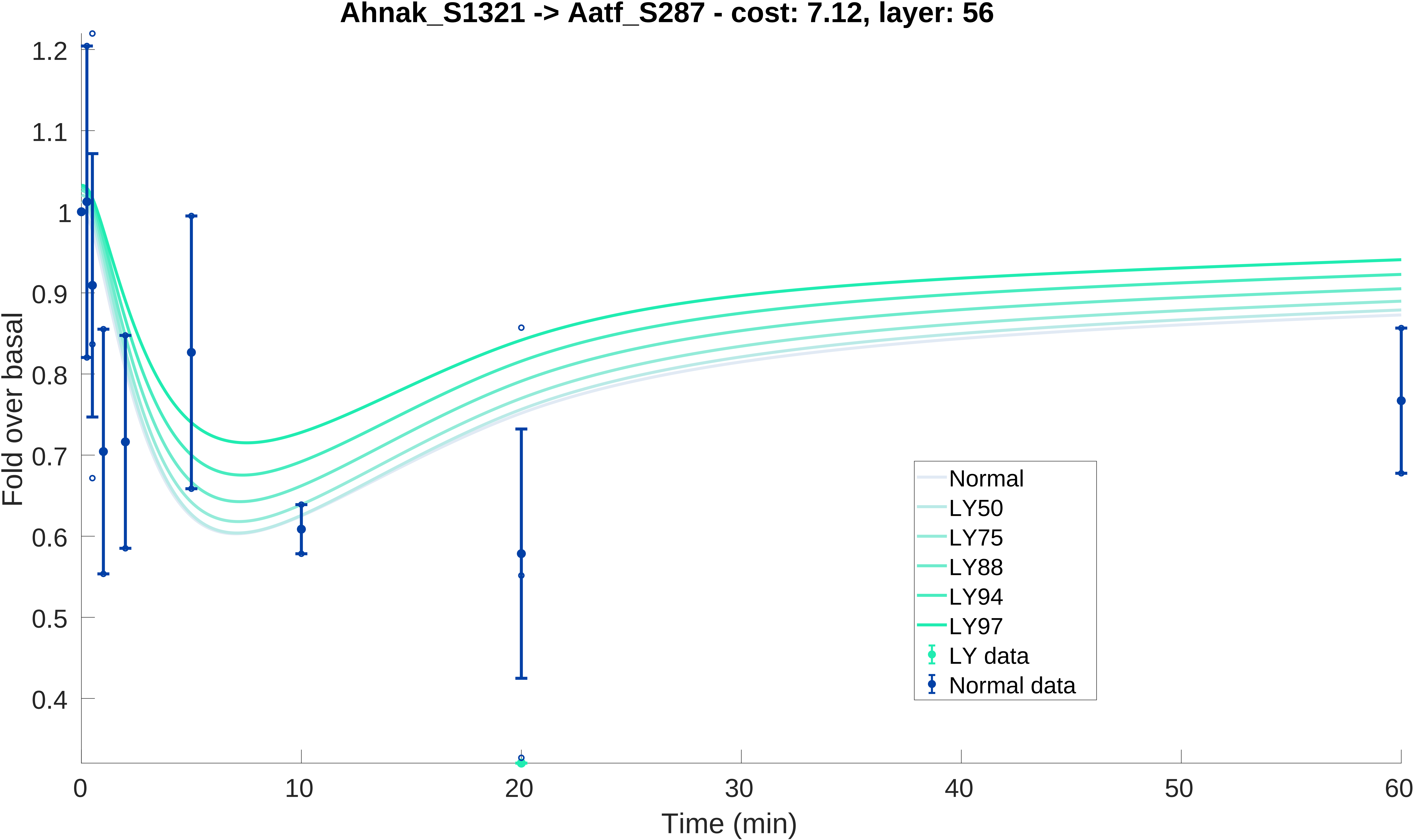

Ahnak_S1321 -> Aatf_S287 - cost: 7.12, layer: 56
Fold over basal
1.2
1.1
1
0.9
0.8
0.7
0.6
0.5
0.4
0
10
20
30
40
50
60
Time (min)
Normal
LY50
LY75
LY88
LY94
LY97
LY data
Normal data

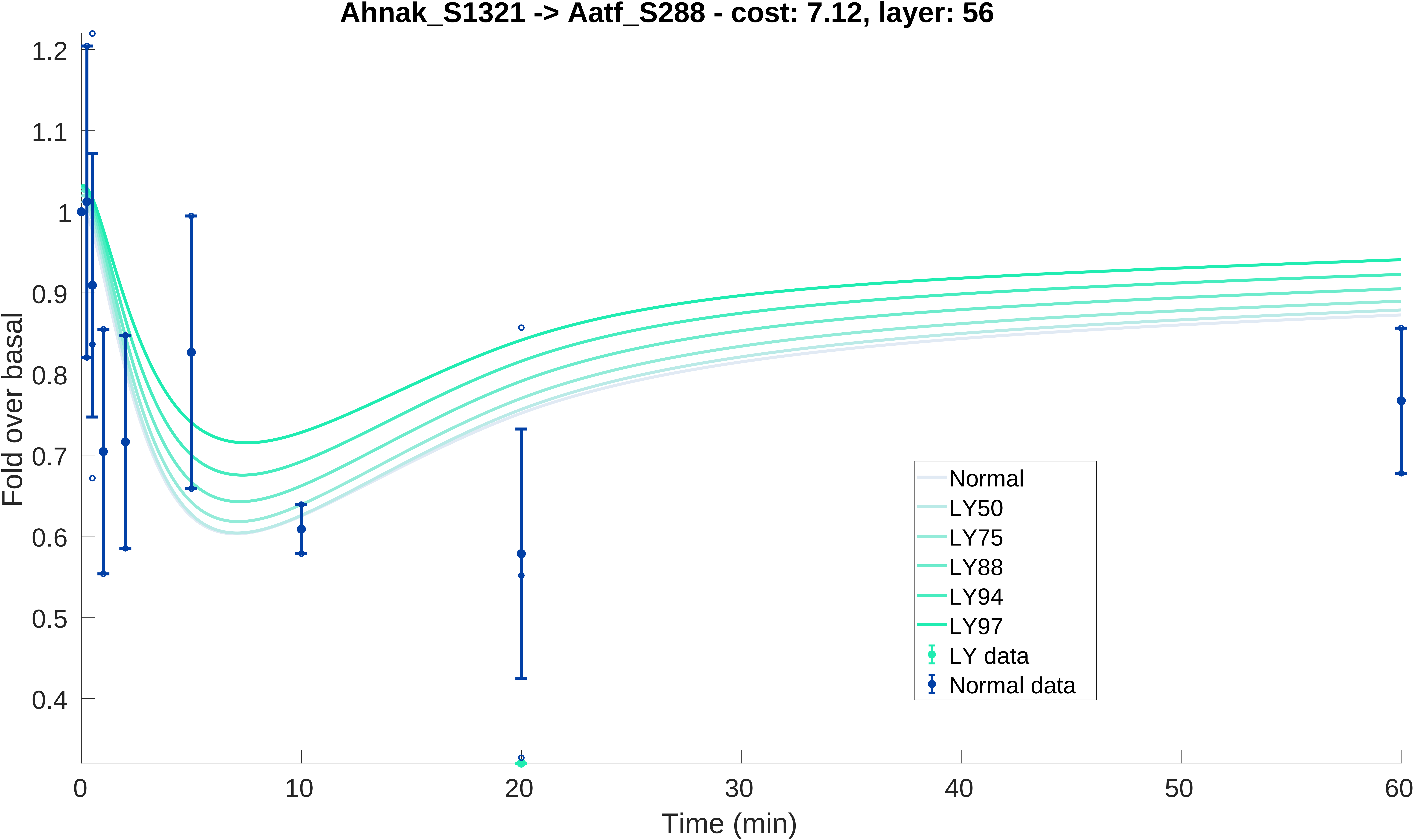

Ahnak_S1321 -> Aatf_S288 - cost: 7.12, layer: 56
Fold over basal
Time (min)
0.4
0.5
0.6
0.7
0.8
0.9
1
1.1
1.2
0
10
20
30
40
50
60
Normal
LY50
LY75
LY88
LY94
LY97
LY data
Normal data

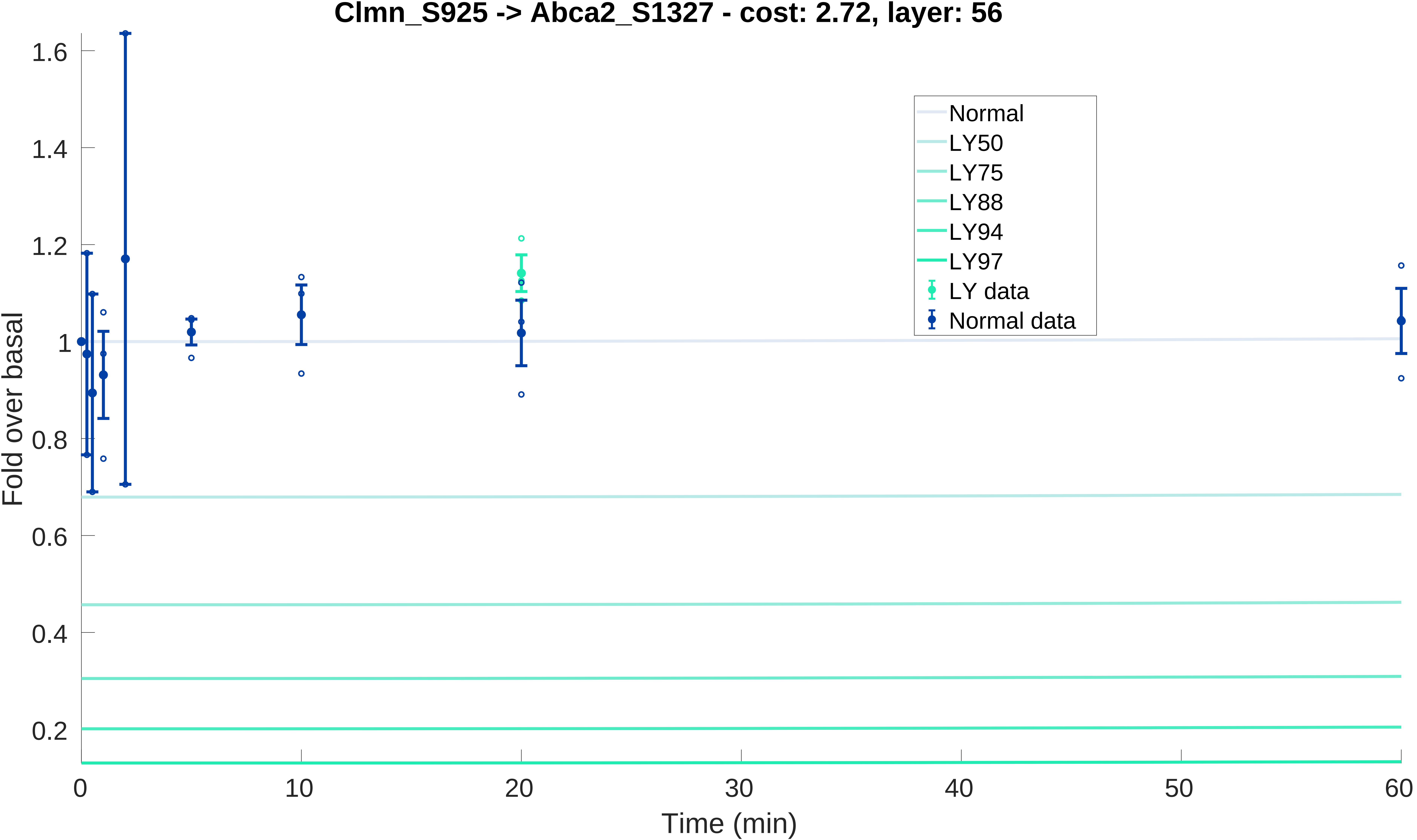
Clmn_S925 -> Abca2_S1327 - cost: 2.72, layer: 56
Fold over basal
Time (min)
0.2
0.4
0.6
0.8
1
1.2
1.4
1.6
0
10
20
30
40
50
60
Normal
LY50
LY75
LY88
LY94
LY97
LY data
Normal data

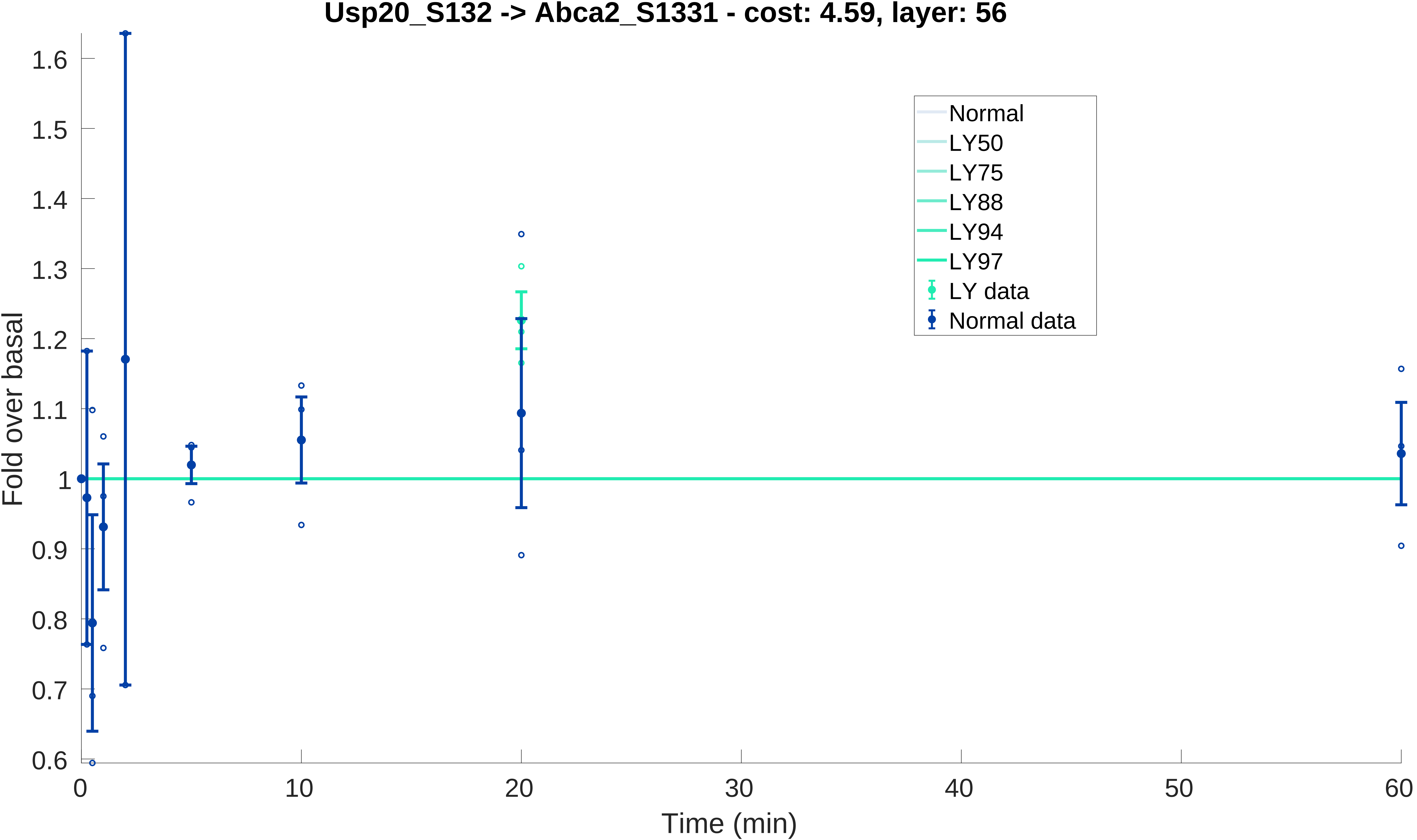

Usp20_S132 -> Abca2_S1331 - cost: 4.59, layer: 56
Fold over basal
Time (min)
1.6
1.5
1.4
1.3
1.2
1.1
1
0.9
0.8
0.7
0.6
0
10
20
30
40
50
60
Normal
LY50
LY75
LY88
LY94
LY97
LY data
Normal data

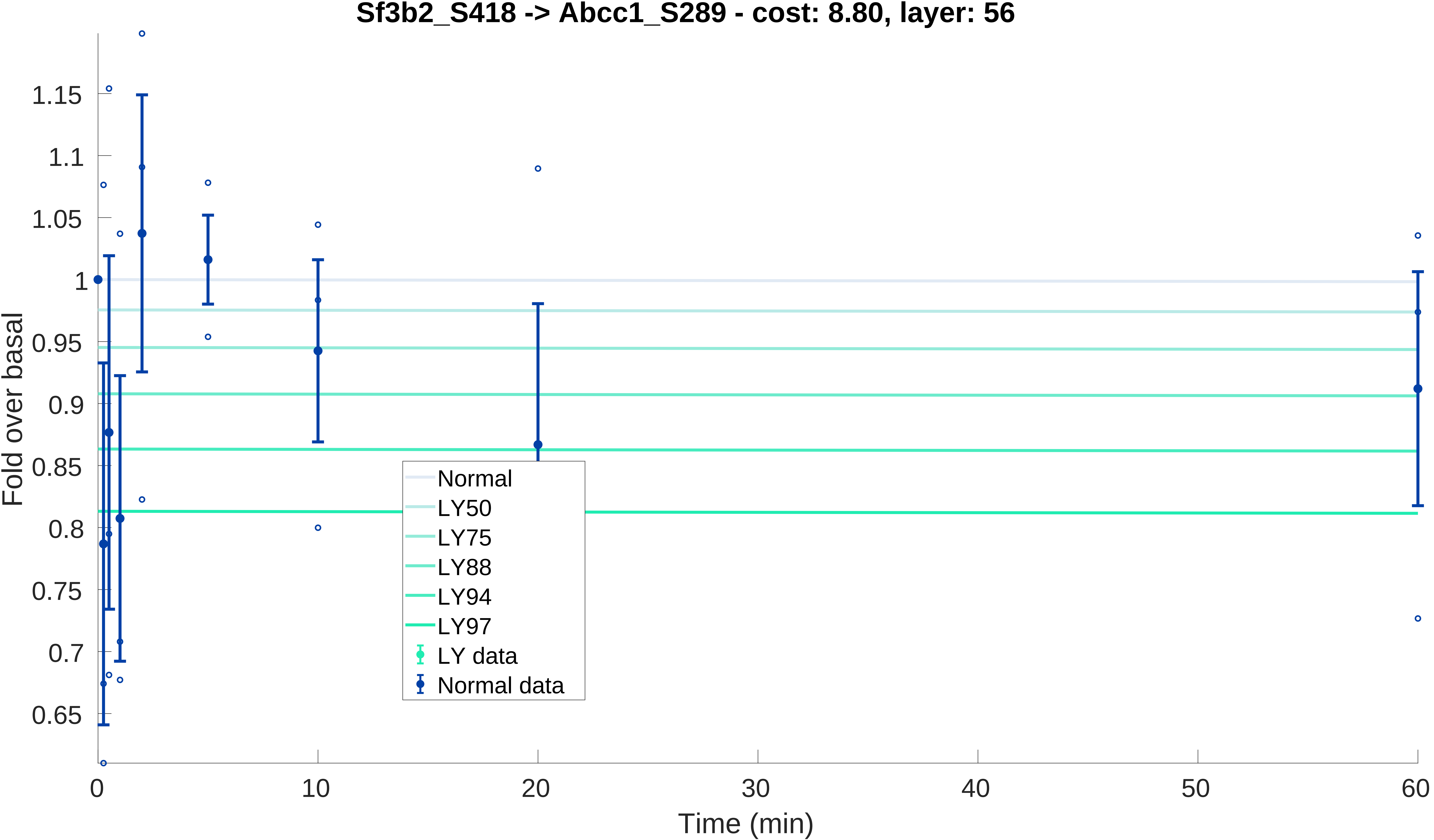

Sf3b2_S418 -> Abcc1_S289 - cost: 8.80, layer: 56
1.15
1.1
1.05
1
0.95
0.9
0.85
0.8
0.75
0.7
0.65
Fold over basal
0
10
20
30
40
50
60
Time (min)
Normal
LY50
LY75
LY88
LY94
LY97
LY data
Normal data

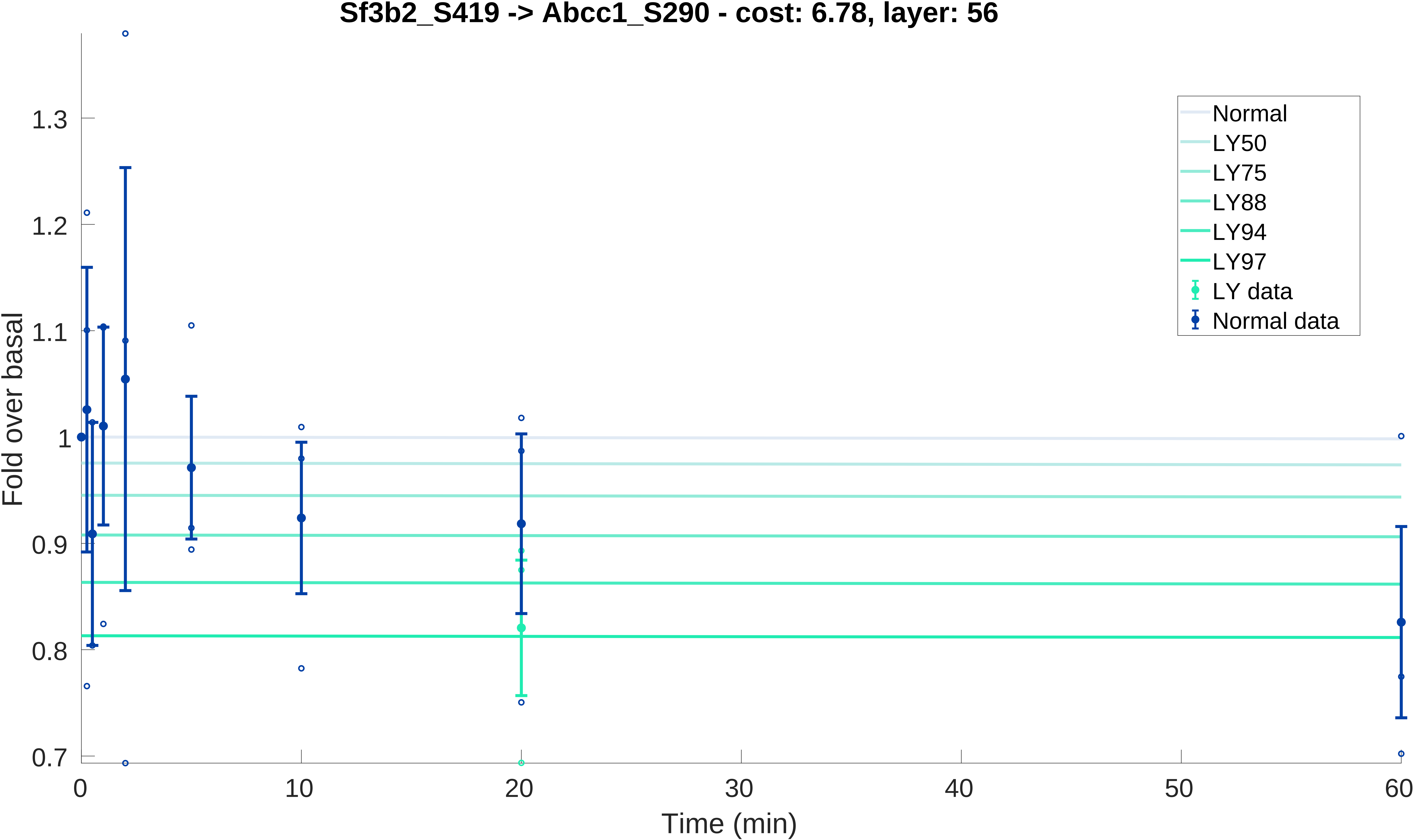

Sf3b2_S419 -> Abcc1_S290 - cost: 6.78, layer: 56
Fold over basal
1.3
1.2
1.1
1
0.9
0.8
0.7
0
10
20
30
40
50
60
Time (min)
Normal
LY50
LY75
LY88
LY94
LY97
LY data
Normal data

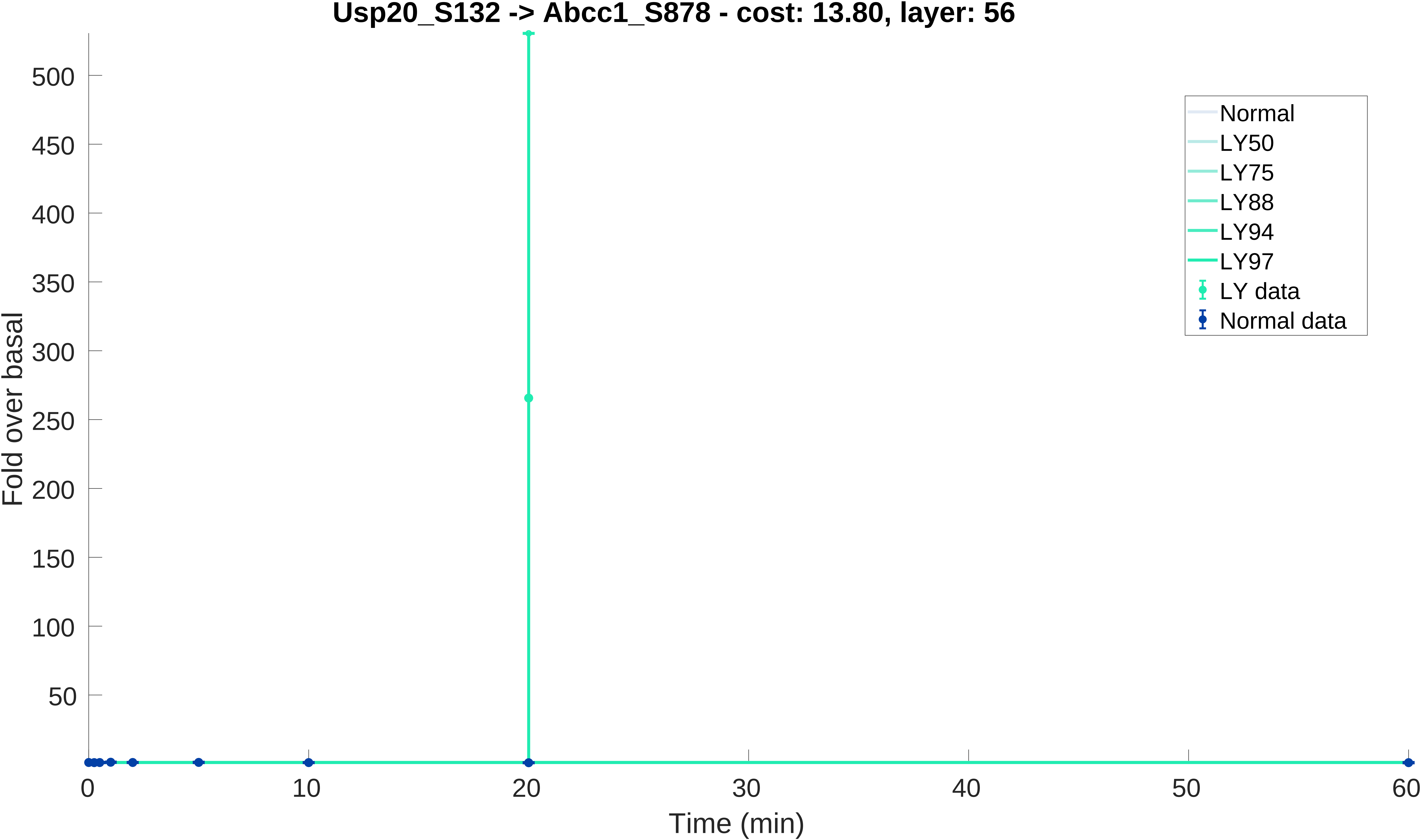
Usp20_S132 -> Abcc1_S878 - cost: 13.80, layer: 56
Fold over basal
500
450
400
350
300
250
200
150
100
50
0
10
20
30
40
50
60
Time (min)
Normal
LY50
LY75
LY88
LY94
LY97
LY data
Normal data

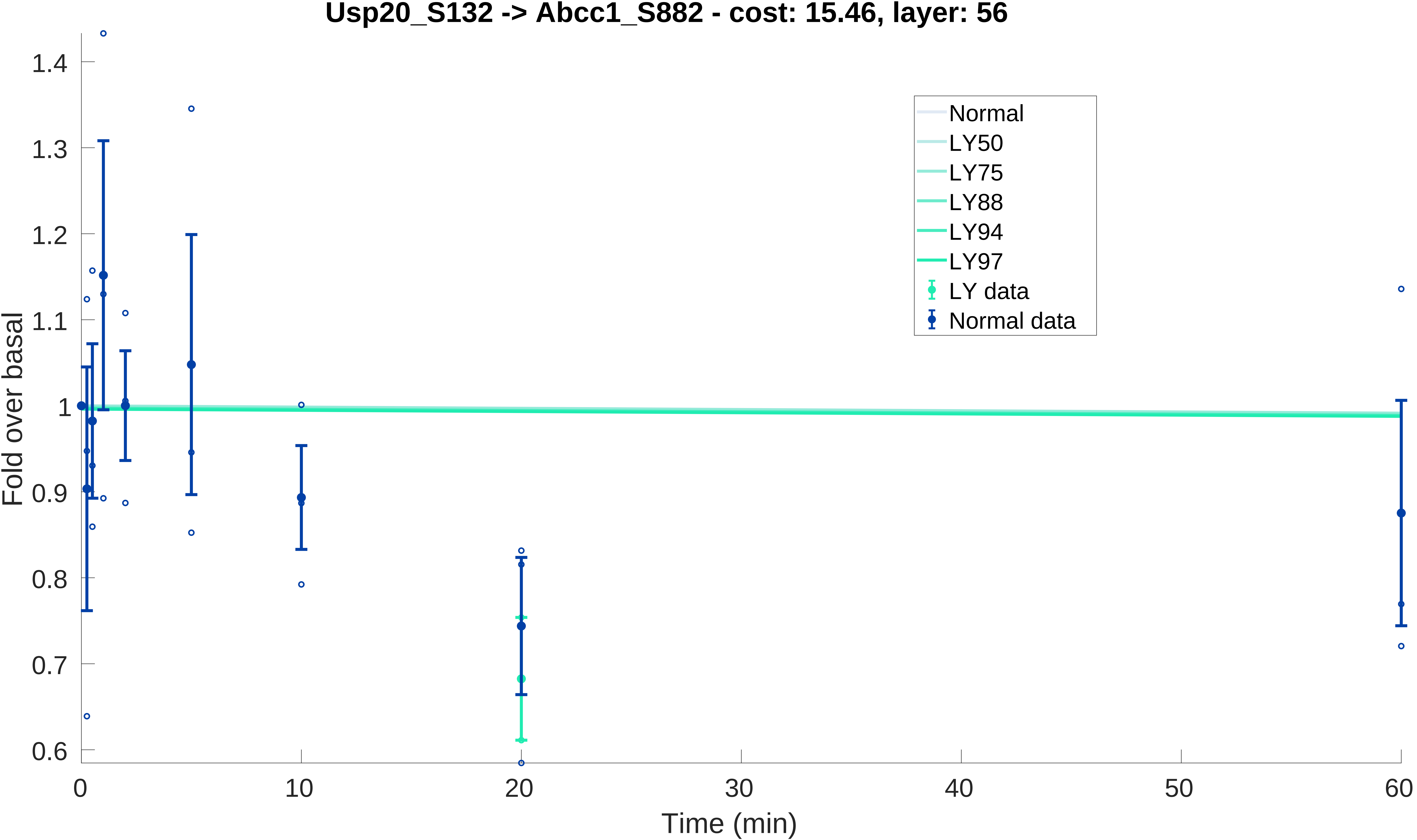
Usp20_S132 -> Abcc1_S882 - cost: 15.46, layer: 56
Fold over basal
Time (min)
0
10
20
30
40
50
60
0.6
0.7
0.8
0.9
1
1.1
1.2
1.3
1.4
Normal
LY50
LY75
LY88
LY94
LY97
LY data
Normal data

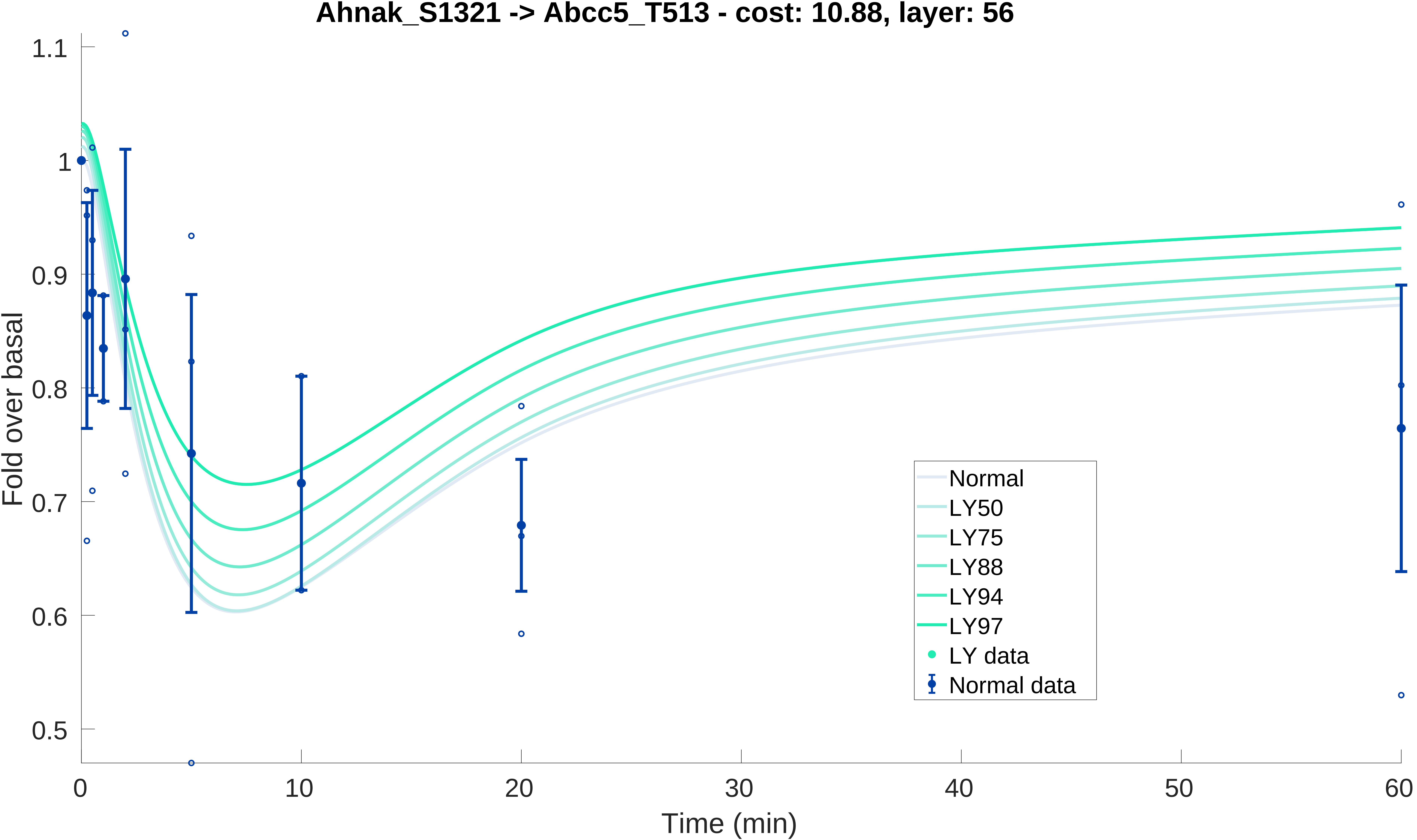

Ahnak_S1321 -> Abcc5_T513 - cost: 10.88, layer: 56
Fold over basal
Time (min)
1.1
1
0.9
0.8
0.7
0.6
0.5
0
10
20
30
40
50
60
Normal
LY50
LY75
LY88
LY94
LY97
LY data
Normal data

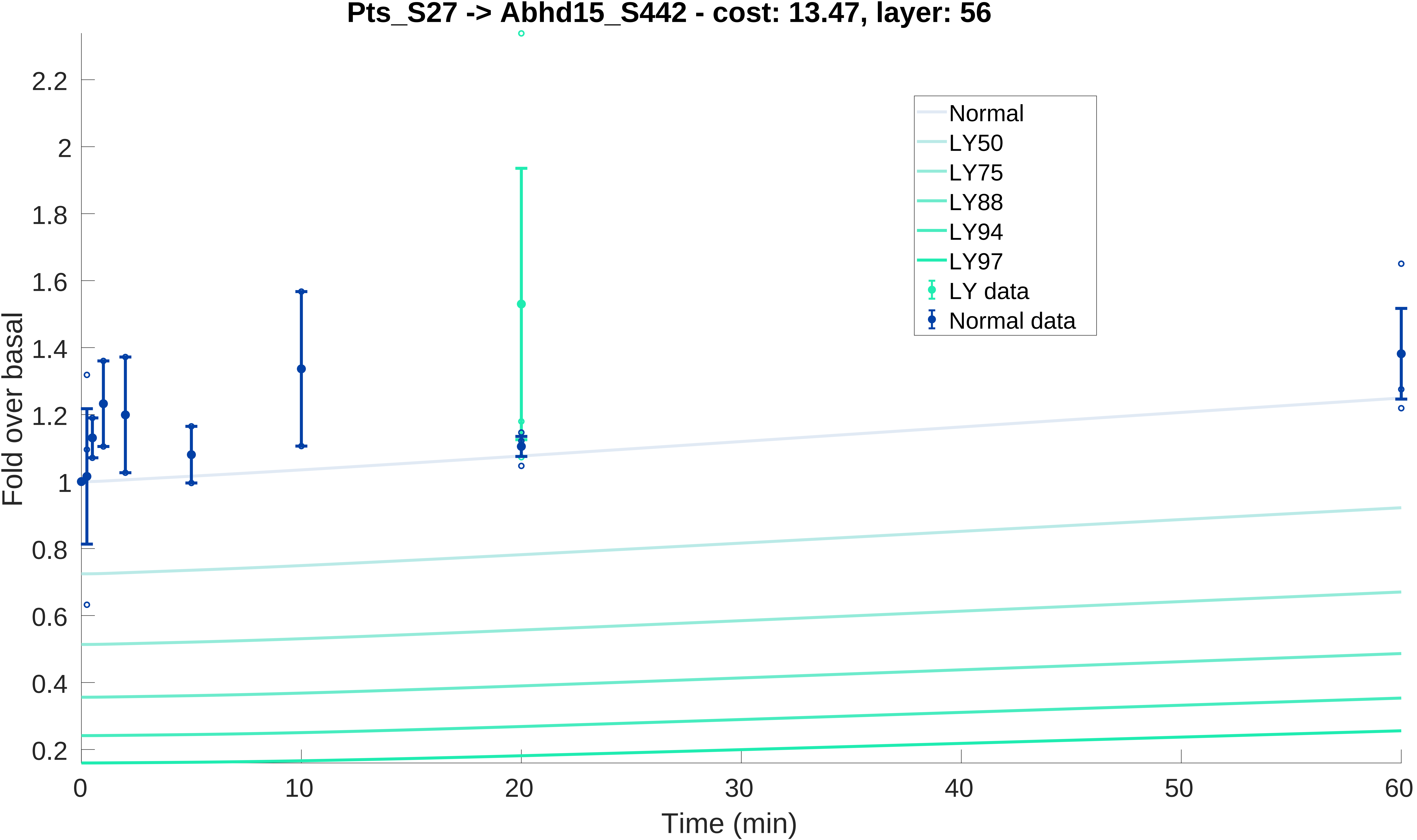

Pts_S27 -> Abhd15_S442 - cost: 13.47, layer: 56
Normal
LY50
LY75
LY88
LY94
LY97
LY data
Normal data
Fold over basal
Time (min)
0.2
0.4
0.6
0.8
1
1.2
1.4
1.6
1.8
2
2.2
0
10
20
30
40
50
60

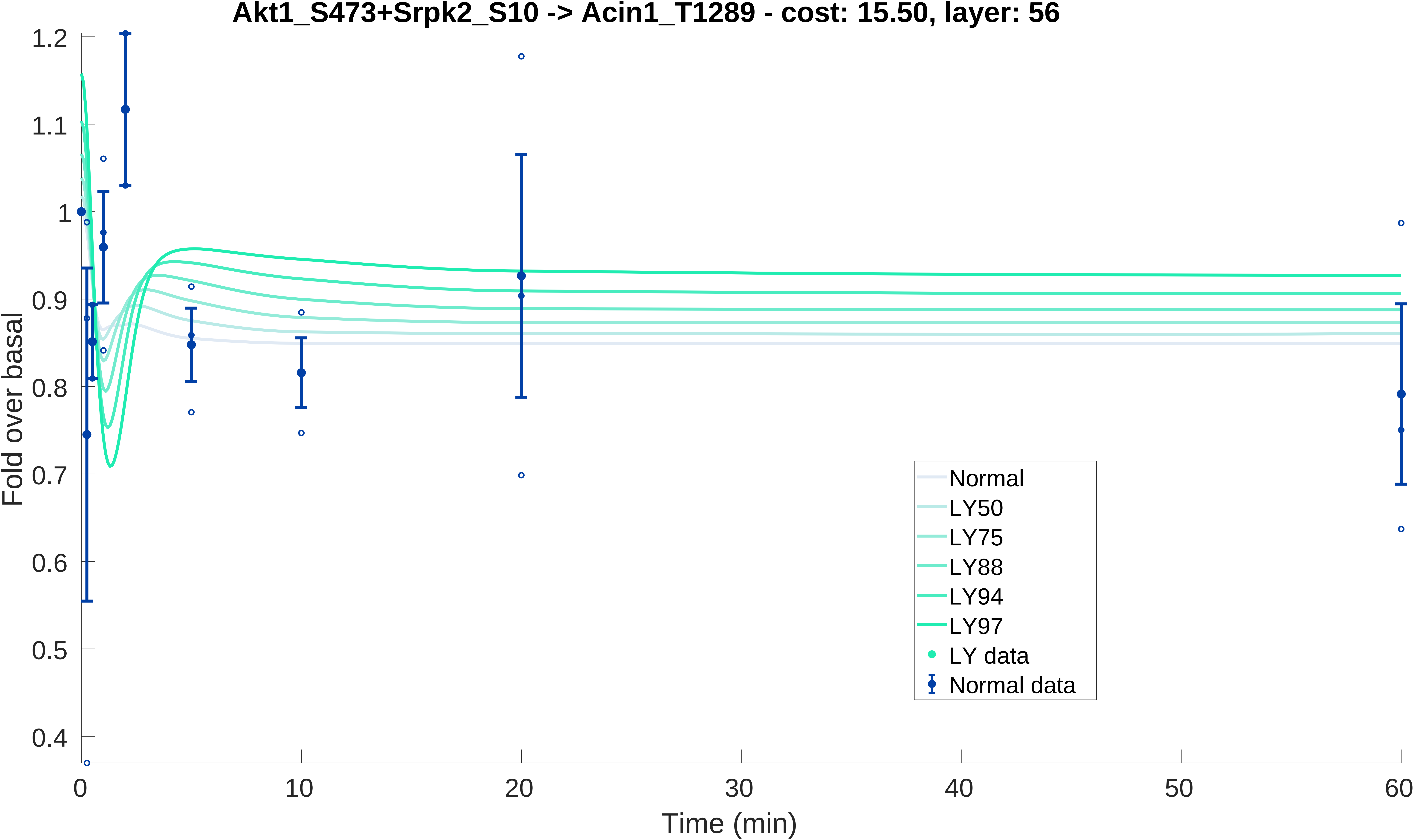

Akt1_S473+Srpk2_S10 -> Acin1_T1289 - cost: 15.50, layer: 56
Fold over basal
Time (min)
1.2
1.1
1
0.9
0.8
0.7
0.6
0.5
0.4
0
10
20
30
40
50
60
Normal
LY50
LY75
LY88
LY94
LY97
LY data
Normal data

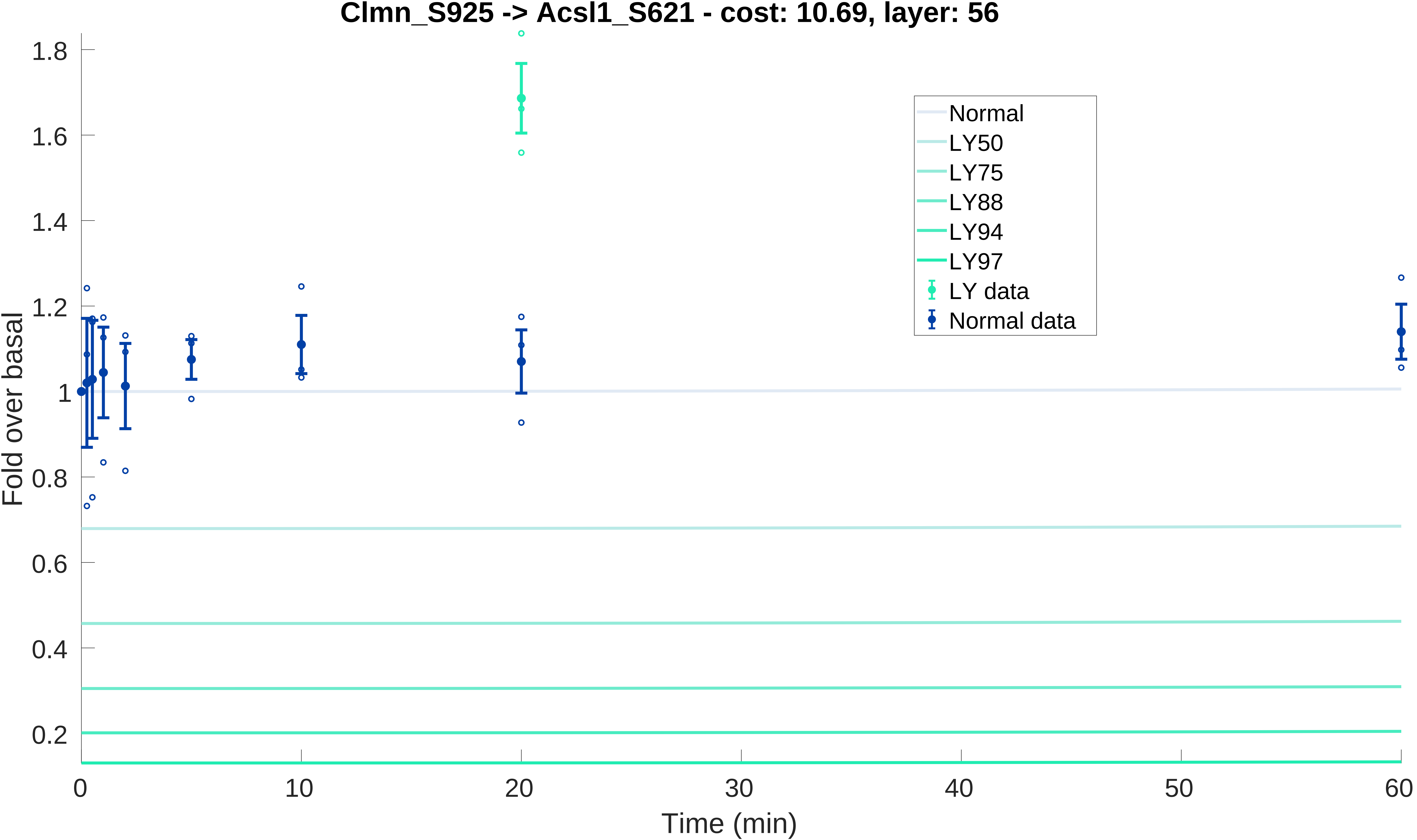

Clmn_S925 -> Acsl1_S621 - cost: 10.69, layer: 56
Fold over basal
Time (min)
1.8
1.6
1.4
1.2
1
0.8
0.6
0.4
0.2
0
10
20
30
40
50
60
Normal
LY50
LY75
LY88
LY94
LY97
LY data
Normal data

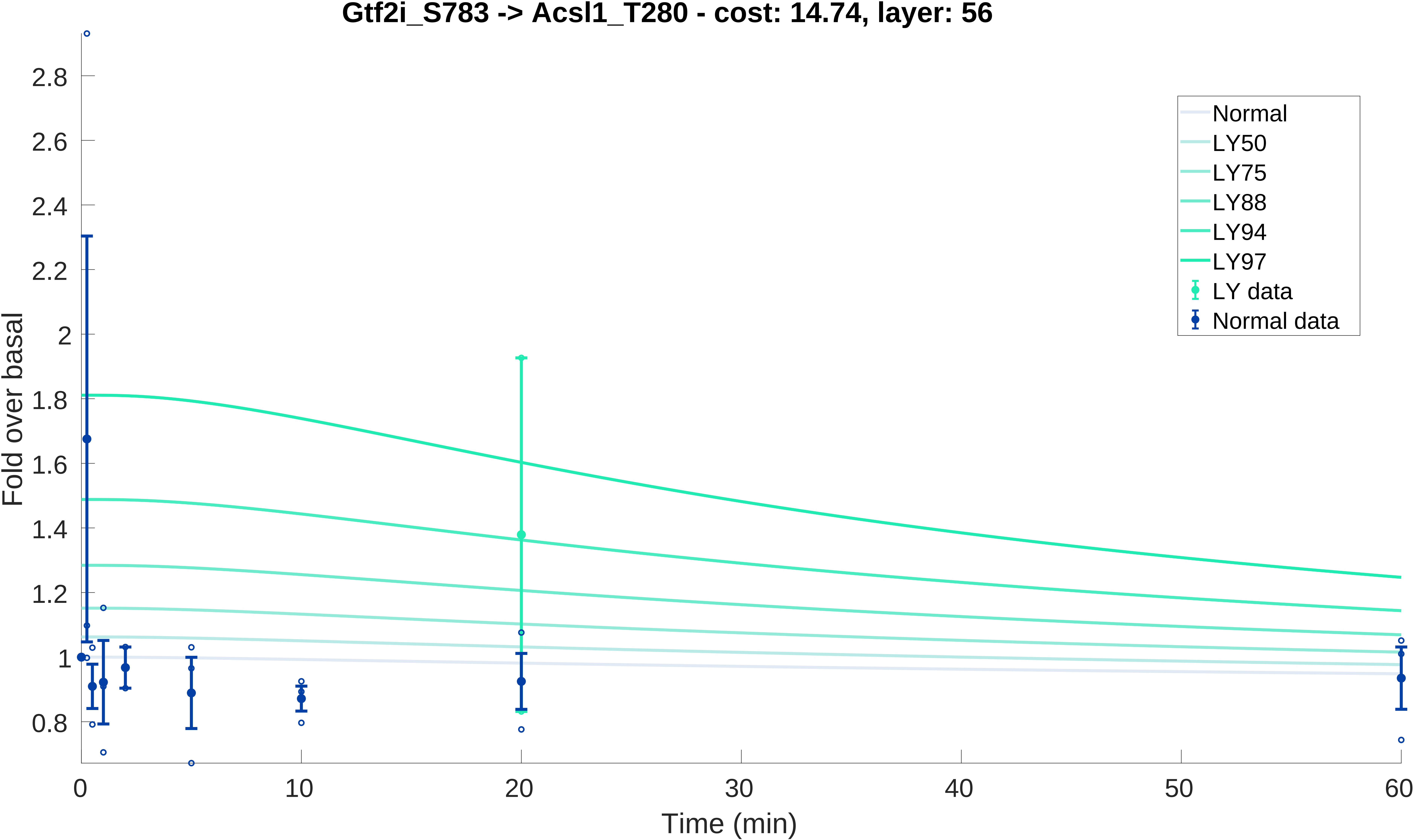

Gtf2i_S783 -> Acsl1_T280 - cost: 14.74, layer: 56
Fold over basal
2.8
2.6
2.4
2.2
2
1.8
1.6
1.4
1.2
1
0.8
0
10
20
30
40
50
60
Time (min)
Normal
LY50
LY75
LY88
LY94
LY97
LY data
Normal data

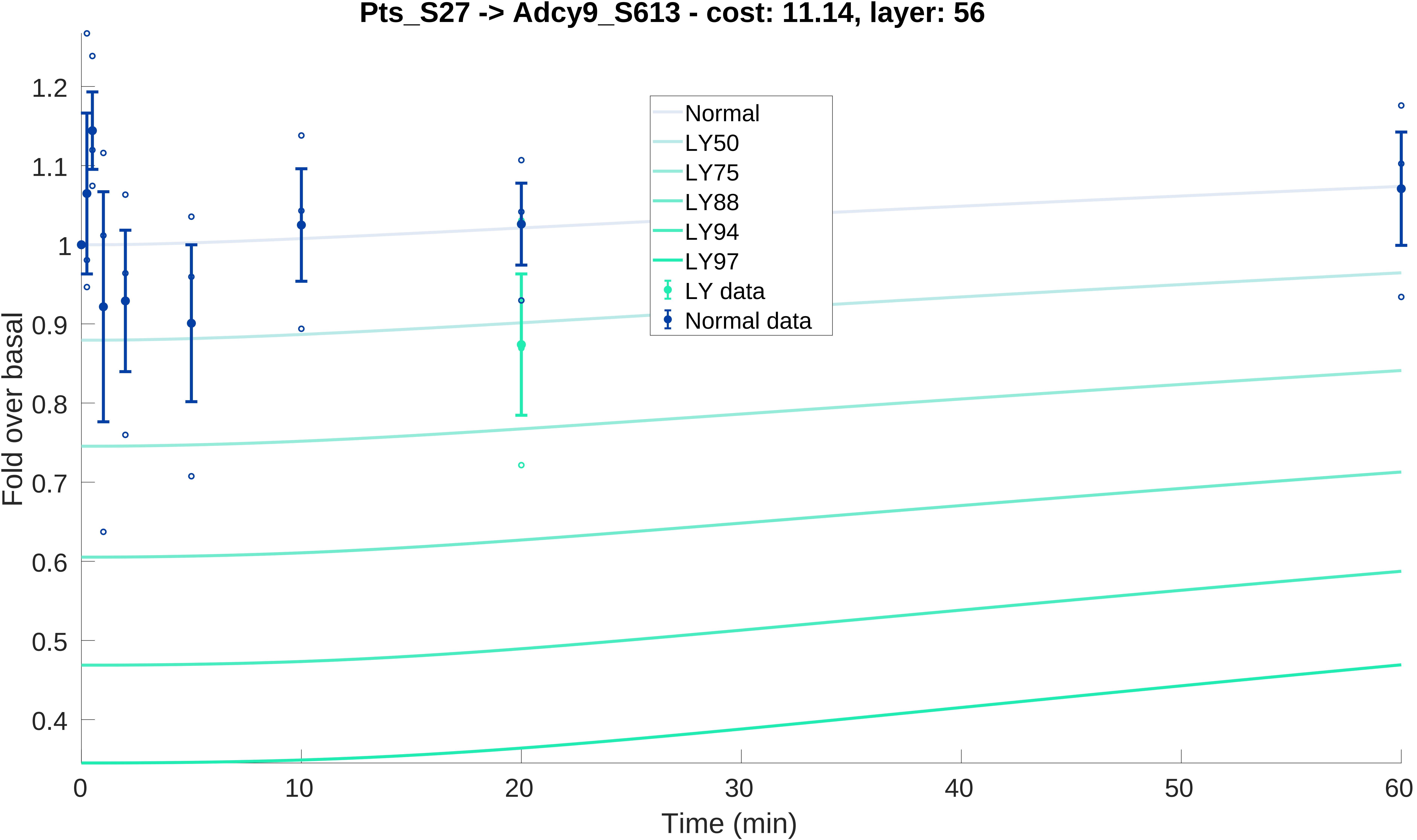

Pts_S27 -> Adcy9_S613 - cost: 11.14, layer: 56
Normal
LY50
LY75
LY88
LY94
LY97
LY data
Normal data
Fold over basal
Time (min)
0
10
20
30
40
50
60
0.4
0.5
0.6
0.7
0.8
0.9
1
1.1
1.2

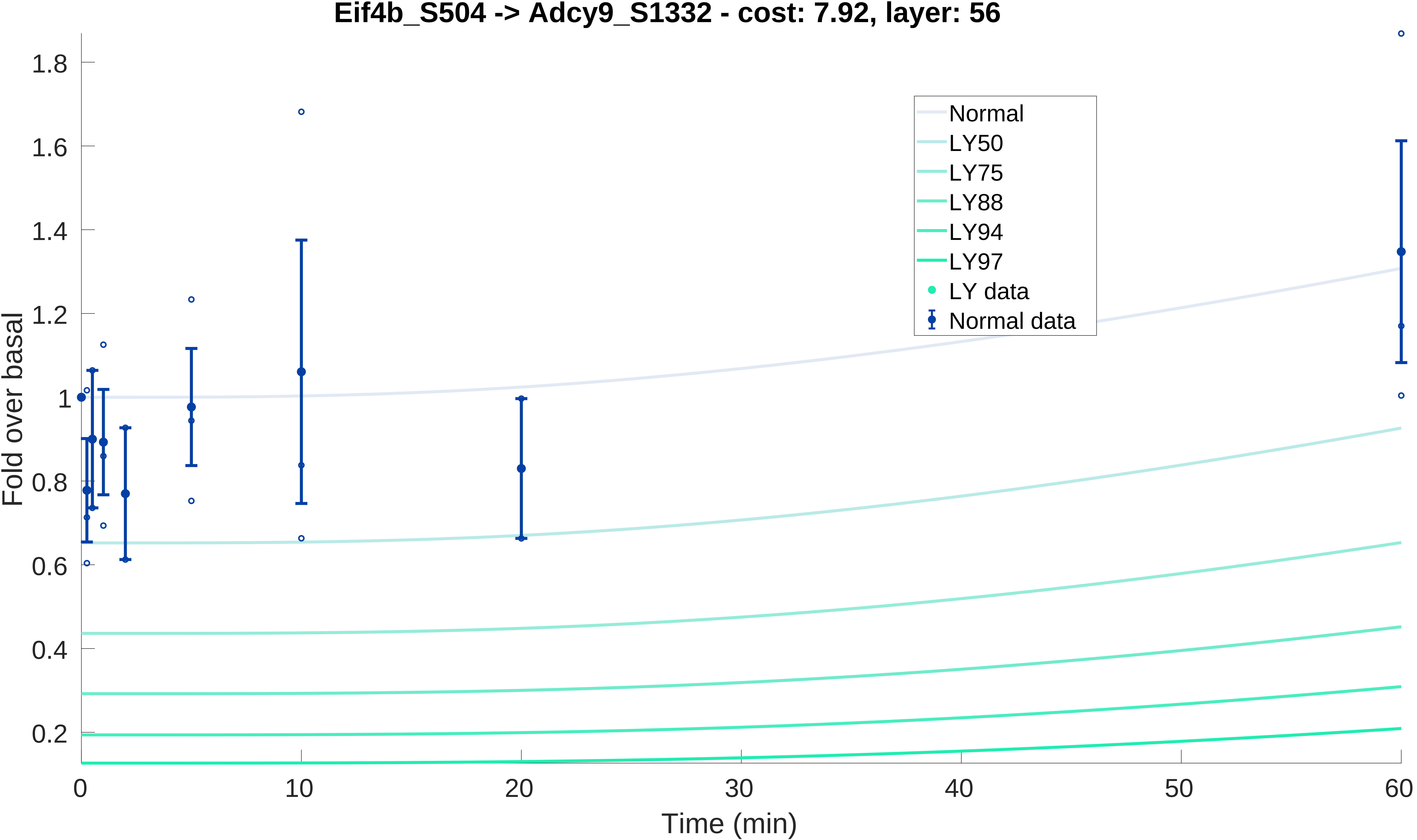
Eif4b_S504 -> Adcy9_S1332 - cost: 7.92, layer: 56
Fold over basal
Time (min)
Normal
LY50
LY75
LY88
LY94
LY97
LY data
Normal data
0.2
0.4
0.6
0.8
1
1.2
1.4
1.6
1.8
0
10
20
30
40
50
60

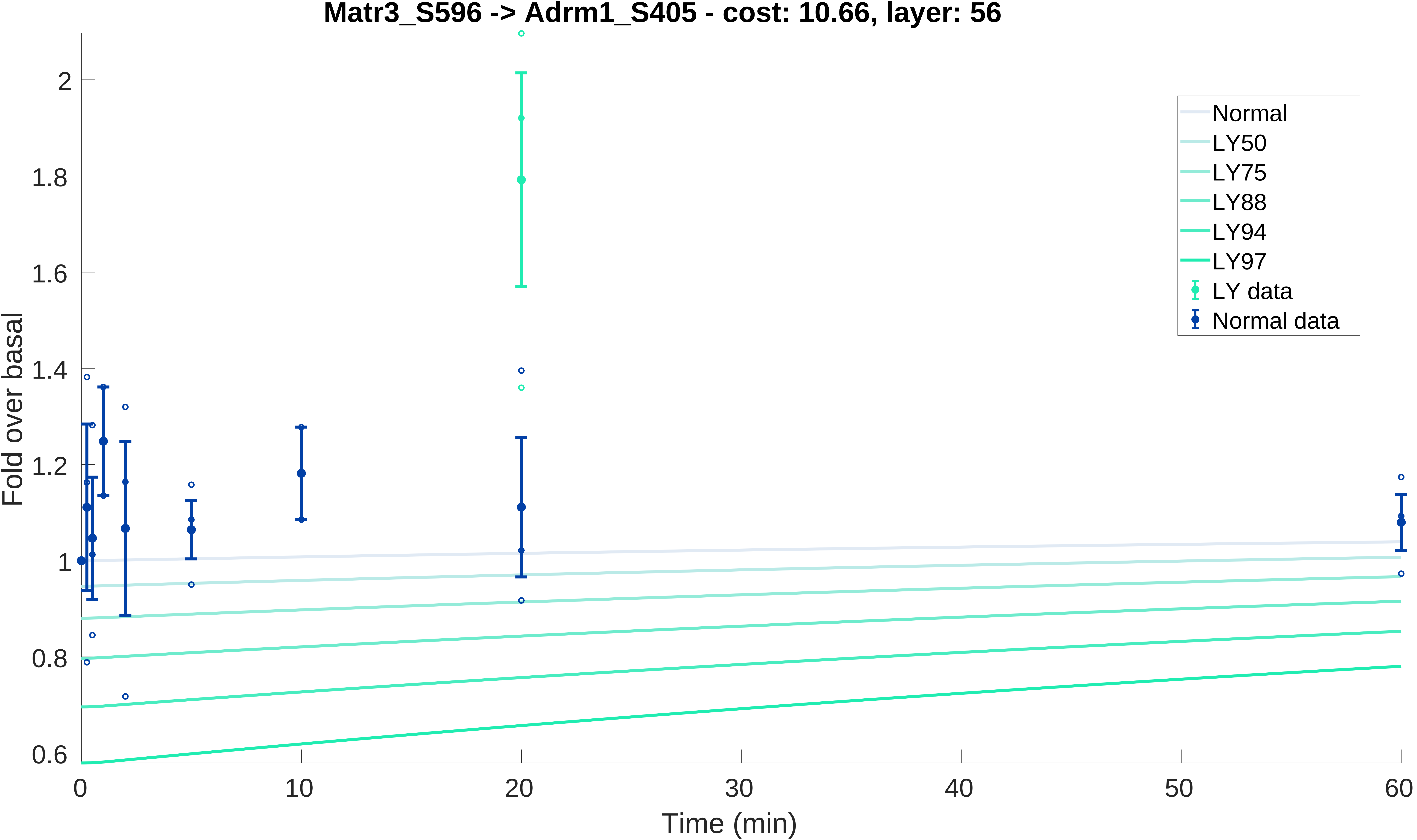

Matr3_S596 -> Adrm1_S405 - cost: 10.66, layer: 56
Fold over basal
Time (min)
0.6
0.8
1
1.2
1.4
1.6
1.8
2
0
10
20
30
40
50
60
Normal
LY50
LY75
LY88
LY94
LY97
LY data
Normal data

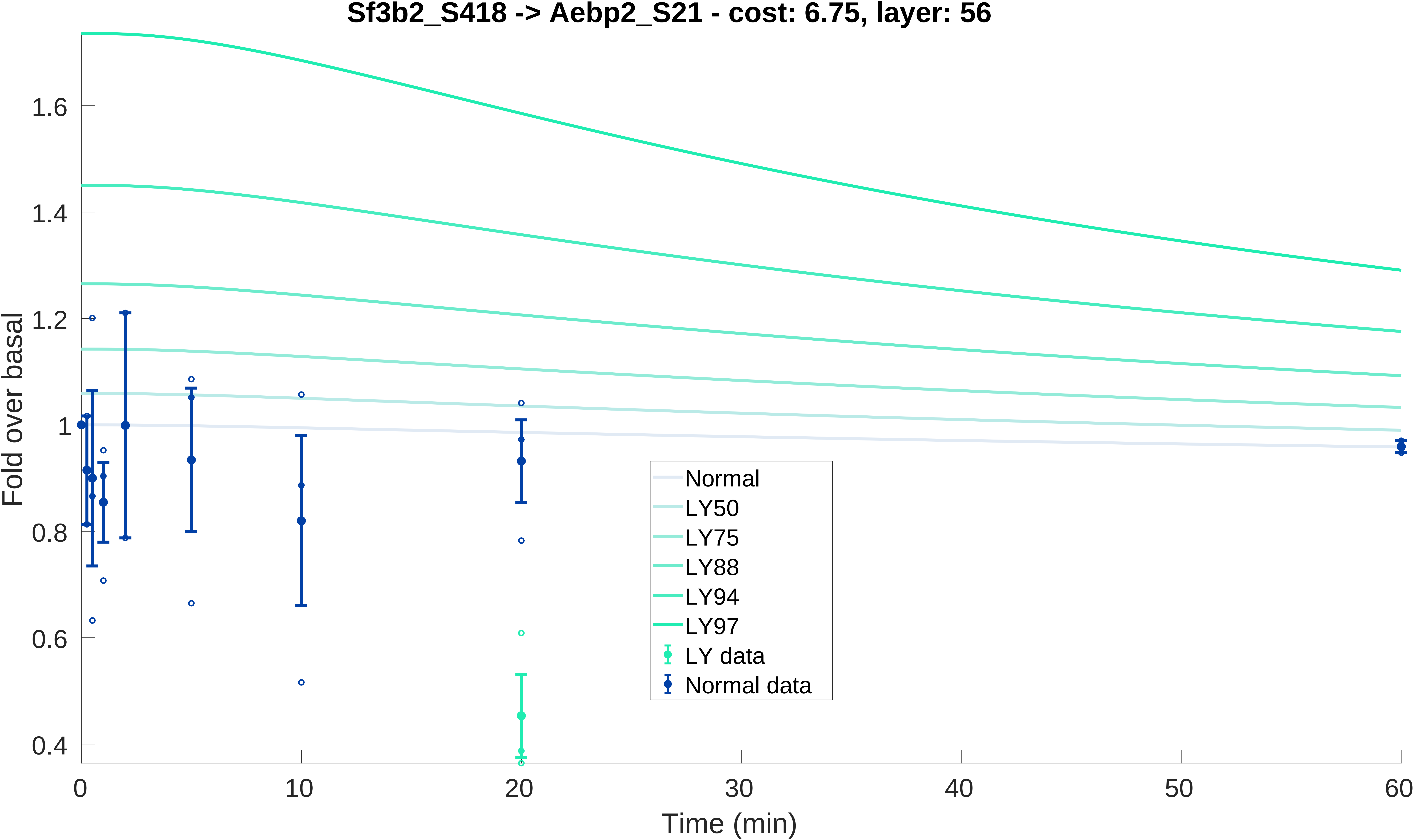

Sf3b2_S418 -> Aebp2_S21 - cost: 6.75, layer: 56
Fold over basal
Time (min)
0
10
20
30
40
50
60
0.4
0.6
0.8
1
1.2
1.4
1.6
Normal
LY50
LY75
LY88
LY94
LY97
LY data
Normal data

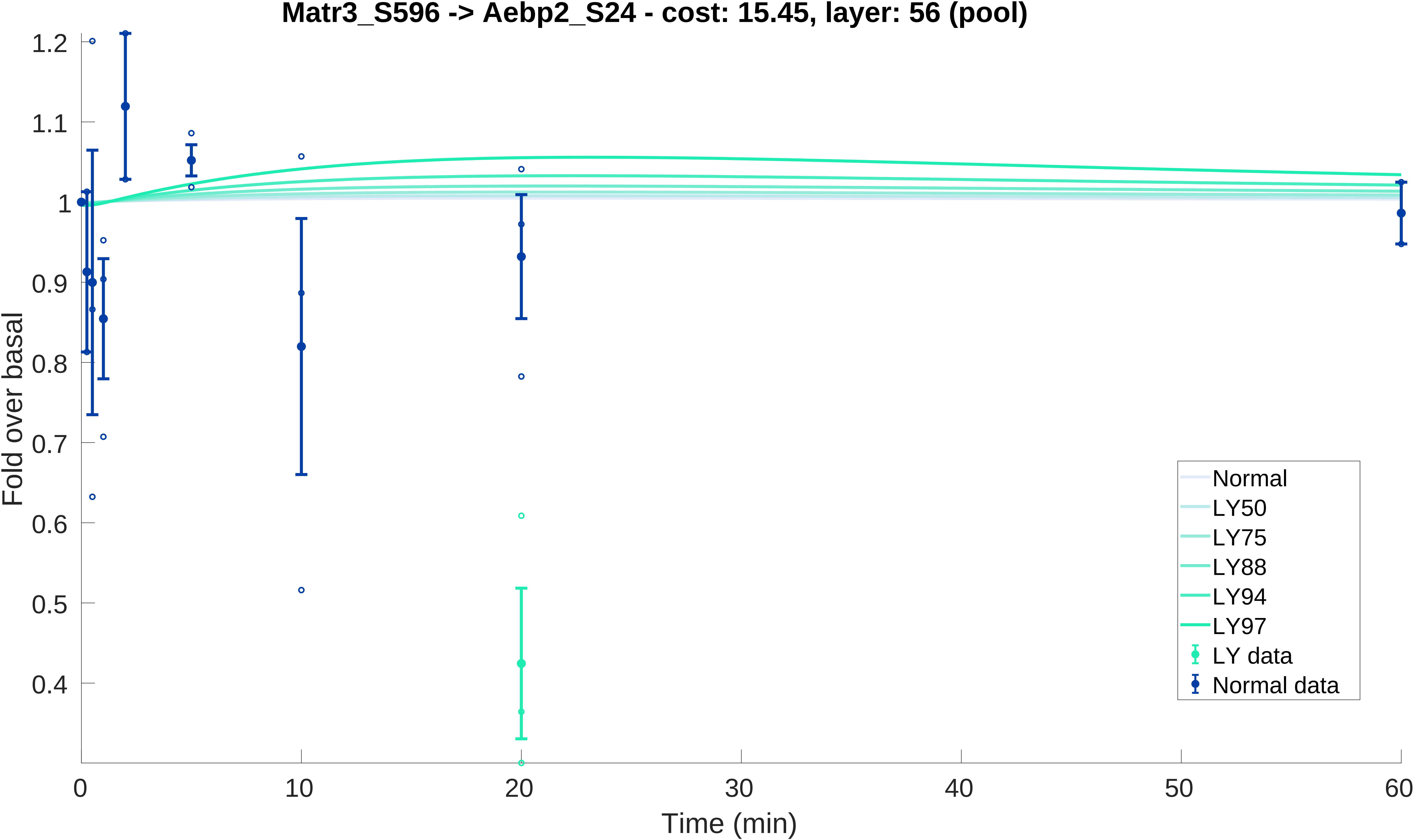
Matr3_S596 -> Aebp2_S24 - cost: 15.45, layer: 56 (pool)
Fold over basal
Time (min)
1.2
1.1
1
0.9
0.8
0.7
0.6
0.5
0.4
0
10
20
30
40
50
60
Normal
LY50
LY75
LY88
LY94
LY97
LY data
Normal data

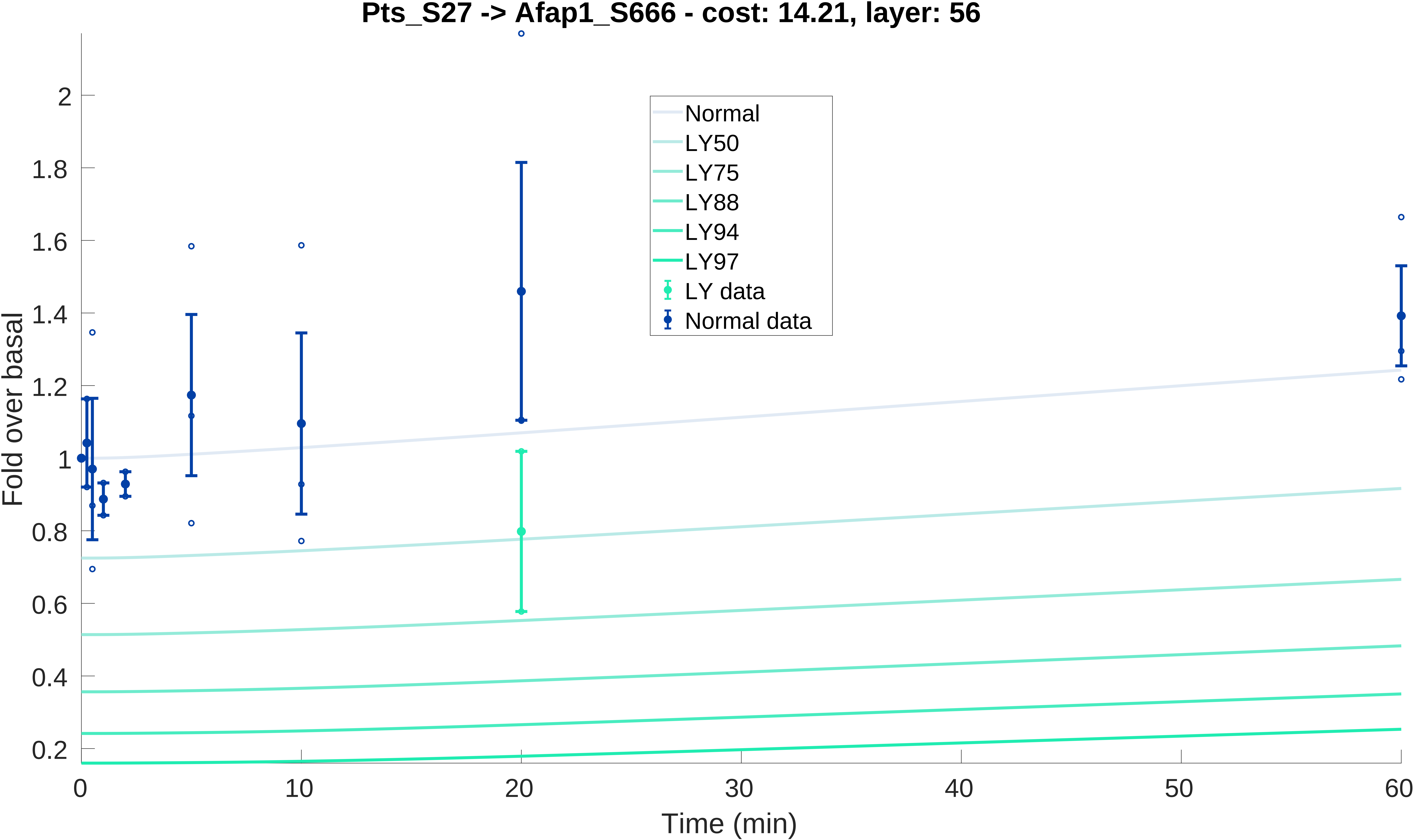

Pts_S27 -> Afap1_S666 - cost: 14.21, layer: 56
Normal
LY50
LY75
LY88
LY94
LY97
LY data
Normal data
Fold over basal
Time (min)
0.2
0.4
0.6
0.8
1
1.2
1.4
1.6
1.8
2
0
10
20
30
40
50
60

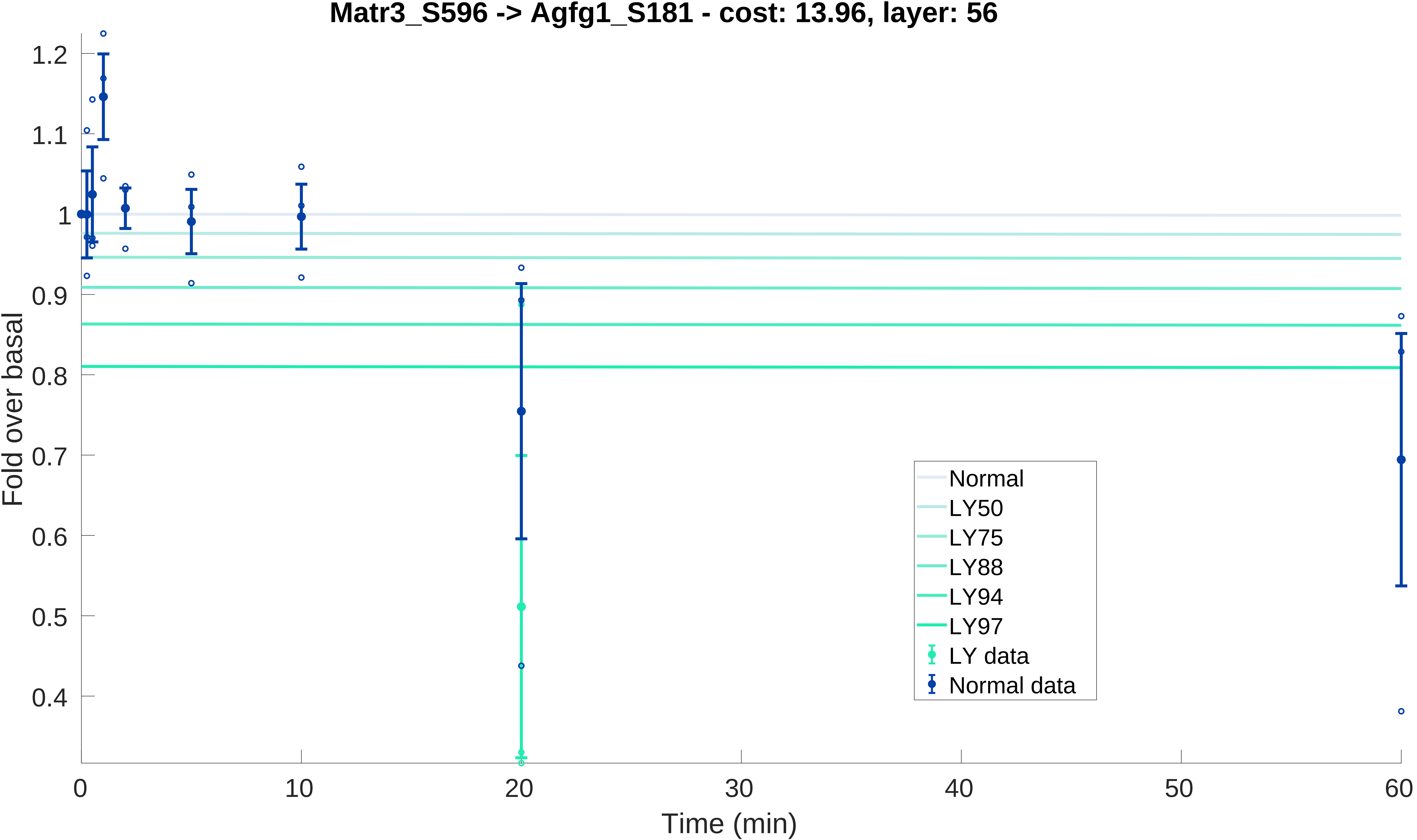

Matr3_S596 -> Agfg1_S181 - cost: 13.96, layer: 56
Fold over basal
Time (min)
0
10
20
30
40
50
60
1.2
1.1
1
0.9
0.8
0.7
0.6
0.5
0.4
Normal
LY50
LY75
LY88
LY94
LY97
LY data
Normal data

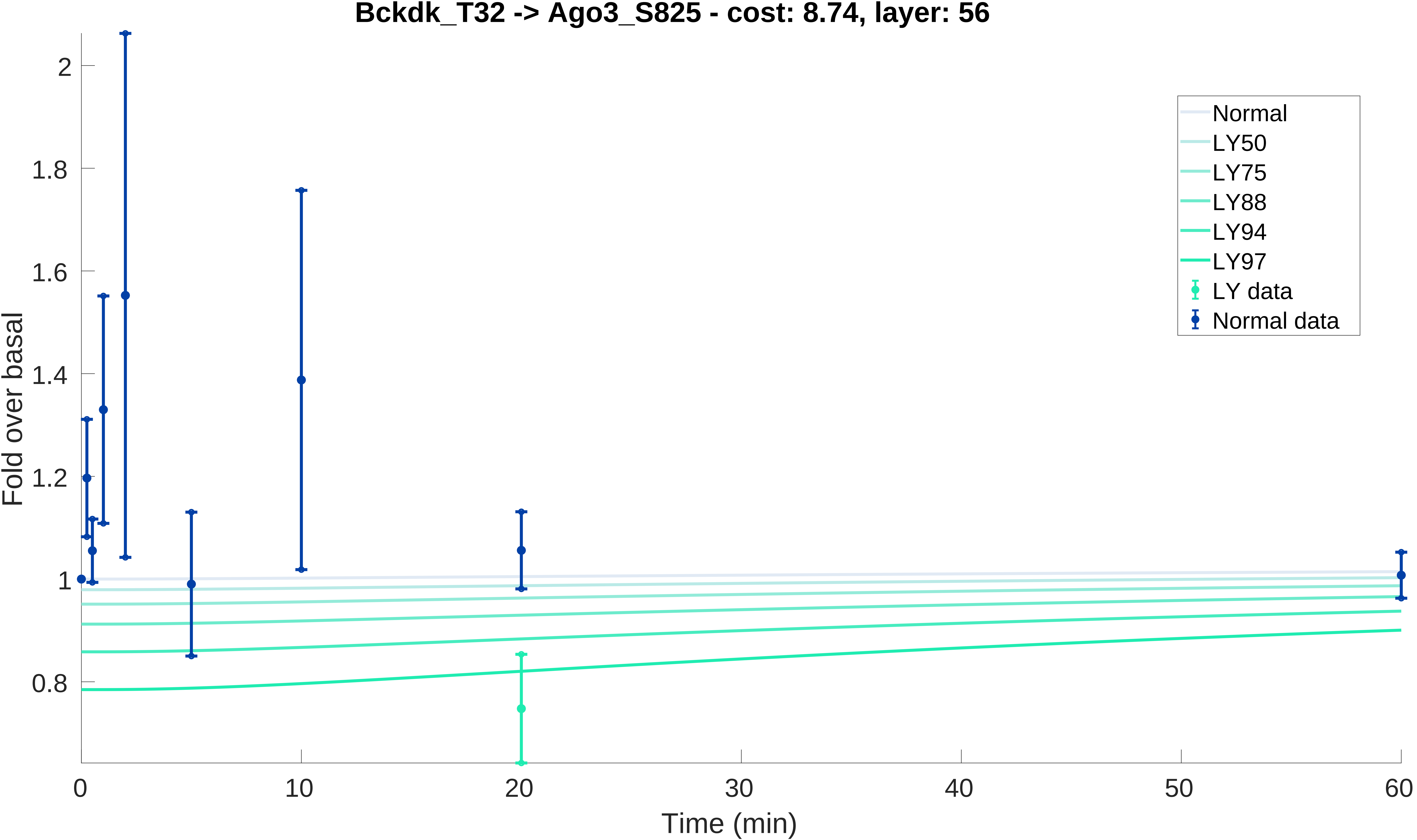
Bckdk_T32 -> Ago3_S825 - cost: 8.74, layer: 56
Fold over basal
Time (min)
0.8
1
1.2
1.4
1.6
1.8
2
0
10
20
30
40
50
60
Normal
LY50
LY75
LY88
LY94
LY97
LY data
Normal data

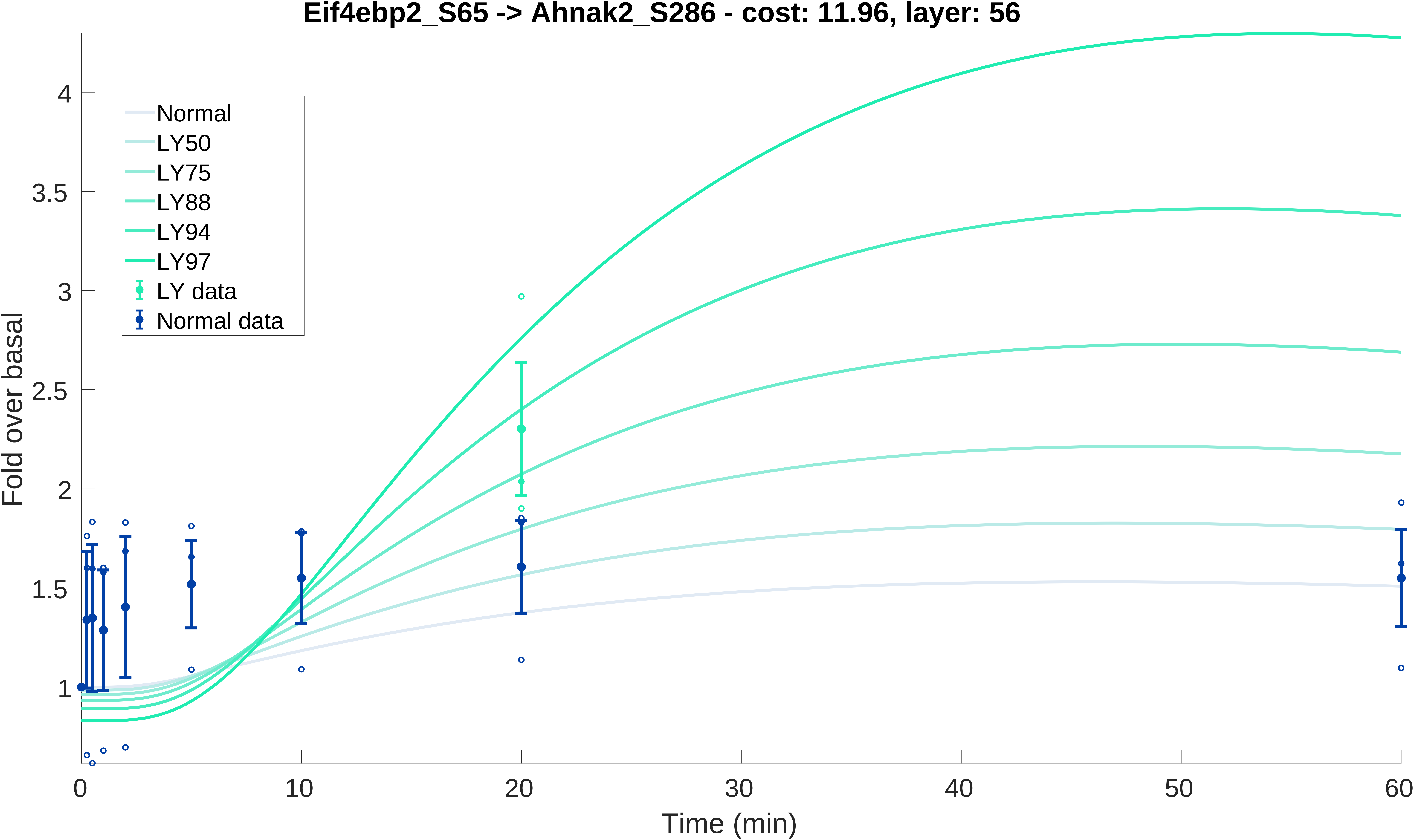
Eif4ebp2_S65 -> Ahnak2_S286 - cost: 11.96, layer: 56
Normal
LY50
LY75
LY88
LY94
LY97
LY data
Normal data
Fold over basal
Time (min)
4
3.5
3
2.5
2
1.5
1
0
10
20
30
40
50
60

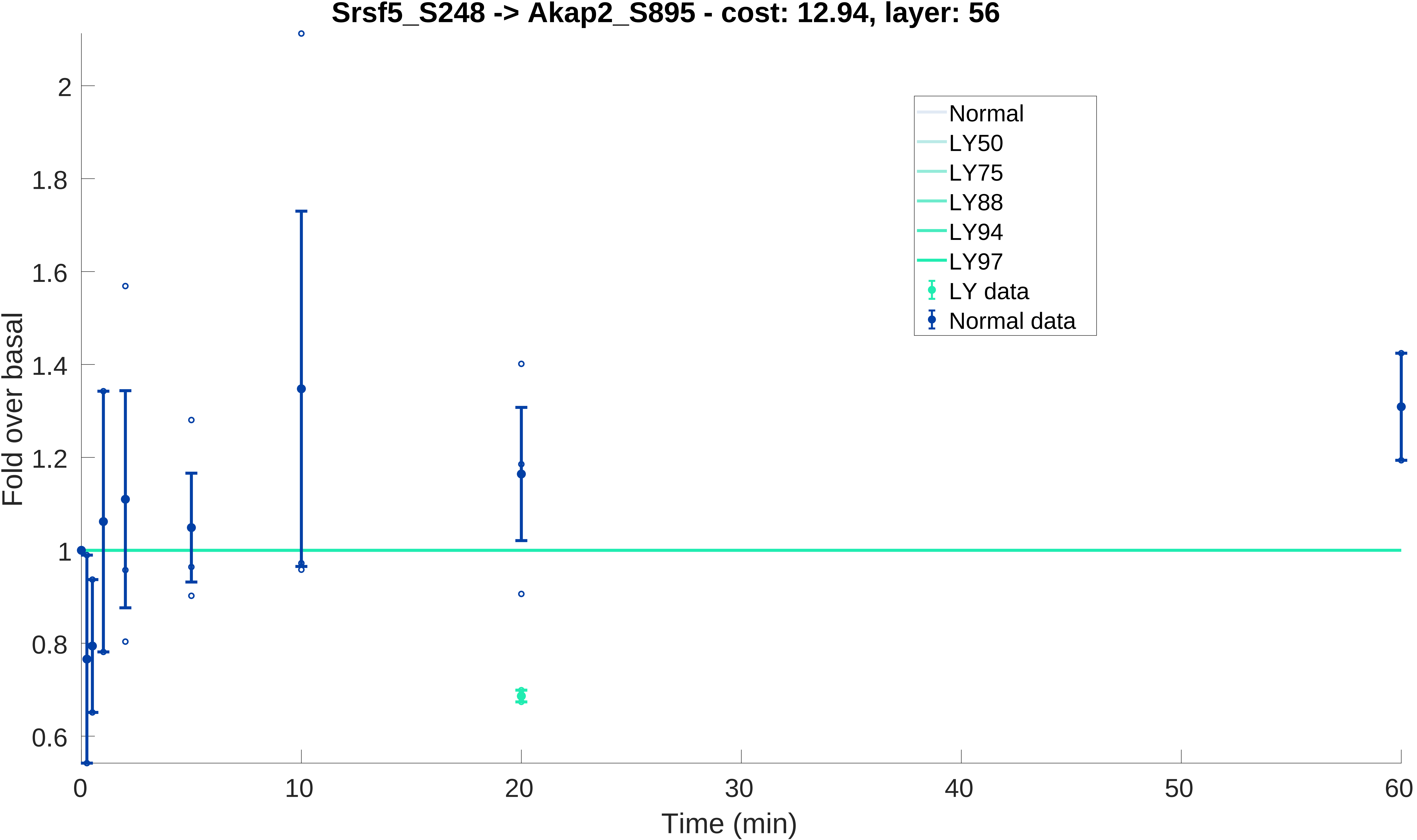

Srsf5_S248 -> Akap2_S895 - cost: 12.94, layer: 56
Fold over basal
Time (min)
Normal
LY50
LY75
LY88
LY94
LY97
LY data
Normal data
0.6
0.8
1
1.2
1.4
1.6
1.8
2
0
10
20
30
40
50
60

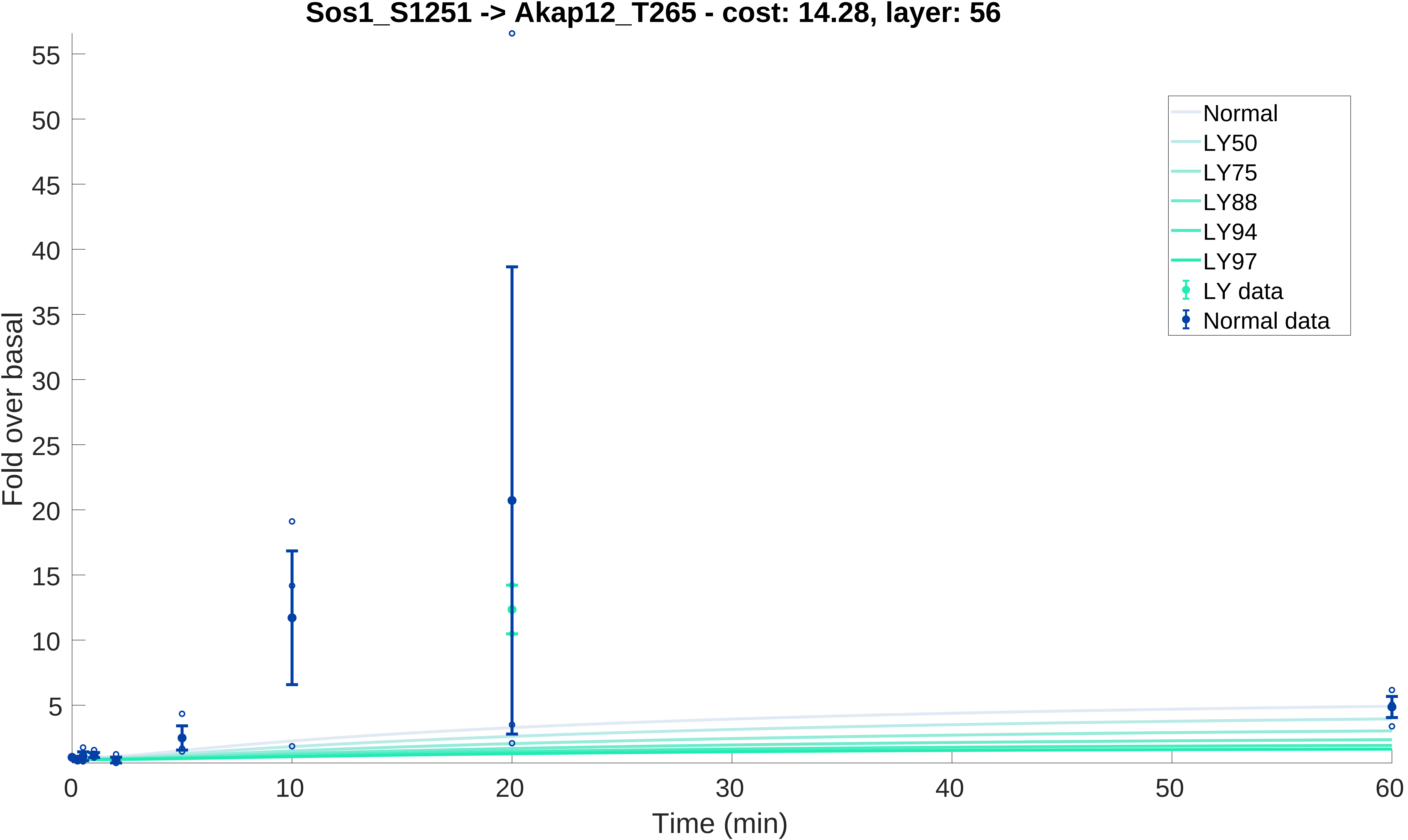

Sos1_S1251 -> Akap12_T265 - cost: 14.28, layer: 56
Fold over basal
55
50
45
40
35
30
25
20
15
10
5
0
10
20
30
40
50
60
Time (min)
Normal
LY50
LY75
LY88
LY94
LY97
LY data
Normal data

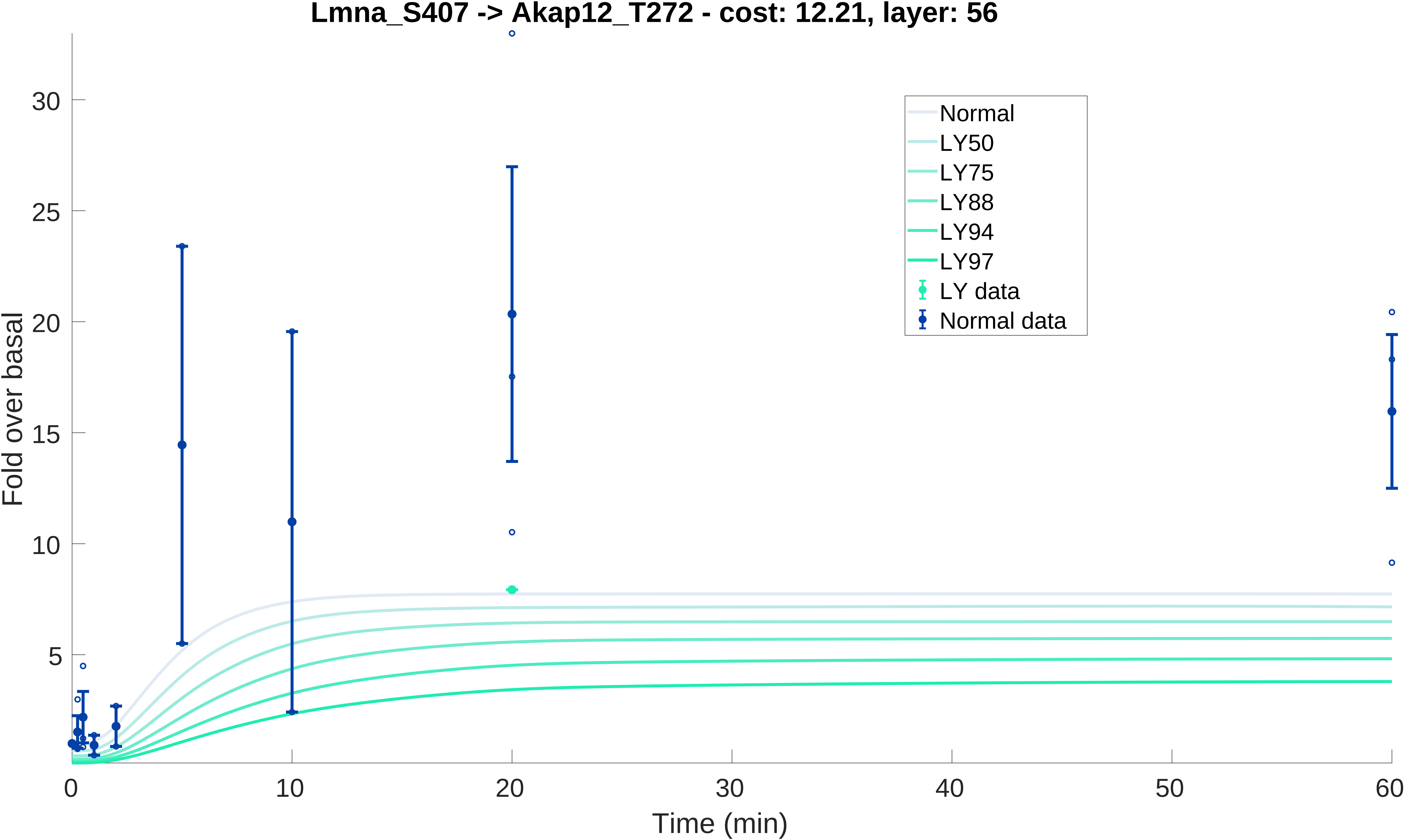

Lmna_S407 -> Akap12_T272 - cost: 12.21, layer: 56
Fold over basal
Time (min)
0
10
20
30
40
50
60
5
10
15
20
25
30
Normal
LY50
LY75
LY88
LY94
LY97
LY data
Normal data

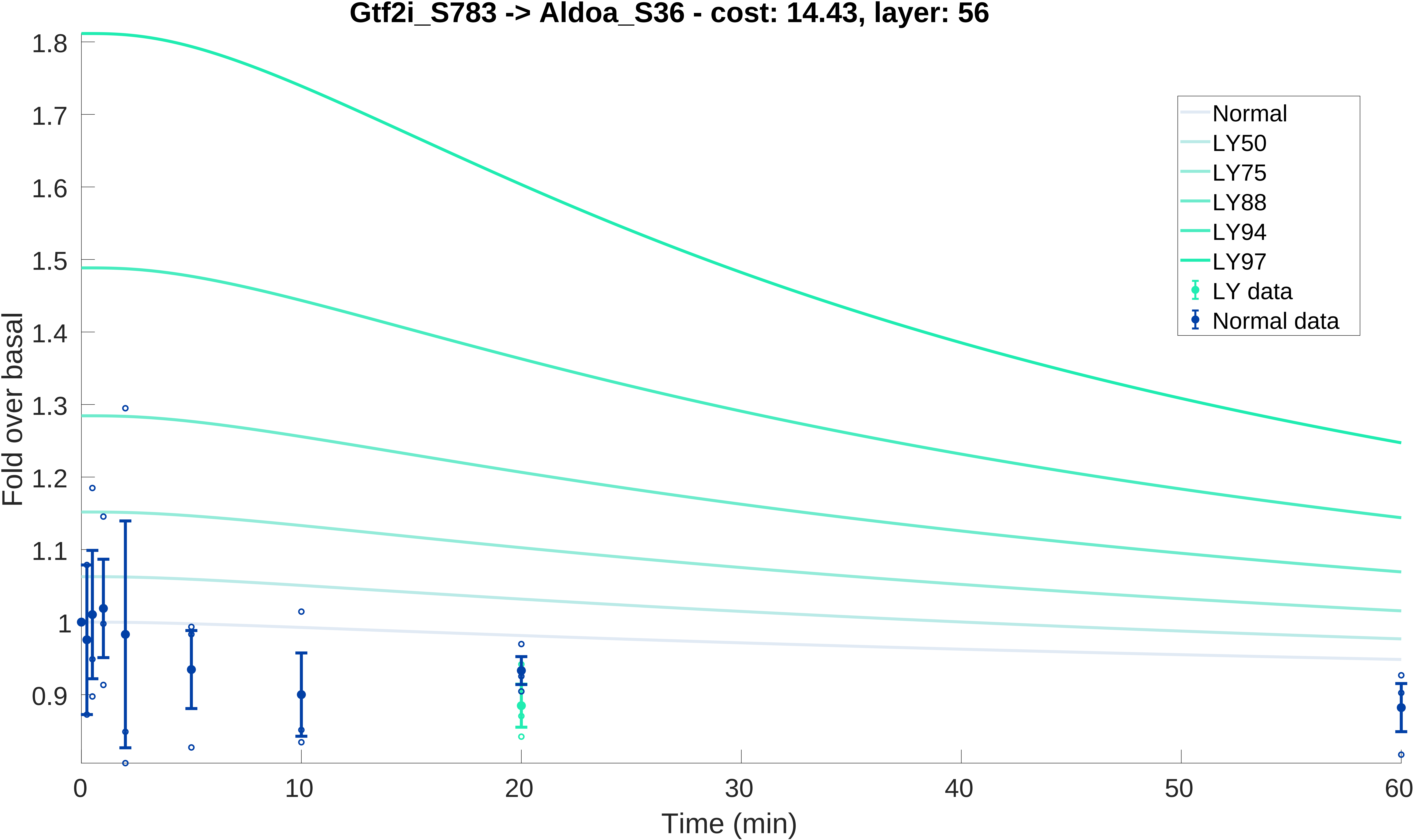

Gtf2i_S783 -> Aldoa_S36 - cost: 14.43, layer: 56
Fold over basal
Time (min)
1.8
1.7
1.6
1.5
1.4
1.3
1.2
1.1
1
0.9
0
10
20
30
40
50
60
Normal
LY50
LY75
LY88
LY94
LY97
LY data
Normal data

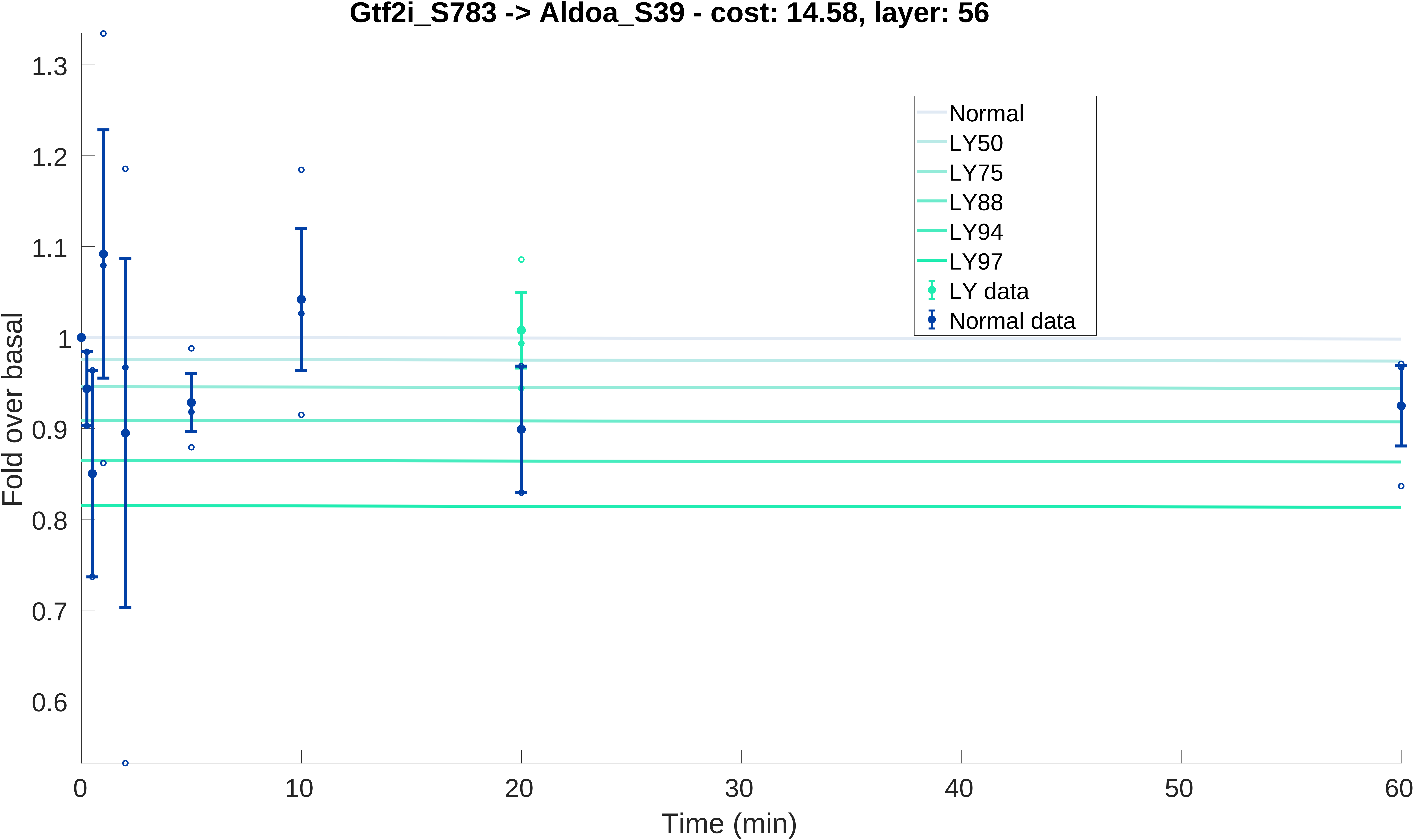
Gtf2i_S783 -> Aldoa_S39 - cost: 14.58, layer: 56
Normal
LY50
LY75
LY88
LY94
LY97
LY data
Normal data
Fold over basal
Time (min)
1.3
1.2
1.1
1
0.9
0.8
0.7
0.6
0
10
20
30
40
50
60

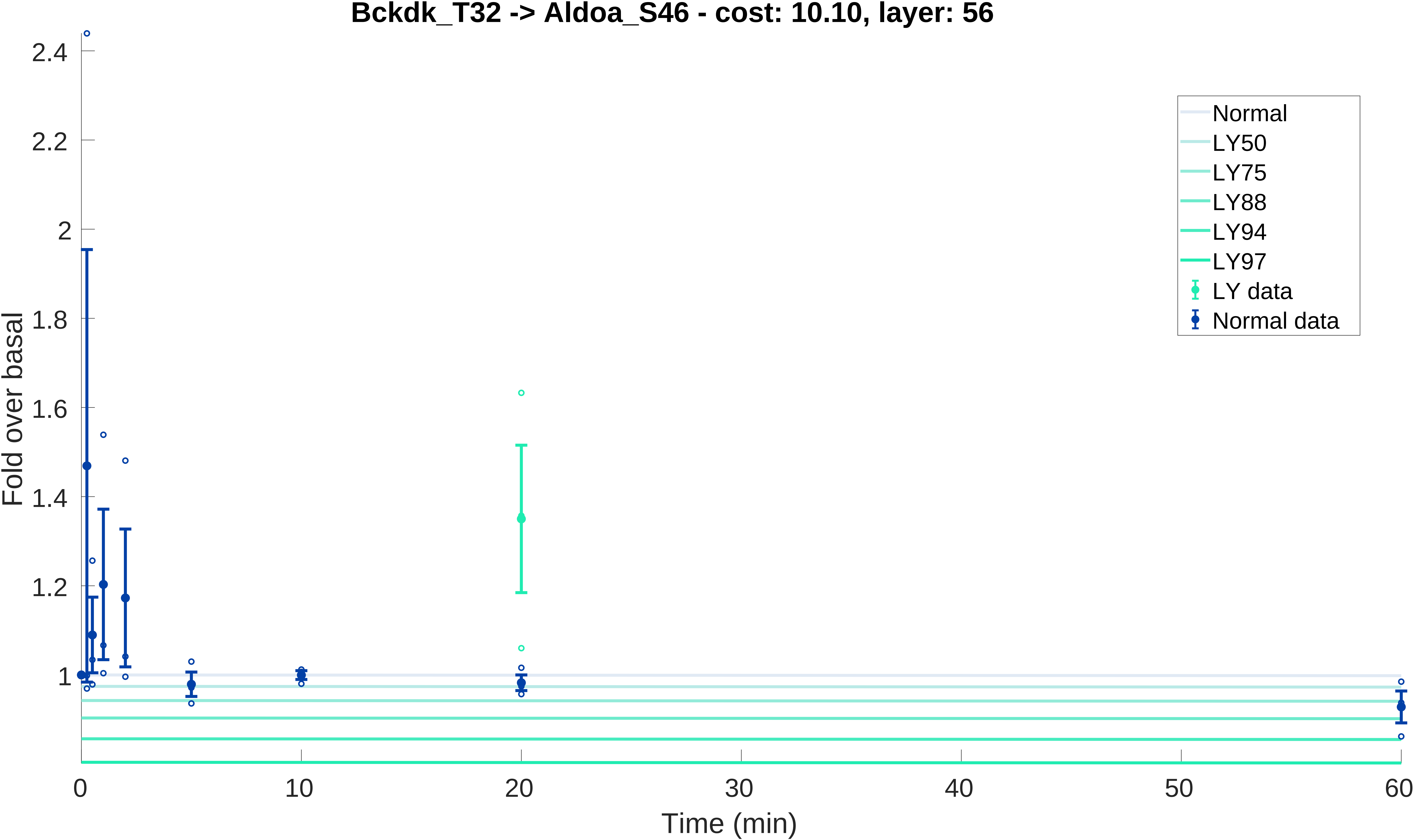

Bckdk_T32 -> Aldoa_S46 - cost: 10.10, layer: 56
Fold over basal
Time (min)
2.4
2.2
2
1.8
1.6
1.4
1.2
1
0
10
20
30
40
50
60
Normal
LY50
LY75
LY88
LY94
LY97
LY data
Normal data

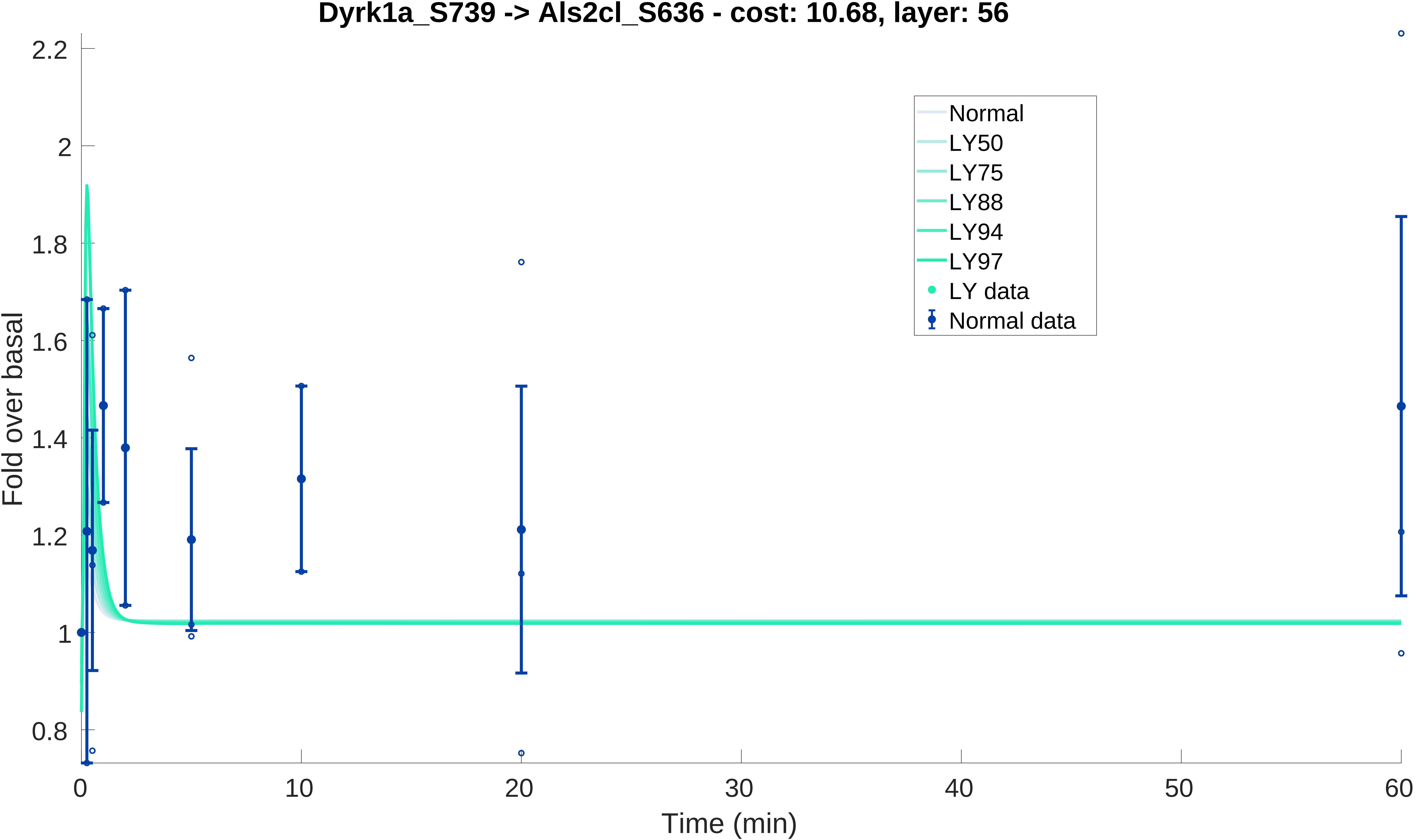

Dyrk1a_S739 -> Als2cl_S636 - cost: 10.68, layer: 56
Fold over basal
Time (min)
0.8
1
1.2
1.4
1.6
1.8
2
2.2
0
10
20
30
40
50
60
Normal
LY50
LY75
LY88
LY94
LY97
LY data
Normal data

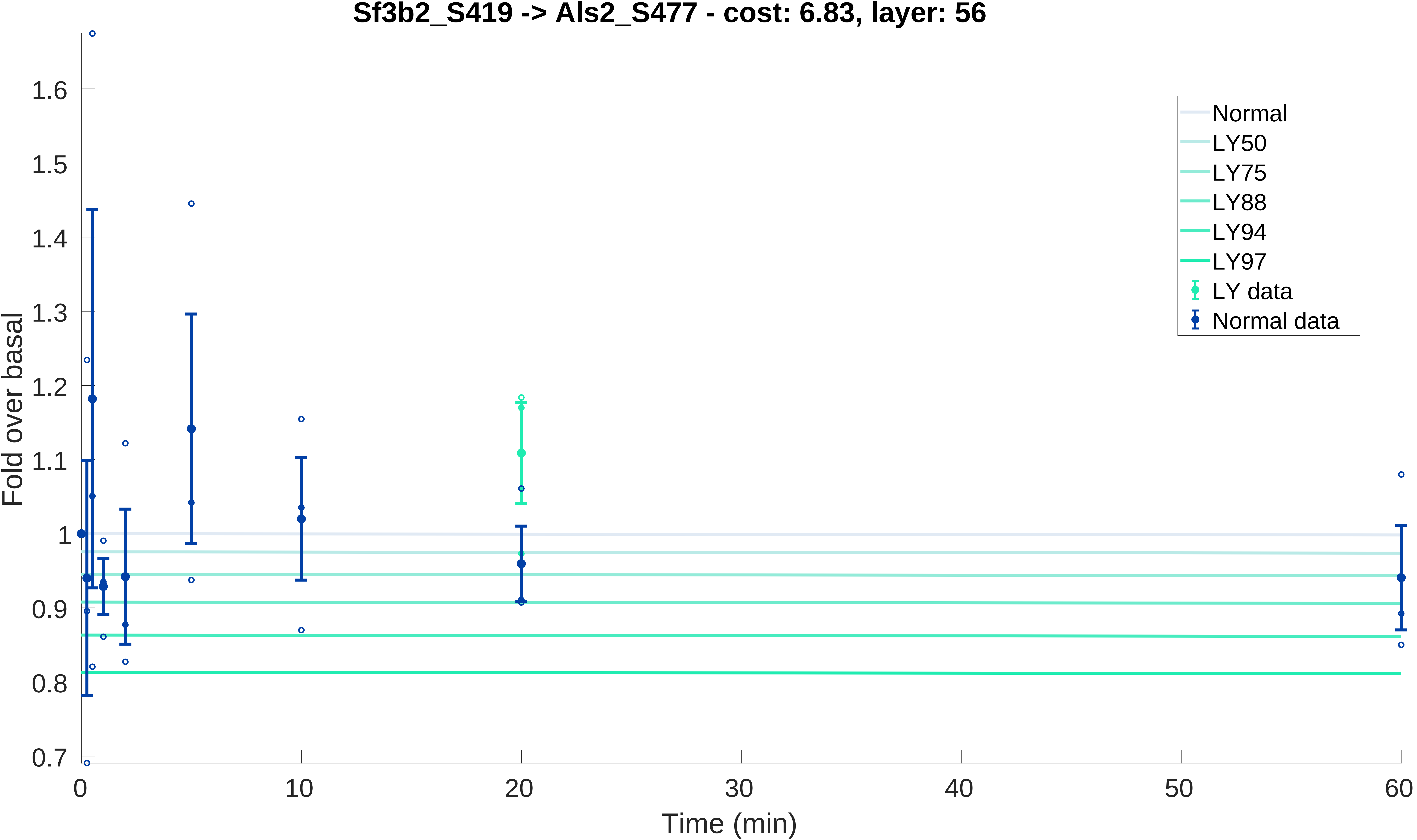

Sf3b2_S419 -> Als2_S477 - cost: 6.83, layer: 56
Fold over basal
Time (min)
0.7
0.8
0.9
1
1.1
1.2
1.3
1.4
1.5
1.6
0
10
20
30
40
50
60
Normal
LY50
LY75
LY88
LY94
LY97
LY data
Normal data

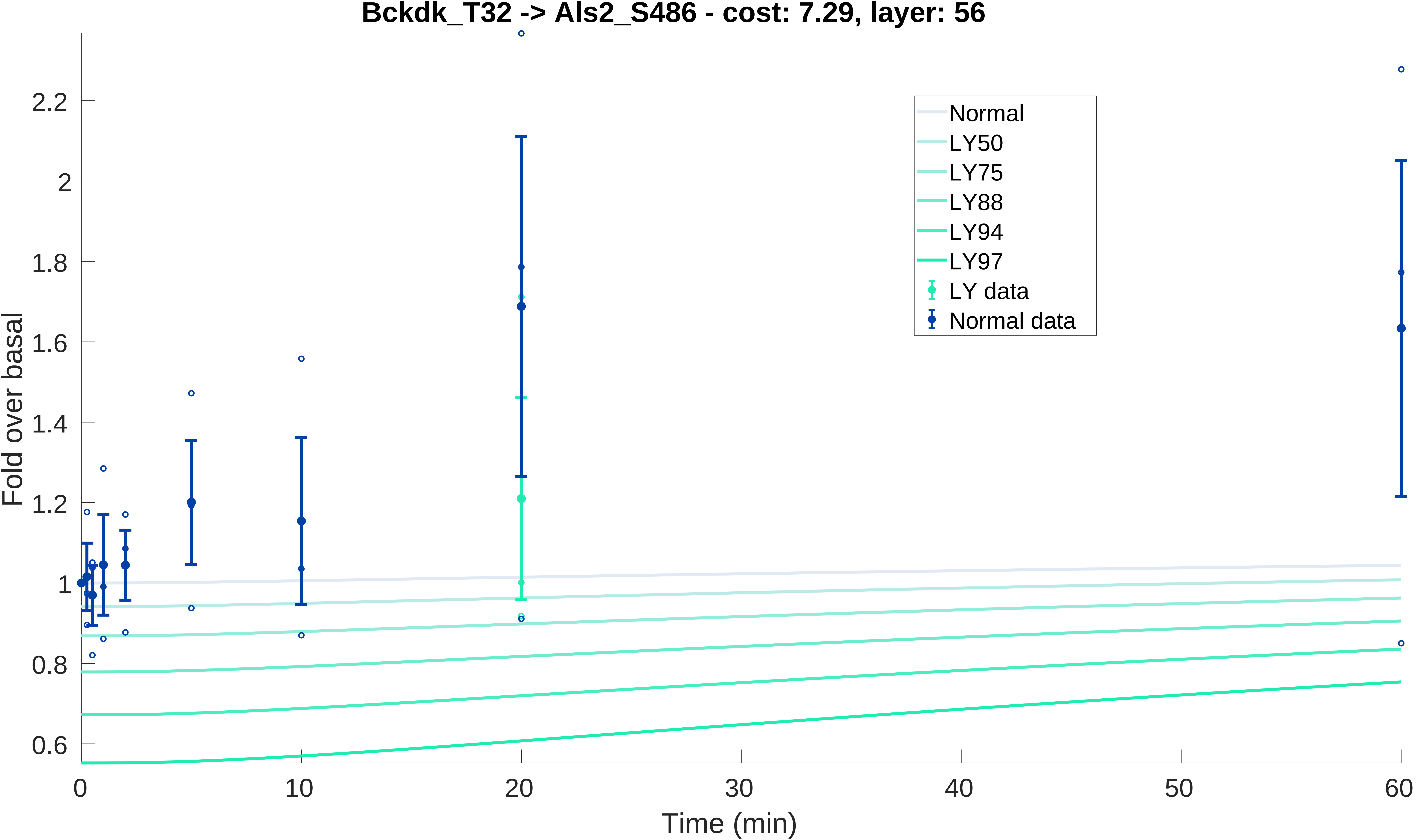
Bckdk_T32 -> Als2_S486 - cost: 7.29, layer: 56
Fold over basal
Time (min)
Normal
LY50
LY75
LY88
LY94
LY97
LY data
Normal data
0.6
0.8
1
1.2
1.4
1.6
1.8
2
2.2
0
10
20
30
40
50
60

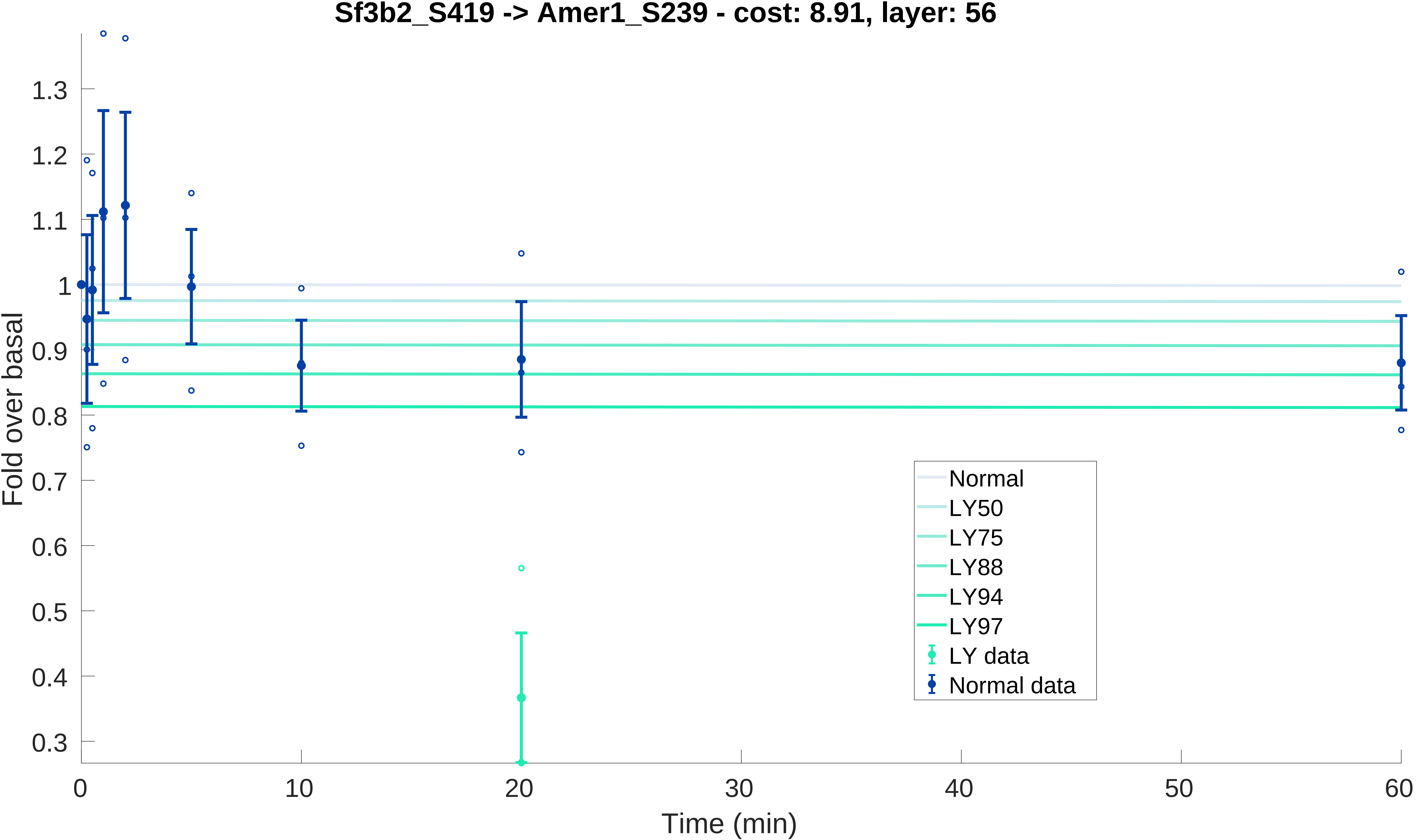

Sf3b2_S419 -> Amer1_S239 - cost: 8.91, layer: 56
Fold over basal
Time (min)
1.3
1.2
1.1
1
0.9
0.8
0.7
0.6
0.5
0.4
0.3
0
10
20
30
40
50
60
Normal
LY50
LY75
LY88
LY94
LY97
LY data
Normal data

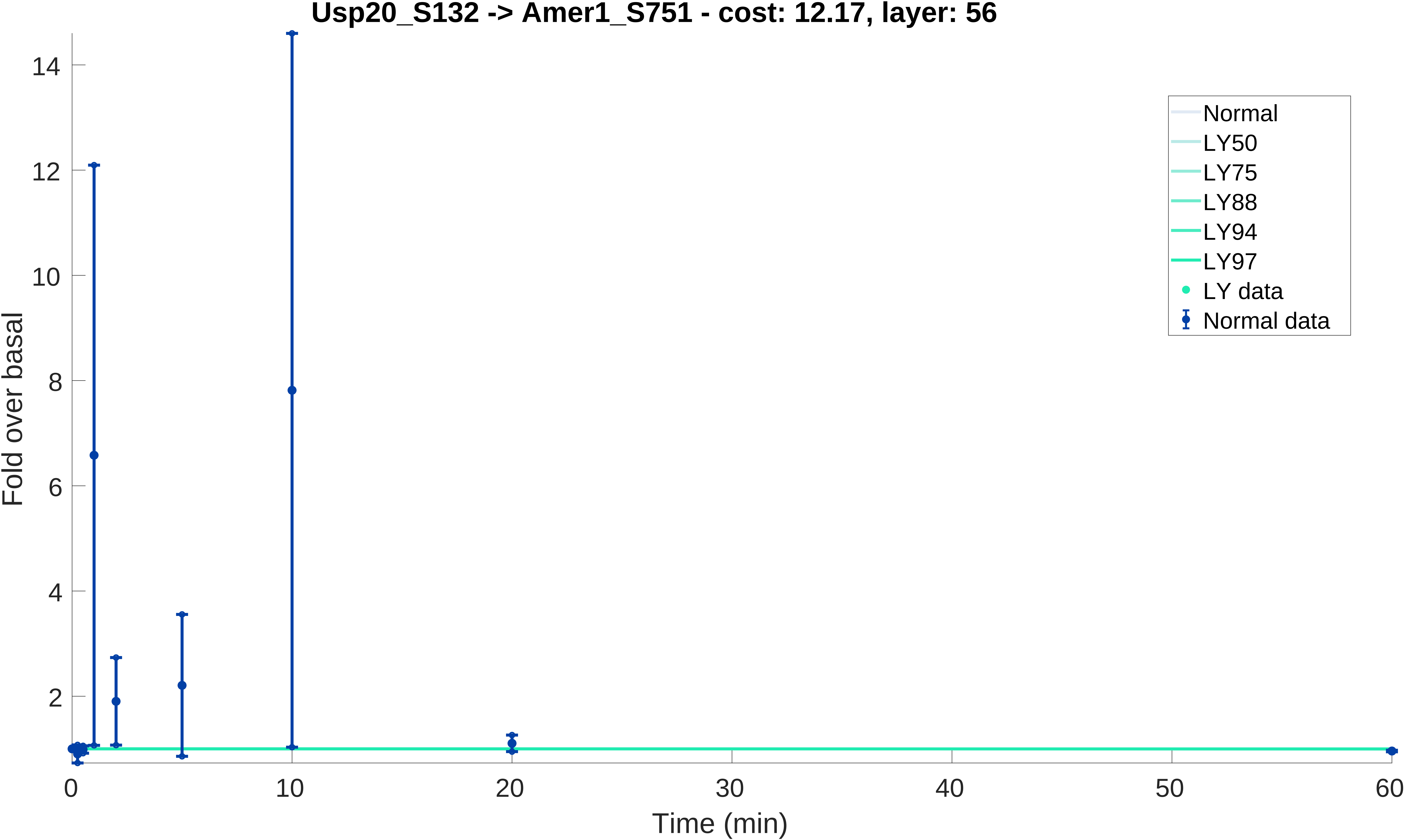

Usp20_S132 -> Amer1_S751 - cost: 12.17, layer: 56
Fold over basal
Time (min)
Normal
LY50
LY75
LY88
LY94
LY97
LY data
Normal data

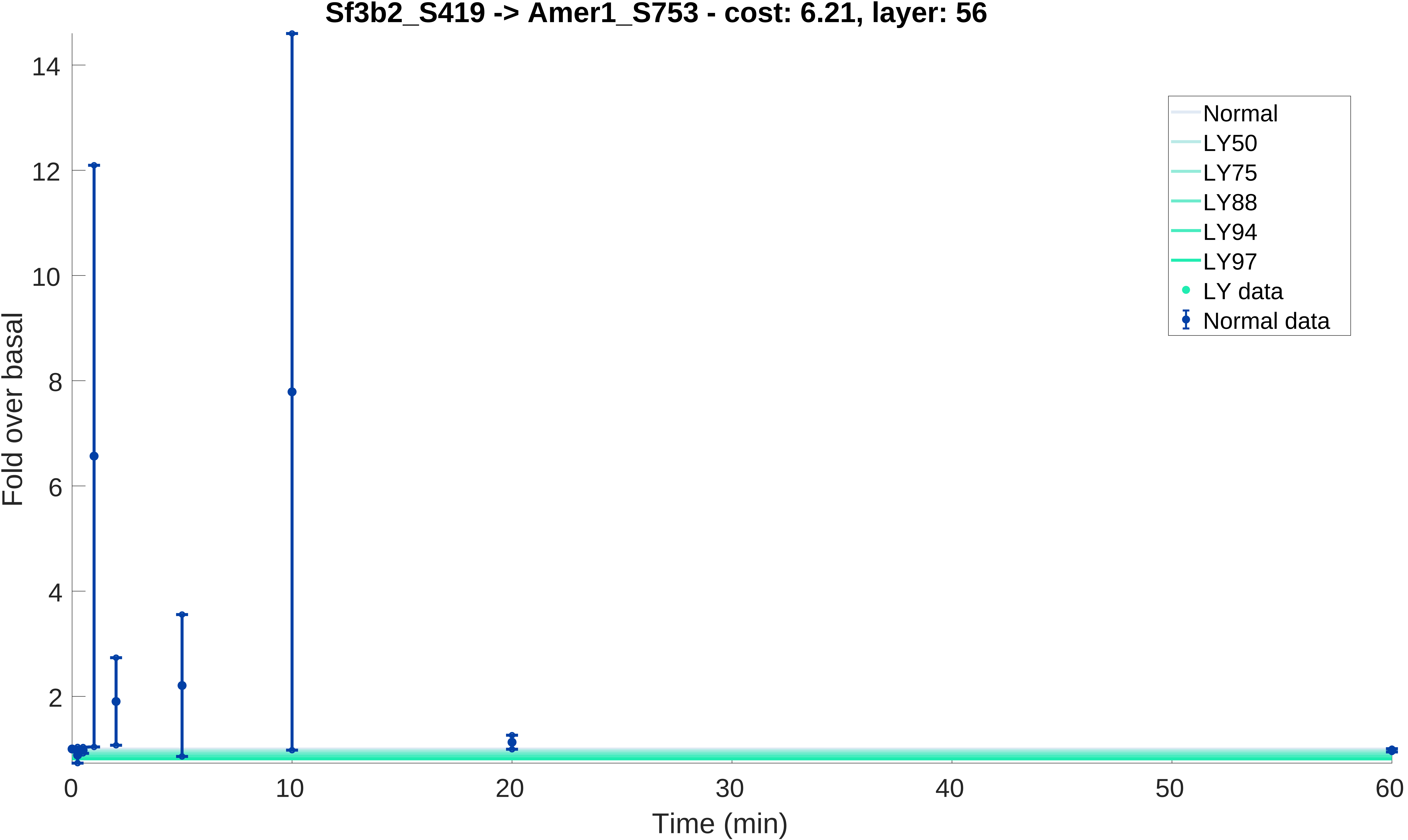

Sf3b2_S419 -> Amer1_S753 - cost: 6.21, layer: 56
Fold over basal
Time (min)
0
10
20
30
40
50
60
2
4
6
8
10
12
14
Normal
LY50
LY75
LY88
LY94
LY97
LY data
Normal data

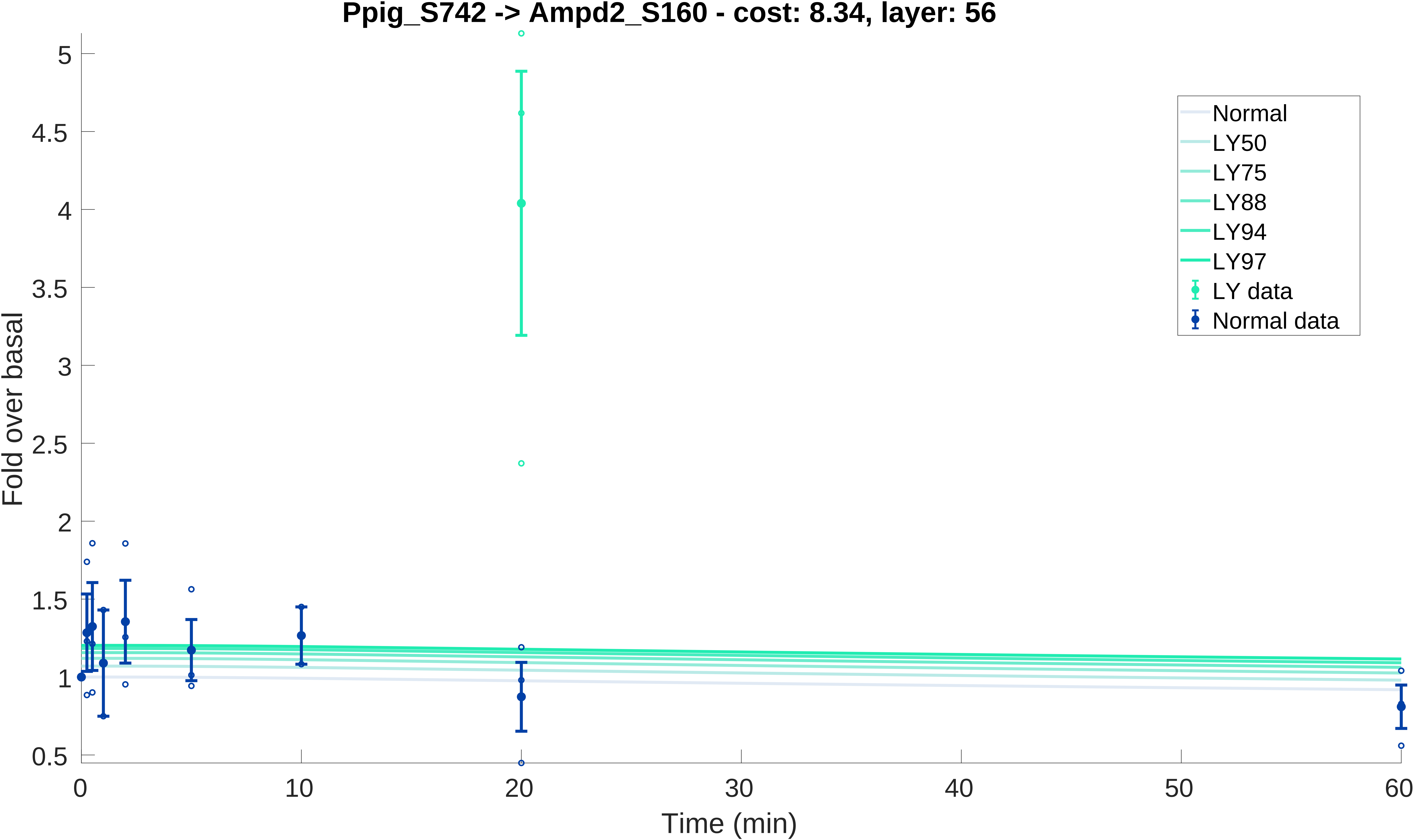
Ppig_S742 -> Ampd2_S160 - cost: 8.34, layer: 56
Fold over basal
Time (min)
Normal
LY50
LY75
LY88
LY94
LY97
LY data
Normal data
0.5
1
1.5
2
2.5
3
3.5
4
4.5
5
0
10
20
30
40
50
60

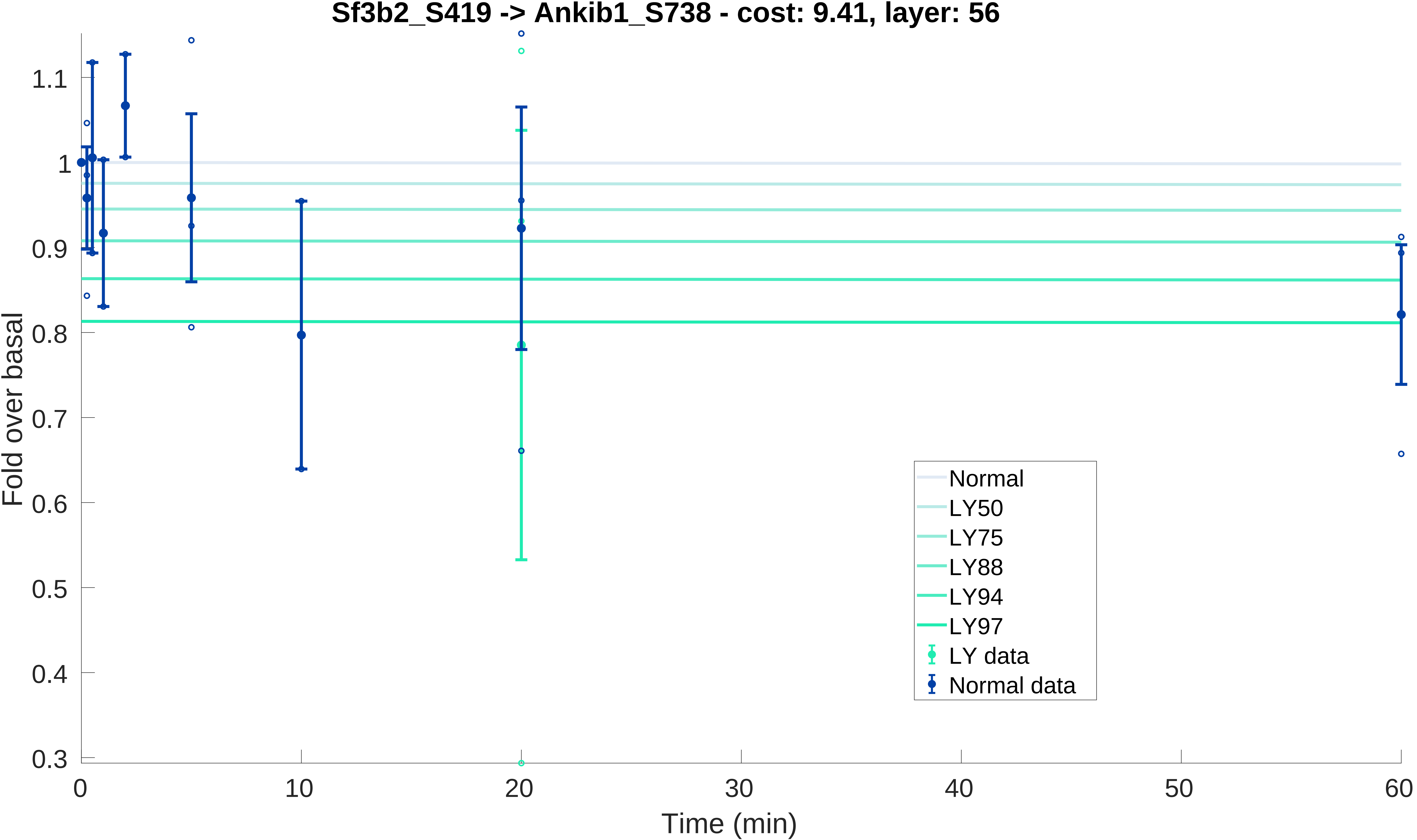

Sf3b2_S419 -> Ankib1_S738 - cost: 9.41, layer: 56
Fold over basal
1.1
1
0.9
0.8
0.7
0.6
0.5
0.4
0.3
0
10
20
30
40
50
60
Time (min)
Normal
LY50
LY75
LY88
LY94
LY97
LY data
Normal data

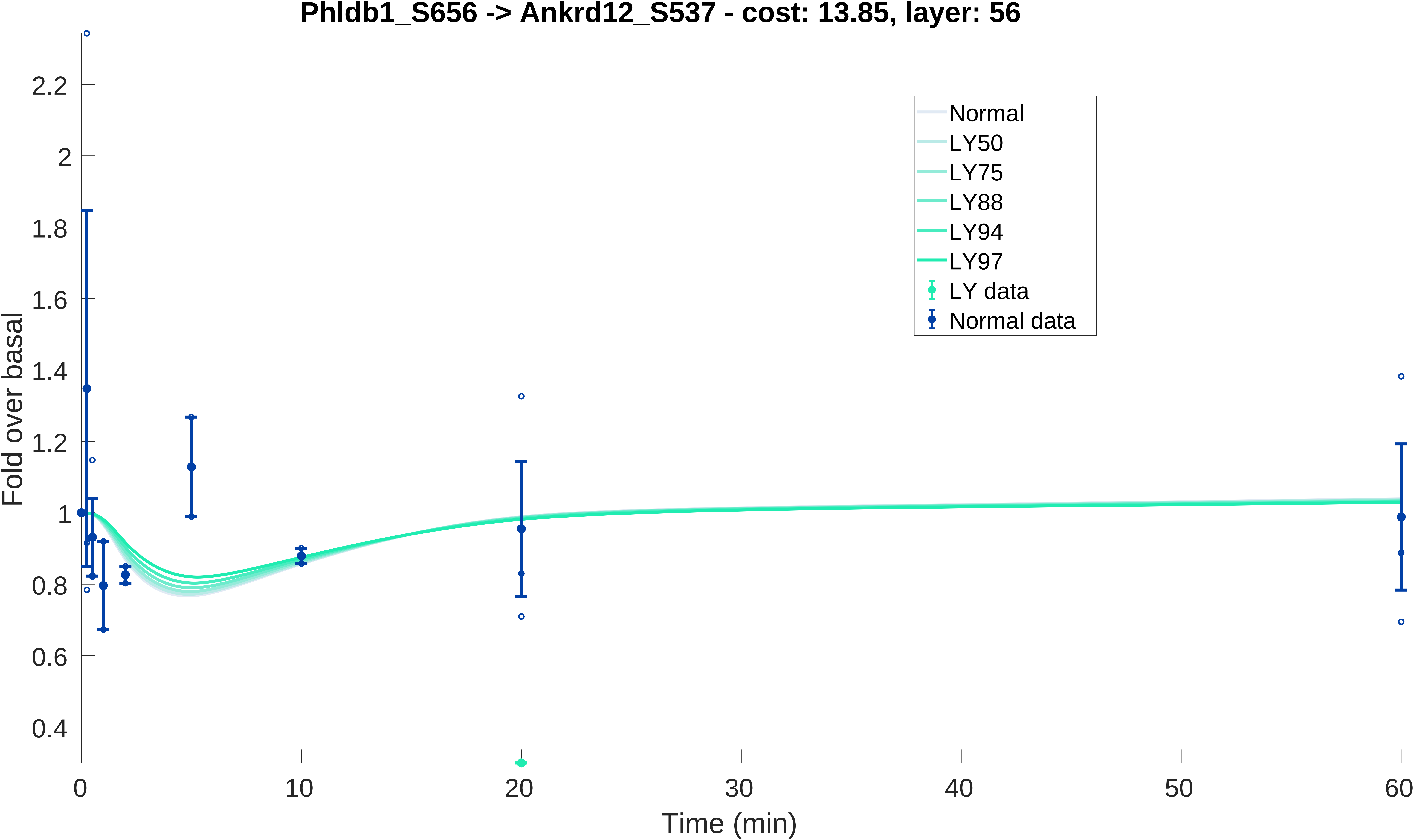

Phldb1_S656 -> Ankrd12_S537 - cost: 13.85, layer: 56
Fold over basal
Time (min)
2.2
2
1.8
1.6
1.4
1.2
1
0.8
0.6
0.4
0
10
20
30
40
50
60
Normal
LY50
LY75
LY88
LY94
LY97
LY data
Normal data

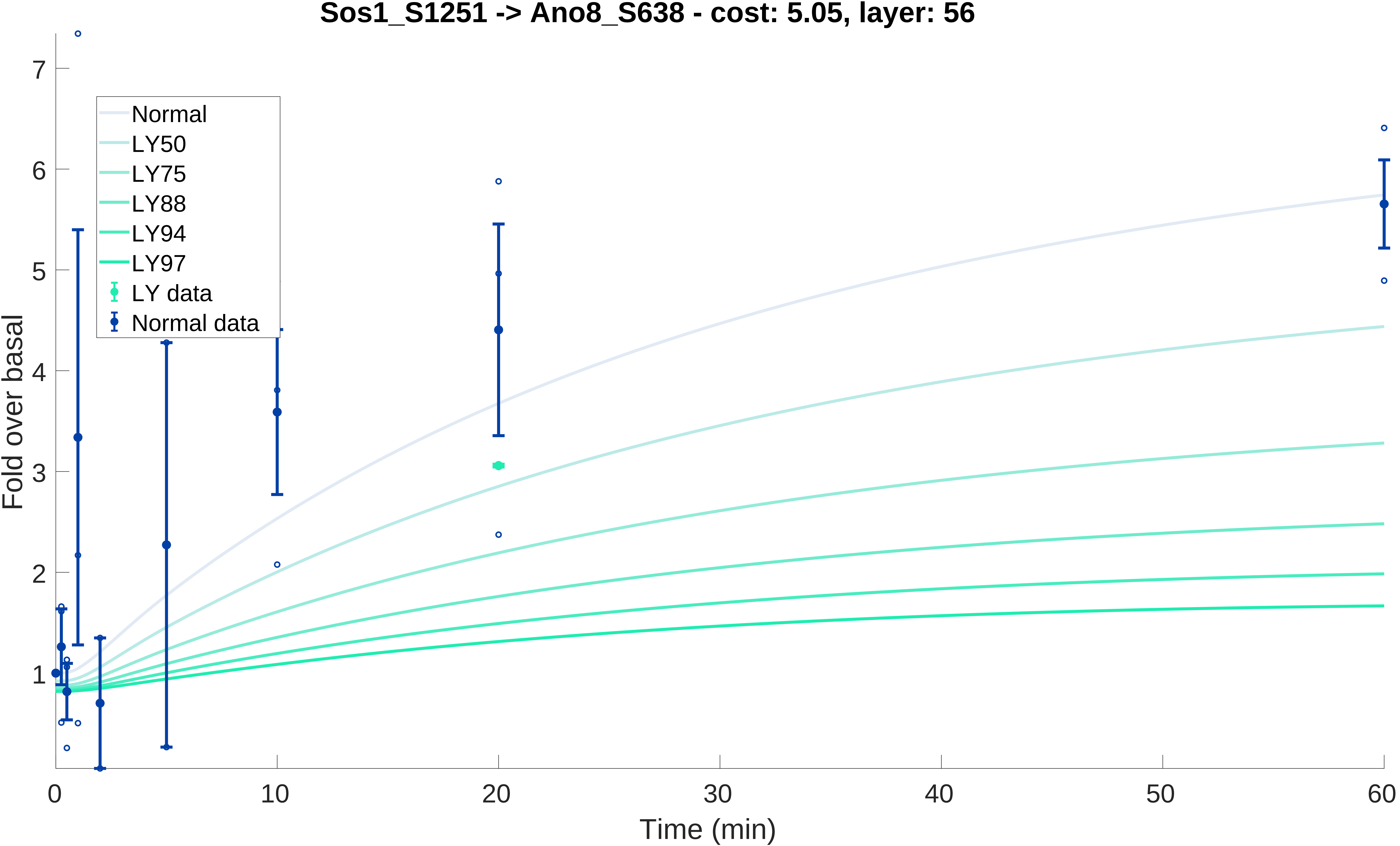

Sos1_S1251 -> Ano8_S638 - cost: 5.05, layer: 56
Fold over basal
Time (min)
1
2
3
4
5
6
7
0
10
20
30
40
50
60
Normal
LY50
LY75
LY88
LY94
LY97
LY data
Normal data

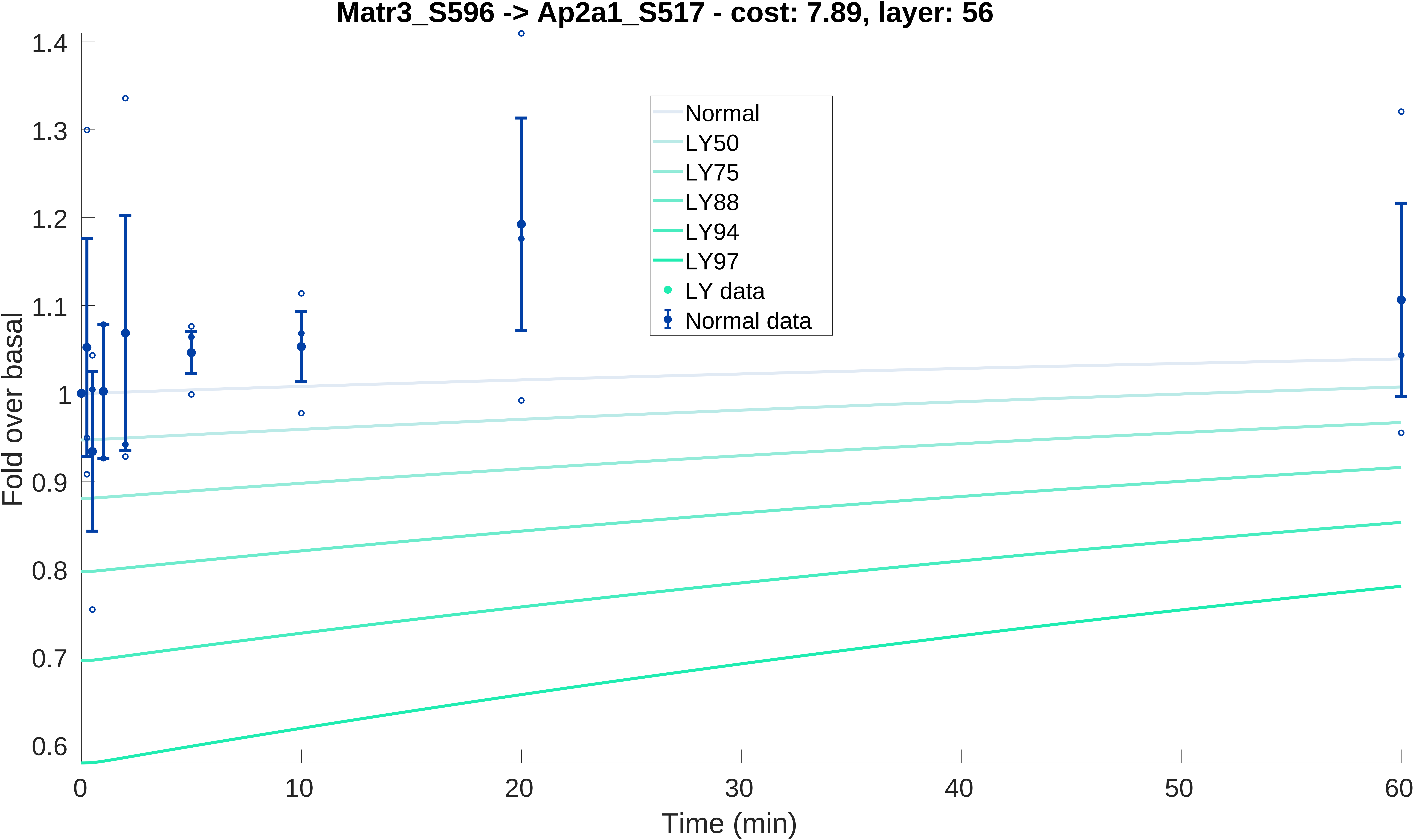
Matr3_S596 -> Ap2a1_S517 - cost: 7.89, layer: 56
Fold over basal
Time (min)
0.6
0.7
0.8
0.9
1
1.1
1.2
1.3
1.4
0
10
20
30
40
50
60
Normal
LY50
LY75
LY88
LY94
LY97
LY data
Normal data

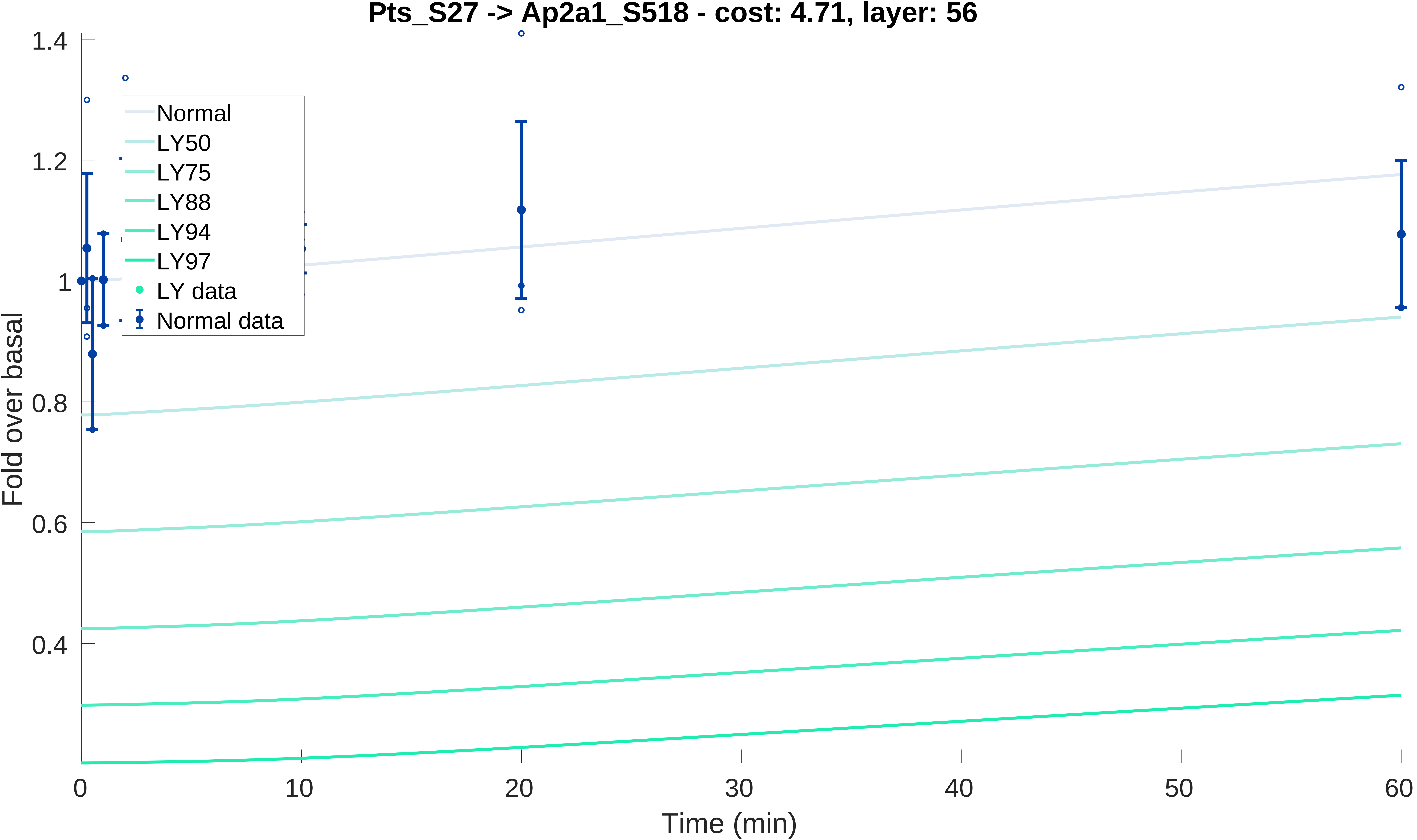

Pts_S27 -> Ap2a1_S518 - cost: 4.71, layer: 56
Fold over basal
Time (min)
1.4
1.2
1
0.8
0.6
0.4
0
10
20
30
40
50
60
Normal
LY50
LY75
LY88
LY94
LY97
LY data
Normal data

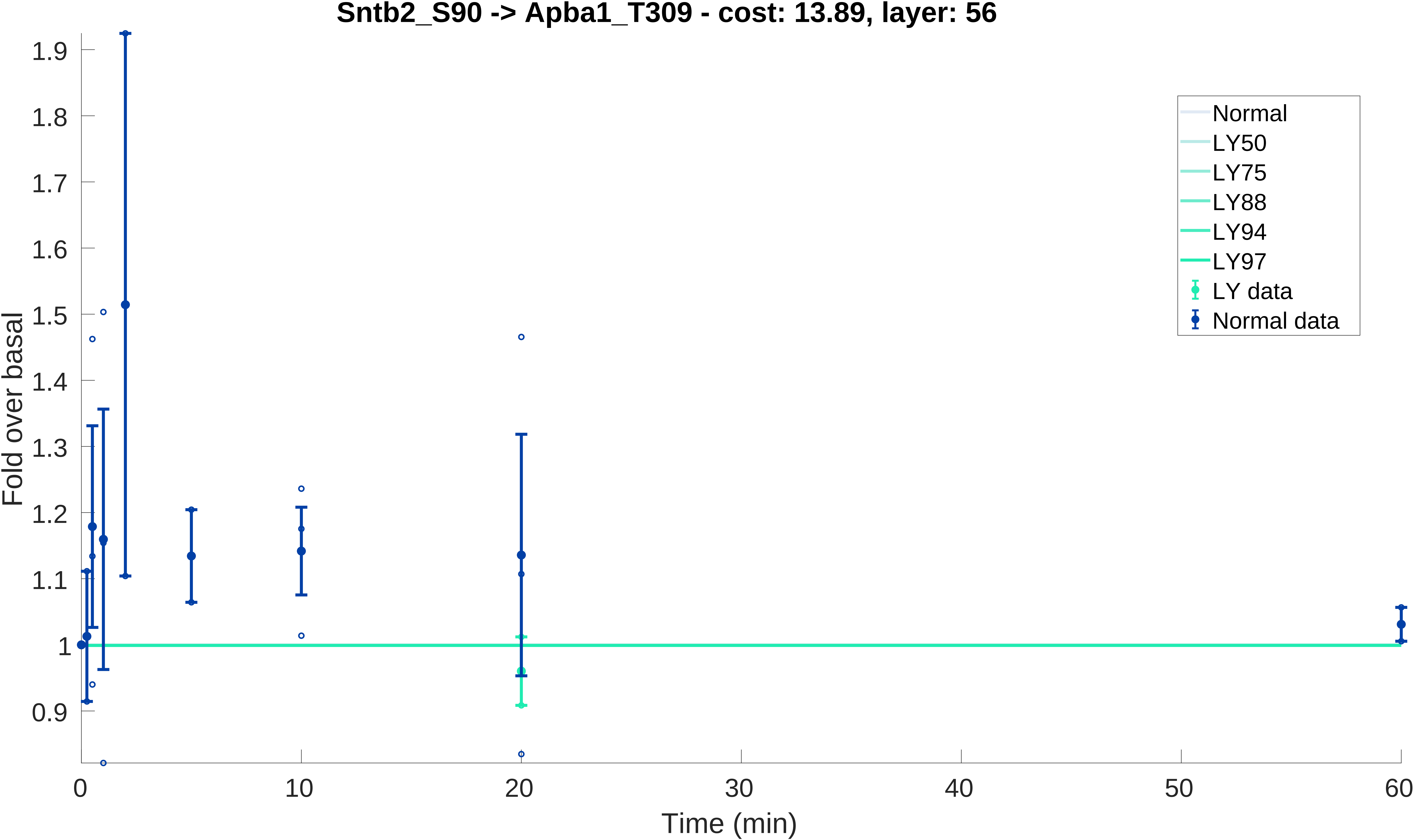

Sntb2_S90 -> Apba1_T309 - cost: 13.89, layer: 56
Fold over basal
Time (min)
Normal
LY50
LY75
LY88
LY94
LY97
LY data
Normal data
0.9
1
1.1
1.2
1.3
1.4
1.5
1.6
1.7
1.8
1.9
0
10
20
30
40
50
60

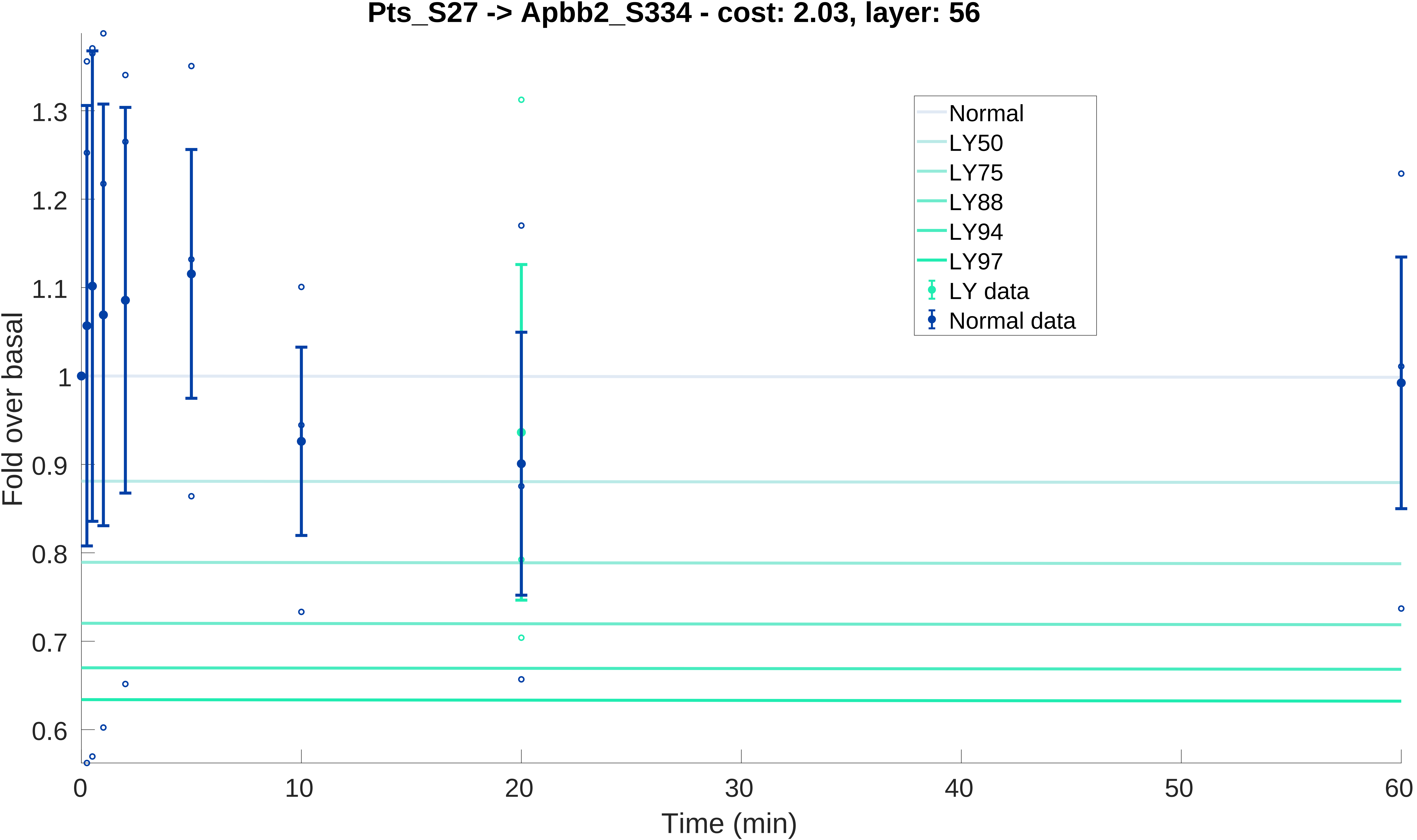

Pts_S27 -> Apbb2_S334 - cost: 2.03, layer: 56
Fold over basal
1.3
1.2
1.1
1
0.9
0.8
0.7
0.6
0
10
20
30
40
50
60
Time (min)
Normal
LY50
LY75
LY88
LY94
LY97
LY data
Normal data

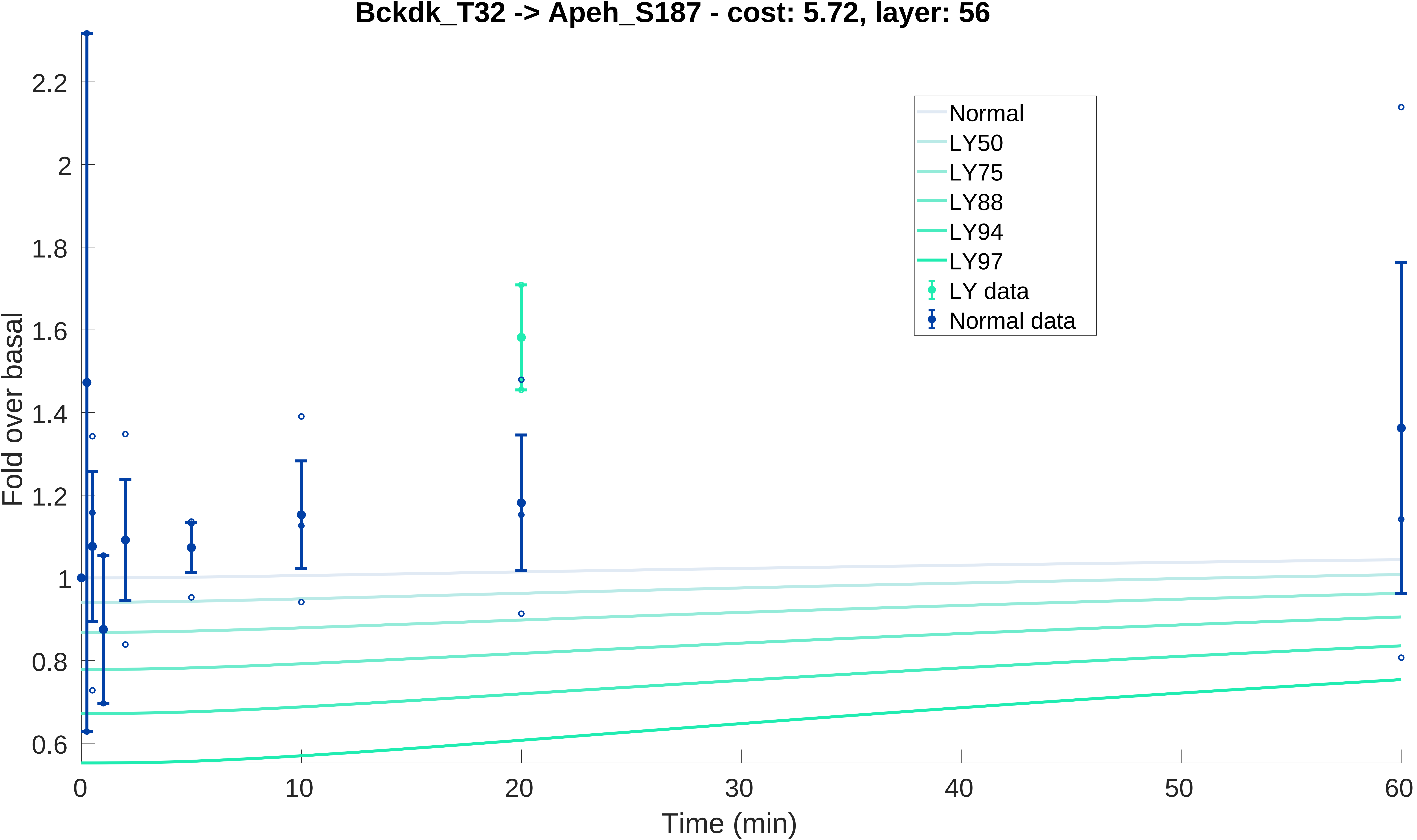

Bckdk_T32 -> Apeh_S187 - cost: 5.72, layer: 56
Fold over basal
0.6
0.8
1
1.2
1.4
1.6
1.8
2
2.2
Time (min)
0
10
20
30
40
50
60
Normal
LY50
LY75
LY88
LY94
LY97
LY data
Normal data

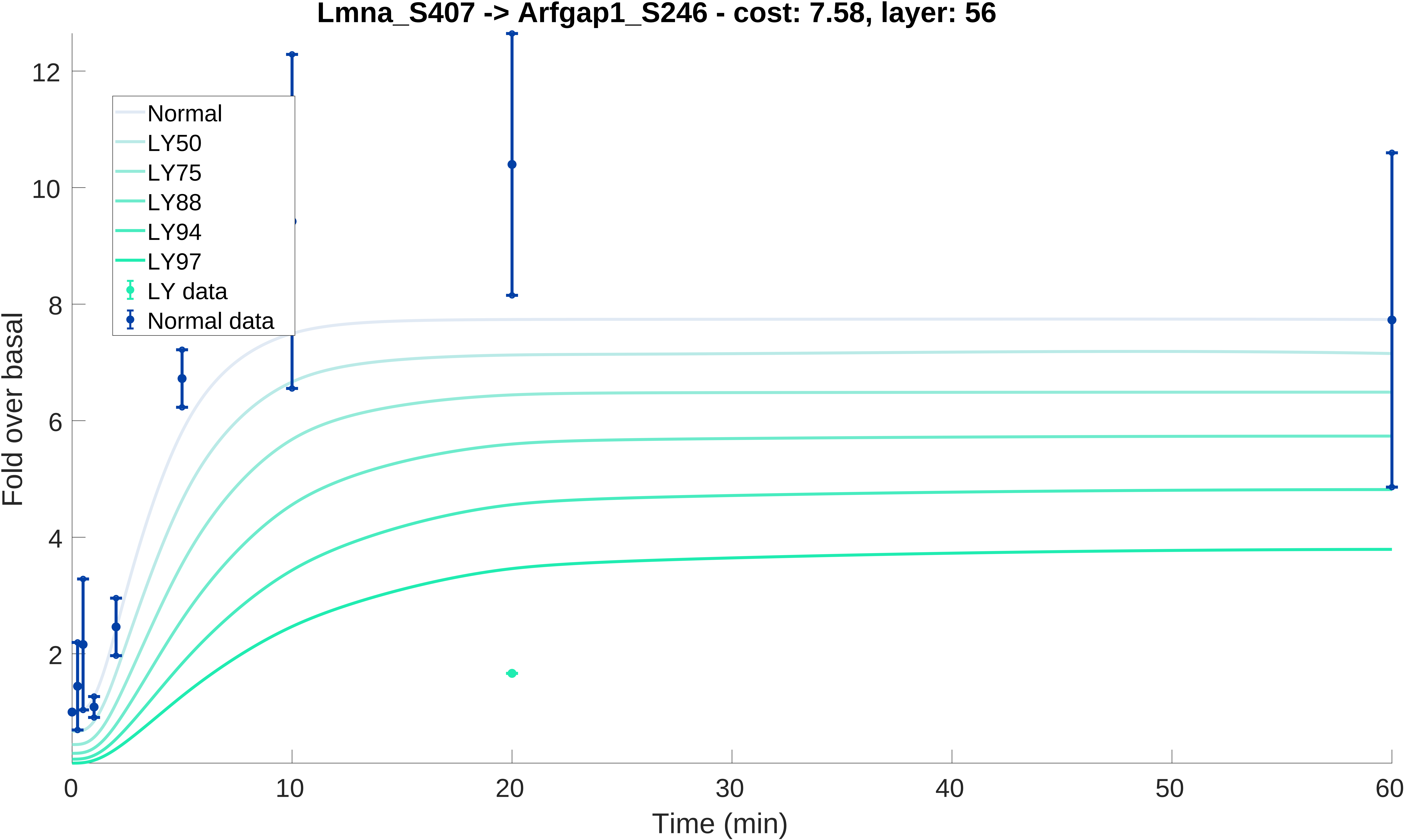

Lmna_S407 -> Arfgap1_S246 - cost: 7.58, layer: 56
Fold over basal
Time (min)
0
10
20
30
40
50
60
2
4
6
8
10
12
Normal
LY50
LY75
LY88
LY94
LY97
LY data
Normal data

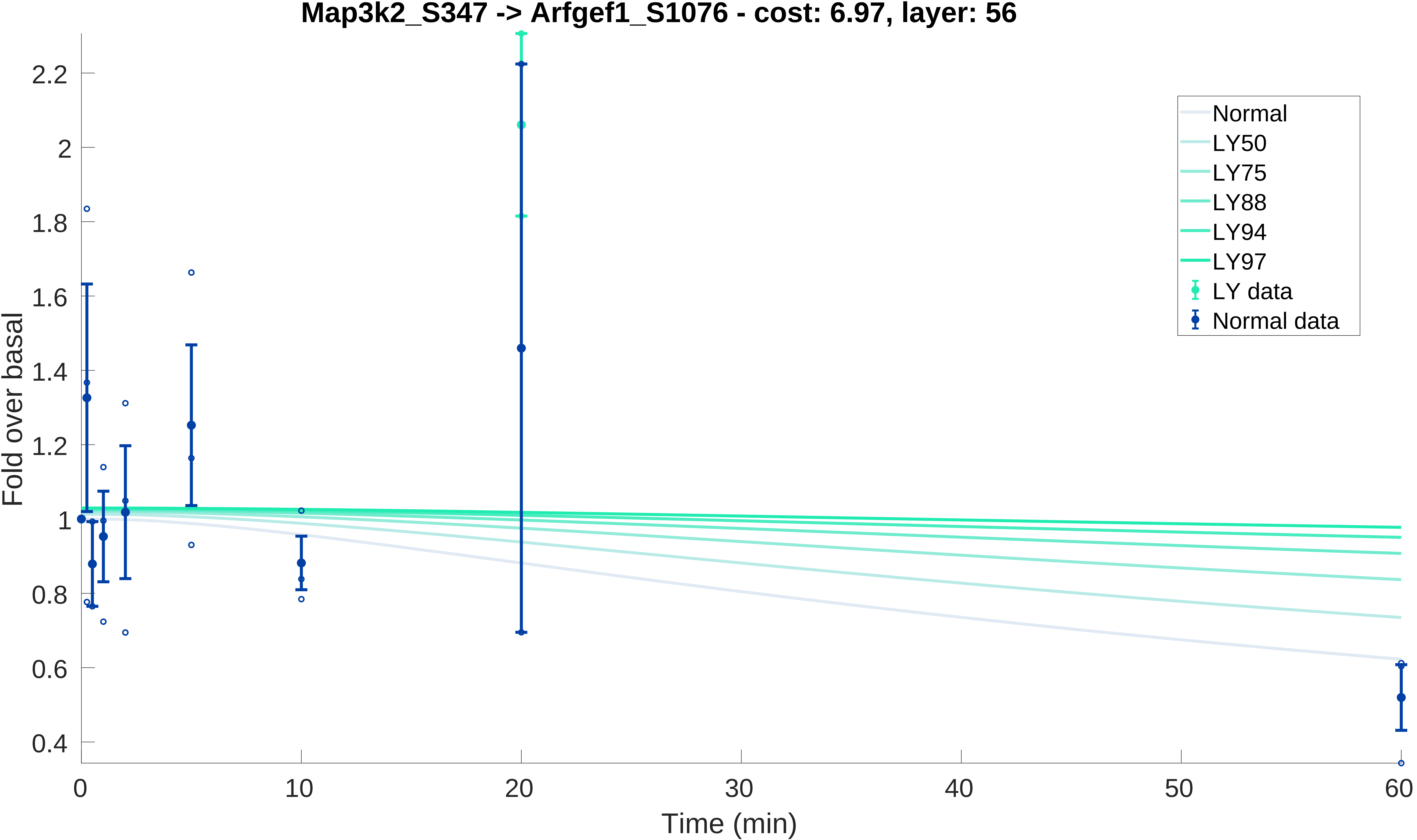

Map3k2_S347 -> Arfgef1_S1076 - cost: 6.97, layer: 56
Fold over basal
Time (min)
2.2
2
1.8
1.6
1.4
1.2
1
0.8
0.6
0.4
0
10
20
30
40
50
60
Normal
LY50
LY75
LY88
LY94
LY97
LY data
Normal data

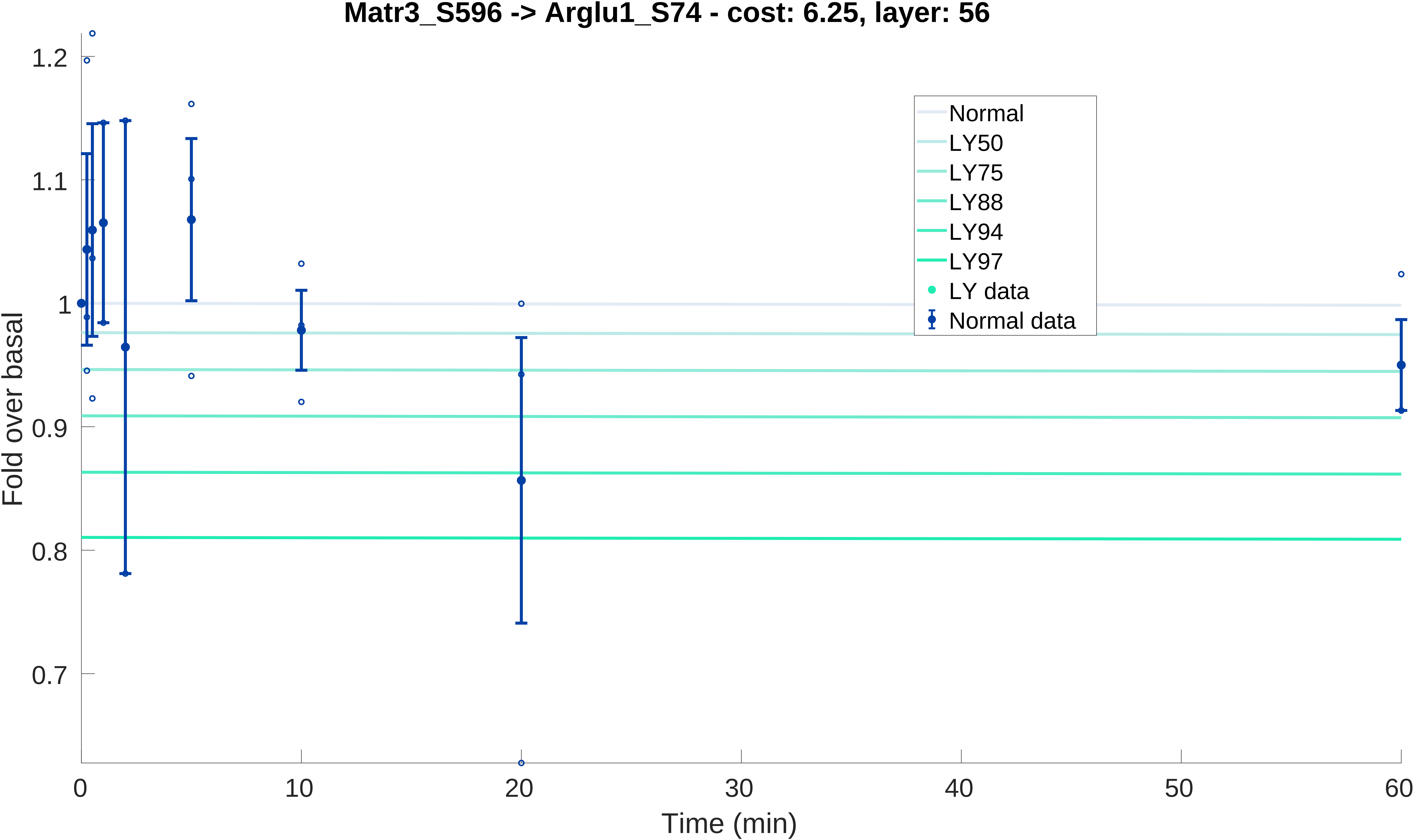

Matr3_S596 -> Arglu1_S74 - cost: 6.25, layer: 56
Normal
LY50
LY75
LY88
LY94
LY97
LY data
Normal data
Fold over basal
1.2
1.1
1
0.9
0.8
0.7
0
10
20
30
40
50
60
Time (min)

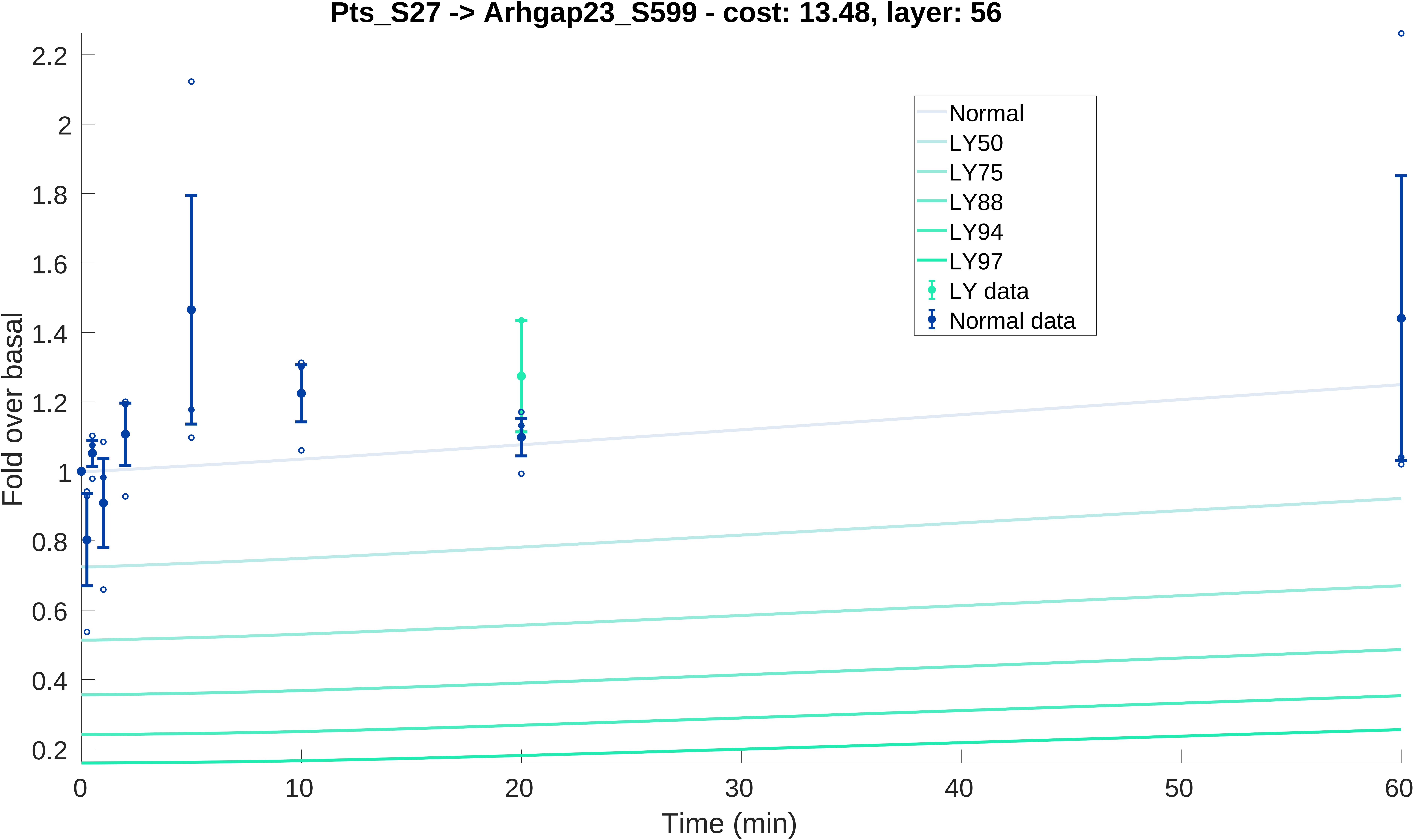

Pts_S27 -> Arhgap23_S599 - cost: 13.48, layer: 56
Fold over basal
Time (min)
Normal
LY50
LY75
LY88
LY94
LY97
LY data
Normal data
2.2
2
1.8
1.6
1.4
1.2
1
0.8
0.6
0.4
0.2
0
10
20
30
40
50
60

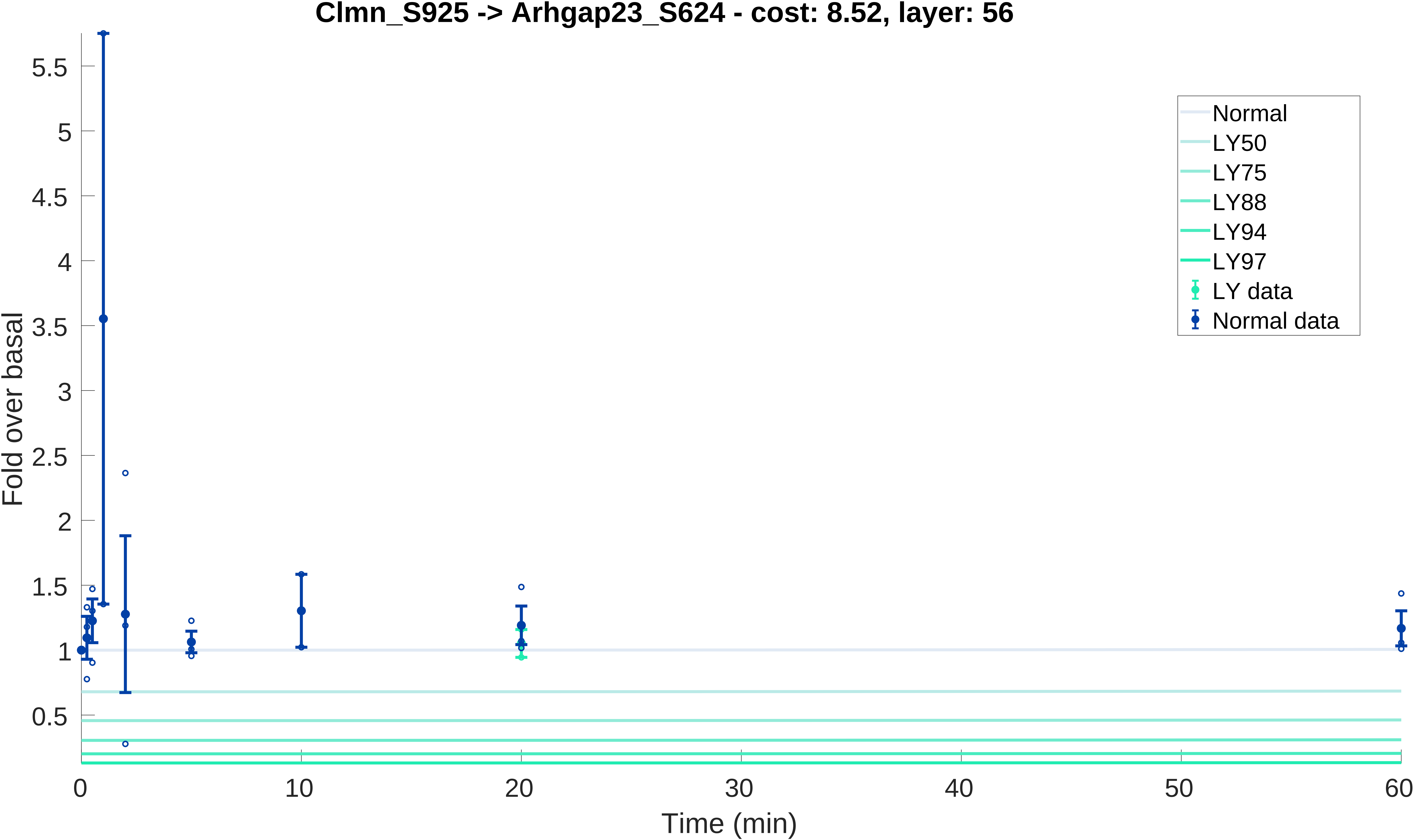
Clmn_S925 -> Arhgap23_S624 - cost: 8.52, layer: 56
Fold over basal
Time (min)
0
10
20
30
40
50
60
0.5
1
1.5
2
2.5
3
3.5
4
4.5
5
5.5
Normal
LY50
LY75
LY88
LY94
LY97
LY data
Normal data

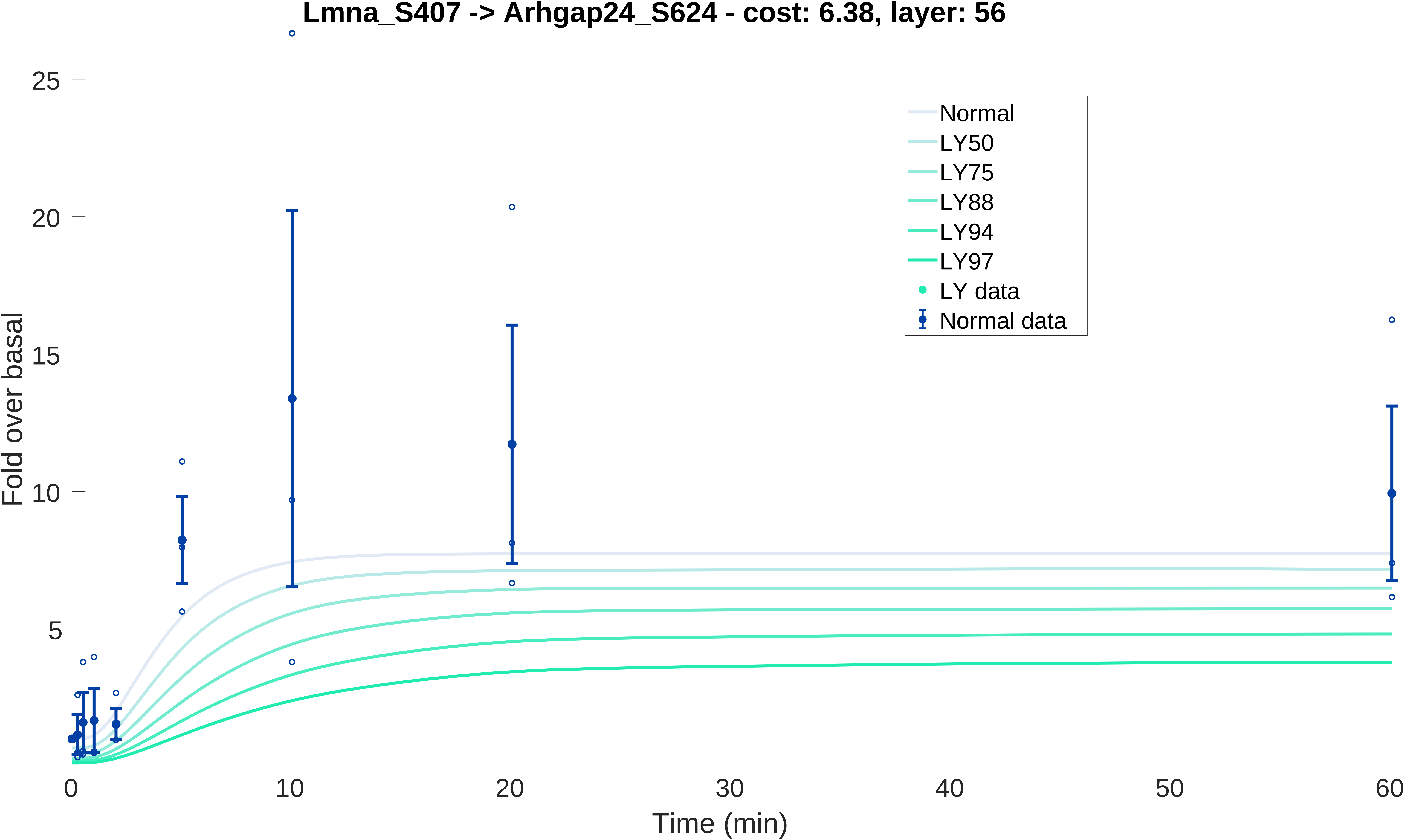

Lmna_S407 -> Arhgap24_S624 - cost: 6.38, layer: 56
Fold over basal
Time (min)
0
5
10
15
20
25
0
10
20
30
40
50
60
Normal
LY50
LY75
LY88
LY94
LY97
LY data
Normal data

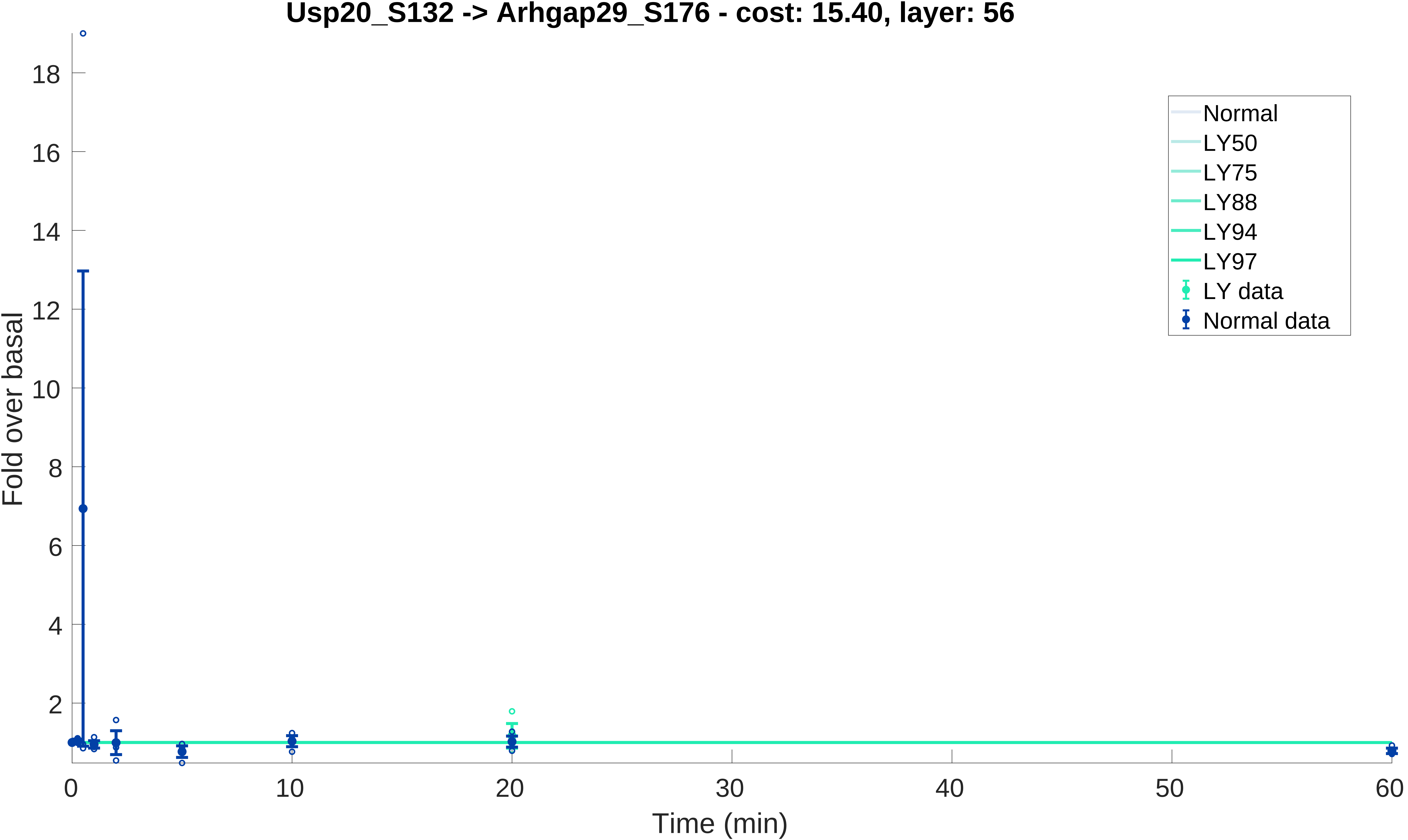

Usp20_S132 -> Arhgap29_S176 - cost: 15.40, layer: 56
Fold over basal
Time (min)
0
10
20
30
40
50
60
2
4
6
8
10
12
14
16
18
Normal
LY50
LY75
LY88
LY94
LY97
LY data
Normal data

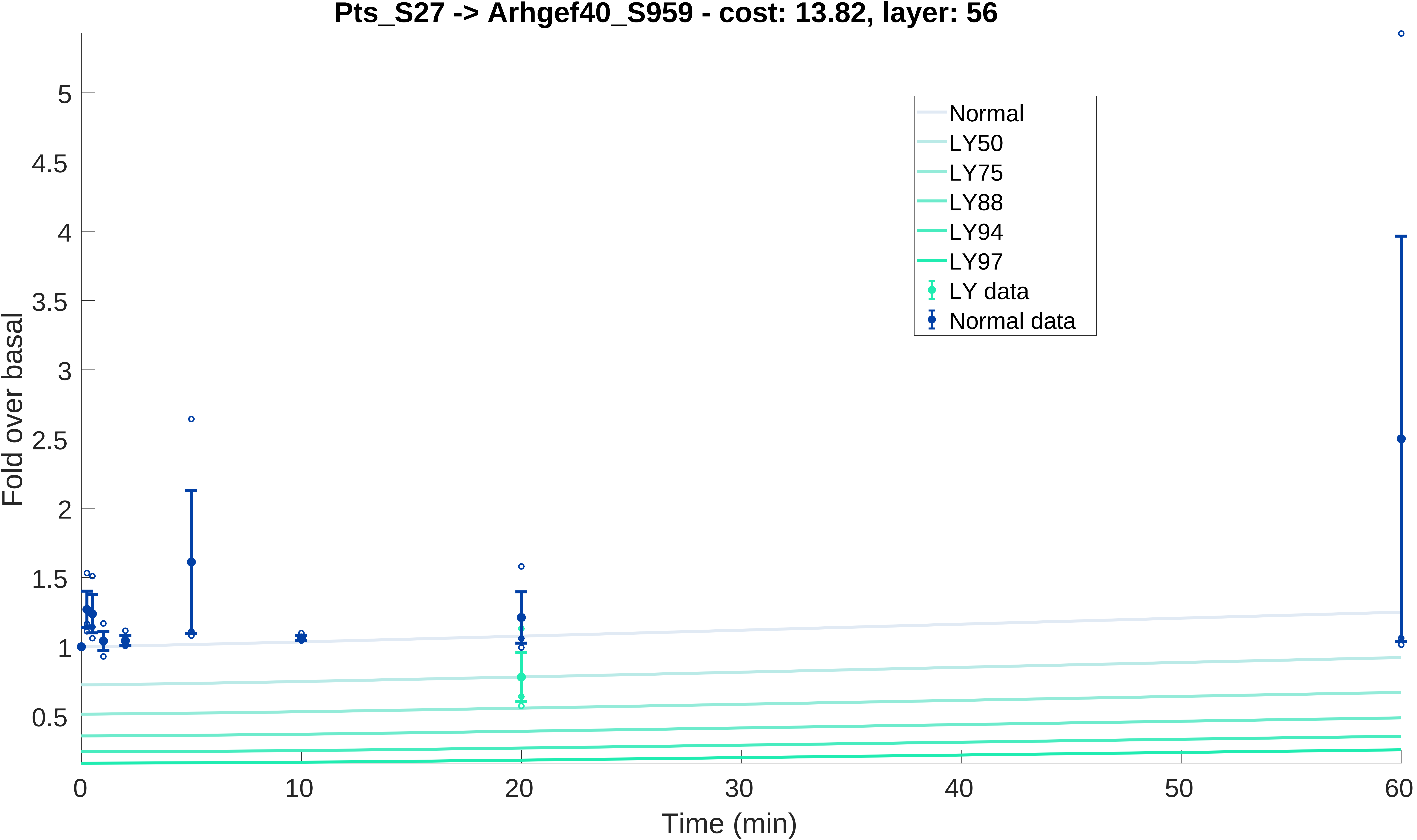

Pts_S27 -> Arhgef40_S959 - cost: 13.82, layer: 56
Fold over basal
Time (min)
0
10
20
30
40
50
60
0.5
1
1.5
2
2.5
3
3.5
4
4.5
5
Normal
LY50
LY75
LY88
LY94
LY97
LY data
Normal data

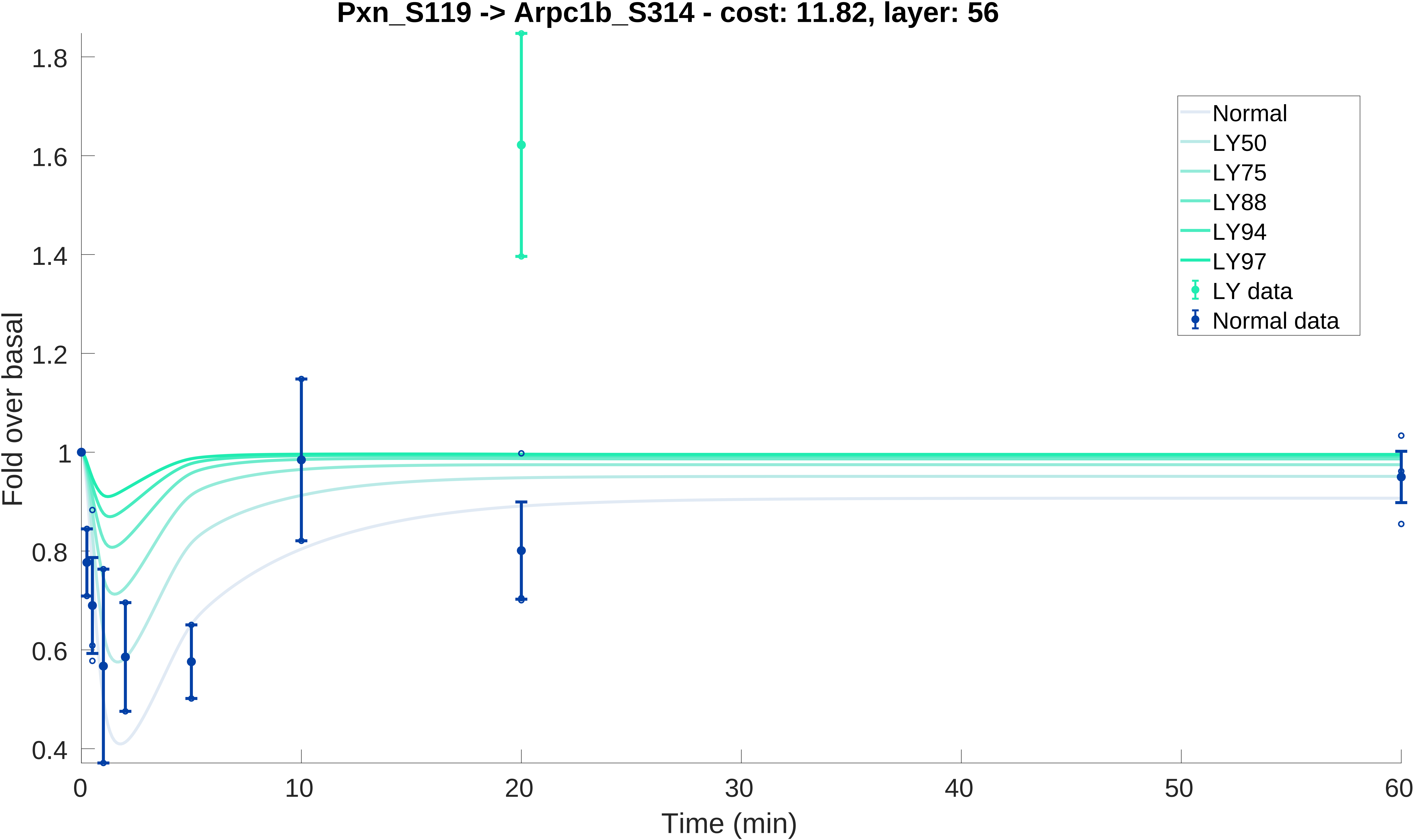
Pxn_S119 -> Arpc1b_S314 - cost: 11.82, layer: 56
Fold over basal
Time (min)
Normal
LY50
LY75
LY88
LY94
LY97
LY data
Normal data
1.8
1.6
1.4
1.2
1
0.8
0.6
0.4
0
10
20
30
40
50
60

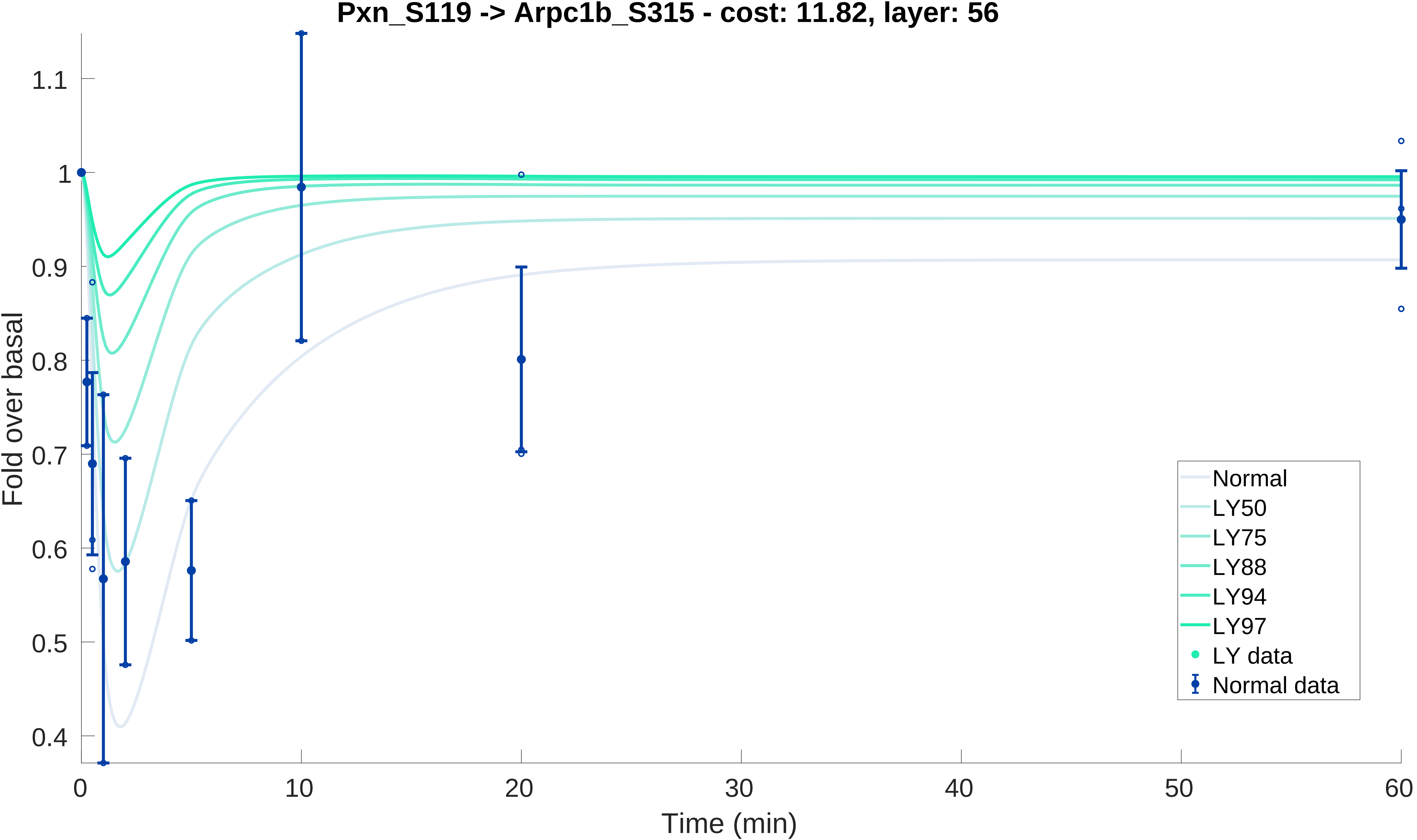

Pxn_S119 -> Arpc1b_S315 - cost: 11.82, layer: 56
Fold over basal
Time (min)
1.1
1
0.9
0.8
0.7
0.6
0.5
0.4
0
10
20
30
40
50
60
Normal
LY50
LY75
LY88
LY94
LY97
LY data
Normal data

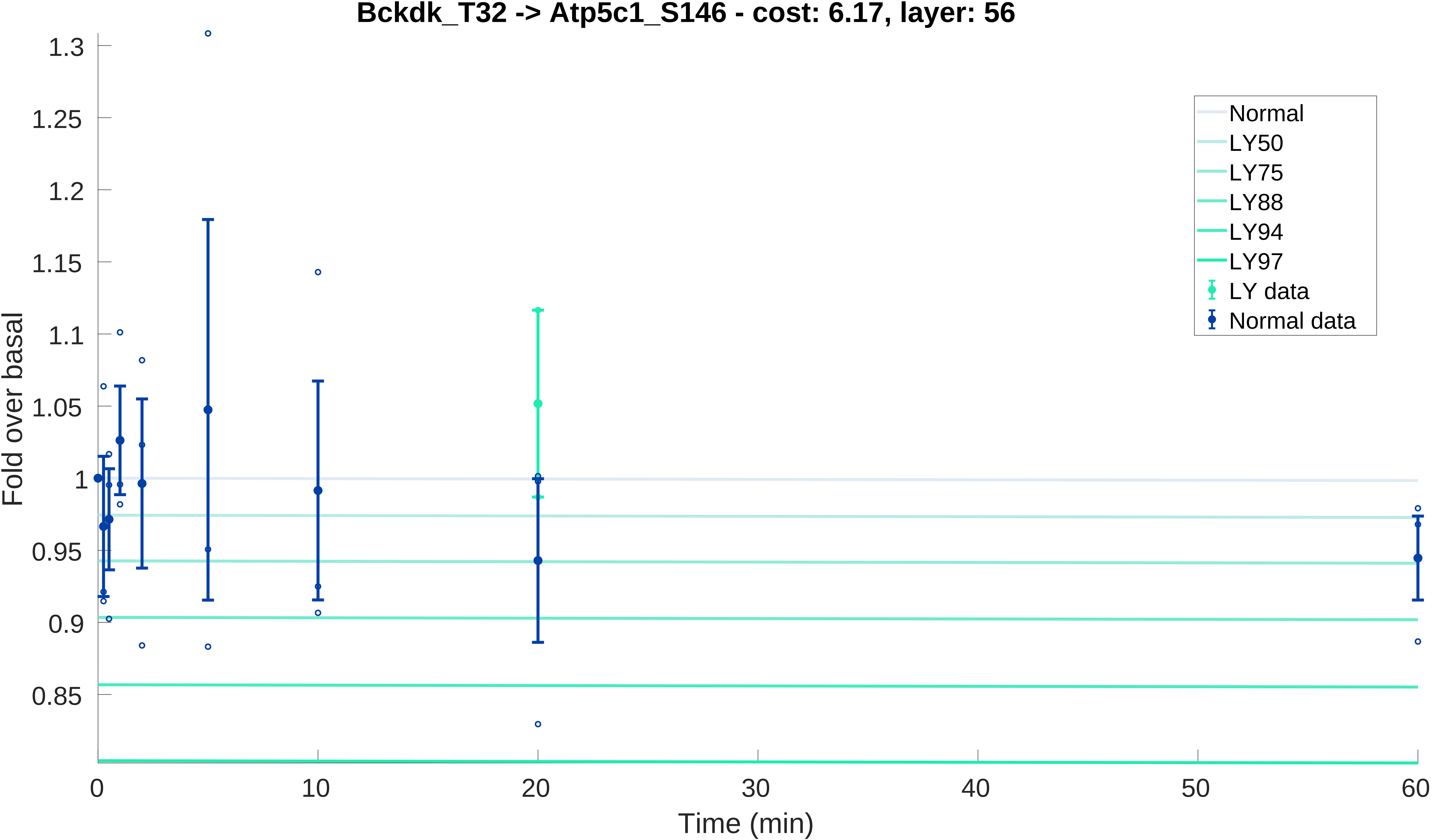
Bckdk_T32 -> Atp5c1_S146 - cost: 6.17, layer: 56
Fold over basal
Time (min)
1.3
1.25
1.2
1.15
1.1
1.05
1
0.95
0.9
0.85
0
10
20
30
40
50
60
Normal
LY50
LY75
LY88
LY94
LY97
LY data
Normal data

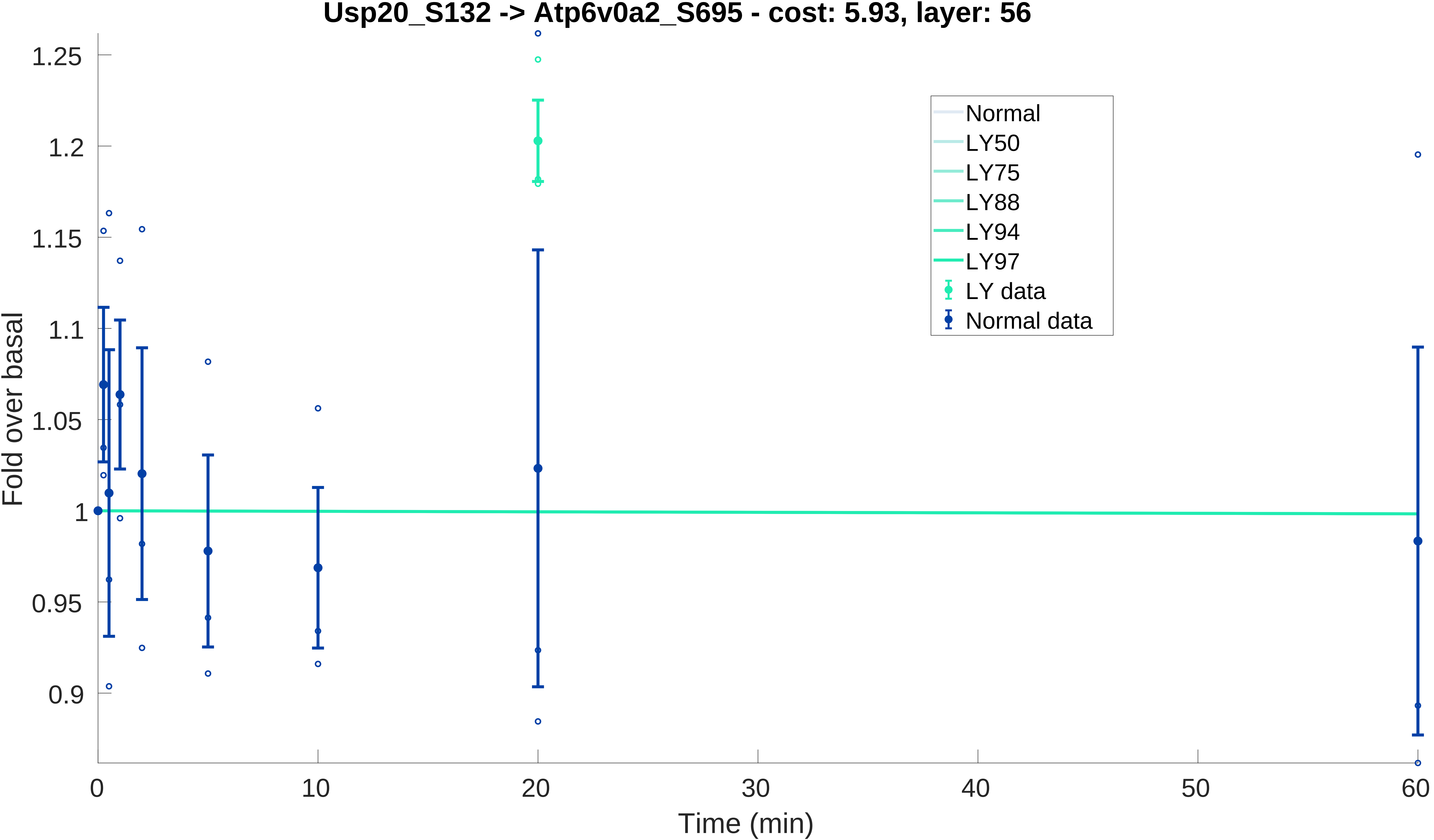

Usp20_S132 -> Atp6v0a2_S695 - cost: 5.93, layer: 56
Fold over basal
Time (min)
1.25
1.2
1.15
1.1
1.05
1
0.95
0.9
0
10
20
30
40
50
60
Normal
LY50
LY75
LY88
LY94
LY97
LY data
Normal data

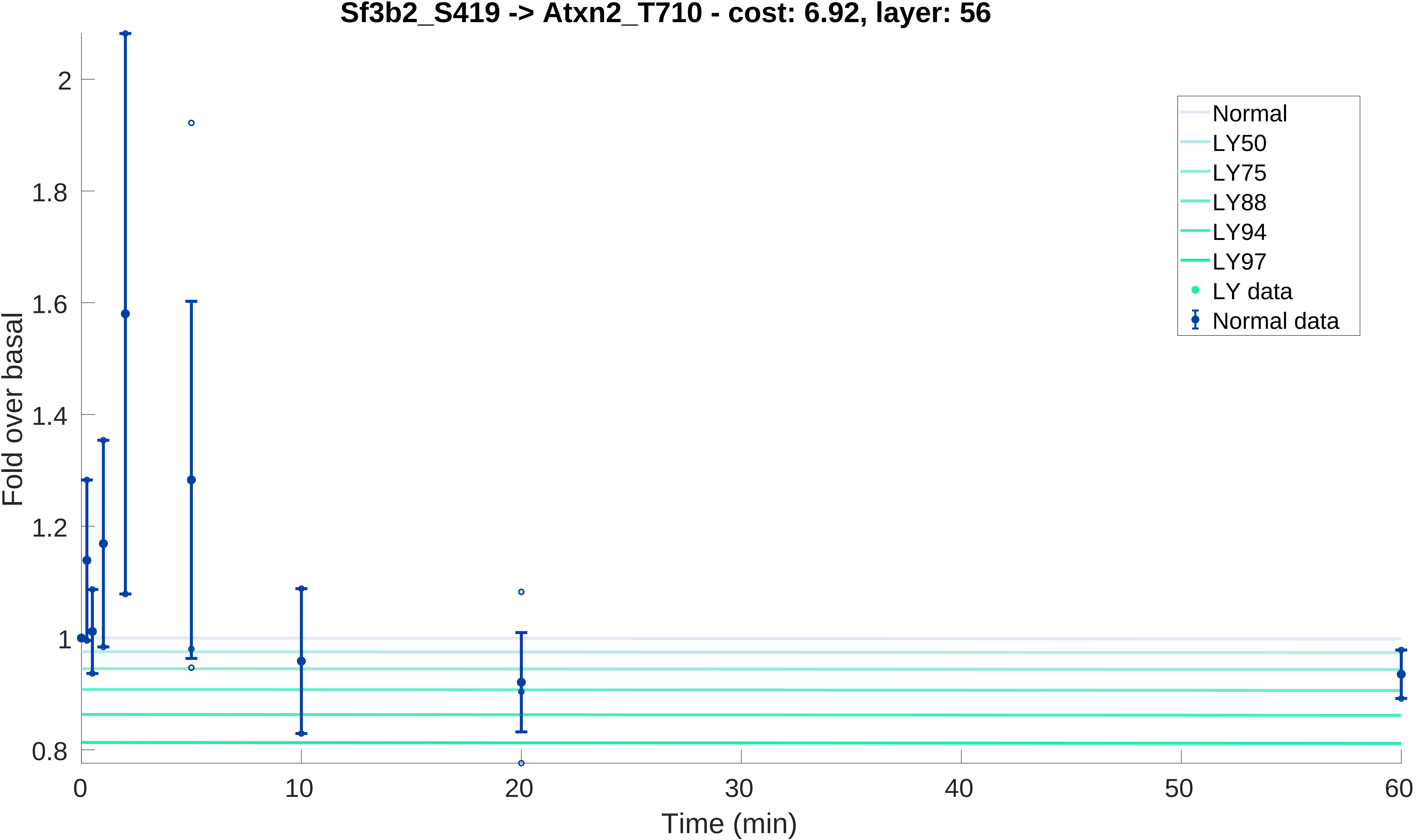
Sf3b2_S419 -> Atxn2_T710 - cost: 6.92, layer: 56
Fold over basal
Time (min)
0
10
20
30
40
50
60
0.8
1
1.2
1.4
1.6
1.8
2
Normal
LY50
LY75
LY88
LY94
LY97
LY data
Normal data

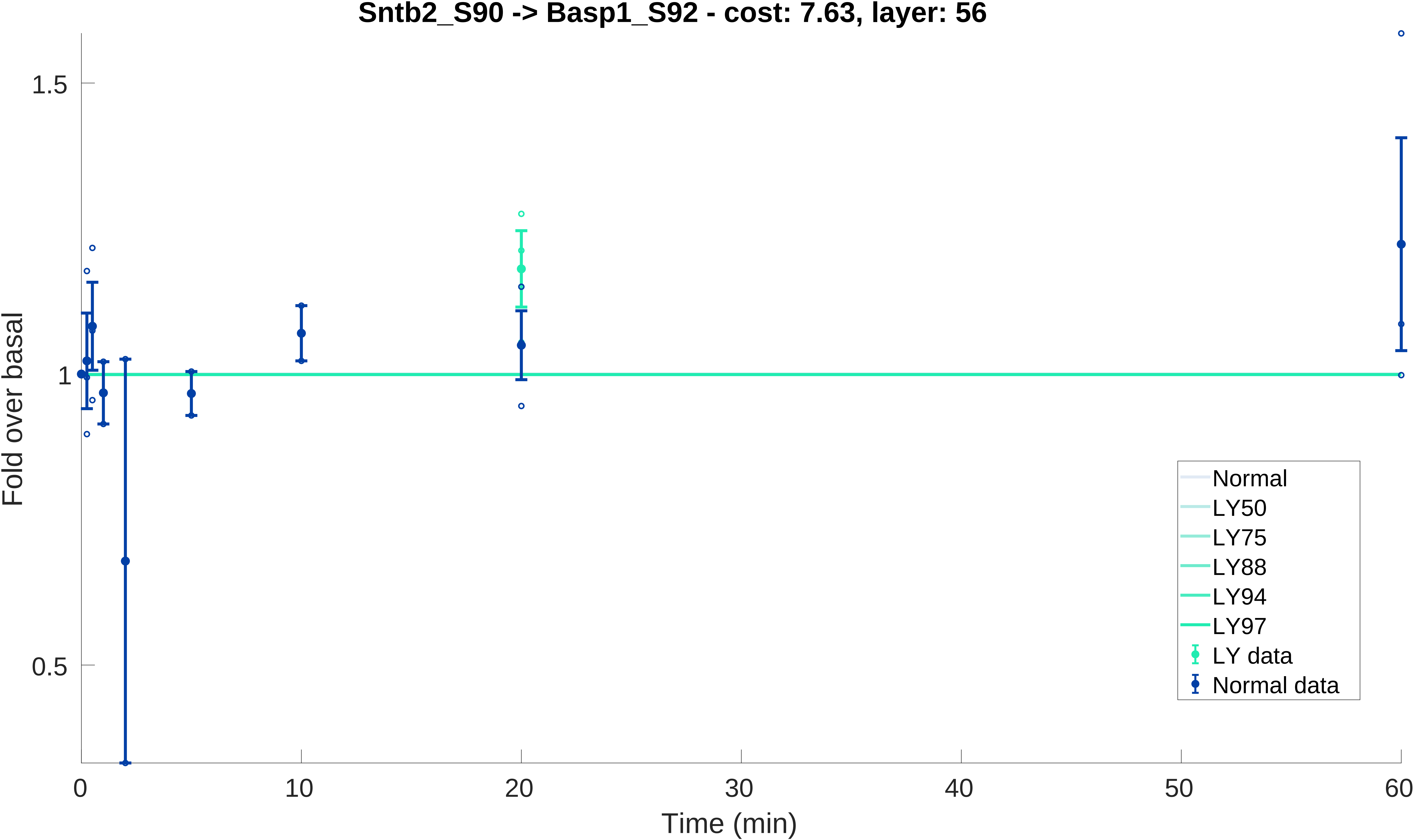
Sntb2_S90 -> Basp1_S92 - cost: 7.63, layer: 56
Fold over basal
1.5
1
0.5
0
10
20
30
40
50
60
Time (min)
Normal
LY50
LY75
LY88
LY94
LY97
LY data
Normal data

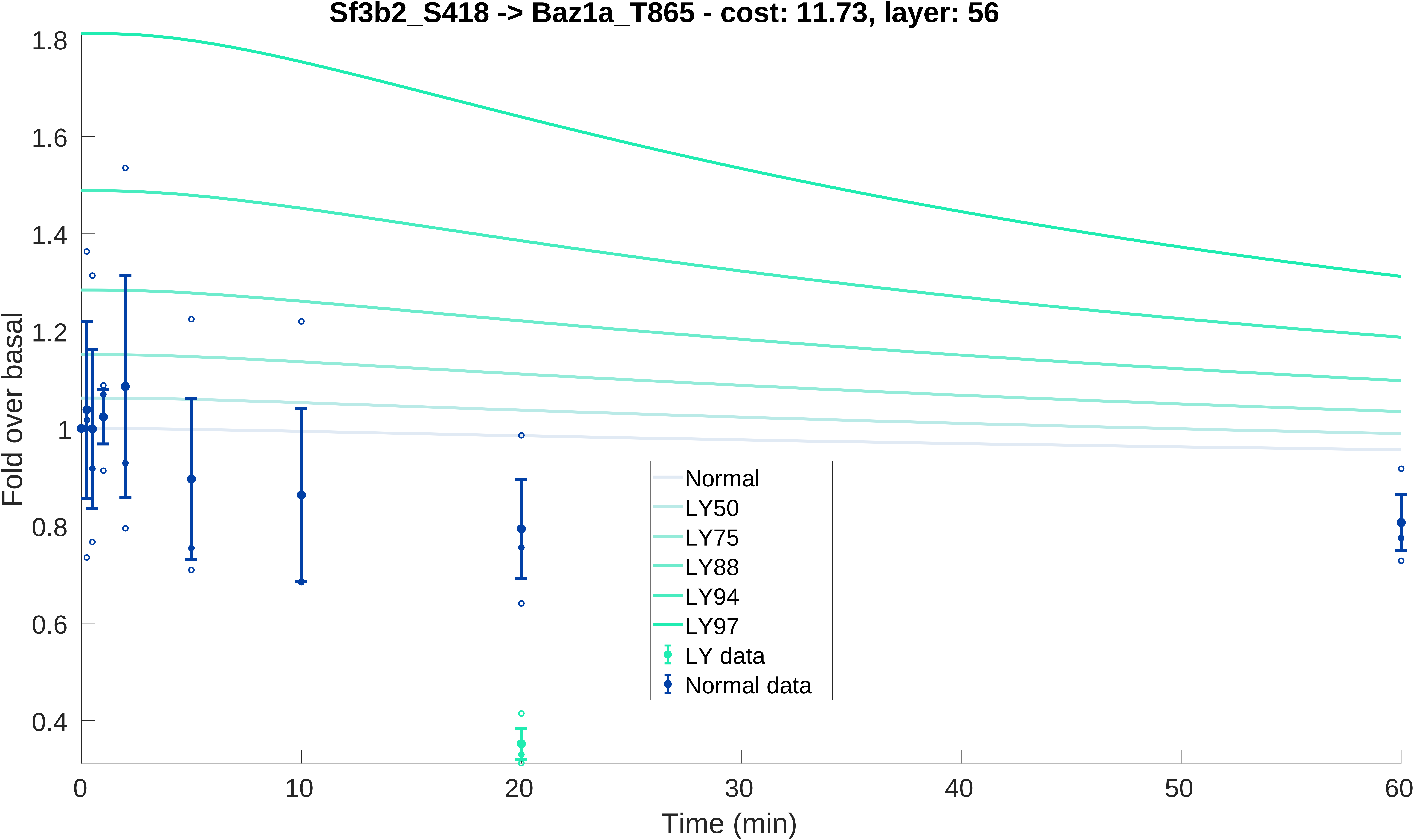
Sf3b2_S418 -> Baz1a_T865 - cost: 11.73, layer: 56
Fold over basal
1.8
1.6
1.4
1.2
1
0.8
0.6
0.4
0
10
20
30
40
50
60
Time (min)
Normal
LY50
LY75
LY88
LY94
LY97
LY data
Normal data

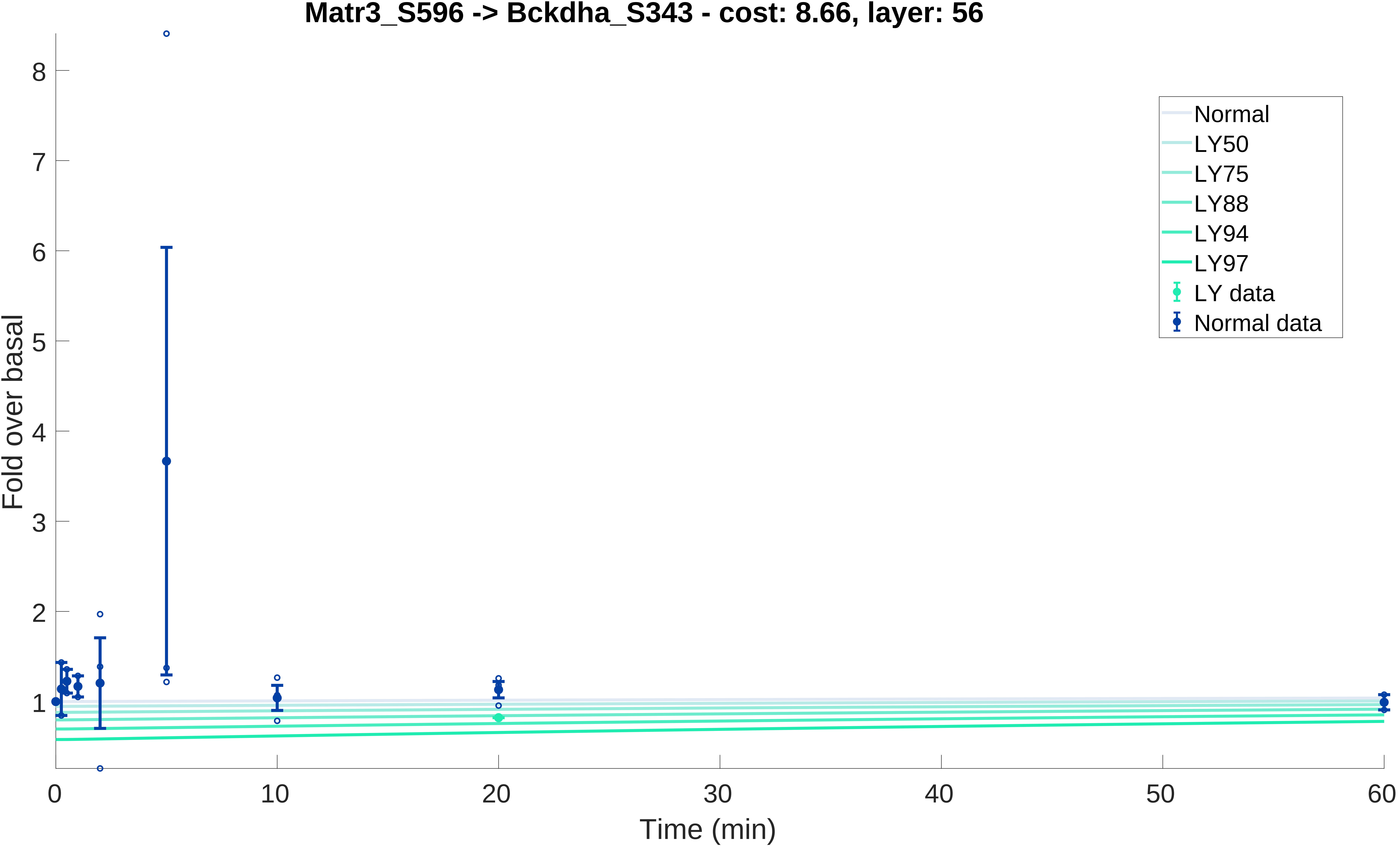

Matr3_S596 -> Bckdha_S343 - cost: 8.66, layer: 56
Fold over basal
8
7
6
5
4
3
2
1
0
10
20
30
40
50
60
Time (min)
Normal
LY50
LY75
LY88
LY94
LY97
LY data
Normal data

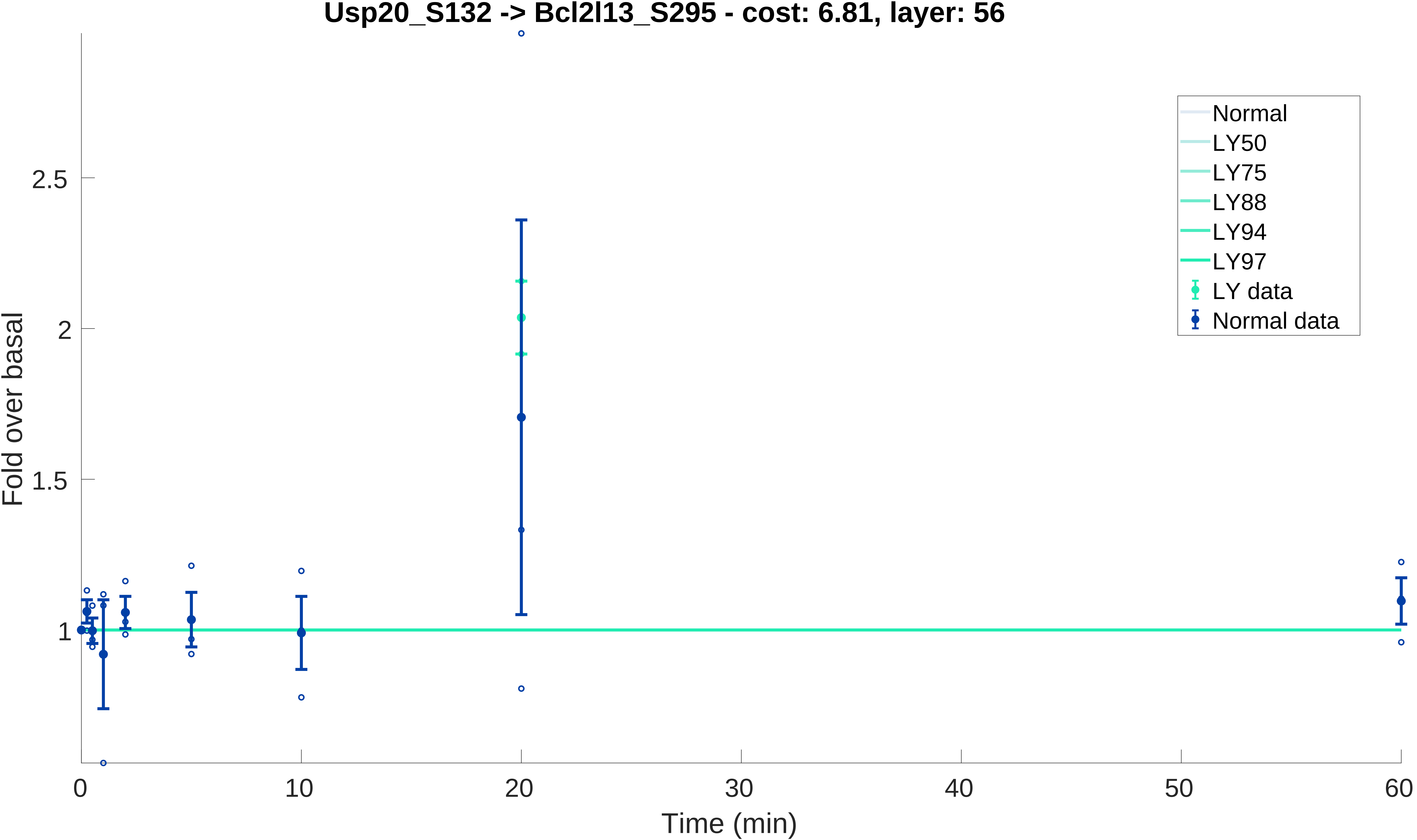
Usp20_S132 -> Bcl2l13_S295 - cost: 6.81, layer: 56
Fold over basal
2.5
2
1.5
1
0
10
20
30
40
50
60
Time (min)
Normal
LY50
LY75
LY88
LY94
LY97
LY data
Normal data

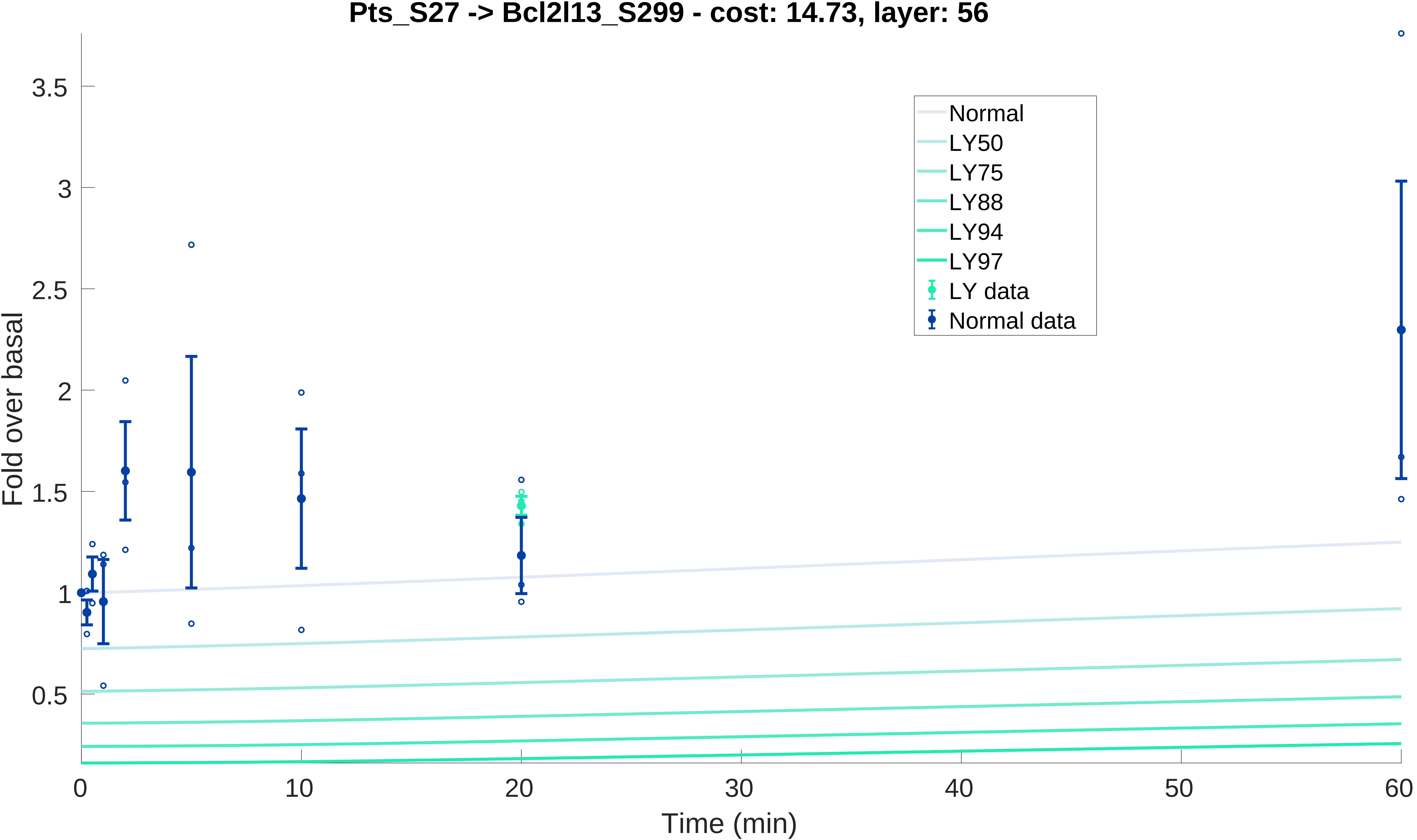

Pts_S27 -> Bcl2l13_S299 - cost: 14.73, layer: 56
Fold over basal
3.5
3
2.5
2
1.5
1
0.5
0
10
20
30
40
50
60
Time (min)
Normal
LY50
LY75
LY88
LY94
LY97
LY data
Normal data

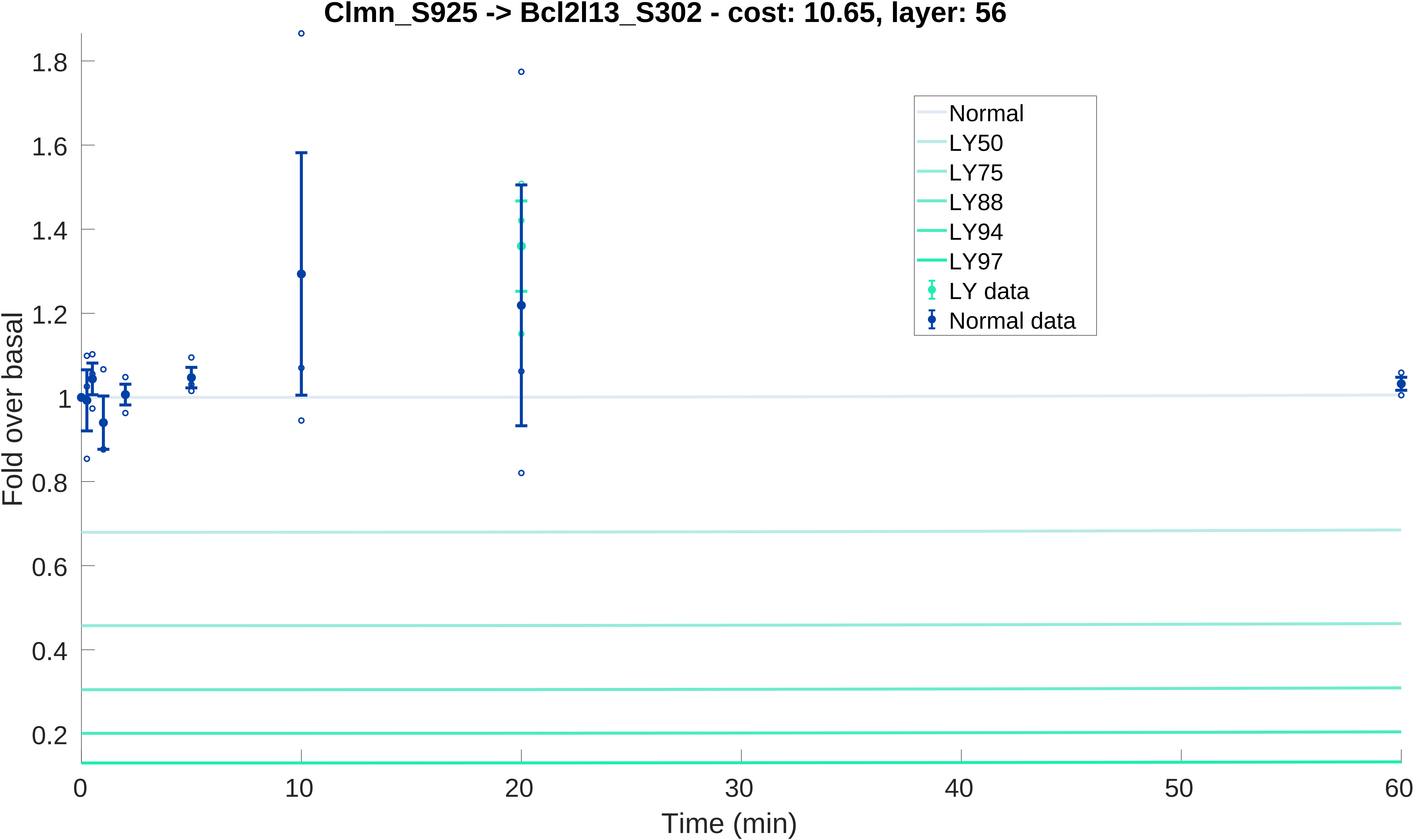

Clmn_S925 -> Bcl2l13_S302 - cost: 10.65, layer: 56
Fold over basal
Time (min)
0
10
20
30
40
50
60
0.2
0.4
0.6
0.8
1
1.2
1.4
1.6
1.8
Normal
LY50
LY75
LY88
LY94
LY97
LY data
Normal data

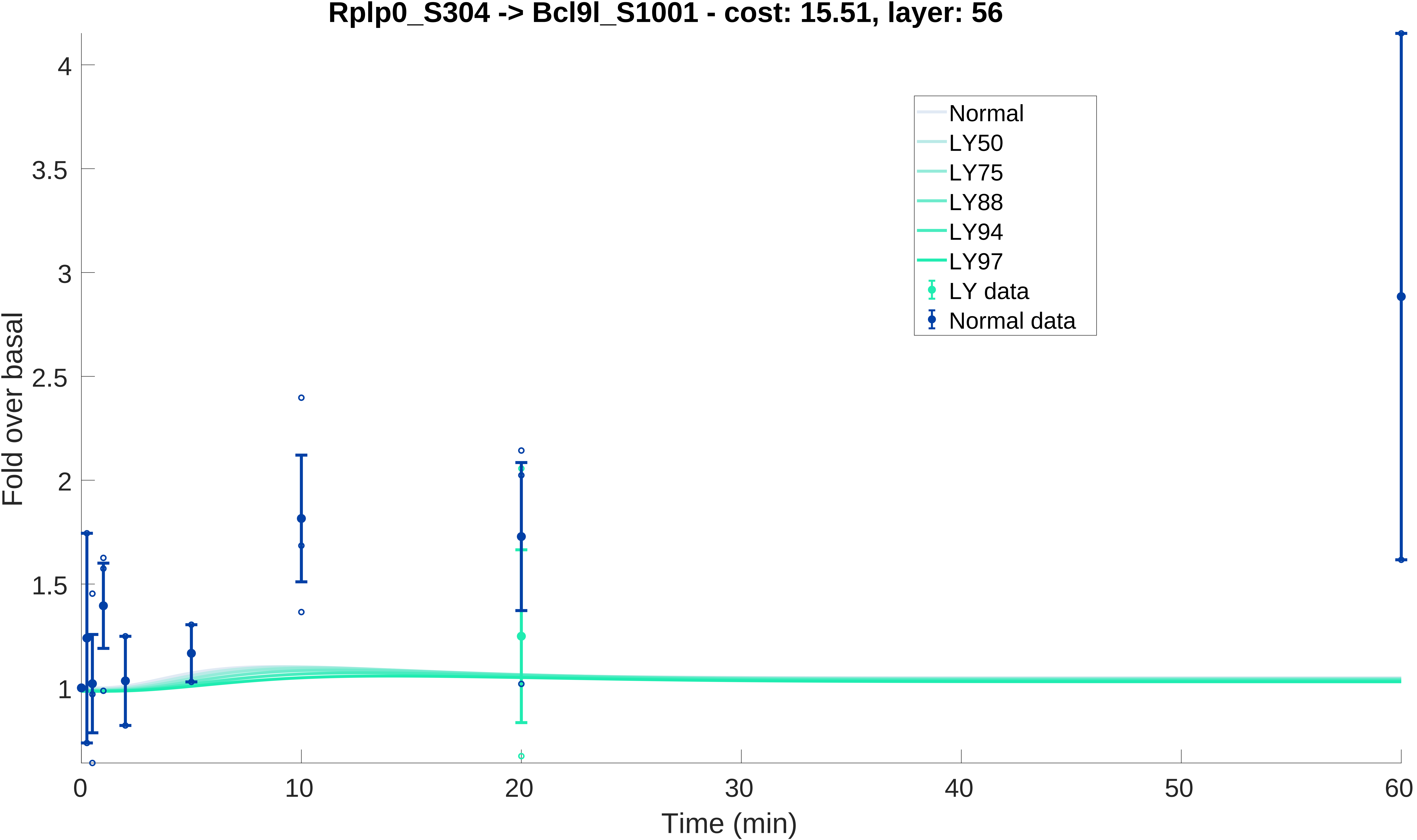

Rplp0_S304 -> Bcl9l_S1001 - cost: 15.51, layer: 56
Fold over basal
Time (min)
4
3.5
3
2.5
2
1.5
1
0
10
20
30
40
50
60
Normal
LY50
LY75
LY88
LY94
LY97
LY data
Normal data

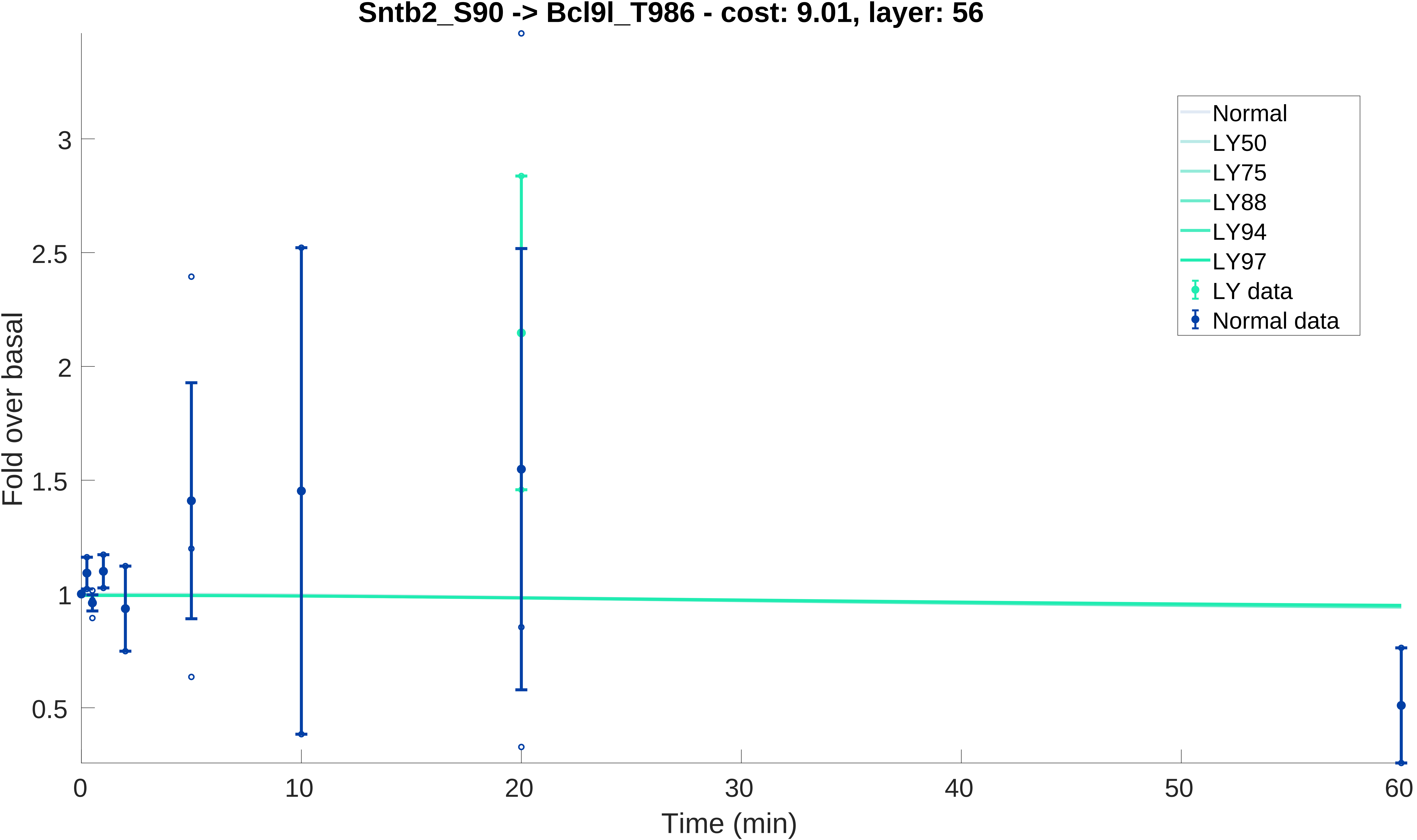
Sntb2_S90 -> Bcl9l_T986 - cost: 9.01, layer: 56
Fold over basal
Time (min)
0.5
1
1.5
2
2.5
3
0
10
20
30
40
50
60
Normal
LY50
LY75
LY88
LY94
LY97
LY data
Normal data

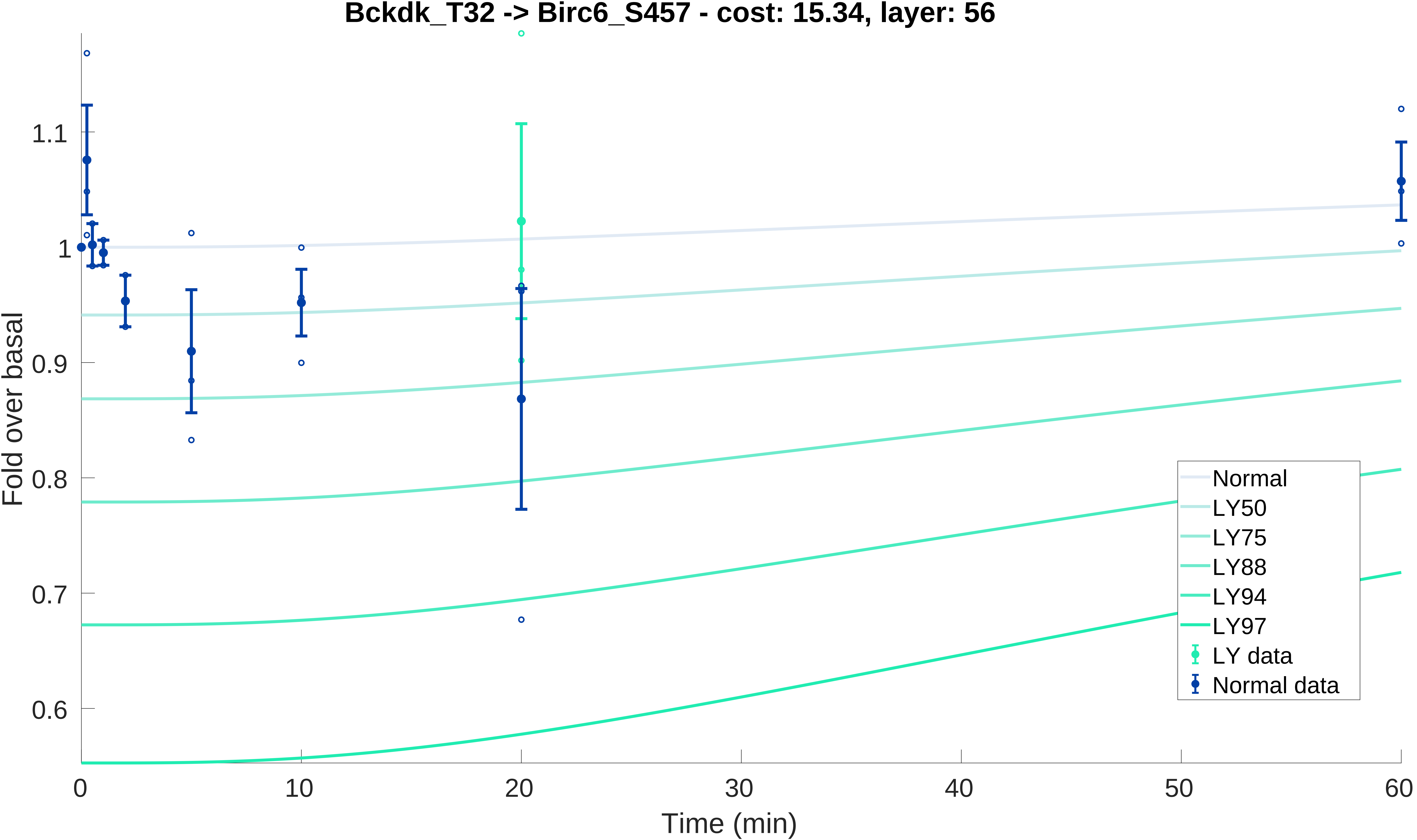

Bckdk_T32 -> Birc6_S457 - cost: 15.34, layer: 56
Fold over basal
1.1
1
0.9
0.8
0.7
0.6
0
10
20
30
40
50
60
Time (min)
Normal
LY50
LY75
LY88
LY94
LY97
LY data
Normal data

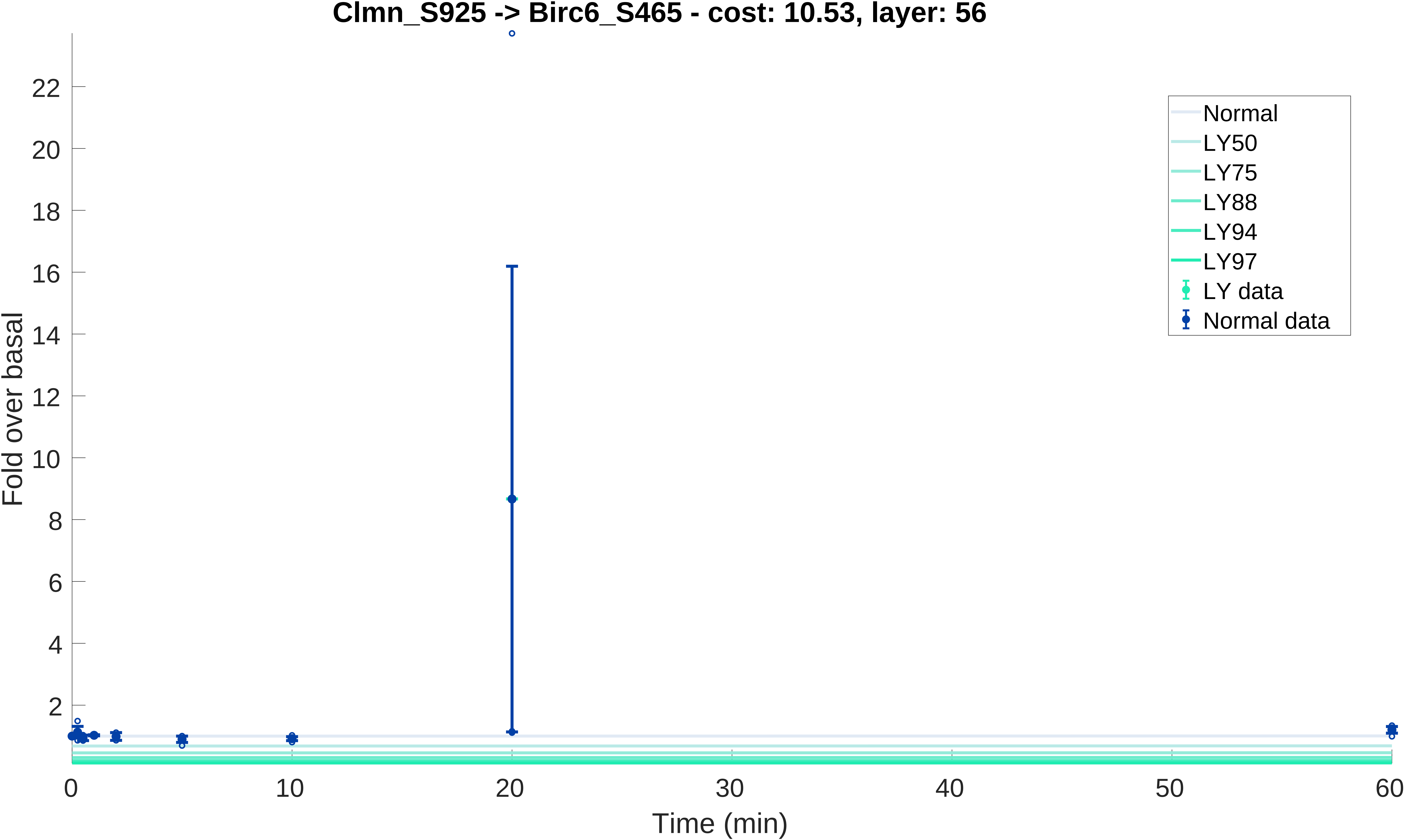

Clmn_S925 -> Birc6_S465 - cost: 10.53, layer: 56
Fold over basal
22
20
18
16
14
12
10
8
6
4
2
0
10
20
30
40
50
60
Time (min)
Normal
LY50
LY75
LY88
LY94
LY97
LY data
Normal data

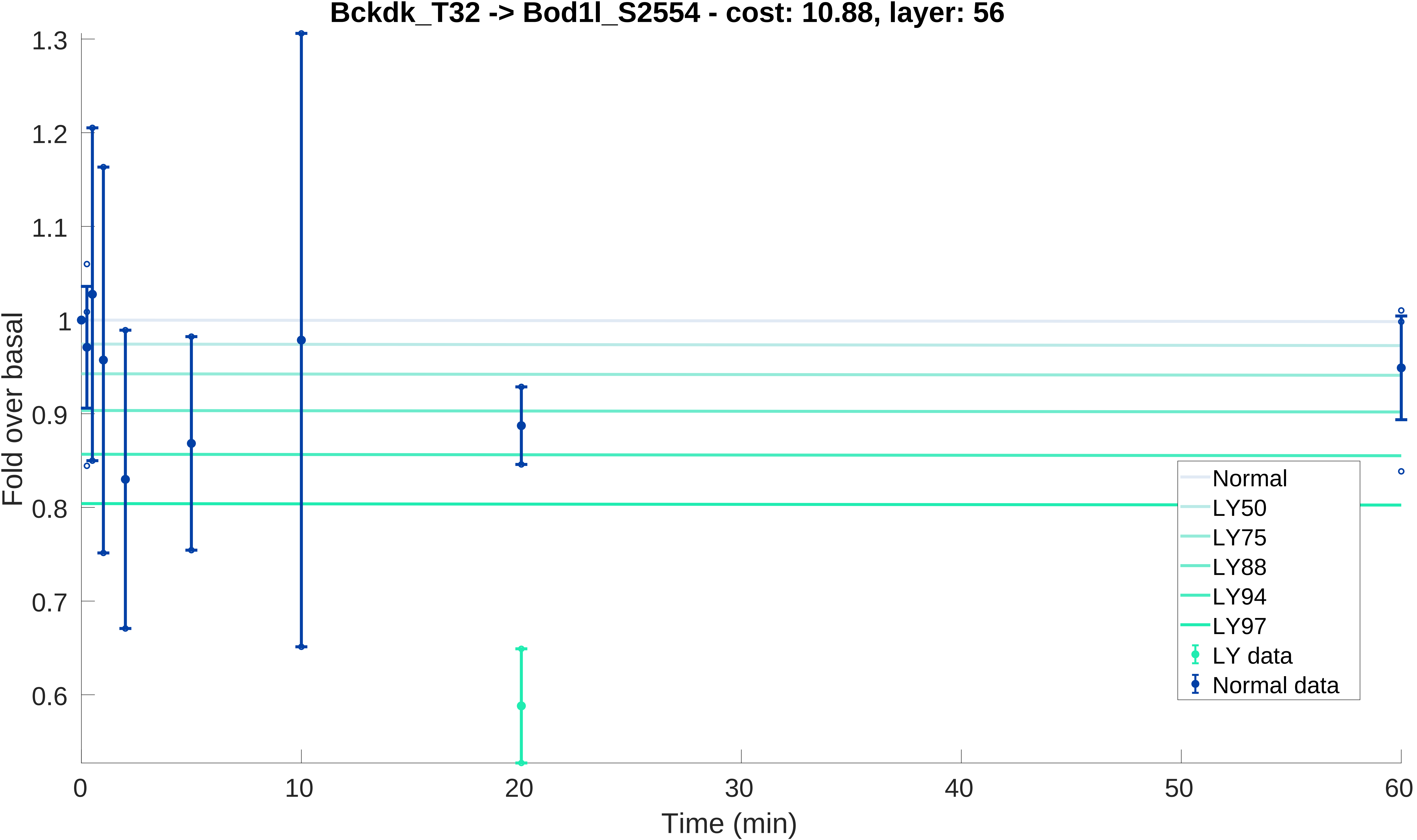

Bckdk_T32 -> Bod1l_S2554 - cost: 10.88, layer: 56
Fold over basal
Time (min)
0.6
0.7
0.8
0.9
1
1.1
1.2
1.3
0
10
20
30
40
50
60
Normal
LY50
LY75
LY88
LY94
LY97
LY data
Normal data

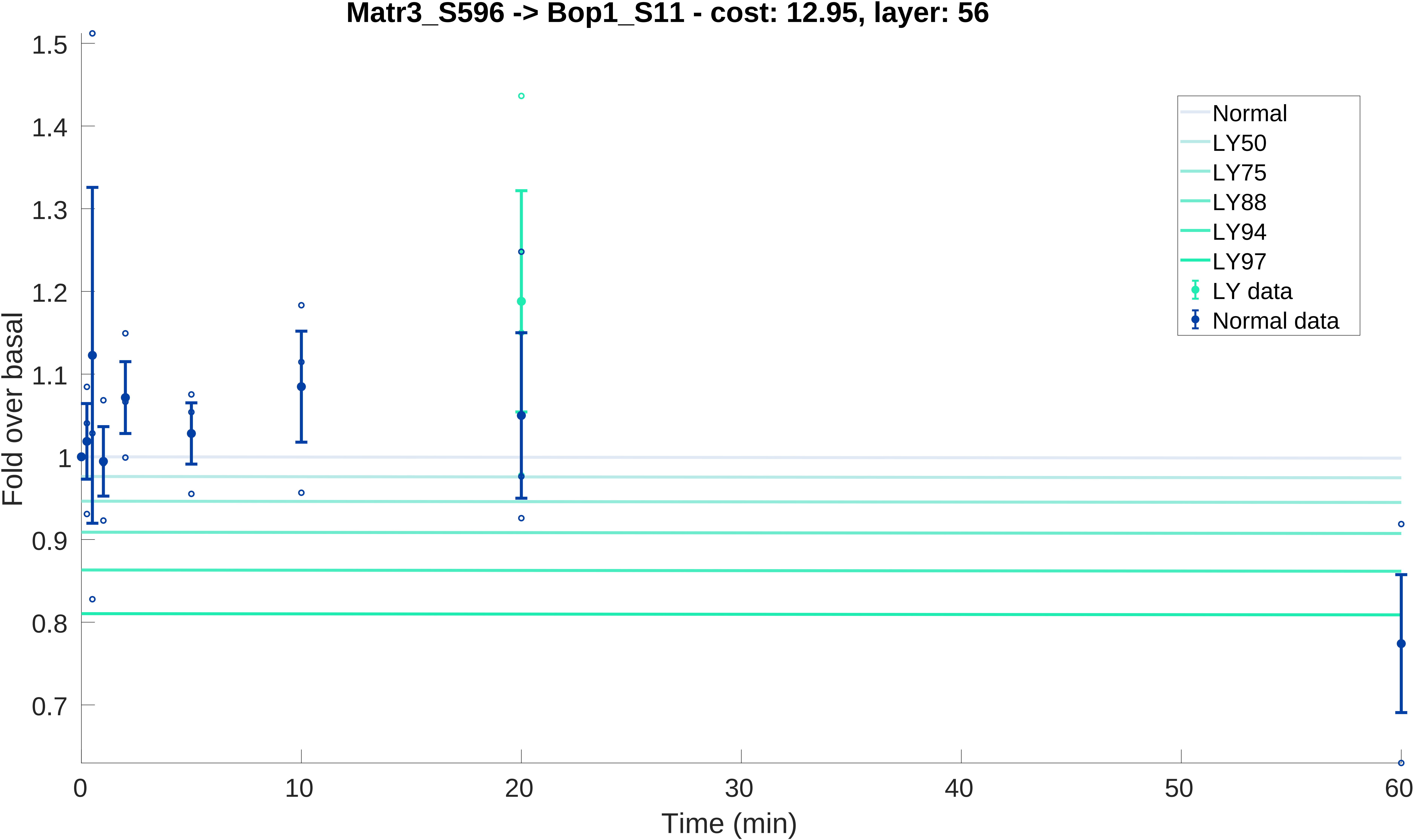
Matr3_S596 -> Bop1_S11 - cost: 12.95, layer: 56
Fold over basal
Time (min)
0
10
20
30
40
50
60
0.7
0.8
0.9
1
1.1
1.2
1.3
1.4
1.5
Normal
LY50
LY75
LY88
LY94
LY97
LY data
Normal data

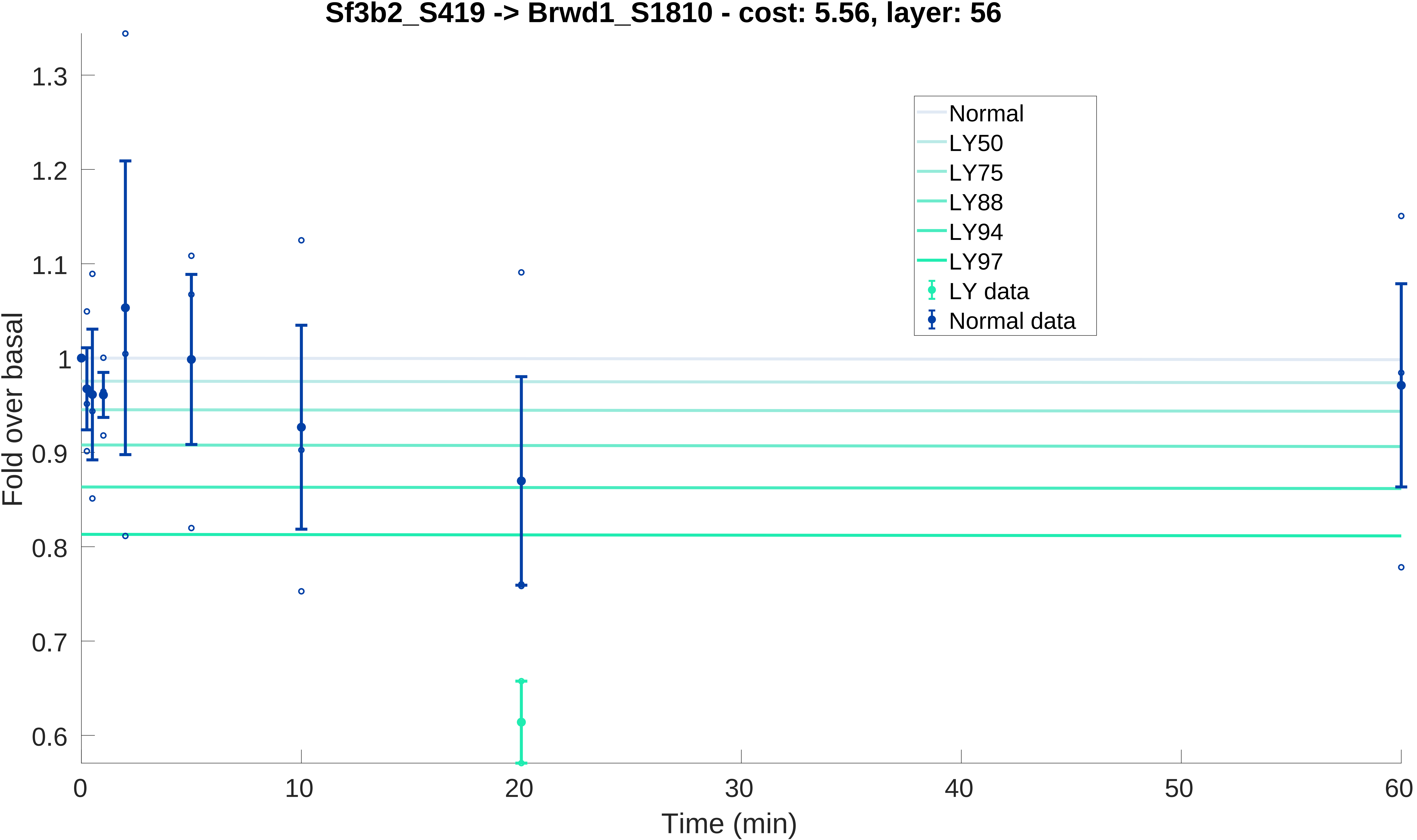

Sf3b2_S419 -> Brwd1_S1810 - cost: 5.56, layer: 56
Fold over basal
Time (min)
Normal
LY50
LY75
LY88
LY94
LY97
LY data
Normal data
1.3
1.2
1.1
1
0.9
0.8
0.7
0.6
0
10
20
30
40
50
60

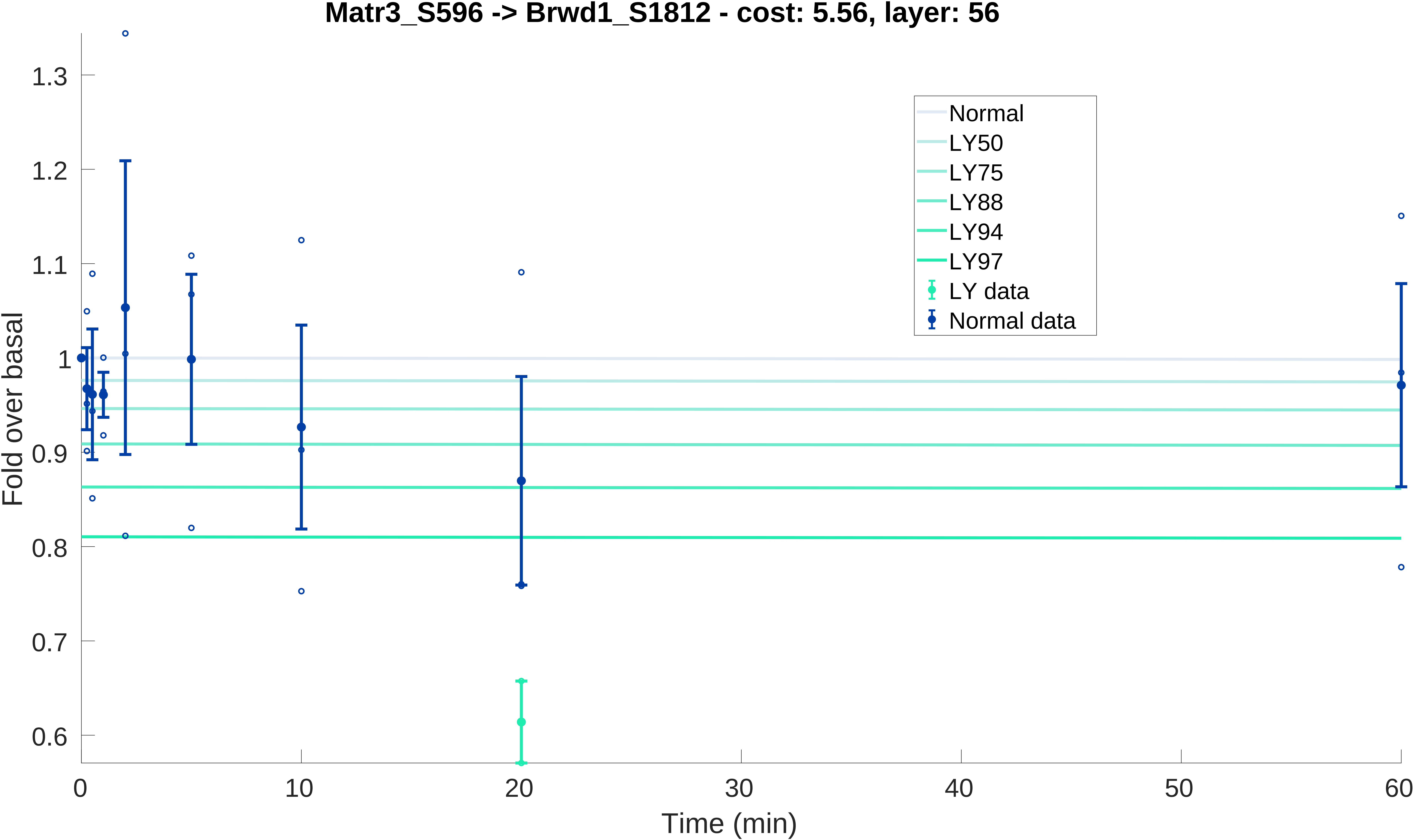

Matr3_S596 -> Brwd1_S1812 - cost: 5.56, layer: 56
Fold over basal
Time (min)
1.3
1.2
1.1
1
0.9
0.8
0.7
0.6
0
10
20
30
40
50
60
Normal
LY50
LY75
LY88
LY94
LY97
LY data
Normal data

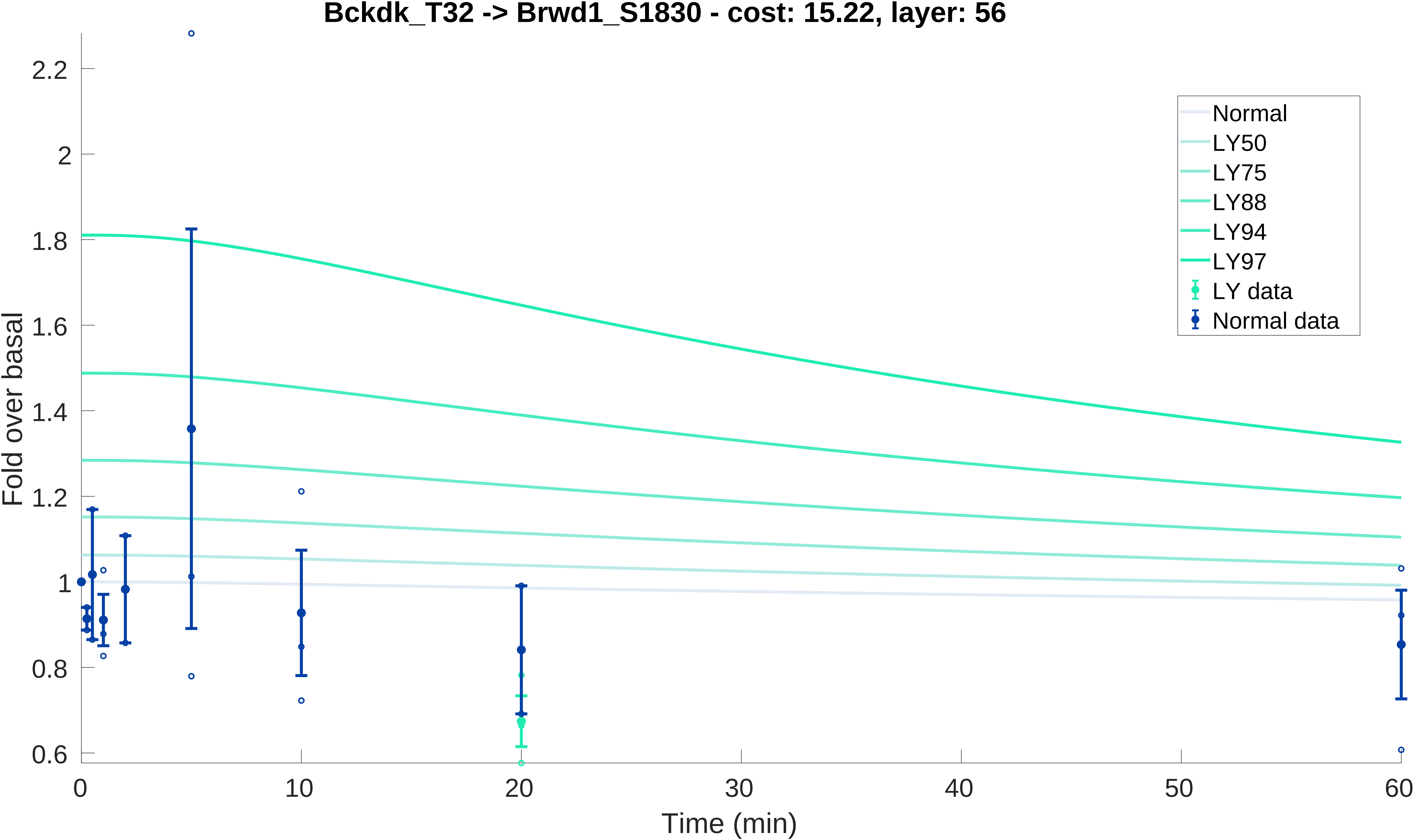

Bckdk_T32 -> Brwd1_S1830 - cost: 15.22, layer: 56
Fold over basal
Time (min)
Normal
LY50
LY75
LY88
LY94
LY97
LY data
Normal data
0.6
0.8
1
1.2
1.4
1.6
1.8
2
2.2
0
10
20
30
40
50
60

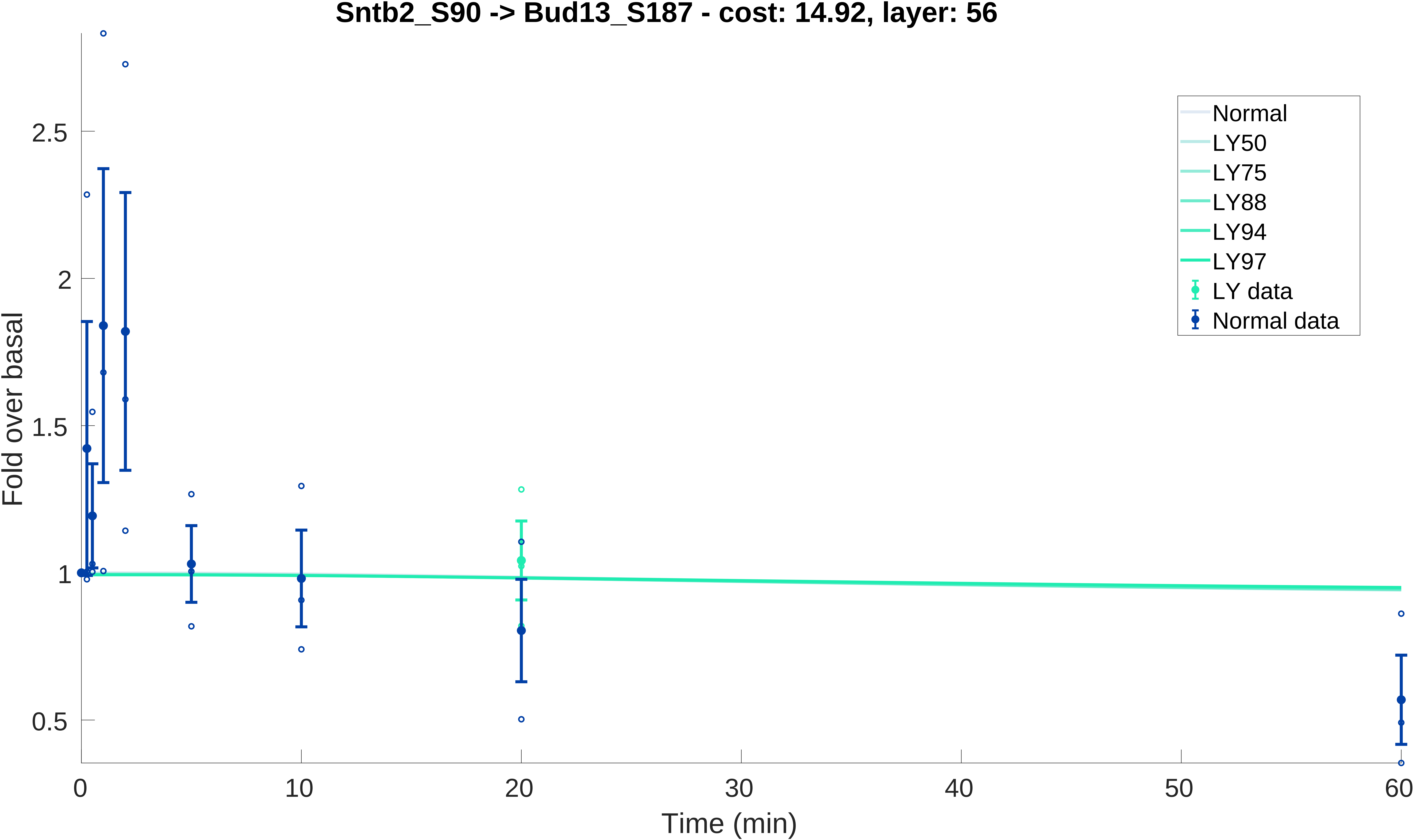

Sntb2_S90 -> Bud13_S187 - cost: 14.92, layer: 56
Fold over basal
Time (min)
0.5
1
1.5
2
2.5
0
10
20
30
40
50
60
Normal
LY50
LY75
LY88
LY94
LY97
LY data
Normal data

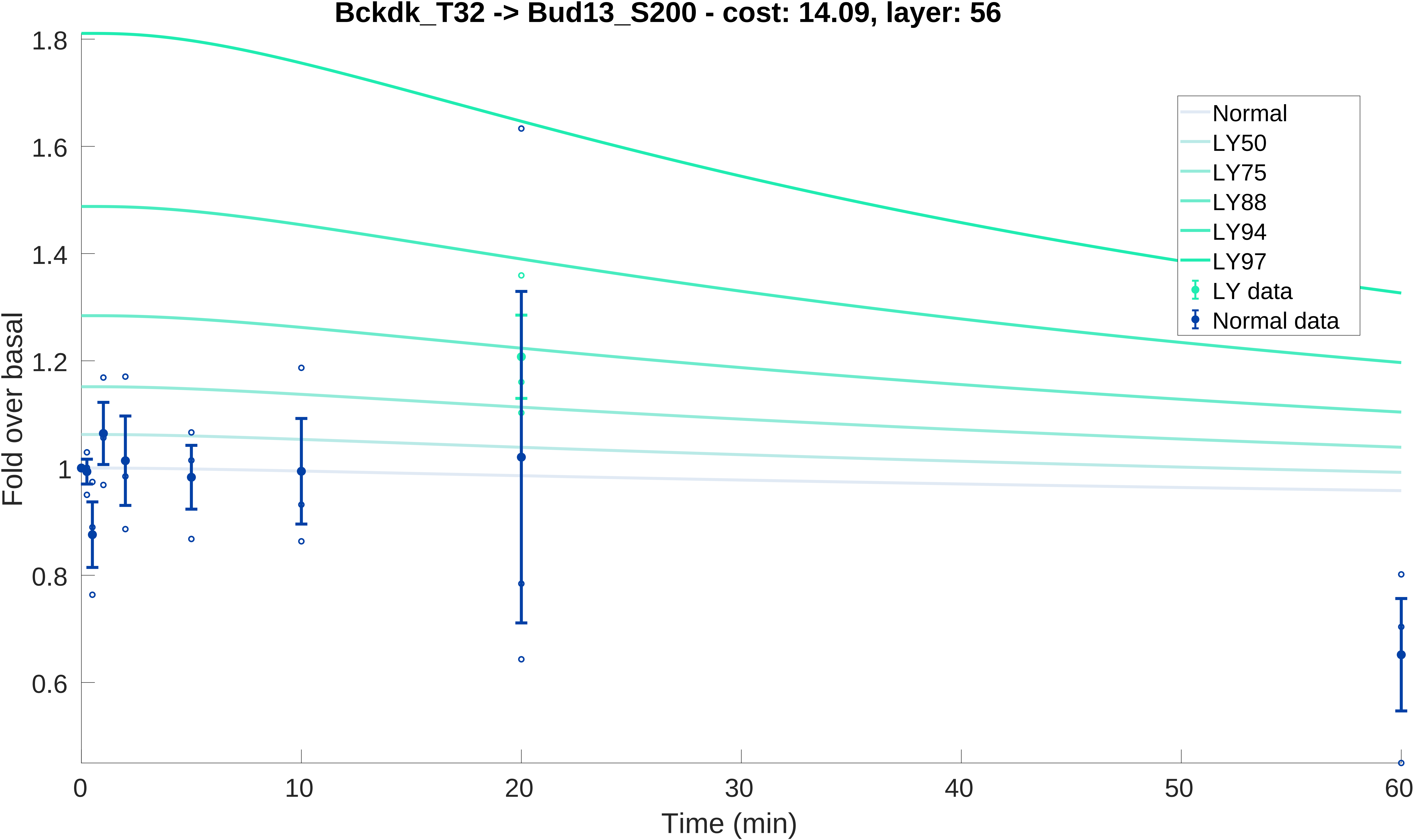

Bckdk_T32 -> Bud13_S200 - cost: 14.09, layer: 56
Fold over basal
Time (min)
1.8
1.6
1.4
1.2
1
0.8
0.6
0
10
20
30
40
50
60
Normal
LY50
LY75
LY88
LY94
LY97
LY data
Normal data

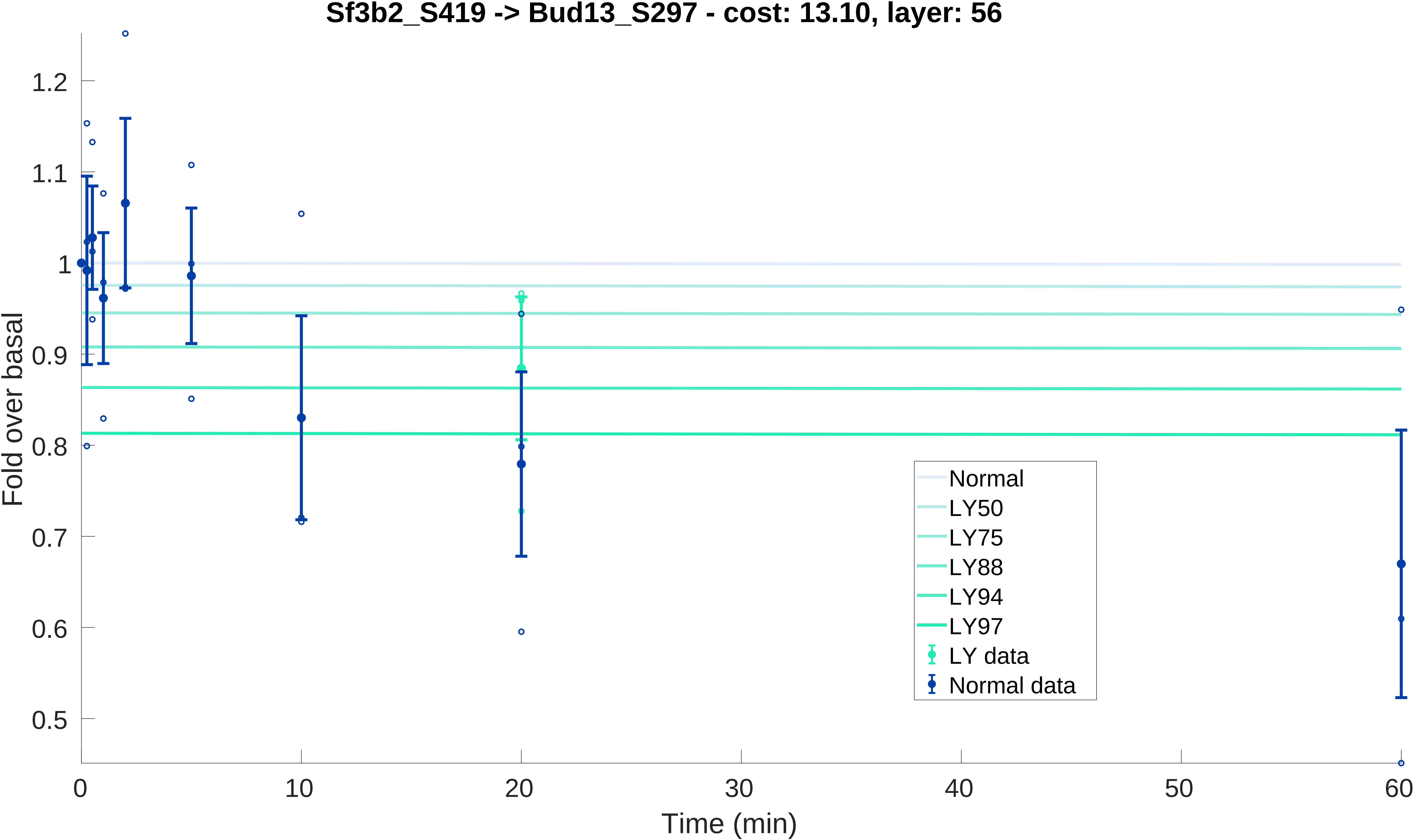

Sf3b2_S419 -> Bud13_S297 - cost: 13.10, layer: 56
Fold over basal
1.2
1.1
1
0.9
0.8
0.7
0.6
0.5
0
10
20
30
40
50
60
Time (min)
Normal
LY50
LY75
LY88
LY94
LY97
LY data
Normal data

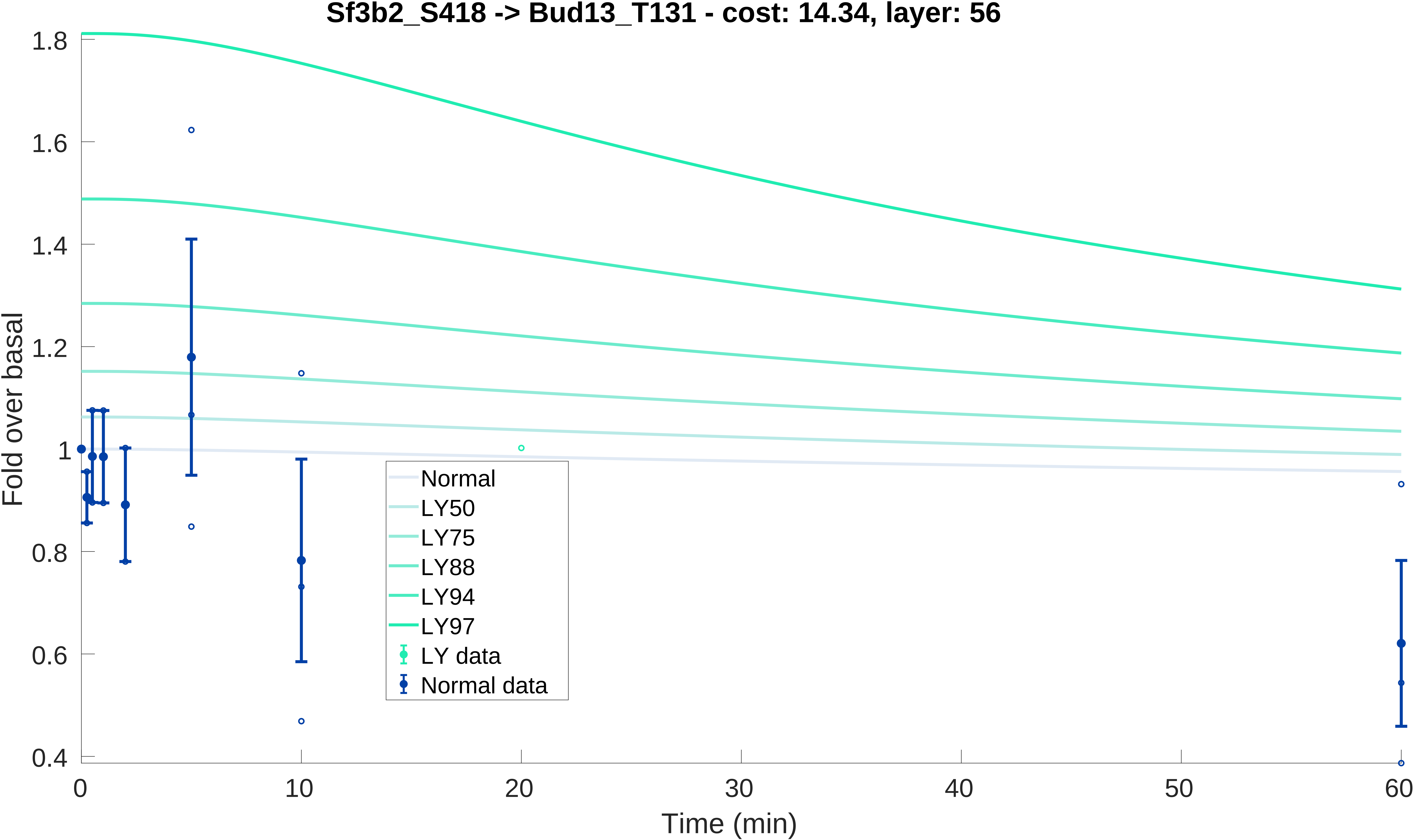

Sf3b2_S418 -> Bud13_T131 - cost: 14.34, layer: 56
Fold over basal
1.8
1.6
1.4
1.2
1
0.8
0.6
0.4
0
10
20
30
40
50
60
Time (min)
Normal
LY50
LY75
LY88
LY94
LY97
LY data
Normal data

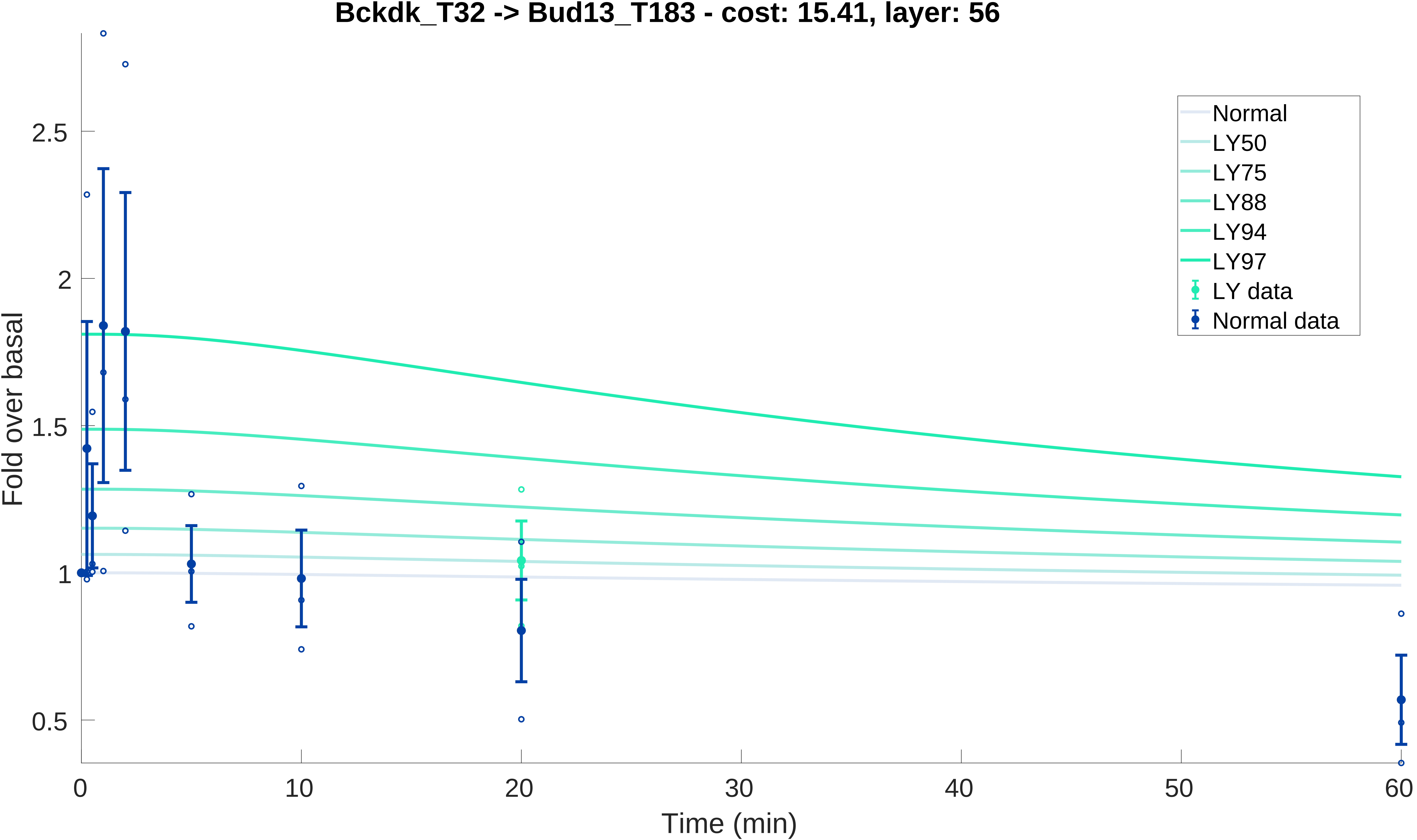
Bckdk_T32 -> Bud13_T183 - cost: 15.41, layer: 56
Fold over basal
Time (min)
0
10
20
30
40
50
60
0.5
1
1.5
2
2.5
Normal
LY50
LY75
LY88
LY94
LY97
LY data
Normal data

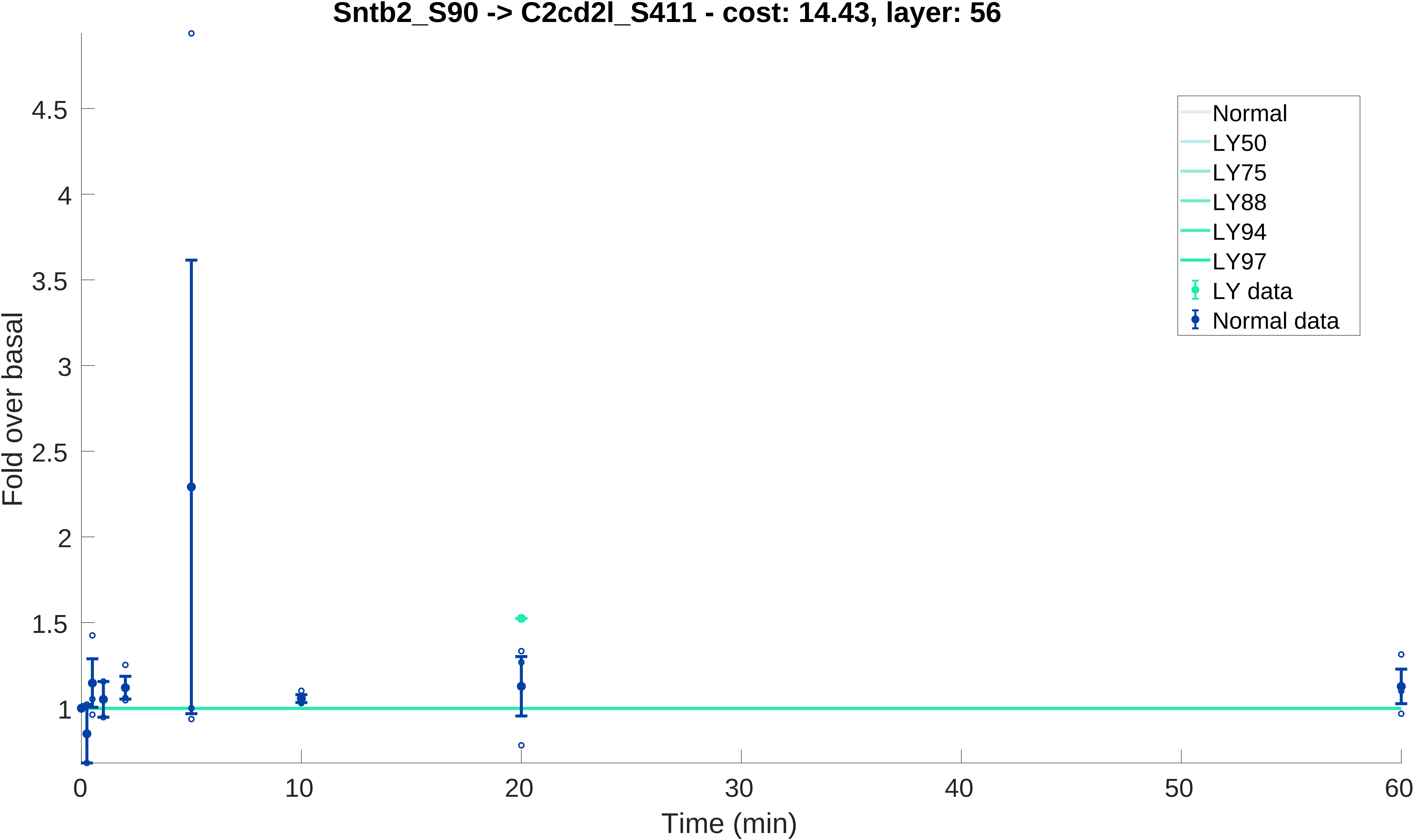

Sntb2_S90 -> C2cd2l_S411 - cost: 14.43, layer: 56
Fold over basal
Time (min)
4.5
4
3.5
3
2.5
2
1.5
1
0
10
20
30
40
50
60
Normal
LY50
LY75
LY88
LY94
LY97
LY data
Normal data

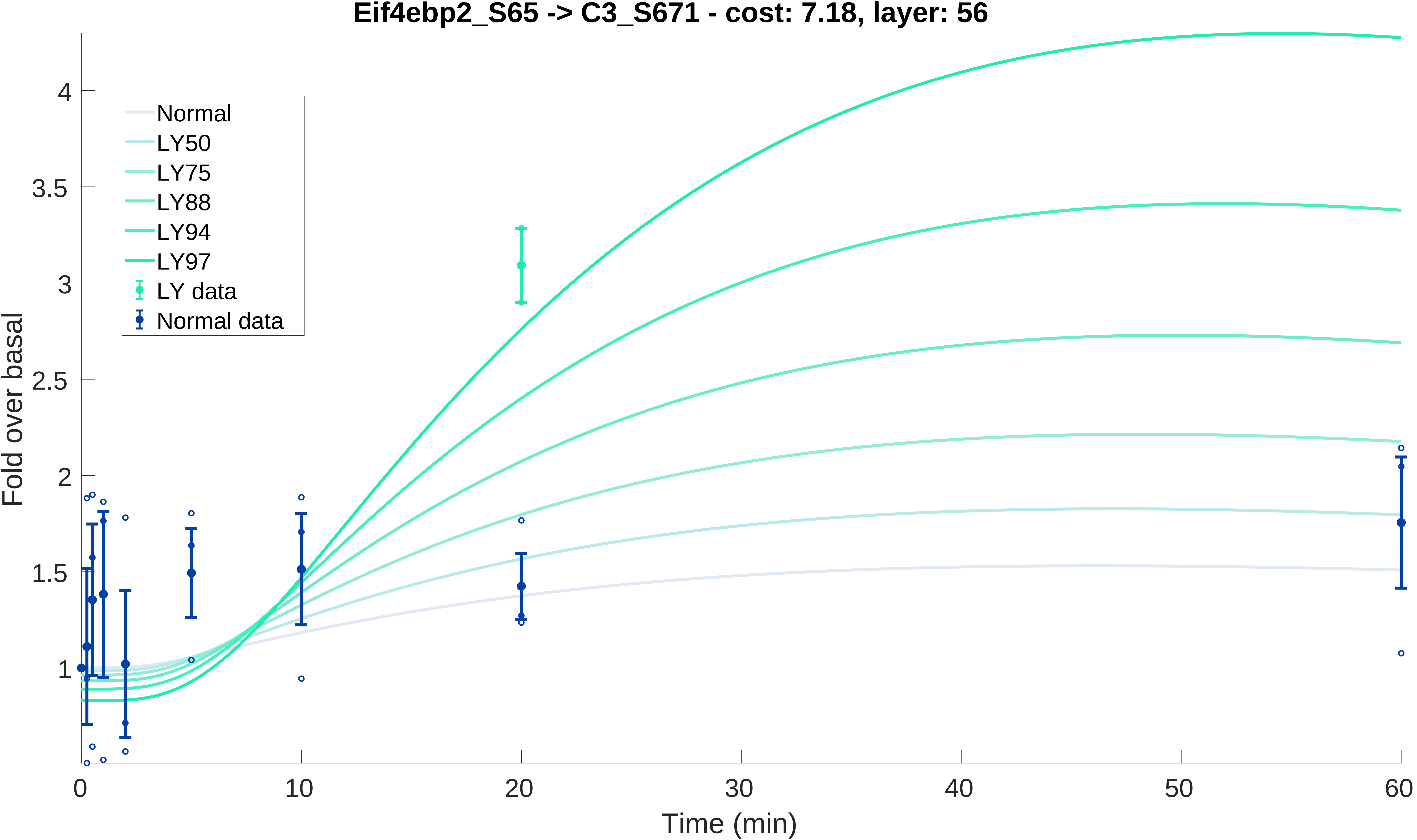
Eif4ebp2_S65 -> C3_S671 - cost: 7.18, layer: 56
Normal
LY50
LY75
LY88
LY94
LY97
LY data
Normal data
Fold over basal
Time (min)
4
3.5
3
2.5
2
1.5
1
0
10
20
30
40
50
60

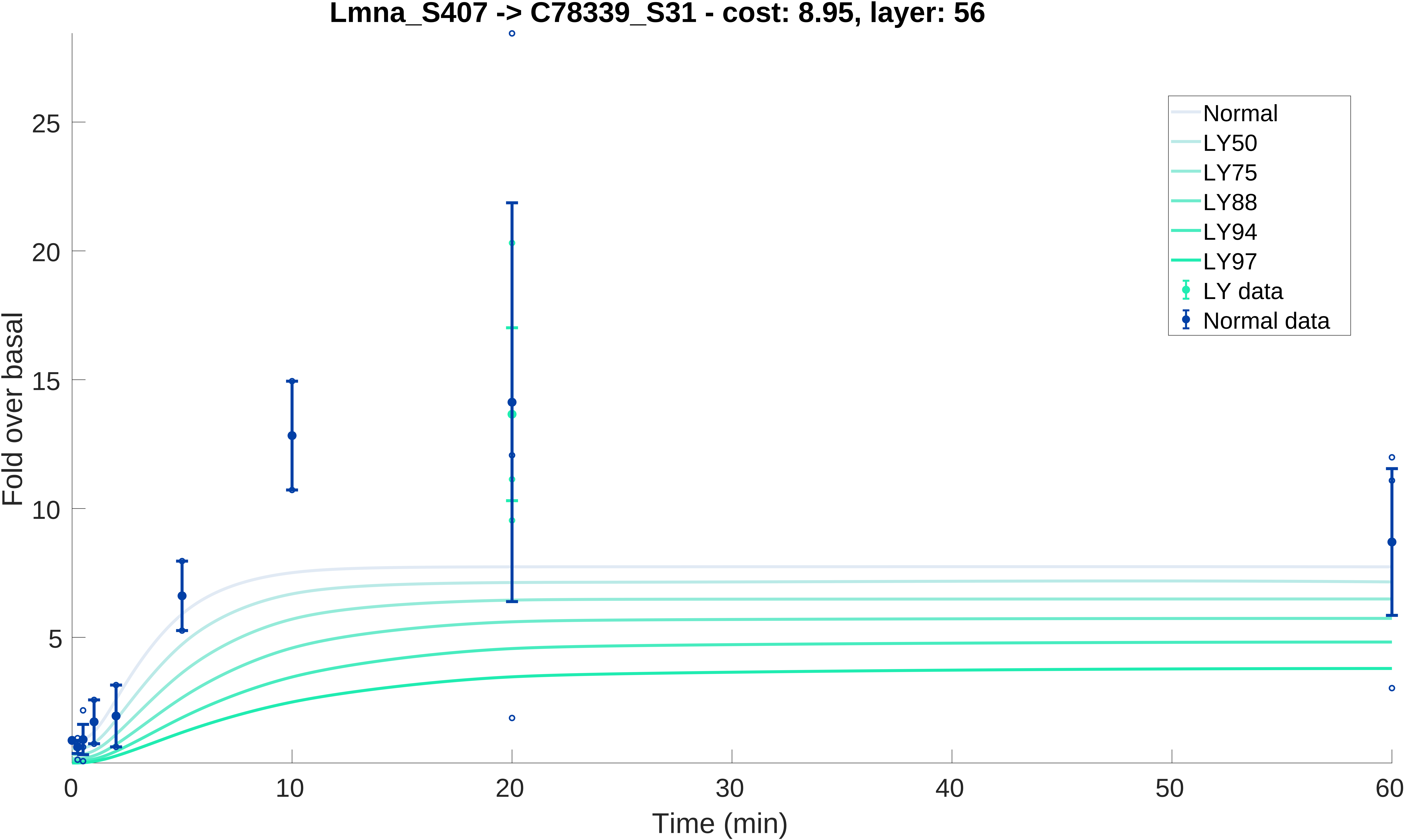

Lmna_S407 -> C78339_S31 - cost: 8.95, layer: 56
Fold over basal
Time (min)
0
10
20
30
40
50
60
5
10
15
20
25
Normal
LY50
LY75
LY88
LY94
LY97
LY data
Normal data

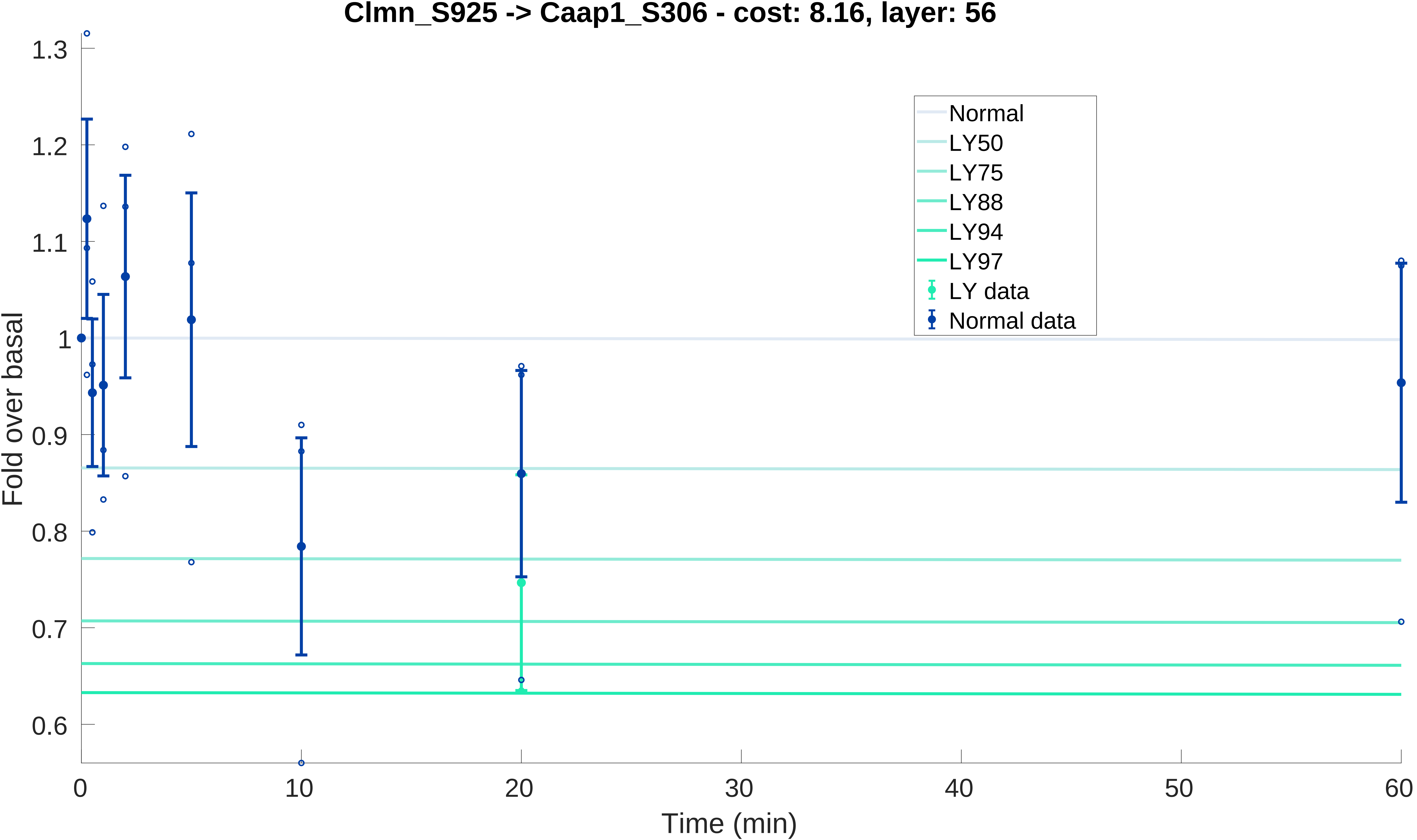

Clmn_S925 -> Caap1_S306 - cost: 8.16, layer: 56
Fold over basal
Time (min)
1.3
1.2
1.1
1
0.9
0.8
0.7
0.6
0
10
20
30
40
50
60
Normal
LY50
LY75
LY88
LY94
LY97
LY data
Normal data

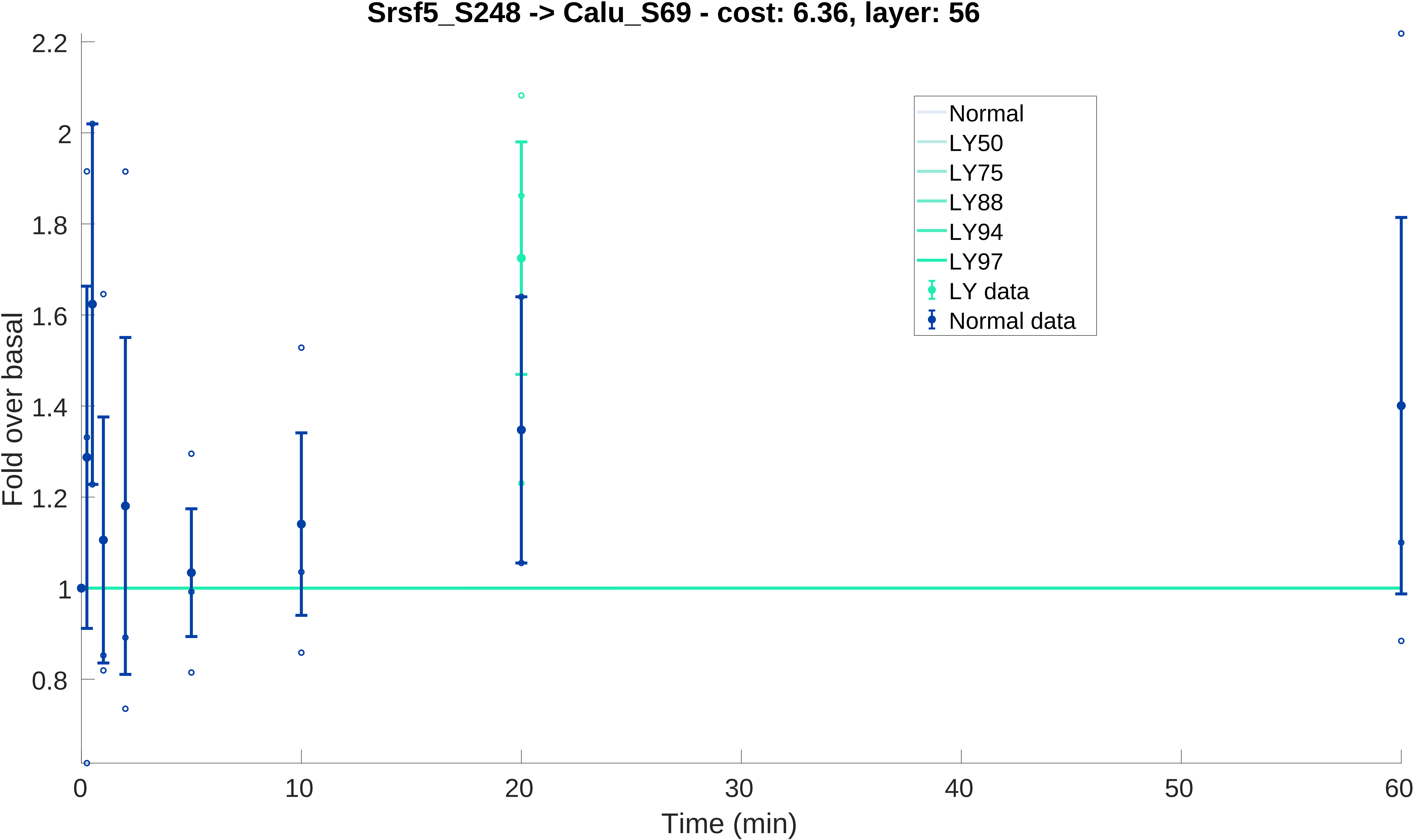

Srsf5_S248 -> Calu_S69 - cost: 6.36, layer: 56
Fold over basal
Time (min)
0
10
20
30
40
50
60
0.8
1
1.2
1.4
1.6
1.8
2
2.2
Normal
LY50
LY75
LY88
LY94
LY97
LY data
Normal data

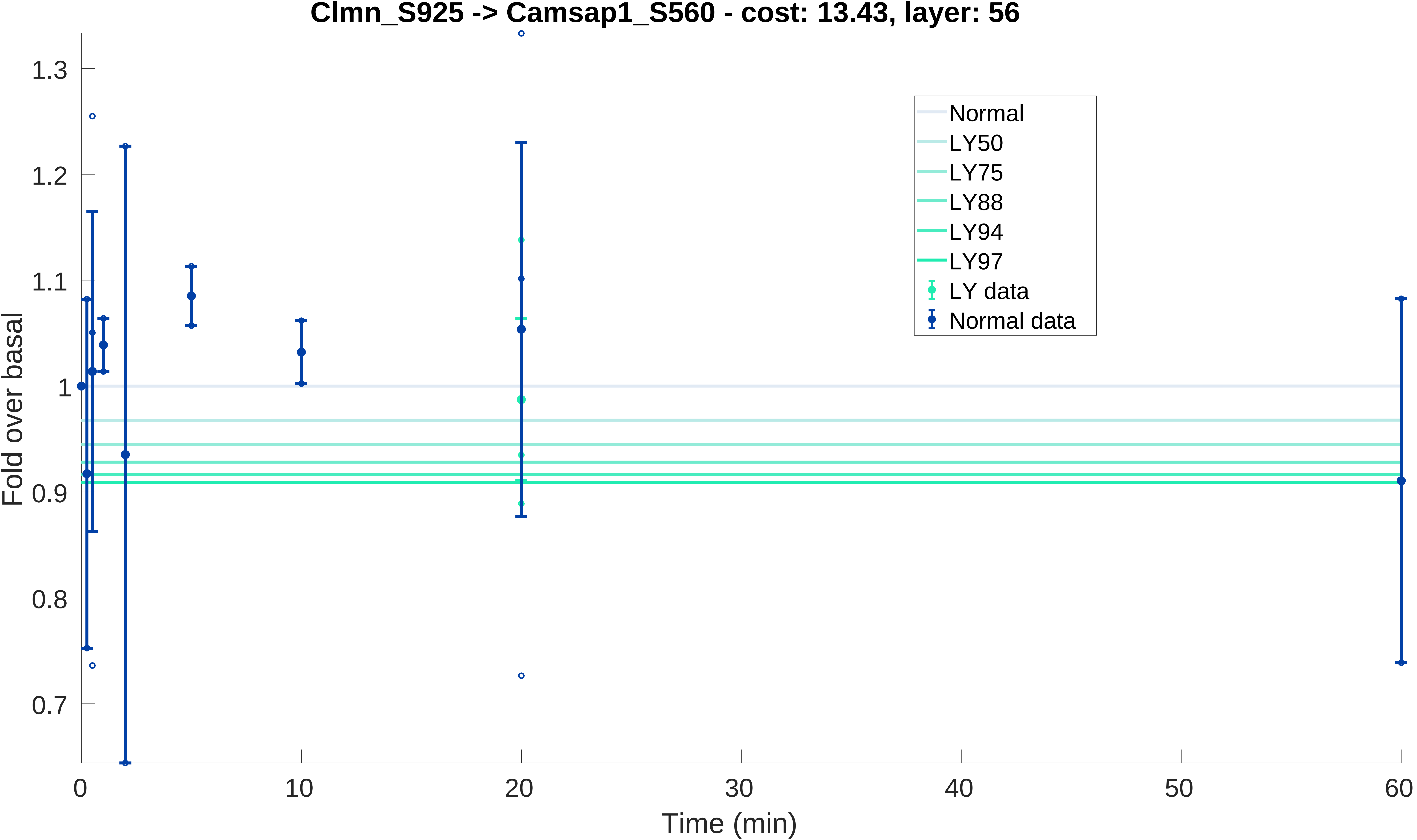

Clmn_S925 -> Camsap1_S560 - cost: 13.43, layer: 56
Fold over basal
Time (min)
1.3
1.2
1.1
1
0.9
0.8
0.7
0
10
20
30
40
50
60
Normal
LY50
LY75
LY88
LY94
LY97
LY data
Normal data

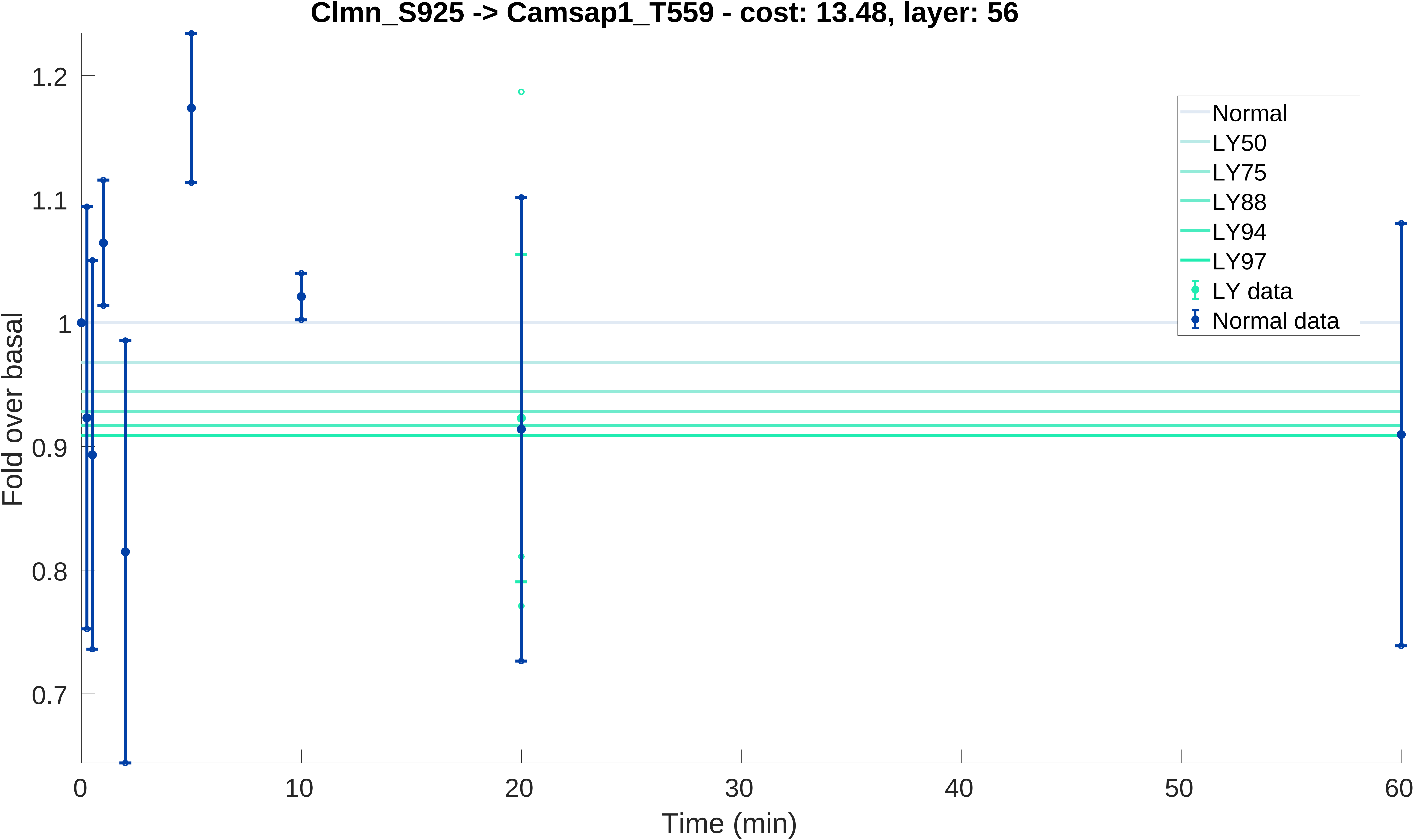

Clmn_S925 -> Camsap1_T559 - cost: 13.48, layer: 56
Fold over basal
Time (min)
1.2
1.1
1
0.9
0.8
0.7
0
10
20
30
40
50
60
Normal
LY50
LY75
LY88
LY94
LY97
LY data
Normal data

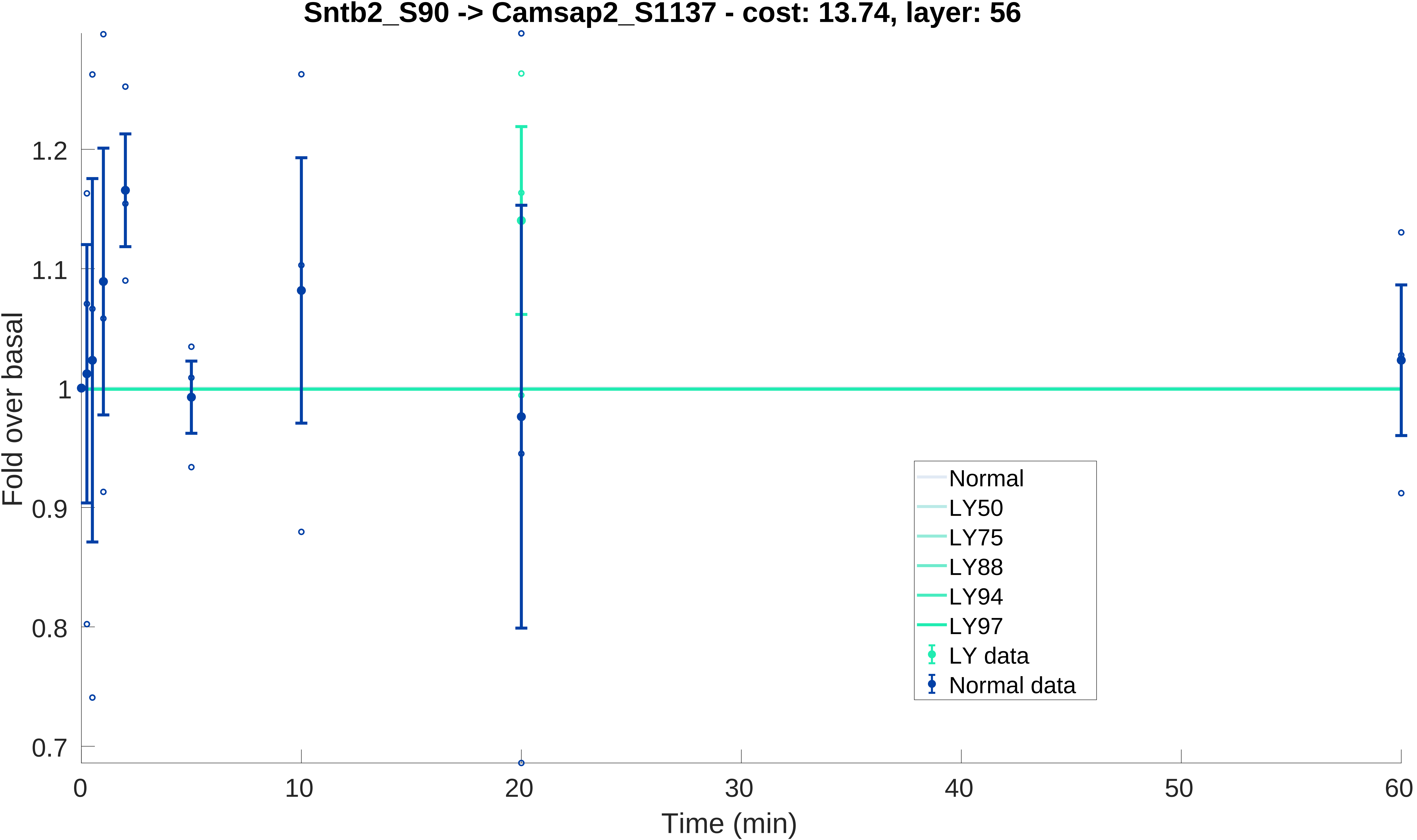

Sntb2_S90 -> Camsap2_S1137 - cost: 13.74, layer: 56
Fold over basal
Time (min)
1.2
1.1
1
0.9
0.8
0.7
0
10
20
30
40
50
60
Normal
LY50
LY75
LY88
LY94
LY97
LY data
Normal data

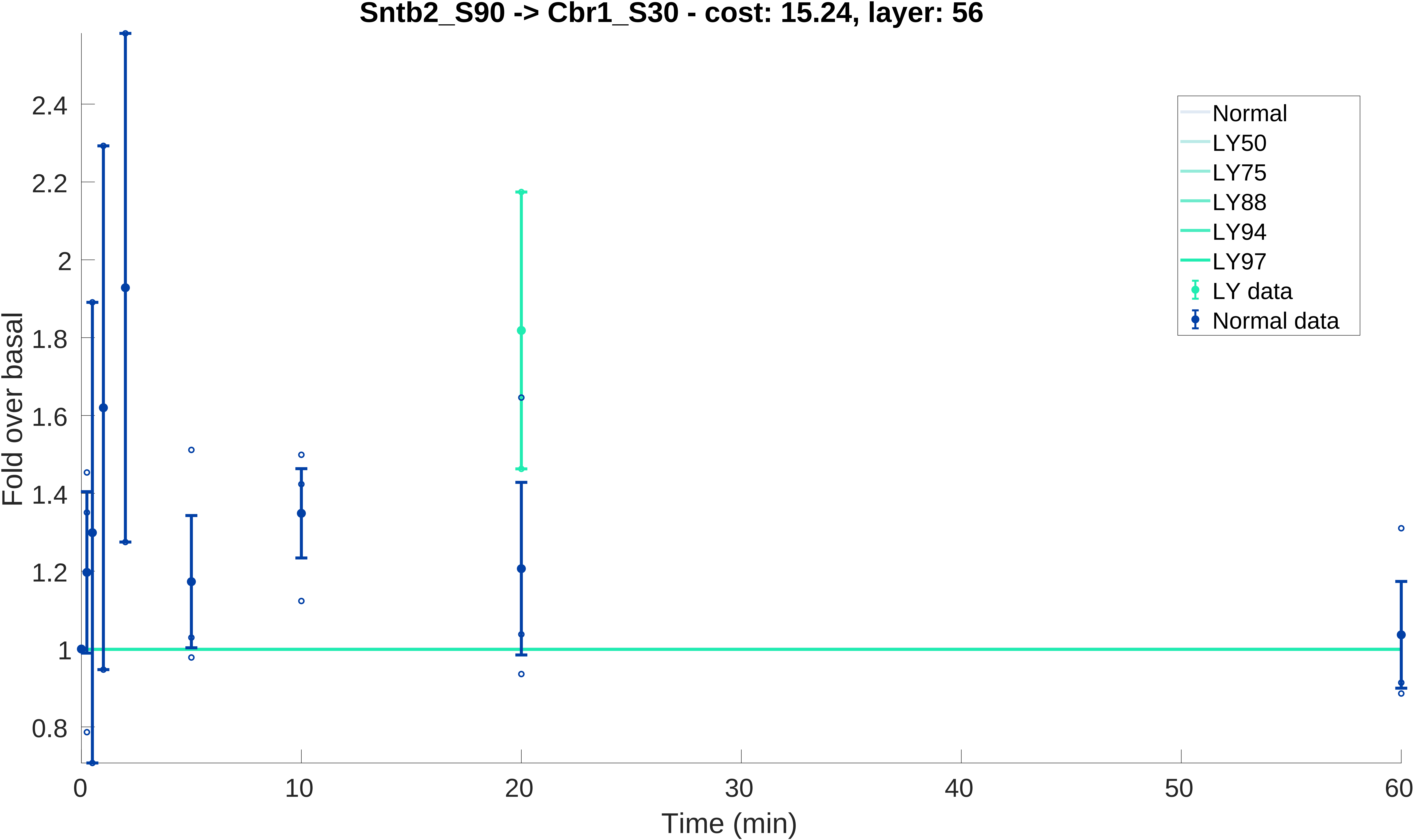

Sntb2_S90 -> Cbr1_S30 - cost: 15.24, layer: 56
Fold over basal
Time (min)
2.4
2.2
2
1.8
1.6
1.4
1.2
1
0.8
0
10
20
30
40
50
60
Normal
LY50
LY75
LY88
LY94
LY97
LY data
Normal data

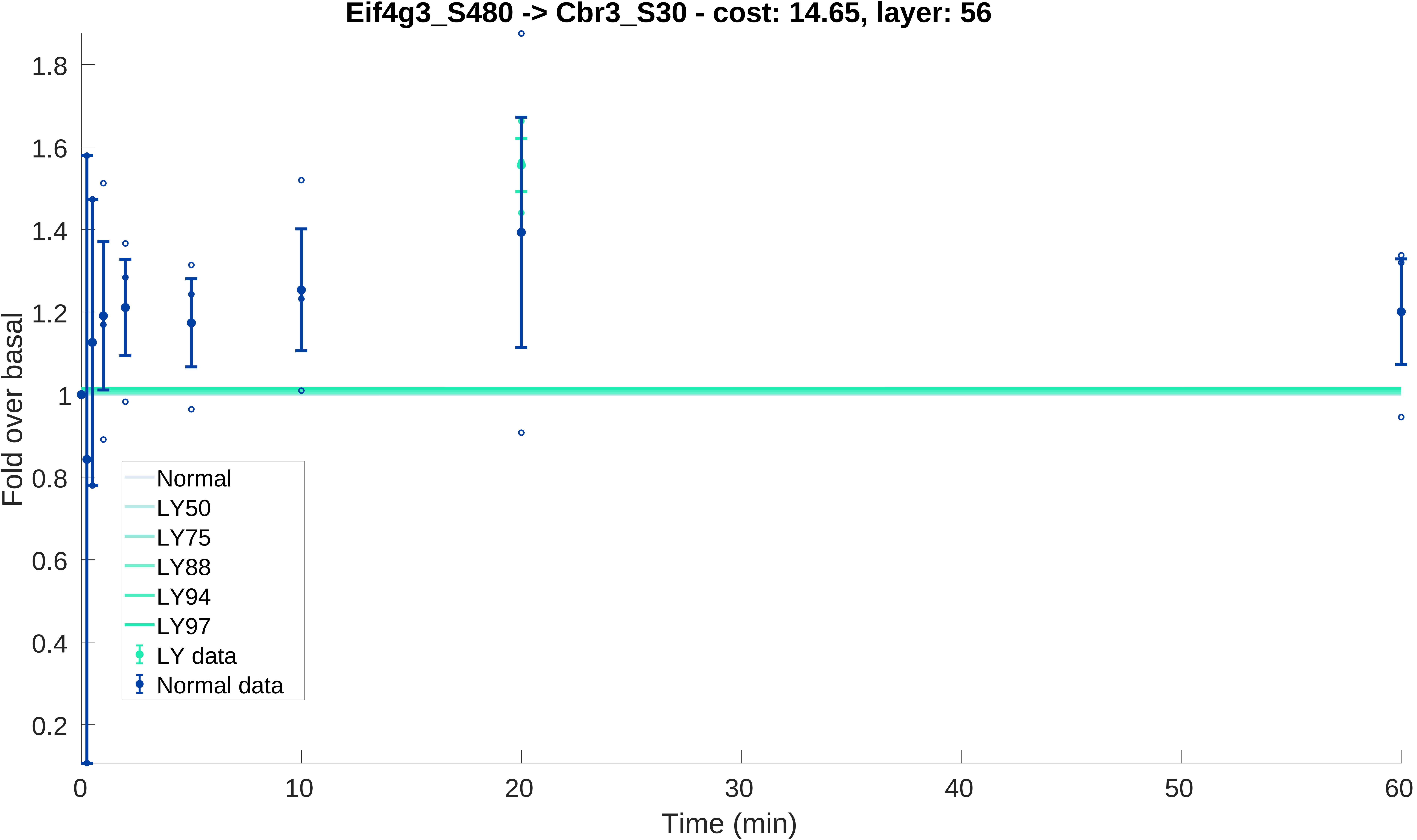

Eif4g3_S480 -> Cbr3_S30 - cost: 14.65, layer: 56
Fold over basal
1.8
1.6
1.4
1.2
1
0.8
0.6
0.4
0.2
0
10
20
30
40
50
60
Time (min)
Normal
LY50
LY75
LY88
LY94
LY97
LY data
Normal data

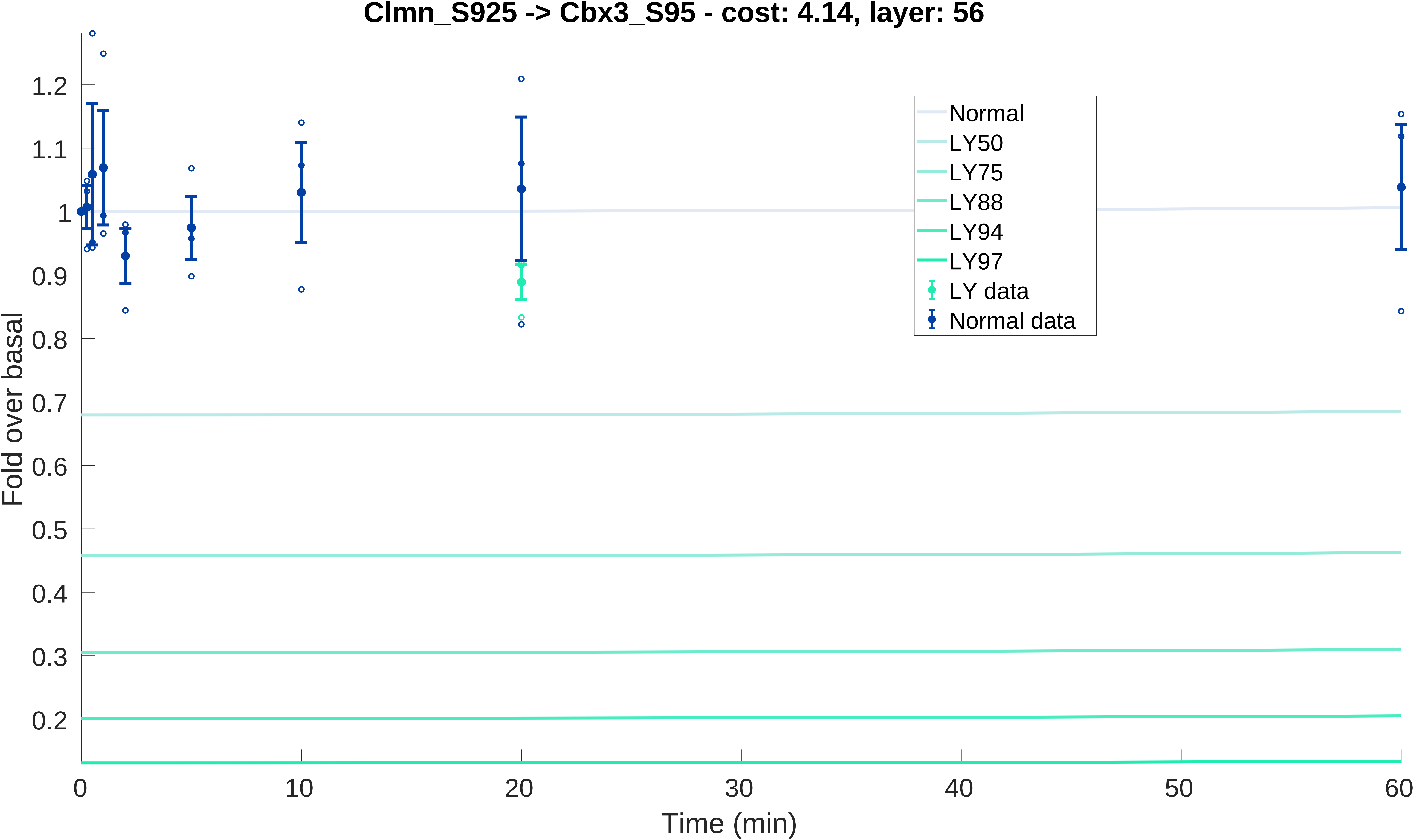

Clmn_S925 -> Cbx3_S95 - cost: 4.14, layer: 56
Fold over basal
Time (min)
0
10
20
30
40
50
60
0.2
0.3
0.4
0.5
0.6
0.7
0.8
0.9
1
1.1
1.2
Normal
LY50
LY75
LY88
LY94
LY97
LY data
Normal data

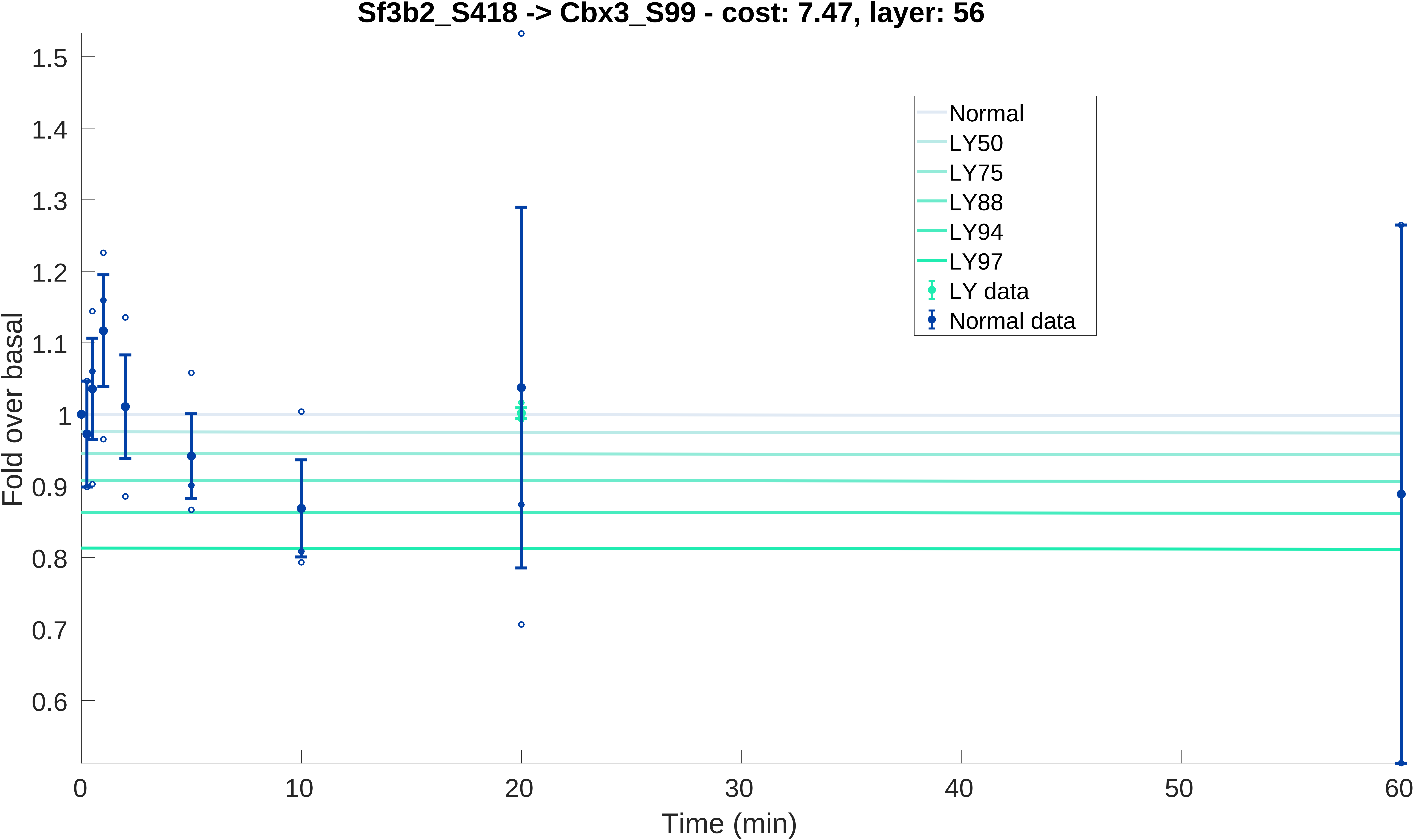
Sf3b2_S418 -> Cbx3_S99 - cost: 7.47, layer: 56
Fold over basal
Time (min)
0.6
0.7
0.8
0.9
1
1.1
1.2
1.3
1.4
1.5
0
10
20
30
40
50
60
Normal
LY50
LY75
LY88
LY94
LY97
LY data
Normal data

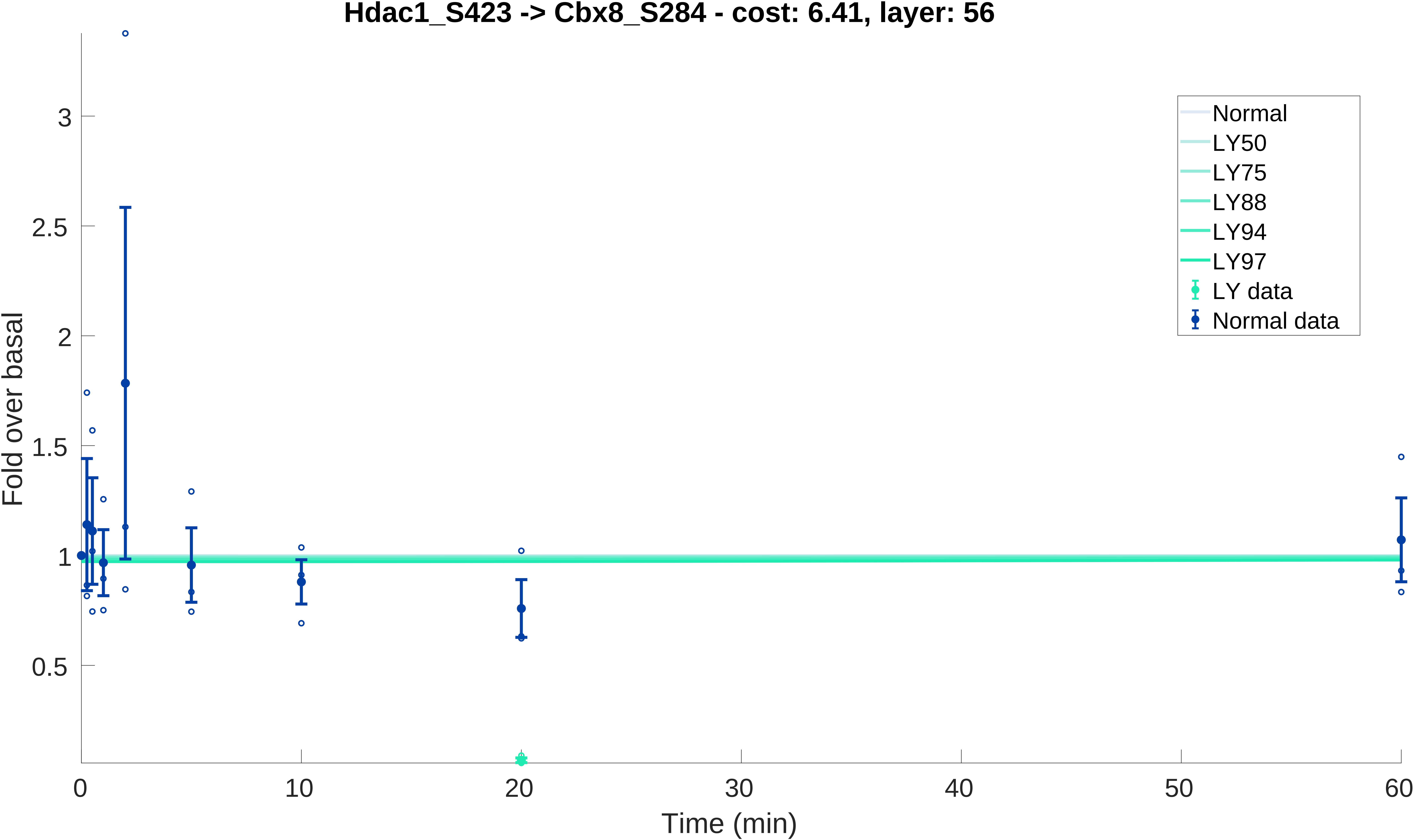

Hdac1_S423 -> Cbx8_S284 - cost: 6.41, layer: 56
Fold over basal
Time (min)
0.5
1
1.5
2
2.5
3
0
10
20
30
40
50
60
Normal
LY50
LY75
LY88
LY94
LY97
LY data
Normal data

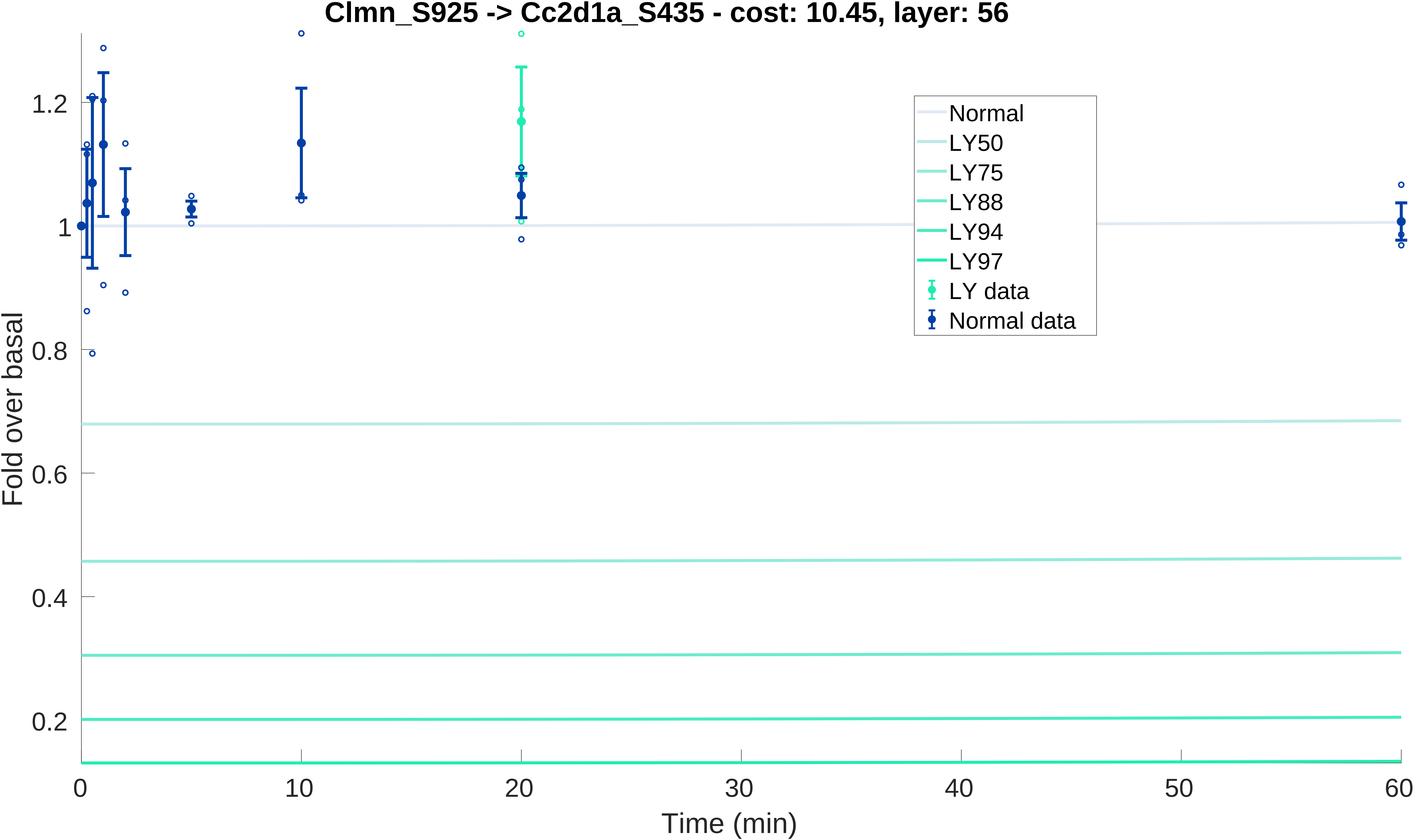

Clmn_S925 -> Cc2d1a_S435 - cost: 10.45, layer: 56
Fold over basal
Time (min)
1.2
1
0.8
0.6
0.4
0.2
0
10
20
30
40
50
60
Normal
LY50
LY75
LY88
LY94
LY97
LY data
Normal data

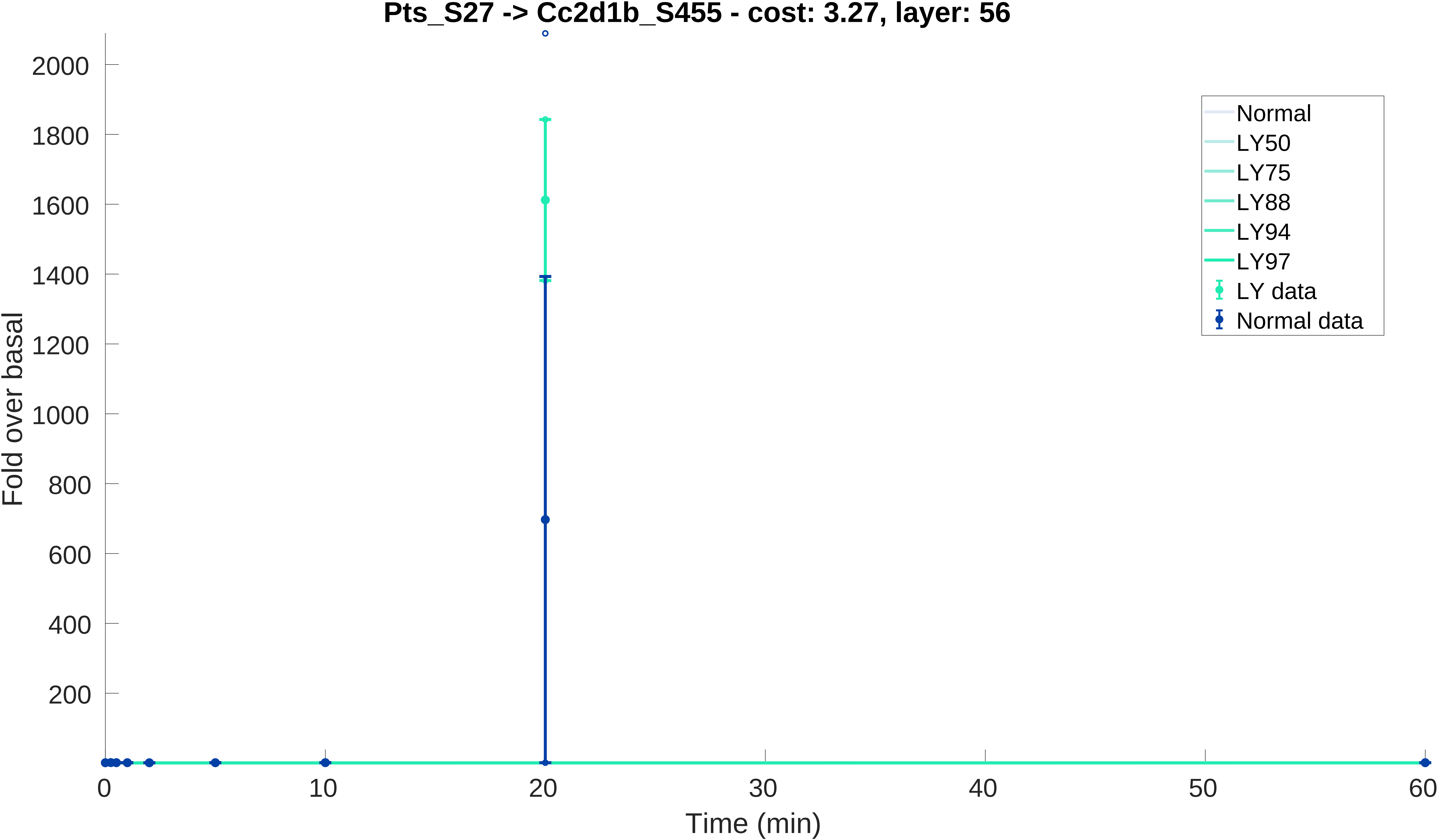

Pts_S27 -> Cc2d1b_S455 - cost: 3.27, layer: 56
Fold over basal
Time (min)
2000
1800
1600
1400
1200
1000
800
600
400
200
0
10
20
30
40
50
60
Normal
LY50
LY75
LY88
LY94
LY97
LY data
Normal data

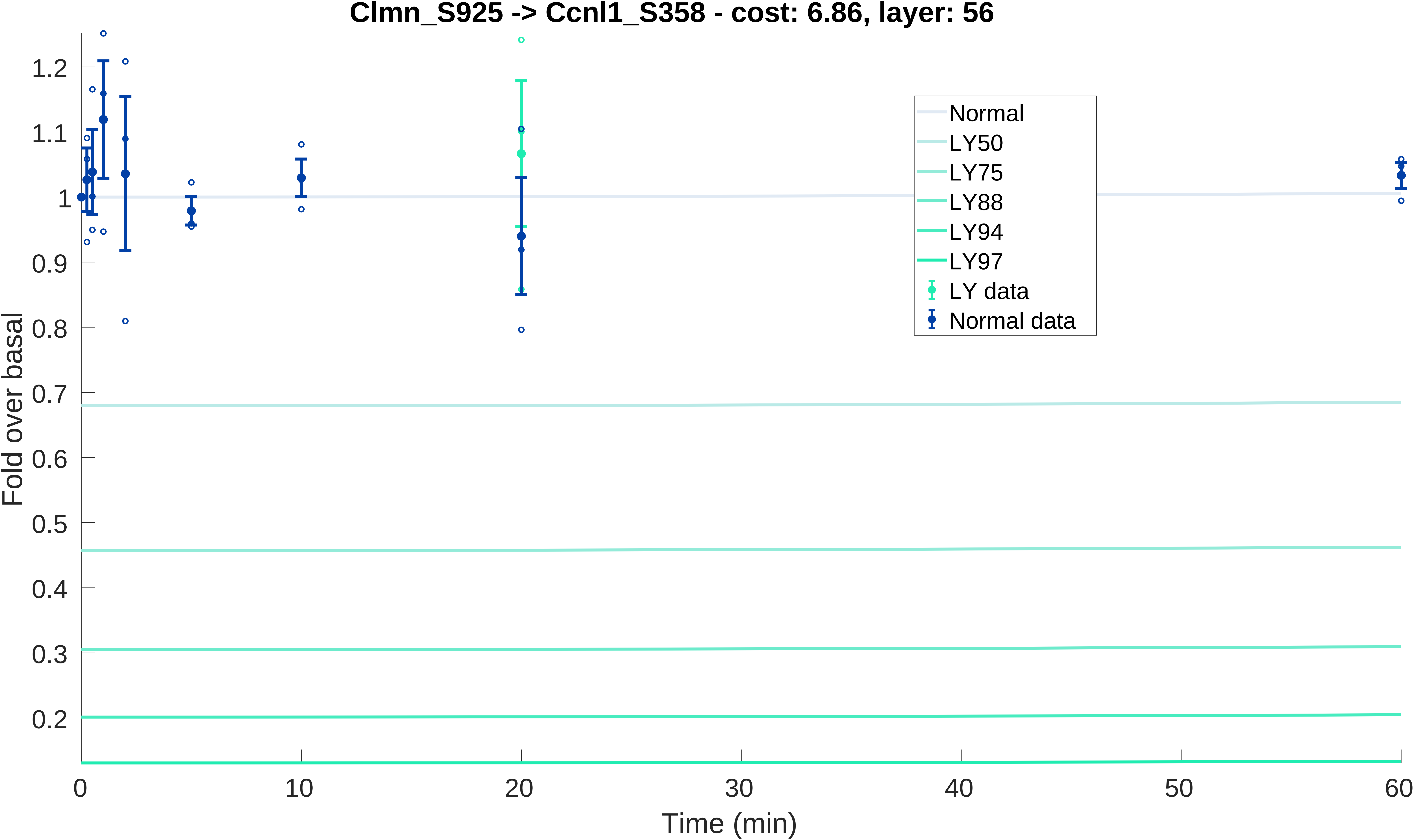

Clmn_S925 -> Ccnl1_S358 - cost: 6.86, layer: 56
Fold over basal
Time (min)
0
10
20
30
40
50
60
0.2
0.3
0.4
0.5
0.6
0.7
0.8
0.9
1
1.1
1.2
Normal
LY50
LY75
LY88
LY94
LY97
LY data
Normal data

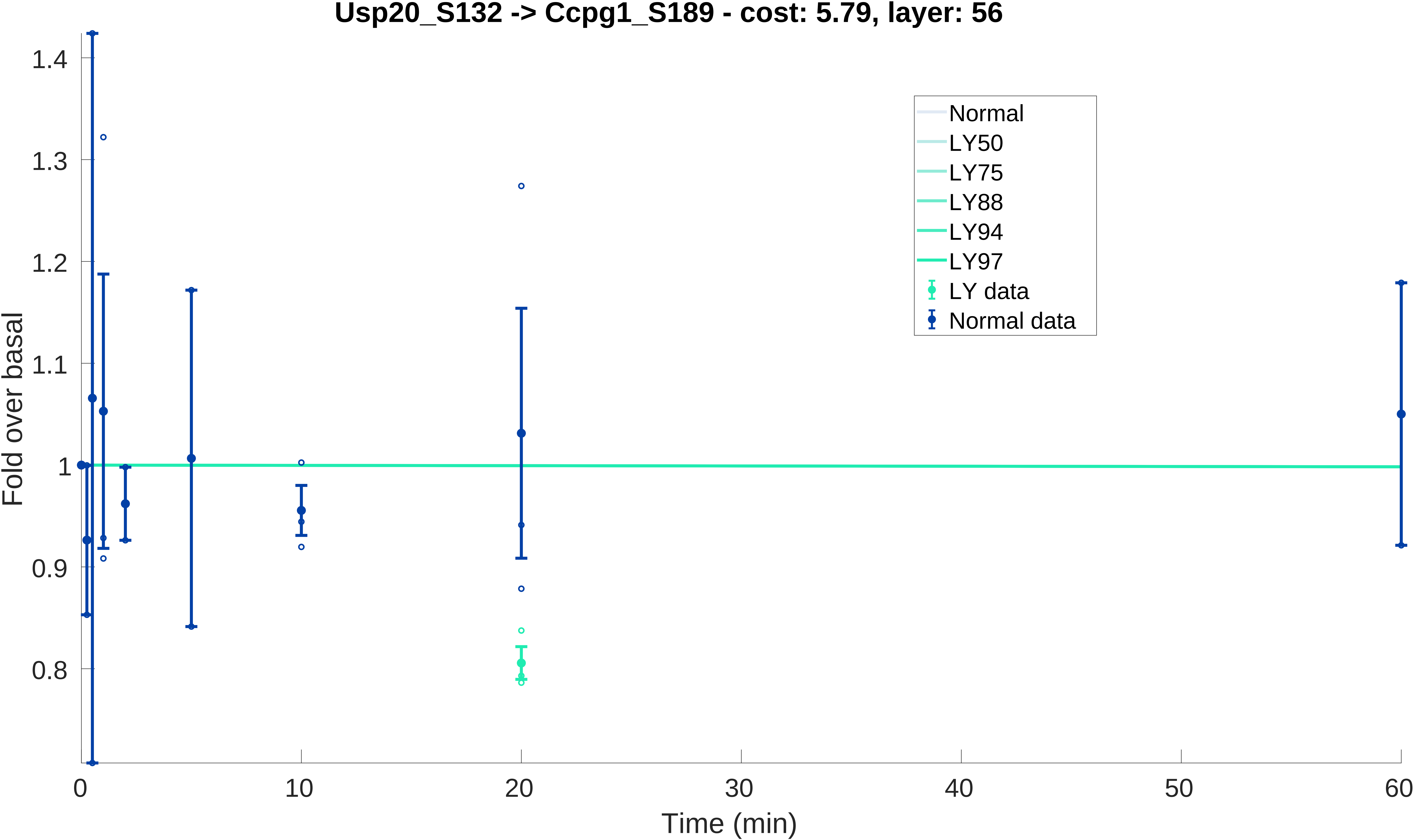

Usp20_S132 -> Ccpg1_S189 - cost: 5.79, layer: 56
Fold over basal
Time (min)
1.4
1.3
1.2
1.1
1
0.9
0.8
0
10
20
30
40
50
60
Normal
LY50
LY75
LY88
LY94
LY97
LY data
Normal data

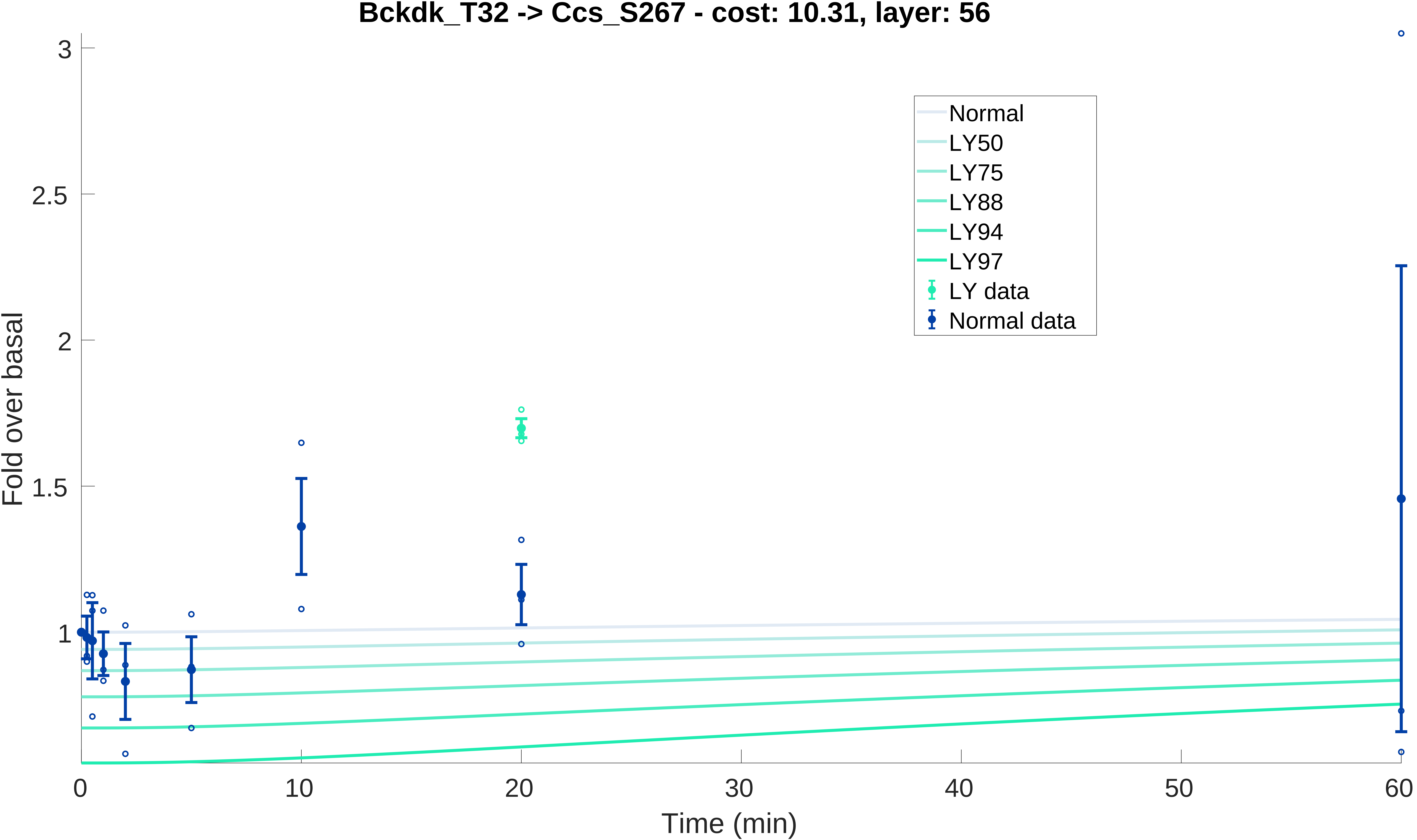
Bckdk_T32 -> Ccs_S267 - cost: 10.31, layer: 56
Fold over basal
3
2.5
2
1.5
1
0
10
20
30
40
50
60
Time (min)
Normal
LY50
LY75
LY88
LY94
LY97
LY data
Normal data

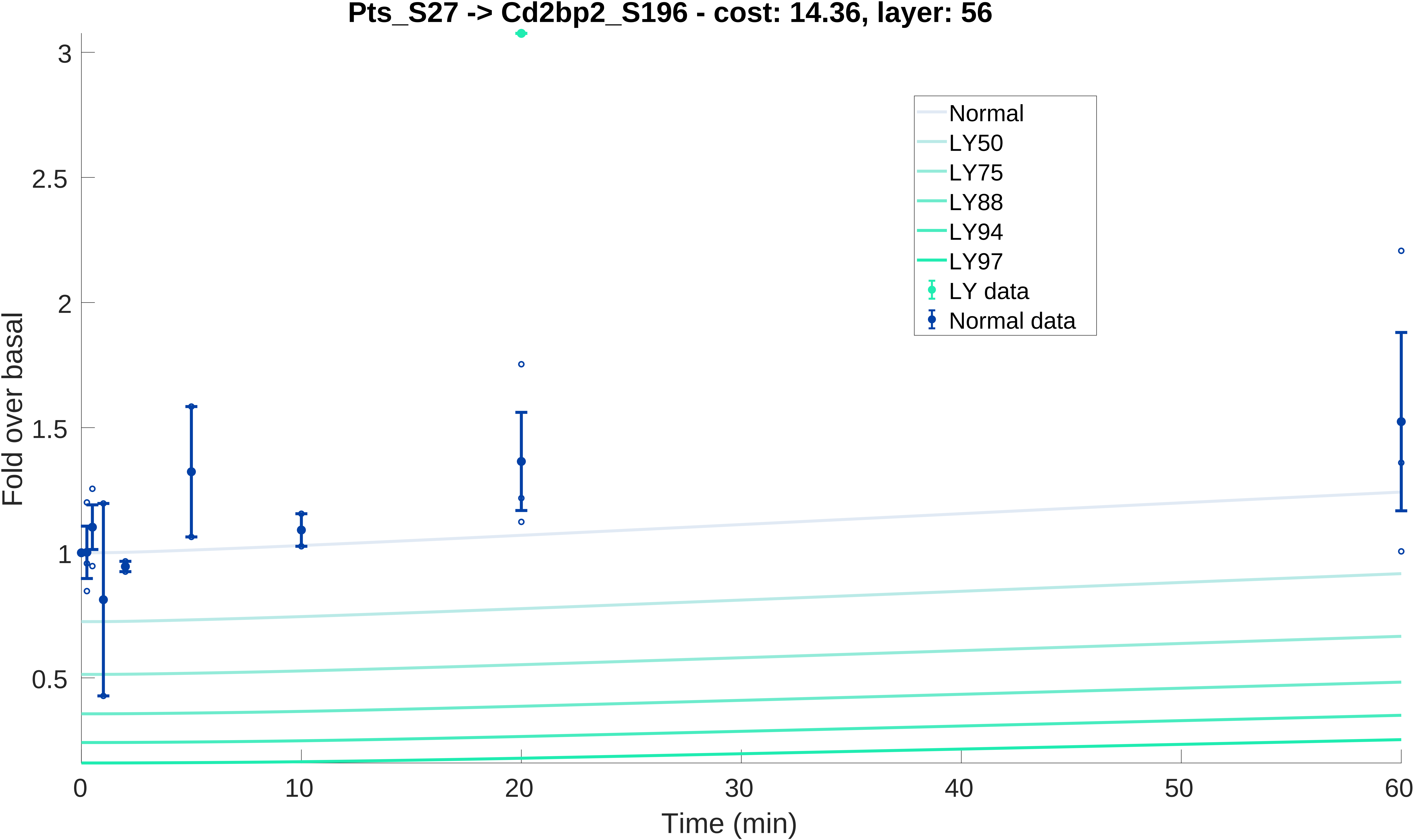
Pts_S27 -> Cd2bp2_S196 - cost: 14.36, layer: 56
Fold over basal
Time (min)
0
10
20
30
40
50
60
0.5
1
1.5
2
2.5
3
Normal
LY50
LY75
LY88
LY94
LY97
LY data
Normal data

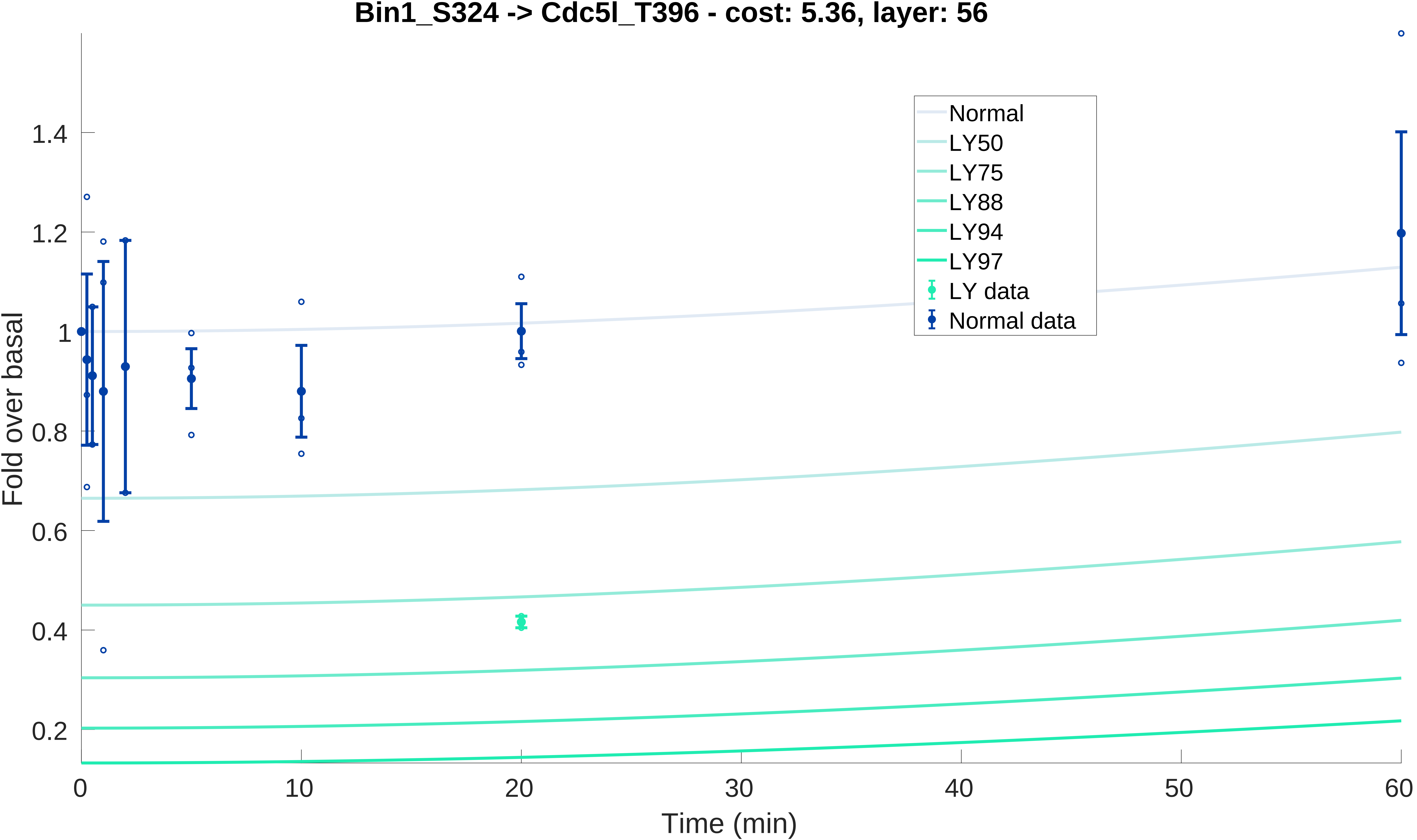

Bin1_S324 -> Cdc5l_T396 - cost: 5.36, layer: 56
Fold over basal
Time (min)
0
10
20
30
40
50
60
0.2
0.4
0.6
0.8
1
1.2
1.4
Normal
LY50
LY75
LY88
LY94
LY97
LY data
Normal data

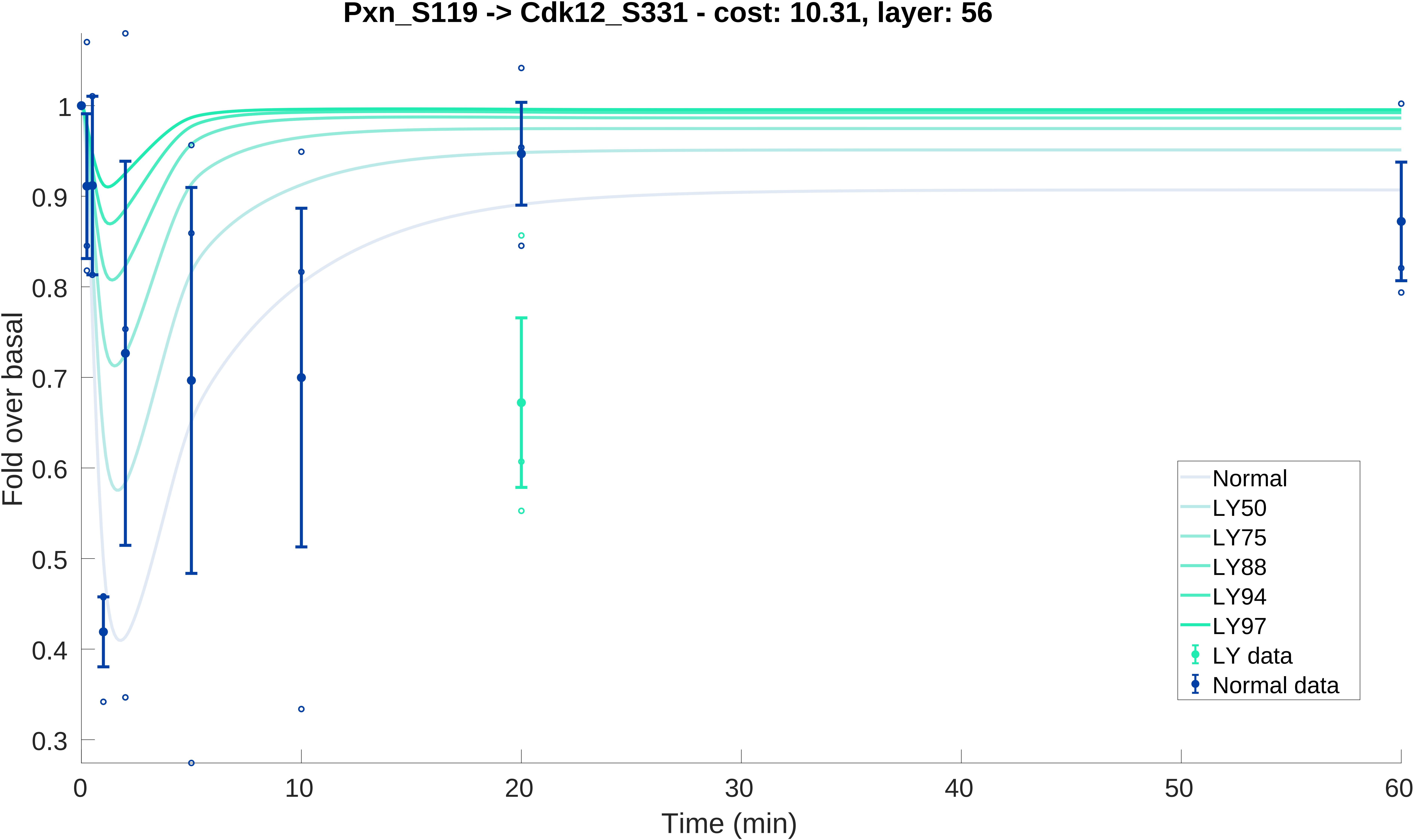

Pxn_S119 -> Cdk12_S331 - cost: 10.31, layer: 56
Fold over basal
Time (min)
0.3
0.4
0.5
0.6
0.7
0.8
0.9
1
0
10
20
30
40
50
60
Normal
LY50
LY75
LY88
LY94
LY97
LY data
Normal data

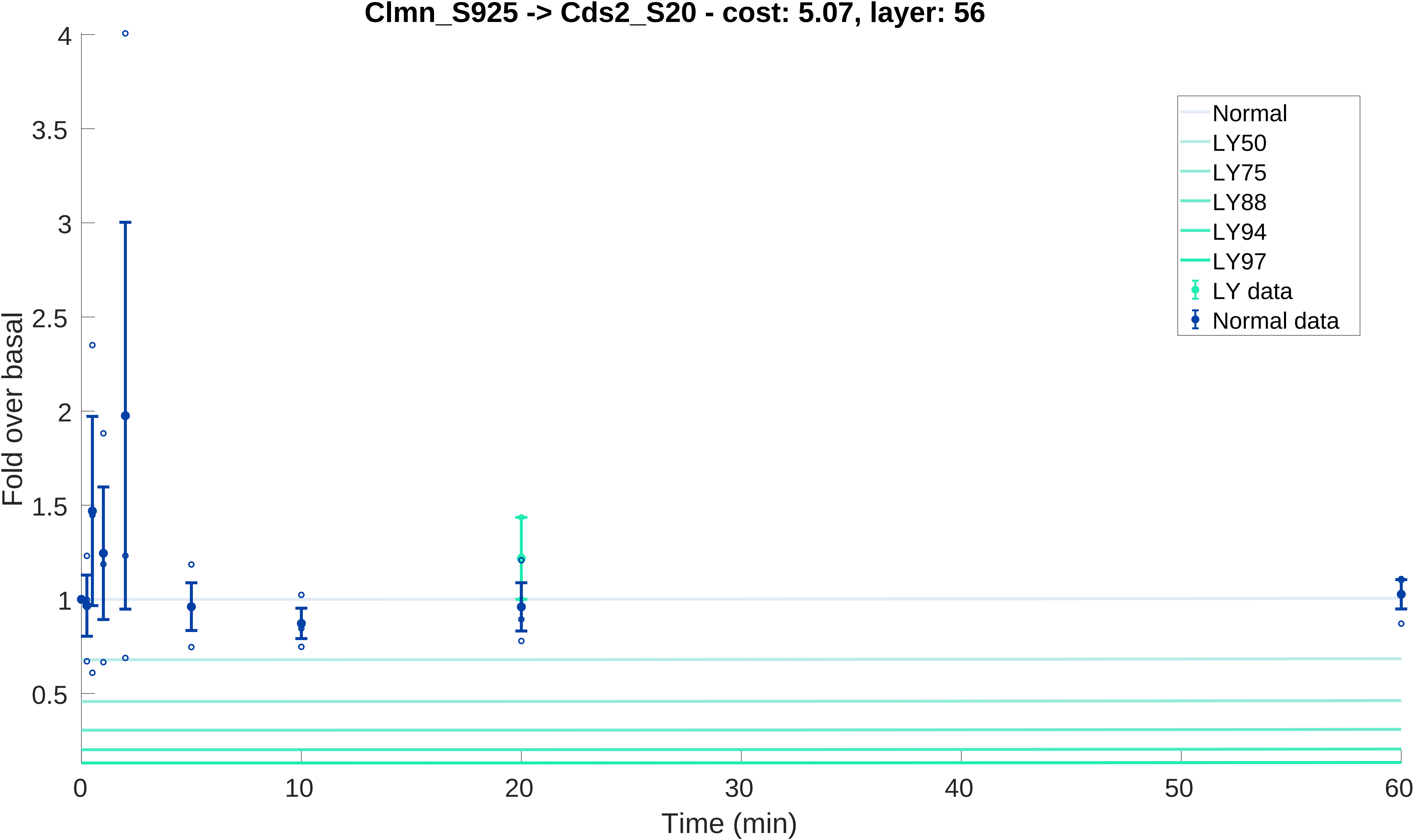

Clmn_S925 -> Cds2_S20 - cost: 5.07, layer: 56
Fold over basal
Time (min)
0.5
1
1.5
2
2.5
3
3.5
4
0
10
20
30
40
50
60
Normal
LY50
LY75
LY88
LY94
LY97
LY data
Normal data

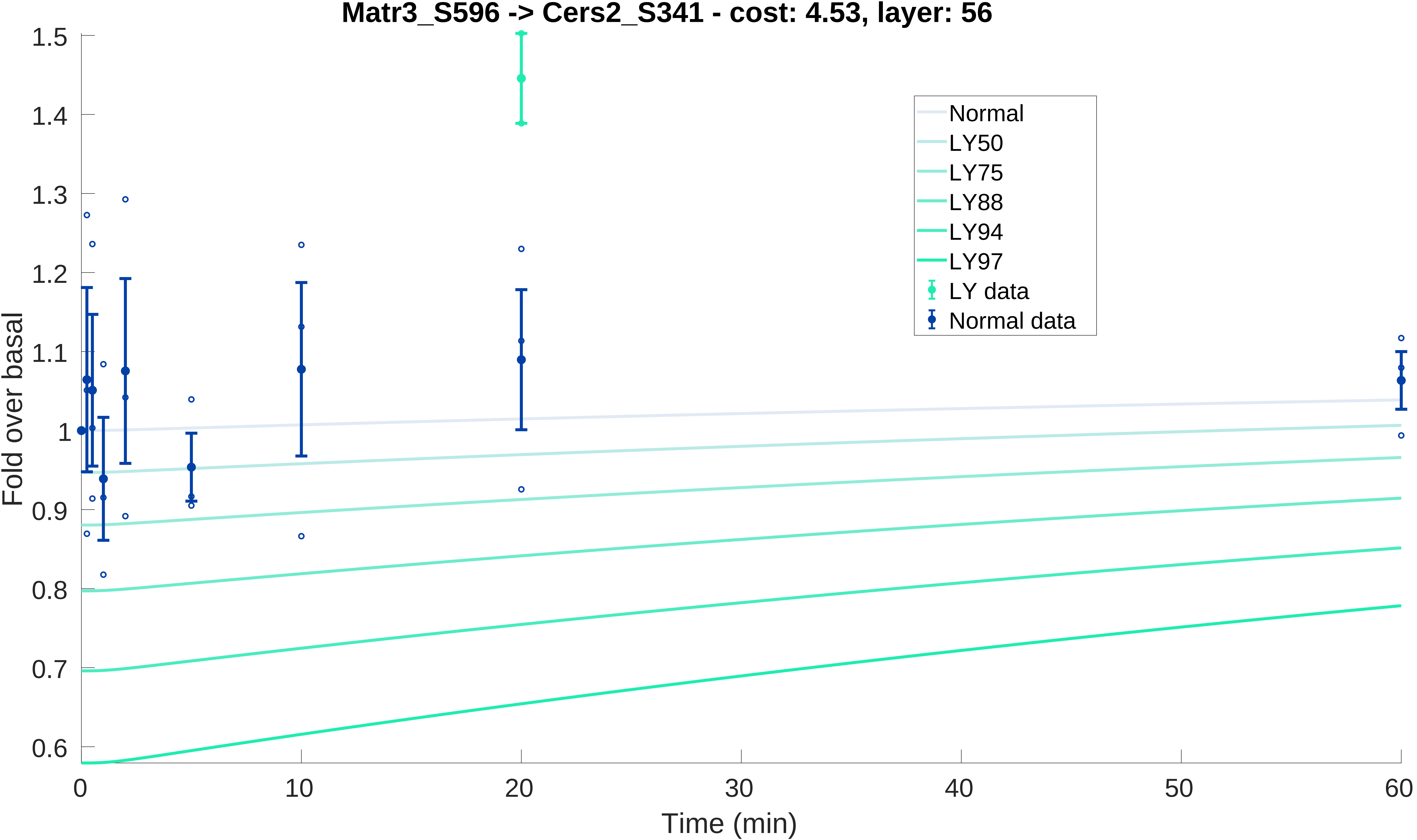
Matr3_S596 -> Cers2_S341 - cost: 4.53, layer: 56
Fold over basal
1.5
1.4
1.3
1.2
1.1
1
0.9
0.8
0.7
0.6
0
10
20
30
40
50
60
Time (min)
Normal
LY50
LY75
LY88
LY94
LY97
LY data
Normal data

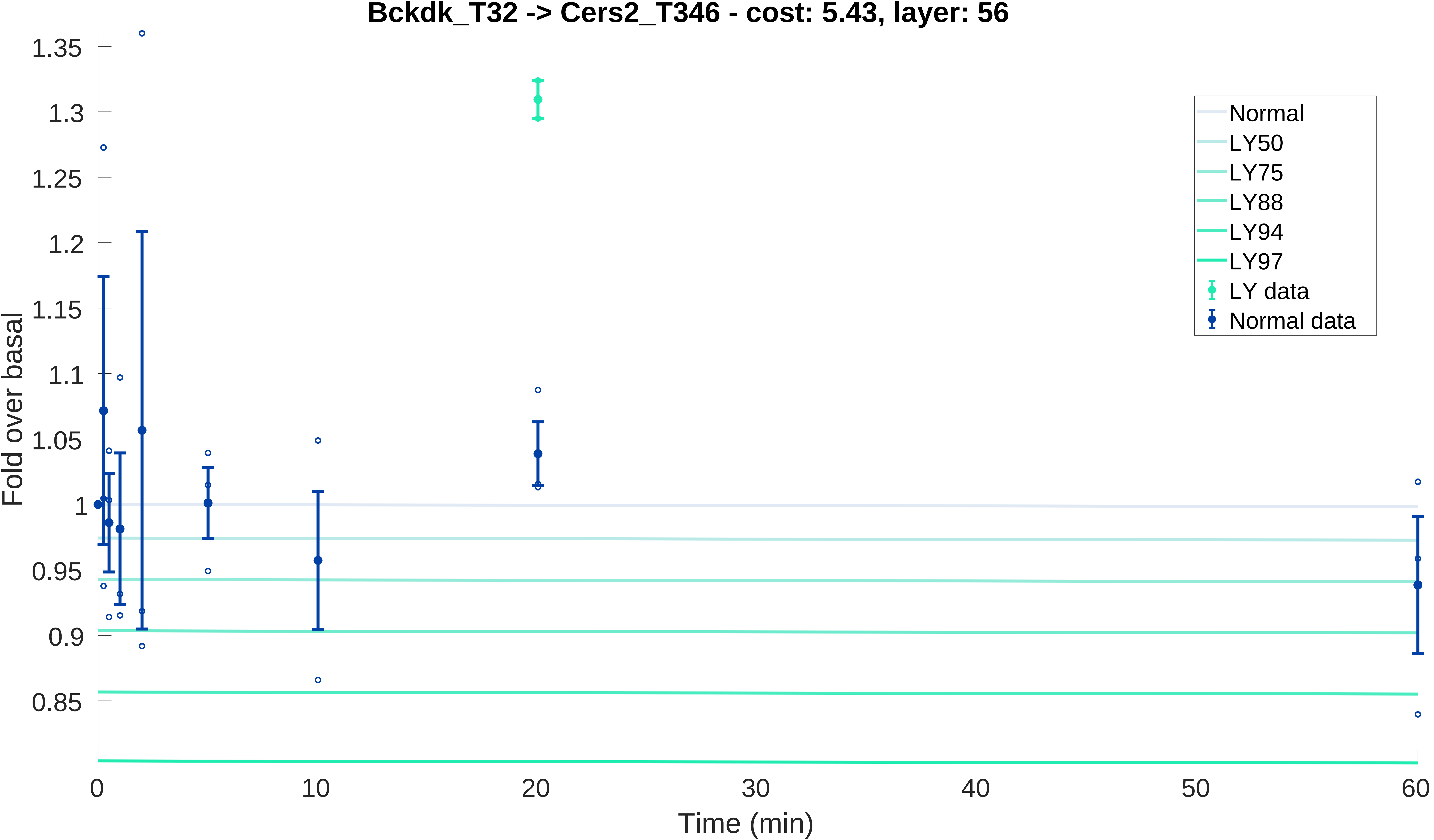

Bckdk_T32 -> Cers2_T346 - cost: 5.43, layer: 56
Fold over basal
Time (min)
1.35
1.3
1.25
1.2
1.15
1.1
1.05
1
0.95
0.9
0.85
0
10
20
30
40
50
60
Normal
LY50
LY75
LY88
LY94
LY97
LY data
Normal data

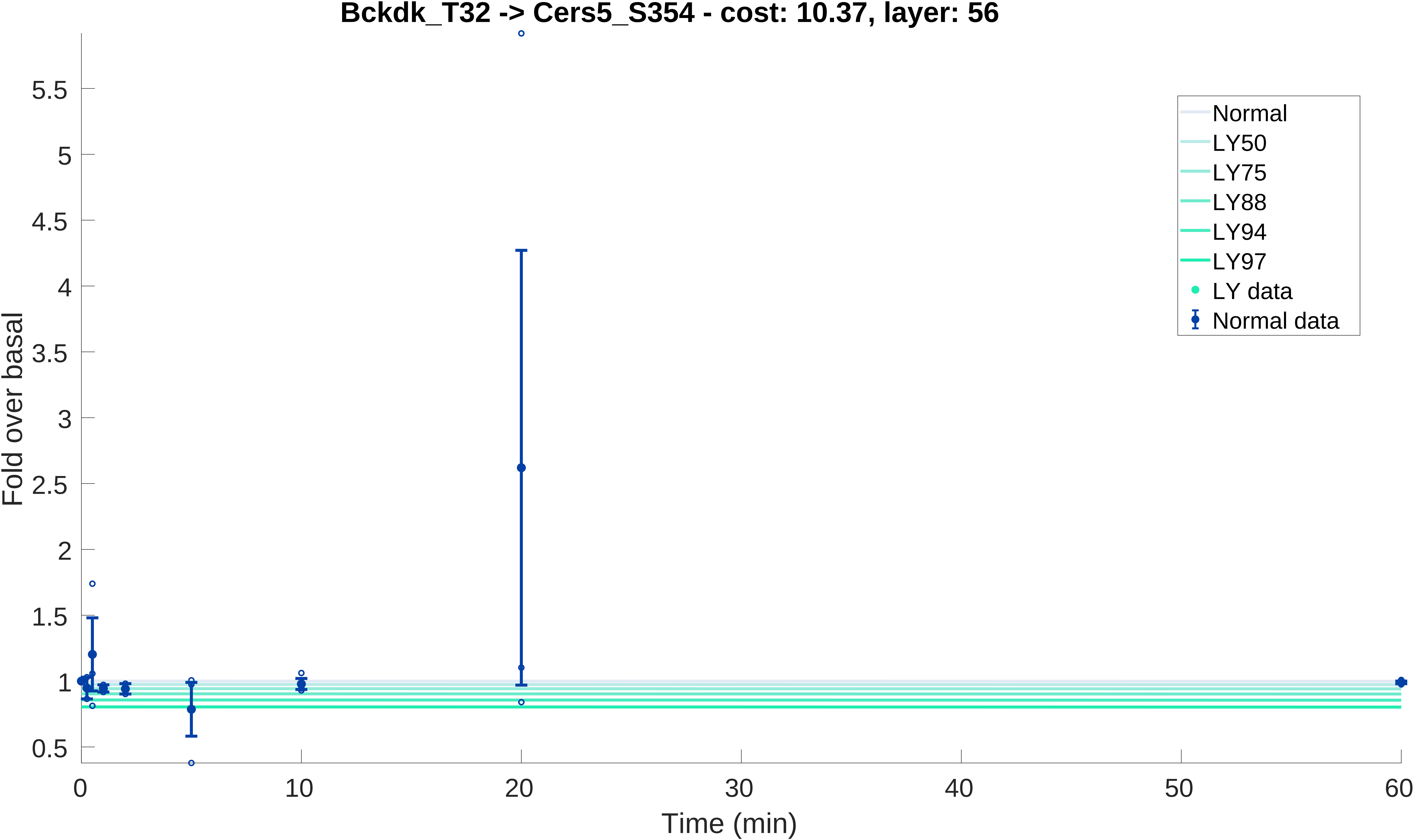
Bckdk_T32 -> Cers5_S354 - cost: 10.37, layer: 56
Fold over basal
Time (min)
0.5
1
1.5
2
2.5
3
3.5
4
4.5
5
5.5
0
10
20
30
40
50
60
Normal
LY50
LY75
LY88
LY94
LY97
LY data
Normal data

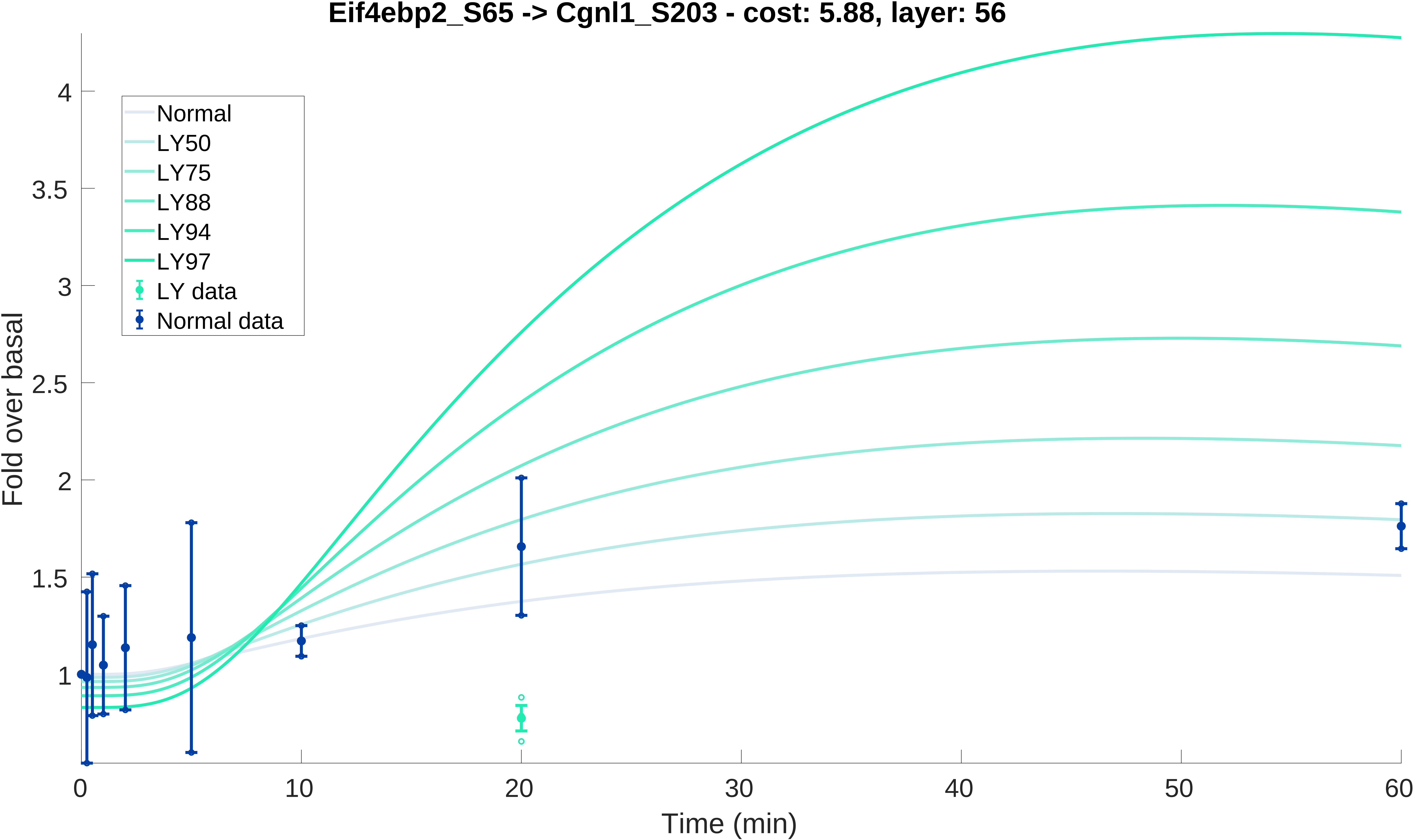
Eif4ebp2_S65 -> Cgnl1_S203 - cost: 5.88, layer: 56
Normal
LY50
LY75
LY88
LY94
LY97
LY data
Normal data
Fold over basal
Time (min)
0
10
20
30
40
50
60
1
1.5
2
2.5
3
3.5
4

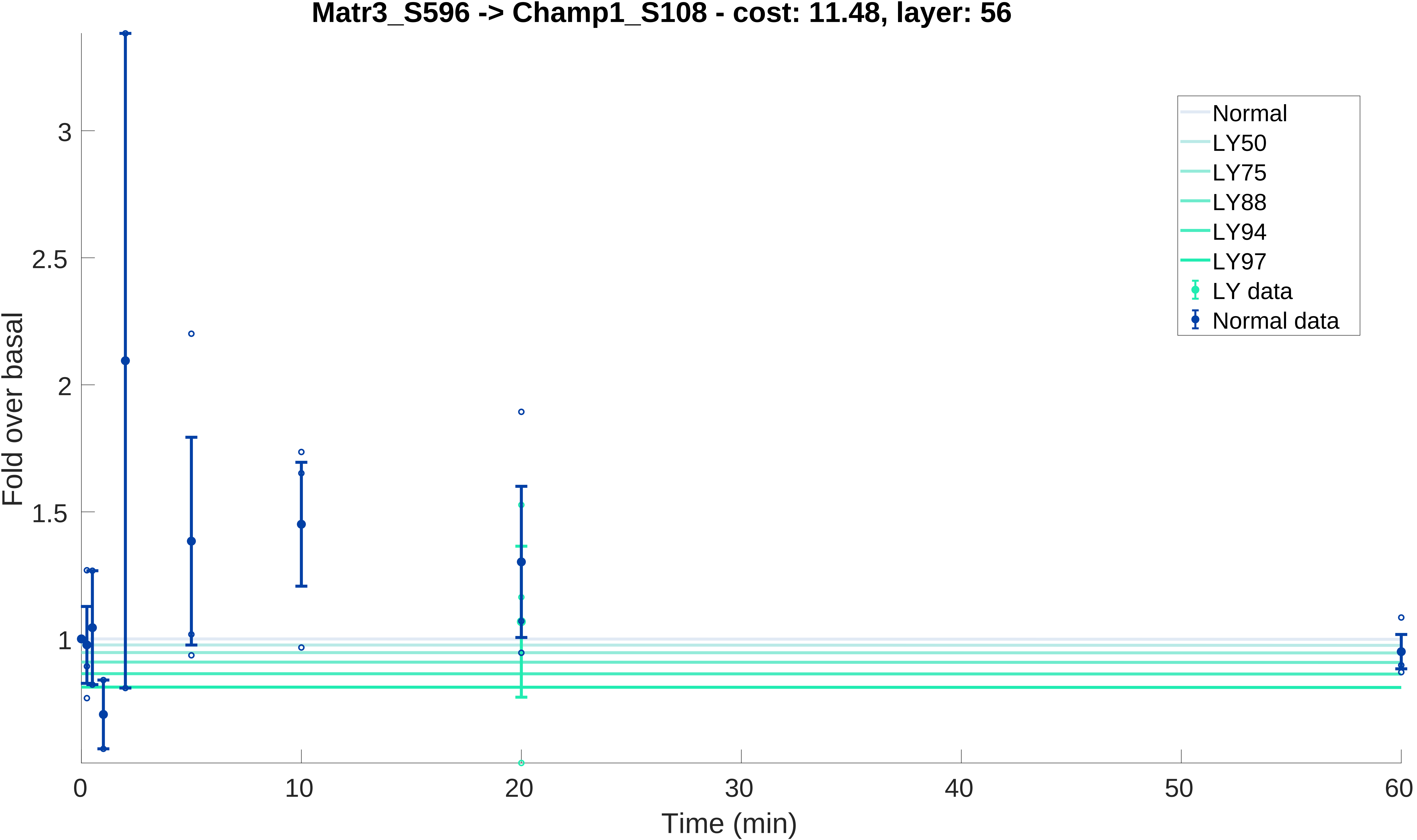

Matr3_S596 -> Champ1_S108 - cost: 11.48, layer: 56
Fold over basal
Time (min)
0
10
20
30
40
50
60
1
1.5
2
2.5
3
Normal
LY50
LY75
LY88
LY94
LY97
LY data
Normal data

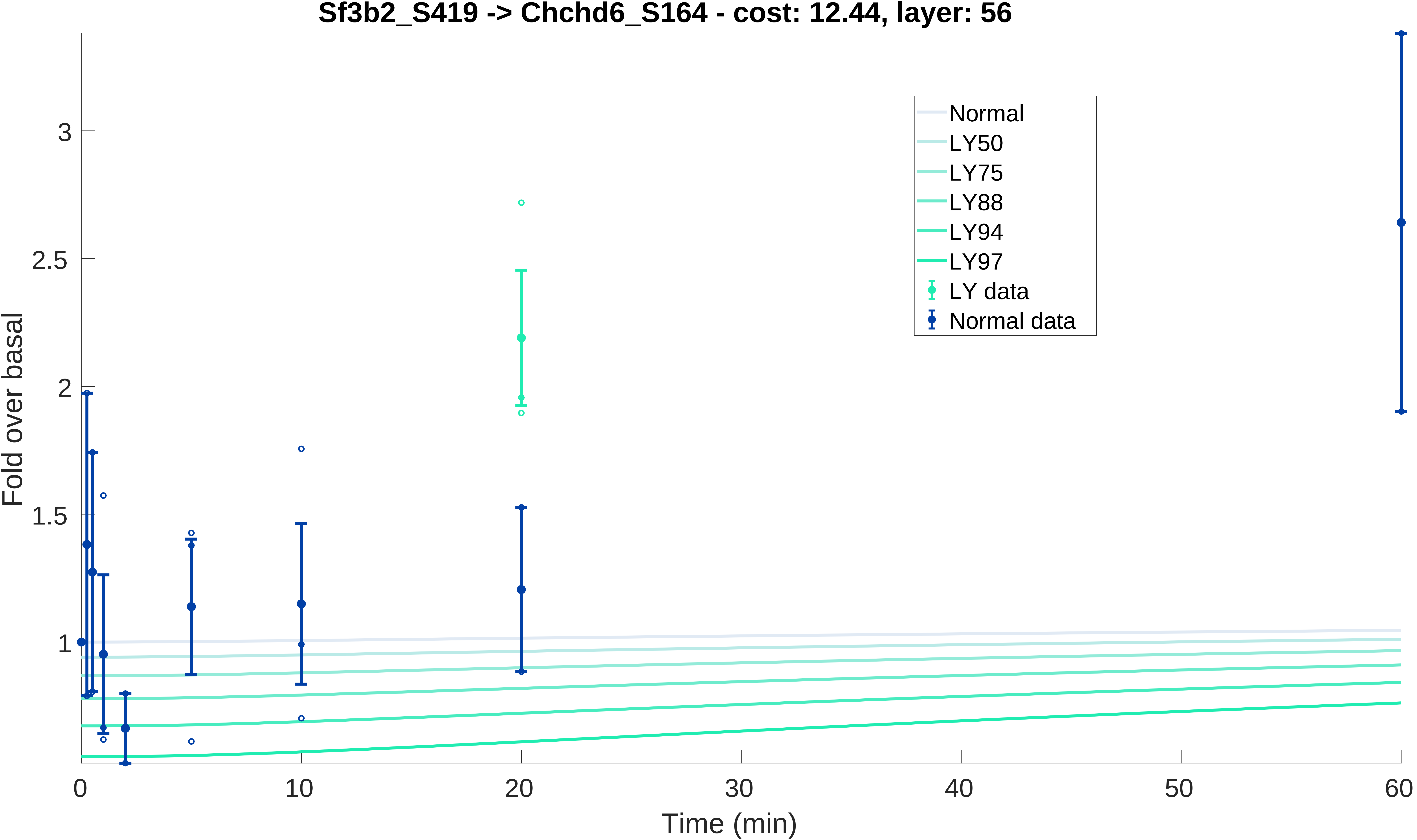
Sf3b2_S419 -> Chchd6_S164 - cost: 12.44, layer: 56
Fold over basal
Time (min)
0
10
20
30
40
50
60
1
1.5
2
2.5
3
Normal
LY50
LY75
LY88
LY94
LY97
LY data
Normal data

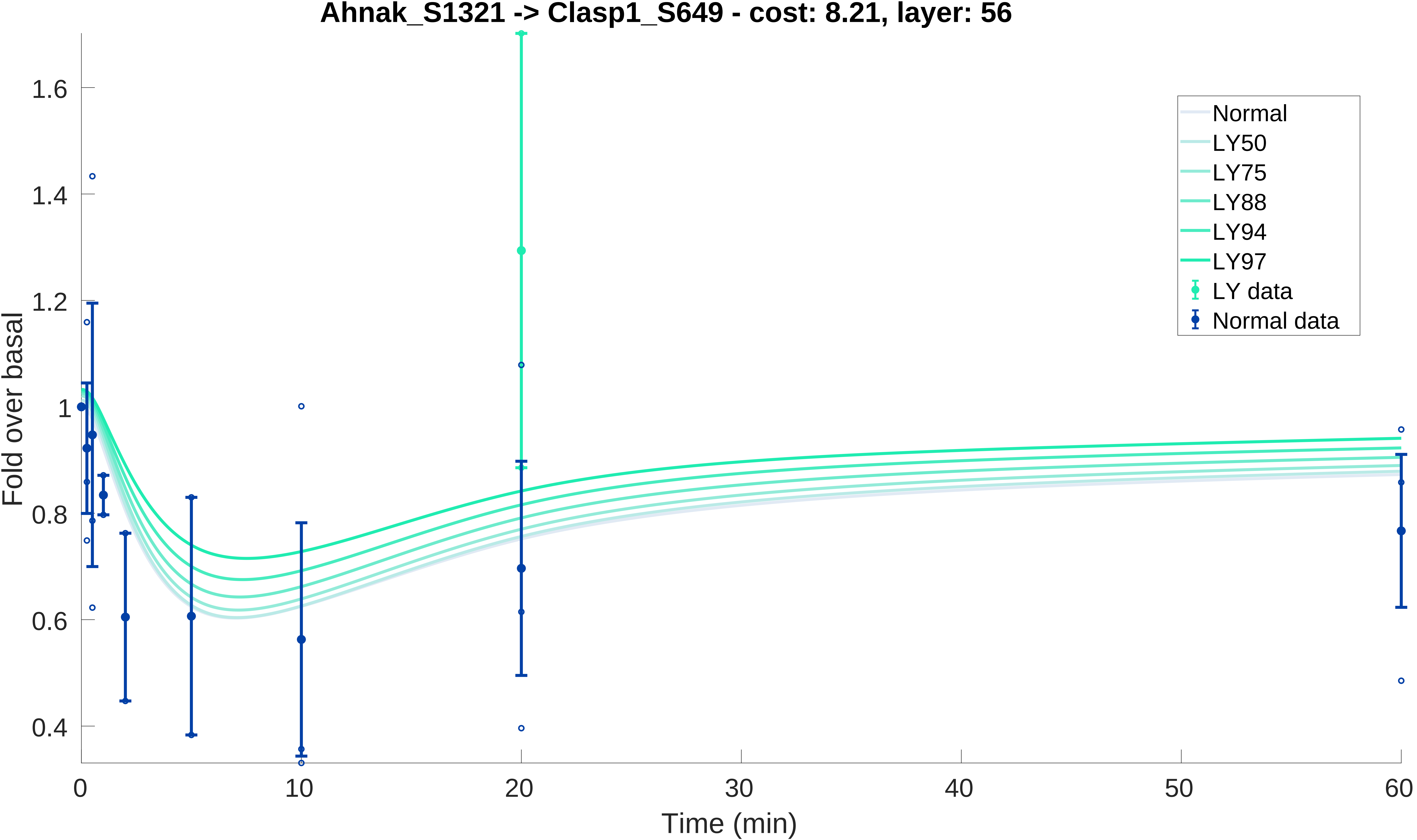

Ahnak_S1321 -> Clasp1_S649 - cost: 8.21, layer: 56
Fold over basal
Time (min)
0.4
0.6
0.8
1
1.2
1.4
1.6
0
10
20
30
40
50
60
Normal
LY50
LY75
LY88
LY94
LY97
LY data
Normal data

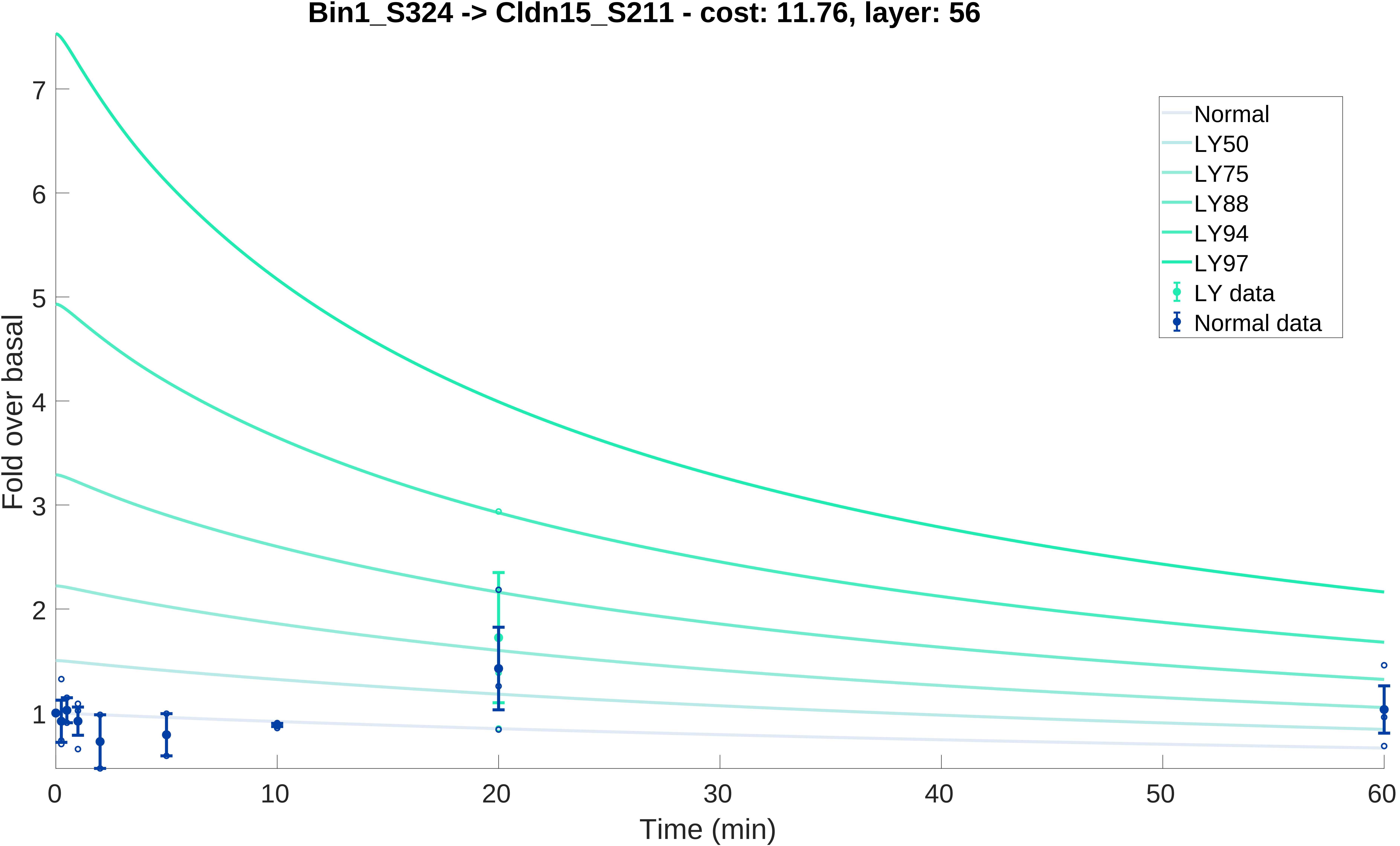

Bin1_S324 -> Cldn15_S211 - cost: 11.76, layer: 56
Fold over basal
Time (min)
Normal
LY50
LY75
LY88
LY94
LY97
LY data
Normal data
0
10
20
30
40
50
60
1
2
3
4
5
6
7

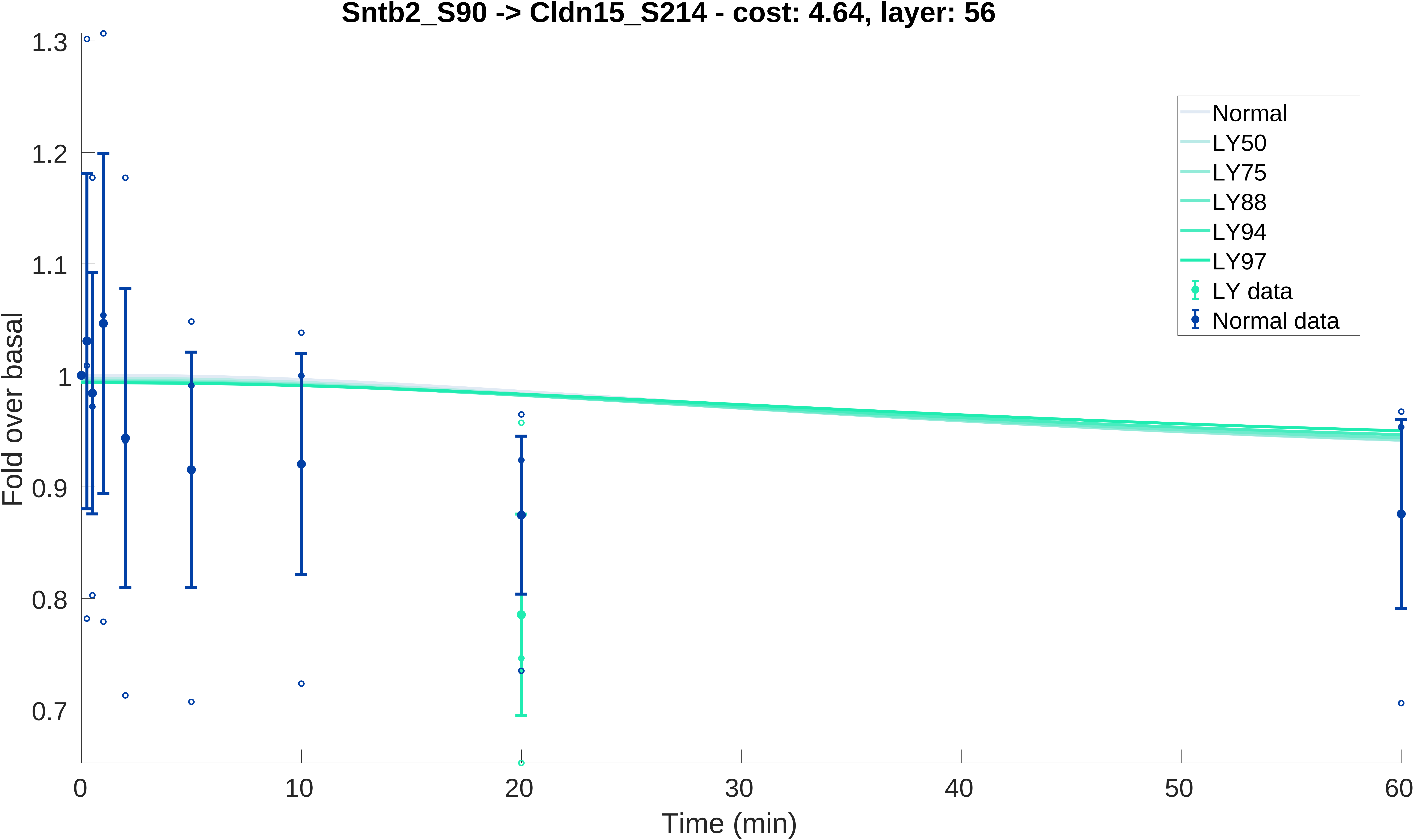

Sntb2_S90 -> Cldn15_S214 - cost: 4.64, layer: 56
Fold over basal
Time (min)
1.3
1.2
1.1
1
0.9
0.8
0.7
0
10
20
30
40
50
60
Normal
LY50
LY75
LY88
LY94
LY97
LY data
Normal data

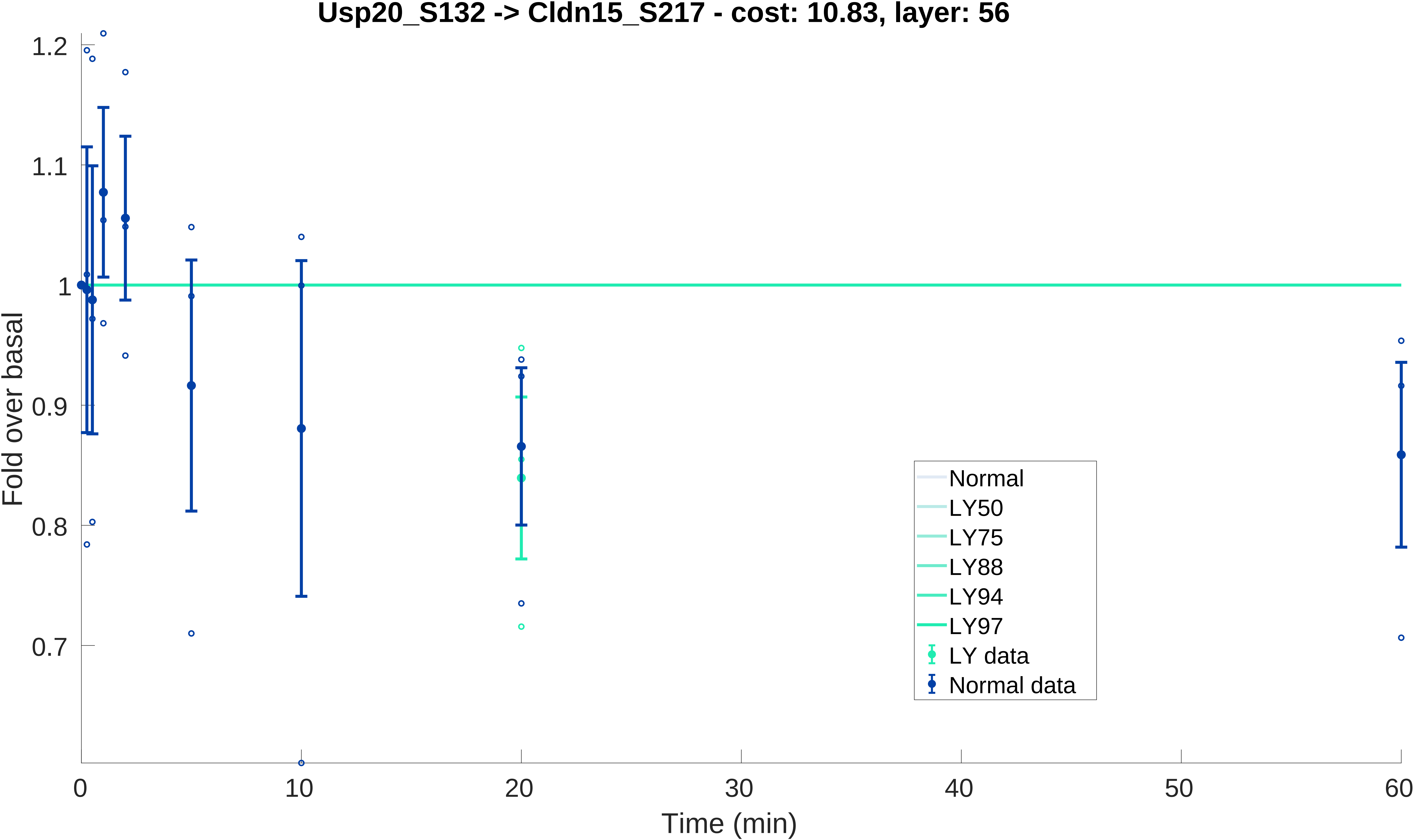
Usp20_S132 -> Cldn15_S217 - cost: 10.83, layer: 56
Fold over basal
Time (min)
1.2
1.1
1
0.9
0.8
0.7
0
10
20
30
40
50
60
Normal
LY50
LY75
LY88
LY94
LY97
LY data
Normal data

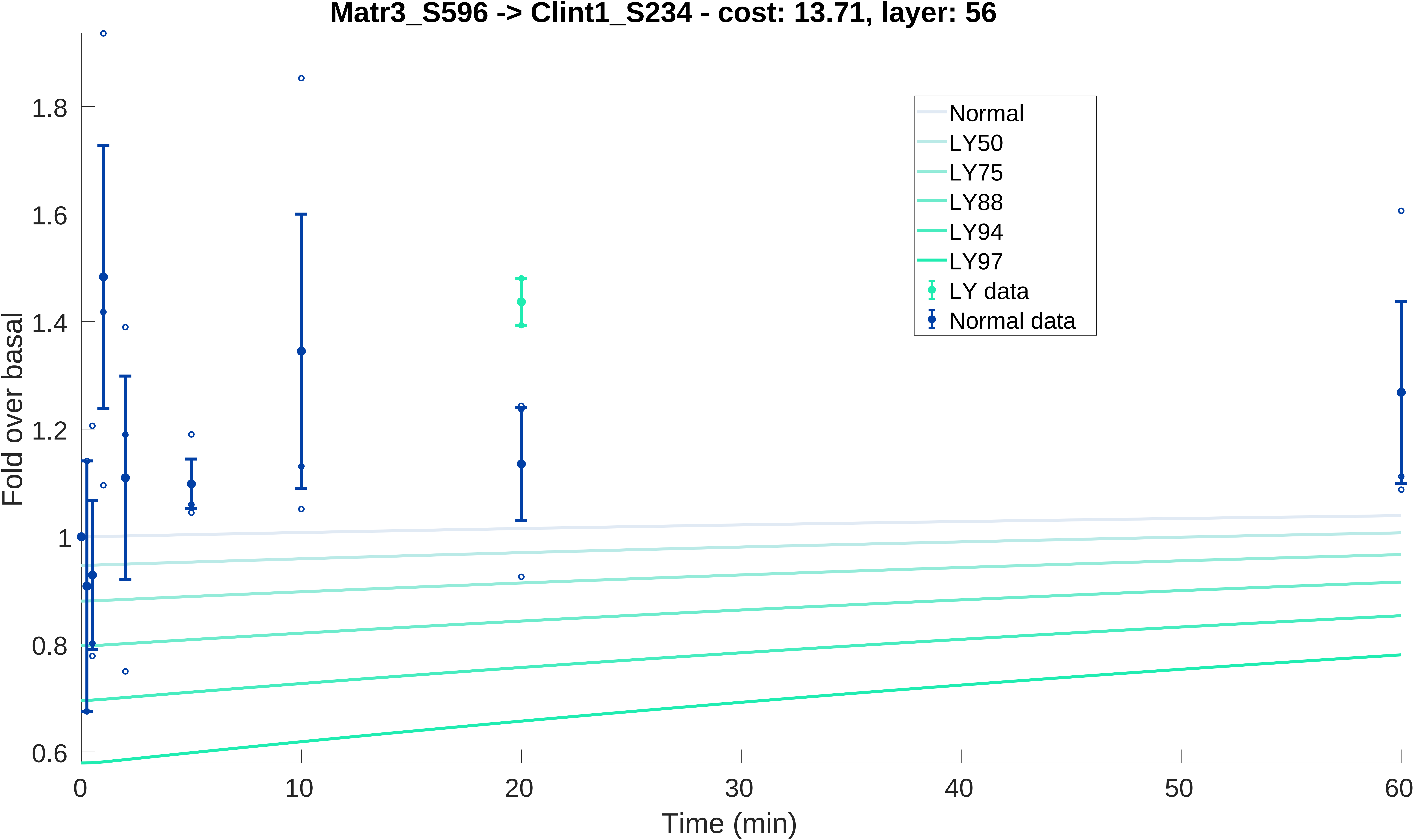
Matr3_S596 -> Clint1_S234 - cost: 13.71, layer: 56
Fold over basal
Time (min)
0.6
0.8
1
1.2
1.4
1.6
1.8
0
10
20
30
40
50
60
Normal
LY50
LY75
LY88
LY94
LY97
LY data
Normal data

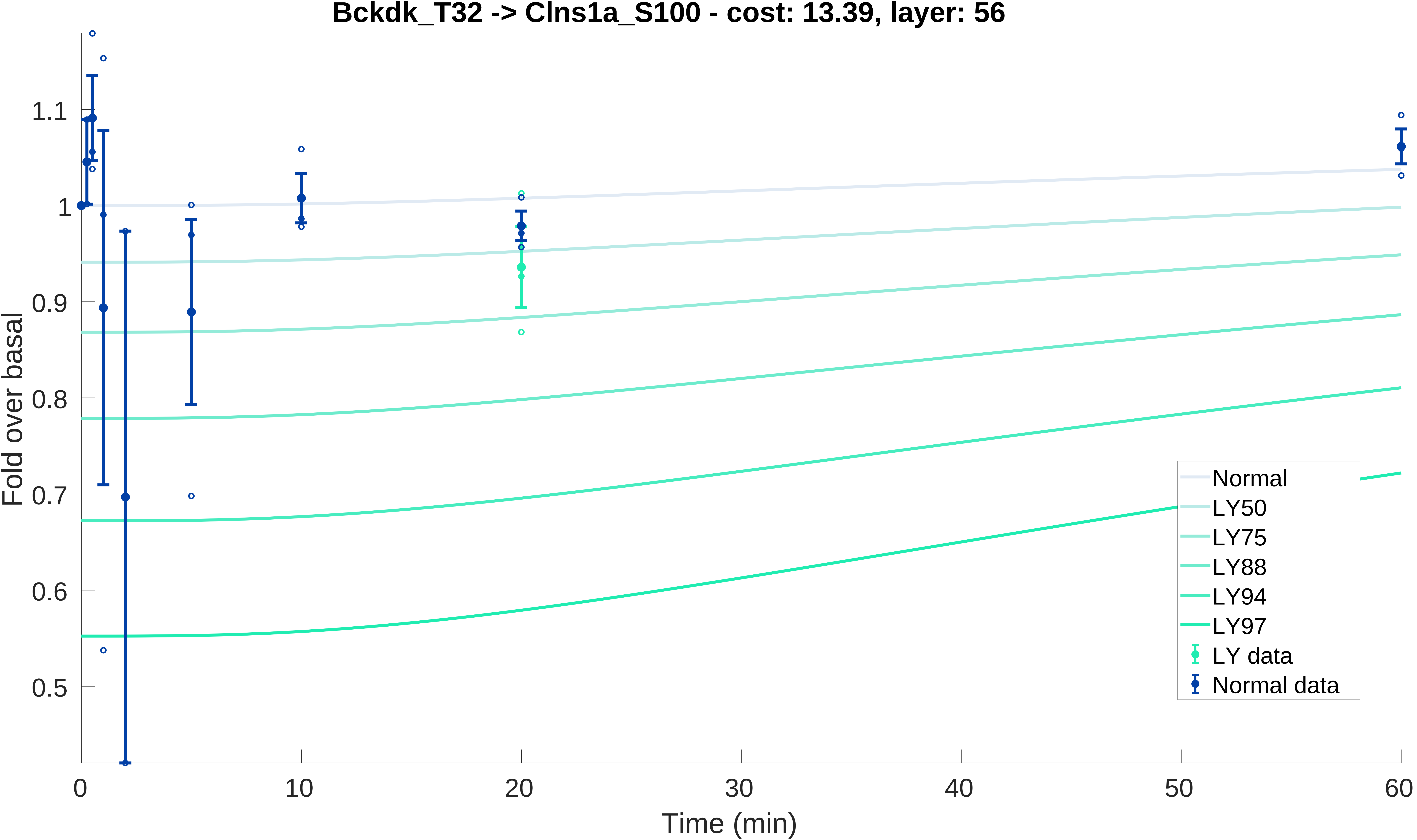

Bckdk_T32 -> Clns1a_S100 - cost: 13.39, layer: 56
Fold over basal
1.1
1
0.9
0.8
0.7
0.6
0.5
0
10
20
30
40
50
60
Time (min)
Normal
LY50
LY75
LY88
LY94
LY97
LY data
Normal data

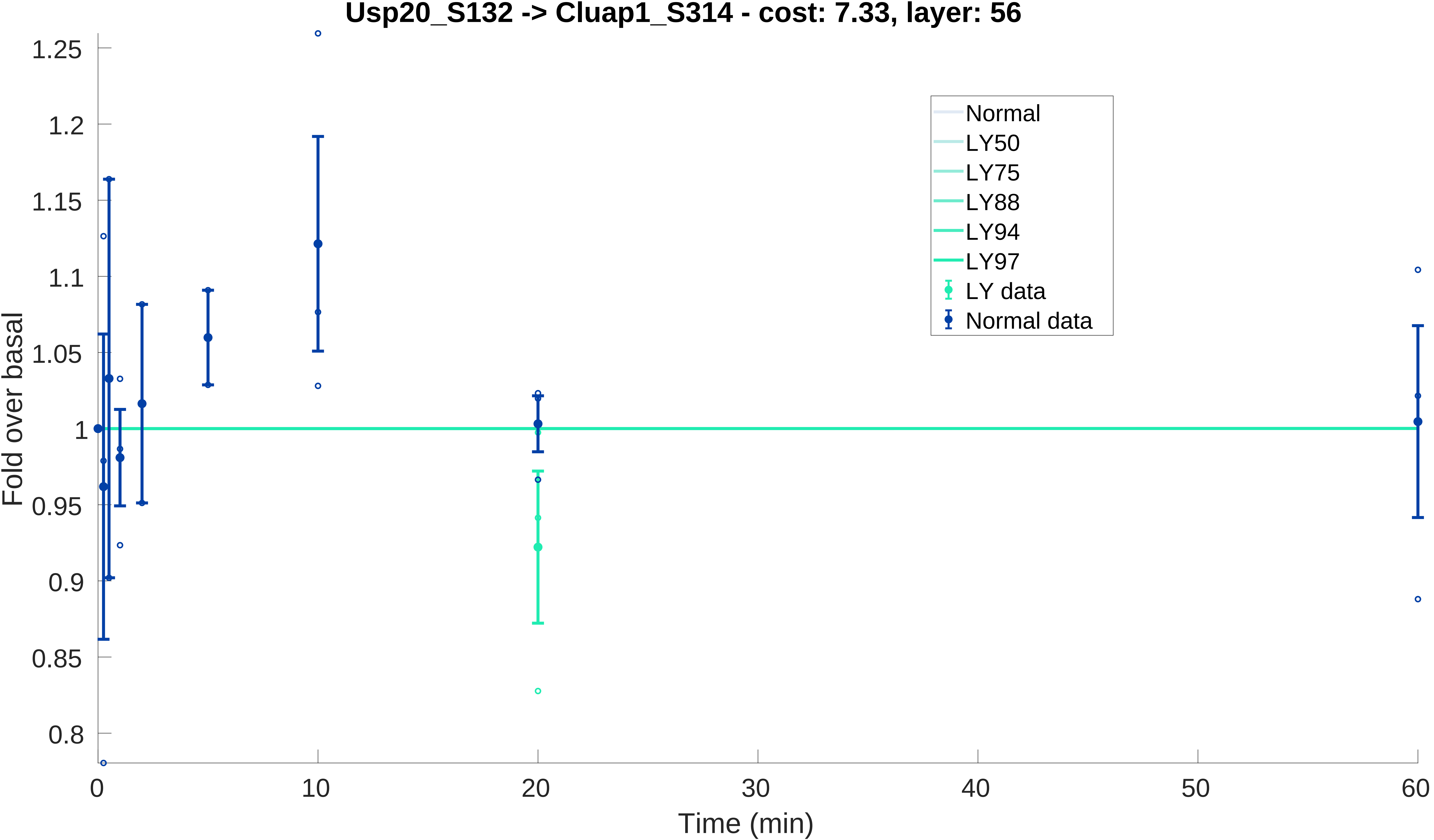

Usp20_S132 -> Cluap1_S314 - cost: 7.33, layer: 56
Normal
LY50
LY75
LY88
LY94
LY97
LY data
Normal data
Fold over basal
Time (min)
1.25
1.2
1.15
1.1
1.05
1
0.95
0.9
0.85
0.8
0
10
20
30
40
50
60

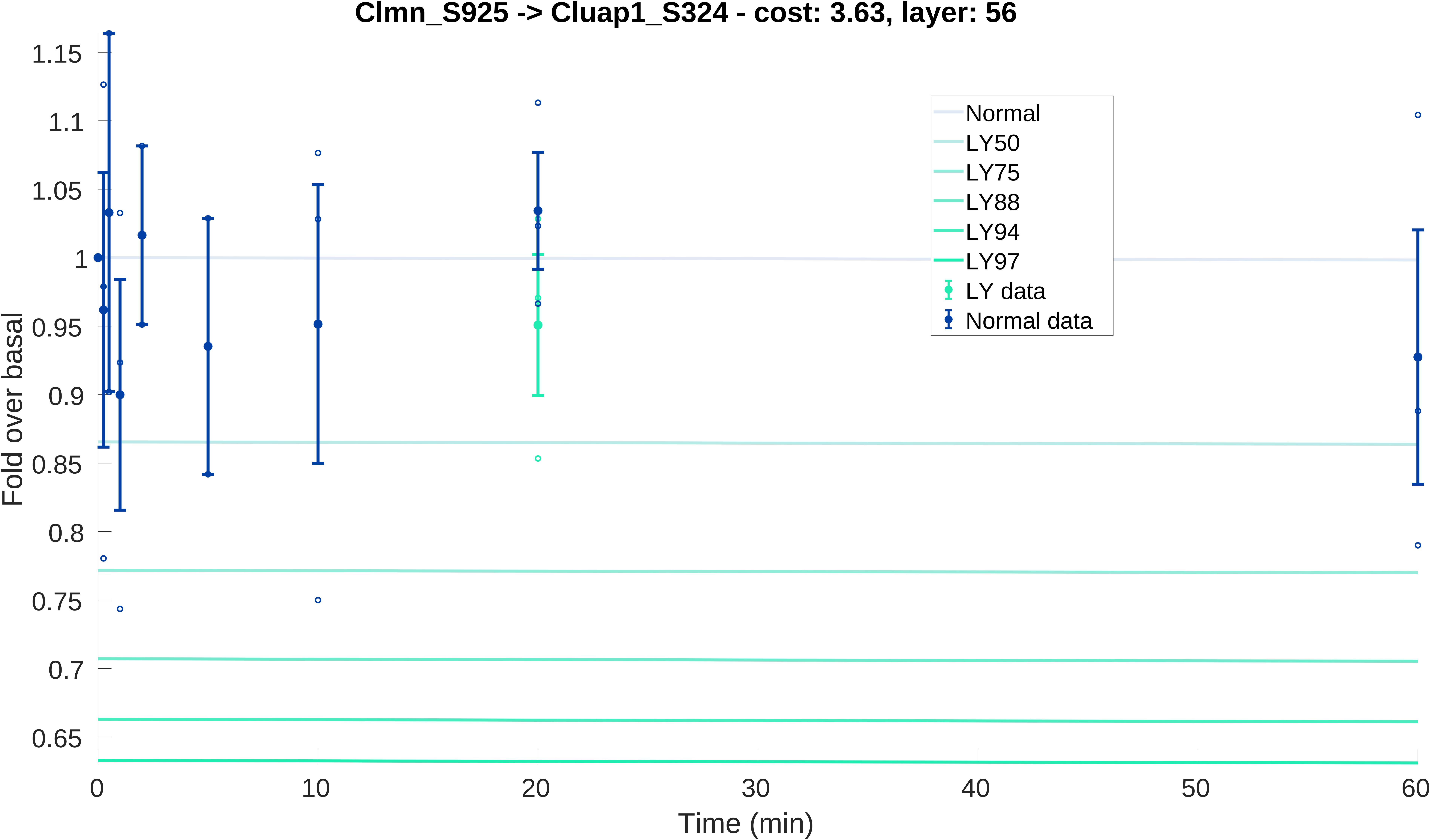

Clmn_S925 -> Cluap1_S324 - cost: 3.63, layer: 56
Fold over basal
Time (min)
1.15
1.1
1.05
1
0.95
0.9
0.85
0.8
0.75
0.7
0.65
0
10
20
30
40
50
60
Normal
LY50
LY75
LY88
LY94
LY97
LY data
Normal data

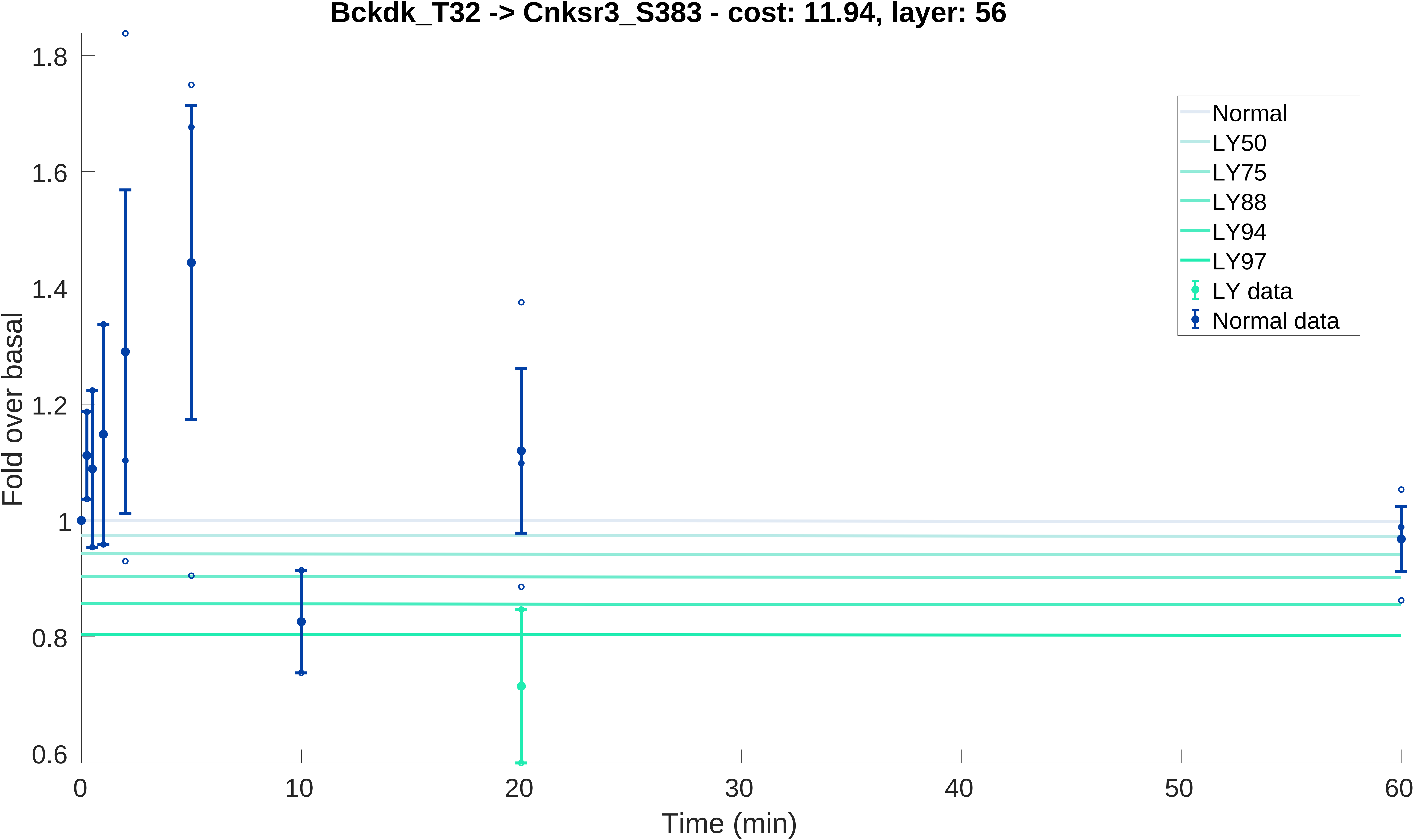

Bckdk_T32 -> Cnksr3_S383 - cost: 11.94, layer: 56
Fold over basal
Time (min)
1.8
1.6
1.4
1.2
1
0.8
0.6
0
10
20
30
40
50
60
Normal
LY50
LY75
LY88
LY94
LY97
LY data
Normal data

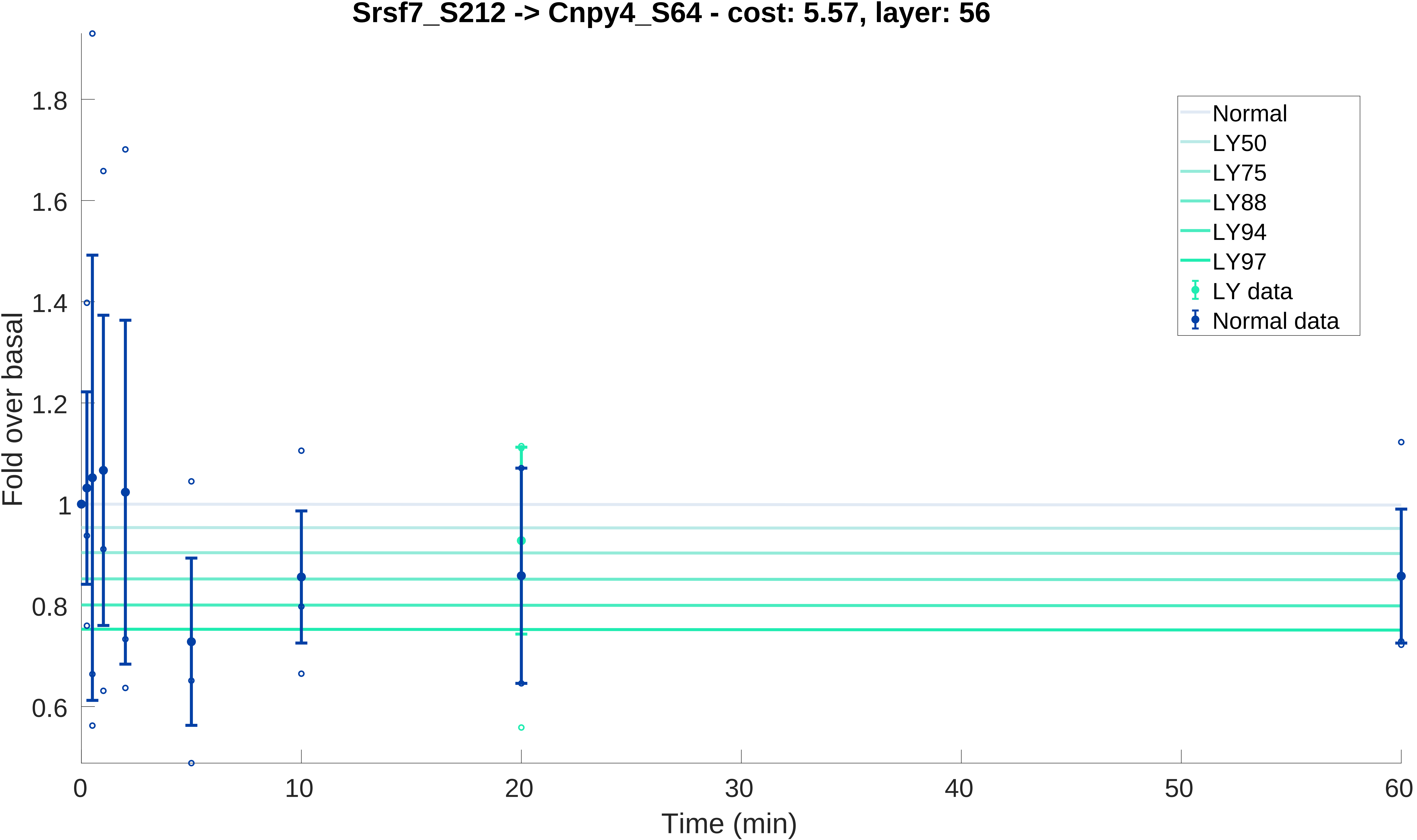

Srsf7_S212 -> Cnpy4_S64 - cost: 5.57, layer: 56
Fold over basal
Time (min)
Normal
LY50
LY75
LY88
LY94
LY97
LY data
Normal data
0.6
0.8
1
1.2
1.4
1.6
1.8
0
10
20
30
40
50
60

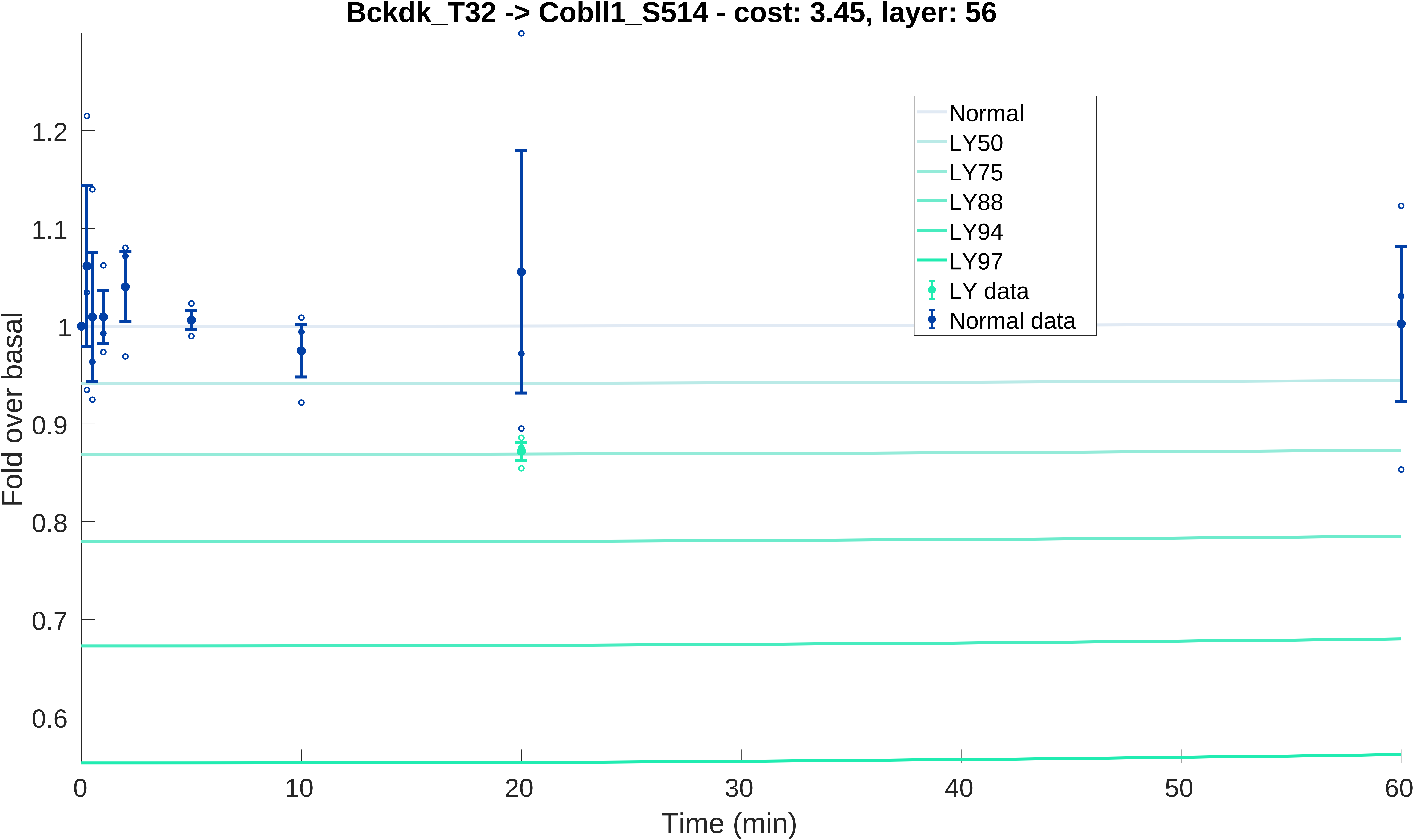
Bckdk_T32 -> Cobll1_S514 - cost: 3.45, layer: 56
Normal
LY50
LY75
LY88
LY94
LY97
LY data
Normal data
Fold over basal
Time (min)
1.2
1.1
1
0.9
0.8
0.7
0.6
0
10
20
30
40
50
60

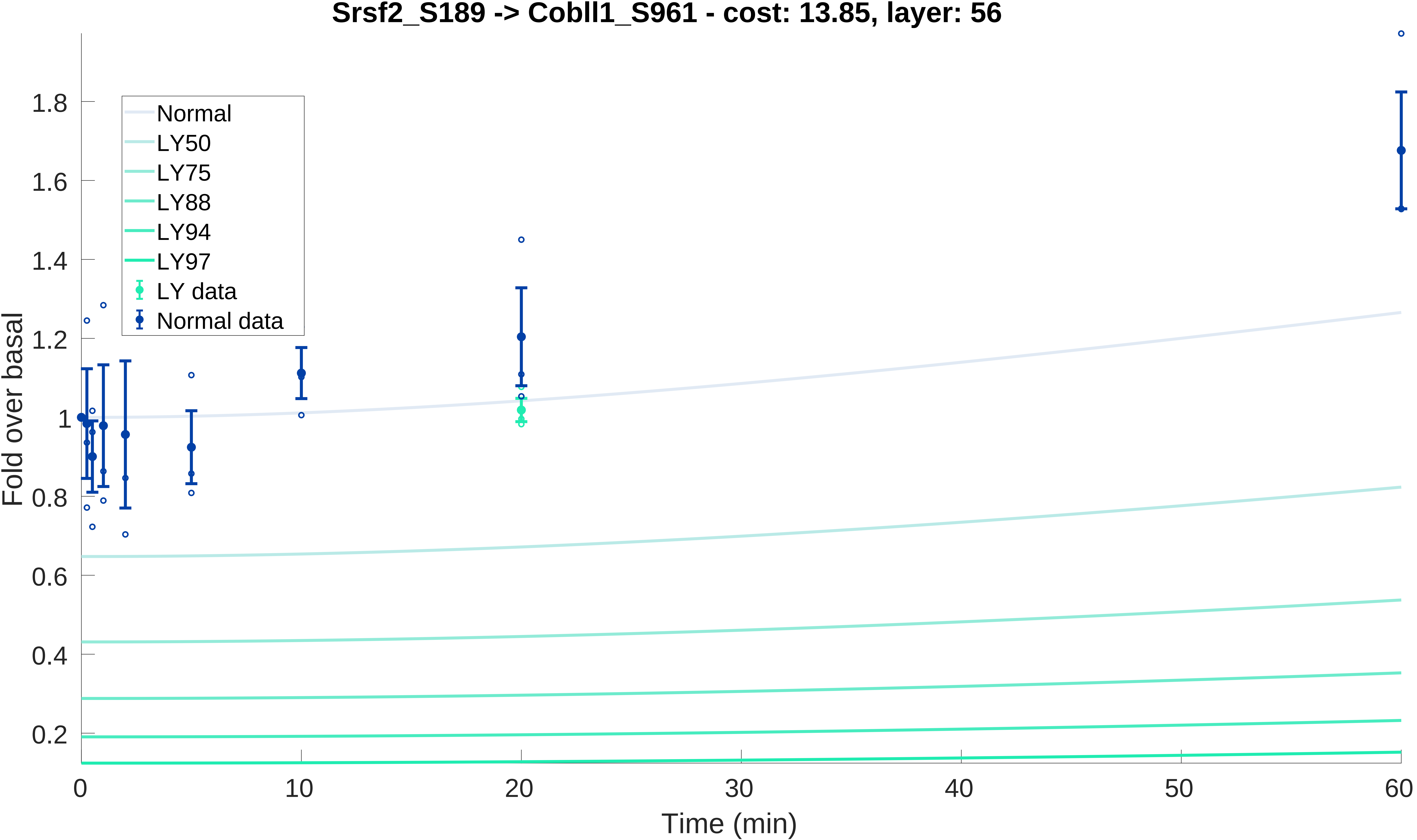

Srsf2_S189 -> Cobll1_S961 - cost: 13.85, layer: 56
Normal
LY50
LY75
LY88
LY94
LY97
LY data
Normal data
Fold over basal
Time (min)
1.8
1.6
1.4
1.2
1
0.8
0.6
0.4
0.2
0
10
20
30
40
50
60

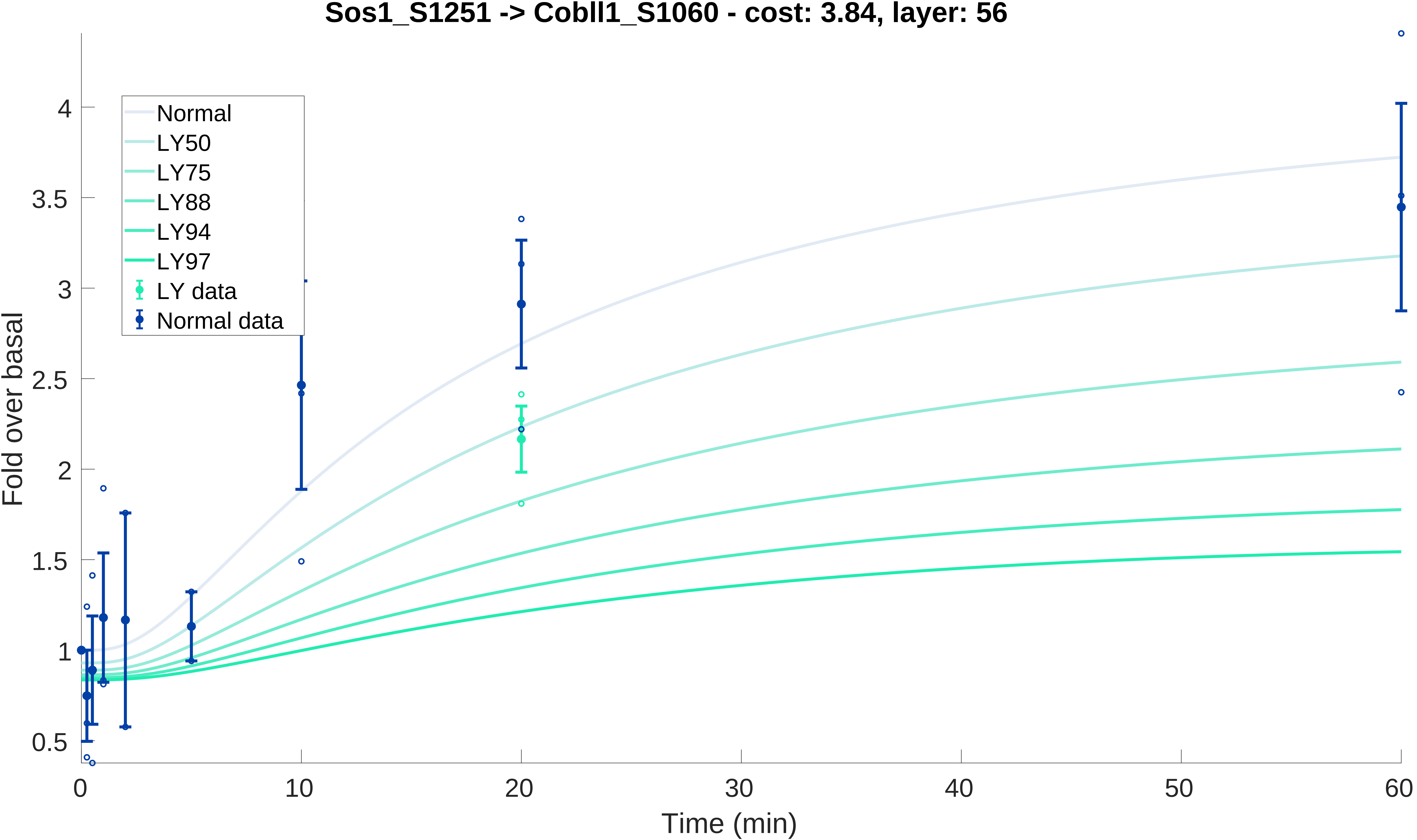

Sos1_S1251 -> Cobll1_S1060 - cost: 3.84, layer: 56
Normal
LY50
LY75
LY88
LY94
LY97
LY data
Normal data
Fold over basal
Time (min)
0.5
1
1.5
2
2.5
3
3.5
4
0
10
20
30
40
50
60

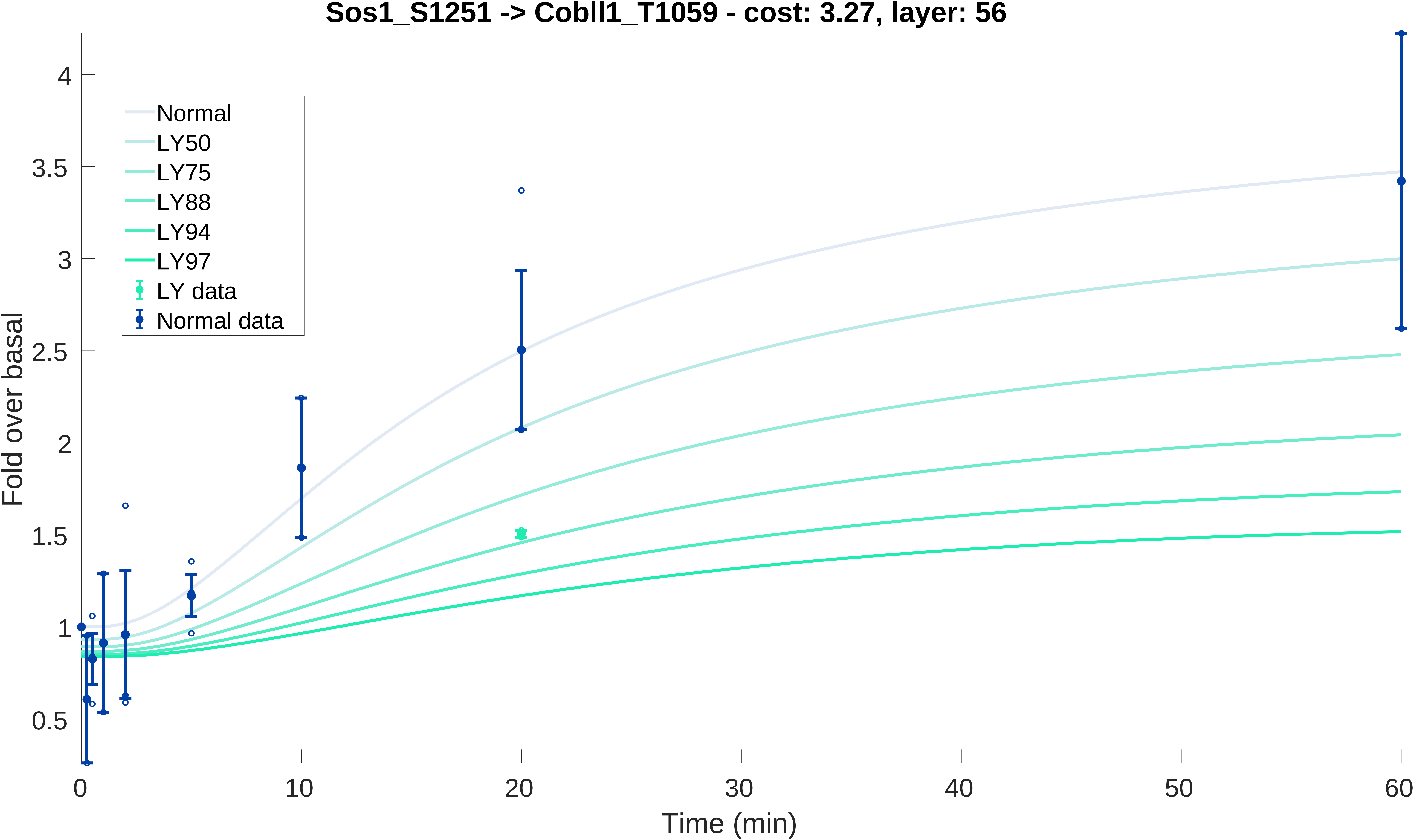

Sos1_S1251 -> Cobll1_T1059 - cost: 3.27, layer: 56
Fold over basal
Time (min)
Normal
LY50
LY75
LY88
LY94
LY97
LY data
Normal data
0.5
1
1.5
2
2.5
3
3.5
4
0
10
20
30
40
50
60

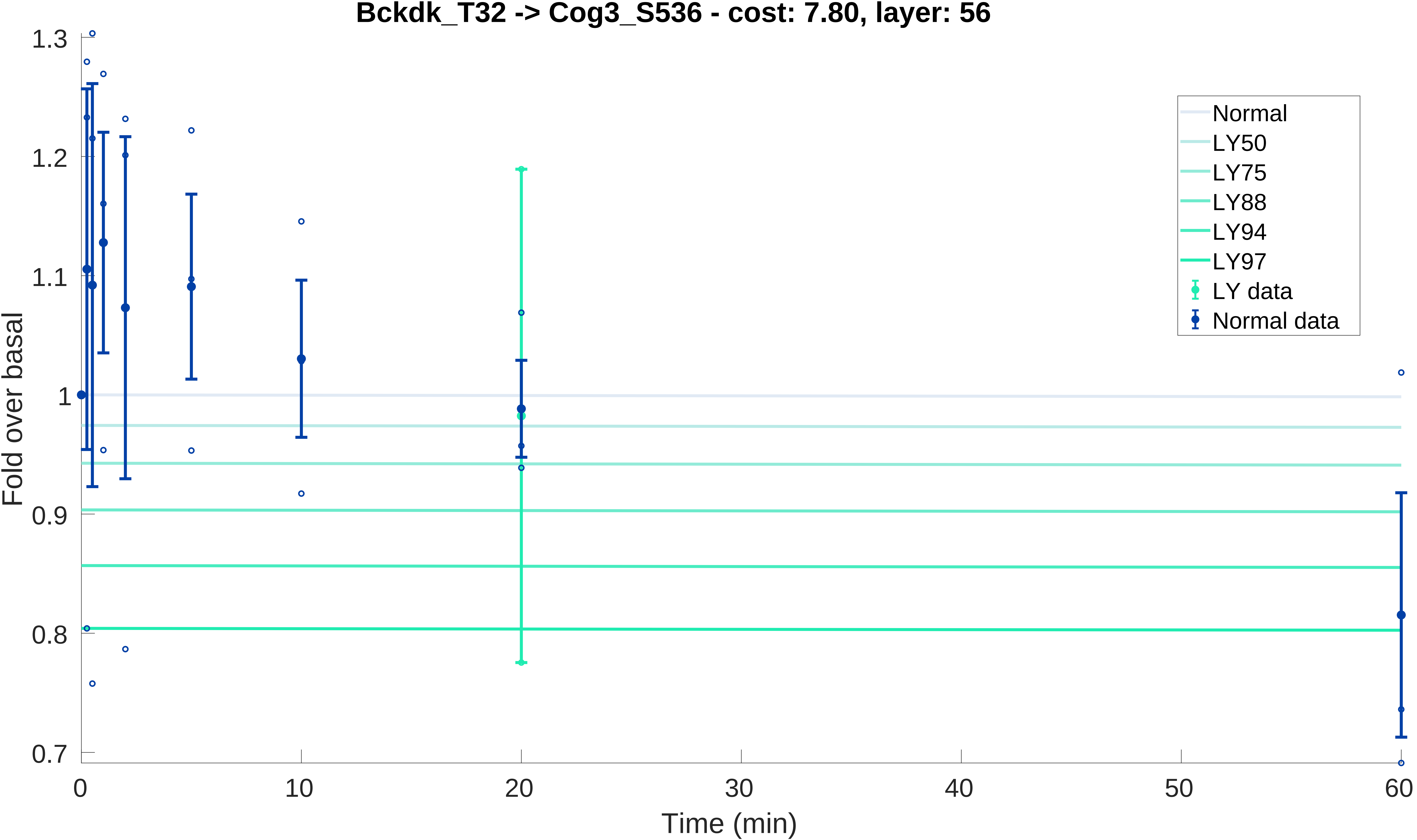

Bckdk_T32 -> Cog3_S536 - cost: 7.80, layer: 56
Fold over basal
1.3
1.2
1.1
1
0.9
0.8
0.7
0
10
20
30
40
50
60
Time (min)
Normal
LY50
LY75
LY88
LY94
LY97
LY data
Normal data

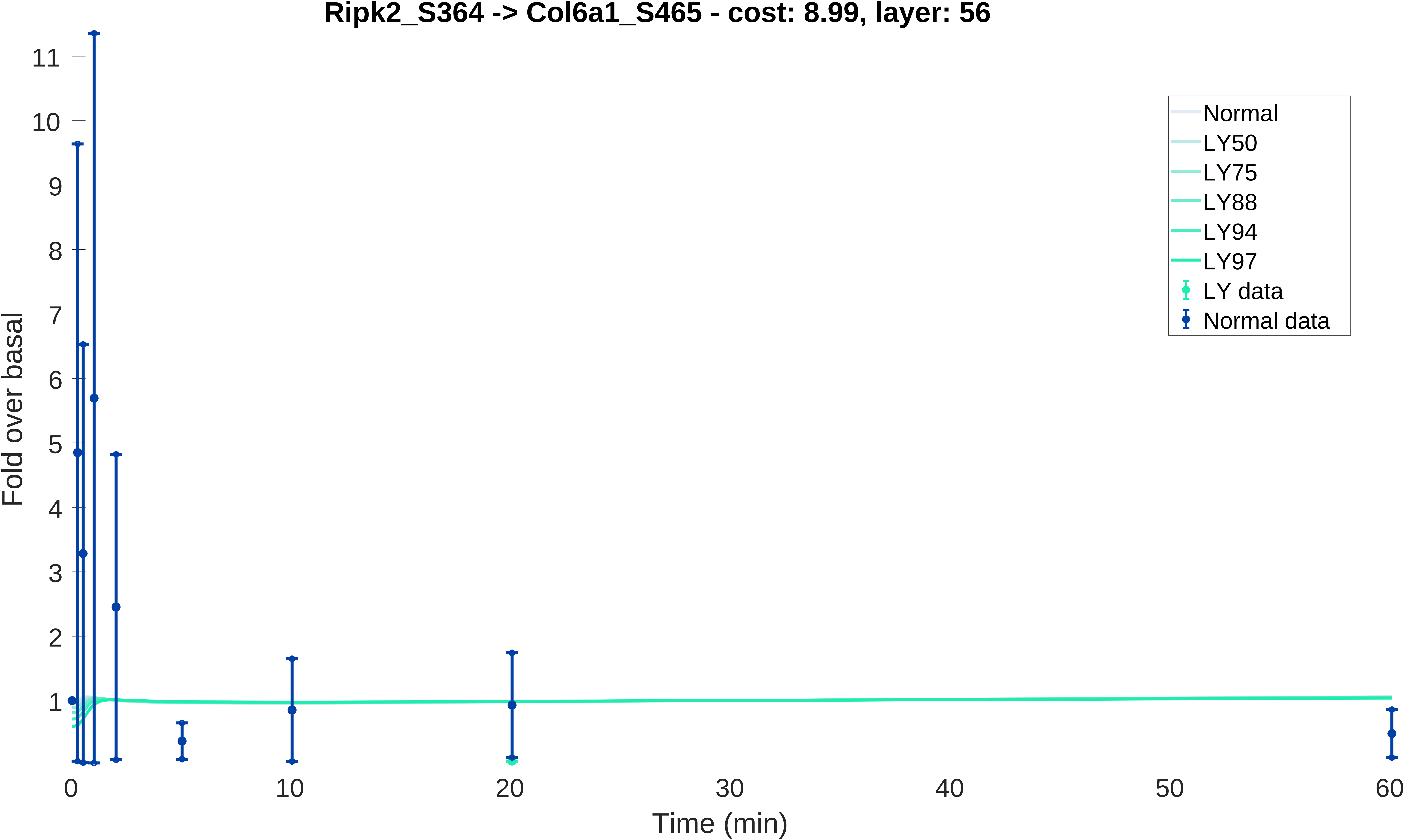

Ripk2_S364 -> Col6a1_S465 - cost: 8.99, layer: 56
Fold over basal
Time (min)
0
10
20
30
40
50
60
1
2
3
4
5
6
7
8
9
10
11
Normal
LY50
LY75
LY88
LY94
LY97
LY data
Normal data

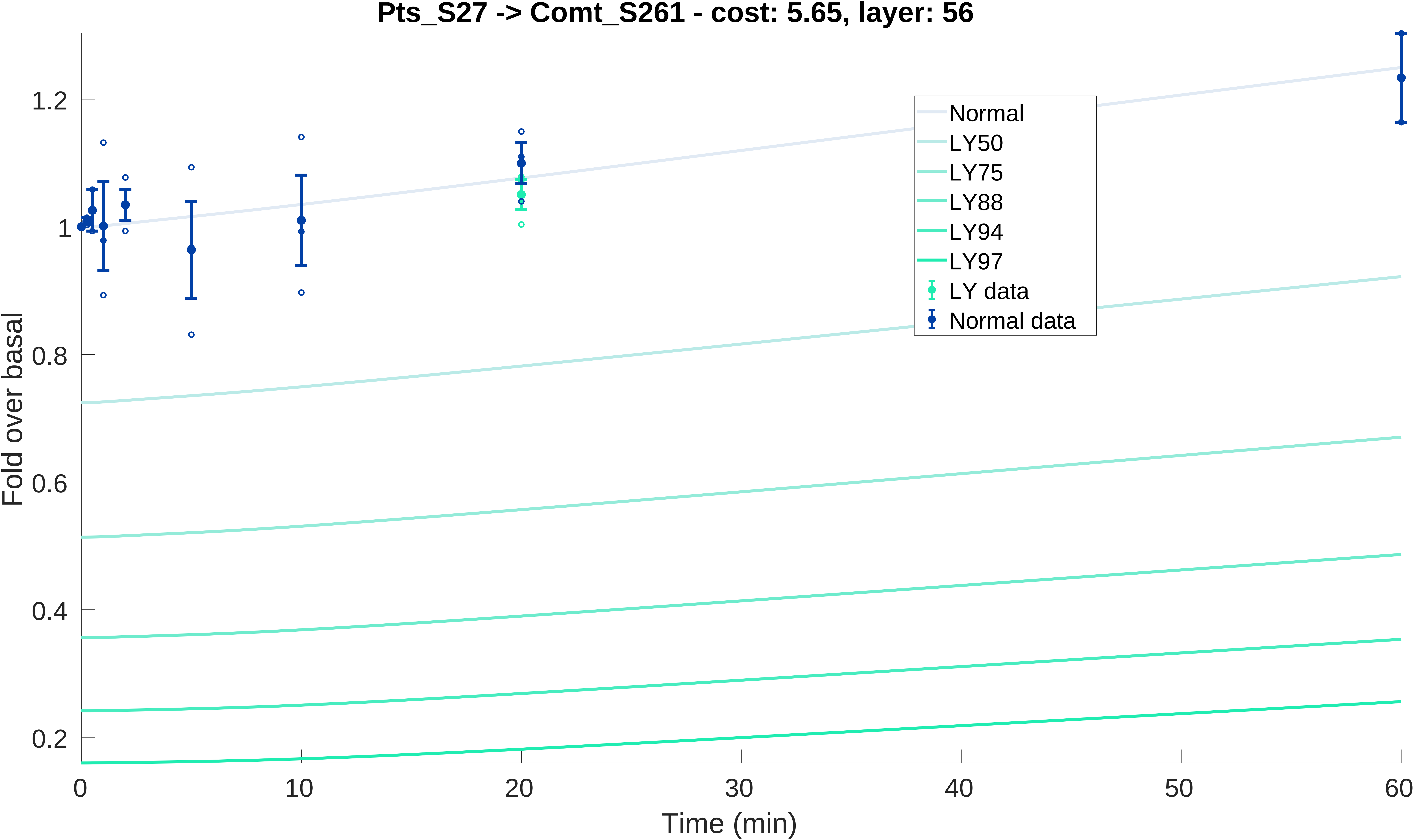

Pts_S27 -> Comt_S261 - cost: 5.65, layer: 56
Fold over basal
Time (min)
1.2
1
0.8
0.6
0.4
0.2
0
10
20
30
40
50
60
Normal
LY50
LY75
LY88
LY94
LY97
LY data
Normal data

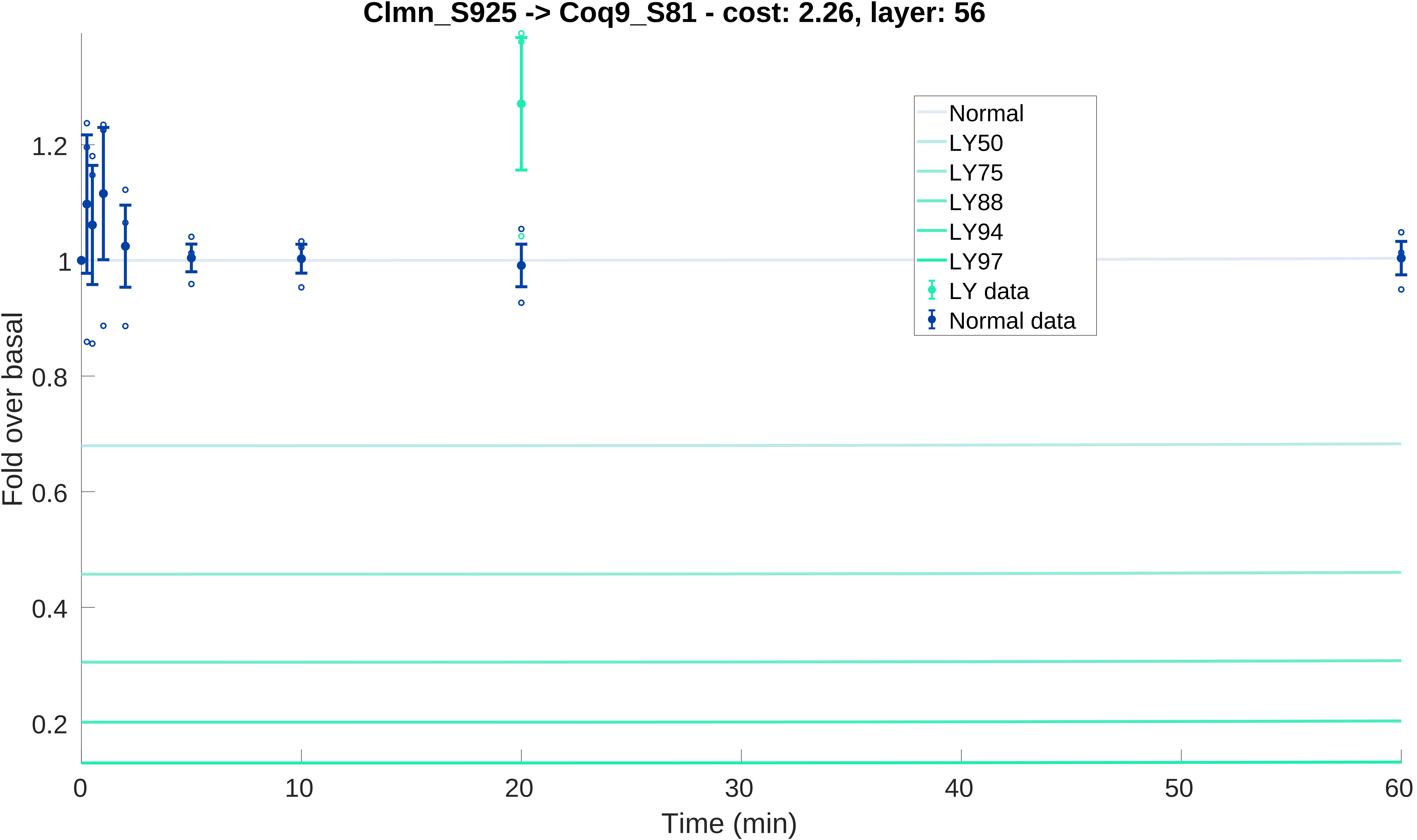
Clmn_S925 -> Coq9_S81 - cost: 2.26, layer: 56
Fold over basal
Time (min)
0
10
20
30
40
50
60
0.2
0.4
0.6
0.8
1
1.2
Normal
LY50
LY75
LY88
LY94
LY97
LY data
Normal data

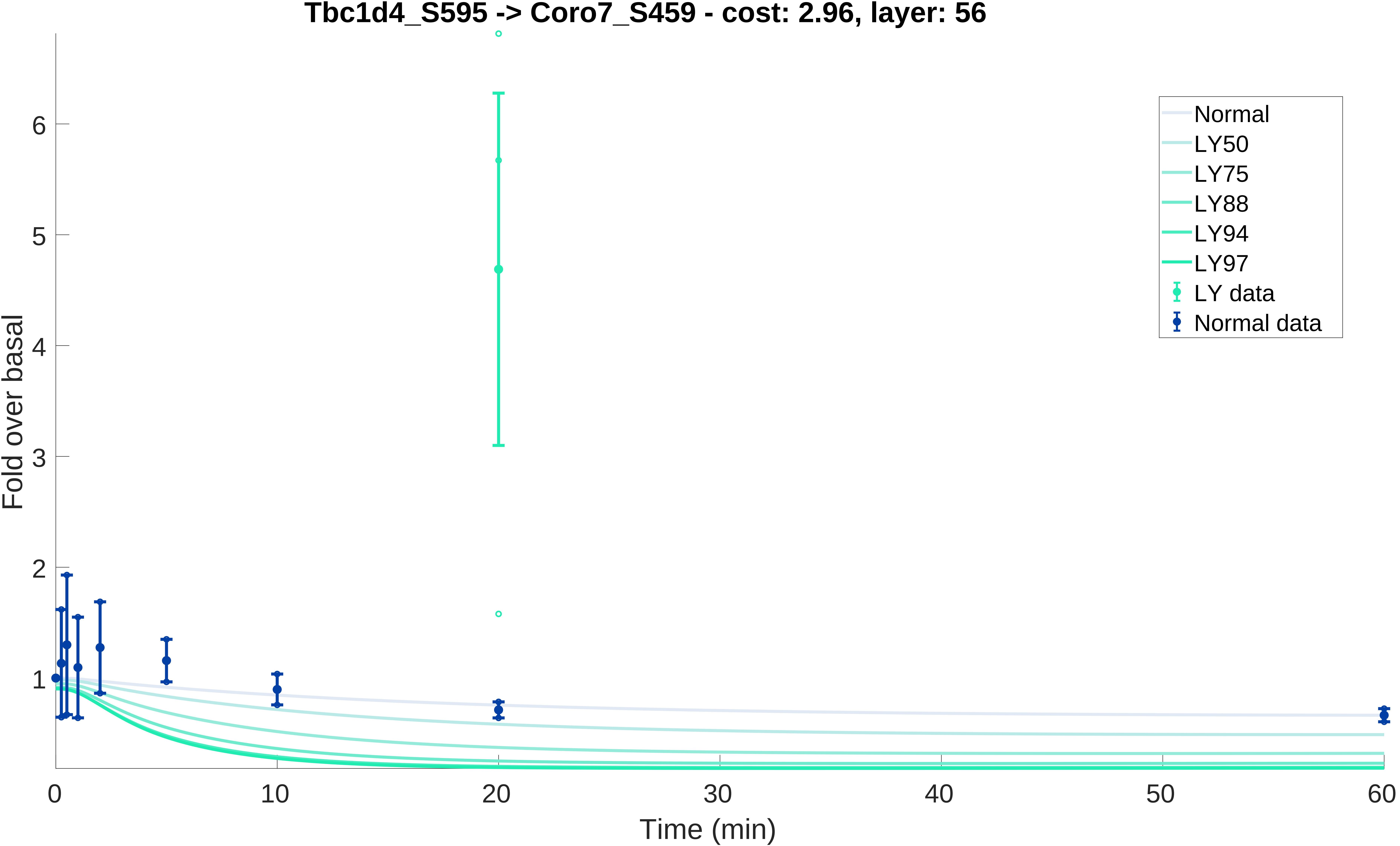

Tbc1d4_S595 -> Coro7_S459 - cost: 2.96, layer: 56
Fold over basal
Time (min)
Normal
LY50
LY75
LY88
LY94
LY97
LY data
Normal data
0
10
20
30
40
50
60
1
2
3
4
5
6

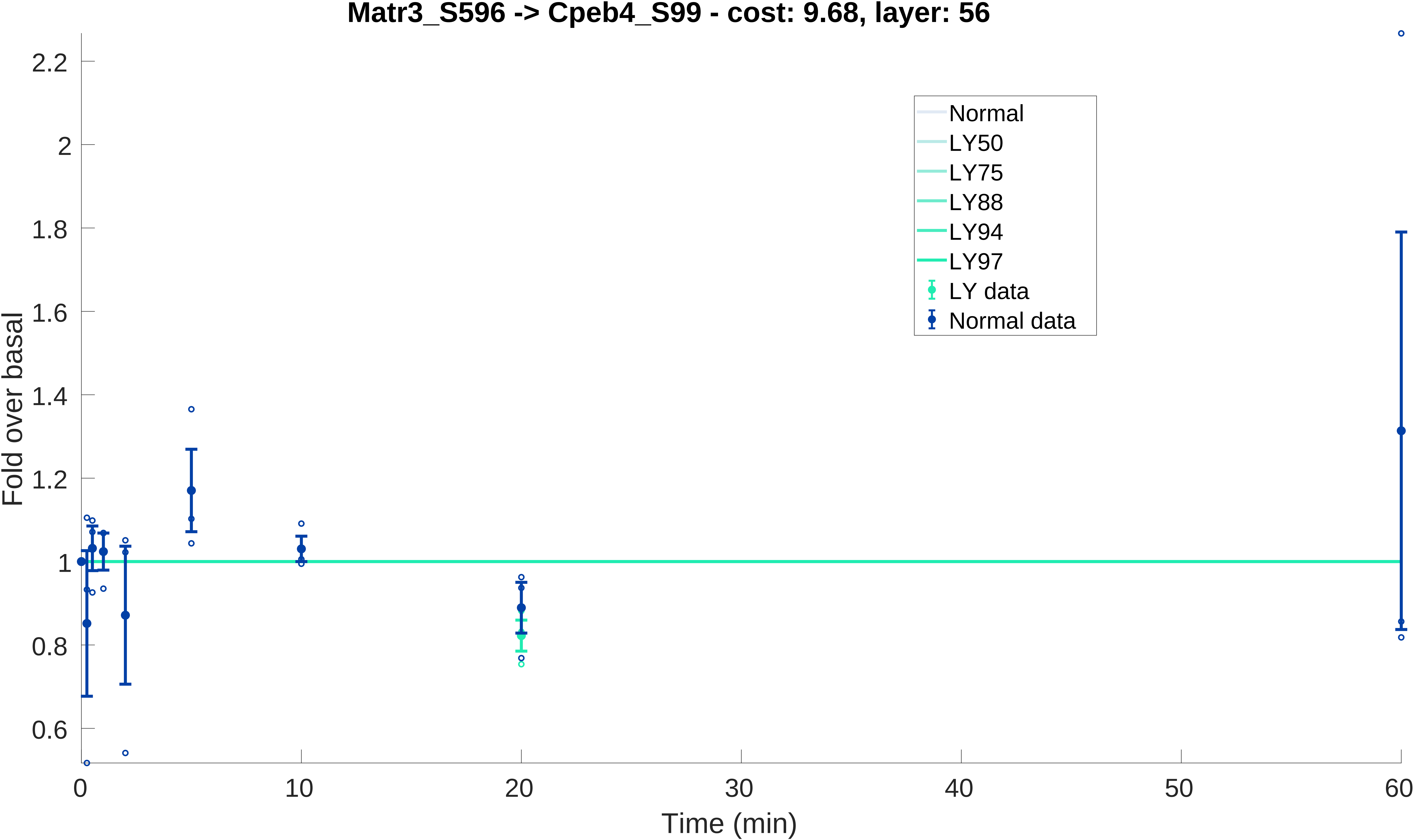

Matr3_S596 -> Cpeb4_S99 - cost: 9.68, layer: 56
Fold over basal
Time (min)
Normal
LY50
LY75
LY88
LY94
LY97
LY data
Normal data
0.6
0.8
1
1.2
1.4
1.6
1.8
2
2.2
0
10
20
30
40
50
60

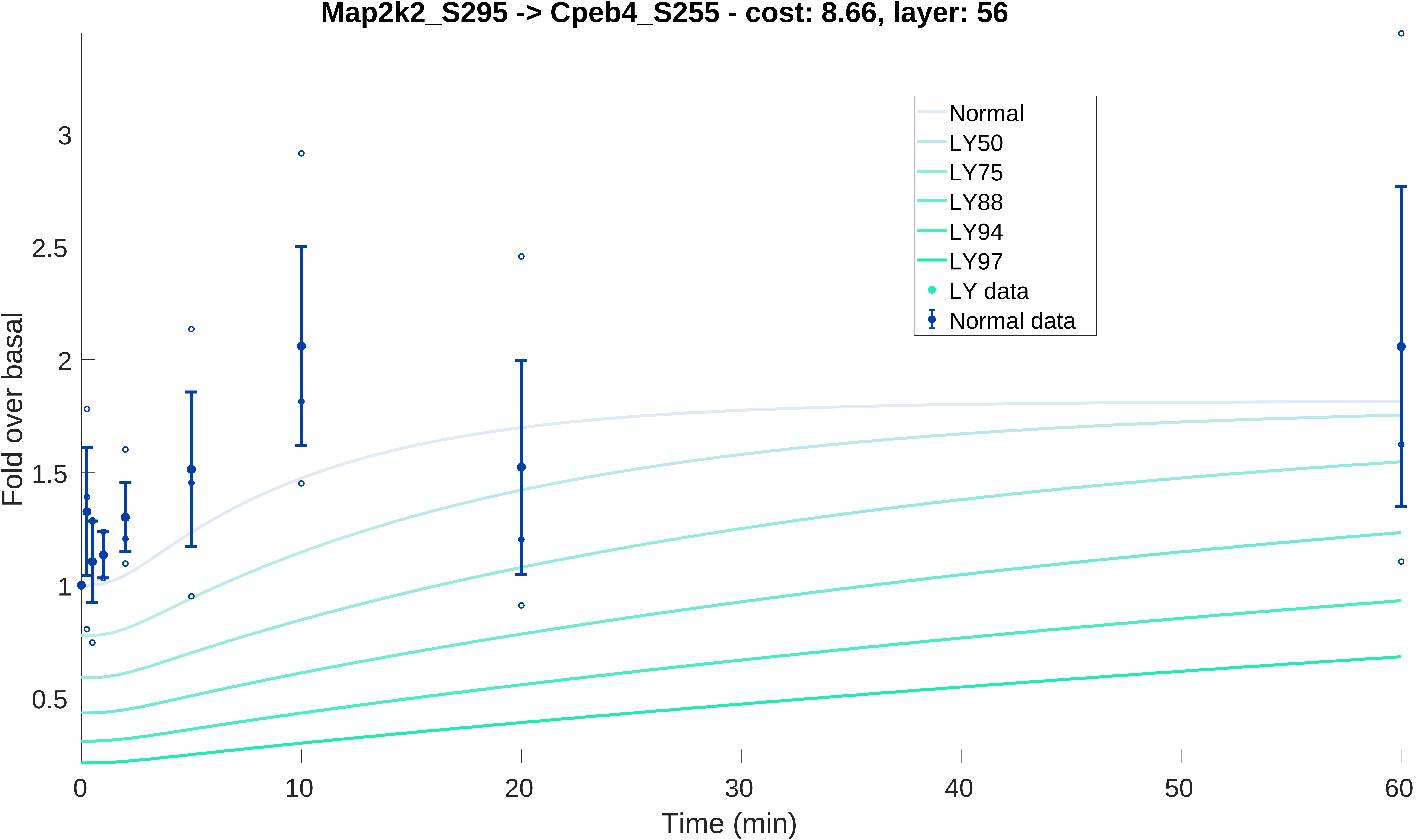

Map2k2_S295 -> Cpeb4_S255 - cost: 8.66, layer: 56
Normal
LY50
LY75
LY88
LY94
LY97
LY data
Normal data
Fold over basal
Time (min)
0
10
20
30
40
50
60
0.5
1
1.5
2
2.5
3

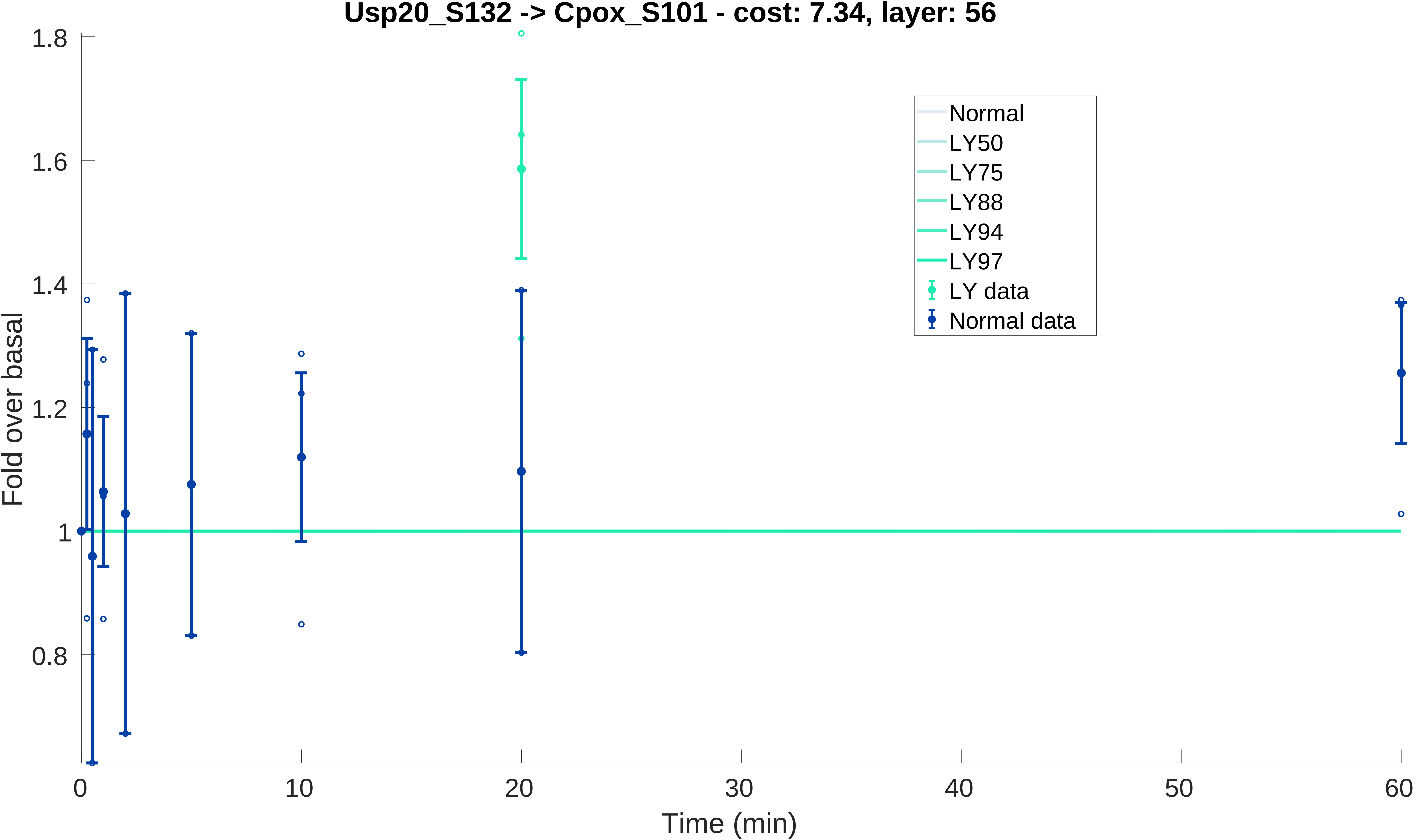
Usp20_S132 -> Cpox_S101 - cost: 7.34, layer: 56
Fold over basal
Time (min)
0
10
20
30
40
50
60
0.8
1
1.2
1.4
1.6
1.8
Normal
LY50
LY75
LY88
LY94
LY97
LY data
Normal data

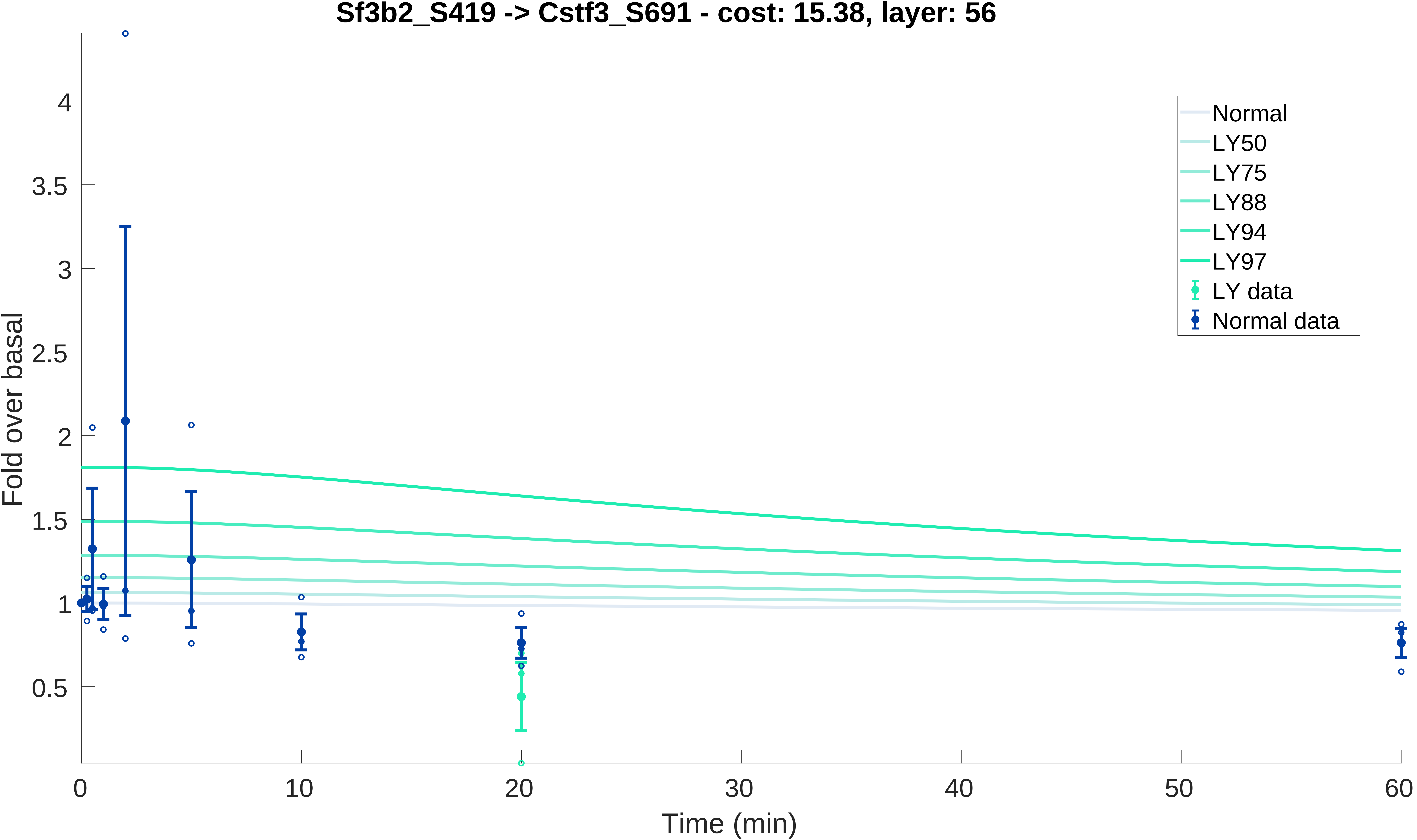

Sf3b2_S419 -> Cstf3_S691 - cost: 15.38, layer: 56
Fold over basal
Time (min)
0.5
1
1.5
2
2.5
3
3.5
4
0
10
20
30
40
50
60
Normal
LY50
LY75
LY88
LY94
LY97
LY data
Normal data

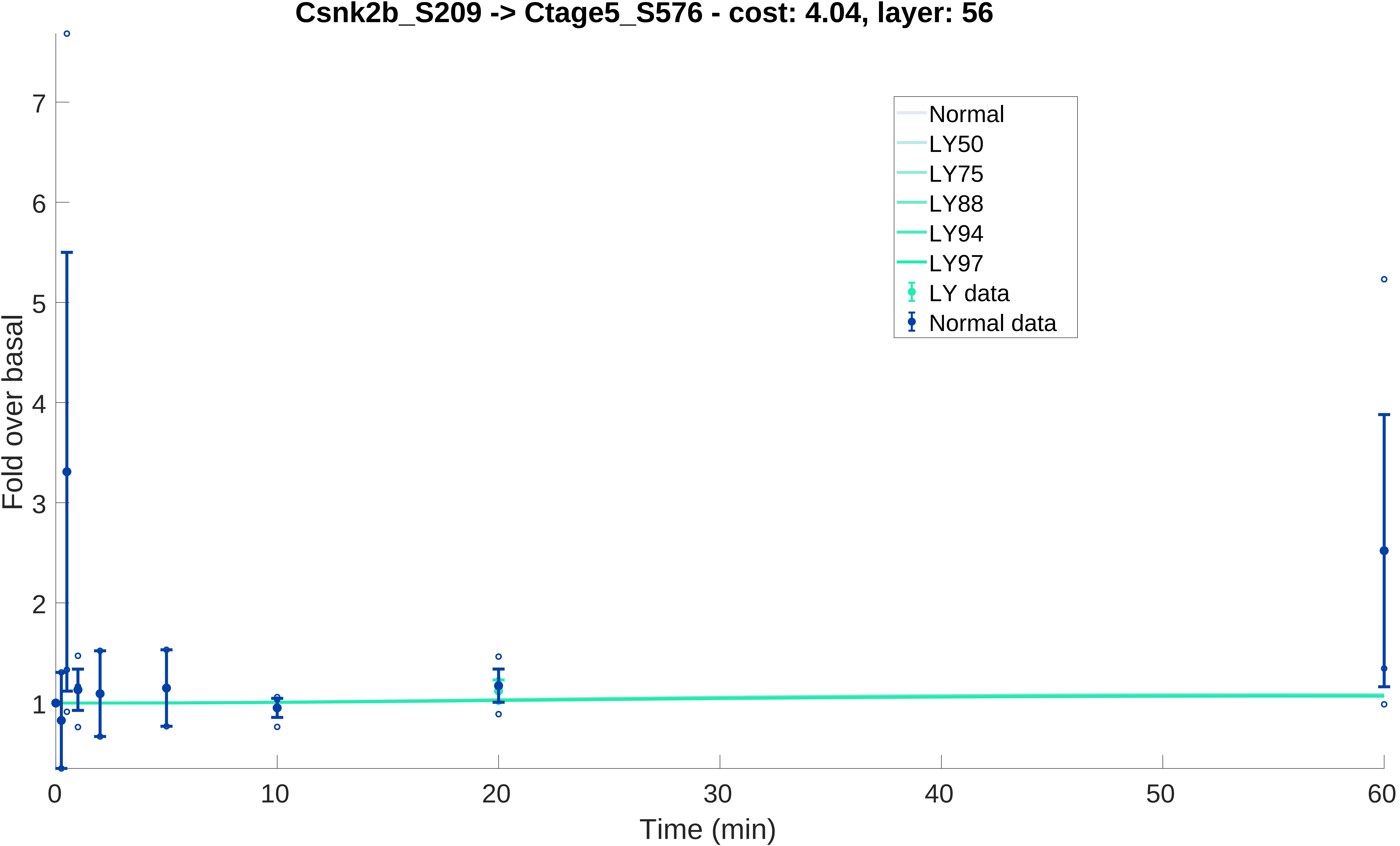

Csnk2b_S209 -> Ctage5_S576 - cost: 4.04, layer: 56
Normal
LY50
LY75
LY88
LY94
LY97
LY data
Normal data
Fold over basal
Time (min)
0
10
20
30
40
50
60
1
2
3
4
5
6
7

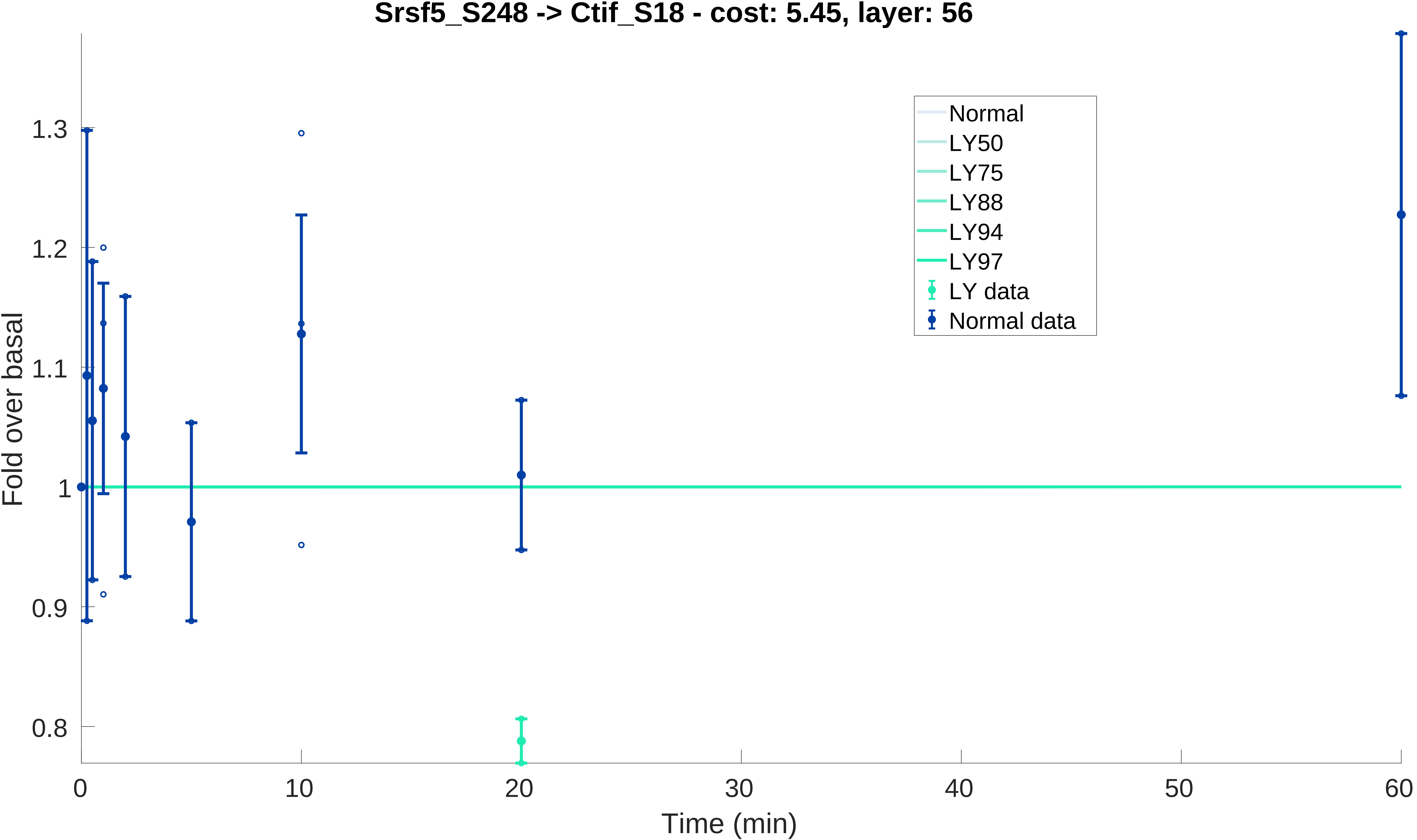

Srsf5_S248 -> Ctif_S18 - cost: 5.45, layer: 56
Normal
LY50
LY75
LY88
LY94
LY97
LY data
Normal data
Fold over basal
1.3
1.2
1.1
1
0.9
0.8
0
10
20
30
40
50
60
Time (min)

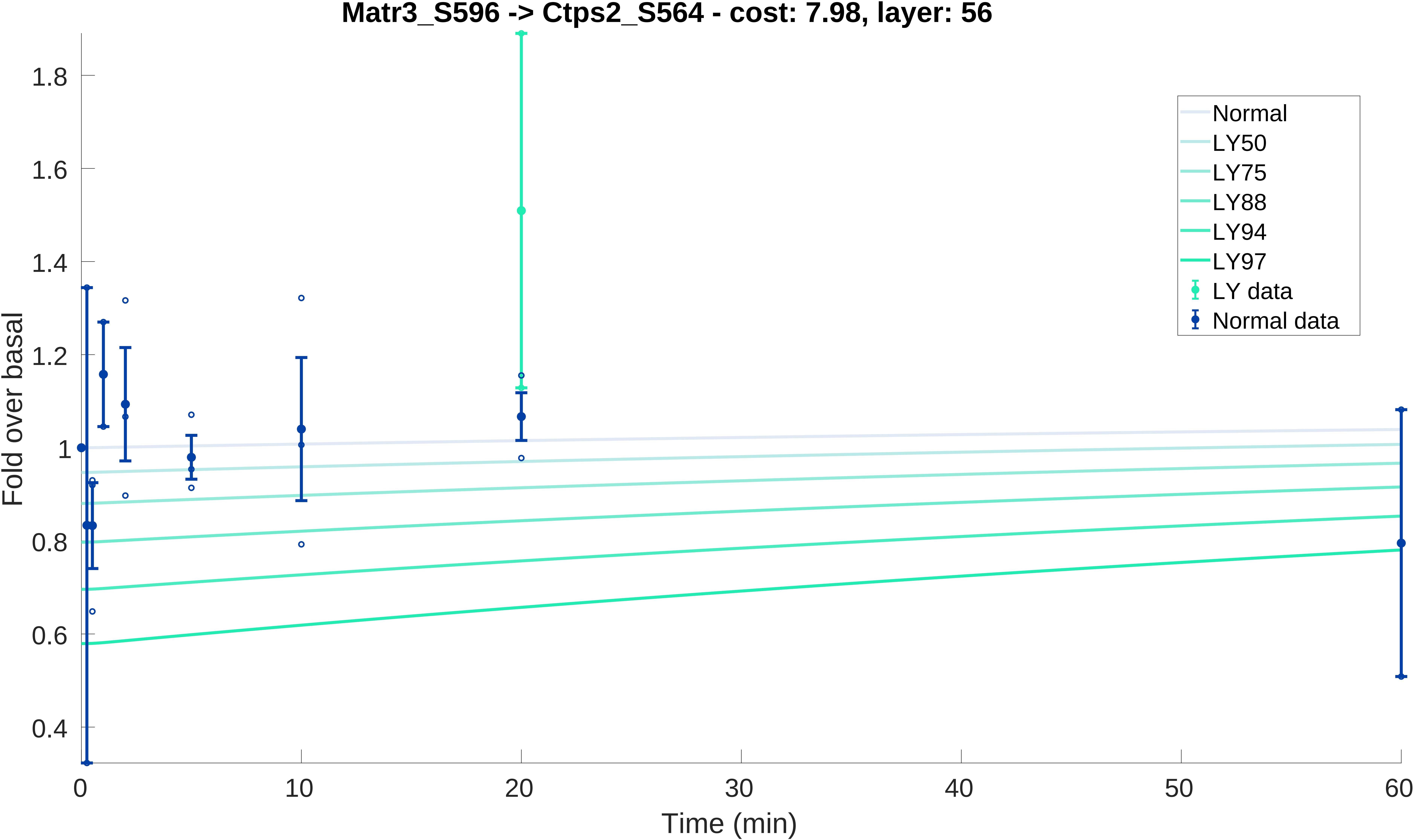

Matr3_S596 -> Ctps2_S564 - cost: 7.98, layer: 56
Fold over basal
Time (min)
1.8
1.6
1.4
1.2
1
0.8
0.6
0.4
0
10
20
30
40
50
60
Normal
LY50
LY75
LY88
LY94
LY97
LY data
Normal data

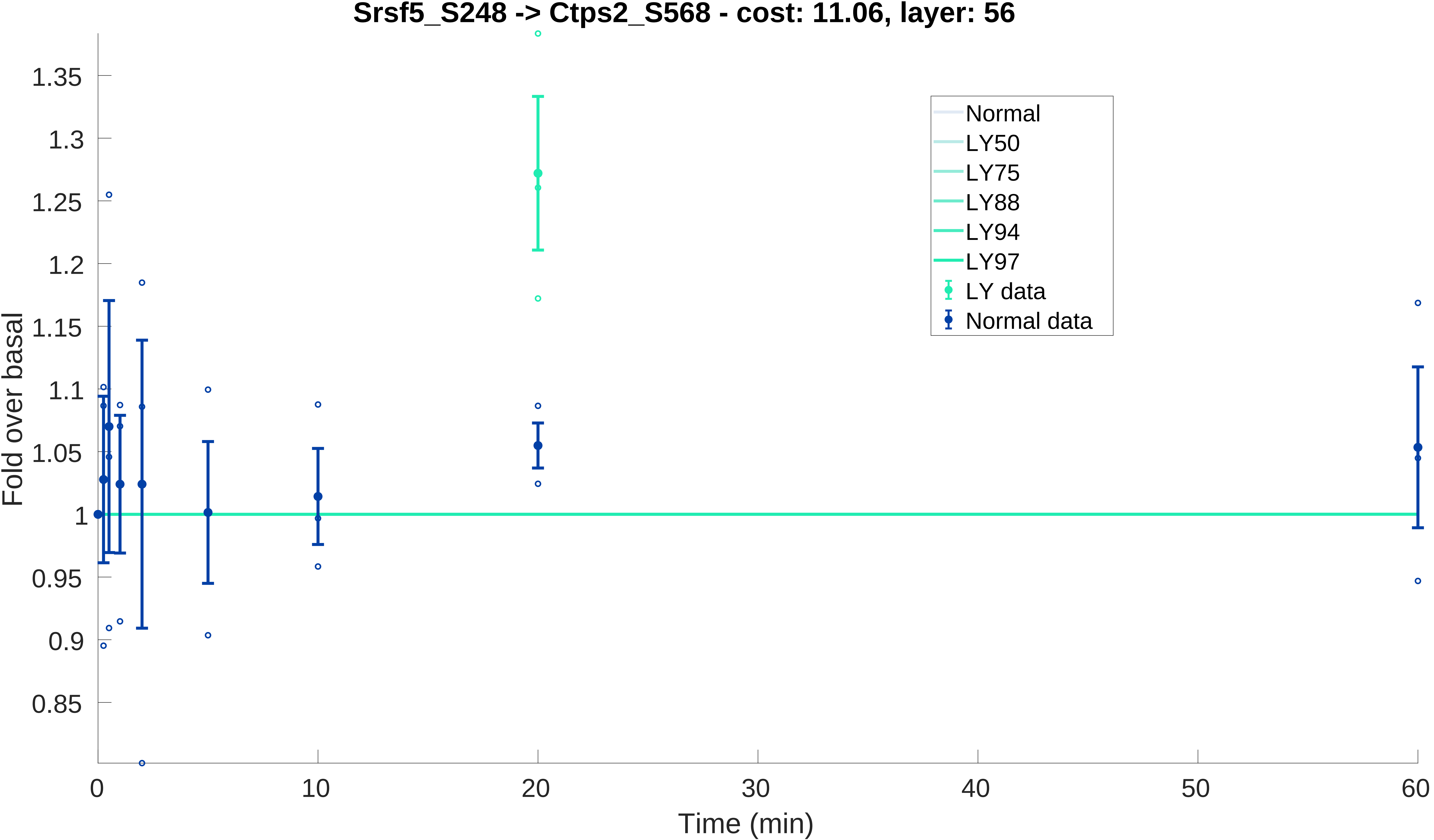

Srsf5_S248 -> Ctps2_S568 - cost: 11.06, layer: 56
Fold over basal
Time (min)
Normal
LY50
LY75
LY88
LY94
LY97
LY data
Normal data
1.35
1.3
1.25
1.2
1.15
1.1
1.05
1
0.95
0.9
0.85
0
10
20
30
40
50
60

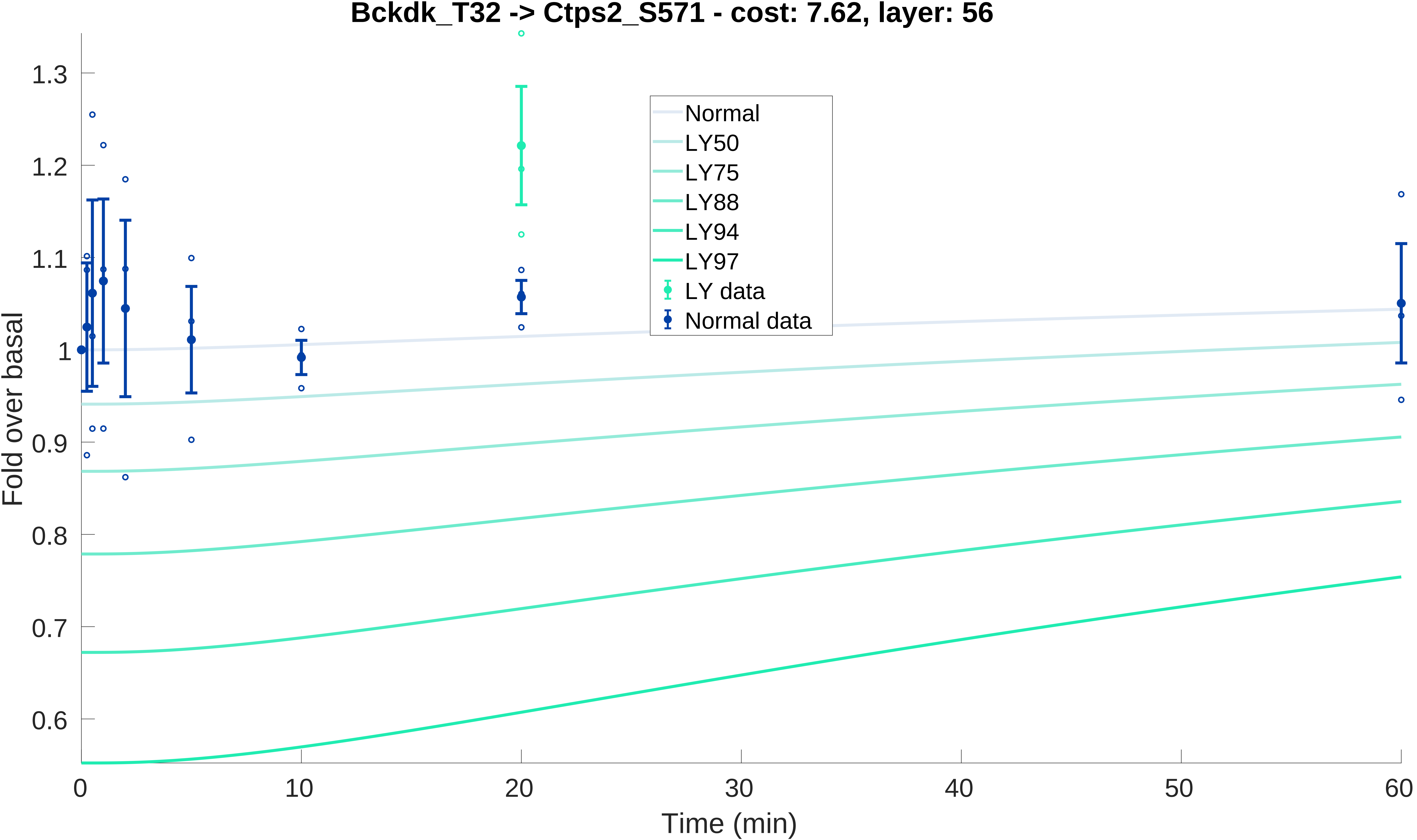

Bckdk_T32 -> Ctps2_S571 - cost: 7.62, layer: 56
Normal
LY50
LY75
LY88
LY94
LY97
LY data
Normal data
Fold over basal
Time (min)
1.3
1.2
1.1
1
0.9
0.8
0.7
0.6
0
10
20
30
40
50
60

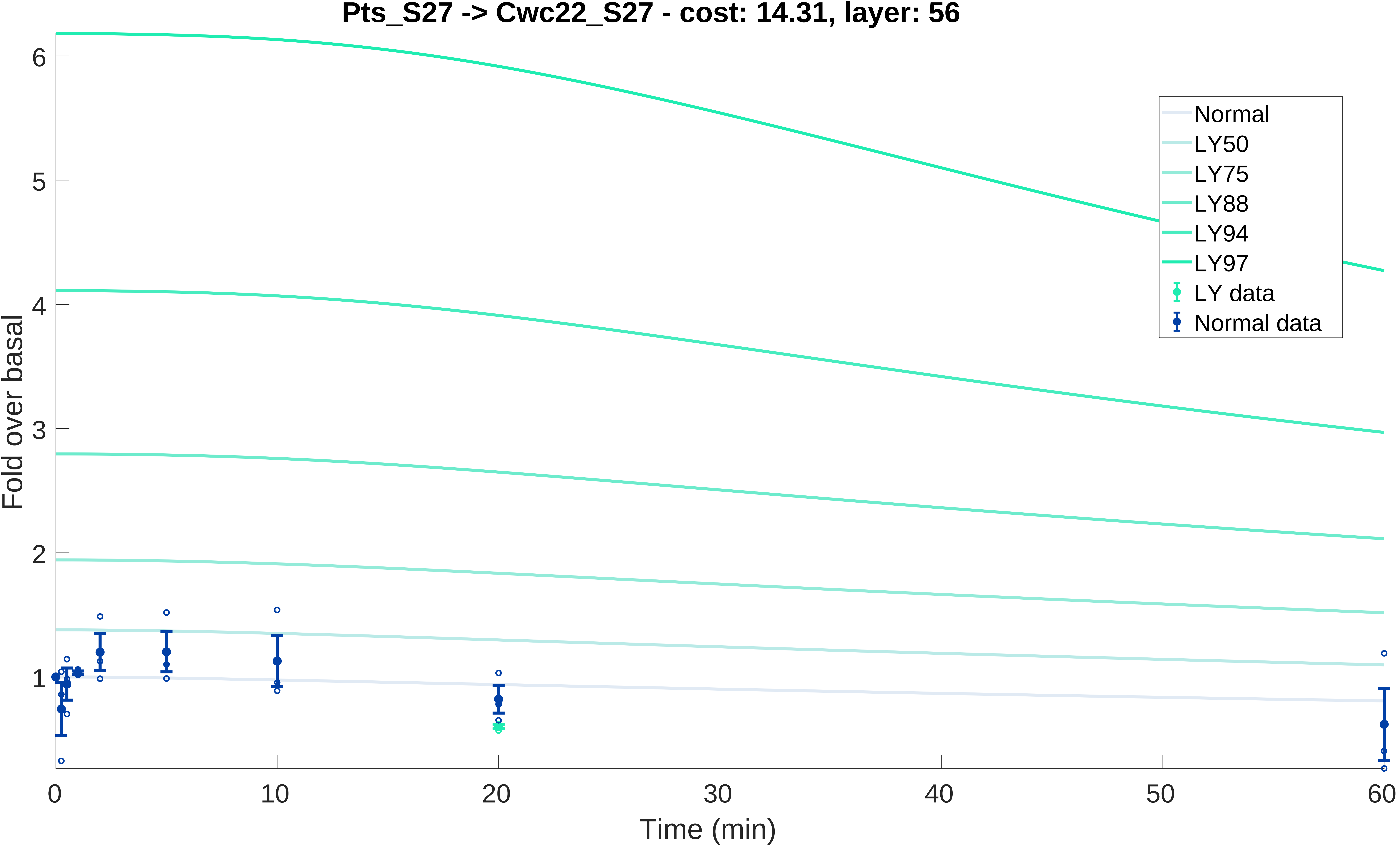
Pts_S27 -> Cwc22_S27 - cost: 14.31, layer: 56
Fold over basal
Time (min)
0
10
20
30
40
50
60
1
2
3
4
5
6
Normal
LY50
LY75
LY88
LY94
LY97
LY data
Normal data

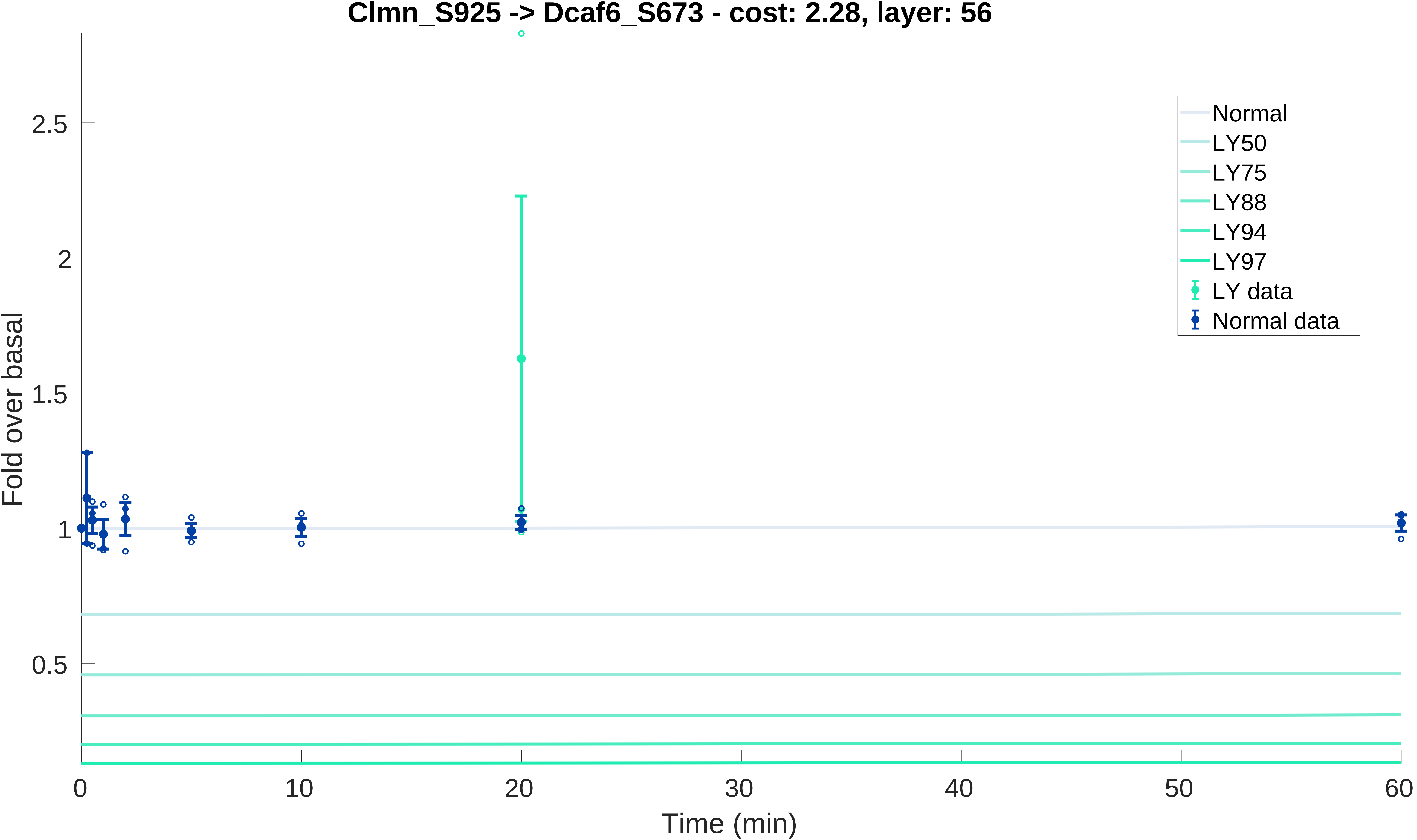

Clmn_S925 -> Dcaf6_S673 - cost: 2.28, layer: 56
Fold over basal
Time (min)
0
10
20
30
40
50
60
0.5
1
1.5
2
2.5
Normal
LY50
LY75
LY88
LY94
LY97
LY data
Normal data

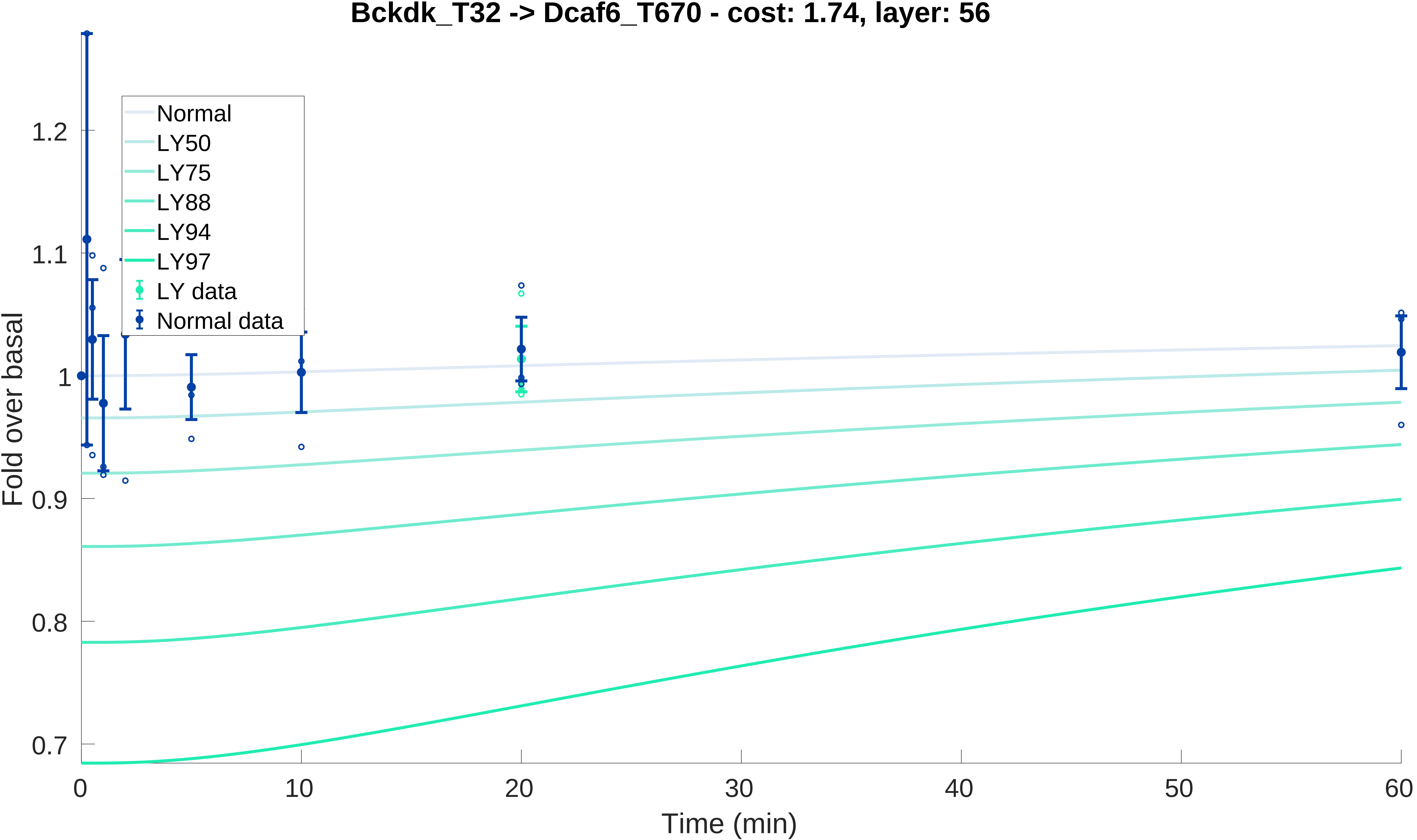

Bckdk_T32 -> Dcaf6_T670 - cost: 1.74, layer: 56
Normal
LY50
LY75
LY88
LY94
LY97
LY data
Normal data
Fold over basal
1.2
1.1
1
0.9
0.8
0.7
0
10
20
30
40
50
60
Time (min)

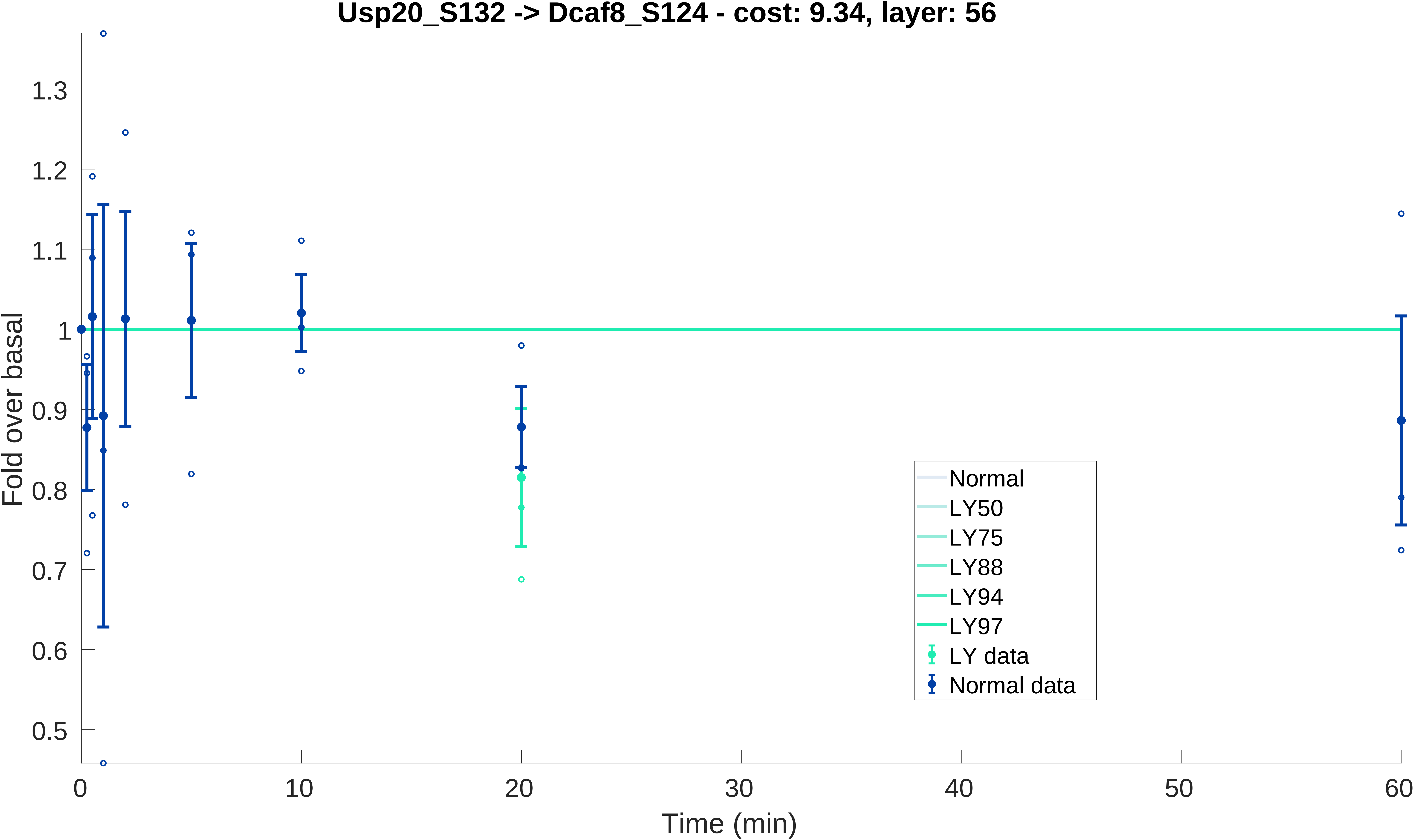

Usp20_S132 -> Dcaf8_S124 - cost: 9.34, layer: 56
Fold over basal
Time (min)
0.5
0.6
0.7
0.8
0.9
1
1.1
1.2
1.3
0
10
20
30
40
50
60
Normal
LY50
LY75
LY88
LY94
LY97
LY data
Normal data

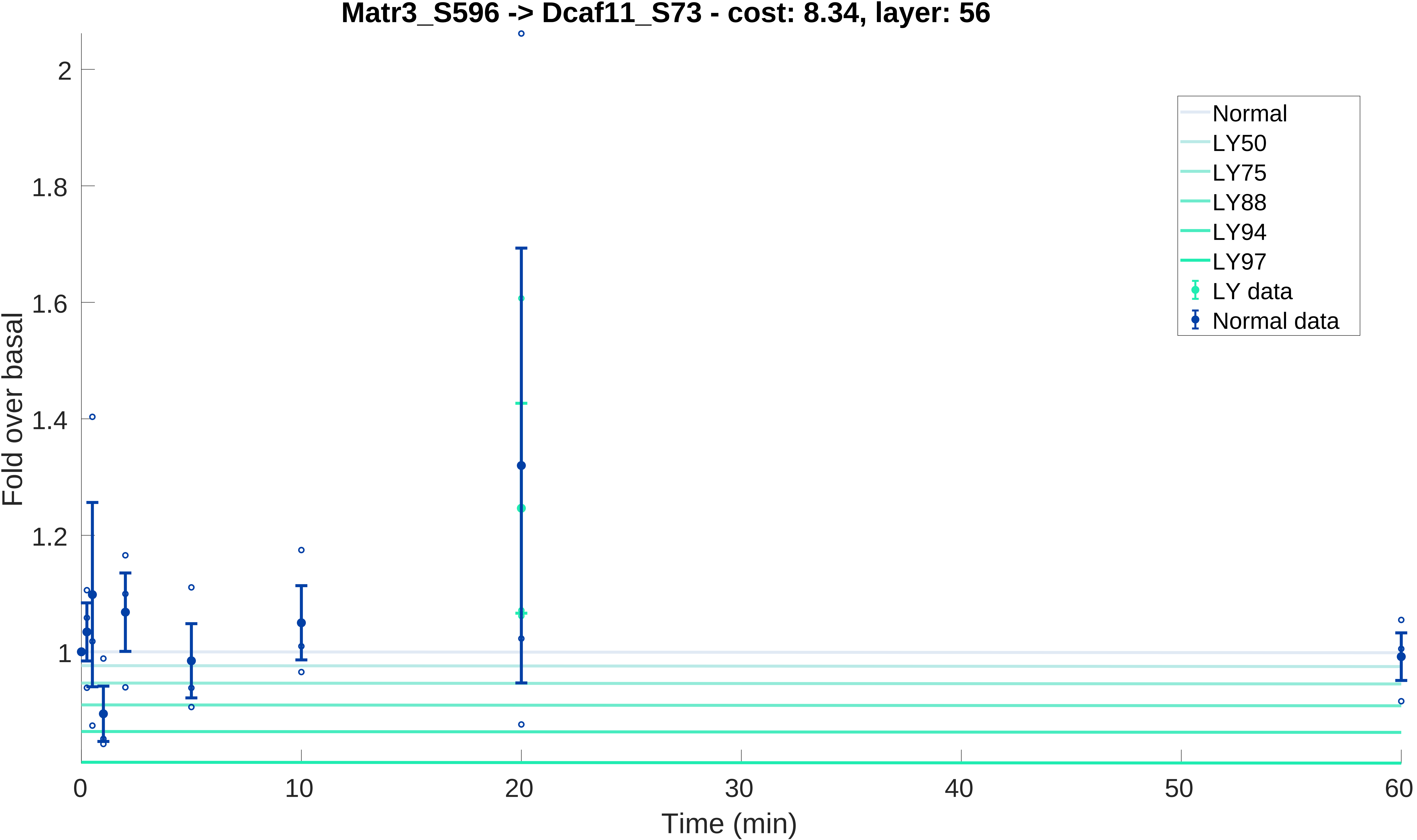

Matr3_S596 -> Dcaf11_S73 - cost: 8.34, layer: 56
Fold over basal
Time (min)
2
1.8
1.6
1.4
1.2
1
0
10
20
30
40
50
60
Normal
LY50
LY75
LY88
LY94
LY97
LY data
Normal data

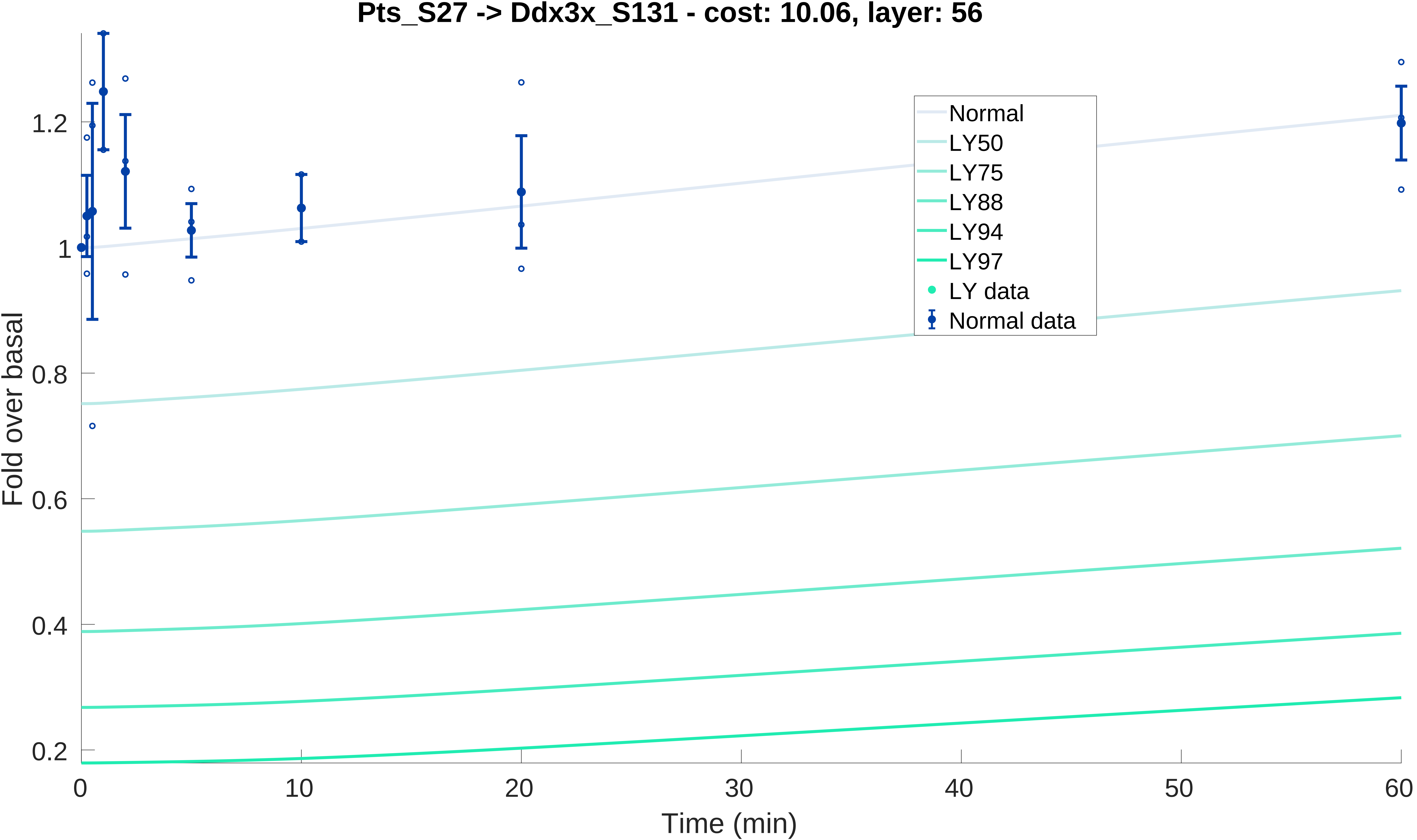

Pts_S27 -> Ddx3x_S131 - cost: 10.06, layer: 56
Normal
LY50
LY75
LY88
LY94
LY97
LY data
Normal data
Fold over basal
Time (min)
0.2
0.4
0.6
0.8
1
1.2
0
10
20
30
40
50
60

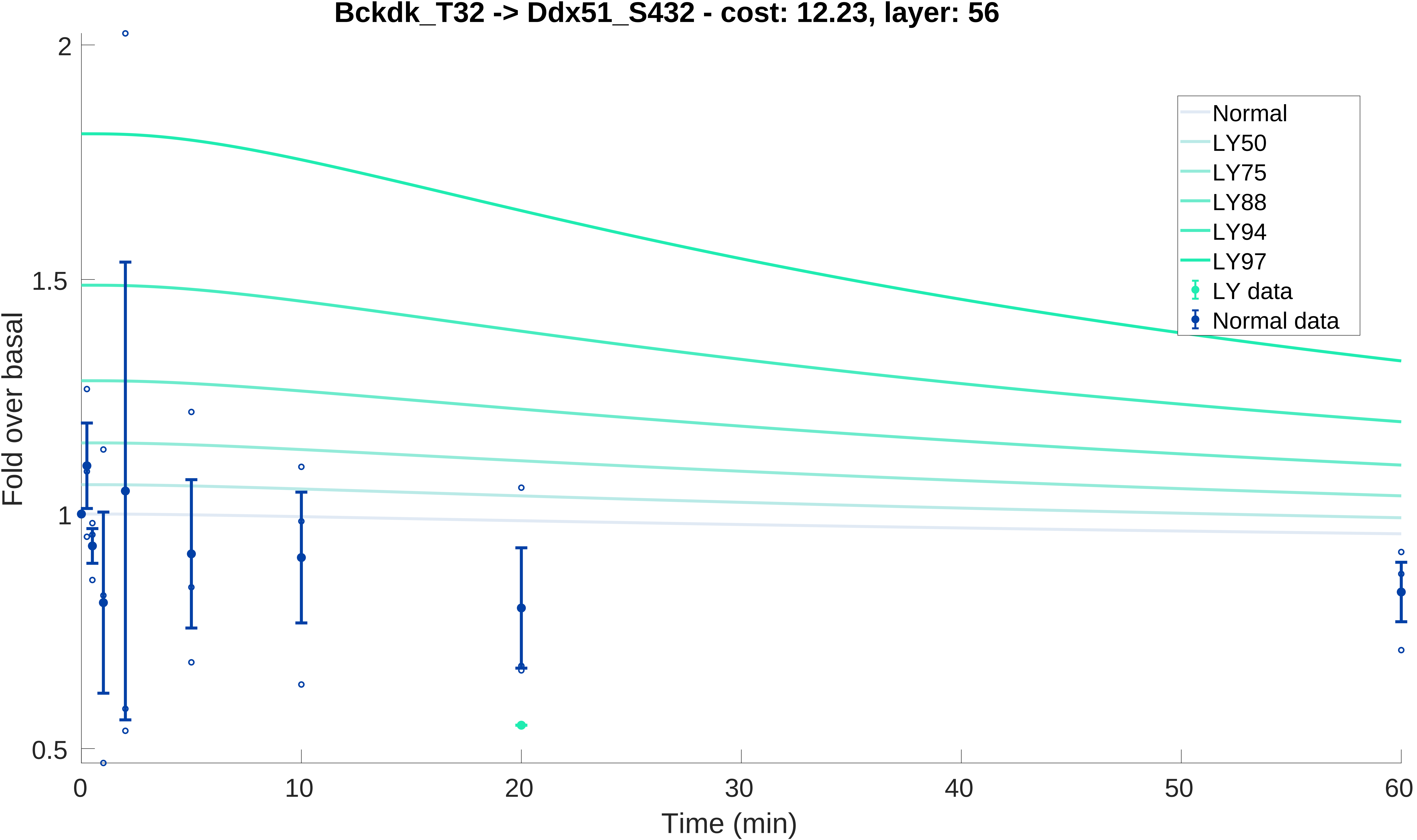
Bckdk_T32 -> Ddx51_S432 - cost: 12.23, layer: 56
Fold over basal
2
1.5
1
0.5
0
10
20
30
40
50
60
Time (min)
Normal
LY50
LY75
LY88
LY94
LY97
LY data
Normal data

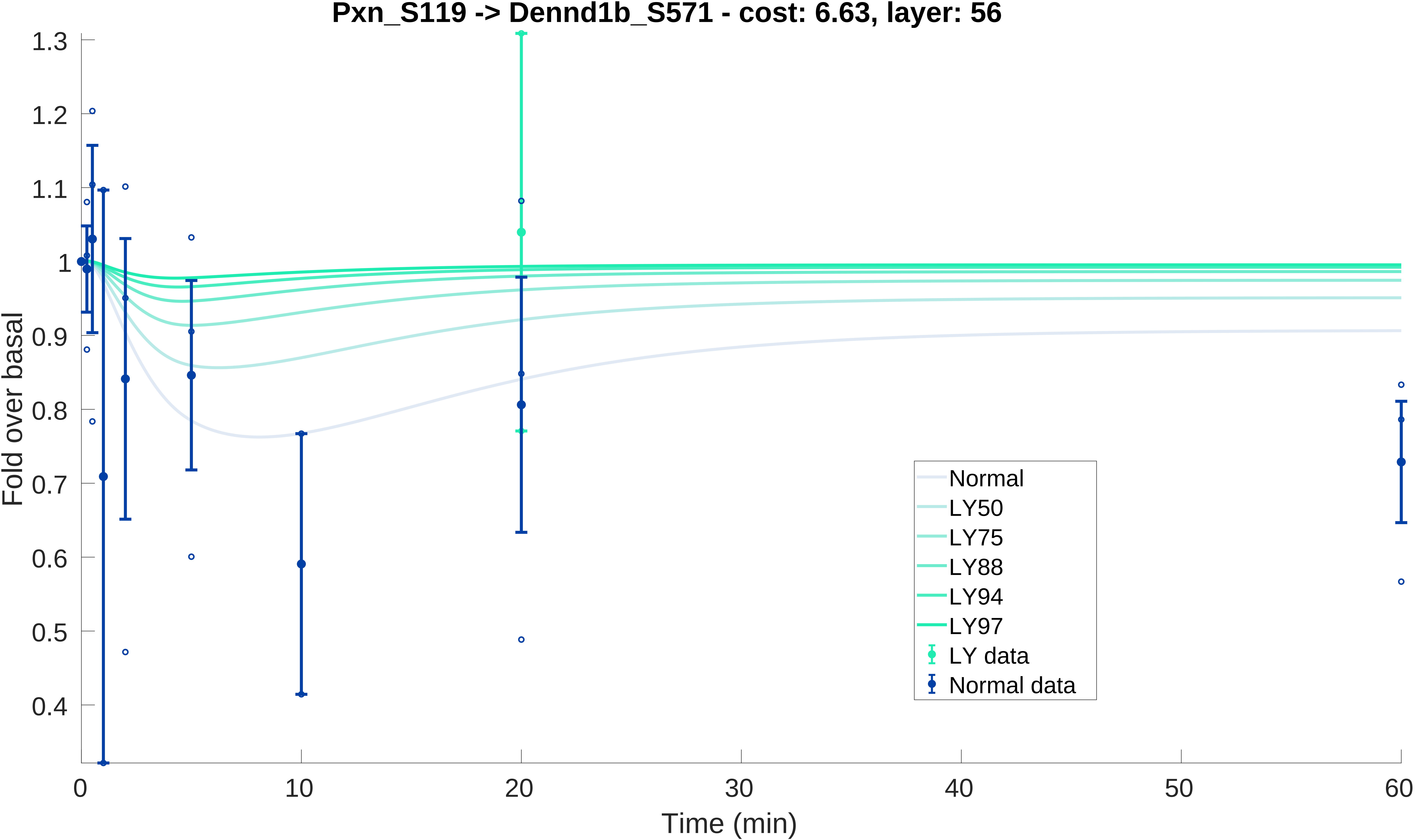

Pxn_S119 -> Dennd1b_S571 - cost: 6.63, layer: 56
Fold over basal
Time (min)
1.3
1.2
1.1
1
0.9
0.8
0.7
0.6
0.5
0.4
0
10
20
30
40
50
60
Normal
LY50
LY75
LY88
LY94
LY97
LY data
Normal data

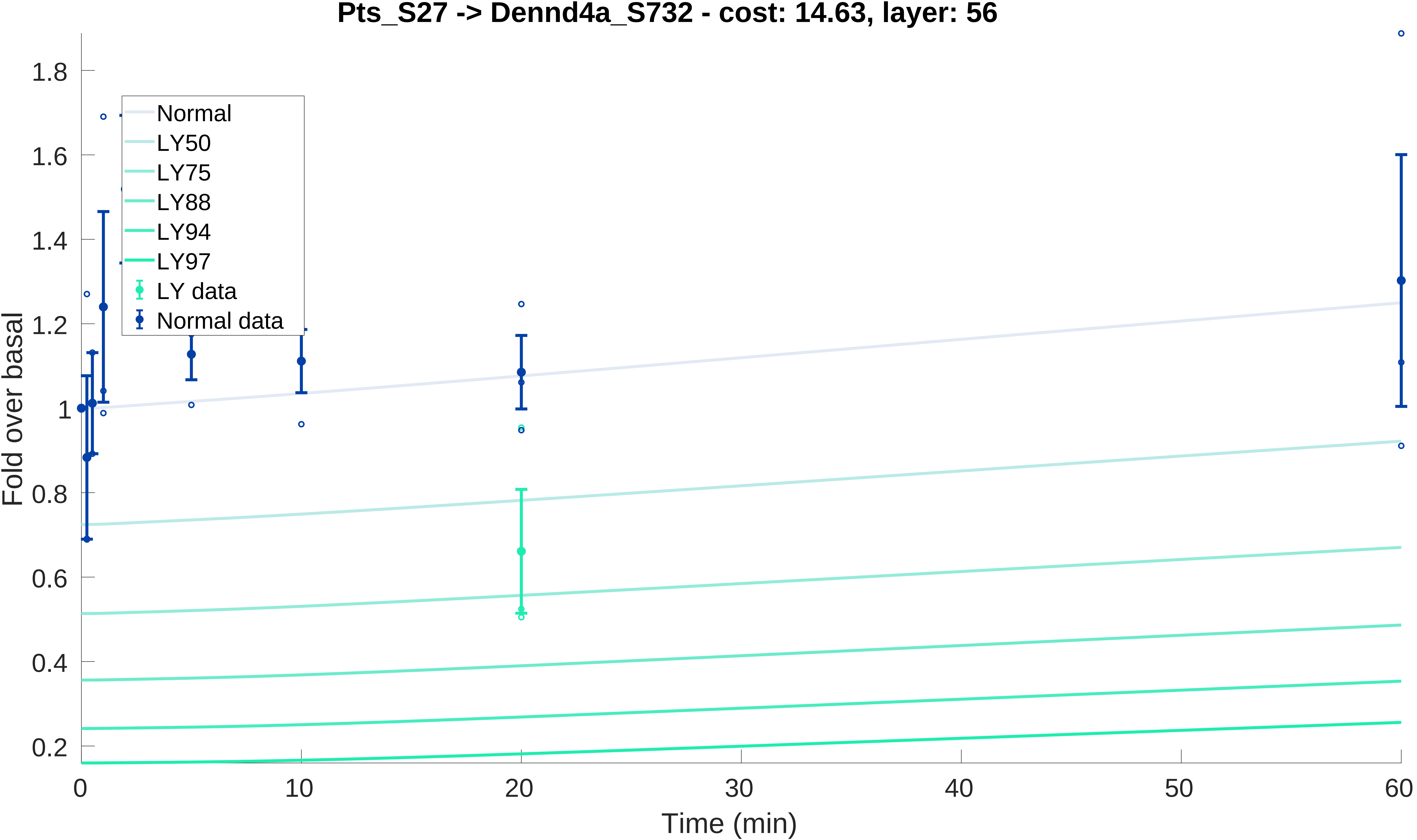

Pts_S27 -> Dennd4a_S732 - cost: 14.63, layer: 56
Normal
LY50
LY75
LY88
LY94
LY97
LY data
Normal data
Fold over basal
Time (min)
0.2
0.4
0.6
0.8
1
1.2
1.4
1.6
1.8
0
10
20
30
40
50
60

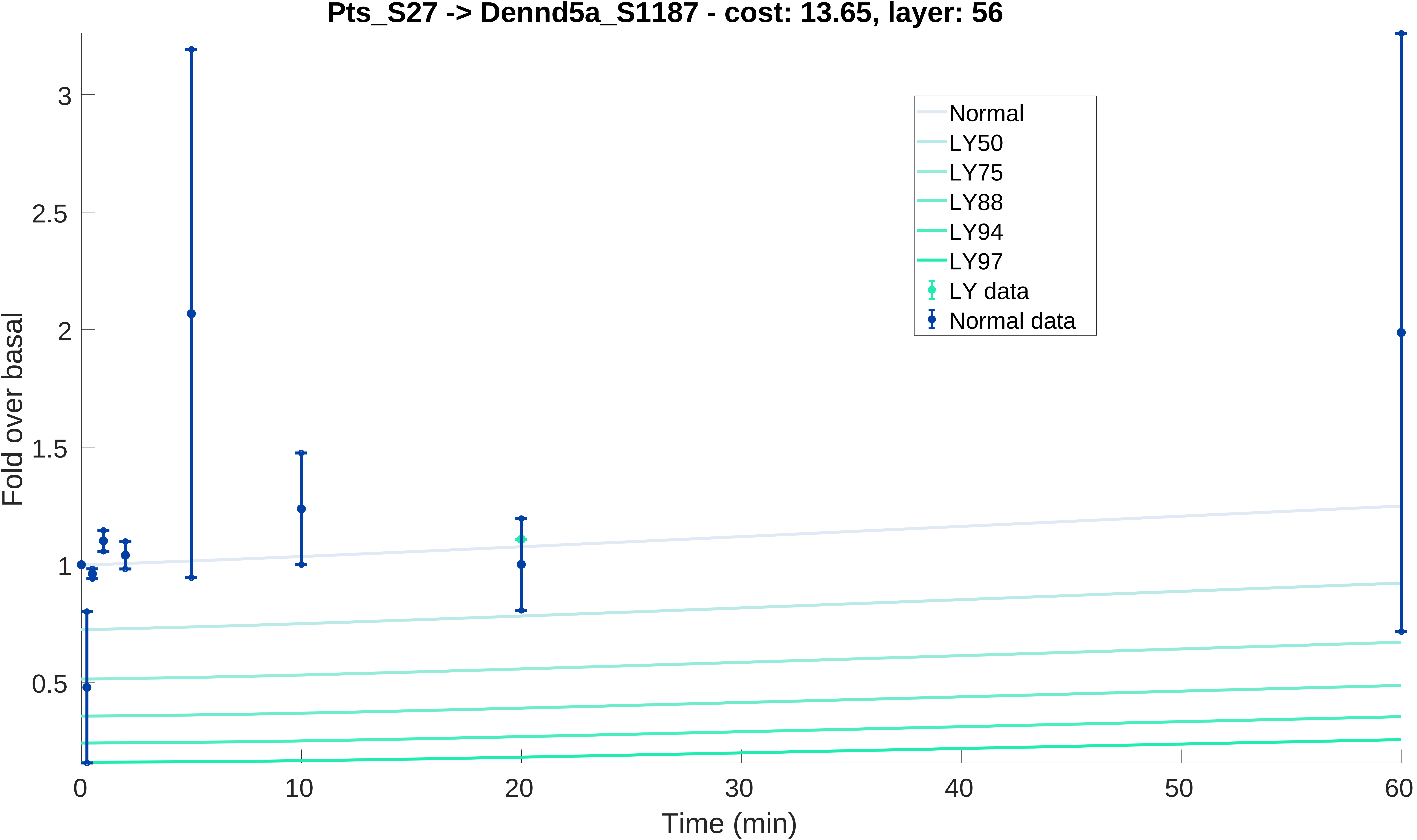

Pts_S27 -> Dennd5a_S1187 - cost: 13.65, layer: 56
Fold over basal
Time (min)
0
10
20
30
40
50
60
0.5
1
1.5
2
2.5
3
Normal
LY50
LY75
LY88
LY94
LY97
LY data
Normal data

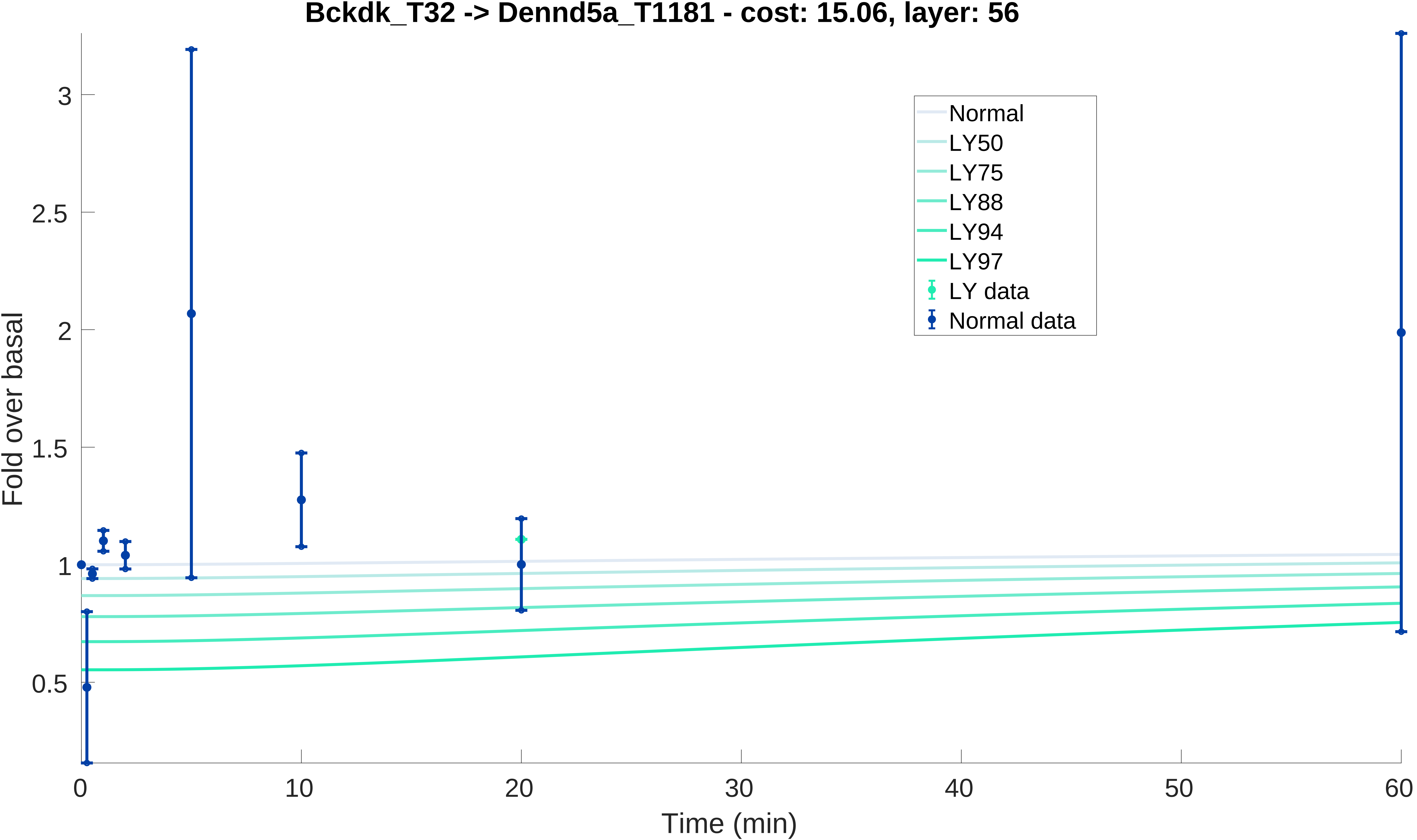

Bckdk_T32 -> Dennd5a_T1181 - cost: 15.06, layer: 56
Fold over basal
Time (min)
Normal
LY50
LY75
LY88
LY94
LY97
LY data
Normal data
0.5
1
1.5
2
2.5
3
0
10
20
30
40
50
60

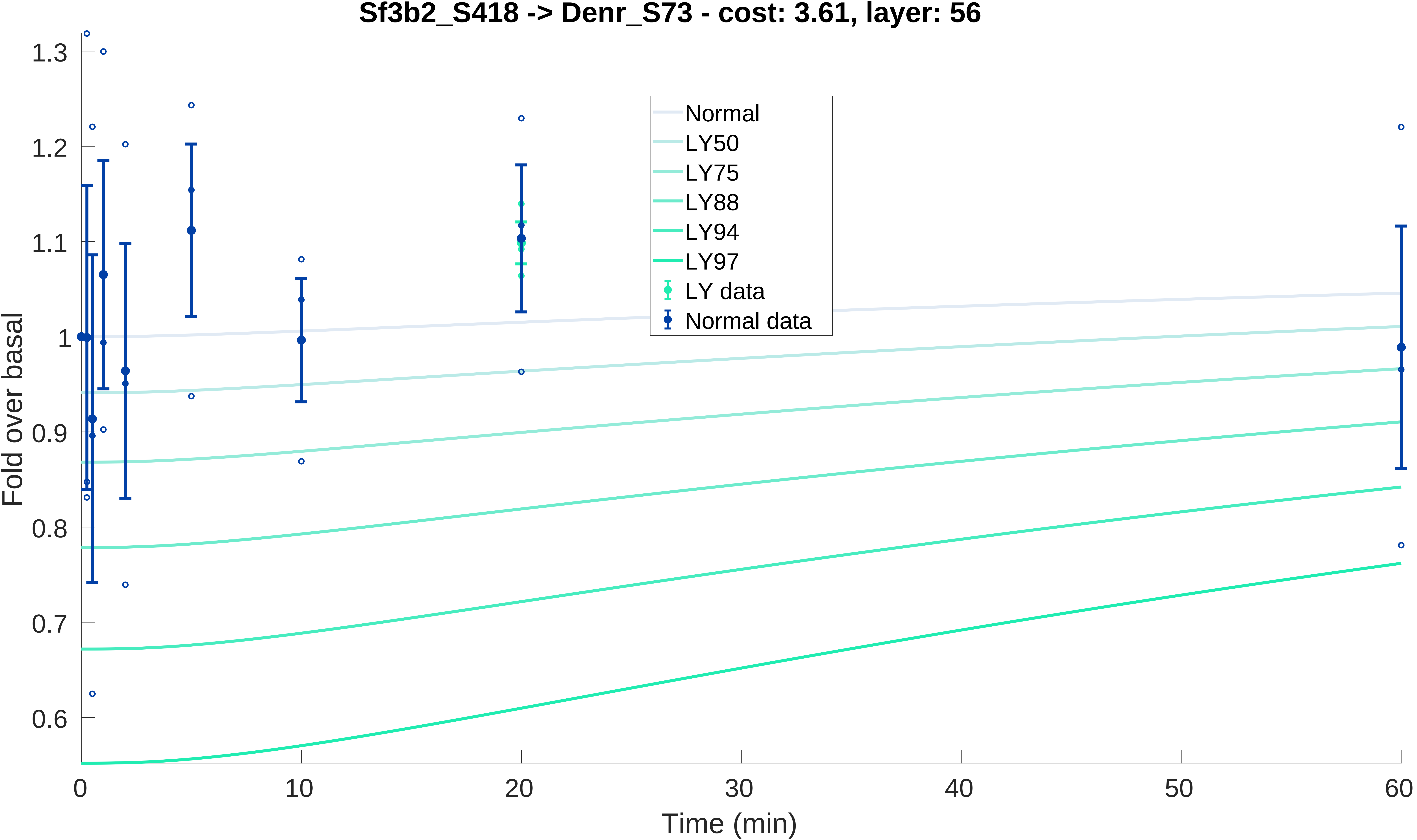

Sf3b2_S418 -> Denr_S73 - cost: 3.61, layer: 56
Fold over basal
Time (min)
Normal
LY50
LY75
LY88
LY94
LY97
LY data
Normal data
1.3
1.2
1.1
1
0.9
0.8
0.7
0.6
0
10
20
30
40
50
60

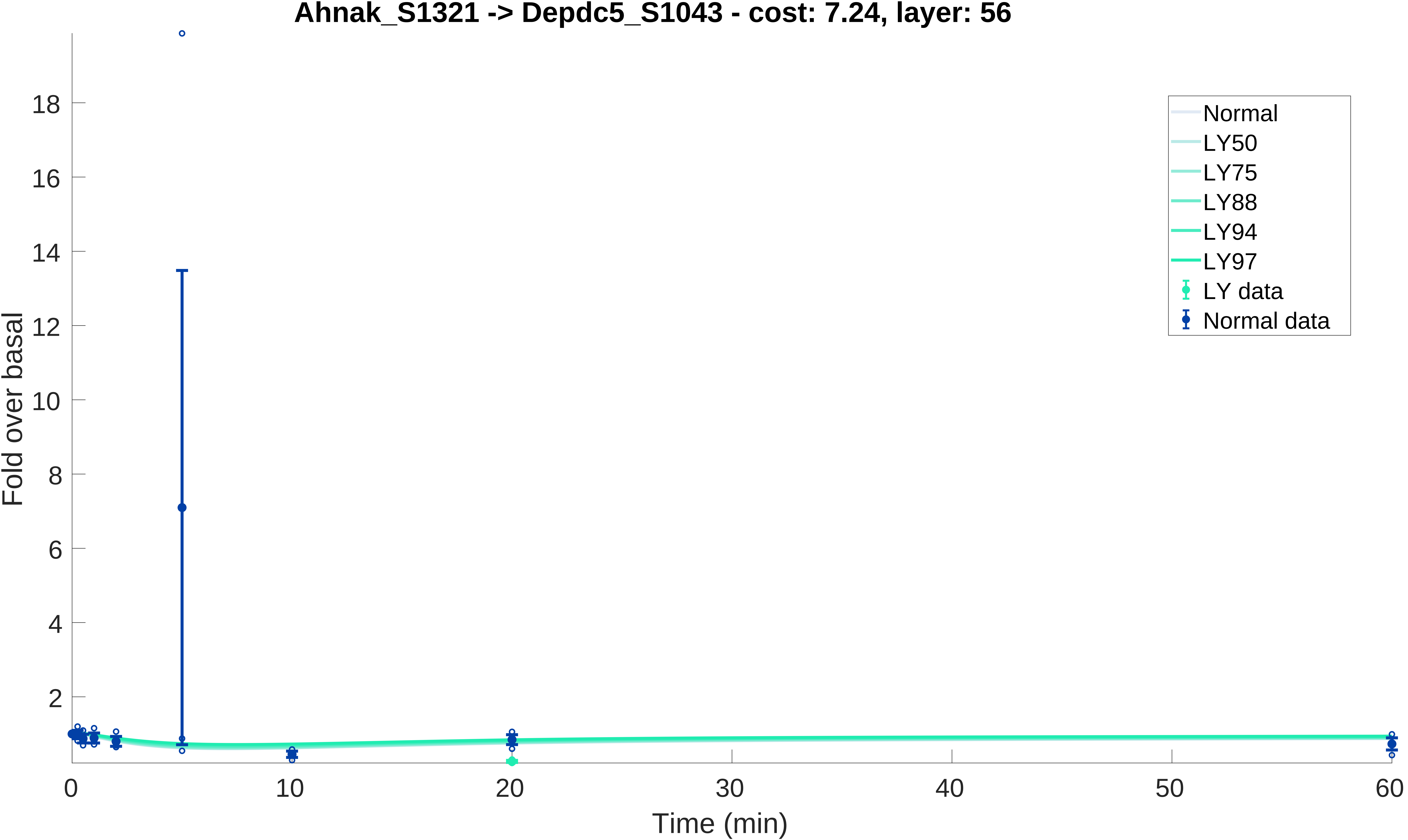
Ahnak_S1321 -> Depdc5_S1043 - cost: 7.24, layer: 56
Fold over basal
Time (min)
0
10
20
30
40
50
60
2
4
6
8
10
12
14
16
18
Normal
LY50
LY75
LY88
LY94
LY97
LY data
Normal data

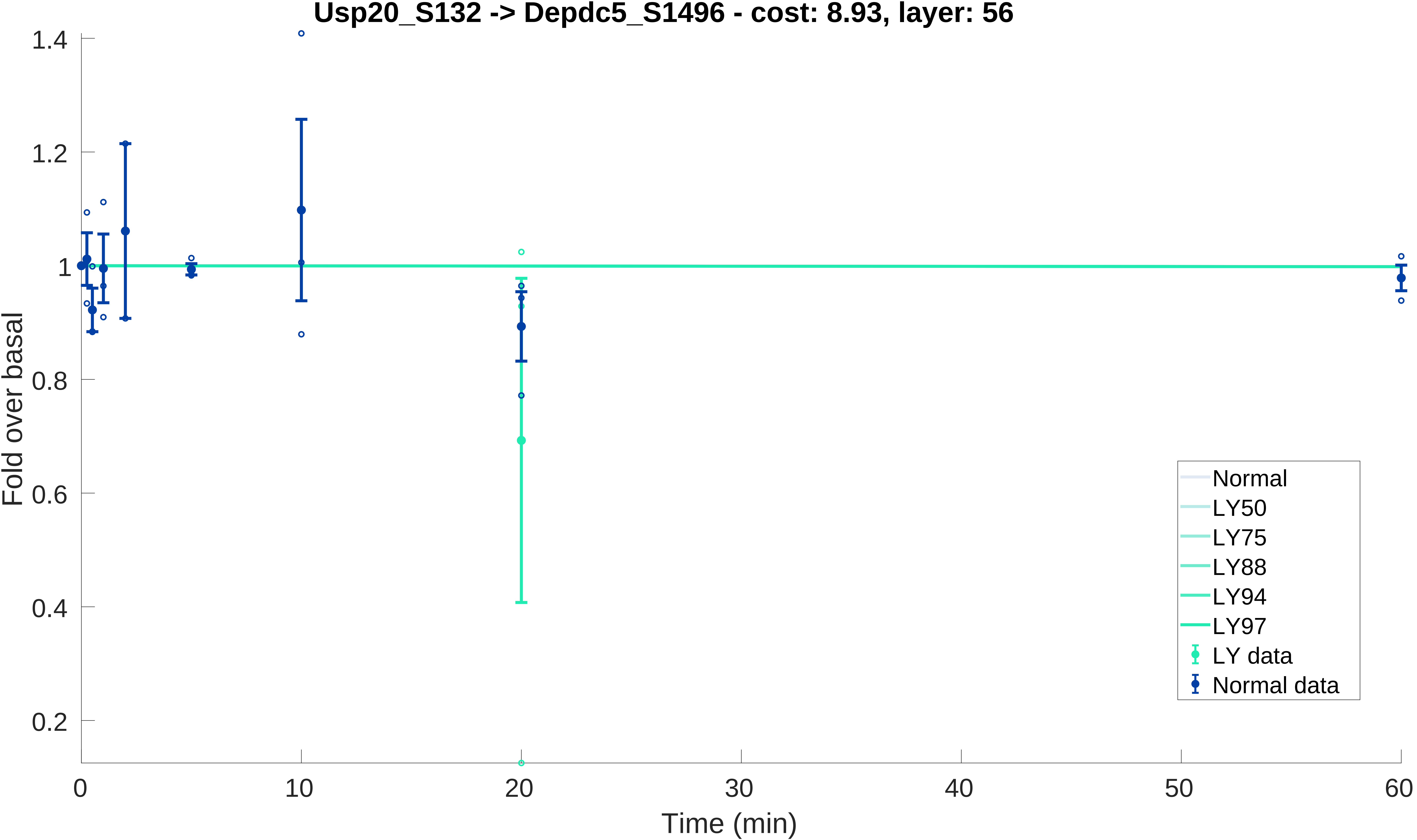
Usp20_S132 -> Depdc5_S1496 - cost: 8.93, layer: 56
Fold over basal
Time (min)
1.4
1.2
1
0.8
0.6
0.4
0.2
0
10
20
30
40
50
60
Normal
LY50
LY75
LY88
LY94
LY97
LY data
Normal data

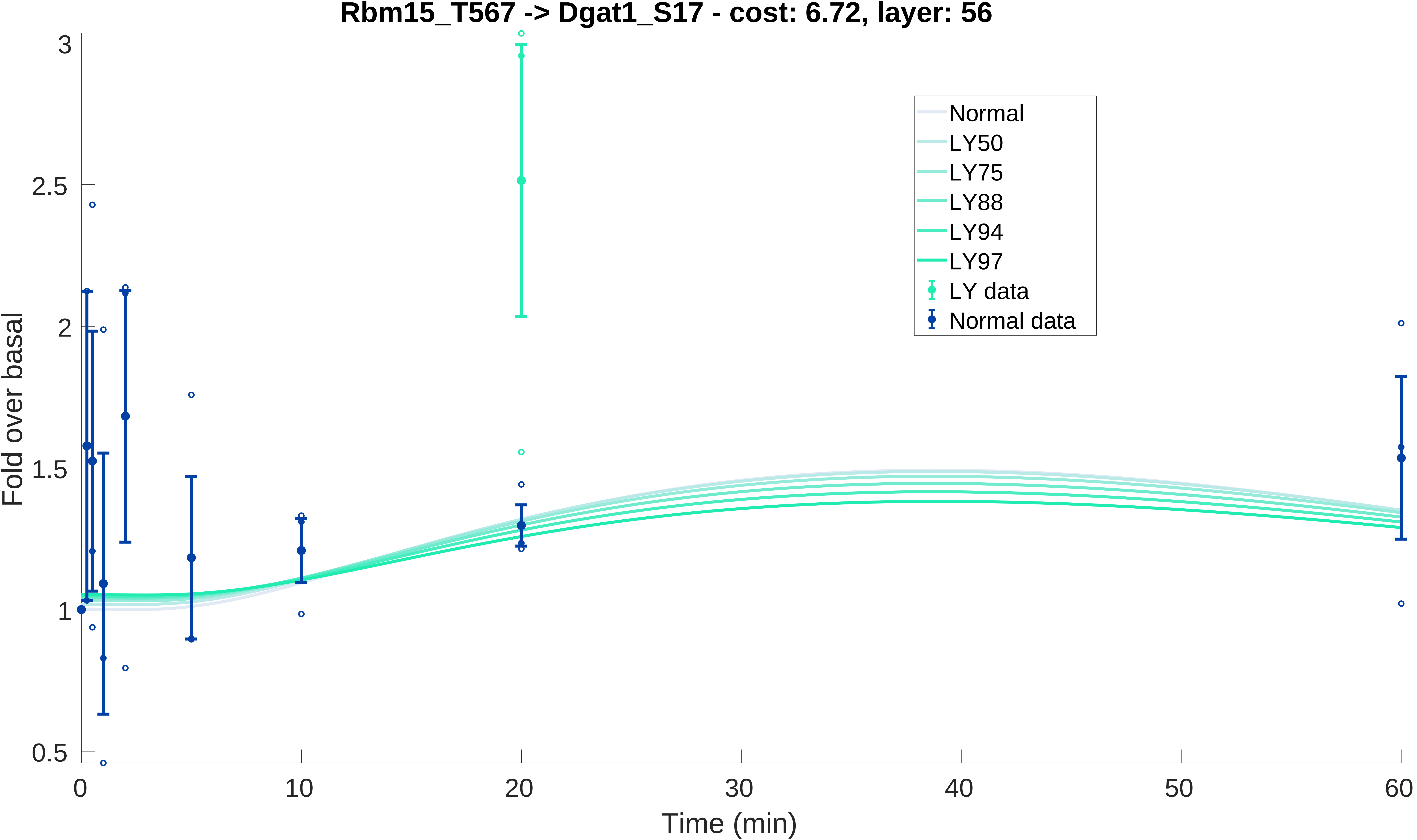

Rbm15_T567 -> Dgat1_S17 - cost: 6.72, layer: 56
Fold over basal
Time (min)
0
10
20
30
40
50
60
0.5
1
1.5
2
2.5
3
Normal
LY50
LY75
LY88
LY94
LY97
LY data
Normal data

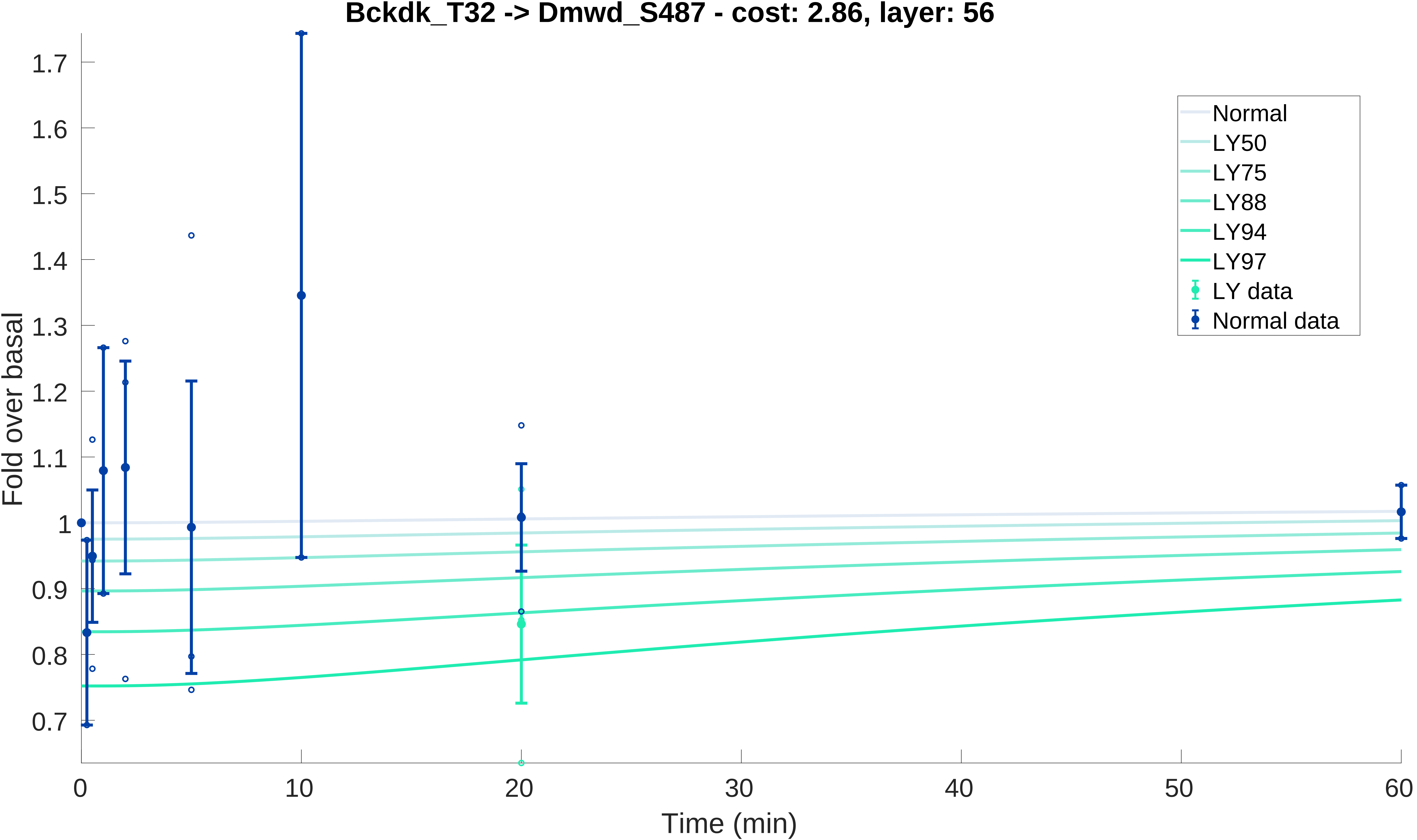
Bckdk_T32 -> Dmwd_S487 - cost: 2.86, layer: 56
Fold over basal
Time (min)
0
10
20
30
40
50
60
0.7
0.8
0.9
1
1.1
1.2
1.3
1.4
1.5
1.6
1.7
Normal
LY50
LY75
LY88
LY94
LY97
LY data
Normal data

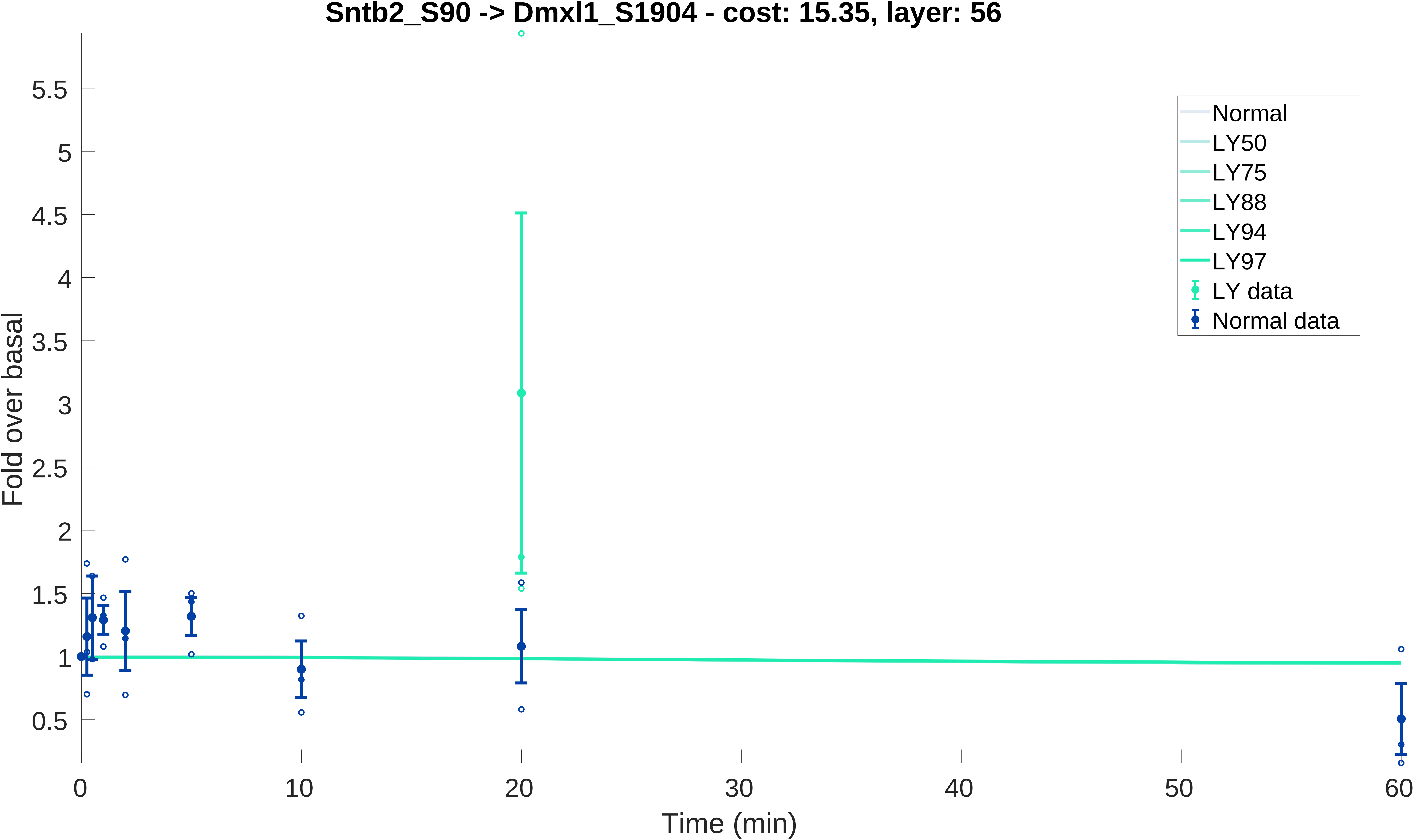
Sntb2_S90 -> Dmxl1_S1904 - cost: 15.35, layer: 56
Fold over basal
5.5
5
4.5
4
3.5
3
2.5
2
1.5
1
0.5
0
10
20
30
40
50
60
Time (min)
Normal
LY50
LY75
LY88
LY94
LY97
LY data
Normal data

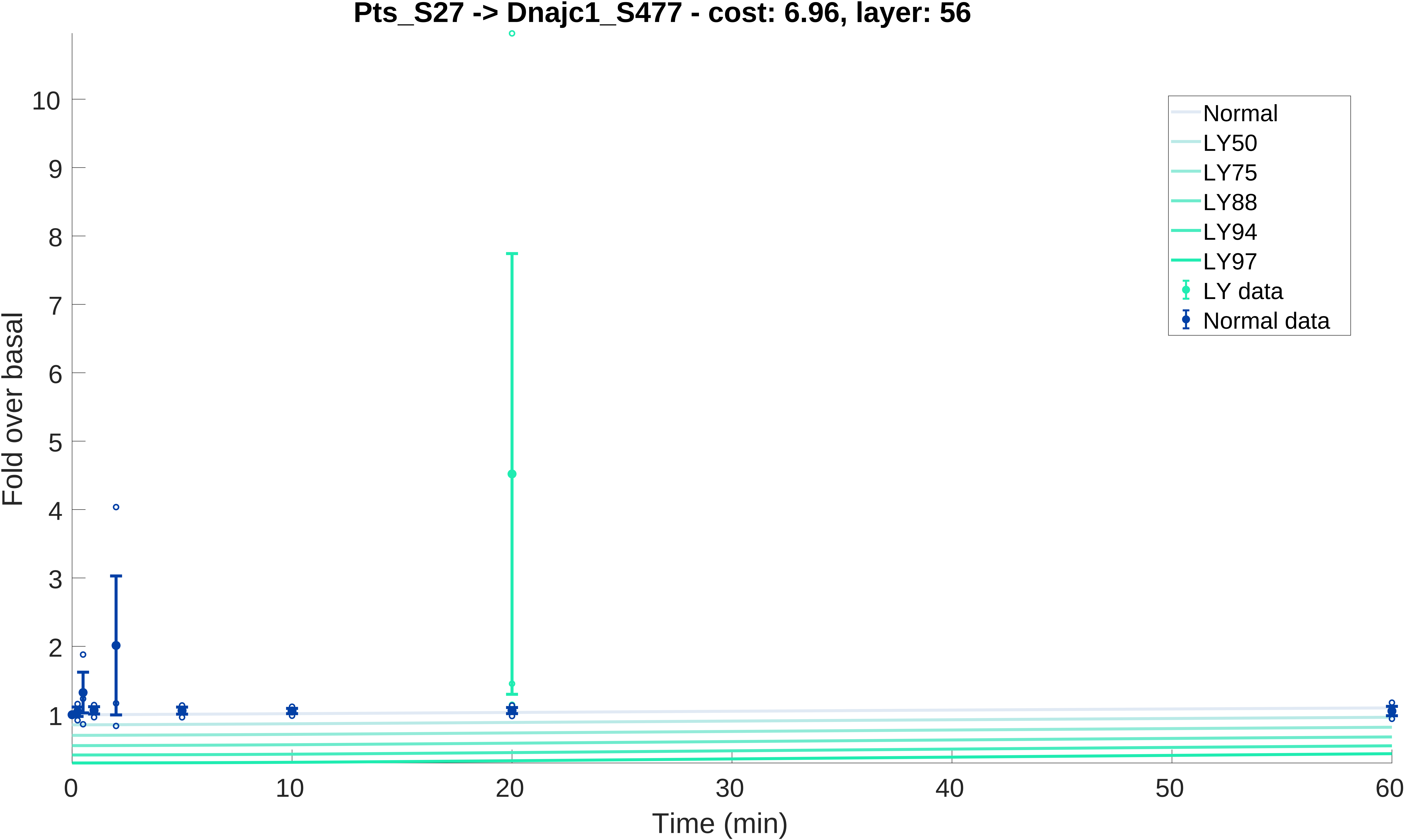
Pts_S27 -> Dnajc1_S477 - cost: 6.96, layer: 56
Fold over basal
Time (min)
0
10
20
30
40
50
60
1
2
3
4
5
6
7
8
9
10
Normal
LY50
LY75
LY88
LY94
LY97
LY data
Normal data

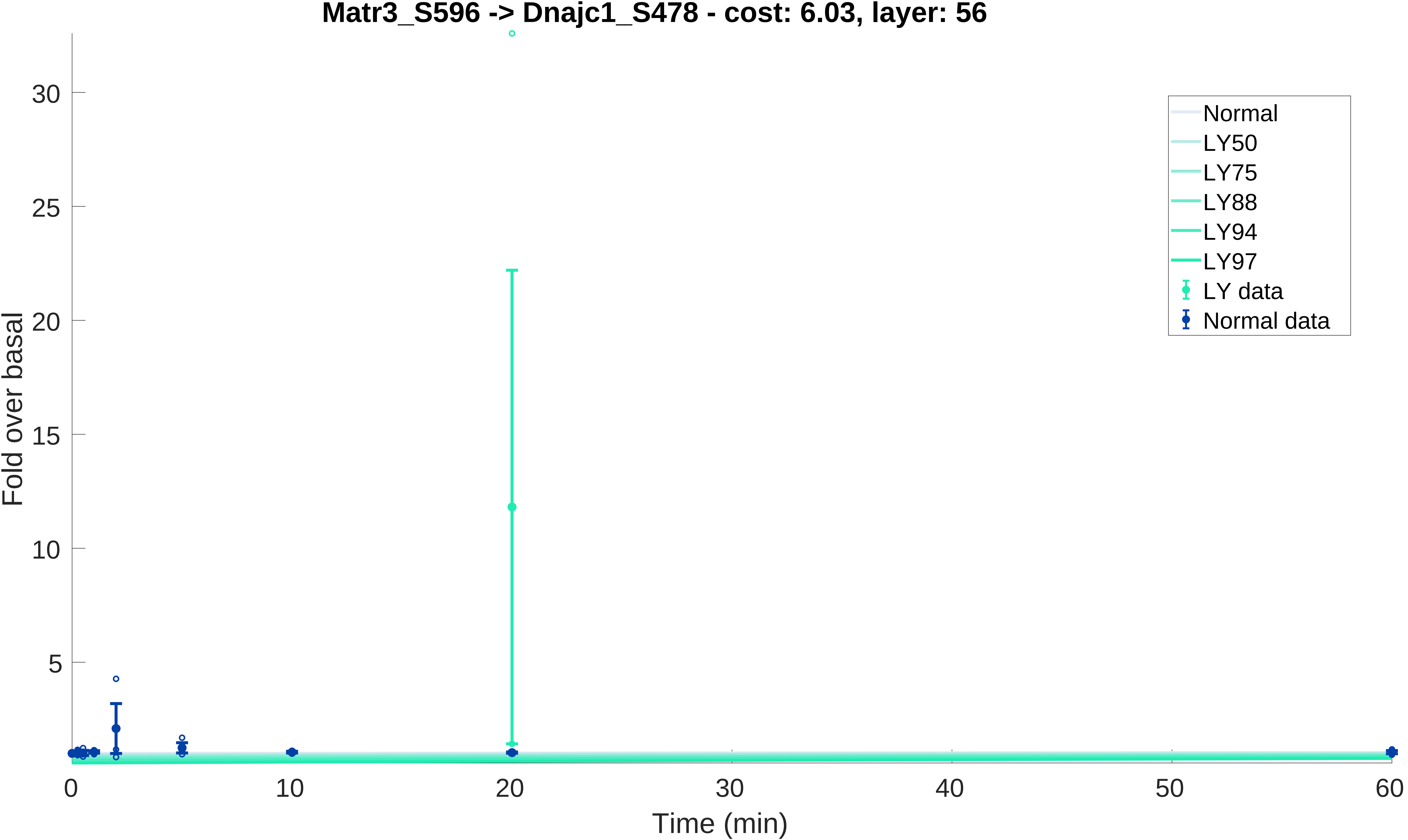

Matr3_S596 -> Dnajc1_S478 - cost: 6.03, layer: 56
Fold over basal
Time (min)
0
10
20
30
40
50
60
5
10
15
20
25
30
Normal
LY50
LY75
LY88
LY94
LY97
LY data
Normal data

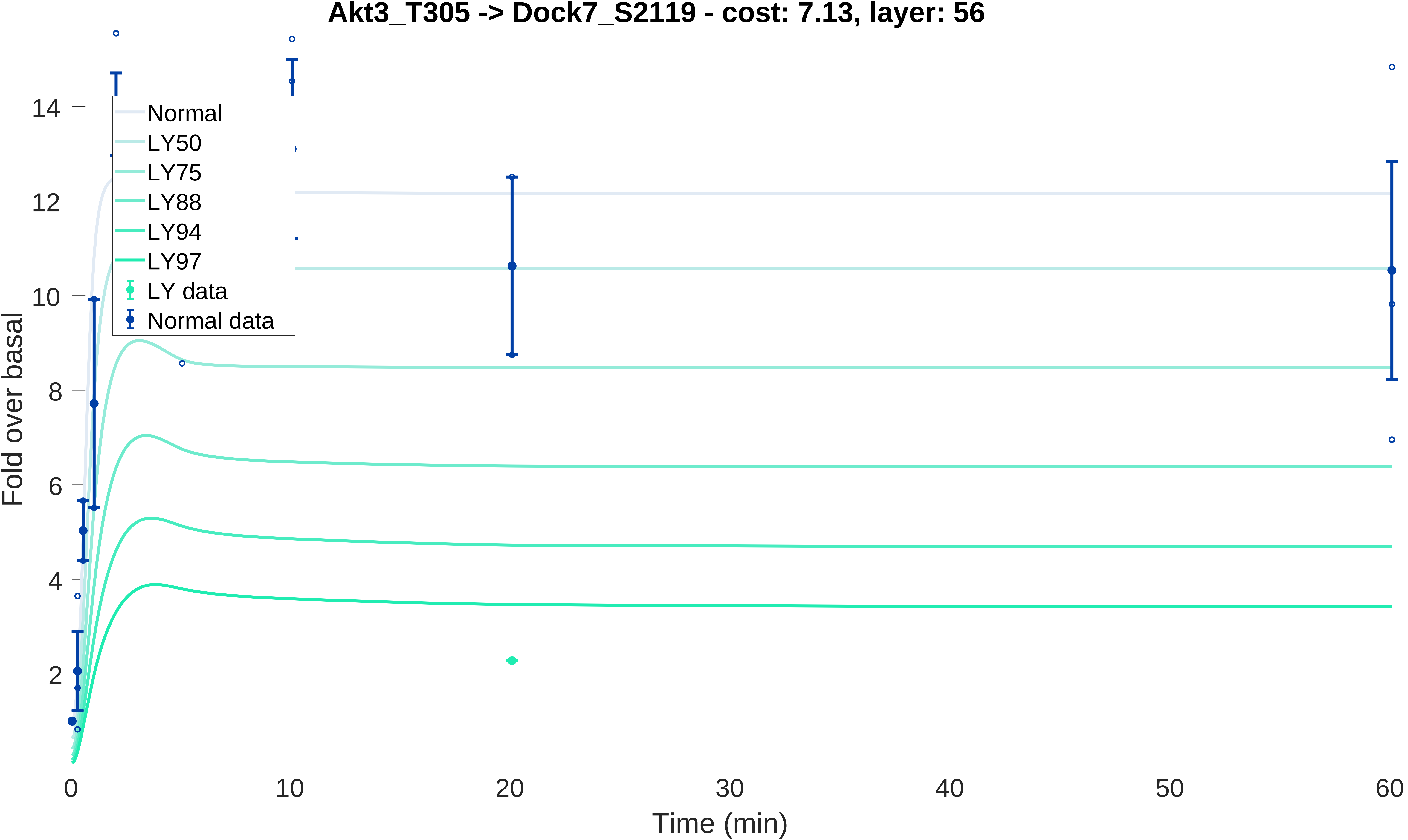

Akt3_T305 -> Dock7_S2119 - cost: 7.13, layer: 56
Fold over basal
Time (min)
Normal
LY50
LY75
LY88
LY94
LY97
LY data
Normal data
0
10
20
30
40
50
60
2
4
6
8
10
12
14

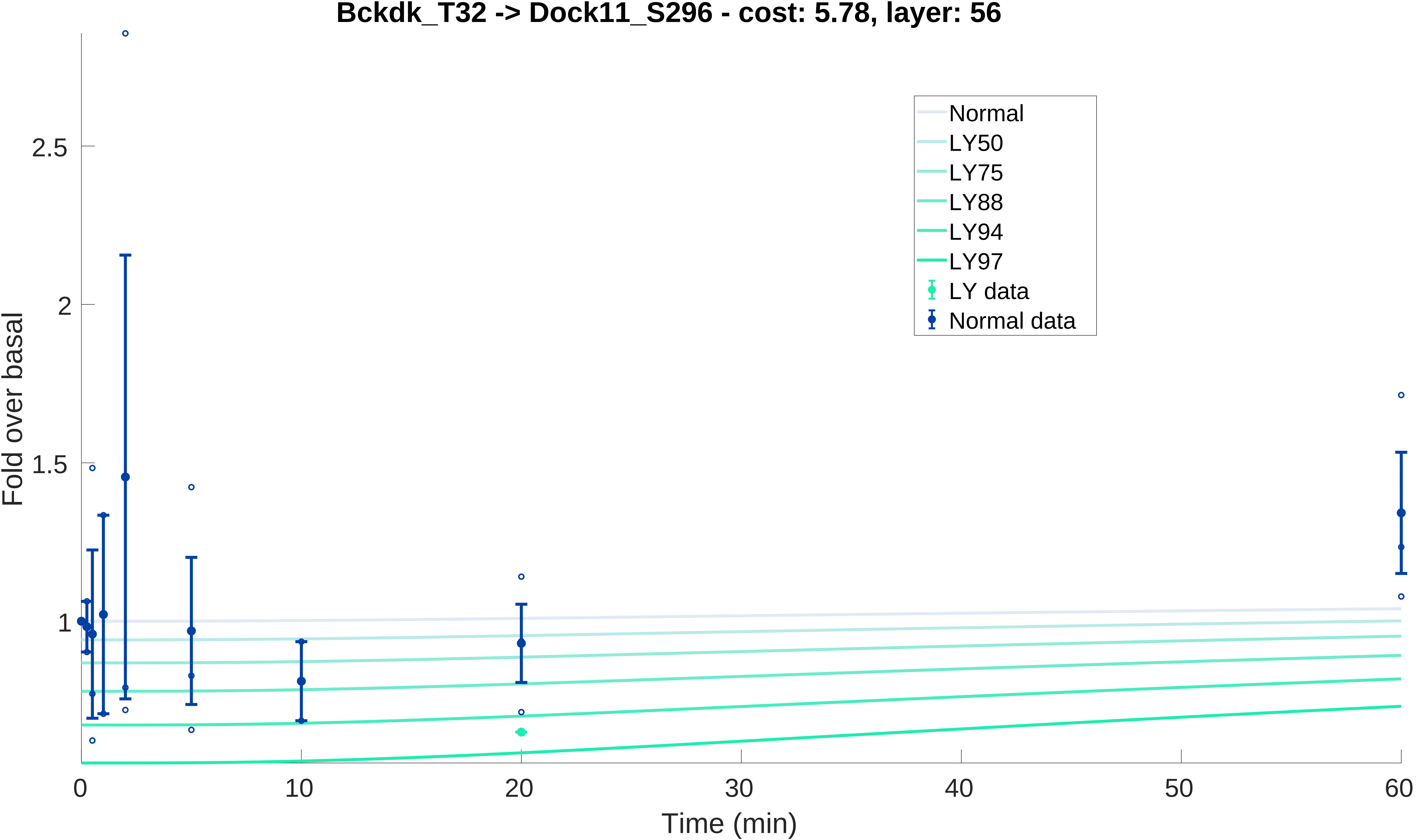

Bckdk_T32 -> Dock11_S296 - cost: 5.78, layer: 56
Normal
LY50
LY75
LY88
LY94
LY97
LY data
Normal data
Fold over basal
Time (min)
2.5
2
1.5
1
0
10
20
30
40
50
60

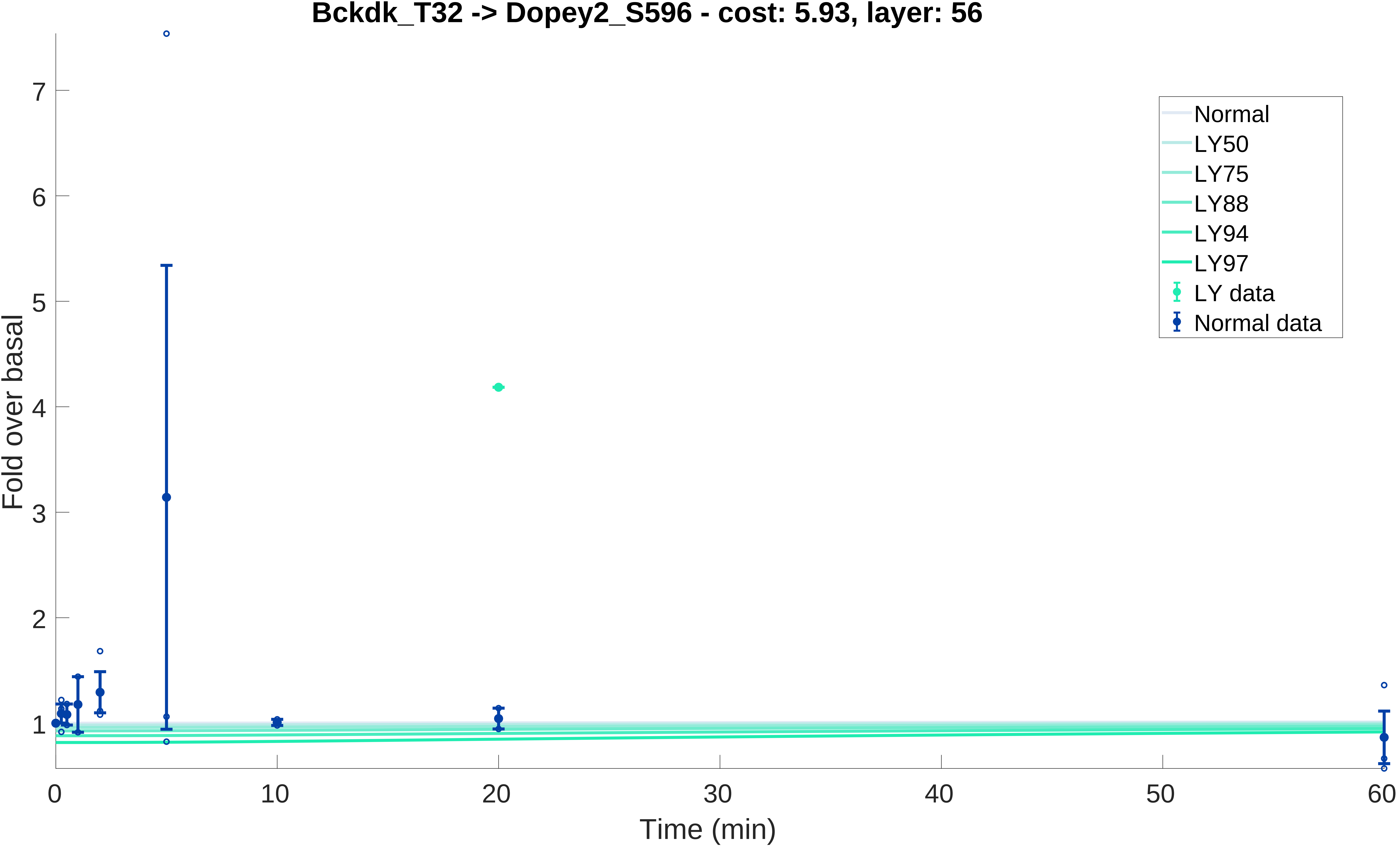

Bckdk_T32 -> Dopey2_S596 - cost: 5.93, layer: 56
Fold over basal
Time (min)
Normal
LY50
LY75
LY88
LY94
LY97
LY data
Normal data
0
10
20
30
40
50
60
1
2
3
4
5
6
7

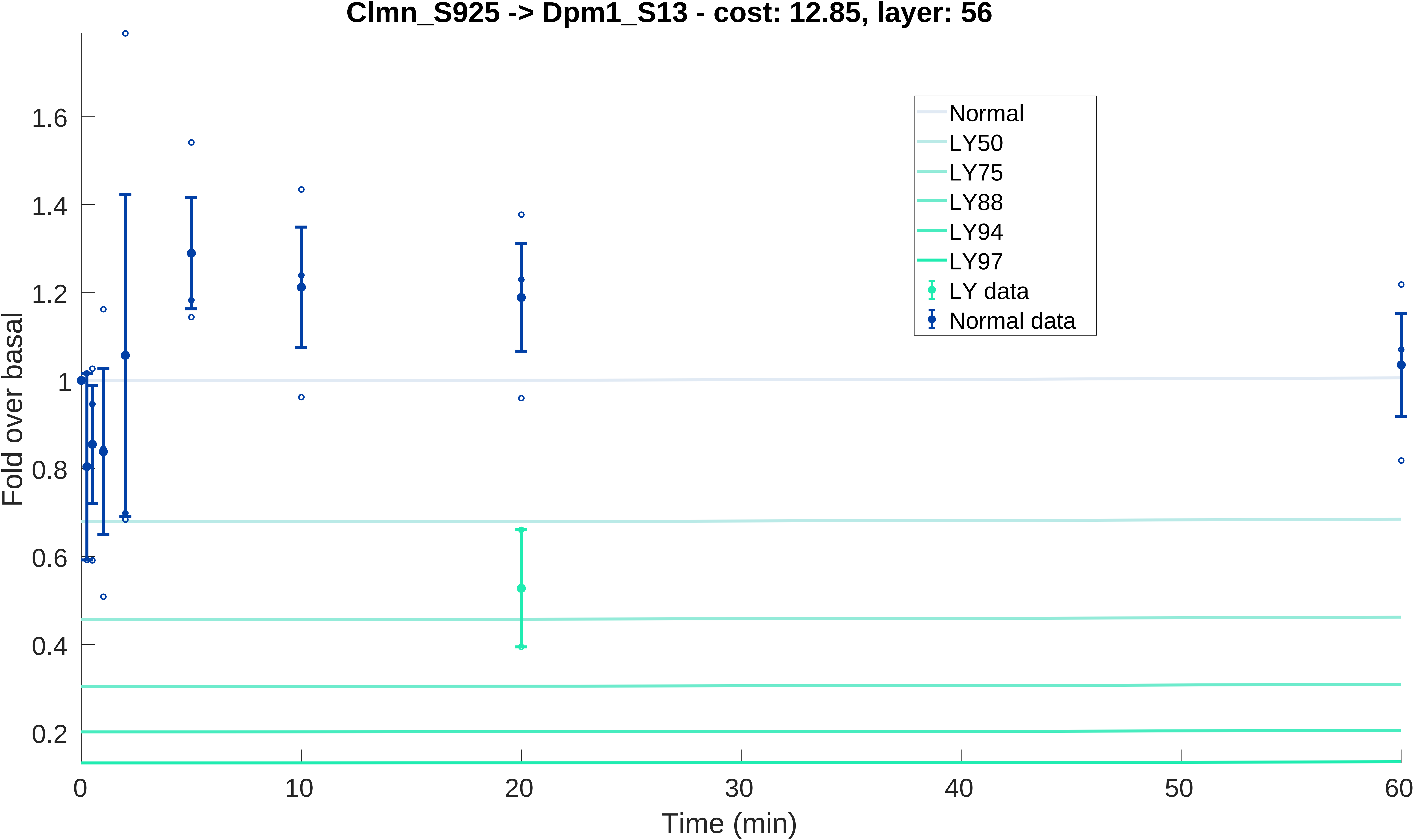
Clmn_S925 -> Dpm1_S13 - cost: 12.85, layer: 56
Fold over basal
Time (min)
0.2
0.4
0.6
0.8
1
1.2
1.4
1.6
0
10
20
30
40
50
60
Normal
LY50
LY75
LY88
LY94
LY97
LY data
Normal data

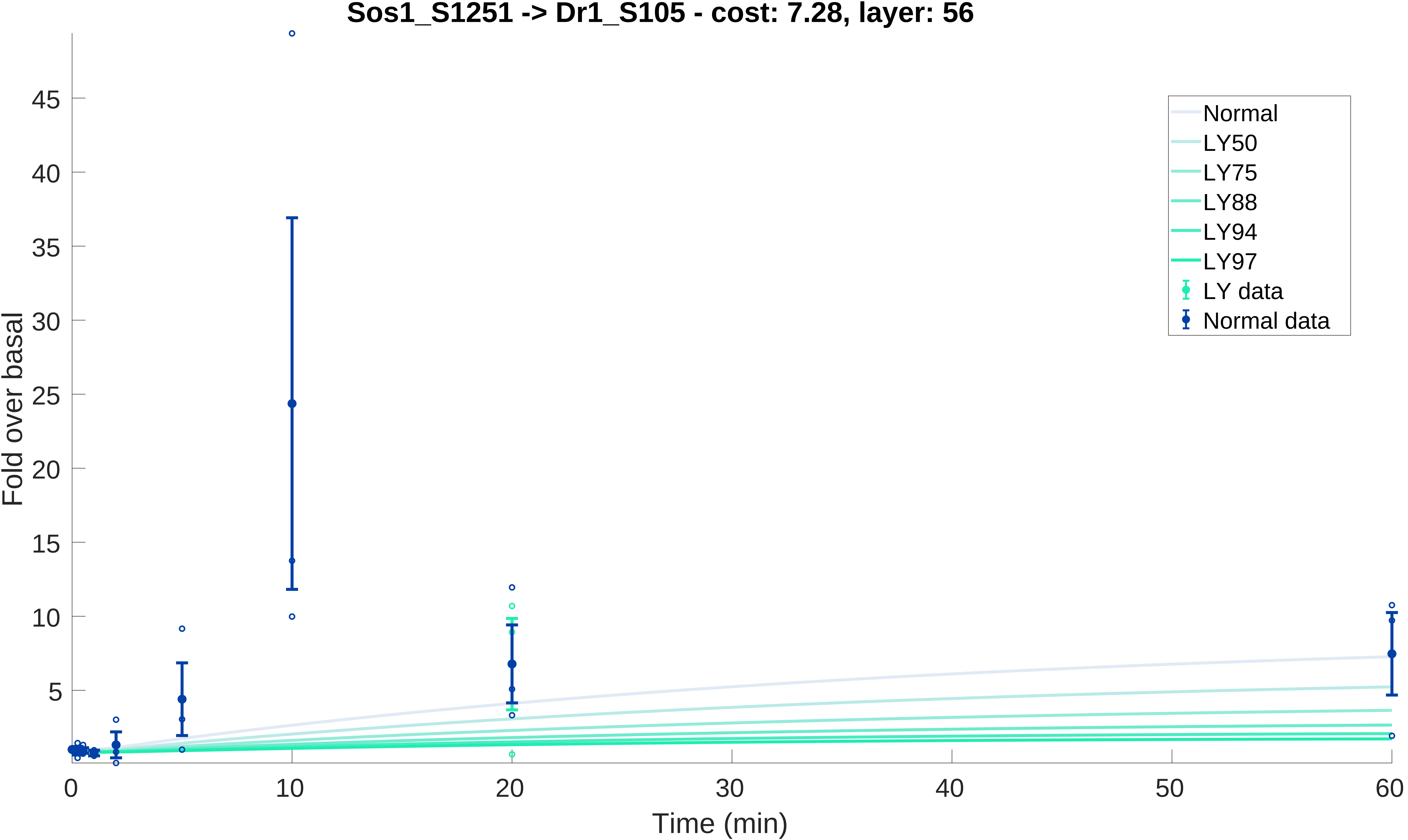
Sos1_S1251 -> Dr1_S105 - cost: 7.28, layer: 56
Fold over basal
Time (min)
0
10
20
30
40
50
60
5
10
15
20
25
30
35
40
45
Normal
LY50
LY75
LY88
LY94
LY97
LY data
Normal data

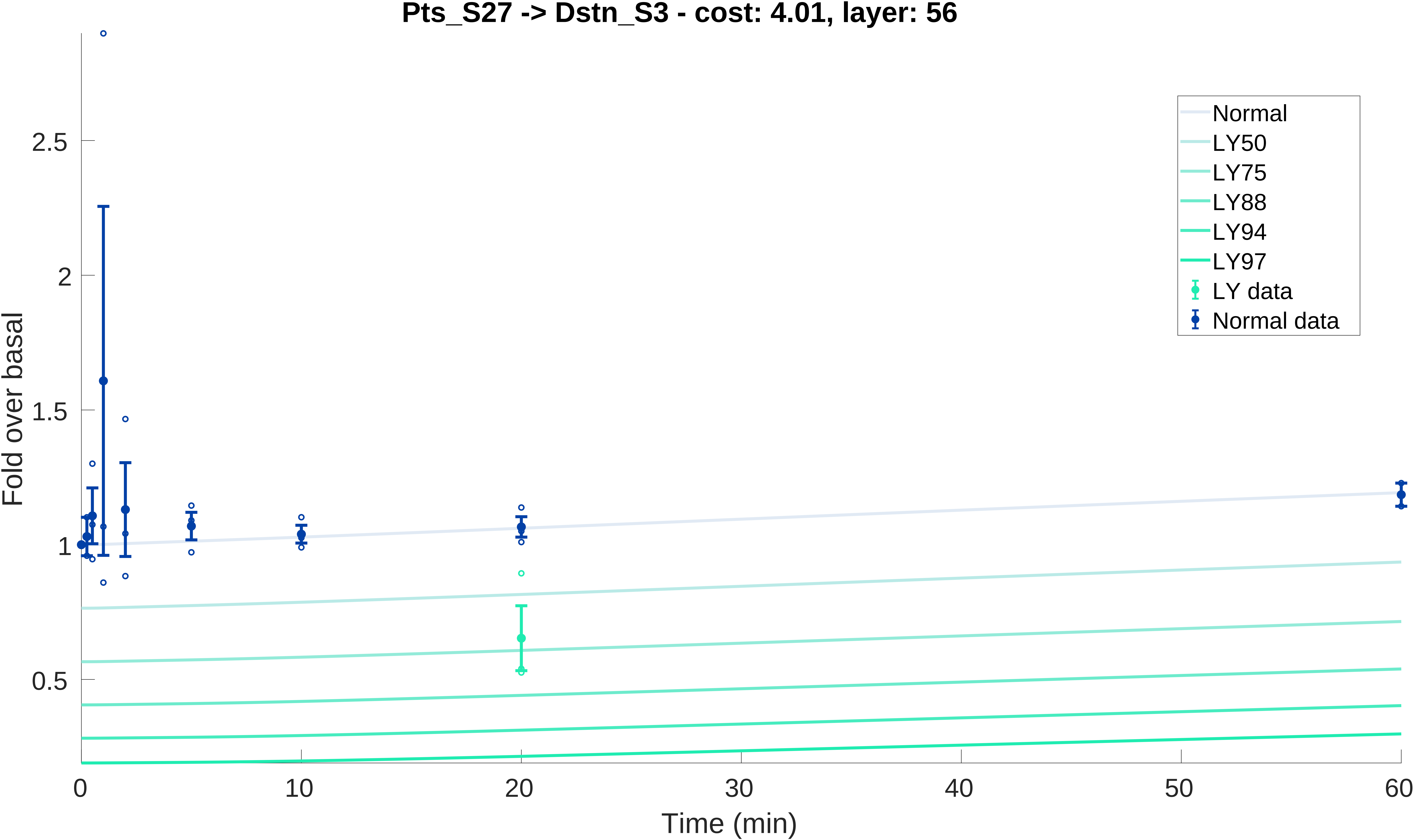

Pts_S27 -> Dstn_S3 - cost: 4.01, layer: 56
Fold over basal
2.5
2
1.5
1
0.5
0
10
20
30
40
50
60
Time (min)
Normal
LY50
LY75
LY88
LY94
LY97
LY data
Normal data

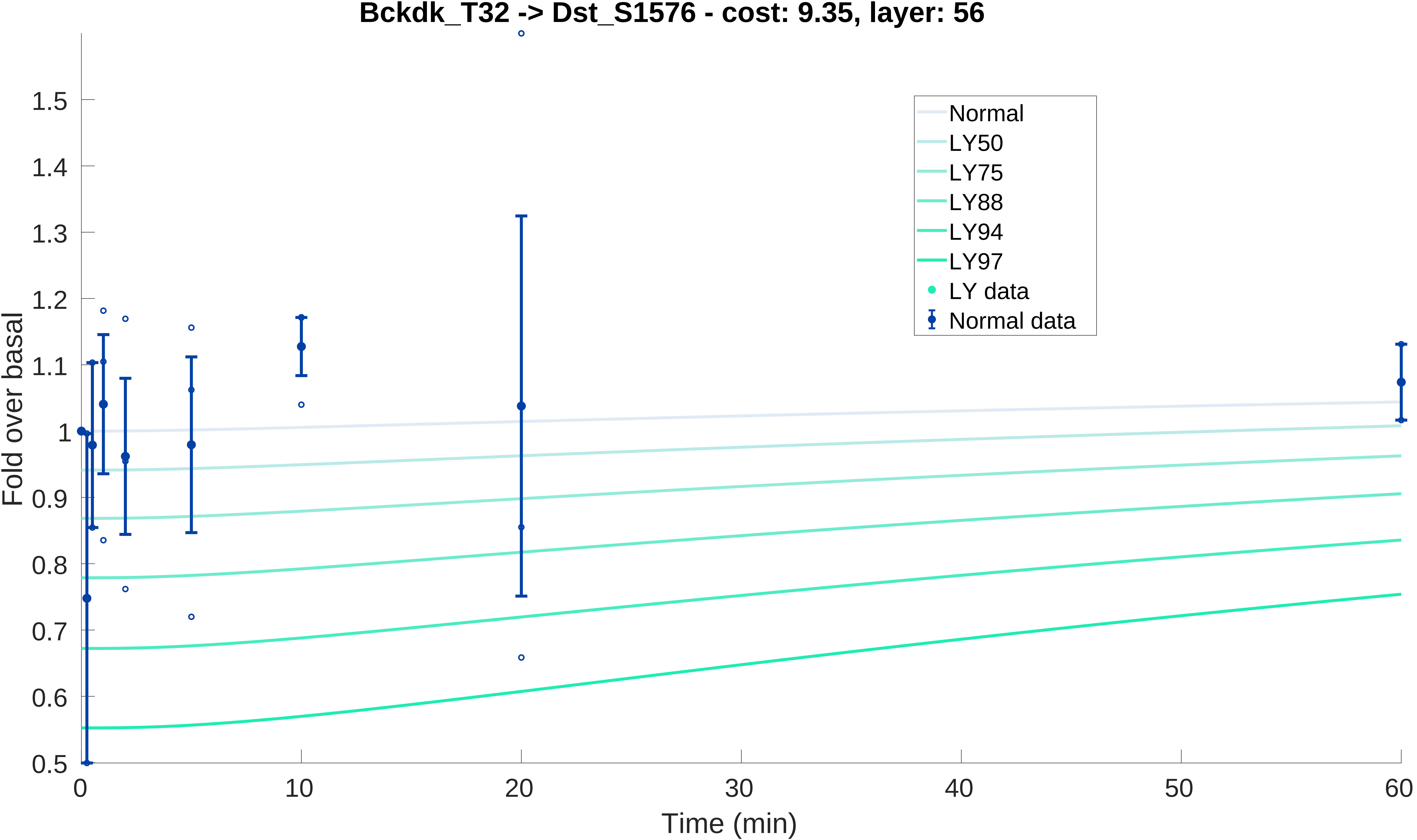

Bckdk_T32 -> Dst_S1576 - cost: 9.35, layer: 56
Normal
LY50
LY75
LY88
LY94
LY97
LY data
Normal data
Fold over basal
Time (min)
0.5
0.6
0.7
0.8
0.9
1
1.1
1.2
1.3
1.4
1.5
0
10
20
30
40
50
60

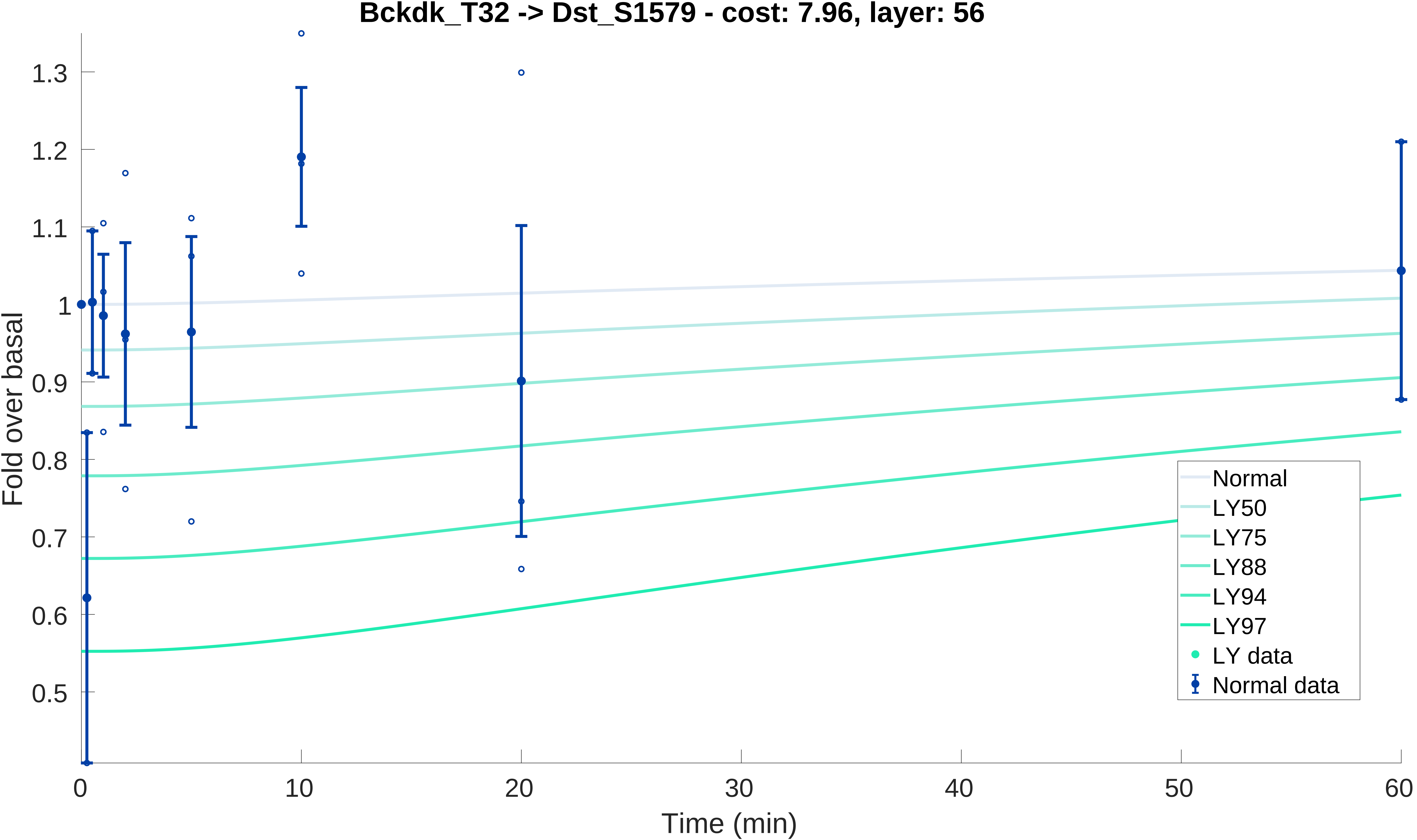

Bckdk_T32 -> Dst_S1579 - cost: 7.96, layer: 56
Fold over basal
1.3
1.2
1.1
1
0.9
0.8
0.7
0.6
0.5
0
10
20
30
40
50
60
Time (min)
Normal
LY50
LY75
LY88
LY94
LY97
LY data
Normal data

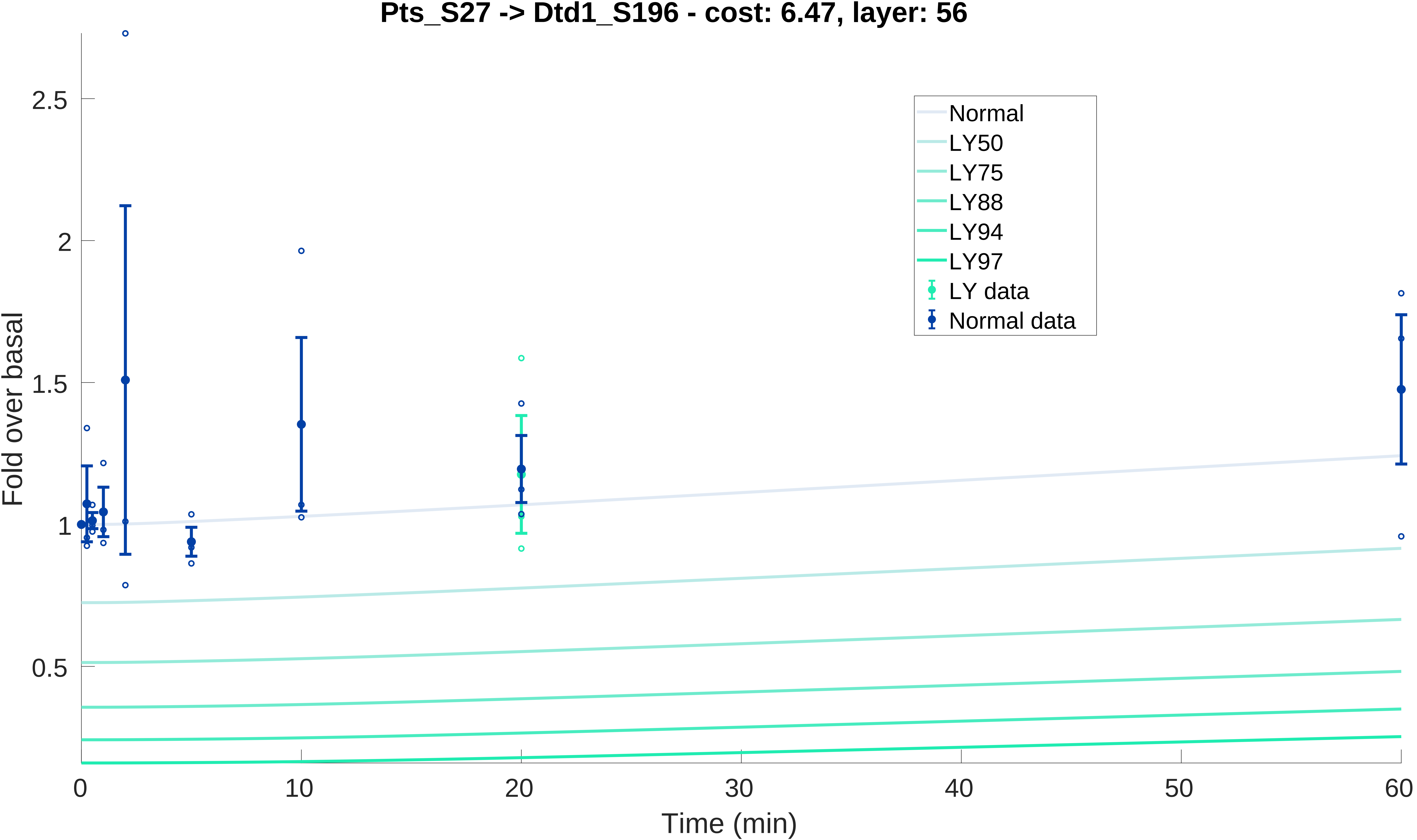

Pts_S27 -> Dtd1_S196 - cost: 6.47, layer: 56
Fold over basal
Time (min)
0.5
1
1.5
2
2.5
0
10
20
30
40
50
60
Normal
LY50
LY75
LY88
LY94
LY97
LY data
Normal data

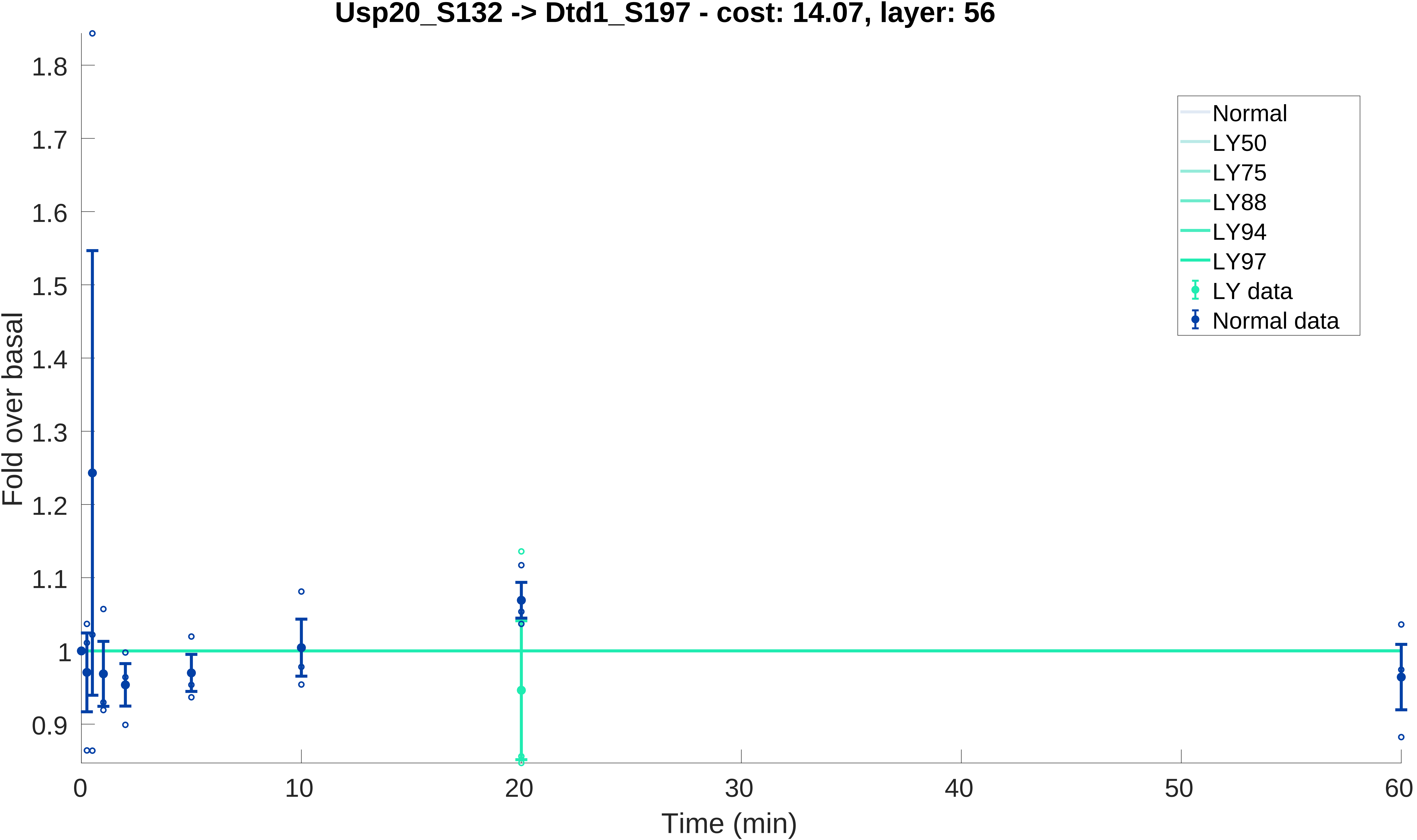

Usp20_S132 -> Dtd1_S197 - cost: 14.07, layer: 56
Fold over basal
1.8
1.7
1.6
1.5
1.4
1.3
1.2
1.1
1
0.9
Time (min)
0
10
20
30
40
50
60
Normal
LY50
LY75
LY88
LY94
LY97
LY data
Normal data

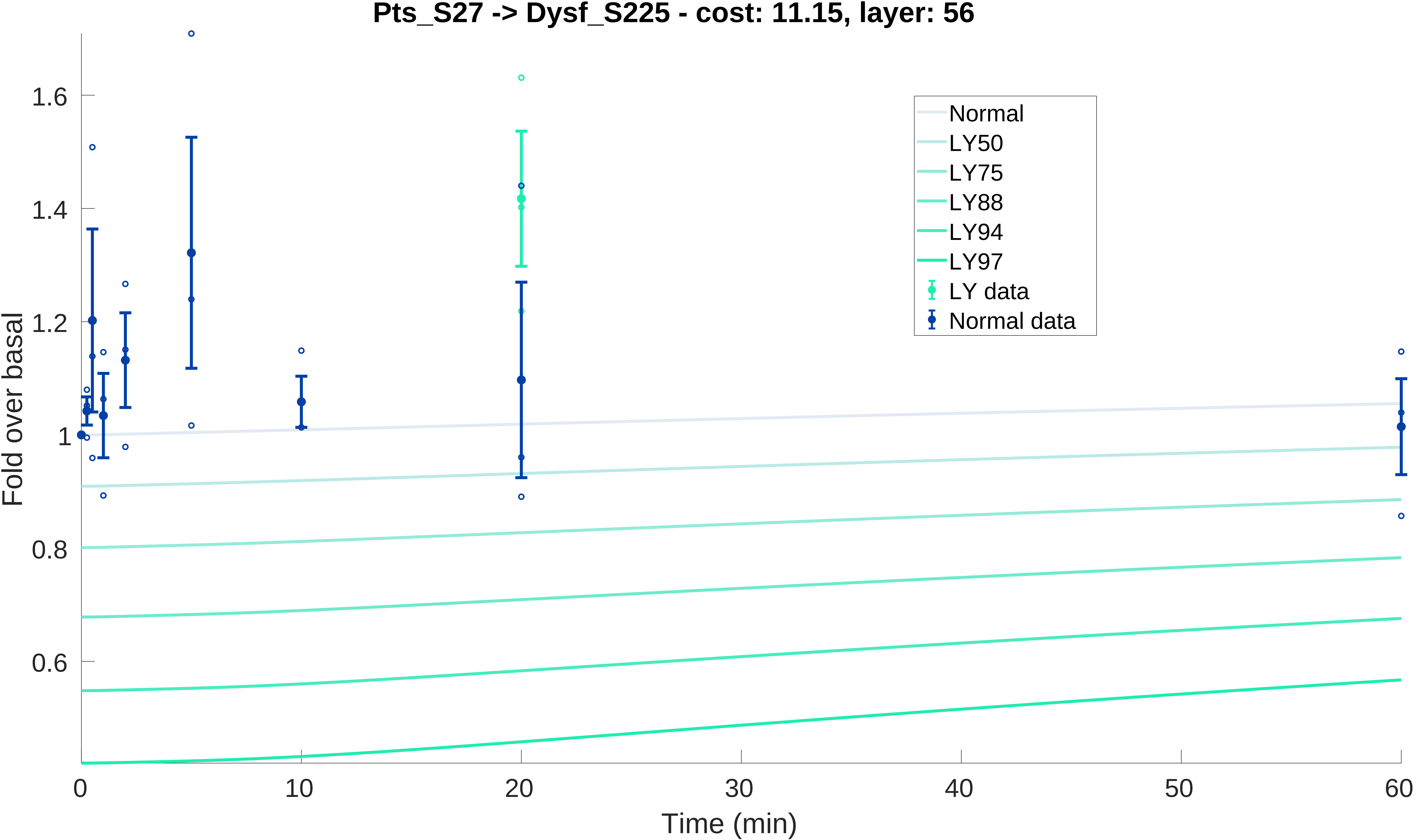

Pts_S27 -> Dysf_S225 - cost: 11.15, layer: 56
Normal
LY50
LY75
LY88
LY94
LY97
LY data
Normal data
Fold over basal
1.6
1.4
1.2
1
0.8
0.6
0
10
20
30
40
50
60
Time (min)

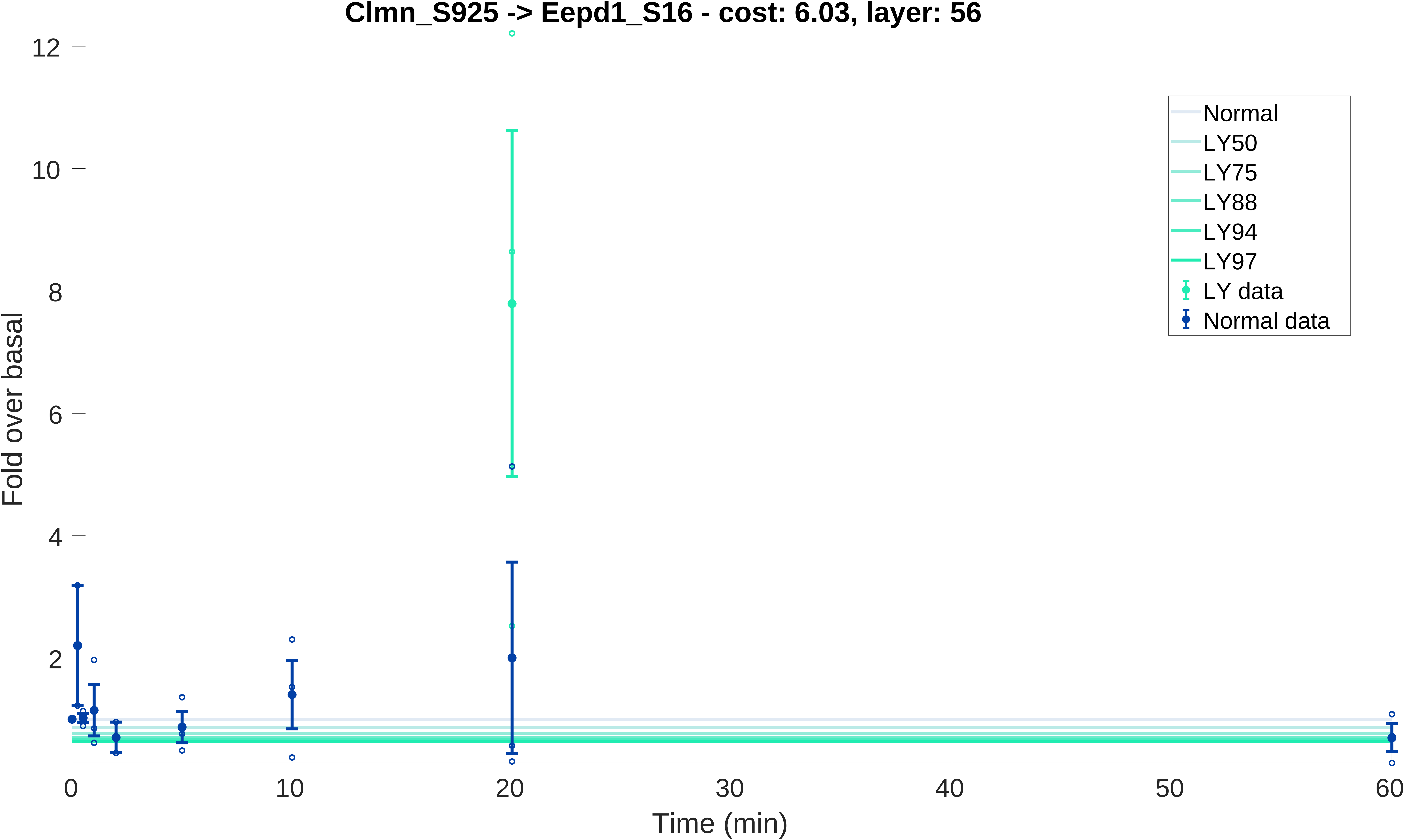
Clmn_S925 -> Eepd1_S16 - cost: 6.03, layer: 56
Fold over basal
Time (min)
0
10
20
30
40
50
60
2
4
6
8
10
12
Normal
LY50
LY75
LY88
LY94
LY97
LY data
Normal data

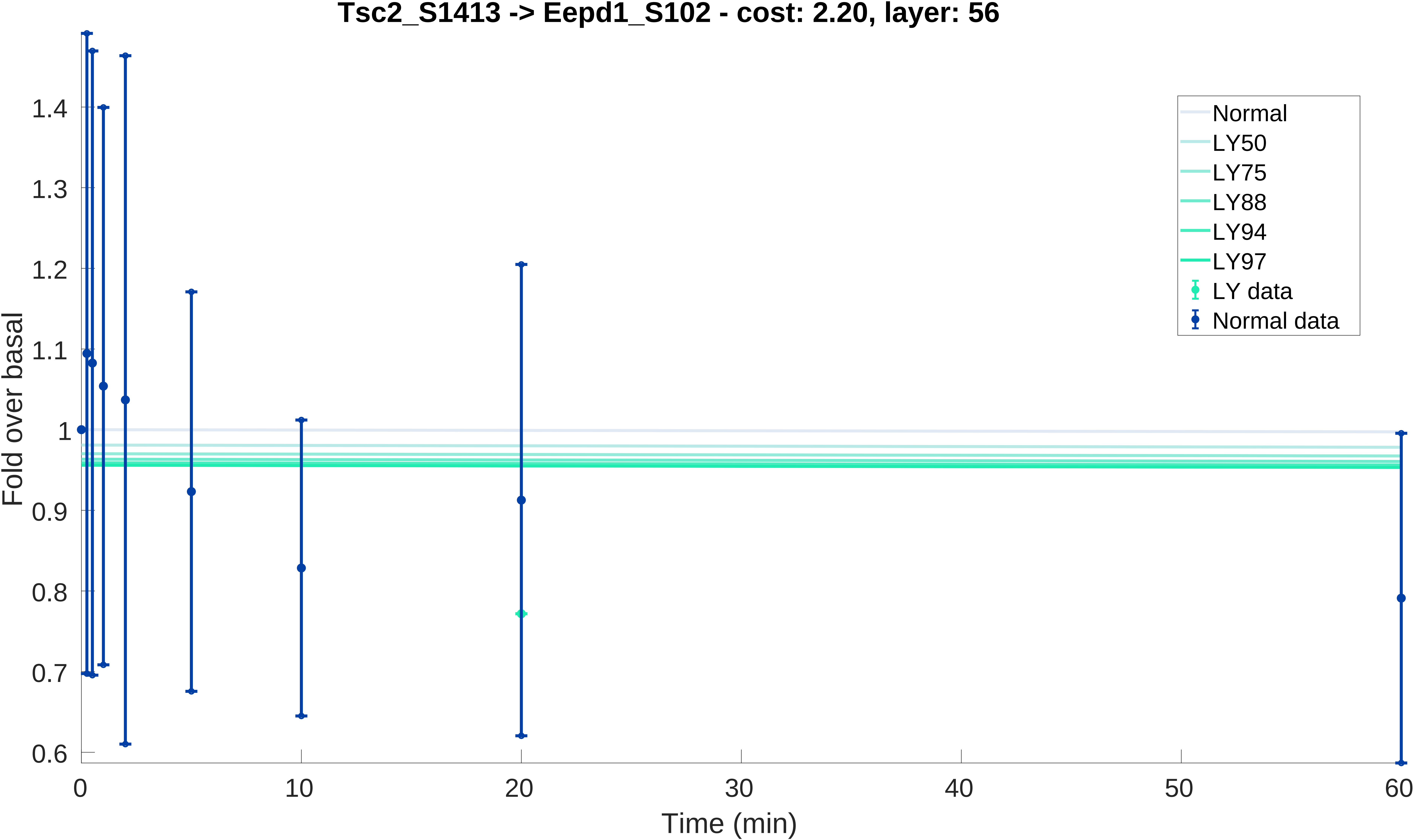
Tsc2_S1413 -> Eepd1_S102 - cost: 2.20, layer: 56
Fold over basal
Time (min)
0.6
0.7
0.8
0.9
1
1.1
1.2
1.3
1.4
0
10
20
30
40
50
60
Normal
LY50
LY75
LY88
LY94
LY97
LY data
Normal data

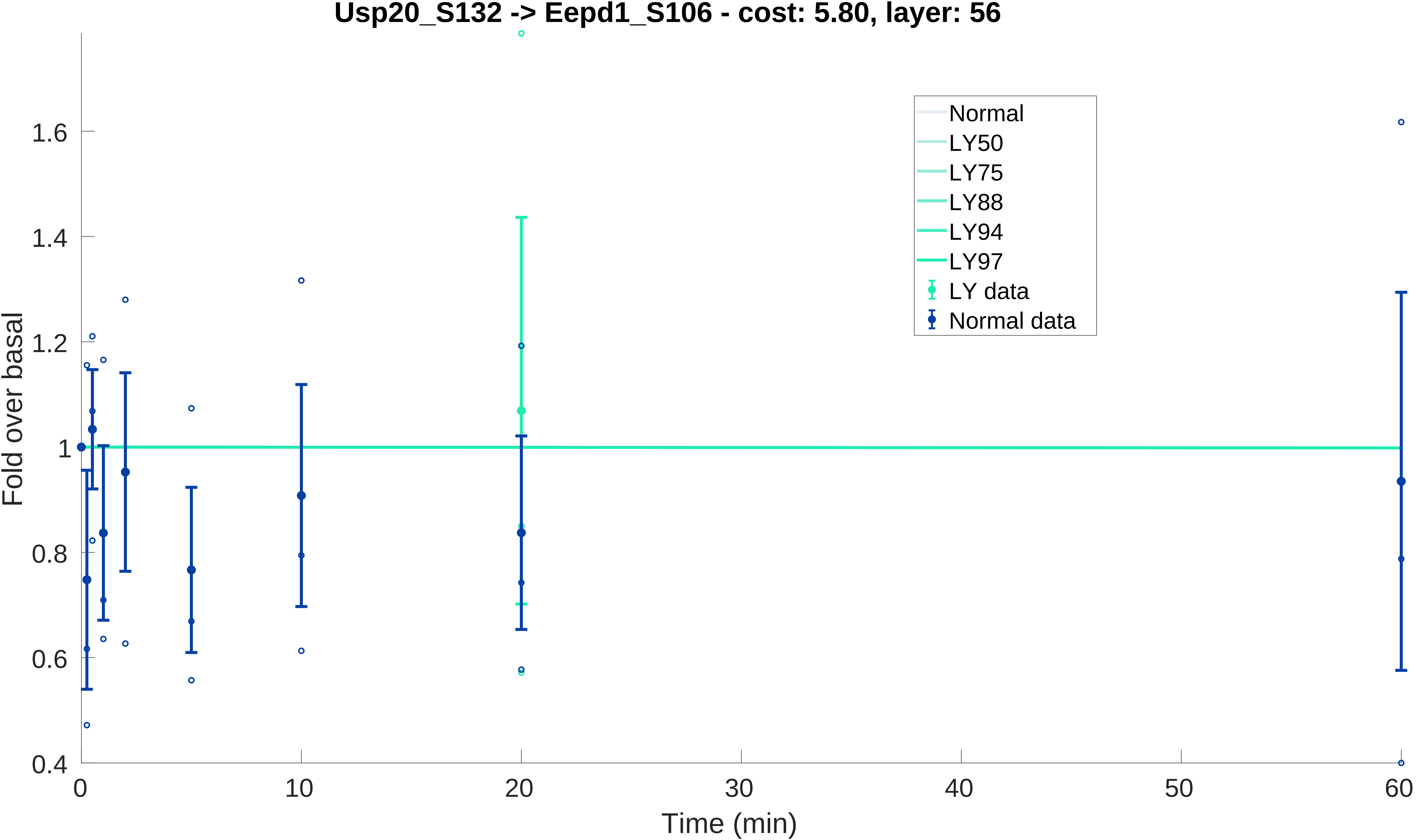

Usp20_S132 -> Eepd1_S106 - cost: 5.80, layer: 56
Normal
LY50
LY75
LY88
LY94
LY97
LY data
Normal data
Fold over basal
1.6
1.4
1.2
1
0.8
0.6
0.4
0
10
20
30
40
50
60
Time (min)

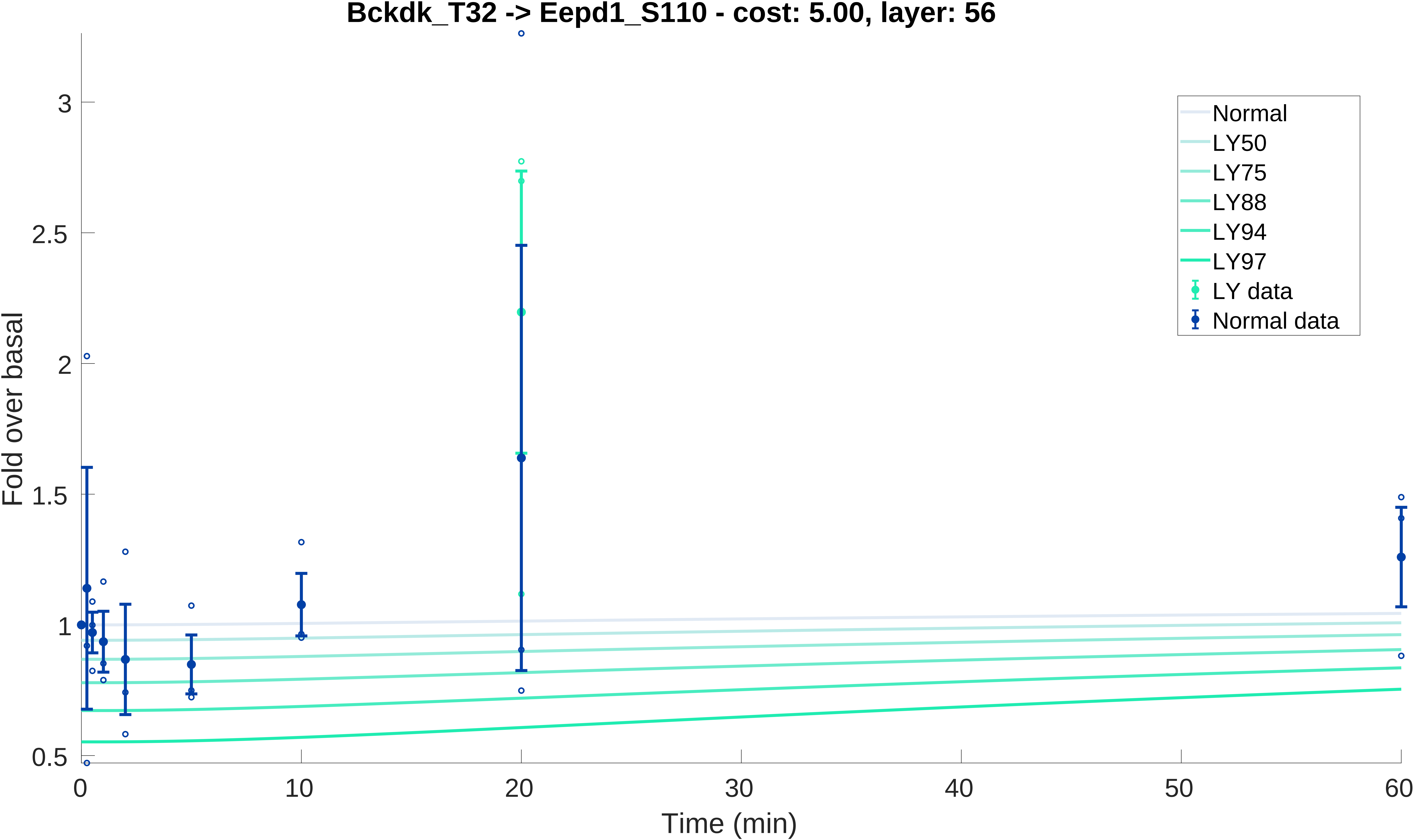

Bckdk_T32 -> Eepd1_S110 - cost: 5.00, layer: 56
Fold over basal
Time (min)
0.5
1
1.5
2
2.5
3
0
10
20
30
40
50
60
Normal
LY50
LY75
LY88
LY94
LY97
LY data
Normal data

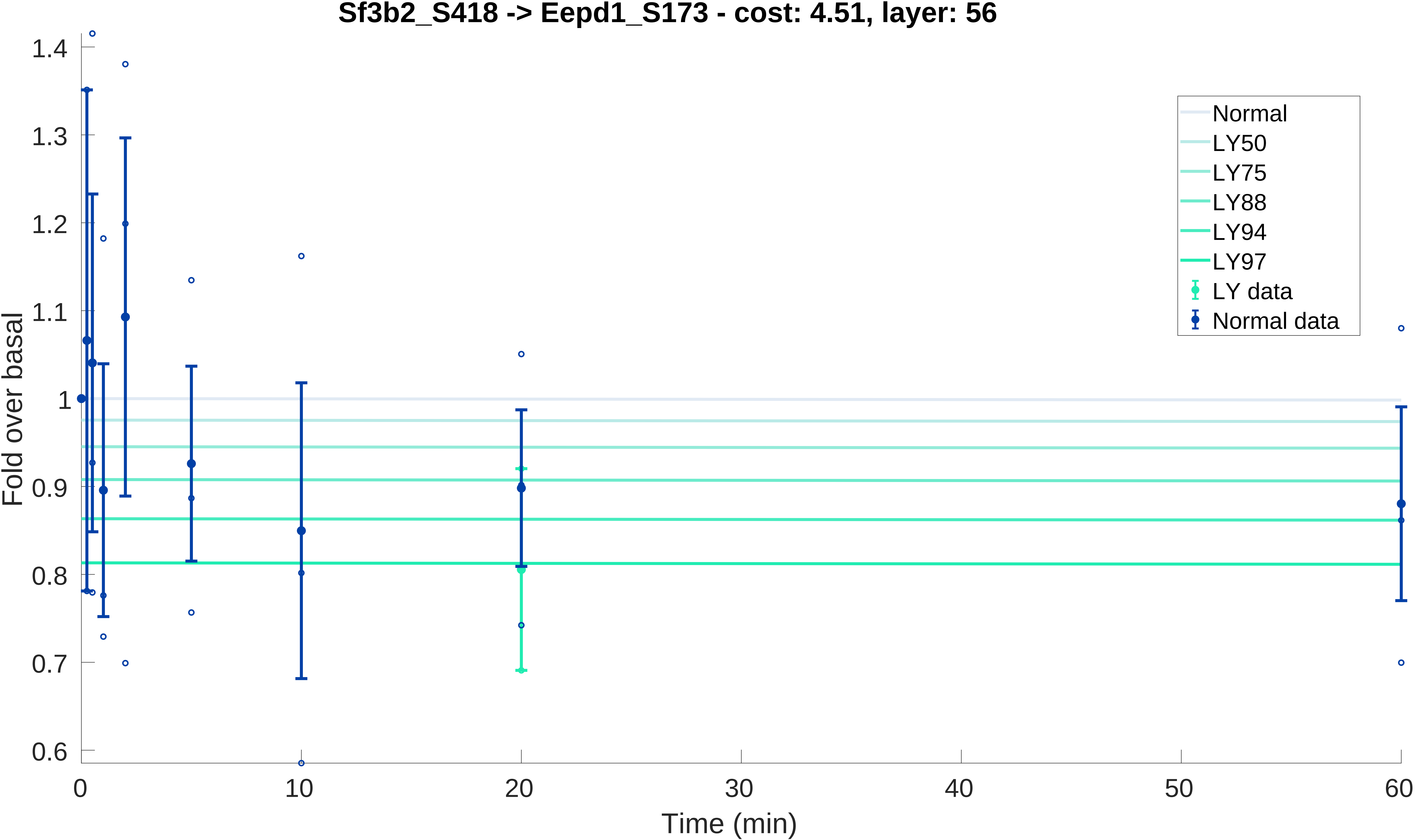
Sf3b2_S418 -> Eepd1_S173 - cost: 4.51, layer: 56
Fold over basal
1.4
1.3
1.2
1.1
1
0.9
0.8
0.7
0.6
0
10
20
30
40
50
60
Time (min)
Normal
LY50
LY75
LY88
LY94
LY97
LY data
Normal data

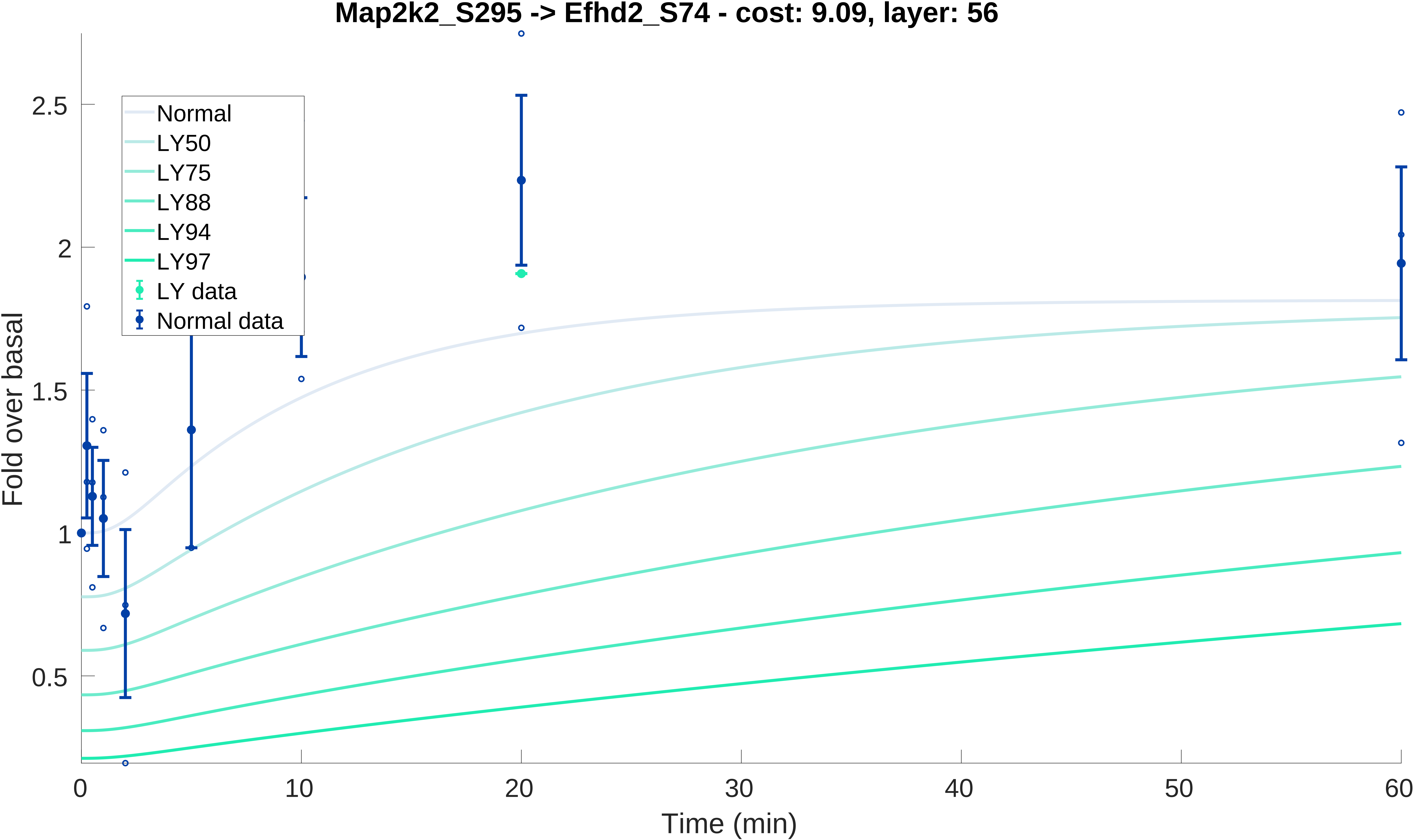

Map2k2_S295 -> Efhd2_S74 - cost: 9.09, layer: 56
Normal
LY50
LY75
LY88
LY94
LY97
LY data
Normal data
Fold over basal
Time (min)
0.5
1
1.5
2
2.5
0
10
20
30
40
50
60

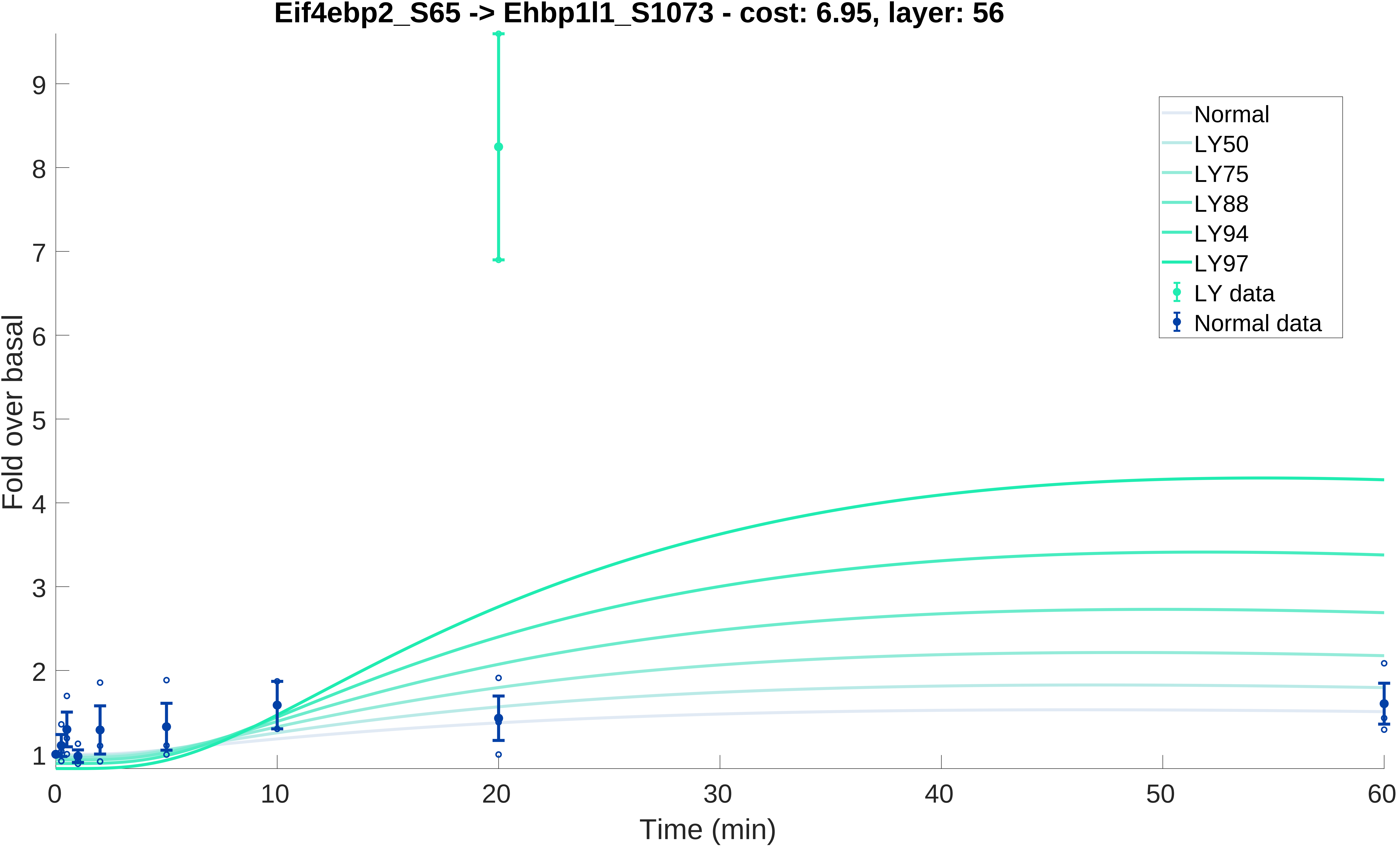

Eif4ebp2_S65 -> Ehbp1l1_S1073 - cost: 6.95, layer: 56
Fold over basal
Time (min)
1
2
3
4
5
6
7
8
9
0
10
20
30
40
50
60
Normal
LY50
LY75
LY88
LY94
LY97
LY data
Normal data

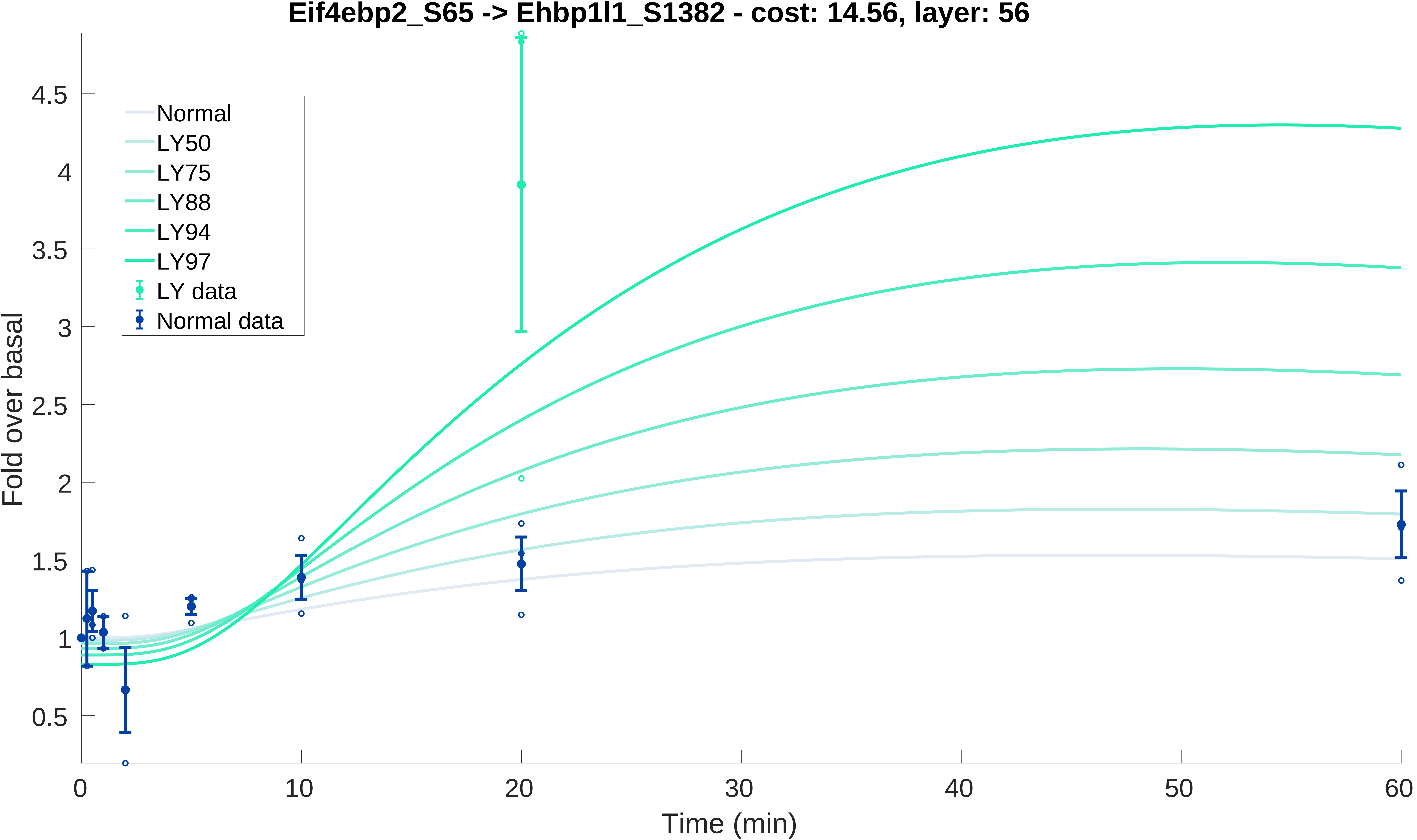

Eif4ebp2_S65 -> Ehbp1l1_S1382 - cost: 14.56, layer: 56
Normal
LY50
LY75
LY88
LY94
LY97
LY data
Normal data
Fold over basal
Time (min)
0
10
20
30
40
50
60
0.5
1
1.5
2
2.5
3
3.5
4
4.5

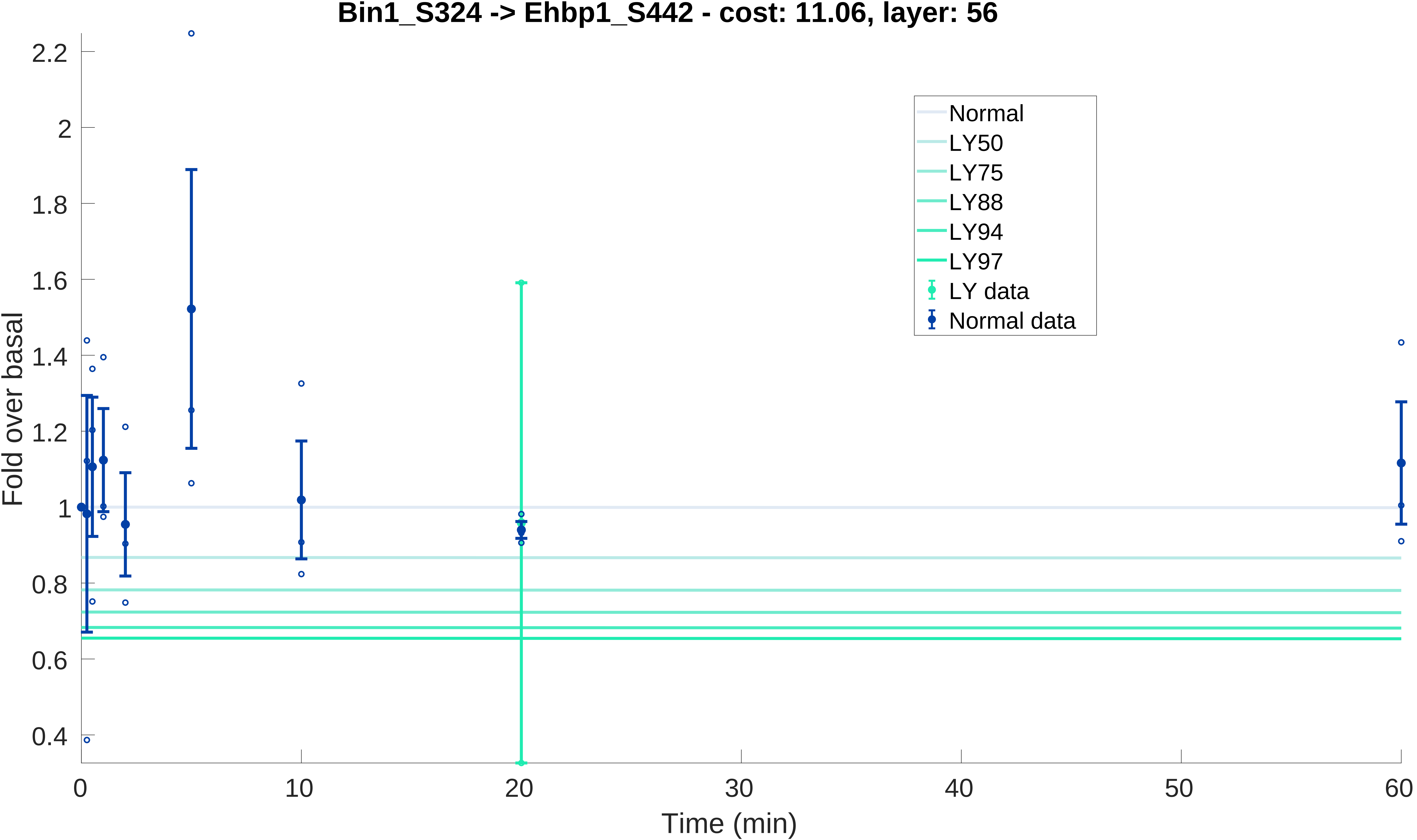

Bin1_S324 -> Ehbp1_S442 - cost: 11.06, layer: 56
Fold over basal
Time (min)
0.4
0.6
0.8
1
1.2
1.4
1.6
1.8
2
2.2
0
10
20
30
40
50
60
Normal
LY50
LY75
LY88
LY94
LY97
LY data
Normal data

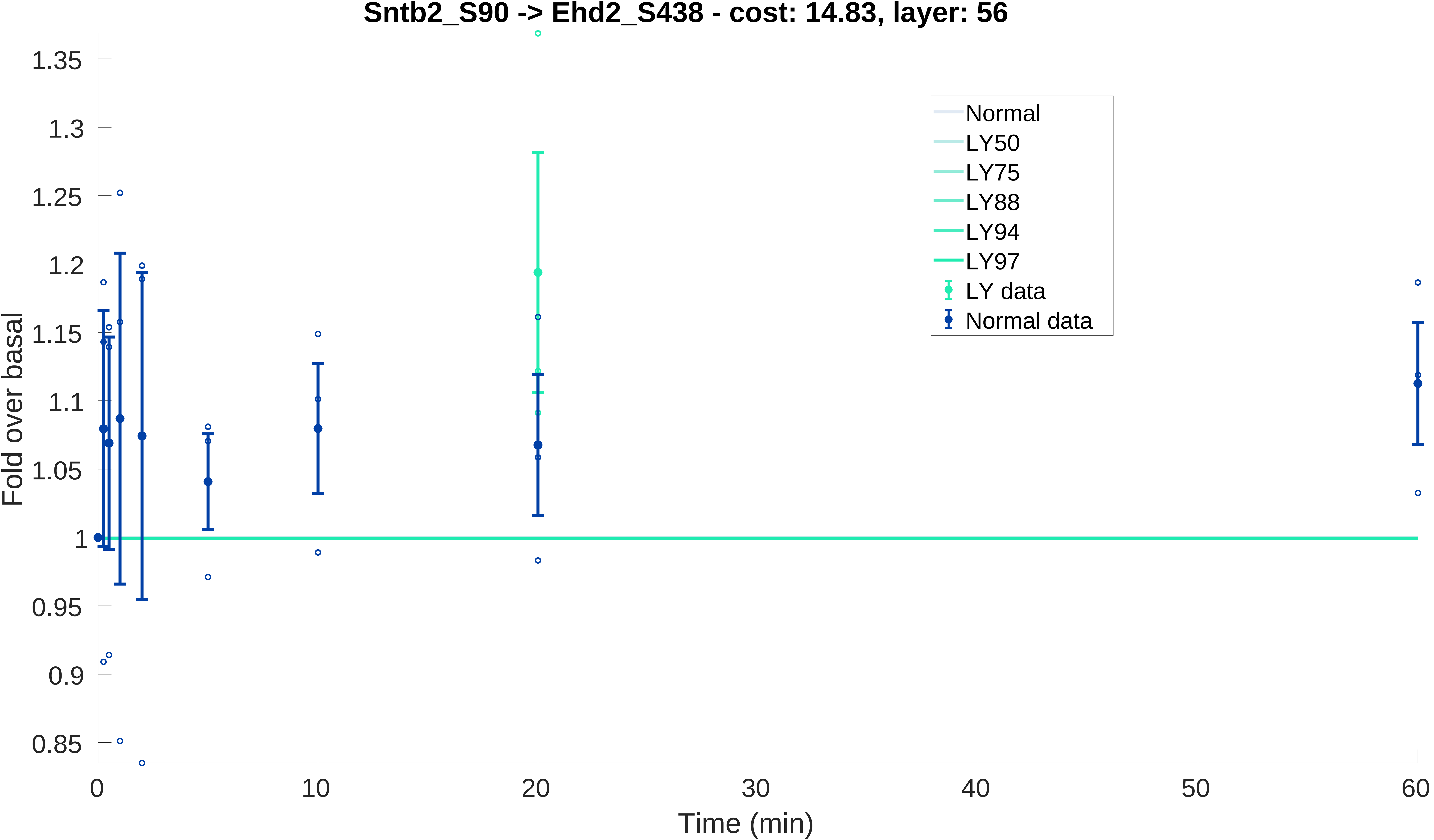

Sntb2_S90 -> Ehd2_S438 - cost: 14.83, layer: 56
Fold over basal
Time (min)
1.35
1.3
1.25
1.2
1.15
1.1
1.05
1
0.95
0.9
0.85
0
10
20
30
40
50
60
Normal
LY50
LY75
LY88
LY94
LY97
LY data
Normal data

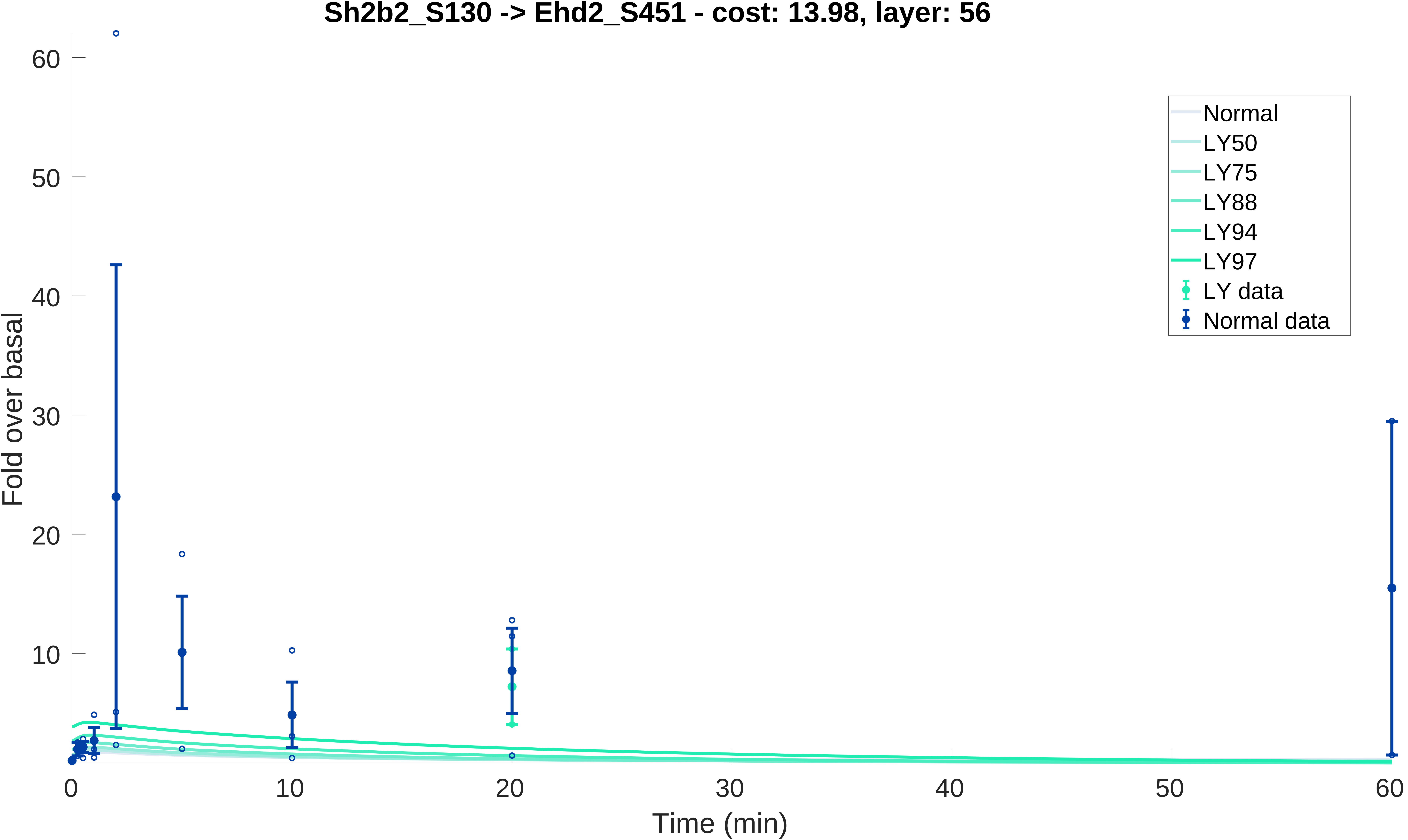

Sh2b2_S130 -> Ehd2_S451 - cost: 13.98, layer: 56
Fold over basal
Time (min)
0
10
20
30
40
50
60
Normal
LY50
LY75
LY88
LY94
LY97
LY data
Normal data

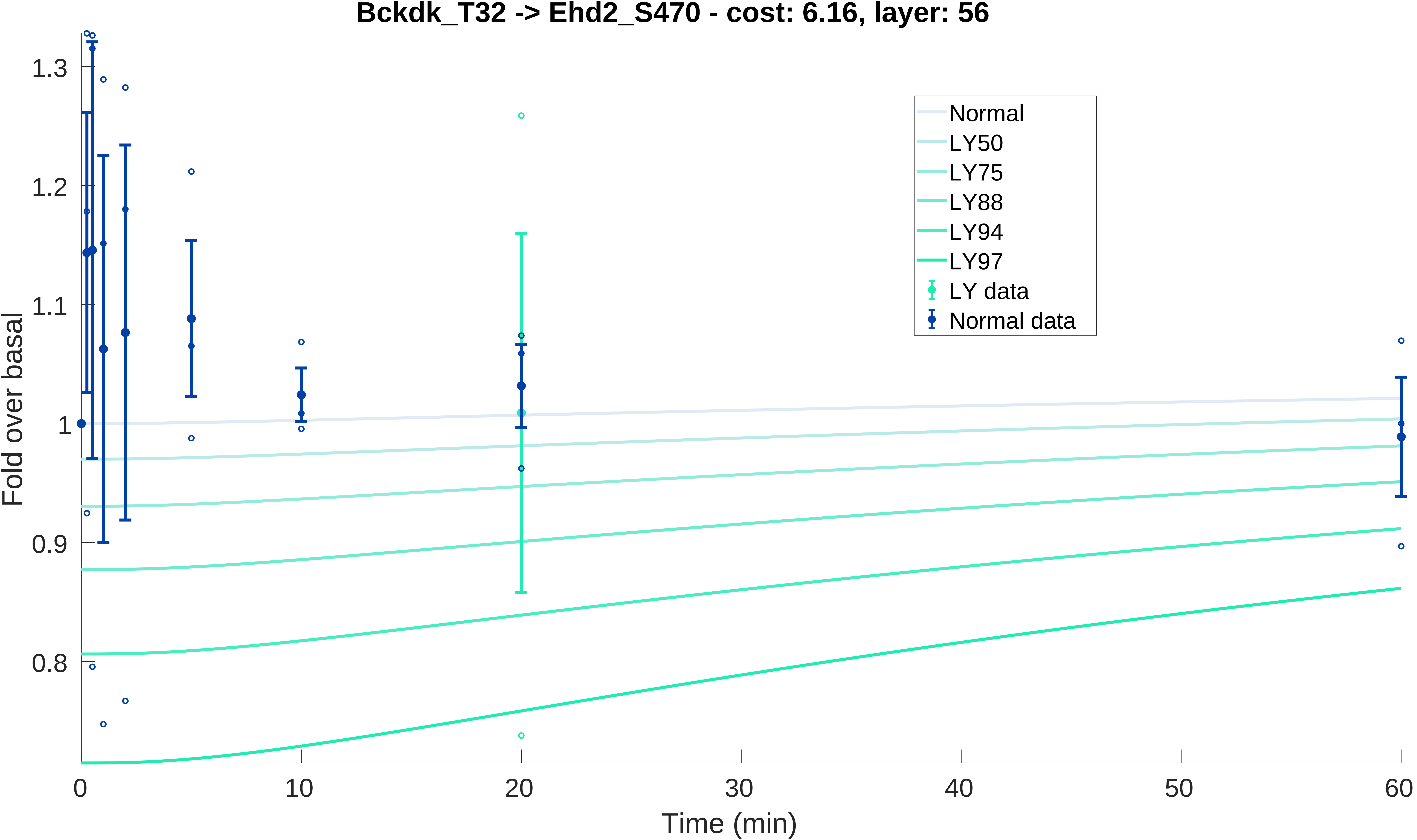
Bckdk_T32 -> Ehd2_S470 - cost: 6.16, layer: 56
Normal
LY50
LY75
LY88
LY94
LY97
LY data
Normal data
Fold over basal
Time (min)
1.3
1.2
1.1
1
0.9
0.8
0
10
20
30
40
50
60

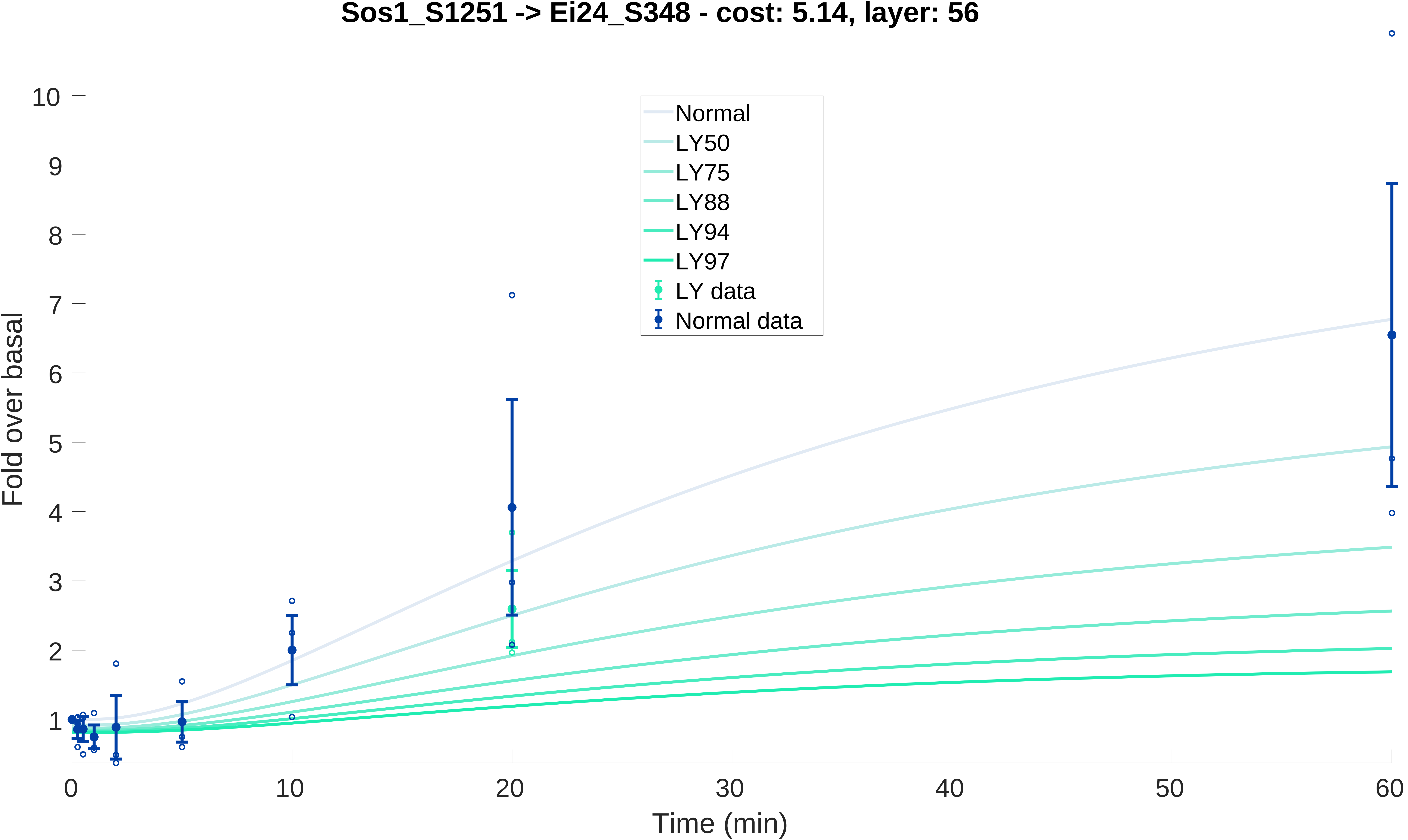

Sos1_S1251 -> Ei24_S348 - cost: 5.14, layer: 56
Normal
LY50
LY75
LY88
LY94
LY97
LY data
Normal data
Fold over basal
Time (min)
1
2
3
4
5
6
7
8
9
10
0
10
20
30
40
50
60

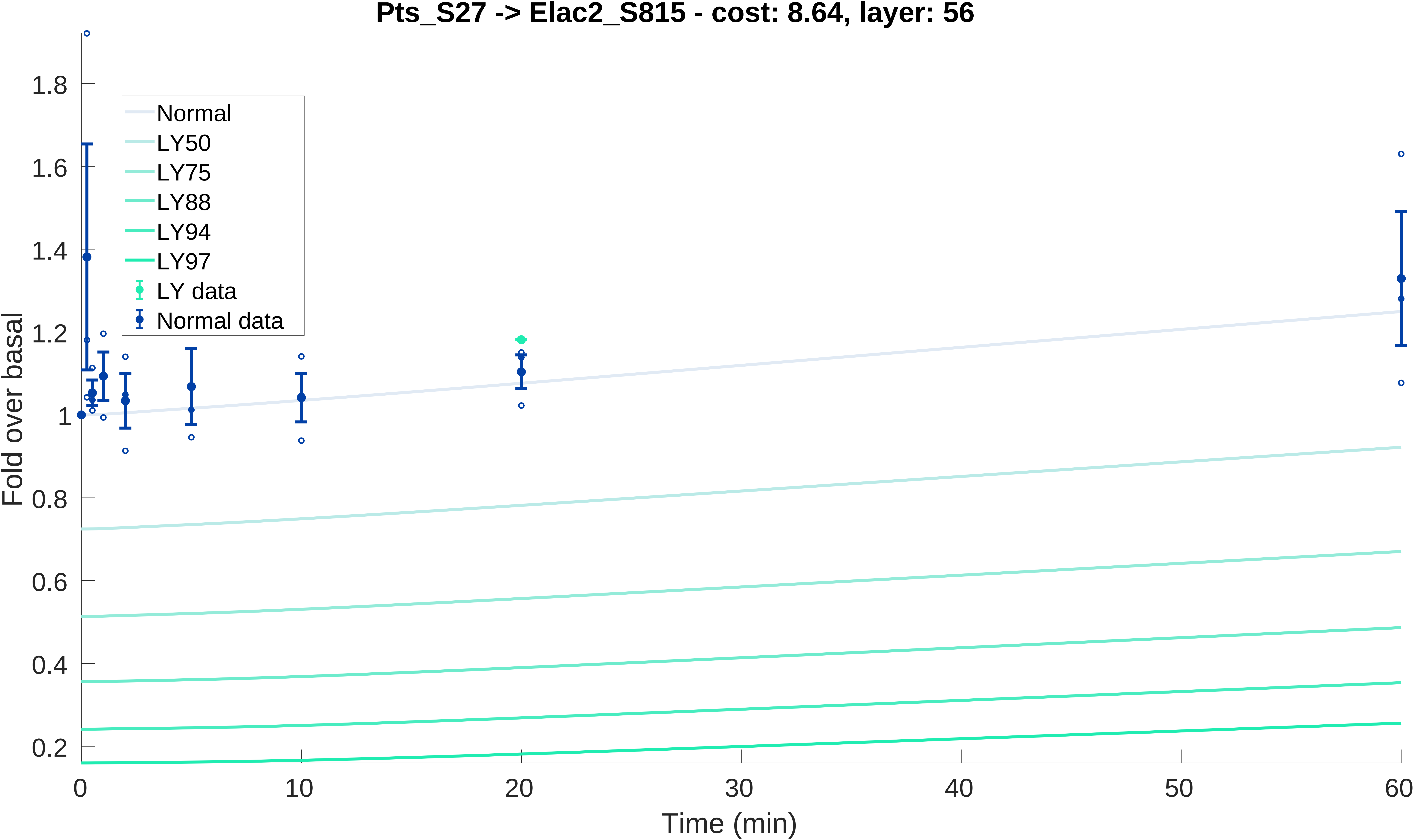
Pts_S27 -> Elac2_S815 - cost: 8.64, layer: 56
Fold over basal
1.8
1.6
1.4
1.2
1
0.8
0.6
0.4
0.2
Normal
LY50
LY75
LY88
LY94
LY97
LY data
Normal data
0
10
20
30
40
50
60
Time (min)

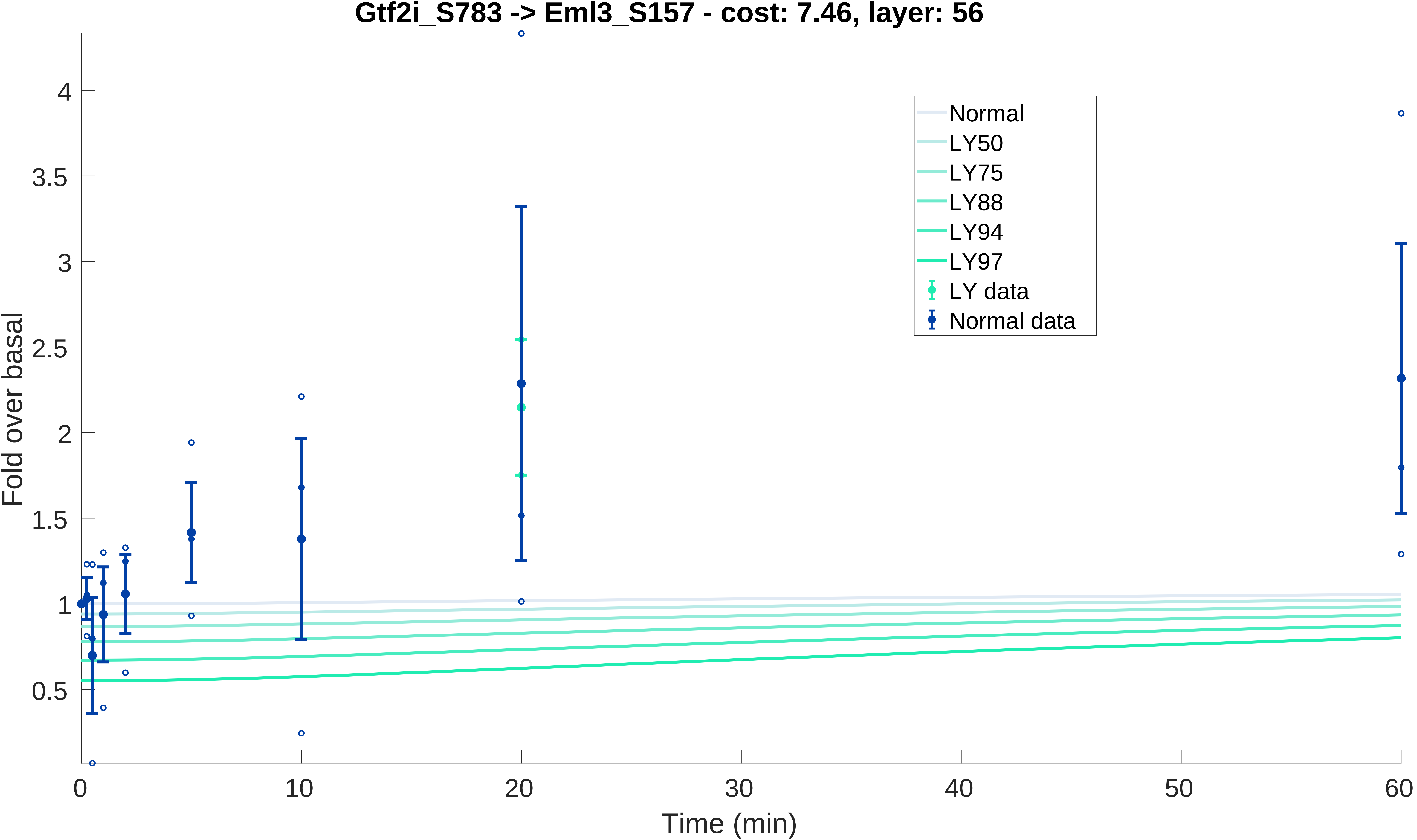

Gtf2i_S783 -> Eml3_S157 - cost: 7.46, layer: 56
Normal
LY50
LY75
LY88
LY94
LY97
LY data
Normal data
Fold over basal
Time (min)
0
10
20
30
40
50
60
0.5
1
1.5
2
2.5
3
3.5
4

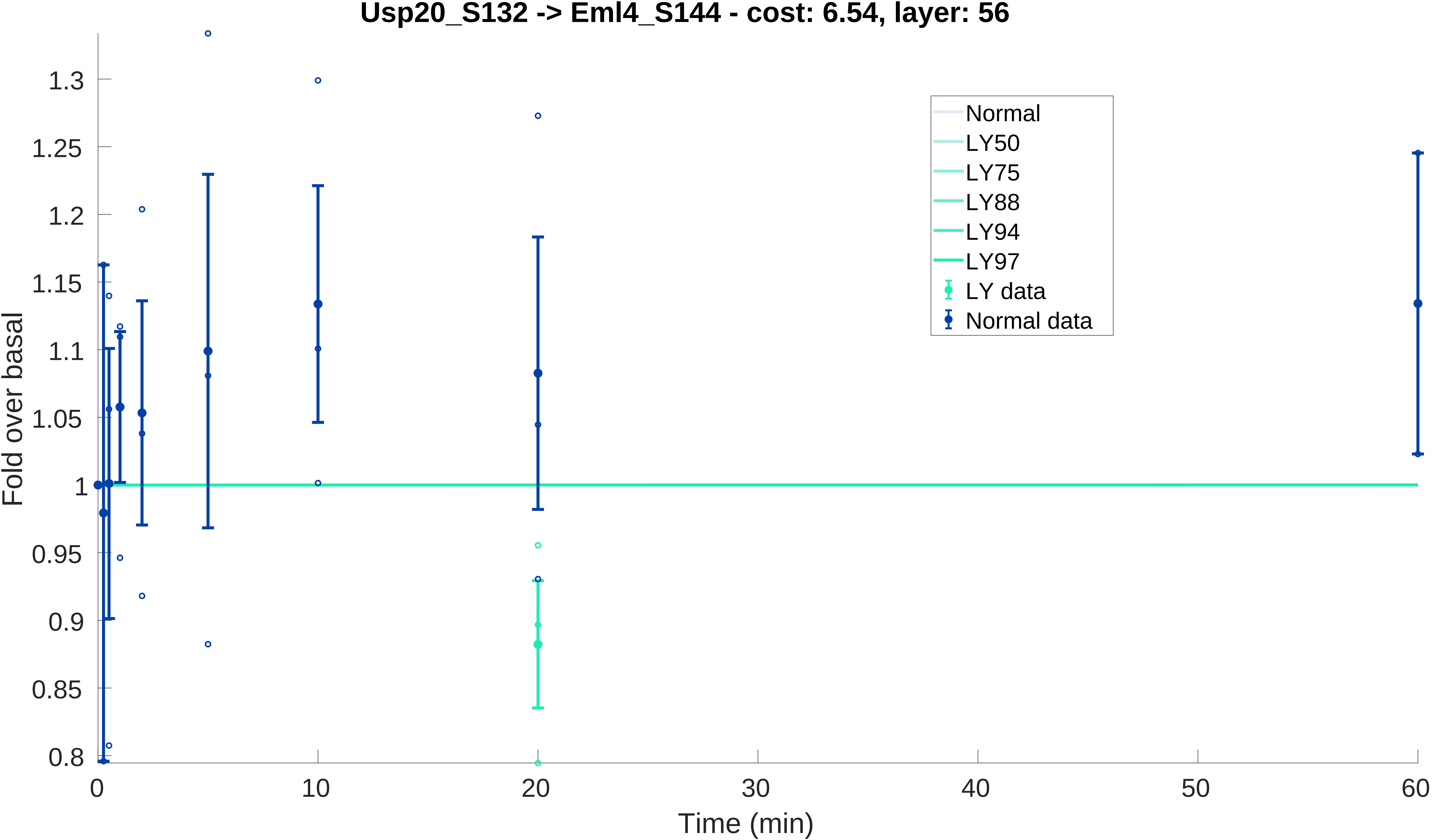

Usp20_S132 -> Eml4_S144 - cost: 6.54, layer: 56
Fold over basal
Time (min)
1.3
1.25
1.2
1.15
1.1
1.05
1
0.95
0.9
0.85
0.8
0
10
20
30
40
50
60
Normal
LY50
LY75
LY88
LY94
LY97
LY data
Normal data

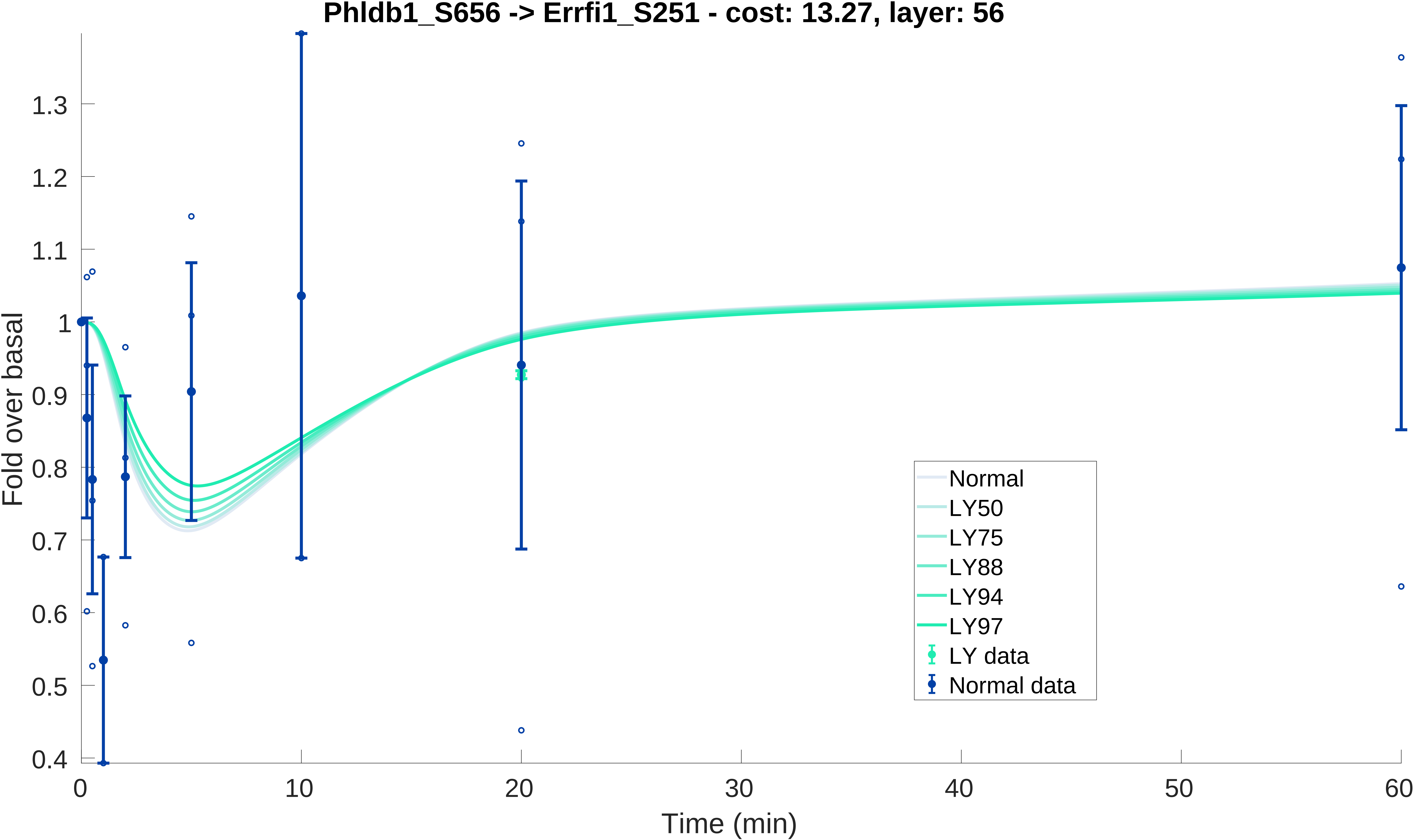

Phldb1_S656 -> Errfi1_S251 - cost: 13.27, layer: 56
Fold over basal
Time (min)
1.3
1.2
1.1
1
0.9
0.8
0.7
0.6
0.5
0.4
0
10
20
30
40
50
60
Normal
LY50
LY75
LY88
LY94
LY97
LY data
Normal data

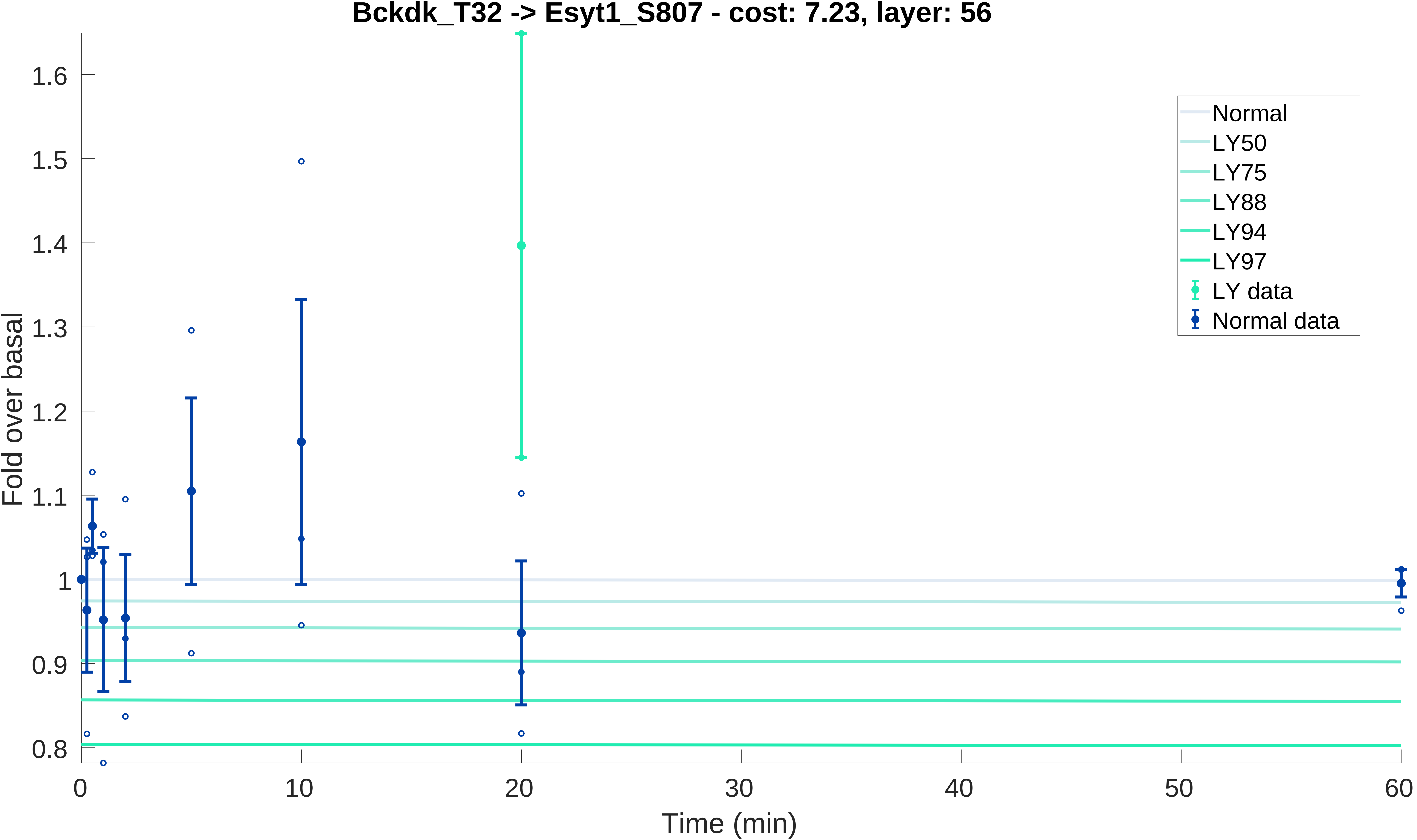

Bckdk_T32 -> Esyt1_S807 - cost: 7.23, layer: 56
Fold over basal
1.6
1.5
1.4
1.3
1.2
1.1
1
0.9
0.8
0
10
20
30
40
50
60
Time (min)
Normal
LY50
LY75
LY88
LY94
LY97
LY data
Normal data

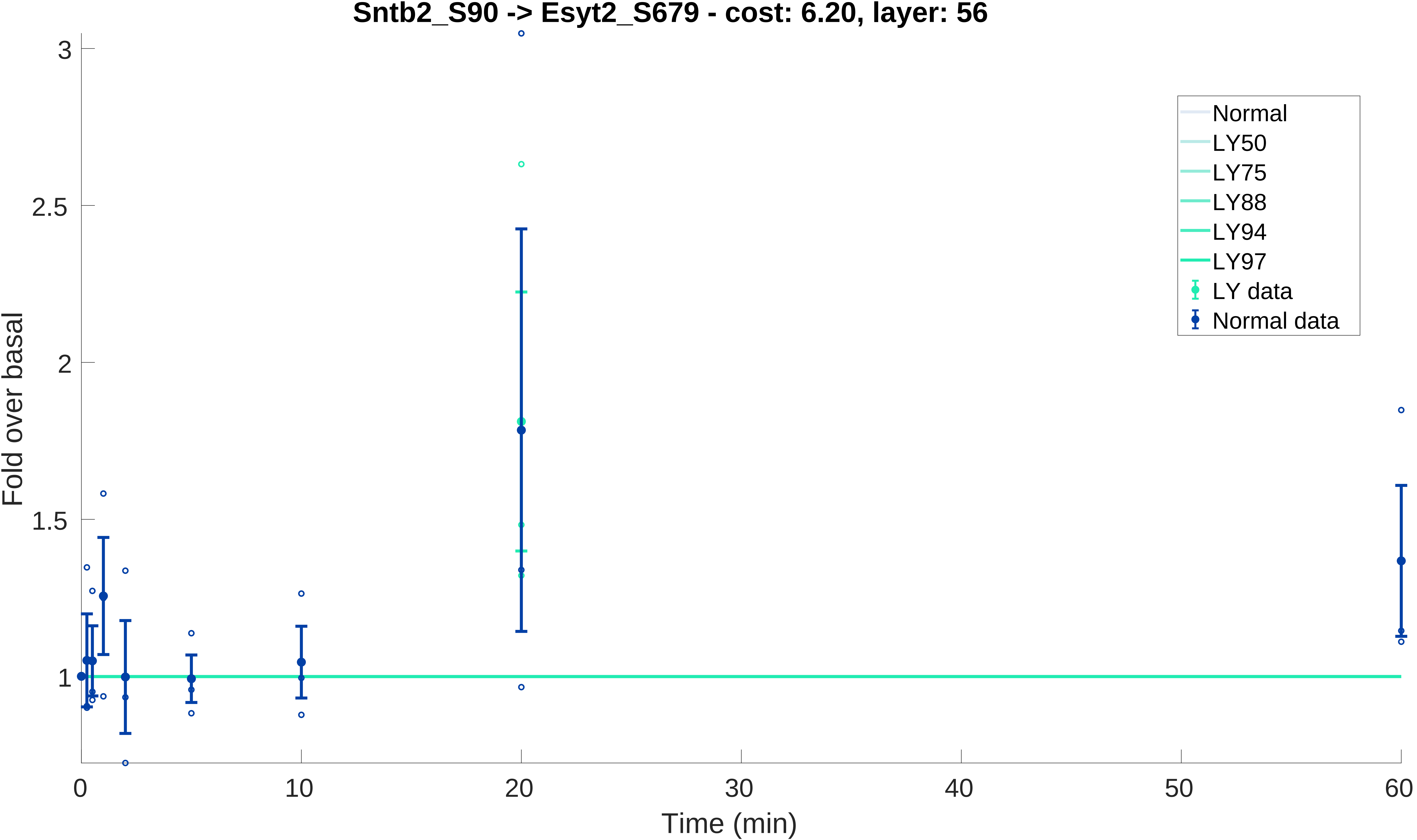

Sntb2_S90 -> Esyt2_S679 - cost: 6.20, layer: 56
Fold over basal
Time (min)
Normal
LY50
LY75
LY88
LY94
LY97
LY data
Normal data

Pts_S27 -> Esyt2_S682 - cost: 3.43, layer: 56

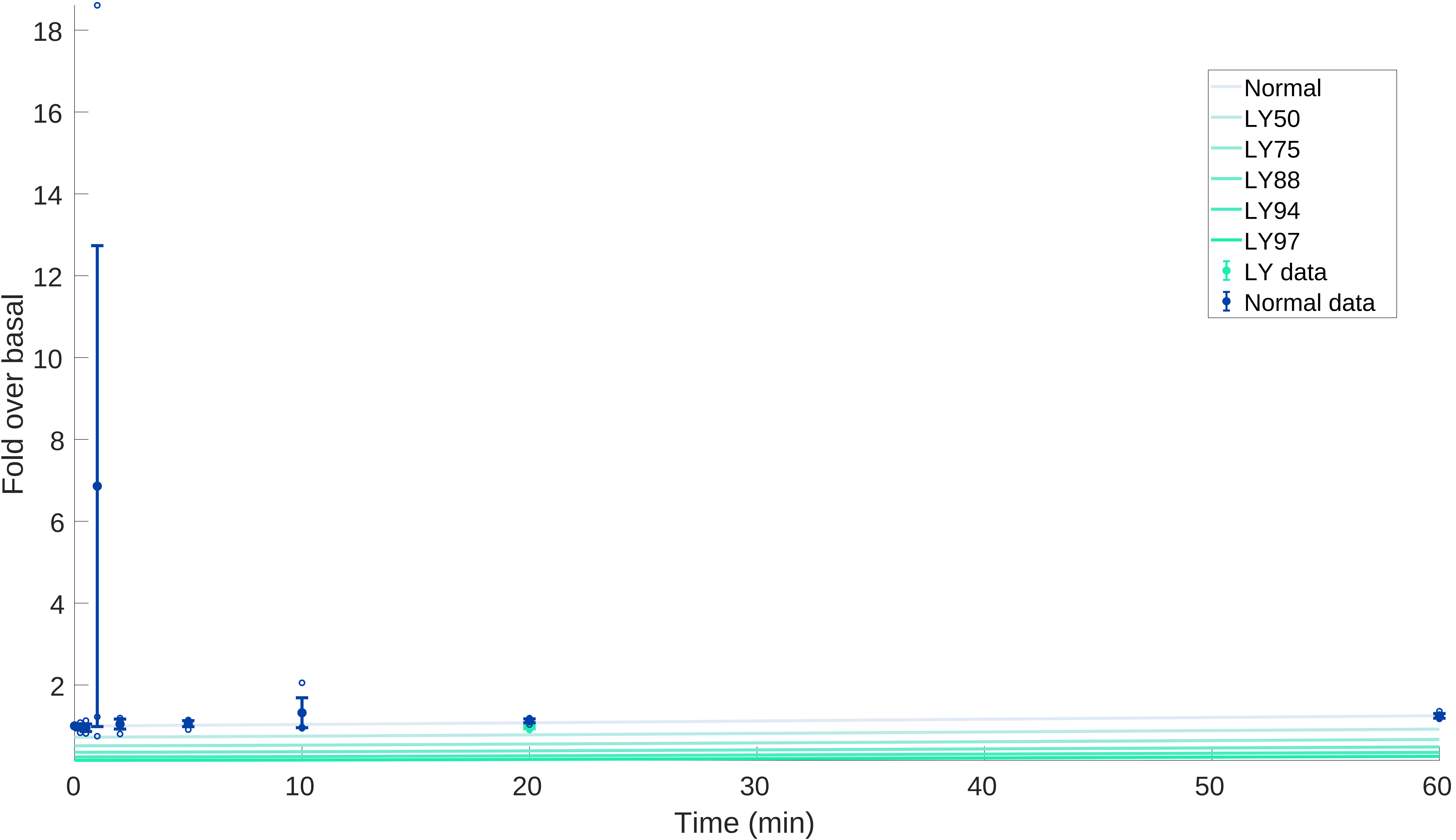

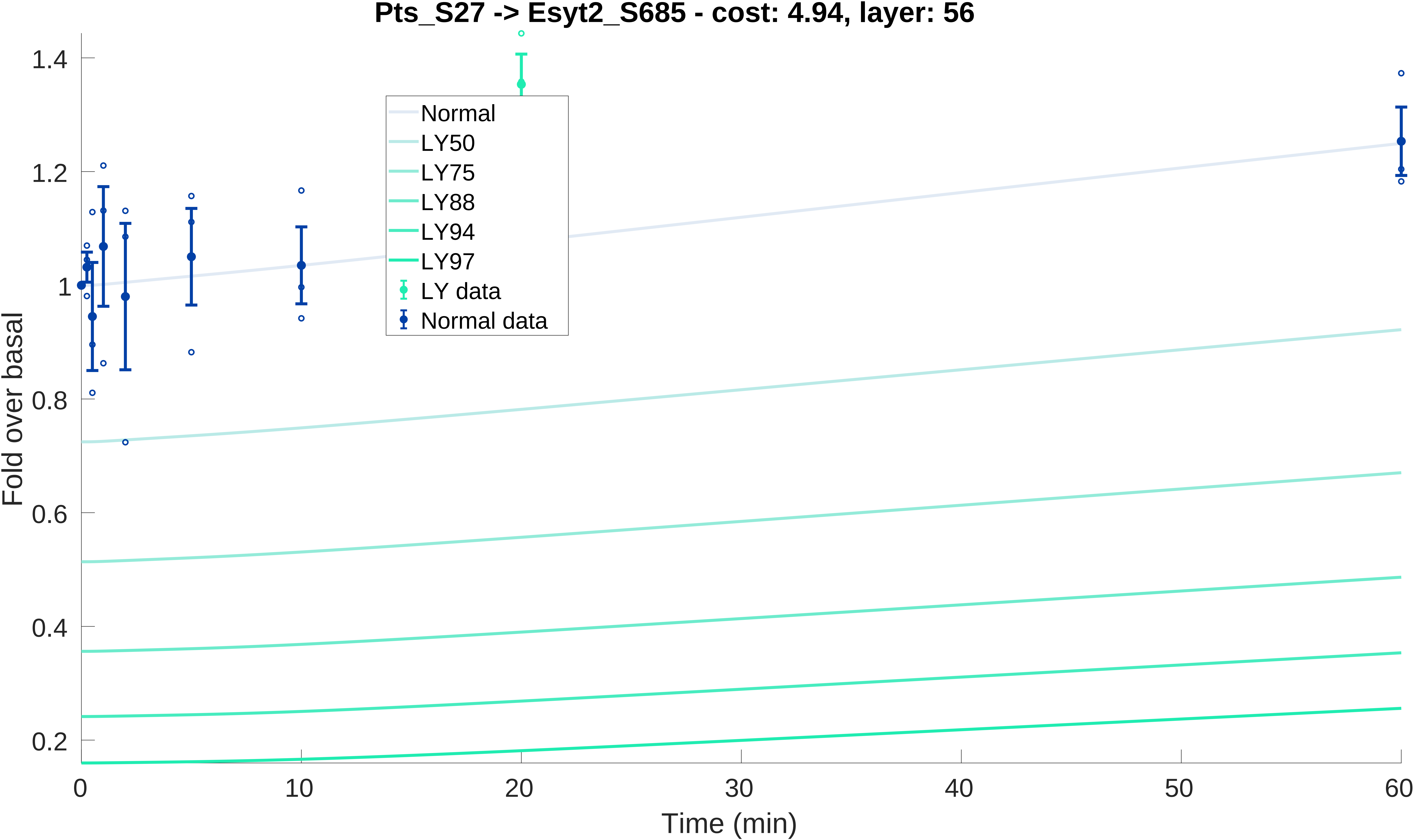

Pts_S27 -> Esyt2_S685 - cost: 4.94, layer: 56
Normal
LY50
LY75
LY88
LY94
LY97
LY data
Normal data
Fold over basal
Time (min)
1.4
1.2
1
0.8
0.6
0.4
0.2
0
10
20
30
40
50
60

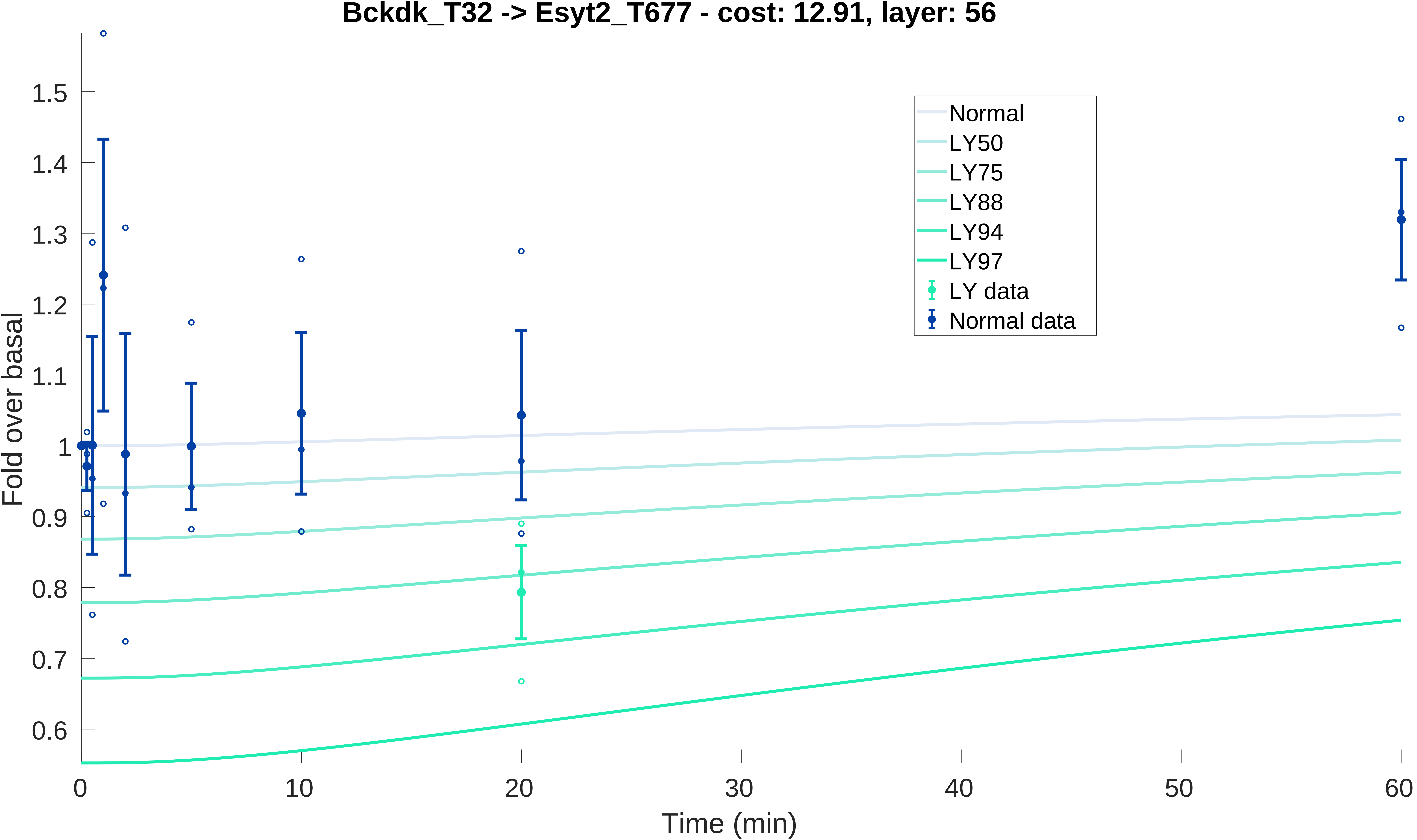

Bckdk_T32 -> Esyt2_T677 - cost: 12.91, layer: 56
Fold over basal
1.5
1.4
1.3
1.2
1.1
1
0.9
0.8
0.7
0.6
0
10
20
30
40
50
60
Time (min)
Normal
LY50
LY75
LY88
LY94
LY97
LY data
Normal data

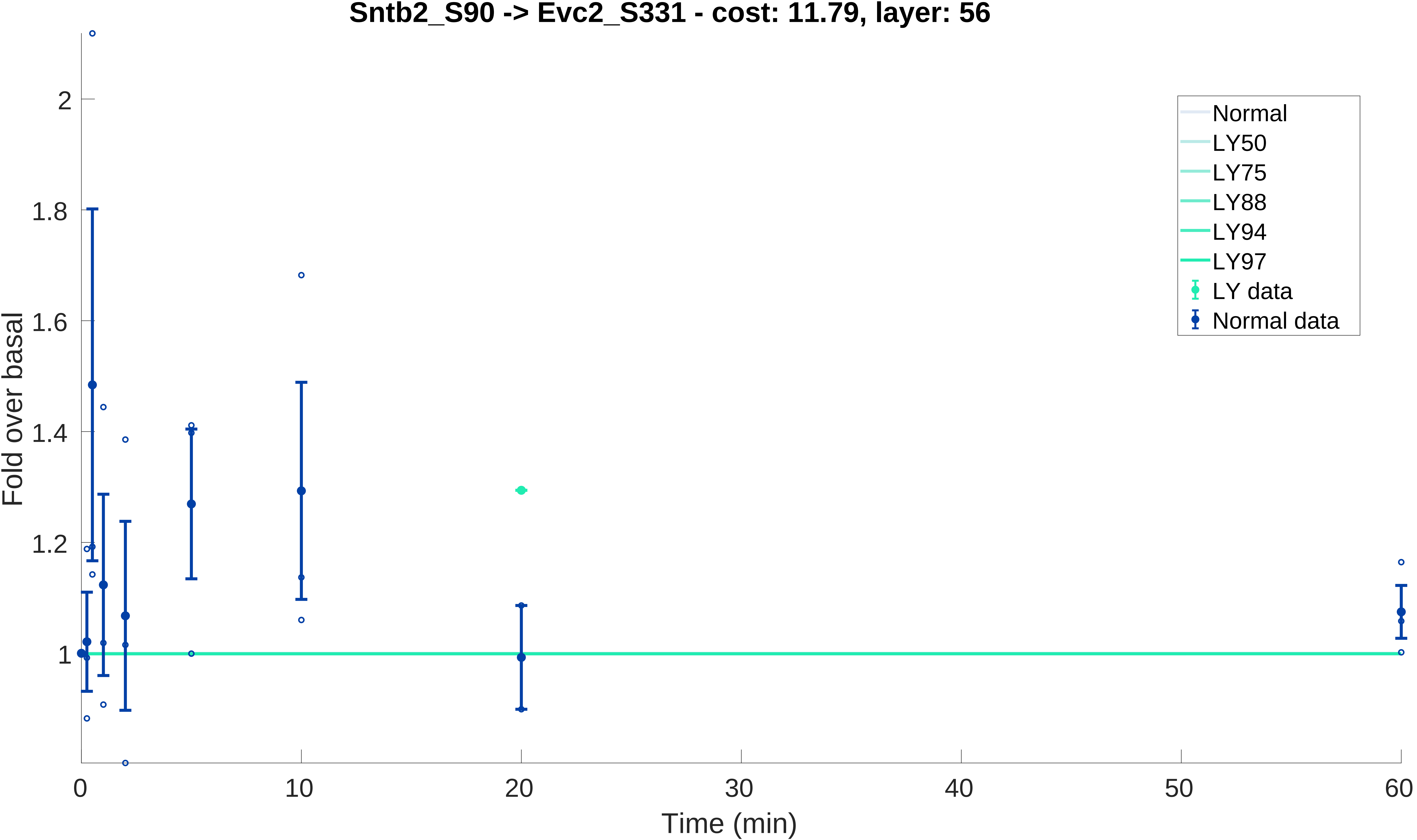

Sntb2_S90 -> Evc2_S331 - cost: 11.79, layer: 56
Fold over basal
Time (min)
Normal
LY50
LY75
LY88
LY94
LY97
LY data
Normal data
2
1.8
1.6
1.4
1.2
1
0
10
20
30
40
50
60

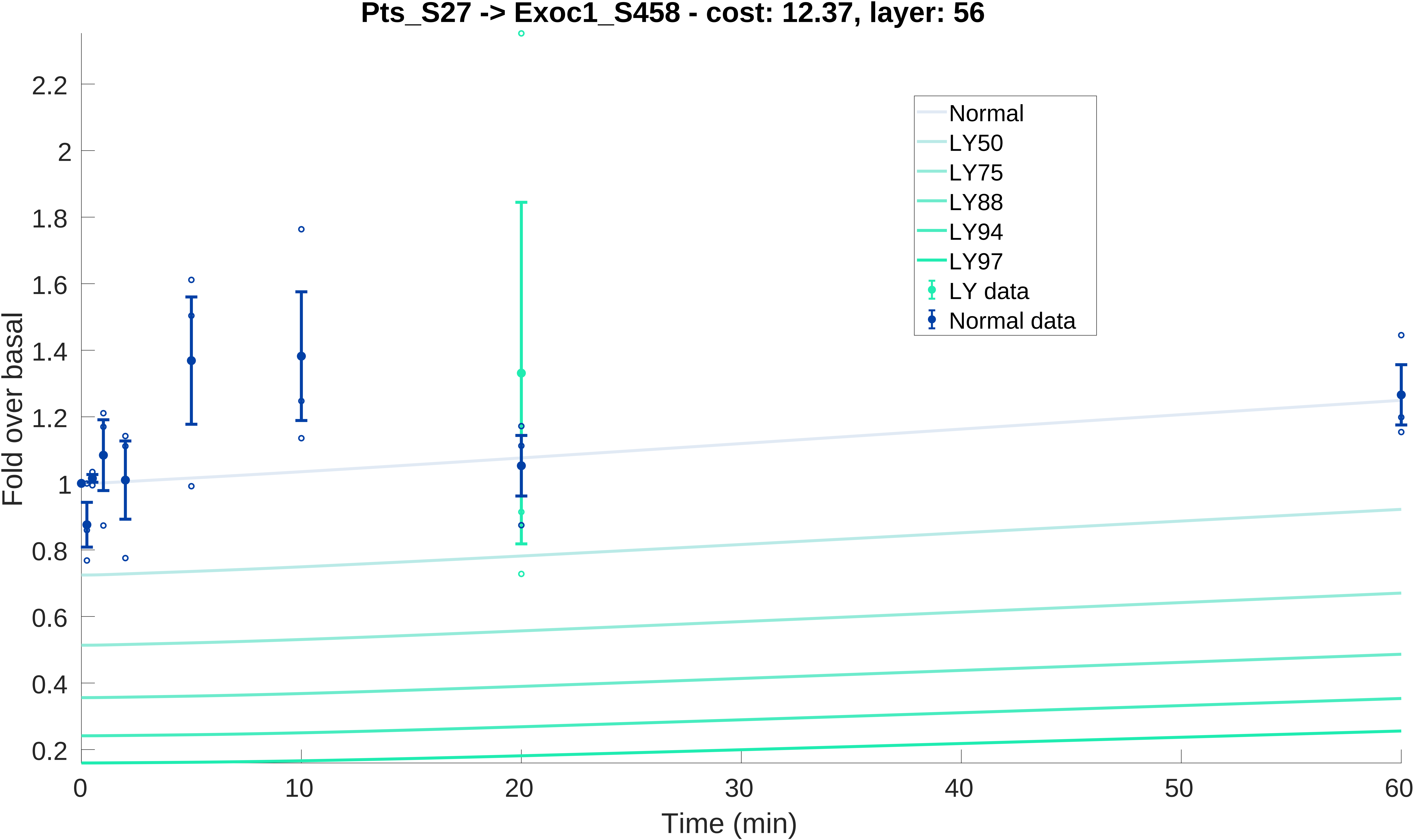

Pts_S27 -> Exoc1_S458 - cost: 12.37, layer: 56
Fold over basal
Time (min)
0.2
0.4
0.6
0.8
1
1.2
1.4
1.6
1.8
2
2.2
0
10
20
30
40
50
60
Normal
LY50
LY75
LY88
LY94
LY97
LY data
Normal data

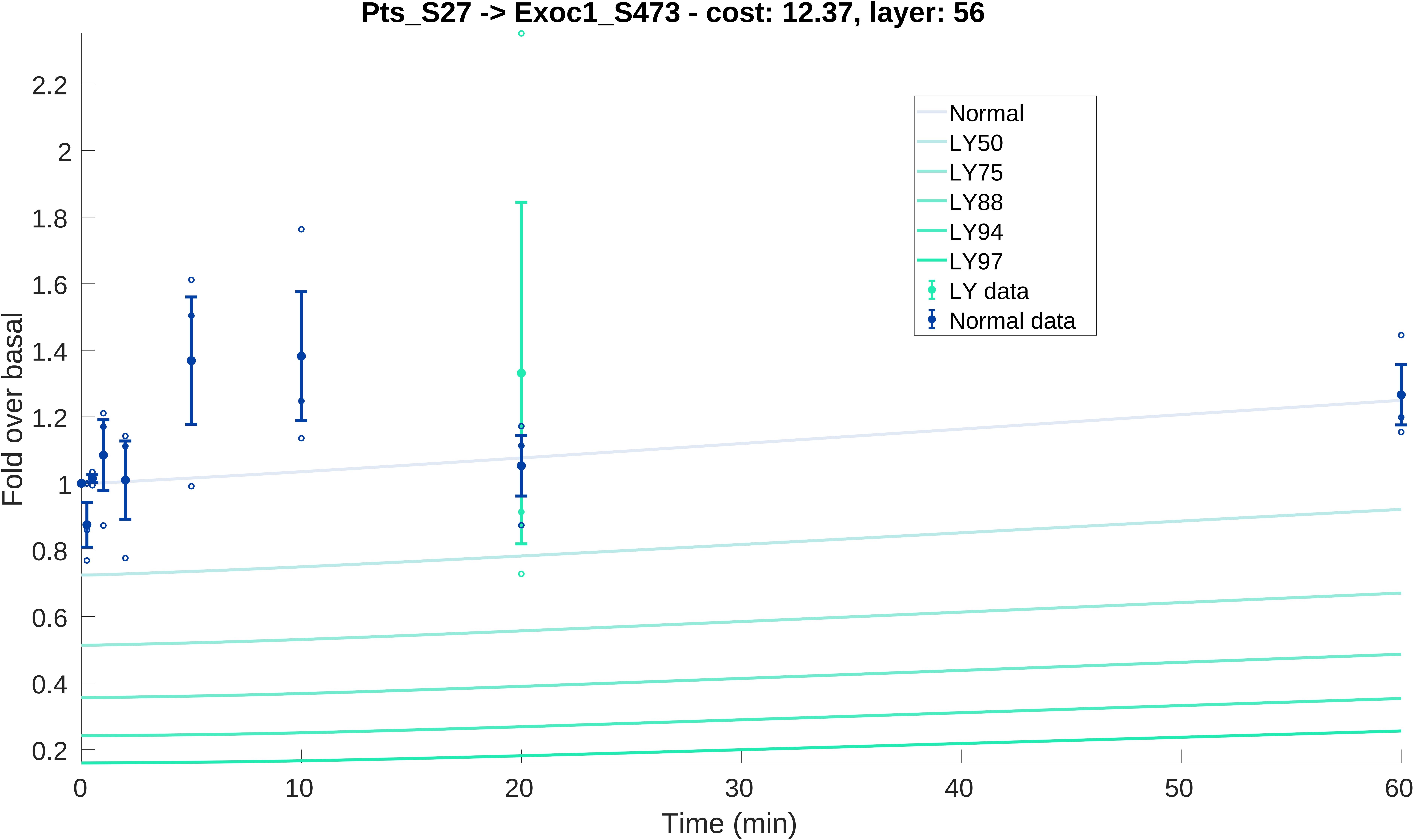

Pts_S27 -> Exoc1_S473 - cost: 12.37, layer: 56
Fold over basal
Time (min)
0.2
0.4
0.6
0.8
1
1.2
1.4
1.6
1.8
2
2.2
0
10
20
30
40
50
60
Normal
LY50
LY75
LY88
LY94
LY97
LY data
Normal data

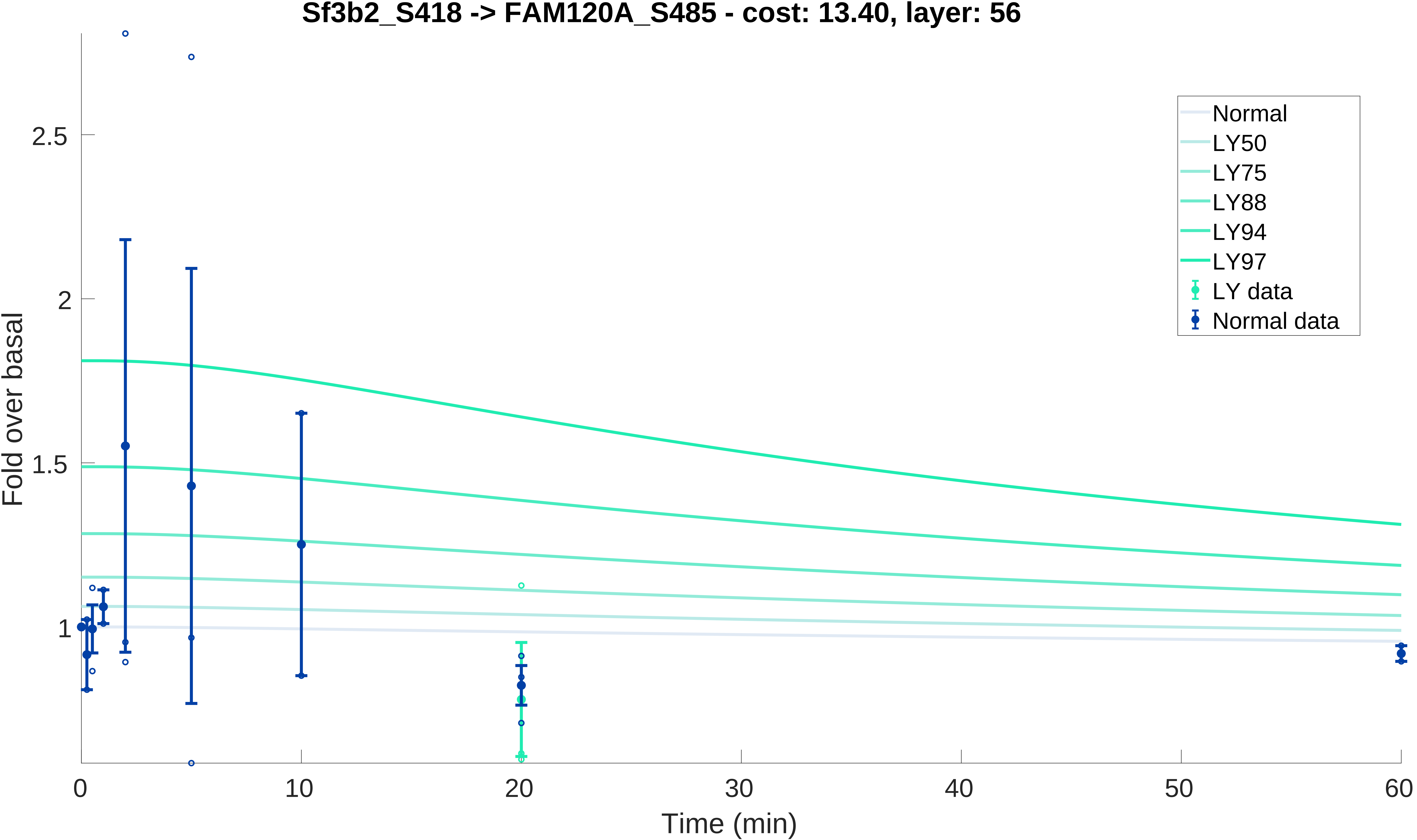

Sf3b2_S418 -> FAM120A_S485 - cost: 13.40, layer: 56
Fold over basal
2.5
2
1.5
1
0
10
20
30
40
50
60
Time (min)
Normal
LY50
LY75
LY88
LY94
LY97
LY data
Normal data

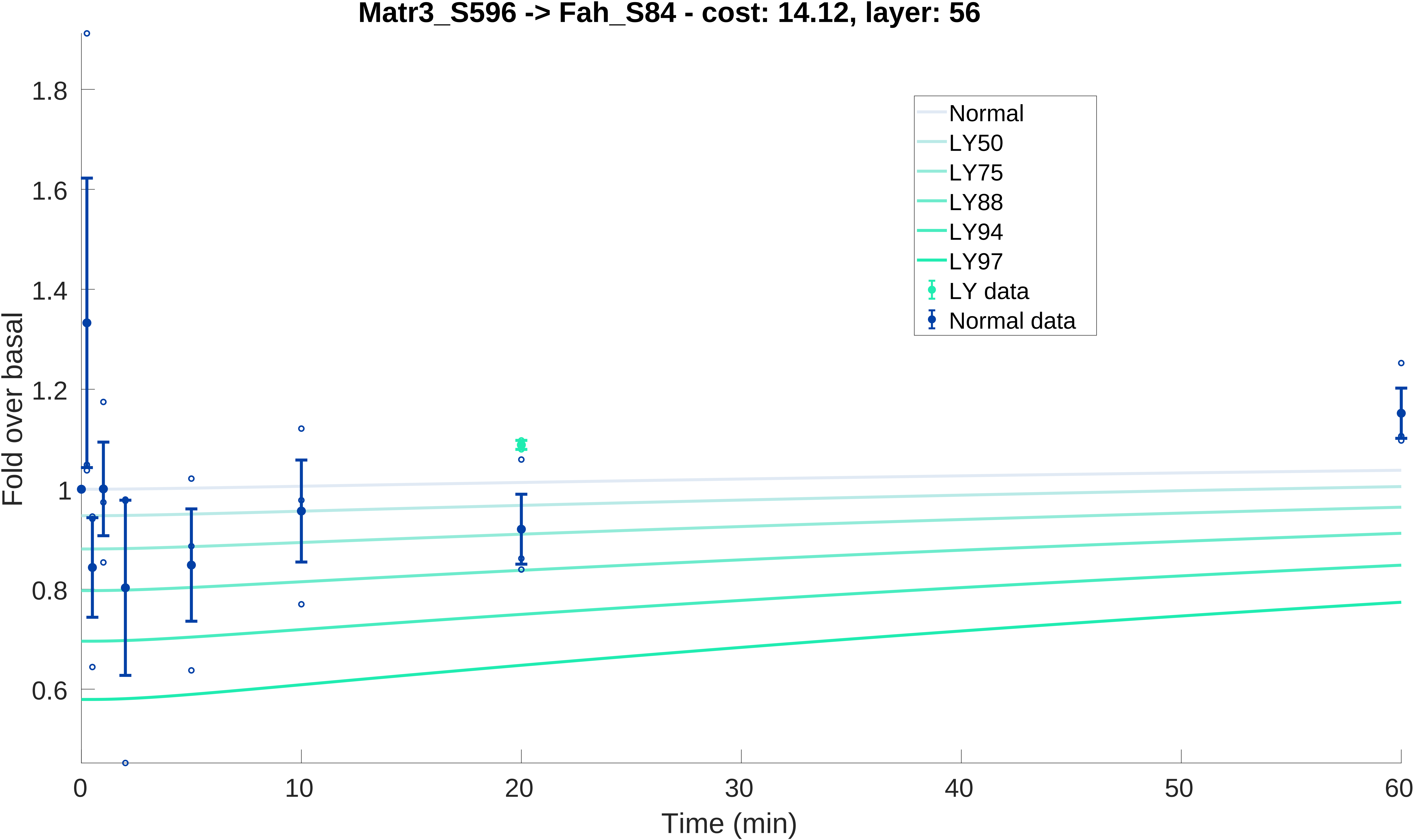

Matr3_S596 -> Fah_S84 - cost: 14.12, layer: 56
Normal
LY50
LY75
LY88
LY94
LY97
LY data
Normal data
Fold over basal
1.8
1.6
1.4
1.2
1
0.8
0.6
0
10
20
30
40
50
60
Time (min)

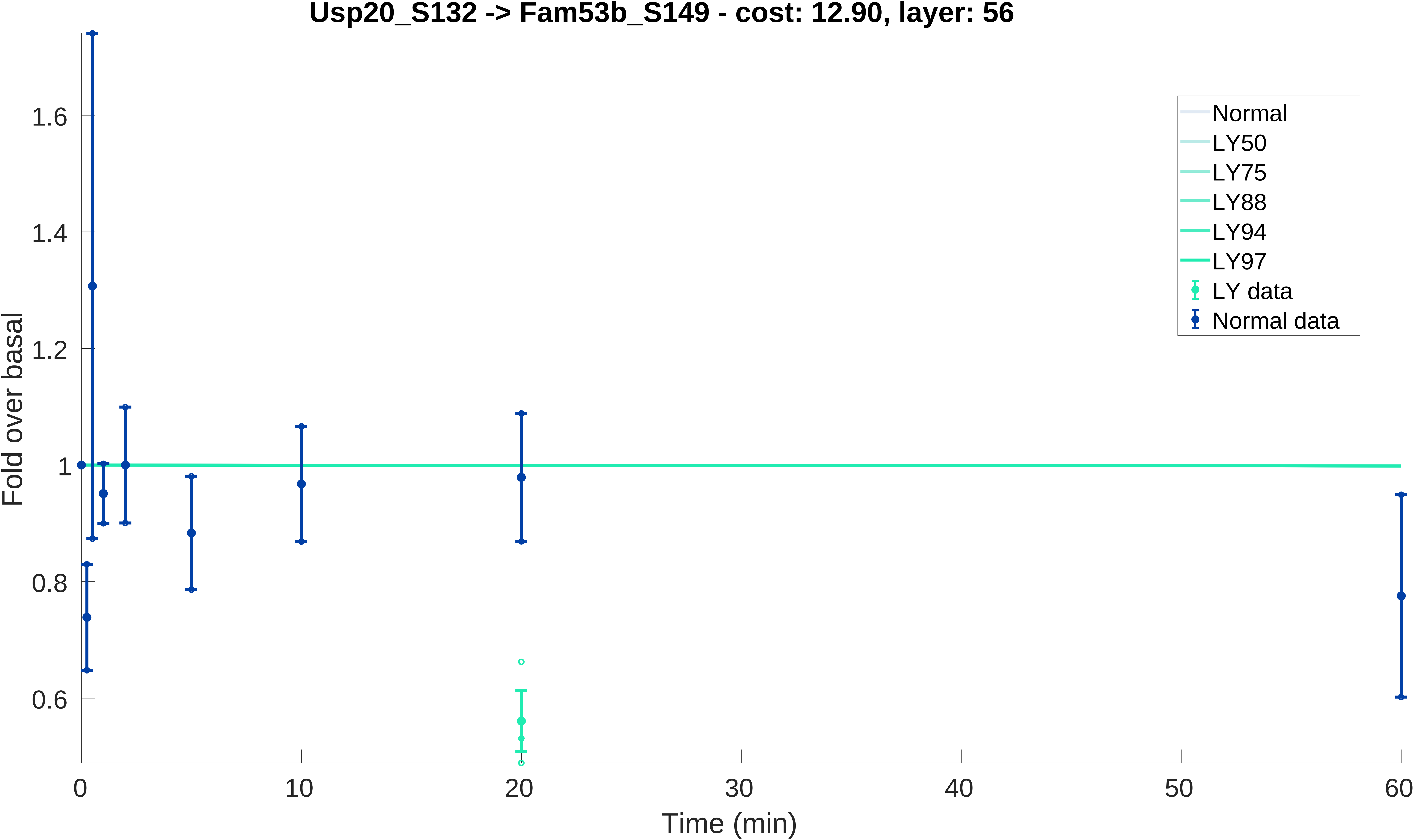
Usp20_S132 -> Fam53b_S149 - cost: 12.90, layer: 56
Fold over basal
Time (min)
0
10
20
30
40
50
60
0.6
0.8
1
1.2
1.4
1.6
Normal
LY50
LY75
LY88
LY94
LY97
LY data
Normal data

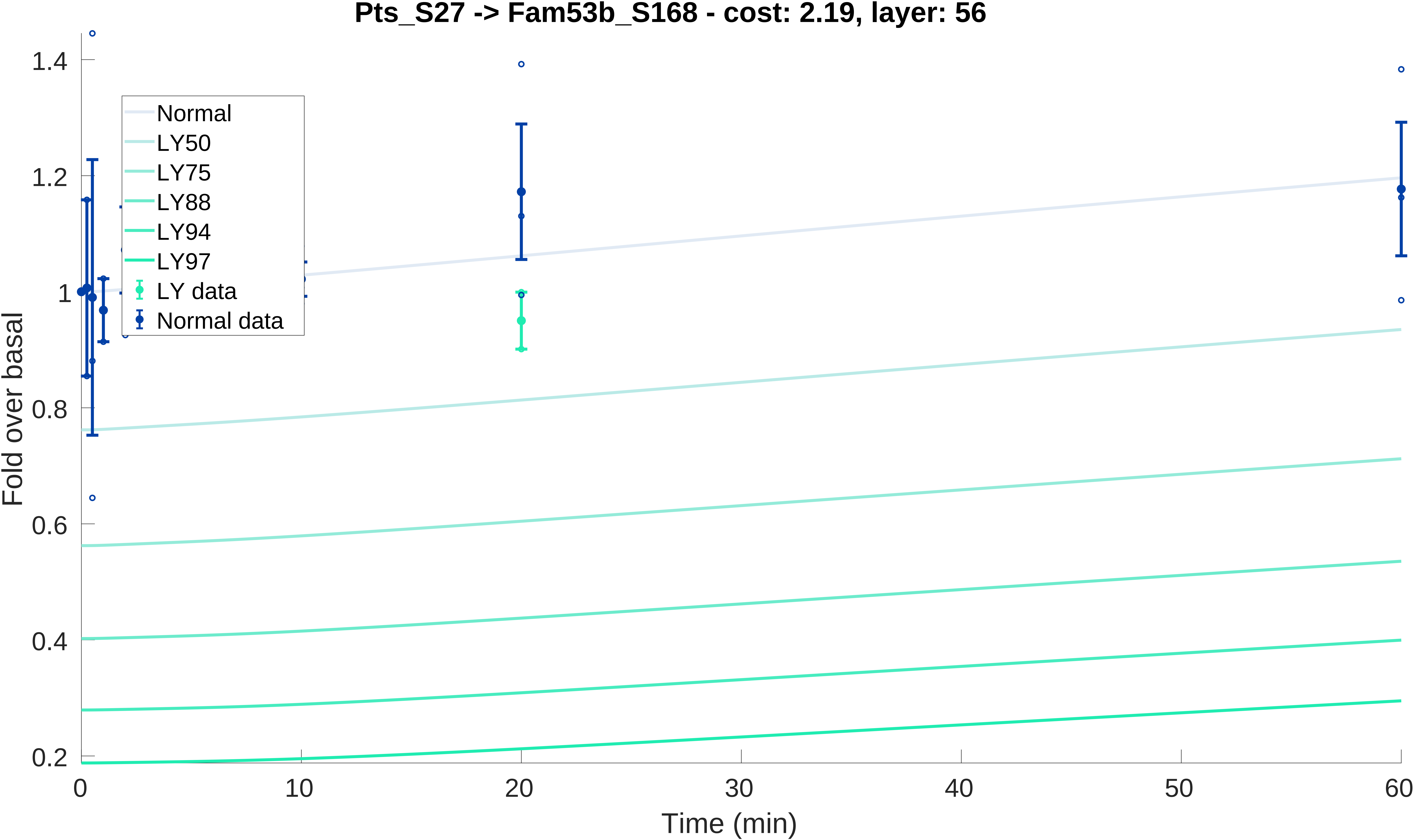

Pts_S27 -> Fam53b_S168 - cost: 2.19, layer: 56
Fold over basal
Time (min)
Normal
LY50
LY75
LY88
LY94
LY97
LY data
Normal data
0.2
0.4
0.6
0.8
1
1.2
1.4
0
10
20
30
40
50
60

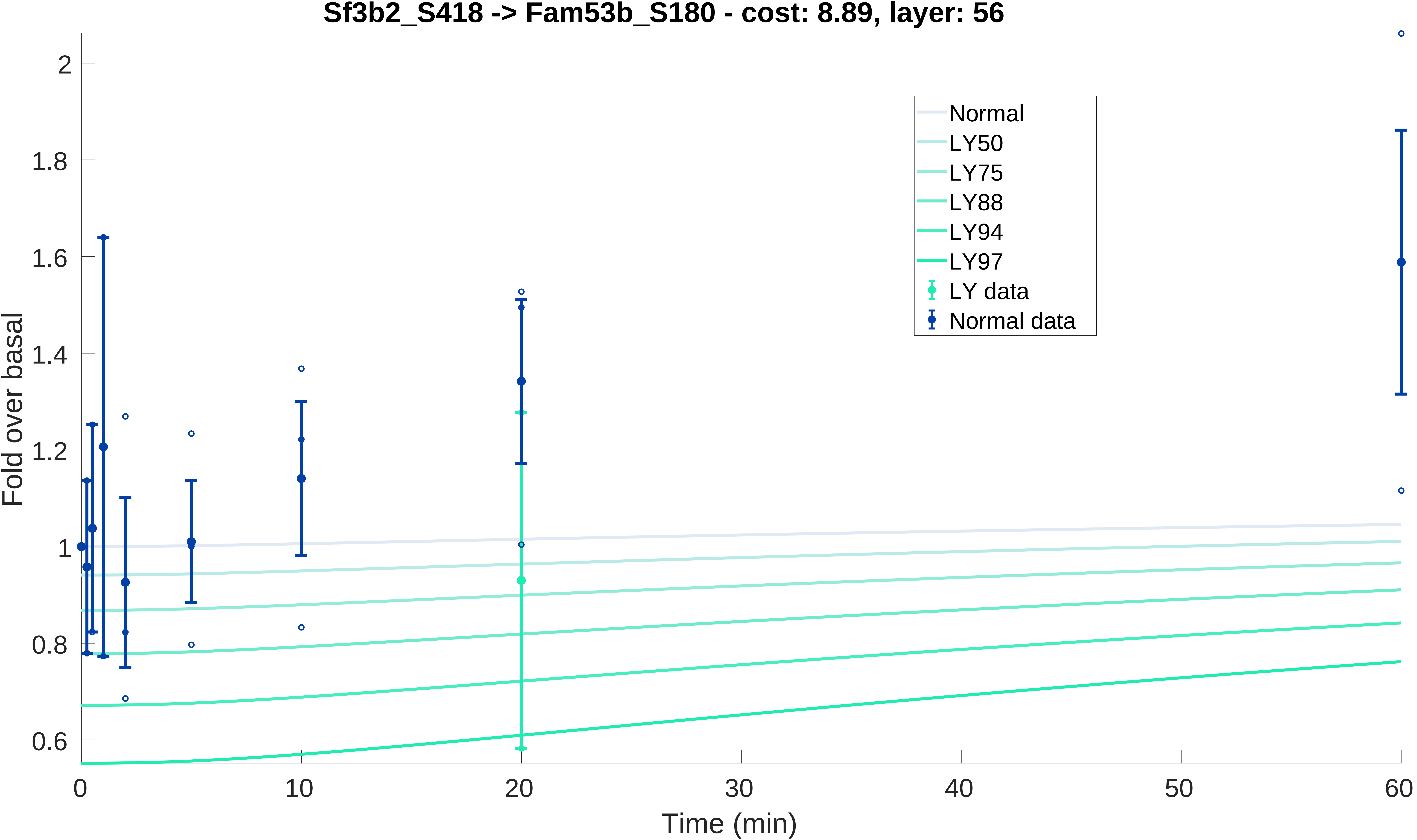

Sf3b2_S418 -> Fam53b_S180 - cost: 8.89, layer: 56
Fold over basal
Time (min)
Normal
LY50
LY75
LY88
LY94
LY97
LY data
Normal data
0.6
0.8
1
1.2
1.4
1.6
1.8
2
0
10
20
30
40
50
60

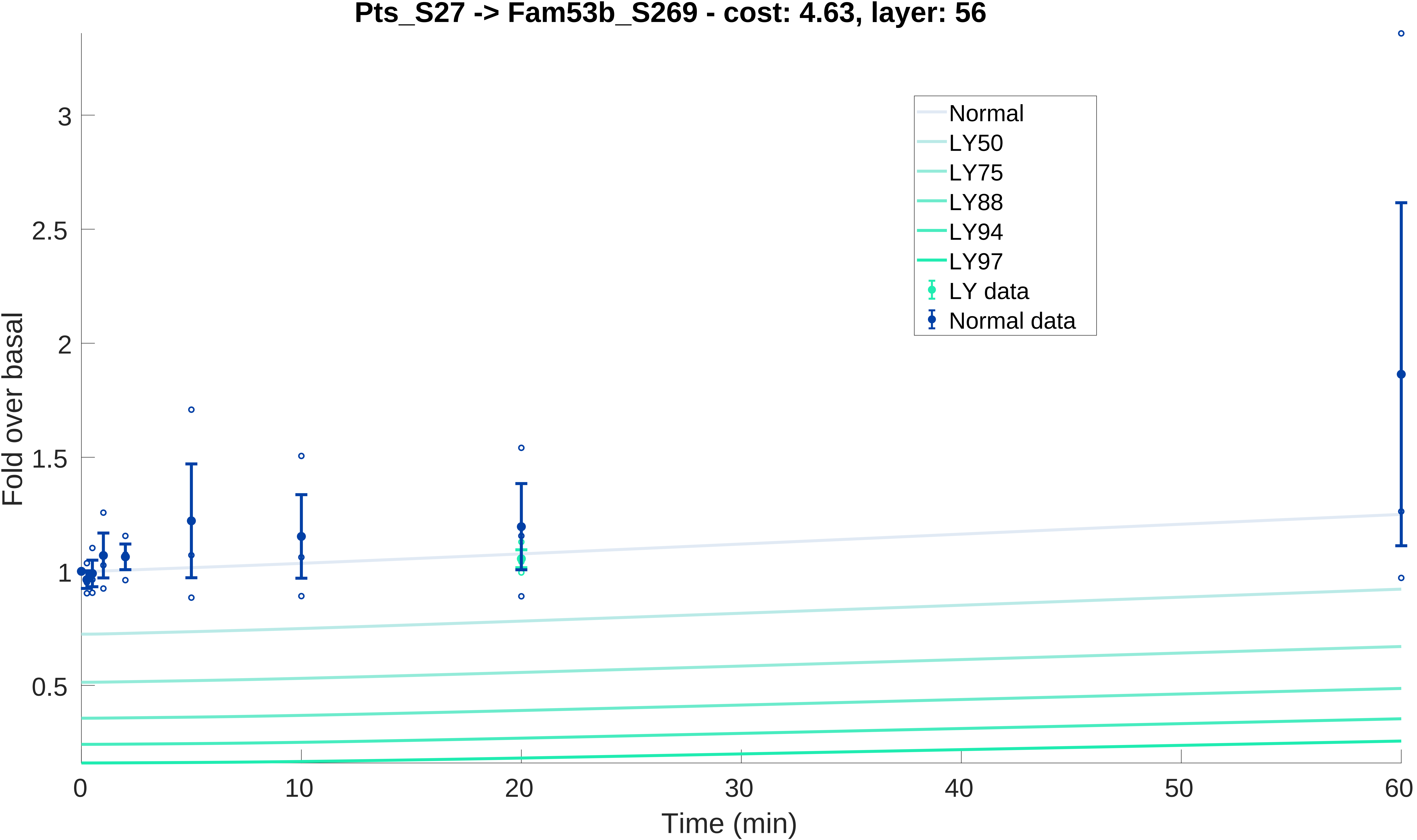

Pts_S27 -> Fam53b_S269 - cost: 4.63, layer: 56
Fold over basal
Time (min)
0
10
20
30
40
50
60
0.5
1
1.5
2
2.5
3
Normal
LY50
LY75
LY88
LY94
LY97
LY data
Normal data

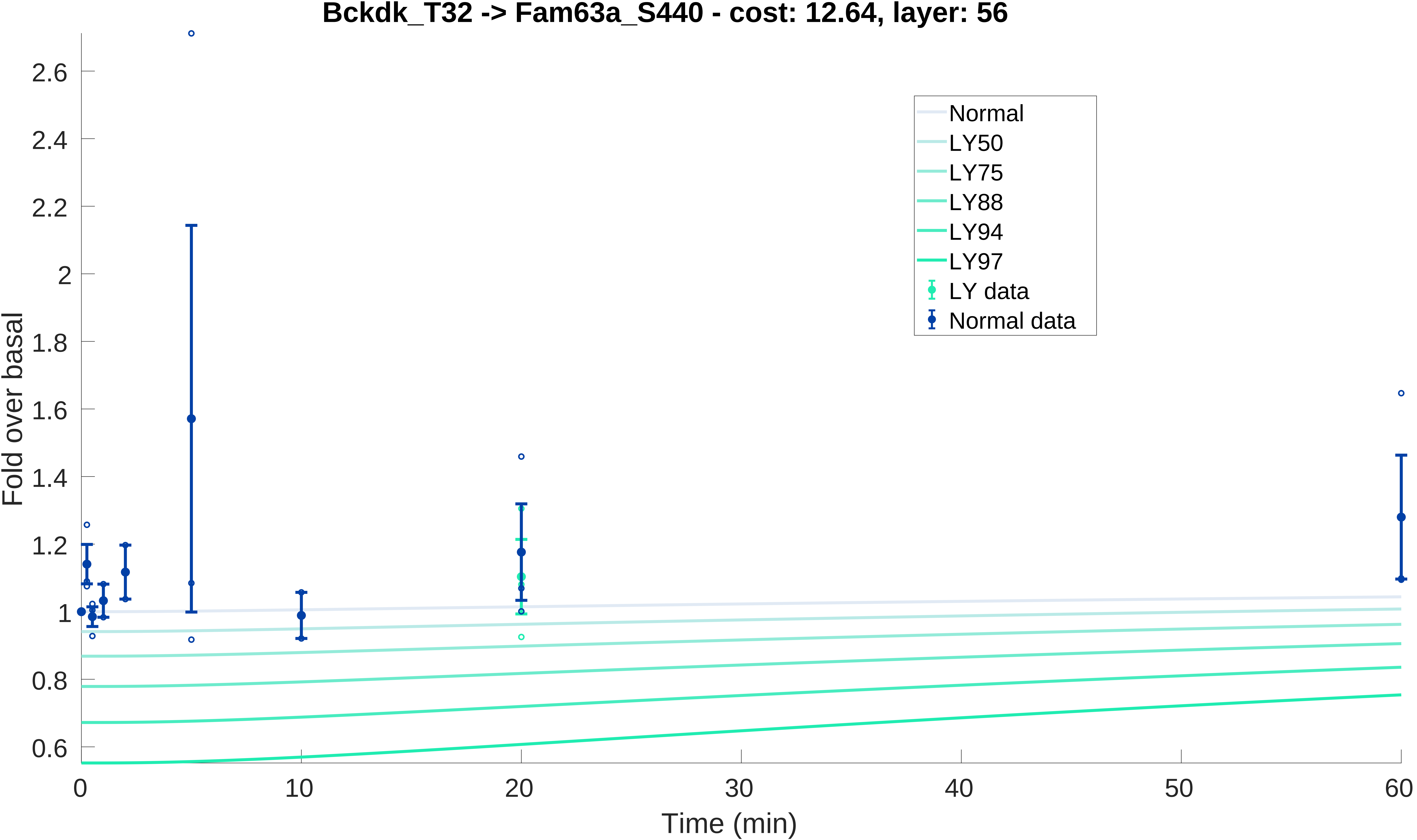
Bckdk_T32 -> Fam63a_S440 - cost: 12.64, layer: 56
Fold over basal
Time (min)
Normal
LY50
LY75
LY88
LY94
LY97
LY data
Normal data
0.6
0.8
1
1.2
1.4
1.6
1.8
2
2.2
2.4
2.6
0
10
20
30
40
50
60

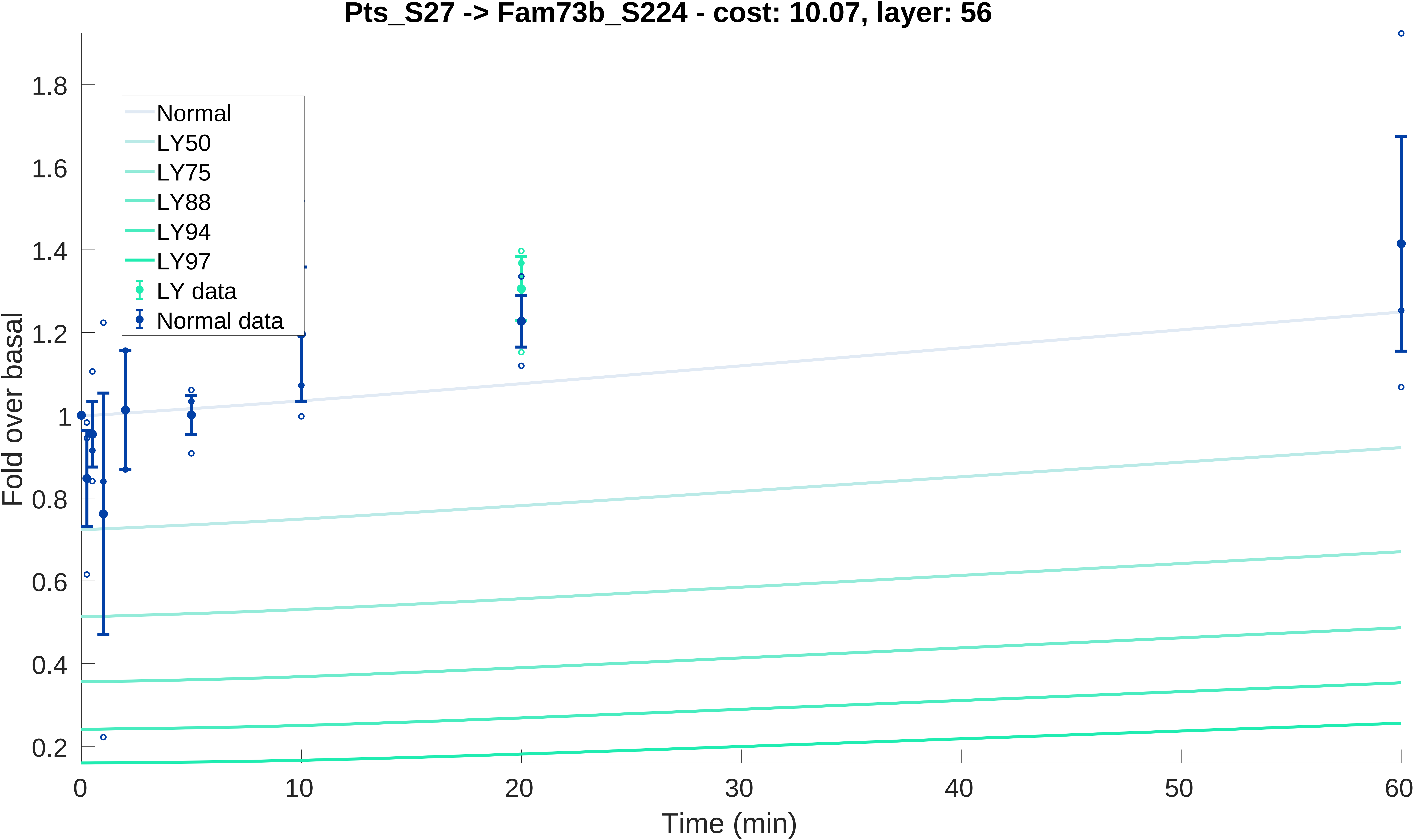

Pts_S27 -> Fam73b_S224 - cost: 10.07, layer: 56
Normal
LY50
LY75
LY88
LY94
LY97
LY data
Normal data
Fold over basal
Time (min)
0
10
20
30
40
50
60
0.2
0.4
0.6
0.8
1
1.2
1.4
1.6
1.8

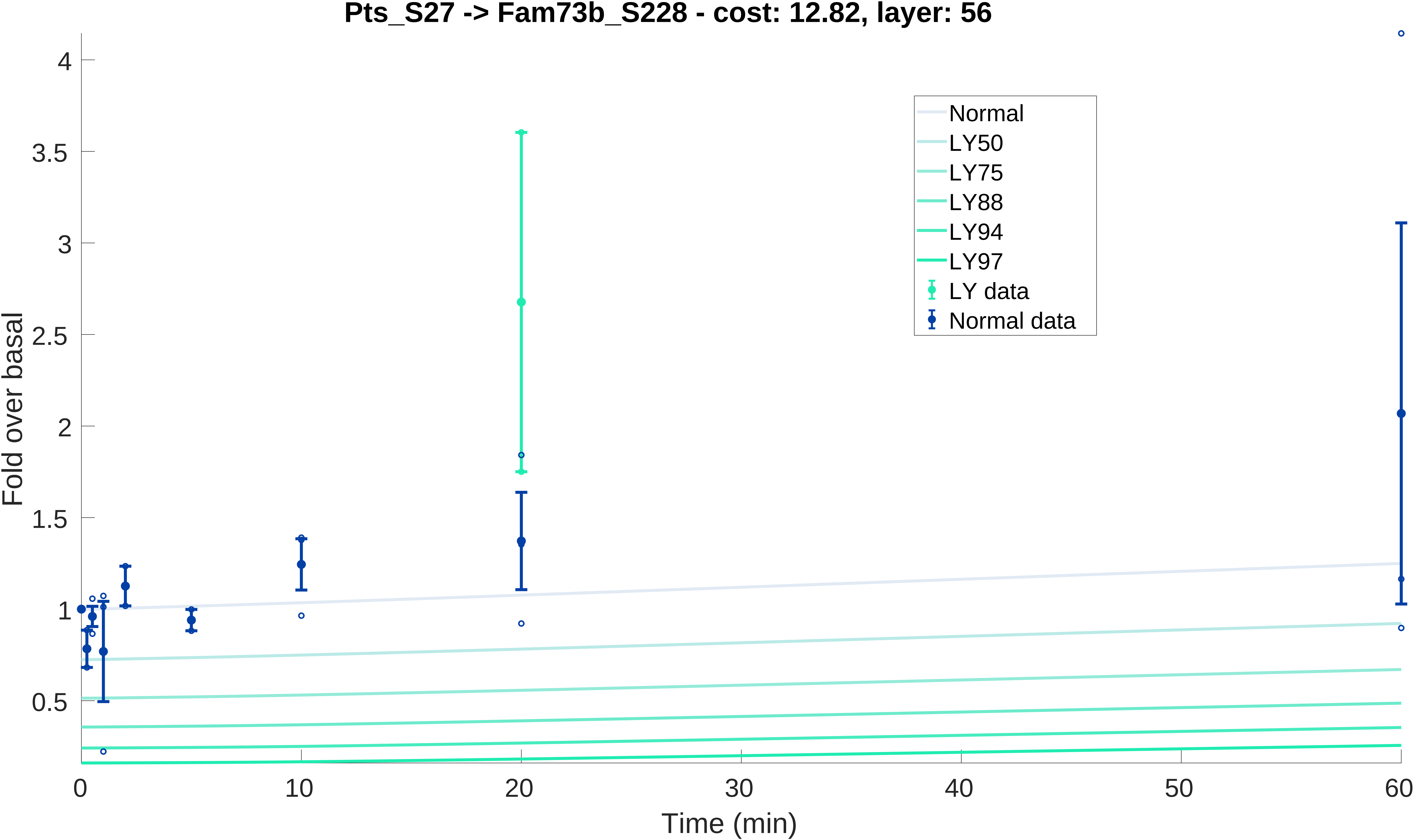
Pts_S27 -> Fam73b_S228 - cost: 12.82, layer: 56
Fold over basal
Time (min)
0
10
20
30
40
50
60
0.5
1
1.5
2
2.5
3
3.5
4
Normal
LY50
LY75
LY88
LY94
LY97
LY data
Normal data

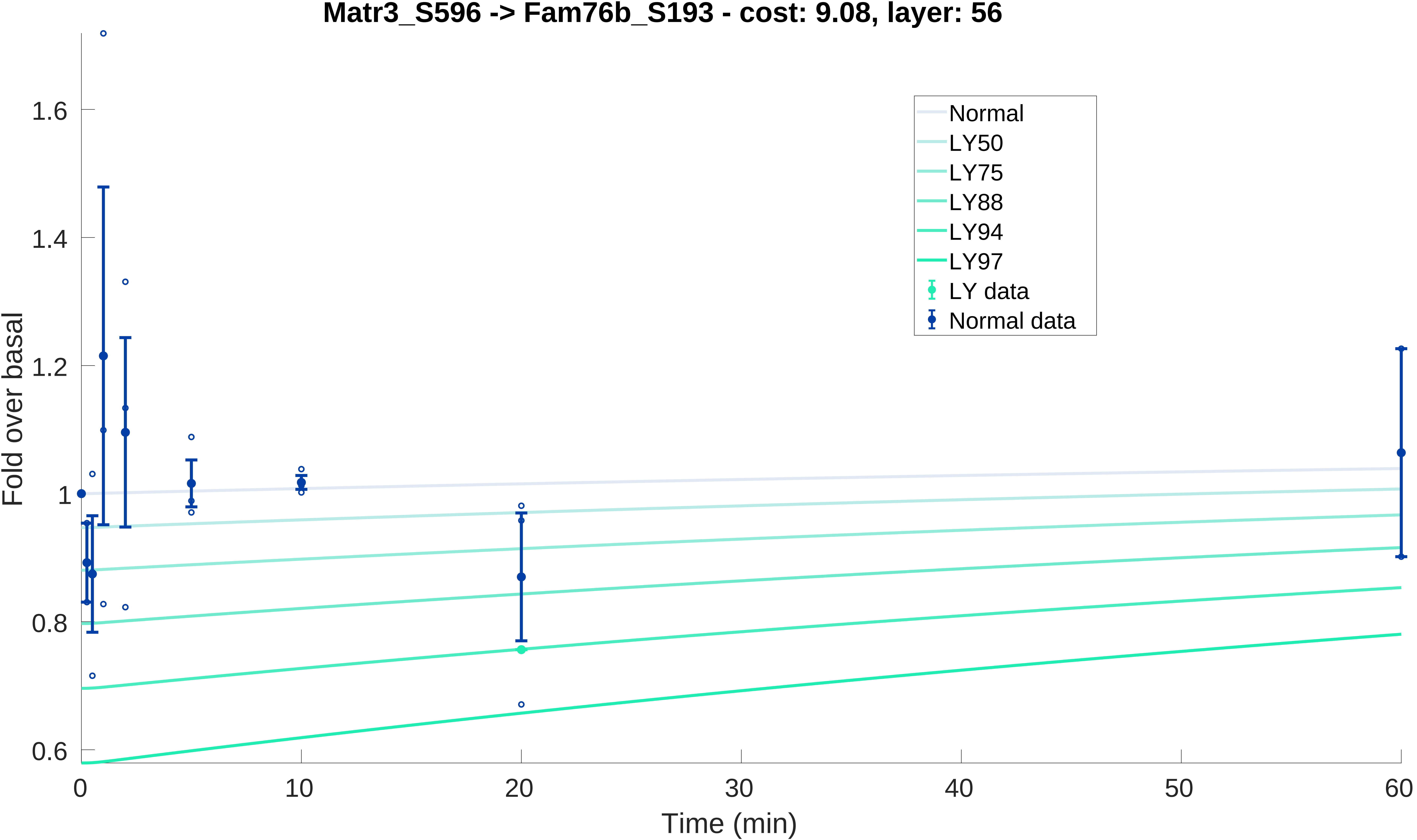

Matr3_S596 -> Fam76b_S193 - cost: 9.08, layer: 56
Fold over basal
1.6
1.4
1.2
1
0.8
0.6
0
10
20
30
40
50
60
Time (min)
Normal
LY50
LY75
LY88
LY94
LY97
LY data
Normal data

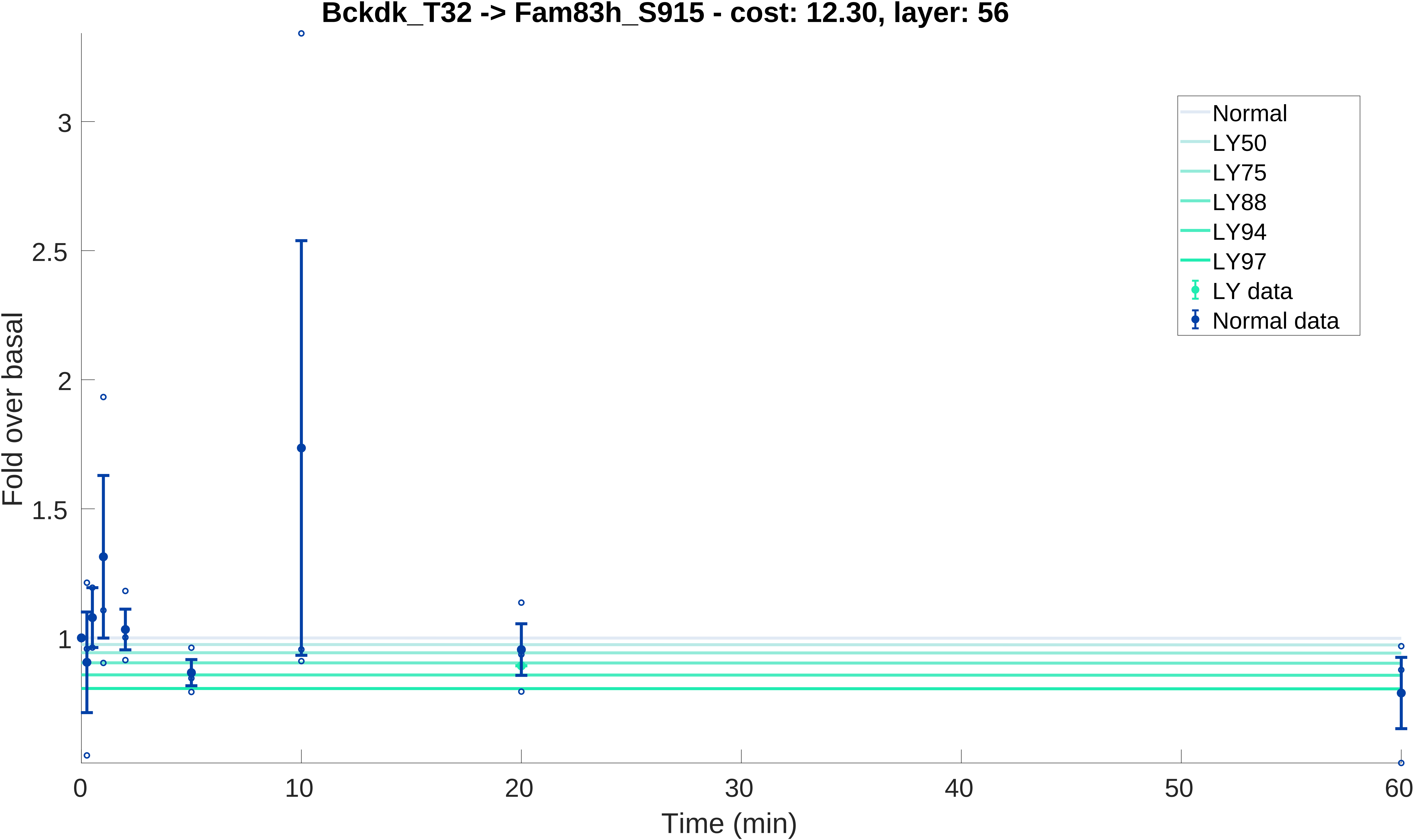

Bckdk_T32 -> Fam83h_S915 - cost: 12.30, layer: 56
Fold over basal
Time (min)
0
10
20
30
40
50
60
1
1.5
2
2.5
3
Normal
LY50
LY75
LY88
LY94
LY97
LY data
Normal data

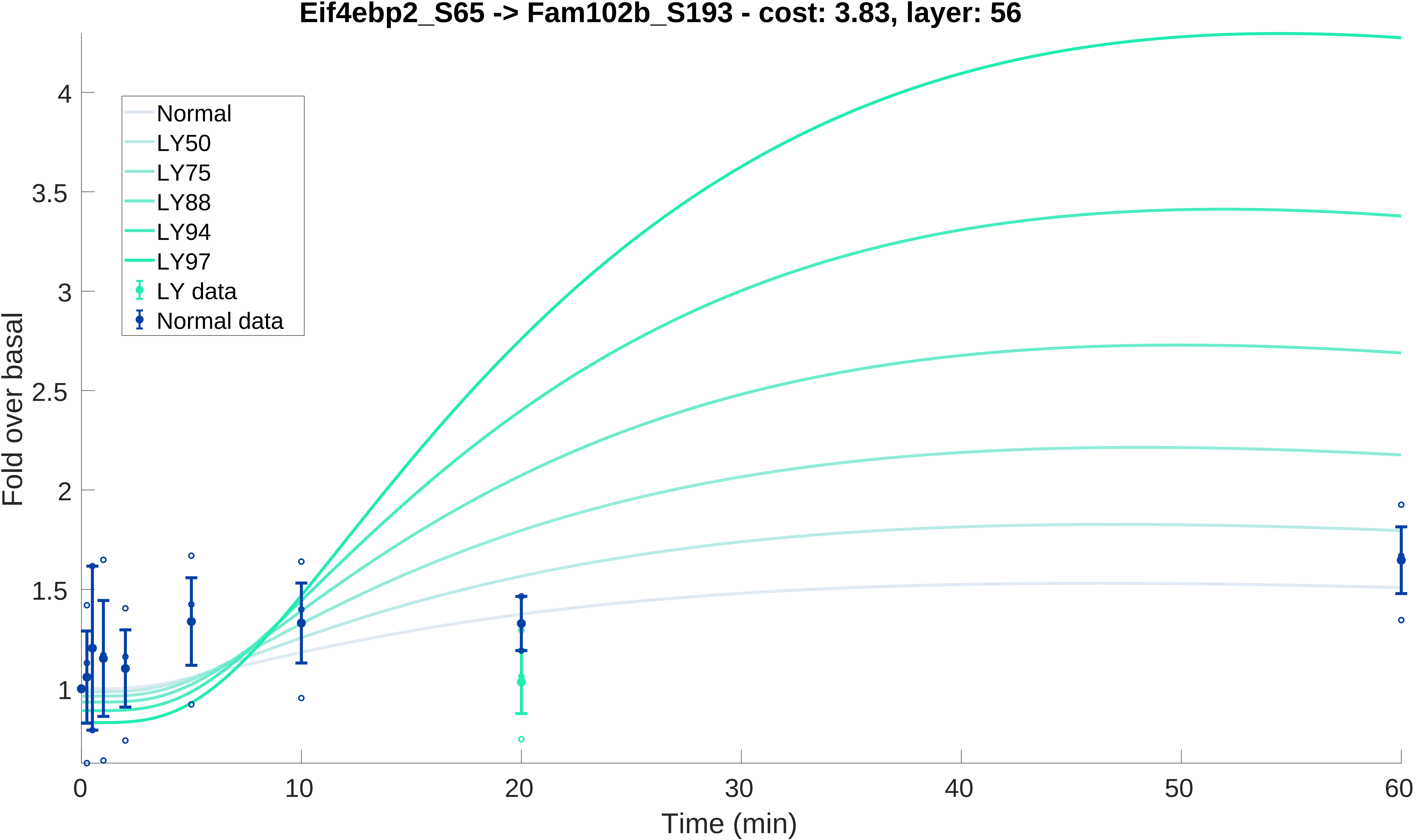

Eif4ebp2_S65 -> Fam102b_S193 - cost: 3.83, layer: 56
Normal
LY50
LY75
LY88
LY94
LY97
LY data
Normal data
Fold over basal
Time (min)
0
10
20
30
40
50
60
1
1.5
2
2.5
3
3.5
4

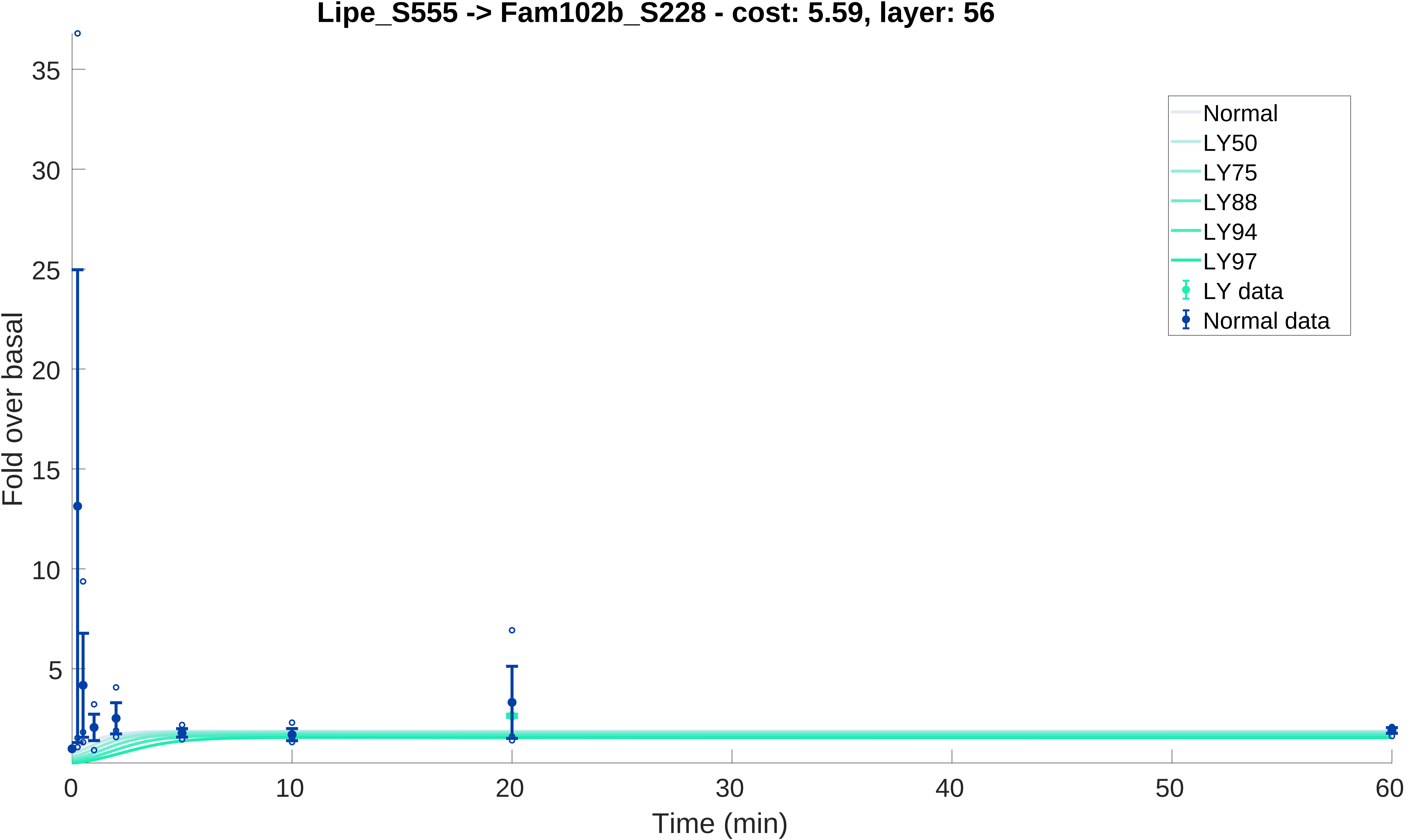

Lipe_S555 -> Fam102b_S228 - cost: 5.59, layer: 56
Fold over basal
Time (min)
0
10
20
30
40
50
60
5
10
15
20
25
30
35
Normal
LY50
LY75
LY88
LY94
LY97
LY data
Normal data

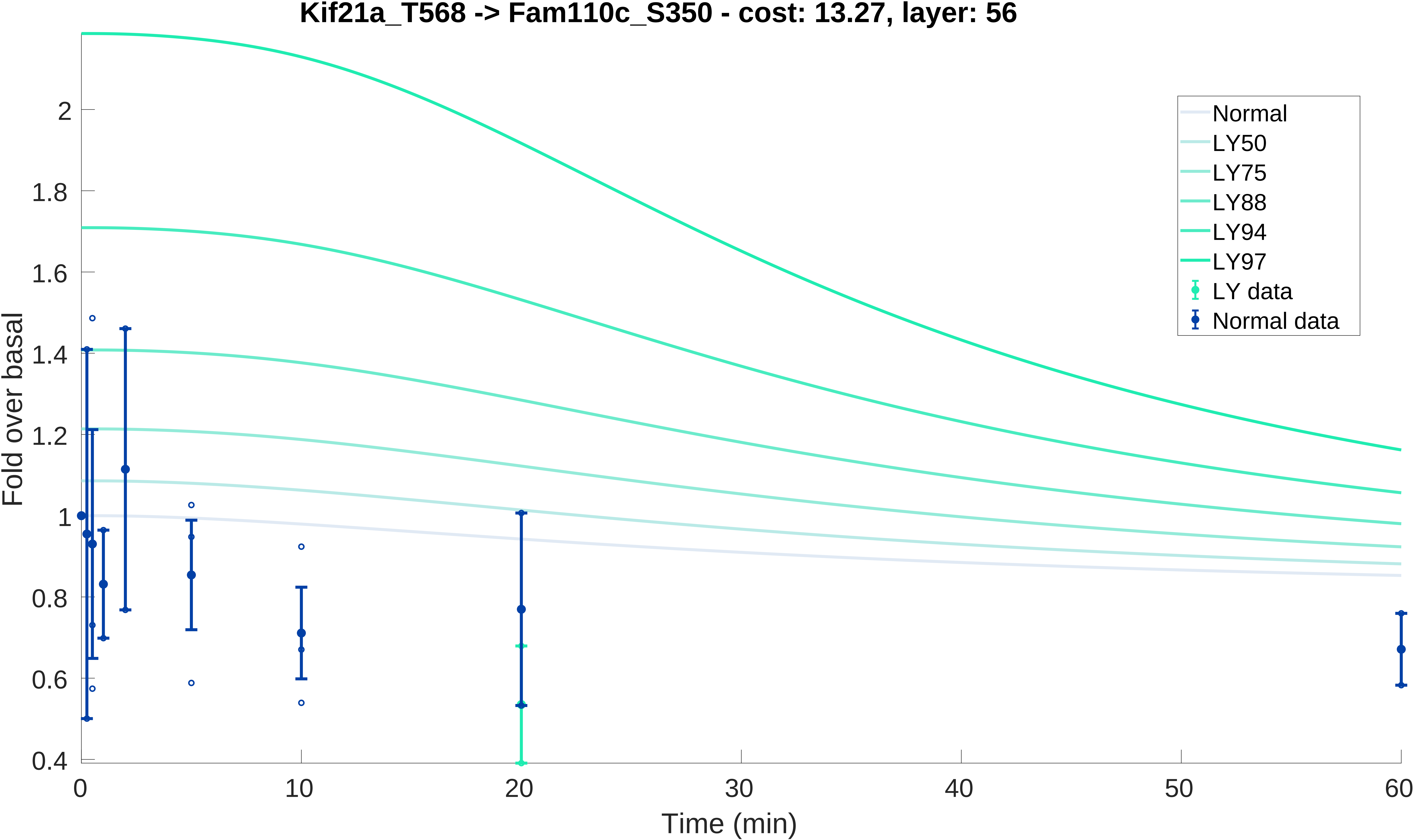

Kif21a_T568 -> Fam110c_S350 - cost: 13.27, layer: 56
Fold over basal
Time (min)
Normal
LY50
LY75
LY88
LY94
LY97
LY data
Normal data
0.4
0.6
0.8
1
1.2
1.4
1.6
1.8
2
0
10
20
30
40
50
60

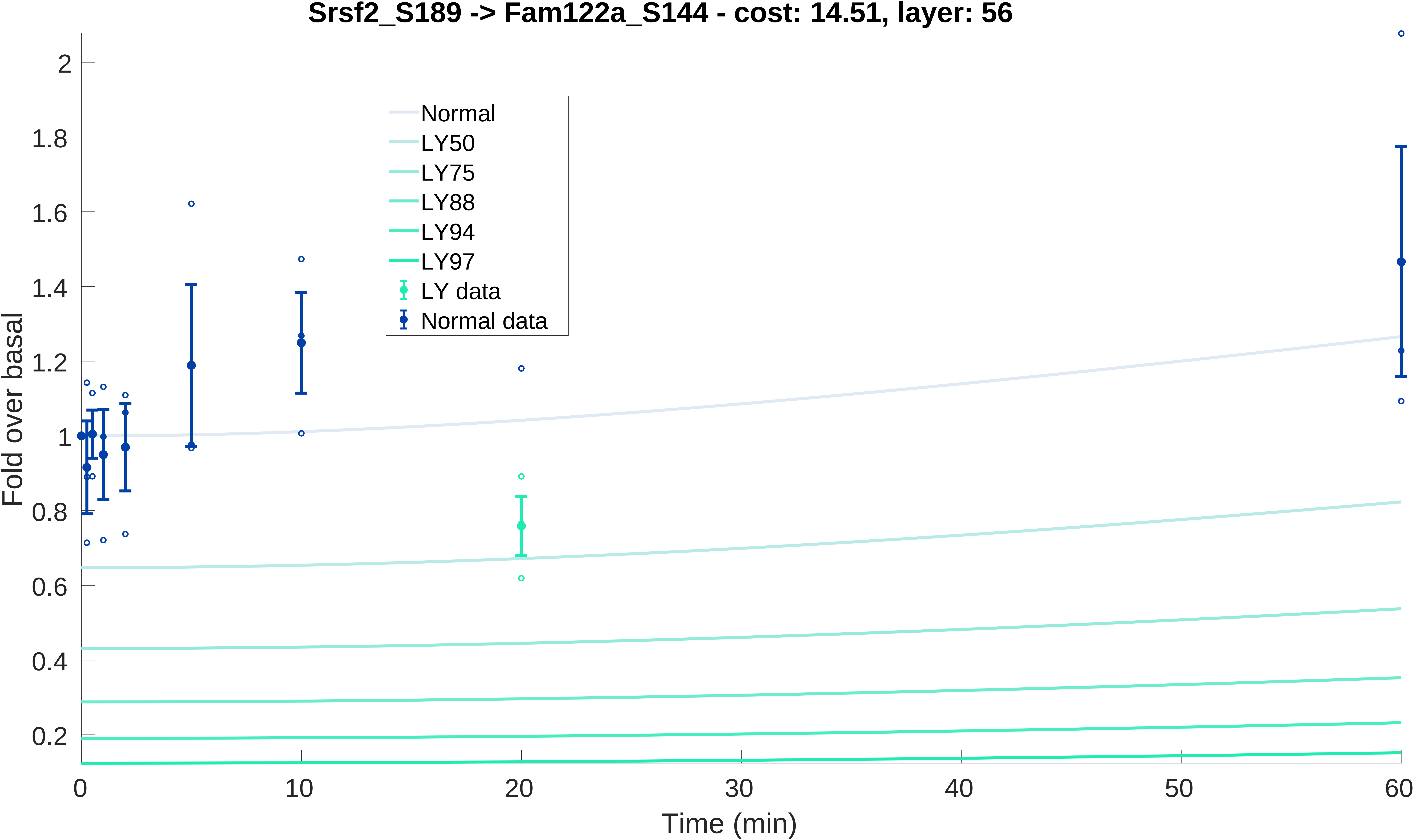

Srsf2_S189 -> Fam122a_S144 - cost: 14.51, layer: 56
Fold over basal
Time (min)
Normal
LY50
LY75
LY88
LY94
LY97
LY data
Normal data
2
1.8
1.6
1.4
1.2
1
0.8
0.6
0.4
0.2
0
10
20
30
40
50
60

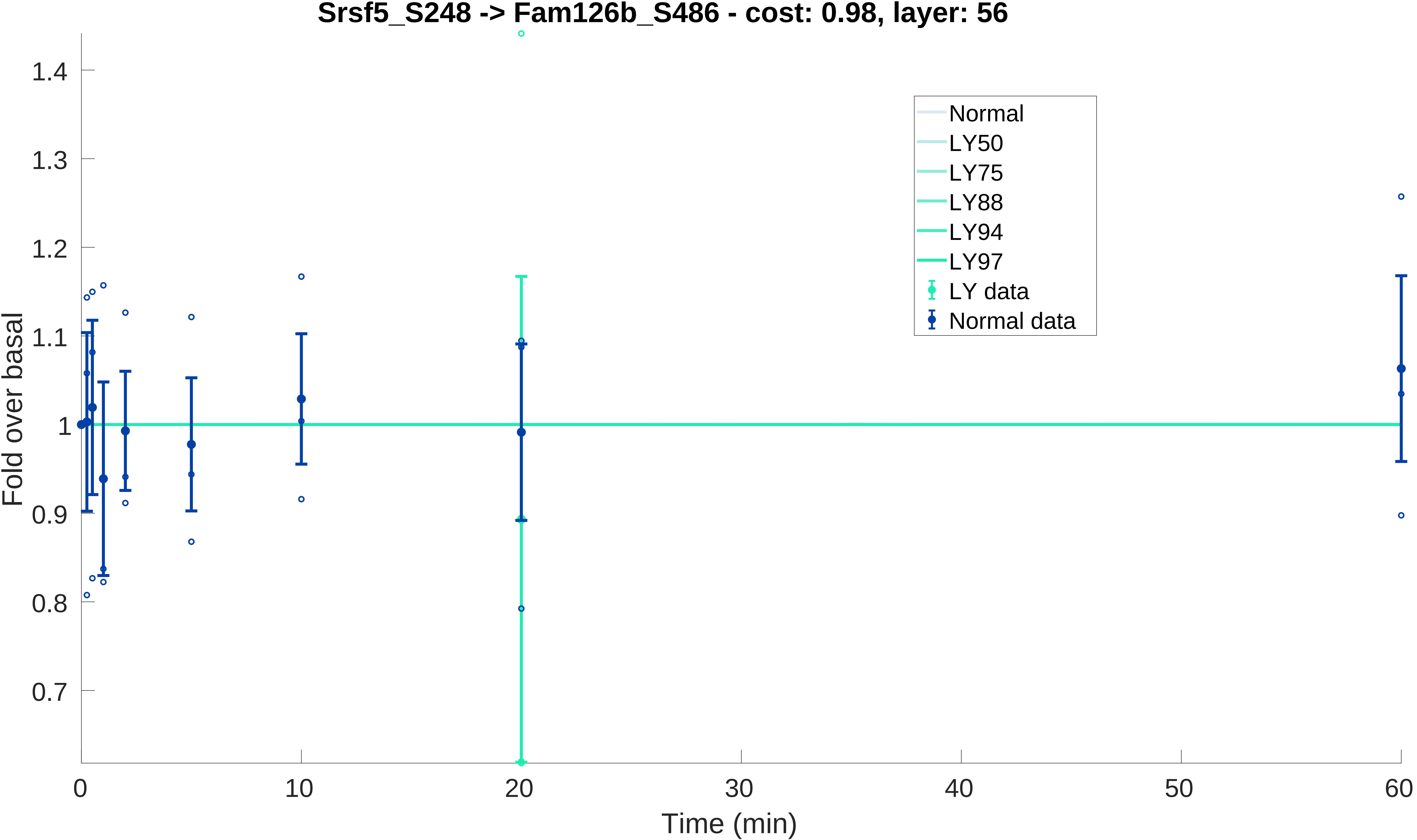

Srsf5_S248 -> Fam126b_S486 - cost: 0.98, layer: 56
Fold over basal
Time (min)
1.4
1.3
1.2
1.1
1
0.9
0.8
0.7
0
10
20
30
40
50
60
Normal
LY50
LY75
LY88
LY94
LY97
LY data
Normal data

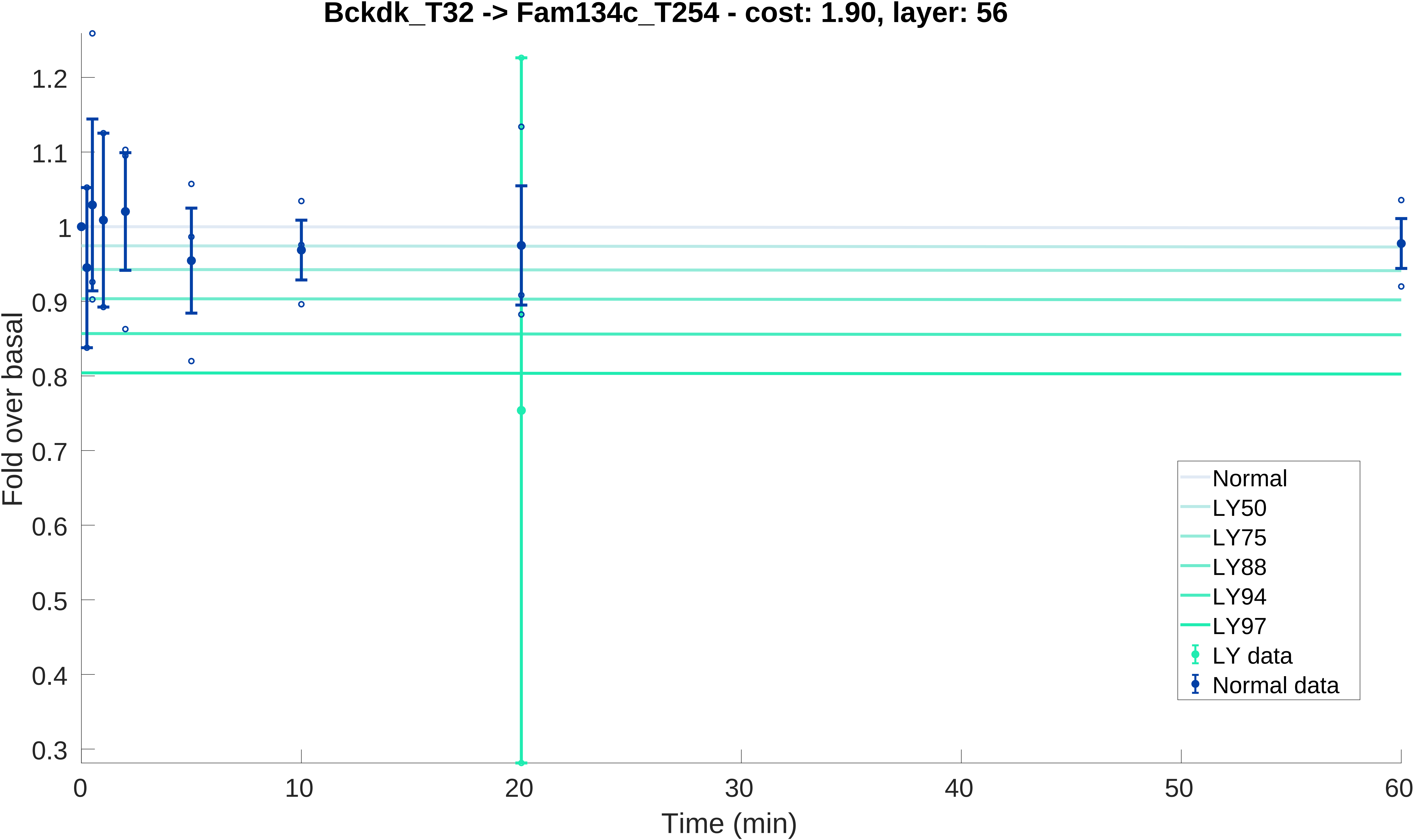

Bckdk_T32 -> Fam134c_T254 - cost: 1.90, layer: 56
Fold over basal
1.2
1.1
1
0.9
0.8
0.7
0.6
0.5
0.4
0.3
0
10
20
30
40
50
60
Time (min)
Normal
LY50
LY75
LY88
LY94
LY97
LY data
Normal data

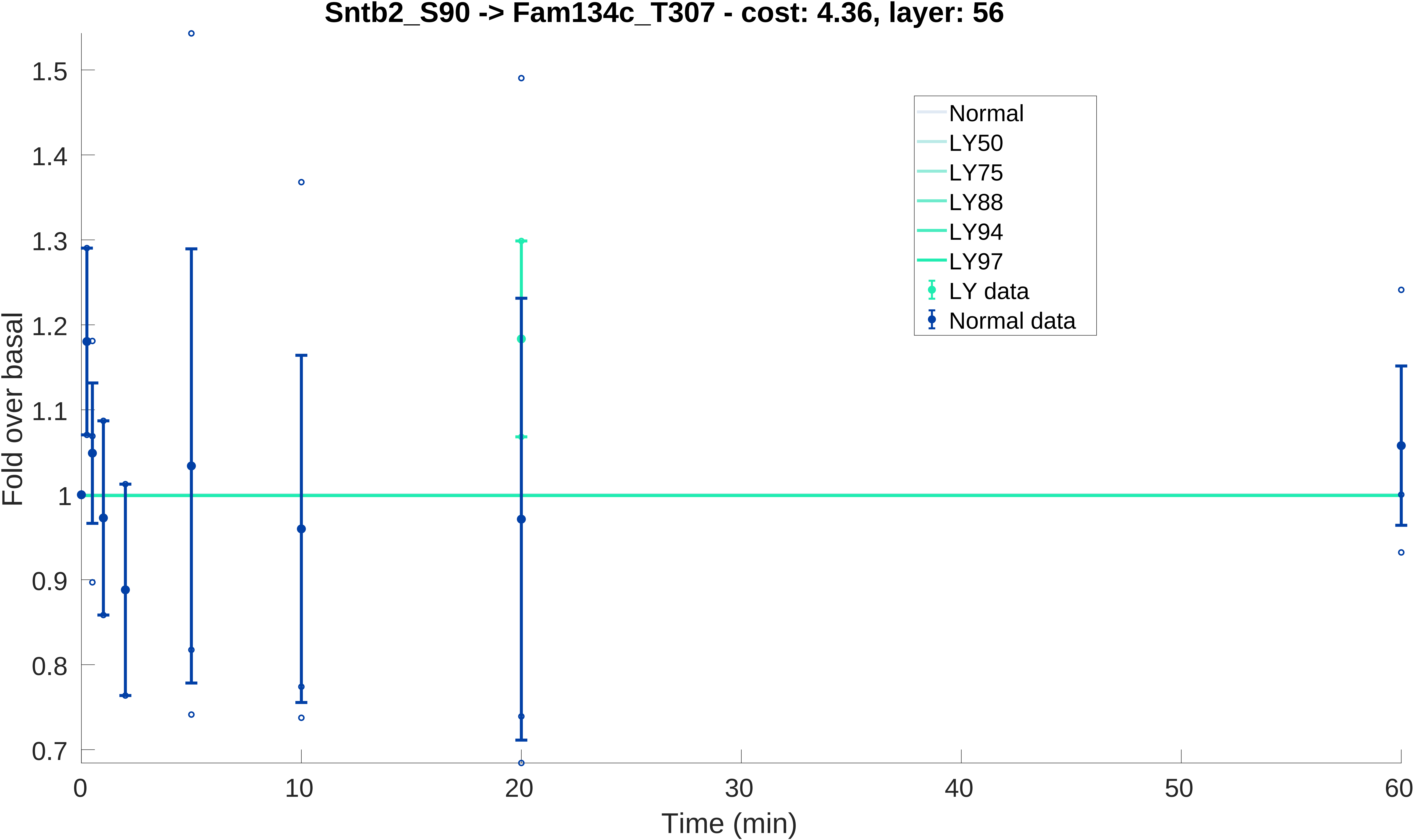

Sntb2_S90 -> Fam134c_T307 - cost: 4.36, layer: 56
Fold over basal
Time (min)
1.5
1.4
1.3
1.2
1.1
1
0.9
0.8
0.7
0
10
20
30
40
50
60
Normal
LY50
LY75
LY88
LY94
LY97
LY data
Normal data

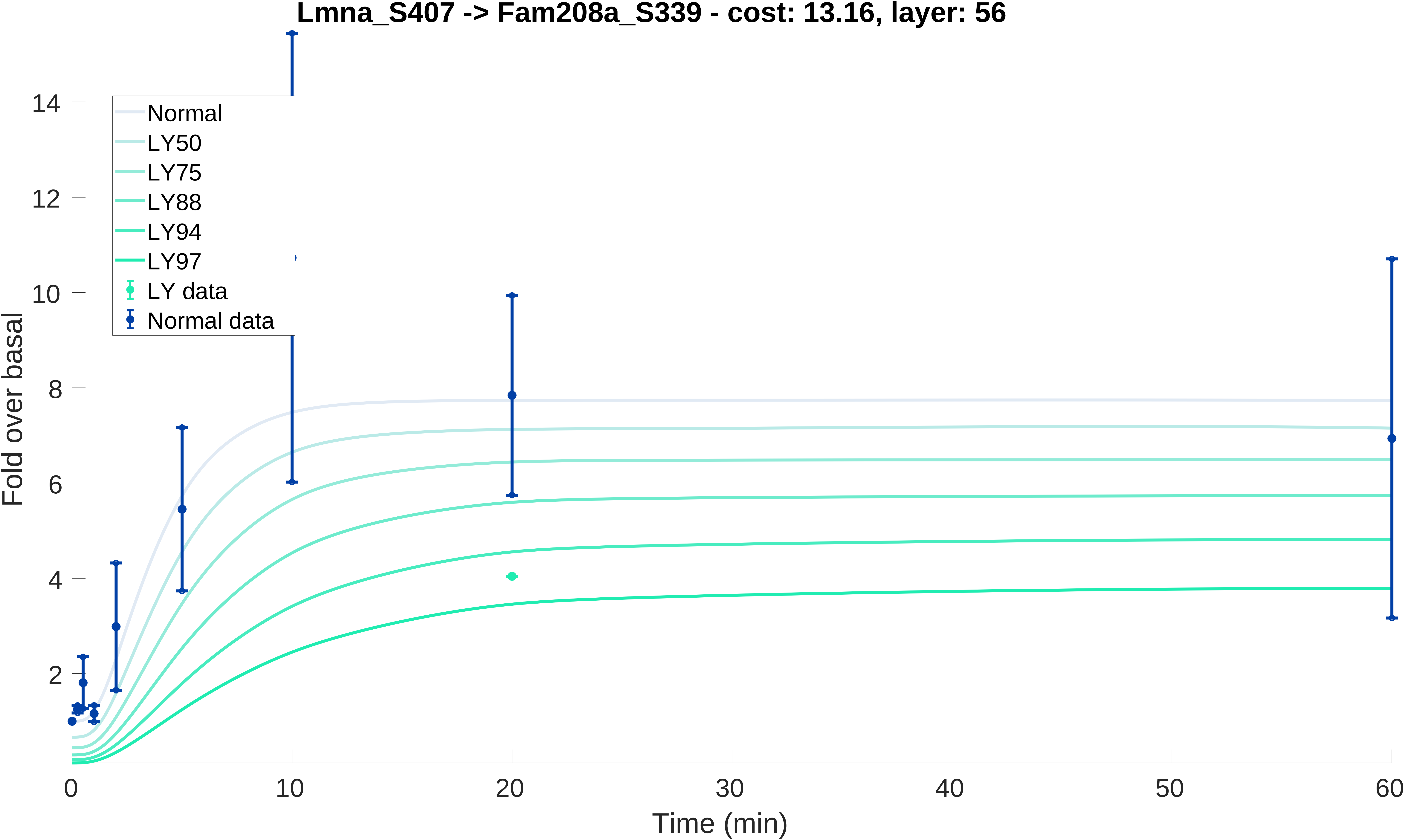

Lmna_S407 -> Fam208a_S339 - cost: 13.16, layer: 56
Normal
LY50
LY75
LY88
LY94
LY97
LY data
Normal data
Fold over basal
Time (min)
0
10
20
30
40
50
60
2
4
6
8
10
12
14

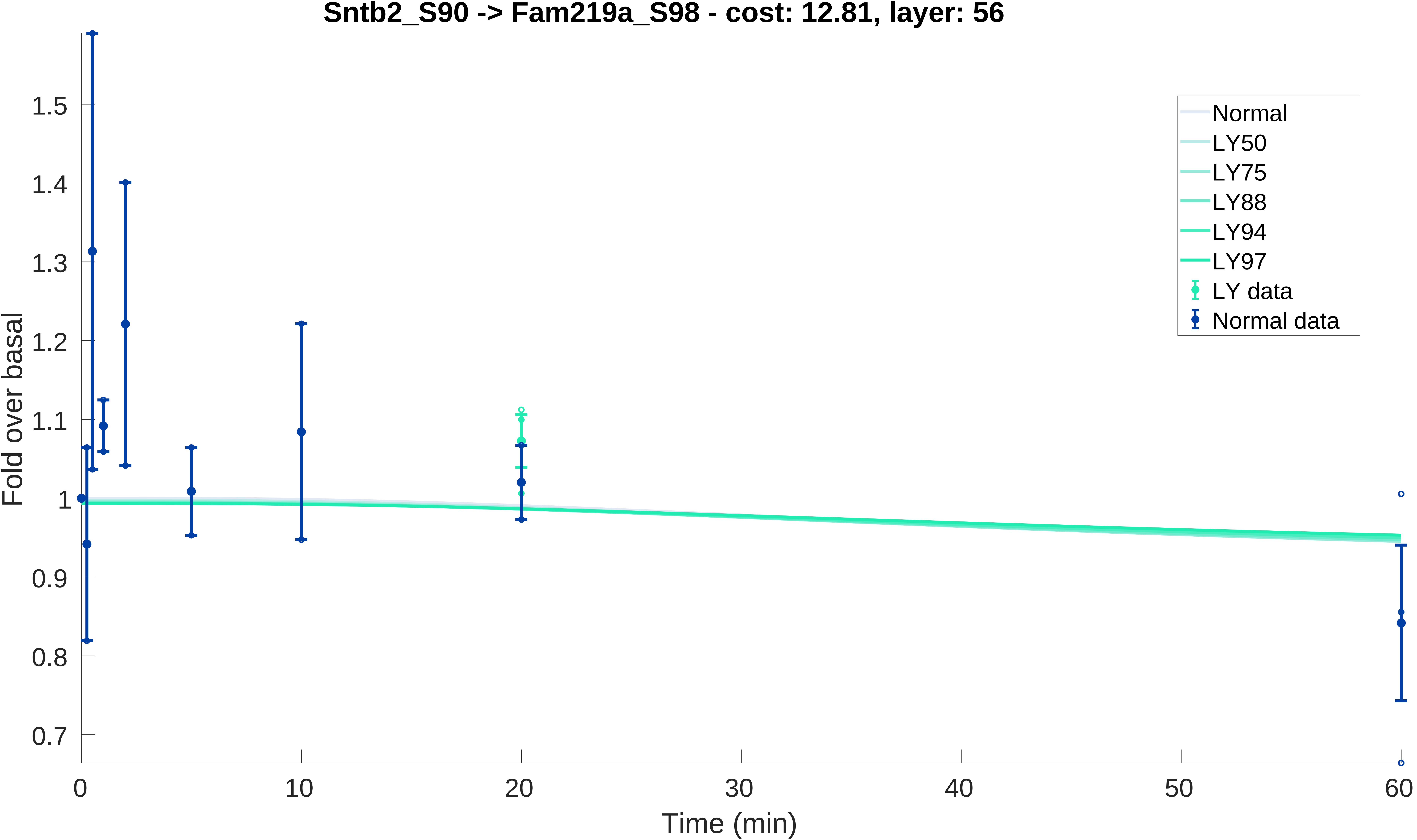

Sntb2_S90 -> Fam219a_S98 - cost: 12.81, layer: 56
Fold over basal
1.5
1.4
1.3
1.2
1.1
1
0.9
0.8
0.7
0
10
20
30
40
50
60
Time (min)
Normal
LY50
LY75
LY88
LY94
LY97
LY data
Normal data

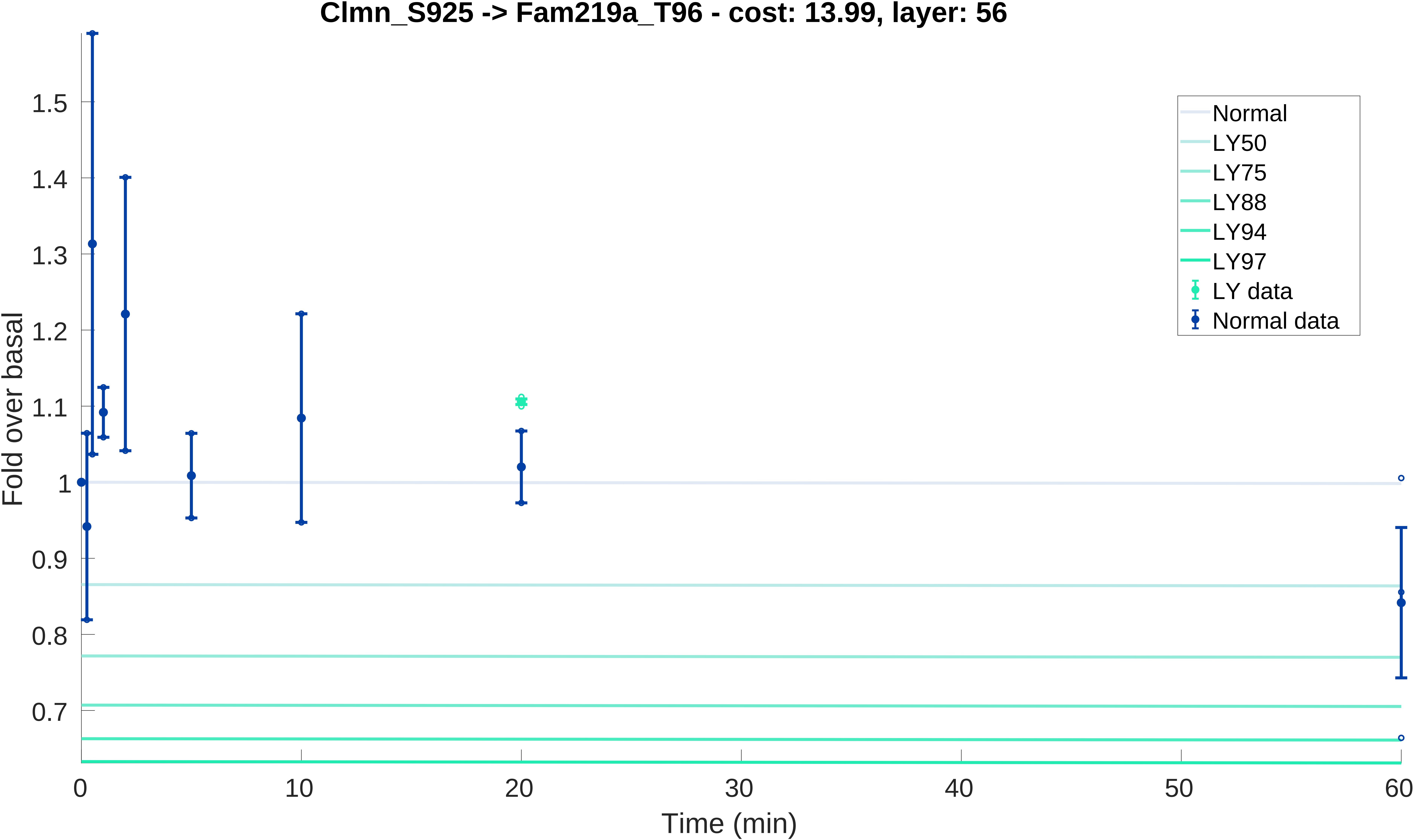

Clmn_S925 -> Fam219a_T96 - cost: 13.99, layer: 56
Fold over basal
Time (min)
Normal
LY50
LY75
LY88
LY94
LY97
LY data
Normal data
1.5
1.4
1.3
1.2
1.1
1
0.9
0.8
0.7
0
10
20
30
40
50
60

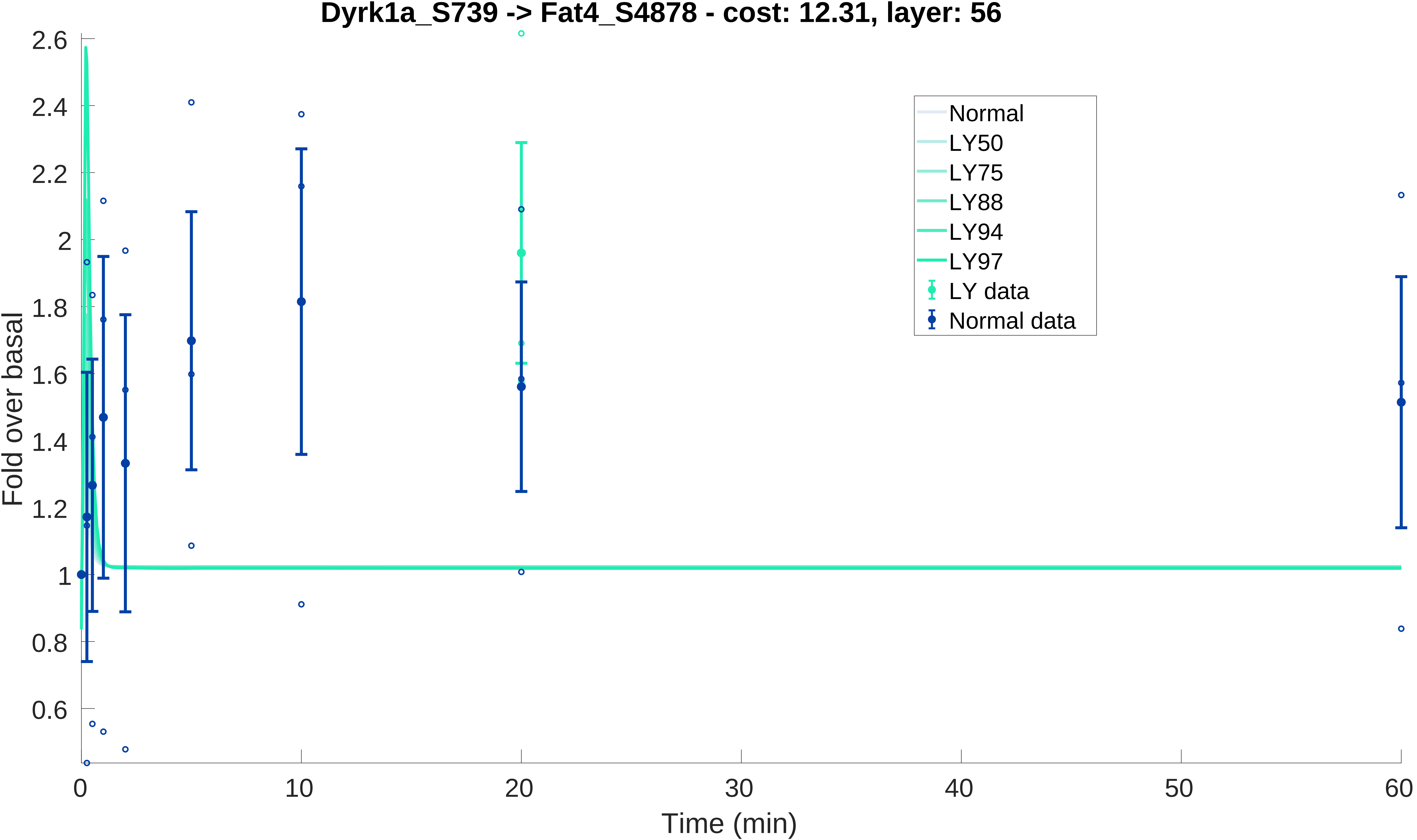

Dyrk1a_S739 -> Fat4_S4878 - cost: 12.31, layer: 56
Fold over basal
Time (min)
0
10
20
30
40
50
60
0.6
0.8
1
1.2
1.4
1.6
1.8
2
2.2
2.4
2.6
Normal
LY50
LY75
LY88
LY94
LY97
LY data
Normal data

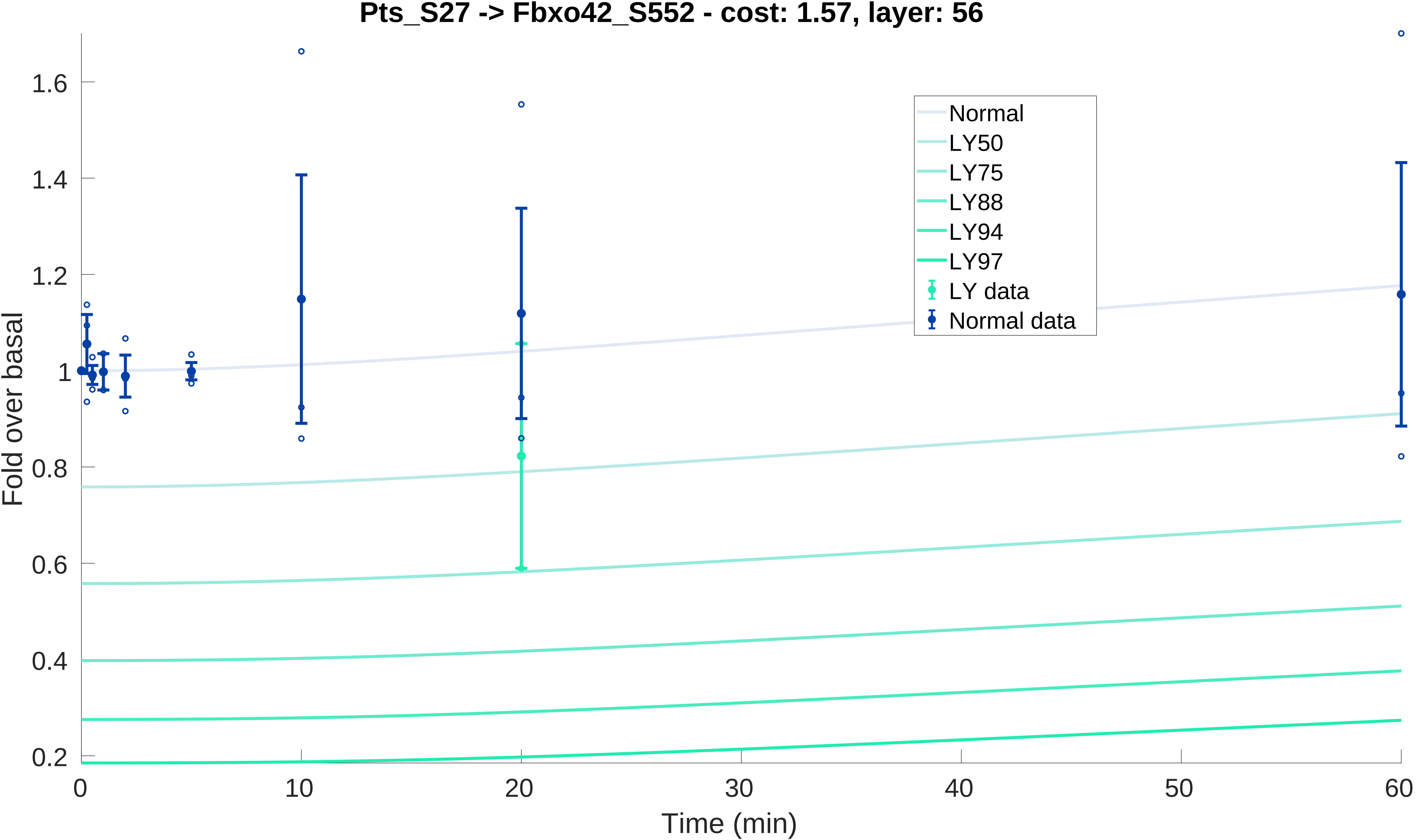

Pts_S27 -> Fbxo42_S552 - cost: 1.57, layer: 56
Fold over basal
Time (min)
0
10
20
30
40
50
60
0.2
0.4
0.6
0.8
1
1.2
1.4
1.6
Normal
LY50
LY75
LY88
LY94
LY97
LY data
Normal data

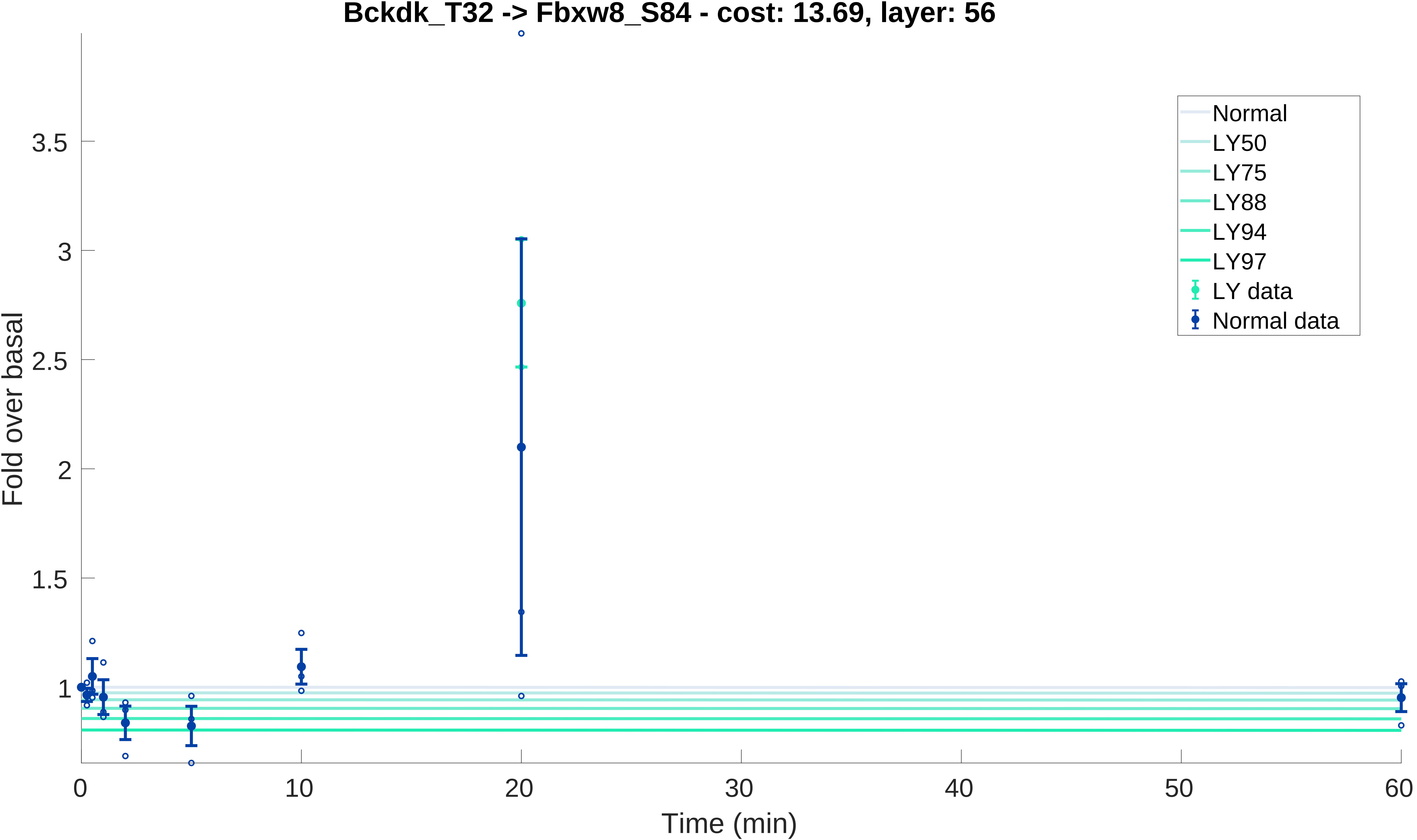

Bckdk_T32 -> Fbxw8_S84 - cost: 13.69, layer: 56
Fold over basal
3.5
3
2.5
2
1.5
1
Time (min)
0
10
20
30
40
50
60
Normal
LY50
LY75
LY88
LY94
LY97
LY data
Normal data

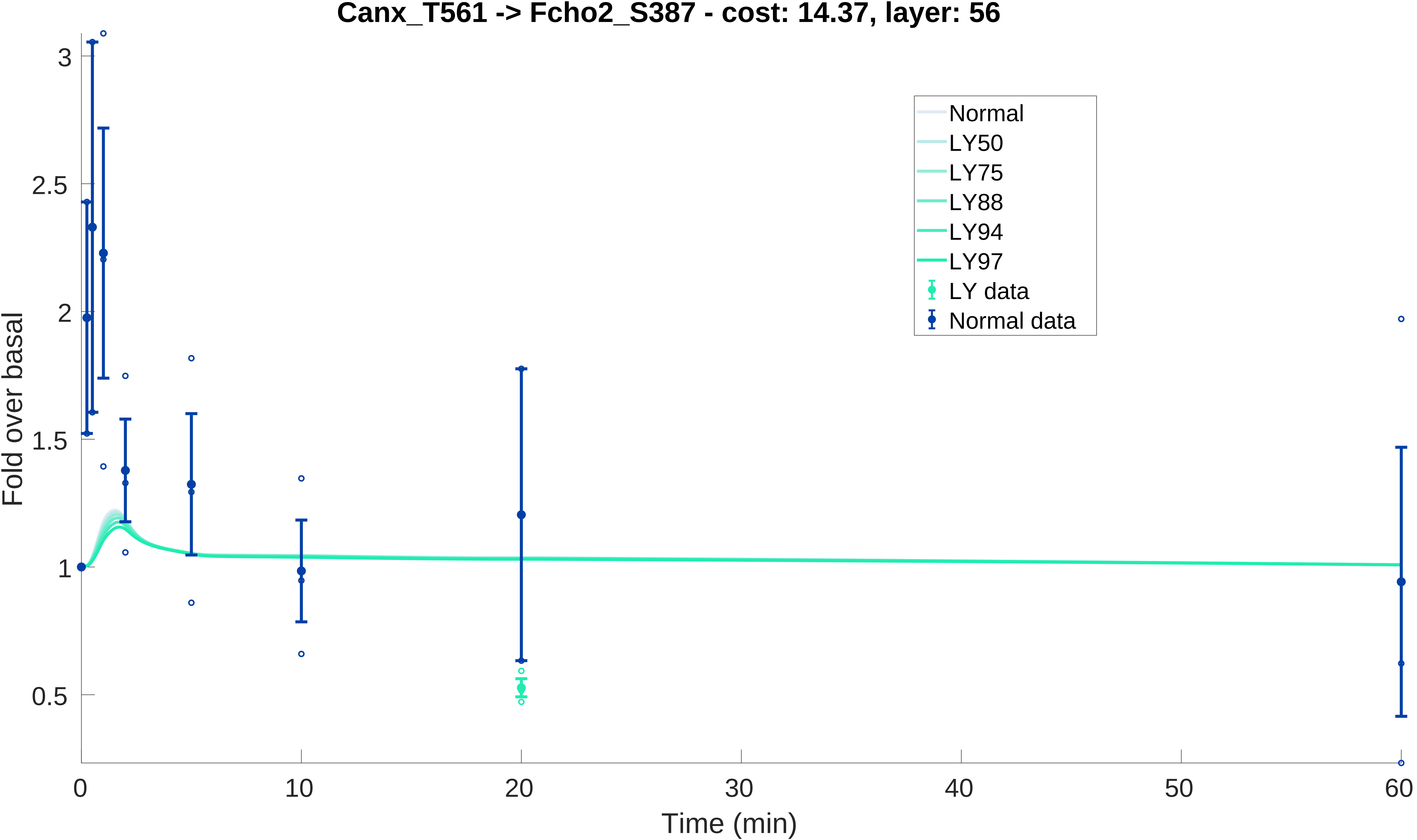
Canx_T561 -> Fcho2_S387 - cost: 14.37, layer: 56
Fold over basal
Time (min)
0.5
1
1.5
2
2.5
3
0
10
20
30
40
50
60
Normal
LY50
LY75
LY88
LY94
LY97
LY data
Normal data

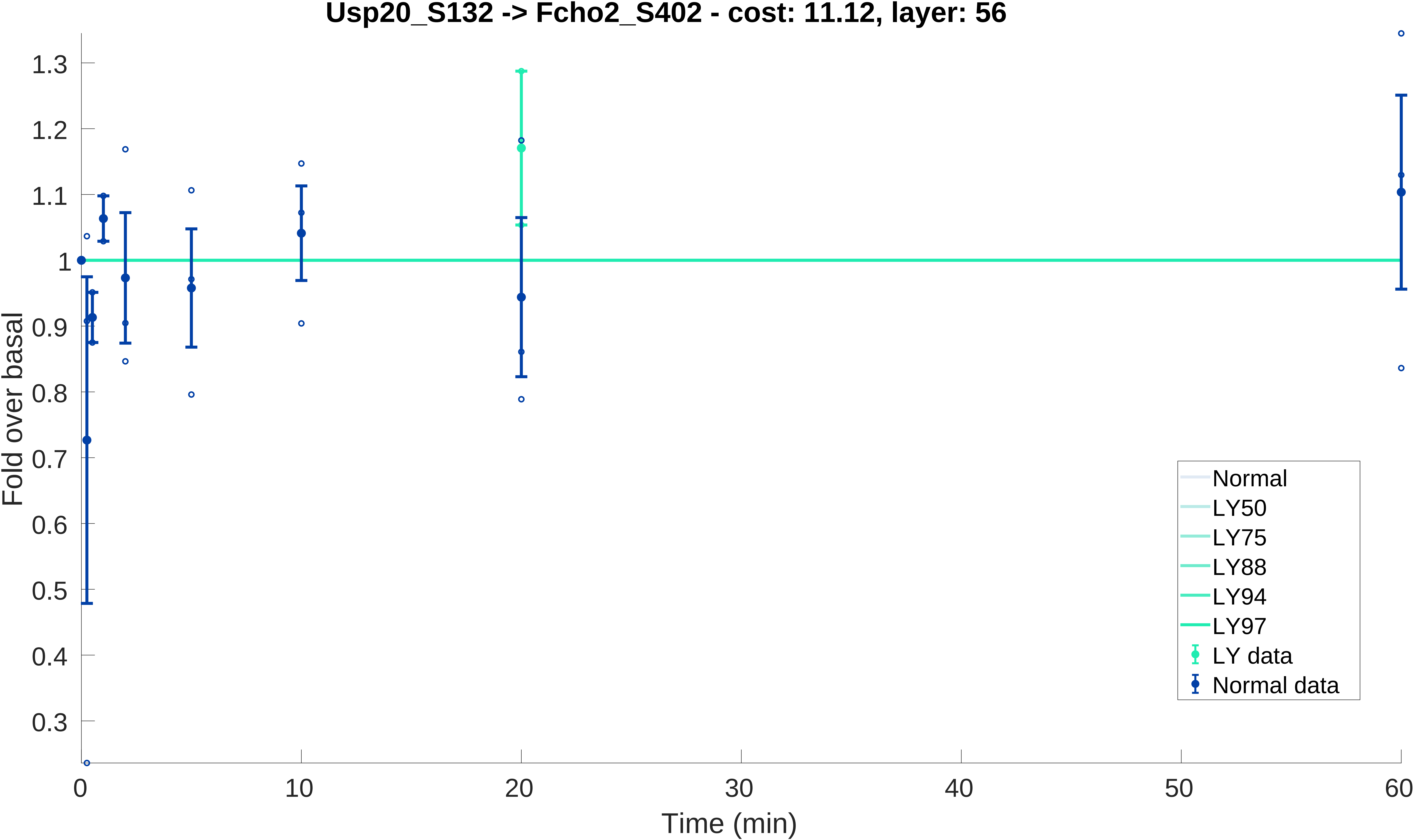

Usp20_S132 -> Fcho2_S402 - cost: 11.12, layer: 56
Fold over basal
1.3
1.2
1.1
1
0.9
0.8
0.7
0.6
0.5
0.4
0.3
0
10
20
30
40
50
60
Time (min)
Normal
LY50
LY75
LY88
LY94
LY97
LY data
Normal data

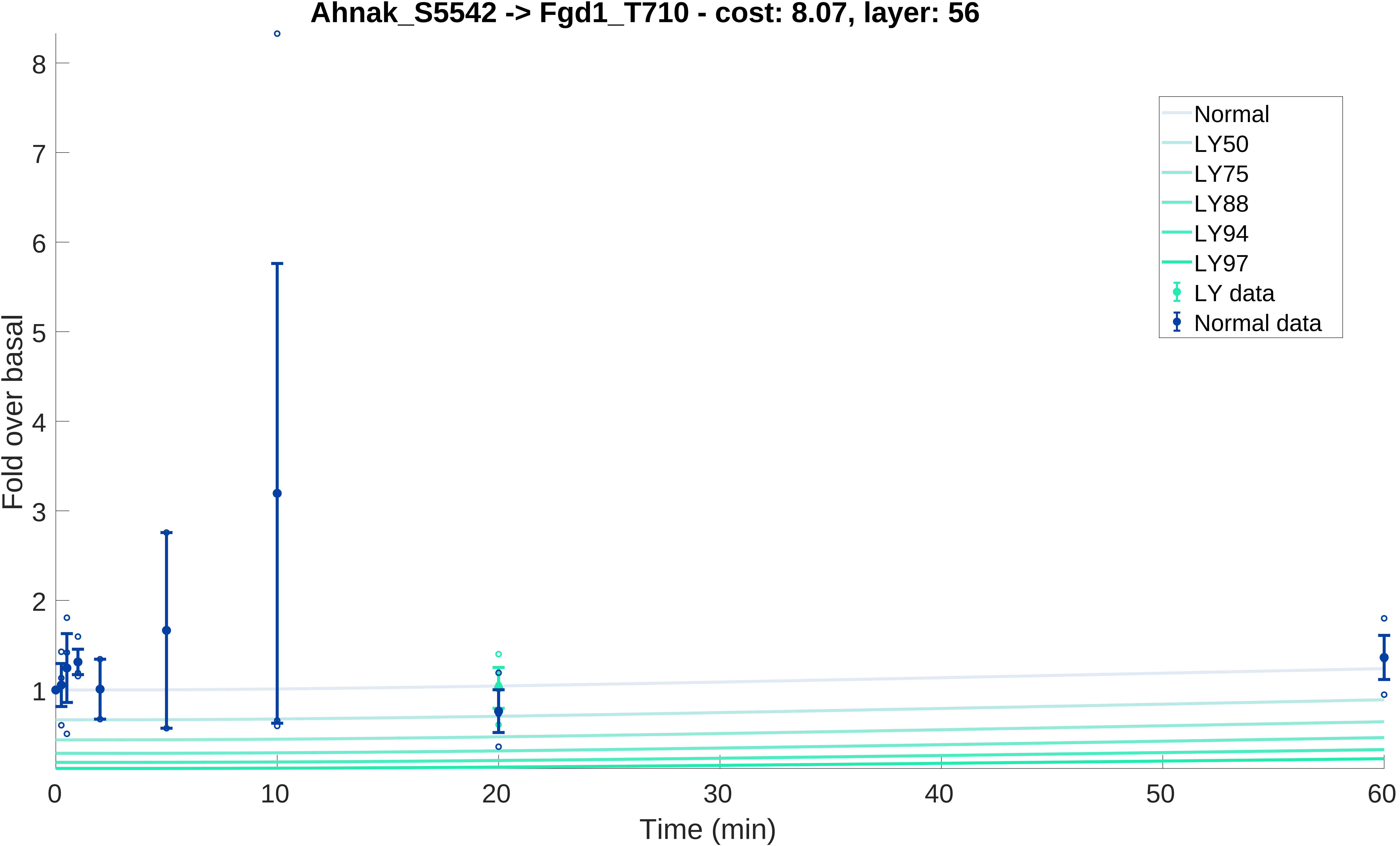

Ahnak_S5542 -> Fgd1_T710 - cost: 8.07, layer: 56
Fold over basal
Time (min)
0
10
20
30
40
50
60
1
2
3
4
5
6
7
8
Normal
LY50
LY75
LY88
LY94
LY97
LY data
Normal data

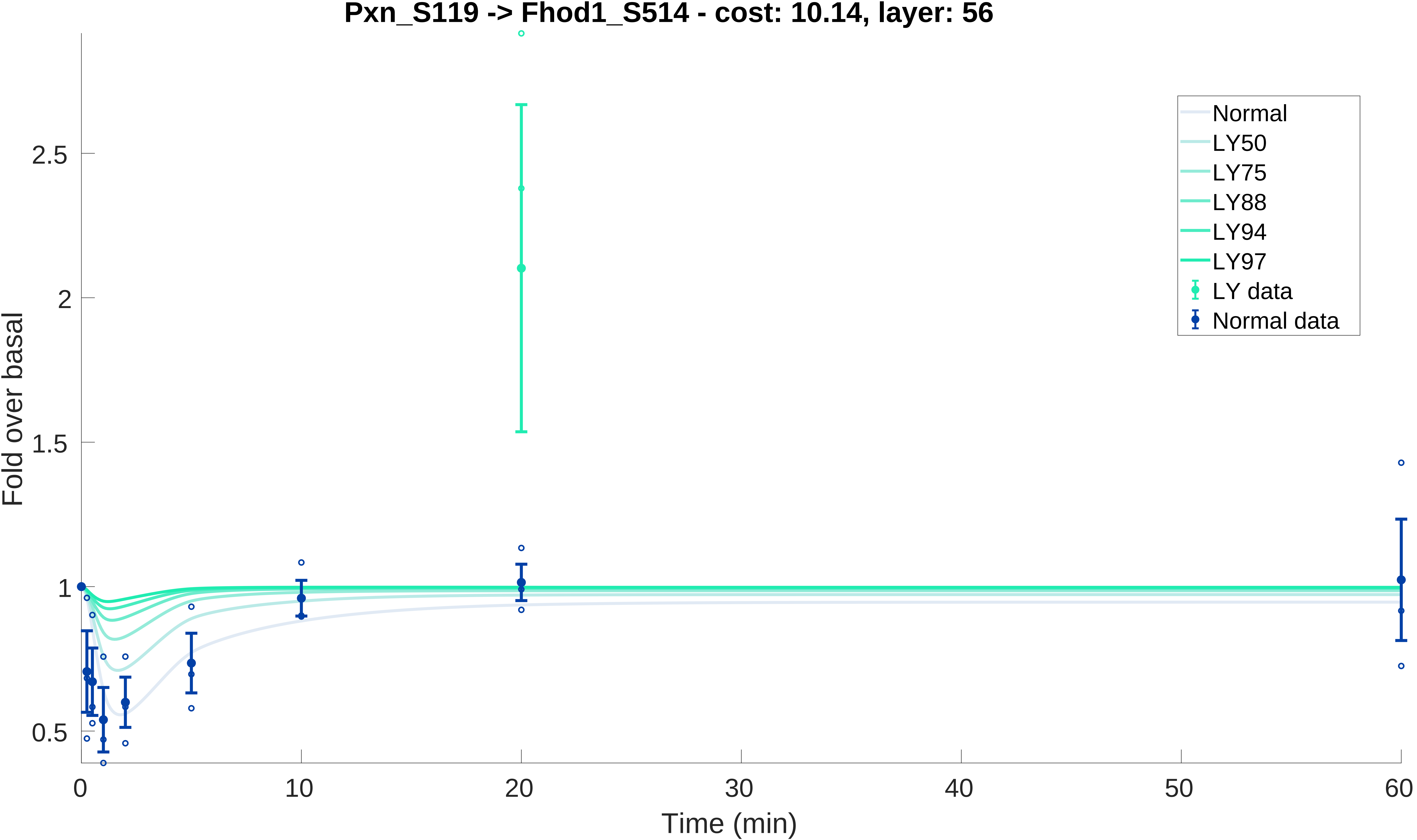

Pxn_S119 -> Fhod1_S514 - cost: 10.14, layer: 56
Fold over basal
Time (min)
0.5
1
1.5
2
2.5
0
10
20
30
40
50
60
Normal
LY50
LY75
LY88
LY94
LY97
LY data
Normal data

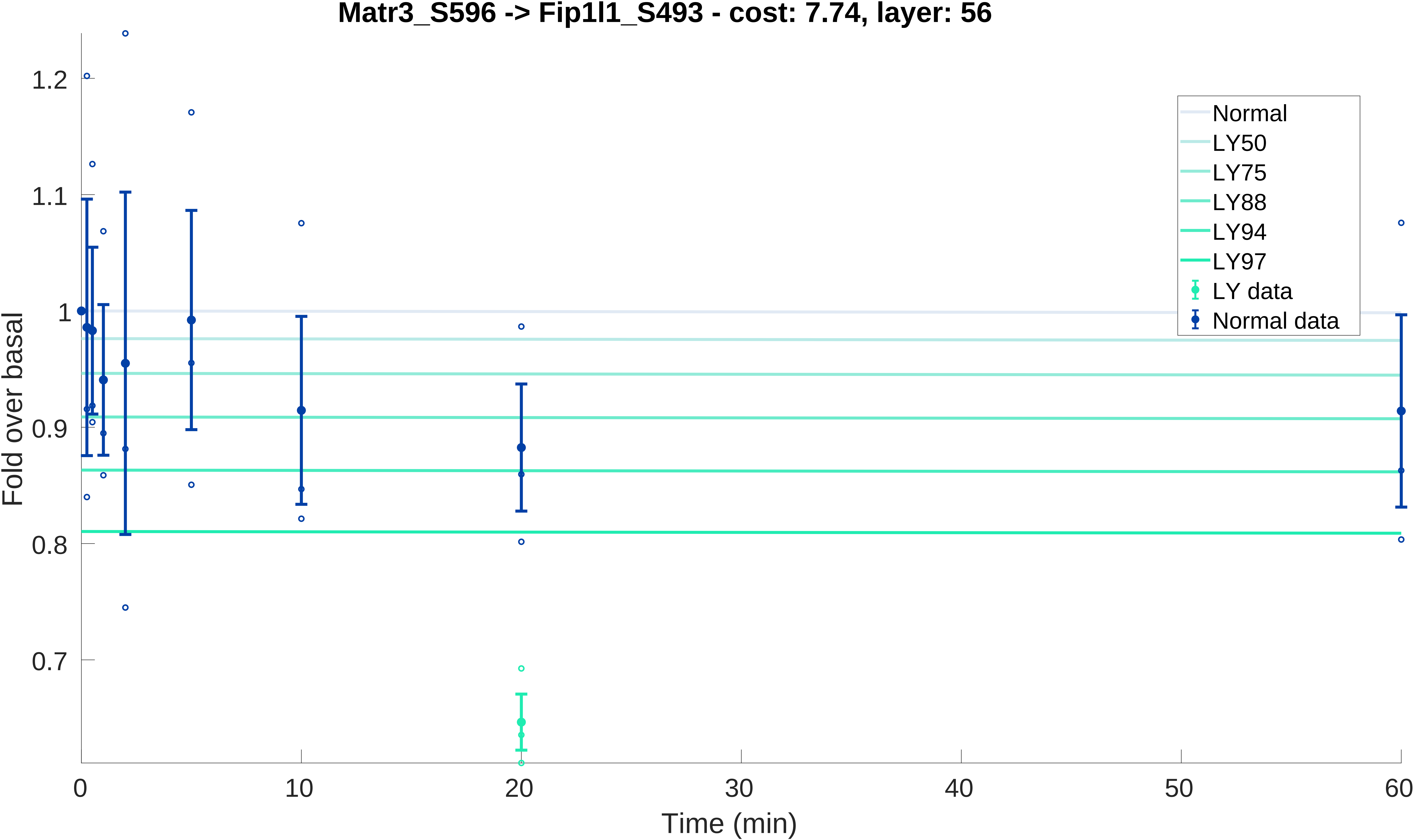
Matr3_S596 -> Fip1l1_S493 - cost: 7.74, layer: 56
Fold over basal
1.2
1.1
1
0.9
0.8
0.7
Time (min)
0
10
20
30
40
50
60
Normal
LY50
LY75
LY88
LY94
LY97
LY data
Normal data

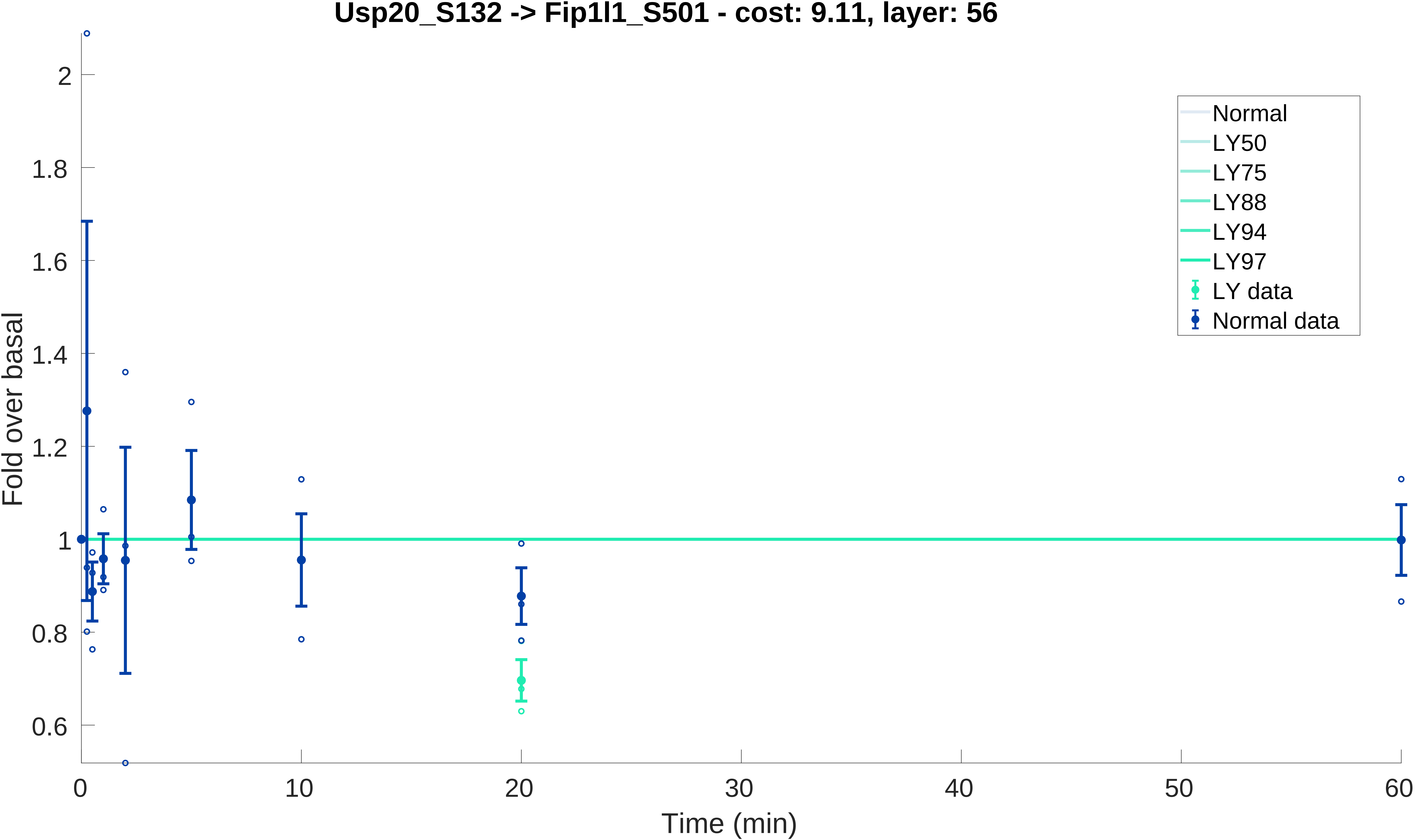

Usp20_S132 -> Fip1l1_S501 - cost: 9.11, layer: 56
Fold over basal
Time (min)
0
10
20
30
40
50
60
0.6
0.8
1
1.2
1.4
1.6
1.8
2
Normal
LY50
LY75
LY88
LY94
LY97
LY data
Normal data

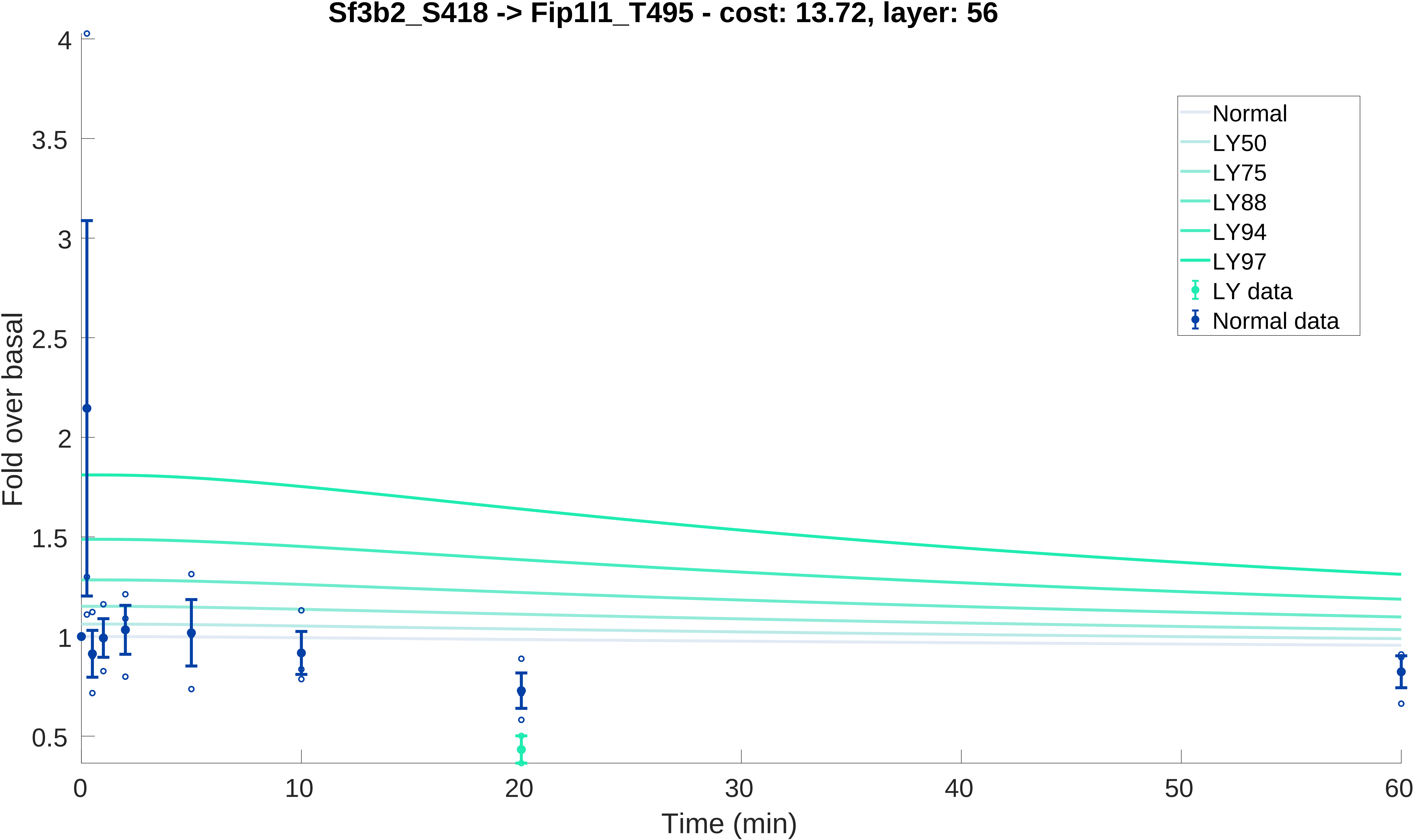
Sf3b2_S418 -> Fip1l1_T495 - cost: 13.72, layer: 56
Fold over basal
Time (min)
0
10
20
30
40
50
60
0.5
1
1.5
2
2.5
3
3.5
4
Normal
LY50
LY75
LY88
LY94
LY97
LY data
Normal data

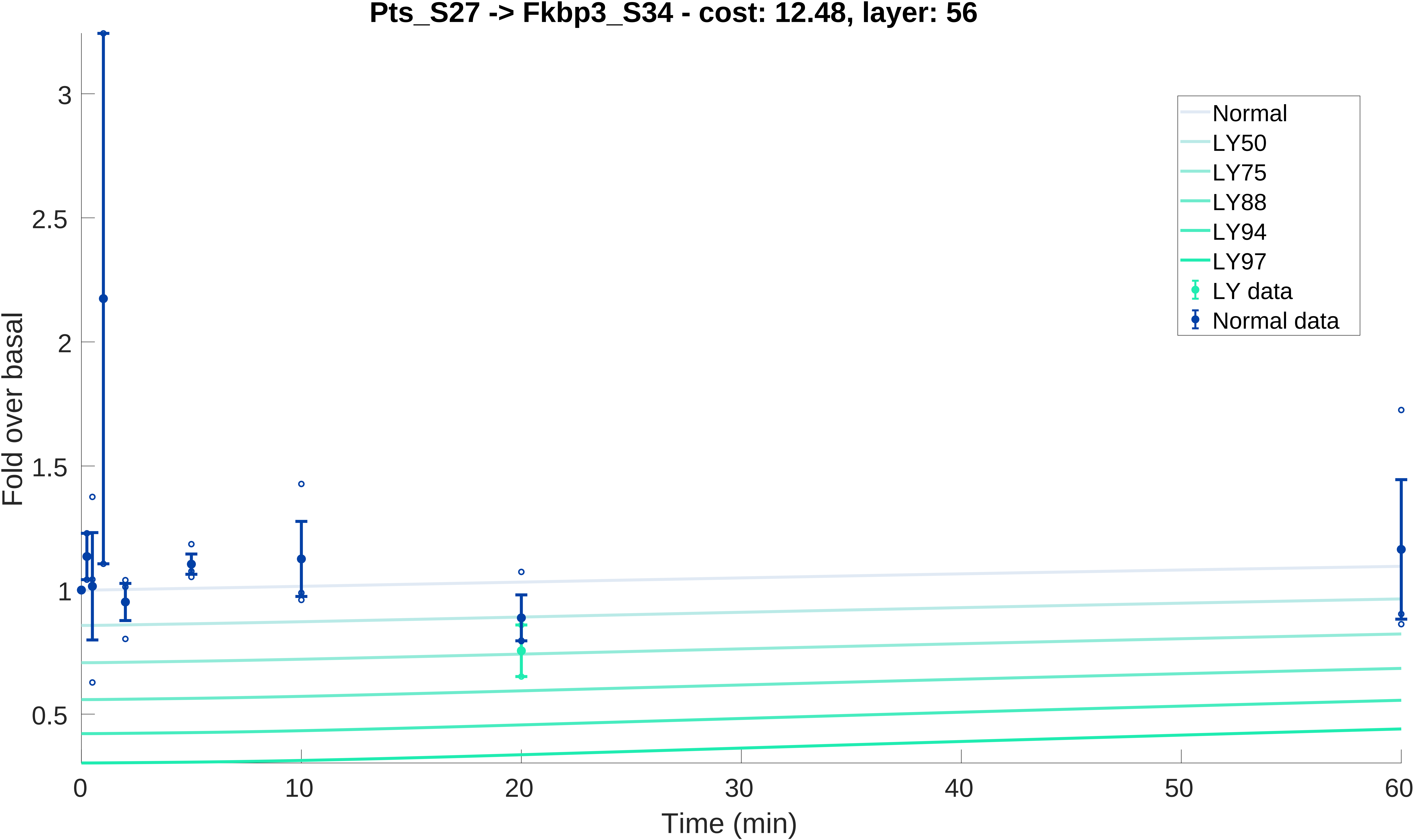
Pts_S27 -> Fkbp3_S34 - cost: 12.48, layer: 56
Fold over basal
3
2.5
2
1.5
1
0.5
0
10
20
30
40
50
60
Time (min)
Normal
LY50
LY75
LY88
LY94
LY97
LY data
Normal data

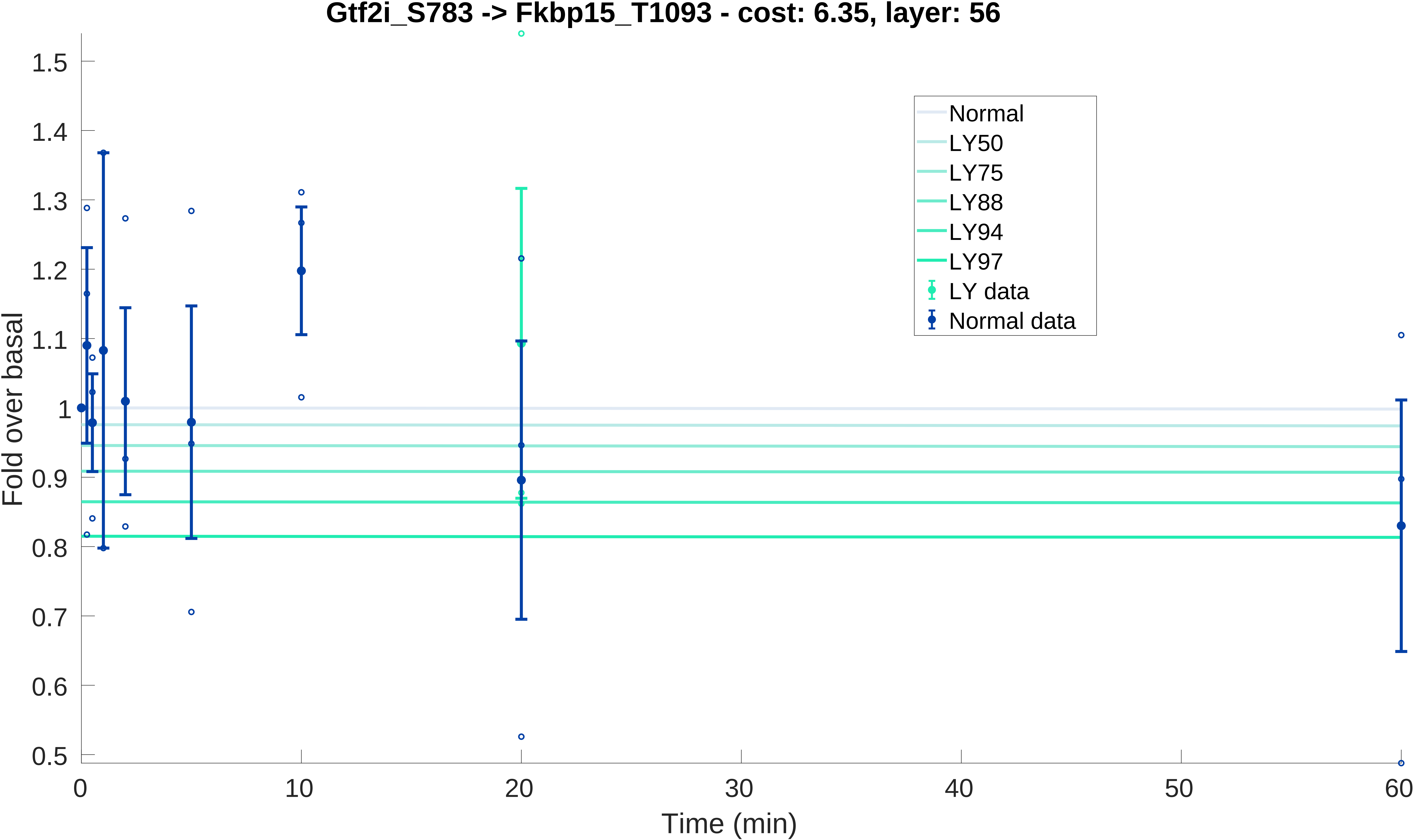

Gtf2i_S783 -> Fkbp15_T1093 - cost: 6.35, layer: 56
Fold over basal
Time (min)
0
10
20
30
40
50
60
0.5
0.6
0.7
0.8
0.9
1
1.1
1.2
1.3
1.4
1.5
Normal
LY50
LY75
LY88
LY94
LY97
LY data
Normal data

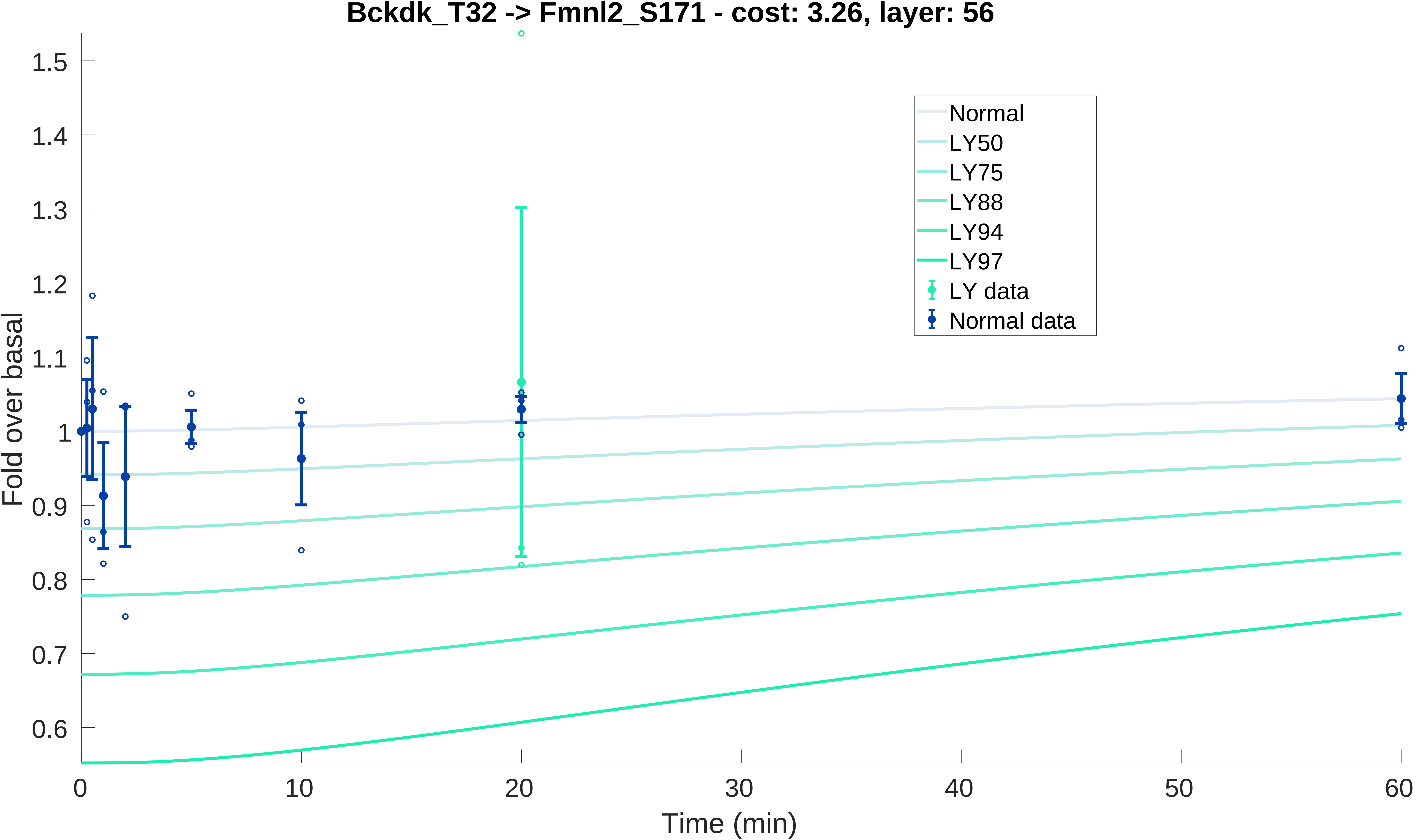

Bckdk_T32 -> Fmnl2_S171 - cost: 3.26, layer: 56
Fold over basal
1.5
1.4
1.3
1.2
1.1
1
0.9
0.8
0.7
0.6
Time (min)
0
10
20
30
40
50
60
Normal
LY50
LY75
LY88
LY94
LY97
LY data
Normal data

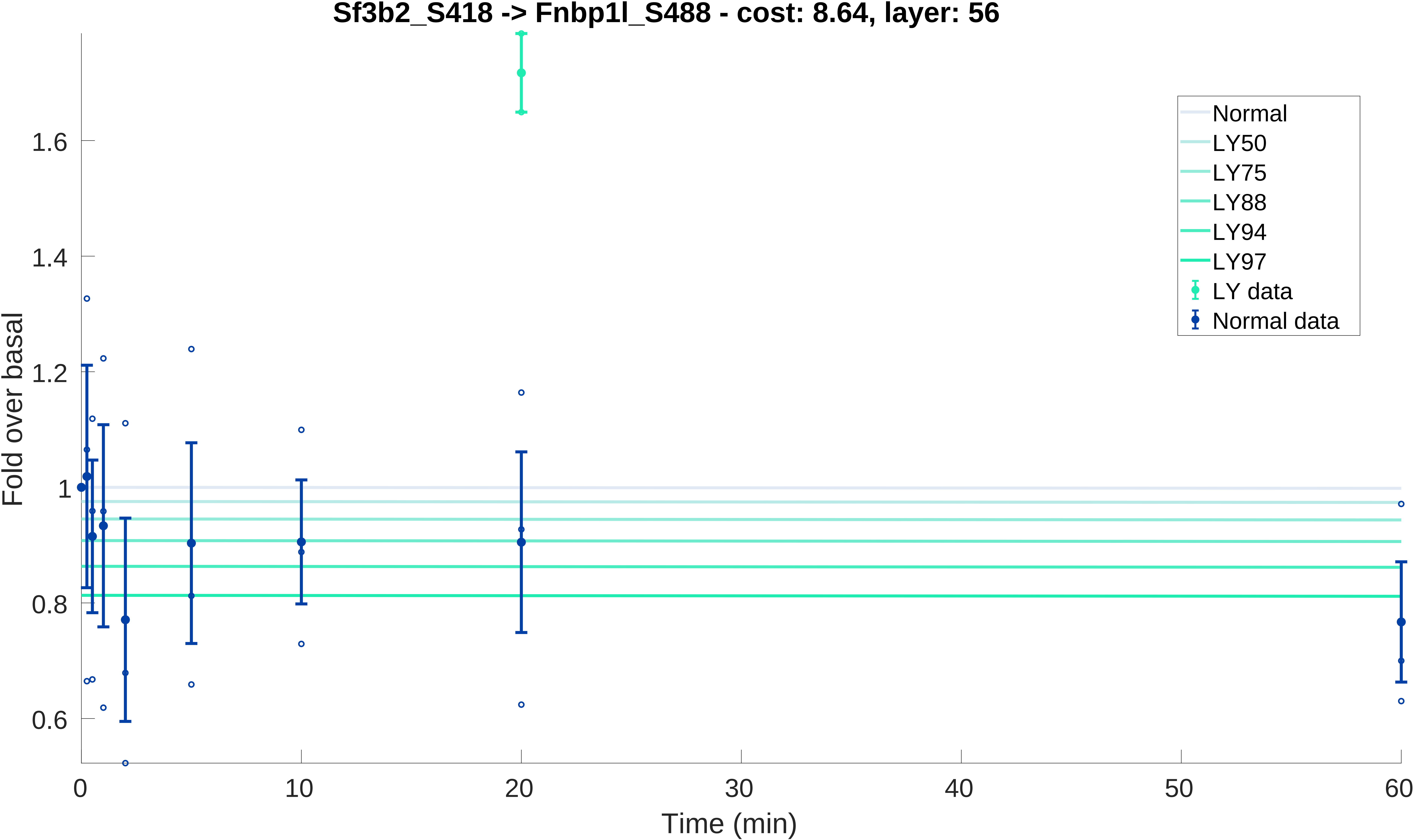

Sf3b2_S418 -> Fnbp1l_S488 - cost: 8.64, layer: 56
Fold over basal
1.6
1.4
1.2
1
0.8
0.6
0
10
20
30
40
50
60
Time (min)
Normal
LY50
LY75
LY88
LY94
LY97
LY data
Normal data

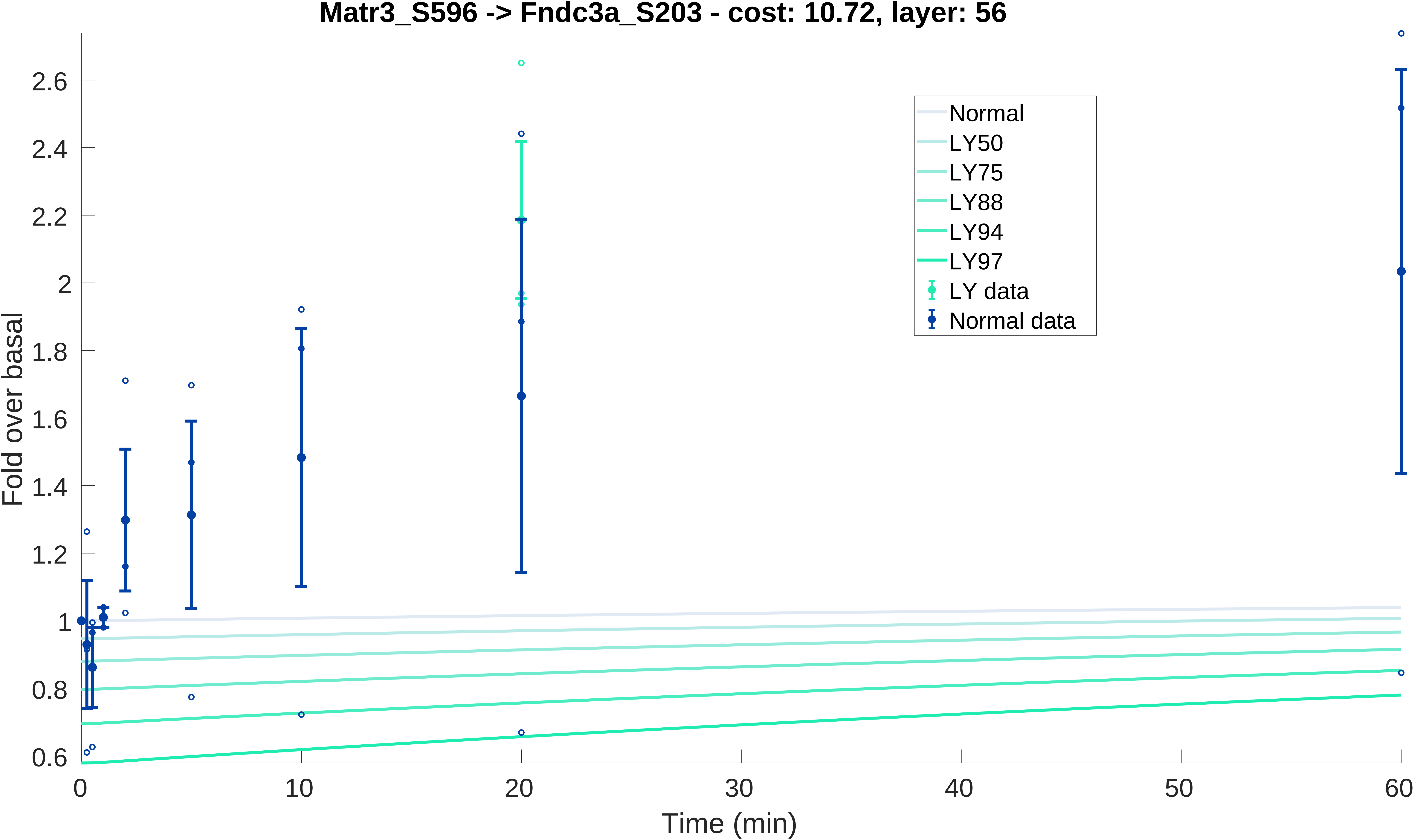
Matr3_S596 -> Fndc3a_S203 - cost: 10.72, layer: 56
Fold over basal
2.6
2.4
2.2
2
1.8
1.6
1.4
1.2
1
0.8
0.6
0
10
20
30
40
50
60
Time (min)
Normal
LY50
LY75
LY88
LY94
LY97
LY data
Normal data

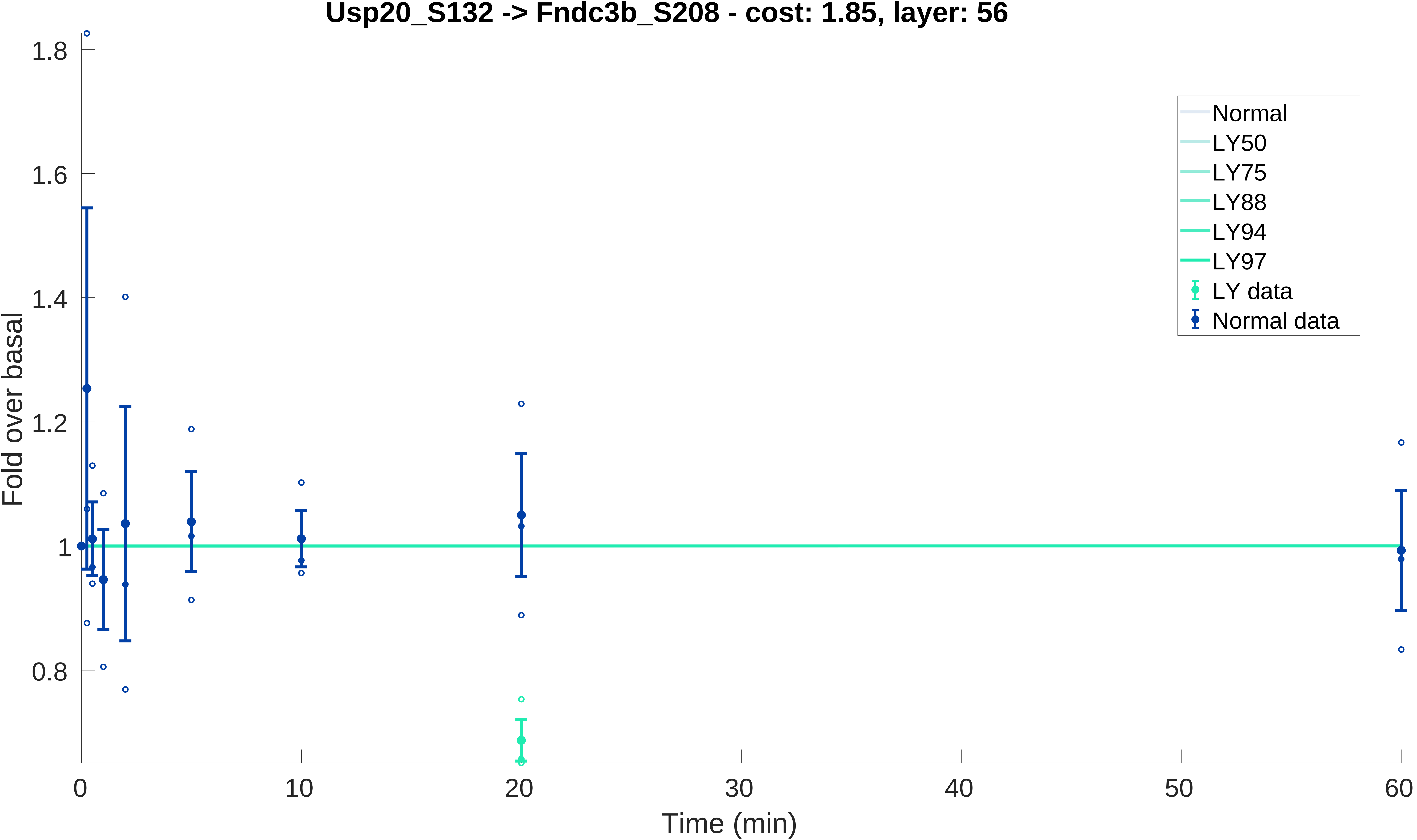
Usp20_S132 -> Fndc3b_S208 - cost: 1.85, layer: 56
Fold over basal
1.8
1.6
1.4
1.2
1
0.8
0
10
20
30
40
50
60
Time (min)
Normal
LY50
LY75
LY88
LY94
LY97
LY data
Normal data

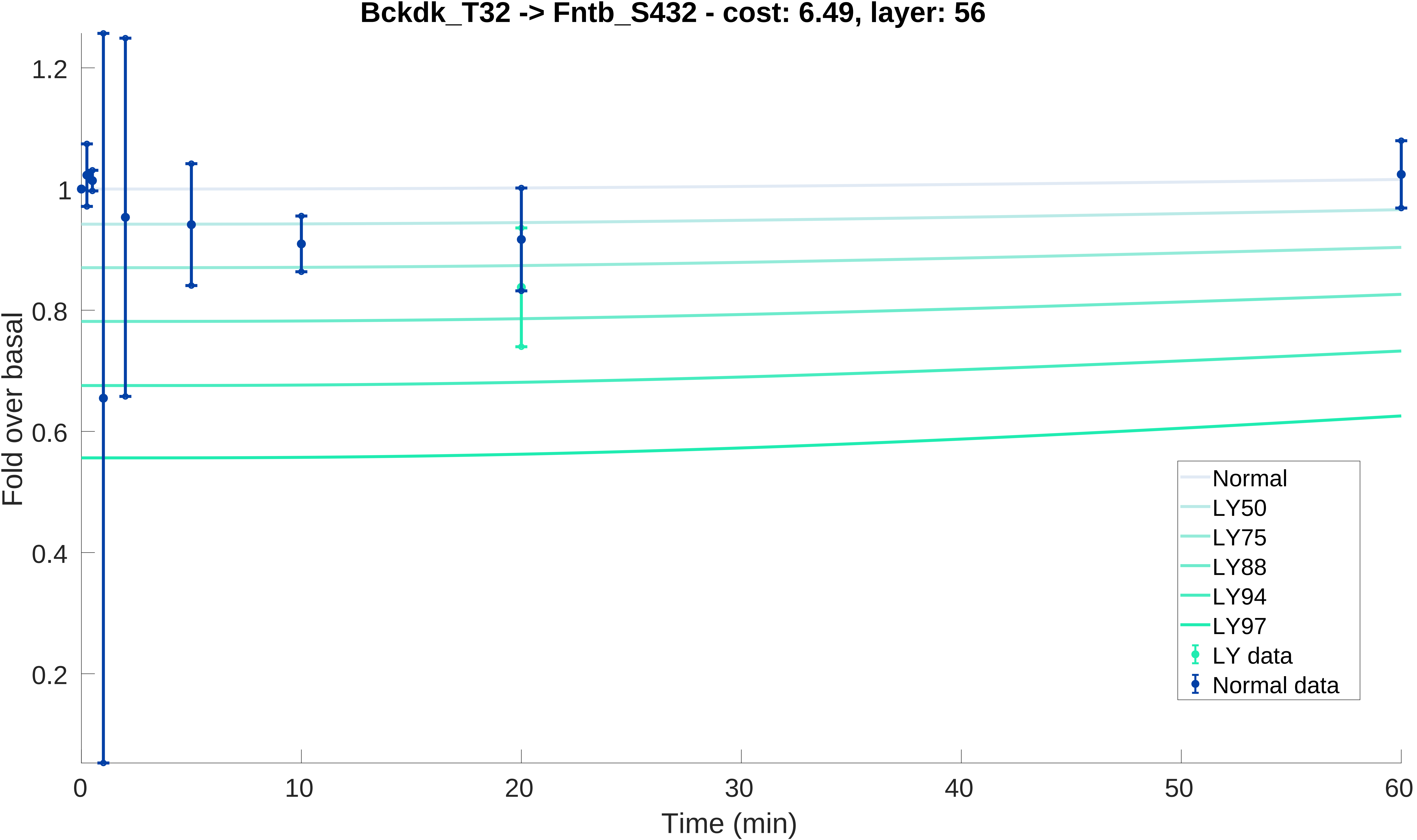
Bckdk_T32 -> Fntb_S432 - cost: 6.49, layer: 56
Fold over basal
Time (min)
1.2
1
0.8
0.6
0.4
0.2
0
10
20
30
40
50
60
Normal
LY50
LY75
LY88
LY94
LY97
LY data
Normal data

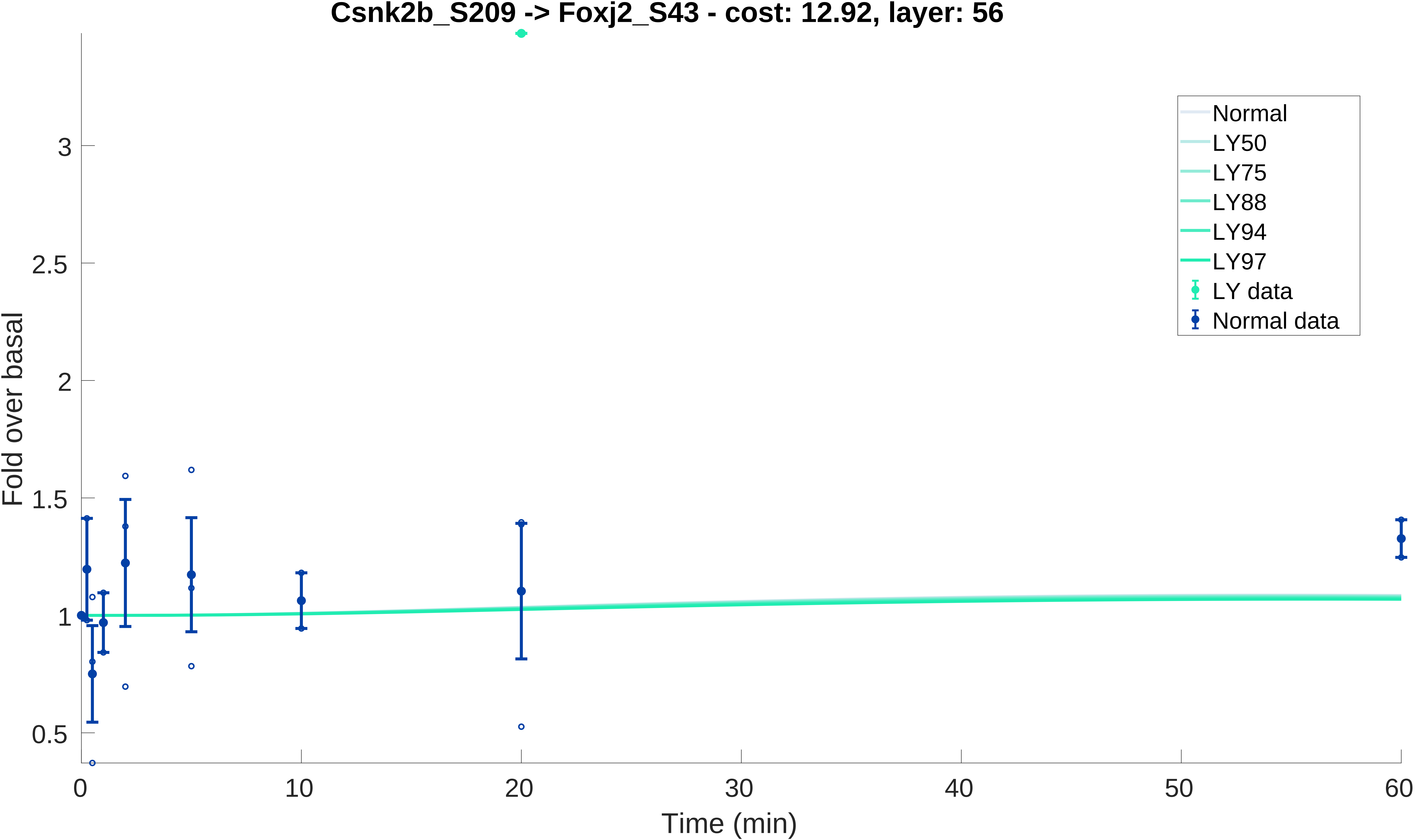
Csnk2b_S209 -> Foxj2_S43 - cost: 12.92, layer: 56
Fold over basal
Time (min)
0
10
20
30
40
50
60
0.5
1
1.5
2
2.5
3
Normal
LY50
LY75
LY88
LY94
LY97
LY data
Normal data

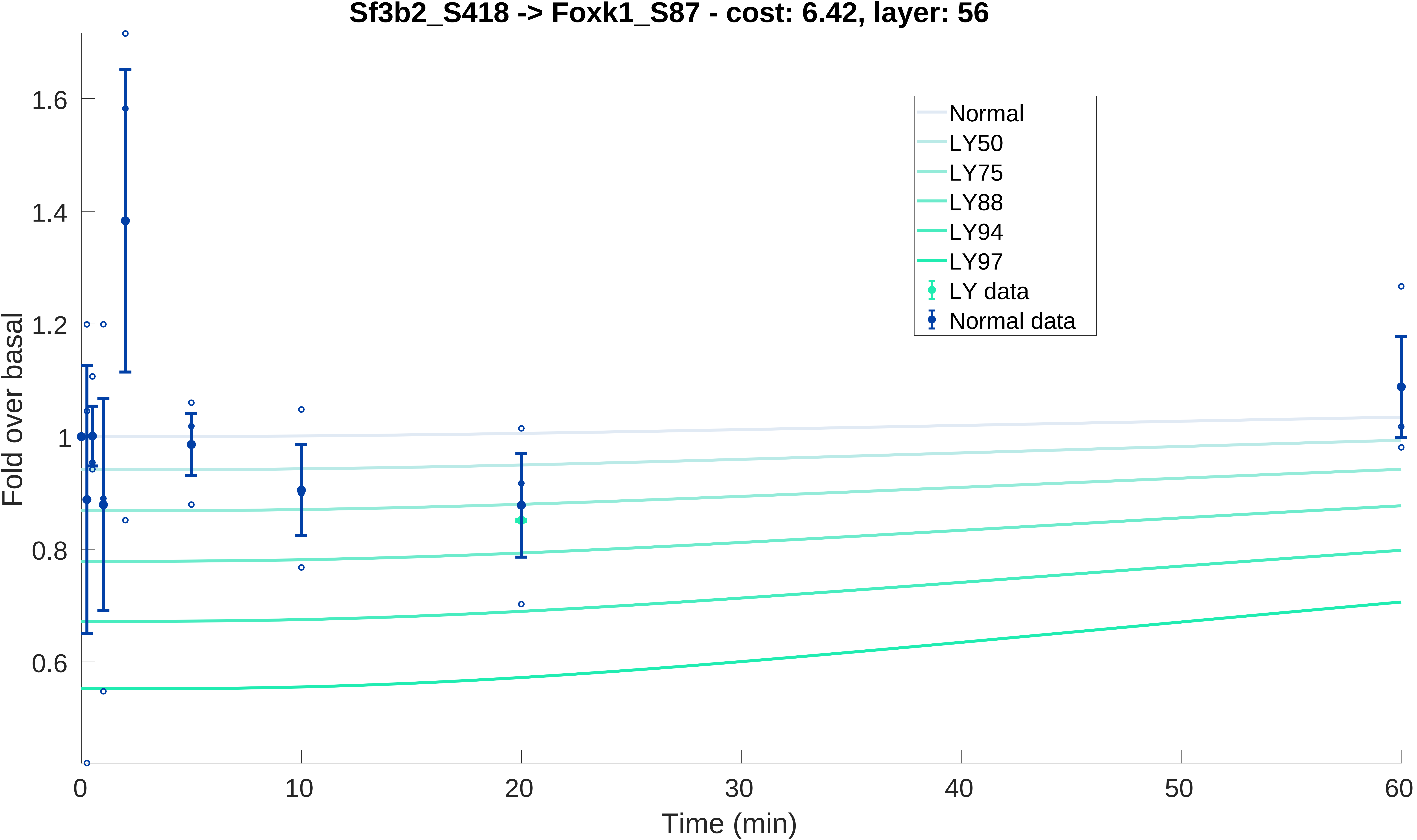

Sf3b2_S418 -> Foxk1_S87 - cost: 6.42, layer: 56
Fold over basal
Time (min)
Normal
LY50
LY75
LY88
LY94
LY97
LY data
Normal data
1.6
1.4
1.2
1
0.8
0.6
0
10
20
30
40
50
60

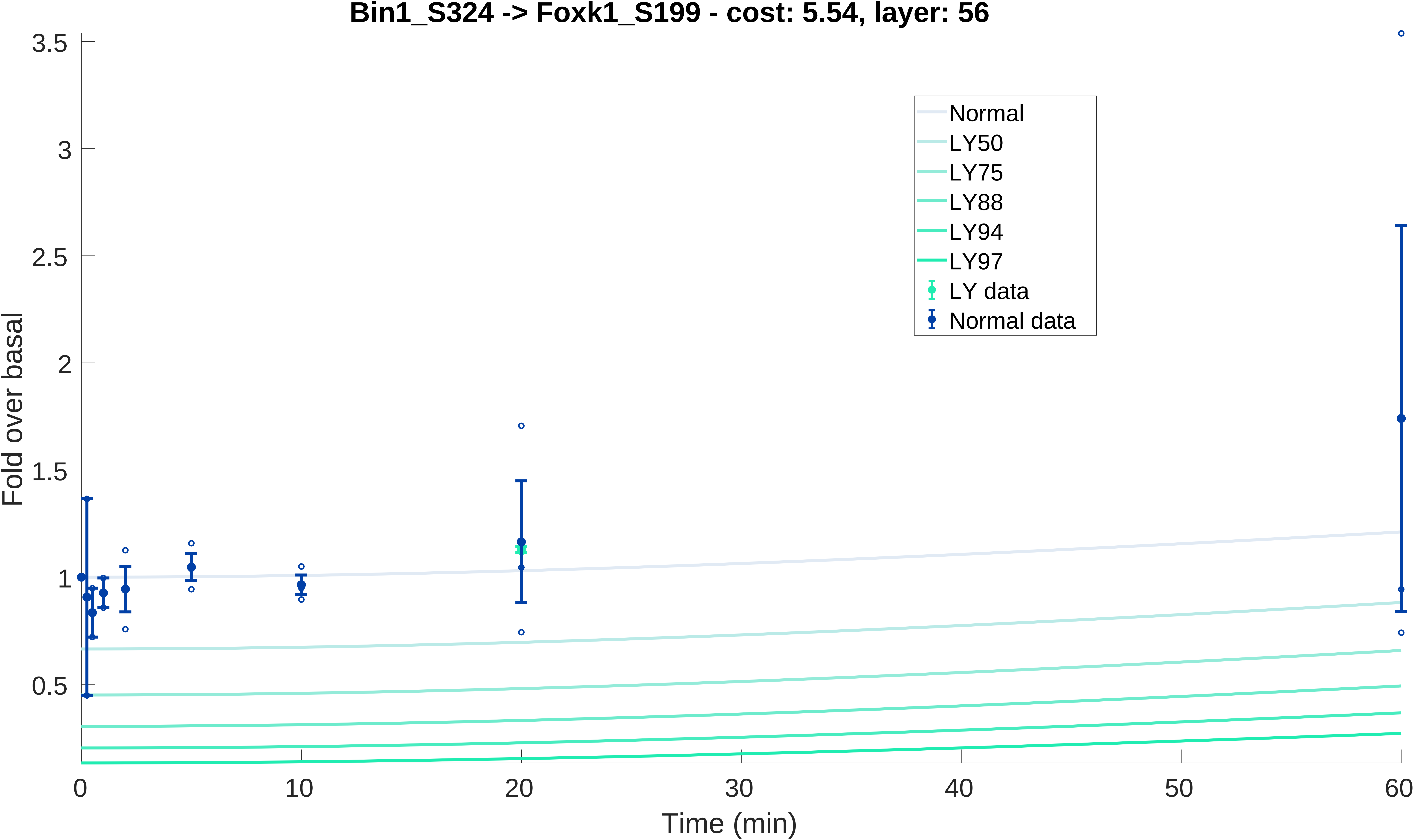

Bin1_S324 -> Foxk1_S199 - cost: 5.54, layer: 56
Fold over basal
3.5
3
2.5
2
1.5
1
0.5
0
10
20
30
40
50
60
Time (min)
Normal
LY50
LY75
LY88
LY94
LY97
LY data
Normal data

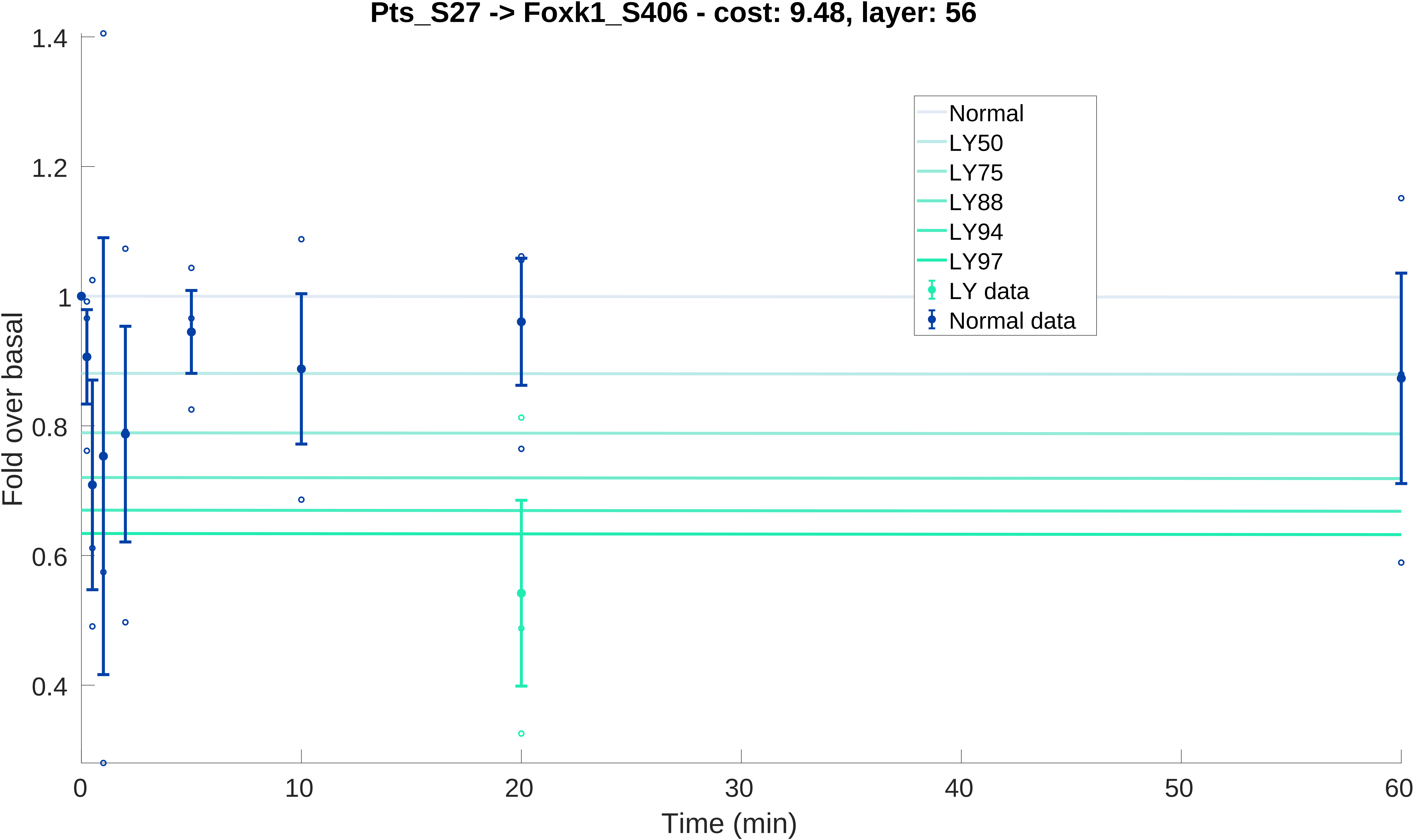
Pts_S27 -> Foxk1_S406 - cost: 9.48, layer: 56
Fold over basal
1.4
1.2
1
0.8
0.6
0.4
0
10
20
30
40
50
60
Time (min)
Normal
LY50
LY75
LY88
LY94
LY97
LY data
Normal data

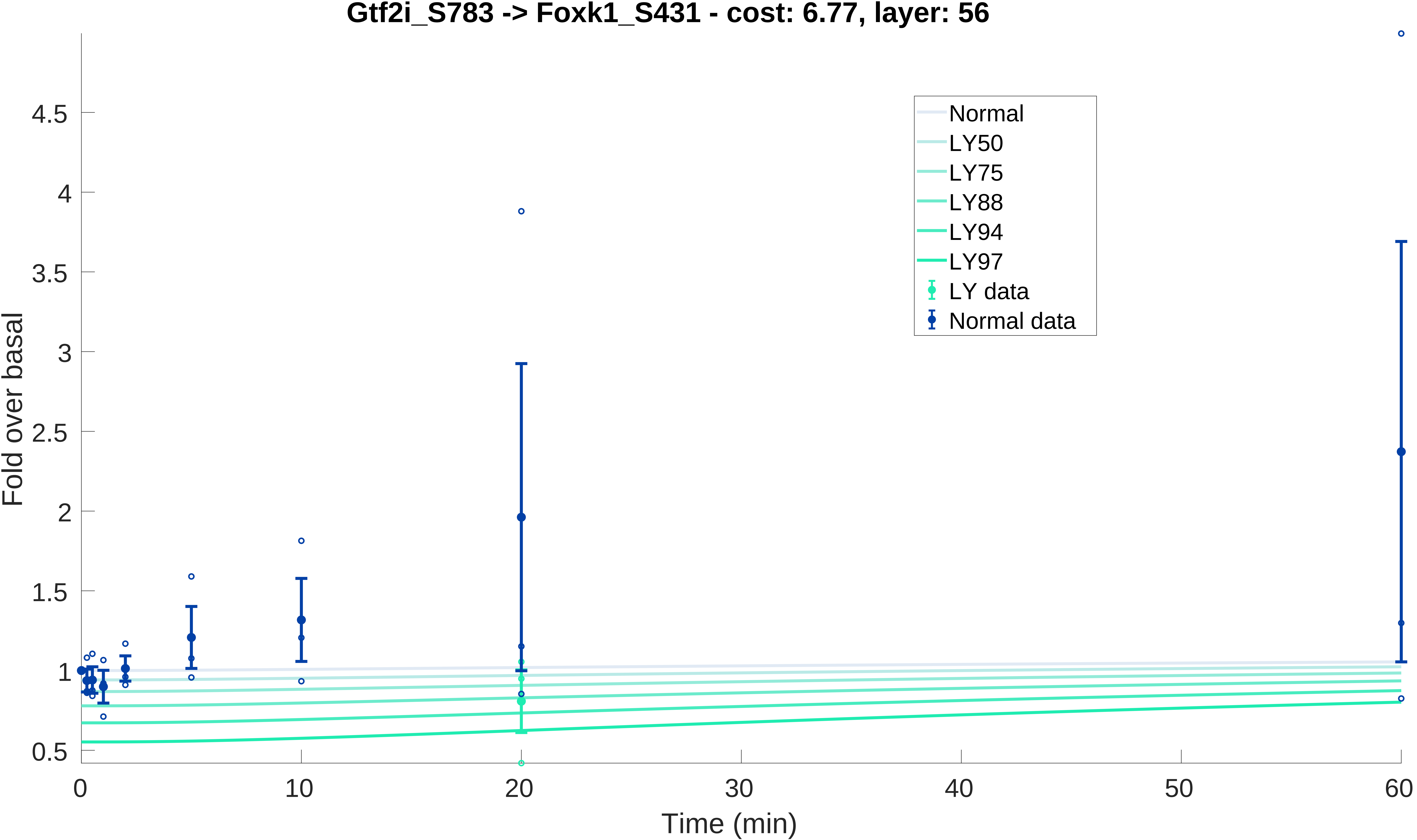

Gtf2i_S783 -> Foxk1_S431 - cost: 6.77, layer: 56
Fold over basal
Time (min)
0.5
1
1.5
2
2.5
3
3.5
4
4.5
0
10
20
30
40
50
60
Normal
LY50
LY75
LY88
LY94
LY97
LY data
Normal data

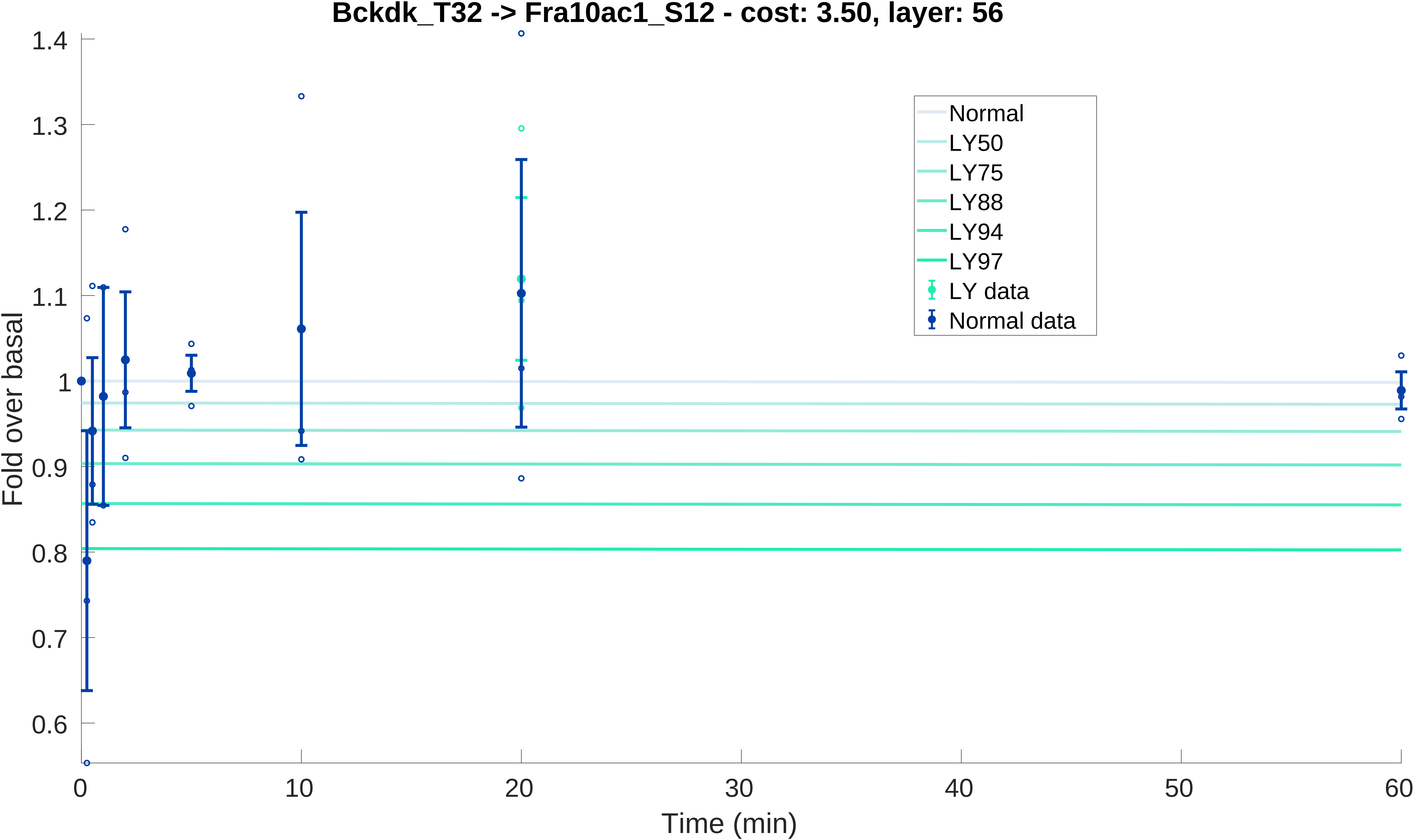

Bckdk_T32 -> Fra10ac1_S12 - cost: 3.50, layer: 56
Fold over basal
Time (min)
1.4
1.3
1.2
1.1
1
0.9
0.8
0.7
0.6
0
10
20
30
40
50
60
Normal
LY50
LY75
LY88
LY94
LY97
LY data
Normal data

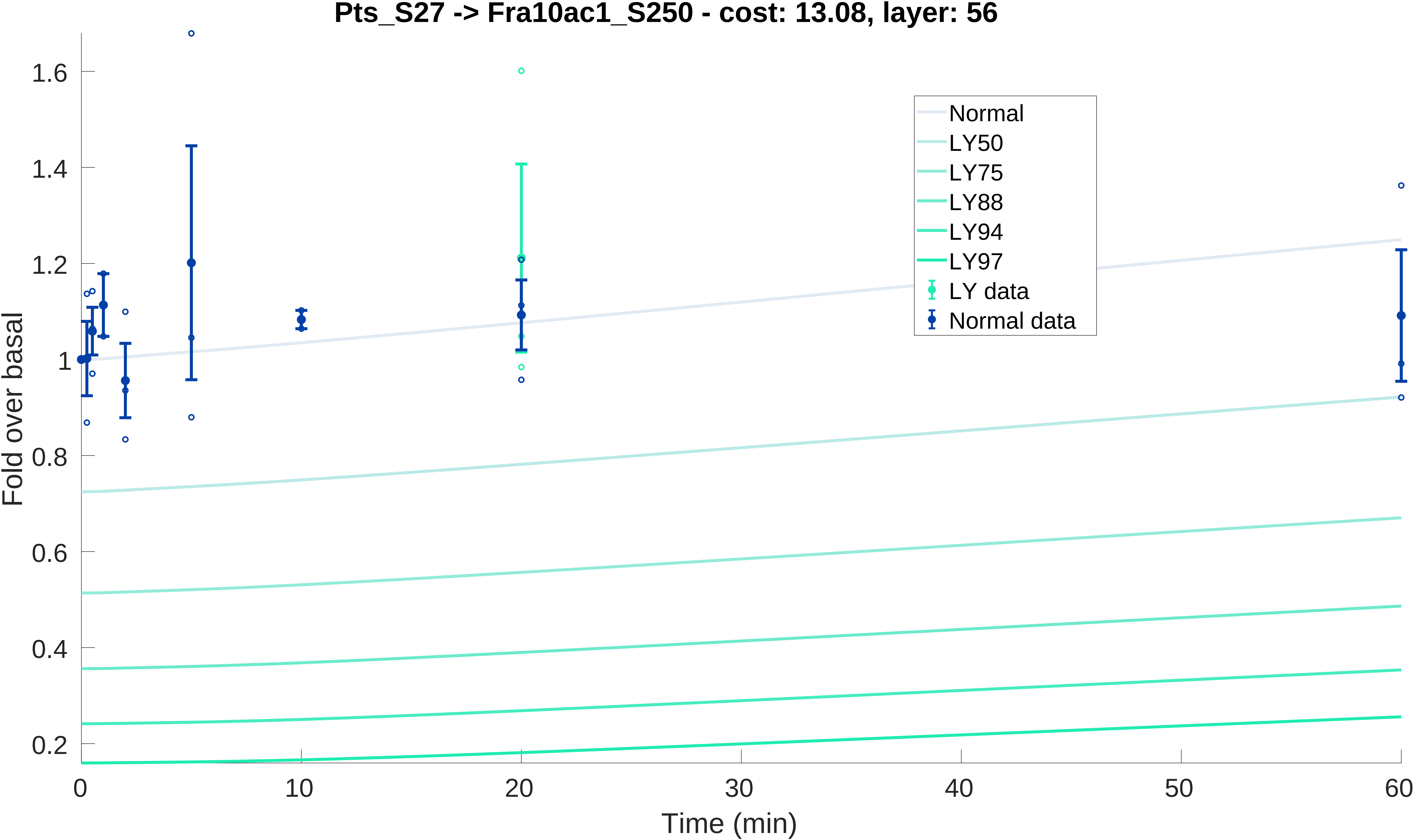

Pts_S27 -> Fra10ac1_S250 - cost: 13.08, layer: 56
Fold over basal
Time (min)
0
10
20
30
40
50
60
0.2
0.4
0.6
0.8
1
1.2
1.4
1.6
Normal
LY50
LY75
LY88
LY94
LY97
LY data
Normal data

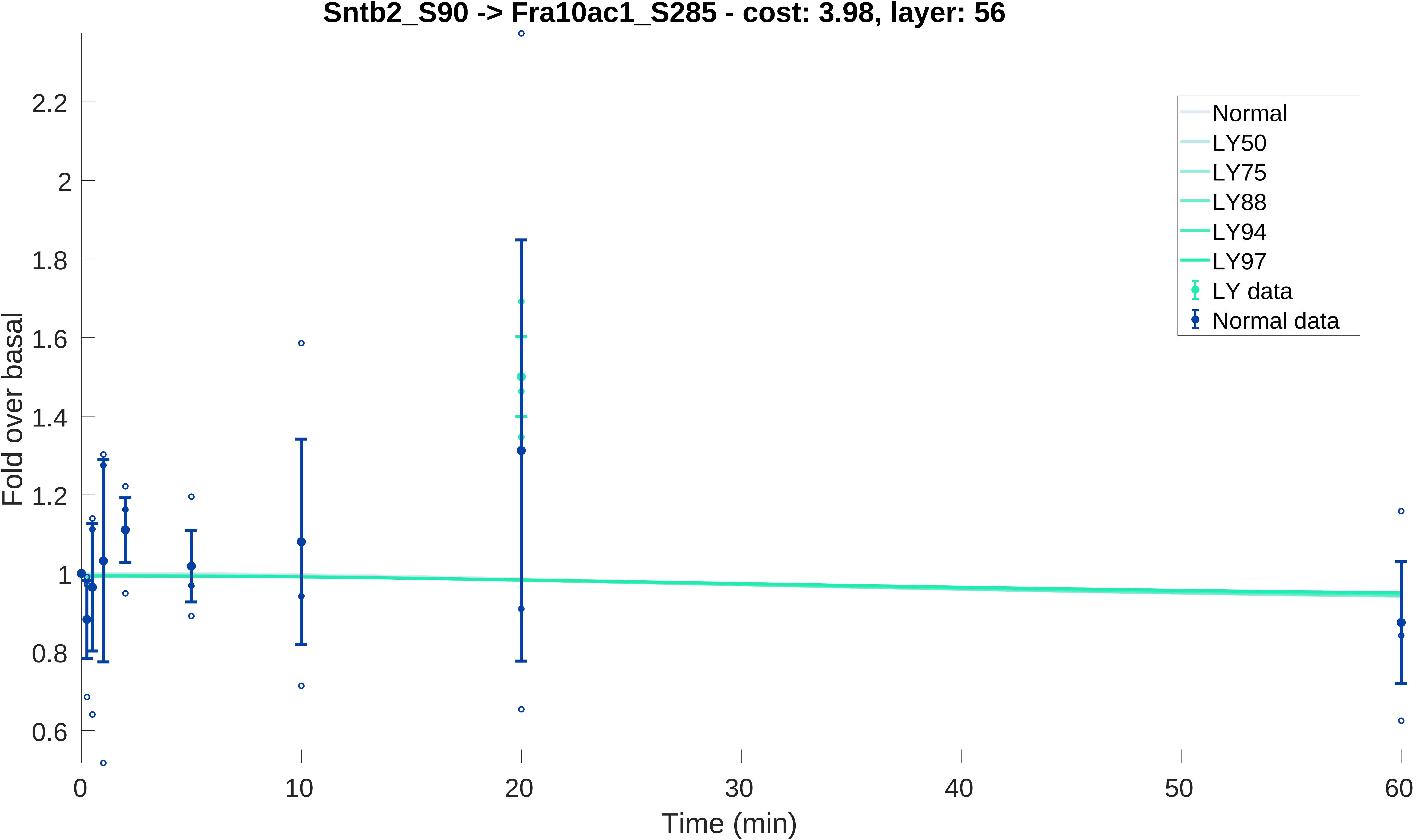

Sntb2_S90 -> Fra10ac1_S285 - cost: 3.98, layer: 56
Fold over basal
Time (min)
Normal
LY50
LY75
LY88
LY94
LY97
LY data
Normal data
0.6
0.8
1
1.2
1.4
1.6
1.8
2
2.2
0
10
20
30
40
50
60

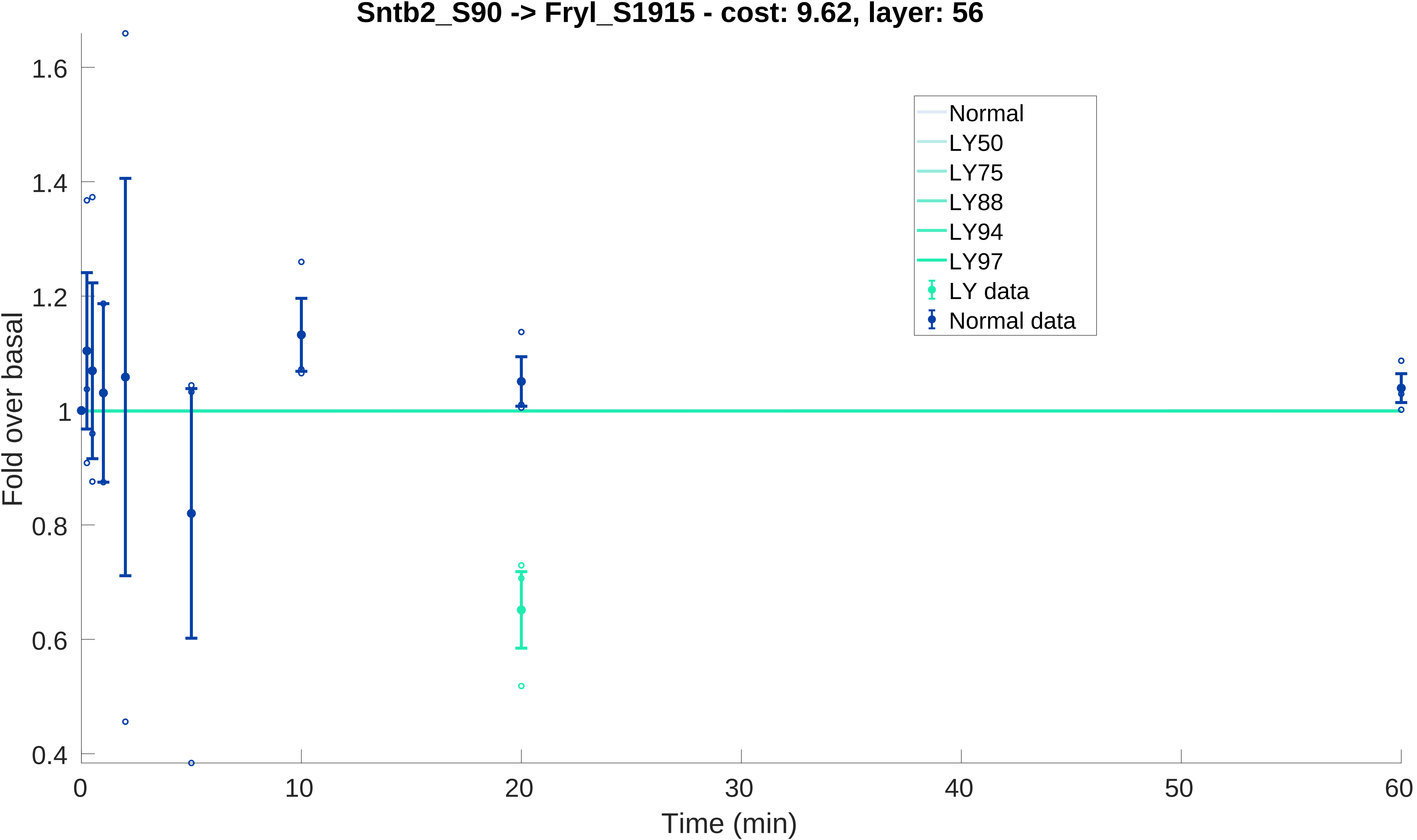

Sntb2_S90 -> Fryl_S1915 - cost: 9.62, layer: 56
Fold over basal
Time (min)
0
10
20
30
40
50
60
0.4
0.6
0.8
1
1.2
1.4
1.6
Normal
LY50
LY75
LY88
LY94
LY97
LY data
Normal data

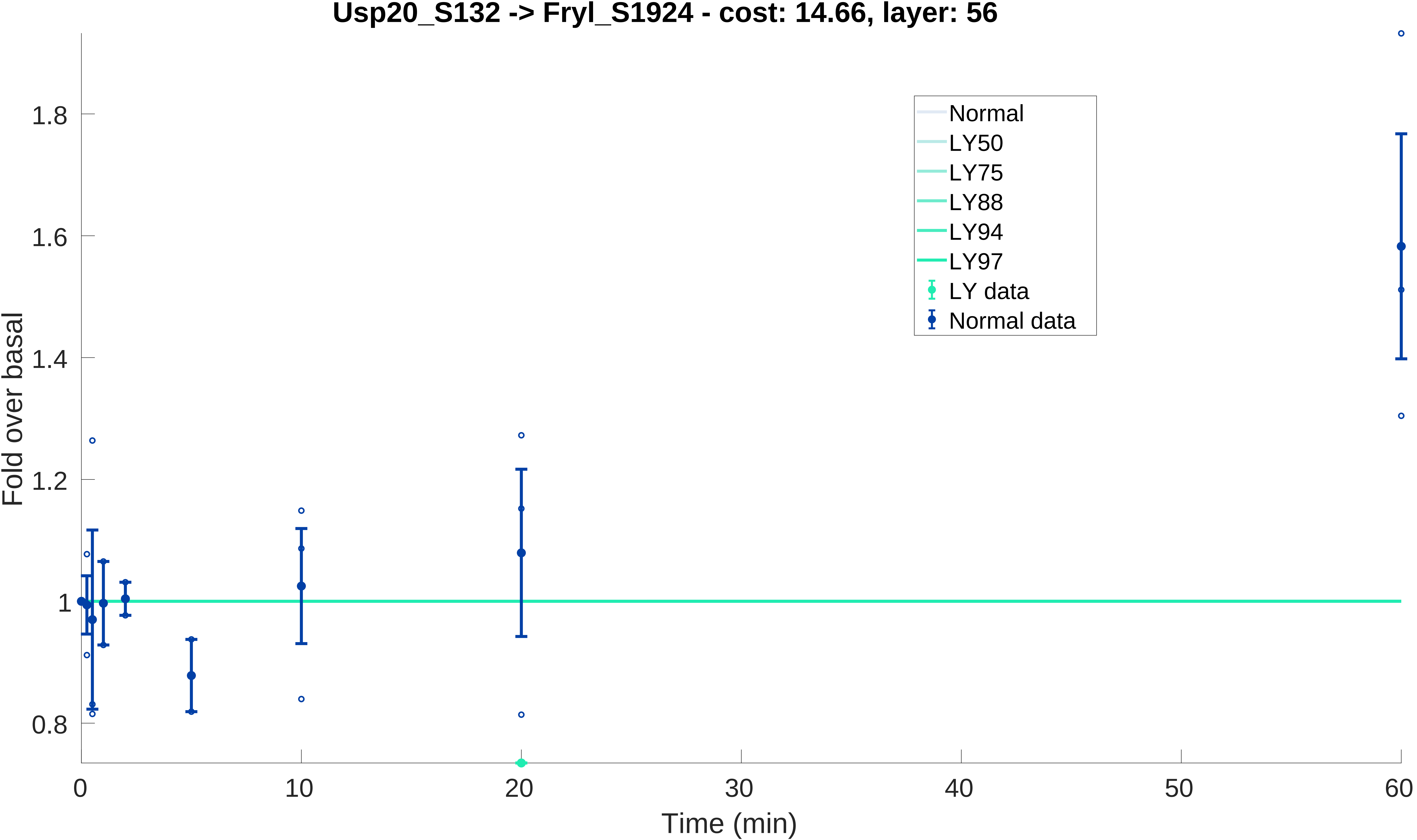
Usp20_S132 -> Fryl_S1924 - cost: 14.66, layer: 56
Fold over basal
Time (min)
0.8
1
1.2
1.4
1.6
1.8
0
10
20
30
40
50
60
Normal
LY50
LY75
LY88
LY94
LY97
LY data
Normal data

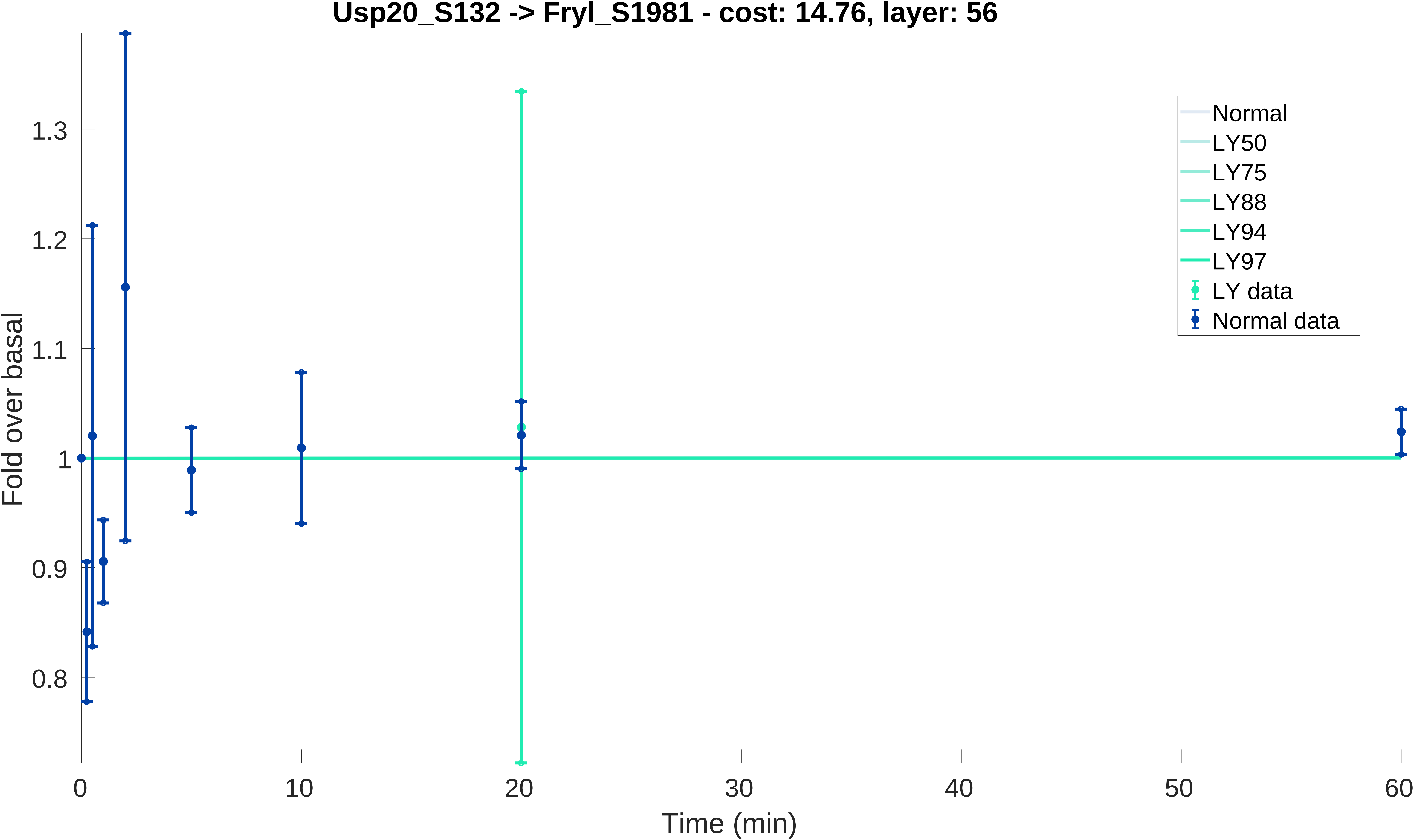
Usp20_S132 -> Fryl_S1981 - cost: 14.76, layer: 56
Normal
LY50
LY75
LY88
LY94
LY97
LY data
Normal data
Fold over basal
1.3
1.2
1.1
1
0.9
0.8
Time (min)
0
10
20
30
40
50
60

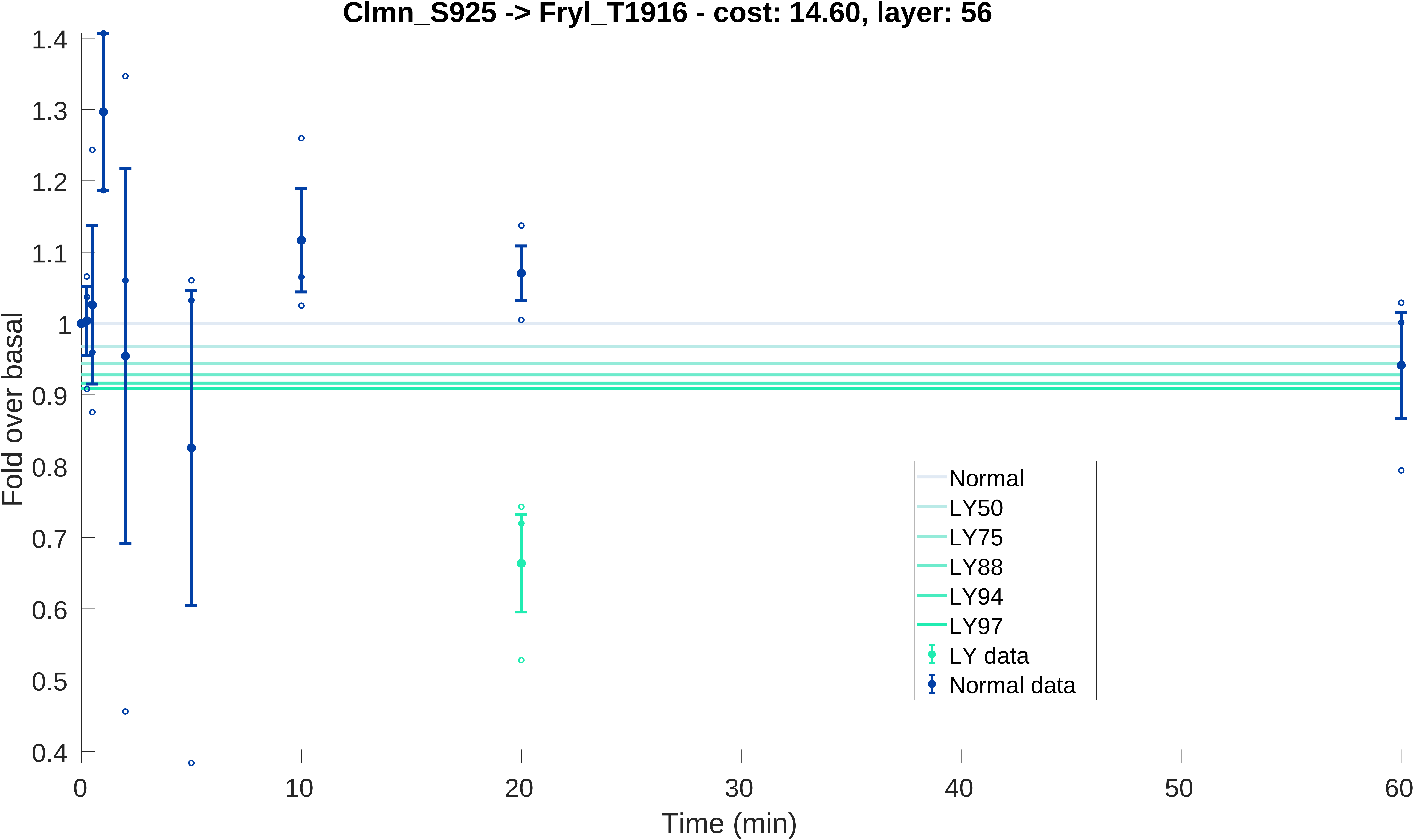
Clmn_S925 -> Fryl_T1916 - cost: 14.60, layer: 56
Fold over basal
Time (min)
0
10
20
30
40
50
60
0.4
0.5
0.6
0.7
0.8
0.9
1
1.1
1.2
1.3
1.4
Normal
LY50
LY75
LY88
LY94
LY97
LY data
Normal data

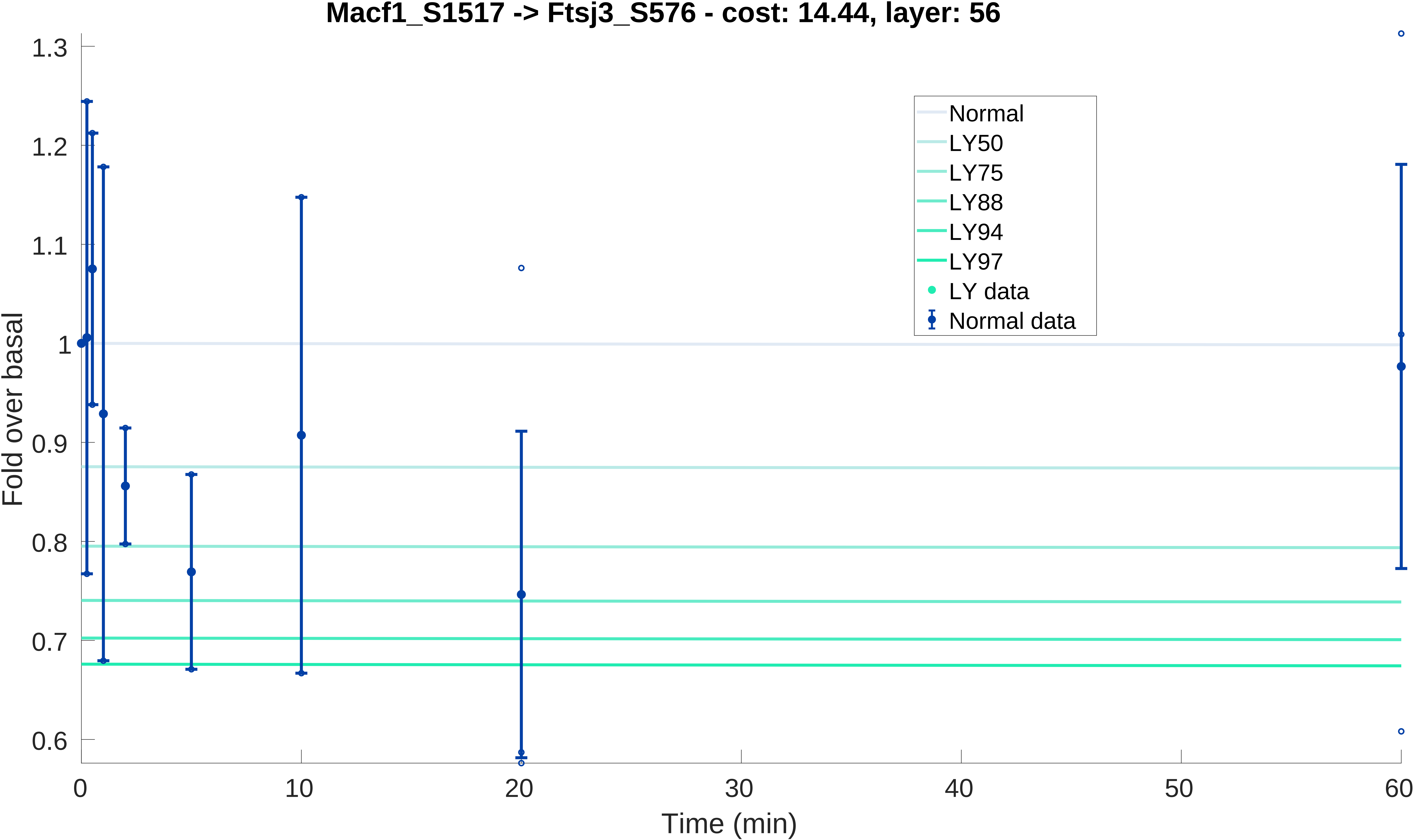

Macf1_S1517 -> Ftsj3_S576 - cost: 14.44, layer: 56
Fold over basal
Time (min)
Normal
LY50
LY75
LY88
LY94
LY97
LY data
Normal data
1.3
1.2
1.1
1
0.9
0.8
0.7
0.6
0
10
20
30
40
50
60

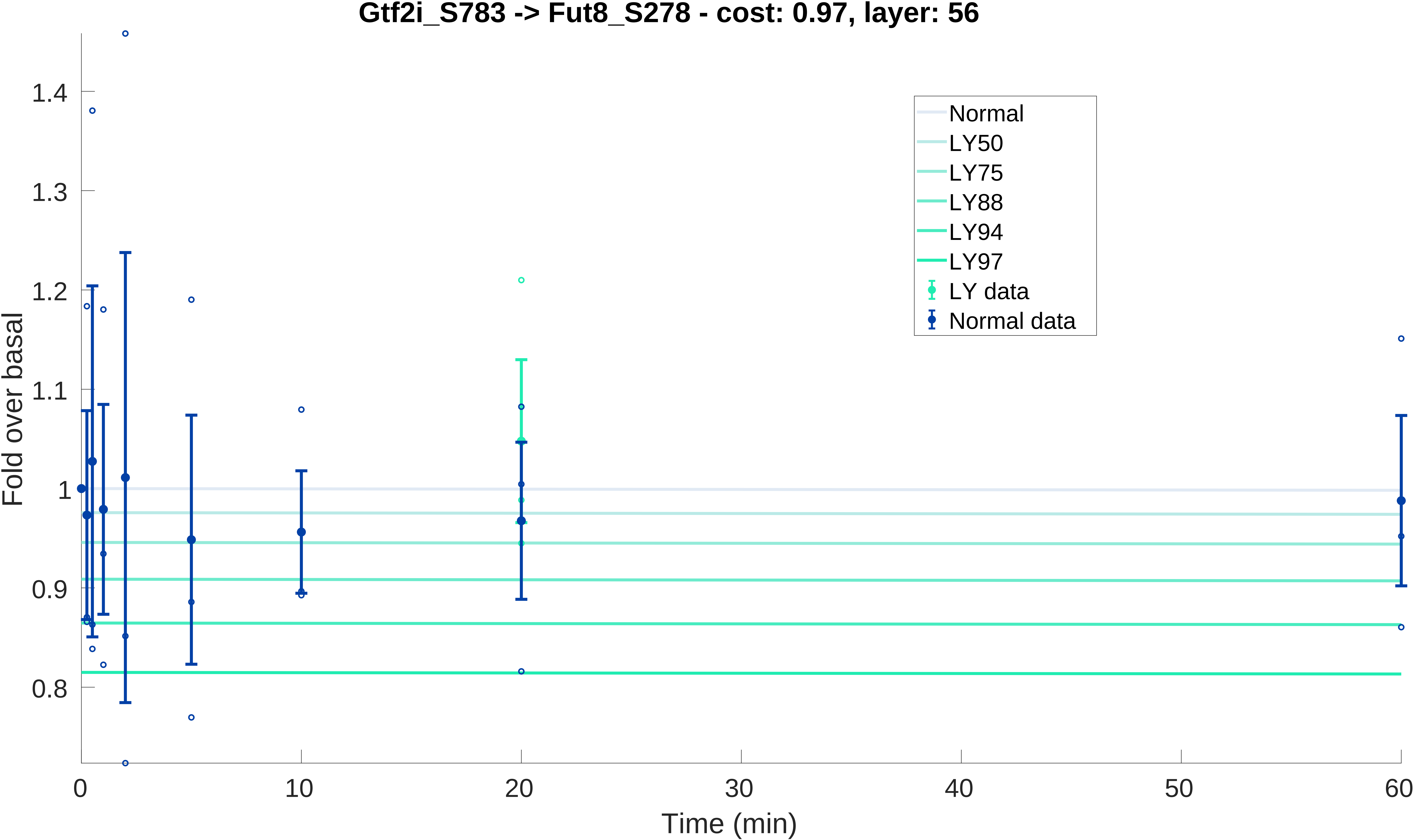

Gtf2i_S783 -> Fut8_S278 - cost: 0.97, layer: 56
Fold over basal
1.4
1.3
1.2
1.1
1
0.9
0.8
0
10
20
30
40
50
60
Time (min)
Normal
LY50
LY75
LY88
LY94
LY97
LY data
Normal data

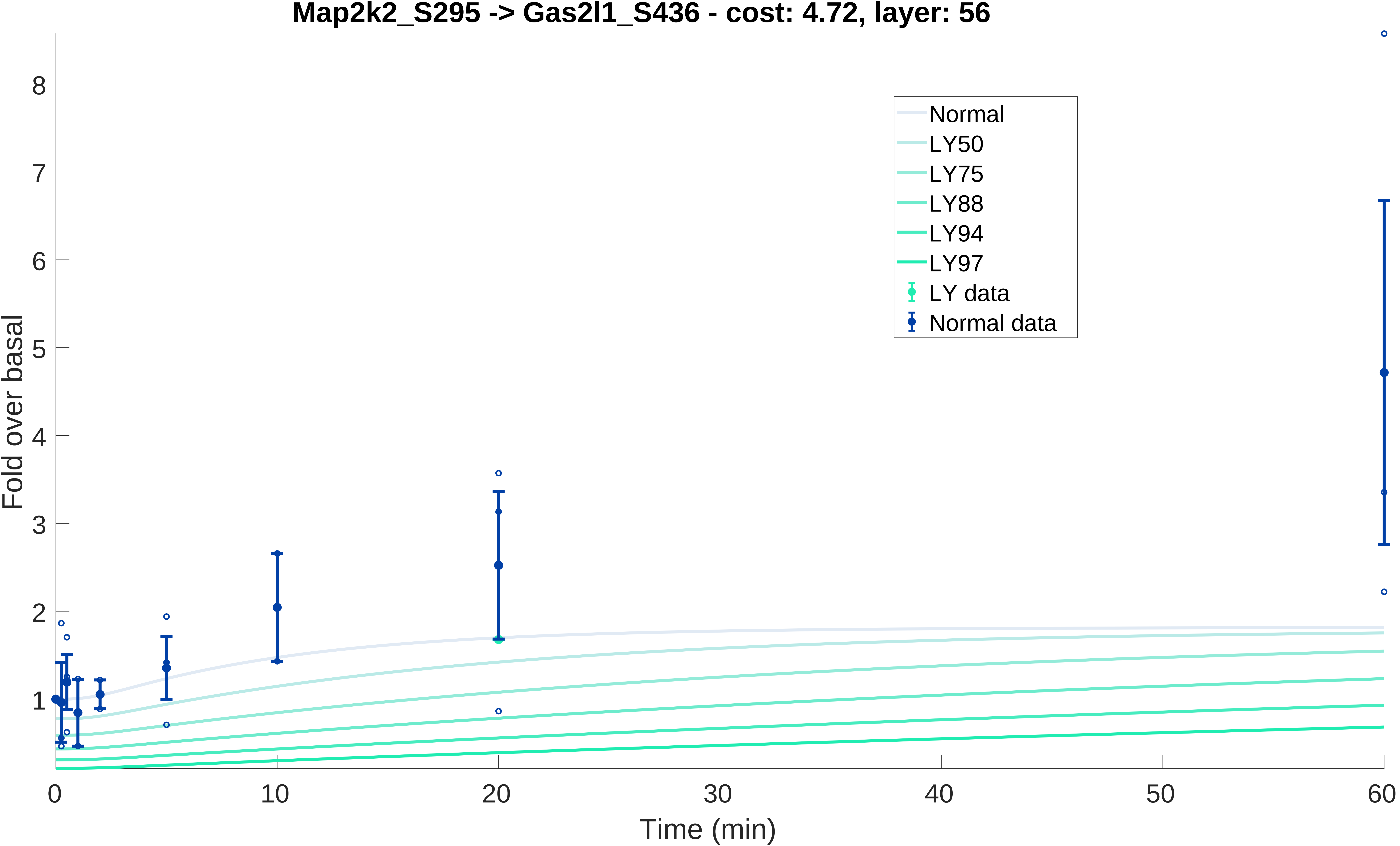

Map2k2_S295 -> Gas2l1_S436 - cost: 4.72, layer: 56
Normal
LY50
LY75
LY88
LY94
LY97
LY data
Normal data
Fold over basal
Time (min)
0
10
20
30
40
50
60
1
2
3
4
5
6
7
8

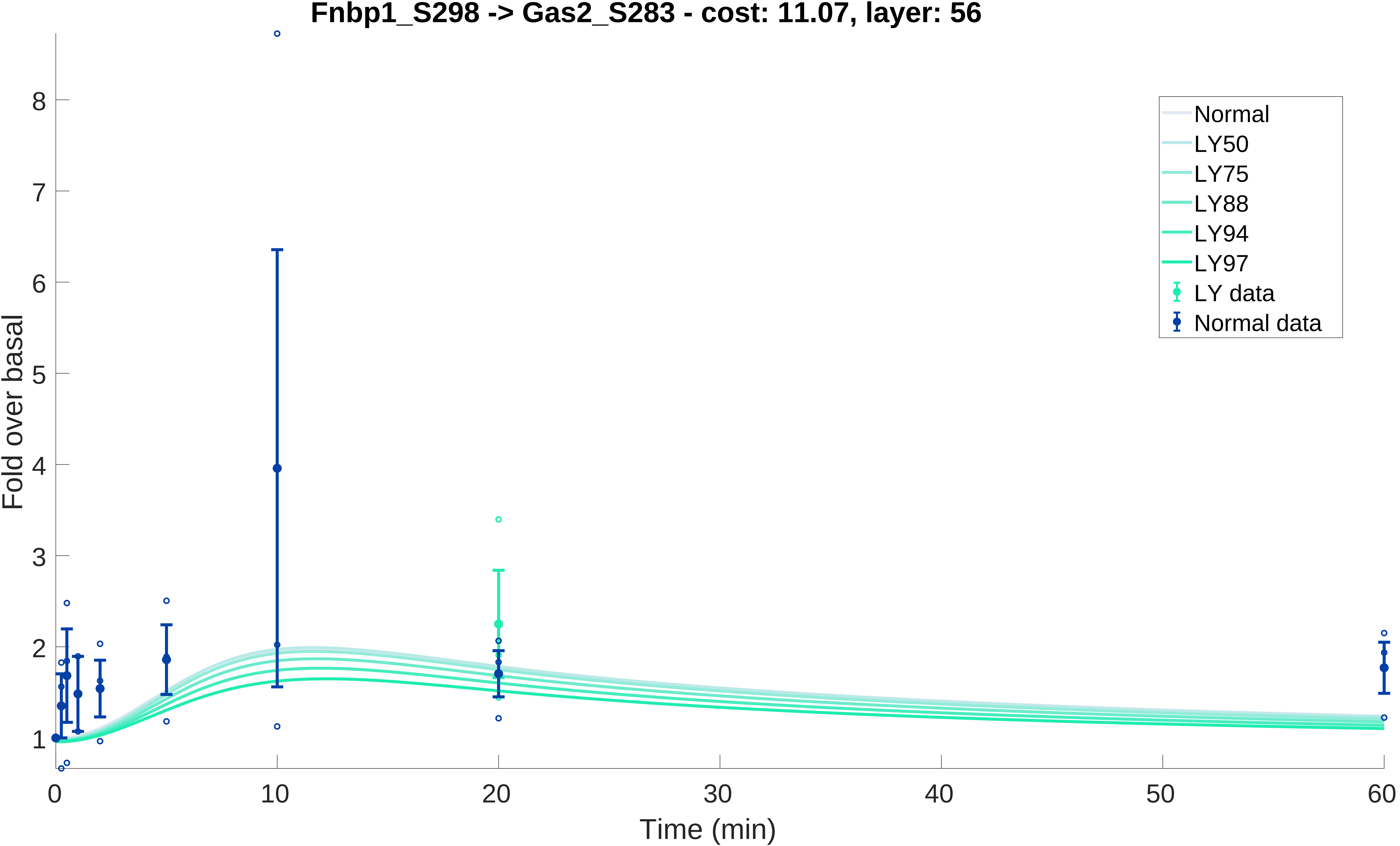

Fnbp1_S298 -> Gas2_S283 - cost: 11.07, layer: 56
Fold over basal
Time (min)
Normal
LY50
LY75
LY88
LY94
LY97
LY data
Normal data
0
10
20
30
40
50
60
1
2
3
4
5
6
7
8

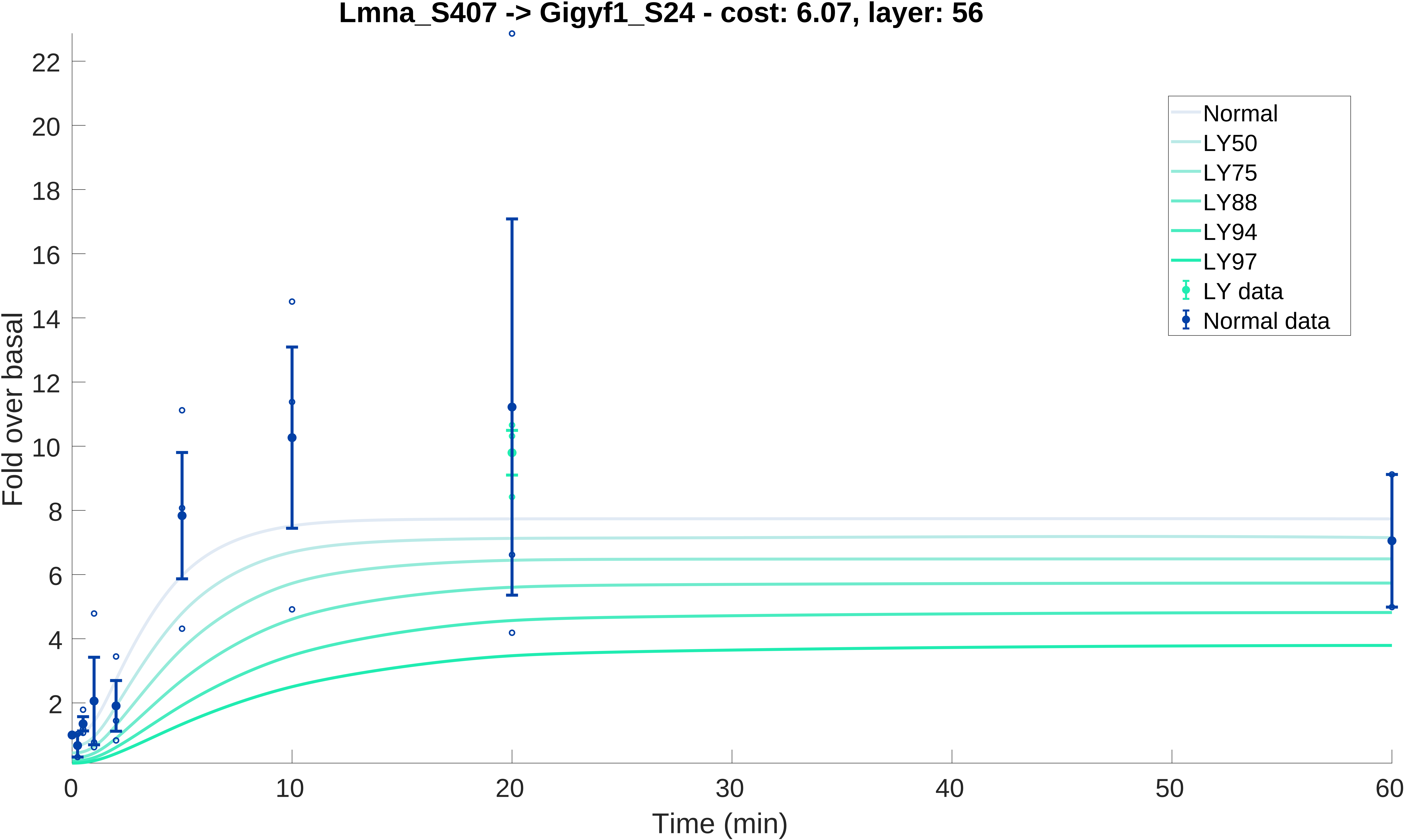

Lmna_S407 -> Gigyf1_S24 - cost: 6.07, layer: 56
Fold over basal
Time (min)
0
10
20
30
40
50
60
2
4
6
8
10
12
14
16
18
20
22
Normal
LY50
LY75
LY88
LY94
LY97
LY data
Normal data

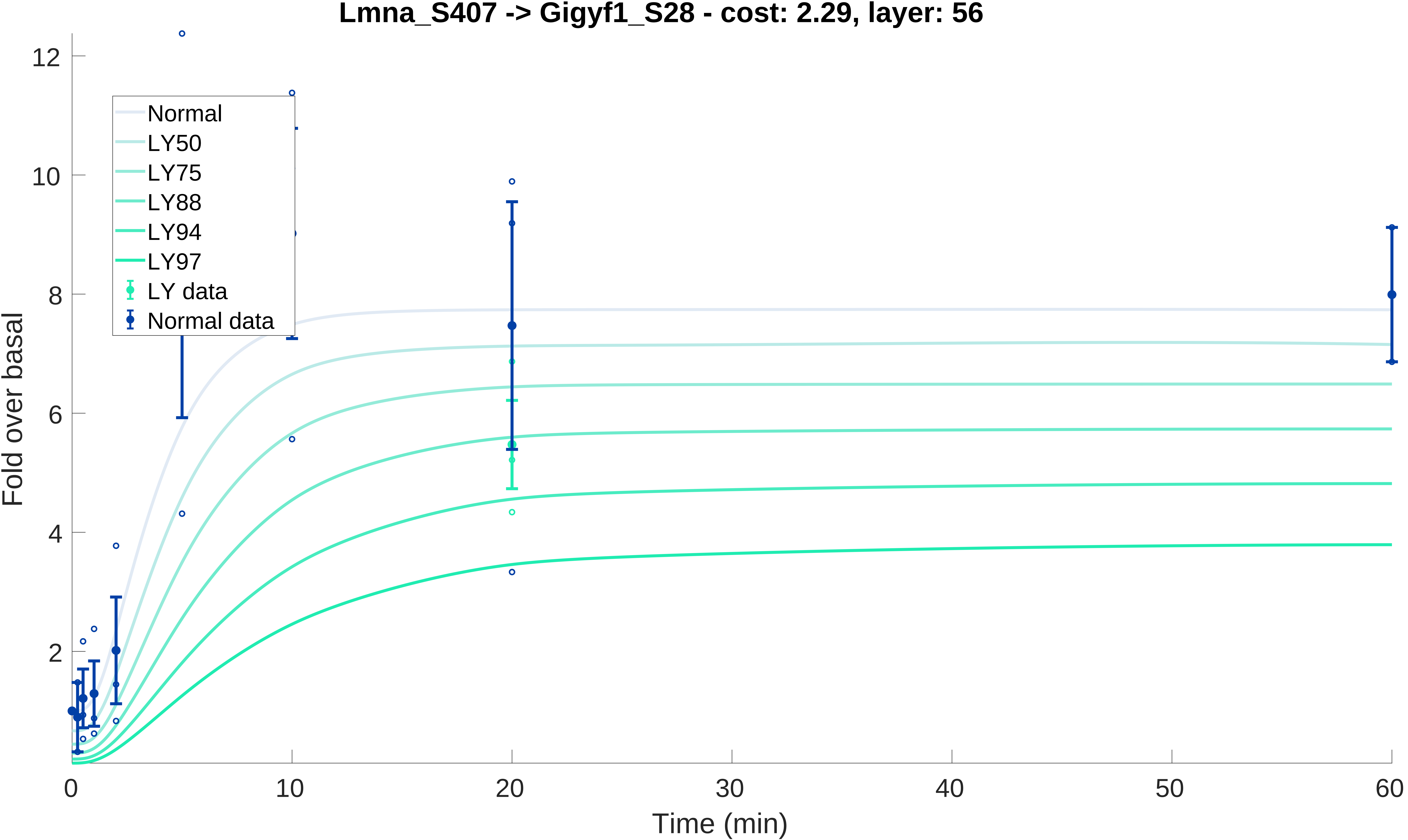

Lmna_S407 -> Gigyf1_S28 - cost: 2.29, layer: 56
Normal
LY50
LY75
LY88
LY94
LY97
LY data
Normal data
Fold over basal
Time (min)
0
10
20
30
40
50
60
2
4
6
8
10
12

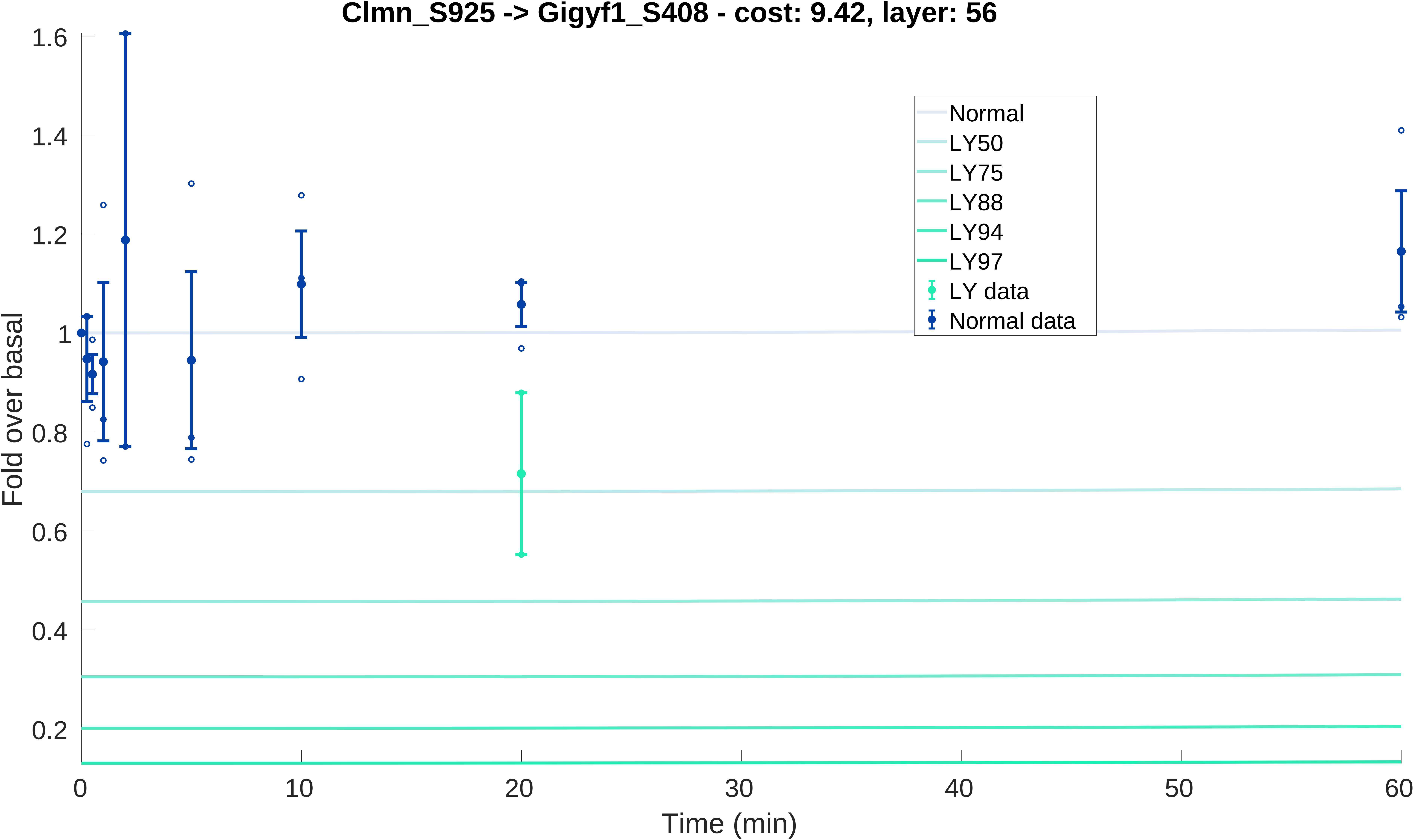
Clmn_S925 -> Gigyf1_S408 - cost: 9.42, layer: 56
Fold over basal
1.6
1.4
1.2
1
0.8
0.6
0.4
0.2
0
10
20
30
40
50
60
Time (min)
Normal
LY50
LY75
LY88
LY94
LY97
LY data
Normal data

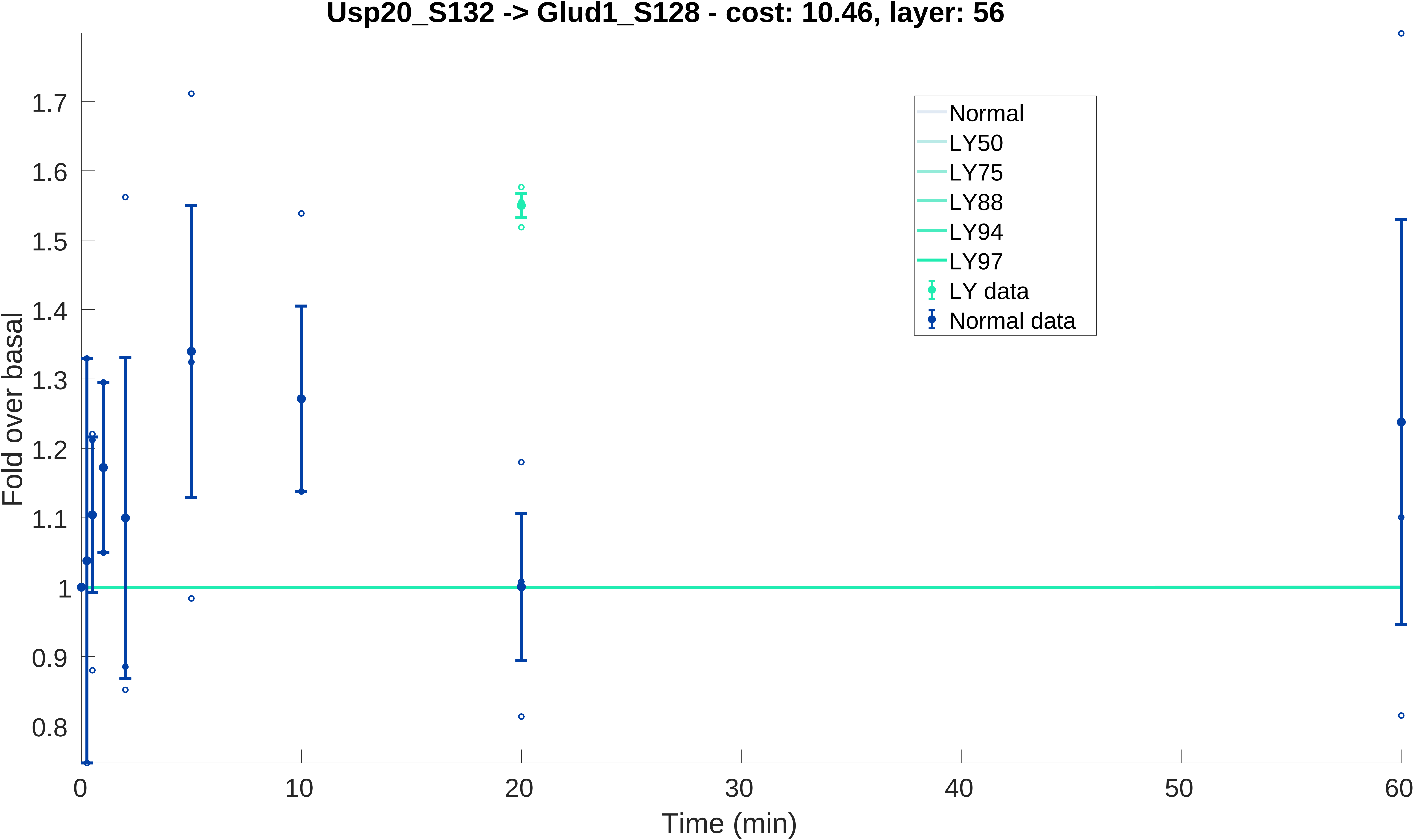

Usp20_S132 -> Glud1_S128 - cost: 10.46, layer: 56
Normal
LY50
LY75
LY88
LY94
LY97
LY data
Normal data
Fold over basal
Time (min)
1.7
1.6
1.5
1.4
1.3
1.2
1.1
1
0.9
0.8
0
10
20
30
40
50
60

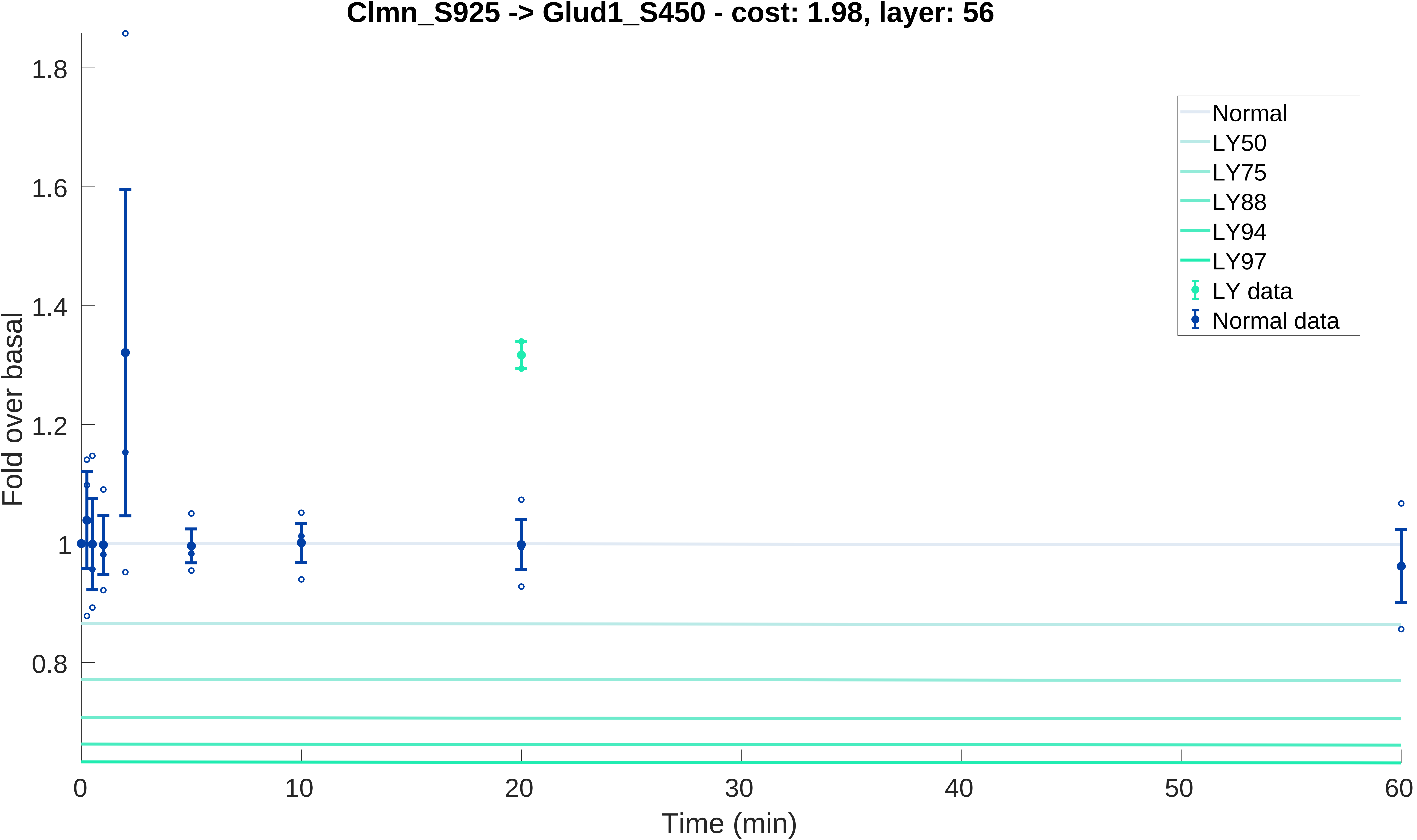

Clmn_S925 -> Glud1_S450 - cost: 1.98, layer: 56
Fold over basal
Time (min)
0.8
1
1.2
1.4
1.6
1.8
0
10
20
30
40
50
60
Normal
LY50
LY75
LY88
LY94
LY97
LY data
Normal data

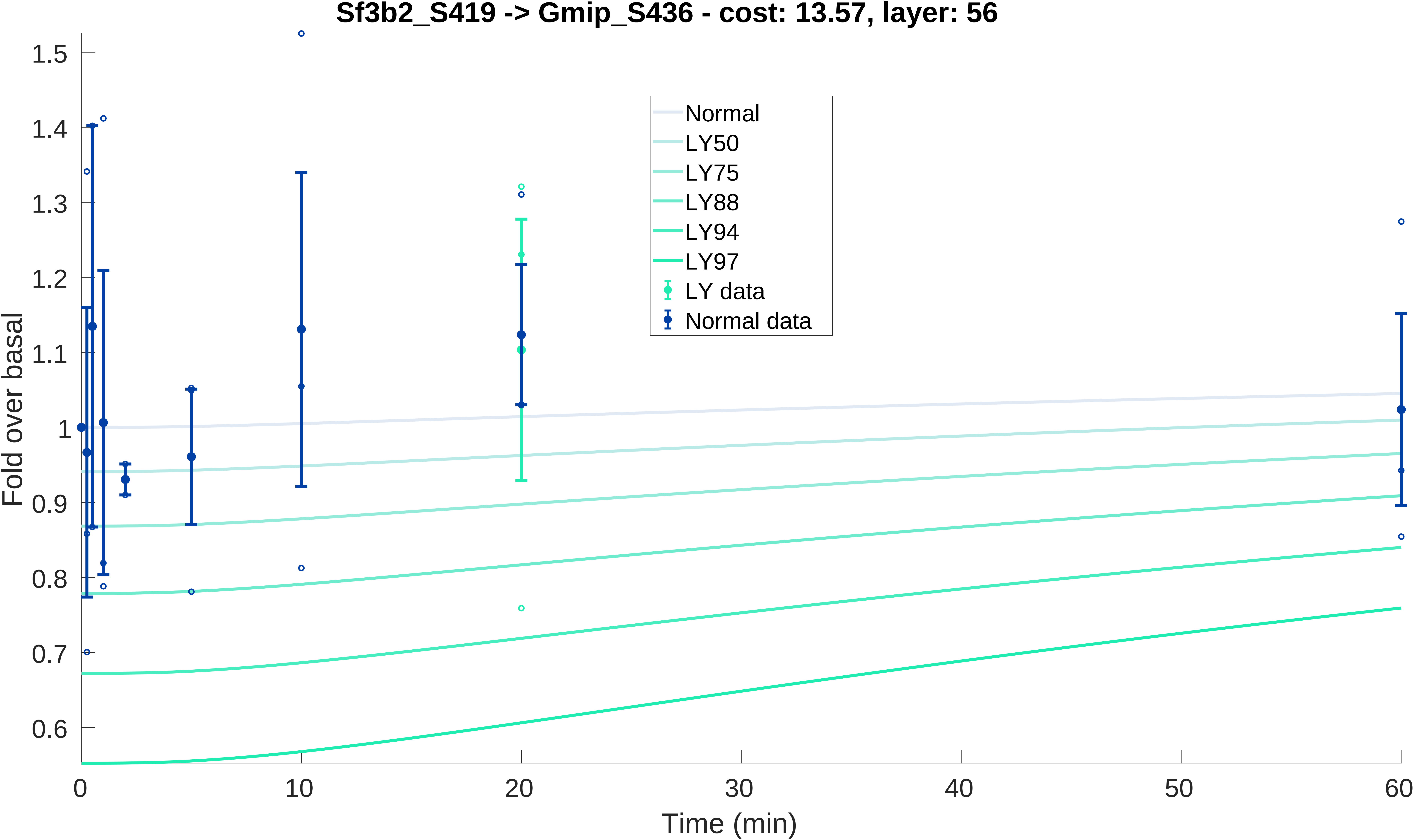
Sf3b2_S419 -> Gmip_S436 - cost: 13.57, layer: 56
Fold over basal
Time (min)
1.5
1.4
1.3
1.2
1.1
1
0.9
0.8
0.7
0.6
0
10
20
30
40
50
60
Normal
LY50
LY75
LY88
LY94
LY97
LY data
Normal data

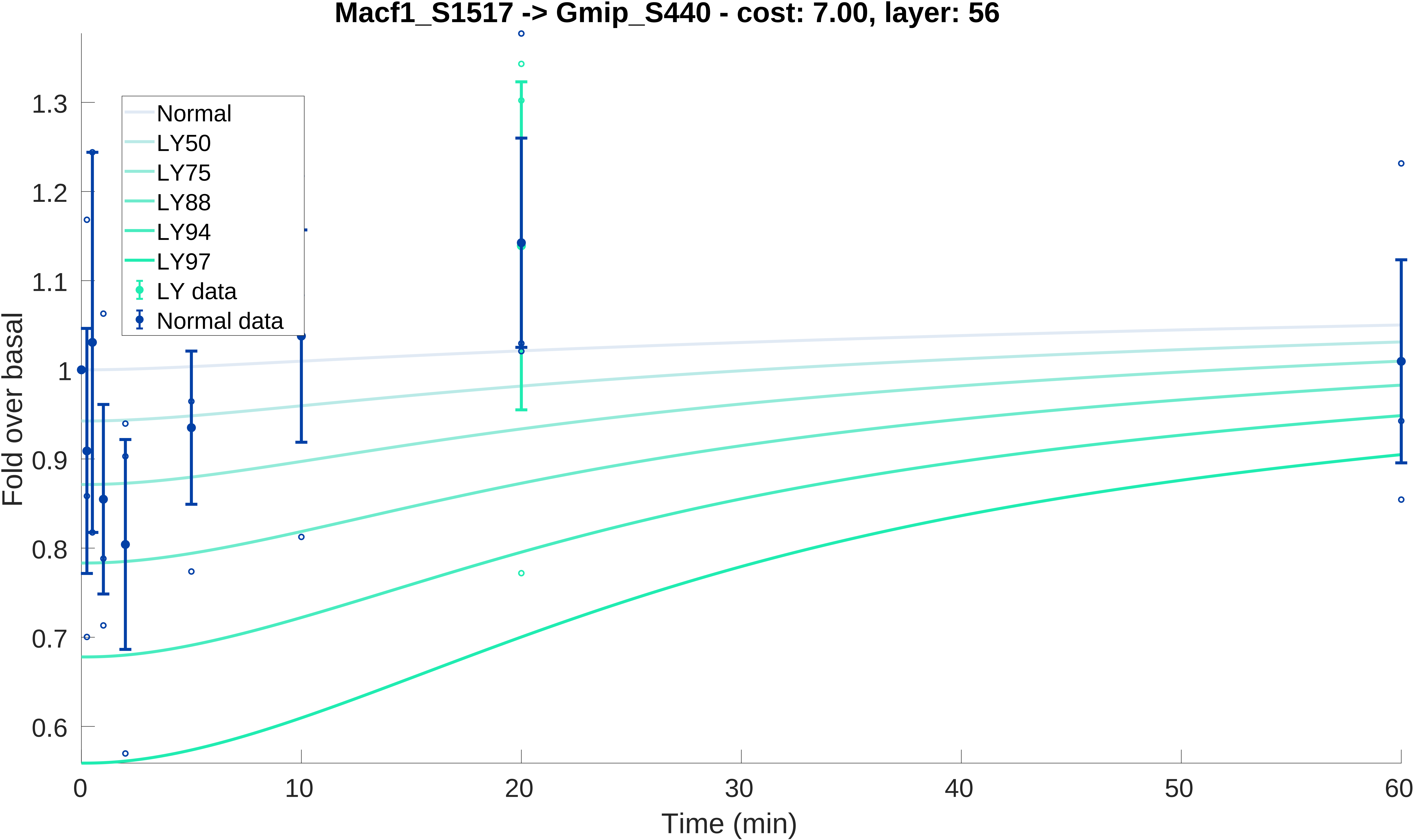

Macf1_S1517 -> Gmip_S440 - cost: 7.00, layer: 56
Normal
LY50
LY75
LY88
LY94
LY97
LY data
Normal data
Fold over basal
Time (min)
0.6
0.7
0.8
0.9
1
1.1
1.2
1.3
0
10
20
30
40
50
60

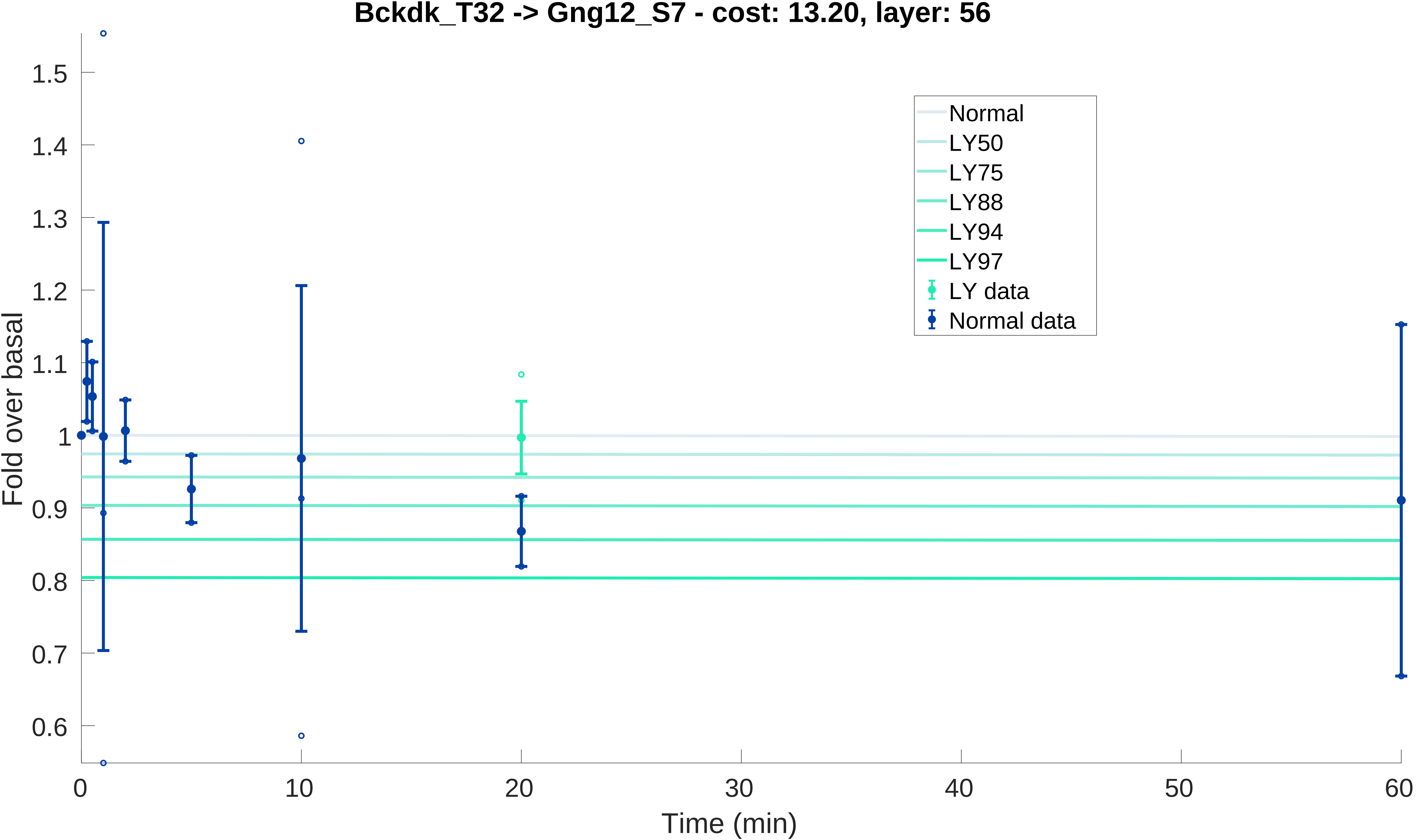

Bckdk_T32 -> Gng12_S7 - cost: 13.20, layer: 56
Fold over basal
Time (min)
1.5
1.4
1.3
1.2
1.1
1
0.9
0.8
0.7
0.6
0
10
20
30
40
50
60
Normal
LY50
LY75
LY88
LY94
LY97
LY data
Normal data

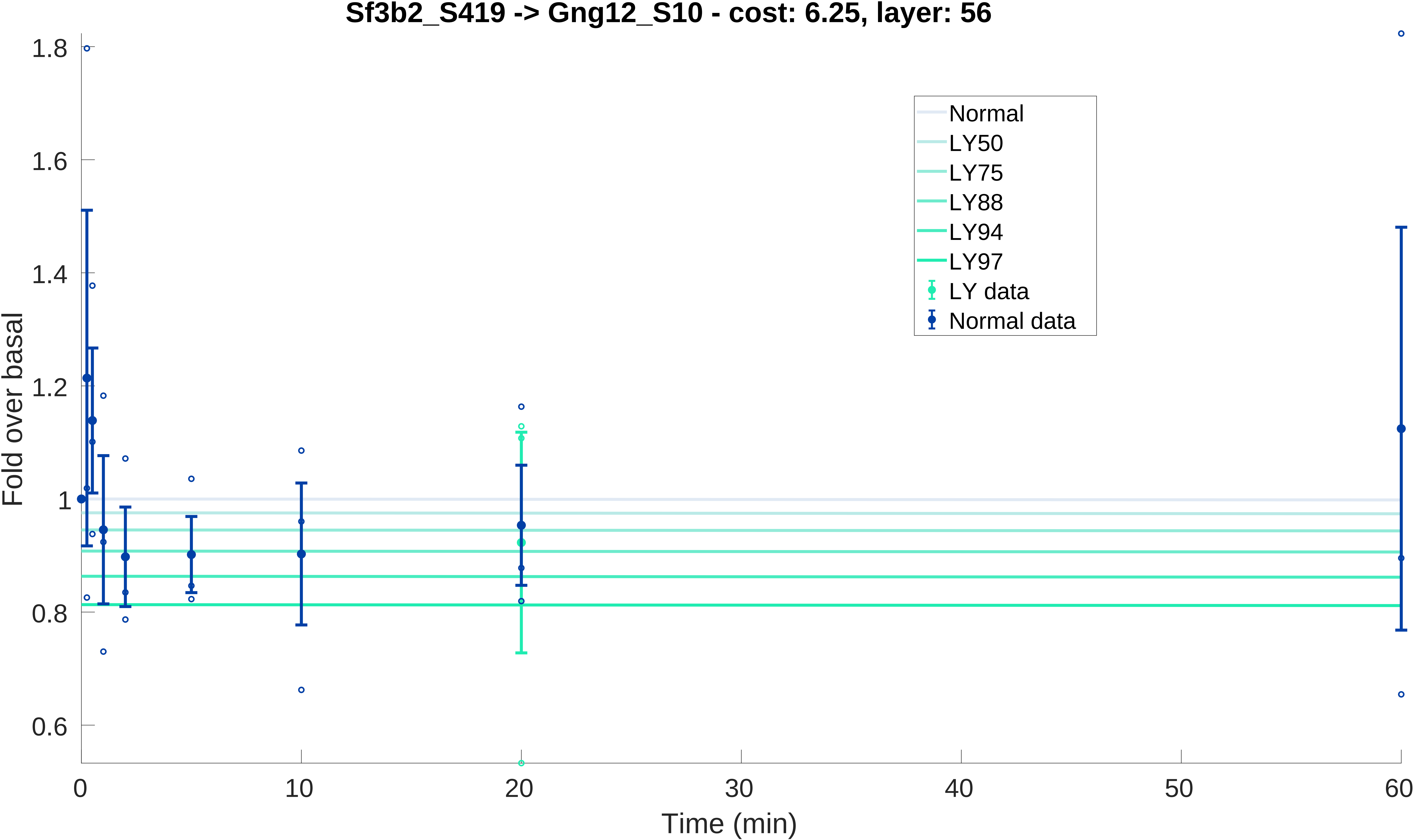
Sf3b2_S419 -> Gng12_S10 - cost: 6.25, layer: 56
Fold over basal
Time (min)
Normal
LY50
LY75
LY88
LY94
LY97
LY data
Normal data
1.8
1.6
1.4
1.2
1
0.8
0.6
0
10
20
30
40
50
60

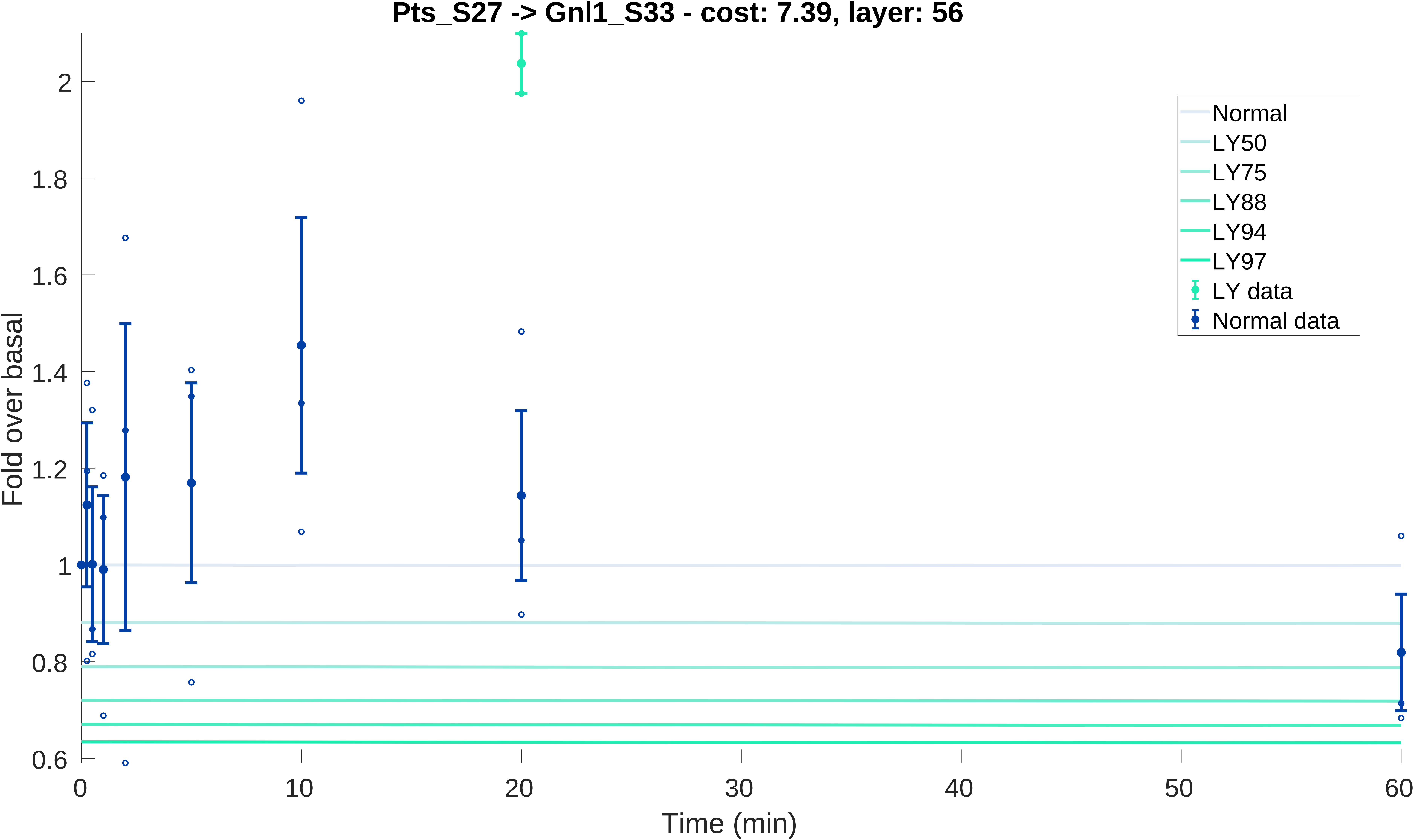

Pts_S27 -> Gnl1_S33 - cost: 7.39, layer: 56
Normal
LY50
LY75
LY88
LY94
LY97
LY data
Normal data
Fold over basal
Time (min)
0.6
0.8
1
1.2
1.4
1.6
1.8
2
0
10
20
30
40
50
60

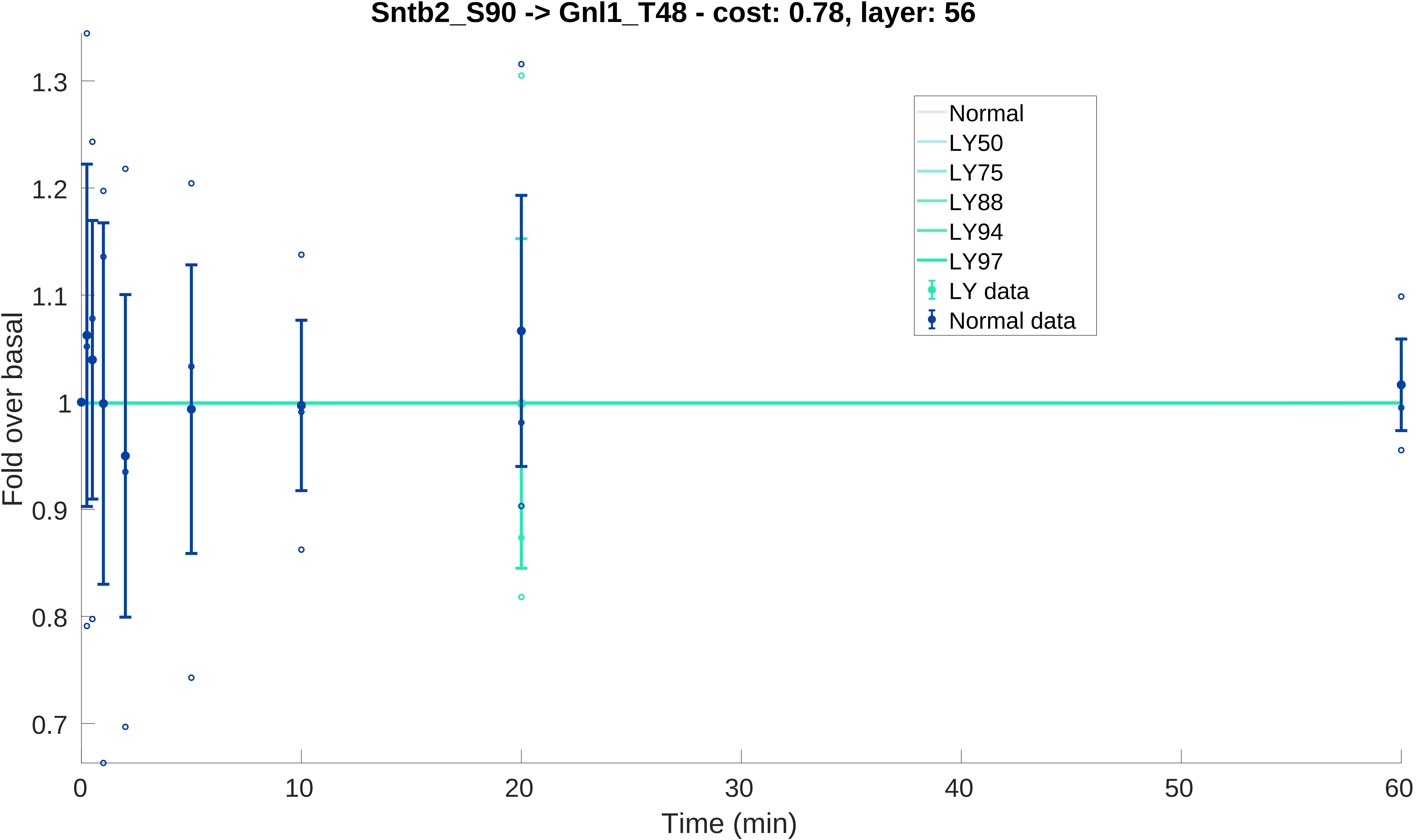

Sntb2_S90 -> Gnl1_T48 - cost: 0.78, layer: 56
Fold over basal
Time (min)
Normal
LY50
LY75
LY88
LY94
LY97
LY data
Normal data
0.7
0.8
0.9
1
1.1
1.2
1.3
0
10
20
30
40
50
60

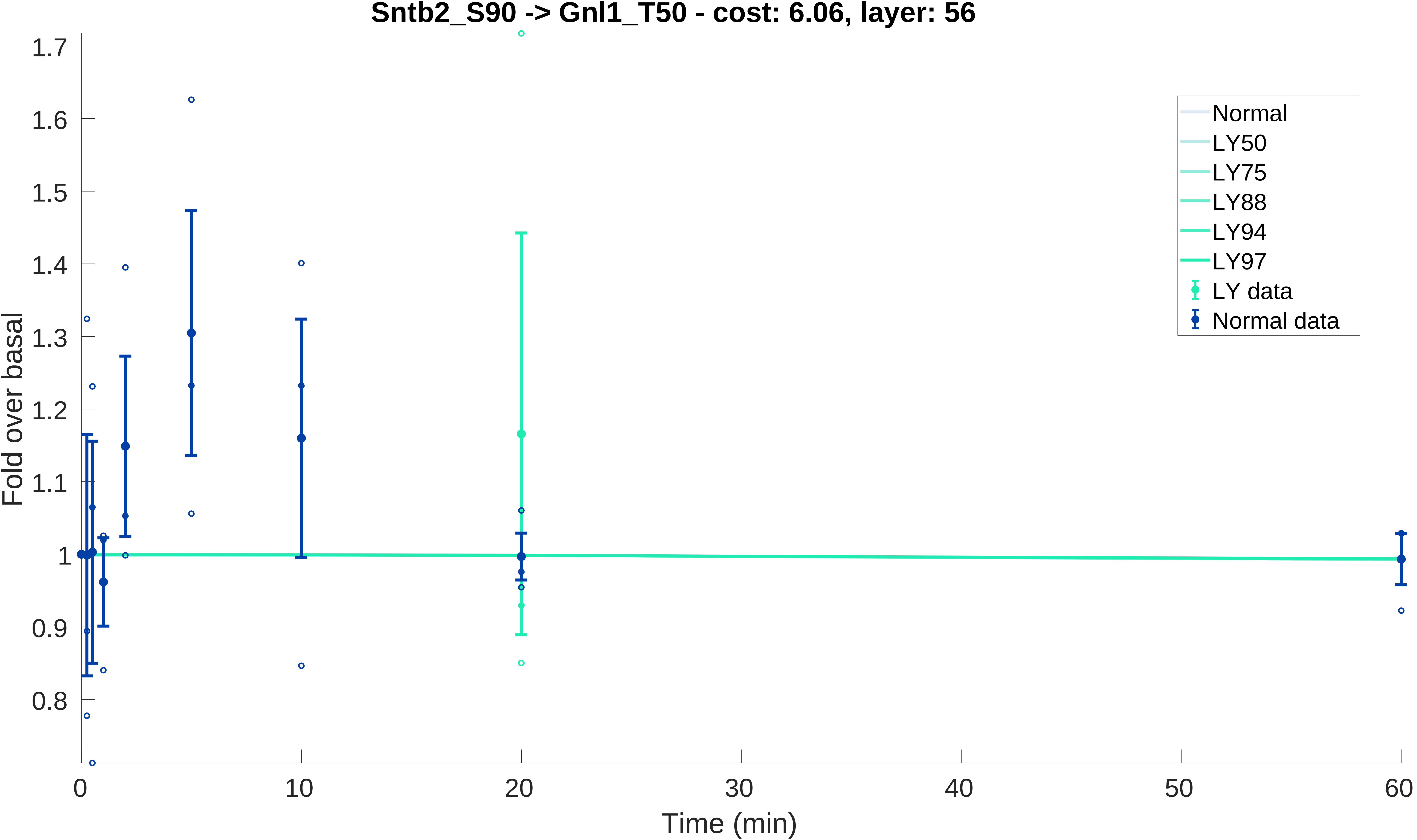

Sntb2_S90 -> Gnl1_T50 - cost: 6.06, layer: 56
Fold over basal
Time (min)
1.7
1.6
1.5
1.4
1.3
1.2
1.1
1
0.9
0.8
0
10
20
30
40
50
60
Normal
LY50
LY75
LY88
LY94
LY97
LY data
Normal data

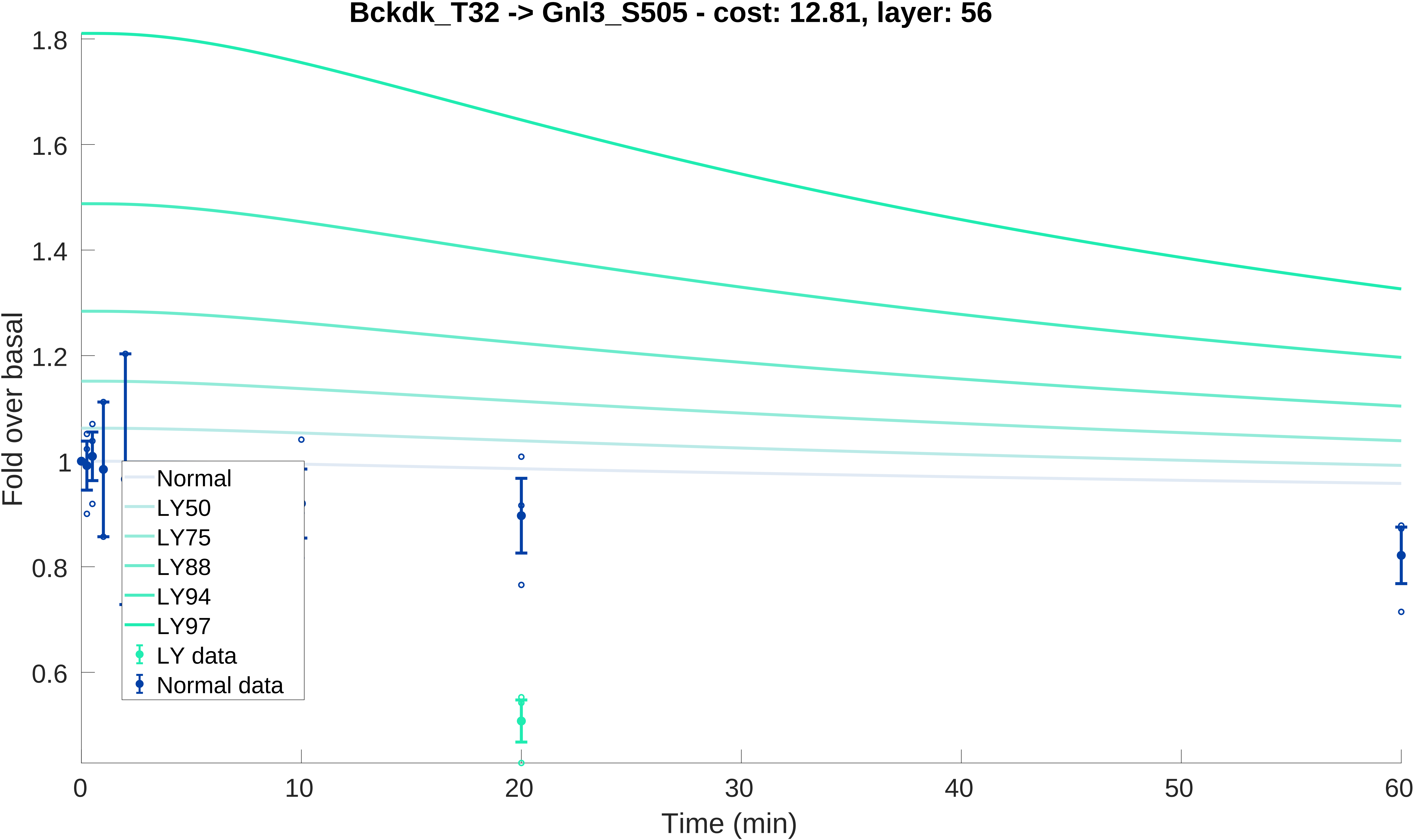
Bckdk_T32 -> Gnl3_S505 - cost: 12.81, layer: 56
Fold over basal
1.8
1.6
1.4
1.2
1
0.8
0.6
Normal
LY50
LY75
LY88
LY94
LY97
LY data
Normal data
0
10
20
30
40
50
60
Time (min)

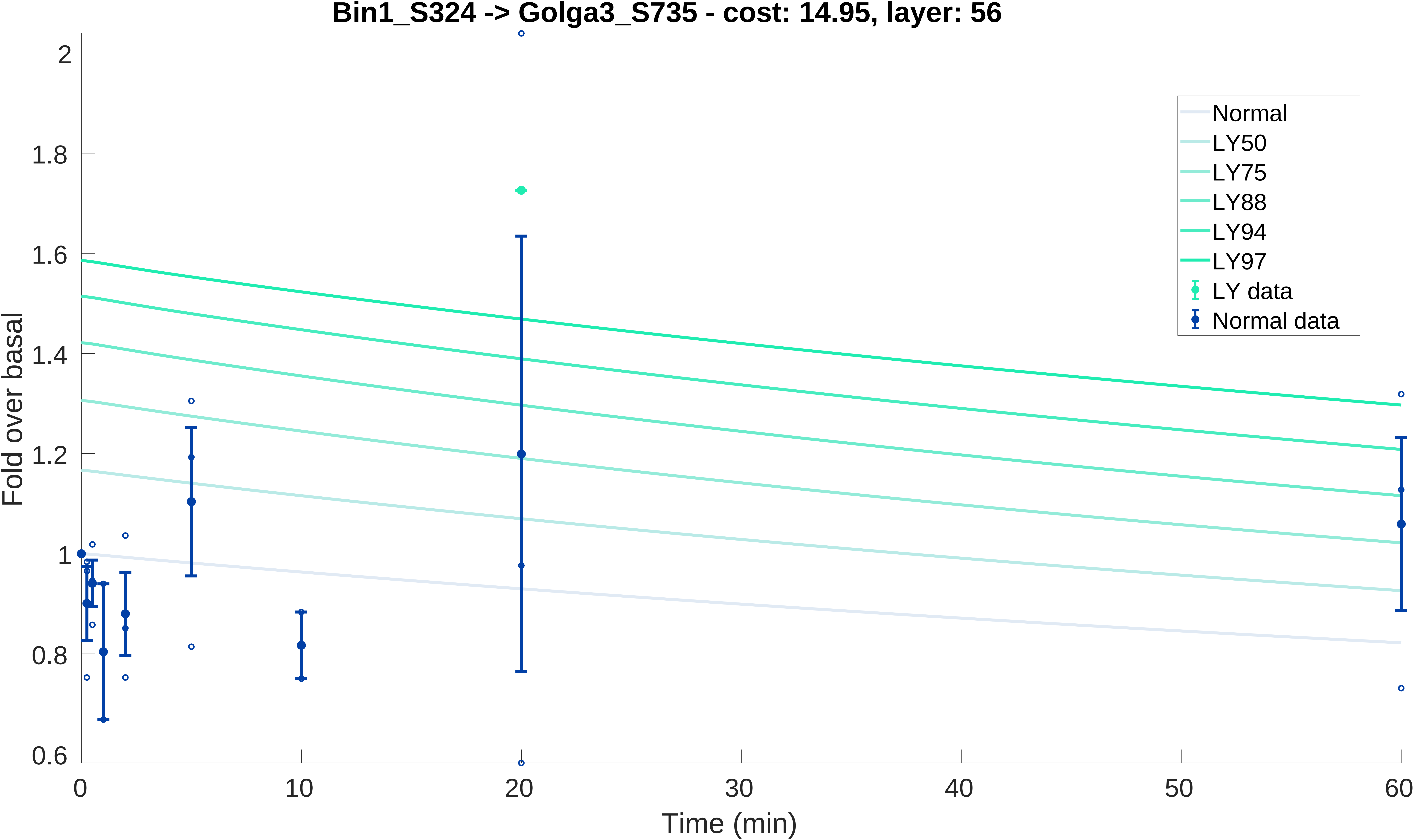
Bin1_S324 -> Golga3_S735 - cost: 14.95, layer: 56
Fold over basal
Time (min)
0.6
0.8
1
1.2
1.4
1.6
1.8
2
0
10
20
30
40
50
60
Normal
LY50
LY75
LY88
LY94
LY97
LY data
Normal data

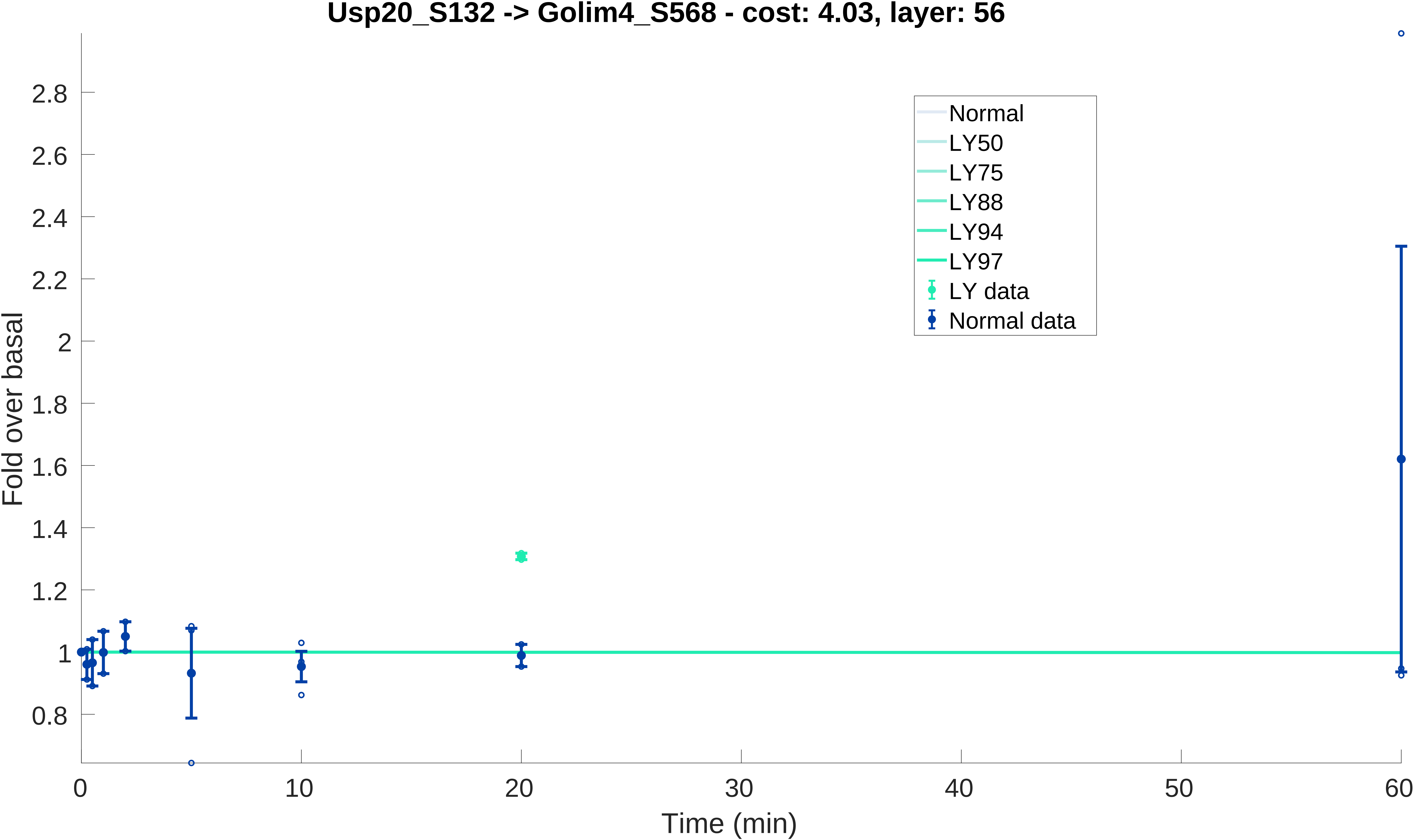
Usp20_S132 -> Golim4_S568 - cost: 4.03, layer: 56
Fold over basal
Time (min)
0
10
20
30
40
50
60
0.8
1
1.2
1.4
1.6
1.8
2
2.2
2.4
2.6
2.8
Normal
LY50
LY75
LY88
LY94
LY97
LY data
Normal data

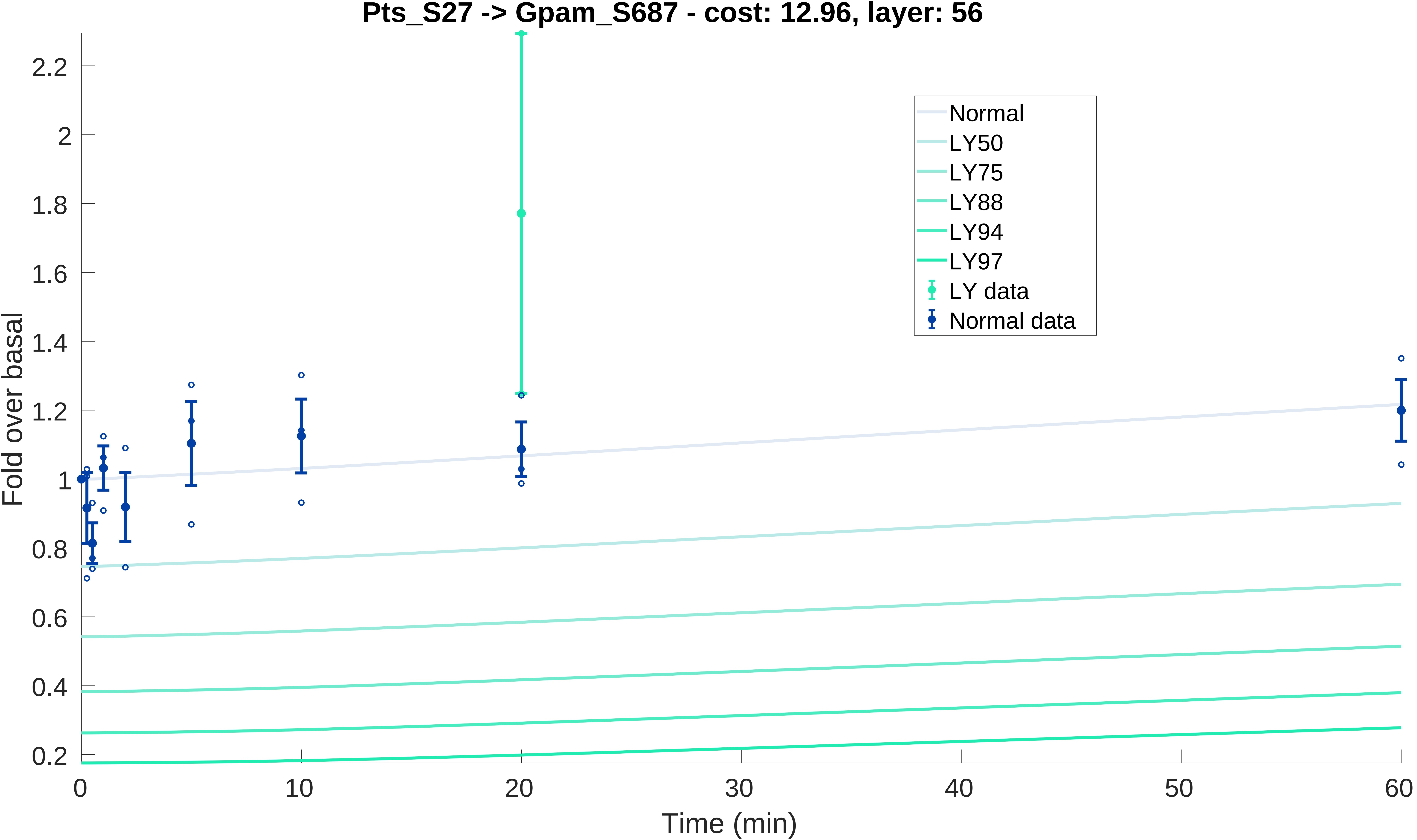

Pts_S27 -> Gpam_S687 - cost: 12.96, layer: 56
Normal
LY50
LY75
LY88
LY94
LY97
LY data
Normal data
Fold over basal
Time (min)
2.2
2
1.8
1.6
1.4
1.2
1
0.8
0.6
0.4
0.2
0
10
20
30
40
50
60

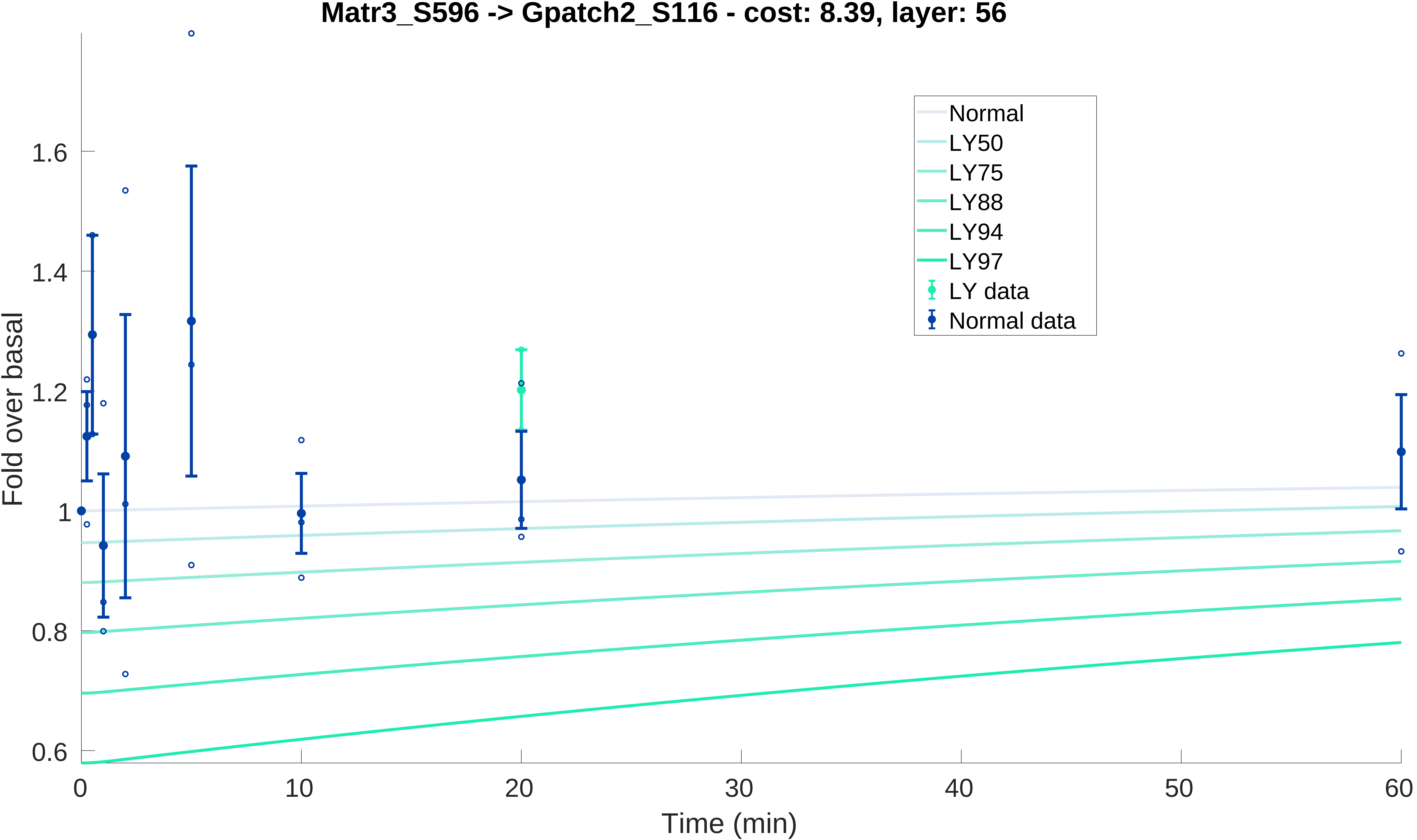

Matr3_S596 -> Gpatch2_S116 - cost: 8.39, layer: 56
Fold over basal
Time (min)
0.6
0.8
1
1.2
1.4
1.6
0
10
20
30
40
50
60
Normal
LY50
LY75
LY88
LY94
LY97
LY data
Normal data

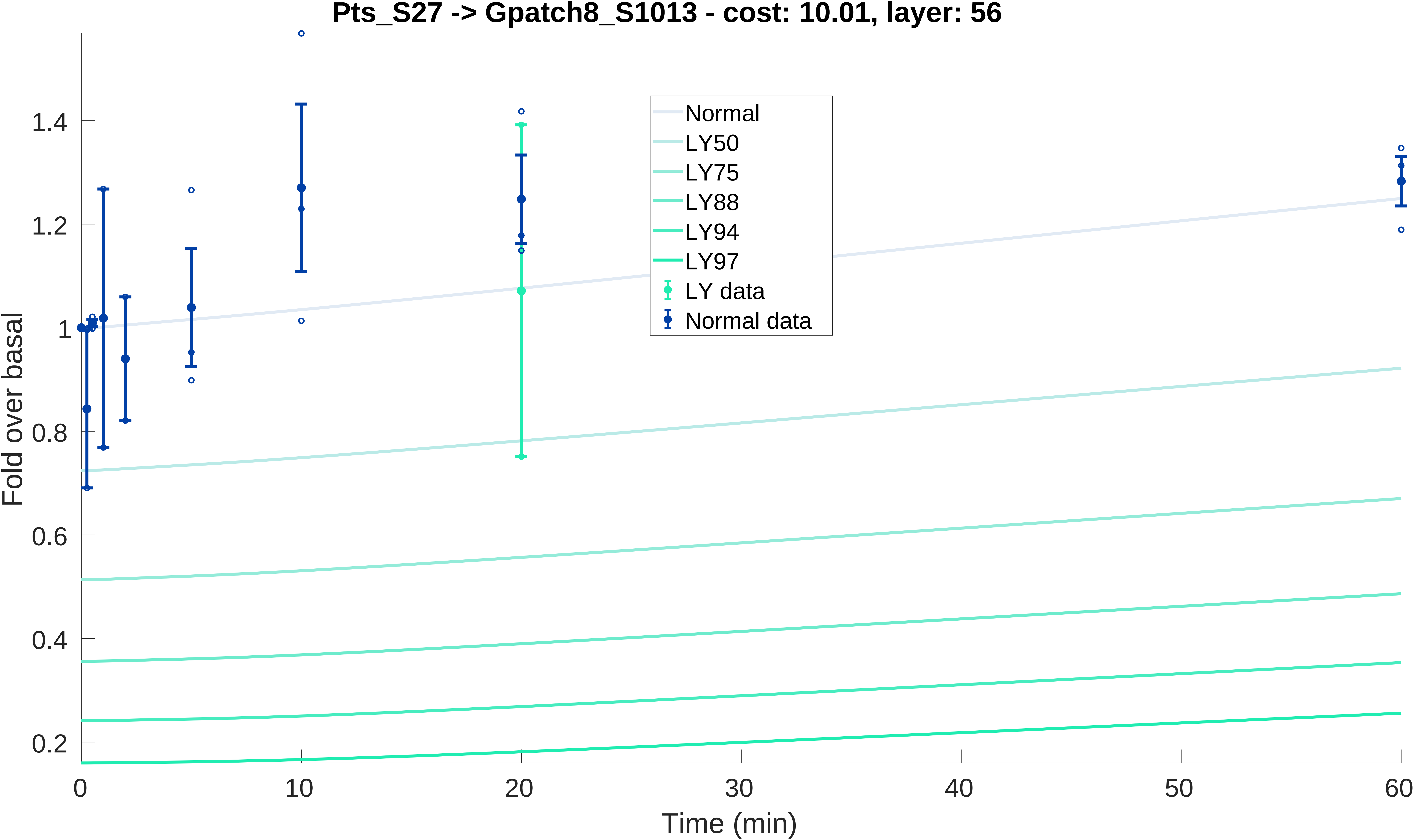

Pts_S27 -> Gpatch8_S1013 - cost: 10.01, layer: 56
Fold over basal
Time (min)
0.2
0.4
0.6
0.8
1
1.2
1.4
0
10
20
30
40
50
60
Normal
LY50
LY75
LY88
LY94
LY97
LY data
Normal data

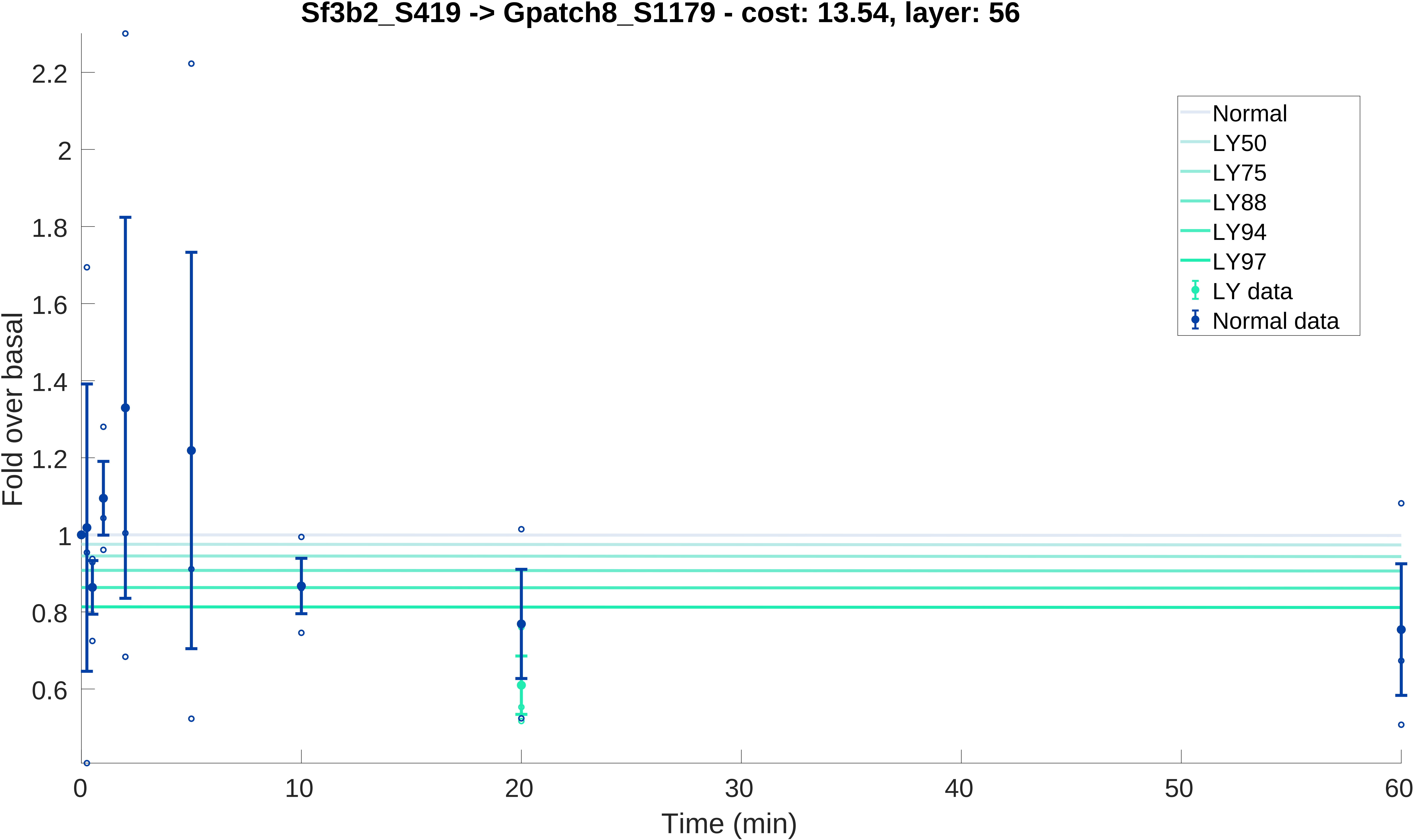

Sf3b2_S419 -> Gpatch8_S1179 - cost: 13.54, layer: 56
Fold over basal
2.2
2
1.8
1.6
1.4
1.2
1
0.8
0.6
0
10
20
30
40
50
60
Time (min)
Normal
LY50
LY75
LY88
LY94
LY97
LY data
Normal data

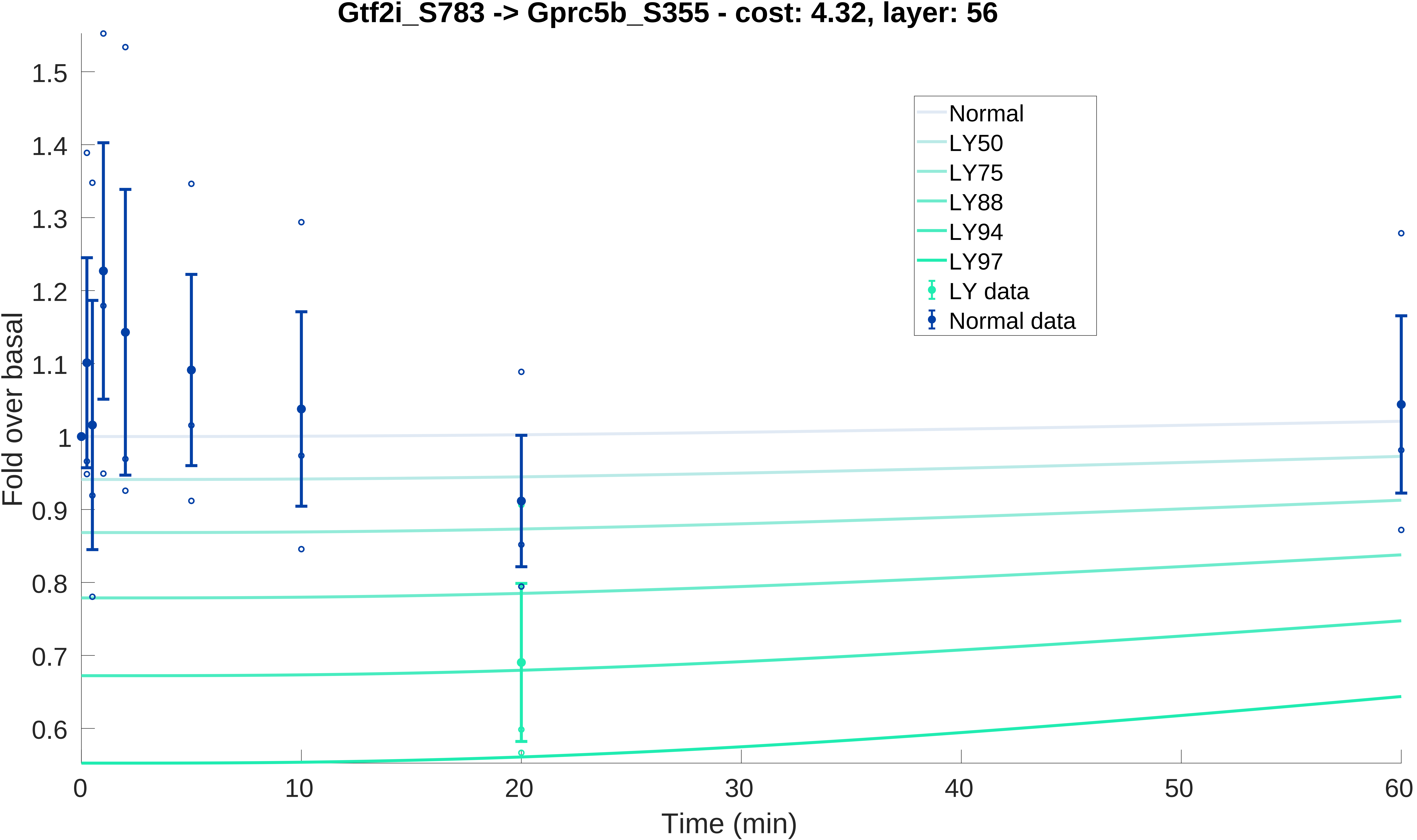

Gtf2i_S783 -> Gprc5b_S355 - cost: 4.32, layer: 56
Fold over basal
Time (min)
0
10
20
30
40
50
60
0.6
0.7
0.8
0.9
1
1.1
1.2
1.3
1.4
1.5
Normal
LY50
LY75
LY88
LY94
LY97
LY data
Normal data

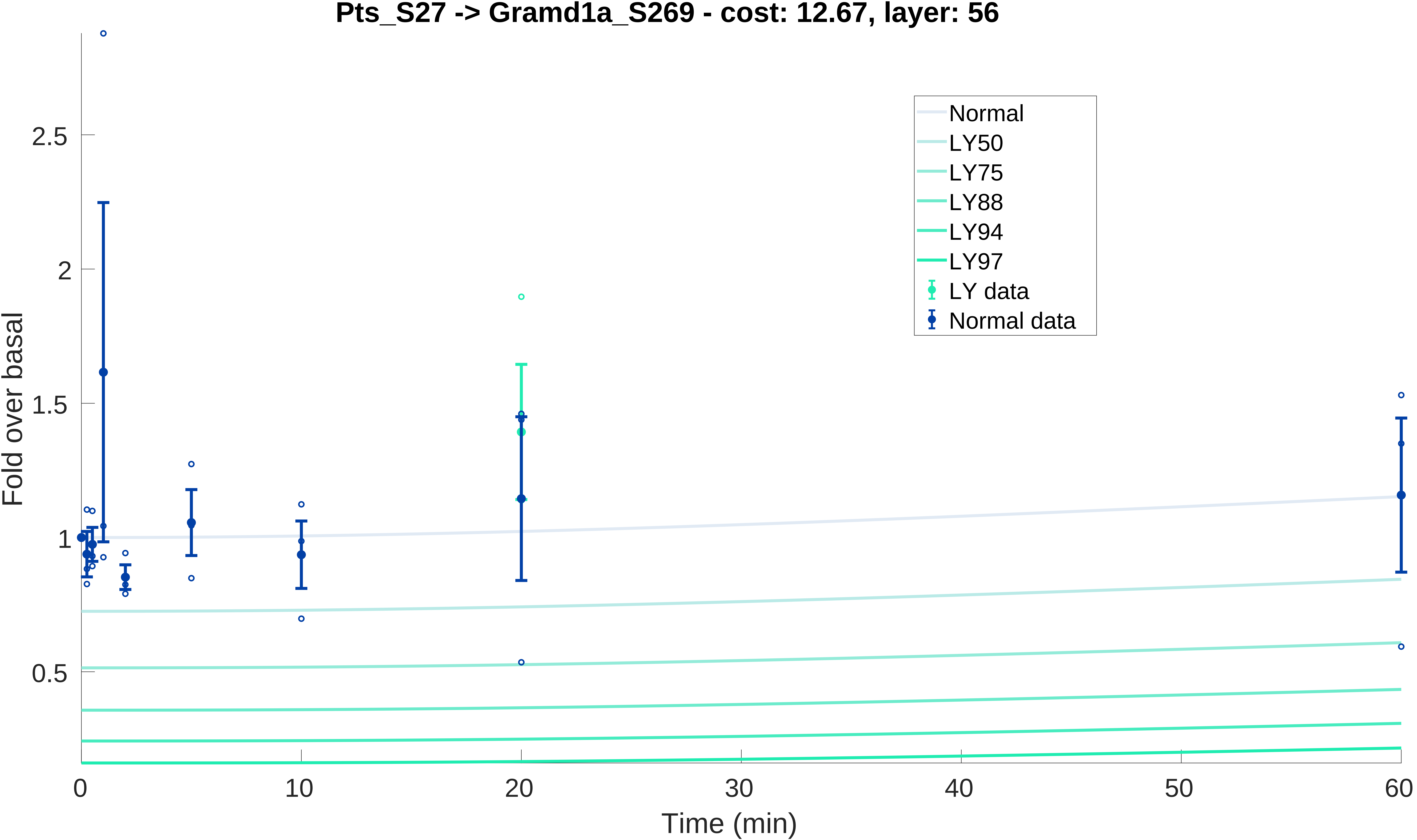

Pts_S27 -> Gramd1a_S269 - cost: 12.67, layer: 56
Normal
LY50
LY75
LY88
LY94
LY97
LY data
Normal data
Fold over basal
Time (min)
0.5
1
1.5
2
2.5
0
10
20
30
40
50
60

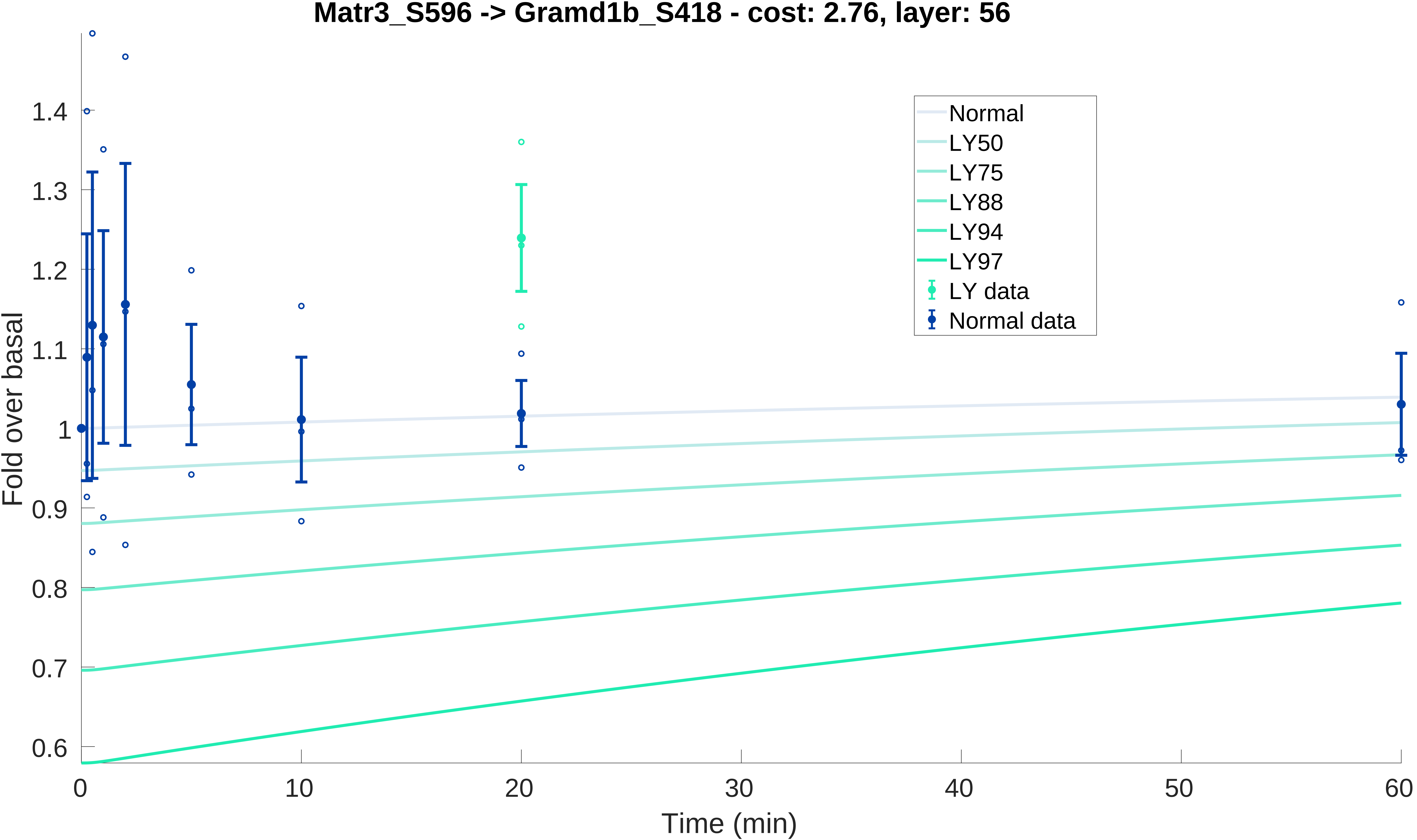
Matr3_S596 -> Gramd1b_S418 - cost: 2.76, layer: 56
Fold over basal
1.4
1.3
1.2
1.1
1
0.9
0.8
0.7
0.6
0
10
20
30
40
50
60
Time (min)
Normal
LY50
LY75
LY88
LY94
LY97
LY data
Normal data

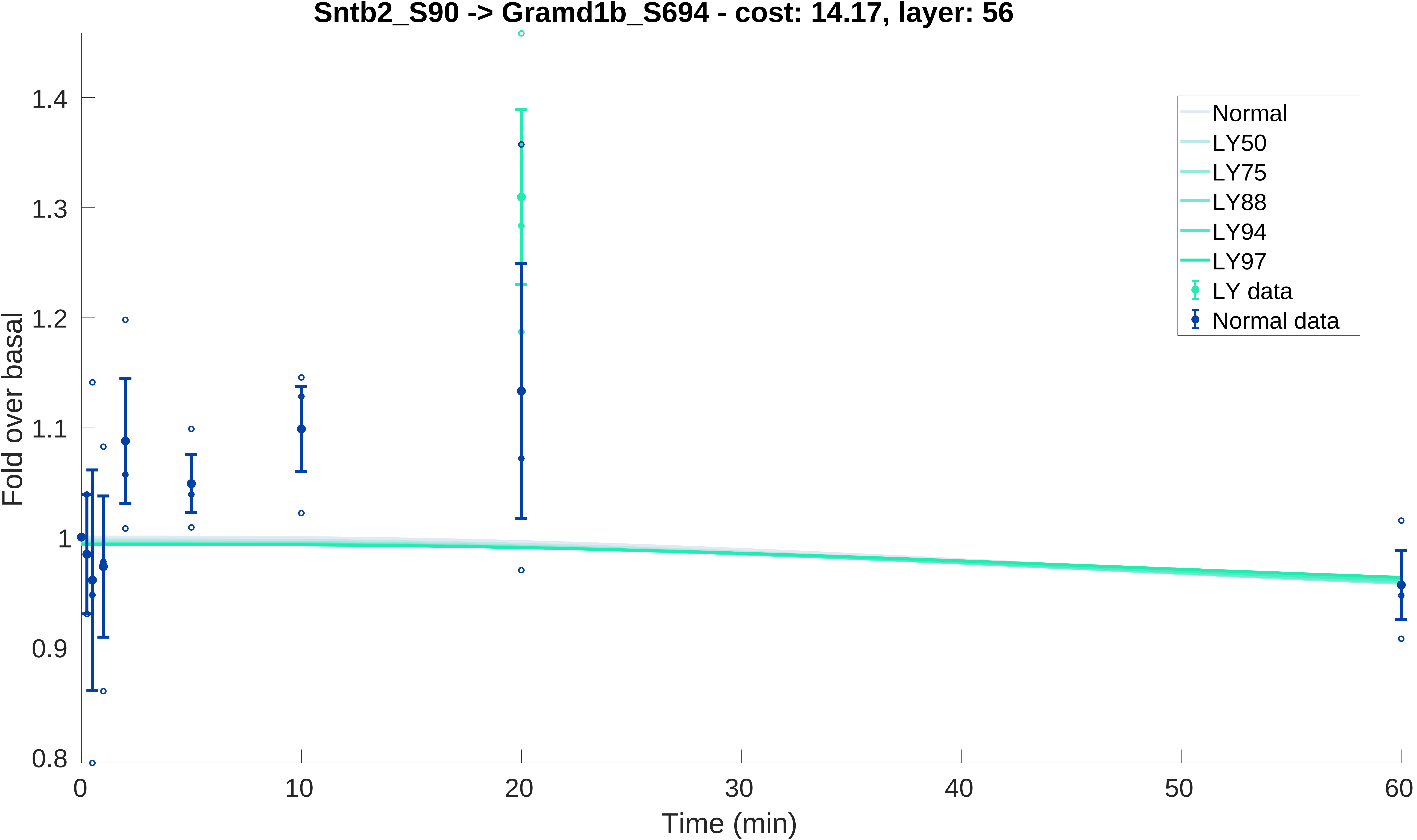
Sntb2_S90 -> Gramd1b_S694 - cost: 14.17, layer: 56
Fold over basal
1.4
1.3
1.2
1.1
1
0.9
0.8
0
10
20
30
40
50
60
Time (min)
Normal
LY50
LY75
LY88
LY94
LY97
LY data
Normal data

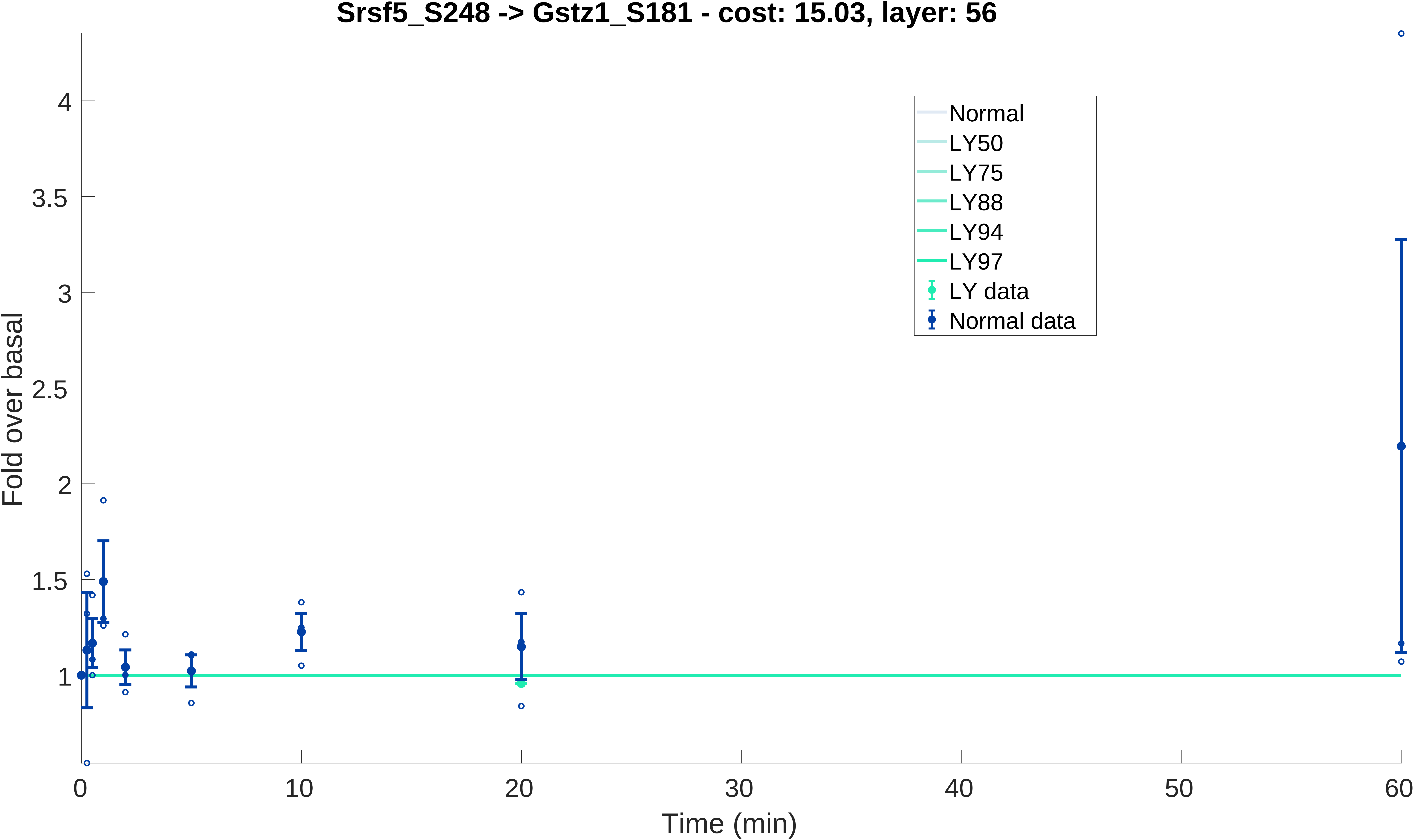

Srsf5_S248 -> Gstz1_S181 - cost: 15.03, layer: 56
Normal
LY50
LY75
LY88
LY94
LY97
LY data
Normal data
Fold over basal
4
3.5
3
2.5
2
1.5
1
0
10
20
30
40
50
60
Time (min)

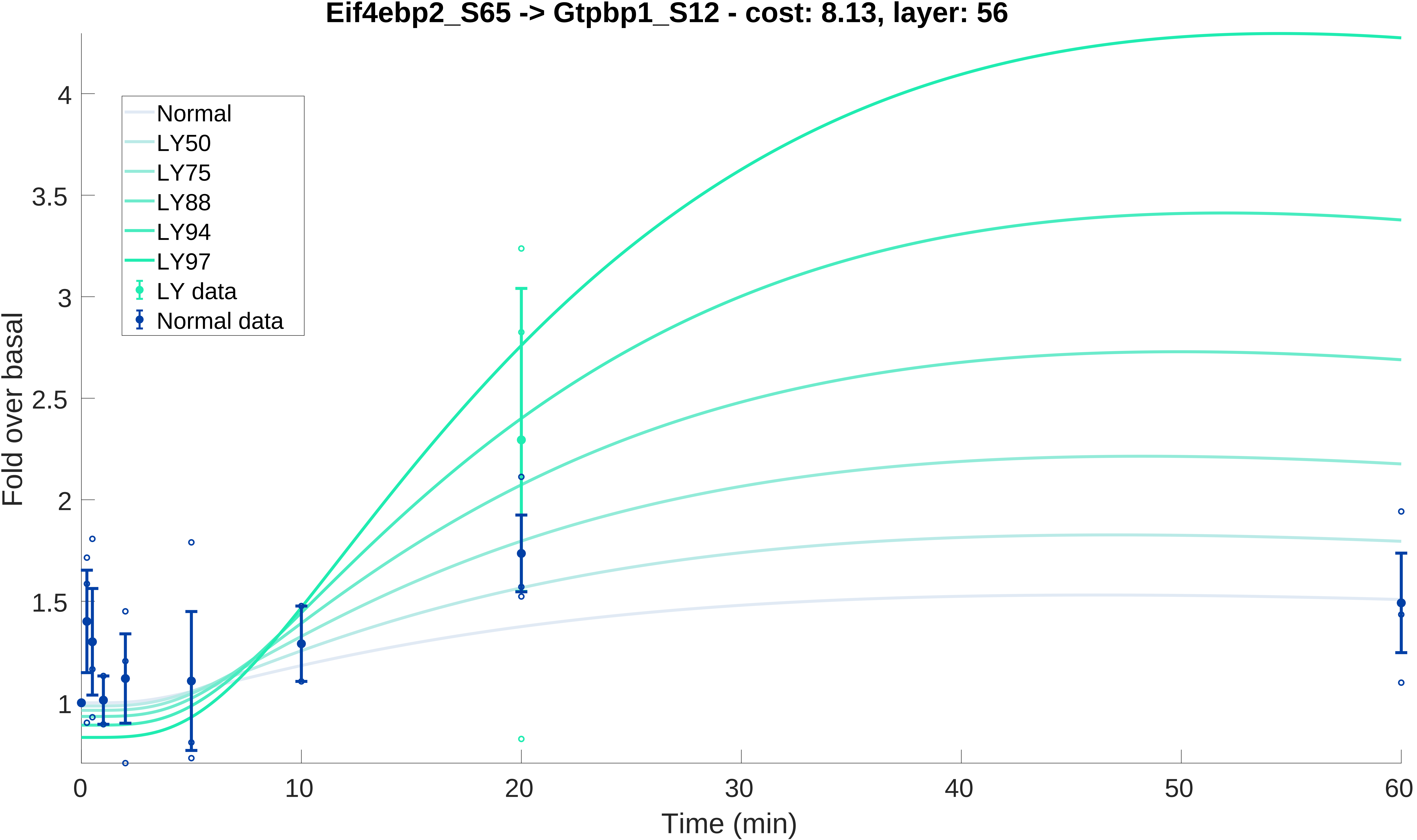

Eif4ebp2_S65 -> Gtpbp1_S12 - cost: 8.13, layer: 56
Normal
LY50
LY75
LY88
LY94
LY97
LY data
Normal data
Fold over basal
Time (min)
4
3.5
3
2.5
2
1.5
1
0
10
20
30
40
50
60

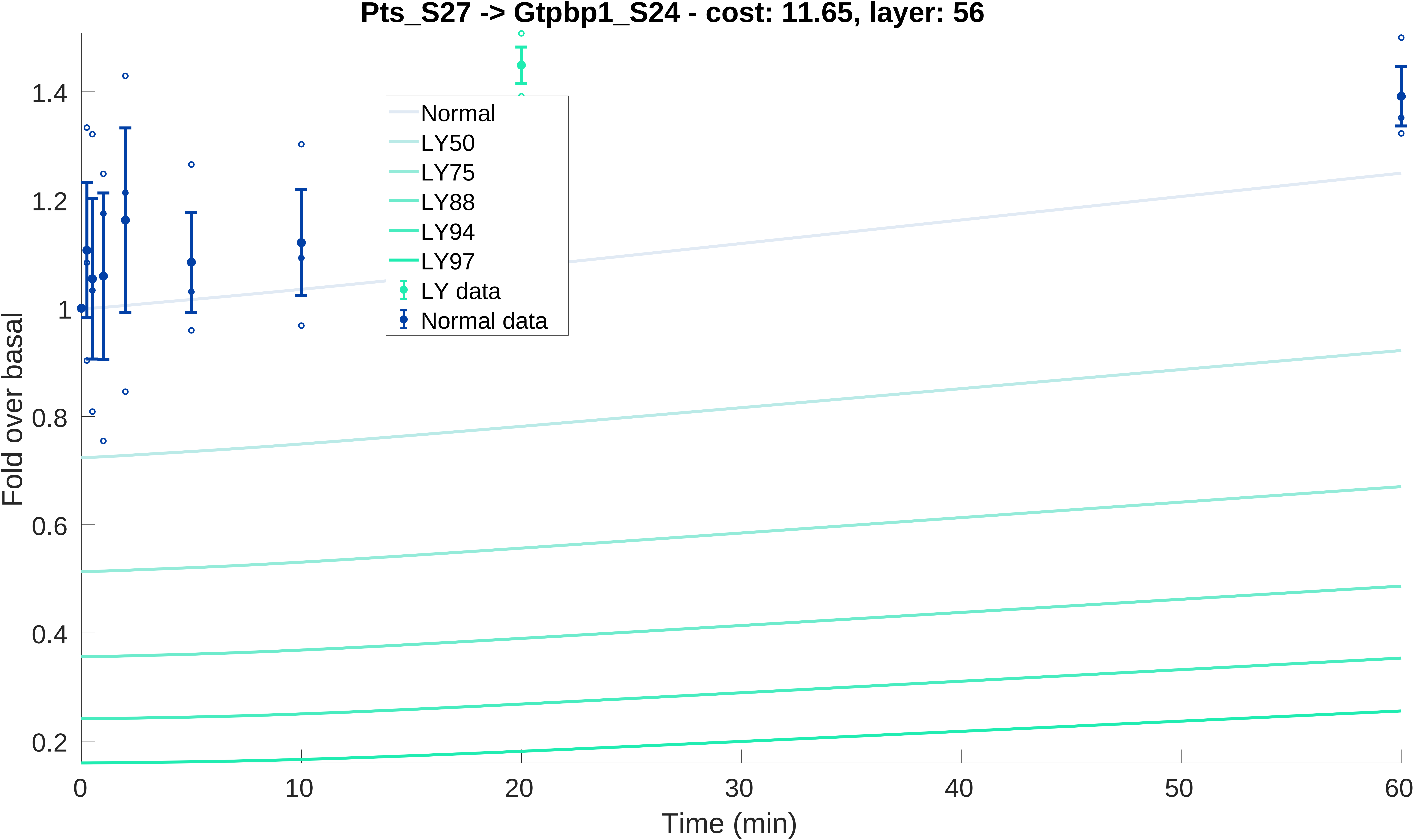

Pts_S27 -> Gtpbp1_S24 - cost: 11.65, layer: 56
Fold over basal
1.4
1.2
1
0.8
0.6
0.4
0.2
0
10
20
30
40
50
60
Time (min)
Normal
LY50
LY75
LY88
LY94
LY97
LY data
Normal data

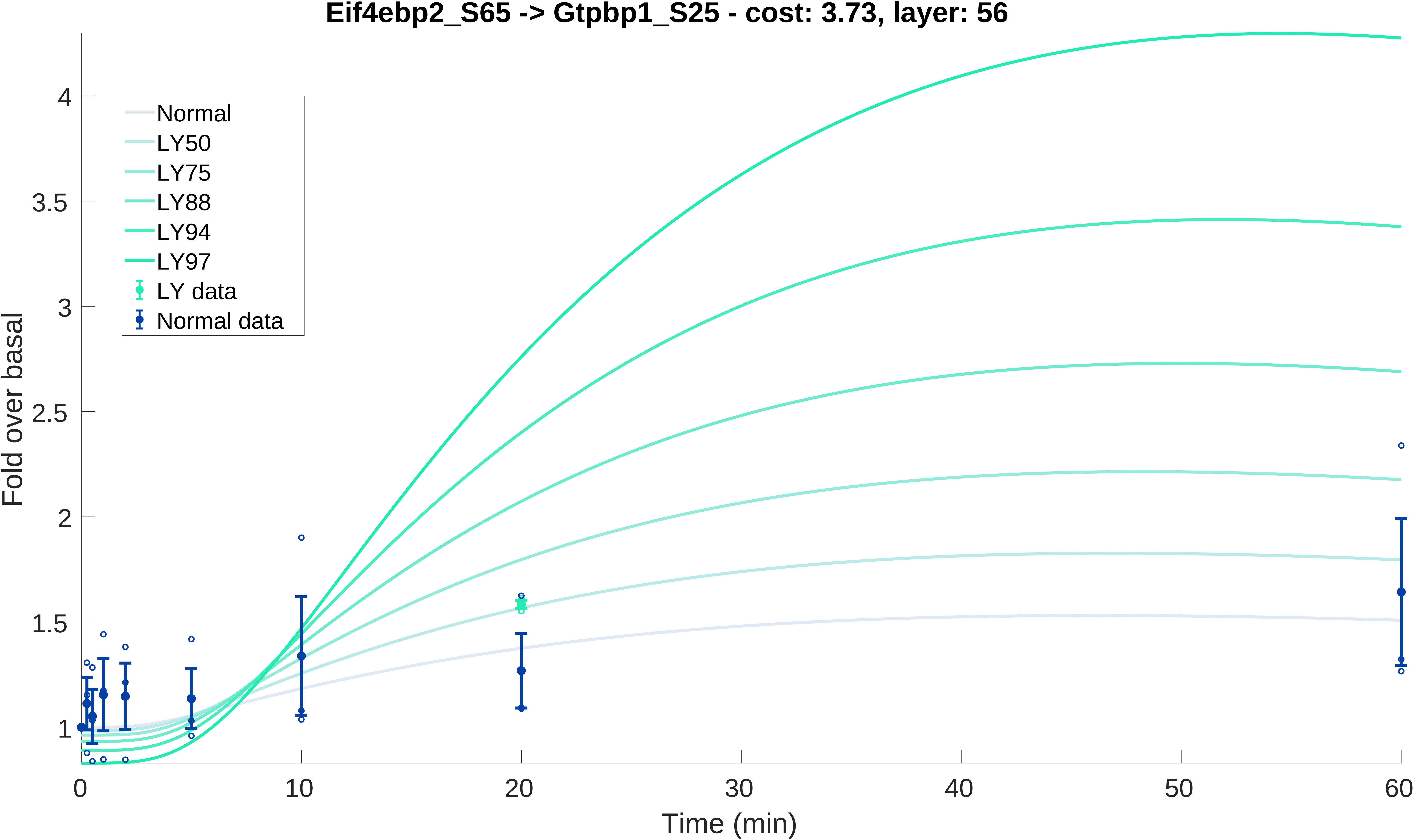
Eif4ebp2_S65 -> Gtpbp1_S25 - cost: 3.73, layer: 56
Normal
LY50
LY75
LY88
LY94
LY97
LY data
Normal data
Fold over basal
Time (min)
1
1.5
2
2.5
3
3.5
4
0
10
20
30
40
50
60

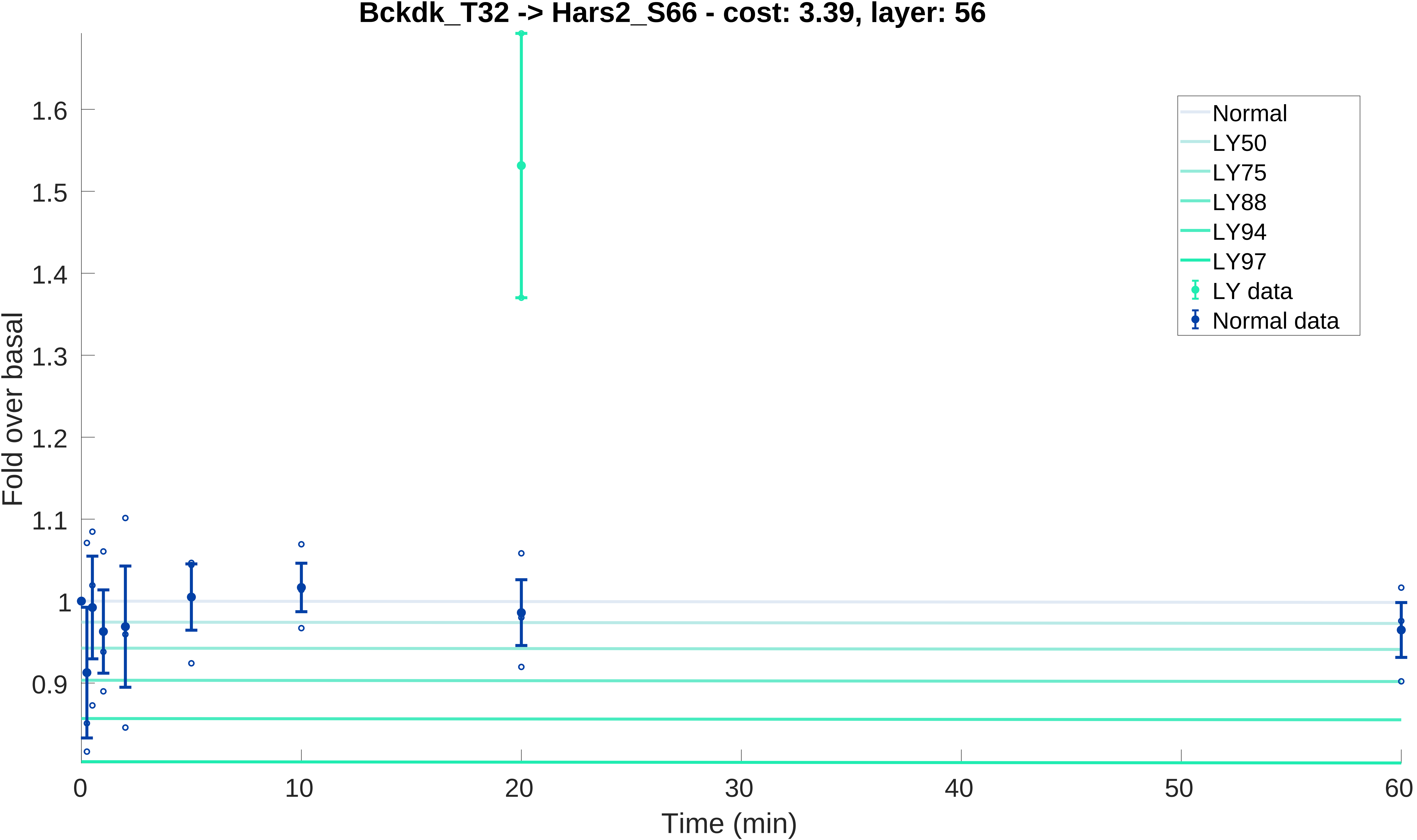

Bckdk_T32 -> Hars2_S66 - cost: 3.39, layer: 56
Fold over basal
Time (min)
1.6
1.5
1.4
1.3
1.2
1.1
1
0.9
0
10
20
30
40
50
60
Normal
LY50
LY75
LY88
LY94
LY97
LY data
Normal data

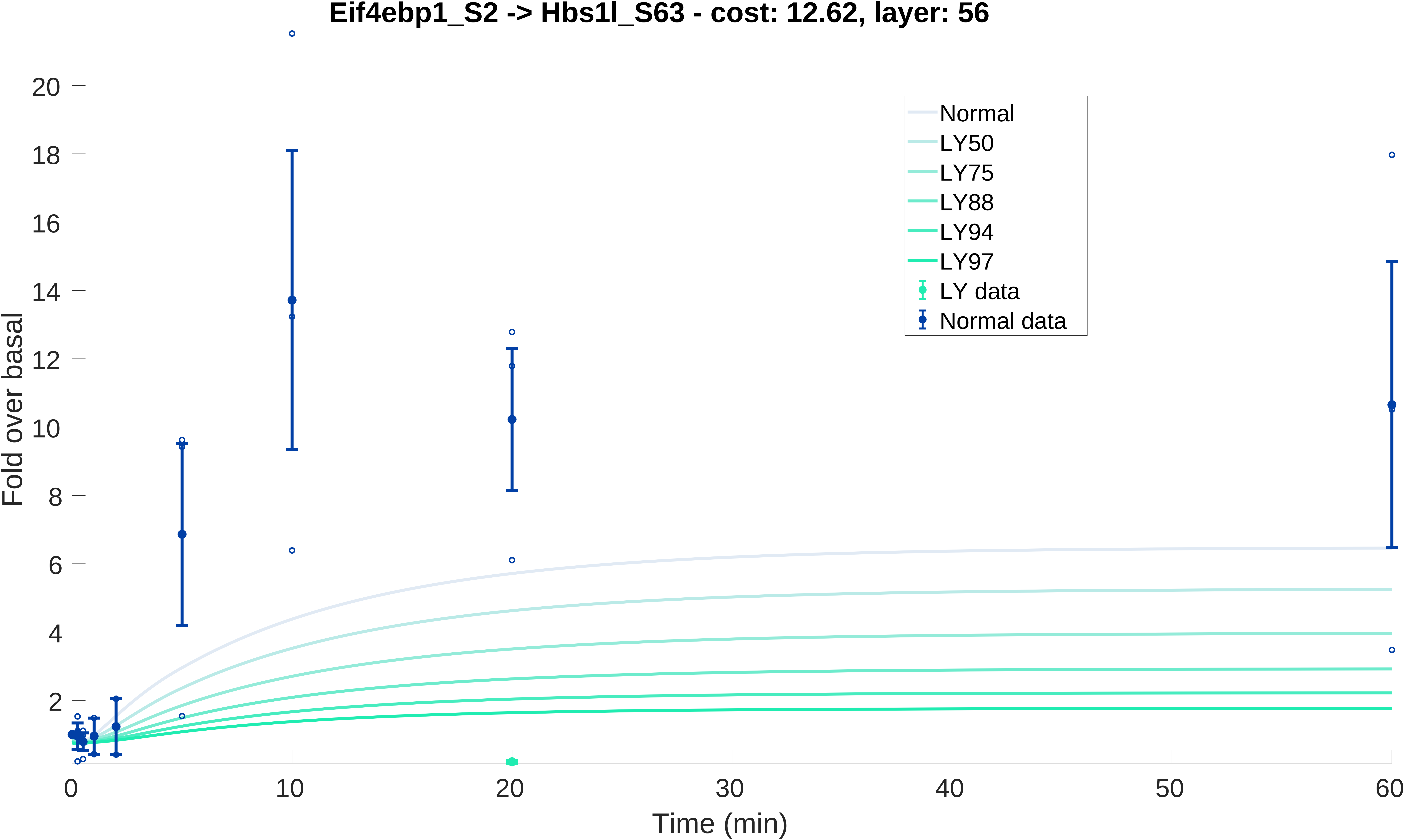

Eif4ebp1_S2 -> Hbs1l_S63 - cost: 12.62, layer: 56
Fold over basal
Time (min)
0
10
20
30
40
50
60
2
4
6
8
10
12
14
16
18
20
Normal
LY50
LY75
LY88
LY94
LY97
LY data
Normal data

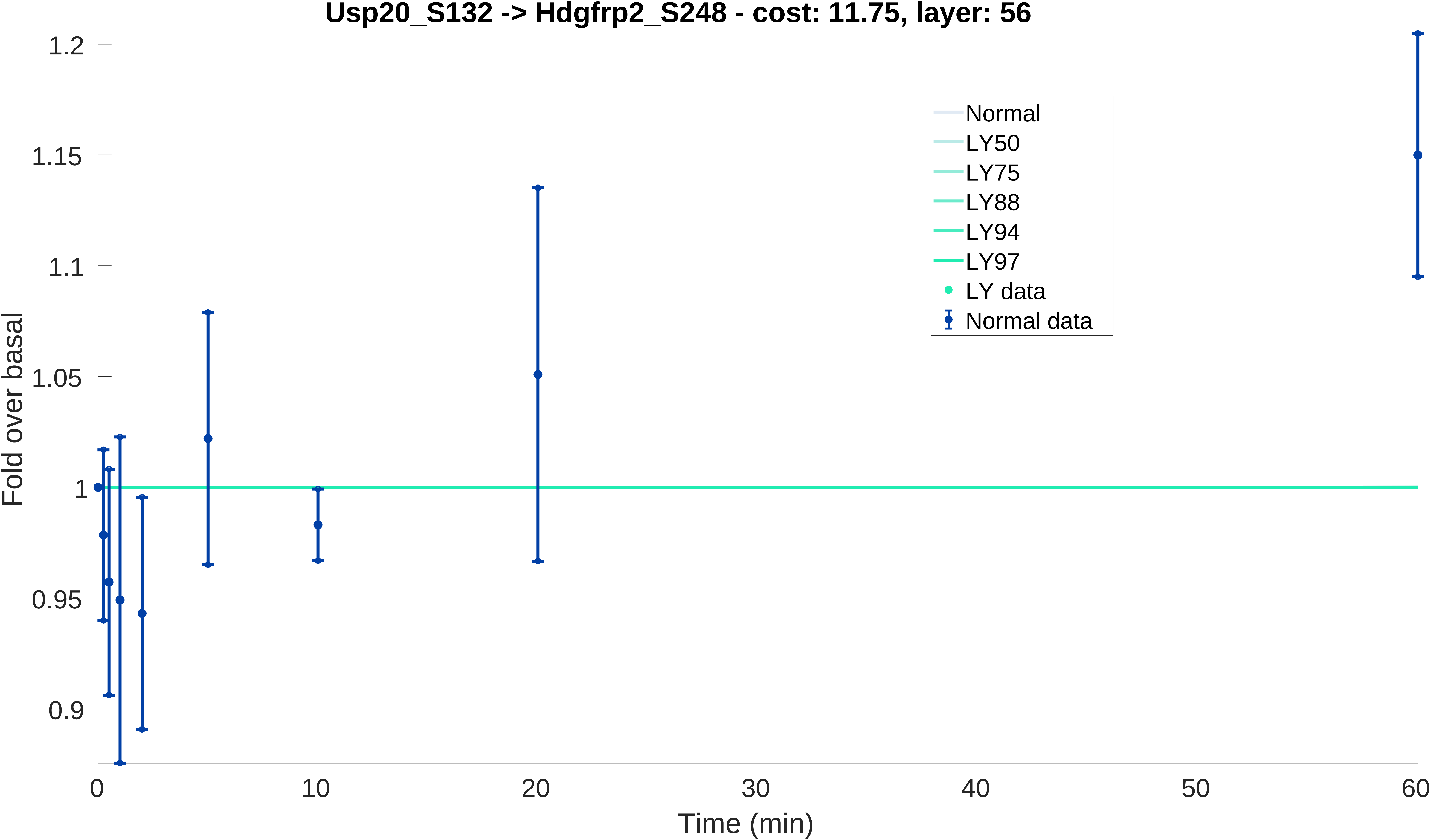

Usp20_S132 -> Hdgfrp2_S248 - cost: 11.75, layer: 56
Fold over basal
1.2
1.15
1.1
1.05
1
0.95
0.9
Time (min)
0
10
20
30
40
50
60
Normal
LY50
LY75
LY88
LY94
LY97
LY data
Normal data

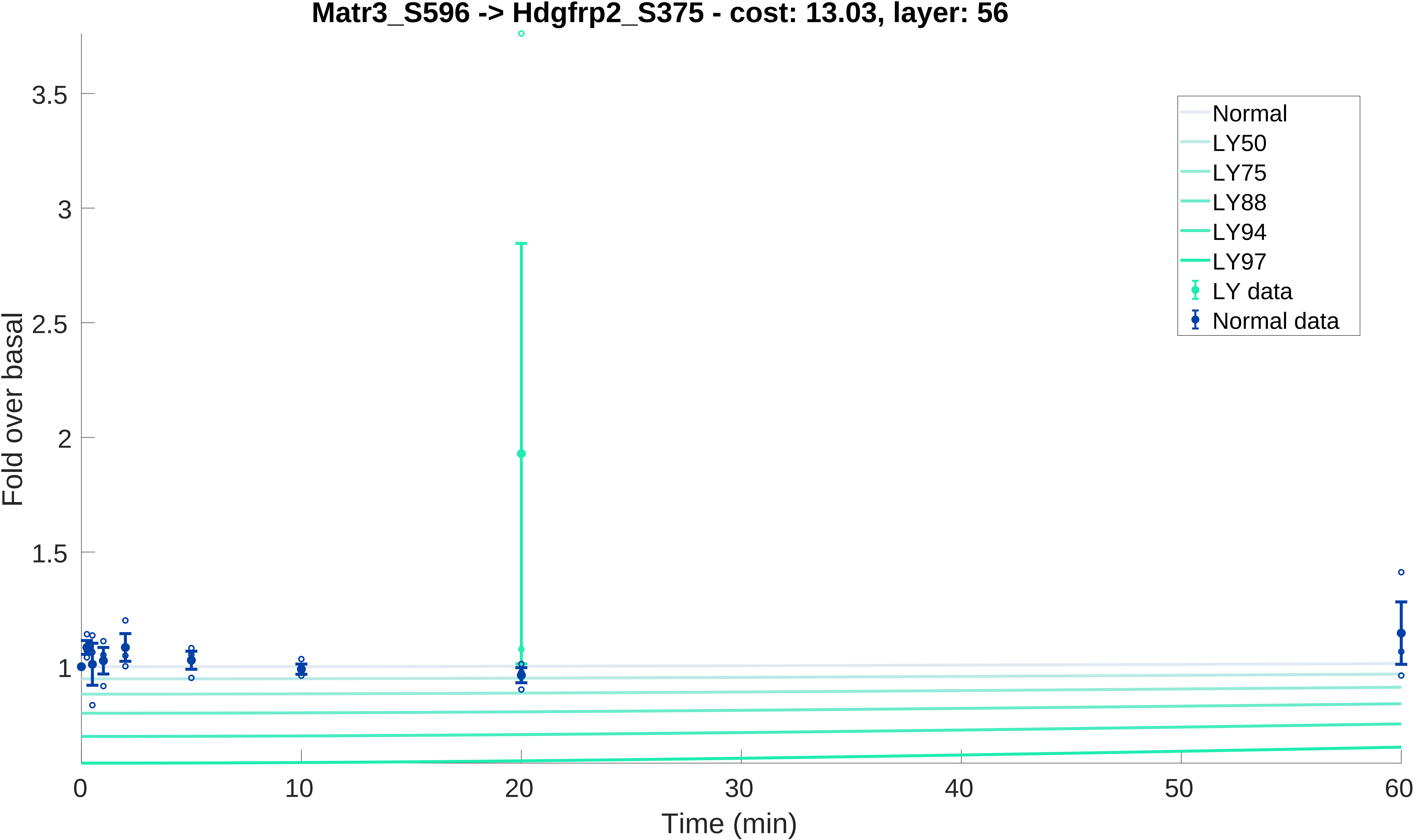
Matr3_S596 -> Hdgfrp2_S375 - cost: 13.03, layer: 56
Normal
LY50
LY75
LY88
LY94
LY97
LY data
Normal data
Fold over basal
3.5
3
2.5
2
1.5
1
0
10
20
30
40
50
60
Time (min)

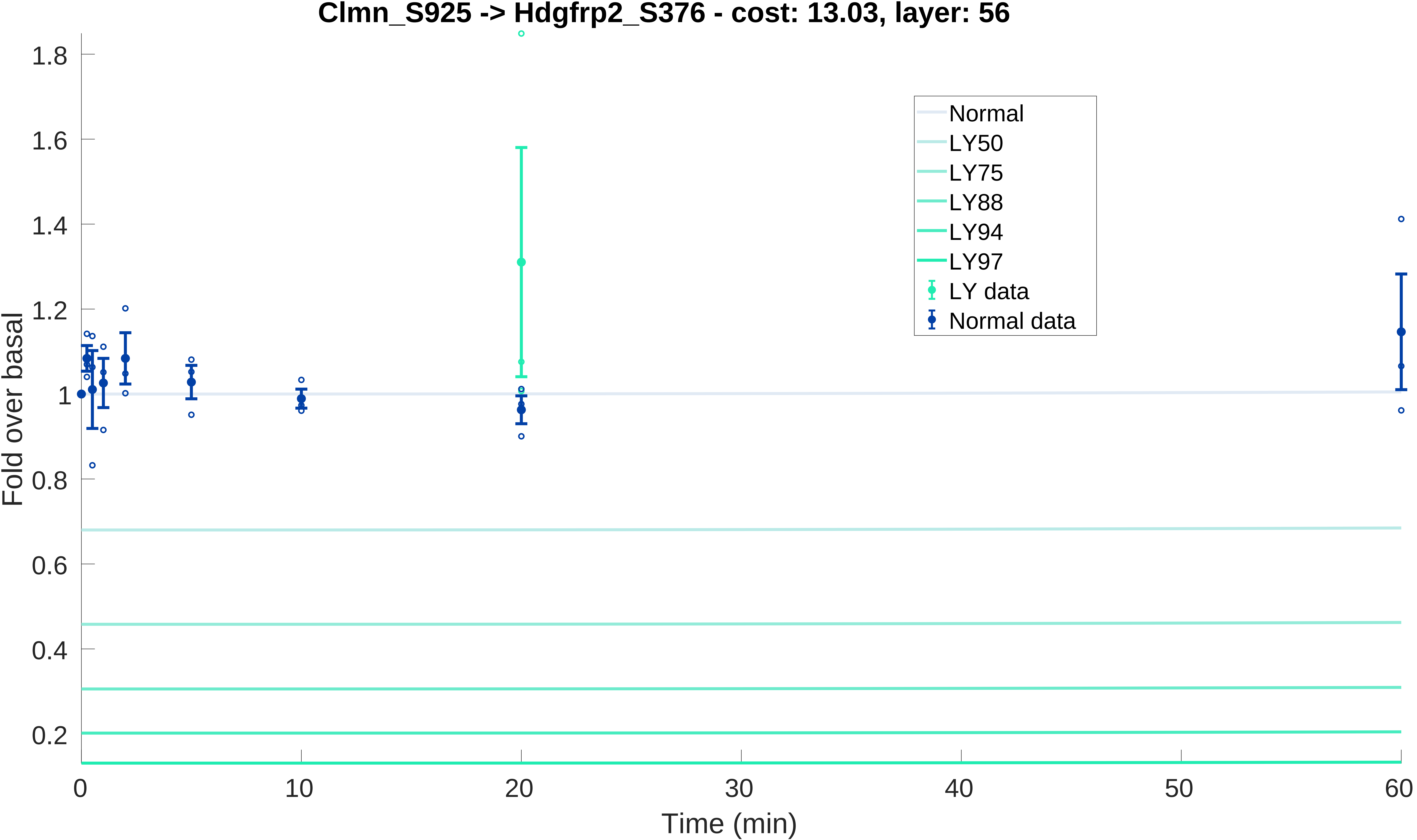

Clmn_S925 -> Hdgfrp2_S376 - cost: 13.03, layer: 56
Fold over basal
Time (min)
1.8
1.6
1.4
1.2
1
0.8
0.6
0.4
0.2
0
10
20
30
40
50
60
Normal
LY50
LY75
LY88
LY94
LY97
LY data
Normal data

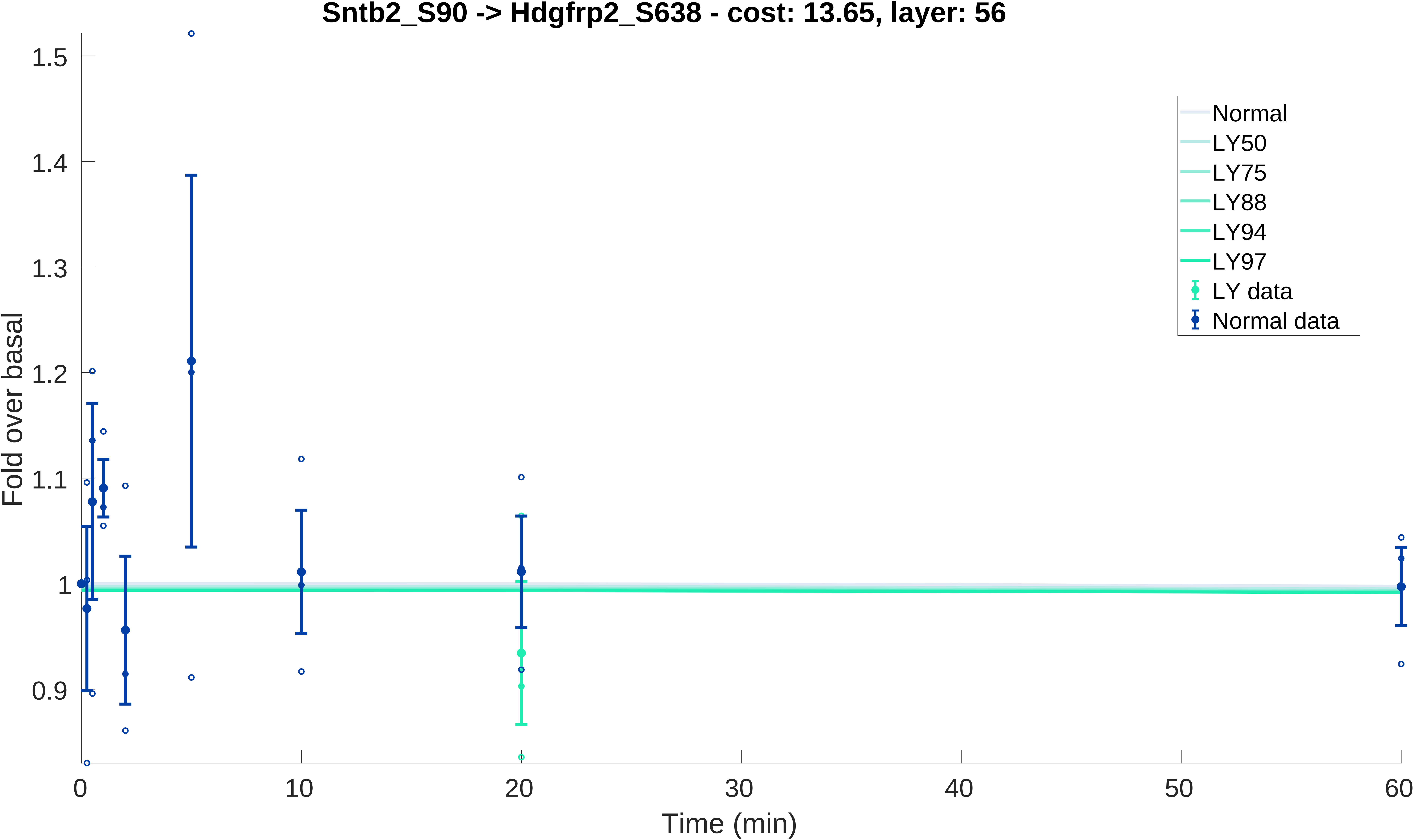

Sntb2_S90 -> Hdgfrp2_S638 - cost: 13.65, layer: 56
Fold over basal
Time (min)
Normal
LY50
LY75
LY88
LY94
LY97
LY data
Normal data
0.9
1
1.1
1.2
1.3
1.4
1.5
0
10
20
30
40
50
60

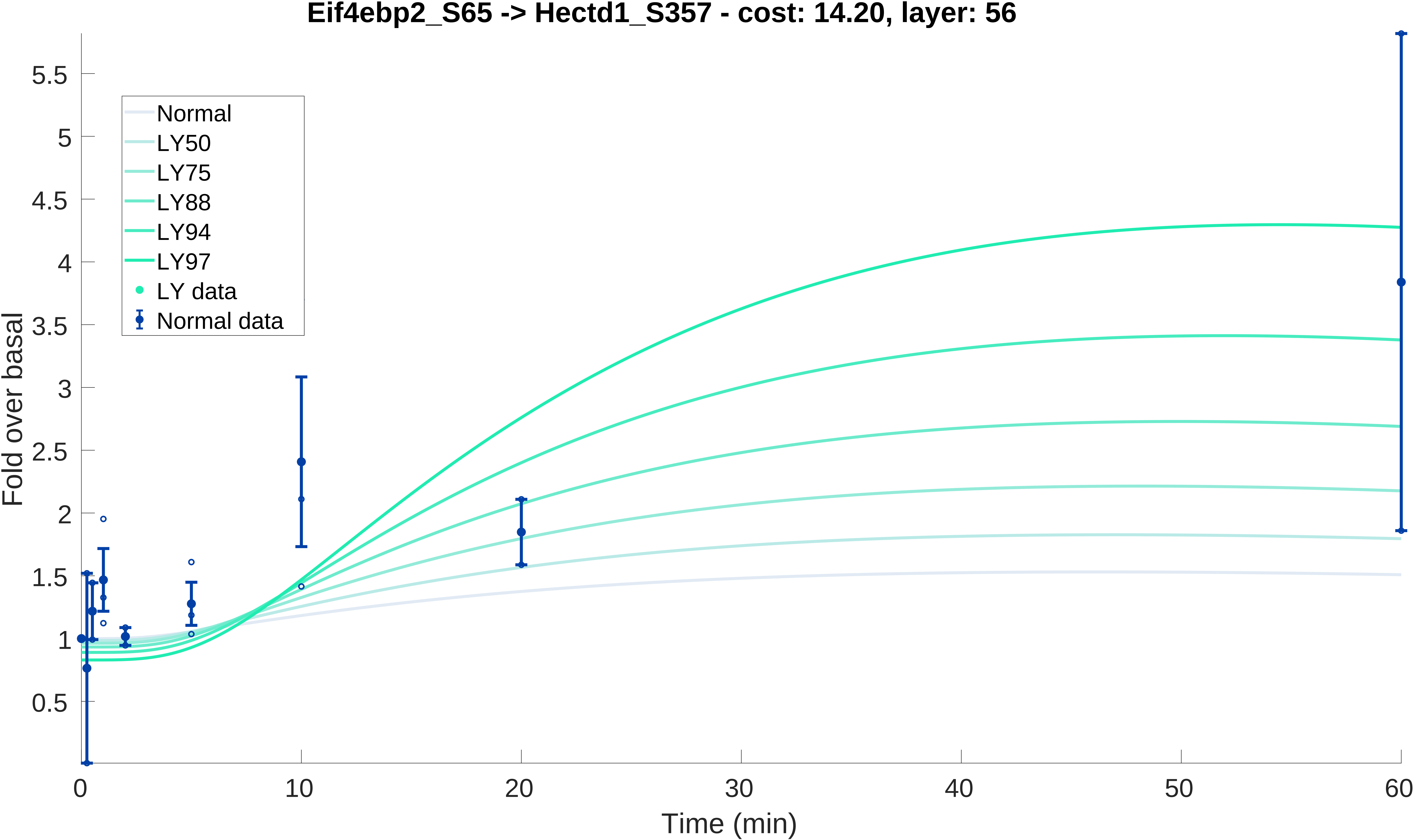

Eif4ebp2_S65 -> Hectd1_S357 - cost: 14.20, layer: 56
Fold over basal
Time (min)
0.5
1
1.5
2
2.5
3
3.5
4
4.5
5
5.5
0
10
20
30
40
50
60
Normal
LY50
LY75
LY88
LY94
LY97
LY data
Normal data

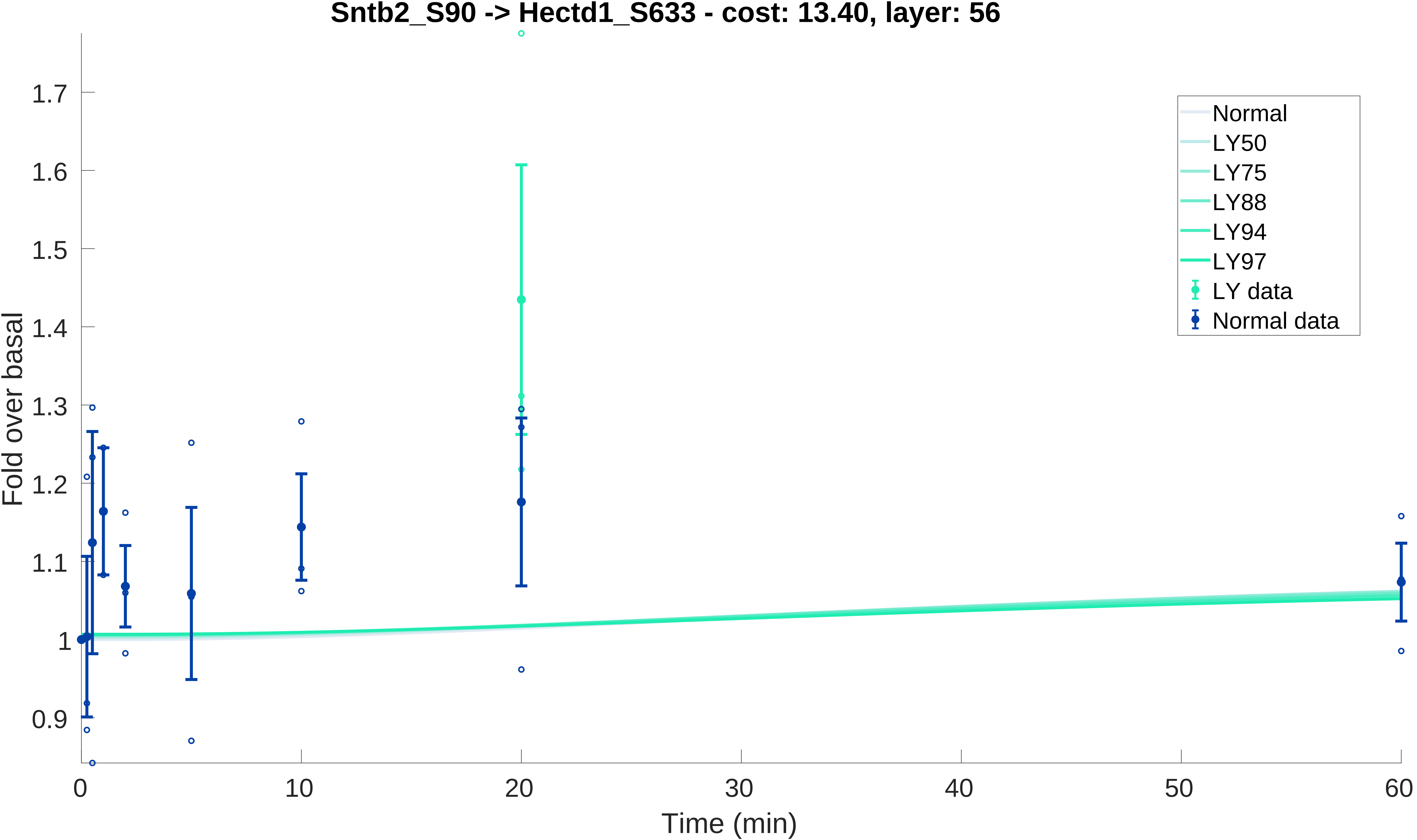

Sntb2_S90 -> Hectd1_S633 - cost: 13.40, layer: 56
Fold over basal
Time (min)
0.9
1
1.1
1.2
1.3
1.4
1.5
1.6
1.7
0
10
20
30
40
50
60
Normal
LY50
LY75
LY88
LY94
LY97
LY data
Normal data

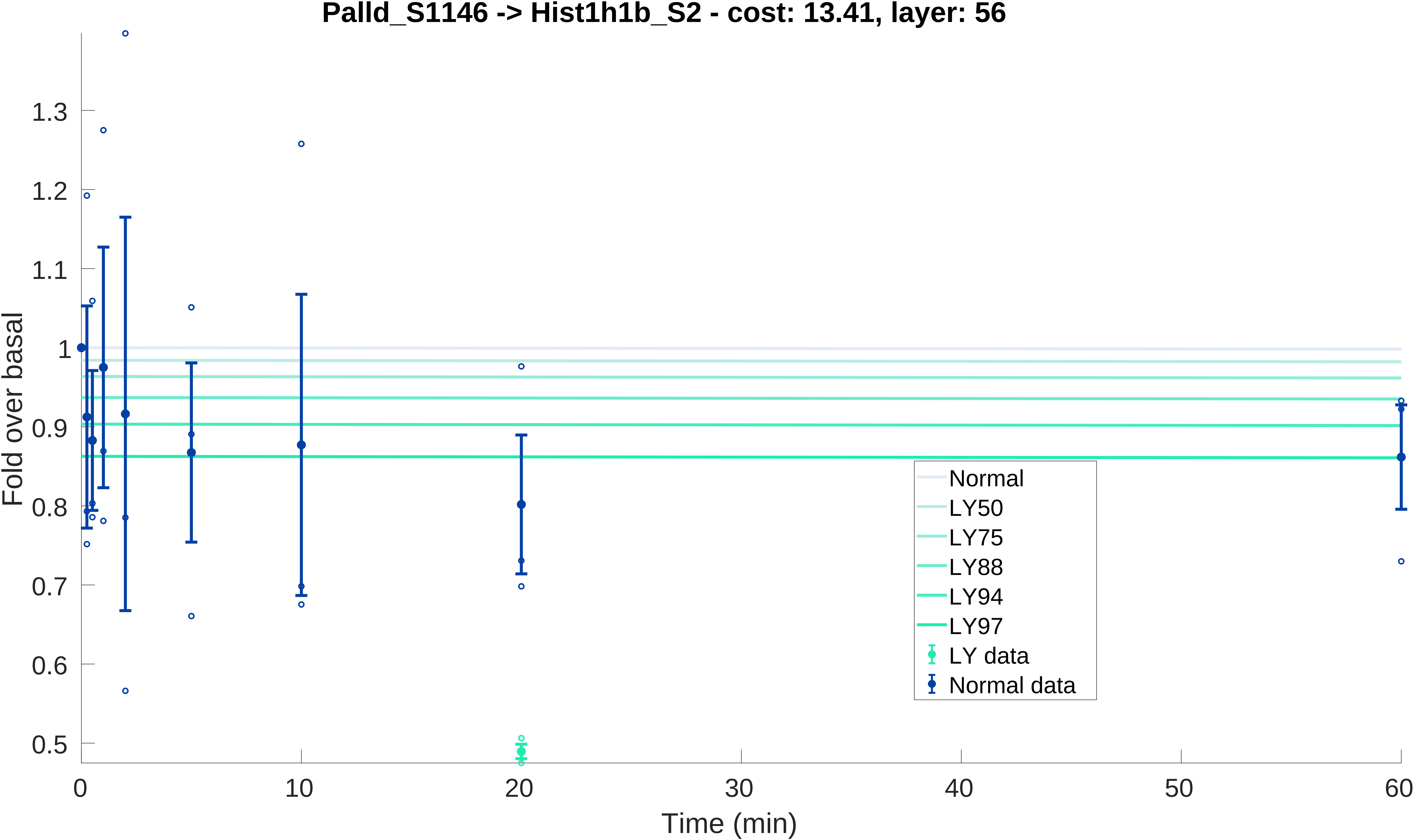
Palld_S1146 -> Hist1h1b_S2 - cost: 13.41, layer: 56
Fold over basal
Time (min)
0
10
20
30
40
50
60
0.5
0.6
0.7
0.8
0.9
1
1.1
1.2
1.3
Normal
LY50
LY75
LY88
LY94
LY97
LY data
Normal data

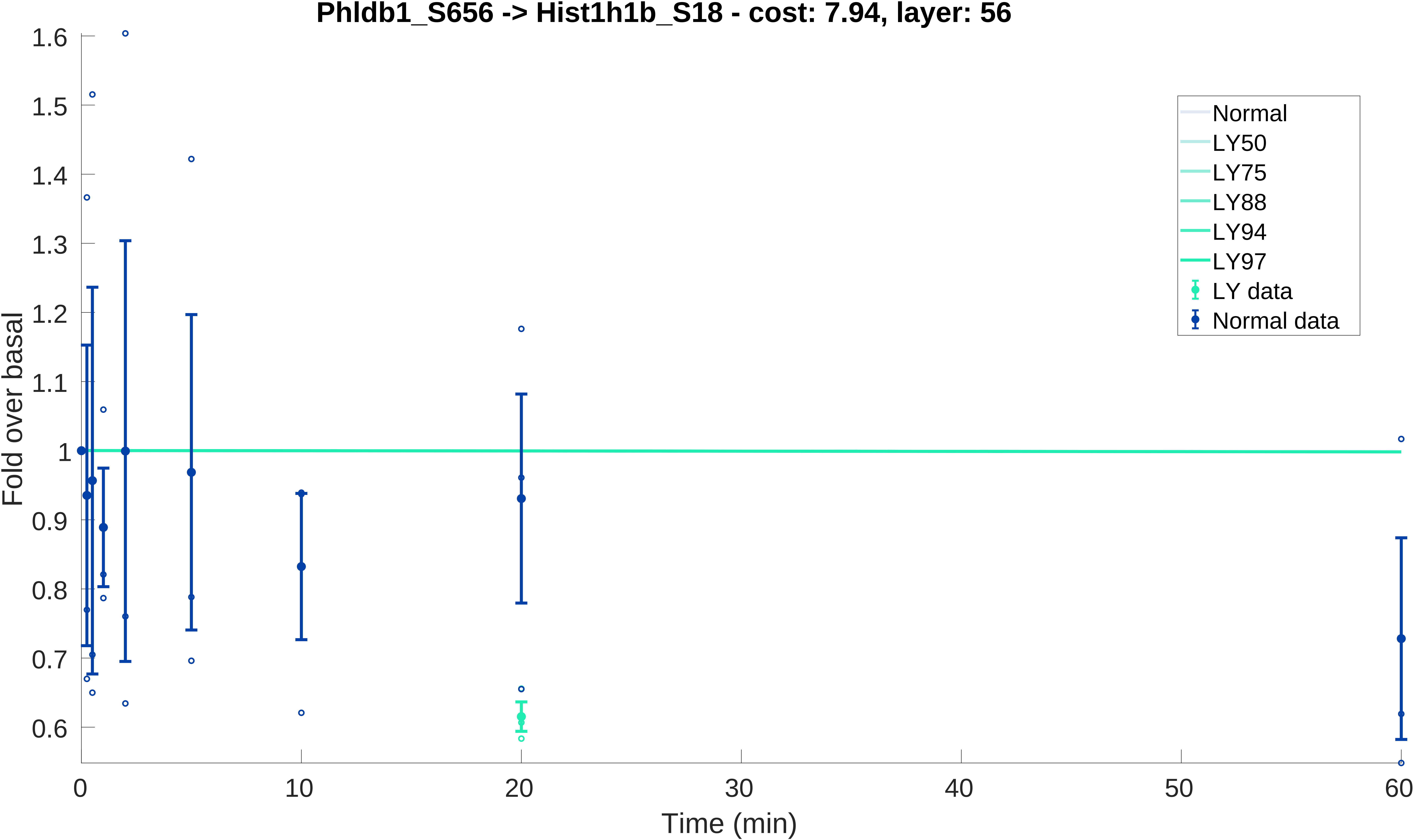
Phldb1_S656 -> Hist1h1b_S18 - cost: 7.94, layer: 56
Fold over basal
Time (min)
Normal
LY50
LY75
LY88
LY94
LY97
LY data
Normal data
1.6
1.5
1.4
1.3
1.2
1.1
1
0.9
0.8
0.7
0.6
0
10
20
30
40
50
60

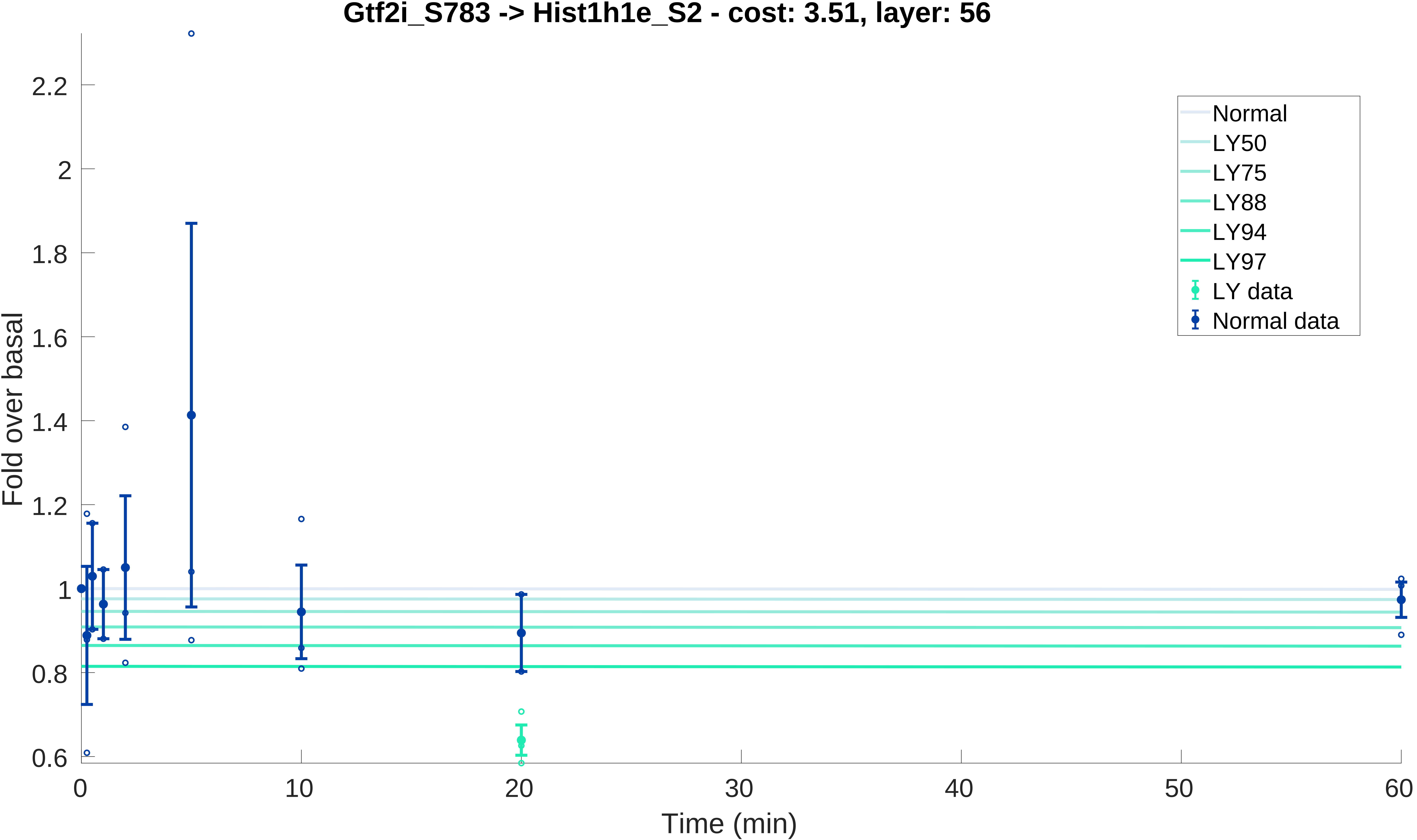

Gtf2i_S783 -> Hist1h1e_S2 - cost: 3.51, layer: 56
Normal
LY50
LY75
LY88
LY94
LY97
LY data
Normal data
Fold over basal
Time (min)
2.2
2
1.8
1.6
1.4
1.2
1
0.8
0.6
0
10
20
30
40
50
60

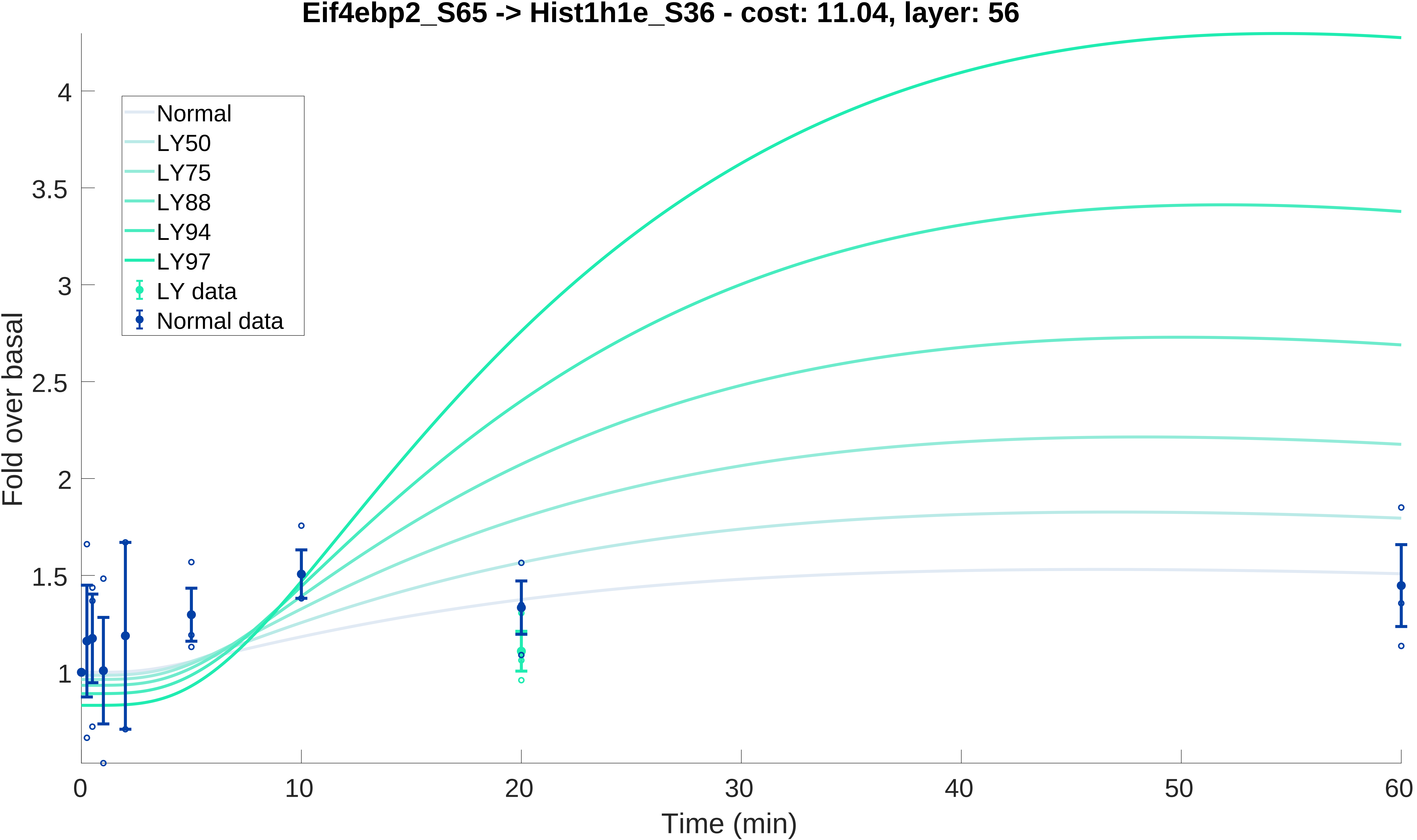
Eif4ebp2_S65 -> Hist1h1e_S36 - cost: 11.04, layer: 56
Normal
LY50
LY75
LY88
LY94
LY97
LY data
Normal data
Fold over basal
Time (min)
4
3.5
3
2.5
2
1.5
1
0
10
20
30
40
50
60

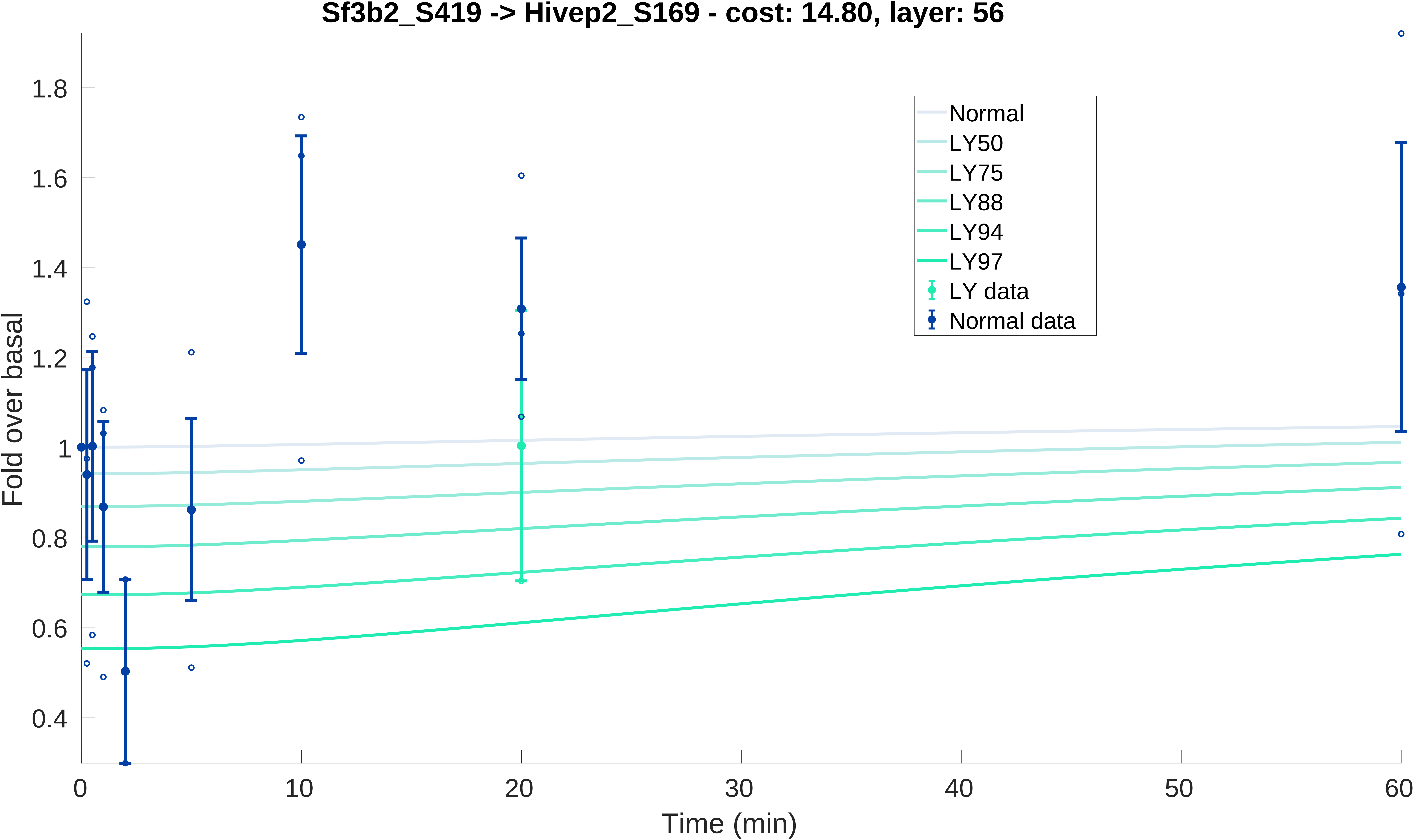

Sf3b2_S419 -> Hivep2_S169 - cost: 14.80, layer: 56
Fold over basal
Time (min)
0.4
0.6
0.8
1
1.2
1.4
1.6
1.8
0
10
20
30
40
50
60
Normal
LY50
LY75
LY88
LY94
LY97
LY data
Normal data

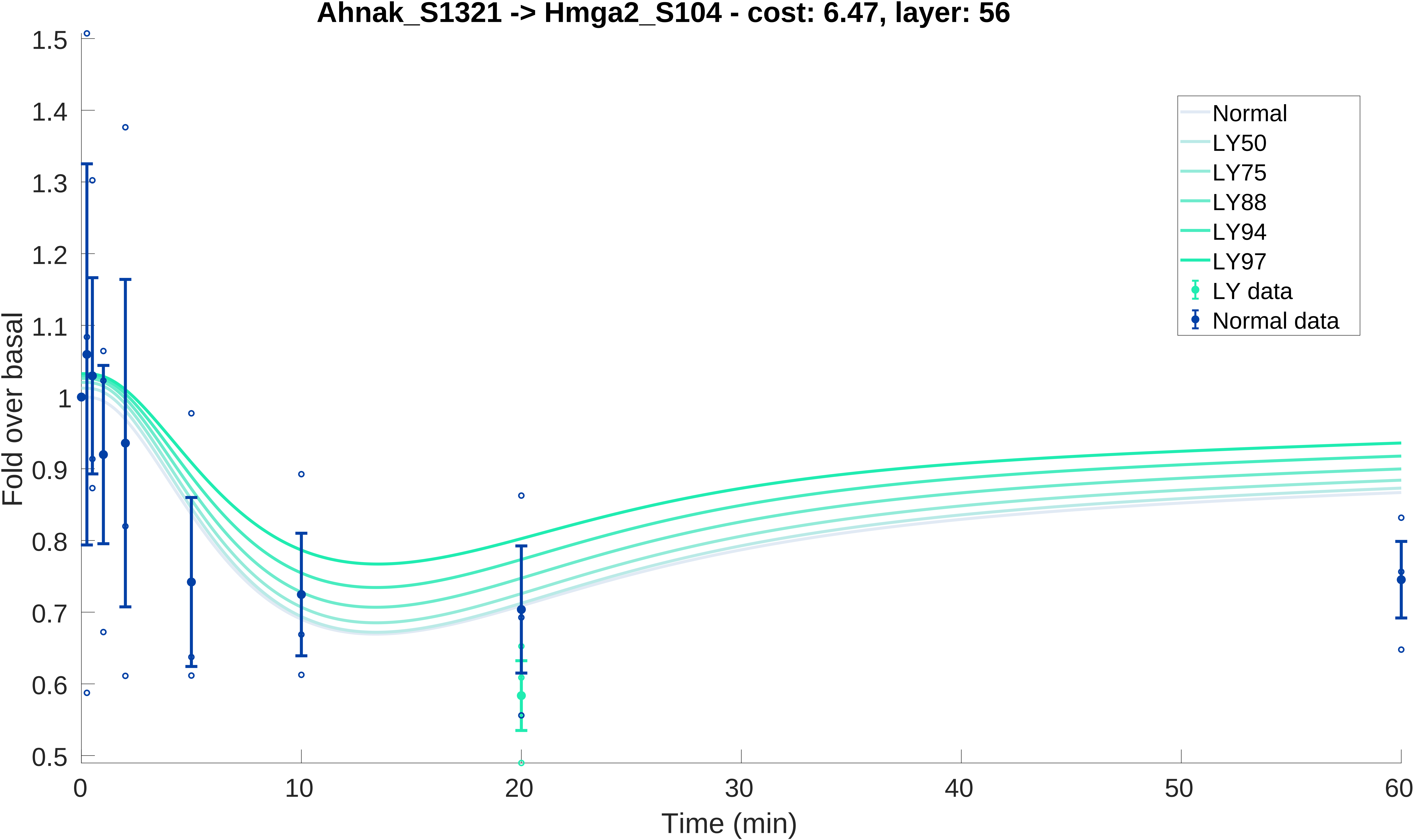

Ahnak_S1321 -> Hmga2_S104 - cost: 6.47, layer: 56
Fold over basal
1.5
1.4
1.3
1.2
1.1
1
0.9
0.8
0.7
0.6
0.5
0
10
20
30
40
50
60
Time (min)
Normal
LY50
LY75
LY88
LY94
LY97
LY data
Normal data

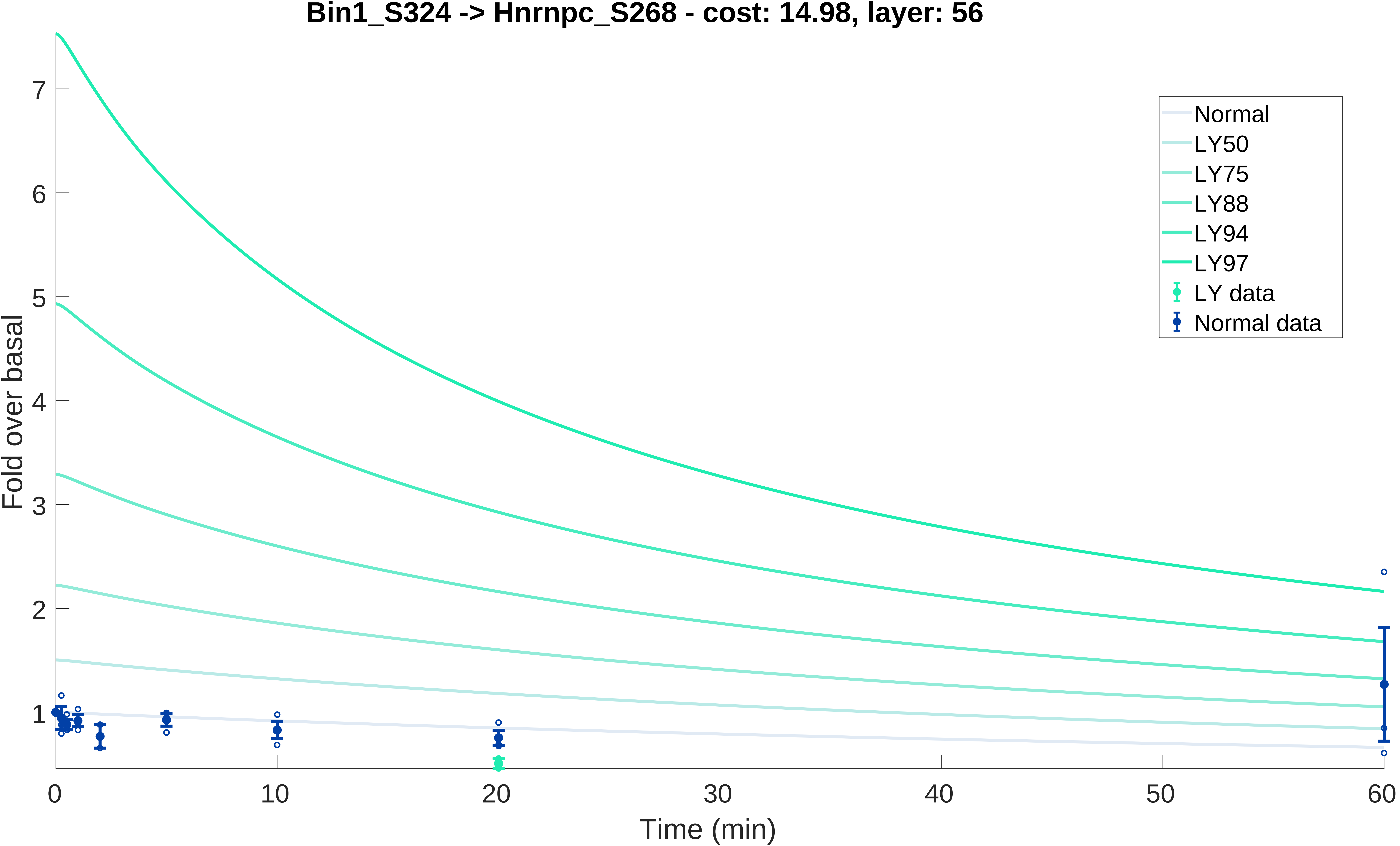
Bin1_S324 -> Hnrnpc_S268 - cost: 14.98, layer: 56
Fold over basal
Time (min)
Normal
LY50
LY75
LY88
LY94
LY97
LY data
Normal data
0
10
20
30
40
50
60
1
2
3
4
5
6
7

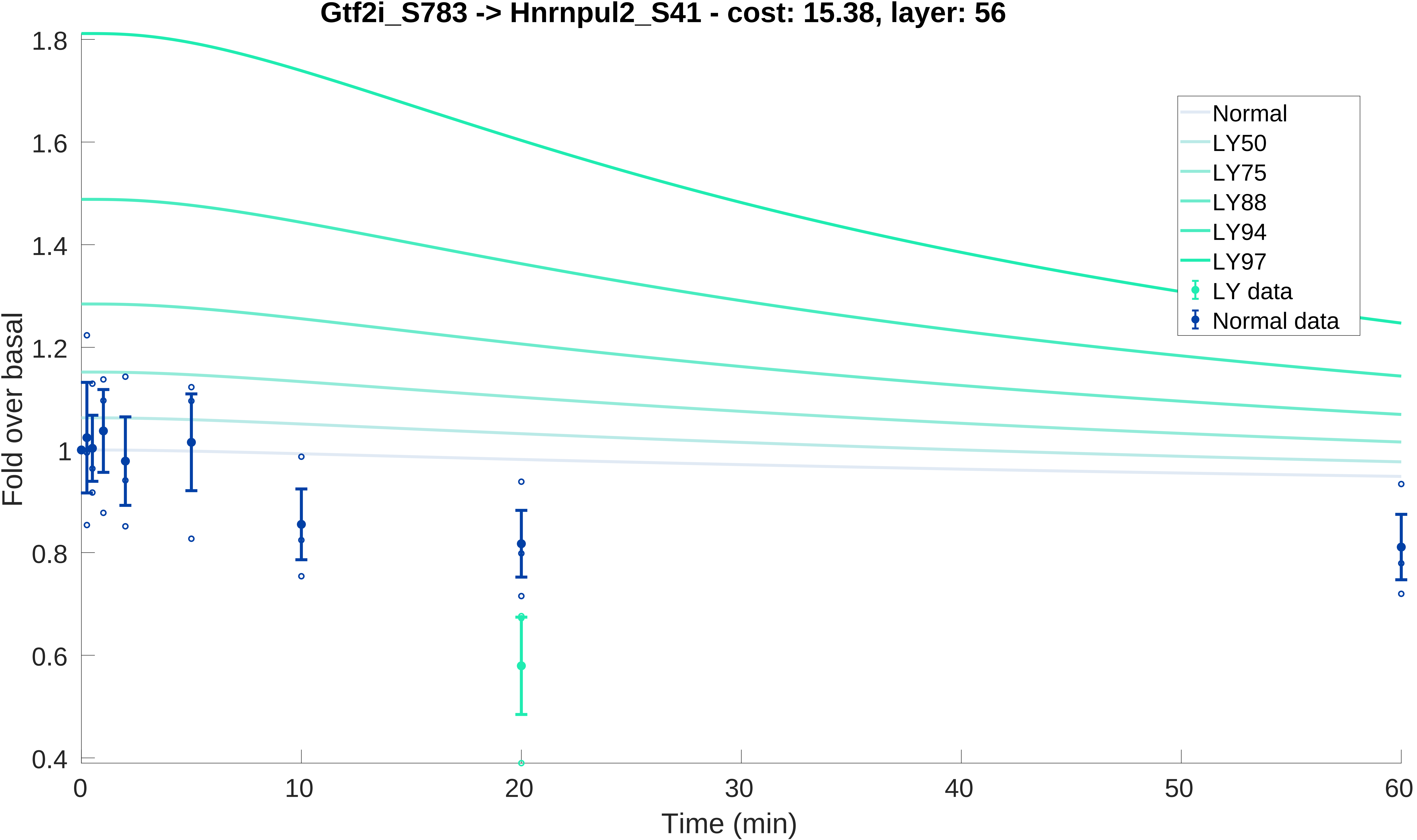
Gtf2i_S783 -> Hnrnpul2_S41 - cost: 15.38, layer: 56
1.8
1.6
1.4
1.2
1
0.8
0.6
0.4
Fold over basal
0
10
20
30
40
50
60
Time (min)
Normal
LY50
LY75
LY88
LY94
LY97
LY data
Normal data

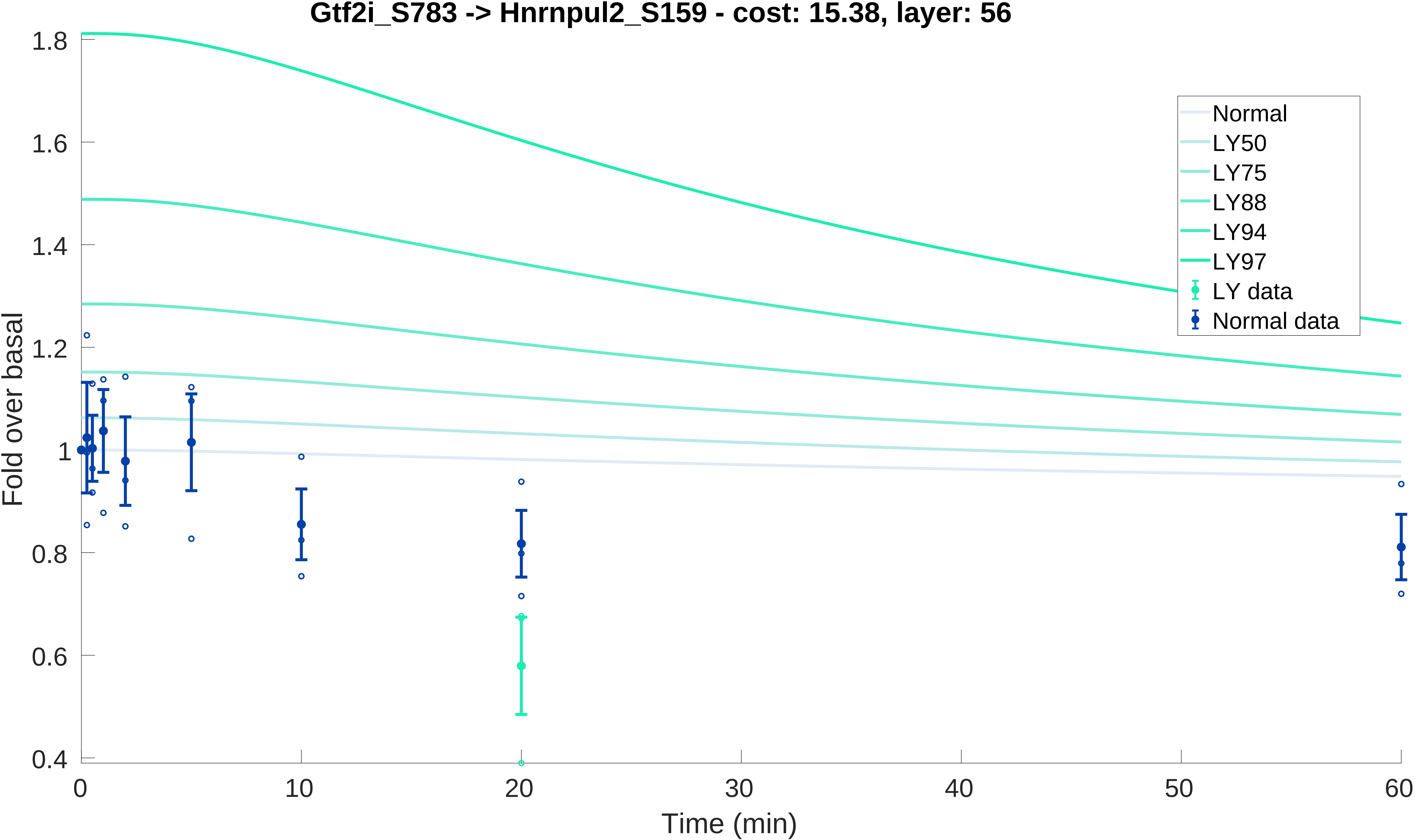
Gtf2i_S783 -> Hnrnpul2_S159 - cost: 15.38, layer: 56
Fold over basal
Time (min)
1.8
1.6
1.4
1.2
1
0.8
0.6
0.4
0
10
20
30
40
50
60
Normal
LY50
LY75
LY88
LY94
LY97
LY data
Normal data

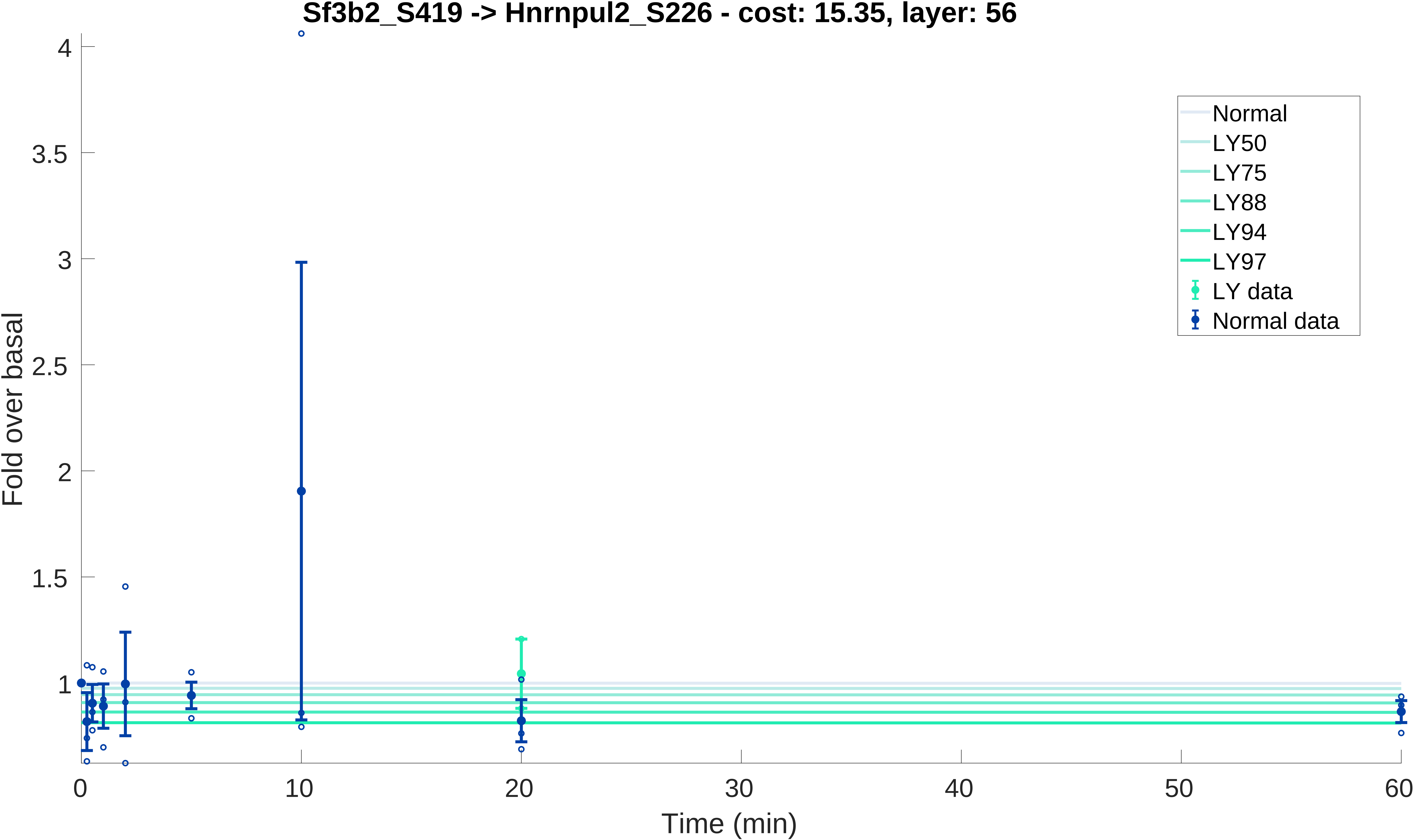

Sf3b2_S419 -> Hnrnpul2_S226 - cost: 15.35, layer: 56
Fold over basal
4
3.5
3
2.5
2
1.5
1
0
10
20
30
40
50
60
Time (min)
Normal
LY50
LY75
LY88
LY94
LY97
LY data
Normal data

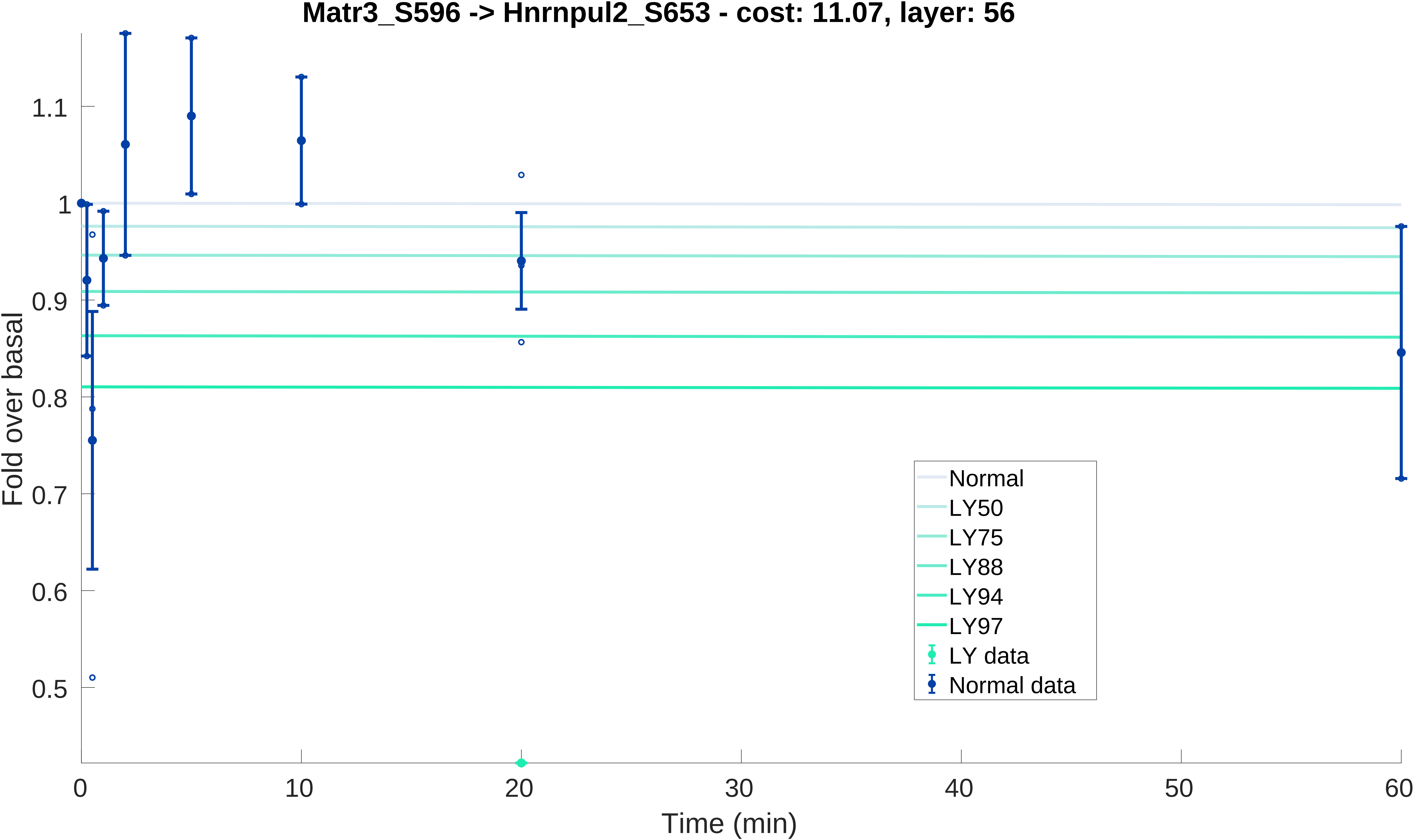

Matr3_S596 -> Hnrnpul2_S653 - cost: 11.07, layer: 56
Fold over basal
Time (min)
1.1
1
0.9
0.8
0.7
0.6
0.5
0
10
20
30
40
50
60
Normal
LY50
LY75
LY88
LY94
LY97
LY data
Normal data

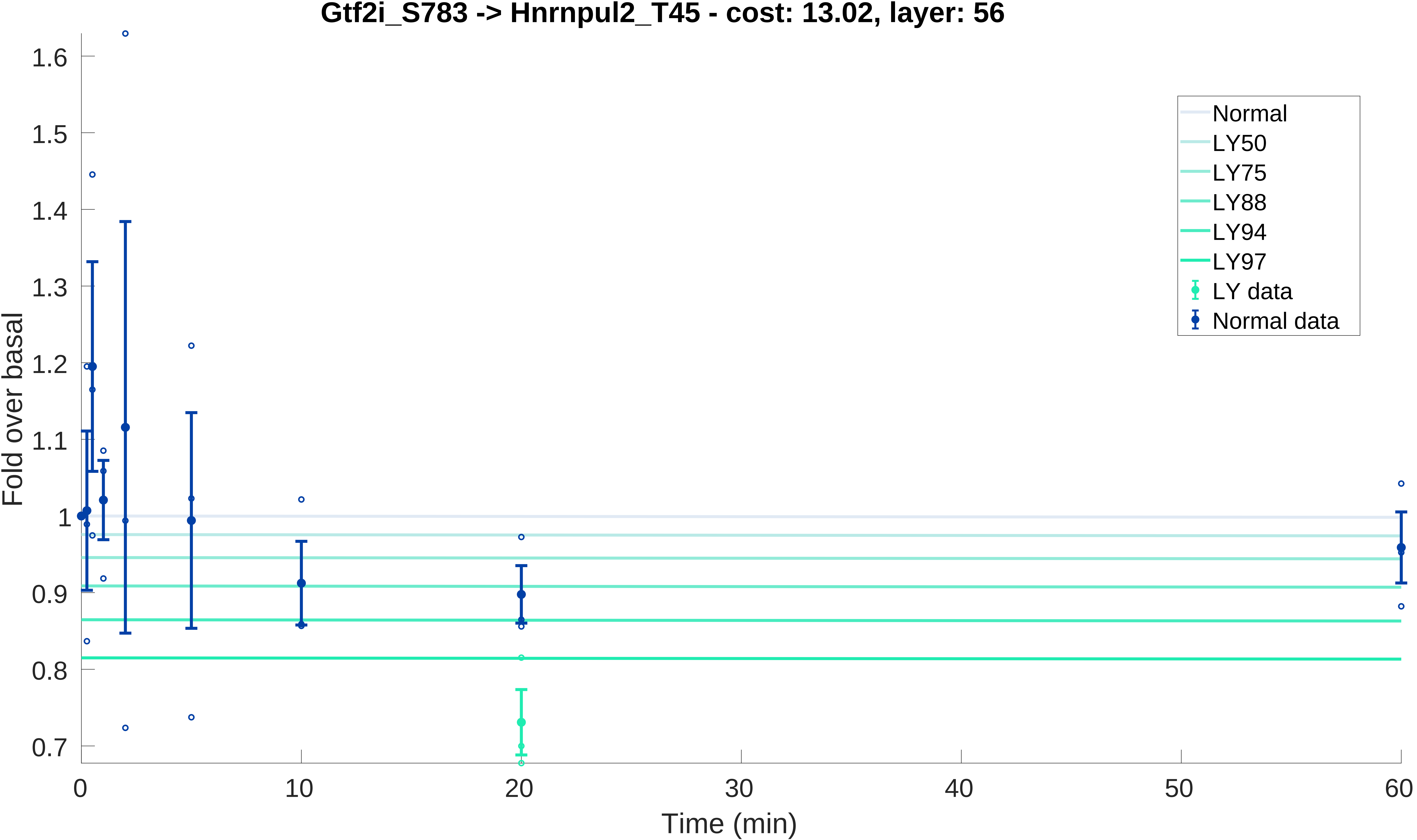

Gtf2i_S783 -> Hnrnpul2_T45 - cost: 13.02, layer: 56
Fold over basal
Time (min)
1.6
1.5
1.4
1.3
1.2
1.1
1
0.9
0.8
0.7
0
10
20
30
40
50
60
Normal
LY50
LY75
LY88
LY94
LY97
LY data
Normal data

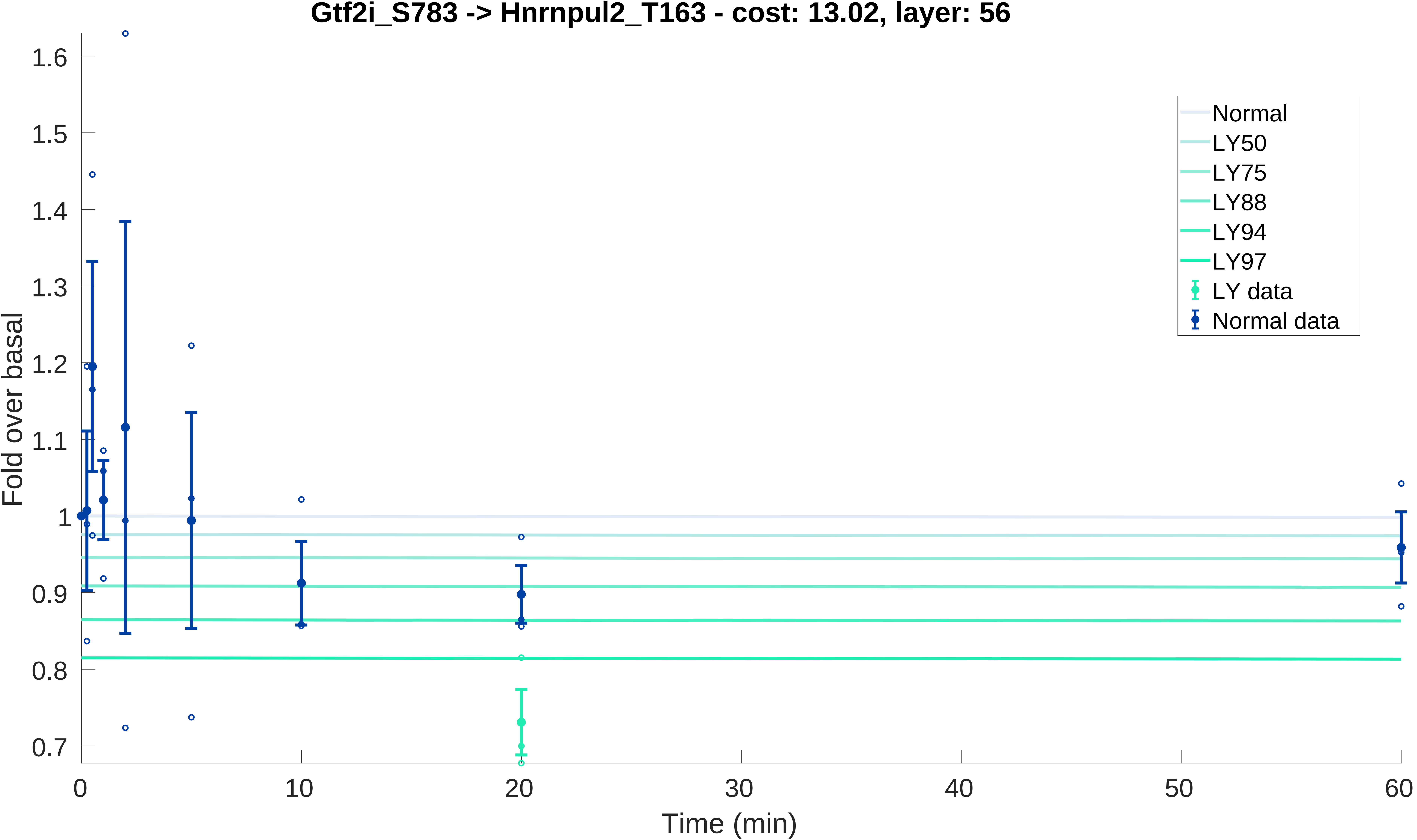

Gtf2i_S783 -> Hnrnpul2_T163 - cost: 13.02, layer: 56
Fold over basal
Time (min)
1.6
1.5
1.4
1.3
1.2
1.1
1
0.9
0.8
0.7
0
10
20
30
40
50
60
Normal
LY50
LY75
LY88
LY94
LY97
LY data
Normal data

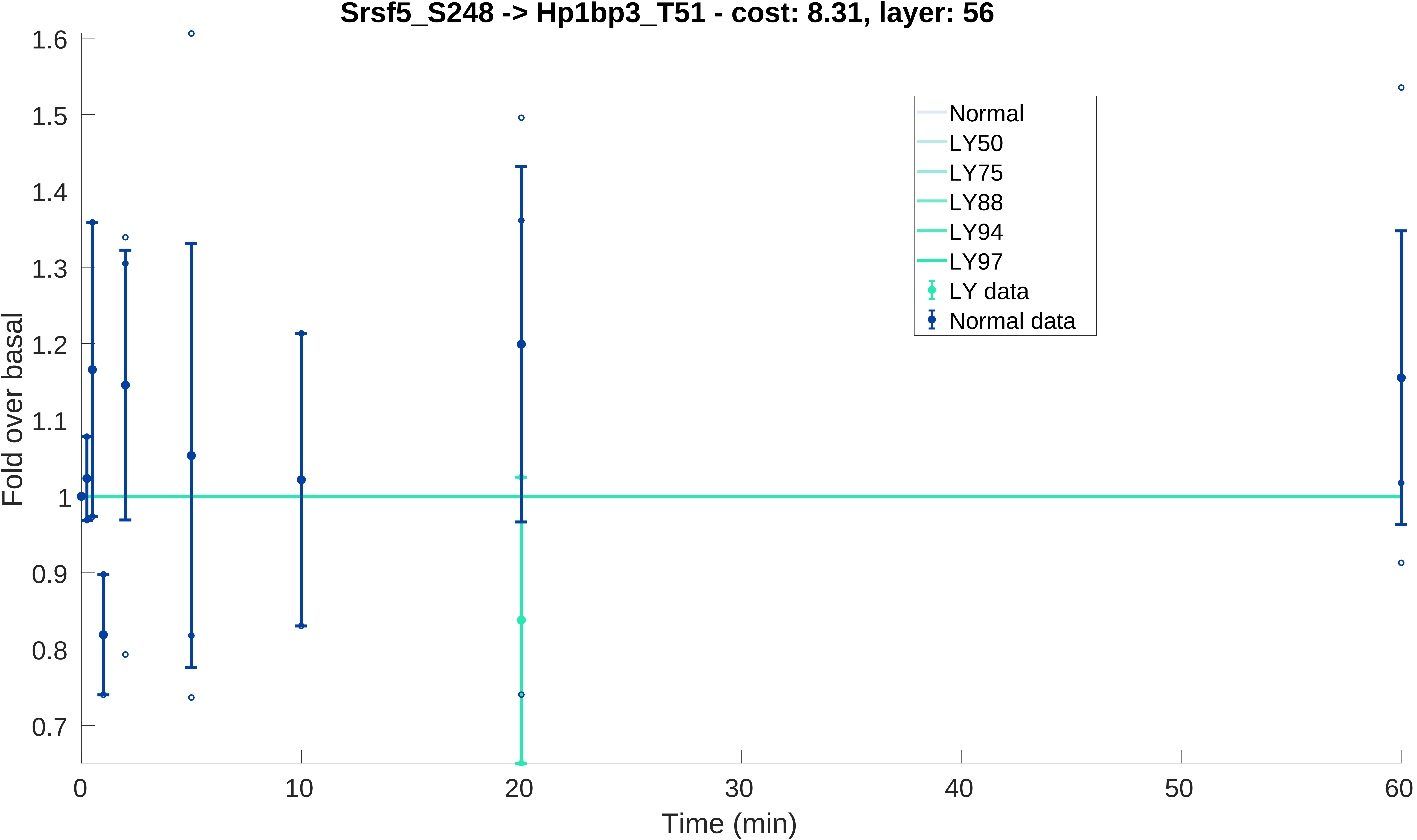

Srsf5_S248 -> Hp1bp3_T51 - cost: 8.31, layer: 56
Fold over basal
1.6
1.5
1.4
1.3
1.2
1.1
1
0.9
0.8
0.7
0
10
20
30
40
50
60
Time (min)
Normal
LY50
LY75
LY88
LY94
LY97
LY data
Normal data

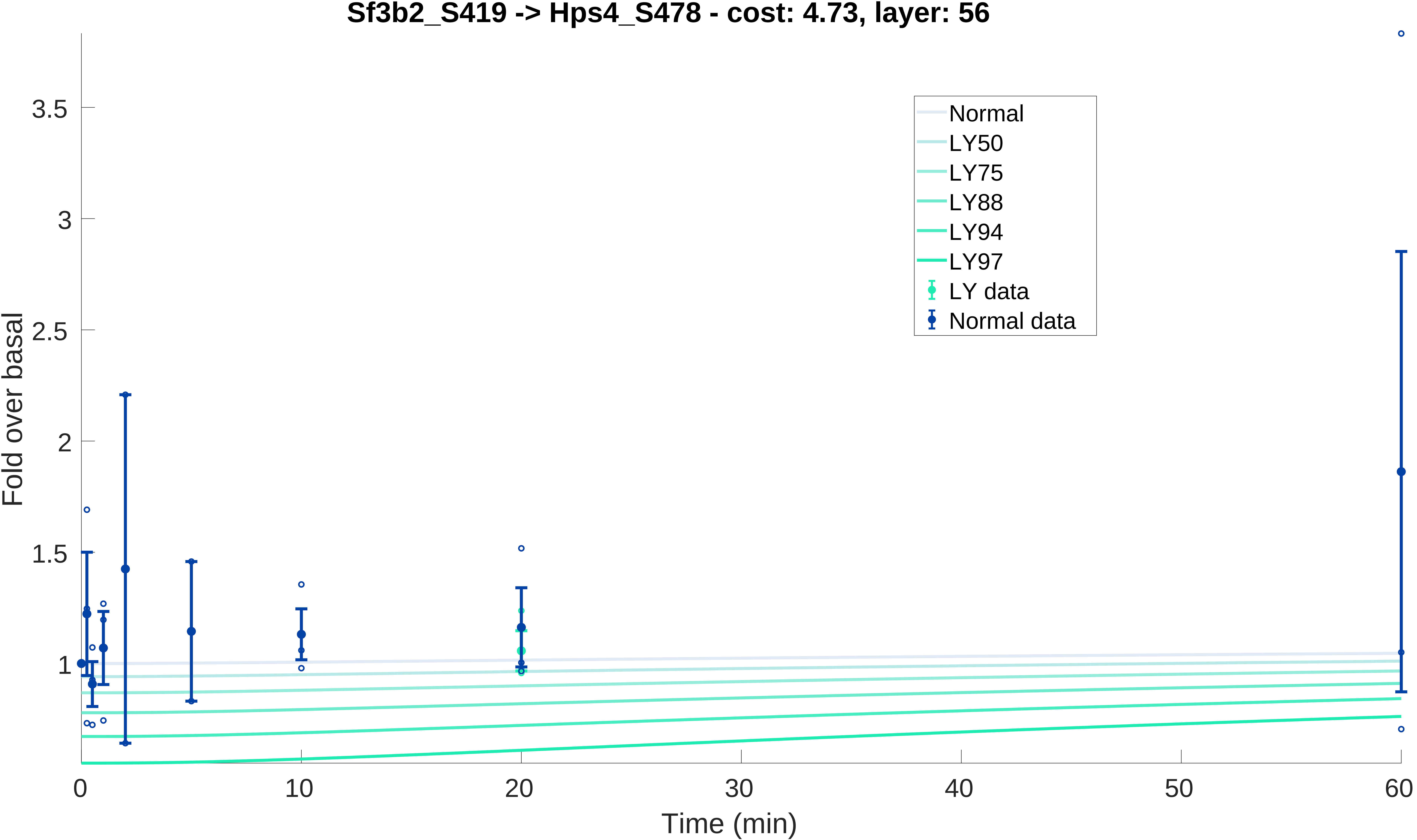

Sf3b2_S419 -> Hps4_S478 - cost: 4.73, layer: 56
Fold over basal
Time (min)
0
10
20
30
40
50
60
1
1.5
2
2.5
3
3.5
Normal
LY50
LY75
LY88
LY94
LY97
LY data
Normal data

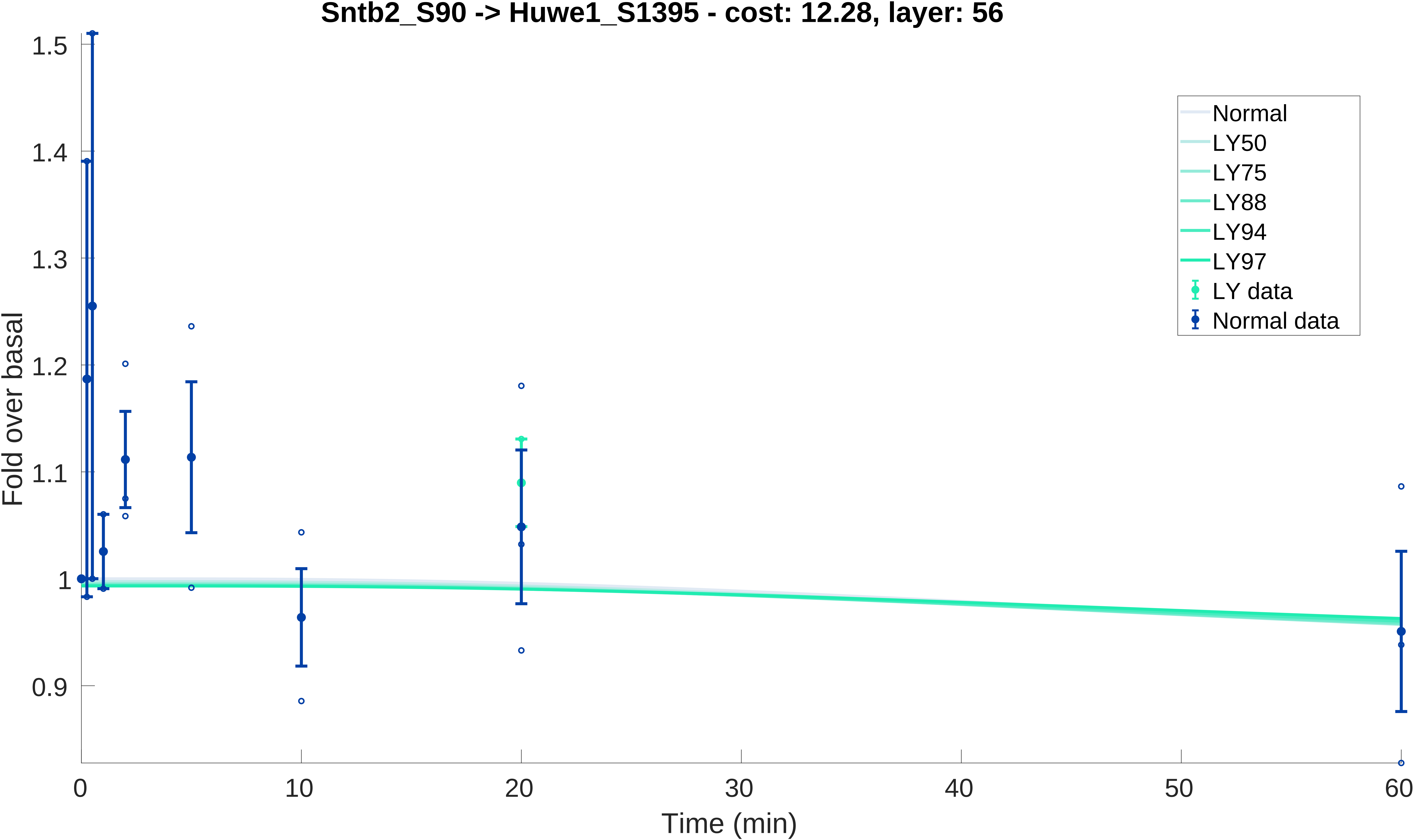
Sntb2_S90 -> Huwe1_S1395 - cost: 12.28, layer: 56
Fold over basal
Time (min)
Normal
LY50
LY75
LY88
LY94
LY97
LY data
Normal data
1.5
1.4
1.3
1.2
1.1
1
0.9
0
10
20
30
40
50
60

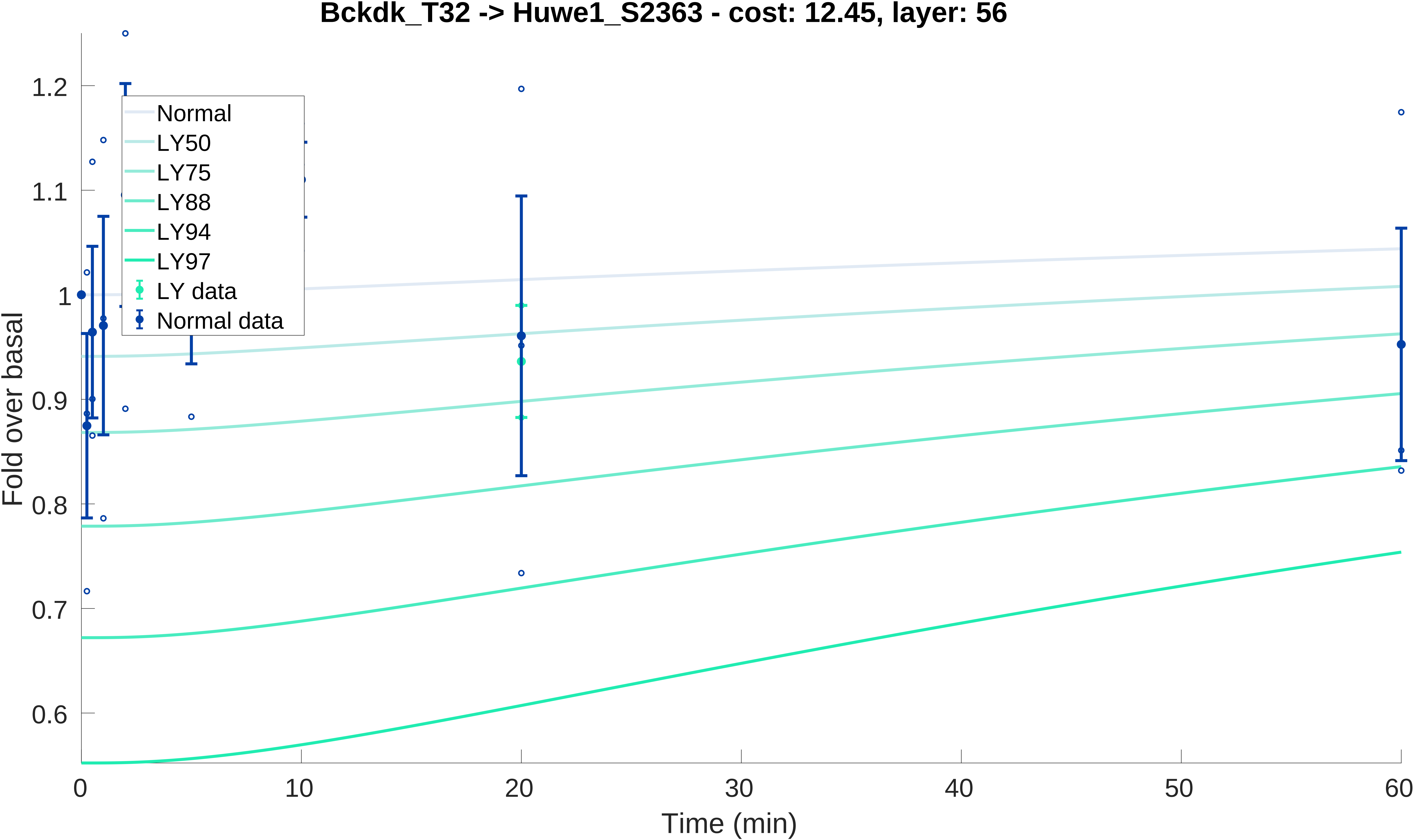

Bckdk_T32 -> Huwe1_S2363 - cost: 12.45, layer: 56
Normal
LY50
LY75
LY88
LY94
LY97
LY data
Normal data
Fold over basal
Time (min)
0.6
0.7
0.8
0.9
1
1.1
1.2
0
10
20
30
40
50
60

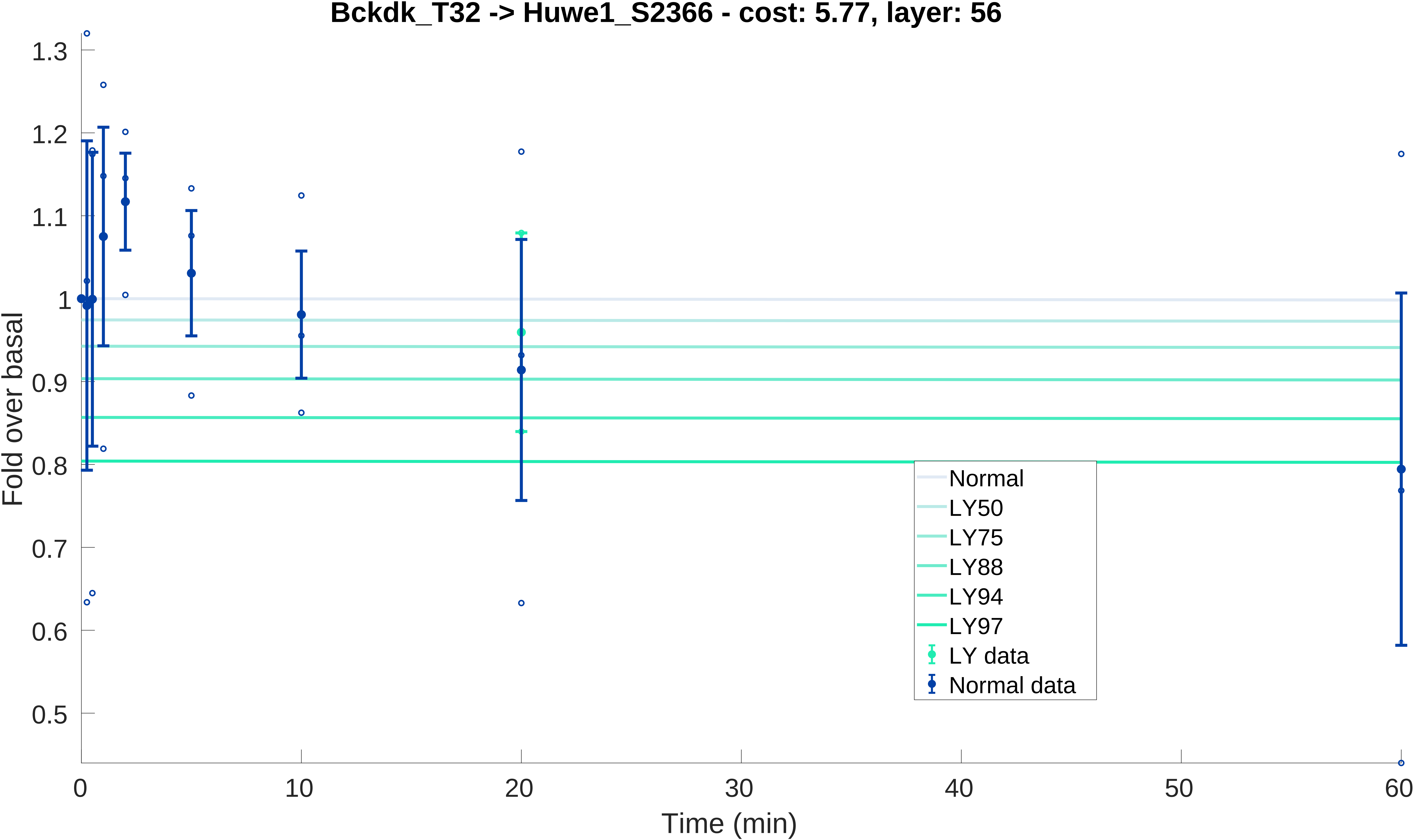

Bckdk_T32 -> Huwe1_S2366 - cost: 5.77, layer: 56
Fold over basal
Time (min)
1.3
1.2
1.1
1
0.9
0.8
0.7
0.6
0.5
0
10
20
30
40
50
60
Normal
LY50
LY75
LY88
LY94
LY97
LY data
Normal data

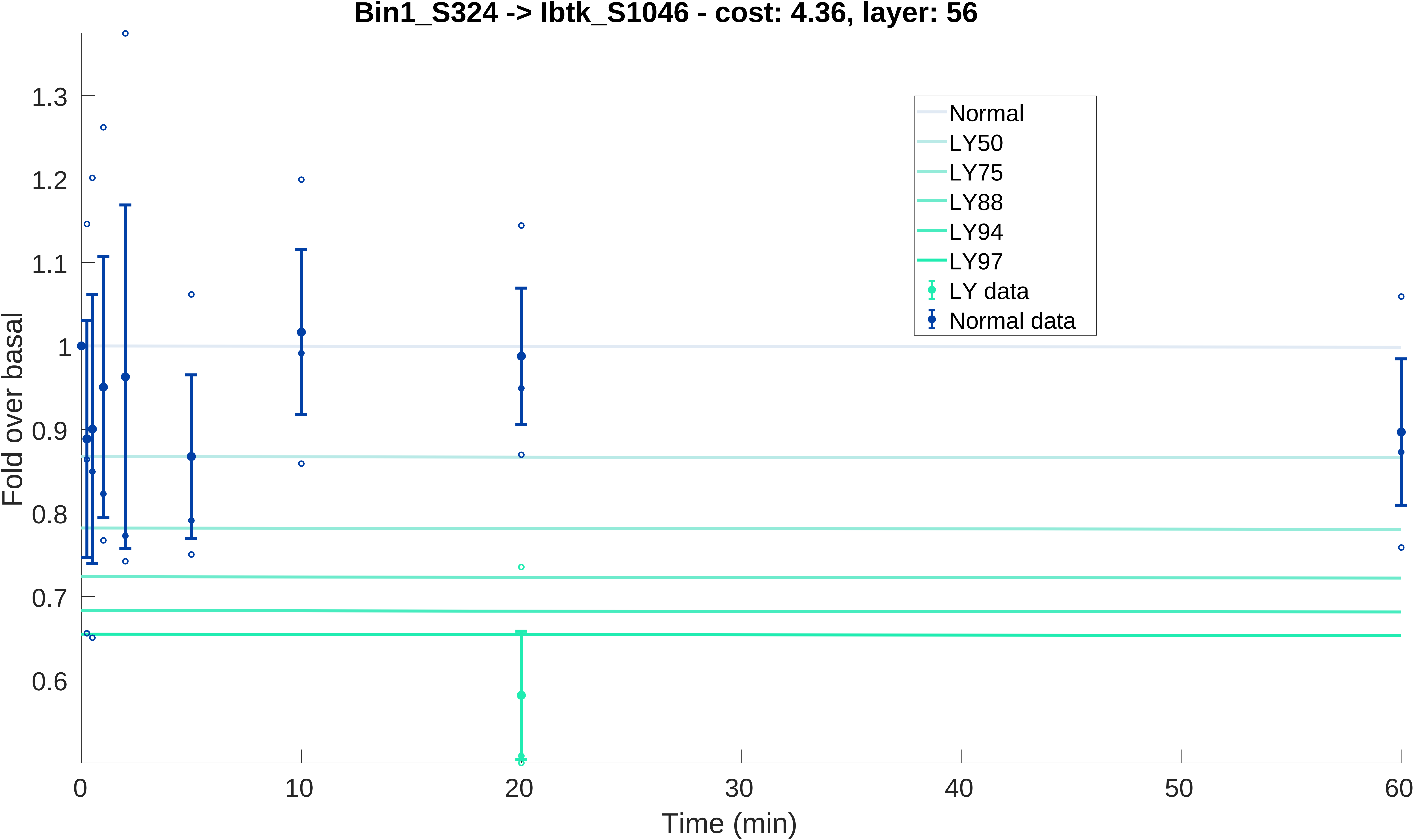

Bin1_S324 -> Ibtk_S1046 - cost: 4.36, layer: 56
Fold over basal
Time (min)
1.3
1.2
1.1
1
0.9
0.8
0.7
0.6
0
10
20
30
40
50
60
Normal
LY50
LY75
LY88
LY94
LY97
LY data
Normal data

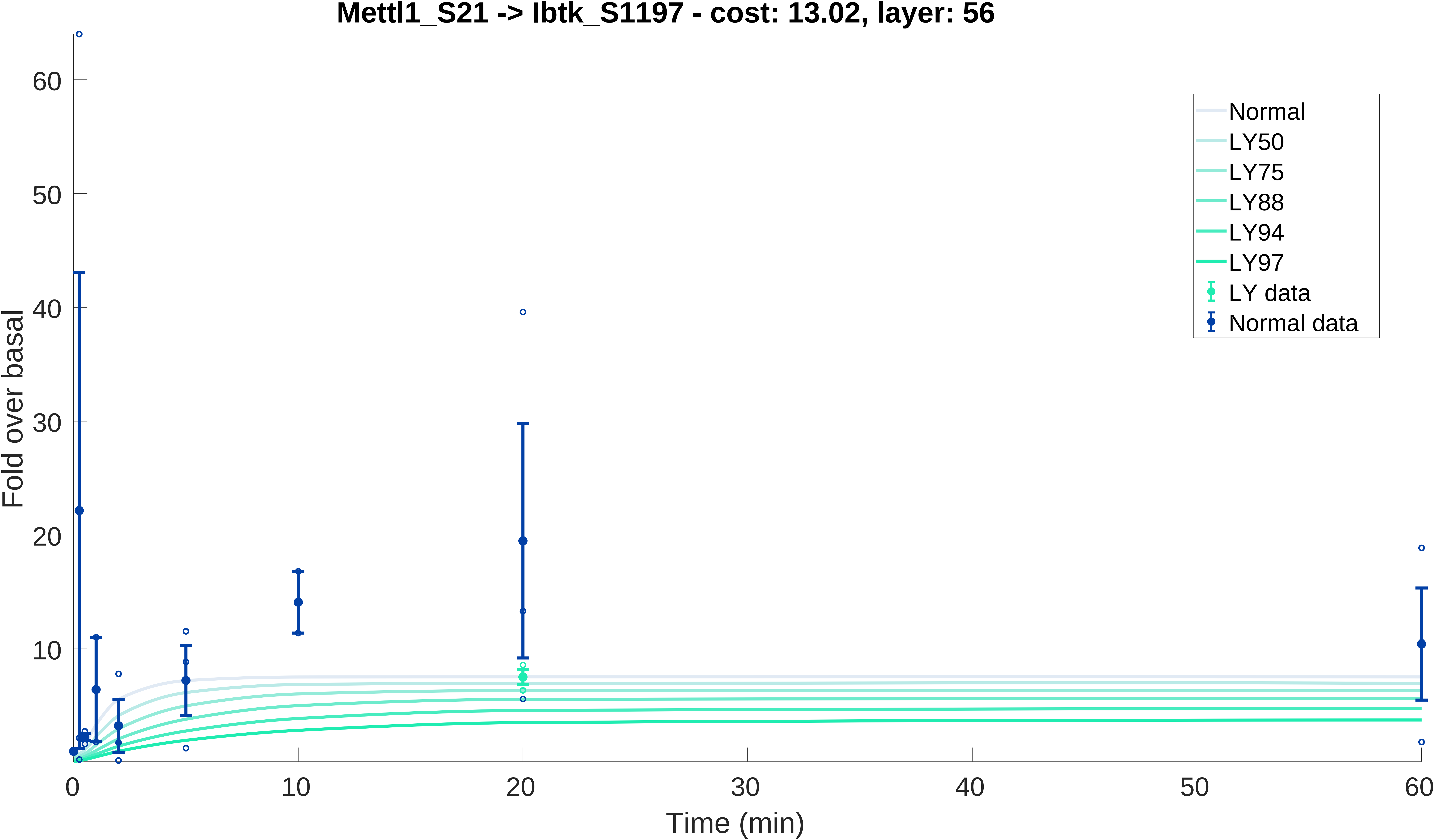

Mettl1_S21 -> Ibtk_S1197 - cost: 13.02, layer: 56
Fold over basal
Time (min)
0
10
20
30
40
50
60
Normal
LY50
LY75
LY88
LY94
LY97
LY data
Normal data

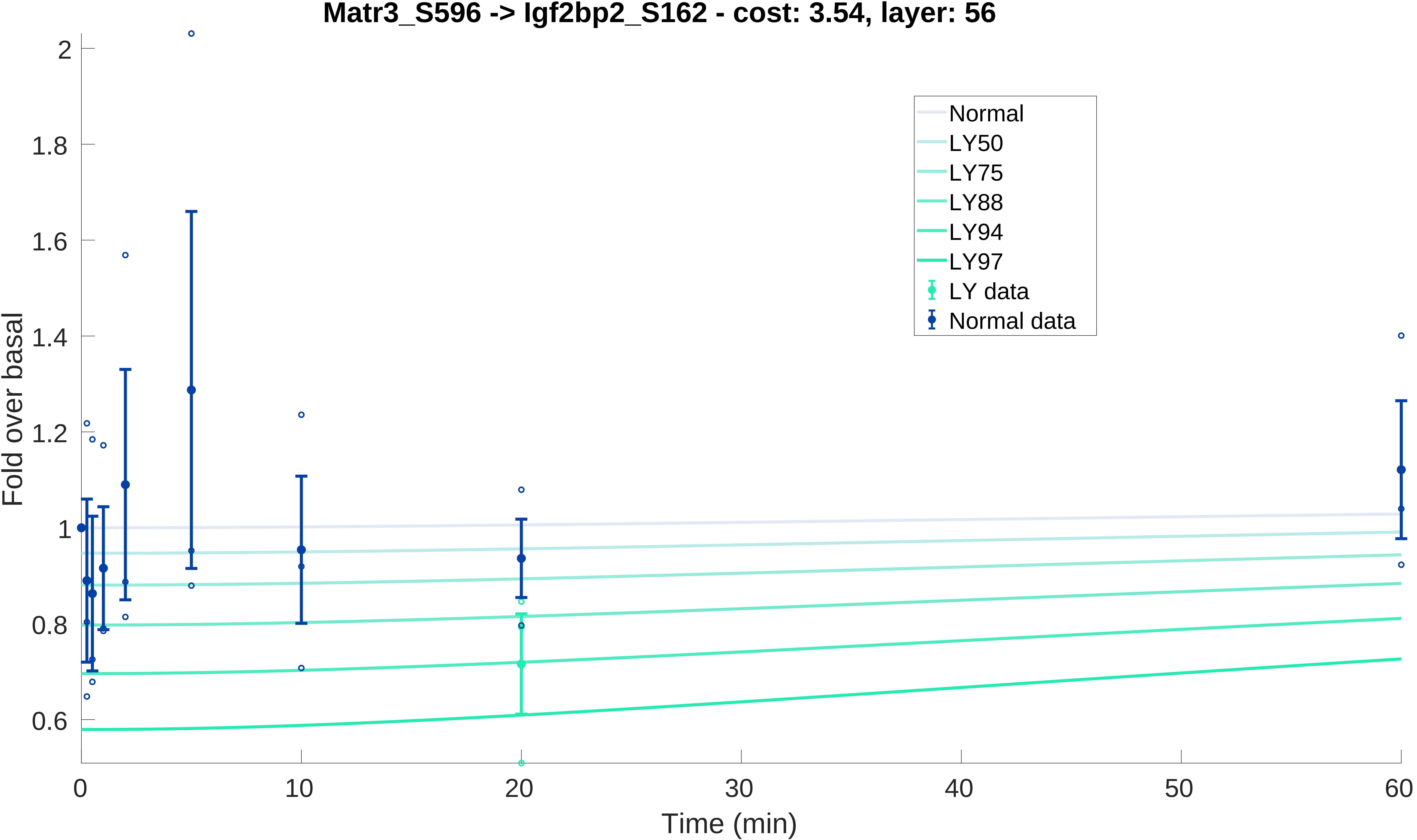
Matr3_S596 -> Igf2bp2_S162 - cost: 3.54, layer: 56
Fold over basal
2
1.8
1.6
1.4
1.2
1
0.8
0.6
0
10
20
30
40
50
60
Time (min)
Normal
LY50
LY75
LY88
LY94
LY97
LY data
Normal data

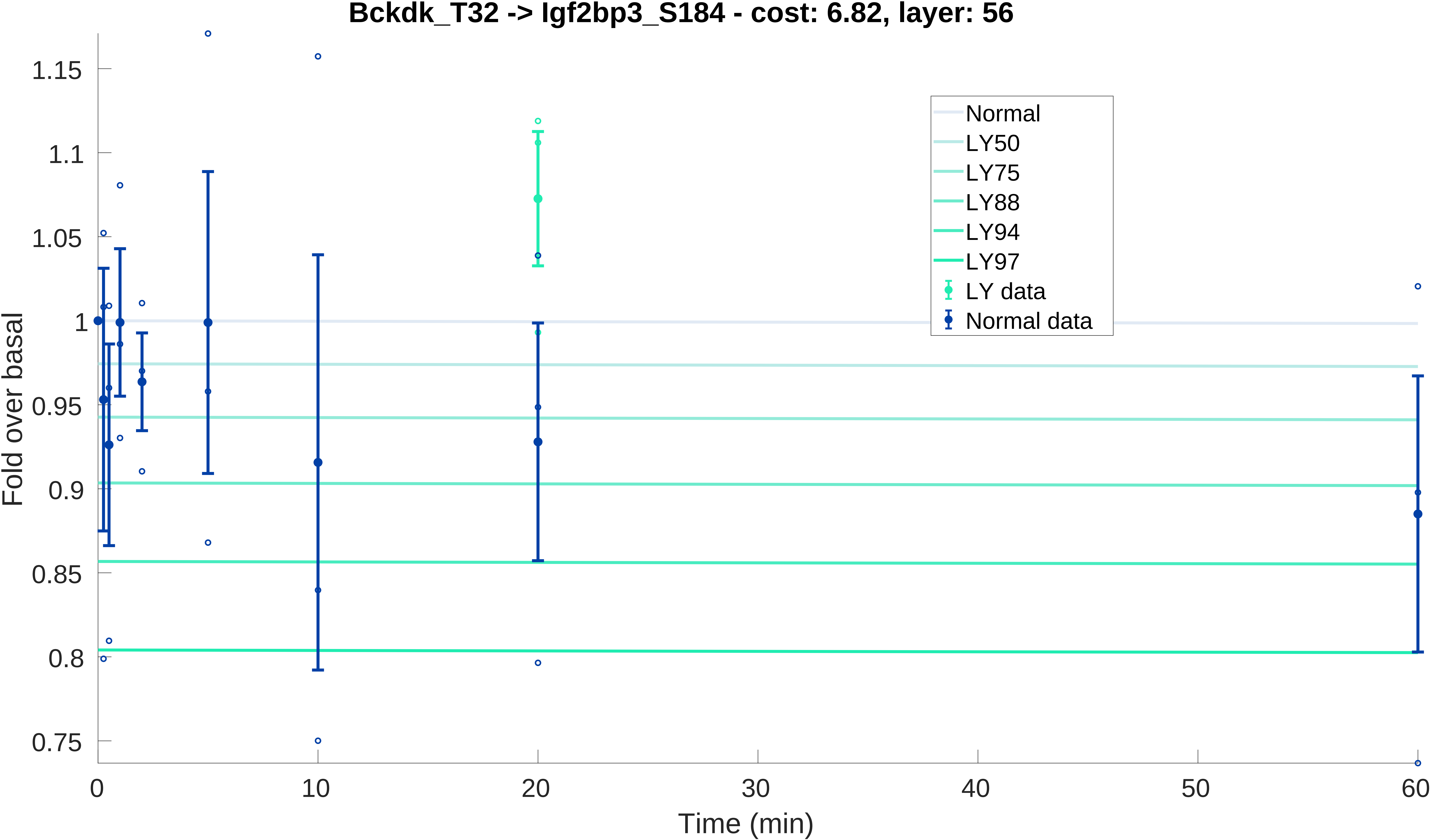

Bckdk_T32 -> Igf2bp3_S184 - cost: 6.82, layer: 56
Fold over basal
Time (min)
1.15
1.1
1.05
1
0.95
0.9
0.85
0.8
0.75
0
10
20
30
40
50
60
Normal
LY50
LY75
LY88
LY94
LY97
LY data
Normal data

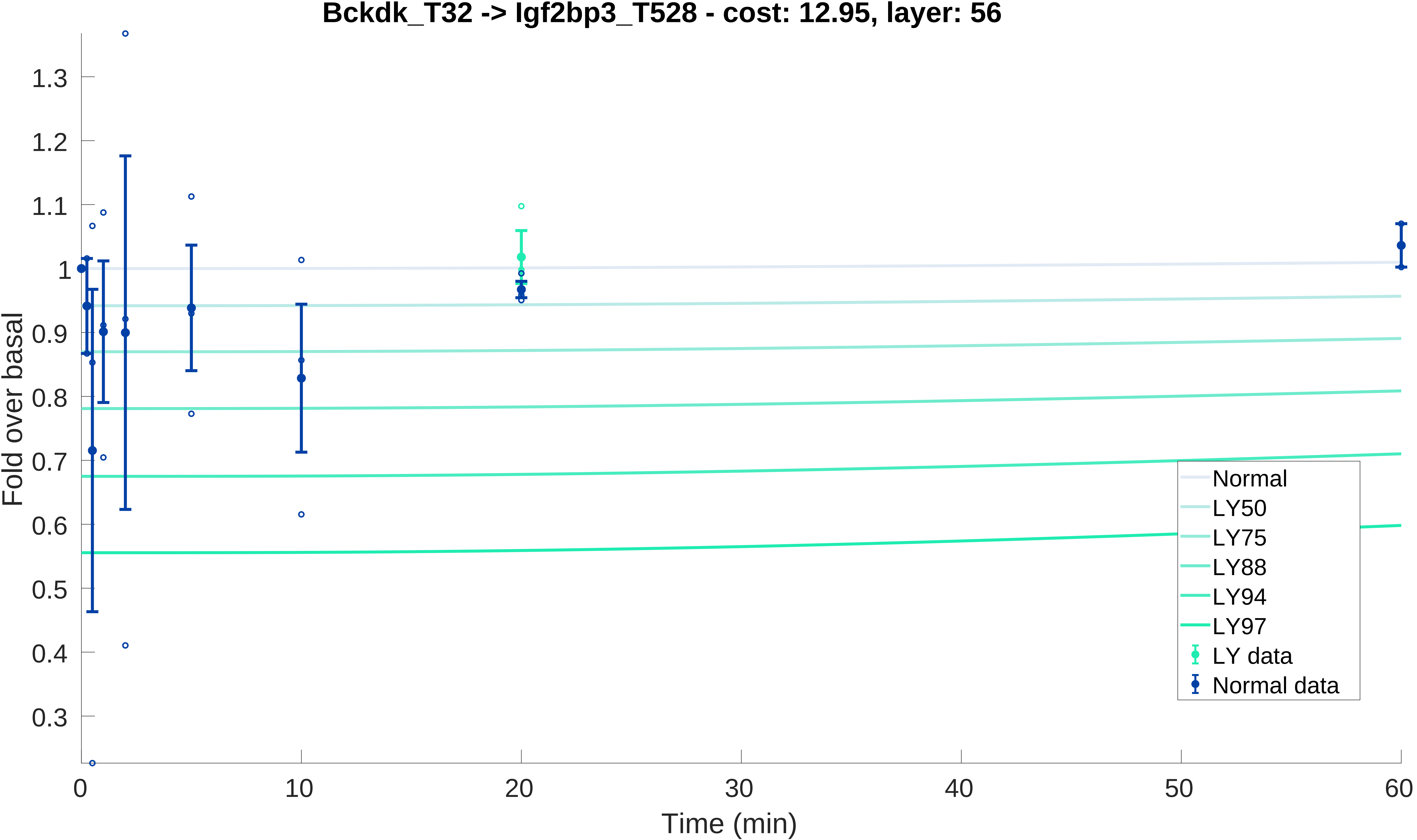

Bckdk_T32 -> Igf2bp3_T528 - cost: 12.95, layer: 56
Fold over basal
Time (min)
0
10
20
30
40
50
60
0.3
0.4
0.5
0.6
0.7
0.8
0.9
1
1.1
1.2
1.3
Normal
LY50
LY75
LY88
LY94
LY97
LY data
Normal data

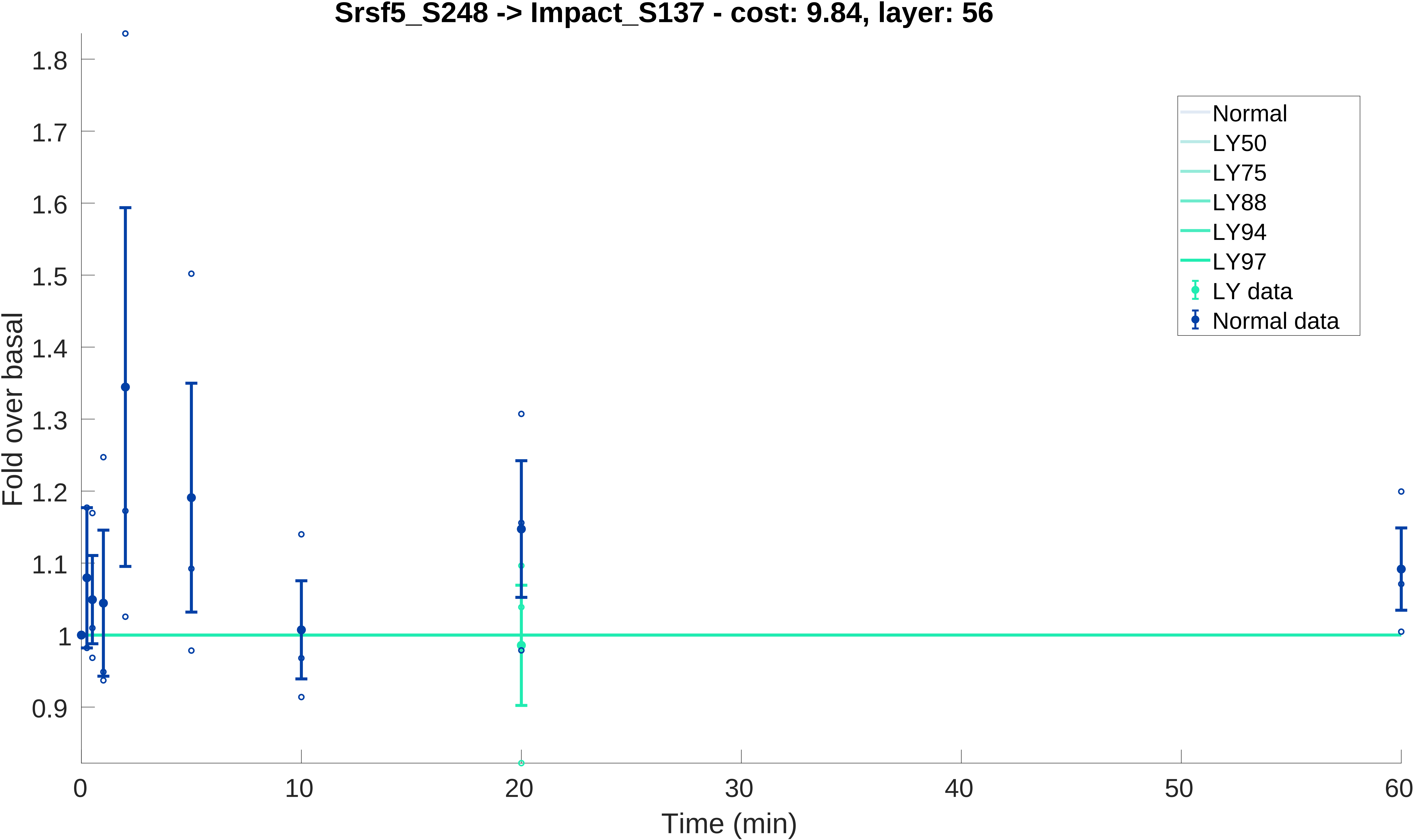

Srsf5_S248 -> Impact_S137 - cost: 9.84, layer: 56
Fold over basal
Time (min)
1.8
1.7
1.6
1.5
1.4
1.3
1.2
1.1
1
0.9
0
10
20
30
40
50
60
Normal
LY50
LY75
LY88
LY94
LY97
LY data
Normal data

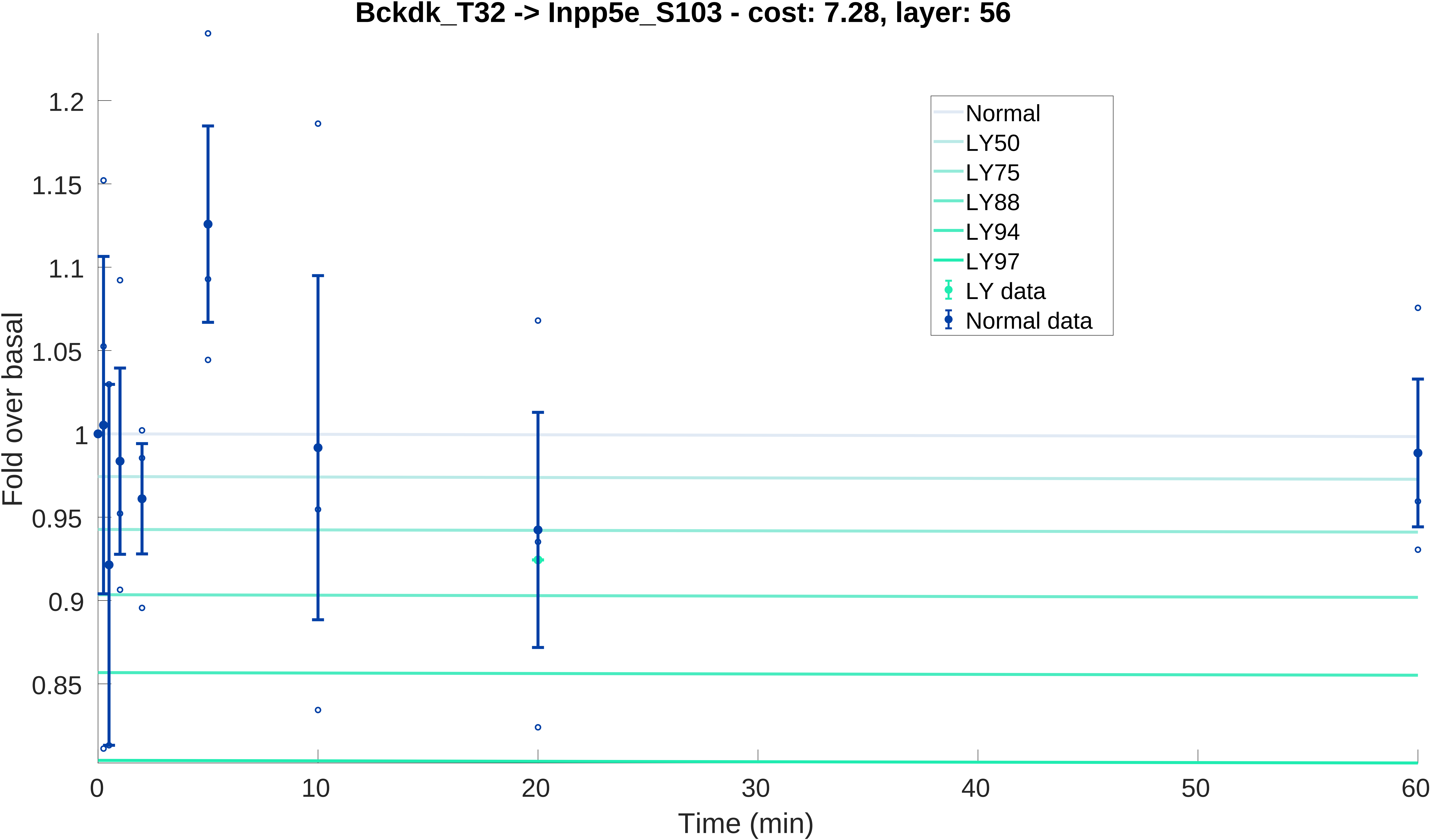
Bckdk_T32 -> Inpp5e_S103 - cost: 7.28, layer: 56
Fold over basal
1.2
1.15
1.1
1.05
1
0.95
0.9
0.85
0
10
20
30
40
50
60
Time (min)
Normal
LY50
LY75
LY88
LY94
LY97
LY data
Normal data

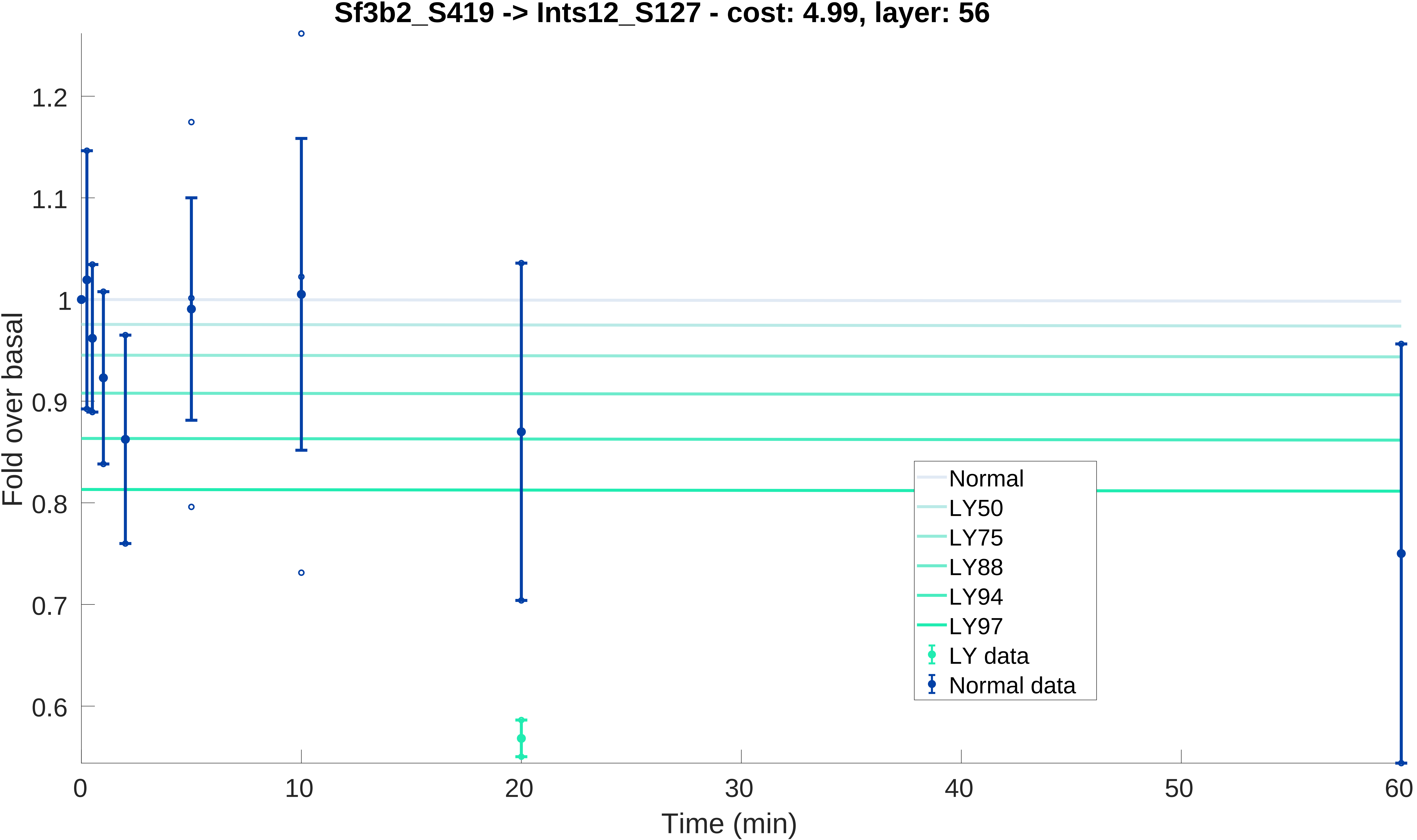

Sf3b2_S419 -> Ints12_S127 - cost: 4.99, layer: 56
Fold over basal
Time (min)
1.2
1.1
1
0.9
0.8
0.7
0.6
0
10
20
30
40
50
60
Normal
LY50
LY75
LY88
LY94
LY97
LY data
Normal data

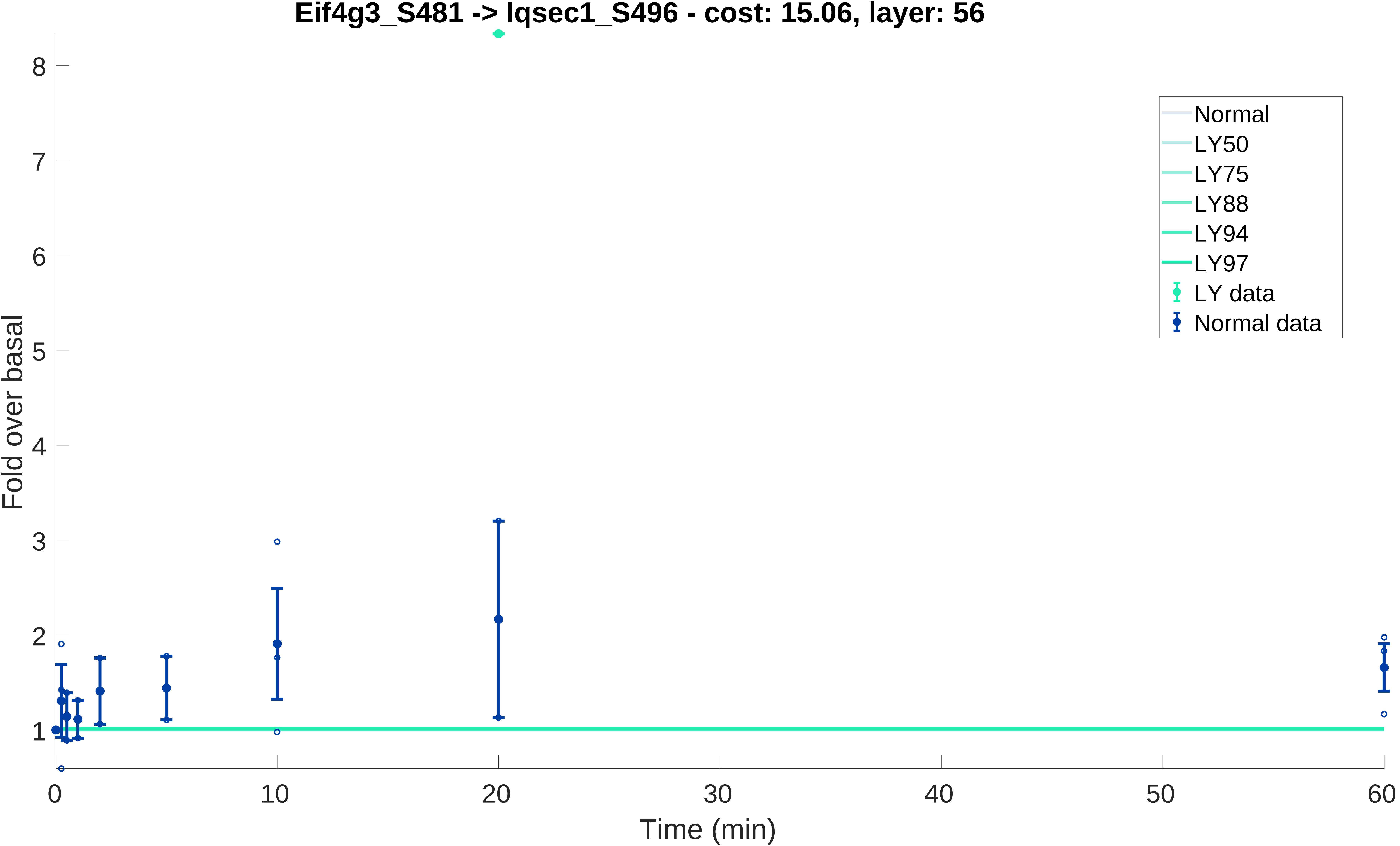
Eif4g3_S481 -> Iqsec1_S496 - cost: 15.06, layer: 56
Fold over basal
Time (min)
1
2
3
4
5
6
7
8
0
10
20
30
40
50
60
Normal
LY50
LY75
LY88
LY94
LY97
LY data
Normal data

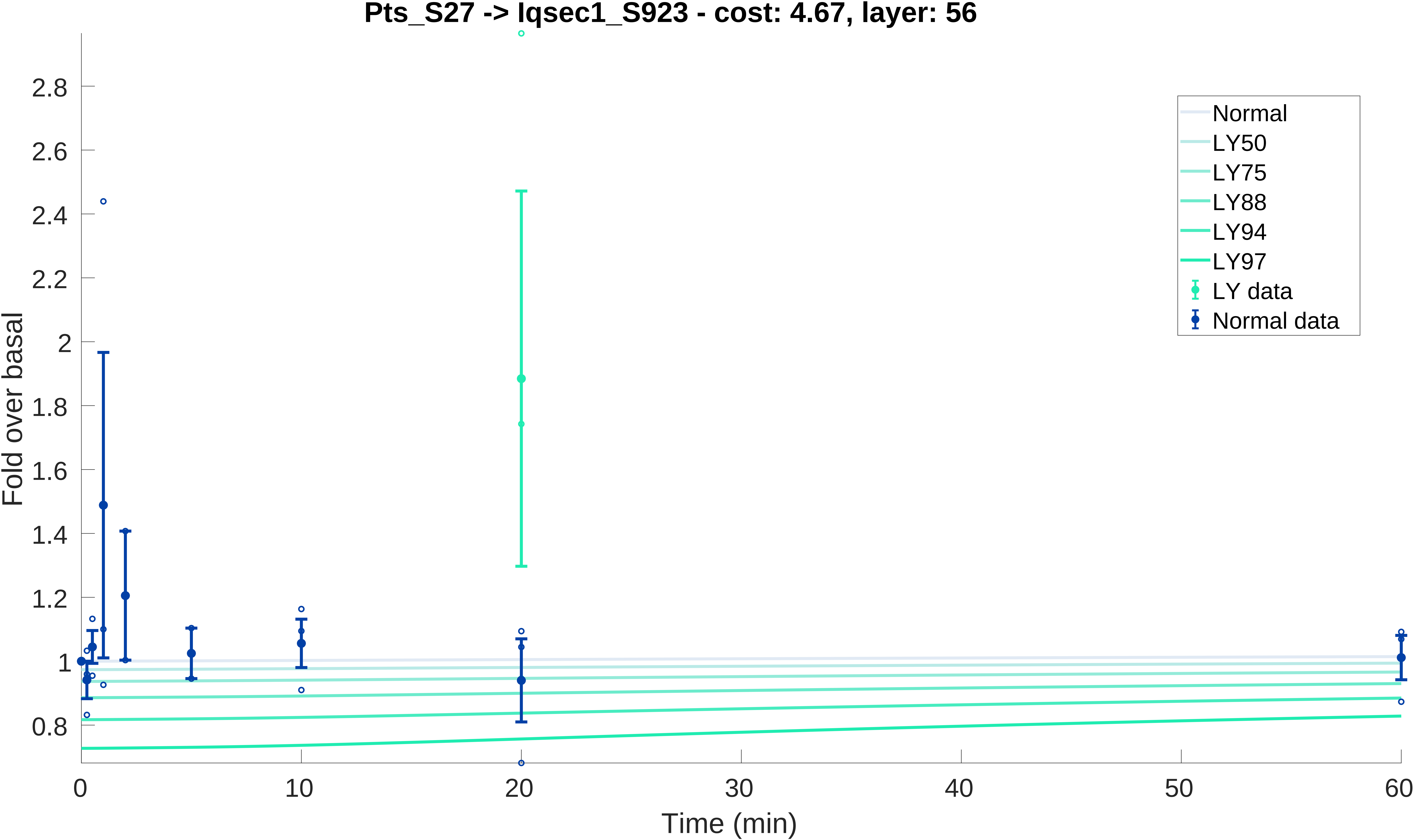
Pts_S27 -> Iqsec1_S923 - cost: 4.67, layer: 56
Fold over basal
2.8
2.6
2.4
2.2
2
1.8
1.6
1.4
1.2
1
0.8
Time (min)
0
10
20
30
40
50
60
Normal
LY50
LY75
LY88
LY94
LY97
LY data
Normal data

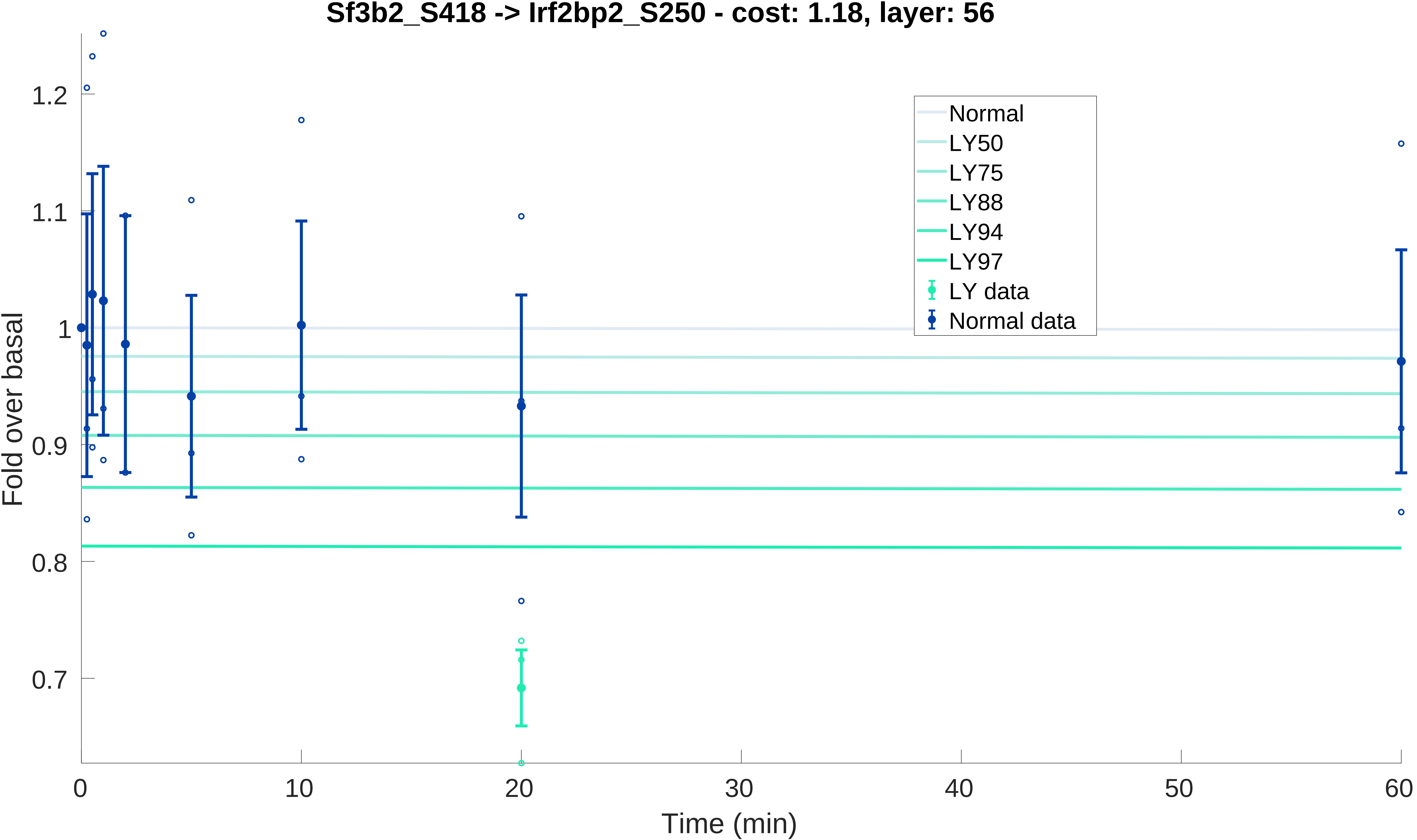

Sf3b2_S418 -> Irf2bp2_S250 - cost: 1.18, layer: 56
Fold over basal
Time (min)
1.2
1.1
1
0.9
0.8
0.7
0
10
20
30
40
50
60
Normal
LY50
LY75
LY88
LY94
LY97
LY data
Normal data

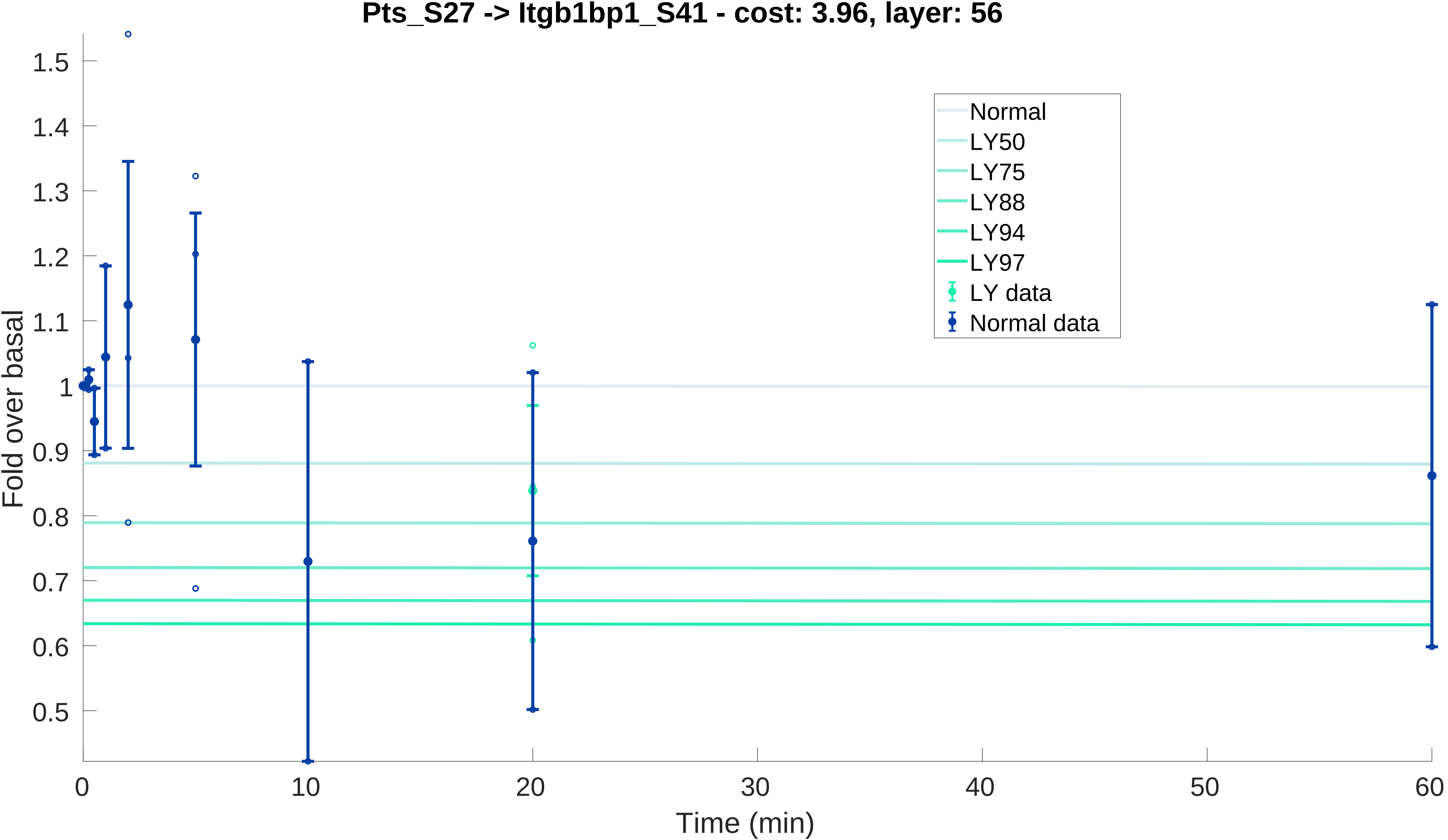

Pts_S27 -> Itgb1bp1_S41 - cost: 3.96, layer: 56
Fold over basal
Time (min)
0
10
20
30
40
50
60
0.5
0.6
0.7
0.8
0.9
1
1.1
1.2
1.3
1.4
1.5
Normal
LY50
LY75
LY88
LY94
LY97
LY data
Normal data

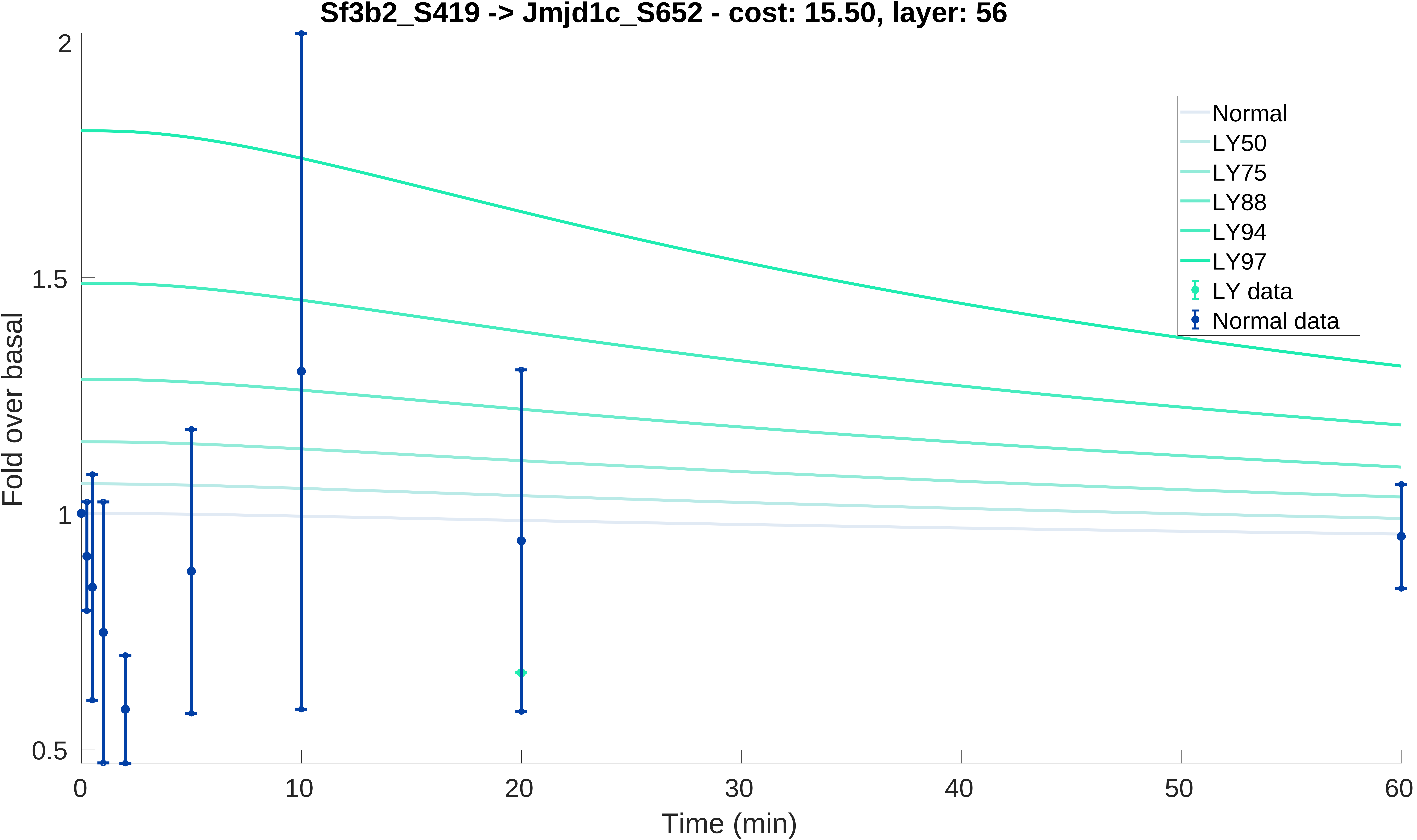

Sf3b2_S419 -> Jmjd1c_S652 - cost: 15.50, layer: 56
Fold over basal
2
1.5
1
0.5
0
10
20
30
40
50
60
Time (min)
Normal
LY50
LY75
LY88
LY94
LY97
LY data
Normal data

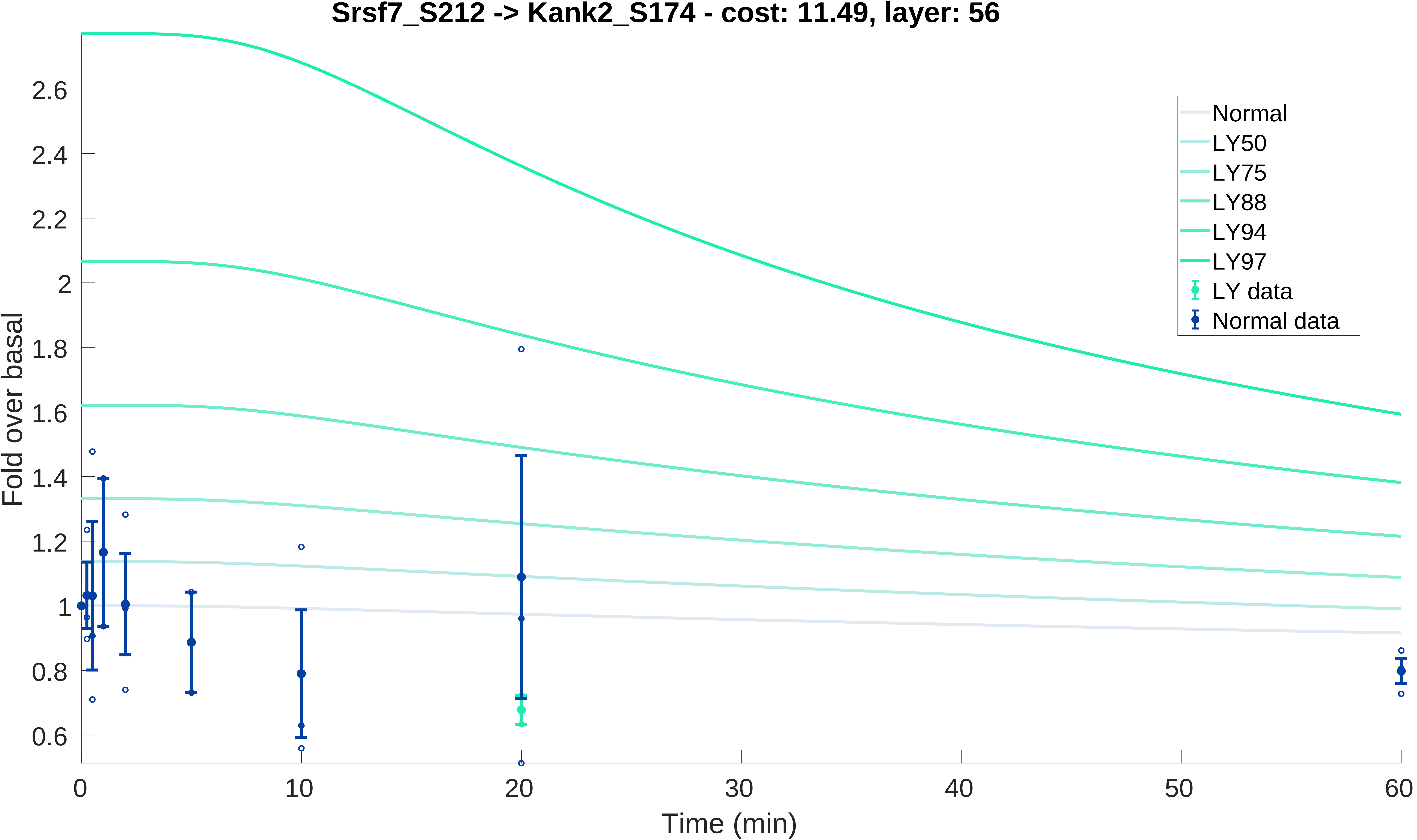
Srsf7_S212 -> Kank2_S174 - cost: 11.49, layer: 56
Fold over basal
Time (min)
2.6
2.4
2.2
2
1.8
1.6
1.4
1.2
1
0.8
0.6
0
10
20
30
40
50
60
Normal
LY50
LY75
LY88
LY94
LY97
LY data
Normal data

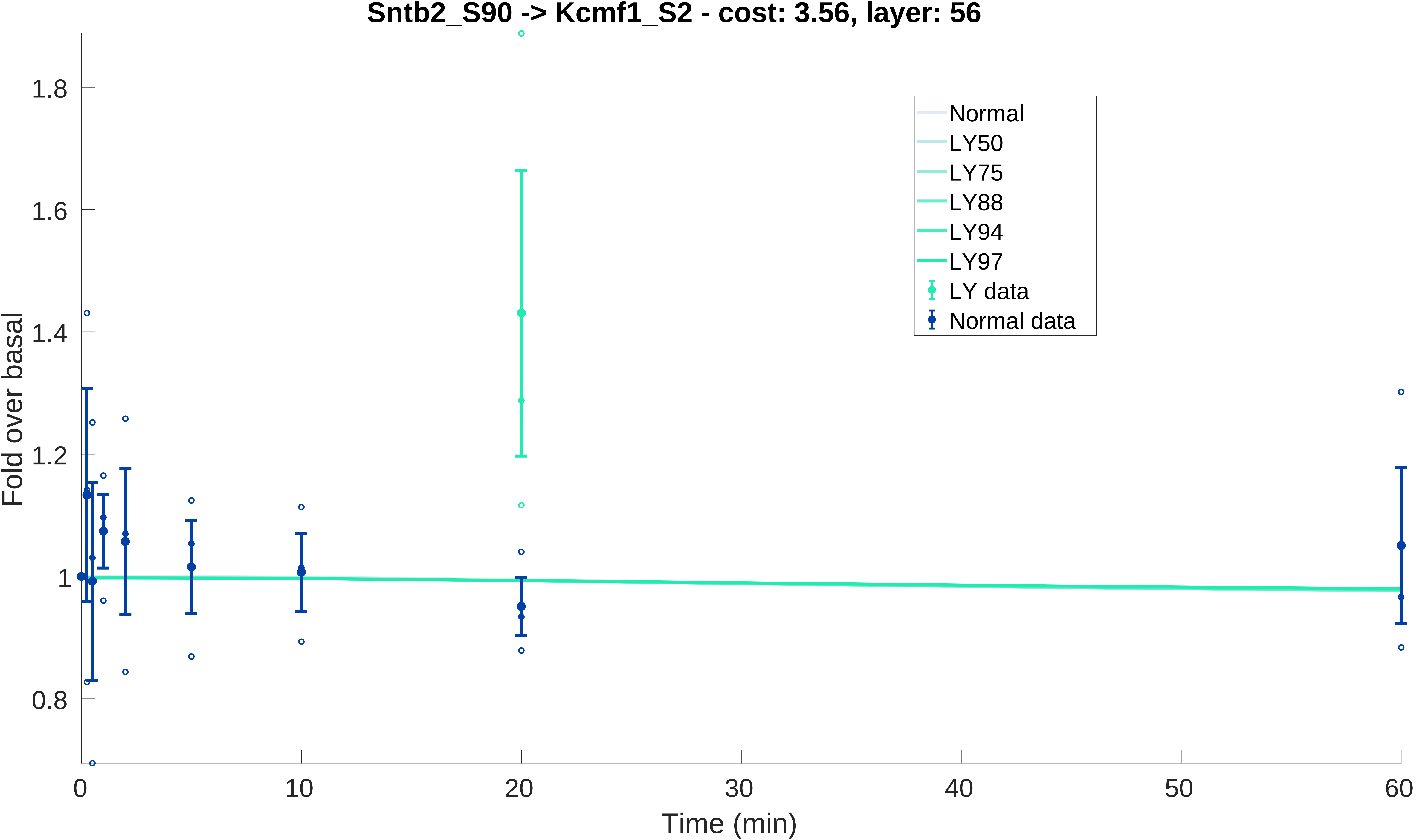

Sntb2_S90 -> Kcmf1_S2 - cost: 3.56, layer: 56
Fold over basal
Time (min)
0.8
1
1.2
1.4
1.6
1.8
0
10
20
30
40
50
60
Normal
LY50
LY75
LY88
LY94
LY97
LY data
Normal data

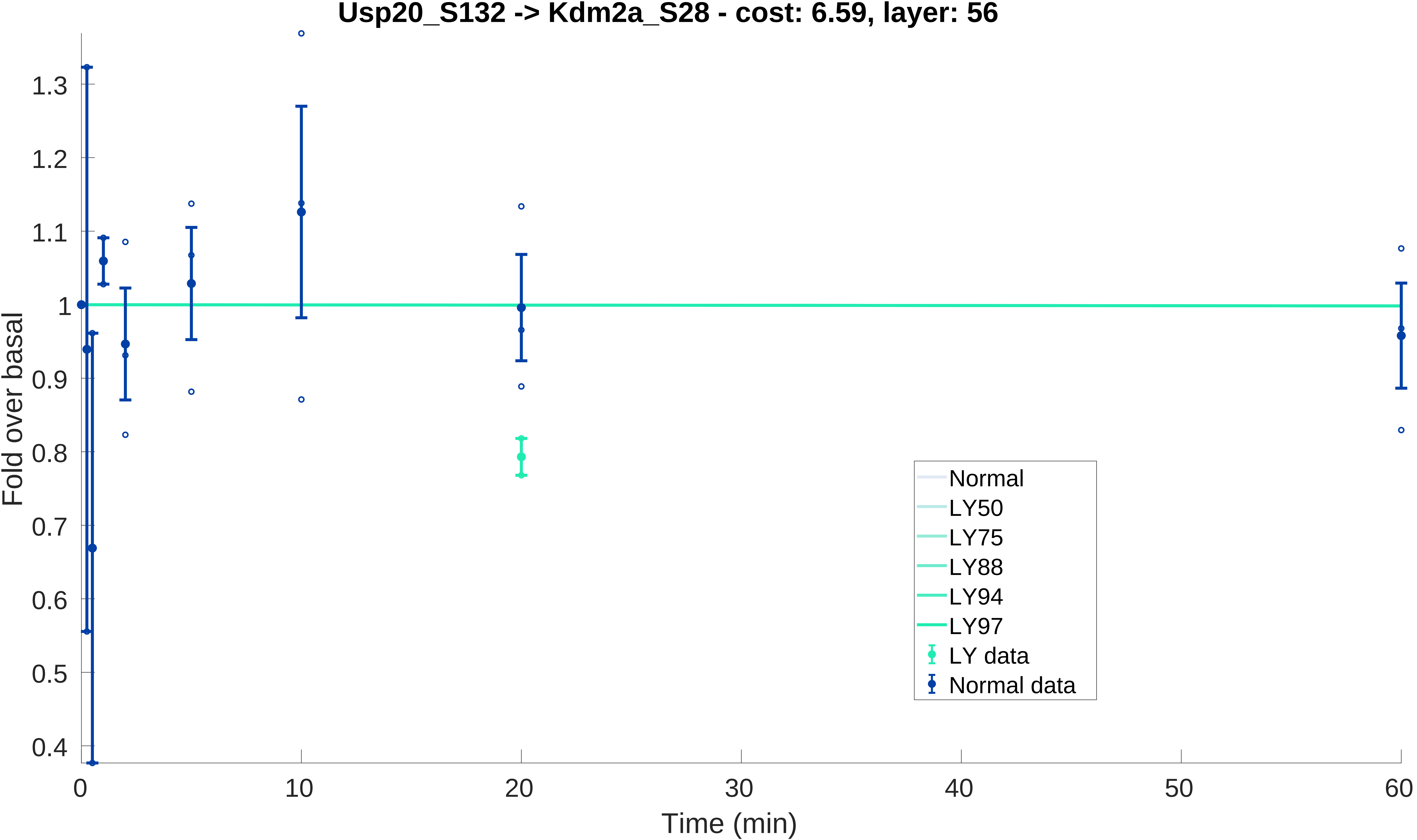

Usp20_S132 -> Kdm2a_S28 - cost: 6.59, layer: 56
Fold over basal
Time (min)
0.4
0.5
0.6
0.7
0.8
0.9
1
1.1
1.2
1.3
0
10
20
30
40
50
60
Normal
LY50
LY75
LY88
LY94
LY97
LY data
Normal data

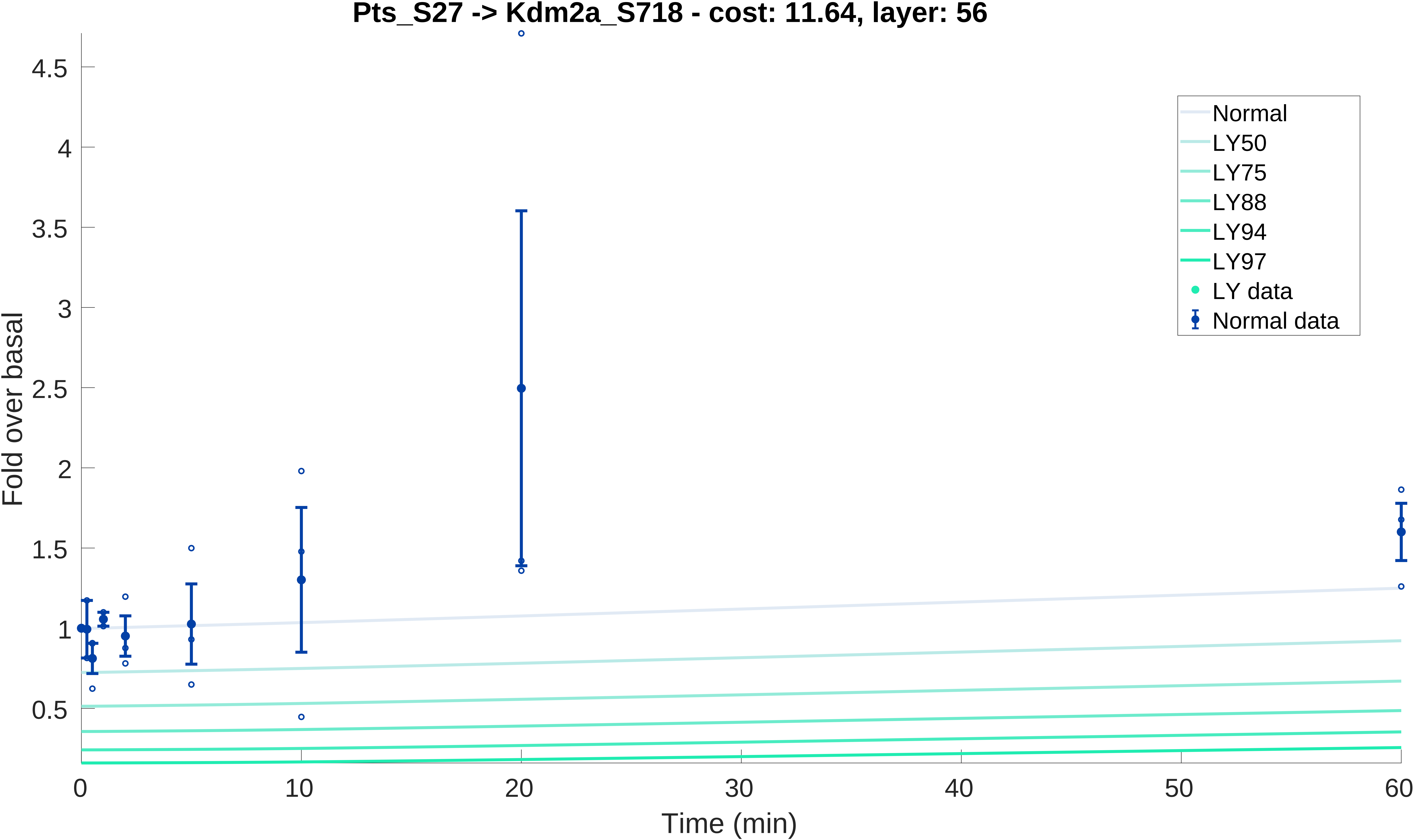
Pts_S27 -> Kdm2a_S718 - cost: 11.64, layer: 56
Fold over basal
Time (min)
0
10
20
30
40
50
60
0.5
1
1.5
2
2.5
3
3.5
4
4.5
Normal
LY50
LY75
LY88
LY94
LY97
LY data
Normal data

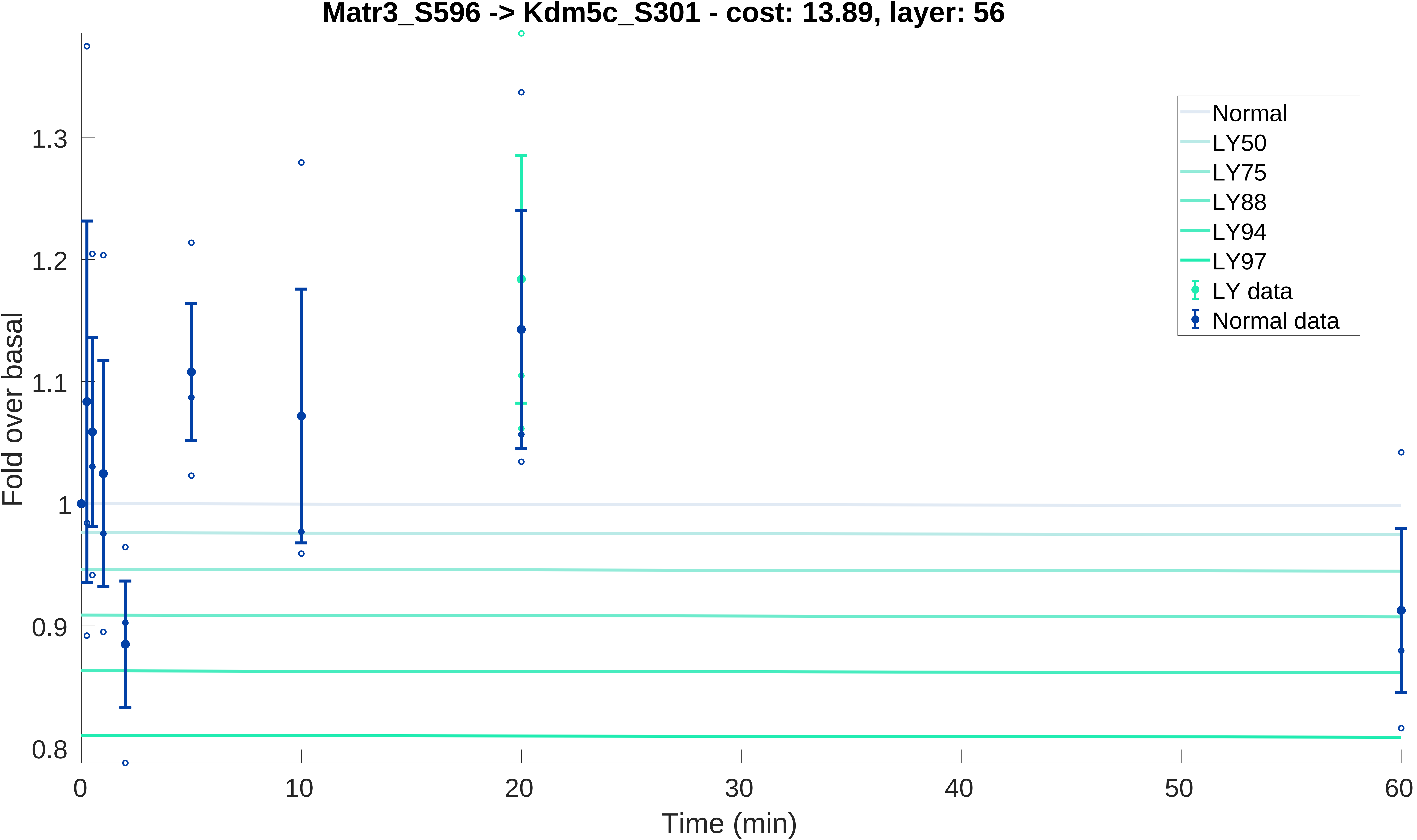
Matr3_S596 -> Kdm5c_S301 - cost: 13.89, layer: 56
Fold over basal
Time (min)
Normal
LY50
LY75
LY88
LY94
LY97
LY data
Normal data
1.3
1.2
1.1
1
0.9
0.8
0
10
20
30
40
50
60

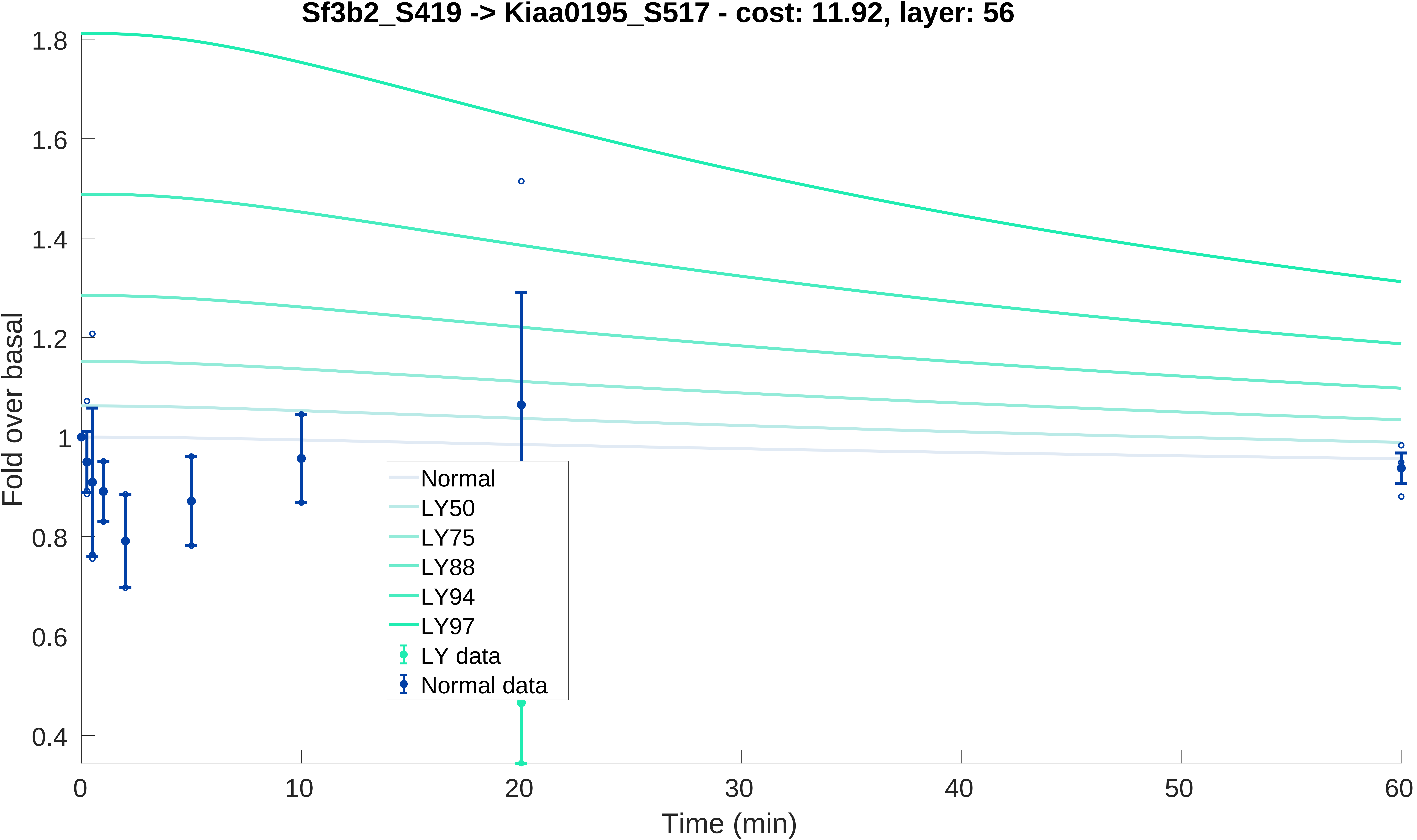

Sf3b2_S419 -> Kiaa0195_S517 - cost: 11.92, layer: 56
Fold over basal
Time (min)
1.8
1.6
1.4
1.2
1
0.8
0.6
0.4
0
10
20
30
40
50
60
Normal
LY50
LY75
LY88
LY94
LY97
LY data
Normal data

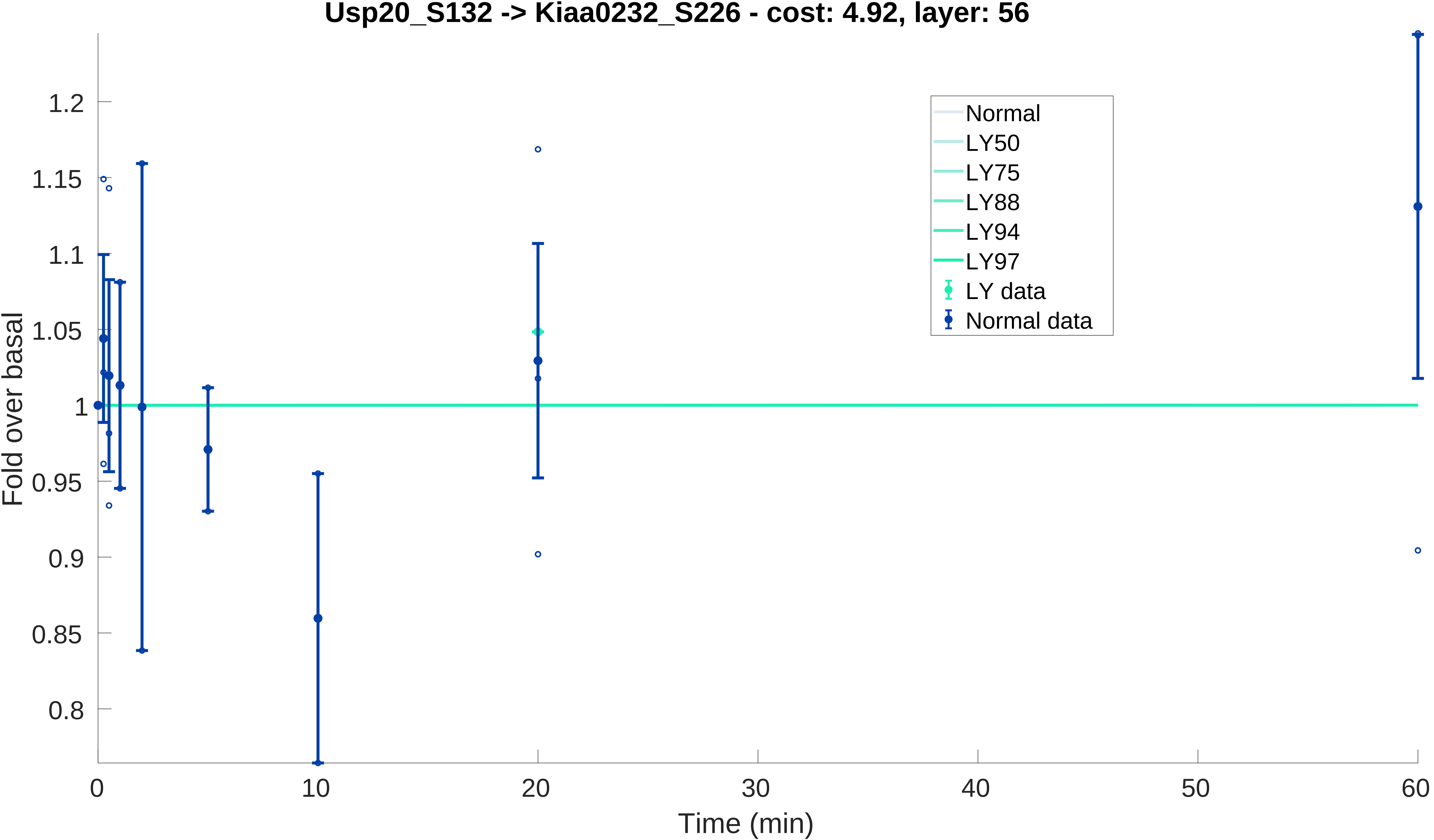

Usp20_S132 -> Kiaa0232_S226 - cost: 4.92, layer: 56
Normal
LY50
LY75
LY88
LY94
LY97
LY data
Normal data
Fold over basal
1.2
1.15
1.1
1.05
1
0.95
0.9
0.85
0.8
0
10
20
30
40
50
60
Time (min)

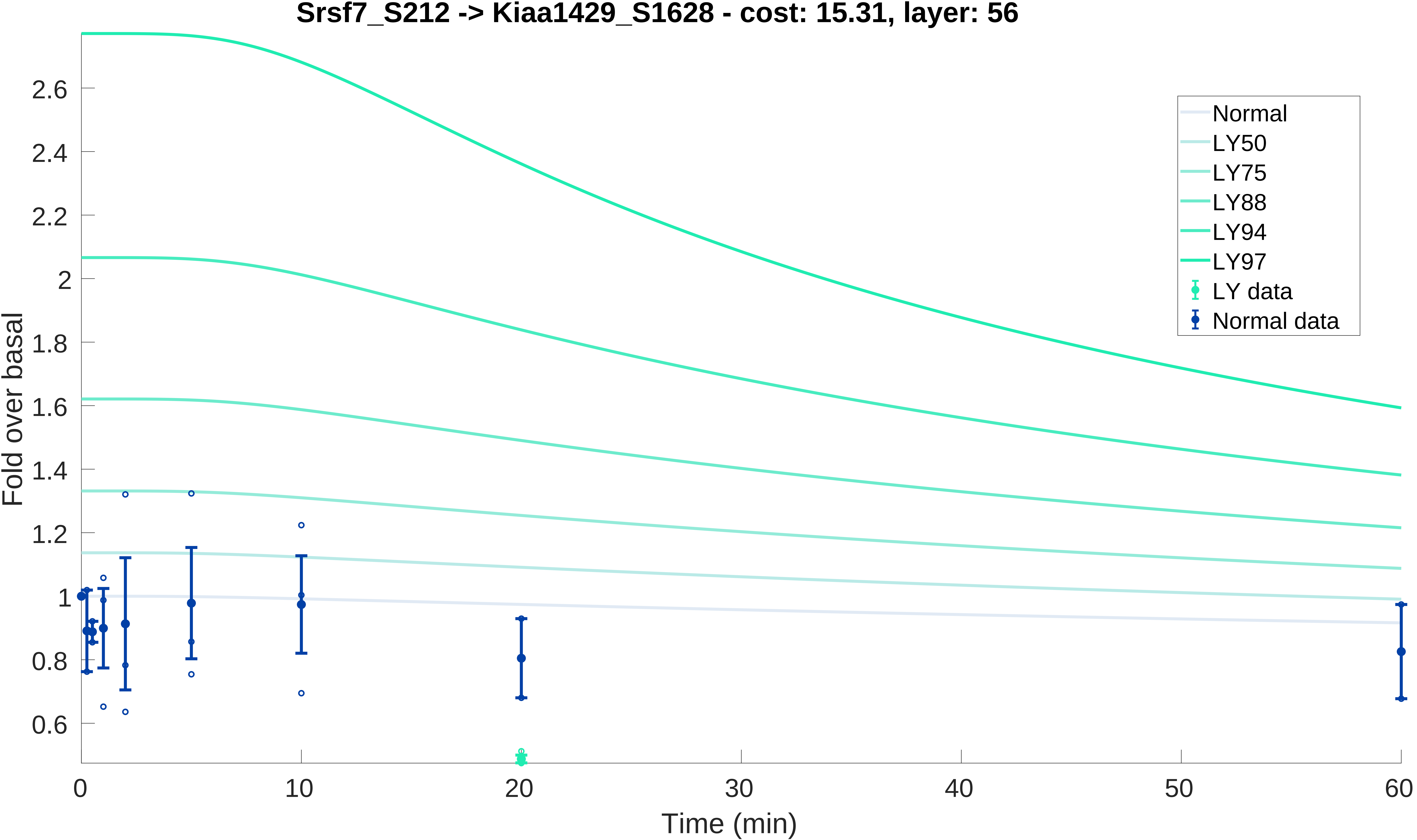

Srsf7_S212 -> Kiaa1429_S1628 - cost: 15.31, layer: 56
Fold over basal
Time (min)
0
10
20
30
40
50
60
0.6
0.8
1
1.2
1.4
1.6
1.8
2
2.2
2.4
2.6
Normal
LY50
LY75
LY88
LY94
LY97
LY data
Normal data

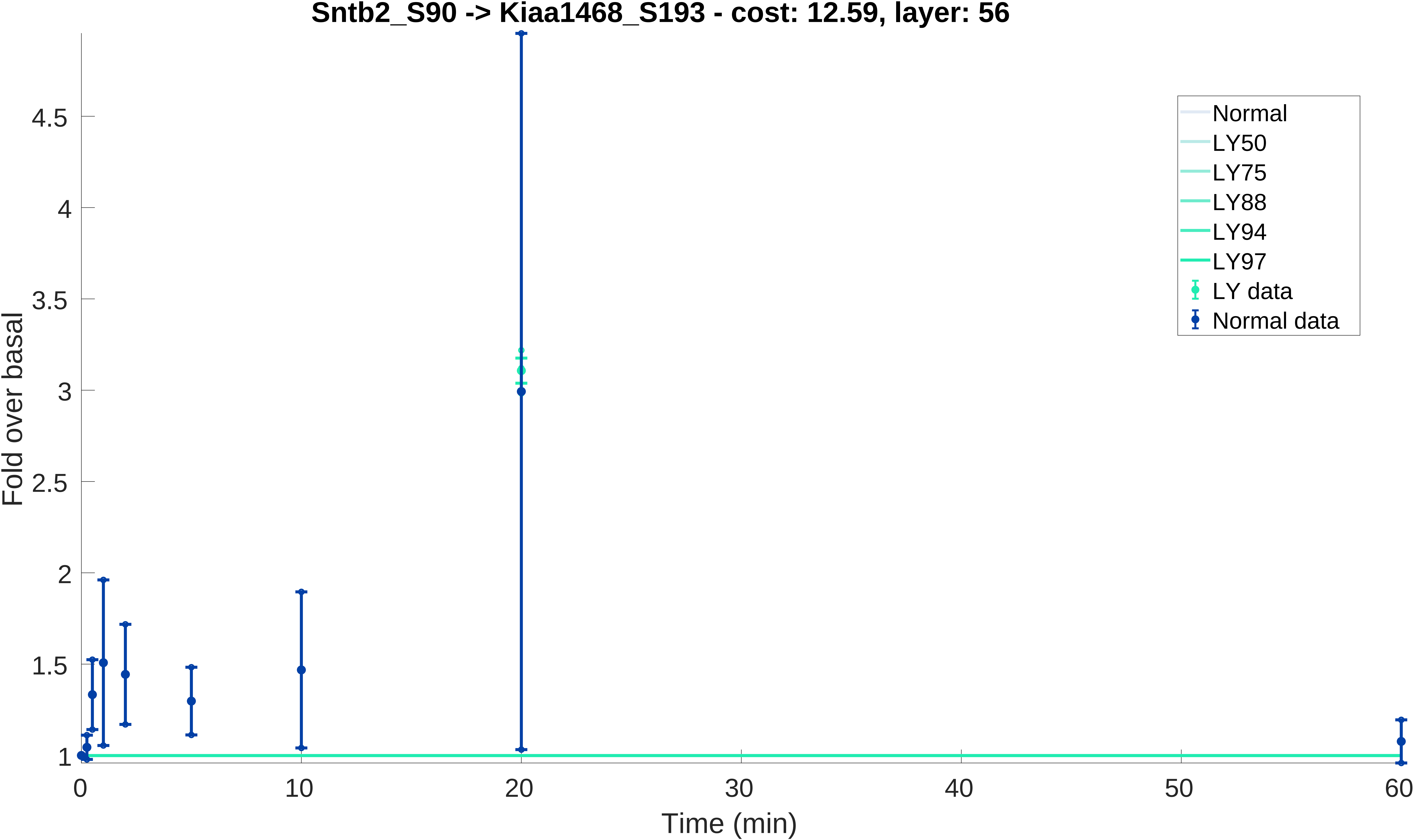

Sntb2_S90 -> Kiaa1468_S193 - cost: 12.59, layer: 56
Fold over basal
Time (min)
4.5
4
3.5
3
2.5
2
1.5
1
0
10
20
30
40
50
60
Normal
LY50
LY75
LY88
LY94
LY97
LY data
Normal data

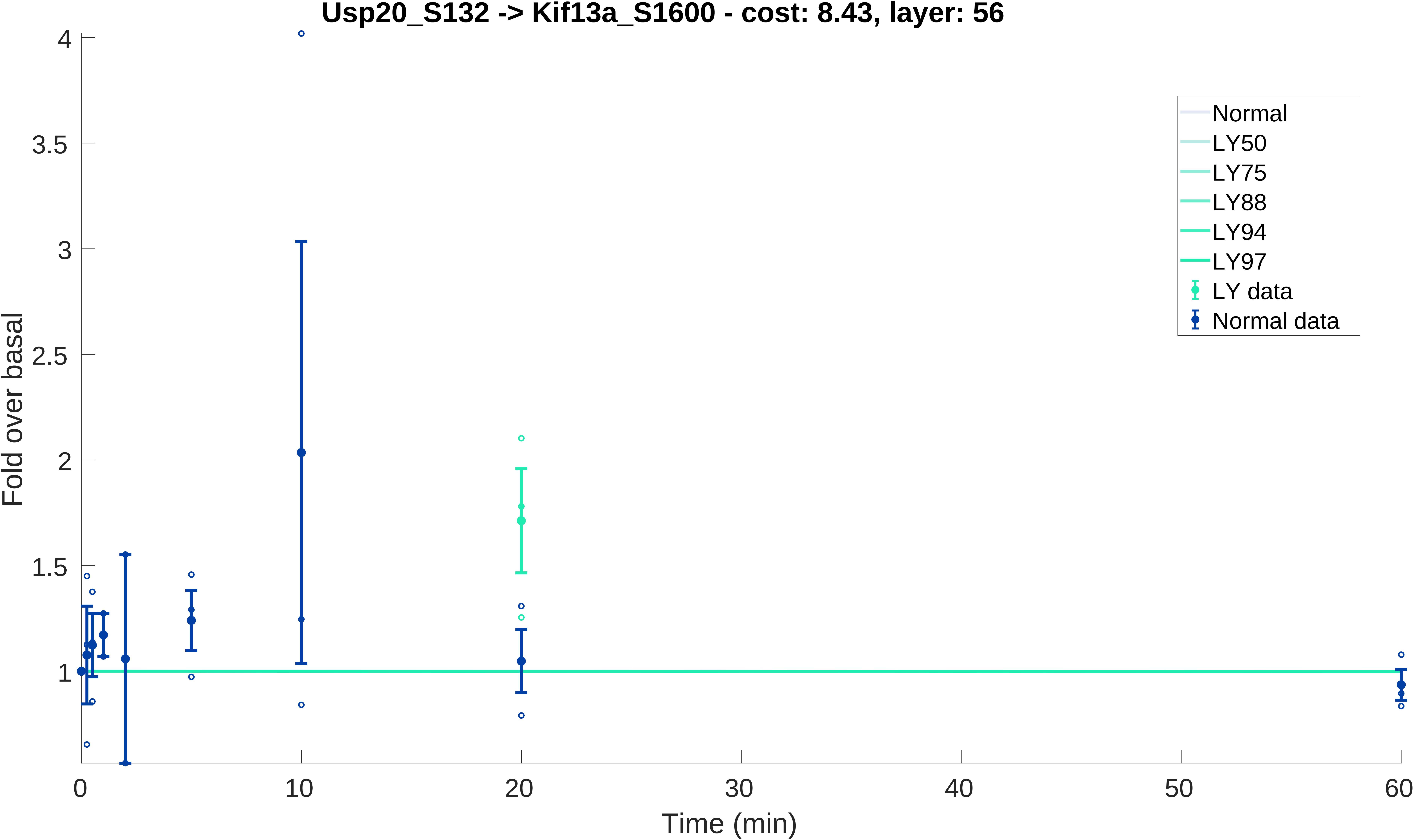

Usp20_S132 -> Kif13a_S1600 - cost: 8.43, layer: 56
Fold over basal
Time (min)
4
3.5
3
2.5
2
1.5
1
0
10
20
30
40
50
60
Normal
LY50
LY75
LY88
LY94
LY97
LY data
Normal data

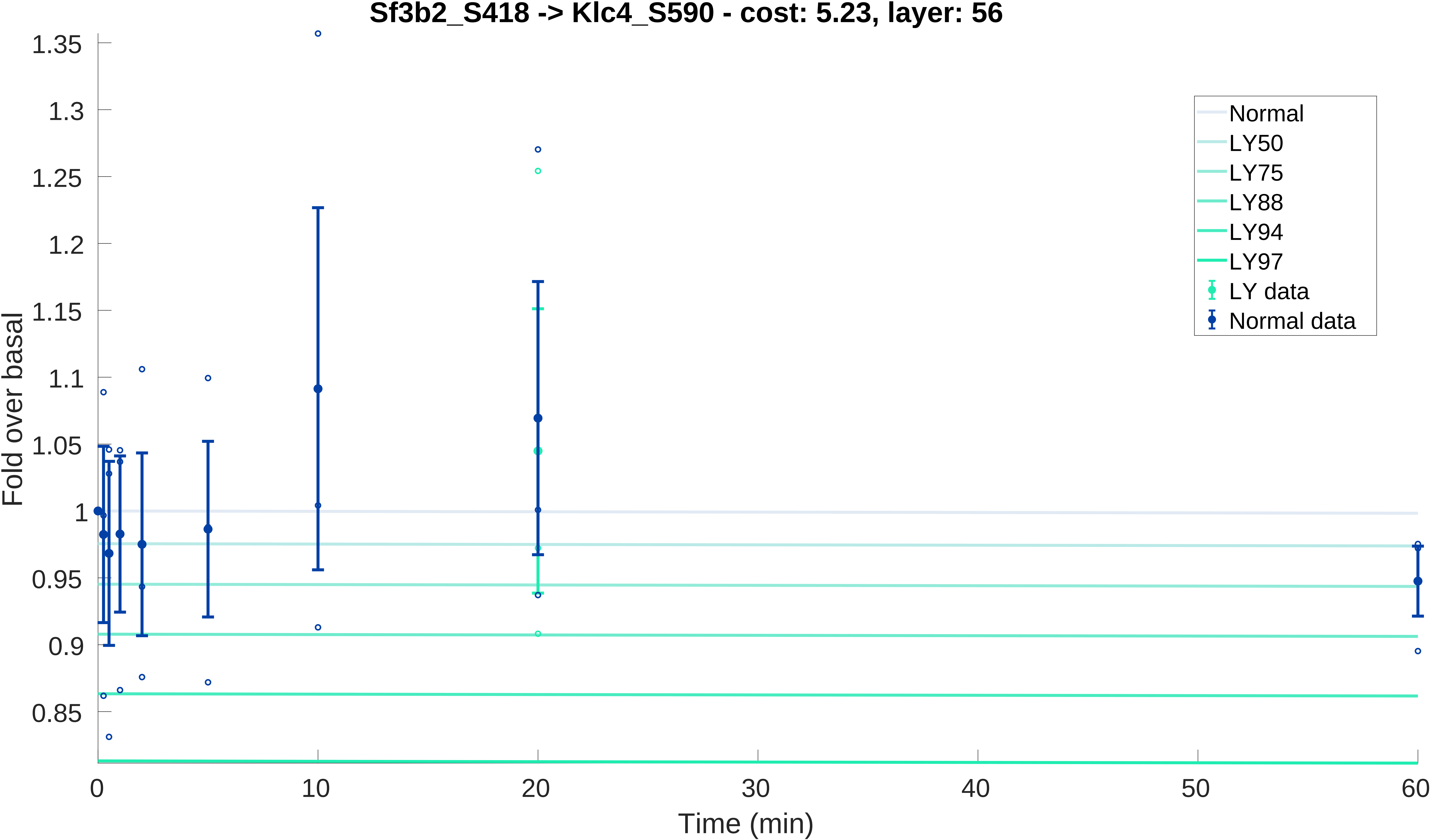
Sf3b2_S418 -> Klc4_S590 - cost: 5.23, layer: 56
Fold over basal
Time (min)
Normal
LY50
LY75
LY88
LY94
LY97
LY data
Normal data
1.35
1.3
1.25
1.2
1.15
1.1
1.05
1
0.95
0.9
0.85
0
10
20
30
40
50
60

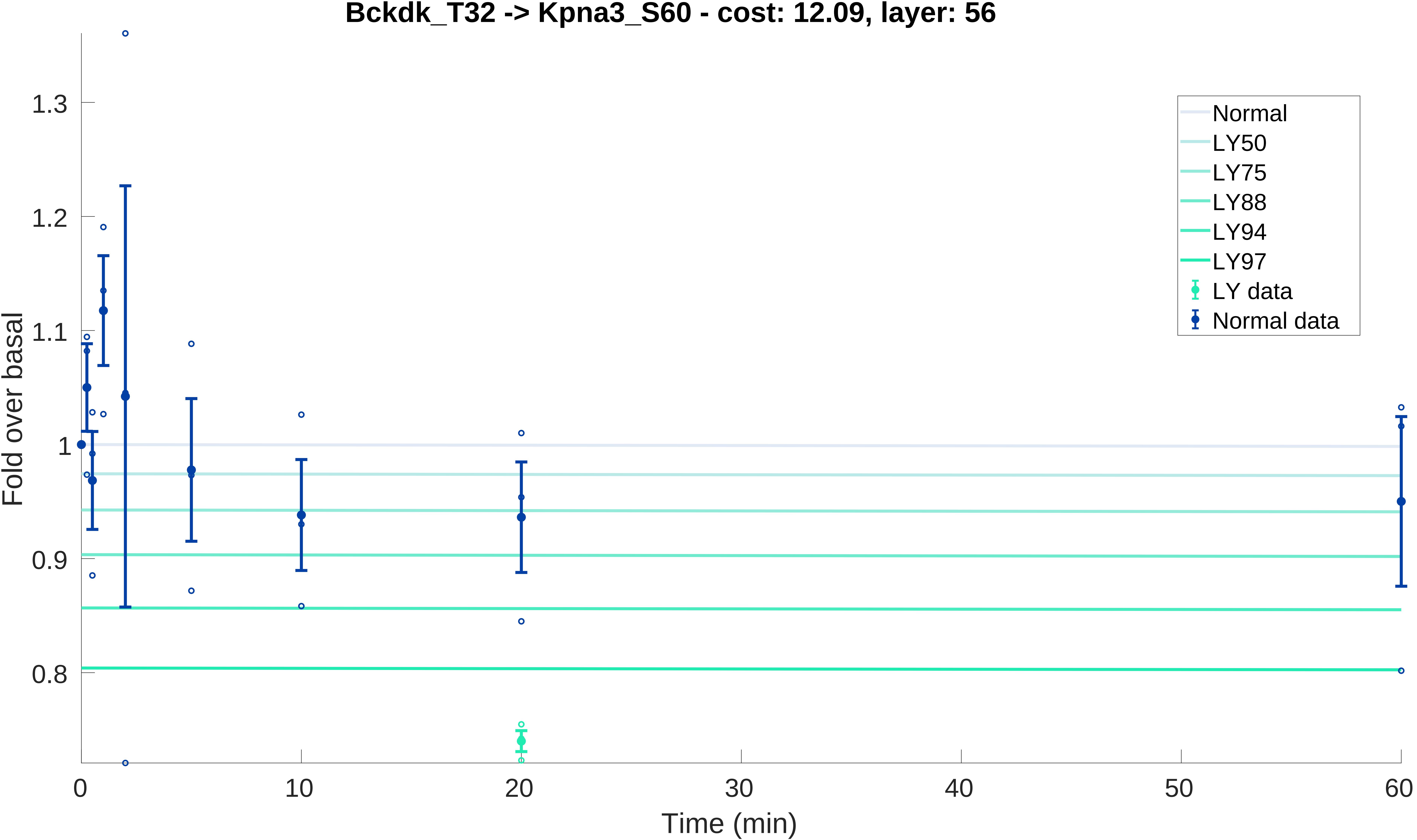
Bckdk_T32 -> Kpna3_S60 - cost: 12.09, layer: 56
Fold over basal
Time (min)
1.3
1.2
1.1
1
0.9
0.8
0
10
20
30
40
50
60
Normal
LY50
LY75
LY88
LY94
LY97
LY data
Normal data

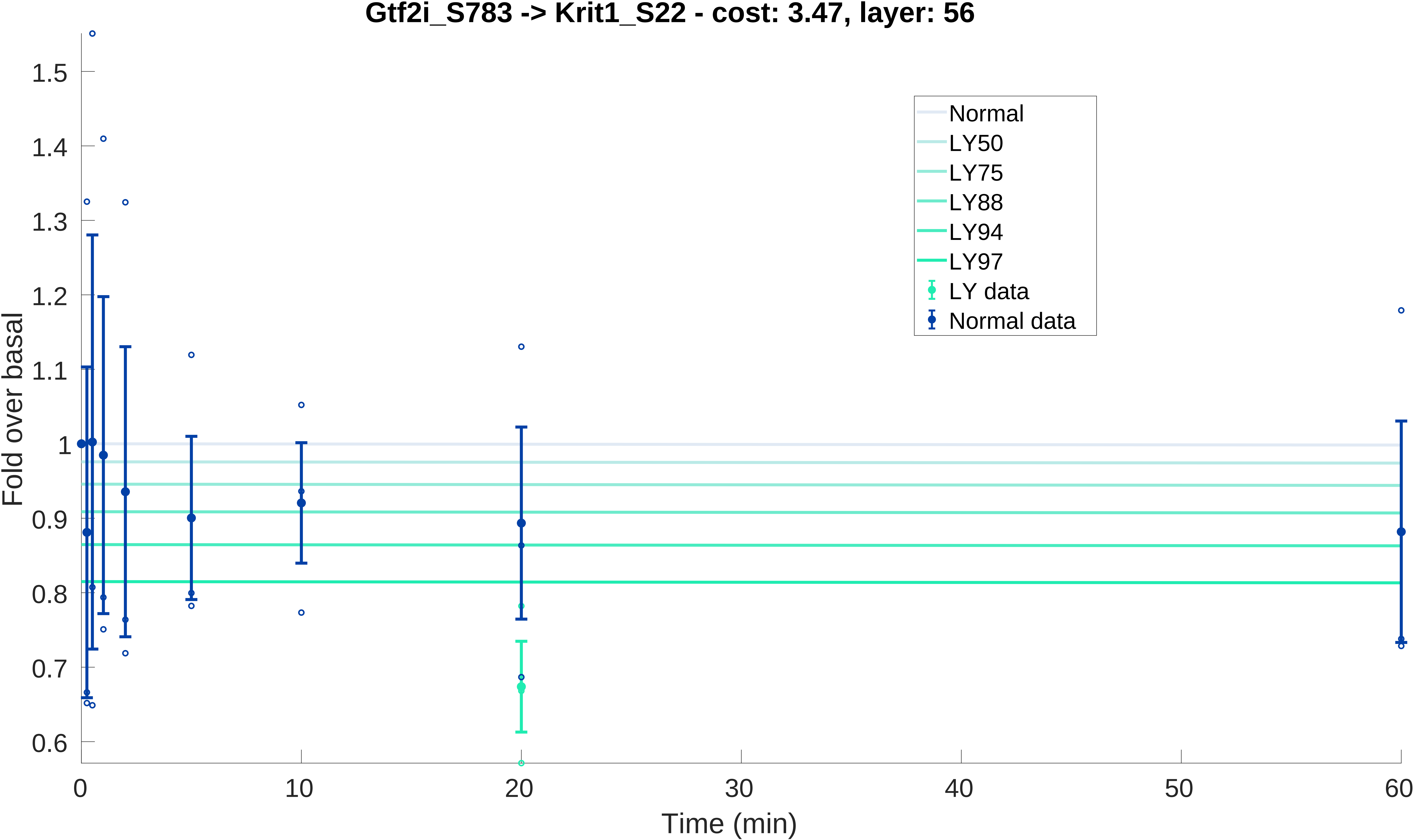

Gtf2i_S783 -> Krit1_S22 - cost: 3.47, layer: 56
Fold over basal
Time (min)
0.6
0.7
0.8
0.9
1
1.1
1.2
1.3
1.4
1.5
0
10
20
30
40
50
60
Normal
LY50
LY75
LY88
LY94
LY97
LY data
Normal data

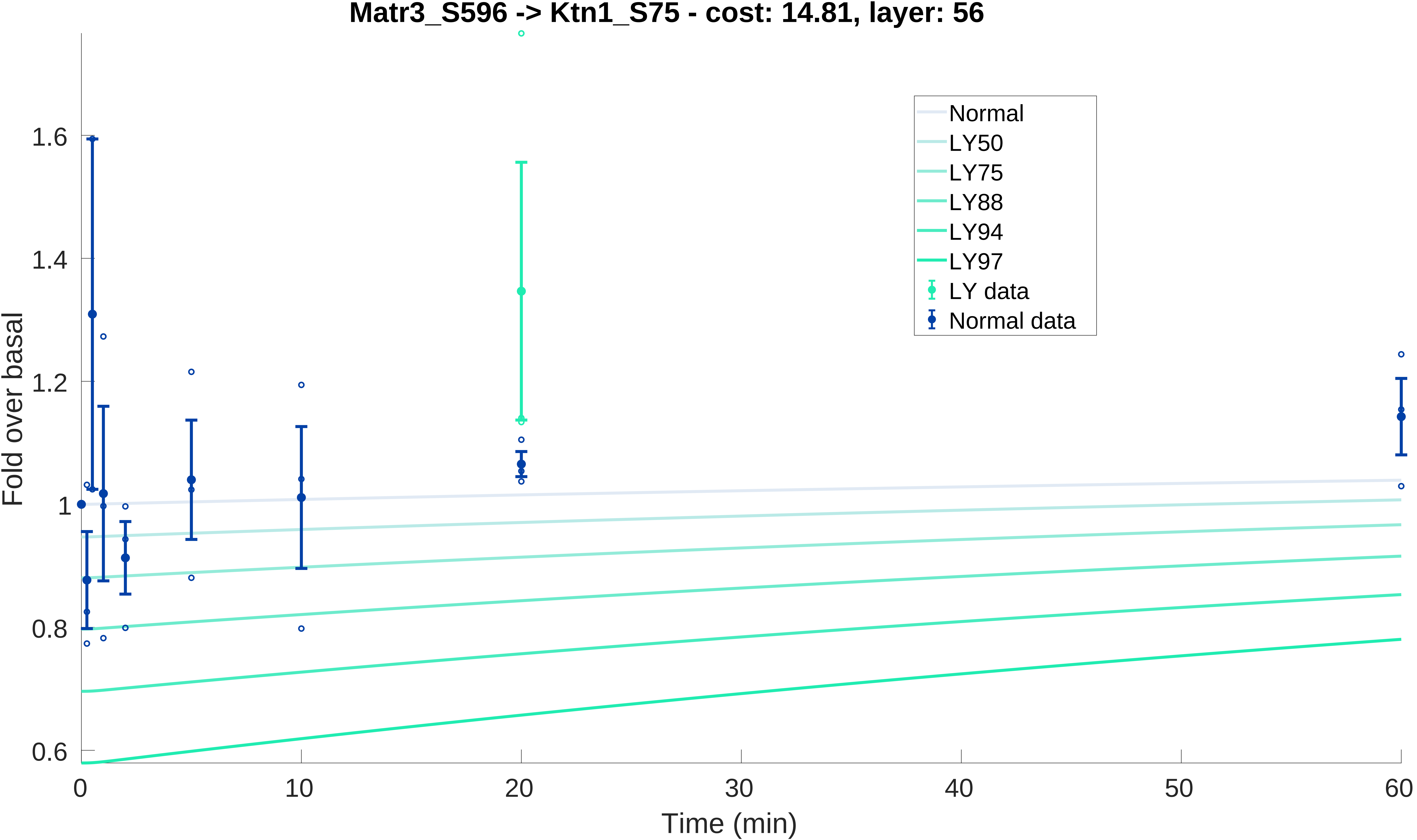

Matr3_S596 -> Ktn1_S75 - cost: 14.81, layer: 56
Fold over basal
Time (min)
0.6
0.8
1
1.2
1.4
1.6
0
10
20
30
40
50
60
Normal
LY50
LY75
LY88
LY94
LY97
LY data
Normal data

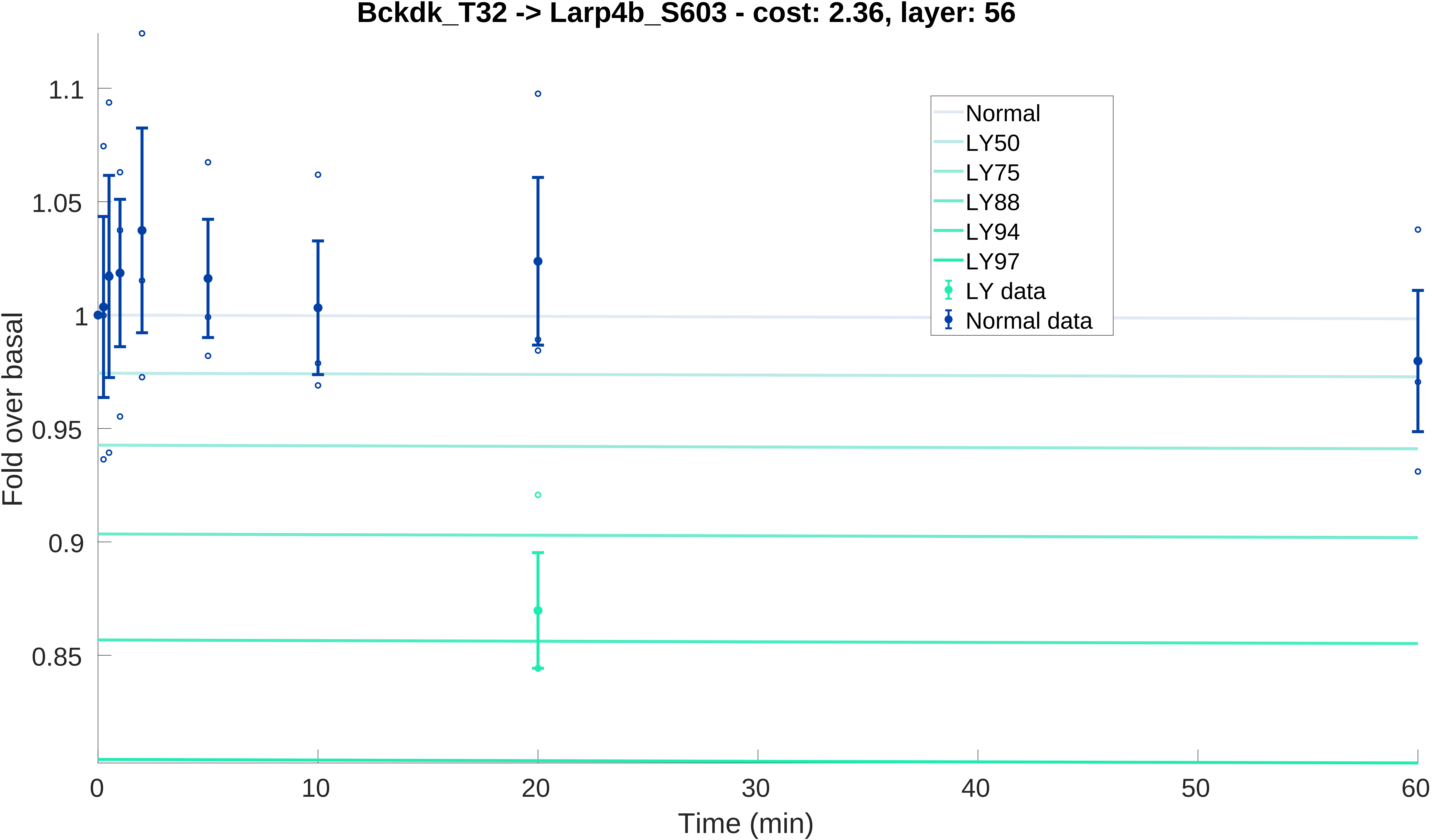

Bckdk_T32 -> Larp4b_S603 - cost: 2.36, layer: 56
1.1
1.05
1
0.95
0.9
0.85
Fold over basal
0
10
20
30
40
50
60
Time (min)
Normal
LY50
LY75
LY88
LY94
LY97
LY data
Normal data

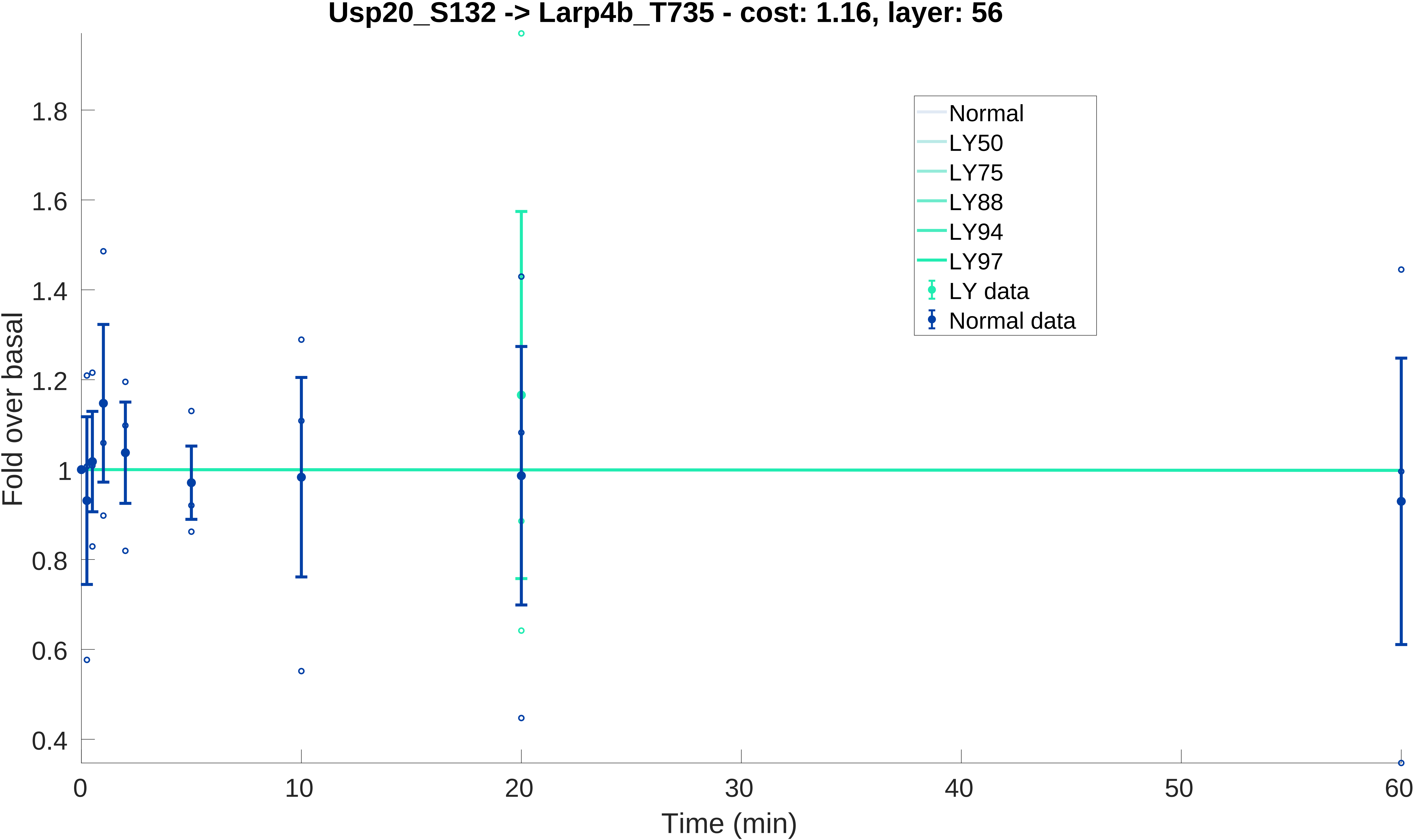

Usp20_S132 -> Larp4b_T735 - cost: 1.16, layer: 56
Fold over basal
0.4
0.6
0.8
1
1.2
1.4
1.6
1.8
Time (min)
0
10
20
30
40
50
60
Normal
LY50
LY75
LY88
LY94
LY97
LY data
Normal data

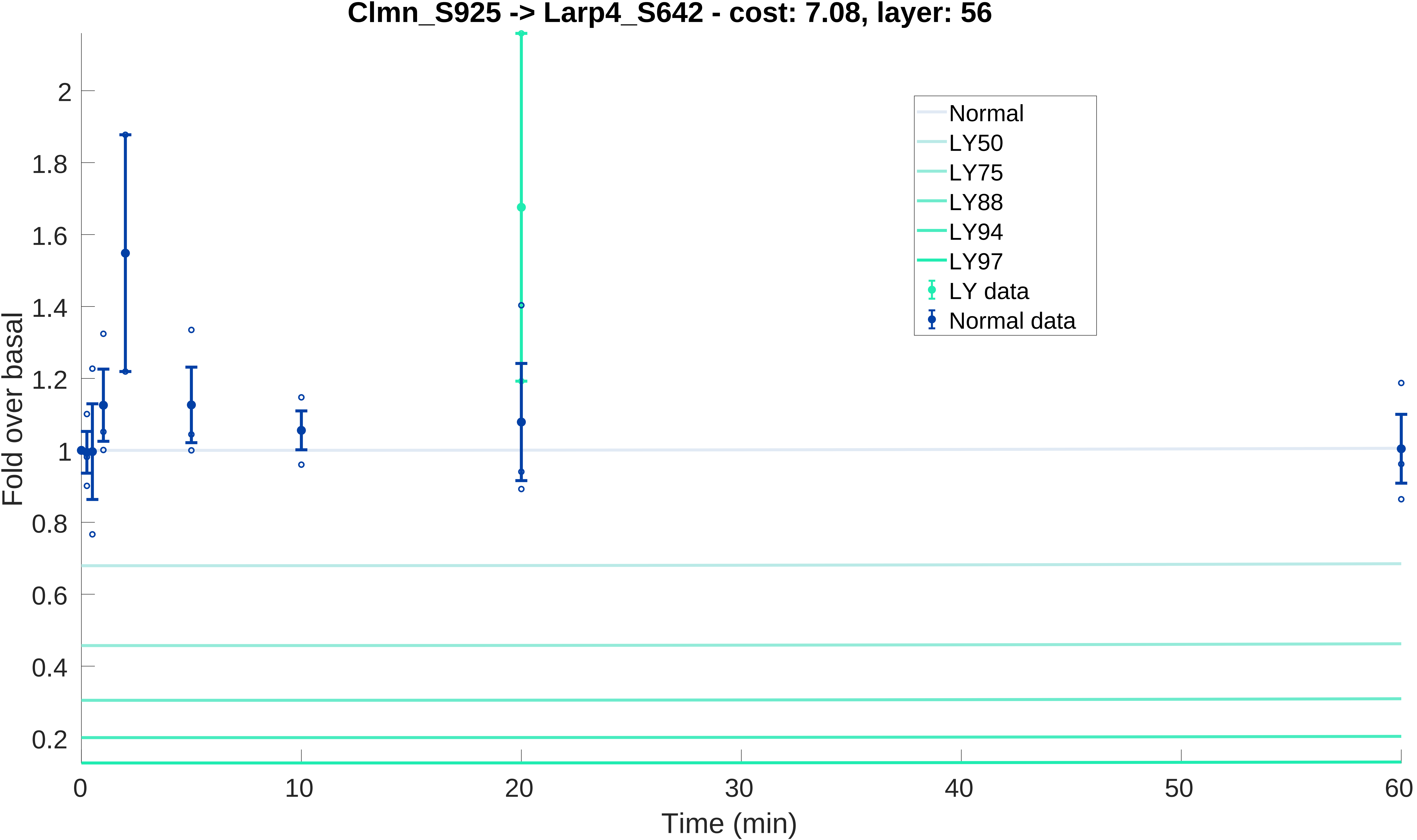
Clmn_S925 -> Larp4_S642 - cost: 7.08, layer: 56
Fold over basal
Time (min)
0
10
20
30
40
50
60
0.2
0.4
0.6
0.8
1
1.2
1.4
1.6
1.8
2
Normal
LY50
LY75
LY88
LY94
LY97
LY data
Normal data

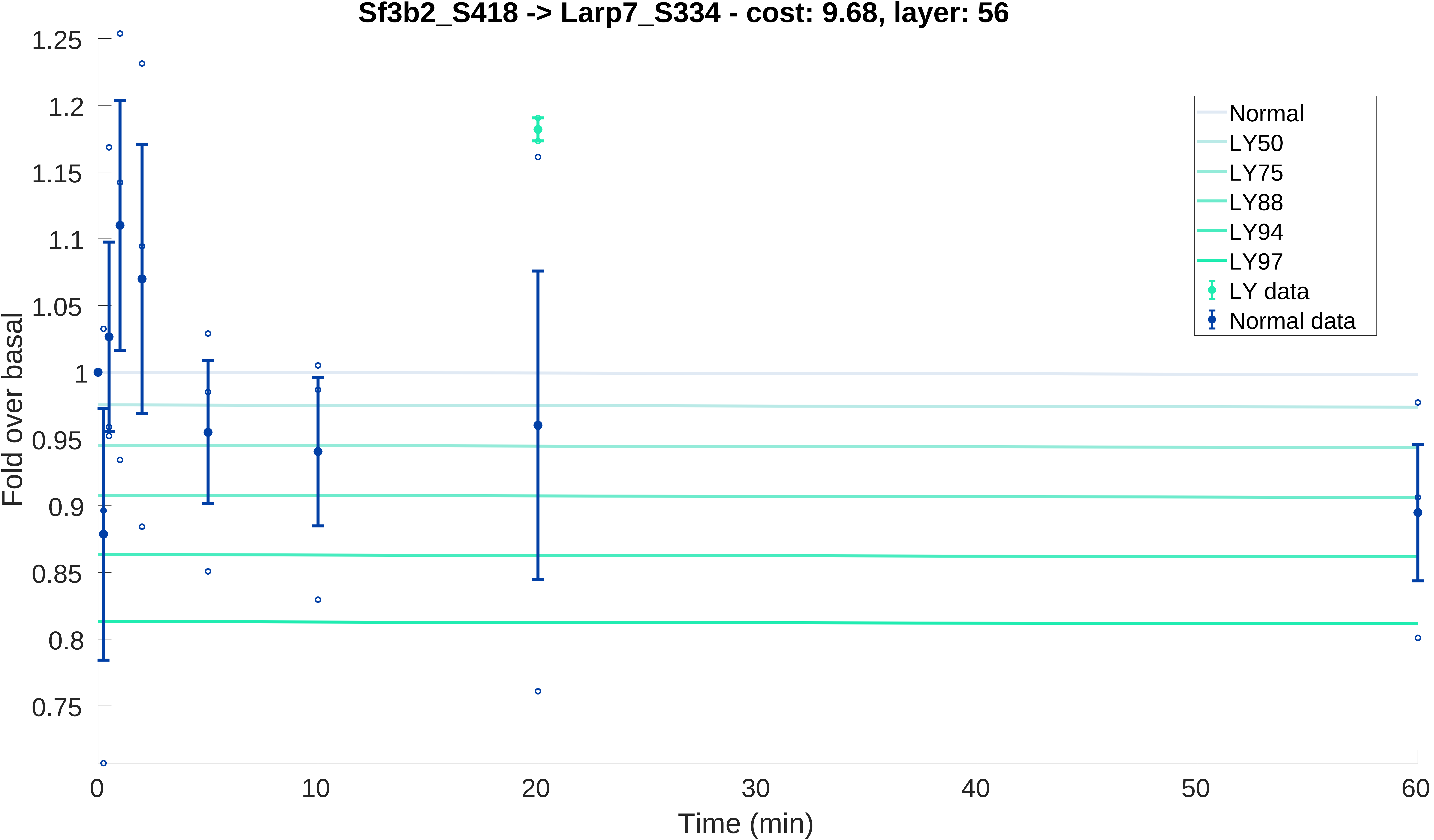

Sf3b2_S418 -> Larp7_S334 - cost: 9.68, layer: 56
Fold over basal
Time (min)
1.25
1.2
1.15
1.1
1.05
1
0.95
0.9
0.85
0.8
0.75
0
10
20
30
40
50
60
Normal
LY50
LY75
LY88
LY94
LY97
LY data
Normal data

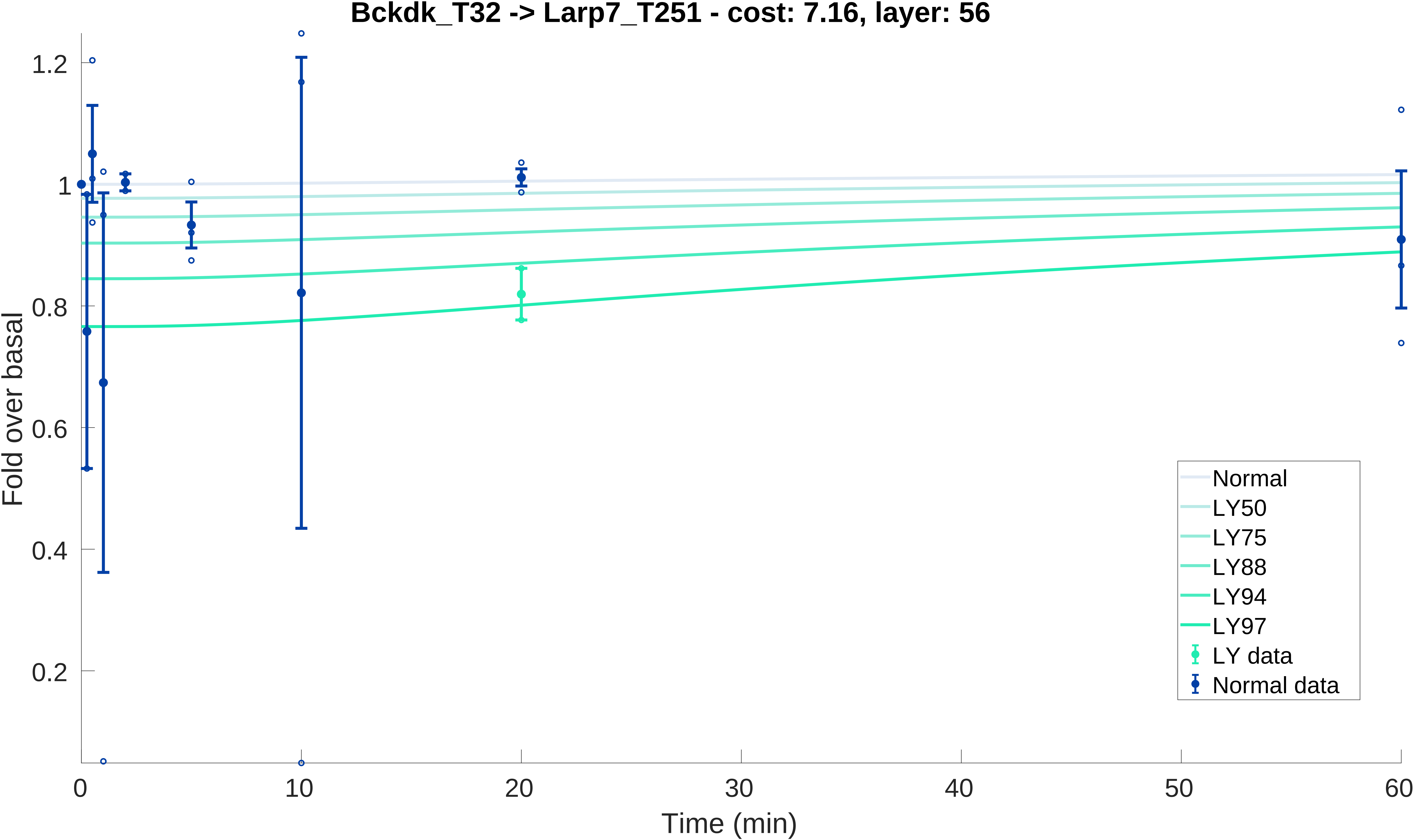
Bckdk_T32 -> Larp7_T251 - cost: 7.16, layer: 56
Fold over basal
Time (min)
1.2
1
0.8
0.6
0.4
0.2
0
10
20
30
40
50
60
Normal
LY50
LY75
LY88
LY94
LY97
LY data
Normal data

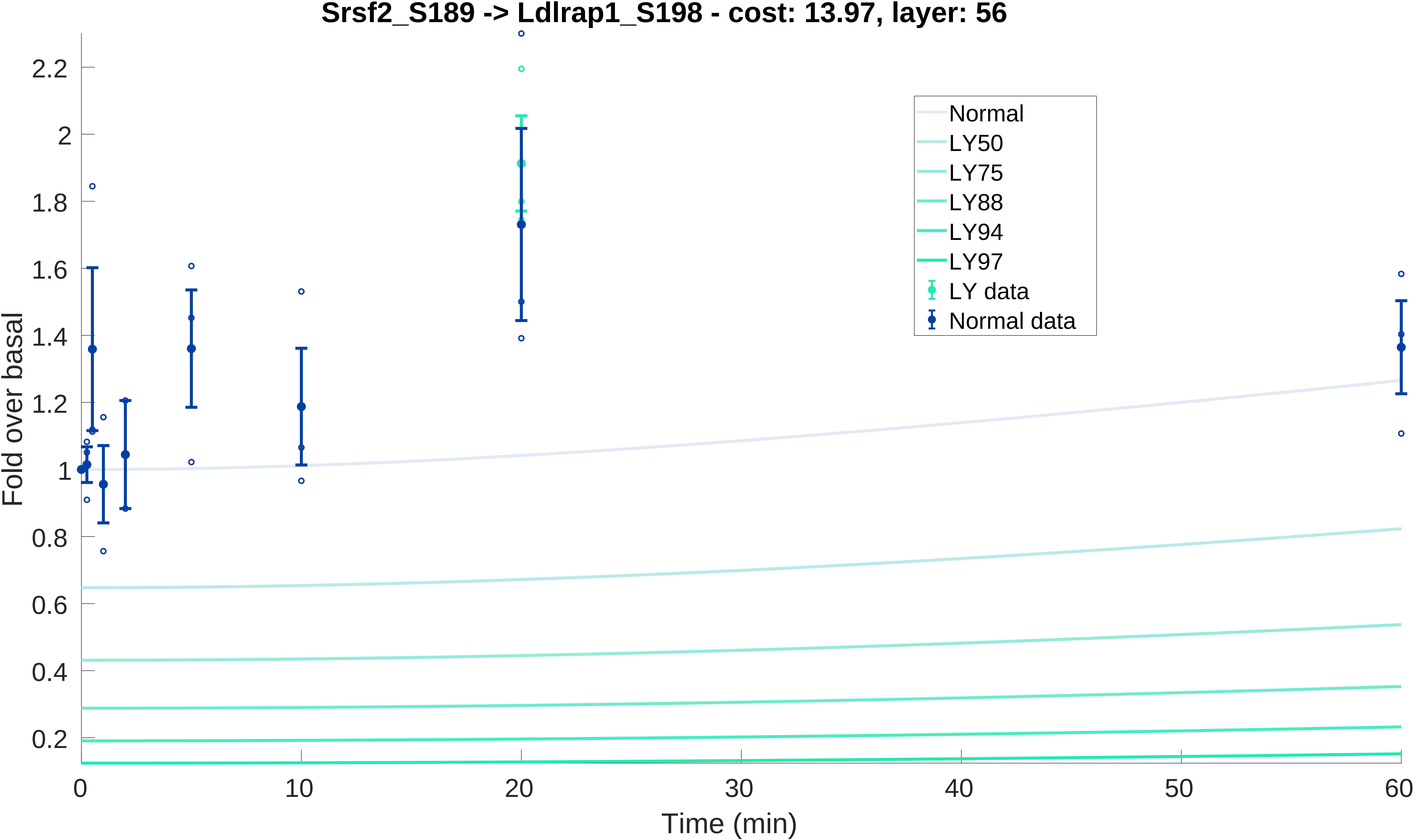

Srsf2_S189 -> Ldlrap1_S198 - cost: 13.97, layer: 56
Fold over basal
Time (min)
0
10
20
30
40
50
60
0.2
0.4
0.6
0.8
1
1.2
1.4
1.6
1.8
2
2.2
Normal
LY50
LY75
LY88
LY94
LY97
LY data
Normal data

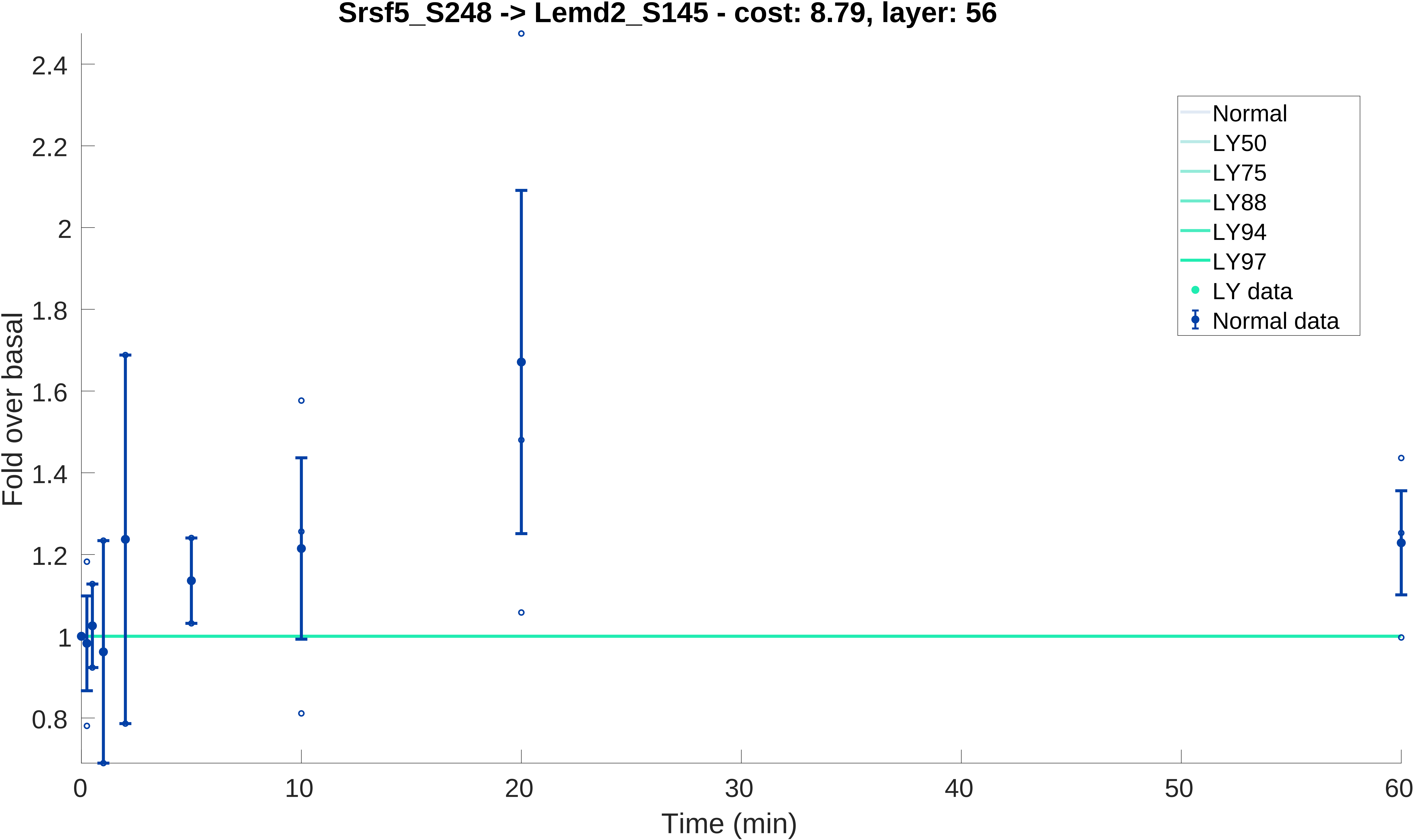

Srsf5_S248 -> Lemd2_S145 - cost: 8.79, layer: 56
Fold over basal
Time (min)
2.4
2.2
2
1.8
1.6
1.4
1.2
1
0.8
0
10
20
30
40
50
60
Normal
LY50
LY75
LY88
LY94
LY97
LY data
Normal data

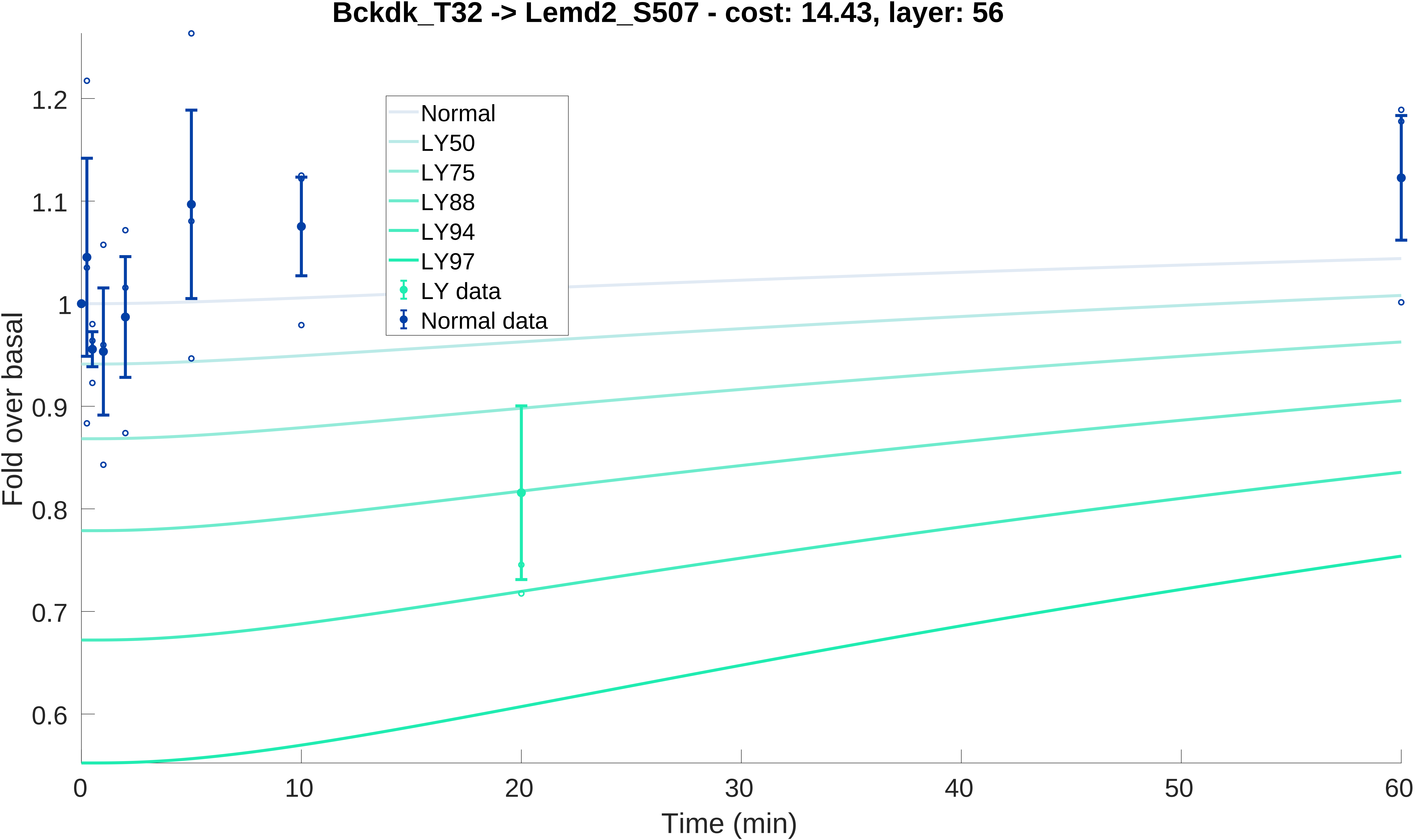

Bckdk_T32 -> Lemd2_S507 - cost: 14.43, layer: 56
Normal
LY50
LY75
LY88
LY94
LY97
LY data
Normal data
Fold over basal
Time (min)
0
10
20
30
40
50
60
0.6
0.7
0.8
0.9
1
1.1
1.2

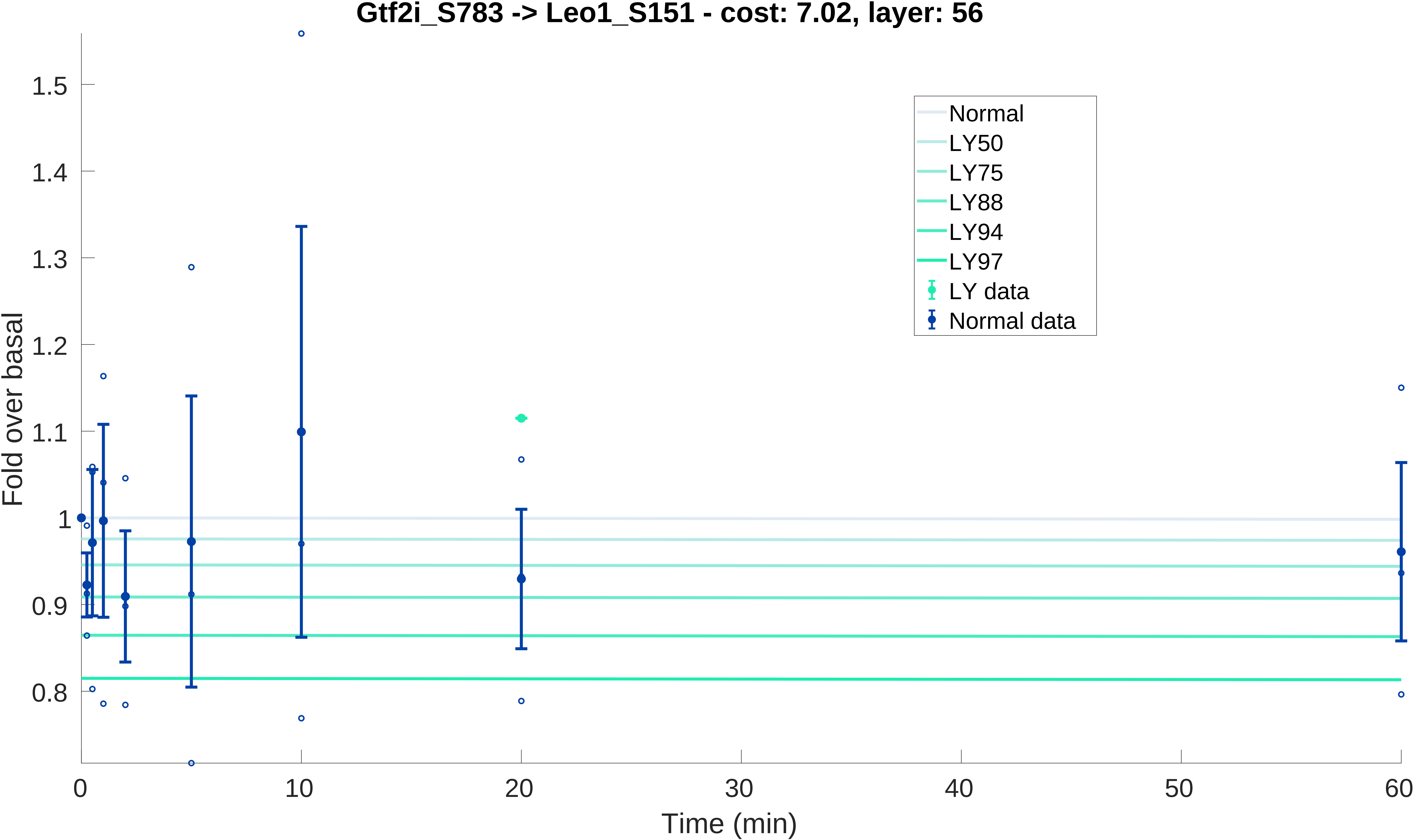
Gtf2i_S783 -> Leo1_S151 - cost: 7.02, layer: 56
Fold over basal
1.5
1.4
1.3
1.2
1.1
1
0.9
0.8
0
10
20
30
40
50
60
Time (min)
Normal
LY50
LY75
LY88
LY94
LY97
LY data
Normal data

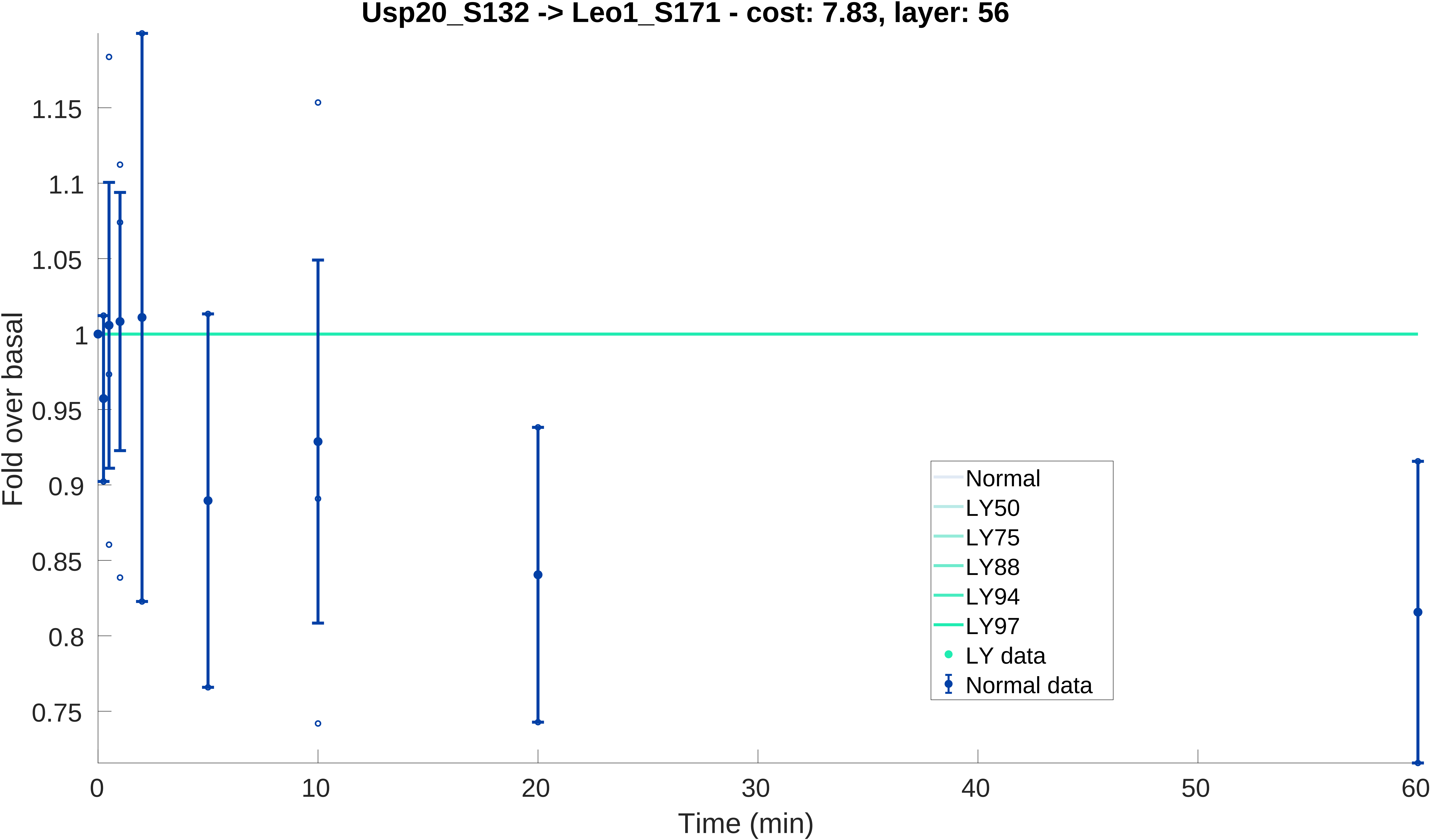

Usp20_S132 -> Leo1_S171 - cost: 7.83, layer: 56
Fold over basal
1.15
1.1
1.05
1
0.95
0.9
0.85
0.8
0.75
0
10
20
30
40
50
60
Time (min)
Normal
LY50
LY75
LY88
LY94
LY97
LY data
Normal data

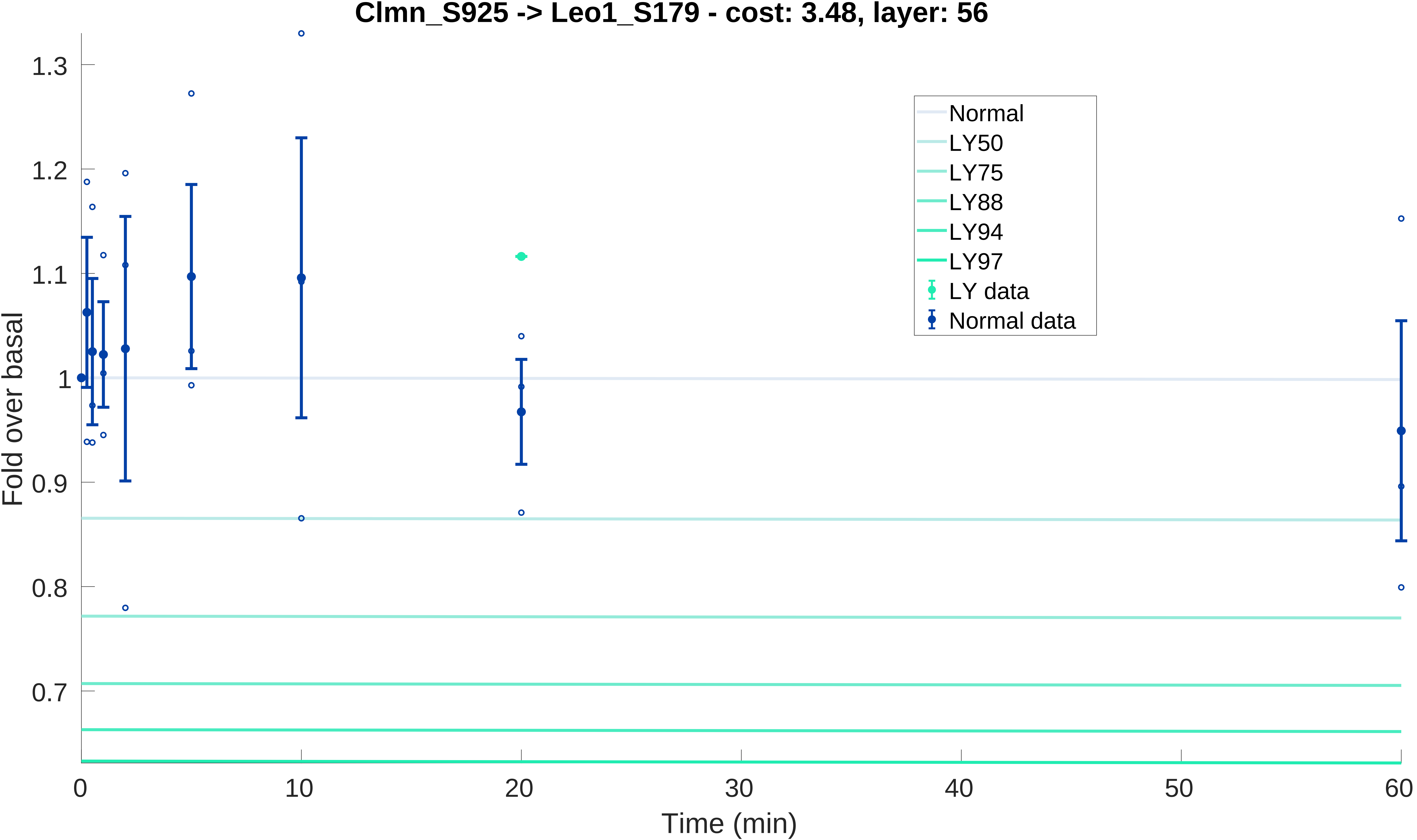

Clmn_S925 -> Leo1_S179 - cost: 3.48, layer: 56
Fold over basal
Time (min)
1.3
1.2
1.1
1
0.9
0.8
0.7
0
10
20
30
40
50
60
Normal
LY50
LY75
LY88
LY94
LY97
LY data
Normal data

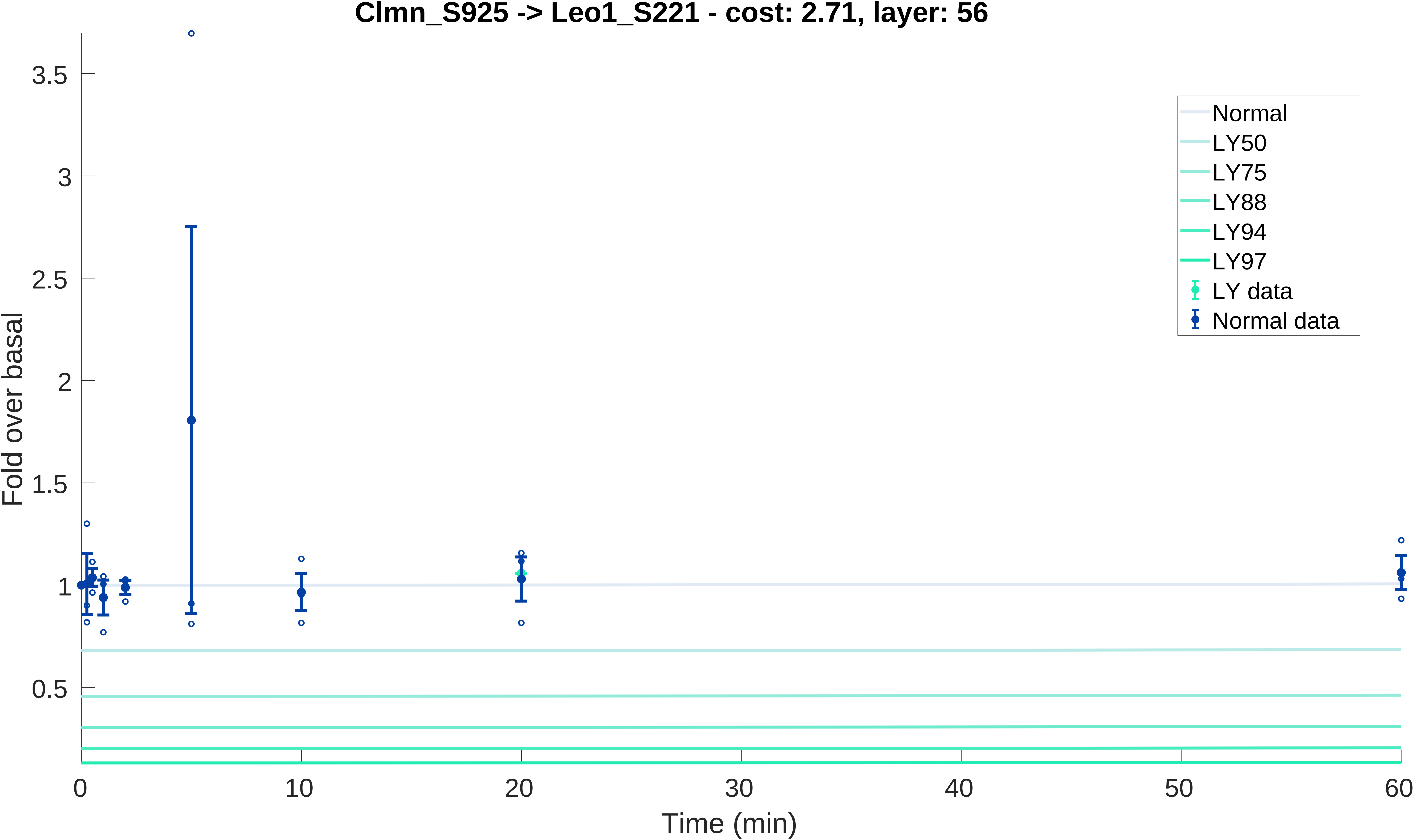

Clmn_S925 -> Leo1_S221 - cost: 2.71, layer: 56
Fold over basal
Time (min)
3.5
3
2.5
2
1.5
1
0.5
0
10
20
30
40
50
60
Normal
LY50
LY75
LY88
LY94
LY97
LY data
Normal data

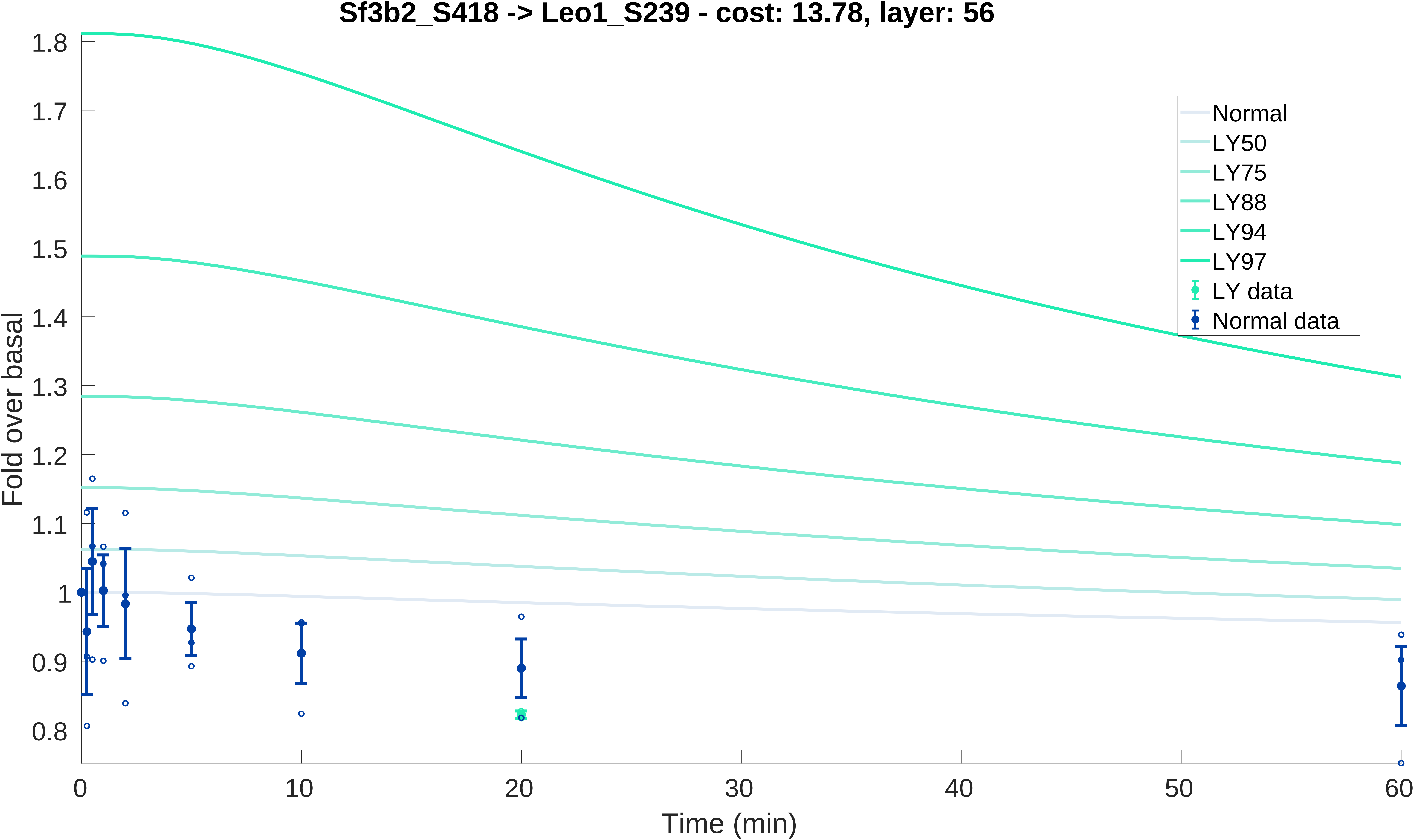

Sf3b2_S418 -> Leo1_S239 - cost: 13.78, layer: 56
Fold over basal
Time (min)
0
10
20
30
40
50
60
0.8
0.9
1
1.1
1.2
1.3
1.4
1.5
1.6
1.7
1.8
Normal
LY50
LY75
LY88
LY94
LY97
LY data
Normal data

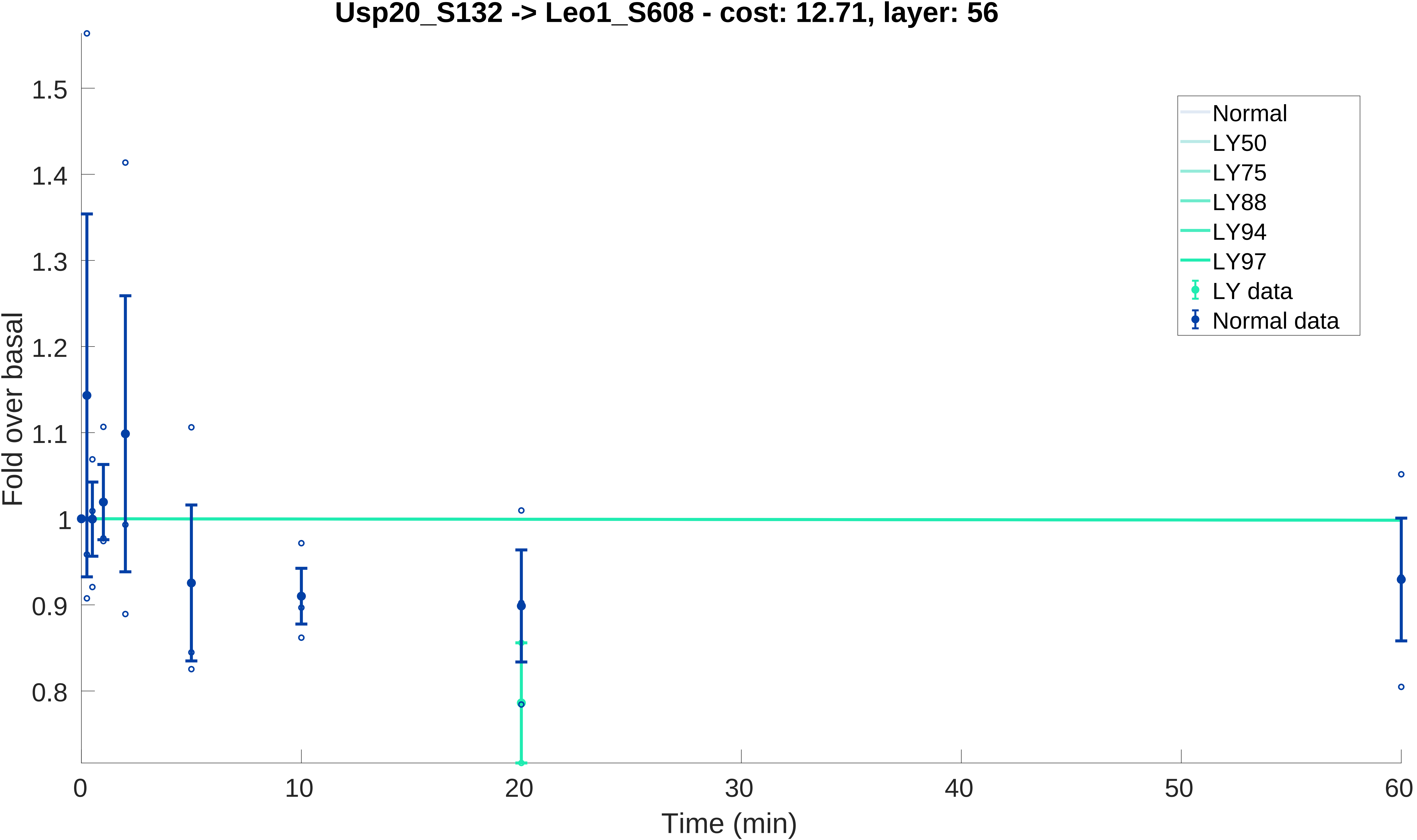

Usp20_S132 -> Leo1_S608 - cost: 12.71, layer: 56
Fold over basal
1.5
1.4
1.3
1.2
1.1
1
0.9
0.8
0
10
20
30
40
50
60
Time (min)
Normal
LY50
LY75
LY88
LY94
LY97
LY data
Normal data

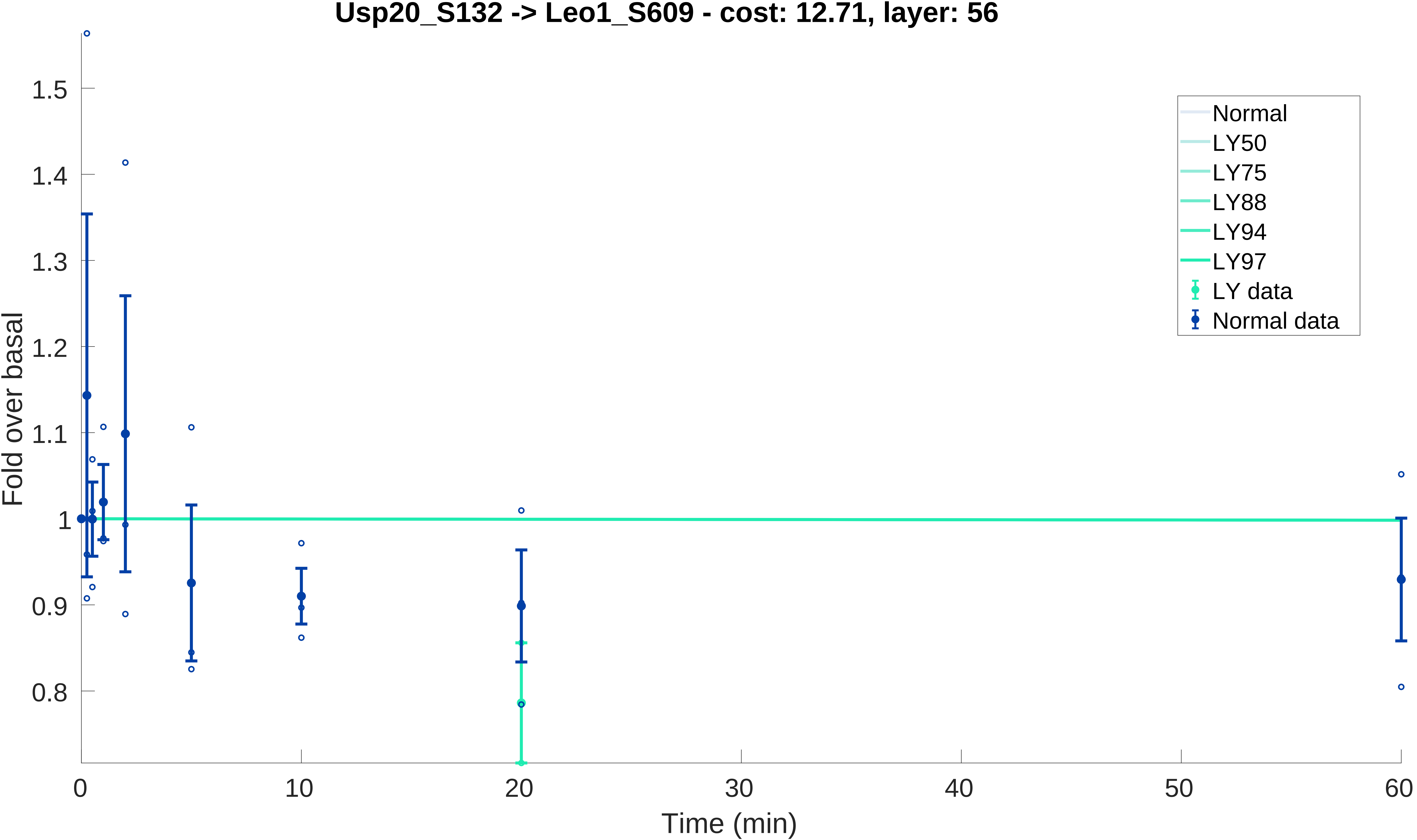

Usp20_S132 -> Leo1_S609 - cost: 12.71, layer: 56
Fold over basal
1.5
1.4
1.3
1.2
1.1
1
0.9
0.8
0
10
20
30
40
50
60
Time (min)
Normal
LY50
LY75
LY88
LY94
LY97
LY data
Normal data

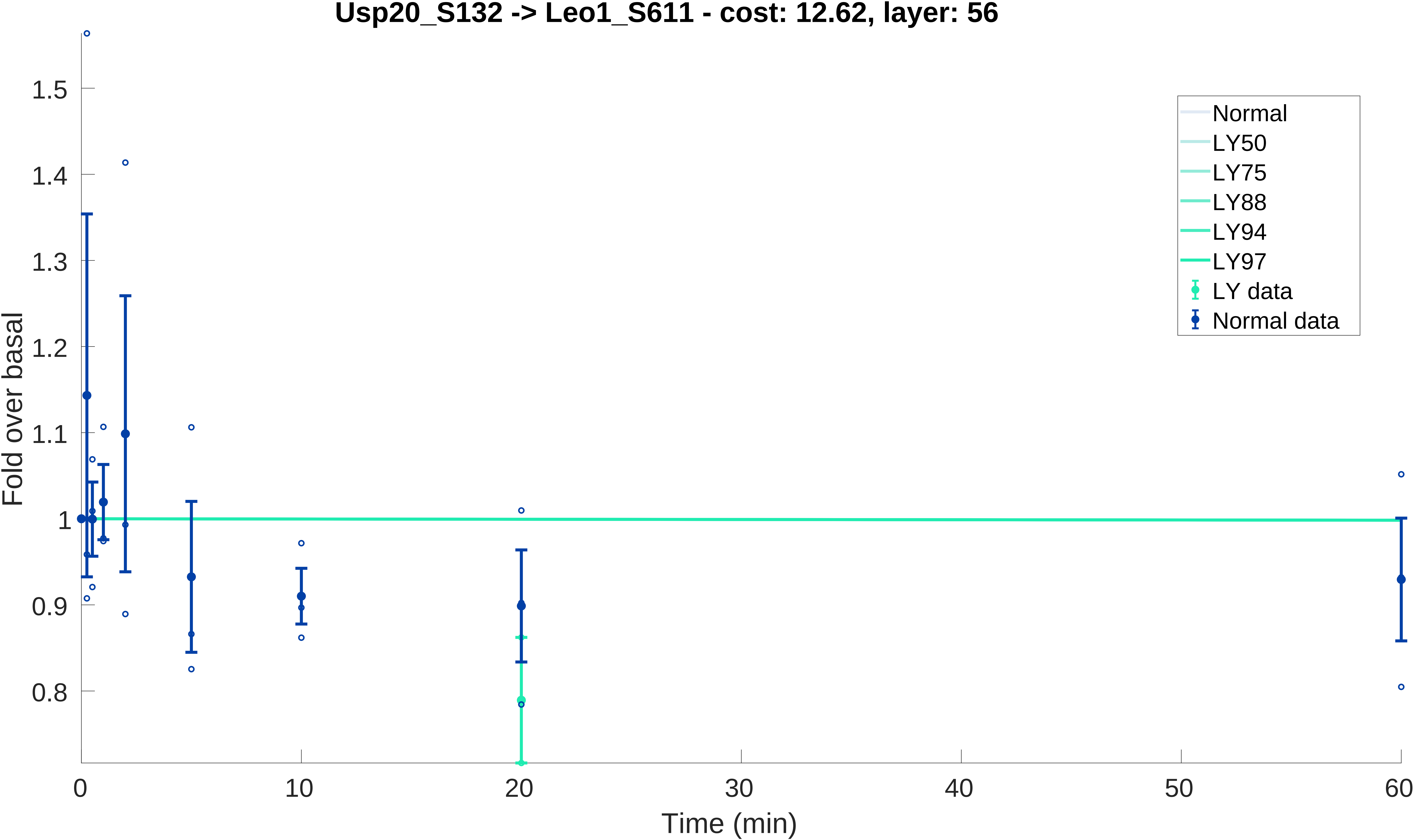
Usp20_S132 -> Leo1_S611 - cost: 12.62, layer: 56
Fold over basal
1.5
1.4
1.3
1.2
1.1
1
0.9
0.8
0
10
20
30
40
50
60
Time (min)
Normal
LY50
LY75
LY88
LY94
LY97
LY data
Normal data

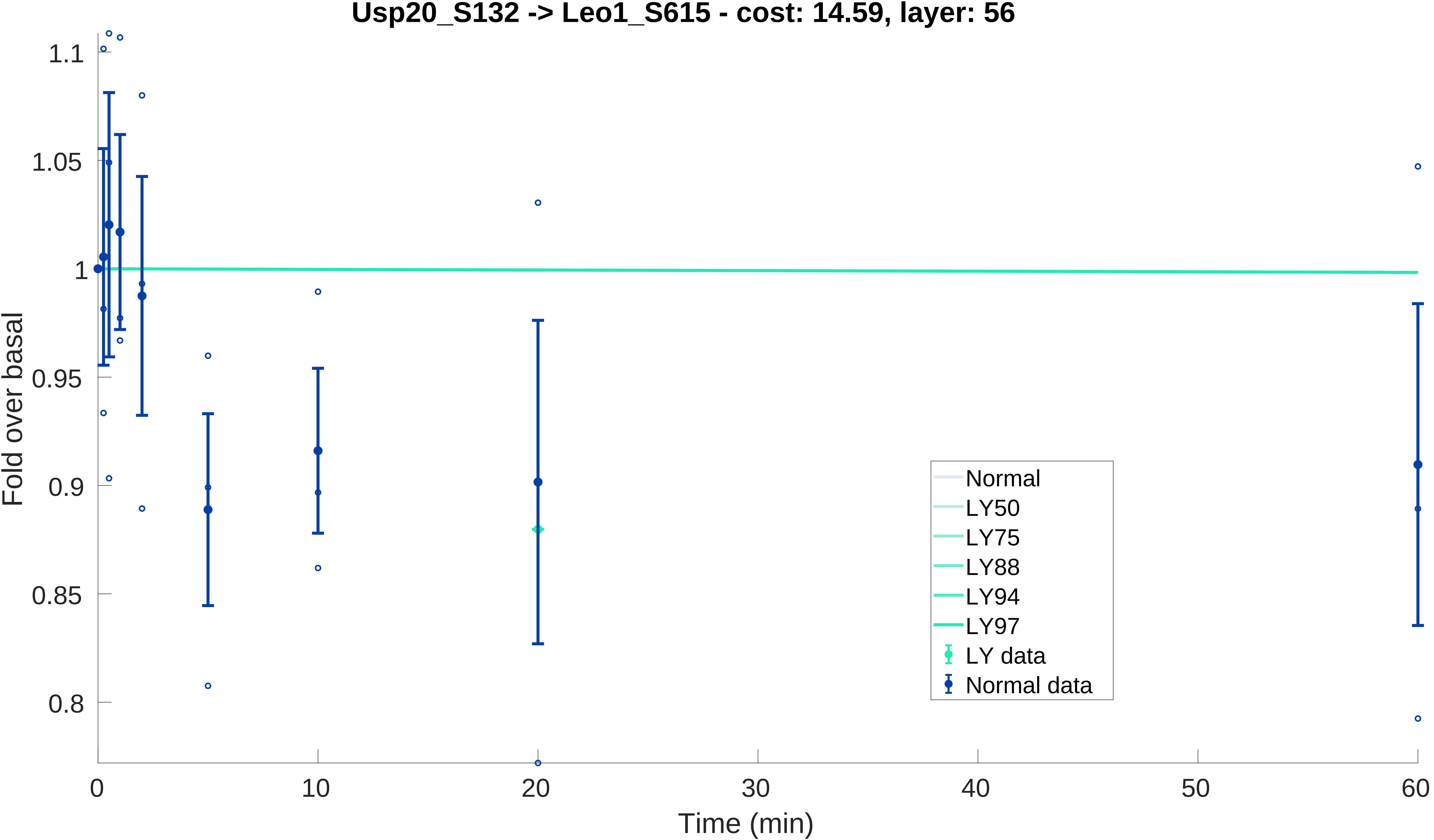

Usp20_S132 -> Leo1_S615 - cost: 14.59, layer: 56
Fold over basal
1.1
1.05
1
0.95
0.9
0.85
0.8
0
10
20
30
40
50
60
Time (min)
Normal
LY50
LY75
LY88
LY94
LY97
LY data
Normal data

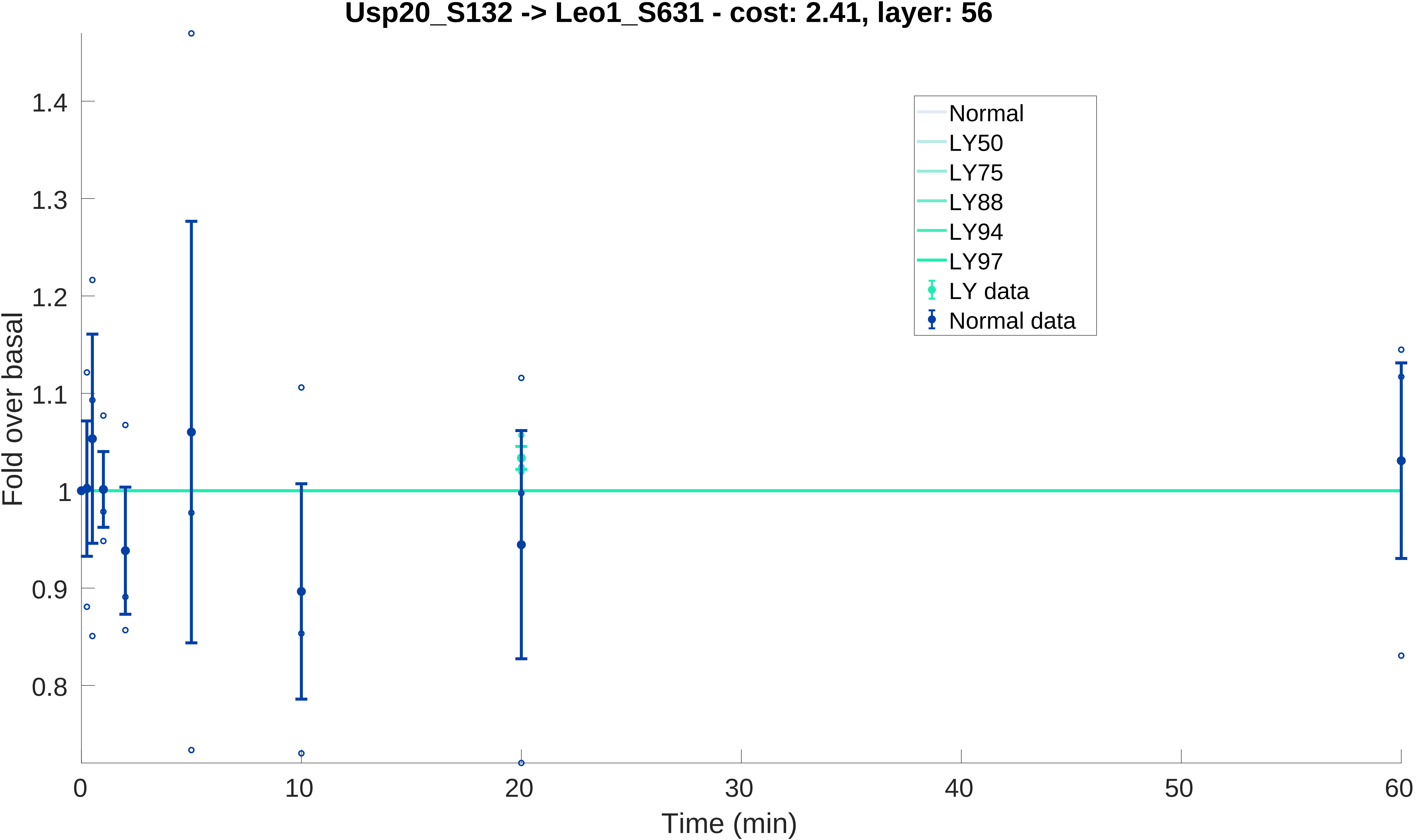
Usp20_S132 -> Leo1_S631 - cost: 2.41, layer: 56
Normal
LY50
LY75
LY88
LY94
LY97
LY data
Normal data
Fold over basal
Time (min)
1.4
1.3
1.2
1.1
1
0.9
0.8
0
10
20
30
40
50
60

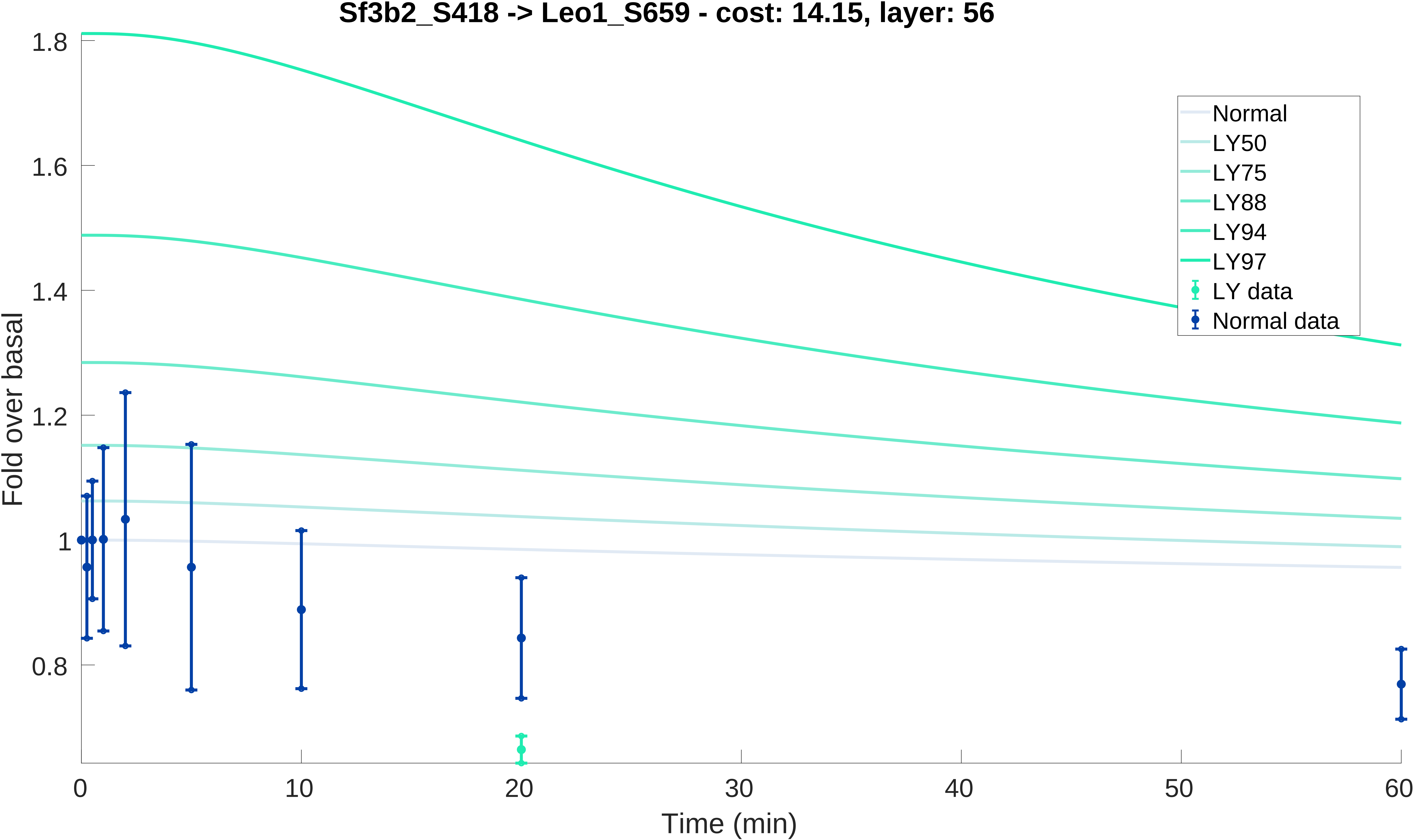

Sf3b2_S418 -> Leo1_S659 - cost: 14.15, layer: 56
Fold over basal
Time (min)
1.8
1.6
1.4
1.2
1
0.8
0
10
20
30
40
50
60
Normal
LY50
LY75
LY88
LY94
LY97
LY data
Normal data

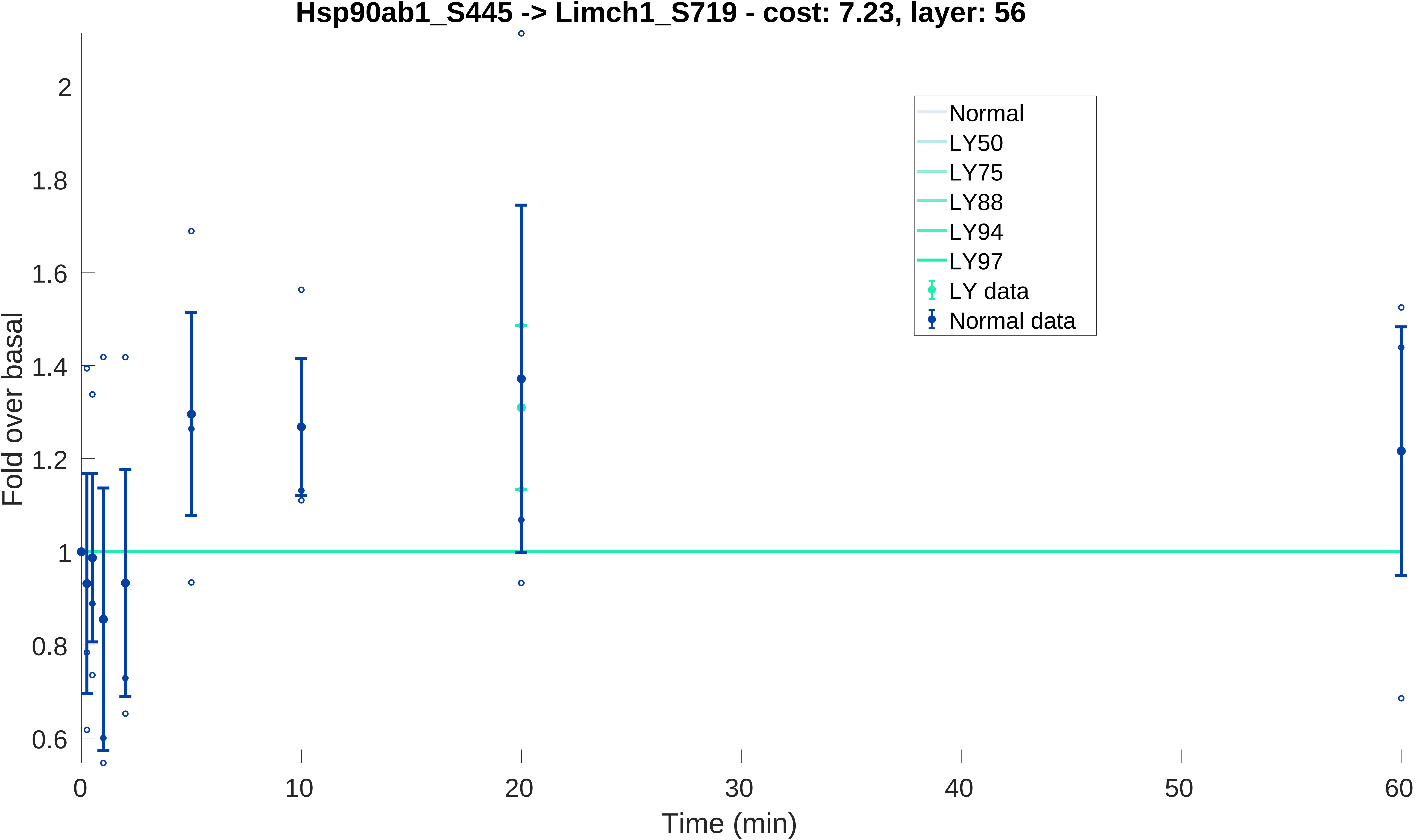

Hsp90ab1_S445 -> Limch1_S719 - cost: 7.23, layer: 56
Fold over basal
Time (min)
0
10
20
30
40
50
60
0.6
0.8
1
1.2
1.4
1.6
1.8
2
Normal
LY50
LY75
LY88
LY94
LY97
LY data
Normal data

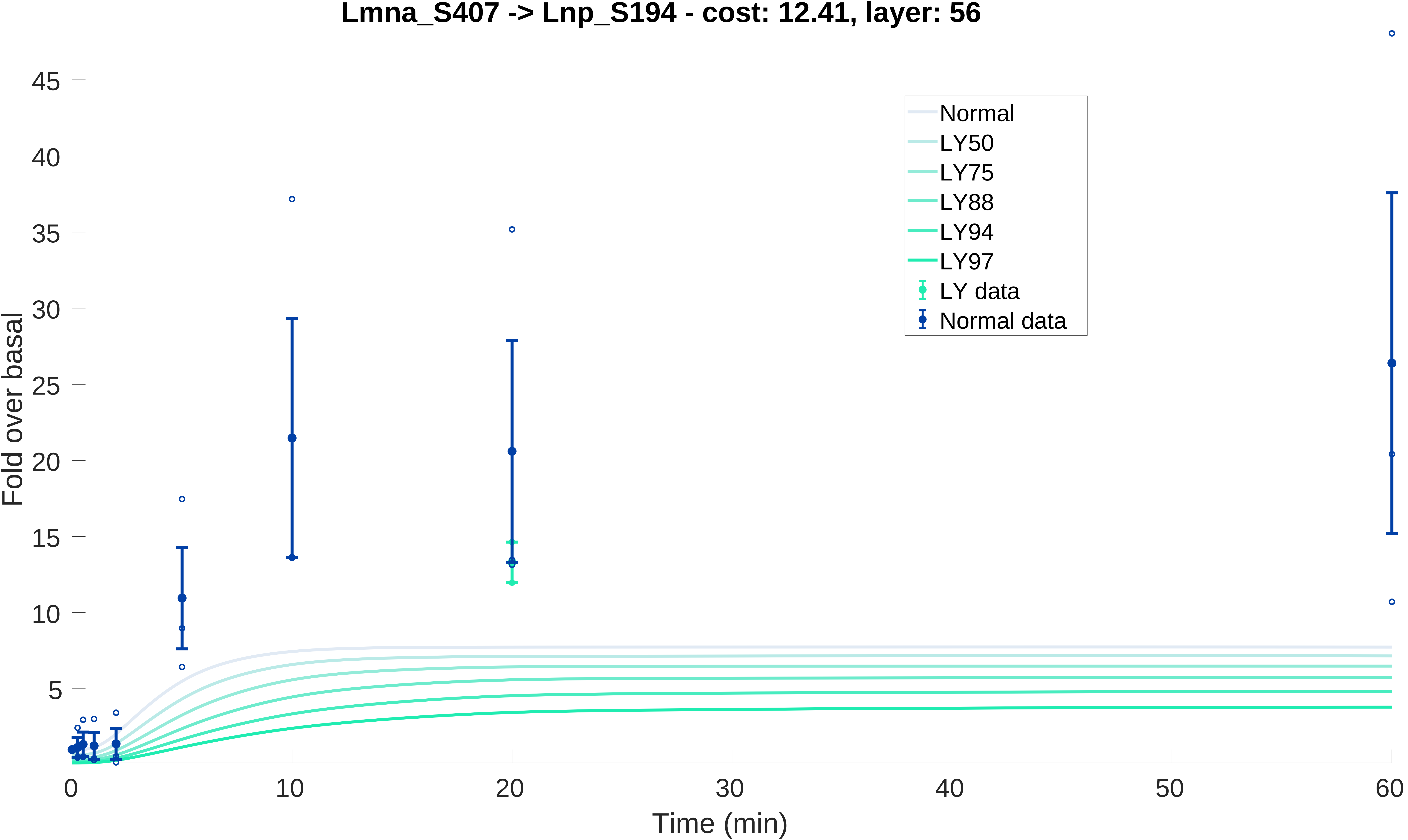
Lmna_S407 -> Lnp_S194 - cost: 12.41, layer: 56
Fold over basal
Time (min)
0
10
20
30
40
50
60
5
10
15
20
25
30
35
40
45
Normal
LY50
LY75
LY88
LY94
LY97
LY data
Normal data

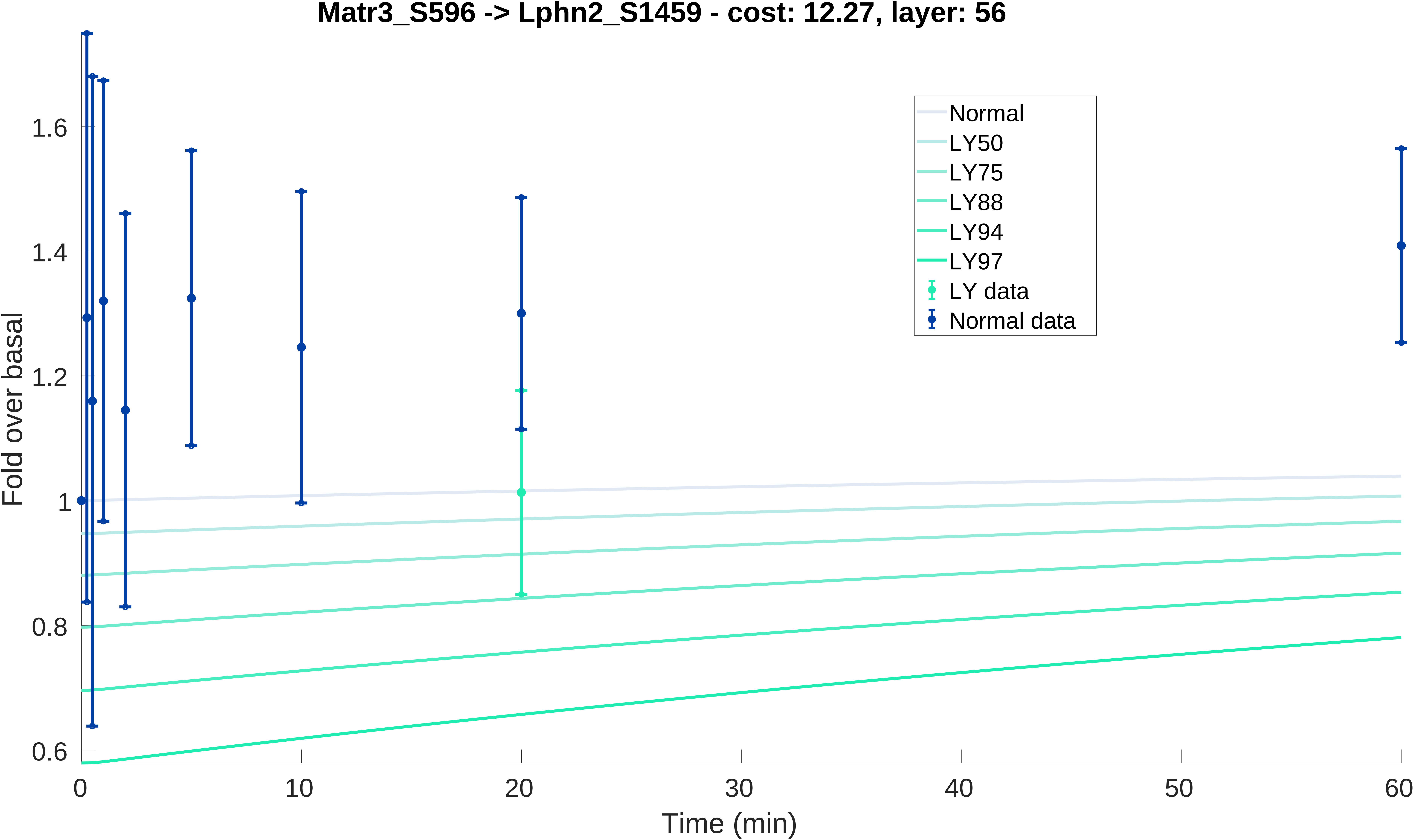

Matr3_S596 -> Lphn2_S1459 - cost: 12.27, layer: 56
Fold over basal
Time (min)
0.6
0.8
1
1.2
1.4
1.6
0
10
20
30
40
50
60
Normal
LY50
LY75
LY88
LY94
LY97
LY data
Normal data

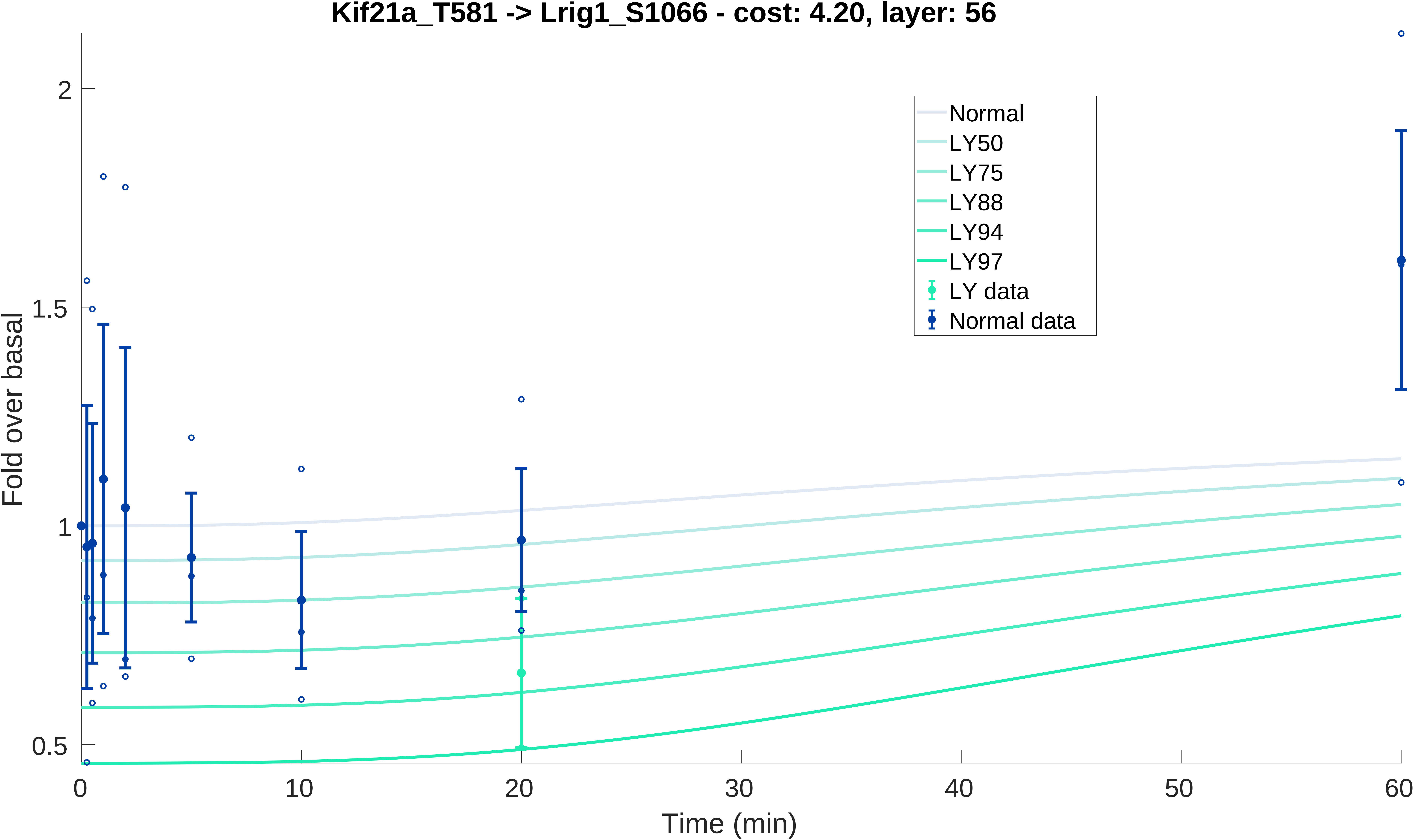

Kif21a_T581 -> Lrig1_S1066 - cost: 4.20, layer: 56
Fold over basal
Time (min)
0.5
1
1.5
2
0
10
20
30
40
50
60
Normal
LY50
LY75
LY88
LY94
LY97
LY data
Normal data

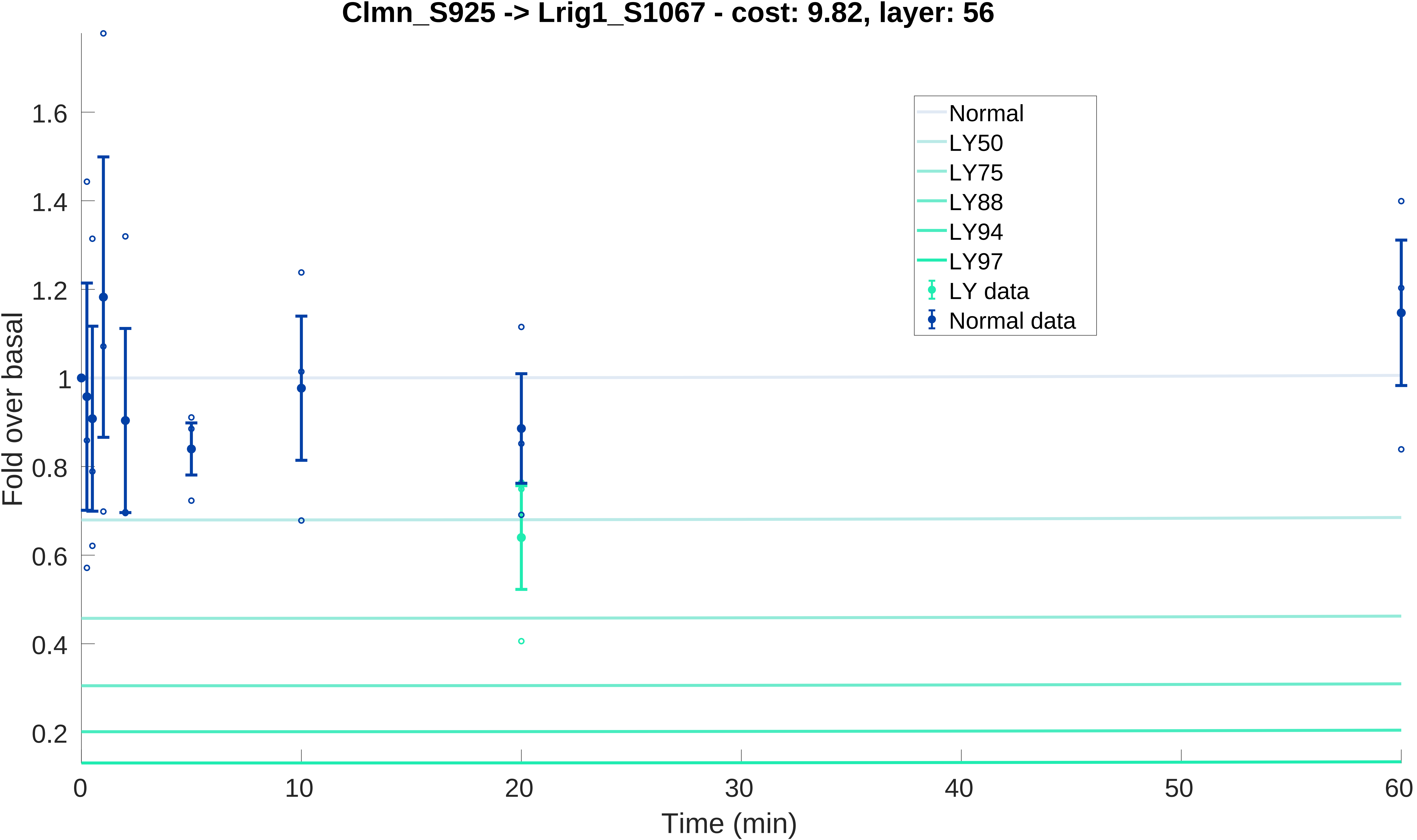
Clmn_S925 -> Lrig1_S1067 - cost: 9.82, layer: 56
Fold over basal
Time (min)
1.6
1.4
1.2
1
0.8
0.6
0.4
0.2
0
10
20
30
40
50
60
Normal
LY50
LY75
LY88
LY94
LY97
LY data
Normal data

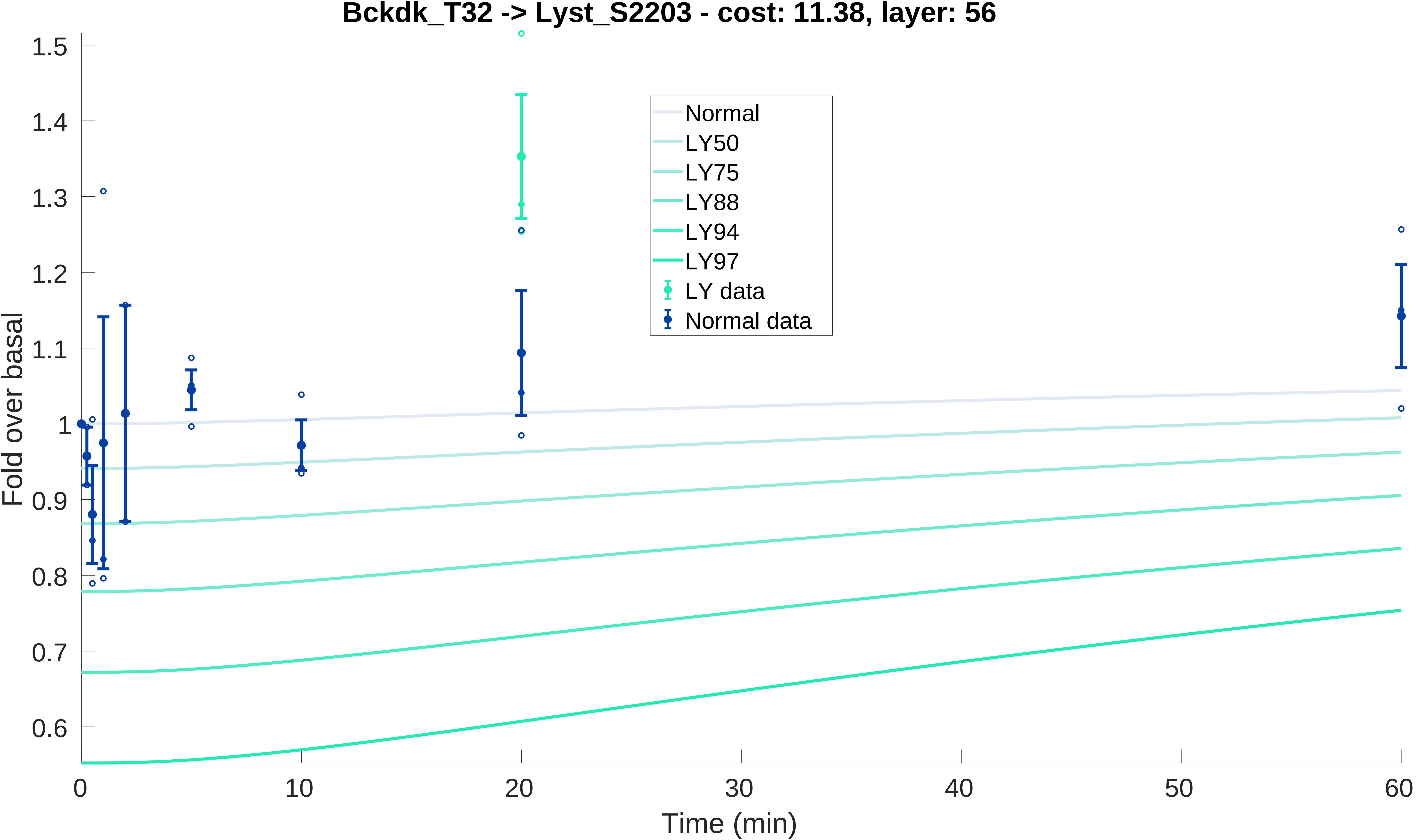

Bckdk_T32 -> Lyst_S2203 - cost: 11.38, layer: 56
Normal
LY50
LY75
LY88
LY94
LY97
LY data
Normal data
Fold over basal
Time (min)
1.5
1.4
1.3
1.2
1.1
1
0.9
0.8
0.7
0.6
0
10
20
30
40
50
60

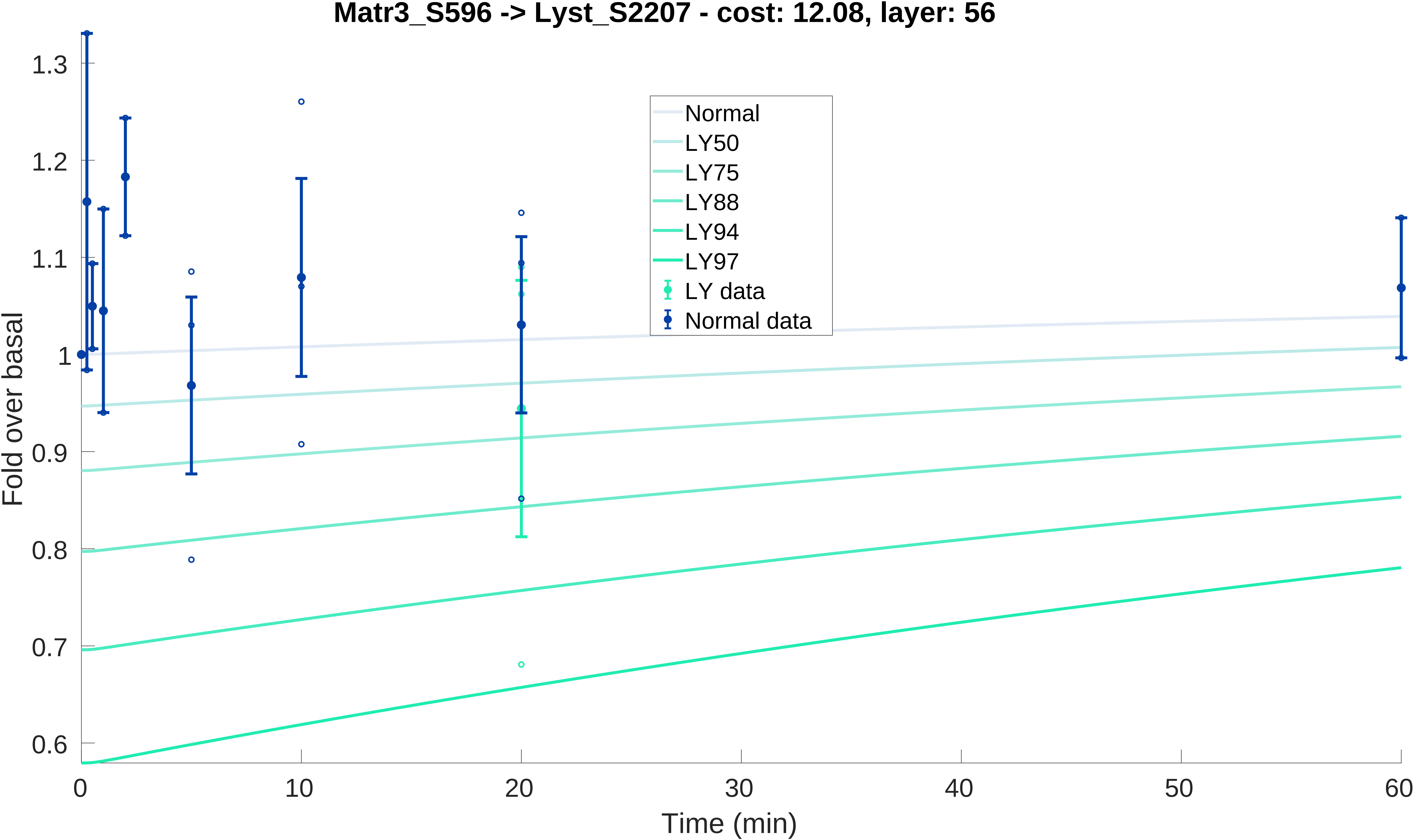
Matr3_S596 -> Lyst_S2207 - cost: 12.08, layer: 56
Fold over basal
Time (min)
Normal
LY50
LY75
LY88
LY94
LY97
LY data
Normal data
1.3
1.2
1.1
1
0.9
0.8
0.7
0.6
0
10
20
30
40
50
60

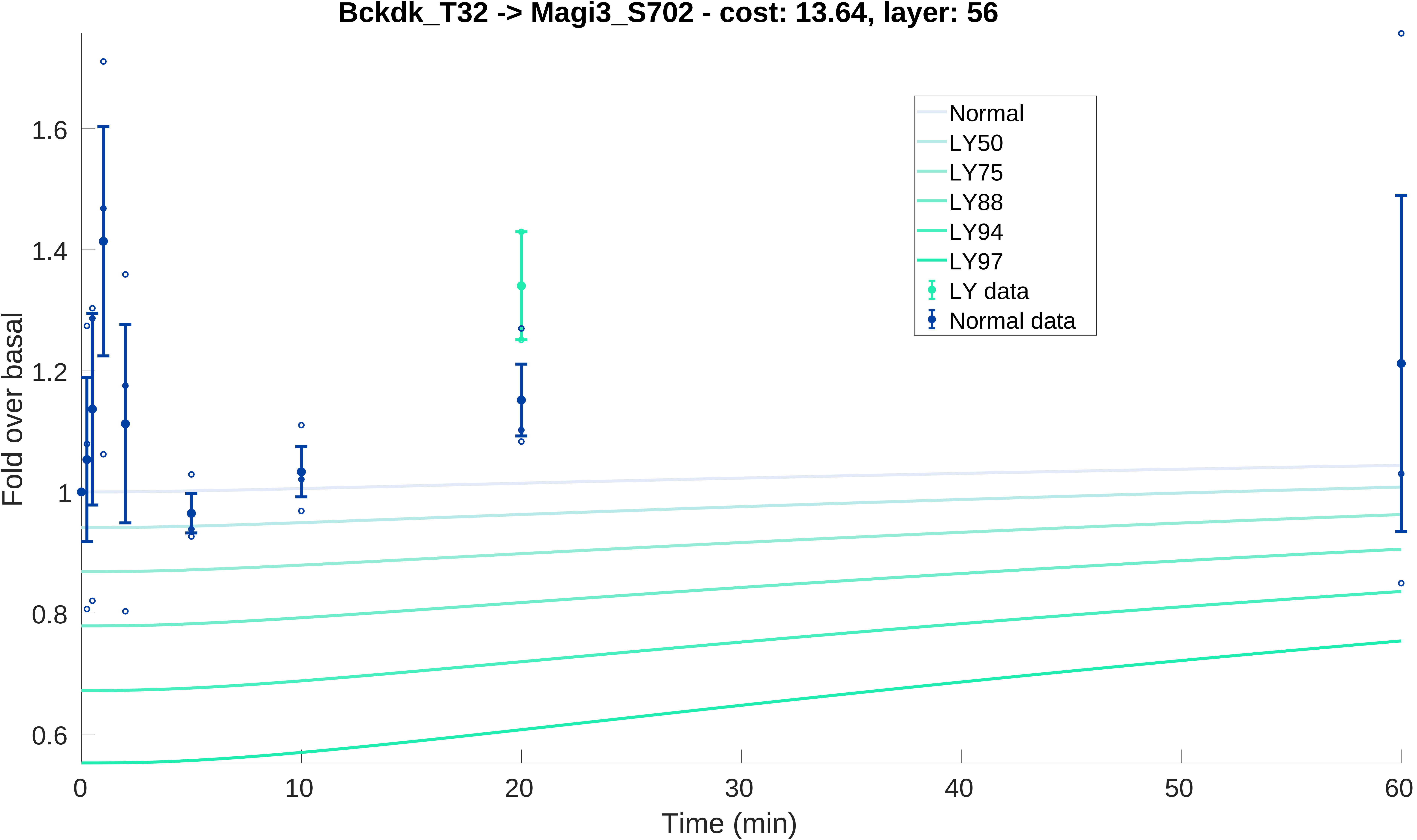

Bckdk_T32 -> Magi3_S702 - cost: 13.64, layer: 56
Normal
LY50
LY75
LY88
LY94
LY97
LY data
Normal data
Fold over basal
1.6
1.4
1.2
1
0.8
0.6
0
10
20
30
40
50
60
Time (min)

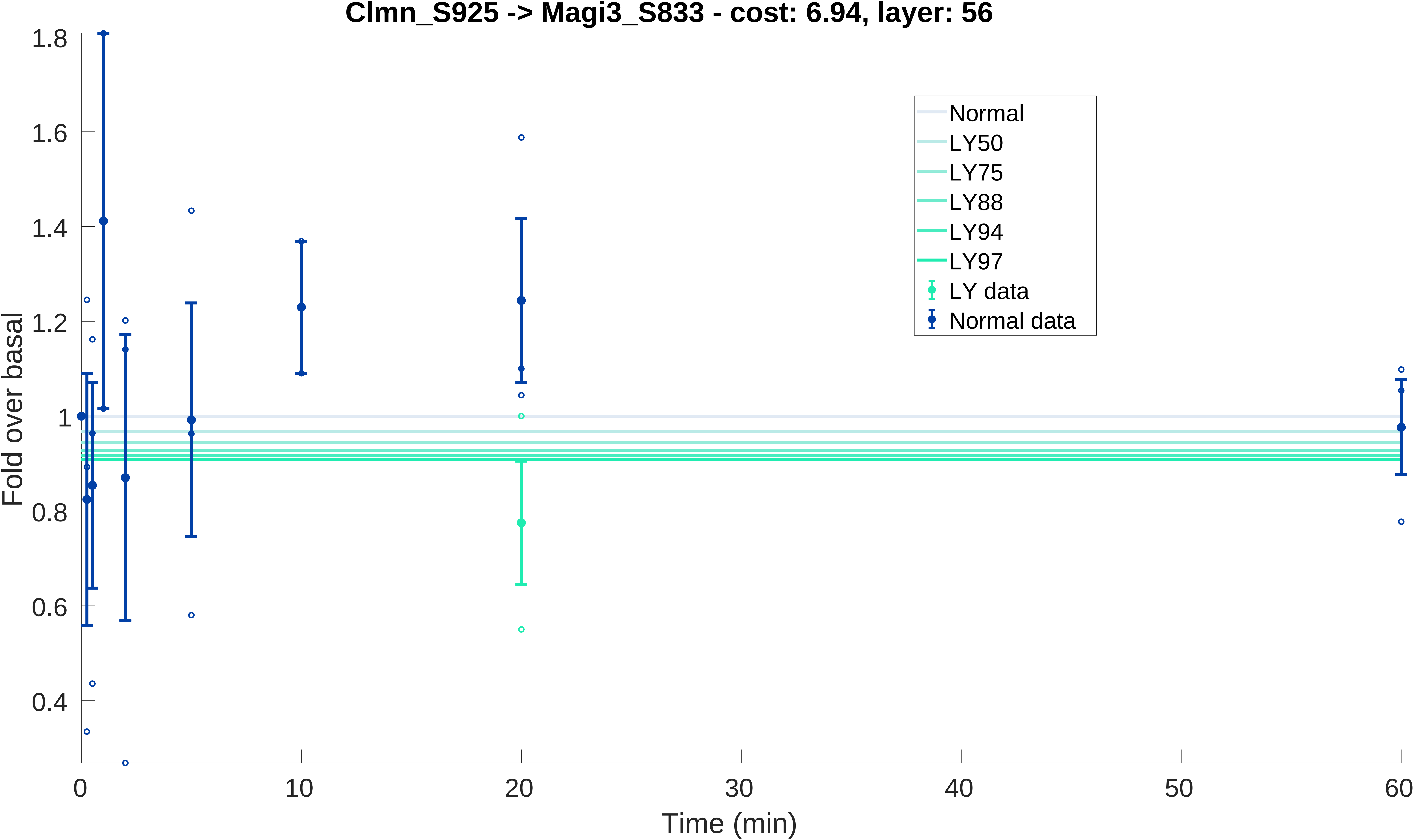
Clmn_S925 -> Magi3_S833 - cost: 6.94, layer: 56
Fold over basal
Time (min)
0
10
20
30
40
50
60
0.4
0.6
0.8
1
1.2
1.4
1.6
1.8
Normal
LY50
LY75
LY88
LY94
LY97
LY data
Normal data

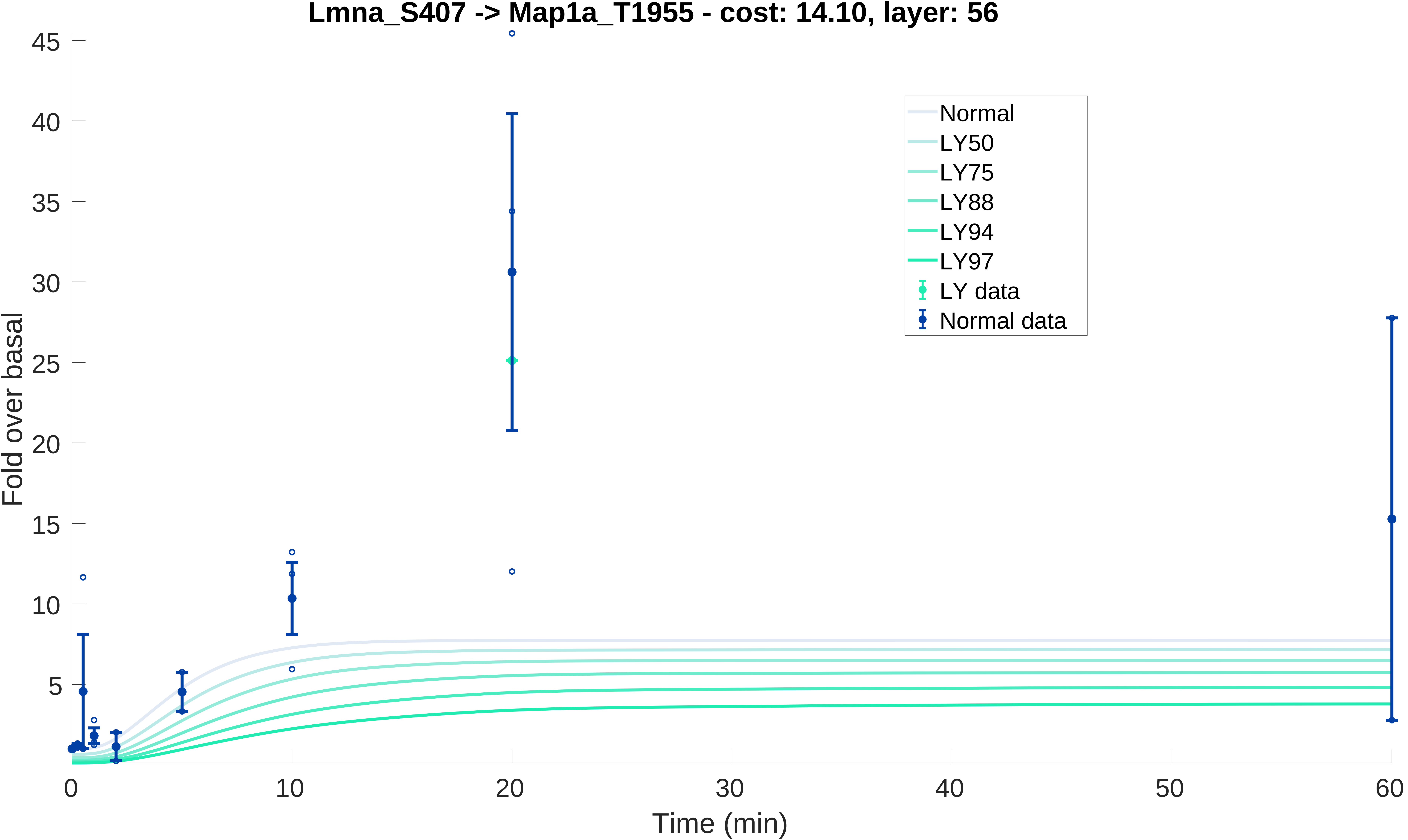

Lmna_S407 -> Map1a_T1955 - cost: 14.10, layer: 56
Fold over basal
Time (min)
0
10
20
30
40
50
60
5
10
15
20
25
30
35
40
45
Normal
LY50
LY75
LY88
LY94
LY97
LY data
Normal data

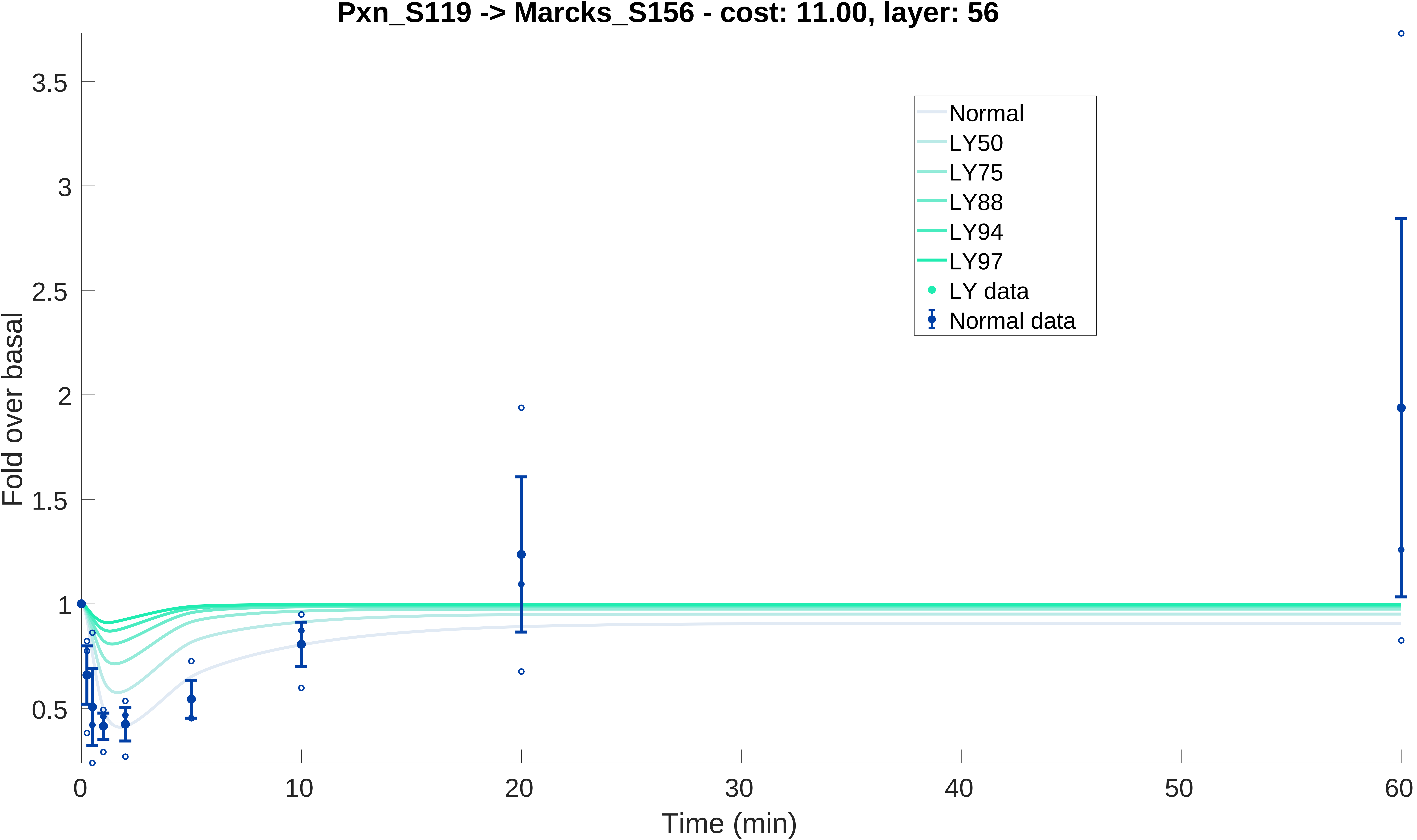

Pxn_S119 -> Marcks_S156 - cost: 11.00, layer: 56
Fold over basal
Time (min)
0.5
1
1.5
2
2.5
3
3.5
0
10
20
30
40
50
60
Normal
LY50
LY75
LY88
LY94
LY97
LY data
Normal data

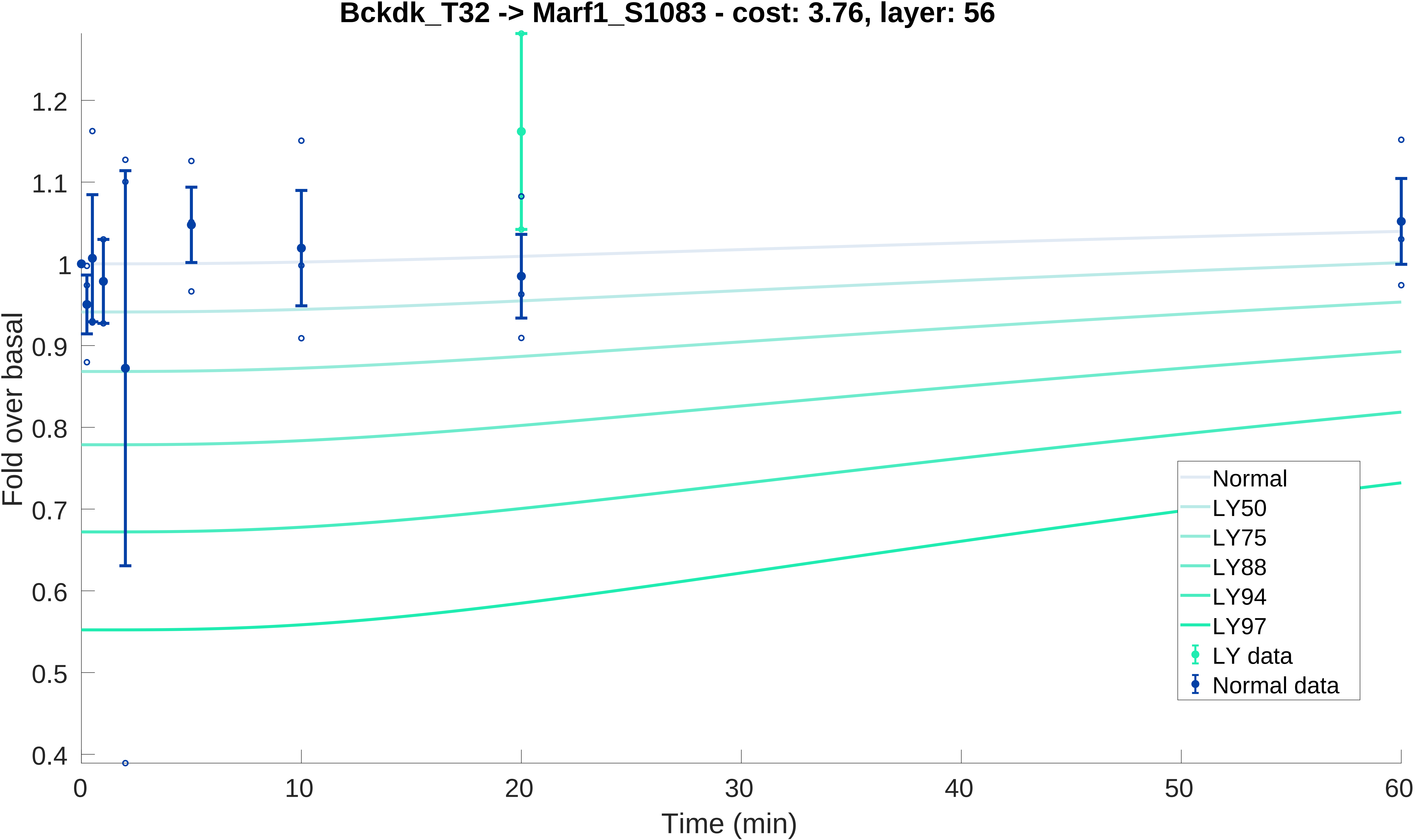
Bckdk_T32 -> Marf1_S1083 - cost: 3.76, layer: 56
Fold over basal
1.2
1.1
1
0.9
0.8
0.7
0.6
0.5
0.4
0
10
20
30
40
50
60
Time (min)
Normal
LY50
LY75
LY88
LY94
LY97
LY data
Normal data

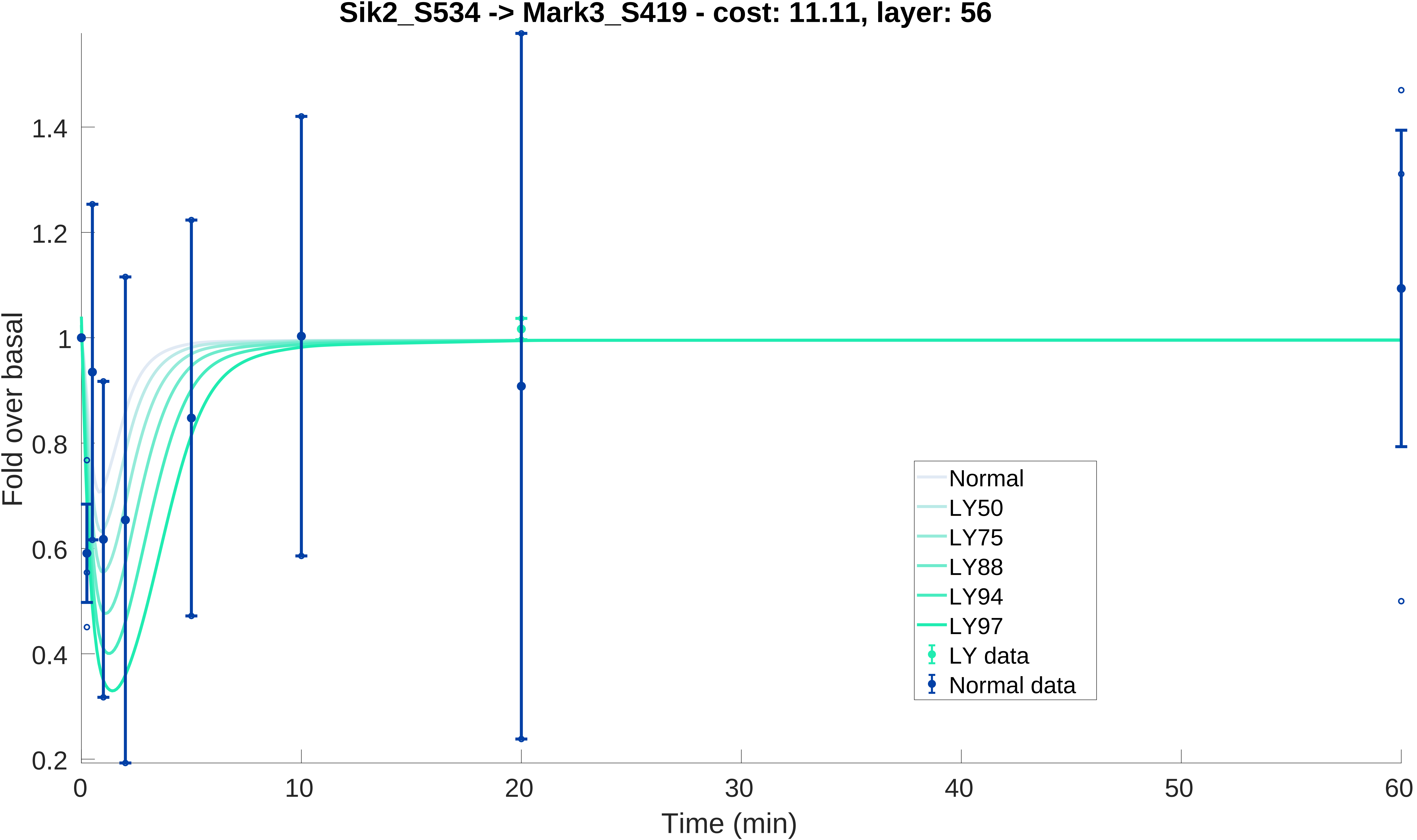

Sik2_S534 -> Mark3_S419 - cost: 11.11, layer: 56
Fold over basal
Time (min)
0.2
0.4
0.6
0.8
1
1.2
1.4
0
10
20
30
40
50
60
Normal
LY50
LY75
LY88
LY94
LY97
LY data
Normal data

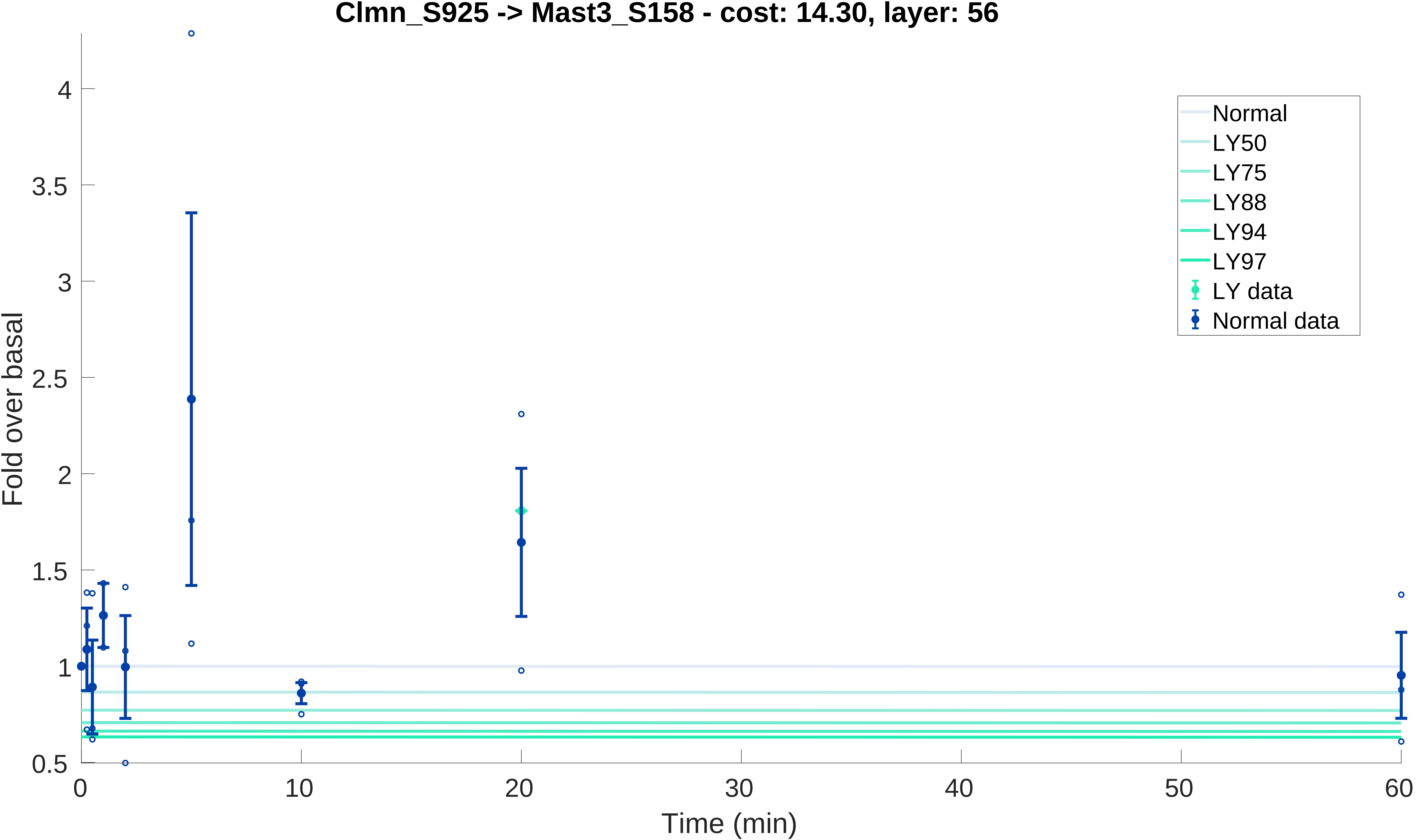

Clmn_S925 -> Mast3_S158 - cost: 14.30, layer: 56
Fold over basal
Time (min)
0.5
1
1.5
2
2.5
3
3.5
4
0
10
20
30
40
50
60
Normal
LY50
LY75
LY88
LY94
LY97
LY data
Normal data

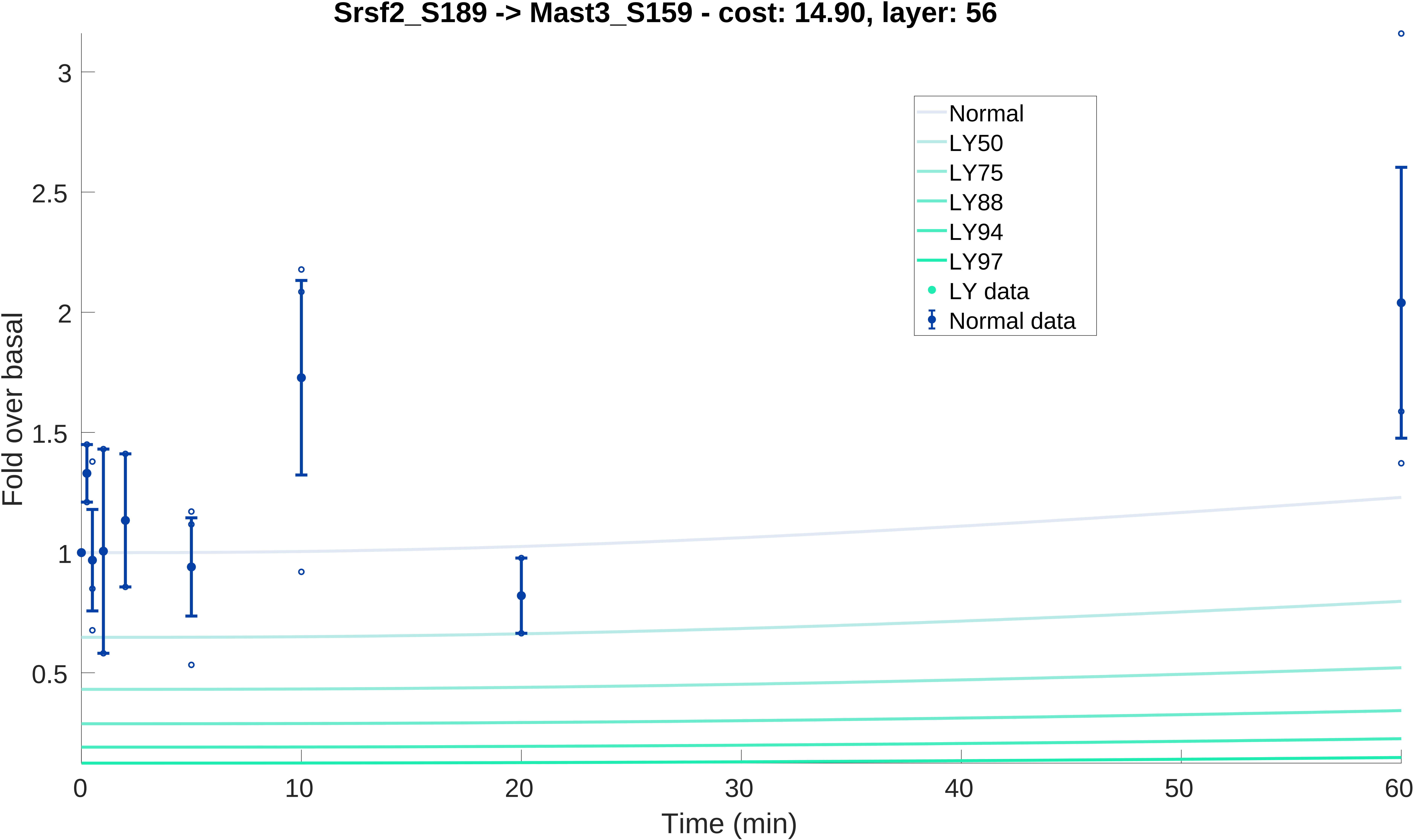

Srsf2_S189 -> Mast3_S159 - cost: 14.90, layer: 56
Fold over basal
Time (min)
0
10
20
30
40
50
60
0.5
1
1.5
2
2.5
3
Normal
LY50
LY75
LY88
LY94
LY97
LY data
Normal data

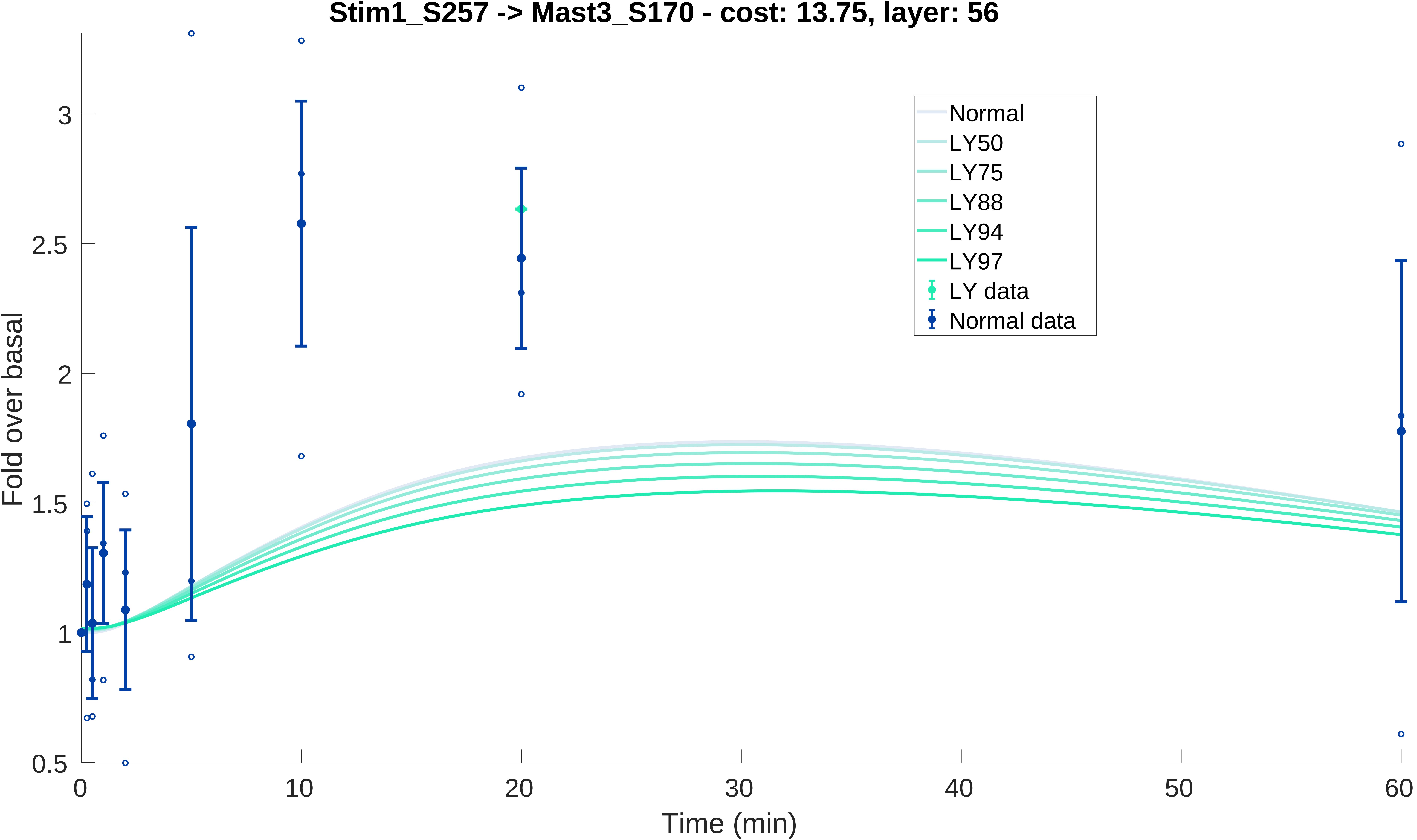

Stim1_S257 -> Mast3_S170 - cost: 13.75, layer: 56
Fold over basal
Time (min)
0.5
1
1.5
2
2.5
3
0
10
20
30
40
50
60
Normal
LY50
LY75
LY88
LY94
LY97
LY data
Normal data

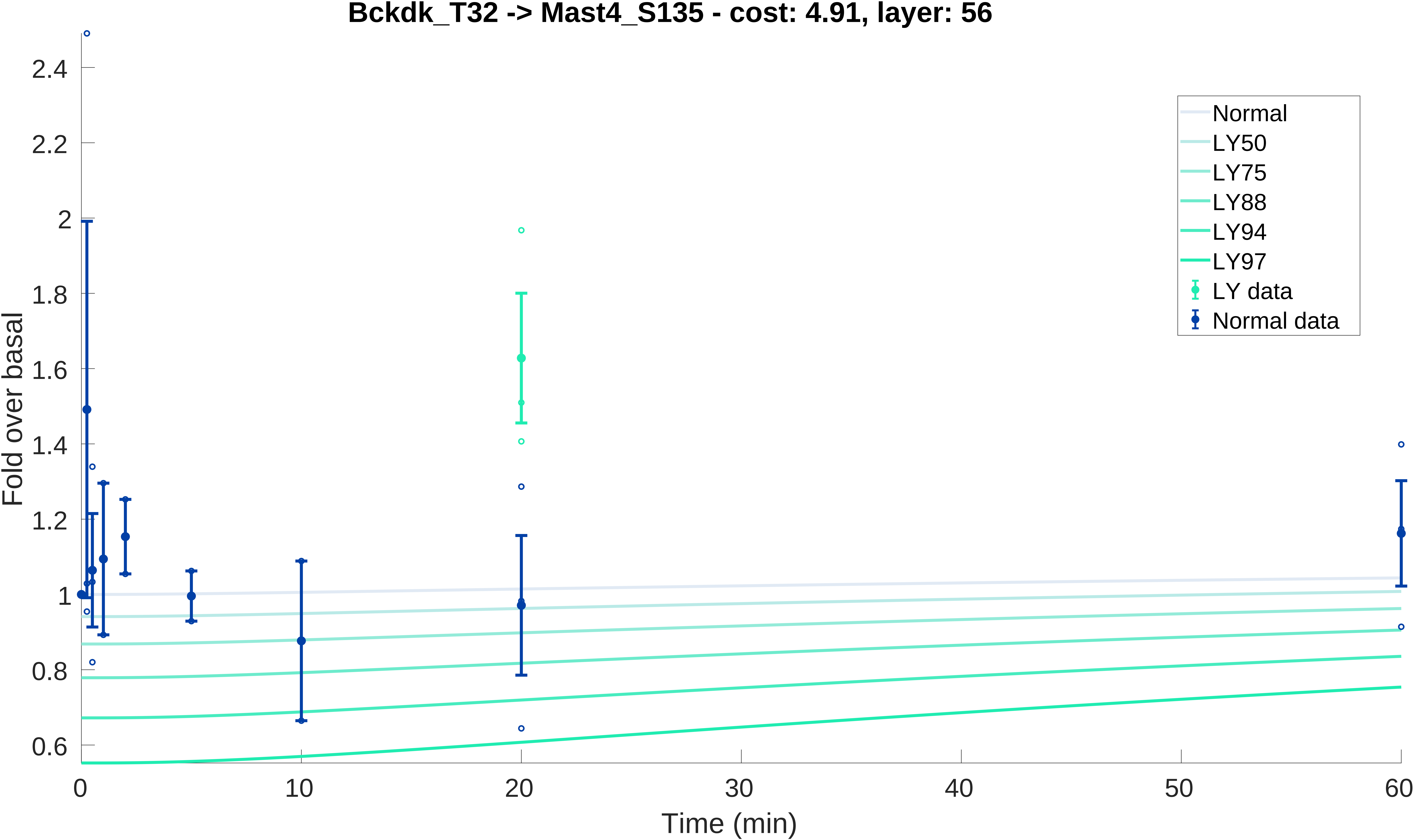
Bckdk_T32 -> Mast4_S135 - cost: 4.91, layer: 56
Fold over basal
Time (min)
2.4
2.2
2
1.8
1.6
1.4
1.2
1
0.8
0.6
0
10
20
30
40
50
60
Normal
LY50
LY75
LY88
LY94
LY97
LY data
Normal data

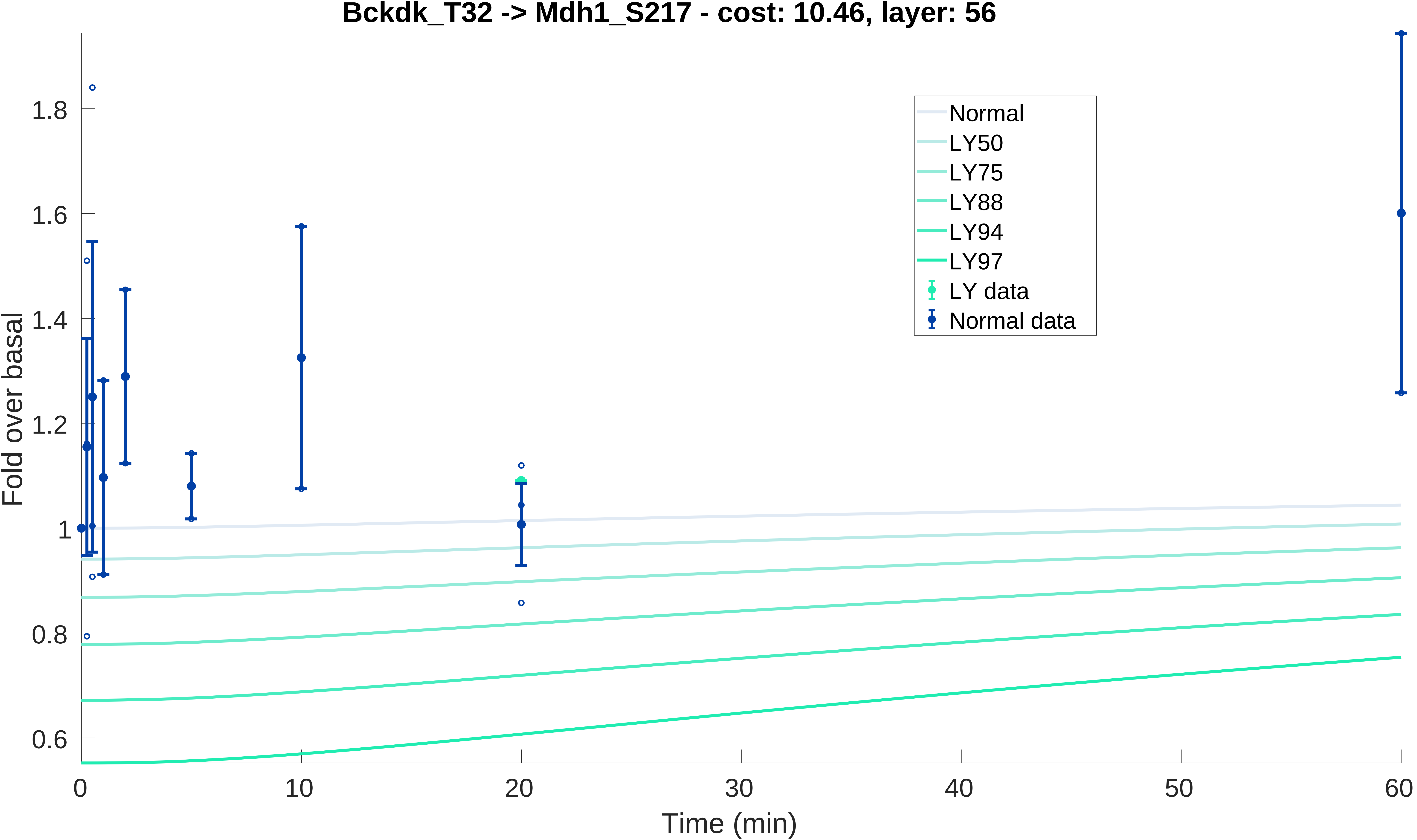

Bckdk_T32 -> Mdh1_S217 - cost: 10.46, layer: 56
Normal
LY50
LY75
LY88
LY94
LY97
LY data
Normal data
Fold over basal
1.8
1.6
1.4
1.2
1
0.8
0.6
0
10
20
30
40
50
60
Time (min)

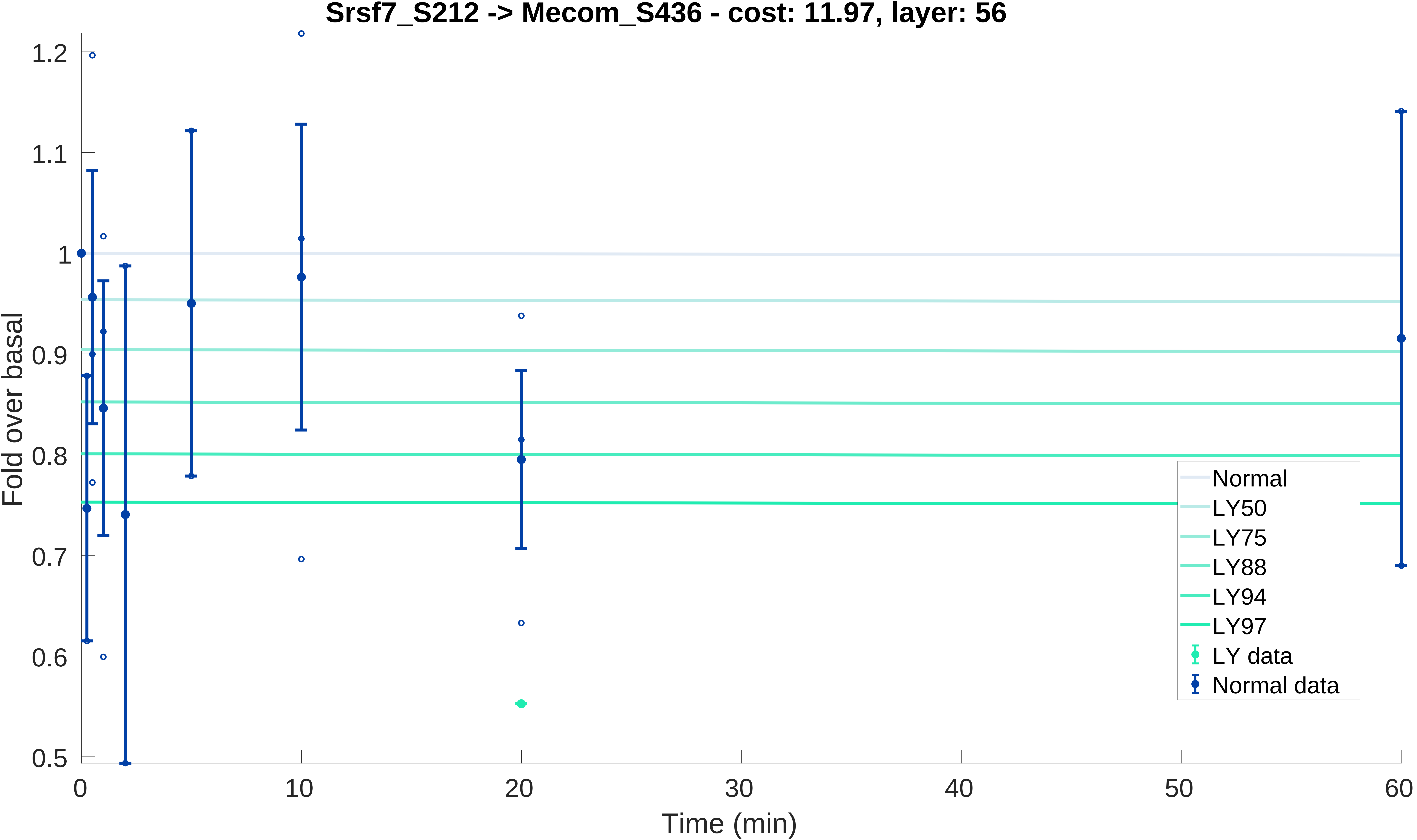
Srsf7_S212 -> Mecom_S436 - cost: 11.97, layer: 56
Fold over basal
Time (min)
0
10
20
30
40
50
60
0.5
0.6
0.7
0.8
0.9
1
1.1
1.2
Normal
LY50
LY75
LY88
LY94
LY97
LY data
Normal data

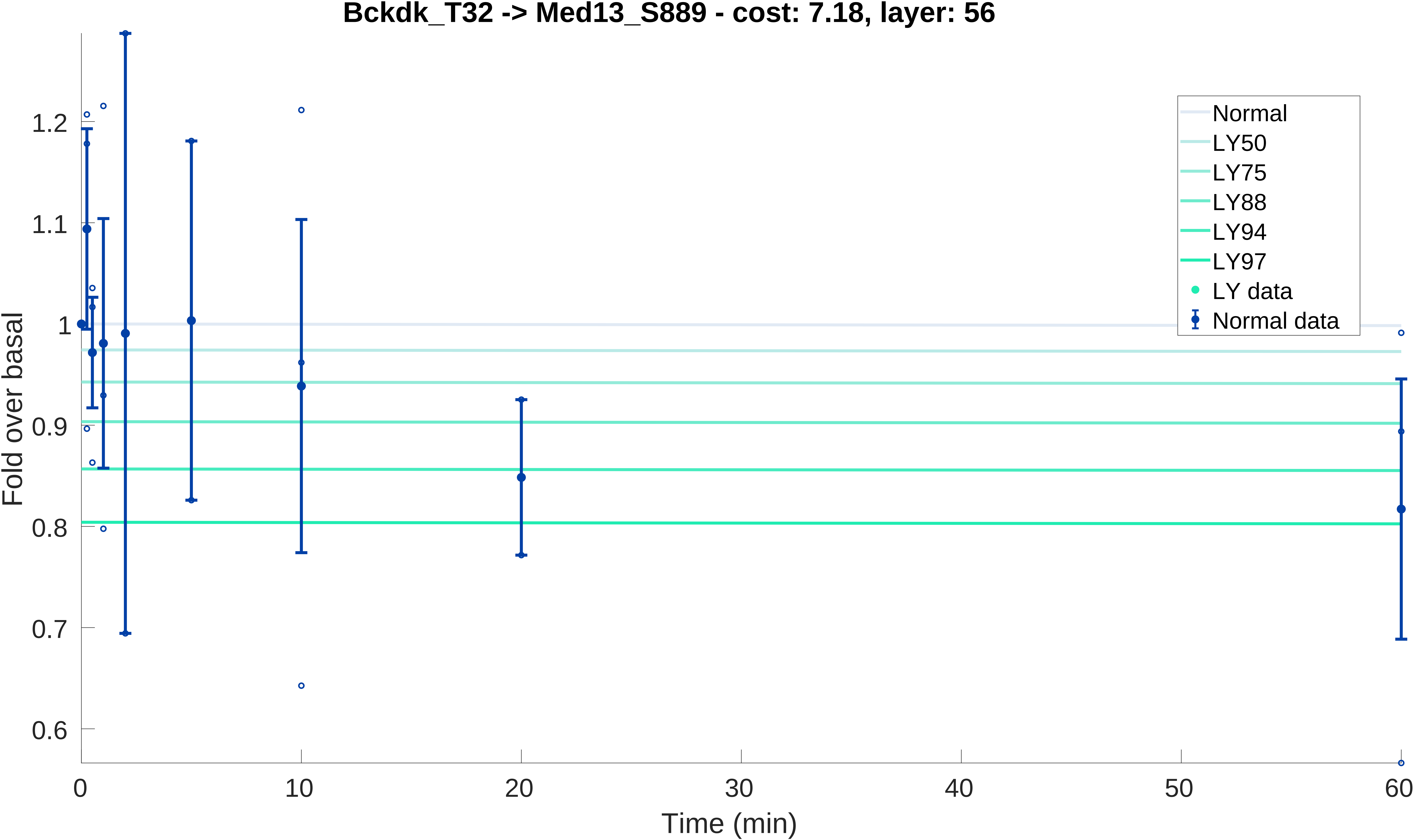
Bckdk_T32 -> Med13_S889 - cost: 7.18, layer: 56
Fold over basal
1.2
1.1
1
0.9
0.8
0.7
0.6
0
10
20
30
40
50
60
Time (min)
Normal
LY50
LY75
LY88
LY94
LY97
LY data
Normal data

Pts_S27 -> Med26_S232 - cost: 6.64, layer: 56

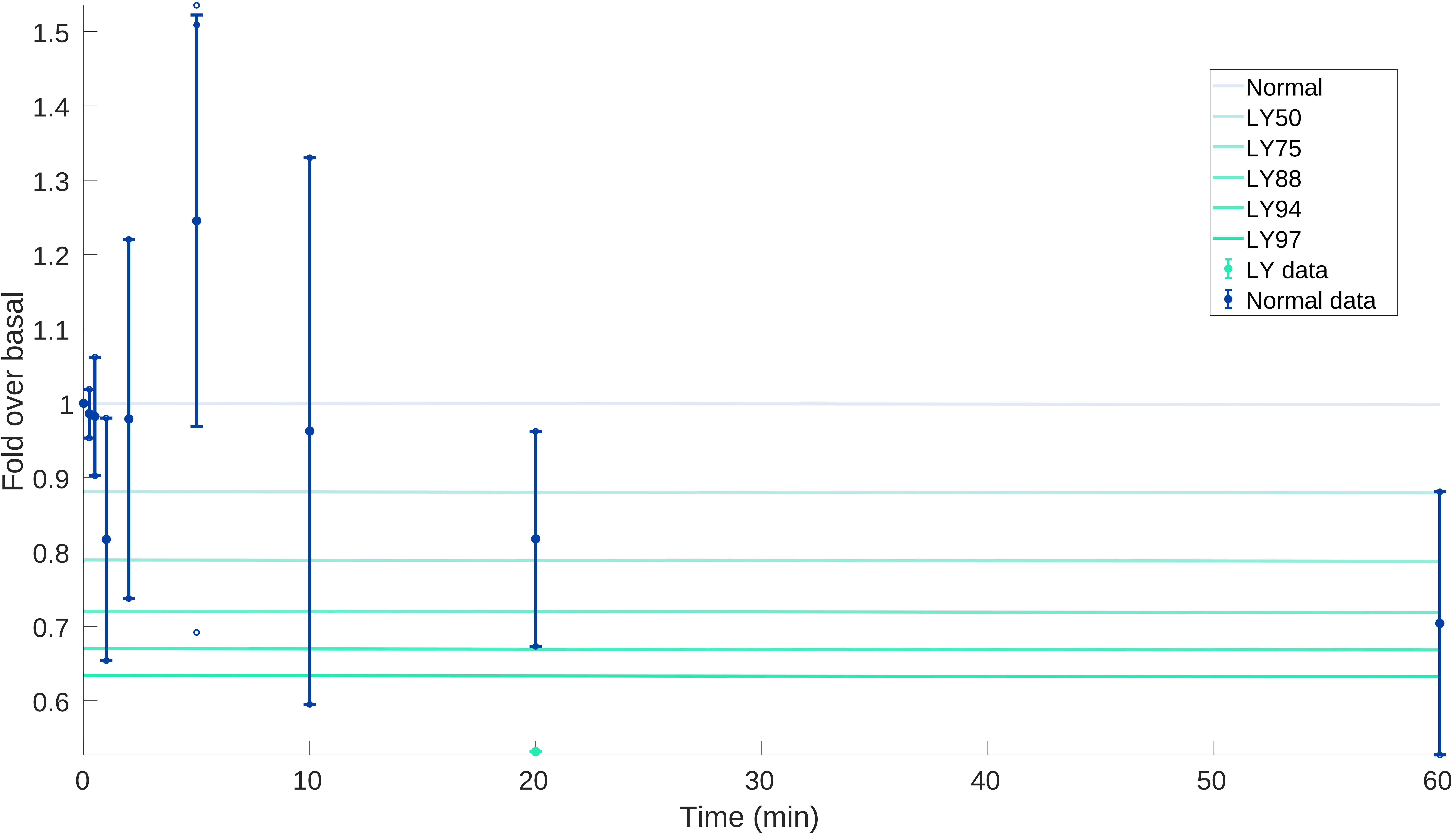

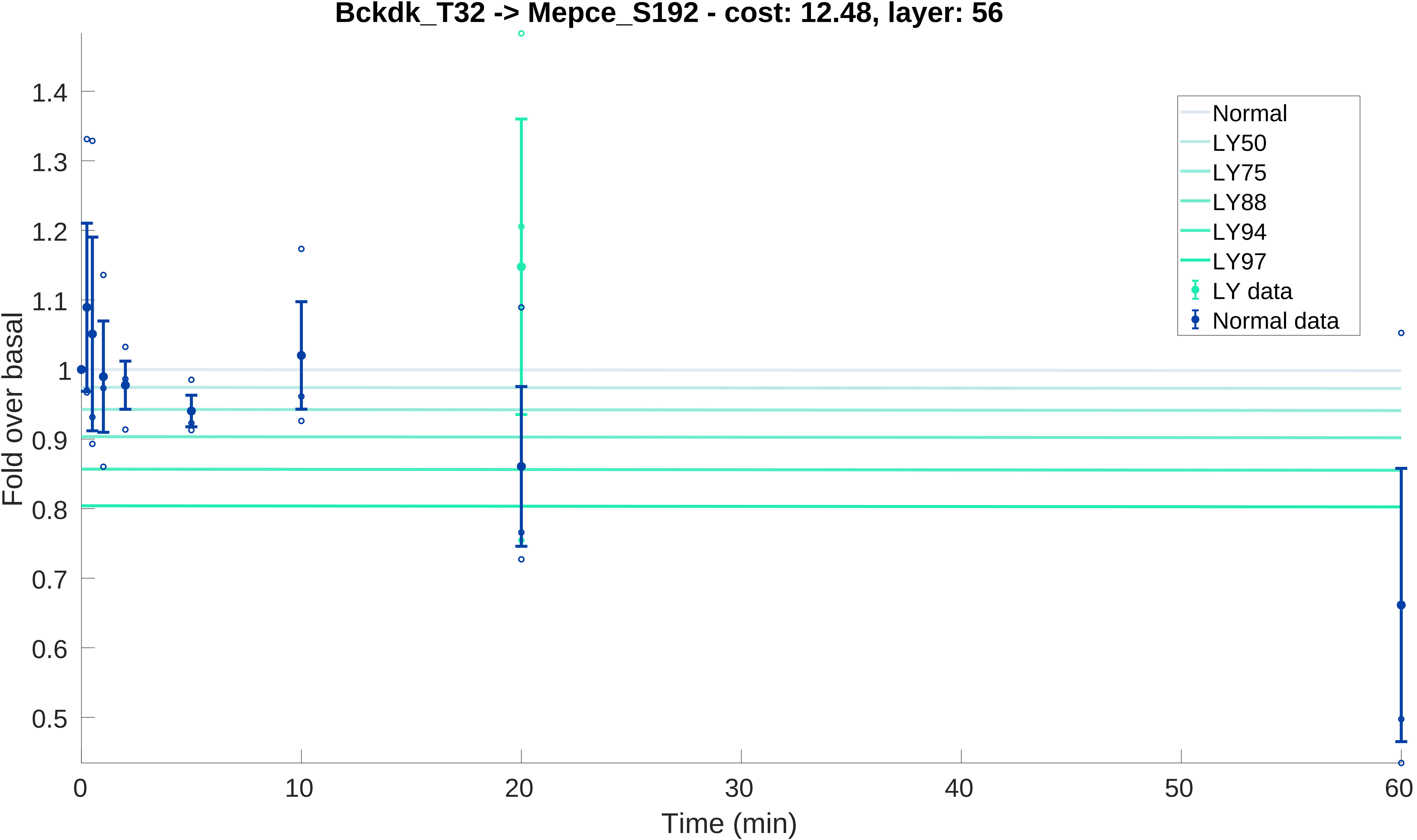

Bckdk_T32 -> Mepce_S192 - cost: 12.48, layer: 56
Fold over basal
Time (min)
1.4
1.3
1.2
1.1
1
0.9
0.8
0.7
0.6
0.5
0
10
20
30
40
50
60
Normal
LY50
LY75
LY88
LY94
LY97
LY data
Normal data

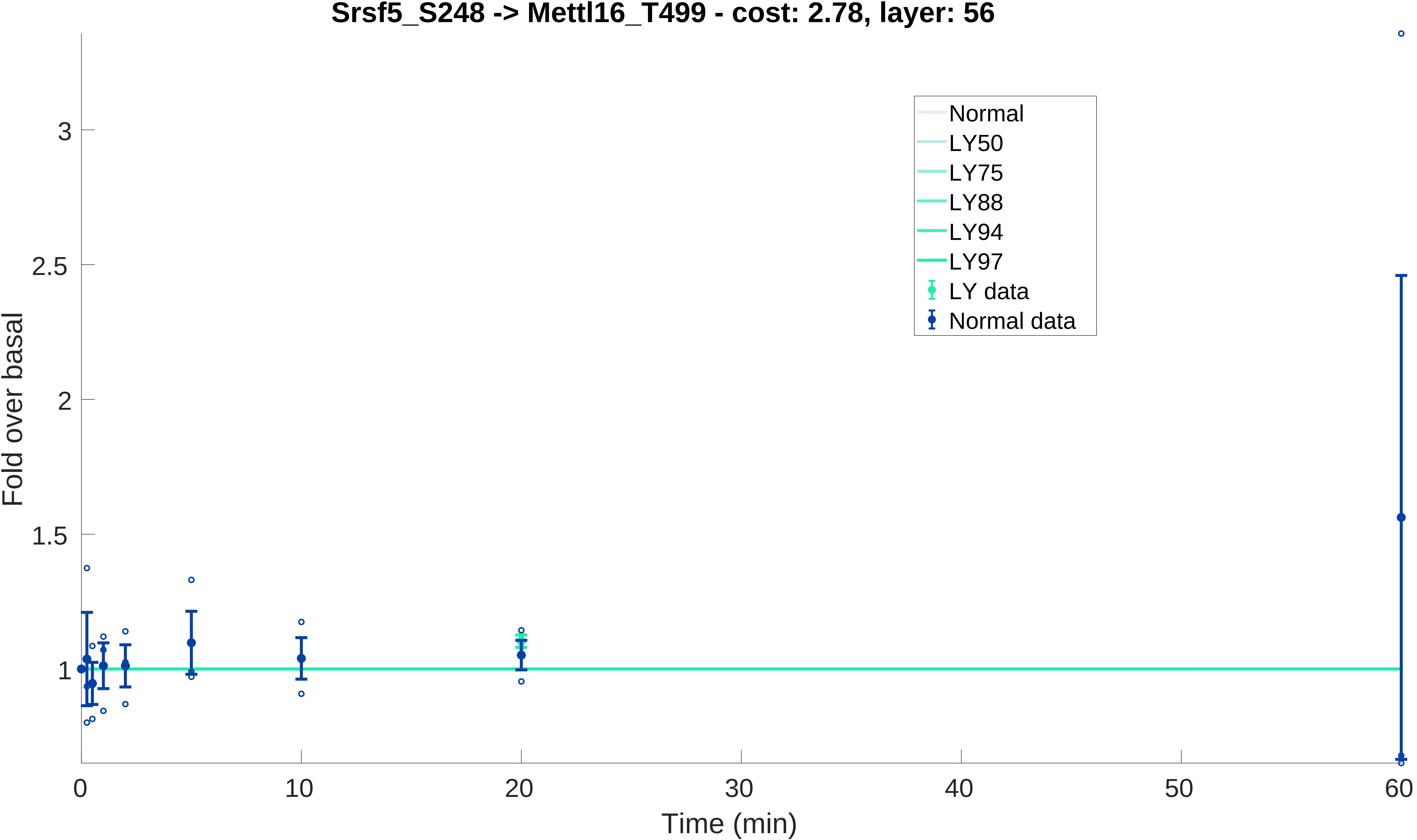

Srsf5_S248 -> Mettl16_T499 - cost: 2.78, layer: 56
Fold over basal
Time (min)
Normal
LY50
LY75
LY88
LY94
LY97
LY data
Normal data
0
10
20
30
40
50
60
1
1.5
2
2.5
3

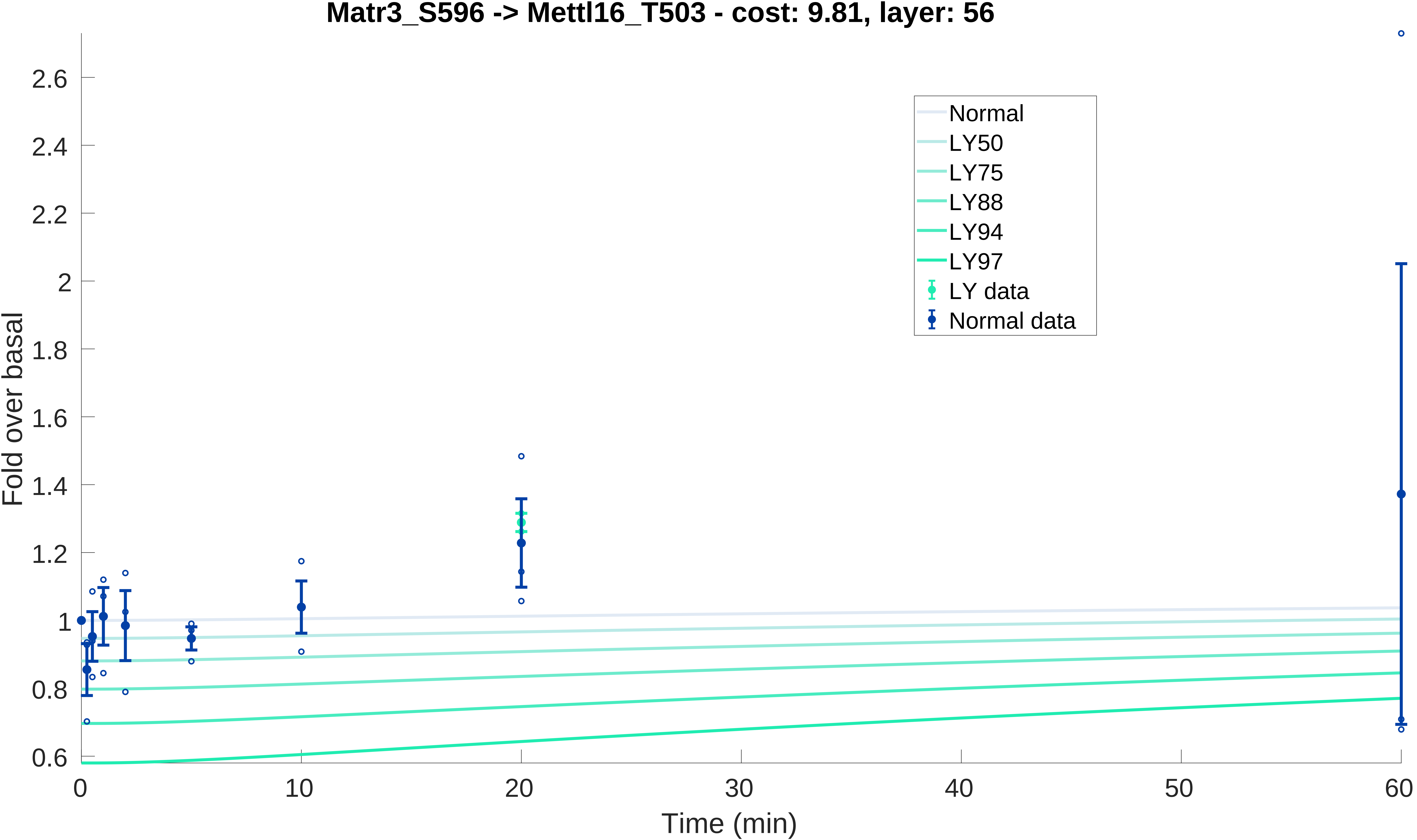

Matr3_S596 -> Mettl16_T503 - cost: 9.81, layer: 56
Fold over basal
2.6
2.4
2.2
2
1.8
1.6
1.4
1.2
1
0.8
0.6
0
10
20
30
40
50
60
Time (min)
Normal
LY50
LY75
LY88
LY94
LY97
LY data
Normal data

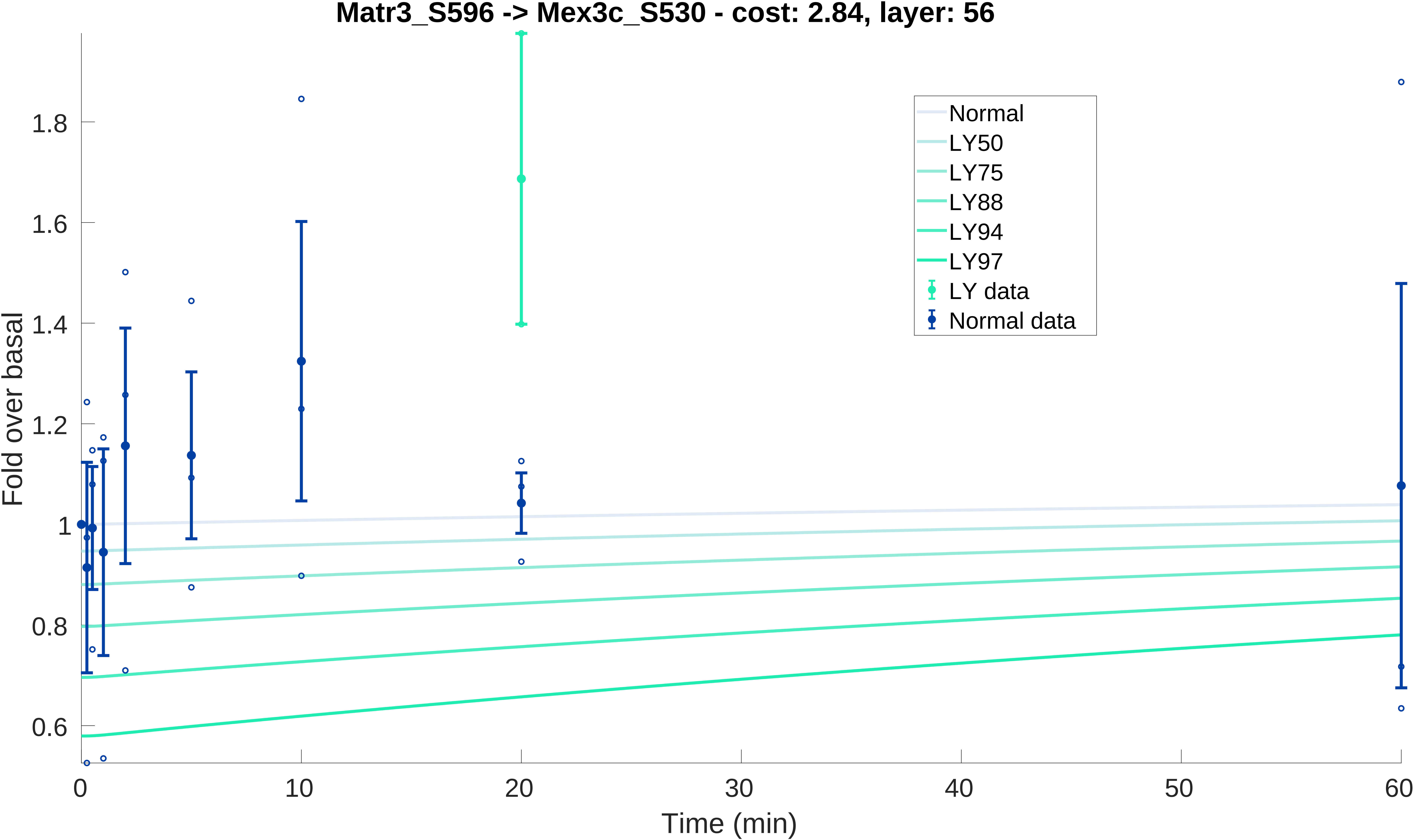

Matr3_S596 -> Mex3c_S530 - cost: 2.84, layer: 56
Normal
LY50
LY75
LY88
LY94
LY97
LY data
Normal data
Fold over basal
1.8
1.6
1.4
1.2
1
0.8
0.6
0
10
20
30
40
50
60
Time (min)

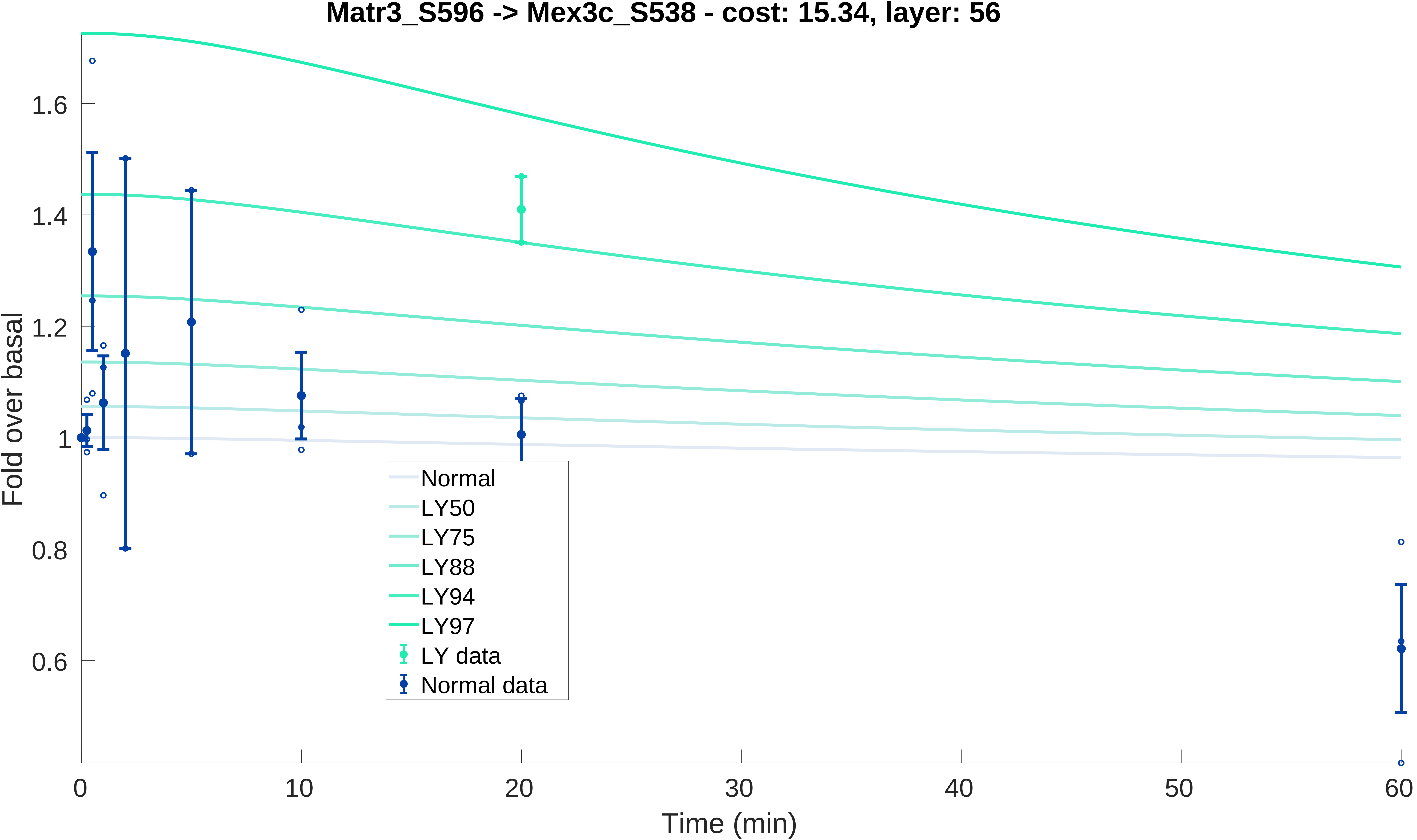
Matr3_S596 -> Mex3c_S538 - cost: 15.34, layer: 56
Fold over basal
Time (min)
1.6
1.4
1.2
1
0.8
0.6
0
10
20
30
40
50
60
Normal
LY50
LY75
LY88
LY94
LY97
LY data
Normal data

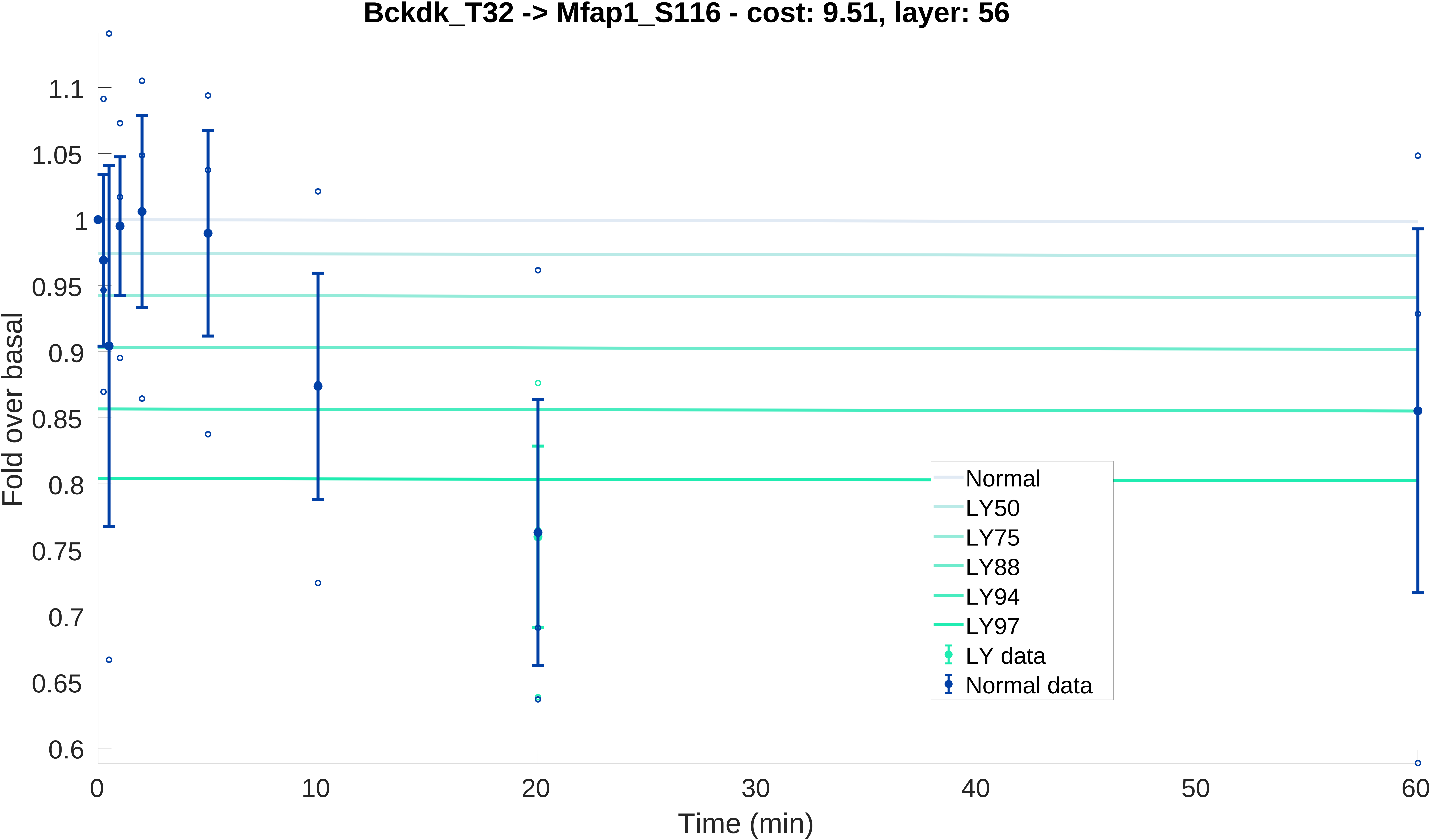

Bckdk_T32 -> Mfap1_S116 - cost: 9.51, layer: 56
Fold over basal
Time (min)
1.1
1.05
1
0.95
0.9
0.85
0.8
0.75
0.7
0.65
0.6
0
10
20
30
40
50
60
Normal
LY50
LY75
LY88
LY94
LY97
LY data
Normal data

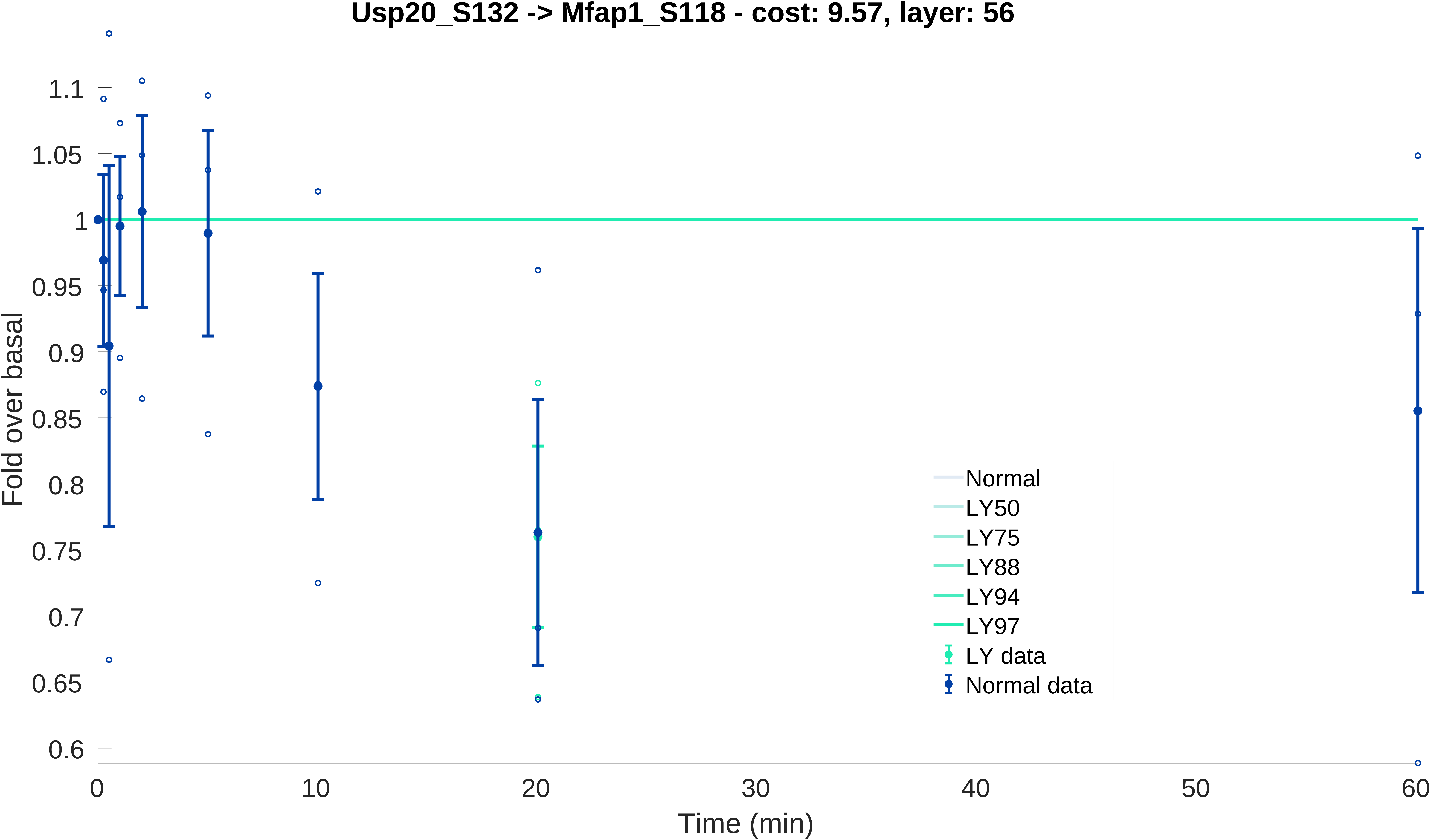

Usp20_S132 -> Mfap1_S118 - cost: 9.57, layer: 56
Fold over basal
Time (min)
1.1
1.05
1
0.95
0.9
0.85
0.8
0.75
0.7
0.65
0.6
0
10
20
30
40
50
60
Normal
LY50
LY75
LY88
LY94
LY97
LY data
Normal data

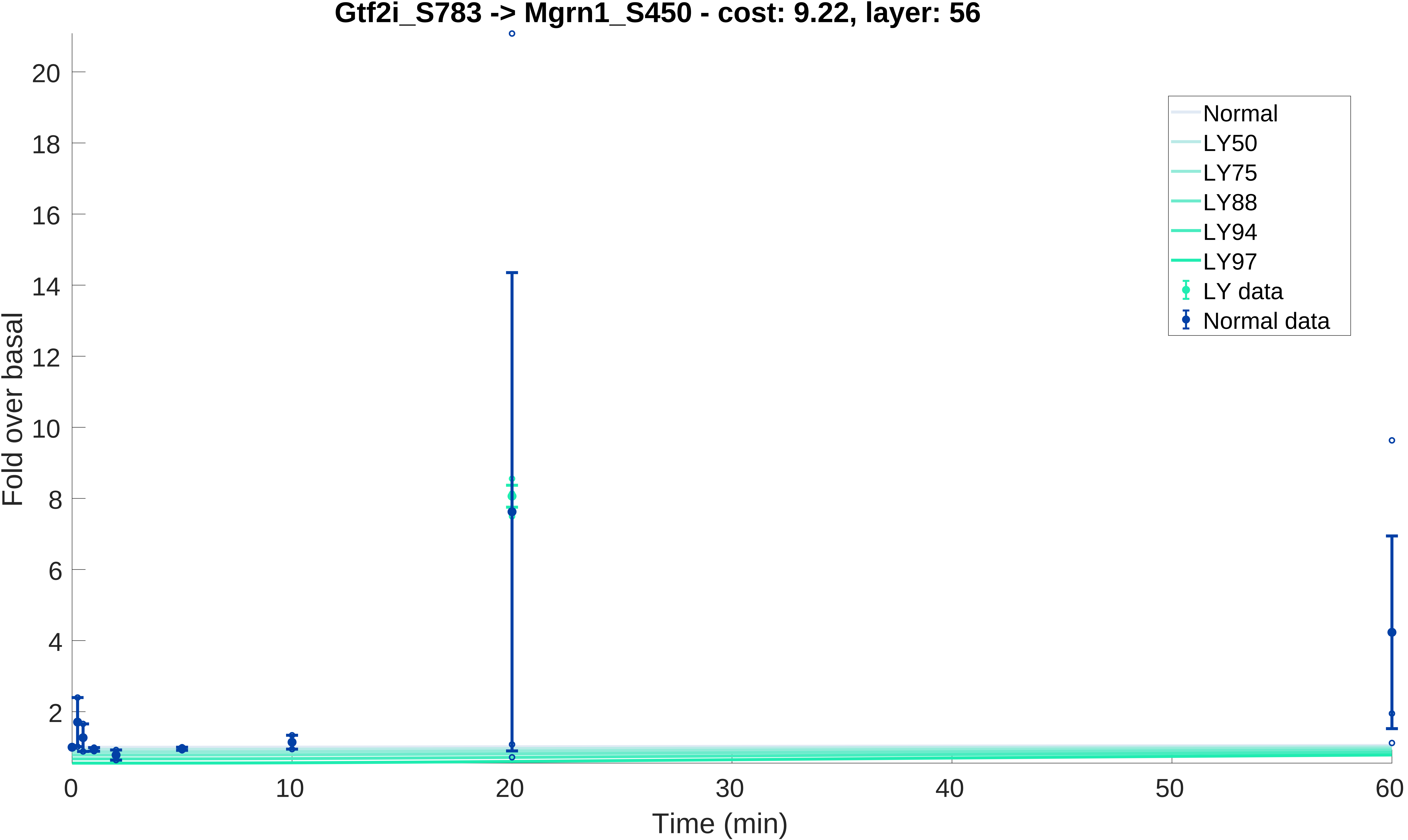

Gtf2i_S783 -> Mgrn1_S450 - cost: 9.22, layer: 56
Fold over basal
Time (min)
0
10
20
30
40
50
60
2
4
6
8
10
12
14
16
18
20
Normal
LY50
LY75
LY88
LY94
LY97
LY data
Normal data

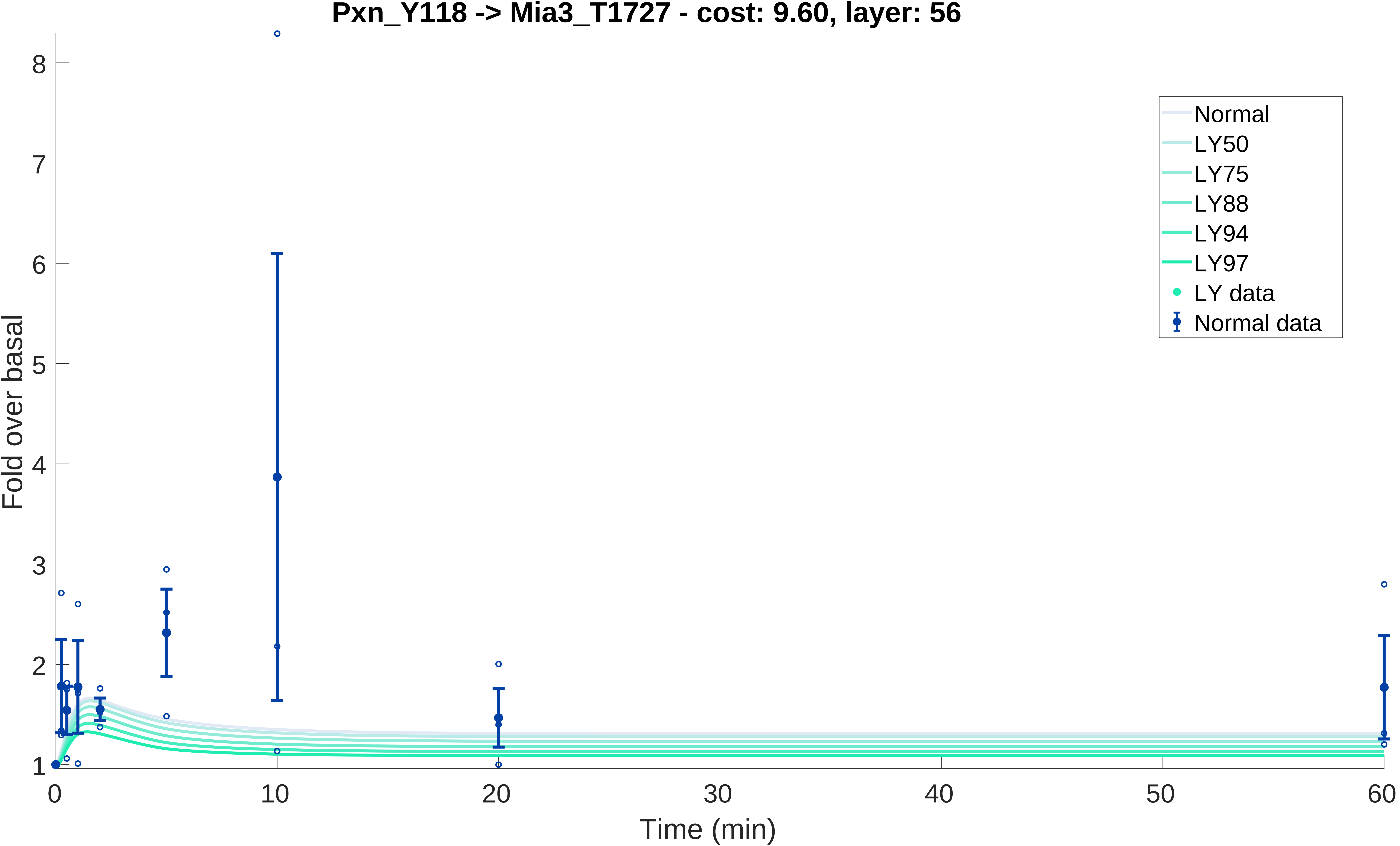

Pxn_Y118 -> Mia3_T1727 - cost: 9.60, layer: 56
Fold over basal
Time (min)
Normal
LY50
LY75
LY88
LY94
LY97
LY data
Normal data
0
10
20
30
40
50
60
1
2
3
4
5
6
7
8

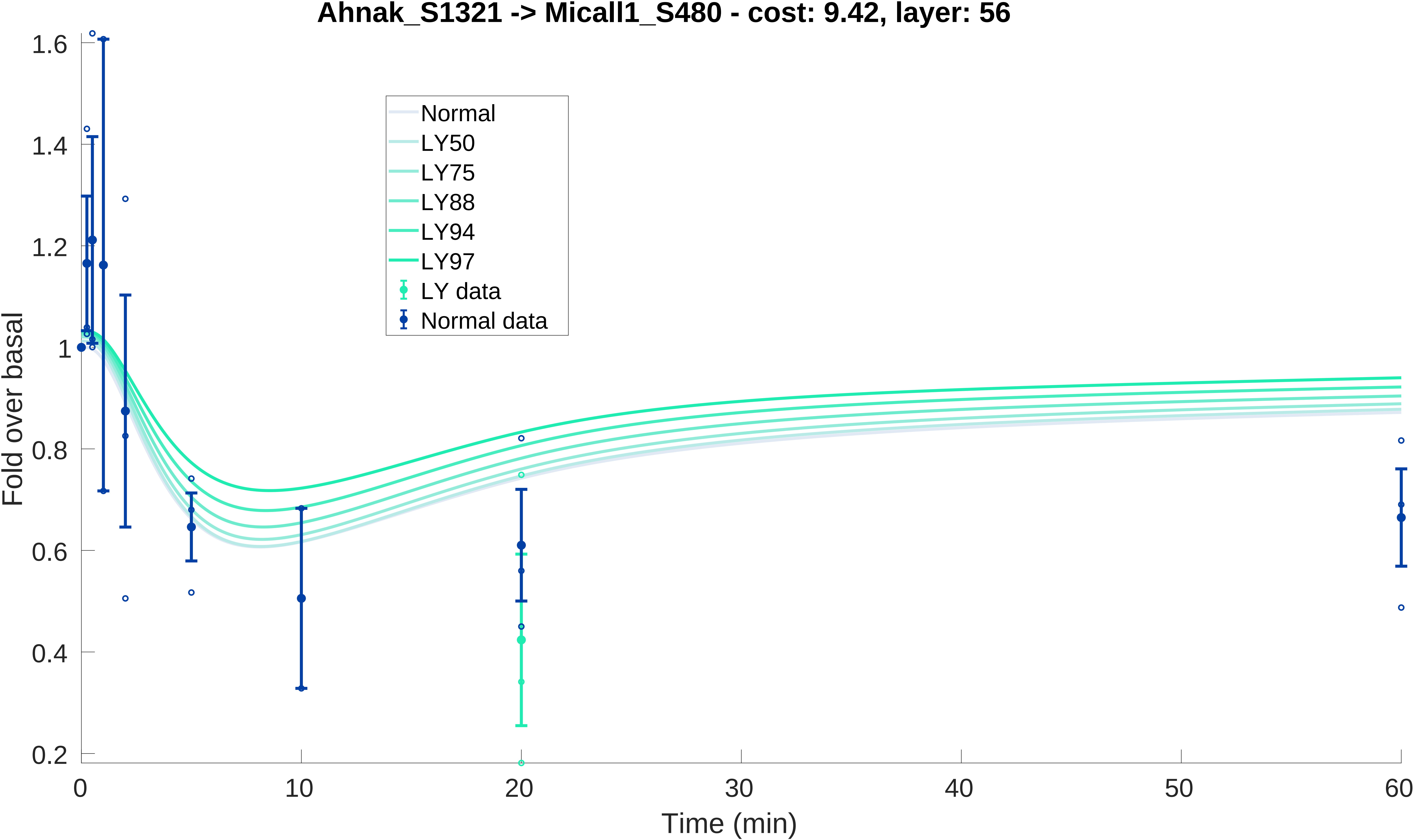

Ahnak_S1321 -> Micall1_S480 - cost: 9.42, layer: 56
Fold over basal
Time (min)
1.6
1.4
1.2
1
0.8
0.6
0.4
0.2
0
10
20
30
40
50
60
Normal
LY50
LY75
LY88
LY94
LY97
LY data
Normal data

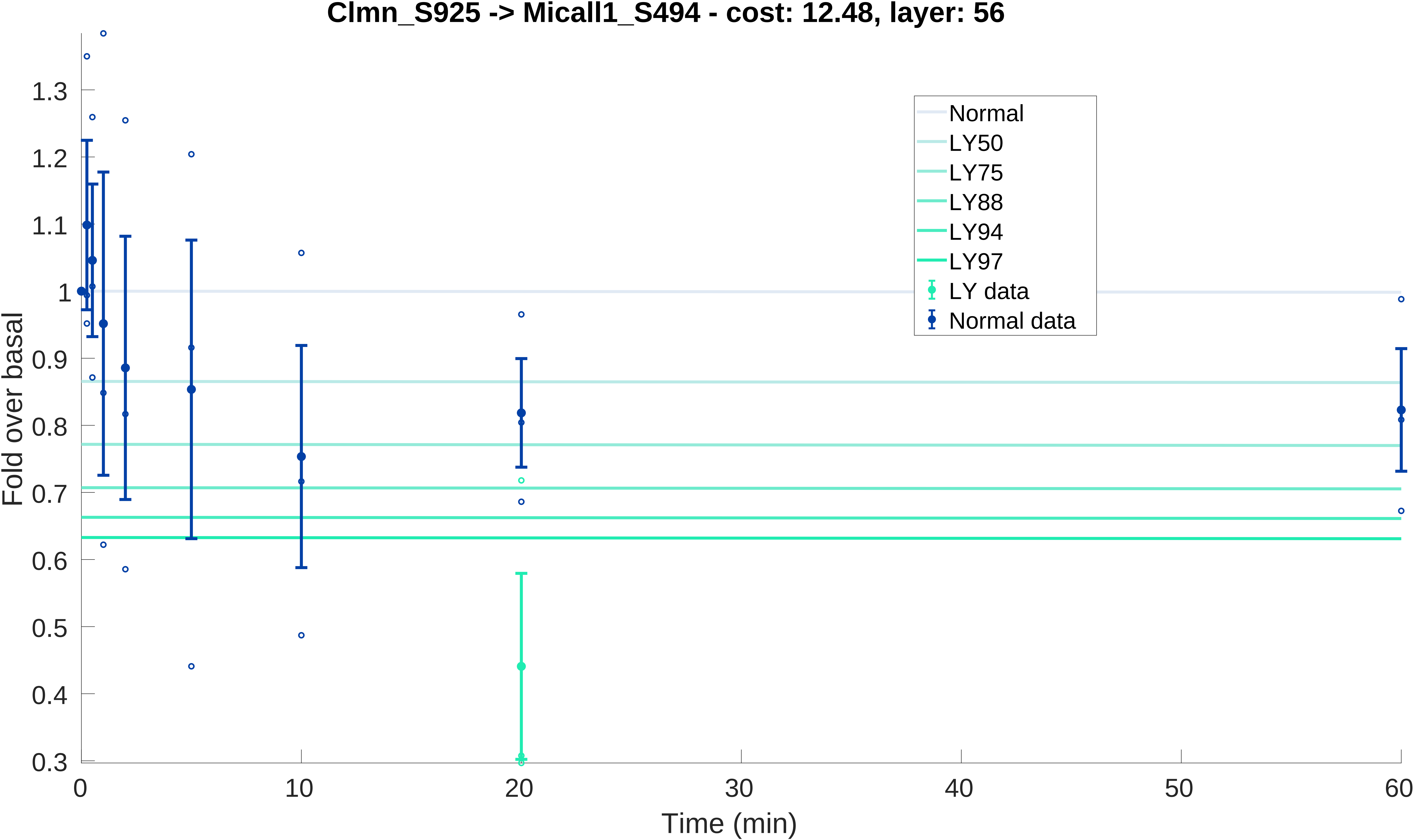

Clmn_S925 -> Micall1_S494 - cost: 12.48, layer: 56
Fold over basal
Time (min)
0.3
0.4
0.5
0.6
0.7
0.8
0.9
1
1.1
1.2
1.3
0
10
20
30
40
50
60
Normal
LY50
LY75
LY88
LY94
LY97
LY data
Normal data

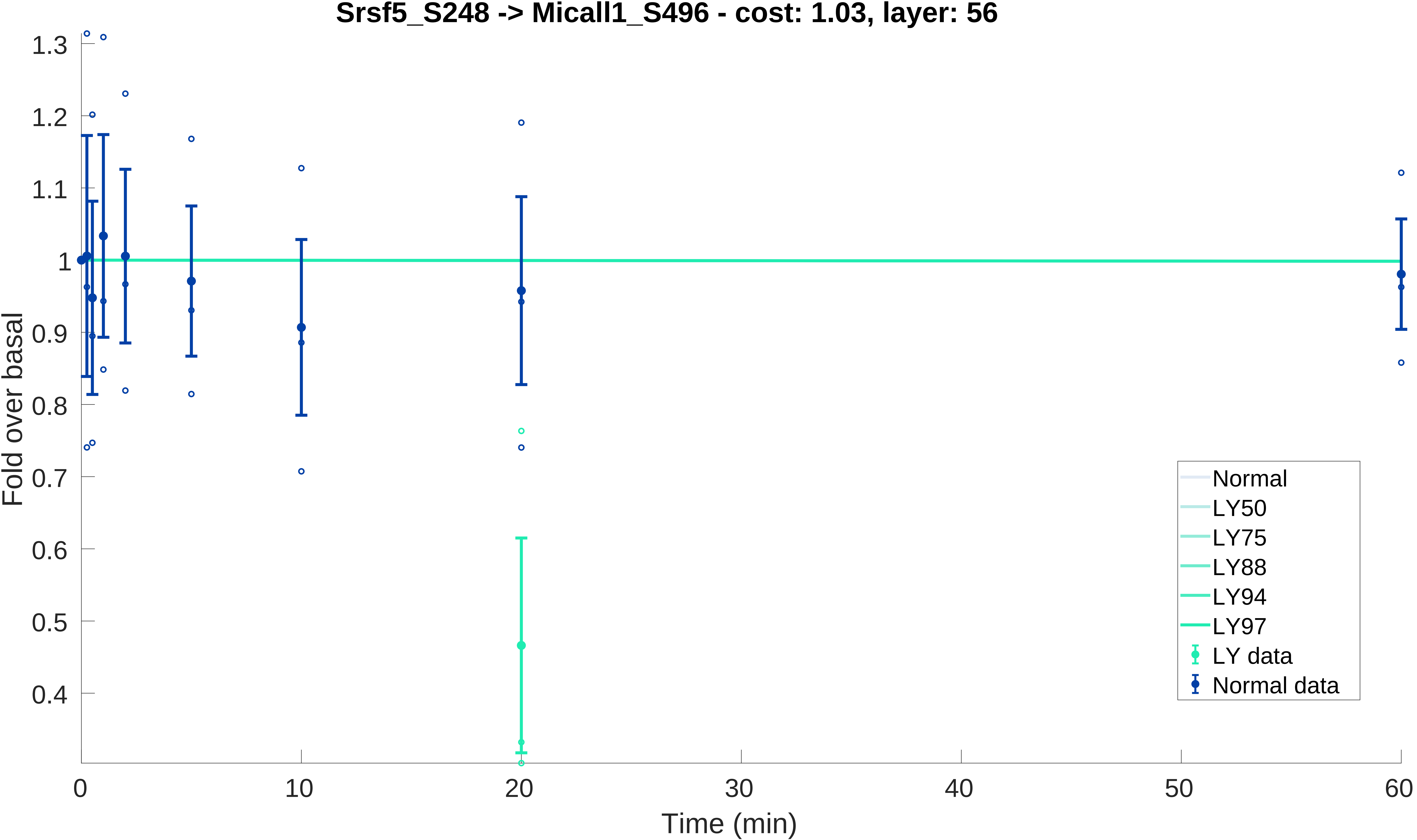
Srsf5_S248 -> Micall1_S496 - cost: 1.03, layer: 56
Fold over basal
Time (min)
1.3
1.2
1.1
1
0.9
0.8
0.7
0.6
0.5
0.4
0
10
20
30
40
50
60
Normal
LY50
LY75
LY88
LY94
LY97
LY data
Normal data

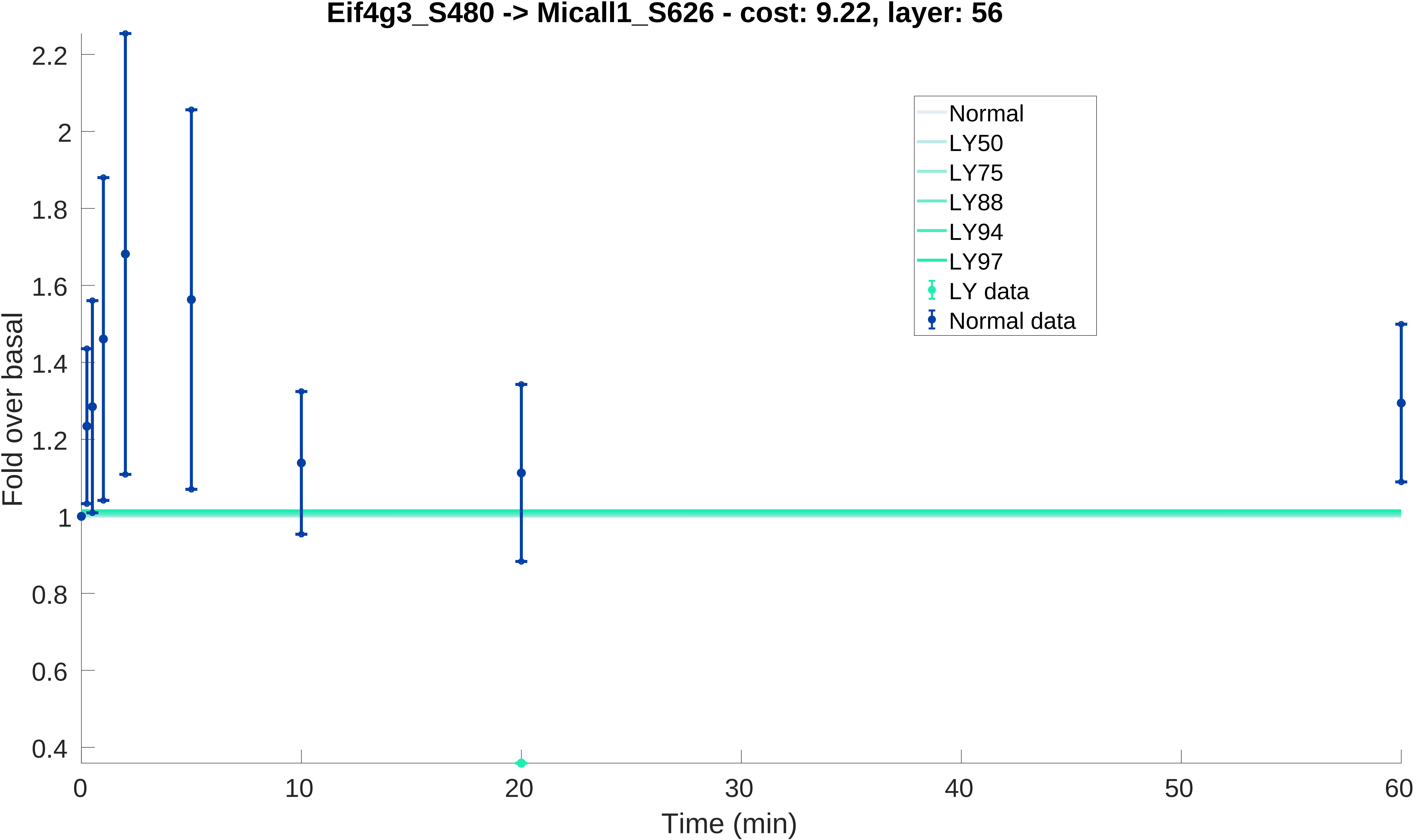

Eif4g3_S480 -> Micall1_S626 - cost: 9.22, layer: 56
Fold over basal
2.2
2
1.8
1.6
1.4
1.2
1
0.8
0.6
0.4
0
10
20
30
40
50
60
Time (min)
Normal
LY50
LY75
LY88
LY94
LY97
LY data
Normal data

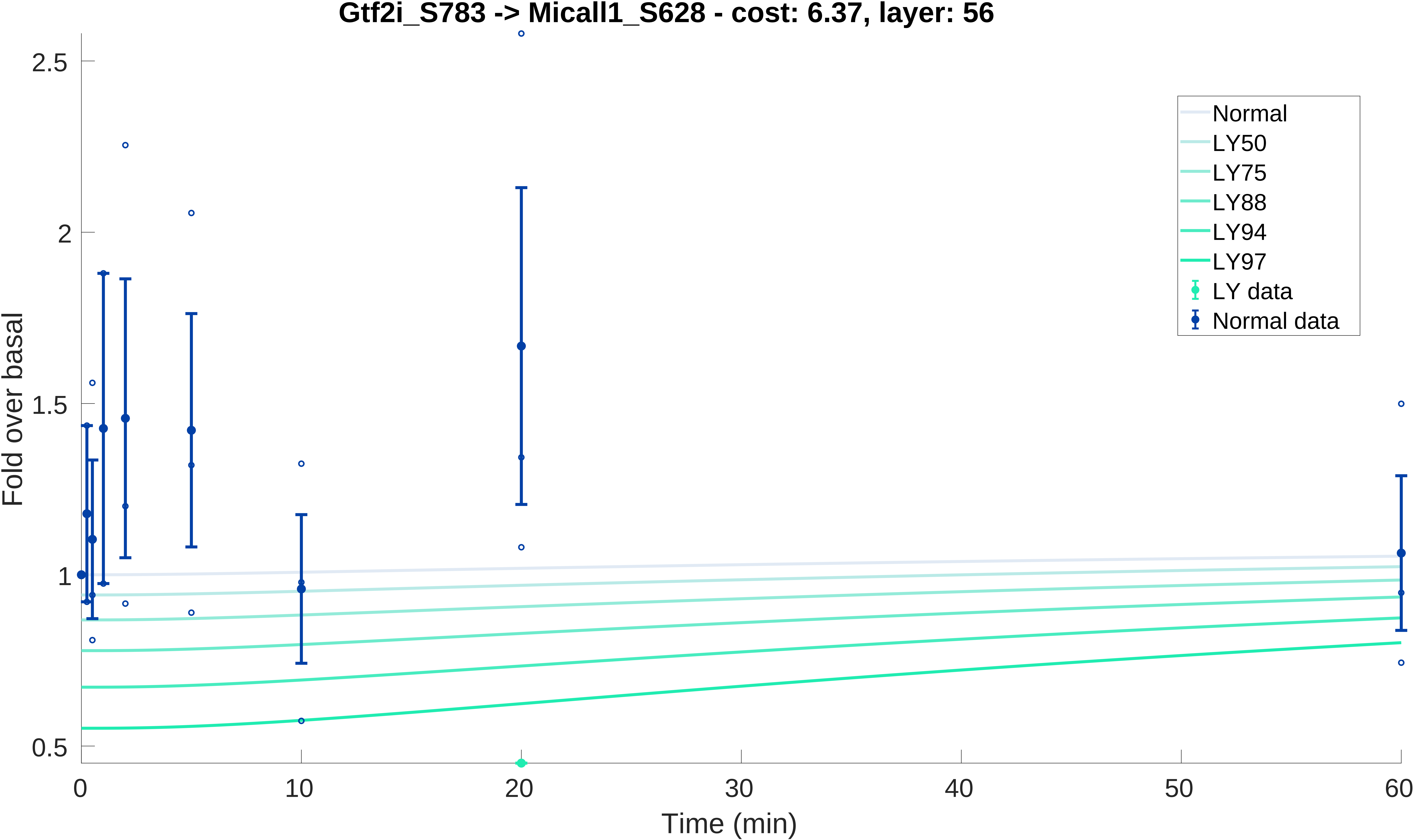

Gtf2i_S783 -> Micall1_S628 - cost: 6.37, layer: 56
Fold over basal
Time (min)
0.5
1
1.5
2
2.5
0
10
20
30
40
50
60
Normal
LY50
LY75
LY88
LY94
LY97
LY data
Normal data

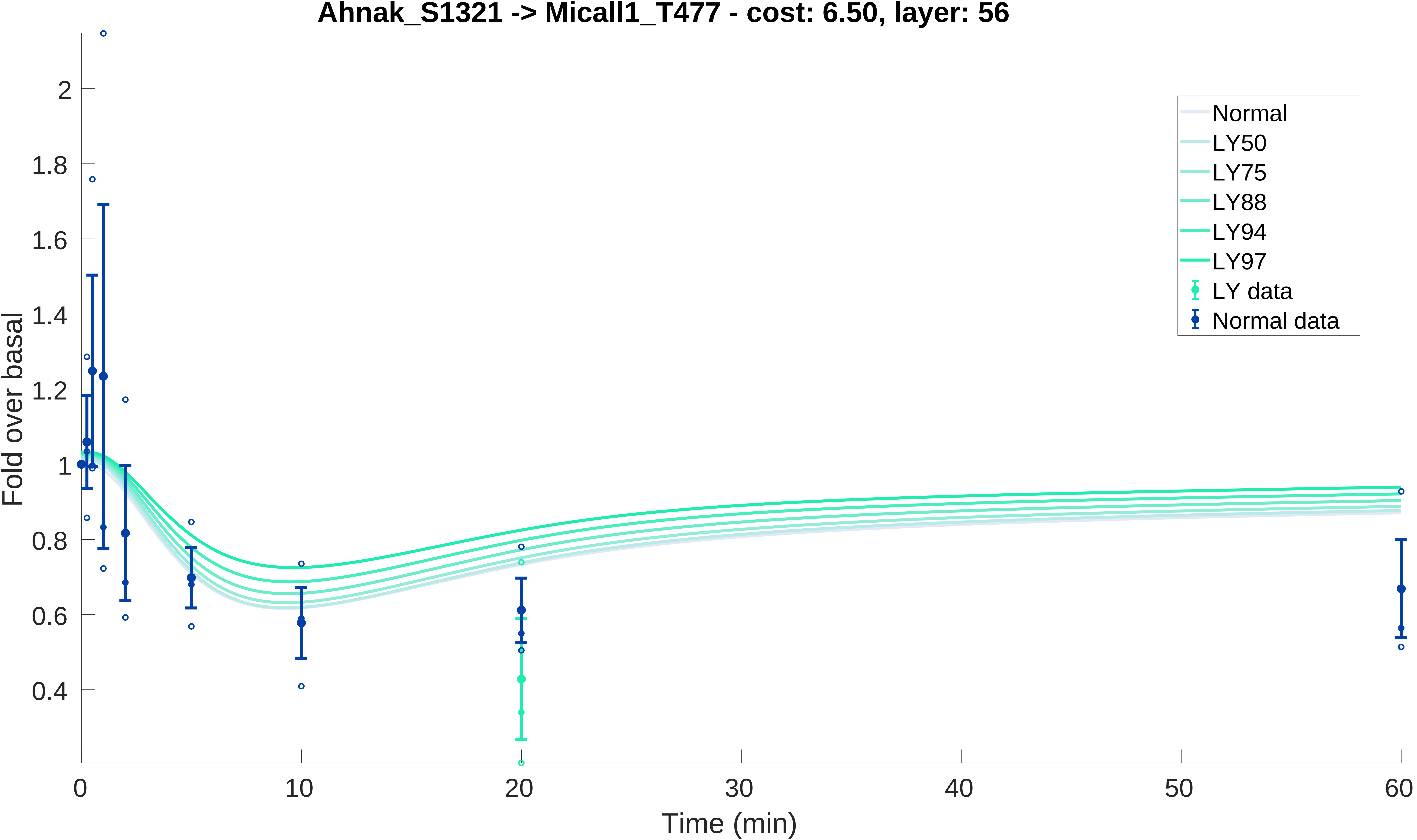

Ahnak_S1321 -> Micall1_T477 - cost: 6.50, layer: 56
Fold over basal
Time (min)
0
10
20
30
40
50
60
0.4
0.6
0.8
1
1.2
1.4
1.6
1.8
2
Normal
LY50
LY75
LY88
LY94
LY97
LY data
Normal data

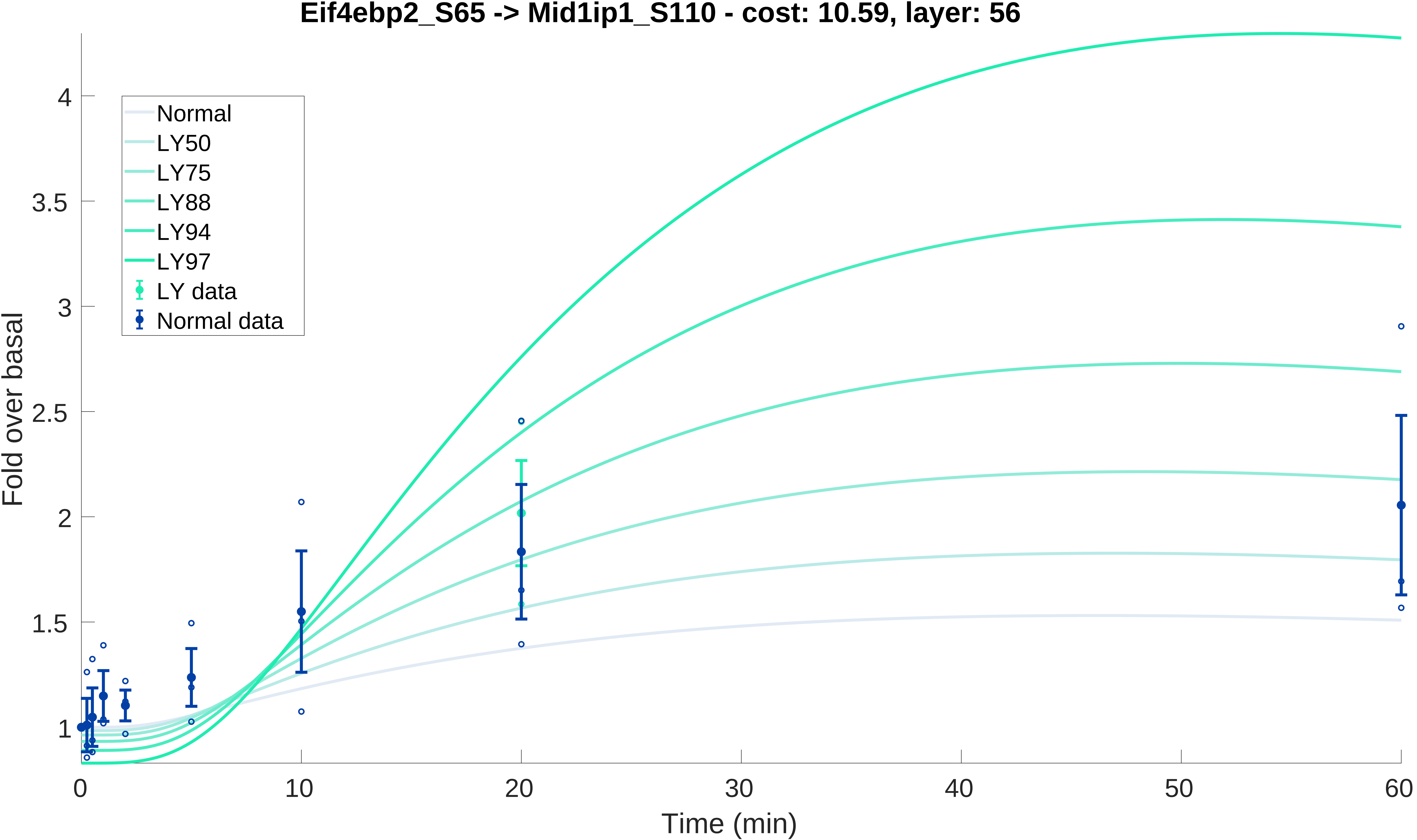

Eif4ebp2_S65 -> Mid1ip1_S110 - cost: 10.59, layer: 56
Normal
LY50
LY75
LY88
LY94
LY97
LY data
Normal data
Fold over basal
Time (min)
4
3.5
3
2.5
2
1.5
1
0
10
20
30
40
50
60

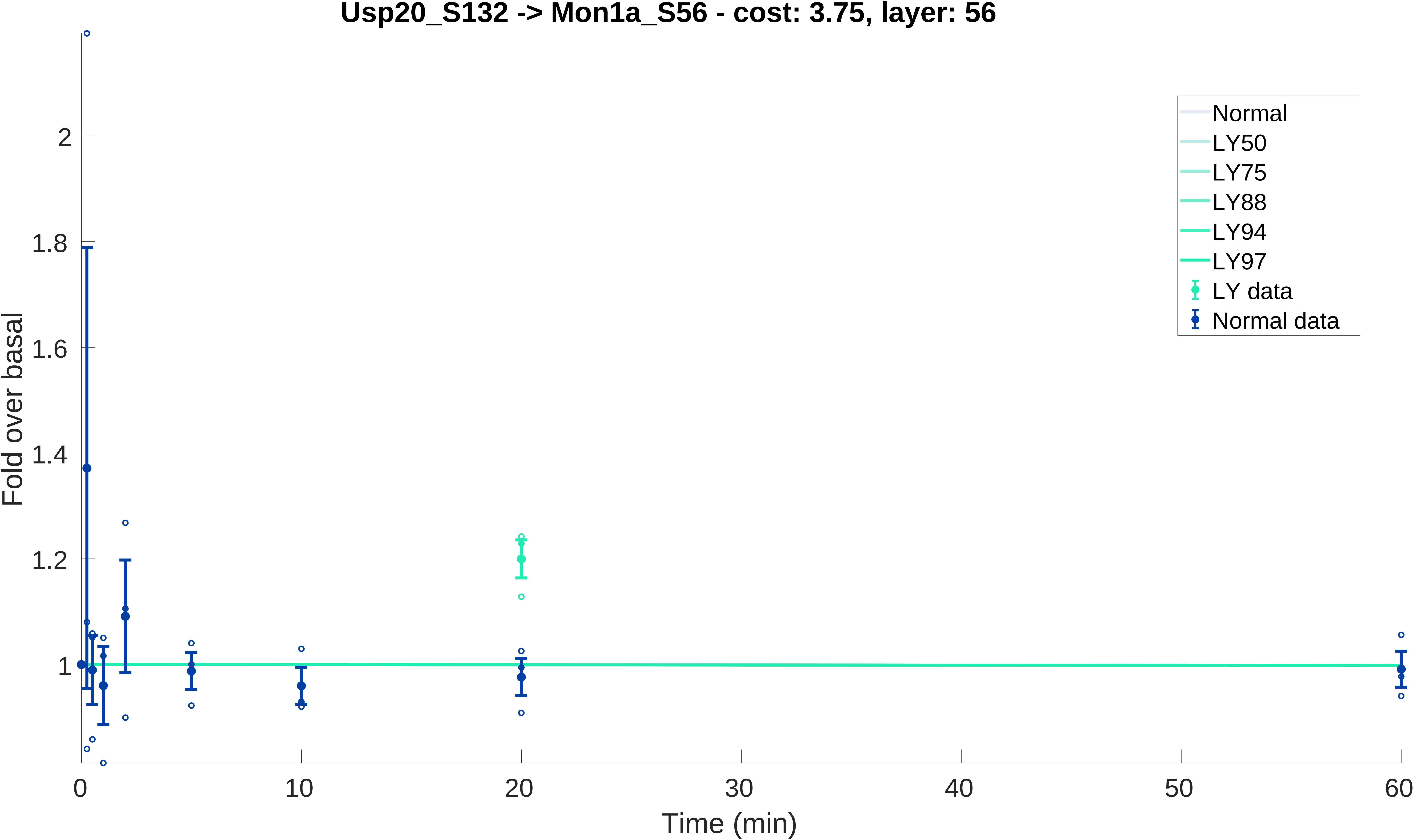

Usp20_S132 -> Mon1a_S56 - cost: 3.75, layer: 56
Fold over basal
Time (min)
2
1.8
1.6
1.4
1.2
1
0
10
20
30
40
50
60
Normal
LY50
LY75
LY88
LY94
LY97
LY data
Normal data

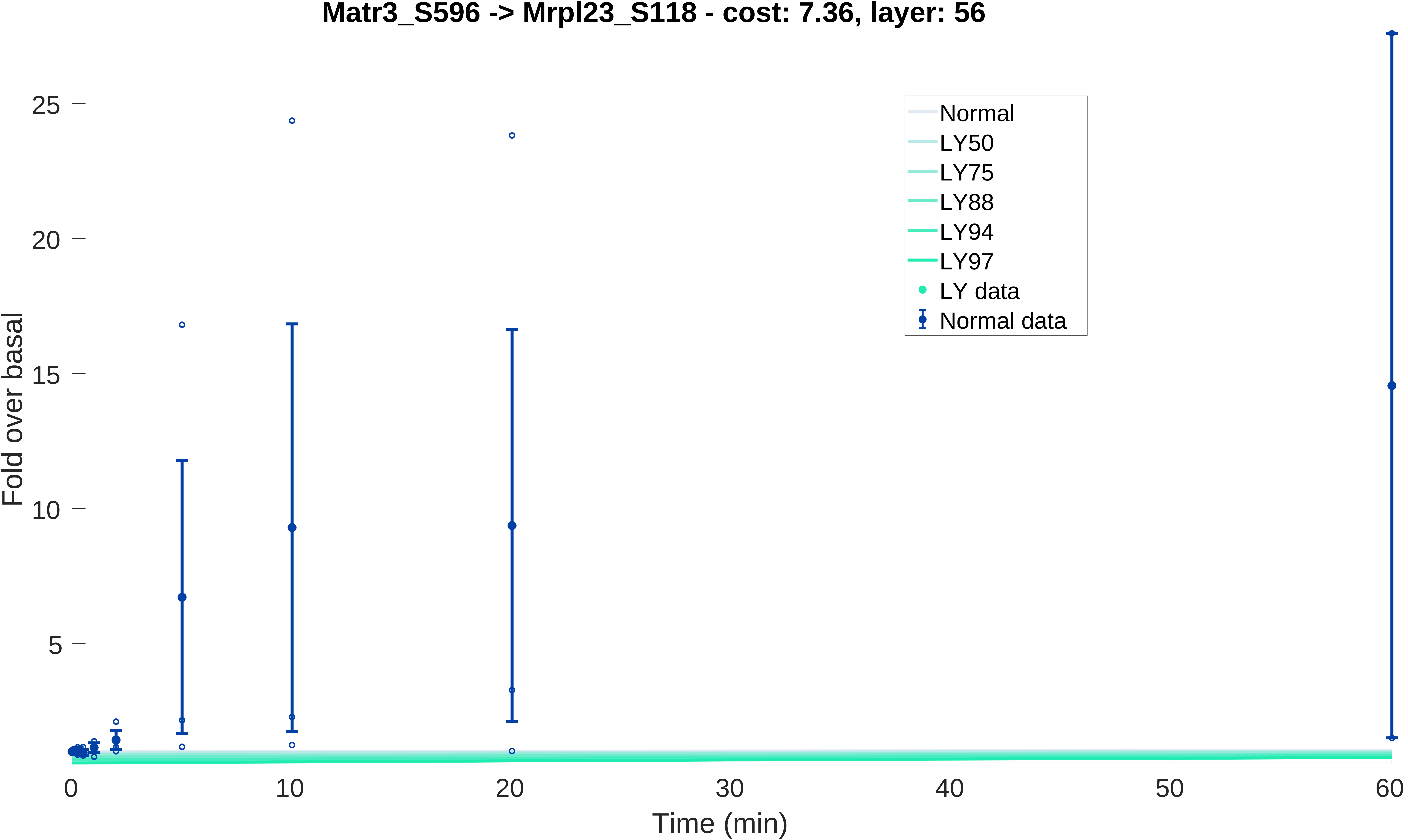

Matr3_S596 -> Mrpl23_S118 - cost: 7.36, layer: 56
Fold over basal
Time (min)
0
10
20
30
40
50
60
5
10
15
20
25
Normal
LY50
LY75
LY88
LY94
LY97
LY data
Normal data

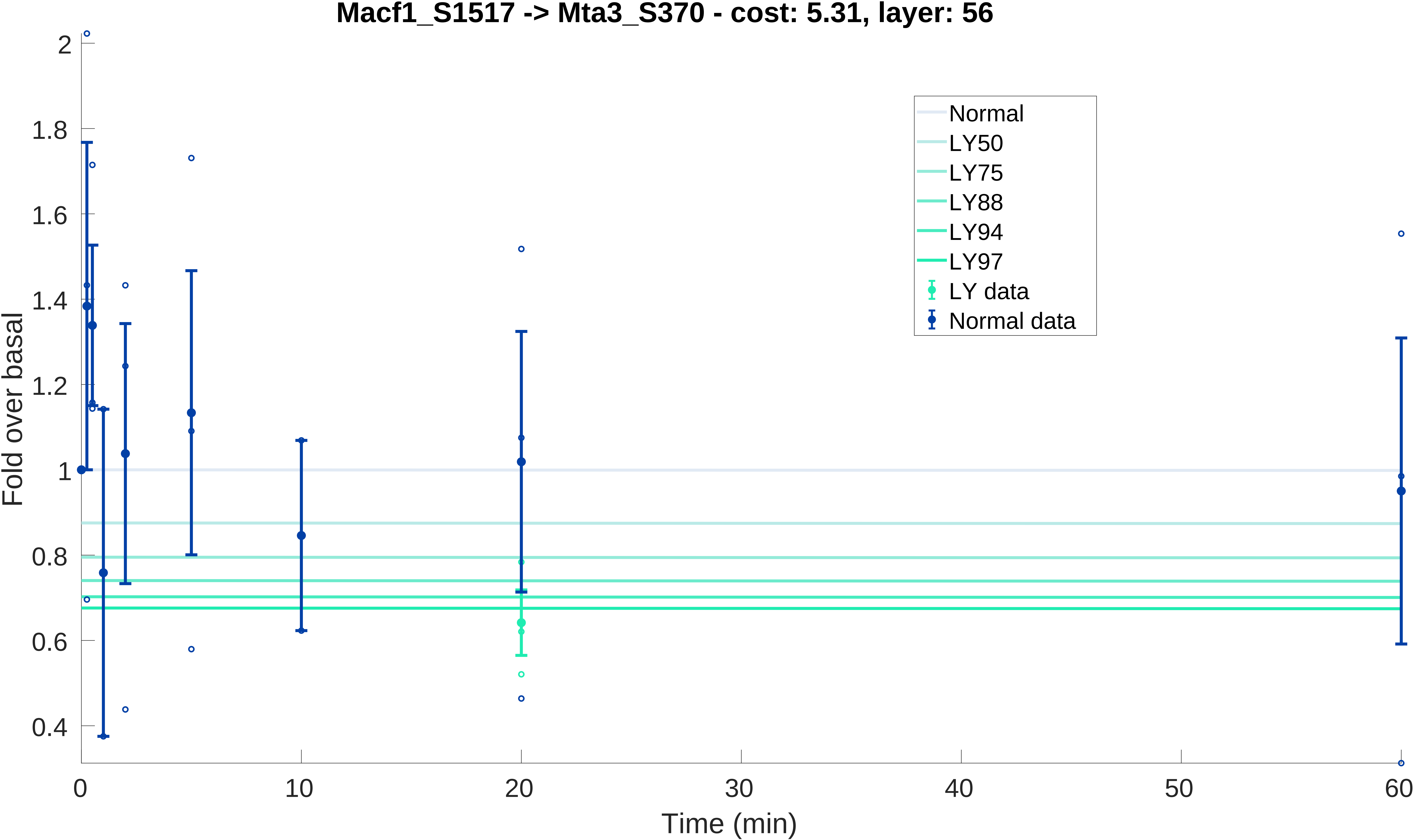

Macf1_S1517 -> Mta3_S370 - cost: 5.31, layer: 56
Fold over basal
Time (min)
2
1.8
1.6
1.4
1.2
1
0.8
0.6
0.4
0
10
20
30
40
50
60
Normal
LY50
LY75
LY88
LY94
LY97
LY data
Normal data

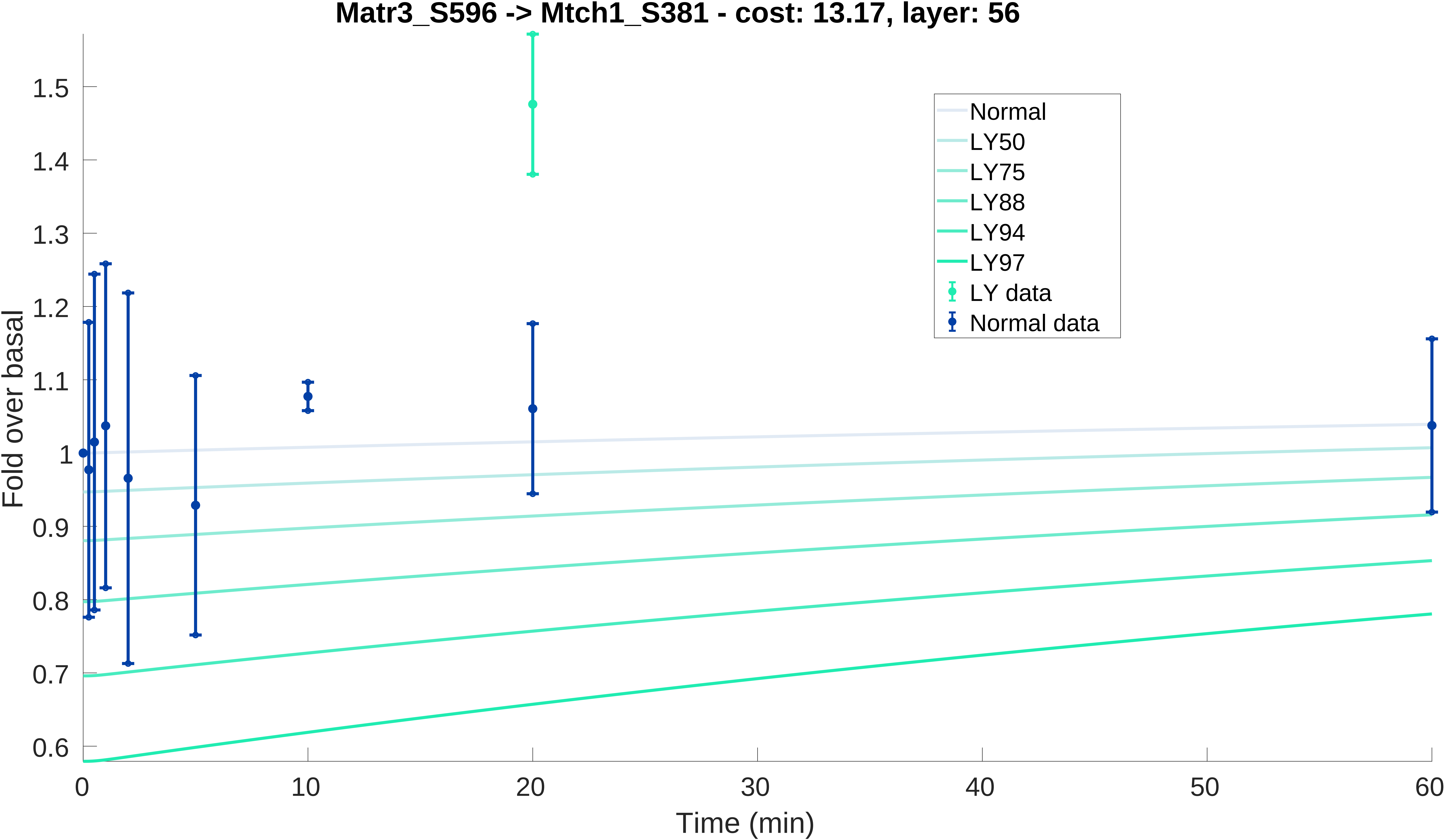

Matr3_S596 -> Mtch1_S381 - cost: 13.17, layer: 56
Fold over basal
Time (min)
0
10
20
30
40
50
60
0.6
0.7
0.8
0.9
1
1.1
1.2
1.3
1.4
1.5
Normal
LY50
LY75
LY88
LY94
LY97
LY data
Normal data

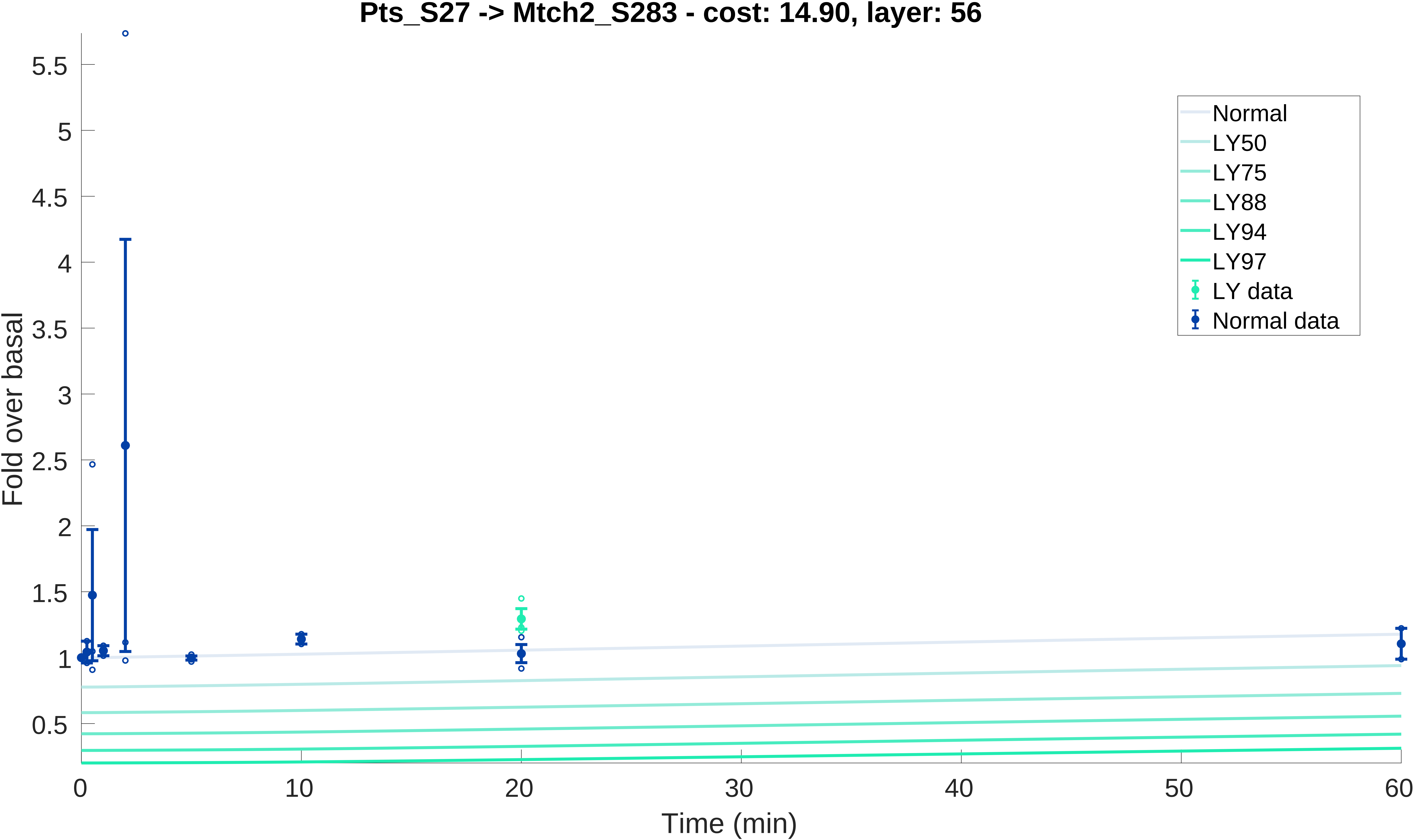

Pts_S27 -> Mtch2_S283 - cost: 14.90, layer: 56
Fold over basal
Time (min)
5.5
5
4.5
4
3.5
3
2.5
2
1.5
1
0.5
0
10
20
30
40
50
60
Normal
LY50
LY75
LY88
LY94
LY97
LY data
Normal data

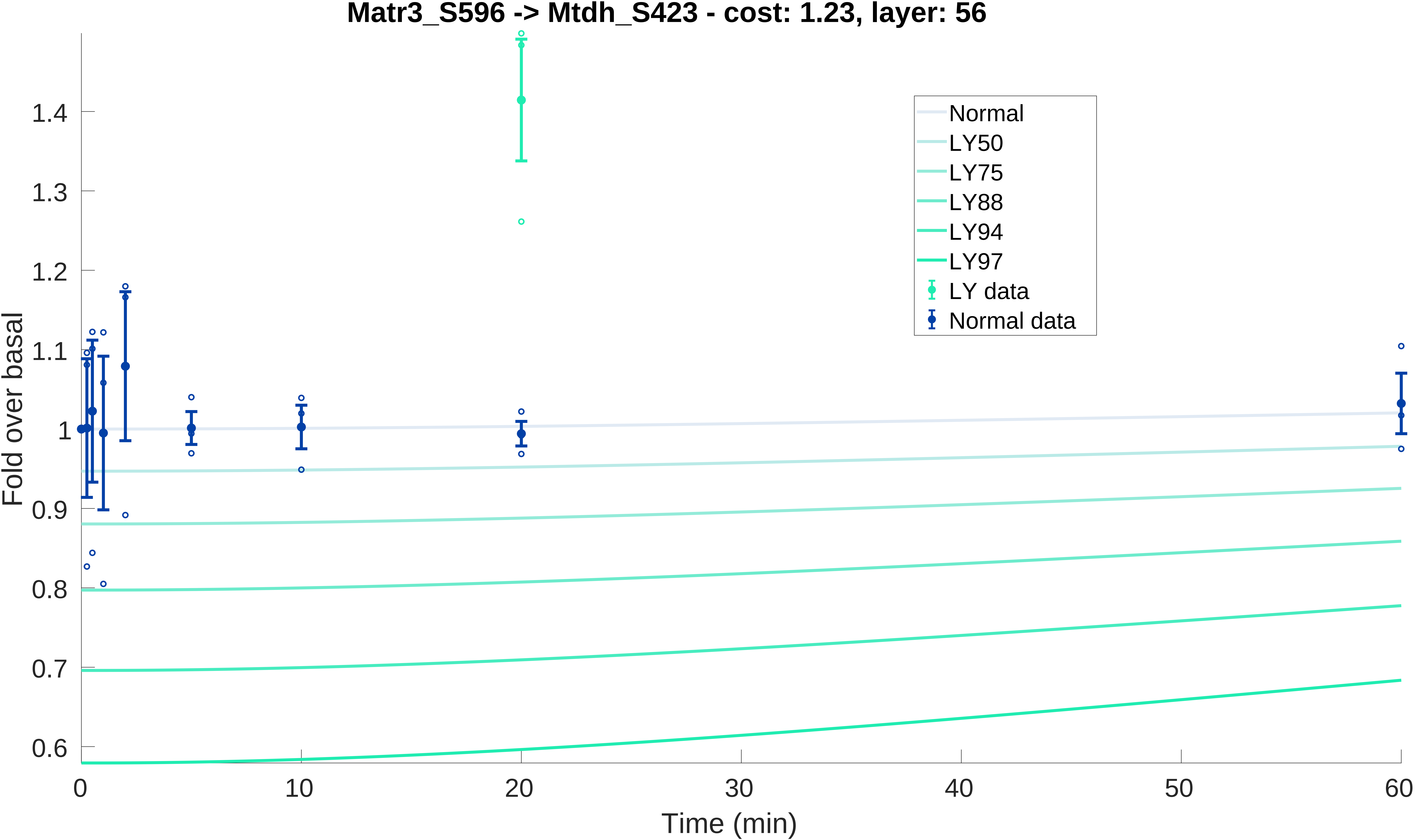

Matr3_S596 -> Mtdh_S423 - cost: 1.23, layer: 56
Fold over basal
Time (min)
1.4
1.3
1.2
1.1
1
0.9
0.8
0.7
0.6
0
10
20
30
40
50
60
Normal
LY50
LY75
LY88
LY94
LY97
LY data
Normal data

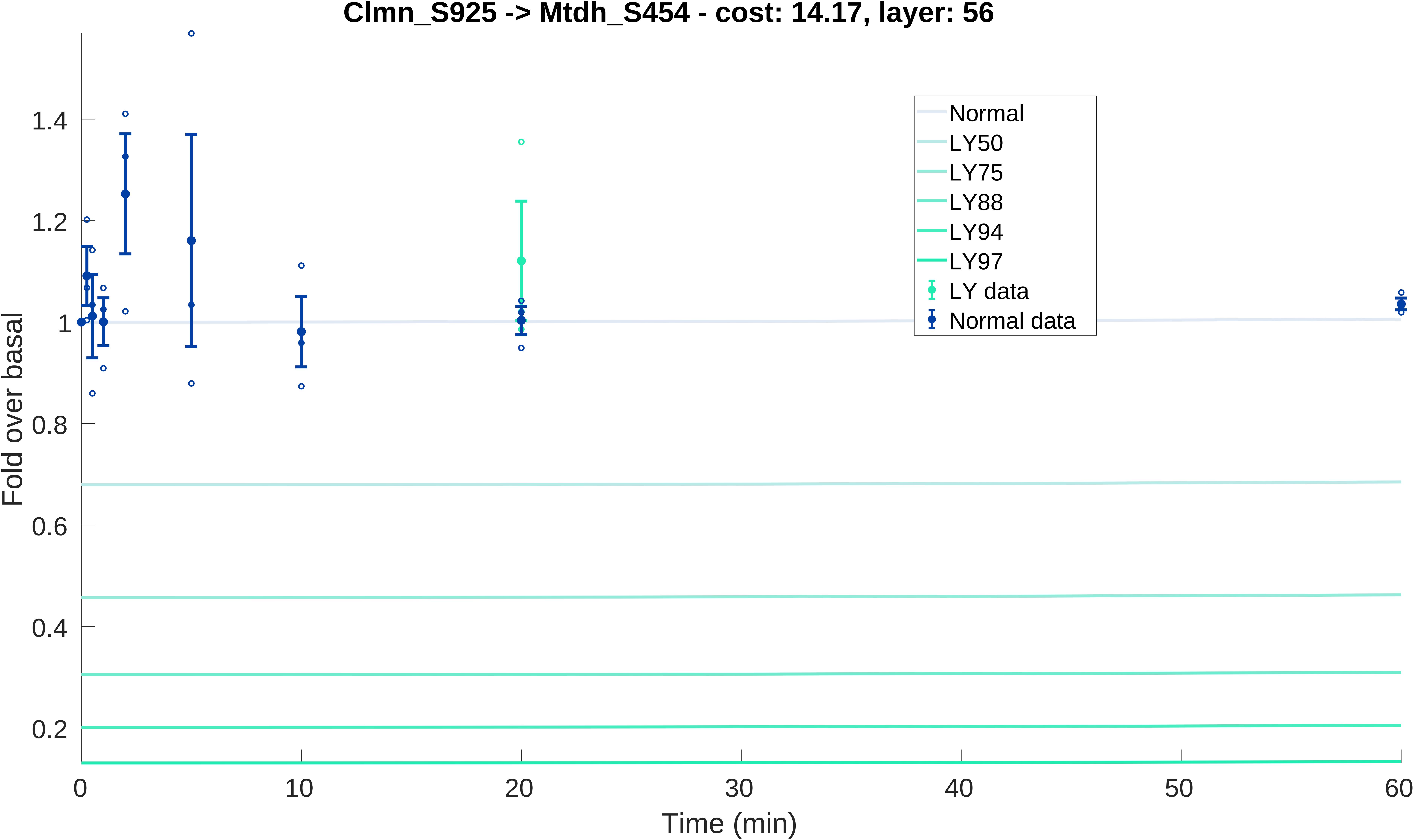

Clmn_S925 -> Mtdh_S454 - cost: 14.17, layer: 56
Fold over basal
Time (min)
0
10
20
30
40
50
60
0.2
0.4
0.6
0.8
1
1.2
1.4
Normal
LY50
LY75
LY88
LY94
LY97
LY data
Normal data

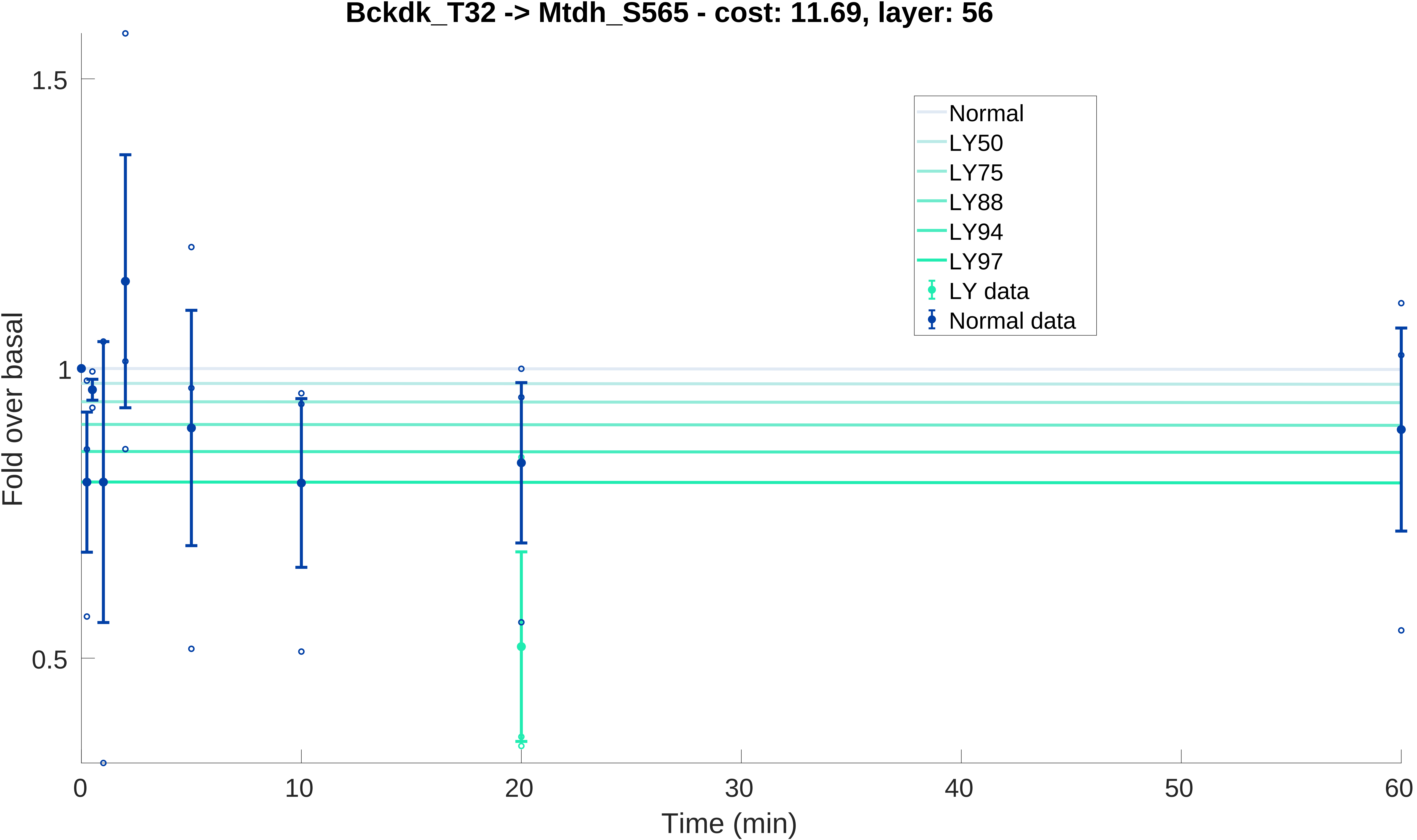
Bckdk_T32 -> Mtdh_S565 - cost: 11.69, layer: 56
Normal
LY50
LY75
LY88
LY94
LY97
LY data
Normal data
Fold over basal
1.5
1
0.5
0
10
20
30
40
50
60
Time (min)

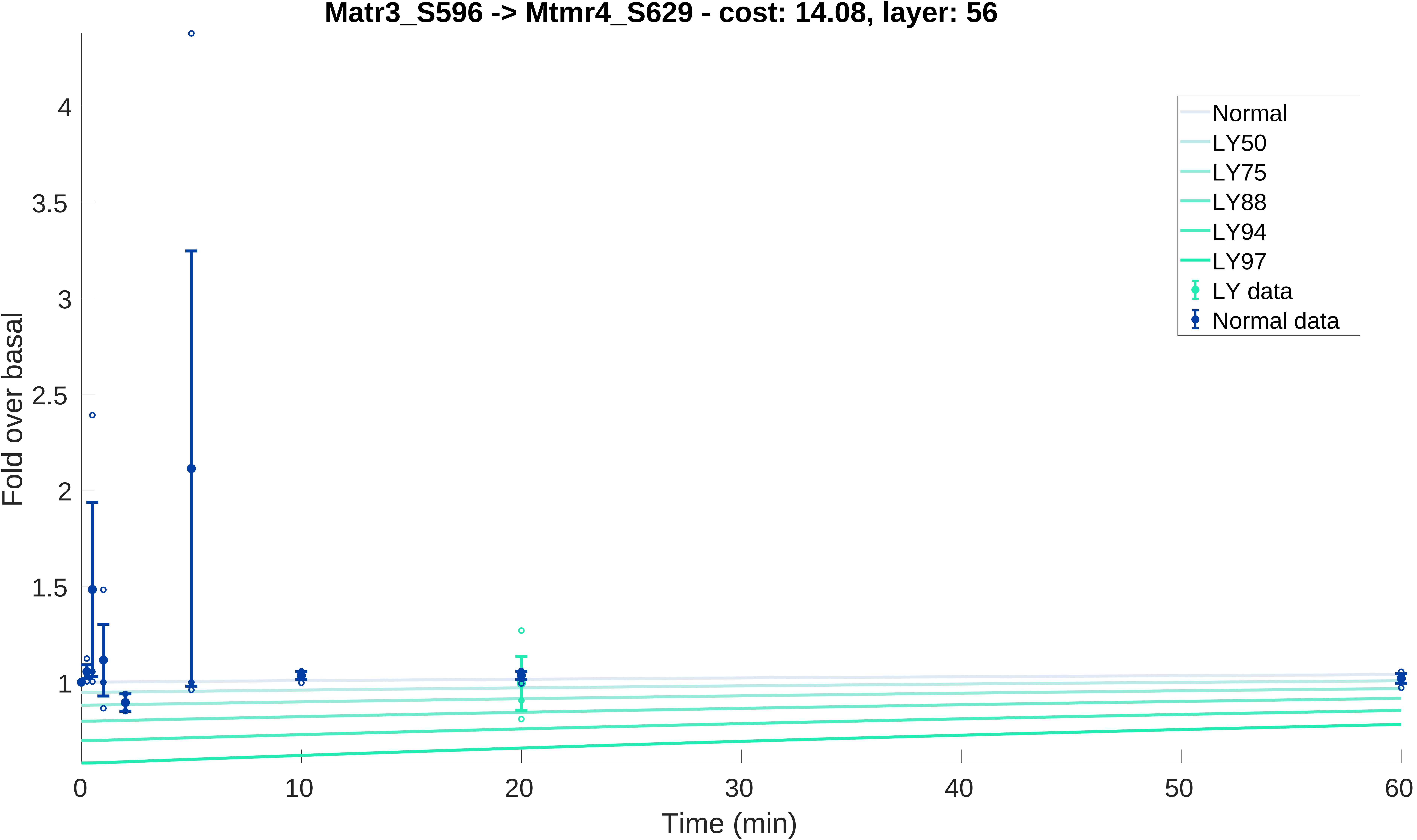

Matr3_S596 -> Mtmr4_S629 - cost: 14.08, layer: 56
Fold over basal
Time (min)
4
3.5
3
2.5
2
1.5
1
0
10
20
30
40
50
60
Normal
LY50
LY75
LY88
LY94
LY97
LY data
Normal data

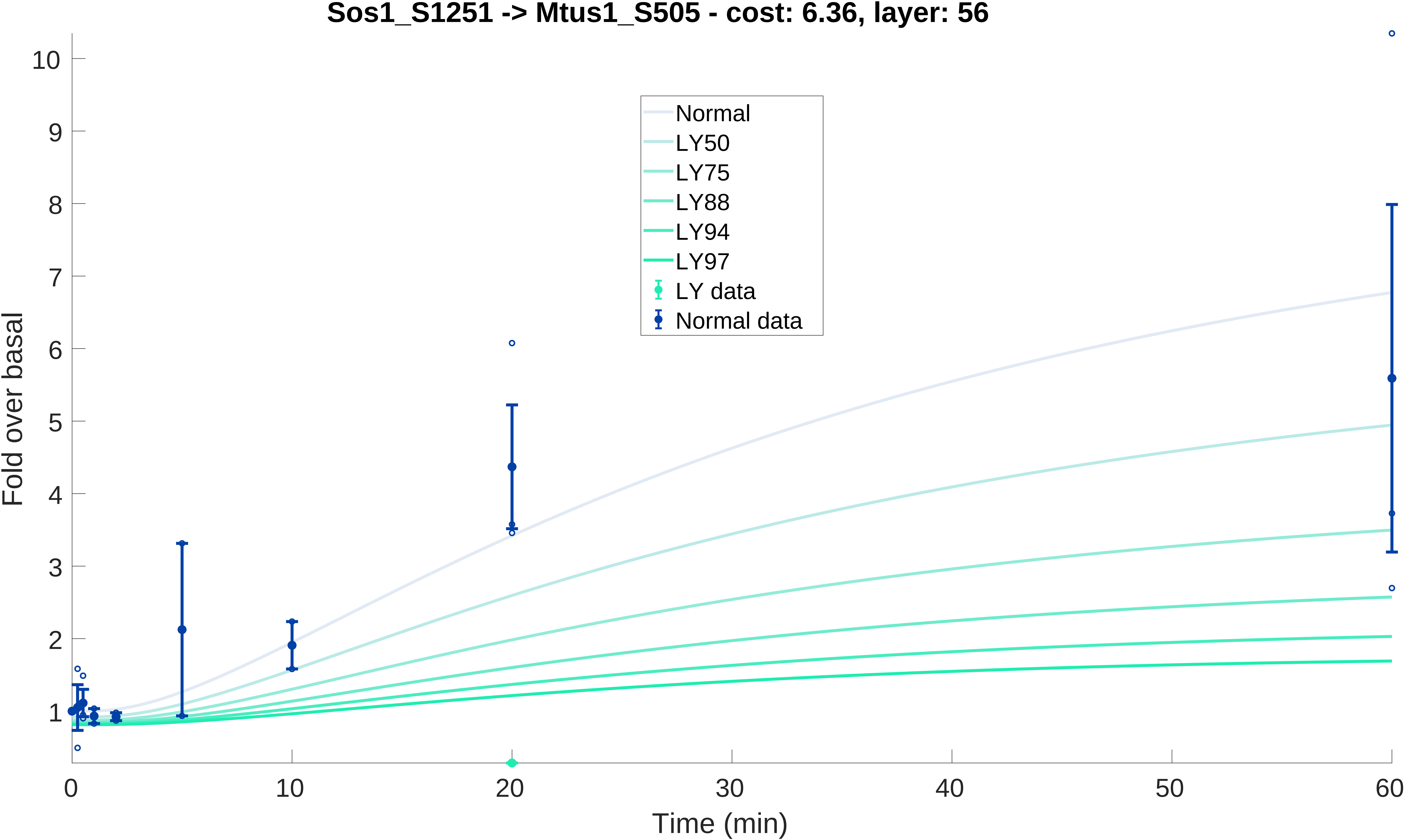
Sos1_S1251 -> Mtus1_S505 - cost: 6.36, layer: 56
Normal
LY50
LY75
LY88
LY94
LY97
LY data
Normal data
Fold over basal
Time (min)
1
2
3
4
5
6
7
8
9
10
0
10
20
30
40
50
60

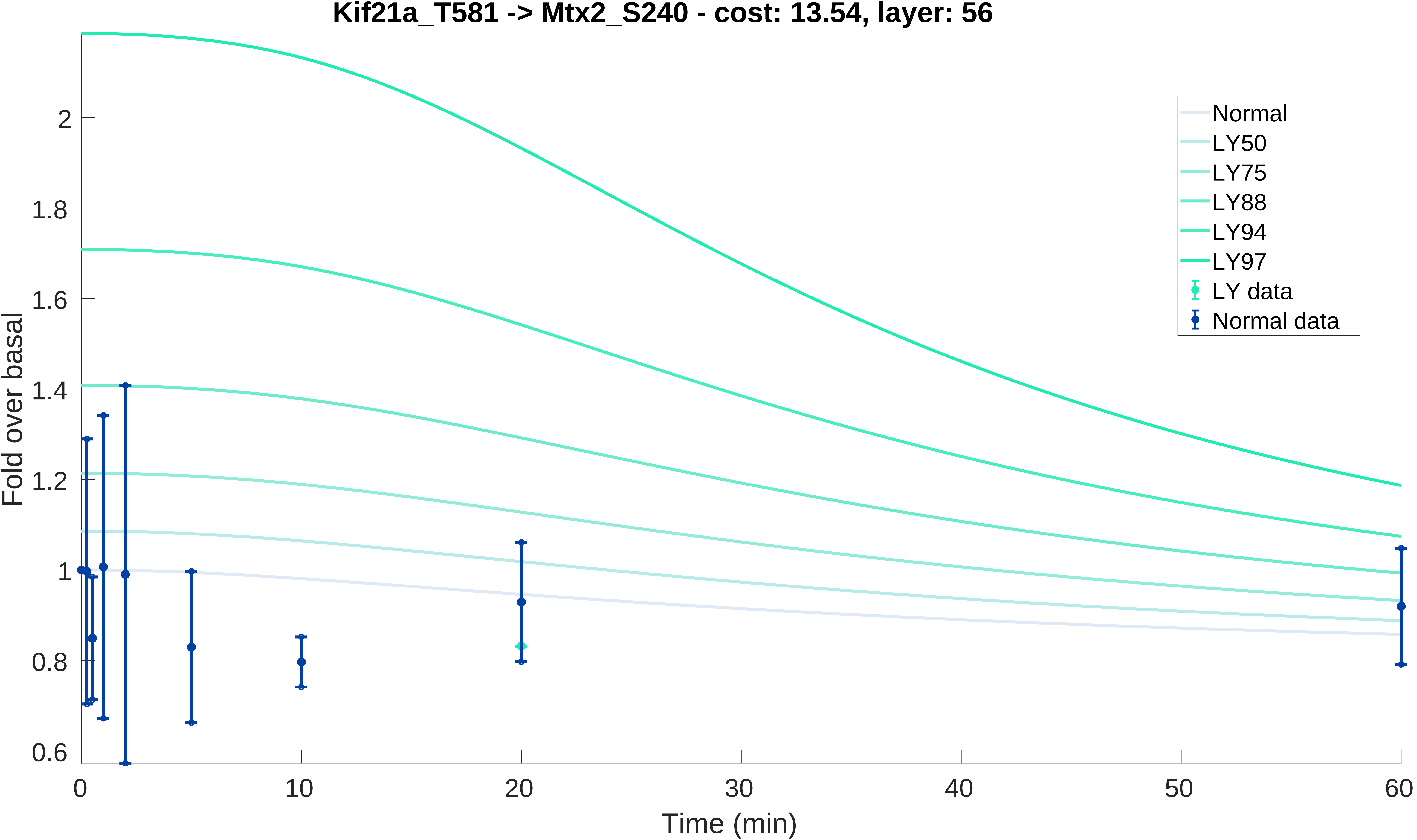
Kif21a_T581 -> Mtx2_S240 - cost: 13.54, layer: 56
Fold over basal
Time (min)
Normal
LY50
LY75
LY88
LY94
LY97
LY data
Normal data
0.6
0.8
1
1.2
1.4
1.6
1.8
2
0
10
20
30
40
50
60

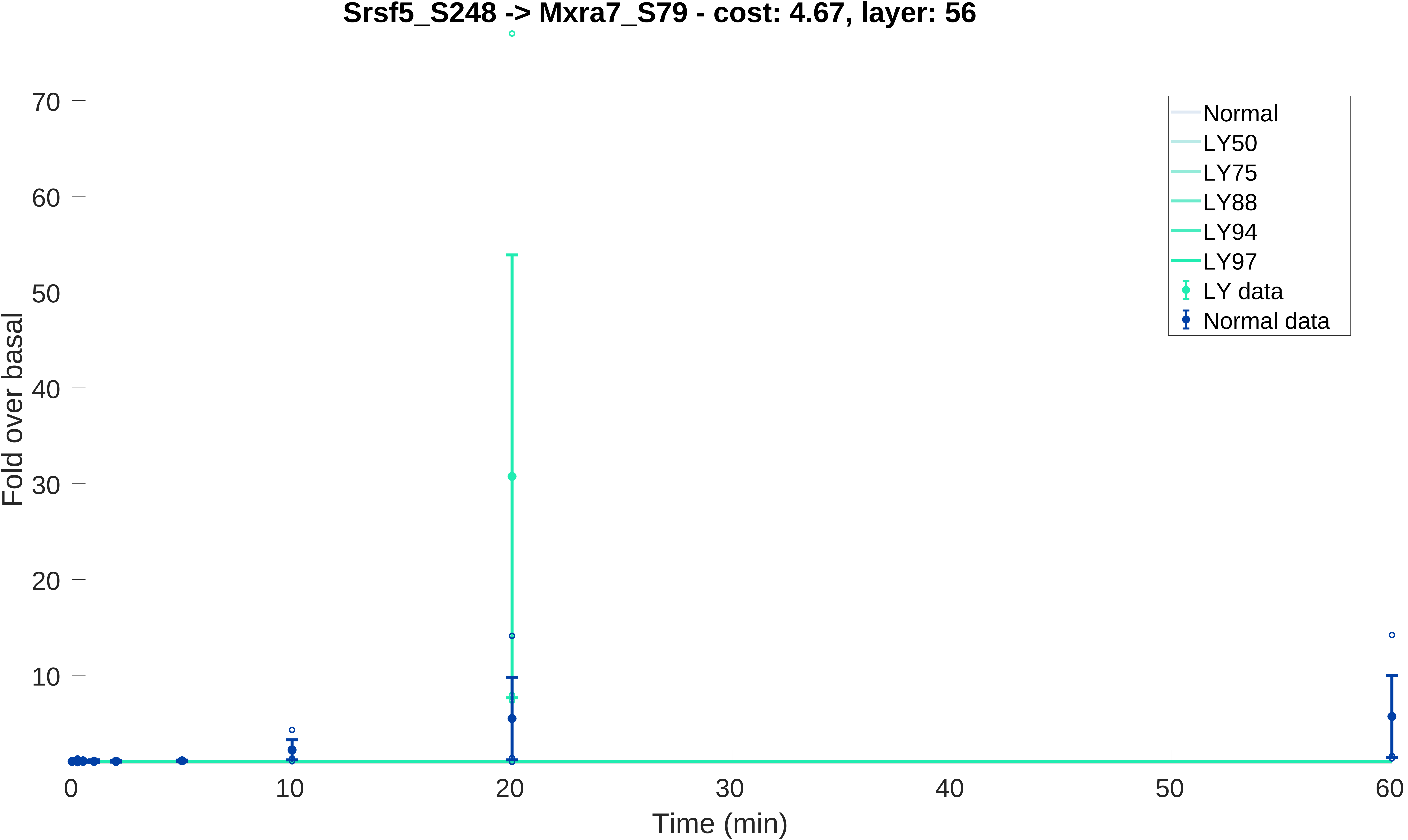
Srsf5_S248 -> Mxra7_S79 - cost: 4.67, layer: 56
Fold over basal
Time (min)
0
10
20
30
40
50
60
70
Normal
LY50
LY75
LY88
LY94
LY97
LY data
Normal data

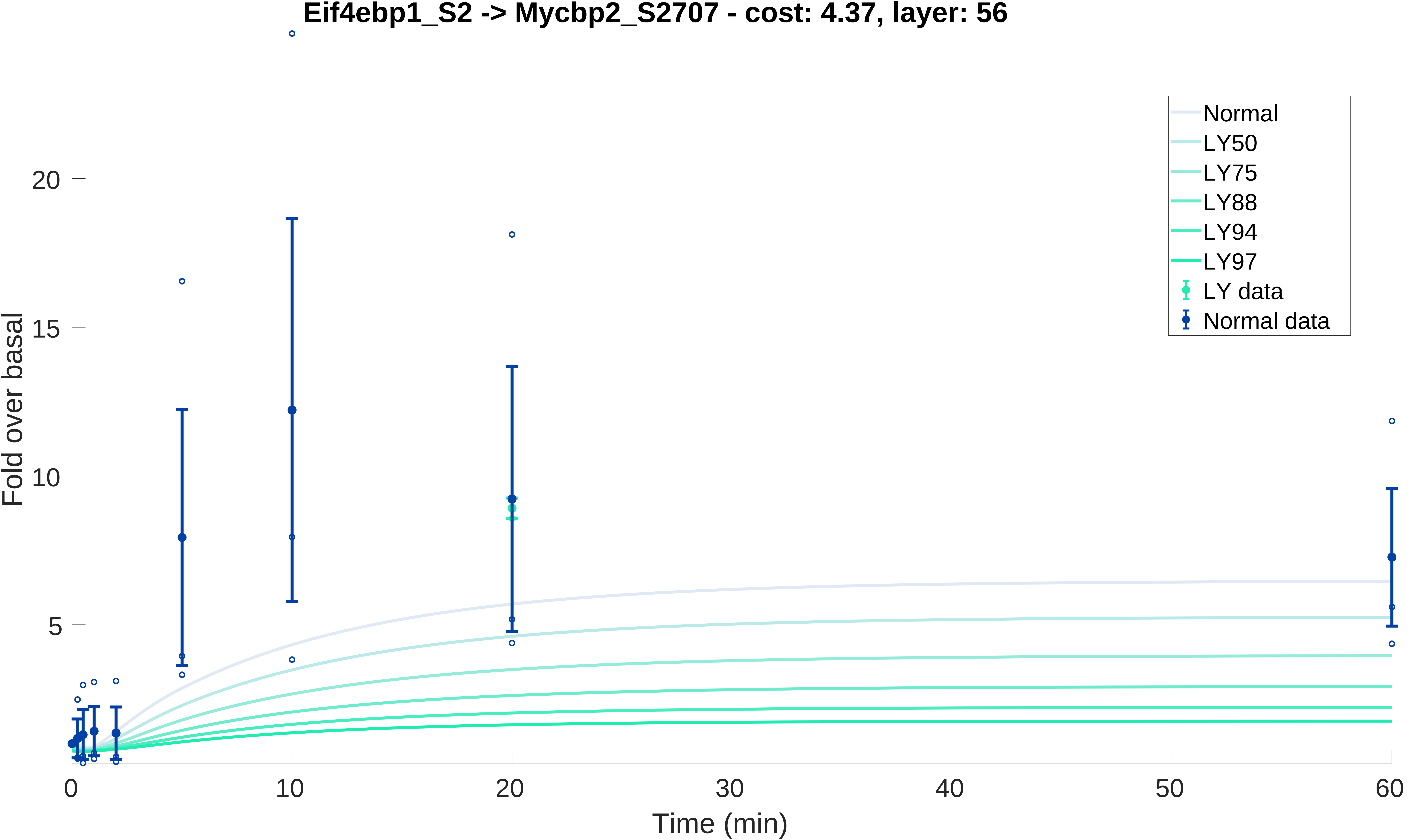

Eif4ebp1_S2 -> Mycbp2_S2707 - cost: 4.37, layer: 56
Fold over basal
Time (min)
0
10
20
30
40
50
60
5
10
15
20
Normal
LY50
LY75
LY88
LY94
LY97
LY data
Normal data

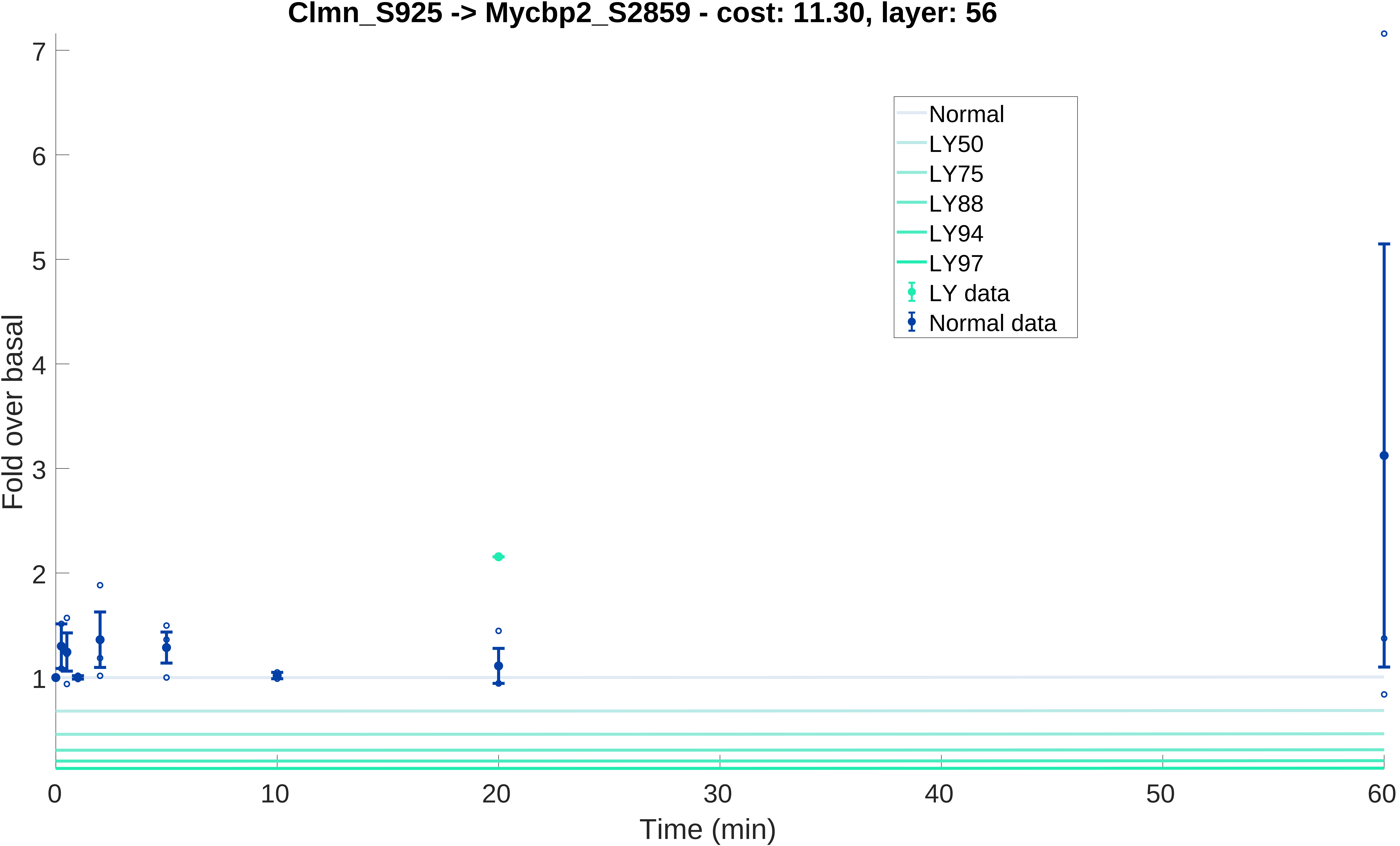

Clmn_S925 -> Mycbp2_S2859 - cost: 11.30, layer: 56
Fold over basal
Time (min)
Normal
LY50
LY75
LY88
LY94
LY97
LY data
Normal data

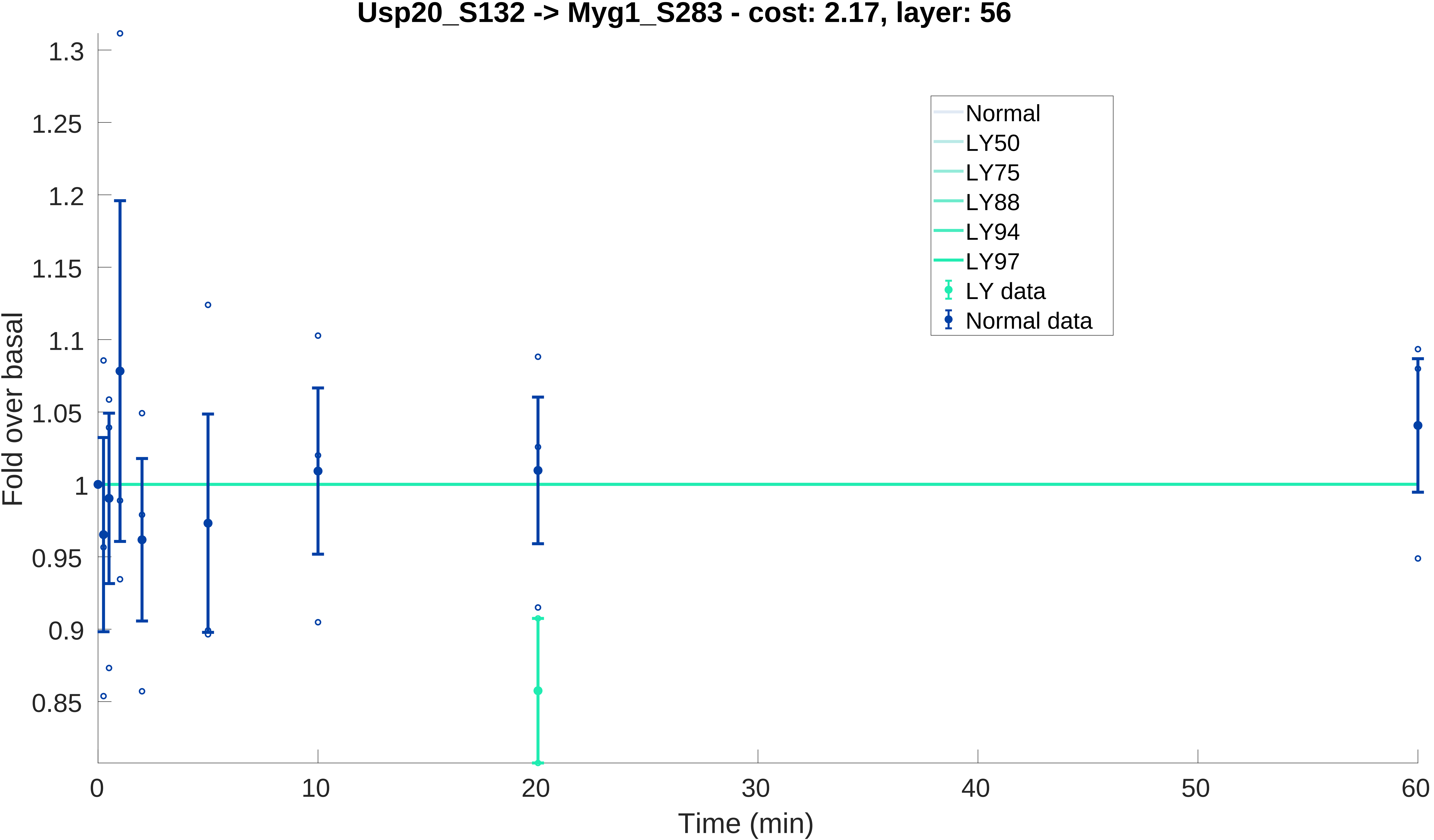
Usp20_S132 -> Myg1_S283 - cost: 2.17, layer: 56
Fold over basal
Time (min)
1.3
1.25
1.2
1.15
1.1
1.05
1
0.95
0.9
0.85
0
10
20
30
40
50
60
Normal
LY50
LY75
LY88
LY94
LY97
LY data
Normal data

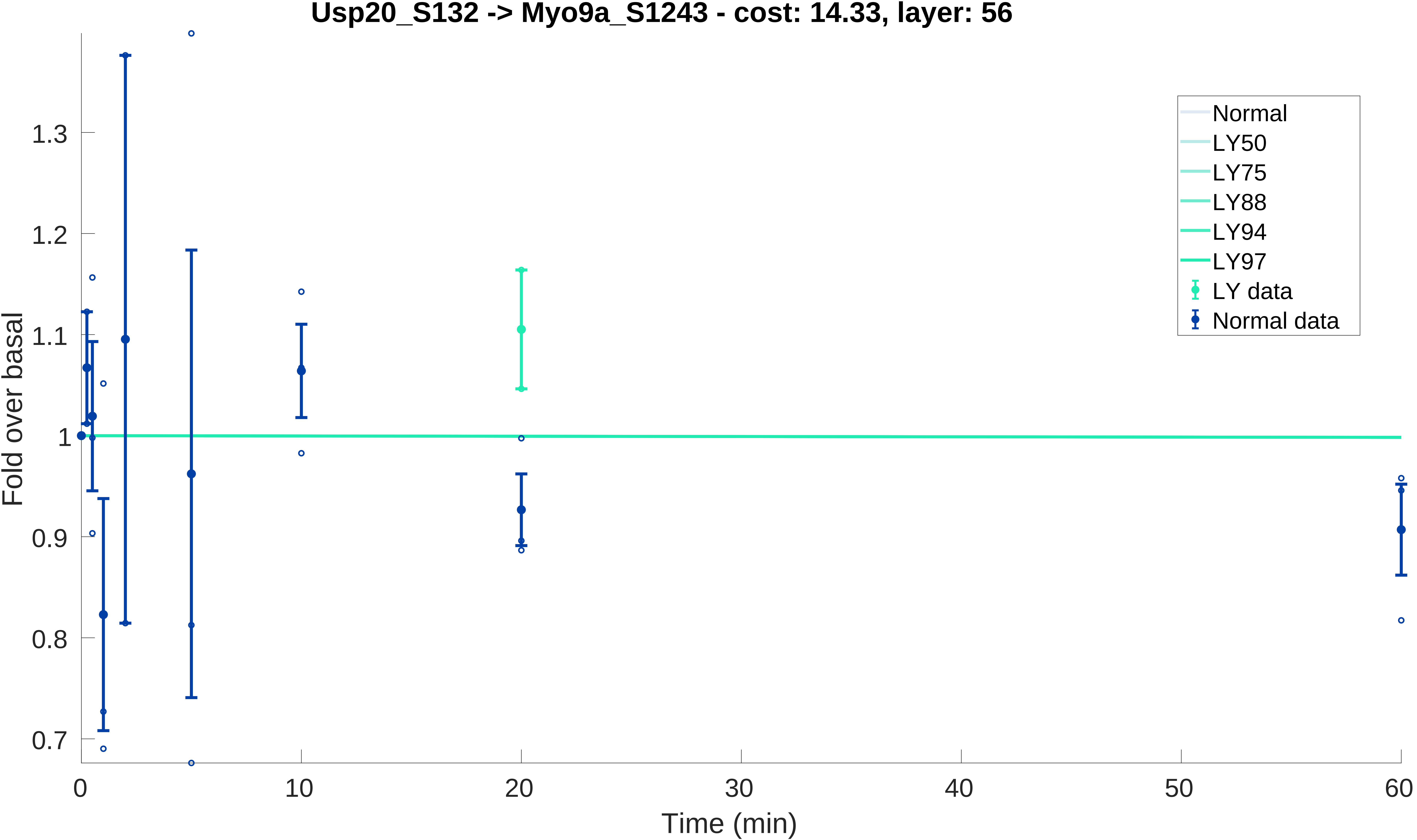
Usp20_S132 -> Myo9a_S1243 - cost: 14.33, layer: 56
Fold over basal
Time (min)
Normal
LY50
LY75
LY88
LY94
LY97
LY data
Normal data
0
10
20
30
40
50
60
0.7
0.8
0.9
1
1.1
1.2
1.3

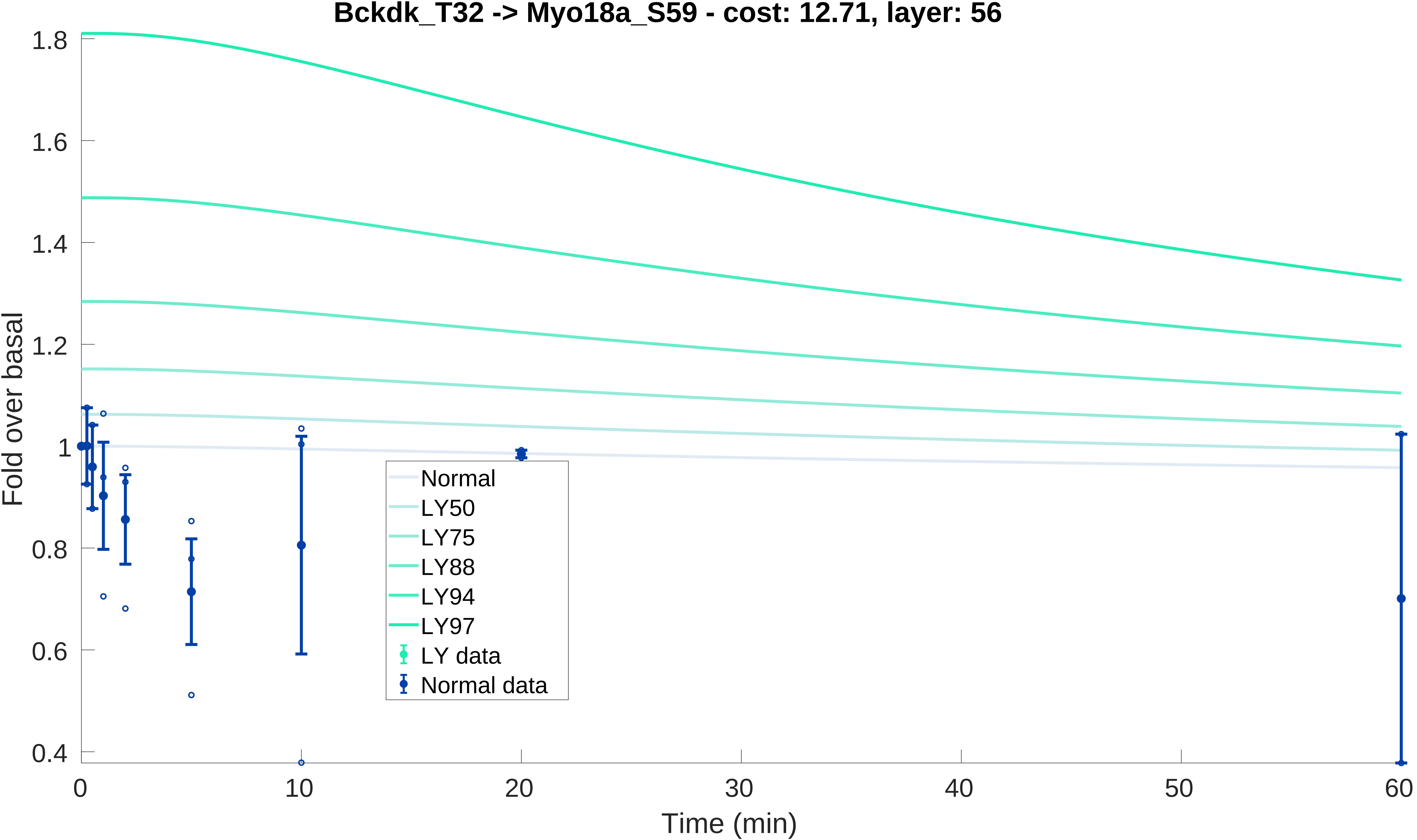

Bckdk_T32 -> Myo18a_S59 - cost: 12.71, layer: 56
Fold over basal
1.8
1.6
1.4
1.2
1
0.8
0.6
0.4
0
10
20
30
40
50
60
Time (min)
Normal
LY50
LY75
LY88
LY94
LY97
LY data
Normal data

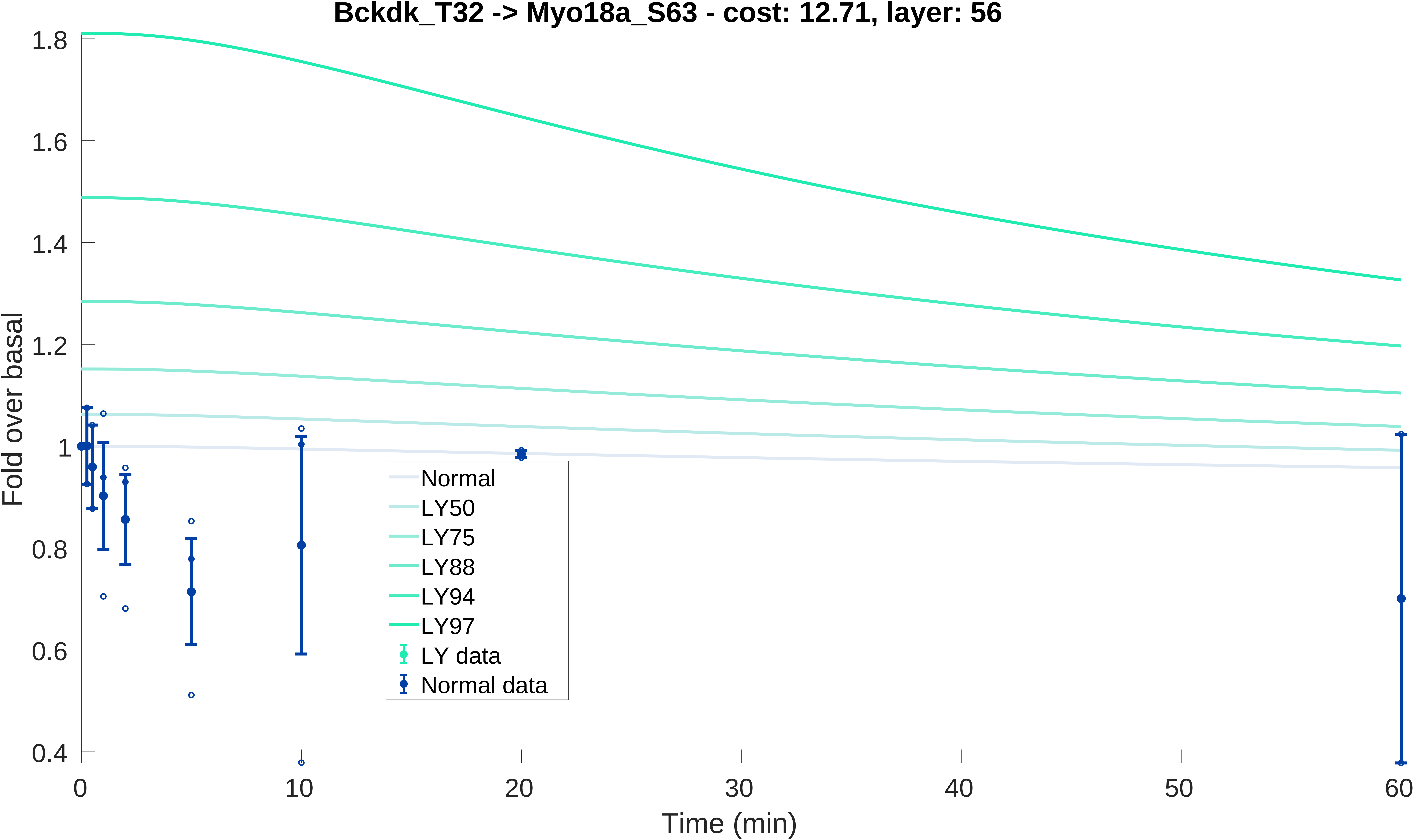
Bckdk_T32 -> Myo18a_S63 - cost: 12.71, layer: 56
Fold over basal
Time (min)
1.8
1.6
1.4
1.2
1
0.8
0.6
0.4
0
10
20
30
40
50
60
Normal
LY50
LY75
LY88
LY94
LY97
LY data
Normal data

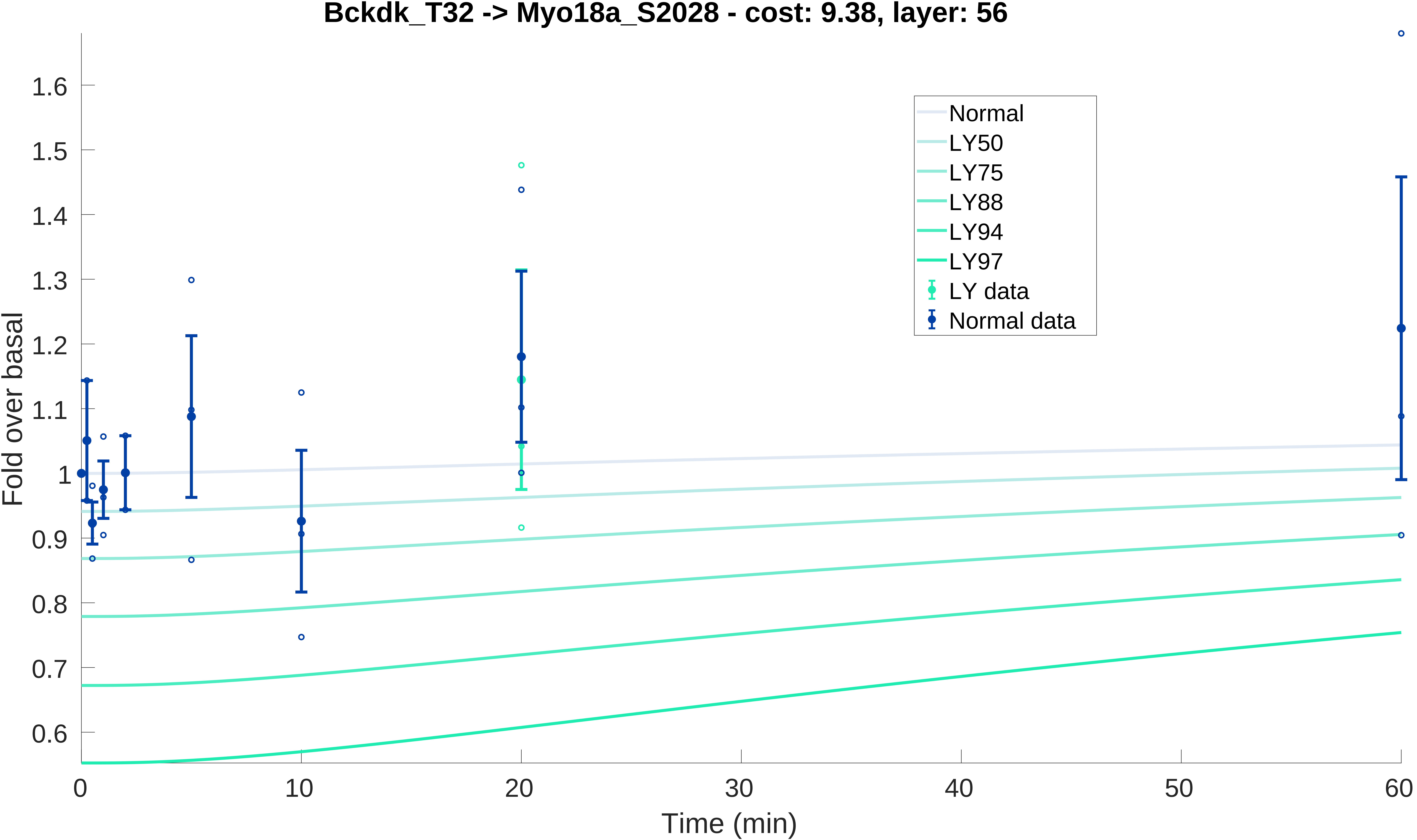

Bckdk_T32 -> Myo18a_S2028 - cost: 9.38, layer: 56
Fold over basal
1.6
1.5
1.4
1.3
1.2
1.1
1
0.9
0.8
0.7
0.6
0
10
20
30
40
50
60
Time (min)
Normal
LY50
LY75
LY88
LY94
LY97
LY data
Normal data

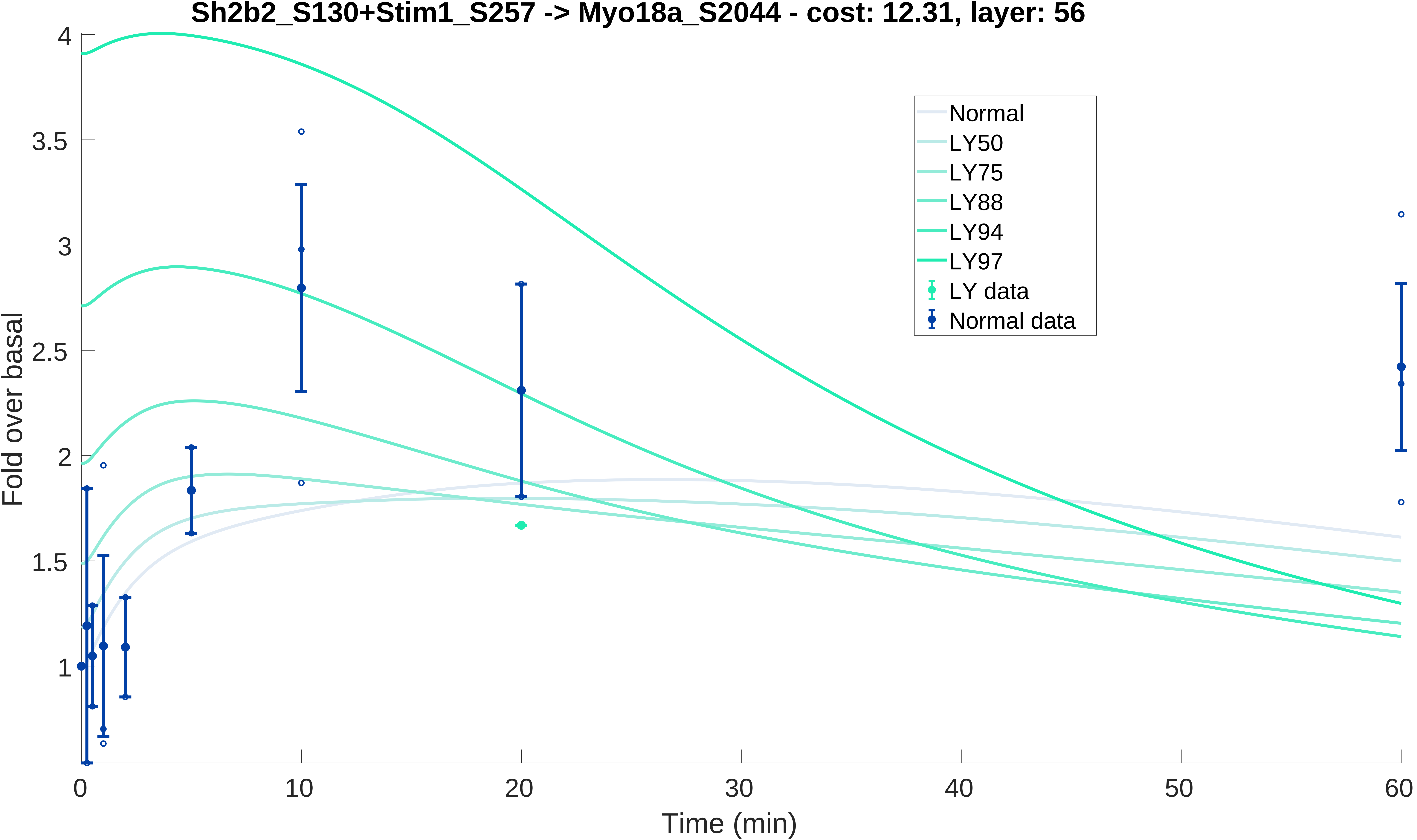

Sh2b2_S130+Stim1_S257 -> Myo18a_S2044 - cost: 12.31, layer: 56
Fold over basal
Time (min)
0
10
20
30
40
50
60
1
1.5
2
2.5
3
3.5
4
Normal
LY50
LY75
LY88
LY94
LY97
LY data
Normal data

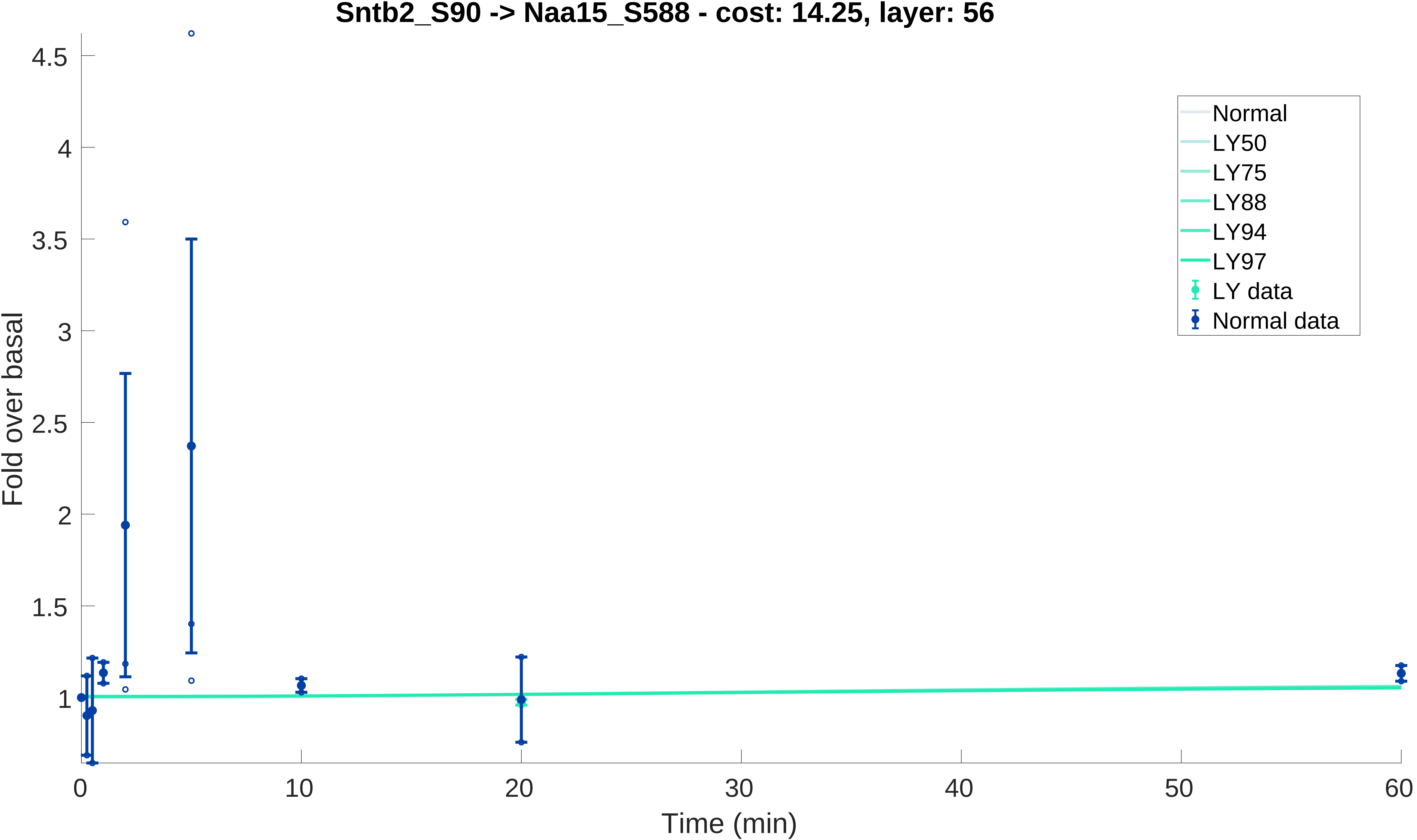

Sntb2_S90 -> Naa15_S588 - cost: 14.25, layer: 56
Fold over basal
Time (min)
4.5
4
3.5
3
2.5
2
1.5
1
0
10
20
30
40
50
60
Normal
LY50
LY75
LY88
LY94
LY97
LY data
Normal data

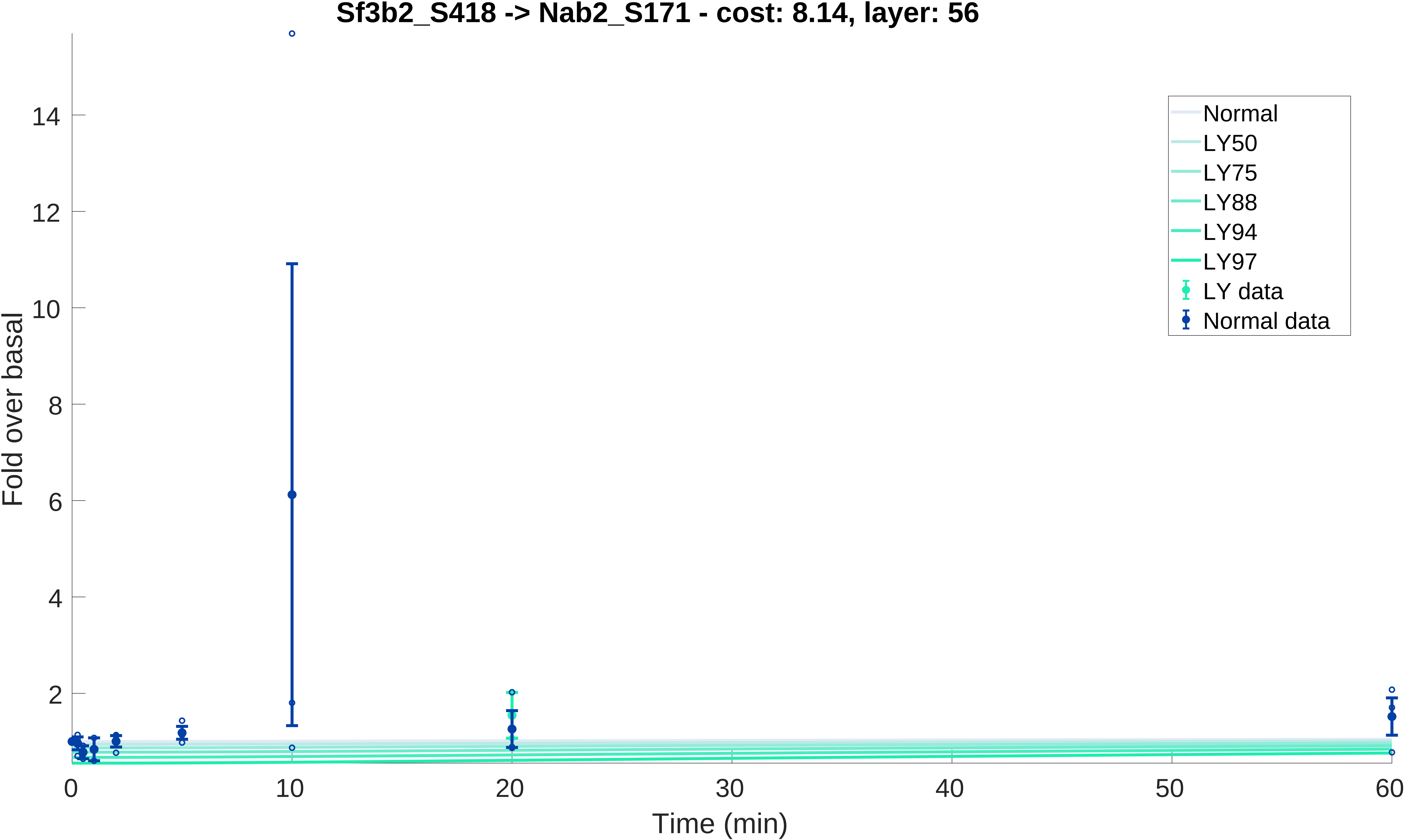

Sf3b2_S418 -> Nab2_S171 - cost: 8.14, layer: 56
Fold over basal
Time (min)
0
10
20
30
40
50
60
2
4
6
8
10
12
14
Normal
LY50
LY75
LY88
LY94
LY97
LY data
Normal data

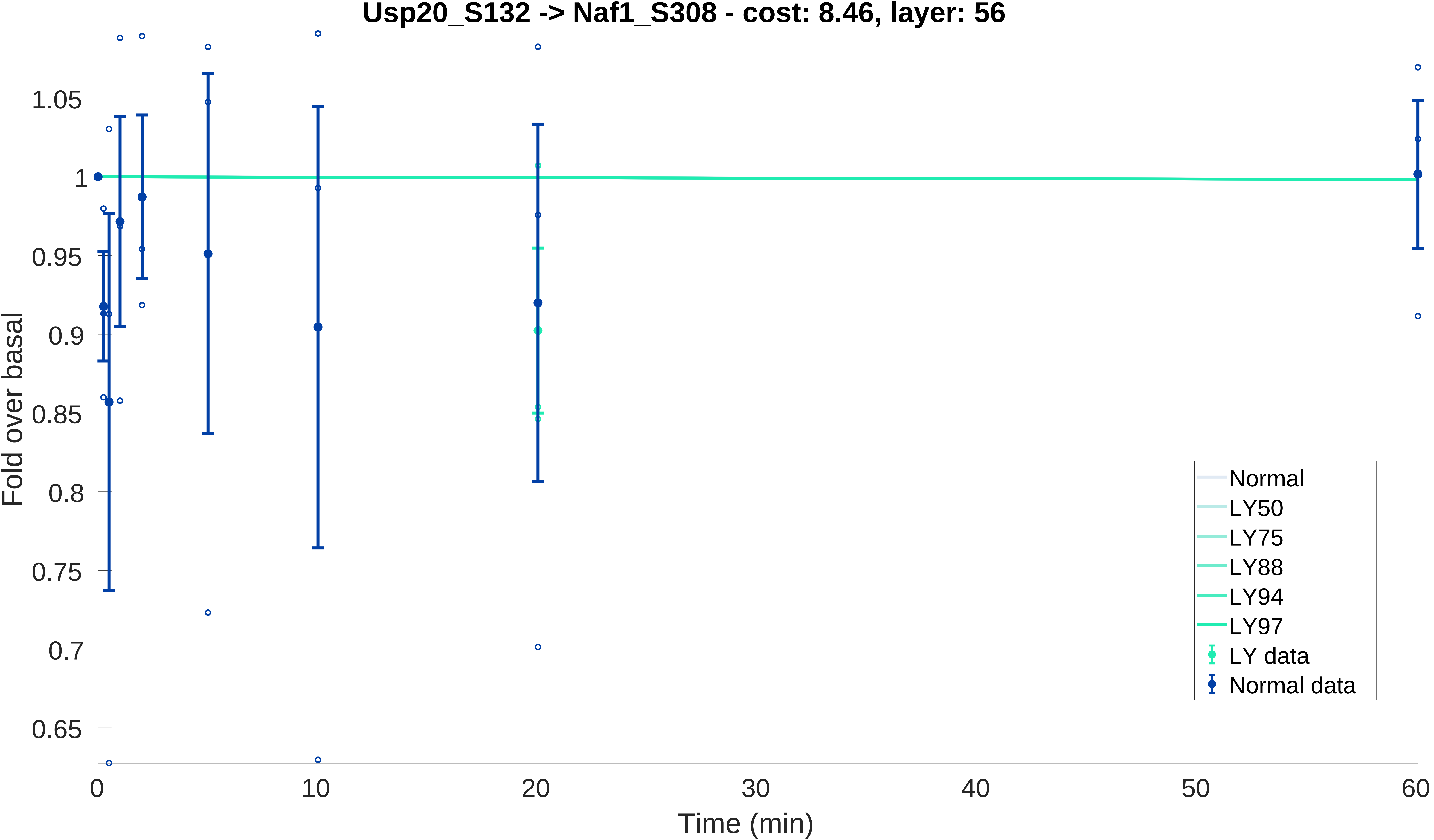

Usp20_S132 -> Naf1_S308 - cost: 8.46, layer: 56
Fold over basal
1.05
1
0.95
0.9
0.85
0.8
0.75
0.7
0.65
0
10
20
30
40
50
60
Time (min)
Normal
LY50
LY75
LY88
LY94
LY97
LY data
Normal data

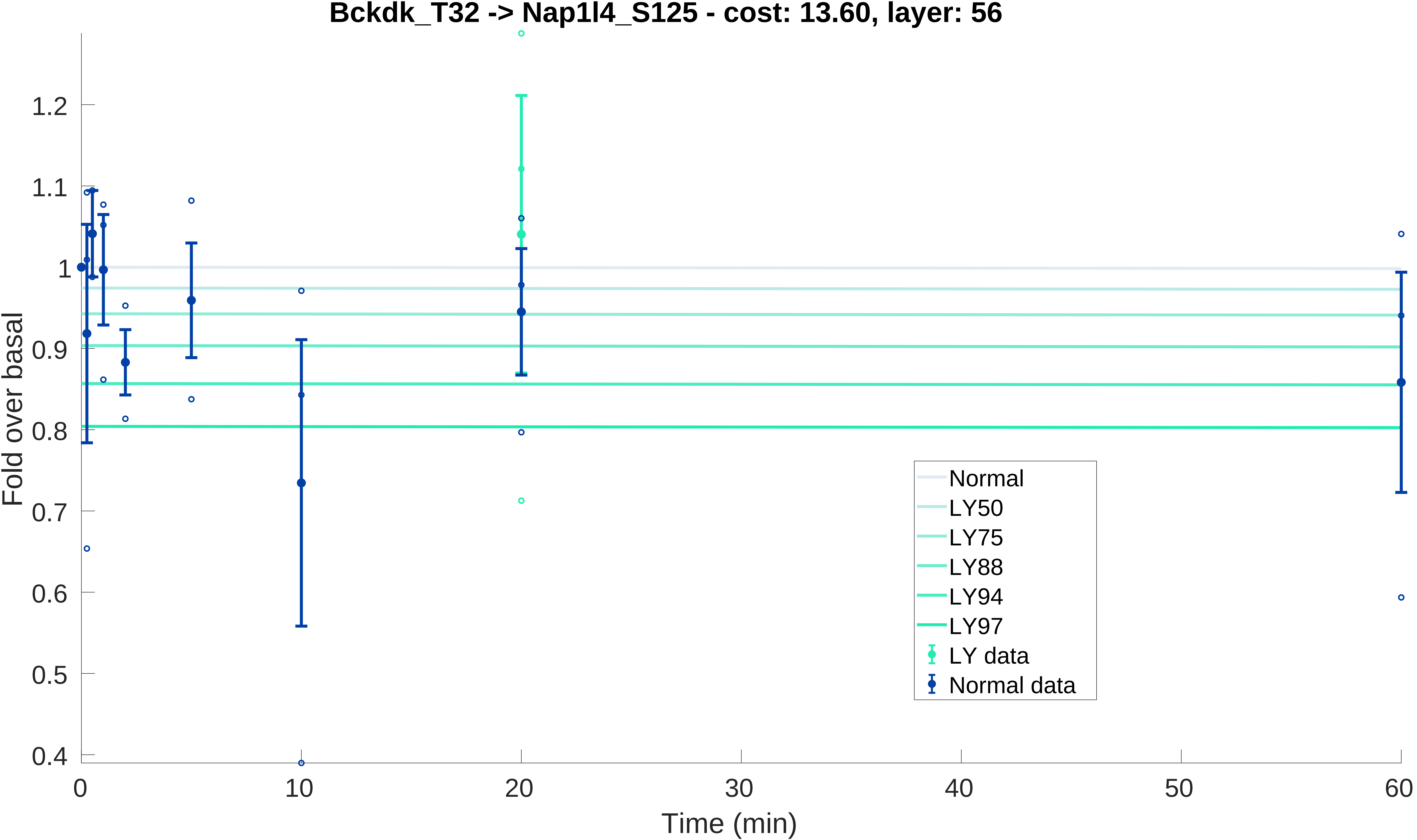
Bckdk_T32 -> Nap1l4_S125 - cost: 13.60, layer: 56
Fold over basal
Time (min)
1.2
1.1
1
0.9
0.8
0.7
0.6
0.5
0.4
0
10
20
30
40
50
60
Normal
LY50
LY75
LY88
LY94
LY97
LY data
Normal data

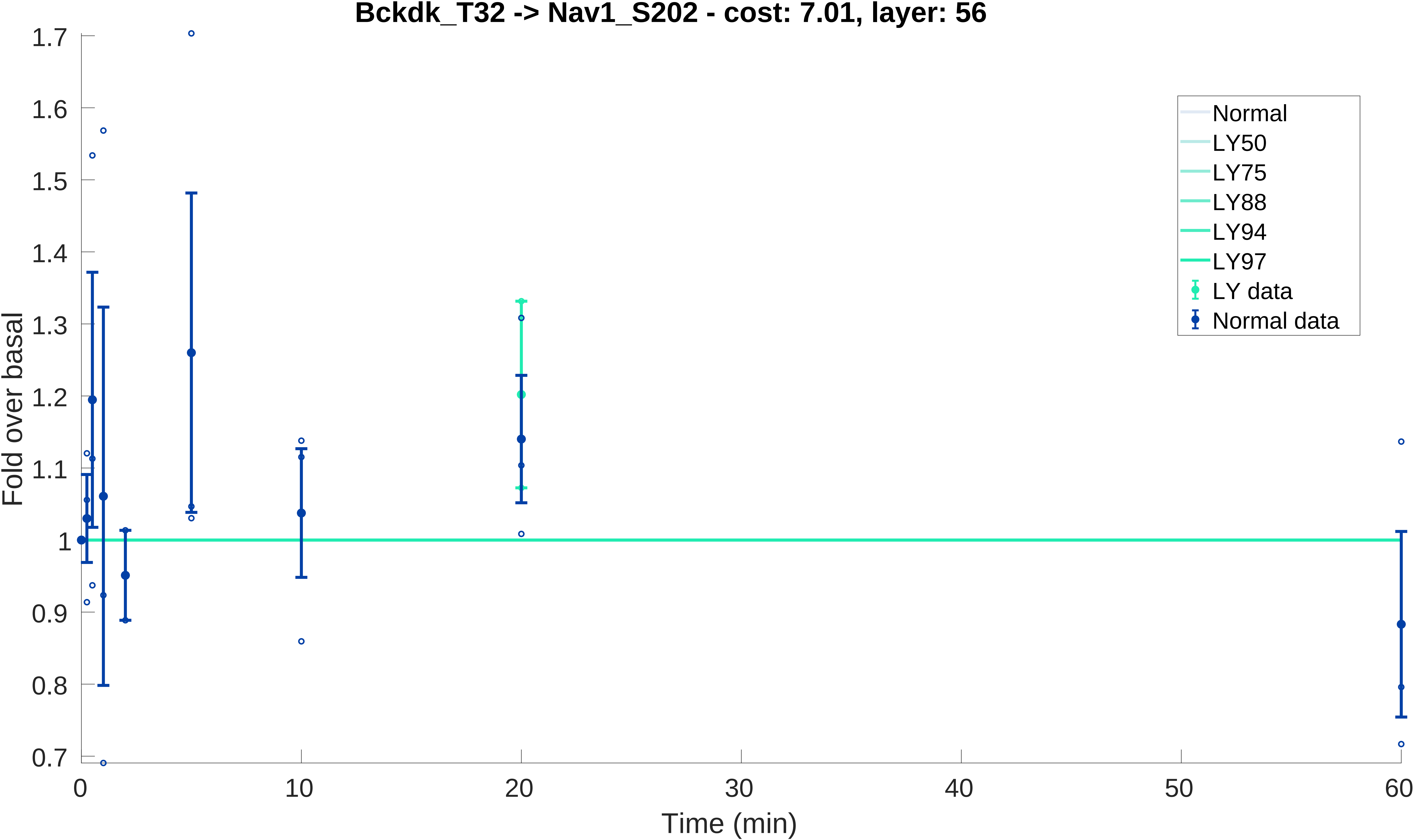
Bckdk_T32 -> Nav1_S202 - cost: 7.01, layer: 56
Fold over basal
Time (min)
1.7
1.6
1.5
1.4
1.3
1.2
1.1
1
0.9
0.8
0.7
0
10
20
30
40
50
60
Normal
LY50
LY75
LY88
LY94
LY97
LY data
Normal data

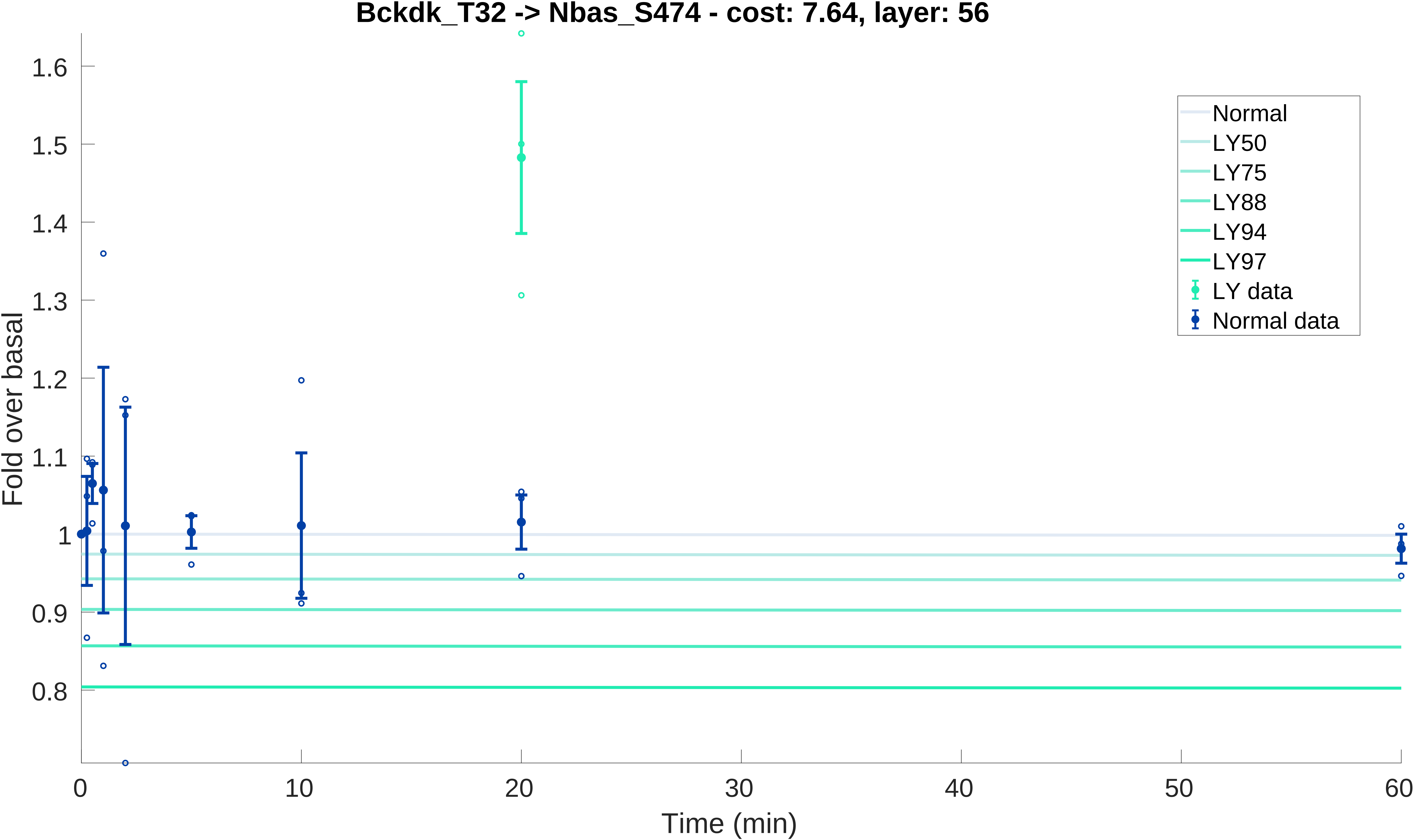
Bckdk_T32 -> Nbas_S474 - cost: 7.64, layer: 56
Fold over basal
Time (min)
1.6
1.5
1.4
1.3
1.2
1.1
1
0.9
0.8
0
10
20
30
40
50
60
Normal
LY50
LY75
LY88
LY94
LY97
LY data
Normal data

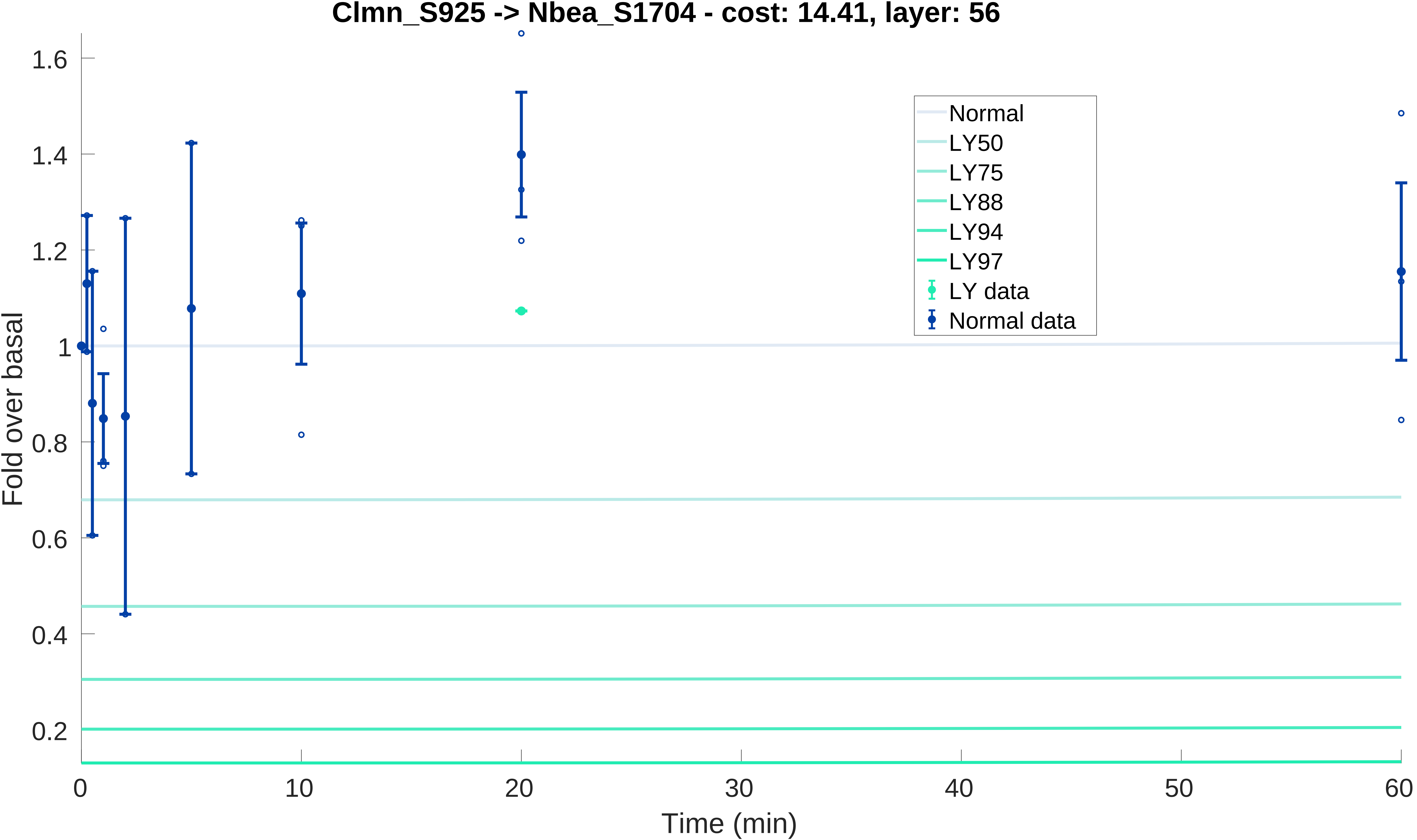

Clmn_S925 -> Nbea_S1704 - cost: 14.41, layer: 56
Fold over basal
0.2
0.4
0.6
0.8
1
1.2
1.4
1.6
Time (min)
0
10
20
30
40
50
60
Normal
LY50
LY75
LY88
LY94
LY97
LY data
Normal data

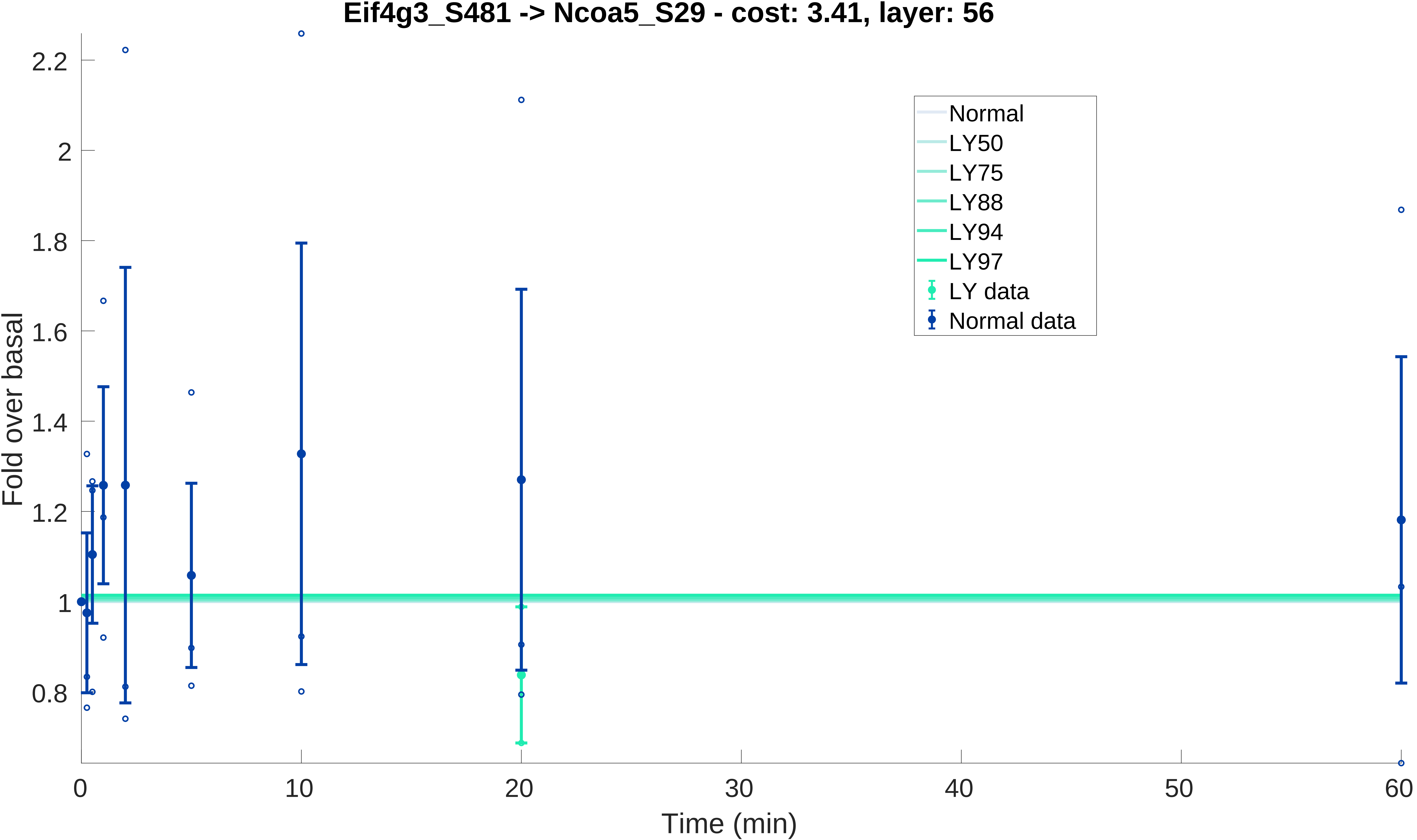

Eif4g3_S481 -> Ncoa5_S29 - cost: 3.41, layer: 56
Fold over basal
Time (min)
2.2
2
1.8
1.6
1.4
1.2
1
0.8
0
10
20
30
40
50
60
Normal
LY50
LY75
LY88
LY94
LY97
LY data
Normal data

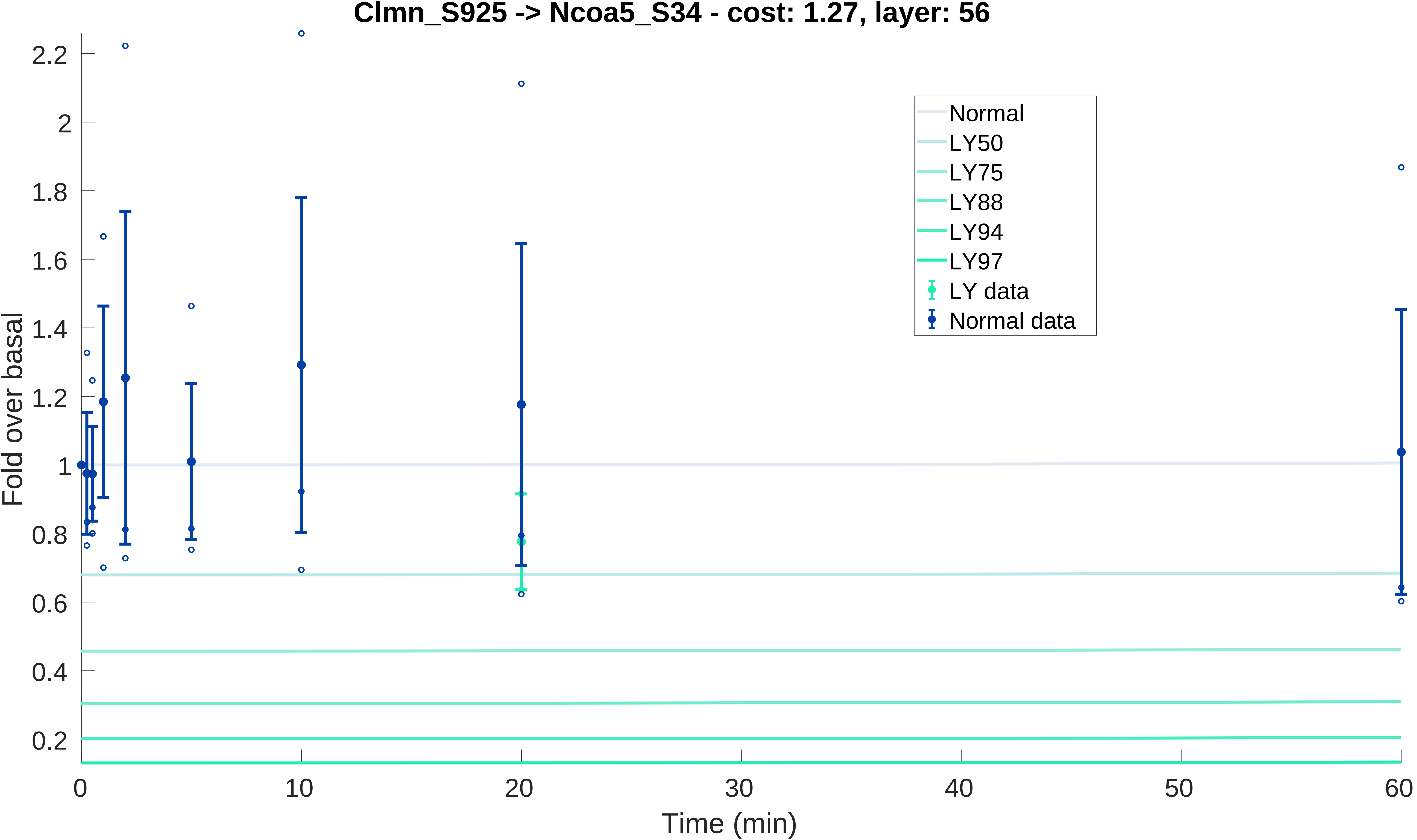

Clmn_S925 -> Ncoa5_S34 - cost: 1.27, layer: 56
Fold over basal
Time (min)
0.2
0.4
0.6
0.8
1
1.2
1.4
1.6
1.8
2
2.2
0
10
20
30
40
50
60
Normal
LY50
LY75
LY88
LY94
LY97
LY data
Normal data

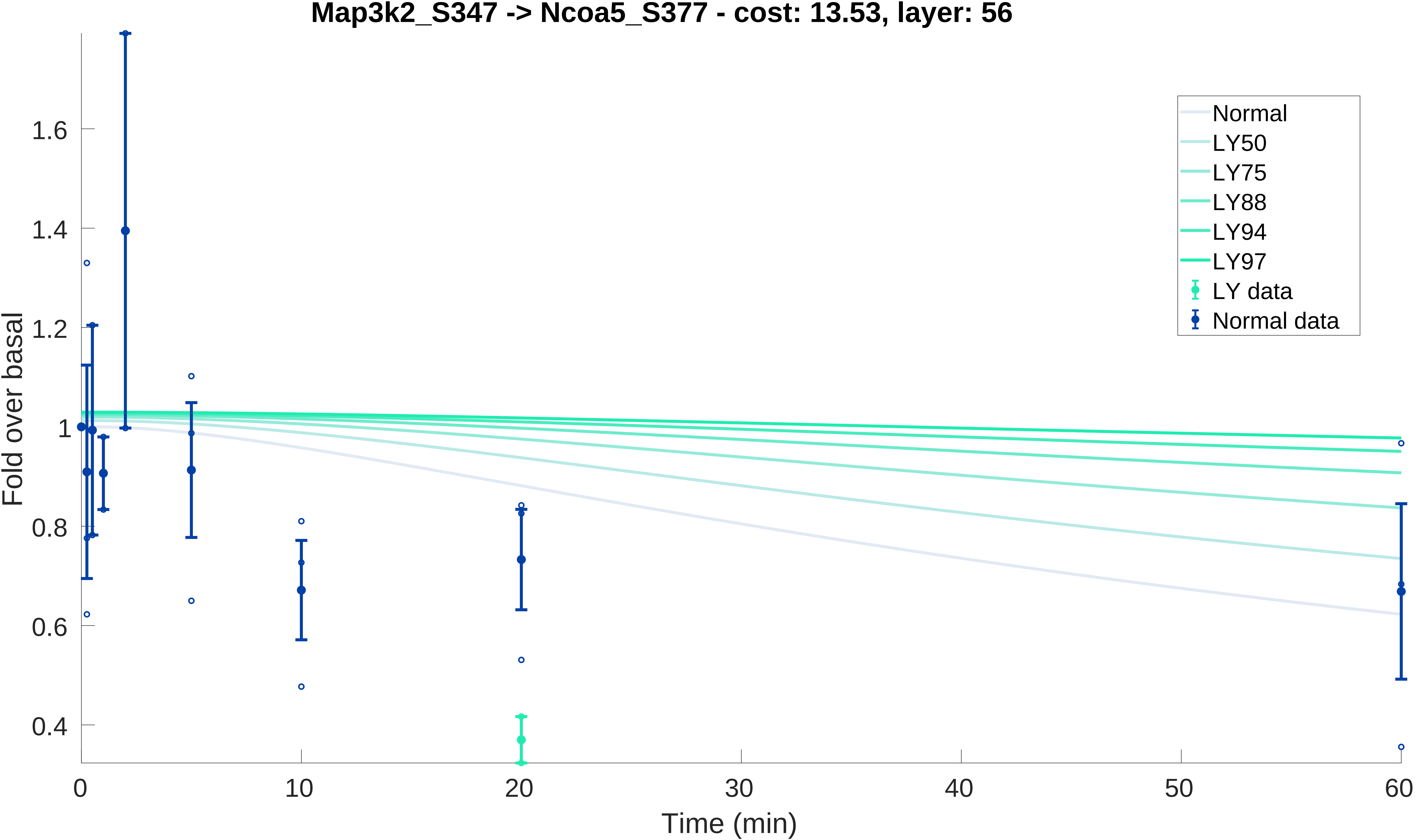

Map3k2_S347 -> Ncoa5_S377 - cost: 13.53, layer: 56
Fold over basal
Time (min)
1.6
1.4
1.2
1
0.8
0.6
0.4
0
10
20
30
40
50
60
Normal
LY50
LY75
LY88
LY94
LY97
LY data
Normal data

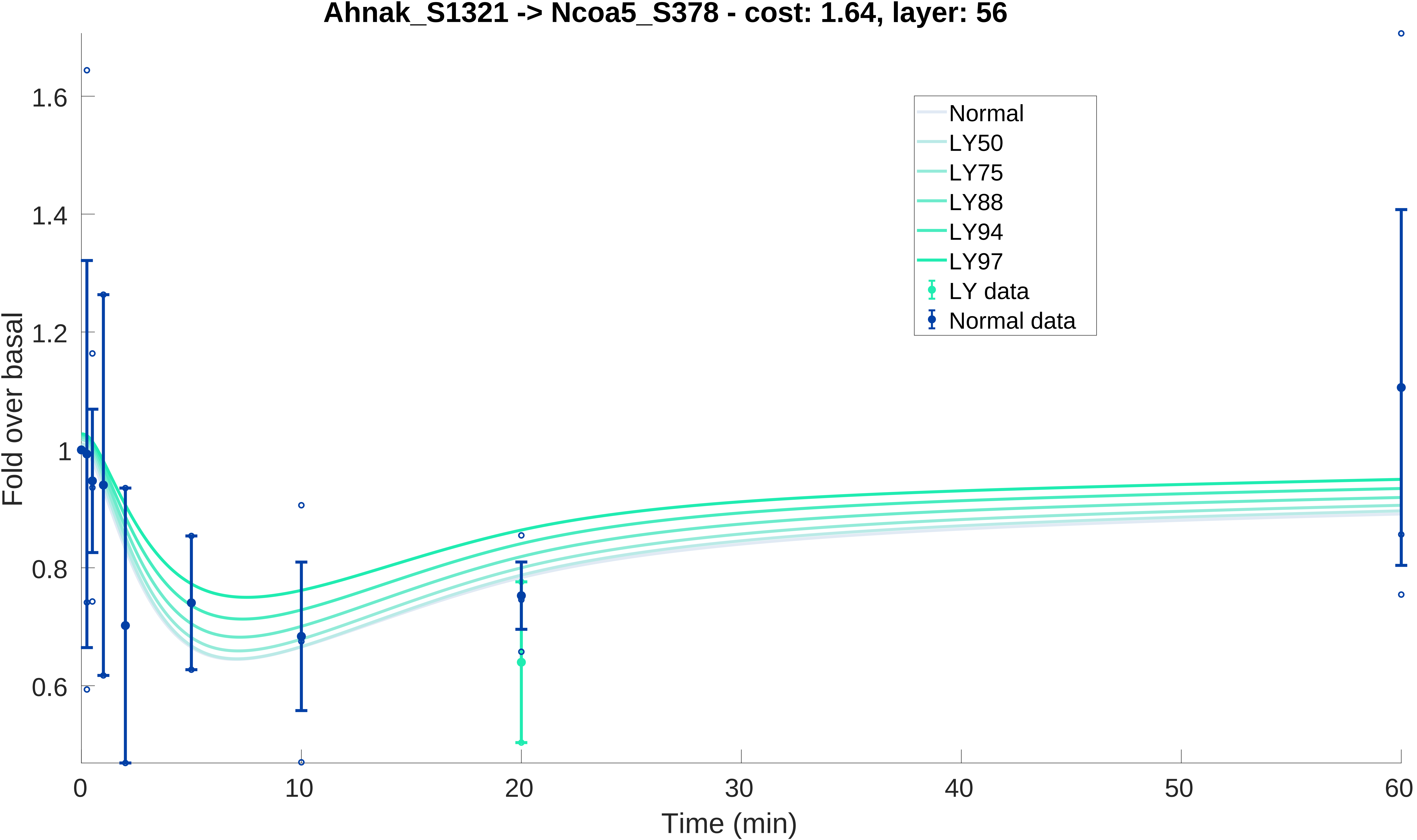

Ahnak_S1321 -> Ncoa5_S378 - cost: 1.64, layer: 56
Fold over basal
1.6
1.4
1.2
1
0.8
0.6
0
10
20
30
40
50
60
Time (min)
Normal
LY50
LY75
LY88
LY94
LY97
LY data
Normal data

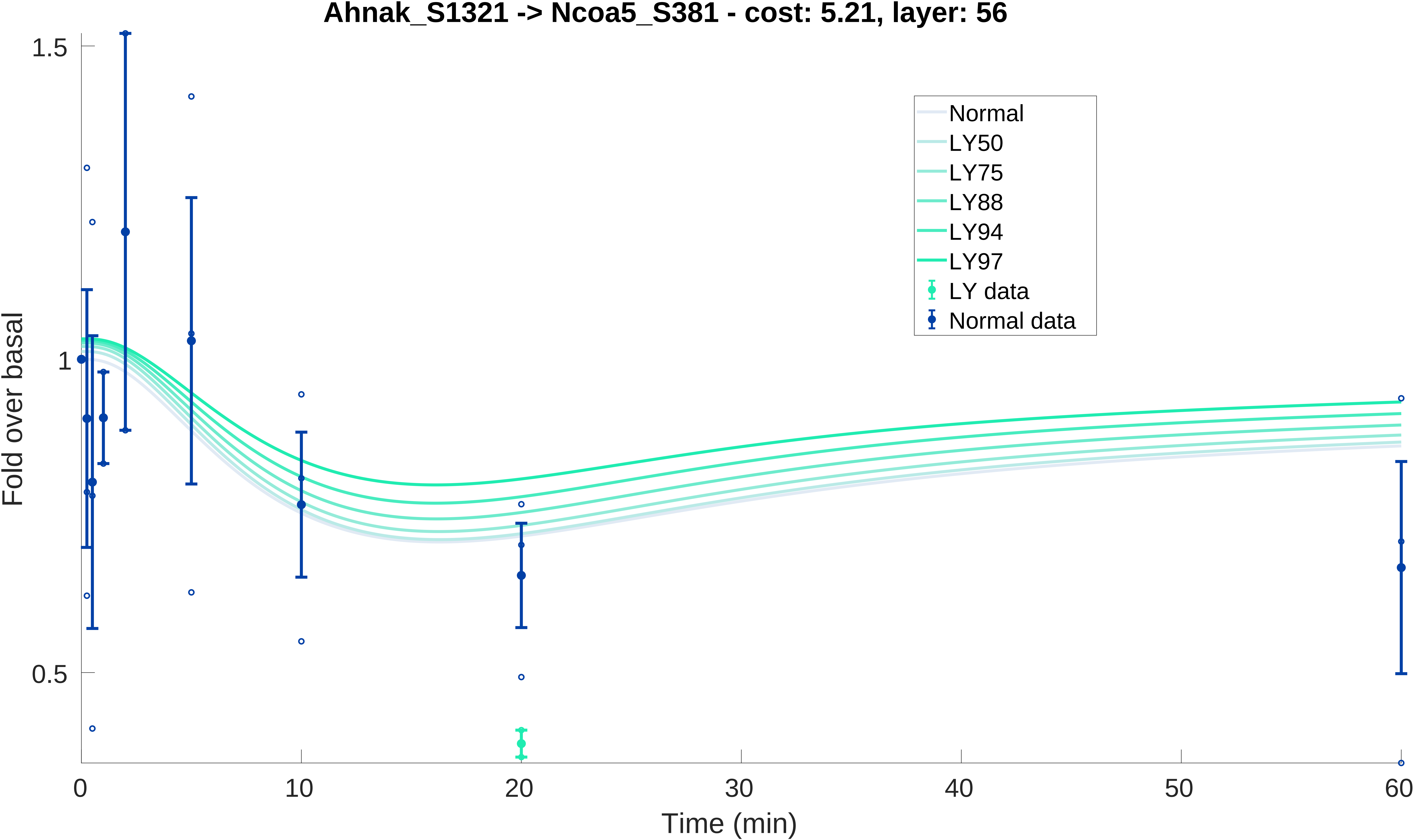

Ahnak_S1321 -> Ncoa5_S381 - cost: 5.21, layer: 56
Fold over basal
Time (min)
1.5
1
0.5
0
10
20
30
40
50
60
Normal
LY50
LY75
LY88
LY94
LY97
LY data
Normal data

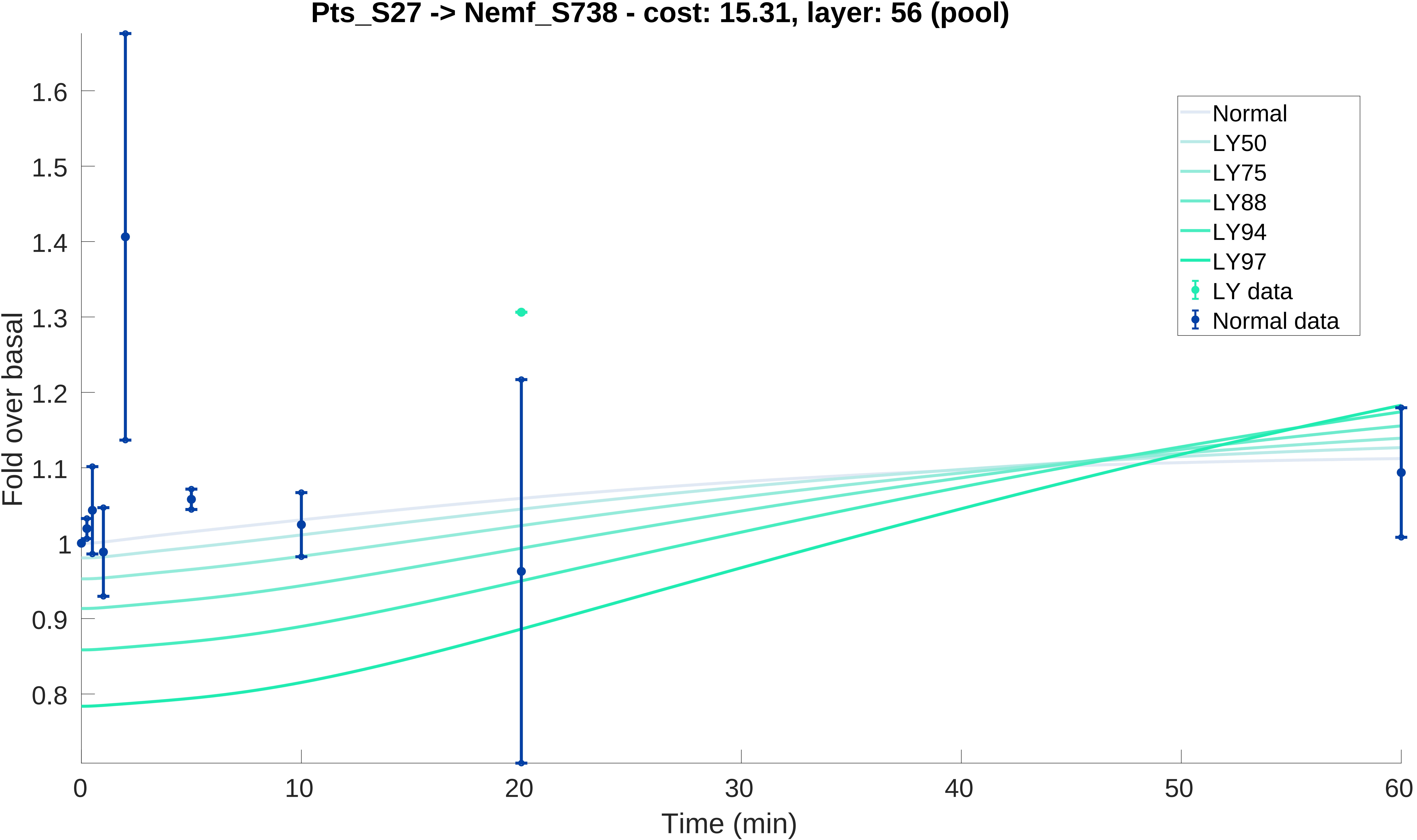
Pts_S27 -> Nemf_S738 - cost: 15.31, layer: 56 (pool)
Fold over basal
Time (min)
0
10
20
30
40
50
60
0.8
0.9
1
1.1
1.2
1.3
1.4
1.5
1.6
Normal
LY50
LY75
LY88
LY94
LY97
LY data
Normal data

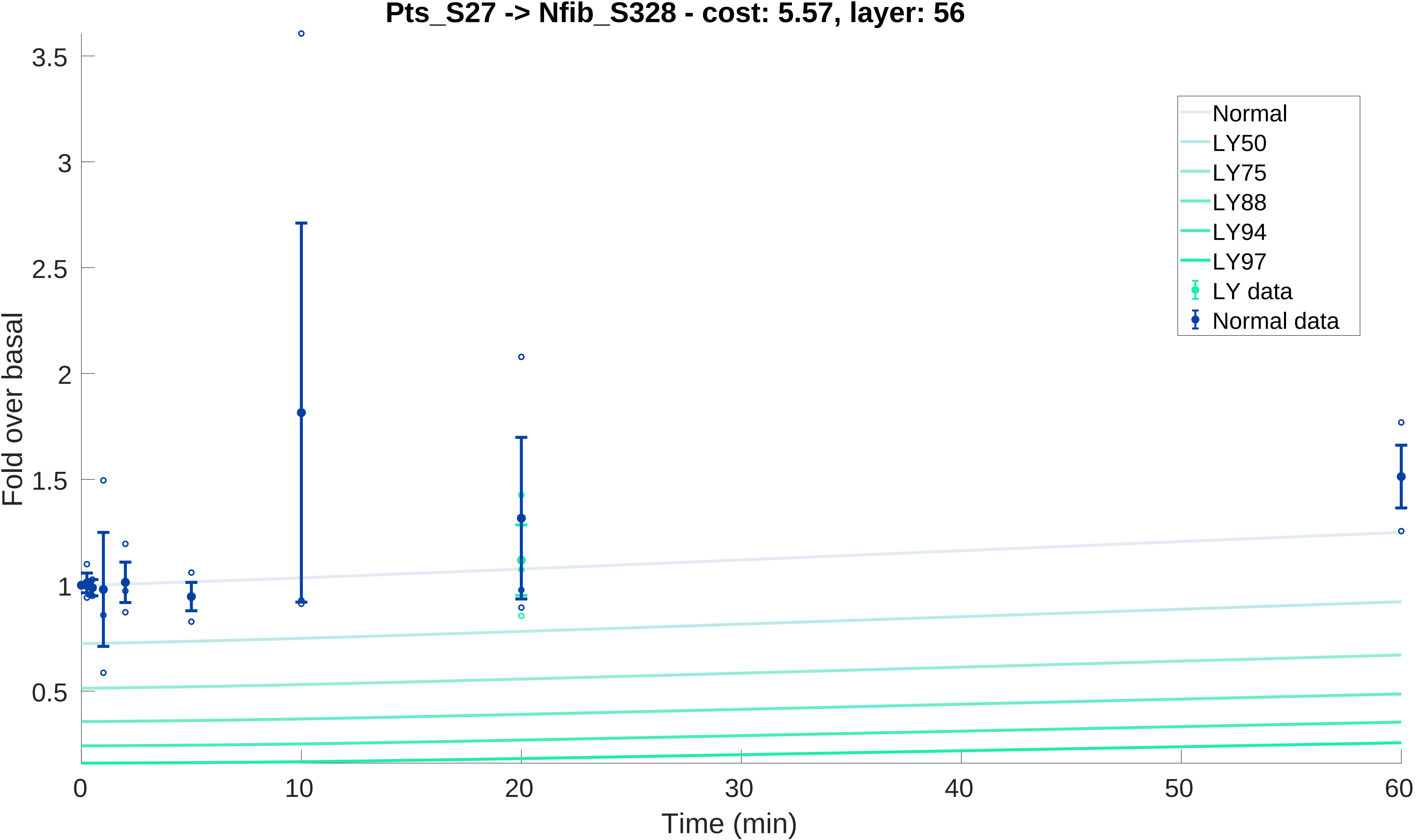

Pts_S27 -> Nfib_S328 - cost: 5.57, layer: 56
Fold over basal
Time (min)
0
10
20
30
40
50
60
0.5
1
1.5
2
2.5
3
3.5
Normal
LY50
LY75
LY88
LY94
LY97
LY data
Normal data

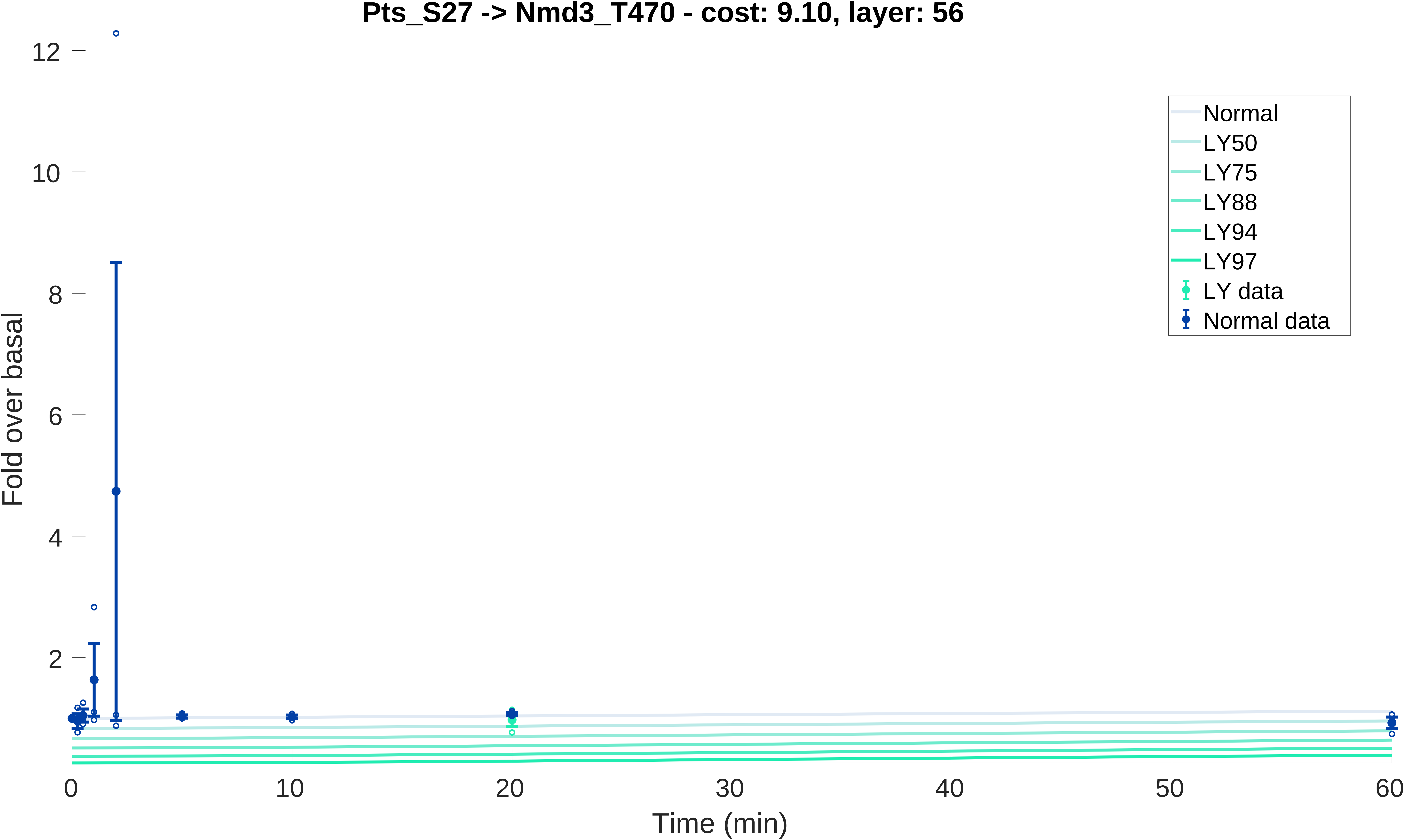

Pts_S27 -> Nmd3_T470 - cost: 9.10, layer: 56
Fold over basal
Time (min)
0
2
4
6
8
10
12
0
10
20
30
40
50
60
Normal
LY50
LY75
LY88
LY94
LY97
LY data
Normal data

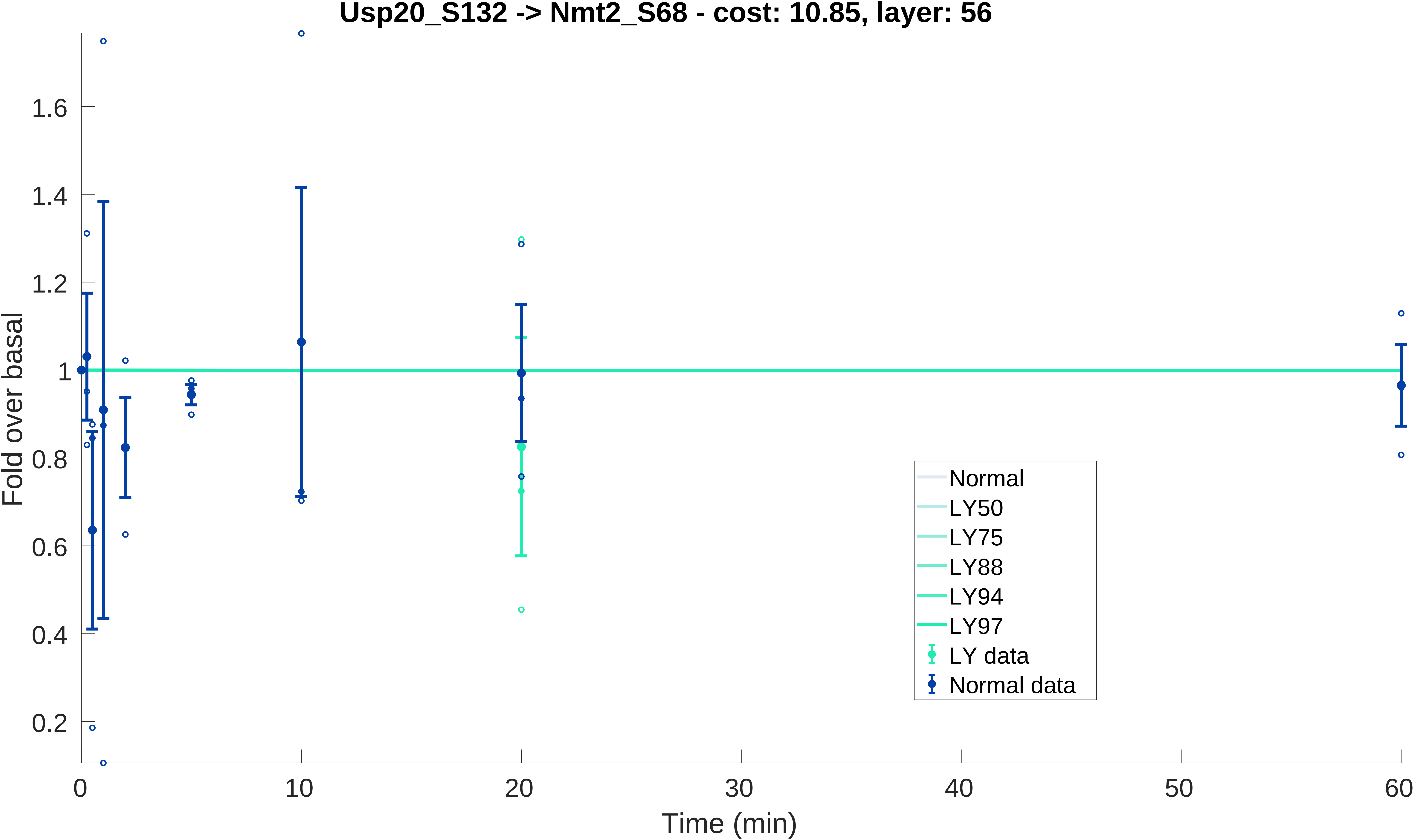
Usp20_S132 -> Nmt2_S68 - cost: 10.85, layer: 56
Fold over basal
Time (min)
0.2
0.4
0.6
0.8
1
1.2
1.4
1.6
0
10
20
30
40
50
60
Normal
LY50
LY75
LY88
LY94
LY97
LY data
Normal data

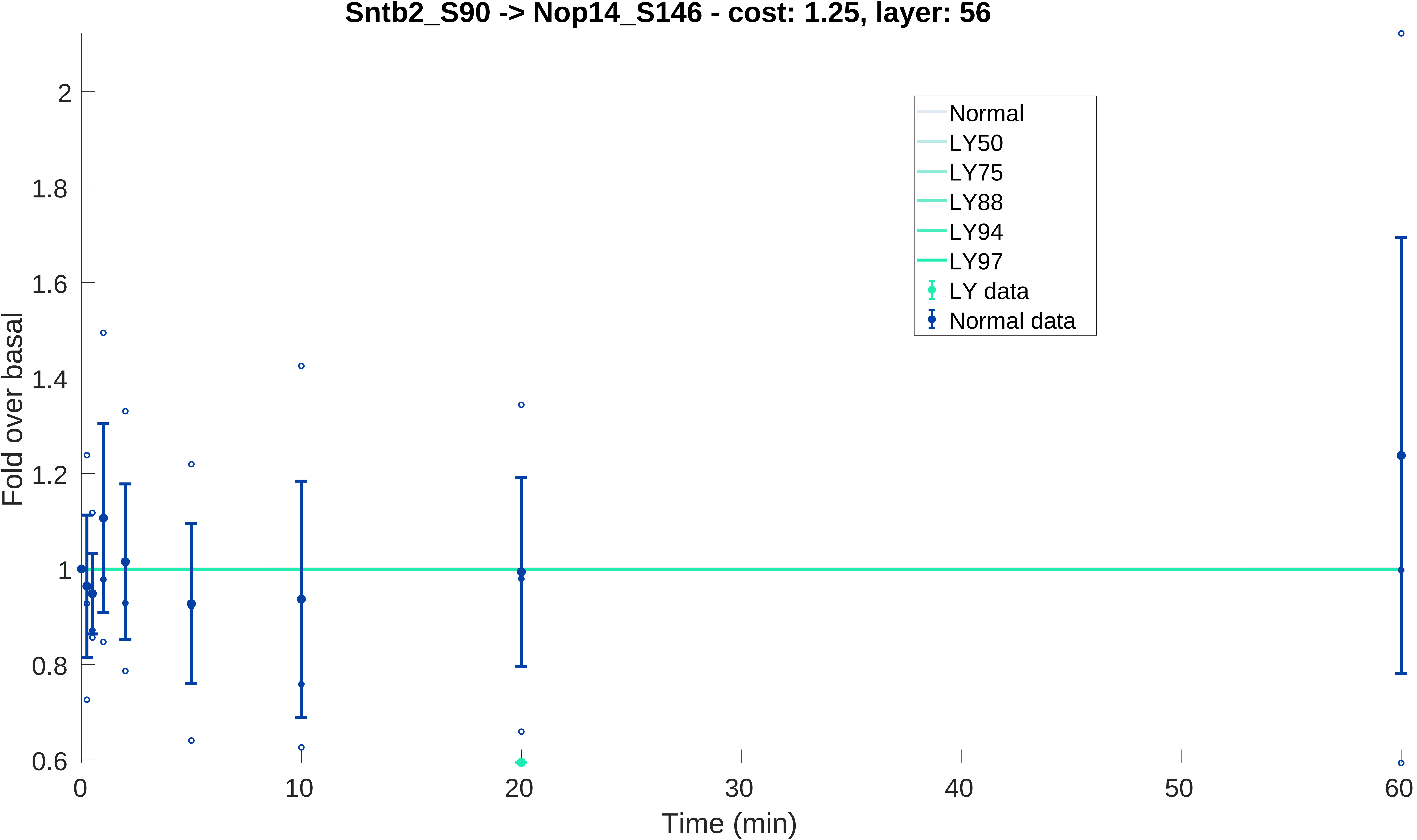

Sntb2_S90 -> Nop14_S146 - cost: 1.25, layer: 56
Normal
LY50
LY75
LY88
LY94
LY97
LY data
Normal data
Fold over basal
Time (min)
0.6
0.8
1
1.2
1.4
1.6
1.8
2
0
10
20
30
40
50
60

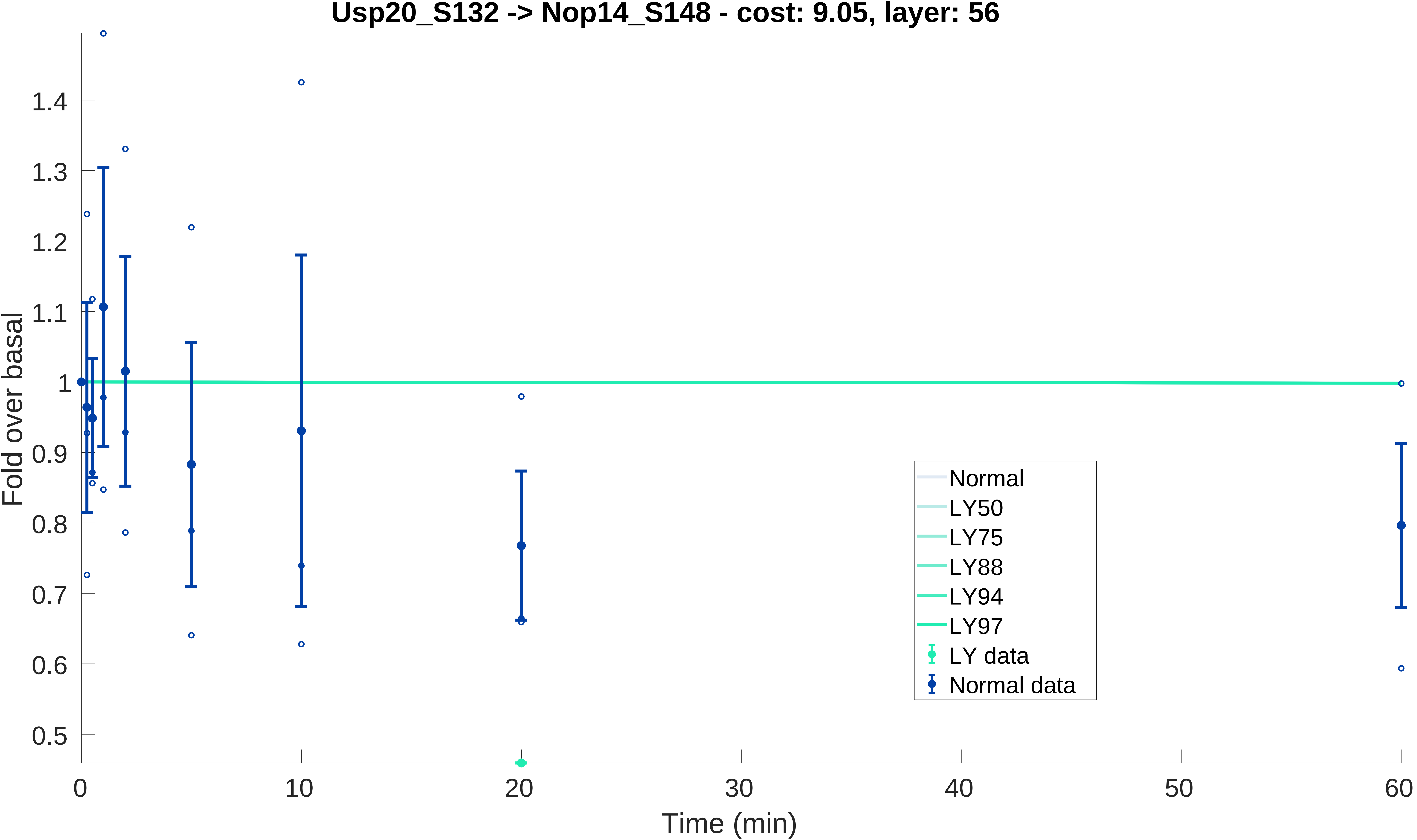
Usp20_S132 -> Nop14_S148 - cost: 9.05, layer: 56
Fold over basal
Time (min)
0.5
0.6
0.7
0.8
0.9
1
1.1
1.2
1.3
1.4
0
10
20
30
40
50
60
Normal
LY50
LY75
LY88
LY94
LY97
LY data
Normal data

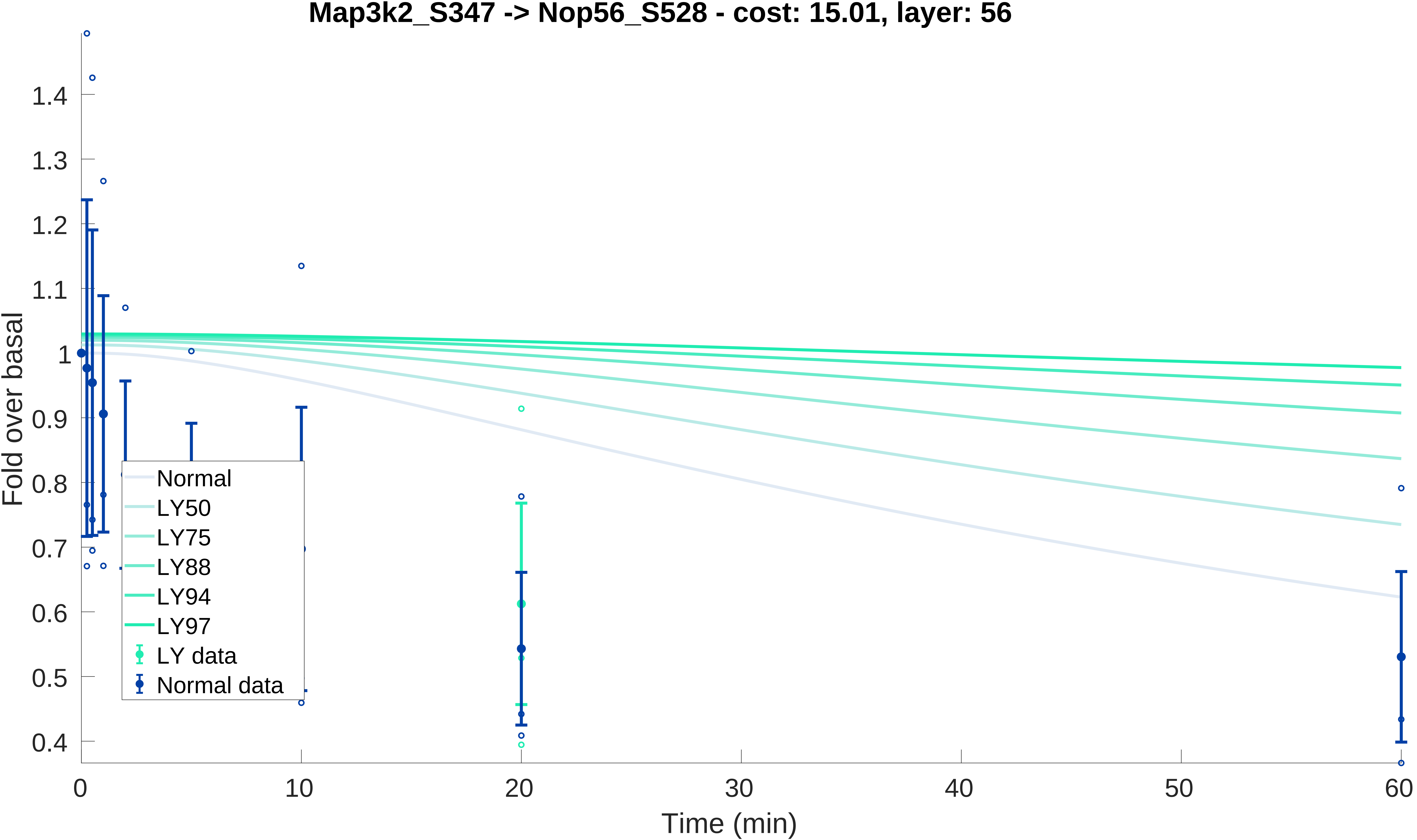
Map3k2_S347 -> Nop56_S528 - cost: 15.01, layer: 56
Fold over basal
Time (min)
Normal
LY50
LY75
LY88
LY94
LY97
LY data
Normal data
0
10
20
30
40
50
60
0.4
0.5
0.6
0.7
0.8
0.9
1
1.1
1.2
1.3
1.4

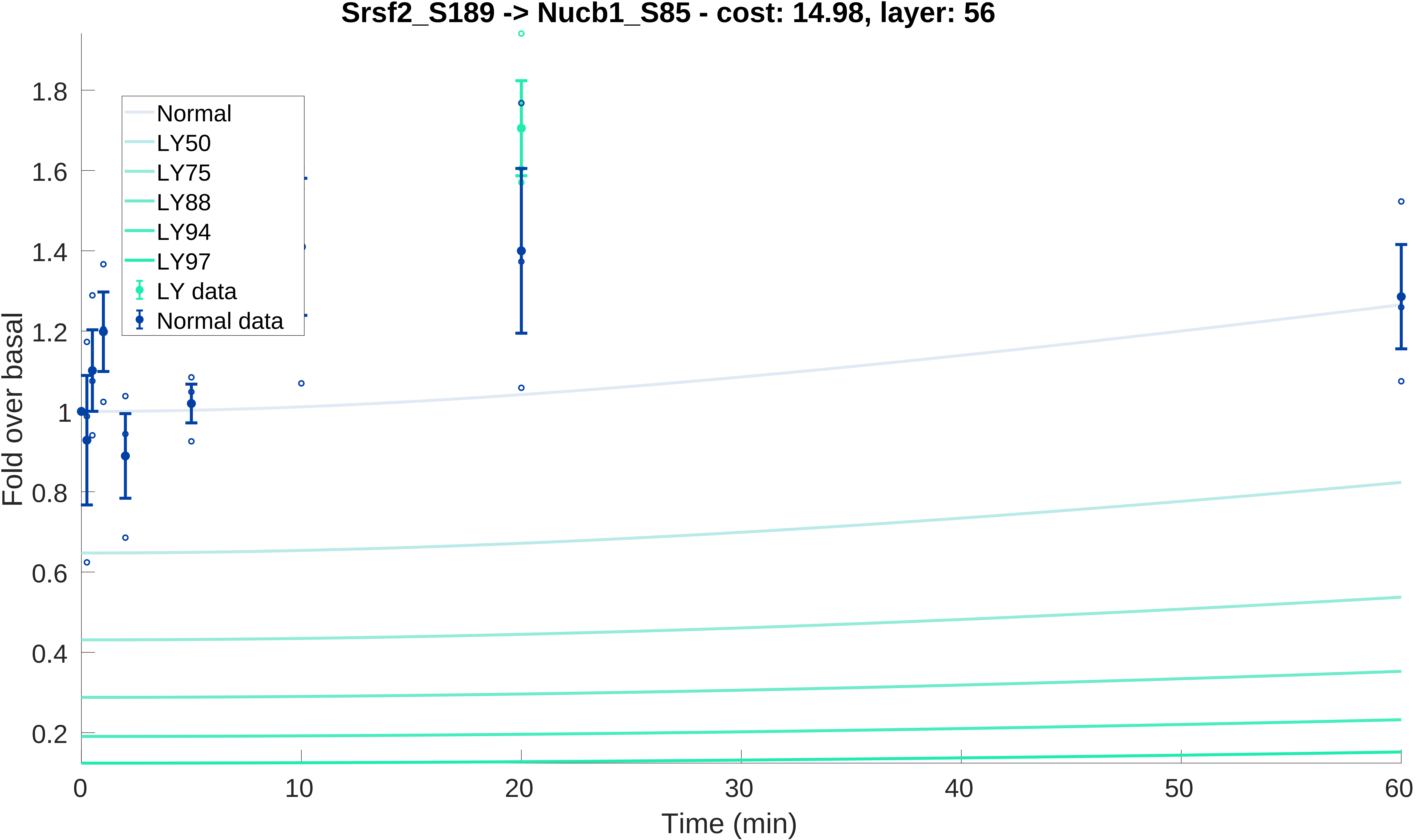

Srsf2_S189 -> Nucb1_S85 - cost: 14.98, layer: 56
Normal
LY50
LY75
LY88
LY94
LY97
LY data
Normal data
Fold over basal
Time (min)
0.2
0.4
0.6
0.8
1
1.2
1.4
1.6
1.8
0
10
20
30
40
50
60

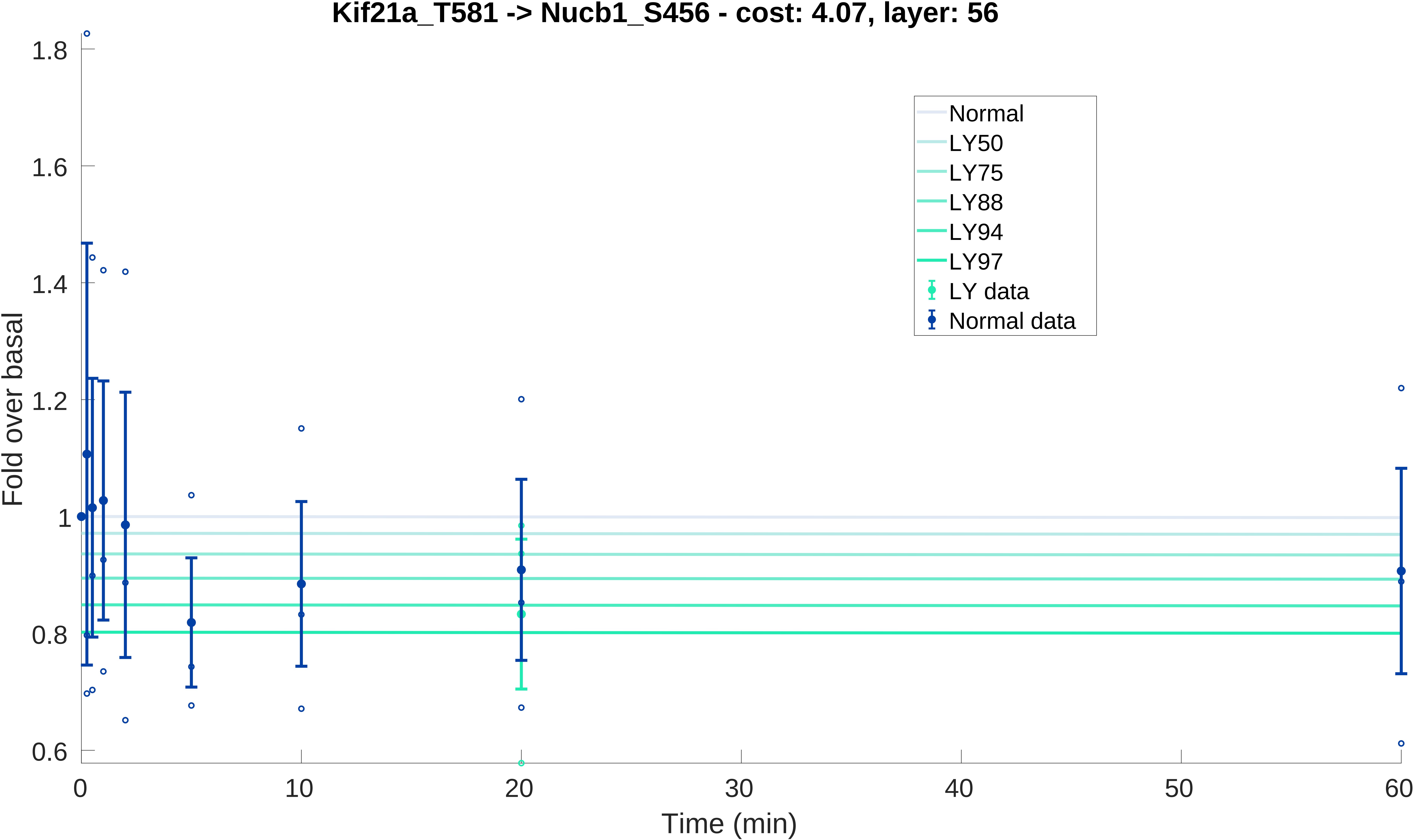
Kif21a_T581 -> Nucb1_S456 - cost: 4.07, layer: 56
Fold over basal
Time (min)
1.8
1.6
1.4
1.2
1
0.8
0.6
0
10
20
30
40
50
60
Normal
LY50
LY75
LY88
LY94
LY97
LY data
Normal data

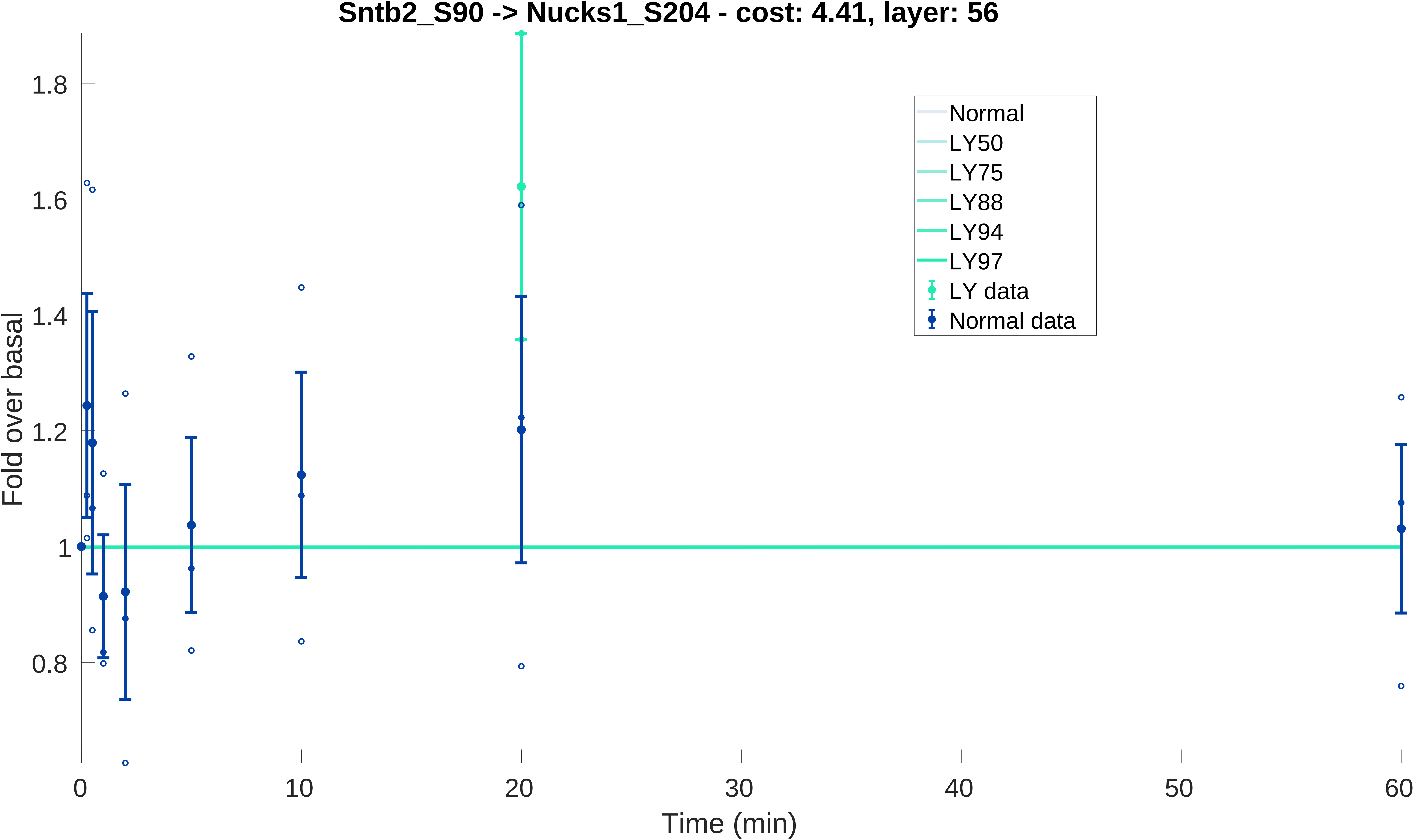

Sntb2_S90 -> Nucks1_S204 - cost: 4.41, layer: 56
Fold over basal
Time (min)
1.8
1.6
1.4
1.2
1
0.8
0
10
20
30
40
50
60
Normal
LY50
LY75
LY88
LY94
LY97
LY data
Normal data

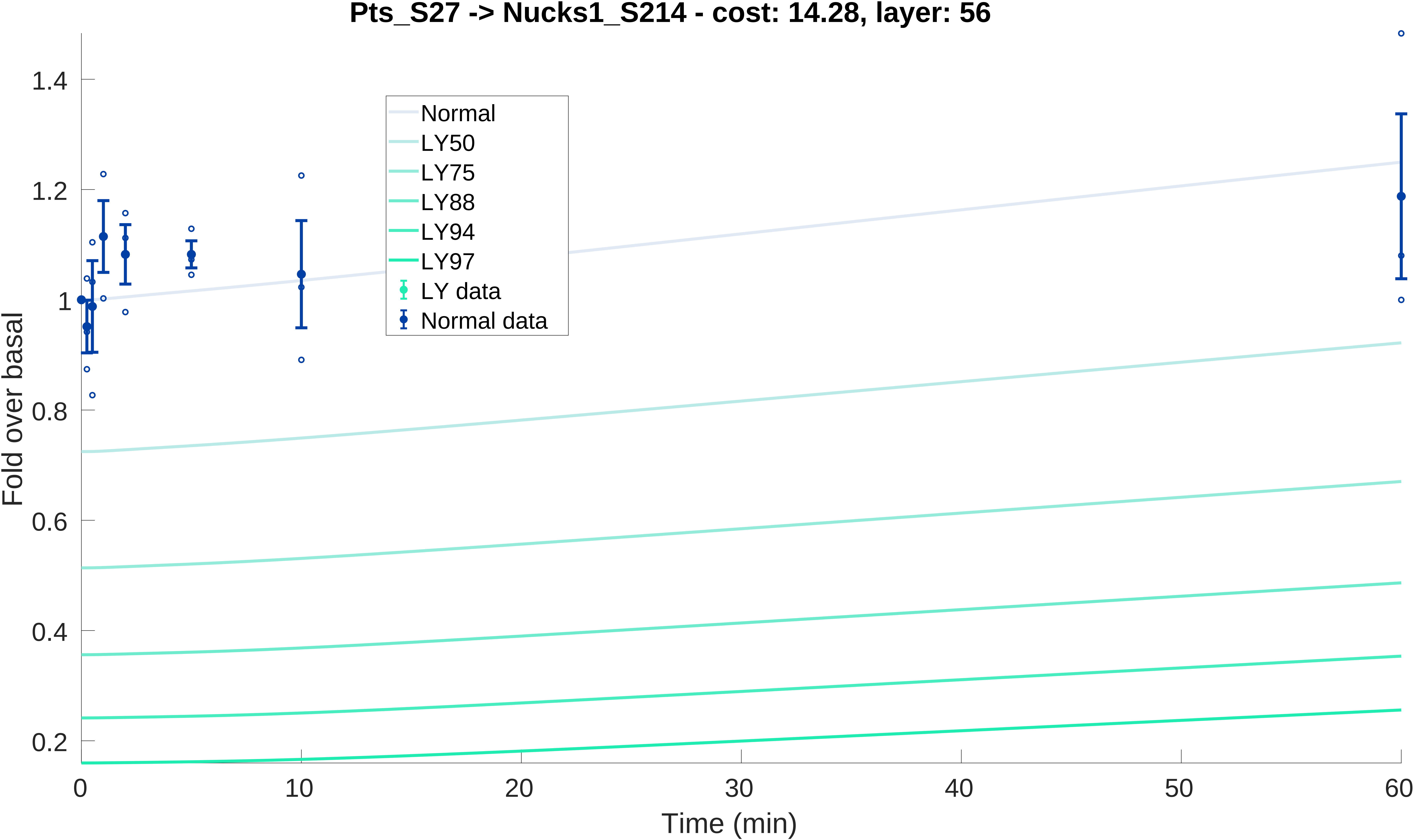

Pts_S27 -> Nucks1_S214 - cost: 14.28, layer: 56
Fold over basal
Time (min)
0
10
20
30
40
50
60
0.2
0.4
0.6
0.8
1
1.2
1.4
Normal
LY50
LY75
LY88
LY94
LY97
LY data
Normal data

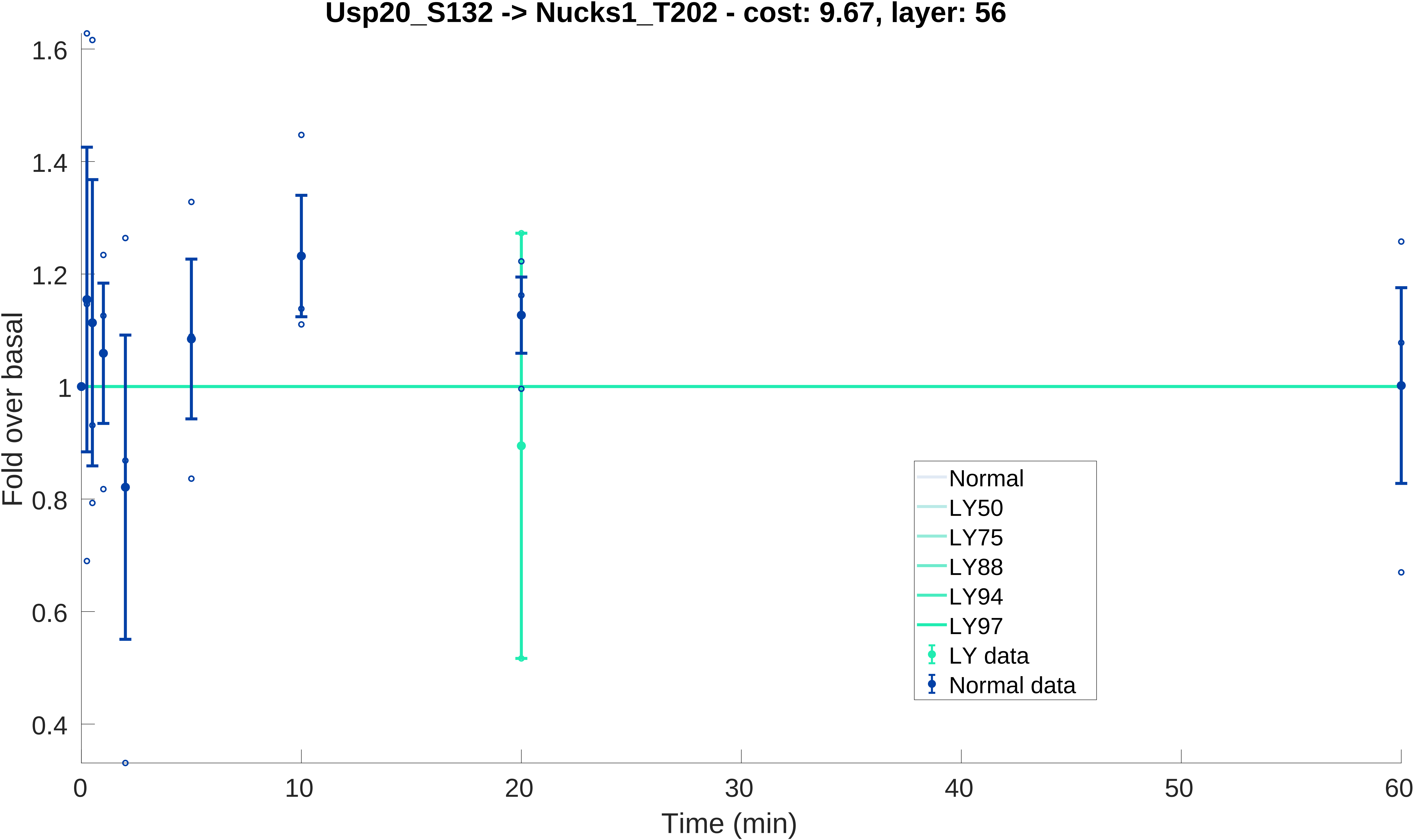

Usp20_S132 -> Nucks1_T202 - cost: 9.67, layer: 56
Fold over basal
Time (min)
0.4
0.6
0.8
1
1.2
1.4
1.6
0
10
20
30
40
50
60
Normal
LY50
LY75
LY88
LY94
LY97
LY data
Normal data

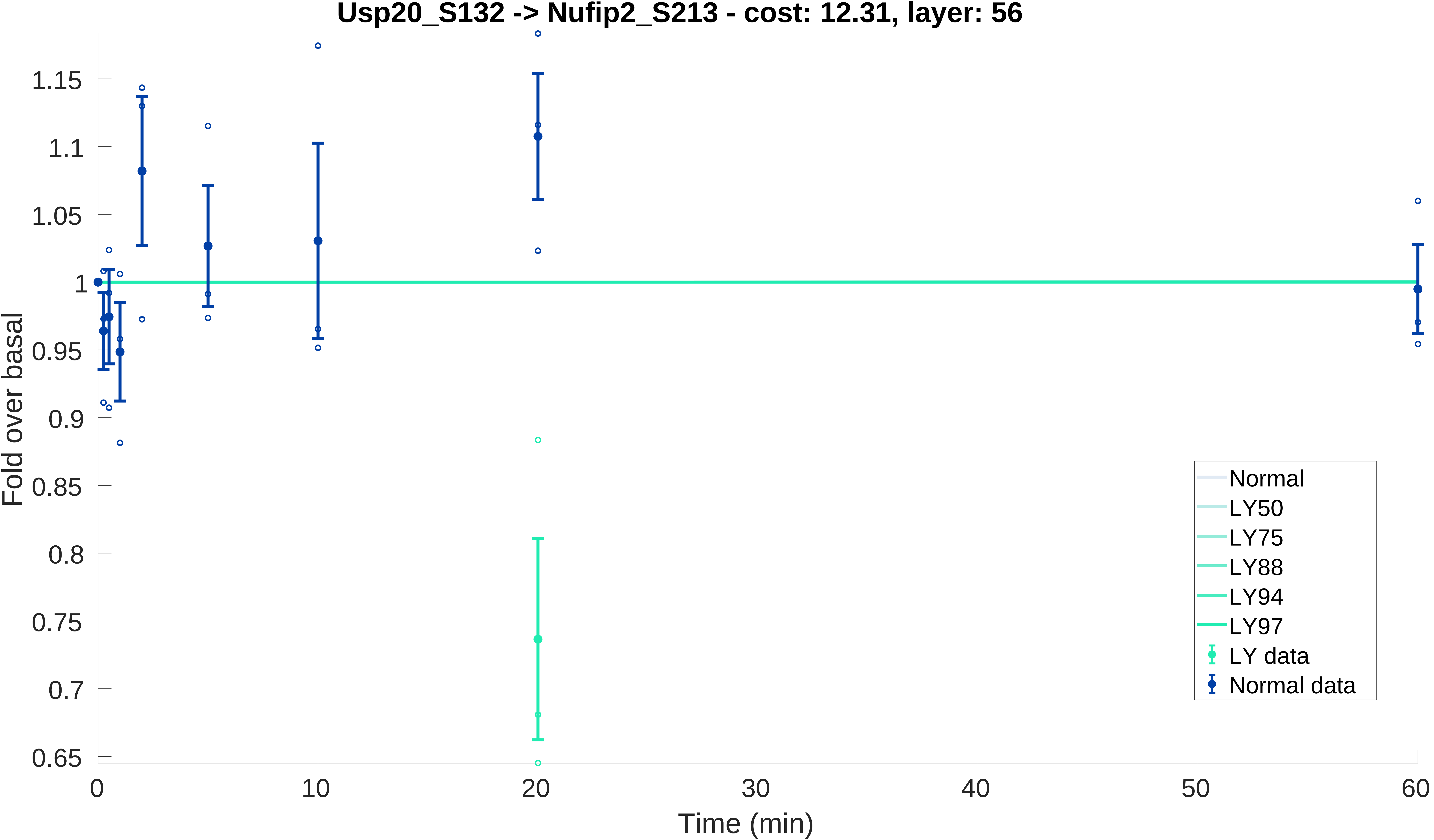
Usp20_S132 -> Nufip2_S213 - cost: 12.31, layer: 56
Fold over basal
Time (min)
1.15
1.1
1.05
1
0.95
0.9
0.85
0.8
0.75
0.7
0.65
0
10
20
30
40
50
60
Normal
LY50
LY75
LY88
LY94
LY97
LY data
Normal data

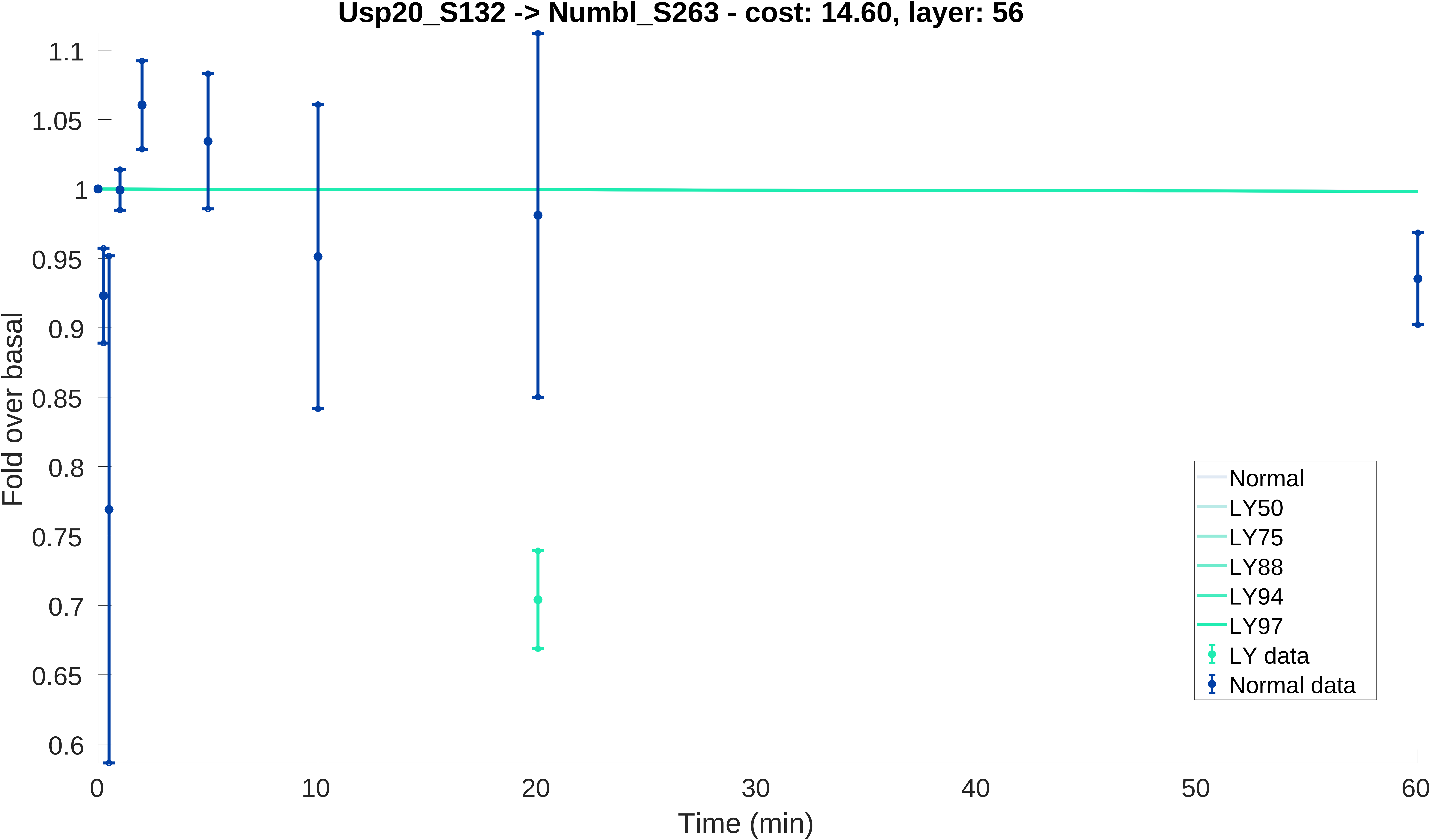
Usp20_S132 -> Numbl_S263 - cost: 14.60, layer: 56
Fold over basal
1.1
1.05
1
0.95
0.9
0.85
0.8
0.75
0.7
0.65
0.6
0
10
20
30
40
50
60
Time (min)
Normal
LY50
LY75
LY88
LY94
LY97
LY data
Normal data

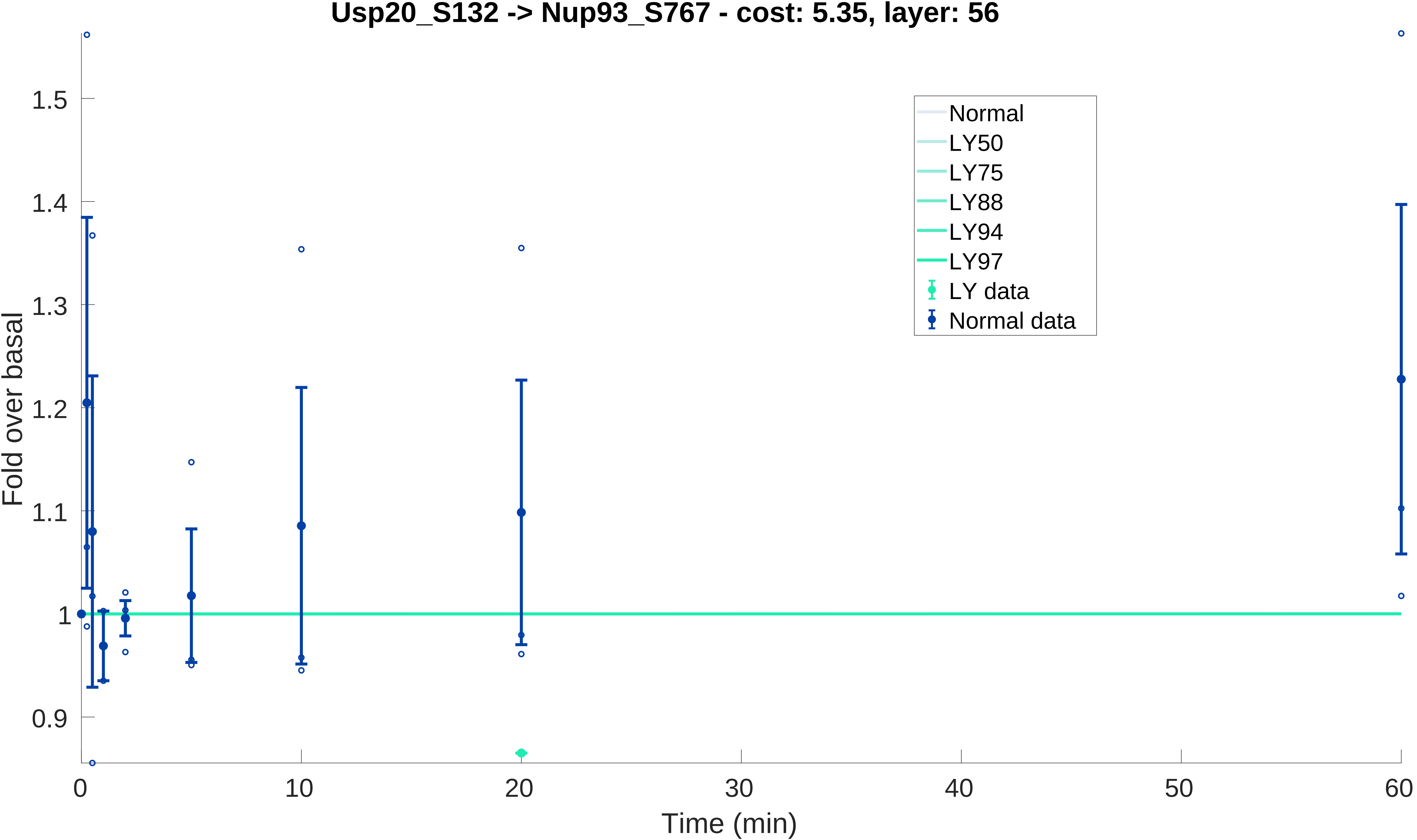

Usp20_S132 -> Nup93_S767 - cost: 5.35, layer: 56
Fold over basal
Time (min)
0.9
1
1.1
1.2
1.3
1.4
1.5
0
10
20
30
40
50
60
Normal
LY50
LY75
LY88
LY94
LY97
LY data
Normal data

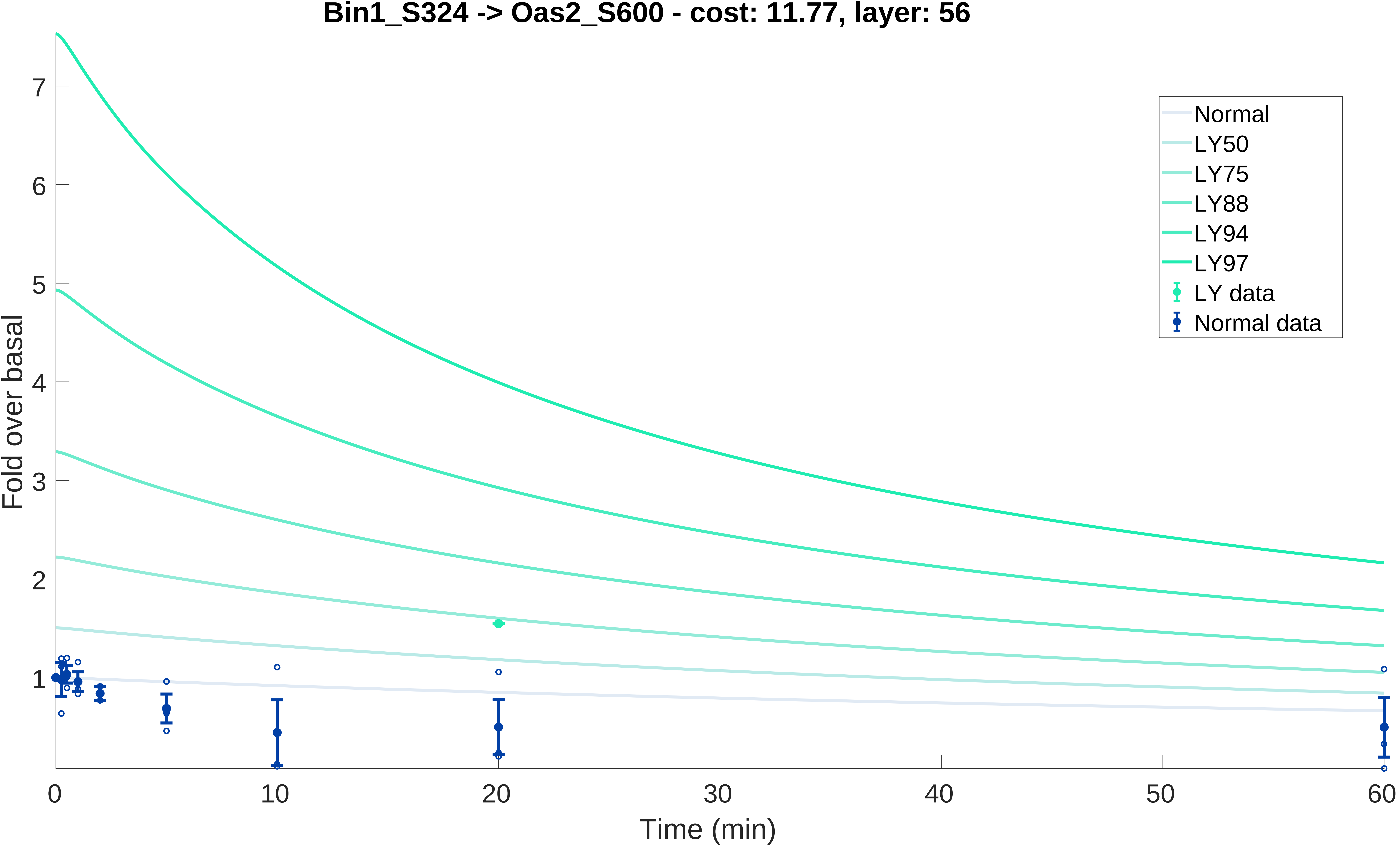
Bin1_S324 -> Oas2_S600 - cost: 11.77, layer: 56
Fold over basal
Time (min)
Normal
LY50
LY75
LY88
LY94
LY97
LY data
Normal data
0
10
20
30
40
50
60
1
2
3
4
5
6
7

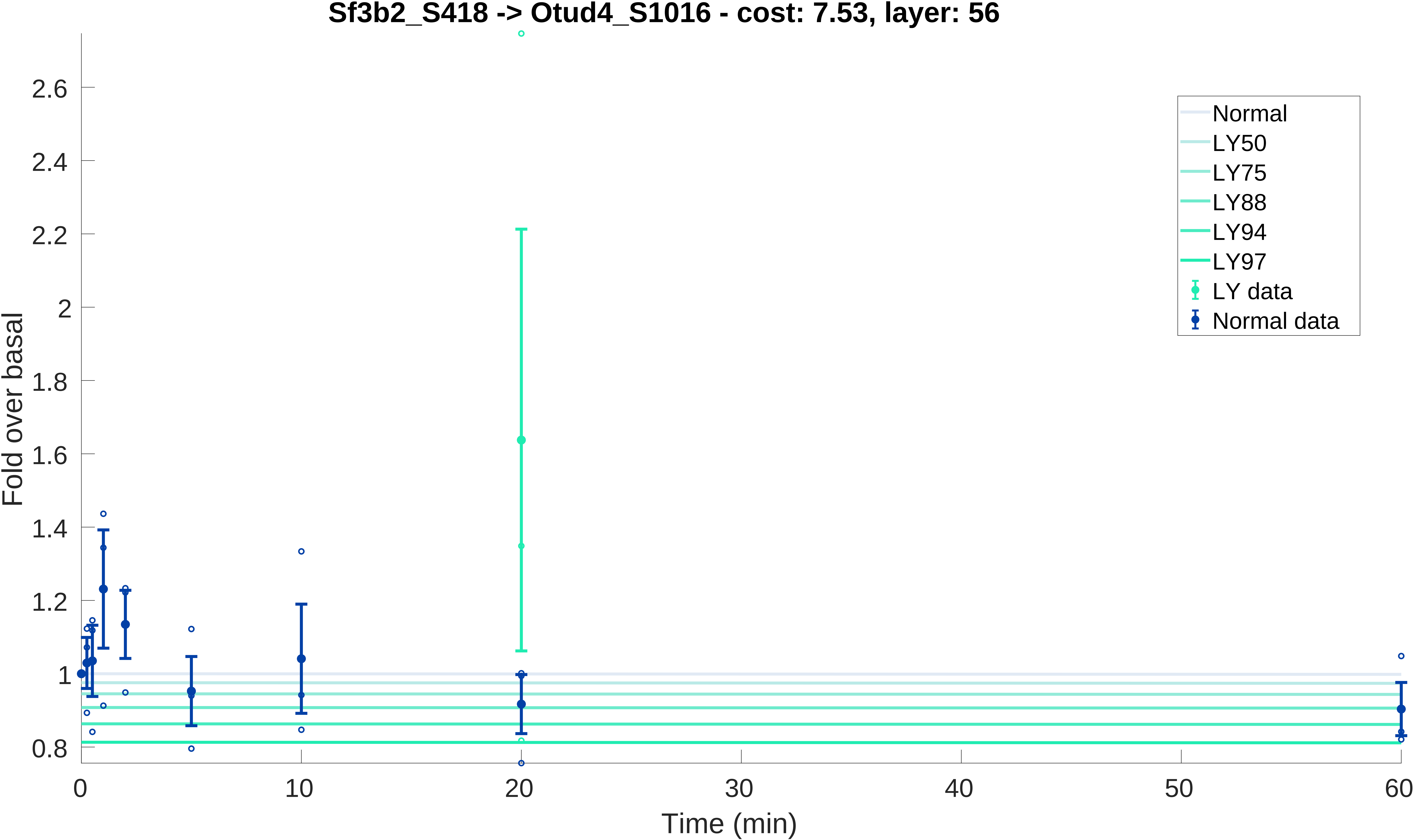

Sf3b2_S418 -> Otud4_S1016 - cost: 7.53, layer: 56
Fold over basal
Time (min)
2.6
2.4
2.2
2
1.8
1.6
1.4
1.2
1
0.8
0
10
20
30
40
50
60
Normal
LY50
LY75
LY88
LY94
LY97
LY data
Normal data

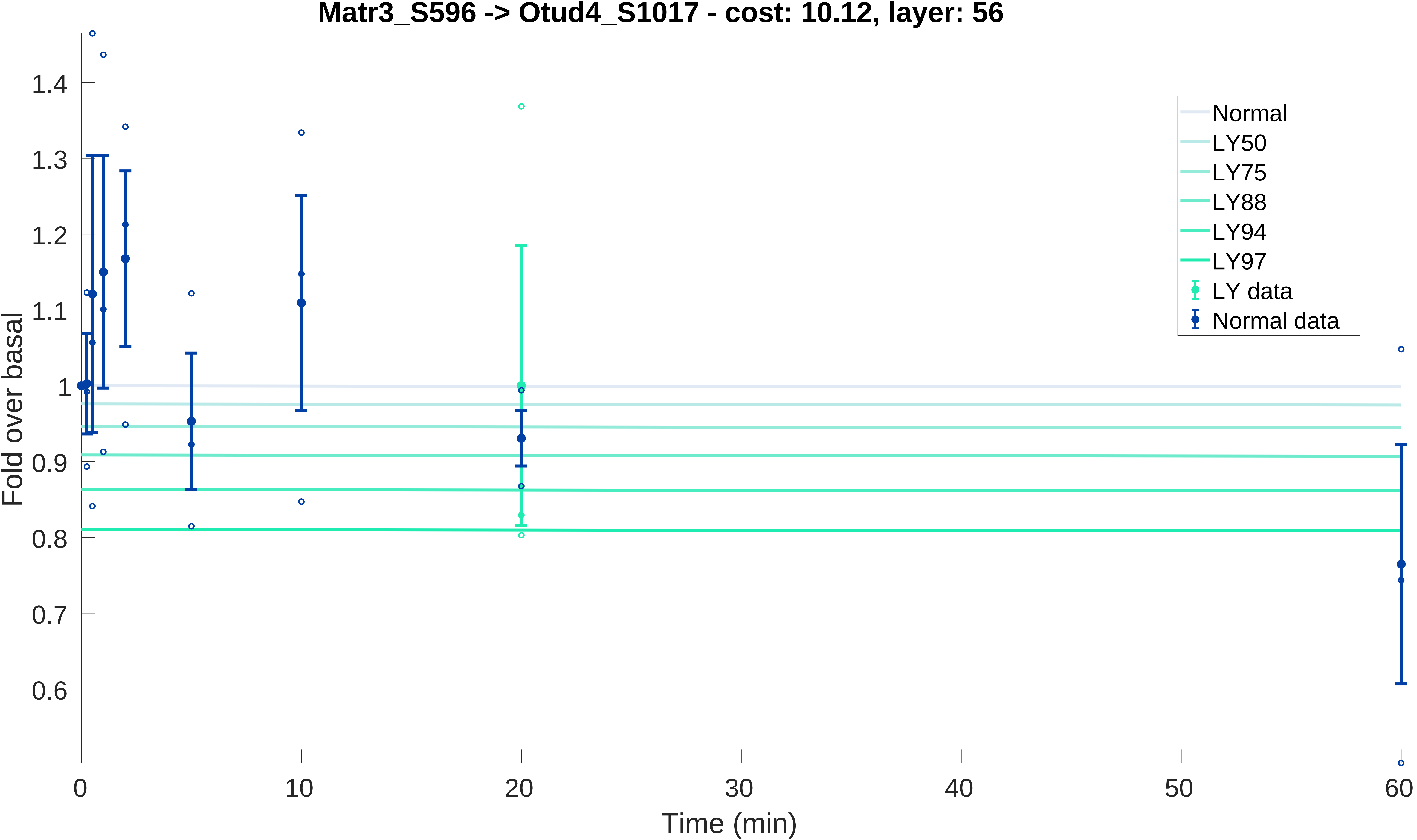

Matr3_S596 -> Otud4_S1017 - cost: 10.12, layer: 56
Fold over basal
Time (min)
0
10
20
30
40
50
60
0.6
0.7
0.8
0.9
1
1.1
1.2
1.3
1.4
Normal
LY50
LY75
LY88
LY94
LY97
LY data
Normal data

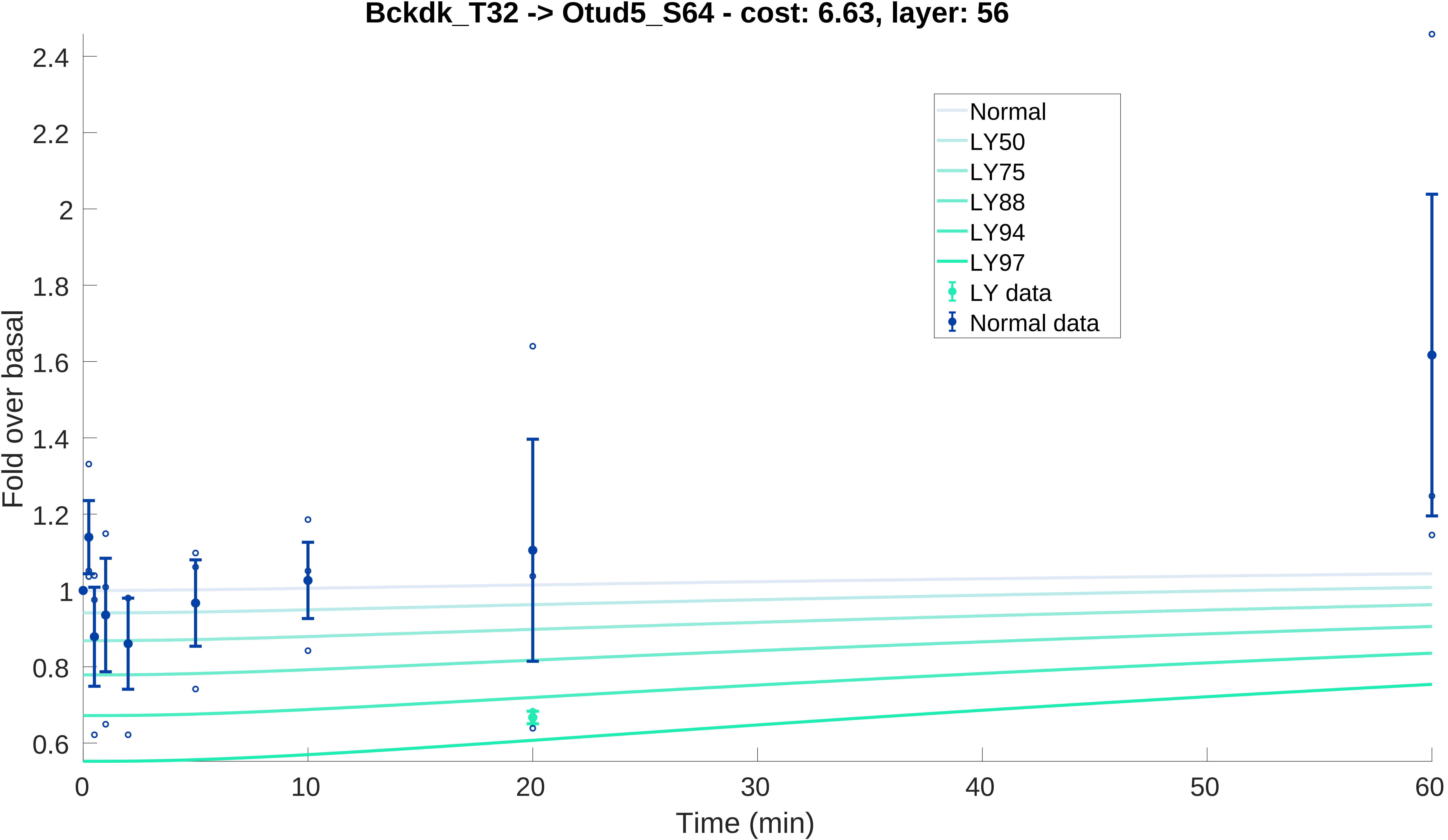

Bckdk_T32 -> Otud5_S64 - cost: 6.63, layer: 56
Fold over basal
Time (min)
0
10
20
30
40
50
60
0.6
0.8
1
1.2
1.4
1.6
1.8
2
2.2
2.4
Normal
LY50
LY75
LY88
LY94
LY97
LY data
Normal data

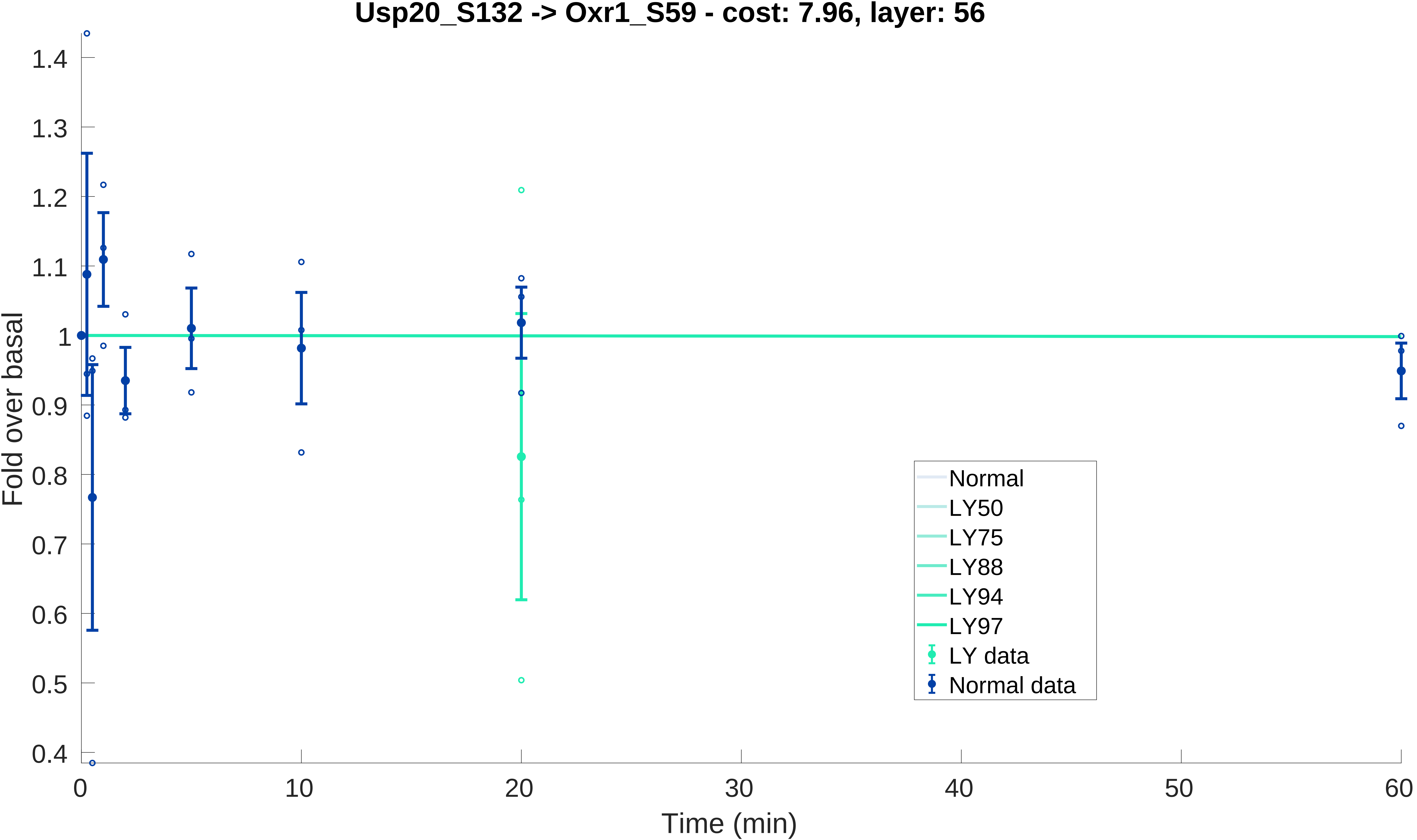
Usp20_S132 -> Oxr1_S59 - cost: 7.96, layer: 56
Fold over basal
1.4
1.3
1.2
1.1
1
0.9
0.8
0.7
0.6
0.5
0.4
0
10
20
30
40
50
60
Time (min)
Normal
LY50
LY75
LY88
LY94
LY97
LY data
Normal data

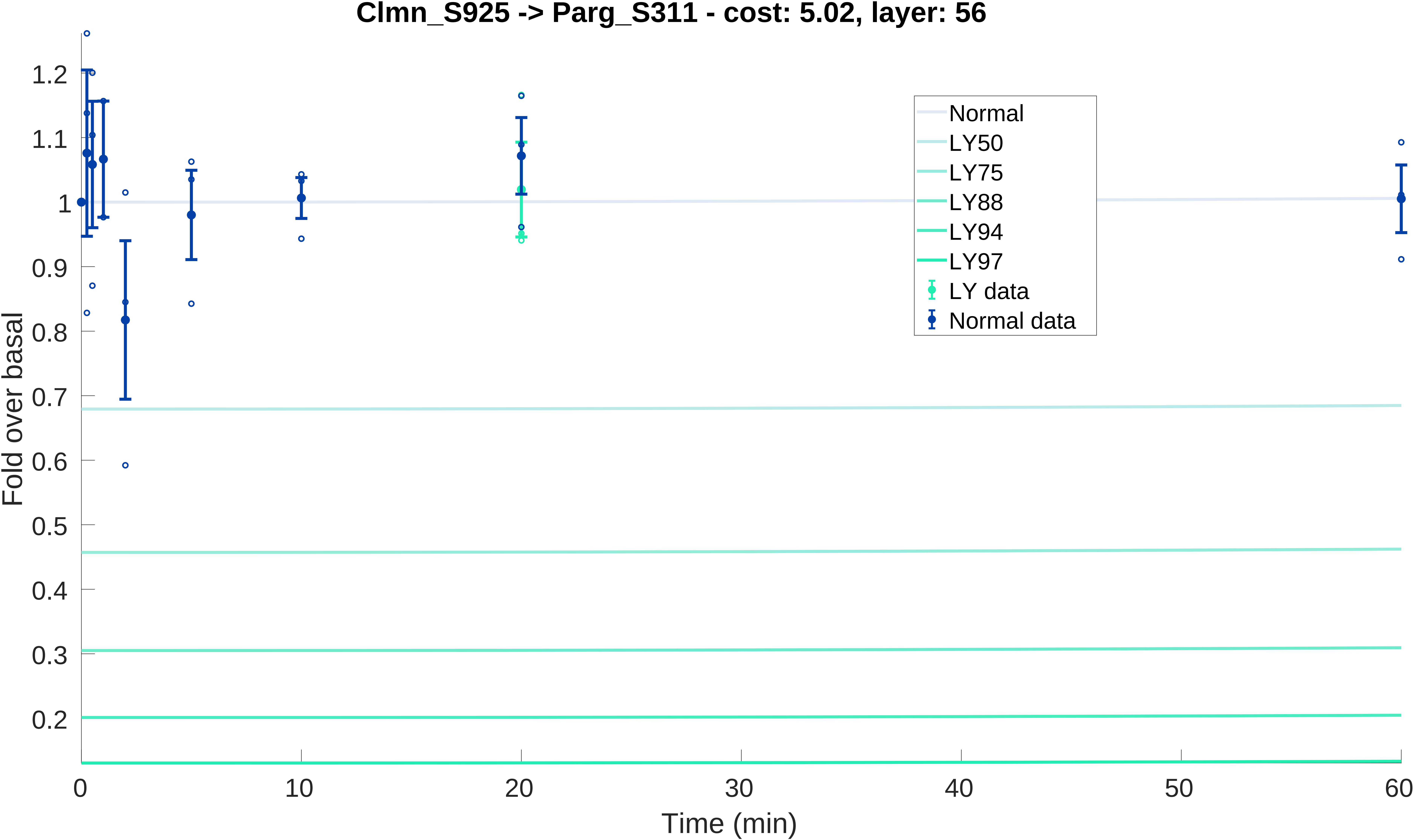
Clmn_S925 -> Parg_S311 - cost: 5.02, layer: 56
Normal
LY50
LY75
LY88
LY94
LY97
LY data
Normal data
Fold over basal
Time (min)
1.2
1.1
1
0.9
0.8
0.7
0.6
0.5
0.4
0.3
0.2
0
10
20
30
40
50
60

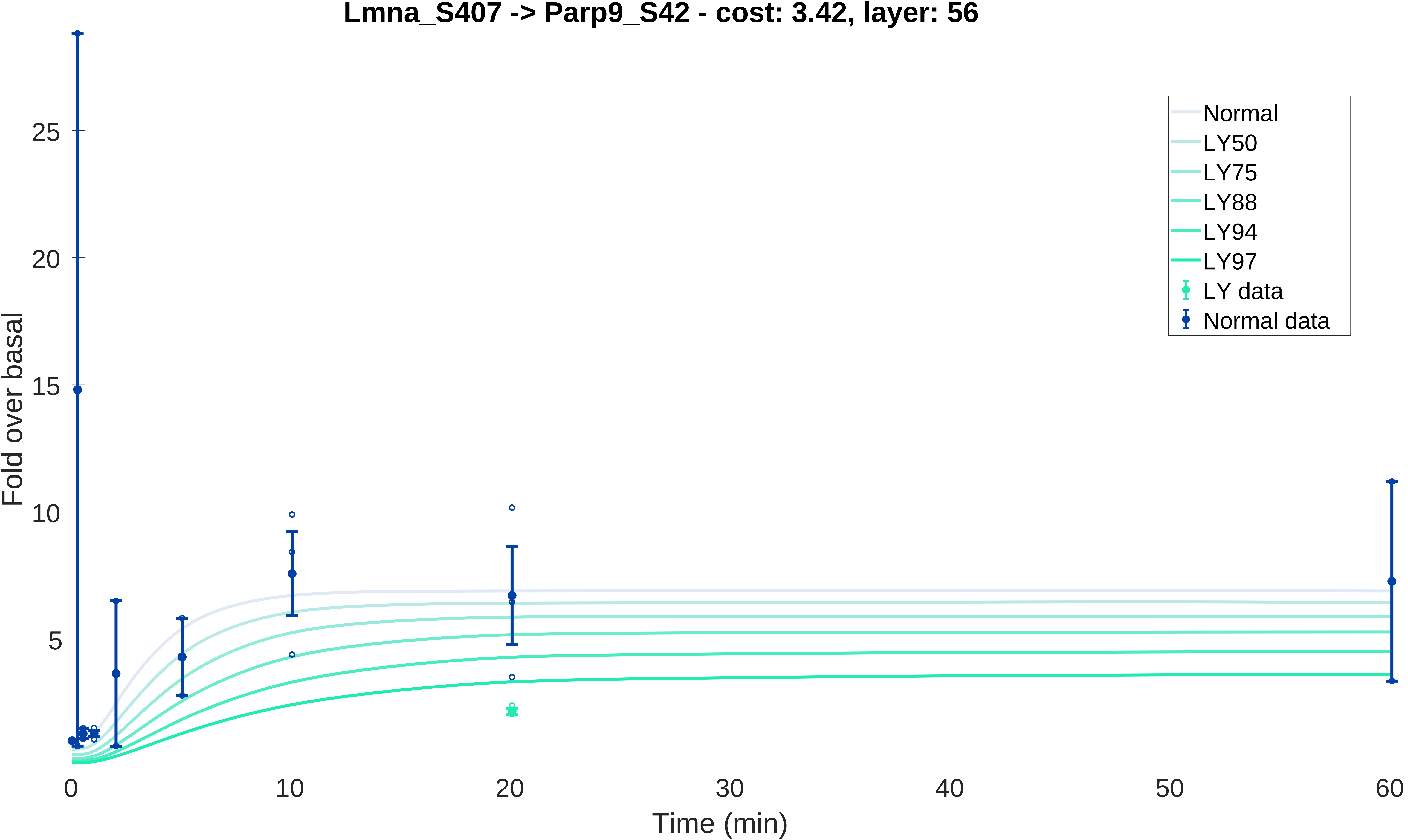

Lmna_S407 -> Parp9_S42 - cost: 3.42, layer: 56
Fold over basal
Time (min)
0
10
20
30
40
50
60
5
10
15
20
25
Normal
LY50
LY75
LY88
LY94
LY97
LY data
Normal data

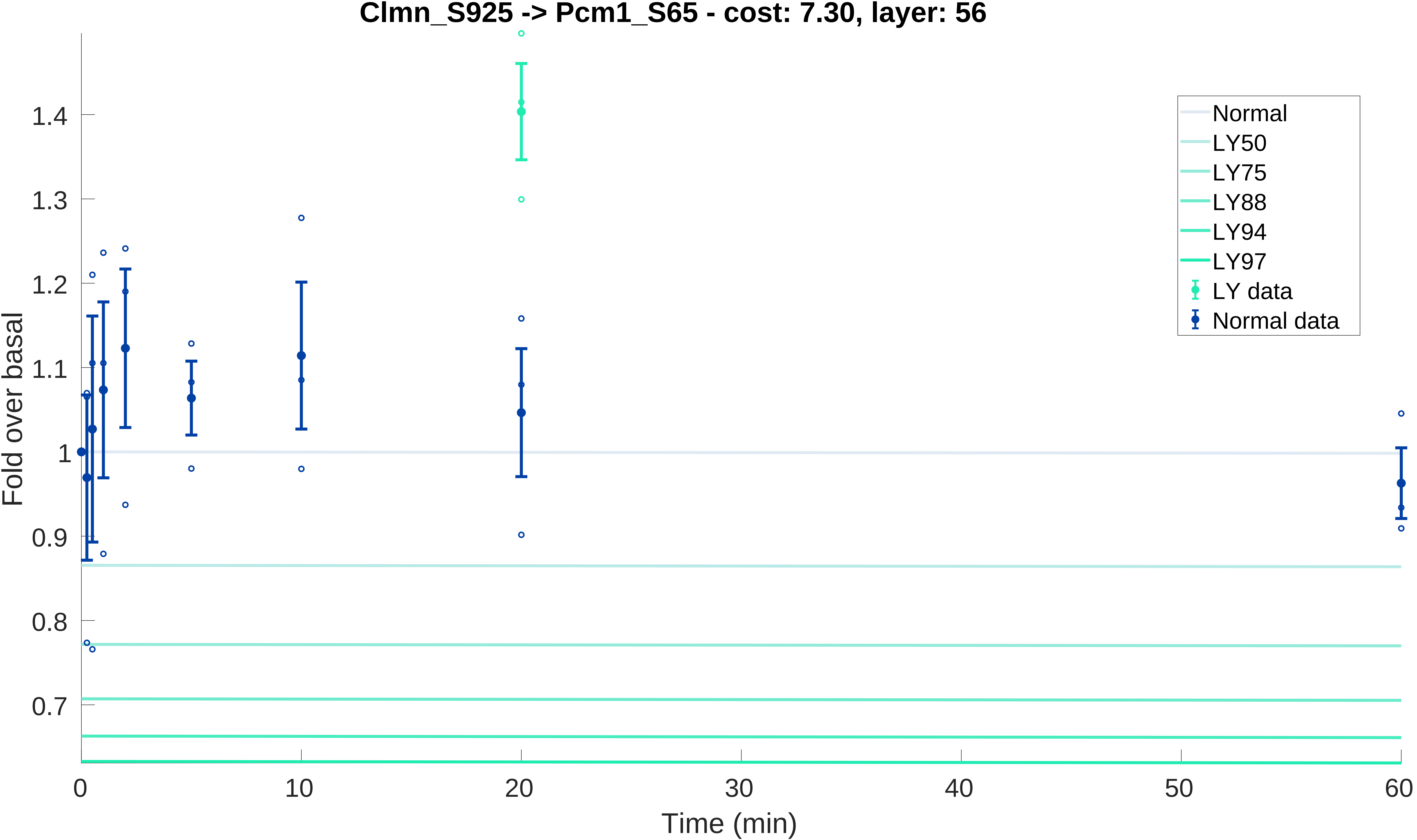

Clmn_S925 -> Pcm1_S65 - cost: 7.30, layer: 56
Fold over basal
Time (min)
0.7
0.8
0.9
1
1.1
1.2
1.3
1.4
0
10
20
30
40
50
60
Normal
LY50
LY75
LY88
LY94
LY97
LY data
Normal data

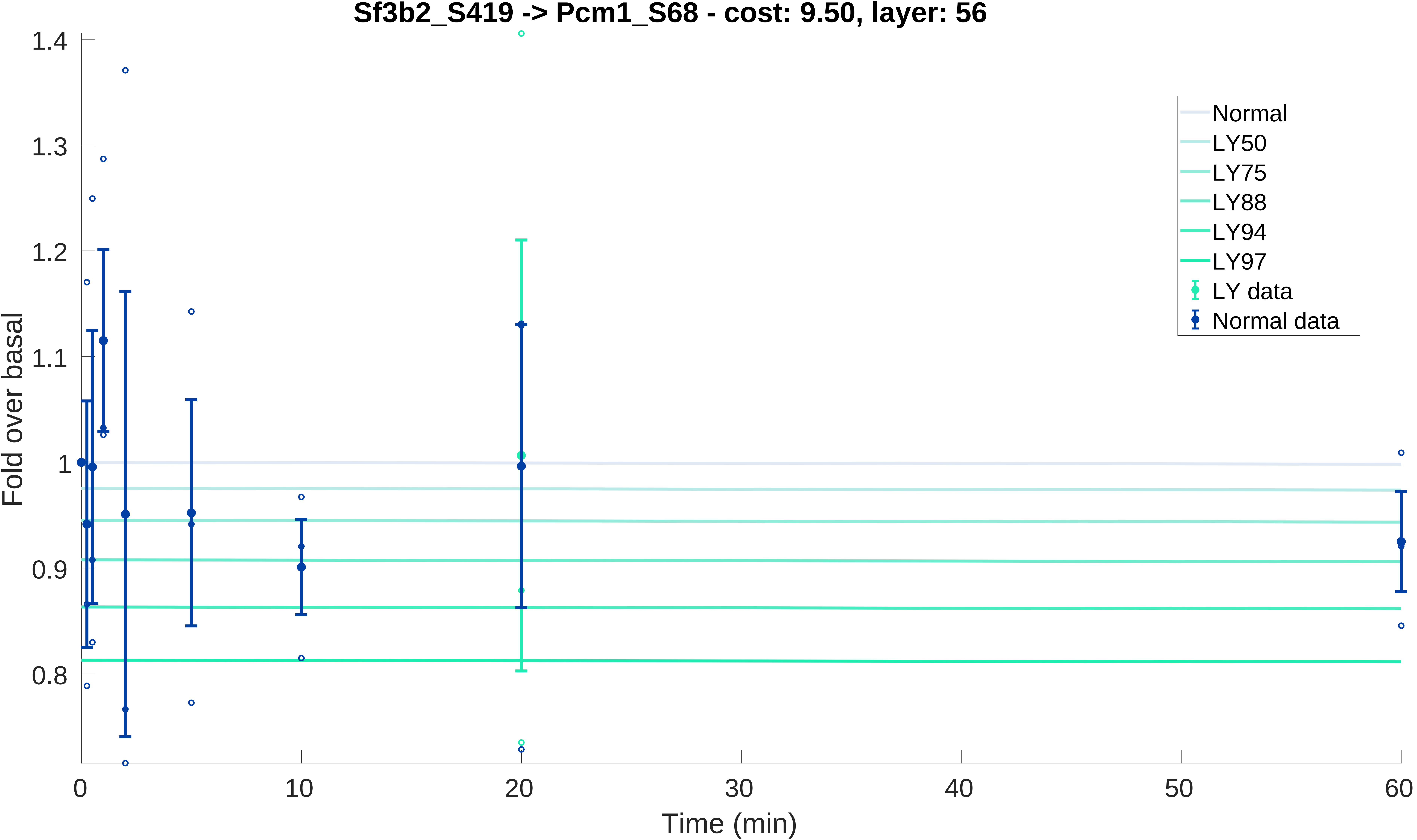

Sf3b2_S419 -> Pcm1_S68 - cost: 9.50, layer: 56
Fold over basal
Time (min)
1.4
1.3
1.2
1.1
1
0.9
0.8
0
10
20
30
40
50
60
Normal
LY50
LY75
LY88
LY94
LY97
LY data
Normal data

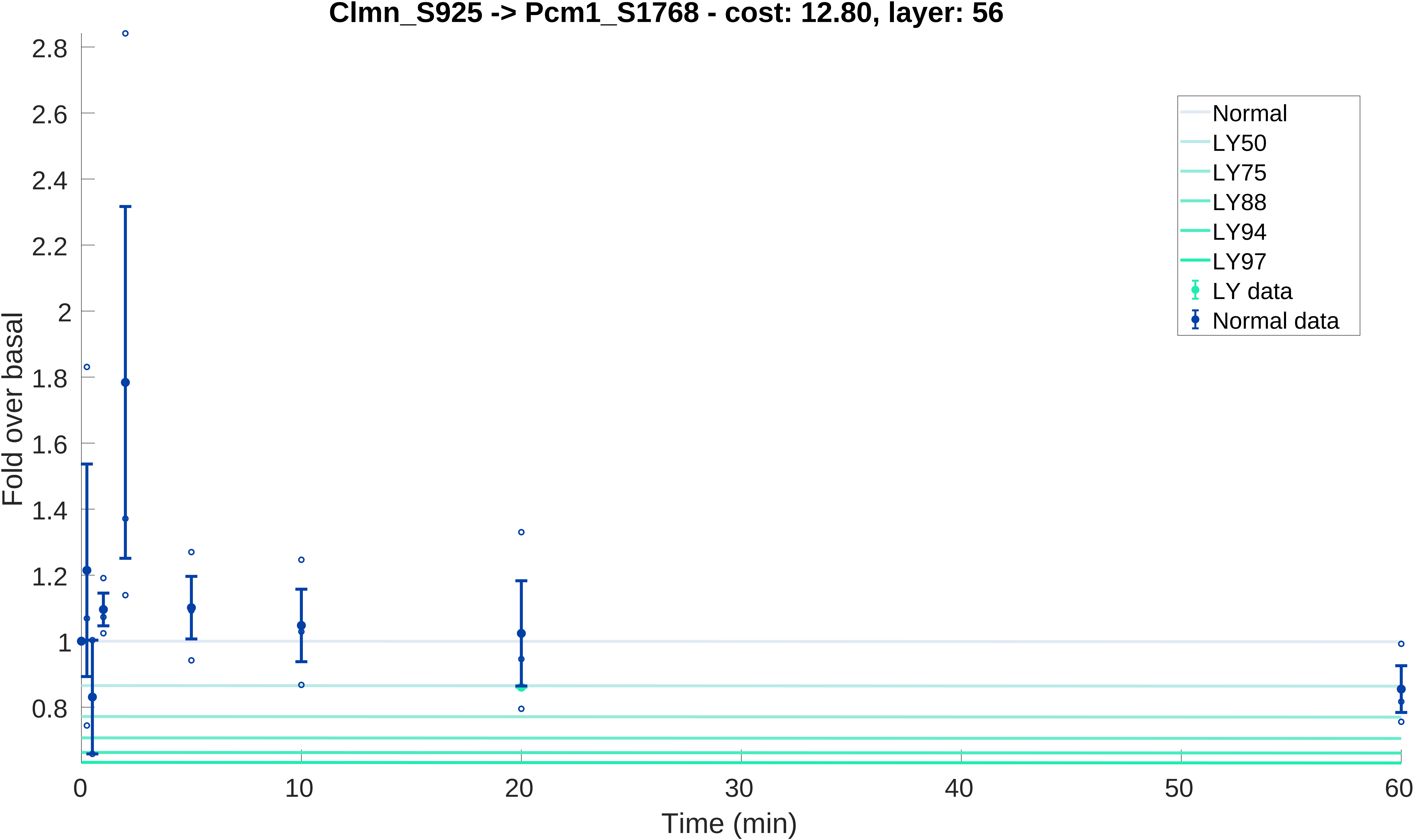

Clmn_S925 -> Pcm1_S1768 - cost: 12.80, layer: 56
Fold over basal
Time (min)
2.8
2.6
2.4
2.2
2
1.8
1.6
1.4
1.2
1
0.8
0
10
20
30
40
50
60
Normal
LY50
LY75
LY88
LY94
LY97
LY data
Normal data

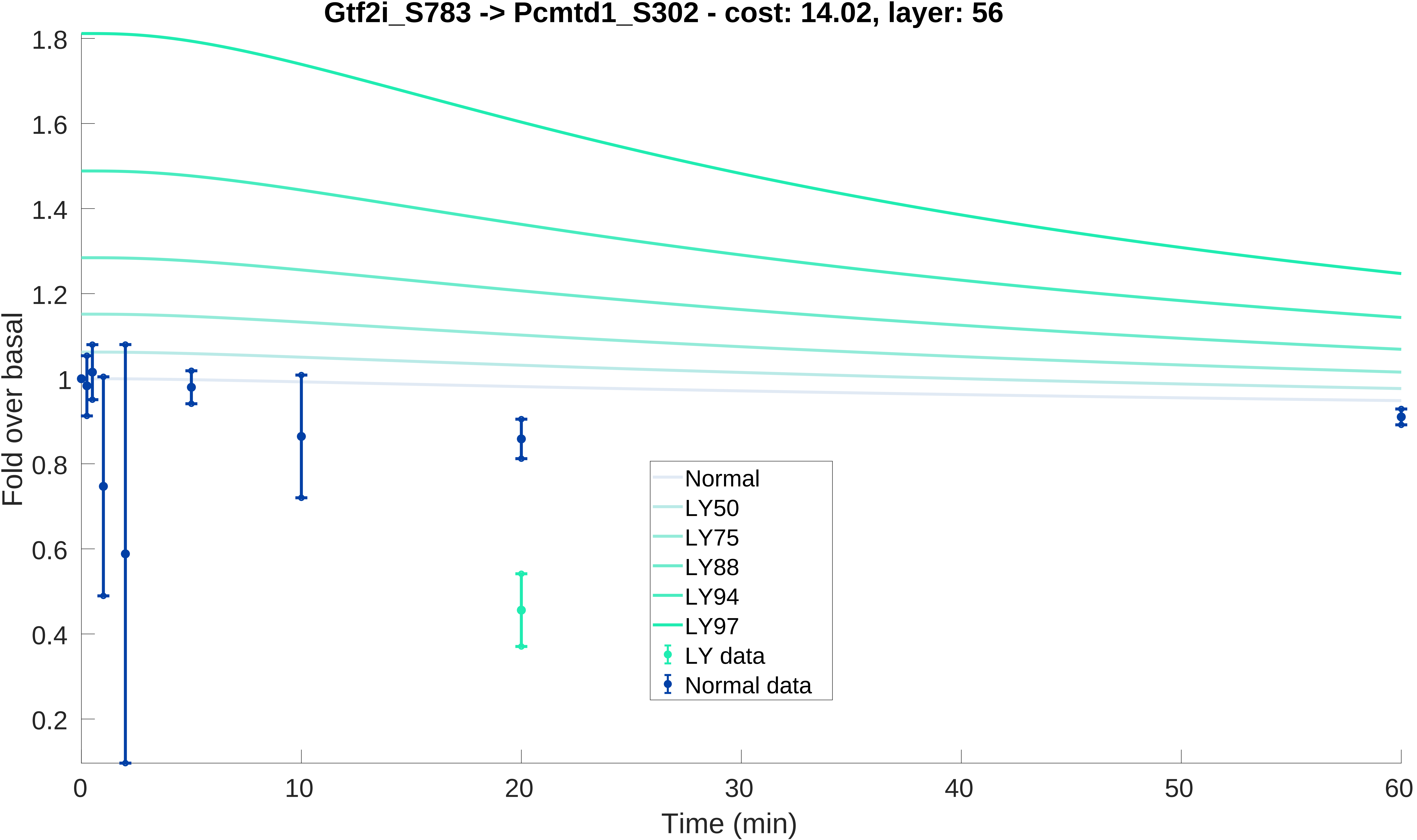

Gtf2i_S783 -> Pcmtd1_S302 - cost: 14.02, layer: 56
Fold over basal
1.8
1.6
1.4
1.2
1
0.8
0.6
0.4
0.2
Time (min)
0
10
20
30
40
50
60
Normal
LY50
LY75
LY88
LY94
LY97
LY data
Normal data

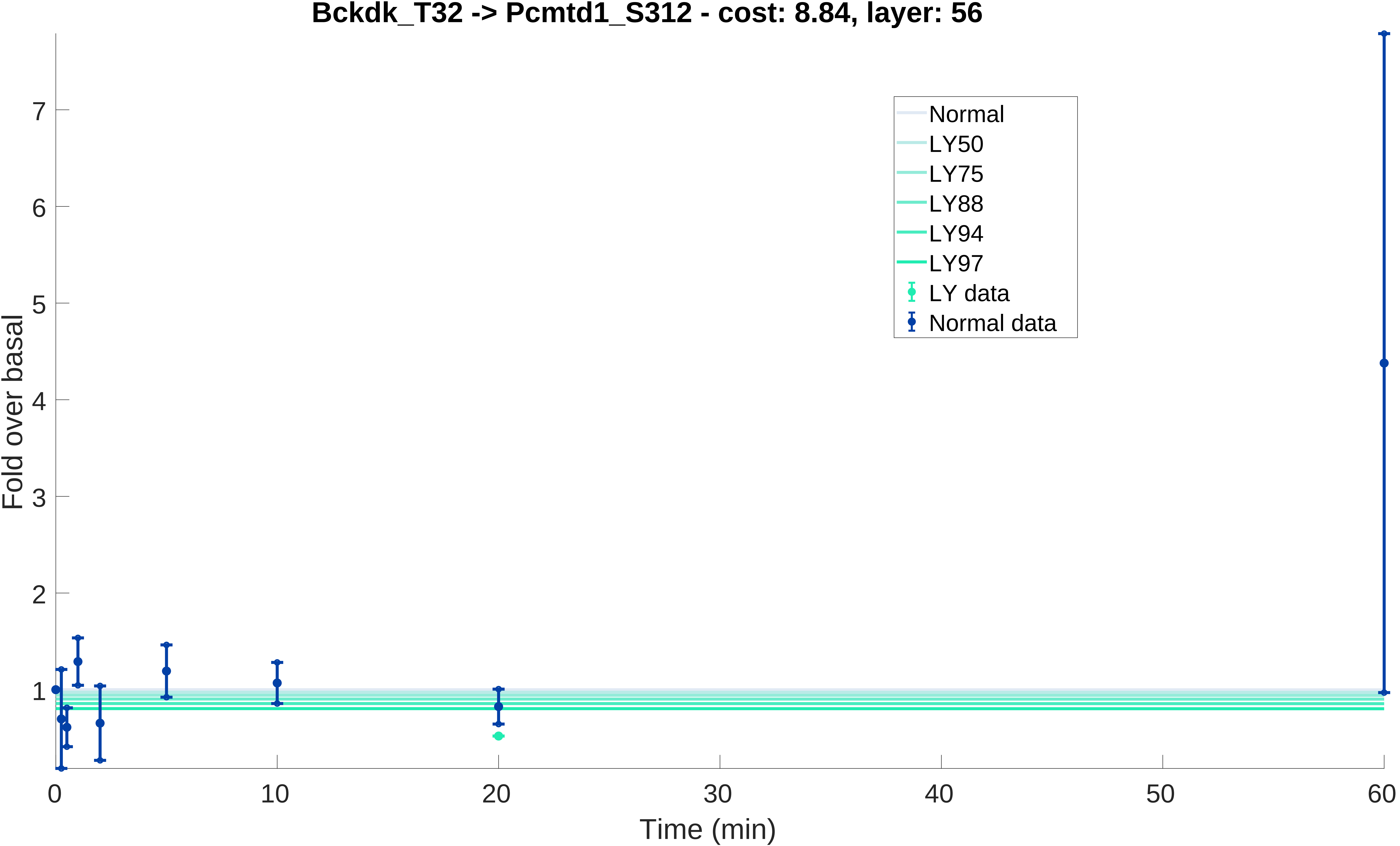

Bckdk_T32 -> Pcmtd1_S312 - cost: 8.84, layer: 56
Fold over basal
Time (min)
0
10
20
30
40
50
60
1
2
3
4
5
6
7
Normal
LY50
LY75
LY88
LY94
LY97
LY data
Normal data

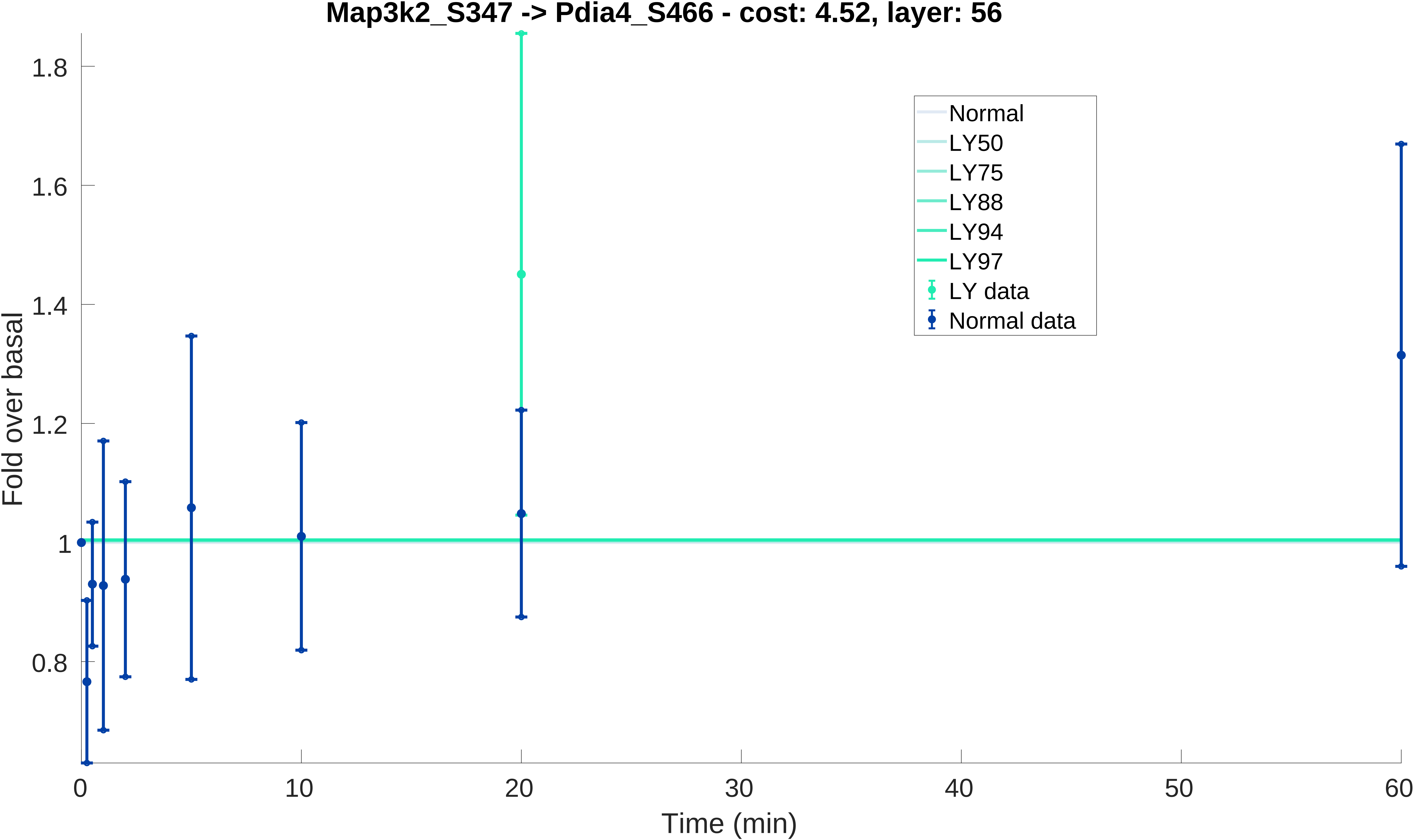
Map3k2_S347 -> Pdia4_S466 - cost: 4.52, layer: 56
Fold over basal
1.8
1.6
1.4
1.2
1
0.8
0
10
20
30
40
50
60
Time (min)
Normal
LY50
LY75
LY88
LY94
LY97
LY data
Normal data

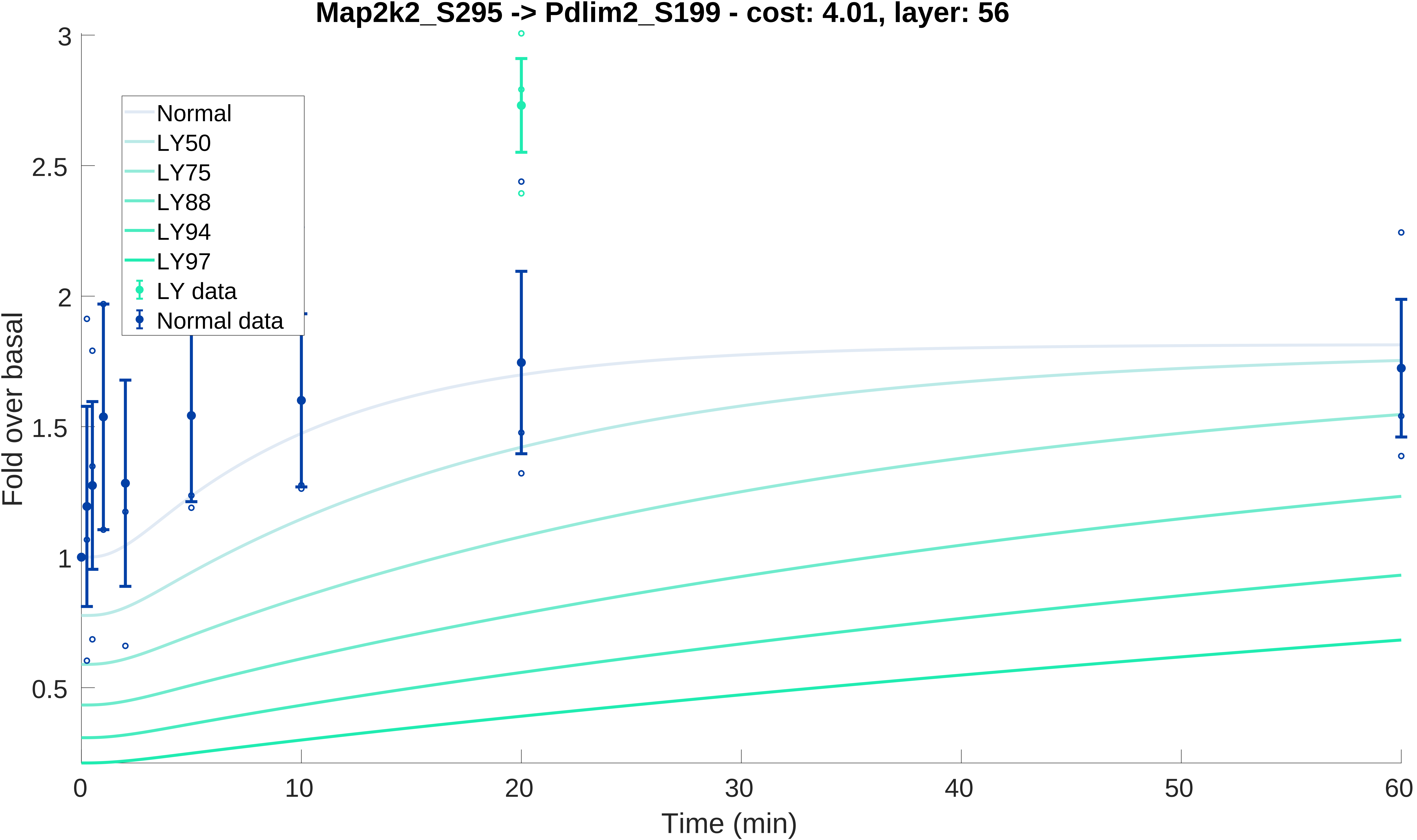

Map2k2_S295 -> Pdlim2_S199 - cost: 4.01, layer: 56
Normal
LY50
LY75
LY88
LY94
LY97
LY data
Normal data
Fold over basal
Time (min)
0.5
1
1.5
2
2.5
3
0
10
20
30
40
50
60

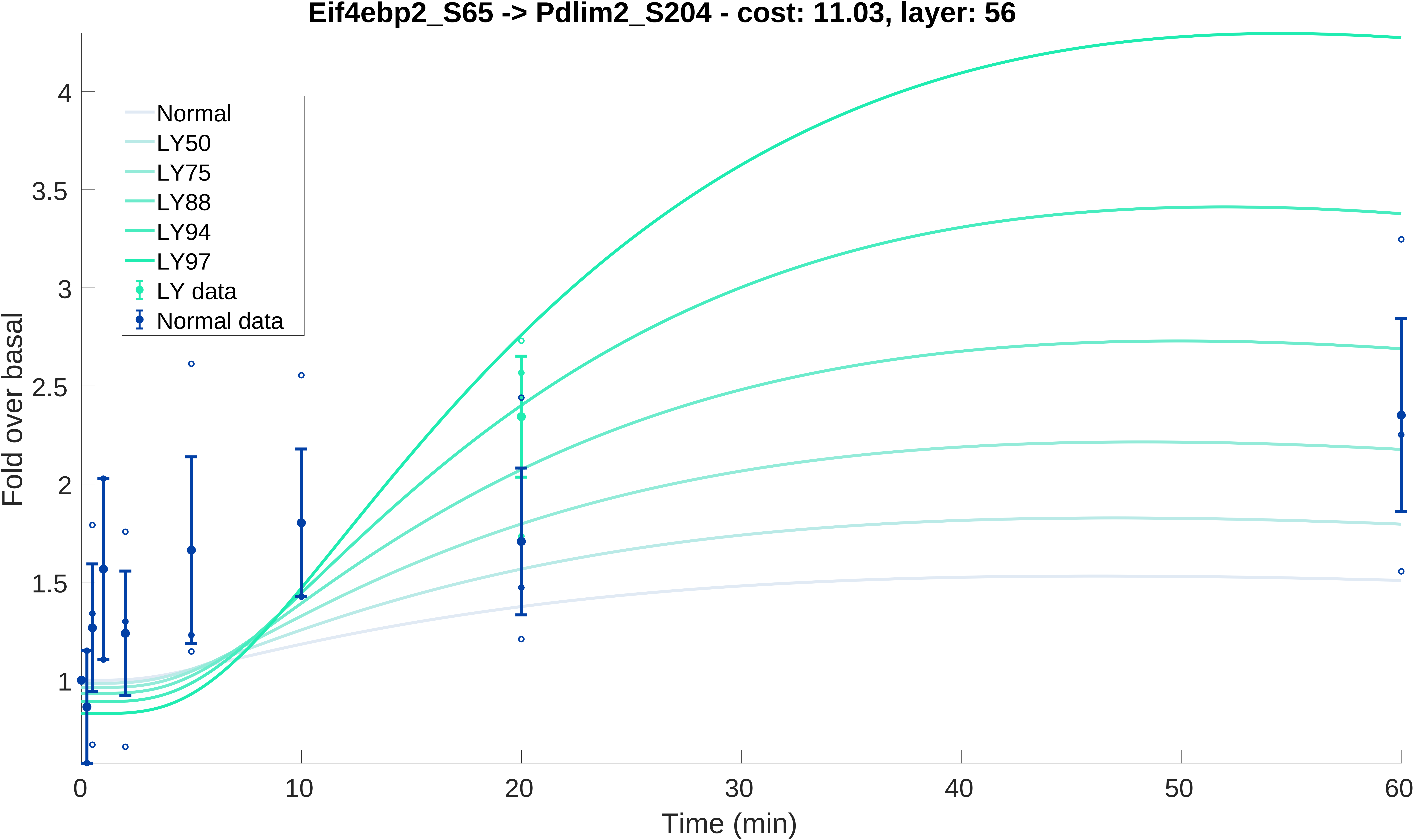

Eif4ebp2_S65 -> Pdlim2_S204 - cost: 11.03, layer: 56
Normal
LY50
LY75
LY88
LY94
LY97
LY data
Normal data
Fold over basal
Time (min)
4
3.5
3
2.5
2
1.5
1
0
10
20
30
40
50
60

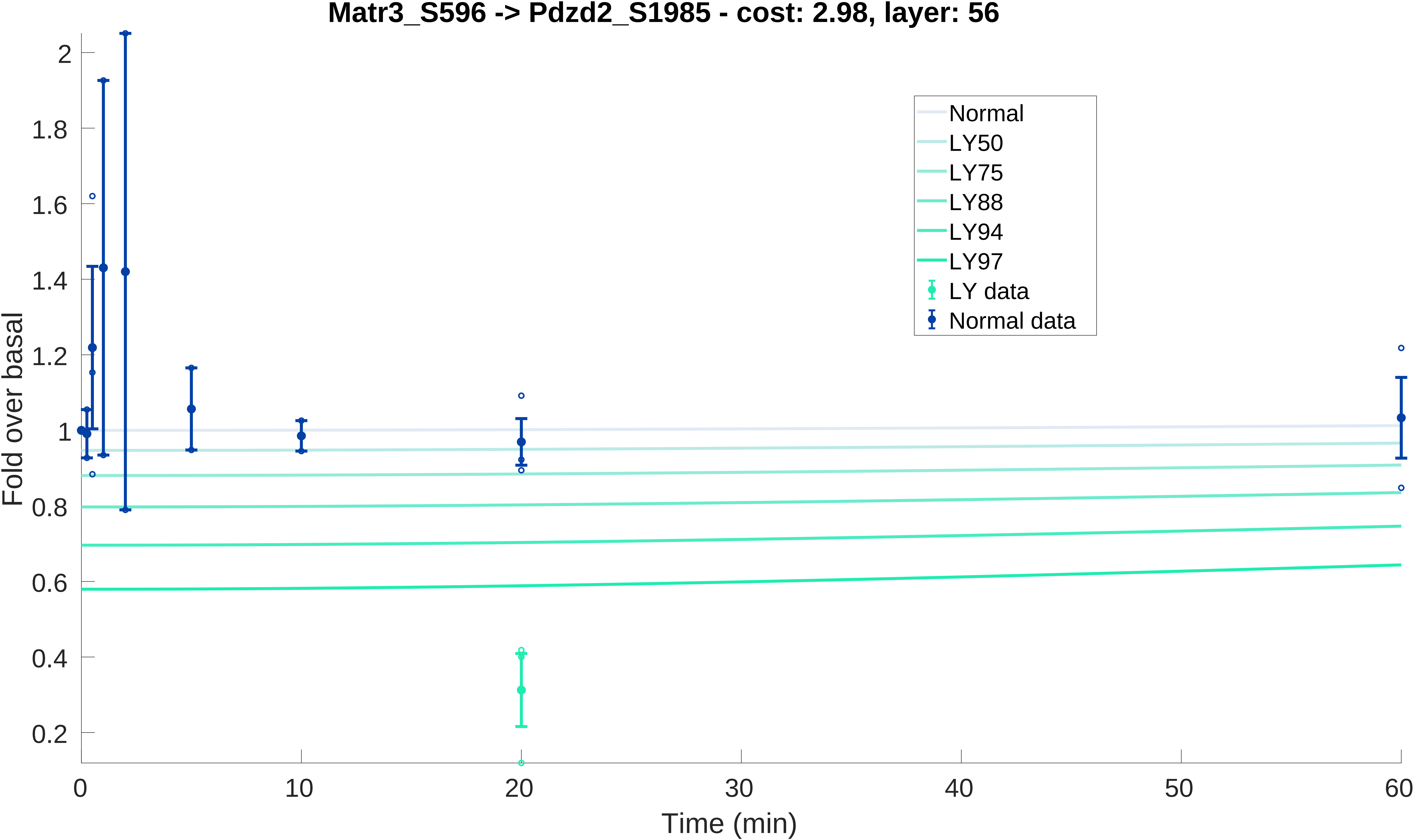

Matr3_S596 -> Pdzd2_S1985 - cost: 2.98, layer: 56
Fold over basal
Time (min)
0.2
0.4
0.6
0.8
1
1.2
1.4
1.6
1.8
2
0
10
20
30
40
50
60
Normal
LY50
LY75
LY88
LY94
LY97
LY data
Normal data

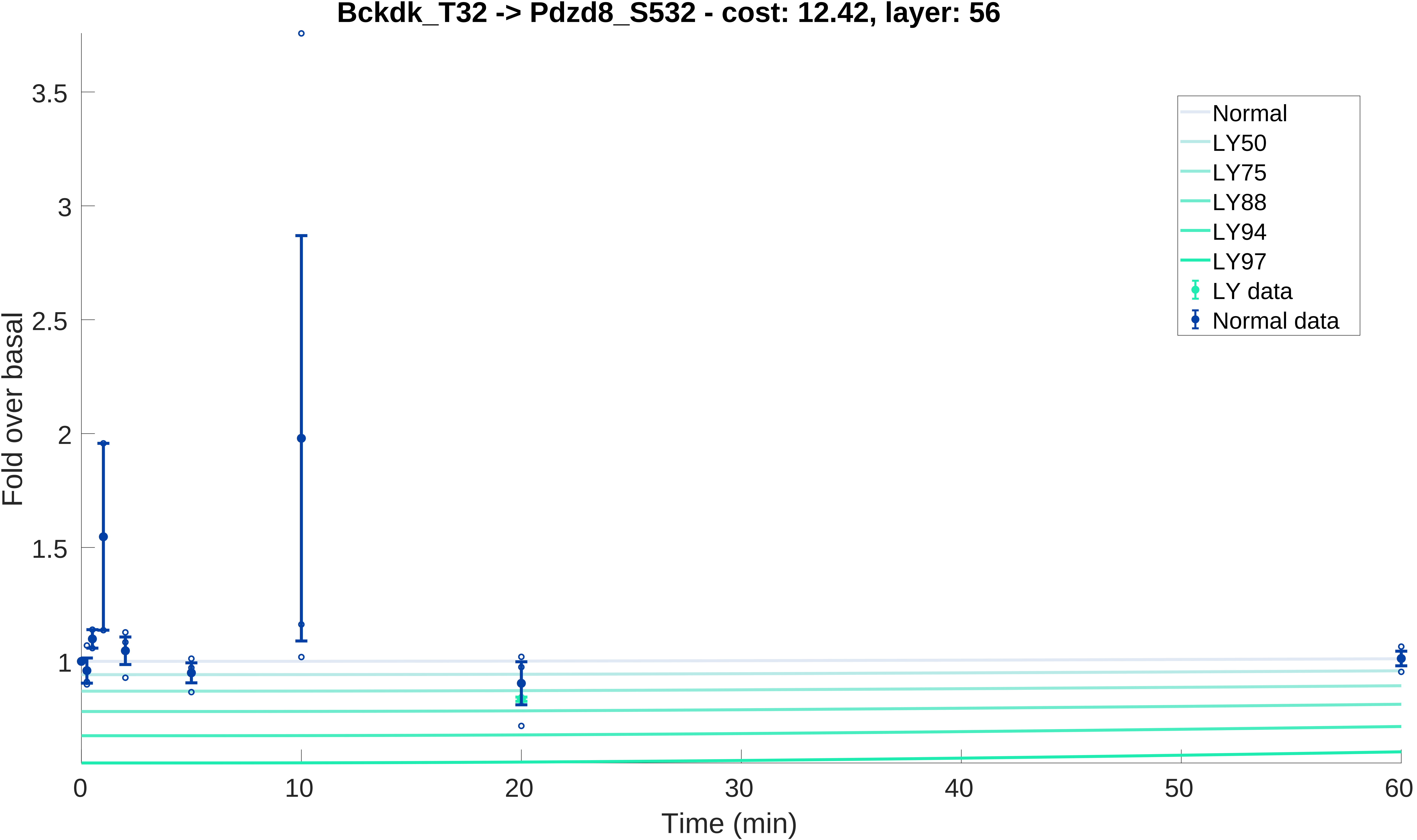

Bckdk_T32 -> Pdzd8_S532 - cost: 12.42, layer: 56
Fold over basal
3.5
3
2.5
2
1.5
1
0
10
20
30
40
50
60
Time (min)
Normal
LY50
LY75
LY88
LY94
LY97
LY data
Normal data

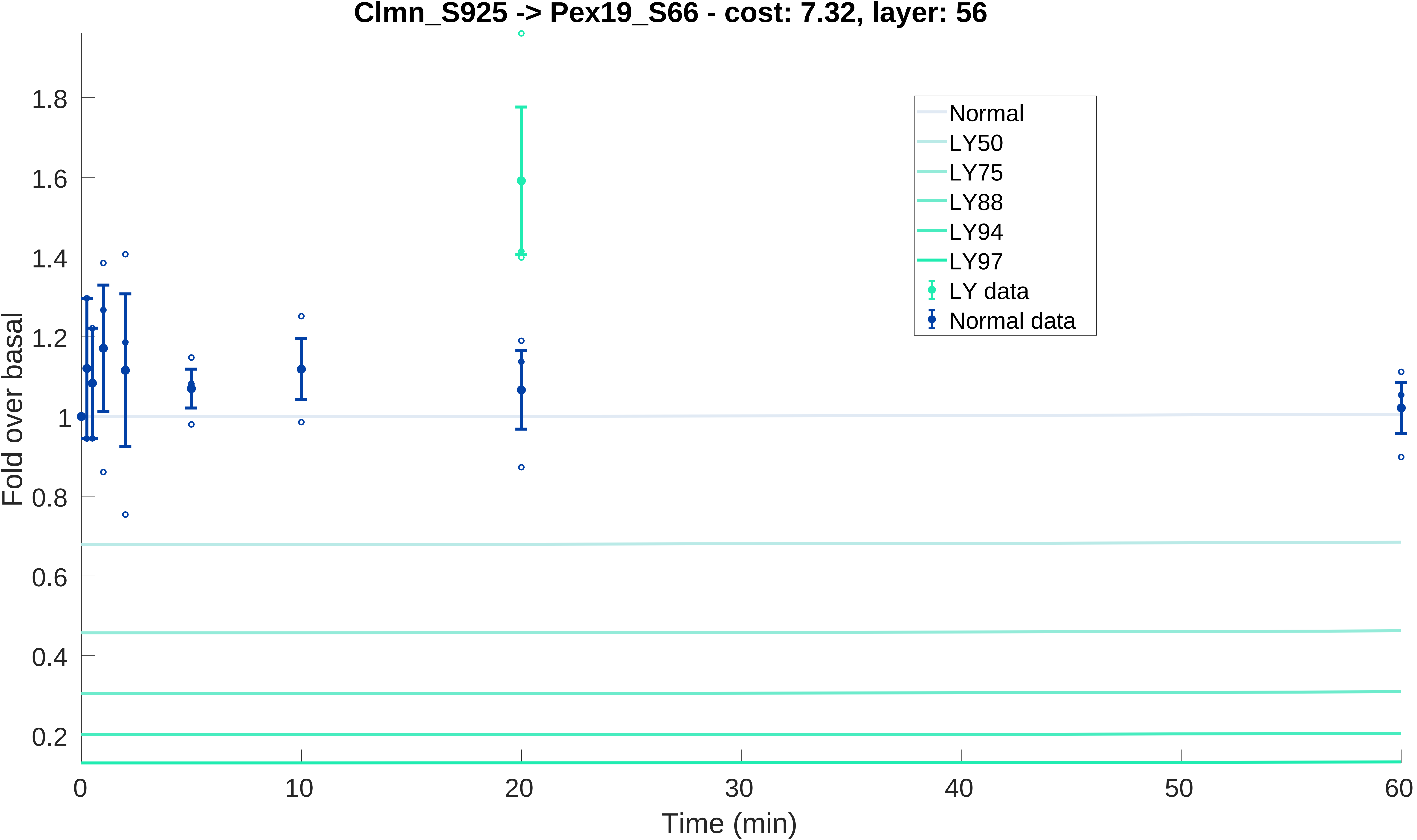
Clmn_S925 -> Pex19_S66 - cost: 7.32, layer: 56
Normal
LY50
LY75
LY88
LY94
LY97
LY data
Normal data
Fold over basal
Time (min)
1.8
1.6
1.4
1.2
1
0.8
0.6
0.4
0.2
0
10
20
30
40
50
60

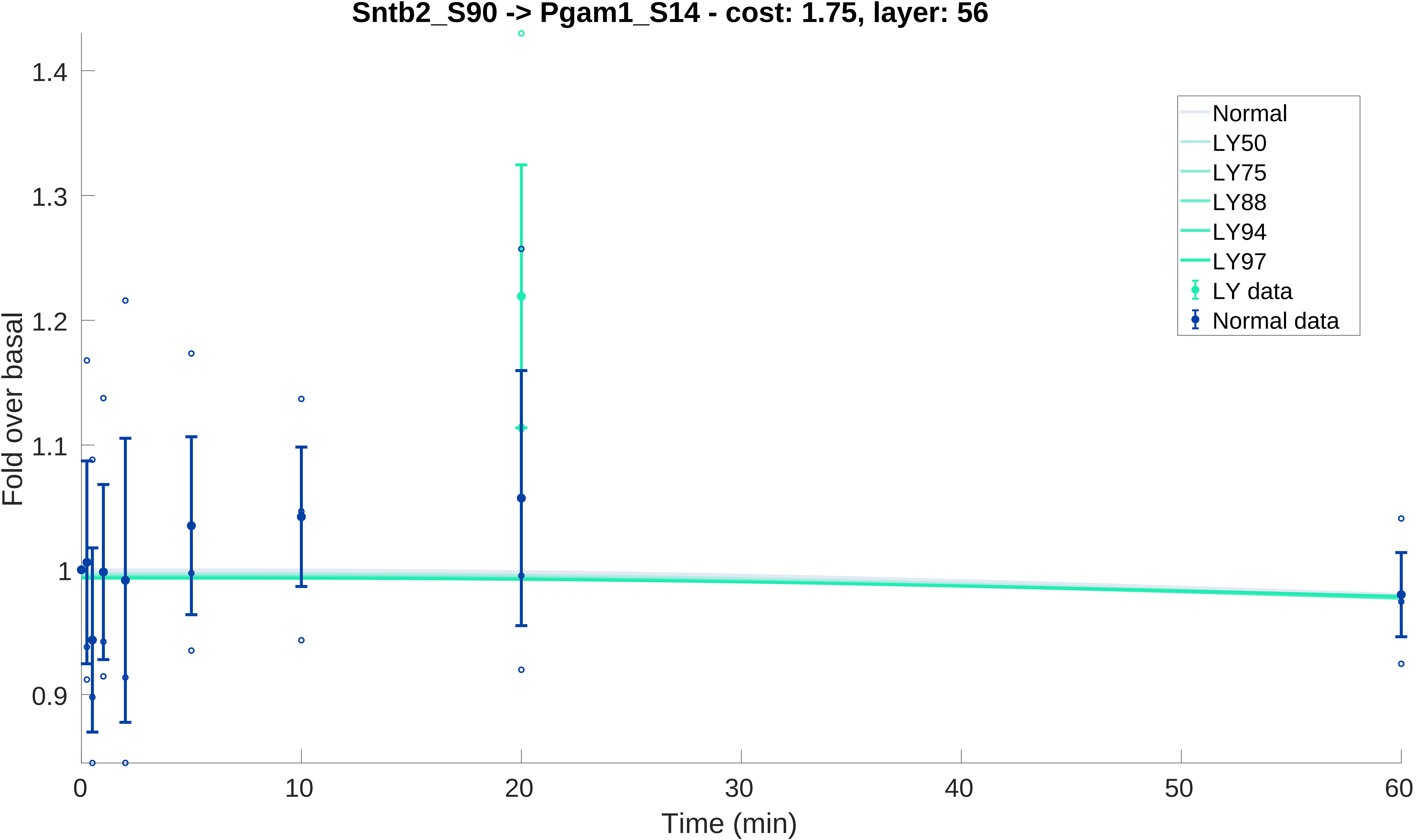

Sntb2_S90 -> Pgam1_S14 - cost: 1.75, layer: 56
Fold over basal
Time (min)
1.4
1.3
1.2
1.1
1
0.9
0
10
20
30
40
50
60
Normal
LY50
LY75
LY88
LY94
LY97
LY data
Normal data

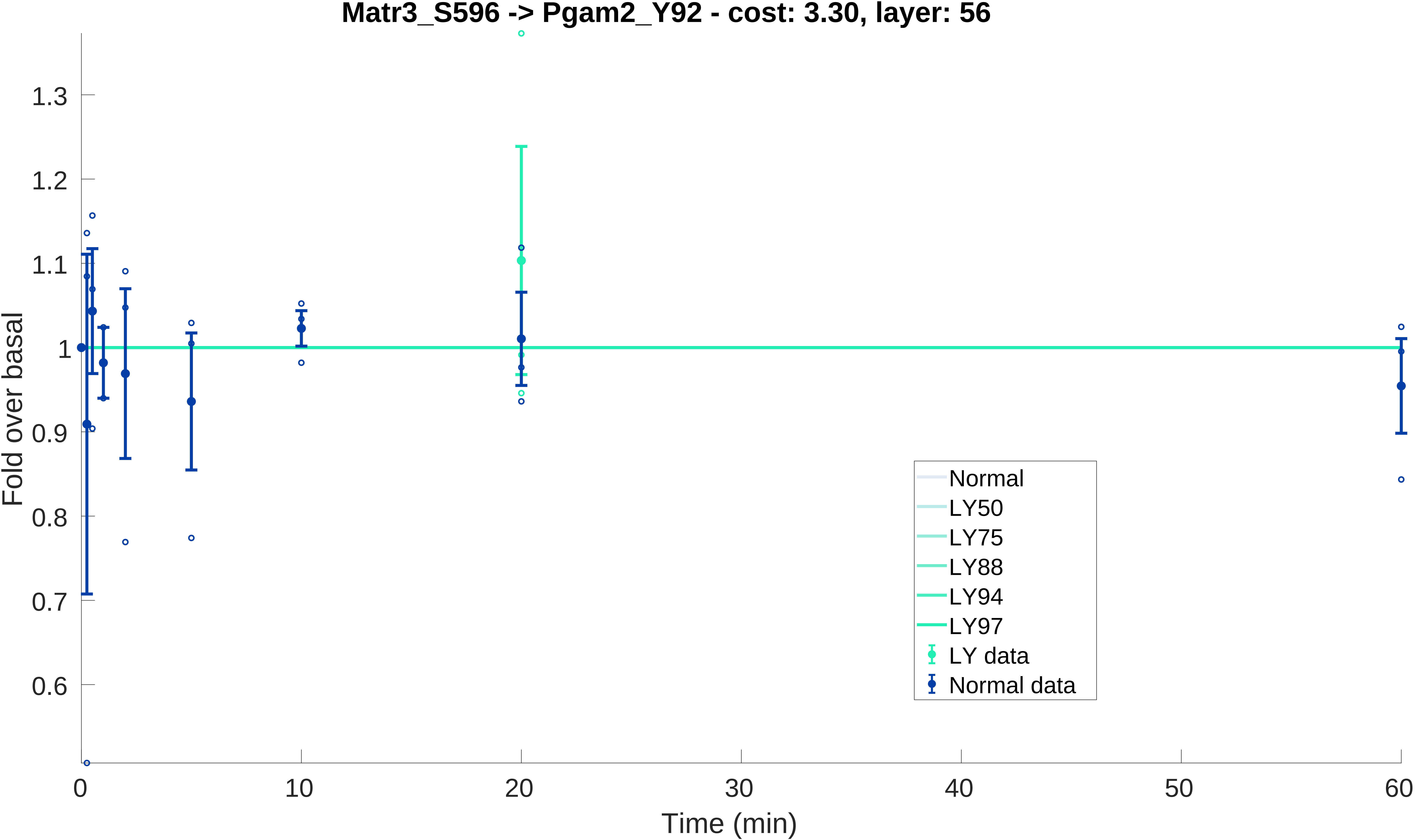

Matr3_S596 -> Pgam2_Y92 - cost: 3.30, layer: 56
Fold over basal
1.3
1.2
1.1
1
0.9
0.8
0.7
0.6
0
10
20
30
40
50
60
Time (min)
Normal
LY50
LY75
LY88
LY94
LY97
LY data
Normal data

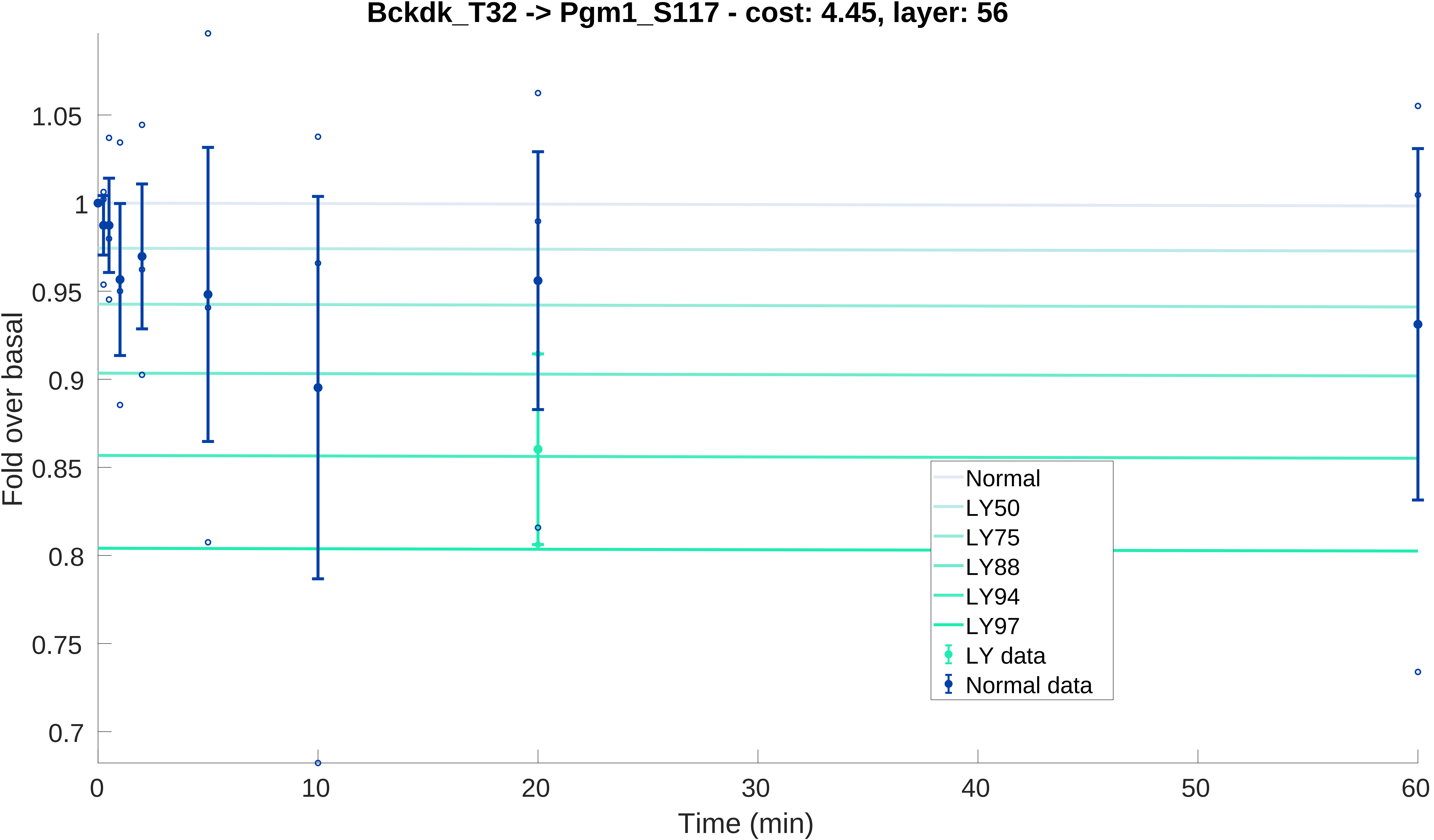
Bckdk_T32 -> Pgm1_S117 - cost: 4.45, layer: 56
Fold over basal
Time (min)
1.05
1
0.95
0.9
0.85
0.8
0.75
0.7
0
10
20
30
40
50
60
Normal
LY50
LY75
LY88
LY94
LY97
LY data
Normal data

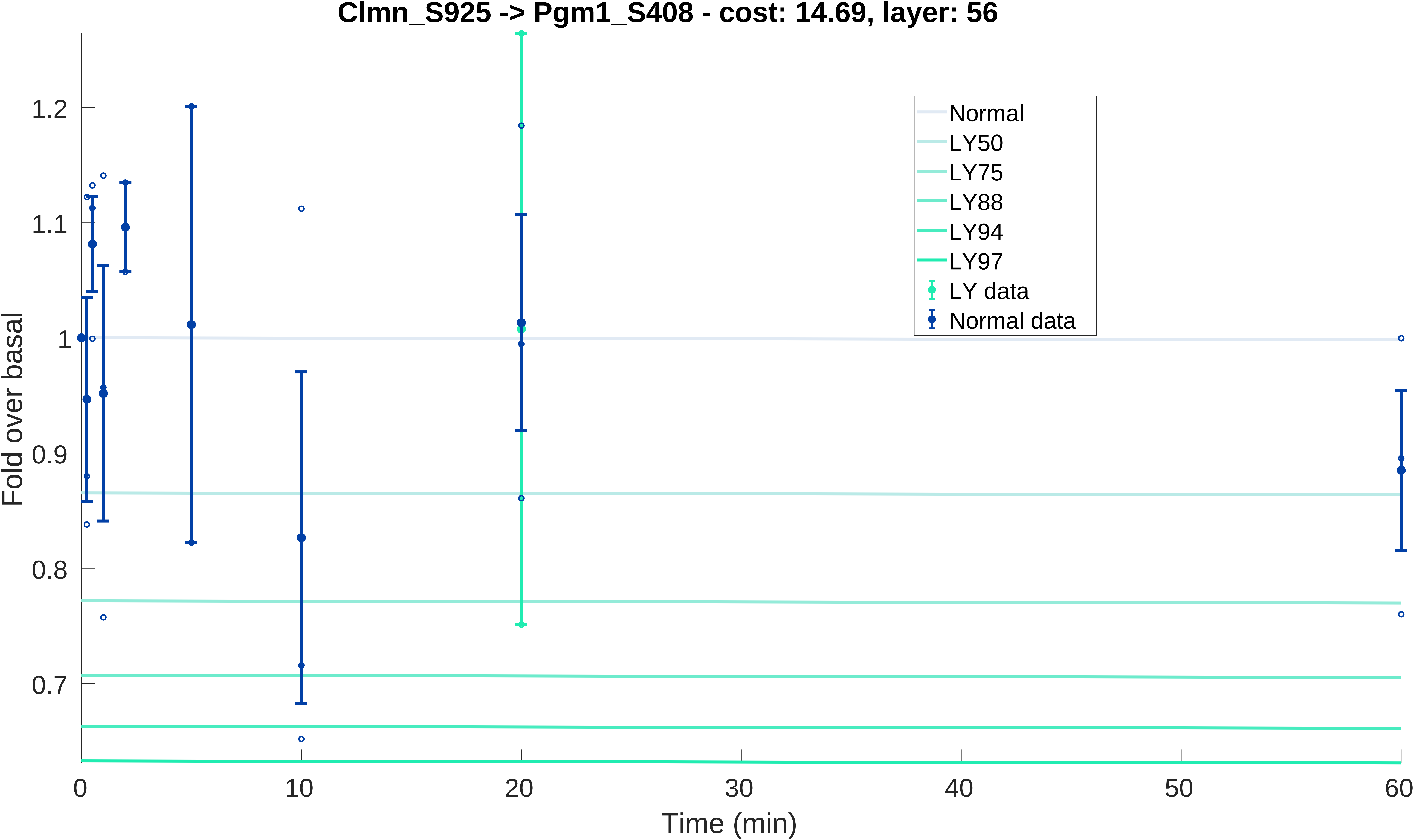
Clmn_S925 -> Pgm1_S408 - cost: 14.69, layer: 56
Fold over basal
Time (min)
0
10
20
30
40
50
60
0.7
0.8
0.9
1
1.1
1.2
Normal
LY50
LY75
LY88
LY94
LY97
LY data
Normal data

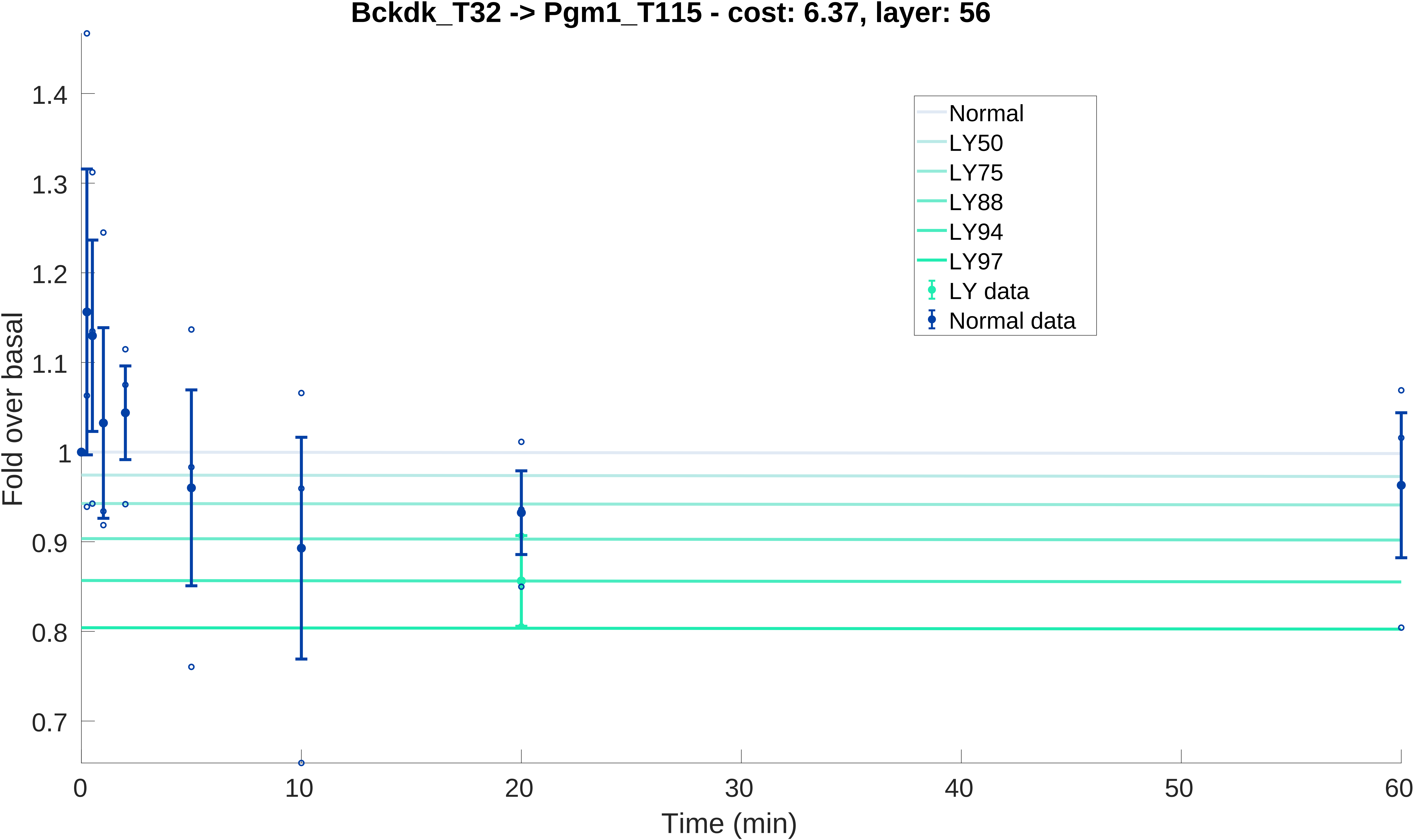
Bckdk_T32 -> Pgm1_T115 - cost: 6.37, layer: 56
Fold over basal
Time (min)
0.7
0.8
0.9
1
1.1
1.2
1.3
1.4
0
10
20
30
40
50
60
Normal
LY50
LY75
LY88
LY94
LY97
LY data
Normal data

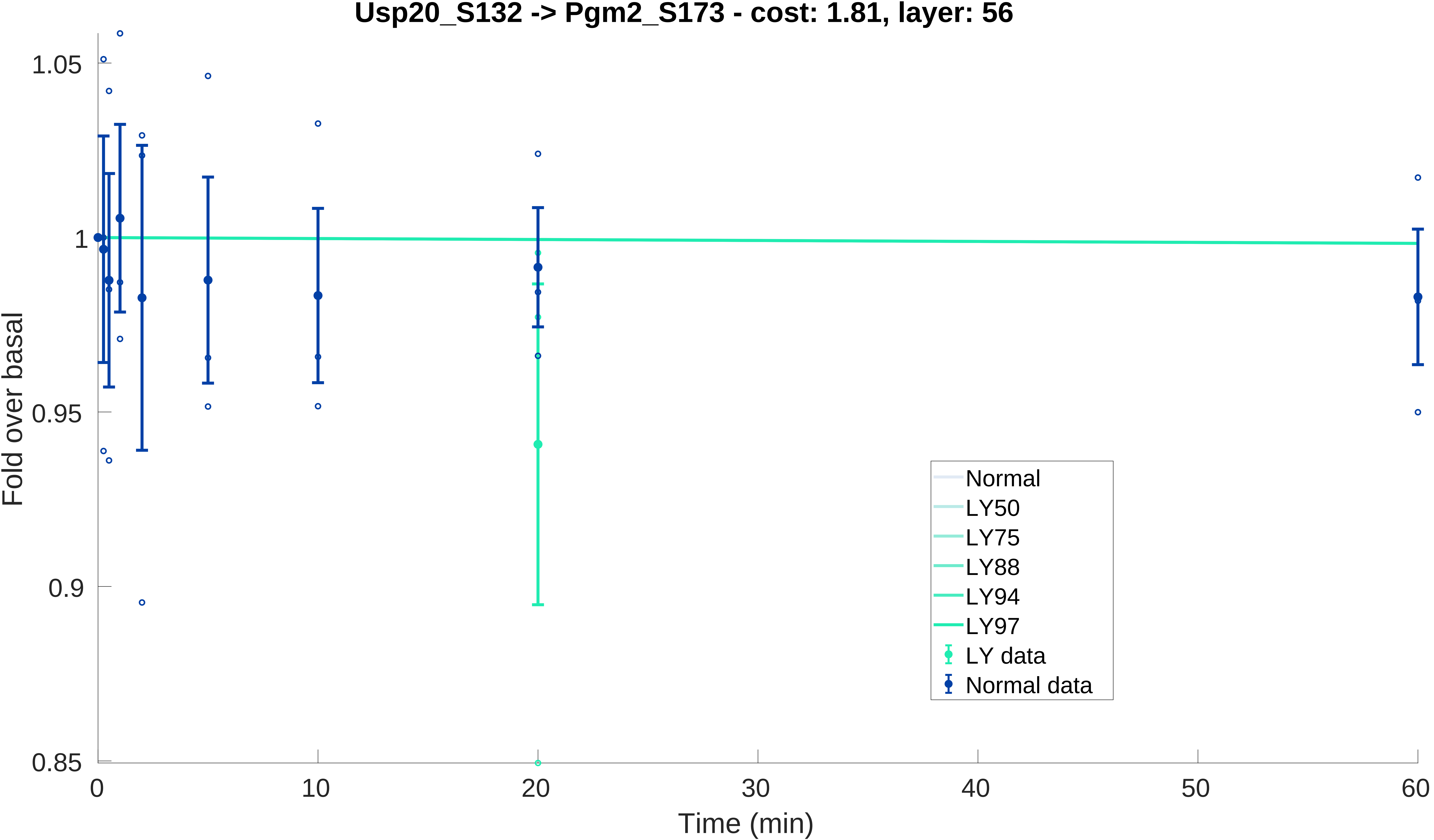

Usp20_S132 -> Pgm2_S173 - cost: 1.81, layer: 56
Fold over basal
1.05
1
0.95
0.9
0.85
0
10
20
30
40
50
60
Time (min)
Normal
LY50
LY75
LY88
LY94
LY97
LY data
Normal data

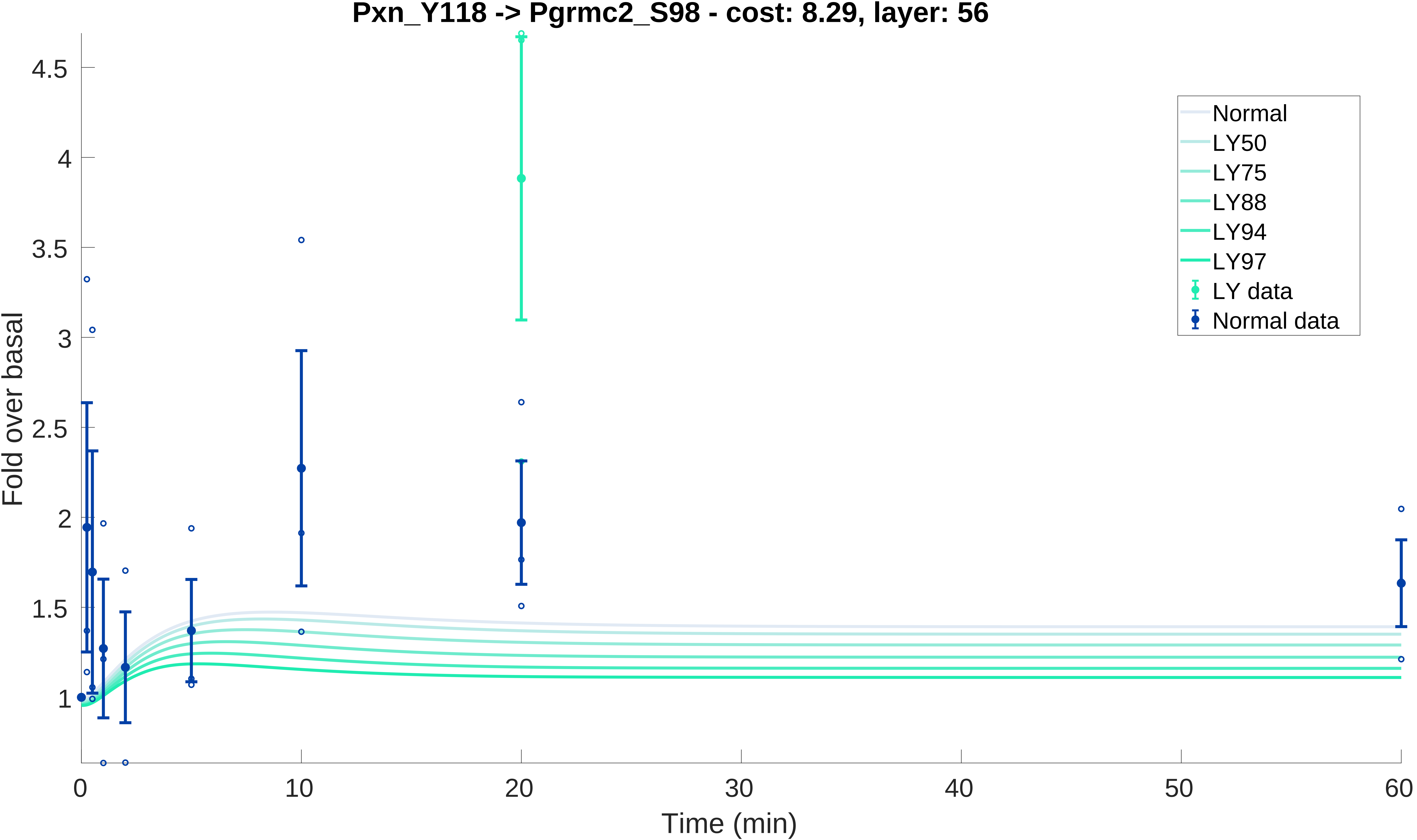

Pxn_Y118 -> Pgrmc2_S98 - cost: 8.29, layer: 56
Fold over basal
Time (min)
4.5
4
3.5
3
2.5
2
1.5
1
0
10
20
30
40
50
60
Normal
LY50
LY75
LY88
LY94
LY97
LY data
Normal data

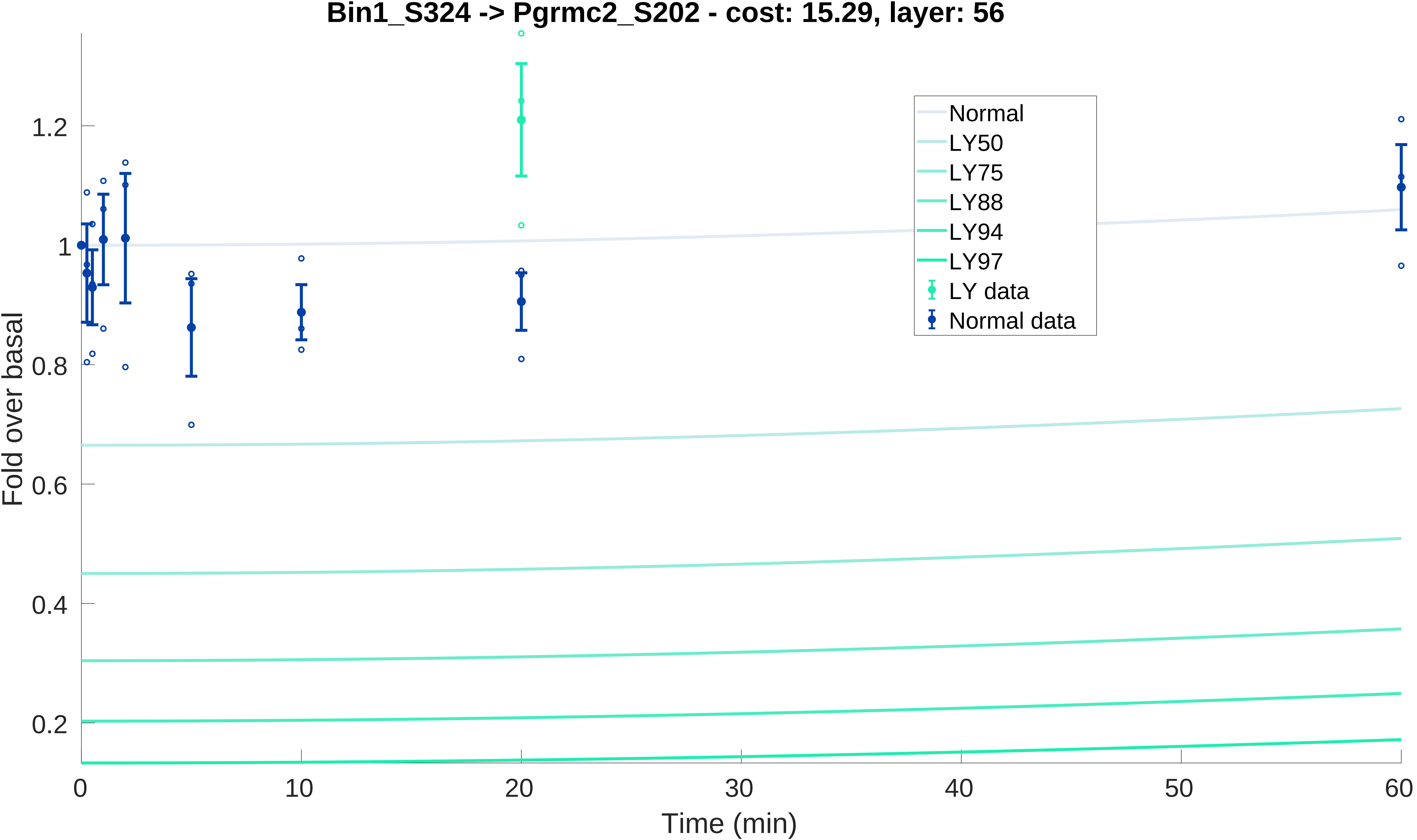
Bin1_S324 -> Pgrmc2_S202 - cost: 15.29, layer: 56
Fold over basal
0.2
0.4
0.6
0.8
1
1.2
0
10
20
30
40
50
60
Time (min)
Normal
LY50
LY75
LY88
LY94
LY97
LY data
Normal data

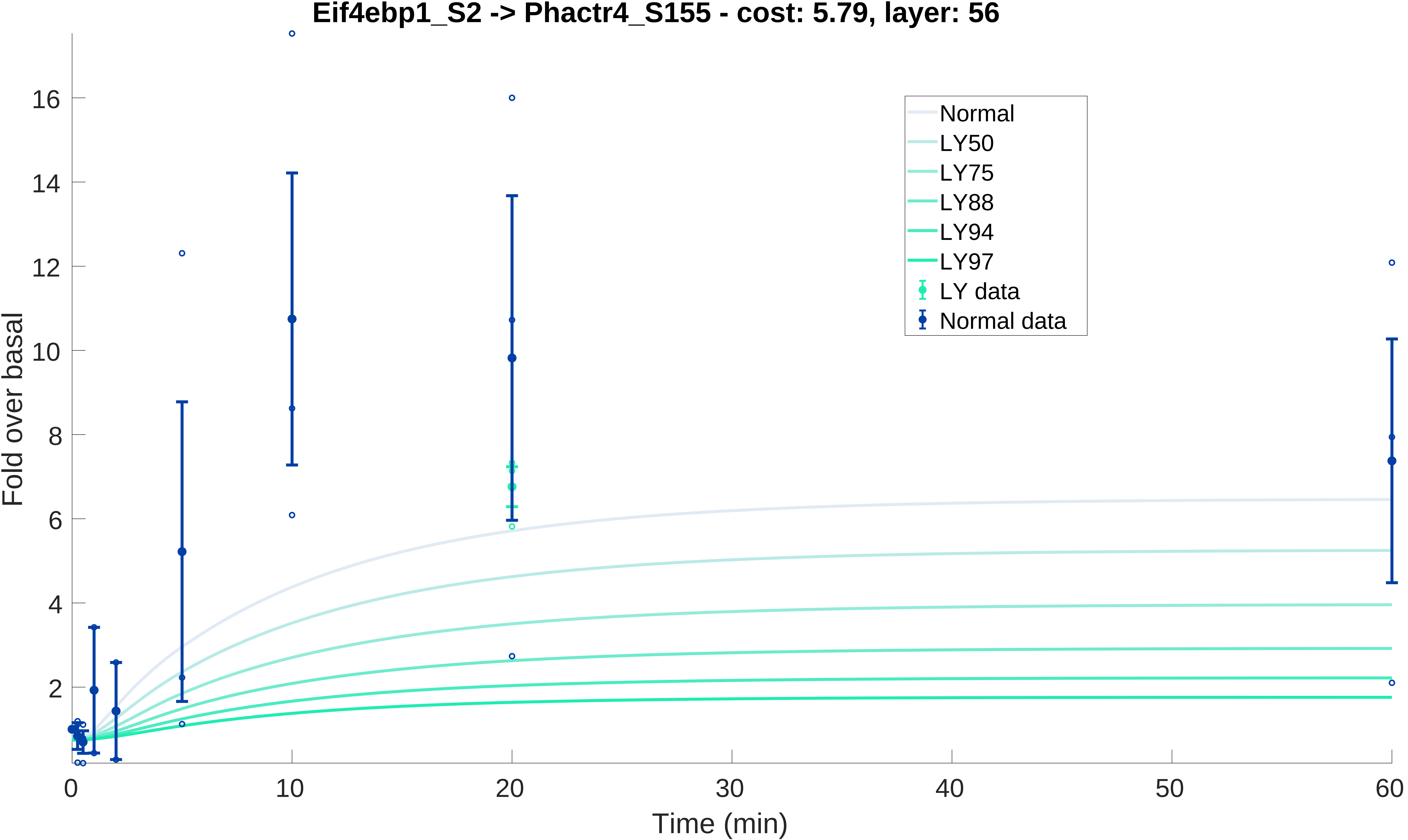

Eif4ebp1_S2 -> Phactr4_S155 - cost: 5.79, layer: 56
Fold over basal
Time (min)
0
10
20
30
40
50
60
2
4
6
8
10
12
14
16
Normal
LY50
LY75
LY88
LY94
LY97
LY data
Normal data

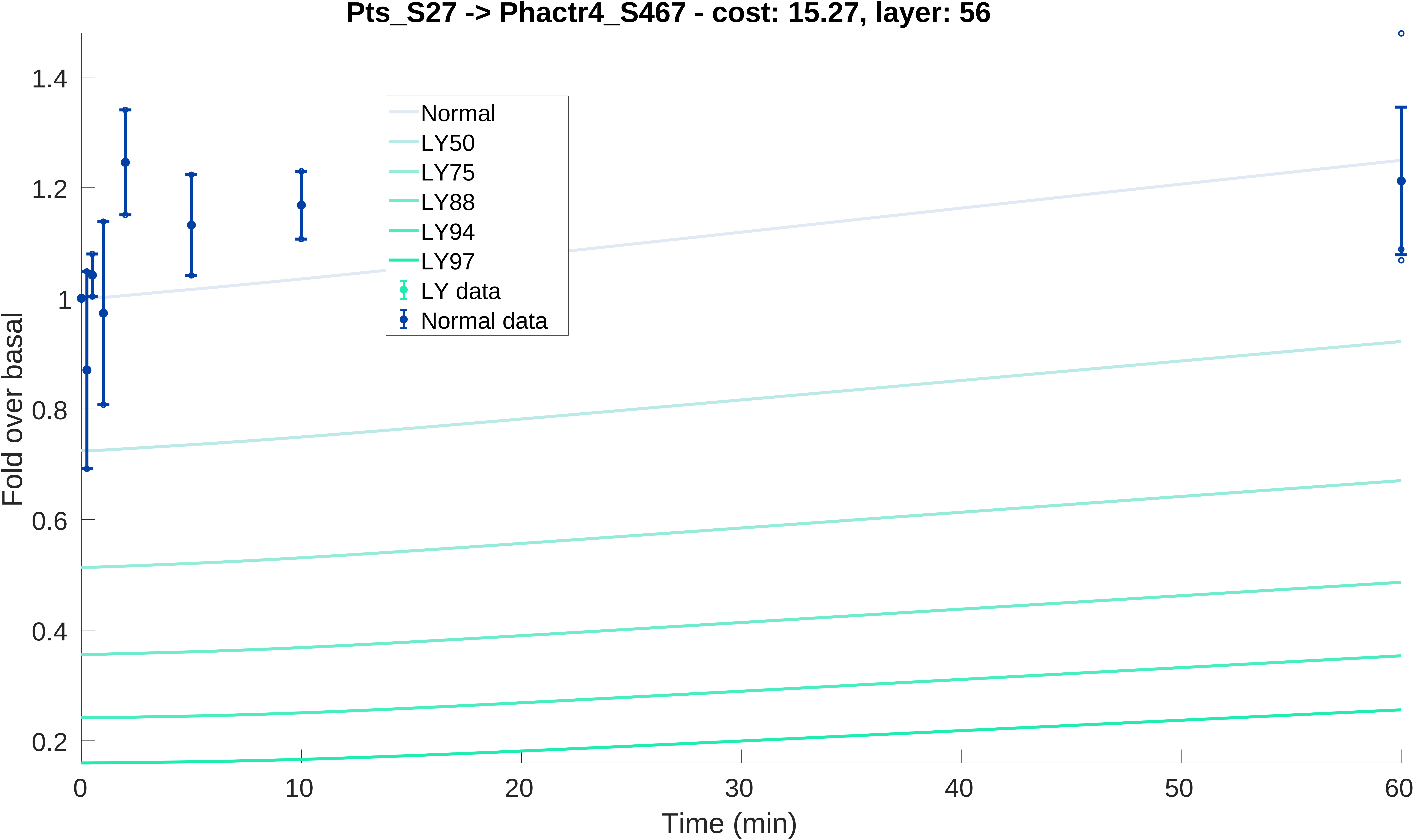
Pts_S27 -> Phactr4_S467 - cost: 15.27, layer: 56
Fold over basal
1.4
1.2
1
0.8
0.6
0.4
0.2
0
10
20
30
40
50
60
Time (min)
Normal
LY50
LY75
LY88
LY94
LY97
LY data
Normal data

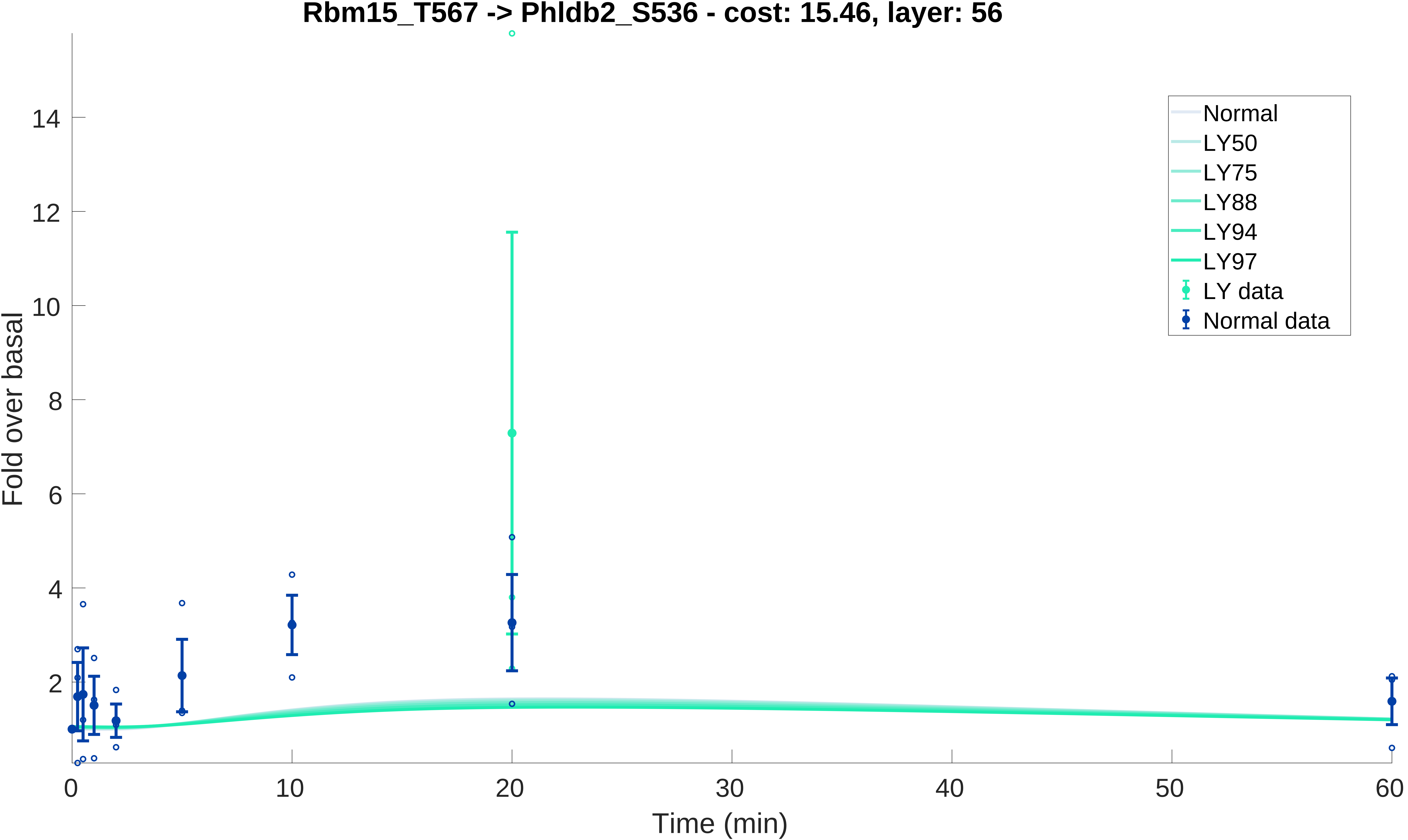
Rbm15_T567 -> Phldb2_S536 - cost: 15.46, layer: 56
Fold over basal
Time (min)
0
10
20
30
40
50
60
2
4
6
8
10
12
14
Normal
LY50
LY75
LY88
LY94
LY97
LY data
Normal data

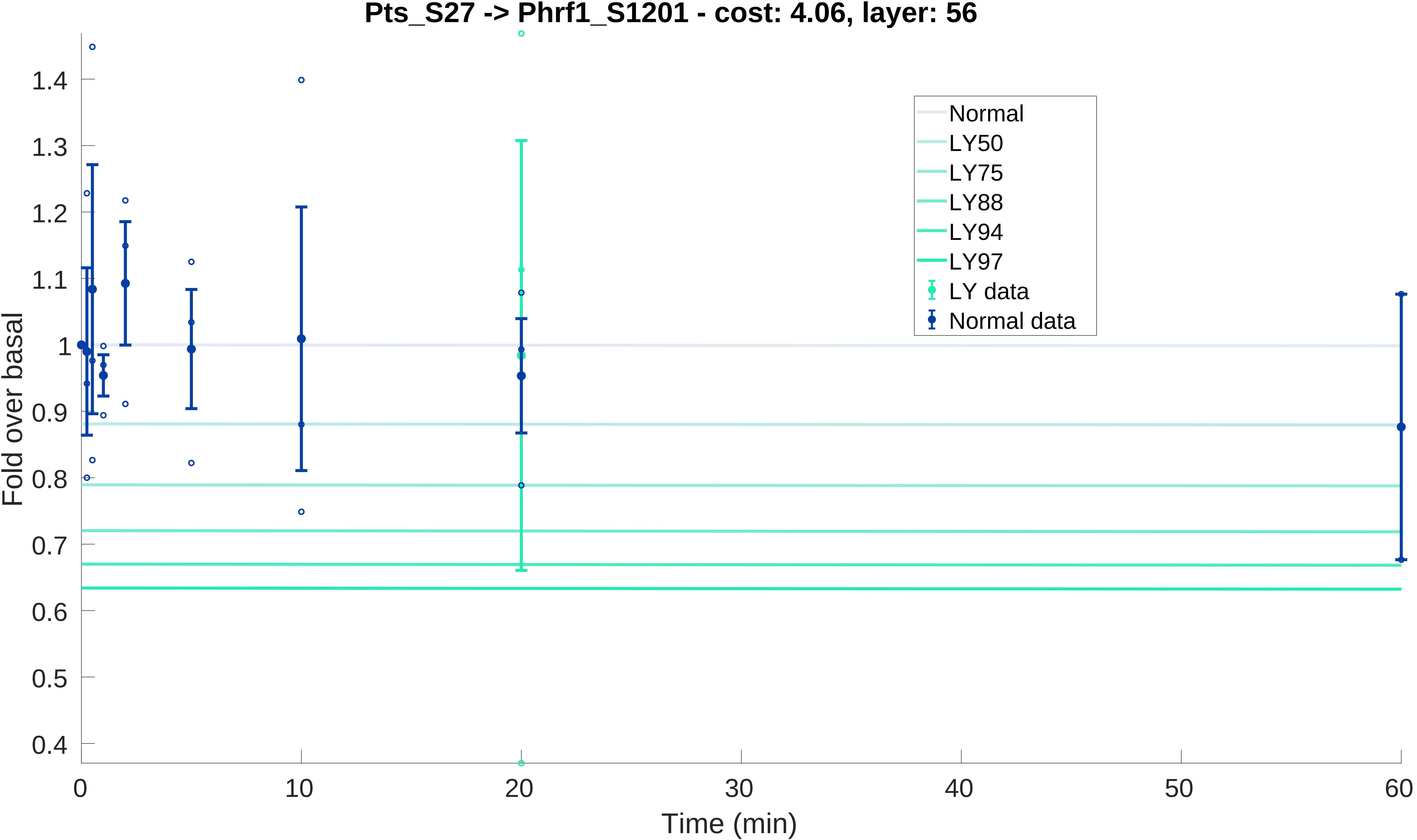
Pts_S27 -> Phrf1_S1201 - cost: 4.06, layer: 56
Fold over basal
Time (min)
0
10
20
30
40
50
60
0.4
0.5
0.6
0.7
0.8
0.9
1
1.1
1.2
1.3
1.4
Normal
LY50
LY75
LY88
LY94
LY97
LY data
Normal data

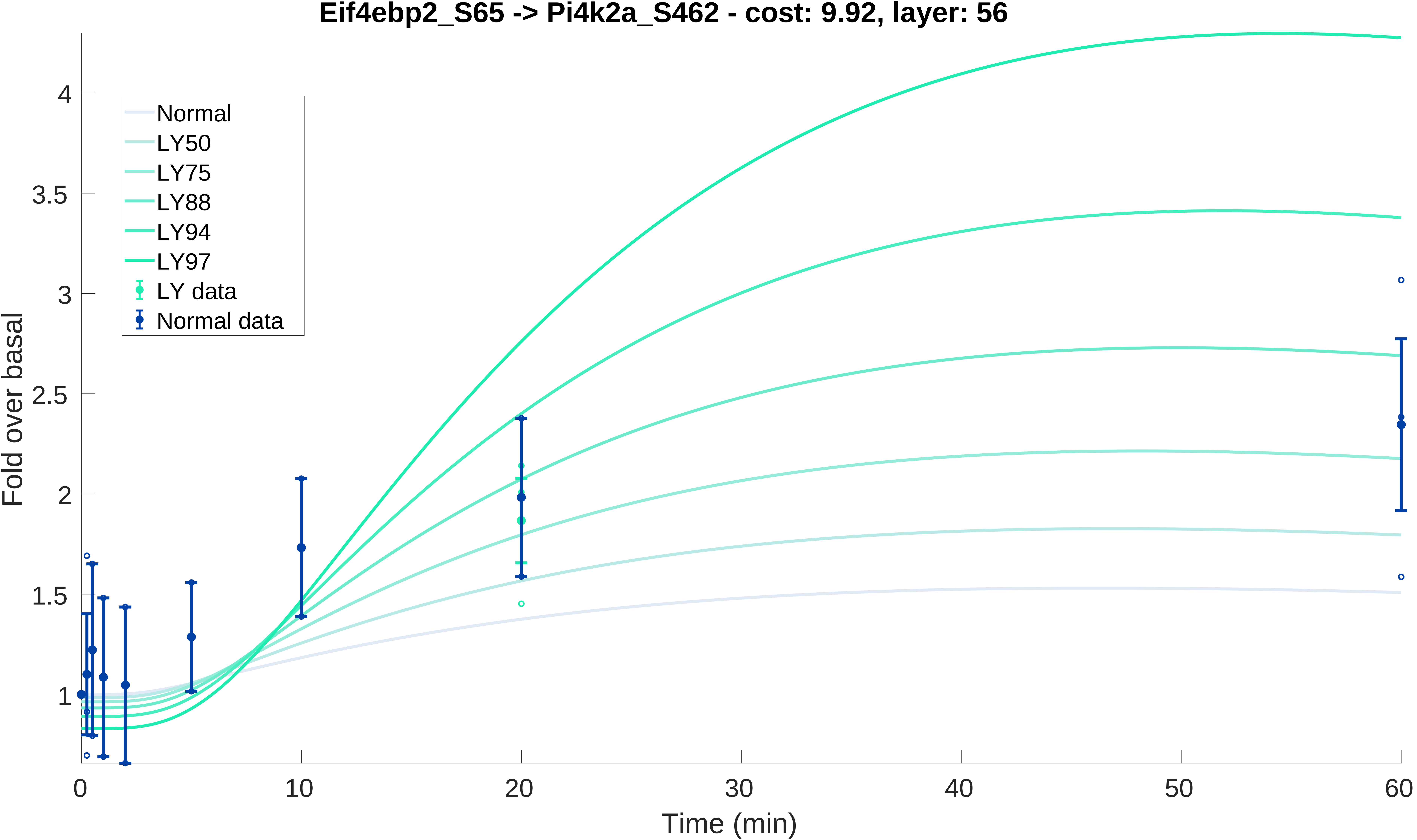
Eif4ebp2_S65 -> Pi4k2a_S462 - cost: 9.92, layer: 56
Normal
LY50
LY75
LY88
LY94
LY97
LY data
Normal data
Fold over basal
Time (min)
1
1.5
2
2.5
3
3.5
4
0
10
20
30
40
50
60

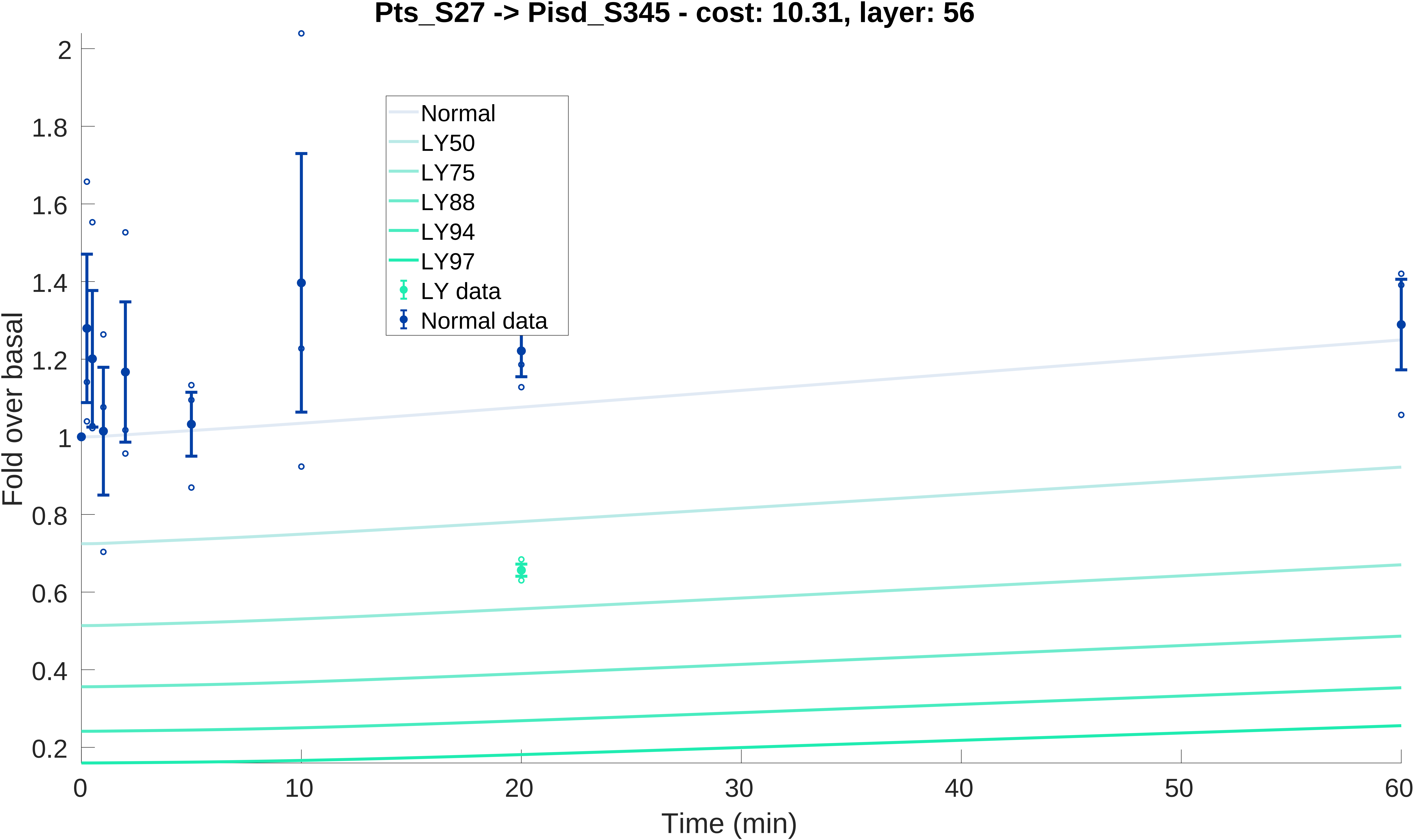
Pts_S27 -> Pisd_S345 - cost: 10.31, layer: 56
Fold over basal
Time (min)
Normal
LY50
LY75
LY88
LY94
LY97
LY data
Normal data
0
10
20
30
40
50
60
0.2
0.4
0.6
0.8
1
1.2
1.4
1.6
1.8
2

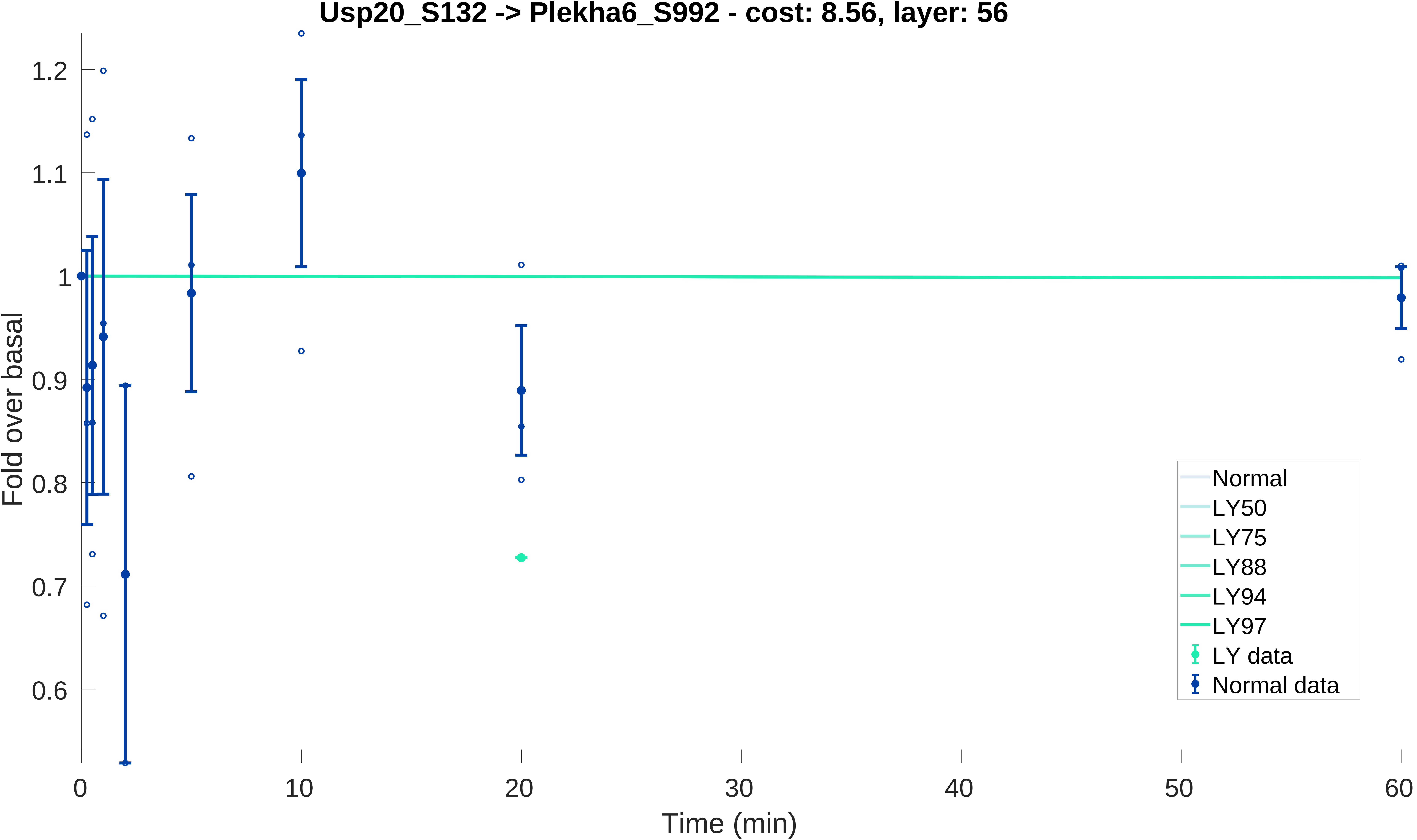

Usp20_S132 -> Plekha6_S992 - cost: 8.56, layer: 56
Fold over basal
1.2
1.1
1
0.9
0.8
0.7
0.6
0
10
20
30
40
50
60
Time (min)
Normal
LY50
LY75
LY88
LY94
LY97
LY data
Normal data

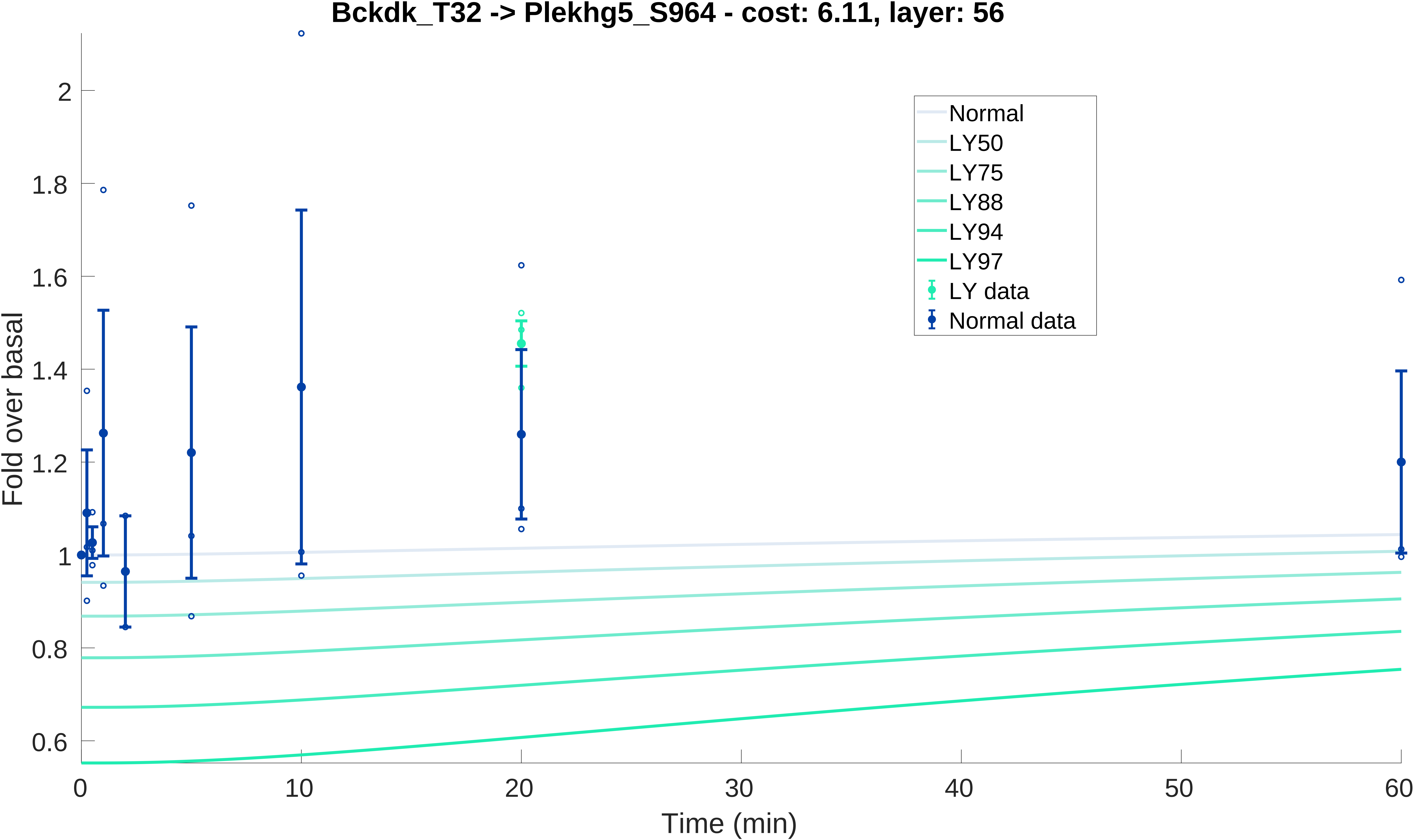
Bckdk_T32 -> Plekhg5_S964 - cost: 6.11, layer: 56
Fold over basal
Time (min)
0.6
0.8
1
1.2
1.4
1.6
1.8
2
0
10
20
30
40
50
60
Normal
LY50
LY75
LY88
LY94
LY97
LY data
Normal data

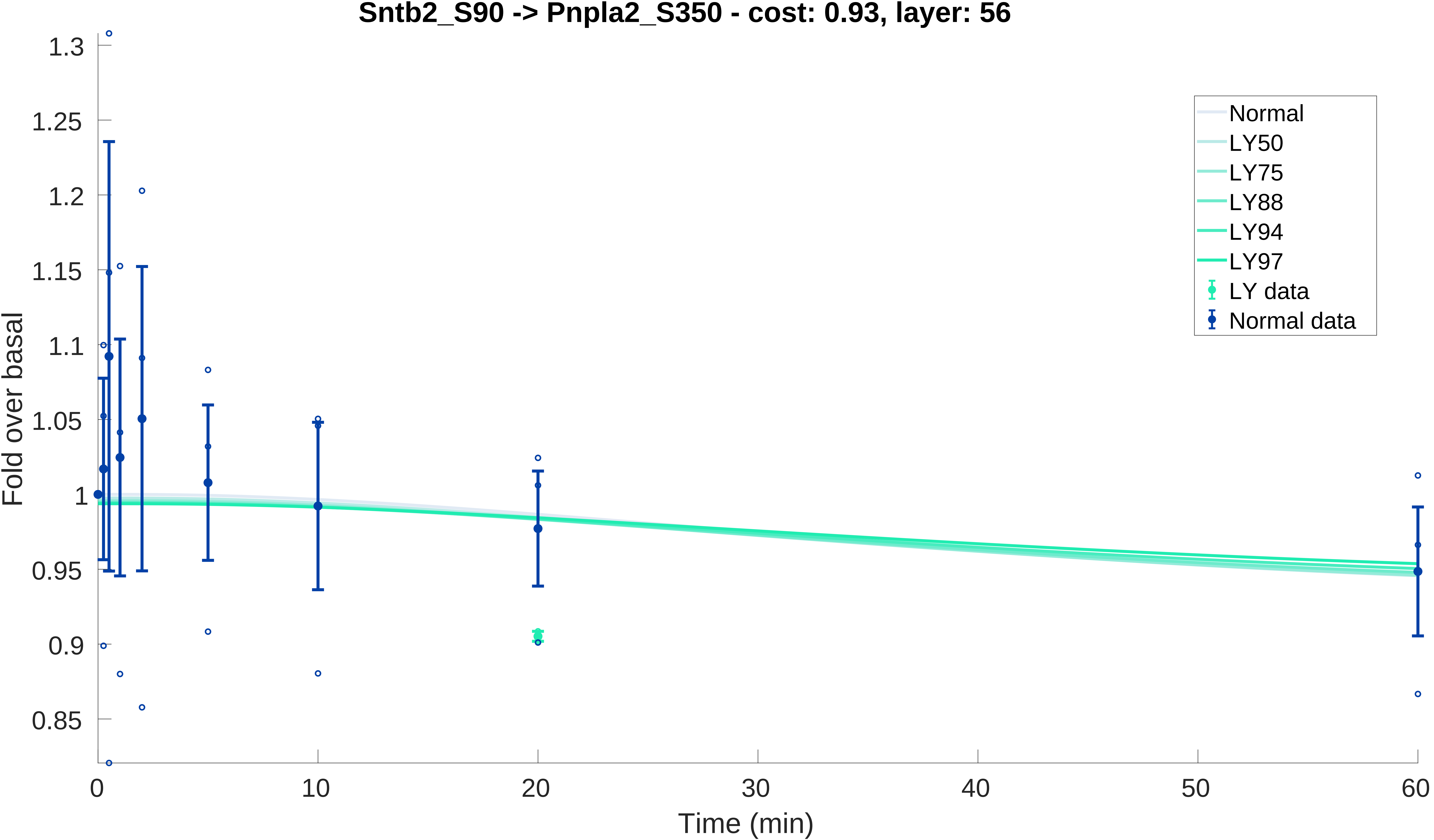

Sntb2_S90 -> Pnpla2_S350 - cost: 0.93, layer: 56
Fold over basal
1.3
1.25
1.2
1.15
1.1
1.05
1
0.95
0.9
0.85
0
10
20
30
40
50
60
Time (min)
Normal
LY50
LY75
LY88
LY94
LY97
LY data
Normal data

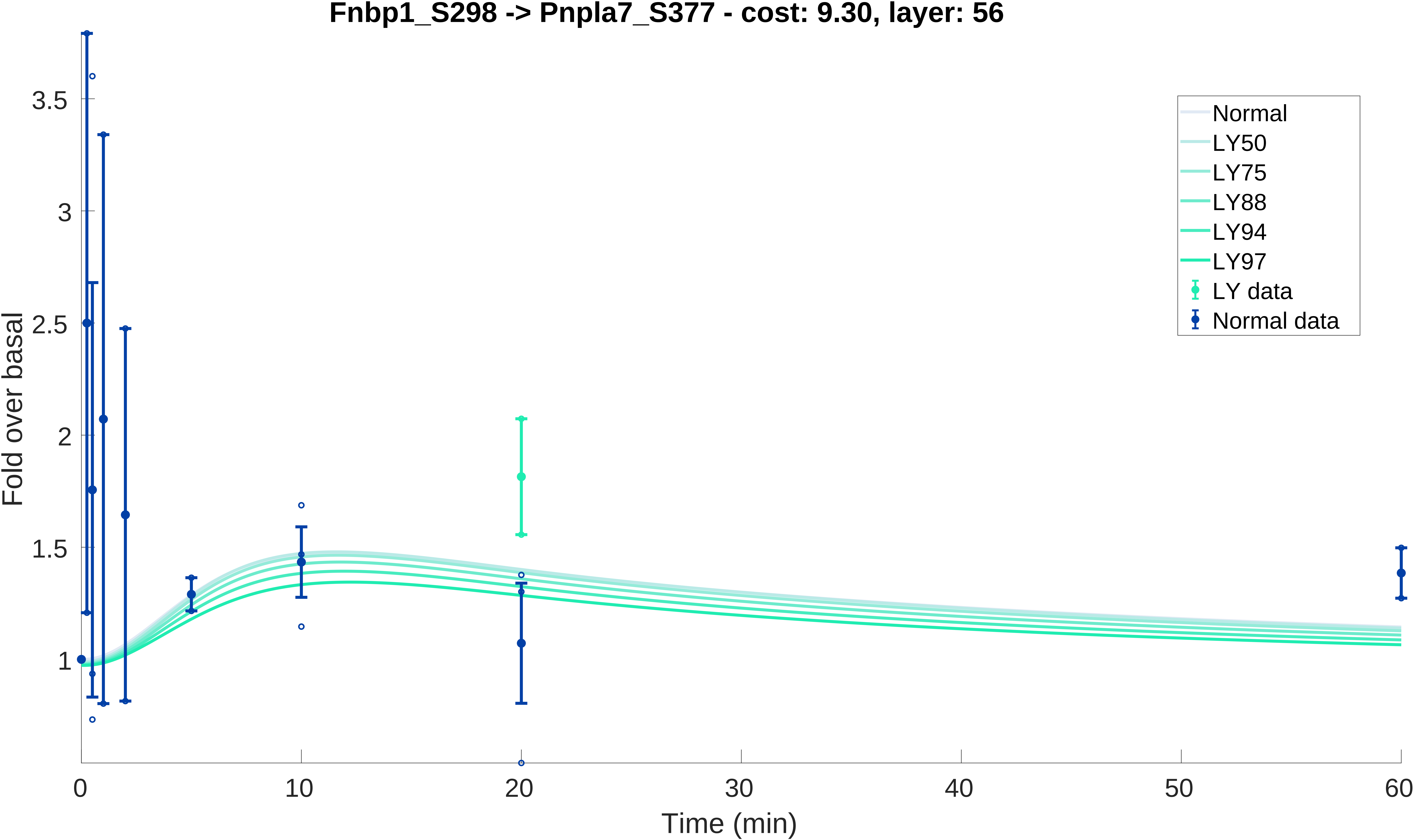
Fnbp1_S298 -> Pnpla7_S377 - cost: 9.30, layer: 56
Fold over basal
Time (min)
Normal
LY50
LY75
LY88
LY94
LY97
LY data
Normal data
3.5
3
2.5
2
1.5
1
0
10
20
30
40
50
60

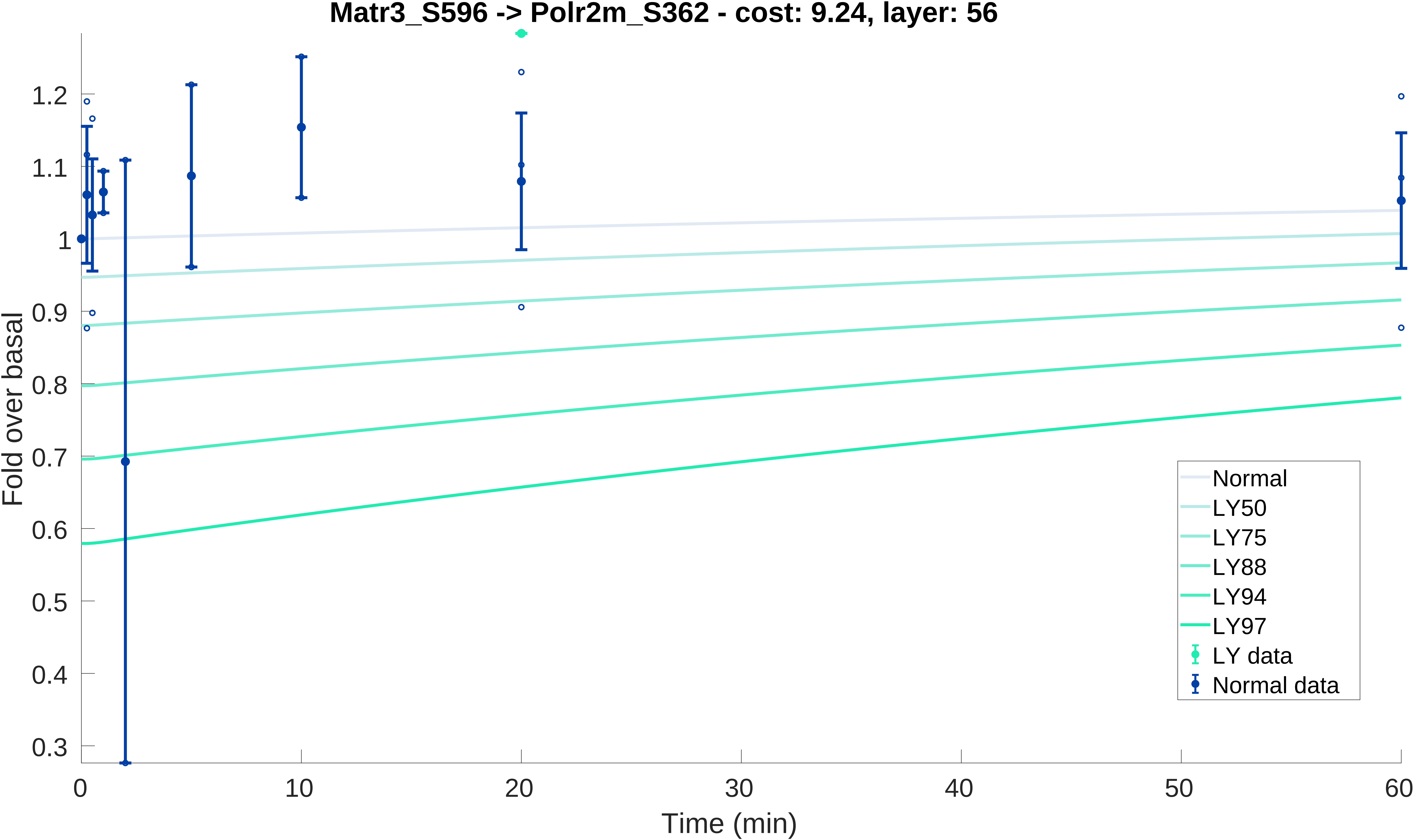
Matr3_S596 -> Polr2m_S362 - cost: 9.24, layer: 56
Fold over basal
Time (min)
0
10
20
30
40
50
60
0.3
0.4
0.5
0.6
0.7
0.8
0.9
1
1.1
1.2
Normal
LY50
LY75
LY88
LY94
LY97
LY data
Normal data

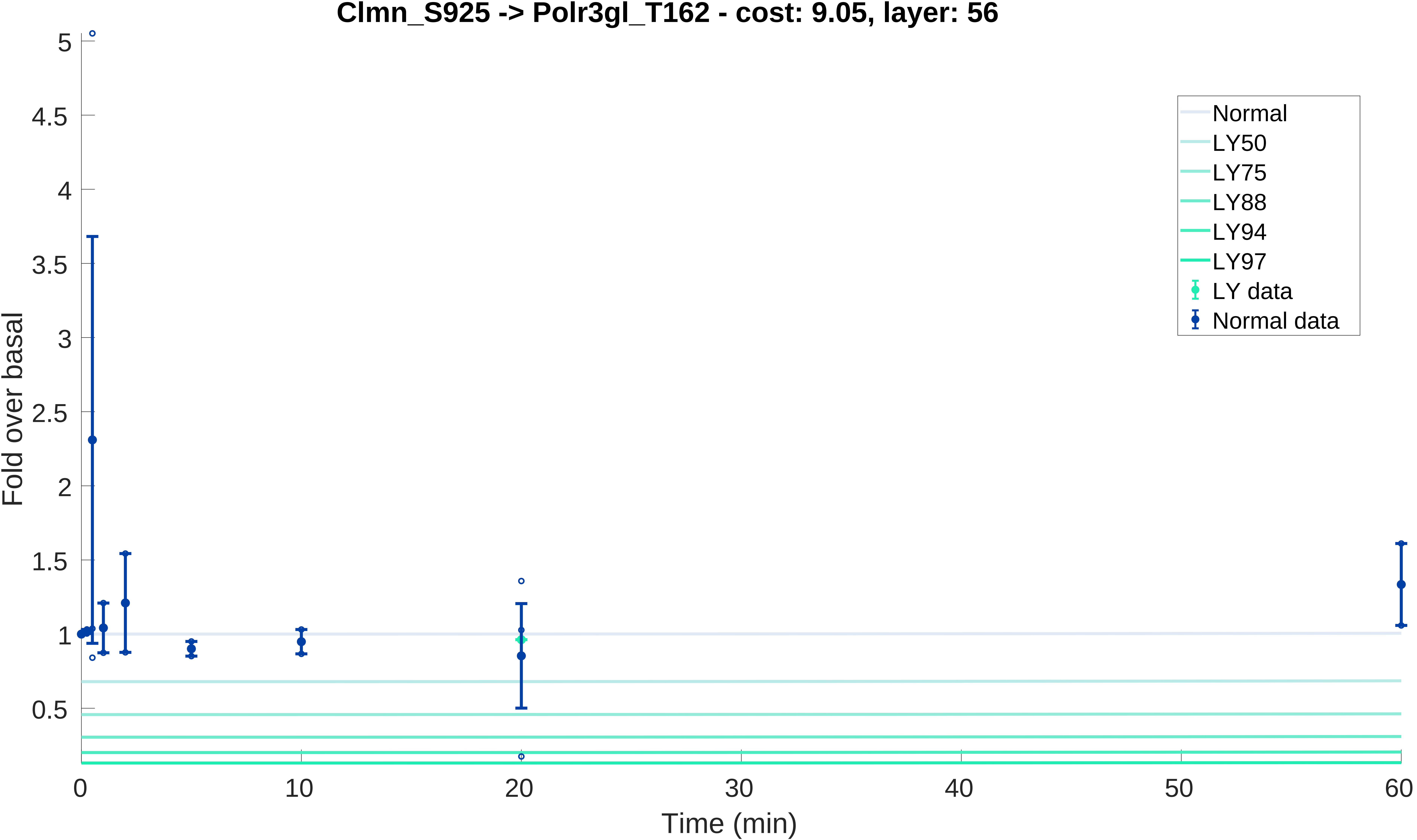

Clmn_S925 -> Polr3gl_T162 - cost: 9.05, layer: 56
Fold over basal
Time (min)
0
10
20
30
40
50
60
0.5
1
1.5
2
2.5
3
3.5
4
4.5
5
Normal
LY50
LY75
LY88
LY94
LY97
LY data
Normal data

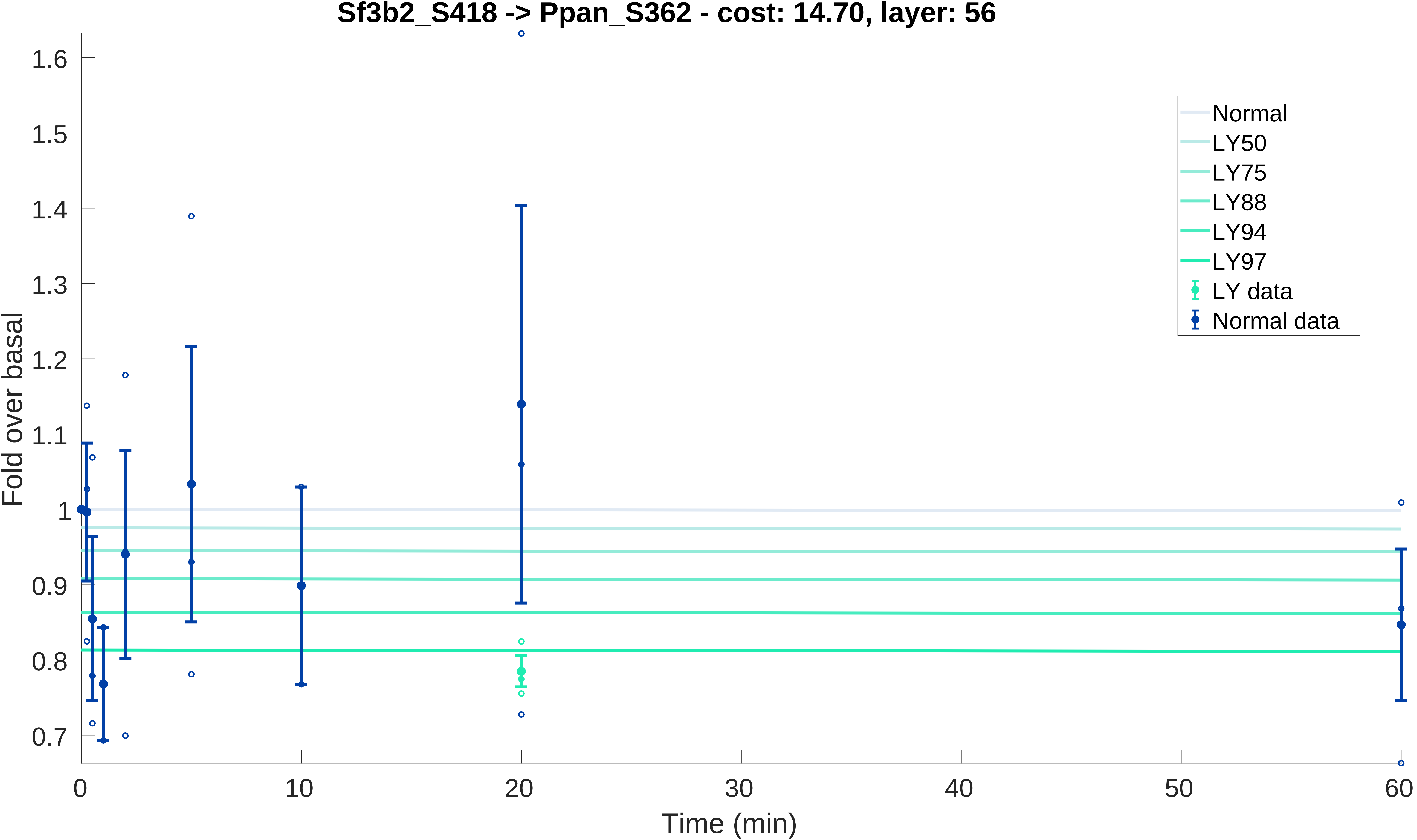

Sf3b2_S418 -> Ppan_S362 - cost: 14.70, layer: 56
Fold over basal
1.6
1.5
1.4
1.3
1.2
1.1
1
0.9
0.8
0.7
Time (min)
0
10
20
30
40
50
60
Normal
LY50
LY75
LY88
LY94
LY97
LY data
Normal data

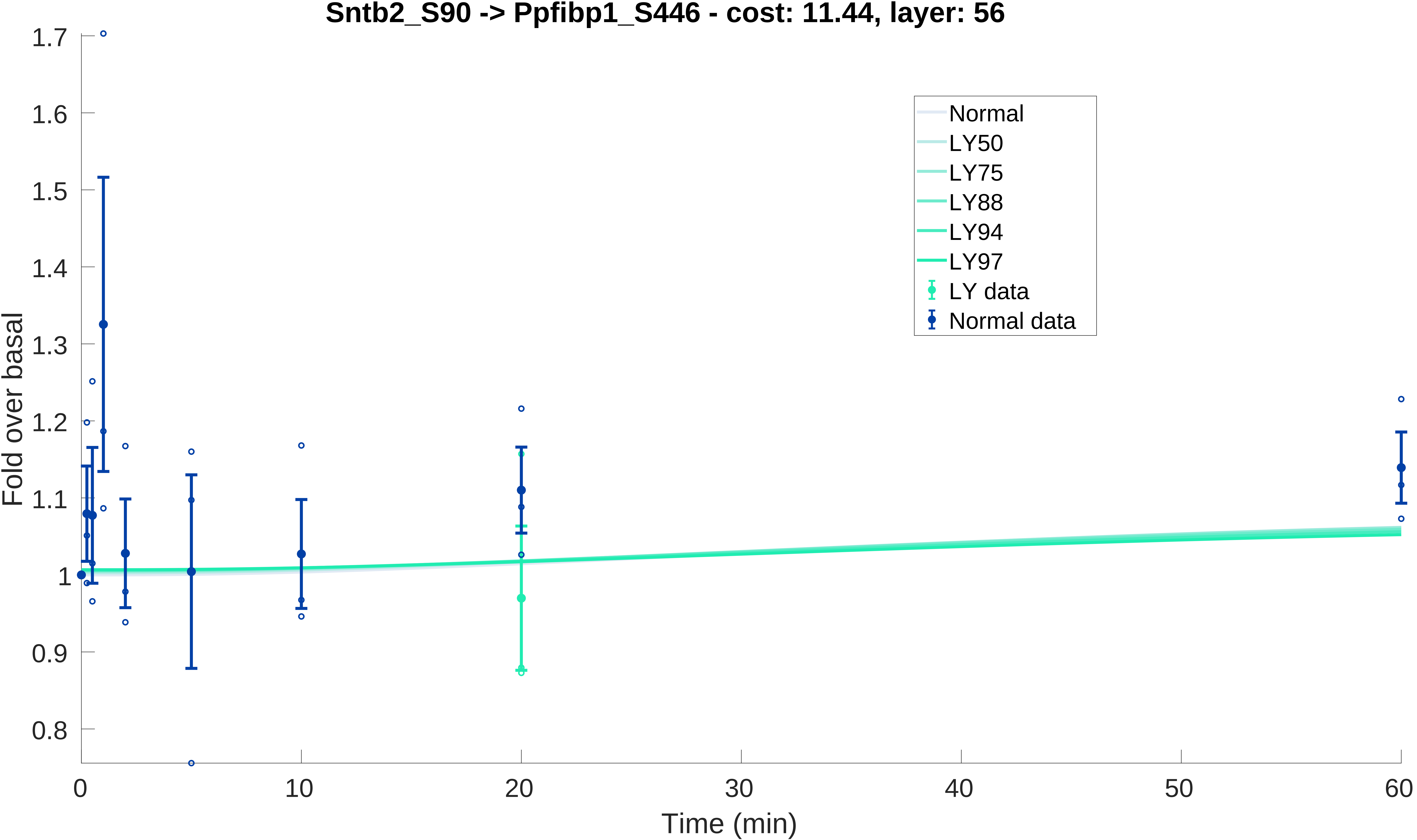
Sntb2_S90 -> Ppfibp1_S446 - cost: 11.44, layer: 56
Fold over basal
Time (min)
1.7
1.6
1.5
1.4
1.3
1.2
1.1
1
0.9
0.8
0
10
20
30
40
50
60
Normal
LY50
LY75
LY88
LY94
LY97
LY data
Normal data

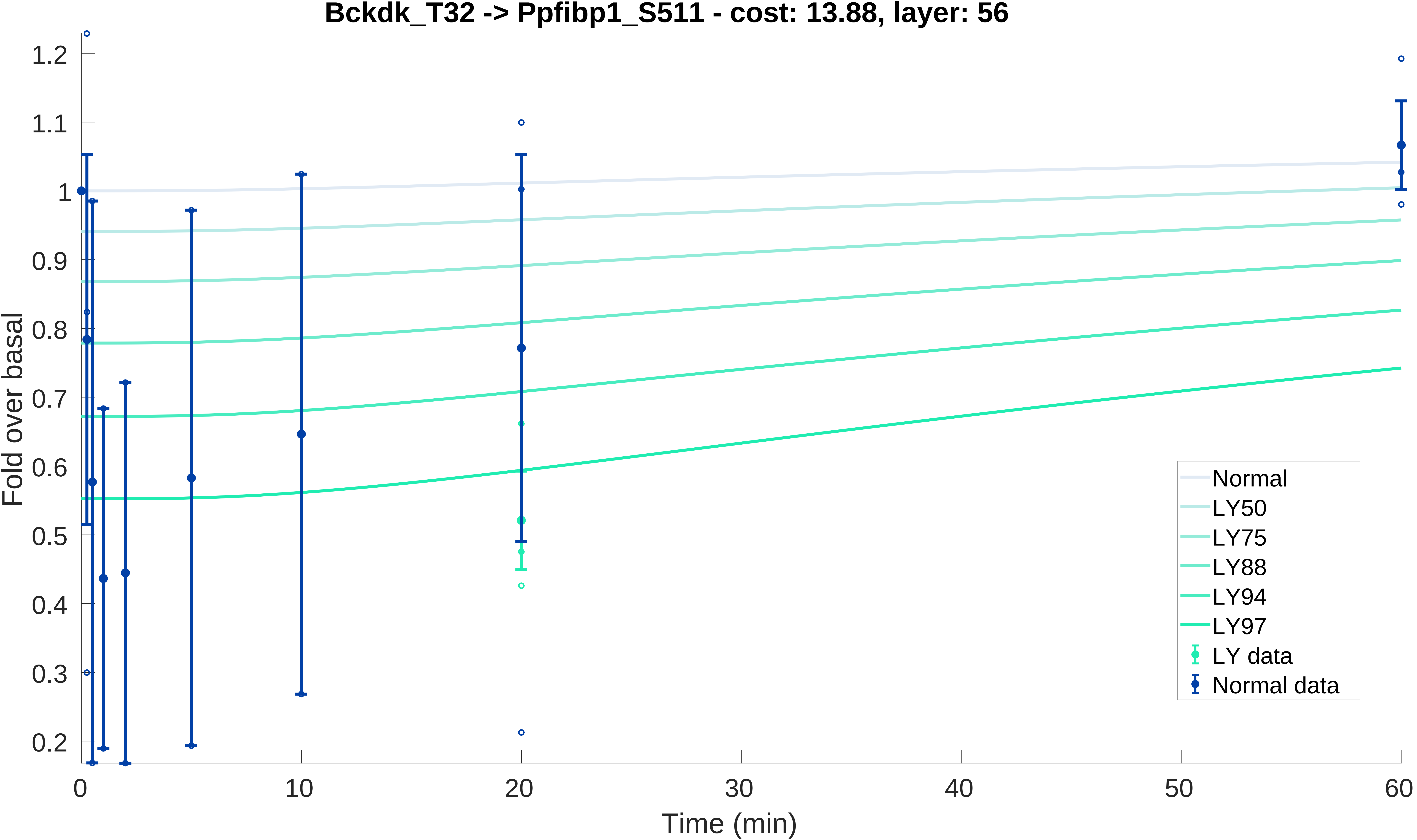

Bckdk_T32 -> Ppfibp1_S511 - cost: 13.88, layer: 56
Fold over basal
1.2
1.1
1
0.9
0.8
0.7
0.6
0.5
0.4
0.3
0.2
0
10
20
30
40
50
60
Time (min)
Normal
LY50
LY75
LY88
LY94
LY97
LY data
Normal data

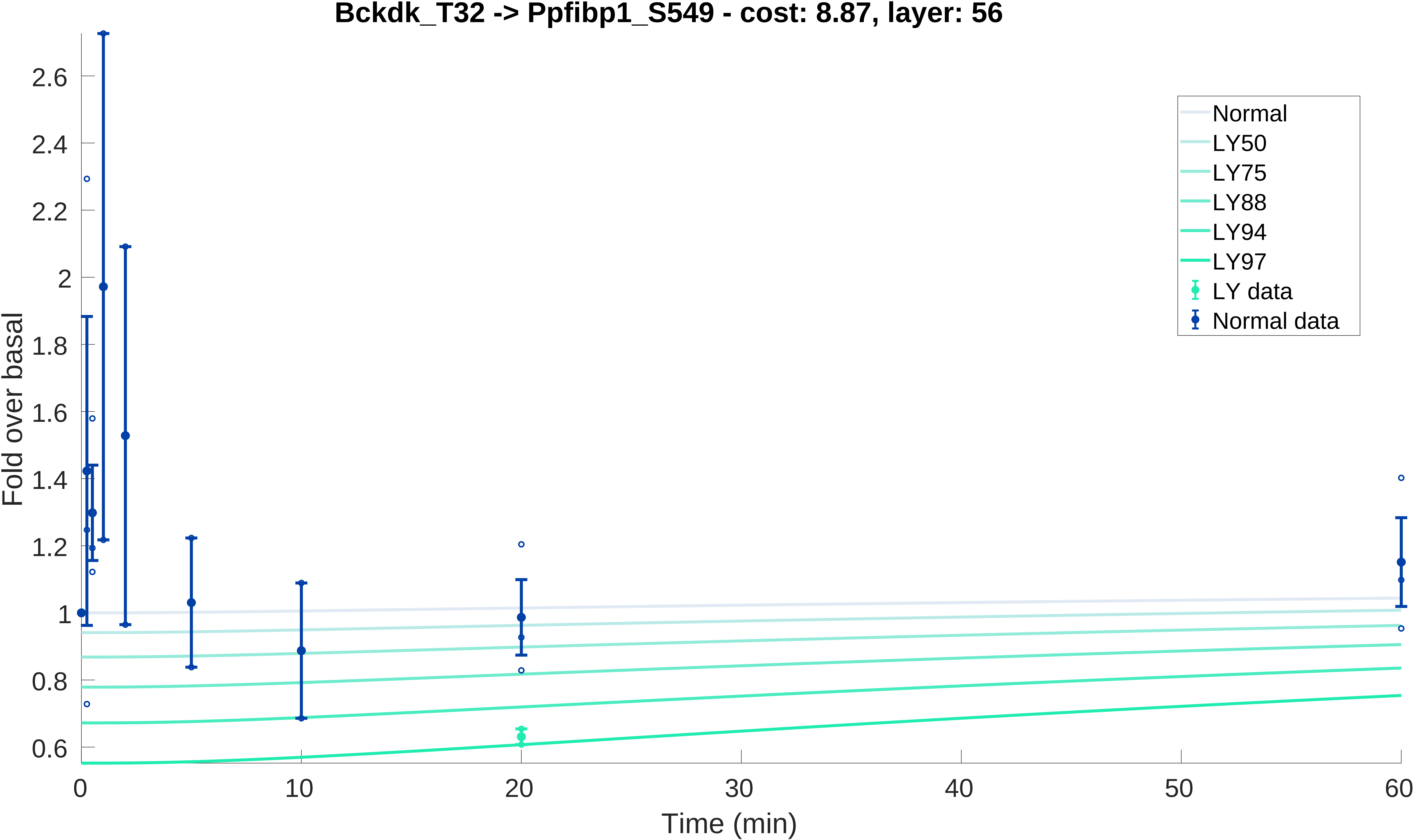

Bckdk_T32 -> Ppfibp1_S549 - cost: 8.87, layer: 56
Fold over basal
Time (min)
0
10
20
30
40
50
60
0.6
0.8
1
1.2
1.4
1.6
1.8
2
2.2
2.4
2.6
Normal
LY50
LY75
LY88
LY94
LY97
LY data
Normal data

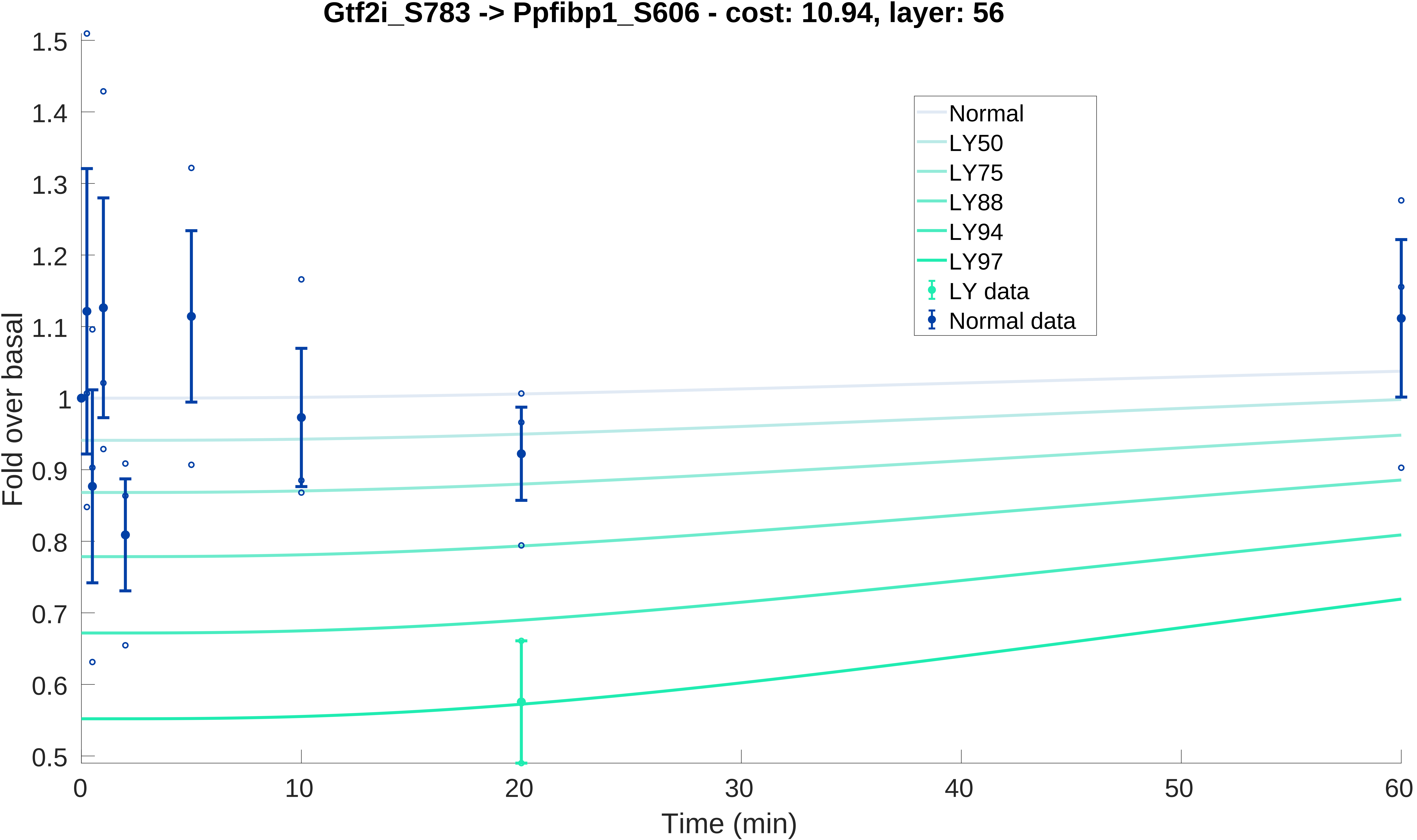

Gtf2i_S783 -> Ppfibp1_S606 - cost: 10.94, layer: 56
Fold over basal
Time (min)
0.5
0.6
0.7
0.8
0.9
1
1.1
1.2
1.3
1.4
1.5
0
10
20
30
40
50
60
Normal
LY50
LY75
LY88
LY94
LY97
LY data
Normal data

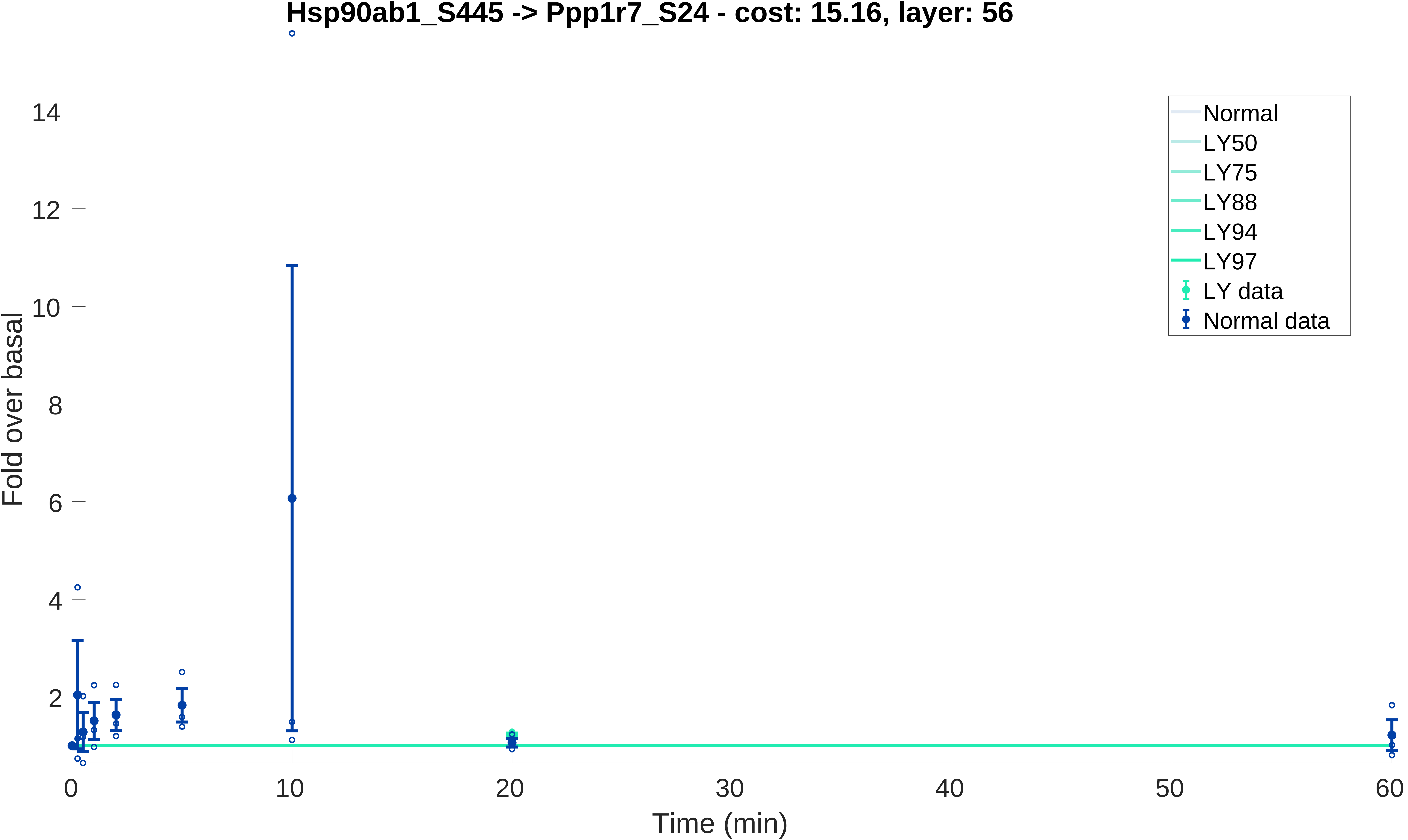

Hsp90ab1_S445 -> Ppp1r7_S24 - cost: 15.16, layer: 56
Fold over basal
Time (min)
0
10
20
30
40
50
60
2
4
6
8
10
12
14
Normal
LY50
LY75
LY88
LY94
LY97
LY data
Normal data

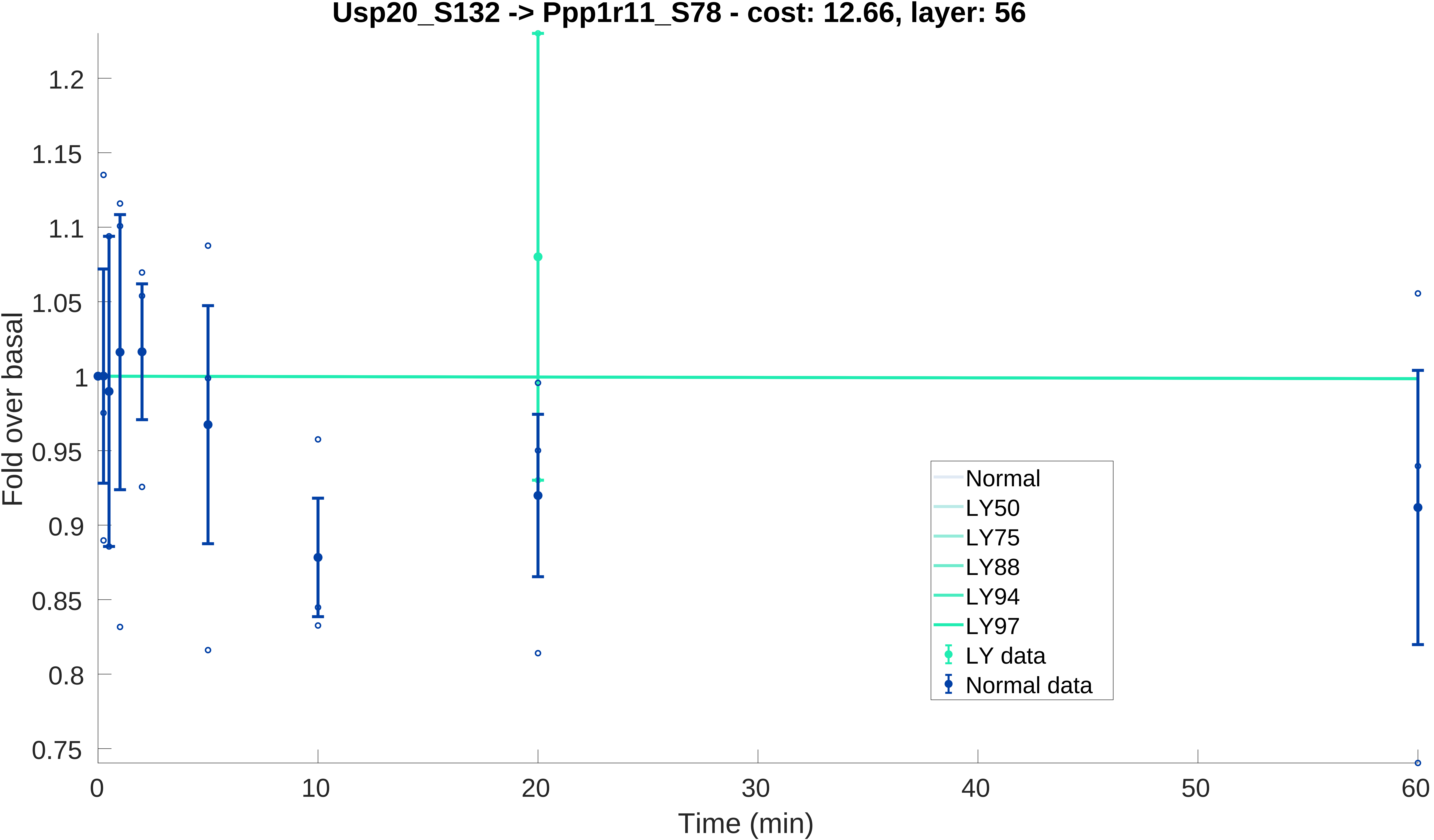

Usp20_S132 -> Ppp1r11_S78 - cost: 12.66, layer: 56
Fold over basal
1.2
1.15
1.1
1.05
1
0.95
0.9
0.85
0.8
0.75
0
10
20
30
40
50
60
Time (min)
Normal
LY50
LY75
LY88
LY94
LY97
LY data
Normal data

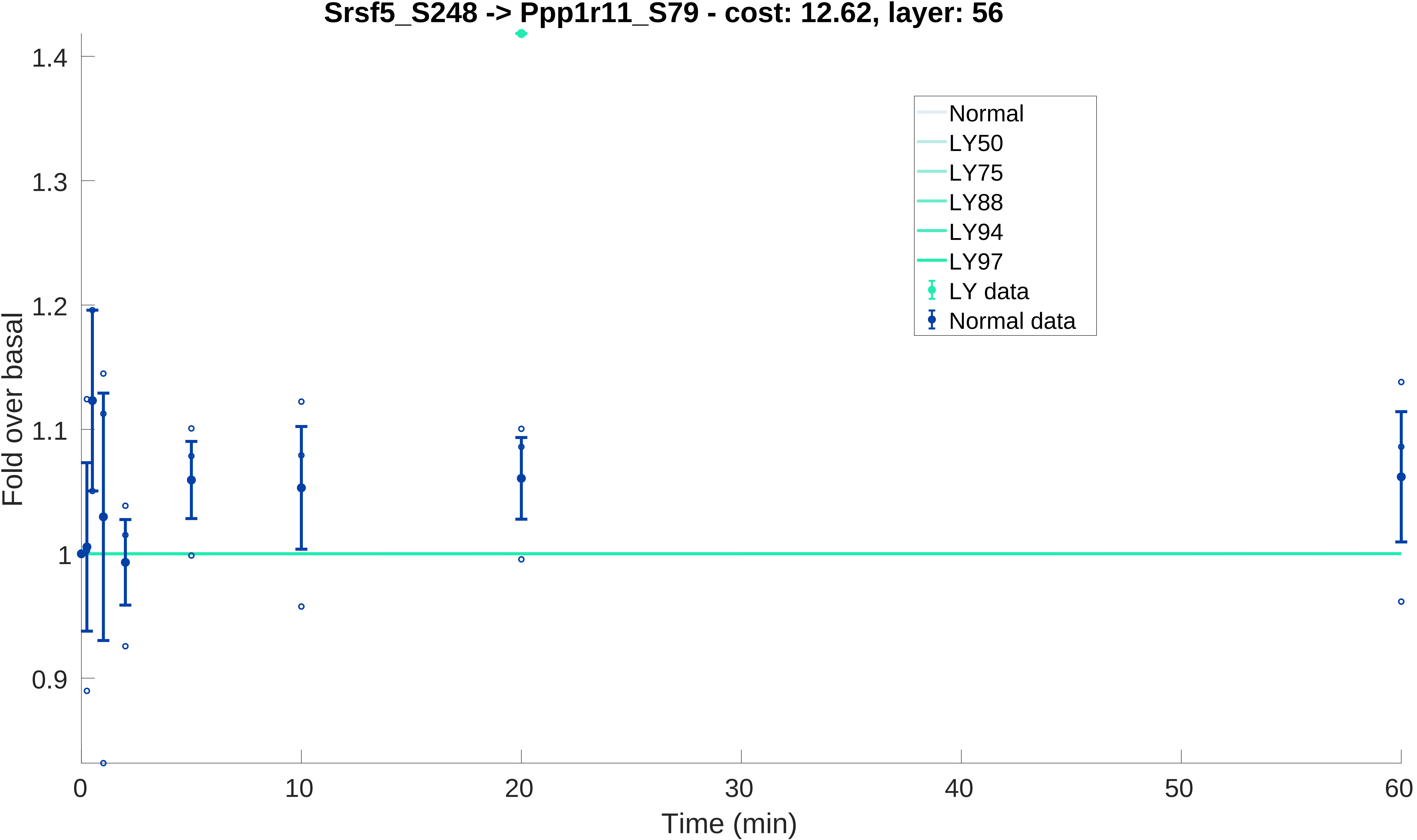

Srsf5_S248 -> Ppp1r11_S79 - cost: 12.62, layer: 56
Normal
LY50
LY75
LY88
LY94
LY97
LY data
Normal data
Fold over basal
Time (min)
1.4
1.3
1.2
1.1
1
0.9
0
10
20
30
40
50
60

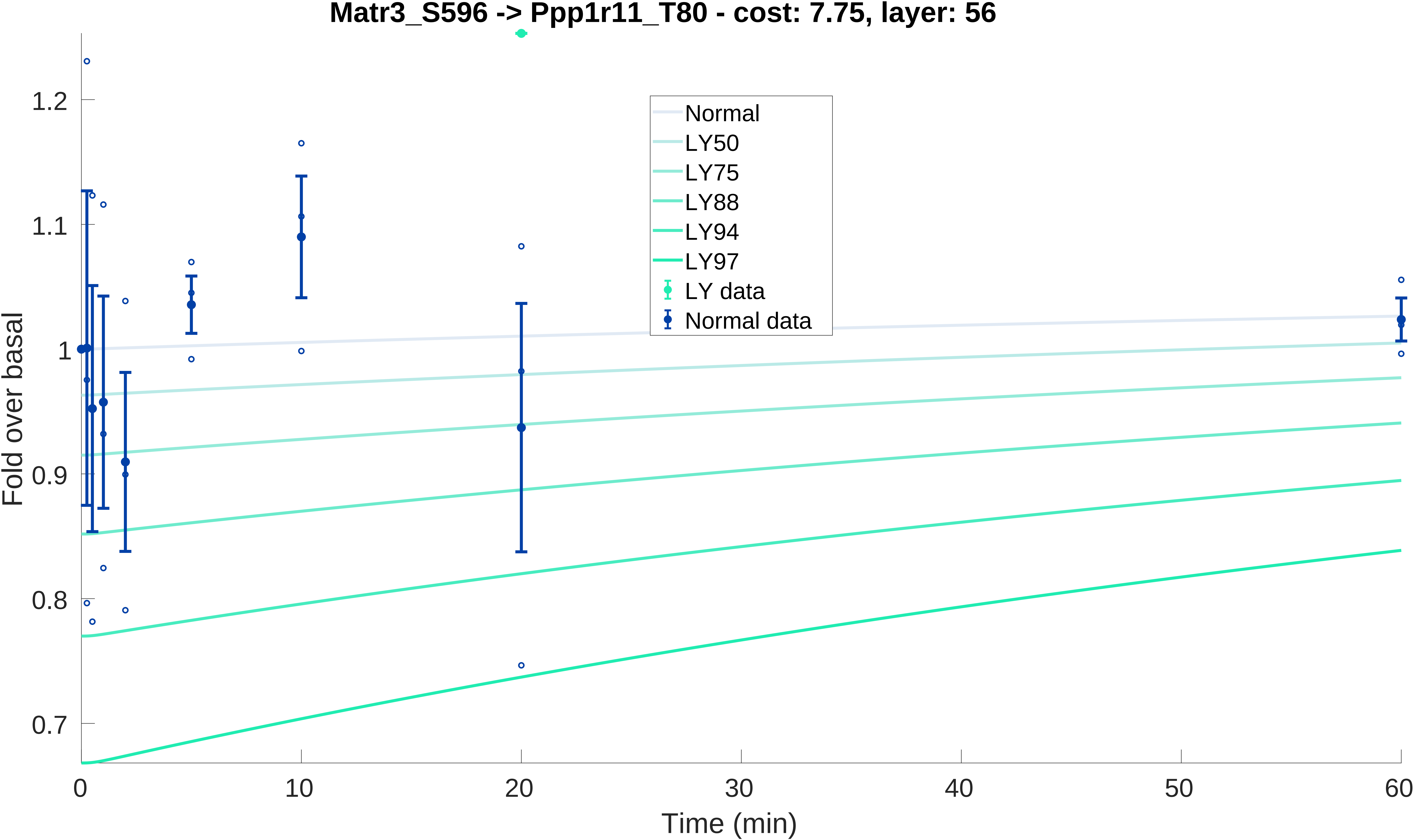

Matr3_S596 -> Ppp1r11_T80 - cost: 7.75, layer: 56
Normal
LY50
LY75
LY88
LY94
LY97
LY data
Normal data
Fold over basal
Time (min)
1.2
1.1
1
0.9
0.8
0.7
0
10
20
30
40
50
60

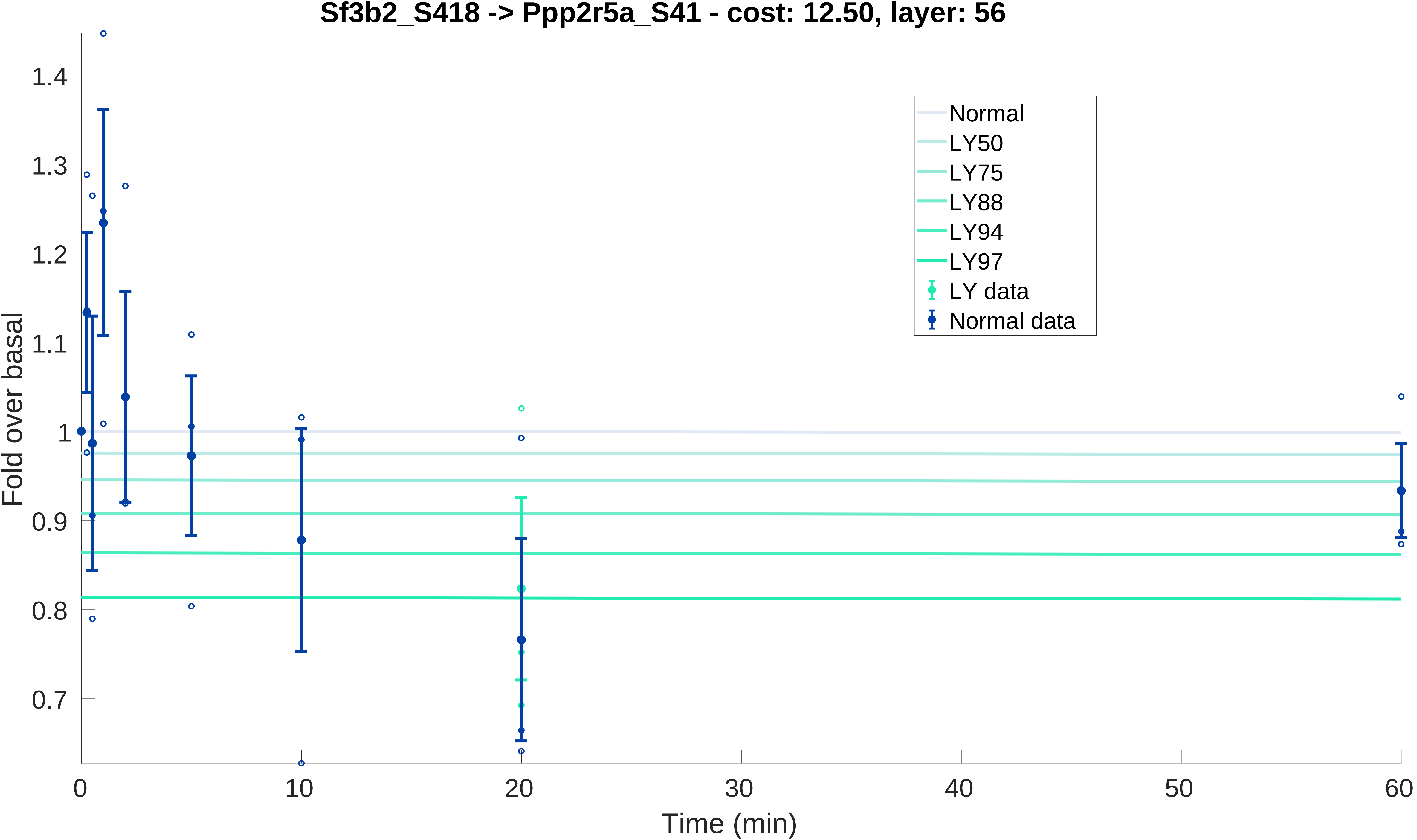

Sf3b2_S418 -> Ppp2r5a_S41 - cost: 12.50, layer: 56
Fold over basal
1.4
1.3
1.2
1.1
1
0.9
0.8
0.7
0
10
20
30
40
50
60
Time (min)
Normal
LY50
LY75
LY88
LY94
LY97
LY data
Normal data

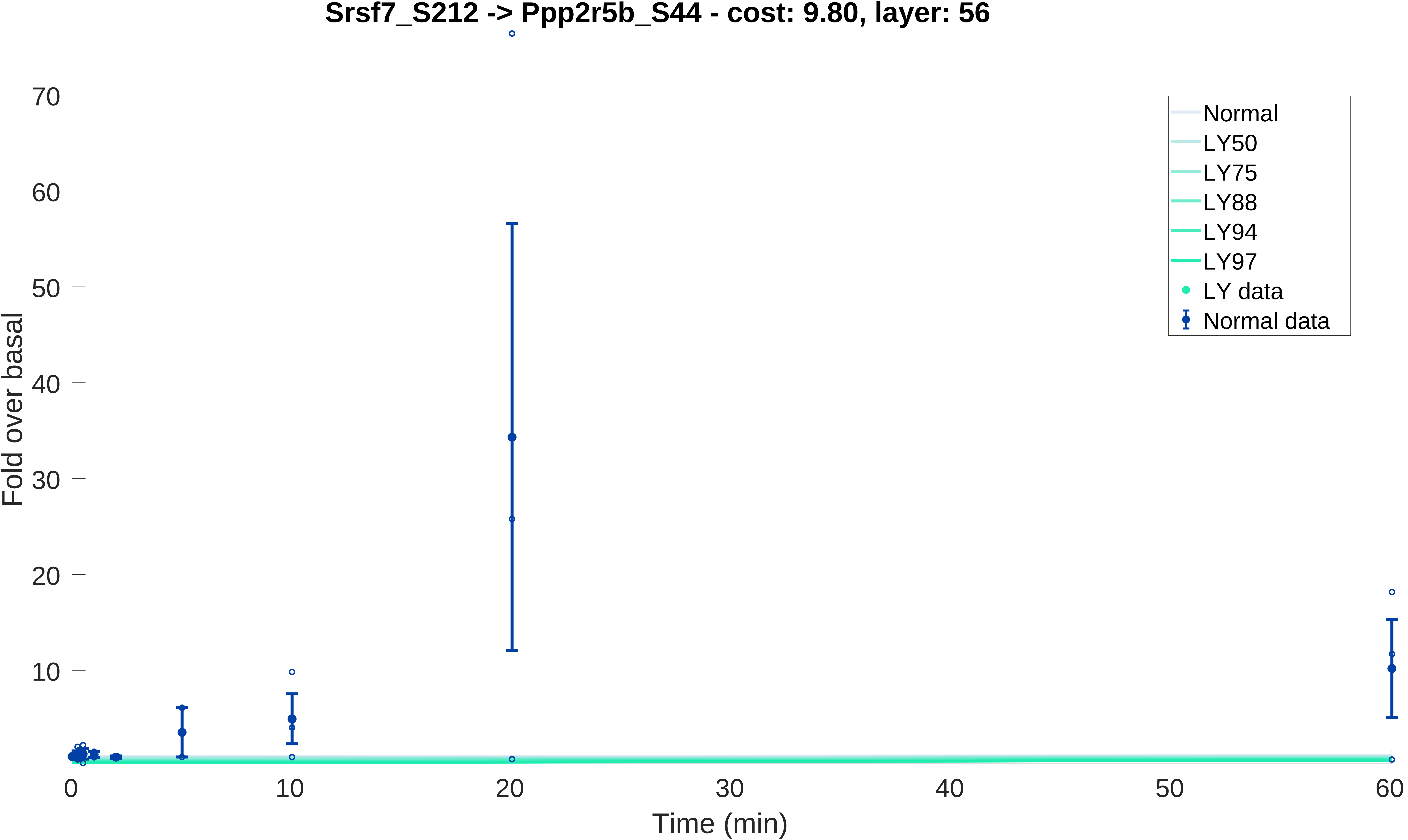

Srsf7_S212 -> Ppp2r5b_S44 - cost: 9.80, layer: 56
Fold over basal
Time (min)
0
10
20
30
40
50
60
70
Normal
LY50
LY75
LY88
LY94
LY97
LY data
Normal data

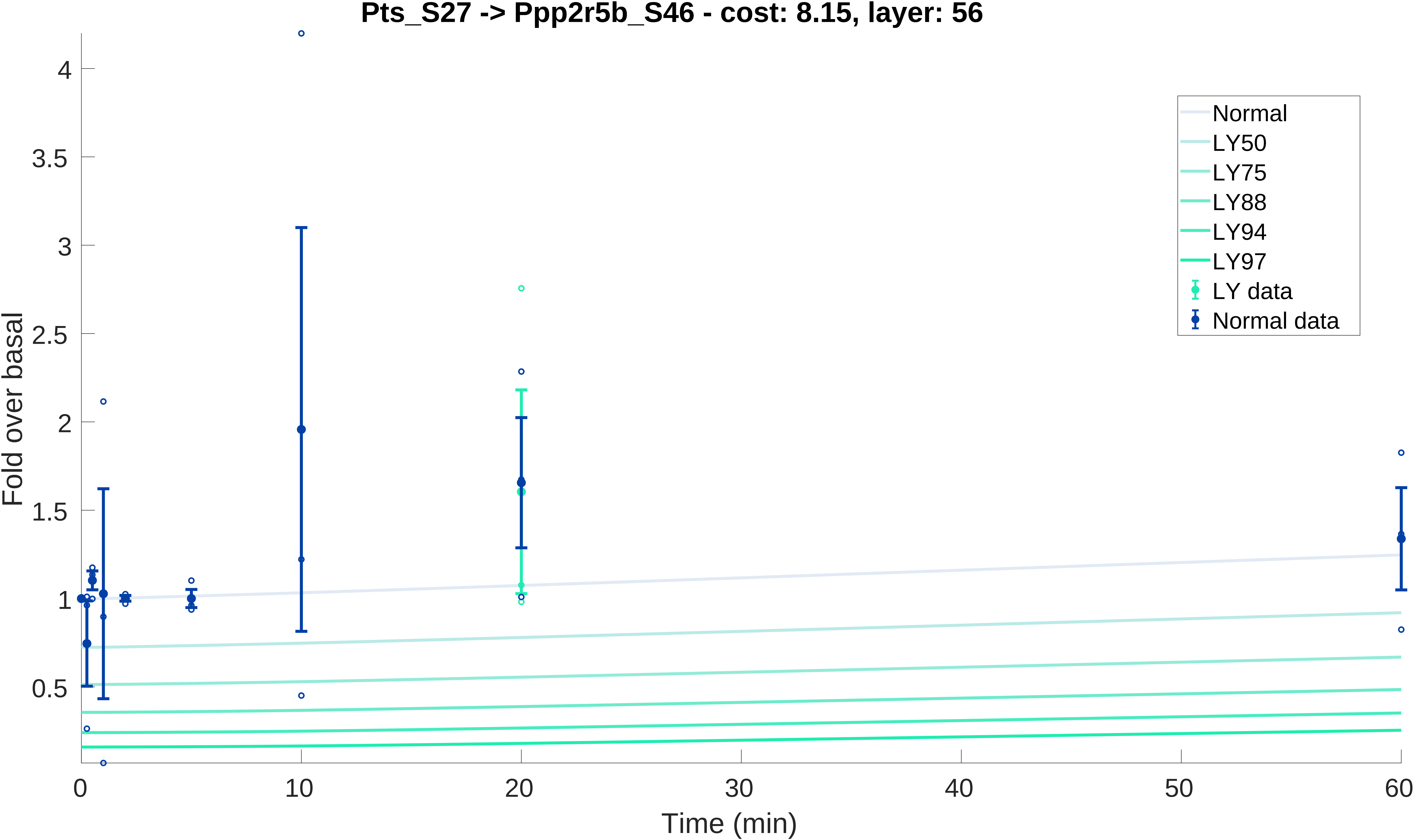
Pts_S27 -> Ppp2r5b_S46 - cost: 8.15, layer: 56
Fold over basal
Time (min)
0
10
20
30
40
50
60
0.5
1
1.5
2
2.5
3
3.5
4
Normal
LY50
LY75
LY88
LY94
LY97
LY data
Normal data

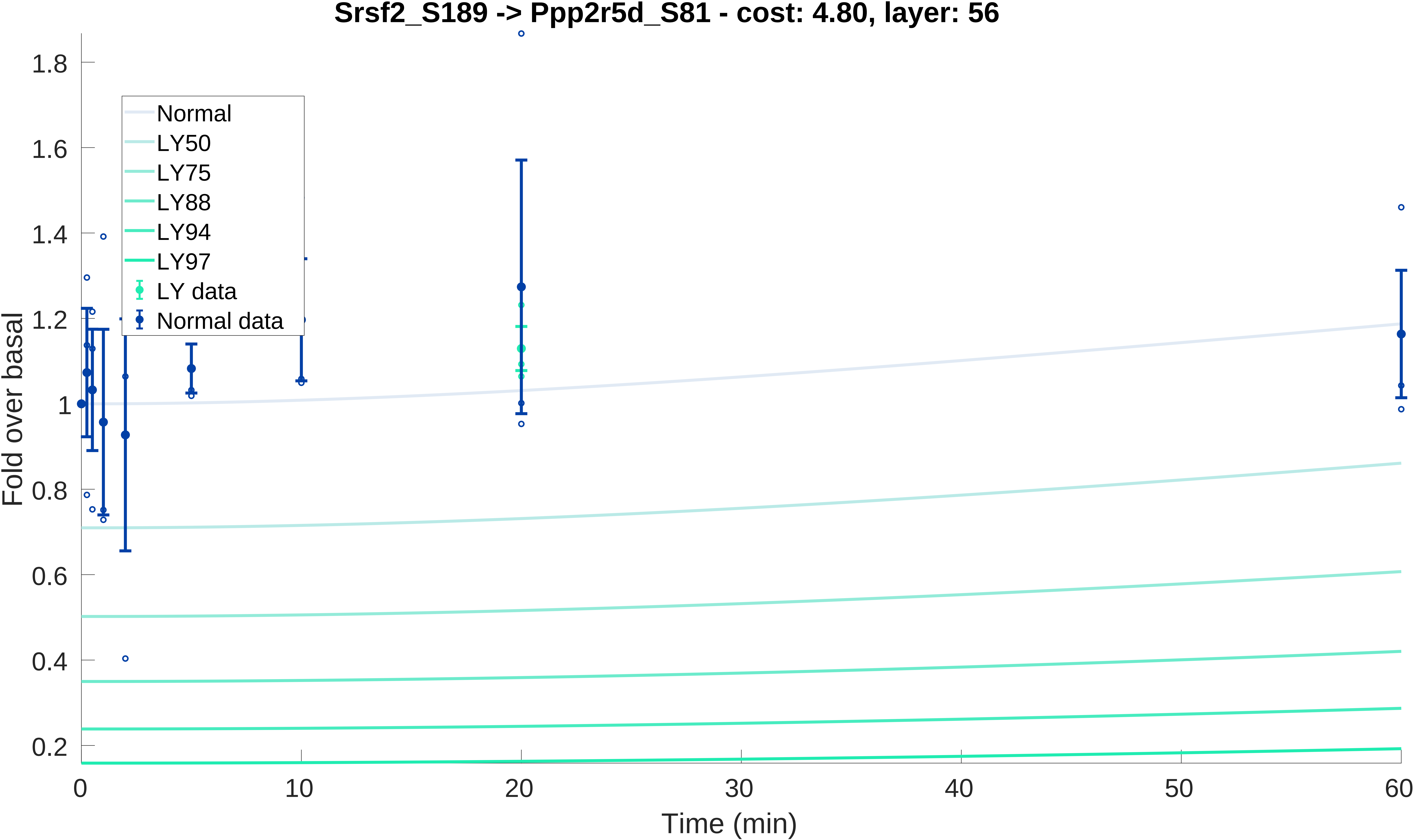
Srsf2_S189 -> Ppp2r5d_S81 - cost: 4.80, layer: 56
Normal
LY50
LY75
LY88
LY94
LY97
LY data
Normal data
Fold over basal
Time (min)
0
10
20
30
40
50
60
0.2
0.4
0.6
0.8
1
1.2
1.4
1.6
1.8

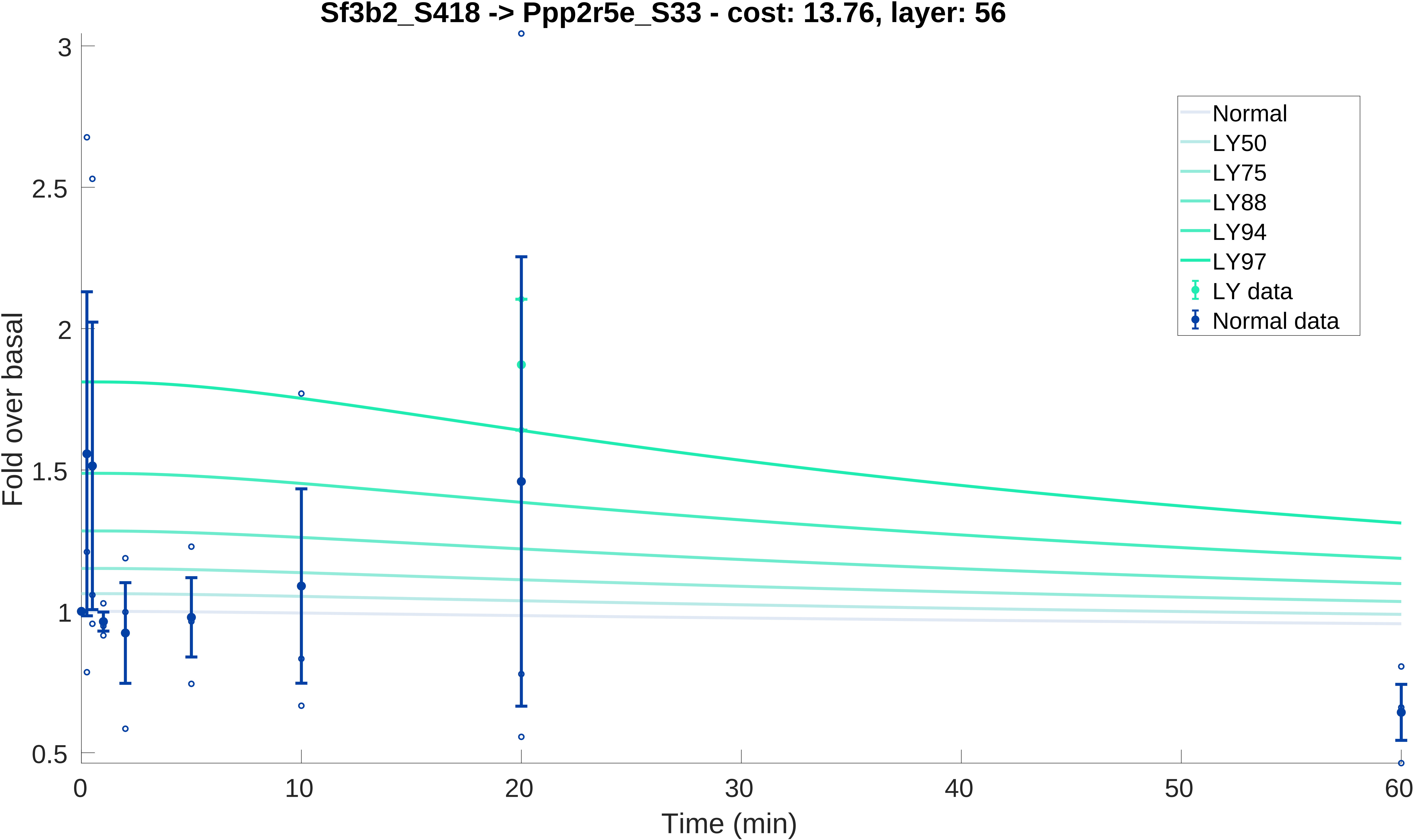

Sf3b2_S418 -> Ppp2r5e_S33 - cost: 13.76, layer: 56
Fold over basal
Time (min)
0.5
1
1.5
2
2.5
3
0
10
20
30
40
50
60
Normal
LY50
LY75
LY88
LY94
LY97
LY data
Normal data

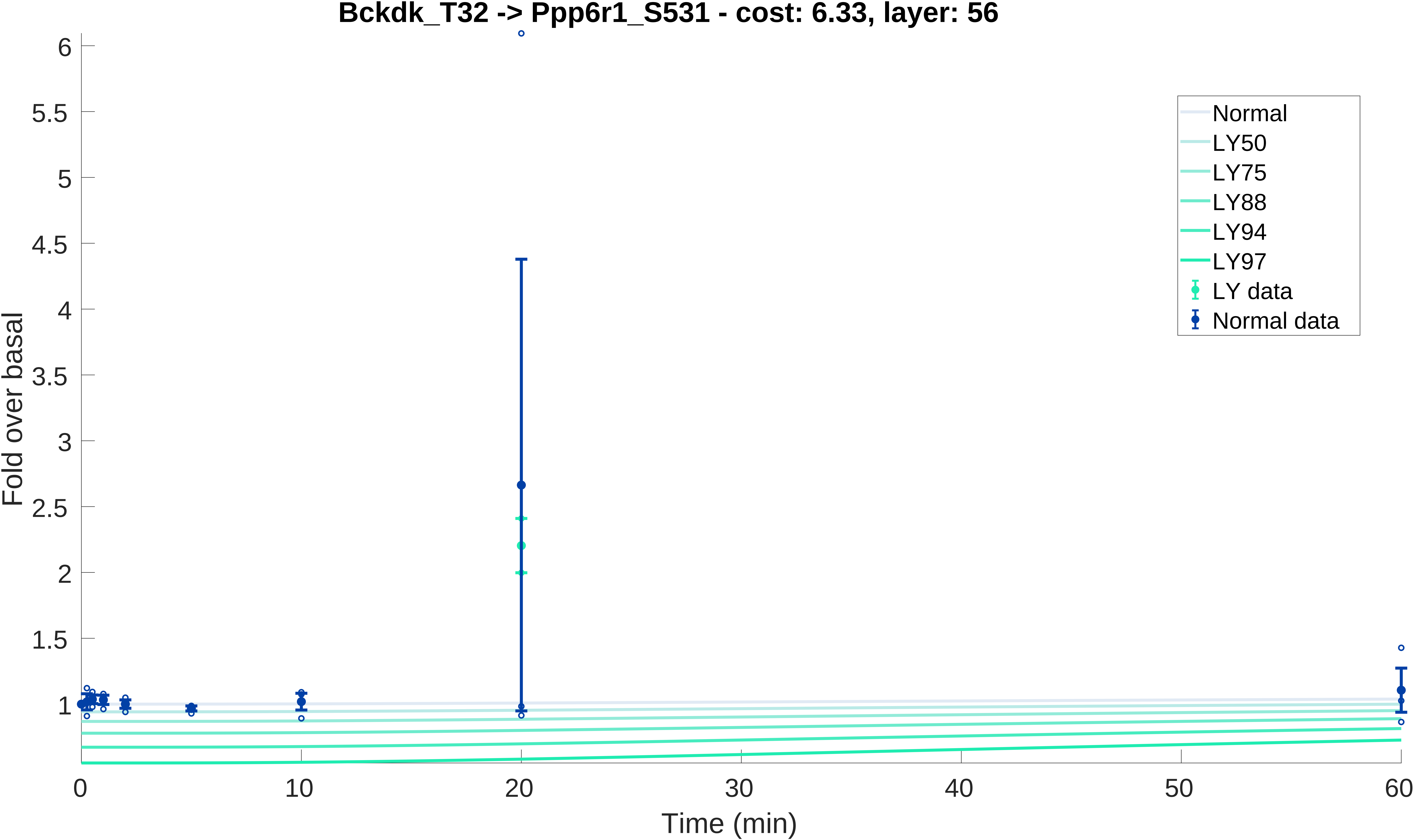

Bckdk_T32 -> Ppp6r1_S531 - cost: 6.33, layer: 56
Fold over basal
Time (min)
6
5.5
5
4.5
4
3.5
3
2.5
2
1.5
1
0
10
20
30
40
50
60
Normal
LY50
LY75
LY88
LY94
LY97
LY data
Normal data

Clmn_S925 -> Ppp6r3_S617 - cost: 6.47, layer: 56

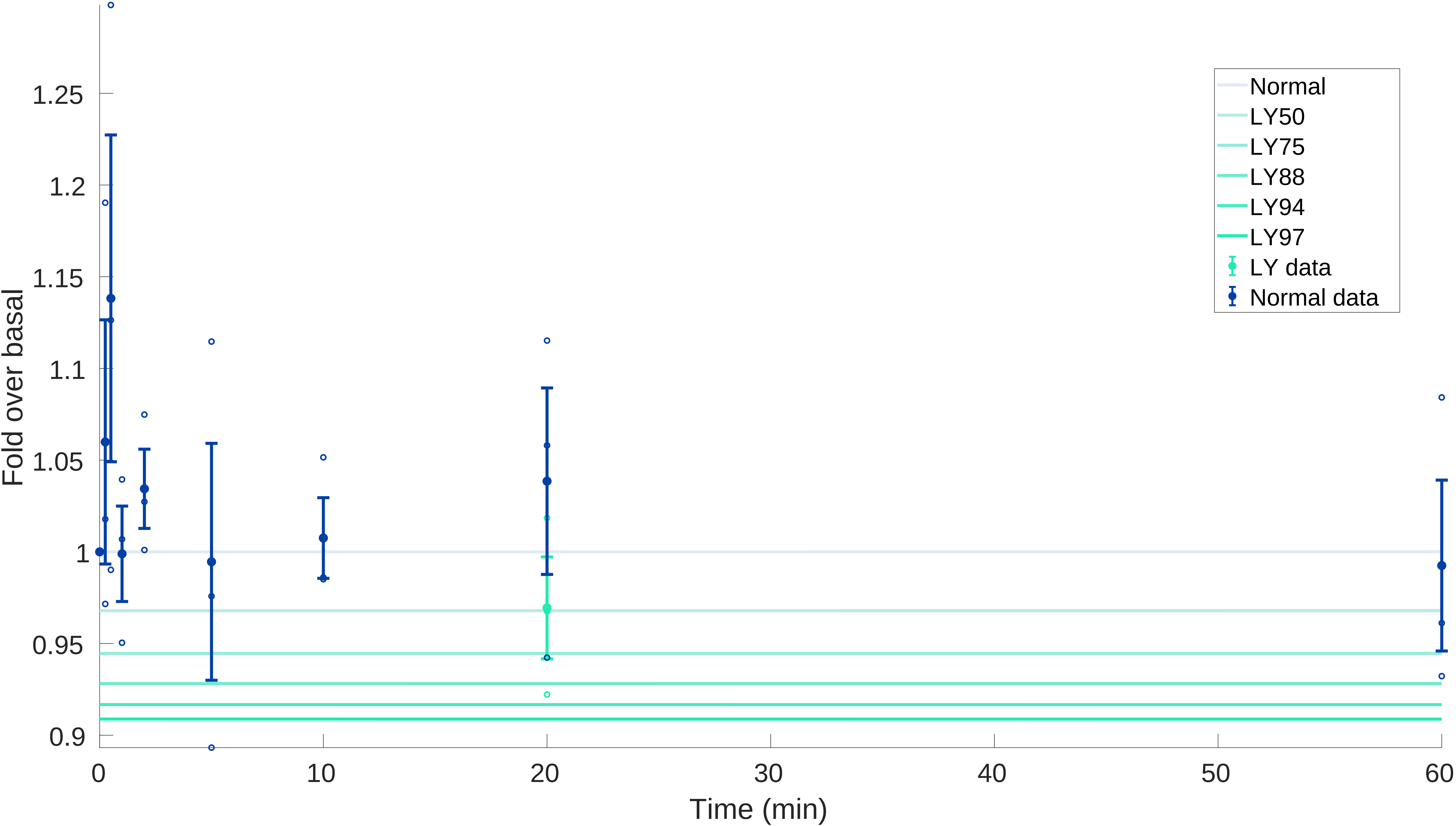

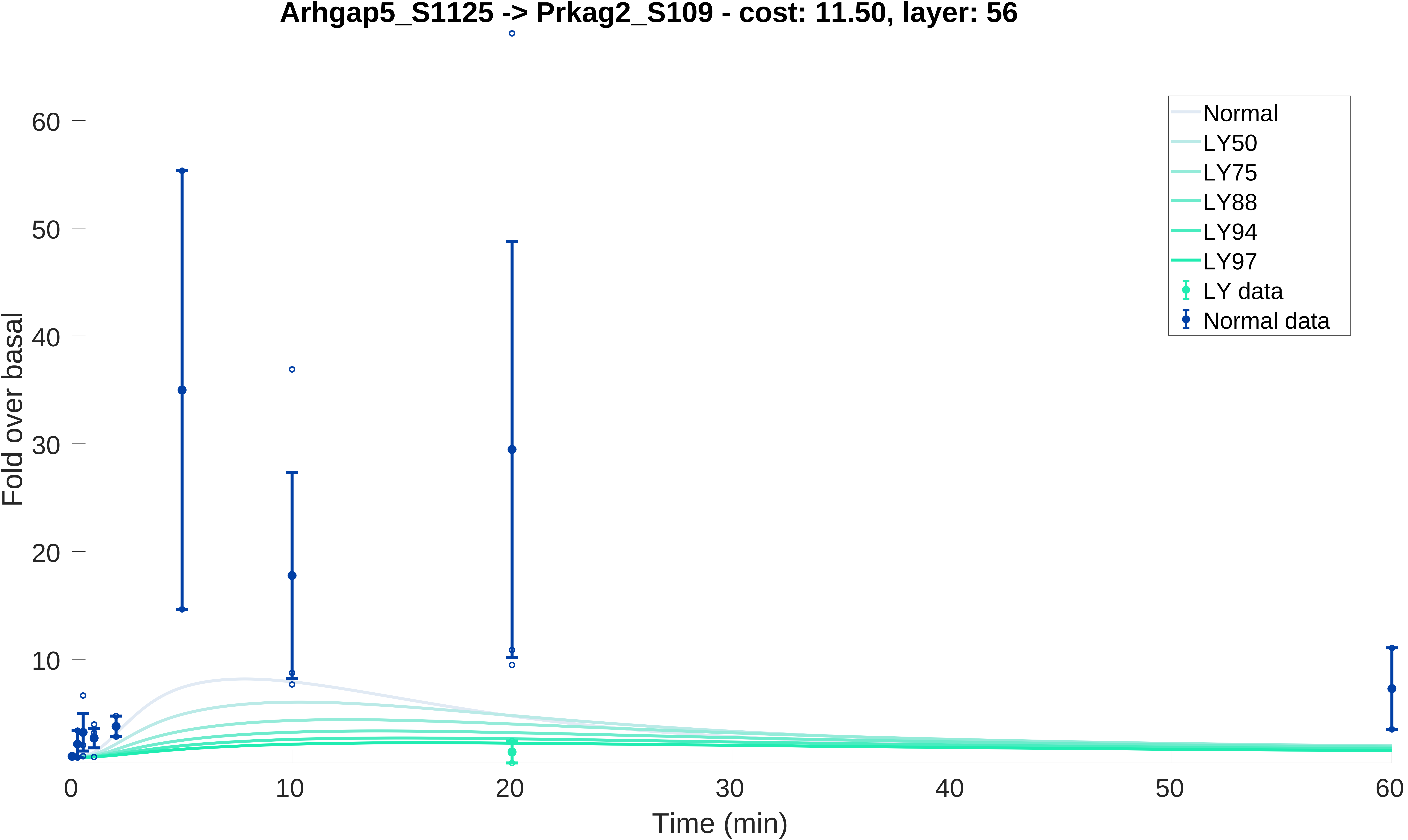

Arhgap5_S1125 -> Prkag2_S109 - cost: 11.50, layer: 56
Fold over basal
Time (min)
0
10
20
30
40
50
60
Normal
LY50
LY75
LY88
LY94
LY97
LY data
Normal data

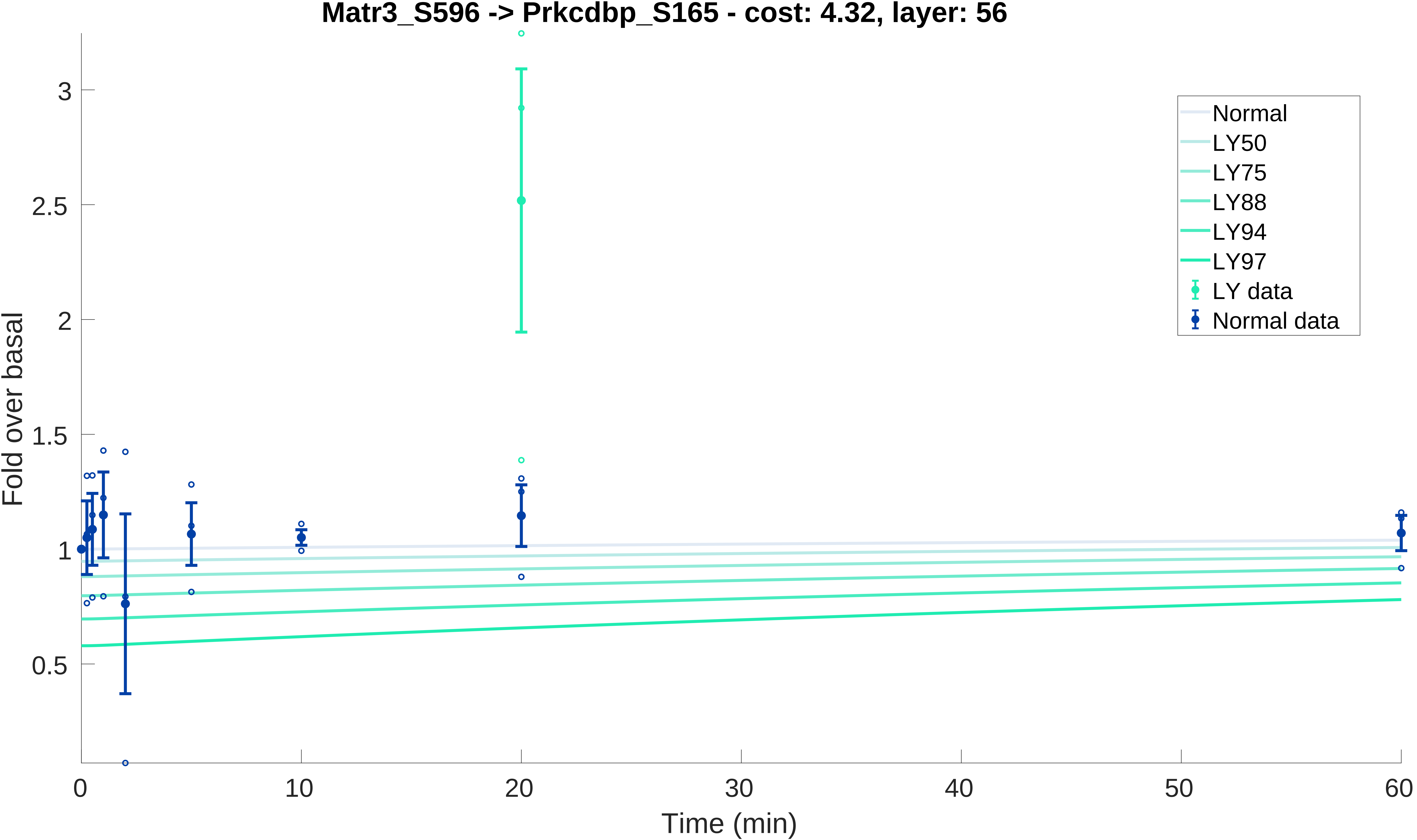
Matr3_S596 -> Prkcdbp_S165 - cost: 4.32, layer: 56
Fold over basal
Time (min)
Normal
LY50
LY75
LY88
LY94
LY97
LY data
Normal data
0.5
1
1.5
2
2.5
3
0
10
20
30
40
50
60

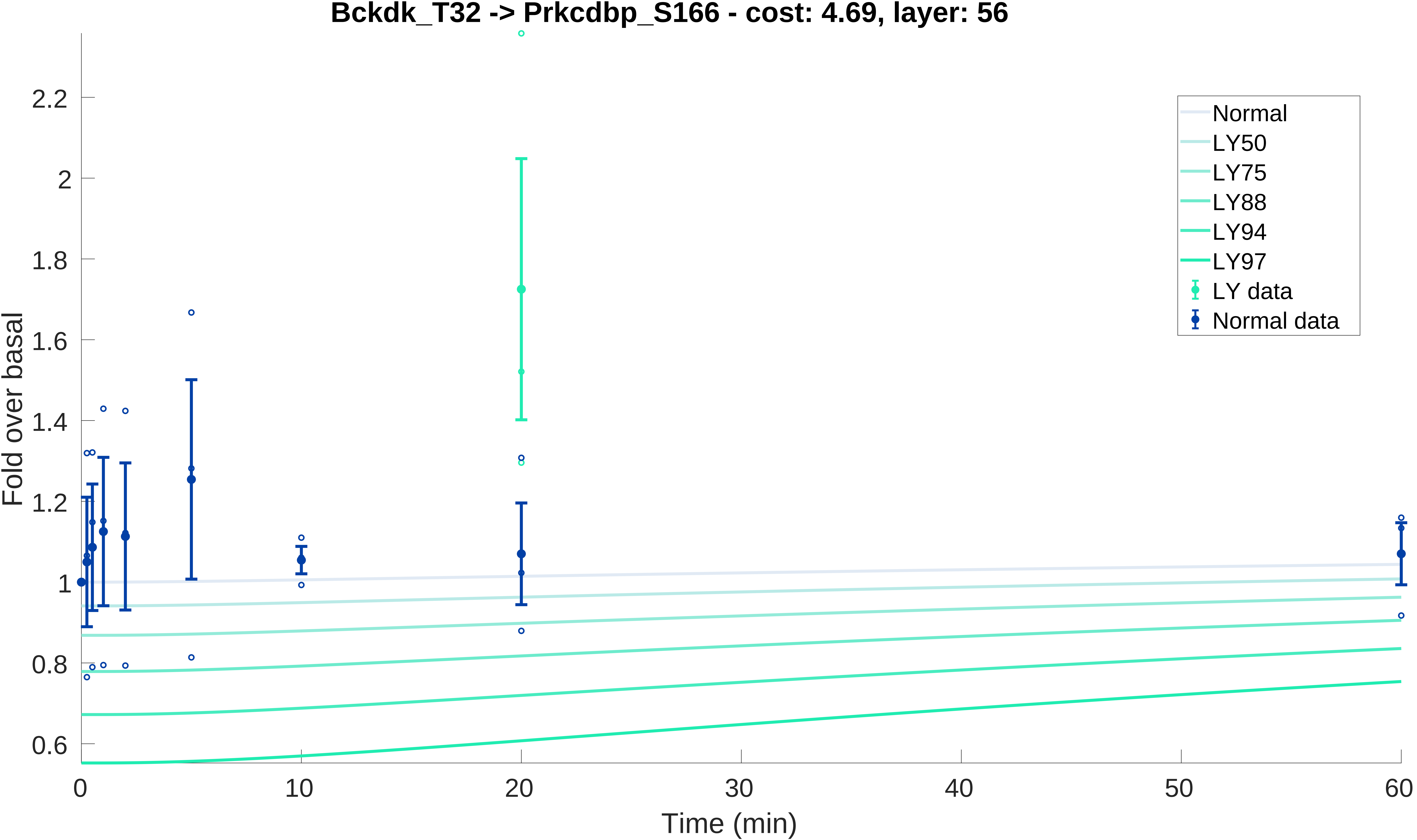
Bckdk_T32 -> Prkcdbp_S166 - cost: 4.69, layer: 56
Fold over basal
Time (min)
0.6
0.8
1
1.2
1.4
1.6
1.8
2
2.2
0
10
20
30
40
50
60
Normal
LY50
LY75
LY88
LY94
LY97
LY data
Normal data

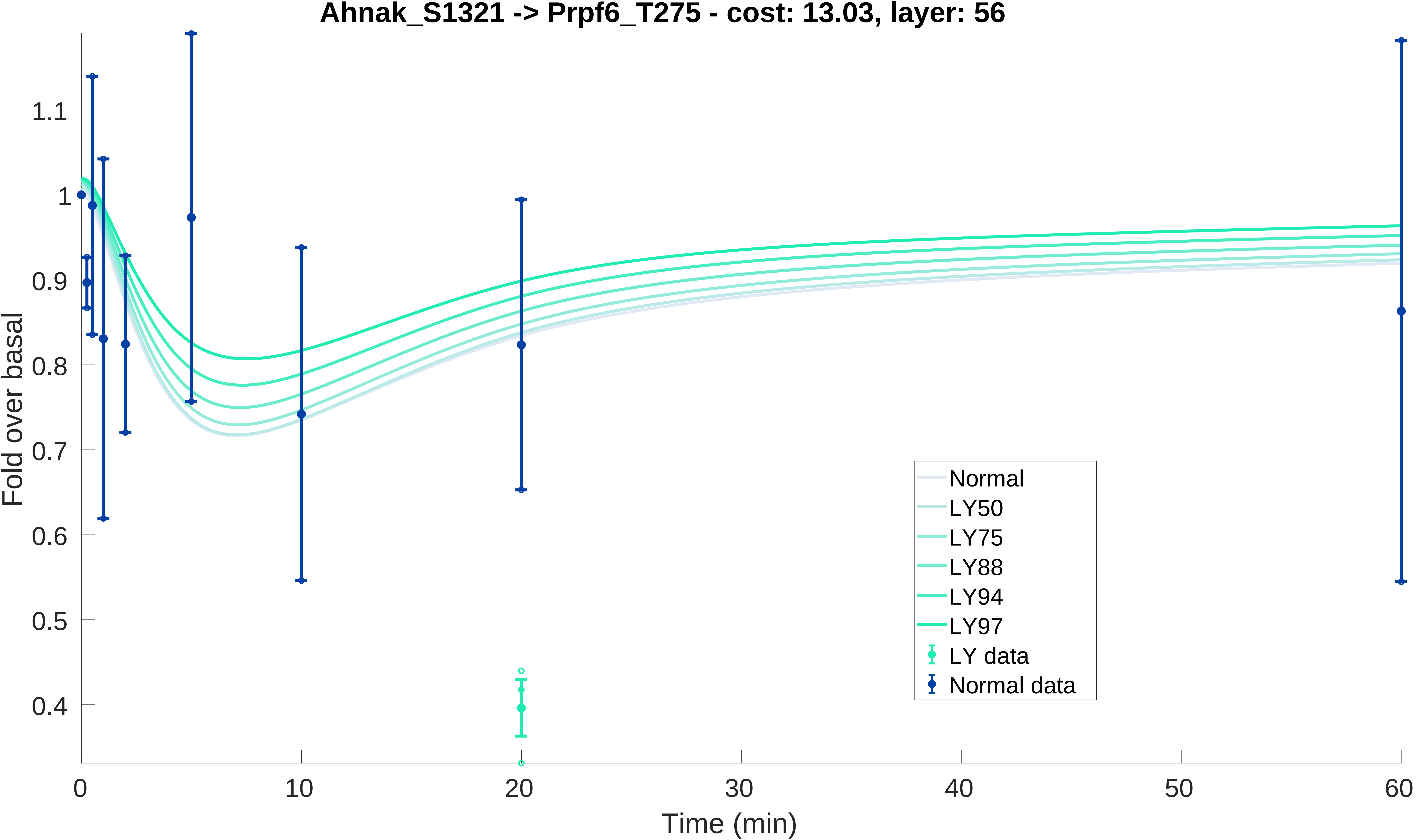

Ahnak_S1321 -> Prpf6_T275 - cost: 13.03, layer: 56
Fold over basal
1.1
1
0.9
0.8
0.7
0.6
0.5
0.4
0
10
20
30
40
50
60
Time (min)
Normal
LY50
LY75
LY88
LY94
LY97
LY data
Normal data

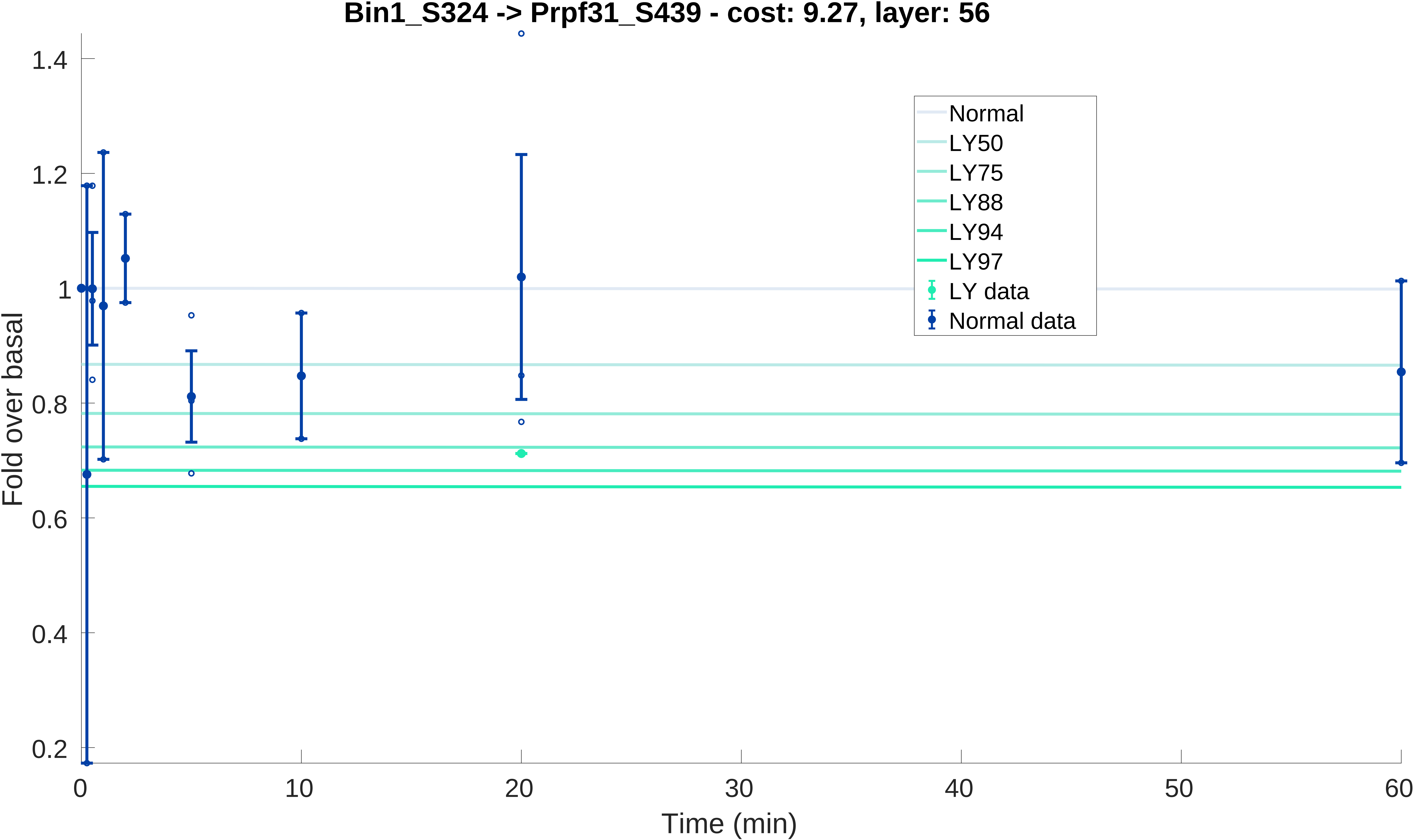

Bin1_S324 -> Prpf31_S439 - cost: 9.27, layer: 56
Fold over basal
Time (min)
1.4
1.2
1
0.8
0.6
0.4
0.2
0
10
20
30
40
50
60
Normal
LY50
LY75
LY88
LY94
LY97
LY data
Normal data

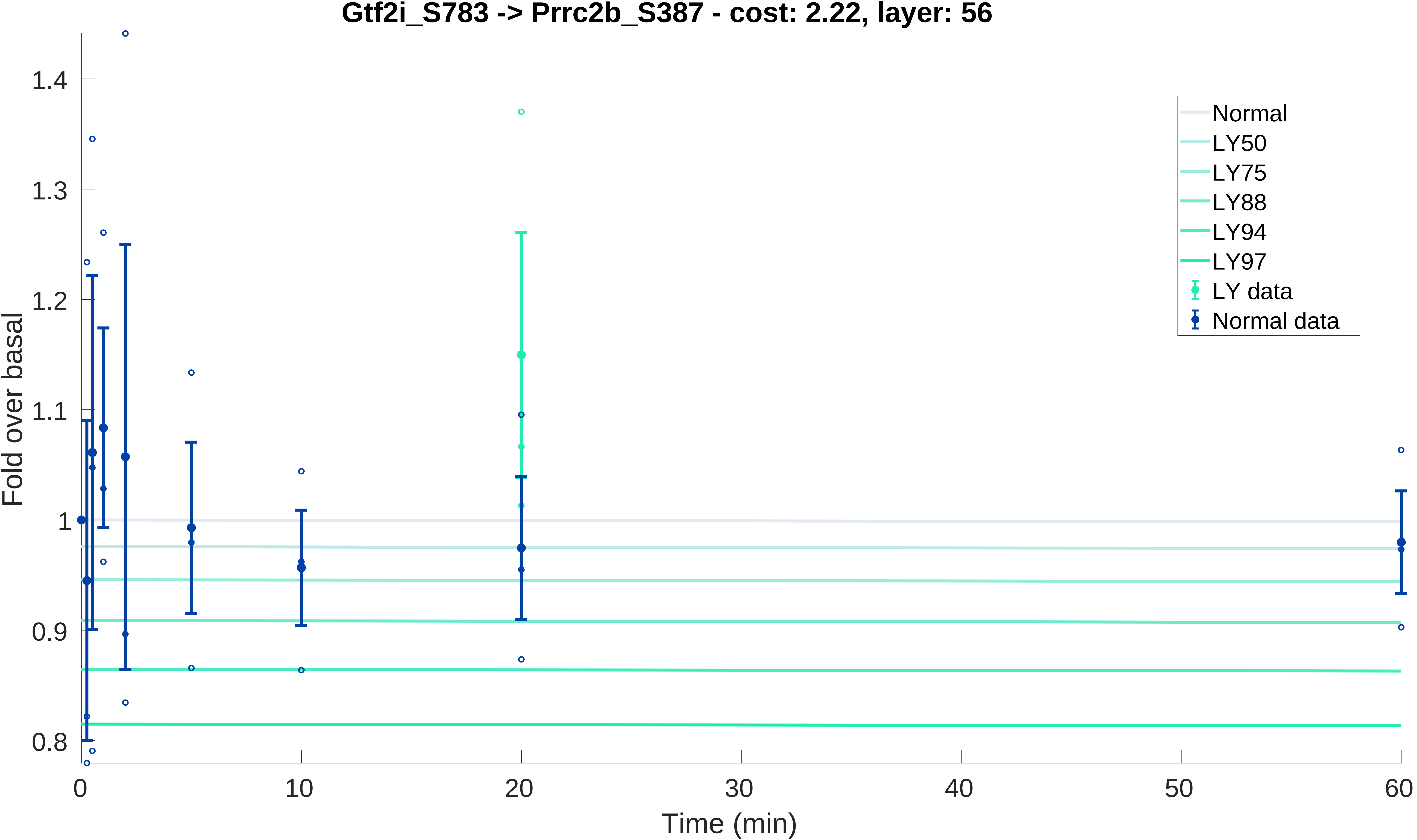

Gtf2i_S783 -> Prrc2b_S387 - cost: 2.22, layer: 56
Fold over basal
Time (min)
1.4
1.3
1.2
1.1
1
0.9
0.8
0
10
20
30
40
50
60
Normal
LY50
LY75
LY88
LY94
LY97
LY data
Normal data

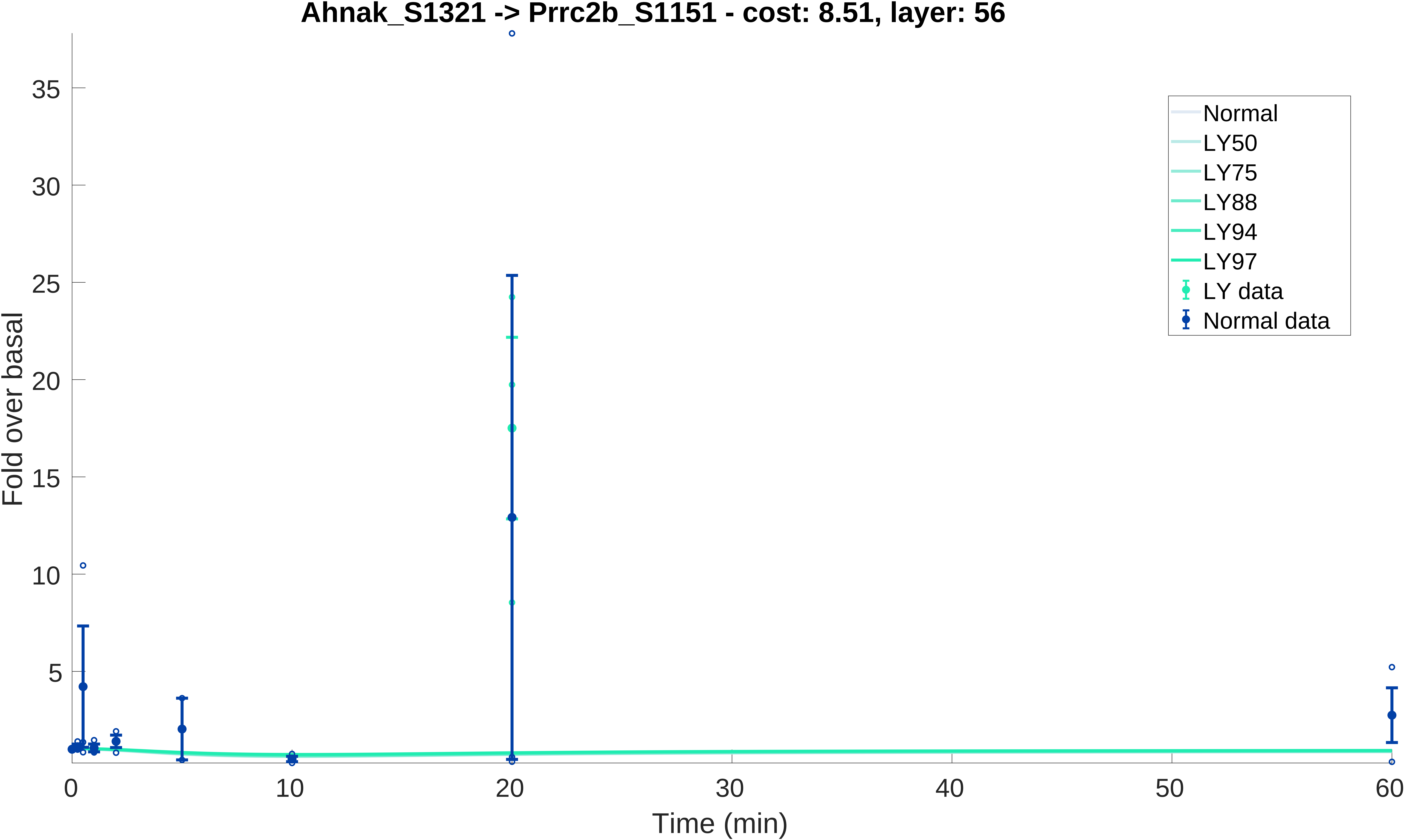

Ahnak_S1321 -> Prrc2b_S1151 - cost: 8.51, layer: 56
Fold over basal
Time (min)
0
10
20
30
40
50
60
5
10
15
20
25
30
35
Normal
LY50
LY75
LY88
LY94
LY97
LY data
Normal data

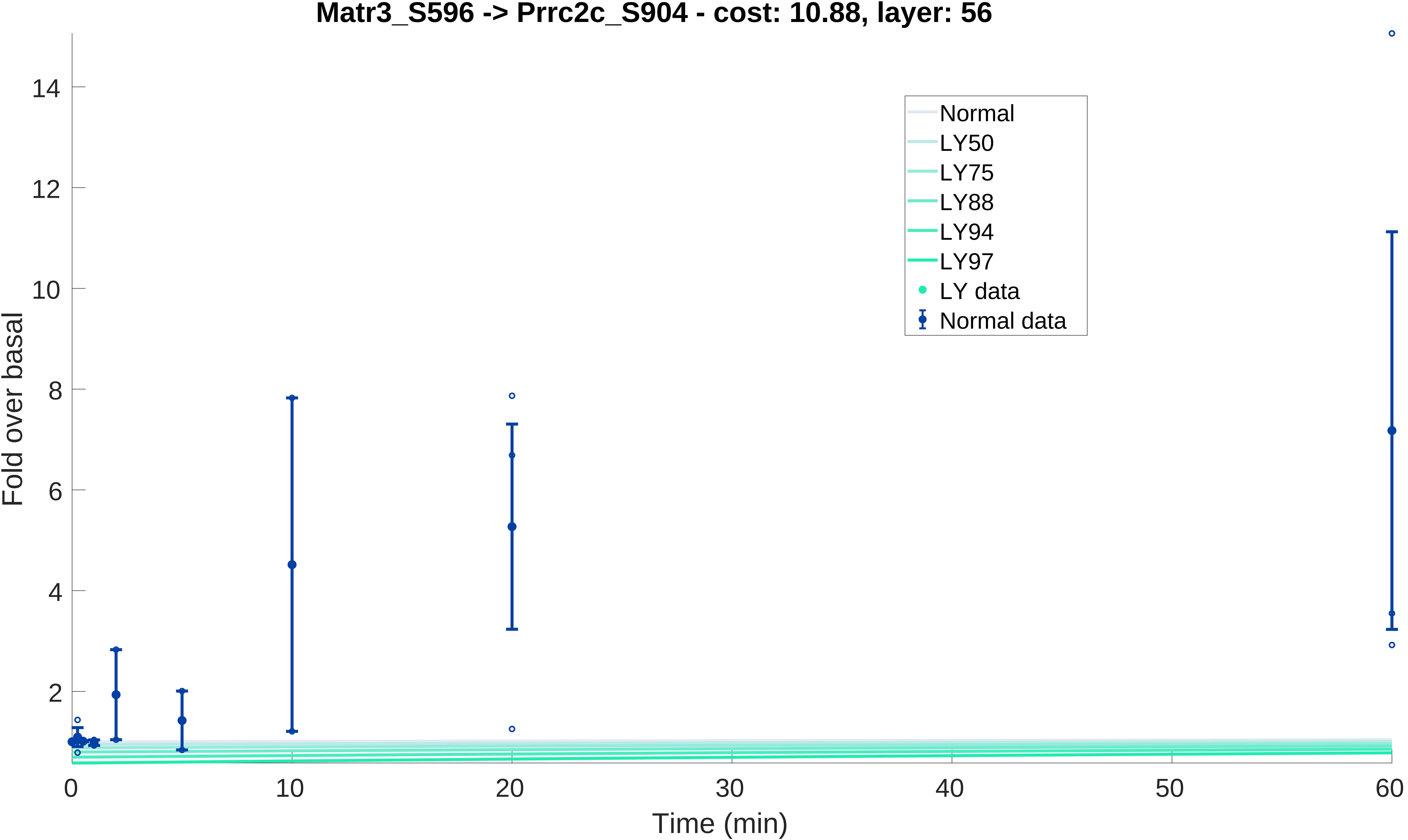

Matr3_S596 -> Prrc2c_S904 - cost: 10.88, layer: 56
Fold over basal
Time (min)
0
10
20
30
40
50
60
2
4
6
8
10
12
14
Normal
LY50
LY75
LY88
LY94
LY97
LY data
Normal data

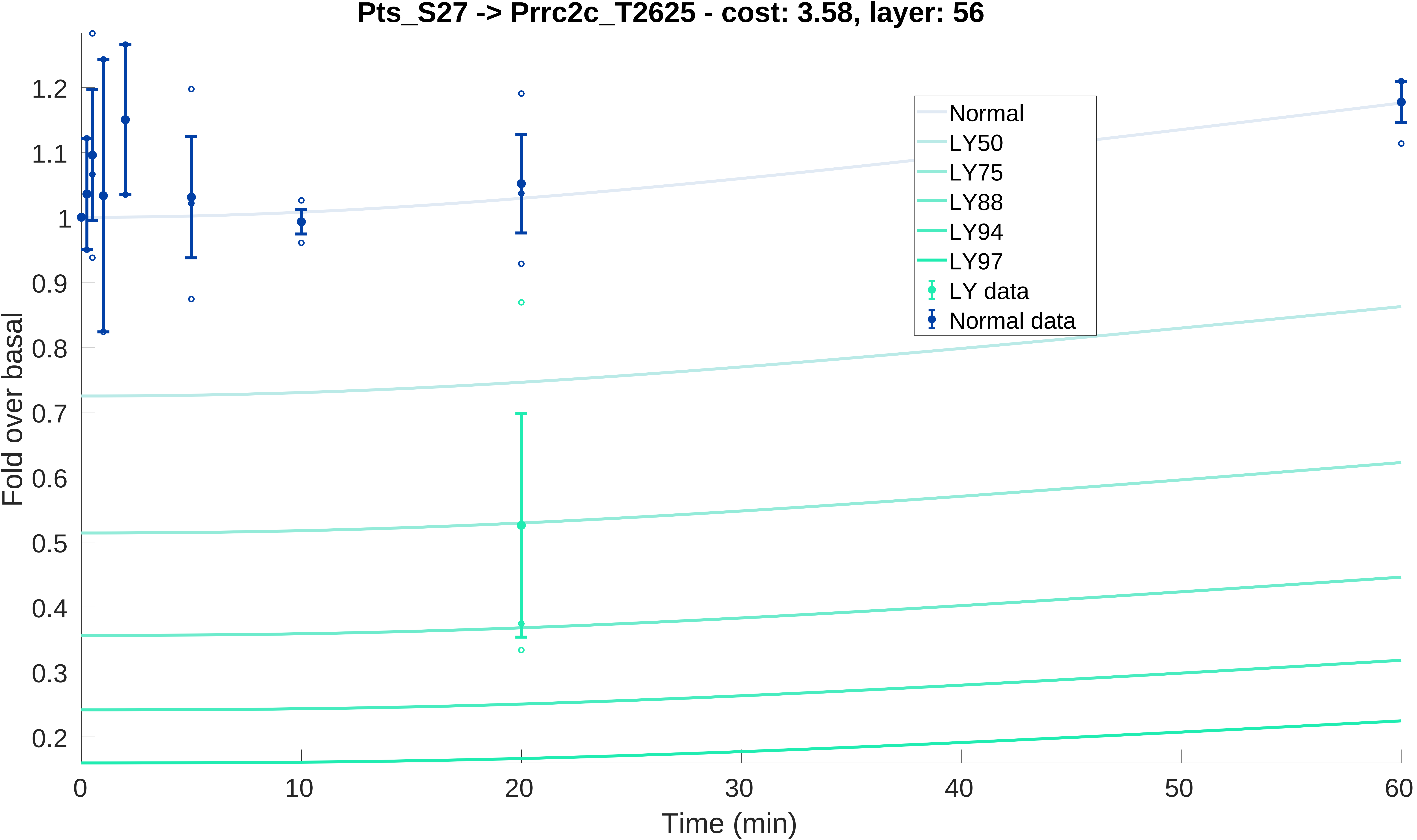

Pts_S27 -> Prrc2c_T2625 - cost: 3.58, layer: 56
Normal
LY50
LY75
LY88
LY94
LY97
LY data
Normal data
Fold over basal
Time (min)
0
10
20
30
40
50
60
0.2
0.3
0.4
0.5
0.6
0.7
0.8
0.9
1
1.1
1.2

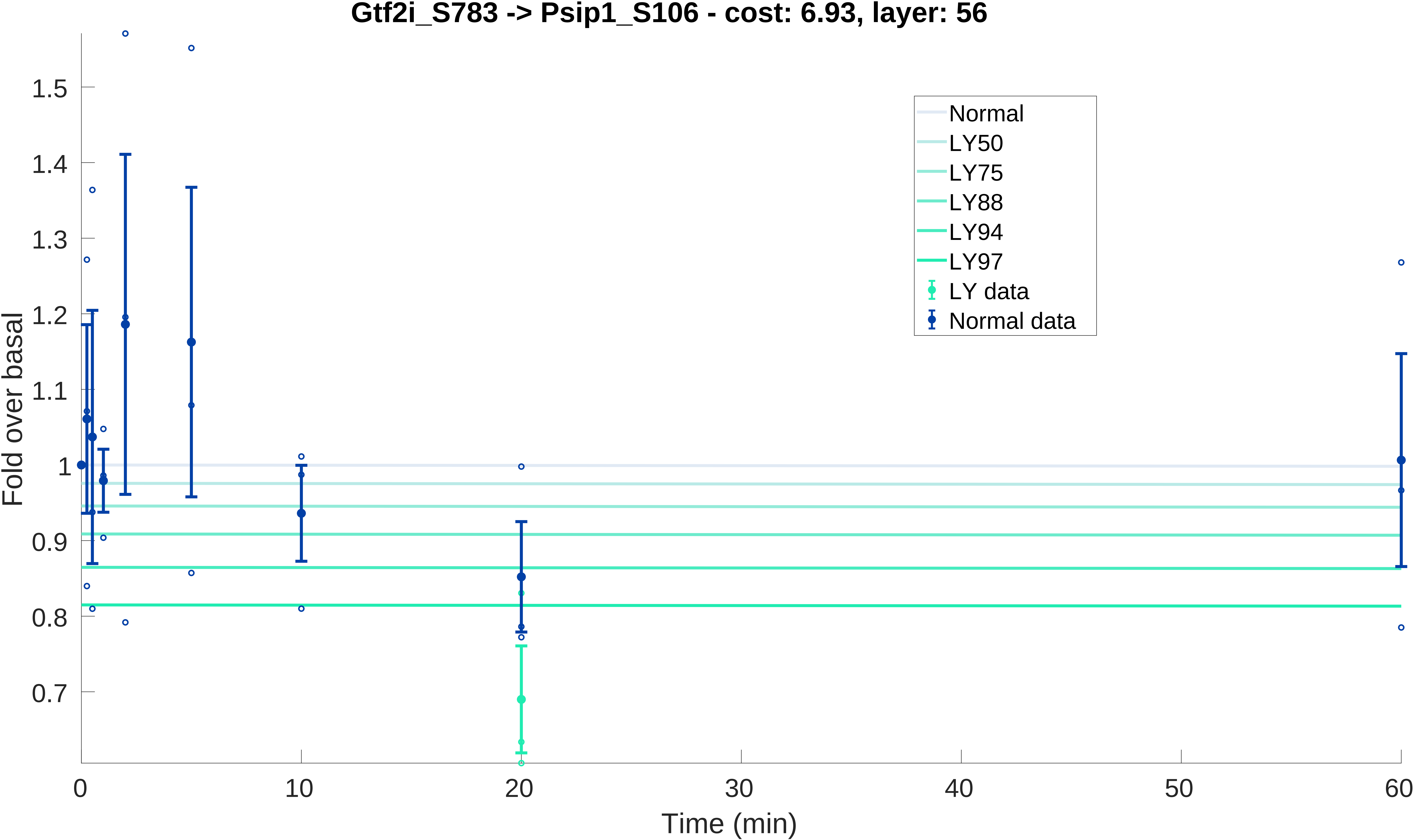

Gtf2i_S783 -> Psip1_S106 - cost: 6.93, layer: 56
Normal
LY50
LY75
LY88
LY94
LY97
LY data
Normal data
Fold over basal
Time (min)
0
10
20
30
40
50
60
0.7
0.8
0.9
1
1.1
1.2
1.3
1.4
1.5

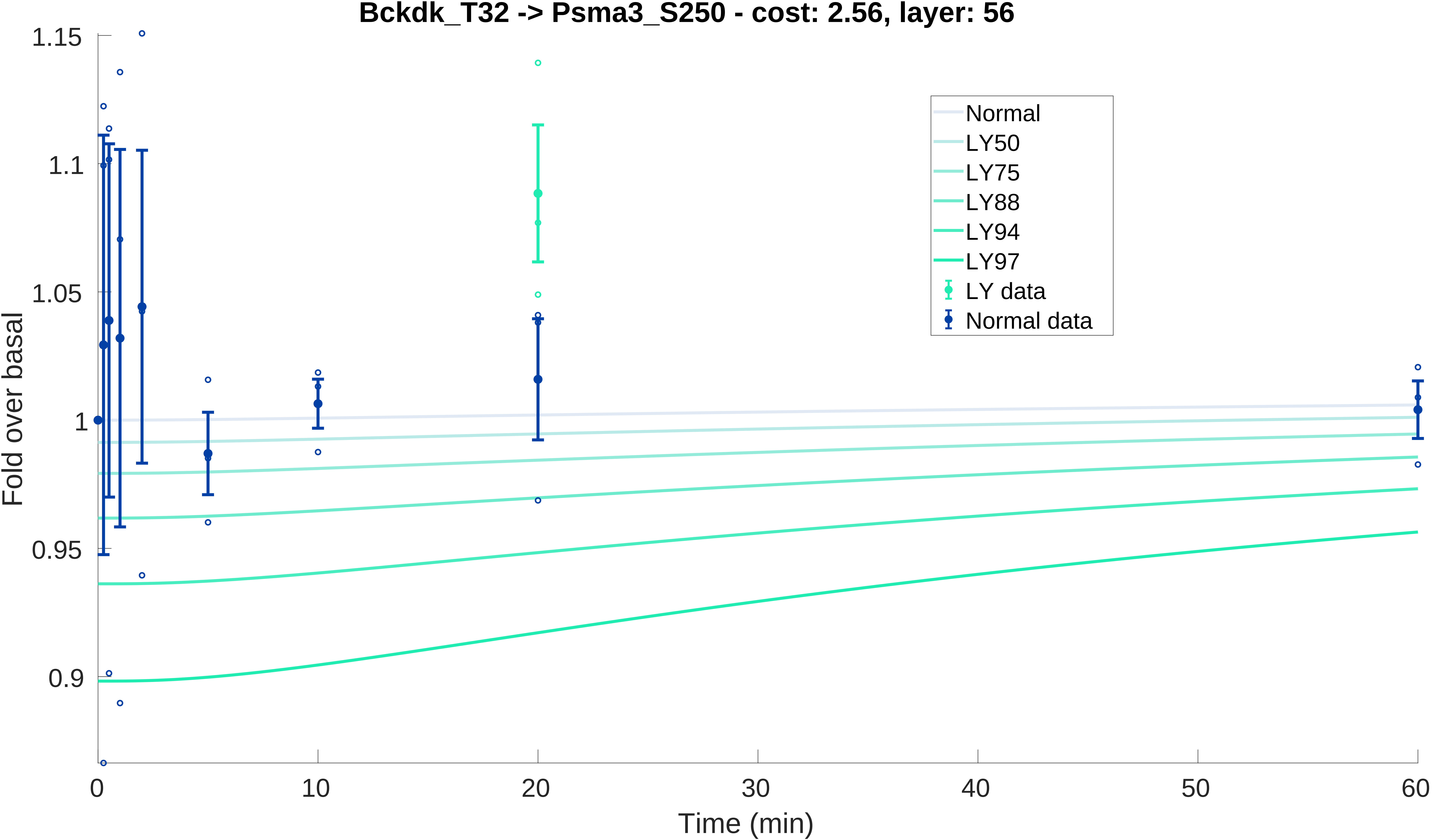

Bckdk_T32 -> Psma3_S250 - cost: 2.56, layer: 56
Fold over basal
Time (min)
1.15
1.1
1.05
1
0.95
0.9
0
10
20
30
40
50
60
Normal
LY50
LY75
LY88
LY94
LY97
LY data
Normal data

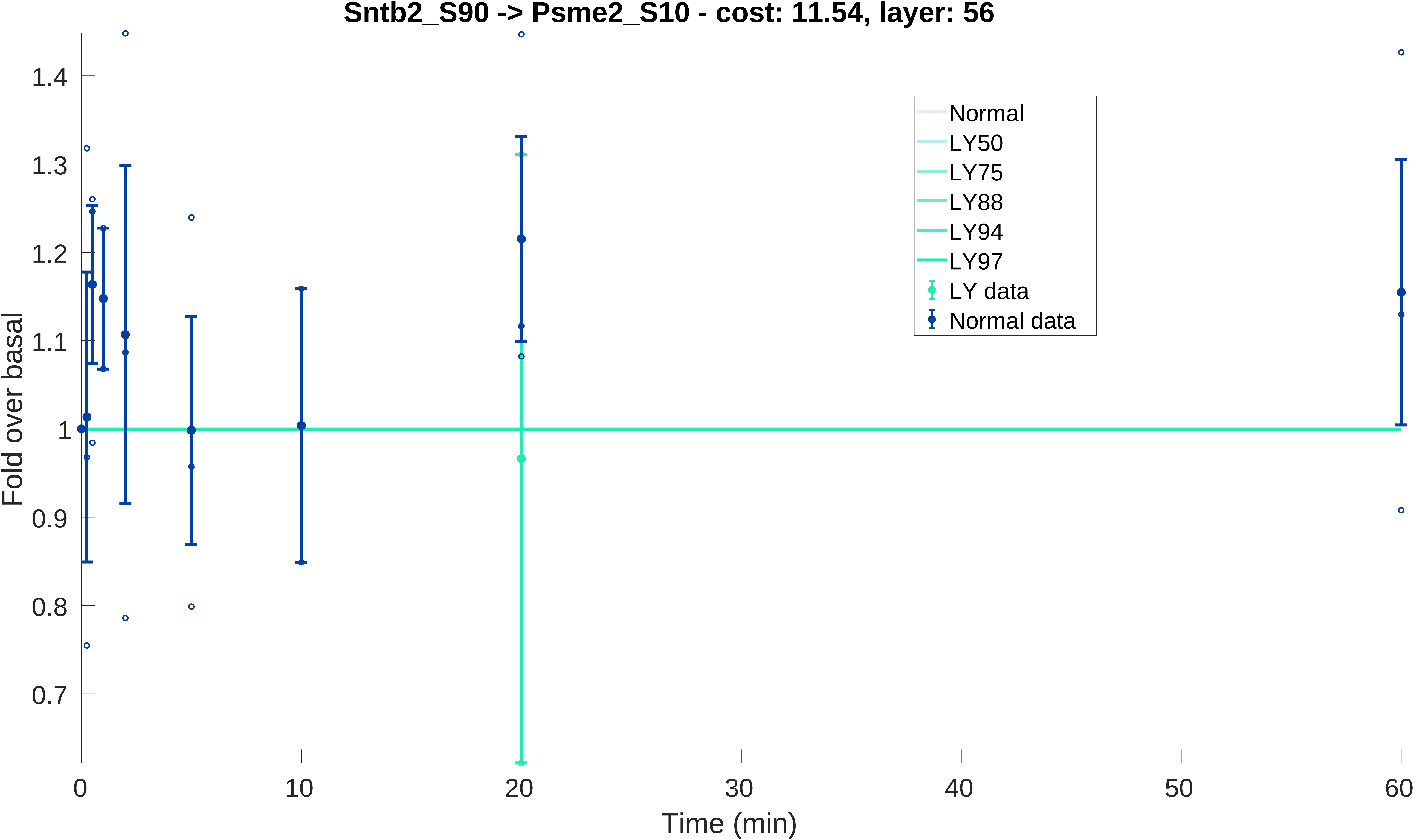

Sntb2_S90 -> Psme2_S10 - cost: 11.54, layer: 56
Fold over basal
Time (min)
0.7
0.8
0.9
1
1.1
1.2
1.3
1.4
0
10
20
30
40
50
60
Normal
LY50
LY75
LY88
LY94
LY97
LY data
Normal data

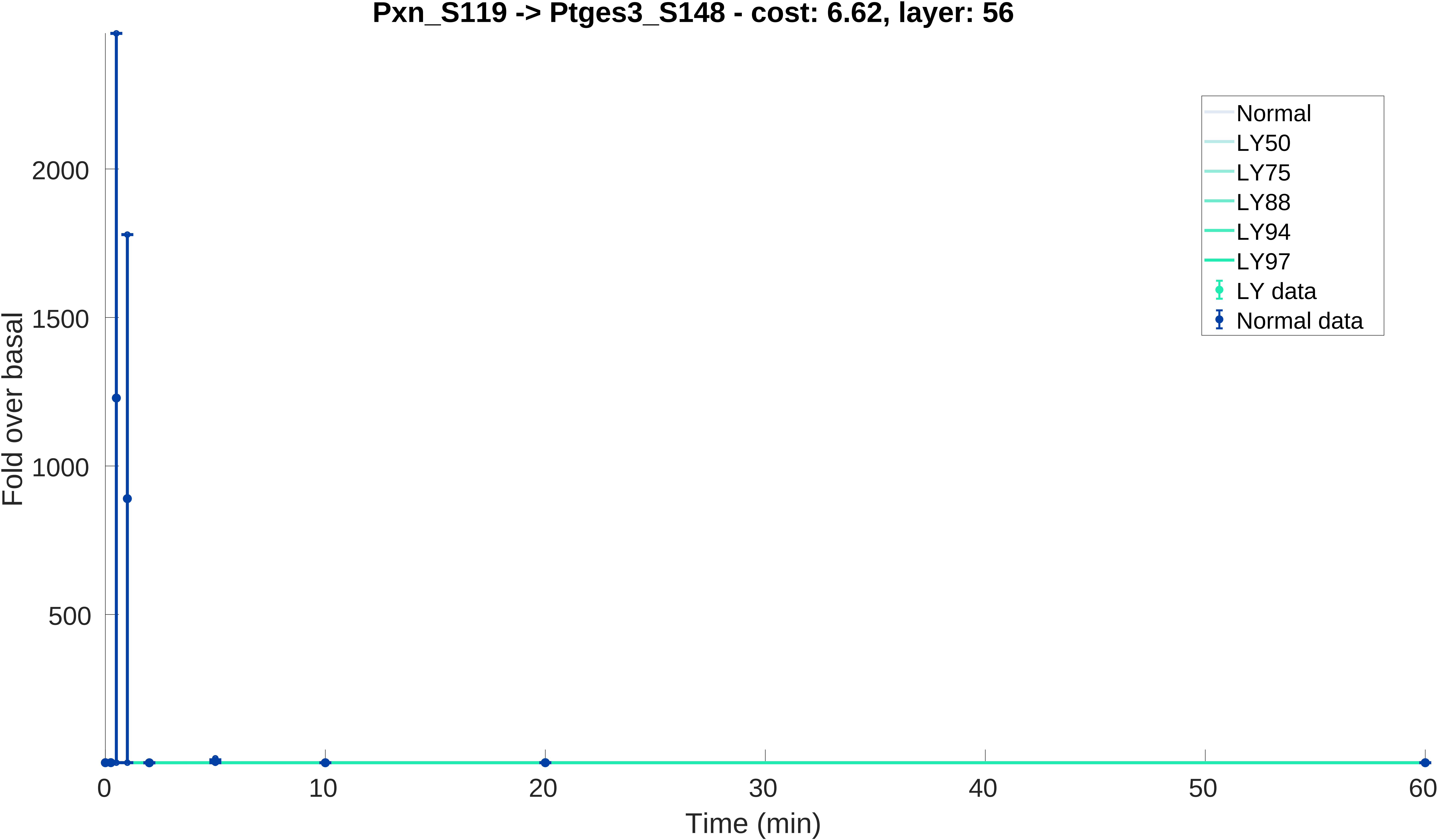

Pxn_S119 -> Ptges3_S148 - cost: 6.62, layer: 56
Fold over basal
500
1000
1500
2000
Time (min)
0
10
20
30
40
50
60
Normal
LY50
LY75
LY88
LY94
LY97
LY data
Normal data

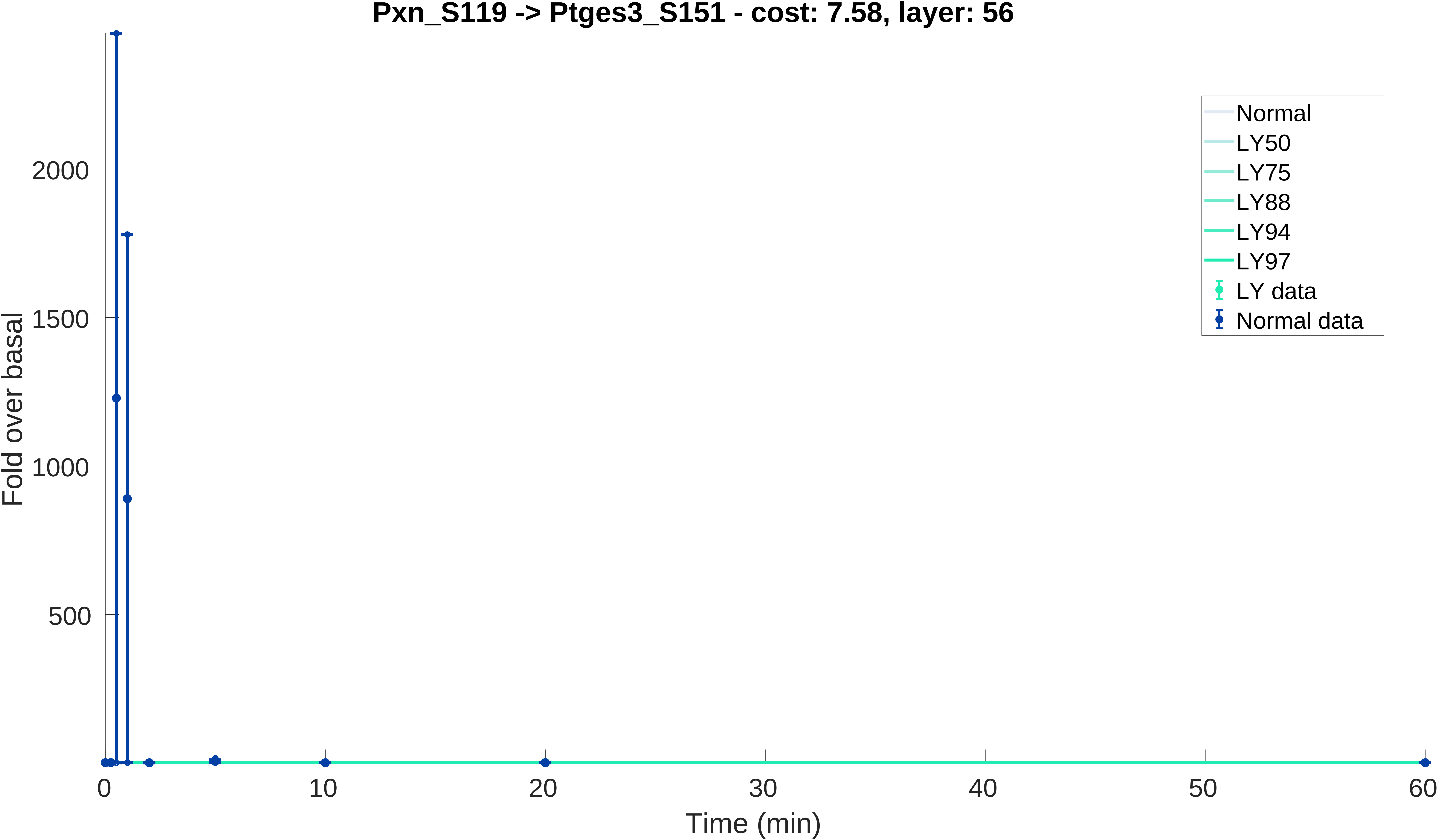

Pxn_S119 -> Ptges3_S151 - cost: 7.58, layer: 56
Fold over basal
2000
1500
1000
500
0
10
20
30
40
50
60
Time (min)
Normal
LY50
LY75
LY88
LY94
LY97
LY data
Normal data

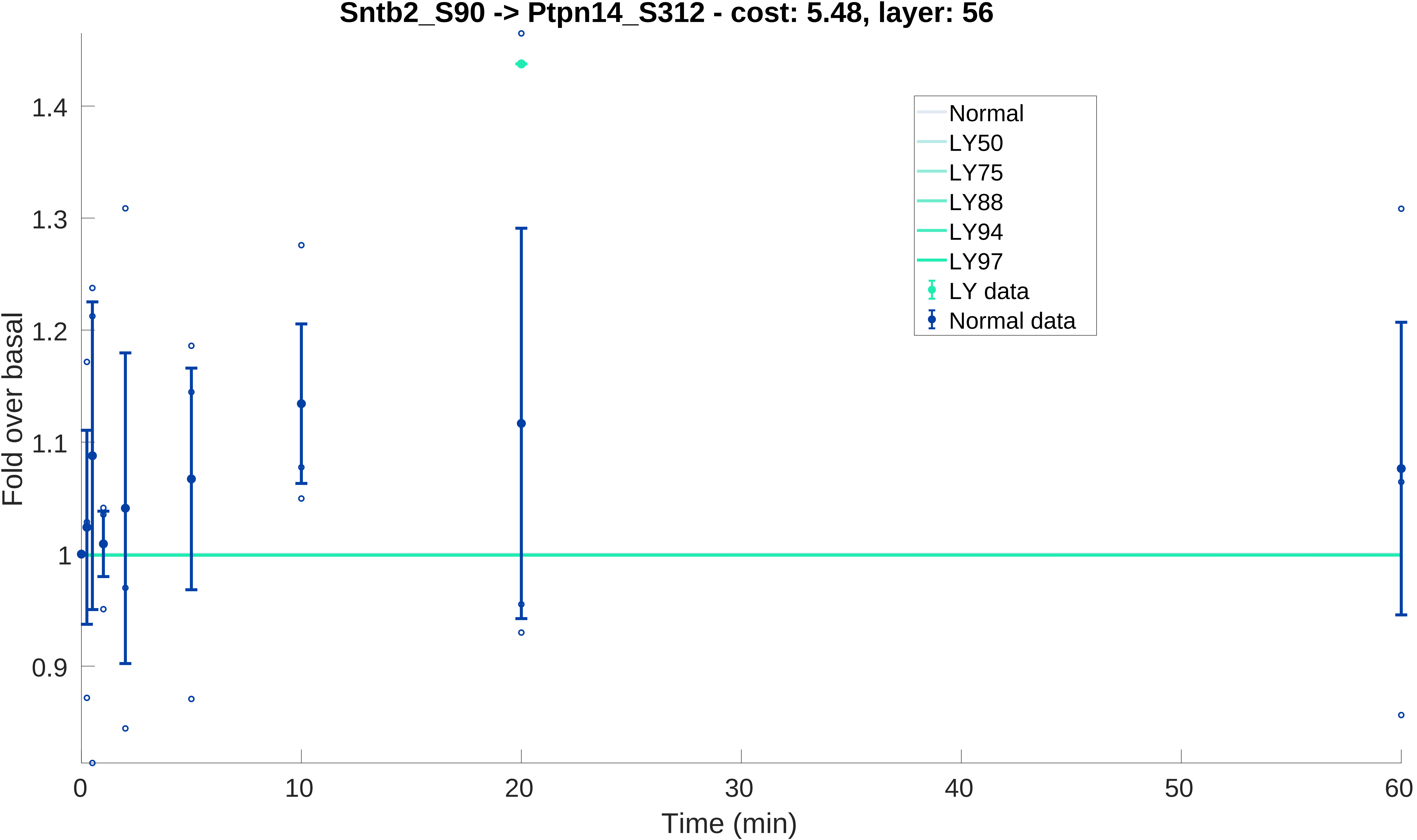
Sntb2_S90 -> Ptpn14_S312 - cost: 5.48, layer: 56
Fold over basal
1.4
1.3
1.2
1.1
1
0.9
0
10
20
30
40
50
60
Time (min)
Normal
LY50
LY75
LY88
LY94
LY97
LY data
Normal data

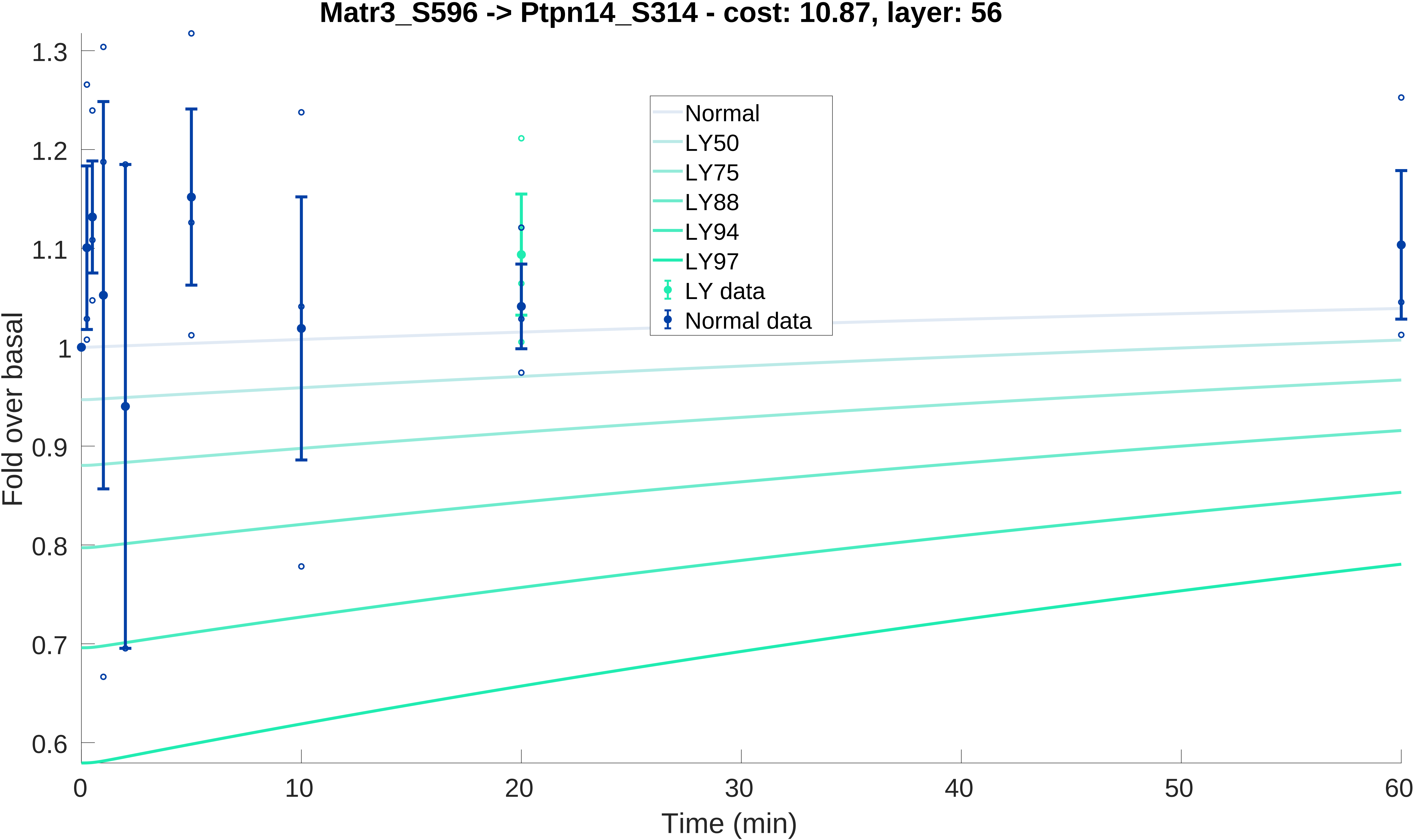

Matr3_S596 -> Ptpn14_S314 - cost: 10.87, layer: 56
Normal
LY50
LY75
LY88
LY94
LY97
LY data
Normal data
Fold over basal
Time (min)
1.3
1.2
1.1
1
0.9
0.8
0.7
0.6
0
10
20
30
40
50
60

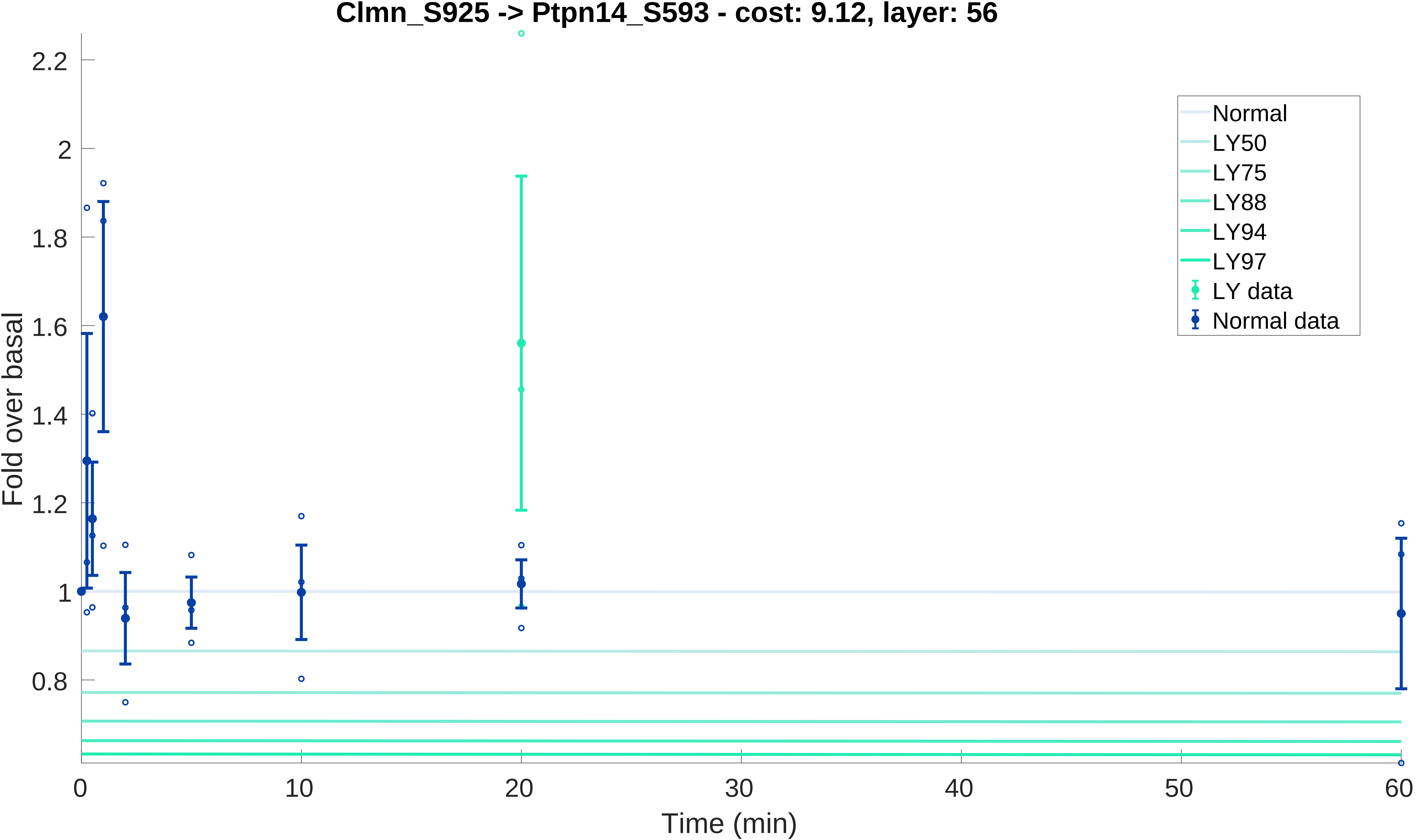

Clmn_S925 -> Ptpn14_S593 - cost: 9.12, layer: 56
Fold over basal
Time (min)
Normal
LY50
LY75
LY88
LY94
LY97
LY data
Normal data
0.8
1
1.2
1.4
1.6
1.8
2
2.2
0
10
20
30
40
50
60

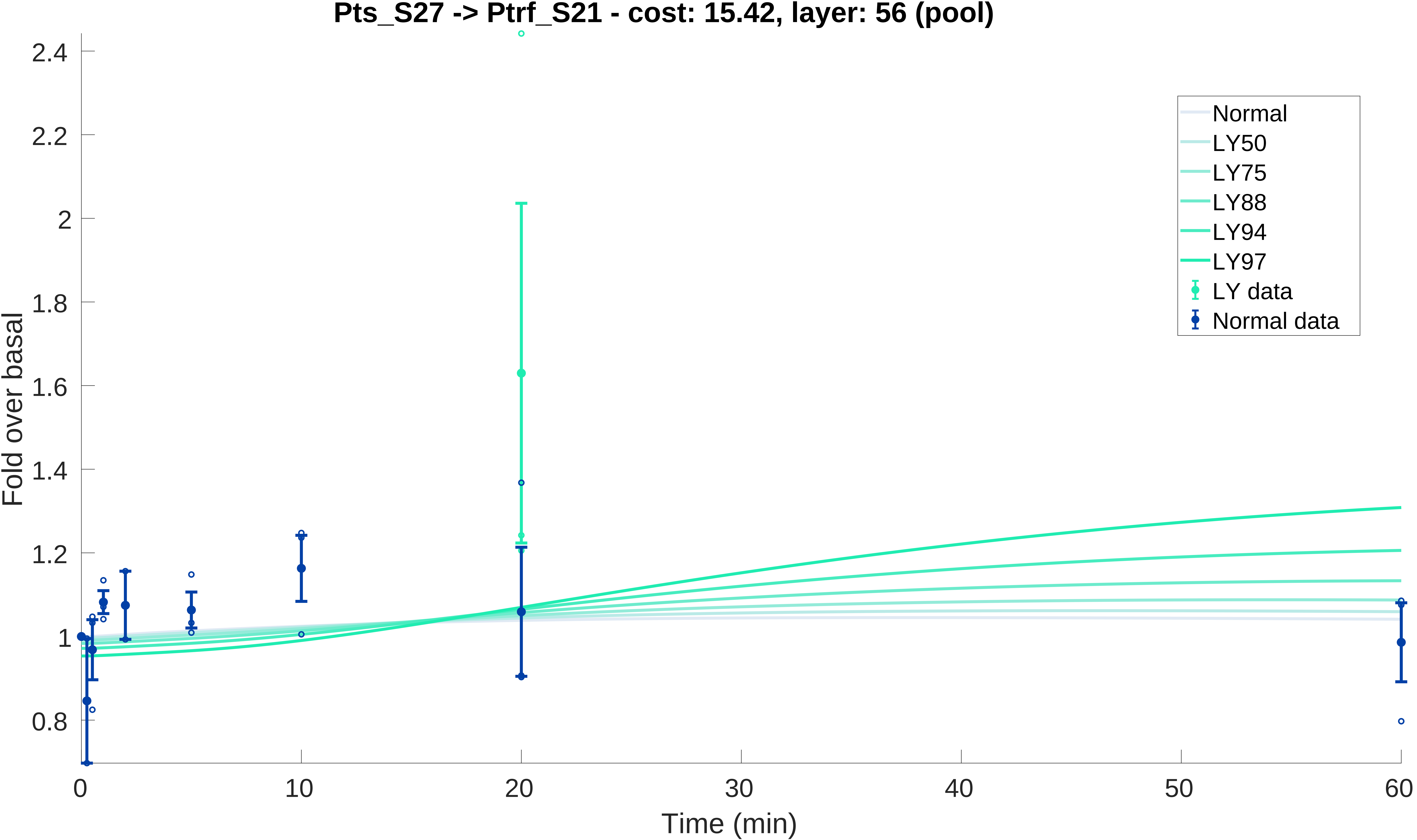

Pts_S27 -> Ptrf_S21 - cost: 15.42, layer: 56 (pool)
Fold over basal
Time (min)
0.8
1
1.2
1.4
1.6
1.8
2
2.2
2.4
0
10
20
30
40
50
60
Normal
LY50
LY75
LY88
LY94
LY97
LY data
Normal data

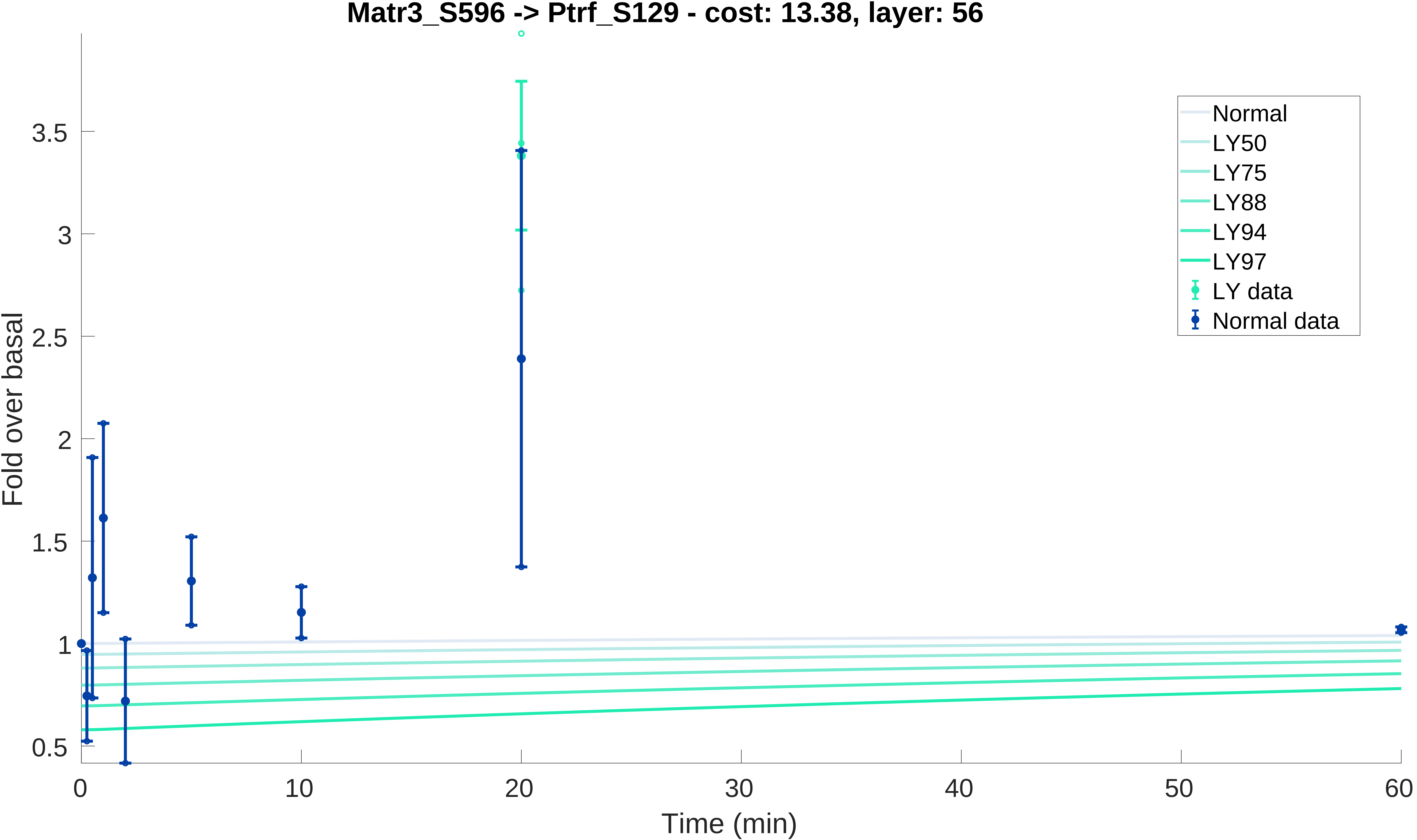

Matr3_S596 -> Ptrf_S129 - cost: 13.38, layer: 56
Fold over basal
Time (min)
0.5
1
1.5
2
2.5
3
3.5
0
10
20
30
40
50
60
Normal
LY50
LY75
LY88
LY94
LY97
LY data
Normal data

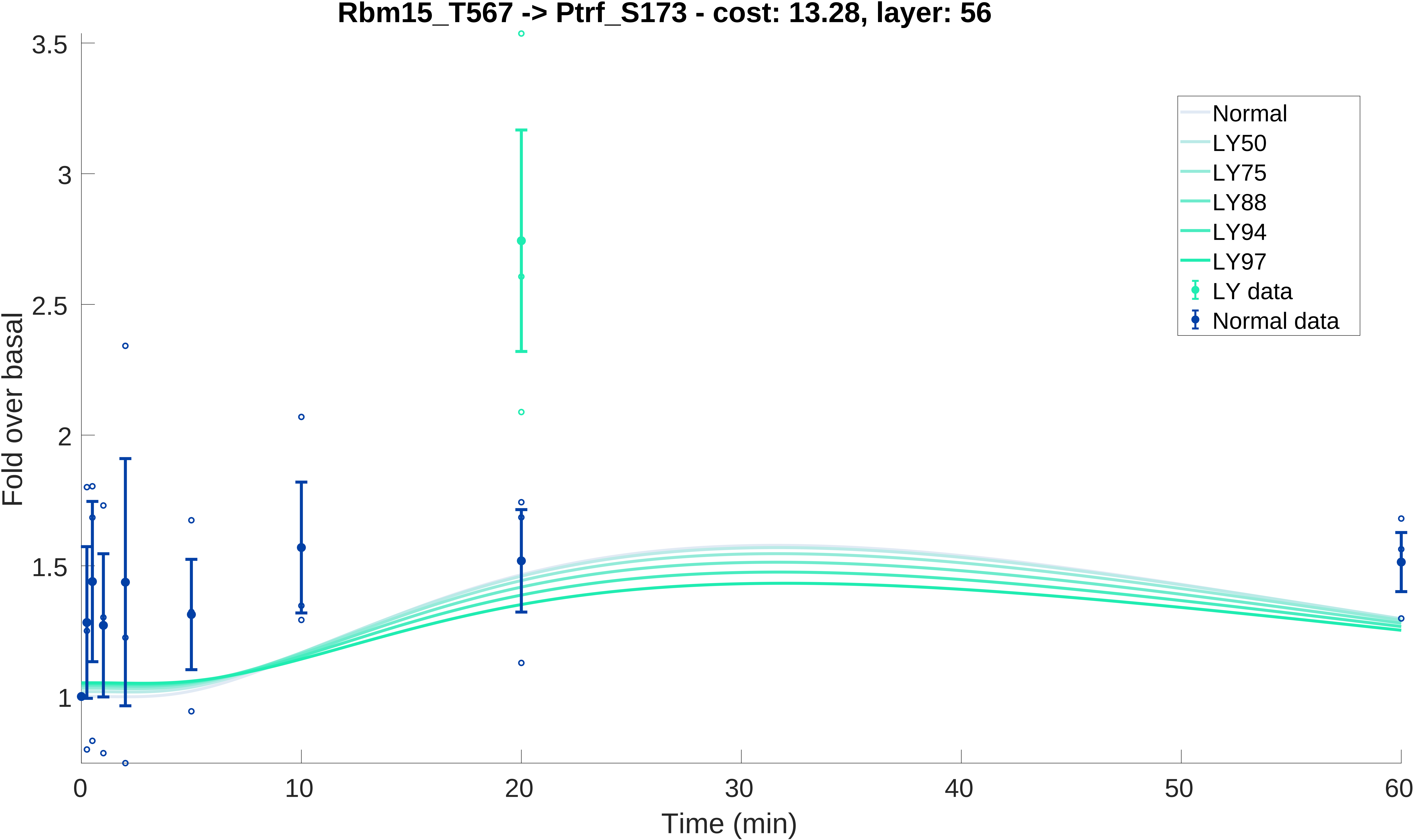

Rbm15_T567 -> Ptrf_S173 - cost: 13.28, layer: 56
Fold over basal
Time (min)
Normal
LY50
LY75
LY88
LY94
LY97
LY data
Normal data
3.5
3
2.5
2
1.5
1
0
10
20
30
40
50
60

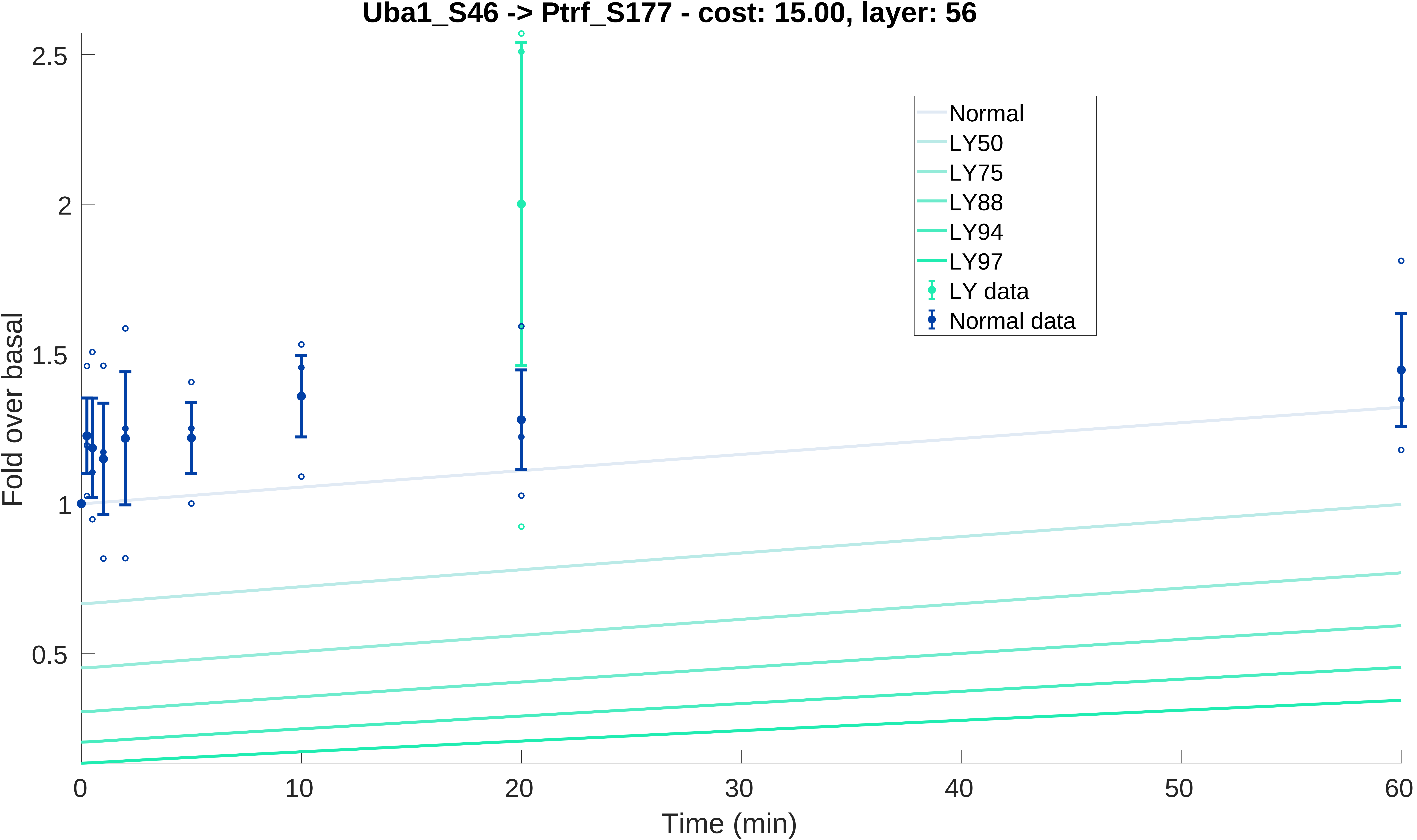
Uba1_S46 -> Ptrf_S177 - cost: 15.00, layer: 56
Fold over basal
2.5
2
1.5
1
0.5
0
10
20
30
40
50
60
Time (min)
Normal
LY50
LY75
LY88
LY94
LY97
LY data
Normal data

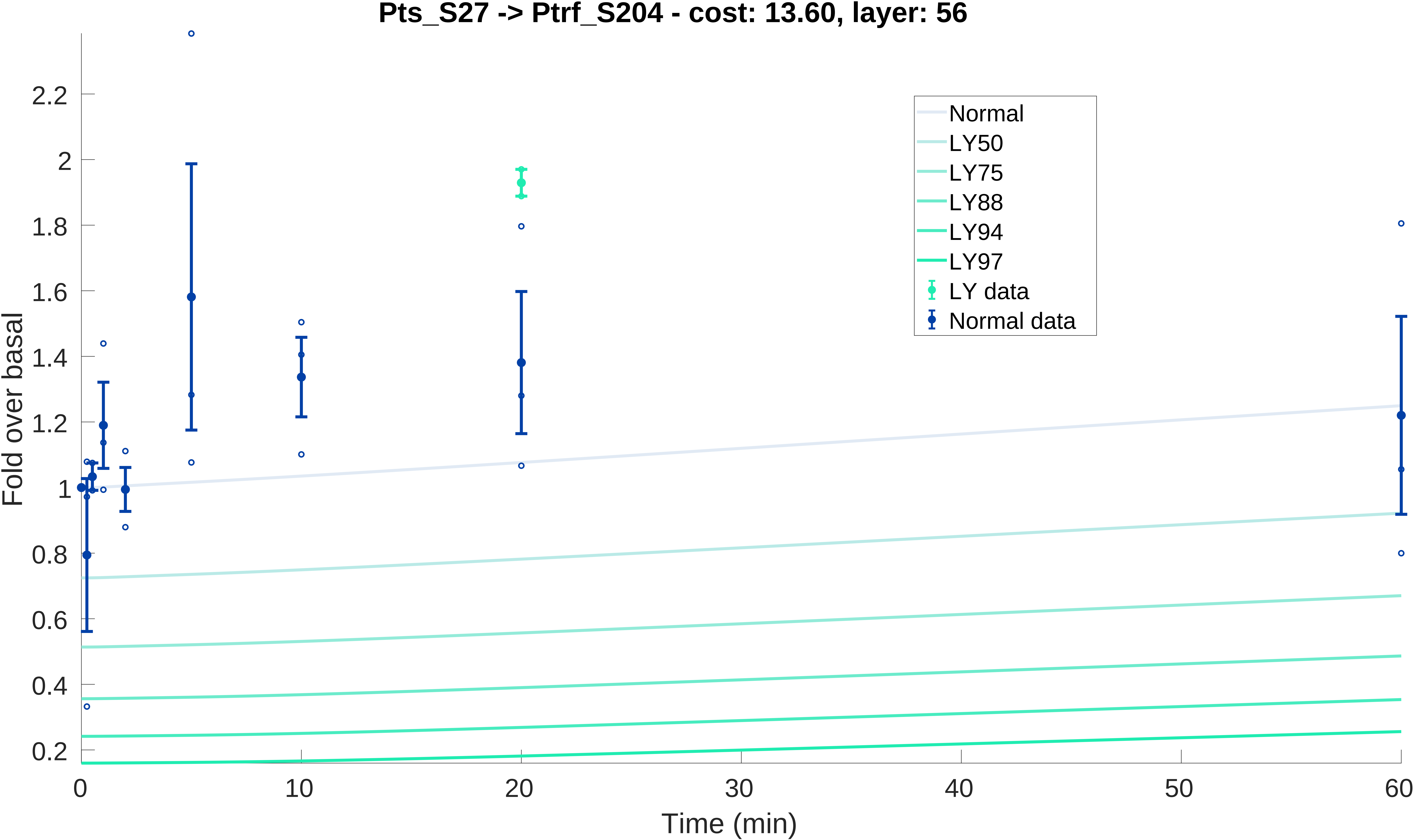

Pts_S27 -> Ptrf_S204 - cost: 13.60, layer: 56
Fold over basal
Time (min)
0
10
20
30
40
50
60
0.2
0.4
0.6
0.8
1
1.2
1.4
1.6
1.8
2
2.2
Normal
LY50
LY75
LY88
LY94
LY97
LY data
Normal data

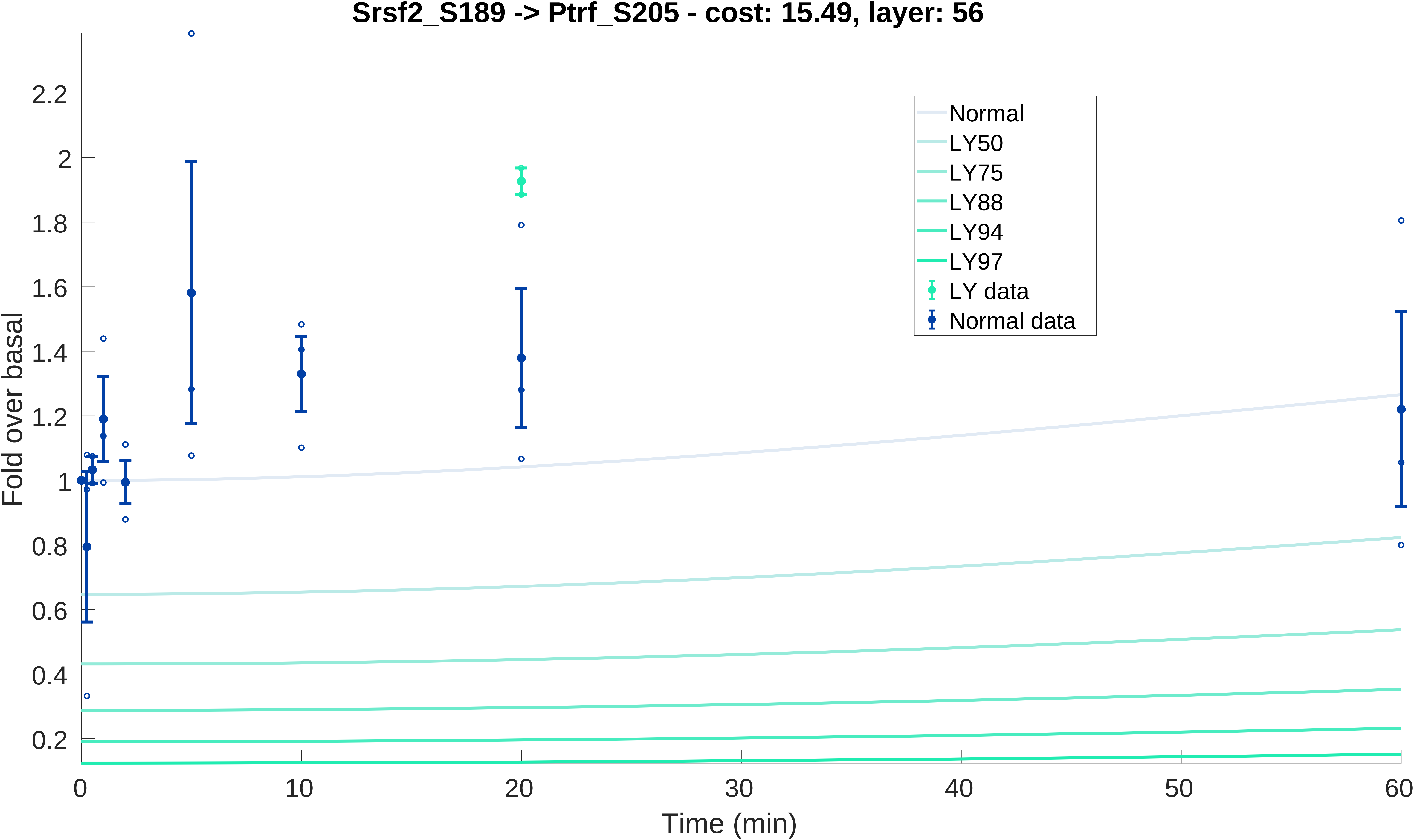

Srsf2_S189 -> Ptrf_S205 - cost: 15.49, layer: 56
Fold over basal
Time (min)
0.2
0.4
0.6
0.8
1
1.2
1.4
1.6
1.8
2
2.2
0
10
20
30
40
50
60
Normal
LY50
LY75
LY88
LY94
LY97
LY data
Normal data

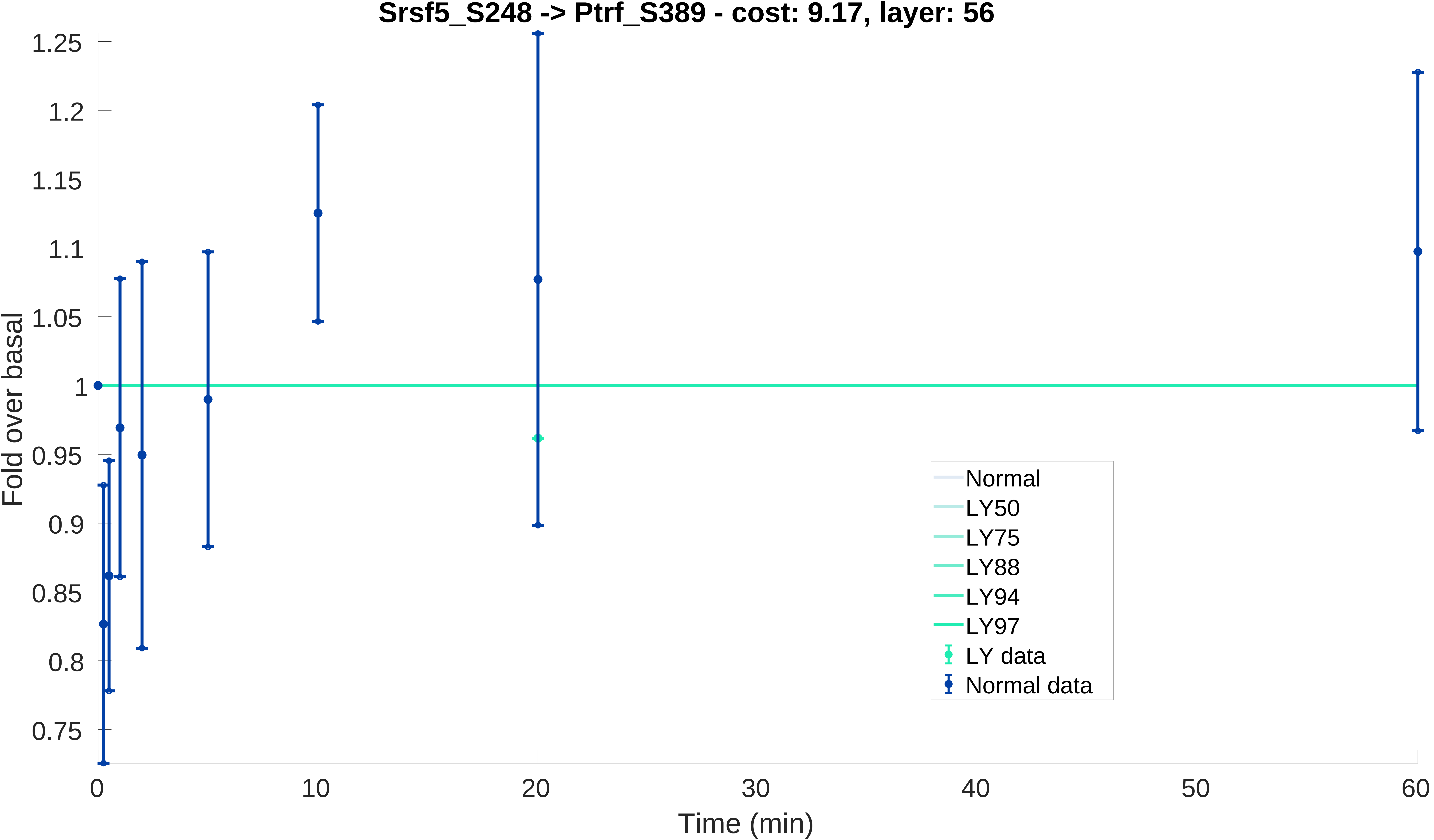

Srsf5_S248 -> Ptrf_S389 - cost: 9.17, layer: 56
Fold over basal
Time (min)
1.25
1.2
1.15
1.1
1.05
1
0.95
0.9
0.85
0.8
0.75
0
10
20
30
40
50
60
Normal
LY50
LY75
LY88
LY94
LY97
LY data
Normal data

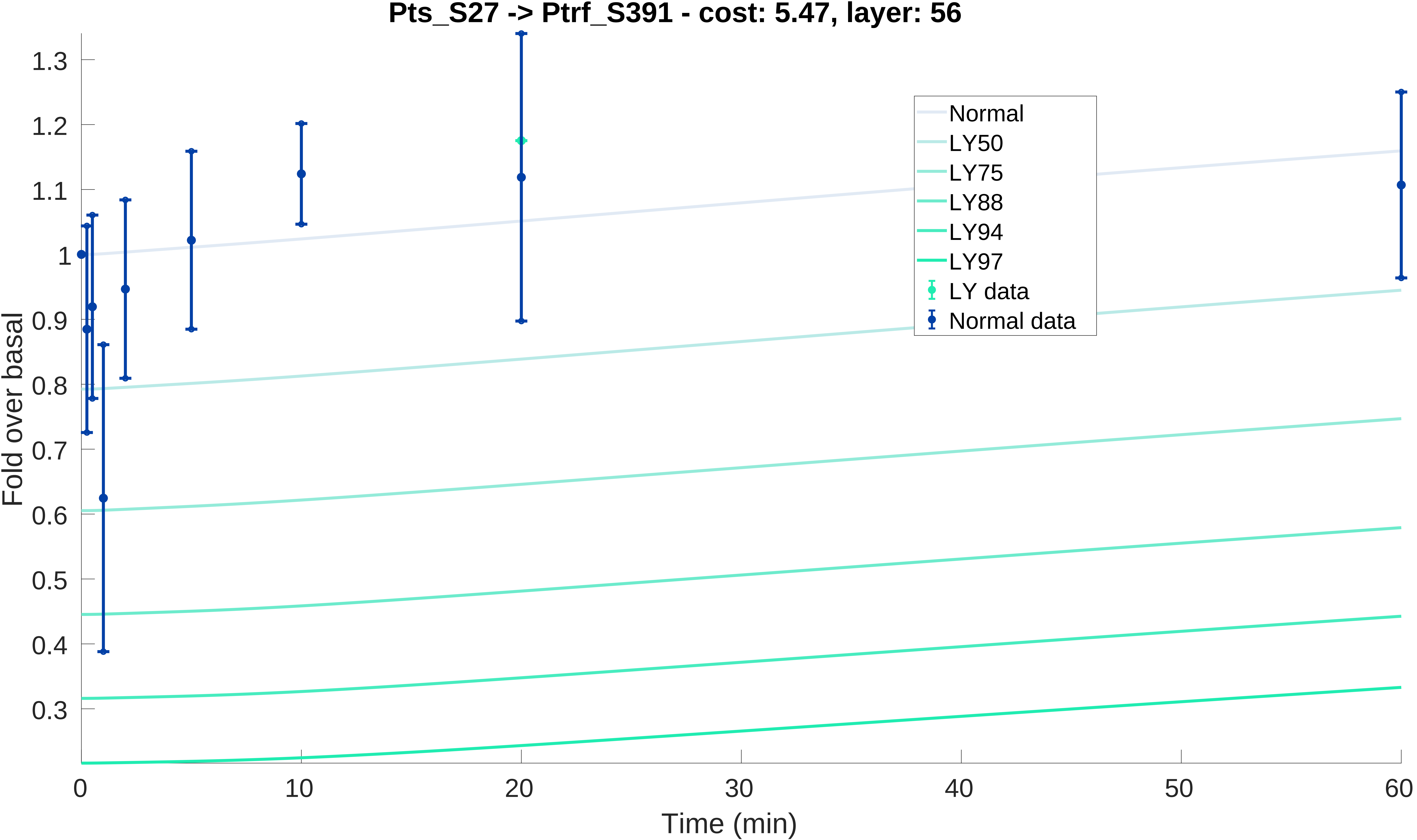
Pts_S27 -> Ptrf_S391 - cost: 5.47, layer: 56
Fold over basal
Time (min)
0
10
20
30
40
50
60
0.3
0.4
0.5
0.6
0.7
0.8
0.9
1
1.1
1.2
1.3
Normal
LY50
LY75
LY88
LY94
LY97
LY data
Normal data

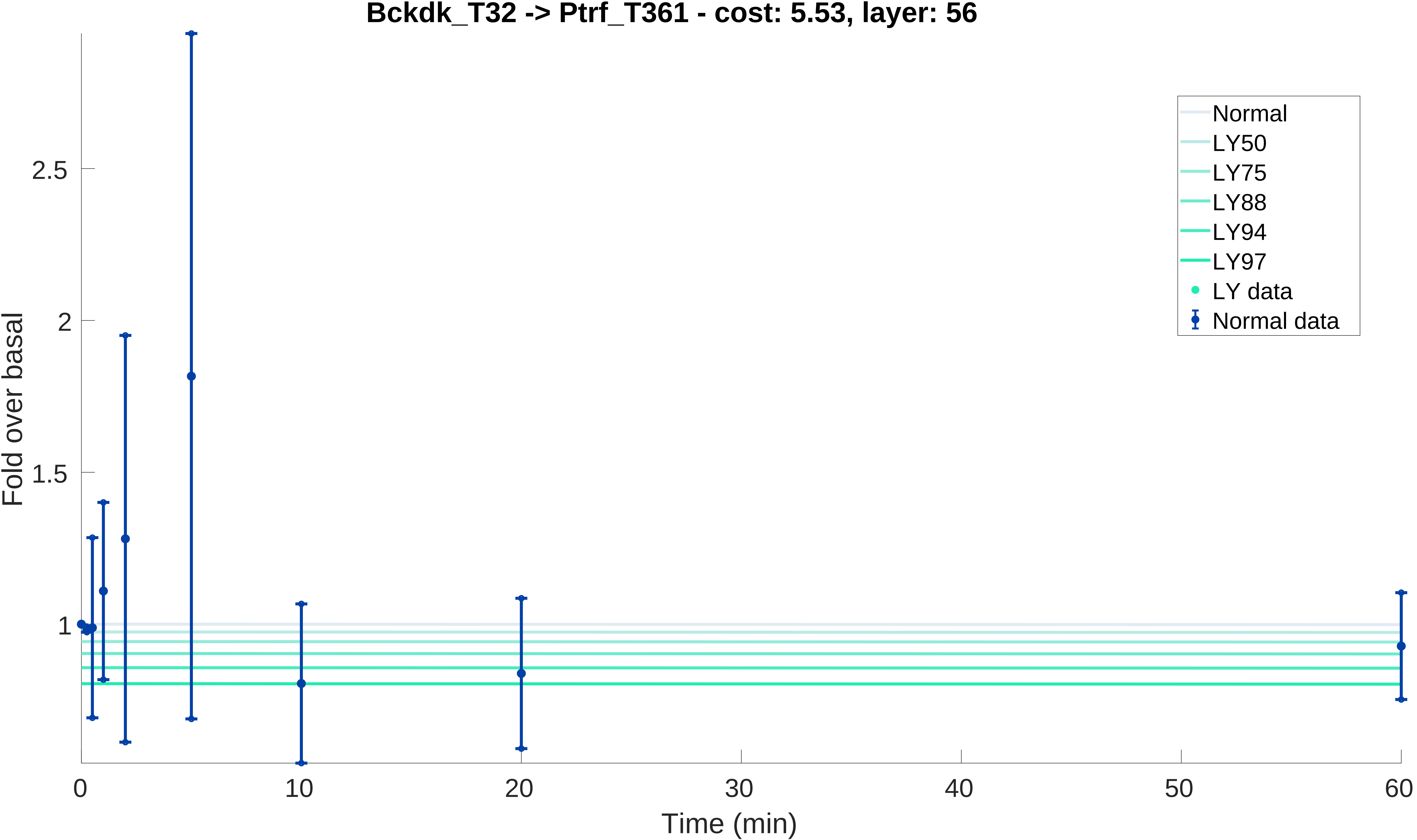
Bckdk_T32 -> Ptrf_T361 - cost: 5.53, layer: 56
Normal
LY50
LY75
LY88
LY94
LY97
LY data
Normal data
Fold over basal
2.5
2
1.5
1
0
10
20
30
40
50
60
Time (min)

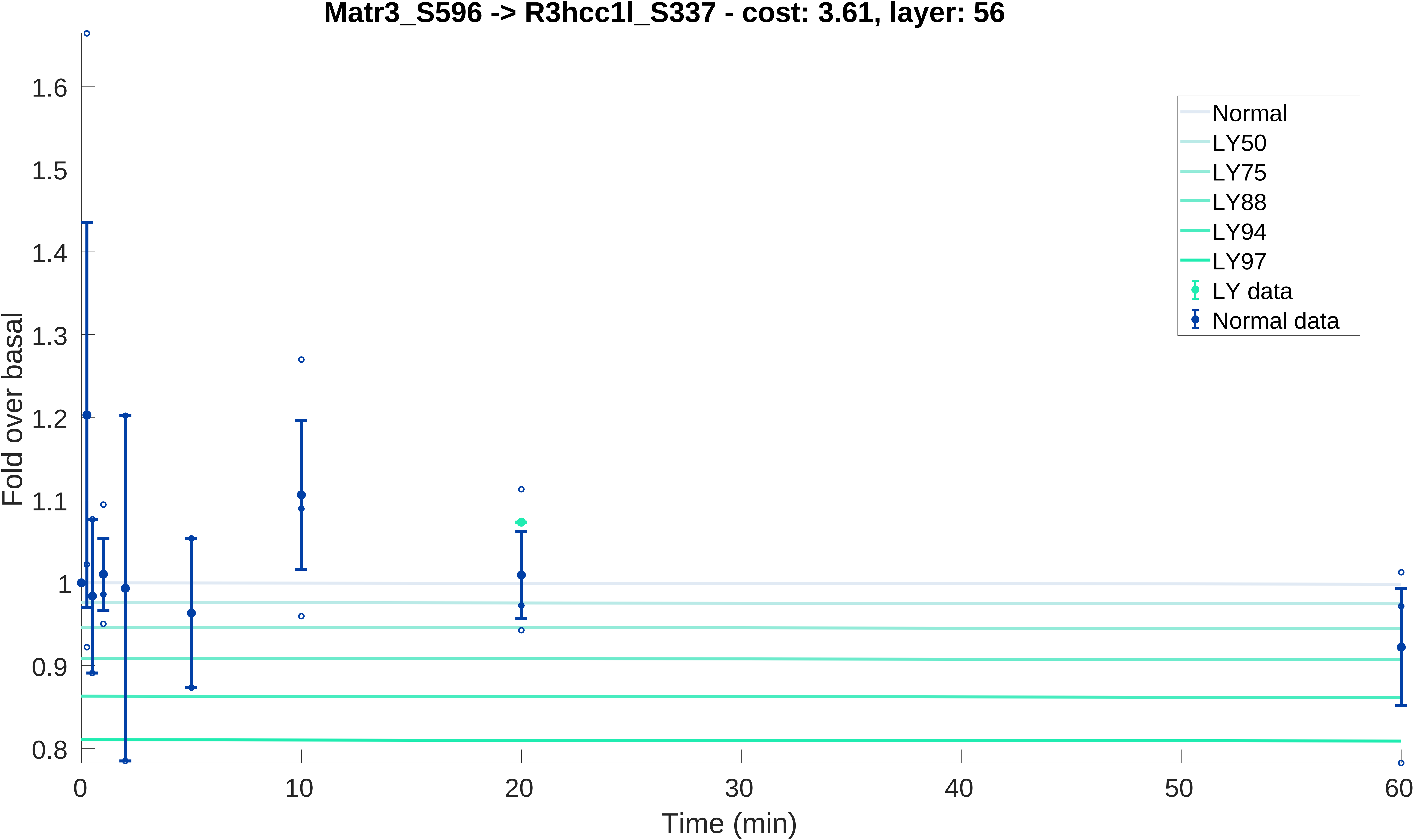

Matr3_S596 -> R3hcc1l_S337 - cost: 3.61, layer: 56
Fold over basal
1.6
1.5
1.4
1.3
1.2
1.1
1
0.9
0.8
0
10
20
30
40
50
60
Time (min)
Normal
LY50
LY75
LY88
LY94
LY97
LY data
Normal data

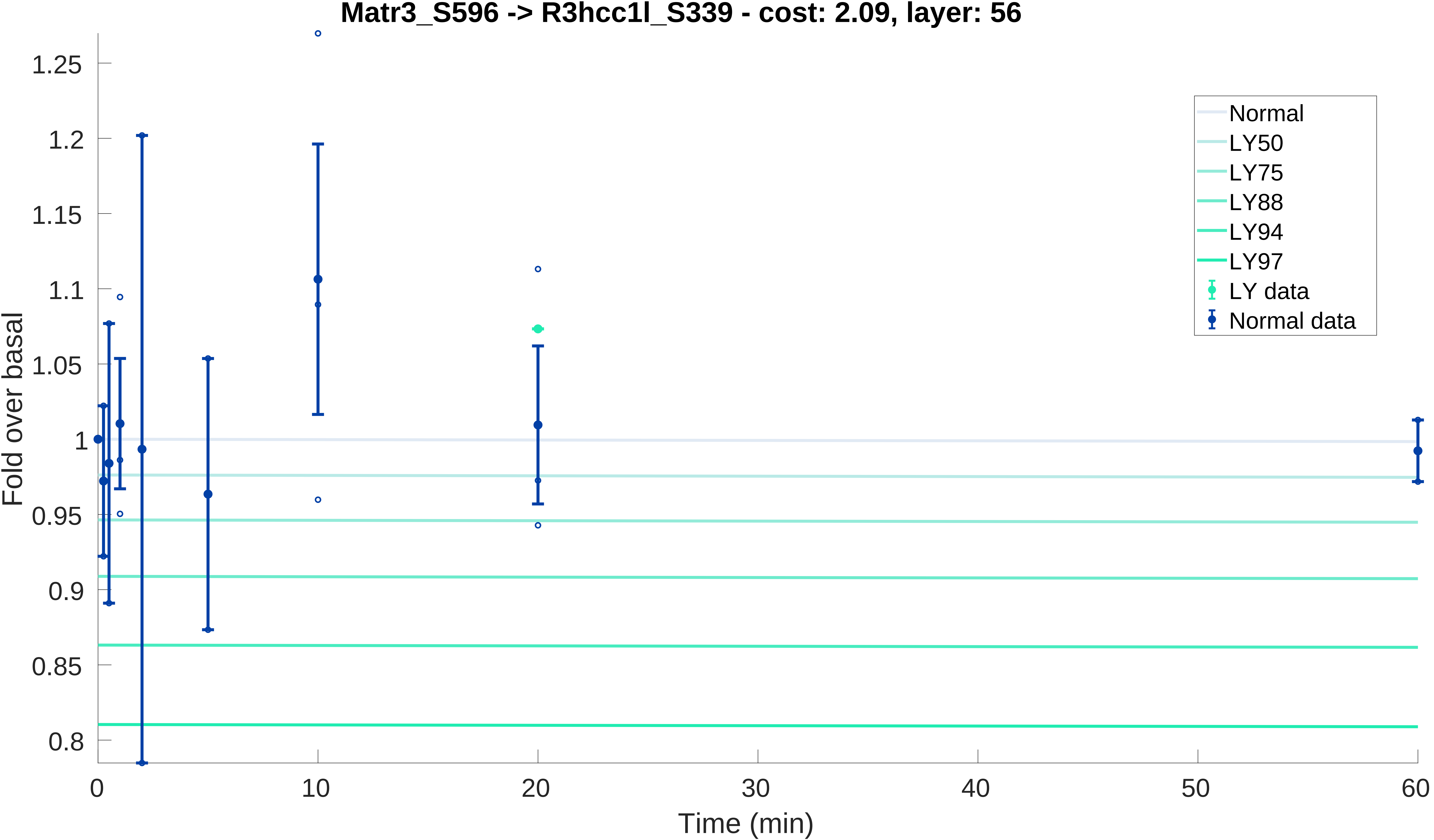

Matr3_S596 -> R3hcc1l_S339 - cost: 2.09, layer: 56
Fold over basal
Time (min)
1.25
1.2
1.15
1.1
1.05
1
0.95
0.9
0.85
0.8
0
10
20
30
40
50
60
Normal
LY50
LY75
LY88
LY94
LY97
LY data
Normal data

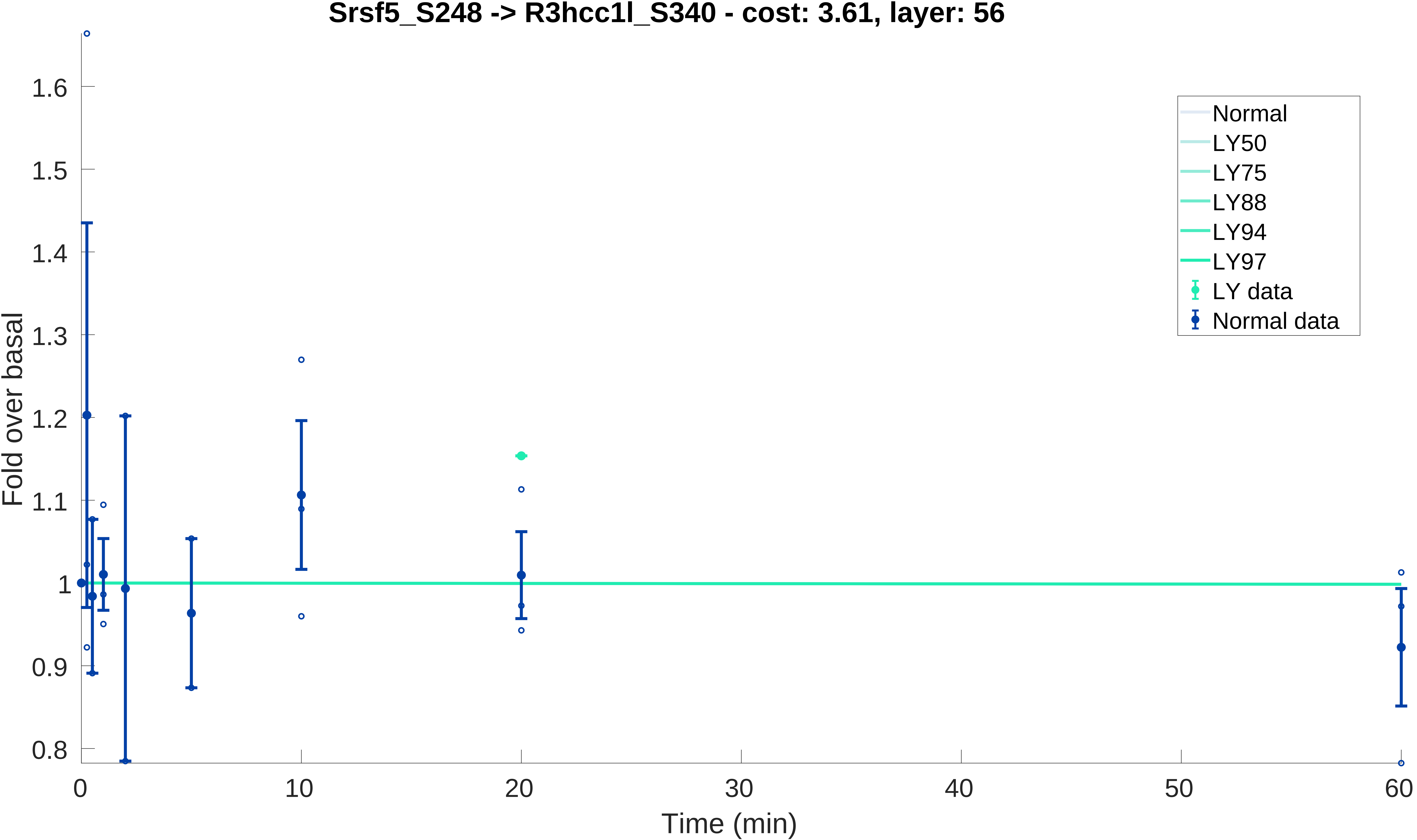
Srsf5_S248 -> R3hcc1l_S340 - cost: 3.61, layer: 56
Fold over basal
Time (min)
0.8
0.9
1
1.1
1.2
1.3
1.4
1.5
1.6
0
10
20
30
40
50
60
Normal
LY50
LY75
LY88
LY94
LY97
LY data
Normal data

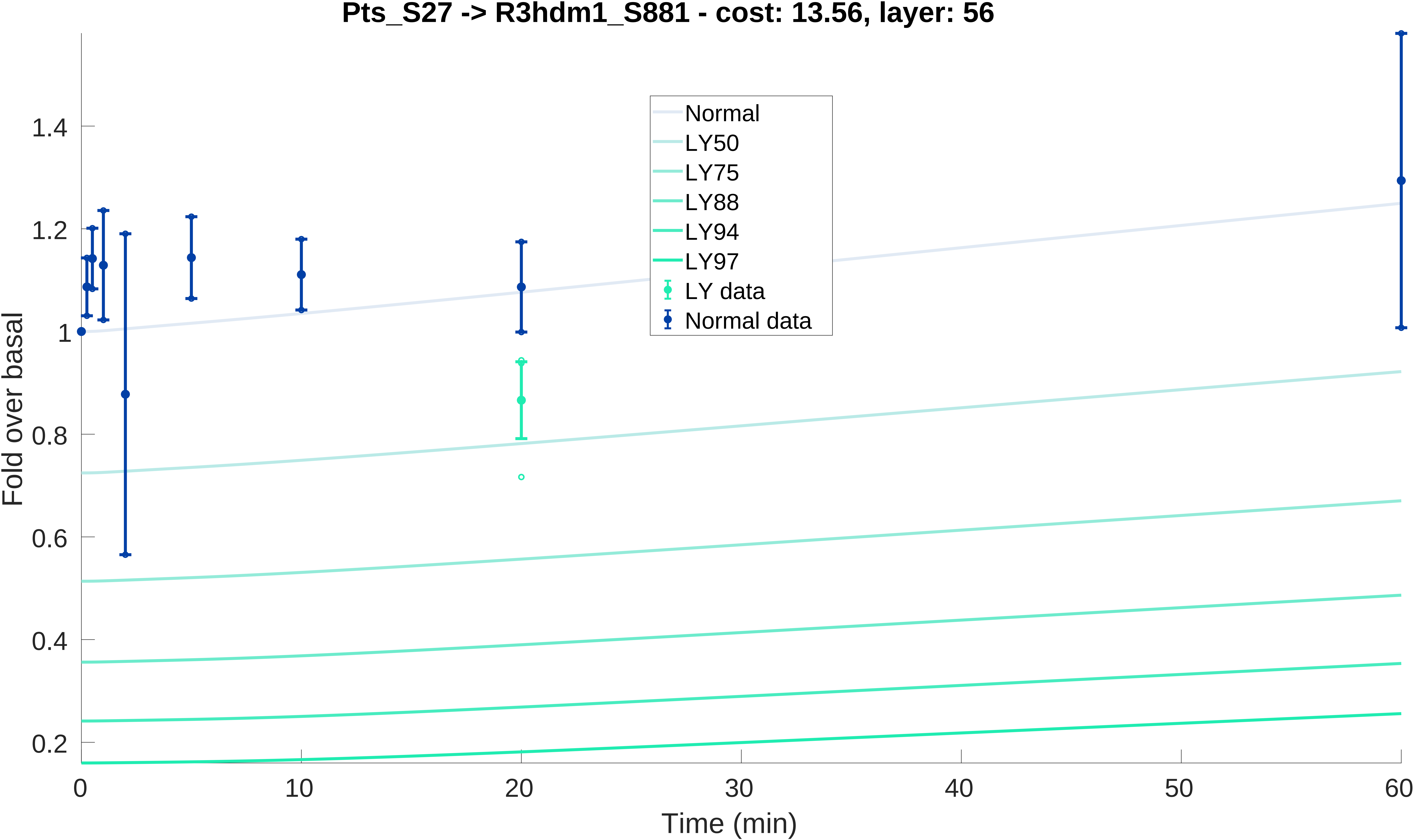

Pts_S27 -> R3hdm1_S881 - cost: 13.56, layer: 56
Fold over basal
Time (min)
Normal
LY50
LY75
LY88
LY94
LY97
LY data
Normal data
1.4
1.2
1
0.8
0.6
0.4
0.2
0
10
20
30
40
50
60

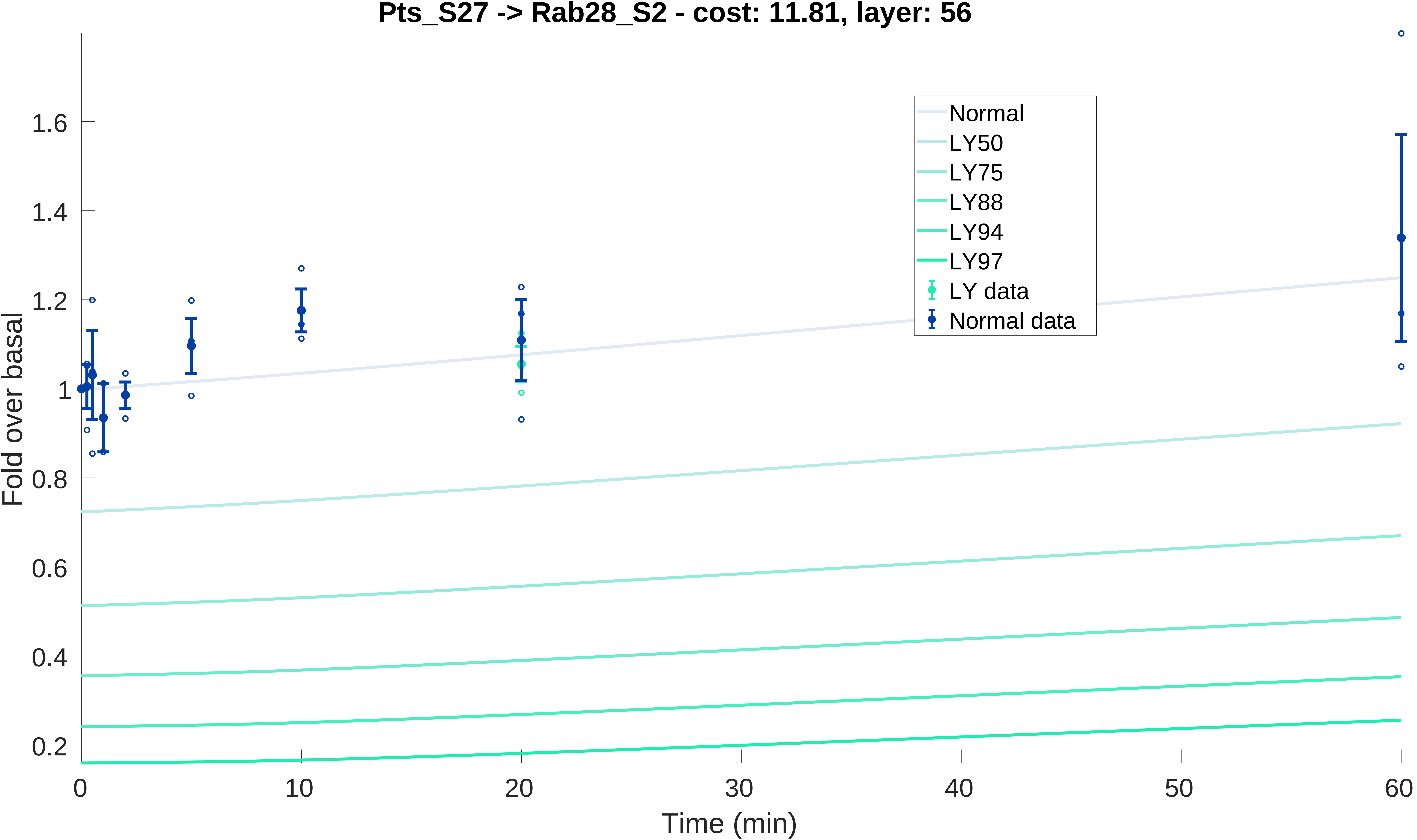

Pts_S27 -> Rab28_S2 - cost: 11.81, layer: 56
Fold over basal
Time (min)
0.2
0.4
0.6
0.8
1
1.2
1.4
1.6
0
10
20
30
40
50
60
Normal
LY50
LY75
LY88
LY94
LY97
LY data
Normal data

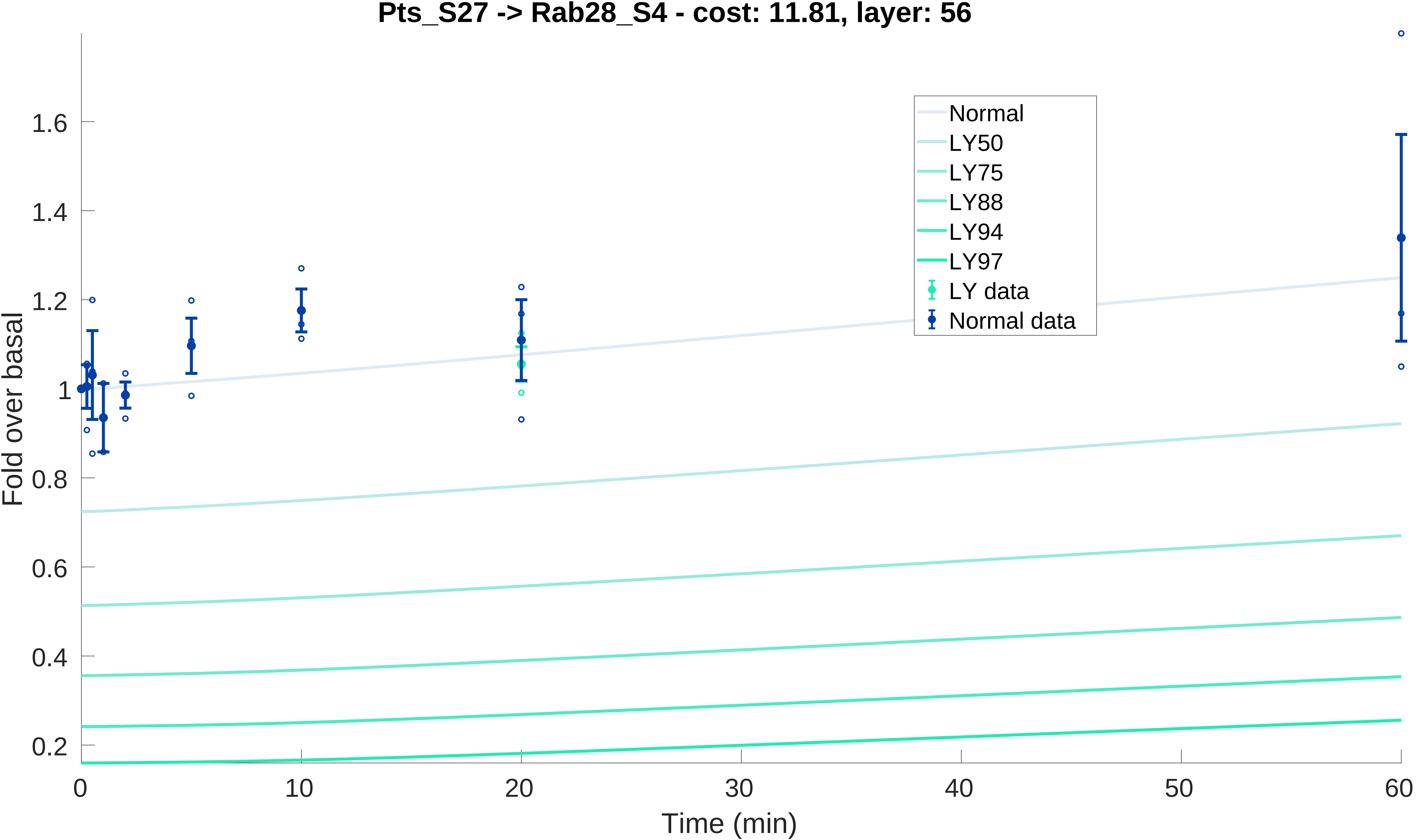

Pts_S27 -> Rab28_S4 - cost: 11.81, layer: 56
Fold over basal
Time (min)
1.6
1.4
1.2
1
0.8
0.6
0.4
0.2
0
10
20
30
40
50
60
Normal
LY50
LY75
LY88
LY94
LY97
LY data
Normal data

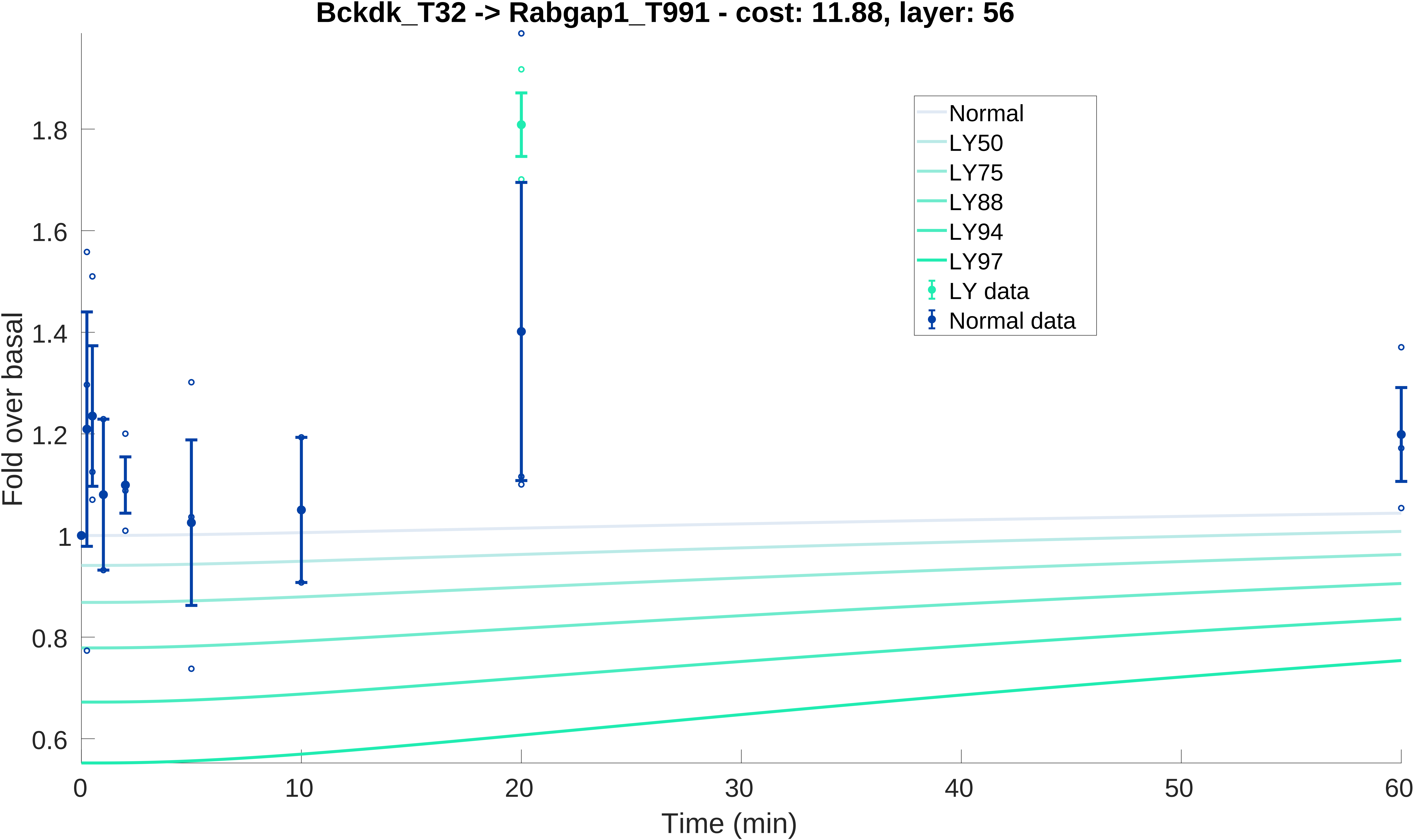

Bckdk_T32 -> Rabgap1_T991 - cost: 11.88, layer: 56
Fold over basal
Time (min)
0.6
0.8
1
1.2
1.4
1.6
1.8
0
10
20
30
40
50
60
Normal
LY50
LY75
LY88
LY94
LY97
LY data
Normal data

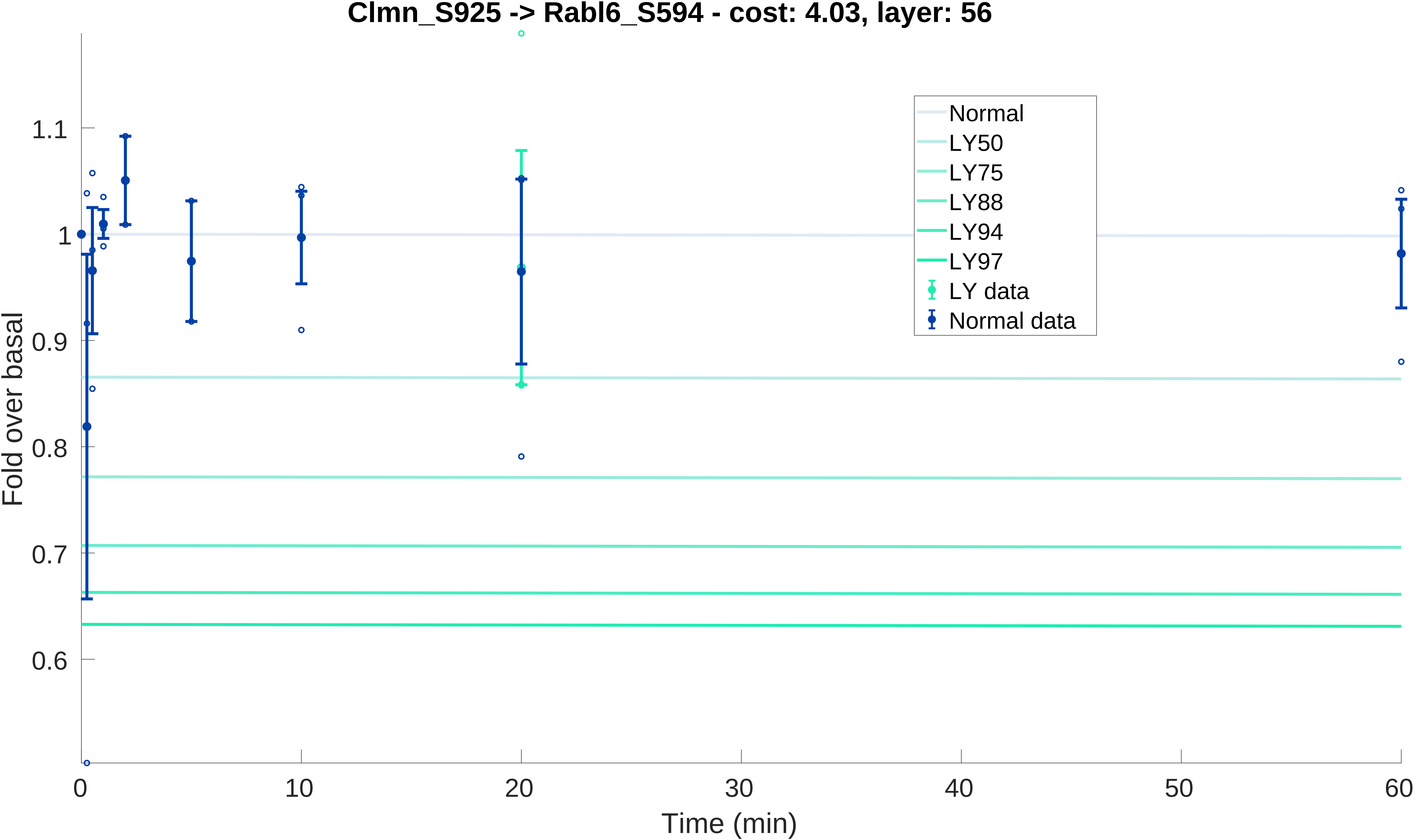

Clmn_S925 -> Rabl6_S594 - cost: 4.03, layer: 56
Fold over basal
Time (min)
1.1
1
0.9
0.8
0.7
0.6
0
10
20
30
40
50
60
Normal
LY50
LY75
LY88
LY94
LY97
LY data
Normal data

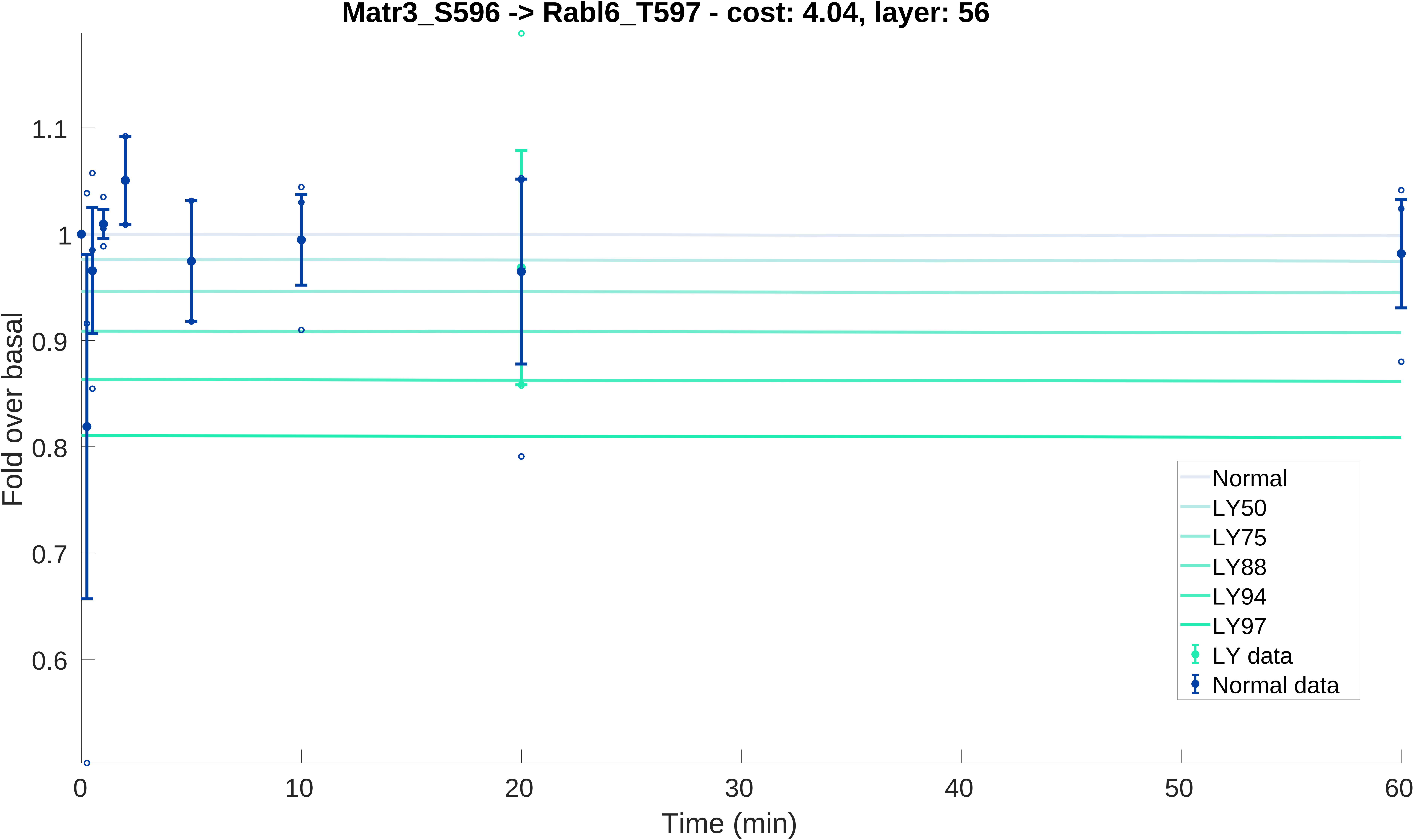

Matr3_S596 -> Rabl6_T597 - cost: 4.04, layer: 56
Fold over basal
1.1
1
0.9
0.8
0.7
0.6
0
10
20
30
40
50
60
Time (min)
Normal
LY50
LY75
LY88
LY94
LY97
LY data
Normal data

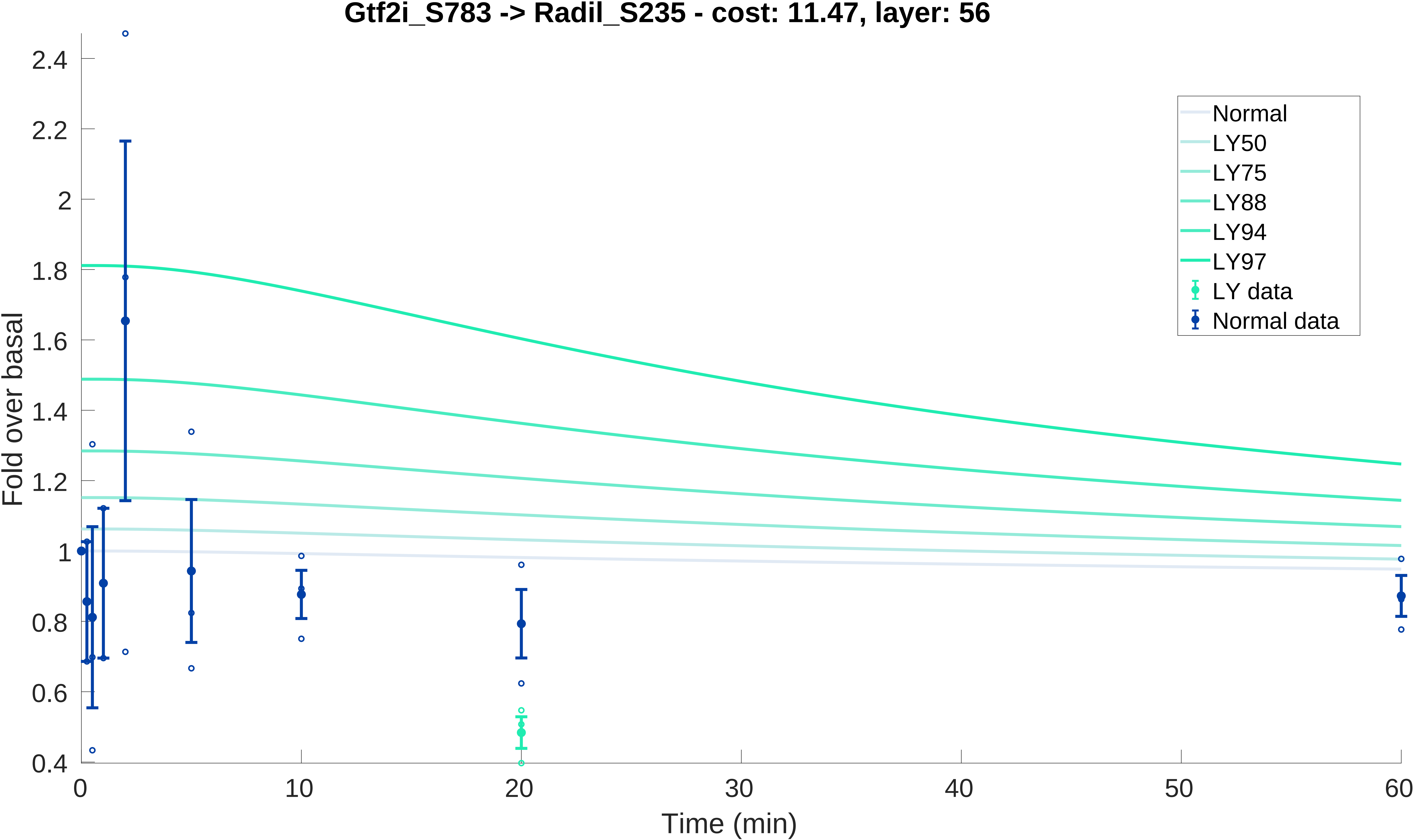

Gtf2i_S783 -> Radil_S235 - cost: 11.47, layer: 56
Fold over basal
Time (min)
0.4
0.6
0.8
1
1.2
1.4
1.6
1.8
2
2.2
2.4
0
10
20
30
40
50
60
Normal
LY50
LY75
LY88
LY94
LY97
LY data
Normal data

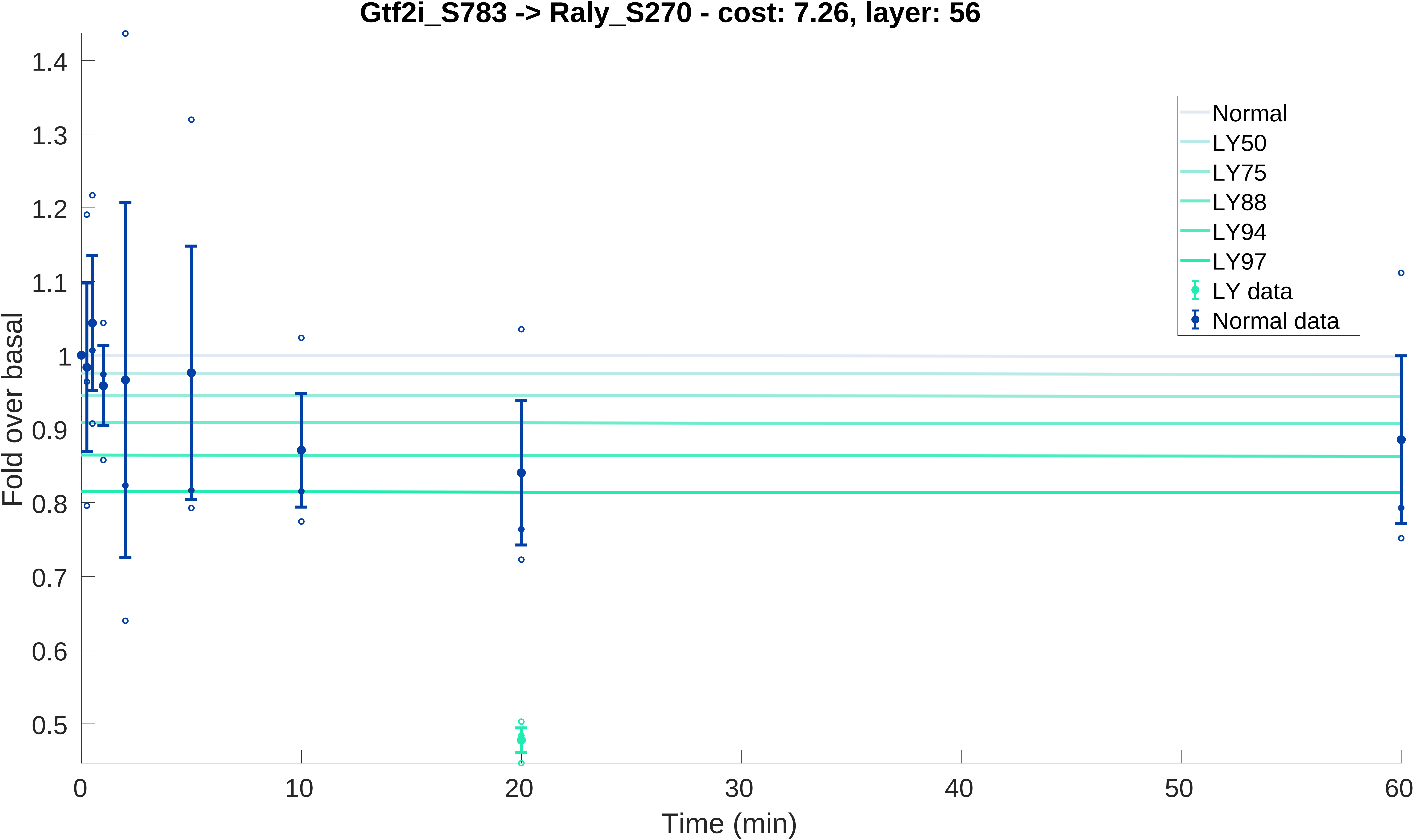
Gtf2i_S783 -> Raly_S270 - cost: 7.26, layer: 56
Fold over basal
Time (min)
1.4
1.3
1.2
1.1
1
0.9
0.8
0.7
0.6
0.5
0
10
20
30
40
50
60
Normal
LY50
LY75
LY88
LY94
LY97
LY data
Normal data

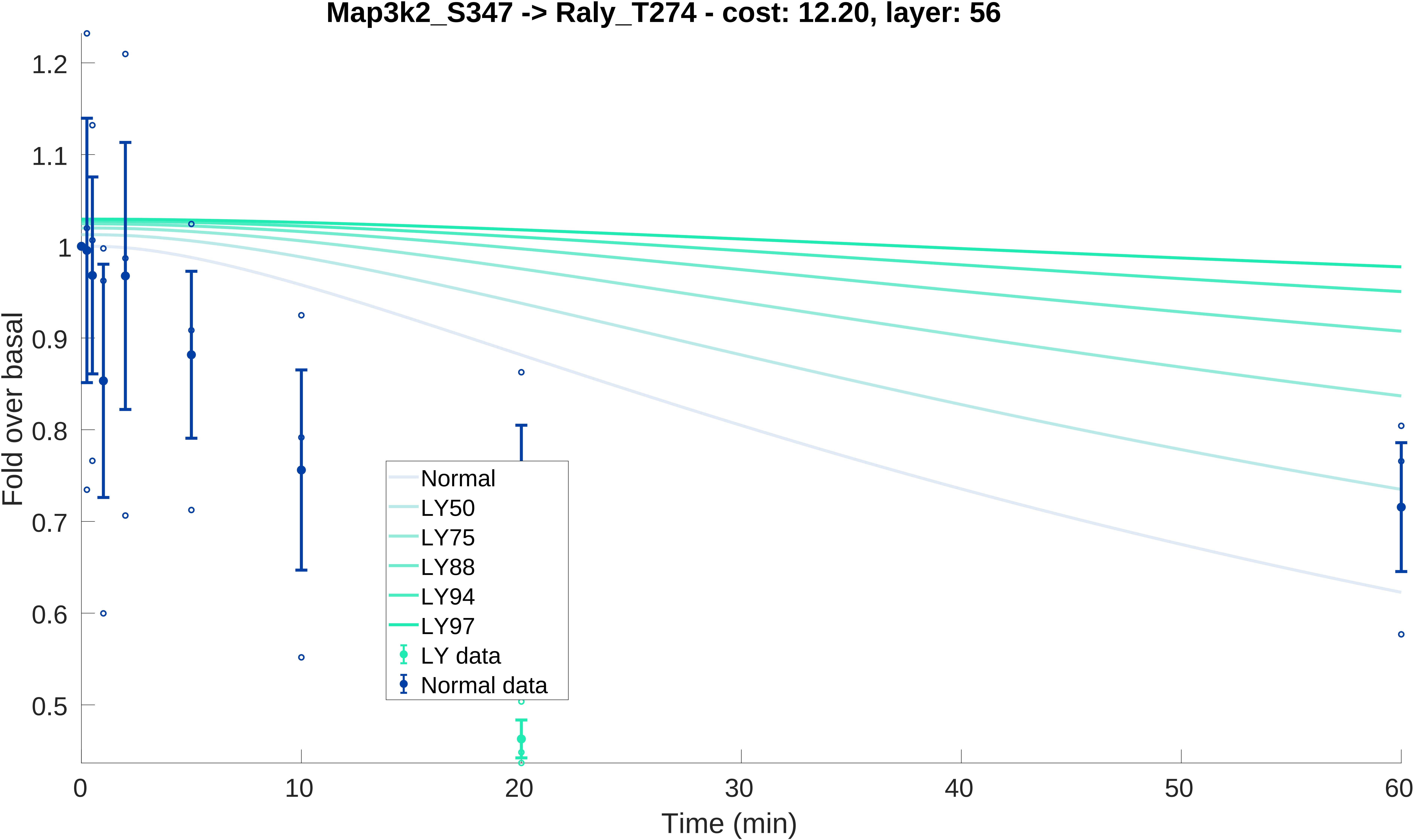
Map3k2_S347 -> Raly_T274 - cost: 12.20, layer: 56
Fold over basal
Time (min)
1.2
1.1
1
0.9
0.8
0.7
0.6
0.5
0
10
20
30
40
50
60
Normal
LY50
LY75
LY88
LY94
LY97
LY data
Normal data

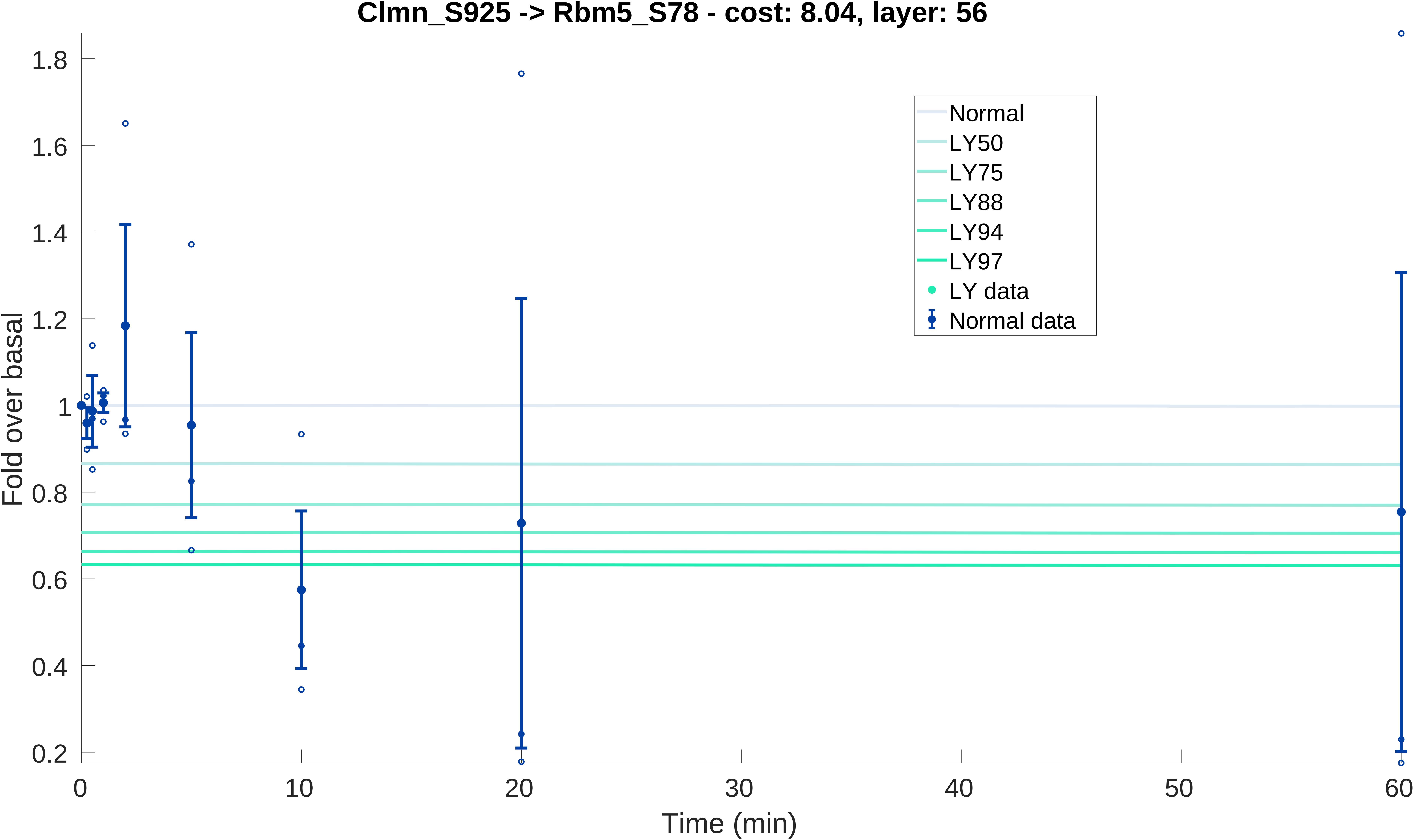

Clmn_S925 -> Rbm5_S78 - cost: 8.04, layer: 56
Fold over basal
Time (min)
Normal
LY50
LY75
LY88
LY94
LY97
LY data
Normal data
0.2
0.4
0.6
0.8
1
1.2
1.4
1.6
1.8
0
10
20
30
40
50
60

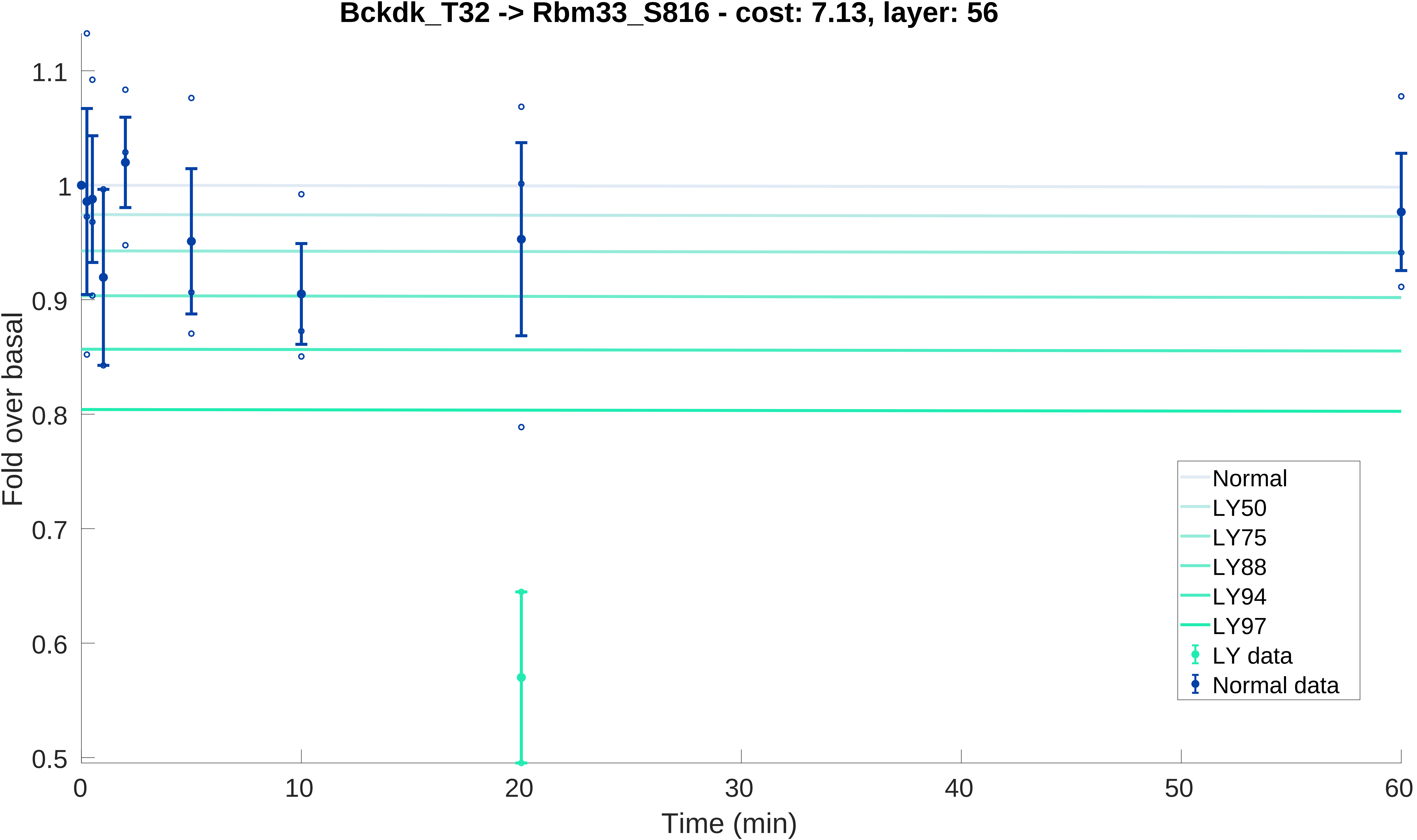

Bckdk_T32 -> Rbm33_S816 - cost: 7.13, layer: 56
Fold over basal
Time (min)
0.5
0.6
0.7
0.8
0.9
1
1.1
0
10
20
30
40
50
60
Normal
LY50
LY75
LY88
LY94
LY97
LY data
Normal data

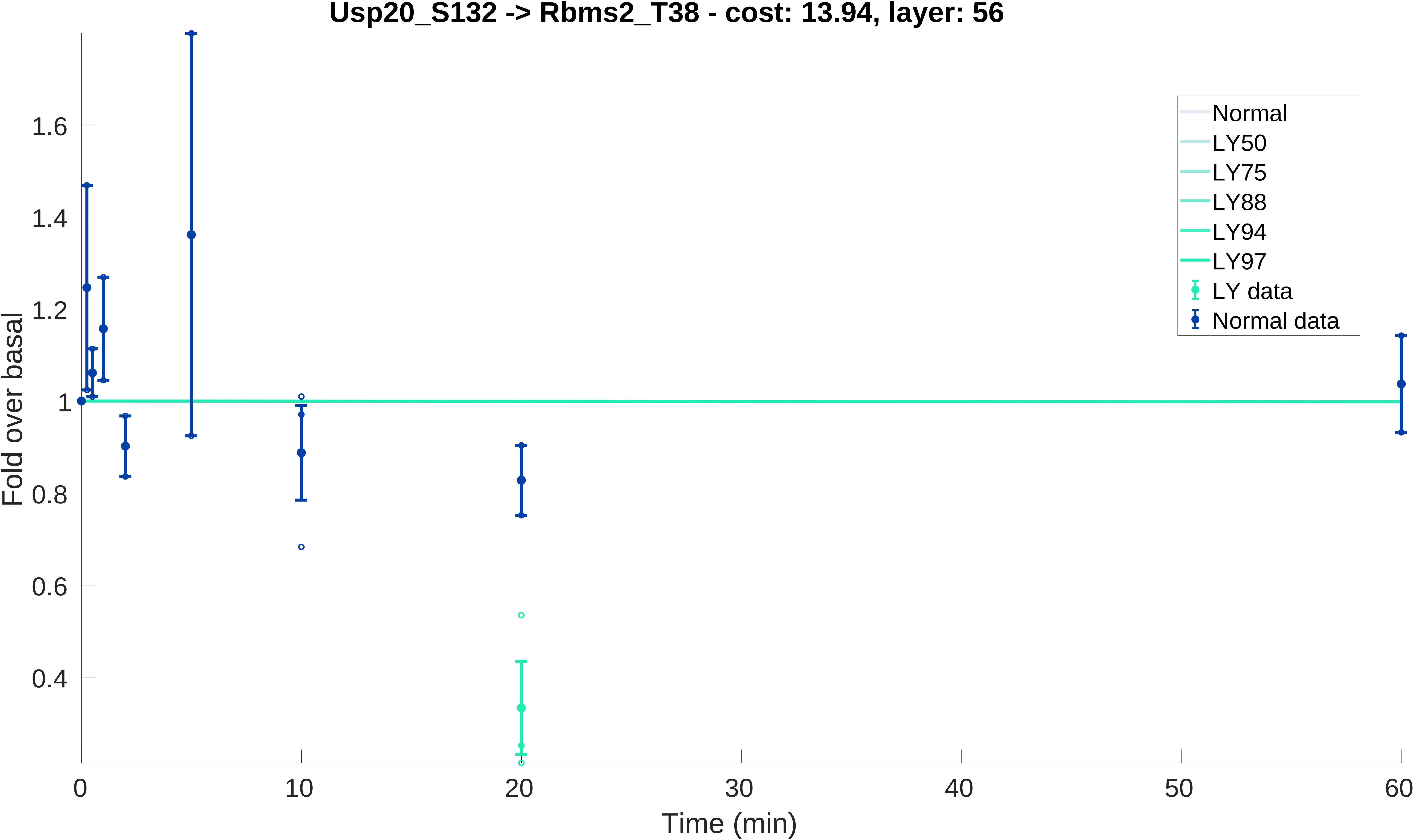
Usp20_S132 -> Rbms2_T38 - cost: 13.94, layer: 56
Fold over basal
1.6
1.4
1.2
1
0.8
0.6
0.4
0
10
20
30
40
50
60
Time (min)
Normal
LY50
LY75
LY88
LY94
LY97
LY data
Normal data

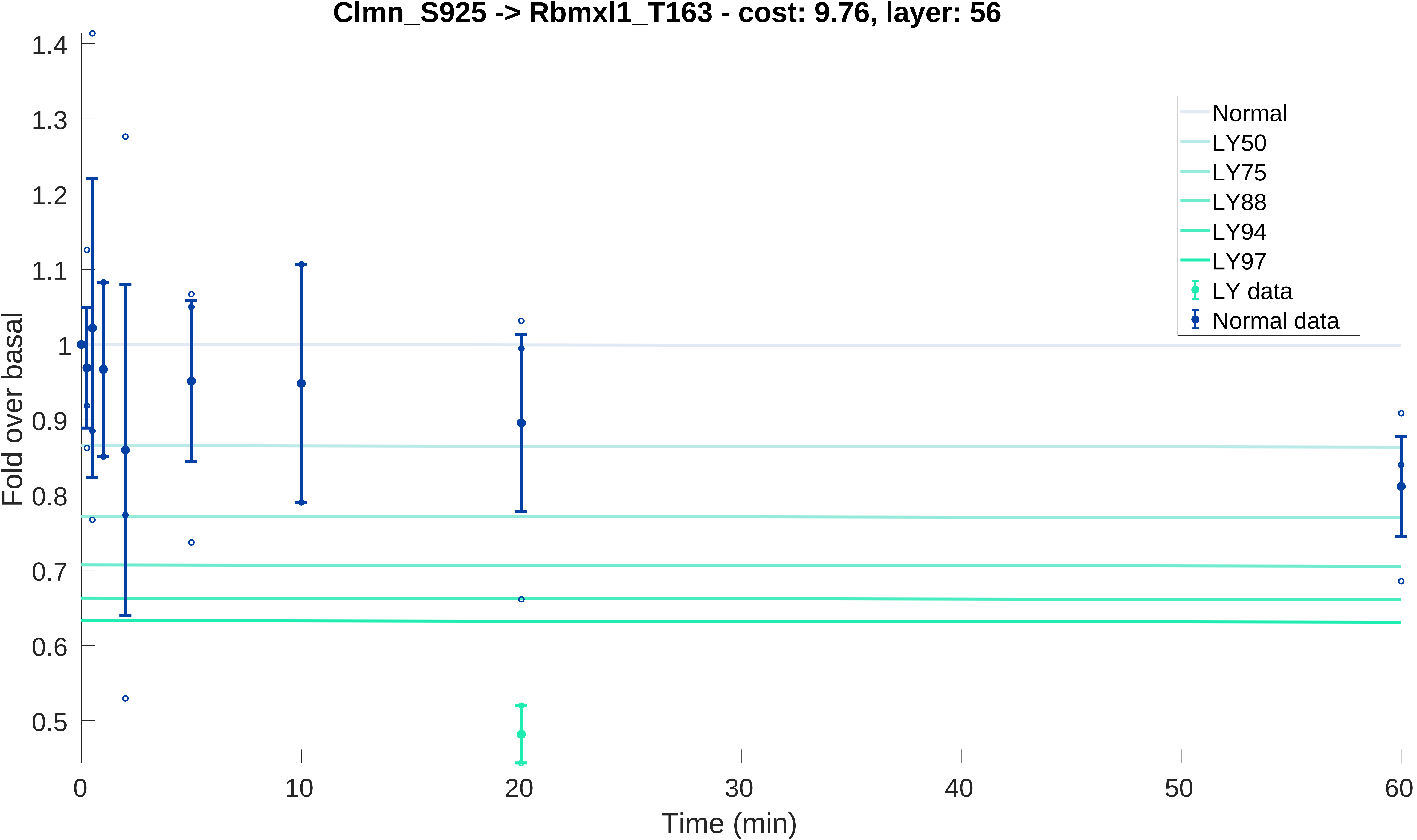

Clmn_S925 -> Rbmxl1_T163 - cost: 9.76, layer: 56
Fold over basal
Time (min)
0
10
20
30
40
50
60
0.5
0.6
0.7
0.8
0.9
1
1.1
1.2
1.3
1.4
Normal
LY50
LY75
LY88
LY94
LY97
LY data
Normal data

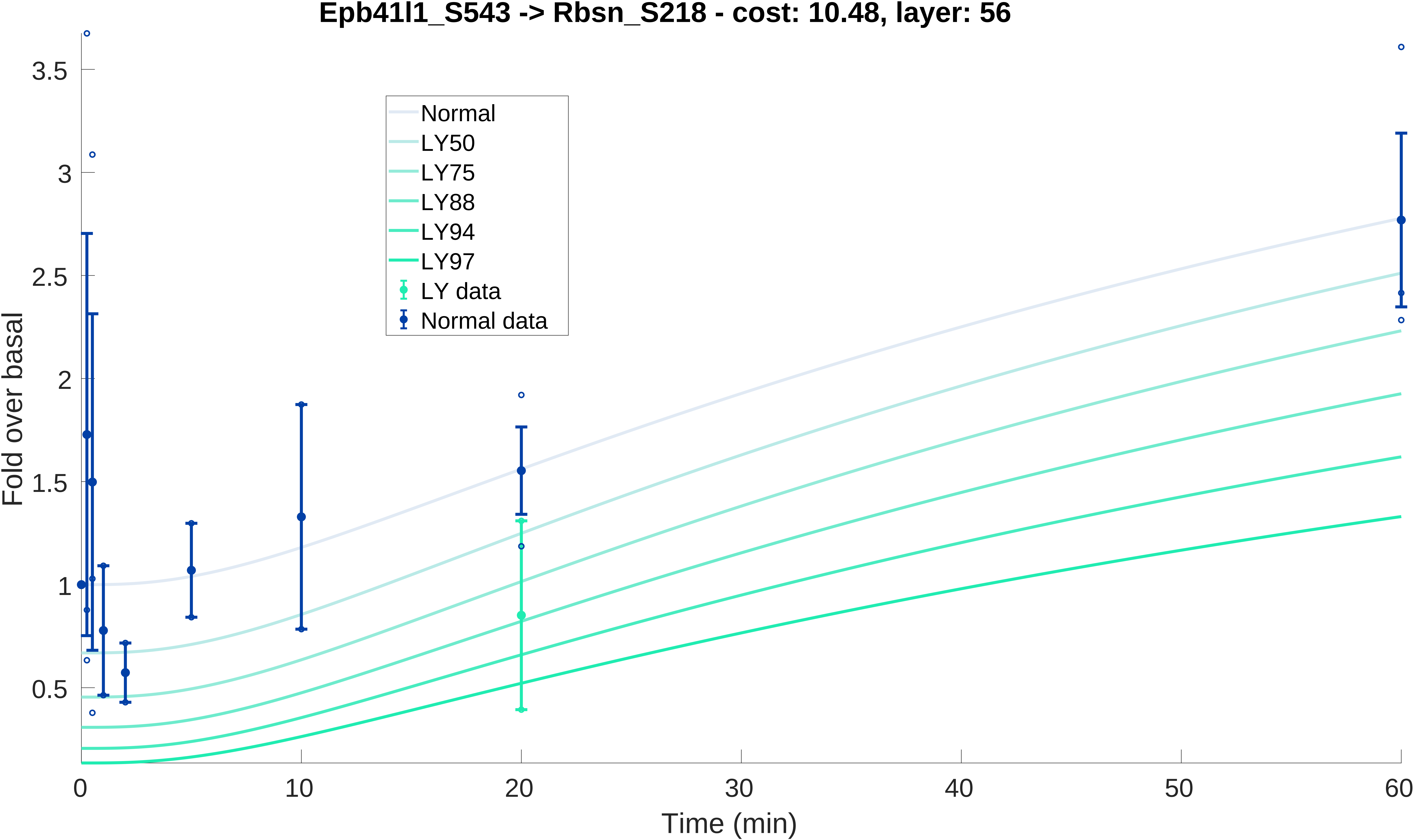

Epb41l1_S543 -> Rbsn_S218 - cost: 10.48, layer: 56
Normal
LY50
LY75
LY88
LY94
LY97
LY data
Normal data
Fold over basal
Time (min)
0.5
1
1.5
2
2.5
3
3.5
0
10
20
30
40
50
60

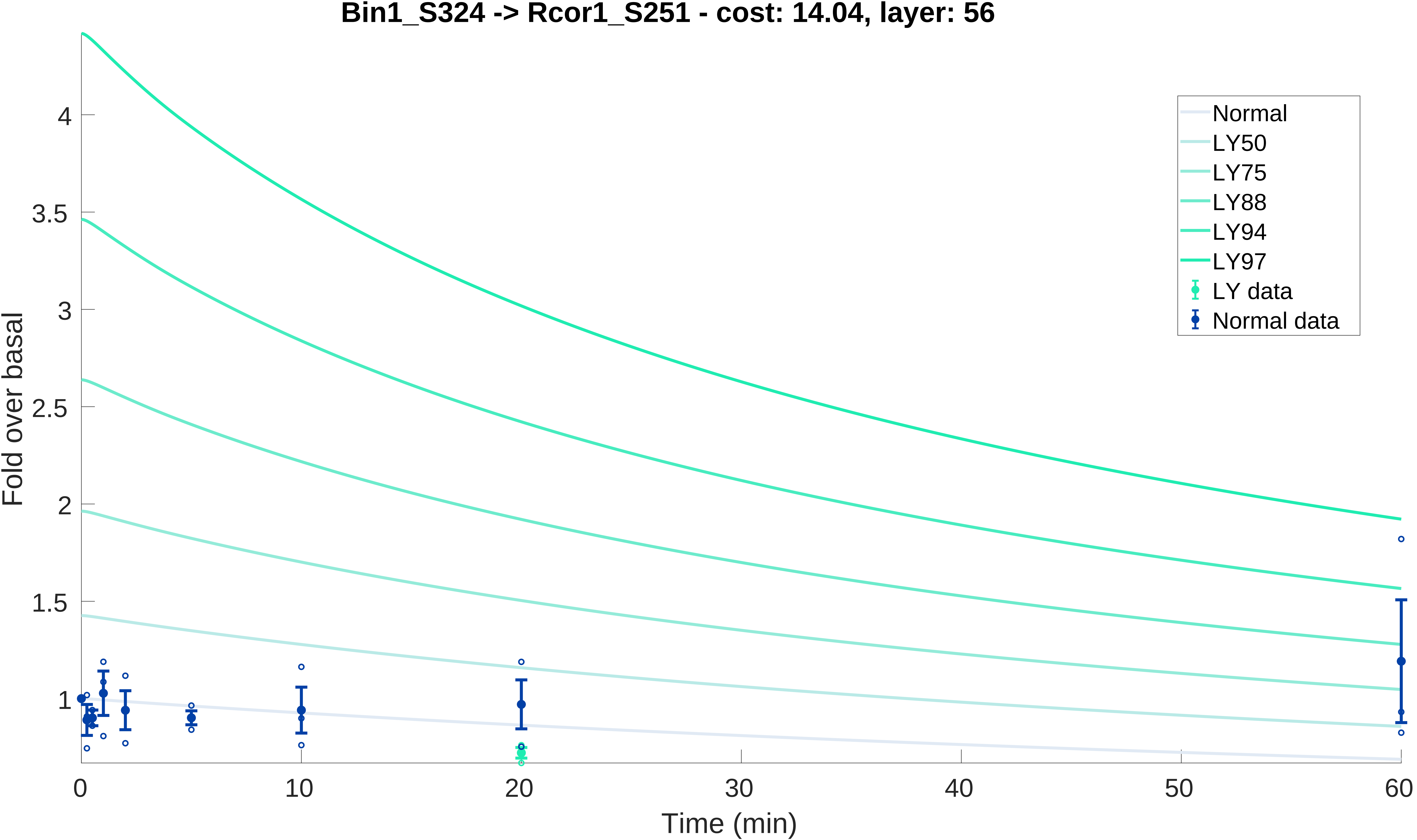

Bin1_S324 -> Rcor1_S251 - cost: 14.04, layer: 56
Fold over basal
4
3.5
3
2.5
2
1.5
1
0
10
20
30
40
50
60
Time (min)
Normal
LY50
LY75
LY88
LY94
LY97
LY data
Normal data

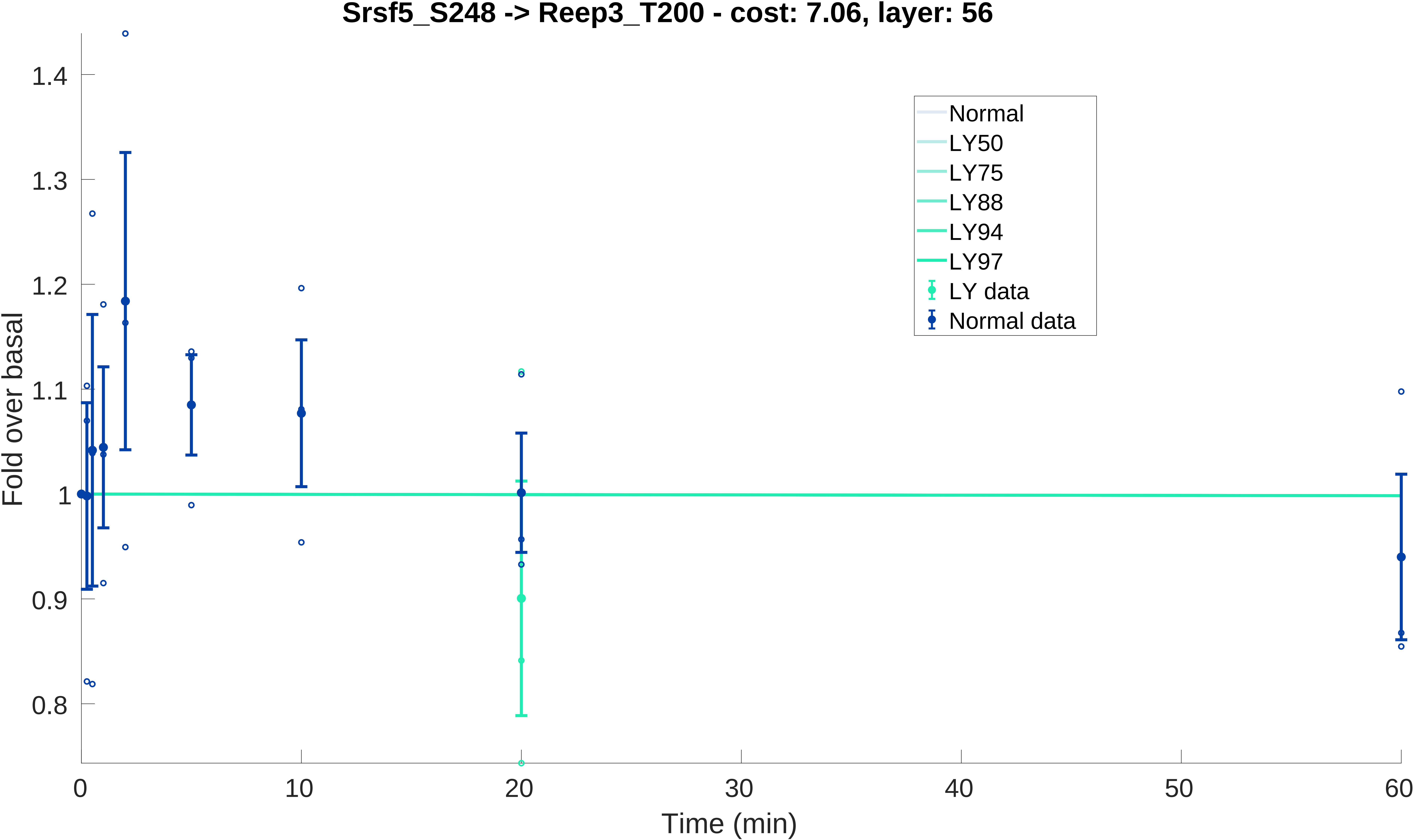

Srsf5_S248 -> Reep3_T200 - cost: 7.06, layer: 56
Fold over basal
1.4
1.3
1.2
1.1
1
0.9
0.8
0
10
20
30
40
50
60
Time (min)
Normal
LY50
LY75
LY88
LY94
LY97
LY data
Normal data

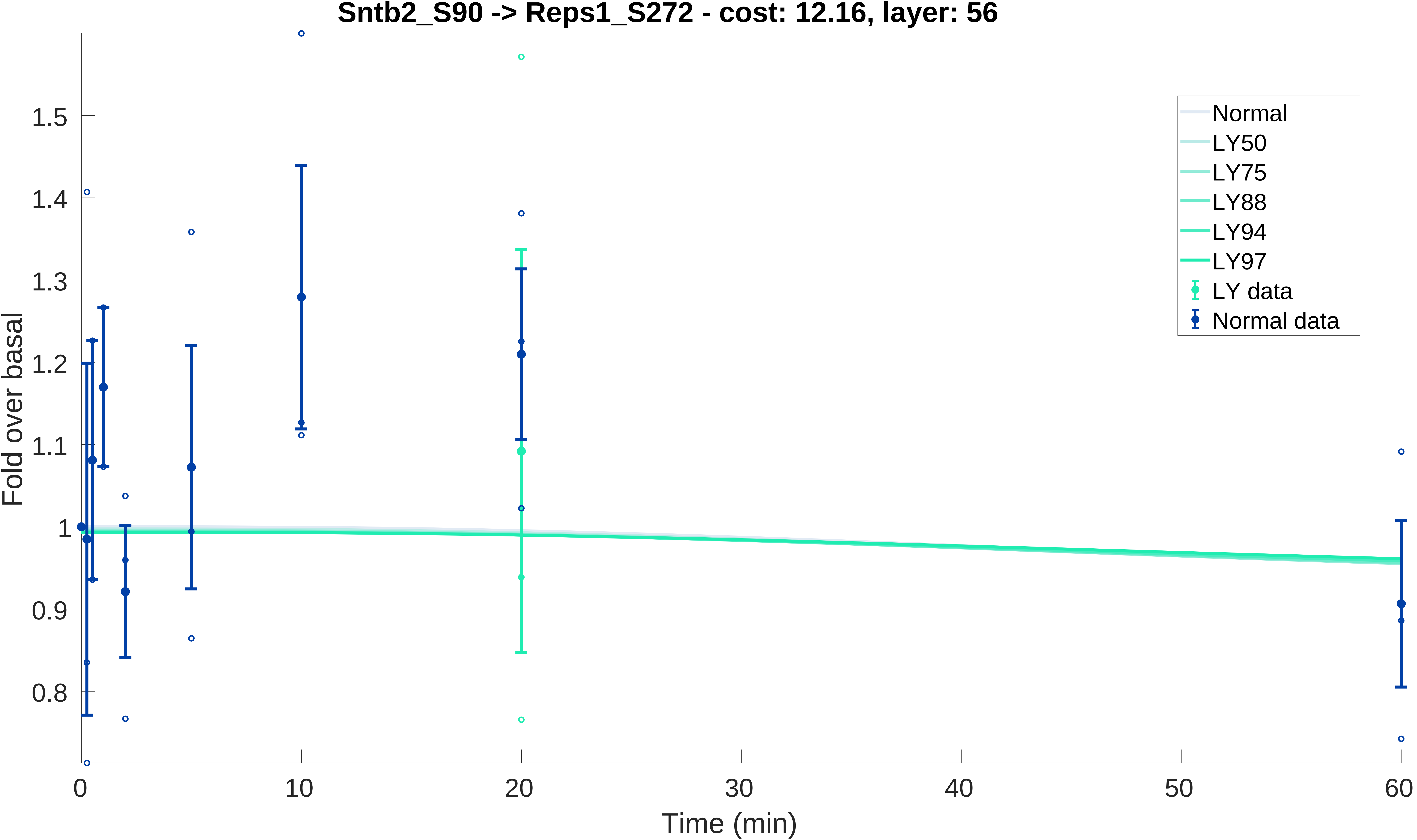

Sntb2_S90 -> Reps1_S272 - cost: 12.16, layer: 56
Fold over basal
Time (min)
1.5
1.4
1.3
1.2
1.1
1
0.9
0.8
0
10
20
30
40
50
60
Normal
LY50
LY75
LY88
LY94
LY97
LY data
Normal data

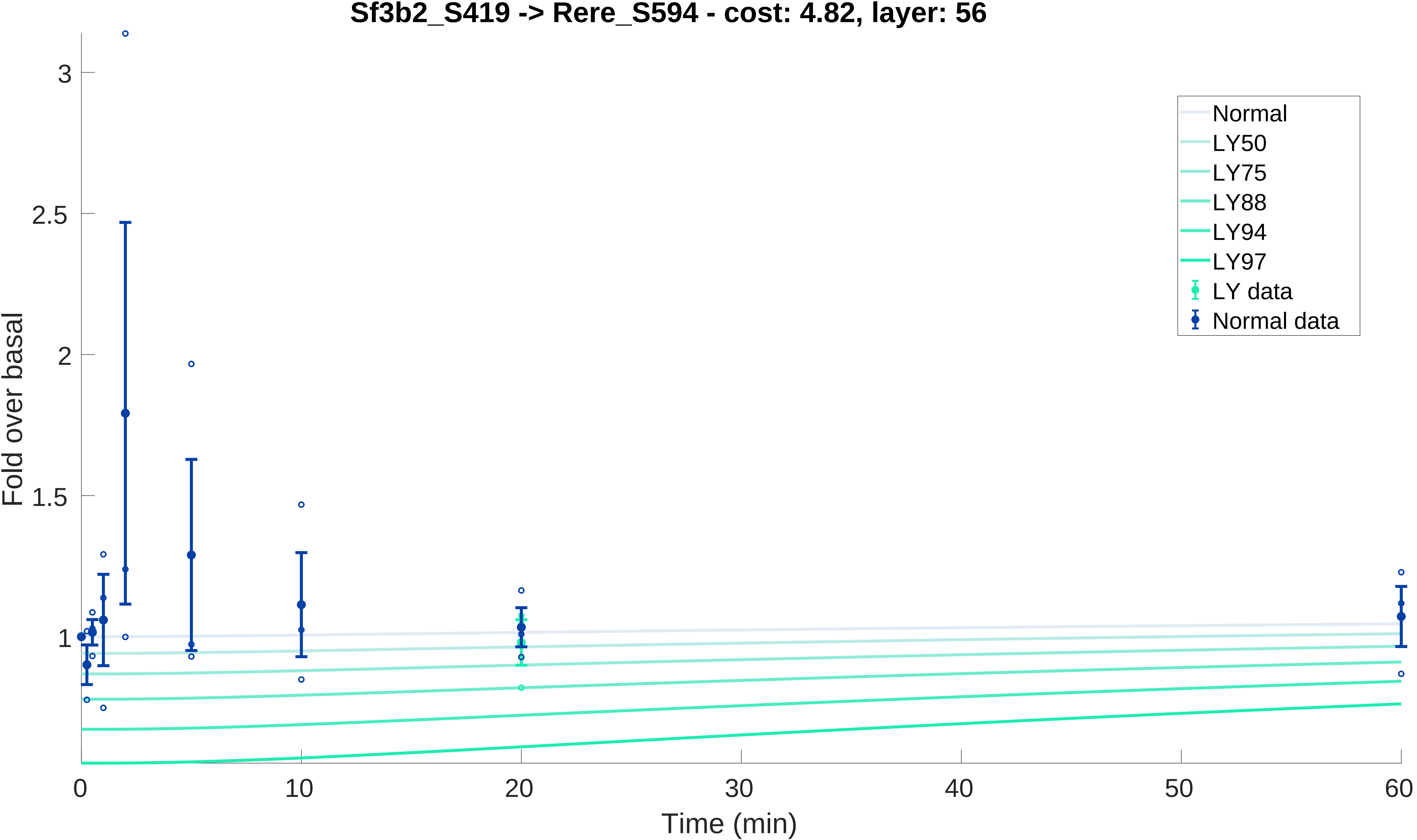
Sf3b2_S419 -> Rere_S594 - cost: 4.82, layer: 56
Fold over basal
Time (min)
Normal
LY50
LY75
LY88
LY94
LY97
LY data
Normal data
0
10
20
30
40
50
60
1
1.5
2
2.5
3

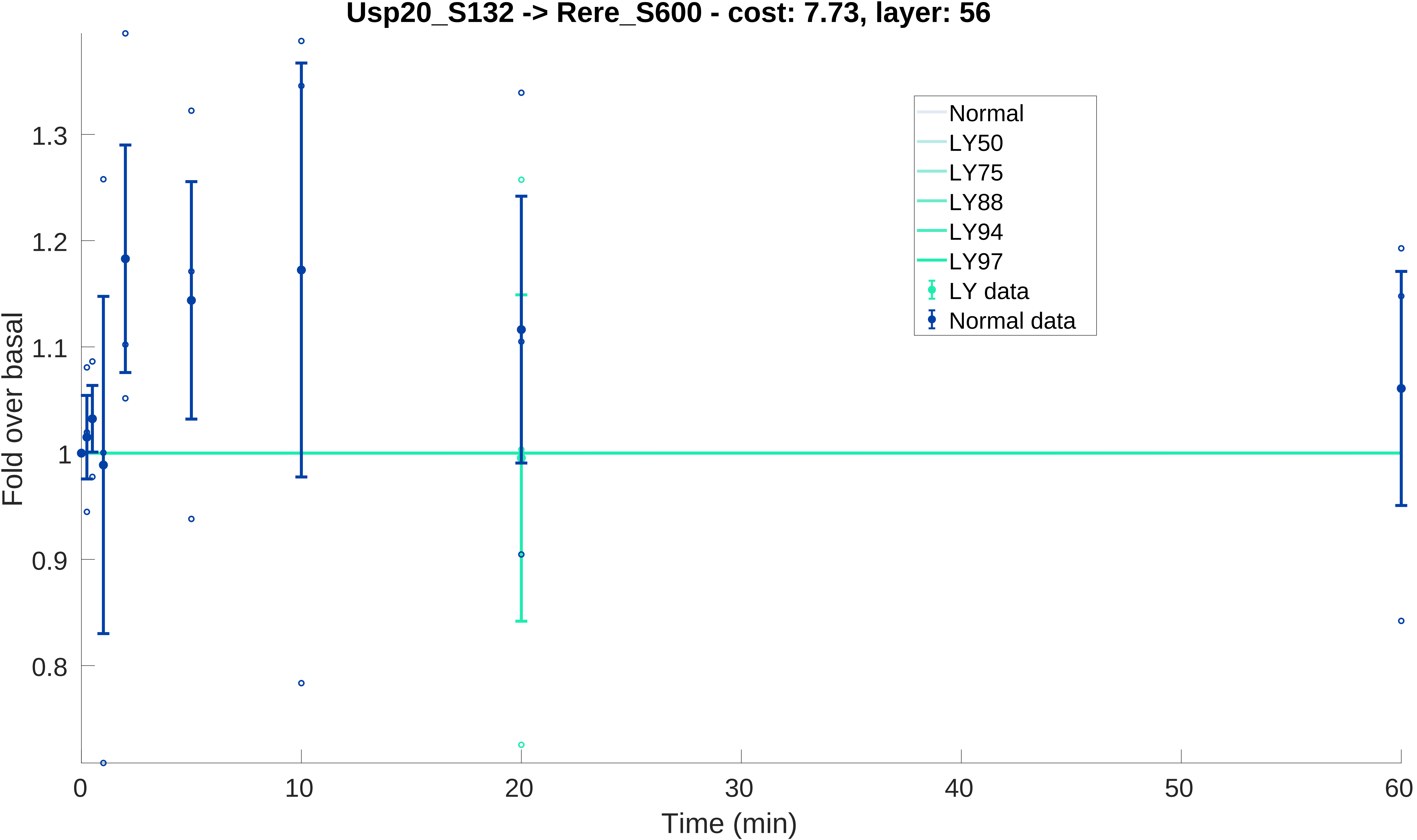

Usp20_S132 -> Rere_S600 - cost: 7.73, layer: 56
Fold over basal
Time (min)
1.3
1.2
1.1
1
0.9
0.8
0
10
20
30
40
50
60
Normal
LY50
LY75
LY88
LY94
LY97
LY data
Normal data

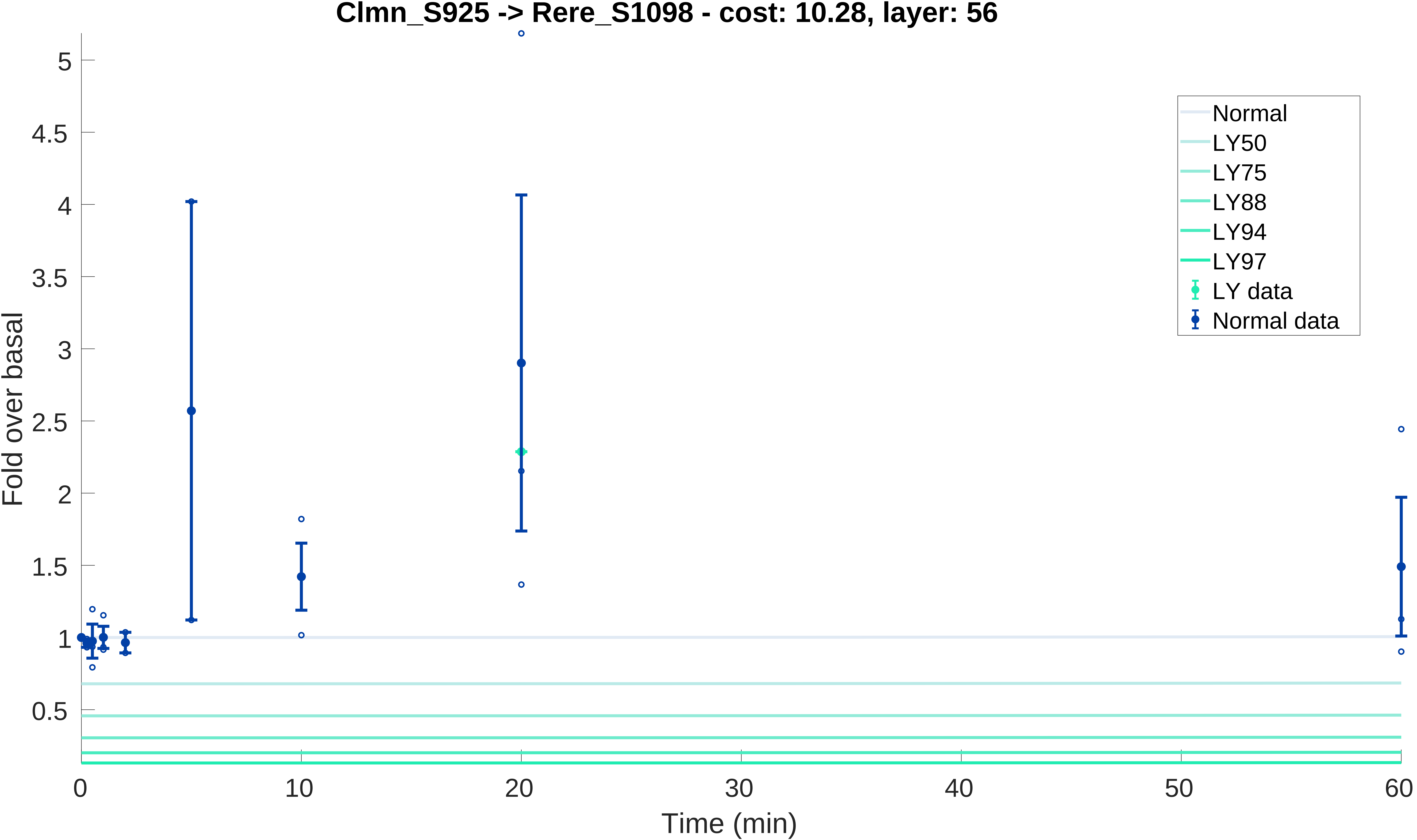

Clmn_S925 -> Rere_S1098 - cost: 10.28, layer: 56
Fold over basal
Time (min)
0
10
20
30
40
50
60
0.5
1
1.5
2
2.5
3
3.5
4
4.5
5
Normal
LY50
LY75
LY88
LY94
LY97
LY data
Normal data

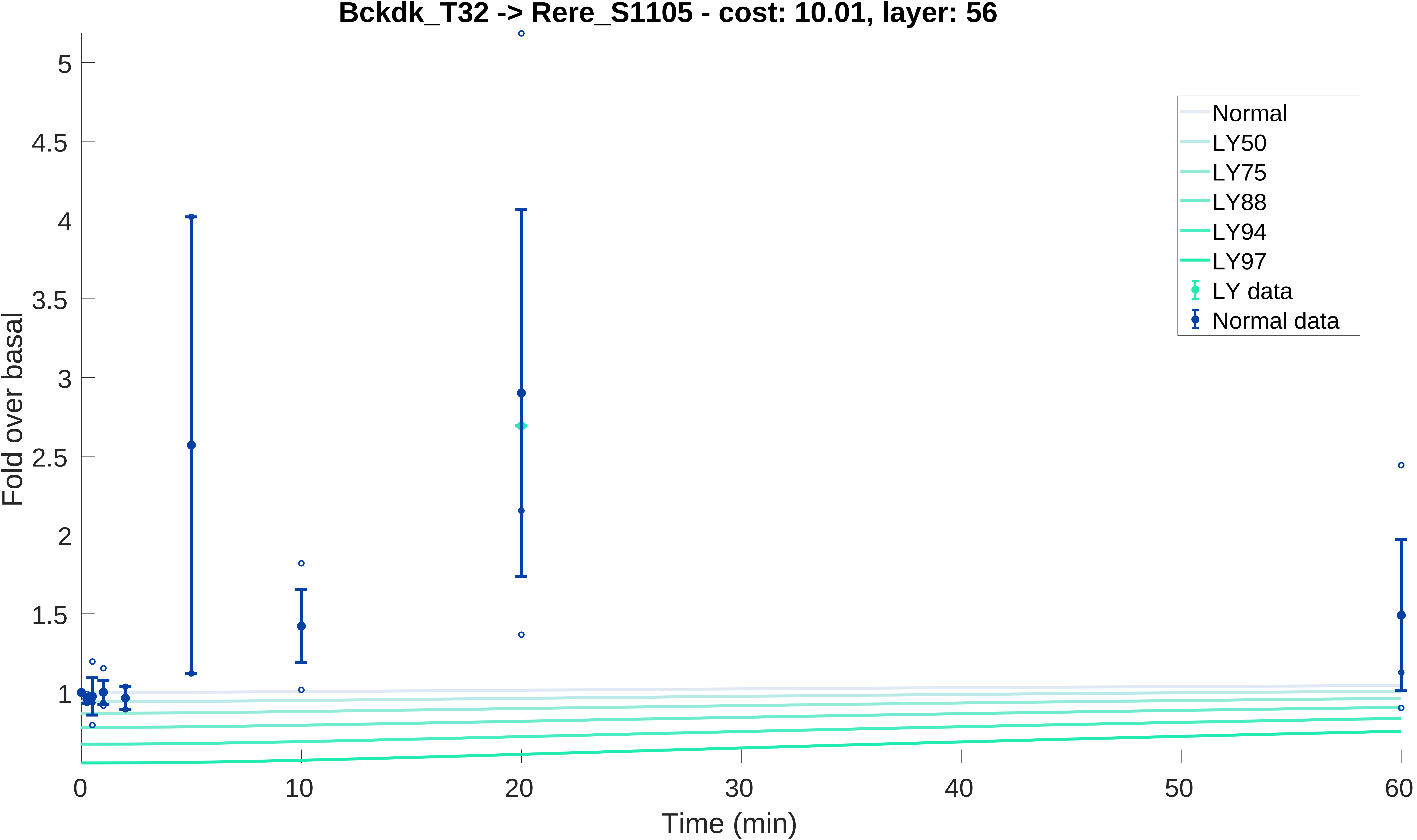

Bckdk_T32 -> Rere_S1105 - cost: 10.01, layer: 56
Fold over basal
Time (min)
0
10
20
30
40
50
60
1
1.5
2
2.5
3
3.5
4
4.5
5
Normal
LY50
LY75
LY88
LY94
LY97
LY data
Normal data

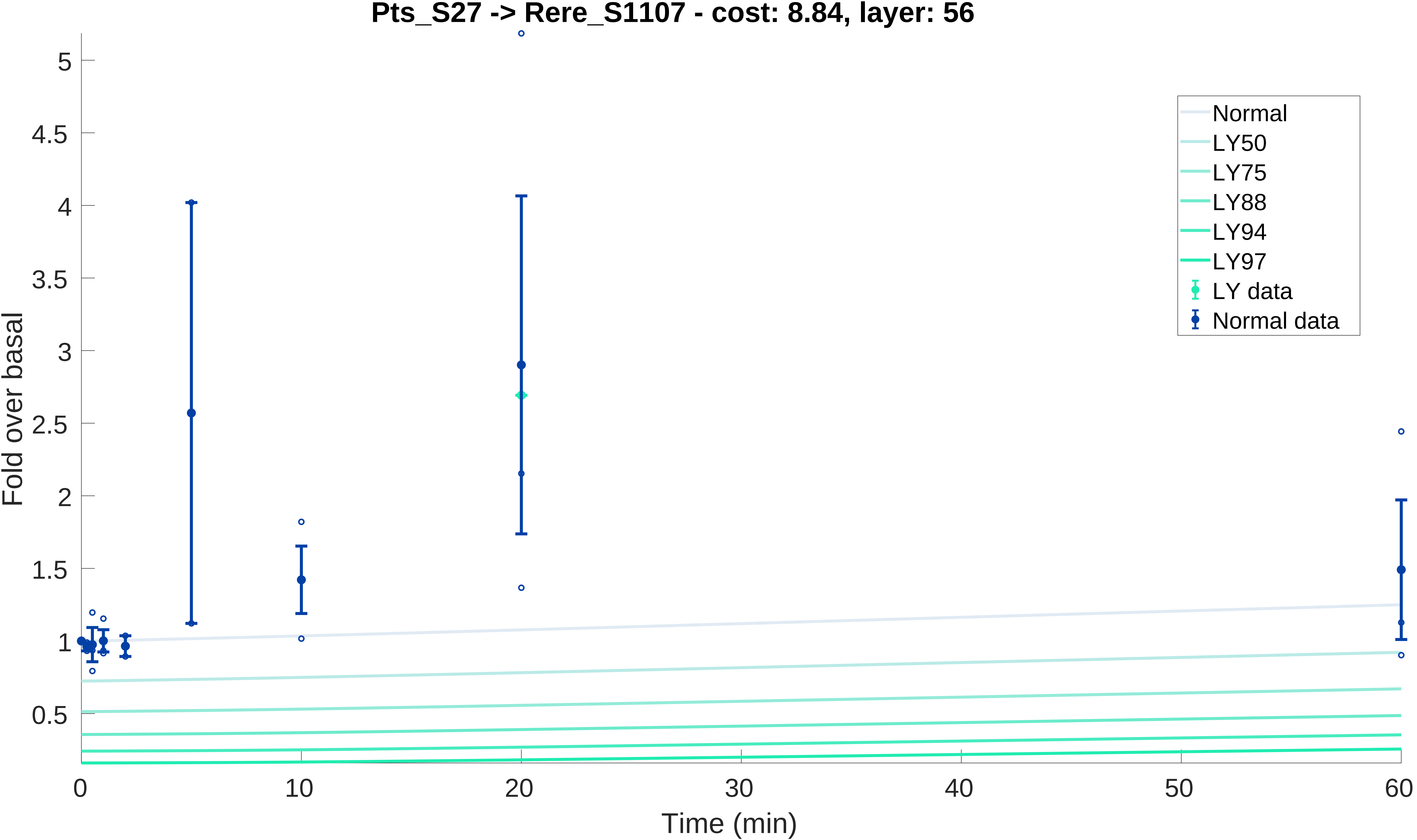

Pts_S27 -> Rere_S1107 - cost: 8.84, layer: 56
Fold over basal
Time (min)
0
10
20
30
40
50
60
0.5
1
1.5
2
2.5
3
3.5
4
4.5
5
Normal
LY50
LY75
LY88
LY94
LY97
LY data
Normal data

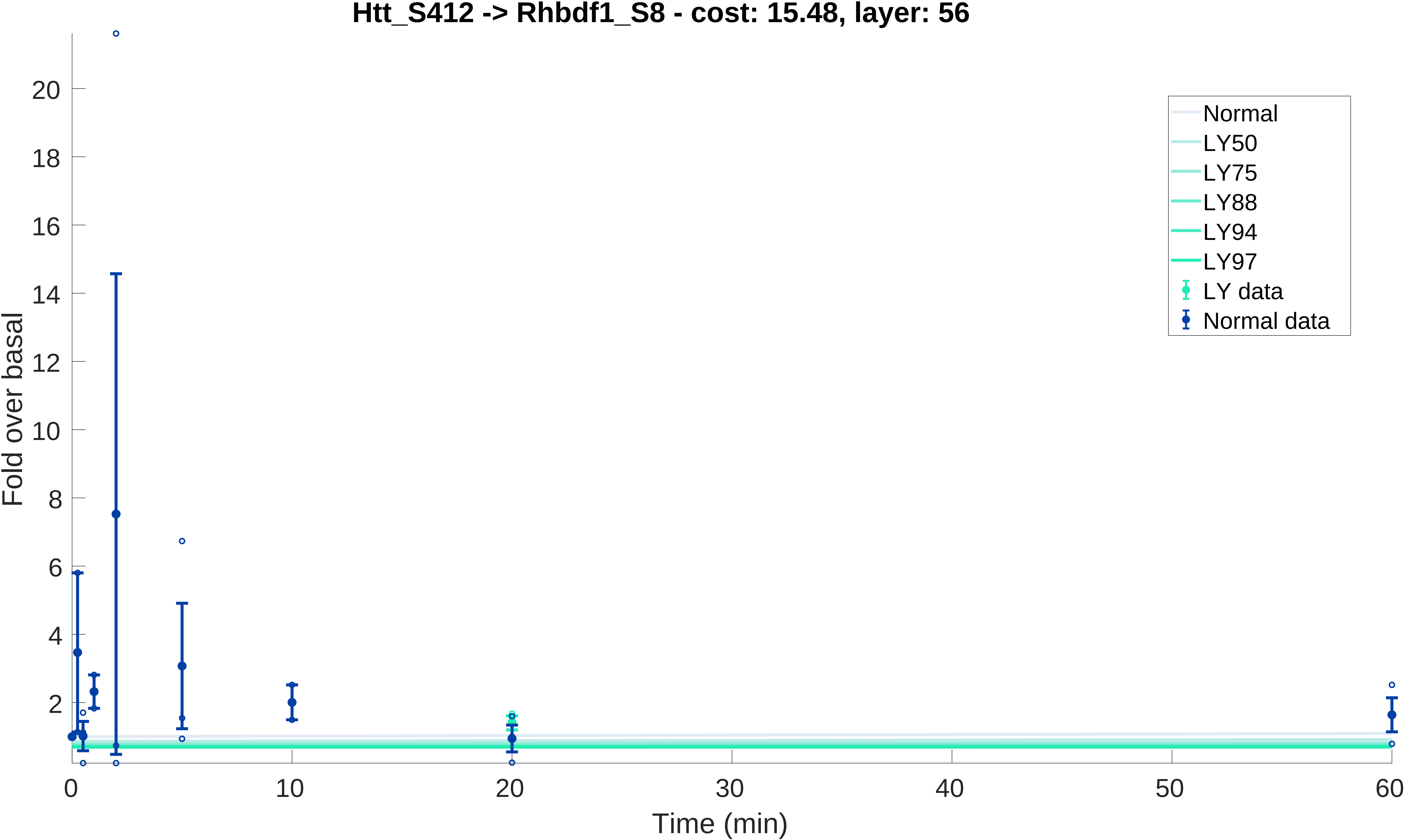

Htt_S412 -> Rhbdf1_S8 - cost: 15.48, layer: 56
Fold over basal
Time (min)
Normal
LY50
LY75
LY88
LY94
LY97
LY data
Normal data
0
10
20
30
40
50
60
2
4
6
8
10
12
14
16
18
20

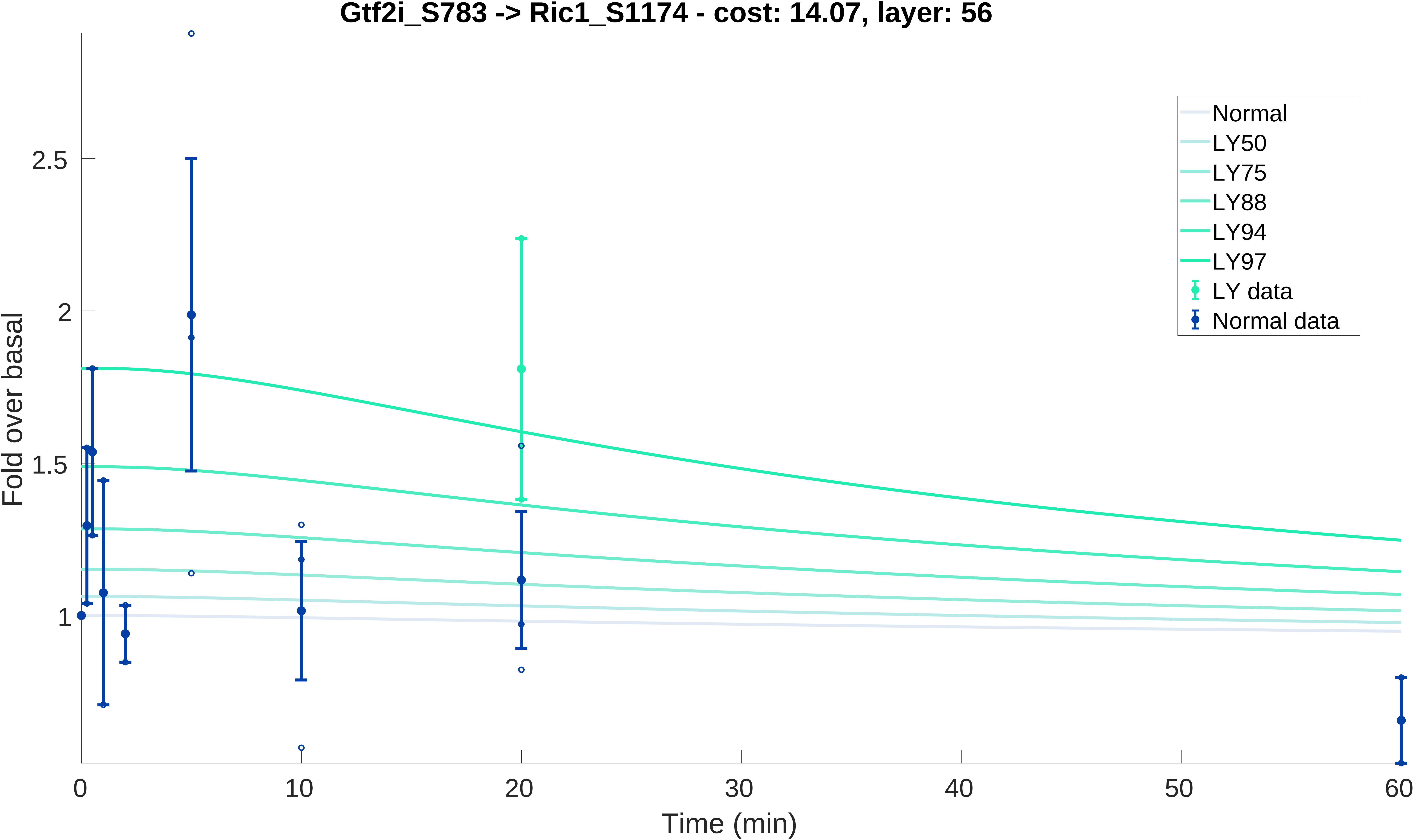
Gtf2i_S783 -> Ric1_S1174 - cost: 14.07, layer: 56
Fold over basal
2.5
2
1.5
1
0
10
20
30
40
50
60
Time (min)
Normal
LY50
LY75
LY88
LY94
LY97
LY data
Normal data

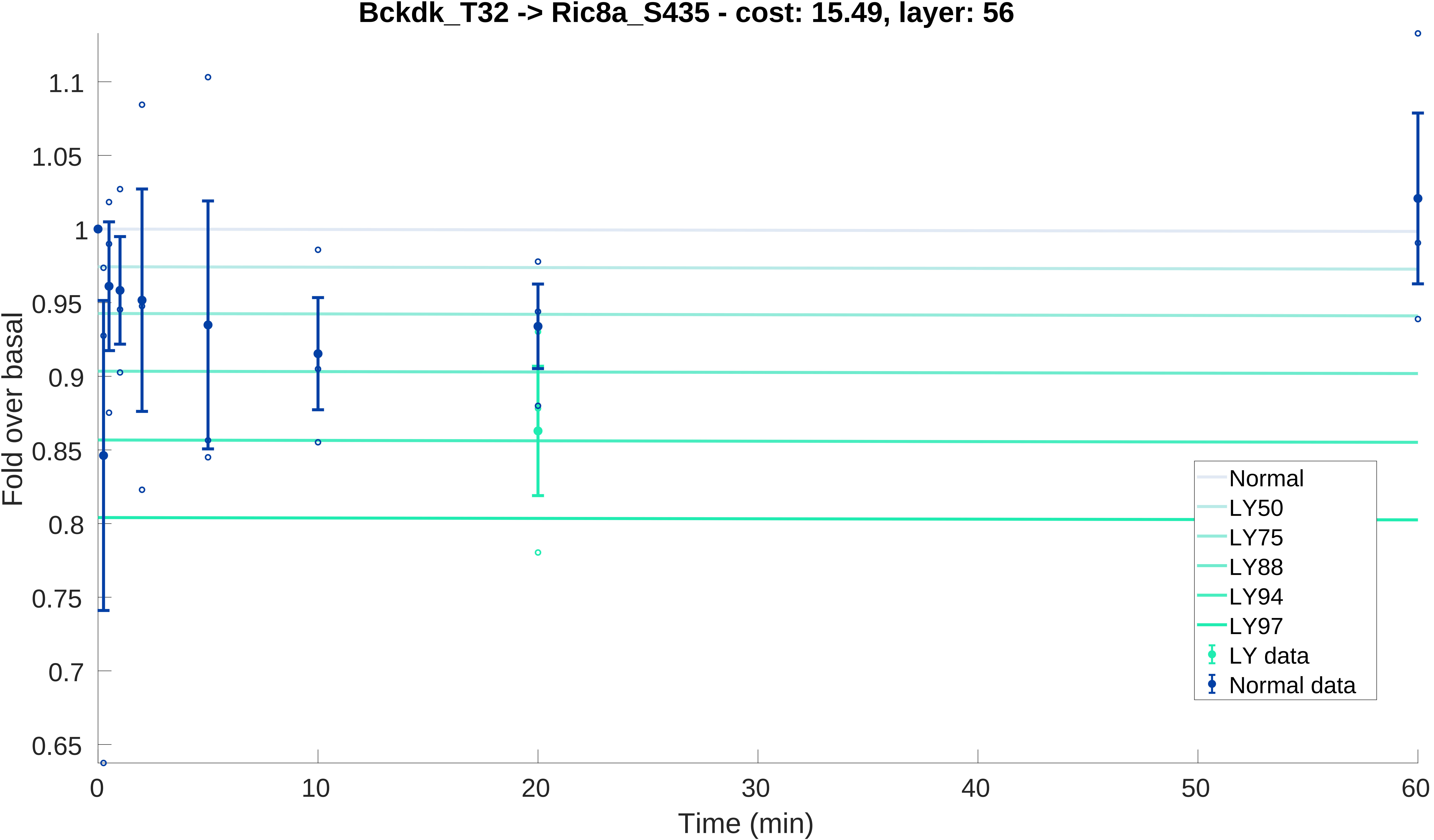

Bckdk_T32 -> Ric8a_S435 - cost: 15.49, layer: 56
Fold over basal
1.1
1.05
1
0.95
0.9
0.85
0.8
0.75
0.7
0.65
0
10
20
30
40
50
60
Time (min)
Normal
LY50
LY75
LY88
LY94
LY97
LY data
Normal data

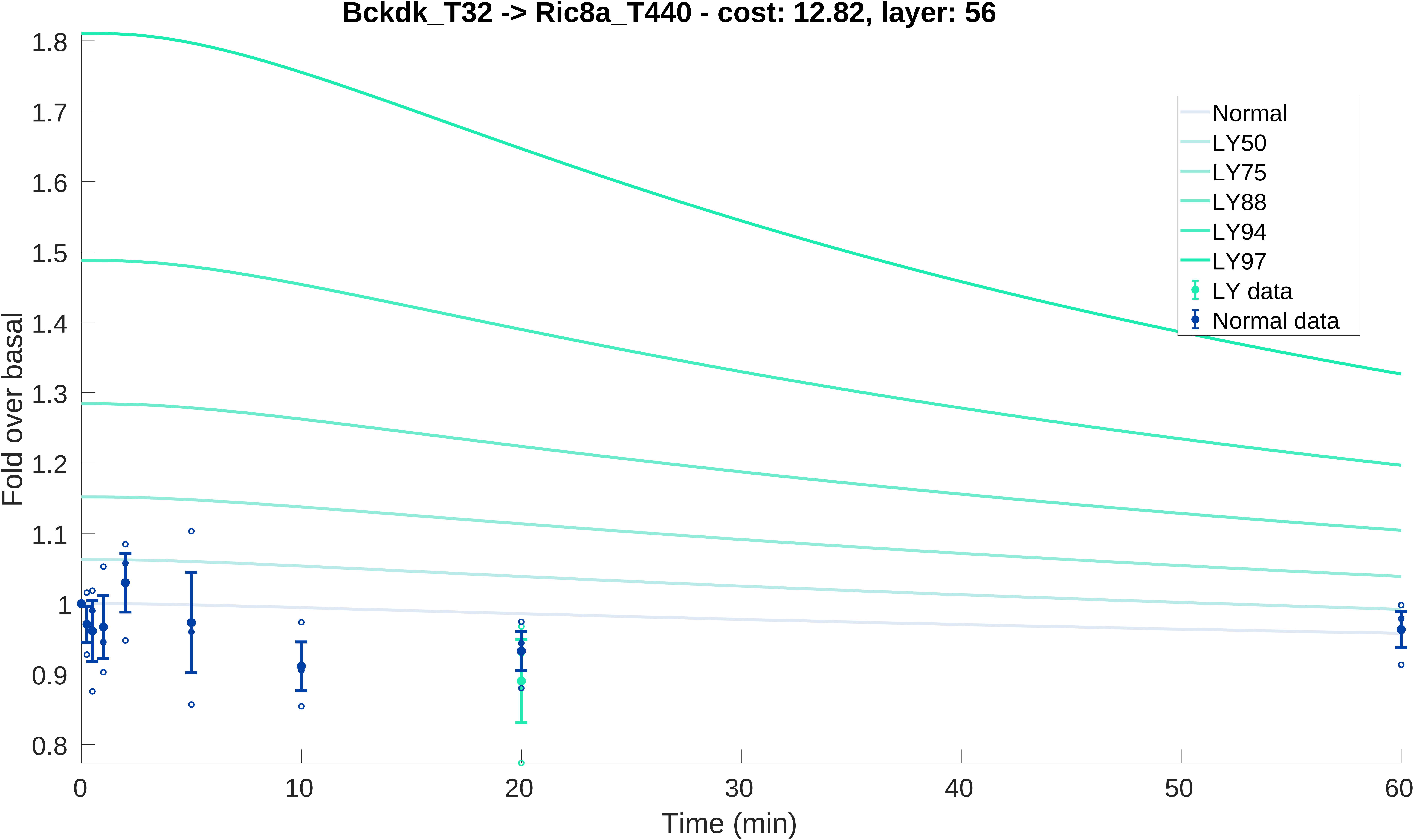
Bckdk_T32 -> Ric8a_T440 - cost: 12.82, layer: 56
Fold over basal
1.8
1.7
1.6
1.5
1.4
1.3
1.2
1.1
1
0.9
0.8
0
10
20
30
40
50
60
Time (min)
Normal
LY50
LY75
LY88
LY94
LY97
LY data
Normal data

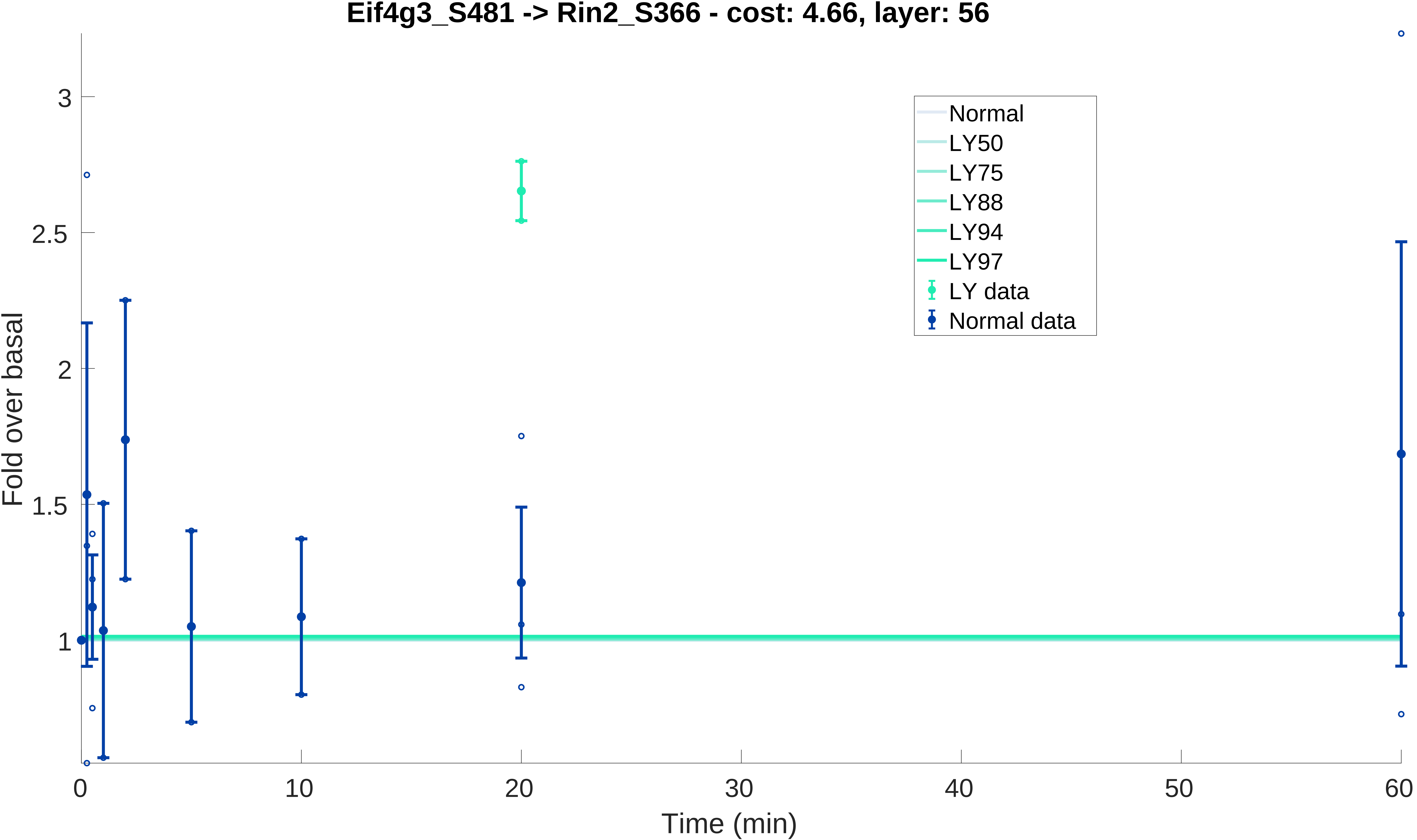

Eif4g3_S481 -> Rin2_S366 - cost: 4.66, layer: 56
Normal
LY50
LY75
LY88
LY94
LY97
LY data
Normal data
Fold over basal
Time (min)
0
10
20
30
40
50
60
1
1.5
2
2.5
3

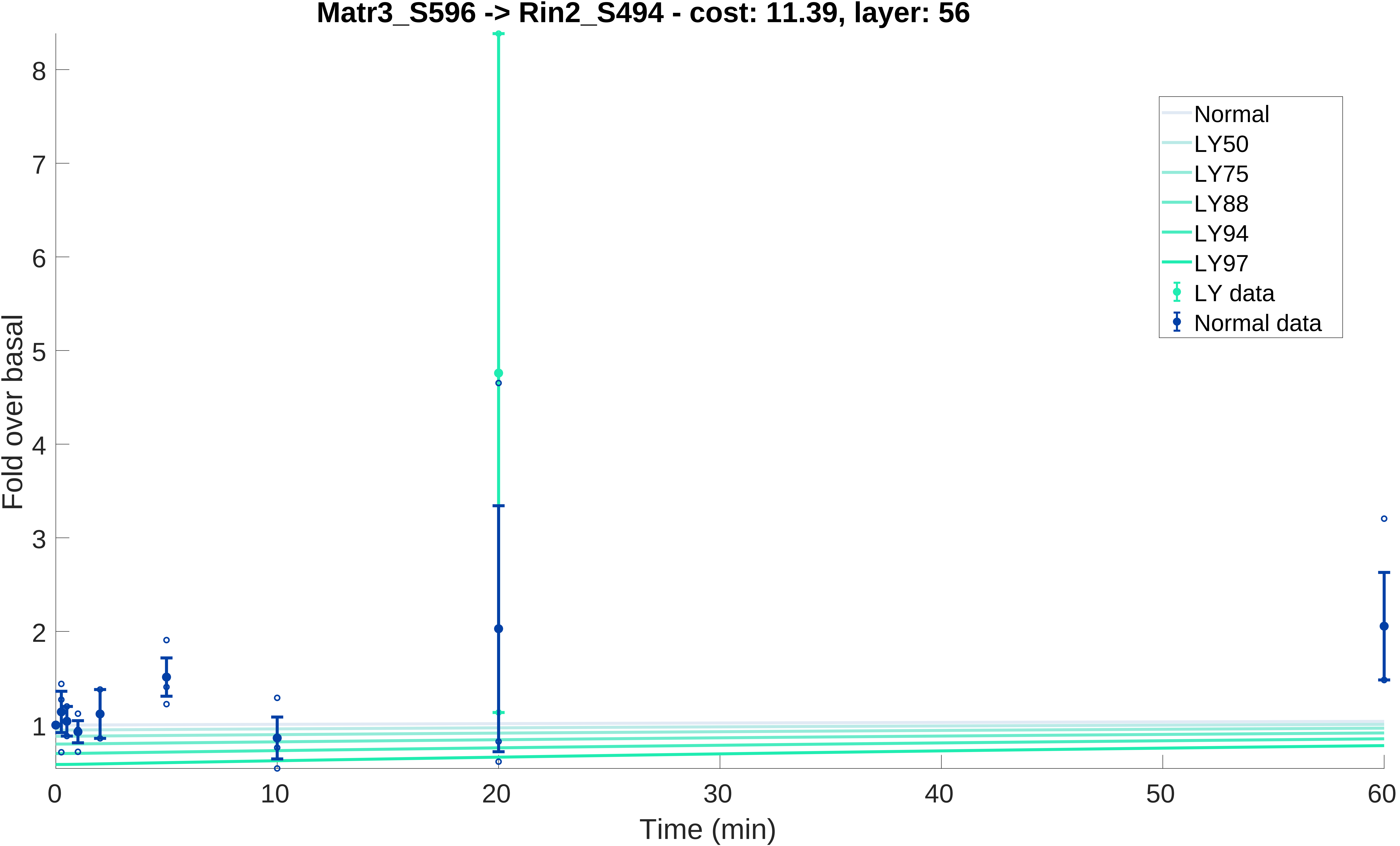
Matr3_S596 -> Rin2_S494 - cost: 11.39, layer: 56
Fold over basal
1
2
3
4
5
6
7
8
0
10
20
30
40
50
60
Time (min)
Normal
LY50
LY75
LY88
LY94
LY97
LY data
Normal data

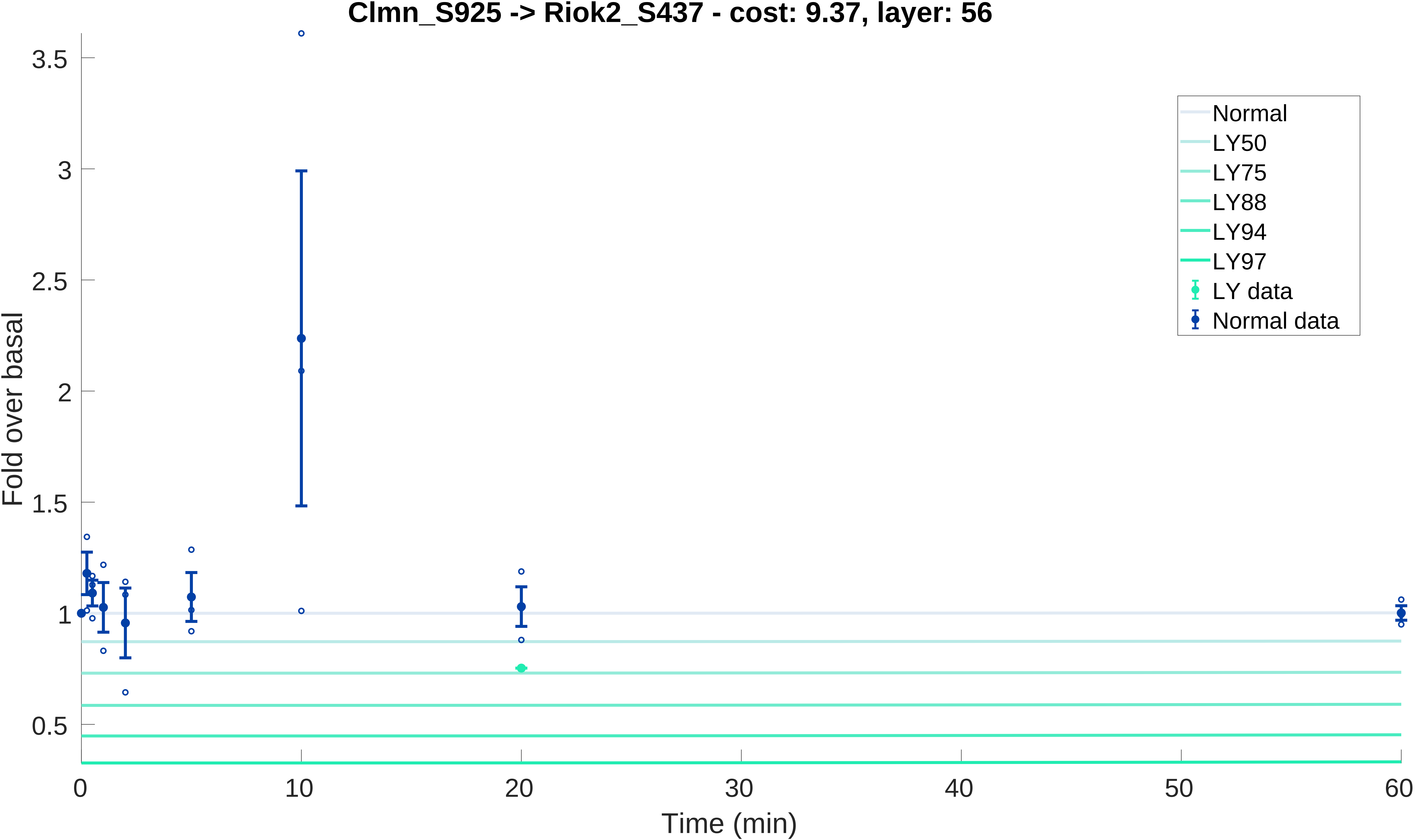

Clmn_S925 -> Riok2_S437 - cost: 9.37, layer: 56
Fold over basal
Time (min)
0
10
20
30
40
50
60
0.5
1
1.5
2
2.5
3
3.5
Normal
LY50
LY75
LY88
LY94
LY97
LY data
Normal data

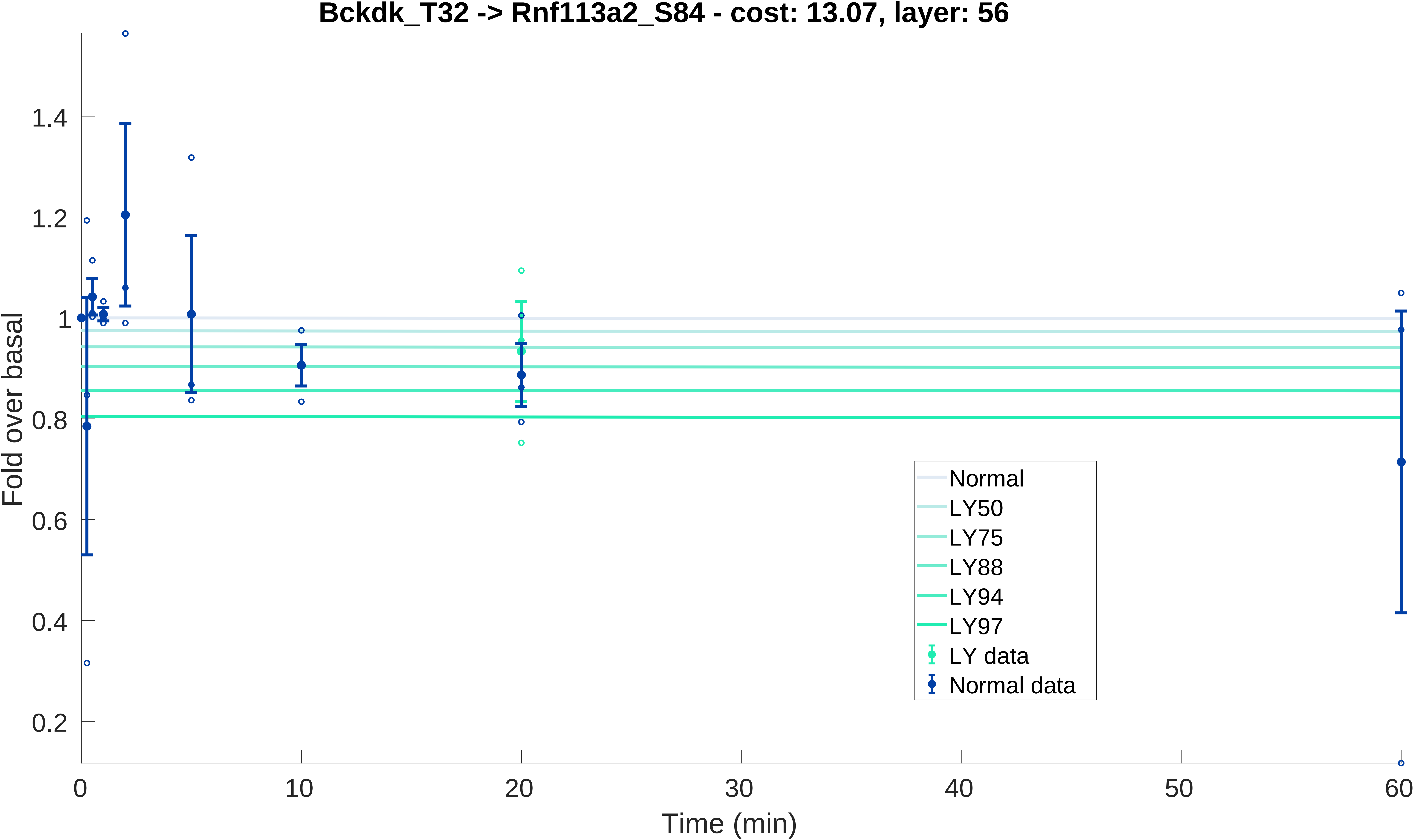

Bckdk_T32 -> Rnf113a2_S84 - cost: 13.07, layer: 56
Fold over basal
Time (min)
0.2
0.4
0.6
0.8
1
1.2
1.4
0
10
20
30
40
50
60
Normal
LY50
LY75
LY88
LY94
LY97
LY data
Normal data

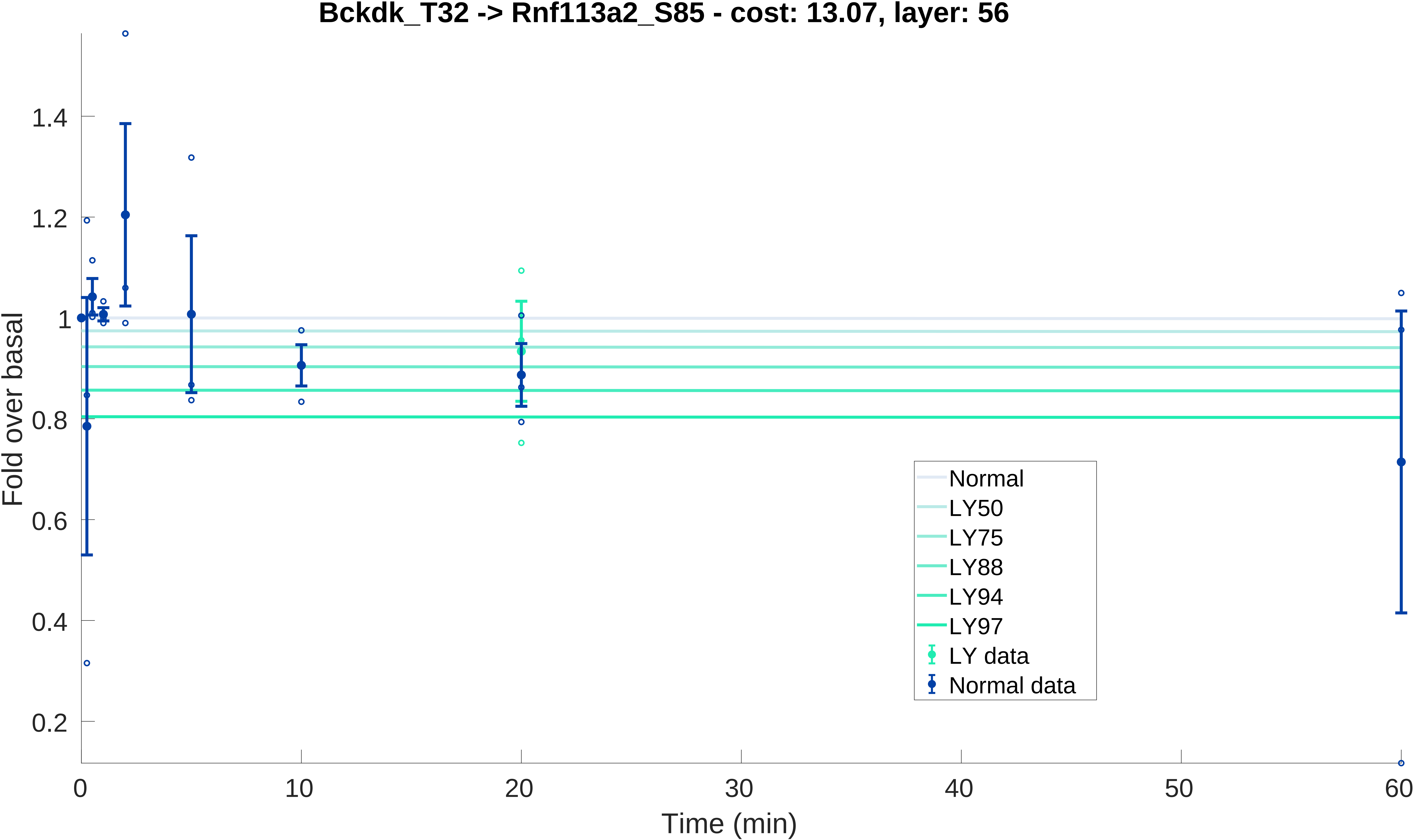
Bckdk_T32 -> Rnf113a2_S85 - cost: 13.07, layer: 56
Fold over basal
1.4
1.2
1
0.8
0.6
0.4
0.2
0
10
20
30
40
50
60
Time (min)
Normal
LY50
LY75
LY88
LY94
LY97
LY data
Normal data

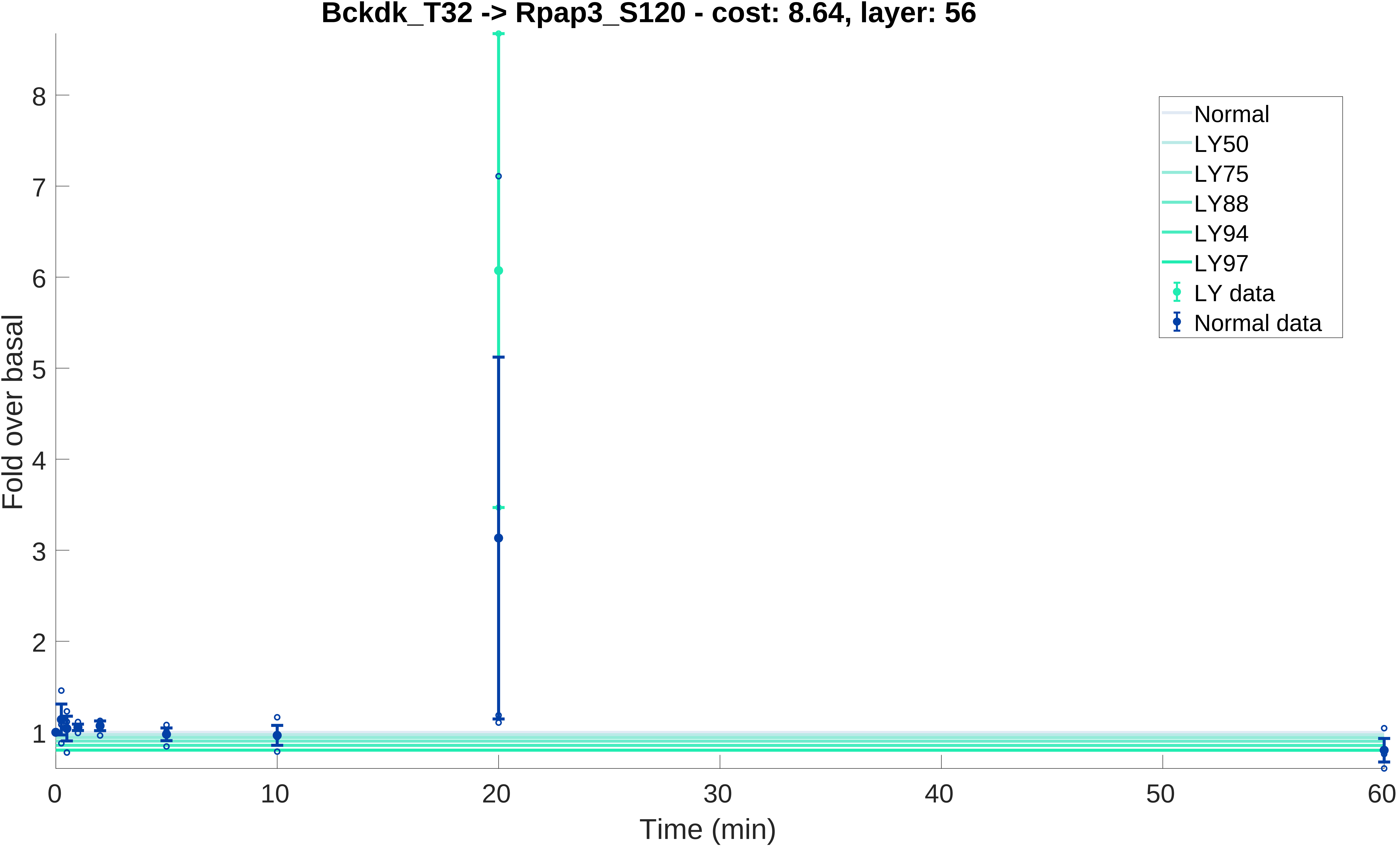

Bckdk_T32 -> Rpap3_S120 - cost: 8.64, layer: 56
Fold over basal
Time (min)
Normal
LY50
LY75
LY88
LY94
LY97
LY data
Normal data

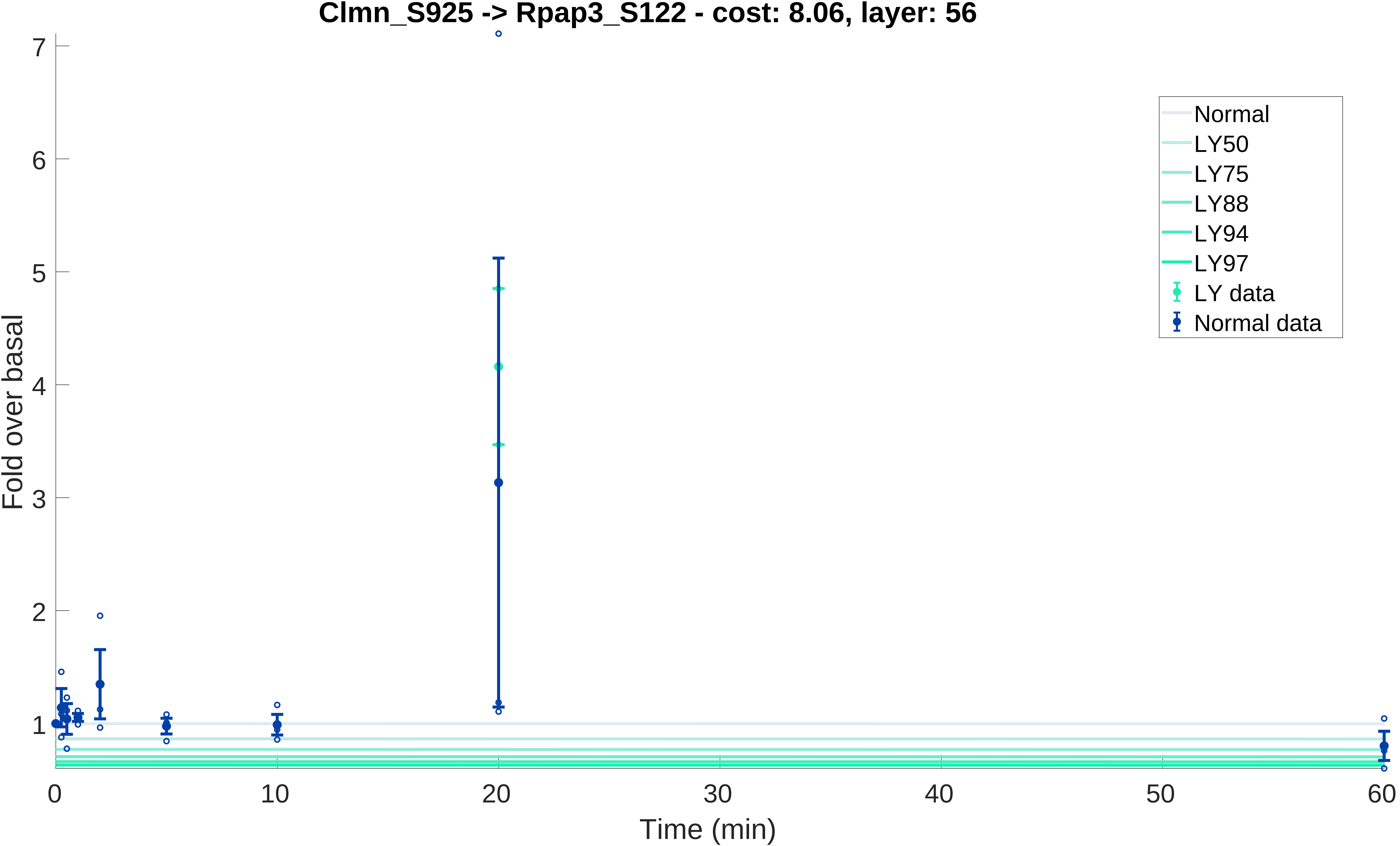

Clmn_S925 -> Rpap3_S122 - cost: 8.06, layer: 56
Fold over basal
Time (min)
0
10
20
30
40
50
60
1
2
3
4
5
6
7
Normal
LY50
LY75
LY88
LY94
LY97
LY data
Normal data

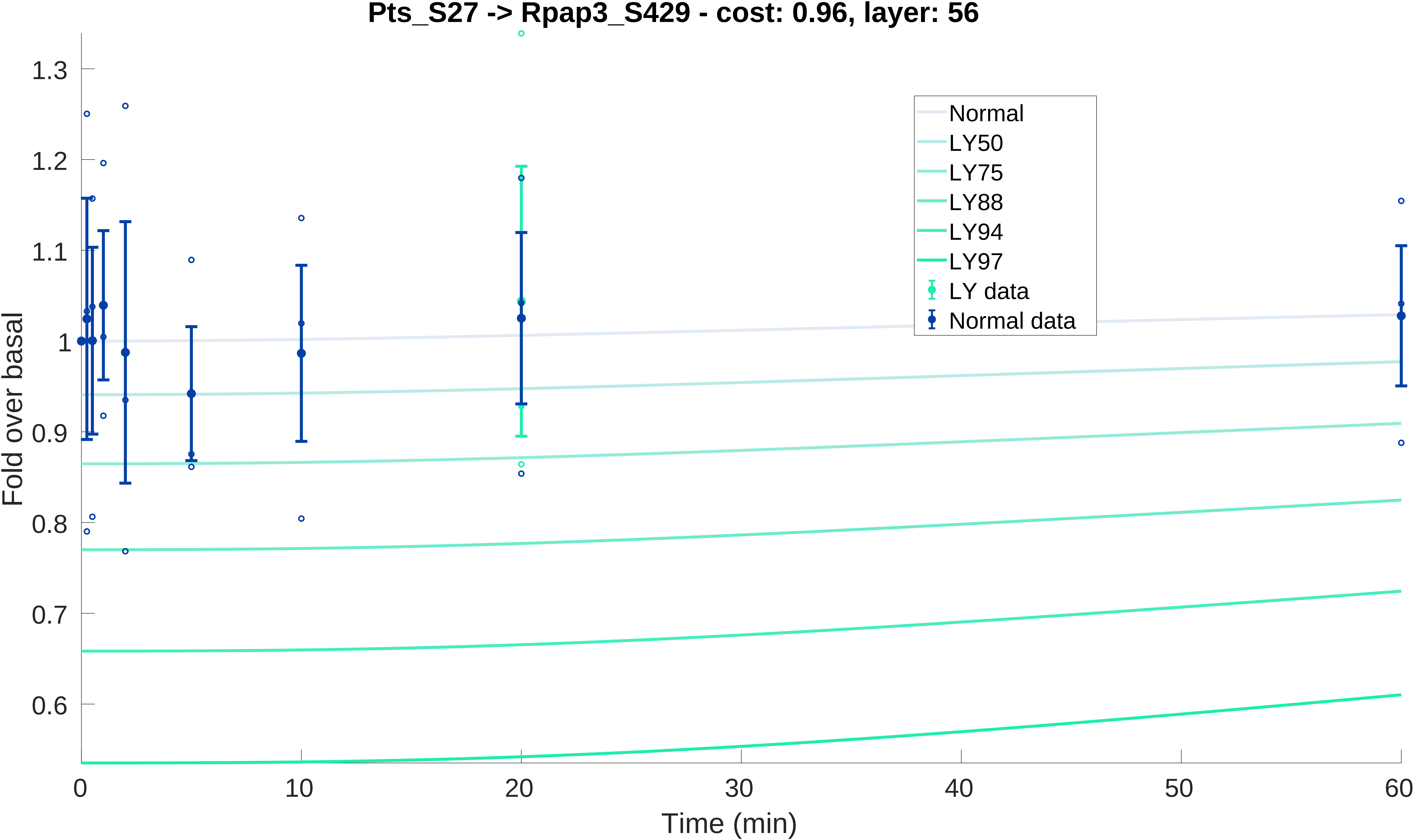

Pts_S27 -> Rpap3_S429 - cost: 0.96, layer: 56
Fold over basal
Time (min)
1.3
1.2
1.1
1
0.9
0.8
0.7
0.6
0
10
20
30
40
50
60
Normal
LY50
LY75
LY88
LY94
LY97
LY data
Normal data

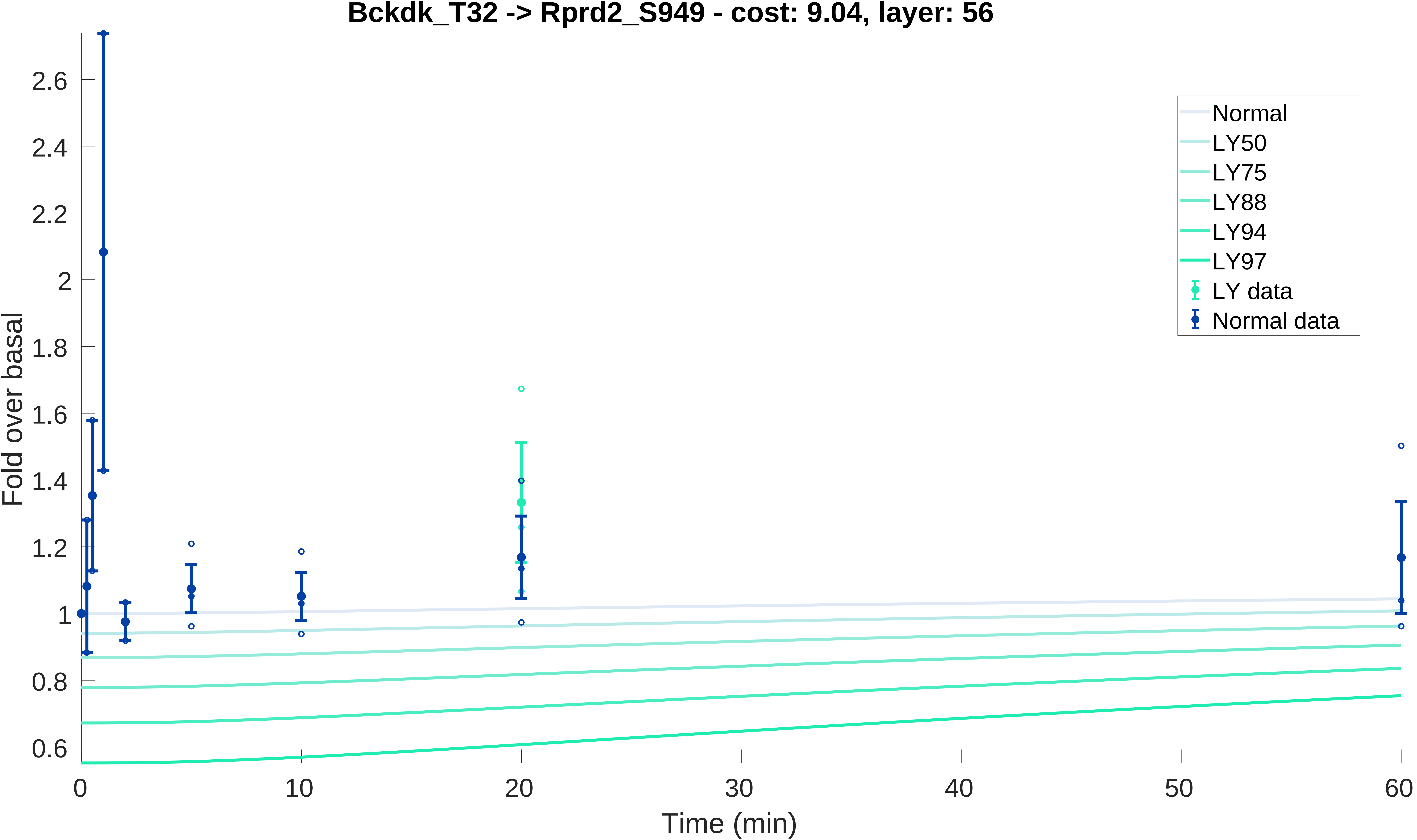
Bckdk_T32 -> Rprd2_S949 - cost: 9.04, layer: 56
Fold over basal
Time (min)
0.6
0.8
1
1.2
1.4
1.6
1.8
2
2.2
2.4
2.6
0
10
20
30
40
50
60
Normal
LY50
LY75
LY88
LY94
LY97
LY data
Normal data

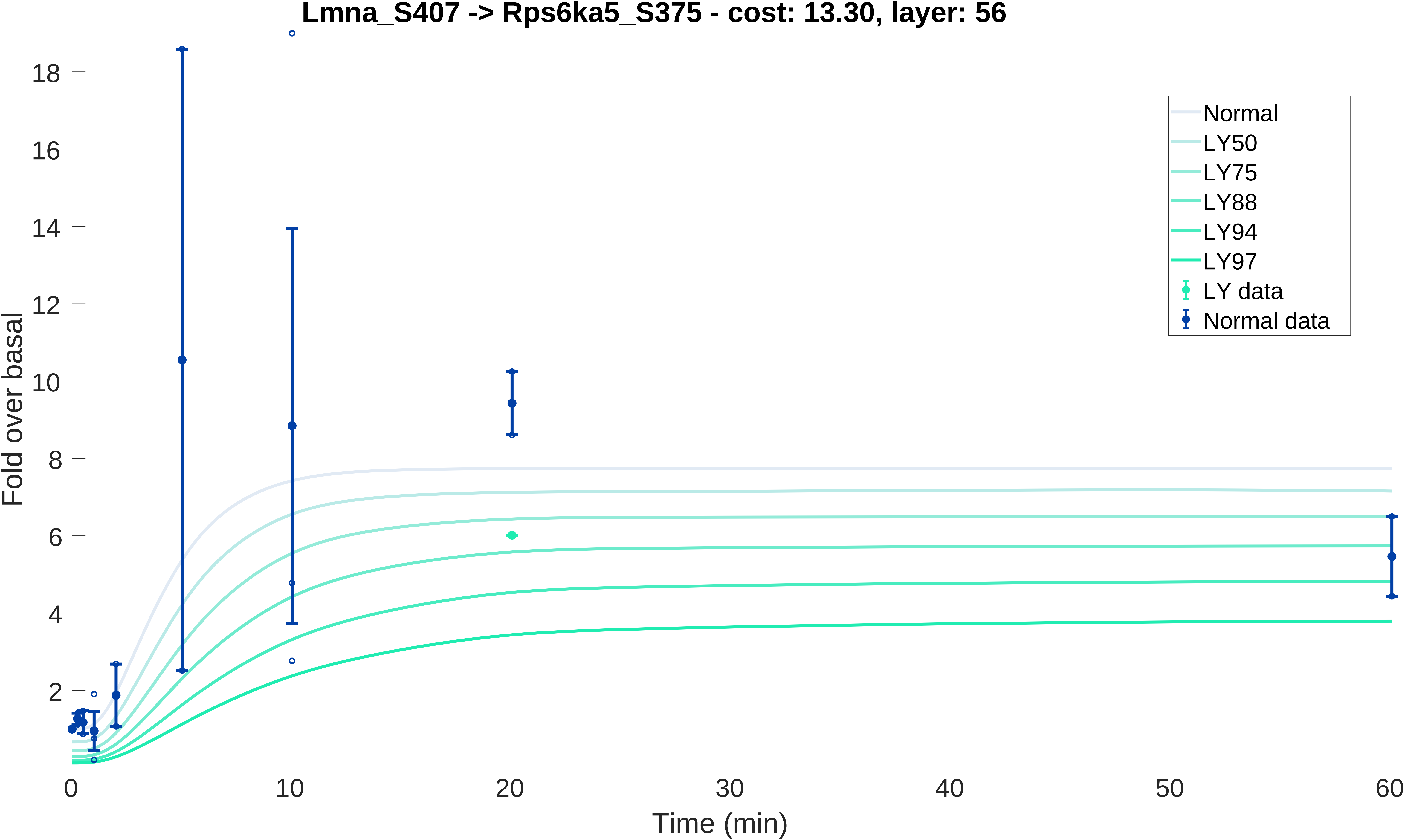

Lmna_S407 -> Rps6ka5_S375 - cost: 13.30, layer: 56
Fold over basal
Time (min)
0
10
20
30
40
50
60
2
4
6
8
10
12
14
16
18
Normal
LY50
LY75
LY88
LY94
LY97
LY data
Normal data

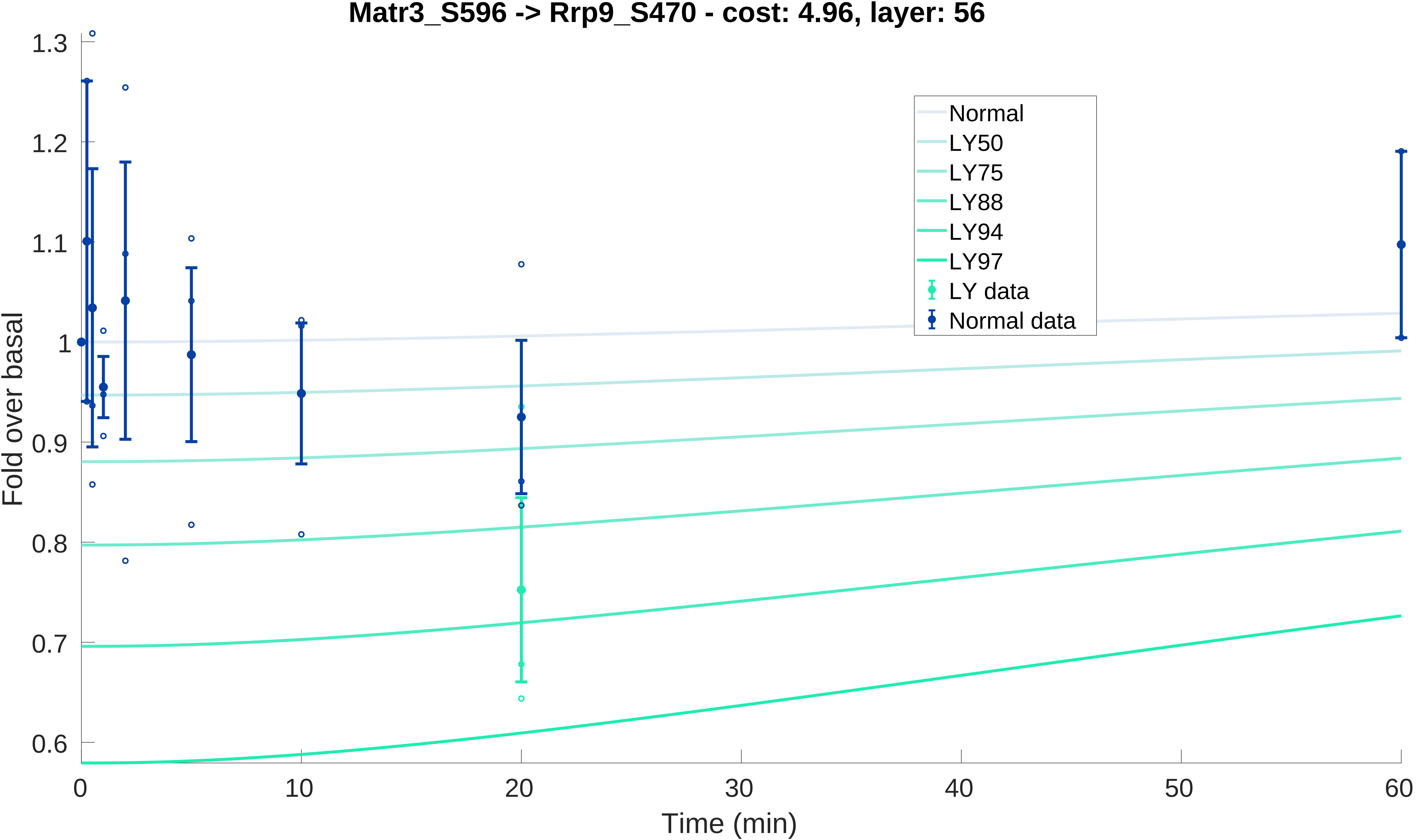

Matr3_S596 -> Rrp9_S470 - cost: 4.96, layer: 56
Fold over basal
Time (min)
0.6
0.7
0.8
0.9
1
1.1
1.2
1.3
0
10
20
30
40
50
60
Normal
LY50
LY75
LY88
LY94
LY97
LY data
Normal data

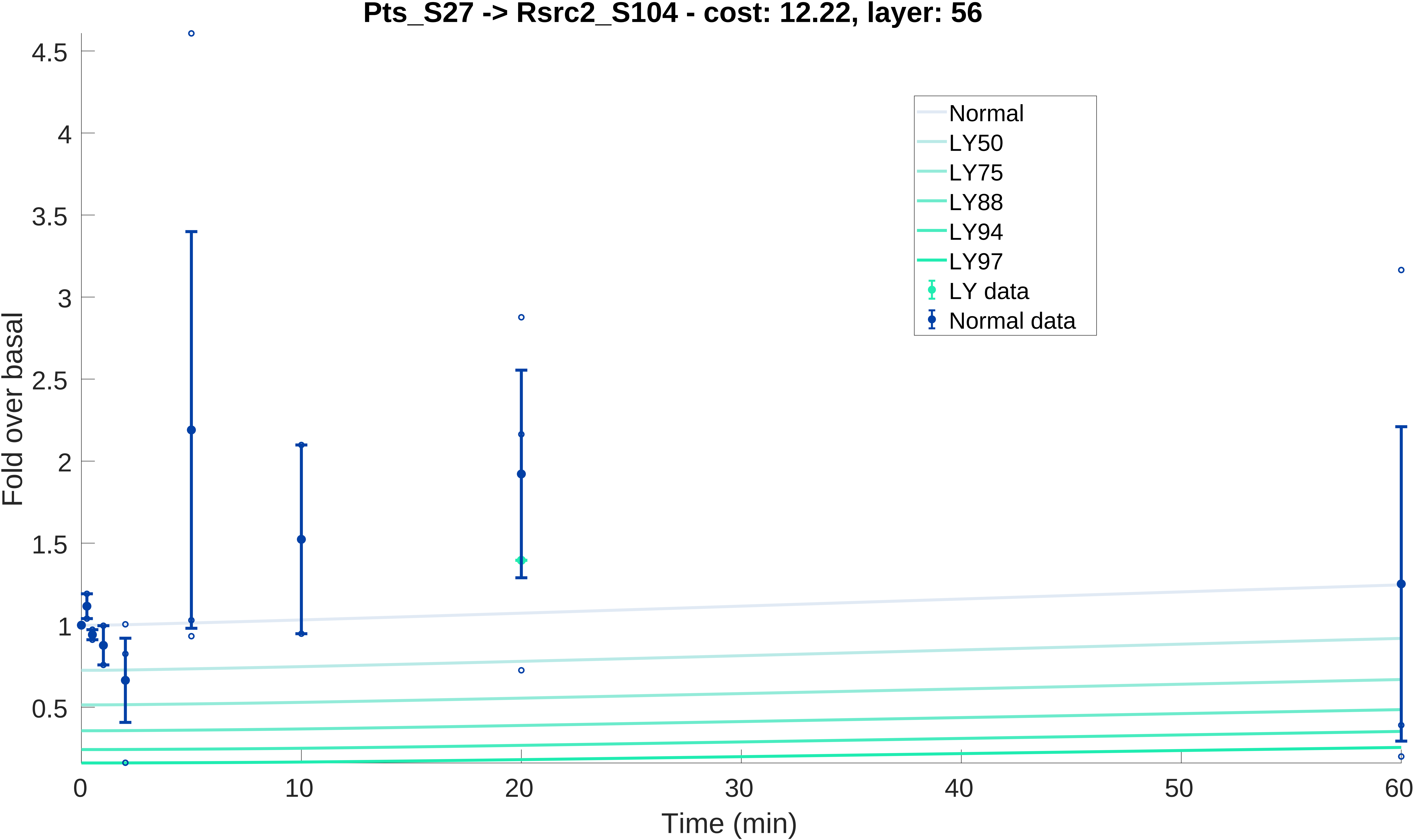

Pts_S27 -> Rsrc2_S104 - cost: 12.22, layer: 56
Fold over basal
Time (min)
0
10
20
30
40
50
60
0.5
1
1.5
2
2.5
3
3.5
4
4.5
Normal
LY50
LY75
LY88
LY94
LY97
LY data
Normal data

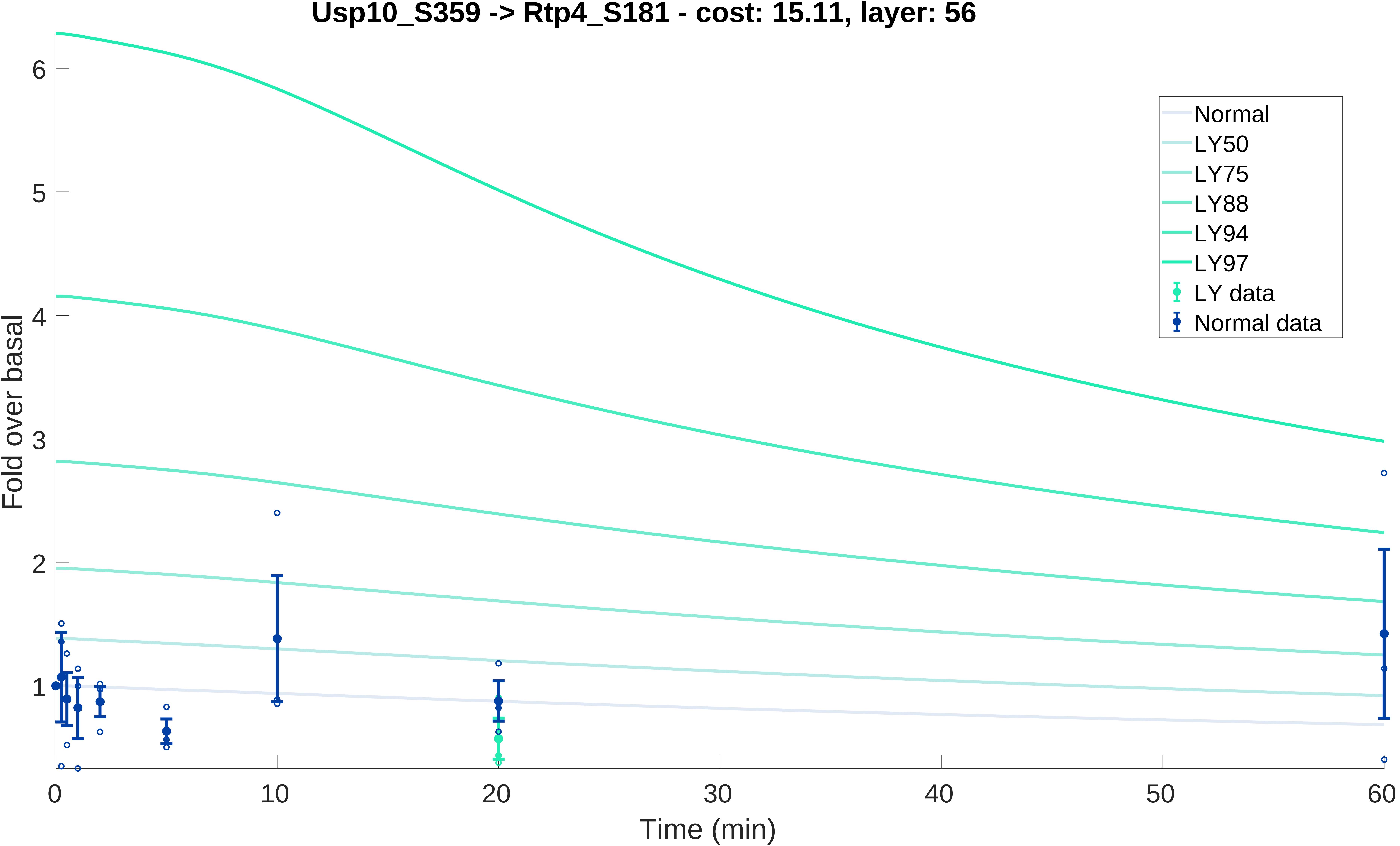
Usp10_S359 -> Rtp4_S181 - cost: 15.11, layer: 56
Fold over basal
Time (min)
0
10
20
30
40
50
60
1
2
3
4
5
6
Normal
LY50
LY75
LY88
LY94
LY97
LY data
Normal data

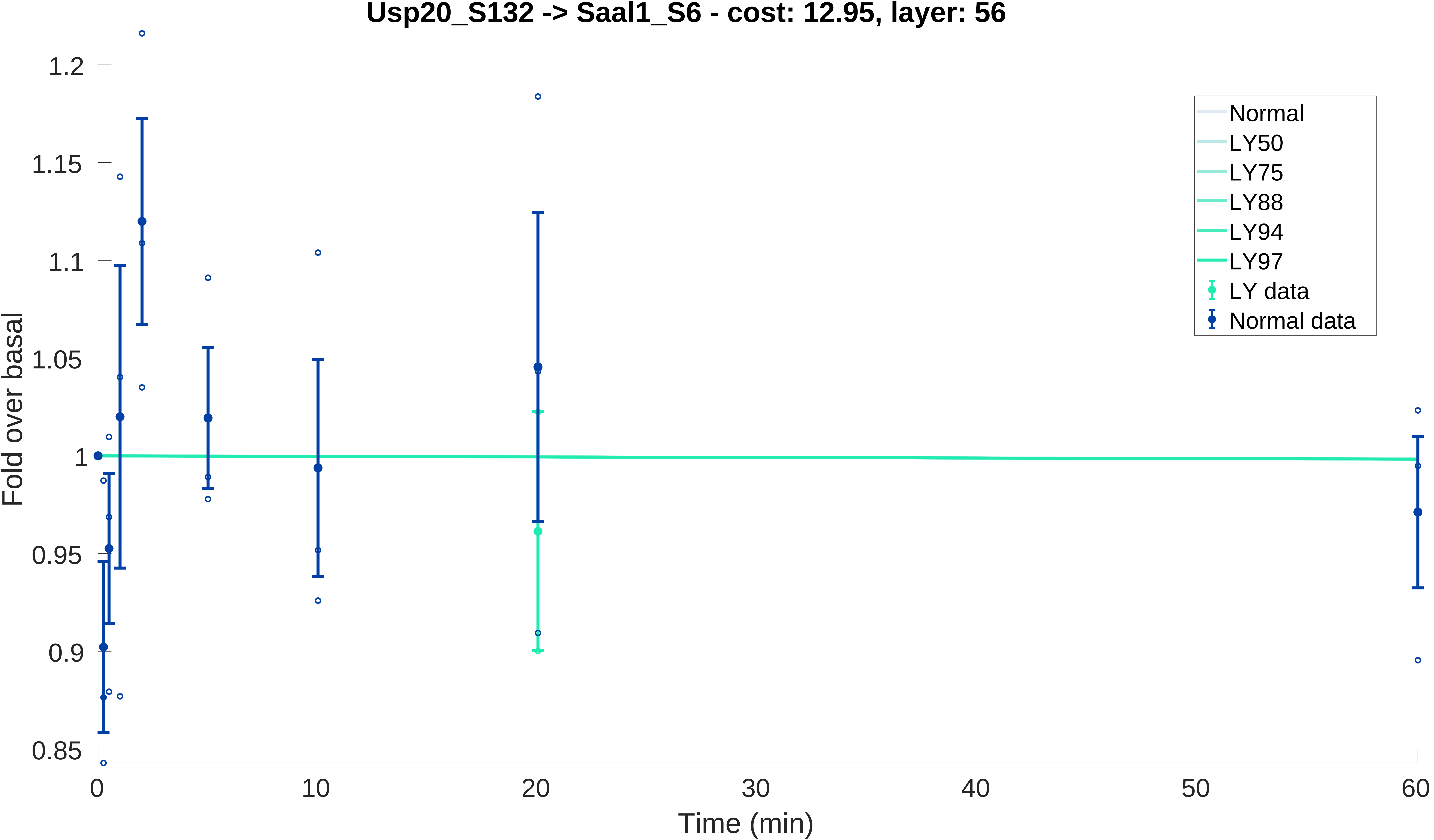

Usp20_S132 -> Saal1_S6 - cost: 12.95, layer: 56
Fold over basal
Time (min)
1.2
1.15
1.1
1.05
1
0.95
0.9
0.85
0
10
20
30
40
50
60
Normal
LY50
LY75
LY88
LY94
LY97
LY data
Normal data

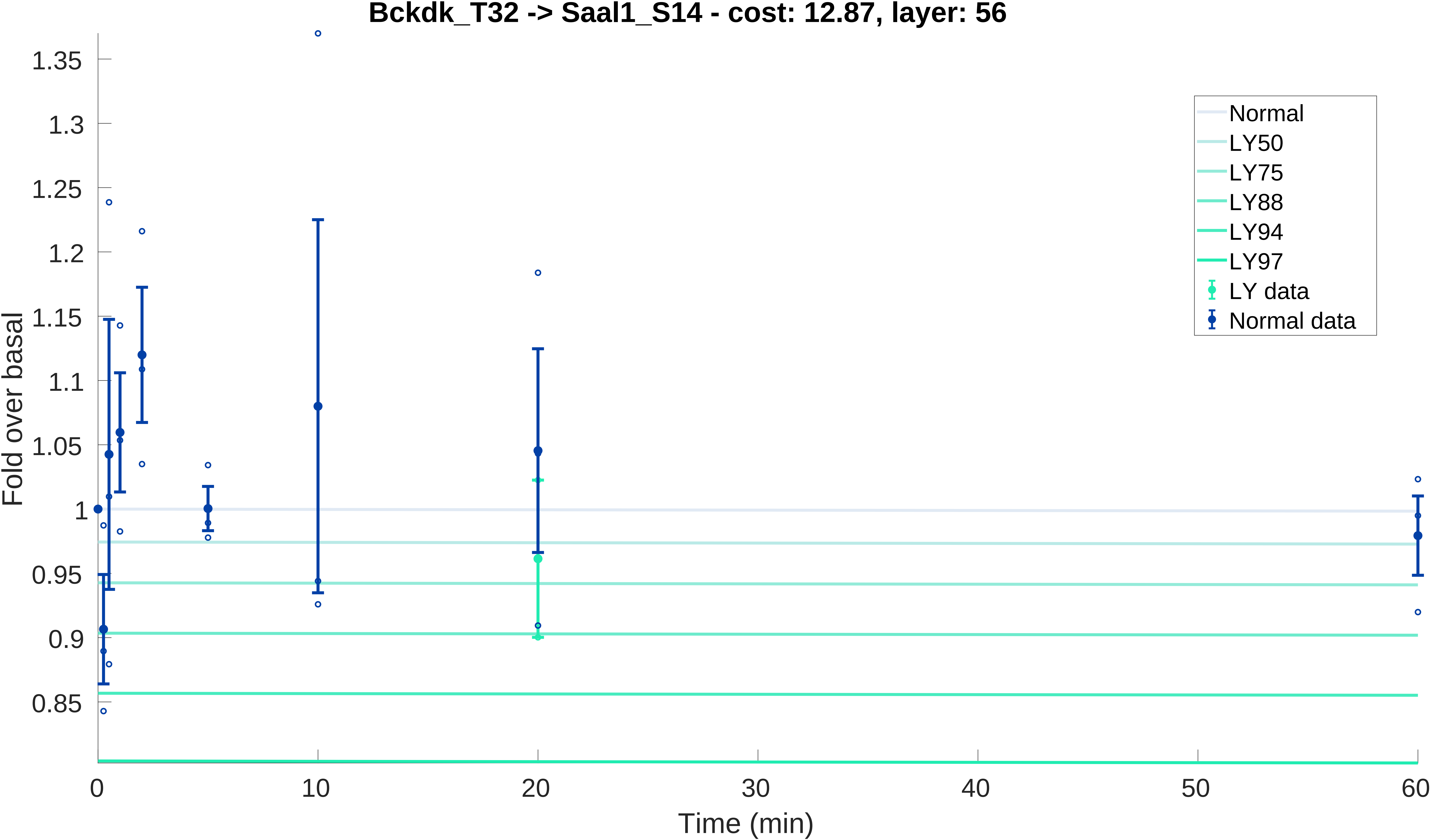

Bckdk_T32 -> Saal1_S14 - cost: 12.87, layer: 56
Fold over basal
Time (min)
1.35
1.3
1.25
1.2
1.15
1.1
1.05
1
0.95
0.9
0.85
0
10
20
30
40
50
60
Normal
LY50
LY75
LY88
LY94
LY97
LY data
Normal data

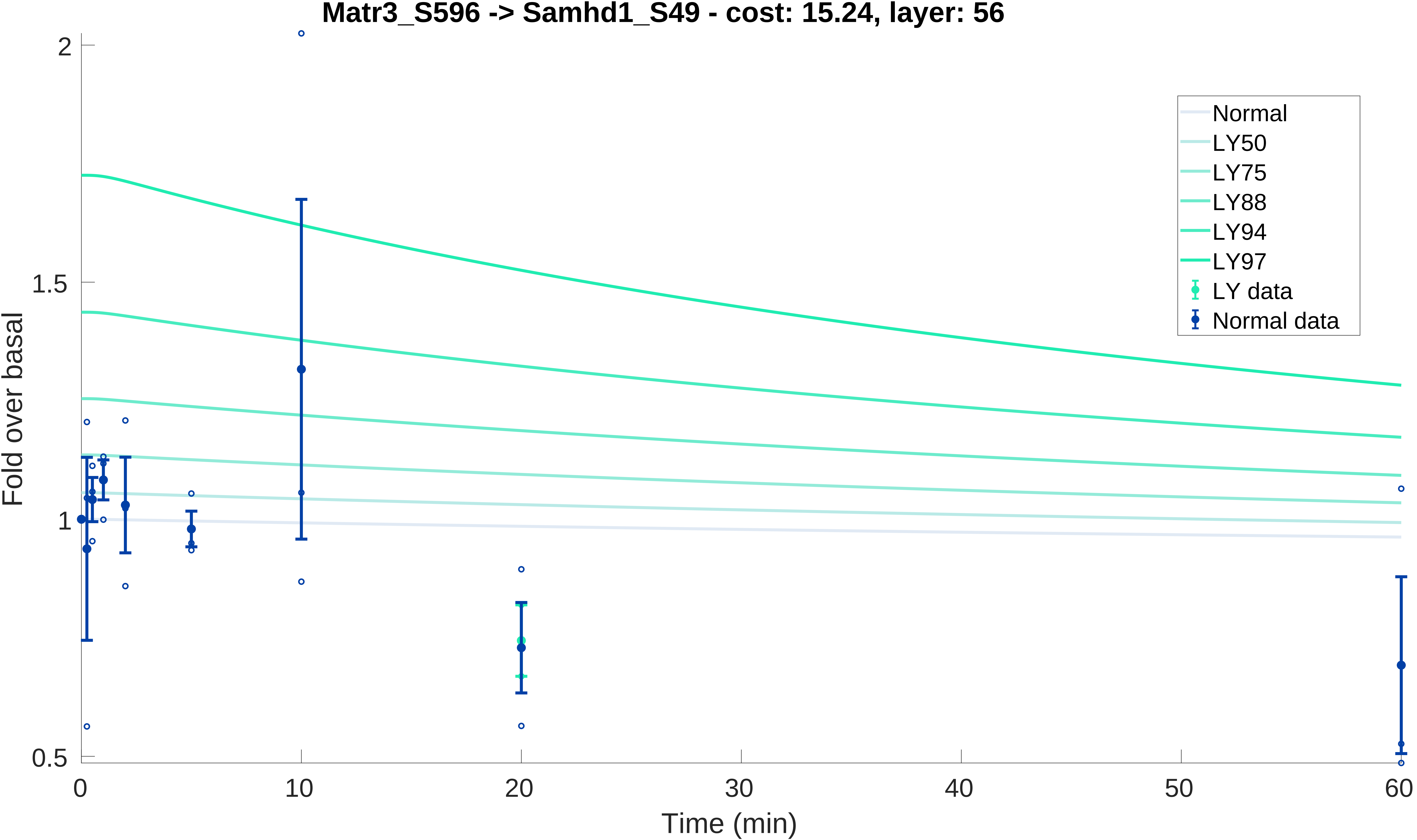

Matr3_S596 -> Samhd1_S49 - cost: 15.24, layer: 56
Fold over basal
Time (min)
Normal
LY50
LY75
LY88
LY94
LY97
LY data
Normal data
0.5
1
1.5
2
0
10
20
30
40
50
60

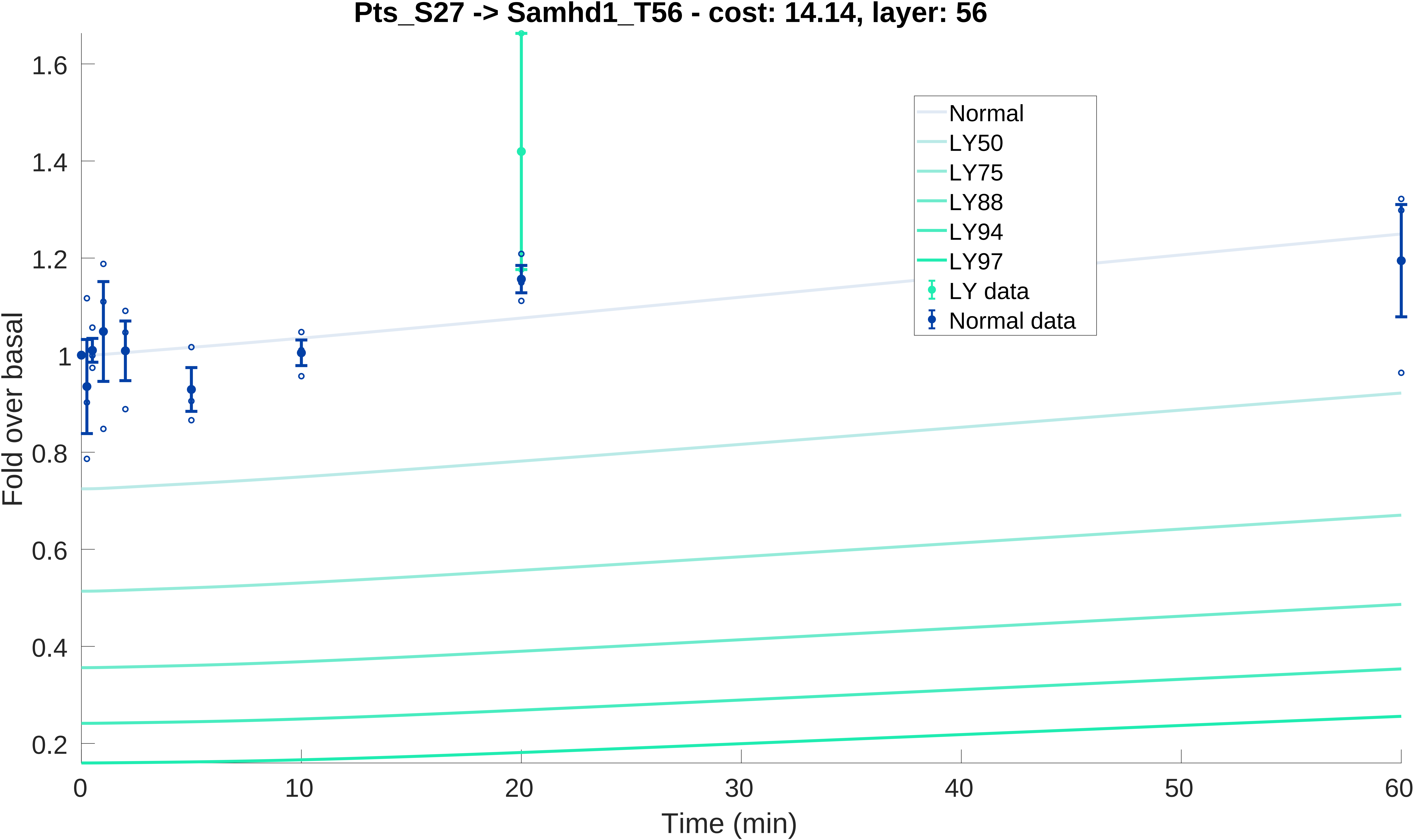

Pts_S27 -> Samhd1_T56 - cost: 14.14, layer: 56
Fold over basal
Time (min)
Normal
LY50
LY75
LY88
LY94
LY97
LY data
Normal data
0.2
0.4
0.6
0.8
1
1.2
1.4
1.6
0
10
20
30
40
50
60

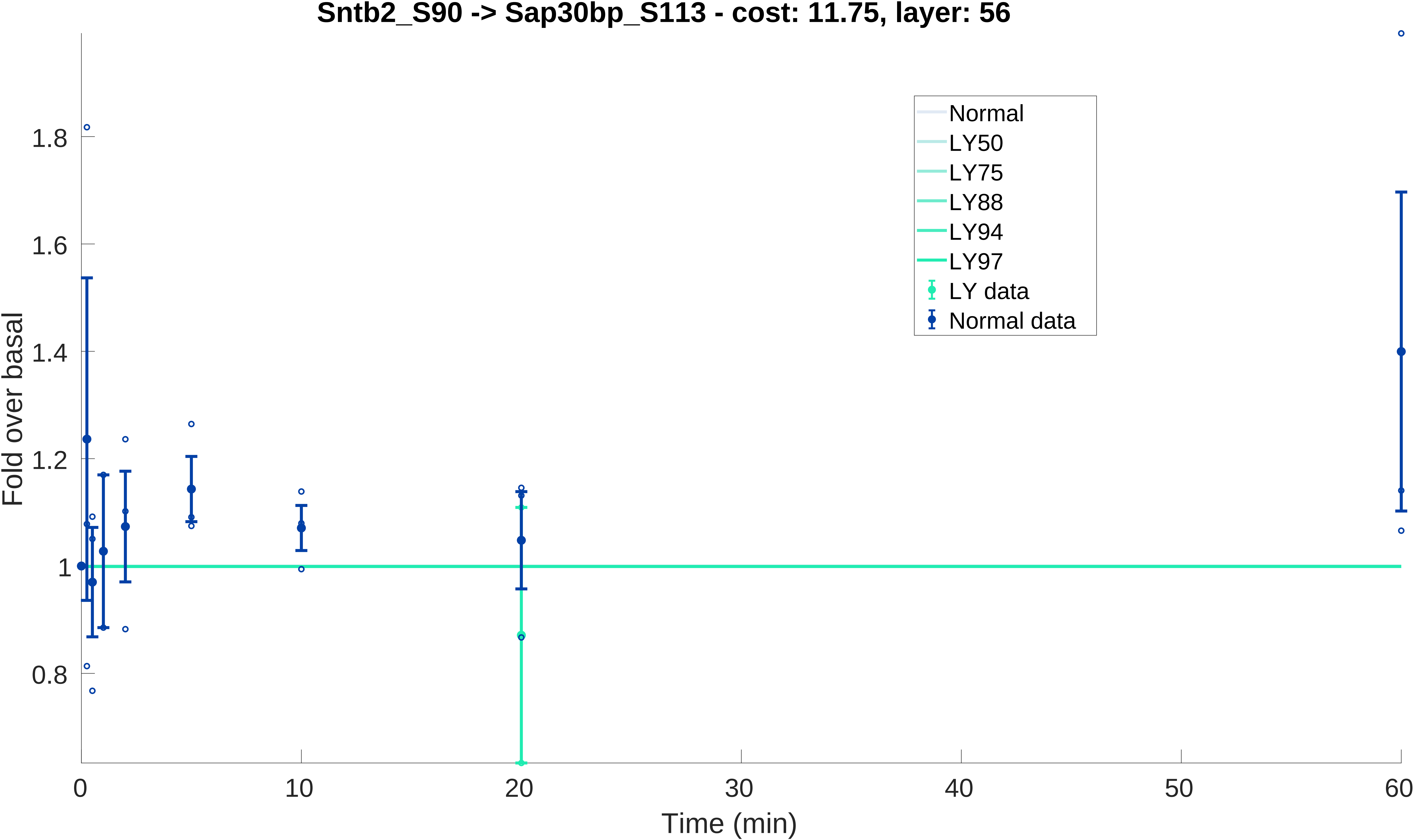
Sntb2_S90 -> Sap30bp_S113 - cost: 11.75, layer: 56
Fold over basal
Time (min)
Normal
LY50
LY75
LY88
LY94
LY97
LY data
Normal data
0.8
1
1.2
1.4
1.6
1.8
0
10
20
30
40
50
60

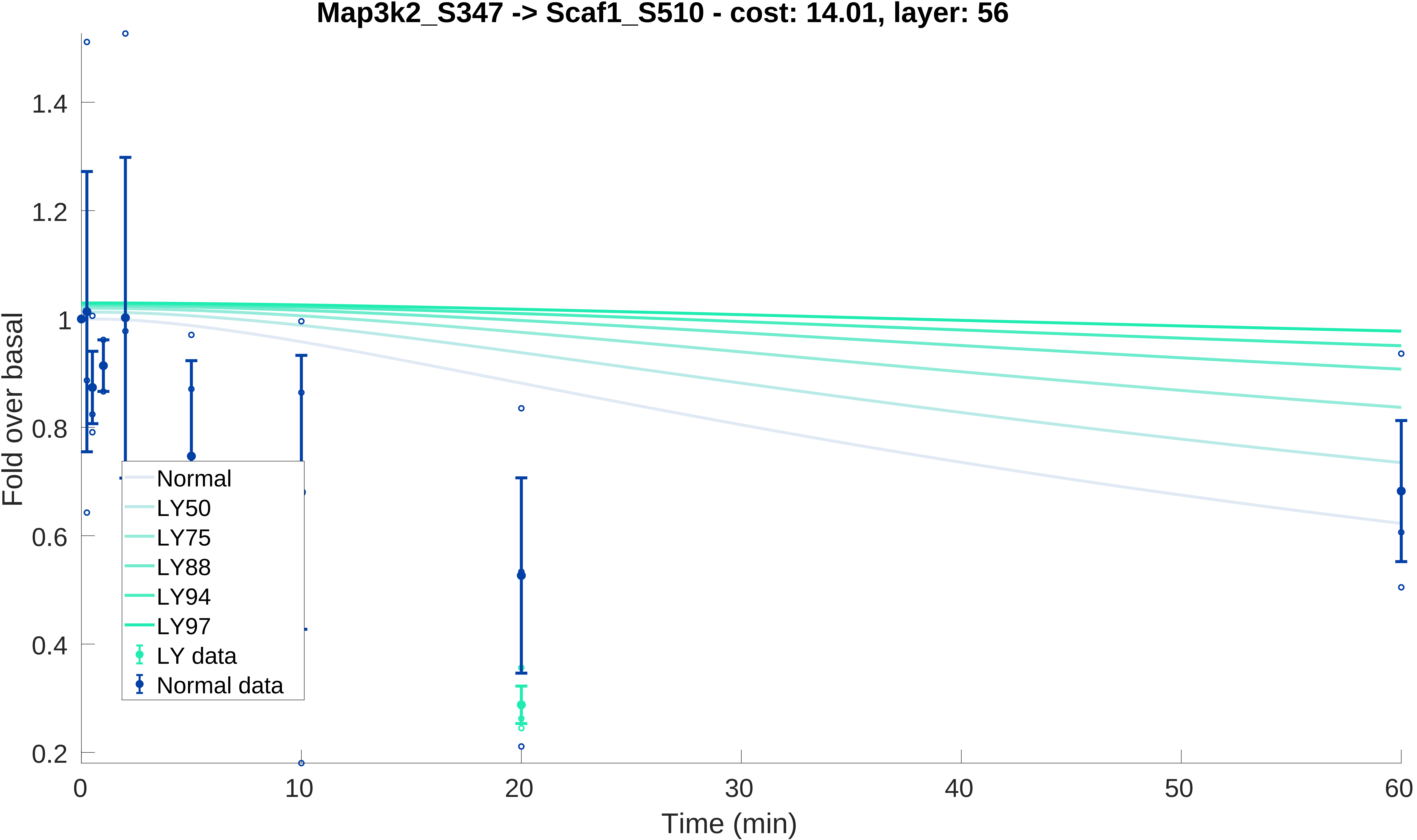

Map3k2_S347 -> Scaf1_S510 - cost: 14.01, layer: 56
Fold over basal
1.4
1.2
1
0.8
0.6
0.4
0.2
0
10
20
30
40
50
60
Time (min)
Normal
LY50
LY75
LY88
LY94
LY97
LY data
Normal data

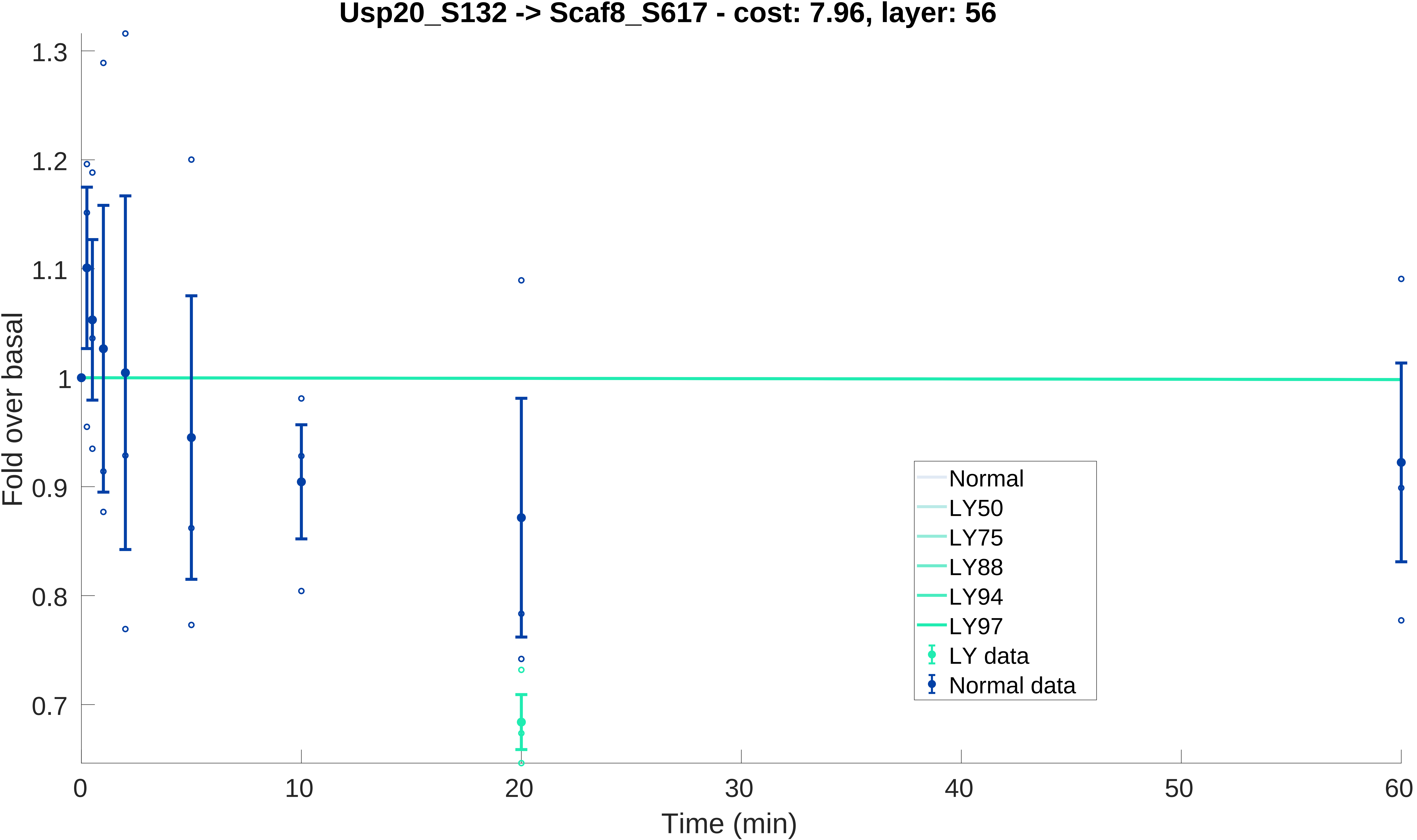

Usp20_S132 -> Scaf8_S617 - cost: 7.96, layer: 56
Fold over basal
1.3
1.2
1.1
1
0.9
0.8
0.7
0
10
20
30
40
50
60
Time (min)
Normal
LY50
LY75
LY88
LY94
LY97
LY data
Normal data

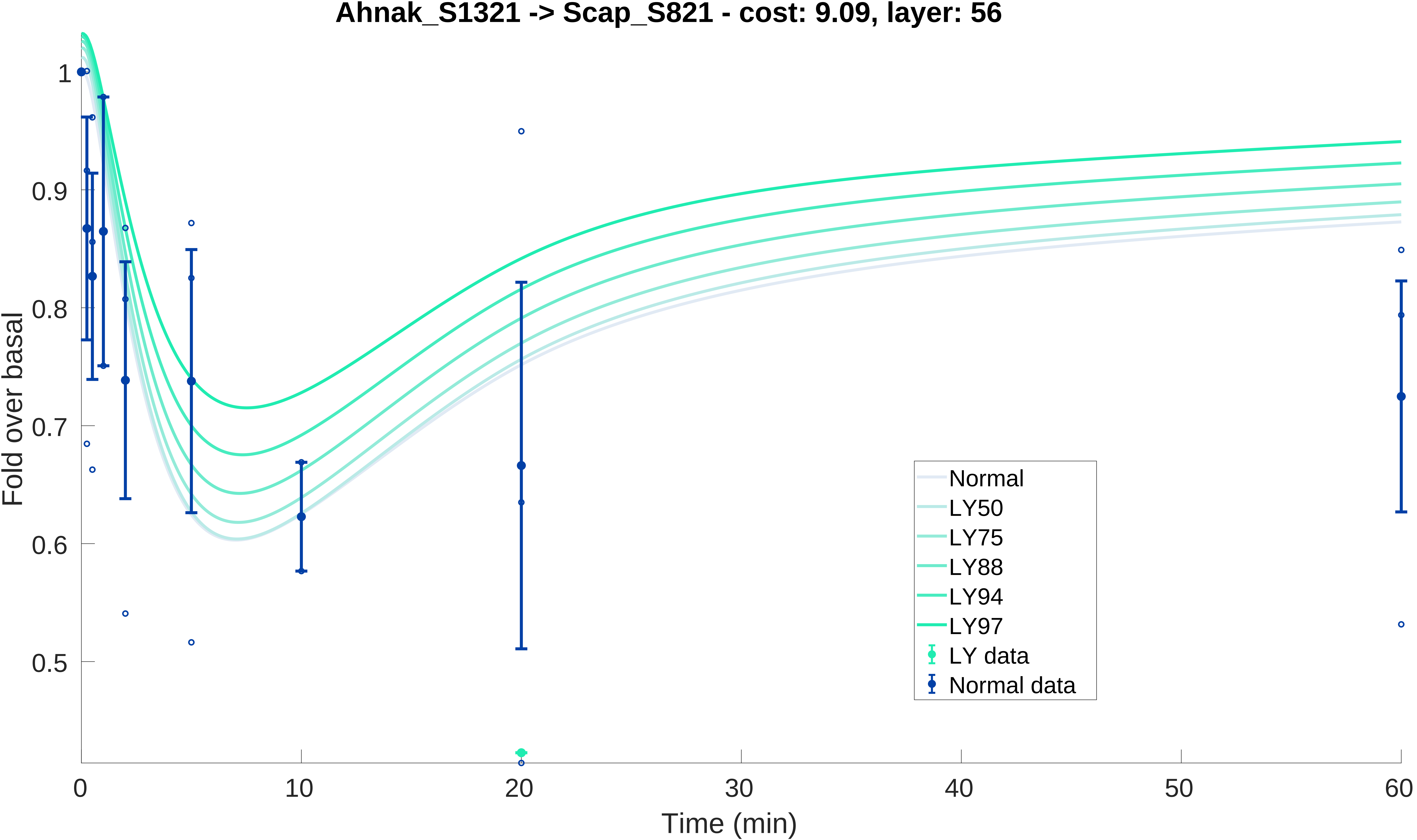

Ahnak_S1321 -> Scap_S821 - cost: 9.09, layer: 56
Fold over basal
1
0.9
0.8
0.7
0.6
0.5
0
10
20
30
40
50
60
Time (min)
Normal
LY50
LY75
LY88
LY94
LY97
LY data
Normal data

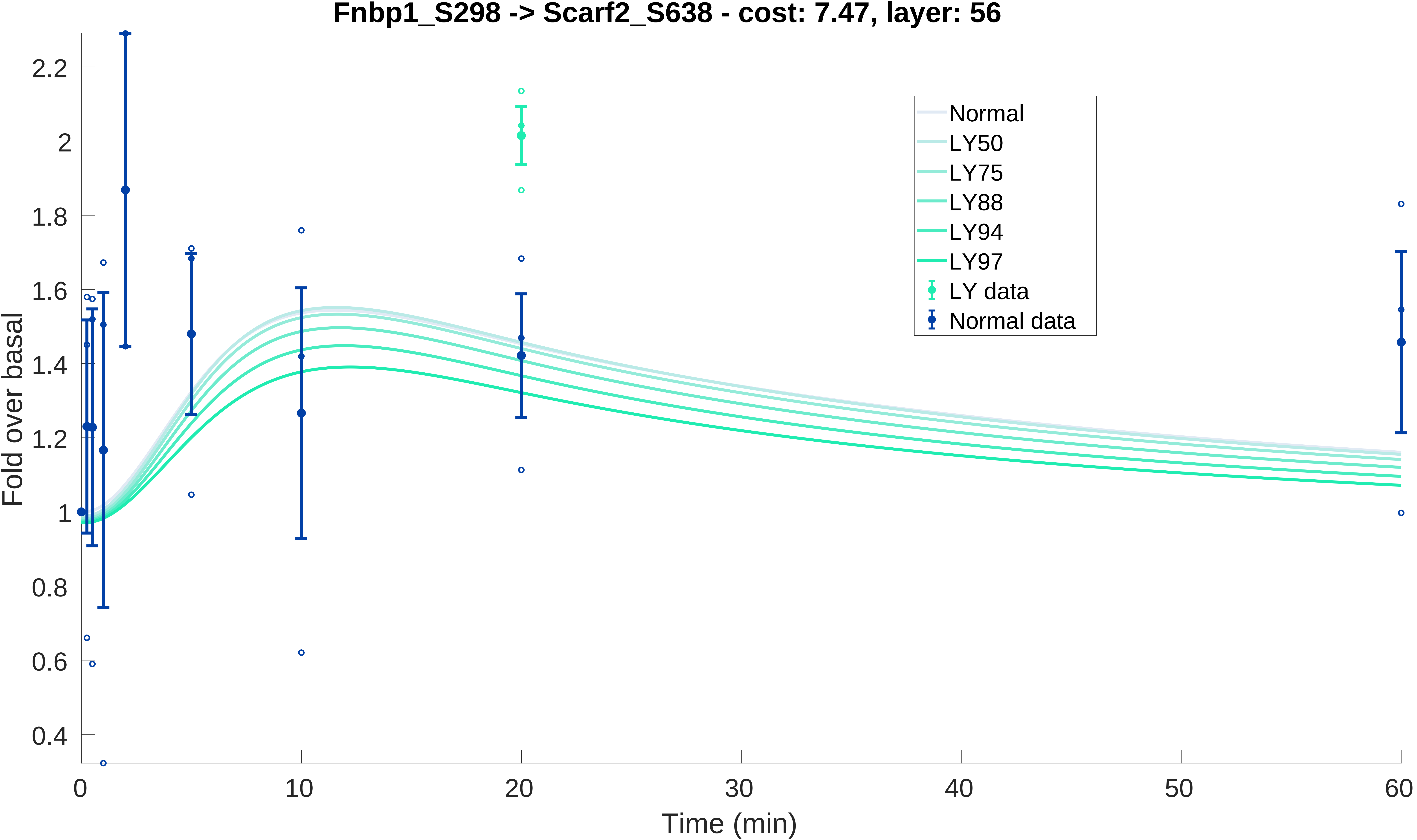

Fnbp1_S298 -> Scarf2_S638 - cost: 7.47, layer: 56
Fold over basal
Time (min)
0.4
0.6
0.8
1
1.2
1.4
1.6
1.8
2
2.2
0
10
20
30
40
50
60
Normal
LY50
LY75
LY88
LY94
LY97
LY data
Normal data

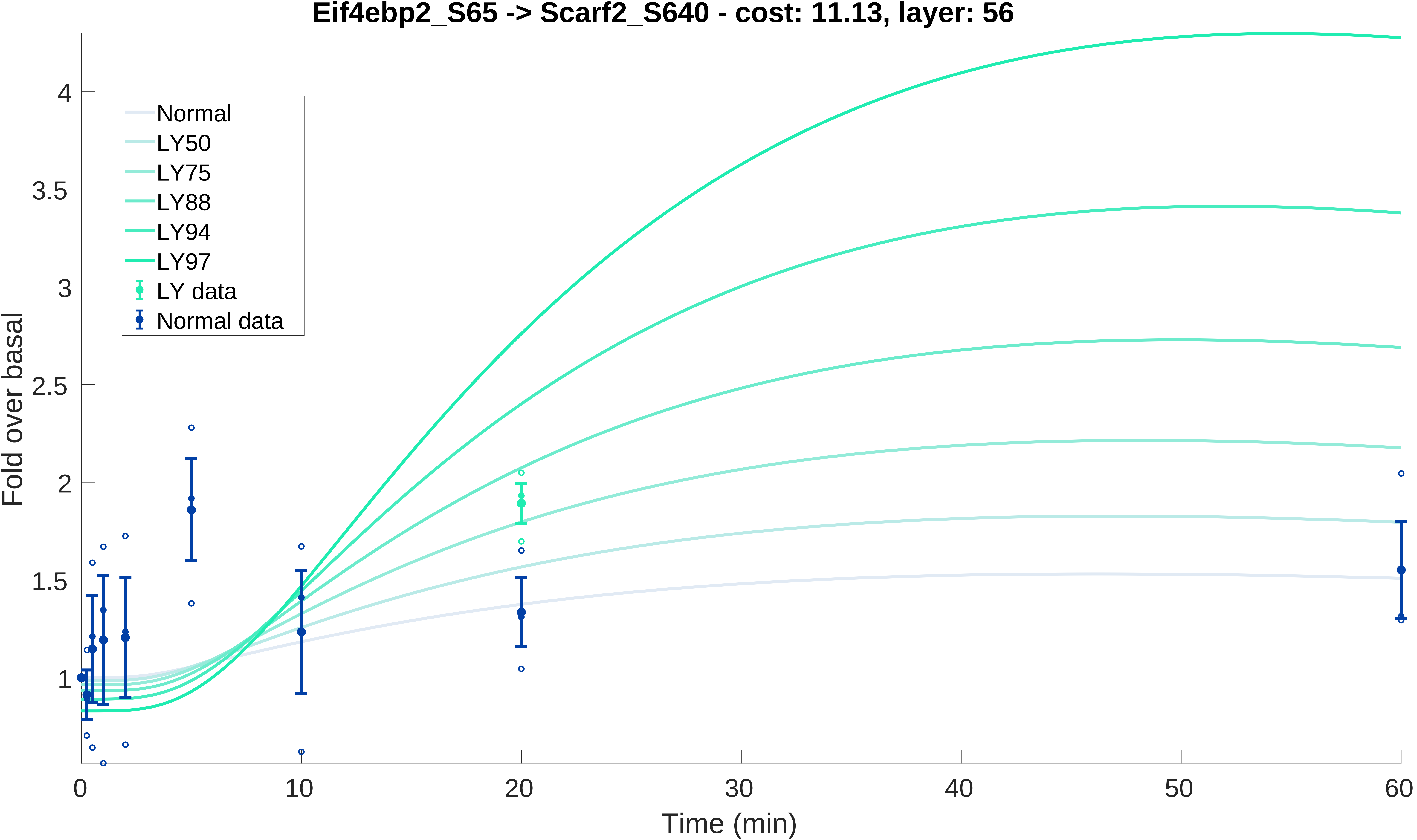

Eif4ebp2_S65 -> Scarf2_S640 - cost: 11.13, layer: 56
Normal
LY50
LY75
LY88
LY94
LY97
LY data
Normal data
Fold over basal
Time (min)
4
3.5
3
2.5
2
1.5
1
0
10
20
30
40
50
60

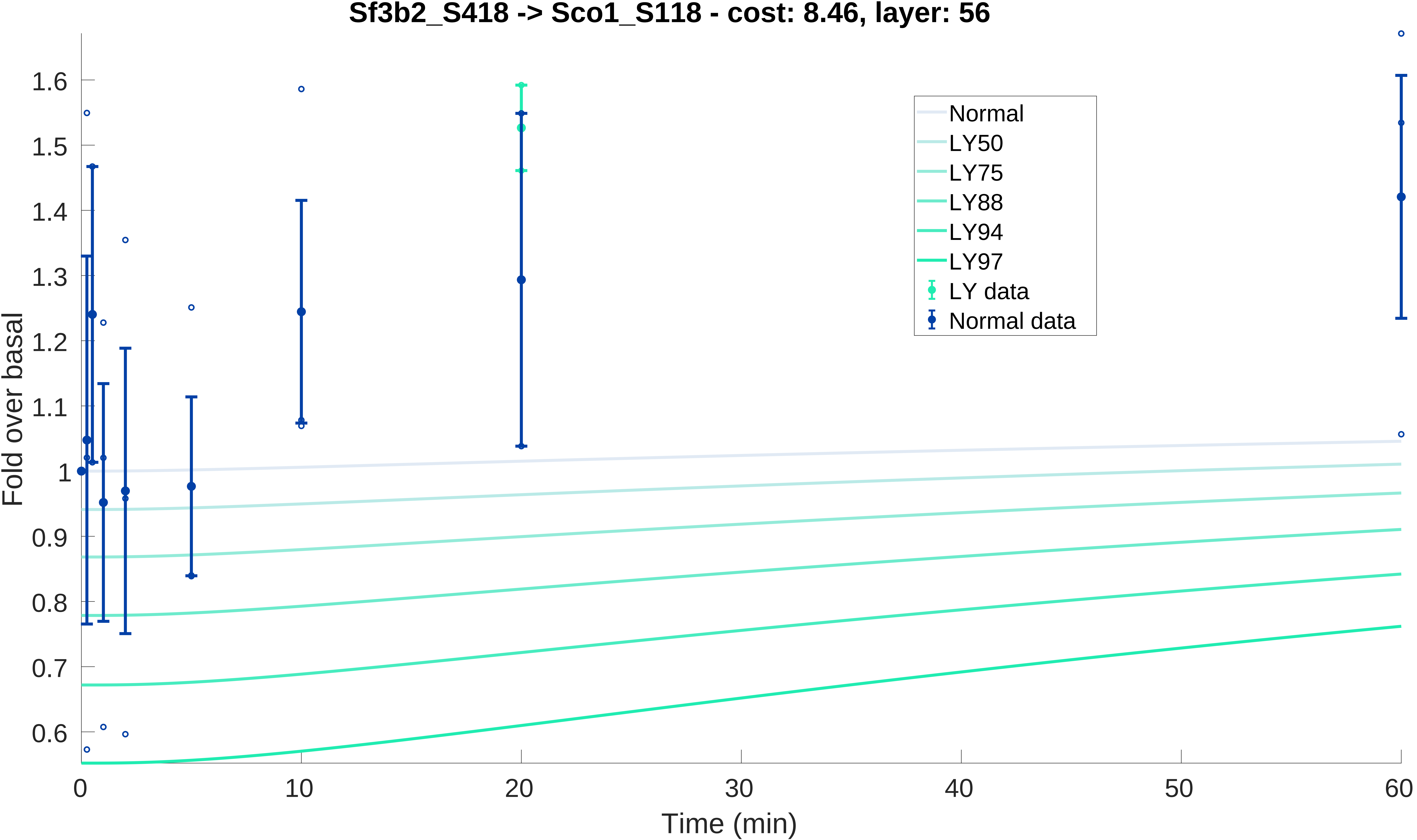

Sf3b2_S418 -> Sco1_S118 - cost: 8.46, layer: 56
Fold over basal
Time (min)
0
10
20
30
40
50
60
0.6
0.7
0.8
0.9
1
1.1
1.2
1.3
1.4
1.5
1.6
Normal
LY50
LY75
LY88
LY94
LY97
LY data
Normal data

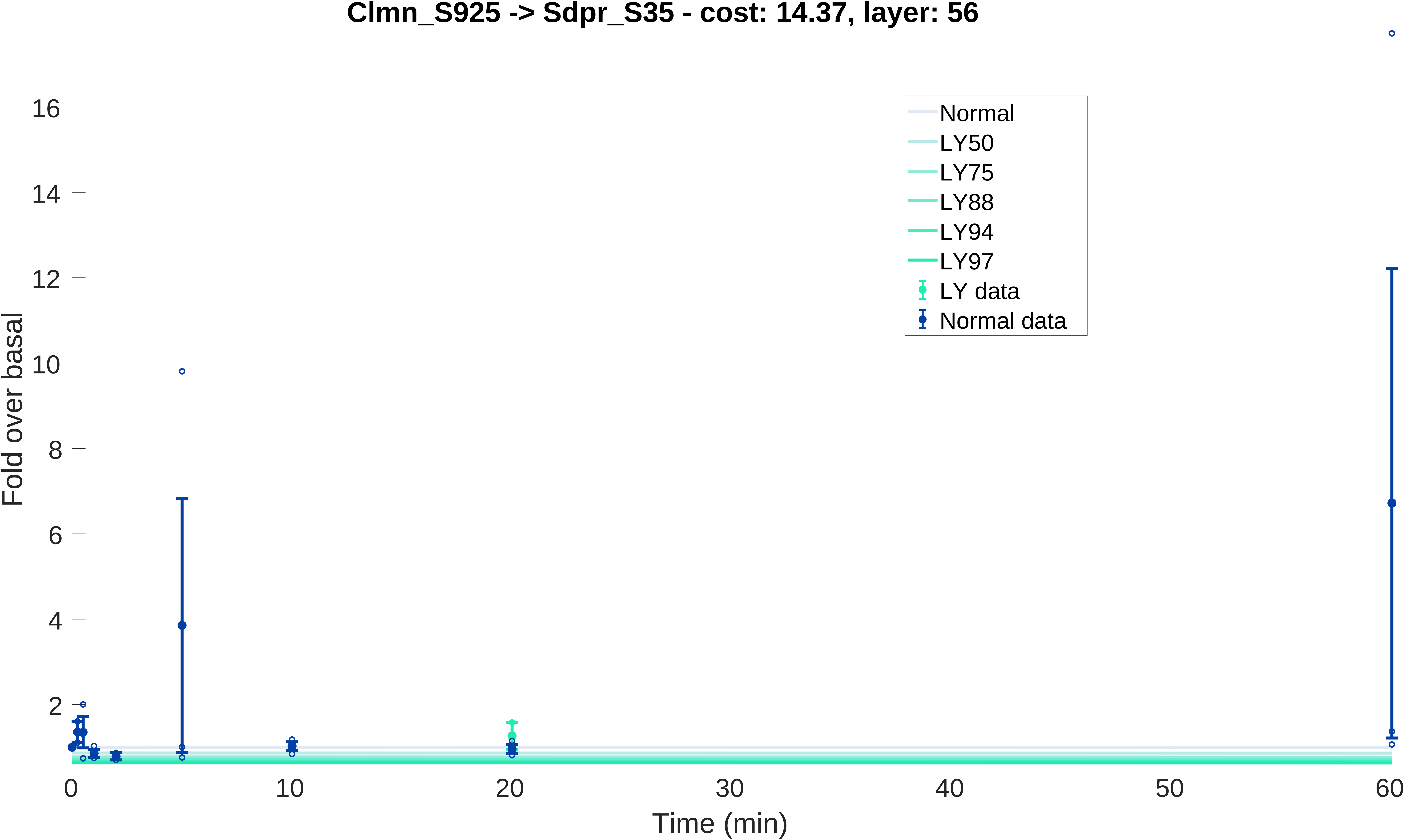
Clmn_S925 -> Sdpr_S35 - cost: 14.37, layer: 56
Fold over basal
Time (min)
0
10
20
30
40
50
60
2
4
6
8
10
12
14
16
Normal
LY50
LY75
LY88
LY94
LY97
LY data
Normal data

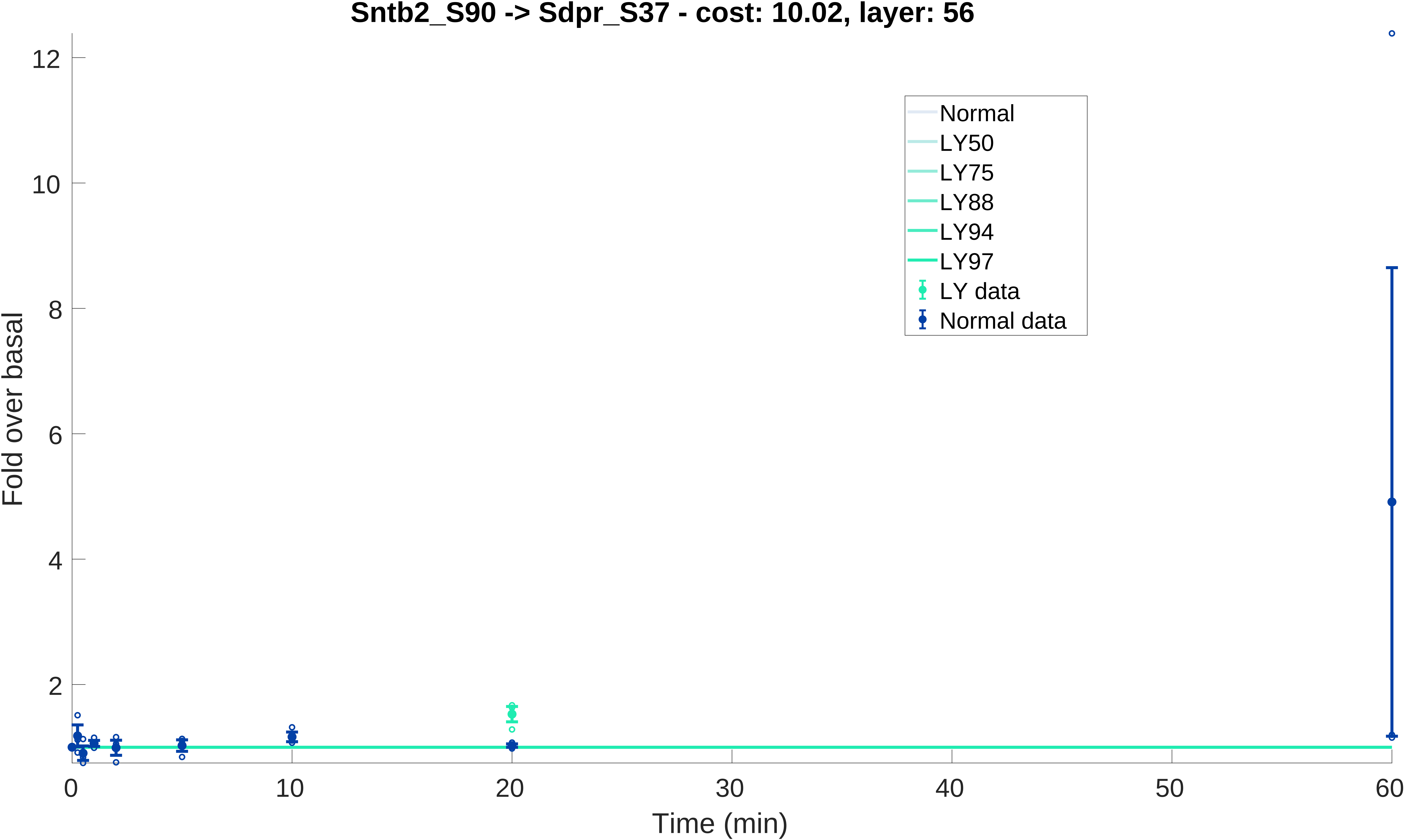

Sntb2_S90 -> Sdpr_S37 - cost: 10.02, layer: 56
Fold over basal
Time (min)
0
10
20
30
40
50
60
2
4
6
8
10
12
Normal
LY50
LY75
LY88
LY94
LY97
LY data
Normal data

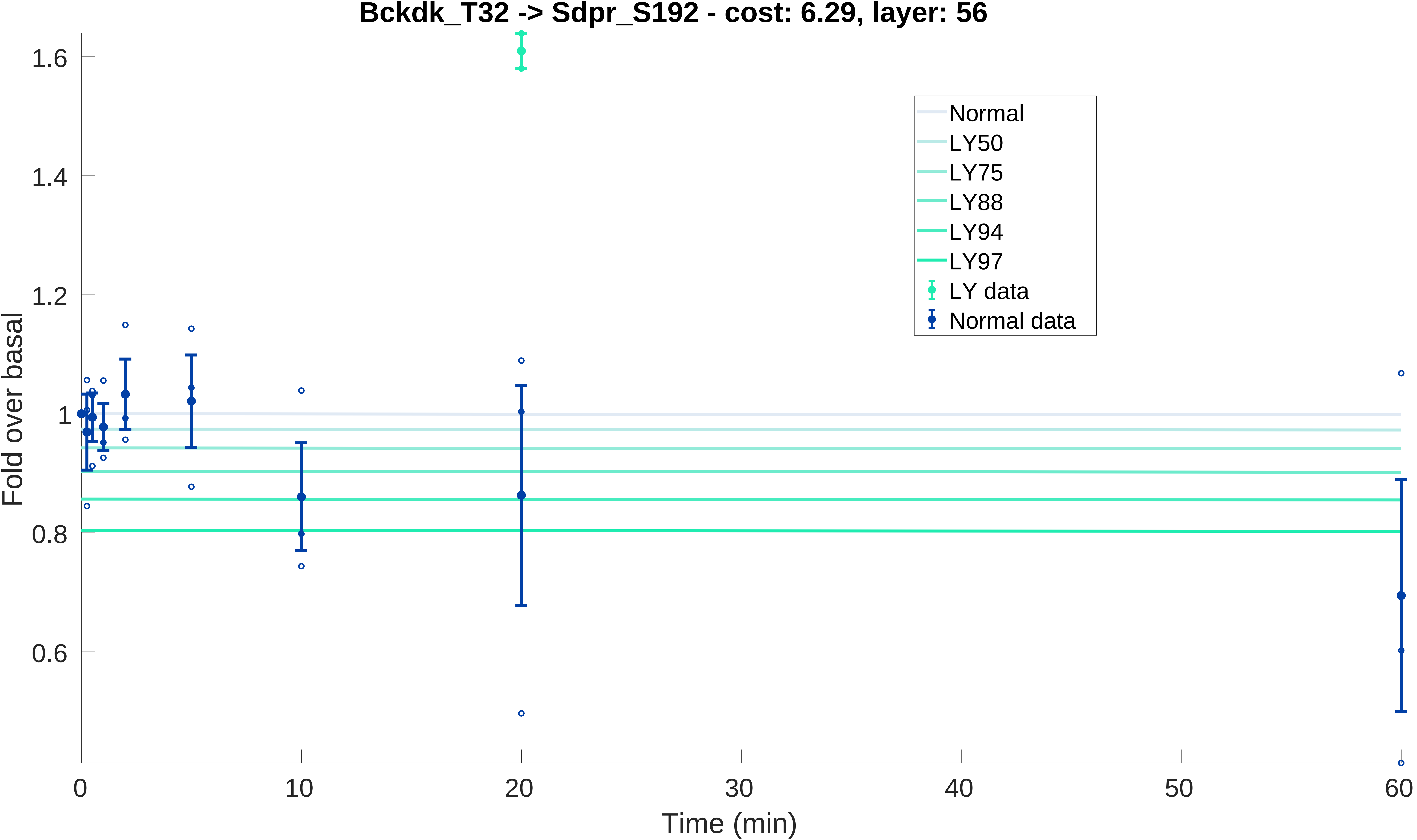

Bckdk_T32 -> Sdpr_S192 - cost: 6.29, layer: 56
Normal
LY50
LY75
LY88
LY94
LY97
LY data
Normal data
Fold over basal
Time (min)
0
10
20
30
40
50
60
0.6
0.8
1
1.2
1.4
1.6

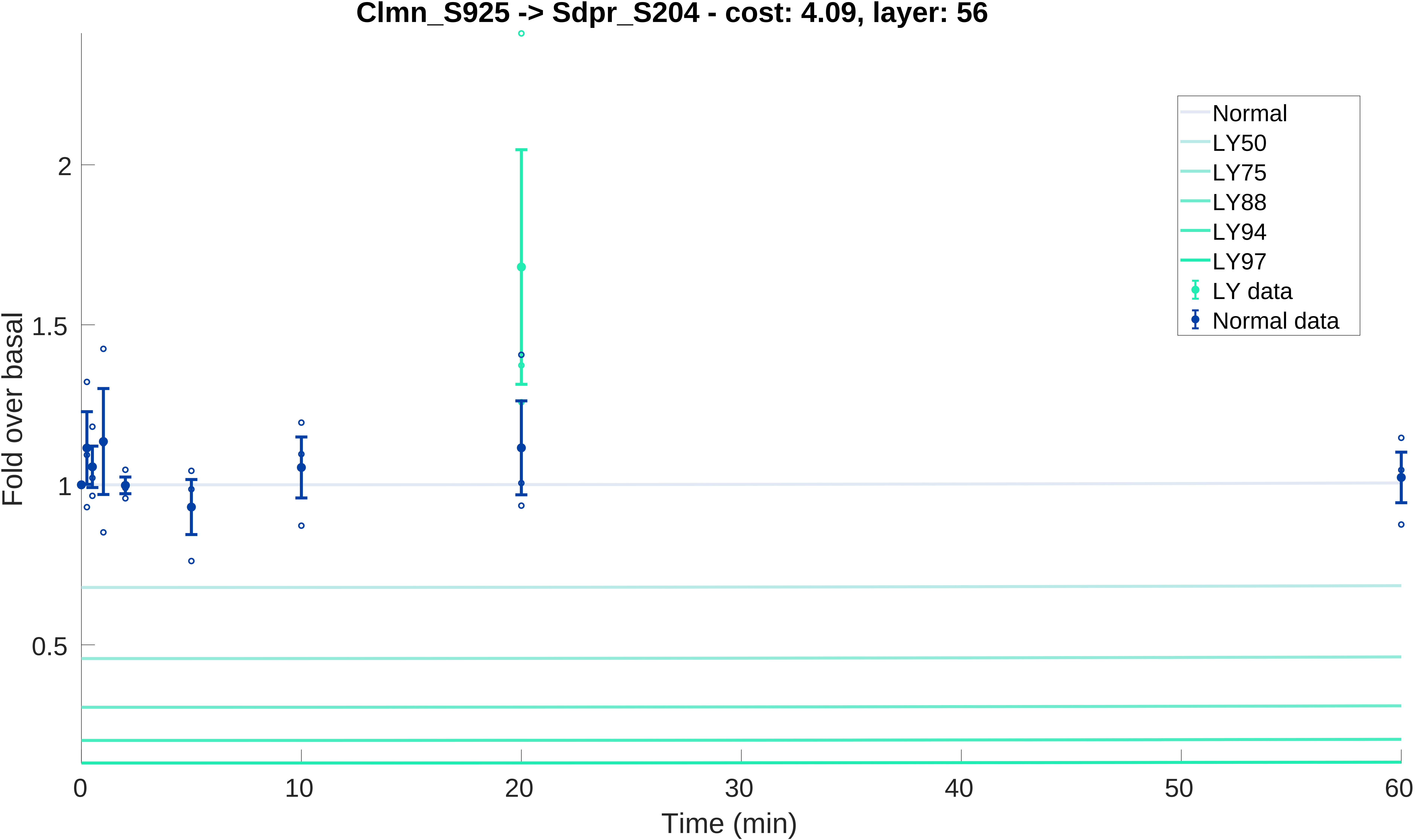

Clmn_S925 -> Sdpr_S204 - cost: 4.09, layer: 56
Fold over basal
Time (min)
0
10
20
30
40
50
60
0.5
1
1.5
2
Normal
LY50
LY75
LY88
LY94
LY97
LY data
Normal data

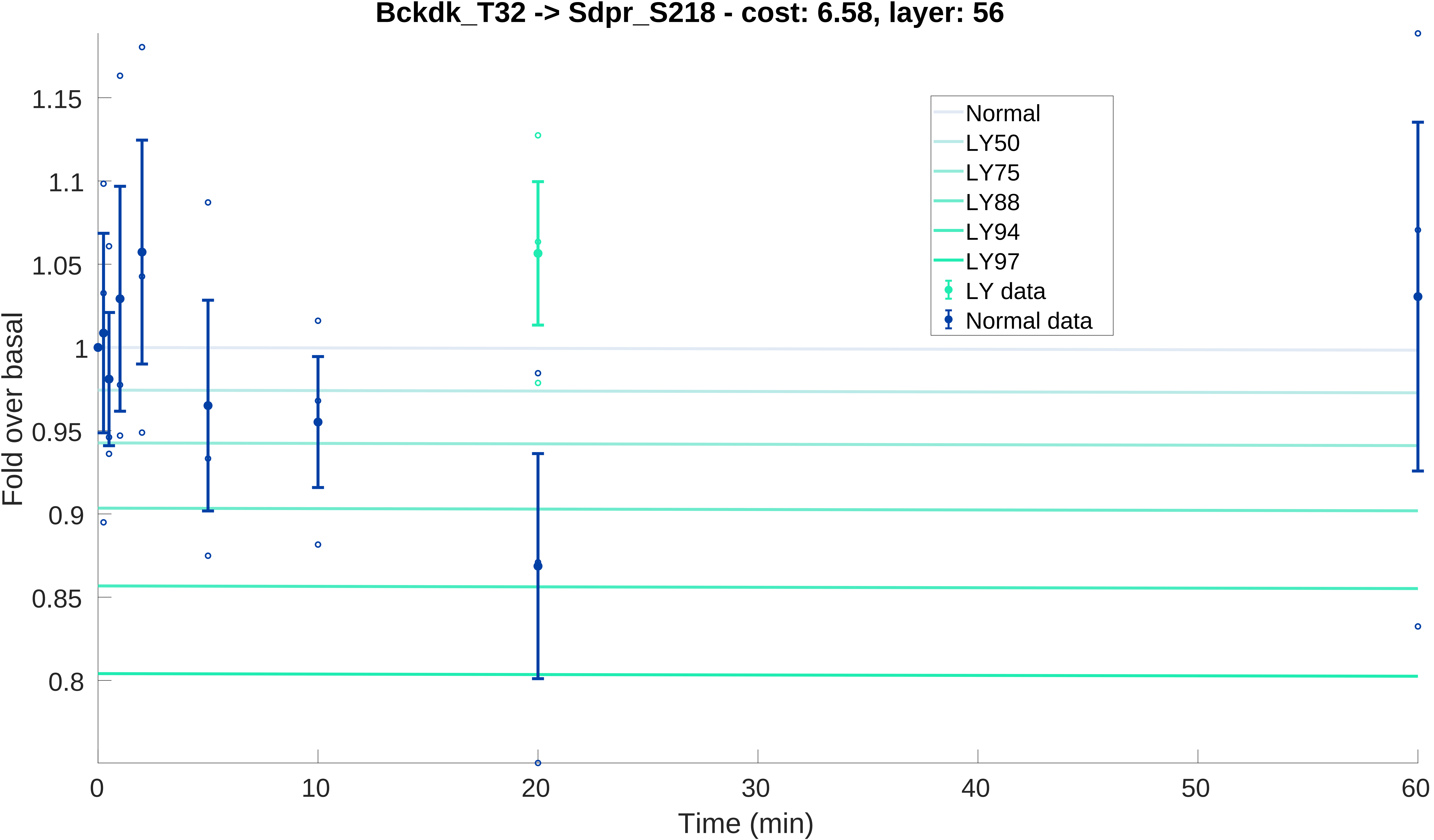
Bckdk_T32 -> Sdpr_S218 - cost: 6.58, layer: 56
Normal
LY50
LY75
LY88
LY94
LY97
LY data
Normal data
Fold over basal
Time (min)
1.15
1.1
1.05
1
0.95
0.9
0.85
0.8
0
10
20
30
40
50
60

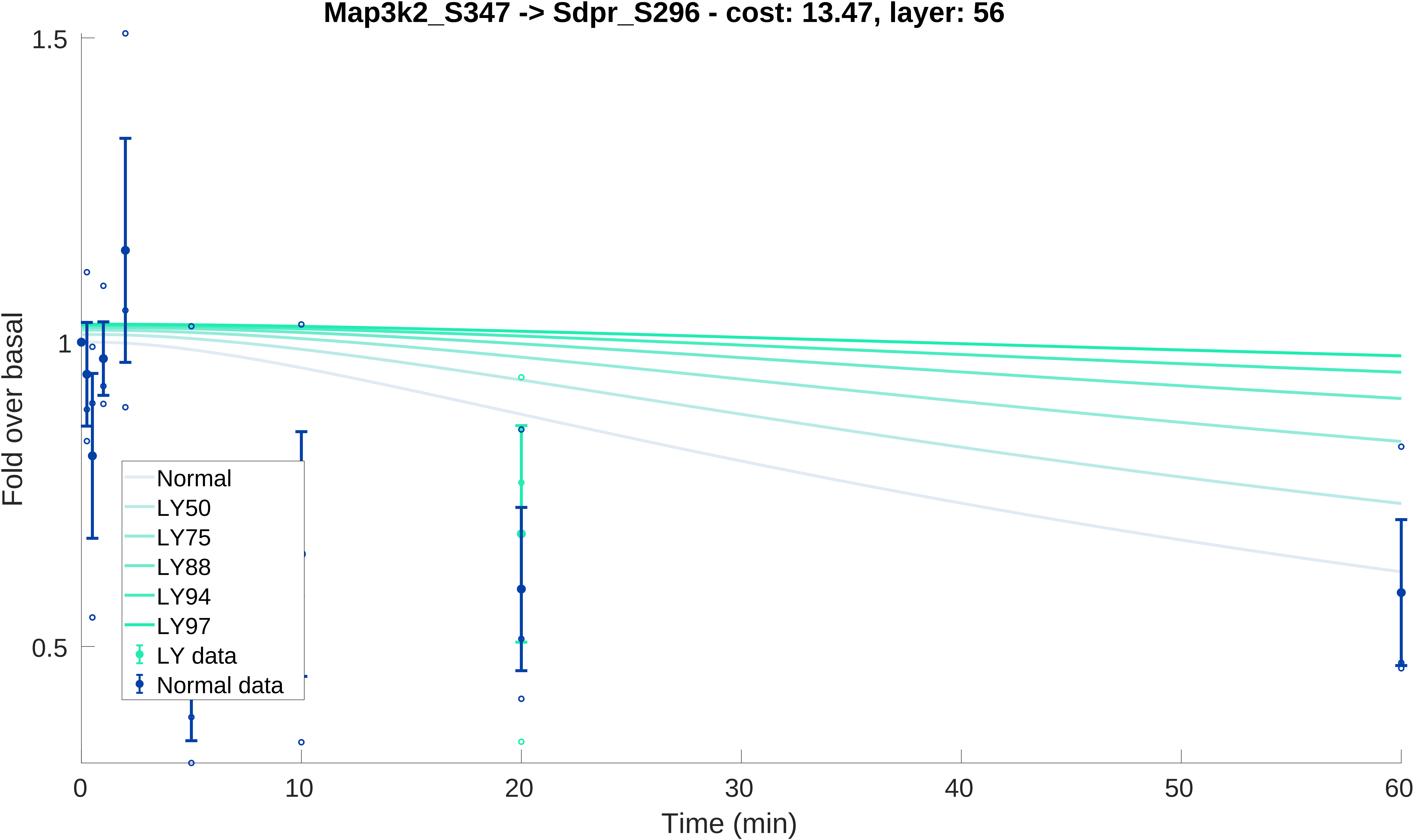

Map3k2_S347 -> Sdpr_S296 - cost: 13.47, layer: 56
Fold over basal
1.5
1
0.5
0
10
20
30
40
50
60
Time (min)
Normal
LY50
LY75
LY88
LY94
LY97
LY data
Normal data

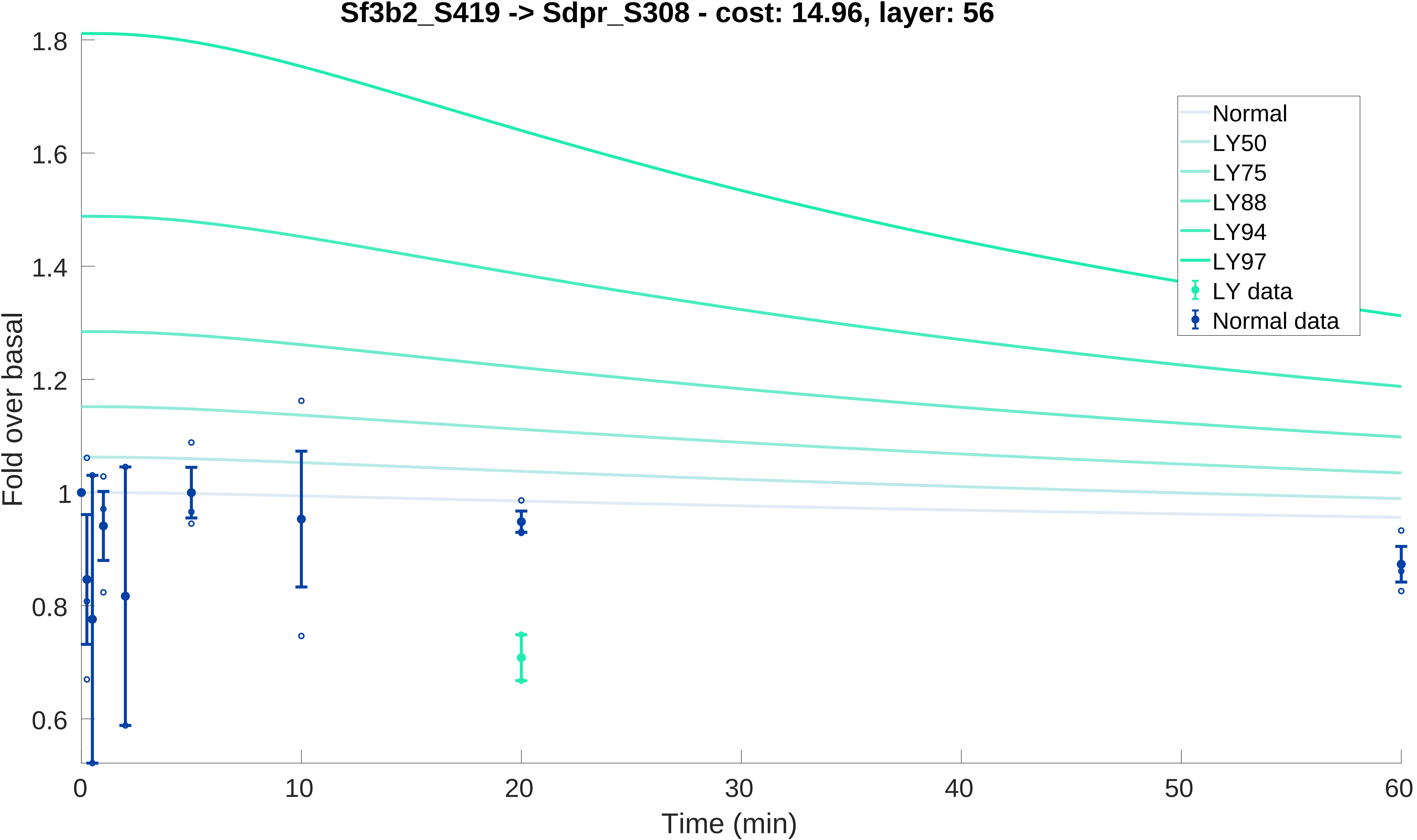

Sf3b2_S419 -> Sdpr_S308 - cost: 14.96, layer: 56
Fold over basal
Time (min)
1.8
1.6
1.4
1.2
1
0.8
0.6
0
10
20
30
40
50
60
Normal
LY50
LY75
LY88
LY94
LY97
LY data
Normal data

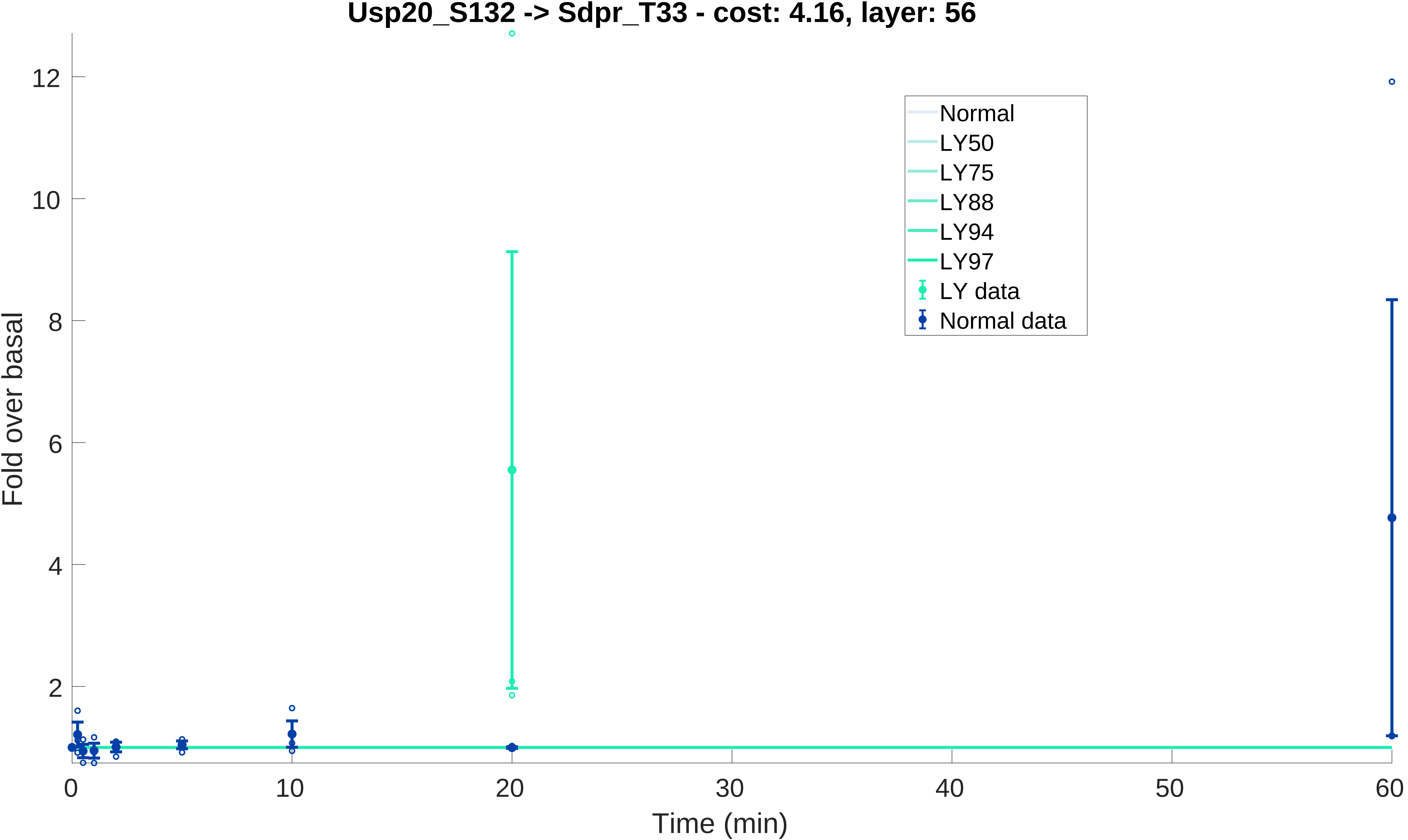

Usp20_S132 -> Sdpr_T33 - cost: 4.16, layer: 56
Fold over basal
Time (min)
Normal
LY50
LY75
LY88
LY94
LY97
LY data
Normal data
0
10
20
30
40
50
60
2
4
6
8
10
12

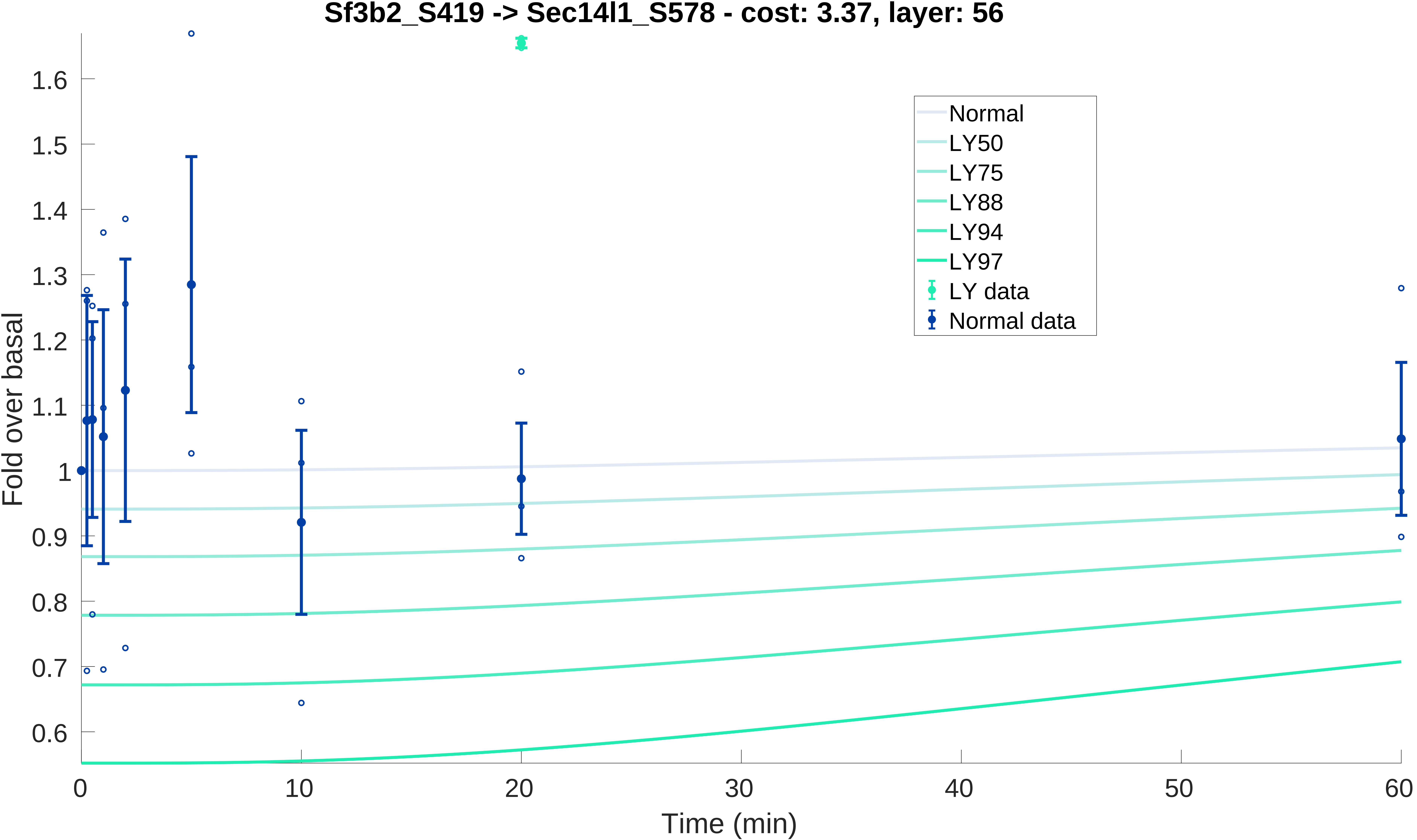

Sf3b2_S419 -> Sec14l1_S578 - cost: 3.37, layer: 56
Normal
LY50
LY75
LY88
LY94
LY97
LY data
Normal data
Fold over basal
Time (min)
1.6
1.5
1.4
1.3
1.2
1.1
1
0.9
0.8
0.7
0.6
0
10
20
30
40
50
60

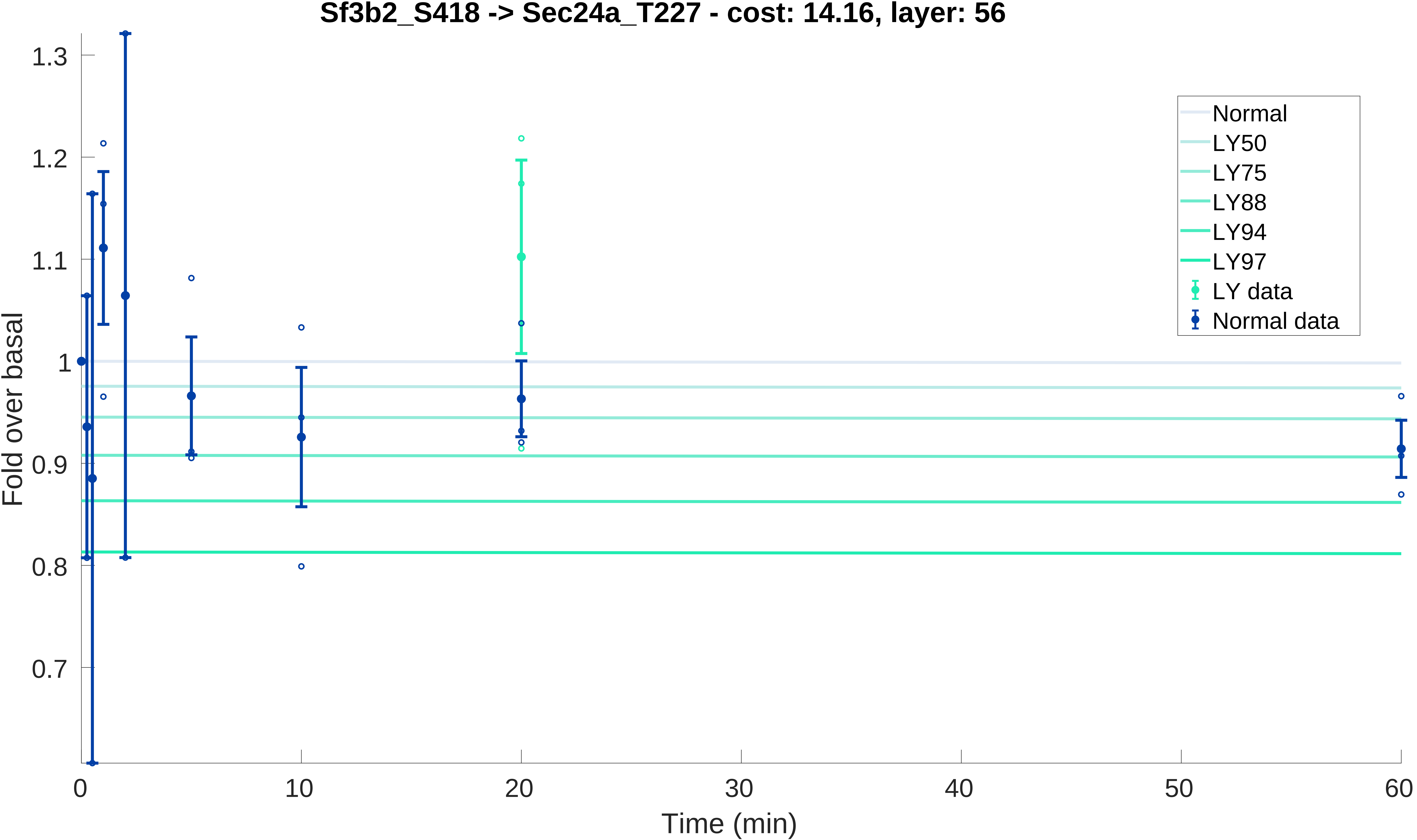
Sf3b2_S418 -> Sec24a_T227 - cost: 14.16, layer: 56
Fold over basal
Time (min)
1.3
1.2
1.1
1
0.9
0.8
0.7
0
10
20
30
40
50
60
Normal
LY50
LY75
LY88
LY94
LY97
LY data
Normal data

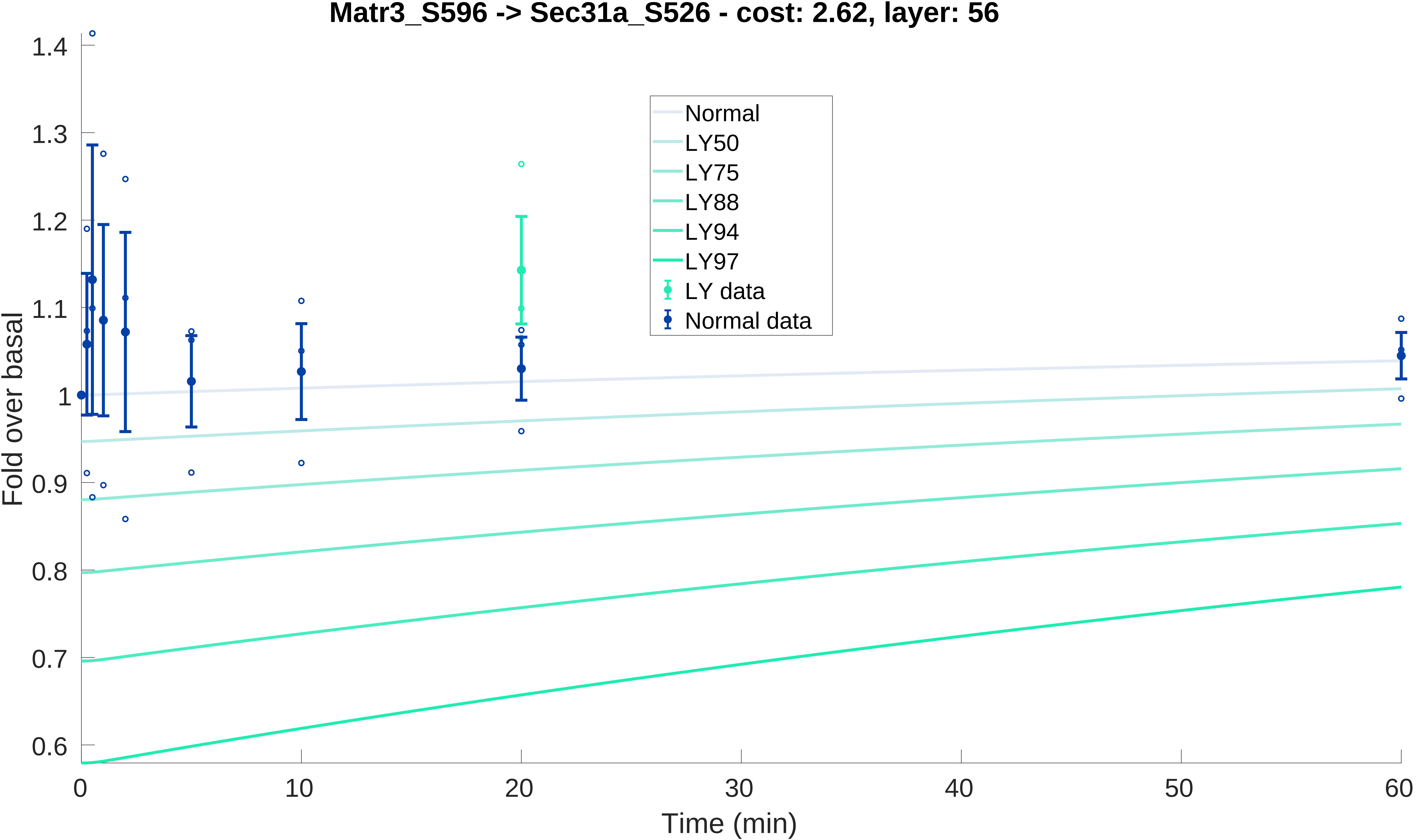

Matr3_S596 -> Sec31a_S526 - cost: 2.62, layer: 56
Fold over basal
Time (min)
Normal
LY50
LY75
LY88
LY94
LY97
LY data
Normal data
1.4
1.3
1.2
1.1
1
0.9
0.8
0.7
0.6
0
10
20
30
40
50
60

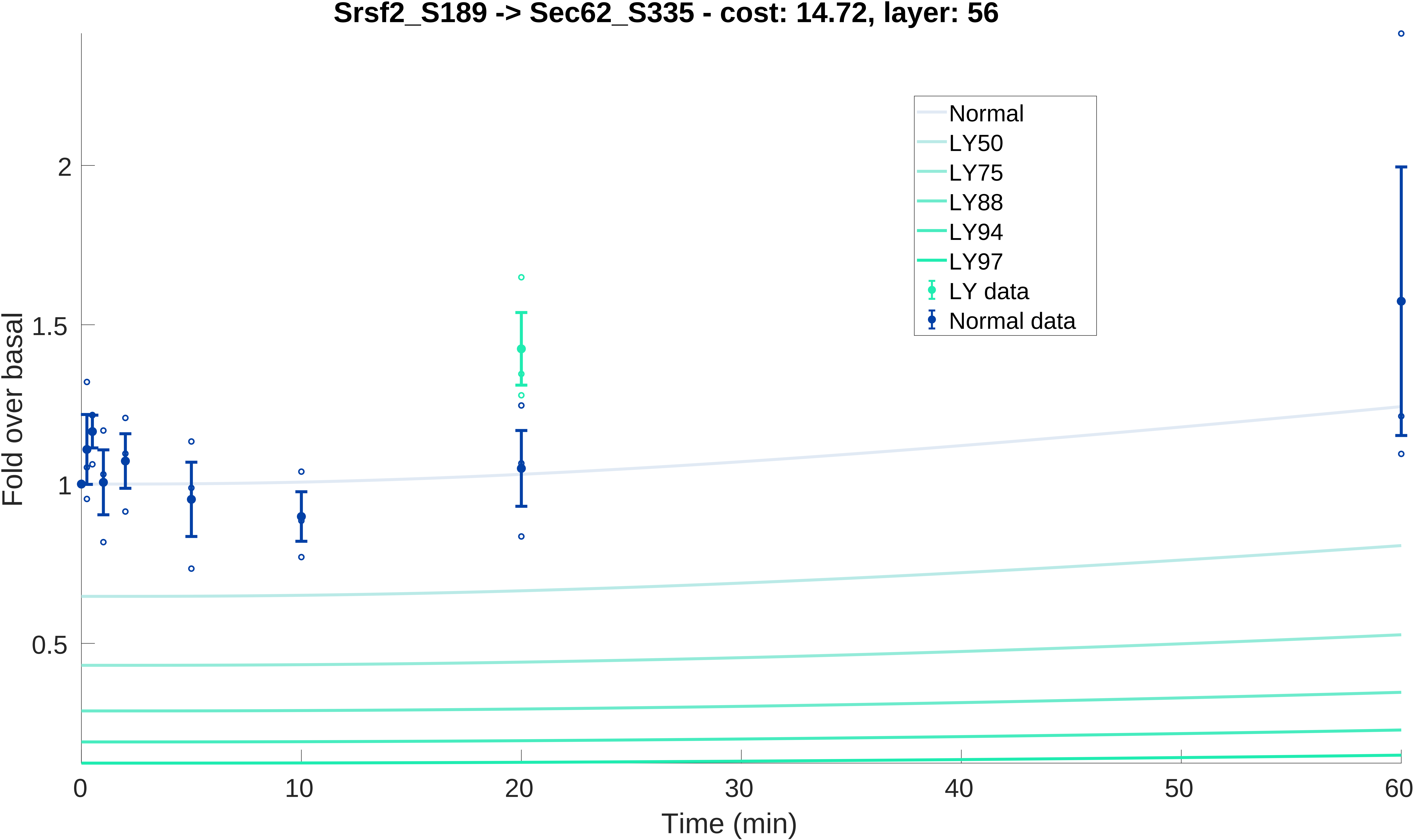

Srsf2_S189 -> Sec62_S335 - cost: 14.72, layer: 56
Fold over basal
0.5
1
1.5
2
0
10
20
30
40
50
60
Time (min)
Normal
LY50
LY75
LY88
LY94
LY97
LY data
Normal data

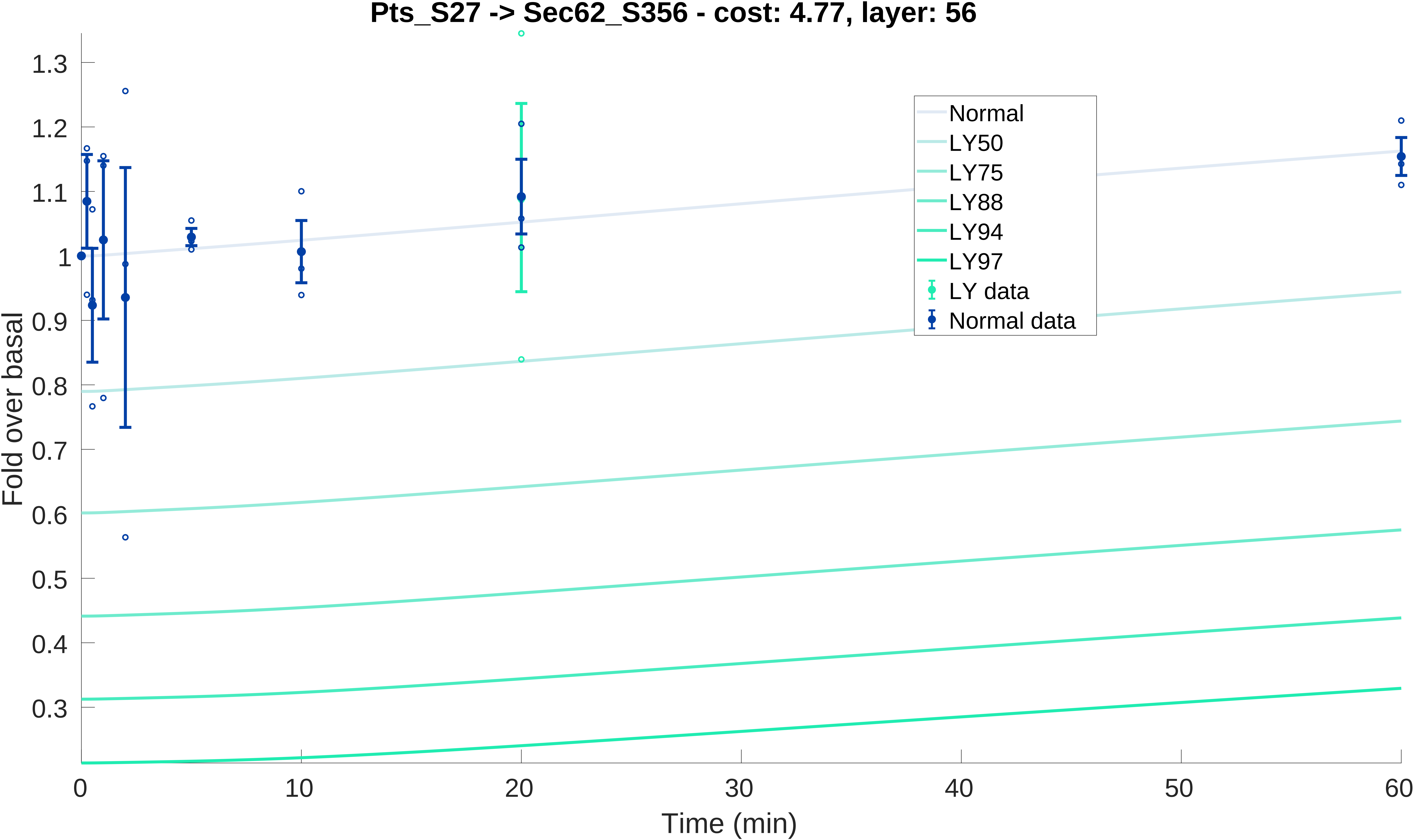
Pts_S27 -> Sec62_S356 - cost: 4.77, layer: 56
Fold over basal
Time (min)
1.3
1.2
1.1
1
0.9
0.8
0.7
0.6
0.5
0.4
0.3
0
10
20
30
40
50
60
Normal
LY50
LY75
LY88
LY94
LY97
LY data
Normal data

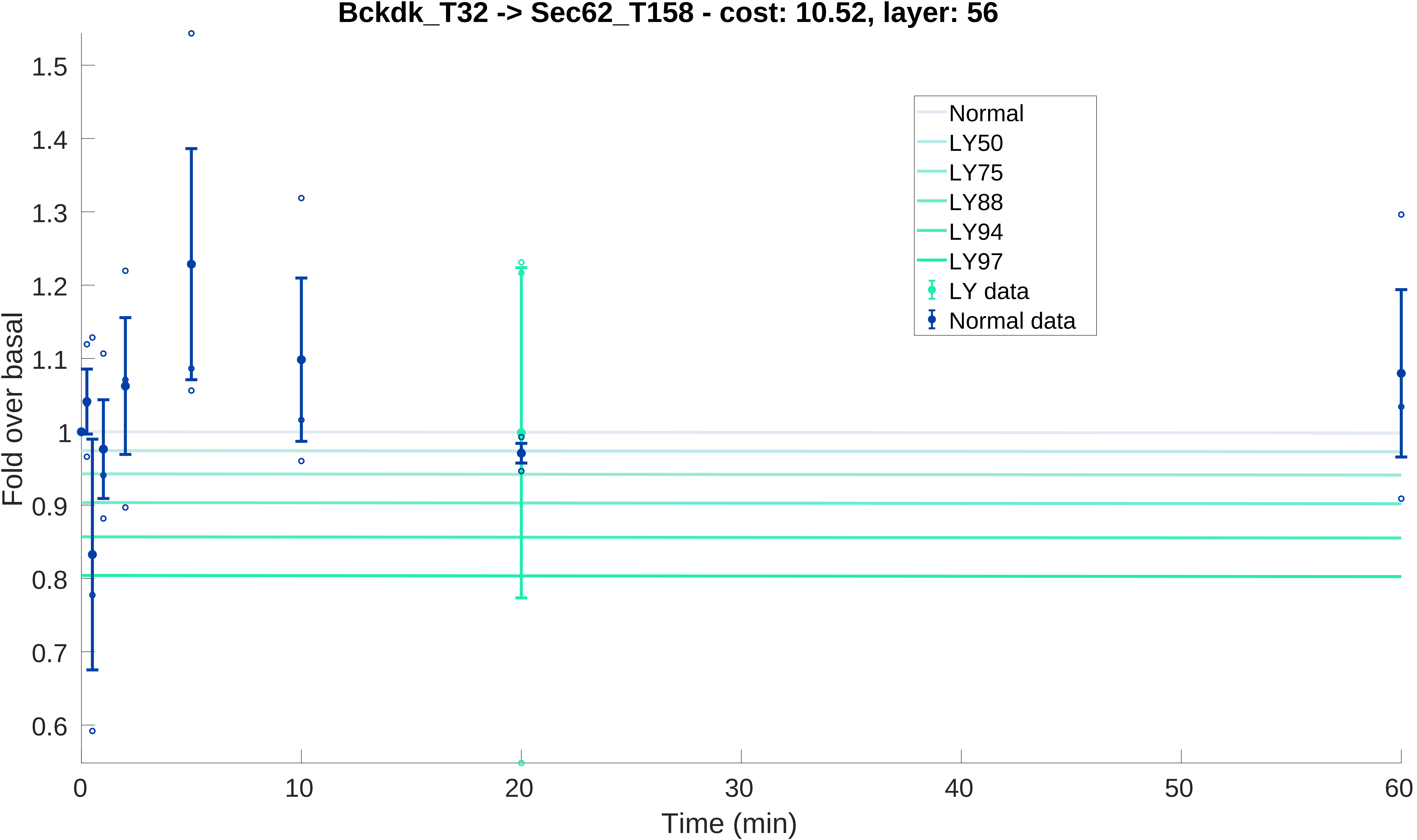

Bckdk_T32 -> Sec62_T158 - cost: 10.52, layer: 56
Fold over basal
Time (min)
1.5
1.4
1.3
1.2
1.1
1
0.9
0.8
0.7
0.6
0
10
20
30
40
50
60
Normal
LY50
LY75
LY88
LY94
LY97
LY data
Normal data

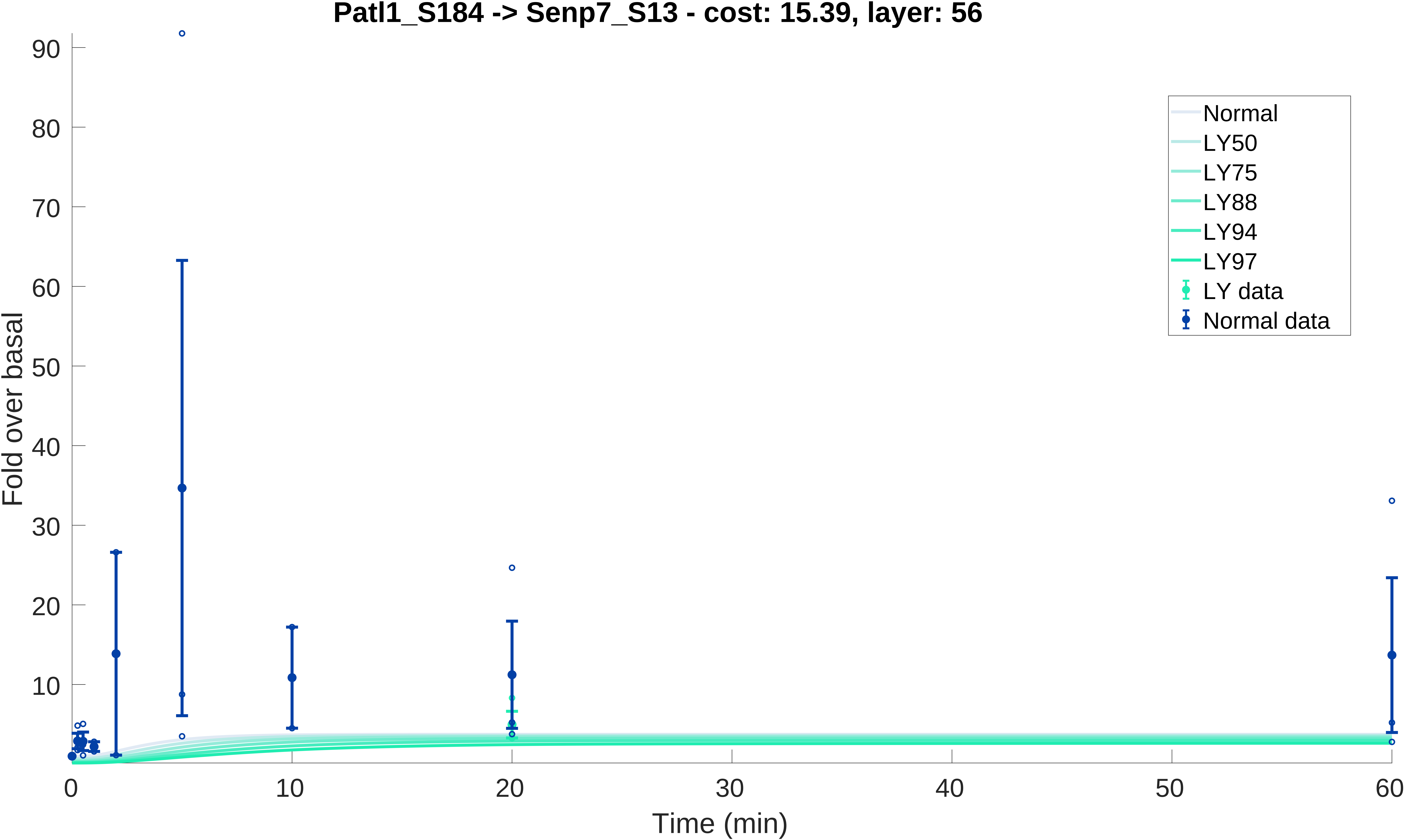

Patl1_S184 -> Senp7_S13 - cost: 15.39, layer: 56
Fold over basal
Time (min)
0
10
20
30
40
50
60
70
80
90
Normal
LY50
LY75
LY88
LY94
LY97
LY data
Normal data

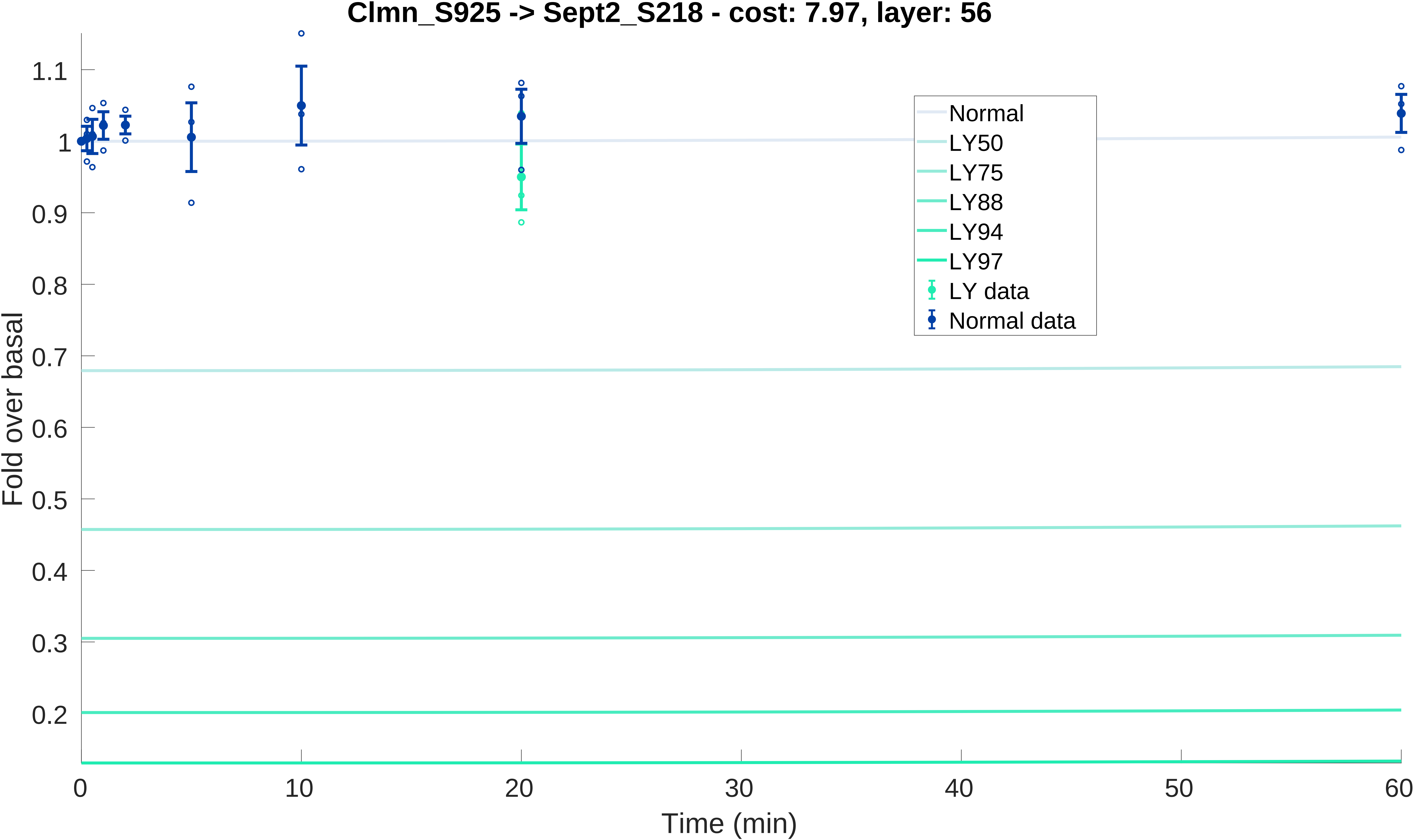

Clmn_S925 -> Sept2_S218 - cost: 7.97, layer: 56
Fold over basal
Time (min)
1.1
1
0.9
0.8
0.7
0.6
0.5
0.4
0.3
0.2
0
10
20
30
40
50
60
Normal
LY50
LY75
LY88
LY94
LY97
LY data
Normal data

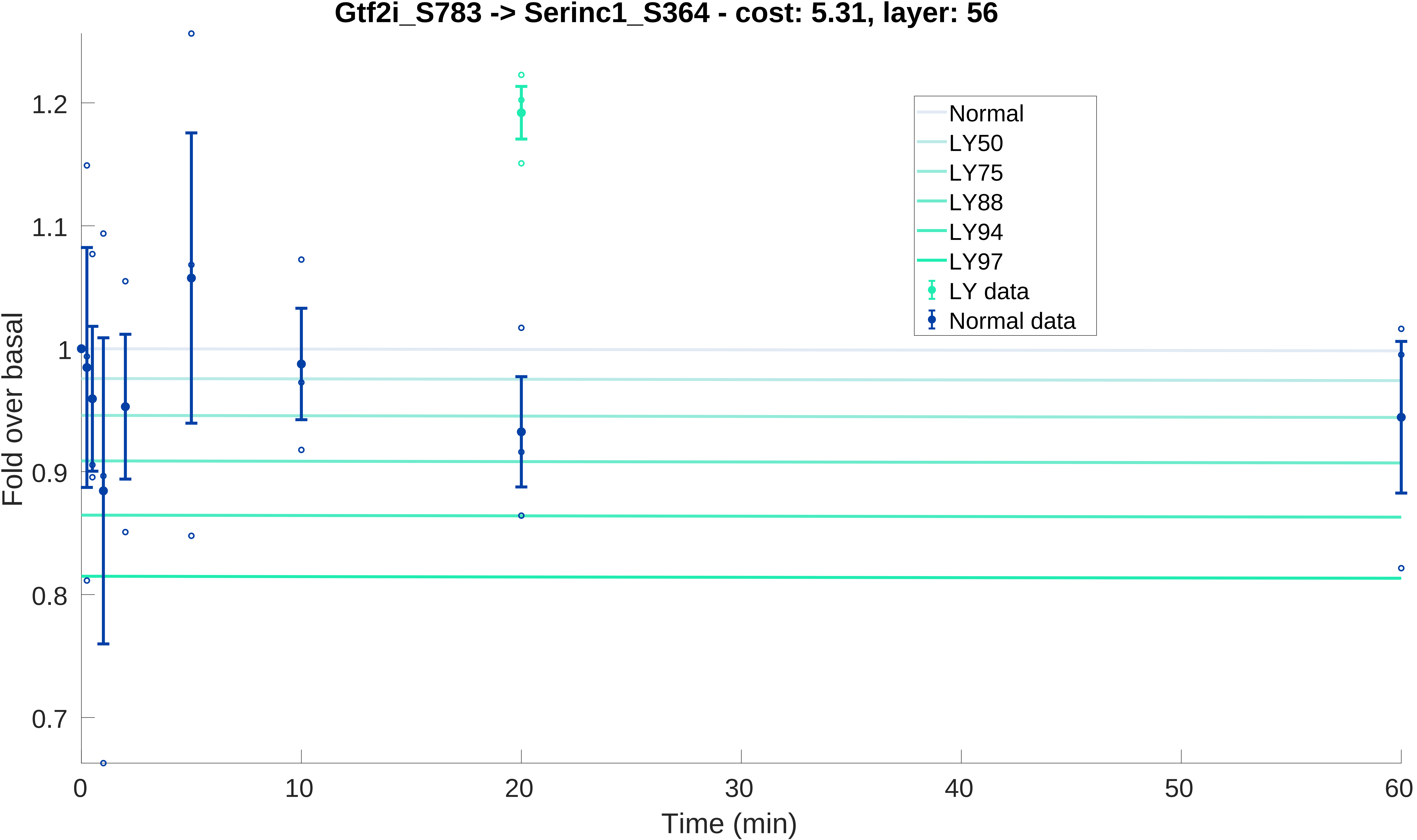

Gtf2i_S783 -> Serinc1_S364 - cost: 5.31, layer: 56
Fold over basal
Time (min)
0.7
0.8
0.9
1
1.1
1.2
0
10
20
30
40
50
60
Normal
LY50
LY75
LY88
LY94
LY97
LY data
Normal data

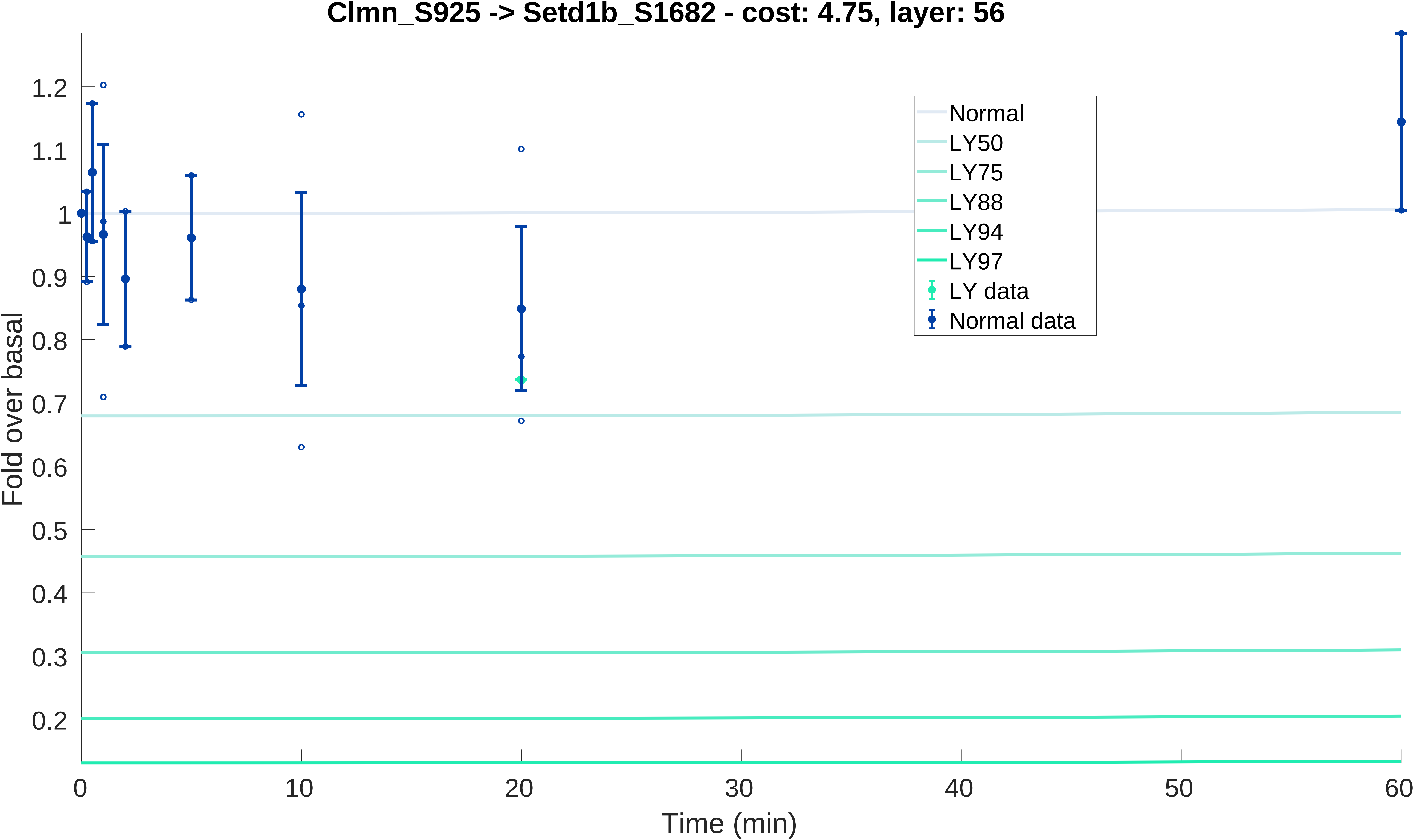
Clmn_S925 -> Setd1b_S1682 - cost: 4.75, layer: 56
Fold over basal
Time (min)
1.2
1.1
1
0.9
0.8
0.7
0.6
0.5
0.4
0.3
0.2
0
10
20
30
40
50
60
Normal
LY50
LY75
LY88
LY94
LY97
LY data
Normal data

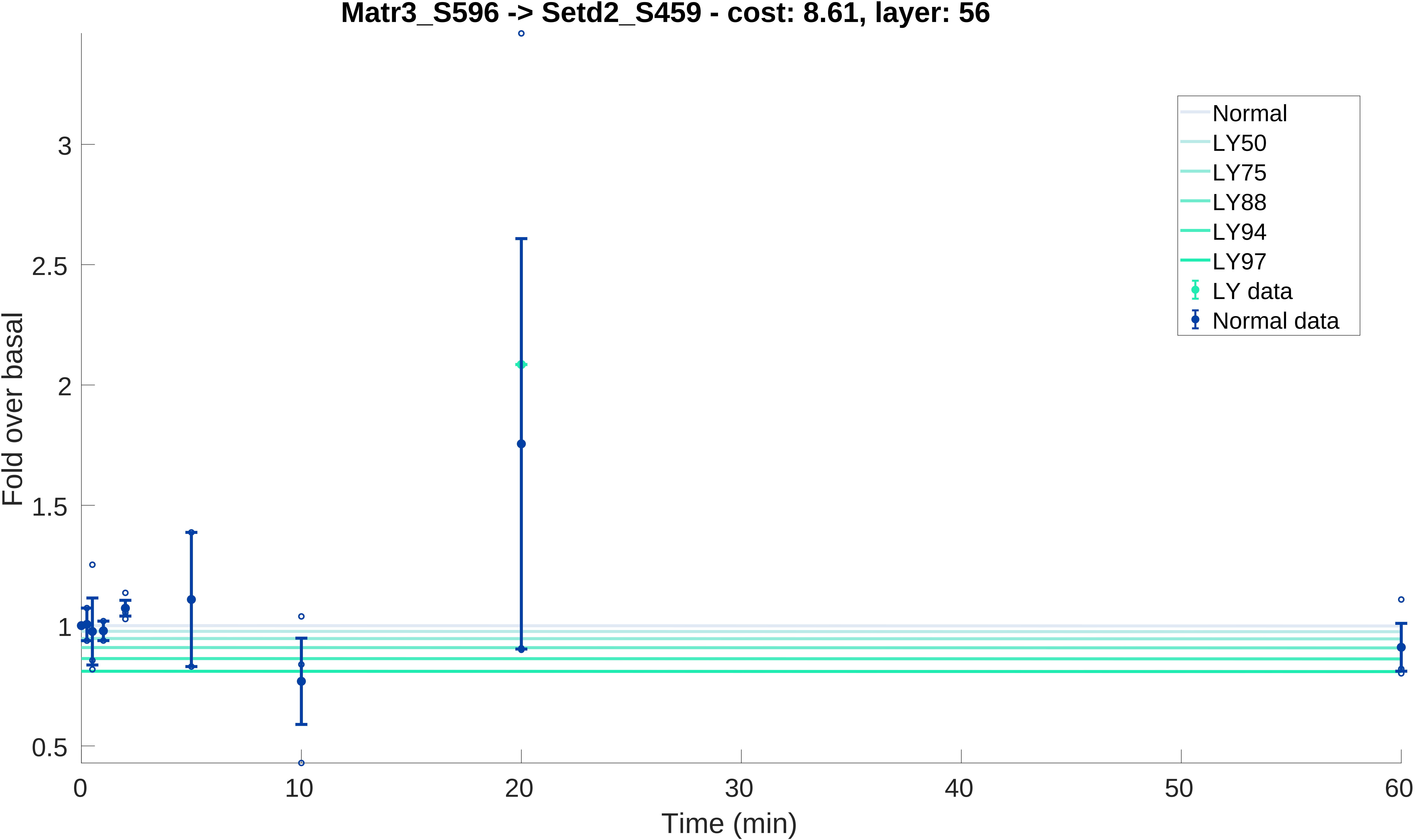
Matr3_S596 -> Setd2_S459 - cost: 8.61, layer: 56
Fold over basal
Time (min)
0.5
1
1.5
2
2.5
3
0
10
20
30
40
50
60
Normal
LY50
LY75
LY88
LY94
LY97
LY data
Normal data

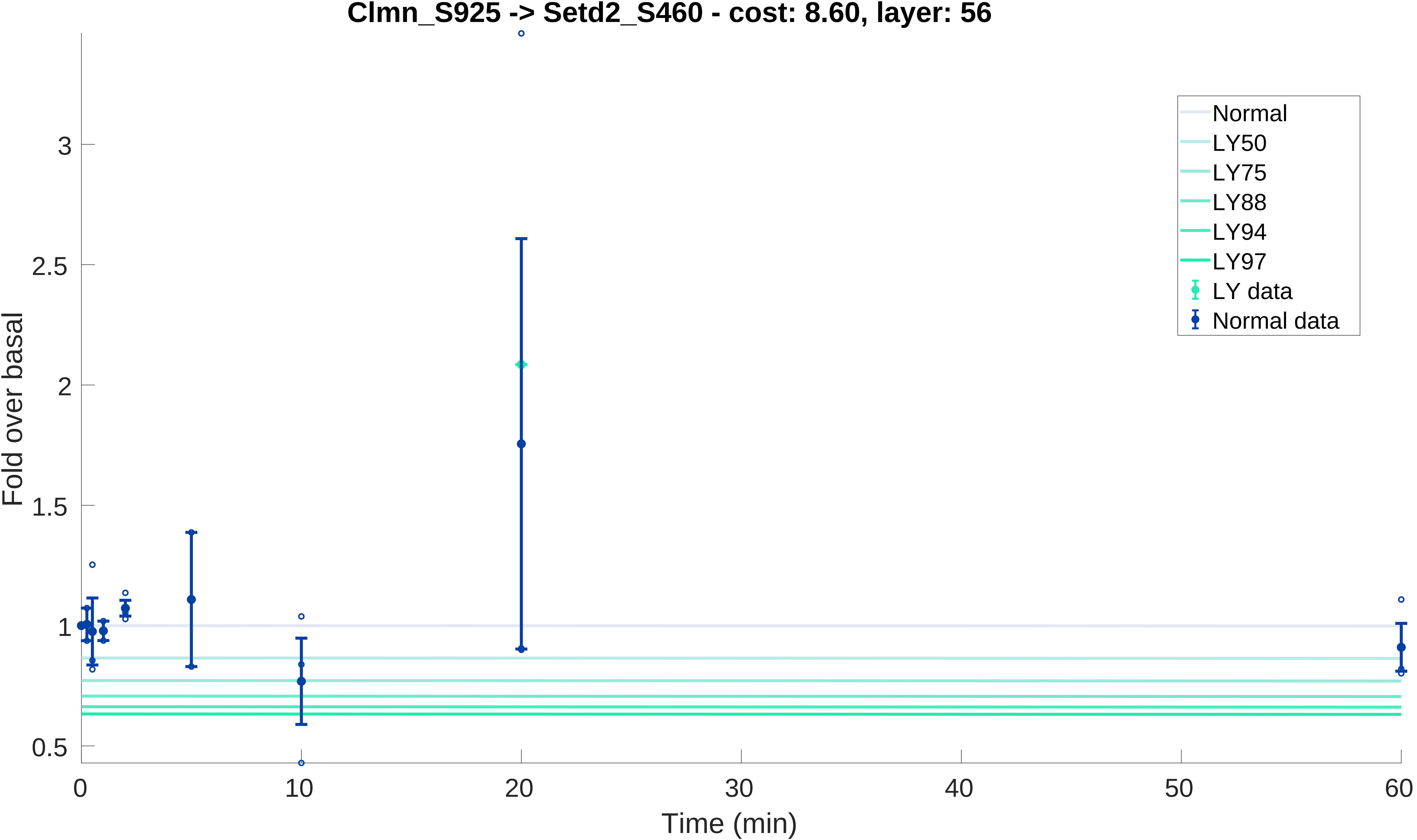

Clmn_S925 -> Setd2_S460 - cost: 8.60, layer: 56
Fold over basal
Time (min)
0.5
1
1.5
2
2.5
3
0
10
20
30
40
50
60
Normal
LY50
LY75
LY88
LY94
LY97
LY data
Normal data

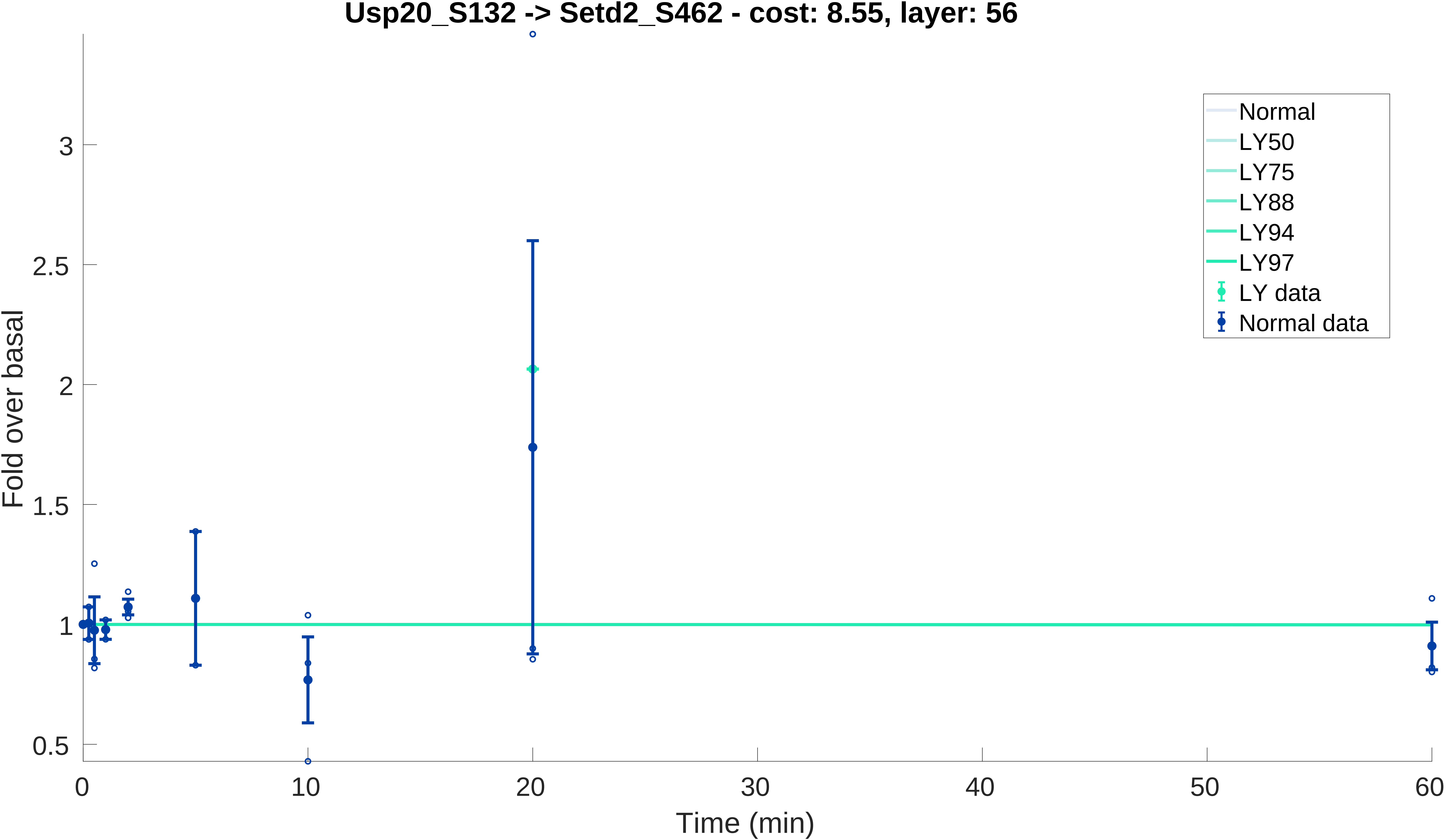

Usp20_S132 -> Setd2_S462 - cost: 8.55, layer: 56
Fold over basal
0.5
1
1.5
2
2.5
3
Time (min)
0
10
20
30
40
50
60
Normal
LY50
LY75
LY88
LY94
LY97
LY data
Normal data

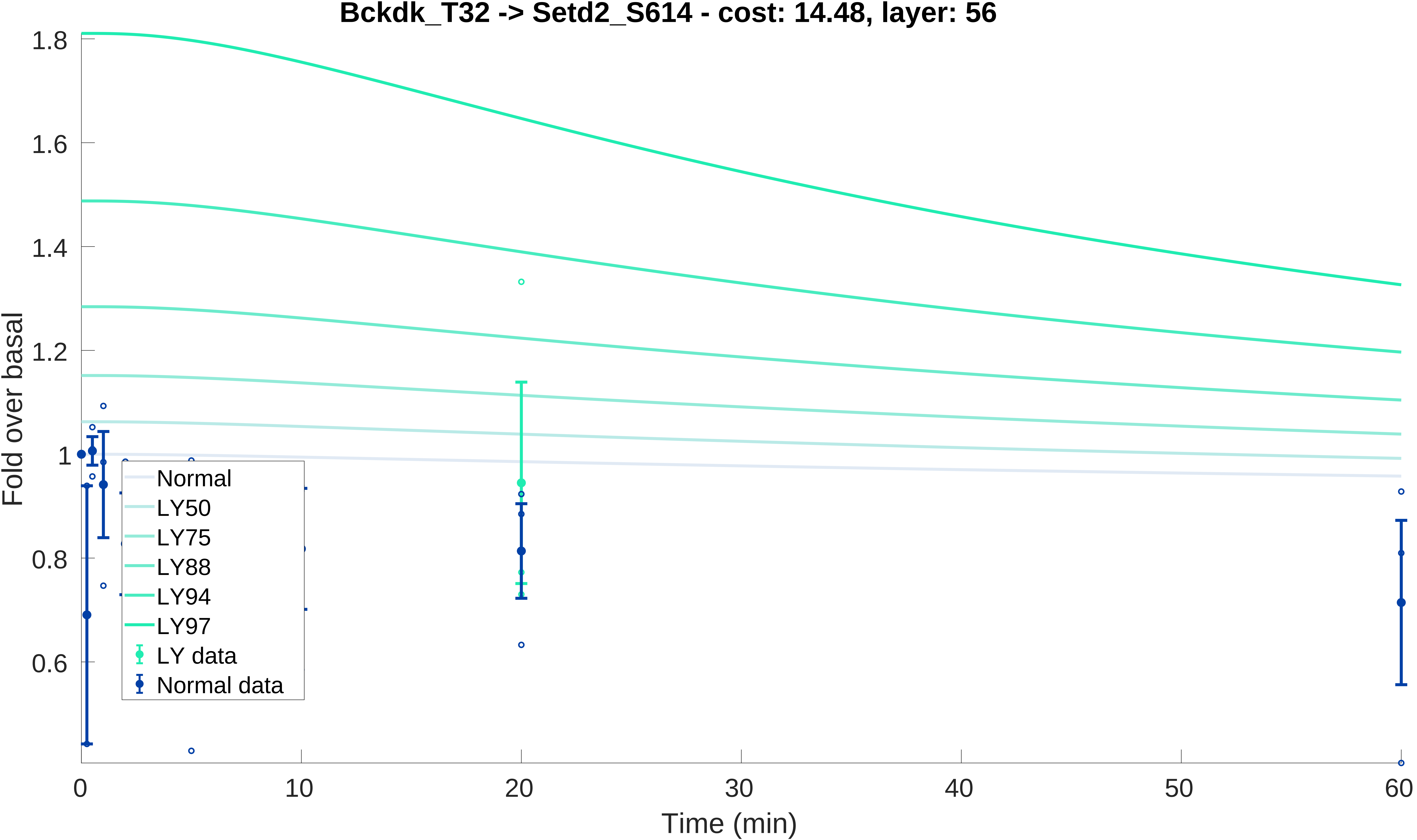

Bckdk_T32 -> Setd2_S614 - cost: 14.48, layer: 56
1.8
1.6
1.4
1.2
1
0.8
0.6
Fold over basal
0
10
20
30
40
50
60
Time (min)
Normal
LY50
LY75
LY88
LY94
LY97
LY data
Normal data

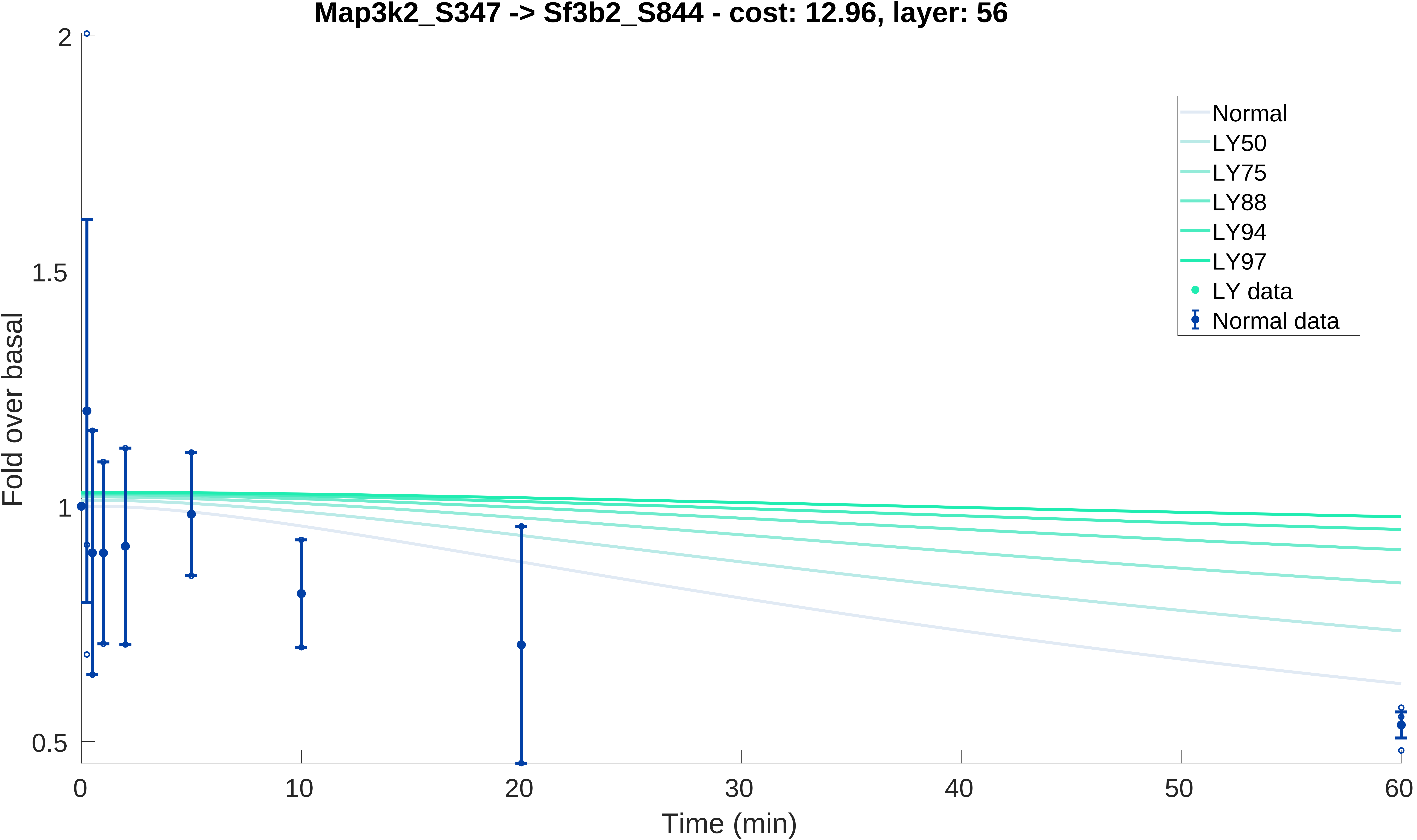

Map3k2_S347 -> Sf3b2_S844 - cost: 12.96, layer: 56
Fold over basal
Time (min)
2
1.5
1
0.5
0
10
20
30
40
50
60
Normal
LY50
LY75
LY88
LY94
LY97
LY data
Normal data

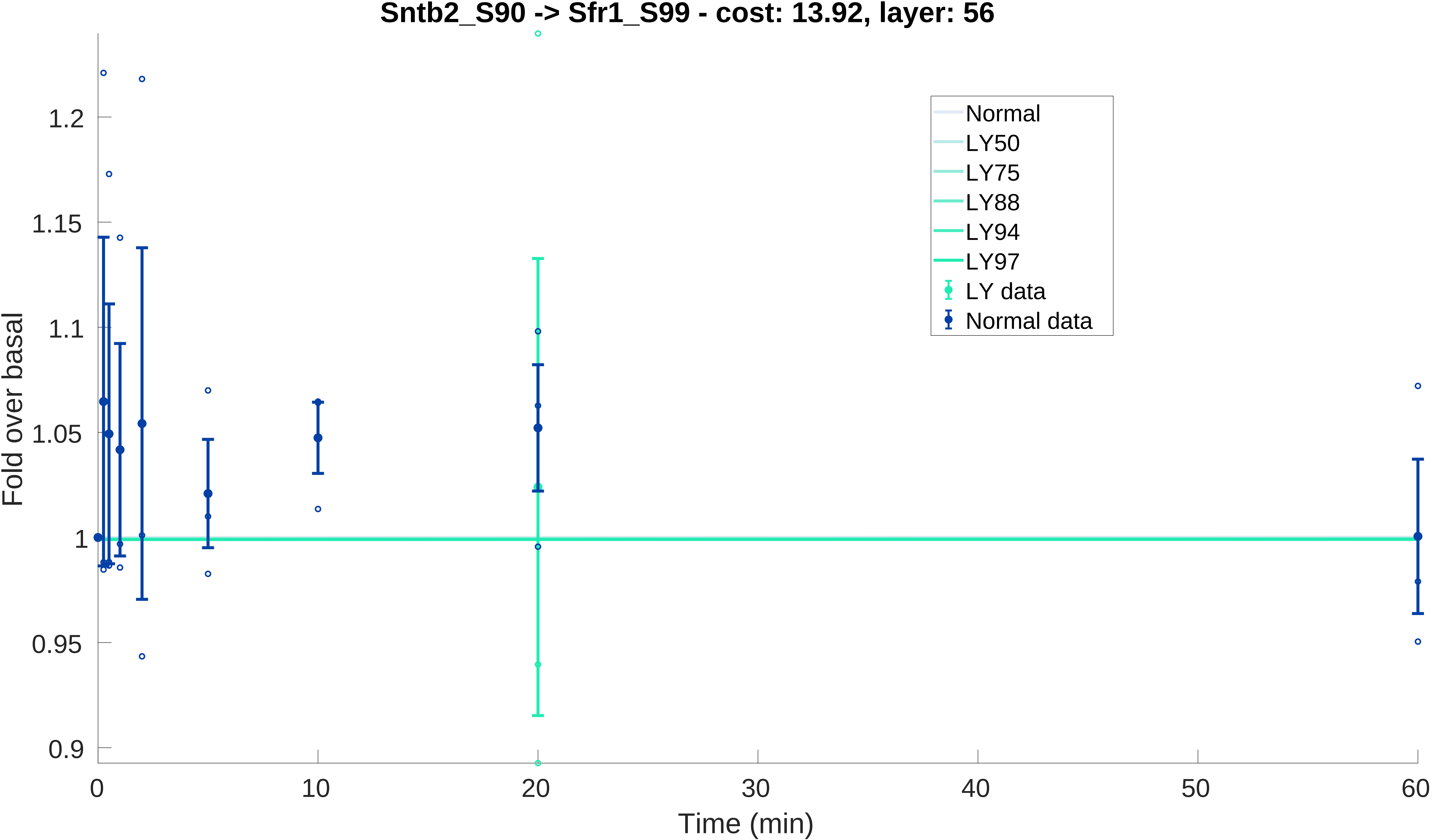

Sntb2_S90 -> Sfr1_S99 - cost: 13.92, layer: 56
Normal
LY50
LY75
LY88
LY94
LY97
LY data
Normal data
Fold over basal
1.2
1.15
1.1
1.05
1
0.95
0.9
0
10
20
30
40
50
60
Time (min)

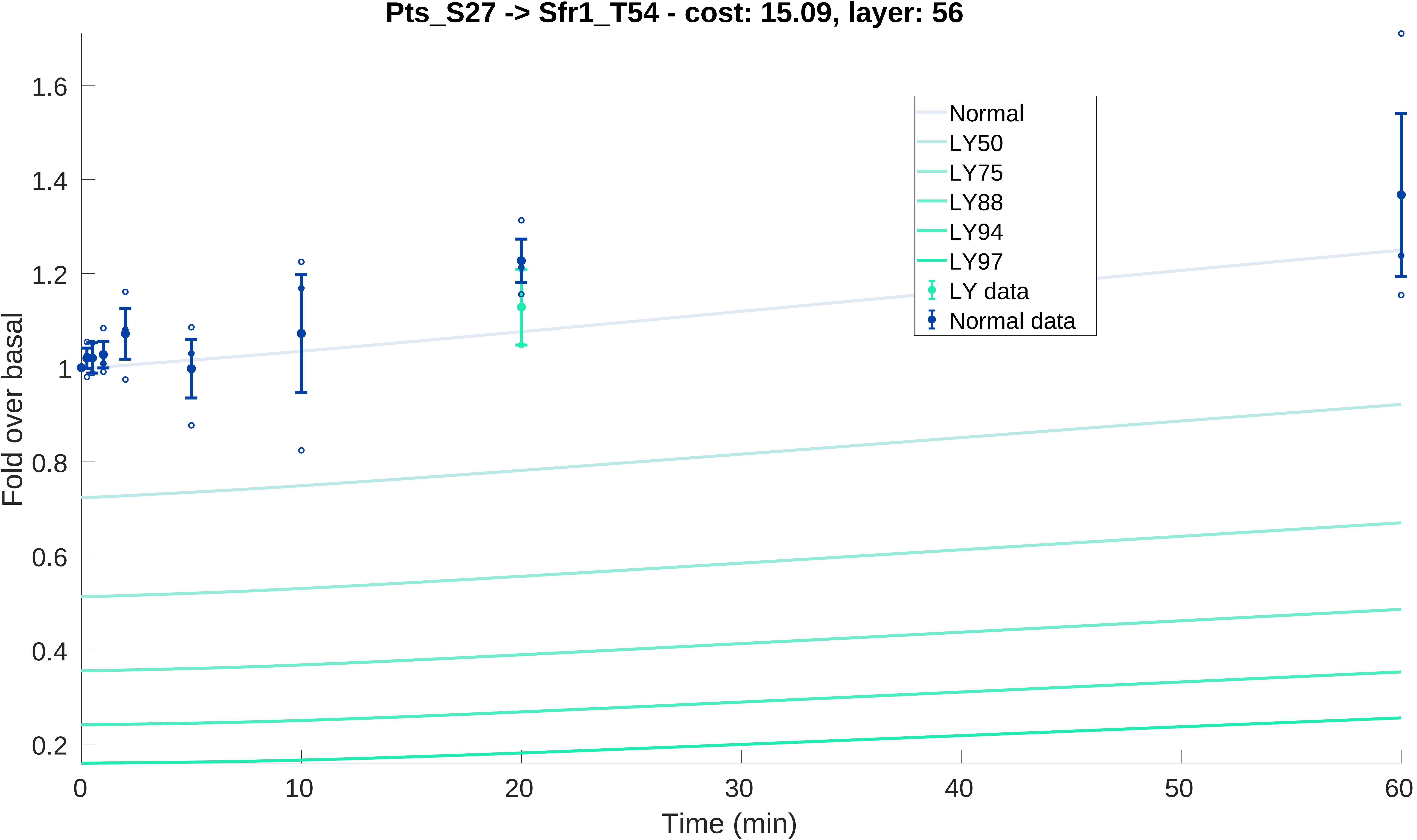

Pts_S27 -> Sfr1_T54 - cost: 15.09, layer: 56
Fold over basal
1.6
1.4
1.2
1
0.8
0.6
0.4
0.2
0
10
20
30
40
50
60
Time (min)
Normal
LY50
LY75
LY88
LY94
LY97
LY data
Normal data

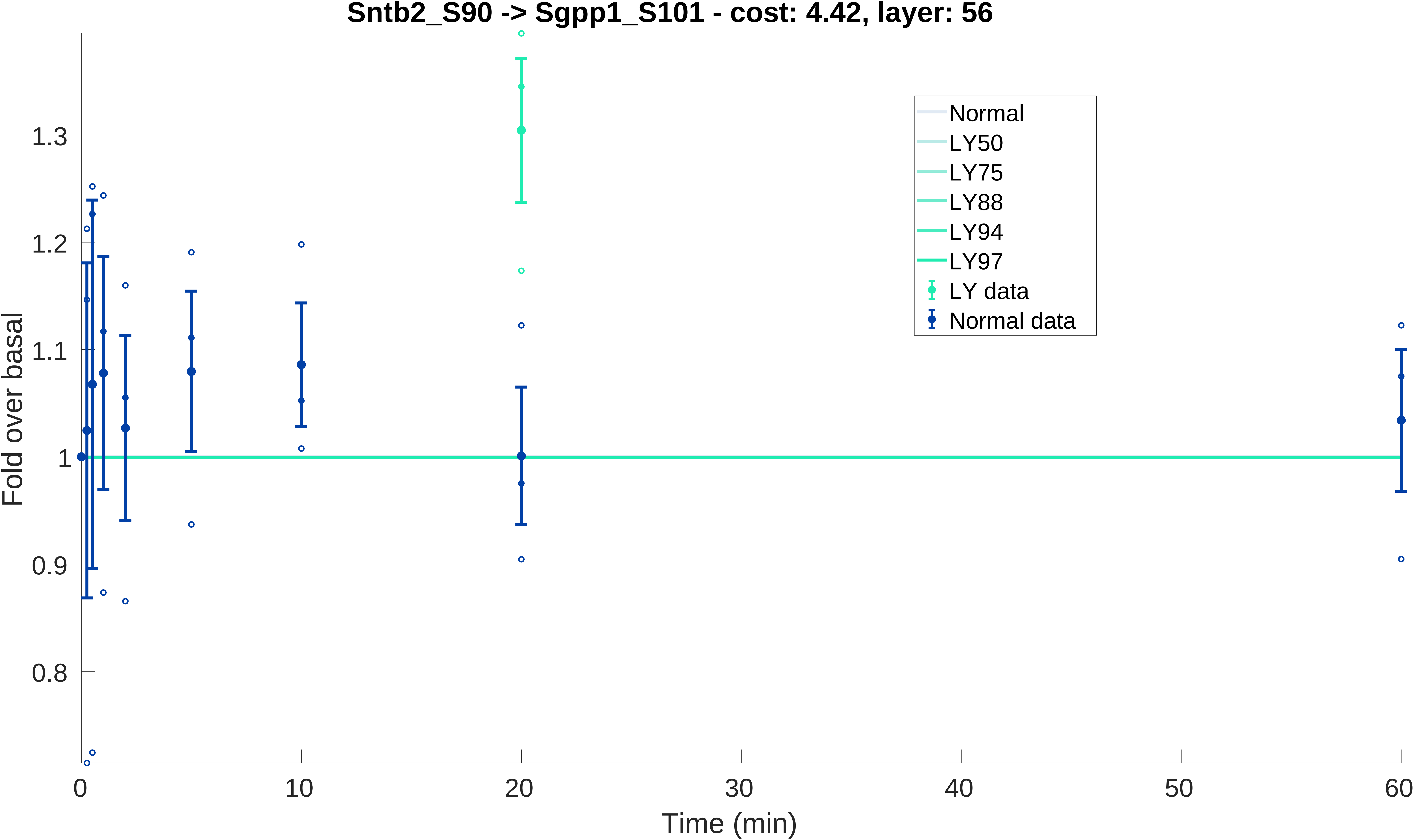
Sntb2_S90 -> Sgpp1_S101 - cost: 4.42, layer: 56
Normal
LY50
LY75
LY88
LY94
LY97
LY data
Normal data
Fold over basal
1.3
1.2
1.1
1
0.9
0.8
0
10
20
30
40
50
60
Time (min)

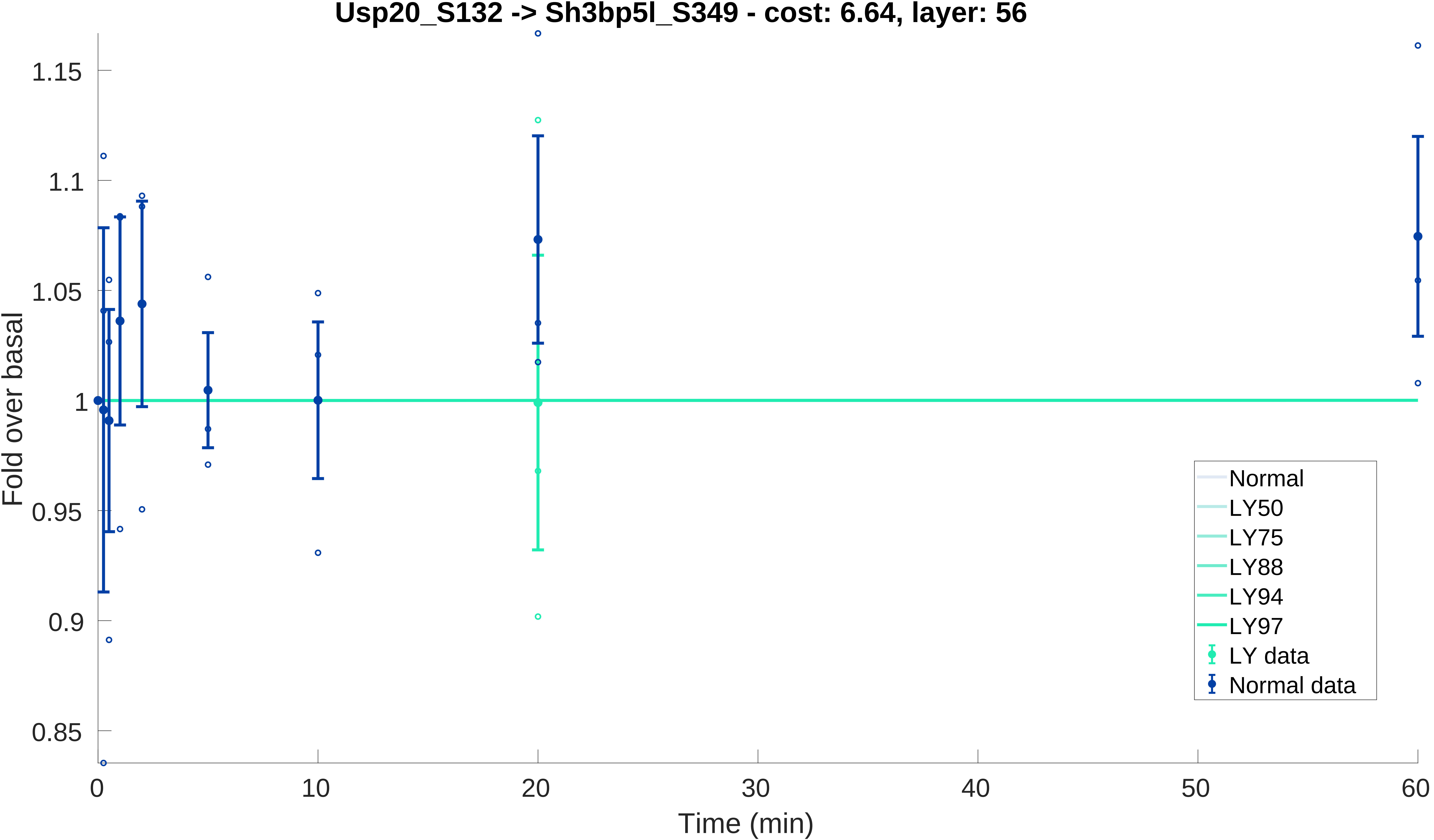

Usp20_S132 -> Sh3bp5l_S349 - cost: 6.64, layer: 56
Fold over basal
1.15
1.1
1.05
1
0.95
0.9
0.85
0
10
20
30
40
50
60
Time (min)
Normal
LY50
LY75
LY88
LY94
LY97
LY data
Normal data

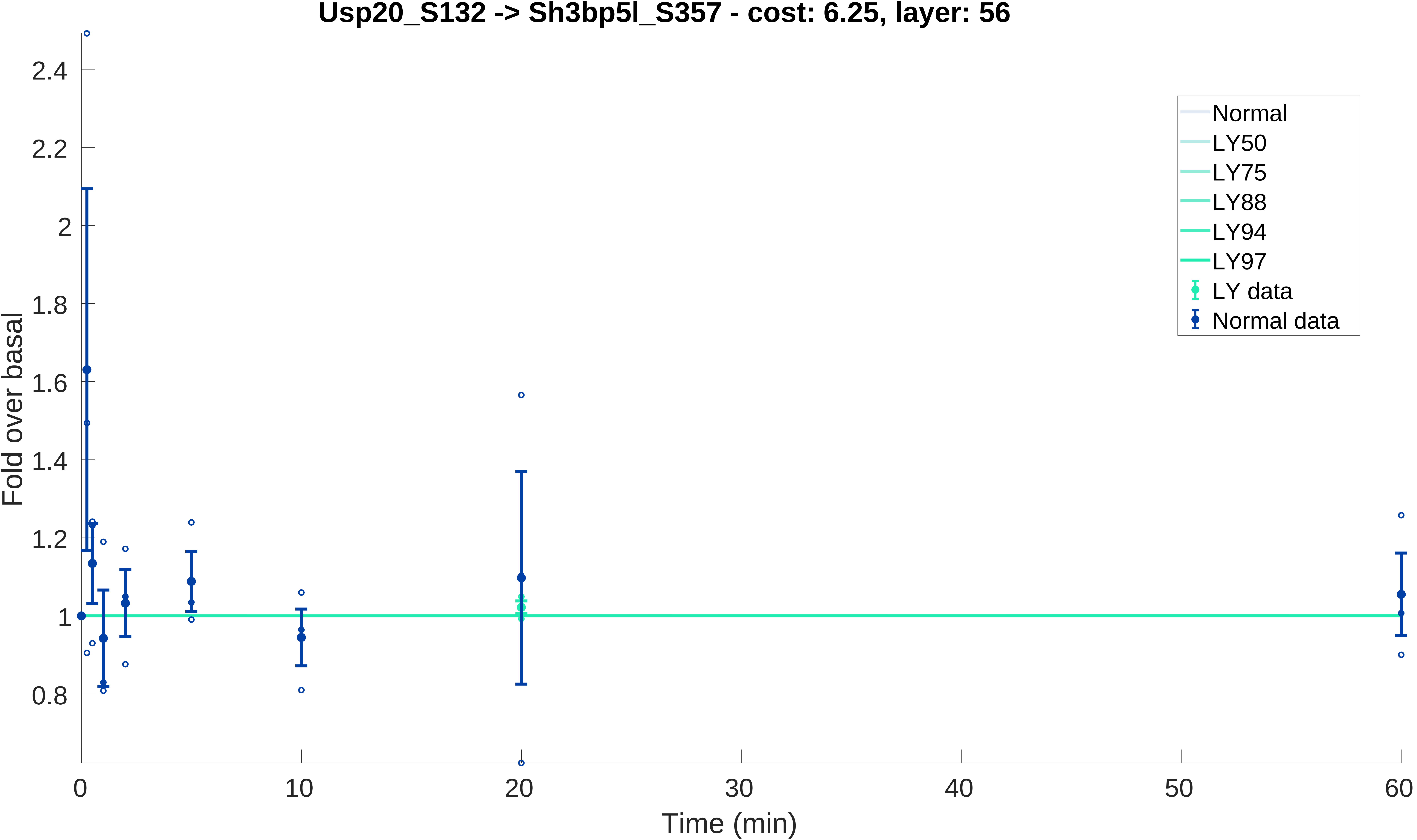
Usp20_S132 -> Sh3bp5l_S357 - cost: 6.25, layer: 56
Fold over basal
Time (min)
0.8
1
1.2
1.4
1.6
1.8
2
2.2
2.4
0
10
20
30
40
50
60
Normal
LY50
LY75
LY88
LY94
LY97
LY data
Normal data

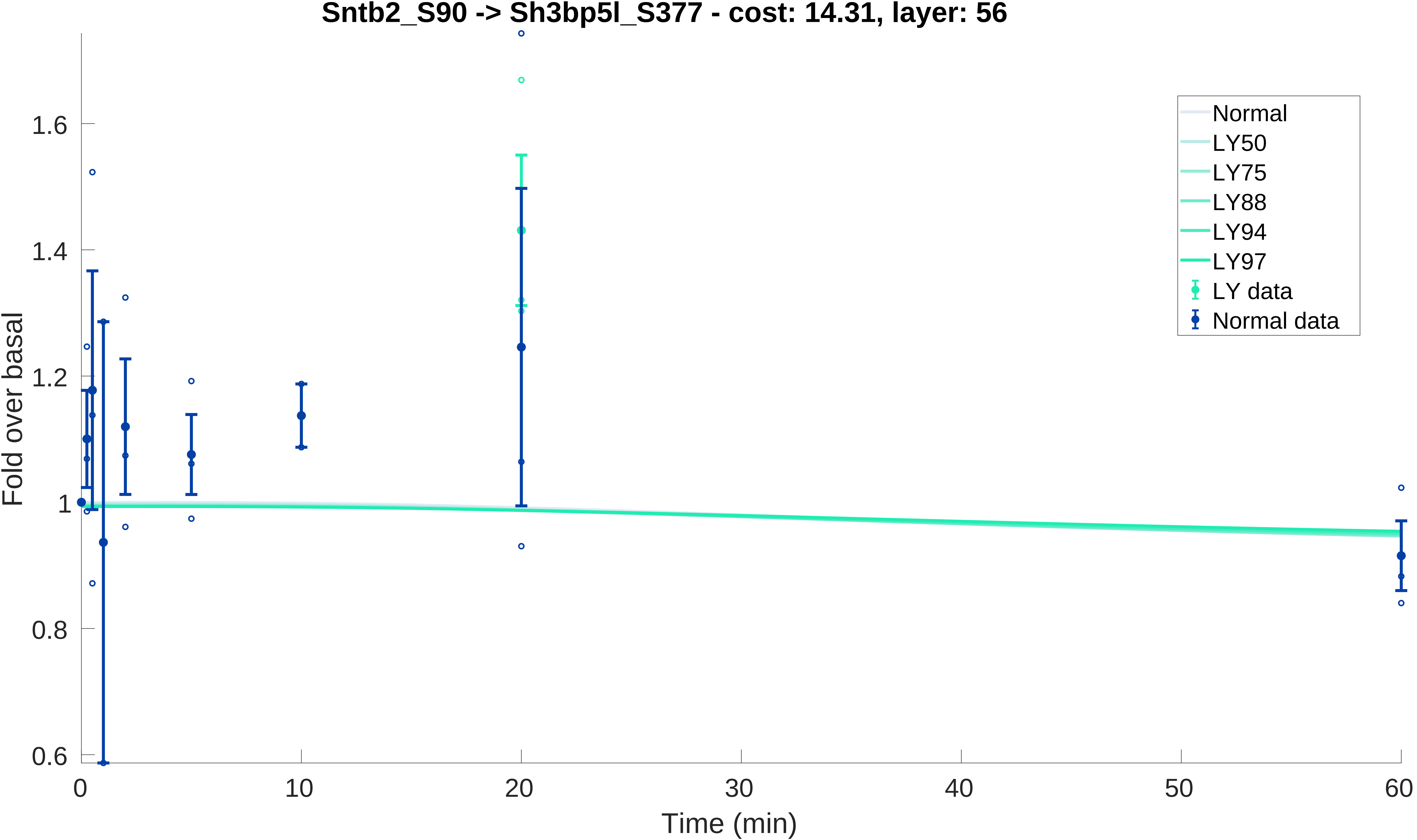

Sntb2_S90 -> Sh3bp5l_S377 - cost: 14.31, layer: 56
Fold over basal
Time (min)
0.6
0.8
1
1.2
1.4
1.6
0
10
20
30
40
50
60
Normal
LY50
LY75
LY88
LY94
LY97
LY data
Normal data

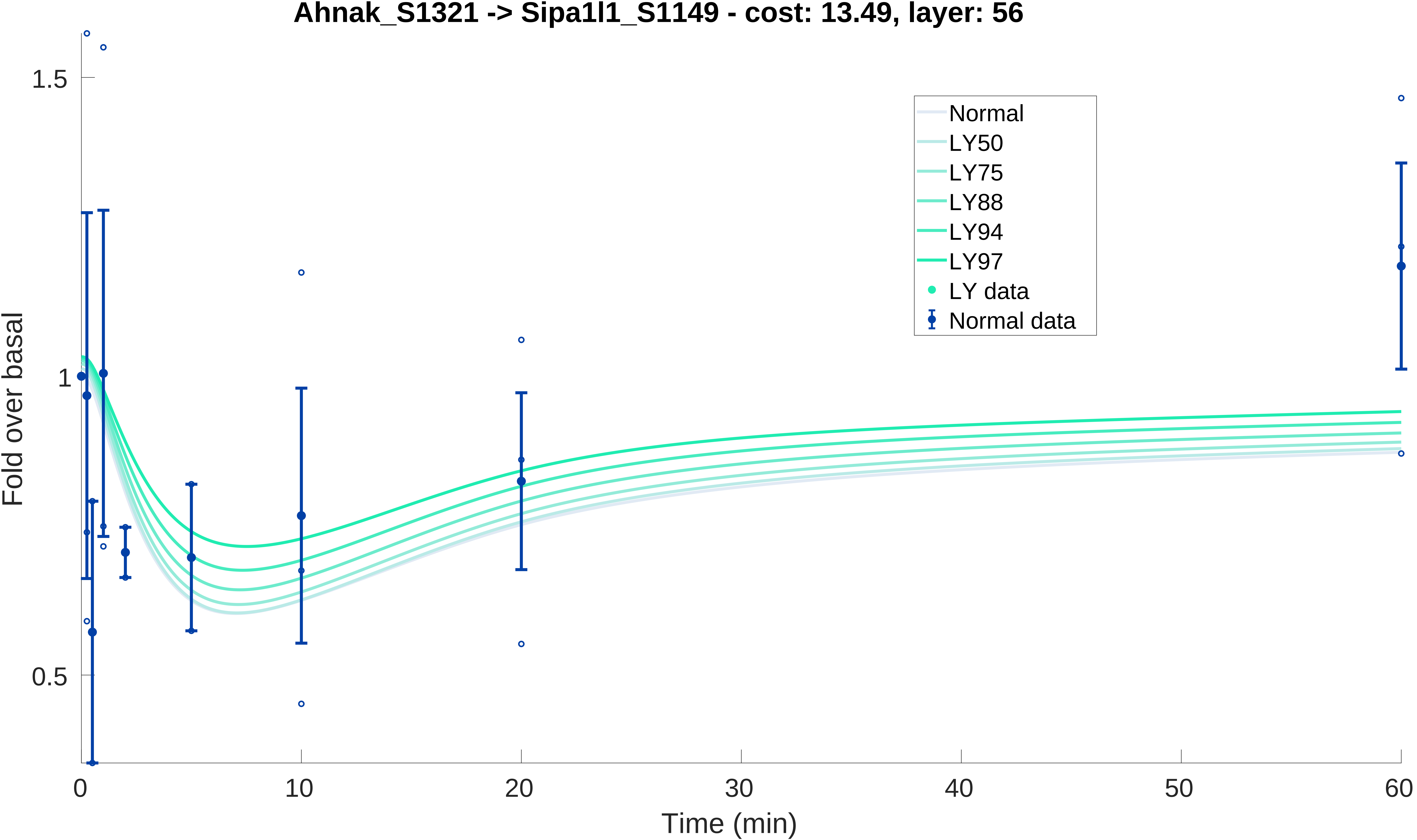

Ahnak_S1321 -> Sipa1l1_S1149 - cost: 13.49, layer: 56
Fold over basal
1.5
1
0.5
0
10
20
30
40
50
60
Time (min)
Normal
LY50
LY75
LY88
LY94
LY97
LY data
Normal data

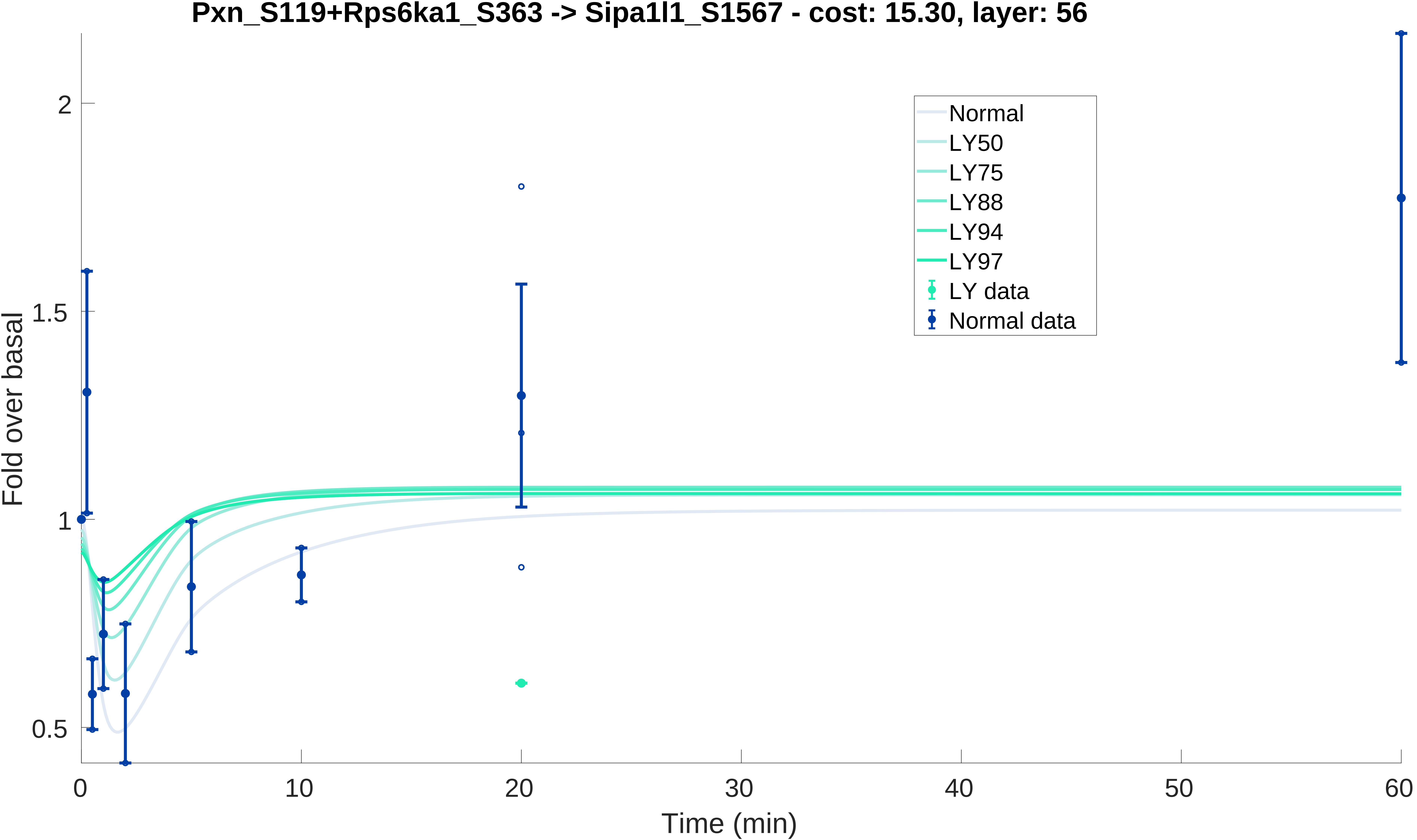

Pxn_S119+Rps6ka1_S363 -> Sipa1l1_S1567 - cost: 15.30, layer: 56
Fold over basal
2
1.5
1
0.5
0
10
20
30
40
50
60
Time (min)
Normal
LY50
LY75
LY88
LY94
LY97
LY data
Normal data

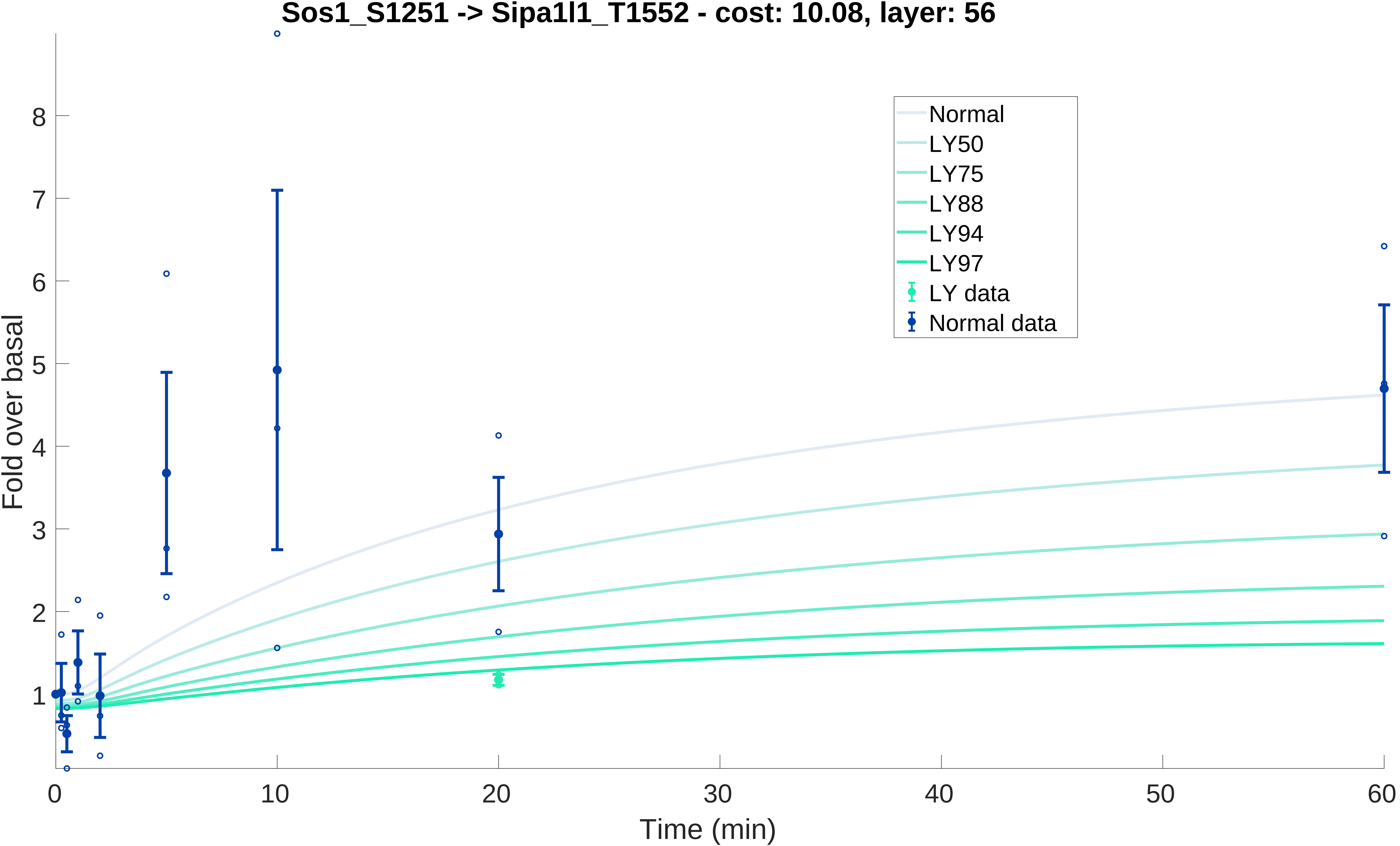

Sos1_S1251 -> Sipa1l1_T1552 - cost: 10.08, layer: 56
Normal
LY50
LY75
LY88
LY94
LY97
LY data
Normal data
Fold over basal
Time (min)
0
10
20
30
40
50
60
1
2
3
4
5
6
7
8

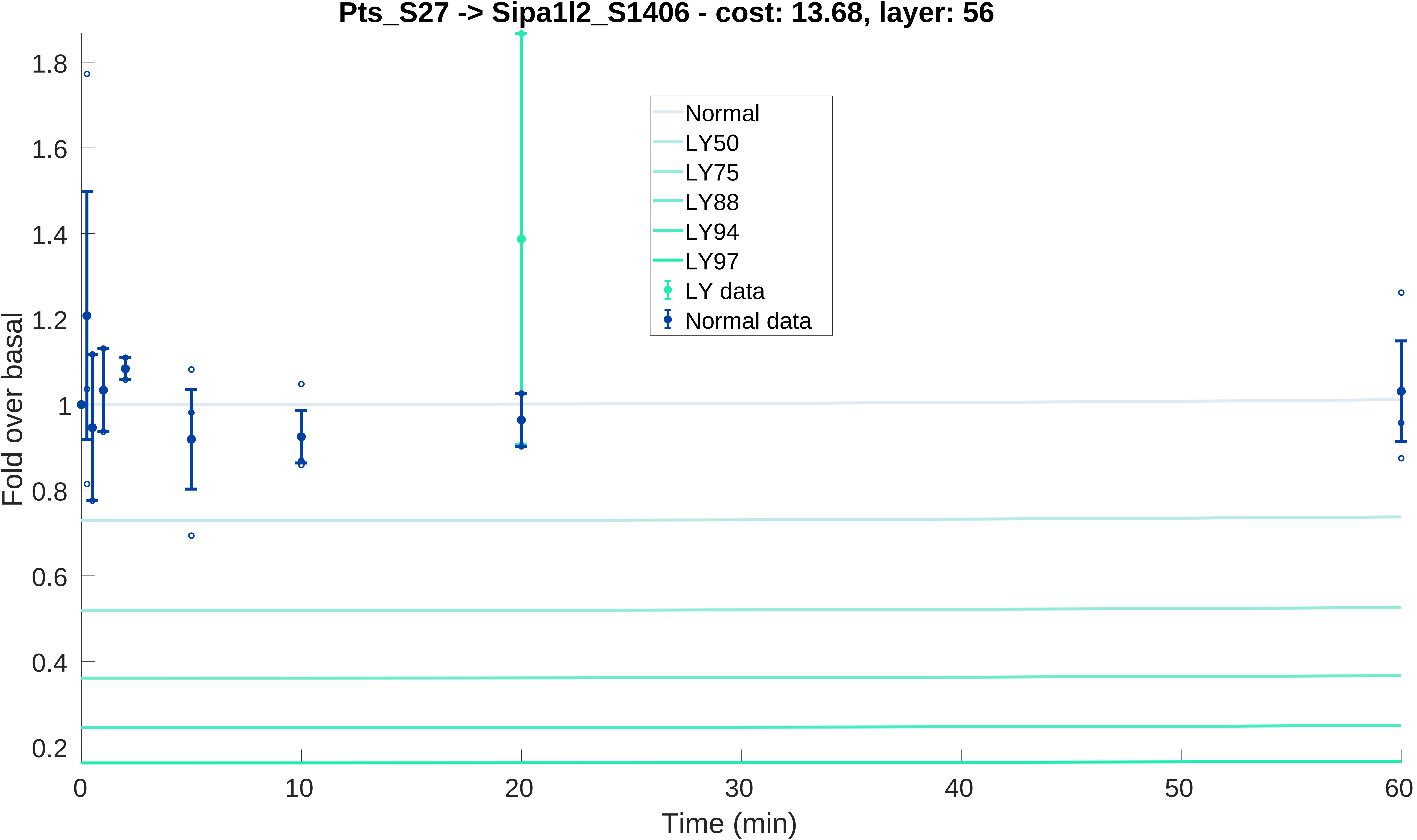
Pts_S27 -> Sipa1l2_S1406 - cost: 13.68, layer: 56
Fold over basal
Time (min)
Normal
LY50
LY75
LY88
LY94
LY97
LY data
Normal data
0.2
0.4
0.6
0.8
1
1.2
1.4
1.6
1.8
0
10
20
30
40
50
60

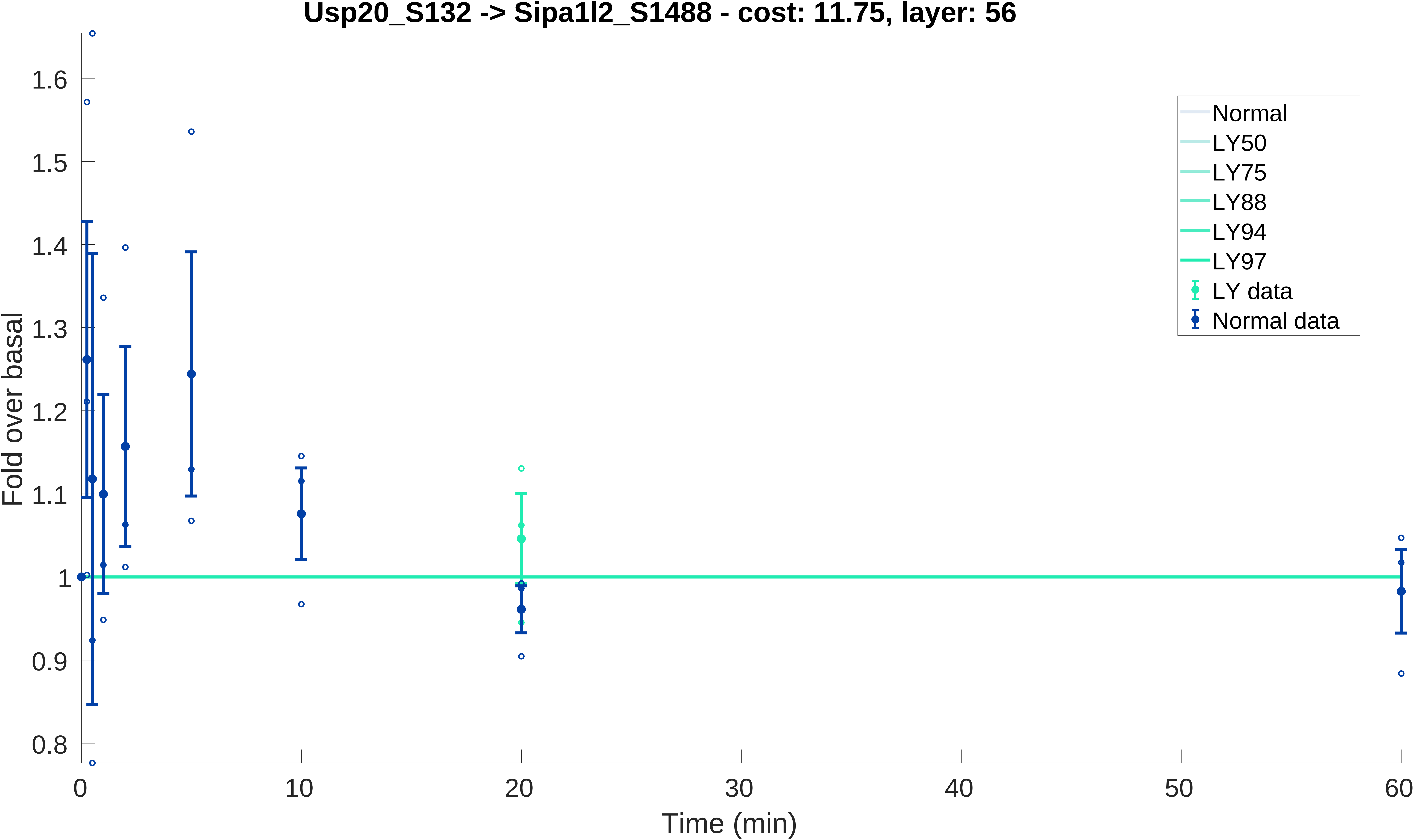

Usp20_S132 -> Sipa1l2_S1488 - cost: 11.75, layer: 56
Fold over basal
1.6
1.5
1.4
1.3
1.2
1.1
1
0.9
0.8
0
10
20
30
40
50
60
Time (min)
Normal
LY50
LY75
LY88
LY94
LY97
LY data
Normal data

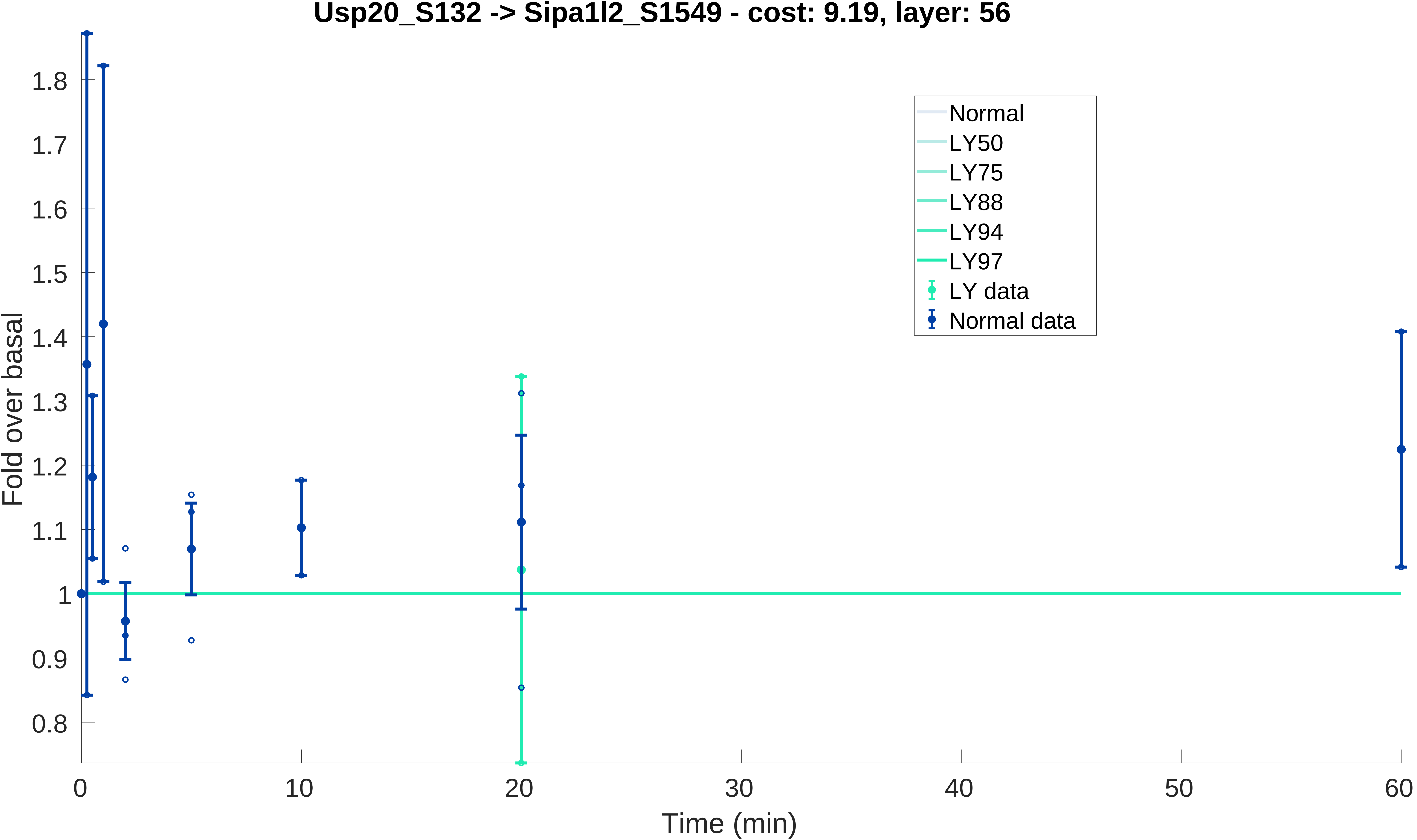

Usp20_S132 -> Sipa1l2_S1549 - cost: 9.19, layer: 56
Fold over basal
1.8
1.7
1.6
1.5
1.4
1.3
1.2
1.1
1
0.9
0.8
Time (min)
0
10
20
30
40
50
60
Normal
LY50
LY75
LY88
LY94
LY97
LY data
Normal data

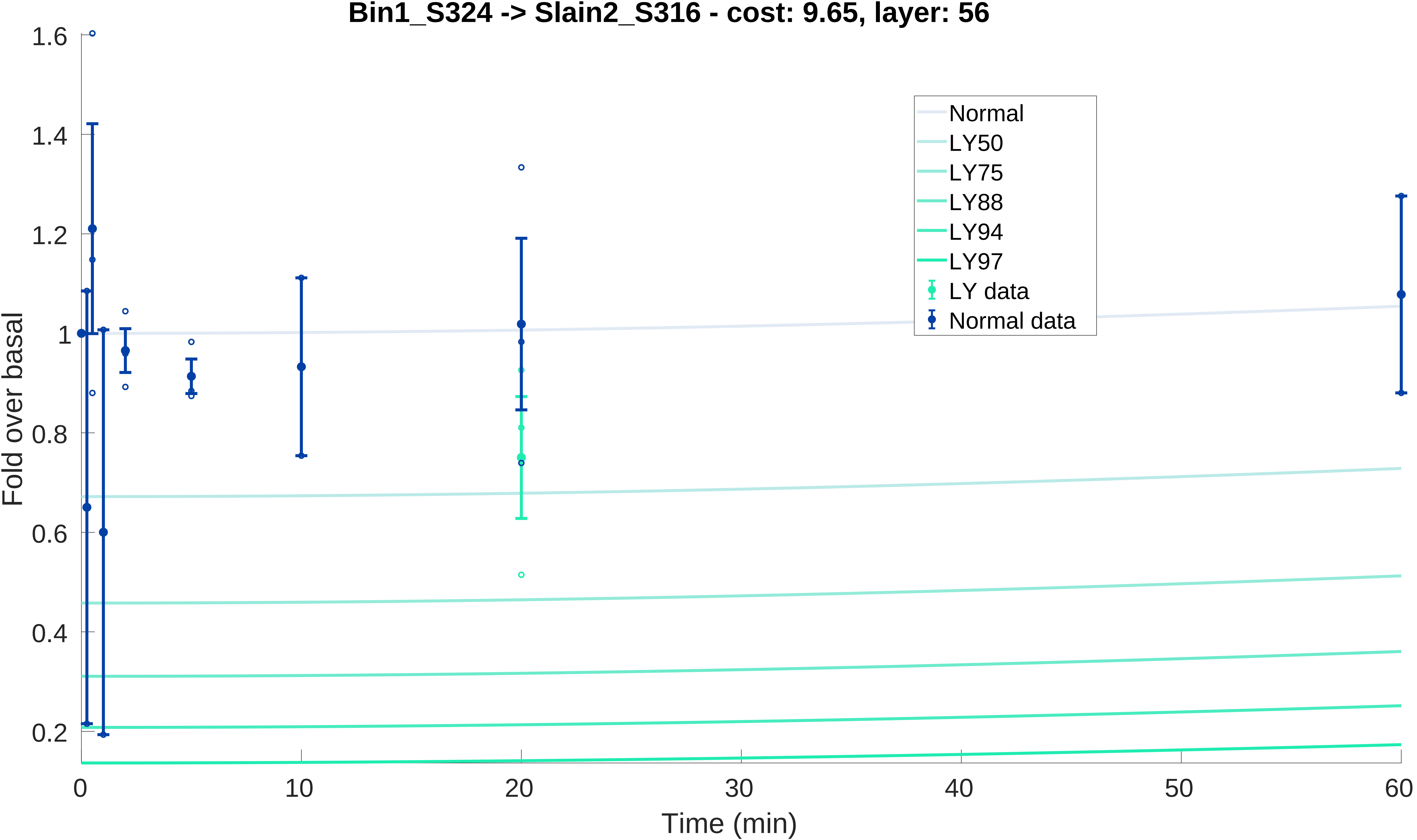

Bin1_S324 -> Slain2_S316 - cost: 9.65, layer: 56
Fold over basal
Time (min)
1.6
1.4
1.2
1
0.8
0.6
0.4
0.2
0
10
20
30
40
50
60
Normal
LY50
LY75
LY88
LY94
LY97
LY data
Normal data

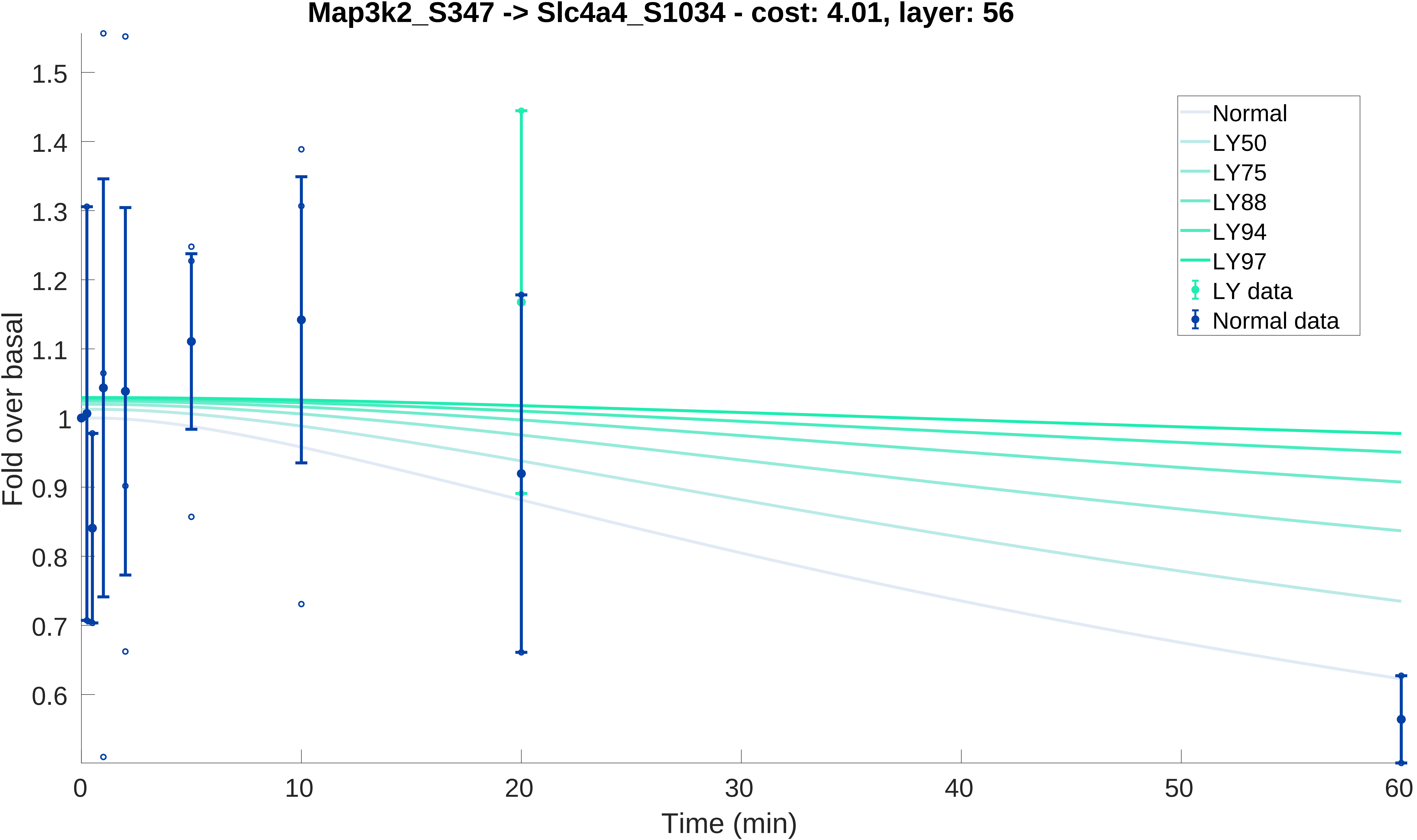

Map3k2_S347 -> Slc4a4_S1034 - cost: 4.01, layer: 56
Fold over basal
Time (min)
1.5
1.4
1.3
1.2
1.1
1
0.9
0.8
0.7
0.6
0
10
20
30
40
50
60
Normal
LY50
LY75
LY88
LY94
LY97
LY data
Normal data

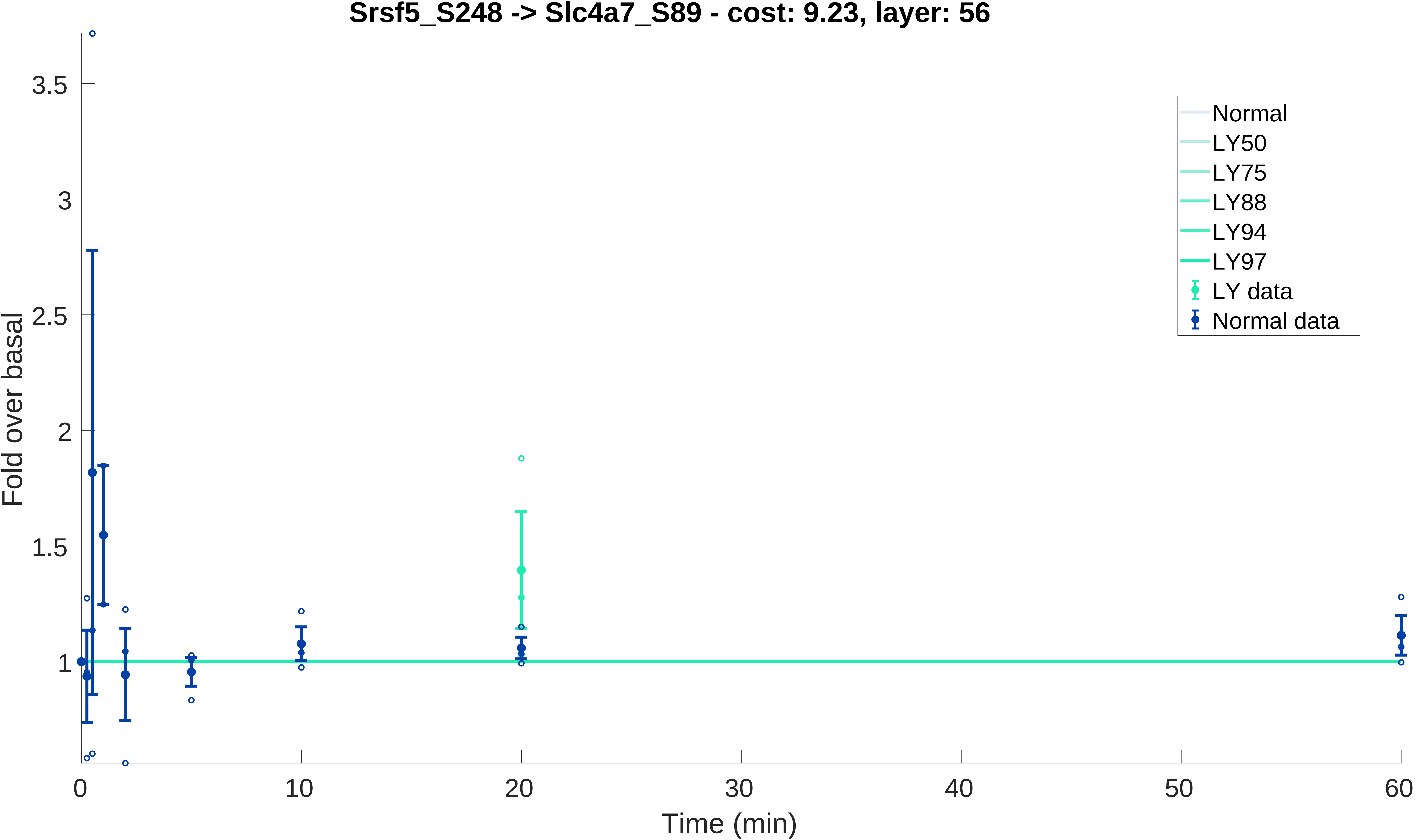
Srsf5_S248 -> Slc4a7_S89 - cost: 9.23, layer: 56
Fold over basal
Time (min)
0
10
20
30
40
50
60
1
1.5
2
2.5
3
3.5
Normal
LY50
LY75
LY88
LY94
LY97
LY data
Normal data

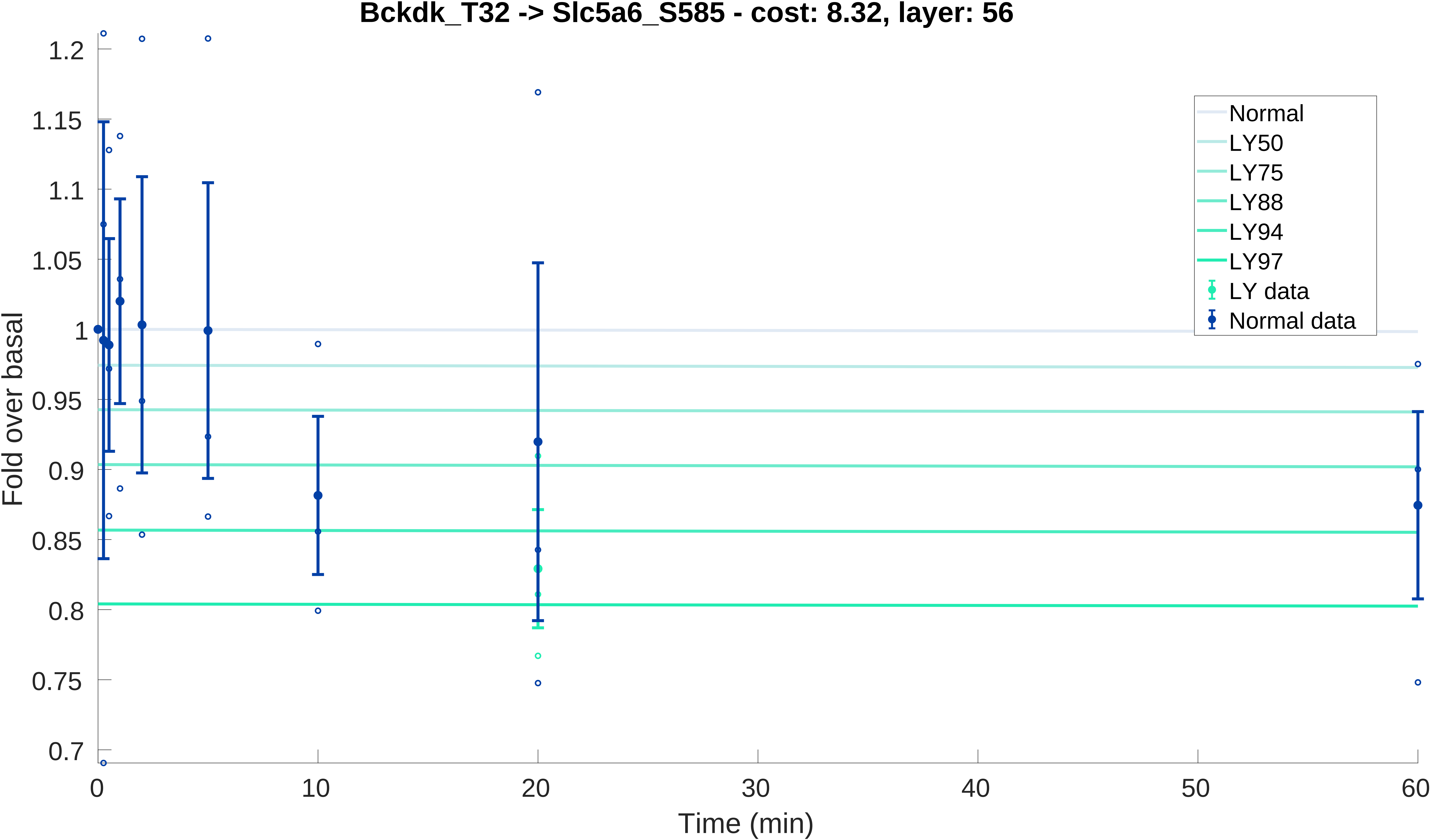
Bckdk_T32 -> Slc5a6_S585 - cost: 8.32, layer: 56
Fold over basal
1.2
1.15
1.1
1.05
1
0.95
0.9
0.85
0.8
0.75
0.7
0
10
20
30
40
50
60
Time (min)
Normal
LY50
LY75
LY88
LY94
LY97
LY data
Normal data

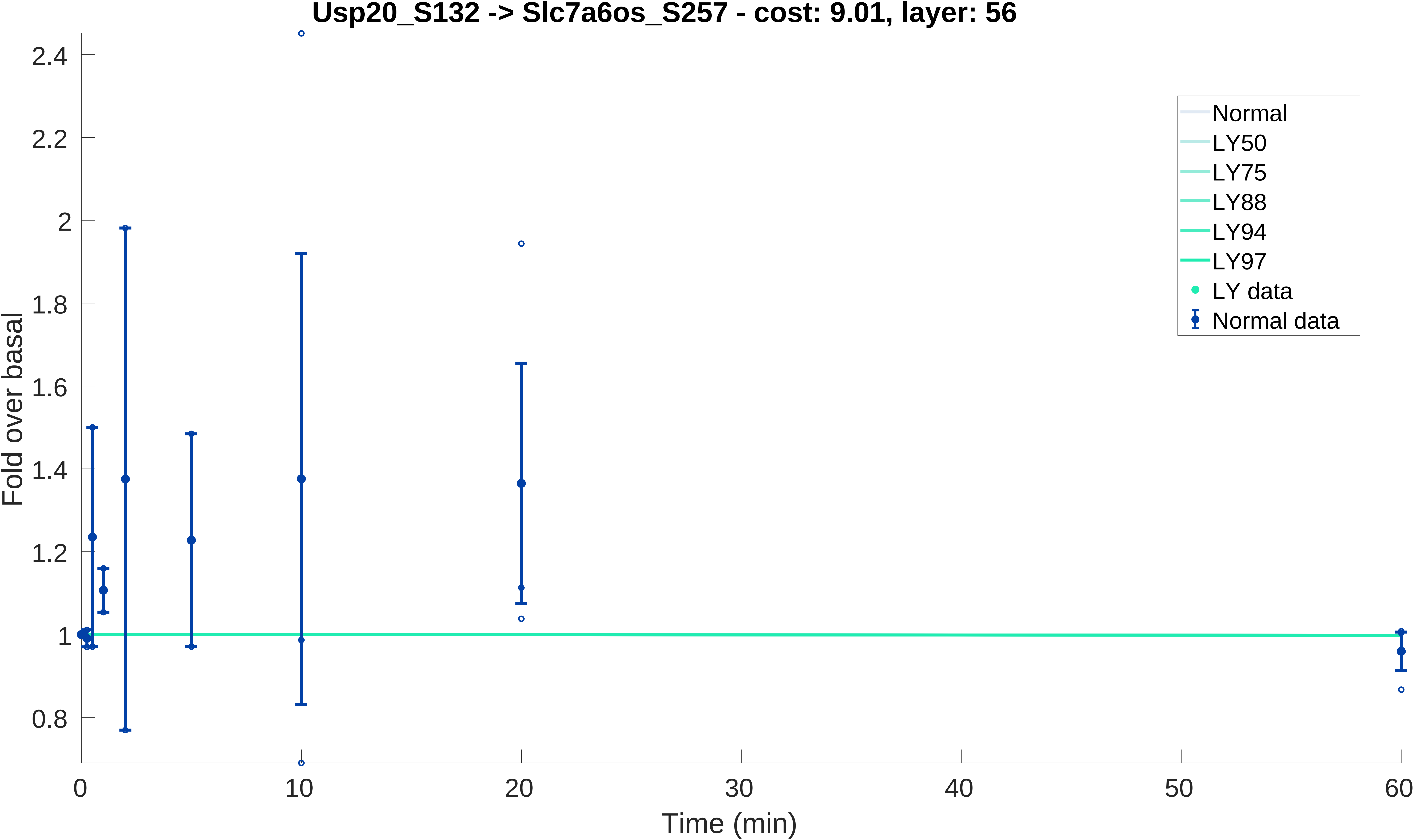
Usp20_S132 -> Slc7a6os_S257 - cost: 9.01, layer: 56
Fold over basal
Time (min)
2.4
2.2
2
1.8
1.6
1.4
1.2
1
0.8
0
10
20
30
40
50
60
Normal
LY50
LY75
LY88
LY94
LY97
LY data
Normal data

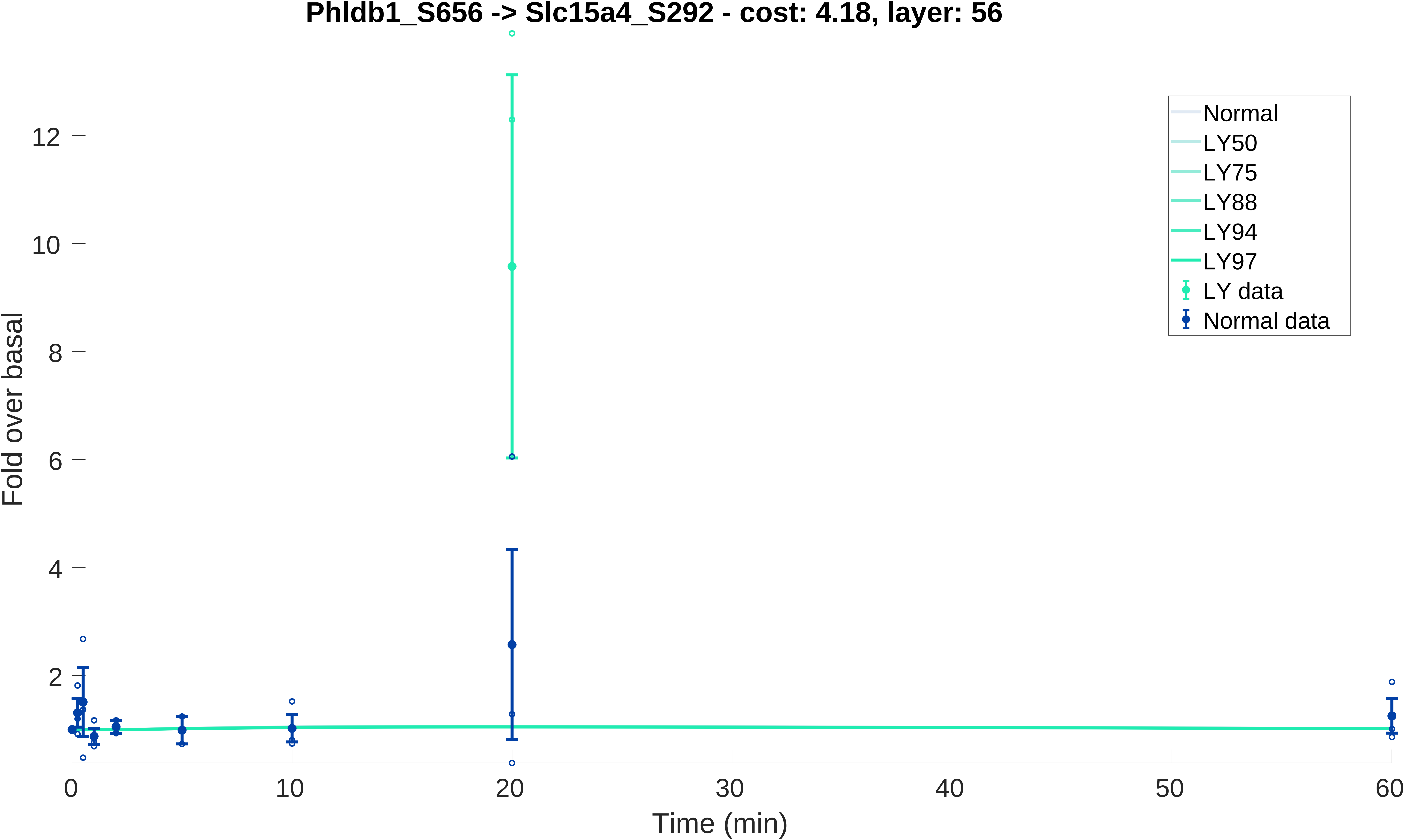
Phldb1_S656 -> Slc15a4_S292 - cost: 4.18, layer: 56
Fold over basal
Time (min)
0
2
4
6
8
10
12
0
10
20
30
40
50
60
Normal
LY50
LY75
LY88
LY94
LY97
LY data
Normal data

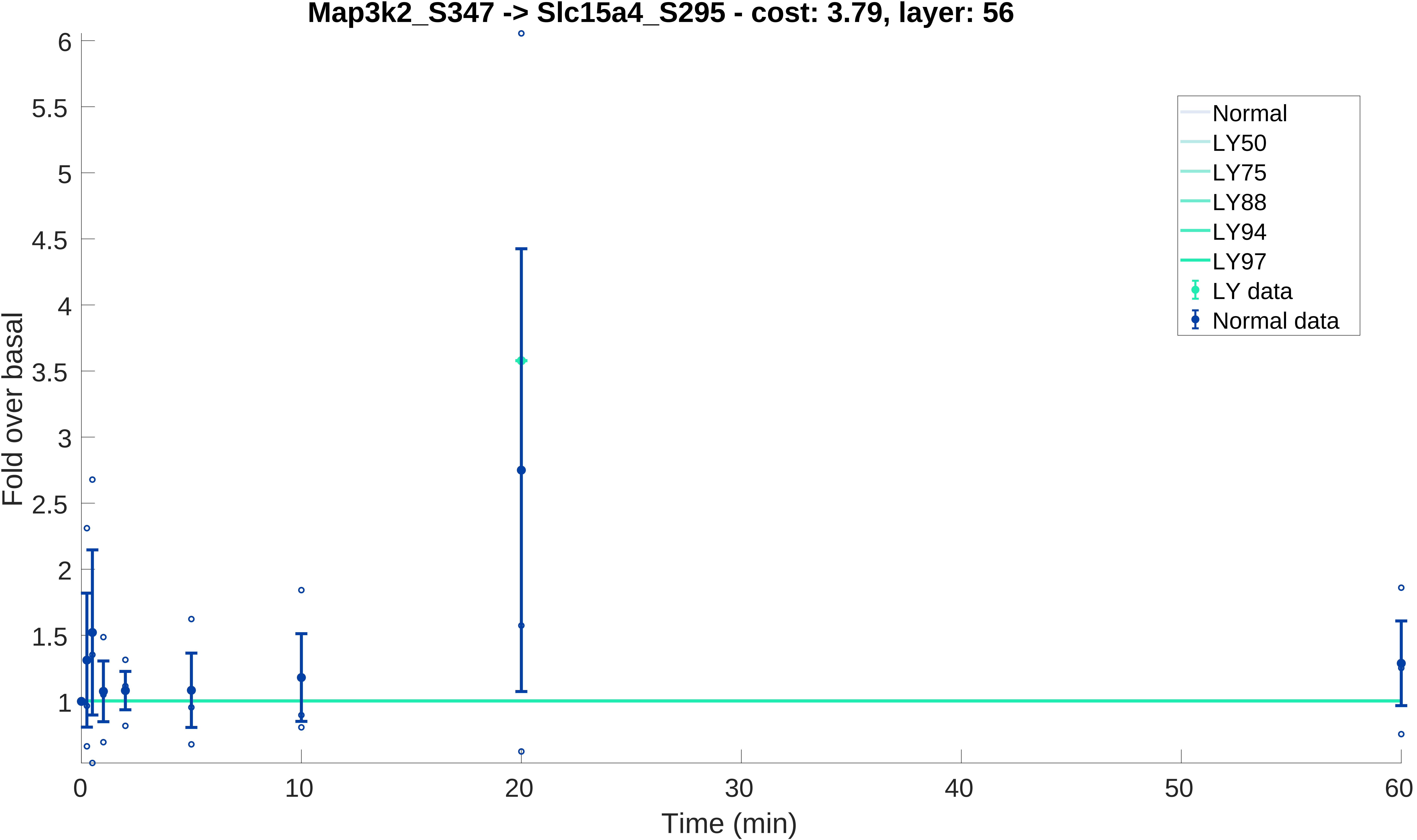

Map3k2_S347 -> Slc15a4_S295 - cost: 3.79, layer: 56
Fold over basal
Time (min)
Normal
LY50
LY75
LY88
LY94
LY97
LY data
Normal data
0
10
20
30
40
50
60
1
1.5
2
2.5
3
3.5
4
4.5
5
5.5
6

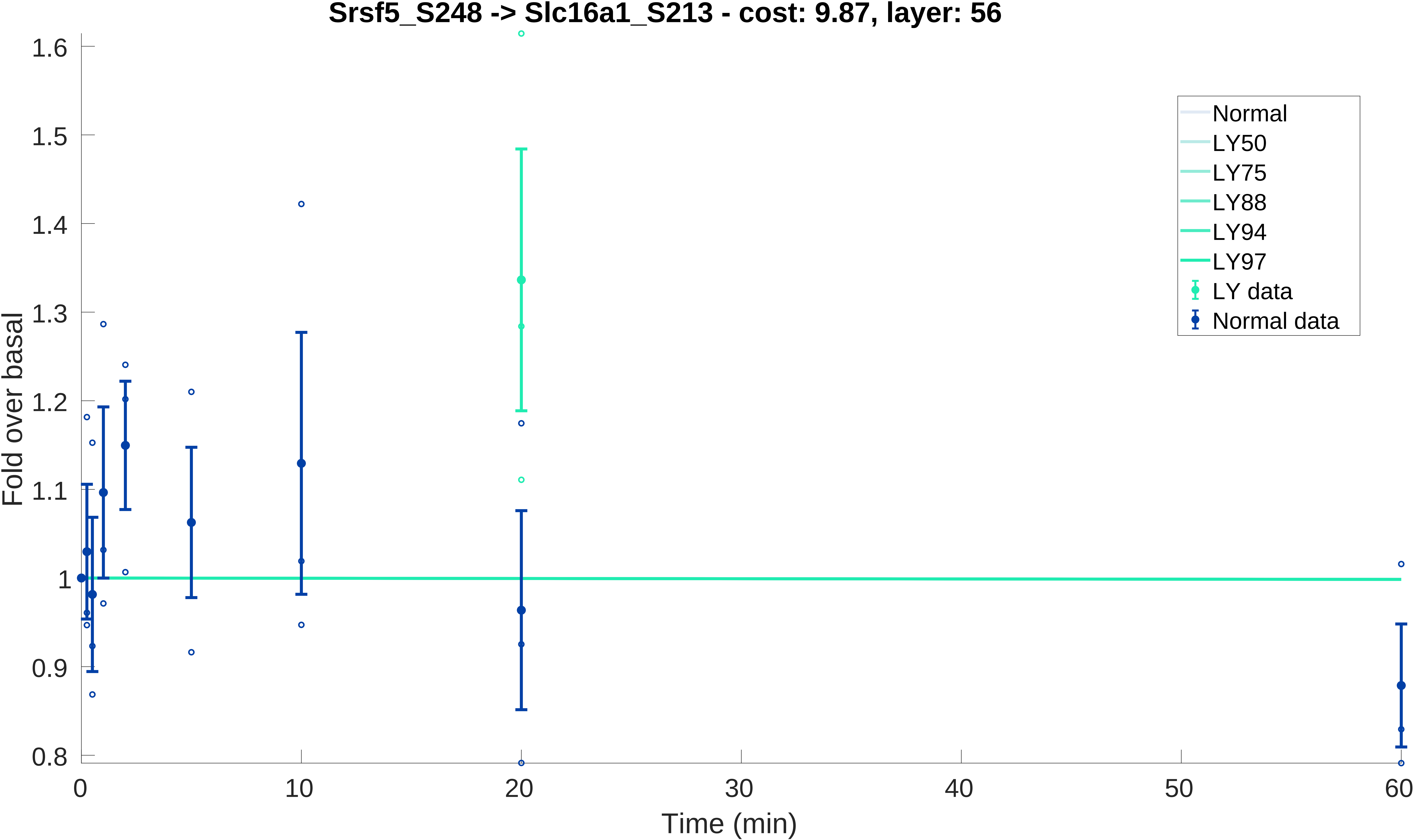

Srsf5_S248 -> Slc16a1_S213 - cost: 9.87, layer: 56
Fold over basal
1.6
1.5
1.4
1.3
1.2
1.1
1
0.9
0.8
0
10
20
30
40
50
60
Time (min)
Normal
LY50
LY75
LY88
LY94
LY97
LY data
Normal data

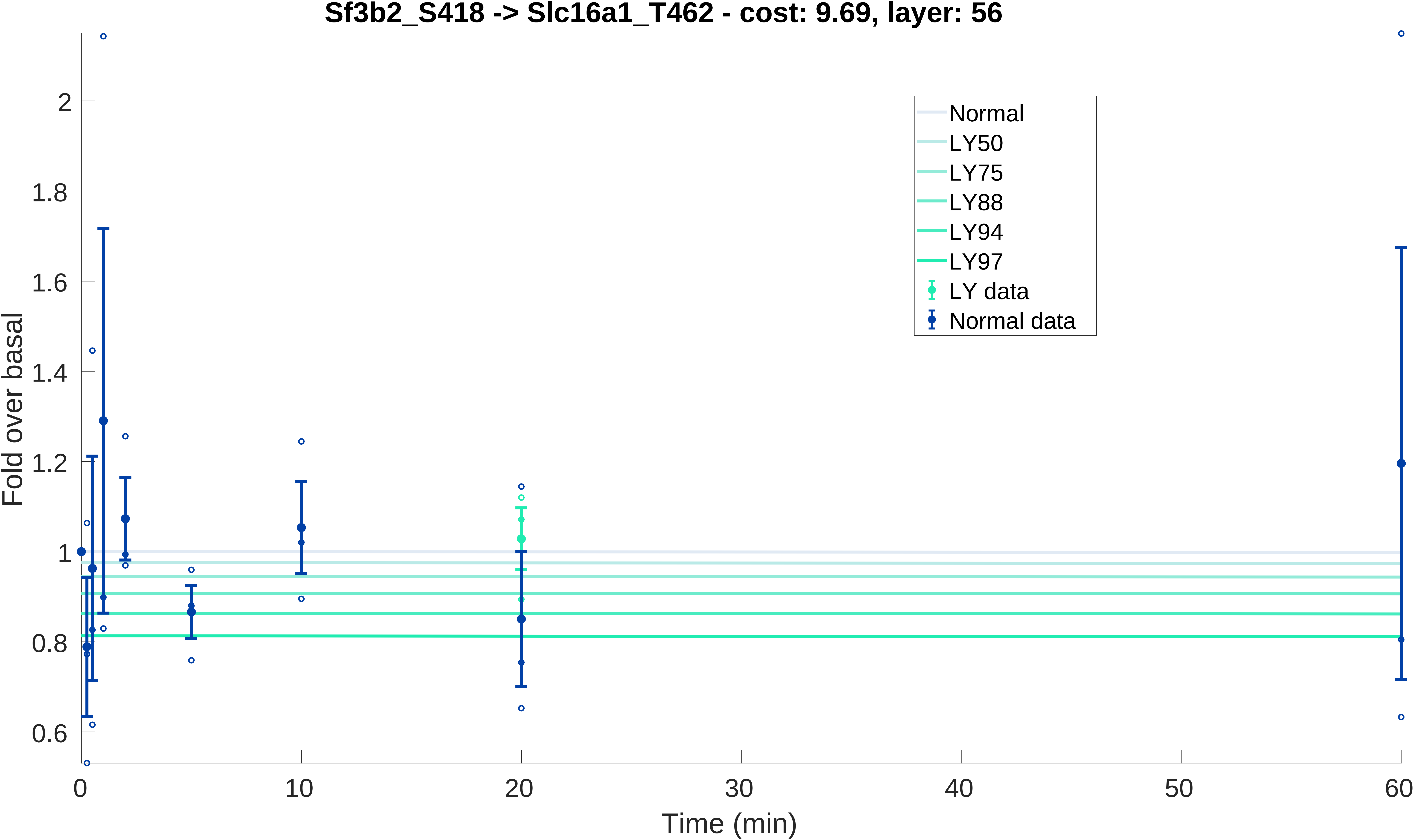
Sf3b2_S418 -> Slc16a1_T462 - cost: 9.69, layer: 56
Fold over basal
Time (min)
0
10
20
30
40
50
60
0.6
0.8
1
1.2
1.4
1.6
1.8
2
Normal
LY50
LY75
LY88
LY94
LY97
LY data
Normal data

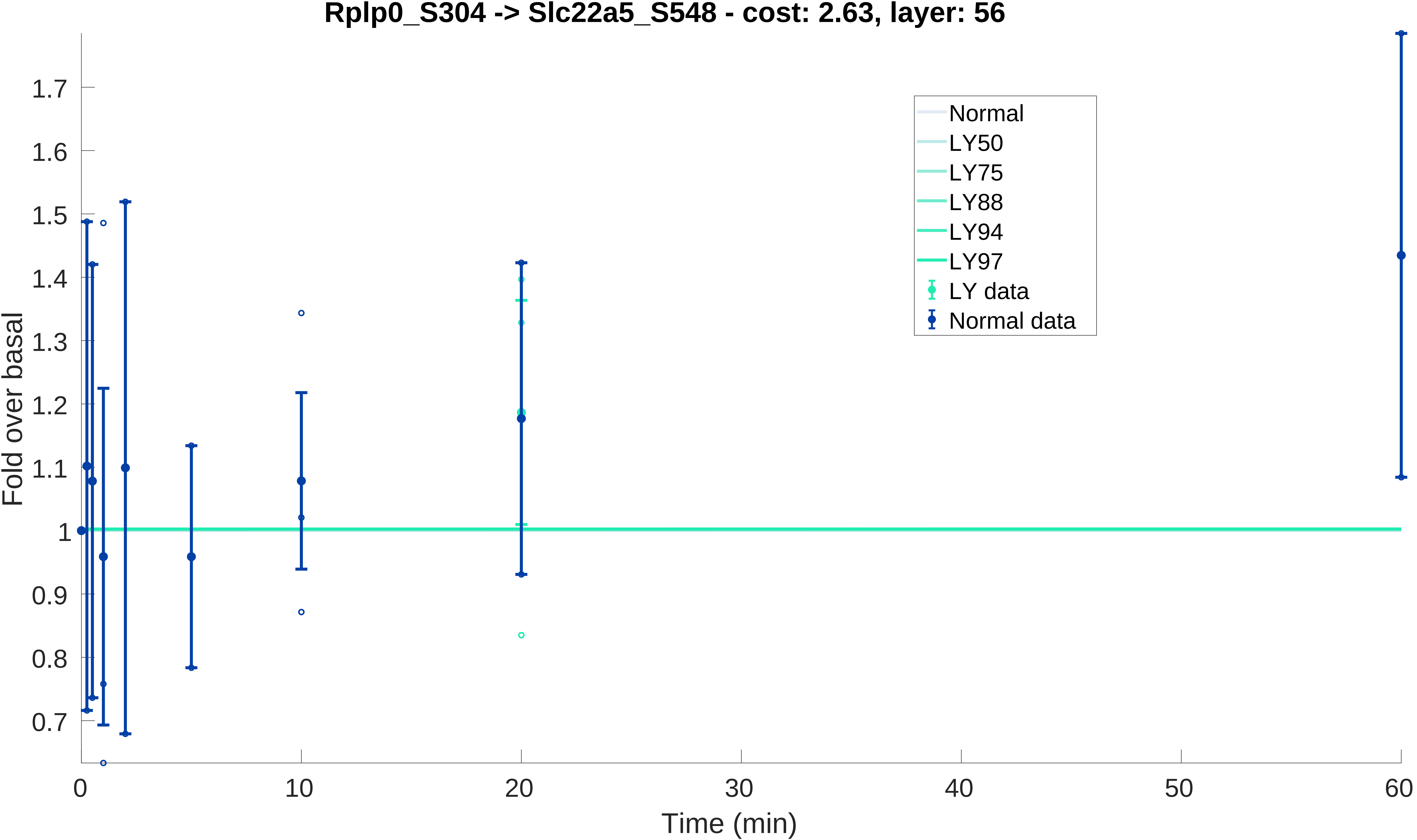
Rplp0_S304 -> Slc22a5_S548 - cost: 2.63, layer: 56
Normal
LY50
LY75
LY88
LY94
LY97
LY data
Normal data
Fold over basal
Time (min)
0.7
0.8
0.9
1
1.1
1.2
1.3
1.4
1.5
1.6
1.7
0
10
20
30
40
50
60

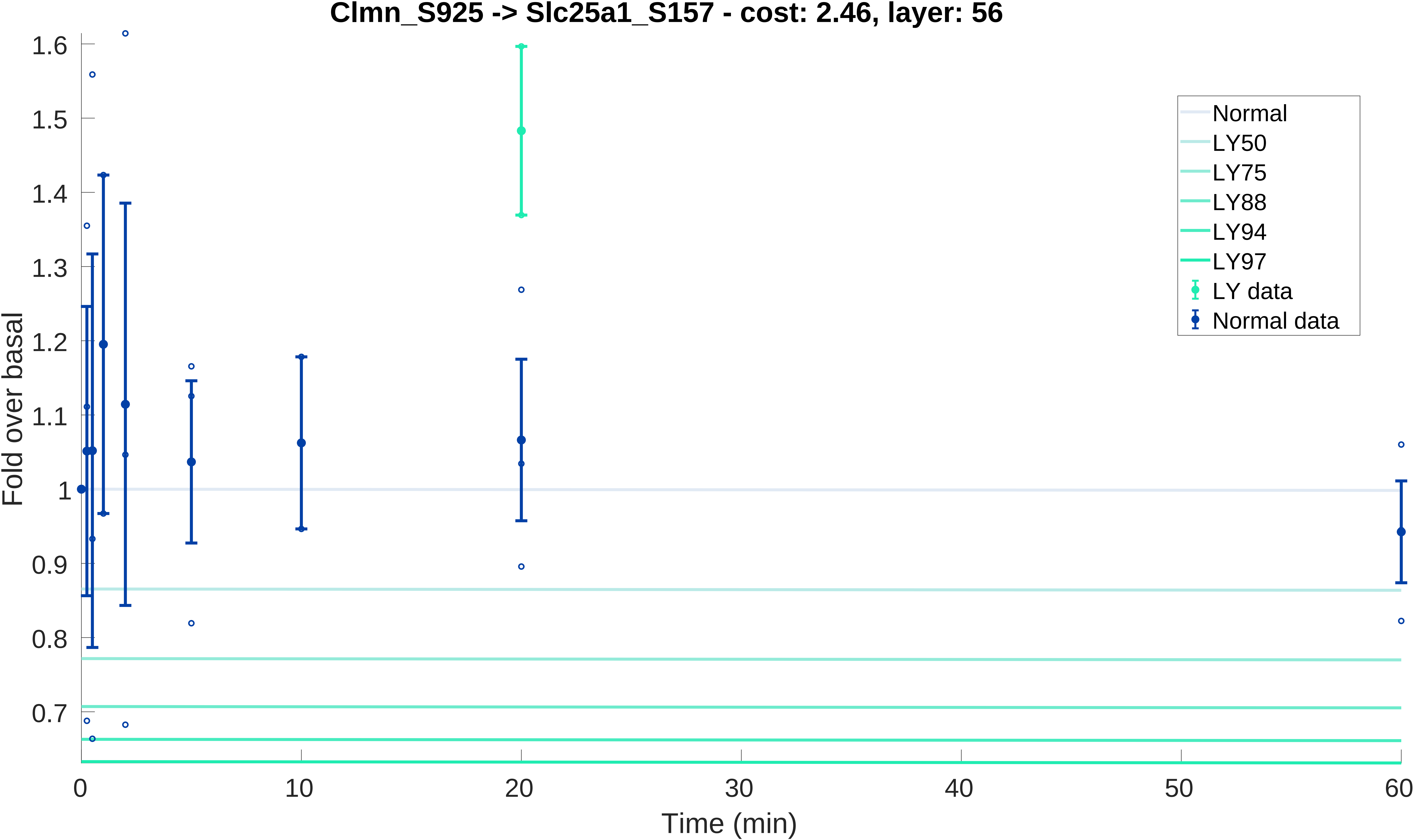

Clmn_S925 -> Slc25a1_S157 - cost: 2.46, layer: 56
Fold over basal
Time (min)
1.6
1.5
1.4
1.3
1.2
1.1
1
0.9
0.8
0.7
0
10
20
30
40
50
60
Normal
LY50
LY75
LY88
LY94
LY97
LY data
Normal data

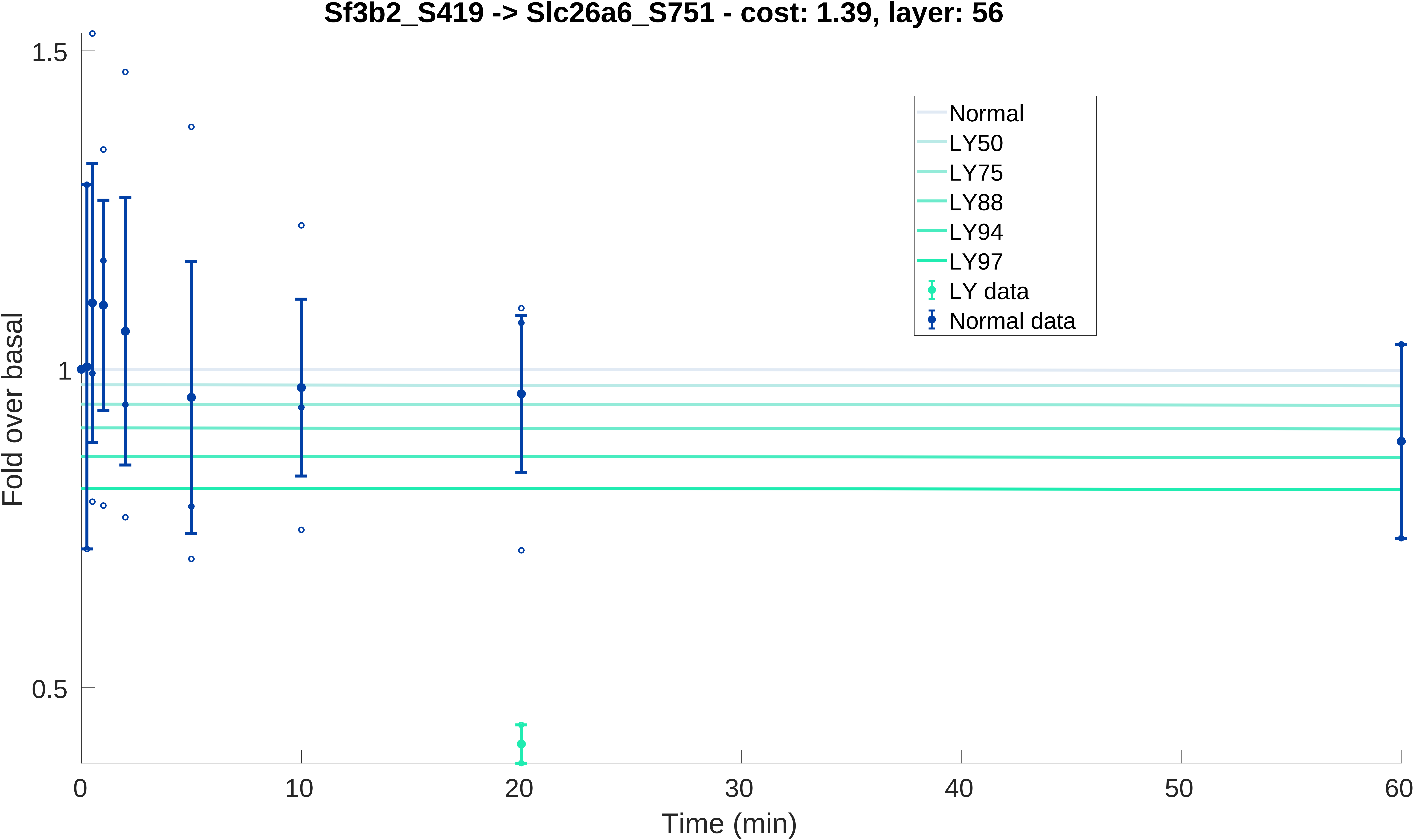

Sf3b2_S419 -> Slc26a6_S751 - cost: 1.39, layer: 56
Fold over basal
1.5
1
0.5
Time (min)
0
10
20
30
40
50
60
Normal
LY50
LY75
LY88
LY94
LY97
LY data
Normal data

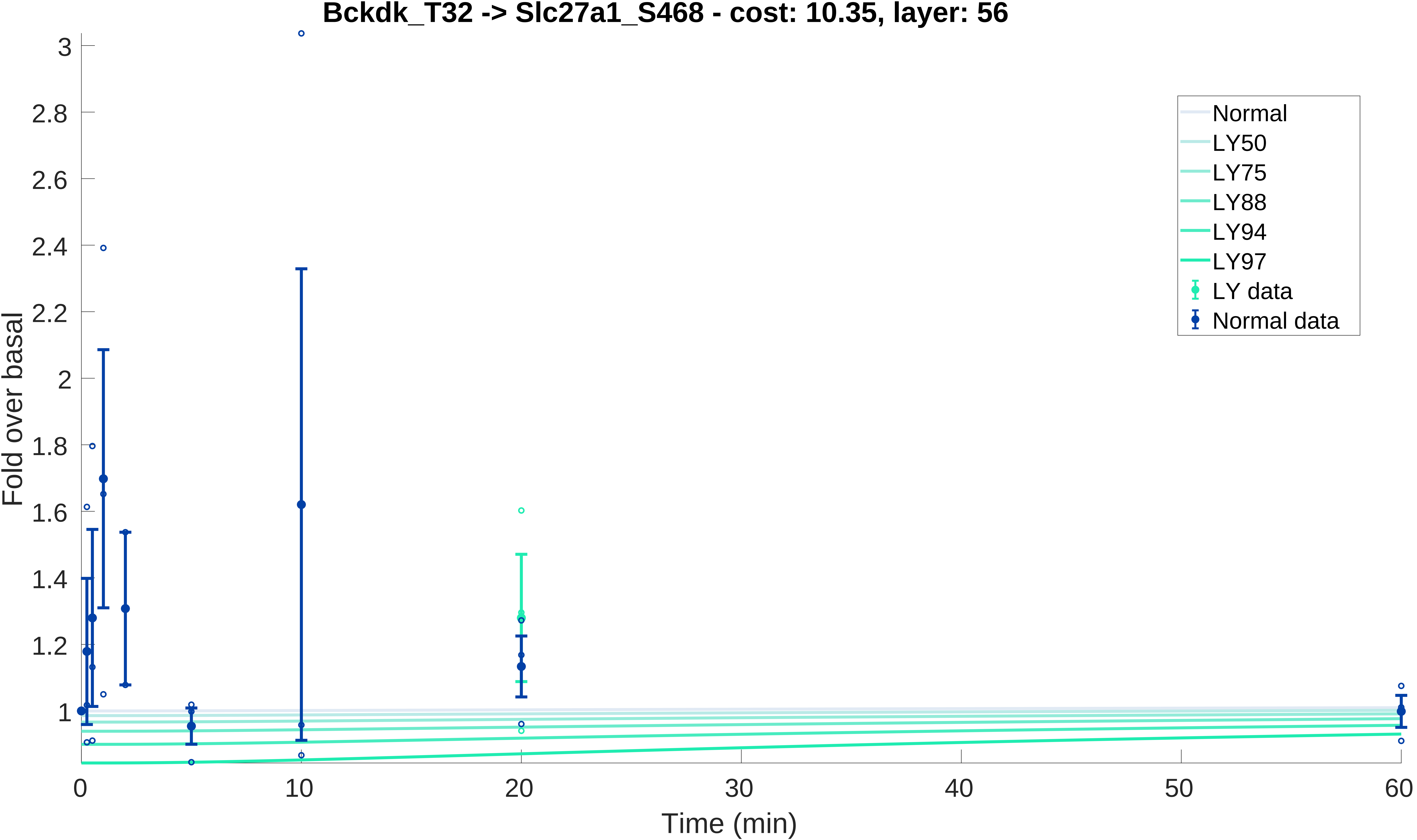

Bckdk_T32 -> Slc27a1_S468 - cost: 10.35, layer: 56
Fold over basal
3
2.8
2.6
2.4
2.2
2
1.8
1.6
1.4
1.2
1
0
10
20
30
40
50
60
Time (min)
Normal
LY50
LY75
LY88
LY94
LY97
LY data
Normal data

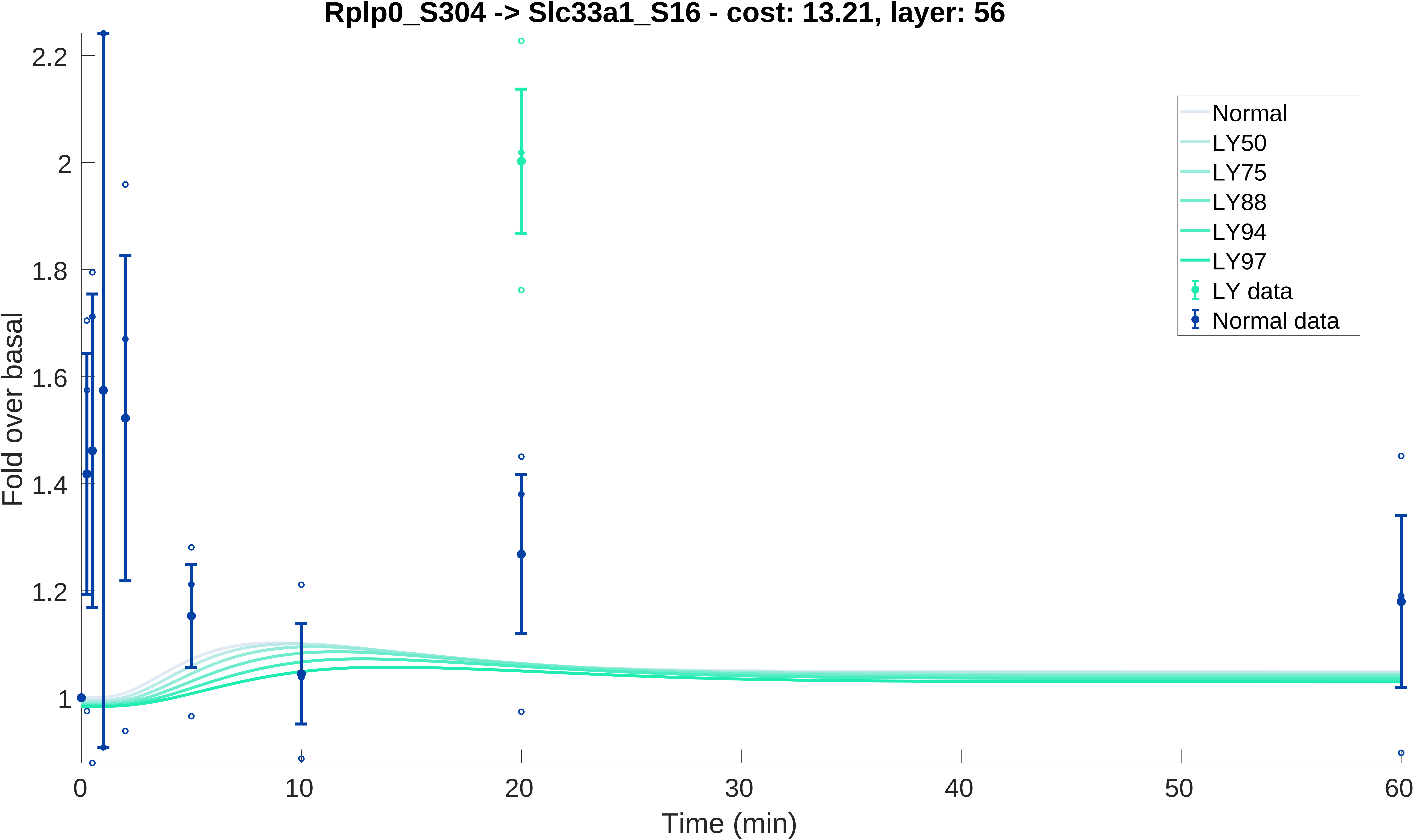

Rplp0_S304 -> Slc33a1_S16 - cost: 13.21, layer: 56
Fold over basal
Time (min)
2.2
2
1.8
1.6
1.4
1.2
1
0
10
20
30
40
50
60
Normal
LY50
LY75
LY88
LY94
LY97
LY data
Normal data

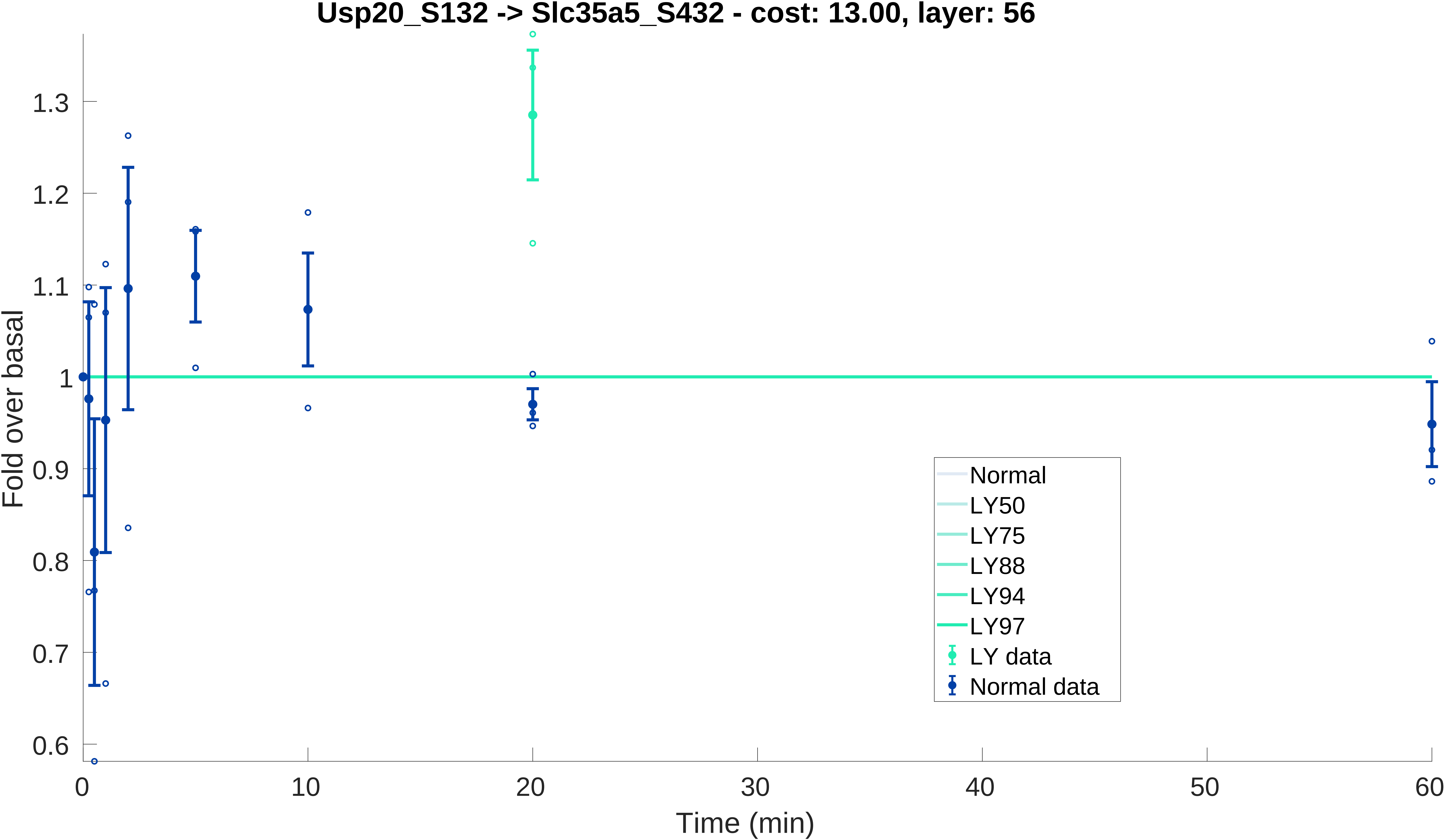
Usp20_S132 -> Slc35a5_S432 - cost: 13.00, layer: 56
Fold over basal
1.3
1.2
1.1
1
0.9
0.8
0.7
0.6
0
10
20
30
40
50
60
Time (min)
Normal
LY50
LY75
LY88
LY94
LY97
LY data
Normal data

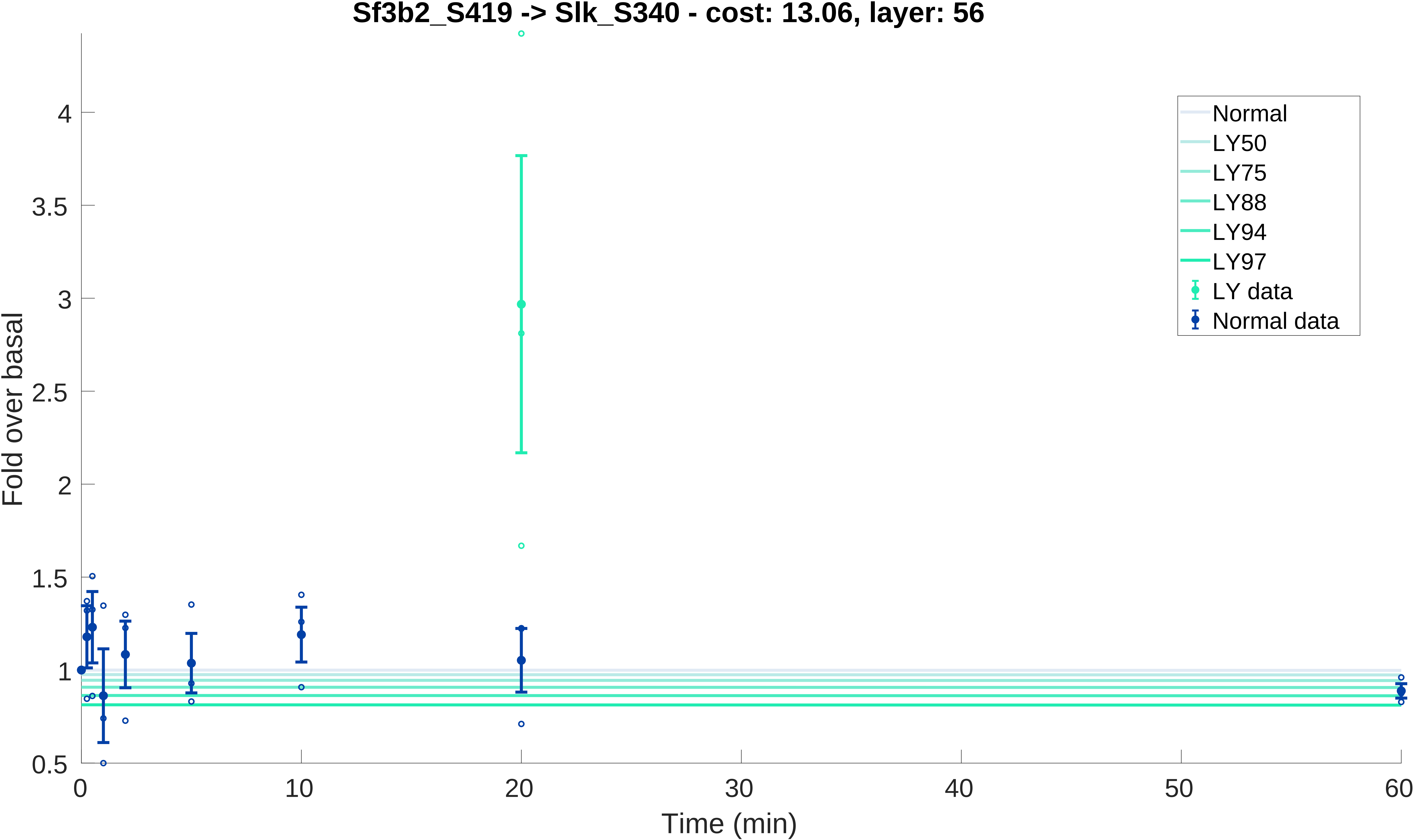
Sf3b2_S419 -> Slk_S340 - cost: 13.06, layer: 56
Fold over basal
Time (min)
0.5
1
1.5
2
2.5
3
3.5
4
0
10
20
30
40
50
60
Normal
LY50
LY75
LY88
LY94
LY97
LY data
Normal data

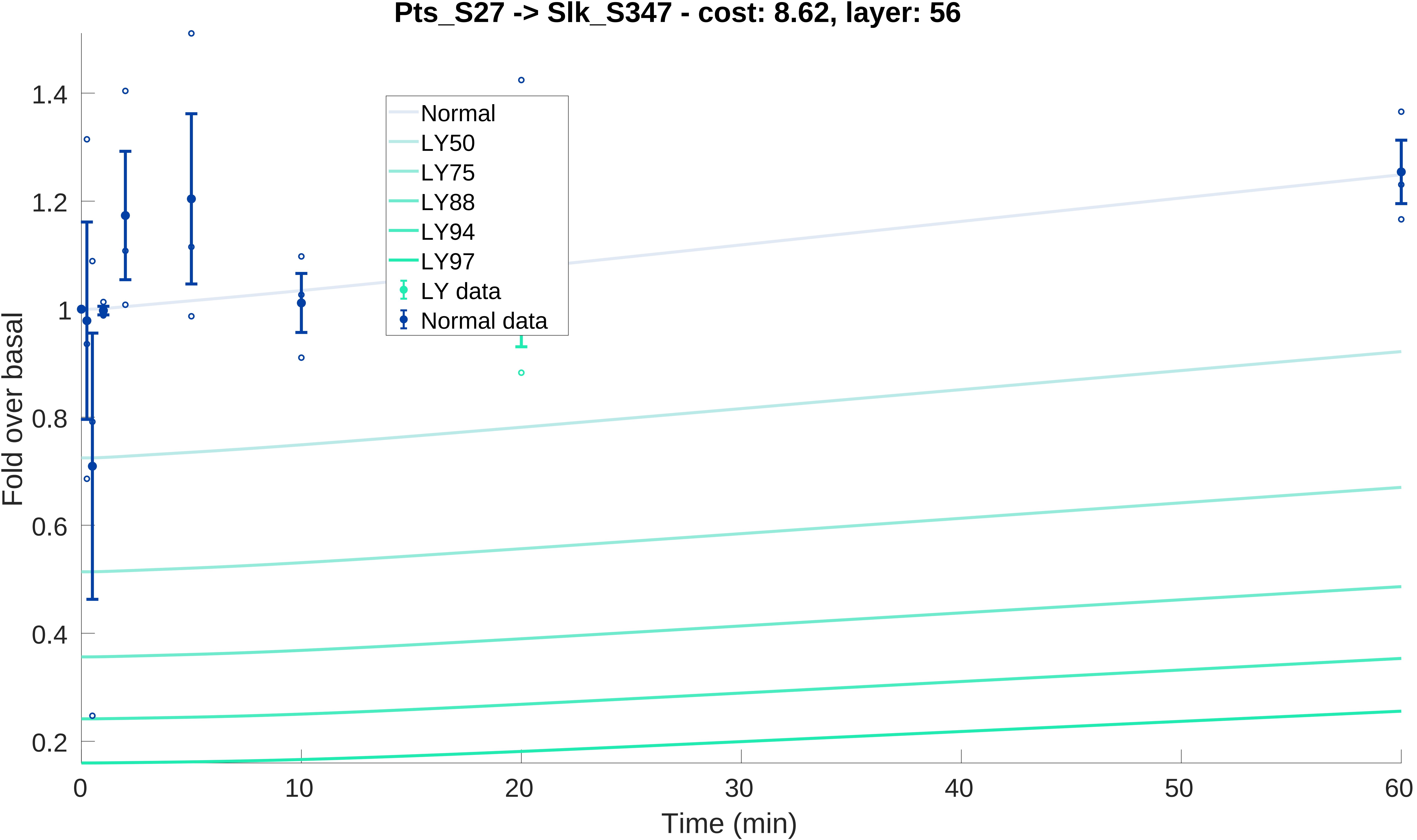

Pts_S27 -> Slk_S347 - cost: 8.62, layer: 56
Fold over basal
Time (min)
0
10
20
30
40
50
60
0.2
0.4
0.6
0.8
1
1.2
1.4
Normal
LY50
LY75
LY88
LY94
LY97
LY data
Normal data

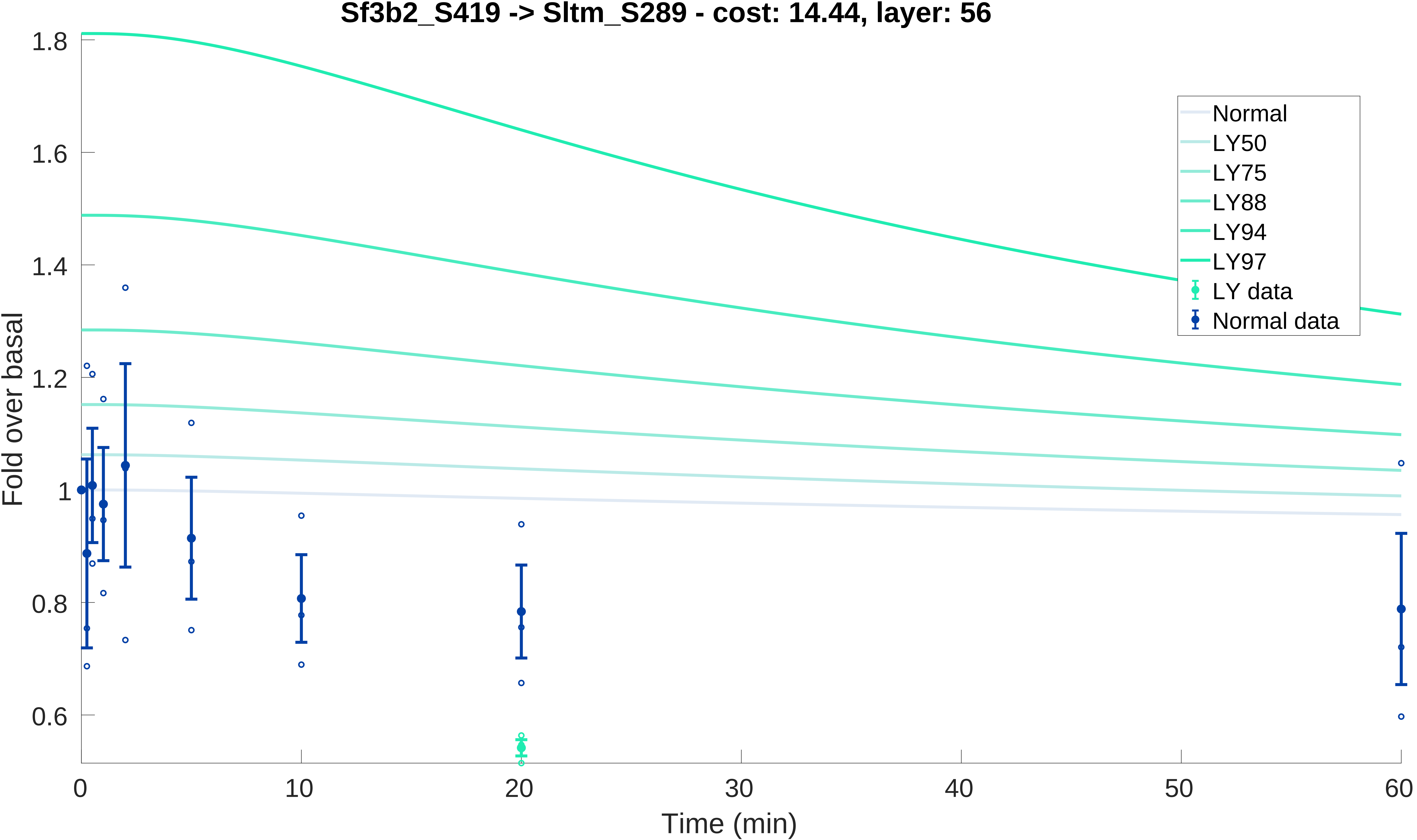
Sf3b2_S419 -> Sltm_S289 - cost: 14.44, layer: 56
Fold over basal
Time (min)
1.8
1.6
1.4
1.2
1
0.8
0.6
0
10
20
30
40
50
60
Normal
LY50
LY75
LY88
LY94
LY97
LY data
Normal data

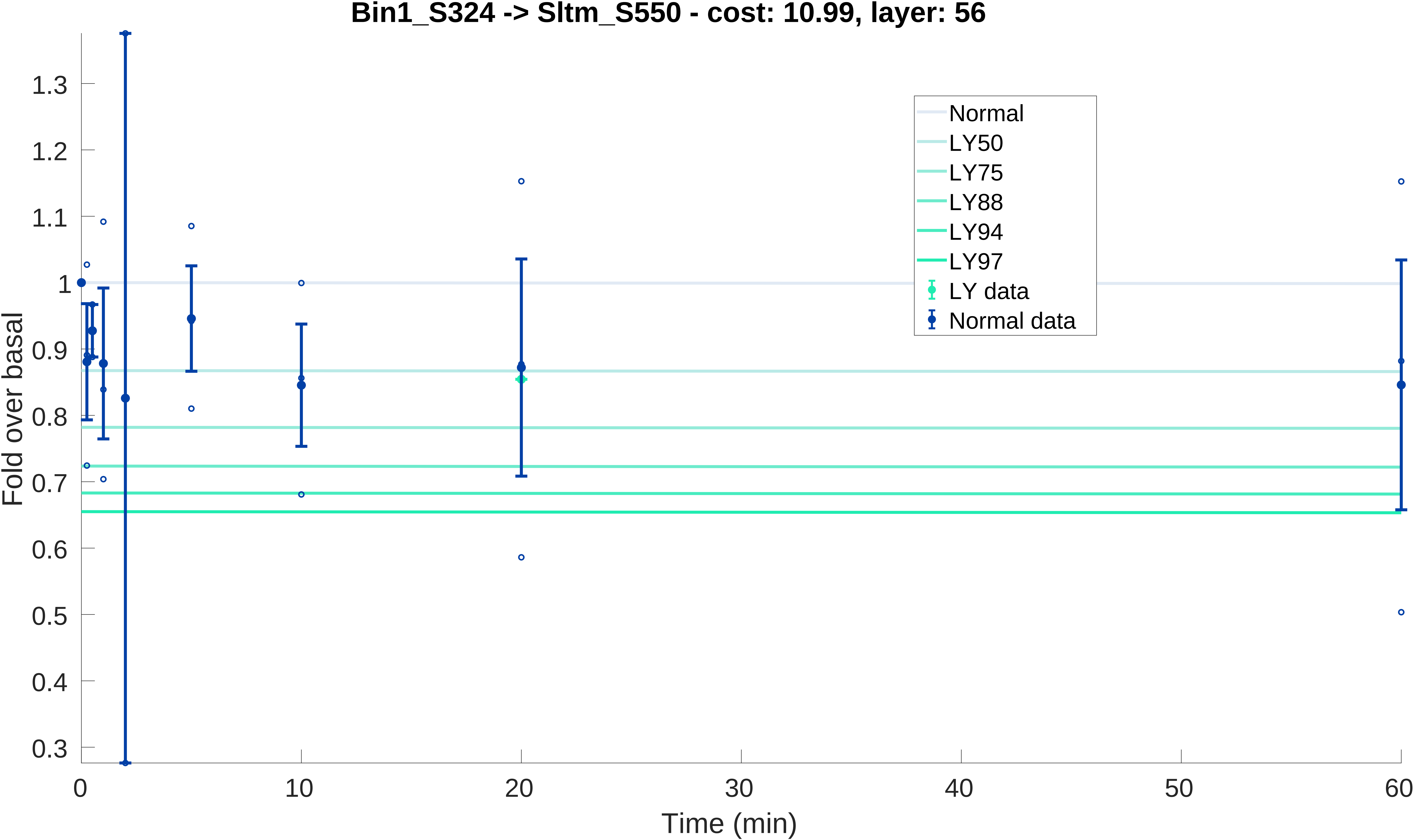

Bin1_S324 -> Sltm_S550 - cost: 10.99, layer: 56
Normal
LY50
LY75
LY88
LY94
LY97
LY data
Normal data
Fold over basal
Time (min)
1.3
1.2
1.1
1
0.9
0.8
0.7
0.6
0.5
0.4
0.3
0
10
20
30
40
50
60

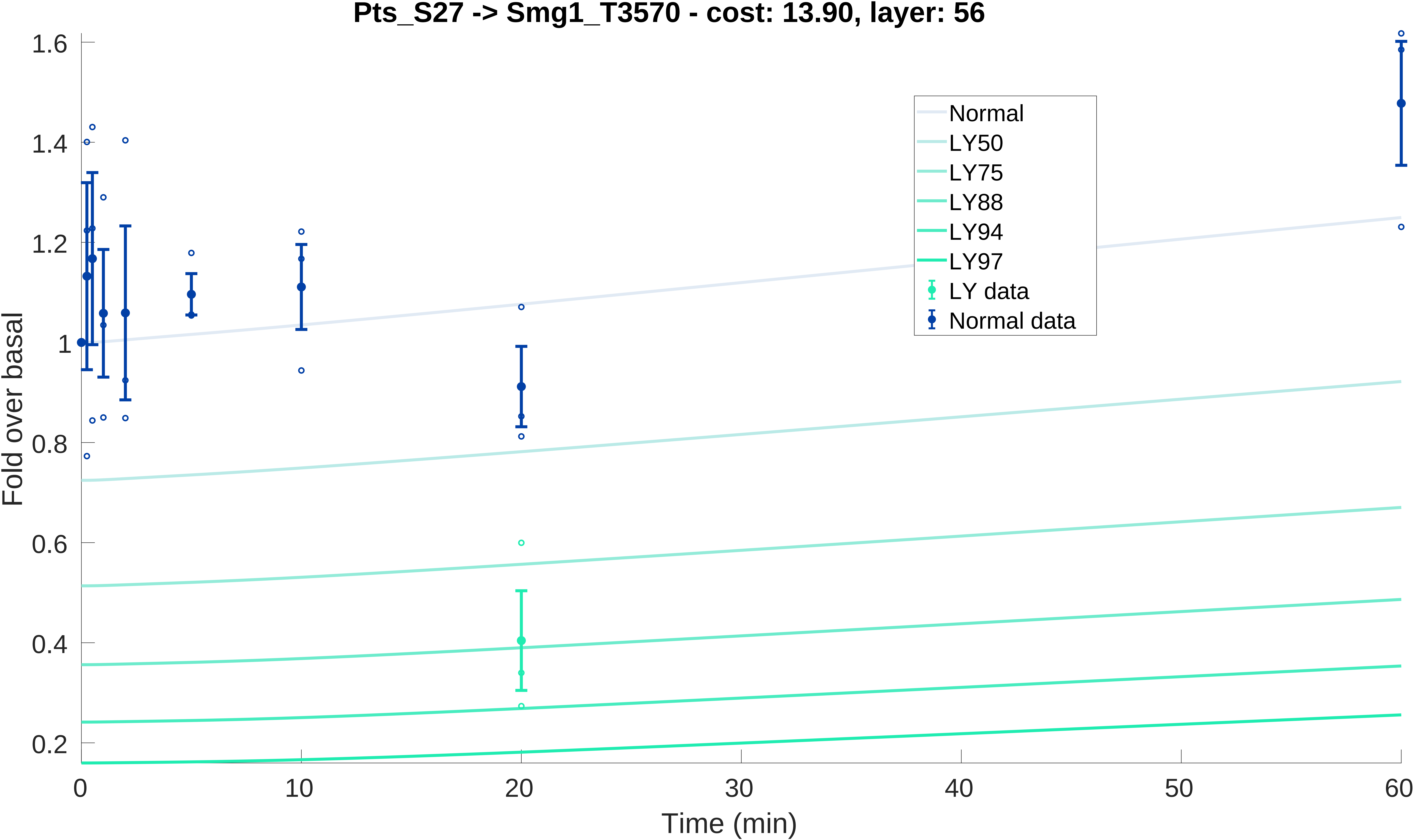

Pts_S27 -> Smg1_T3570 - cost: 13.90, layer: 56
Fold over basal
Time (min)
Normal
LY50
LY75
LY88
LY94
LY97
LY data
Normal data
0.2
0.4
0.6
0.8
1
1.2
1.4
1.6
0
10
20
30
40
50
60

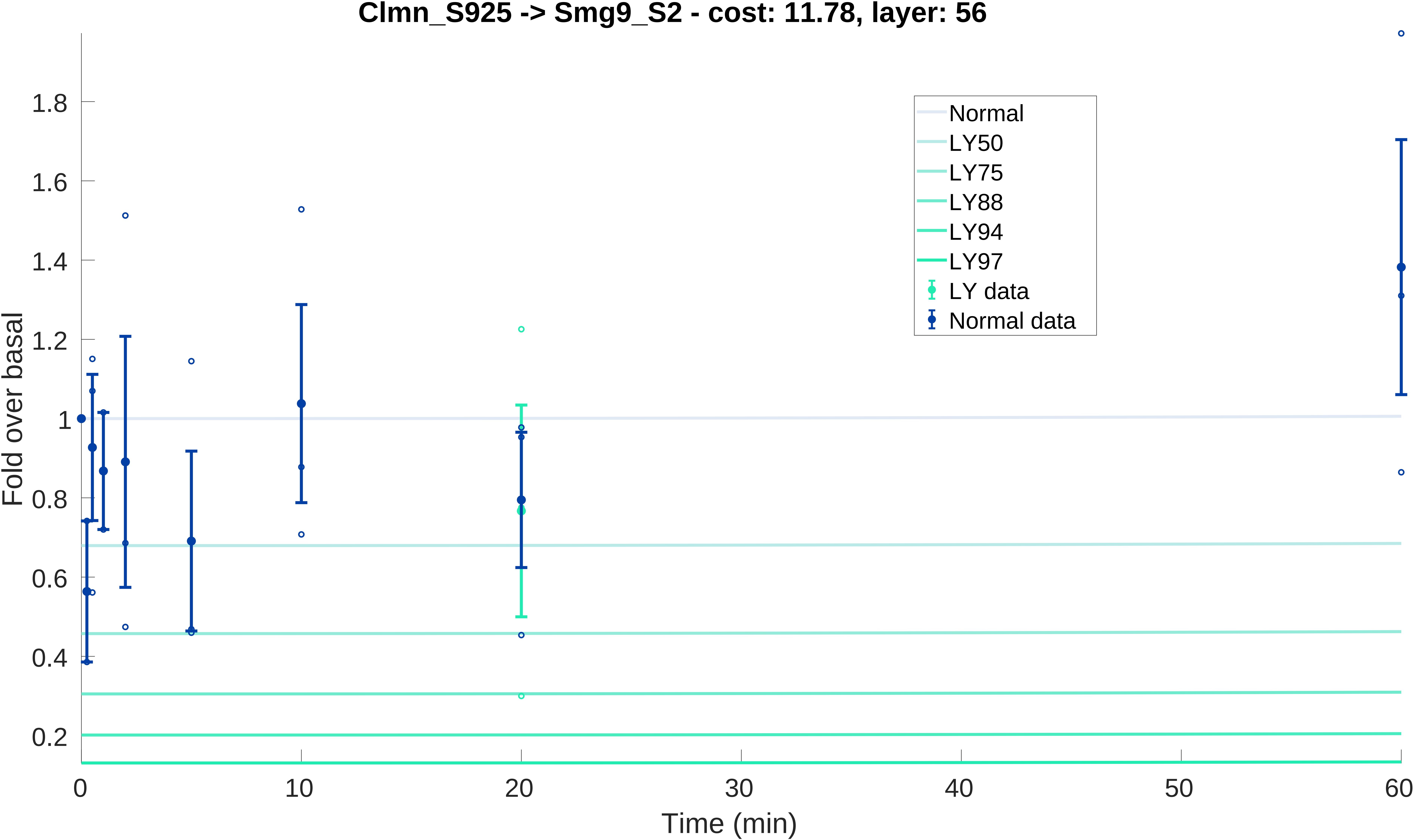
Clmn_S925 -> Smg9_S2 - cost: 11.78, layer: 56
Fold over basal
Time (min)
Normal
LY50
LY75
LY88
LY94
LY97
LY data
Normal data
0.2
0.4
0.6
0.8
1
1.2
1.4
1.6
1.8
0
10
20
30
40
50
60

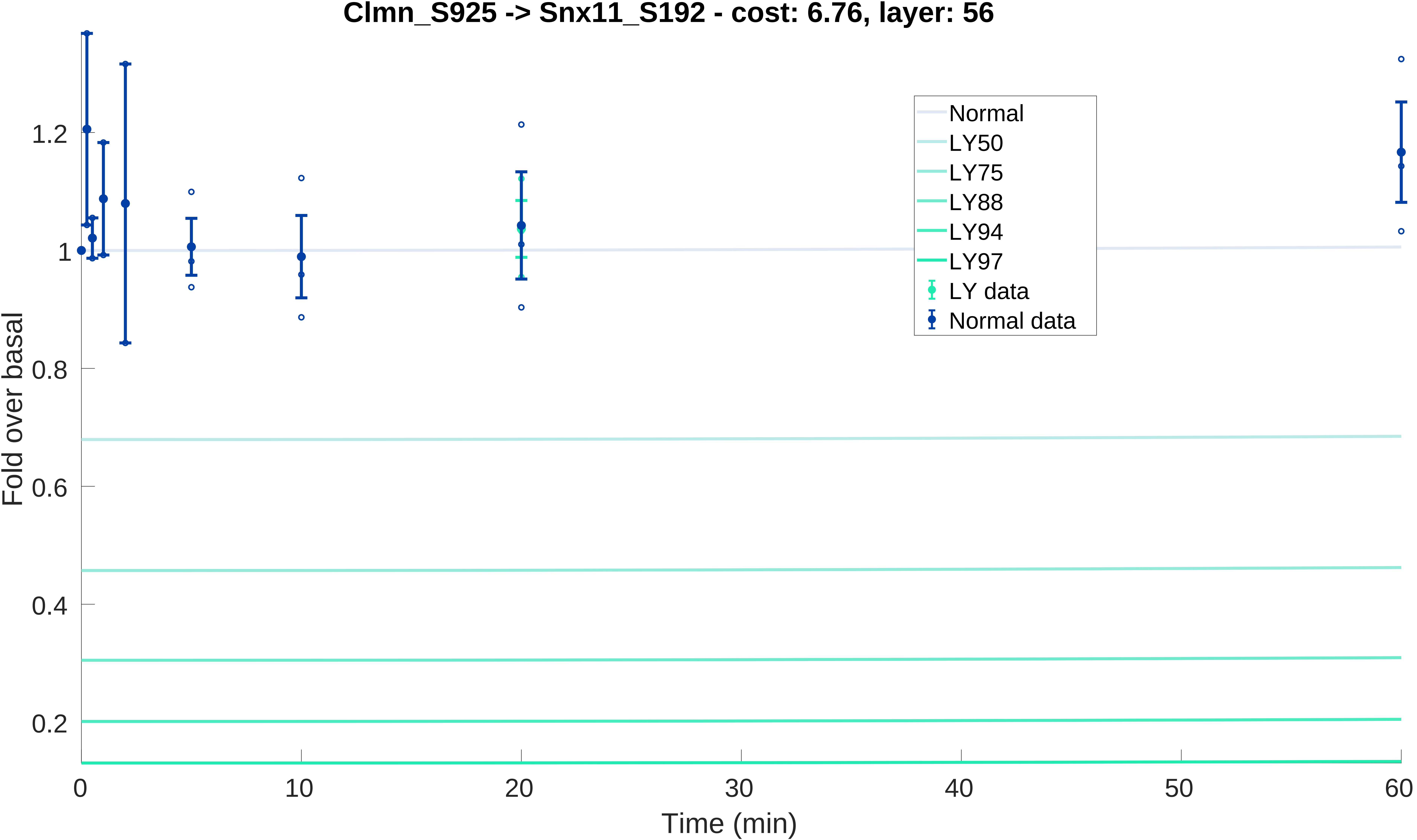

Clmn_S925 -> Snx11_S192 - cost: 6.76, layer: 56
Normal
LY50
LY75
LY88
LY94
LY97
LY data
Normal data
Fold over basal
Time (min)
0.2
0.4
0.6
0.8
1
1.2
0
10
20
30
40
50
60

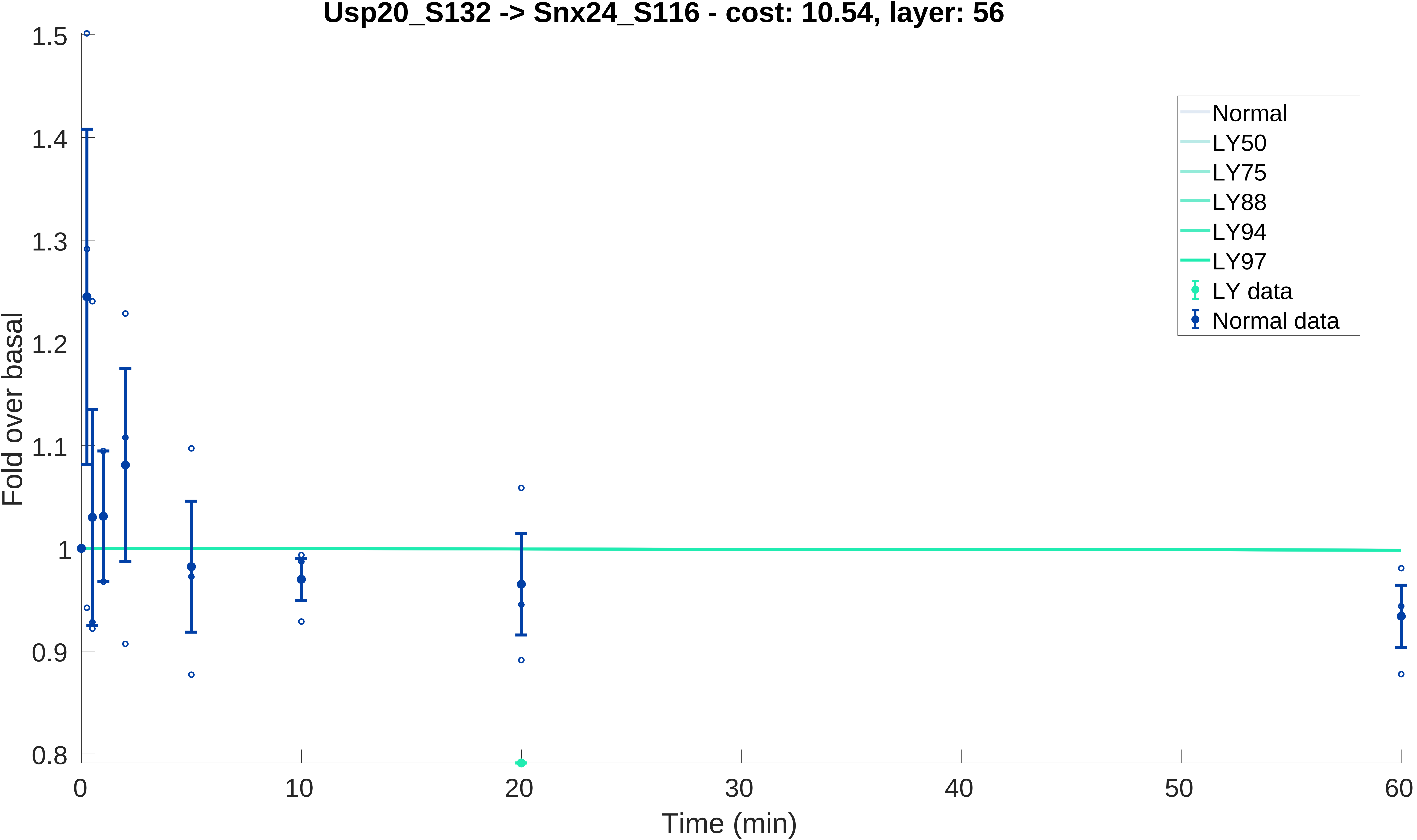
Usp20_S132 -> Snx24_S116 - cost: 10.54, layer: 56
Fold over basal
1.5
1.4
1.3
1.2
1.1
1
0.9
0.8
0
10
20
30
40
50
60
Time (min)
Normal
LY50
LY75
LY88
LY94
LY97
LY data
Normal data

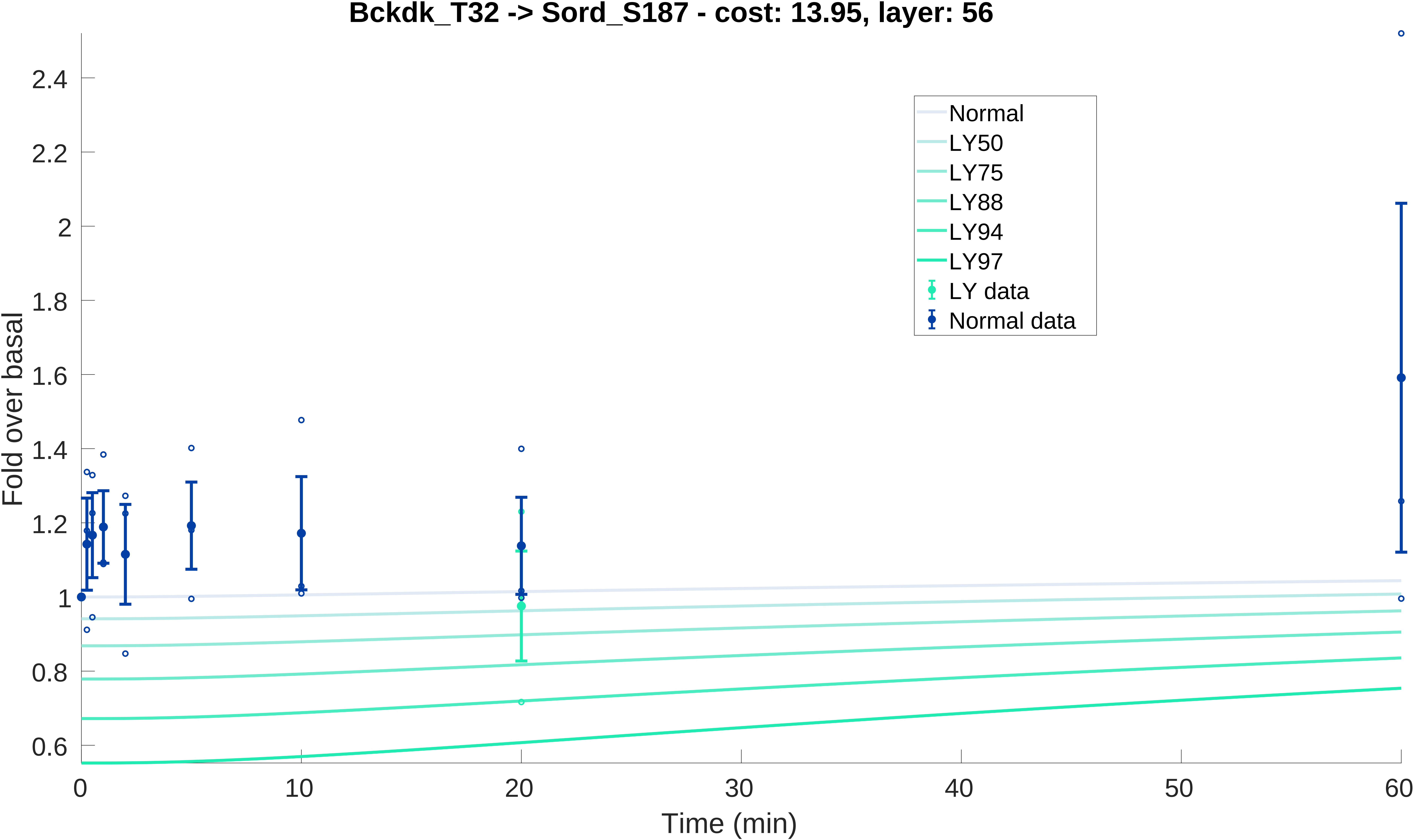

Bckdk_T32 -> Sord_S187 - cost: 13.95, layer: 56
Fold over basal
Time (min)
0
10
20
30
40
50
60
0.6
0.8
1
1.2
1.4
1.6
1.8
2
2.2
2.4
Normal
LY50
LY75
LY88
LY94
LY97
LY data
Normal data

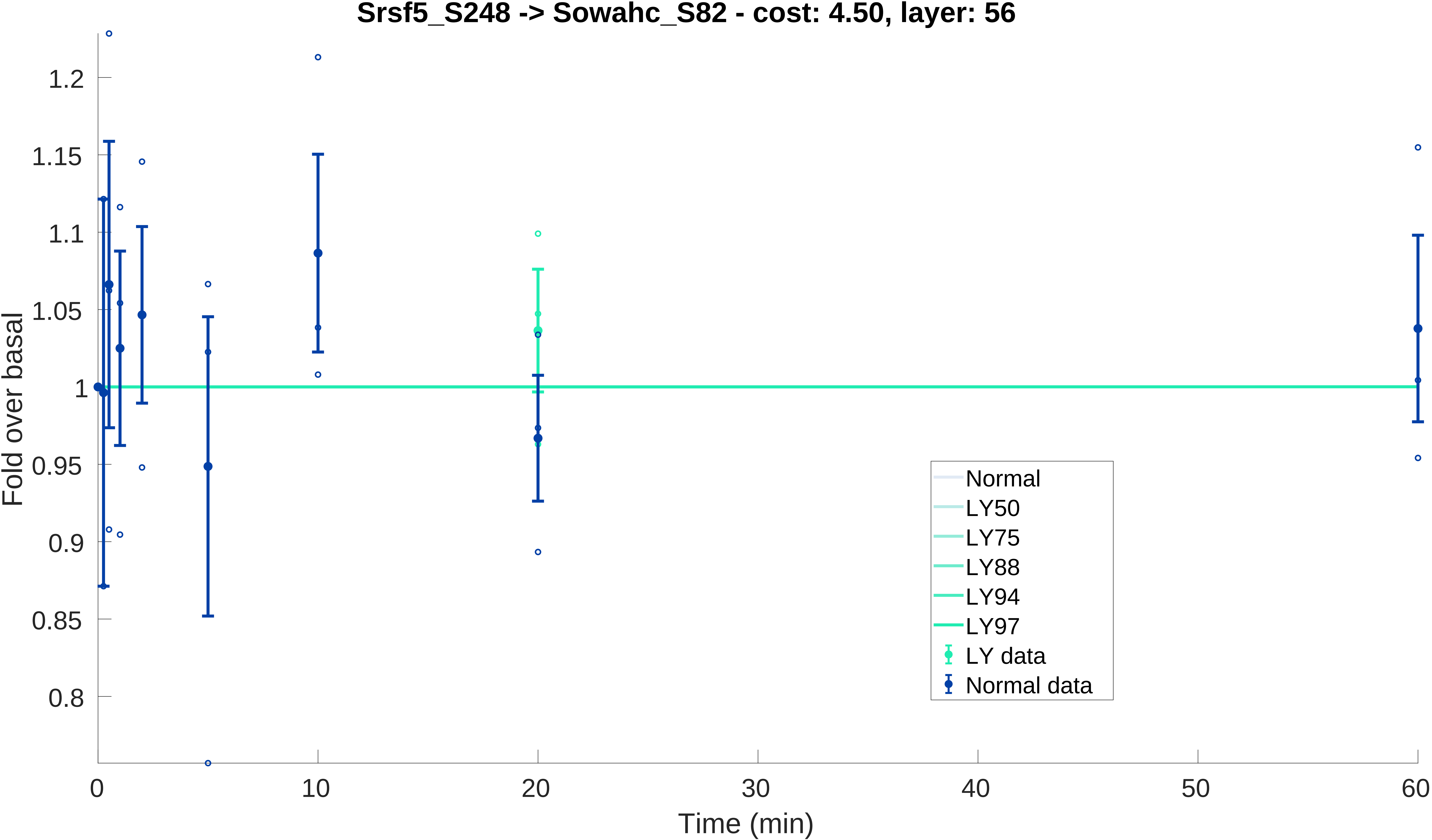

Srsf5_S248 -> Sowahc_S82 - cost: 4.50, layer: 56
Fold over basal
1.2
1.15
1.1
1.05
1
0.95
0.9
0.85
0.8
0
10
20
30
40
50
60
Time (min)
Normal
LY50
LY75
LY88
LY94
LY97
LY data
Normal data

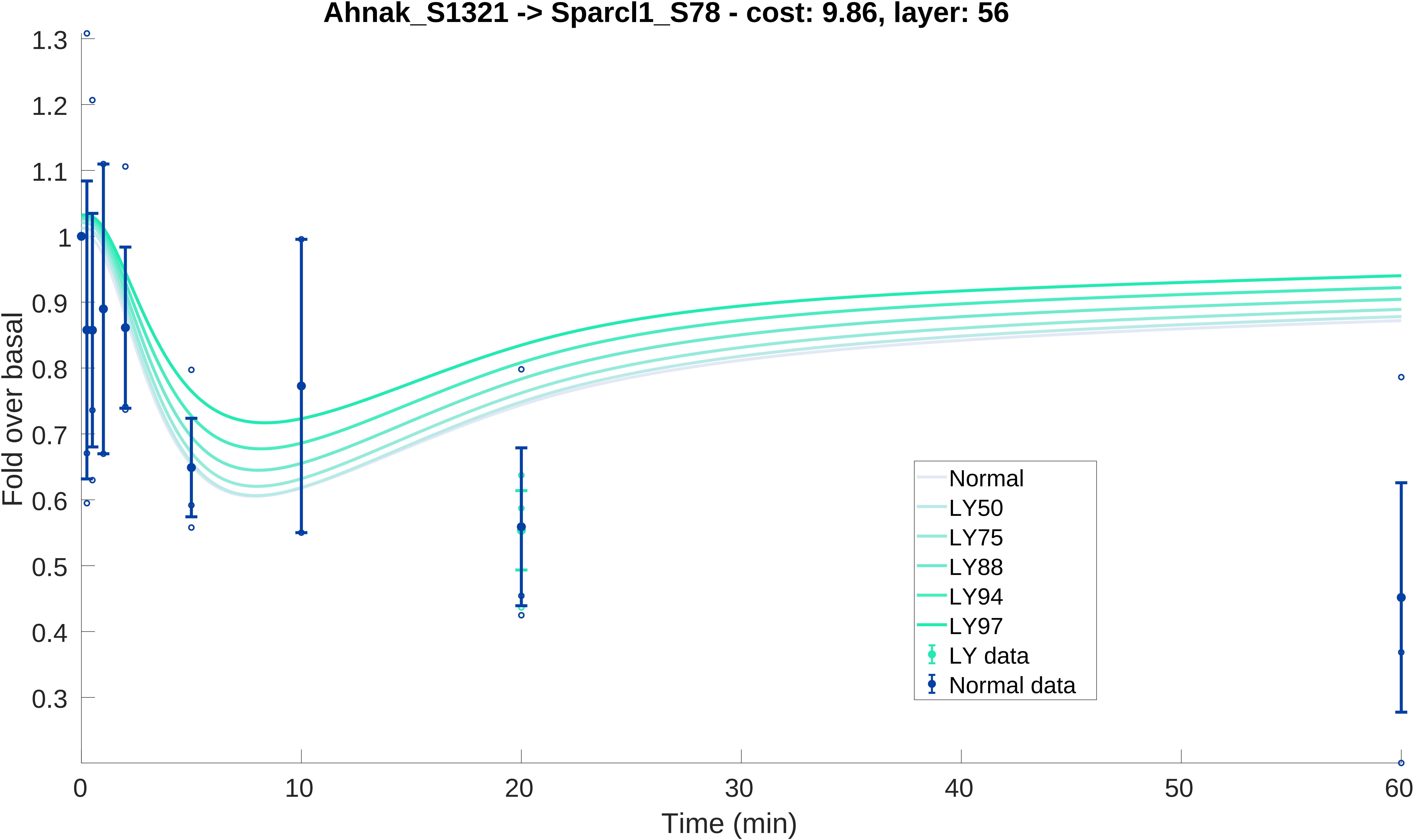

Ahnak_S1321 -> Sparcl1_S78 - cost: 9.86, layer: 56
Fold over basal
1.3
1.2
1.1
1
0.9
0.8
0.7
0.6
0.5
0.4
0.3
0
10
20
30
40
50
60
Time (min)
Normal
LY50
LY75
LY88
LY94
LY97
LY data
Normal data

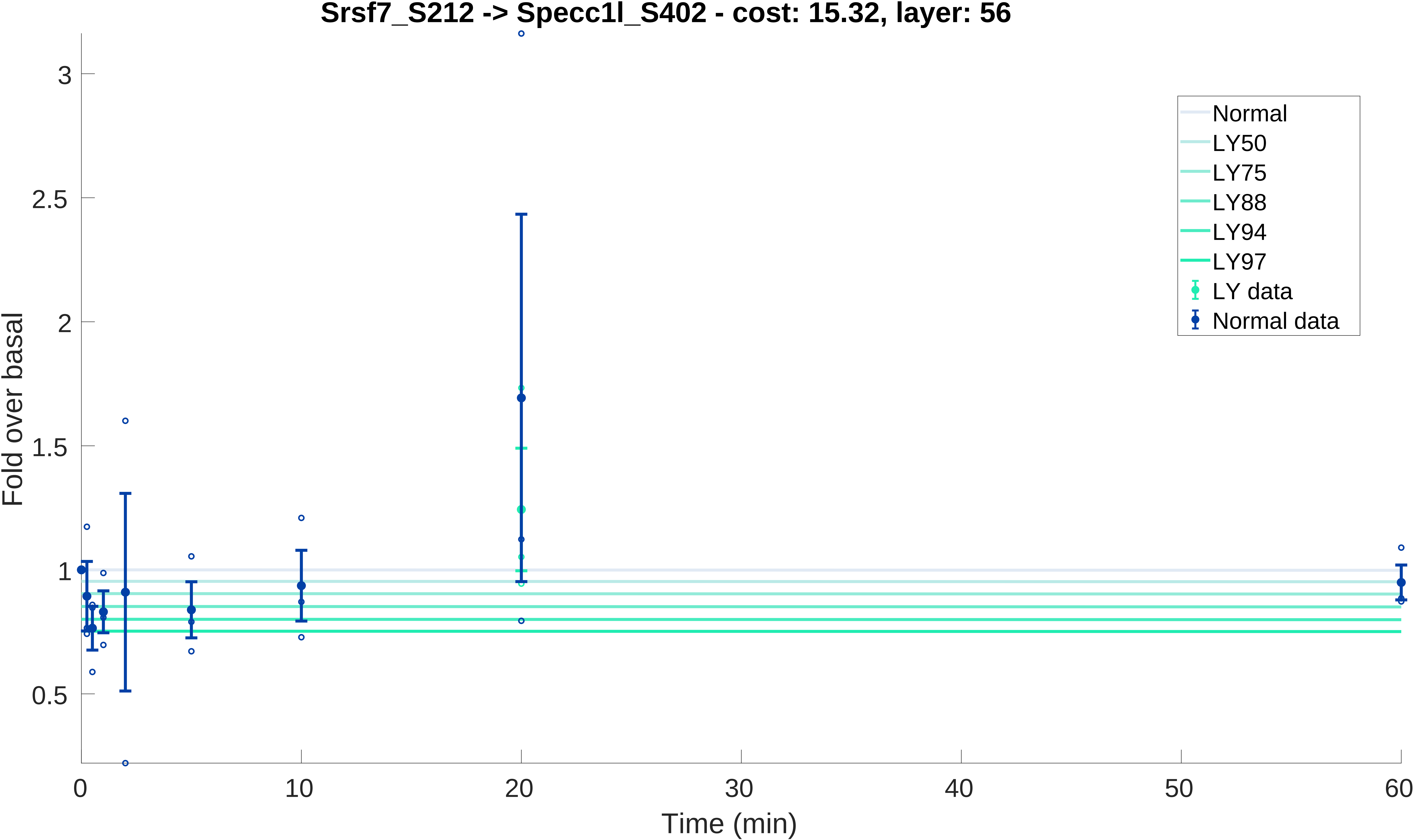

Srsf7_S212 -> Specc1l_S402 - cost: 15.32, layer: 56
Fold over basal
Time (min)
0.5
1
1.5
2
2.5
3
0
10
20
30
40
50
60
Normal
LY50
LY75
LY88
LY94
LY97
LY data
Normal data

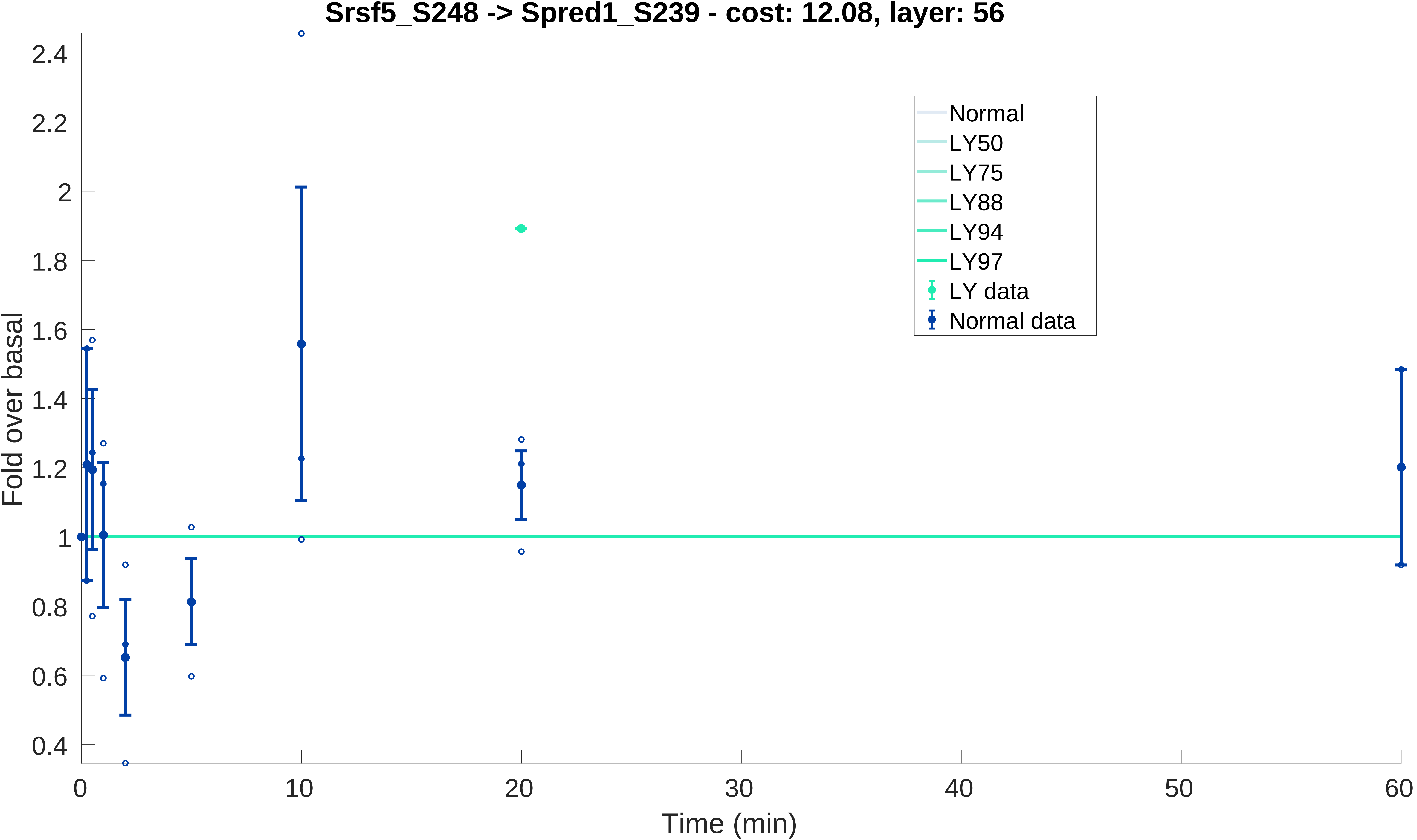
Srsf5_S248 -> Spred1_S239 - cost: 12.08, layer: 56
Normal
LY50
LY75
LY88
LY94
LY97
LY data
Normal data
Fold over basal
Time (min)
0.4
0.6
0.8
1
1.2
1.4
1.6
1.8
2
2.2
2.4
0
10
20
30
40
50
60

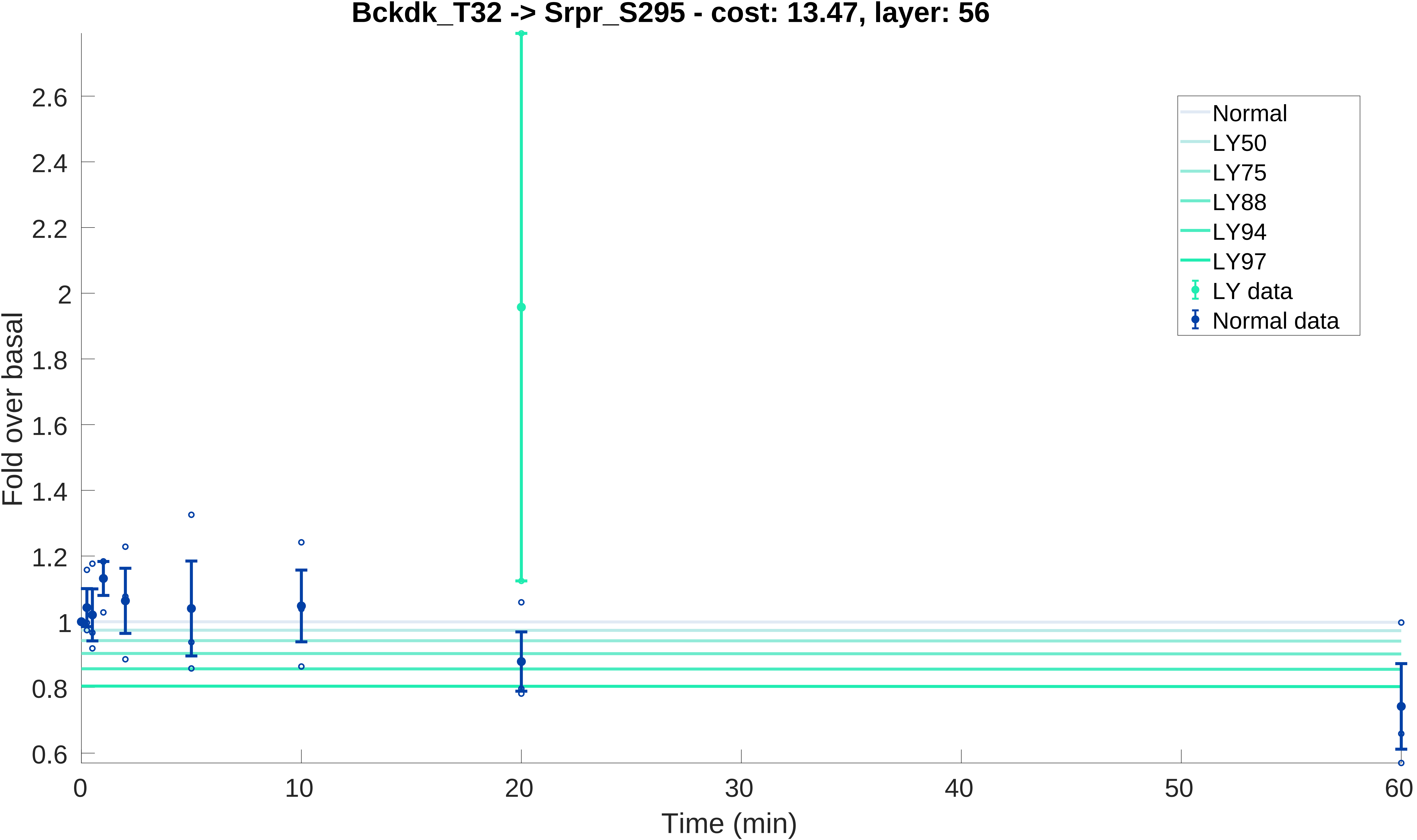

Bckdk_T32 -> Srpr_S295 - cost: 13.47, layer: 56
Fold over basal
2.6
2.4
2.2
2
1.8
1.6
1.4
1.2
1
0.8
0.6
0
10
20
30
40
50
60
Time (min)
Normal
LY50
LY75
LY88
LY94
LY97
LY data
Normal data

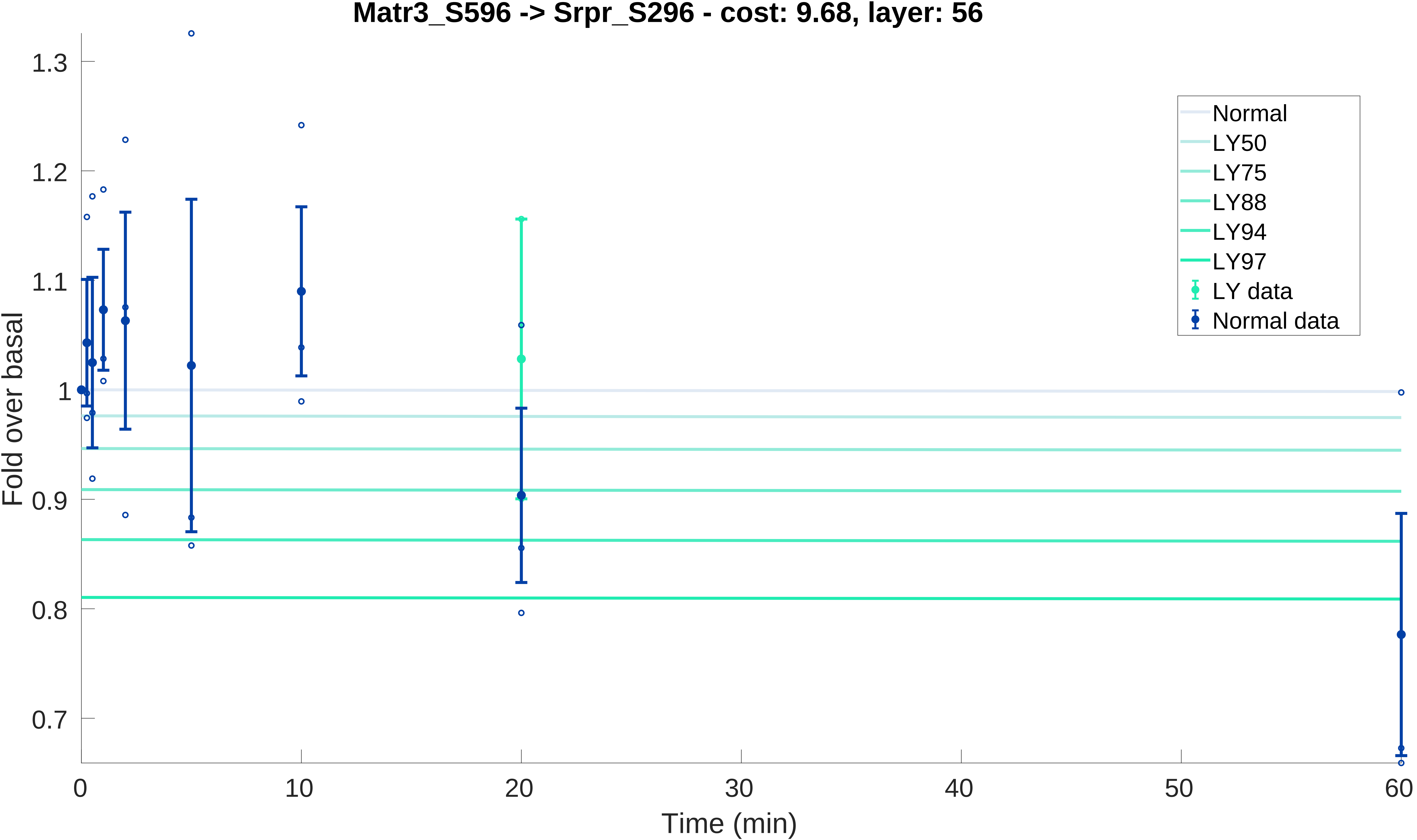

Matr3_S596 -> Srpr_S296 - cost: 9.68, layer: 56
Fold over basal
Time (min)
1.3
1.2
1.1
1
0.9
0.8
0.7
0
10
20
30
40
50
60
Normal
LY50
LY75
LY88
LY94
LY97
LY data
Normal data

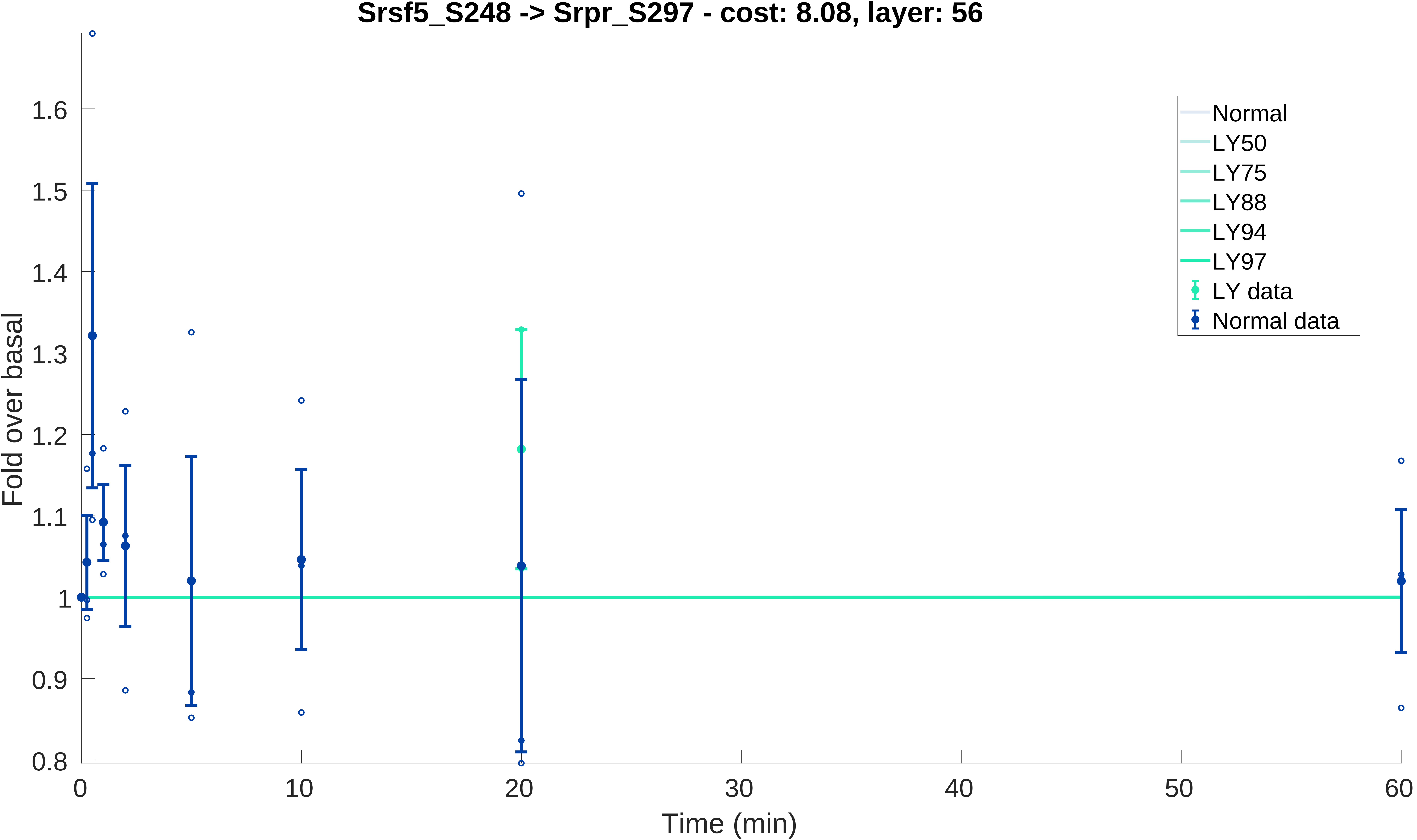
Srsf5_S248 -> Srpr_S297 - cost: 8.08, layer: 56
Normal
LY50
LY75
LY88
LY94
LY97
LY data
Normal data
Fold over basal
Time (min)
0.8
0.9
1
1.1
1.2
1.3
1.4
1.5
1.6
0
10
20
30
40
50
60

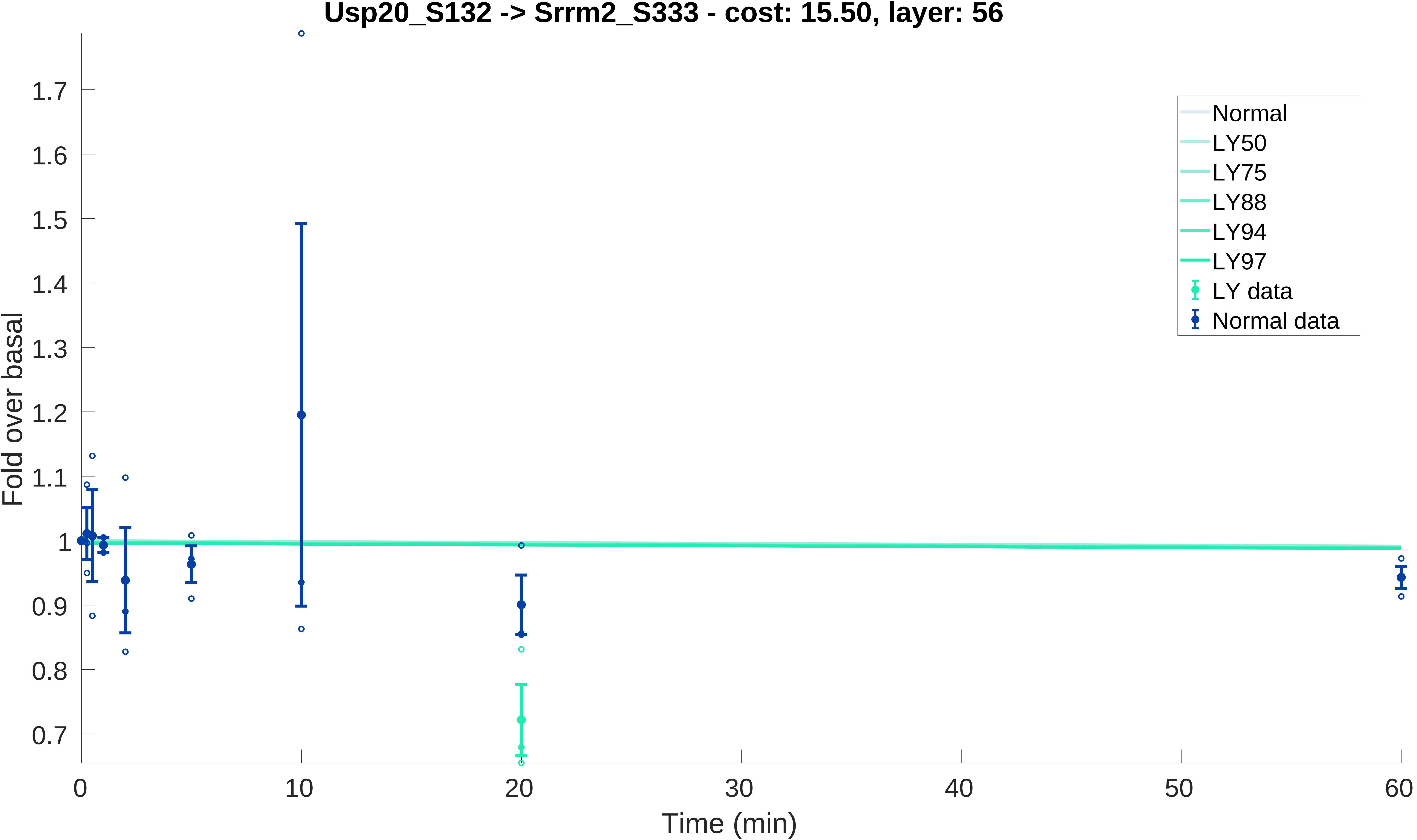

Usp20_S132 -> Srrm2_S333 - cost: 15.50, layer: 56
Fold over basal
Time (min)
1.7
1.6
1.5
1.4
1.3
1.2
1.1
1
0.9
0.8
0.7
0
10
20
30
40
50
60
Normal
LY50
LY75
LY88
LY94
LY97
LY data
Normal data

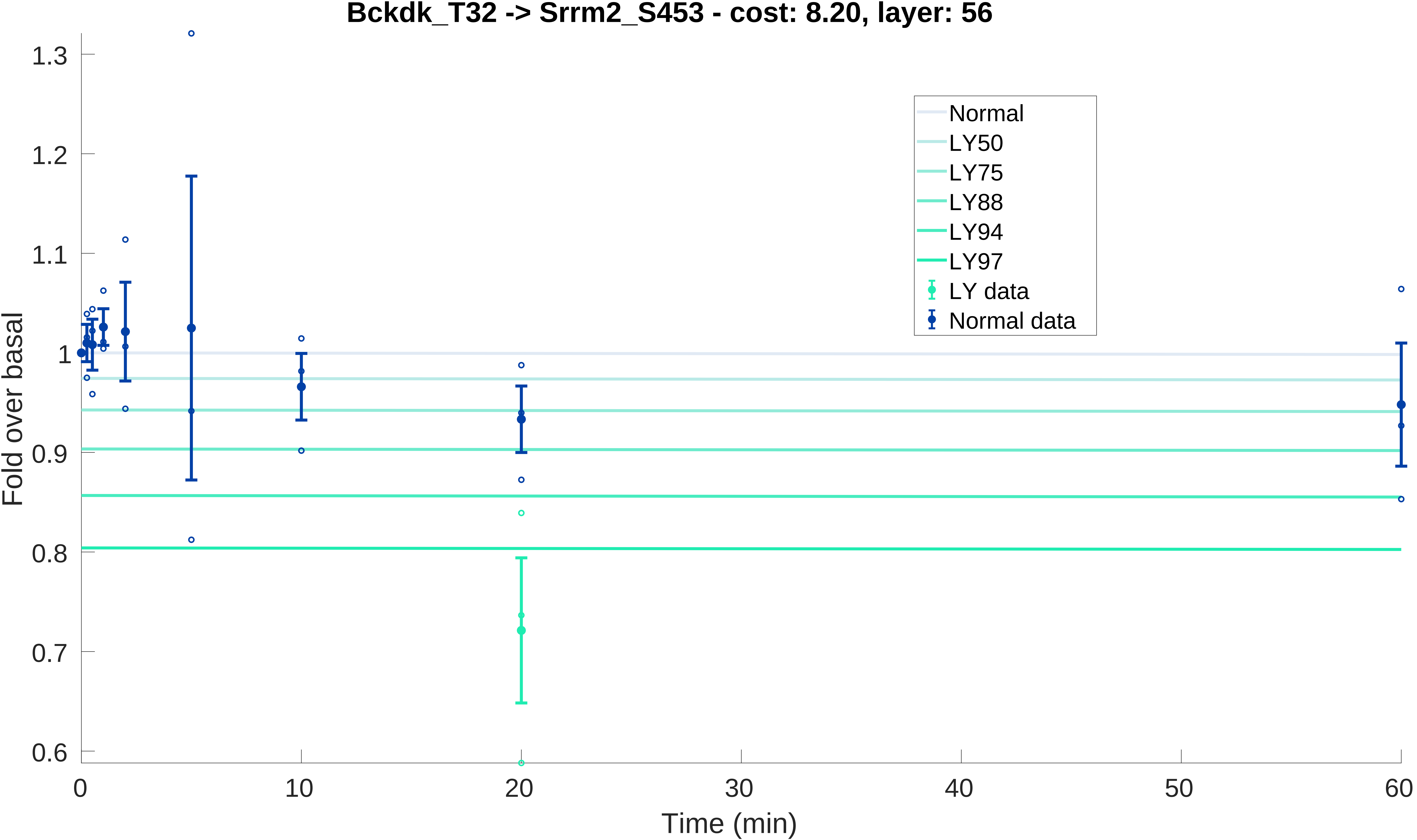

Bckdk_T32 -> Srrm2_S453 - cost: 8.20, layer: 56
Fold over basal
Time (min)
1.3
1.2
1.1
1
0.9
0.8
0.7
0.6
0
10
20
30
40
50
60
Normal
LY50
LY75
LY88
LY94
LY97
LY data
Normal data

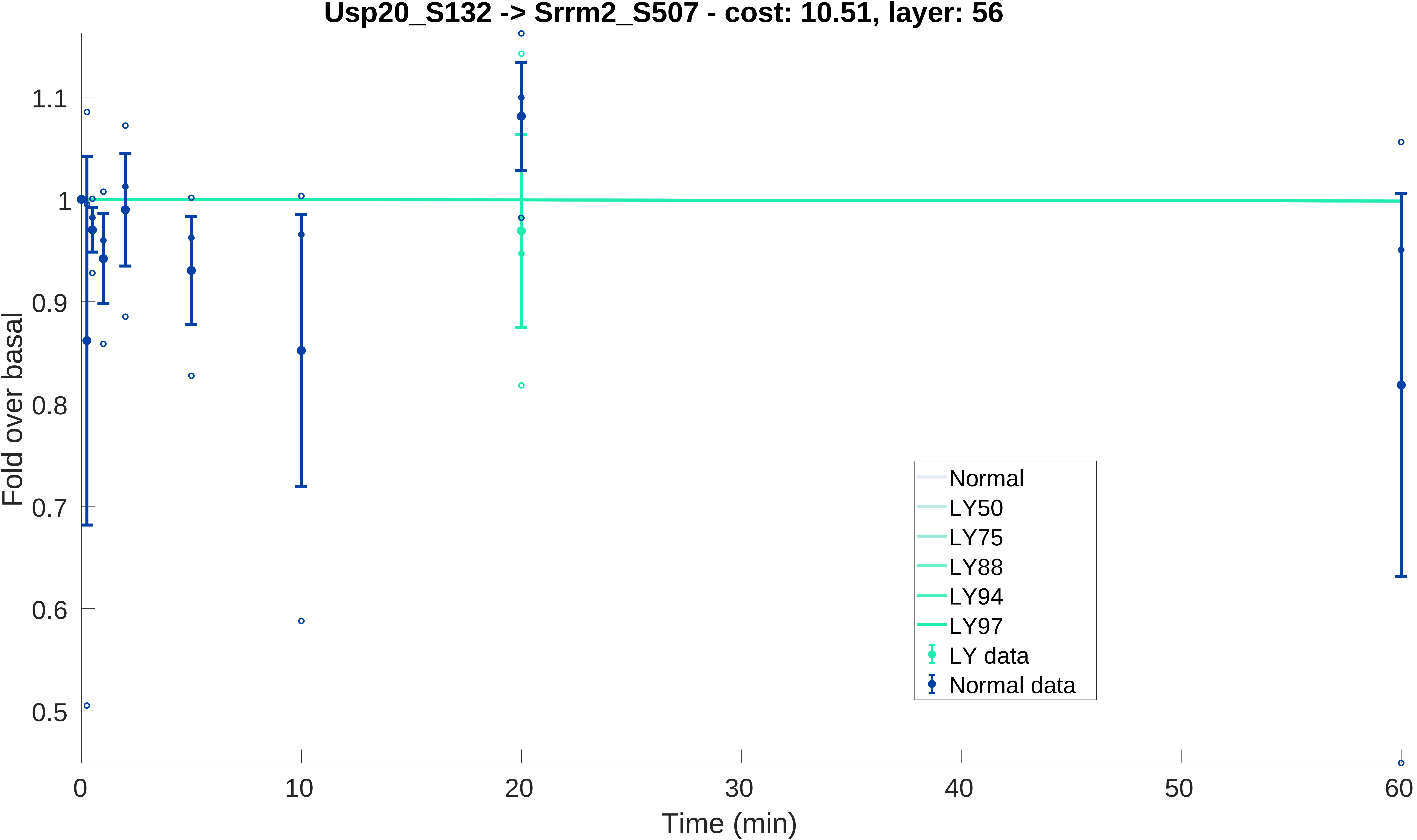
Usp20_S132 -> Srrm2_S507 - cost: 10.51, layer: 56
Fold over basal
Time (min)
0.5
0.6
0.7
0.8
0.9
1
1.1
0
10
20
30
40
50
60
Normal
LY50
LY75
LY88
LY94
LY97
LY data
Normal data

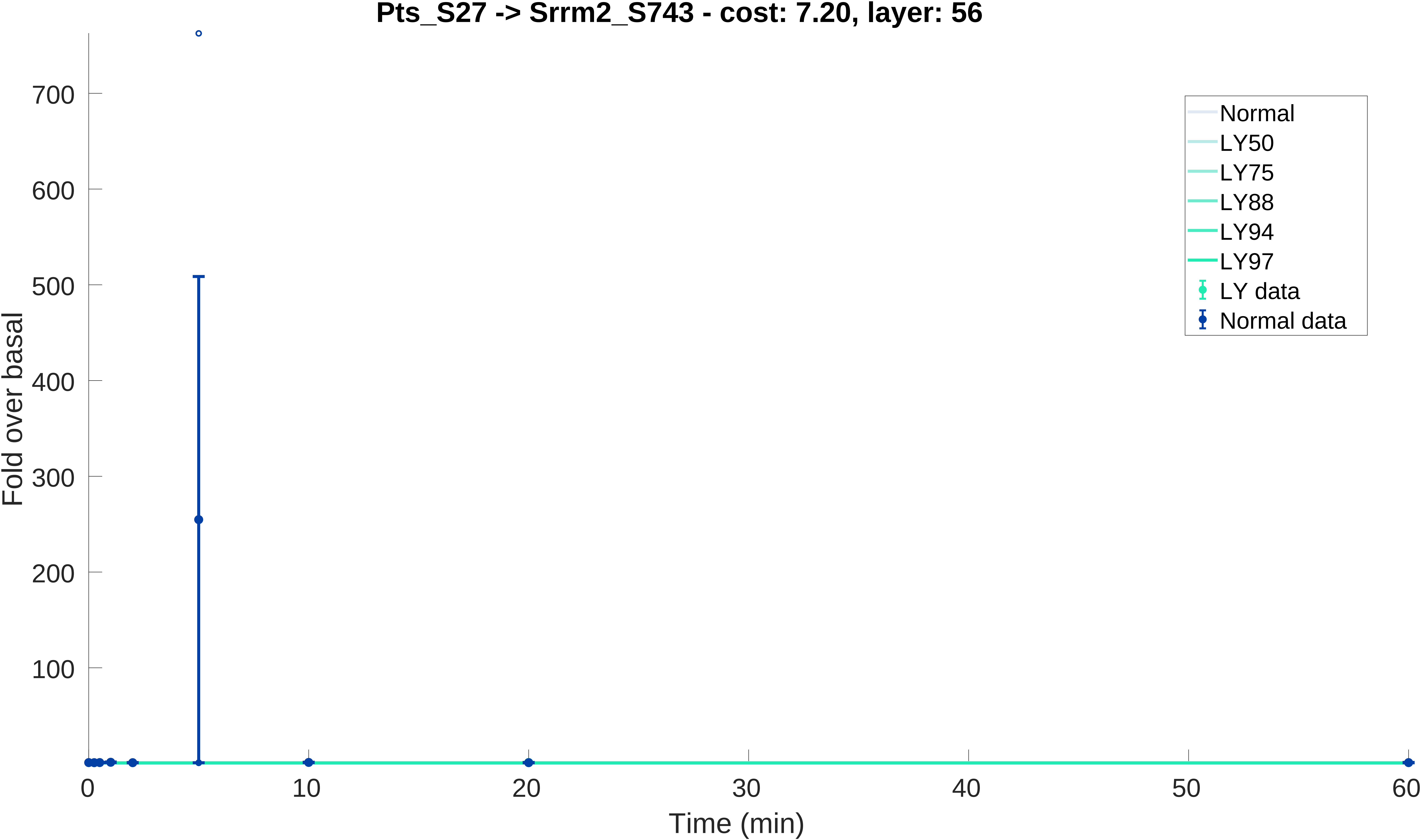

Pts_S27 -> Srrm2_S743 - cost: 7.20, layer: 56
Fold over basal
Time (min)
100
200
300
400
500
600
700
0
10
20
30
40
50
60
Normal
LY50
LY75
LY88
LY94
LY97
LY data
Normal data

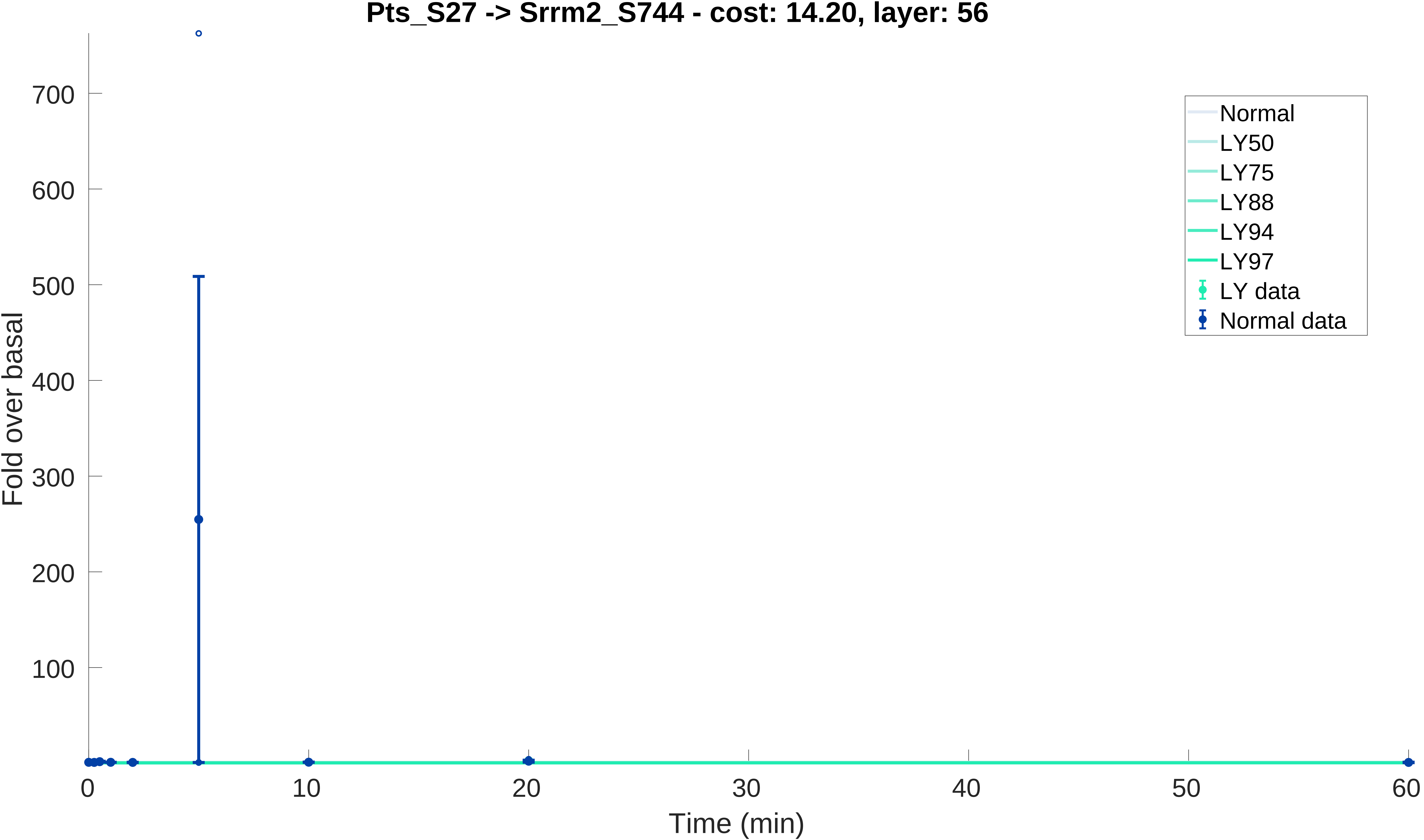

Pts_S27 -> Srrm2_S744 - cost: 14.20, layer: 56
Fold over basal
Time (min)
100
200
300
400
500
600
700
0
10
20
30
40
50
60
Normal
LY50
LY75
LY88
LY94
LY97
LY data
Normal data

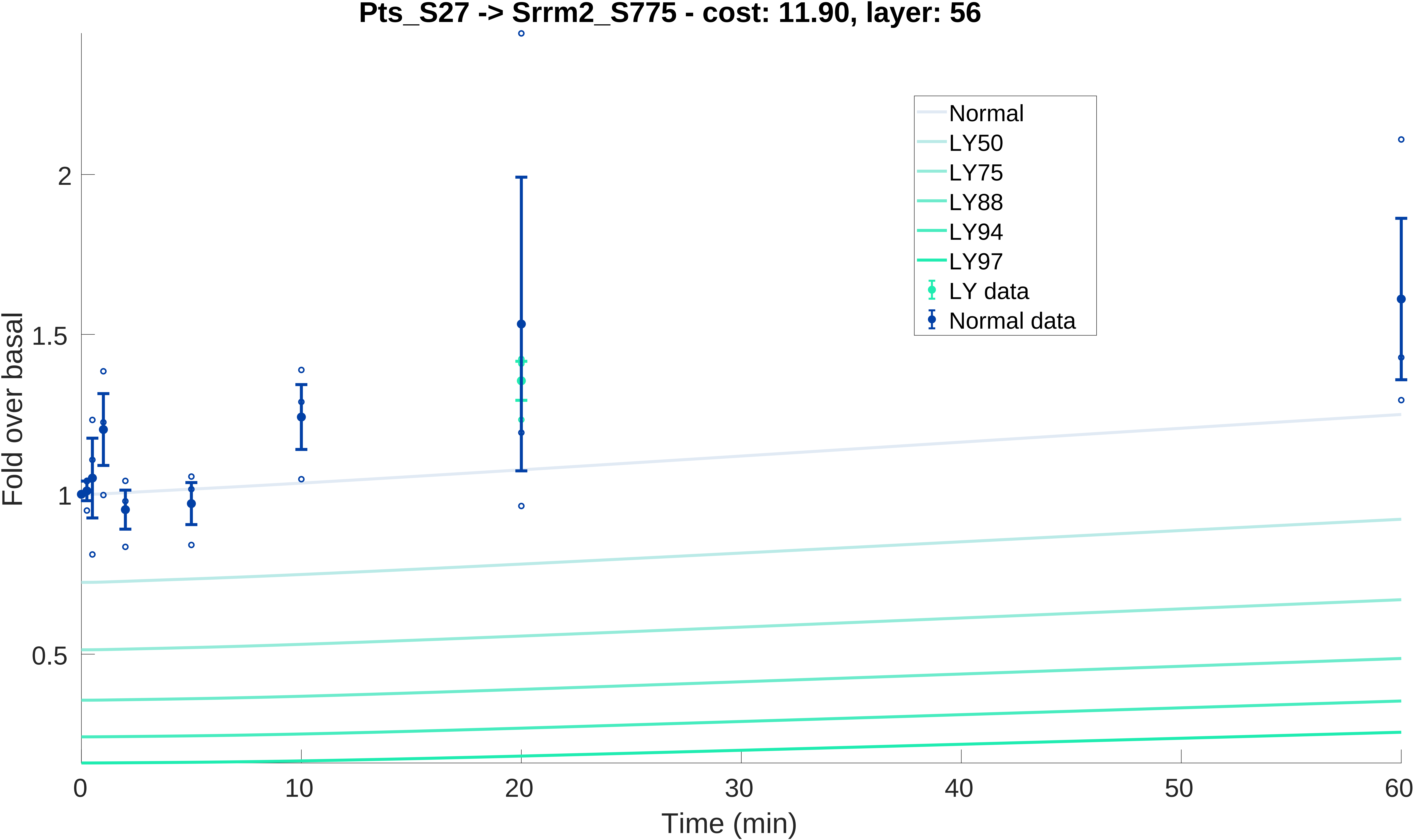

Pts_S27 -> Srrm2_S775 - cost: 11.90, layer: 56
Normal
LY50
LY75
LY88
LY94
LY97
LY data
Normal data
Fold over basal
2
1.5
1
0.5
0
10
20
30
40
50
60
Time (min)

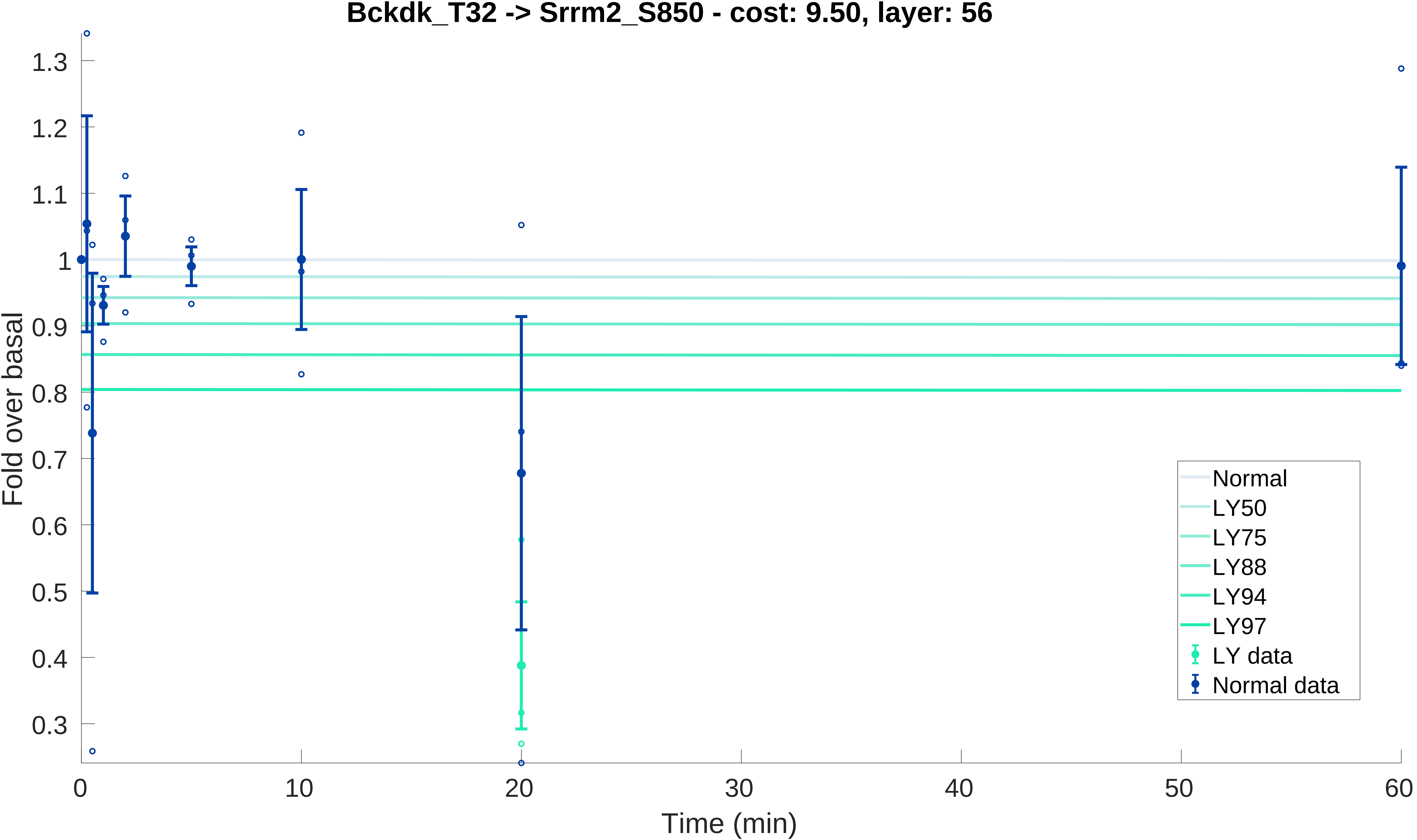

Bckdk_T32 -> Srrm2_S850 - cost: 9.50, layer: 56
Fold over basal
Time (min)
1.3
1.2
1.1
1
0.9
0.8
0.7
0.6
0.5
0.4
0.3
0
10
20
30
40
50
60
Normal
LY50
LY75
LY88
LY94
LY97
LY data
Normal data

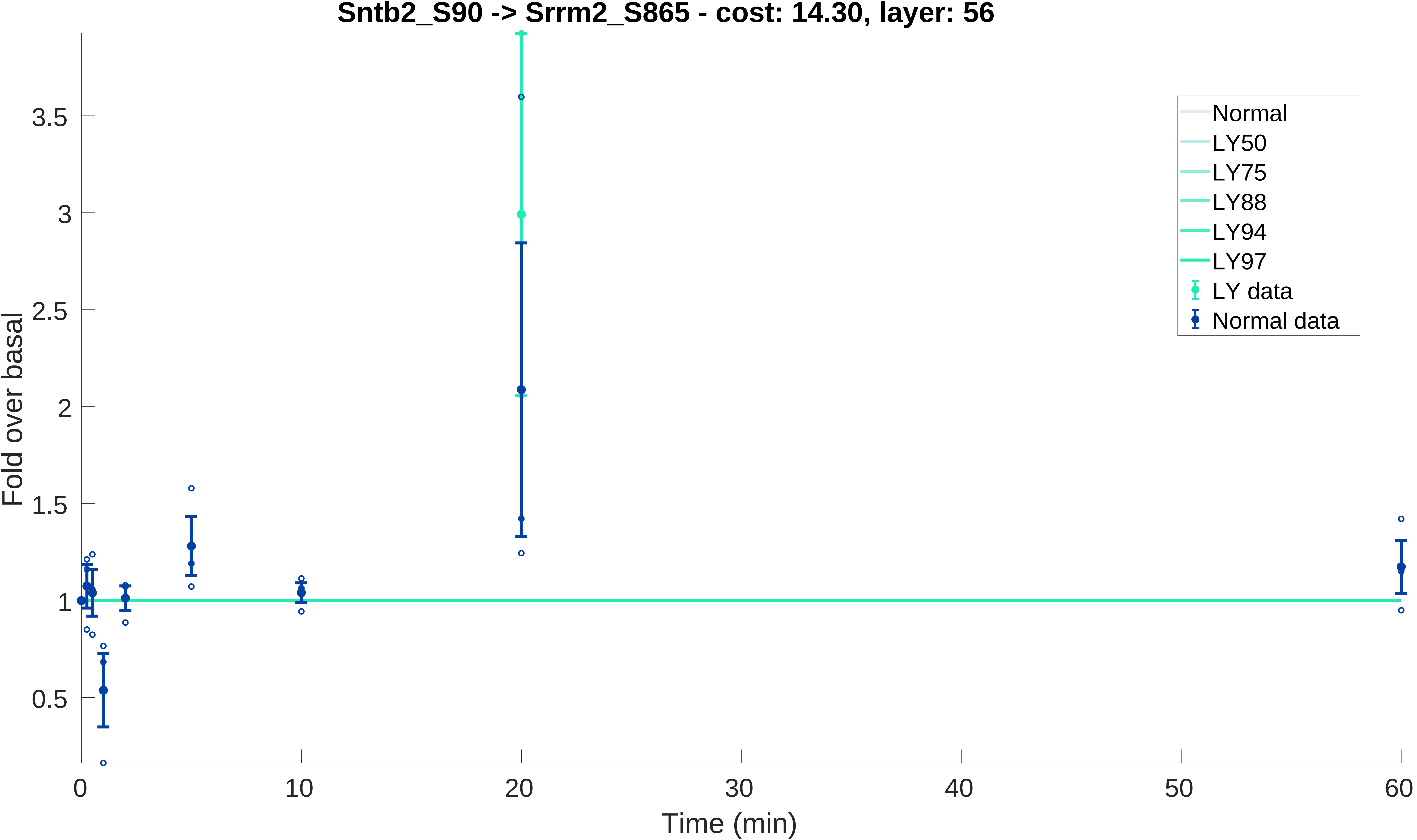

Sntb2_S90 -> Srrm2_S865 - cost: 14.30, layer: 56
Fold over basal
Time (min)
0.5
1
1.5
2
2.5
3
3.5
0
10
20
30
40
50
60
Normal
LY50
LY75
LY88
LY94
LY97
LY data
Normal data

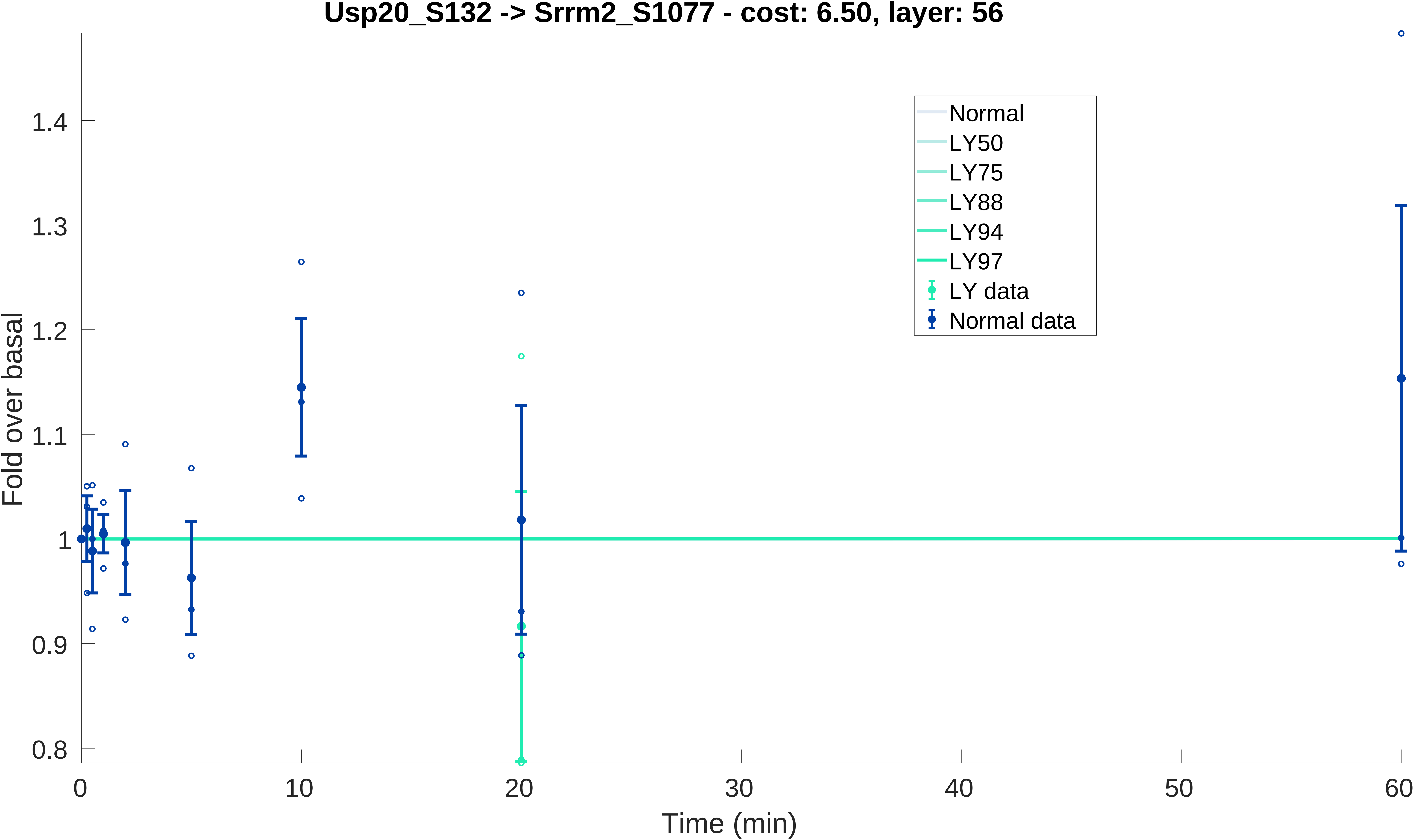
Usp20_S132 -> Srrm2_S1077 - cost: 6.50, layer: 56
Fold over basal
Time (min)
0.8
0.9
1
1.1
1.2
1.3
1.4
0
10
20
30
40
50
60
Normal
LY50
LY75
LY88
LY94
LY97
LY data
Normal data

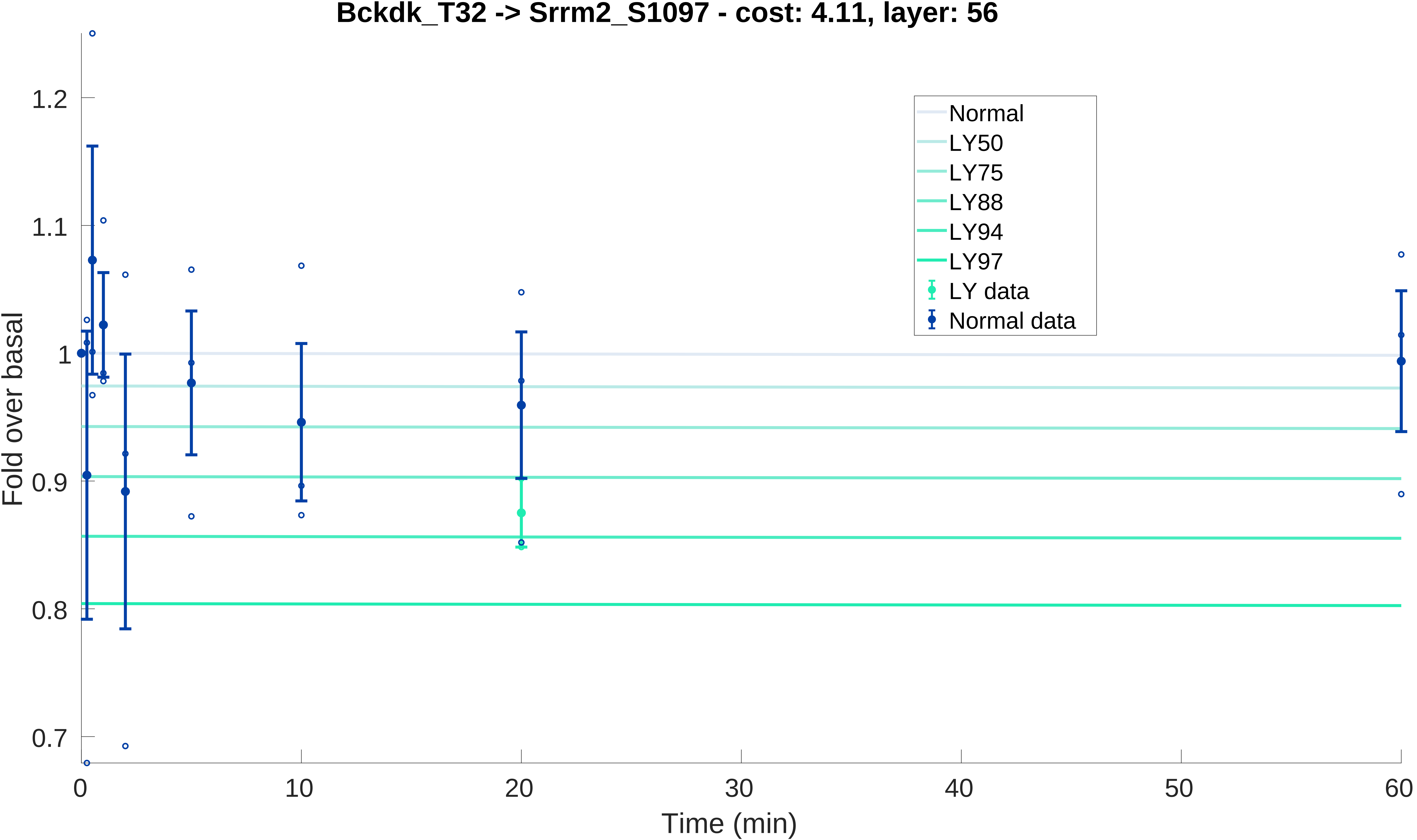
Bckdk_T32 -> Srrm2_S1097 - cost: 4.11, layer: 56
Fold over basal
Time (min)
1.2
1.1
1
0.9
0.8
0.7
0
10
20
30
40
50
60
Normal
LY50
LY75
LY88
LY94
LY97
LY data
Normal data

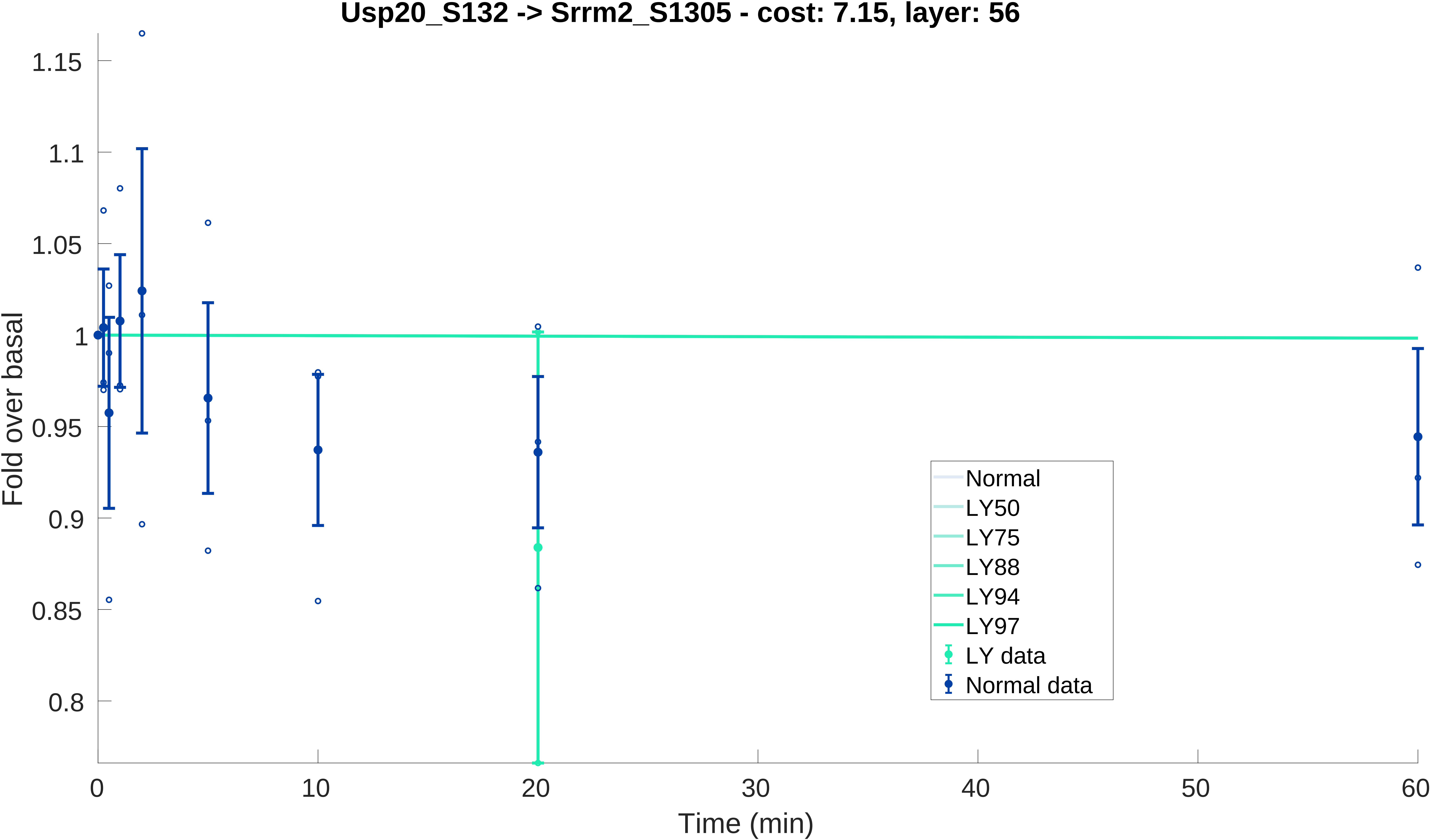

Usp20_S132 -> Srrm2_S1305 - cost: 7.15, layer: 56
Fold over basal
1.15
1.1
1.05
1
0.95
0.9
0.85
0.8
0
10
20
30
40
50
60
Time (min)
Normal
LY50
LY75
LY88
LY94
LY97
LY data
Normal data

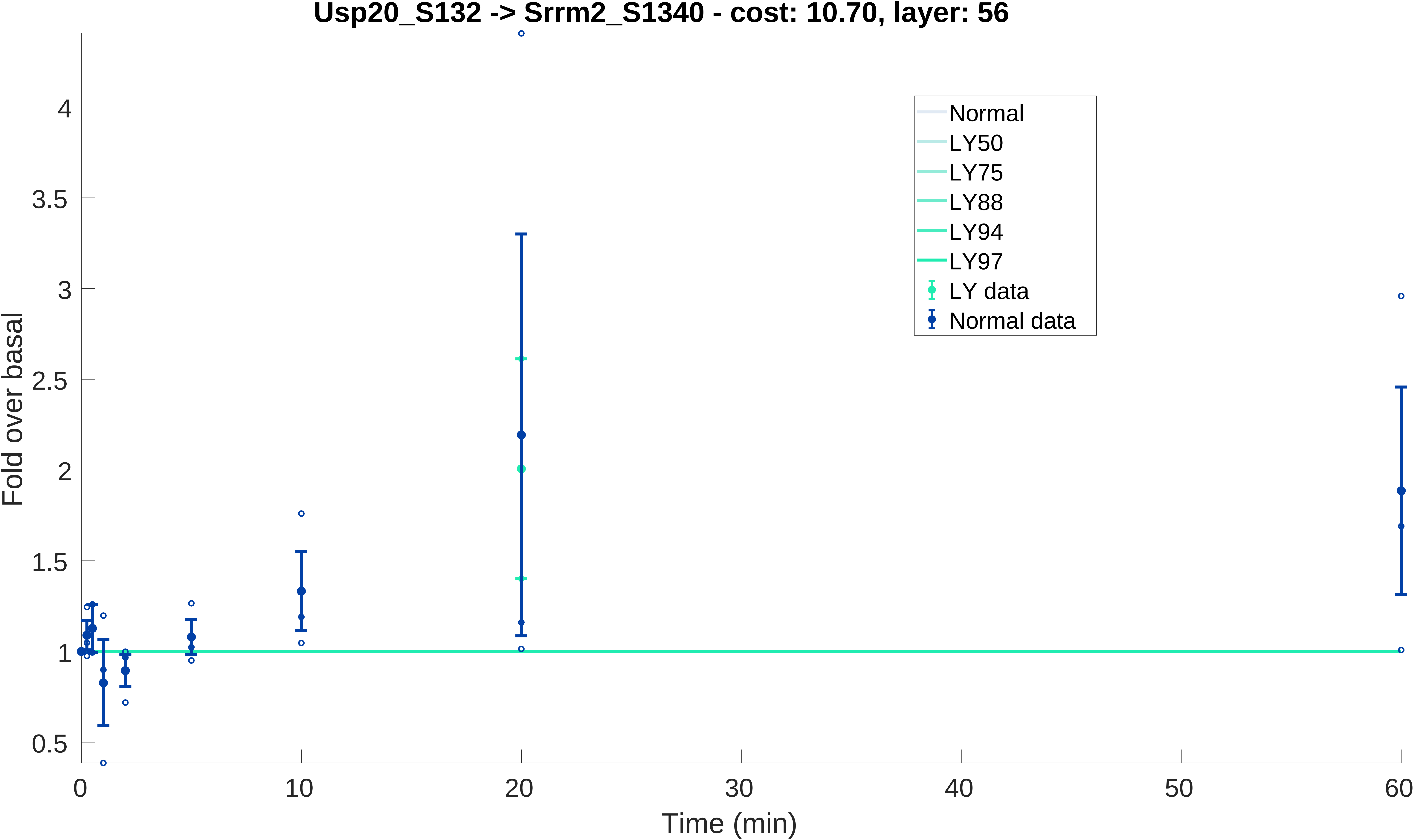
Usp20_S132 -> Srrm2_S1340 - cost: 10.70, layer: 56
Fold over basal
Time (min)
0.5
1
1.5
2
2.5
3
3.5
4
0
10
20
30
40
50
60
Normal
LY50
LY75
LY88
LY94
LY97
LY data
Normal data

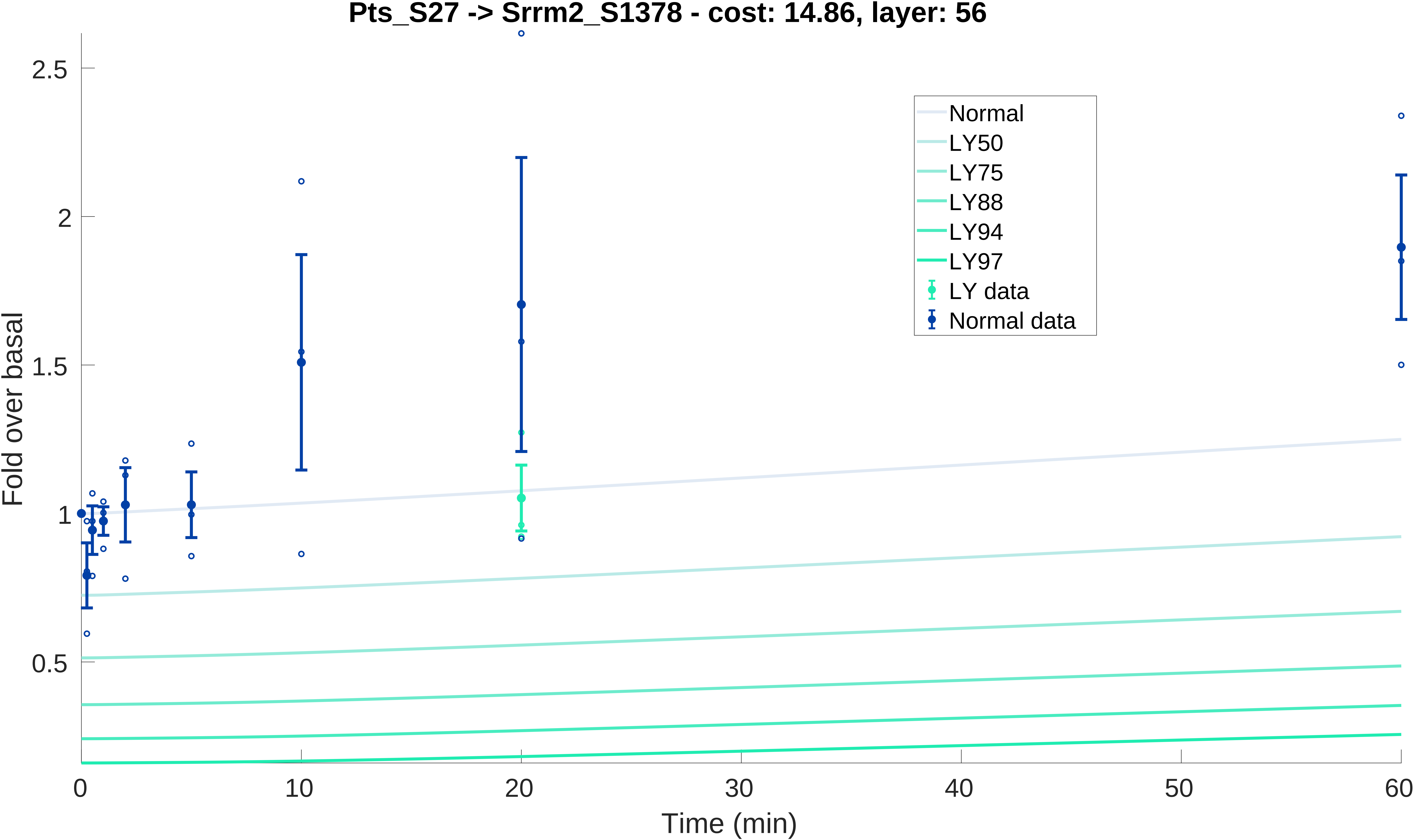

Pts_S27 -> Srrm2_S1378 - cost: 14.86, layer: 56
Normal
LY50
LY75
LY88
LY94
LY97
LY data
Normal data
Fold over basal
Time (min)
0
10
20
30
40
50
60
0.5
1
1.5
2
2.5

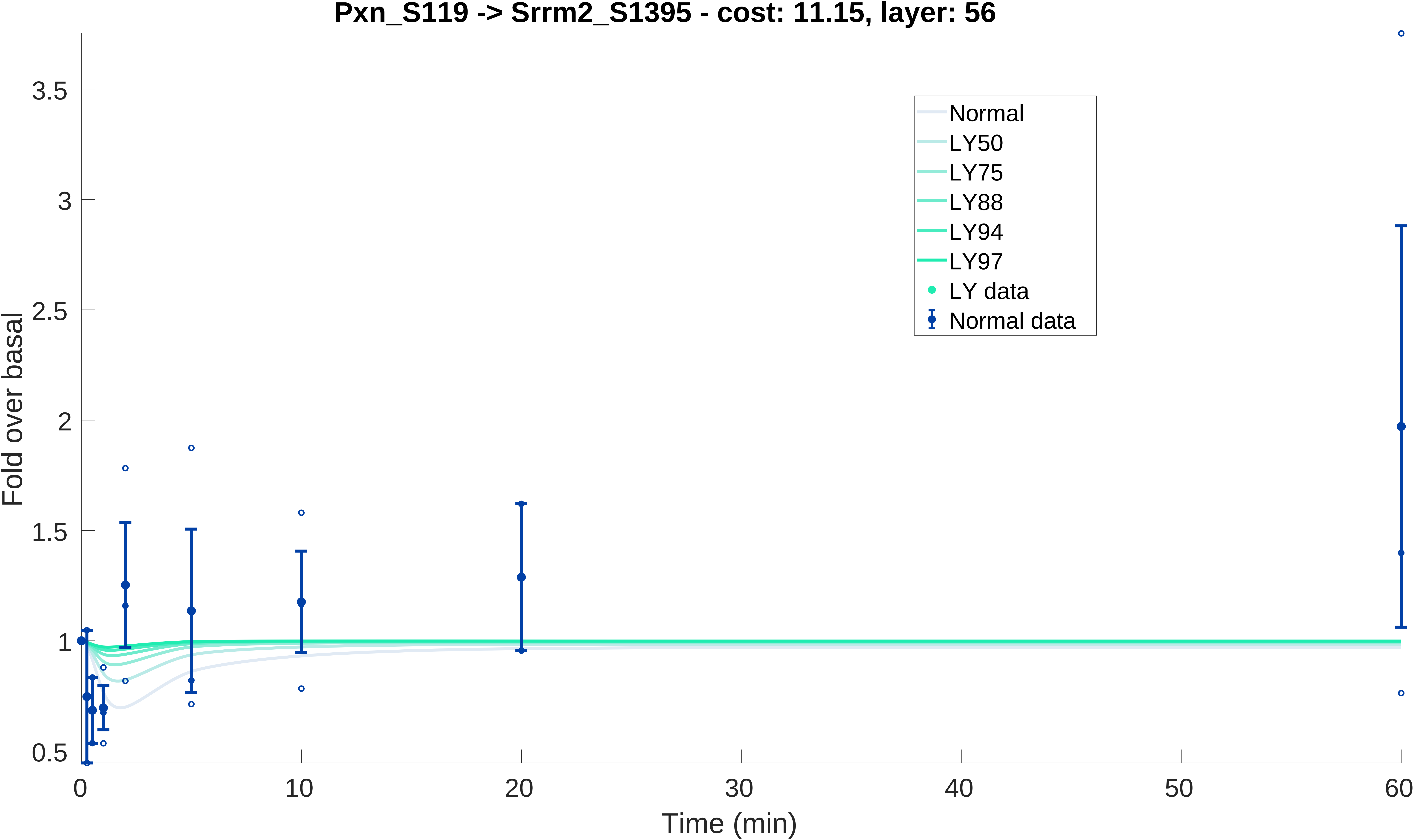
Pxn_S119 -> Srrm2_S1395 - cost: 11.15, layer: 56
Fold over basal
Time (min)
Normal
LY50
LY75
LY88
LY94
LY97
LY data
Normal data
0.5
1
1.5
2
2.5
3
3.5
0
10
20
30
40
50
60

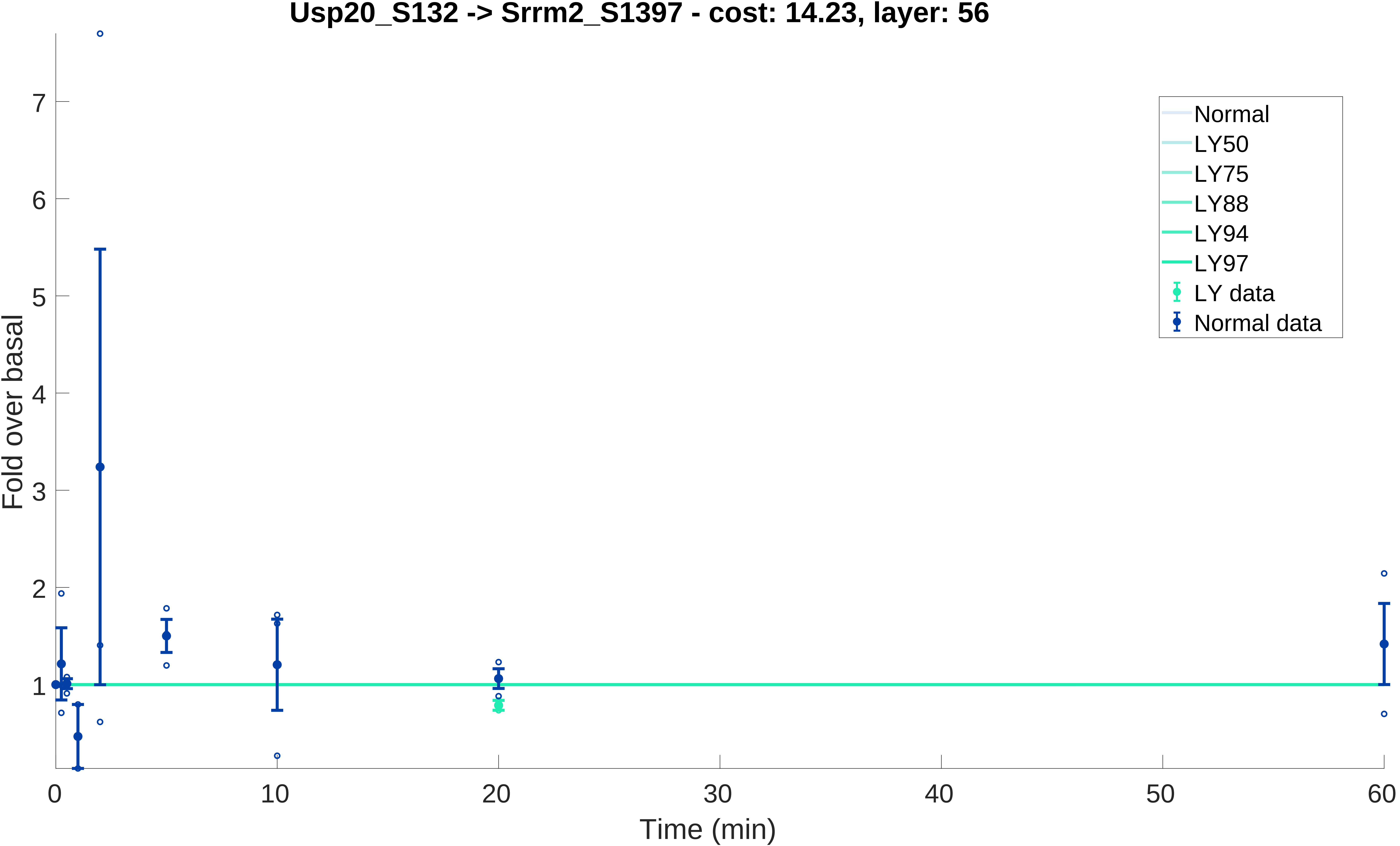

Usp20_S132 -> Srrm2_S1397 - cost: 14.23, layer: 56
Fold over basal
Time (min)
0
10
20
30
40
50
60
1
2
3
4
5
6
7
Normal
LY50
LY75
LY88
LY94
LY97
LY data
Normal data

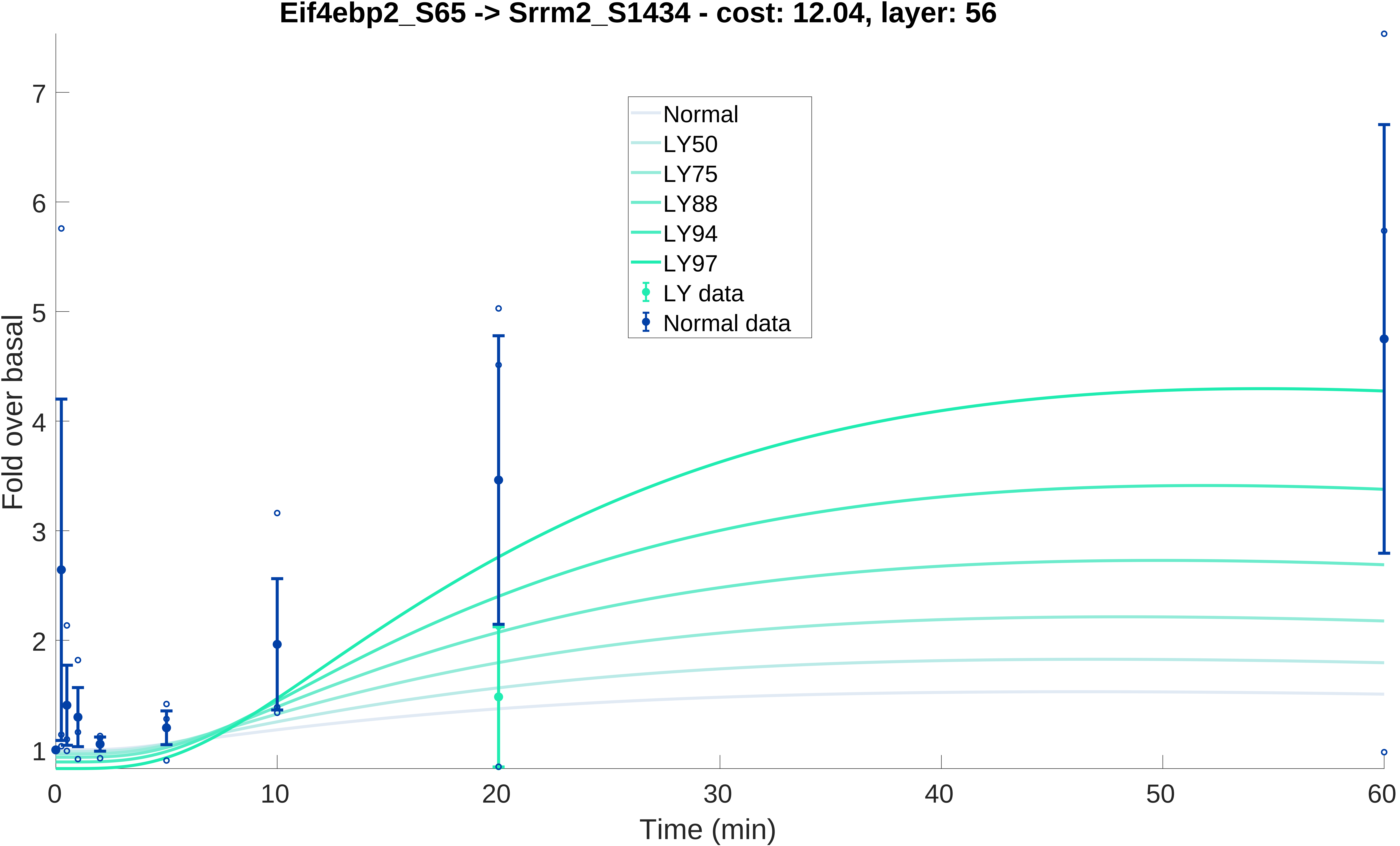

Eif4ebp2_S65 -> Srrm2_S1434 - cost: 12.04, layer: 56
Normal
LY50
LY75
LY88
LY94
LY97
LY data
Normal data
Fold over basal
Time (min)
1
2
3
4
5
6
7
0
10
20
30
40
50
60

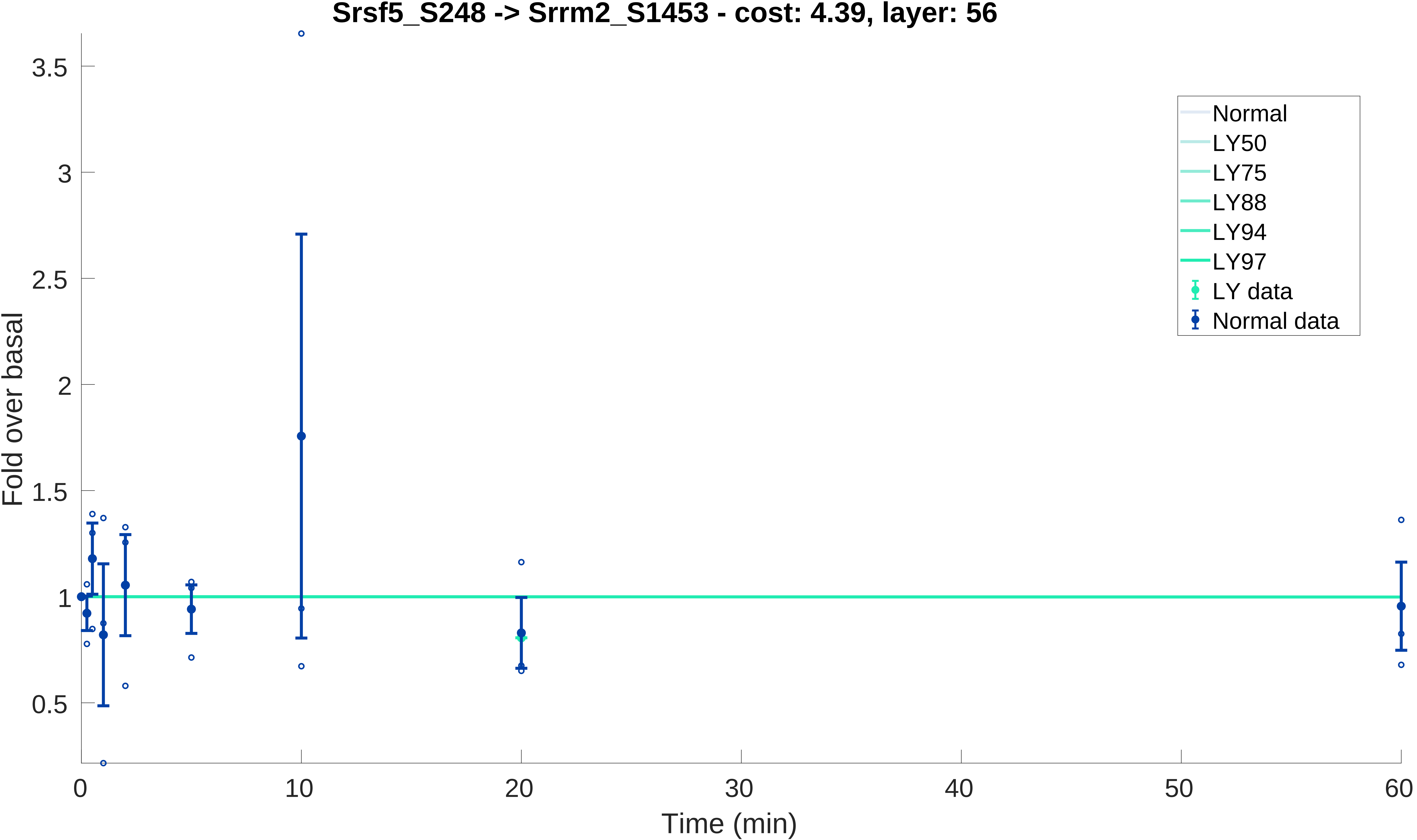
Srsf5_S248 -> Srrm2_S1453 - cost: 4.39, layer: 56
Fold over basal
Time (min)
0.5
1
1.5
2
2.5
3
3.5
0
10
20
30
40
50
60
Normal
LY50
LY75
LY88
LY94
LY97
LY data
Normal data

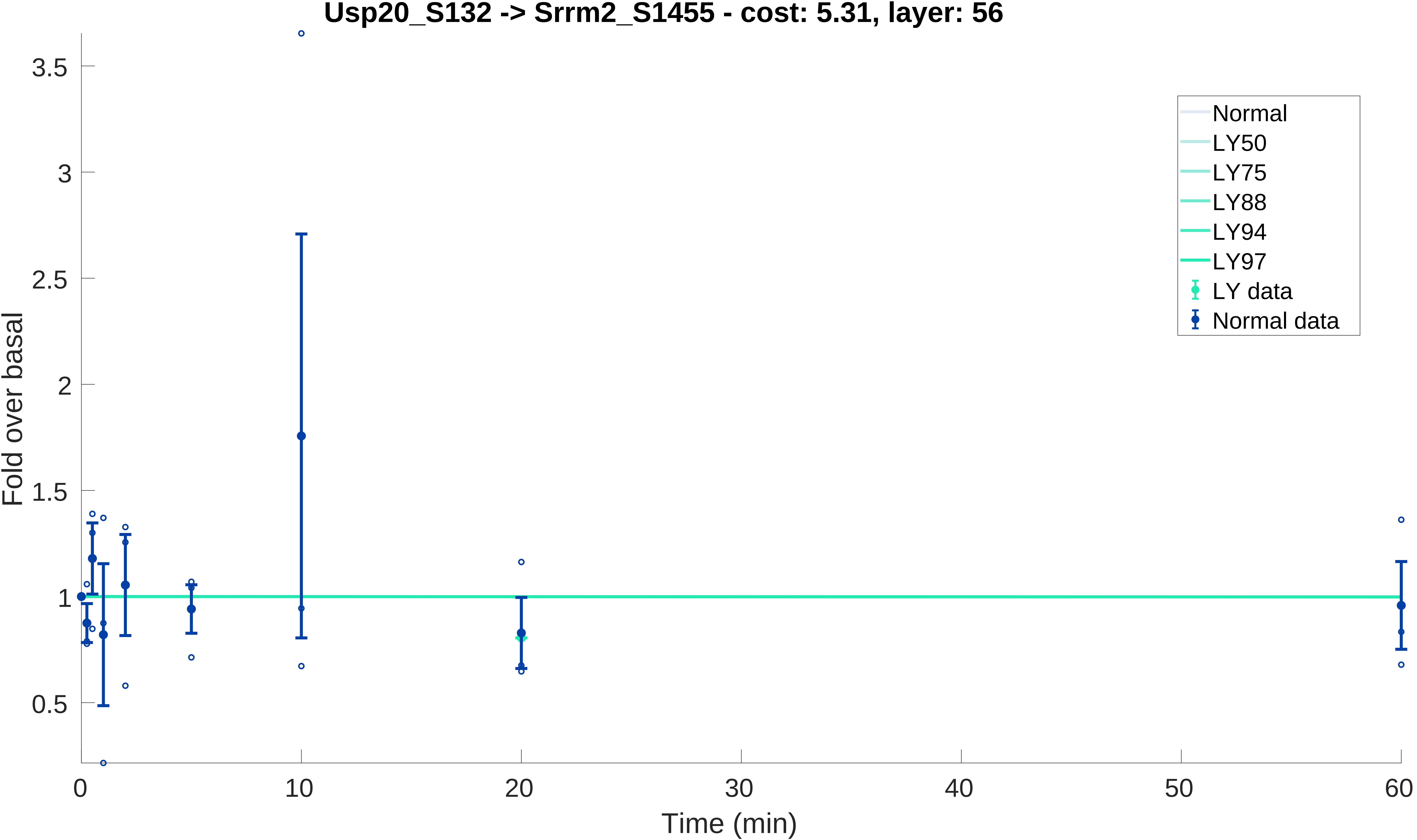

Usp20_S132 -> Srrm2_S1455 - cost: 5.31, layer: 56
Fold over basal
Time (min)
0.5
1
1.5
2
2.5
3
3.5
0
10
20
30
40
50
60
Normal
LY50
LY75
LY88
LY94
LY97
LY data
Normal data

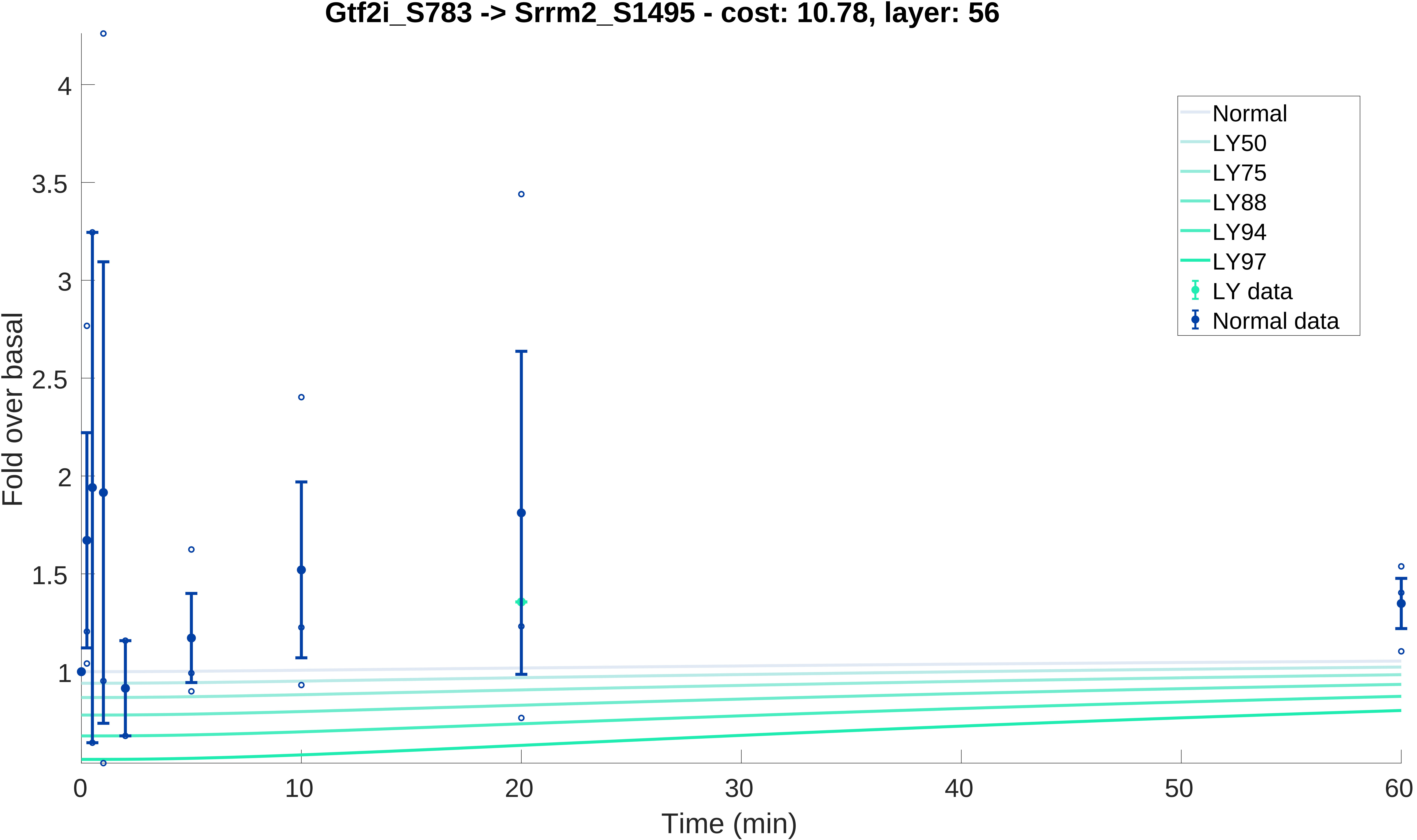

Gtf2i_S783 -> Srrm2_S1495 - cost: 10.78, layer: 56
Fold over basal
Time (min)
Normal
LY50
LY75
LY88
LY94
LY97
LY data
Normal data
4
3.5
3
2.5
2
1.5
1
0
10
20
30
40
50
60

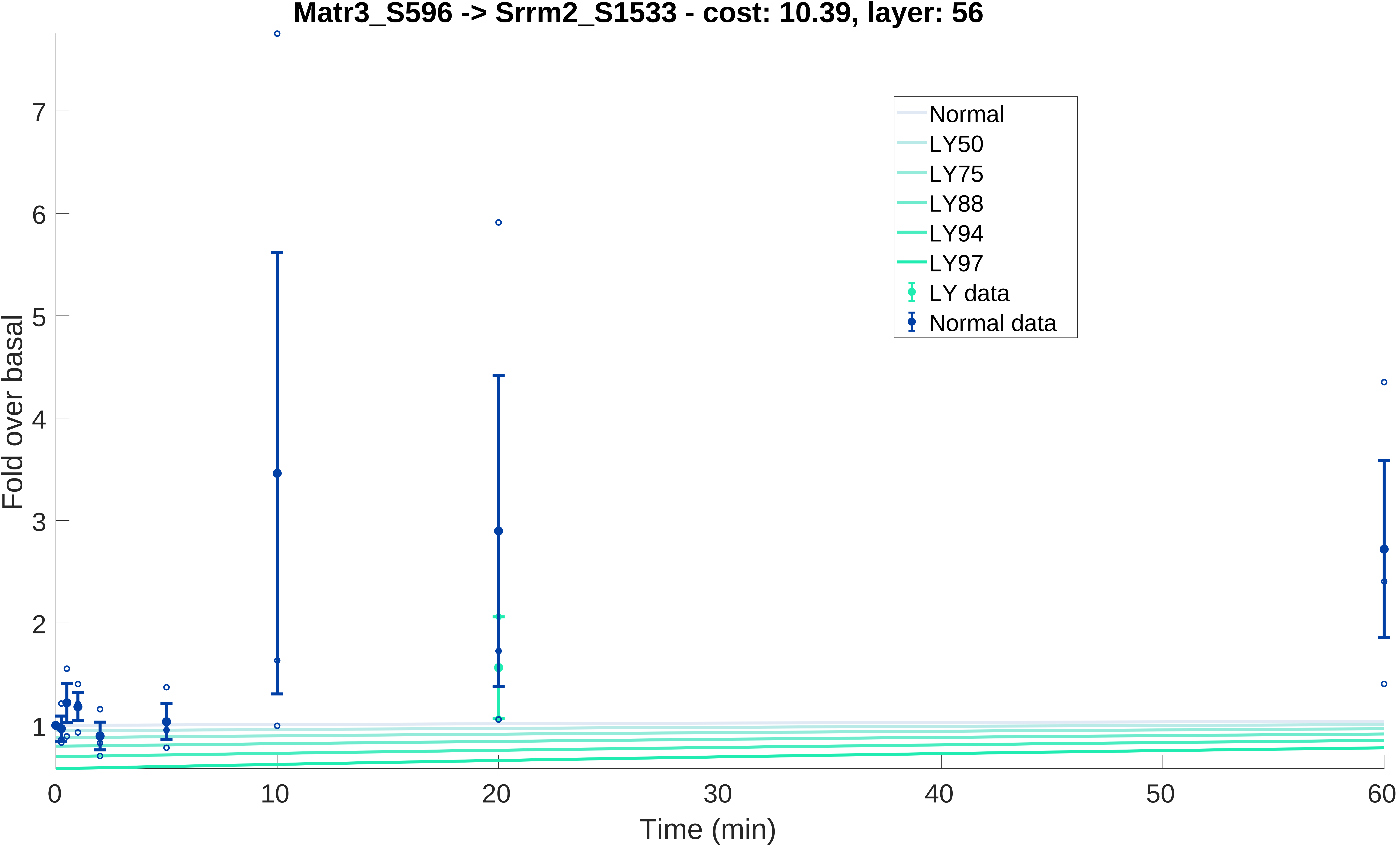

Matr3_S596 -> Srrm2_S1533 - cost: 10.39, layer: 56
Fold over basal
Time (min)
Normal
LY50
LY75
LY88
LY94
LY97
LY data
Normal data
0
10
20
30
40
50
60
1
2
3
4
5
6
7

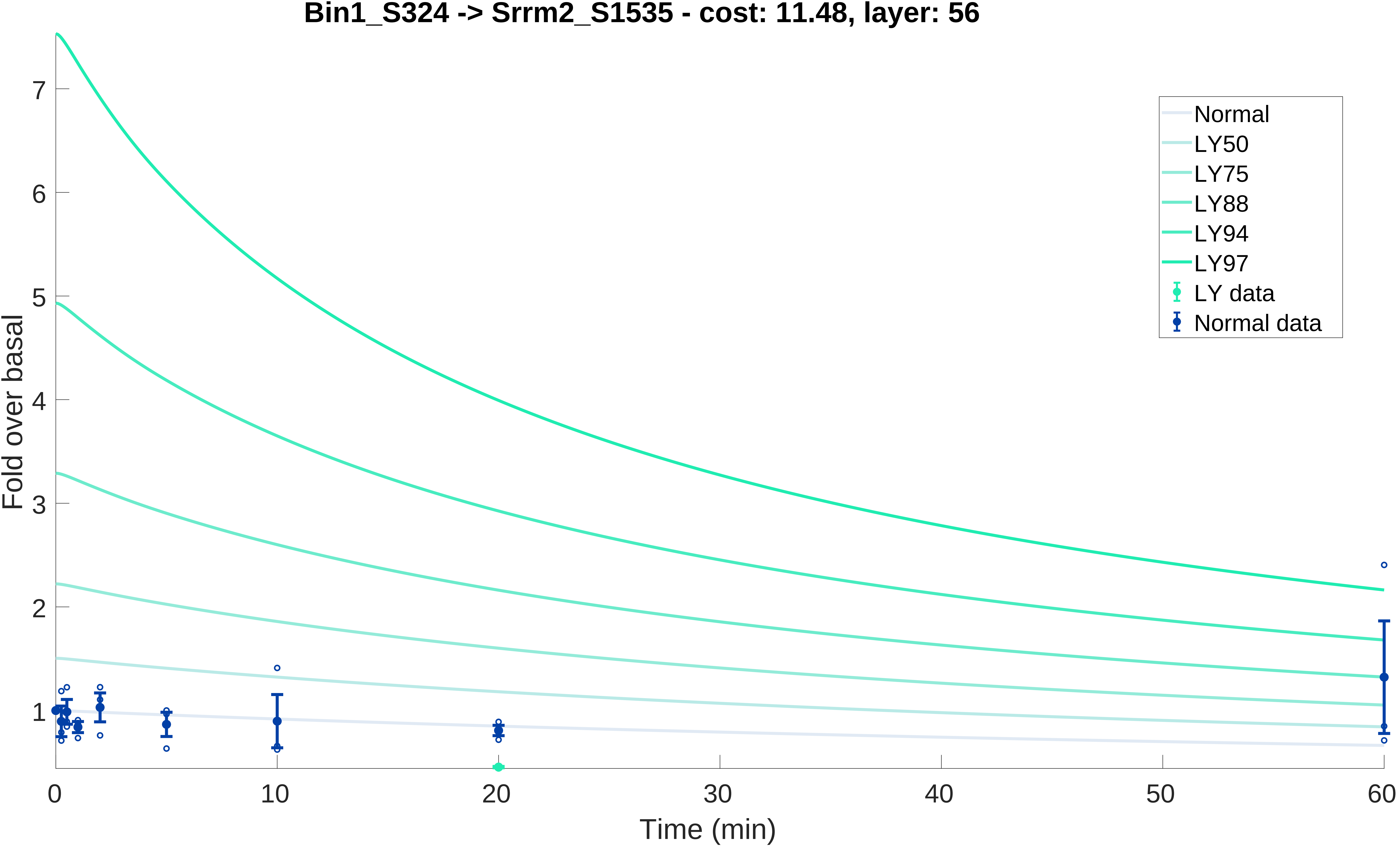

Bin1_S324 -> Srrm2_S1535 - cost: 11.48, layer: 56
Fold over basal
Time (min)
1
2
3
4
5
6
7
0
10
20
30
40
50
60
Normal
LY50
LY75
LY88
LY94
LY97
LY data
Normal data

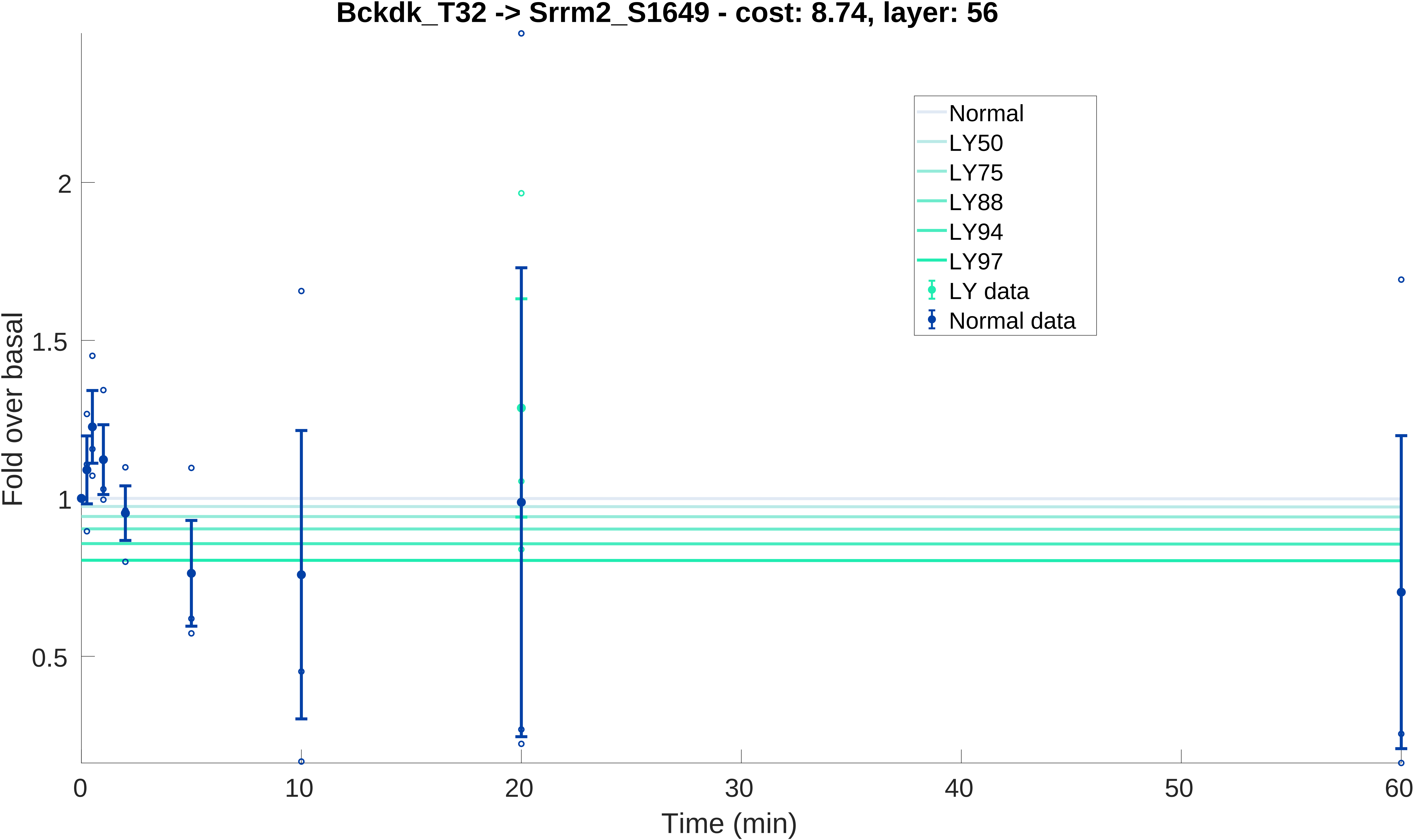

Bckdk_T32 -> Srrm2_S1649 - cost: 8.74, layer: 56
Fold over basal
Time (min)
0.5
1
1.5
2
0
10
20
30
40
50
60
Normal
LY50
LY75
LY88
LY94
LY97
LY data
Normal data

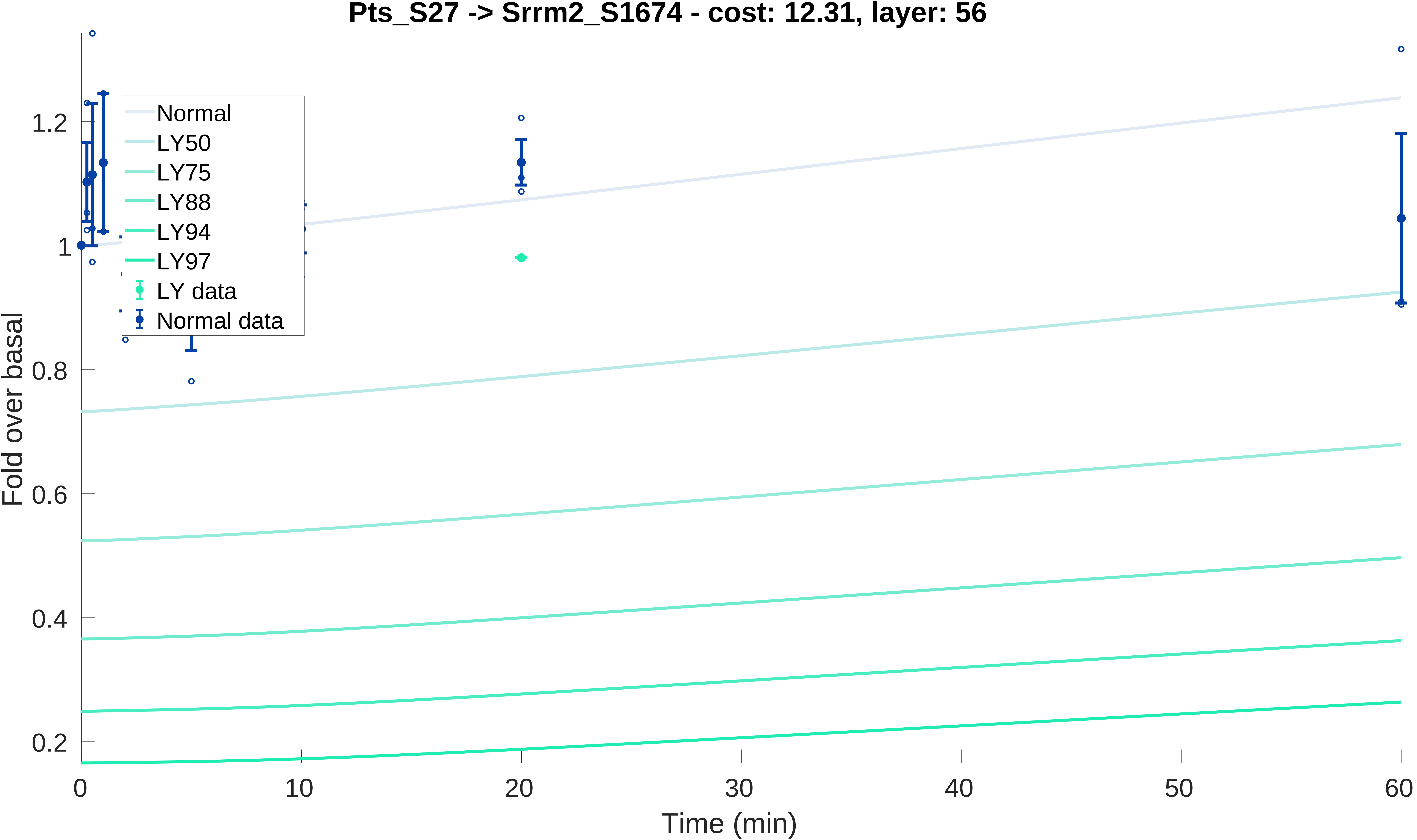

Pts_S27 -> Srrm2_S1674 - cost: 12.31, layer: 56
Normal
LY50
LY75
LY88
LY94
LY97
LY data
Normal data
Fold over basal
Time (min)
0.2
0.4
0.6
0.8
1
1.2
0
10
20
30
40
50
60

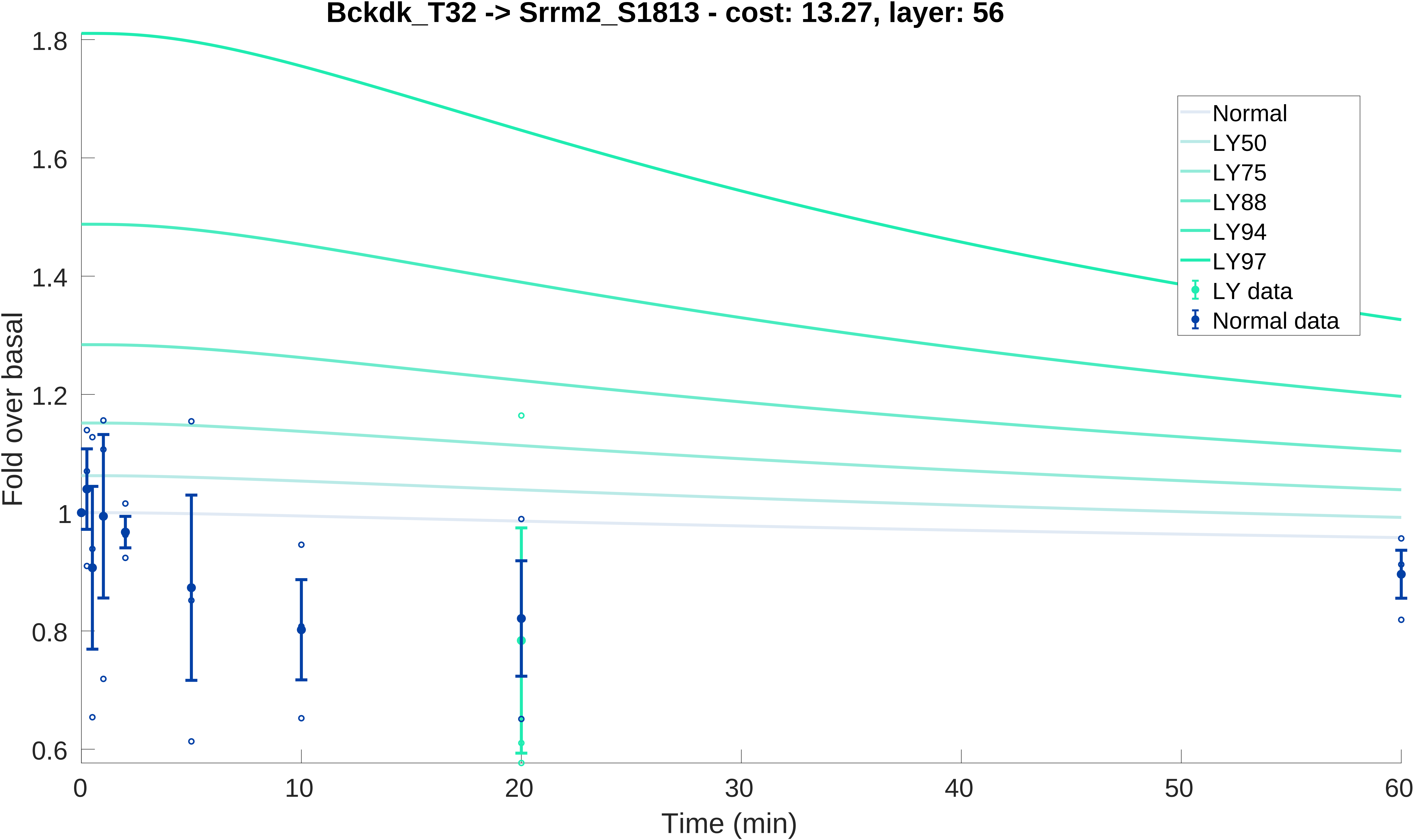

Bckdk_T32 -> Srrm2_S1813 - cost: 13.27, layer: 56
Fold over basal
Time (min)
1.8
1.6
1.4
1.2
1
0.8
0.6
0
10
20
30
40
50
60
Normal
LY50
LY75
LY88
LY94
LY97
LY data
Normal data

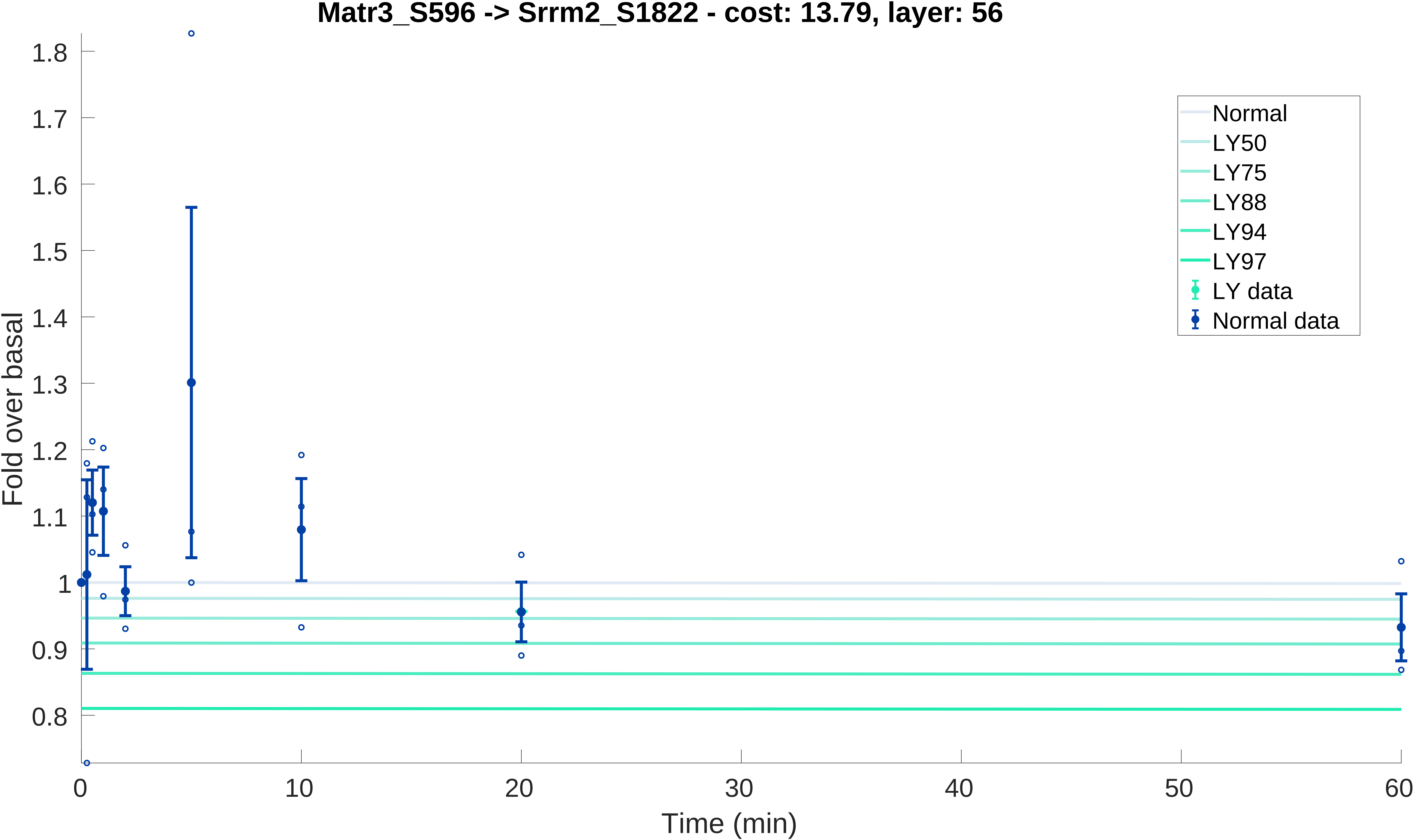

Matr3_S596 -> Srrm2_S1822 - cost: 13.79, layer: 56
Fold over basal
1.8
1.7
1.6
1.5
1.4
1.3
1.2
1.1
1
0.9
0.8
0
10
20
30
40
50
60
Time (min)
Normal
LY50
LY75
LY88
LY94
LY97
LY data
Normal data

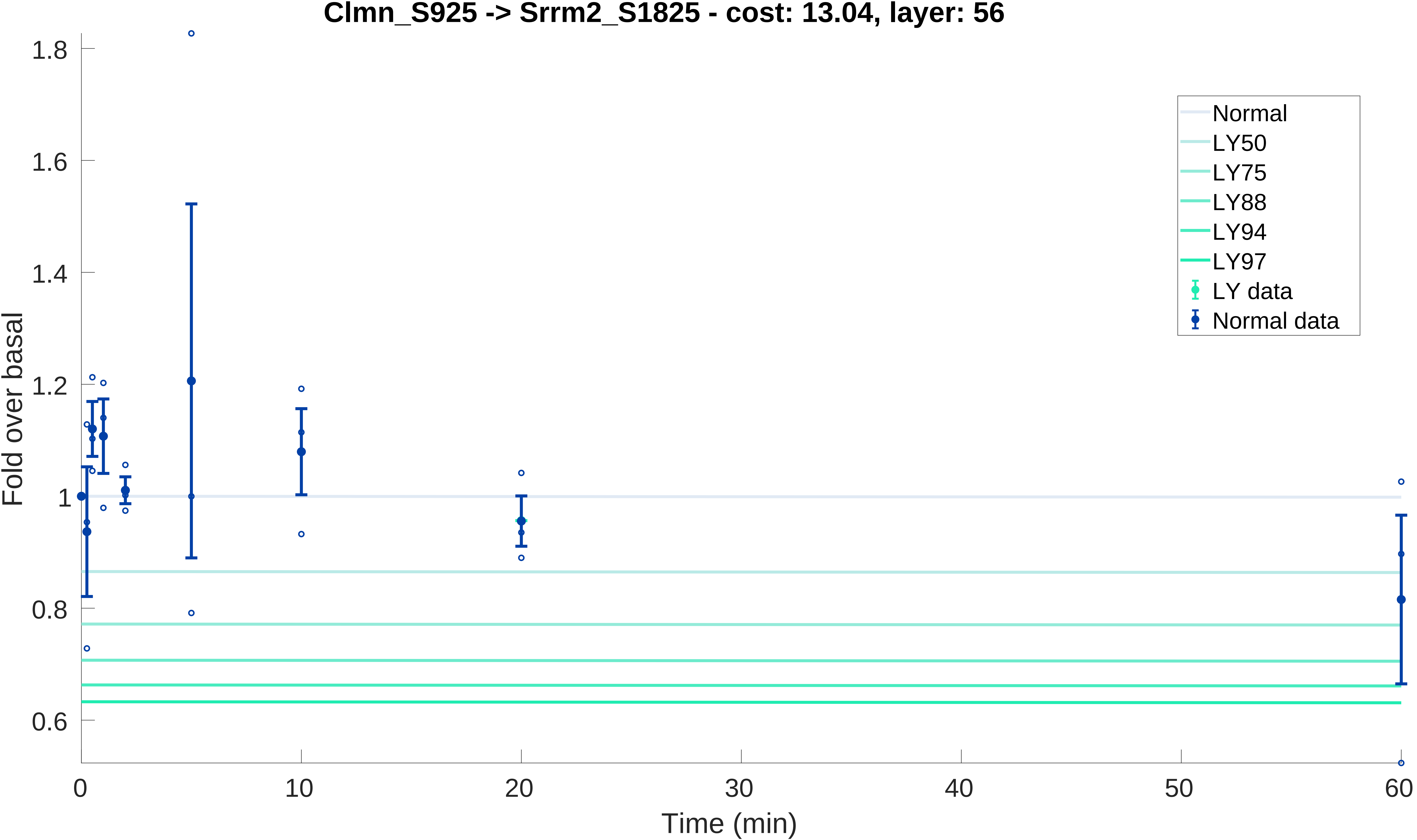

Clmn_S925 -> Srrm2_S1825 - cost: 13.04, layer: 56
Fold over basal
1.8
1.6
1.4
1.2
1
0.8
0.6
0
10
20
30
40
50
60
Time (min)
Normal
LY50
LY75
LY88
LY94
LY97
LY data
Normal data

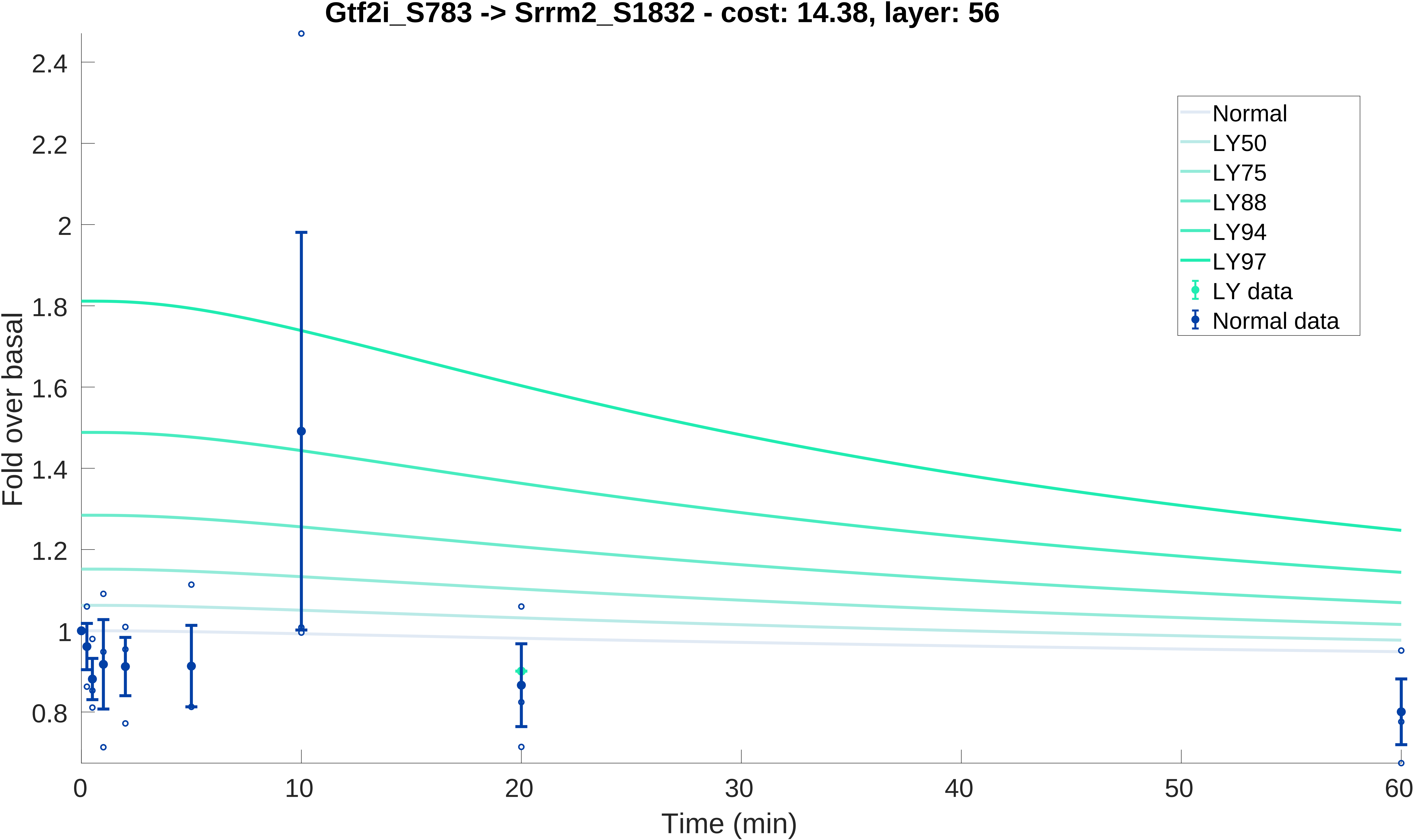
Gtf2i_S783 -> Srrm2_S1832 - cost: 14.38, layer: 56
Fold over basal
2.4
2.2
2
1.8
1.6
1.4
1.2
1
0.8
0
10
20
30
40
50
60
Time (min)
Normal
LY50
LY75
LY88
LY94
LY97
LY data
Normal data

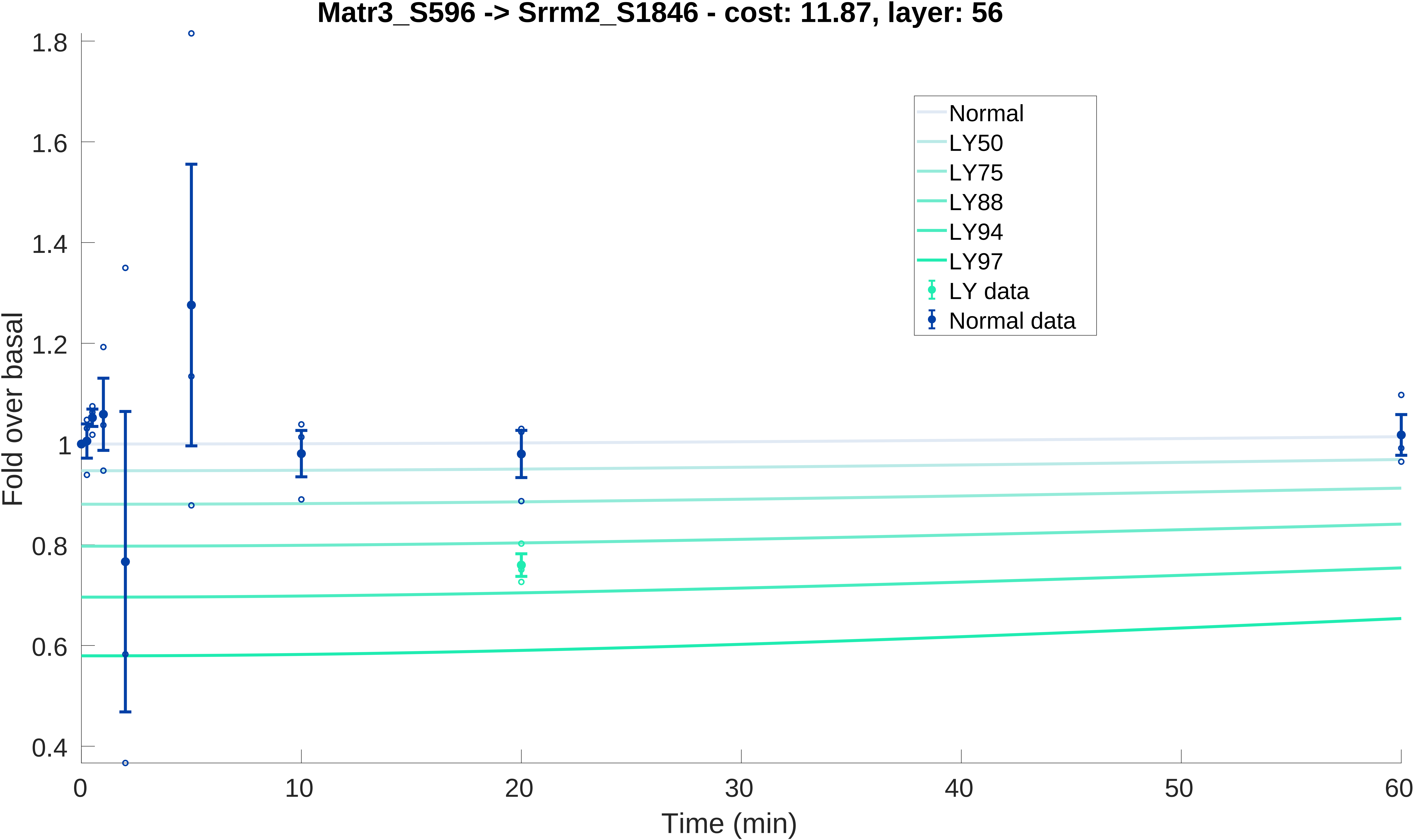

Matr3_S596 -> Srrm2_S1846 - cost: 11.87, layer: 56
Fold over basal
Time (min)
1.8
1.6
1.4
1.2
1
0.8
0.6
0.4
0
10
20
30
40
50
60
Normal
LY50
LY75
LY88
LY94
LY97
LY data
Normal data

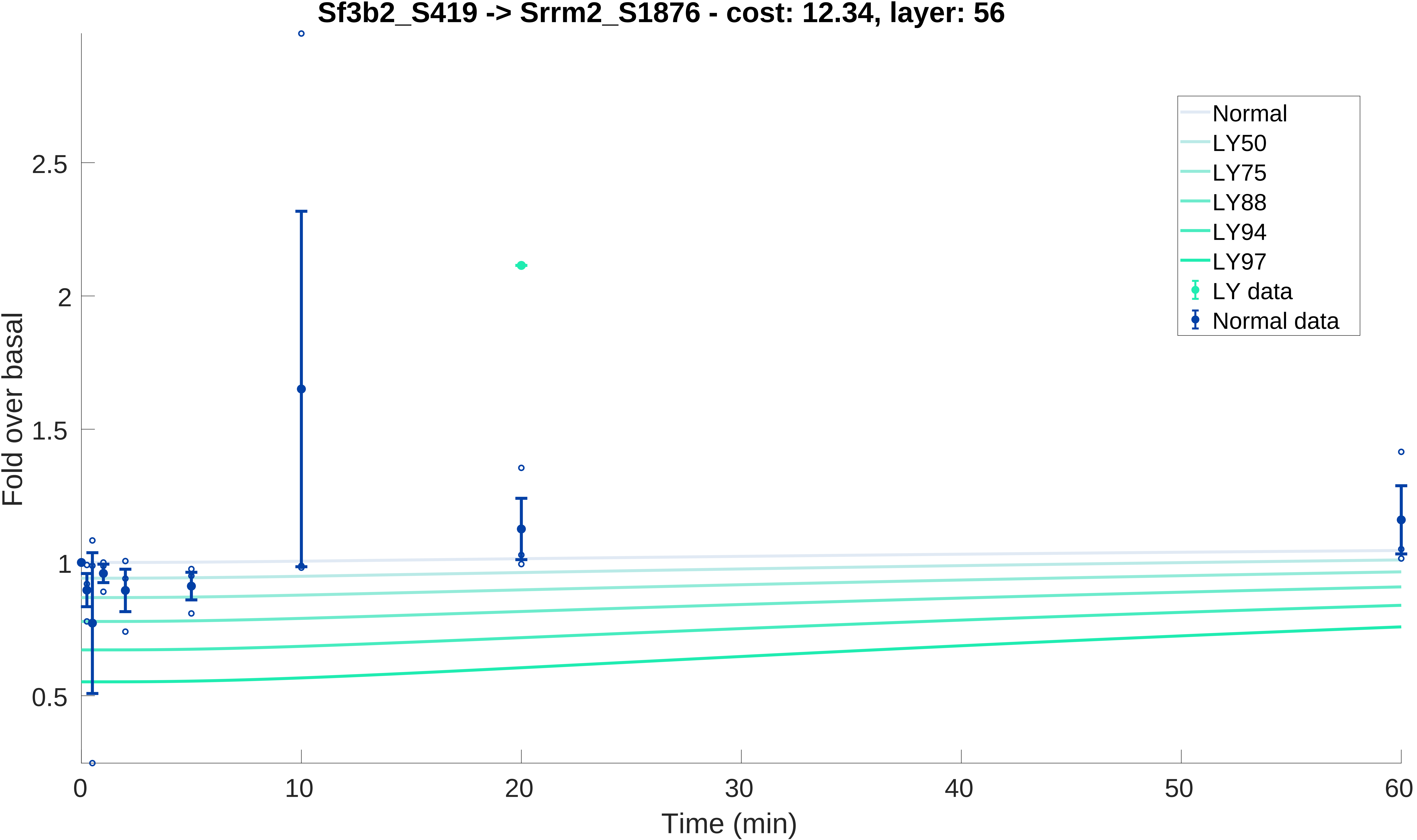

Sf3b2_S419 -> Srrm2_S1876 - cost: 12.34, layer: 56
Fold over basal
Time (min)
0.5
1
1.5
2
2.5
0
10
20
30
40
50
60
Normal
LY50
LY75
LY88
LY94
LY97
LY data
Normal data

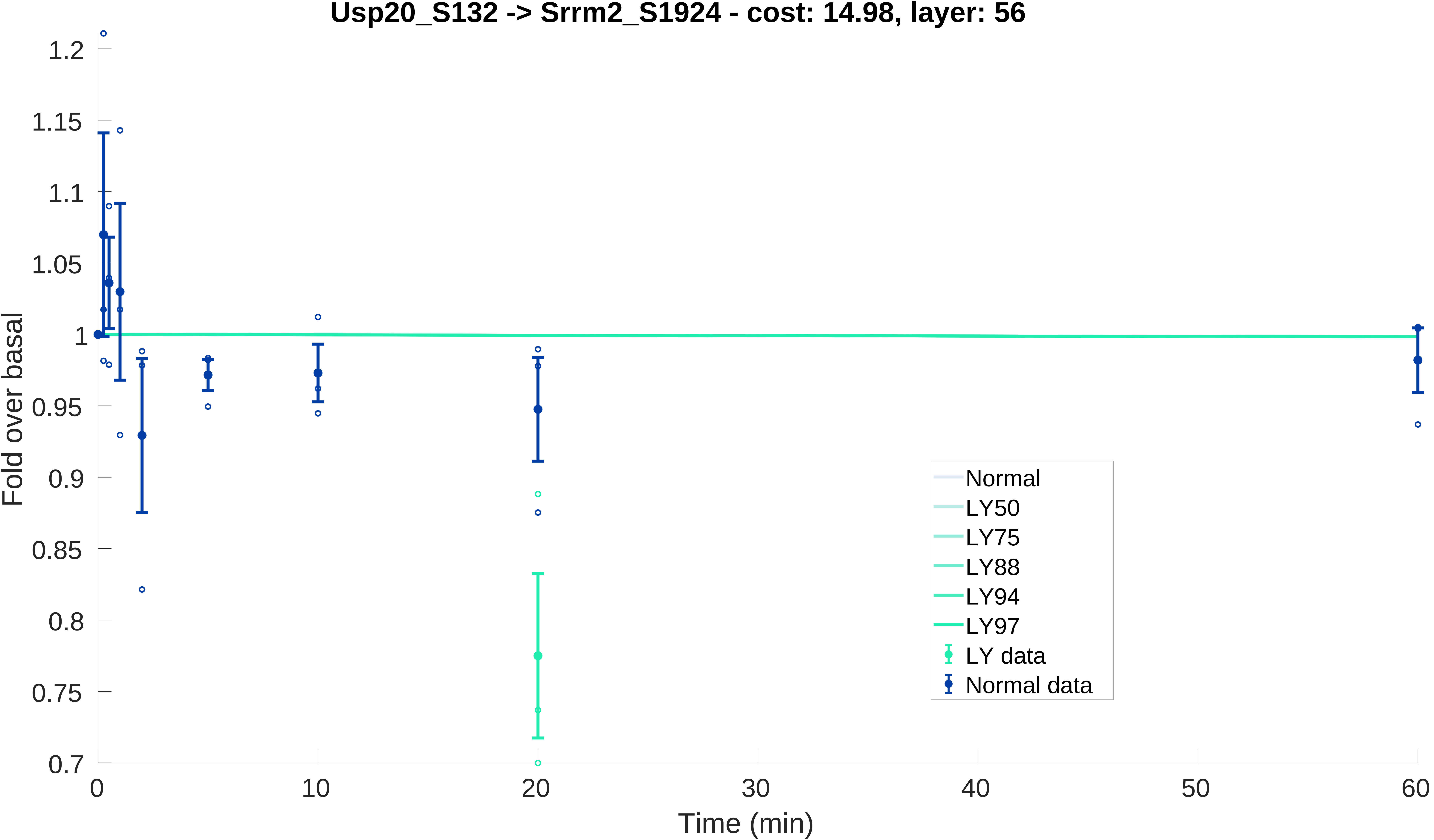

Usp20_S132 -> Srrm2_S1924 - cost: 14.98, layer: 56
Fold over basal
Time (min)
1.2
1.15
1.1
1.05
1
0.95
0.9
0.85
0.8
0.75
0.7
0
10
20
30
40
50
60
Normal
LY50
LY75
LY88
LY94
LY97
LY data
Normal data

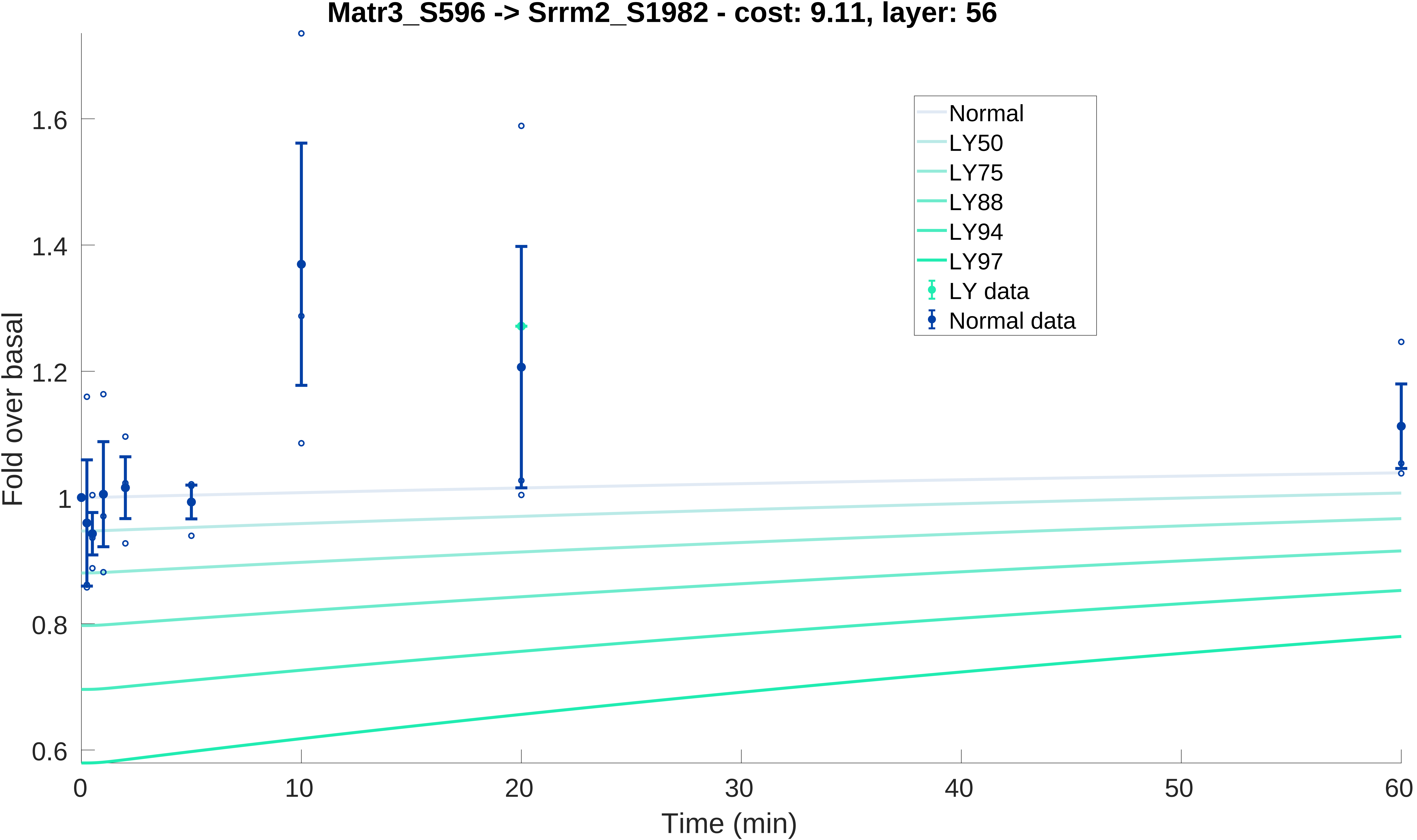

Matr3_S596 -> Srrm2_S1982 - cost: 9.11, layer: 56
Fold over basal
Time (min)
0.6
0.8
1
1.2
1.4
1.6
0
10
20
30
40
50
60
Normal
LY50
LY75
LY88
LY94
LY97
LY data
Normal data

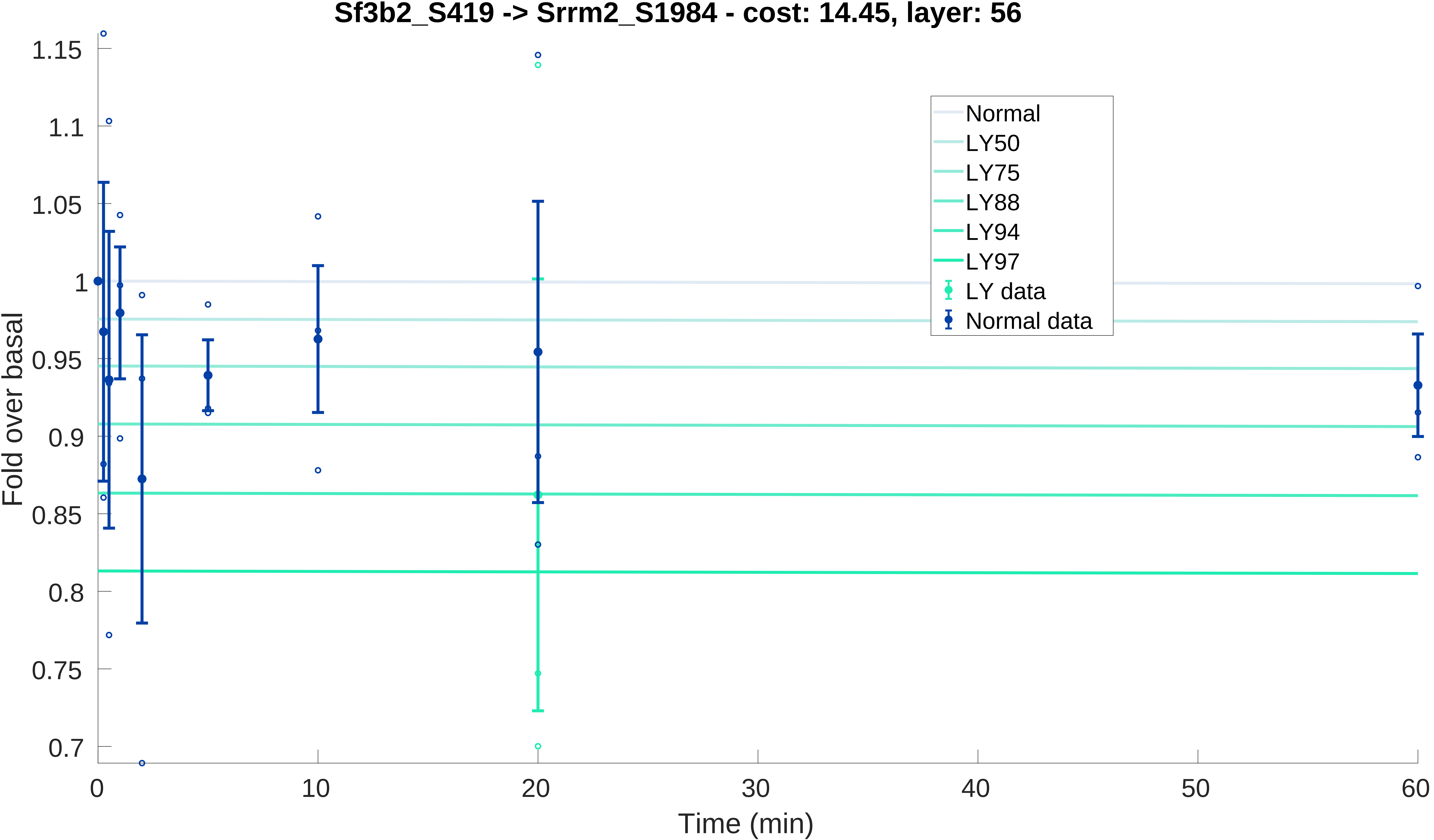
Sf3b2_S419 -> Srrm2_S1984 - cost: 14.45, layer: 56
Fold over basal
Time (min)
Normal
LY50
LY75
LY88
LY94
LY97
LY data
Normal data
1.15
1.1
1.05
1
0.95
0.9
0.85
0.8
0.75
0.7
0
10
20
30
40
50
60

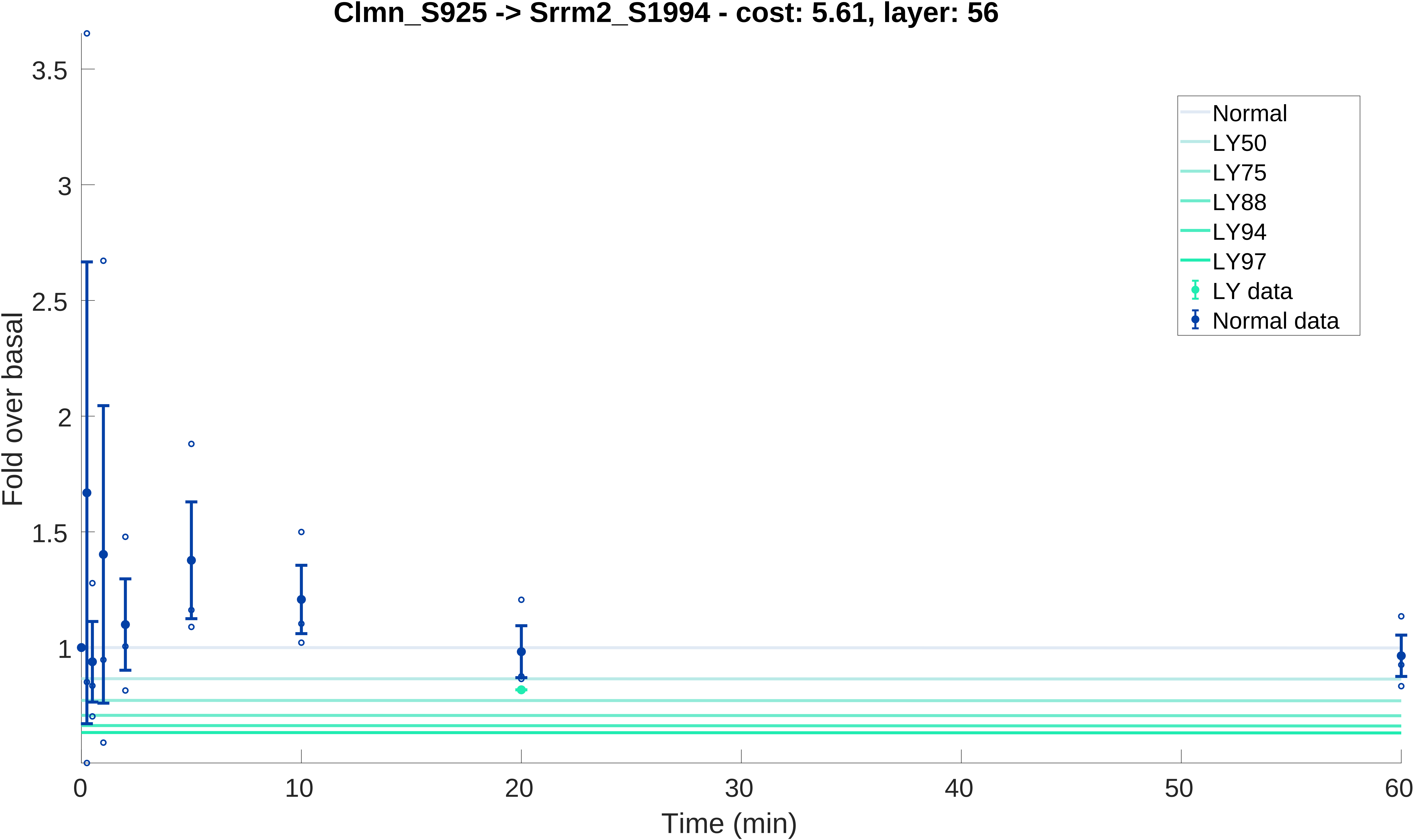
Clmn_S925 -> Srrm2_S1994 - cost: 5.61, layer: 56
Fold over basal
3.5
3
2.5
2
1.5
1
0
10
20
30
40
50
60
Time (min)
Normal
LY50
LY75
LY88
LY94
LY97
LY data
Normal data

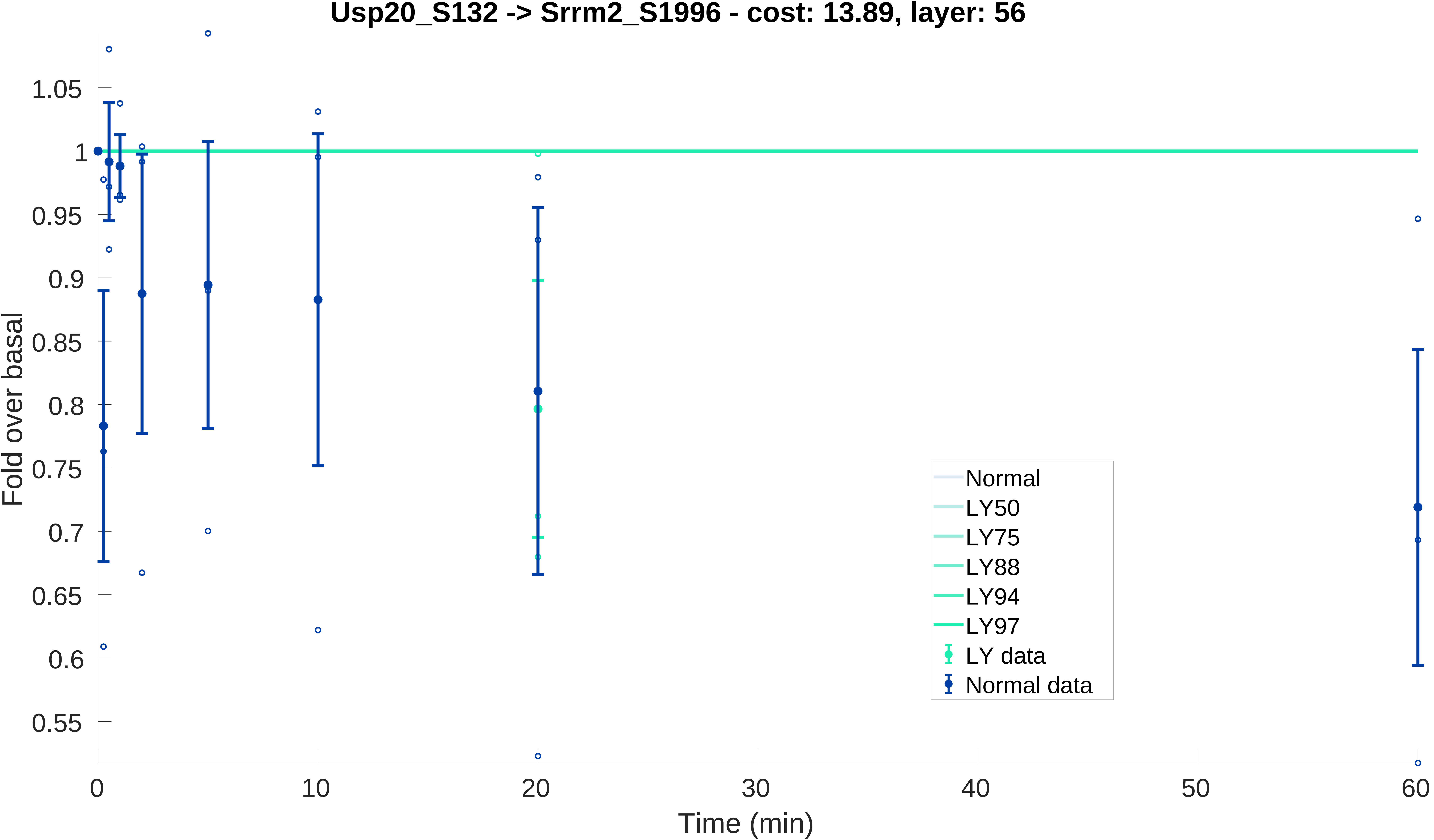

Usp20_S132 -> Srrm2_S1996 - cost: 13.89, layer: 56
Fold over basal
Time (min)
1.05
1
0.95
0.9
0.85
0.8
0.75
0.7
0.65
0.6
0.55
0
10
20
30
40
50
60
Normal
LY50
LY75
LY88
LY94
LY97
LY data
Normal data

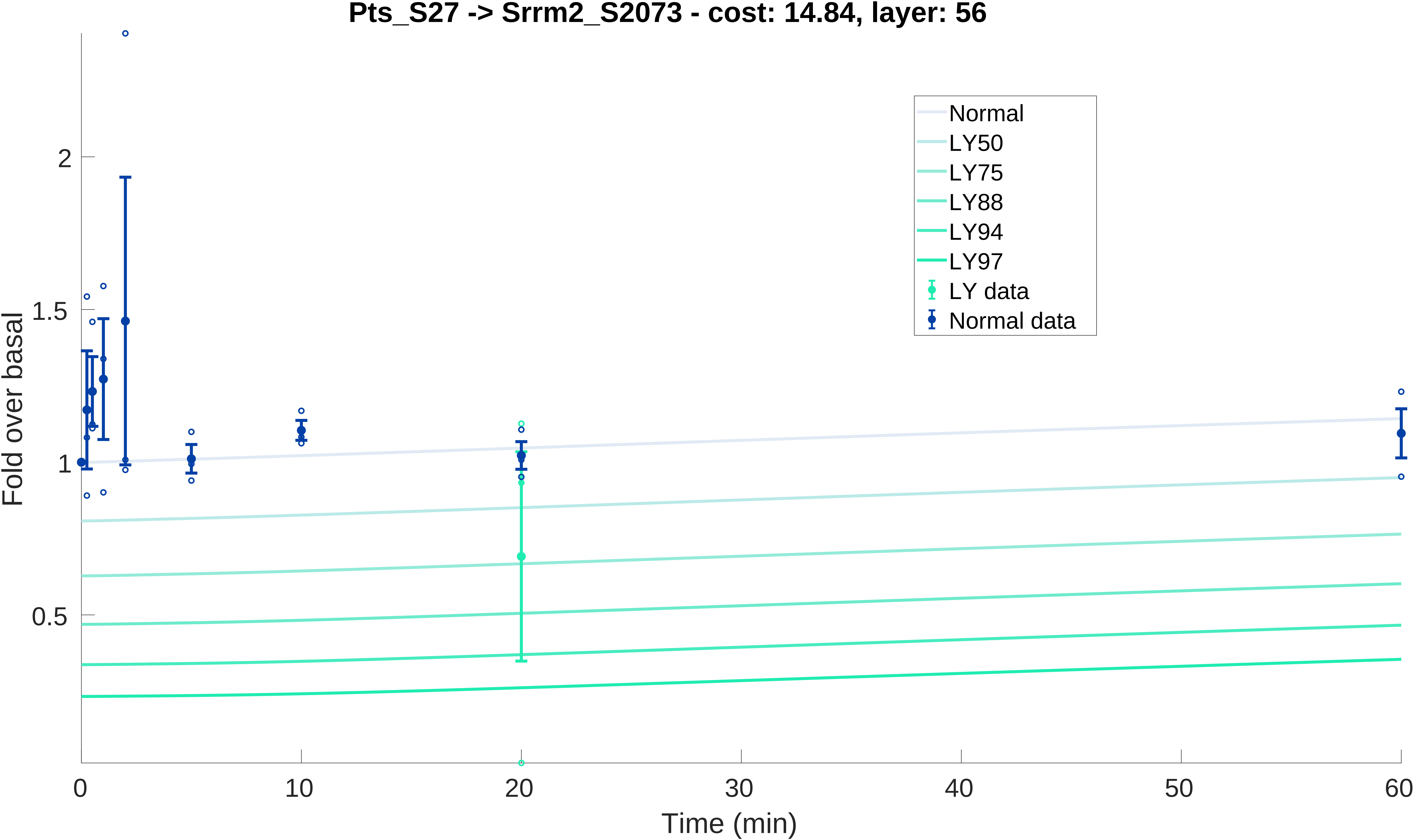
Pts_S27 -> Srrm2_S2073 - cost: 14.84, layer: 56
Fold over basal
0.5
1
1.5
2
0
10
20
30
40
50
60
Time (min)
Normal
LY50
LY75
LY88
LY94
LY97
LY data
Normal data

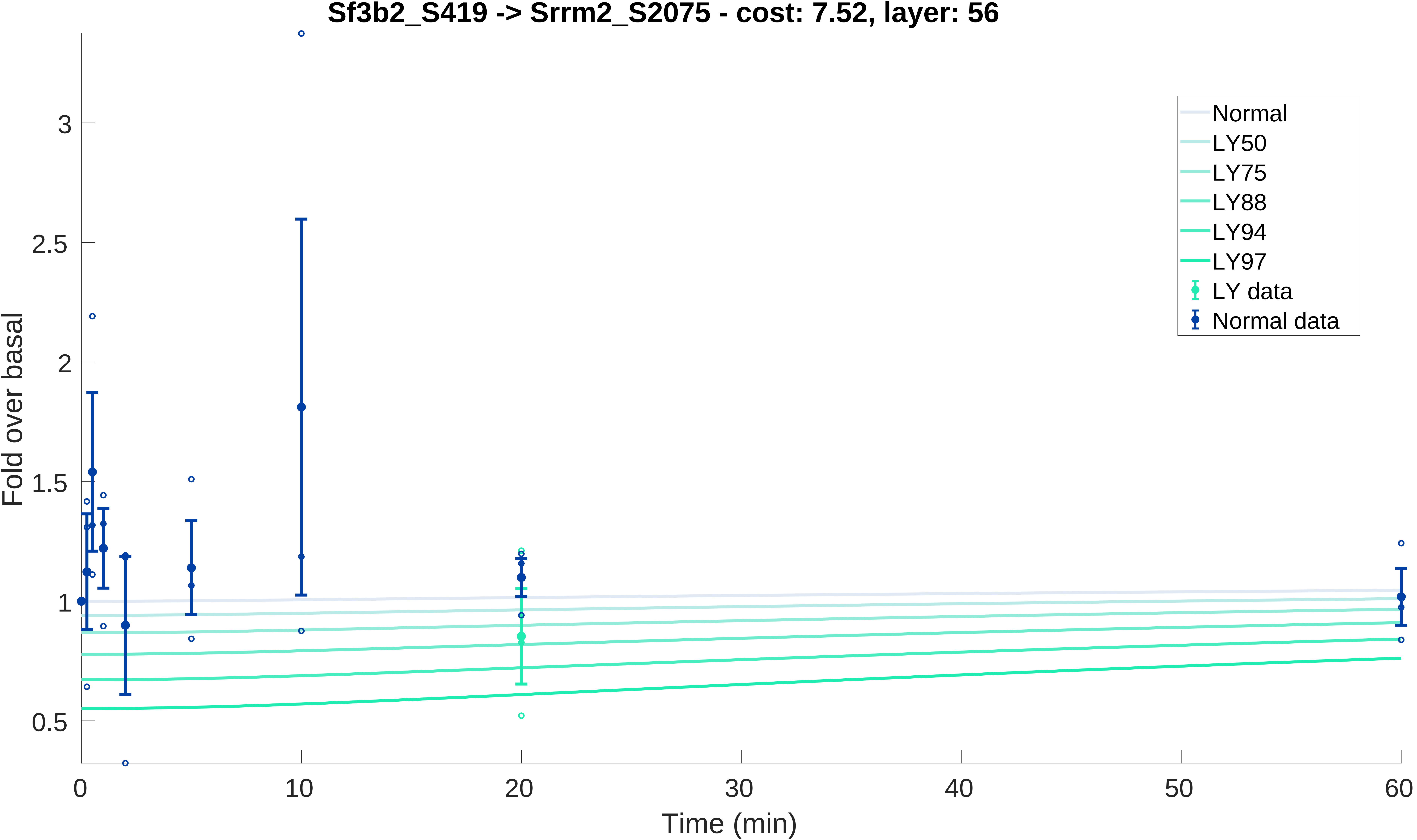
Sf3b2_S419 -> Srrm2_S2075 - cost: 7.52, layer: 56
Fold over basal
Time (min)
0.5
1
1.5
2
2.5
3
0
10
20
30
40
50
60
Normal
LY50
LY75
LY88
LY94
LY97
LY data
Normal data

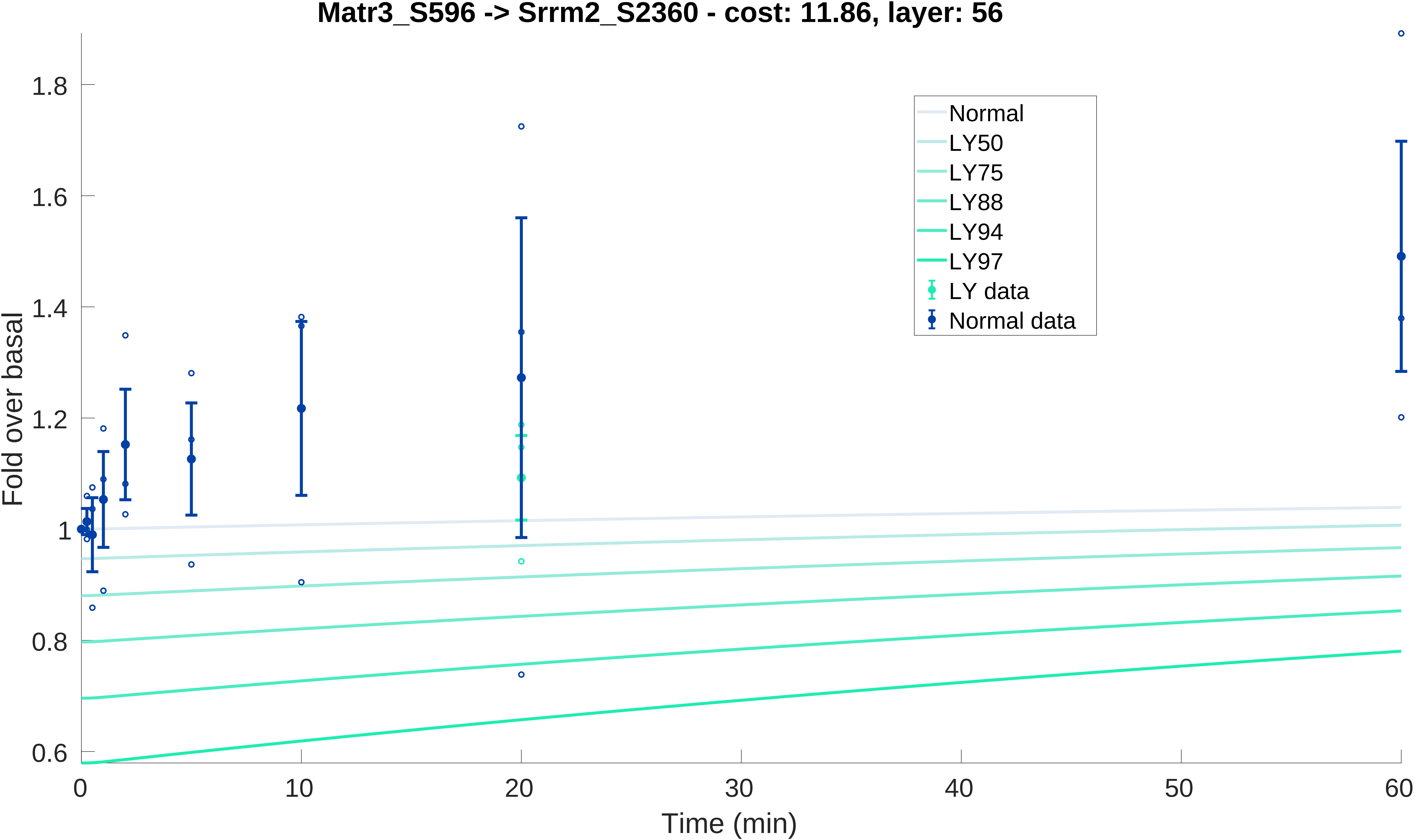

Matr3_S596 -> Srrm2_S2360 - cost: 11.86, layer: 56
Fold over basal
Time (min)
0.6
0.8
1
1.2
1.4
1.6
1.8
0
10
20
30
40
50
60
Normal
LY50
LY75
LY88
LY94
LY97
LY data
Normal data

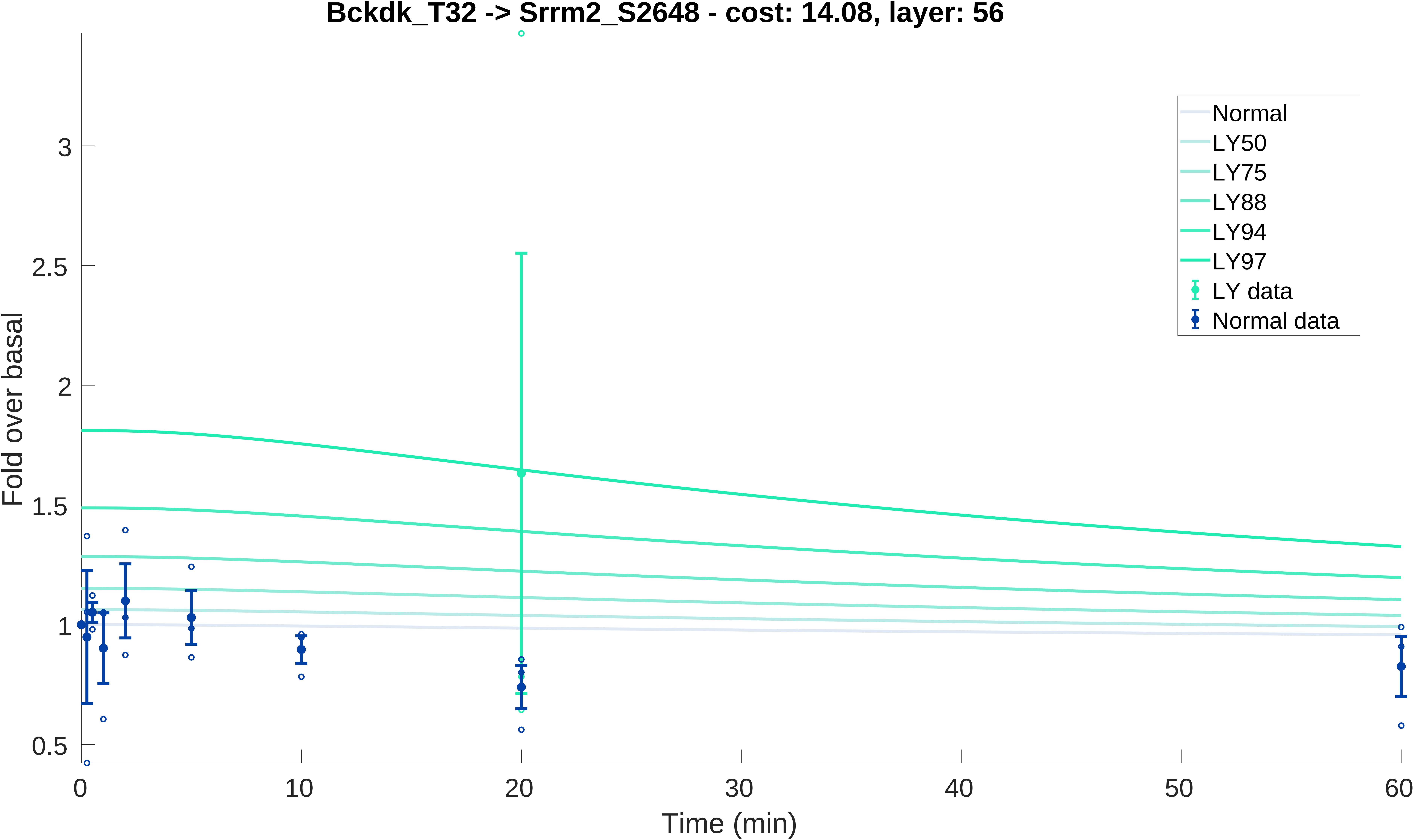
Bckdk_T32 -> Srrm2_S2648 - cost: 14.08, layer: 56
Fold over basal
Time (min)
0.5
1
1.5
2
2.5
3
0
10
20
30
40
50
60
Normal
LY50
LY75
LY88
LY94
LY97
LY data
Normal data

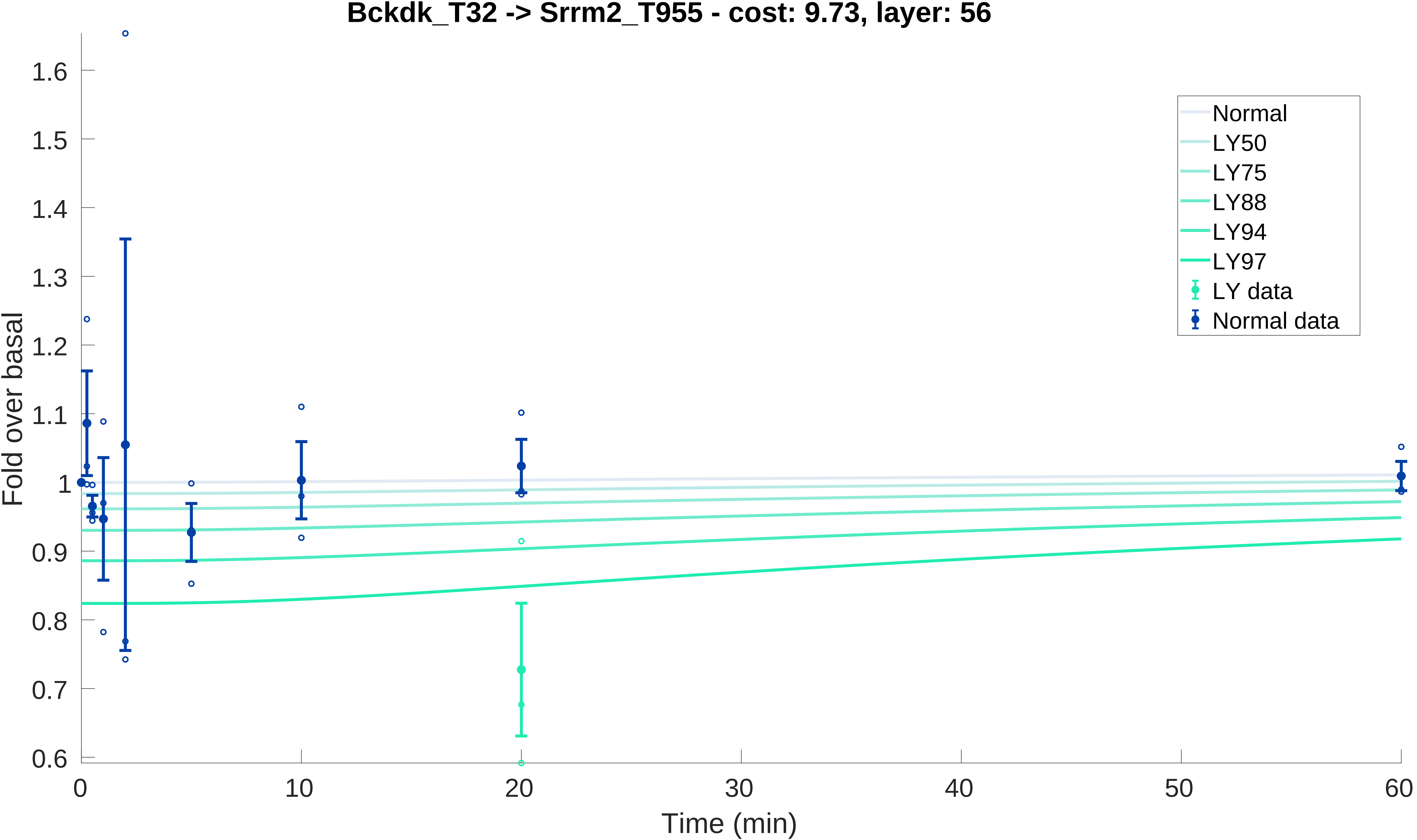

Bckdk_T32 -> Srrm2_T955 - cost: 9.73, layer: 56
Fold over basal
1.6
1.5
1.4
1.3
1.2
1.1
1
0.9
0.8
0.7
0.6
0
10
20
30
40
50
60
Time (min)
Normal
LY50
LY75
LY88
LY94
LY97
LY data
Normal data

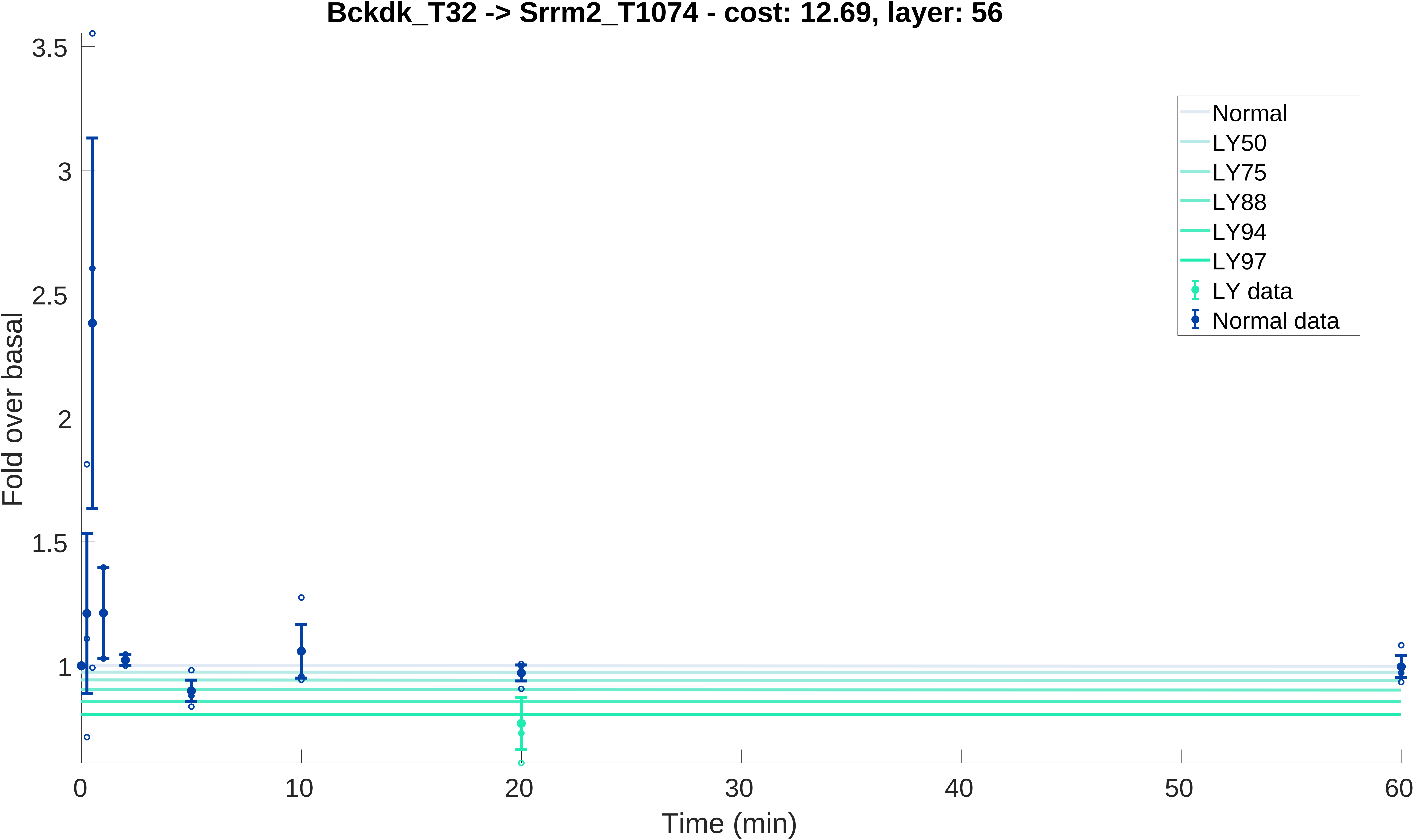

Bckdk_T32 -> Srrm2_T1074 - cost: 12.69, layer: 56
Fold over basal
Time (min)
3.5
3
2.5
2
1.5
1
0
10
20
30
40
50
60
Normal
LY50
LY75
LY88
LY94
LY97
LY data
Normal data

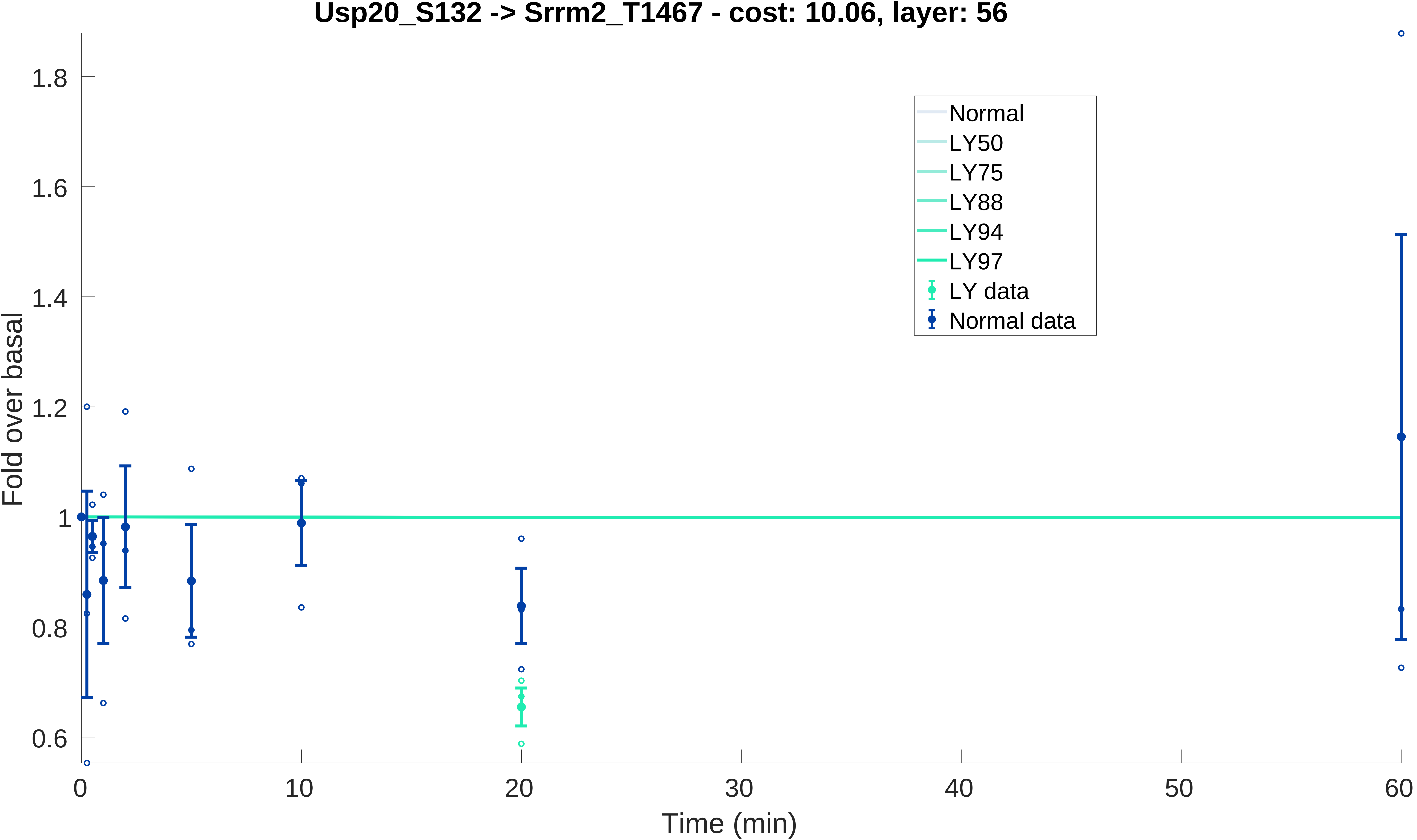
Usp20_S132 -> Srrm2_T1467 - cost: 10.06, layer: 56
Fold over basal
Time (min)
1.8
1.6
1.4
1.2
1
0.8
0.6
0
10
20
30
40
50
60
Normal
LY50
LY75
LY88
LY94
LY97
LY data
Normal data

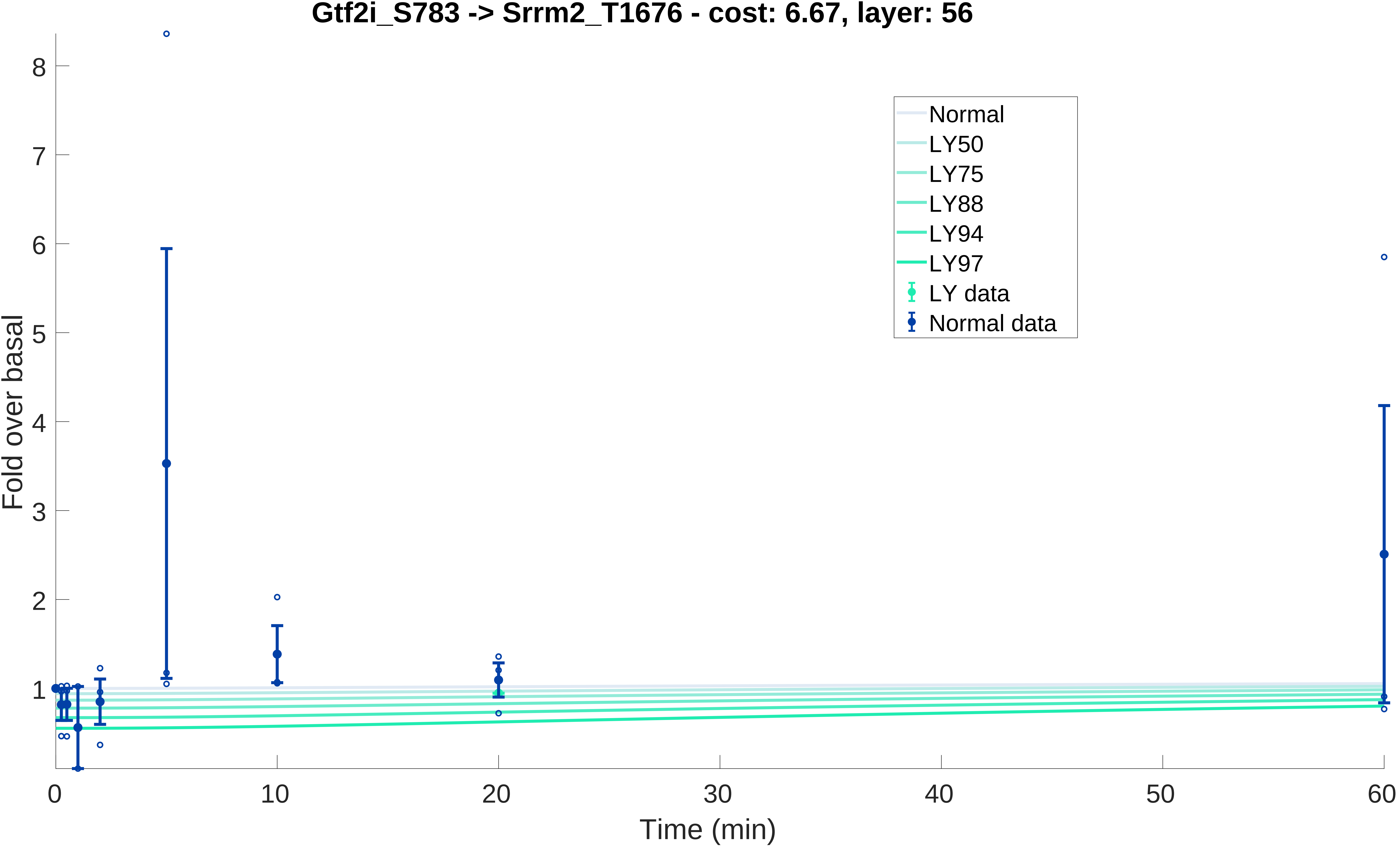
Gtf2i_S783 -> Srrm2_T1676 - cost: 6.67, layer: 56
Fold over basal
Time (min)
0
10
20
30
40
50
60
1
2
3
4
5
6
7
8
Normal
LY50
LY75
LY88
LY94
LY97
LY data
Normal data

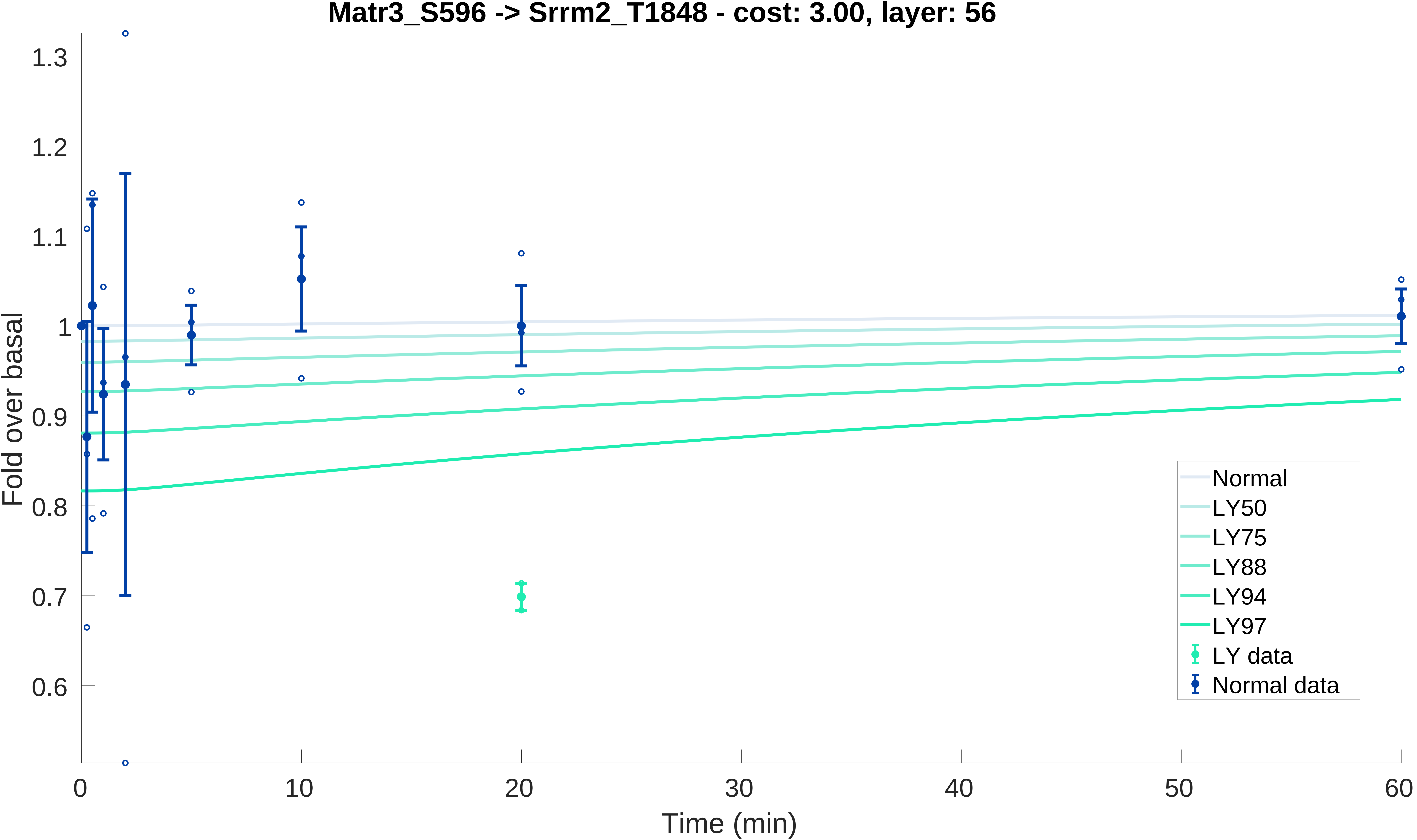

Matr3_S596 -> Srrm2_T1848 - cost: 3.00, layer: 56
Fold over basal
Time (min)
1.3
1.2
1.1
1
0.9
0.8
0.7
0.6
0
10
20
30
40
50
60
Normal
LY50
LY75
LY88
LY94
LY97
LY data
Normal data

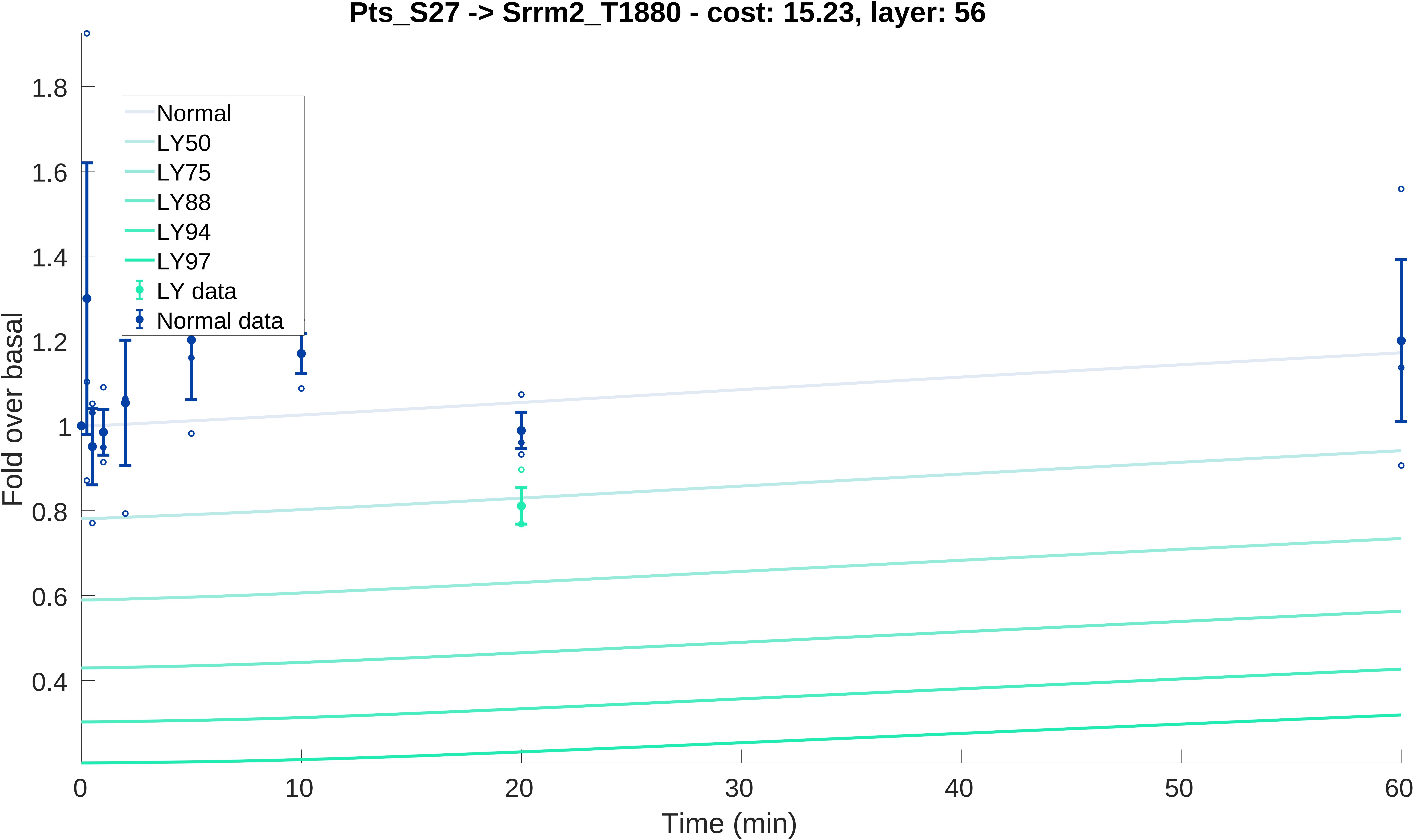
Pts_S27 -> Srrm2_T1880 - cost: 15.23, layer: 56
Normal
LY50
LY75
LY88
LY94
LY97
LY data
Normal data
Fold over basal
Time (min)
0
10
20
30
40
50
60
0.4
0.6
0.8
1
1.2
1.4
1.6
1.8

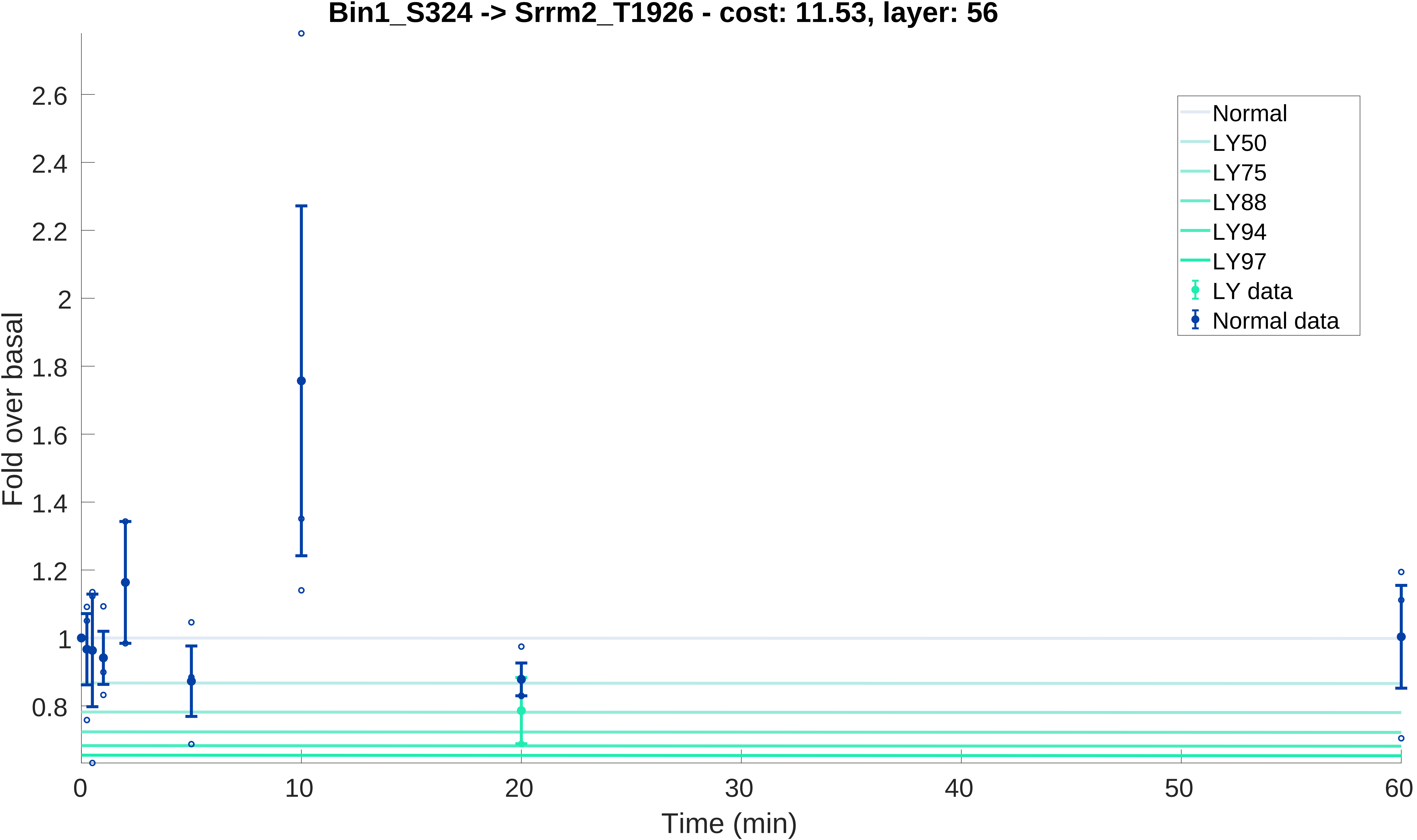
Bin1_S324 -> Srrm2_T1926 - cost: 11.53, layer: 56
Fold over basal
Time (min)
Normal
LY50
LY75
LY88
LY94
LY97
LY data
Normal data
2.6
2.4
2.2
2
1.8
1.6
1.4
1.2
1
0.8
0
10
20
30
40
50
60

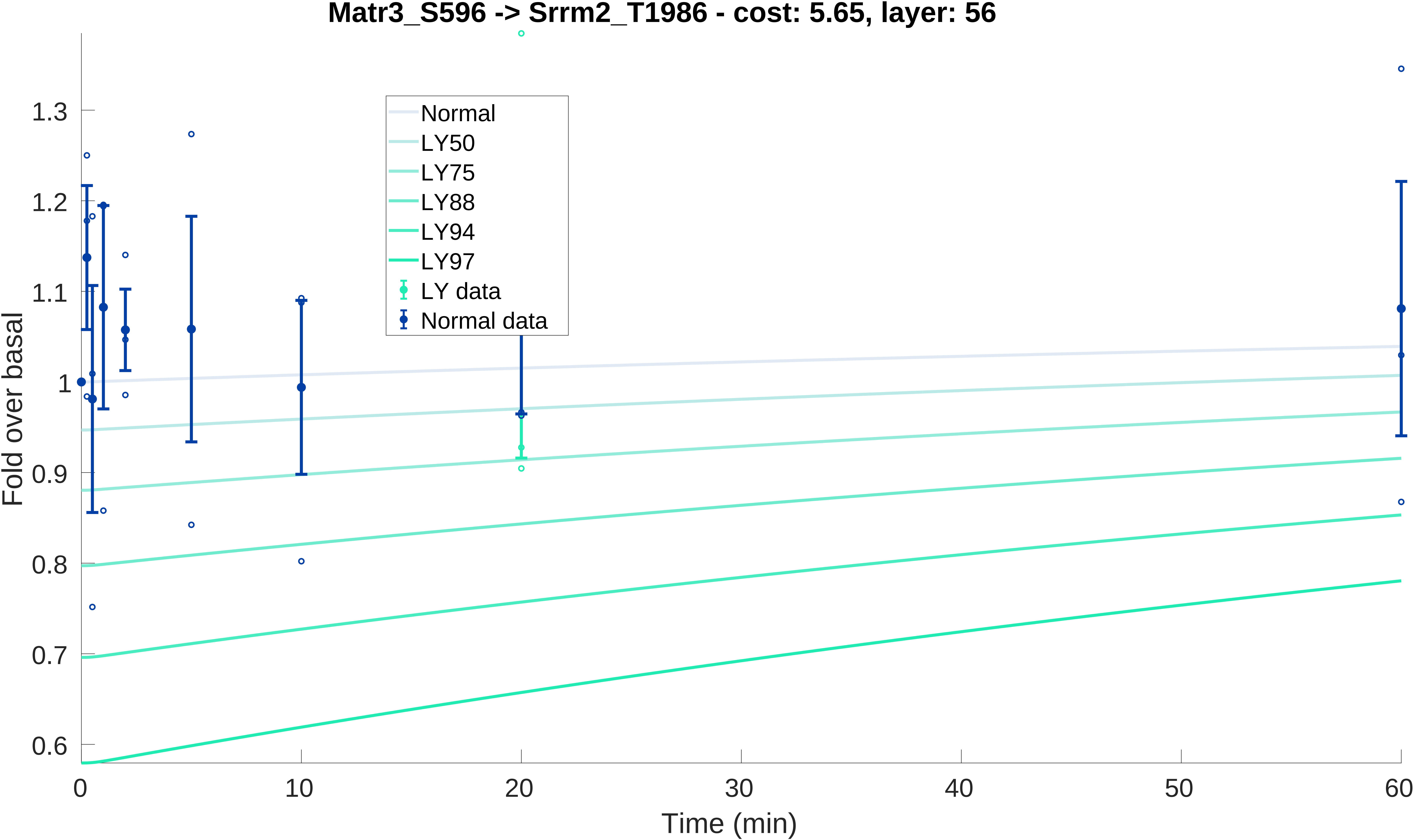

Matr3_S596 -> Srrm2_T1986 - cost: 5.65, layer: 56
Normal
LY50
LY75
LY88
LY94
LY97
LY data
Normal data
Fold over basal
Time (min)
1.3
1.2
1.1
1
0.9
0.8
0.7
0.6
0
10
20
30
40
50
60

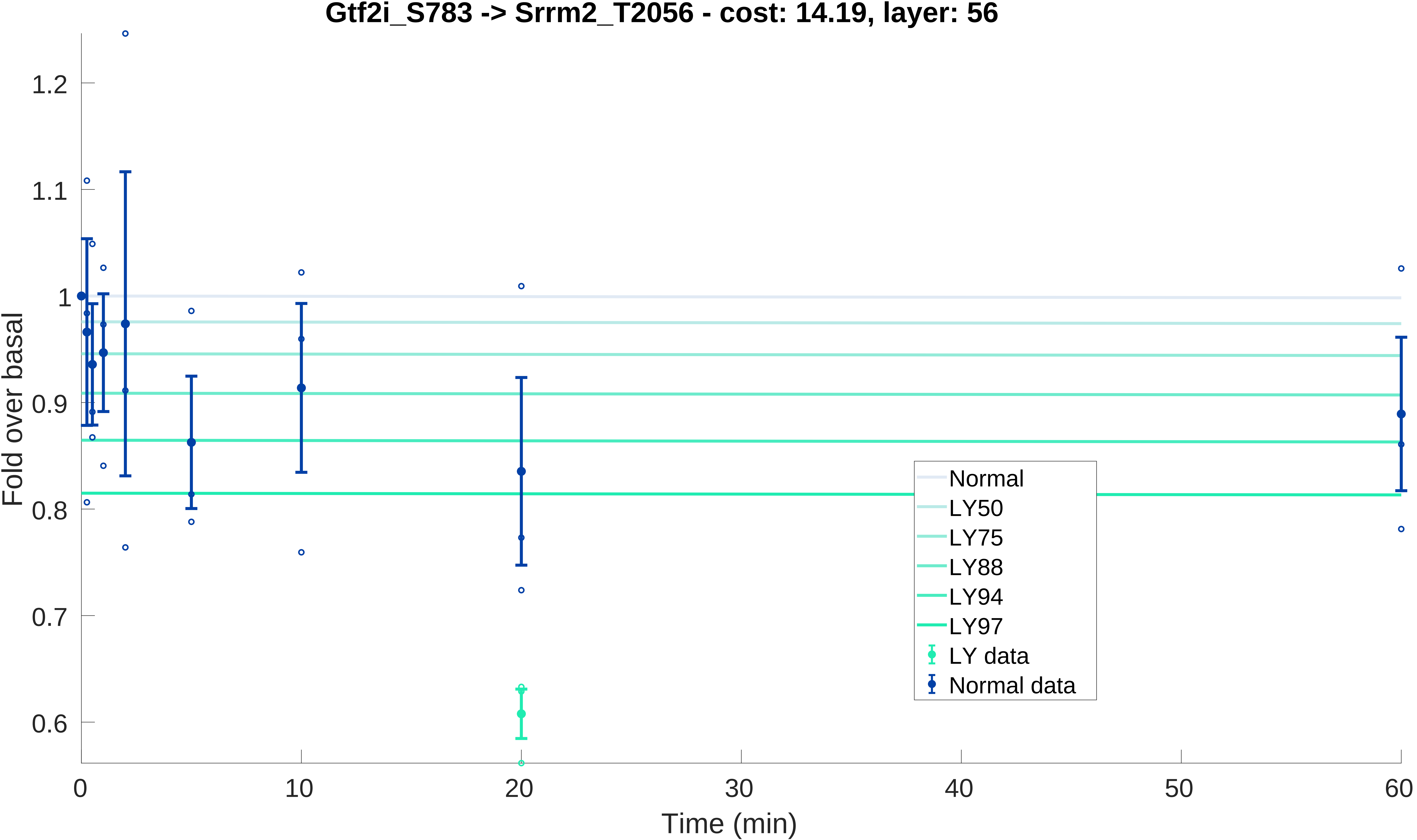

Gtf2i_S783 -> Srrm2_T2056 - cost: 14.19, layer: 56
Fold over basal
Time (min)
1.2
1.1
1
0.9
0.8
0.7
0.6
0
10
20
30
40
50
60
Normal
LY50
LY75
LY88
LY94
LY97
LY data
Normal data

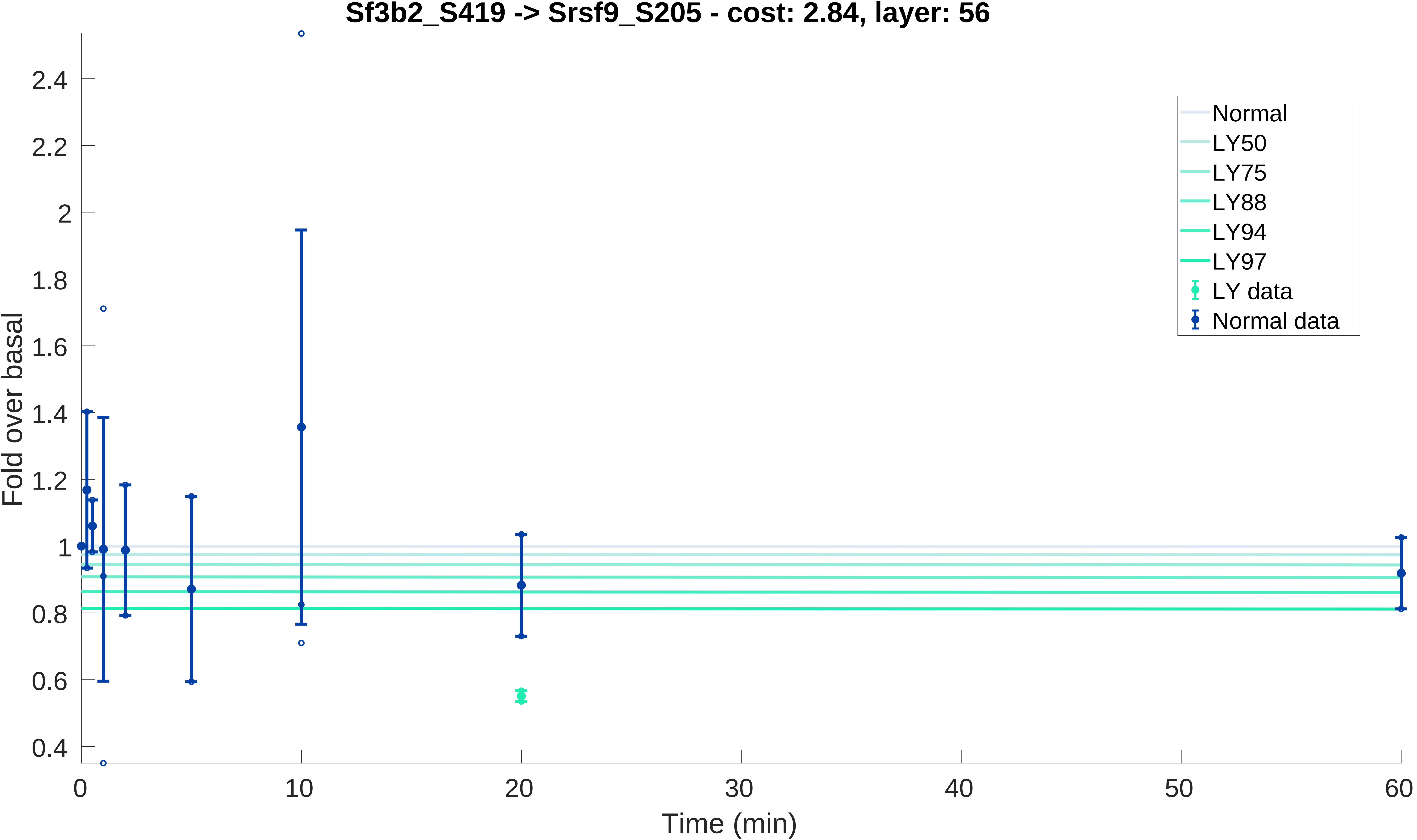
Sf3b2_S419 -> Srsf9_S205 - cost: 2.84, layer: 56
Fold over basal
Time (min)
2.4
2.2
2
1.8
1.6
1.4
1.2
1
0.8
0.6
0.4
0
10
20
30
40
50
60
Normal
LY50
LY75
LY88
LY94
LY97
LY data
Normal data

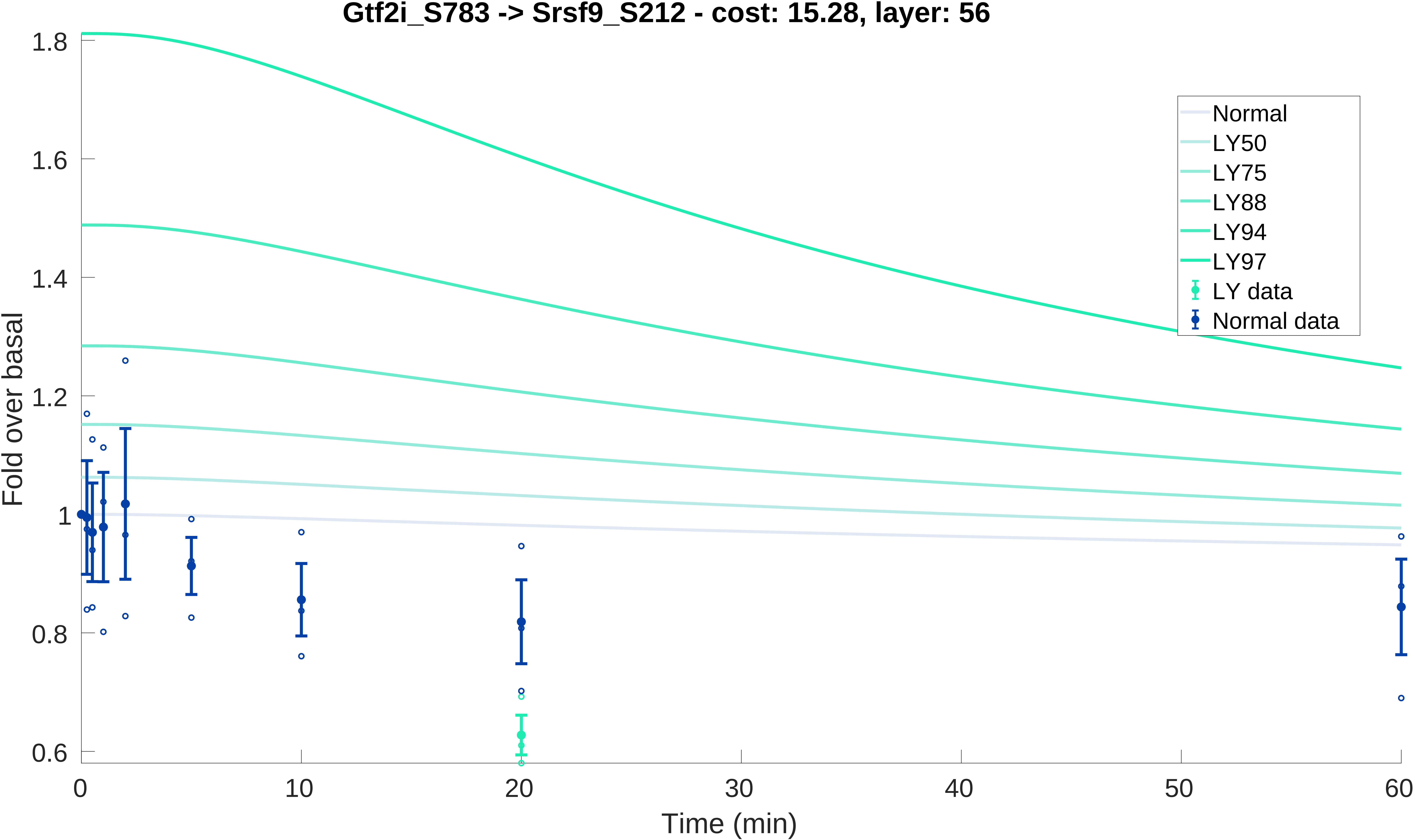

Gtf2i_S783 -> Srsf9_S212 - cost: 15.28, layer: 56
Normal
LY50
LY75
LY88
LY94
LY97
LY data
Normal data
Fold over basal
Time (min)
1.8
1.6
1.4
1.2
1
0.8
0.6
0
10
20
30
40
50
60

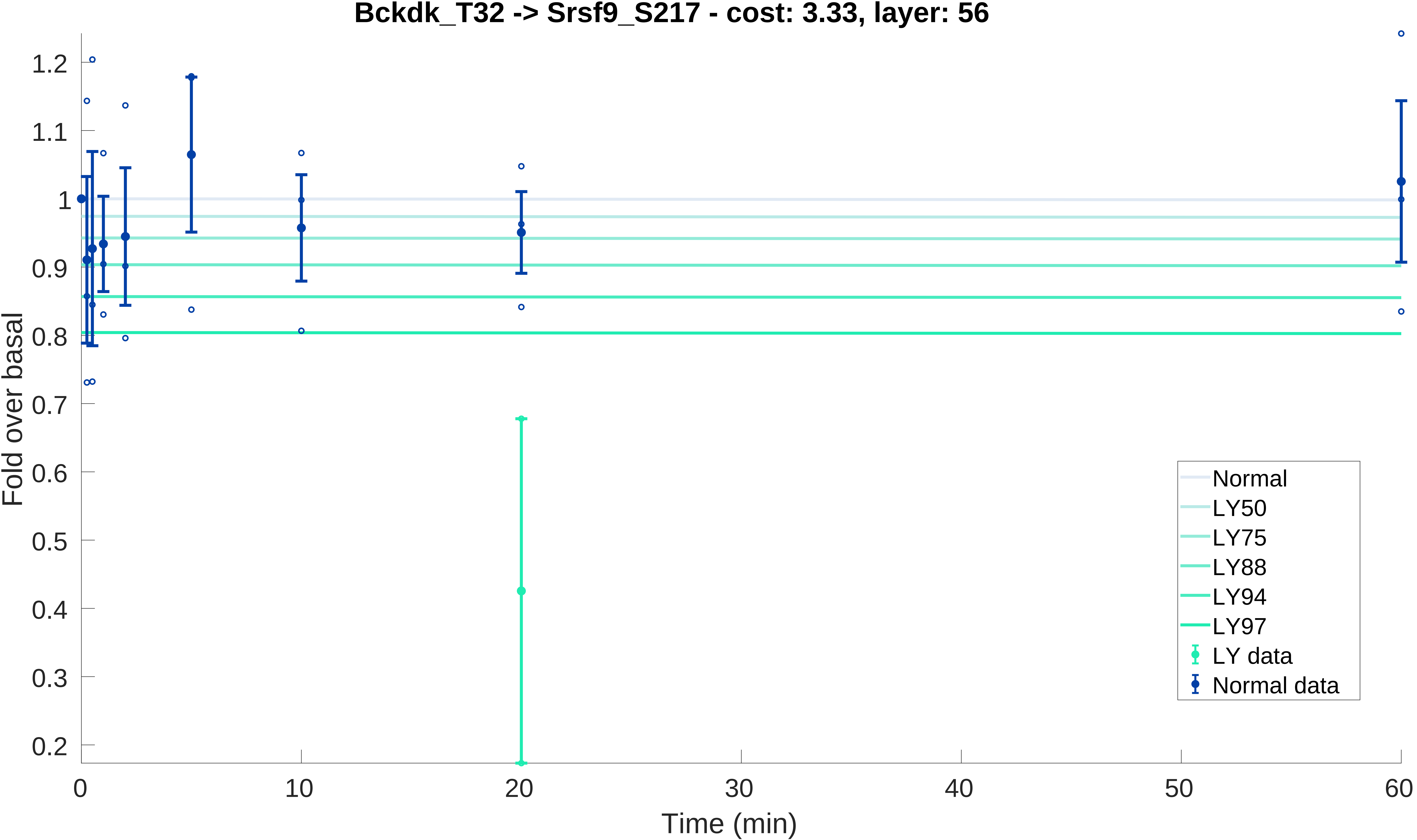

Bckdk_T32 -> Srsf9_S217 - cost: 3.33, layer: 56
Fold over basal
Time (min)
0.2
0.3
0.4
0.5
0.6
0.7
0.8
0.9
1
1.1
1.2
0
10
20
30
40
50
60
Normal
LY50
LY75
LY88
LY94
LY97
LY data
Normal data

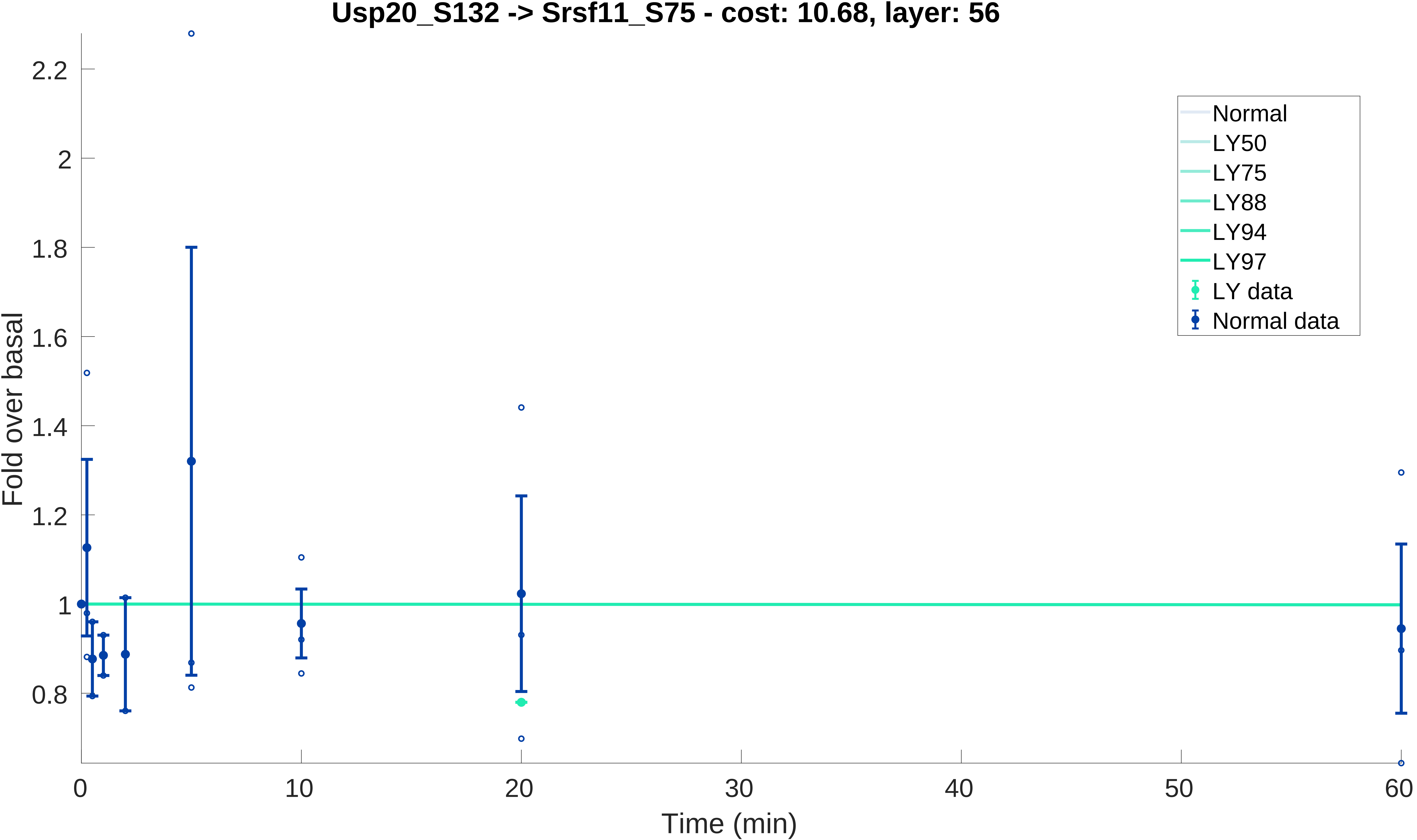

Usp20_S132 -> Srsf11_S75 - cost: 10.68, layer: 56
Fold over basal
Time (min)
2.2
2
1.8
1.6
1.4
1.2
1
0.8
0
10
20
30
40
50
60
Normal
LY50
LY75
LY88
LY94
LY97
LY data
Normal data

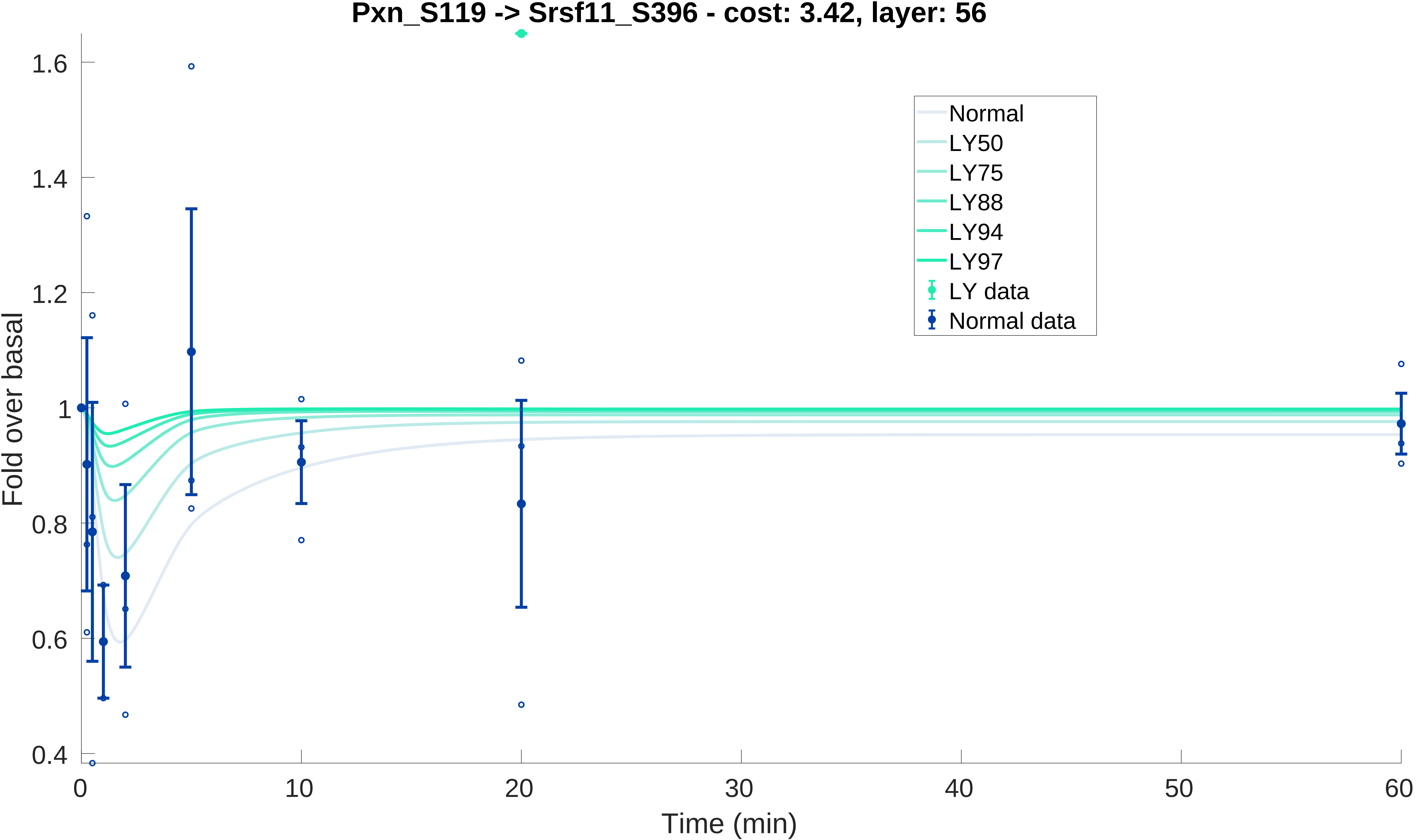

Pxn_S119 -> Srsf11_S396 - cost: 3.42, layer: 56
Fold over basal
1.6
1.4
1.2
1
0.8
0.6
0.4
0
10
20
30
40
50
60
Time (min)
Normal
LY50
LY75
LY88
LY94
LY97
LY data
Normal data

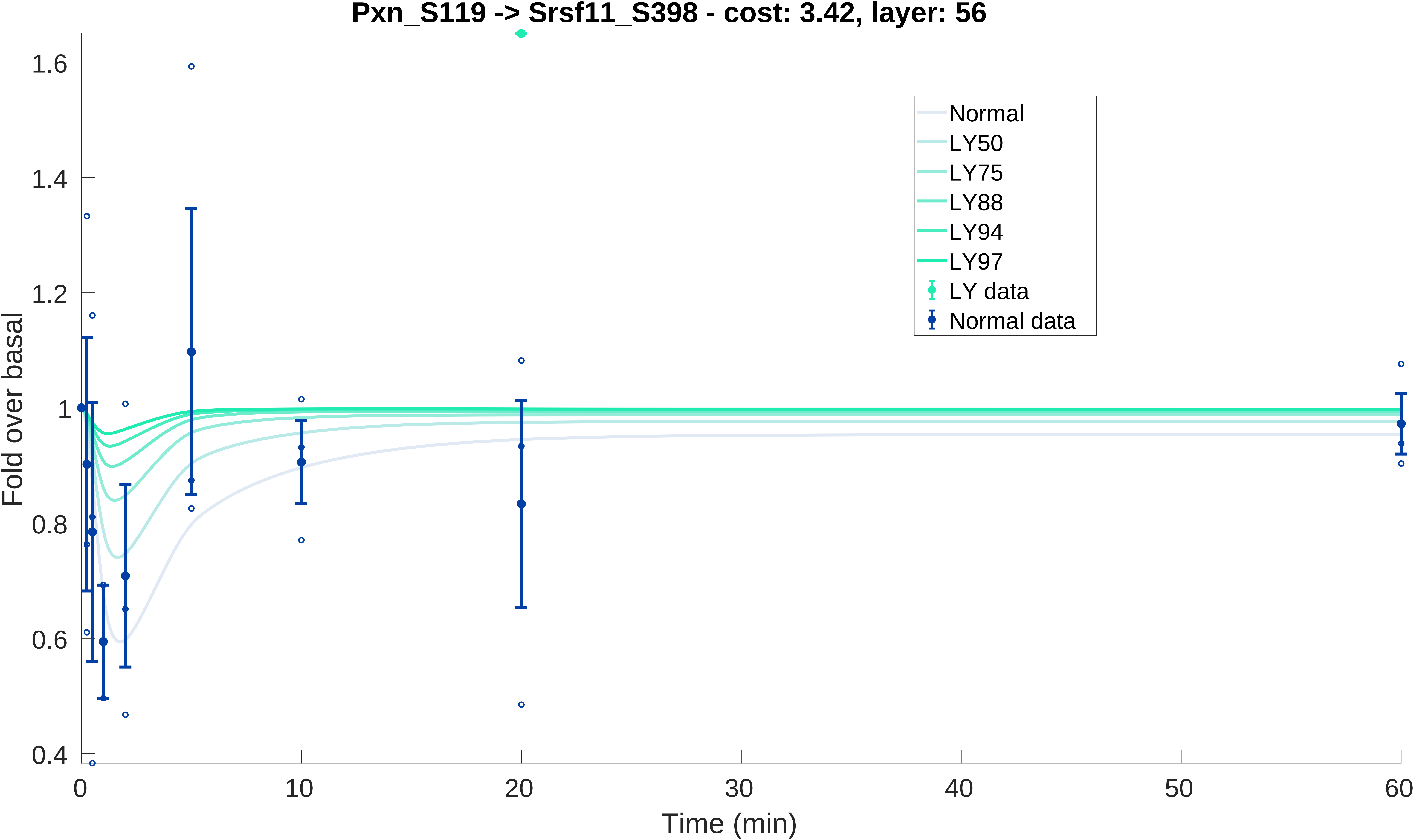

Pxn_S119 -> Srsf11_S398 - cost: 3.42, layer: 56
Fold over basal
Time (min)
Normal
LY50
LY75
LY88
LY94
LY97
LY data
Normal data
1.6
1.4
1.2
1
0.8
0.6
0.4
0
10
20
30
40
50
60

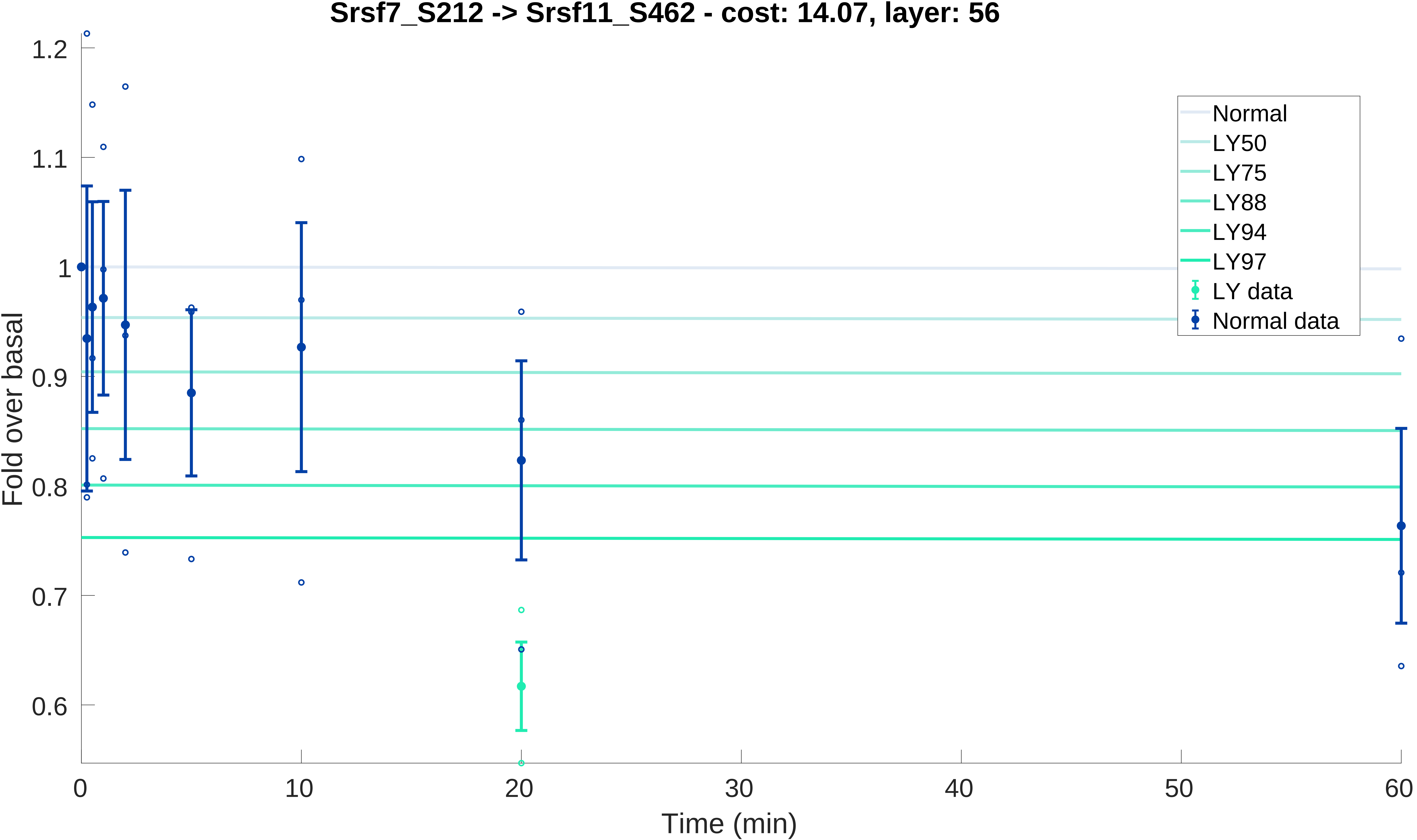

Srsf7_S212 -> Srsf11_S462 - cost: 14.07, layer: 56
Fold over basal
1.2
1.1
1
0.9
0.8
0.7
0.6
0
10
20
30
40
50
60
Time (min)
Normal
LY50
LY75
LY88
LY94
LY97
LY data
Normal data

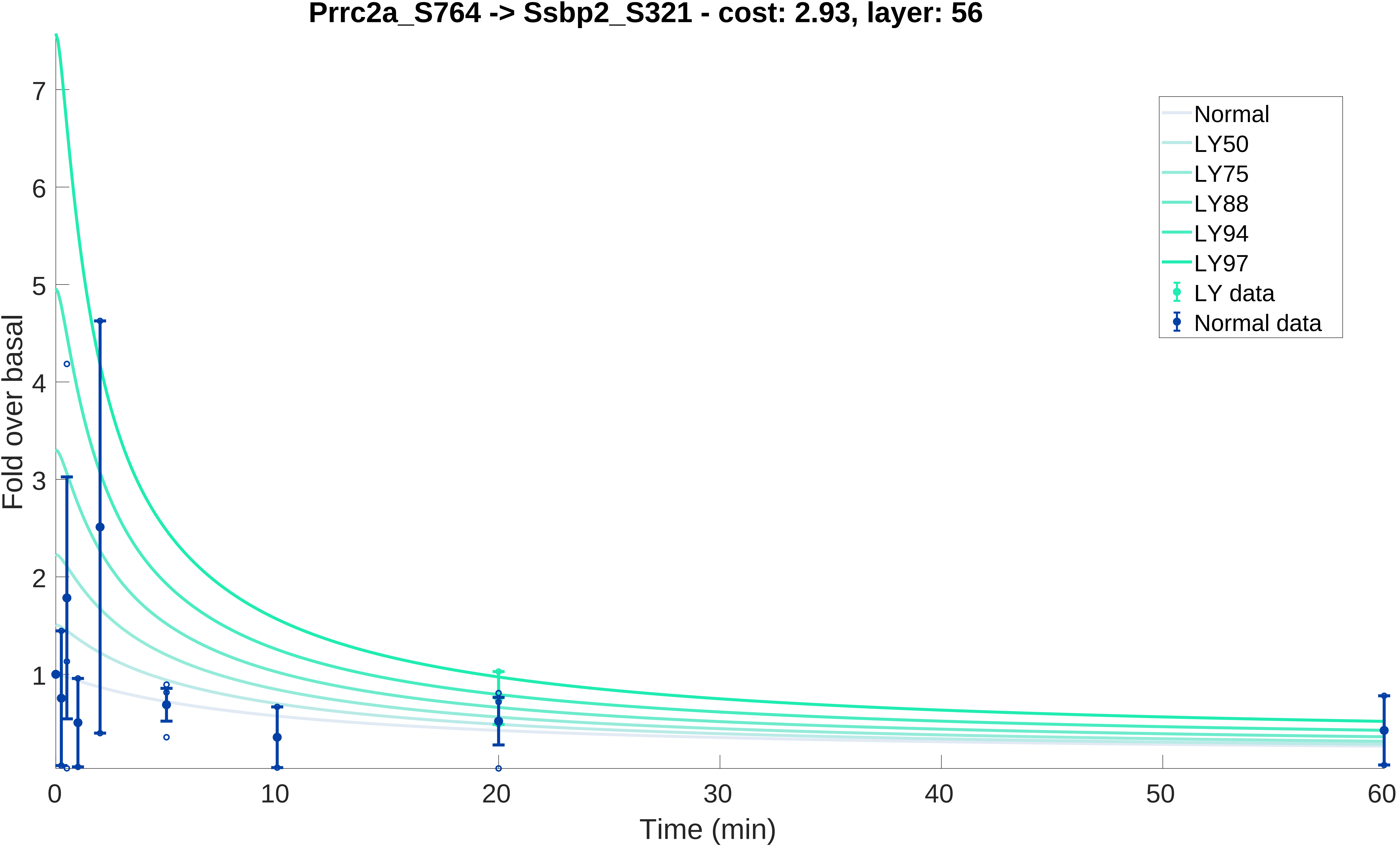

Prrc2a_S764 -> Ssbp2_S321 - cost: 2.93, layer: 56
Fold over basal
Time (min)
0
10
20
30
40
50
60
1
2
3
4
5
6
7
Normal
LY50
LY75
LY88
LY94
LY97
LY data
Normal data

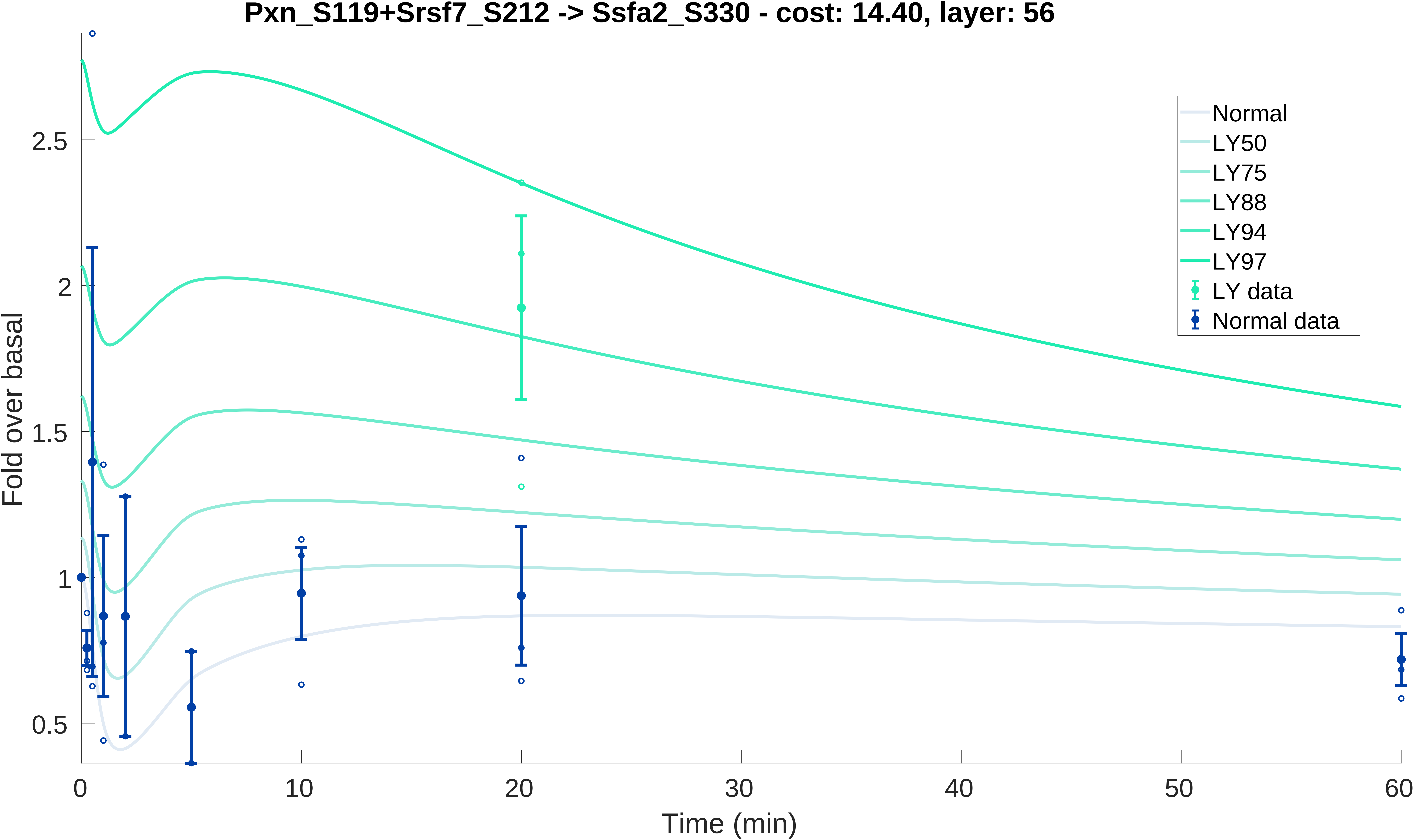

Pxn_S119+Srsf7_S212 -> Ssfa2_S330 - cost: 14.40, layer: 56
Fold over basal
2.5
2
1.5
1
0.5
0
10
20
30
40
50
60
Time (min)
Normal
LY50
LY75
LY88
LY94
LY97
LY data
Normal data

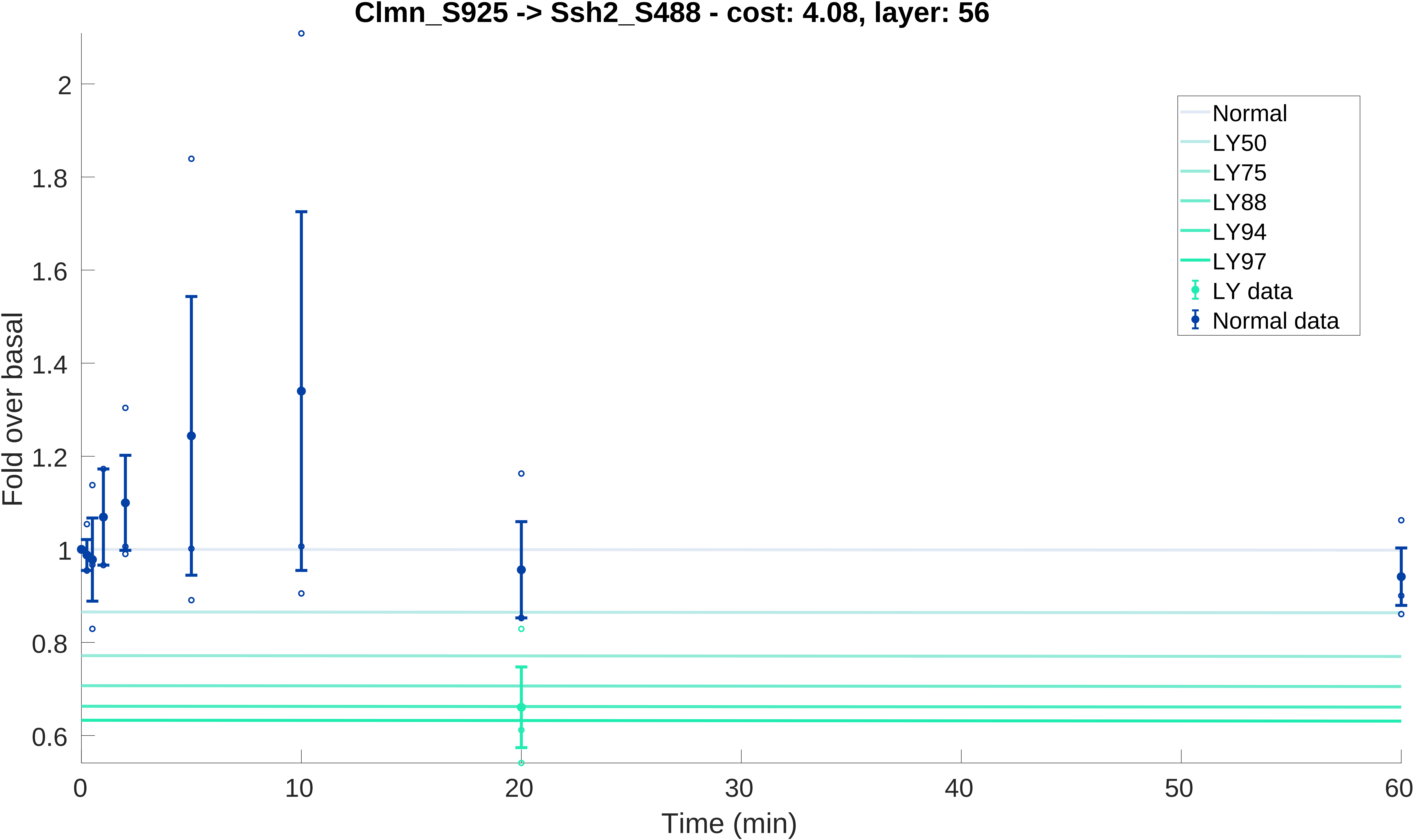

Clmn_S925 -> Ssh2_S488 - cost: 4.08, layer: 56
Fold over basal
Time (min)
0.6
0.8
1
1.2
1.4
1.6
1.8
2
0
10
20
30
40
50
60
Normal
LY50
LY75
LY88
LY94
LY97
LY data
Normal data

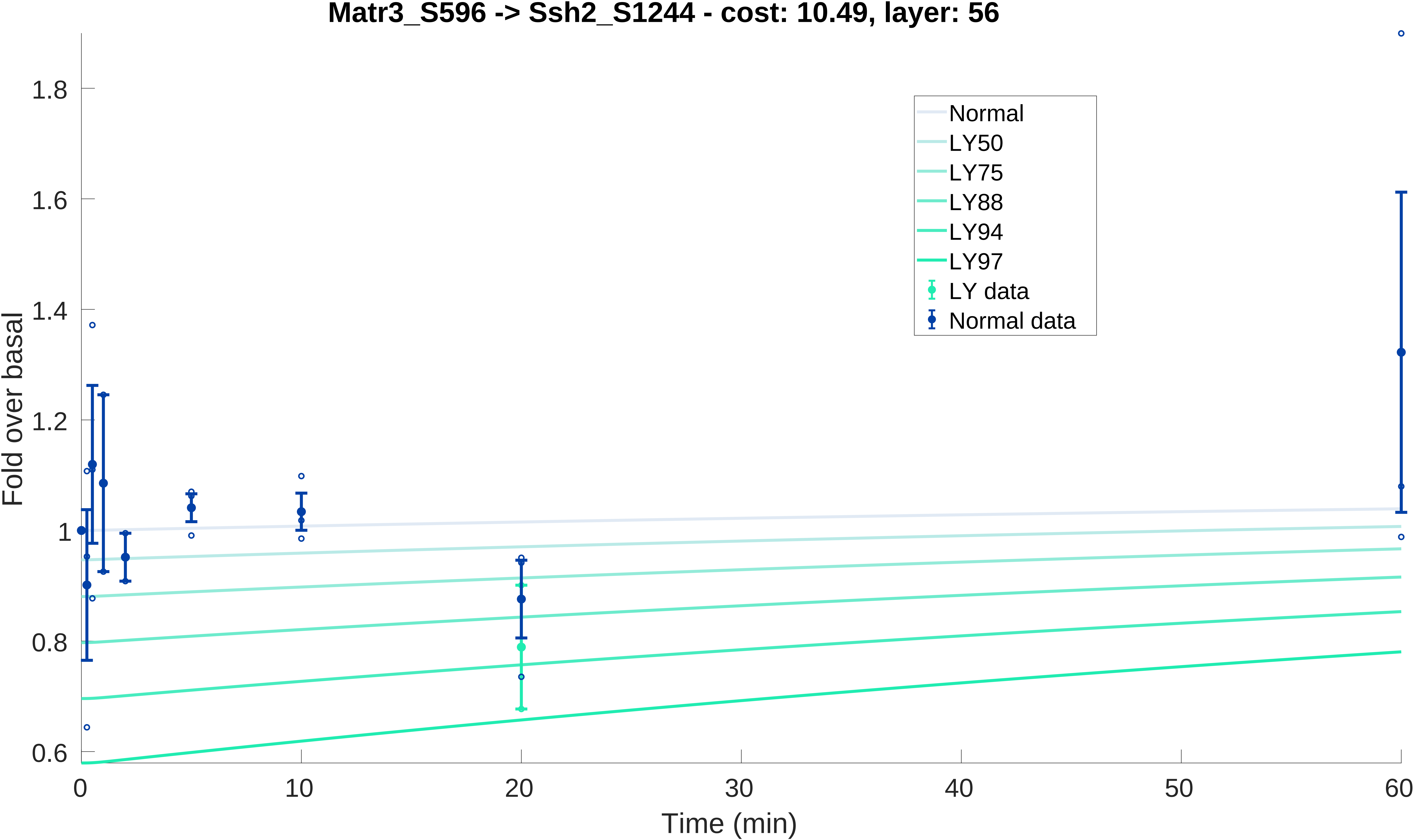

Matr3_S596 -> Ssh2_S1244 - cost: 10.49, layer: 56
Fold over basal
Time (min)
Normal
LY50
LY75
LY88
LY94
LY97
LY data
Normal data
1.8
1.6
1.4
1.2
1
0.8
0.6
0
10
20
30
40
50
60

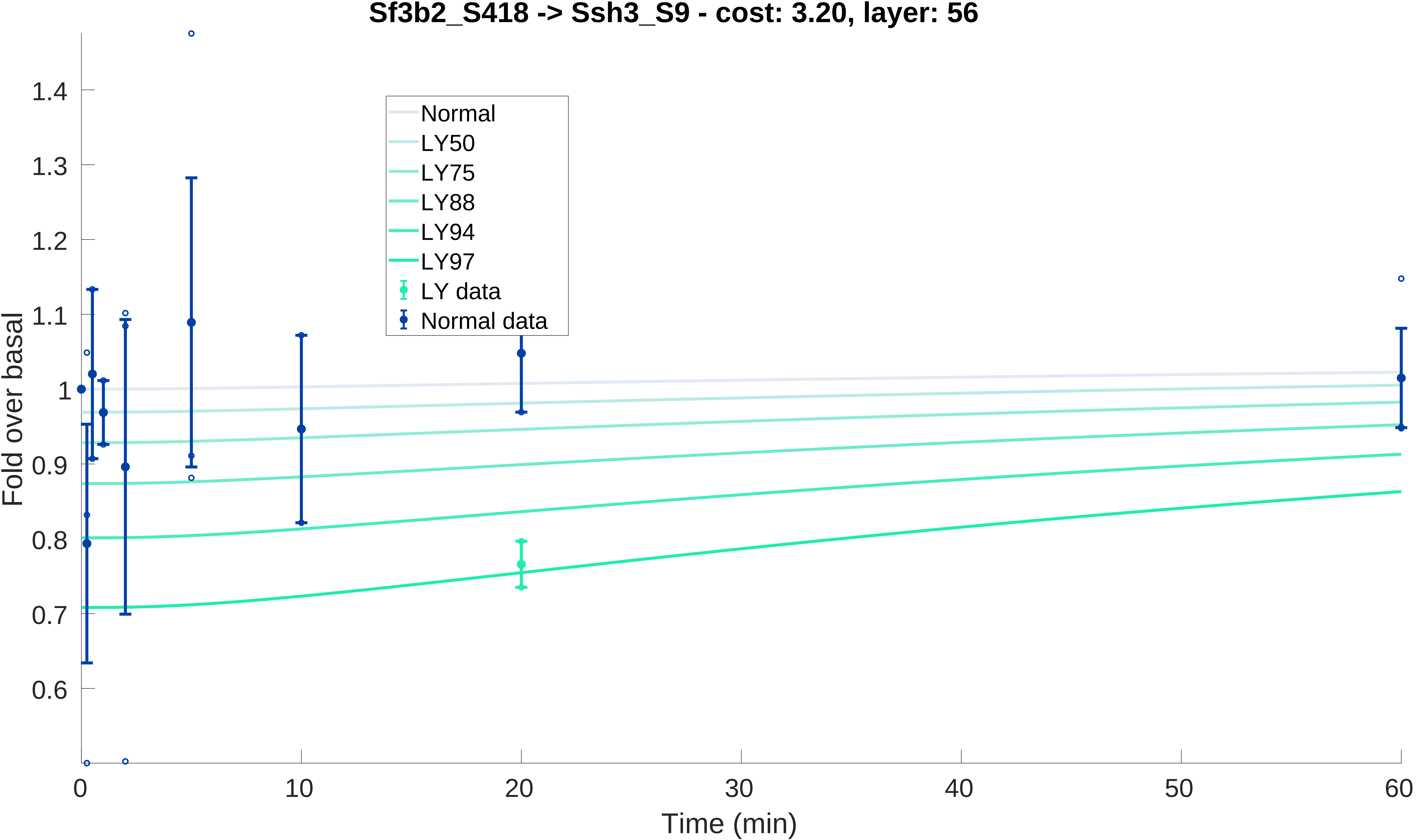

Sf3b2_S418 -> Ssh3_S9 - cost: 3.20, layer: 56
Normal
LY50
LY75
LY88
LY94
LY97
LY data
Normal data
Fold over basal
Time (min)
1.4
1.3
1.2
1.1
1
0.9
0.8
0.7
0.6
0
10
20
30
40
50
60

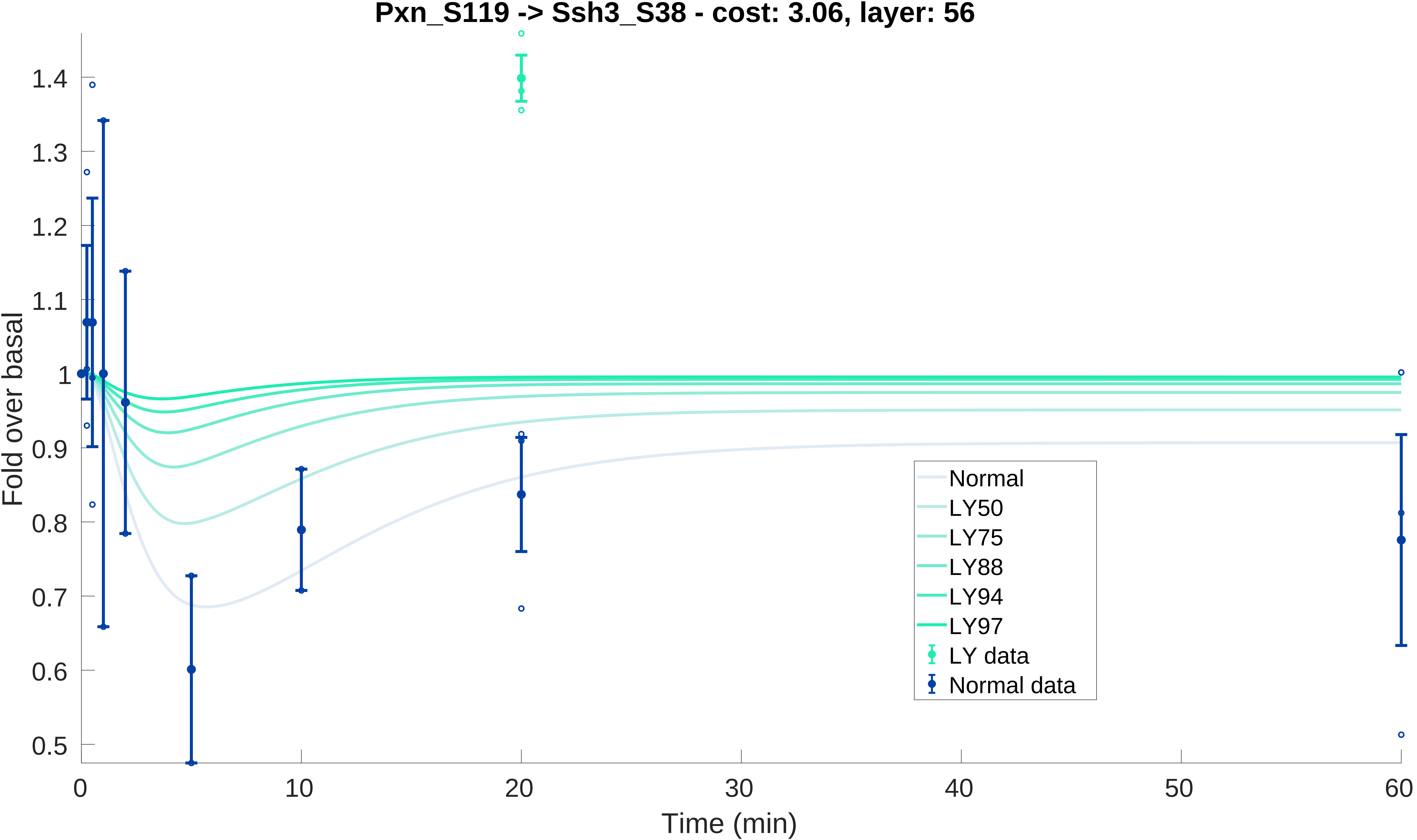

Pxn_S119 -> Ssh3_S38 - cost: 3.06, layer: 56
Fold over basal
Time (min)
0.5
0.6
0.7
0.8
0.9
1
1.1
1.2
1.3
1.4
0
10
20
30
40
50
60
Normal
LY50
LY75
LY88
LY94
LY97
LY data
Normal data

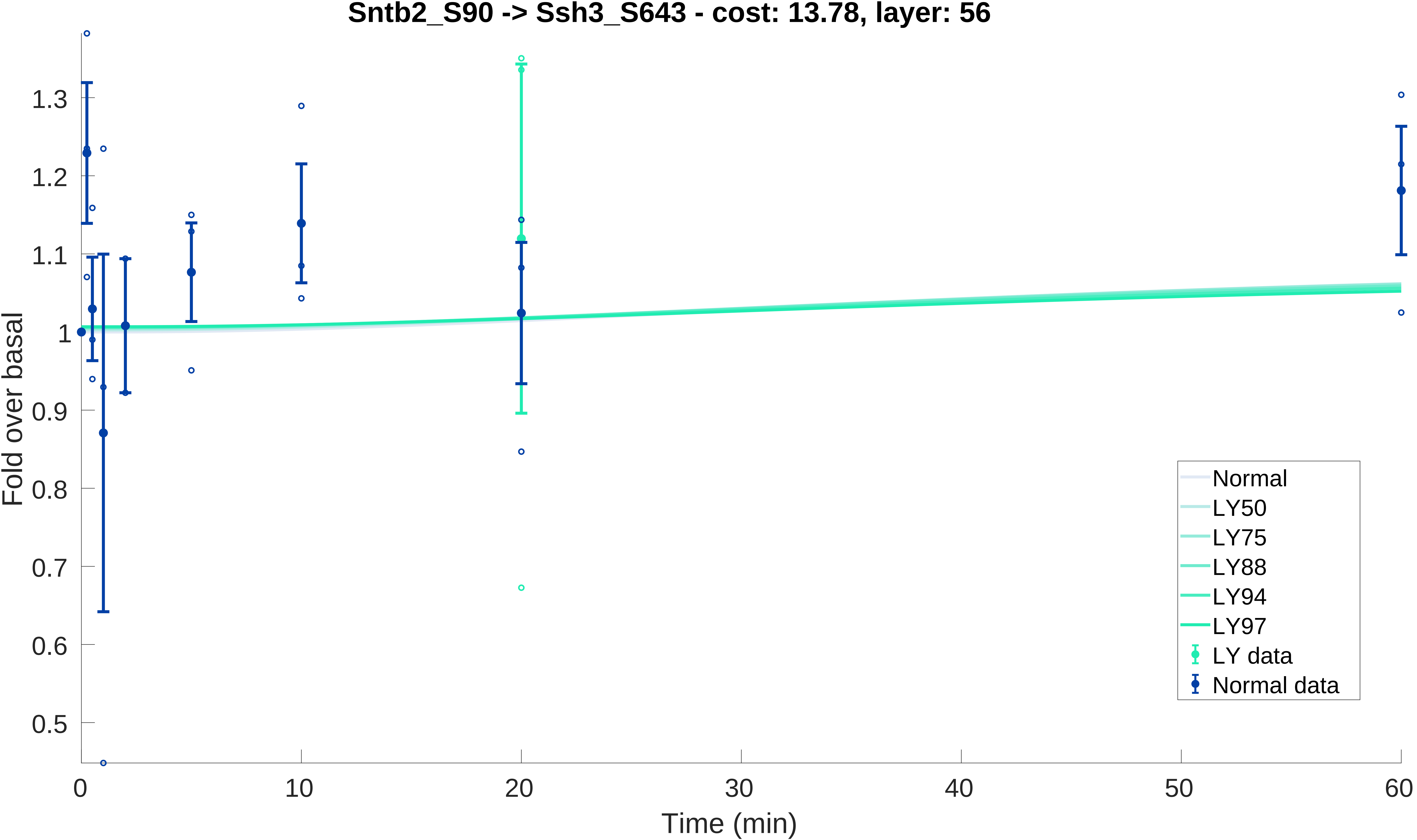

Sntb2_S90 -> Ssh3_S643 - cost: 13.78, layer: 56
Fold over basal
1.3
1.2
1.1
1
0.9
0.8
0.7
0.6
0.5
0
10
20
30
40
50
60
Time (min)
Normal
LY50
LY75
LY88
LY94
LY97
LY data
Normal data

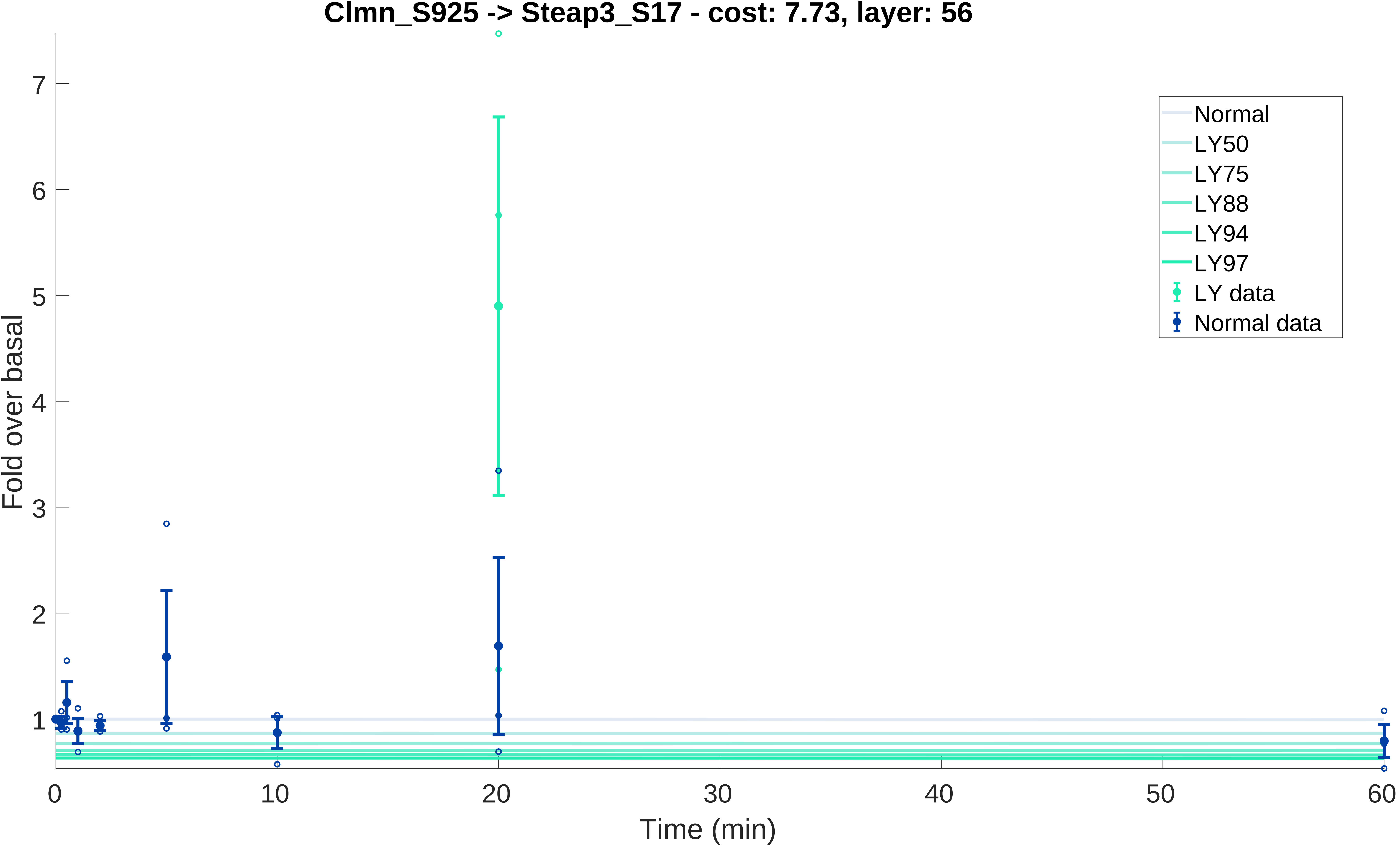
Clmn_S925 -> Steap3_S17 - cost: 7.73, layer: 56
Fold over basal
Time (min)
Normal
LY50
LY75
LY88
LY94
LY97
LY data
Normal data
0
10
20
30
40
50
60
1
2
3
4
5
6
7

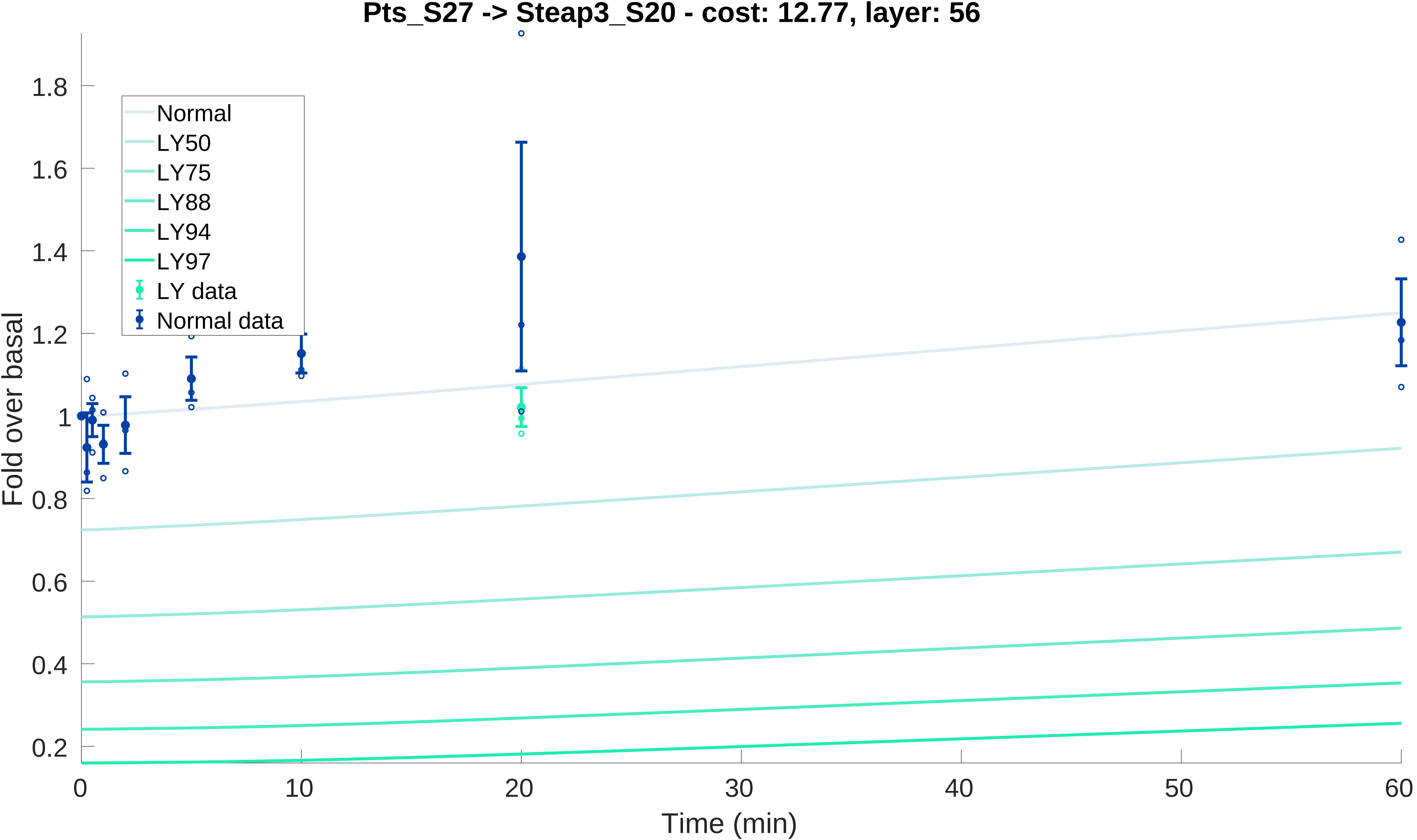

Pts_S27 -> Steap3_S20 - cost: 12.77, layer: 56
Normal
LY50
LY75
LY88
LY94
LY97
LY data
Normal data
Fold over basal
Time (min)
1.8
1.6
1.4
1.2
1
0.8
0.6
0.4
0.2
0
10
20
30
40
50
60

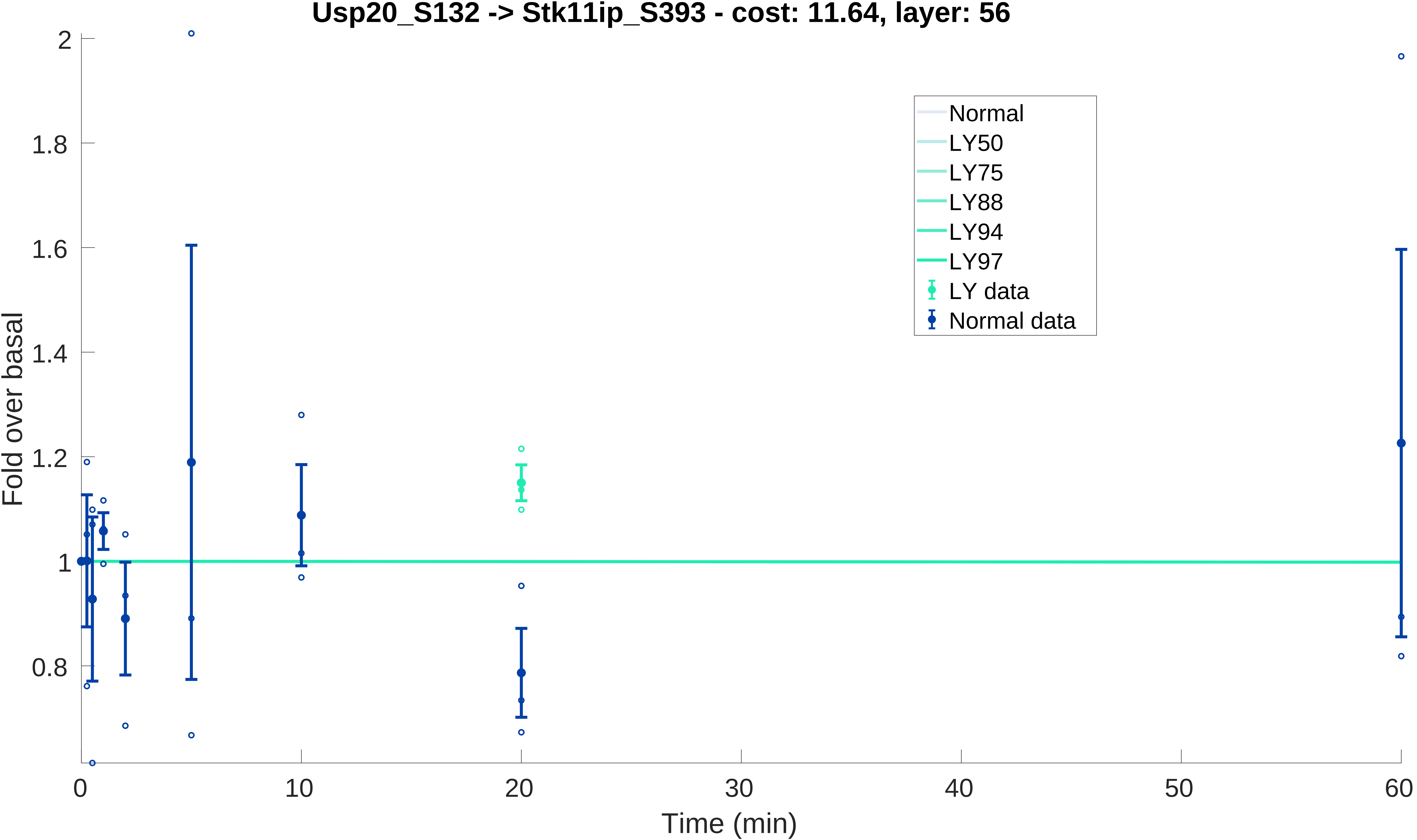

Usp20_S132 -> Stk11ip_S393 - cost: 11.64, layer: 56
Fold over basal
Time (min)
0
10
20
30
40
50
60
0.8
1
1.2
1.4
1.6
1.8
2
Normal
LY50
LY75
LY88
LY94
LY97
LY data
Normal data

Srsf2_S189 -> Strip1_S335 - cost: 7.94, layer: 56

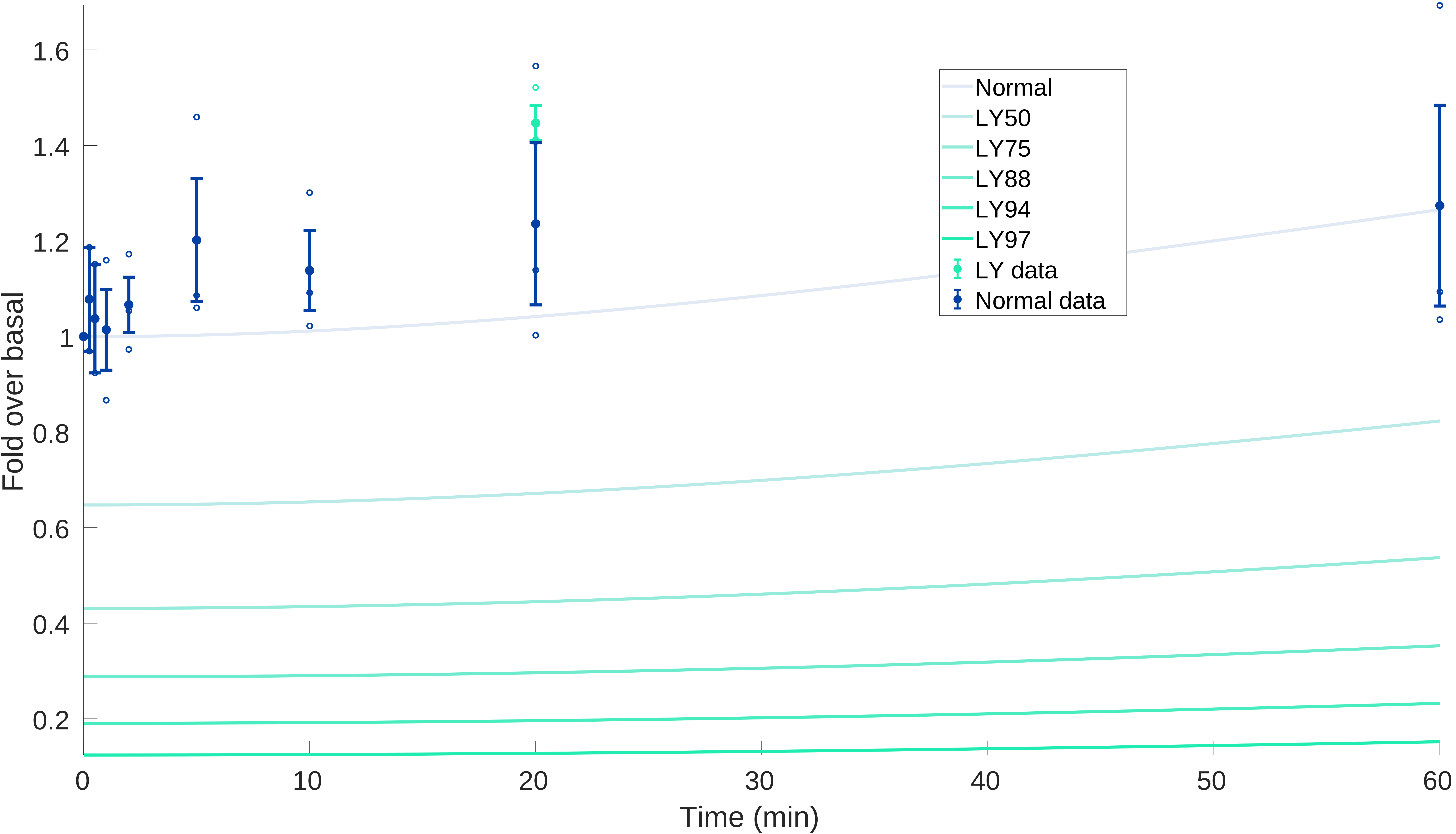

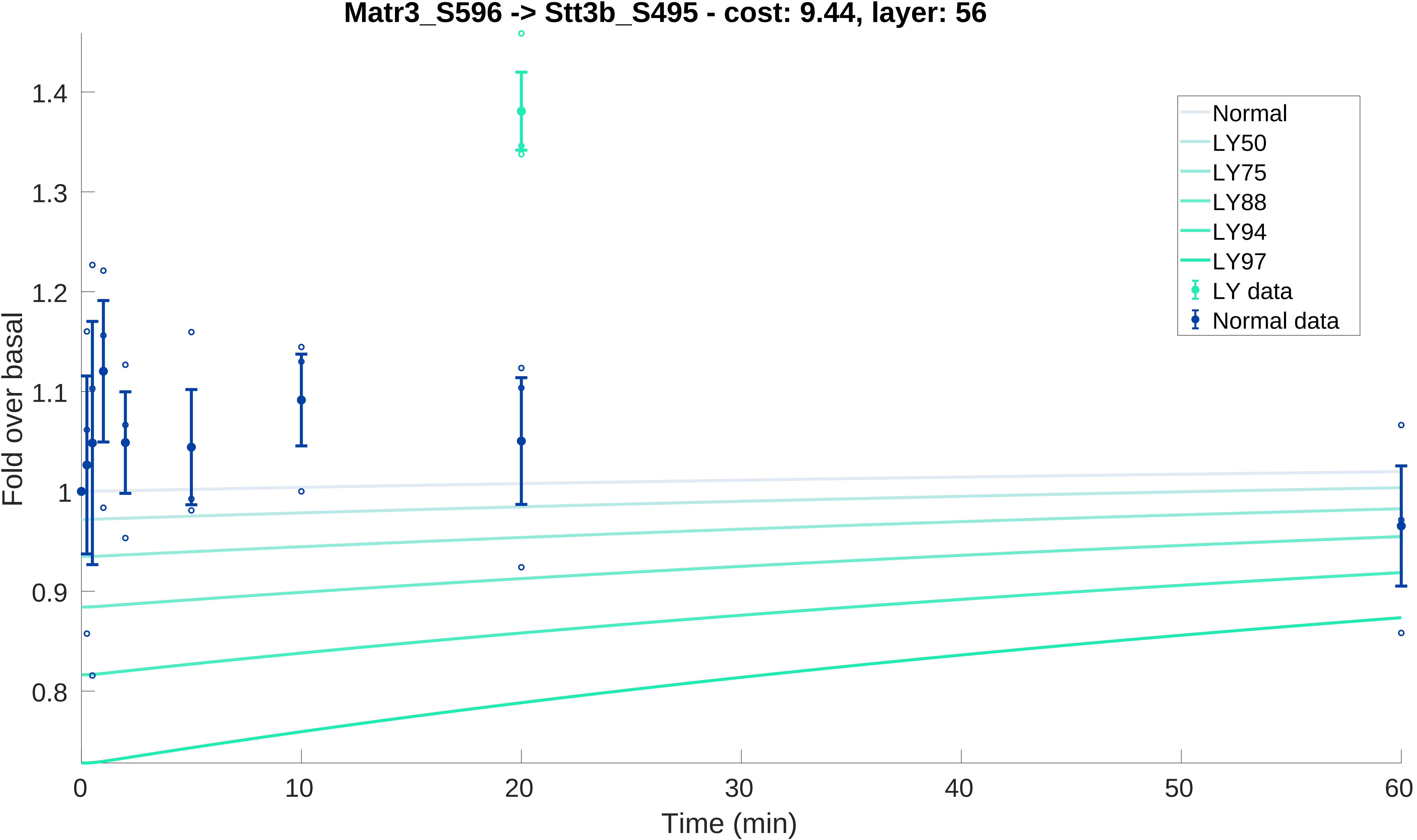

Matr3_S596 -> Stt3b_S495 - cost: 9.44, layer: 56
Fold over basal
Time (min)
0
10
20
30
40
50
60
0.8
0.9
1
1.1
1.2
1.3
1.4
Normal
LY50
LY75
LY88
LY94
LY97
LY data
Normal data

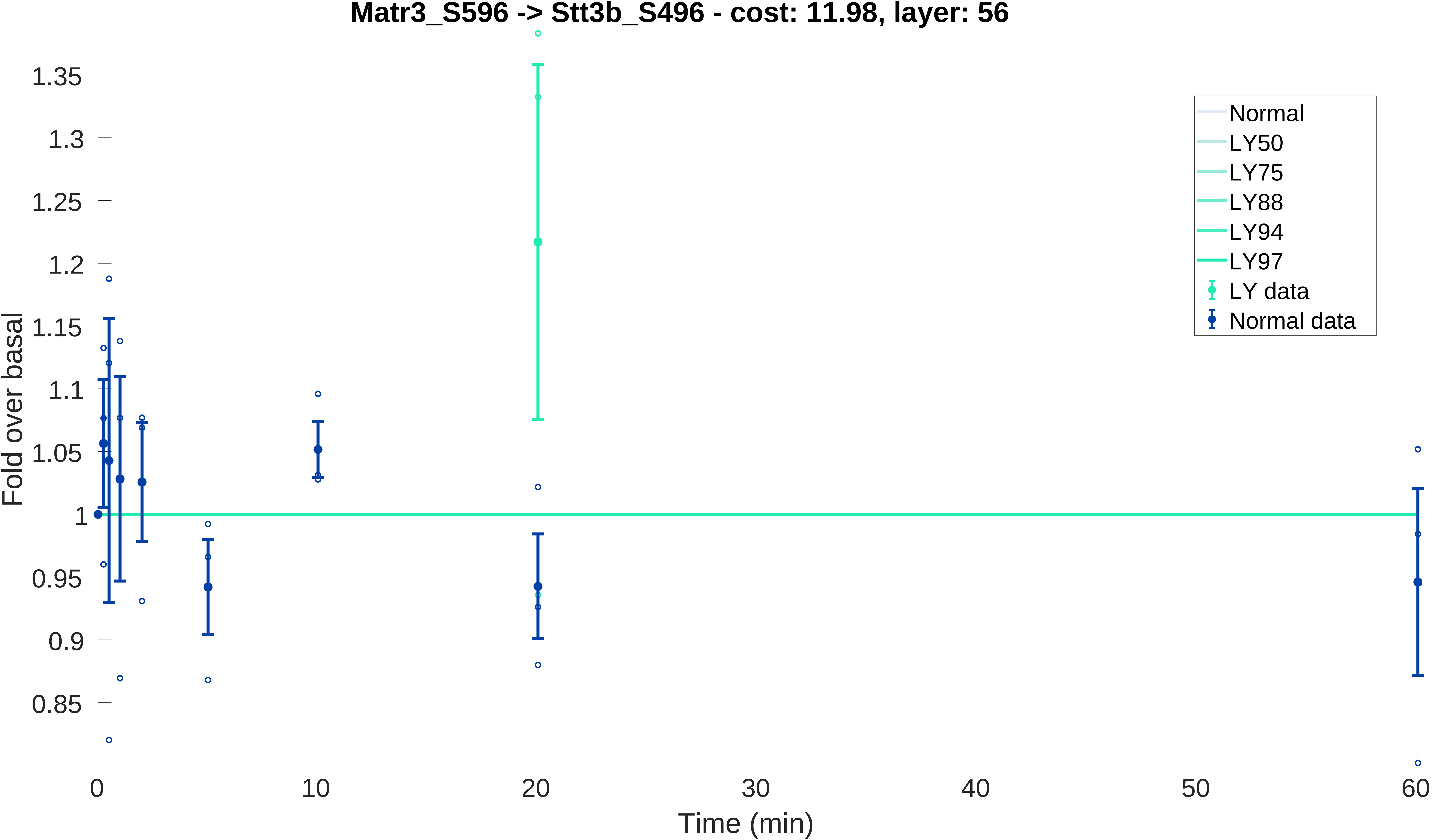
Matr3_S596 -> Stt3b_S496 - cost: 11.98, layer: 56
Fold over basal
1.35
1.3
1.25
1.2
1.15
1.1
1.05
1
0.95
0.9
0.85
0
10
20
30
40
50
60
Time (min)
Normal
LY50
LY75
LY88
LY94
LY97
LY data
Normal data

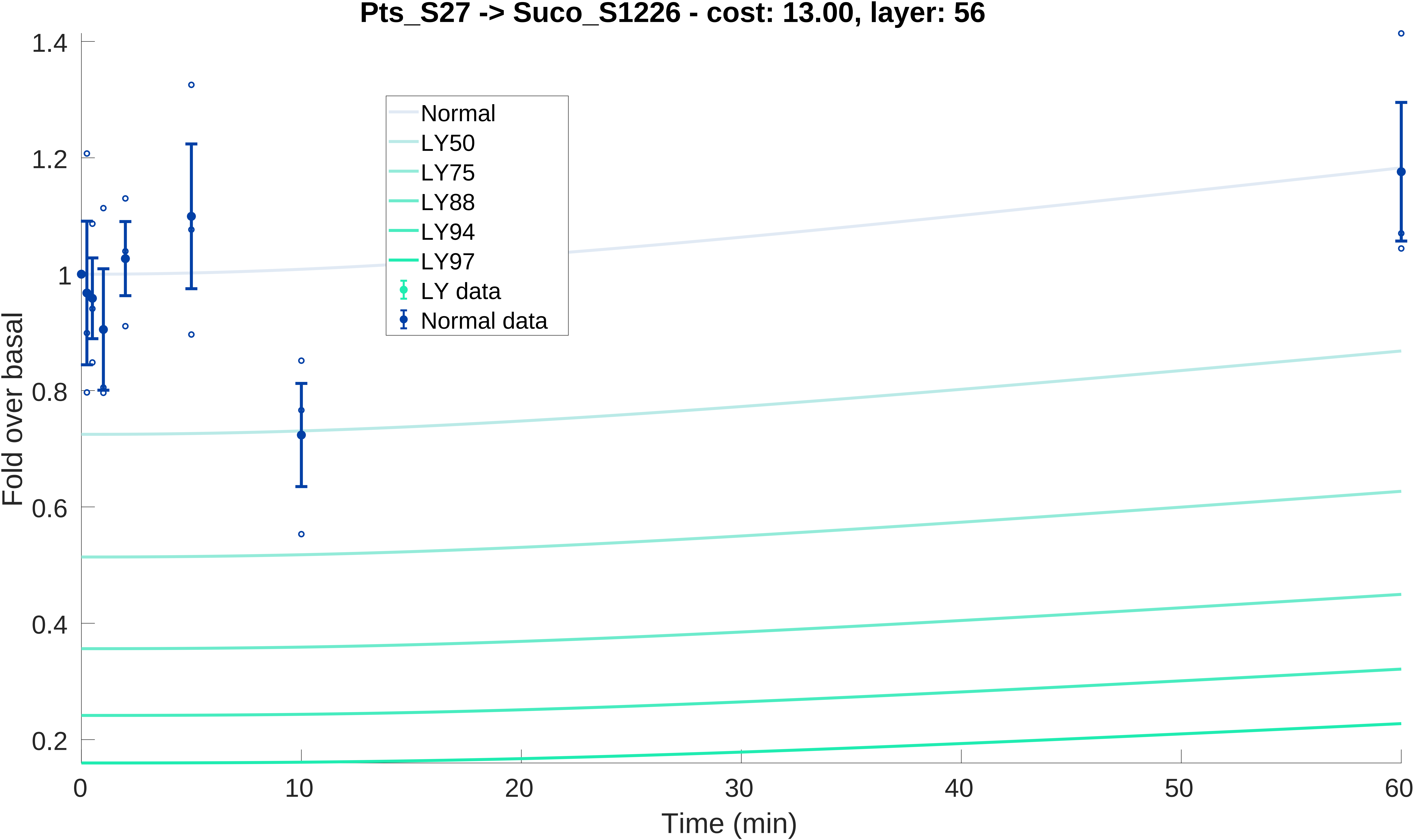

Pts_S27 -> Suco_S1226 - cost: 13.00, layer: 56
Normal
LY50
LY75
LY88
LY94
LY97
LY data
Normal data
Fold over basal
Time (min)
0
10
20
30
40
50
60
0.2
0.4
0.6
0.8
1
1.2
1.4

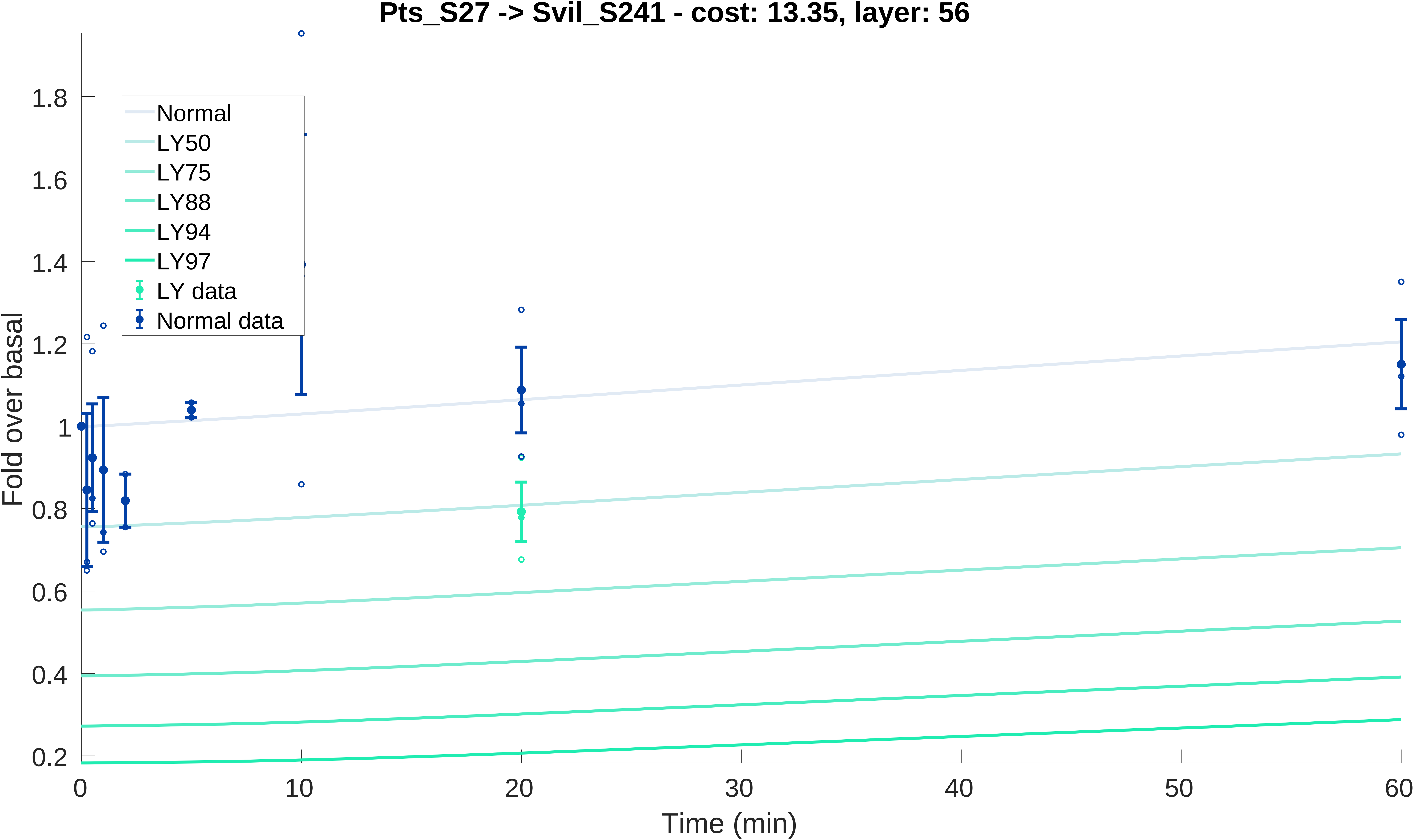

Pts_S27 -> Svil_S241 - cost: 13.35, layer: 56
Fold over basal
Time (min)
Normal
LY50
LY75
LY88
LY94
LY97
LY data
Normal data
1.8
1.6
1.4
1.2
1
0.8
0.6
0.4
0.2
0
10
20
30
40
50
60

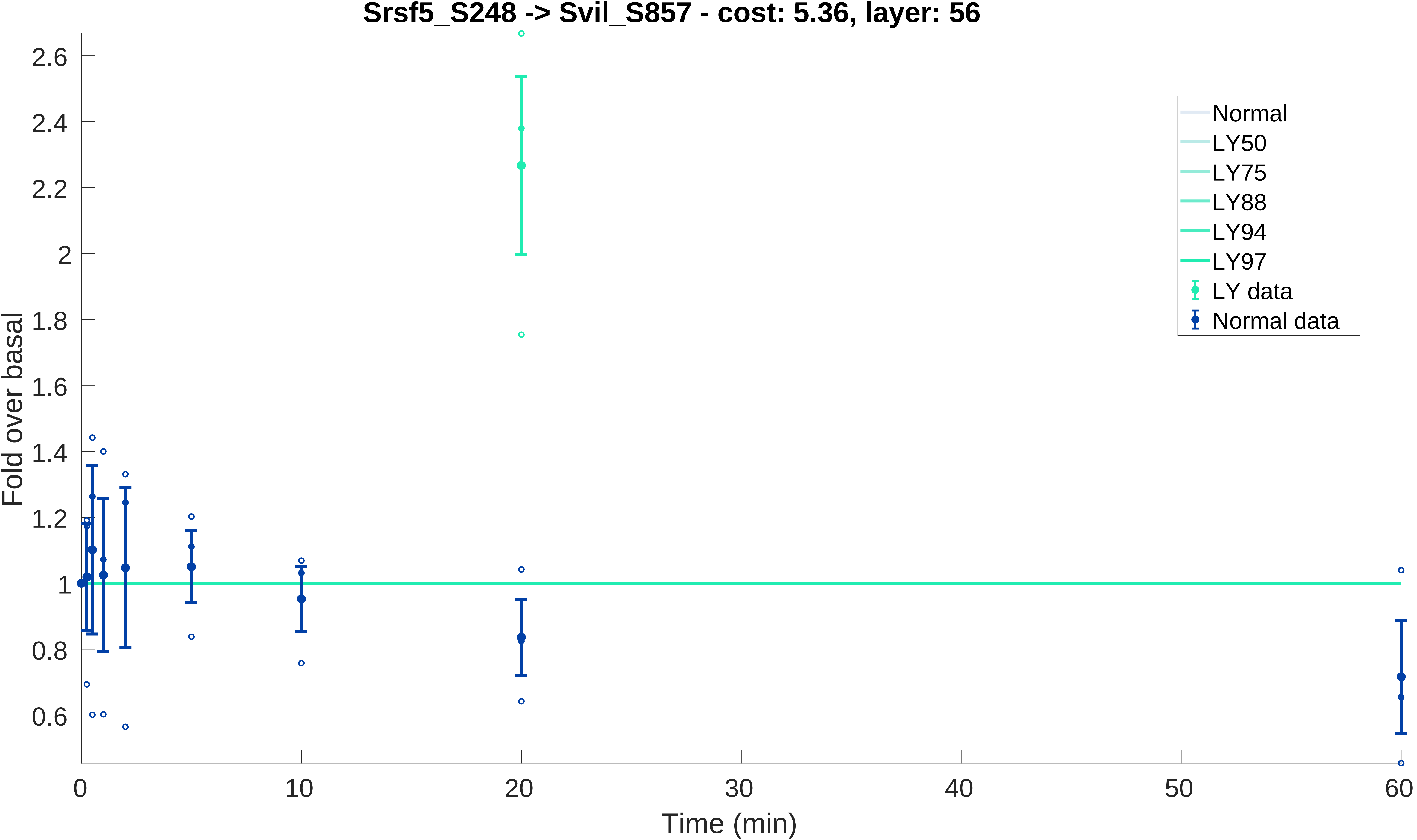
Srsf5_S248 -> Svil_S857 - cost: 5.36, layer: 56
Fold over basal
Time (min)
Normal
LY50
LY75
LY88
LY94
LY97
LY data
Normal data
2.6
2.4
2.2
2
1.8
1.6
1.4
1.2
1
0.8
0.6
0
10
20
30
40
50
60

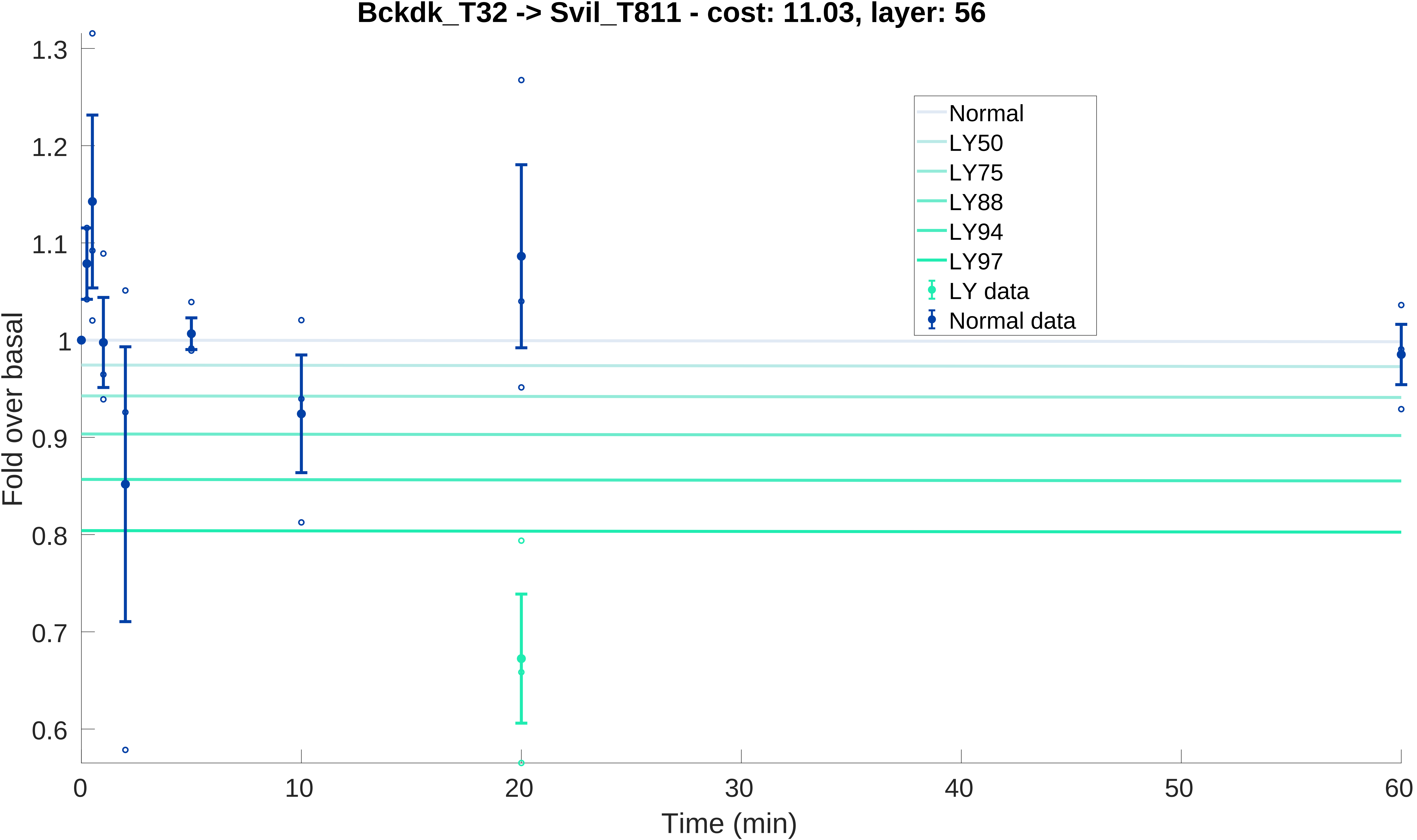

Bckdk_T32 -> Svil_T811 - cost: 11.03, layer: 56
Normal
LY50
LY75
LY88
LY94
LY97
LY data
Normal data
Fold over basal
Time (min)
0
10
20
30
40
50
60
0.6
0.7
0.8
0.9
1
1.1
1.2
1.3

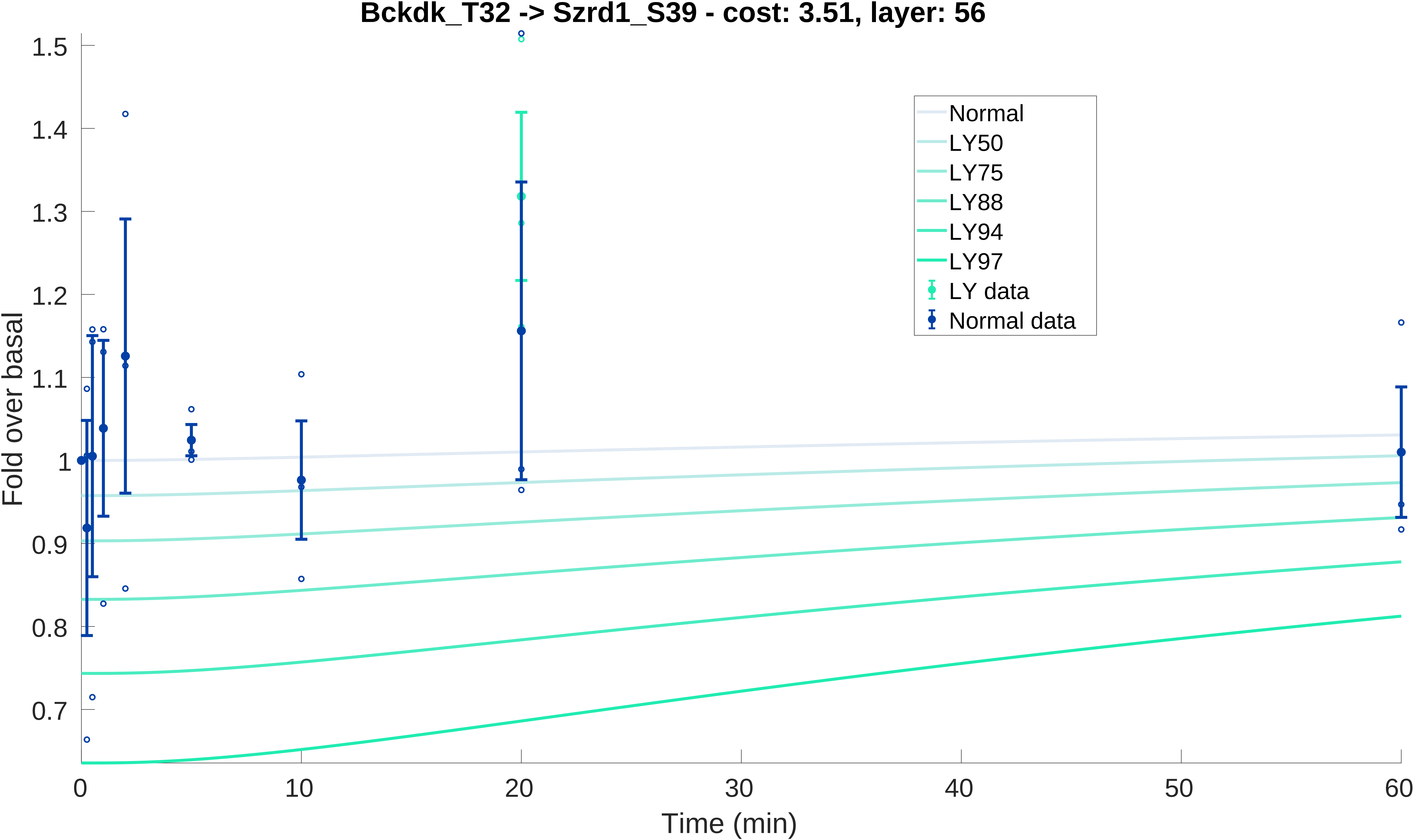

Bckdk_T32 -> Szrd1_S39 - cost: 3.51, layer: 56
Fold over basal
Time (min)
Normal
LY50
LY75
LY88
LY94
LY97
LY data
Normal data
1.5
1.4
1.3
1.2
1.1
1
0.9
0.8
0.7
0
10
20
30
40
50
60

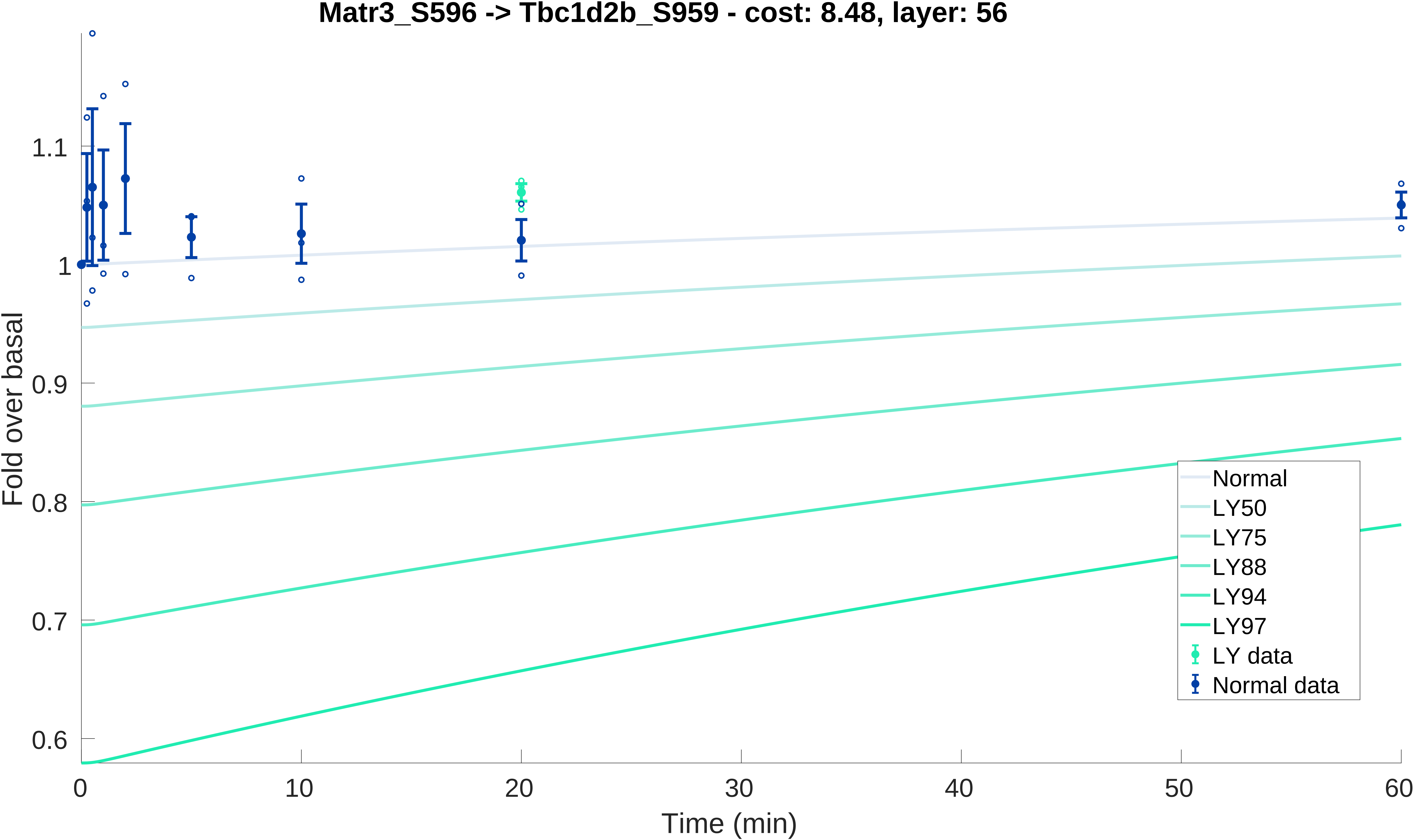

Matr3_S596 -> Tbc1d2b_S959 - cost: 8.48, layer: 56
Fold over basal
Time (min)
1.1
1
0.9
0.8
0.7
0.6
0
10
20
30
40
50
60
Normal
LY50
LY75
LY88
LY94
LY97
LY data
Normal data

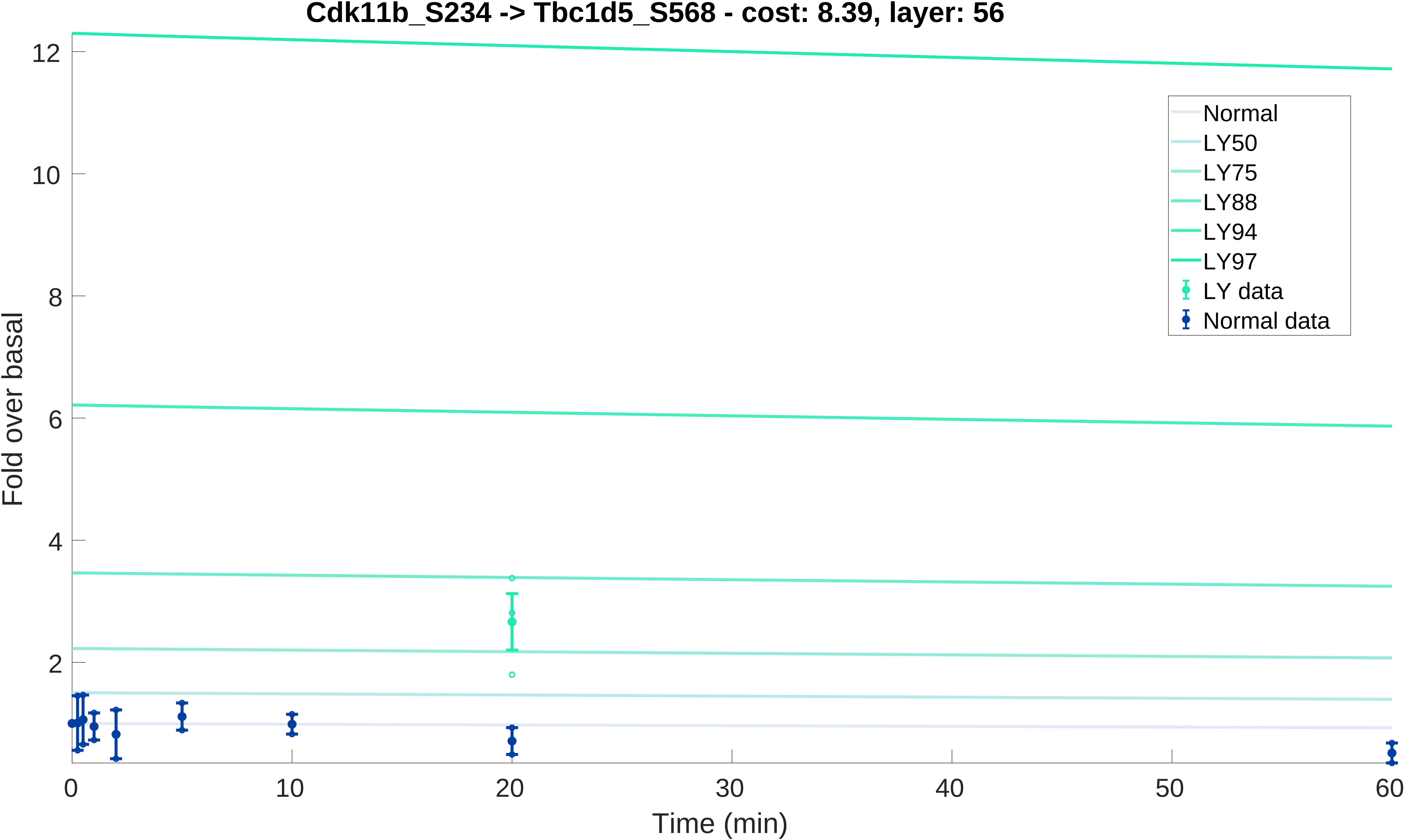

Cdk11b_S234 -> Tbc1d5_S568 - cost: 8.39, layer: 56
Fold over basal
Time (min)
0
10
20
30
40
50
60
2
4
6
8
10
12
Normal
LY50
LY75
LY88
LY94
LY97
LY data
Normal data

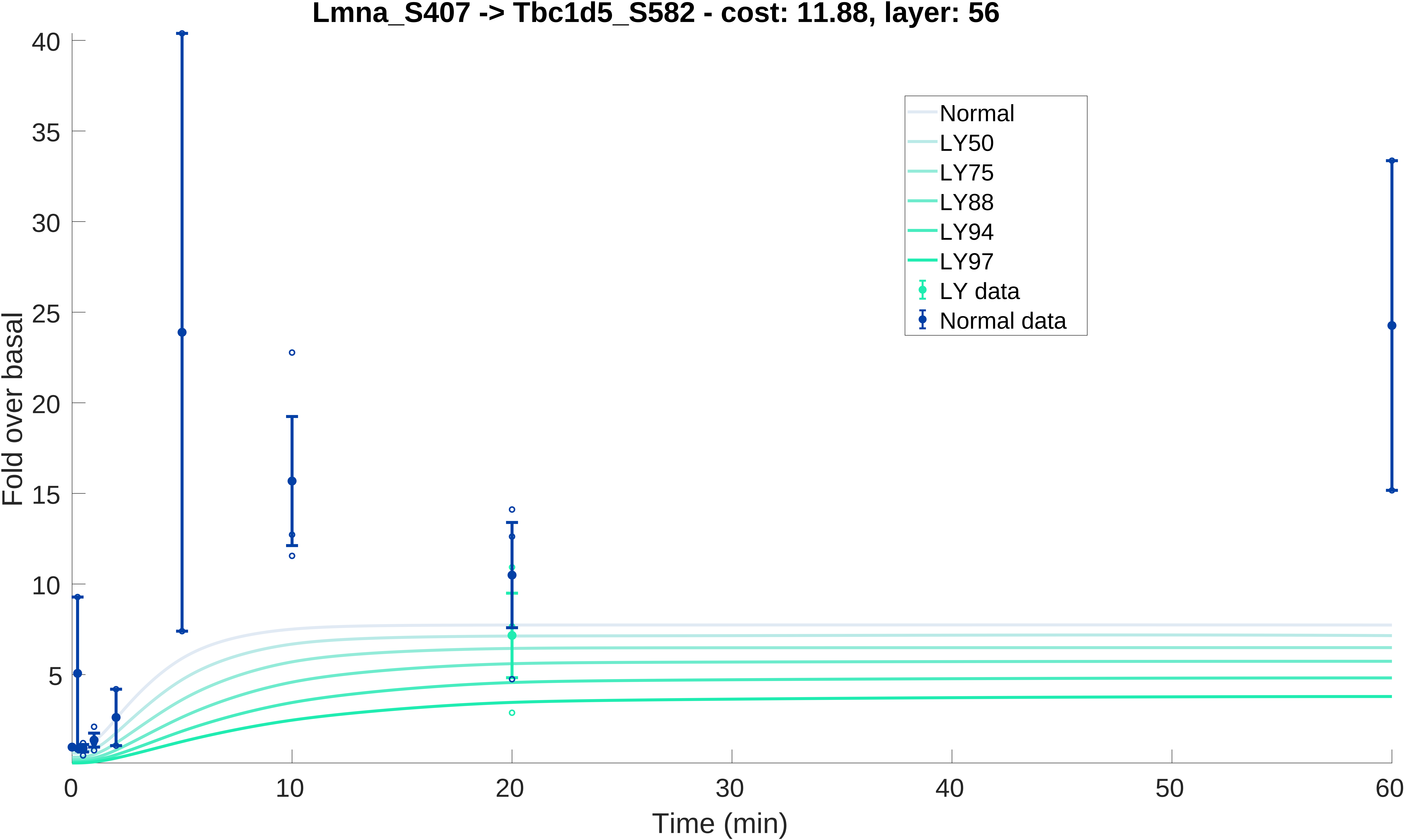

Lmna_S407 -> Tbc1d5_S582 - cost: 11.88, layer: 56
Fold over basal
Time (min)
0
10
20
30
40
50
60
5
10
15
20
25
30
35
40
Normal
LY50
LY75
LY88
LY94
LY97
LY data
Normal data

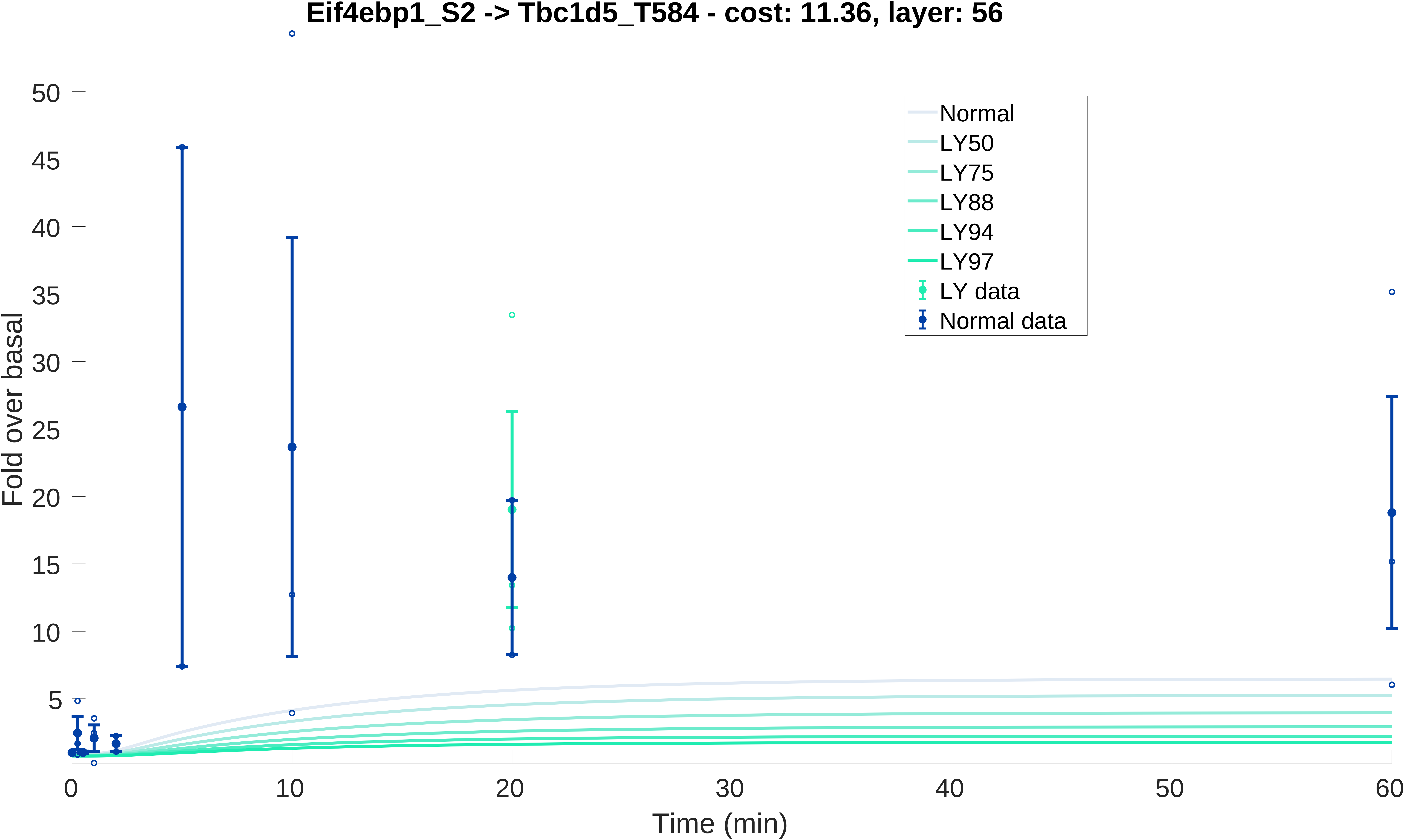

Eif4ebp1_S2 -> Tbc1d5_T584 - cost: 11.36, layer: 56
Normal
LY50
LY75
LY88
LY94
LY97
LY data
Normal data
Fold over basal
5
10
15
20
25
30
35
40
45
50
0
10
20
30
40
50
60
Time (min)

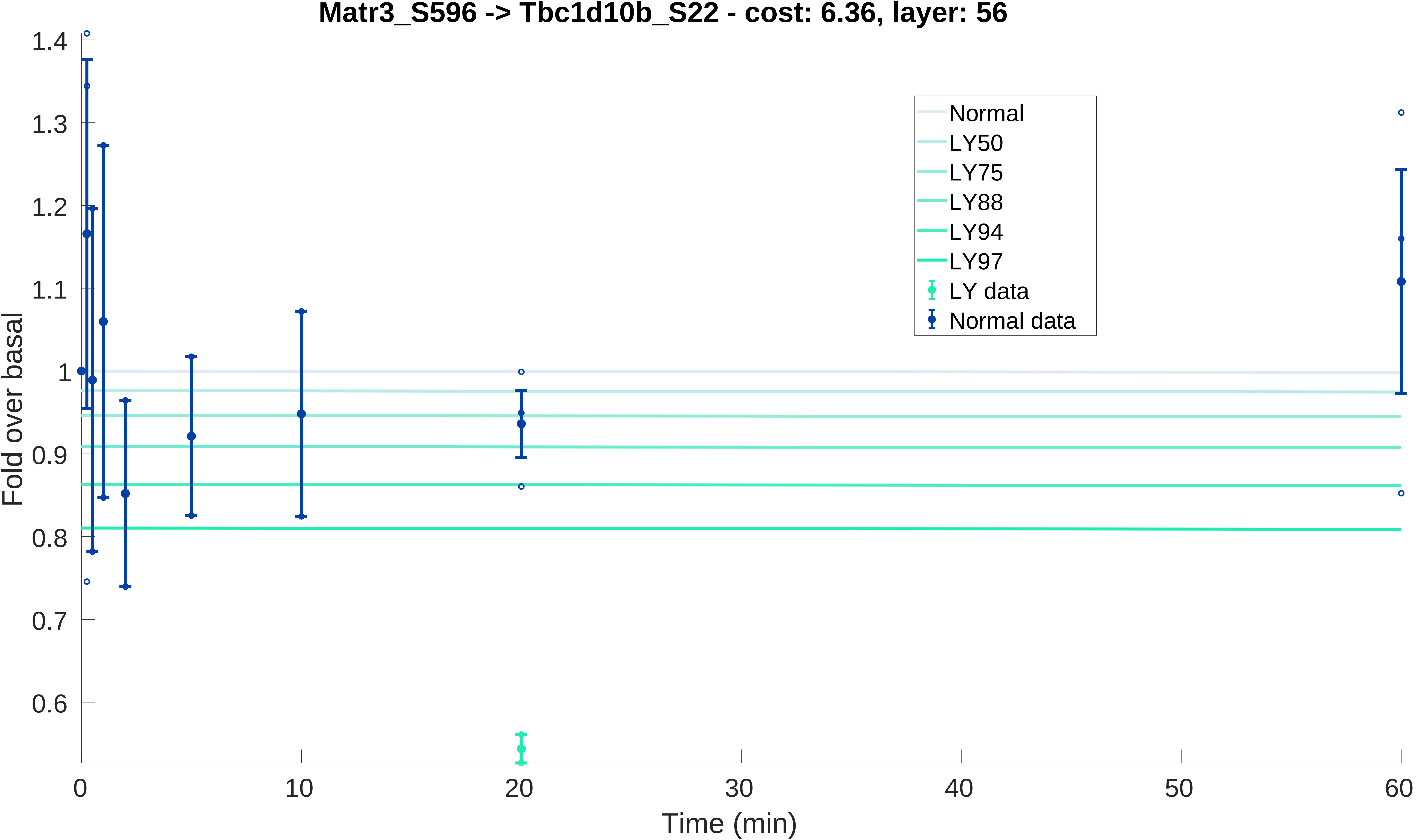
Matr3_S596 -> Tbc1d10b_S22 - cost: 6.36, layer: 56
Fold over basal
1.4
1.3
1.2
1.1
1
0.9
0.8
0.7
0.6
0
10
20
30
40
50
60
Time (min)
Normal
LY50
LY75
LY88
LY94
LY97
LY data
Normal data

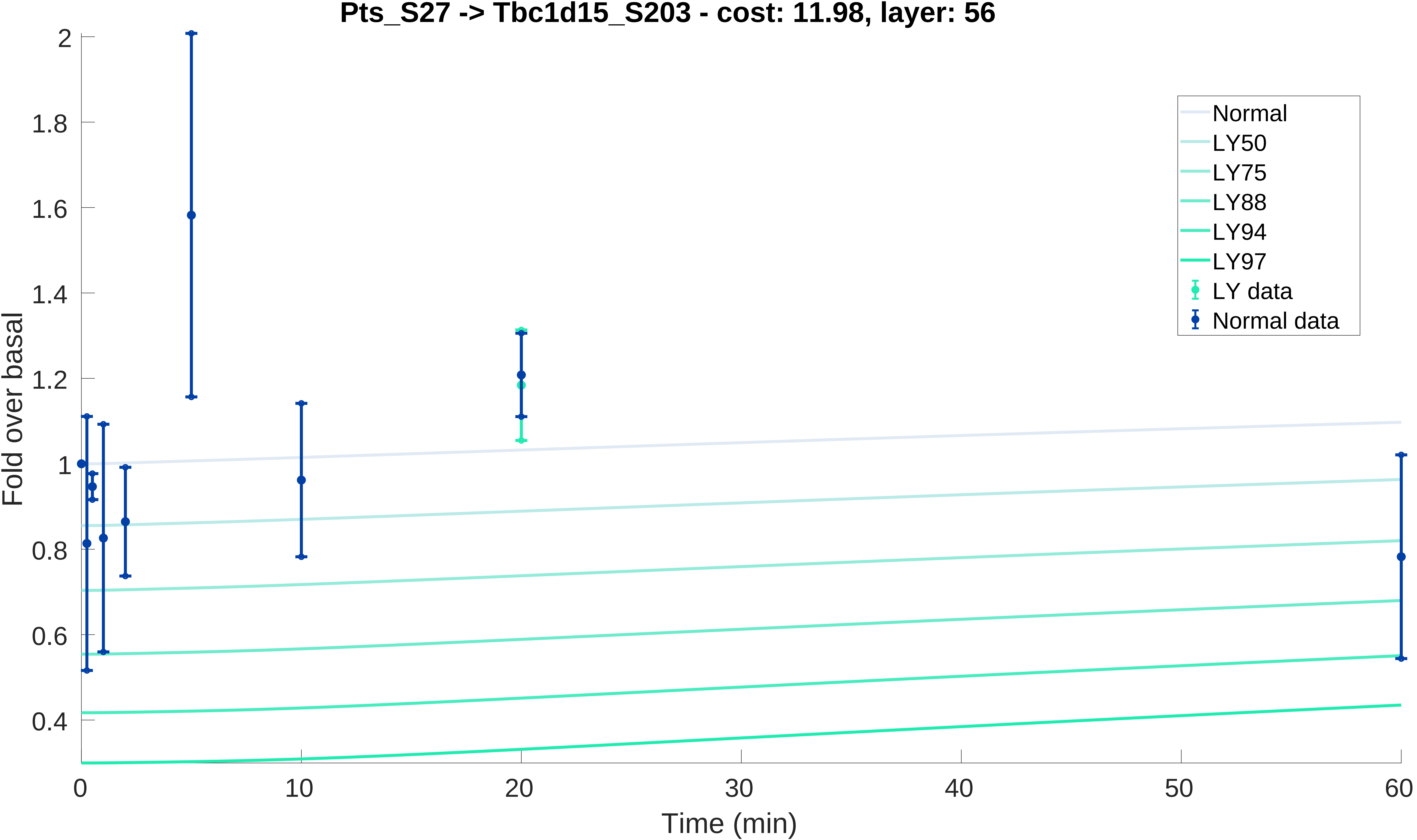

Pts_S27 -> Tbc1d15_S203 - cost: 11.98, layer: 56
Fold over basal
Time (min)
0
10
20
30
40
50
60
0.4
0.6
0.8
1
1.2
1.4
1.6
1.8
2
Normal
LY50
LY75
LY88
LY94
LY97
LY data
Normal data

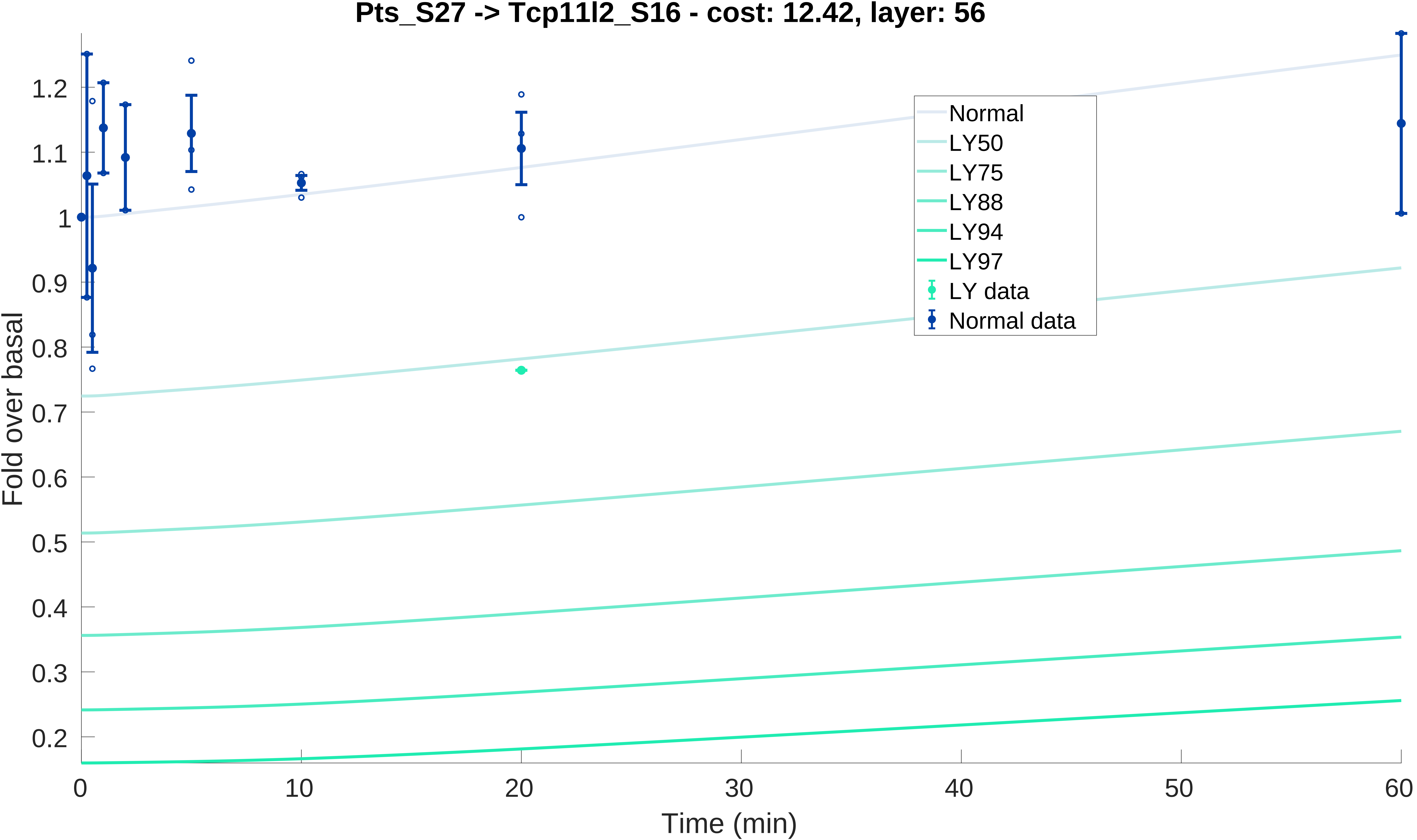

Pts_S27 -> Tcp11l2_S16 - cost: 12.42, layer: 56
Fold over basal
1.2
1.1
1
0.9
0.8
0.7
0.6
0.5
0.4
0.3
0.2
0
10
20
30
40
50
60
Time (min)
Normal
LY50
LY75
LY88
LY94
LY97
LY data
Normal data

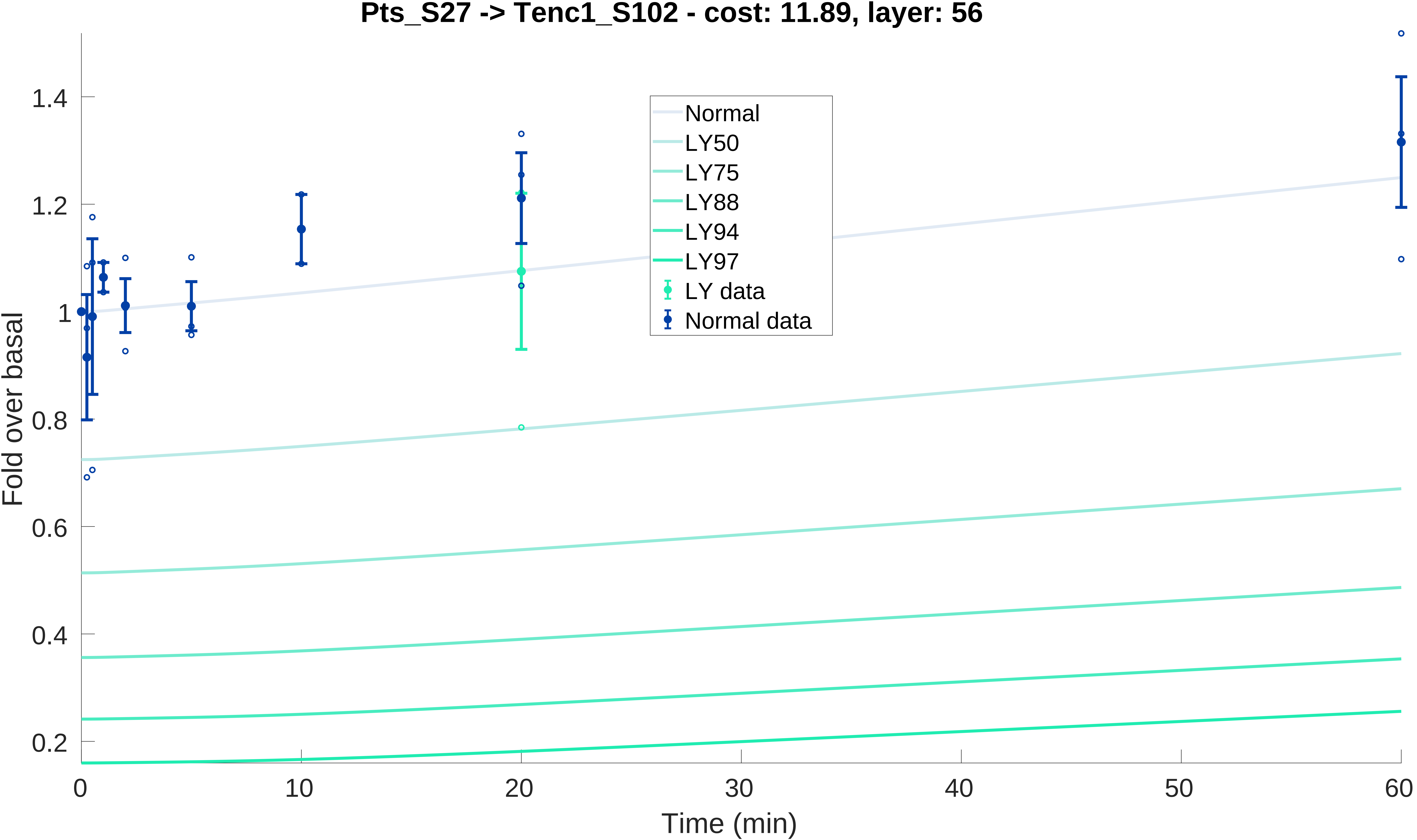
Pts_S27 -> Tenc1_S102 - cost: 11.89, layer: 56
Normal
LY50
LY75
LY88
LY94
LY97
LY data
Normal data
Fold over basal
Time (min)
0.2
0.4
0.6
0.8
1
1.2
1.4
0
10
20
30
40
50
60

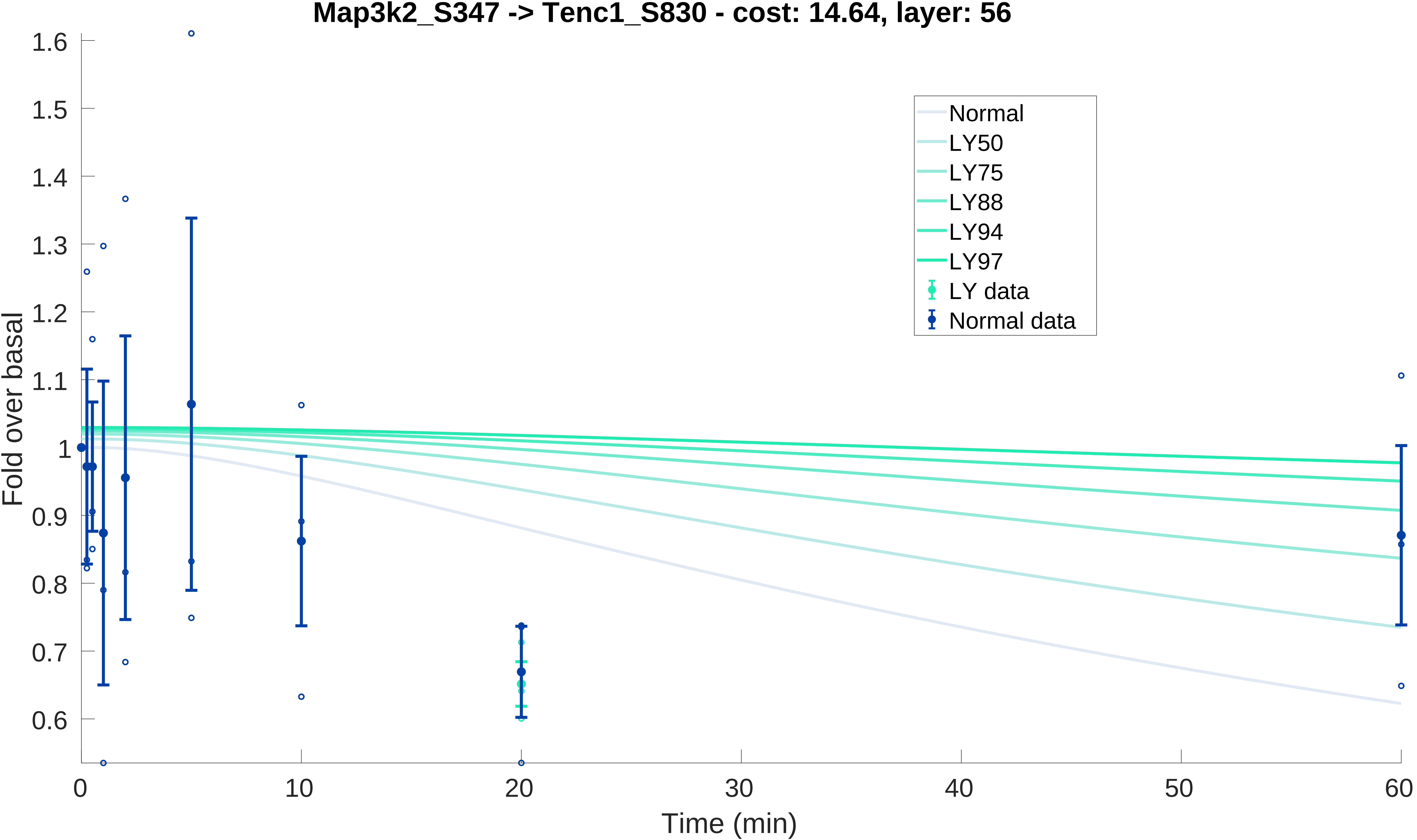

Map3k2_S347 -> Tenc1_S830 - cost: 14.64, layer: 56
Normal
LY50
LY75
LY88
LY94
LY97
LY data
Normal data
Fold over basal
Time (min)
1.6
1.5
1.4
1.3
1.2
1.1
1
0.9
0.8
0.7
0.6
0
10
20
30
40
50
60

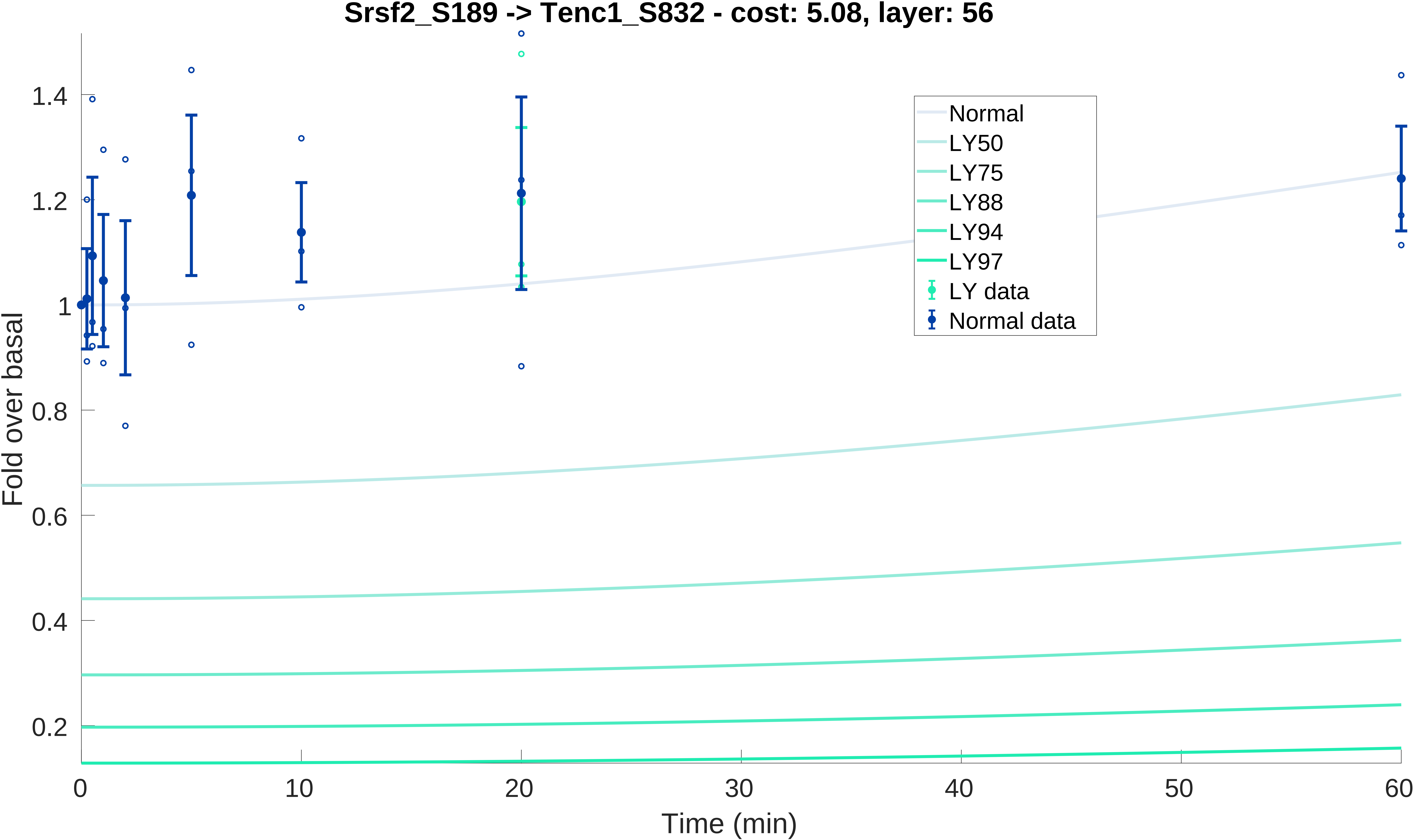

Srsf2_S189 -> Tenc1_S832 - cost: 5.08, layer: 56
Fold over basal
Time (min)
0
10
20
30
40
50
60
0.2
0.4
0.6
0.8
1
1.2
1.4
Normal
LY50
LY75
LY88
LY94
LY97
LY data
Normal data

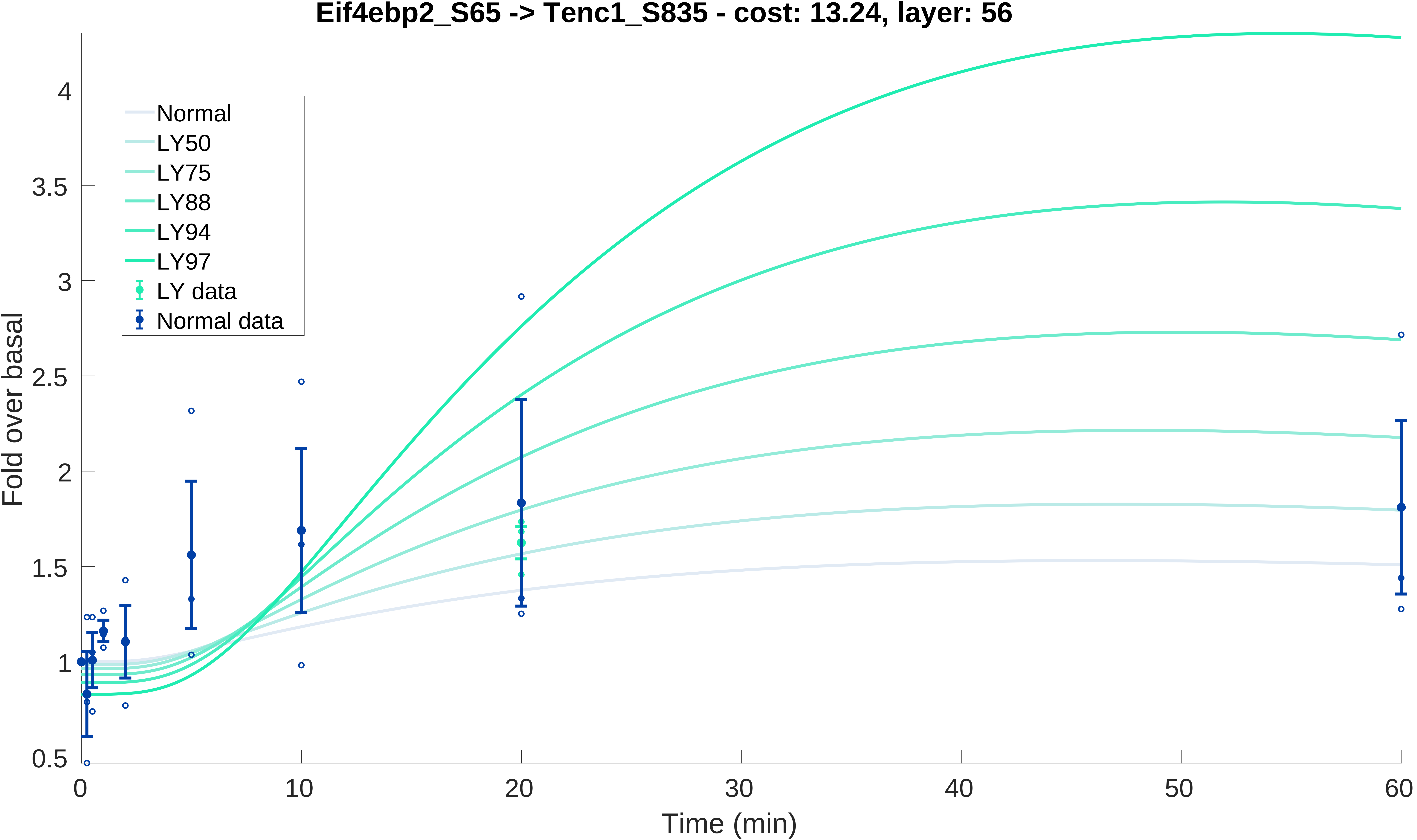
Eif4ebp2_S65 -> Tenc1_S835 - cost: 13.24, layer: 56
Normal
LY50
LY75
LY88
LY94
LY97
LY data
Normal data
Fold over basal
Time (min)
0.5
1
1.5
2
2.5
3
3.5
4
0
10
20
30
40
50
60

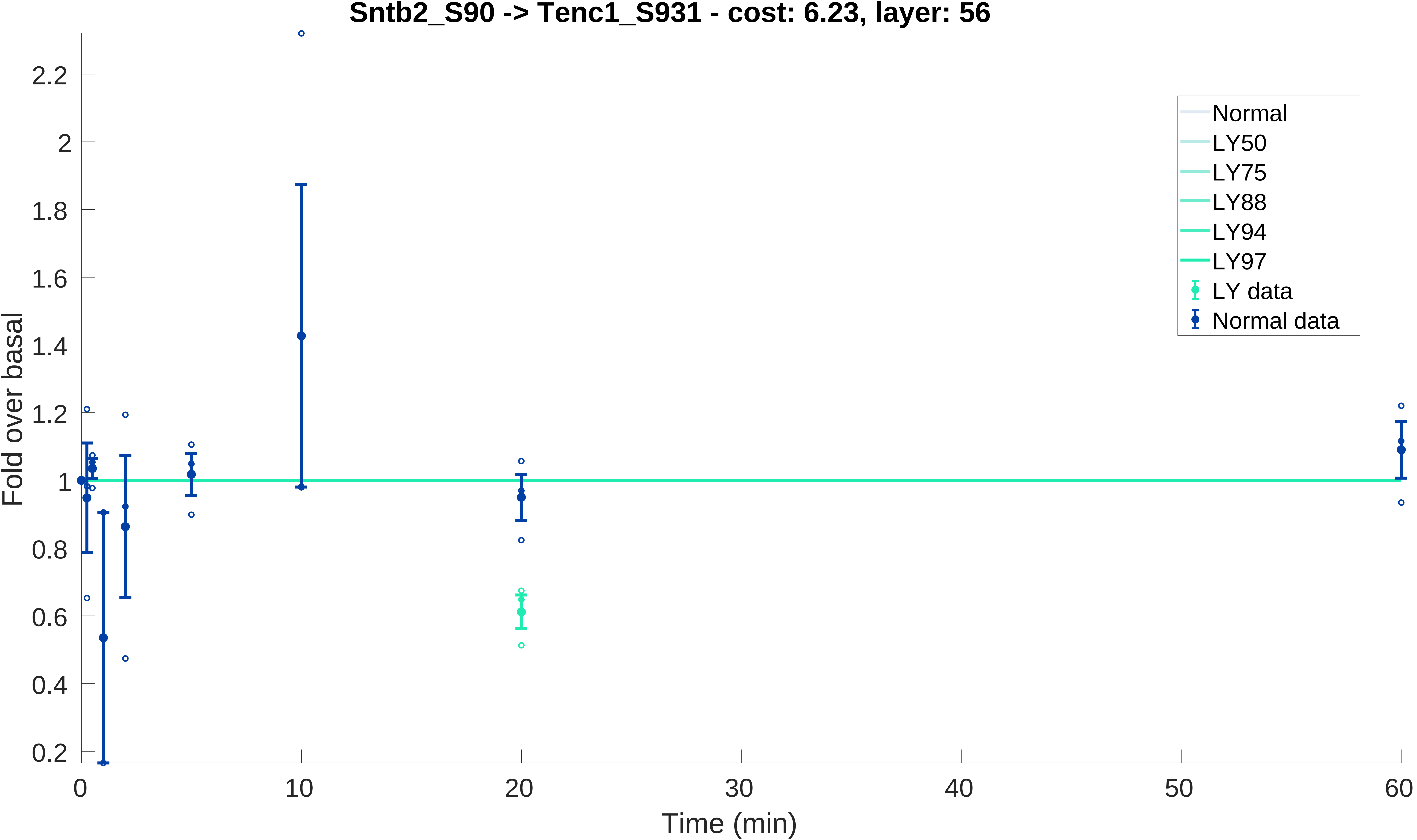

Sntb2_S90 -> Tenc1_S931 - cost: 6.23, layer: 56
Fold over basal
Time (min)
0.2
0.4
0.6
0.8
1
1.2
1.4
1.6
1.8
2
2.2
0
10
20
30
40
50
60
Normal
LY50
LY75
LY88
LY94
LY97
LY data
Normal data

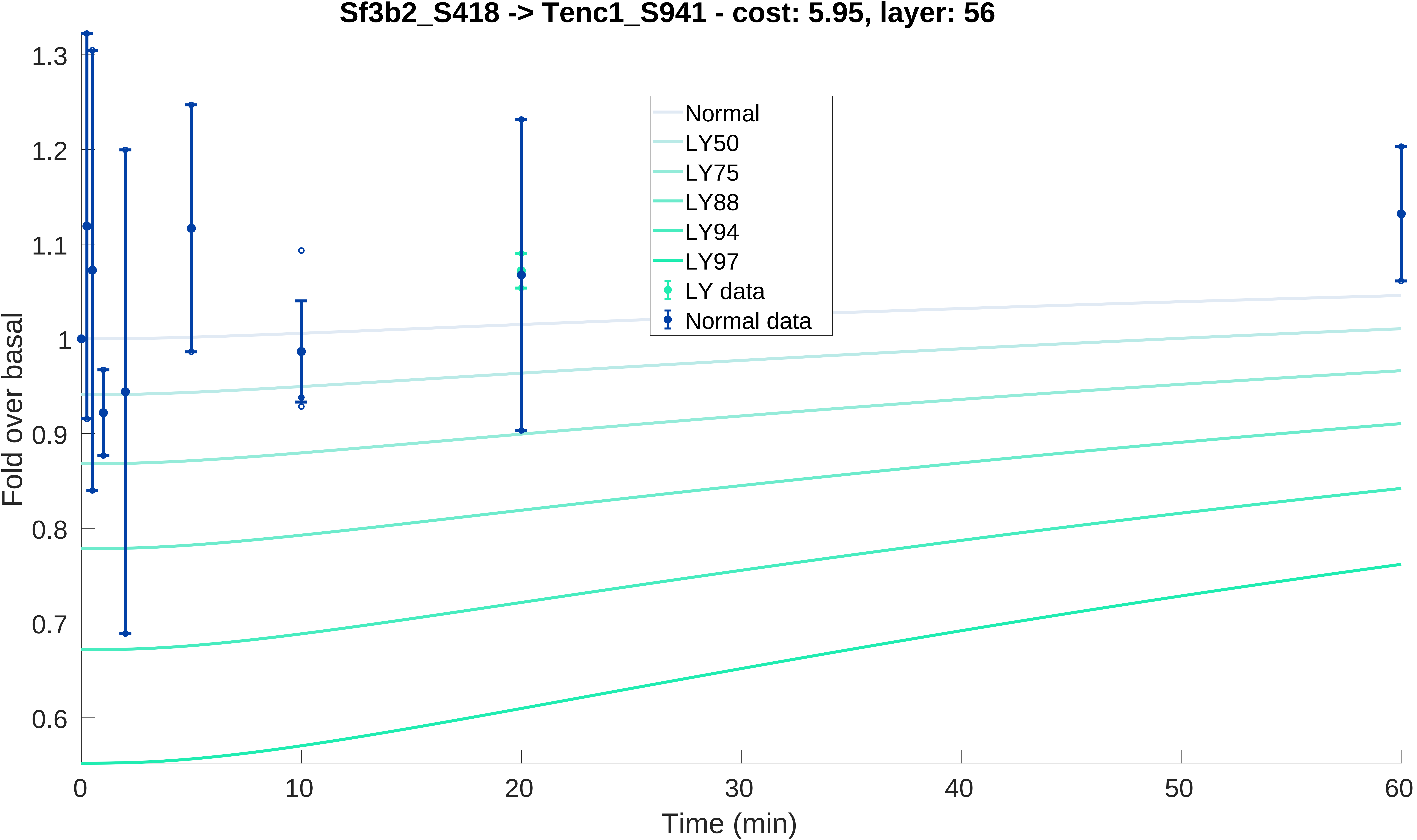

Sf3b2_S418 -> Tenc1_S941 - cost: 5.95, layer: 56
Fold over basal
Time (min)
1.3
1.2
1.1
1
0.9
0.8
0.7
0.6
0
10
20
30
40
50
60
Normal
LY50
LY75
LY88
LY94
LY97
LY data
Normal data

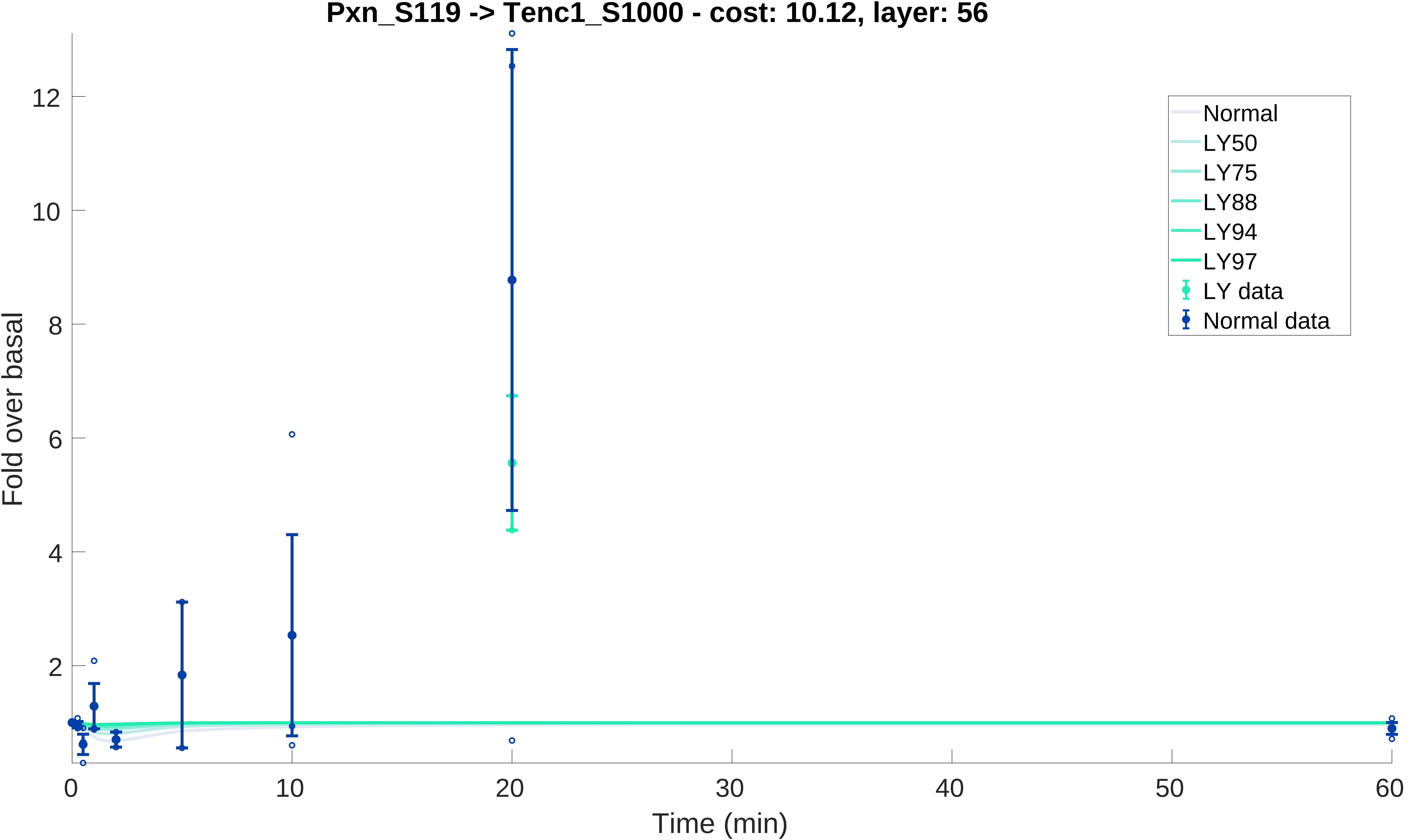

Pxn_S119 -> Tenc1_S1000 - cost: 10.12, layer: 56
Fold over basal
Time (min)
0
10
20
30
40
50
60
2
4
6
8
10
12
Normal
LY50
LY75
LY88
LY94
LY97
LY data
Normal data

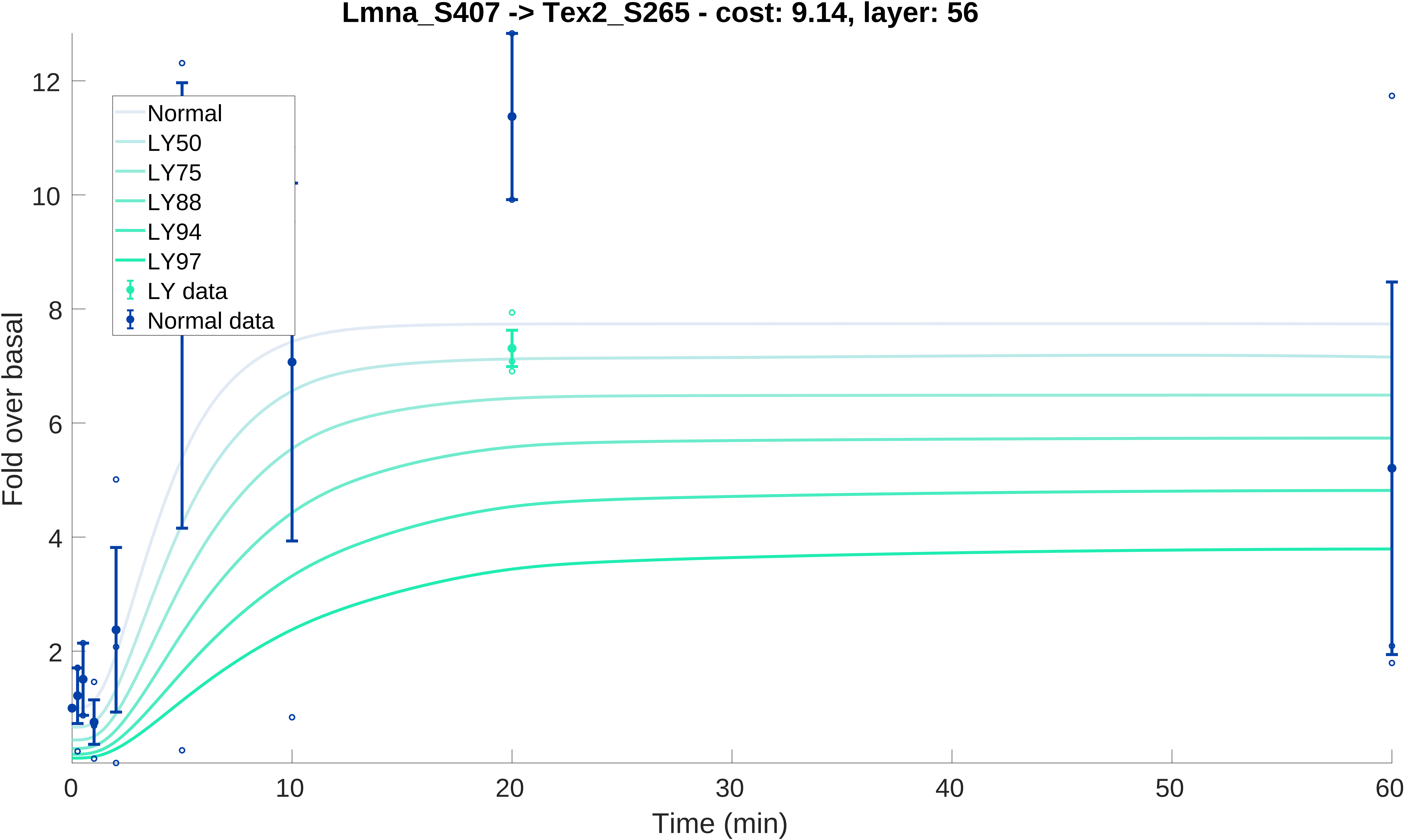

Lmna_S407 -> Tex2_S265 - cost: 9.14, layer: 56
Normal
LY50
LY75
LY88
LY94
LY97
LY data
Normal data
Fold over basal
Time (min)
0
10
20
30
40
50
60
2
4
6
8
10
12

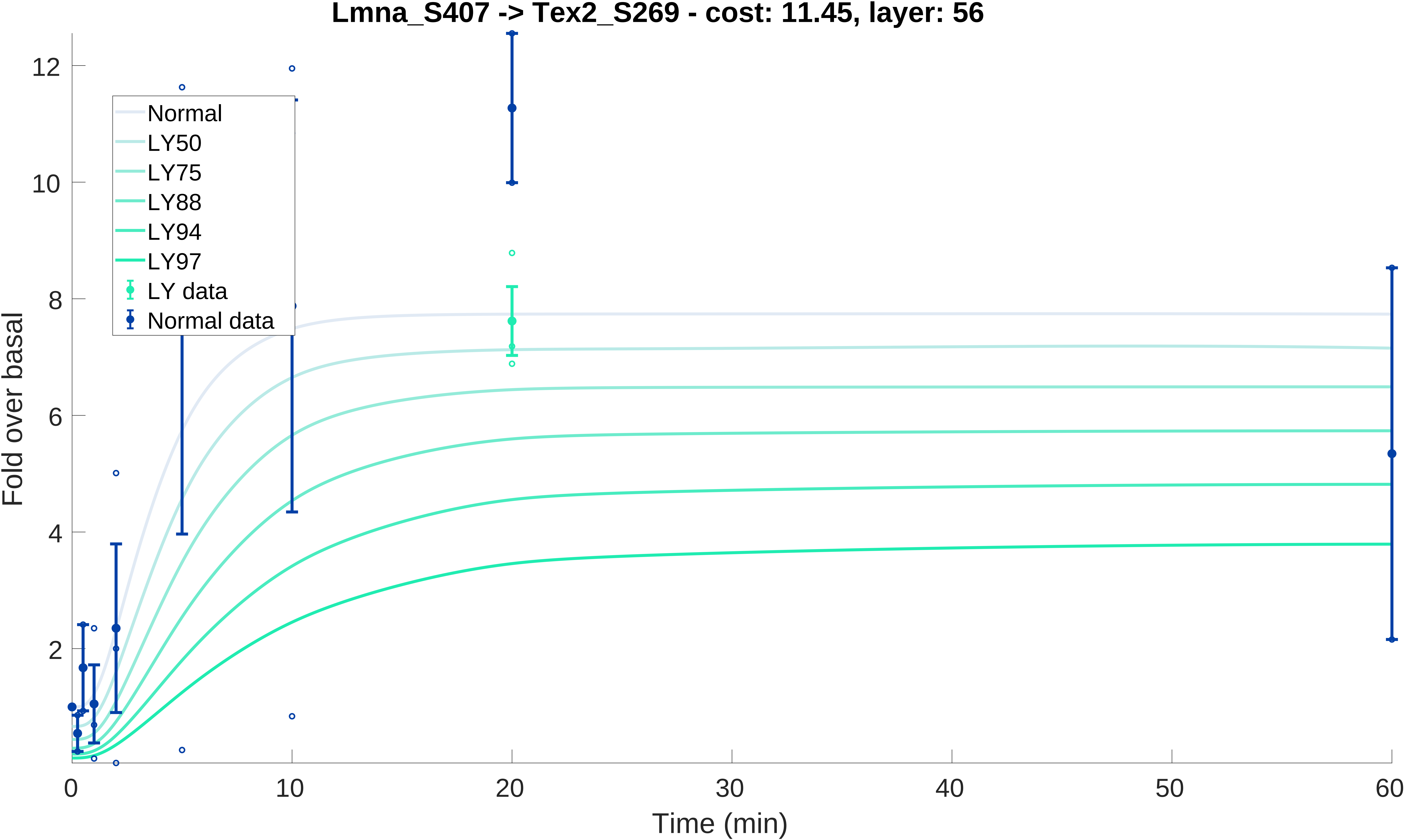

Lmna_S407 -> Tex2_S269 - cost: 11.45, layer: 56
Normal
LY50
LY75
LY88
LY94
LY97
LY data
Normal data
Fold over basal
Time (min)
0
10
20
30
40
50
60
2
4
6
8
10
12

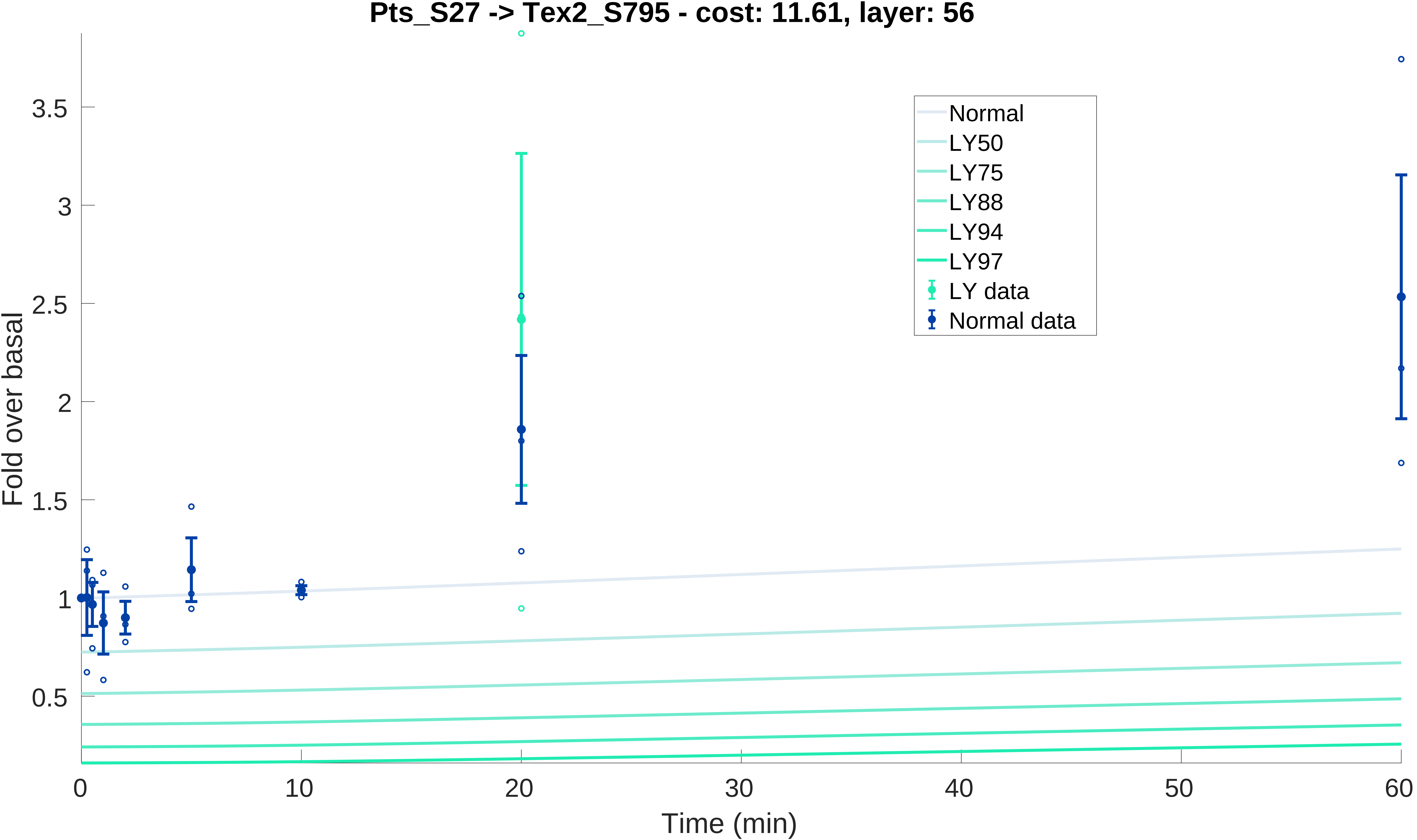

Pts_S27 -> Tex2_S795 - cost: 11.61, layer: 56
Fold over basal
Time (min)
Normal
LY50
LY75
LY88
LY94
LY97
LY data
Normal data
0.5
1
1.5
2
2.5
3
3.5
0
10
20
30
40
50
60

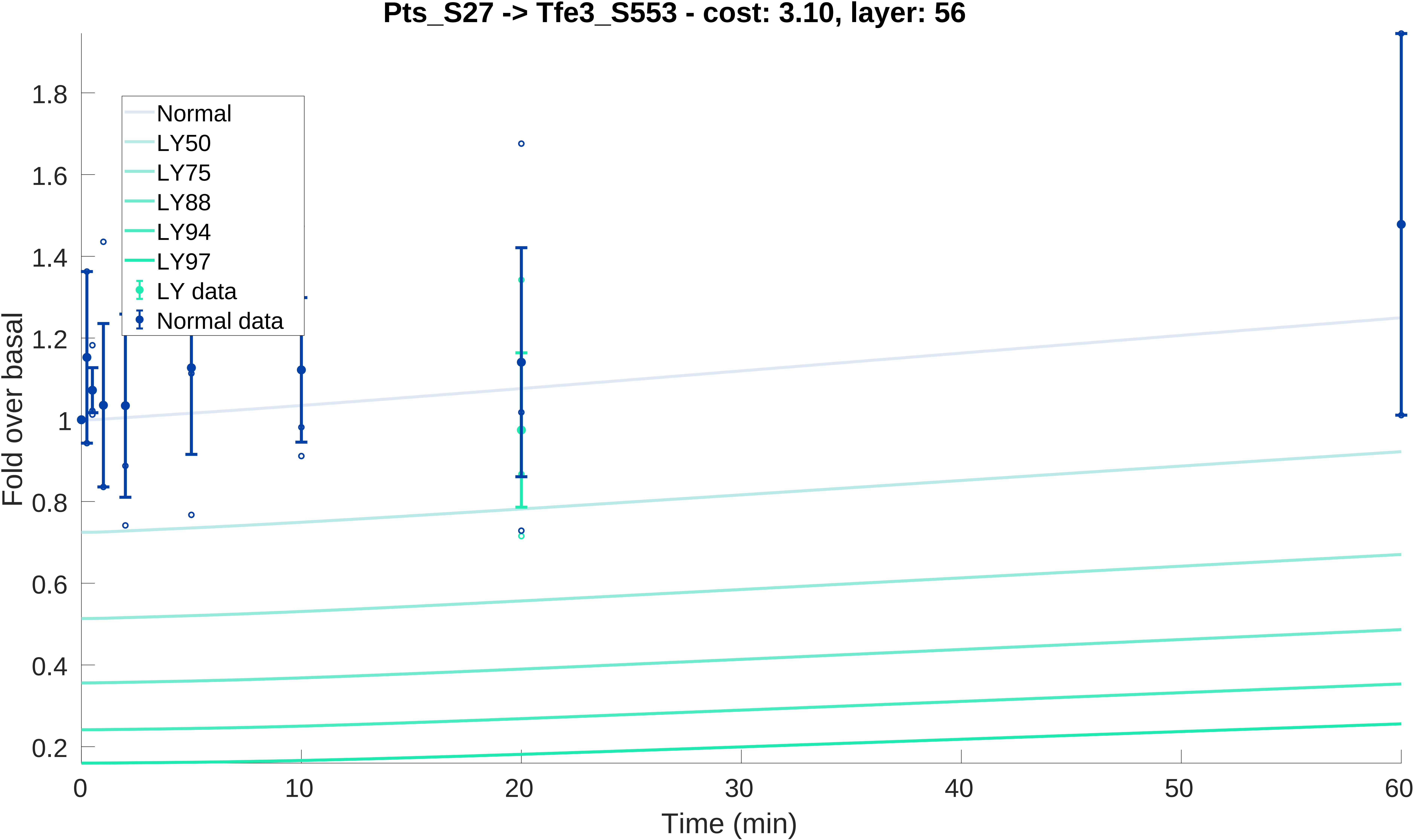

Pts_S27 -> Tfe3_S553 - cost: 3.10, layer: 56
Normal
LY50
LY75
LY88
LY94
LY97
LY data
Normal data
Fold over basal
1.8
1.6
1.4
1.2
1
0.8
0.6
0.4
0.2
0
10
20
30
40
50
60
Time (min)

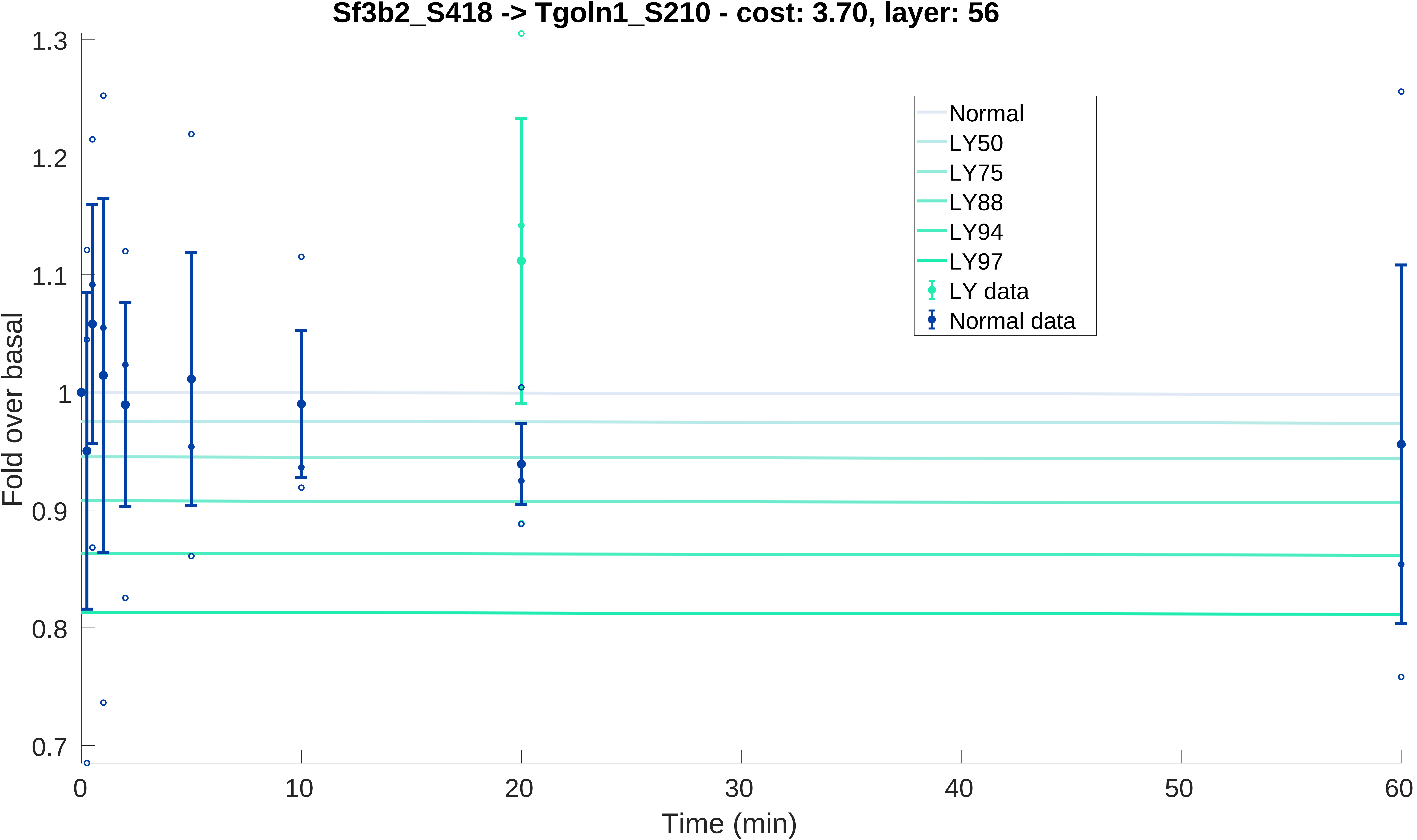

Sf3b2_S418 -> Tgoln1_S210 - cost: 3.70, layer: 56
Fold over basal
Time (min)
1.3
1.2
1.1
1
0.9
0.8
0.7
0
10
20
30
40
50
60
Normal
LY50
LY75
LY88
LY94
LY97
LY data
Normal data

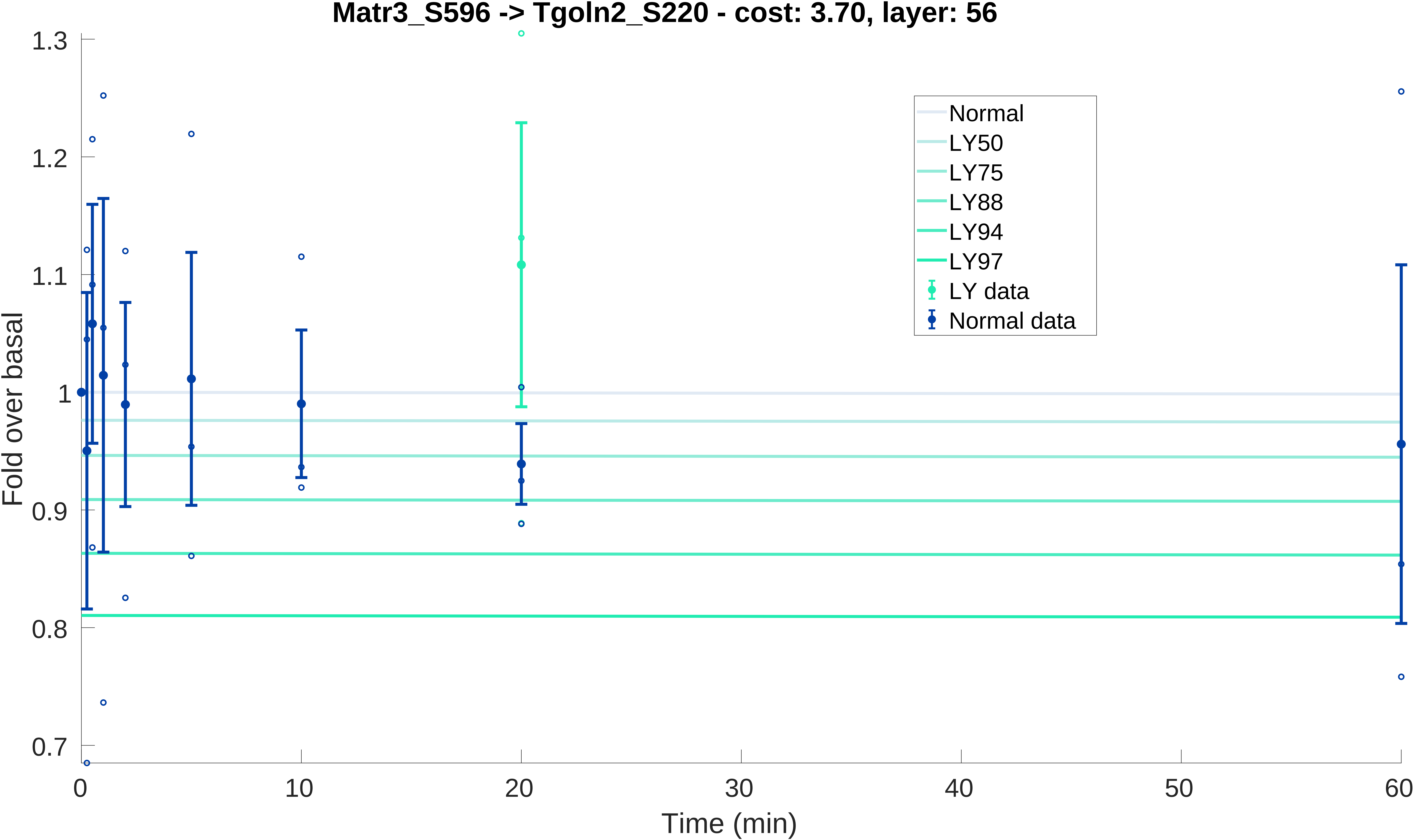
Matr3_S596 -> Tgoln2_S220 - cost: 3.70, layer: 56
Fold over basal
1.3
1.2
1.1
1
0.9
0.8
0.7
0
10
20
30
40
50
60
Time (min)
Normal
LY50
LY75
LY88
LY94
LY97
LY data
Normal data

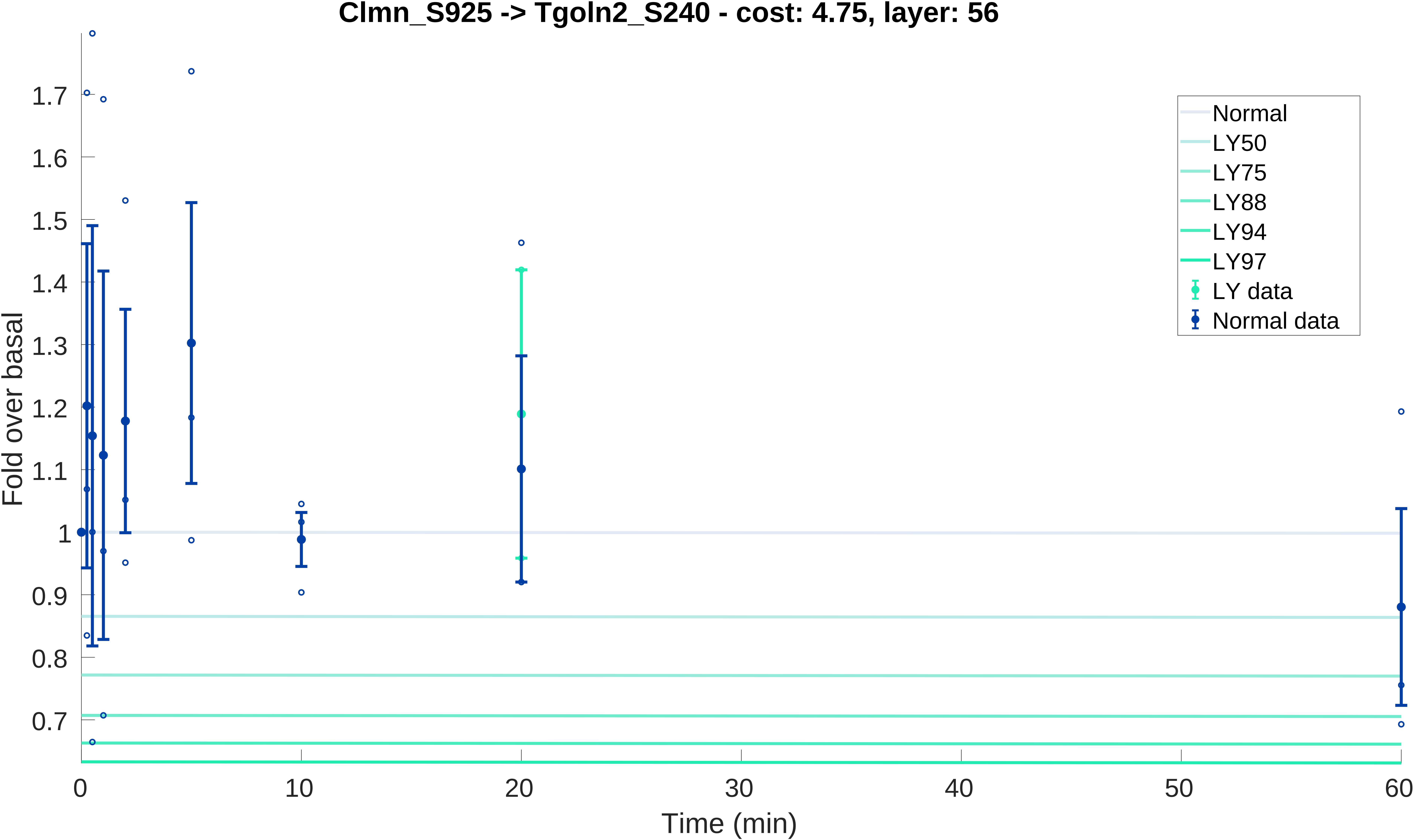
Clmn_S925 -> Tgoln2_S240 - cost: 4.75, layer: 56
Fold over basal
Time (min)
Normal
LY50
LY75
LY88
LY94
LY97
LY data
Normal data
0.7
0.8
0.9
1
1.1
1.2
1.3
1.4
1.5
1.6
1.7
0
10
20
30
40
50
60

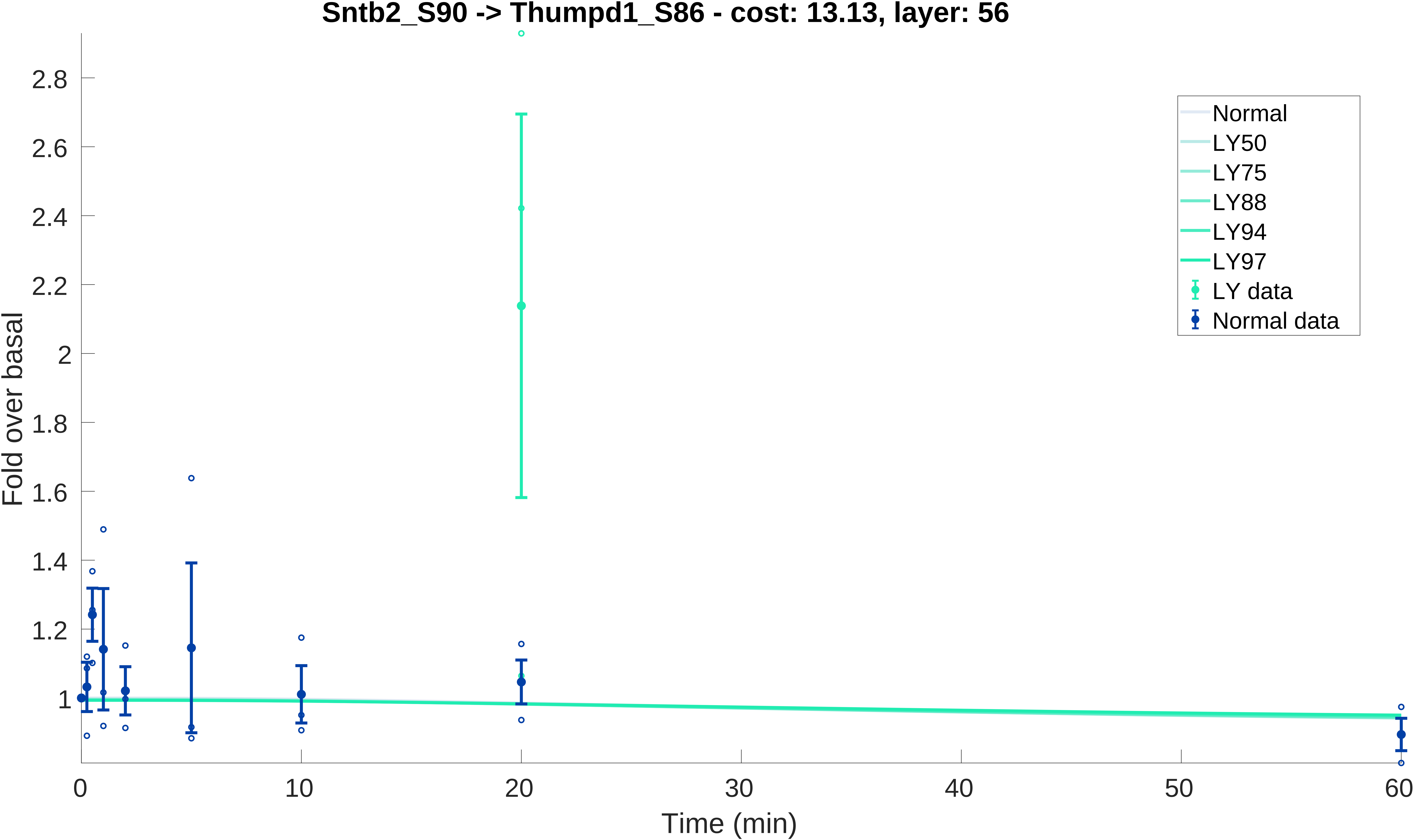
Sntb2_S90 -> Thumpd1_S86 - cost: 13.13, layer: 56
Fold over basal
2.8
2.6
2.4
2.2
2
1.8
1.6
1.4
1.2
1
Time (min)
0
10
20
30
40
50
60
Normal
LY50
LY75
LY88
LY94
LY97
LY data
Normal data

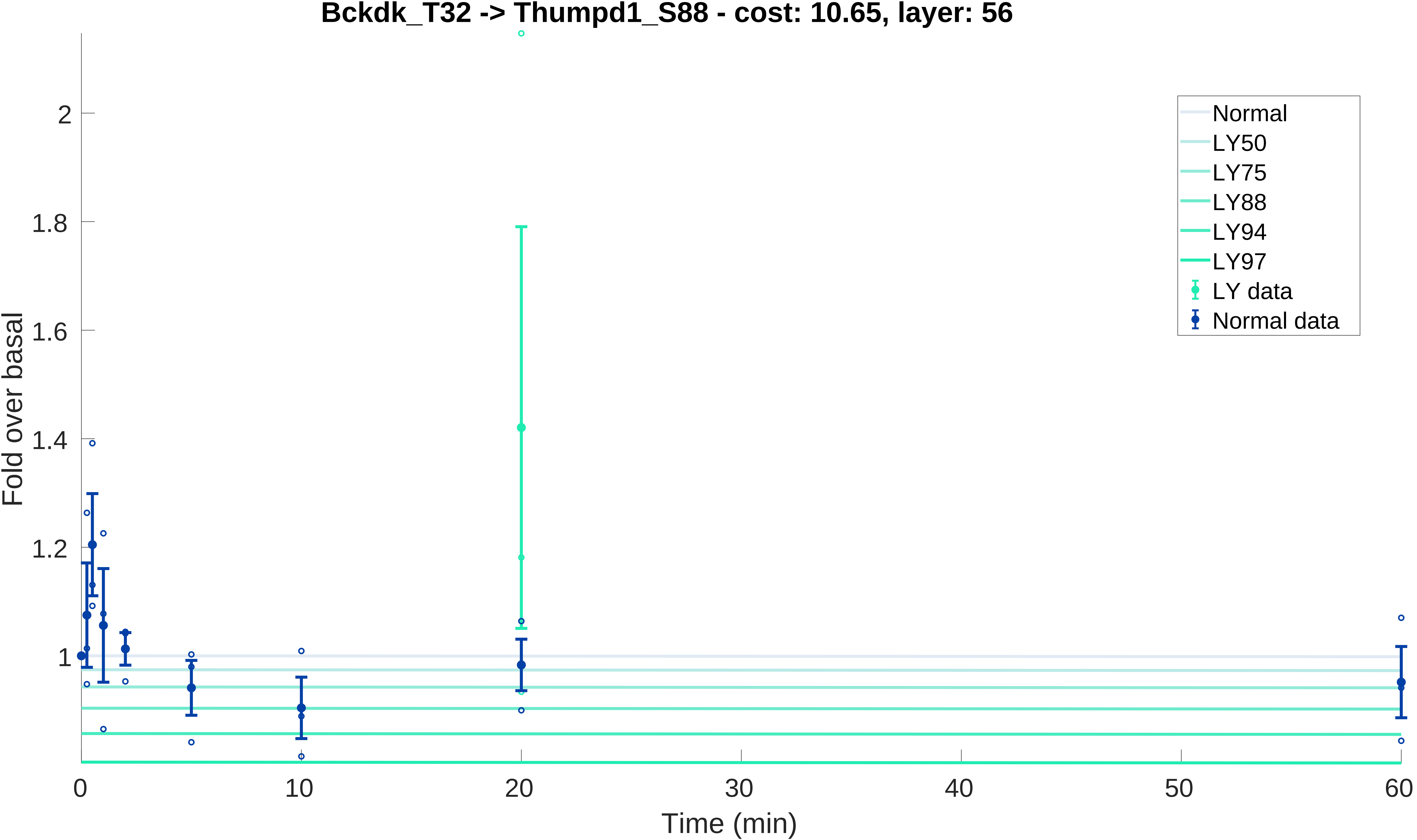
Bckdk_T32 -> Thumpd1_S88 - cost: 10.65, layer: 56
Fold over basal
Time (min)
Normal
LY50
LY75
LY88
LY94
LY97
LY data
Normal data
2
1.8
1.6
1.4
1.2
1
0
10
20
30
40
50
60

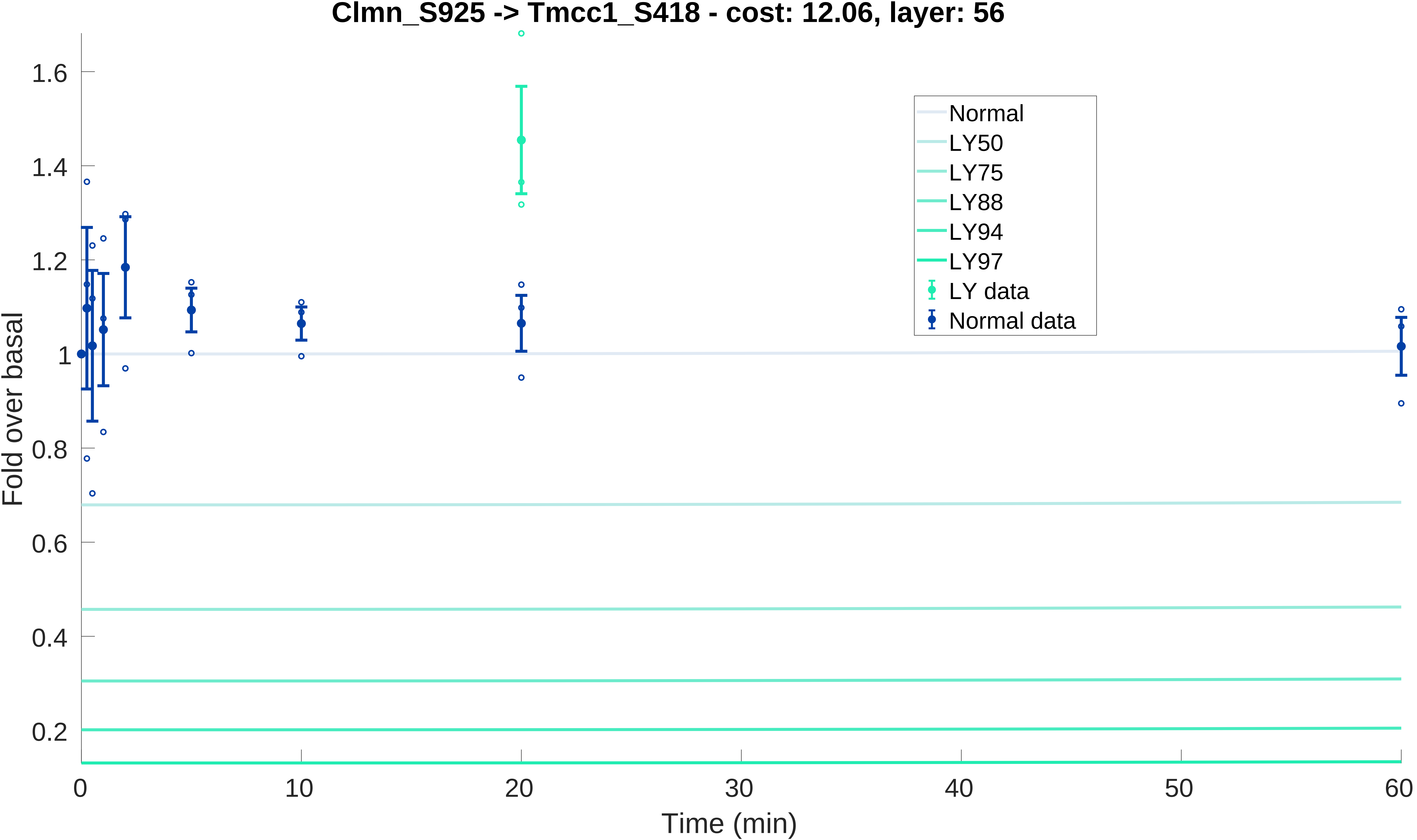

Clmn_S925 -> Tmcc1_S418 - cost: 12.06, layer: 56
Fold over basal
Time (min)
0.2
0.4
0.6
0.8
1
1.2
1.4
1.6
0
10
20
30
40
50
60
Normal
LY50
LY75
LY88
LY94
LY97
LY data
Normal data

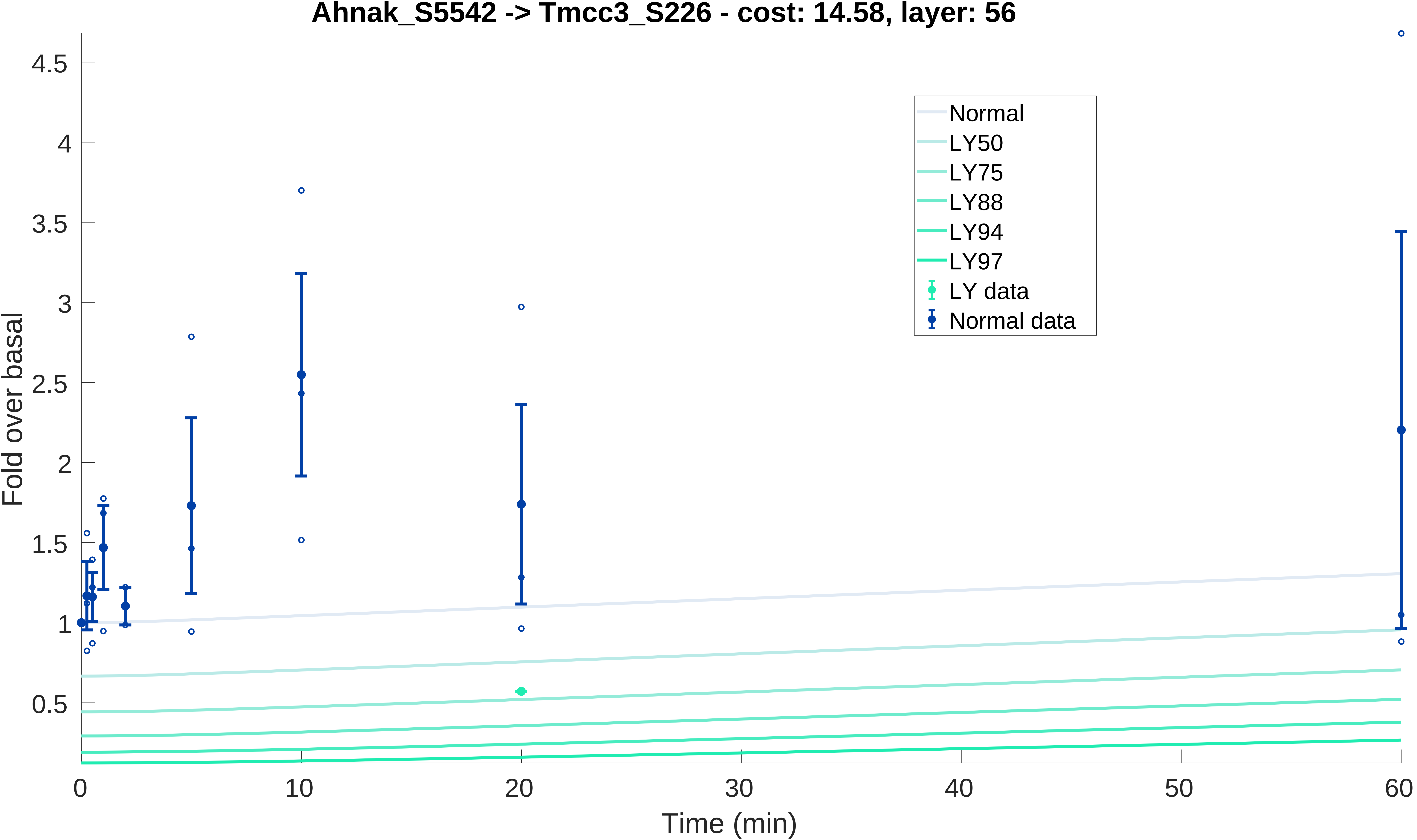

Ahnak_S5542 -> Tmcc3_S226 - cost: 14.58, layer: 56
Fold over basal
Time (min)
0.5
1
1.5
2
2.5
3
3.5
4
4.5
0
10
20
30
40
50
60
Normal
LY50
LY75
LY88
LY94
LY97
LY data
Normal data

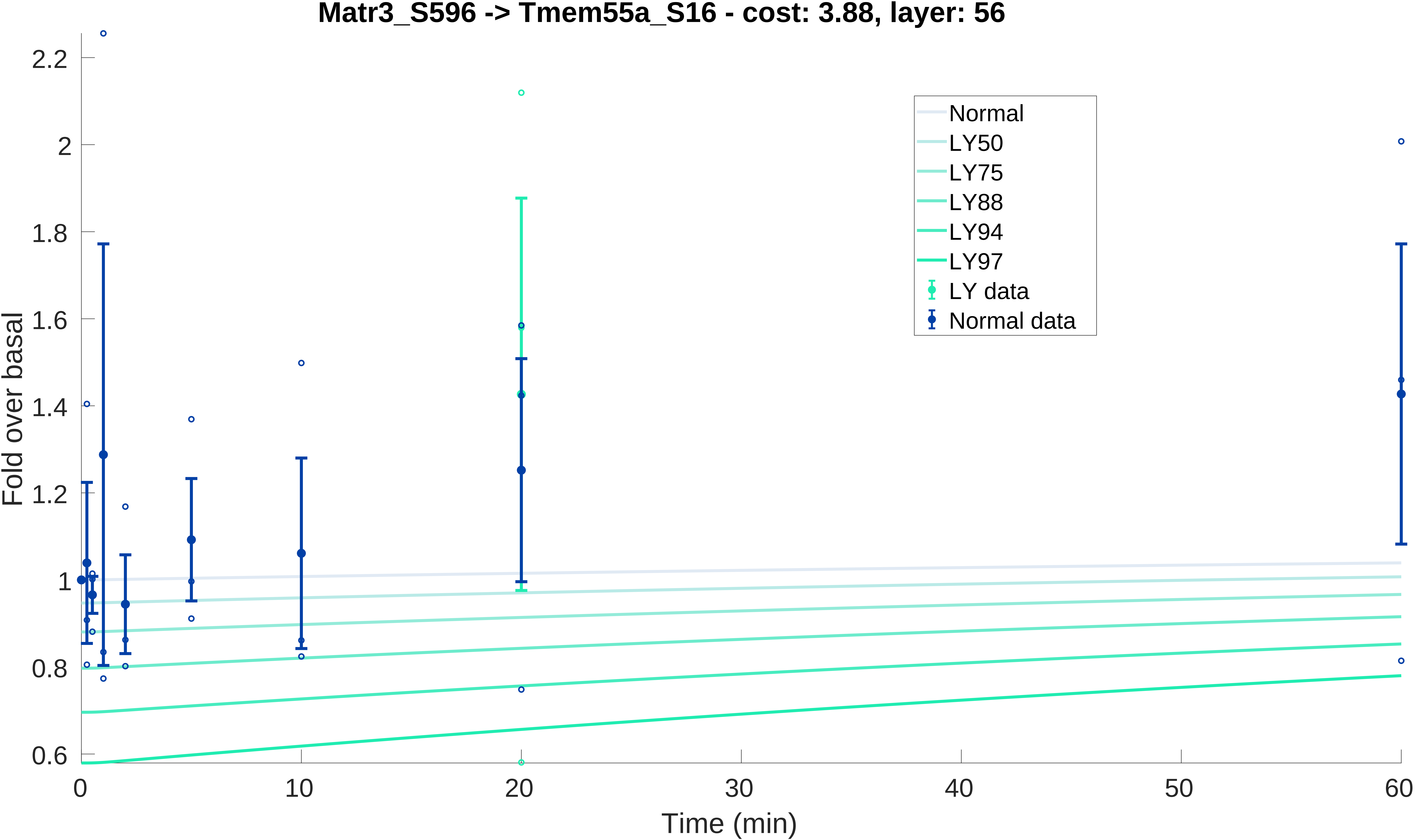

Matr3_S596 -> Tmem55a_S16 - cost: 3.88, layer: 56
Fold over basal
Time (min)
0.6
0.8
1
1.2
1.4
1.6
1.8
2
2.2
0
10
20
30
40
50
60
Normal
LY50
LY75
LY88
LY94
LY97
LY data
Normal data

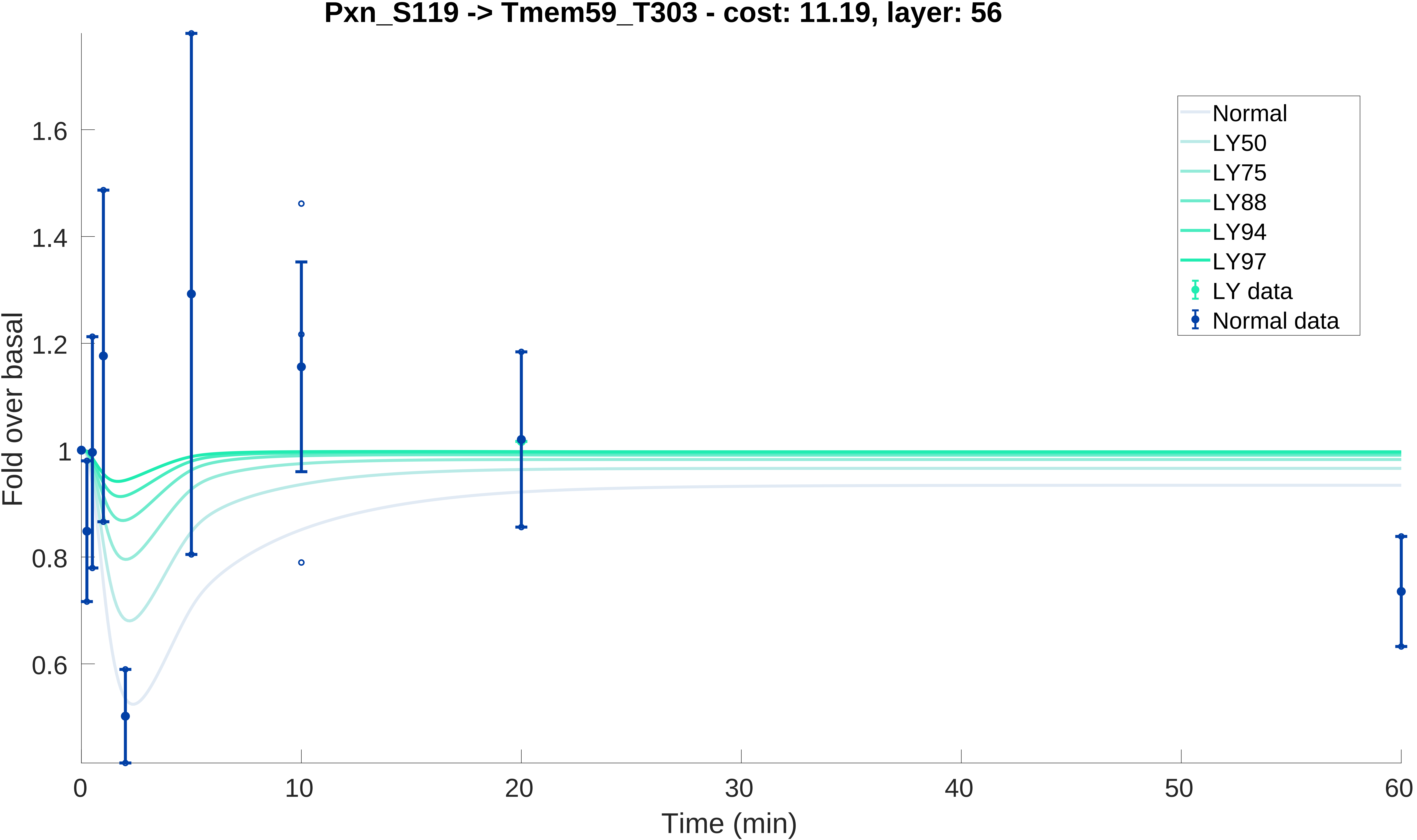

Pxn_S119 -> Tmem59_T303 - cost: 11.19, layer: 56
Fold over basal
1.6
1.4
1.2
1
0.8
0.6
0
10
20
30
40
50
60
Time (min)
Normal
LY50
LY75
LY88
LY94
LY97
LY data
Normal data

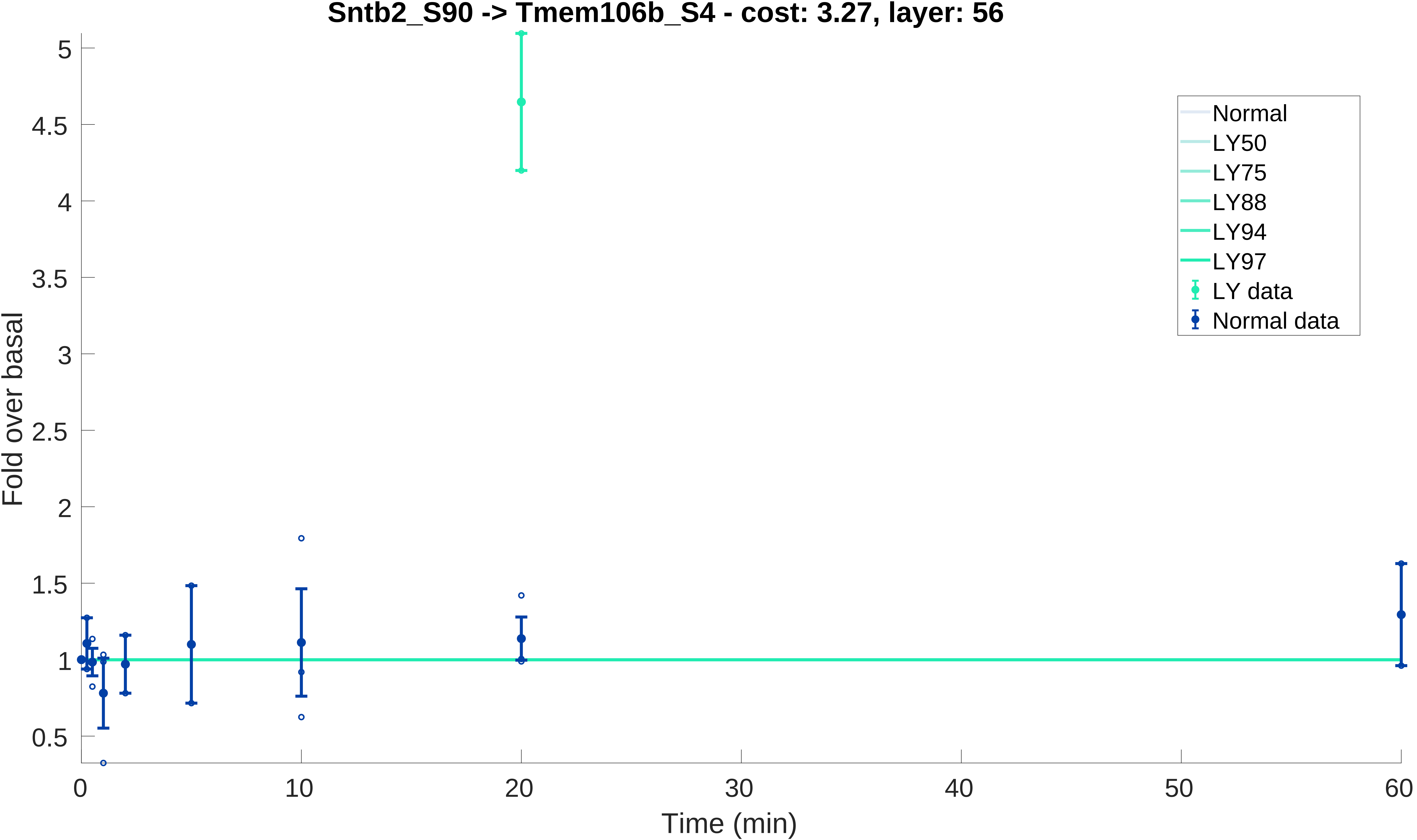

Sntb2_S90 -> Tmem106b_S4 - cost: 3.27, layer: 56
Fold over basal
Time (min)
0.5
1
1.5
2
2.5
3
3.5
4
4.5
5
0
10
20
30
40
50
60
Normal
LY50
LY75
LY88
LY94
LY97
LY data
Normal data

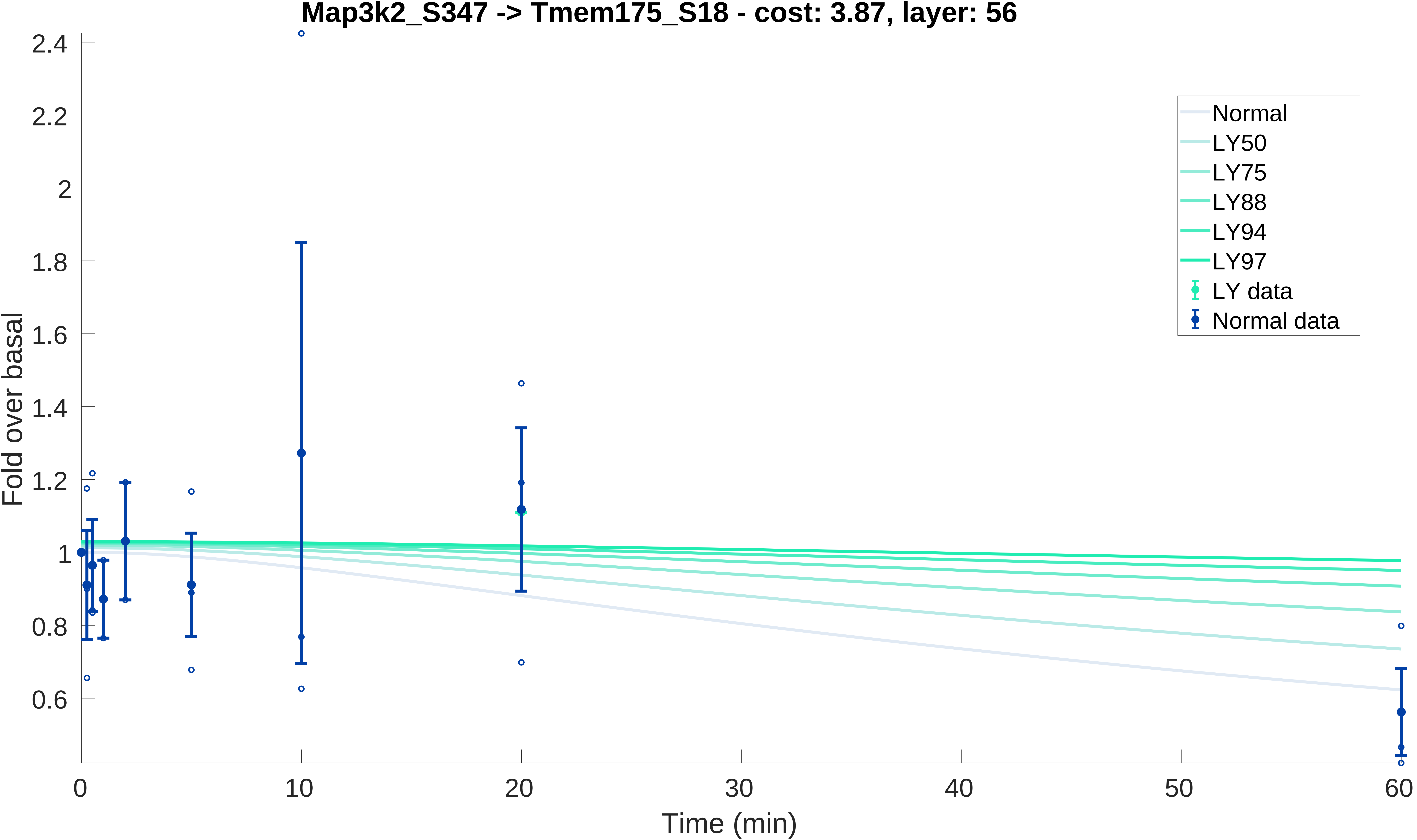

Map3k2_S347 -> Tmem175_S18 - cost: 3.87, layer: 56
Fold over basal
Time (min)
0
10
20
30
40
50
60
0.6
0.8
1
1.2
1.4
1.6
1.8
2
2.2
2.4
Normal
LY50
LY75
LY88
LY94
LY97
LY data
Normal data

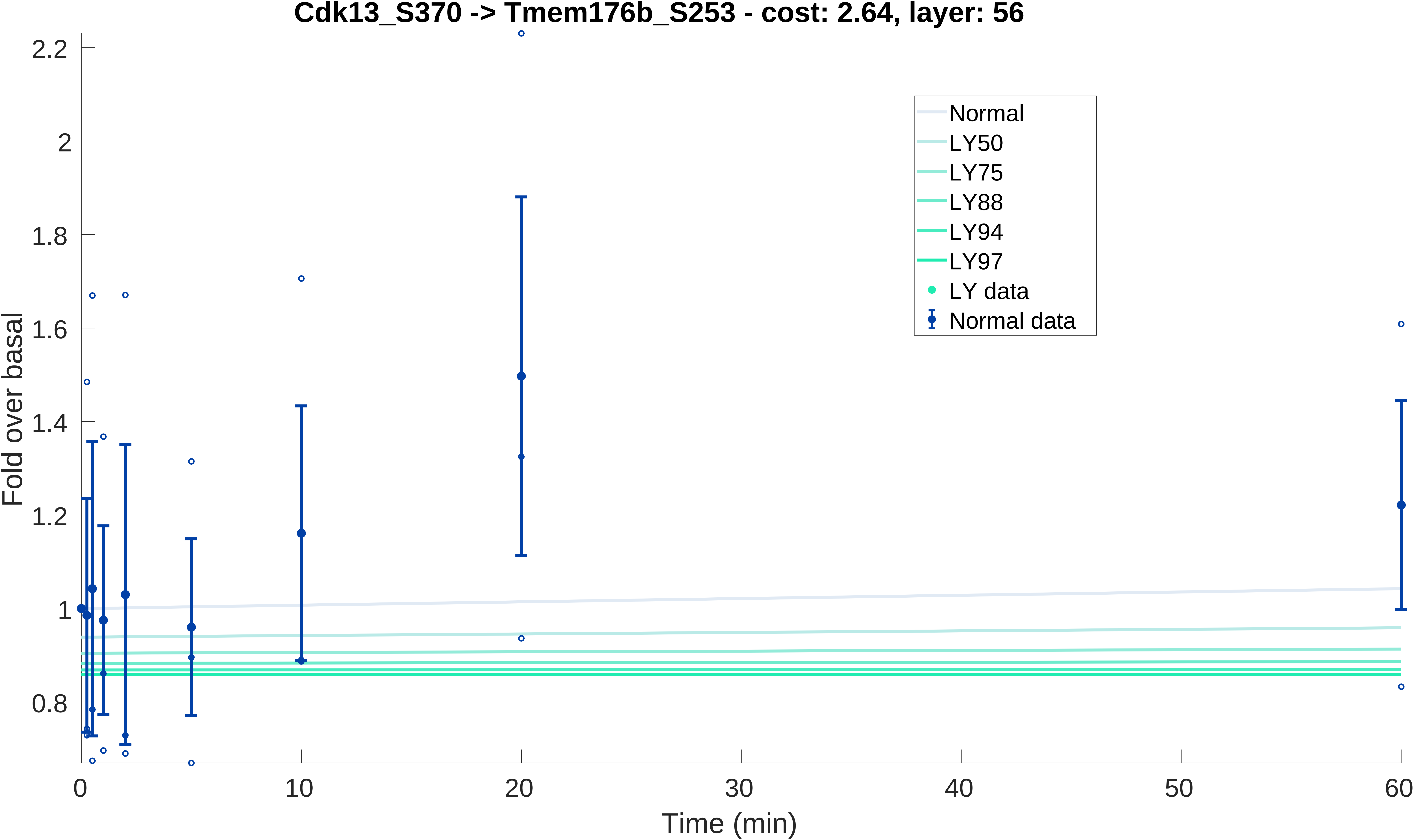

Cdk13_S370 -> Tmem176b_S253 - cost: 2.64, layer: 56
Fold over basal
Time (min)
2.2
2
1.8
1.6
1.4
1.2
1
0.8
0
10
20
30
40
50
60
Normal
LY50
LY75
LY88
LY94
LY97
LY data
Normal data

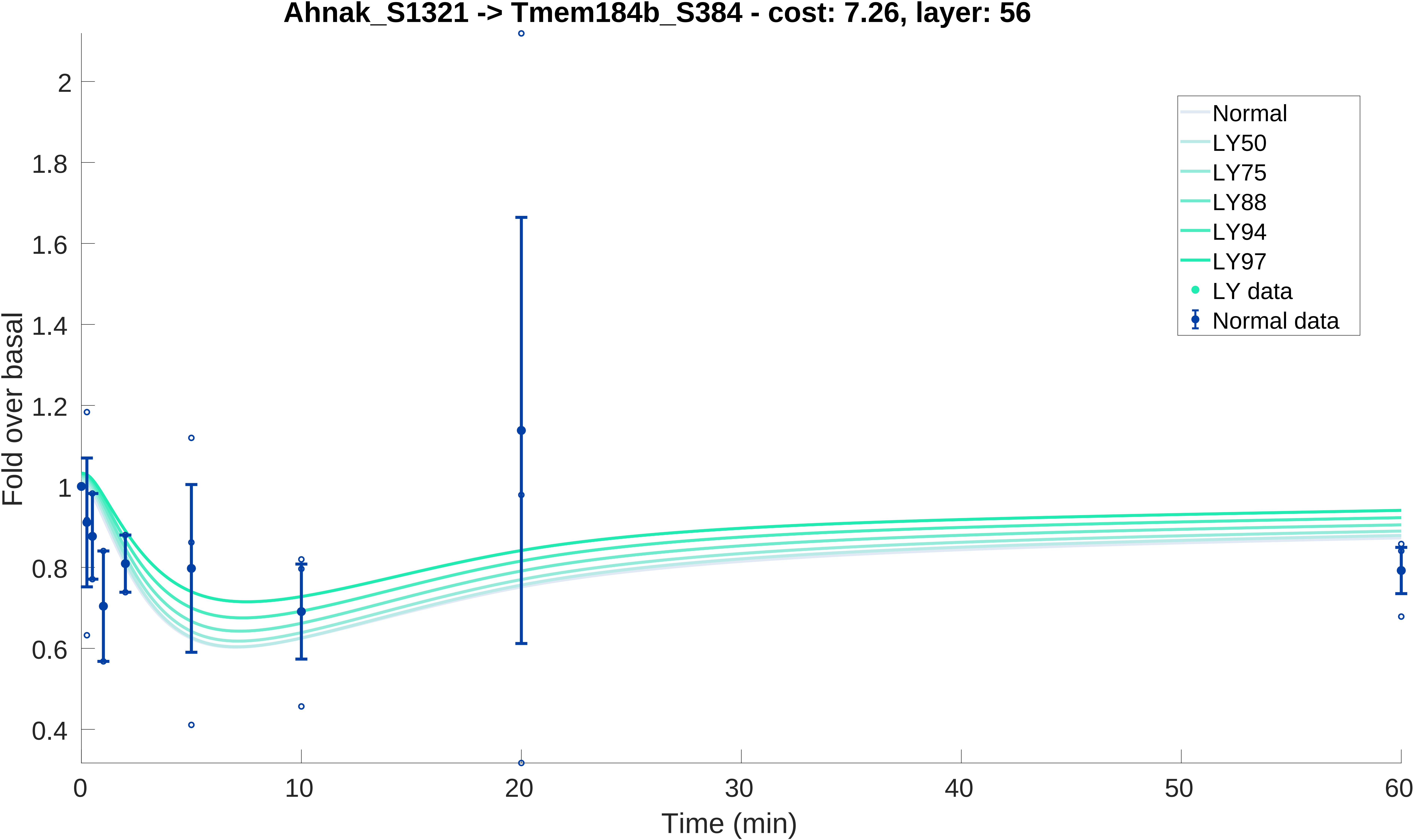

Ahnak_S1321 -> Tmem184b_S384 - cost: 7.26, layer: 56
Fold over basal
Time (min)
Normal
LY50
LY75
LY88
LY94
LY97
LY data
Normal data
0.4
0.6
0.8
1
1.2
1.4
1.6
1.8
2
0
10
20
30
40
50
60

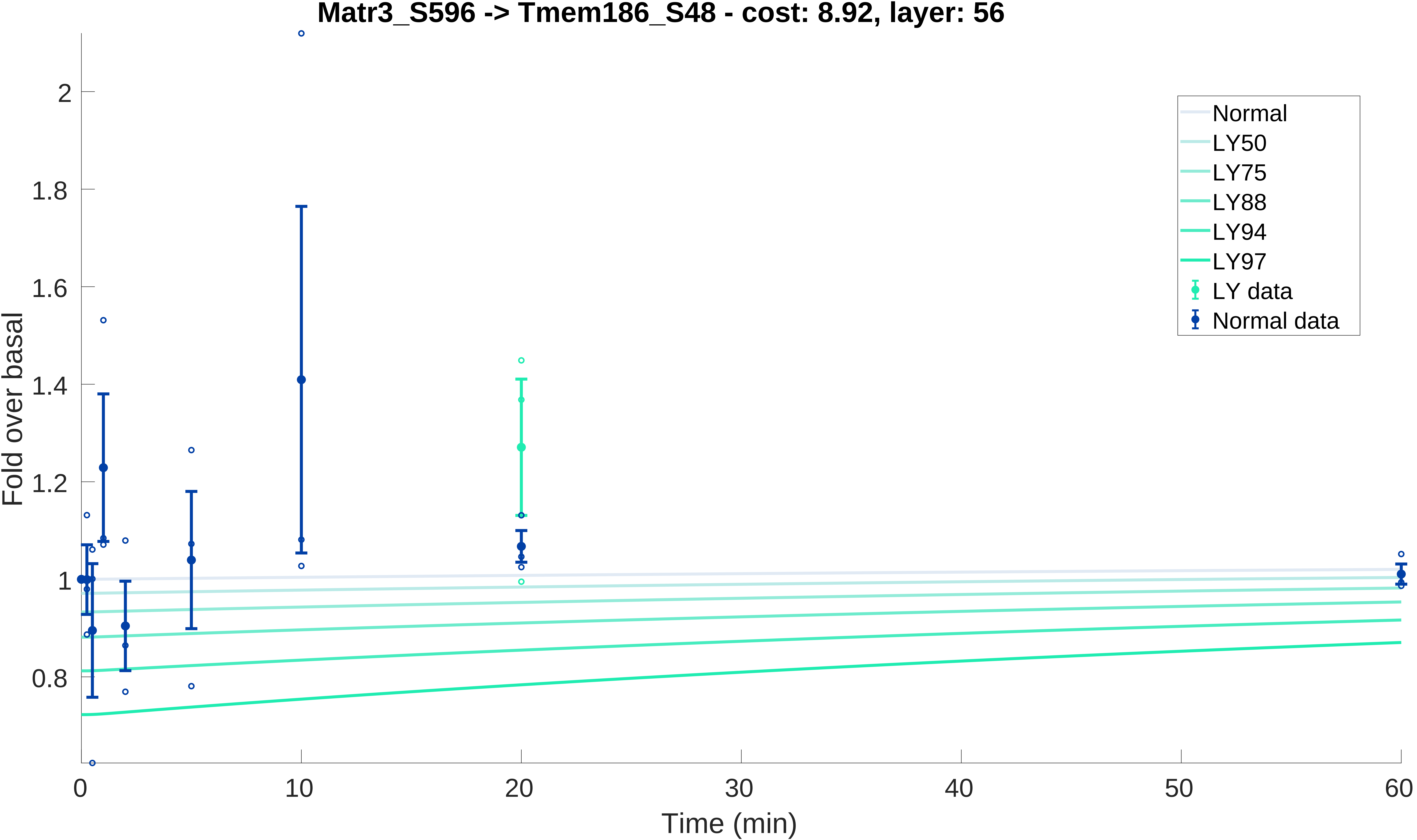

Matr3_S596 -> Tmem186_S48 - cost: 8.92, layer: 56
Fold over basal
Time (min)
0.8
1
1.2
1.4
1.6
1.8
2
0
10
20
30
40
50
60
Normal
LY50
LY75
LY88
LY94
LY97
LY data
Normal data

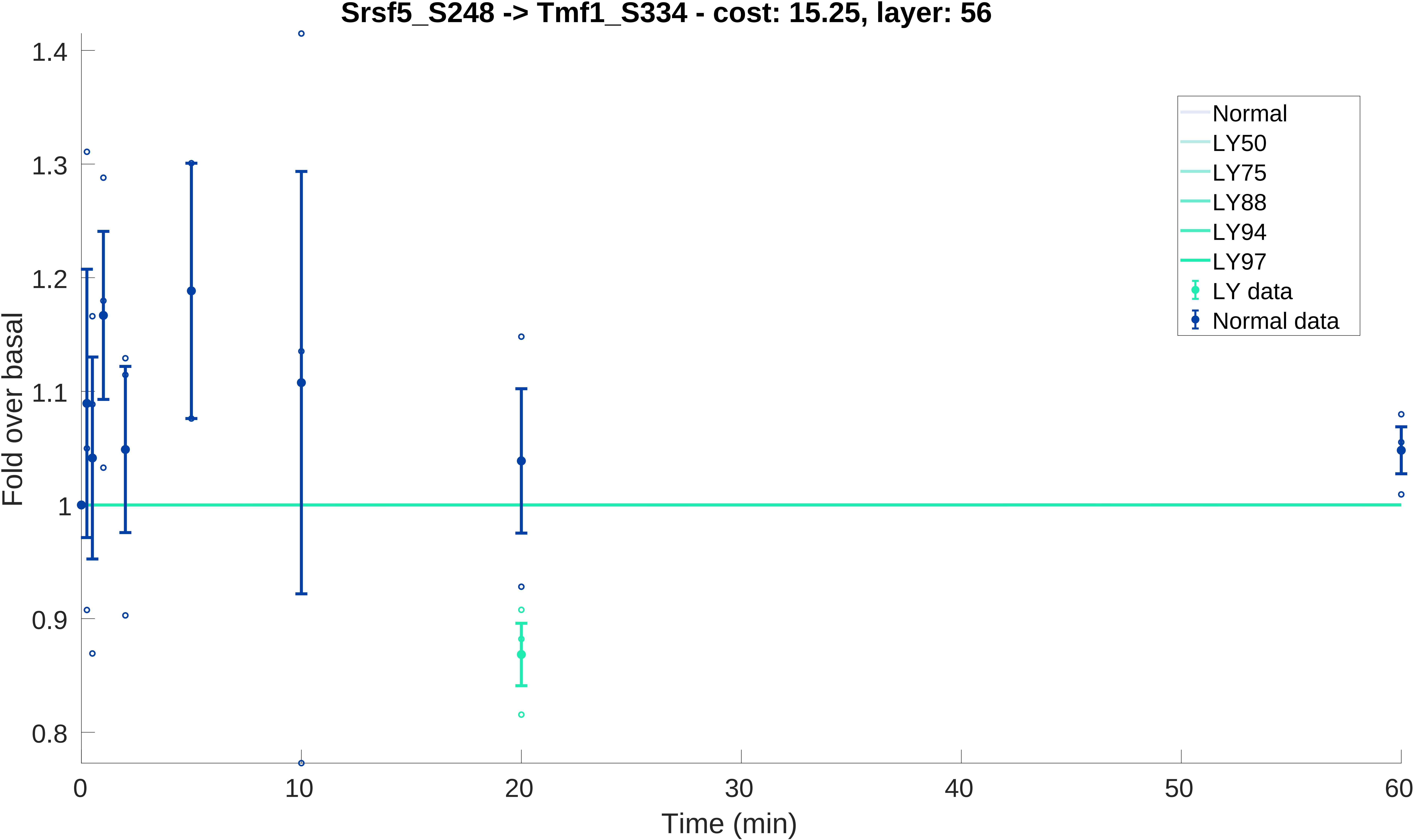

Srsf5_S248 -> Tmf1_S334 - cost: 15.25, layer: 56
Fold over basal
Time (min)
Normal
LY50
LY75
LY88
LY94
LY97
LY data
Normal data
1.4
1.3
1.2
1.1
1
0.9
0.8
0
10
20
30
40
50
60

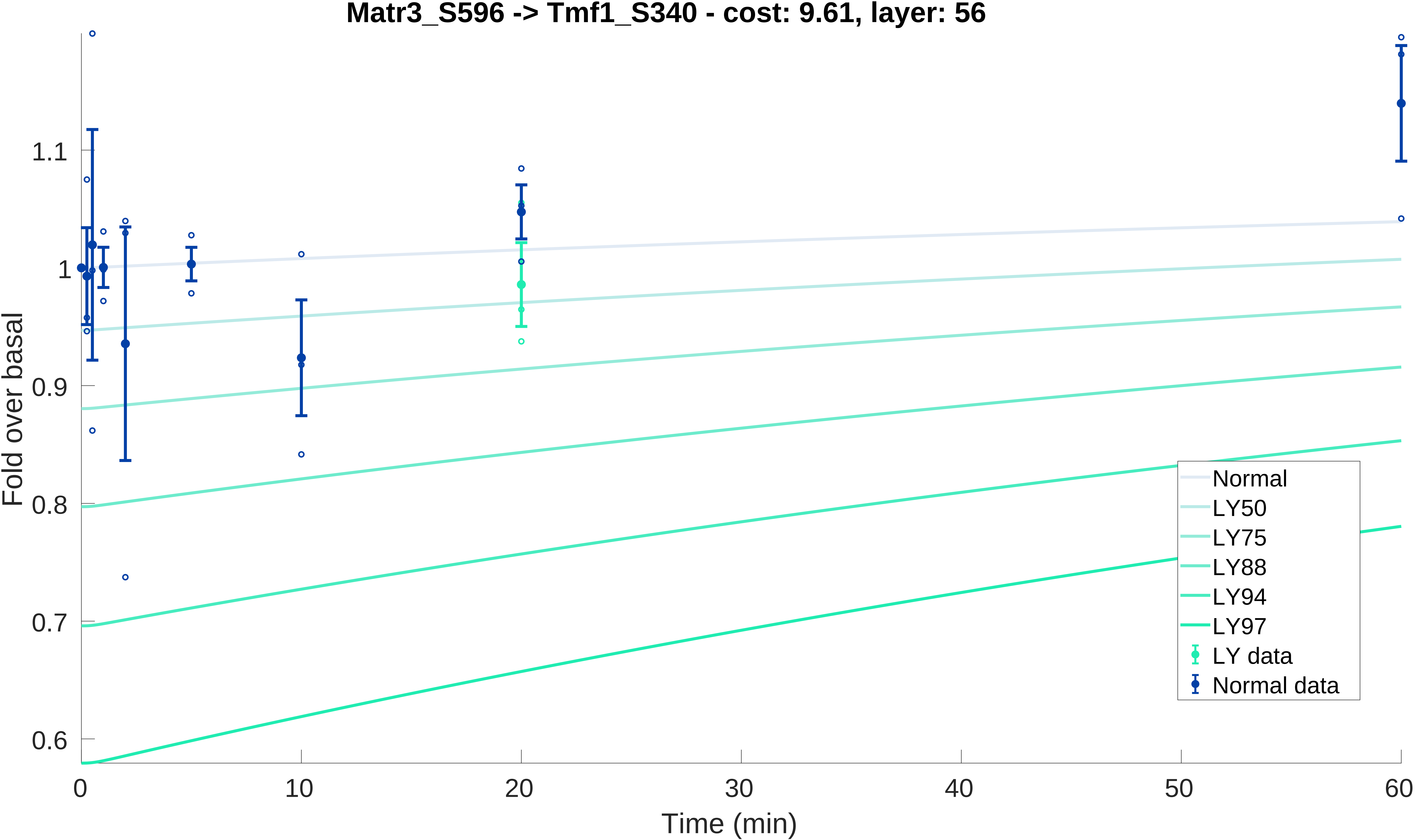

Matr3_S596 -> Tmf1_S340 - cost: 9.61, layer: 56
Fold over basal
1.1
1
0.9
0.8
0.7
0.6
0
10
20
30
40
50
60
Time (min)
Normal
LY50
LY75
LY88
LY94
LY97
LY data
Normal data

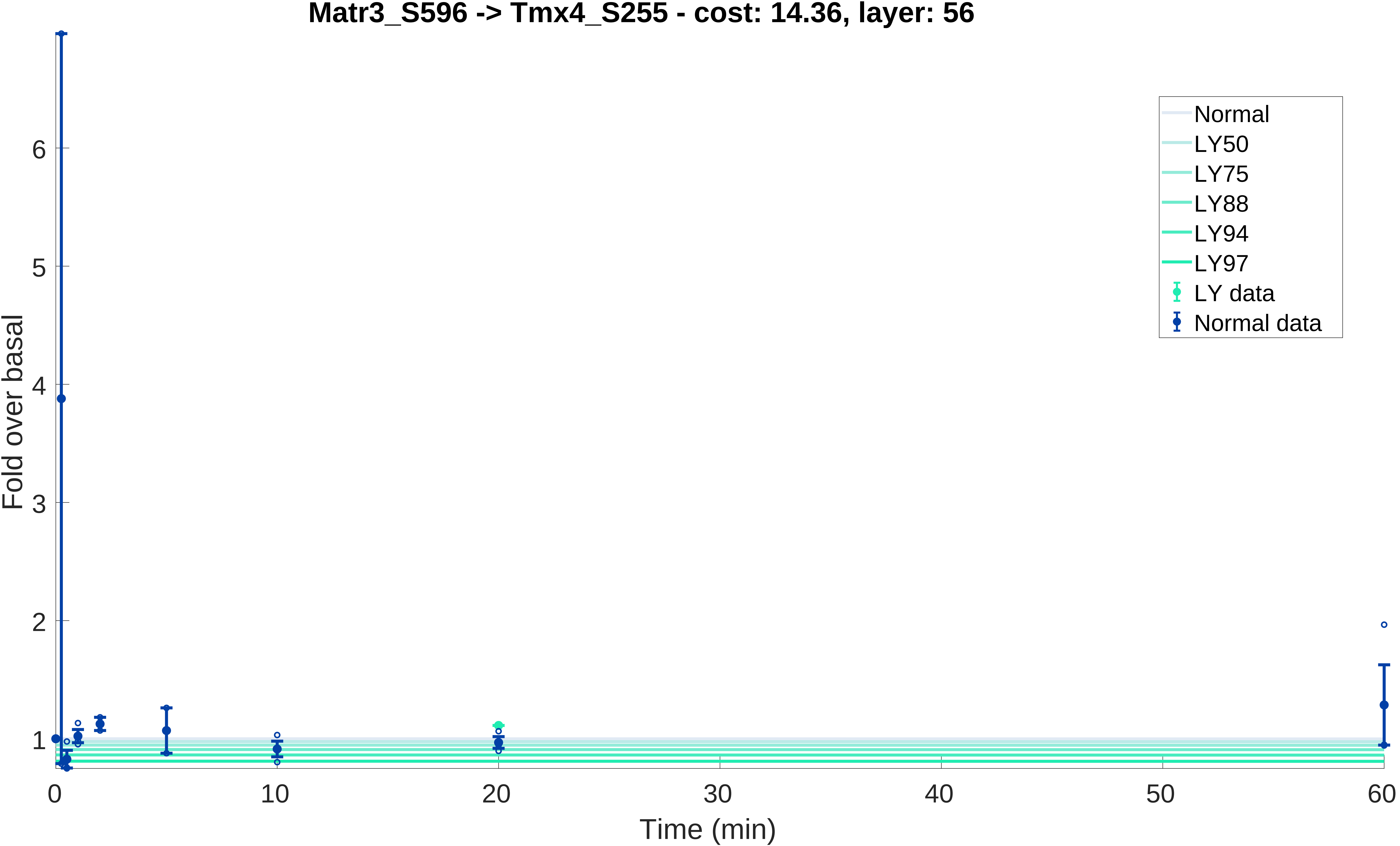

Matr3_S596 -> Tmx4_S255 - cost: 14.36, layer: 56
Fold over basal
Time (min)
0
10
20
30
40
50
60
1
2
3
4
5
6
Normal
LY50
LY75
LY88
LY94
LY97
LY data
Normal data

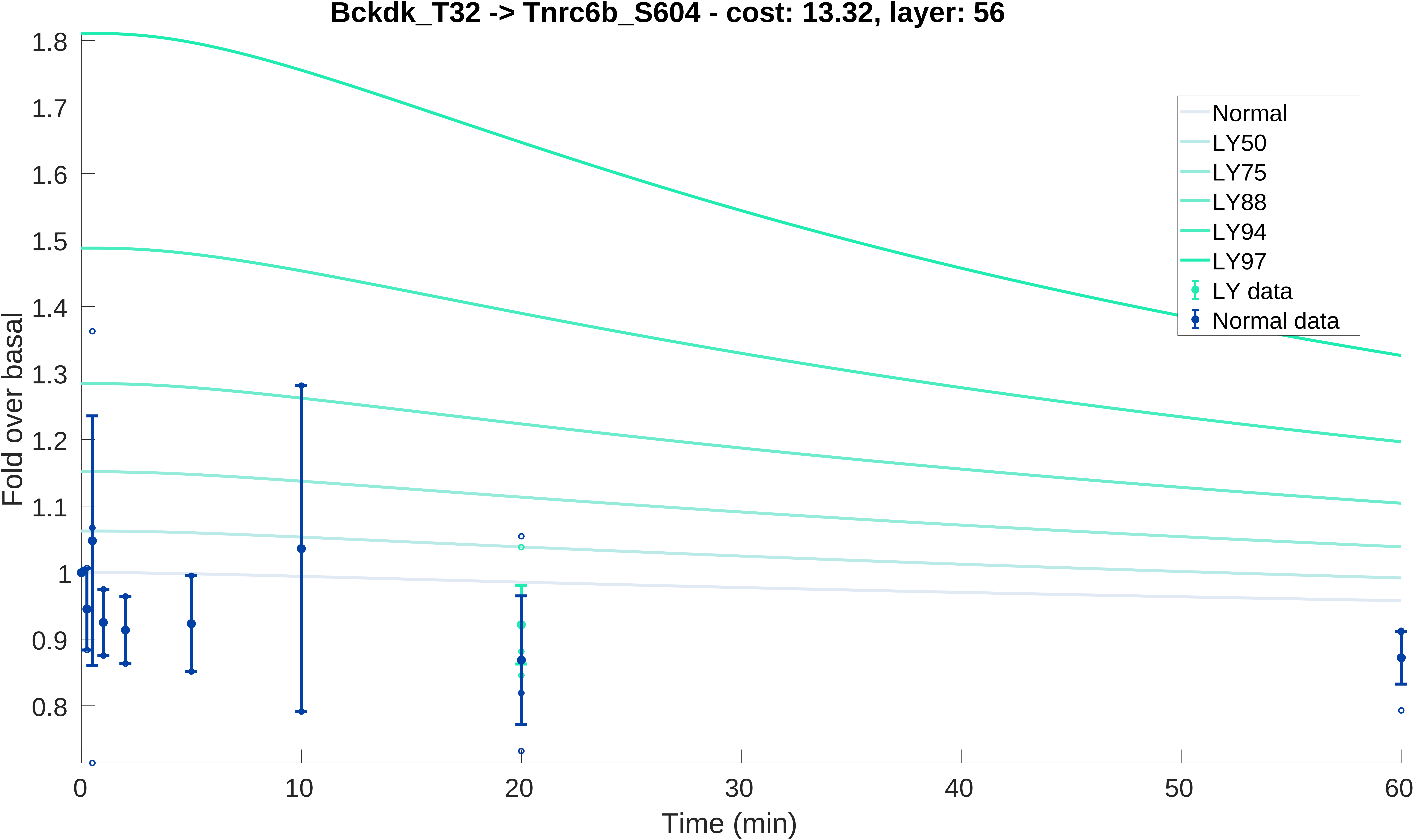

Bckdk_T32 -> Tnrc6b_S604 - cost: 13.32, layer: 56
Fold over basal
Time (min)
1.8
1.7
1.6
1.5
1.4
1.3
1.2
1.1
1
0.9
0.8
0
10
20
30
40
50
60
Normal
LY50
LY75
LY88
LY94
LY97
LY data
Normal data

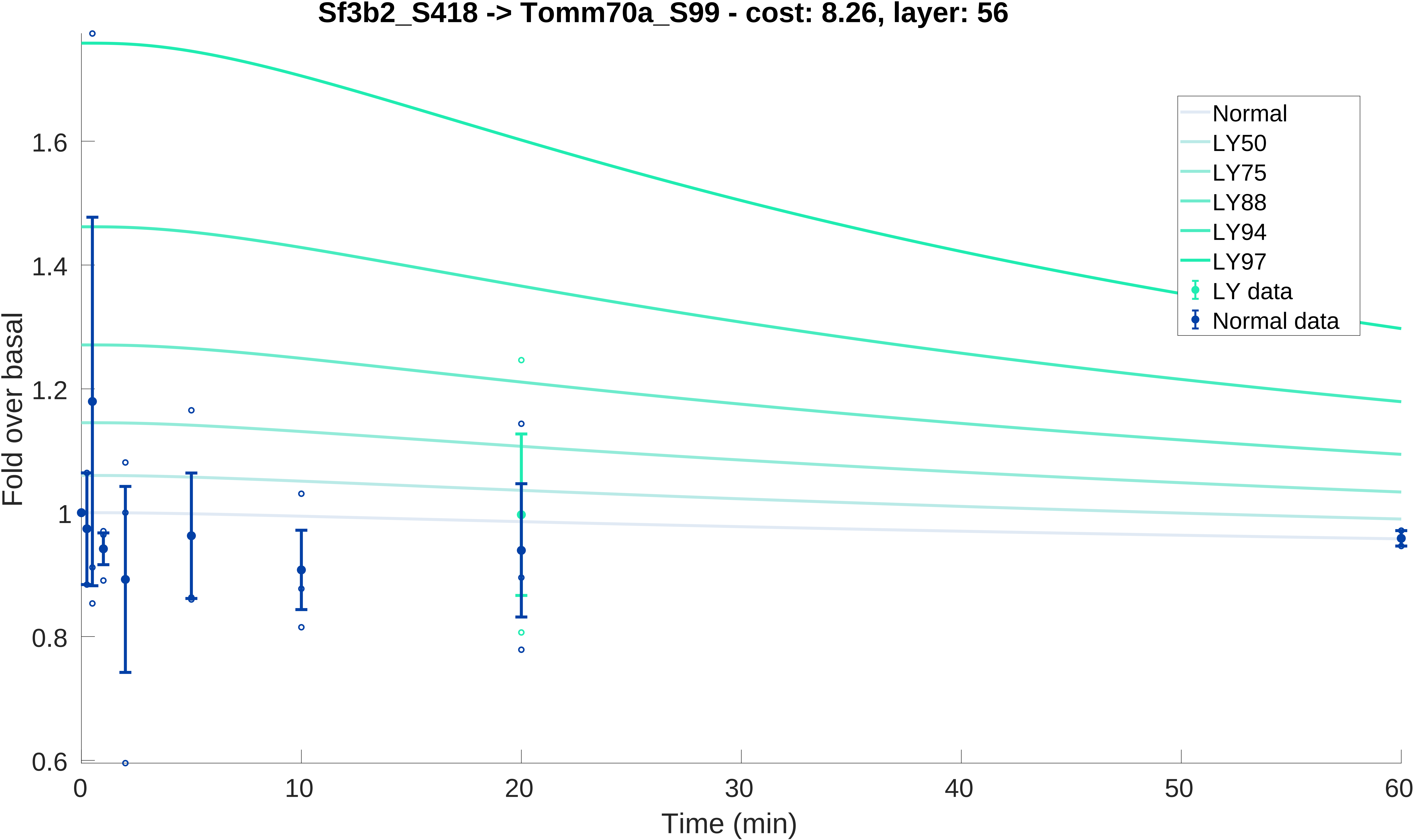
Sf3b2_S418 -> Tomm70a_S99 - cost: 8.26, layer: 56
Fold over basal
Time (min)
0.6
0.8
1
1.2
1.4
1.6
0
10
20
30
40
50
60
Normal
LY50
LY75
LY88
LY94
LY97
LY data
Normal data

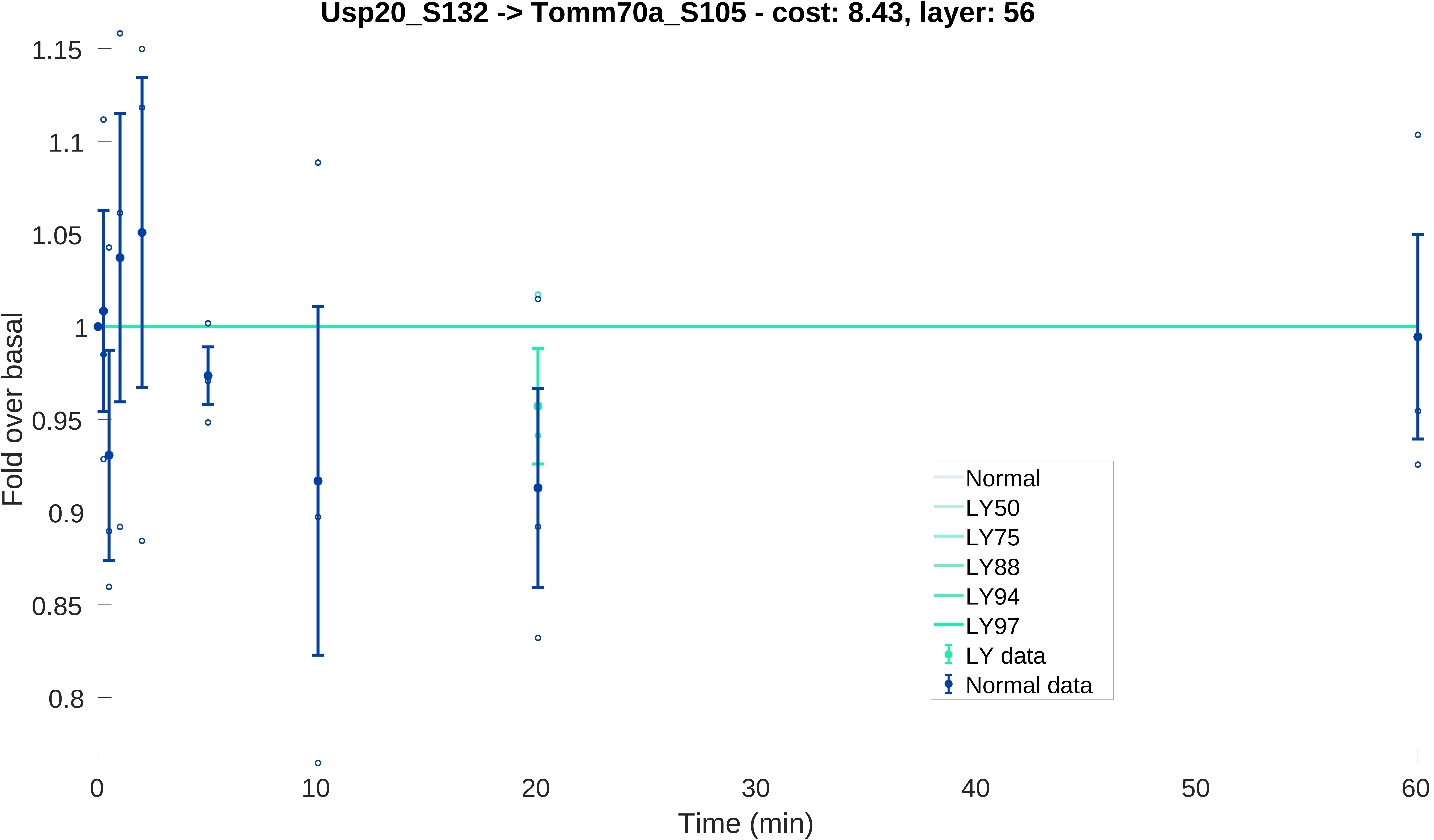

Usp20_S132 -> Tomm70a_S105 - cost: 8.43, layer: 56
Fold over basal
Time (min)
1.15
1.1
1.05
1
0.95
0.9
0.85
0.8
0
10
20
30
40
50
60
Normal
LY50
LY75
LY88
LY94
LY97
LY data
Normal data

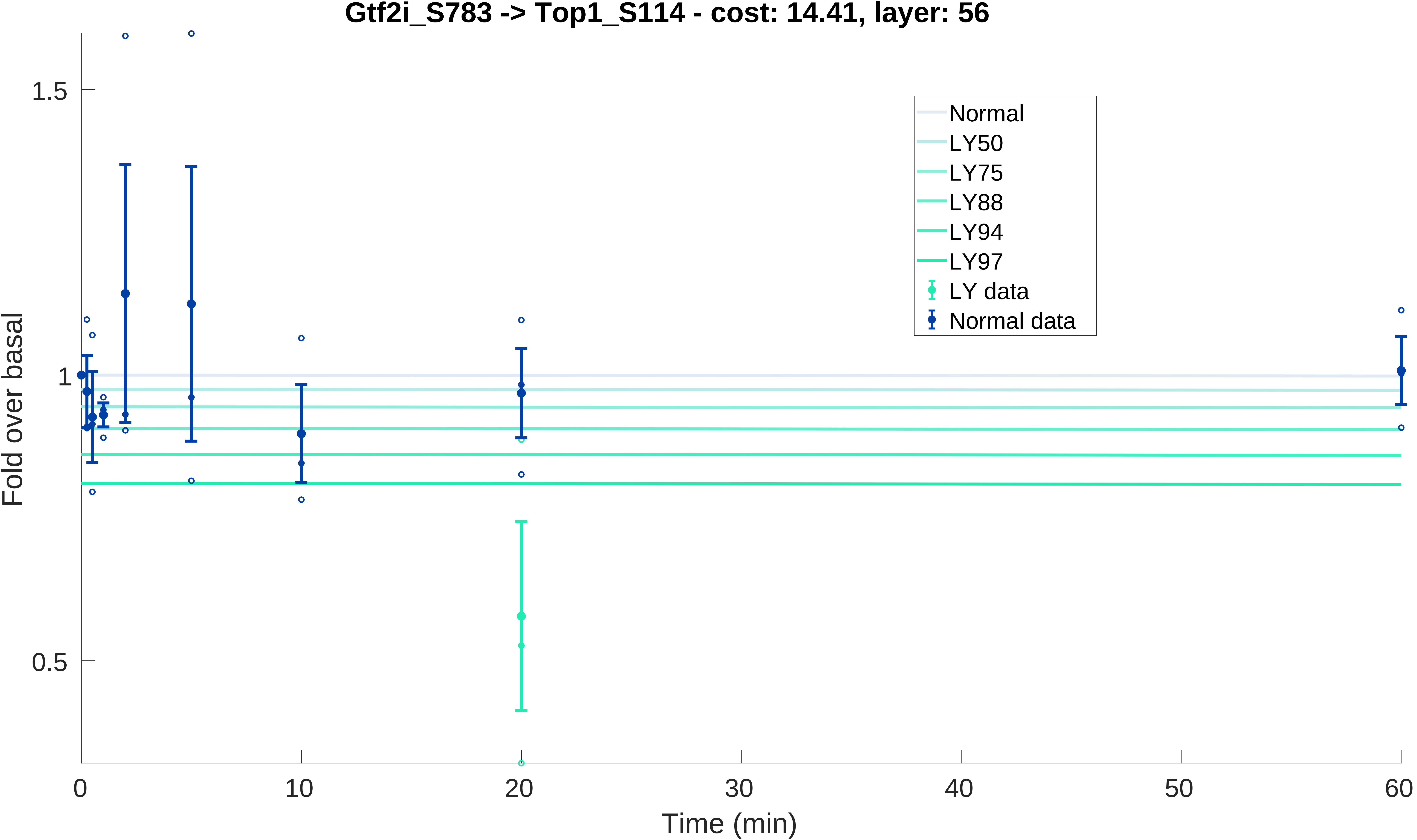

Gtf2i_S783 -> Top1_S114 - cost: 14.41, layer: 56
Fold over basal
1.5
1
0.5
0
10
20
30
40
50
60
Time (min)
Normal
LY50
LY75
LY88
LY94
LY97
LY data
Normal data

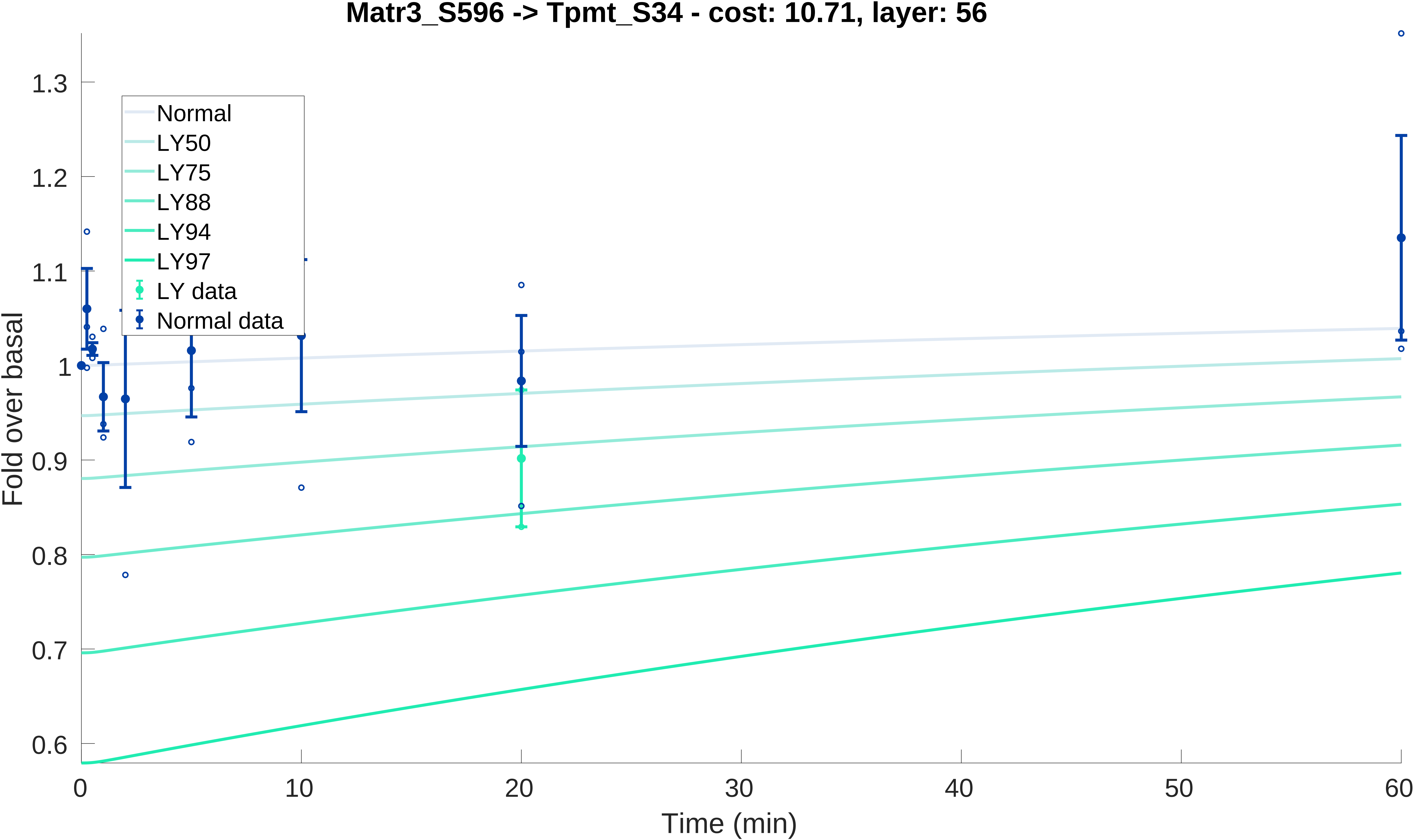

Matr3_S596 -> Tpmt_S34 - cost: 10.71, layer: 56
Normal
LY50
LY75
LY88
LY94
LY97
LY data
Normal data
Fold over basal
Time (min)
0.6
0.7
0.8
0.9
1
1.1
1.2
1.3
0
10
20
30
40
50
60

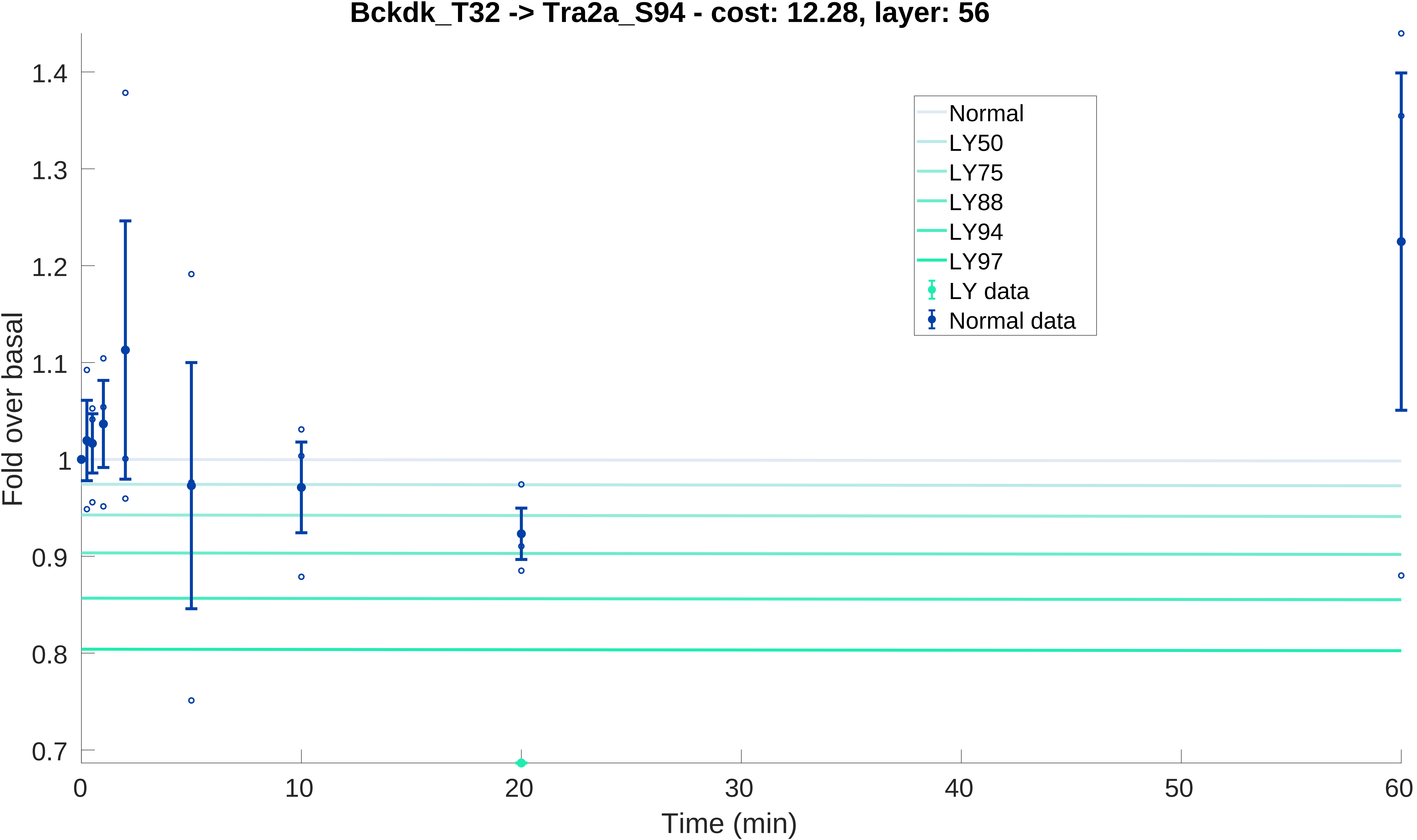
Bckdk_T32 -> Tra2a_S94 - cost: 12.28, layer: 56
Fold over basal
Time (min)
1.4
1.3
1.2
1.1
1
0.9
0.8
0.7
0
10
20
30
40
50
60
Normal
LY50
LY75
LY88
LY94
LY97
LY data
Normal data

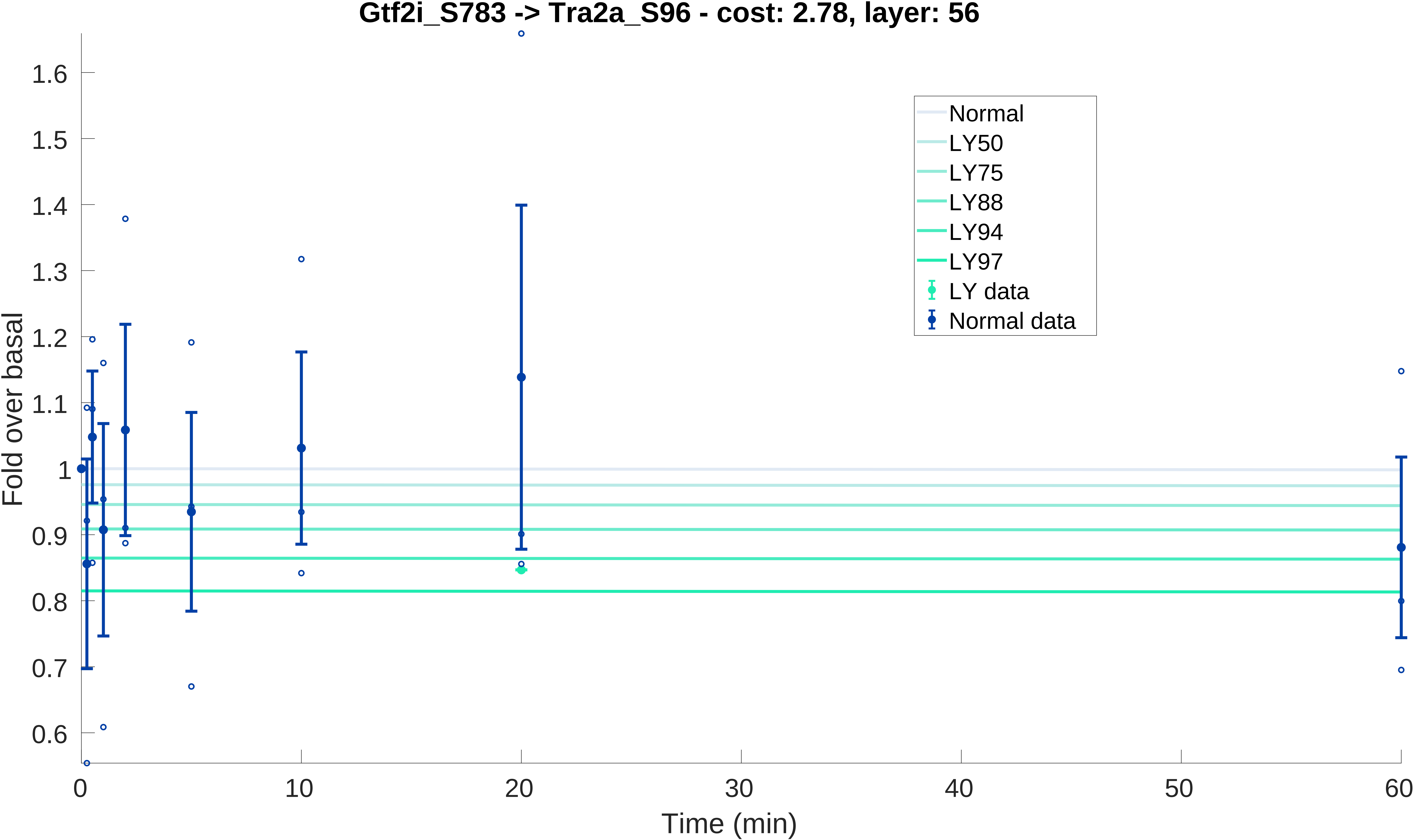

Gtf2i_S783 -> Tra2a_S96 - cost: 2.78, layer: 56
Fold over basal
Time (min)
Normal
LY50
LY75
LY88
LY94
LY97
LY data
Normal data
1.6
1.5
1.4
1.3
1.2
1.1
1
0.9
0.8
0.7
0.6
0
10
20
30
40
50
60

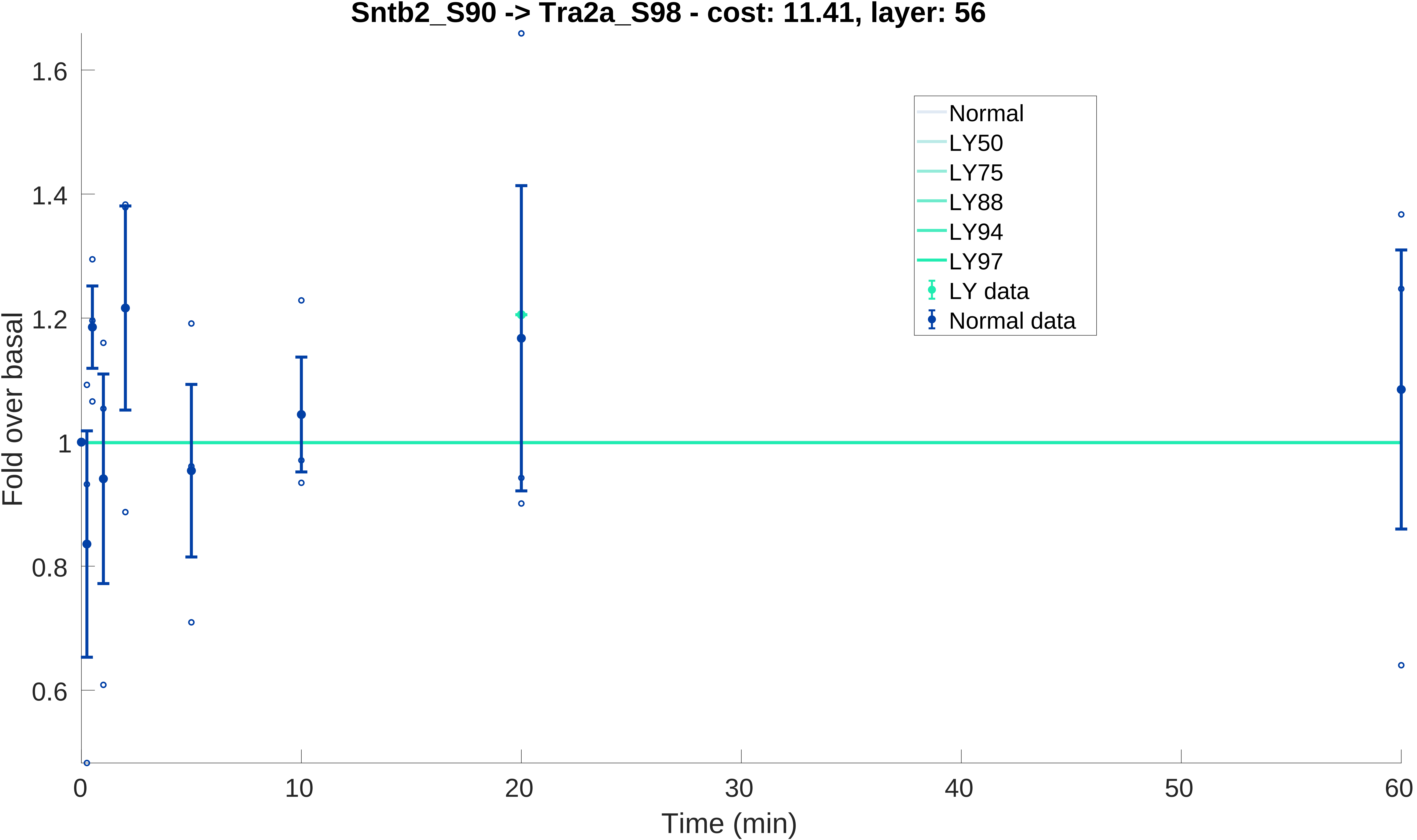

Sntb2_S90 -> Tra2a_S98 - cost: 11.41, layer: 56
Normal
LY50
LY75
LY88
LY94
LY97
LY data
Normal data
Fold over basal
Time (min)
1.6
1.4
1.2
1
0.8
0.6
0
10
20
30
40
50
60

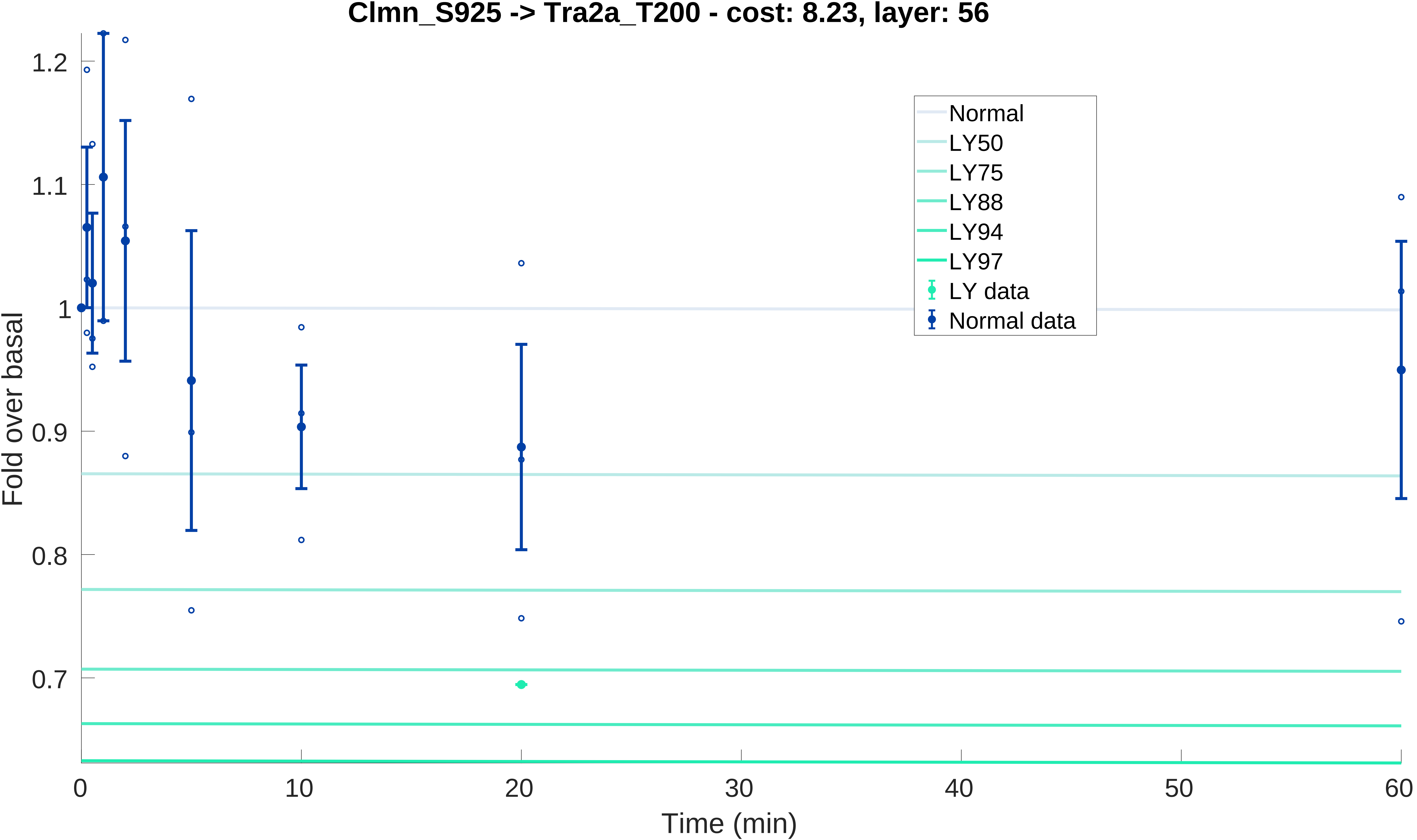
Clmn_S925 -> Tra2a_T200 - cost: 8.23, layer: 56
Fold over basal
1.2
1.1
1
0.9
0.8
0.7
0
10
20
30
40
50
60
Time (min)
Normal
LY50
LY75
LY88
LY94
LY97
LY data
Normal data

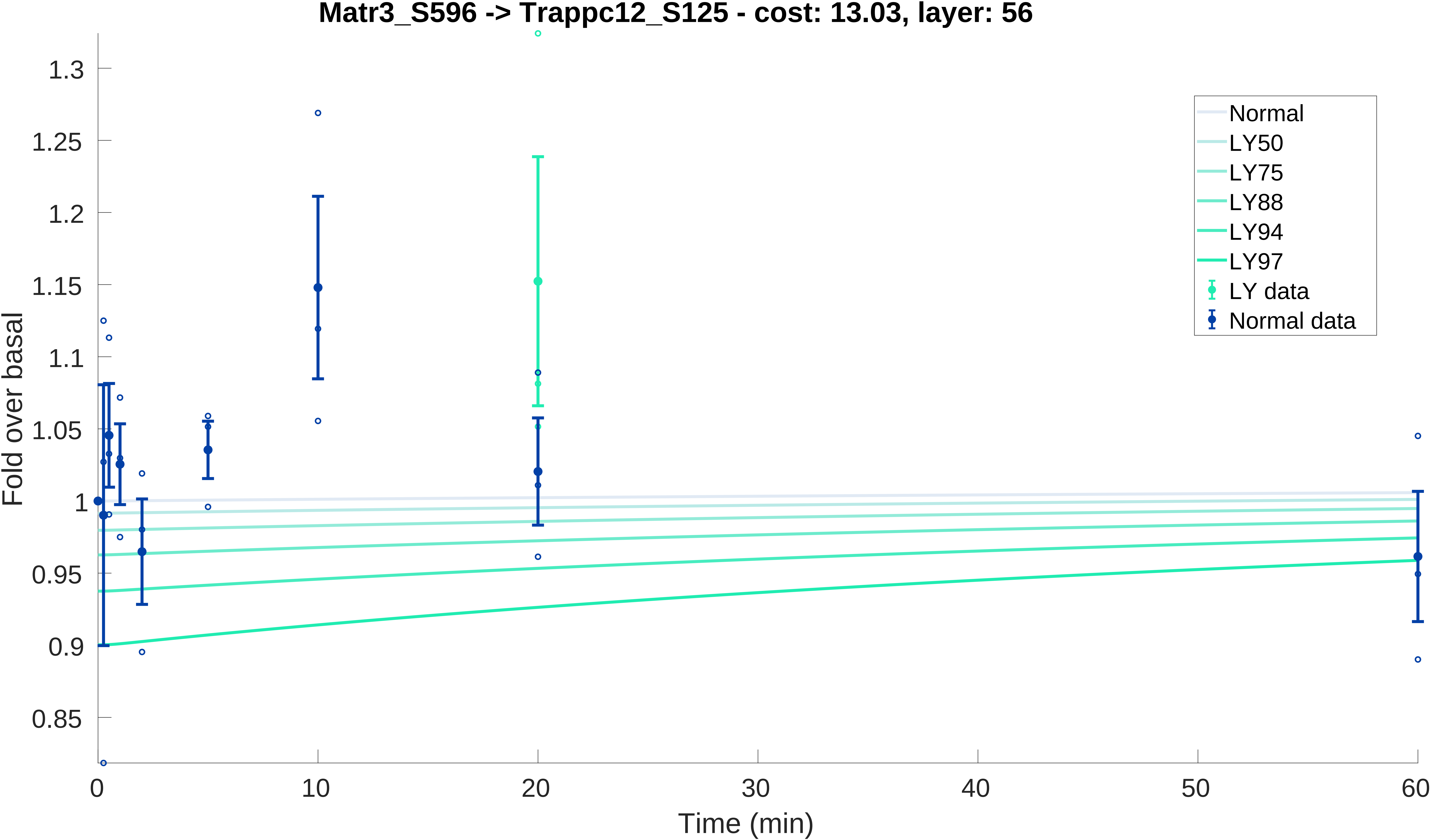

Matr3_S596 -> Trappc12_S125 - cost: 13.03, layer: 56
Fold over basal
1.3
1.25
1.2
1.15
1.1
1.05
1
0.95
0.9
0.85
0
10
20
30
40
50
60
Time (min)
Normal
LY50
LY75
LY88
LY94
LY97
LY data
Normal data

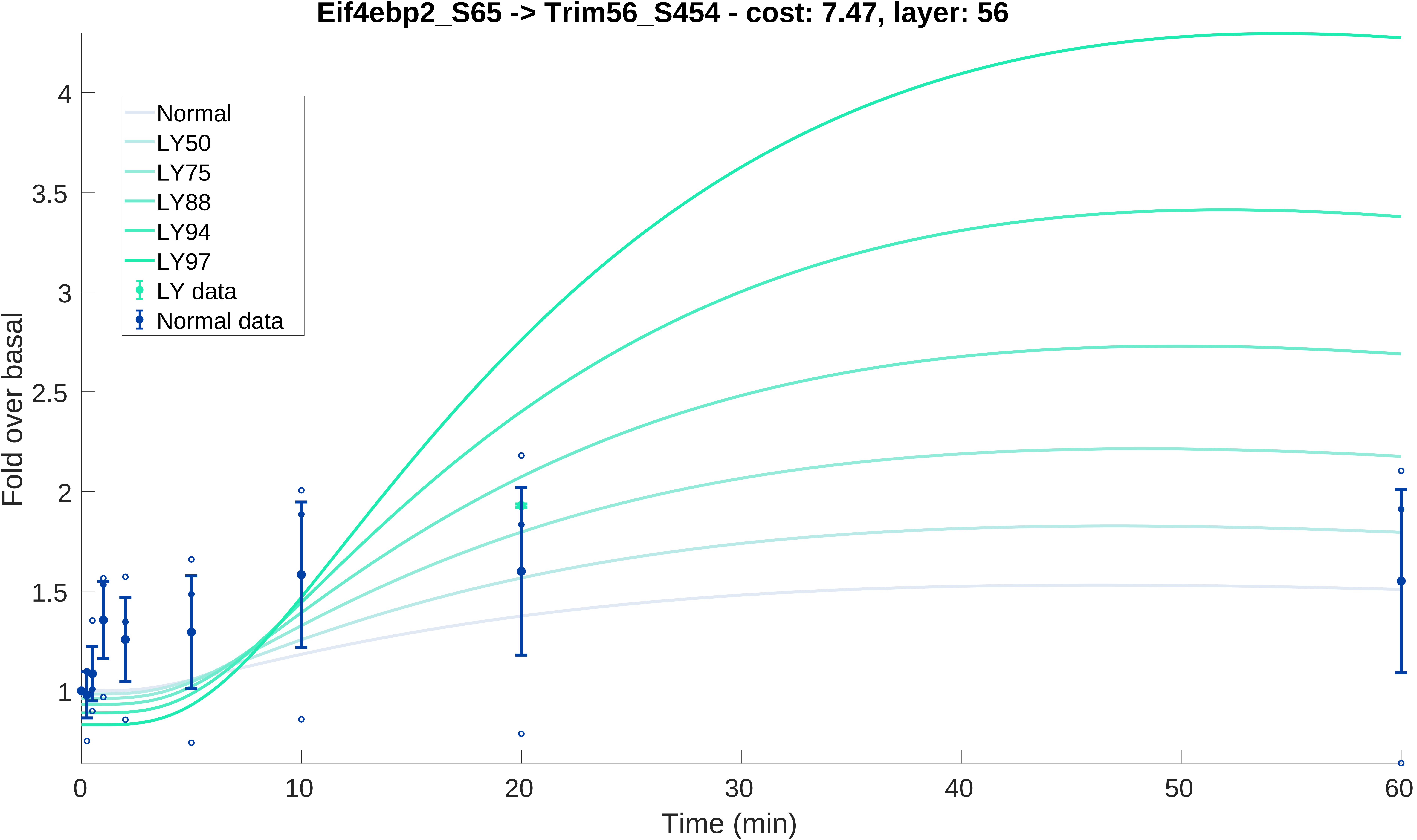

Eif4ebp2_S65 -> Trim56_S454 - cost: 7.47, layer: 56
Normal
LY50
LY75
LY88
LY94
LY97
LY data
Normal data
Fold over basal
Time (min)
0
10
20
30
40
50
60
1
1.5
2
2.5
3
3.5
4

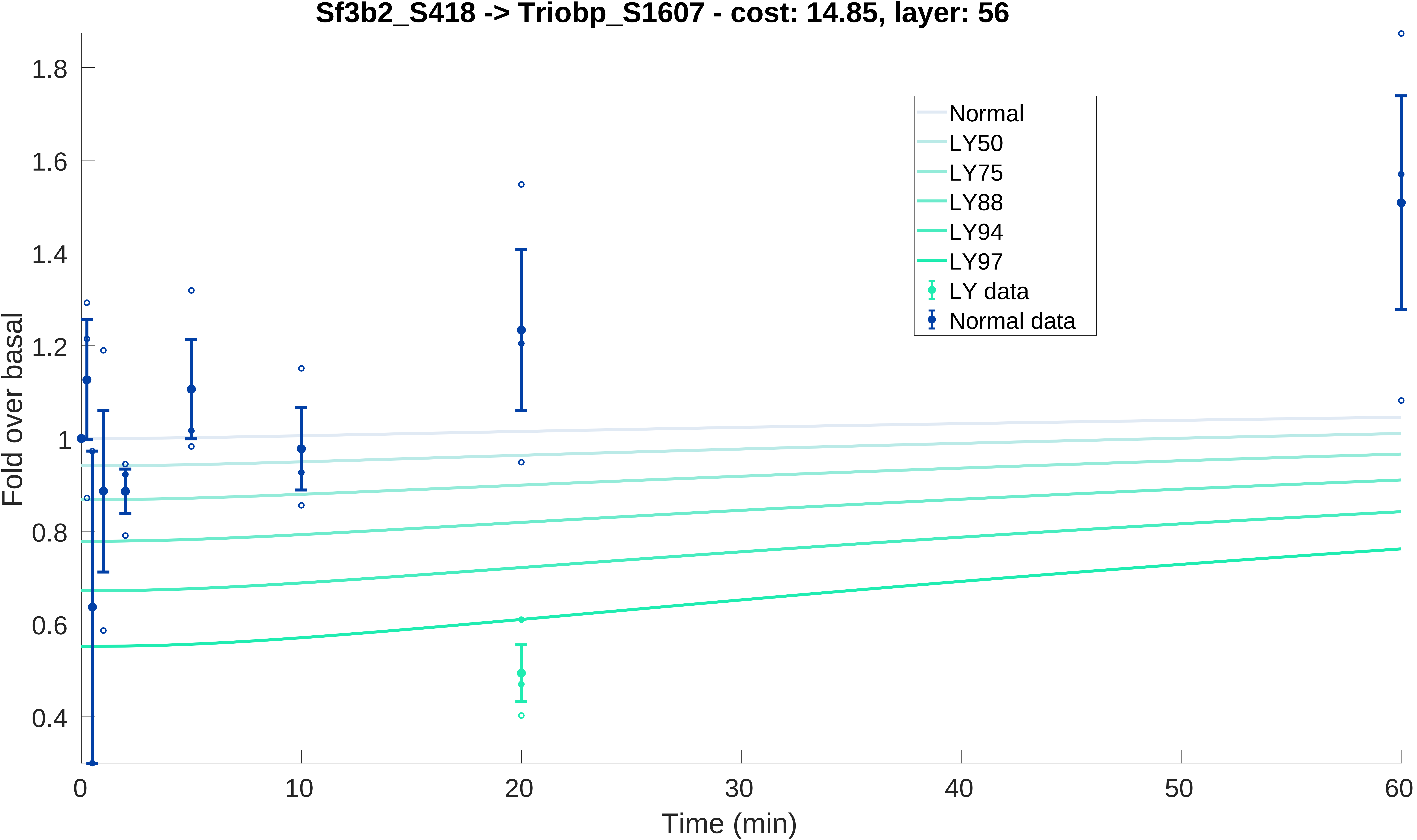

Sf3b2_S418 -> Triobp_S1607 - cost: 14.85, layer: 56
Fold over basal
Time (min)
Normal
LY50
LY75
LY88
LY94
LY97
LY data
Normal data

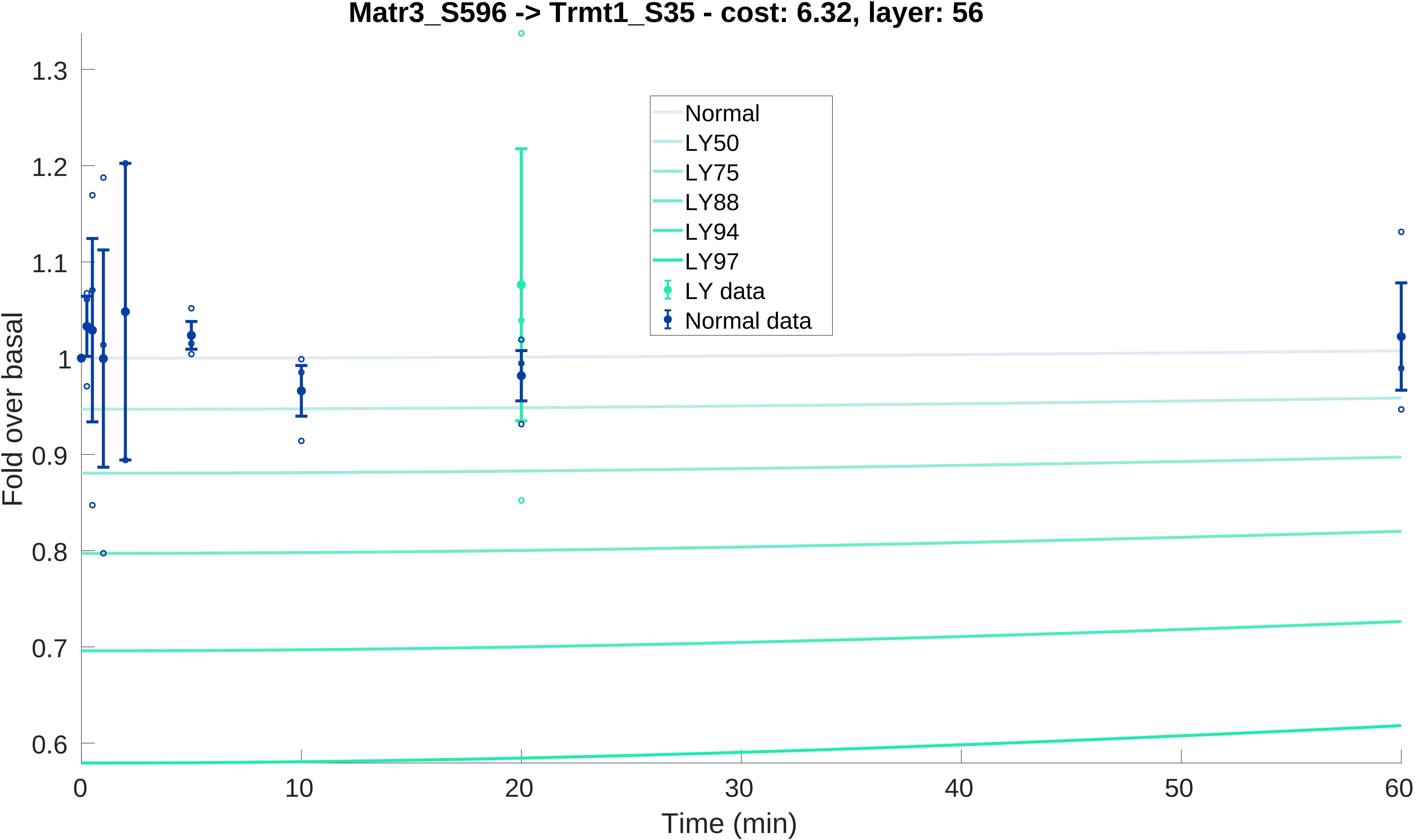

Matr3_S596 -> Trmt1_S35 - cost: 6.32, layer: 56
Normal
LY50
LY75
LY88
LY94
LY97
LY data
Normal data
Fold over basal
1.3
1.2
1.1
1
0.9
0.8
0.7
0.6
0
10
20
30
40
50
60
Time (min)

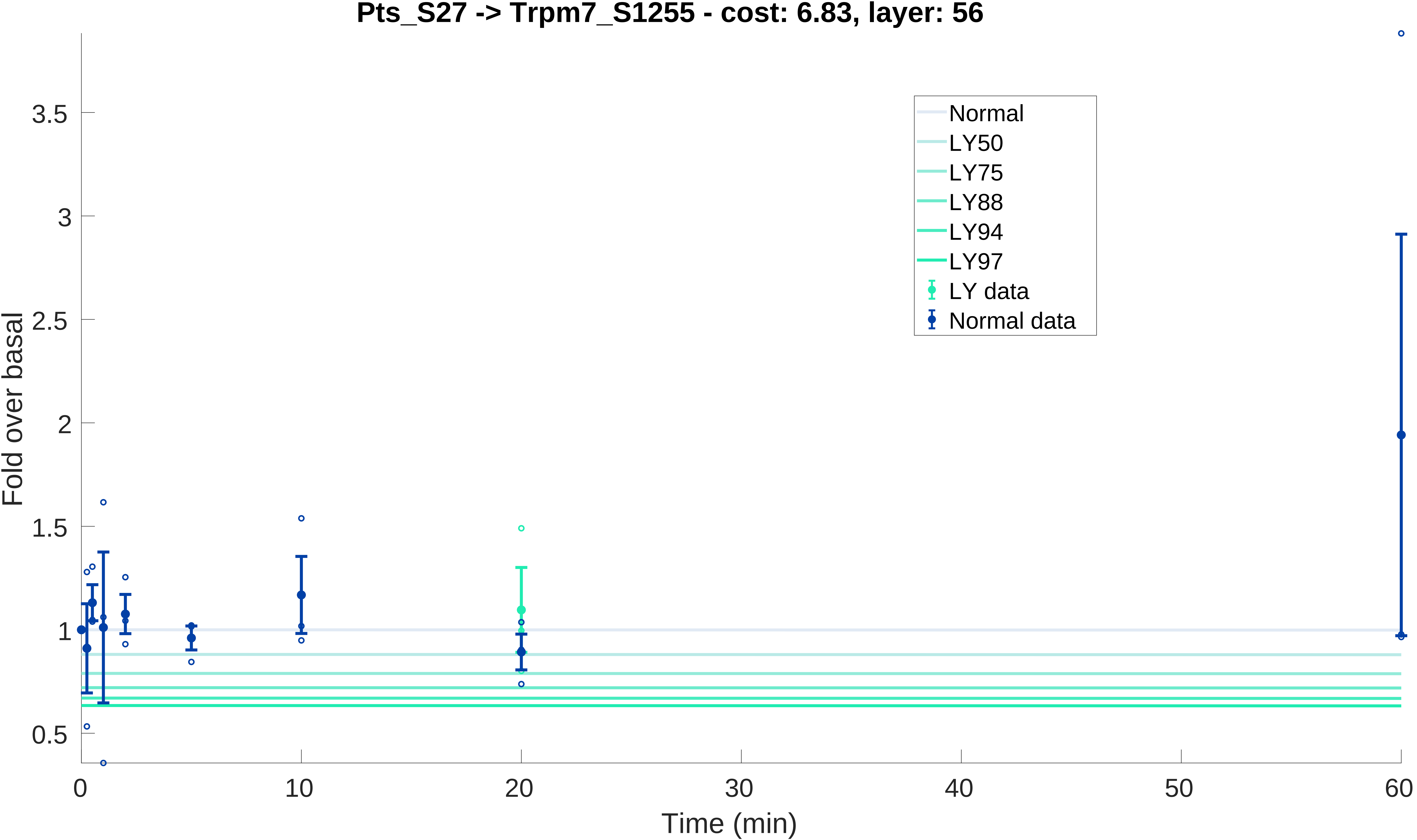

Pts_S27 -> Trpm7_S1255 - cost: 6.83, layer: 56
Fold over basal
Time (min)
Normal
LY50
LY75
LY88
LY94
LY97
LY data
Normal data
0.5
1
1.5
2
2.5
3
3.5
0
10
20
30
40
50
60

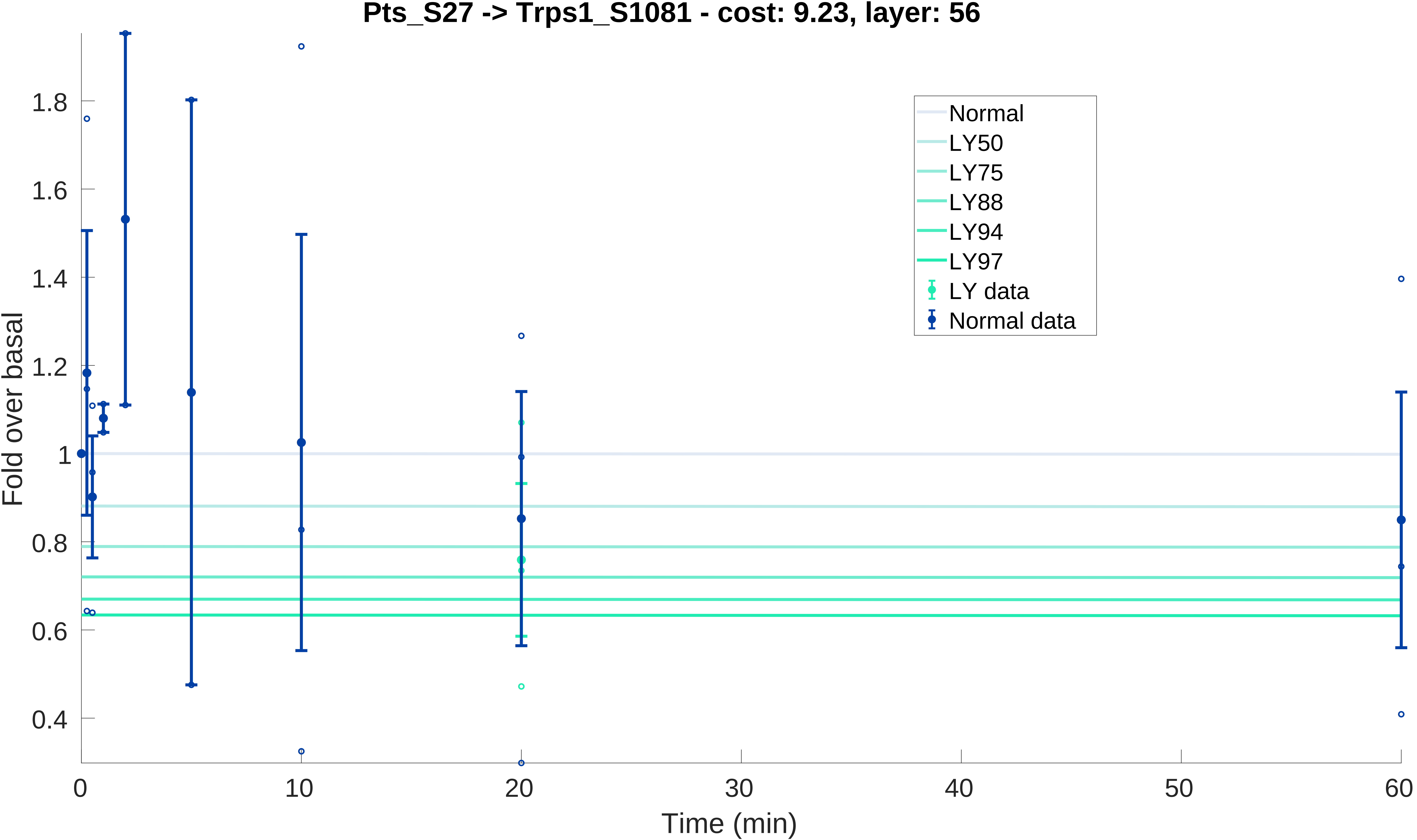

Pts_S27 -> Trps1_S1081 - cost: 9.23, layer: 56
Normal
LY50
LY75
LY88
LY94
LY97
LY data
Normal data
Fold over basal
1.8
1.6
1.4
1.2
1
0.8
0.6
0.4
0
10
20
30
40
50
60
Time (min)

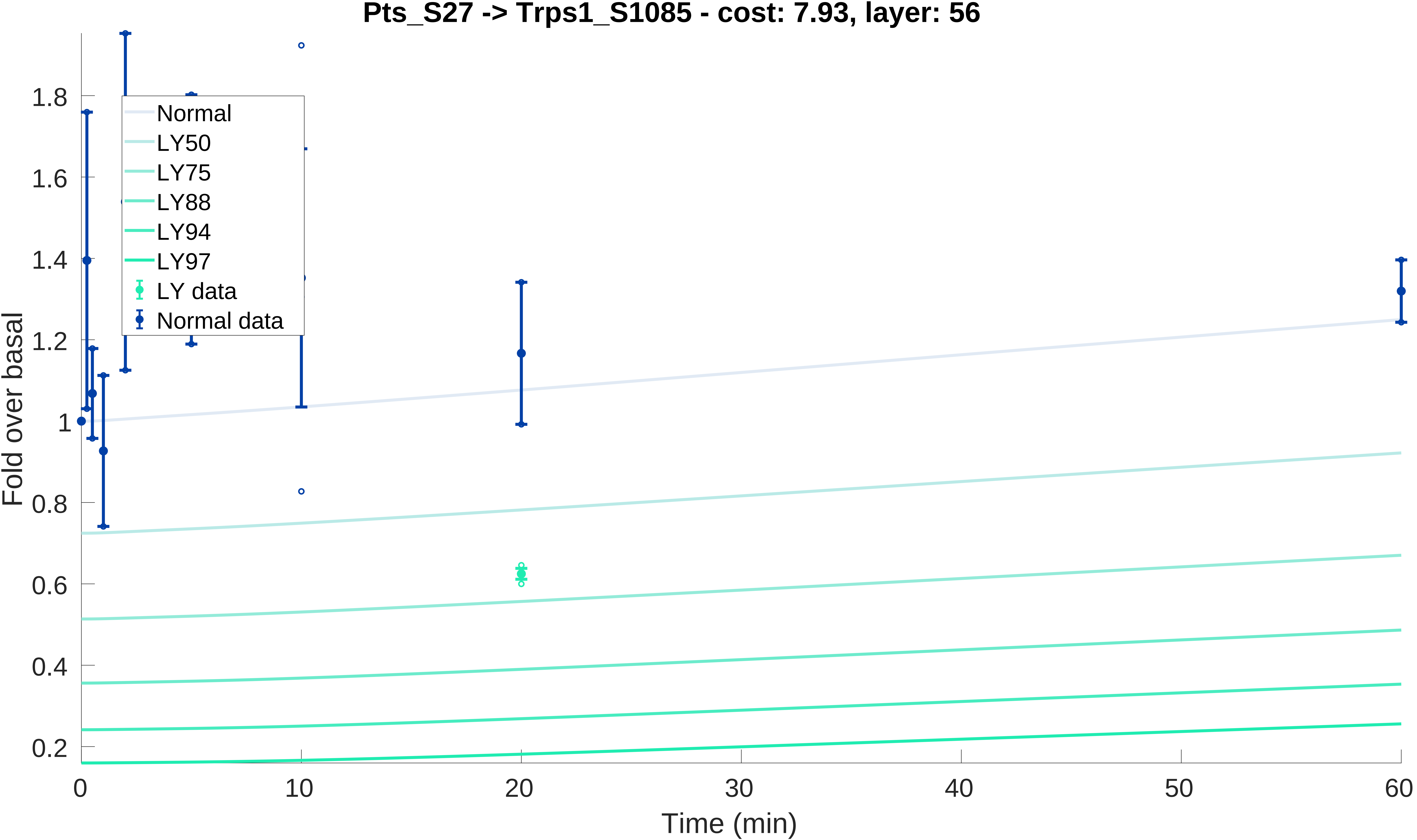

Pts_S27 -> Trps1_S1085 - cost: 7.93, layer: 56
Normal
LY50
LY75
LY88
LY94
LY97
LY data
Normal data
Fold over basal
Time (min)
0
10
20
30
40
50
60
0.2
0.4
0.6
0.8
1
1.2
1.4
1.6
1.8

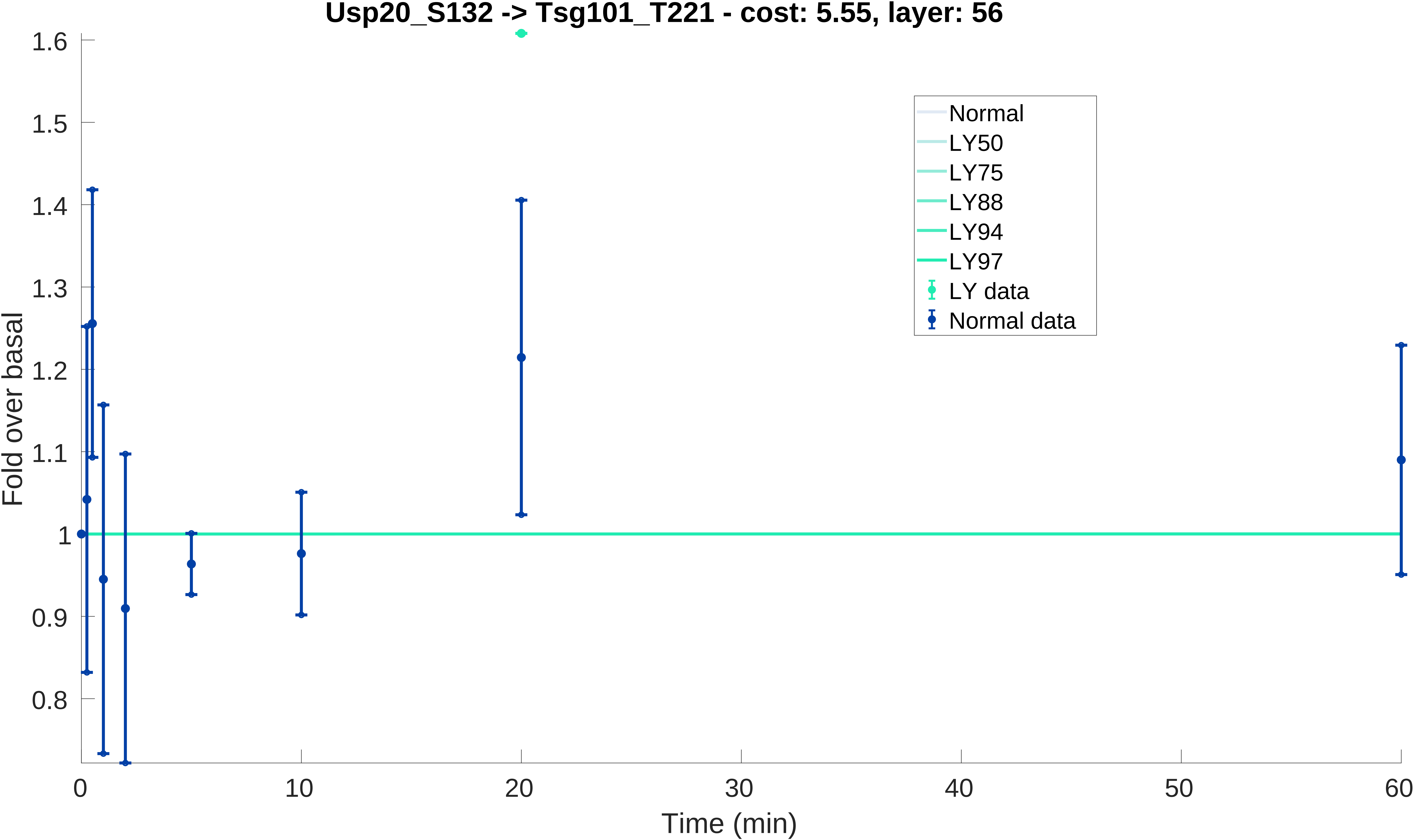

Usp20_S132 -> Tsg101_T221 - cost: 5.55, layer: 56
Fold over basal
Time (min)
1.6
1.5
1.4
1.3
1.2
1.1
1
0.9
0.8
0
10
20
30
40
50
60
Normal
LY50
LY75
LY88
LY94
LY97
LY data
Normal data

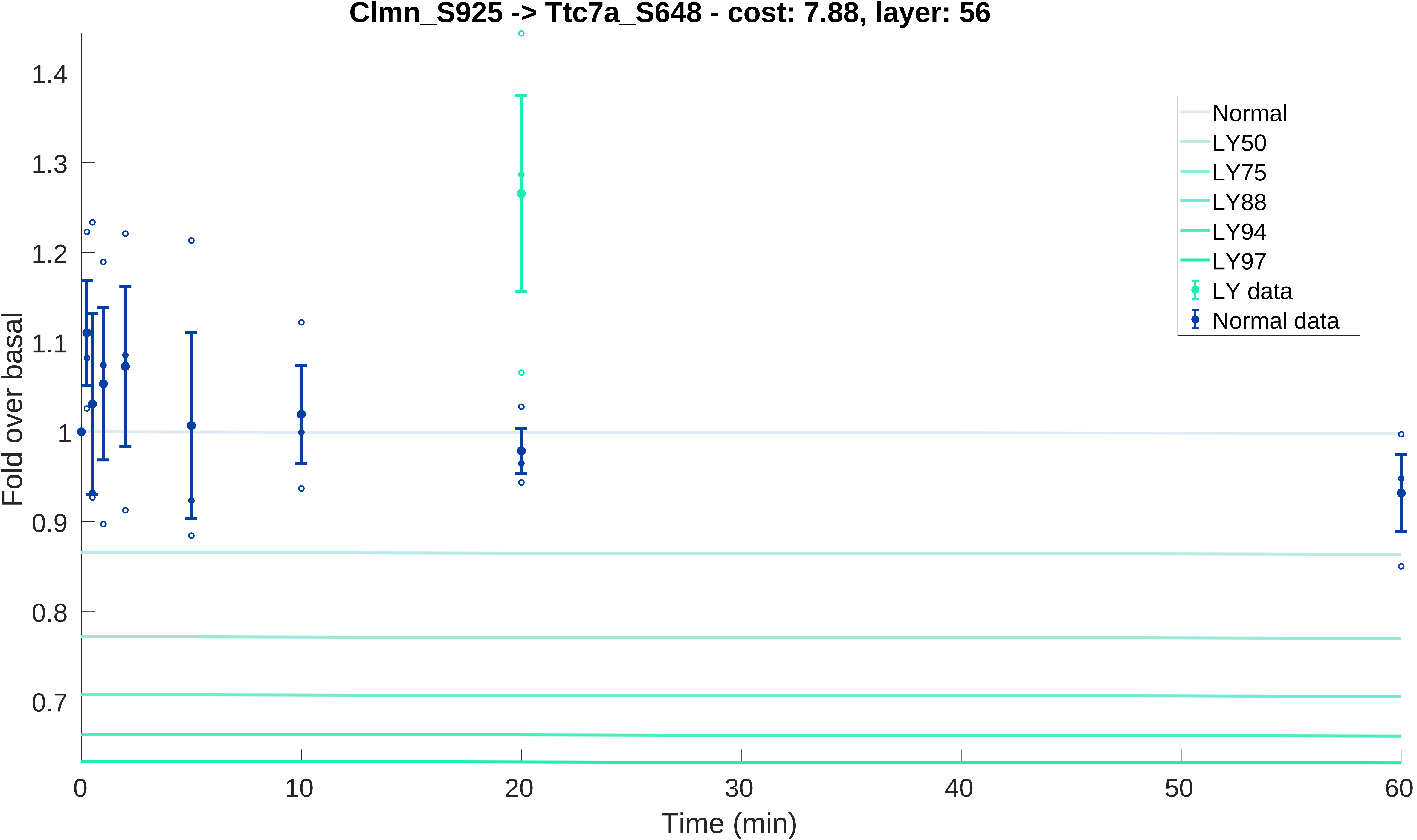

Clmn_S925 -> Ttc7a_S648 - cost: 7.88, layer: 56
Fold over basal
1.4
1.3
1.2
1.1
1
0.9
0.8
0.7
0
10
20
30
40
50
60
Time (min)
Normal
LY50
LY75
LY88
LY94
LY97
LY data
Normal data

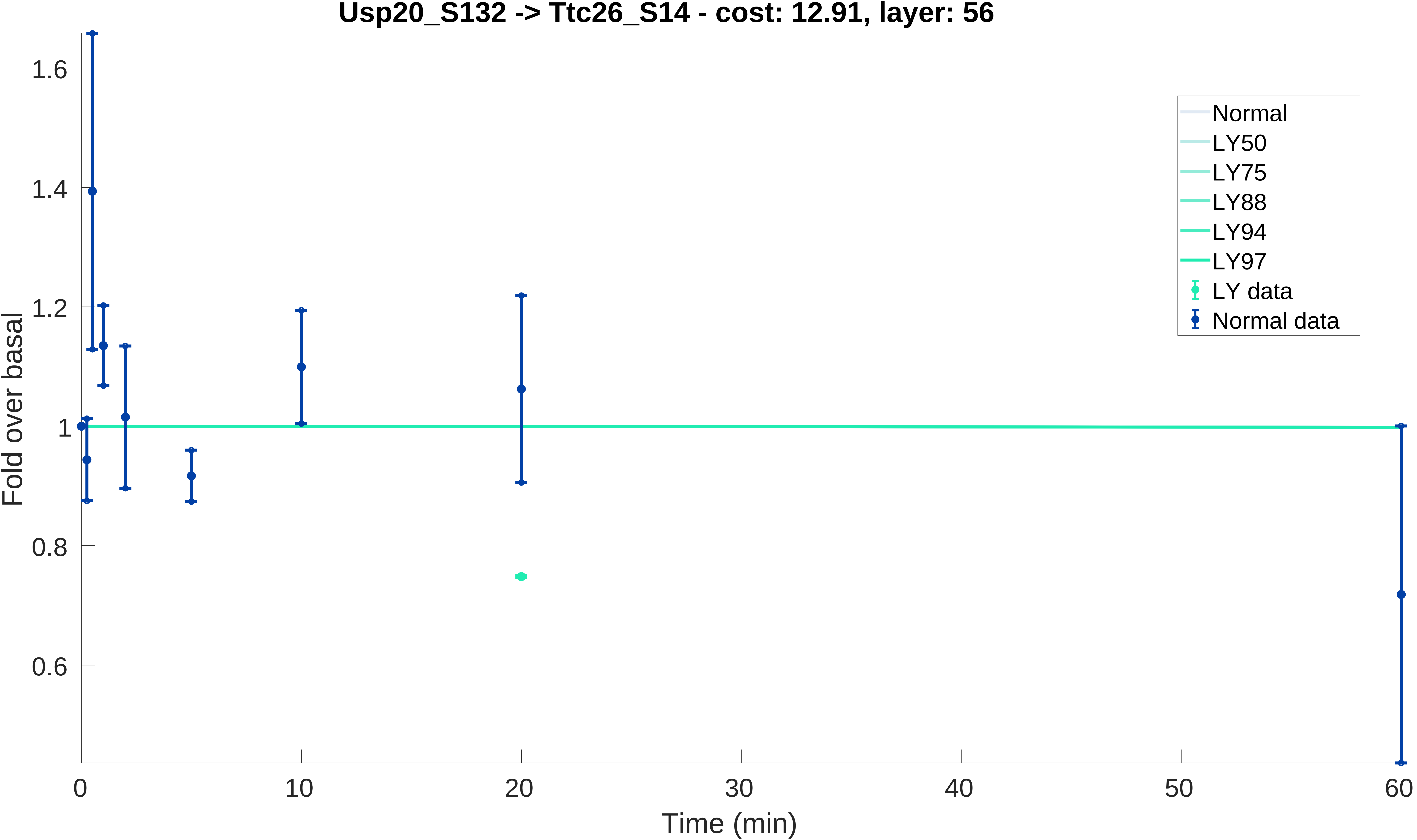

Usp20_S132 -> Ttc26_S14 - cost: 12.91, layer: 56
1.6
1.4
1.2
1
0.8
0.6
Fold over basal
0
10
20
30
40
50
60
Time (min)
Normal
LY50
LY75
LY88
LY94
LY97
LY data
Normal data

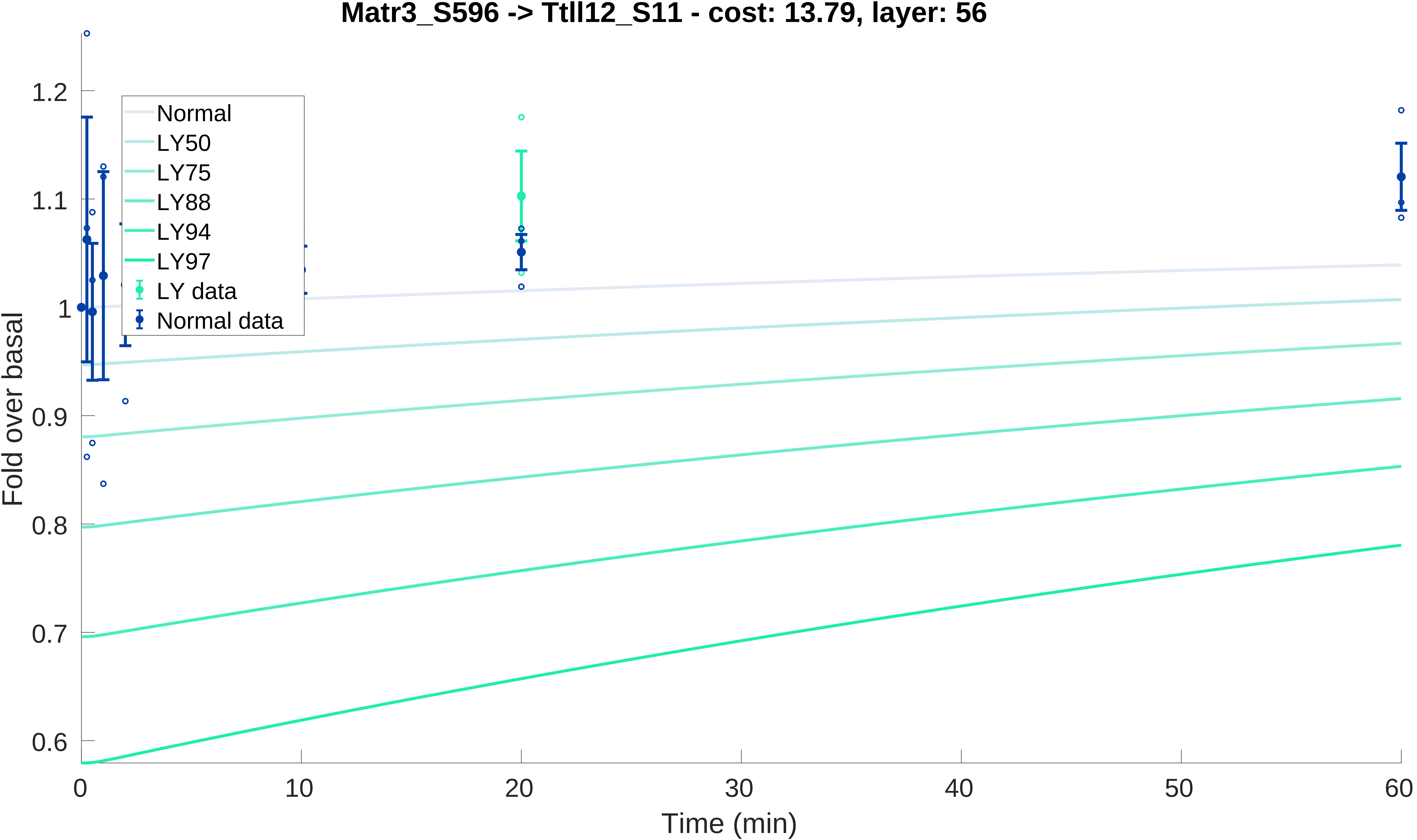

Matr3_S596 -> Ttll12_S11 - cost: 13.79, layer: 56
Fold over basal
Time (min)
1.2
1.1
1
0.9
0.8
0.7
0.6
0
10
20
30
40
50
60
Normal
LY50
LY75
LY88
LY94
LY97
LY data
Normal data

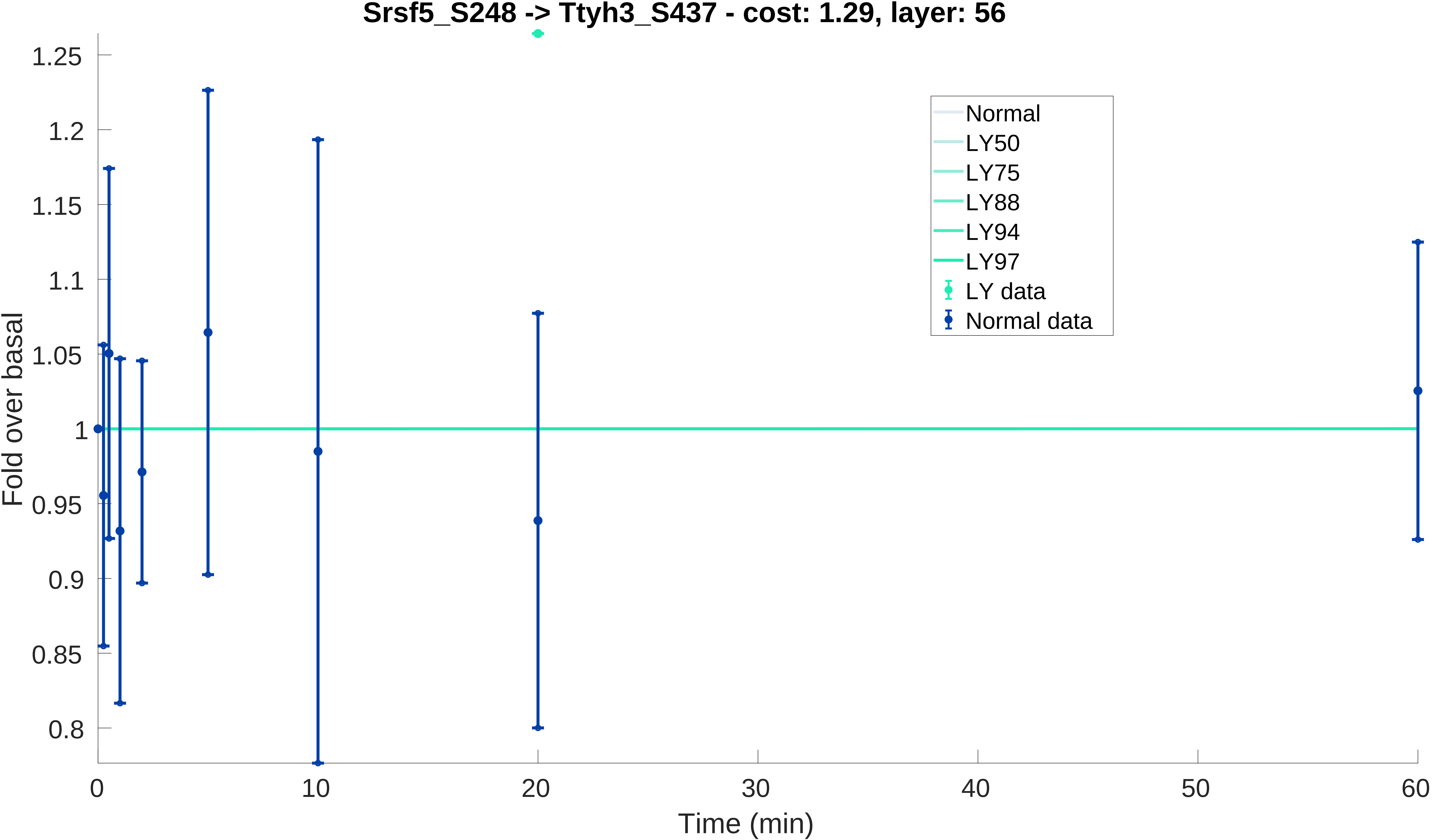

Srsf5_S248 -> Ttyh3_S437 - cost: 1.29, layer: 56
Fold over basal
1.25
1.2
1.15
1.1
1.05
1
0.95
0.9
0.85
0.8
Normal
LY50
LY75
LY88
LY94
LY97
LY data
Normal data
0
10
20
30
40
50
60
Time (min)

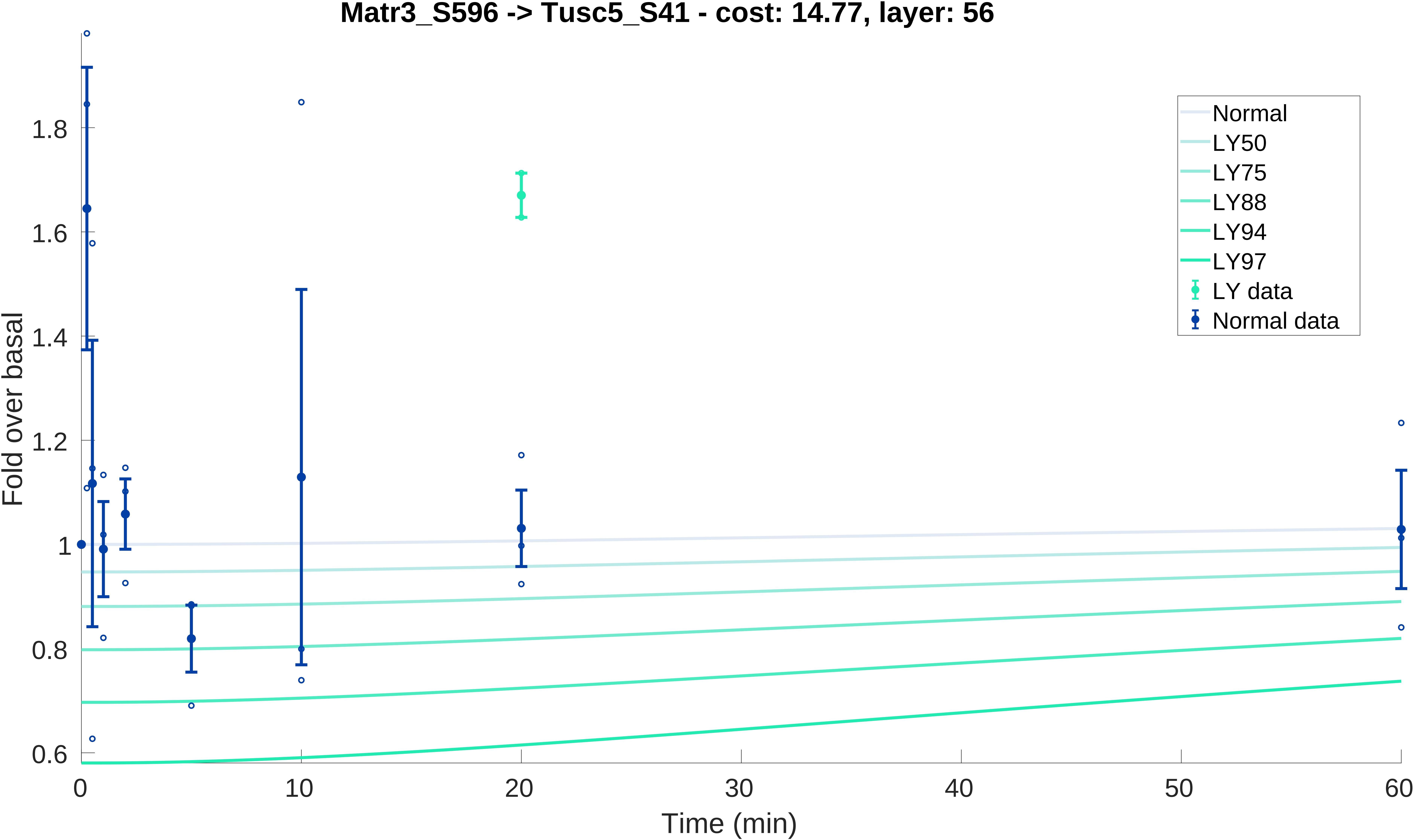

Matr3_S596 -> Tusc5_S41 - cost: 14.77, layer: 56
Fold over basal
Time (min)
0.6
0.8
1
1.2
1.4
1.6
1.8
0
10
20
30
40
50
60
Normal
LY50
LY75
LY88
LY94
LY97
LY data
Normal data

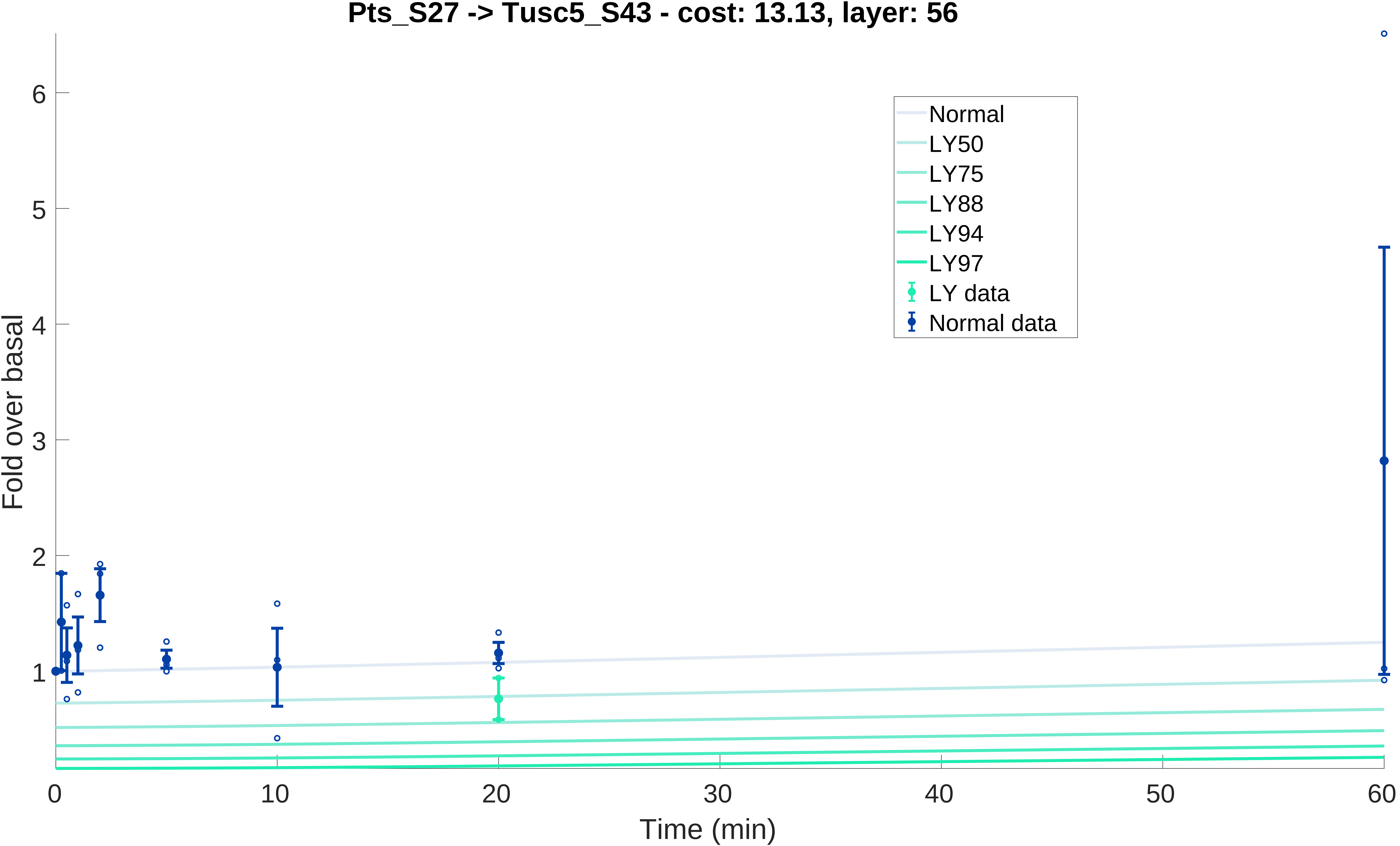
Pts_S27 -> Tusc5_S43 - cost: 13.13, layer: 56
Fold over basal
Time (min)
0
10
20
30
40
50
60
1
2
3
4
5
6
Normal
LY50
LY75
LY88
LY94
LY97
LY data
Normal data

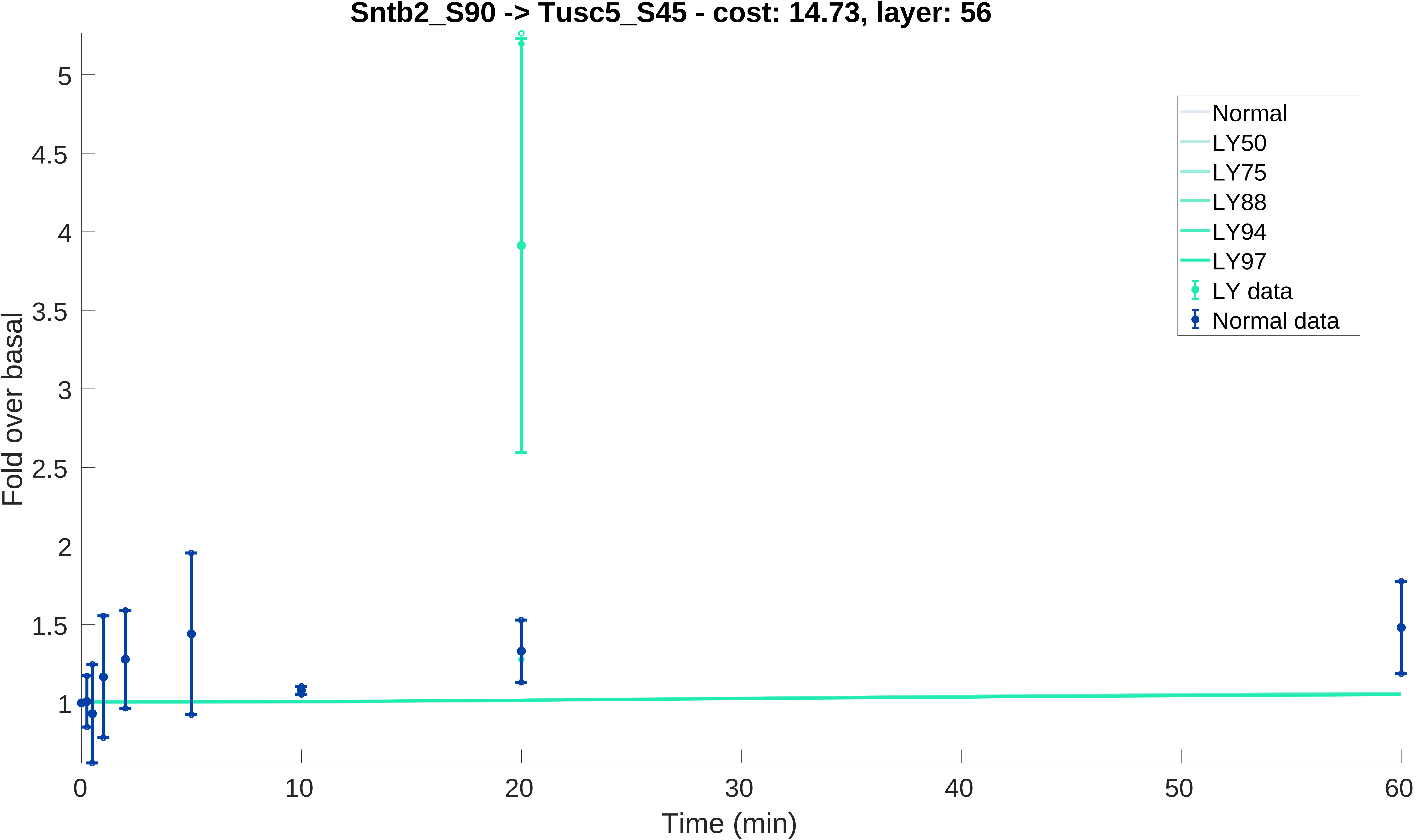

Sntb2_S90 -> Tusc5_S45 - cost: 14.73, layer: 56
Fold over basal
Time (min)
5
4.5
4
3.5
3
2.5
2
1.5
1
0
10
20
30
40
50
60
Normal
LY50
LY75
LY88
LY94
LY97
LY data
Normal data

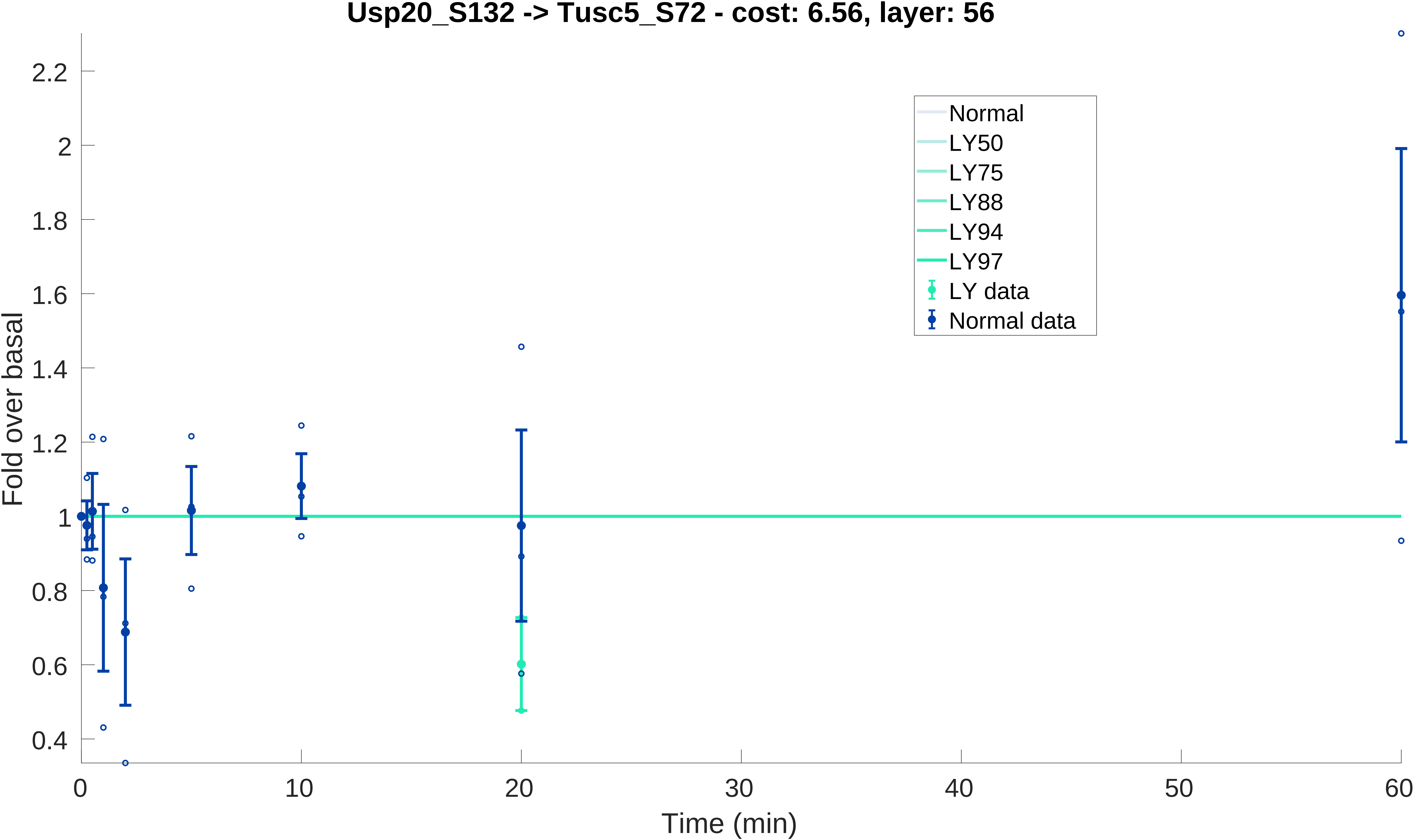

Usp20_S132 -> Tusc5_S72 - cost: 6.56, layer: 56
Fold over basal
Time (min)
0.4
0.6
0.8
1
1.2
1.4
1.6
1.8
2
2.2
0
10
20
30
40
50
60
Normal
LY50
LY75
LY88
LY94
LY97
LY data
Normal data

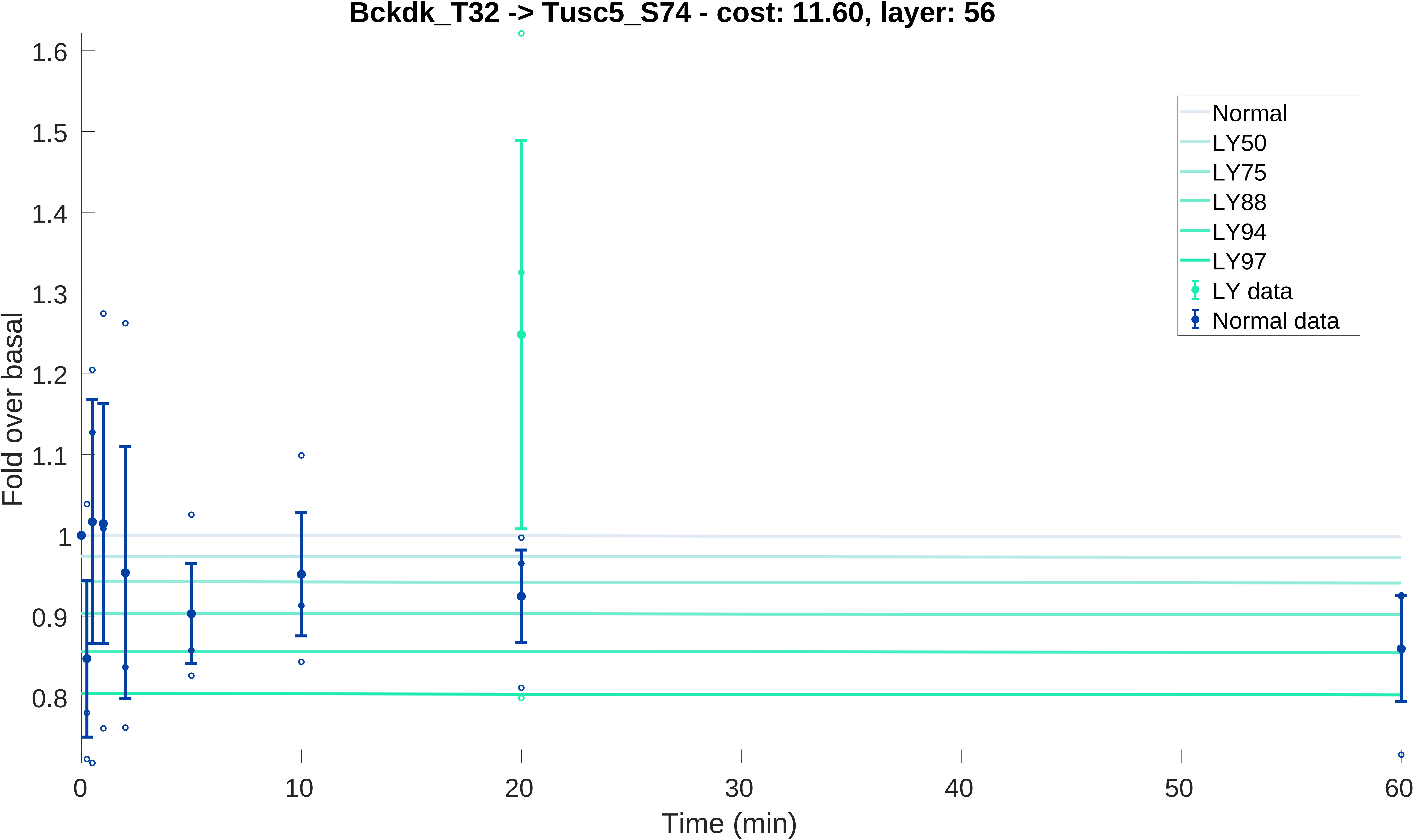

Bckdk_T32 -> Tusc5_S74 - cost: 11.60, layer: 56
Fold over basal
1.6
1.5
1.4
1.3
1.2
1.1
1
0.9
0.8
0
10
20
30
40
50
60
Time (min)
Normal
LY50
LY75
LY88
LY94
LY97
LY data
Normal data

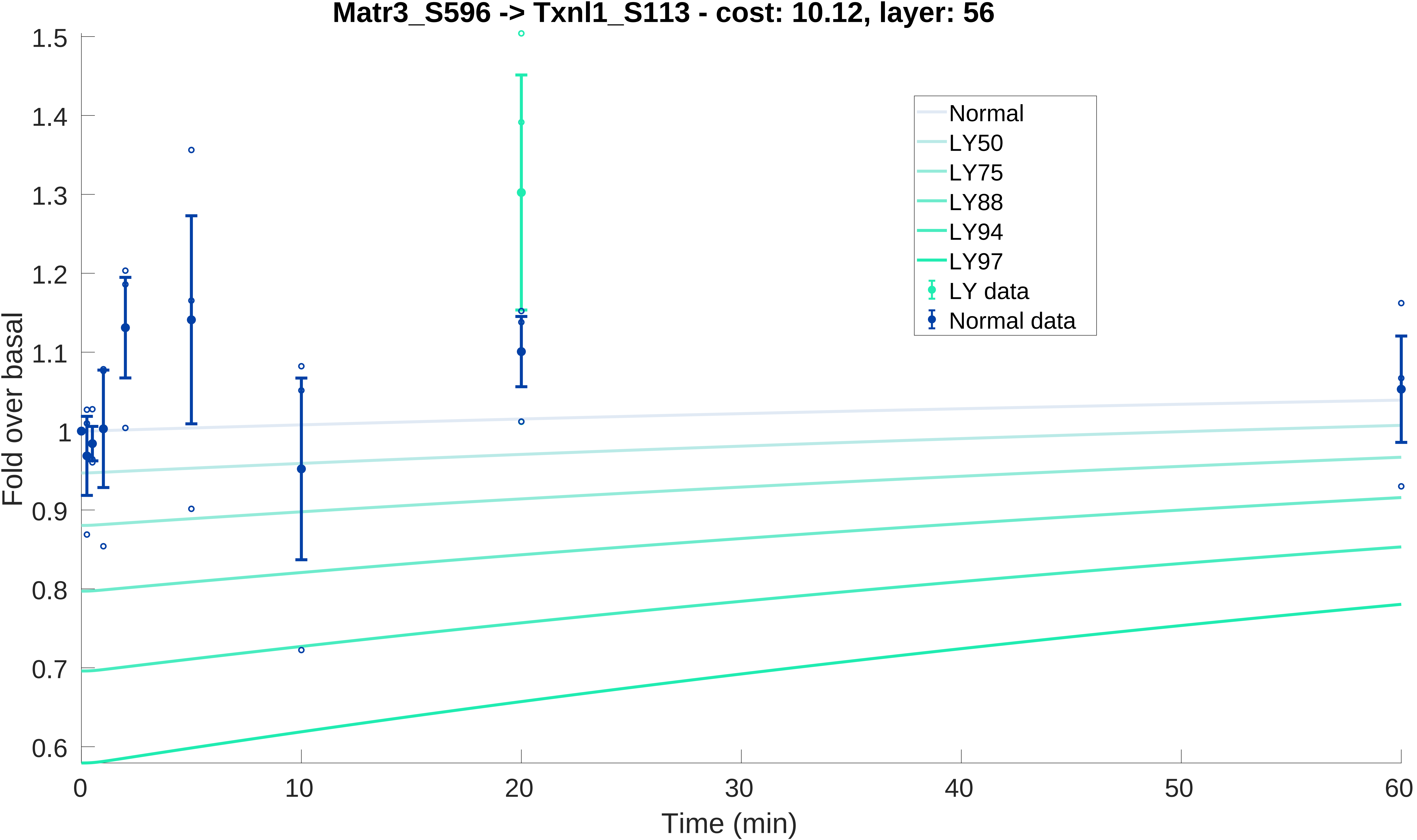

Matr3_S596 -> Txnl1_S113 - cost: 10.12, layer: 56
Fold over basal
Time (min)
0.6
0.7
0.8
0.9
1
1.1
1.2
1.3
1.4
1.5
0
10
20
30
40
50
60
Normal
LY50
LY75
LY88
LY94
LY97
LY data
Normal data

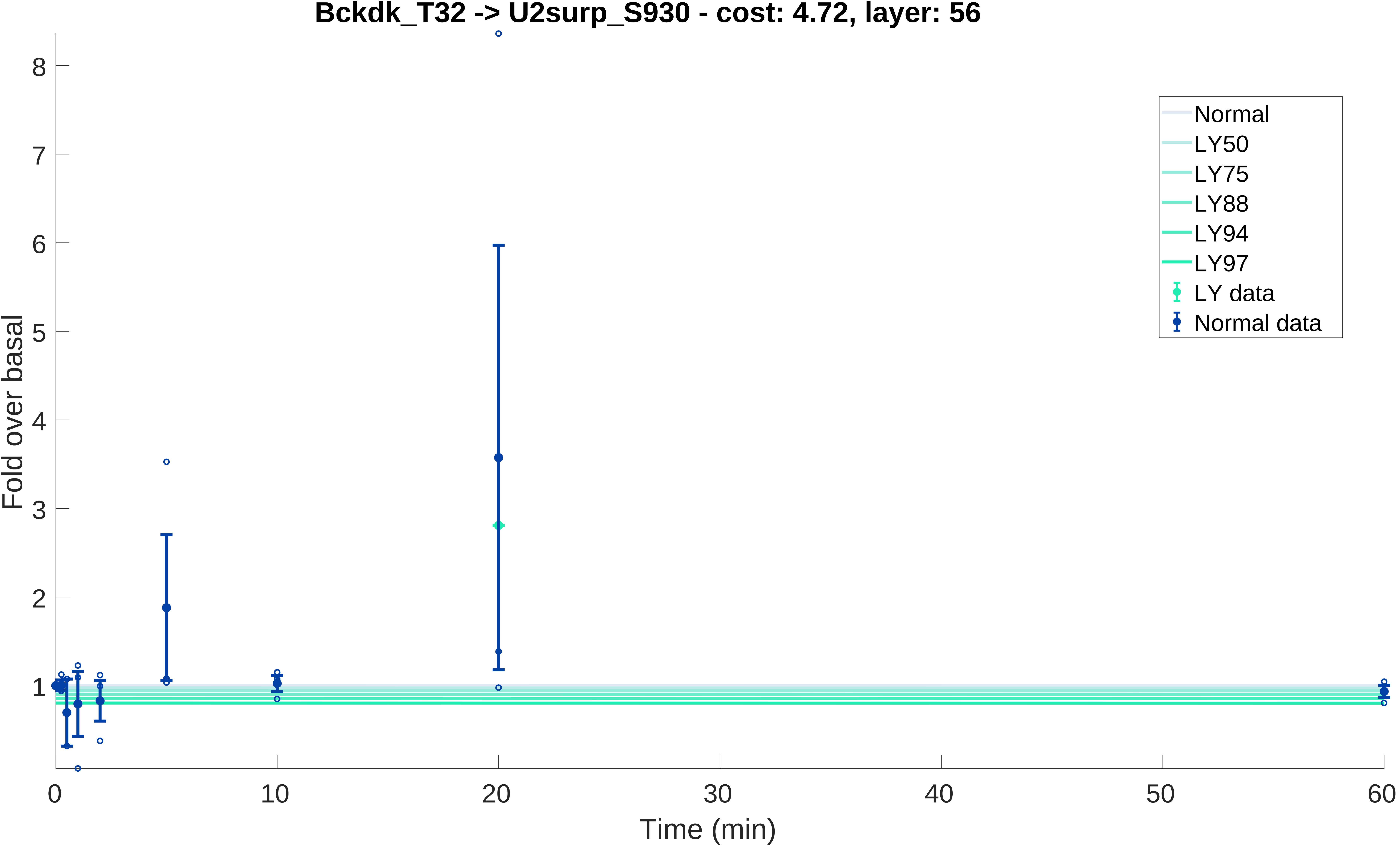

Bckdk_T32 -> U2surp_S930 - cost: 4.72, layer: 56
Fold over basal
8
7
6
5
4
3
2
1
0
10
20
30
40
50
60
Time (min)
Normal
LY50
LY75
LY88
LY94
LY97
LY data
Normal data

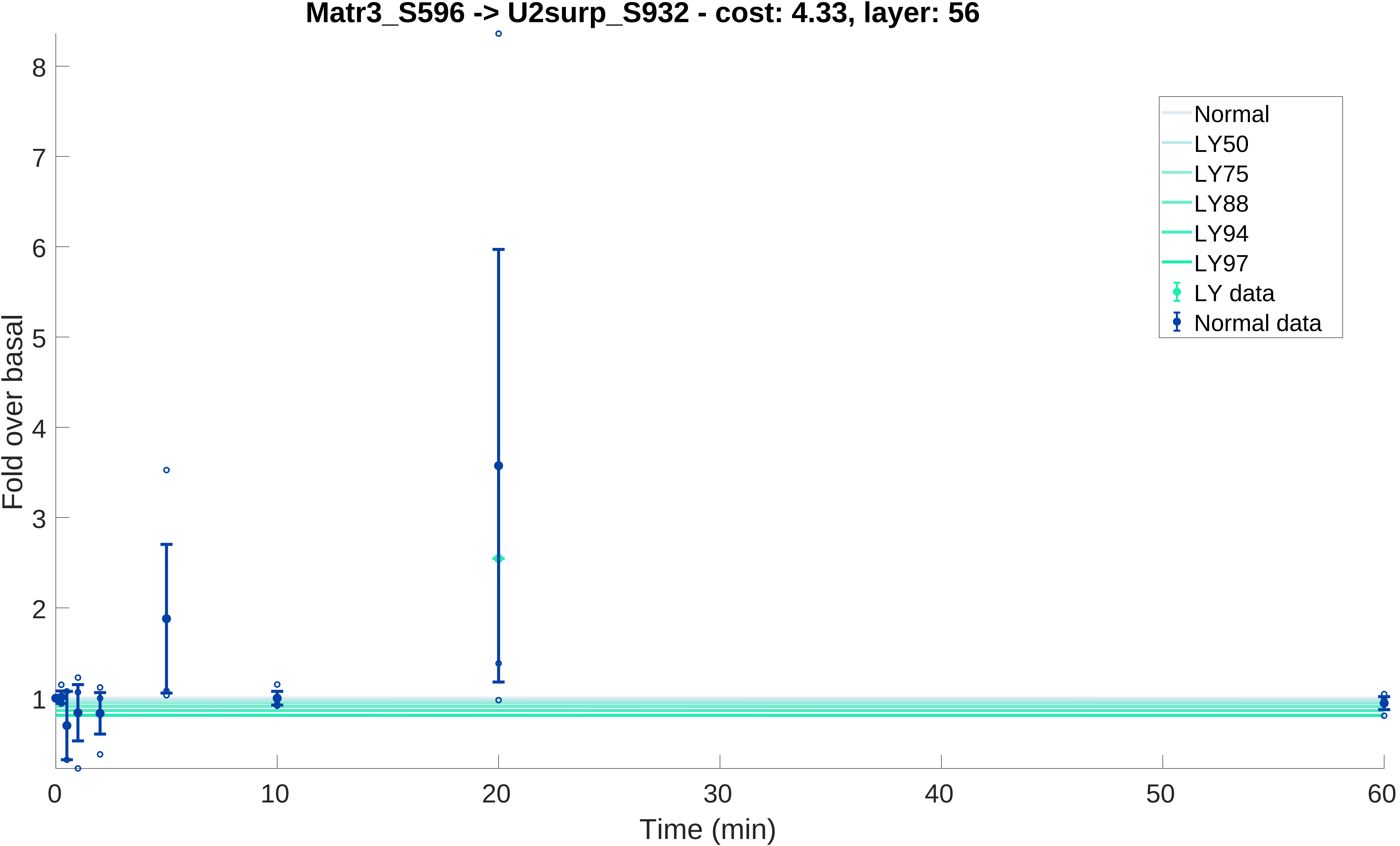

Matr3_S596 -> U2surp_S932 - cost: 4.33, layer: 56
Fold over basal
Time (min)
0
10
20
30
40
50
60
1
2
3
4
5
6
7
8
Normal
LY50
LY75
LY88
LY94
LY97
LY data
Normal data

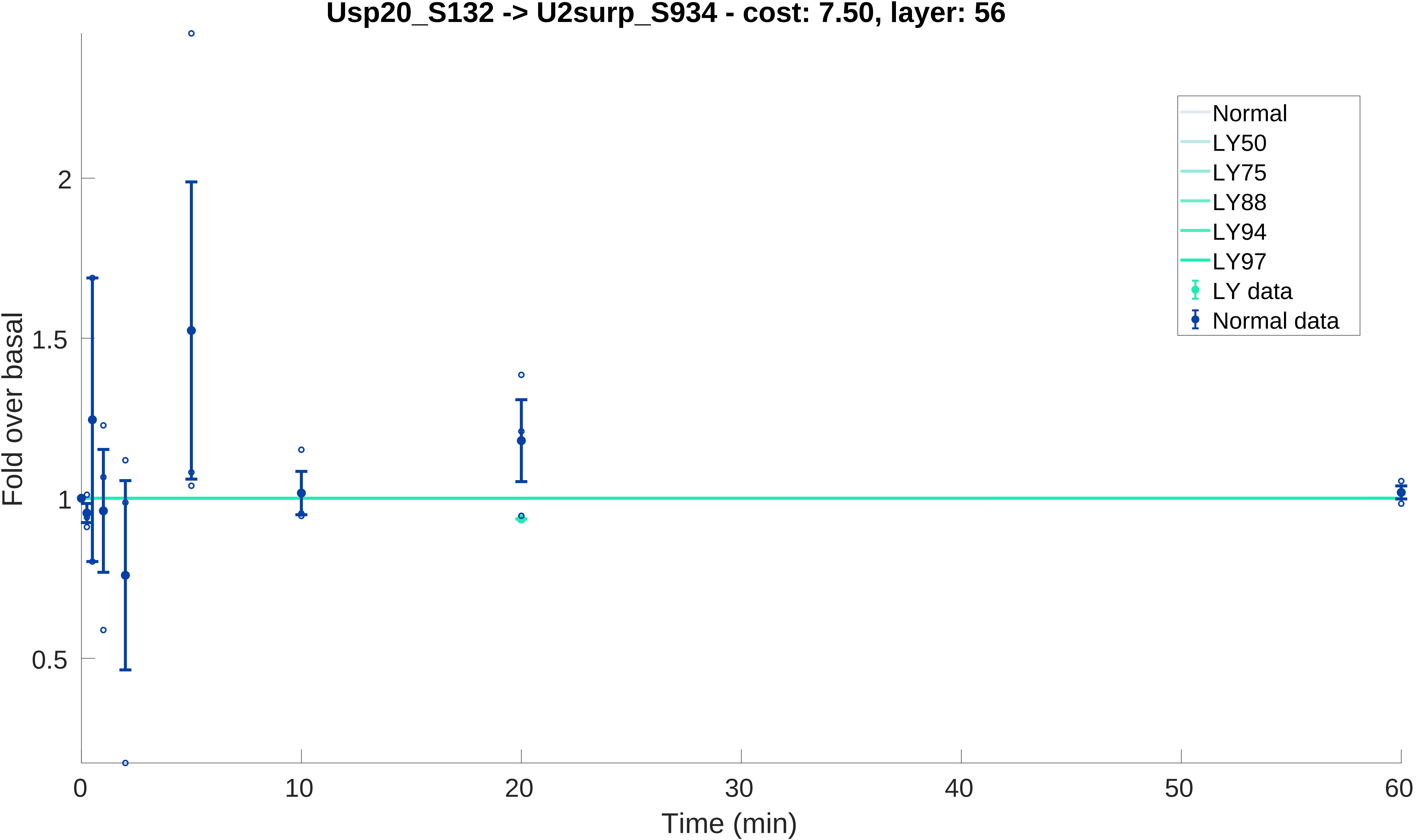

Usp20_S132 -> U2surp_S934 - cost: 7.50, layer: 56
Fold over basal
Time (min)
0.5
1
1.5
2
0
10
20
30
40
50
60
Normal
LY50
LY75
LY88
LY94
LY97
LY data
Normal data

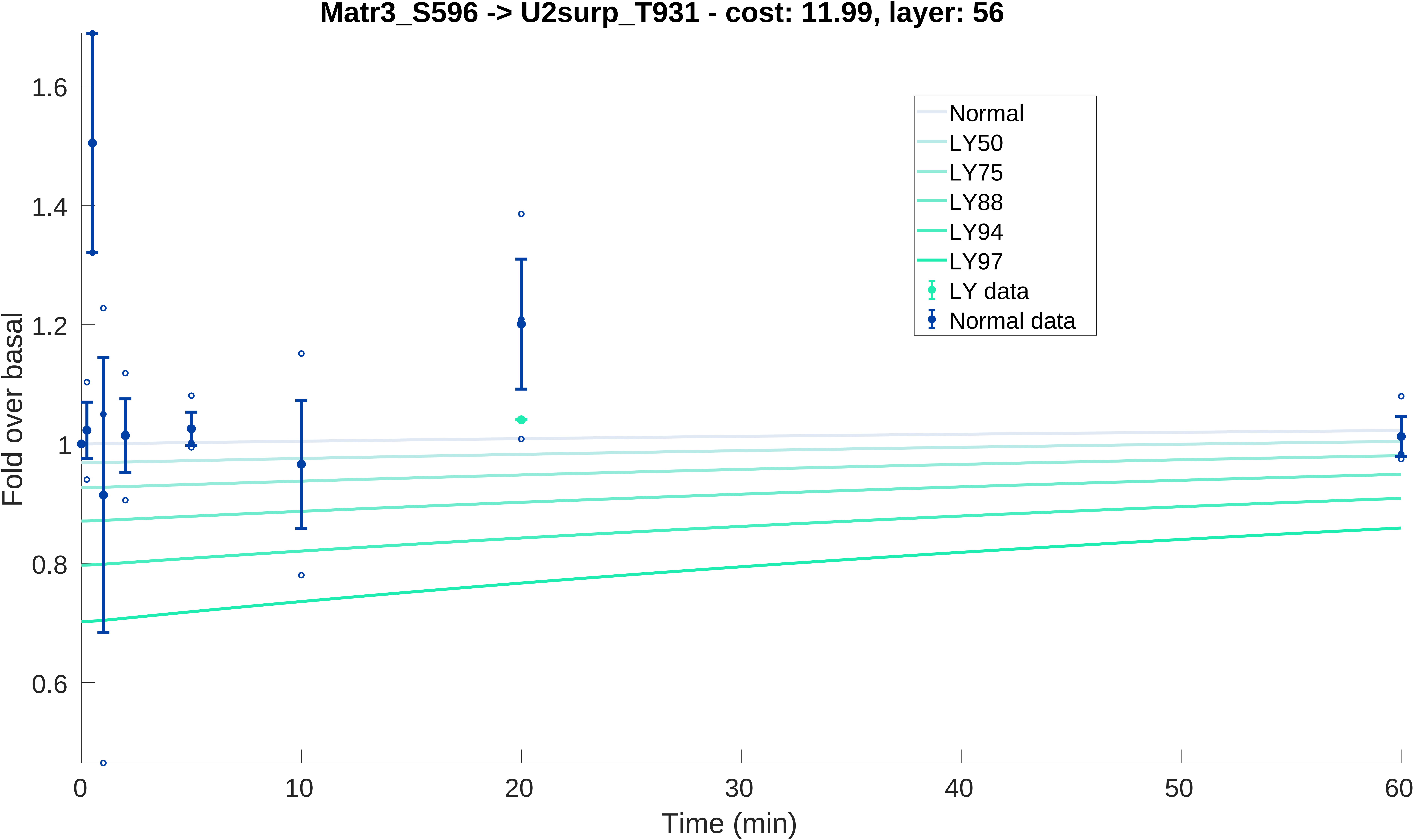

Matr3_S596 -> U2surp_T931 - cost: 11.99, layer: 56
Fold over basal
Time (min)
1.6
1.4
1.2
1
0.8
0.6
0
10
20
30
40
50
60
Normal
LY50
LY75
LY88
LY94
LY97
LY data
Normal data

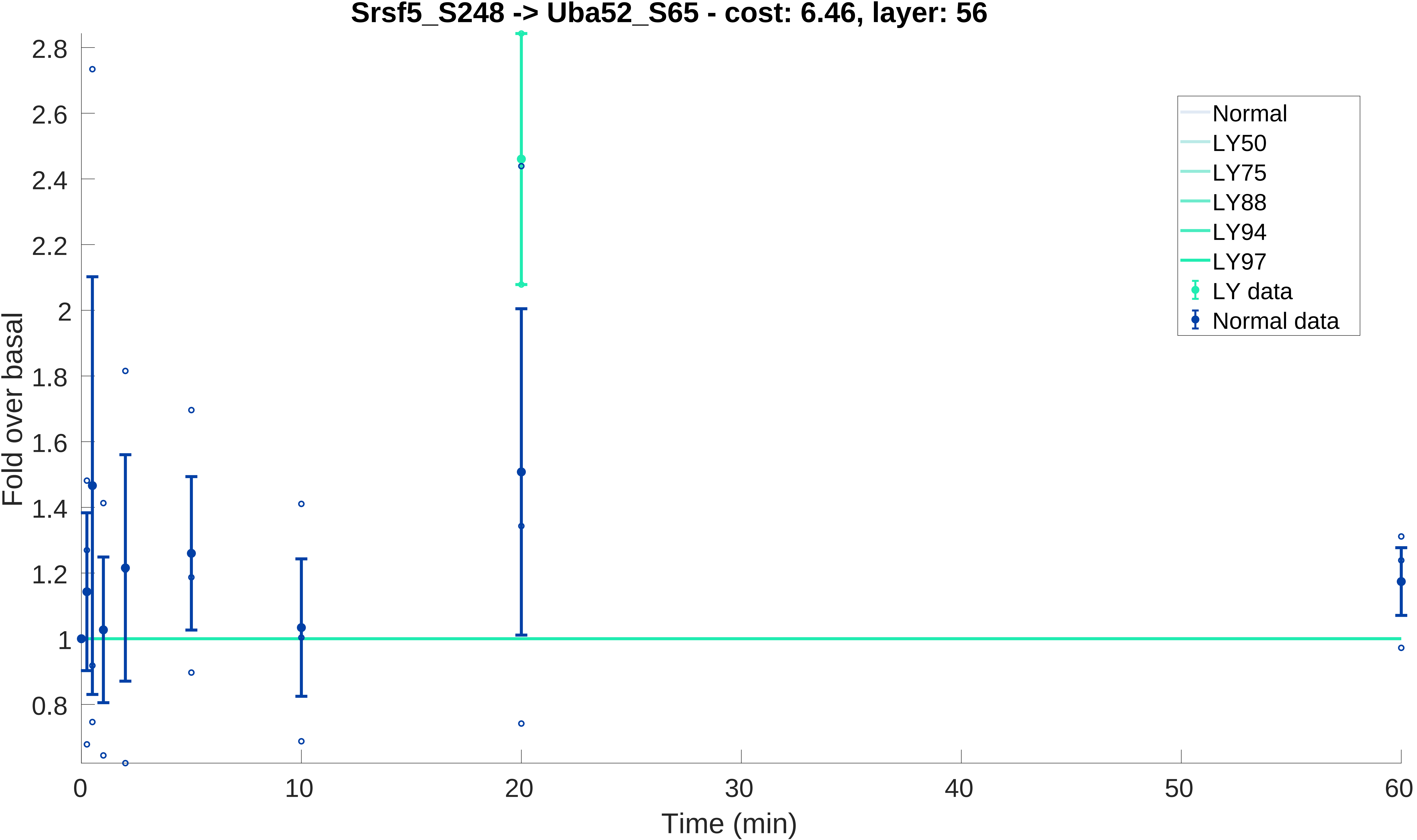
Srsf5_S248 -> Uba52_S65 - cost: 6.46, layer: 56
Fold over basal
Time (min)
0
10
20
30
40
50
60
0.8
1
1.2
1.4
1.6
1.8
2
2.2
2.4
2.6
2.8
Normal
LY50
LY75
LY88
LY94
LY97
LY data
Normal data

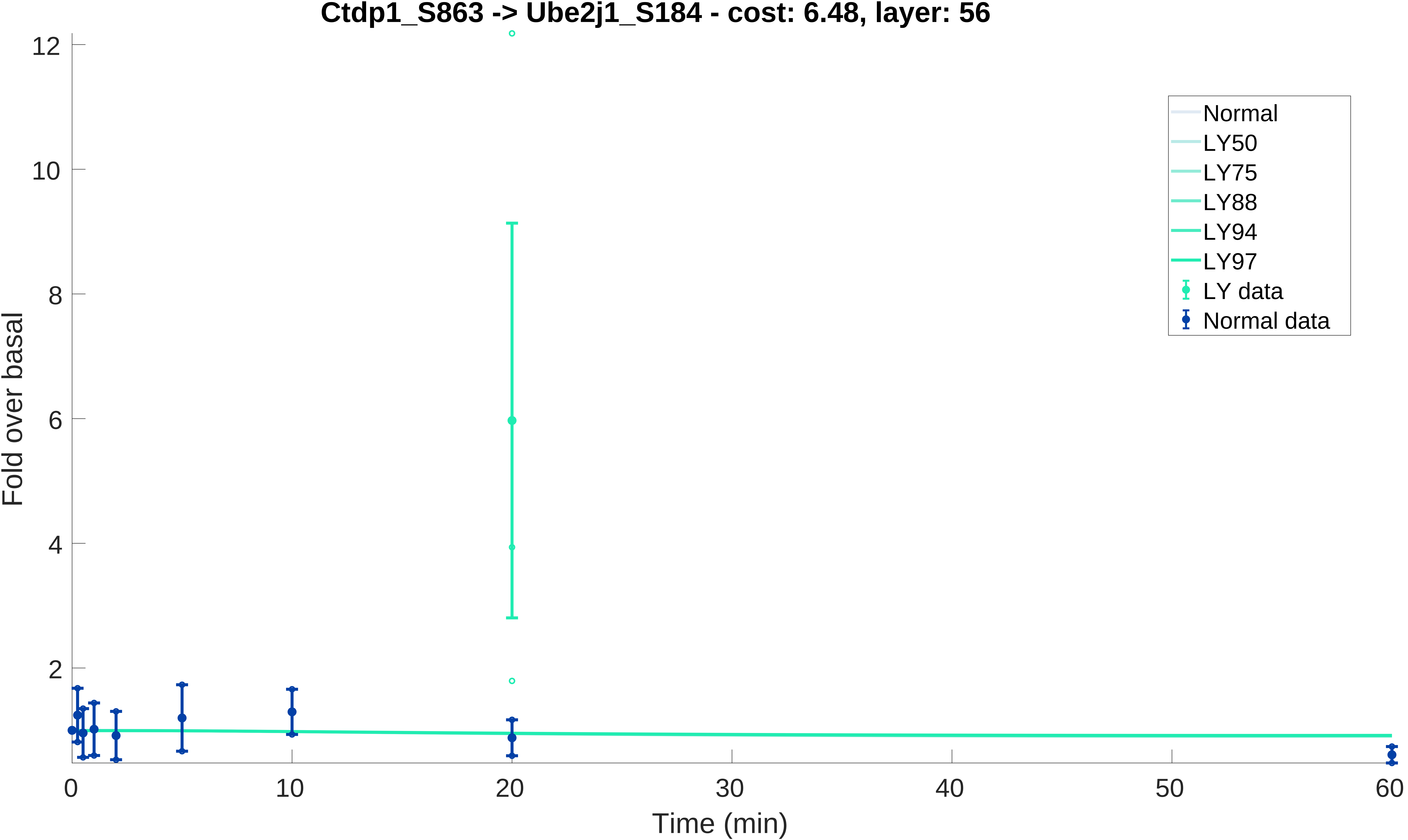

Ctdp1_S863 -> Ube2j1_S184 - cost: 6.48, layer: 56
Fold over basal
Time (min)
0
10
20
30
40
50
60
2
4
6
8
10
12
Normal
LY50
LY75
LY88
LY94
LY97
LY data
Normal data

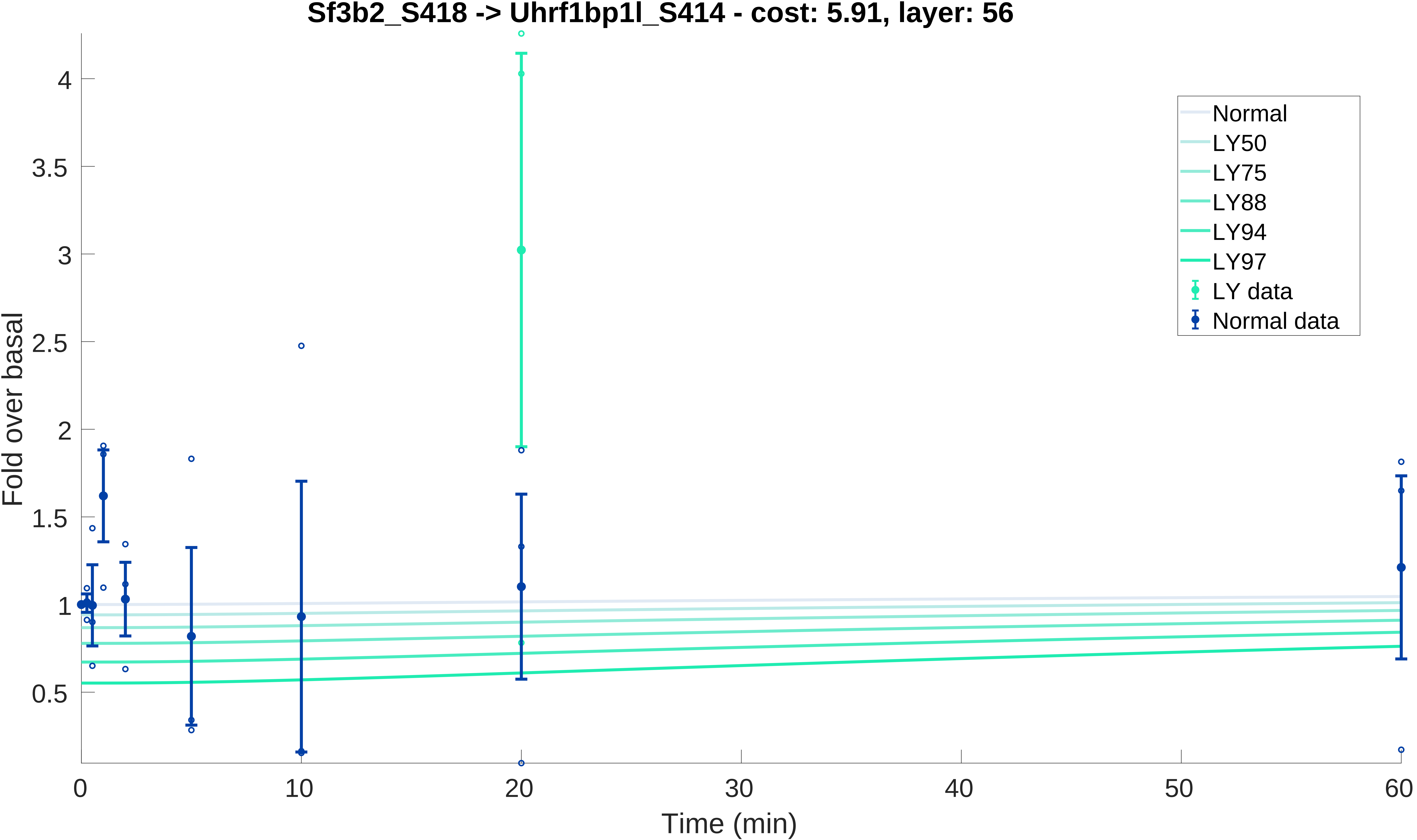

Sf3b2_S418 -> Uhrf1bp1l_S414 - cost: 5.91, layer: 56
Fold over basal
Time (min)
0
10
20
30
40
50
60
0.5
1
1.5
2
2.5
3
3.5
4
Normal
LY50
LY75
LY88
LY94
LY97
LY data
Normal data

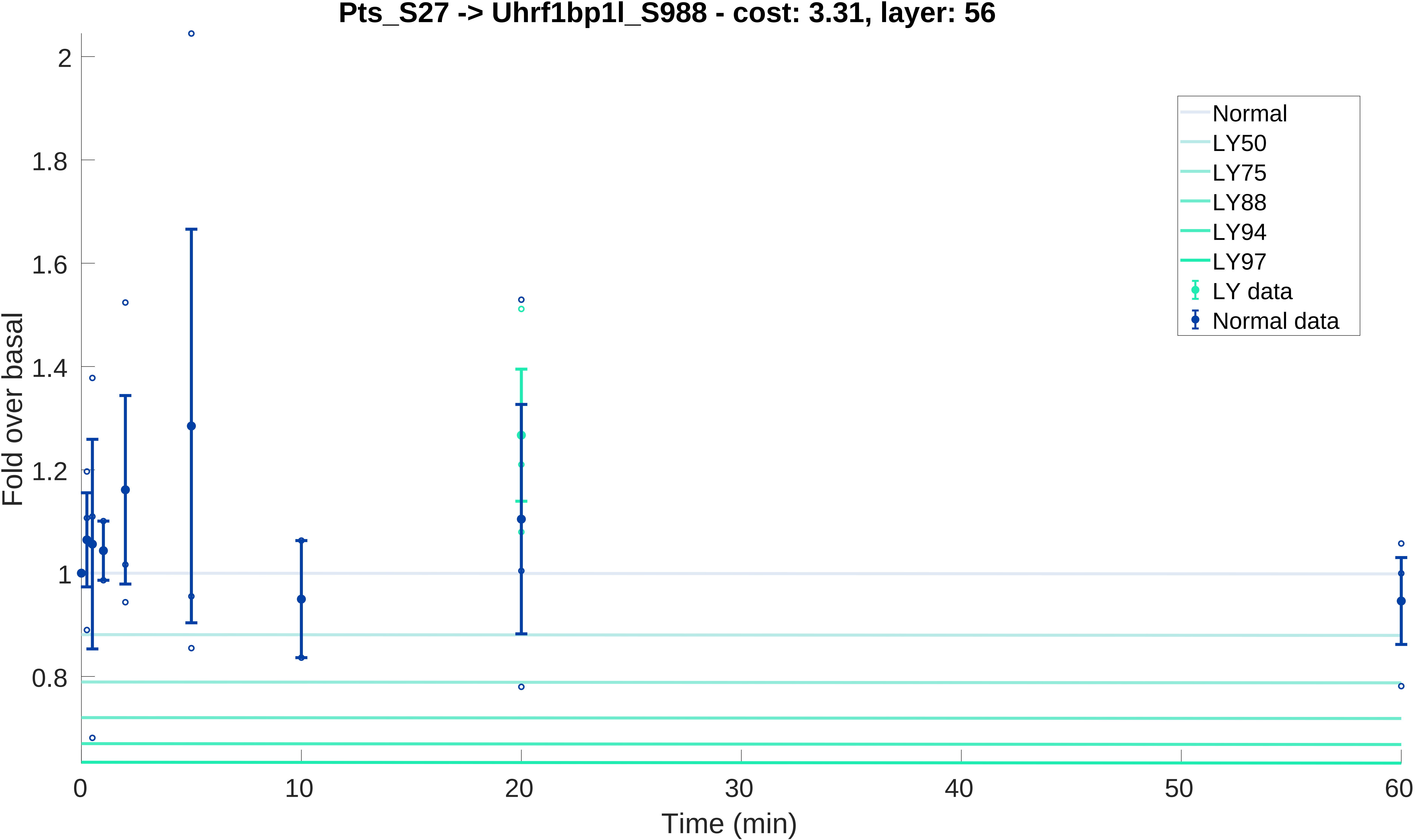

Pts_S27 -> Uhrf1bp1l_S988 - cost: 3.31, layer: 56
Fold over basal
Time (min)
0
10
20
30
40
50
60
0.8
1
1.2
1.4
1.6
1.8
2
Normal
LY50
LY75
LY88
LY94
LY97
LY data
Normal data

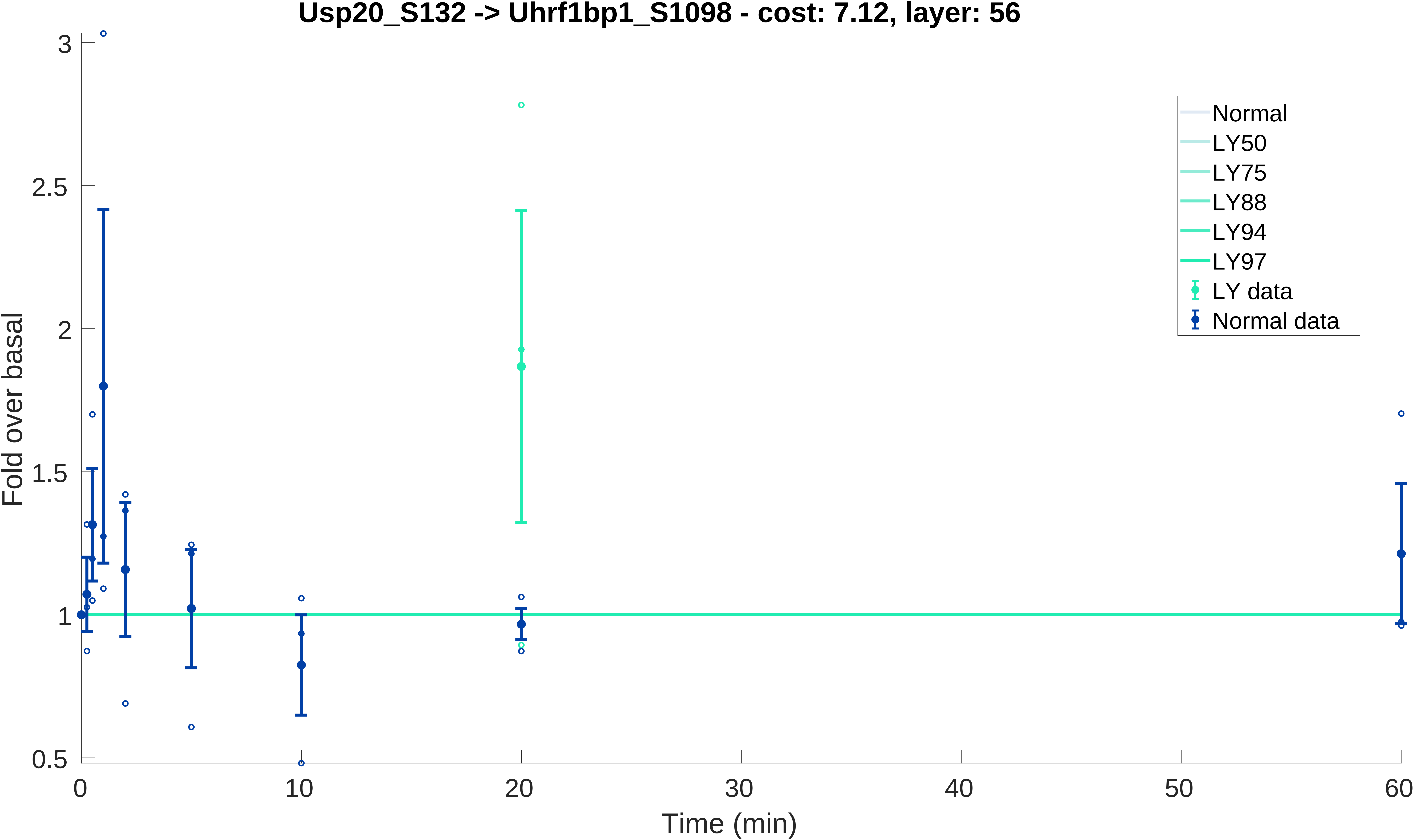
Usp20_S132 -> Uhrf1bp1_S1098 - cost: 7.12, layer: 56
Fold over basal
Time (min)
Normal
LY50
LY75
LY88
LY94
LY97
LY data
Normal data
0.5
1
1.5
2
2.5
3
0
10
20
30
40
50
60

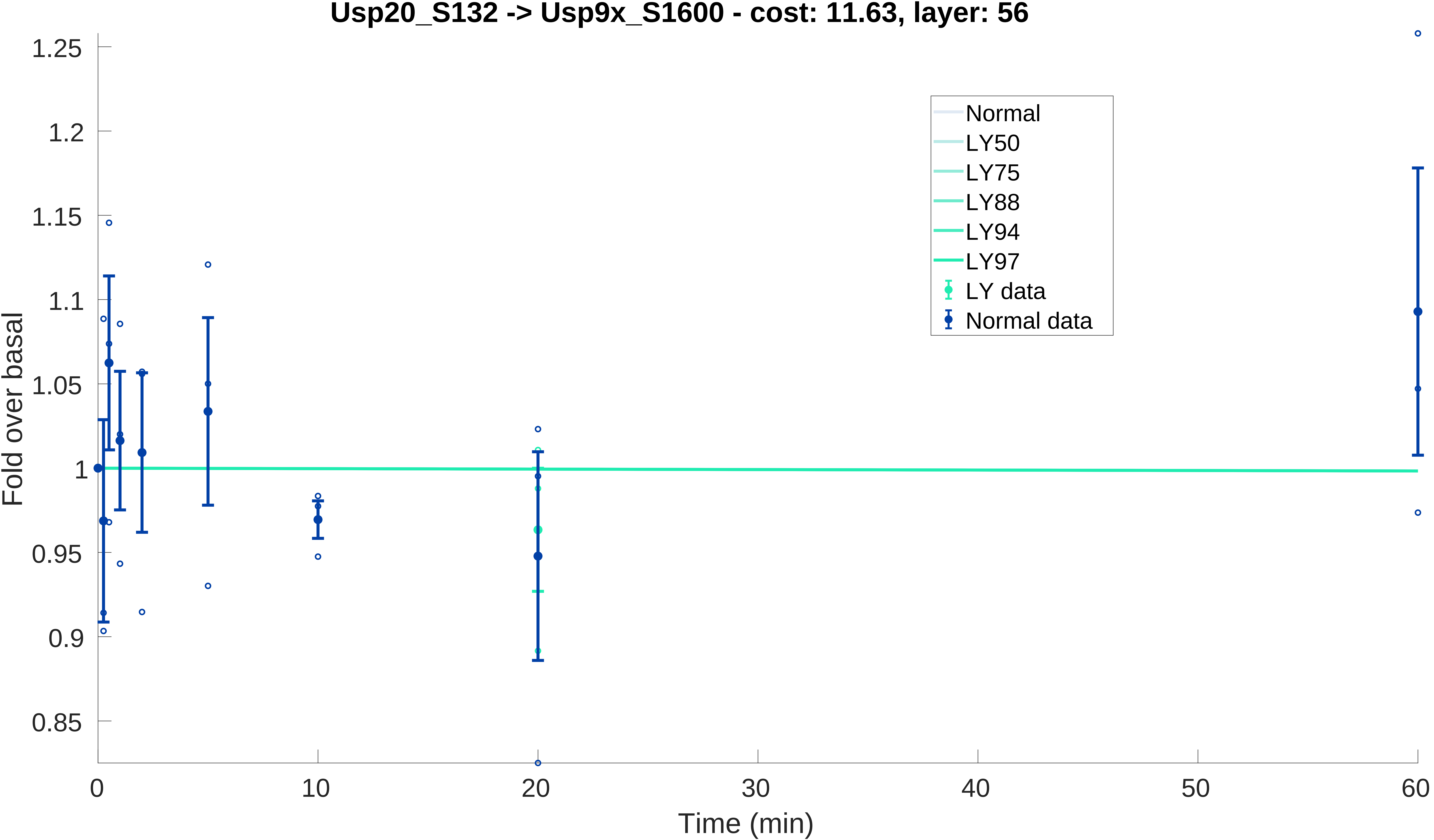

Usp20_S132 -> Usp9x_S1600 - cost: 11.63, layer: 56
Fold over basal
1.25
1.2
1.15
1.1
1.05
1
0.95
0.9
0.85
0
10
20
30
40
50
60
Time (min)
Normal
LY50
LY75
LY88
LY94
LY97
LY data
Normal data

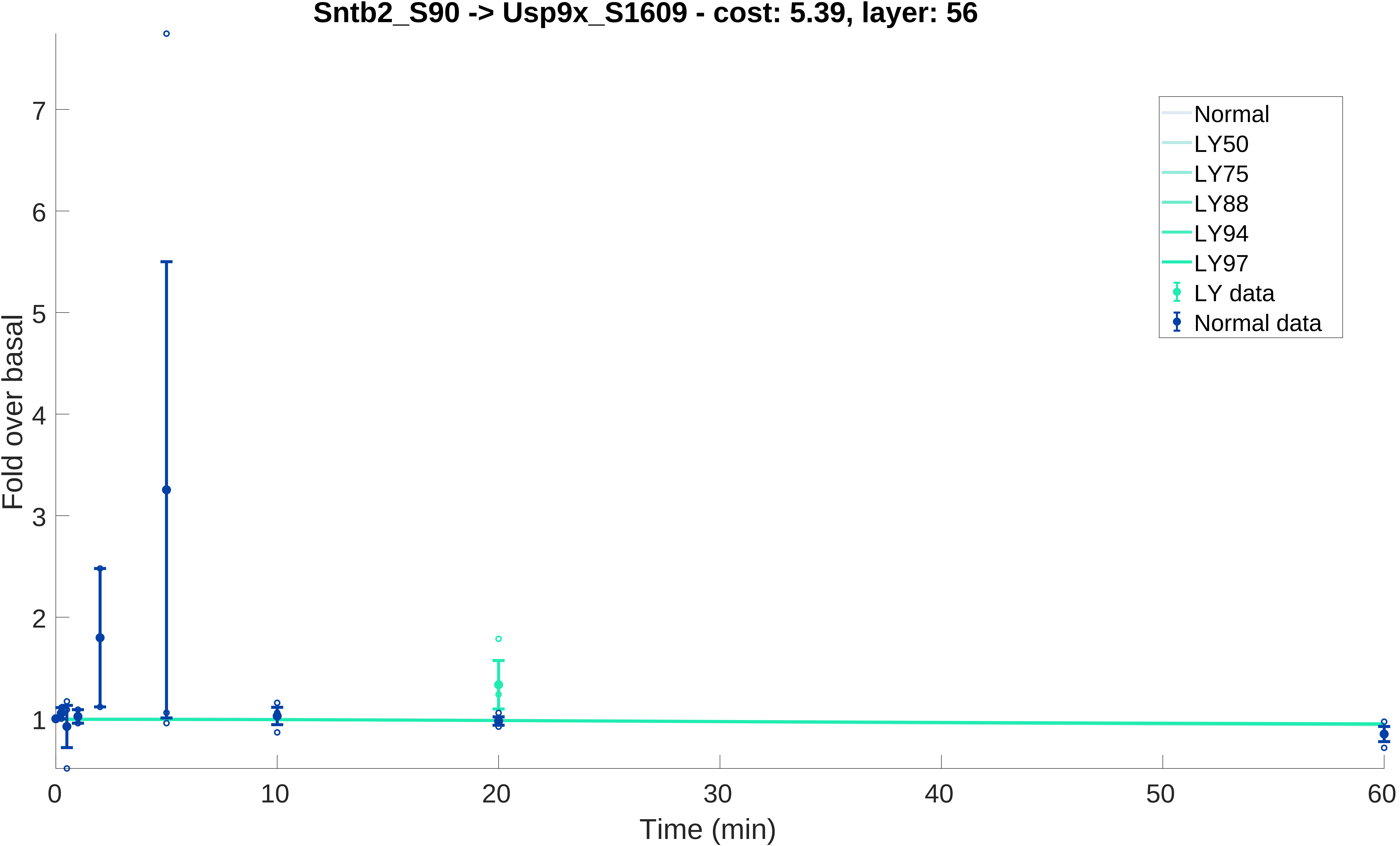

Sntb2_S90 -> Usp9x_S1609 - cost: 5.39, layer: 56
Fold over basal
Time (min)
0
10
20
30
40
50
60
1
2
3
4
5
6
7
Normal
LY50
LY75
LY88
LY94
LY97
LY data
Normal data

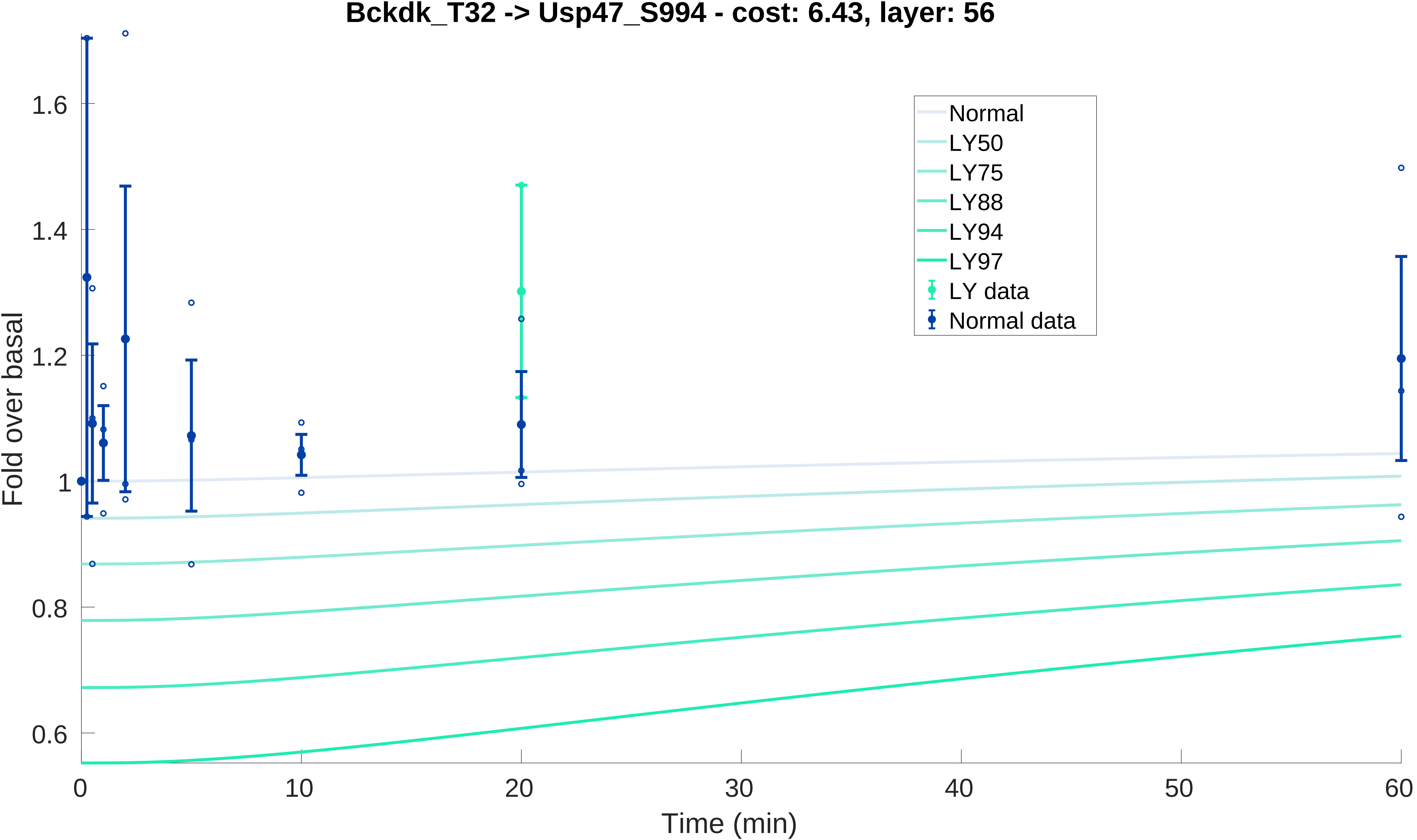

Bckdk_T32 -> Usp47_S994 - cost: 6.43, layer: 56
Fold over basal
1.6
1.4
1.2
1
0.8
0.6
0
10
20
30
40
50
60
Time (min)
Normal
LY50
LY75
LY88
LY94
LY97
LY data
Normal data

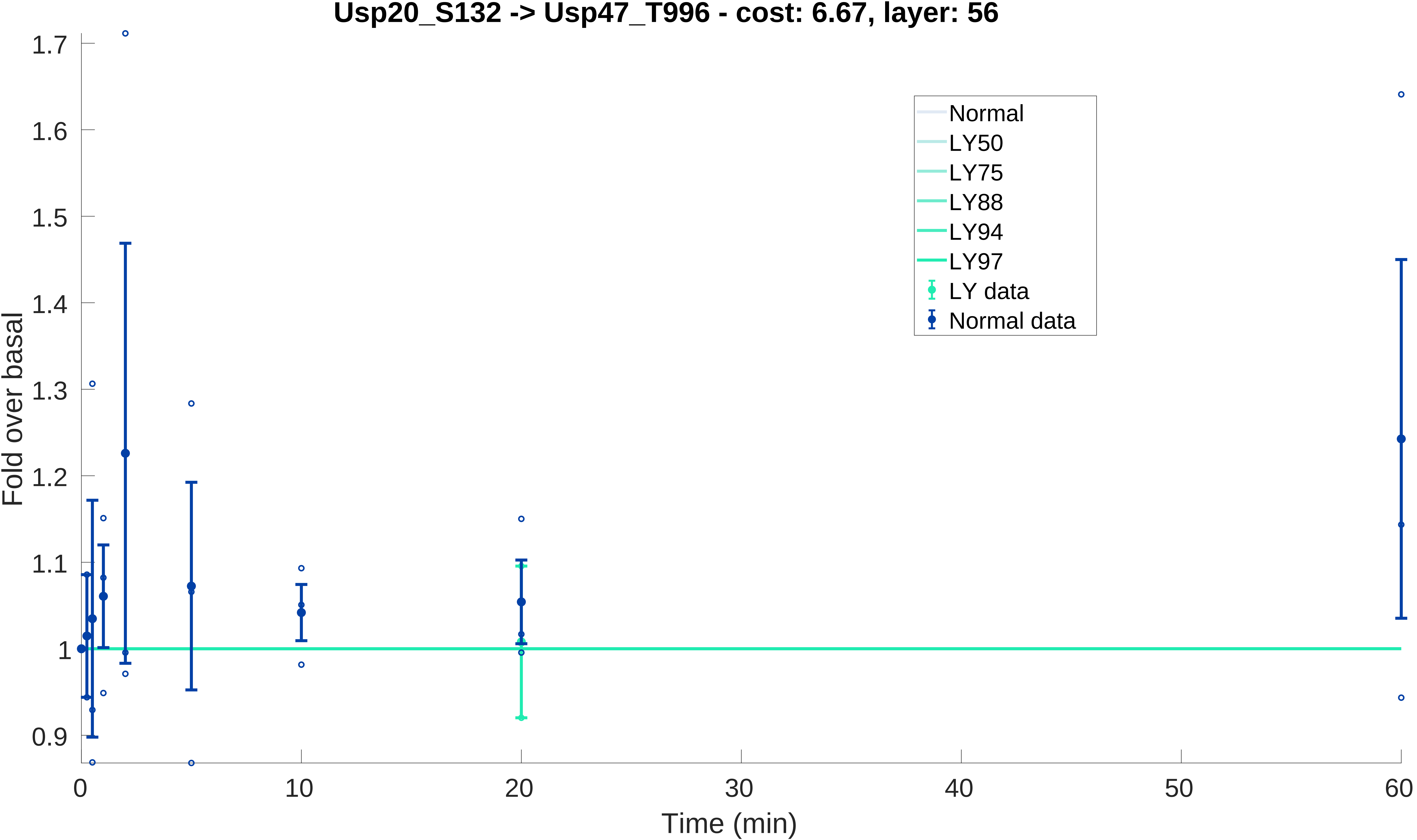
Usp20_S132 -> Usp47_T996 - cost: 6.67, layer: 56
Fold over basal
Time (min)
0.9
1
1.1
1.2
1.3
1.4
1.5
1.6
1.7
0
10
20
30
40
50
60
Normal
LY50
LY75
LY88
LY94
LY97
LY data
Normal data

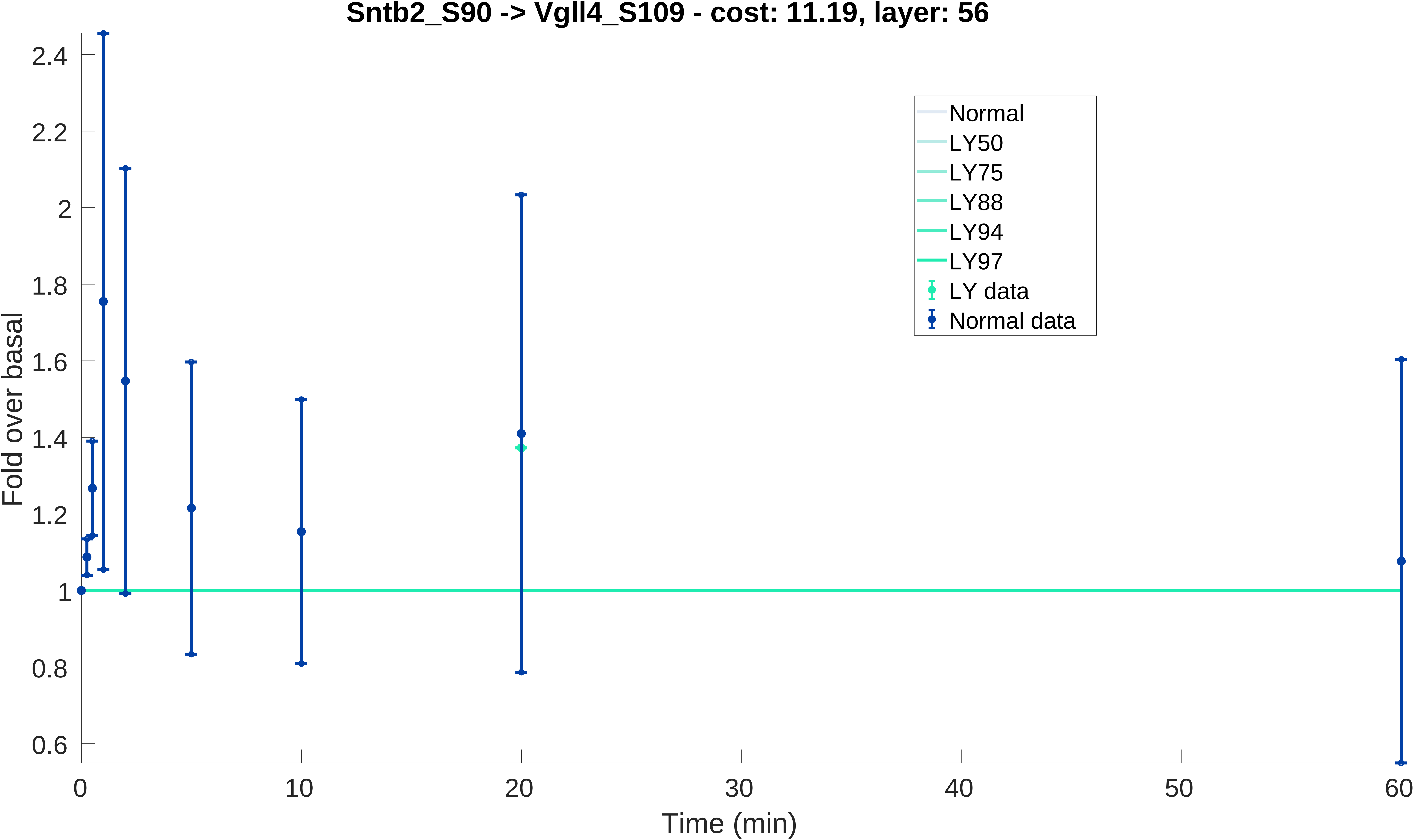
Sntb2_S90 -> Vgll4_S109 - cost: 11.19, layer: 56
Fold over basal
Time (min)
2.4
2.2
2
1.8
1.6
1.4
1.2
1
0.8
0.6
0
10
20
30
40
50
60
Normal
LY50
LY75
LY88
LY94
LY97
LY data
Normal data

Clmn_S925 -> Vps4a_S95 - cost: 9.19, layer: 56

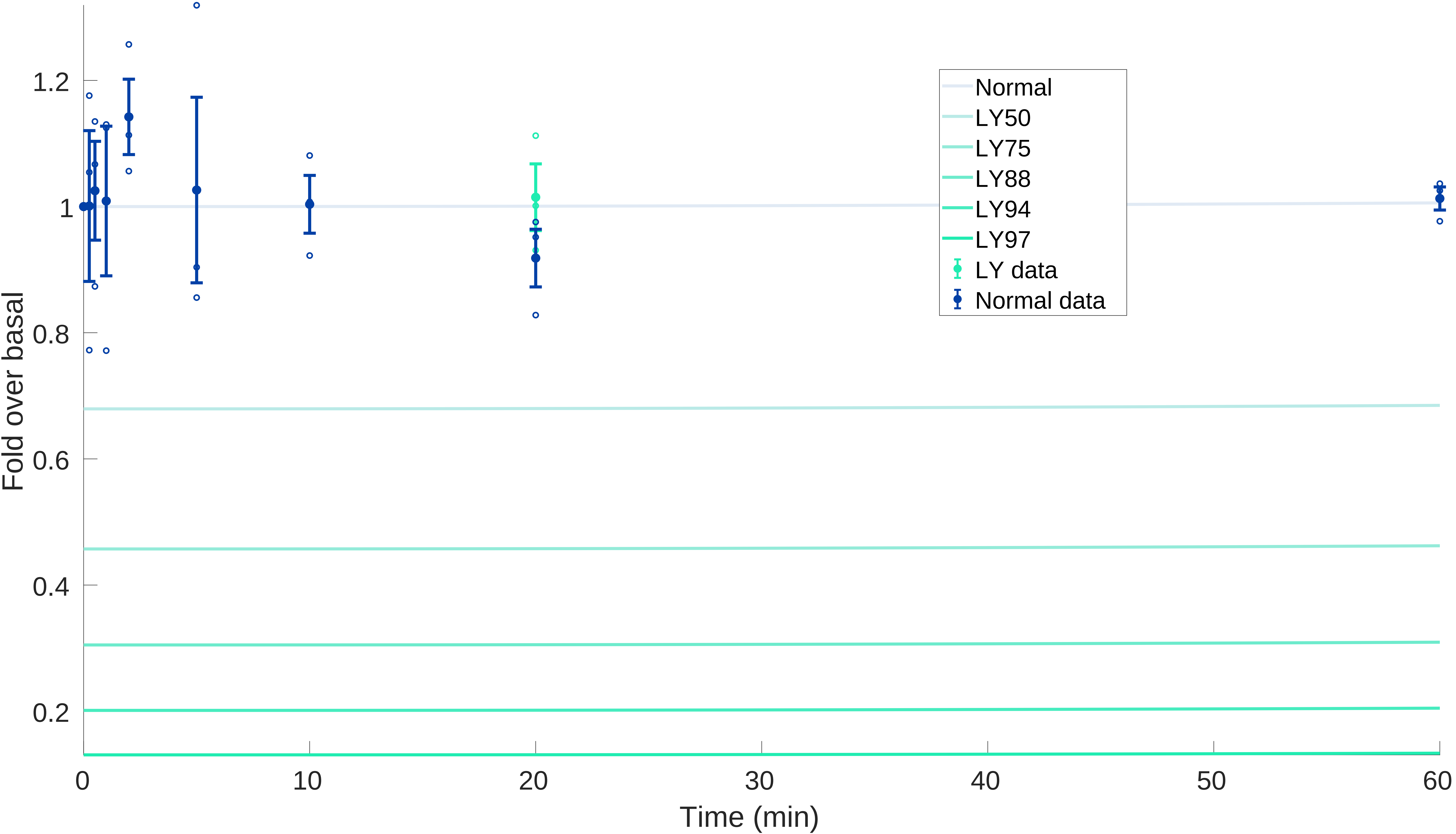

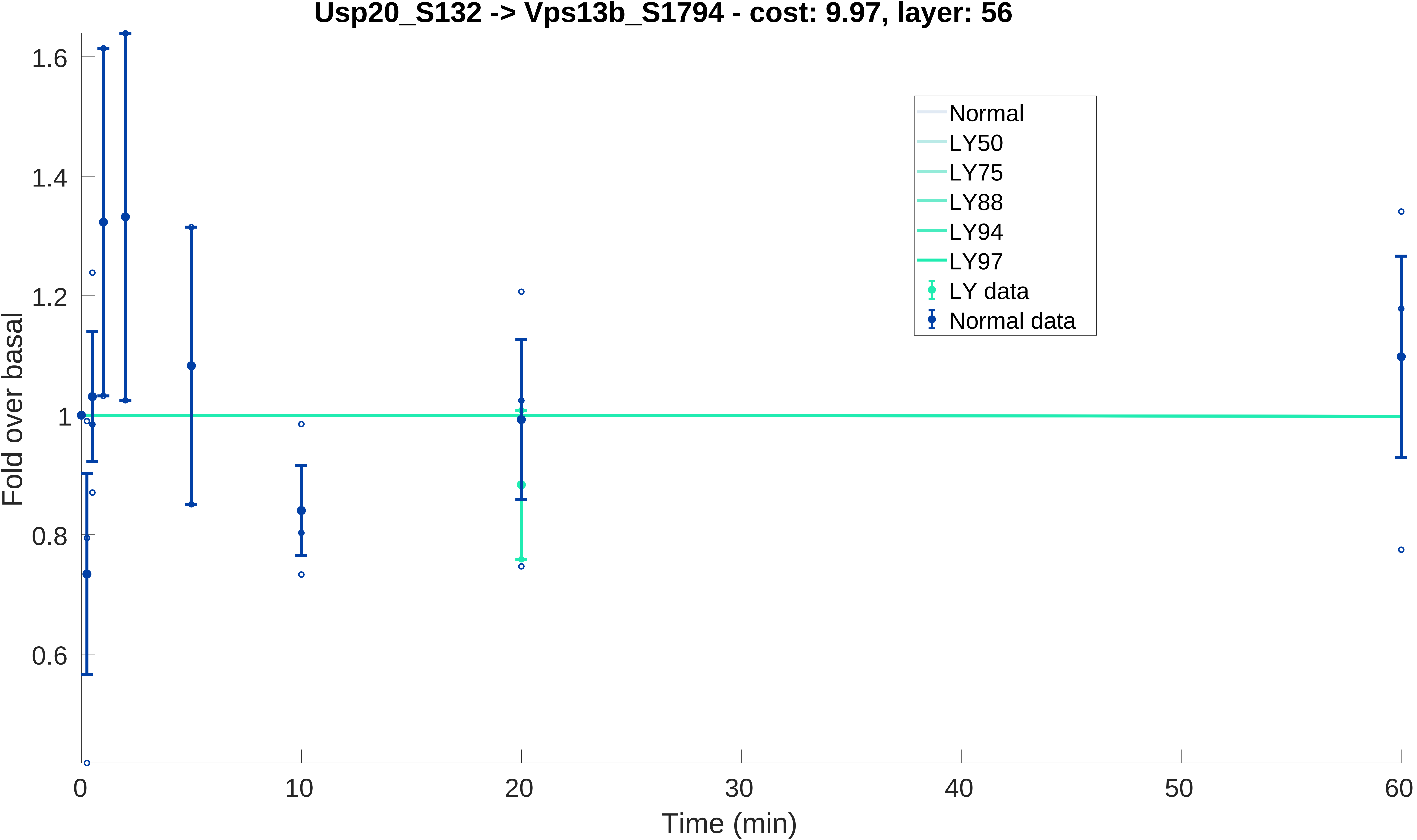
Usp20_S132 -> Vps13b_S1794 - cost: 9.97, layer: 56
Fold over basal
1.6
1.4
1.2
1
0.8
0.6
0
10
20
30
40
50
60
Time (min)
Normal
LY50
LY75
LY88
LY94
LY97
LY data
Normal data

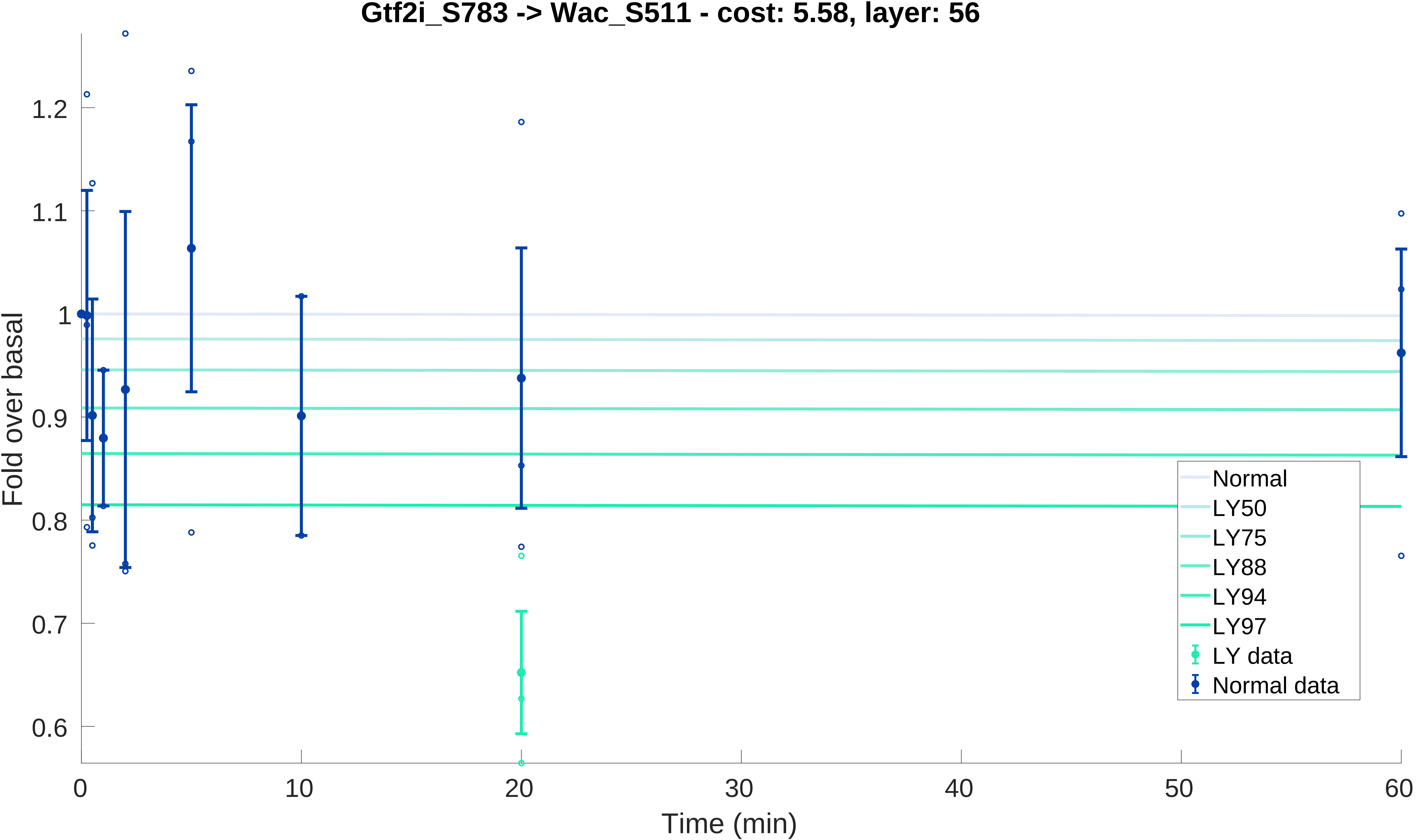
Gtf2i_S783 -> Wac_S511 - cost: 5.58, layer: 56
Fold over basal
Time (min)
0
10
20
30
40
50
60
0.6
0.7
0.8
0.9
1
1.1
1.2
Normal
LY50
LY75
LY88
LY94
LY97
LY data
Normal data

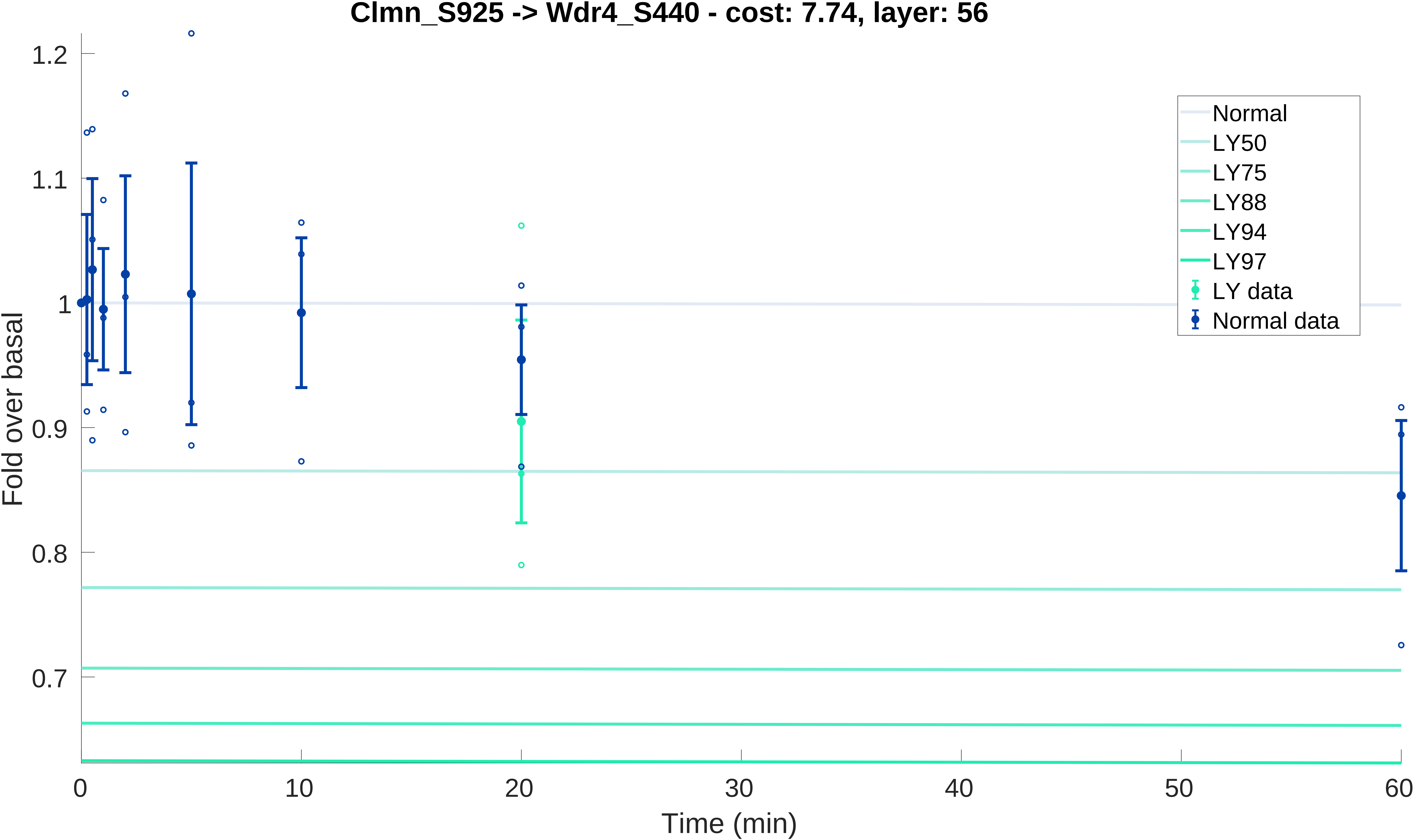

Clmn_S925 -> Wdr4_S440 - cost: 7.74, layer: 56
Fold over basal
1.2
1.1
1
0.9
0.8
0.7
Time (min)
0
10
20
30
40
50
60
Normal
LY50
LY75
LY88
LY94
LY97
LY data
Normal data

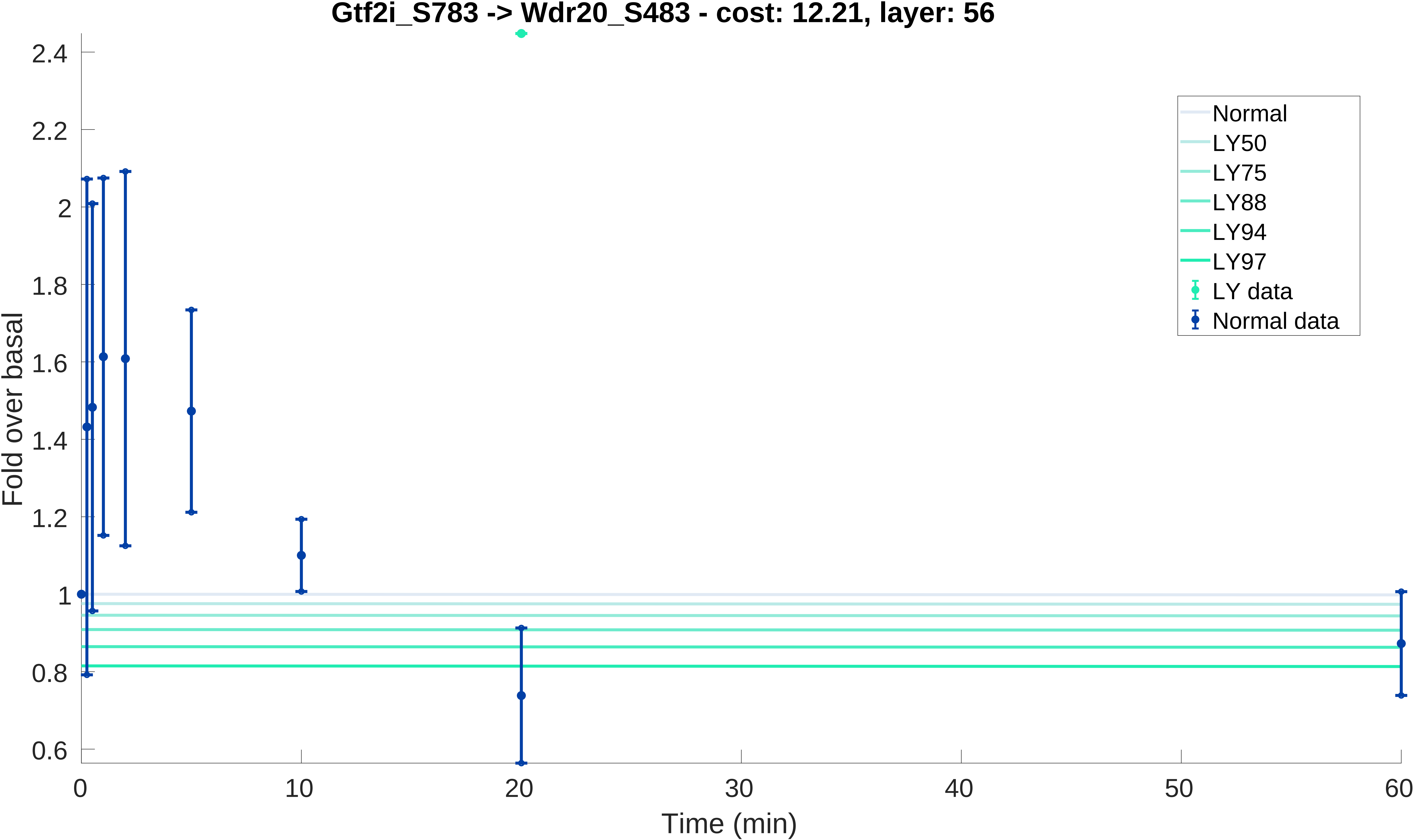

Gtf2i_S783 -> Wdr20_S483 - cost: 12.21, layer: 56
Fold over basal
2.4
2.2
2
1.8
1.6
1.4
1.2
1
0.8
0.6
0
10
20
30
40
50
60
Time (min)
Normal
LY50
LY75
LY88
LY94
LY97
LY data
Normal data

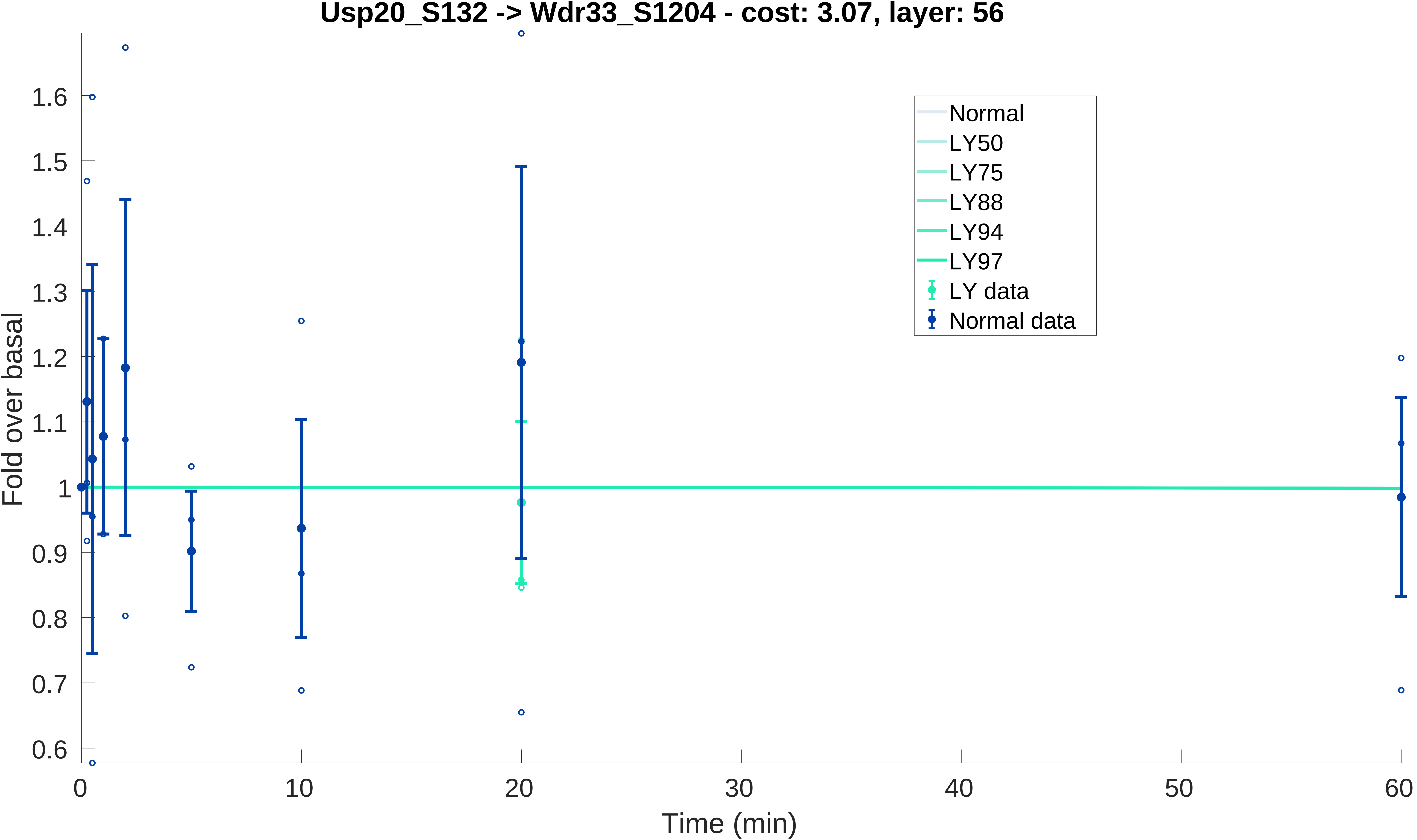

Usp20_S132 -> Wdr33_S1204 - cost: 3.07, layer: 56
Normal
LY50
LY75
LY88
LY94
LY97
LY data
Normal data
Fold over basal
Time (min)
1.6
1.5
1.4
1.3
1.2
1.1
1
0.9
0.8
0.7
0.6
0
10
20
30
40
50
60

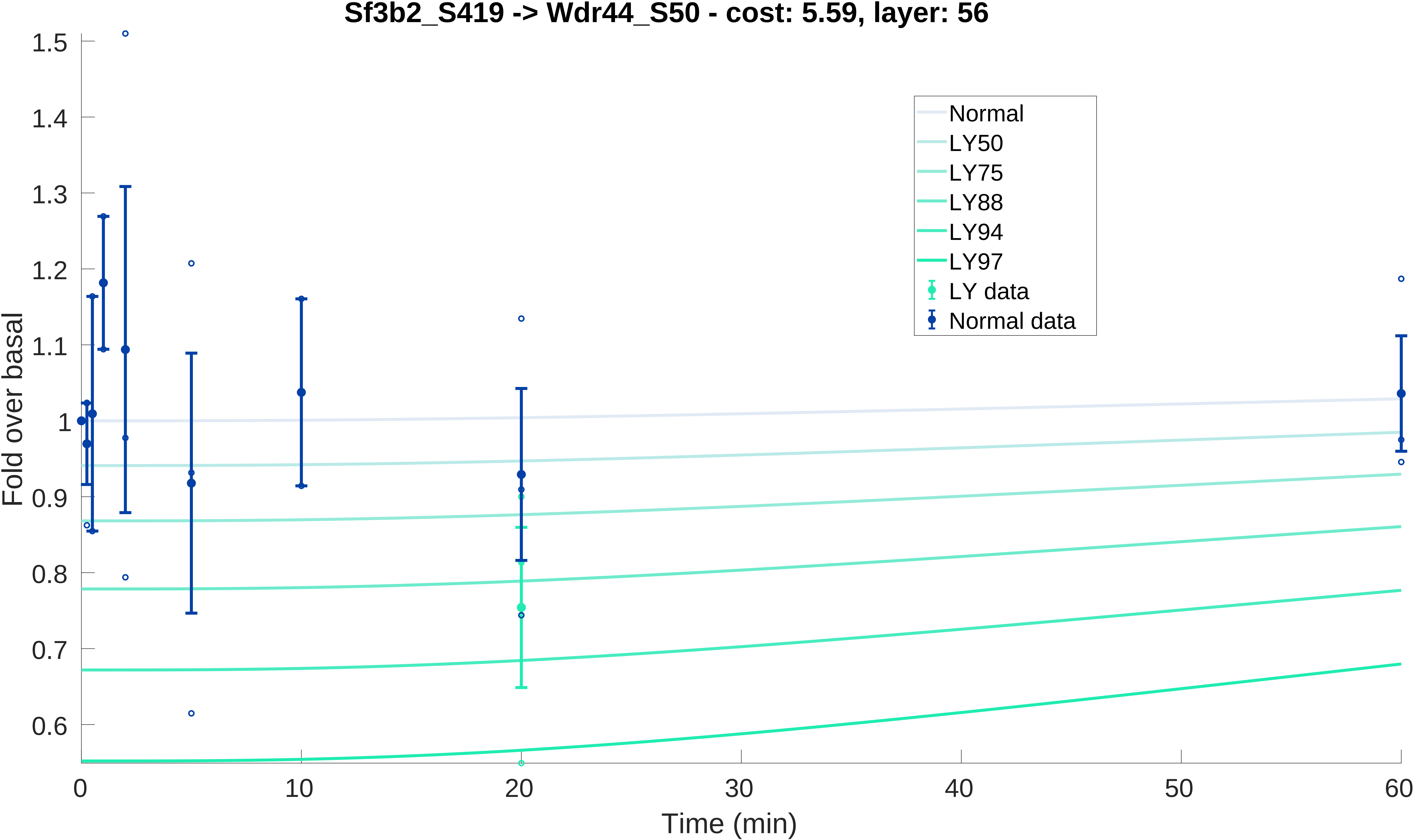
Sf3b2_S419 -> Wdr44_S50 - cost: 5.59, layer: 56
Fold over basal
1.5
1.4
1.3
1.2
1.1
1
0.9
0.8
0.7
0.6
0
10
20
30
40
50
60
Time (min)
Normal
LY50
LY75
LY88
LY94
LY97
LY data
Normal data

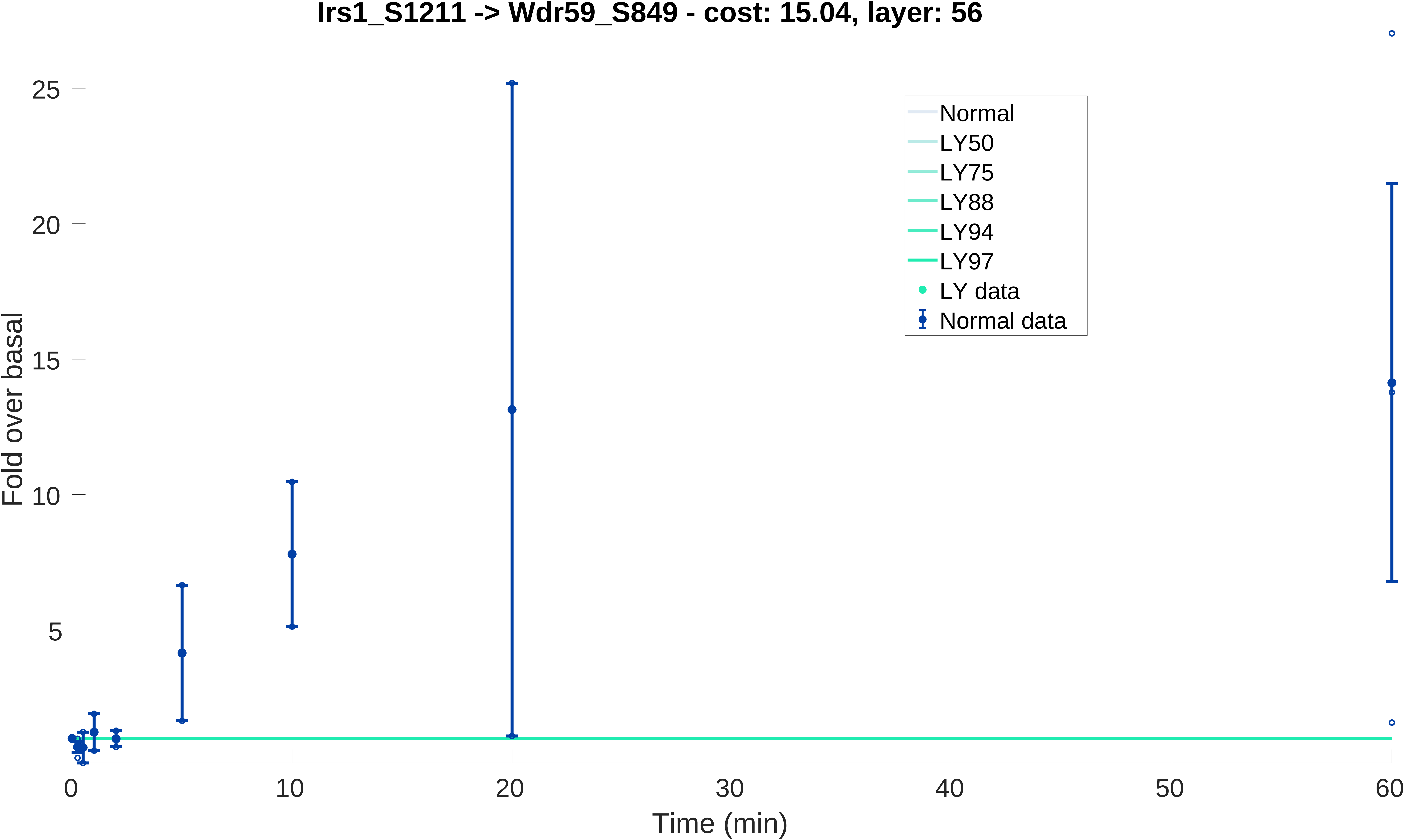

Irs1_S1211 -> Wdr59_S849 - cost: 15.04, layer: 56
Fold over basal
Time (min)
0
5
10
15
20
25
30
40
50
60
Normal
LY50
LY75
LY88
LY94
LY97
LY data
Normal data

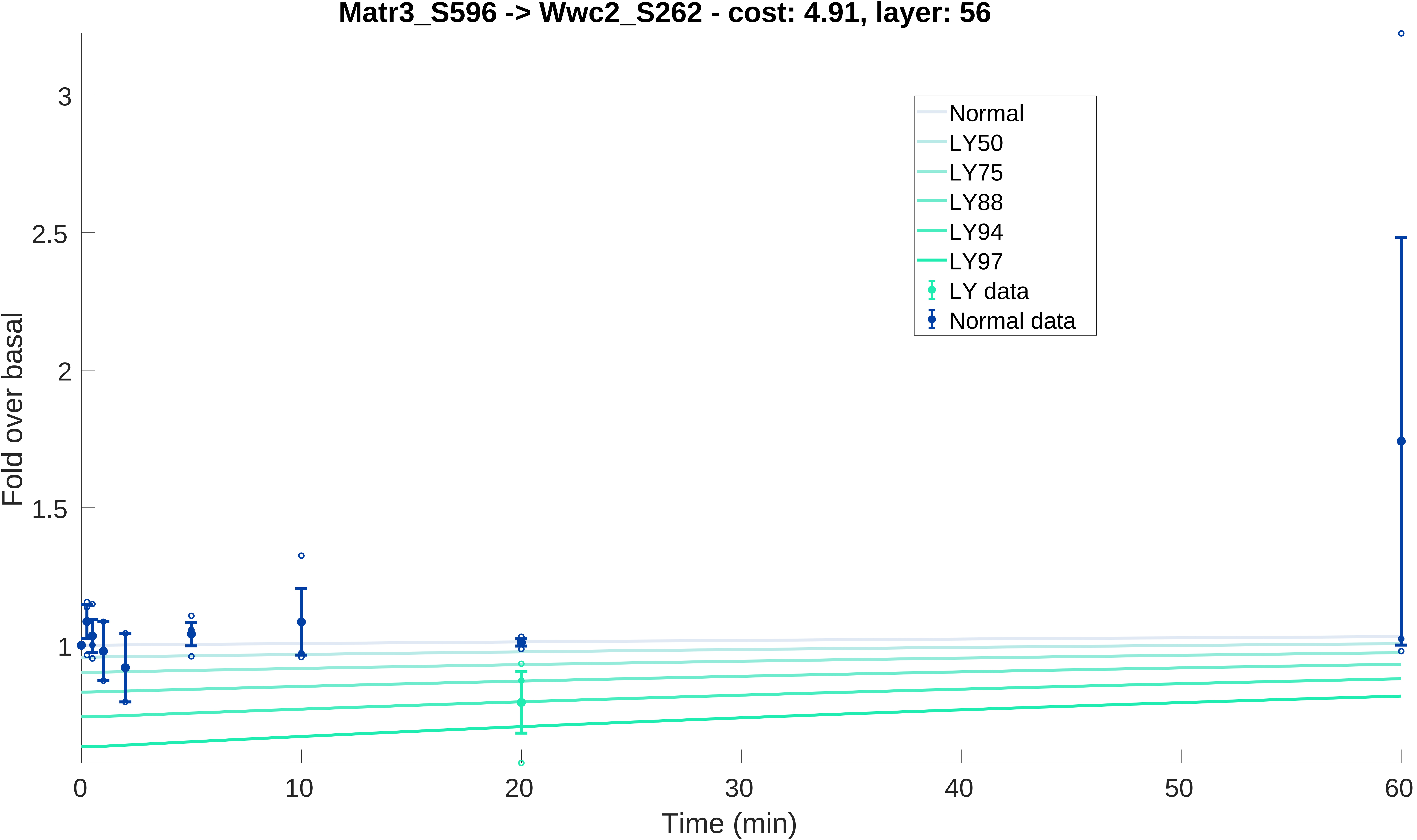

Matr3_S596 -> Wwc2_S262 - cost: 4.91, layer: 56
Fold over basal
Time (min)
3
2.5
2
1.5
1
0
10
20
30
40
50
60
Normal
LY50
LY75
LY88
LY94
LY97
LY data
Normal data

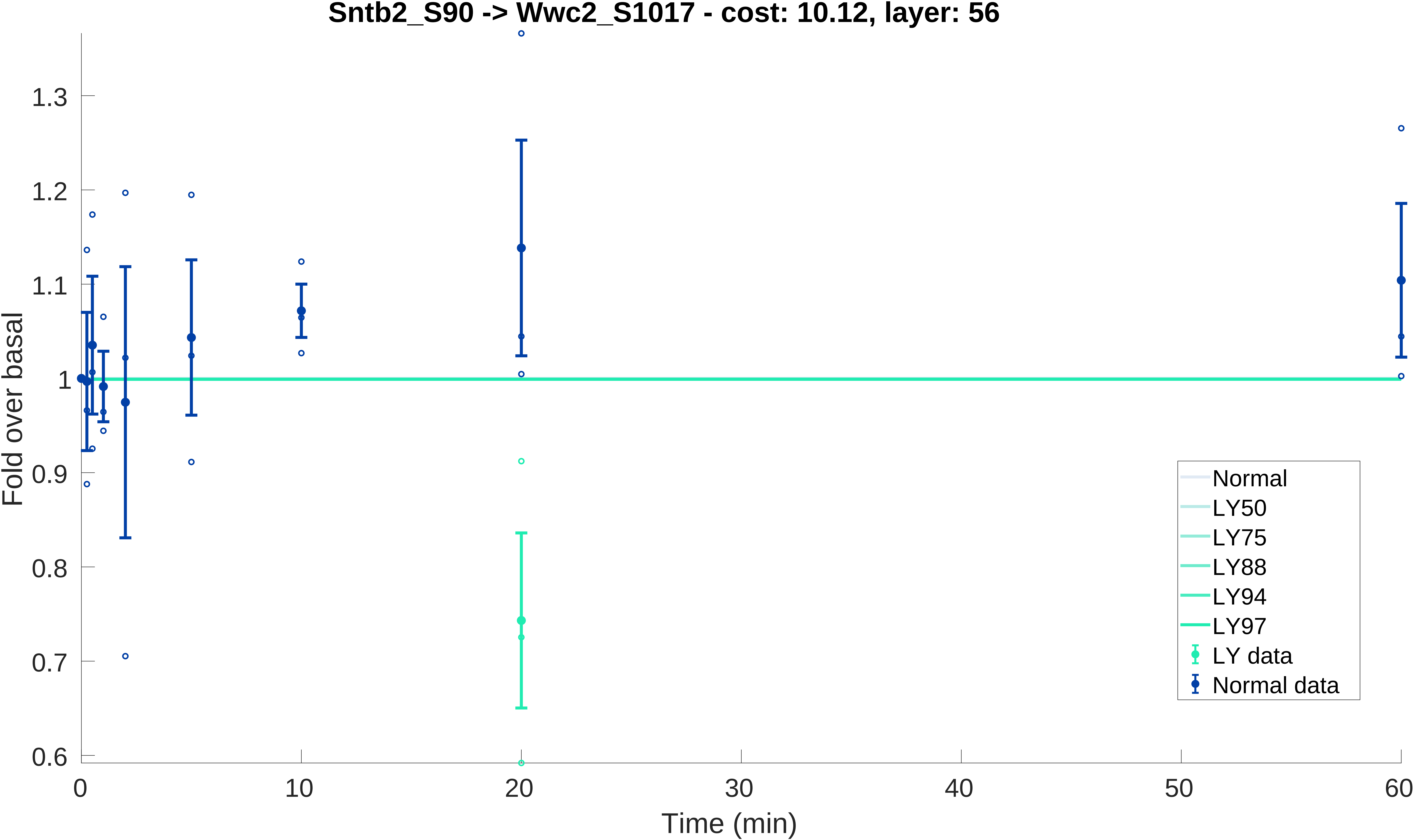

Sntb2_S90 -> Wwc2_S1017 - cost: 10.12, layer: 56
Fold over basal
1.3
1.2
1.1
1
0.9
0.8
0.7
0.6
0
10
20
30
40
50
60
Time (min)
Normal
LY50
LY75
LY88
LY94
LY97
LY data
Normal data

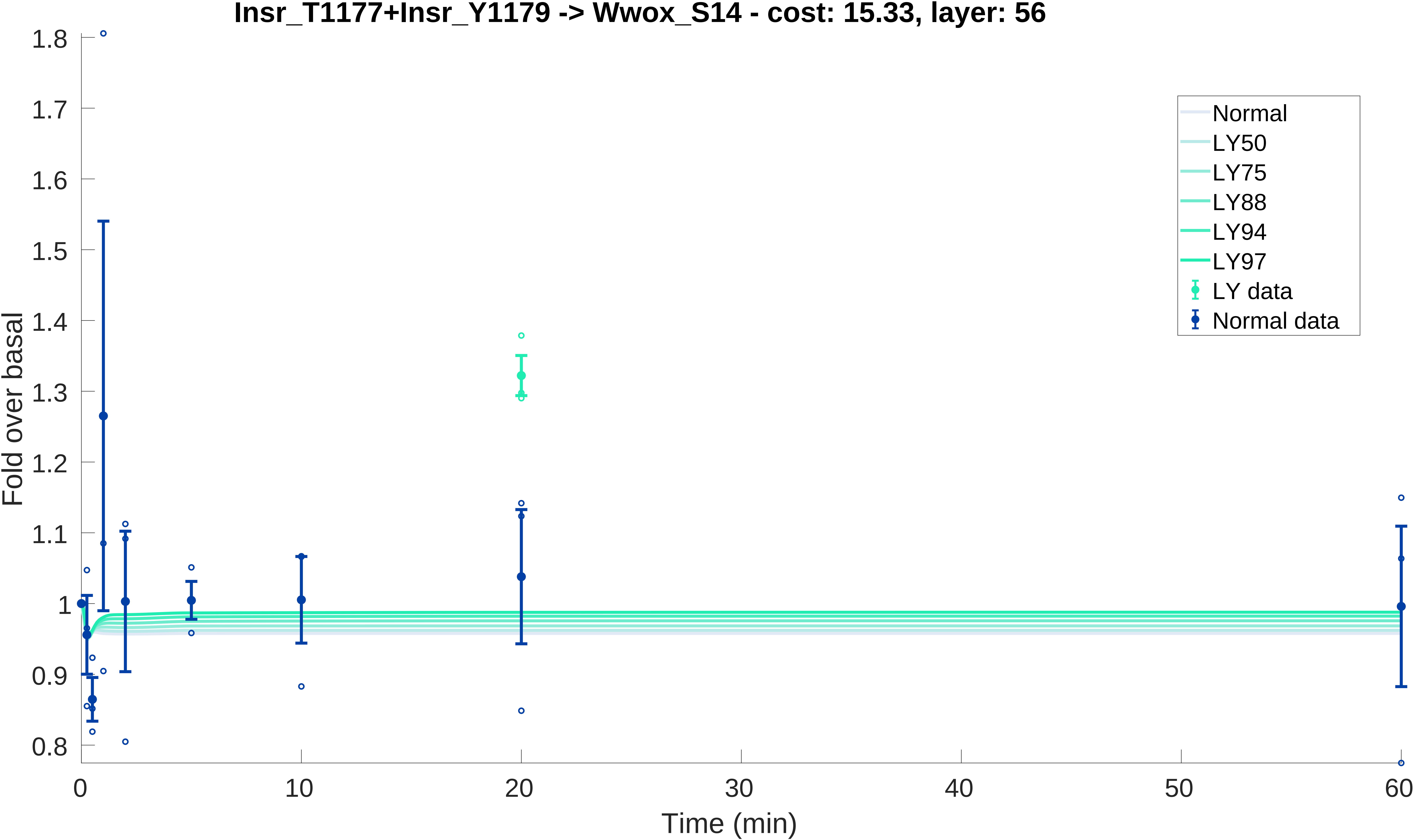
Insr_T1177+Insr_Y1179 -> Wwox_S14 - cost: 15.33, layer: 56
Fold over basal
Time (min)
1.8
1.7
1.6
1.5
1.4
1.3
1.2
1.1
1
0.9
0.8
0
10
20
30
40
50
60
Normal
LY50
LY75
LY88
LY94
LY97
LY data
Normal data

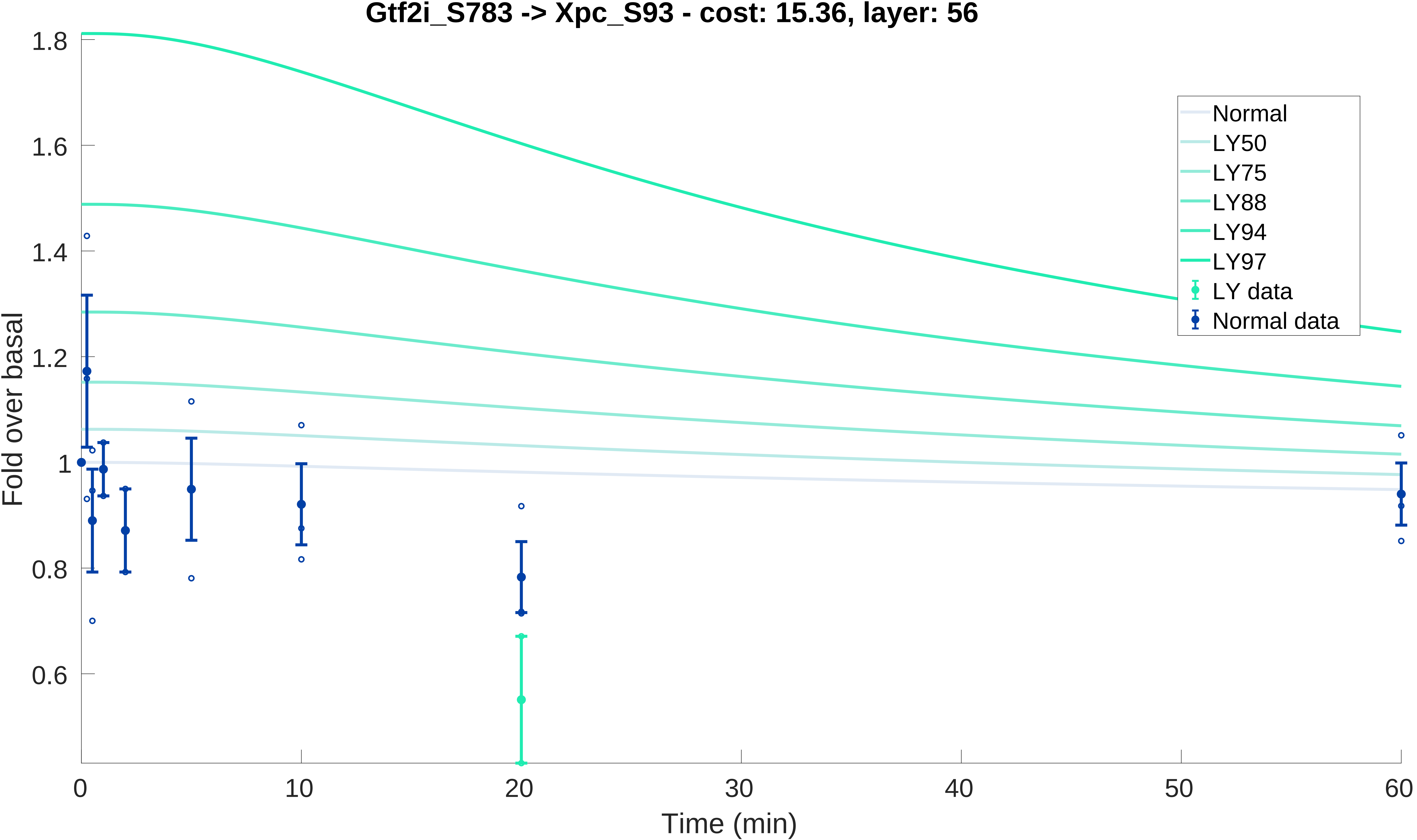

Gtf2i_S783 -> Xpc_S93 - cost: 15.36, layer: 56
Fold over basal
Time (min)
1.8
1.6
1.4
1.2
1
0.8
0.6
0
10
20
30
40
50
60
Normal
LY50
LY75
LY88
LY94
LY97
LY data
Normal data

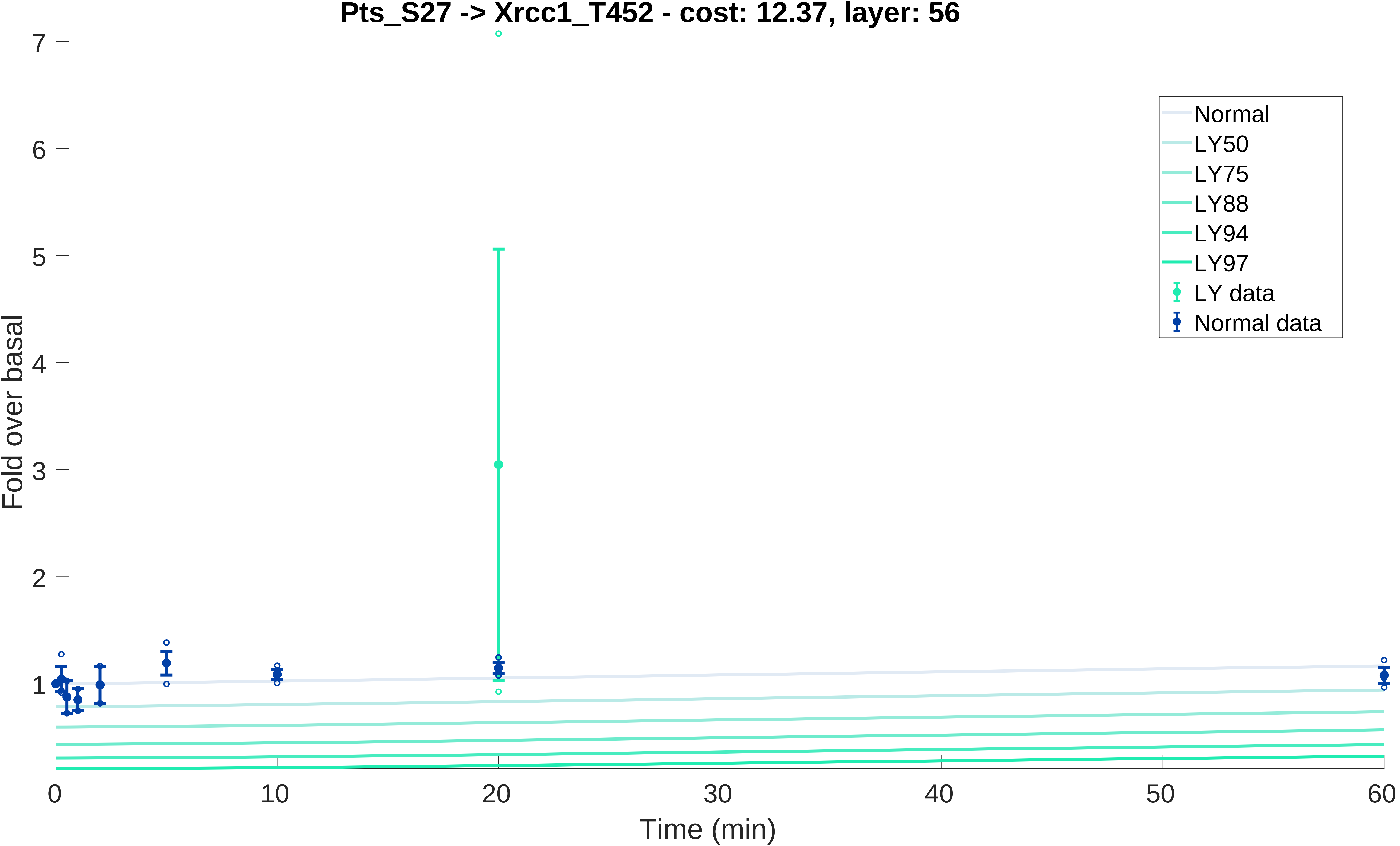

Pts_S27 -> Xrcc1_T452 - cost: 12.37, layer: 56
Fold over basal
Time (min)
0
10
20
30
40
50
60
1
2
3
4
5
6
7
Normal
LY50
LY75
LY88
LY94
LY97
LY data
Normal data

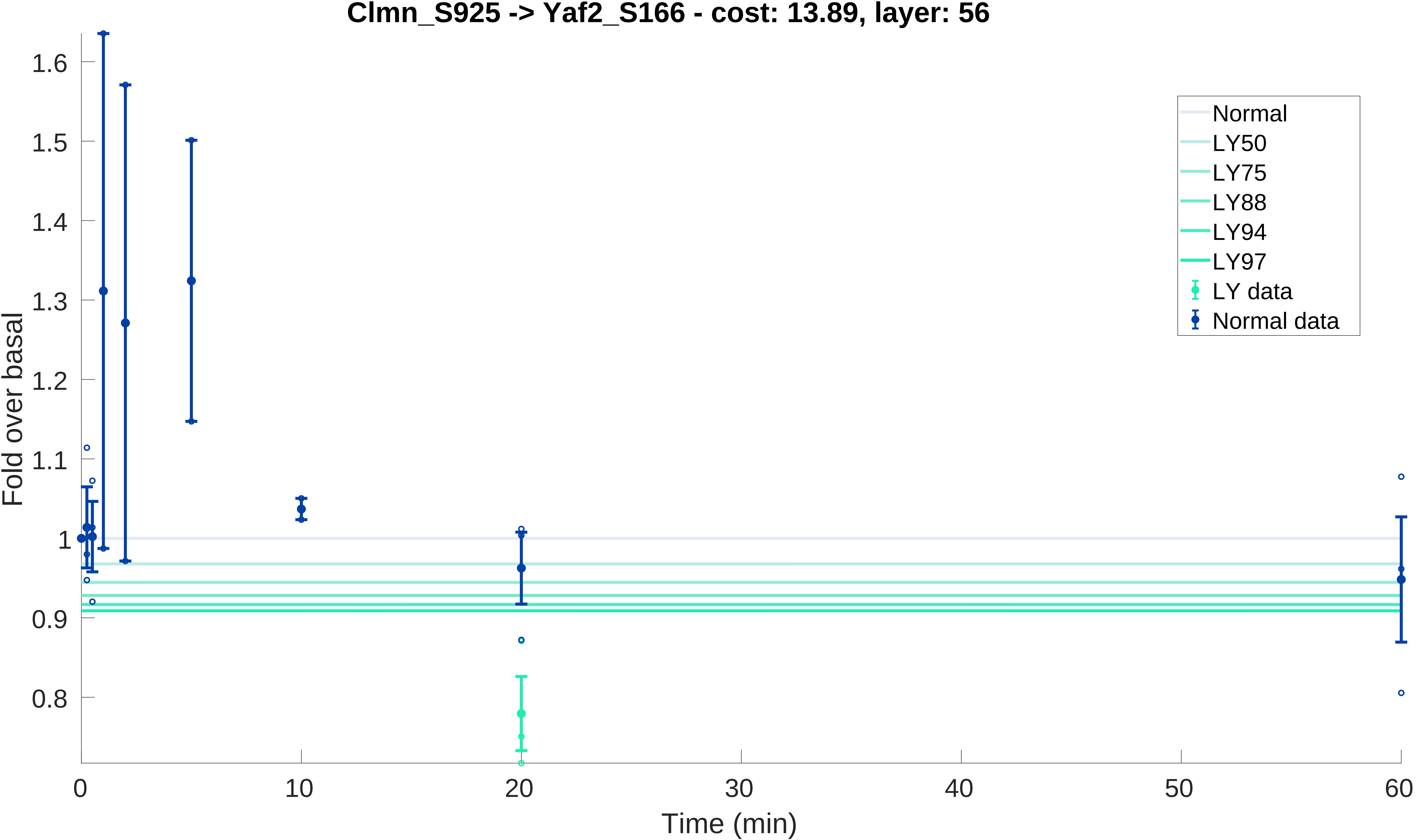

Clmn_S925 -> Yaf2_S166 - cost: 13.89, layer: 56
Fold over basal
1.6
1.5
1.4
1.3
1.2
1.1
1
0.9
0.8
0
10
20
30
40
50
60
Time (min)
Normal
LY50
LY75
LY88
LY94
LY97
LY data
Normal data

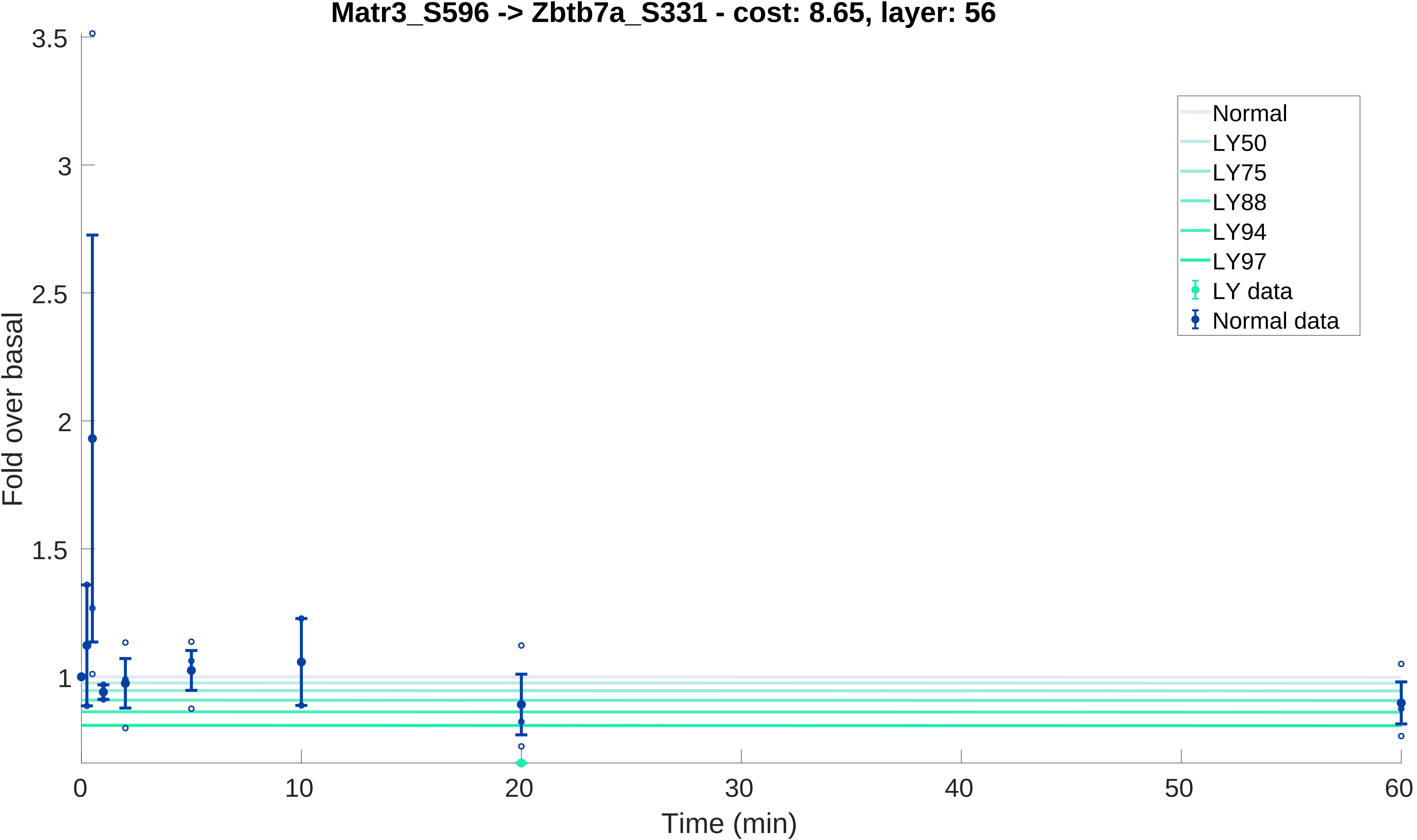
Matr3_S596 -> Zbtb7a_S331 - cost: 8.65, layer: 56
Fold over basal
Time (min)
3.5
3
2.5
2
1.5
1
0
10
20
30
40
50
60
Normal
LY50
LY75
LY88
LY94
LY97
LY data
Normal data

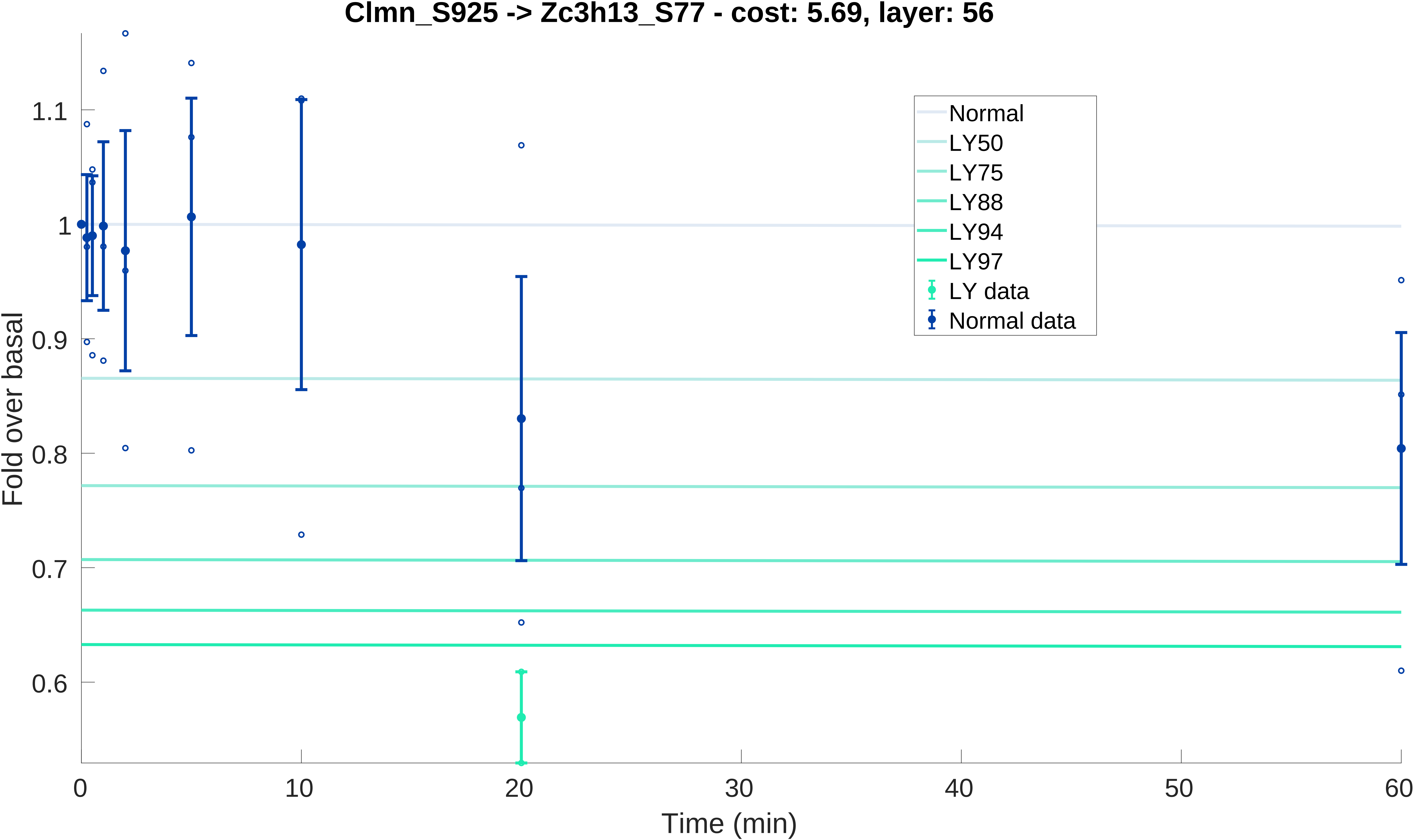

Clmn_S925 -> Zc3h13_S77 - cost: 5.69, layer: 56
Fold over basal
Time (min)
0
10
20
30
40
50
60
0.6
0.7
0.8
0.9
1
1.1
Normal
LY50
LY75
LY88
LY94
LY97
LY data
Normal data

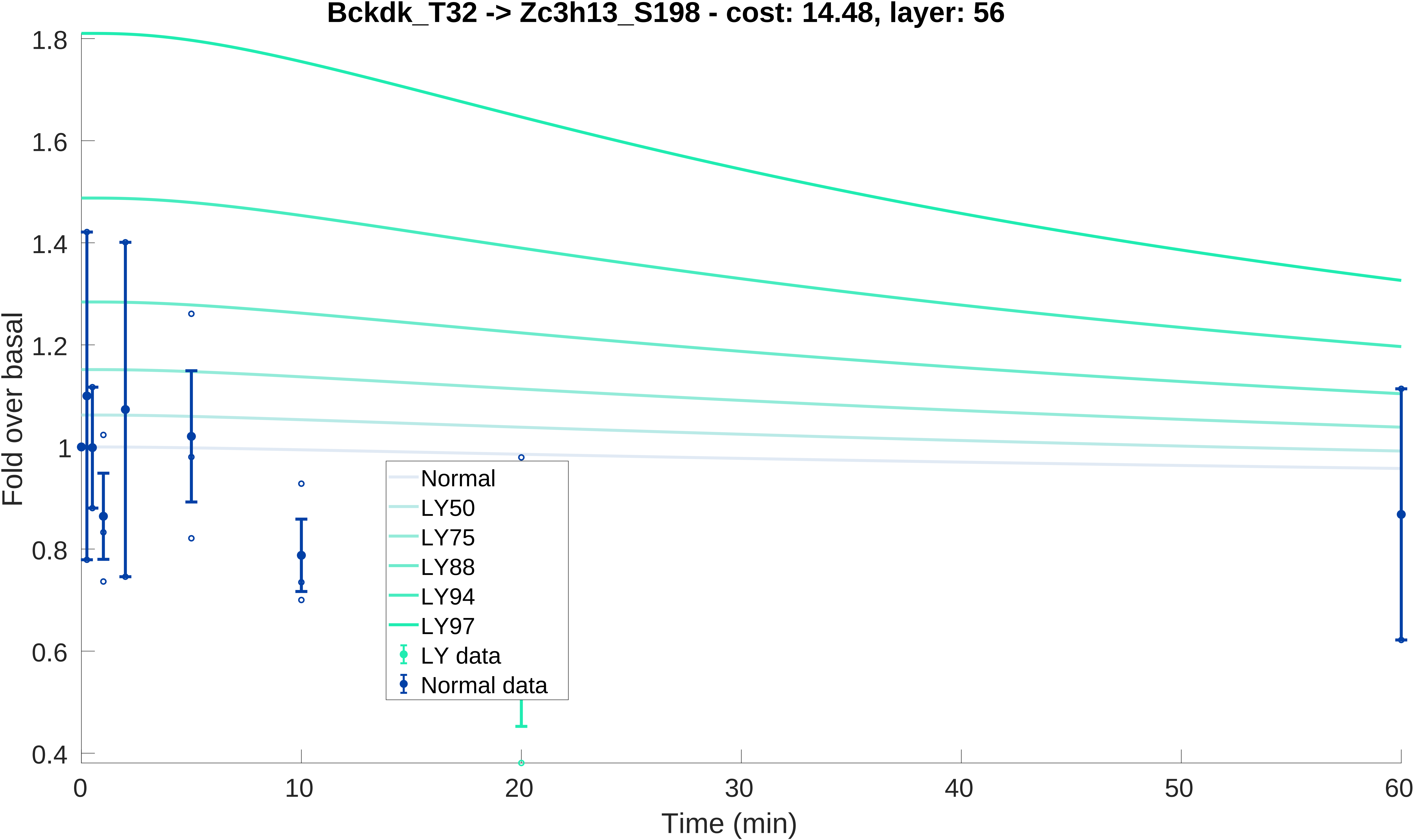

Bckdk_T32 -> Zc3h13_S198 - cost: 14.48, layer: 56
Fold over basal
Time (min)
1.8
1.6
1.4
1.2
1
0.8
0.6
0.4
0
10
20
30
40
50
60
Normal
LY50
LY75
LY88
LY94
LY97
LY data
Normal data

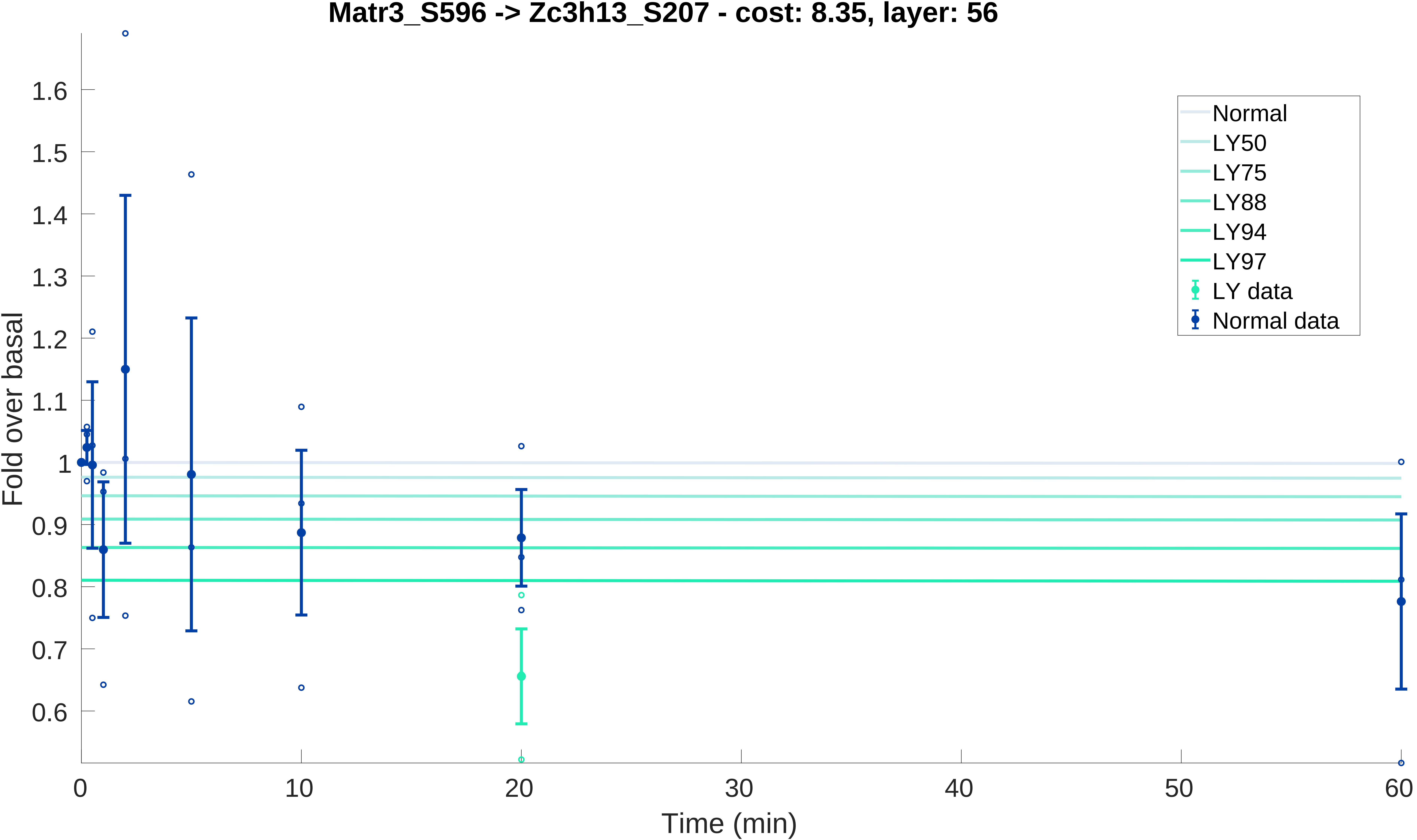
Matr3_S596 -> Zc3h13_S207 - cost: 8.35, layer: 56
Fold over basal
Time (min)
0.6
0.7
0.8
0.9
1
1.1
1.2
1.3
1.4
1.5
1.6
0
10
20
30
40
50
60
Normal
LY50
LY75
LY88
LY94
LY97
LY data
Normal data

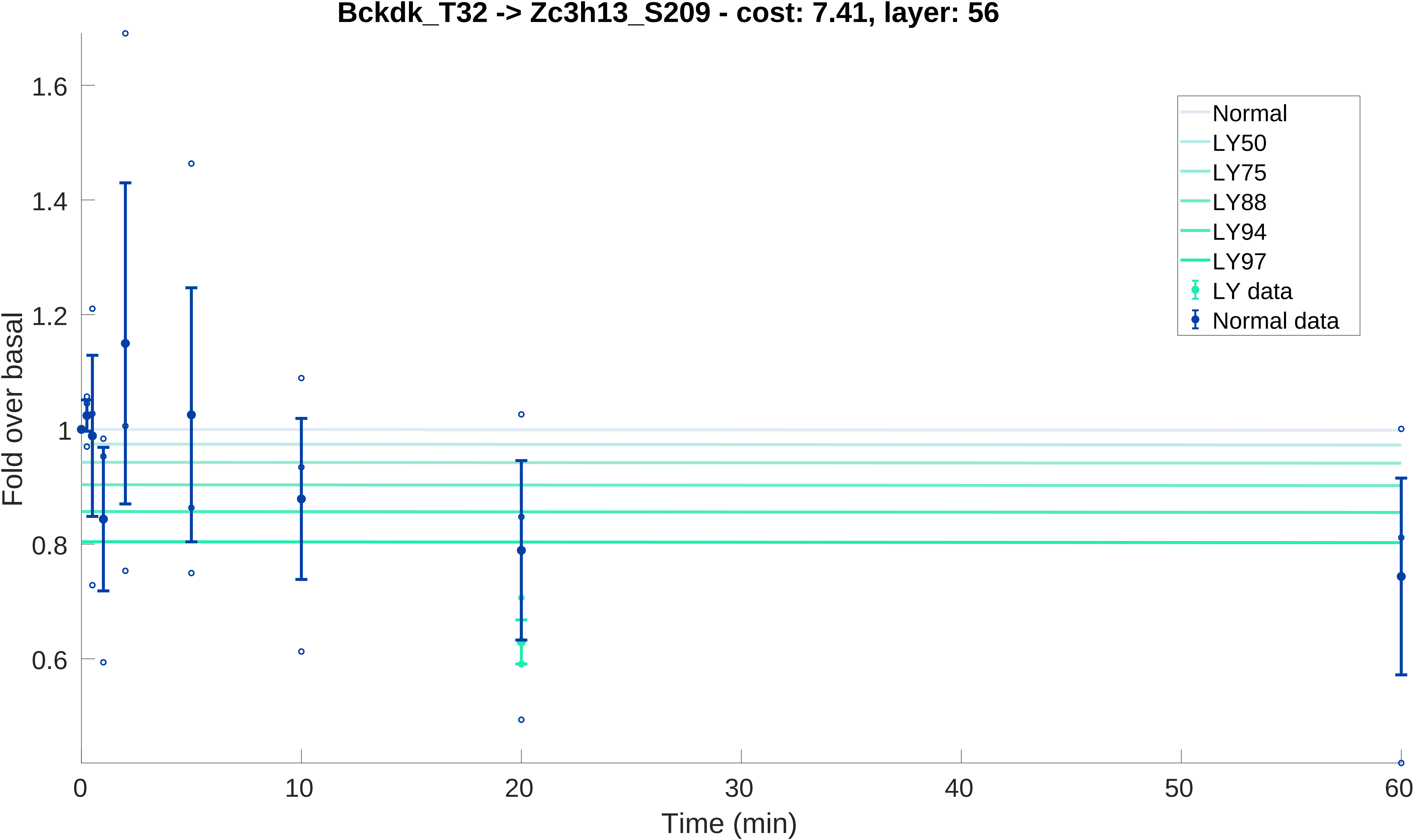

Bckdk_T32 -> Zc3h13_S209 - cost: 7.41, layer: 56
Fold over basal
1.6
1.4
1.2
1
0.8
0.6
0
10
20
30
40
50
60
Time (min)
Normal
LY50
LY75
LY88
LY94
LY97
LY data
Normal data

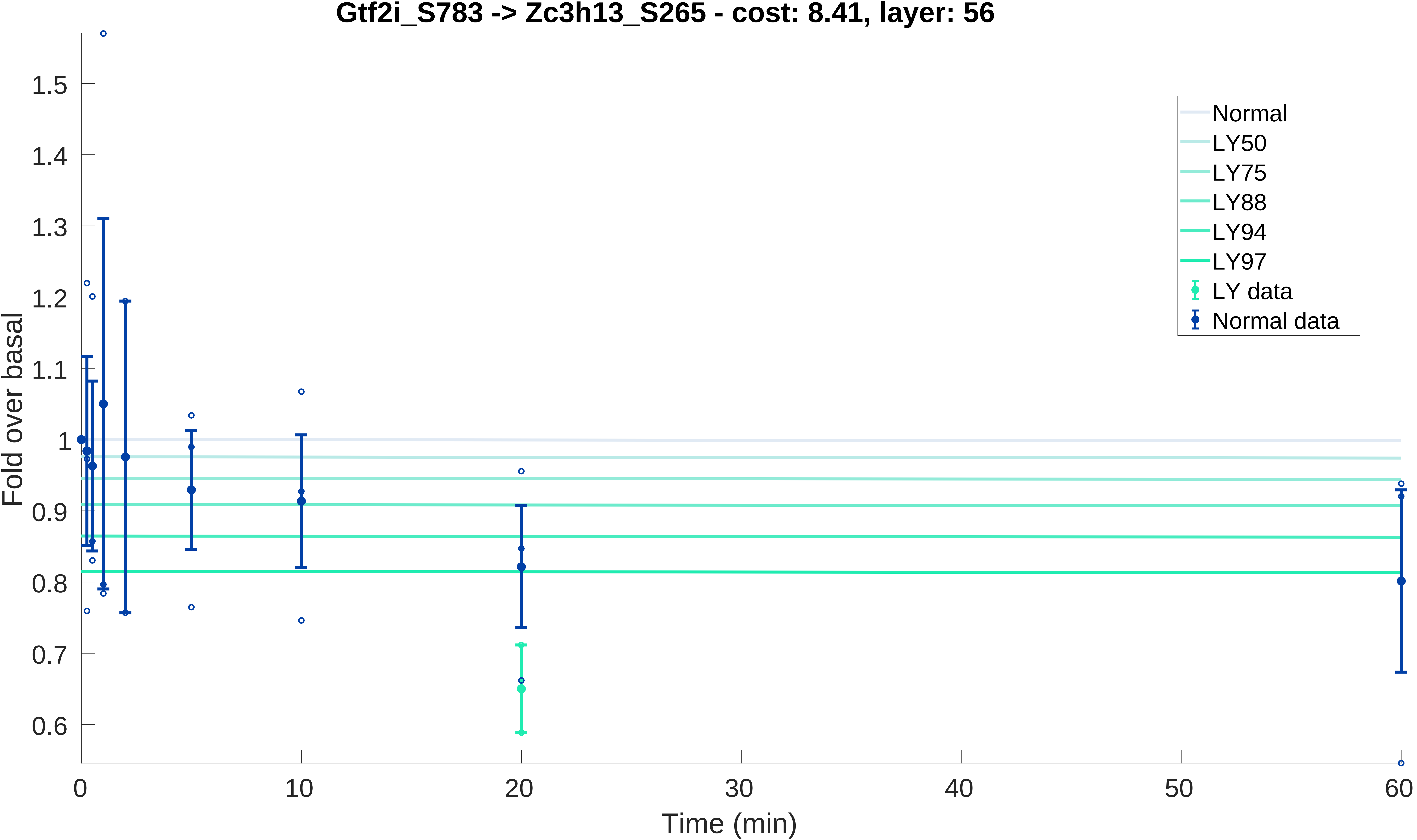

Gtf2i_S783 -> Zc3h13_S265 - cost: 8.41, layer: 56
Fold over basal
Time (min)
0.6
0.7
0.8
0.9
1
1.1
1.2
1.3
1.4
1.5
0
10
20
30
40
50
60
Normal
LY50
LY75
LY88
LY94
LY97
LY data
Normal data

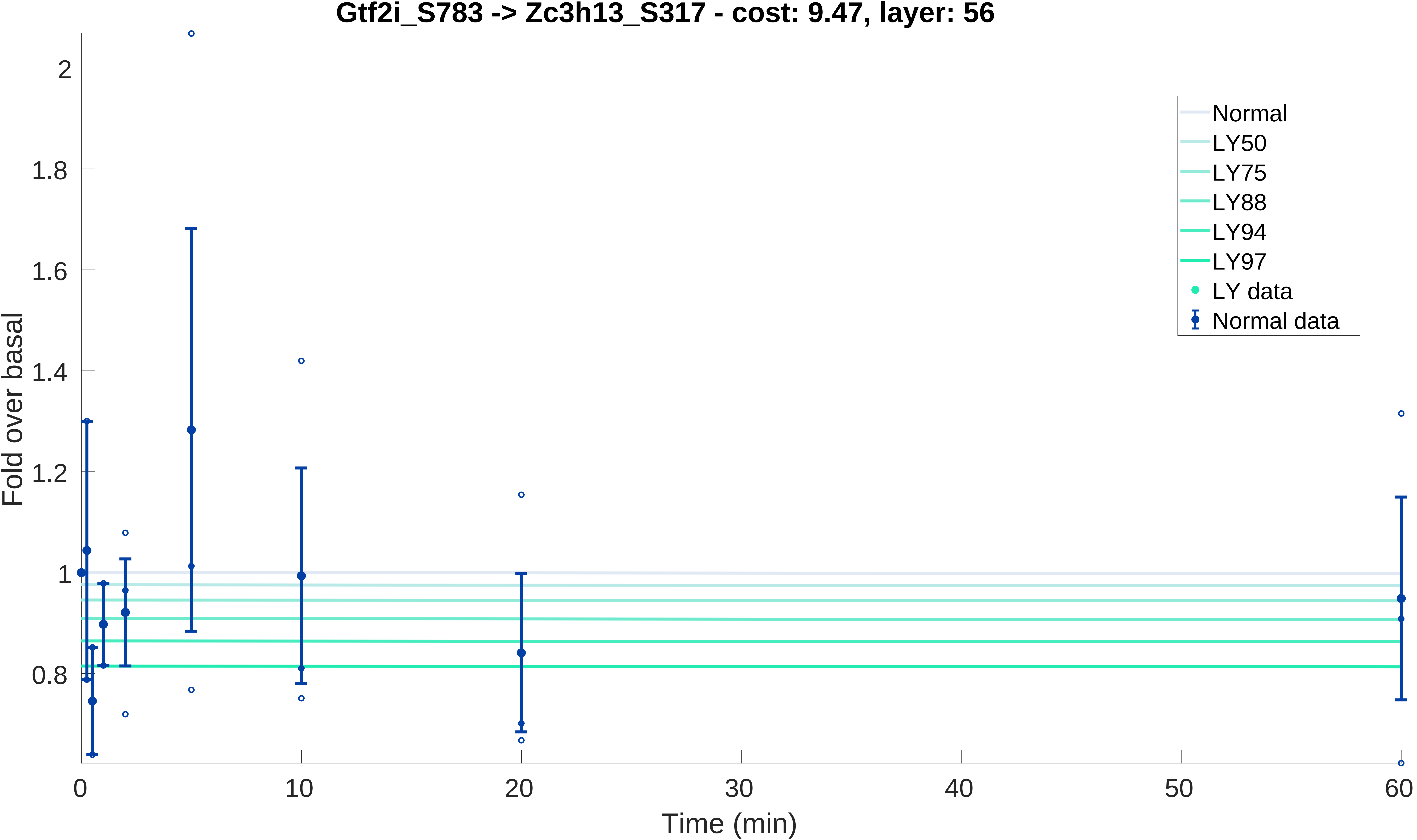

Gtf2i_S783 -> Zc3h13_S317 - cost: 9.47, layer: 56
Fold over basal
2
1.8
1.6
1.4
1.2
1
0.8
0
10
20
30
40
50
60
Time (min)
Normal
LY50
LY75
LY88
LY94
LY97
LY data
Normal data

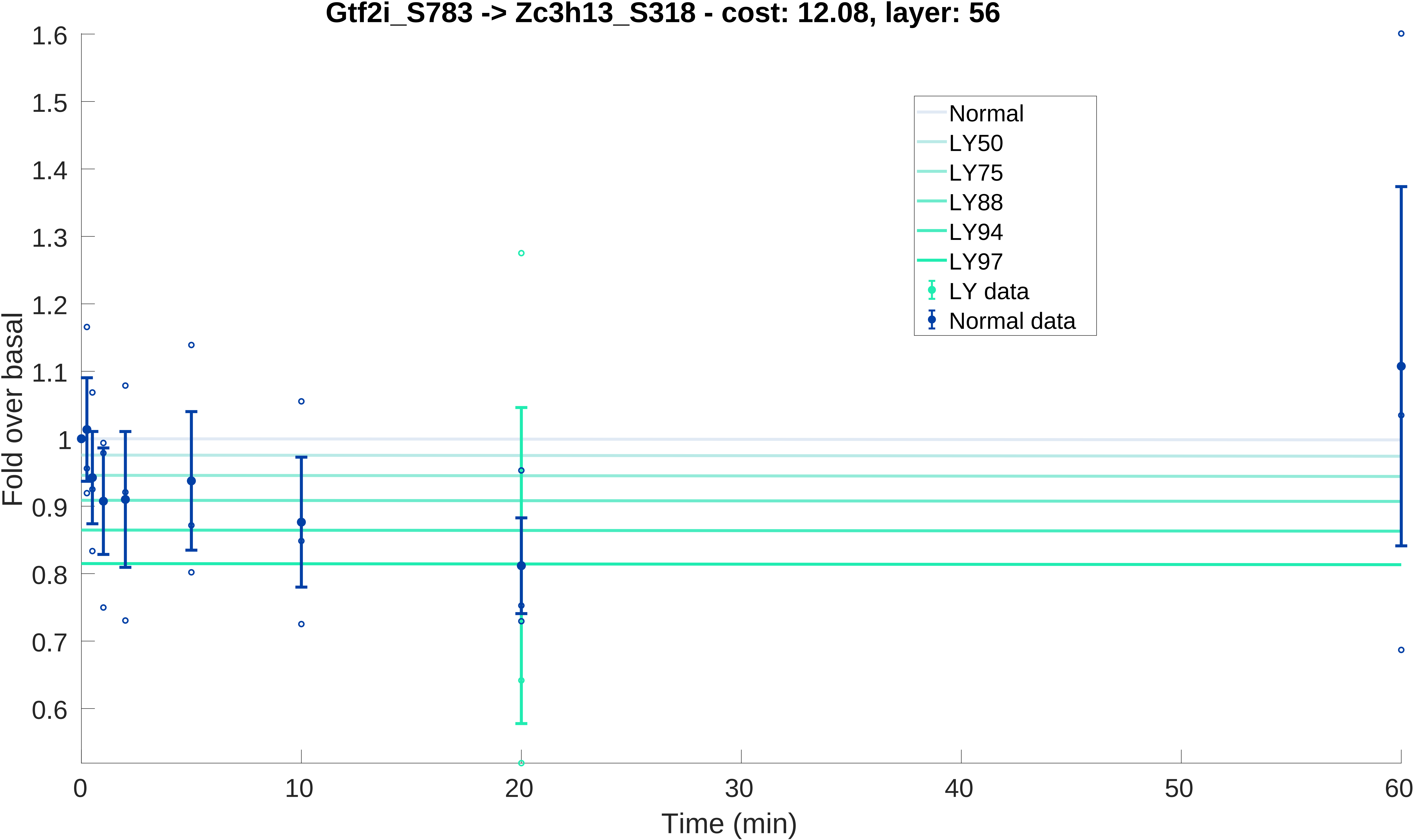

Gtf2i_S783 -> Zc3h13_S318 - cost: 12.08, layer: 56
Fold over basal
Time (min)
1.6
1.5
1.4
1.3
1.2
1.1
1
0.9
0.8
0.7
0.6
0
10
20
30
40
50
60
Normal
LY50
LY75
LY88
LY94
LY97
LY data
Normal data

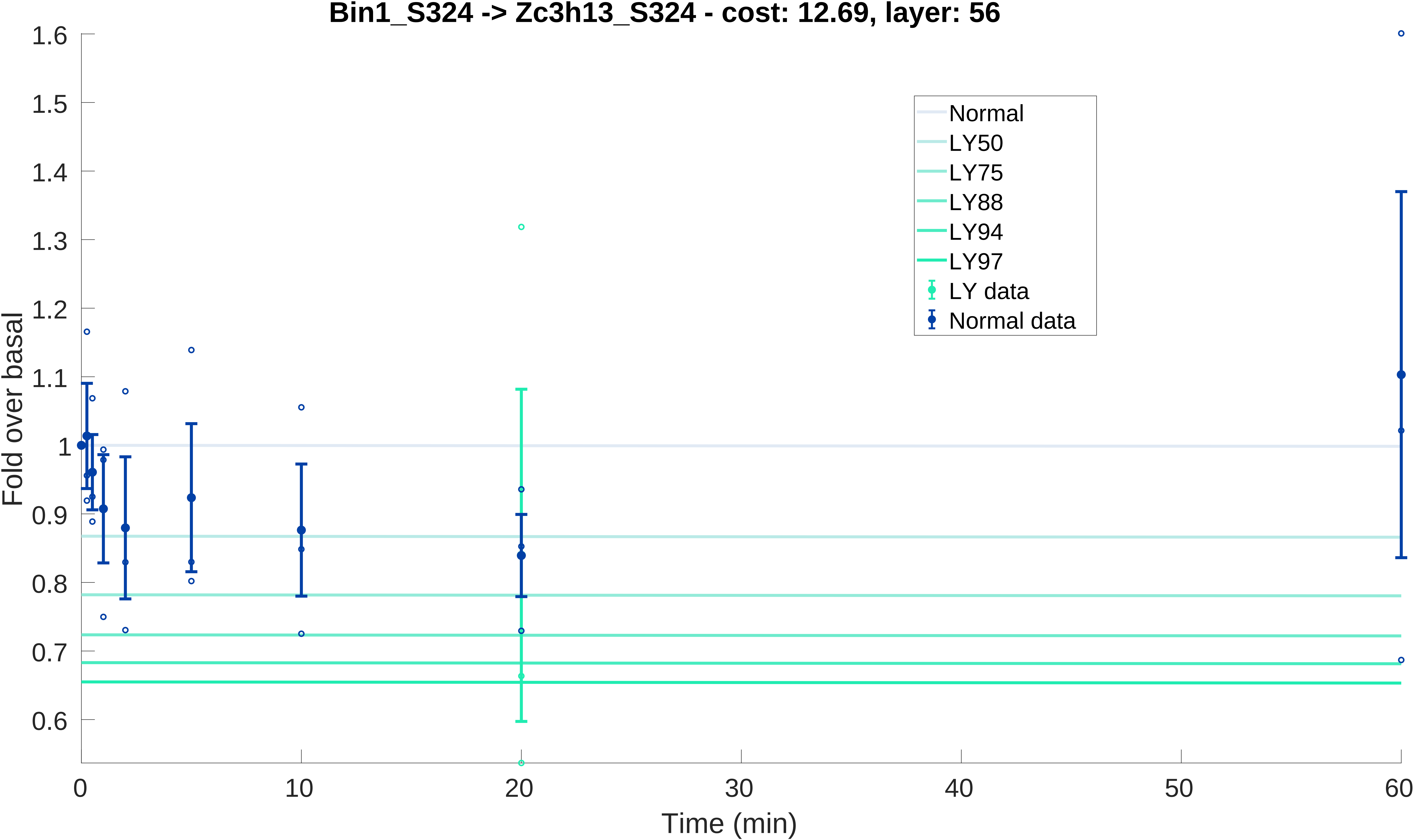

Bin1_S324 -> Zc3h13_S324 - cost: 12.69, layer: 56
Fold over basal
Time (min)
Normal
LY50
LY75
LY88
LY94
LY97
LY data
Normal data
1.6
1.5
1.4
1.3
1.2
1.1
1
0.9
0.8
0.7
0.6
0
10
20
30
40
50
60

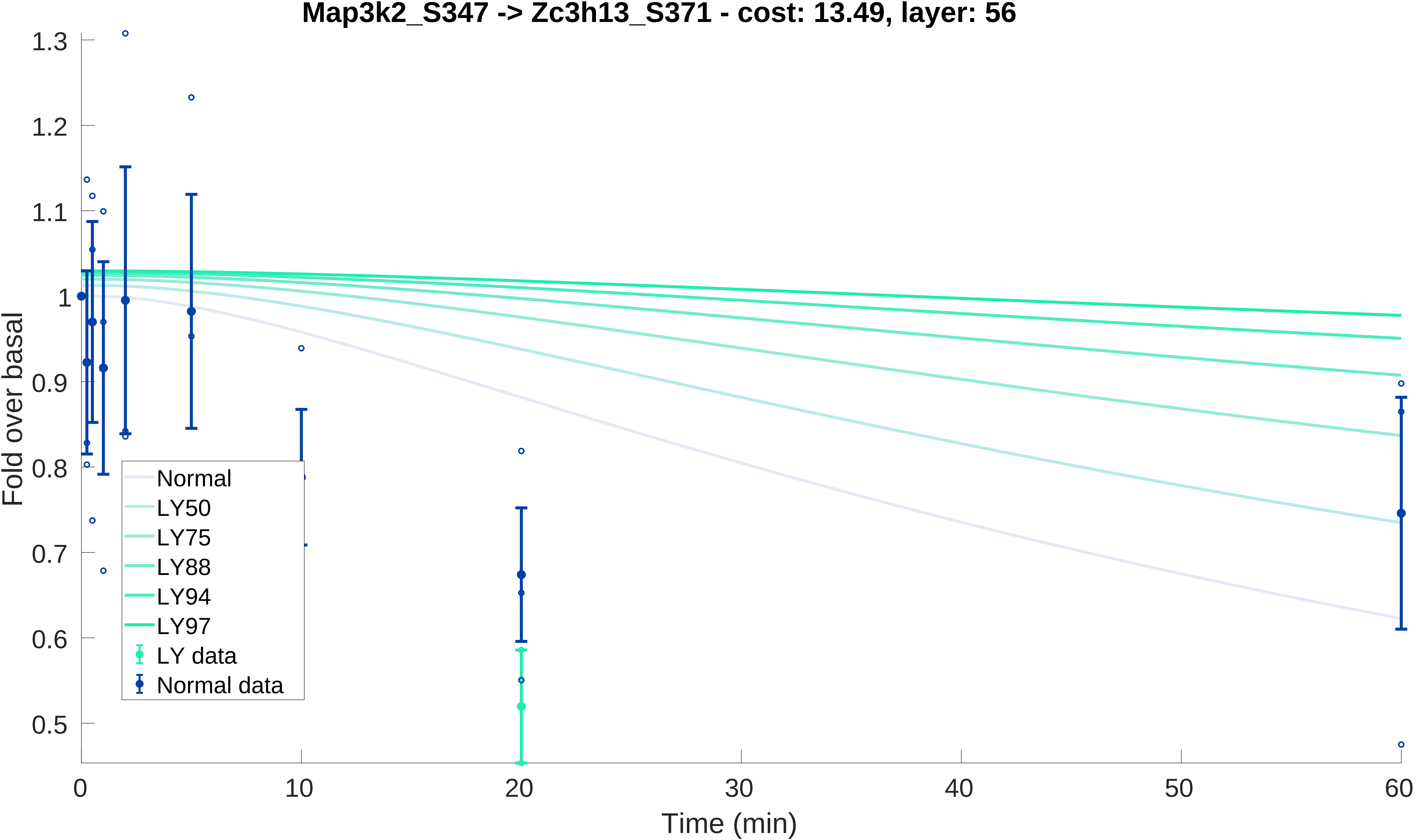

Map3k2_S347 -> Zc3h13_S371 - cost: 13.49, layer: 56
Fold over basal
Time (min)
1.3
1.2
1.1
1
0.9
0.8
0.7
0.6
0.5
0
10
20
30
40
50
60
Normal
LY50
LY75
LY88
LY94
LY97
LY data
Normal data

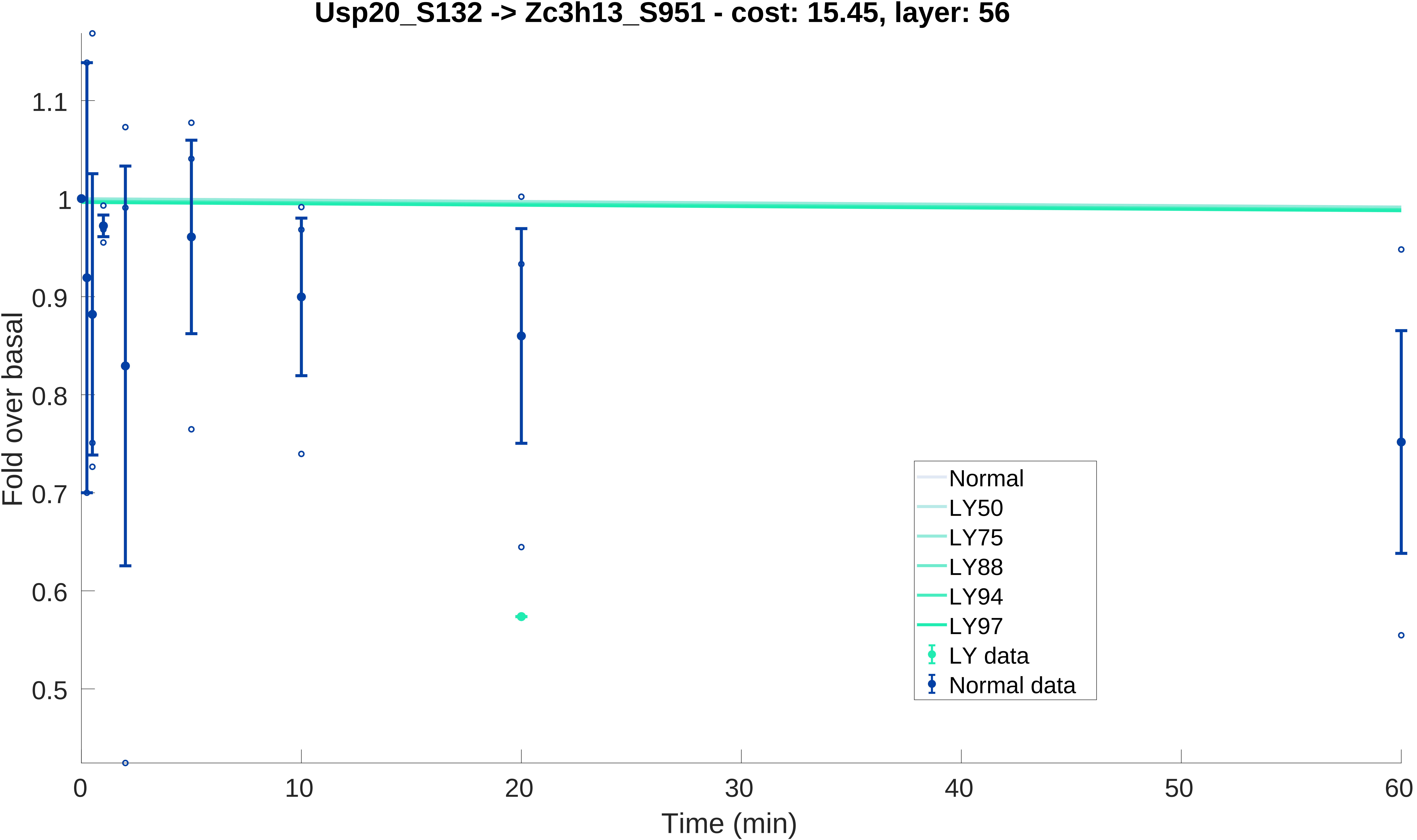
Usp20_S132 -> Zc3h13_S951 - cost: 15.45, layer: 56
Fold over basal
Time (min)
1.1
1
0.9
0.8
0.7
0.6
0.5
0
10
20
30
40
50
60
Normal
LY50
LY75
LY88
LY94
LY97
LY data
Normal data

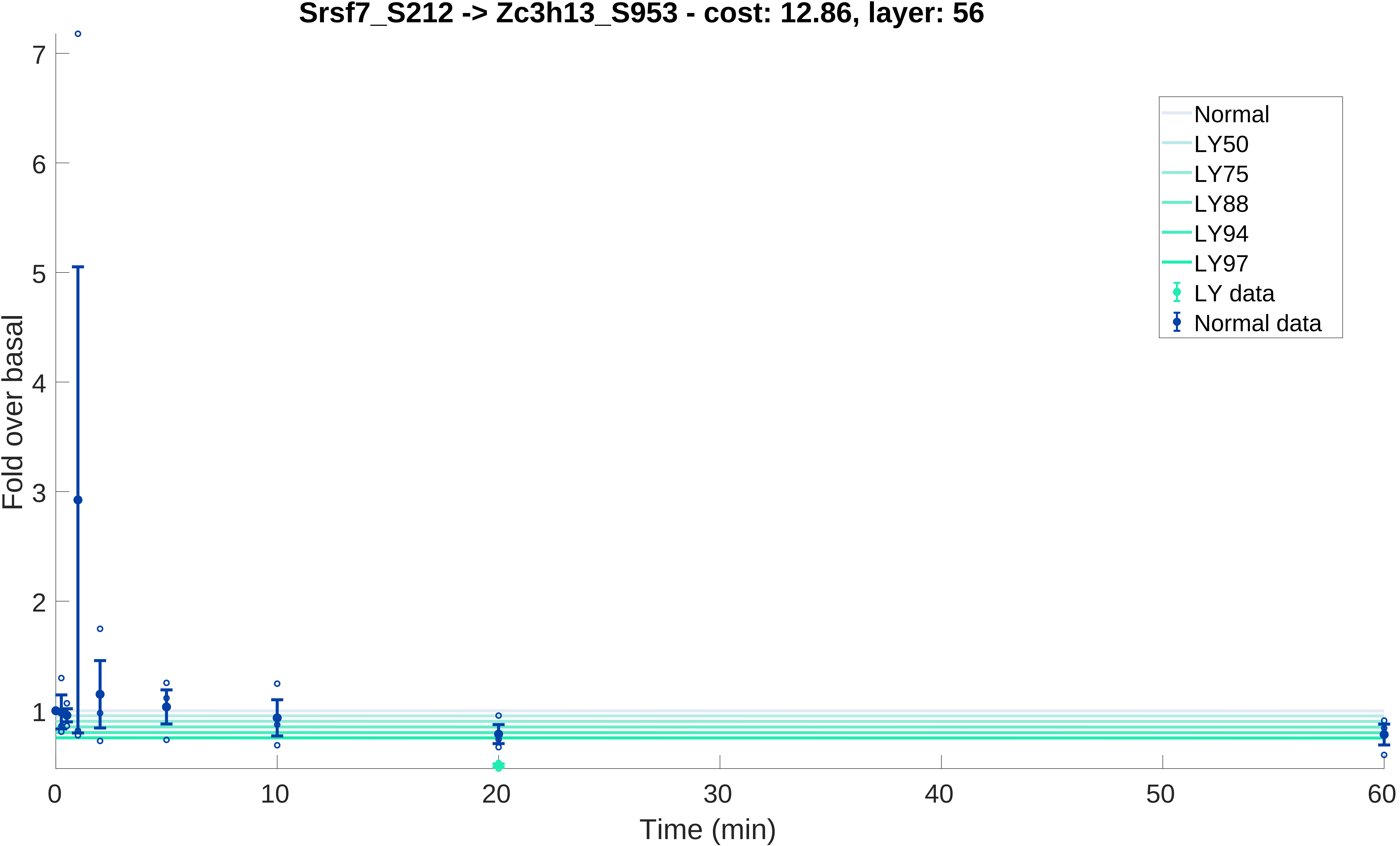

Srsf7_S212 -> Zc3h13_S953 - cost: 12.86, layer: 56
Fold over basal
Time (min)
0
10
20
30
40
50
60
1
2
3
4
5
6
7
Normal
LY50
LY75
LY88
LY94
LY97
LY data
Normal data

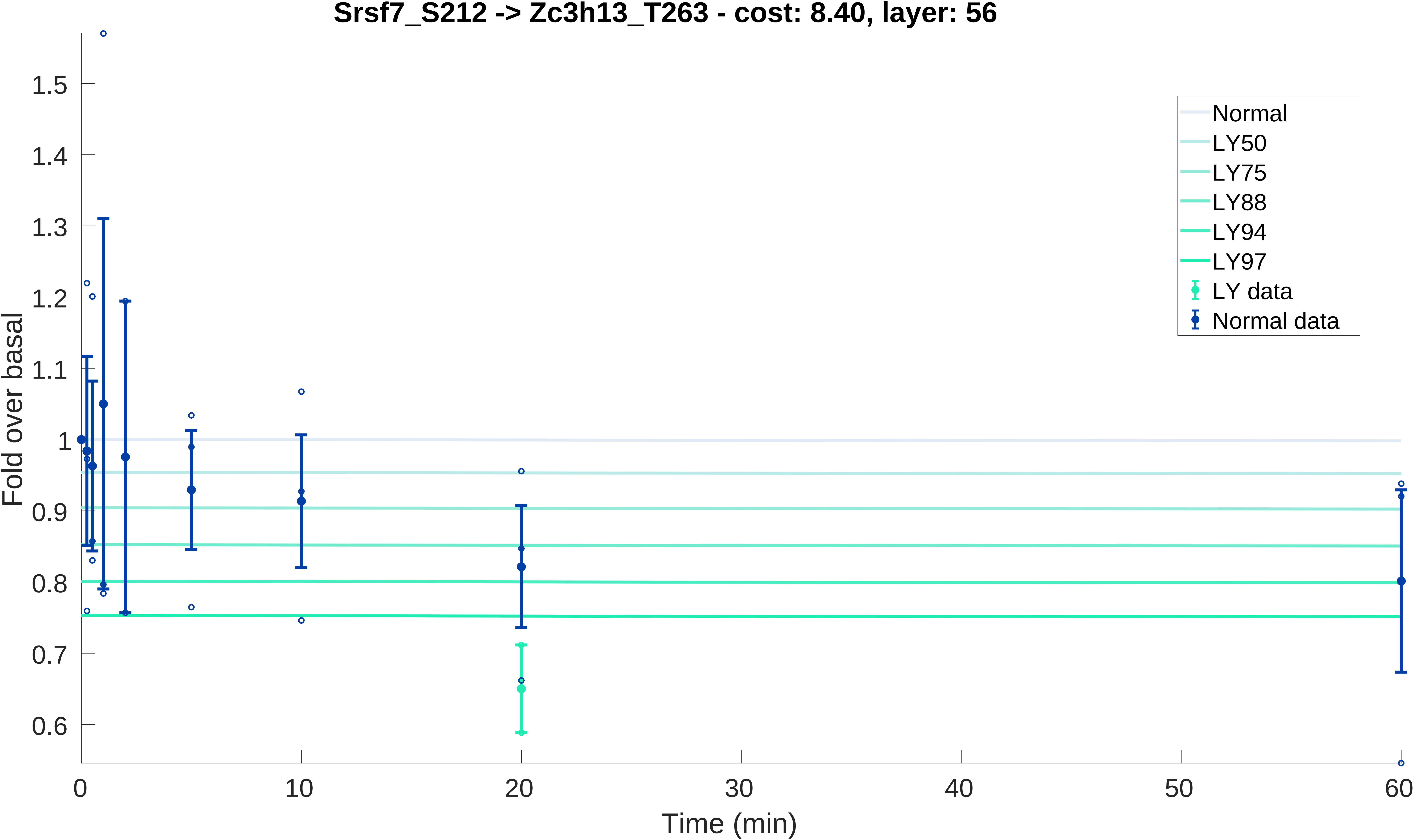

Srsf7_S212 -> Zc3h13_T263 - cost: 8.40, layer: 56
Fold over basal
Time (min)
1.5
1.4
1.3
1.2
1.1
1
0.9
0.8
0.7
0.6
0
10
20
30
40
50
60
Normal
LY50
LY75
LY88
LY94
LY97
LY data
Normal data

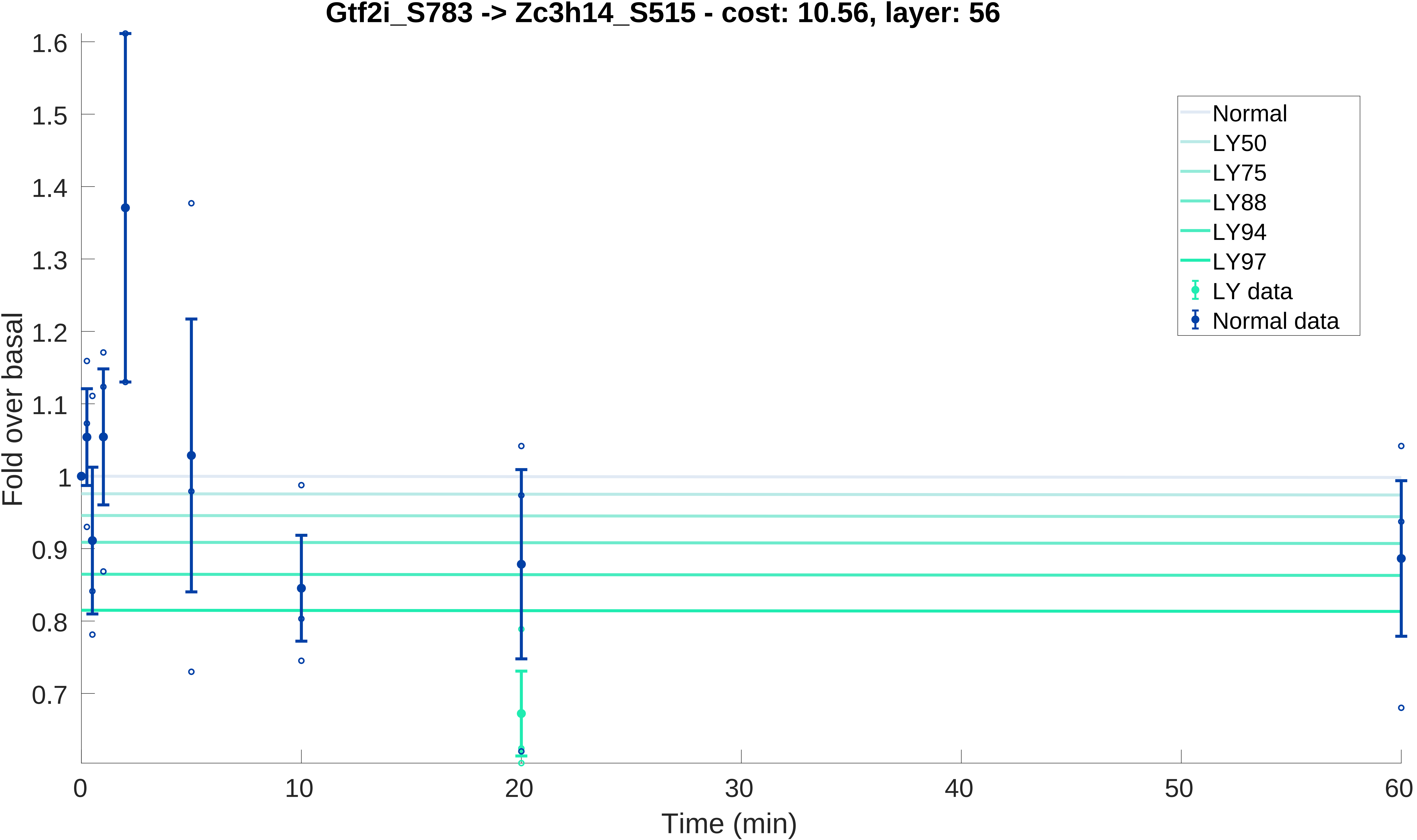

Gtf2i_S783 -> Zc3h14_S515 - cost: 10.56, layer: 56
Fold over basal
Time (min)
0
10
20
30
40
50
60
0.7
0.8
0.9
1
1.1
1.2
1.3
1.4
1.5
1.6
Normal
LY50
LY75
LY88
LY94
LY97
LY data
Normal data

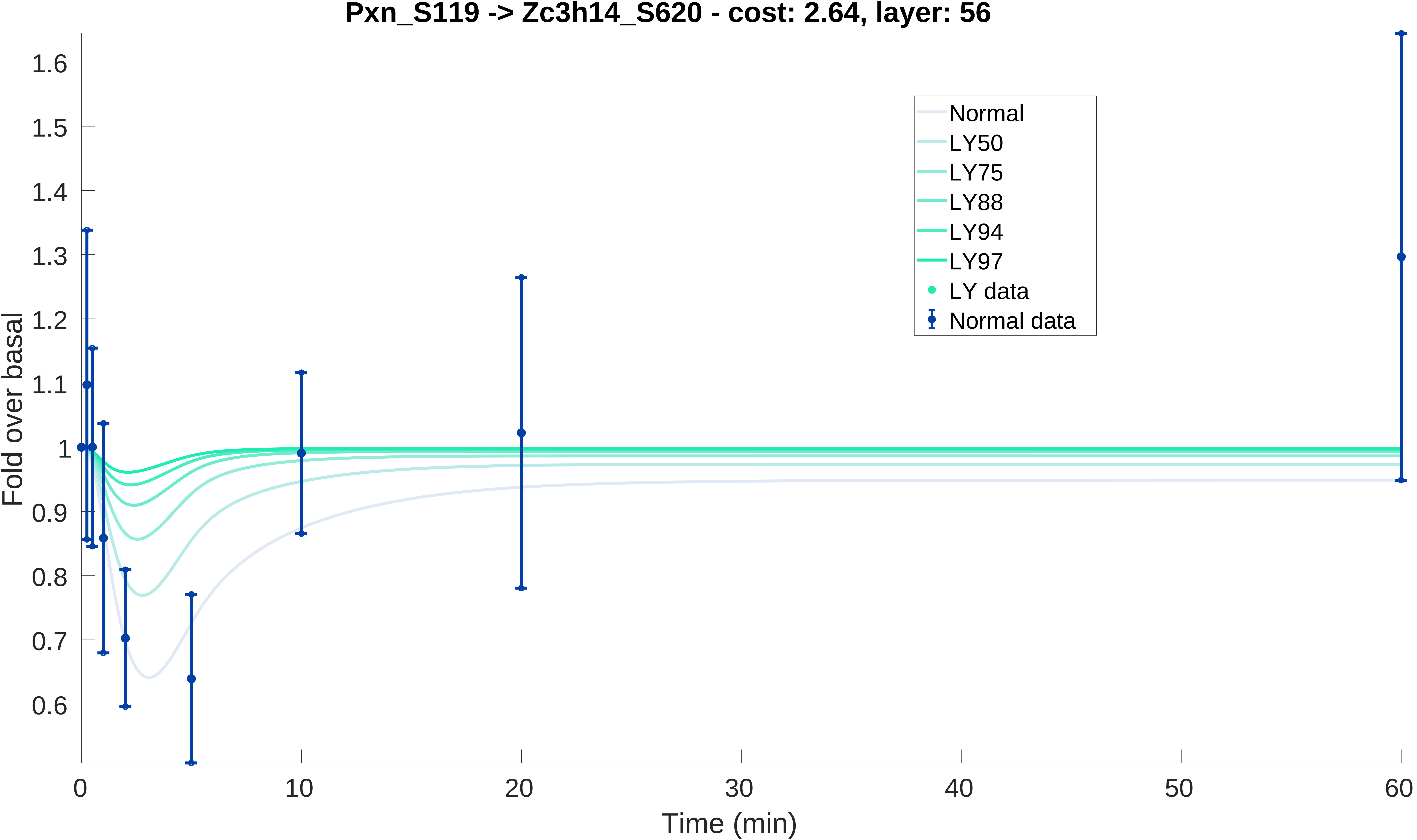

Pxn_S119 -> Zc3h14_S620 - cost: 2.64, layer: 56
Fold over basal
1.6
1.5
1.4
1.3
1.2
1.1
1
0.9
0.8
0.7
0.6
0
10
20
30
40
50
60
Time (min)
Normal
LY50
LY75
LY88
LY94
LY97
LY data
Normal data

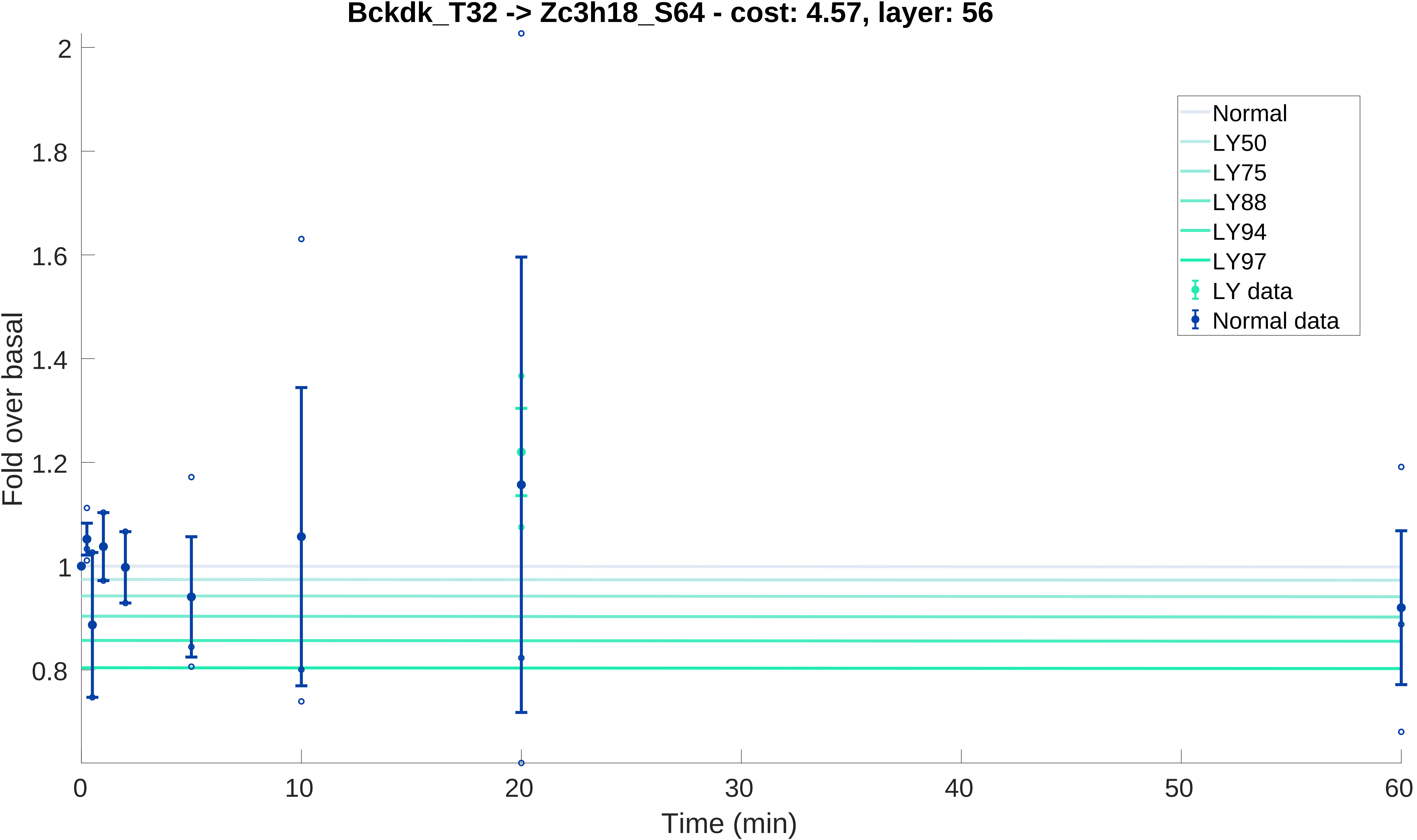

Bckdk_T32 -> Zc3h18_S64 - cost: 4.57, layer: 56
Fold over basal
Time (min)
Normal
LY50
LY75
LY88
LY94
LY97
LY data
Normal data
2
1.8
1.6
1.4
1.2
1
0.8
0
10
20
30
40
50
60

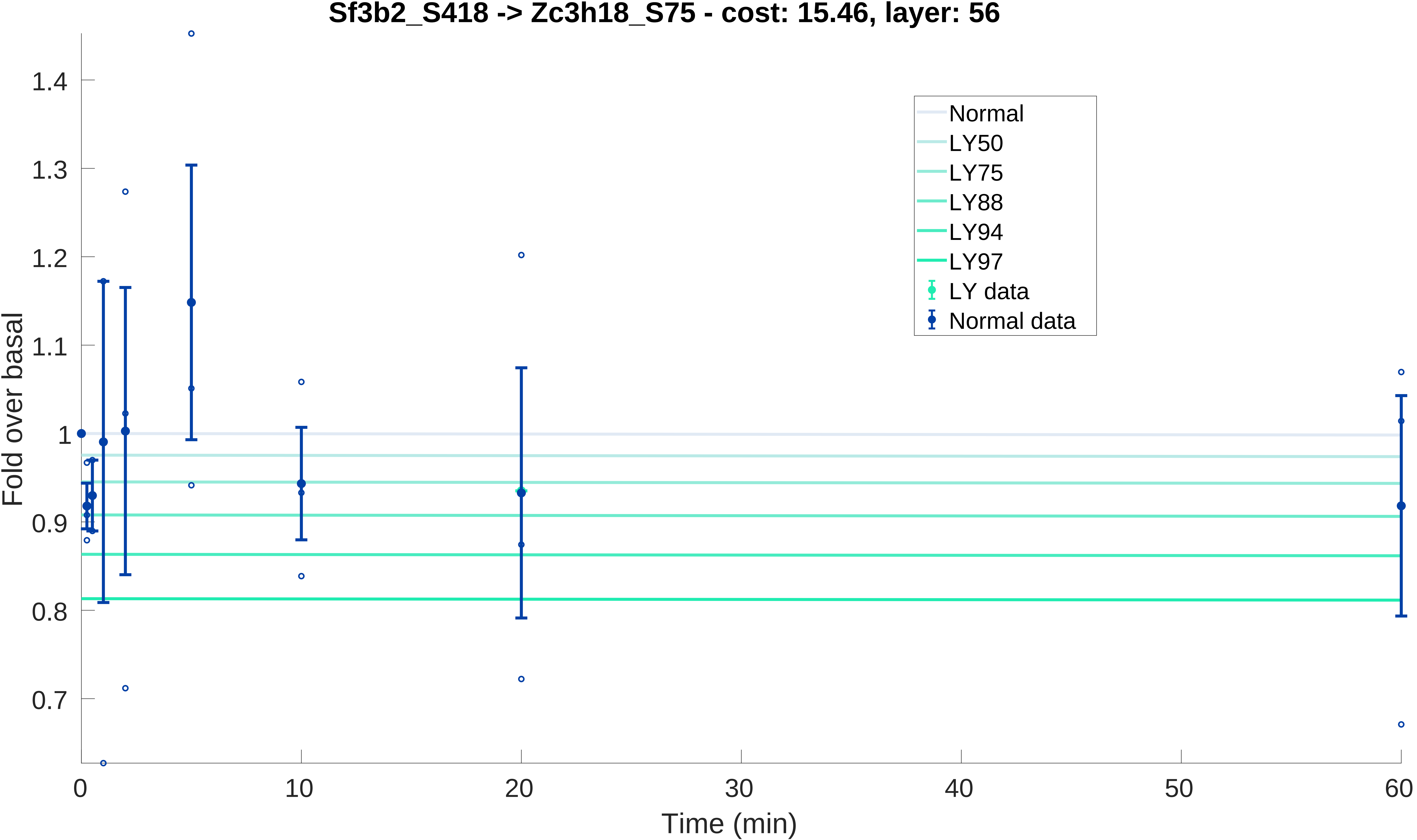

Sf3b2_S418 -> Zc3h18_S75 - cost: 15.46, layer: 56
Fold over basal
Time (min)
Normal
LY50
LY75
LY88
LY94
LY97
LY data
Normal data
1.4
1.3
1.2
1.1
1
0.9
0.8
0.7
0
10
20
30
40
50
60

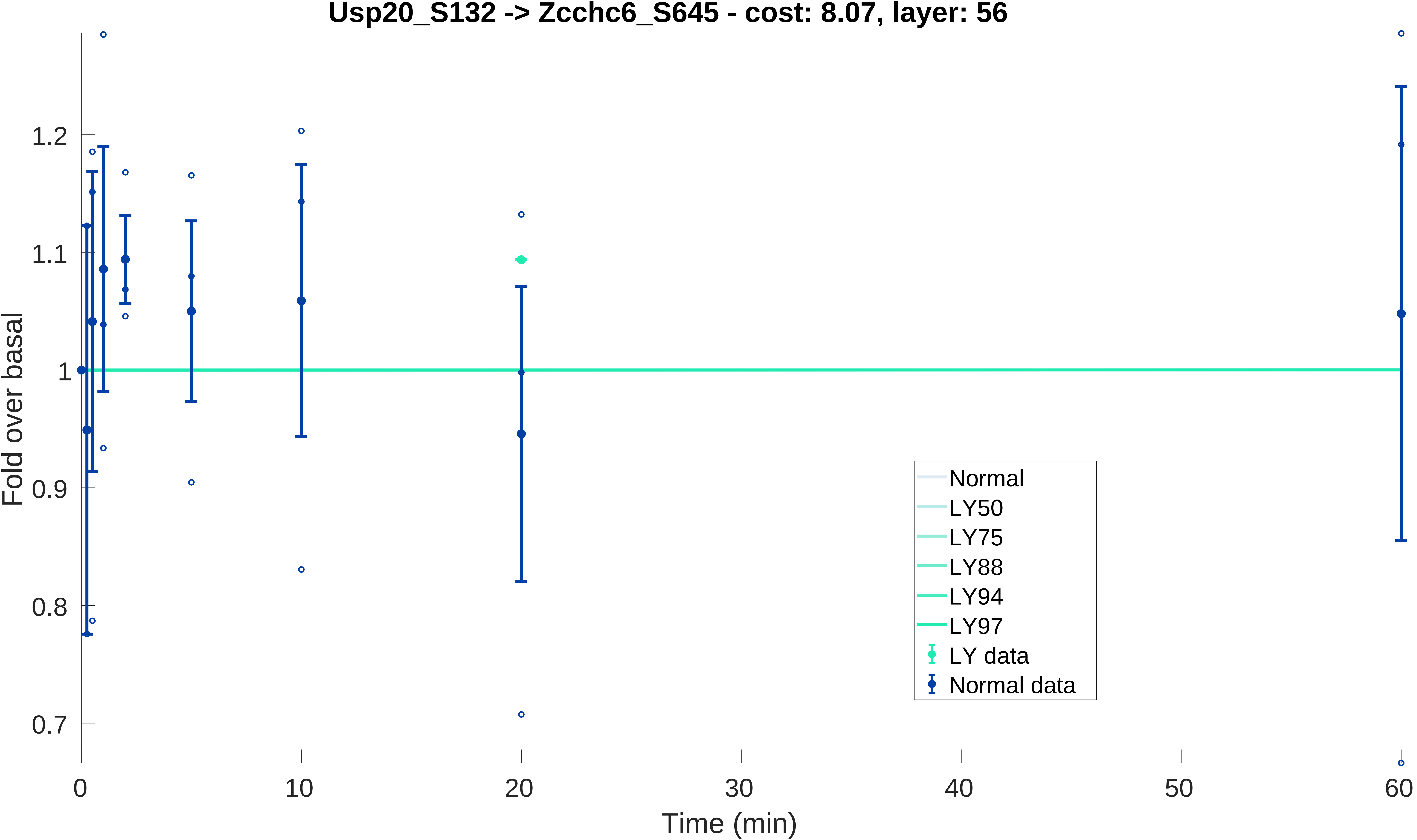

Usp20_S132 -> Zcchc6_S645 - cost: 8.07, layer: 56
Fold over basal
Time (min)
0.7
0.8
0.9
1
1.1
1.2
0
10
20
30
40
50
60
Normal
LY50
LY75
LY88
LY94
LY97
LY data
Normal data

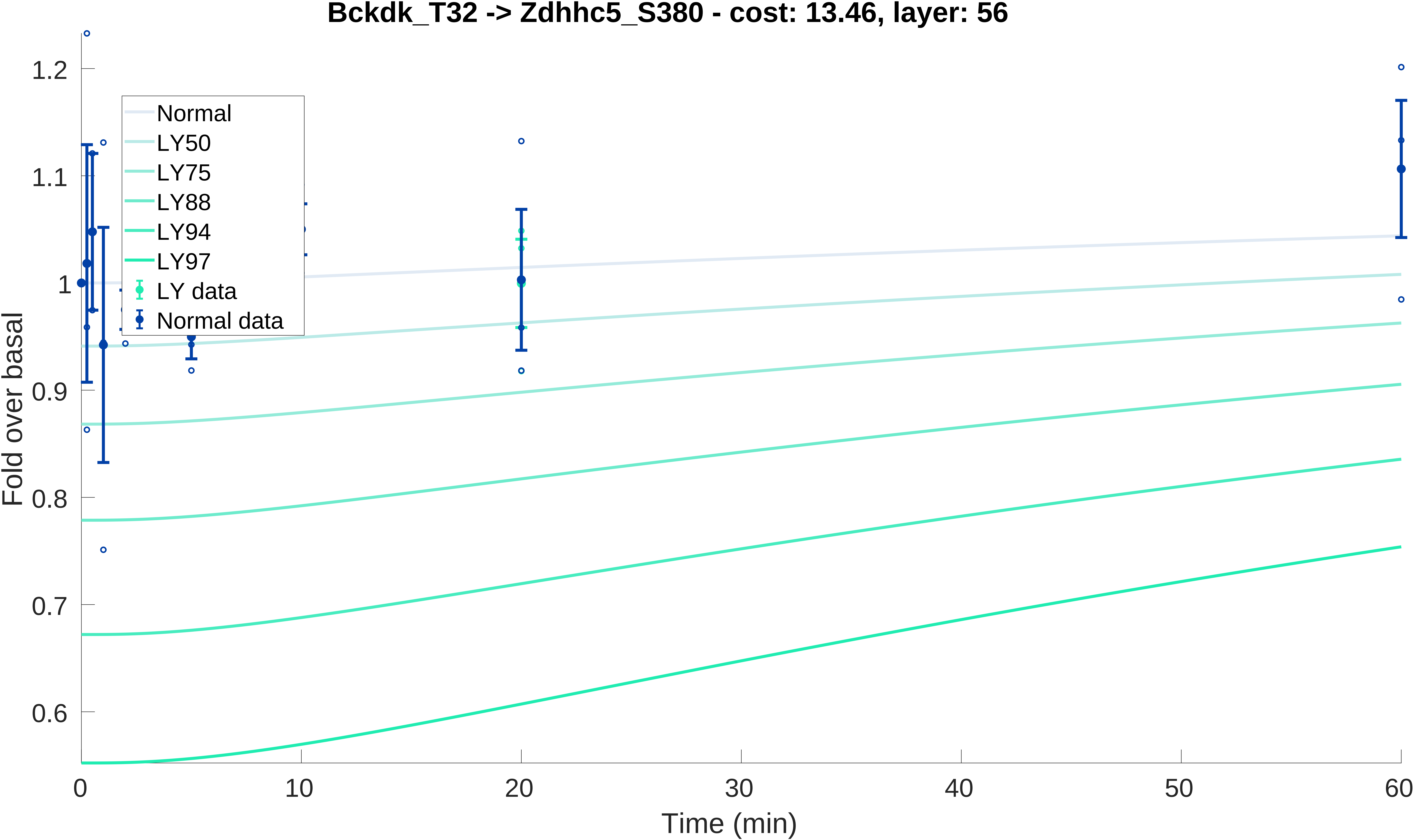

Bckdk_T32 -> Zdhhc5_S380 - cost: 13.46, layer: 56
Normal
LY50
LY75
LY88
LY94
LY97
LY data
Normal data
Fold over basal
Time (min)
1.2
1.1
1
0.9
0.8
0.7
0.6
0
10
20
30
40
50
60

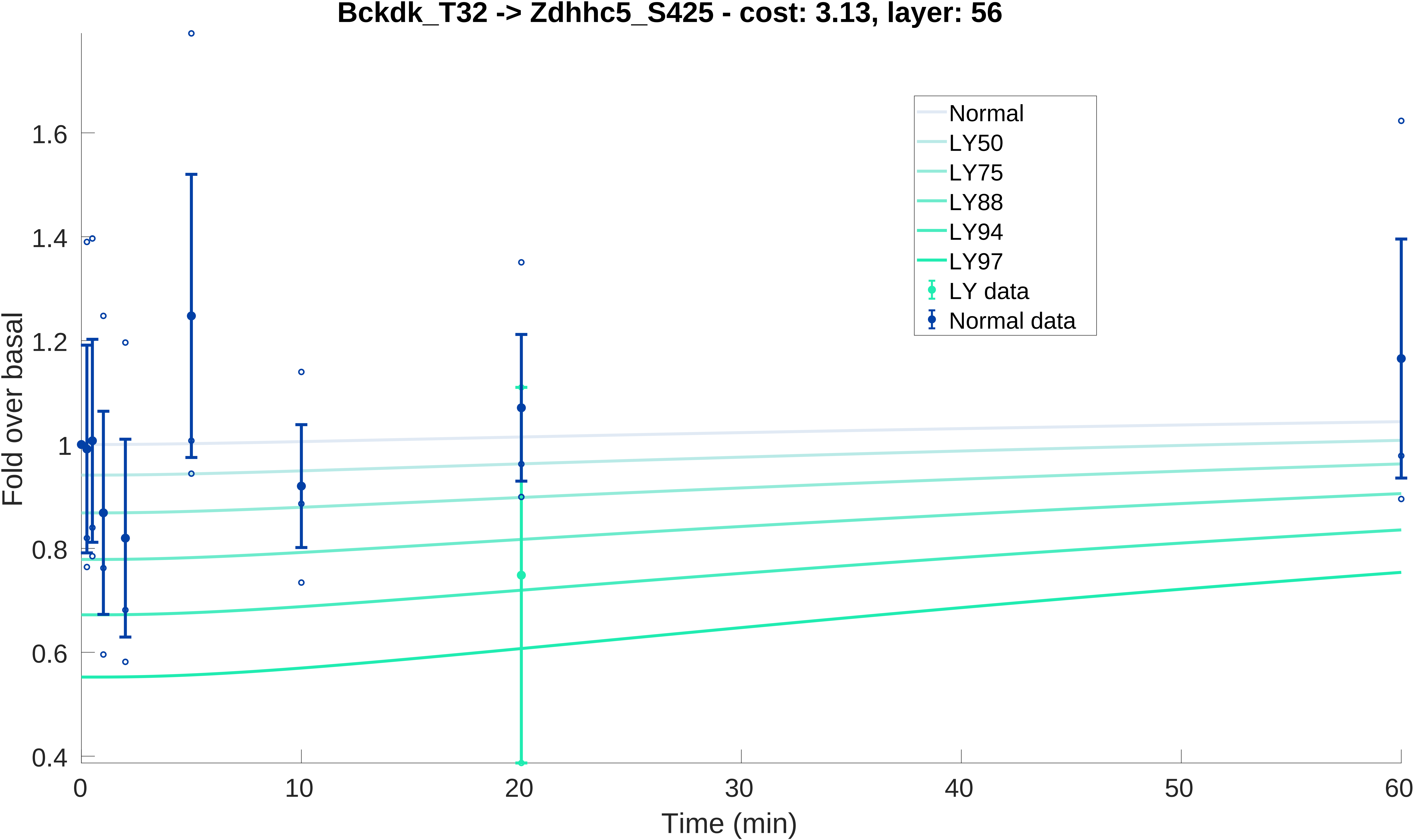
Bckdk_T32 -> Zdhhc5_S425 - cost: 3.13, layer: 56
Fold over basal
Time (min)
0.4
0.6
0.8
1
1.2
1.4
1.6
0
10
20
30
40
50
60
Normal
LY50
LY75
LY88
LY94
LY97
LY data
Normal data

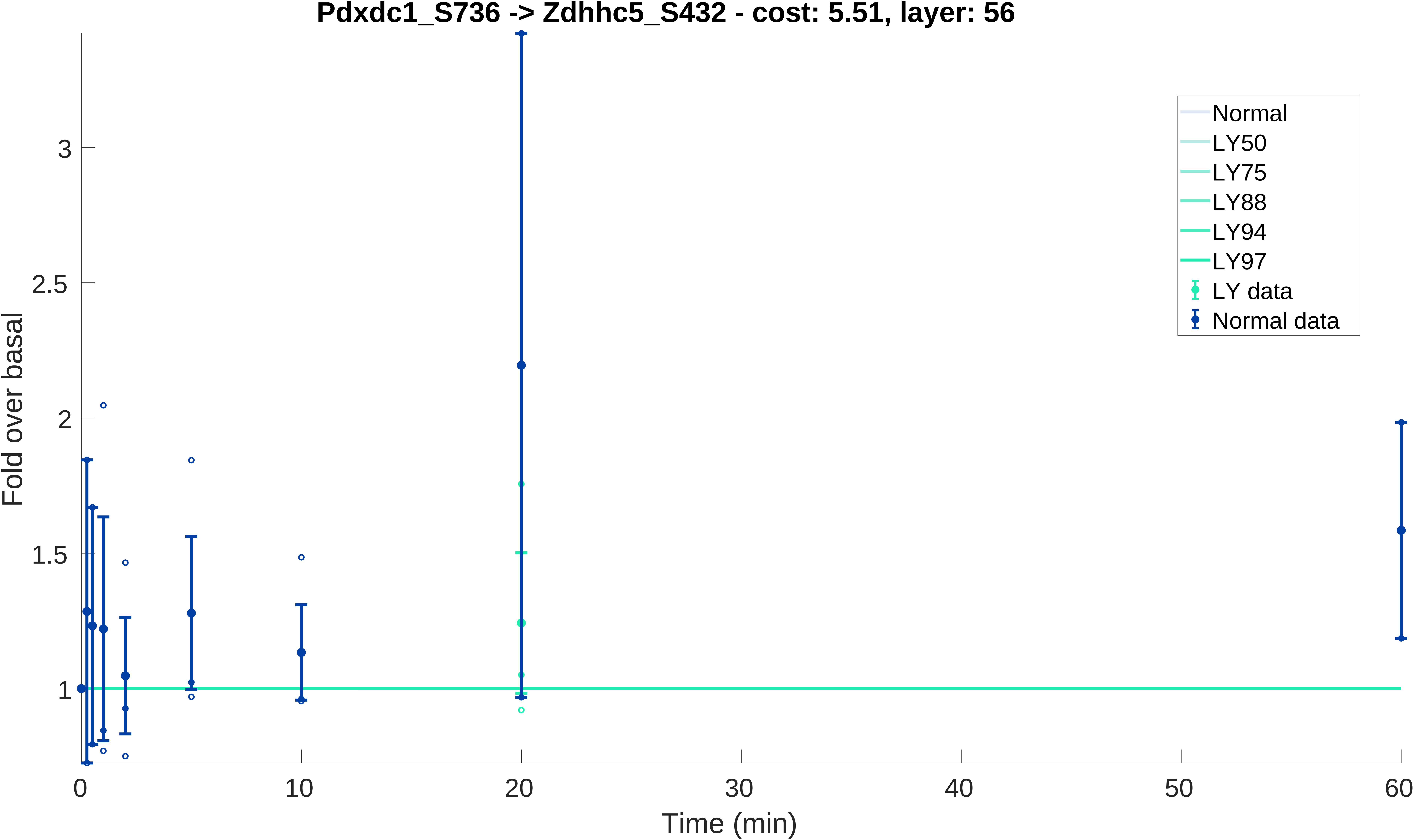

Pdxdc1_S736 -> Zdhhc5_S432 - cost: 5.51, layer: 56
Fold over basal
Time (min)
Normal
LY50
LY75
LY88
LY94
LY97
LY data
Normal data
3
2.5
2
1.5
1
0
10
20
30
40
50
60

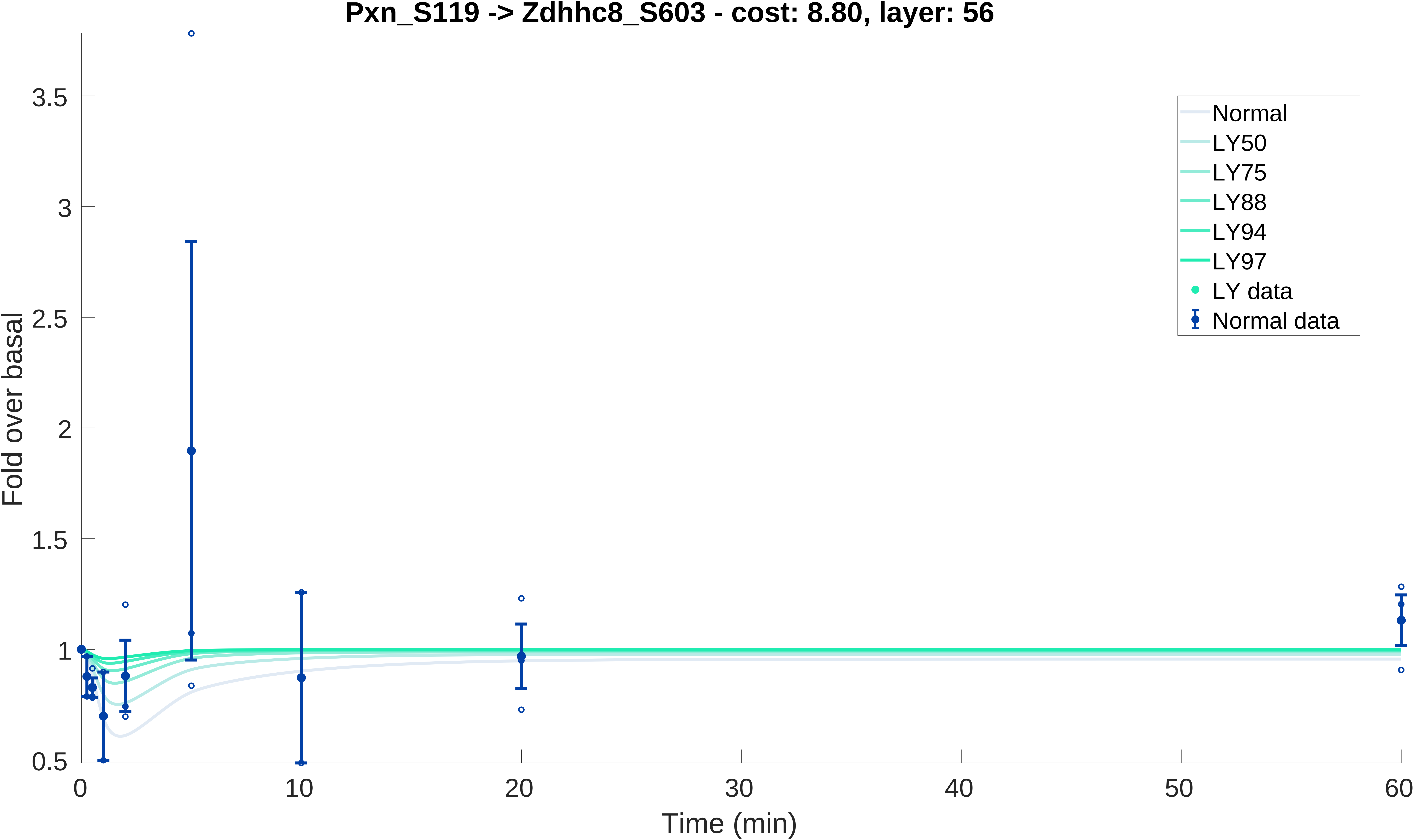

Pxn_S119 -> Zdhhc8_S603 - cost: 8.80, layer: 56
Fold over basal
Time (min)
0.5
1
1.5
2
2.5
3
3.5
0
10
20
30
40
50
60
Normal
LY50
LY75
LY88
LY94
LY97
LY data
Normal data

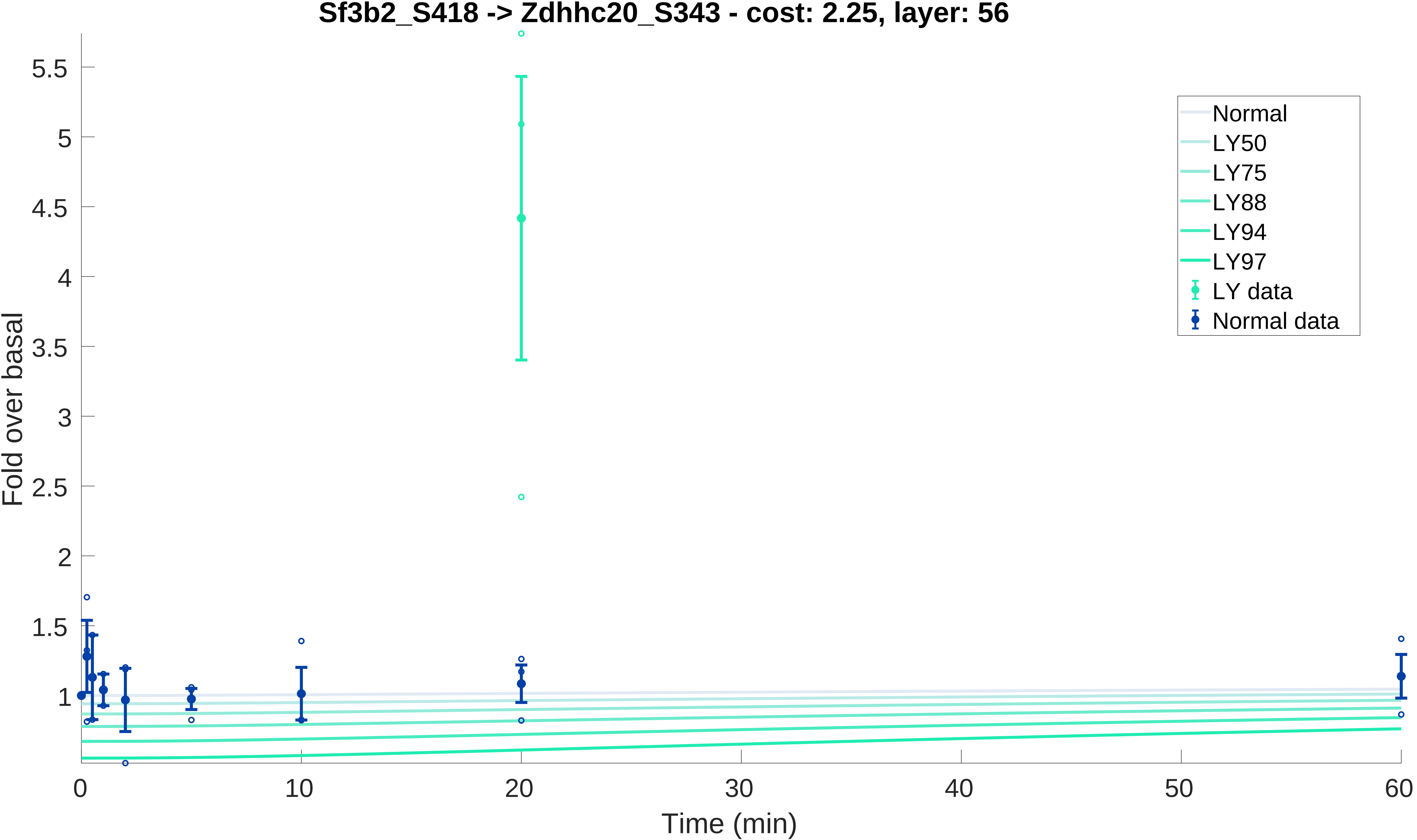
Sf3b2_S418 -> Zdhhc20_S343 - cost: 2.25, layer: 56
Fold over basal
Time (min)
Normal
LY50
LY75
LY88
LY94
LY97
LY data
Normal data
5.5
5
4.5
4
3.5
3
2.5
2
1.5
1
0
10
20
30
40
50
60

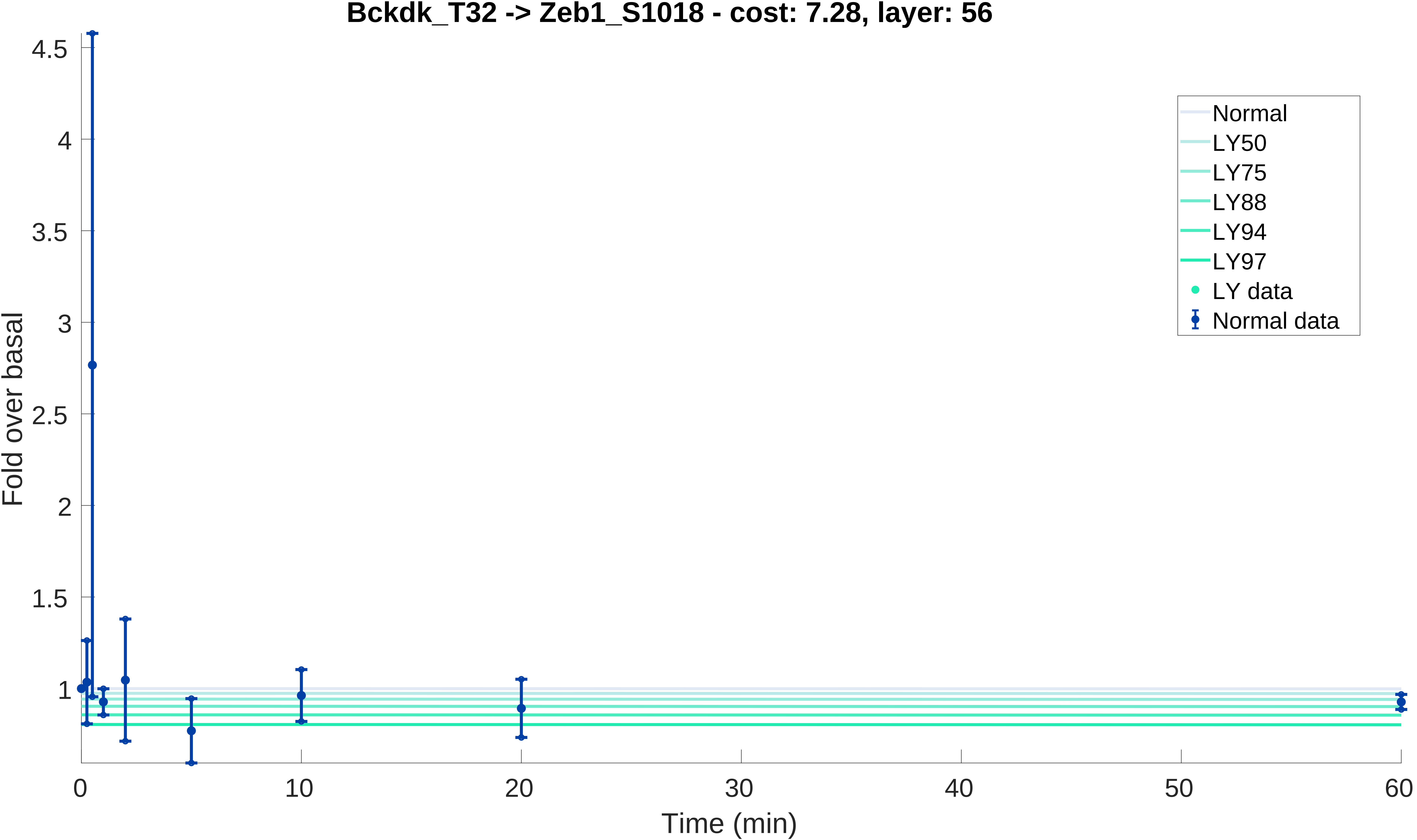
Bckdk_T32 -> Zeb1_S1018 - cost: 7.28, layer: 56
Fold over basal
Time (min)
Normal
LY50
LY75
LY88
LY94
LY97
LY data
Normal data
4.5
4
3.5
3
2.5
2
1.5
1
0
10
20
30
40
50
60

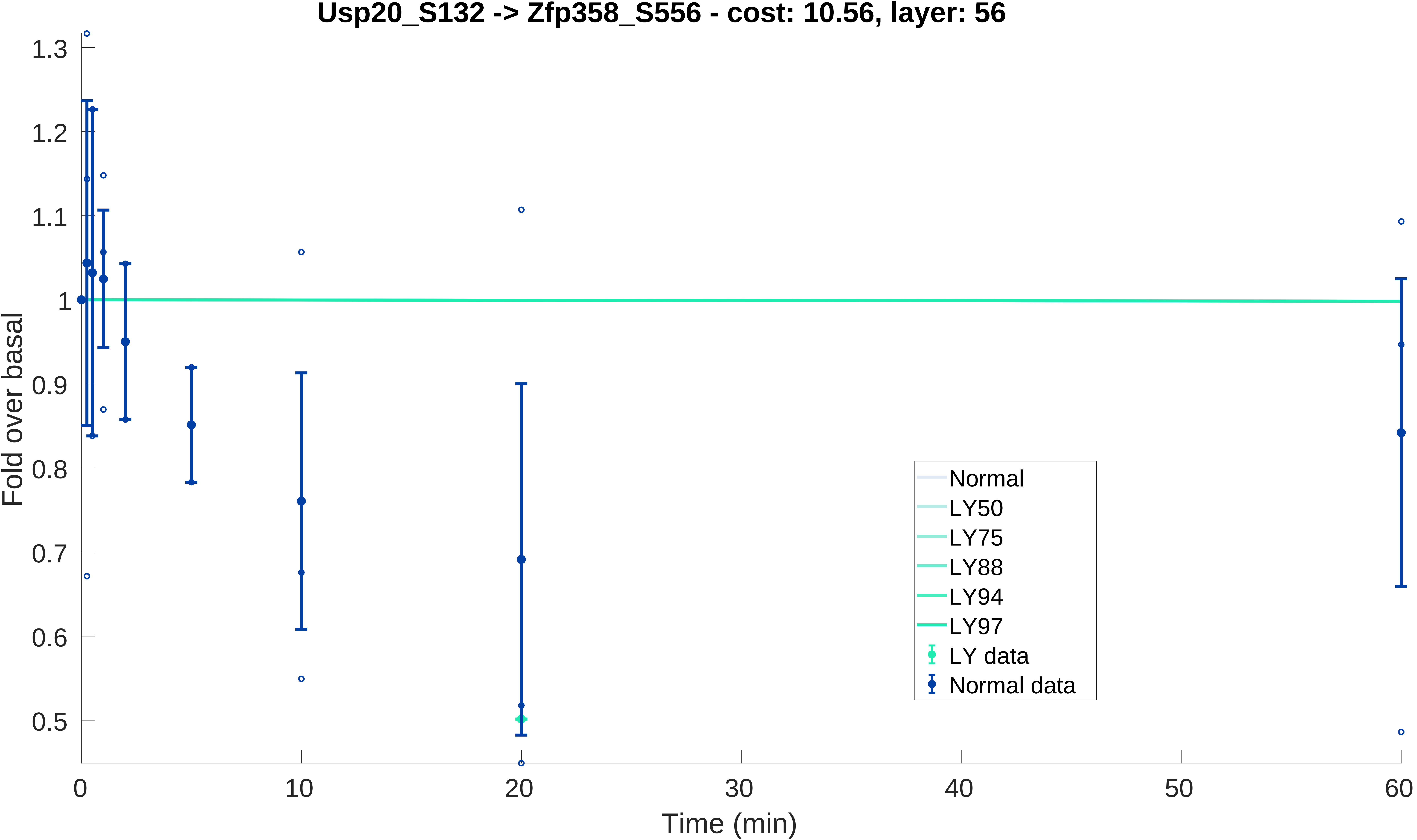

Usp20_S132 -> Zfp358_S556 - cost: 10.56, layer: 56
Fold over basal
Time (min)
1.3
1.2
1.1
1
0.9
0.8
0.7
0.6
0.5
0
10
20
30
40
50
60
Normal
LY50
LY75
LY88
LY94
LY97
LY data
Normal data

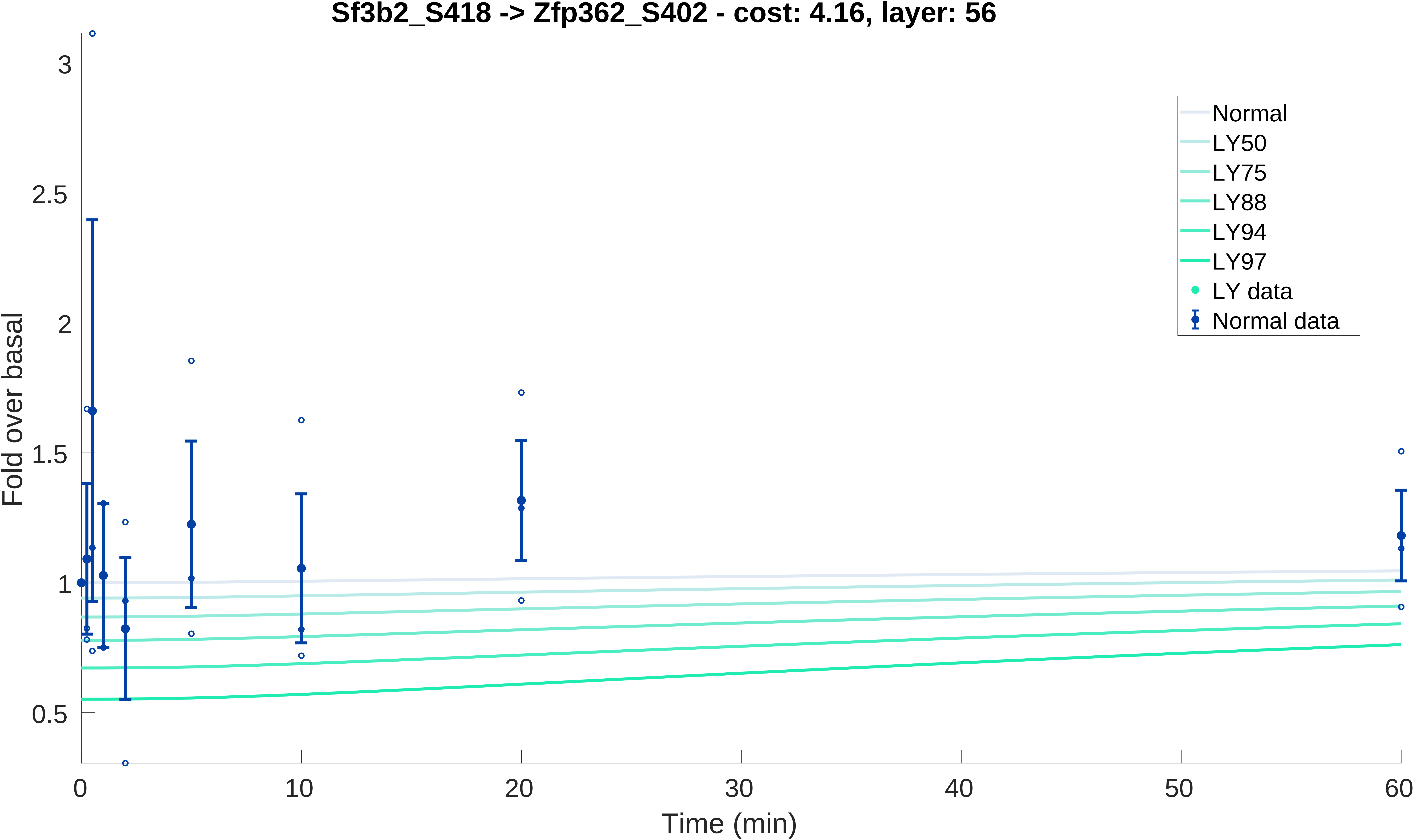

Sf3b2_S418 -> Zfp362_S402 - cost: 4.16, layer: 56
Fold over basal
Time (min)
0.5
1
1.5
2
2.5
3
0
10
20
30
40
50
60
Normal
LY50
LY75
LY88
LY94
LY97
LY data
Normal data

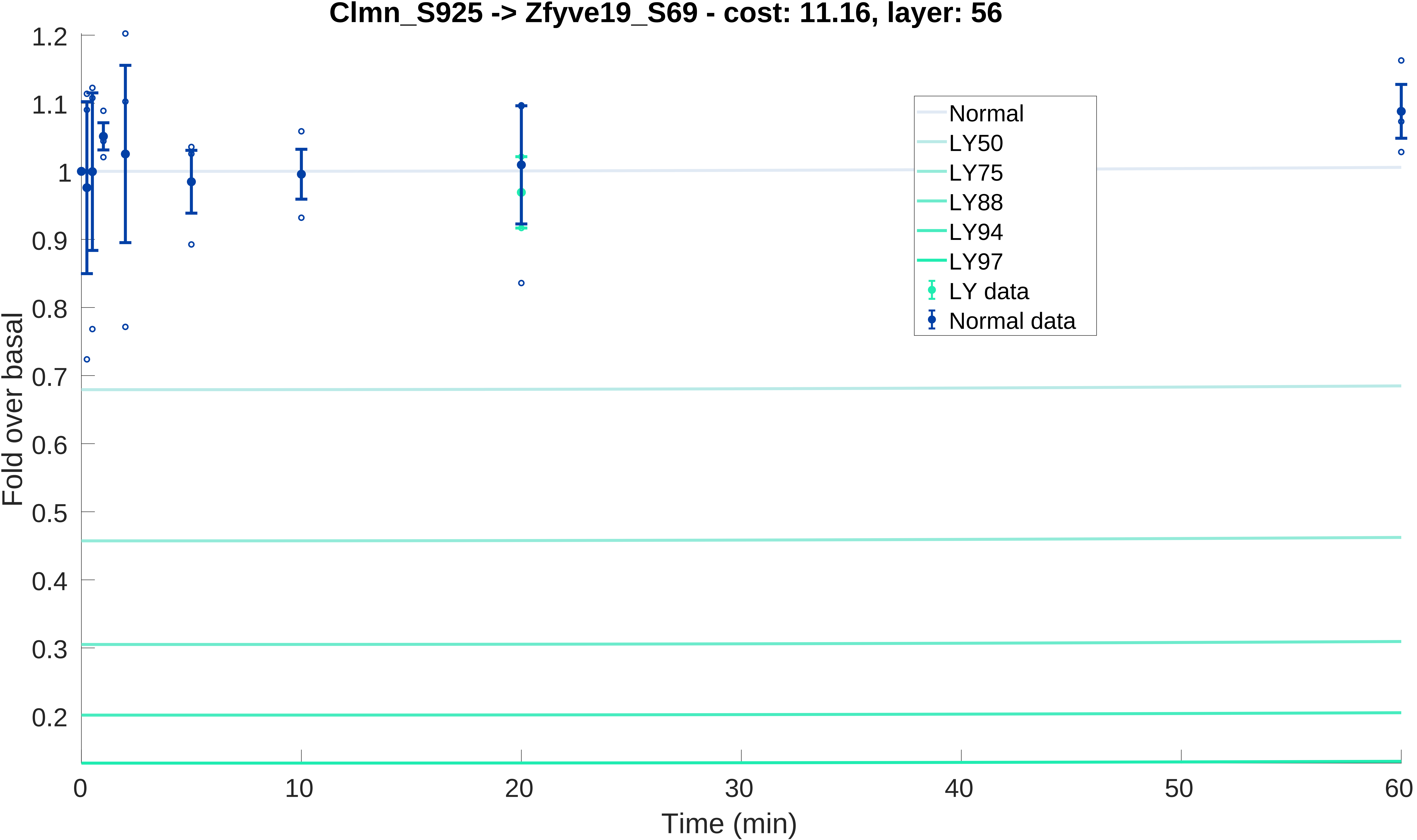

Clmn_S925 -> Zfyve19_S69 - cost: 11.16, layer: 56
Fold over basal
Time (min)
0
10
20
30
40
50
60
0.2
0.3
0.4
0.5
0.6
0.7
0.8
0.9
1
1.1
1.2
Normal
LY50
LY75
LY88
LY94
LY97
LY data
Normal data

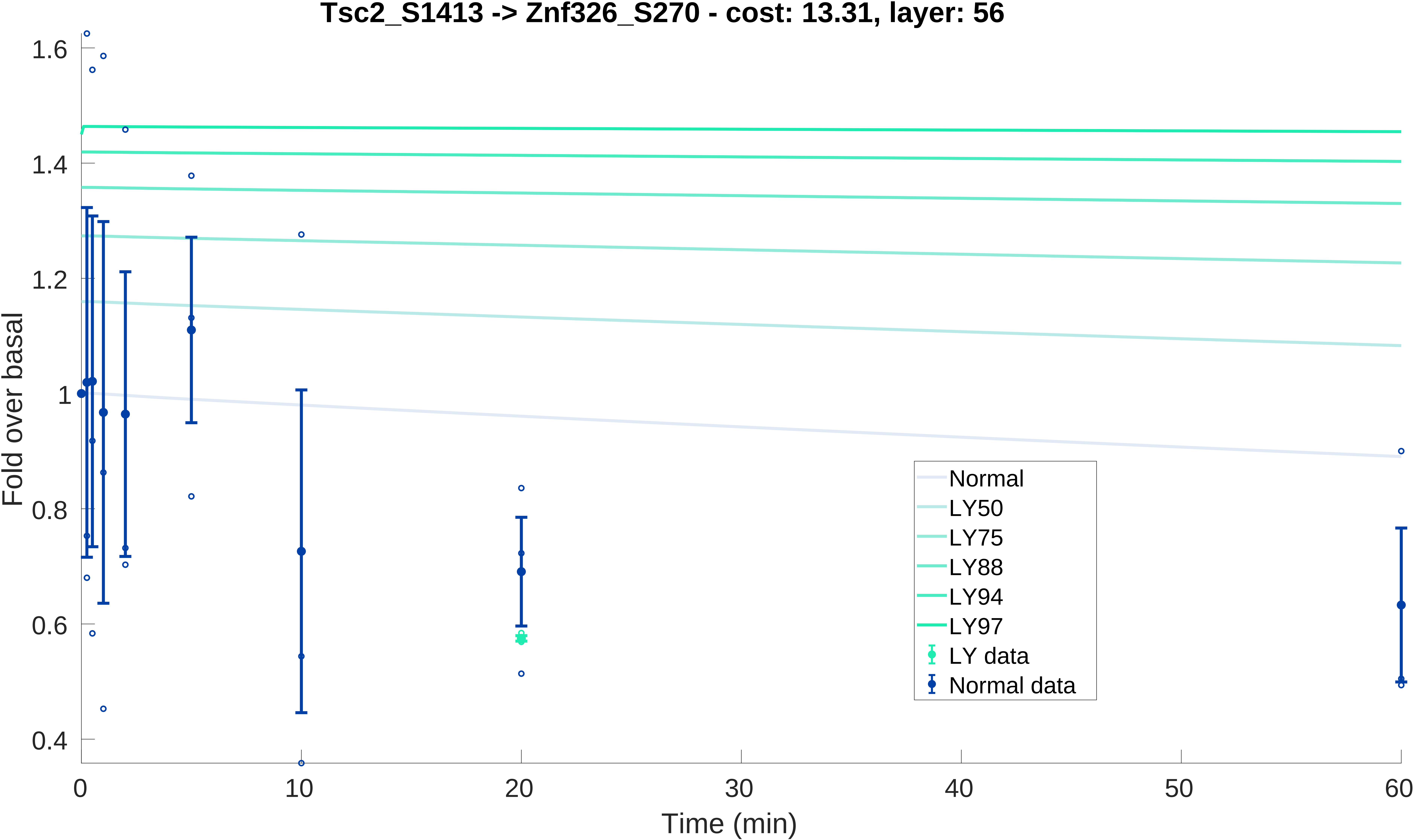

Tsc2_S1413 -> Znf326_S270 - cost: 13.31, layer: 56
Fold over basal
1.6
1.4
1.2
1
0.8
0.6
0.4
0
10
20
30
40
50
60
Time (min)
Normal
LY50
LY75
LY88
LY94
LY97
LY data
Normal data

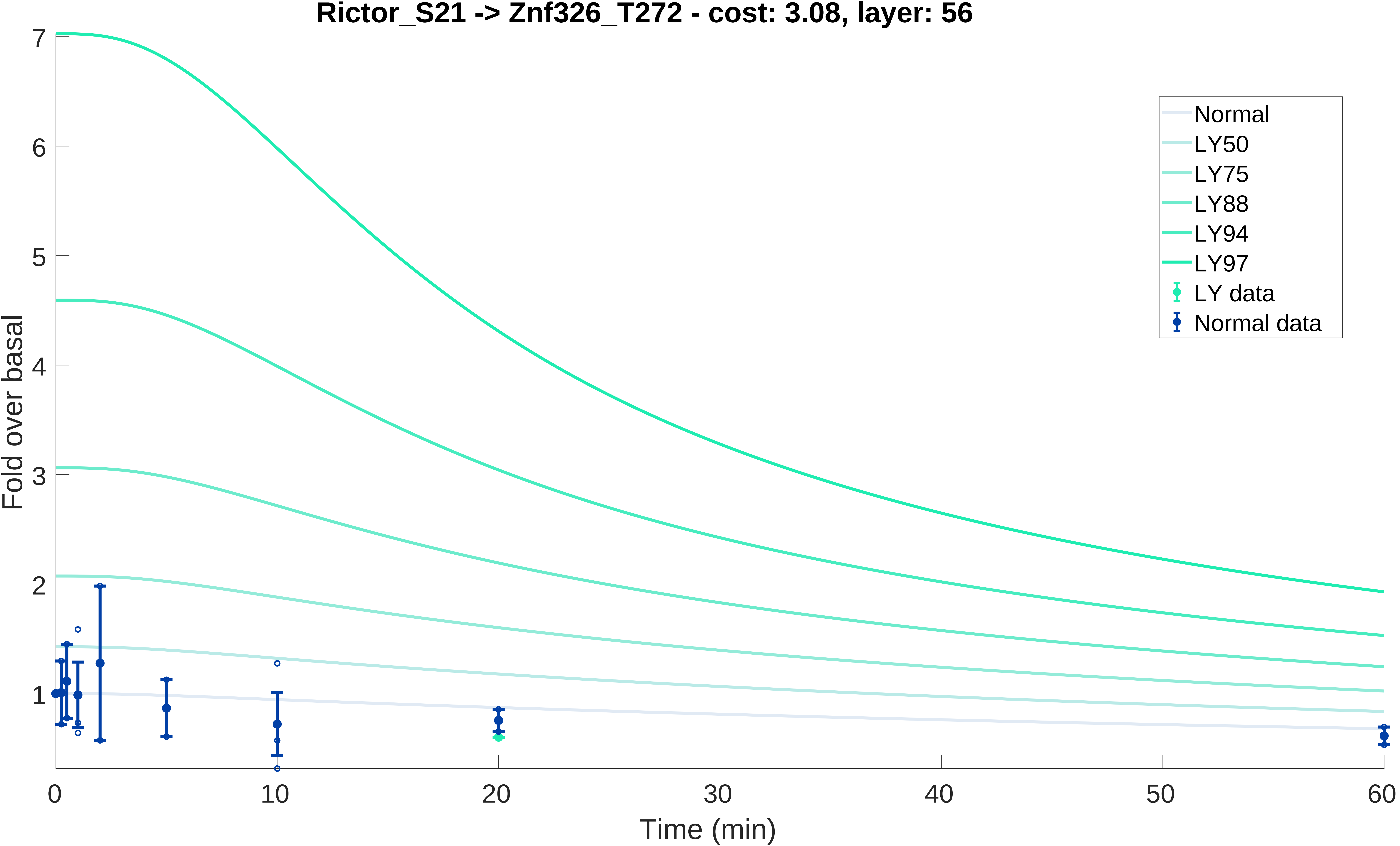
Rictor_S21 -> Znf326_T272 - cost: 3.08, layer: 56
Fold over basal
Time (min)
Normal
LY50
LY75
LY88
LY94
LY97
LY data
Normal data
0
10
20
30
40
50
60
1
2
3
4
5
6
7

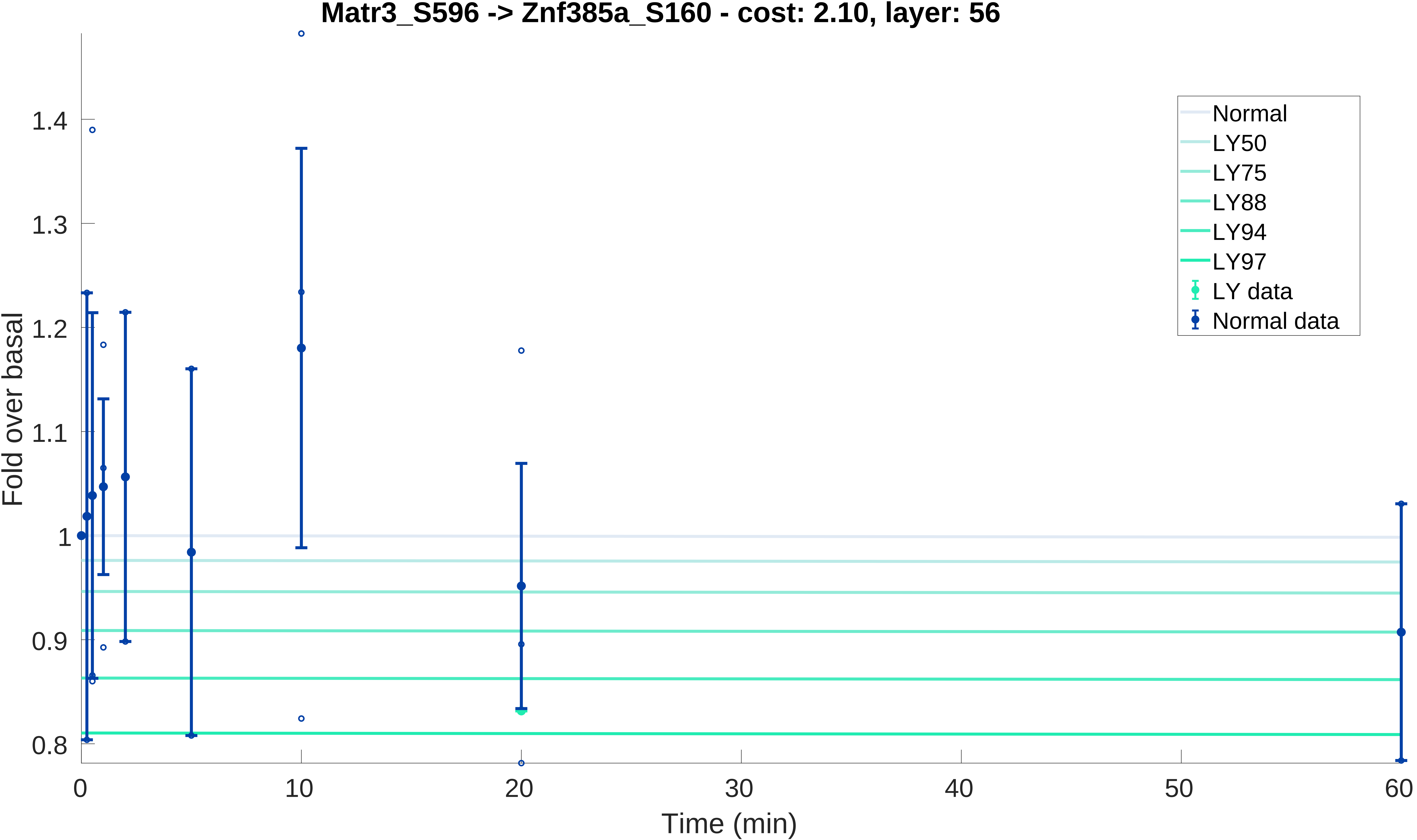
Matr3_S596 -> Znf385a_S160 - cost: 2.10, layer: 56
Fold over basal
Time (min)
0.8
0.9
1
1.1
1.2
1.3
1.4
0
10
20
30
40
50
60
Normal
LY50
LY75
LY88
LY94
LY97
LY data
Normal data

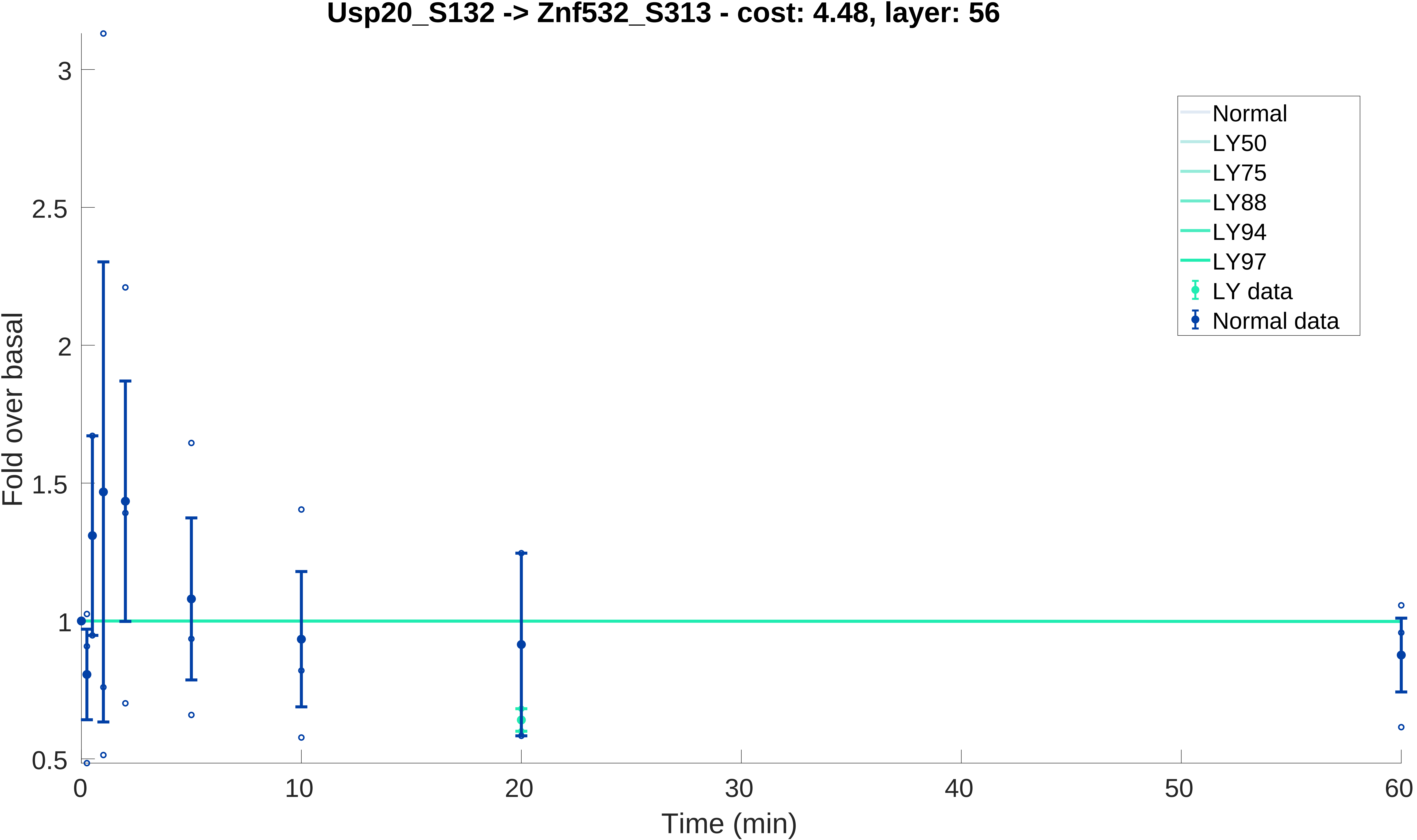

Usp20_S132 -> Znf532_S313 - cost: 4.48, layer: 56
Fold over basal
Time (min)
0.5
1
1.5
2
2.5
3
0
10
20
30
40
50
60
Normal
LY50
LY75
LY88
LY94
LY97
LY data
Normal data

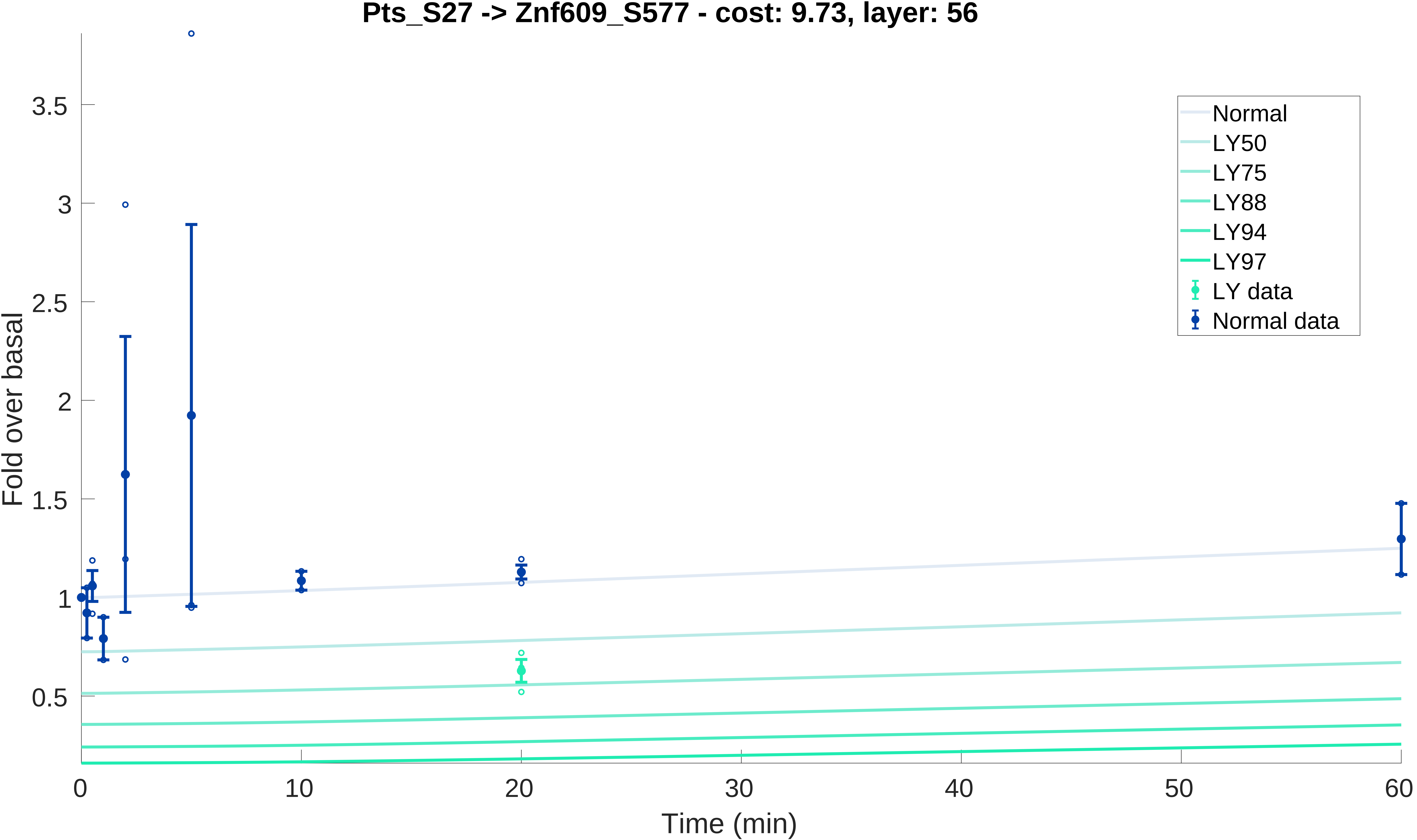

Pts_S27 -> Znf609_S577 - cost: 9.73, layer: 56
Fold over basal
Time (min)
0.5
1
1.5
2
2.5
3
3.5
0
10
20
30
40
50
60
Normal
LY50
LY75
LY88
LY94
LY97
LY data
Normal data

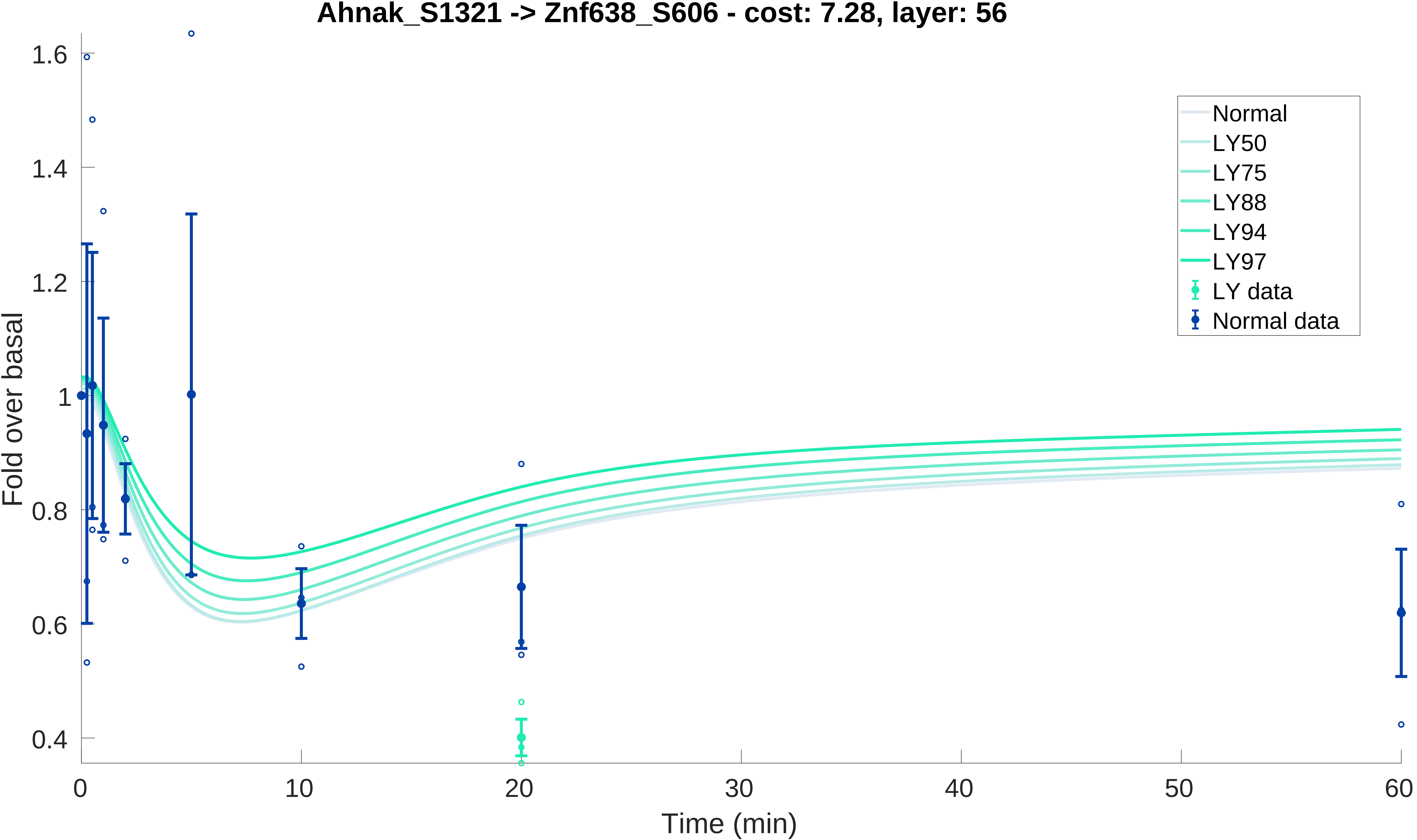
Ahnak_S1321 -> Znf638_S606 - cost: 7.28, layer: 56
Fold over basal
Time (min)
1.6
1.4
1.2
1
0.8
0.6
0.4
0
10
20
30
40
50
60
Normal
LY50
LY75
LY88
LY94
LY97
LY data
Normal data

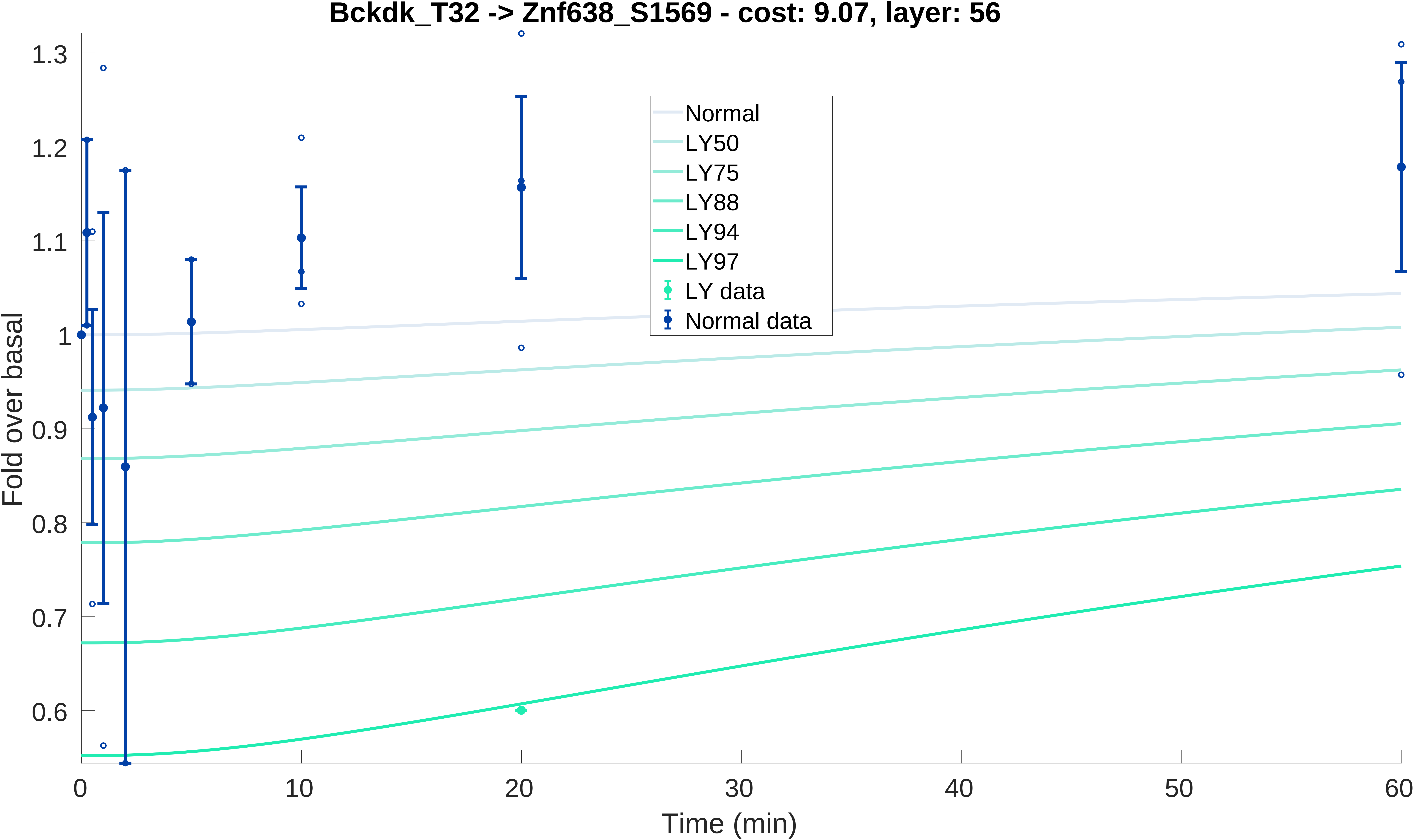
Bckdk_T32 -> Znf638_S1569 - cost: 9.07, layer: 56
Fold over basal
Time (min)
0
10
20
30
40
50
60
1.3
1.2
1.1
1
0.9
0.8
0.7
0.6
Normal
LY50
LY75
LY88
LY94
LY97
LY data
Normal data

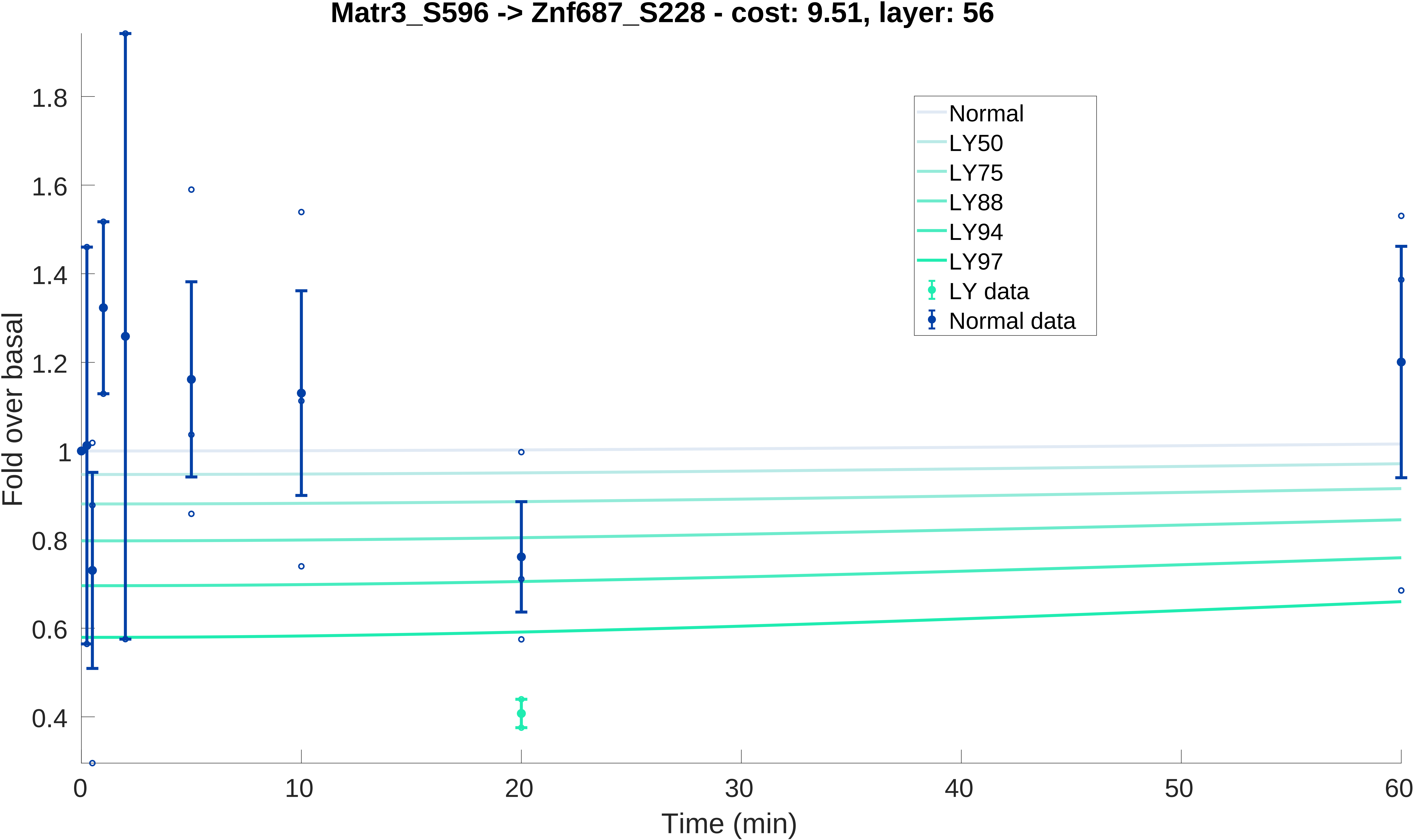

Matr3_S596 -> Znf687_S228 - cost: 9.51, layer: 56
Fold over basal
Time (min)
0.4
0.6
0.8
1
1.2
1.4
1.6
1.8
0
10
20
30
40
50
60
Normal
LY50
LY75
LY88
LY94
LY97
LY data
Normal data

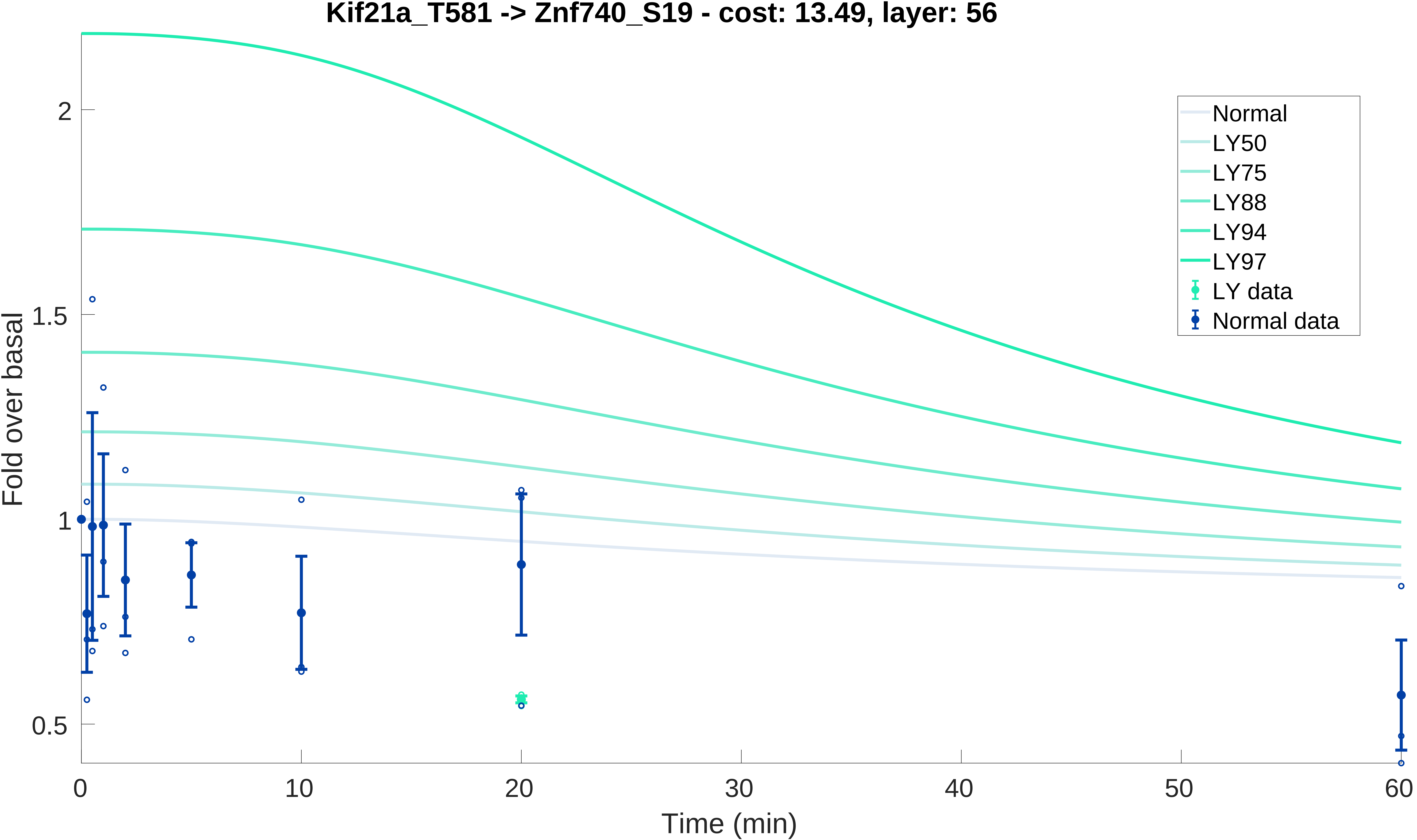

Kif21a_T581 -> Znf740_S19 - cost: 13.49, layer: 56
Fold over basal
Time (min)
0.5
1
1.5
2
0
10
20
30
40
50
60
Normal
LY50
LY75
LY88
LY94
LY97
LY data
Normal data

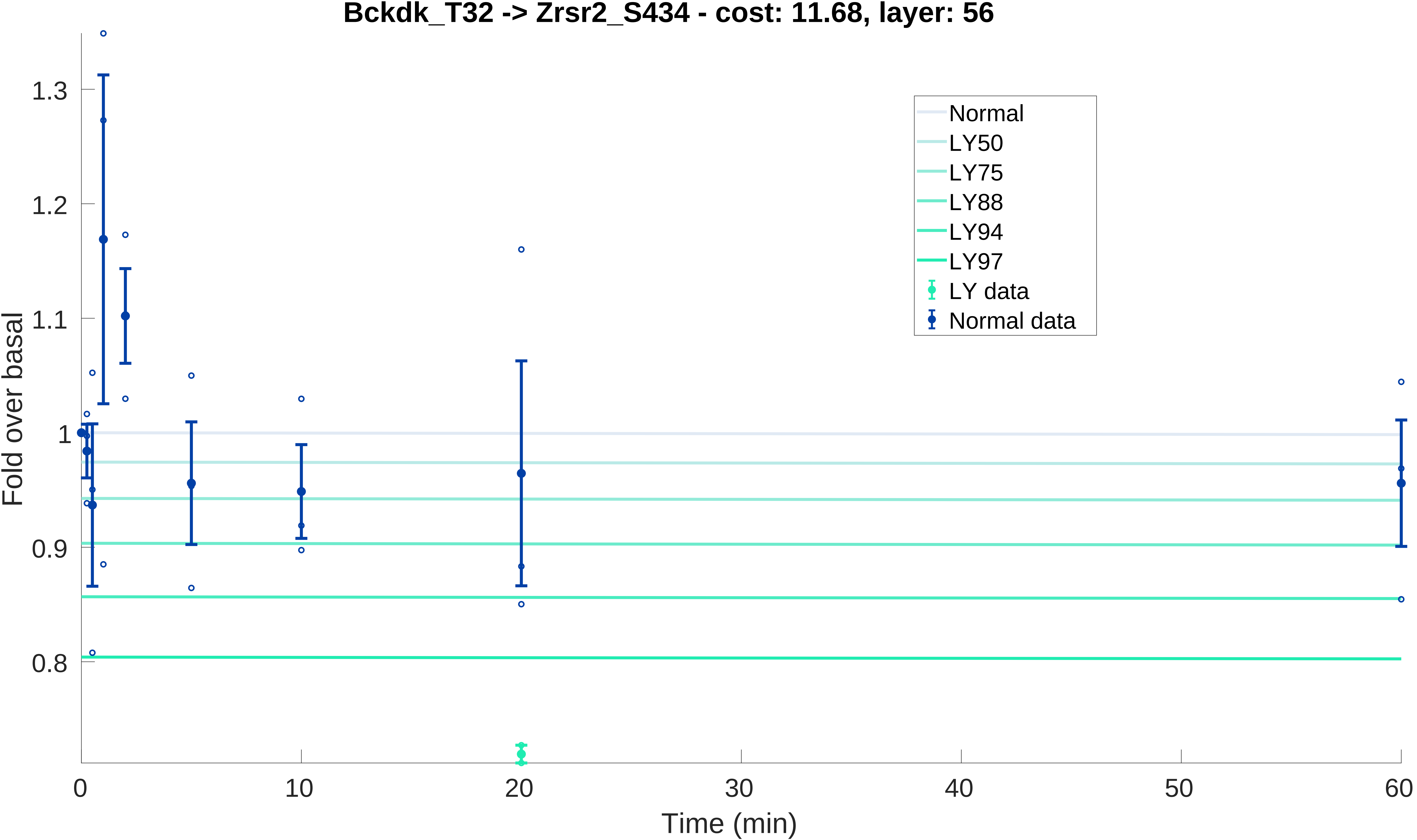

Bckdk_T32 -> Zrsr2_S434 - cost: 11.68, layer: 56
Normal
LY50
LY75
LY88
LY94
LY97
LY data
Normal data
Fold over basal
Time (min)
1.3
1.2
1.1
1
0.9
0.8
0
10
20
30
40
50
60

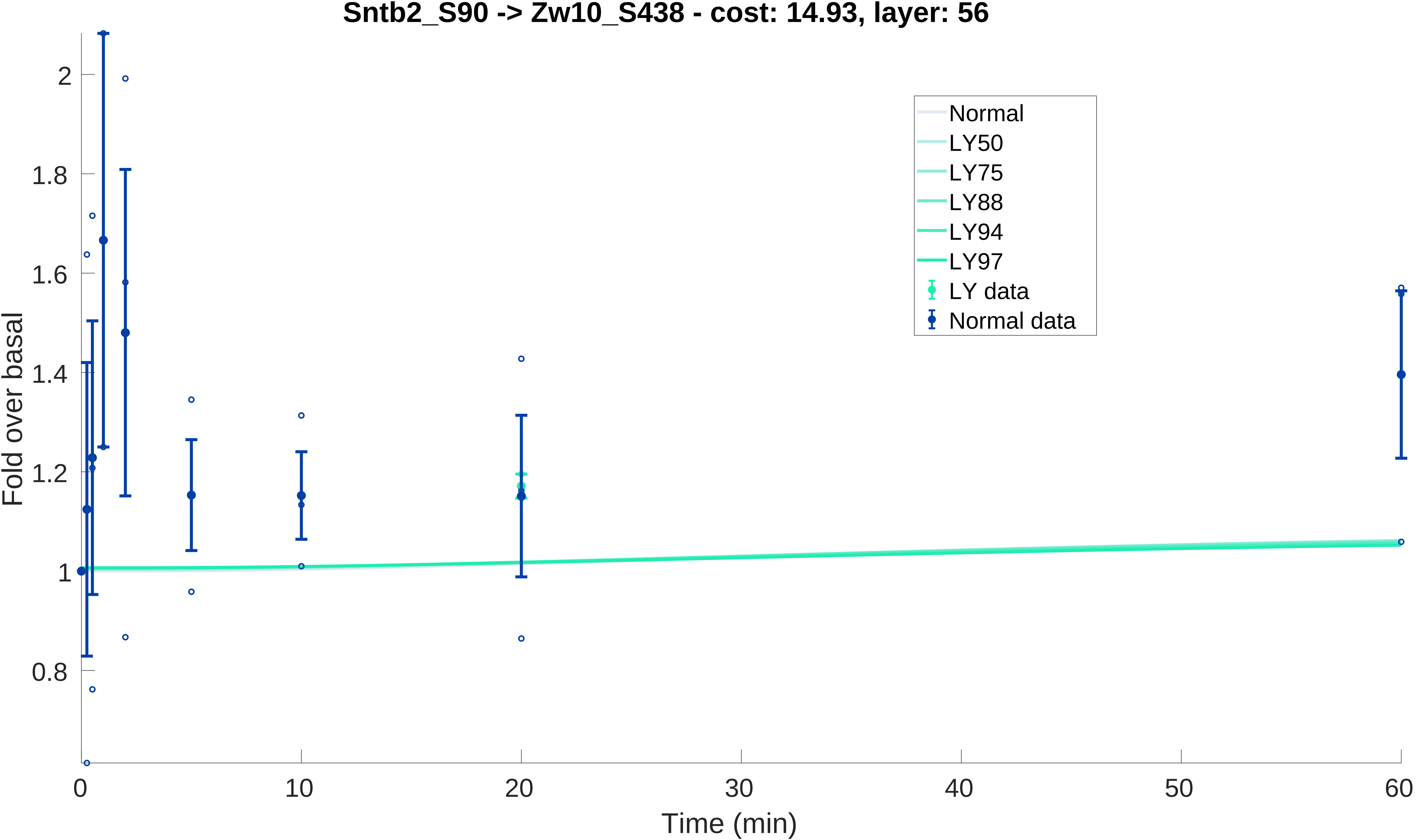
Sntb2_S90 -> Zw10_S438 - cost: 14.93, layer: 56
Fold over basal
Time (min)
2
1.8
1.6
1.4
1.2
1
0.8
0
10
20
30
40
50
60
Normal
LY50
LY75
LY88
LY94
LY97
LY data
Normal data

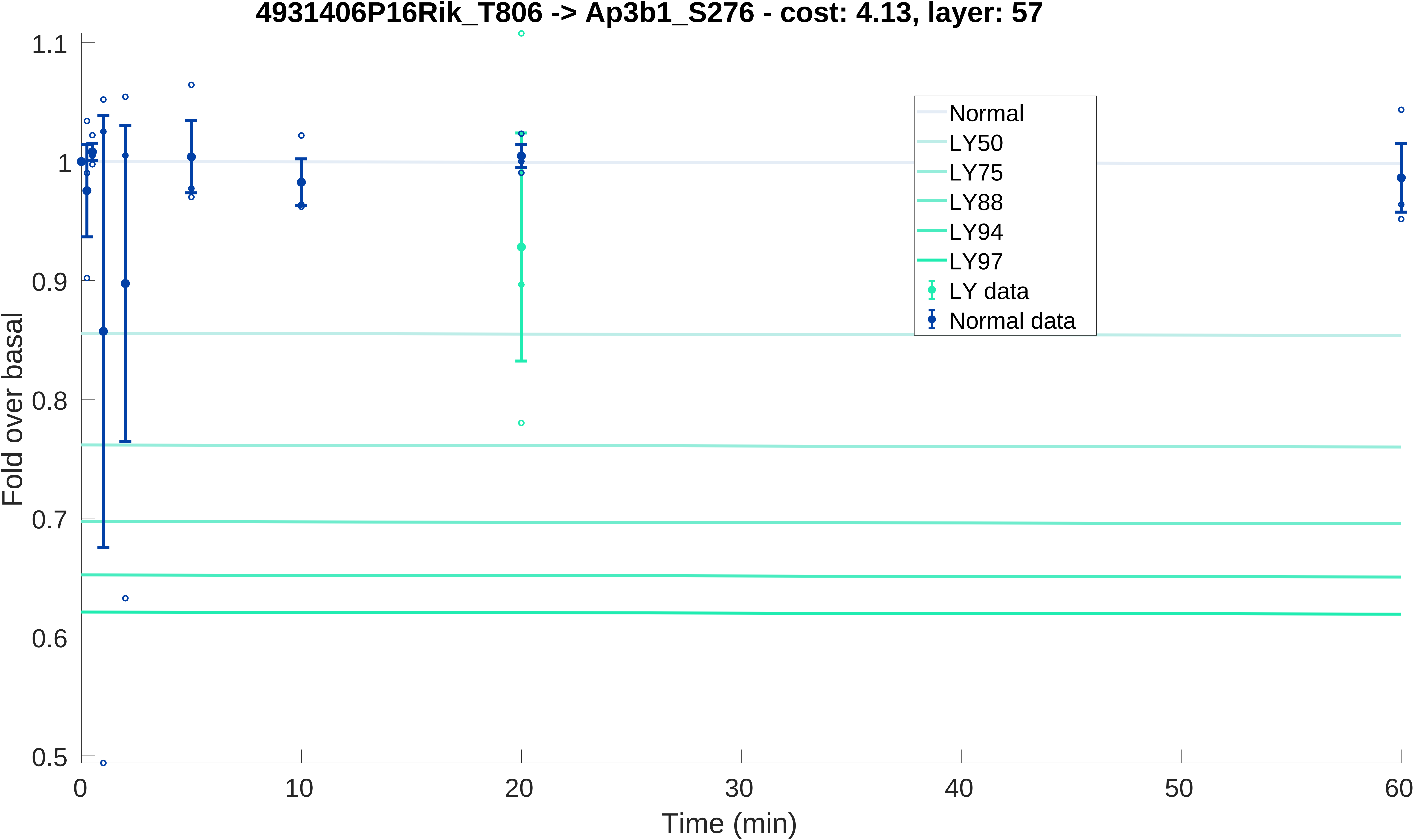
4931406P16Rik_T806 -> Ap3b1_S276 - cost: 4.13, layer: 57
Fold over basal
Time (min)
1.1
1
0.9
0.8
0.7
0.6
0.5
0
10
20
30
40
50
60
Normal
LY50
LY75
LY88
LY94
LY97
LY data
Normal data

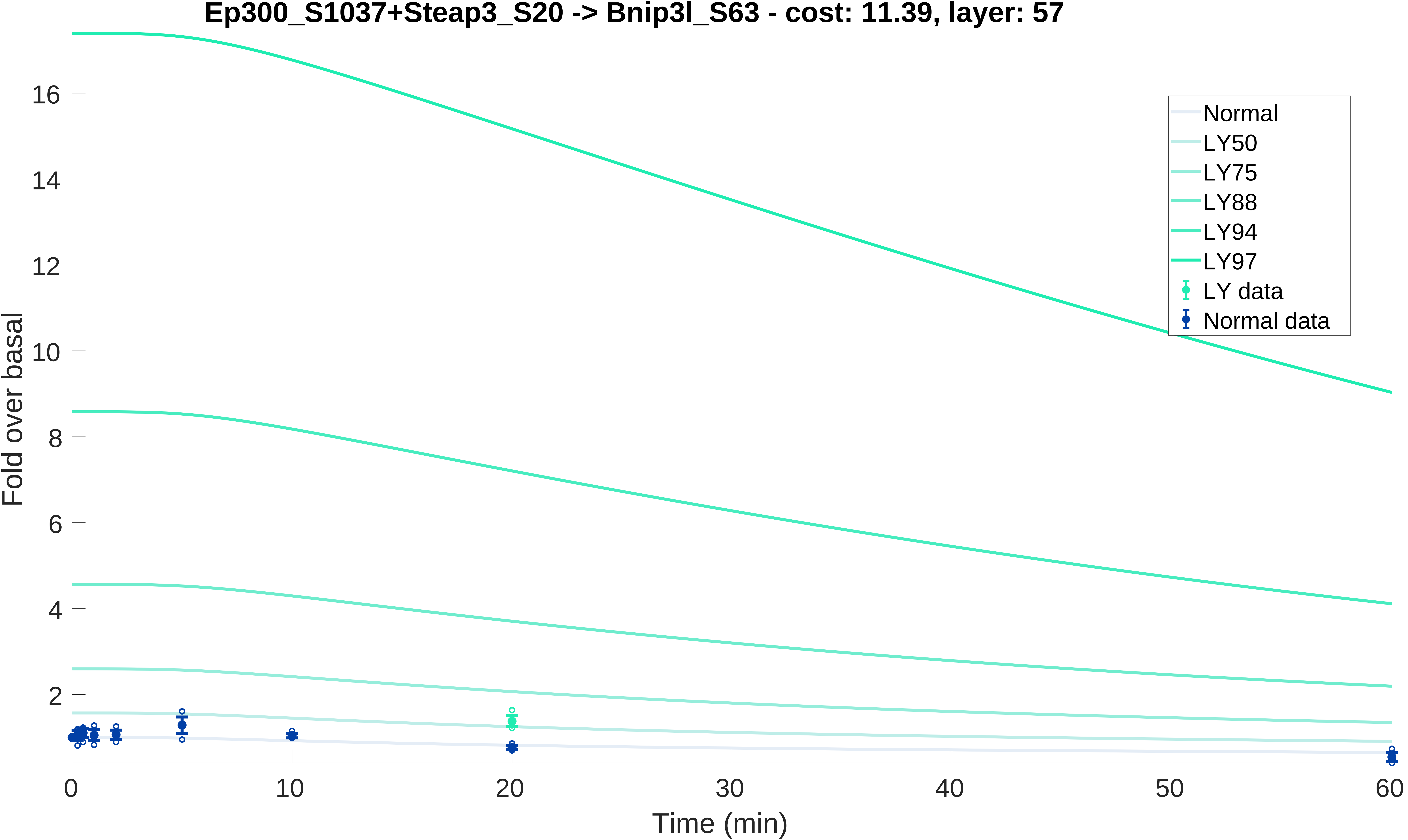

Ep300_S1037+Steap3_S20 -> Bnip3l_S63 - cost: 11.39, layer: 57
Fold over basal
16
14
12
10
8
6
4
2
0
10
20
30
40
50
60
Time (min)
Normal
LY50
LY75
LY88
LY94
LY97
LY data
Normal data

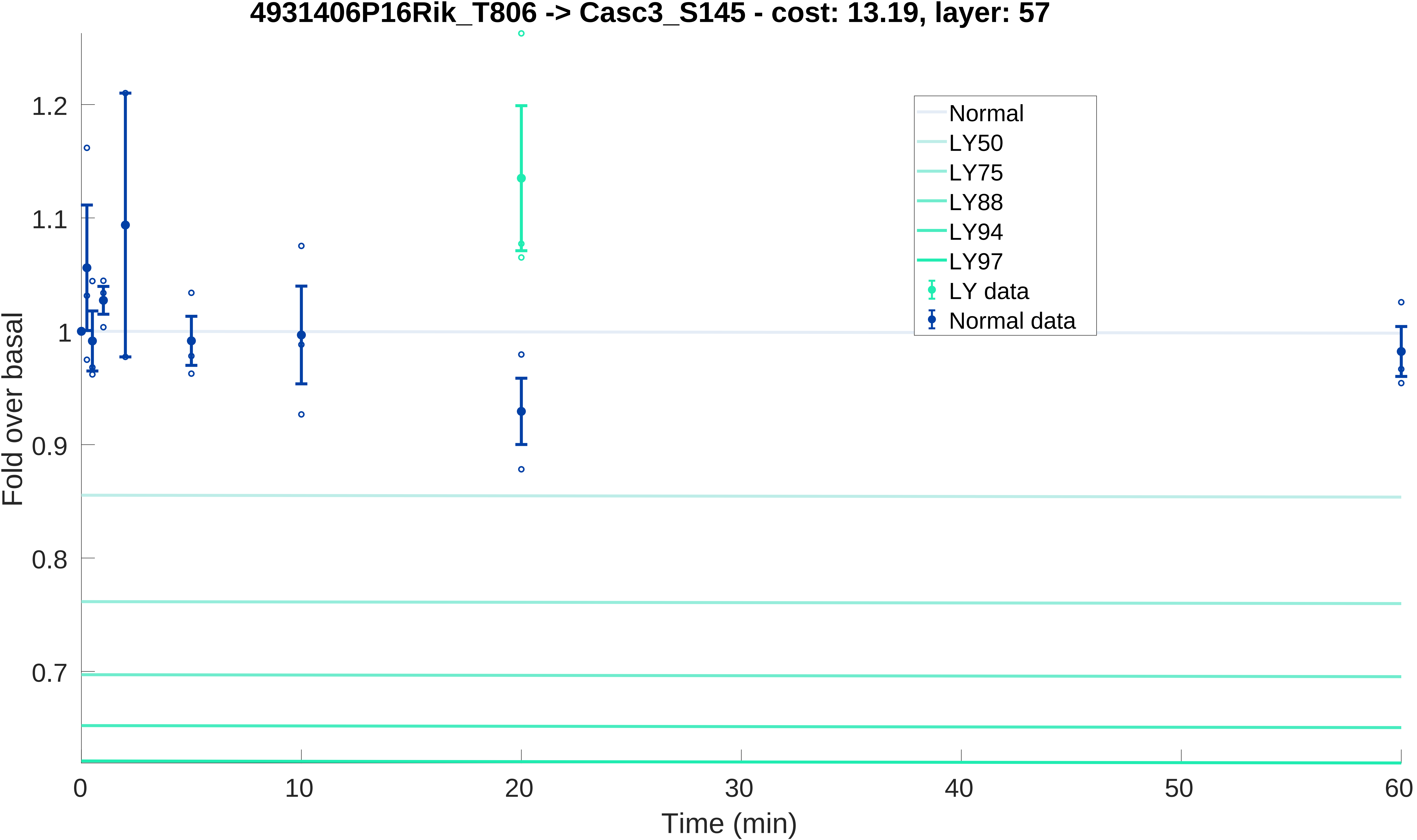

4931406P16Rik_T806 -> Casc3_S145 - cost: 13.19, layer: 57
Fold over basal
Time (min)
Normal
LY50
LY75
LY88
LY94
LY97
LY data
Normal data
1.2
1.1
1
0.9
0.8
0.7
0
10
20
30
40
50
60

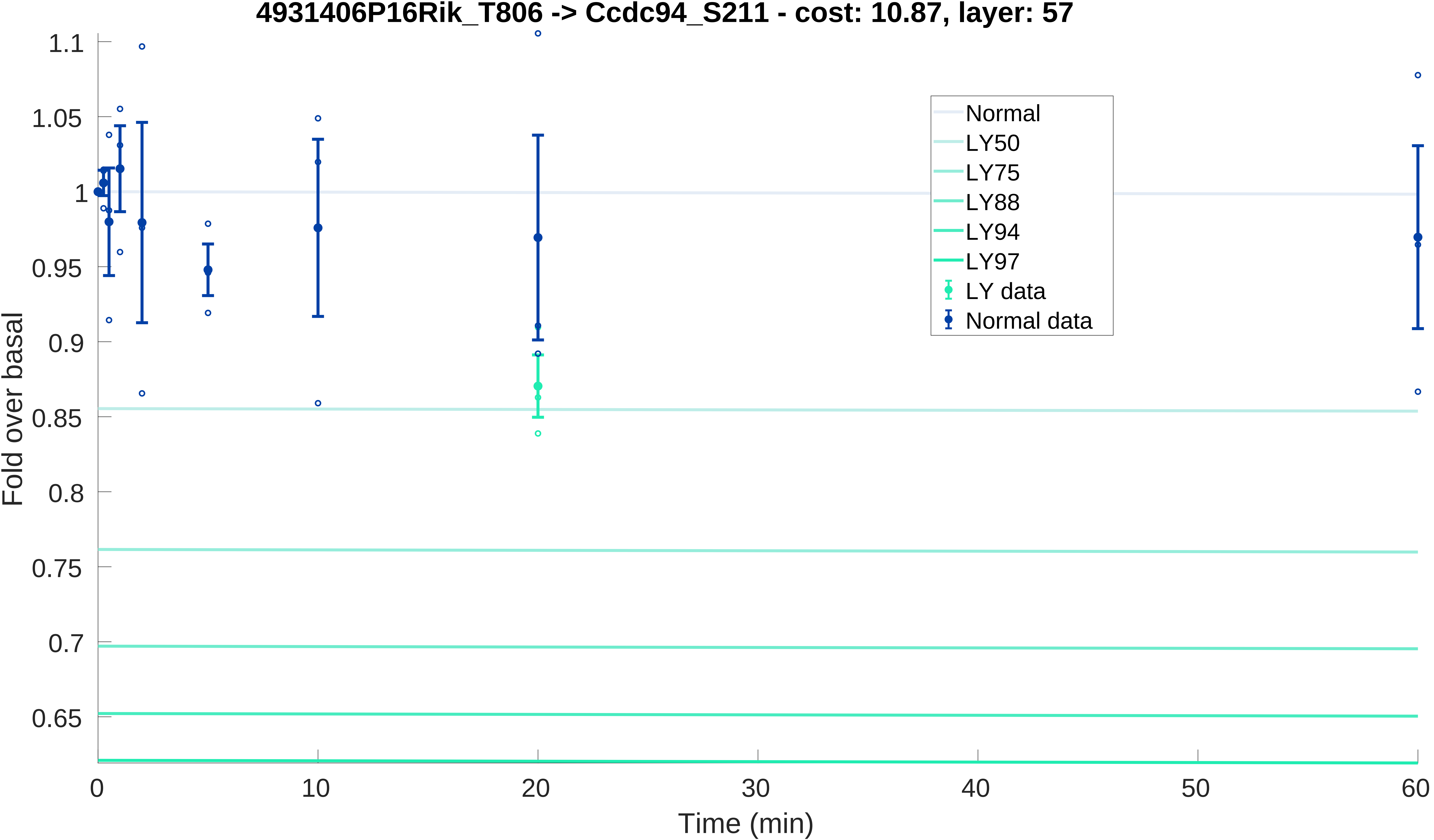

4931406P16Rik_T806 -> Ccdc94_S211 - cost: 10.87, layer: 57
Fold over basal
Time (min)
1.1
1.05
1
0.95
0.9
0.85
0.8
0.75
0.7
0.65
0
10
20
30
40
50
60
Normal
LY50
LY75
LY88
LY94
LY97
LY data
Normal data

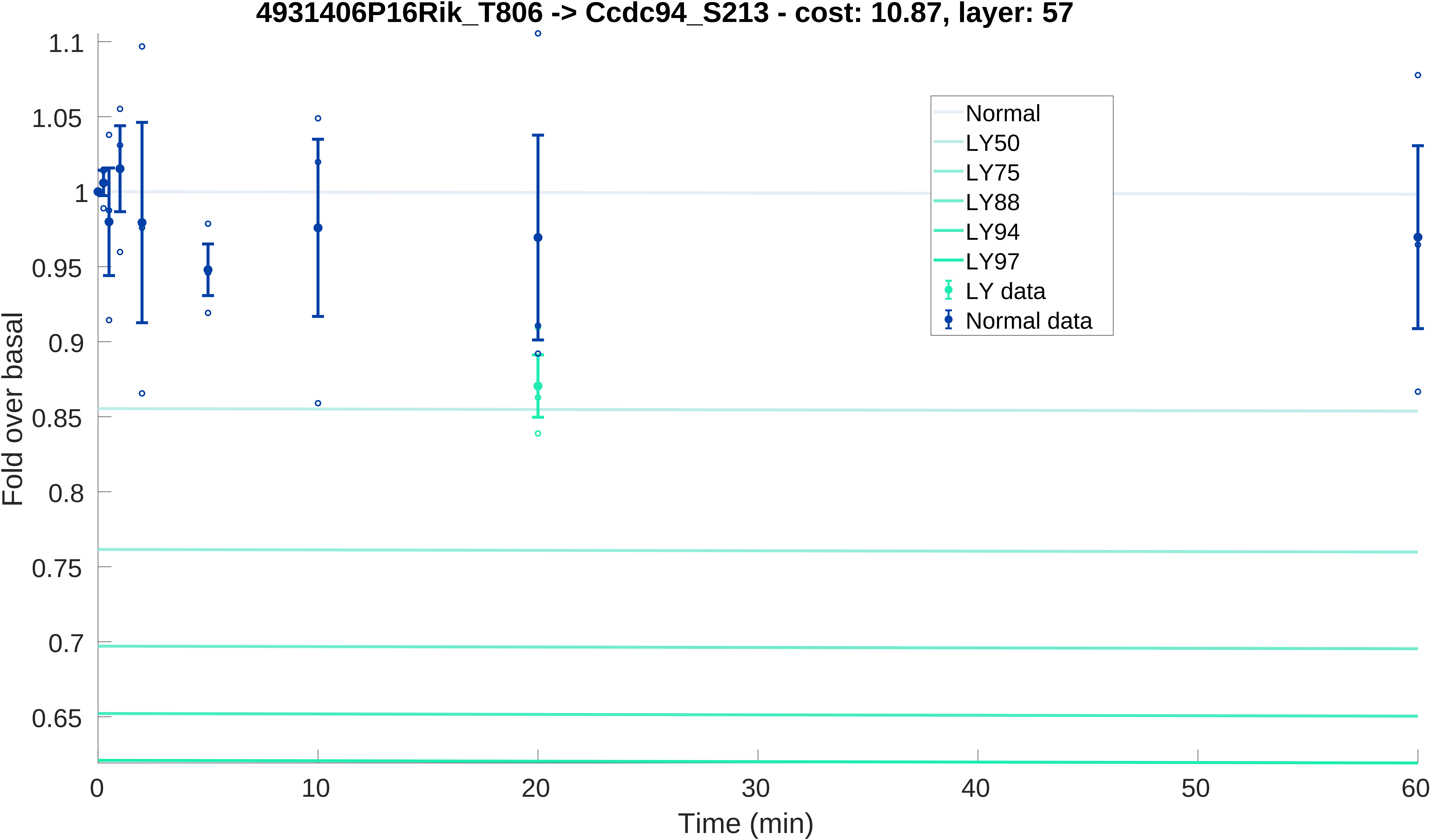

4931406P16Rik_T806 -> Ccdc94_S213 - cost: 10.87, layer: 57
Fold over basal
Time (min)
1.1
1.05
1
0.95
0.9
0.85
0.8
0.75
0.7
0.65
0
10
20
30
40
50
60
Normal
LY50
LY75
LY88
LY94
LY97
LY data
Normal data

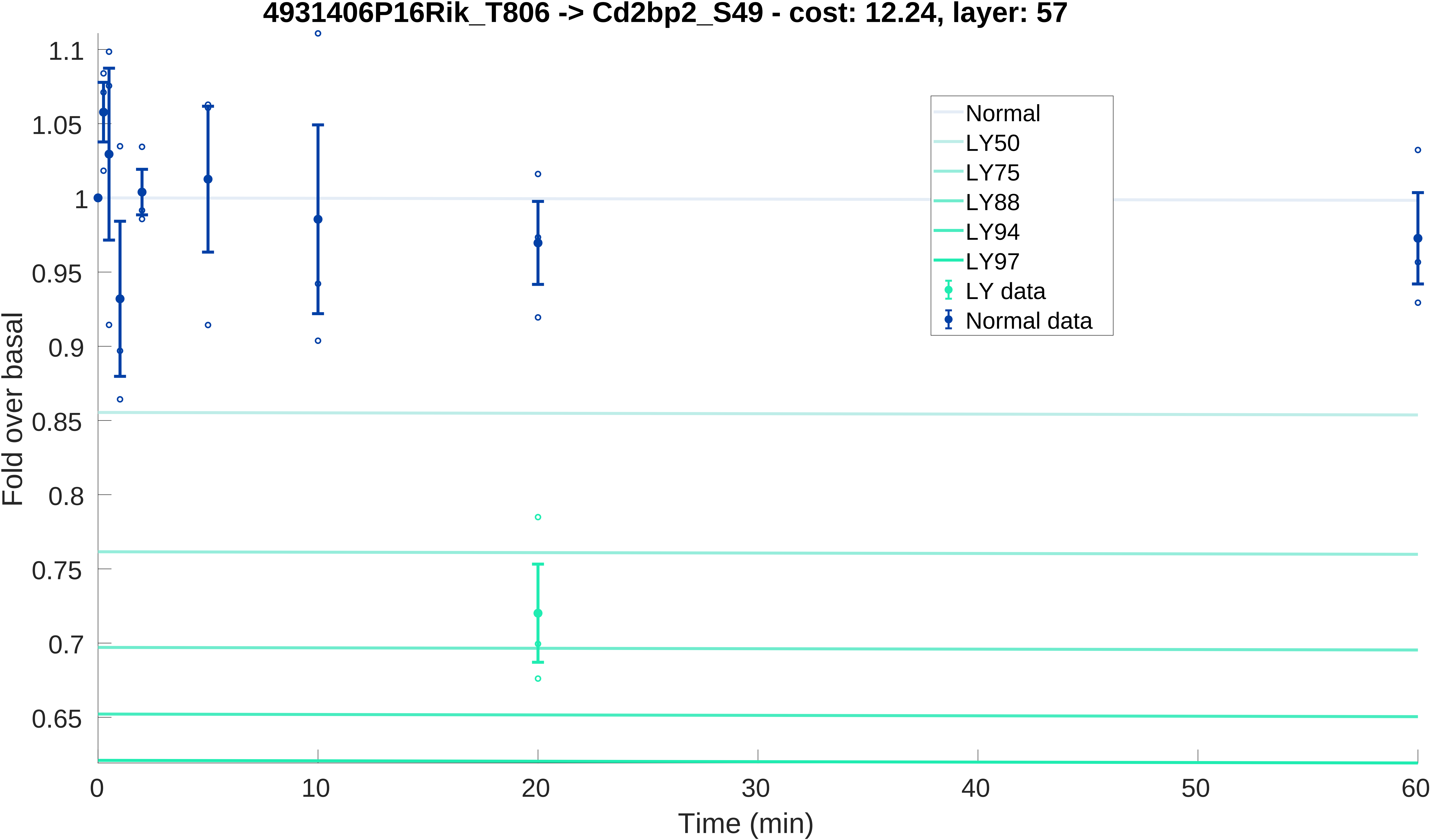
4931406P16Rik_T806 -> Cd2bp2_S49 - cost: 12.24, layer: 57
Fold over basal
1.1
1.05
1
0.95
0.9
0.85
0.8
0.75
0.7
0.65
0
10
20
30
40
50
60
Time (min)
Normal
LY50
LY75
LY88
LY94
LY97
LY data
Normal data

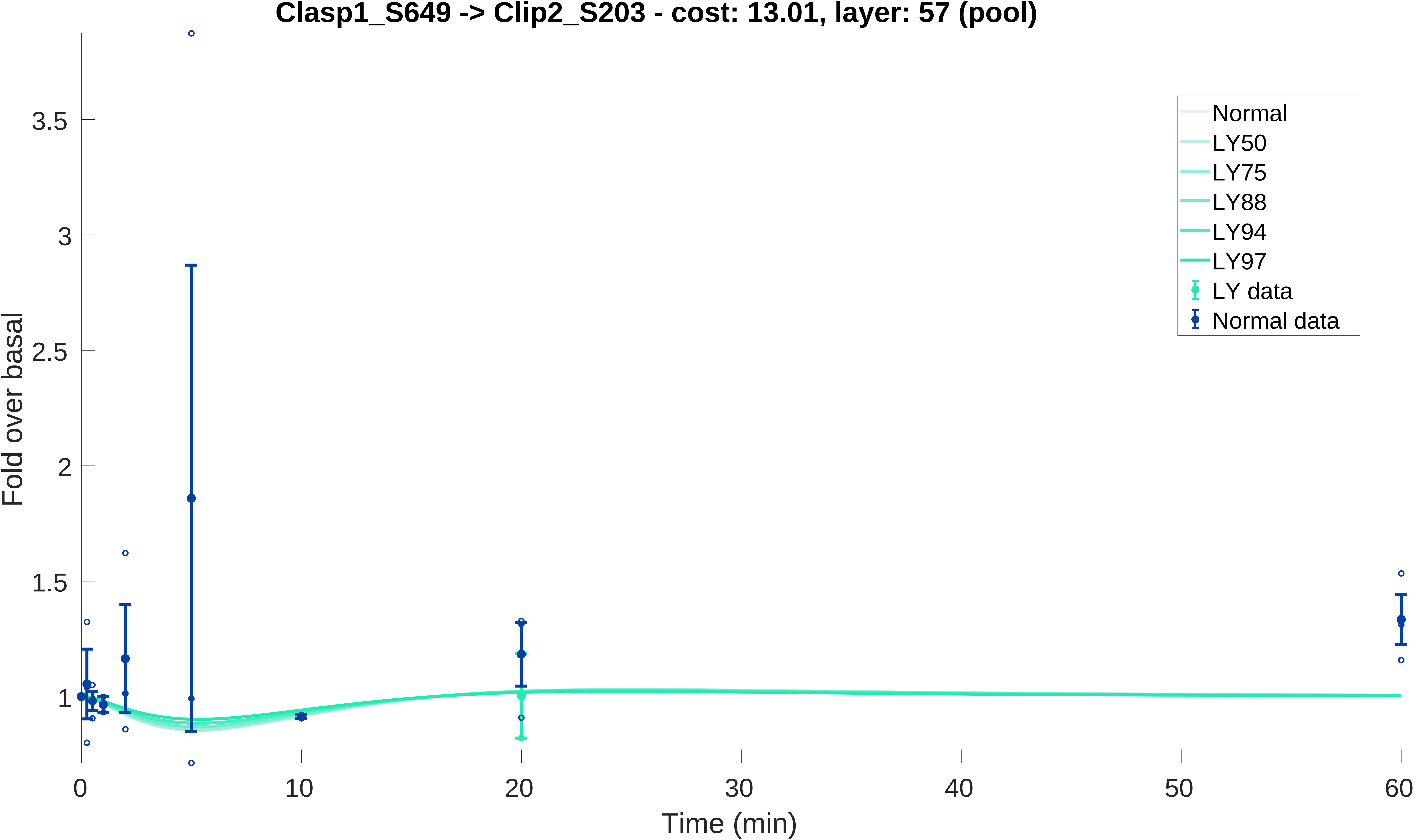
Clasp1_S649 -> Clip2_S203 - cost: 13.01, layer: 57 (pool)
Normal
LY50
LY75
LY88
LY94
LY97
LY data
Normal data
Fold over basal
3.5
3
2.5
2
1.5
1
0
10
20
30
40
50
60
Time (min)

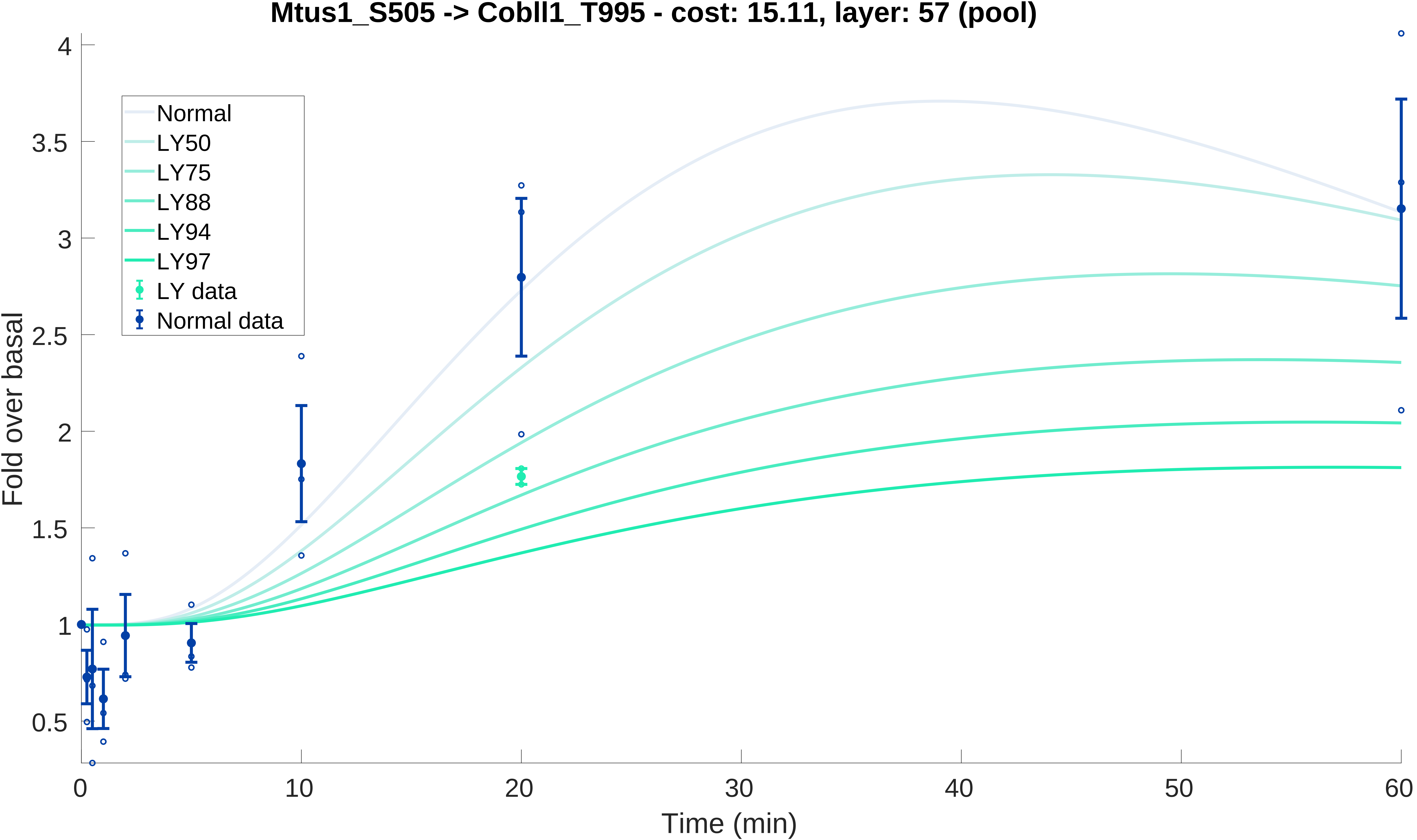

Mtus1_S505 -> Cobll1_T995 - cost: 15.11, layer: 57 (pool)
Normal
LY50
LY75
LY88
LY94
LY97
LY data
Normal data
Fold over basal
Time (min)
4
3.5
3
2.5
2
1.5
1
0.5
0
10
20
30
40
50
60

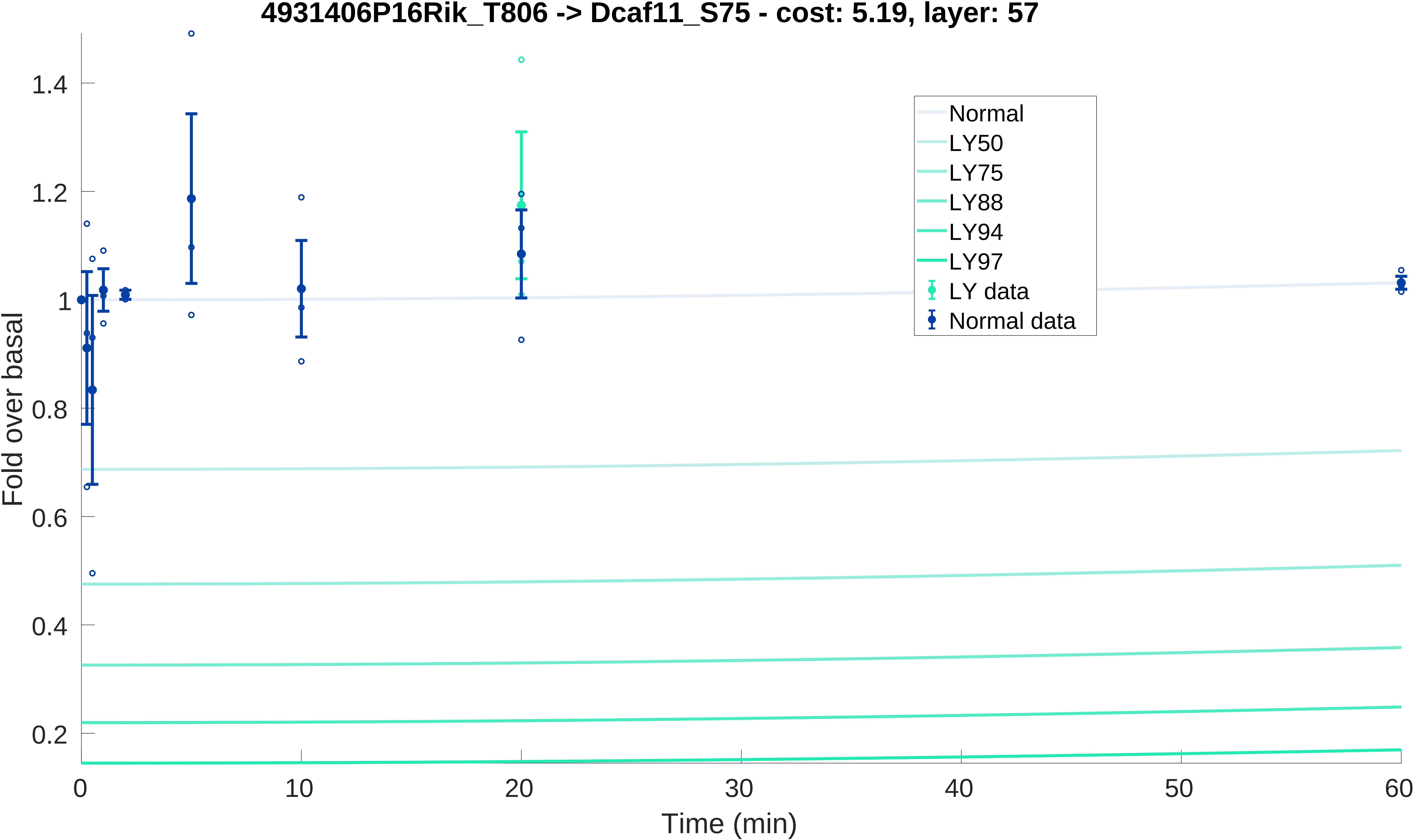
4931406P16Rik_T806 -> Dcaf11_S75 - cost: 5.19, layer: 57
Fold over basal
Time (min)
0
10
20
30
40
50
60
0.2
0.4
0.6
0.8
1
1.2
1.4
Normal
LY50
LY75
LY88
LY94
LY97
LY data
Normal data

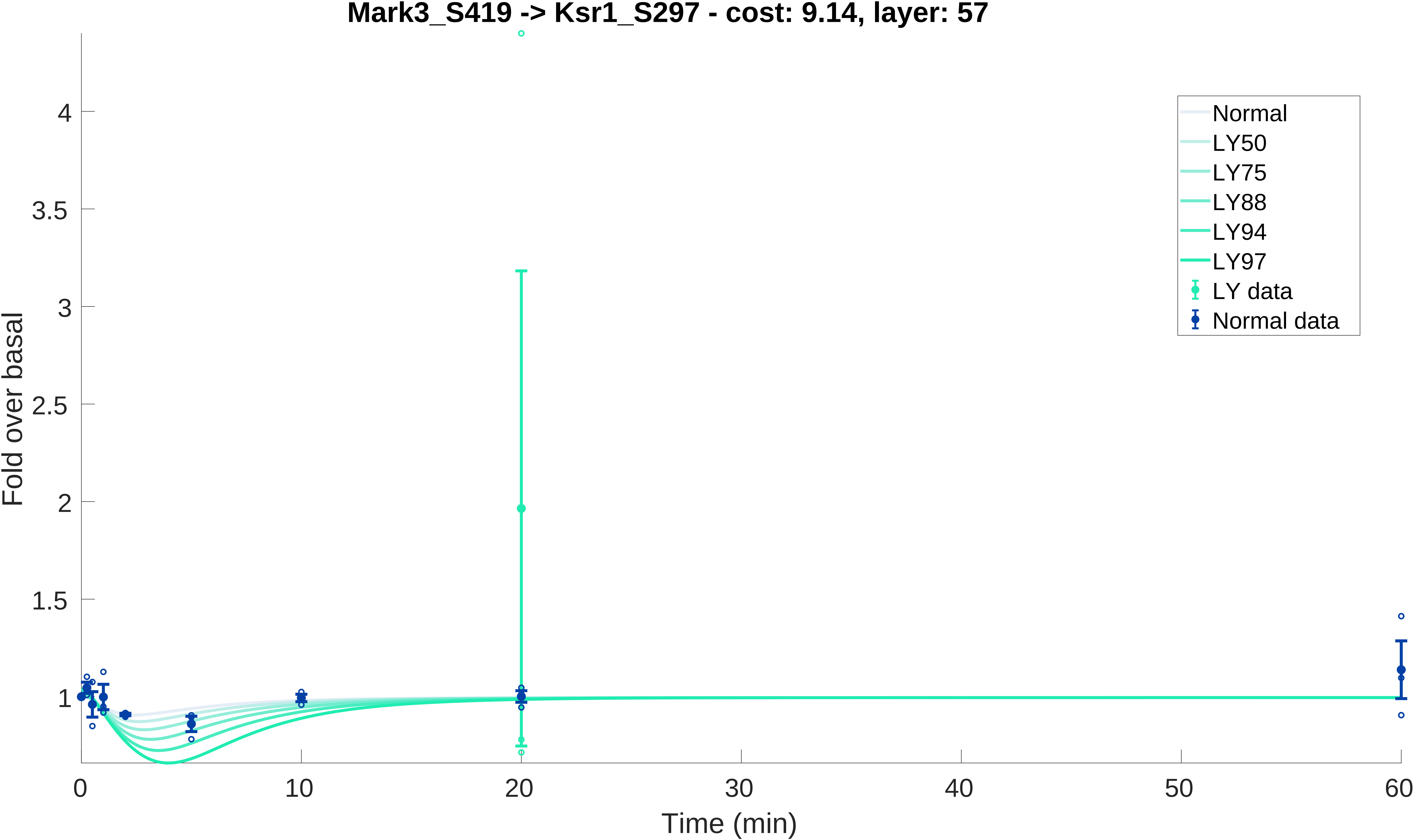

Mark3_S419 -> Ksr1_S297 - cost: 9.14, layer: 57
Fold over basal
Time (min)
Normal
LY50
LY75
LY88
LY94
LY97
LY data
Normal data
4
3.5
3
2.5
2
1.5
1
0
10
20
30
40
50
60

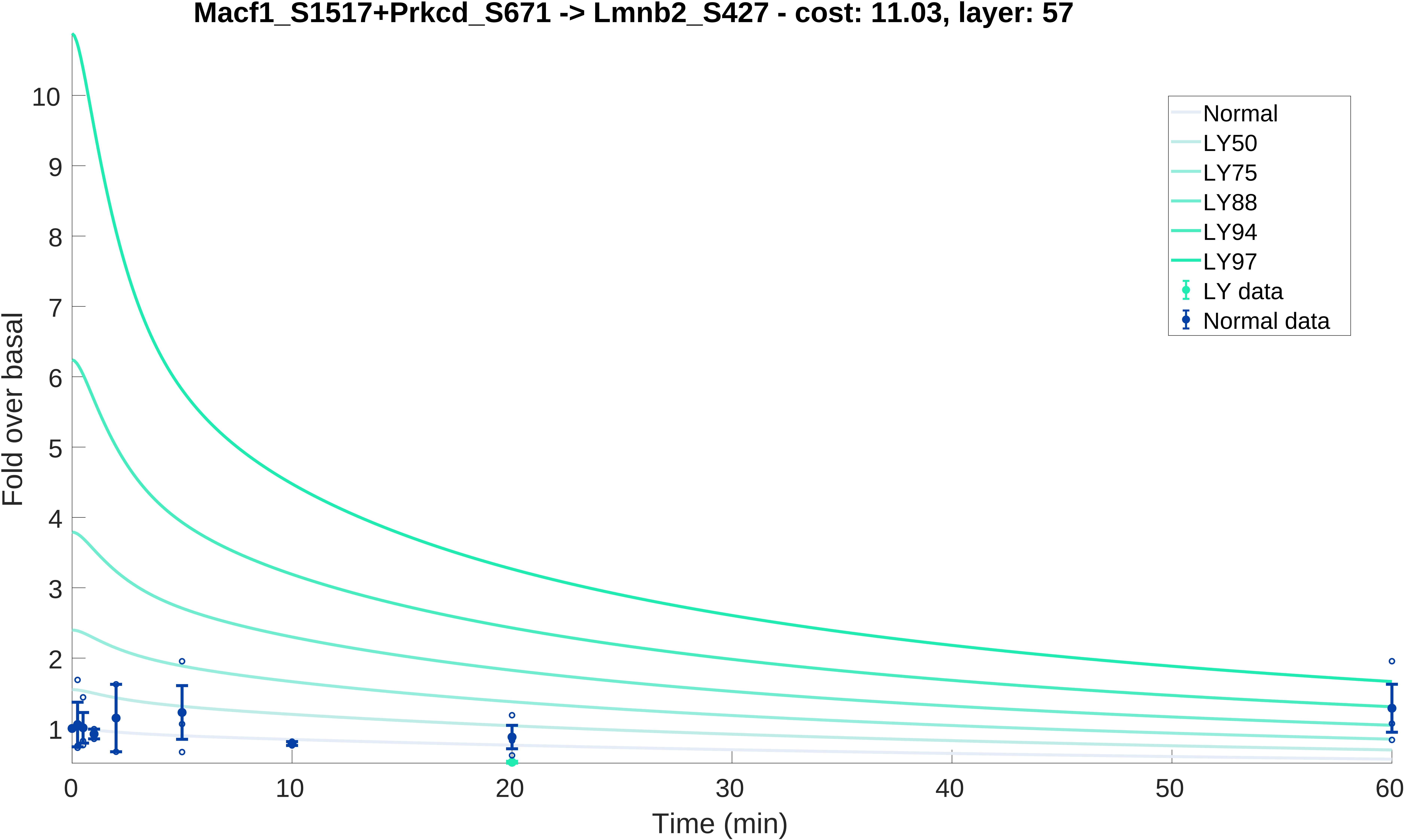

Macf1_S1517+Prkcd_S671 -> Lmnb2_S427 - cost: 11.03, layer: 57
Fold over basal
Time (min)
Normal
LY50
LY75
LY88
LY94
LY97
LY data
Normal data
0
10
20
30
40
50
60
1
2
3
4
5
6
7
8
9
10

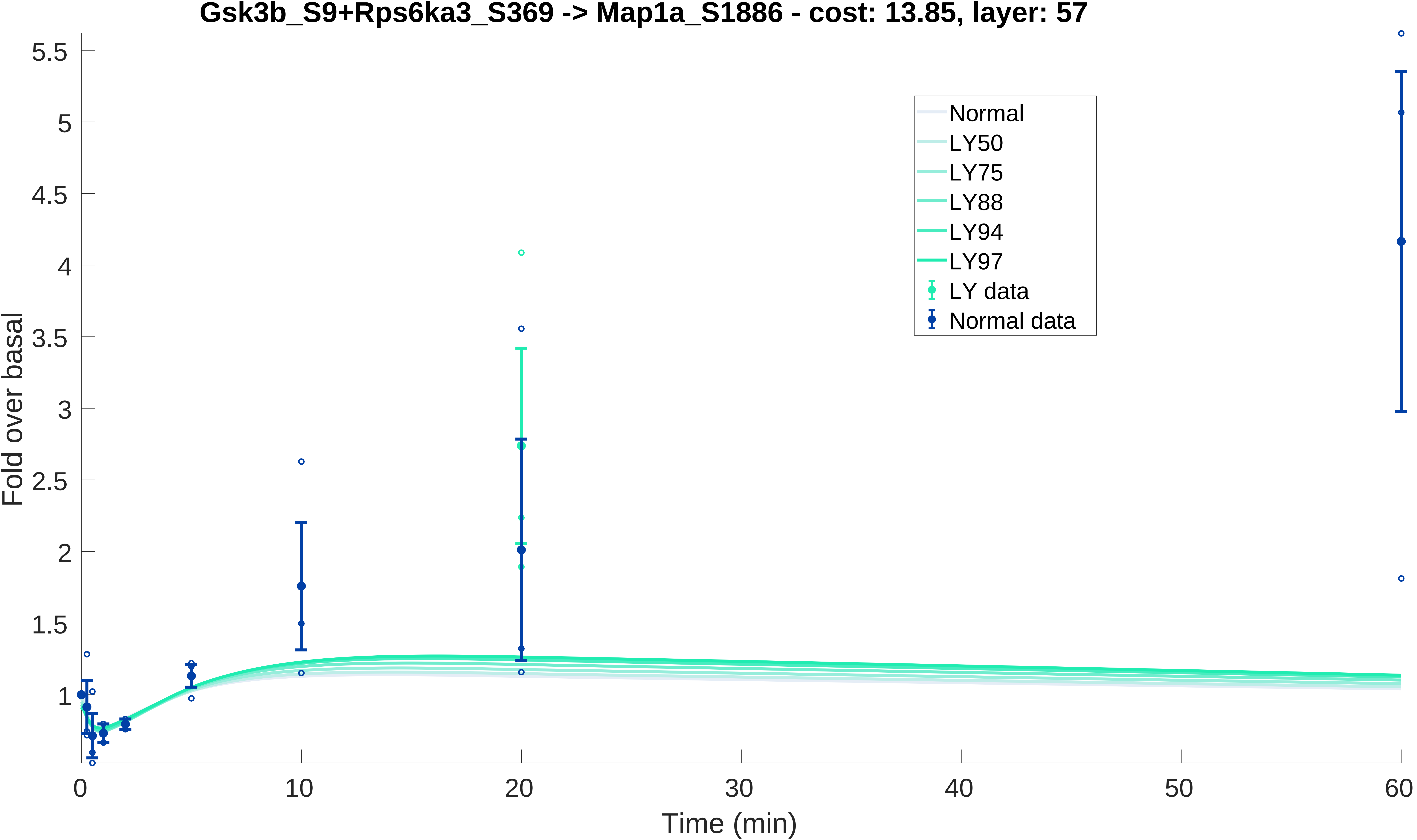
Gsk3b_S9+Rps6ka3_S369 -> Map1a_S1886 - cost: 13.85, layer: 57
Fold over basal
Time (min)
Normal
LY50
LY75
LY88
LY94
LY97
LY data
Normal data
5.5
5
4.5
4
3.5
3
2.5
2
1.5
1
0
10
20
30
40
50
60

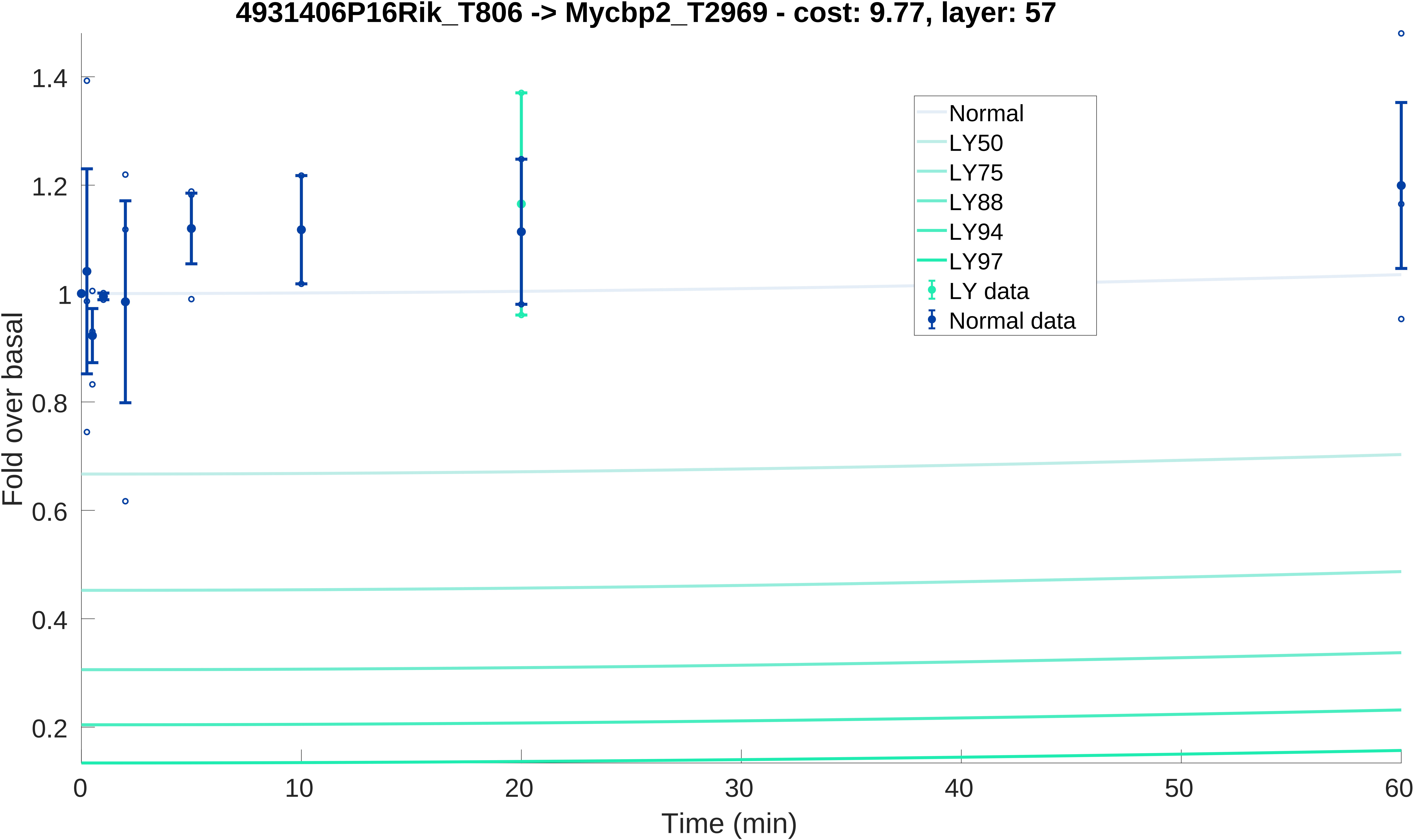

4931406P16Rik_T806 -> Mycbp2_T2969 - cost: 9.77, layer: 57
Fold over basal
Time (min)
0
10
20
30
40
50
60
0.2
0.4
0.6
0.8
1
1.2
1.4
Normal
LY50
LY75
LY88
LY94
LY97
LY data
Normal data

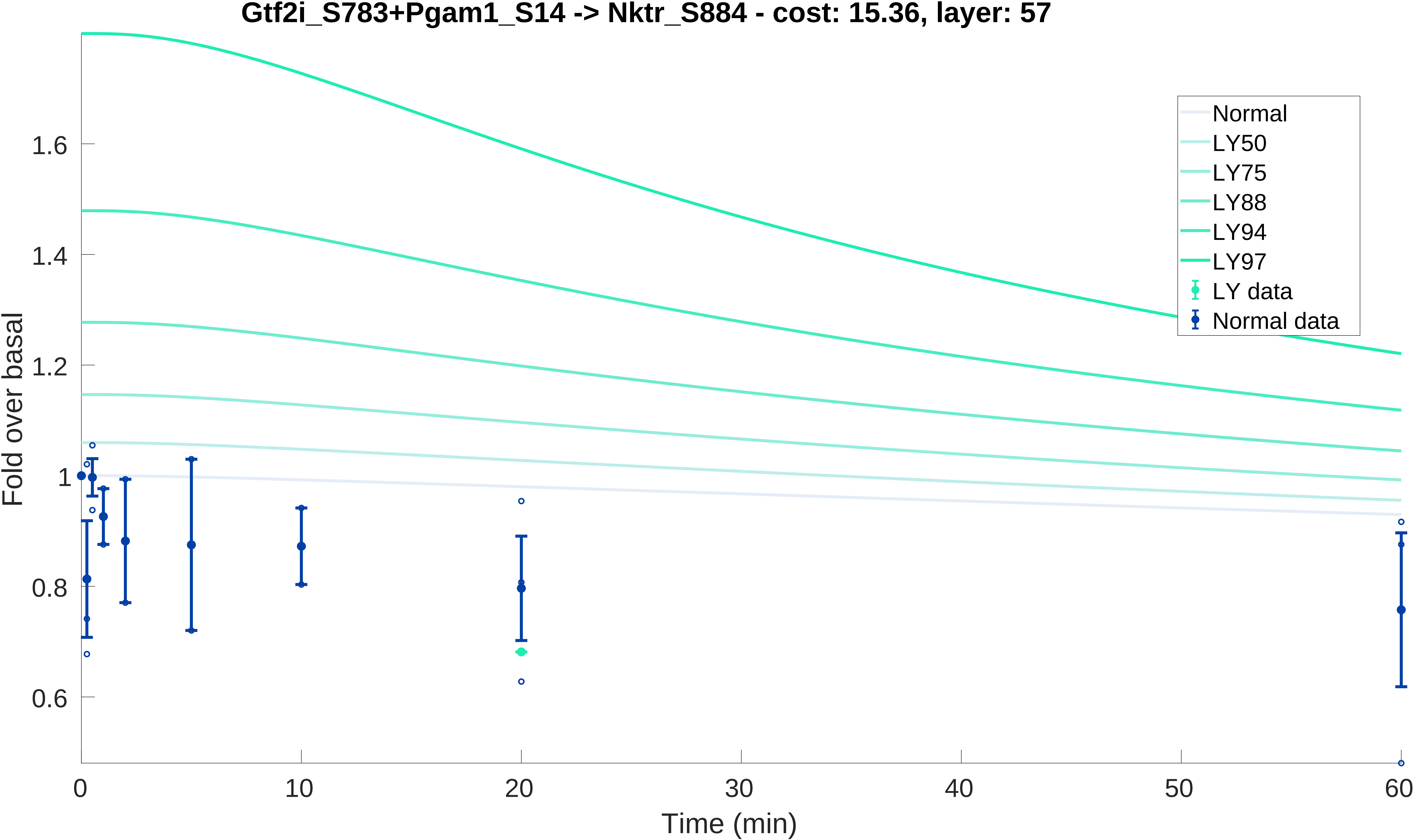

Gtf2i_S783+Pgam1_S14 -> Nktr_S884 - cost: 15.36, layer: 57
Fold over basal
Time (min)
0.6
0.8
1
1.2
1.4
1.6
0
10
20
30
40
50
60
Normal
LY50
LY75
LY88
LY94
LY97
LY data
Normal data

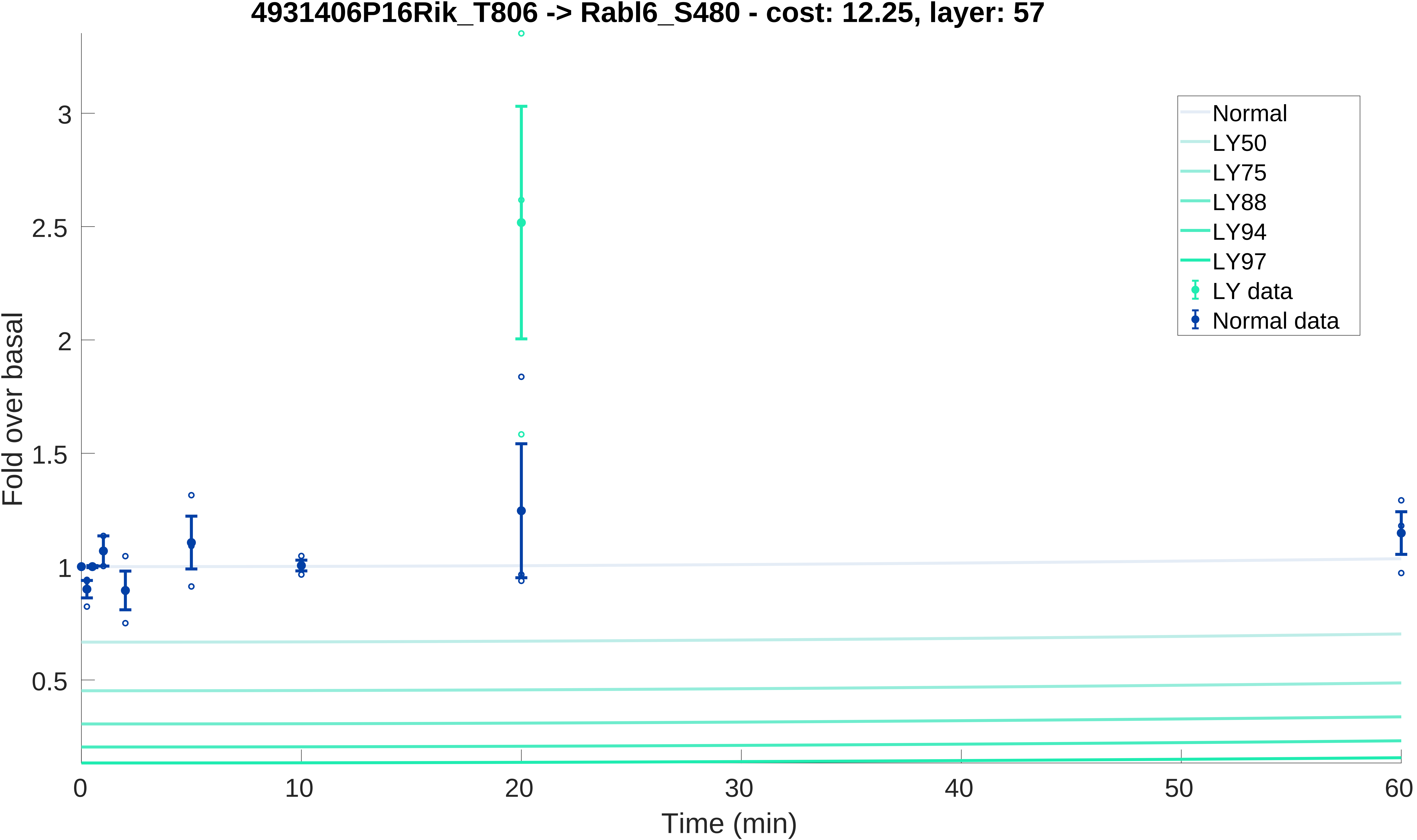

4931406P16Rik_T806 -> Rabl6_S480 - cost: 12.25, layer: 57
Fold over basal
Time (min)
0
10
20
30
40
50
60
0.5
1
1.5
2
2.5
3
Normal
LY50
LY75
LY88
LY94
LY97
LY data
Normal data

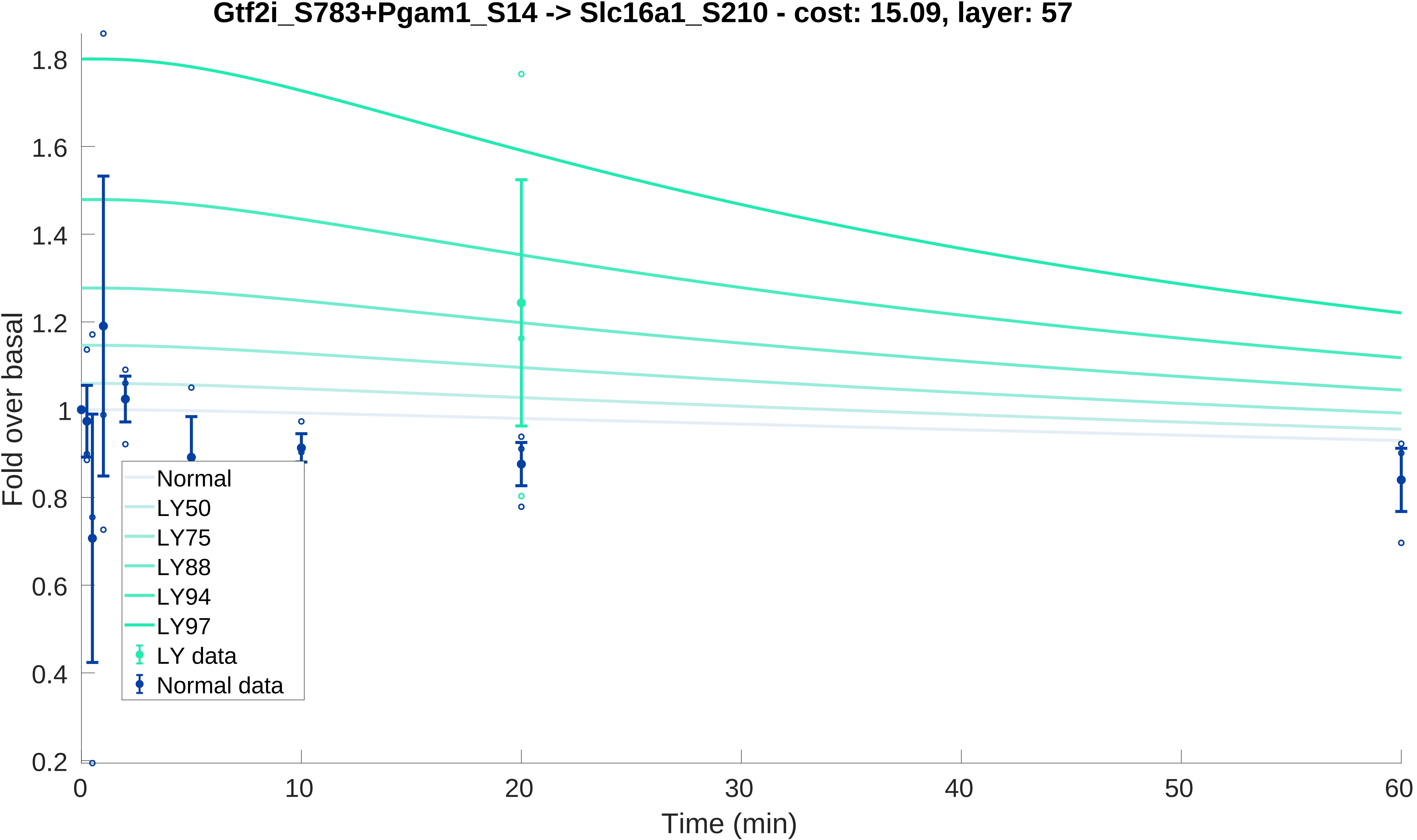

Gtf2i_S783+Pgam1_S14 -> Slc16a1_S210 - cost: 15.09, layer: 57
Fold over basal
Time (min)
0.2
0.4
0.6
0.8
1
1.2
1.4
1.6
1.8
0
10
20
30
40
50
60
Normal
LY50
LY75
LY88
LY94
LY97
LY data
Normal data

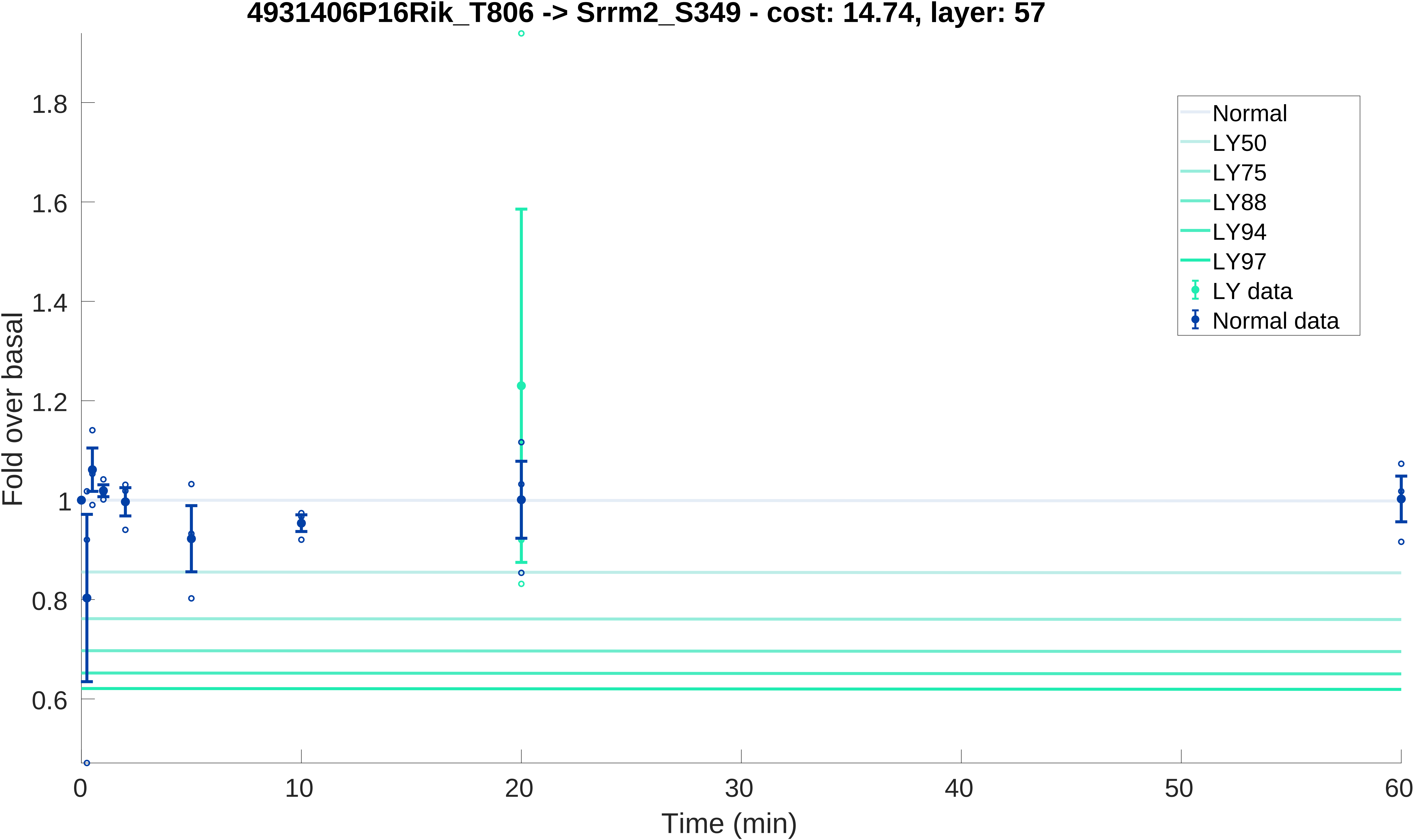
4931406P16Rik_T806 -> Srrm2_S349 - cost: 14.74, layer: 57
Fold over basal
1.8
1.6
1.4
1.2
1
0.8
0.6
0
10
20
30
40
50
60
Time (min)
Normal
LY50
LY75
LY88
LY94
LY97
LY data
Normal data

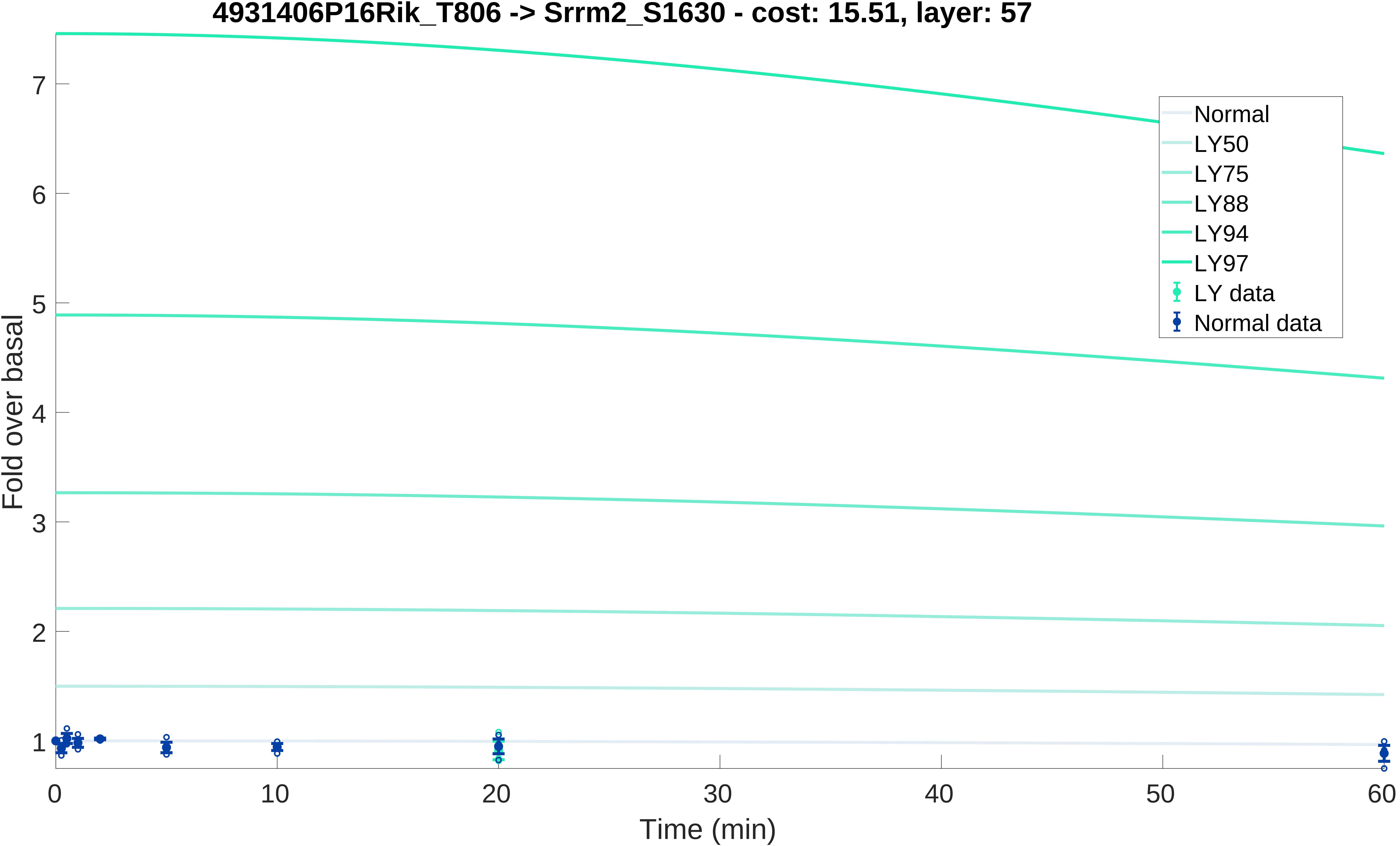
4931406P16Rik_T806 -> Srrm2_S1630 - cost: 15.51, layer: 57
Fold over basal
Time (min)
0
10
20
30
40
50
60
1
2
3
4
5
6
7
Normal
LY50
LY75
LY88
LY94
LY97
LY data
Normal data

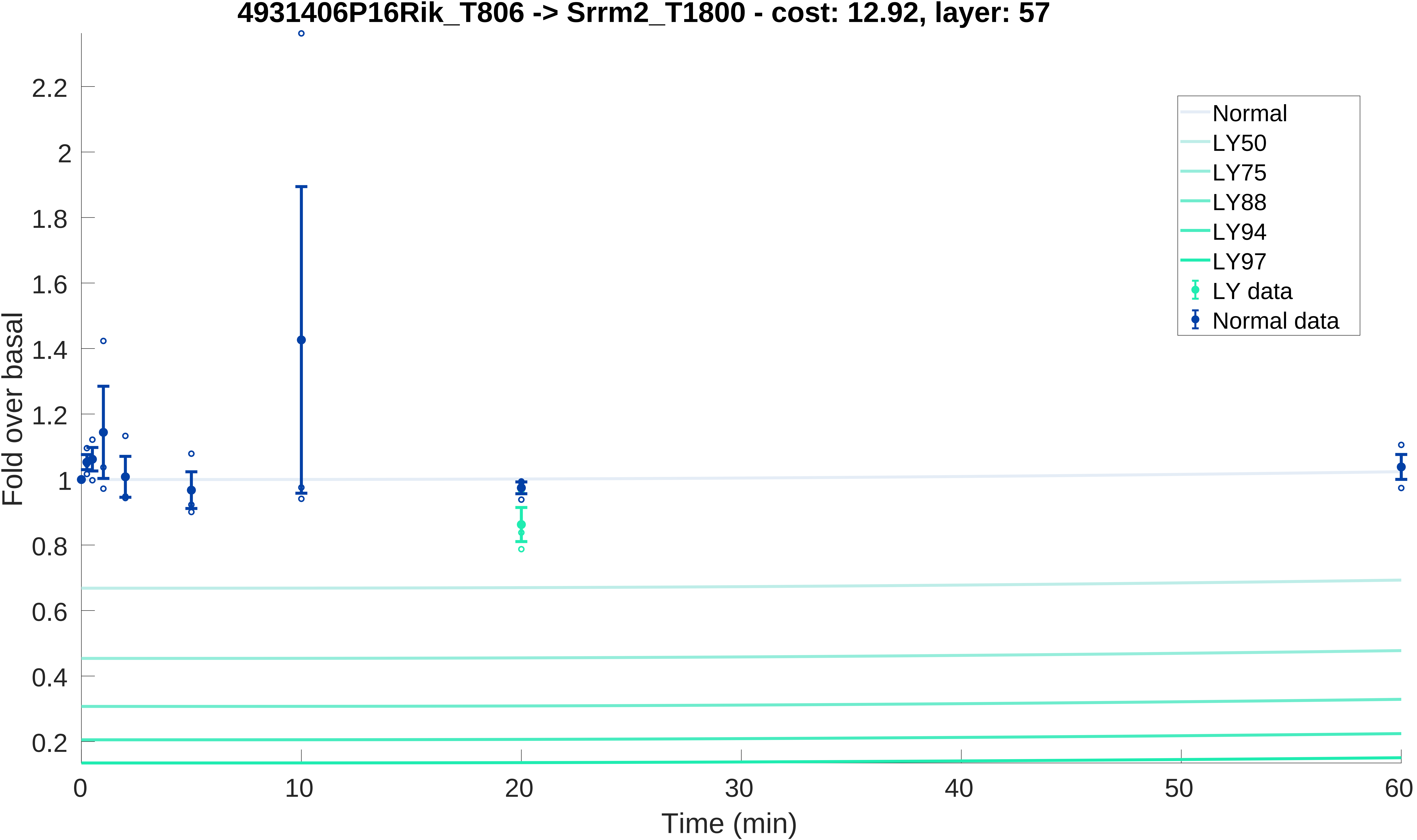

4931406P16Rik_T806 -> Srrm2_T1800 - cost: 12.92, layer: 57
Fold over basal
Time (min)
0
10
20
30
40
50
60
0.2
0.4
0.6
0.8
1
1.2
1.4
1.6
1.8
2
2.2
Normal
LY50
LY75
LY88
LY94
LY97
LY data
Normal data

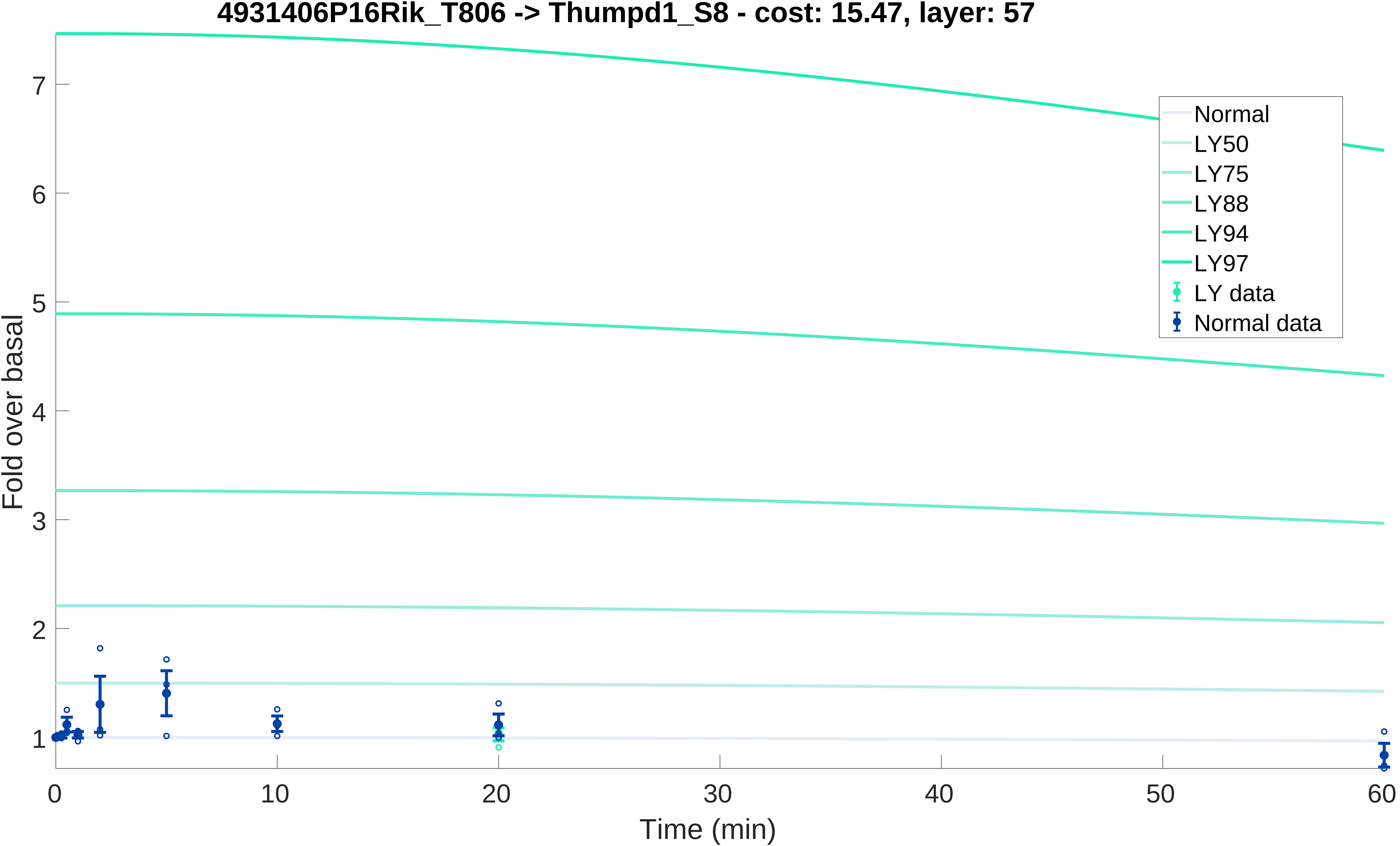
4931406P16Rik_T806 -> Thumpd1_S8 - cost: 15.47, layer: 57
Fold over basal
Time (min)
1
2
3
4
5
6
7
0
10
20
30
40
50
60
Normal
LY50
LY75
LY88
LY94
LY97
LY data
Normal data

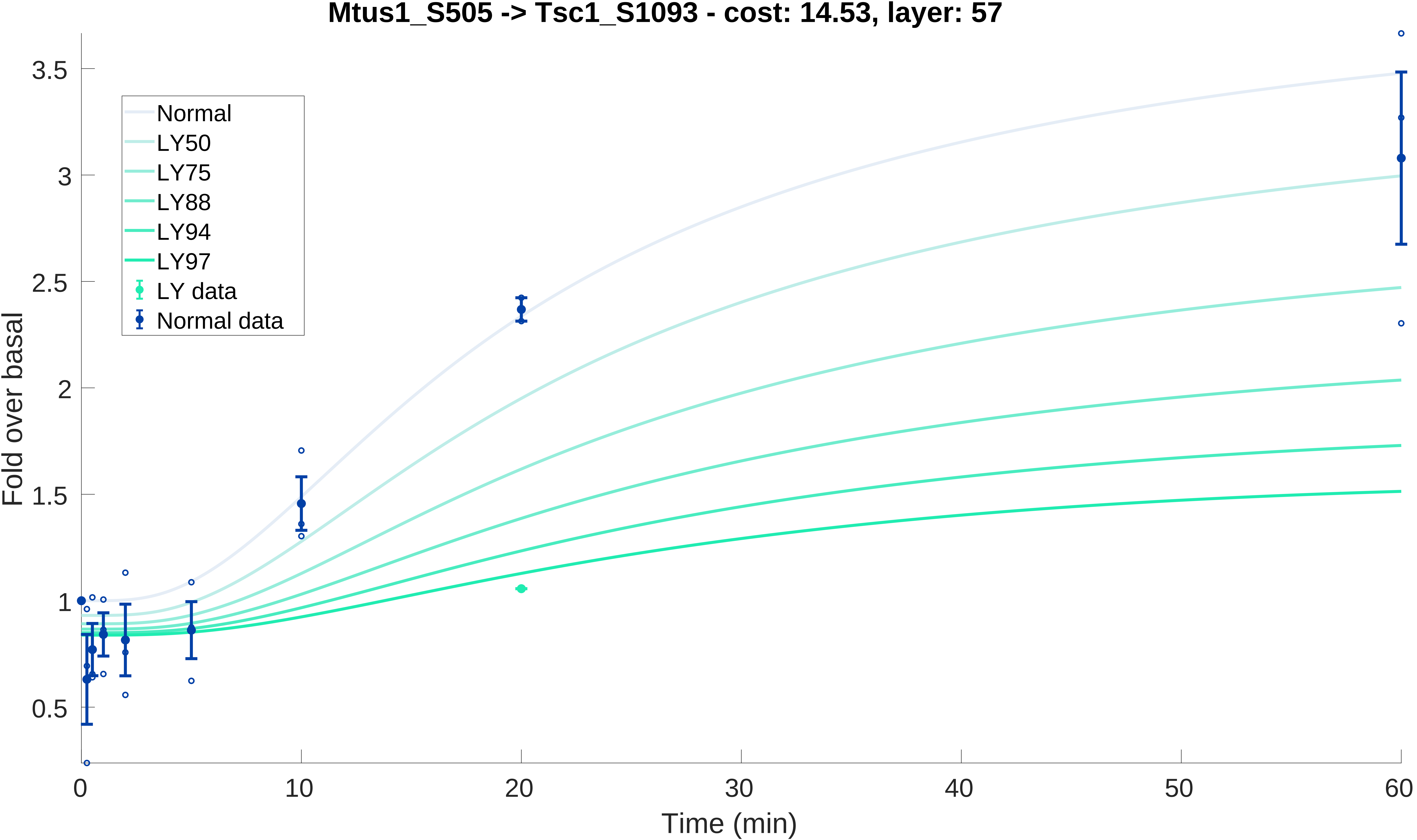

Mtus1_S505 -> Tsc1_S1093 - cost: 14.53, layer: 57
Normal
LY50
LY75
LY88
LY94
LY97
LY data
Normal data
Fold over basal
Time (min)
0.5
1
1.5
2
2.5
3
3.5
0
10
20
30
40
50
60

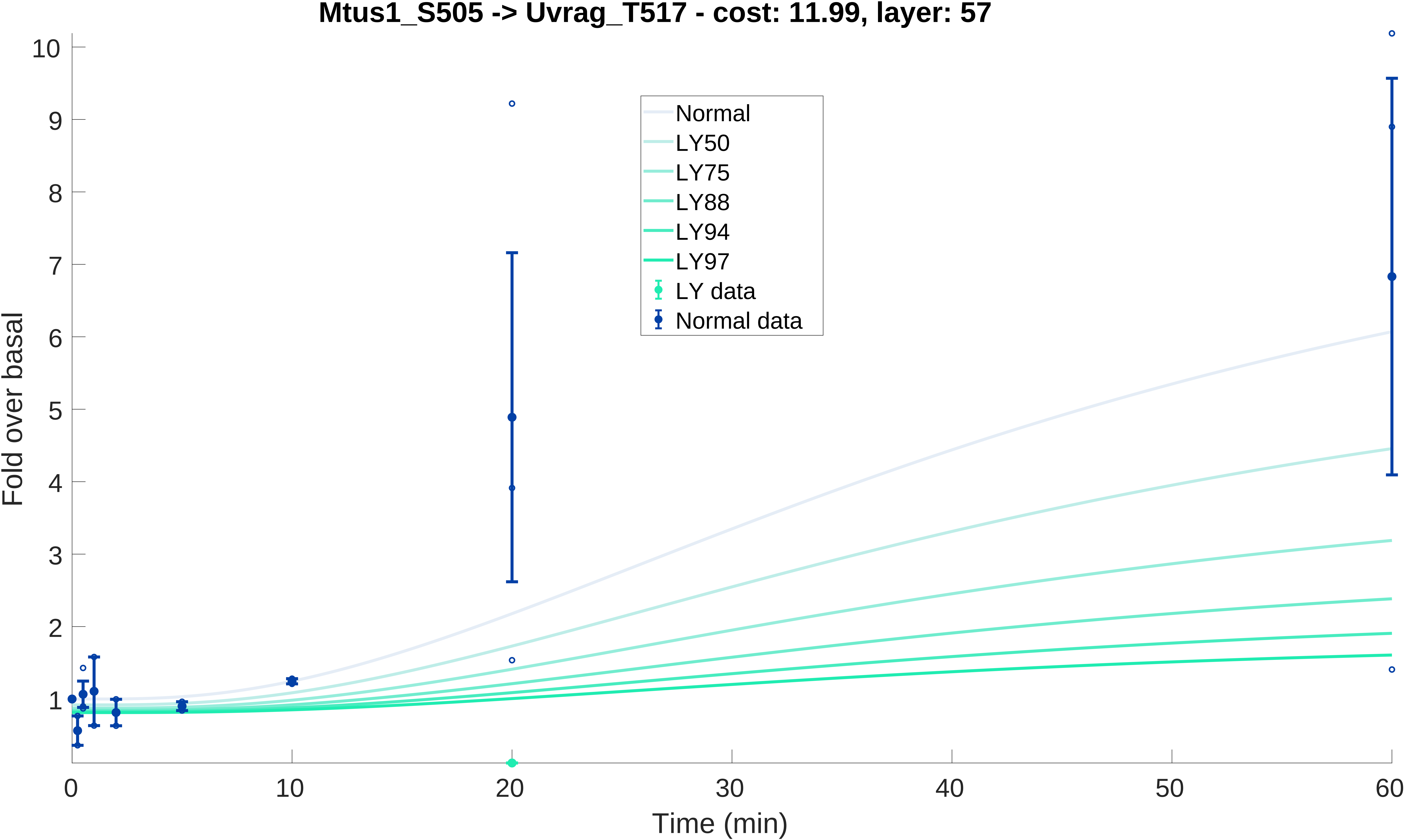

Mtus1_S505 -> Uvrag_T517 - cost: 11.99, layer: 57
Fold over basal
Time (min)
Normal
LY50
LY75
LY88
LY94
LY97
LY data
Normal data
0
10
20
30
40
50
60
1
2
3
4
5
6
7
8
9
10

Supplementary References

1. Lövfors, W. *et al.* A Systems Biology Analysis of Lipolysis and Fatty Acid Release from Adipocytes in Vitro and from Adipose Tissue in Vivo. *PLOS ONE* **16,** e0261681 (Dec. 31, 2021).
2. Rajan, M. R., Nyman, E., Kjølhede, P., Cedersund, G. & Strålfors, P. Systems-Wide Experimental and Modeling Analysis of Insulin Signaling through Forkhead Box Protein O1 (FOXO1) in Human Adipocytes, Normally and in Type 2 Diabetes. *Journal of Biological Chemistry* **291,** 15806–15819 (July 22, 2016).
3. Brännmark, C. *et al.* Insulin Signaling in Type 2 Diabetes: Experimental and Modeling Analyses Reveal Mechanisms of Insulin Resistance in Human Adipocytes. *Journal of Biological Chemistry* **288,** 9867–9880 (Apr. 5, 2013).
4. Jönsson, C., Castor Batista, A. P., Kjølhede, P. & Strålfors, P. Insulin and β-Adrenergic Receptors Mediate Lipolytic and Anti-Lipolytic Signalling That Is Not Altered by Type 2 Diabetes in Human Adipocytes. *Biochemical Journal* **476,** 2883–2908 (Oct. 15, 2019).
5. Stich, V. *et al.* Activation of A2-Adrenergic Receptors Blunts Epinephrine-Induced Lipolysis in Subcutaneous Adipose Tissue during a Hyperinsulinemic Euglycemic Clamp in Men. *American Journal of Physiology-Endocrinology and Metabolism* **285,** E599–E607 (Sept. 1, 2003).
6. Lövfors, W. *et al.* A Systems Biology Analysis of Adrenergically Stimulated Adiponectin Exocytosis in White Adipocytes. *Journal of Biological Chemistry* **297** (Nov. 1, 2021).
7. Nyman, E., Fagerholm, S., Jullesson, D., Strålfors, P. & Cedersund, G. Mechanistic Explanations for Counter-Intuitive Phosphorylation Dynamics of the Insulin Receptor and Insulin Receptor Substrate-1 in Response to Insulin in Murine Adipocytes. *The FEBS journal* **279,** 987–999 (Mar. 2012).
